# Insights into the regulation of human CNV-miRNAs from the view of their target genes

**DOI:** 10.1186/1471-2164-13-707

**Published:** 2012-12-18

**Authors:** Xudong Wu, Dinglin Zhang, Guohui Li

**Affiliations:** 1 Laboratory of Molecular Modeling and Design, State key Laboratory of Molecular Reaction Dynamics, Dalian Institute of Chemical Physics, Chinese Academy of Sciences, 457 Zhongshan Rd., Dalian, 116023, PR China

**Keywords:** Copy number variation, miRNA, Expression variation, HapMap ethnic population

## Abstract

**Background:**

microRNAs (miRNAs) represent a class of small (typically 22 nucleotides in length) non-coding RNAs that can degrade their target mRNAs or block their translation. Recent research showed that copy number alterations of miRNAs and their target genes are highly prevalent in cancers; however, the evolutionary and biological functions of naturally existing copy number variable miRNAs (CNV-miRNAs) among individuals have not been studied extensively throughout the genome.

**Results:**

In this study, we comprehensively analyzed the properties of genes regulated by CNV-miRNAs, and found that CNV-miRNAs tend to target a higher average number of genes and prefer to synergistically regulate the same genes; further, the targets of CNV-miRNAs tend to have higher variability of expression within and between populations. Finally, we found the targets of CNV-miRNAs are more likely to be differentially expressed among tissues and developmental stages, and participate in a wide range of cellular responses.

**Conclusions:**

Our analyses of CNV-miRNAs provide new insights into the impact of copy number variations on miRNA-mediated post-transcriptional networks. The deeper interpretation of patterns of gene expression variation and the functional characterization of CNV-miRNAs will help to broaden the current understanding of the molecular basis of human phenotypic diversity.

## Background

miRNAs are a class of small non-coding RNAs, which act through binding in a sequence-specific manner to the 3^′^UTR of target genes [1]. Each miRNA can potentially regulate many transcripts and at least one-third of human genes are estimated to be miRNA targets. miRNAs participate in posttranscriptional gene regulation by repressing the expression of their target genes through inhibition of translation or cleavage of mRNAs [2-6]. miRNAs also contribute to genetic buffering of the gene expression variation, and play an important role in maintaining the identity of mature tissues through a feed-forward loop regulatory architecture [7,8], such as the relationship between *miR-9a* and *E(spl)* in *Drosophila*[9,10] and the regulation of *E2F1* by *miR-17* in human [11].

A primary goal in medical and evolutionary genomics is to understand the genetic mechanisms of natural variation in gene expression [12-16]. The structure of the human genome is highly variable and the copy number variations (CNVs) refer to alterations of genomic segments of more than 1,000 nucleotides that are present at significant frequencies within a population [17-19]. Many studies showed that CNVs can expand dosage variation of the associated genes, leading to the under-representation of dosage-sensitive protein-coding functional units such as transcription factors and members of protein complexes [20,21]. CNVs can be discovered by cytogenetic techniques, such as fluorescent *in situ* hybridization, comparative genomic hybridization, array comparative genomic hybridization, and next-generation sequencing [22-24]. In humans, more than 30,000 genomic regions with segmental duplications have been recognized by systematic comparative genomic hybridizations on the DNA of healthy human subjects; however, the CNVs of other animals were far less studied (see http://projects.tcag.ca/variation). For example, only about 2,000 CNVs have been identified in *Pan troglodytes*[25] and about 4,000 CNVs in inbred *Mus musculus*[26,27].

Recent studies revealed a high frequency in copy number abnormality of miRNA processing genes, such as *Dicer1* and *Argonature2*, in breast and ovarian cancers [28,29]. Although copy number alterations of miRNAs and their regulatory genes were frequently investigated in oncogenesis [28-30], the evolutionary and functional impact of CNV-miRNAs on the human genome has not been studied extensively. Based on the human genomic structure variations, Marcinkowska *et al.* recently detected about 30% miRNAs located in the human CNV-regions, indicating that non-coding RNAs also have potential functional variants [31].

In this study, we comprehensively analyzed the properties of genes regulated by CNV-miRNAs and explored the potential involvement of CNV-miRNAs in the expression variability of their targets within and between populations. Our analysis revealed significant functional differences between the targets of CNV-miRNAs and the targets of non-CNV-miRNAs. The involvement of CNV-miRNAs in a wide range of cellular responses provided us with valuable information of the impact of CNVs on the post-transcriptional network.

## Results

### Characterization of the regulation of CNV-miRNAs from the view of their target genes

We first compiled the genes regulated by CNV-miRNAs using the targets from TargetScan5.1 [32], which predicts miRNA targets based on sequence complementarities, sequence context information and binding energy. Because of its high confidence, TargetScan5.1 has been widely used in a variety of “omics” studies (see Methods) [32-34]. From among the miRNA-Target associations that were obtained, the representative miRNA for each family with the lowest total context score was presented, but all other miRNAs from the same family were considered to target the same gene at the same target sites [34]. To study the non-redundant miRNA binding sites directly, we replaced the miRNAs by their miRNA-family ID. Finally, 63,428 regulatory relationships were constructed comprising 541 miRNA-families and 9,174 targets (see Additional file 1).

According to the study by Marcinkowska *et al.*[31], a total of 209 miRNAs were found to locate in the human CNV-regions. These miRNAs belong to 172 families (see Additional file 2); the remaining 369 miRNA-families had no members in the CNV-regions. In the following analysis, these two types were referred to as CNV-miRNAs and non-CNV-miRNAs, respectively.

We investigated target genes of the non-CNV-miRNAs and CNV-miRNAs and classified them into three groups (see Additional file 3). The first group contains a total of 1,134 target genes that are regulated exclusively by CNV-miRNAs, 823 of the genes are regulated by one CNV-miRNA, 211 by two CNV-miRNAs, 67 by three CNV-miRNAs, 22 by four CNV-miRNAs, and 11 by ≥ 5 CNV-miRNAs. The second group contains a total of 5,710 target genes that are regulated by non-CNV-miRNAs and at least one CNV-miRNA. The third group consists of 2,330 target genes that are regulated exclusively by non-CNV-miRNAs, 1,408 of the genes are regulated by one non-CNV-miRNA, 515 by two non-CNV-miRNAs, 207 by three non-CNV-miRNAs, 95 by four non-CNV-miRNAs and 105 by ≥ 5 non-CNV-miRNAs.

**Figure 1:**
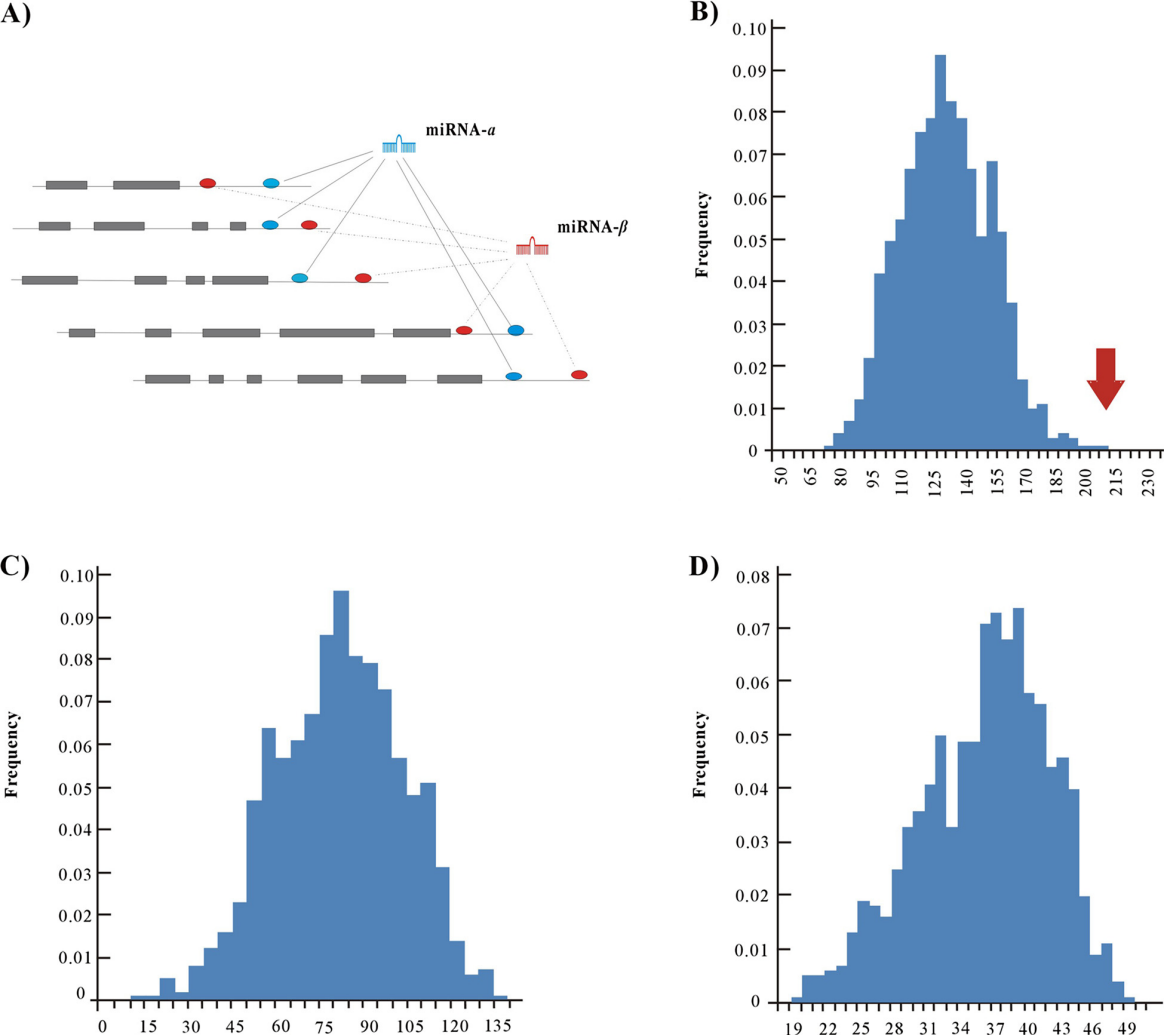
**Synergistic regulation of CNV-miRNAs.** (**A**) Schematic representation of five genes regulated by both CNV-miRNA-*α* and CNV-miRNA-*β*. (**B**) Distribution of genes affected by two pseudo-CNV-miRNAs in 1000 simulations. The arrow on the right hand side represents the observed number of targets of two CNV-miRNAs. (**C**) Distribution of genes targeted by the two non-independent CNV-miRNAs in 1,000 random simulations. (**D**) Distribution of CNV-miRNAs as a result of dosage-balance from synergistic regulation.

To explore the target-recognition preference of CNV-miRNAs and non-CNV-miRNAs, we devised a sampling method to investigate whether the observed number of target genes for each regulatory type could be expected from random sampling. The simulation analysis involved two steps: (a) 172 miRNAs were selected randomly from the 541 miRNAs, and assumed to be pseudo-CNV-miRNAs; (b) in the miRNA-target regulatory network (see Additional file 1), the edges connecting genes and pseudo-CNV-miRNAs, and the edges connecting genes and pseudo-non-CNV-miRNAs were marked, respectively; the number of target genes (*k*) was recorded for each type. The steps (a) and (b) were repeated 1,000 times, and resulted in normal distributions of target genes for each type of miRNA regulation. The Z-scores and their transformed p-values (calculated by NORMDIST function in Microsoft Excel) were then used to assess the statistical significance of whether the observed number deviated significantly from random expectation. The simulations provide clues to the regulatory patterns of CNV-miRNAs. As shown in Table 1, the number of genes regulated exclusively by one CNV-miRNA (823 genes were regulated by 137 CNV-miRNAs, approximately 6 target genes per CNV-miRNA) was significantly higher than the number expected from random simulations (p~0.05). In contrast, the number of genes regulated exclusively by one non-CNV-miRNA (1,408 genes were regulated by 280 non-CNV-miRNAs, approximately 5 target genes per non-CNV-miRNA) was significantly lower than the number expected from random simulations (p~0.05). Thus CNV-miRNAs tend to target a higher average number of genes compared with non-CNV-miRNAs. Besides, two and more CNV-miRNAs tend to synergistically regulate the same genes; that is, these genes are preferentially targeted by a combination of CNV-miRNAs in which directional selection may be involved in increasing the frequency of CNV-miRNAs in the human genome [35-37]. Obviously, the copy number variation of miRNAs is not independent of copy number variation of the other miRNAs if their binding sites are co-located in the same untranslated regions (UTRs) and regulate the same genes. As shown in Figure 1A for this type of co-regulation, miRNA-*α*<−>miRNA-*β*, the copy number alteration of miRNA-*α* could influence copy number alteration of miRNA-*β*, or vice versa. Theoretically, it is required that dosage of miRNA-*α* and miRNA-*β* should be balanced in synergistically regulating the same genes, which may promote the simultaneous retention of concurrent CNV-miRNAs and finally increase reciprocally the number of genes regulated by CNV-miRNAs. To verify this speculation, we analyzed 211 target genes that were regulated exclusively by two CNV-miRNAs, this dataset contained 422 interactions among 211 genes and 113 CNV-miRNAs (see Additional file 3). If CNV-miRNAs were retained or occurred independently, the number of target genes should follow a normal distribution of N(134,22) (see Table 1 and Figure 1B). Therefore, the number of genes affected by non-independent CNV-miRNAs can be estimated as 211-N(134,22) = N(77,22) (see Figure 1C). To investigate how many of the CNV-miRNAs were caused by the dosage-balance in co-regulation of the same genes, we (a) removed the information of CNV-miRNAs and then drew a number (*m*) from a normal distribution N(77, 22), (b) randomly assigned *m* genes to the miRNA-target regulatory network (see Additional file 1), miRNAs which targeted the selected genes were marked, and their number (*f*) was recorded. The two steps, (a) and (b), were repeated 1,000 times. *f* followed a normal distribution as N(74, 14) and was then divided by 2 to give N(37, 7). Thus, the miRNA-target recognition retained about 37 CNV- miRNAs with the standard error of 7 (see Figure 1D); at least one-third (calculated by *37/113*) of the CNV-miRNAs were attributable to the requirement of dosage-balance for synergistic regulation.

**Table 1:** Simulation analysis to explore the target-recognition preference of CNV-miRNAs and non-CNV-miRNAs

	**The numberof regulatory miRNAs**	**Mean of 1,000 simulations**	**Std. dev of 1,000 simulations**	**Observed number**	**p-values**
**Genes regulated exclusively by CNV miRNAs**	**1 CNV-miRNA**	716.479	67.633	**823**	0.0576
	**2 CNV-miRNAs**	134.428	22.810	**211**	0.000392
	**3 CNV-miRNAs**	28.597	8.115	**67**	0.000001
	**4 CNV-miRNAs**	7.119	3.421	**22**	0.000006
	≥**5 CNV-miRNAs**	2.809	1.846	**11**	0.0000004
**Genes regulated exclusively by non-CNV miRNAs**	**1 non-CNV-miRNA**	1514.503	67.633	**1,408**	0.0576
	**2 non-CNV-miRNAs**	609.760	45.420	**515**	0.0184
	**3 non-CNV-miRNAs**	277.837	31.374	**207**	0.0119
	**4 non-CNV-miRNAs**	145.793	22.981	**95**	0.0135
	≥**5 non-CNV-miRNAs**	185.483	42.717	**105**	0.0297

The p-values were obtained from the Z-score, which was calculated as (observed number – mean number of 1000 simulations) / standard deviation of 1000 simulations.

### Target genes of CNV-miRNAs tend to be differentially expressed among individuals within a population

Intuitively, CNVs of miRNA genes can dramatically change their dosage, and this would then affect the expression levels of the target genes in the corresponding individuals [5,15]. Recently, a series of genome-wide gene expression profiles have been measured in four HapMap ethnic populations, *CEU* (U.S. residents with Northern and Western European ancestry), *YRI* (Yoruba people of Ibadan, Nigeria), *CHB* (Chinese Han in Beijing) and *JPT* (Japanese from Tokyo). We calculated the coefficient of variation (CV) for each protein-coding gene across individuals in the four populations to quantify the within-population expression variability of each of the genes (see Methods). Briefly, the CV is the ratio of the standard deviation of gene’s expression to its mean intensity, which is considered to be an unbiased and comprehensive metric to measure the regulation diversity at the expression level among individuals [38] (see Additional file 4).

**Figure 2:**
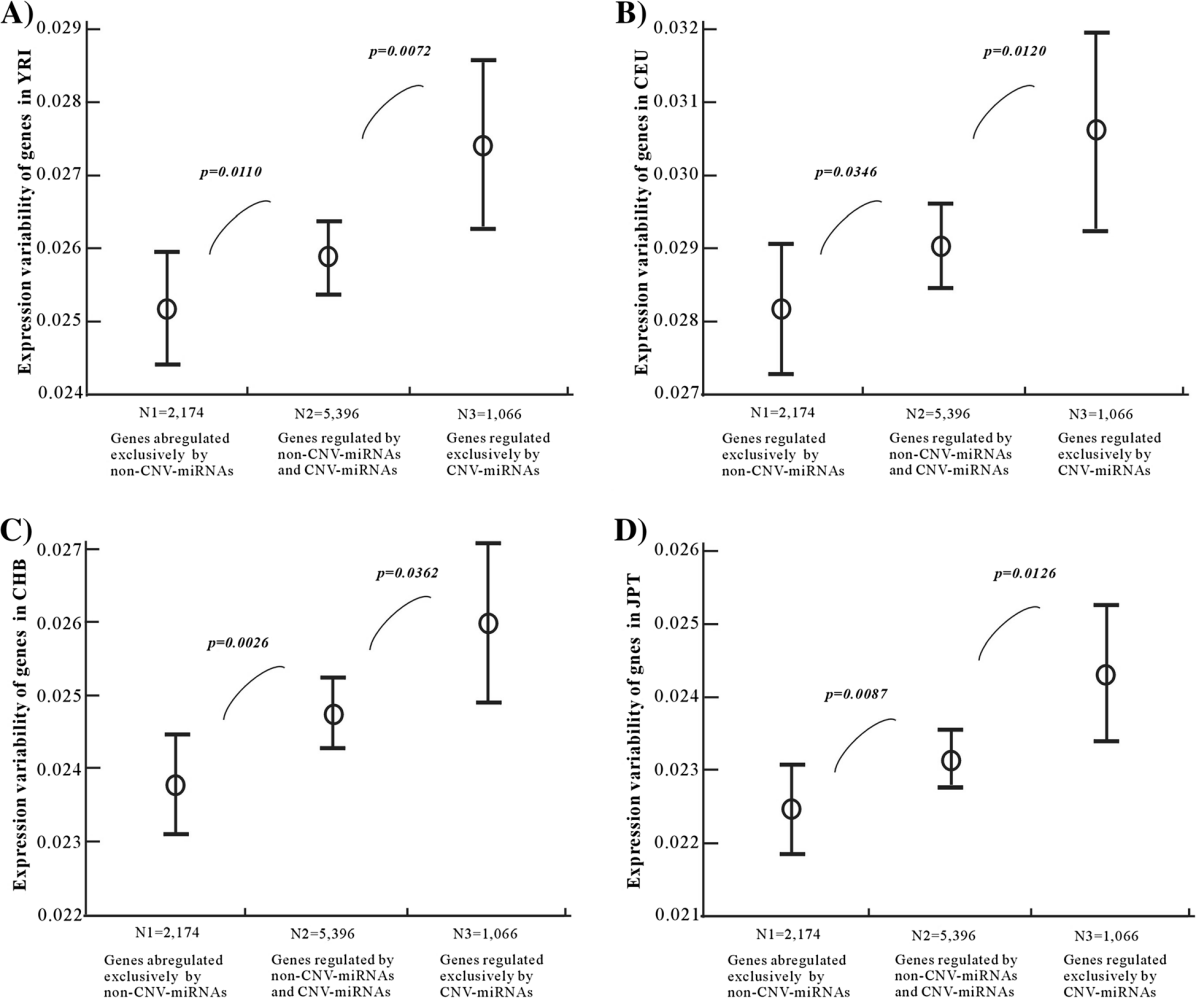
**Expression variability of target genes within four human populations.** This figure shows the comparison of the coefficient of variation calculated from the gene expression profiles in (**A**) *YRI*, (**B**) *CEU*, (**C**) *CHB*, and (**D**) *JPT* populations.

As shown in Figure 2A for the YRI population, the mean CV was 0.0251 for target genes regulated exclusively by non-CNV-miRNAs and increased to 0.0258 for target genes regulated by both CNV-miRNAs and non-CNV-miRNAs (p=0.0110, *Mann–Whitney U, two-tail test*), the mean CV was further increased to 0.0274 for target genes regulated exclusively by CNV-miRNAs (p=0.0072, *Mann–Whitney U, two-tail test*). Using the CVs calculated in CEU (Figure 2B), CHB (Figure 2C) and JPT (Figure 2D) populations, we obtained similar results.

**Figure 3:**
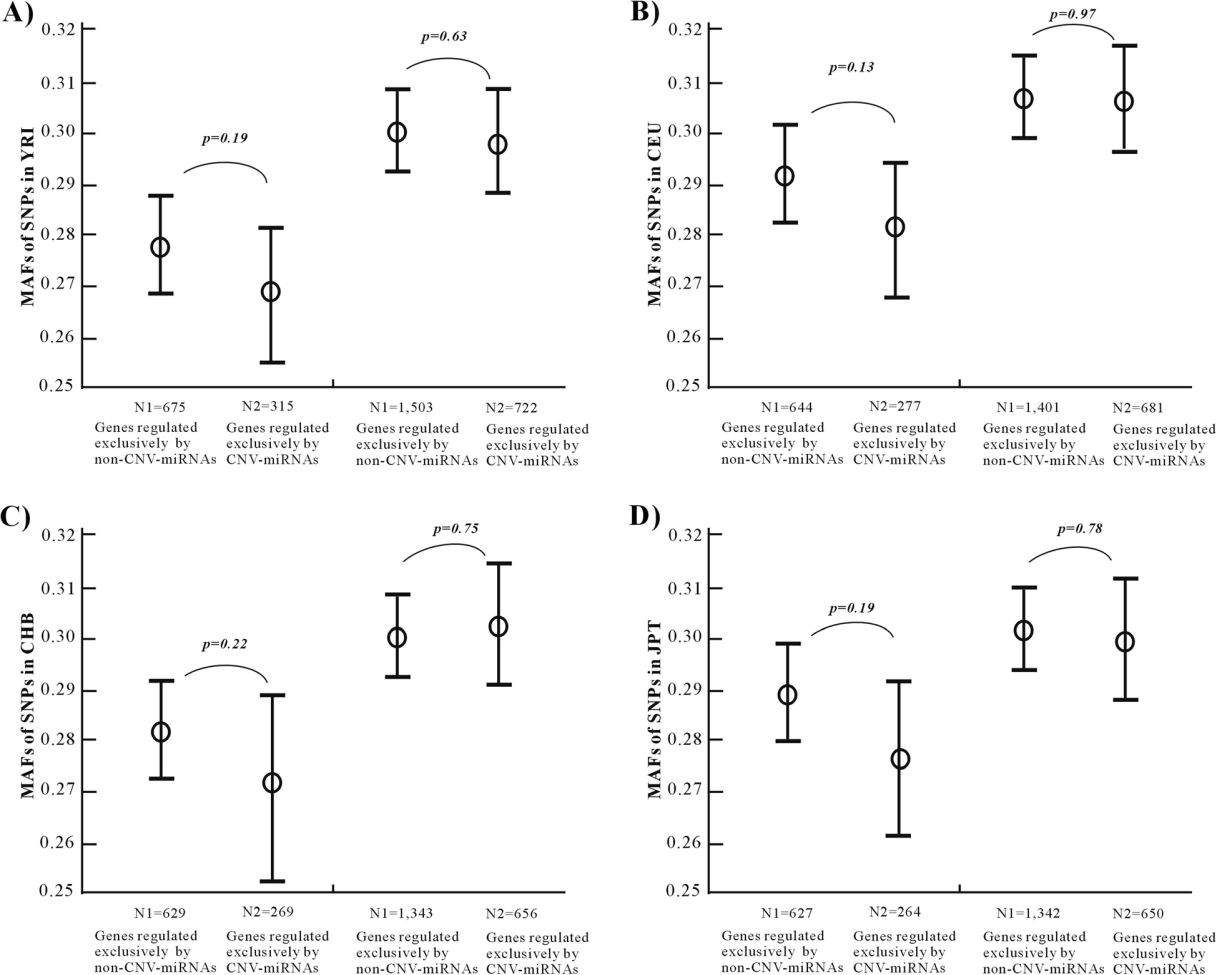
**MAFs of SNPs in UTRs of target genes regulated exclusively by CNV-miRNAs or non-CNV-miRNAs.** This figure shows the comparison of MAFs of SNPs in (**A**) YRI, (**B**) CEU, (**C**) CHB, and (**D**) JPT populations. In each sub-figure, the left panel shows the comparison of MAFs in the 5^′^UTRs, the right panel shows the comparison of MAFs in the 3^′^UTRs.

The associated sequence variants, such as causative bi-allelic SNPs, could also lead to the different expression variability [12-14,39], we explored whether the minor allele frequencies (MAFs) of SNPs in the target genes of the CNV-miRNAs were significantly different from target genes of non-CNV-miRNAs. The 5^′^UTR and 3^′^UTR sequences of human Ensembl genes were downloaded using BioMart [40], and then the HapMap Phase III SNPs (retrieved from http://hgdownload.cse.ucsc.edu/goldenPath/hg19/database/) [41] were mapped onto the sequences (see Methods and Additional file 5). As shown in Figure 3, genes regulated exclusively by either non-CNV-miRNAs or CNV-miRNAs have similar proportions of genes that have SNPs in 5^′^UTRs and 3^′^UTRs; furthermore, the SNPs in the 5^′^UTRs and 3^′^UTRs have similar MAFs in each of four HapMap populations (p-values range from 0.13 to 0.97, two-tailed t-test). Because genome-wide association and regression analyses have mainly used the MAFs to infer statistical correlations of SNPs with a trait; similar MAFs often indicate that the corresponding SNPs have similar probability to be detected. Therefore, the *cis*-elements of 5^′^UTRs and 3^′^UTRs may contain less information than *trans*-elements in explaining gene expression variations, it is possible that the regulation of some CNV-miRNAs adds a more diversifying control and promotes the differential expression of their target genes among individuals.

### Target genes of CNV-miRNAs are more likely to be differentially expressed between populations

A good study has demonstrated that the within-population expression variability of genes can influence the propensity of their differential expression levels between populations [42]. Here, some CNV-miRNAs may live in different populations; thus, the genes targeted by these CNV-miRNAs are likely to be differentially expressed among individuals within a population and also between different populations.

**Figure 4:**
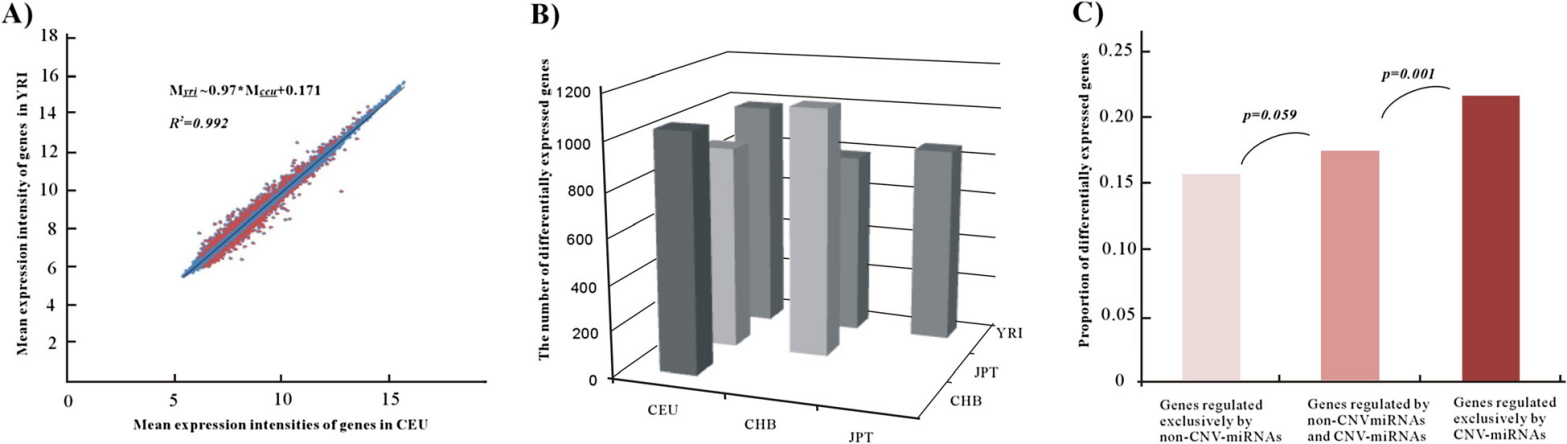
**Expression variability of target genes between four human populations.** (**A**) The plot of expression intensities of the genes in the YRI and CEU population, the red dots represent the genes that show CEU- and YRI-specific significantly differential expression intensities. (**B**) Number distribution of the differentially expressed genes in six population pairs selected from four ethnic populations. (**C**) Comparison of proportion of genes differentially expressed in the genes that are regulated exclusively by either non-CNV-miRNAs, non-CNV-miRNAs and CNV-miRNAs, or CNV-miRNAs, respectively.

To verify this prediction, we identified the genes that were differentially expressed between any two of the four populations. Taking the CEU and YRI populations as example, we first (a) regress average gene expression intensity, M*yri*, in YRI and M*ceu*, in CEU reciprocally; (b) using M*yri* as the explanatory variable, a liner model was derived by minimizing the square errors between the observed M*yri* and the predicted values (^^^M*yri*); (c) the residues, r = M*yri* - ^^^M*yri*, were transformed by a quartile normalization and studentized to ^^^r, the outliers were detected according to their ^^^r away from the calculated 95% confidence intervals of the t-distribution (see details in *lm* and *rstudent* functions of stats R package http://www.r-project.org/); (d) using M*ceu*, as the explanatory variable, the two steps (b) and (c) were repeated. As shown in Figure 4A, the mean expression intensities of the genes in the CEU and YRI populations were compared; the red dots in the plot of M*yri* and M*ceu* represent genes showing *CEU*- and *YRI*-specific variation of expression intensity.

Using the method described above, we identified genes that were differentially expressed in at least one of the four ethnic populations (see Additional file 6). As shown in Figure 4B, a similar number of genes were differentially expressed among six population pairs selected from the four ethnic populations. We then investigated whether genes targeted by CNV-miRNAs were over-represented in these differentially expressed genes. As shown in Figure 4C, the proportion of differentially expressed genes was 15.7% for targets regulated exclusively by non-CNV-miRNAs, 17.4% for targets regulated by both CNV-miRNAs and non-CNV-miRNAs (p=0.060, *Chi-square, two-tail test*), the proportion increased further to 21.7% for targets regulated exclusively by CNV-miRNAs (p=0.001, *Chi-square, two-tail test*).

### Target genes of CNV-miRNAs tend to be differentially expressed across tissues and developmental stages

**Figure 5:**
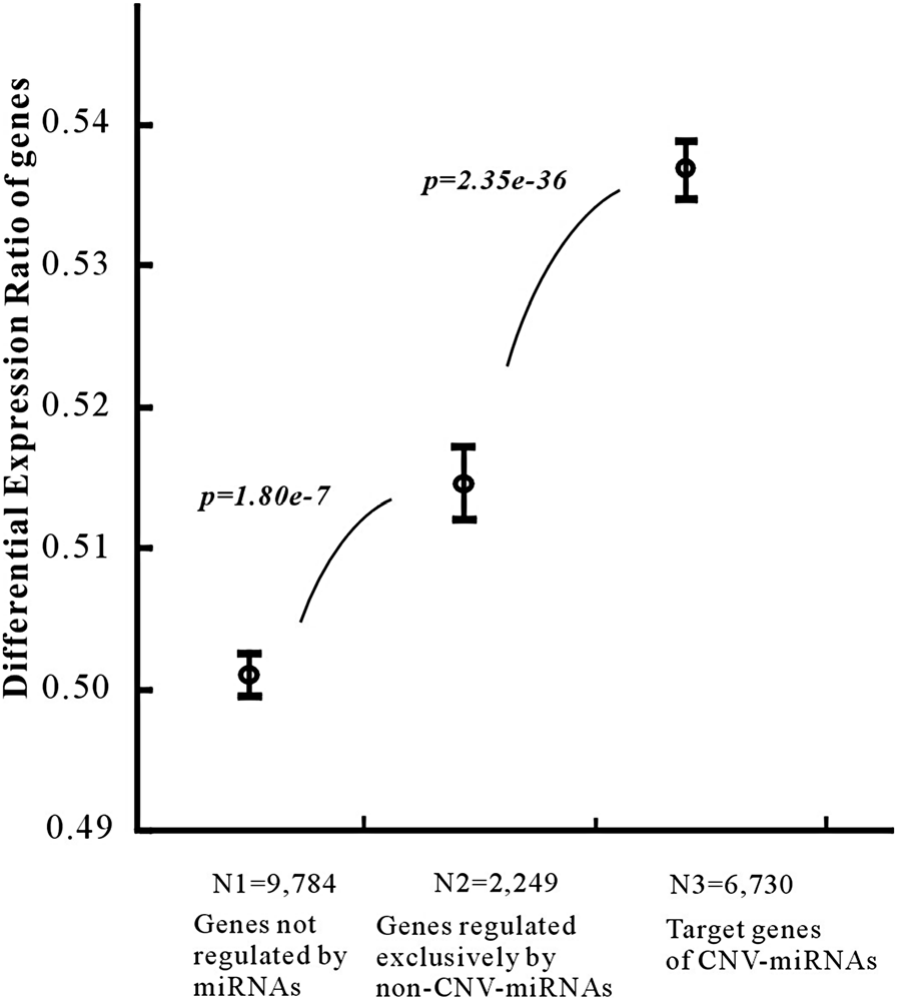
**Comparison of the differential expression ratios of human genes.** Expression variation was measured across 4,877 subset-versus-subset comparisons.

For miRNAs that are specifically expressed in a particular tissue or at a particular developmental stage, the copy number duplication or deletion of miRNAs may lead to either weaker or stronger expression of their target genes in the corresponding tissue and developmental stage. For each human gene, we obtained its Differential Expression Ratio (DER) from the FitSNPs [43]. This DER value was a measure of the frequency of differential expression of the gene in multiple microarray studies across thousands of samples (see Methods). Because the *DER* is derived from all available human microarray datasets deposited in NCBI’s GEO database (http://www.ncbi.nlm.nih.gov/geo/), it provides a comprehensive metric to measure the regulation diversity of genes at the expression level [44]. As shown in Figure 5, the mean DER was 0.506 for 9,784 genes that are not regulated by miRNAs, 0.514 for 2,249 target genes regulated exclusively by non-CNV-miRNAs (p=1.81E-7, *Mann–Whitney U, two-tail test*), and increased further to 0.535 for 6,730 target genes of CNV-miRNAs (p=2.36E-36, *Mann–Whitney U, two-tail test*), which include 5,626 targets regulated by non-CNV-miRNAs and CNV-miRNAs, and 1,104 targets regulated exclusively by CNV-miRNAs (see Additional file 7). Therefore, CNV-miRNAs indeed add a more diversifying and complex regulation control to their targets and contribute to an increased likelihood of differential expression among different tissues, cell types, developmental and disease stages.

### Functional differences between target genes regulated exclusively by CNV-miRNAs and target genes regulated exclusively by non-CNV-miRNAs

**Figure 6:**
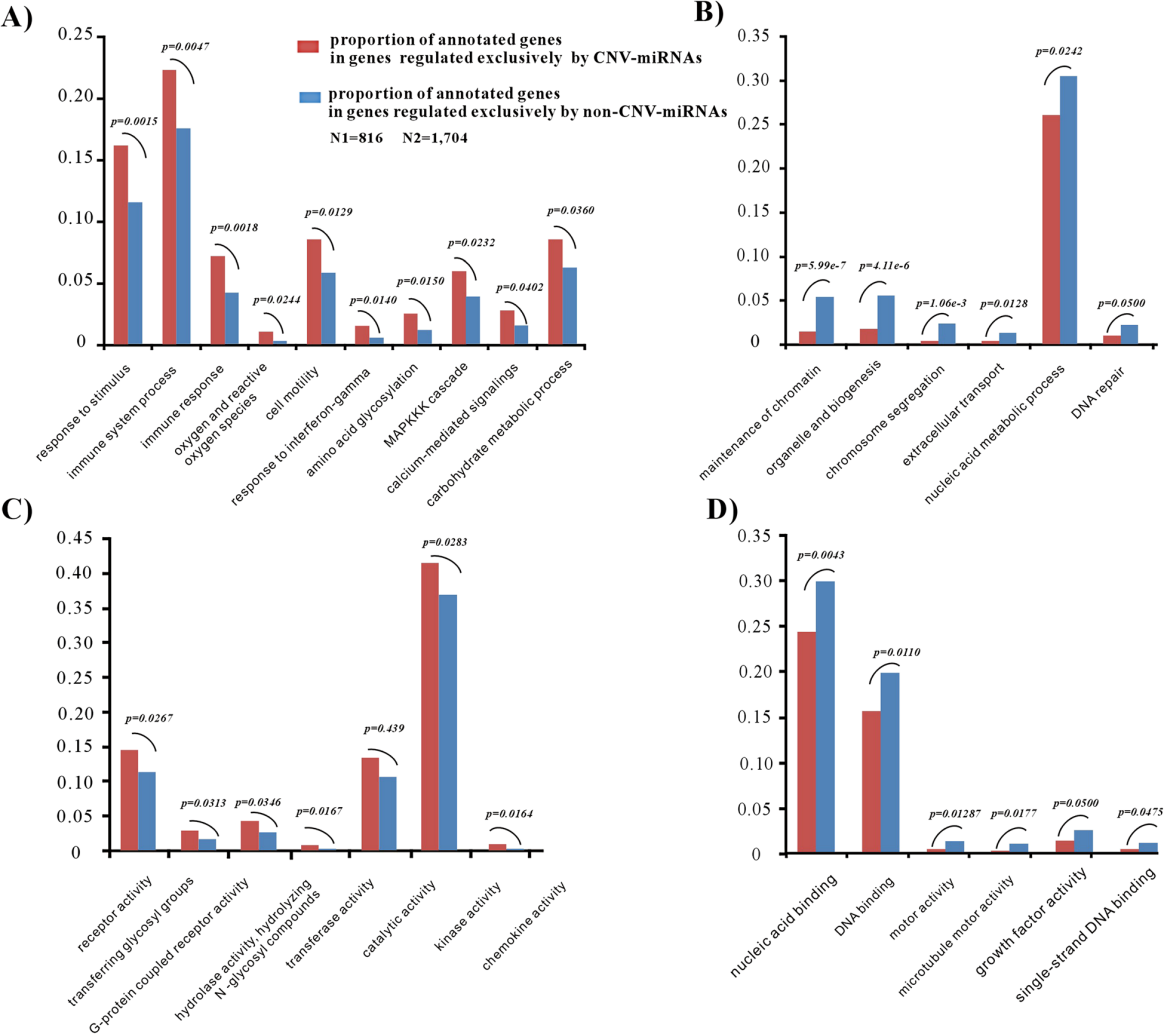
**Functional differences based on GO terms between the genes regulated exclusively by CNV-miRNAs and the genes regulated exclusively by non-CNV-miRNAs.** (**A**) Biological process groups that show a significantly higher percent of genes regulated exclusively by CNV-miRNAs. (**B**) Biological process groups that show a significantly higher percent of genes regulated exclusively by non-CNV-miRNAs. (**C**) Molecular function groups that show a significantly higher percent of genes regulated exclusively by CNV-miRNAs (**D**) Molecular function groups that show a significantly higher percent of genes regulated exclusively by non-CNV-miRNAs. N1 represents the number of genes with GO-annotation in genes exclusively regulated by CNV-miRNAs, N2 represents the number of genes with GO-annotation in genes exclusively regulated by non-CNV-miRNAs. P-values were calculated by Fisher’s exact two tailed test.

The Gene Ontology annotation system [45] contained 190,525 associations among 14,117 human genes and 412 GO terms. This data was downloaded and intersected with the 9,174 miRNA target genes that were identified using TargetScan5.1. We obtained GO terms for 6,952 miRNA targets and sought to determine whether the genes that were regulated exclusively by CNV-miRNAs encode proteins that have specific molecular functions or that are involved in particular biological processes (see Methods). As shown in Figure 6B and 6D, targets regulated exclusively by non-CNV-miRNAs were significantly enriched for fundamental biological processes such as maintenance of chromatin, organelle and biogenesis, chromosome segregation, extracellular transport and nucleic acid metabolic process. These processes are known to be essential and dosage-sensitive and their radical fluctuation usually reduces an organism’s fitness. In contrast, targets regulated exclusively by CNV-miRNAs are enriched for processes responsible for stimulus response, immune response, amino acid glycosylation and the MAPKKK cascade (Figure 6A and 6C). These processes were environment-oriented and transduce a large variety of external signals, leading to a wide range of cellular responses such as growth, differentiation, inflammation and apoptosis. The flexible regulation for these processes is required and generally provides positive selectiveness to an organism’s survival.

## Discussion

It is interesting to know whether or not the orthologs of human CNV-miRNAs were also located in CNV-regions of other animals. We compiled the available CNVs of *Pan troglodytes*[25] and *Mus musculus*[26,27], and then intersected the location of their miRNAs with the coordinates of the CNVs. The results showed that only 21 and eight miRNA-families have members located in CNV-regions in *Pan troglodytes* and inbred *Mus musculus*, respectively (see Additional file 8). Hence, the human genome contained the highest proportion of CNV-miRNAs, making it the best model to detect the mechanisms and function of CNV-miRNAs.

Animal genomes have the characteristics of dynamics and plasticity, giving them the ability to adapt to changing environmental conditions. Mobile and evolving elements such as telomeres, transposons, and copy number variants have been studied in investigations into the potential effect of environment on genomes. For example, Haasl and Payseur designed a mathematical model to study microsatellite variations, such as the expected distribution of repeat sizes, and the expected squared difference in repeat size among samples; their simulations revealed that microsatellites, especially triplet repeats, provided adaptation facilitators for beneficial evolution of genomes [46]. miRNAs are relatively newly discovered genomic elements, but their post-transcriptional regulation is present early on in metazoan evolution [47]. The number of miRNAs in a genome correlates with the morphological complexity of the animal, indicating that they play roles in evolutionary changes of body structure [48]. It is now widely accepted that an increase in the complexity of gene regulatory mechanisms, at both the genomic and transcriptomic level, drives the appearance of more complex organisms. Two distinct mechanisms of increasing complexity of gene expression, namely, the co-evolution between CNVs and miRNAs, have been recently recognized and studied. Marcinkowska *et al.* compared the fractions of miRNA loci and the fraction of genome covered by CNVs, and reported that the CNV purification effect was insignificant [31]. Felekkis *et al.* demonstrated that the number of distinct miRNA types and the average number of miRNA binding sites in genes in CNV regions were significantly higher than genes in non-CNV regions [37]. In this study, we proposed the miRNA-target recognition may play important roles in escape from purification of the CNV-miRNAs that target the same genes. Further analysis revealed that “targeting by CNV-miRNAs” seems to be favored and that the target genes participate in a wide-range of cellular responses to environmental factors. For target genes regulated by one miRNA, CNV-miRNAs tend to target a higher average number of genes than non-CNV-miRNAs. From an evolutionary viewpoint, if the CNV-miRNAs were deleterious and only remained in the genome because they were difficult to remove, then we might expect them to have a tendency to target, on average, a lesser number of genes than non-CNV-miRNAs; furthermore, if the CNV-miRNAs were neutral and their retention attributed to random genetic drift, the CNV-miRNAs and non-CNV-miRNAs should target a similar average number of genes. Therefore, some CNV-miRNAs seems to be beneficial to the organism and “targeting by CNV-miRNAs” may provide positive selective pressure to their target genes.

**Figure 7:**
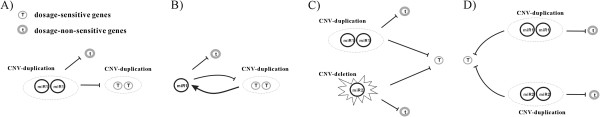
**Schematic representation of the four paradigms used to explain co-evolution between CNVs and miRNAs.** (**A**) A simple repression motif is involved where a miRNA reduces the expression of its target (T), the increased dosage due to CNV-duplication of the target (T) is balanced by the corresponding CNV-duplication of miRNA. (**B**) miRNA and its target (T) mutually buffer each other’s expression from perturbation in a negative feedback loop, the increased dosage due to CNV-duplication of the target (T) is buffered by the expression variation of the miRNA. (**C**) The CNV-duplication of some miRNAs compensate for the CNV-deletion of other miRNAs in balancing the dosage of their common target (T). (**D**) The common target (T) of two miRNAs is up-regulated in the cellular response to environmental factors; the intrinsic dosage-sensitivity of the target (T) makes the CNV-duplication of both the miRNA1 and miRNA2 become favorable. miRNA1 and miRNA2 represent two different miRNAs.

From a biological view, four paradigms could be used to explain the co-evolutionary relationship between CNVs and miRNAs. In the first paradigm, a simple repression motif is involved where miRNA reduces the expression of its target (T), and the increased dosage due to CNV-duplication of the target (T) is balanced by the corresponding CNV-duplication of miRNA (Figure 7A). In the second paradigm, a miRNA and its target (T) mutually buffer each other’s expression from perturbation in a negative feedback loop, the increased dosage due to CNV-duplication of the target (T) is buffered by the expression variation of the miRNA [49] (Figure 7B). In the third paradigm, the CNV-duplication of some miRNAs can compensate for the CNV-deletion of other miRNAs in balancing the dosage variation of their common target (T) (Figure 7C). In the final paradigm, the common target (T) of two miRNAs is up-regulated in the cellular response to environmental factors, the intrinsic dosage-sensitivity of the target (T) makes the CNV-duplication of both the miRNAs favorable (Figure 7D). Obviously, CNVs and miRNAs must have co-evolved complementarily in a tradeoff between maintaining the balance of the dosage-sensitive genes and the increasing diversity of dosage-non-sensitive genes [50]. With genomic plasticity being controlled, CNV-miRNAs provide the possibility of increasing regulatory complexity and the evolvability of genomes.

Our analyses revealed pervasive impacts of CNV on the miRNA-mediated post-transcription regulatory network. Previous studies demonstrated that miRNAs preferentially regulated the hubs of protein interaction [51] and metabolic networks [52]. We here propose that the CNV of miRNAs may fluctuate the dosage balance of signal transduction pathways, metabolic flux or protein complexes [53,54], leading eventually to individuals of the same population or different populations having different susceptibility to diseases [55]. Although it is difficult to identify these CNV-miRNAs without a comprehensive investigation of health risks among human populations, recent experimental studies have discovered CNV-causing dysregulation of miRNAs that confirmed their roles in disease occurrence. In one study, next-generation sequencing technology was used to explore CNV as a potential mechanism of miRNA mis-expression, the affected miRNA loci were consistently found to be either lost or gained, and their candidate mRNA targets were coordinately dysregulated; the authors demonstrated the structure variation of the miRNA loci clearly characterized the pre-invasive stage of breast cancer [56]. In another study, genetic networks were inferred from miRNA expression in normal and cancer tissues, and cancer networks built from disjointed sub-networks were found to accompany miRNA copy number alterations, such as the amplification of the hsa-miR-17/92 family, the deletion of the hsa-miR-143/145 cluster, and the physical alteration of the hsa-miR-204/30 at the DNA copy number level [57]. The results of these studies clearly demonstrate the feasibility of using the dysregulation of CNV-miRNAs as biological markers for disease screening; indicating that CNV-miRNAs and their targets should be given more attention in studies of human health.

## Conclusions

To the best of our knowledge, this is the first genome-wide integrative analysis among human CNVs, miRNAs, their targets and expression variations. Our results will pave the way for future studies for the functional characterization of CNV-miRNAs. This study reveals more clear roles of CNV-miRNAs and is valuable for studying the impact of CNVs on human health.

## Methods

### Compilation of human miRNA target genes

The miRNAs and their predicted targets were taken from TargetScan (http://www.targetscan.org version 5.1) [32,33]. Targets with a total context score of −0.3 or lower were ignored, where the score quantitatively measure the overall target efficacy [58]. A total of 9,174 targets with at least one conserved 7-mer or 8-mer were selected as reliable miRNA targets [59] (see Additional file 1).

### Analysis of human gene expression data

The microarray-based gene expression profiles were derived from lymphoblastic cell lines of 270 HapMap individuals (http://www.sanger.ac.uk/humgen/genevar, GSE6536), including 90 samples of YRI (Yoruba people of Ibadan, Nigeria), 90 samples of CEU (U.S. residents with northern and western European ancestry), 45 samples of CHB (Chinese Han in Beijing) and 45 samples of JPT (Japanese from Tokyo) [60,61]. The annotation table was retrieved from http://www.ncbi.nlm.nih.gov/projects/geo/query/acc.cgi?acc=GPL2507. The RefSeq identifiers were transformed to Ensembl Gene ID through BioMart [40]. Finally, the expression profiles of 16,686 human genes (including 8,636 miRNA targets) across four HapMap populations were complied.

The following formulas were adopted to calculate the coefficient of variation (CV) of gene *i* in each ethnic population.

The mean intensity *M*_*i*_ calculated by $M_{i}=\frac{\sum_{j=1}^{n}S_{\mathrm{ij}}}{n}$

The standard deviation *σ*_*i*_ calculated by $\sigma_{i}=\sqrt{\frac{{\left(S_{\mathrm{ij}}-M_{i}\right)}^{2}}{n-1}}$,

The coefficient of variation *CV*_*i*_ calculated by $CV_{i}=\frac{\sigma_{i}}{M_{i}}$

Where *j*=1 to *n*, *n* represents the number of samples in a population, *S*_*ij*_ represents the expression signal of gene *i* in sample *j*. Greater CV implies higher expression variability of a gene across individuals within the corresponding population (see Additional file 4).

### Calculation of MAFs of SNPs in UTRs of human genes

Minor allele frequency (MAF) refers to the frequency at which the less common allele occurs in a given population. SNPs with a minor allele frequency of 5% or greater were targeted by the HapMap project and have been widely employed in Genome Wide Association Studies for complex traits (GWAS) [62,63].

For a SNP *A/a*, the minor allele frequency was calculated by the following formula

(1) $$\mathrm{MAF}=\frac{\min\left(2N_{\mathrm{AA}}+N_{\mathrm{Aa}},2N_{\mathrm{aa}}+N_{\mathrm{Aa}}\right)}{\left(2N_{\mathrm{AA}}+2N_{\mathrm{aa}}+N_{\mathrm{Aa}}\right)}$$

Where N_*aa*_ represents the count of individuals who are homozygous for allele1, N_*Aa*_ represents the count of individuals who are heterozygous, N_*aa*_ represents the count of individuals who are homozygous for allele2.

### Compilation of DERs of human genes

The differential expression ratios (DER) of human genes were obtained from the study by Chen *et al*. (FitSNPs, http://fitsnps.stanford.edu/download.php) [43]. Briefly, the authors downloaded 476 human GEO datasets from the NCBI Gene Expression Omnibus and categorized each GEO dataset into 24 types of comparisons, such as disease state, cell type, metabolism and so on. A total of 4,877 subset-versus-subset comparisons were performed to identify differentially expressed genes with a cutoff of *q value* ≤ 0.05 by *SAM* package [44]. For each human gene, the count of GEO datasets in which it was differentially expressed was divided by the count of its measured GEO.

The gene symbols and EntrezGene IDs were transformed to their Ensembl gene IDs using the BioMart program [40].The Ensembl genes with available DERs were then intersected with the genes that were used for TargetScan5.1 prediction. Finally, the DER values of 9,784 genes that are not regulated by miRNAs and 8,979 target genes of miRNAs were obtained.

### Functional analysis of human genes based on gene ontology

The Gene Ontology (GO) has developed three structured controlled vocabularies to describe gene products in terms of their associated biological processes, cellular components and molecular functions [45]. The human gene association file was downloaded from http://www.geneontology.org/gene-associations/. For each GO term, the proportion of annotated genes was compared between the genes regulated exclusively by CNV-miRNAs and the genes regulated exclusively by non-CNV-miRNAs. The p-value was estimated by Fisher’s exact two-tailed test*,* and a cutoff of p ≤ 0.05 was used to identify the over-represented or under-represented GO terms among the genes that are regulated exclusively by CNV-miRNAs.

### Computational environment

The project was started and completed in Dalian Institute of chemical Physics. Computations were performed on a Linux cluster with 50 nodes (Intel 5130, 2.0 GHz CPU, 4G memory, Laboratory of Molecular Modeling and Design, Dalian Institute of Chemical Physics, Chinese Academy of Sciences). Perl (http://perl.org) and R (http://www.r-project.org/) scripts were used for analysis, and can be obtained on request.

## Abbreviations

CNV: Copy number variation; CNV-miRNA: miRNA that is located in copy number variation regions; non-CNV-miRNA: miRNA that is not located in copy number variation regions; CEU: U.S. residents with northern and western European ancestry; YRI: Yoruba people of Ibadan, Nigeria; CHB: Chinese Han in Beijing; JPT: Japanese from Tokyo; CV: The coefficient of variation ratio; MAF: Minor allele frequency; GO: Gene Ontology.

## Competing interests

The authors declare that they have no competing interests.

## Authors’ contributions

XW and GL conceived and designed the study, XW performed the experiments, XW, DZ and GL analyzed the data, XW, DZ and GL wrote the paper. All authors read and approved the final manuscript.

## Supplementary Material

Additional file 1 The 63,428 regulatory relationships among 541 miRNA families and 9,174 target genes. Click here for file

Additional file 2 The 172 human CNV-miRNA-families and their encoding members. Click here for file

Additional file 3 **List of targets genes with three regulatory patterns of miRNAs.** The numbers in parenthesis represent the total number of regulatory miRNAs and the number of CNV-miRNAs, respectively. Click here for file

Additional file 4 Coefficient of variation (CV) of human protein-coding genes in four HapMap ethnic populations. Click here for file

Additional file 5 **Minor allele frequencies (MAFs) of 5**^**′**^**UTR- and 3**^**′**^**UTR-SNPs in four HapMap ethnic populations.** Click here for file

Additional file 6 List of 2,624 differentially expressed genes among six population pairs comparisons selected from four HapMap ethnic populations. Click here for file

Additional file 7 The differential expression ratios (DERs) of 1,8763 human genes that were used for TargetScan5.1 prediction. Click here for file

Additional file 8 **The 21 and eight CNV-miRNA-families of *Pan troglodytes *and *Mus musculus,*respectively.** As there are no miRNA targets from the TargetScan prediction for *Pan troglodyte*, the miRNA-family IDs were represented by Rfam identifiers (http://rfam.sanger.ac.uk/). Click here for file
