# Supplementary material for: Insights into the regulation of human CNV-miRNAs from the view of their target genes
Source: BMC Genomics. 2012 Dec 18;13:707. doi: 10.1186/1471-2164-13-707 (PMC3582595; doi:10.1186/1471-2164-13-707)
Supplement: Additional file 2 — The 172 human CNV-miRNA-families and their encoding members. [file 1471-2164-13-707-S2.pdf]

miRNA Family ID	miRNA
**GCUGGUG**	*hsa-miR-138;*
**CAGUCCA**	*hsa-miR-455-3p;*
**GGACGGA**	*hsa-miR-184;*
**GUAUUCU**	*hsa-miR-630;*
**UCACAUU**	*hsa-miR-23a;*
**CUCCCAA**	*hsa-miR-150;*
**CUGGCUC**	*hsa-miR-149;*
**UCCCUUU**	*hsa-miR-211;*
**UUGGUCC**	*hsa-miR-133a;*
**AGAUAAA**	*hsa-miR-577;*
**AUUGCAC**	*hsa-miR-25; hsa-miR-92b;*
**CACAGUG**	*hsa-miR-128;*
**CGUUUGC**	*hsa-miR-1282;*
**UUGGCAA**	*hsa-miR-182;*
**CCAGUGC**	*hsa-miR-1825;*
**ACAUUCA**	*hsa-miR-181c; hsa-miR-181d;*
**GCAGGAC**	*hsa-miR-1286;*
**UGCAUAU**	*hsa-miR-448;*
**AAUCUCU**	*hsa-miR-216b;*
**UCAACGG**	*hsa-miR-95;*
**CGUGGCC**	*hsa-miR-1204;*
**ACAAUAU**	*hsa-miR-338-5p;*
**GGGGUCC**	*hsa-miR-615-5p;*
**UGCAGCU**	*hsa-miR-1301;*
**GGAUGAG**	*hsa-miR-1255b;*
**UAAGACU**	*hsa-miR-499-5p;*
**AGGUAGU**	*hsa-miR-196a;*
**UCUCACC**	*hsa-miR-1229;*
**CCUUCUU**	*hsa-miR-1248;*
**GCCUGGG**	*hsa-miR-661;*
**AGCCCUU**	*hsa-miR-129-3p;*
**UGGGACA**	*hsa-miR-1302;*
**CAGCAGA**	*hsa-miR-922;*
**GAGAAGA**	*hsa-miR-1253;*
**GGCAGUG**	*hsa-miR-34a;*
**ACCACAG**	*hsa-miR-140-3p;*
**AACCUGU**	*hsa-miR-649;*
**GGAGGCA**	*hsa-miR-650;*
**CCAGUGU**	*hsa-miR-199a-5p;*
**CUCUUCC**	*hsa-miR-1236;*
**AAGGCAC**	*hsa-miR-124; hsa-miR-506;*
**GGGUAAG**	*hsa-miR-555;*
**AGUGGUU**	*hsa-miR-140-5p;*
**CUGGGCA**	*hsa-miR-612;*
**CCCUGAG**	*hsa-miR-125a-5p;*
**UGUCUUU**	*hsa-miR-511;*
**GAUUGUA**	*hsa-miR-508-3p;*

**GGCUGCG**	*hsa-miR-604;*
**CACAGGG**	*hsa-miR-220c;*
**GGGUCGG**	*hsa-miR-886-5p;*
**ACUGCAG**	*hsa-miR-509-5p;*
**UCAUUCG**	*hsa-miR-1298;*
**CCCACCU**	*hsa-miR-1224-3p;*
**AAUACUG**	*hsa-miR-200b; hsa-miR-429;*
**UCACAAG**	*hsa-miR-513b;*
**GGCGCCU**	*hsa-miR-566;*
**AGUAGUU**	*hsa-miR-1244;*
**AAGUCUU**	*hsa-miR-1264;*
**UUAGGAU**	*hsa-miR-651;*
**ACUGUAG**	*hsa-miR-1183;*
**UUGUGUC**	*hsa-miR-599;*
**AGGAUGU**	*hsa-miR-1265;*
**UGGCUGC**	*hsa-miR-647;*
**ACUCAGC**	*hsa-miR-512-5p;*
**CGUACCG**	*hsa-miR-126;*
**CCGAGCC**	*hsa-miR-615-3p;*
**GGGAUCG**	*hsa-miR-638;*
**AUGGCUU**	*hsa-miR-135a;*
**UUUUUGC**	*hsa-miR-129-5p;*
**UGAAAUG**	*hsa-miR-203;*
**AAGACAU**	*hsa-miR-641;*
**CAGACAG**	*hsa-miR-1324;*
**AUGGCAC**	*hsa-miR-183;*
**GCCUGGA**	*hsa-miR-1254;*
**GAGGUAG**	*hsa-let-7a; hsa-let-7b; hsa-let-7d; hsa-let-7e; hsa-let-7f; hsa-let-7g; hsa-let-7i; hsa-miR-98;*
**GUGGCCC**	*hsa-miR-663b;*
**GCAGGUU**	*hsa-miR-657;*
**ACCUGGA**	*hsa-miR-619;*
**UGGGUAC**	*hsa-miR-1225-5p;*
**GUCAUUG**	*hsa-miR-616;*
**CAGUUAC**	*hsa-miR-935;*
**CGUUGGC**	*hsa-miR-1306;*
**AGGCAGG**	*hsa-miR-940;*
**GGGGUGG**	*hsa-miR-608;*
**ACUCCAG**	*hsa-miR-508-5p;*
**UCAAGUA**	*hsa-miR-26a;*
**CUCUAGC**	*hsa-miR-1251;*
**ACUCAGG**	*hsa-miR-510;*
**CAGUAGU**	*hsa-miR-199a-3p;*
**UGUGCGU**	*hsa-miR-210;*
**GUGAAUG**	*hsa-miR-1257;*
**CCAGUAC**	*hsa-miR-770-5p;*
**GUGCCAC**	*hsa-miR-1227;*
**GGAGAGA**	*hsa-miR-185;*

**UCUCAAG**	*hsa-miR-513c;*
**CCAGCAU**	*hsa-miR-338-3p;*
**UAUUACC**	*hsa-miR-556-3p;*
**GGAAGAC**	*hsa-miR-7;*
**GCGGAGG**	*hsa-miR-658;*
**ACAAGGU**	*hsa-miR-624;*
**UCACAGU**	*hsa-miR-27a;*
**UCACAGG**	*hsa-miR-513a-5p;*
**GGCGGGG**	*hsa-miR-663;*
**UUUGCAC**	*hsa-miR-507; hsa-miR-557;*
**UGGUACC**	*hsa-miR-1263;*
**AGCUGCC**	*hsa-miR-22;*
**GGGCGUG**	*hsa-miR-1268;*
**GCCCUUA**	*hsa-miR-938;*
**AAAGUAA**	*hsa-miR-548d-5p; hsa-miR-548h; hsa-miR-548i; hsa-miR-548j;*
**GAAAACA**	*hsa-miR-570;*
**CGACCCA**	*hsa-miR-551a;*
**UGGAGAU**	*hsa-miR-1270; hsa-miR-620;*
**GUAGUGU**	*hsa-miR-142-3p;*
**GGCAAGA**	*hsa-miR-31;*
**AUGUGCC**	*hsa-miR-455-5p;*
**GAGCUGC**	*hsa-miR-558;*
**AAAGAGG**	*hsa-miR-583;*
**AUACCUC**	*hsa-miR-875-5p;*
**CUGCAGC**	*hsa-miR-1184;*
**UUGGUUC**	*hsa-miR-659;*
**AACUACU**	*hsa-miR-606;*
**GAGCCCC**	*hsa-miR-1225-3p;*
**AGCCUGC**	*hsa-miR-596;*
**GCGGGUG**	*hsa-miR-886-3p;*
**GGUGCGG**	*hsa-miR-675;*
**UCAUUUG**	*hsa-miR-579;*
**UUGAUCA**	*hsa-miR-1826;*
**CGGCCUG**	*hsa-miR-1234;*
**ACCCGUA**	*hsa-miR-99b;*
**UUCCGGC**	*hsa-miR-1180;*
**AUGAGCU**	*hsa-miR-556-5p;*
**GAGUUGG**	*hsa-miR-571;*
**AAAUUUC**	*hsa-miR-513a-3p;*
**GCUGGAU**	*hsa-miR-1287;*
**ACCCUGU**	*hsa-miR-10a;*
**ACUGCAU**	*hsa-miR-217;*
**GGACCUG**	*hsa-miR-492;*
**AGUGCUG**	*hsa-miR-512-3p;*
**CAAAACU**	*hsa-miR-548o;*
**AAAGUGC**	*hsa-miR-106b; hsa-miR-93;*
**UGUGCUU**	*hsa-miR-218;*
**GAUCAGA**	*hsa-miR-383;*

**GAGAACC**	*hsa-miR-589;*
**CGGUGCU**	*hsa-miR-1250;*
**AGUGCAA**	*hsa-miR-130a; hsa-miR-301a; hsa-miR-454;*
**GAGGUAU**	*hsa-miR-202;*
**GGGGAGC**	*hsa-miR-939;*
**CAGUCUG**	*hsa-miR-622;*
**AAUCUCA**	*hsa-miR-216a;*
**CGCCCUU**	*hsa-miR-1249;*
**ACCCGGC**	*hsa-miR-941;*
**UUGGCAC**	*hsa-miR-96;*
**GGCUCAG**	*hsa-miR-24;*
**CUGGAAA**	*hsa-miR-875-3p;*
**AACACUG**	*hsa-miR-200a;*
**UGAGGAC**	*hsa-miR-1224-5p;*
**AAAACUG**	*hsa-miR-548f;*
**UGAGUCU**	*hsa-miR-627;*
**UGCAUAG**	*hsa-miR-153;*
**AUAAAGU**	*hsa-miR-142-5p;*
**AAAGCUG**	*hsa-miR-320a; hsa-miR-320b; hsa-miR-320d;*
**ACAUCAC**	*hsa-miR-499-3p;*
**GAUUGGU**	*hsa-miR-509-3p;*
**UUGACAC**	*hsa-miR-514;*
**GGAAUGU**	*hsa-miR-1;*
**CCCACGU**	*hsa-miR-662;*
**UCUGGAA**	*hsa-miR-1299;*
**CCCACCG**	*hsa-miR-1280;*
**UUCCUAG**	*hsa-miR-384;*
**CAGGCUC**	*hsa-miR-484;*
**GAGCCCU**	*hsa-miR-1233;*
**CAGGUGA**	*hsa-miR-125a-3p;*
