# Supplementary material for: Insights into the regulation of human CNV-miRNAs from the view of their target genes
Source: BMC Genomics. 2012 Dec 18;13:707. doi: 10.1186/1471-2164-13-707 (PMC3582595; doi:10.1186/1471-2164-13-707)
Supplement: Additional file 3 — List of targets genes with three regulatory patterns of miRNAs. The numbers in parenthesis represent the total number of regulatory miRNAs and the number of CNV-miRNAs, respectively. [file 1471-2164-13-707-S3.pdf]

| Genes regulated exclusively by CNV-miRNAs | Genes regulated by non-CNV-miRNAs and at least one CNV-miRNA | Genes regulated exclusively by non-CNV-miRNAs |
|---|---|---|
| ENSG00000001460(1, 1) | ENSG00000003249(2, 1) | ENSG00000000005(1, 0) |
| ENSG00000001630(1, 1) | ENSG00000003400(2, 1) | ENSG00000000938(1, 0) |
| ENSG00000002330(1, 1) | ENSG00000003987(2, 1) | ENSG00000002016(1, 0) |
| ENSG00000002919(1, 1) | ENSG00000004939(2, 1) | ENSG00000004779(1, 0) |
| ENSG00000004776(1, 1) | ENSG00000005844(2, 1) | ENSG00000004864(1, 0) |
| ENSG00000004961(1, 1) | ENSG00000006530(2, 1) | ENSG00000005001(1, 0) |
| ENSG00000006075(1, 1) | ENSG00000006715(2, 1) | ENSG00000005108(1, 0) |
| ENSG00000006695(1, 1) | ENSG00000011260(2, 1) | ENSG00000005194(1, 0) |
| ENSG00000006712(1, 1) | ENSG00000011332(2, 1) | ENSG00000005700(1, 0) |
| ENSG00000007376(1, 1) | ENSG00000011465(2, 1) | ENSG00000005961(1, 0) |
| ENSG00000007952(1, 1) | ENSG00000018510(2, 1) | ENSG00000006015(1, 0) |
| ENSG00000008853(1, 1) | ENSG00000020922(2, 1) | ENSG00000006118(1, 0) |
| ENSG00000010318(1, 1) | ENSG00000025039(2, 1) | ENSG00000006194(1, 0) |
| ENSG00000010610(1, 1) | ENSG00000030582(2, 1) | ENSG00000006652(1, 0) |
| ENSG00000011007(1, 1) | ENSG00000031691(2, 1) | ENSG00000006757(1, 0) |
| ENSG00000011198(1, 1) | ENSG00000039523(2, 1) | ENSG00000007174(1, 0) |
| ENSG00000011478(1, 1) | ENSG00000039600(2, 1) | ENSG00000007312(1, 0) |
| ENSG00000012983(1, 1) | ENSG00000040608(2, 1) | ENSG00000007350(1, 0) |
| ENSG00000013293(1, 1) | ENSG00000043514(2, 1) | ENSG00000007516(1, 0) |
| ENSG00000015475(1, 1) | ENSG00000047346(2, 1) | ENSG00000008277(1, 0) |
| ENSG00000015479(1, 1) | ENSG00000049323(2, 1) | ENSG00000008513(1, 0) |
| ENSG00000018236(1, 1) | ENSG00000049768(2, 1) | ENSG00000008516(1, 0) |
| ENSG00000019549(1, 1) | ENSG00000055732(2, 1) | ENSG00000009950(1, 0) |
| ENSG00000021852(1, 1) | ENSG00000057294(2, 1) | ENSG00000010072(1, 0) |
| ENSG00000023228(1, 1) | ENSG00000057608(2, 1) | ENSG00000010361(1, 0) |
| ENSG00000023892(1, 1) | ENSG00000059378(2, 1) | ENSG00000010671(1, 0) |
| ENSG00000025434(1, 1) | ENSG00000060762(2, 1) | ENSG00000010704(1, 0) |
| ENSG00000025796(1, 1) | ENSG00000063601(2, 1) | ENSG00000011376(1, 0) |
| ENSG00000029639(1, 1) | ENSG00000064547(2, 1) | ENSG00000013503(1, 0) |
| ENSG00000031698(1, 1) | ENSG00000065029(2, 1) | ENSG00000013573(1, 0) |
| ENSG00000032389(1, 1) | ENSG00000065485(2, 1) | ENSG00000014824(1, 0) |
| ENSG00000036530(1, 1) | ENSG00000065675(2, 1) | ENSG00000016402(1, 0) |
| ENSG00000037280(1, 1) | ENSG00000066027(2, 1) | ENSG00000016864(1, 0) |
| ENSG00000038427(1, 1) | ENSG00000066455(2, 1) | ENSG00000019186(1, 0) |
| ENSG00000039987(1, 1) | ENSG00000068971(2, 1) | ENSG00000020256(1, 0) |
| ENSG00000040341(1, 1) | ENSG00000069122(2, 1) | ENSG00000022976(1, 0) |
| ENSG00000041357(1, 1) | ENSG00000071794(2, 1) | ENSG00000023572(1, 0) |
| ENSG00000046651(1, 1) | ENSG00000072042(2, 1) | ENSG00000024526(1, 0) |
| ENSG00000047621(1, 1) | ENSG00000072201(2, 1) | ENSG00000025423(1, 0) |
| ENSG00000047634(1, 1) | ENSG00000072210(2, 1) | ENSG00000027847(1, 0) |
| ENSG00000049449(1, 1) | ENSG00000072315(2, 1) | ENSG00000033170(1, 0) |
| ENSG00000050327(1, 1) | ENSG00000072778(2, 1) | ENSG00000033800(1, 0) |
| ENSG00000050820(1, 1) | ENSG00000073169(2, 1) | ENSG00000035141(1, 0) |
| ENSG00000052126(1, 1) | ENSG00000074319(2, 1) | ENSG00000037749(1, 0) |
| ENSG00000053702(1, 1) | ENSG00000074621(2, 1) | ENSG00000040731(1, 0) |
| ENSG00000054611(1, 1) | ENSG00000075151(2, 1) | ENSG00000042445(1, 0) |
| ENSG00000054803(1, 1) | ENSG00000075388(2, 1) | ENSG00000048052(1, 0) |
| ENSG00000054983(1, 1) | ENSG00000076864(2, 1) | ENSG00000048140(1, 0) |
| ENSG00000055483(1, 1) | ENSG00000077327(2, 1) | ENSG00000048544(1, 0) |

ENSG00000056998(1, 1)
ENSG00000062524(1, 1)
ENSG00000063245(1, 1)
ENSG00000063438(1, 1)
ENSG00000064313(1, 1)
ENSG00000064490(1, 1)
ENSG00000065618(1, 1)
ENSG00000066056(1, 1)
ENSG00000066248(1, 1)
ENSG00000066294(1, 1)
ENSG00000067064(1, 1)
ENSG00000067225(1, 1)
ENSG00000067606(1, 1)
ENSG00000068724(1, 1)
ENSG00000068885(1, 1)
ENSG00000069275(1, 1)
ENSG00000069493(1, 1)
ENSG00000069974(1, 1)
ENSG00000070061(1, 1)
ENSG00000070404(1, 1)
ENSG00000070770(1, 1)
ENSG00000071205(1, 1)
ENSG00000073536(1, 1)
ENSG00000074803(1, 1)
ENSG00000075826(1, 1)
ENSG00000076555(1, 1)
ENSG00000077063(1, 1)
ENSG00000077080(1, 1)
ENSG00000078246(1, 1)
ENSG00000078618(1, 1)
ENSG00000079739(1, 1)
ENSG00000081051(1, 1)
ENSG00000081985(1, 1)
ENSG00000082515(1, 1)
ENSG00000082701(1, 1)
ENSG00000082781(1, 1)
ENSG00000084754(1, 1)
ENSG00000085377(1, 1)
ENSG00000085491(1, 1)
ENSG00000087086(1, 1)
ENSG00000087111(1, 1)
ENSG00000088682(1, 1)
ENSG00000088826(1, 1)
ENSG00000089006(1, 1)
ENSG00000089685(1, 1)
ENSG00000089916(1, 1)
ENSG00000090104(1, 1)
ENSG00000090432(1, 1)
ENSG00000091127(1, 1)
ENSG00000092439(1, 1)
ENSG00000093167(1, 1)
ENSG00000093183(1, 1)
ENSG00000094804(1, 1)
ENSG00000095066(1, 1)
ENSG00000077514(2, 1)
ENSG00000078269(2, 1)
ENSG00000079156(2, 1)
ENSG00000079337(2, 1)
ENSG00000079385(2, 1)
ENSG00000079435(2, 1)
ENSG00000079691(2, 1)
ENSG00000080546(2, 1)
ENSG00000080644(2, 1)
ENSG00000080822(2, 1)
ENSG00000080824(2, 1)
ENSG00000081277(2, 1)
ENSG00000082497(2, 1)
ENSG00000083444(2, 1)
ENSG00000084463(2, 1)
ENSG00000085511(2, 1)
ENSG00000085998(2, 1)
ENSG00000086991(2, 1)
ENSG00000087903(2, 1)
ENSG00000088387(2, 1)
ENSG00000088448(2, 1)
ENSG00000088451(2, 1)
ENSG00000088992(2, 1)
ENSG00000089041(2, 1)
ENSG00000089289(2, 1)
ENSG00000090238(2, 1)
ENSG00000090470(2, 1)
ENSG00000091164(2, 1)
ENSG00000091986(2, 1)
ENSG00000092098(2, 1)
ENSG00000092345(2, 1)
ENSG00000095303(2, 1)
ENSG00000095564(2, 1)
ENSG00000096093(2, 1)
ENSG00000099246(2, 1)
ENSG00000099282(2, 1)
ENSG00000099331(2, 1)
ENSG00000099998(2, 1)
ENSG00000100060(2, 1)
ENSG00000100139(2, 1)
ENSG00000100206(2, 1)
ENSG00000100241(2, 1)
ENSG00000100243(2, 1)
ENSG00000100350(2, 1)
ENSG00000100412(2, 1)
ENSG00000100478(2, 1)
ENSG00000100916(2, 1)
ENSG00000100934(2, 1)
ENSG00000101115(2, 1)
ENSG00000101144(2, 1)
ENSG00000101321(2, 1)
ENSG00000101624(2, 1)
ENSG00000101825(2, 1)
ENSG00000101868(2, 1)
ENSG00000048991(1, 0)
ENSG00000049167(1, 0)
ENSG00000049239(1, 0)
ENSG00000049247(1, 0)
ENSG00000050748(1, 0)
ENSG00000053372(1, 0)
ENSG00000053918(1, 0)
ENSG00000054277(1, 0)
ENSG00000054938(1, 0)
ENSG00000055955(1, 0)
ENSG00000057252(1, 0)
ENSG00000059573(1, 0)
ENSG00000060339(1, 0)
ENSG00000060718(1, 0)
ENSG00000062038(1, 0)
ENSG00000063241(1, 0)
ENSG00000064102(1, 0)
ENSG00000064115(1, 0)
ENSG00000064218(1, 0)
ENSG00000064726(1, 0)
ENSG00000064961(1, 0)
ENSG00000064995(1, 0)
ENSG00000066322(1, 0)
ENSG00000066336(1, 0)
ENSG00000066583(1, 0)
ENSG00000066827(1, 0)
ENSG00000067704(1, 0)
ENSG00000068001(1, 0)
ENSG00000068028(1, 0)
ENSG00000068793(1, 0)
ENSG00000069431(1, 0)
ENSG00000069998(1, 0)
ENSG00000070190(1, 0)
ENSG00000070423(1, 0)
ENSG00000070731(1, 0)
ENSG00000070950(1, 0)
ENSG00000071462(1, 0)
ENSG00000072135(1, 0)
ENSG00000072571(1, 0)
ENSG00000072736(1, 0)
ENSG00000072958(1, 0)
ENSG00000073067(1, 0)
ENSG00000074201(1, 0)
ENSG00000074266(1, 0)
ENSG00000075223(1, 0)
ENSG00000075340(1, 0)
ENSG00000075407(1, 0)
ENSG00000075568(1, 0)
ENSG00000075624(1, 0)
ENSG00000075856(1, 0)
ENSG00000075945(1, 0)
ENSG00000076067(1, 0)
ENSG00000076248(1, 0)
ENSG00000076685(1, 0)

ENSG00000095209(1, 1)
ENSG00000095261(1, 1)
ENSG00000095321(1, 1)
ENSG00000096092(1, 1)
ENSG00000099377(1, 1)
ENSG00000099800(1, 1)
ENSG00000099957(1, 1)
ENSG00000100023(1, 1)
ENSG00000100228(1, 1)
ENSG00000100258(1, 1)
ENSG00000100280(1, 1)
ENSG00000100433(1, 1)
ENSG00000100528(1, 1)
ENSG00000100599(1, 1)
ENSG00000100629(1, 1)
ENSG00000100931(1, 1)
ENSG00000100994(1, 1)
ENSG00000101138(1, 1)
ENSG00000101280(1, 1)
ENSG00000101310(1, 1)
ENSG00000101335(1, 1)
ENSG00000101452(1, 1)
ENSG00000101474(1, 1)
ENSG00000101574(1, 1)
ENSG00000101680(1, 1)
ENSG00000101843(1, 1)
ENSG00000101882(1, 1)
ENSG00000101977(1, 1)
ENSG00000102104(1, 1)
ENSG00000102119(1, 1)
ENSG00000102125(1, 1)
ENSG00000102172(1, 1)
ENSG00000102181(1, 1)
ENSG00000102316(1, 1)
ENSG00000102359(1, 1)
ENSG00000102471(1, 1)
ENSG00000102743(1, 1)
ENSG00000102805(1, 1)
ENSG00000102897(1, 1)
ENSG00000102984(1, 1)
ENSG00000103043(1, 1)
ENSG00000103061(1, 1)
ENSG00000103226(1, 1)
ENSG00000103248(1, 1)
ENSG00000103269(1, 1)
ENSG00000103512(1, 1)
ENSG00000103534(1, 1)
ENSG00000104218(1, 1)
ENSG00000104299(1, 1)
ENSG00000104388(1, 1)
ENSG00000104783(1, 1)
ENSG00000104808(1, 1)
ENSG00000104856(1, 1)
ENSG00000104870(1, 1)
ENSG00000101911(2, 1)
ENSG00000101955(2, 1)
ENSG00000102024(2, 1)
ENSG00000102158(2, 1)
ENSG00000102796(2, 1)
ENSG00000102934(2, 1)
ENSG00000102978(2, 1)
ENSG00000103111(2, 1)
ENSG00000103257(2, 1)
ENSG00000103264(2, 1)
ENSG00000103426(2, 1)
ENSG00000103502(2, 1)
ENSG00000103522(2, 1)
ENSG00000104093(2, 1)
ENSG00000104177(2, 1)
ENSG00000104375(2, 1)
ENSG00000104497(2, 1)
ENSG00000104812(2, 1)
ENSG00000104936(2, 1)
ENSG00000104960(2, 1)
ENSG00000105176(2, 1)
ENSG00000105255(2, 1)
ENSG00000105329(2, 1)
ENSG00000105559(2, 1)
ENSG00000105583(2, 1)
ENSG00000105851(2, 1)
ENSG00000105879(2, 1)
ENSG00000106069(2, 1)
ENSG00000106077(2, 1)
ENSG00000106278(2, 1)
ENSG00000106341(2, 1)
ENSG00000106443(2, 1)
ENSG00000106692(2, 1)
ENSG00000106993(2, 1)
ENSG00000107140(2, 1)
ENSG00000107175(2, 1)
ENSG00000107186(2, 1)
ENSG00000107295(2, 1)
ENSG00000107551(2, 1)
ENSG00000107736(2, 1)
ENSG00000107816(2, 1)
ENSG00000107829(2, 1)
ENSG00000107872(2, 1)
ENSG00000108064(2, 1)
ENSG00000108100(2, 1)
ENSG00000108423(2, 1)
ENSG00000108932(2, 1)
ENSG00000108961(2, 1)
ENSG00000109103(2, 1)
ENSG00000109339(2, 1)
ENSG00000109832(2, 1)
ENSG00000110046(2, 1)
ENSG00000110944(2, 1)
ENSG00000111199(2, 1)
ENSG00000076706(1, 0)
ENSG00000077044(1, 0)
ENSG00000077522(1, 0)
ENSG00000078098(1, 0)
ENSG00000079246(1, 0)
ENSG00000079557(1, 0)
ENSG00000079689(1, 0)
ENSG00000079931(1, 0)
ENSG00000081237(1, 0)
ENSG00000081800(1, 0)
ENSG00000082438(1, 0)
ENSG00000083635(1, 0)
ENSG00000083844(1, 0)
ENSG00000085644(1, 0)
ENSG00000085741(1, 0)
ENSG00000086189(1, 0)
ENSG00000086506(1, 0)
ENSG00000086666(1, 0)
ENSG00000086730(1, 0)
ENSG00000086758(1, 0)
ENSG00000087074(1, 0)
ENSG00000087263(1, 0)
ENSG00000087302(1, 0)
ENSG00000087495(1, 0)
ENSG00000087586(1, 0)
ENSG00000087916(1, 0)
ENSG00000088827(1, 0)
ENSG00000088876(1, 0)
ENSG00000088986(1, 0)
ENSG00000089050(1, 0)
ENSG00000089091(1, 0)
ENSG00000089116(1, 0)
ENSG00000089123(1, 0)
ENSG00000089163(1, 0)
ENSG00000089220(1, 0)
ENSG00000089356(1, 0)
ENSG00000089682(1, 0)
ENSG00000089692(1, 0)
ENSG00000090269(1, 0)
ENSG00000090520(1, 0)
ENSG00000090581(1, 0)
ENSG00000090661(1, 0)
ENSG00000090924(1, 0)
ENSG00000090971(1, 0)
ENSG00000091128(1, 0)
ENSG00000091482(1, 0)
ENSG00000091651(1, 0)
ENSG00000091664(1, 0)
ENSG00000092020(1, 0)
ENSG00000092108(1, 0)
ENSG00000092208(1, 0)
ENSG00000092978(1, 0)
ENSG00000093072(1, 0)
ENSG00000094796(1, 0)

ENSG00000104881(1, 1)
ENSG00000104903(1, 1)
ENSG00000104946(1, 1)
ENSG00000104957(1, 1)
ENSG00000104964(1, 1)
ENSG00000105281(1, 1)
ENSG00000105419(1, 1)
ENSG00000105514(1, 1)
ENSG00000105707(1, 1)
ENSG00000105825(1, 1)
ENSG00000105829(1, 1)
ENSG00000105948(1, 1)
ENSG00000106013(1, 1)
ENSG00000106034(1, 1)
ENSG00000106302(1, 1)
ENSG00000106397(1, 1)
ENSG00000106789(1, 1)
ENSG00000107147(1, 1)
ENSG00000107290(1, 1)
ENSG00000107651(1, 1)
ENSG00000107731(1, 1)
ENSG00000107796(1, 1)
ENSG00000107815(1, 1)
ENSG00000108187(1, 1)
ENSG00000108370(1, 1)
ENSG00000108405(1, 1)
ENSG00000108688(1, 1)
ENSG00000108771(1, 1)
ENSG00000108839(1, 1)
ENSG00000109062(1, 1)
ENSG00000109133(1, 1)
ENSG00000109458(1, 1)
ENSG00000109738(1, 1)
ENSG00000109762(1, 1)
ENSG00000109790(1, 1)
ENSG00000110060(1, 1)
ENSG00000110148(1, 1)
ENSG00000110244(1, 1)
ENSG00000110514(1, 1)
ENSG00000110711(1, 1)
ENSG00000110934(1, 1)
ENSG00000111271(1, 1)
ENSG00000111331(1, 1)
ENSG00000111361(1, 1)
ENSG00000111859(1, 1)
ENSG00000112038(1, 1)
ENSG00000112394(1, 1)
ENSG00000112425(1, 1)
ENSG00000112759(1, 1)
ENSG00000113073(1, 1)
ENSG00000113211(1, 1)
ENSG00000113231(1, 1)
ENSG00000113638(1, 1)
ENSG00000113645(1, 1)
ENSG00000111276(2, 1)
ENSG00000111321(2, 1)
ENSG00000111554(2, 1)
ENSG00000111816(2, 1)
ENSG00000111817(2, 1)
ENSG00000111860(2, 1)
ENSG00000111897(2, 1)
ENSG00000112214(2, 1)
ENSG00000112246(2, 1)
ENSG00000112414(2, 1)
ENSG00000112685(2, 1)
ENSG00000112796(2, 1)
ENSG00000112992(2, 1)
ENSG00000113368(2, 1)
ENSG00000113389(2, 1)
ENSG00000113621(2, 1)
ENSG00000113649(2, 1)
ENSG00000113734(2, 1)
ENSG00000113971(2, 1)
ENSG00000114125(2, 1)
ENSG00000114638(2, 1)
ENSG00000114698(2, 1)
ENSG00000114744(2, 1)
ENSG00000114867(2, 1)
ENSG00000115073(2, 1)
ENSG00000115252(2, 1)
ENSG00000115266(2, 1)
ENSG00000115526(2, 1)
ENSG00000115556(2, 1)
ENSG00000115665(2, 1)
ENSG00000115944(2, 1)
ENSG00000115946(2, 1)
ENSG00000116031(2, 1)
ENSG00000116095(2, 1)
ENSG00000116337(2, 1)
ENSG00000116478(2, 1)
ENSG00000116514(2, 1)
ENSG00000116580(2, 1)
ENSG00000117069(2, 1)
ENSG00000117174(2, 1)
ENSG00000117472(2, 1)
ENSG00000117586(2, 1)
ENSG00000118245(2, 1)
ENSG00000118257(2, 1)
ENSG00000118518(2, 1)
ENSG00000118526(2, 1)
ENSG00000118729(2, 1)
ENSG00000119321(2, 1)
ENSG00000119725(2, 1)
ENSG00000119969(2, 1)
ENSG00000120129(2, 1)
ENSG00000120159(2, 1)
ENSG00000120708(2, 1)
ENSG00000120949(2, 1)
ENSG00000095627(1, 0)
ENSG00000096063(1, 0)
ENSG00000096264(1, 0)
ENSG00000096384(1, 0)
ENSG00000096968(1, 0)
ENSG00000097046(1, 0)
ENSG00000099203(1, 0)
ENSG00000099290(1, 0)
ENSG00000099814(1, 0)
ENSG00000099860(1, 0)
ENSG00000099864(1, 0)
ENSG00000099901(1, 0)
ENSG00000099949(1, 0)
ENSG00000099994(1, 0)
ENSG00000100012(1, 0)
ENSG00000100101(1, 0)
ENSG00000100219(1, 0)
ENSG00000100292(1, 0)
ENSG00000100319(1, 0)
ENSG00000100359(1, 0)
ENSG00000100362(1, 0)
ENSG00000100372(1, 0)
ENSG00000100385(1, 0)
ENSG00000100413(1, 0)
ENSG00000100417(1, 0)
ENSG00000100442(1, 0)
ENSG00000100523(1, 0)
ENSG00000100557(1, 0)
ENSG00000100979(1, 0)
ENSG00000101000(1, 0)
ENSG00000101134(1, 0)
ENSG00000101146(1, 0)
ENSG00000101190(1, 0)
ENSG00000101198(1, 0)
ENSG00000101343(1, 0)
ENSG00000101448(1, 0)
ENSG00000101457(1, 0)
ENSG00000101464(1, 0)
ENSG00000101544(1, 0)
ENSG00000101842(1, 0)
ENSG00000102054(1, 0)
ENSG00000102078(1, 0)
ENSG00000102178(1, 0)
ENSG00000102387(1, 0)
ENSG00000102452(1, 0)
ENSG00000102543(1, 0)
ENSG00000102763(1, 0)
ENSG00000102780(1, 0)
ENSG00000102878(1, 0)
ENSG00000103021(1, 0)
ENSG00000103091(1, 0)
ENSG00000103150(1, 0)
ENSG00000103254(1, 0)
ENSG00000103266(1, 0)

ENSG00000114354(1, 1)
ENSG00000114503(1, 1)
ENSG00000114529(1, 1)
ENSG00000114626(1, 1)
ENSG00000114779(1, 1)
ENSG00000114948(1, 1)
ENSG00000115216(1, 1)
ENSG00000115415(1, 1)
ENSG00000115457(1, 1)
ENSG00000115474(1, 1)
ENSG00000115541(1, 1)
ENSG00000115839(1, 1)
ENSG00000115956(1, 1)
ENSG00000115963(1, 1)
ENSG00000116096(1, 1)
ENSG00000116489(1, 1)
ENSG00000116649(1, 1)
ENSG00000116663(1, 1)
ENSG00000116668(1, 1)
ENSG00000116685(1, 1)
ENSG00000116704(1, 1)
ENSG00000116830(1, 1)
ENSG00000116857(1, 1)
ENSG00000117009(1, 1)
ENSG00000117020(1, 1)
ENSG00000117215(1, 1)
ENSG00000117305(1, 1)
ENSG00000117308(1, 1)
ENSG00000117395(1, 1)
ENSG00000117643(1, 1)
ENSG00000118217(1, 1)
ENSG00000118402(1, 1)
ENSG00000118407(1, 1)
ENSG00000118960(1, 1)
ENSG00000119227(1, 1)
ENSG00000119411(1, 1)
ENSG00000119514(1, 1)
ENSG00000119729(1, 1)
ENSG00000119801(1, 1)
ENSG00000119922(1, 1)
ENSG00000120057(1, 1)
ENSG00000120158(1, 1)
ENSG00000120278(1, 1)
ENSG00000120280(1, 1)
ENSG00000120334(1, 1)
ENSG00000120539(1, 1)
ENSG00000120694(1, 1)
ENSG00000120784(1, 1)
ENSG00000121210(1, 1)
ENSG00000121594(1, 1)
ENSG00000121848(1, 1)
ENSG00000121900(1, 1)
ENSG00000121966(1, 1)
ENSG00000122012(1, 1)
ENSG00000121083(2, 1)
ENSG00000121578(2, 1)
ENSG00000121742(2, 1)
ENSG00000121749(2, 1)
ENSG00000122085(2, 1)
ENSG00000122121(2, 1)
ENSG00000122223(2, 1)
ENSG00000123240(2, 1)
ENSG00000123243(2, 1)
ENSG00000123358(2, 1)
ENSG00000123395(2, 1)
ENSG00000123454(2, 1)
ENSG00000124243(2, 1)
ENSG00000124615(2, 1)
ENSG00000125246(2, 1)
ENSG00000125780(2, 1)
ENSG00000125868(2, 1)
ENSG00000126106(2, 1)
ENSG00000126353(2, 1)
ENSG00000126562(2, 1)
ENSG00000126858(2, 1)
ENSG00000126903(2, 1)
ENSG00000127481(2, 1)
ENSG00000127948(2, 1)
ENSG00000128298(2, 1)
ENSG00000128652(2, 1)
ENSG00000129038(2, 1)
ENSG00000129355(2, 1)
ENSG00000129534(2, 1)
ENSG00000129682(2, 1)
ENSG00000129946(2, 1)
ENSG00000129990(2, 1)
ENSG00000131094(2, 1)
ENSG00000131238(2, 1)
ENSG00000131408(2, 1)
ENSG00000131669(2, 1)
ENSG00000131724(2, 1)
ENSG00000131910(2, 1)
ENSG00000132394(2, 1)
ENSG00000132406(2, 1)
ENSG00000132561(2, 1)
ENSG00000132879(2, 1)
ENSG00000133111(2, 1)
ENSG00000133392(2, 1)
ENSG00000133997(2, 1)
ENSG00000134186(2, 1)
ENSG00000134259(2, 1)
ENSG00000134333(2, 1)
ENSG00000134955(2, 1)
ENSG00000135338(2, 1)
ENSG00000135414(2, 1)
ENSG00000135541(2, 1)
ENSG00000135549(2, 1)
ENSG00000135605(2, 1)
ENSG00000103356(1, 0)
ENSG00000103657(1, 0)
ENSG00000103671(1, 0)
ENSG00000103707(1, 0)
ENSG00000103740(1, 0)
ENSG00000103932(1, 0)
ENSG00000104112(1, 0)
ENSG00000104133(1, 0)
ENSG00000104142(1, 0)
ENSG00000104341(1, 0)
ENSG00000104408(1, 0)
ENSG00000104415(1, 0)
ENSG00000104435(1, 0)
ENSG00000104472(1, 0)
ENSG00000104613(1, 0)
ENSG00000104814(1, 0)
ENSG00000104826(1, 0)
ENSG00000104938(1, 0)
ENSG00000104983(1, 0)
ENSG00000105053(1, 0)
ENSG00000105135(1, 0)
ENSG00000105202(1, 0)
ENSG00000105220(1, 0)
ENSG00000105325(1, 0)
ENSG00000105369(1, 0)
ENSG00000105401(1, 0)
ENSG00000105447(1, 0)
ENSG00000105516(1, 0)
ENSG00000105519(1, 0)
ENSG00000105549(1, 0)
ENSG00000105613(1, 0)
ENSG00000105643(1, 0)
ENSG00000105649(1, 0)
ENSG00000105695(1, 0)
ENSG00000105726(1, 0)
ENSG00000105737(1, 0)
ENSG00000105819(1, 0)
ENSG00000105821(1, 0)
ENSG00000105854(1, 0)
ENSG00000105928(1, 0)
ENSG00000106006(1, 0)
ENSG00000106100(1, 0)
ENSG00000106123(1, 0)
ENSG00000106290(1, 0)
ENSG00000106328(1, 0)
ENSG00000106336(1, 0)
ENSG00000106633(1, 0)
ENSG00000106638(1, 0)
ENSG00000106686(1, 0)
ENSG00000106714(1, 0)
ENSG00000107201(1, 0)
ENSG00000107223(1, 0)
ENSG00000107282(1, 0)
ENSG00000107554(1, 0)

| | | |
|---|---|---|
| ENSG00000122122(1, 1) | ENSG00000135723(2, 1) | ENSG00000107581(1, 0) |
| ENSG00000122335(1, 1) | ENSG00000135919(2, 1) | ENSG00000107745(1, 0) |
| ENSG00000122435(1, 1) | ENSG00000135924(2, 1) | ENSG00000107819(1, 0) |
| ENSG00000122641(1, 1) | ENSG00000136122(2, 1) | ENSG00000107831(1, 0) |
| ENSG00000122779(1, 1) | ENSG00000136379(2, 1) | ENSG00000107874(1, 0) |
| ENSG00000122783(1, 1) | ENSG00000136643(2, 1) | ENSG00000107937(1, 0) |
| ENSG00000122958(1, 1) | ENSG00000136881(2, 1) | ENSG00000108106(1, 0) |
| ENSG00000123146(1, 1) | ENSG00000136888(2, 1) | ENSG00000108107(1, 0) |
| ENSG00000123576(1, 1) | ENSG00000137204(2, 1) | ENSG00000108176(1, 0) |
| ENSG00000123607(1, 1) | ENSG00000137266(2, 1) | ENSG00000108588(1, 0) |
| ENSG00000123892(1, 1) | ENSG00000137331(2, 1) | ENSG00000108733(1, 0) |
| ENSG00000124422(1, 1) | ENSG00000137672(2, 1) | ENSG00000108950(1, 0) |
| ENSG00000124875(1, 1) | ENSG00000138074(2, 1) | ENSG00000108963(1, 0) |
| ENSG00000125414(1, 1) | ENSG00000138136(2, 1) | ENSG00000108984(1, 0) |
| ENSG00000125505(1, 1) | ENSG00000138279(2, 1) | ENSG00000109047(1, 0) |
| ENSG00000125656(1, 1) | ENSG00000138495(2, 1) | ENSG00000109083(1, 0) |
| ENSG00000125657(1, 1) | ENSG00000138587(2, 1) | ENSG00000109107(1, 0) |
| ENSG00000125816(1, 1) | ENSG00000138614(2, 1) | ENSG00000109805(1, 0) |
| ENSG00000126088(1, 1) | ENSG00000138722(2, 1) | ENSG00000110079(1, 0) |
| ENSG00000126453(1, 1) | ENSG00000139324(2, 1) | ENSG00000110172(1, 0) |
| ENSG00000126458(1, 1) | ENSG00000139437(2, 1) | ENSG00000110200(1, 0) |
| ENSG00000126773(1, 1) | ENSG00000139445(2, 1) | ENSG00000110375(1, 0) |
| ENSG00000127249(1, 1) | ENSG00000139620(2, 1) | ENSG00000110435(1, 0) |
| ENSG00000127452(1, 1) | ENSG00000139629(2, 1) | ENSG00000110442(1, 0) |
| ENSG00000127511(1, 1) | ENSG00000139636(2, 1) | ENSG00000110844(1, 0) |
| ENSG00000127951(1, 1) | ENSG00000139719(2, 1) | ENSG00000110848(1, 0) |
| ENSG00000128242(1, 1) | ENSG00000139737(2, 1) | ENSG00000110871(1, 0) |
| ENSG00000128254(1, 1) | ENSG00000139832(2, 1) | ENSG00000111012(1, 0) |
| ENSG00000128283(1, 1) | ENSG00000139899(2, 1) | ENSG00000111046(1, 0) |
| ENSG00000128563(1, 1) | ENSG00000140279(2, 1) | ENSG00000111145(1, 0) |
| ENSG00000128709(1, 1) | ENSG00000140463(2, 1) | ENSG00000111181(1, 0) |
| ENSG00000128731(1, 1) | ENSG00000140553(2, 1) | ENSG00000111218(1, 0) |
| ENSG00000129048(1, 1) | ENSG00000140650(2, 1) | ENSG00000111241(1, 0) |
| ENSG00000129194(1, 1) | ENSG00000140743(2, 1) | ENSG00000111261(1, 0) |
| ENSG00000129354(1, 1) | ENSG00000140943(2, 1) | ENSG00000111319(1, 0) |
| ENSG00000129566(1, 1) | ENSG00000140983(2, 1) | ENSG00000111665(1, 0) |
| ENSG00000129625(1, 1) | ENSG00000141349(2, 1) | ENSG00000111674(1, 0) |
| ENSG00000129667(1, 1) | ENSG00000141480(2, 1) | ENSG00000111716(1, 0) |
| ENSG00000129691(1, 1) | ENSG00000141526(2, 1) | ENSG00000111863(1, 0) |
| ENSG00000130037(1, 1) | ENSG00000141570(2, 1) | ENSG00000111877(1, 0) |
| ENSG00000130066(1, 1) | ENSG00000142347(2, 1) | ENSG00000112039(1, 0) |
| ENSG00000130222(1, 1) | ENSG00000142606(2, 1) | ENSG00000112116(1, 0) |
| ENSG00000130244(1, 1) | ENSG00000142655(2, 1) | ENSG00000112118(1, 0) |
| ENSG00000130254(1, 1) | ENSG00000142892(2, 1) | ENSG00000112167(1, 0) |
| ENSG00000130304(1, 1) | ENSG00000143156(2, 1) | ENSG00000112195(1, 0) |
| ENSG00000130307(1, 1) | ENSG00000143297(2, 1) | ENSG00000112282(1, 0) |
| ENSG00000130529(1, 1) | ENSG00000143514(2, 1) | ENSG00000112293(1, 0) |
| ENSG00000130741(1, 1) | ENSG00000143554(2, 1) | ENSG00000112297(1, 0) |
| ENSG00000130779(1, 1) | ENSG00000143756(2, 1) | ENSG00000112312(1, 0) |
| ENSG00000130958(1, 1) | ENSG00000143801(2, 1) | ENSG00000112335(1, 0) |
| ENSG00000130985(1, 1) | ENSG00000143858(2, 1) | ENSG00000112473(1, 0) |
| ENSG00000131323(1, 1) | ENSG00000144426(2, 1) | ENSG00000112695(1, 0) |
| ENSG00000131471(1, 1) | ENSG00000144712(2, 1) | ENSG00000112699(1, 0) |
| ENSG00000131686(1, 1) | ENSG00000144747(2, 1) | ENSG00000112727(1, 0) |

ENSG00000131828(1, 1)
ENSG00000131969(1, 1)
ENSG00000132286(1, 1)
ENSG00000132305(1, 1)
ENSG00000132313(1, 1)
ENSG00000132464(1, 1)
ENSG00000132465(1, 1)
ENSG00000132522(1, 1)
ENSG00000132622(1, 1)
ENSG00000132763(1, 1)
ENSG00000132773(1, 1)
ENSG00000132792(1, 1)
ENSG00000132911(1, 1)
ENSG00000133027(1, 1)
ENSG00000133048(1, 1)
ENSG00000133112(1, 1)
ENSG00000133116(1, 1)
ENSG00000133131(1, 1)
ENSG00000133134(1, 1)
ENSG00000133265(1, 1)
ENSG00000133731(1, 1)
ENSG00000133739(1, 1)
ENSG00000134056(1, 1)
ENSG00000134077(1, 1)
ENSG00000134086(1, 1)
ENSG00000134108(1, 1)
ENSG00000134222(1, 1)
ENSG00000134258(1, 1)
ENSG00000134371(1, 1)
ENSG00000134489(1, 1)
ENSG00000134504(1, 1)
ENSG00000134590(1, 1)
ENSG00000134775(1, 1)
ENSG00000134996(1, 1)
ENSG00000135407(1, 1)
ENSG00000135452(1, 1)
ENSG00000135617(1, 1)
ENSG00000135686(1, 1)
ENSG00000135698(1, 1)
ENSG00000135951(1, 1)
ENSG00000136052(1, 1)
ENSG00000136108(1, 1)
ENSG00000136156(1, 1)
ENSG00000136161(1, 1)
ENSG00000136319(1, 1)
ENSG00000136404(1, 1)
ENSG00000136878(1, 1)
ENSG00000137040(1, 1)
ENSG00000137054(1, 1)
ENSG00000137261(1, 1)
ENSG00000137275(1, 1)
ENSG00000137312(1, 1)
ENSG00000137404(1, 1)
ENSG00000137656(1, 1)
ENSG00000144852(2, 1)
ENSG00000145022(2, 1)
ENSG00000145439(2, 1)
ENSG00000145476(2, 1)
ENSG00000145623(2, 1)
ENSG00000146038(2, 1)
ENSG00000146090(2, 1)
ENSG00000146263(2, 1)
ENSG00000146386(2, 1)
ENSG00000146950(2, 1)
ENSG00000147894(2, 1)
ENSG00000148143(2, 1)
ENSG00000148200(2, 1)
ENSG00000148483(2, 1)
ENSG00000149599(2, 1)
ENSG00000150054(2, 1)
ENSG00000150093(2, 1)
ENSG00000150281(2, 1)
ENSG00000150394(2, 1)
ENSG00000150630(2, 1)
ENSG00000151292(2, 1)
ENSG00000151746(2, 1)
ENSG00000151773(2, 1)
ENSG00000152503(2, 1)
ENSG00000152818(2, 1)
ENSG00000152953(2, 1)
ENSG00000153443(2, 1)
ENSG00000153487(2, 1)
ENSG00000154144(2, 1)
ENSG00000154162(2, 1)
ENSG00000154429(2, 1)
ENSG00000154493(2, 1)
ENSG00000155016(2, 1)
ENSG00000155052(2, 1)
ENSG00000156052(2, 1)
ENSG00000156298(2, 1)
ENSG00000156535(2, 1)
ENSG00000156853(2, 1)
ENSG00000157214(2, 1)
ENSG00000157259(2, 1)
ENSG00000157353(2, 1)
ENSG00000158156(2, 1)
ENSG00000158163(2, 1)
ENSG00000158201(2, 1)
ENSG00000158435(2, 1)
ENSG00000158825(2, 1)
ENSG00000158850(2, 1)
ENSG00000159423(2, 1)
ENSG00000160013(2, 1)
ENSG00000160087(2, 1)
ENSG00000160570(2, 1)
ENSG00000160712(2, 1)
ENSG00000161573(2, 1)
ENSG00000162624(2, 1)
ENSG00000112855(1, 0)
ENSG00000113083(1, 0)
ENSG00000113088(1, 0)
ENSG00000113119(1, 0)
ENSG00000113262(1, 0)
ENSG00000113312(1, 0)
ENSG00000113456(1, 0)
ENSG00000113522(1, 0)
ENSG00000113597(1, 0)
ENSG00000113600(1, 0)
ENSG00000113812(1, 0)
ENSG00000113838(1, 0)
ENSG00000113851(1, 0)
ENSG00000114316(1, 0)
ENSG00000114395(1, 0)
ENSG00000114439(1, 0)
ENSG00000114455(1, 0)
ENSG00000114646(1, 0)
ENSG00000114654(1, 0)
ENSG00000114656(1, 0)
ENSG00000114686(1, 0)
ENSG00000114784(1, 0)
ENSG00000115009(1, 0)
ENSG00000115053(1, 0)
ENSG00000115255(1, 0)
ENSG00000115310(1, 0)
ENSG00000115421(1, 0)
ENSG00000115425(1, 0)
ENSG00000115514(1, 0)
ENSG00000115758(1, 0)
ENSG00000115947(1, 0)
ENSG00000116032(1, 0)
ENSG00000116127(1, 0)
ENSG00000116213(1, 0)
ENSG00000116218(1, 0)
ENSG00000116260(1, 0)
ENSG00000116329(1, 0)
ENSG00000116459(1, 0)
ENSG00000116521(1, 0)
ENSG00000116670(1, 0)
ENSG00000116688(1, 0)
ENSG00000116701(1, 0)
ENSG00000116717(1, 0)
ENSG00000116761(1, 0)
ENSG00000116793(1, 0)
ENSG00000116957(1, 0)
ENSG00000117036(1, 0)
ENSG00000117090(1, 0)
ENSG00000117122(1, 0)
ENSG00000117245(1, 0)
ENSG00000117280(1, 0)
ENSG00000117408(1, 0)
ENSG00000117410(1, 0)
ENSG00000117632(1, 0)

| | | |
|---|---|---|
| ENSG00000137825(1, 1) | ENSG00000162627(2, 1) | ENSG00000117640(1, 0) |
| ENSG00000137857(1, 1) | ENSG00000162694(2, 1) | ENSG00000117748(1, 0) |
| ENSG00000138028(1, 1) | ENSG00000162777(2, 1) | ENSG00000117862(1, 0) |
| ENSG00000138029(1, 1) | ENSG00000162804(2, 1) | ENSG00000117899(1, 0) |
| ENSG00000138080(1, 1) | ENSG00000163346(2, 1) | ENSG00000118420(1, 0) |
| ENSG00000138095(1, 1) | ENSG00000163735(2, 1) | ENSG00000118702(1, 0) |
| ENSG00000138152(1, 1) | ENSG00000163811(2, 1) | ENSG00000118804(1, 0) |
| ENSG00000138207(1, 1) | ENSG00000163817(2, 1) | ENSG00000118873(1, 0) |
| ENSG00000138398(1, 1) | ENSG00000163827(2, 1) | ENSG00000119004(1, 0) |
| ENSG00000138459(1, 1) | ENSG00000163879(2, 1) | ENSG00000119392(1, 0) |
| ENSG00000138496(1, 1) | ENSG00000163933(2, 1) | ENSG00000119421(1, 0) |
| ENSG00000138606(1, 1) | ENSG00000163935(2, 1) | ENSG00000119446(1, 0) |
| ENSG00000138613(1, 1) | ENSG00000163964(2, 1) | ENSG00000119509(1, 0) |
| ENSG00000138623(1, 1) | ENSG00000164007(2, 1) | ENSG00000119638(1, 0) |
| ENSG00000138688(1, 1) | ENSG00000164031(2, 1) | ENSG00000119685(1, 0) |
| ENSG00000138744(1, 1) | ENSG00000164070(2, 1) | ENSG00000119714(1, 0) |
| ENSG00000138764(1, 1) | ENSG00000164111(2, 1) | ENSG00000119718(1, 0) |
| ENSG00000138796(1, 1) | ENSG00000164144(2, 1) | ENSG00000119878(1, 0) |
| ENSG00000139211(1, 1) | ENSG00000164258(2, 1) | ENSG00000119938(1, 0) |
| ENSG00000139263(1, 1) | ENSG00000164338(2, 1) | ENSG00000120087(1, 0) |
| ENSG00000139350(1, 1) | ENSG00000164458(2, 1) | ENSG00000120215(1, 0) |
| ENSG00000139880(1, 1) | ENSG00000164615(2, 1) | ENSG00000120217(1, 0) |
| ENSG00000140280(1, 1) | ENSG00000164849(2, 1) | ENSG00000120253(1, 0) |
| ENSG00000140287(1, 1) | ENSG00000164930(2, 1) | ENSG00000120254(1, 0) |
| ENSG00000140326(1, 1) | ENSG00000164932(2, 1) | ENSG00000120586(1, 0) |
| ENSG00000140391(1, 1) | ENSG00000164933(2, 1) | ENSG00000120675(1, 0) |
| ENSG00000140455(1, 1) | ENSG00000165071(2, 1) | ENSG00000120686(1, 0) |
| ENSG00000140474(1, 1) | ENSG00000165379(2, 1) | ENSG00000120690(1, 0) |
| ENSG00000140545(1, 1) | ENSG00000165478(2, 1) | ENSG00000120696(1, 0) |
| ENSG00000140798(1, 1) | ENSG00000165490(2, 1) | ENSG00000120802(1, 0) |
| ENSG00000141376(1, 1) | ENSG00000165637(2, 1) | ENSG00000120903(1, 0) |
| ENSG00000141431(1, 1) | ENSG00000165671(2, 1) | ENSG00000120925(1, 0) |
| ENSG00000141576(1, 1) | ENSG00000165806(2, 1) | ENSG00000121067(1, 0) |
| ENSG00000141756(1, 1) | ENSG00000165821(2, 1) | ENSG00000121101(1, 0) |
| ENSG00000142166(1, 1) | ENSG00000166411(2, 1) | ENSG00000121236(1, 0) |
| ENSG00000142168(1, 1) | ENSG00000166444(2, 1) | ENSG00000121361(1, 0) |
| ENSG00000142197(1, 1) | ENSG00000166452(2, 1) | ENSG00000121775(1, 0) |
| ENSG00000142539(1, 1) | ENSG00000166825(2, 1) | ENSG00000121864(1, 0) |
| ENSG00000142556(1, 1) | ENSG00000166828(2, 1) | ENSG00000121895(1, 0) |
| ENSG00000142657(1, 1) | ENSG00000167105(2, 1) | ENSG00000121897(1, 0) |
| ENSG00000143162(1, 1) | ENSG00000167264(2, 1) | ENSG00000122008(1, 0) |
| ENSG00000143340(1, 1) | ENSG00000167325(2, 1) | ENSG00000122304(1, 0) |
| ENSG00000143341(1, 1) | ENSG00000167549(2, 1) | ENSG00000122375(1, 0) |
| ENSG00000143368(1, 1) | ENSG00000167619(2, 1) | ENSG00000122484(1, 0) |
| ENSG00000143450(1, 1) | ENSG00000167797(2, 1) | ENSG00000122507(1, 0) |
| ENSG00000143499(1, 1) | ENSG00000168079(2, 1) | ENSG00000122642(1, 0) |
| ENSG00000143546(1, 1) | ENSG00000168090(2, 1) | ENSG00000122705(1, 0) |
| ENSG00000143653(1, 1) | ENSG00000168356(2, 1) | ENSG00000122861(1, 0) |
| ENSG00000143851(1, 1) | ENSG00000168476(2, 1) | ENSG00000122862(1, 0) |
| ENSG00000143891(1, 1) | ENSG00000168528(2, 1) | ENSG00000122870(1, 0) |
| ENSG00000144381(1, 1) | ENSG00000168612(2, 1) | ENSG00000123144(1, 0) |
| ENSG00000144476(1, 1) | ENSG00000168924(2, 1) | ENSG00000123444(1, 0) |
| ENSG00000144834(1, 1) | ENSG00000168959(2, 1) | ENSG00000123989(1, 0) |
| ENSG00000144868(1, 1) | ENSG00000169221(2, 1) | ENSG00000124067(1, 0) |

ENSG00000144895(1, 1)
ENSG00000145020(1, 1)
ENSG00000145332(1, 1)
ENSG00000145416(1, 1)
ENSG00000145868(1, 1)
ENSG00000145907(1, 1)
ENSG00000146049(1, 1)
ENSG00000146063(1, 1)
ENSG00000146376(1, 1)
ENSG00000146648(1, 1)
ENSG00000146670(1, 1)
ENSG00000147113(1, 1)
ENSG00000147123(1, 1)
ENSG00000147231(1, 1)
ENSG00000147251(1, 1)
ENSG00000147454(1, 1)
ENSG00000148180(1, 1)
ENSG00000148341(1, 1)
ENSG00000148408(1, 1)
ENSG00000148680(1, 1)
ENSG00000148735(1, 1)
ENSG00000148840(1, 1)
ENSG00000149262(1, 1)
ENSG00000149328(1, 1)
ENSG00000149541(1, 1)
ENSG00000149557(1, 1)
ENSG00000149600(1, 1)
ENSG00000149761(1, 1)
ENSG00000149923(1, 1)
ENSG00000149932(1, 1)
ENSG00000150403(1, 1)
ENSG00000150760(1, 1)
ENSG00000150977(1, 1)
ENSG00000151552(1, 1)
ENSG00000151778(1, 1)
ENSG00000151892(1, 1)
ENSG00000152207(1, 1)
ENSG00000152428(1, 1)
ENSG00000152952(1, 1)
ENSG00000152990(1, 1)
ENSG00000153179(1, 1)
ENSG00000153233(1, 1)
ENSG00000153551(1, 1)
ENSG00000153774(1, 1)
ENSG00000153789(1, 1)
ENSG00000153898(1, 1)
ENSG00000153902(1, 1)
ENSG00000154134(1, 1)
ENSG00000154153(1, 1)
ENSG00000154654(1, 1)
ENSG00000155158(1, 1)
ENSG00000155816(1, 1)
ENSG00000155827(1, 1)
ENSG00000155886(1, 1)
ENSG00000169314(2, 1)
ENSG00000169509(2, 1)
ENSG00000169710(2, 1)
ENSG00000169895(2, 1)
ENSG00000170085(2, 1)
ENSG00000170381(2, 1)
ENSG00000170482(2, 1)
ENSG00000170537(2, 1)
ENSG00000170633(2, 1)
ENSG00000170703(2, 1)
ENSG00000170745(2, 1)
ENSG00000170777(2, 1)
ENSG00000170852(2, 1)
ENSG00000171680(2, 1)
ENSG00000171813(2, 1)
ENSG00000171848(2, 1)
ENSG00000172296(2, 1)
ENSG00000172458(2, 1)
ENSG00000172661(2, 1)
ENSG00000172782(2, 1)
ENSG00000172977(2, 1)
ENSG00000172992(2, 1)
ENSG00000173077(2, 1)
ENSG00000173208(2, 1)
ENSG00000173320(2, 1)
ENSG00000173486(2, 1)
ENSG00000173599(2, 1)
ENSG00000173930(2, 1)
ENSG00000174243(2, 1)
ENSG00000174721(2, 1)
ENSG00000174928(2, 1)
ENSG00000174943(2, 1)
ENSG00000175216(2, 1)
ENSG00000175267(2, 1)
ENSG00000175556(2, 1)
ENSG00000175699(2, 1)
ENSG00000175707(2, 1)
ENSG00000175826(2, 1)
ENSG00000175854(2, 1)
ENSG00000175874(2, 1)
ENSG00000176410(2, 1)
ENSG00000176641(2, 1)
ENSG00000176946(2, 1)
ENSG00000177106(2, 1)
ENSG00000177519(2, 1)
ENSG00000177613(2, 1)
ENSG00000178149(2, 1)
ENSG00000178538(2, 1)
ENSG00000178776(2, 1)
ENSG00000178826(2, 1)
ENSG00000178919(2, 1)
ENSG00000179088(2, 1)
ENSG00000179104(2, 1)
ENSG00000179165(2, 1)
ENSG00000124092(1, 0)
ENSG00000124134(1, 0)
ENSG00000124429(1, 0)
ENSG00000124459(1, 0)
ENSG00000124523(1, 0)
ENSG00000124529(1, 0)
ENSG00000124535(1, 0)
ENSG00000124574(1, 0)
ENSG00000124575(1, 0)
ENSG00000124610(1, 0)
ENSG00000124641(1, 0)
ENSG00000124721(1, 0)
ENSG00000124767(1, 0)
ENSG00000124784(1, 0)
ENSG00000124786(1, 0)
ENSG00000124831(1, 0)
ENSG00000124939(1, 0)
ENSG00000125124(1, 0)
ENSG00000125434(1, 0)
ENSG00000125462(1, 0)
ENSG00000125691(1, 0)
ENSG00000125730(1, 0)
ENSG00000125775(1, 0)
ENSG00000125813(1, 0)
ENSG00000125821(1, 0)
ENSG00000125835(1, 0)
ENSG00000125841(1, 0)
ENSG00000125991(1, 0)
ENSG00000126001(1, 0)
ENSG00000126010(1, 0)
ENSG00000126457(1, 0)
ENSG00000126883(1, 0)
ENSG00000127129(1, 0)
ENSG00000127241(1, 0)
ENSG00000127325(1, 0)
ENSG00000127337(1, 0)
ENSG00000127554(1, 0)
ENSG00000127564(1, 0)
ENSG00000127914(1, 0)
ENSG00000128203(1, 0)
ENSG00000128294(1, 0)
ENSG00000128340(1, 0)
ENSG00000128346(1, 0)
ENSG00000128463(1, 0)
ENSG00000128578(1, 0)
ENSG00000128656(1, 0)
ENSG00000128791(1, 0)
ENSG00000128886(1, 0)
ENSG00000129003(1, 0)
ENSG00000129071(1, 0)
ENSG00000129173(1, 0)
ENSG00000129219(1, 0)
ENSG00000129235(1, 0)
ENSG00000129270(1, 0)

ENSG00000155962(1, 1)
ENSG00000155970(1, 1)
ENSG00000156150(1, 1)
ENSG00000156172(1, 1)
ENSG00000156711(1, 1)
ENSG00000156920(1, 1)
ENSG00000156990(1, 1)
ENSG00000157020(1, 1)
ENSG00000157349(1, 1)
ENSG00000157765(1, 1)
ENSG00000157800(1, 1)
ENSG00000157992(1, 1)
ENSG00000158092(1, 1)
ENSG00000158104(1, 1)
ENSG00000158483(1, 1)
ENSG00000158526(1, 1)
ENSG00000158711(1, 1)
ENSG00000158714(1, 1)
ENSG00000158786(1, 1)
ENSG00000158882(1, 1)
ENSG00000159110(1, 1)
ENSG00000159200(1, 1)
ENSG00000159210(1, 1)
ENSG00000159588(1, 1)
ENSG00000159625(1, 1)
ENSG00000159723(1, 1)
ENSG00000160113(1, 1)
ENSG00000160179(1, 1)
ENSG00000160185(1, 1)
ENSG00000160209(1, 1)
ENSG00000160294(1, 1)
ENSG00000160410(1, 1)
ENSG00000160654(1, 1)
ENSG00000160753(1, 1)
ENSG00000160791(1, 1)
ENSG00000161179(1, 1)
ENSG00000161180(1, 1)
ENSG00000162069(1, 1)
ENSG00000162300(1, 1)
ENSG00000162341(1, 1)
ENSG00000162384(1, 1)
ENSG00000162433(1, 1)
ENSG00000162512(1, 1)
ENSG00000162642(1, 1)
ENSG00000162676(1, 1)
ENSG00000162688(1, 1)
ENSG00000162746(1, 1)
ENSG00000162813(1, 1)
ENSG00000162817(1, 1)
ENSG00000163026(1, 1)
ENSG00000163154(1, 1)
ENSG00000163214(1, 1)
ENSG00000163382(1, 1)
ENSG00000163466(1, 1)
ENSG00000179774(2, 1)
ENSG00000179862(2, 1)
ENSG00000180233(2, 1)
ENSG00000180616(2, 1)
ENSG00000181392(2, 1)
ENSG00000181513(2, 1)
ENSG00000181585(2, 1)
ENSG00000181619(2, 1)
ENSG00000181924(2, 1)
ENSG00000182504(2, 1)
ENSG00000182512(2, 1)
ENSG00000182575(2, 1)
ENSG00000182591(2, 1)
ENSG00000182872(2, 1)
ENSG00000182902(2, 1)
ENSG00000182944(2, 1)
ENSG00000182952(2, 1)
ENSG00000183160(2, 1)
ENSG00000183475(2, 1)
ENSG00000183570(2, 1)
ENSG00000183770(2, 1)
ENSG00000183918(2, 1)
ENSG00000184489(2, 1)
ENSG00000184575(2, 1)
ENSG00000184828(2, 1)
ENSG00000184867(2, 1)
ENSG00000184939(2, 1)
ENSG00000184990(2, 1)
ENSG00000185000(2, 1)
ENSG00000185088(2, 1)
ENSG00000185432(2, 1)
ENSG00000185504(2, 1)
ENSG00000185670(2, 1)
ENSG00000185774(2, 1)
ENSG00000185875(2, 1)
ENSG00000186143(2, 1)
ENSG00000186442(2, 1)
ENSG00000186998(2, 1)
ENSG00000187122(2, 1)
ENSG00000187175(2, 1)
ENSG00000187191(2, 1)
ENSG00000187231(2, 1)
ENSG00000187531(2, 1)
ENSG00000187676(2, 1)
ENSG00000187714(2, 1)
ENSG00000187736(2, 1)
ENSG00000188120(2, 1)
ENSG00000188227(2, 1)
ENSG00000188487(2, 1)
ENSG00000188725(2, 1)
ENSG00000188827(2, 1)
ENSG00000189043(2, 1)
ENSG00000196072(2, 1)
ENSG00000196155(2, 1)
ENSG00000129277(1, 0)
ENSG00000129636(1, 0)
ENSG00000129696(1, 0)
ENSG00000129968(1, 0)
ENSG00000130299(1, 0)
ENSG00000130311(1, 0)
ENSG00000130313(1, 0)
ENSG00000130332(1, 0)
ENSG00000130427(1, 0)
ENSG00000130517(1, 0)
ENSG00000130528(1, 0)
ENSG00000130545(1, 0)
ENSG00000130592(1, 0)
ENSG00000130707(1, 0)
ENSG00000130713(1, 0)
ENSG00000130720(1, 0)
ENSG00000130726(1, 0)
ENSG00000130803(1, 0)
ENSG00000130988(1, 0)
ENSG00000131013(1, 0)
ENSG00000131061(1, 0)
ENSG00000131116(1, 0)
ENSG00000131153(1, 0)
ENSG00000131398(1, 0)
ENSG00000131462(1, 0)
ENSG00000131470(1, 0)
ENSG00000131747(1, 0)
ENSG00000131779(1, 0)
ENSG00000131845(1, 0)
ENSG00000131979(1, 0)
ENSG00000132004(1, 0)
ENSG00000132109(1, 0)
ENSG00000132256(1, 0)
ENSG00000132321(1, 0)
ENSG00000132329(1, 0)
ENSG00000132382(1, 0)
ENSG00000132541(1, 0)
ENSG00000132549(1, 0)
ENSG00000132623(1, 0)
ENSG00000132801(1, 0)
ENSG00000132824(1, 0)
ENSG00000133101(1, 0)
ENSG00000133107(1, 0)
ENSG00000133110(1, 0)
ENSG00000133138(1, 0)
ENSG00000133142(1, 0)
ENSG00000133256(1, 0)
ENSG00000133488(1, 0)
ENSG00000133636(1, 0)
ENSG00000133687(1, 0)
ENSG00000133706(1, 0)
ENSG00000133872(1, 0)
ENSG00000133895(1, 0)
ENSG00000133937(1, 0)

ENSG00000163485(1, 1)
ENSG00000163520(1, 1)
ENSG00000163605(1, 1)
ENSG00000163606(1, 1)
ENSG00000163644(1, 1)
ENSG00000163734(1, 1)
ENSG00000163739(1, 1)
ENSG00000163793(1, 1)
ENSG00000163823(1, 1)
ENSG00000163872(1, 1)
ENSG00000163938(1, 1)
ENSG00000164011(1, 1)
ENSG00000164032(1, 1)
ENSG00000164124(1, 1)
ENSG00000164161(1, 1)
ENSG00000164185(1, 1)
ENSG00000164220(1, 1)
ENSG00000164251(1, 1)
ENSG00000164411(1, 1)
ENSG00000164414(1, 1)
ENSG00000164512(1, 1)
ENSG00000164591(1, 1)
ENSG00000164808(1, 1)
ENSG00000164885(1, 1)
ENSG00000164920(1, 1)
ENSG00000165055(1, 1)
ENSG00000165060(1, 1)
ENSG00000165102(1, 1)
ENSG00000165275(1, 1)
ENSG00000165282(1, 1)
ENSG00000165283(1, 1)
ENSG00000165338(1, 1)
ENSG00000165417(1, 1)
ENSG00000165672(1, 1)
ENSG00000165678(1, 1)
ENSG00000165685(1, 1)
ENSG00000165795(1, 1)
ENSG00000165807(1, 1)
ENSG00000165914(1, 1)
ENSG00000166169(1, 1)
ENSG00000166313(1, 1)
ENSG00000166341(1, 1)
ENSG00000166478(1, 1)
ENSG00000166569(1, 1)
ENSG00000166794(1, 1)
ENSG00000166823(1, 1)
ENSG00000166925(1, 1)
ENSG00000167112(1, 1)
ENSG00000167186(1, 1)
ENSG00000167842(1, 1)
ENSG00000167861(1, 1)
ENSG00000168000(1, 1)
ENSG00000168077(1, 1)
ENSG00000168092(1, 1)
ENSG00000196449(2, 1)
ENSG00000196507(2, 1)
ENSG00000196535(2, 1)
ENSG00000196544(2, 1)
ENSG00000196715(2, 1)
ENSG00000196781(2, 1)
ENSG00000196924(2, 1)
ENSG00000197019(2, 1)
ENSG00000197070(2, 1)
ENSG00000197142(2, 1)
ENSG00000197217(2, 1)
ENSG00000197312(2, 1)
ENSG00000197324(2, 1)
ENSG00000197329(2, 1)
ENSG00000197417(2, 1)
ENSG00000197444(2, 1)
ENSG00000197467(2, 1)
ENSG00000197576(2, 1)
ENSG00000197646(2, 1)
ENSG00000197746(2, 1)
ENSG00000198018(2, 1)
ENSG00000198324(2, 1)
ENSG00000198380(2, 1)
ENSG00000198455(2, 1)
ENSG00000198570(2, 1)
ENSG00000198807(2, 1)
ENSG00000198838(2, 1)
ENSG00000198939(2, 1)
ENSG00000198951(2, 1)
ENSG00000198959(2, 1)
ENSG00000203778(2, 1)
ENSG00000203784(2, 1)
ENSG00000204003(2, 1)
ENSG00000204371(2, 1)
ENSG00000204839(2, 1)
ENSG00000204899(2, 1)
ENSG00000205208(2, 1)
ENSG00000205279(2, 1)
ENSG00000205336(2, 1)
ENSG00000205426(2, 1)
ENSG00000205838(2, 1)
ENSG00000205916(2, 1)
ENSG00000205944(2, 1)
ENSG00000206418(2, 1)
ENSG00000211450(2, 1)
ENSG00000211456(2, 1)
ENSG00000213015(2, 1)
ENSG00000213171(2, 1)
ENSG00000213420(2, 1)
ENSG00000213901(2, 1)
ENSG00000214063(2, 1)
ENSG00000215193(2, 1)
ENSG00000215455(2, 1)
ENSG00000226979(2, 1)
ENSG00000134057(1, 0)
ENSG00000134072(1, 0)
ENSG00000134146(1, 0)
ENSG00000134153(1, 0)
ENSG00000134202(1, 0)
ENSG00000134253(1, 0)
ENSG00000134321(1, 0)
ENSG00000134326(1, 0)
ENSG00000134375(1, 0)
ENSG00000134438(1, 0)
ENSG00000134440(1, 0)
ENSG00000134443(1, 0)
ENSG00000134551(1, 0)
ENSG00000134594(1, 0)
ENSG00000134755(1, 0)
ENSG00000134757(1, 0)
ENSG00000134765(1, 0)
ENSG00000134825(1, 0)
ENSG00000134851(1, 0)
ENSG00000134899(1, 0)
ENSG00000134940(1, 0)
ENSG00000135018(1, 0)
ENSG00000135070(1, 0)
ENSG00000135094(1, 0)
ENSG00000135218(1, 0)
ENSG00000135222(1, 0)
ENSG00000135317(1, 0)
ENSG00000135378(1, 0)
ENSG00000135424(1, 0)
ENSG00000135457(1, 0)
ENSG00000135506(1, 0)
ENSG00000135624(1, 0)
ENSG00000135636(1, 0)
ENSG00000135744(1, 0)
ENSG00000135828(1, 0)
ENSG00000135838(1, 0)
ENSG00000135926(1, 0)
ENSG00000136021(1, 0)
ENSG00000136104(1, 0)
ENSG00000136141(1, 0)
ENSG00000136146(1, 0)
ENSG00000136197(1, 0)
ENSG00000136297(1, 0)
ENSG00000136327(1, 0)
ENSG00000136444(1, 0)
ENSG00000136689(1, 0)
ENSG00000136695(1, 0)
ENSG00000136717(1, 0)
ENSG00000136732(1, 0)
ENSG00000136819(1, 0)
ENSG00000136869(1, 0)
ENSG00000136875(1, 0)
ENSG00000136877(1, 0)
ENSG00000136999(1, 0)

| | | |
|---|---|---|
| ENSG00000168118(1, 1) | ENSG00000234127(2, 1) | ENSG00000137177(1, 0) |
| ENSG00000168214(1, 1) | ENSG00000240065(2, 1) | ENSG00000137259(1, 0) |
| ENSG00000168234(1, 1) | ENSG00000241973(2, 1) | ENSG00000137267(1, 0) |
| ENSG00000168259(1, 1) | ENSG00000244486(2, 1) | ENSG00000137500(1, 0) |
| ENSG00000168282(1, 1) | ENSG00000244754(2, 1) | ENSG00000137501(1, 0) |
| ENSG00000168334(1, 1) | ENSG00000253958(2, 1) | ENSG00000137692(1, 0) |
| ENSG00000168438(1, 1) | ENSG00000000457(3, 2) | ENSG00000137764(1, 0) |
| ENSG00000168461(1, 1) | ENSG00000003147(3, 1) | ENSG00000137812(1, 0) |
| ENSG00000168502(1, 1) | ENSG00000004866(3, 1) | ENSG00000137824(1, 0) |
| ENSG00000168564(1, 1) | ENSG00000004975(3, 2) | ENSG00000137871(1, 0) |
| ENSG00000168778(1, 1) | ENSG00000005102(3, 1) | ENSG00000137941(1, 0) |
| ENSG00000168806(1, 1) | ENSG00000005513(3, 2) | ENSG00000138030(1, 0) |
| ENSG00000168830(1, 1) | ENSG00000005882(3, 1) | ENSG00000138035(1, 0) |
| ENSG00000168843(1, 1) | ENSG00000006451(3, 2) | ENSG00000138073(1, 0) |
| ENSG00000169131(1, 1) | ENSG00000007384(3, 1) | ENSG00000138100(1, 0) |
| ENSG00000169230(1, 1) | ENSG00000007392(3, 1) | ENSG00000138303(1, 0) |
| ENSG00000169299(1, 1) | ENSG00000008118(3, 1) | ENSG00000138308(1, 0) |
| ENSG00000169744(1, 1) | ENSG00000008130(3, 1) | ENSG00000138356(1, 0) |
| ENSG00000169756(1, 1) | ENSG00000008197(3, 1) | ENSG00000138592(1, 0) |
| ENSG00000169871(1, 1) | ENSG00000008323(3, 2) | ENSG00000138669(1, 0) |
| ENSG00000169896(1, 1) | ENSG00000008710(3, 2) | ENSG00000138821(1, 0) |
| ENSG00000170075(1, 1) | ENSG00000011426(3, 1) | ENSG00000139055(1, 0) |
| ENSG00000170162(1, 1) | ENSG00000014164(3, 2) | ENSG00000139131(1, 0) |
| ENSG00000170180(1, 1) | ENSG00000015532(3, 1) | ENSG00000139291(1, 0) |
| ENSG00000170271(1, 1) | ENSG00000018625(3, 1) | ENSG00000139508(1, 0) |
| ENSG00000170296(1, 1) | ENSG00000021826(3, 1) | ENSG00000139547(1, 0) |
| ENSG00000170323(1, 1) | ENSG00000022567(3, 2) | ENSG00000139725(1, 0) |
| ENSG00000170412(1, 1) | ENSG00000026508(3, 1) | ENSG00000140323(1, 0) |
| ENSG00000170445(1, 1) | ENSG00000035681(3, 1) | ENSG00000140386(1, 0) |
| ENSG00000170549(1, 1) | ENSG00000035928(3, 1) | ENSG00000140451(1, 0) |
| ENSG00000170571(1, 1) | ENSG00000036672(3, 1) | ENSG00000140511(1, 0) |
| ENSG00000170606(1, 1) | ENSG00000036828(3, 1) | ENSG00000140543(1, 0) |
| ENSG00000170836(1, 1) | ENSG00000038210(3, 1) | ENSG00000140691(1, 0) |
| ENSG00000171004(1, 1) | ENSG00000038382(3, 2) | ENSG00000140694(1, 0) |
| ENSG00000171094(1, 1) | ENSG00000042286(3, 1) | ENSG00000140830(1, 0) |
| ENSG00000171604(1, 1) | ENSG00000044524(3, 1) | ENSG00000140848(1, 0) |
| ENSG00000172053(1, 1) | ENSG00000046647(3, 1) | ENSG00000140854(1, 0) |
| ENSG00000172081(1, 1) | ENSG00000048405(3, 1) | ENSG00000140859(1, 0) |
| ENSG00000172209(1, 1) | ENSG00000049319(3, 1) | ENSG00000140931(1, 0) |
| ENSG00000172354(1, 1) | ENSG00000050405(3, 1) | ENSG00000140995(1, 0) |
| ENSG00000172497(1, 1) | ENSG00000051009(3, 1) | ENSG00000141161(1, 0) |
| ENSG00000172733(1, 1) | ENSG00000052850(3, 2) | ENSG00000141219(1, 0) |
| ENSG00000172738(1, 1) | ENSG00000056097(3, 1) | ENSG00000141424(1, 0) |
| ENSG00000172831(1, 1) | ENSG00000058404(3, 1) | ENSG00000141485(1, 0) |
| ENSG00000173124(1, 1) | ENSG00000060138(3, 1) | ENSG00000141579(1, 0) |
| ENSG00000173221(1, 1) | ENSG00000064652(3, 1) | ENSG00000141642(1, 0) |
| ENSG00000173226(1, 1) | ENSG00000065833(3, 2) | ENSG00000142319(1, 0) |
| ENSG00000173349(1, 1) | ENSG00000066032(3, 2) | ENSG00000142449(1, 0) |
| ENSG00000173588(1, 1) | ENSG00000066427(3, 1) | ENSG00000142627(1, 0) |
| ENSG00000173692(1, 1) | ENSG00000066654(3, 1) | ENSG00000142677(1, 0) |
| ENSG00000173826(1, 1) | ENSG00000067057(3, 1) | ENSG00000142856(1, 0) |
| ENSG00000174016(1, 1) | ENSG00000067955(3, 1) | ENSG00000142867(1, 0) |
| ENSG00000174021(1, 1) | ENSG00000069869(3, 2) | ENSG00000142910(1, 0) |
| ENSG00000174083(1, 1) | ENSG00000070214(3, 2) | ENSG00000143032(1, 0) |

| | | |
|---|---|---|
| ENSG00000174132(1, 1) | ENSG00000070495(3, 2) | ENSG00000143107(1, 0) |
| ENSG00000174165(1, 1) | ENSG00000070814(3, 1) | ENSG00000143158(1, 0) |
| ENSG00000174564(1, 1) | ENSG00000073598(3, 2) | ENSG00000143222(1, 0) |
| ENSG00000174595(1, 1) | ENSG00000073849(3, 1) | ENSG00000143315(1, 0) |
| ENSG00000174899(1, 1) | ENSG00000073861(3, 1) | ENSG00000143318(1, 0) |
| ENSG00000174915(1, 1) | ENSG00000075790(3, 2) | ENSG00000143387(1, 0) |
| ENSG00000174939(1, 1) | ENSG00000076053(3, 1) | ENSG00000143458(1, 0) |
| ENSG00000174951(1, 1) | ENSG00000076351(3, 1) | ENSG00000143537(1, 0) |
| ENSG00000175183(1, 1) | ENSG00000077097(3, 1) | ENSG00000143545(1, 0) |
| ENSG00000175197(1, 1) | ENSG00000077713(3, 1) | ENSG00000143621(1, 0) |
| ENSG00000175395(1, 1) | ENSG00000078401(3, 1) | ENSG00000143954(1, 0) |
| ENSG00000175463(1, 1) | ENSG00000079819(3, 1) | ENSG00000143977(1, 0) |
| ENSG00000175544(1, 1) | ENSG00000080603(3, 1) | ENSG00000144026(1, 0) |
| ENSG00000175646(1, 1) | ENSG00000081791(3, 1) | ENSG00000144061(1, 0) |
| ENSG00000175779(1, 1) | ENSG00000082196(3, 1) | ENSG00000144152(1, 0) |
| ENSG00000175906(1, 1) | ENSG00000082397(3, 1) | ENSG00000144339(1, 0) |
| ENSG00000176058(1, 1) | ENSG00000082482(3, 2) | ENSG00000144369(1, 0) |
| ENSG00000176153(1, 1) | ENSG00000083123(3, 2) | ENSG00000144468(1, 0) |
| ENSG00000176170(1, 1) | ENSG00000083290(3, 2) | ENSG00000144485(1, 0) |
| ENSG00000176371(1, 1) | ENSG00000084636(3, 1) | ENSG00000144785(1, 0) |
| ENSG00000176903(1, 1) | ENSG00000085063(3, 2) | ENSG00000144857(1, 0) |
| ENSG00000177150(1, 1) | ENSG00000085433(3, 2) | ENSG00000145103(1, 0) |
| ENSG00000177272(1, 1) | ENSG00000086102(3, 1) | ENSG00000145147(1, 0) |
| ENSG00000177548(1, 1) | ENSG00000087157(3, 2) | ENSG00000145192(1, 0) |
| ENSG00000177556(1, 1) | ENSG00000087510(3, 2) | ENSG00000145309(1, 0) |
| ENSG00000177666(1, 1) | ENSG00000089250(3, 1) | ENSG00000145358(1, 0) |
| ENSG00000177674(1, 1) | ENSG00000089280(3, 1) | ENSG00000145365(1, 0) |
| ENSG00000178171(1, 1) | ENSG00000089693(3, 1) | ENSG00000145386(1, 0) |
| ENSG00000178425(1, 1) | ENSG00000089723(3, 2) | ENSG00000145536(1, 0) |
| ENSG00000178537(1, 1) | ENSG00000091137(3, 1) | ENSG00000145569(1, 0) |
| ENSG00000178607(1, 1) | ENSG00000091157(3, 2) | ENSG00000145723(1, 0) |
| ENSG00000178732(1, 1) | ENSG00000091844(3, 2) | ENSG00000145734(1, 0) |
| ENSG00000179059(1, 1) | ENSG00000092931(3, 2) | ENSG00000146047(1, 0) |
| ENSG00000179083(1, 1) | ENSG00000093134(3, 1) | ENSG00000146066(1, 0) |
| ENSG00000179087(1, 1) | ENSG00000095752(3, 2) | ENSG00000146143(1, 0) |
| ENSG00000179097(1, 1) | ENSG00000096433(3, 1) | ENSG00000146373(1, 0) |
| ENSG00000179152(1, 1) | ENSG00000099785(3, 1) | ENSG00000146414(1, 0) |
| ENSG00000179163(1, 1) | ENSG00000099804(3, 2) | ENSG00000146426(1, 0) |
| ENSG00000179387(1, 1) | ENSG00000099940(3, 1) | ENSG00000146540(1, 0) |
| ENSG00000179564(1, 1) | ENSG00000099956(3, 1) | ENSG00000146555(1, 0) |
| ENSG00000180353(1, 1) | ENSG00000100028(3, 1) | ENSG00000146587(1, 0) |
| ENSG00000180432(1, 1) | ENSG00000100227(3, 1) | ENSG00000146701(1, 0) |
| ENSG00000180776(1, 1) | ENSG00000100246(3, 2) | ENSG00000146729(1, 0) |
| ENSG00000180787(1, 1) | ENSG00000100296(3, 2) | ENSG00000146731(1, 0) |
| ENSG00000181026(1, 1) | ENSG00000100304(3, 1) | ENSG00000146809(1, 0) |
| ENSG00000181035(1, 1) | ENSG00000100410(3, 1) | ENSG00000146842(1, 0) |
| ENSG00000181481(1, 1) | ENSG00000100503(3, 1) | ENSG00000146859(1, 0) |
| ENSG00000181577(1, 1) | ENSG00000100711(3, 1) | ENSG00000146910(1, 0) |
| ENSG00000181704(1, 1) | ENSG00000100997(3, 1) | ENSG00000147059(1, 0) |
| ENSG00000181781(1, 1) | ENSG00000101076(3, 2) | ENSG00000147180(1, 0) |
| ENSG00000181789(1, 1) | ENSG00000101188(3, 1) | ENSG00000147394(1, 0) |
| ENSG00000181938(1, 1) | ENSG00000101246(3, 1) | ENSG00000147400(1, 0) |
| ENSG00000182093(1, 1) | ENSG00000101255(3, 2) | ENSG00000147408(1, 0) |
| ENSG00000182132(1, 1) | ENSG00000101333(3, 1) | ENSG00000147437(1, 0) |

ENSG00000182240(1, 1)
ENSG00000182255(1, 1)
ENSG00000182742(1, 1)
ENSG00000182866(1, 1)
ENSG00000182870(1, 1)
ENSG00000182896(1, 1)
ENSG00000183207(1, 1)
ENSG00000183287(1, 1)
ENSG00000183484(1, 1)
ENSG00000183513(1, 1)
ENSG00000183527(1, 1)
ENSG00000183735(1, 1)
ENSG00000183778(1, 1)
ENSG00000184012(1, 1)
ENSG00000184160(1, 1)
ENSG00000184343(1, 1)
ENSG00000184368(1, 1)
ENSG00000184517(1, 1)
ENSG00000184584(1, 1)
ENSG00000184702(1, 1)
ENSG00000184886(1, 1)
ENSG00000184988(1, 1)
ENSG00000185053(1, 1)
ENSG00000185122(1, 1)
ENSG00000185149(1, 1)
ENSG00000185164(1, 1)
ENSG00000185278(1, 1)
ENSG00000185345(1, 1)
ENSG00000185386(1, 1)
ENSG00000185418(1, 1)
ENSG00000185519(1, 1)
ENSG00000185610(1, 1)
ENSG00000185630(1, 1)
ENSG00000185928(1, 1)
ENSG00000186020(1, 1)
ENSG00000186074(1, 1)
ENSG00000186150(1, 1)
ENSG00000186470(1, 1)
ENSG00000186523(1, 1)
ENSG00000186642(1, 1)
ENSG00000186648(1, 1)
ENSG00000186815(1, 1)
ENSG00000186897(1, 1)
ENSG00000187033(1, 1)
ENSG00000187145(1, 1)
ENSG00000187510(1, 1)
ENSG00000187566(1, 1)
ENSG00000187689(1, 1)
ENSG00000187715(1, 1)
ENSG00000187742(1, 1)
ENSG00000187824(1, 1)
ENSG00000187889(1, 1)
ENSG00000187942(1, 1)
ENSG00000189182(1, 1)
ENSG00000101363(3, 2)
ENSG00000101442(3, 1)
ENSG00000101670(3, 2)
ENSG00000101846(3, 2)
ENSG00000101997(3, 1)
ENSG00000102007(3, 1)
ENSG00000102468(3, 1)
ENSG00000102755(3, 1)
ENSG00000102893(3, 1)
ENSG00000103005(3, 1)
ENSG00000103342(3, 2)
ENSG00000103415(3, 2)
ENSG00000103429(3, 1)
ENSG00000103479(3, 1)
ENSG00000103769(3, 1)
ENSG00000103978(3, 1)
ENSG00000104131(3, 2)
ENSG00000104432(3, 1)
ENSG00000104522(3, 2)
ENSG00000104765(3, 2)
ENSG00000104880(3, 2)
ENSG00000104967(3, 1)
ENSG00000105204(3, 1)
ENSG00000105227(3, 2)
ENSG00000105701(3, 1)
ENSG00000105877(3, 1)
ENSG00000105880(3, 1)
ENSG00000105953(3, 1)
ENSG00000105991(3, 2)
ENSG00000106078(3, 2)
ENSG00000106080(3, 1)
ENSG00000106144(3, 2)
ENSG00000106263(3, 1)
ENSG00000106330(3, 2)
ENSG00000106462(3, 1)
ENSG00000106483(3, 1)
ENSG00000106588(3, 1)
ENSG00000107614(3, 2)
ENSG00000107954(3, 2)
ENSG00000108039(3, 1)
ENSG00000108439(3, 2)
ENSG00000108506(3, 1)
ENSG00000108551(3, 1)
ENSG00000108639(3, 1)
ENSG00000108788(3, 1)
ENSG00000108813(3, 1)
ENSG00000108852(3, 2)
ENSG00000109111(3, 1)
ENSG00000109572(3, 2)
ENSG00000110090(3, 1)
ENSG00000110108(3, 2)
ENSG00000110274(3, 2)
ENSG00000111077(3, 2)
ENSG00000111344(3, 1)
ENSG00000147443(1, 0)
ENSG00000147465(1, 0)
ENSG00000147509(1, 0)
ENSG00000147576(1, 0)
ENSG00000147601(1, 0)
ENSG00000147679(1, 0)
ENSG00000147689(1, 0)
ENSG00000148090(1, 0)
ENSG00000148187(1, 0)
ENSG00000148219(1, 0)
ENSG00000148229(1, 0)
ENSG00000148297(1, 0)
ENSG00000148337(1, 0)
ENSG00000148773(1, 0)
ENSG00000148826(1, 0)
ENSG00000148835(1, 0)
ENSG00000148908(1, 0)
ENSG00000149016(1, 0)
ENSG00000149257(1, 0)
ENSG00000149311(1, 0)
ENSG00000149357(1, 0)
ENSG00000149397(1, 0)
ENSG00000149474(1, 0)
ENSG00000149503(1, 0)
ENSG00000149527(1, 0)
ENSG00000149534(1, 0)
ENSG00000149922(1, 0)
ENSG00000149925(1, 0)
ENSG00000150076(1, 0)
ENSG00000150768(1, 0)
ENSG00000150782(1, 0)
ENSG00000151033(1, 0)
ENSG00000151136(1, 0)
ENSG00000151348(1, 0)
ENSG00000151503(1, 0)
ENSG00000151640(1, 0)
ENSG00000151702(1, 0)
ENSG00000151704(1, 0)
ENSG00000151715(1, 0)
ENSG00000151834(1, 0)
ENSG00000151849(1, 0)
ENSG00000151876(1, 0)
ENSG00000151881(1, 0)
ENSG00000152214(1, 0)
ENSG00000152217(1, 0)
ENSG00000152254(1, 0)
ENSG00000152348(1, 0)
ENSG00000152359(1, 0)
ENSG00000152455(1, 0)
ENSG00000152484(1, 0)
ENSG00000152700(1, 0)
ENSG00000152763(1, 0)
ENSG00000152936(1, 0)
ENSG00000152944(1, 0)

| | | |
|---|---|---|
| ENSG00000189212(1, 1) | ENSG00000111450(3, 1) | ENSG00000153064(1, 0) |
| ENSG00000189241(1, 1) | ENSG00000111642(3, 1) | ENSG00000153107(1, 0) |
| ENSG00000189298(1, 1) | ENSG00000111731(3, 1) | ENSG00000153147(1, 0) |
| ENSG00000189377(1, 1) | ENSG00000111880(3, 2) | ENSG00000153214(1, 0) |
| ENSG00000196150(1, 1) | ENSG00000112238(3, 1) | ENSG00000153283(1, 0) |
| ENSG00000196187(1, 1) | ENSG00000112276(3, 2) | ENSG00000153485(1, 0) |
| ENSG00000196188(1, 1) | ENSG00000112406(3, 2) | ENSG00000153575(1, 0) |
| ENSG00000196189(1, 1) | ENSG00000112530(3, 1) | ENSG00000153820(1, 0) |
| ENSG00000196405(1, 1) | ENSG00000112541(3, 1) | ENSG00000154027(1, 0) |
| ENSG00000196644(1, 1) | ENSG00000112561(3, 1) | ENSG00000154188(1, 0) |
| ENSG00000196711(1, 1) | ENSG00000112701(3, 1) | ENSG00000154217(1, 0) |
| ENSG00000196724(1, 1) | ENSG00000112874(3, 2) | ENSG00000154330(1, 0) |
| ENSG00000196767(1, 1) | ENSG00000113108(3, 1) | ENSG00000154358(1, 0) |
| ENSG00000196878(1, 1) | ENSG00000113196(3, 1) | ENSG00000154359(1, 0) |
| ENSG00000196981(1, 1) | ENSG00000113327(3, 1) | ENSG00000154370(1, 0) |
| ENSG00000197128(1, 1) | ENSG00000113328(3, 2) | ENSG00000154781(1, 0) |
| ENSG00000197147(1, 1) | ENSG00000113648(3, 1) | ENSG00000154803(1, 0) |
| ENSG00000197448(1, 1) | ENSG00000114013(3, 2) | ENSG00000154914(1, 0) |
| ENSG00000197808(1, 1) | ENSG00000114302(3, 1) | ENSG00000154920(1, 0) |
| ENSG00000197859(1, 1) | ENSG00000114423(3, 1) | ENSG00000154930(1, 0) |
| ENSG00000197894(1, 1) | ENSG00000114735(3, 1) | ENSG00000154975(1, 0) |
| ENSG00000198000(1, 1) | ENSG00000114805(3, 1) | ENSG00000155229(1, 0) |
| ENSG00000198088(1, 1) | ENSG00000115468(3, 1) | ENSG00000155256(1, 0) |
| ENSG00000198183(1, 1) | ENSG00000115486(3, 1) | ENSG00000155363(1, 0) |
| ENSG00000198211(1, 1) | ENSG00000115507(3, 2) | ENSG00000155368(1, 0) |
| ENSG00000198393(1, 1) | ENSG00000115525(3, 1) | ENSG00000155465(1, 0) |
| ENSG00000198440(1, 1) | ENSG00000115694(3, 1) | ENSG00000155660(1, 0) |
| ENSG00000198554(1, 1) | ENSG00000115840(3, 2) | ENSG00000155666(1, 0) |
| ENSG00000198574(1, 1) | ENSG00000115919(3, 1) | ENSG00000155754(1, 0) |
| ENSG00000198746(1, 1) | ENSG00000116005(3, 1) | ENSG00000156050(1, 0) |
| ENSG00000198753(1, 1) | ENSG00000116194(3, 2) | ENSG00000156253(1, 0) |
| ENSG00000198768(1, 1) | ENSG00000116251(3, 2) | ENSG00000156256(1, 0) |
| ENSG00000198824(1, 1) | ENSG00000116741(3, 1) | ENSG00000156269(1, 0) |
| ENSG00000198892(1, 1) | ENSG00000116750(3, 1) | ENSG00000156304(1, 0) |
| ENSG00000198908(1, 1) | ENSG00000116863(3, 1) | ENSG00000156463(1, 0) |
| ENSG00000198946(1, 1) | ENSG00000116903(3, 1) | ENSG00000156575(1, 0) |
| ENSG00000198960(1, 1) | ENSG00000116977(3, 1) | ENSG00000156587(1, 0) |
| ENSG00000203772(1, 1) | ENSG00000117153(3, 2) | ENSG00000156671(1, 0) |
| ENSG00000203883(1, 1) | ENSG00000117362(3, 1) | ENSG00000156858(1, 0) |
| ENSG00000204645(1, 1) | ENSG00000117616(3, 1) | ENSG00000156958(1, 0) |
| ENSG00000204856(1, 1) | ENSG00000118260(3, 2) | ENSG00000157014(1, 0) |
| ENSG00000205155(1, 1) | ENSG00000118369(3, 1) | ENSG00000157045(1, 0) |
| ENSG00000205420(1, 1) | ENSG00000119397(3, 2) | ENSG00000157093(1, 0) |
| ENSG00000205629(1, 1) | ENSG00000119707(3, 1) | ENSG00000157150(1, 0) |
| ENSG00000212747(1, 1) | ENSG00000119760(3, 1) | ENSG00000157168(1, 0) |
| ENSG00000213047(1, 1) | ENSG00000119820(3, 1) | ENSG00000157219(1, 0) |
| ENSG00000213057(1, 1) | ENSG00000120053(3, 1) | ENSG00000157368(1, 0) |
| ENSG00000213064(1, 1) | ENSG00000120314(3, 1) | ENSG00000157379(1, 0) |
| ENSG00000213213(1, 1) | ENSG00000120332(3, 1) | ENSG00000157423(1, 0) |
| ENSG00000213265(1, 1) | ENSG00000120688(3, 1) | ENSG00000157445(1, 0) |
| ENSG00000213347(1, 1) | ENSG00000120875(3, 2) | ENSG00000157796(1, 0) |
| ENSG00000214021(1, 1) | ENSG00000120885(3, 2) | ENSG00000157869(1, 0) |
| ENSG00000214706(1, 1) | ENSG00000121068(3, 2) | ENSG00000158014(1, 0) |
| ENSG00000221946(1, 1) | ENSG00000121075(3, 2) | ENSG00000158022(1, 0) |

| | | |
|---|---|---|
| ENSG00000222028(1, 1) | ENSG00000121380(3, 2) | ENSG00000158402(1, 0) |
| ENSG00000225830(1, 1) | ENSG00000121486(3, 2) | ENSG00000158406(1, 0) |
| ENSG00000231852(1, 1) | ENSG00000121753(3, 1) | ENSG00000158423(1, 0) |
| ENSG00000234186(1, 1) | ENSG00000121879(3, 2) | ENSG00000158458(1, 0) |
| ENSG00000235568(1, 1) | ENSG00000122203(3, 2) | ENSG00000158578(1, 0) |
| ENSG00000239388(1, 1) | ENSG00000122420(3, 1) | ENSG00000158792(1, 0) |
| ENSG00000239697(1, 1) | ENSG00000122591(3, 1) | ENSG00000158865(1, 0) |
| ENSG00000239704(1, 1) | ENSG00000122692(3, 2) | ENSG00000159184(1, 0) |
| ENSG00000241360(1, 1) | ENSG00000123064(3, 2) | ENSG00000159212(1, 0) |
| ENSG00000242616(1, 1) | ENSG00000123091(3, 2) | ENSG00000159256(1, 0) |
| ENSG00000243667(1, 1) | ENSG00000123992(3, 1) | ENSG00000159307(1, 0) |
| ENSG00000244038(1, 1) | ENSG00000124333(3, 1) | ENSG00000159377(1, 0) |
| ENSG00000248485(1, 1) | ENSG00000124356(3, 1) | ENSG00000159445(1, 0) |
| ENSG00000006042(2, 2) | ENSG00000125255(3, 2) | ENSG00000159450(1, 0) |
| ENSG00000008283(2, 2) | ENSG00000125351(3, 2) | ENSG00000159495(1, 0) |
| ENSG00000011132(2, 2) | ENSG00000125378(3, 2) | ENSG00000159648(1, 0) |
| ENSG00000013306(2, 2) | ENSG00000125798(3, 2) | ENSG00000159650(1, 0) |
| ENSG00000013619(2, 2) | ENSG00000125850(3, 2) | ENSG00000159788(1, 0) |
| ENSG00000023041(2, 2) | ENSG00000125871(3, 1) | ENSG00000160161(1, 0) |
| ENSG00000027075(2, 2) | ENSG00000125898(3, 1) | ENSG00000160190(1, 0) |
| ENSG00000034510(2, 2) | ENSG00000125912(3, 1) | ENSG00000160191(1, 0) |
| ENSG00000040487(2, 2) | ENSG00000125944(3, 1) | ENSG00000160201(1, 0) |
| ENSG00000043355(2, 2) | ENSG00000125945(3, 1) | ENSG00000160202(1, 0) |
| ENSG00000054116(2, 2) | ENSG00000126107(3, 1) | ENSG00000160219(1, 0) |
| ENSG00000062194(2, 2) | ENSG00000126524(3, 2) | ENSG00000160563(1, 0) |
| ENSG00000074603(2, 2) | ENSG00000126583(3, 1) | ENSG00000160606(1, 0) |
| ENSG00000074800(2, 2) | ENSG00000126698(3, 1) | ENSG00000160679(1, 0) |
| ENSG00000076928(2, 2) | ENSG00000126768(3, 2) | ENSG00000160803(1, 0) |
| ENSG00000080819(2, 2) | ENSG00000126804(3, 2) | ENSG00000160883(1, 0) |
| ENSG00000081041(2, 2) | ENSG00000127445(3, 2) | ENSG00000160932(1, 0) |
| ENSG00000082213(2, 2) | ENSG00000127995(3, 1) | ENSG00000160991(1, 0) |
| ENSG00000082269(2, 2) | ENSG00000128045(3, 1) | ENSG00000160994(1, 0) |
| ENSG00000085721(2, 2) | ENSG00000128228(3, 1) | ENSG00000160999(1, 0) |
| ENSG00000085872(2, 2) | ENSG00000128564(3, 1) | ENSG00000161649(1, 0) |
| ENSG00000086619(2, 2) | ENSG00000128604(3, 1) | ENSG00000161677(1, 0) |
| ENSG00000092758(2, 2) | ENSG00000128891(3, 1) | ENSG00000161714(1, 0) |
| ENSG00000100483(2, 2) | ENSG00000129472(3, 2) | ENSG00000161955(1, 0) |
| ENSG00000101210(2, 2) | ENSG00000129535(3, 1) | ENSG00000162004(1, 0) |
| ENSG00000101311(2, 2) | ENSG00000129595(3, 2) | ENSG00000162073(1, 0) |
| ENSG00000103187(2, 2) | ENSG00000130032(3, 1) | ENSG00000162148(1, 0) |
| ENSG00000103966(2, 2) | ENSG00000130201(3, 1) | ENSG00000162241(1, 0) |
| ENSG00000104863(2, 2) | ENSG00000130204(3, 1) | ENSG00000162344(1, 0) |
| ENSG00000104886(2, 2) | ENSG00000130227(3, 1) | ENSG00000162377(1, 0) |
| ENSG00000104904(2, 2) | ENSG00000130396(3, 2) | ENSG00000162496(1, 0) |
| ENSG00000105402(2, 2) | ENSG00000130429(3, 1) | ENSG00000162510(1, 0) |
| ENSG00000105939(2, 2) | ENSG00000131016(3, 1) | ENSG00000162511(1, 0) |
| ENSG00000106701(2, 2) | ENSG00000131374(3, 2) | ENSG00000162517(1, 0) |
| ENSG00000108309(2, 2) | ENSG00000132383(3, 1) | ENSG00000162576(1, 0) |
| ENSG00000109163(2, 2) | ENSG00000132470(3, 1) | ENSG00000162591(1, 0) |
| ENSG00000109452(2, 2) | ENSG00000132604(3, 1) | ENSG00000162607(1, 0) |
| ENSG00000109466(2, 2) | ENSG00000132702(3, 1) | ENSG00000162616(1, 0) |
| ENSG00000109943(2, 2) | ENSG00000132823(3, 2) | ENSG00000162620(1, 0) |
| ENSG00000110104(2, 2) | ENSG00000132912(3, 1) | ENSG00000162623(1, 0) |
| ENSG00000111049(2, 2) | ENSG00000133226(3, 1) | ENSG00000162636(1, 0) |

| | | |
|---|---|---|
| ENSG00000111328(2, 2) | ENSG00000133454(3, 1) | ENSG00000162639(1, 0) |
| ENSG00000111850(2, 2) | ENSG00000133627(3, 1) | ENSG00000162687(1, 0) |
| ENSG00000112210(2, 2) | ENSG00000134058(3, 1) | ENSG00000162782(1, 0) |
| ENSG00000112294(2, 2) | ENSG00000134245(3, 1) | ENSG00000162892(1, 0) |
| ENSG00000112679(2, 2) | ENSG00000134283(3, 1) | ENSG00000162949(1, 0) |
| ENSG00000112977(2, 2) | ENSG00000134748(3, 1) | ENSG00000163064(1, 0) |
| ENSG00000113615(2, 2) | ENSG00000134871(3, 2) | ENSG00000163092(1, 0) |
| ENSG00000114738(2, 2) | ENSG00000134873(3, 1) | ENSG00000163162(1, 0) |
| ENSG00000114982(2, 2) | ENSG00000135052(3, 2) | ENSG00000163166(1, 0) |
| ENSG00000115350(2, 2) | ENSG00000135299(3, 1) | ENSG00000163380(1, 0) |
| ENSG00000116299(2, 2) | ENSG00000135473(3, 1) | ENSG00000163406(1, 0) |
| ENSG00000116497(2, 2) | ENSG00000135638(3, 2) | ENSG00000163421(1, 0) |
| ENSG00000116560(2, 2) | ENSG00000135750(3, 2) | ENSG00000163468(1, 0) |
| ENSG00000116874(2, 2) | ENSG00000135903(3, 2) | ENSG00000163516(1, 0) |
| ENSG00000117013(2, 2) | ENSG00000135925(3, 1) | ENSG00000163534(1, 0) |
| ENSG00000117560(2, 2) | ENSG00000136044(3, 1) | ENSG00000163581(1, 0) |
| ENSG00000118322(2, 2) | ENSG00000136068(3, 1) | ENSG00000163630(1, 0) |
| ENSG00000119139(2, 2) | ENSG00000136213(3, 1) | ENSG00000163701(1, 0) |
| ENSG00000119950(2, 2) | ENSG00000136457(3, 1) | ENSG00000163795(1, 0) |
| ENSG00000120341(2, 2) | ENSG00000136478(3, 2) | ENSG00000163833(1, 0) |
| ENSG00000122912(2, 2) | ENSG00000136521(3, 1) | ENSG00000163867(1, 0) |
| ENSG00000124608(2, 2) | ENSG00000136630(3, 2) | ENSG00000163882(1, 0) |
| ENSG00000125869(2, 2) | ENSG00000136931(3, 2) | ENSG00000164039(1, 0) |
| ENSG00000125965(2, 2) | ENSG00000137055(3, 2) | ENSG00000164048(1, 0) |
| ENSG00000126247(2, 2) | ENSG00000137073(3, 1) | ENSG00000164051(1, 0) |
| ENSG00000126653(2, 2) | ENSG00000137090(3, 2) | ENSG00000164077(1, 0) |
| ENSG00000126821(2, 2) | ENSG00000137193(3, 2) | ENSG00000164082(1, 0) |
| ENSG00000127124(2, 2) | ENSG00000137409(3, 1) | ENSG00000164100(1, 0) |
| ENSG00000128596(2, 2) | ENSG00000137413(3, 1) | ENSG00000164283(1, 0) |
| ENSG00000129911(2, 2) | ENSG00000137486(3, 1) | ENSG00000164296(1, 0) |
| ENSG00000130816(2, 2) | ENSG00000137492(3, 1) | ENSG00000164323(1, 0) |
| ENSG00000130822(2, 2) | ENSG00000137494(3, 1) | ENSG00000164326(1, 0) |
| ENSG00000130830(2, 2) | ENSG00000137575(3, 2) | ENSG00000164385(1, 0) |
| ENSG00000130956(2, 2) | ENSG00000137693(3, 2) | ENSG00000164400(1, 0) |
| ENSG00000130997(2, 2) | ENSG00000137745(3, 2) | ENSG00000164406(1, 0) |
| ENSG00000131386(2, 2) | ENSG00000137747(3, 1) | ENSG00000164466(1, 0) |
| ENSG00000131473(2, 2) | ENSG00000137809(3, 1) | ENSG00000164508(1, 0) |
| ENSG00000132518(2, 2) | ENSG00000137817(3, 1) | ENSG00000164530(1, 0) |
| ENSG00000132793(2, 2) | ENSG00000137947(3, 1) | ENSG00000164620(1, 0) |
| ENSG00000133105(2, 2) | ENSG00000138083(3, 1) | ENSG00000164627(1, 0) |
| ENSG00000133740(2, 2) | ENSG00000138413(3, 1) | ENSG00000164683(1, 0) |
| ENSG00000134318(2, 2) | ENSG00000138434(3, 2) | ENSG00000164879(1, 0) |
| ENSG00000134690(2, 2) | ENSG00000138449(3, 1) | ENSG00000164889(1, 0) |
| ENSG00000135740(2, 2) | ENSG00000138769(3, 2) | ENSG00000164949(1, 0) |
| ENSG00000135912(2, 2) | ENSG00000138835(3, 2) | ENSG00000164953(1, 0) |
| ENSG00000136014(2, 2) | ENSG00000139182(3, 2) | ENSG00000165084(1, 0) |
| ENSG00000136026(2, 2) | ENSG00000139537(3, 2) | ENSG00000165120(1, 0) |
| ENSG00000136631(2, 2) | ENSG00000139613(3, 2) | ENSG00000165125(1, 0) |
| ENSG00000136699(2, 2) | ENSG00000139842(3, 1) | ENSG00000165169(1, 0) |
| ENSG00000136770(2, 2) | ENSG00000139970(3, 1) | ENSG00000165233(1, 0) |
| ENSG00000136830(2, 2) | ENSG00000140265(3, 2) | ENSG00000165238(1, 0) |
| ENSG00000136870(2, 2) | ENSG00000140497(3, 2) | ENSG00000165259(1, 0) |
| ENSG00000136908(2, 2) | ENSG00000140519(3, 2) | ENSG00000165325(1, 0) |
| ENSG00000136938(2, 2) | ENSG00000140575(3, 1) | ENSG00000165349(1, 0) |

ENSG00000137168(2, 2)
ENSG00000137831(2, 2)
ENSG00000138061(2, 2)
ENSG00000139132(2, 2)
ENSG00000139173(2, 2)
ENSG00000139220(2, 2)
ENSG00000140092(2, 2)
ENSG00000141026(2, 2)
ENSG00000141458(2, 2)
ENSG00000143155(2, 2)
ENSG00000143179(2, 2)
ENSG00000143434(2, 2)
ENSG00000144354(2, 2)
ENSG00000145040(2, 2)
ENSG00000145863(2, 2)
ENSG00000145990(2, 2)
ENSG00000146232(2, 2)
ENSG00000147224(2, 2)
ENSG00000147364(2, 2)
ENSG00000148153(2, 2)
ENSG00000148154(2, 2)
ENSG00000148935(2, 2)
ENSG00000149591(2, 2)
ENSG00000150995(2, 2)
ENSG00000152137(2, 2)
ENSG00000152154(2, 2)
ENSG00000152409(2, 2)
ENSG00000152778(2, 2)
ENSG00000153048(2, 2)
ENSG00000153574(2, 2)
ENSG00000154620(2, 2)
ENSG00000154767(2, 2)
ENSG00000155087(2, 2)
ENSG00000155329(2, 2)
ENSG00000158008(2, 2)
ENSG00000160233(2, 2)
ENSG00000160310(2, 2)
ENSG00000161671(2, 2)
ENSG00000162366(2, 2)
ENSG00000163249(2, 2)
ENSG00000163558(2, 2)
ENSG00000163902(2, 2)
ENSG00000164023(2, 2)
ENSG00000164244(2, 2)
ENSG00000164506(2, 2)
ENSG00000165030(2, 2)
ENSG00000165185(2, 2)
ENSG00000165689(2, 2)
ENSG00000165775(2, 2)
ENSG00000166289(2, 2)
ENSG00000166448(2, 2)
ENSG00000166484(2, 2)
ENSG00000167123(2, 2)
ENSG00000167207(2, 2)
ENSG00000140853(3, 1)
ENSG00000140941(3, 1)
ENSG00000141198(3, 1)
ENSG00000141401(3, 1)
ENSG00000141905(3, 1)
ENSG00000142192(3, 1)
ENSG00000142875(3, 1)
ENSG00000143093(3, 2)
ENSG00000143178(3, 1)
ENSG00000143294(3, 1)
ENSG00000143319(3, 2)
ENSG00000143337(3, 1)
ENSG00000143507(3, 2)
ENSG00000143603(3, 1)
ENSG00000143624(3, 2)
ENSG00000143632(3, 1)
ENSG00000143815(3, 2)
ENSG00000143971(3, 1)
ENSG00000144366(3, 2)
ENSG00000144840(3, 1)
ENSG00000144847(3, 2)
ENSG00000144935(3, 1)
ENSG00000145029(3, 1)
ENSG00000145191(3, 2)
ENSG00000145242(3, 1)
ENSG00000145354(3, 1)
ENSG00000145451(3, 2)
ENSG00000145632(3, 2)
ENSG00000145681(3, 1)
ENSG00000145708(3, 1)
ENSG00000145725(3, 1)
ENSG00000145779(3, 2)
ENSG00000146576(3, 2)
ENSG00000146858(3, 1)
ENSG00000147010(3, 2)
ENSG00000147099(3, 1)
ENSG00000147439(3, 2)
ENSG00000147459(3, 1)
ENSG00000147570(3, 2)
ENSG00000147573(3, 1)
ENSG00000148730(3, 2)
ENSG00000149218(3, 2)
ENSG00000149485(3, 2)
ENSG00000149554(3, 1)
ENSG00000149972(3, 2)
ENSG00000150316(3, 2)
ENSG00000151789(3, 1)
ENSG00000151812(3, 2)
ENSG00000151835(3, 1)
ENSG00000151914(3, 2)
ENSG00000152128(3, 1)
ENSG00000152256(3, 1)
ENSG00000152413(3, 2)
ENSG00000152620(3, 1)
ENSG00000165383(1, 0)
ENSG00000165462(1, 0)
ENSG00000165525(1, 0)
ENSG00000165526(1, 0)
ENSG00000165555(1, 0)
ENSG00000165629(1, 0)
ENSG00000165646(1, 0)
ENSG00000165704(1, 0)
ENSG00000165794(1, 0)
ENSG00000165887(1, 0)
ENSG00000165949(1, 0)
ENSG00000166006(1, 0)
ENSG00000166033(1, 0)
ENSG00000166049(1, 0)
ENSG00000166165(1, 0)
ENSG00000166228(1, 0)
ENSG00000166292(1, 0)
ENSG00000166342(1, 0)
ENSG00000166401(1, 0)
ENSG00000166428(1, 0)
ENSG00000166432(1, 0)
ENSG00000166508(1, 0)
ENSG00000166523(1, 0)
ENSG00000166526(1, 0)
ENSG00000166562(1, 0)
ENSG00000166592(1, 0)
ENSG00000166595(1, 0)
ENSG00000166598(1, 0)
ENSG00000166813(1, 0)
ENSG00000166848(1, 0)
ENSG00000166928(1, 0)
ENSG00000166997(1, 0)
ENSG00000167094(1, 0)
ENSG00000167107(1, 0)
ENSG00000167110(1, 0)
ENSG00000167113(1, 0)
ENSG00000167196(1, 0)
ENSG00000167208(1, 0)
ENSG00000167230(1, 0)
ENSG00000167258(1, 0)
ENSG00000167384(1, 0)
ENSG00000167397(1, 0)
ENSG00000167468(1, 0)
ENSG00000167550(1, 0)
ENSG00000167595(1, 0)
ENSG00000167645(1, 0)
ENSG00000167653(1, 0)
ENSG00000167658(1, 0)
ENSG00000167747(1, 0)
ENSG00000167785(1, 0)
ENSG00000167851(1, 0)
ENSG00000167881(1, 0)
ENSG00000167889(1, 0)
ENSG00000167984(1, 0)

ENSG00000167332(2, 2)
ENSG00000167419(2, 2)
ENSG00000167723(2, 2)
ENSG00000167930(2, 2)
ENSG00000167996(2, 2)
ENSG00000168497(2, 2)
ENSG00000168542(2, 2)
ENSG00000170270(2, 2)
ENSG00000170425(2, 2)
ENSG00000170448(2, 2)
ENSG00000170523(2, 2)
ENSG00000170638(2, 2)
ENSG00000170903(2, 2)
ENSG00000171119(2, 2)
ENSG00000171208(2, 2)
ENSG00000171450(2, 2)
ENSG00000171700(2, 2)
ENSG00000172216(2, 2)
ENSG00000172590(2, 2)
ENSG00000172638(2, 2)
ENSG00000173171(2, 2)
ENSG00000173264(2, 2)
ENSG00000173621(2, 2)
ENSG00000175595(2, 2)
ENSG00000176563(2, 2)
ENSG00000177464(2, 2)
ENSG00000178075(2, 2)
ENSG00000178966(2, 2)
ENSG00000179583(2, 2)
ENSG00000179965(2, 2)
ENSG00000180190(2, 2)
ENSG00000180329(2, 2)
ENSG00000180626(2, 2)
ENSG00000181885(2, 2)
ENSG00000182173(2, 2)
ENSG00000182901(2, 2)
ENSG00000183726(2, 2)
ENSG00000183773(2, 2)
ENSG00000183780(2, 2)
ENSG00000184058(2, 2)
ENSG00000184117(2, 2)
ENSG00000185340(2, 2)
ENSG00000185933(2, 2)
ENSG00000186047(2, 2)
ENSG00000186106(2, 2)
ENSG00000187650(2, 2)
ENSG00000188338(2, 2)
ENSG00000196305(2, 2)
ENSG00000196542(2, 2)
ENSG00000198026(2, 2)
ENSG00000198920(2, 2)
ENSG00000203782(2, 2)
ENSG00000204175(2, 2)
ENSG00000204248(2, 2)
ENSG00000152969(3, 2)
ENSG00000153132(3, 1)
ENSG00000153721(3, 1)
ENSG00000154096(3, 1)
ENSG00000154319(3, 1)
ENSG00000154511(3, 2)
ENSG00000154783(3, 1)
ENSG00000154928(3, 2)
ENSG00000155366(3, 2)
ENSG00000156017(3, 1)
ENSG00000156026(3, 2)
ENSG00000156194(3, 2)
ENSG00000156374(3, 1)
ENSG00000156384(3, 1)
ENSG00000156398(3, 1)
ENSG00000156471(3, 2)
ENSG00000157184(3, 1)
ENSG00000157224(3, 1)
ENSG00000157510(3, 1)
ENSG00000157734(3, 1)
ENSG00000157895(3, 1)
ENSG00000158079(3, 2)
ENSG00000158125(3, 2)
ENSG00000158258(3, 2)
ENSG00000158292(3, 1)
ENSG00000158417(3, 2)
ENSG00000158545(3, 1)
ENSG00000158710(3, 1)
ENSG00000158856(3, 1)
ENSG00000159214(3, 2)
ENSG00000159322(3, 1)
ENSG00000159363(3, 1)
ENSG00000160307(3, 1)
ENSG00000160539(3, 1)
ENSG00000160888(3, 1)
ENSG00000160917(3, 2)
ENSG00000161243(3, 1)
ENSG00000162065(3, 1)
ENSG00000162144(3, 1)
ENSG00000162490(3, 2)
ENSG00000162614(3, 1)
ENSG00000162896(3, 1)
ENSG00000162909(3, 2)
ENSG00000163393(3, 2)
ENSG00000163472(3, 1)
ENSG00000163479(3, 2)
ENSG00000163482(3, 1)
ENSG00000163624(3, 2)
ENSG00000163950(3, 2)
ENSG00000164054(3, 2)
ENSG00000164088(3, 1)
ENSG00000164163(3, 2)
ENSG00000164252(3, 2)
ENSG00000164305(3, 2)
ENSG00000168032(1, 0)
ENSG00000168096(1, 0)
ENSG00000168135(1, 0)
ENSG00000168242(1, 0)
ENSG00000168269(1, 0)
ENSG00000168291(1, 0)
ENSG00000168297(1, 0)
ENSG00000168298(1, 0)
ENSG00000168394(1, 0)
ENSG00000168404(1, 0)
ENSG00000168418(1, 0)
ENSG00000168447(1, 0)
ENSG00000168484(1, 0)
ENSG00000168491(1, 0)
ENSG00000168505(1, 0)
ENSG00000168522(1, 0)
ENSG00000168538(1, 0)
ENSG00000168661(1, 0)
ENSG00000168676(1, 0)
ENSG00000168734(1, 0)
ENSG00000168772(1, 0)
ENSG00000168899(1, 0)
ENSG00000168904(1, 0)
ENSG00000168918(1, 0)
ENSG00000168938(1, 0)
ENSG00000168958(1, 0)
ENSG00000169035(1, 0)
ENSG00000169067(1, 0)
ENSG00000169085(1, 0)
ENSG00000169174(1, 0)
ENSG00000169442(1, 0)
ENSG00000169490(1, 0)
ENSG00000169519(1, 0)
ENSG00000169570(1, 0)
ENSG00000169750(1, 0)
ENSG00000169764(1, 0)
ENSG00000169860(1, 0)
ENSG00000169918(1, 0)
ENSG00000170191(1, 0)
ENSG00000170266(1, 0)
ENSG00000170290(1, 0)
ENSG00000170419(1, 0)
ENSG00000170430(1, 0)
ENSG00000170454(1, 0)
ENSG00000170486(1, 0)
ENSG00000170498(1, 0)
ENSG00000170627(1, 0)
ENSG00000170748(1, 0)
ENSG00000170791(1, 0)
ENSG00000170819(1, 0)
ENSG00000170854(1, 0)
ENSG00000170967(1, 0)
ENSG00000171121(1, 0)
ENSG00000171126(1, 0)

ENSG00000212864(2, 2)
ENSG00000213865(2, 2)
ENSG00000215262(2, 2)
ENSG00000243284(2, 2)
ENSG00000253313(2, 2)
ENSG00000003436(3, 3)
ENSG00000023902(3, 3)
ENSG00000025772(3, 3)
ENSG00000042781(3, 3)
ENSG00000044115(3, 3)
ENSG00000064225(3, 3)
ENSG00000065802(3, 3)
ENSG00000067177(3, 3)
ENSG00000075651(3, 3)
ENSG00000078967(3, 3)
ENSG00000084444(3, 3)
ENSG00000097007(3, 3)
ENSG00000099625(3, 3)
ENSG00000099822(3, 3)
ENSG00000102034(3, 3)
ENSG00000102218(3, 3)
ENSG00000103549(3, 3)
ENSG00000106392(3, 3)
ENSG00000107331(3, 3)
ENSG00000109689(3, 3)
ENSG00000115234(3, 3)
ENSG00000116161(3, 3)
ENSG00000118705(3, 3)
ENSG00000123191(3, 3)
ENSG00000129749(3, 3)
ENSG00000131966(3, 3)
ENSG00000133961(3, 3)
ENSG00000134575(3, 3)
ENSG00000136261(3, 3)
ENSG00000136807(3, 3)
ENSG00000142920(3, 3)
ENSG00000143921(3, 3)
ENSG00000146828(3, 3)
ENSG00000153395(3, 3)
ENSG00000154556(3, 3)
ENSG00000155093(3, 3)
ENSG00000158286(3, 3)
ENSG00000163673(3, 3)
ENSG00000163932(3, 3)
ENSG00000164114(3, 3)
ENSG00000164402(3, 3)
ENSG00000166669(3, 3)
ENSG00000166912(3, 3)
ENSG00000167034(3, 3)
ENSG00000168872(3, 3)
ENSG00000169255(3, 3)
ENSG00000170265(3, 3)
ENSG00000171823(3, 3)
ENSG00000172113(3, 3)
ENSG00000164398(3, 2)
ENSG00000164543(3, 2)
ENSG00000164548(3, 2)
ENSG00000164631(3, 2)
ENSG00000164691(3, 1)
ENSG00000164929(3, 2)
ENSG00000165138(3, 1)
ENSG00000165359(3, 2)
ENSG00000165527(3, 1)
ENSG00000165832(3, 1)
ENSG00000165917(3, 1)
ENSG00000165934(3, 1)
ENSG00000166090(3, 1)
ENSG00000166188(3, 1)
ENSG00000166197(3, 1)
ENSG00000166233(3, 1)
ENSG00000166261(3, 2)
ENSG00000166275(3, 1)
ENSG00000166340(3, 2)
ENSG00000166407(3, 1)
ENSG00000166415(3, 1)
ENSG00000166450(3, 2)
ENSG00000166454(3, 1)
ENSG00000166507(3, 2)
ENSG00000166734(3, 1)
ENSG00000166887(3, 2)
ENSG00000166961(3, 1)
ENSG00000167088(3, 1)
ENSG00000167114(3, 2)
ENSG00000167173(3, 2)
ENSG00000167395(3, 1)
ENSG00000167535(3, 2)
ENSG00000167772(3, 1)
ENSG00000167799(3, 1)
ENSG00000168071(3, 1)
ENSG00000168267(3, 2)
ENSG00000168314(3, 2)
ENSG00000168495(3, 1)
ENSG00000168556(3, 2)
ENSG00000168887(3, 1)
ENSG00000169045(3, 2)
ENSG00000169062(3, 1)
ENSG00000169129(3, 1)
ENSG00000169213(3, 1)
ENSG00000169258(3, 2)
ENSG00000169359(3, 2)
ENSG00000169436(3, 2)
ENSG00000169504(3, 1)
ENSG00000169689(3, 1)
ENSG00000169856(3, 2)
ENSG00000169955(3, 1)
ENSG00000170043(3, 2)
ENSG00000170088(3, 1)
ENSG00000170340(3, 2)
ENSG00000171204(1, 0)
ENSG00000171224(1, 0)
ENSG00000171227(1, 0)
ENSG00000171307(1, 0)
ENSG00000171310(1, 0)
ENSG00000171403(1, 0)
ENSG00000171466(1, 0)
ENSG00000171471(1, 0)
ENSG00000171492(1, 0)
ENSG00000171551(1, 0)
ENSG00000171574(1, 0)
ENSG00000171596(1, 0)
ENSG00000171703(1, 0)
ENSG00000171720(1, 0)
ENSG00000171873(1, 0)
ENSG00000171928(1, 0)
ENSG00000171951(1, 0)
ENSG00000172262(1, 0)
ENSG00000172382(1, 0)
ENSG00000172469(1, 0)
ENSG00000172663(1, 0)
ENSG00000172752(1, 0)
ENSG00000172803(1, 0)
ENSG00000172828(1, 0)
ENSG00000172867(1, 0)
ENSG00000172901(1, 0)
ENSG00000172932(1, 0)
ENSG00000172940(1, 0)
ENSG00000172986(1, 0)
ENSG00000173207(1, 0)
ENSG00000173209(1, 0)
ENSG00000173214(1, 0)
ENSG00000173230(1, 0)
ENSG00000173338(1, 0)
ENSG00000173442(1, 0)
ENSG00000173457(1, 0)
ENSG00000173546(1, 0)
ENSG00000173548(1, 0)
ENSG00000173567(1, 0)
ENSG00000173812(1, 0)
ENSG00000173825(1, 0)
ENSG00000173838(1, 0)
ENSG00000173915(1, 0)
ENSG00000173947(1, 0)
ENSG00000174059(1, 0)
ENSG00000174123(1, 0)
ENSG00000174225(1, 0)
ENSG00000174231(1, 0)
ENSG00000174255(1, 0)
ENSG00000174371(1, 0)
ENSG00000174652(1, 0)
ENSG00000174740(1, 0)
ENSG00000174744(1, 0)
ENSG00000174775(1, 0)

ENSG00000172340(3, 3)
ENSG00000172466(3, 3)
ENSG00000174799(3, 3)
ENSG00000174938(3, 3)
ENSG00000175868(3, 3)
ENSG00000175928(3, 3)
ENSG00000176533(3, 3)
ENSG00000176928(3, 3)
ENSG00000179562(3, 3)
ENSG00000181804(3, 3)
ENSG00000197982(3, 3)
ENSG00000198168(3, 3)
ENSG00000198853(3, 3)
ENSG00000218891(3, 3)
ENSG00000022267(4, 4)
ENSG00000057704(4, 4)
ENSG00000099204(4, 4)
ENSG00000112559(4, 4)
ENSG00000125844(4, 4)
ENSG00000136011(4, 4)
ENSG00000140464(4, 4)
ENSG00000141682(4, 4)
ENSG00000148331(4, 4)
ENSG00000163032(4, 4)
ENSG00000163281(4, 4)
ENSG00000165156(4, 4)
ENSG00000167604(4, 4)
ENSG00000168397(4, 4)
ENSG00000170522(4, 4)
ENSG00000172115(4, 4)
ENSG00000173404(4, 4)
ENSG00000174840(4, 4)
ENSG00000178904(4, 4)
ENSG00000183808(4, 4)
ENSG00000185090(4, 4)
ENSG00000198108(4, 4)
ENSG00000078674(5, 5)
ENSG00000103174(5, 5)
ENSG00000119242(5, 5)
ENSG00000123307(5, 5)
ENSG00000124466(5, 5)
ENSG00000138032(5, 5)
ENSG00000138111(5, 5)
ENSG00000161653(5, 5)
ENSG00000166886(5, 5)
ENSG00000167565(5, 5)
ENSG00000164715(6, 6)

ENSG00000170515(3, 1)
ENSG00000171017(3, 1)
ENSG00000171055(3, 1)
ENSG00000171067(3, 1)
ENSG00000171109(3, 1)
ENSG00000171223(3, 2)
ENSG00000171302(3, 2)
ENSG00000171867(3, 1)
ENSG00000171877(3, 1)
ENSG00000172403(3, 2)
ENSG00000172840(3, 1)
ENSG00000173262(3, 1)
ENSG00000173391(3, 2)
ENSG00000173511(3, 1)
ENSG00000173846(3, 2)
ENSG00000173917(3, 2)
ENSG00000174007(3, 1)
ENSG00000174137(3, 1)
ENSG00000174640(3, 1)
ENSG00000174718(3, 1)
ENSG00000174796(3, 1)
ENSG00000174807(3, 2)
ENSG00000175318(3, 1)
ENSG00000175538(3, 1)
ENSG00000175550(3, 1)
ENSG00000175567(3, 1)
ENSG00000175793(3, 2)
ENSG00000175832(3, 1)
ENSG00000176102(3, 2)
ENSG00000176428(3, 1)
ENSG00000176531(3, 2)
ENSG00000176783(3, 2)
ENSG00000177303(3, 1)
ENSG00000177426(3, 2)
ENSG00000177575(3, 2)
ENSG00000177697(3, 2)
ENSG00000177728(3, 1)
ENSG00000177917(3, 2)
ENSG00000178033(3, 2)
ENSG00000178252(3, 2)
ENSG00000178726(3, 1)
ENSG00000178741(3, 1)
ENSG00000178802(3, 2)
ENSG00000178860(3, 2)
ENSG00000178965(3, 2)
ENSG00000179071(3, 1)
ENSG00000179111(3, 1)
ENSG00000179222(3, 1)
ENSG00000179262(3, 2)
ENSG00000179292(3, 2)
ENSG00000179449(3, 2)
ENSG00000179520(3, 2)
ENSG00000179604(3, 2)
ENSG00000180900(3, 1)

ENSG00000174953(1, 0)
ENSG00000175097(1, 0)
ENSG00000175115(1, 0)
ENSG00000175193(1, 0)
ENSG00000175265(1, 0)
ENSG00000175305(1, 0)
ENSG00000175311(1, 0)
ENSG00000175344(1, 0)
ENSG00000175356(1, 0)
ENSG00000175374(1, 0)
ENSG00000175573(1, 0)
ENSG00000175602(1, 0)
ENSG00000175782(1, 0)
ENSG00000175984(1, 0)
ENSG00000176022(1, 0)
ENSG00000176108(1, 0)
ENSG00000176208(1, 0)
ENSG00000176225(1, 0)
ENSG00000176340(1, 0)
ENSG00000176358(1, 0)
ENSG00000176731(1, 0)
ENSG00000176845(1, 0)
ENSG00000176915(1, 0)
ENSG00000176973(1, 0)
ENSG00000177058(1, 0)
ENSG00000177082(1, 0)
ENSG00000177096(1, 0)
ENSG00000177105(1, 0)
ENSG00000177239(1, 0)
ENSG00000177301(1, 0)
ENSG00000177354(1, 0)
ENSG00000177427(1, 0)
ENSG00000177465(1, 0)
ENSG00000177688(1, 0)
ENSG00000177879(1, 0)
ENSG00000177951(1, 0)
ENSG00000177994(1, 0)
ENSG00000178301(1, 0)
ENSG00000178645(1, 0)
ENSG00000179008(1, 0)
ENSG00000179010(1, 0)
ENSG00000179041(1, 0)
ENSG00000179284(1, 0)
ENSG00000179299(1, 0)
ENSG00000179300(1, 0)
ENSG00000179397(1, 0)
ENSG00000179528(1, 0)
ENSG00000179542(1, 0)
ENSG00000179546(1, 0)
ENSG00000179593(1, 0)
ENSG00000179600(1, 0)
ENSG00000179639(1, 0)
ENSG00000179674(1, 0)
ENSG00000179761(1, 0)

| | |
|---|---|
| ENSG00000181031(3, 2) | ENSG00000179869(1, 0) |
| ENSG00000181541(3, 2) | ENSG00000180219(1, 0) |
| ENSG00000181790(3, 2) | ENSG00000180251(1, 0) |
| ENSG00000181982(3, 2) | ENSG00000180287(1, 0) |
| ENSG00000182195(3, 1) | ENSG00000180305(1, 0) |
| ENSG00000182318(3, 2) | ENSG00000180440(1, 0) |
| ENSG00000182359(3, 2) | ENSG00000180483(1, 0) |
| ENSG00000182489(3, 2) | ENSG00000180509(1, 0) |
| ENSG00000182500(3, 2) | ENSG00000180772(1, 0) |
| ENSG00000182552(3, 2) | ENSG00000180878(1, 0) |
| ENSG00000182827(3, 1) | ENSG00000180914(1, 0) |
| ENSG00000183638(3, 1) | ENSG00000181045(1, 0) |
| ENSG00000183662(3, 2) | ENSG00000181143(1, 0) |
| ENSG00000183798(3, 1) | ENSG00000181218(1, 0) |
| ENSG00000184162(3, 1) | ENSG00000181222(1, 0) |
| ENSG00000184347(3, 2) | ENSG00000181322(1, 0) |
| ENSG00000184564(3, 2) | ENSG00000181649(1, 0) |
| ENSG00000184743(3, 2) | ENSG00000181666(1, 0) |
| ENSG00000184845(3, 1) | ENSG00000181817(1, 0) |
| ENSG00000185022(3, 2) | ENSG00000182013(1, 0) |
| ENSG00000185046(3, 2) | ENSG00000182035(1, 0) |
| ENSG00000185787(3, 1) | ENSG00000182185(1, 0) |
| ENSG00000185960(3, 1) | ENSG00000182346(1, 0) |
| ENSG00000186184(3, 1) | ENSG00000182379(1, 0) |
| ENSG00000186314(3, 2) | ENSG00000182450(1, 0) |
| ENSG00000186416(3, 2) | ENSG00000182473(1, 0) |
| ENSG00000186501(3, 2) | ENSG00000182508(1, 0) |
| ENSG00000186522(3, 2) | ENSG00000182611(1, 0) |
| ENSG00000186687(3, 1) | ENSG00000182674(1, 0) |
| ENSG00000187210(3, 1) | ENSG00000182676(1, 0) |
| ENSG00000187735(3, 2) | ENSG00000182747(1, 0) |
| ENSG00000187792(3, 1) | ENSG00000182791(1, 0) |
| ENSG00000187961(3, 1) | ENSG00000182795(1, 0) |
| ENSG00000188811(3, 1) | ENSG00000182871(1, 0) |
| ENSG00000189292(3, 2) | ENSG00000182931(1, 0) |
| ENSG00000189325(3, 1) | ENSG00000182957(1, 0) |
| ENSG00000196092(3, 1) | ENSG00000182973(1, 0) |
| ENSG00000196104(3, 1) | ENSG00000183020(1, 0) |
| ENSG00000196169(3, 2) | ENSG00000183072(1, 0) |
| ENSG00000196557(3, 2) | ENSG00000183161(1, 0) |
| ENSG00000196664(3, 1) | ENSG00000183172(1, 0) |
| ENSG00000197024(3, 2) | ENSG00000183379(1, 0) |
| ENSG00000197157(3, 2) | ENSG00000183423(1, 0) |
| ENSG00000197273(3, 2) | ENSG00000183434(1, 0) |
| ENSG00000197321(3, 1) | ENSG00000183520(1, 0) |
| ENSG00000197442(3, 2) | ENSG00000183558(1, 0) |
| ENSG00000197471(3, 2) | ENSG00000183615(1, 0) |
| ENSG00000198105(3, 2) | ENSG00000183655(1, 0) |
| ENSG00000198121(3, 1) | ENSG00000183748(1, 0) |
| ENSG00000198522(3, 1) | ENSG00000183783(1, 0) |
| ENSG00000198624(3, 2) | ENSG00000183785(1, 0) |
| ENSG00000198722(3, 1) | ENSG00000183814(1, 0) |
| ENSG00000198771(3, 2) | ENSG00000183840(1, 0) |
| ENSG00000198879(3, 2) | ENSG00000183862(1, 0) |

| | |
|---|---|
| ENSG00000204130(3, 1) | ENSG00000184047(1, 0) |
| ENSG00000204267(3, 1) | ENSG00000184113(1, 0) |
| ENSG00000204463(3, 1) | ENSG00000184156(1, 0) |
| ENSG00000204564(3, 1) | ENSG00000184182(1, 0) |
| ENSG00000204624(3, 2) | ENSG00000184194(1, 0) |
| ENSG00000204843(3, 1) | ENSG00000184261(1, 0) |
| ENSG00000205339(3, 2) | ENSG00000184292(1, 0) |
| ENSG00000206190(3, 1) | ENSG00000184357(1, 0) |
| ENSG00000213023(3, 1) | ENSG00000184381(1, 0) |
| ENSG00000213186(3, 2) | ENSG00000184560(1, 0) |
| ENSG00000213445(3, 1) | ENSG00000184571(1, 0) |
| ENSG00000213465(3, 1) | ENSG00000184574(1, 0) |
| ENSG00000213578(3, 1) | ENSG00000184613(1, 0) |
| ENSG00000213614(3, 2) | ENSG00000184788(1, 0) |
| ENSG00000213699(3, 1) | ENSG00000184825(1, 0) |
| ENSG00000213741(3, 1) | ENSG00000184979(1, 0) |
| ENSG00000215386(3, 2) | ENSG00000184983(1, 0) |
| ENSG00000215717(3, 2) | ENSG00000184986(1, 0) |
| ENSG00000227345(3, 1) | ENSG00000184992(1, 0) |
| ENSG00000227372(3, 1) | ENSG00000185010(1, 0) |
| ENSG00000237190(3, 1) | ENSG00000185049(1, 0) |
| ENSG00000242732(3, 1) | ENSG00000185085(1, 0) |
| ENSG00000243244(3, 1) | ENSG00000185272(1, 0) |
| ENSG00000249915(3, 2) | ENSG00000185414(1, 0) |
| ENSG00000250510(3, 2) | ENSG00000185436(1, 0) |
| ENSG00000253928(3, 1) | ENSG00000185442(1, 0) |
| ENSG00000000003(4, 2) | ENSG00000185483(1, 0) |
| ENSG00000001561(4, 1) | ENSG00000185499(1, 0) |
| ENSG00000003096(4, 2) | ENSG00000185567(1, 0) |
| ENSG00000004059(4, 2) | ENSG00000185621(1, 0) |
| ENSG00000005955(4, 3) | ENSG00000185825(1, 0) |
| ENSG00000008056(4, 1) | ENSG00000185860(1, 0) |
| ENSG00000009780(4, 1) | ENSG00000185883(1, 0) |
| ENSG00000010404(4, 1) | ENSG00000185905(1, 0) |
| ENSG00000010810(4, 3) | ENSG00000185946(1, 0) |
| ENSG00000011566(4, 3) | ENSG00000185989(1, 0) |
| ENSG00000012822(4, 1) | ENSG00000186007(1, 0) |
| ENSG00000013588(4, 1) | ENSG00000186017(1, 0) |
| ENSG00000024422(4, 2) | ENSG00000186081(1, 0) |
| ENSG00000028203(4, 2) | ENSG00000186153(1, 0) |
| ENSG00000028839(4, 3) | ENSG00000186265(1, 0) |
| ENSG00000033627(4, 1) | ENSG00000186283(1, 0) |
| ENSG00000038295(4, 2) | ENSG00000186335(1, 0) |
| ENSG00000039068(4, 1) | ENSG00000186352(1, 0) |
| ENSG00000039319(4, 2) | ENSG00000186364(1, 0) |
| ENSG00000040633(4, 2) | ENSG00000186439(1, 0) |
| ENSG00000044446(4, 2) | ENSG00000186493(1, 0) |
| ENSG00000047597(4, 2) | ENSG00000186517(1, 0) |
| ENSG00000048392(4, 1) | ENSG00000186577(1, 0) |
| ENSG00000050767(4, 1) | ENSG00000186594(1, 0) |
| ENSG00000054219(4, 3) | ENSG00000186603(1, 0) |
| ENSG00000054598(4, 1) | ENSG00000186652(1, 0) |
| ENSG00000054654(4, 3) | ENSG00000186675(1, 0) |
| ENSG00000056558(4, 3) | ENSG00000186732(1, 0) |

| | |
|---|---|
| ENSG00000061936(4, 3) | ENSG00000186766(1, 0) |
| ENSG00000062716(4, 3) | ENSG00000186767(1, 0) |
| ENSG00000063169(4, 3) | ENSG00000186787(1, 0) |
| ENSG00000063660(4, 3) | ENSG00000187017(1, 0) |
| ENSG00000064601(4, 1) | ENSG00000187049(1, 0) |
| ENSG00000064655(4, 2) | ENSG00000187097(1, 0) |
| ENSG00000065000(4, 2) | ENSG00000187288(1, 0) |
| ENSG00000065357(4, 1) | ENSG00000187398(1, 0) |
| ENSG00000065911(4, 2) | ENSG00000187475(1, 0) |
| ENSG00000066735(4, 3) | ENSG00000187634(1, 0) |
| ENSG00000066855(4, 3) | ENSG00000187837(1, 0) |
| ENSG00000067082(4, 3) | ENSG00000187855(1, 0) |
| ENSG00000068697(4, 2) | ENSG00000187939(1, 0) |
| ENSG00000068796(4, 3) | ENSG00000187957(1, 0) |
| ENSG00000068831(4, 2) | ENSG00000187990(1, 0) |
| ENSG00000069329(4, 2) | ENSG00000188015(1, 0) |
| ENSG00000070371(4, 2) | ENSG00000188021(1, 0) |
| ENSG00000071626(4, 2) | ENSG00000188130(1, 0) |
| ENSG00000072401(4, 3) | ENSG00000188176(1, 0) |
| ENSG00000072849(4, 2) | ENSG00000188386(1, 0) |
| ENSG00000073060(4, 1) | ENSG00000188542(1, 0) |
| ENSG00000074410(4, 1) | ENSG00000188800(1, 0) |
| ENSG00000075415(4, 1) | ENSG00000188816(1, 0) |
| ENSG00000075461(4, 1) | ENSG00000188984(1, 0) |
| ENSG00000076770(4, 2) | ENSG00000188987(1, 0) |
| ENSG00000078061(4, 2) | ENSG00000189050(1, 0) |
| ENSG00000078081(4, 1) | ENSG00000189060(1, 0) |
| ENSG00000078237(4, 2) | ENSG00000189143(1, 0) |
| ENSG00000079215(4, 2) | ENSG00000196176(1, 0) |
| ENSG00000080802(4, 2) | ENSG00000196226(1, 0) |
| ENSG00000083520(4, 2) | ENSG00000196235(1, 0) |
| ENSG00000083937(4, 3) | ENSG00000196236(1, 0) |
| ENSG00000084073(4, 3) | ENSG00000196268(1, 0) |
| ENSG00000084112(4, 3) | ENSG00000196331(1, 0) |
| ENSG00000084693(4, 1) | ENSG00000196358(1, 0) |
| ENSG00000085733(4, 1) | ENSG00000196407(1, 0) |
| ENSG00000086289(4, 2) | ENSG00000196459(1, 0) |
| ENSG00000086544(4, 3) | ENSG00000196476(1, 0) |
| ENSG00000087470(4, 1) | ENSG00000196532(1, 0) |
| ENSG00000088179(4, 3) | ENSG00000196533(1, 0) |
| ENSG00000089225(4, 1) | ENSG00000196642(1, 0) |
| ENSG00000090659(4, 3) | ENSG00000196704(1, 0) |
| ENSG00000091073(4, 2) | ENSG00000196890(1, 0) |
| ENSG00000092531(4, 3) | ENSG00000197061(1, 0) |
| ENSG00000095794(4, 1) | ENSG00000197114(1, 0) |
| ENSG00000099715(4, 2) | ENSG00000197140(1, 0) |
| ENSG00000099995(4, 3) | ENSG00000197153(1, 0) |
| ENSG00000100065(4, 2) | ENSG00000197170(1, 0) |
| ENSG00000100124(4, 2) | ENSG00000197183(1, 0) |
| ENSG00000100146(4, 1) | ENSG00000197409(1, 0) |
| ENSG00000100147(4, 2) | ENSG00000197520(1, 0) |
| ENSG00000100284(4, 3) | ENSG00000197629(1, 0) |
| ENSG00000100422(4, 2) | ENSG00000197635(1, 0) |
| ENSG00000100601(4, 3) | ENSG00000197697(1, 0) |

| | |
|---|---|
| ENSG00000100604(4, 2) | ENSG00000197712(1, 0) |
| ENSG00000101132(4, 2) | ENSG00000197776(1, 0) |
| ENSG00000101150(4, 2) | ENSG00000197933(1, 0) |
| ENSG00000101166(4, 2) | ENSG00000197951(1, 0) |
| ENSG00000101222(4, 2) | ENSG00000197991(1, 0) |
| ENSG00000101224(4, 1) | ENSG00000198046(1, 0) |
| ENSG00000101266(4, 1) | ENSG00000198198(1, 0) |
| ENSG00000101282(4, 1) | ENSG00000198218(1, 0) |
| ENSG00000101346(4, 2) | ENSG00000198221(1, 0) |
| ENSG00000101400(4, 3) | ENSG00000198286(1, 0) |
| ENSG00000101444(4, 1) | ENSG00000198327(1, 0) |
| ENSG00000101654(4, 2) | ENSG00000198366(1, 0) |
| ENSG00000102245(4, 1) | ENSG00000198374(1, 0) |
| ENSG00000102445(4, 1) | ENSG00000198498(1, 0) |
| ENSG00000102781(4, 2) | ENSG00000198518(1, 0) |
| ENSG00000103184(4, 2) | ENSG00000198558(1, 0) |
| ENSG00000103194(4, 1) | ENSG00000198612(1, 0) |
| ENSG00000103202(4, 3) | ENSG00000198673(1, 0) |
| ENSG00000103423(4, 3) | ENSG00000198691(1, 0) |
| ENSG00000103550(4, 3) | ENSG00000198728(1, 0) |
| ENSG00000103994(4, 3) | ENSG00000198736(1, 0) |
| ENSG00000104043(4, 1) | ENSG00000198758(1, 0) |
| ENSG00000104221(4, 1) | ENSG00000198783(1, 0) |
| ENSG00000104369(4, 2) | ENSG00000198812(1, 0) |
| ENSG00000104725(4, 2) | ENSG00000198821(1, 0) |
| ENSG00000104728(4, 1) | ENSG00000198855(1, 0) |
| ENSG00000105223(4, 3) | ENSG00000198873(1, 0) |
| ENSG00000105323(4, 2) | ENSG00000198881(1, 0) |
| ENSG00000105426(4, 2) | ENSG00000198912(1, 0) |
| ENSG00000106331(4, 2) | ENSG00000198919(1, 0) |
| ENSG00000106346(4, 1) | ENSG00000198937(1, 0) |
| ENSG00000106367(4, 2) | ENSG00000203811(1, 0) |
| ENSG00000106477(4, 1) | ENSG00000203812(1, 0) |
| ENSG00000106636(4, 2) | ENSG00000203813(1, 0) |
| ENSG00000106991(4, 1) | ENSG00000203814(1, 0) |
| ENSG00000107758(4, 1) | ENSG00000203852(1, 0) |
| ENSG00000107807(4, 1) | ENSG00000203880(1, 0) |
| ENSG00000107862(4, 1) | ENSG00000203950(1, 0) |
| ENSG00000107938(4, 3) | ENSG00000204033(1, 0) |
| ENSG00000107968(4, 1) | ENSG00000204052(1, 0) |
| ENSG00000108061(4, 2) | ENSG00000204086(1, 0) |
| ENSG00000108342(4, 3) | ENSG00000204176(1, 0) |
| ENSG00000108387(4, 1) | ENSG00000204228(1, 0) |
| ENSG00000108395(4, 1) | ENSG00000204301(1, 0) |
| ENSG00000108556(4, 1) | ENSG00000204335(1, 0) |
| ENSG00000109065(4, 2) | ENSG00000204352(1, 0) |
| ENSG00000109920(4, 3) | ENSG00000204381(1, 0) |
| ENSG00000110025(4, 3) | ENSG00000204388(1, 0) |
| ENSG00000110492(4, 3) | ENSG00000204392(1, 0) |
| ENSG00000110713(4, 2) | ENSG00000204394(1, 0) |
| ENSG00000110768(4, 3) | ENSG00000204531(1, 0) |
| ENSG00000110801(4, 1) | ENSG00000204590(1, 0) |
| ENSG00000110958(4, 1) | ENSG00000204694(1, 0) |
| ENSG00000110975(4, 2) | ENSG00000204713(1, 0) |

| | |
|---|---|
| ENSG00000111266(4, 3) | ENSG00000204923(1, 0) |
| ENSG00000111481(4, 2) | ENSG00000204952(1, 0) |
| ENSG00000111667(4, 3) | ENSG00000205060(1, 0) |
| ENSG00000112339(4, 2) | ENSG00000205352(1, 0) |
| ENSG00000112851(4, 2) | ENSG00000205445(1, 0) |
| ENSG00000113013(4, 3) | ENSG00000205560(1, 0) |
| ENSG00000113161(4, 2) | ENSG00000205581(1, 0) |
| ENSG00000113360(4, 3) | ENSG00000205683(1, 0) |
| ENSG00000113361(4, 1) | ENSG00000205929(1, 0) |
| ENSG00000113552(4, 2) | ENSG00000205981(1, 0) |
| ENSG00000113583(4, 3) | ENSG00000212916(1, 0) |
| ENSG00000113595(4, 2) | ENSG00000213204(1, 0) |
| ENSG00000113761(4, 1) | ENSG00000213551(1, 0) |
| ENSG00000114541(4, 1) | ENSG00000213625(1, 0) |
| ENSG00000114737(4, 1) | ENSG00000213654(1, 0) |
| ENSG00000114745(4, 3) | ENSG00000213676(1, 0) |
| ENSG00000115109(4, 1) | ENSG00000213694(1, 0) |
| ENSG00000115361(4, 1) | ENSG00000213760(1, 0) |
| ENSG00000115594(4, 1) | ENSG00000213983(1, 0) |
| ENSG00000116001(4, 3) | ENSG00000214013(1, 0) |
| ENSG00000116147(4, 1) | ENSG00000214014(1, 0) |
| ENSG00000116525(4, 3) | ENSG00000214022(1, 0) |
| ENSG00000116544(4, 3) | ENSG00000214078(1, 0) |
| ENSG00000116574(4, 2) | ENSG00000214113(1, 0) |
| ENSG00000116641(4, 3) | ENSG00000214128(1, 0) |
| ENSG00000116729(4, 2) | ENSG00000214435(1, 0) |
| ENSG00000116747(4, 2) | ENSG00000214717(1, 0) |
| ENSG00000116819(4, 3) | ENSG00000214872(1, 0) |
| ENSG00000116871(4, 3) | ENSG00000215252(1, 0) |
| ENSG00000116922(4, 2) | ENSG00000215454(1, 0) |
| ENSG00000117289(4, 1) | ENSG00000215568(1, 0) |
| ENSG00000117411(4, 2) | ENSG00000215788(1, 0) |
| ENSG00000117859(4, 2) | ENSG00000216490(1, 0) |
| ENSG00000118007(4, 3) | ENSG00000216937(1, 0) |
| ENSG00000118482(4, 1) | ENSG00000221837(1, 0) |
| ENSG00000118507(4, 3) | ENSG00000221968(1, 0) |
| ENSG00000118564(4, 1) | ENSG00000221986(1, 0) |
| ENSG00000119138(4, 1) | ENSG00000221994(1, 0) |
| ENSG00000119684(4, 1) | ENSG00000222014(1, 0) |
| ENSG00000119782(4, 1) | ENSG00000222047(1, 0) |
| ENSG00000119812(4, 2) | ENSG00000223501(1, 0) |
| ENSG00000119844(4, 3) | ENSG00000225697(1, 0) |
| ENSG00000120265(4, 3) | ENSG00000227802(1, 0) |
| ENSG00000120659(4, 1) | ENSG00000229117(1, 0) |
| ENSG00000120896(4, 1) | ENSG00000231925(1, 0) |
| ENSG00000120899(4, 1) | ENSG00000233034(1, 0) |
| ENSG00000121058(4, 2) | ENSG00000233224(1, 0) |
| ENSG00000121653(4, 2) | ENSG00000233822(1, 0) |
| ENSG00000121743(4, 1) | ENSG00000235718(1, 0) |
| ENSG00000122068(4, 1) | ENSG00000236279(1, 0) |
| ENSG00000122254(4, 1) | ENSG00000237441(1, 0) |
| ENSG00000122257(4, 2) | ENSG00000239779(1, 0) |
| ENSG00000122359(4, 2) | ENSG00000240021(1, 0) |
| ENSG00000122547(4, 2) | ENSG00000240428(1, 0) |

| | |
|---|---|
| ENSG00000122584(4, 1) | ENSG00000241058(1, 0) |
| ENSG00000123338(4, 1) | ENSG00000241685(1, 0) |
| ENSG00000123352(4, 1) | ENSG00000242028(1, 0) |
| ENSG00000123562(4, 1) | ENSG00000242441(1, 0) |
| ENSG00000124194(4, 2) | ENSG00000242689(1, 0) |
| ENSG00000124205(4, 1) | ENSG00000243056(1, 0) |
| ENSG00000124214(4, 1) | ENSG00000243279(1, 0) |
| ENSG00000124228(4, 1) | ENSG00000243414(1, 0) |
| ENSG00000124302(4, 3) | ENSG00000243646(1, 0) |
| ENSG00000124702(4, 1) | ENSG00000243725(1, 0) |
| ENSG00000124749(4, 2) | ENSG00000243789(1, 0) |
| ENSG00000125257(4, 2) | ENSG00000244005(1, 0) |
| ENSG00000125347(4, 3) | ENSG00000248541(1, 0) |
| ENSG00000125492(4, 2) | ENSG00000248871(1, 0) |
| ENSG00000125637(4, 1) | ENSG00000001626(2, 0) |
| ENSG00000125733(4, 2) | ENSG00000004139(2, 0) |
| ENSG00000125895(4, 1) | ENSG00000004838(2, 0) |
| ENSG00000125954(4, 1) | ENSG00000005812(2, 0) |
| ENSG00000125967(4, 1) | ENSG00000006283(2, 0) |
| ENSG00000126261(4, 1) | ENSG00000006327(2, 0) |
| ENSG00000126500(4, 1) | ENSG00000006634(2, 0) |
| ENSG00000127616(4, 3) | ENSG00000008735(2, 0) |
| ENSG00000127804(4, 1) | ENSG00000010322(2, 0) |
| ENSG00000127870(4, 1) | ENSG00000011638(2, 0) |
| ENSG00000127954(4, 1) | ENSG00000012660(2, 0) |
| ENSG00000128059(4, 1) | ENSG00000019991(2, 0) |
| ENSG00000128271(4, 1) | ENSG00000034713(2, 0) |
| ENSG00000128591(4, 2) | ENSG00000036565(2, 0) |
| ENSG00000128683(4, 1) | ENSG00000041988(2, 0) |
| ENSG00000128829(4, 1) | ENSG00000042429(2, 0) |
| ENSG00000128872(4, 2) | ENSG00000044459(2, 0) |
| ENSG00000128881(4, 3) | ENSG00000047249(2, 0) |
| ENSG00000128915(4, 2) | ENSG00000049283(2, 0) |
| ENSG00000128918(4, 2) | ENSG00000051180(2, 0) |
| ENSG00000129347(4, 2) | ENSG00000057757(2, 0) |
| ENSG00000129451(4, 1) | ENSG00000058453(2, 0) |
| ENSG00000129480(4, 2) | ENSG00000061676(2, 0) |
| ENSG00000129933(4, 1) | ENSG00000064787(2, 0) |
| ENSG00000130558(4, 3) | ENSG00000064989(2, 0) |
| ENSG00000130733(4, 1) | ENSG00000065054(2, 0) |
| ENSG00000130734(4, 2) | ENSG00000065413(2, 0) |
| ENSG00000130758(4, 2) | ENSG00000065534(2, 0) |
| ENSG00000131002(4, 2) | ENSG00000066405(2, 0) |
| ENSG00000131446(4, 3) | ENSG00000066926(2, 0) |
| ENSG00000131558(4, 1) | ENSG00000067646(2, 0) |
| ENSG00000132031(4, 3) | ENSG00000069667(2, 0) |
| ENSG00000132294(4, 2) | ENSG00000069966(2, 0) |
| ENSG00000132326(4, 1) | ENSG00000070785(2, 0) |
| ENSG00000132589(4, 1) | ENSG00000072163(2, 0) |
| ENSG00000132842(4, 1) | ENSG00000072832(2, 0) |
| ENSG00000133030(4, 1) | ENSG00000074527(2, 0) |
| ENSG00000133195(4, 1) | ENSG00000075142(2, 0) |
| ENSG00000133247(4, 3) | ENSG00000075884(2, 0) |
| ENSG00000133424(4, 2) | ENSG00000076043(2, 0) |

| | |
|---|---|
| ENSG00000133477(4, 2) | ENSG00000076201(2, 0) |
| ENSG00000133794(4, 2) | ENSG00000078053(2, 0) |
| ENSG00000133805(4, 2) | ENSG00000079112(2, 0) |
| ENSG00000133818(4, 1) | ENSG00000079150(2, 0) |
| ENSG00000134684(4, 1) | ENSG00000080189(2, 0) |
| ENSG00000134762(4, 1) | ENSG00000080345(2, 0) |
| ENSG00000134769(4, 3) | ENSG00000081154(2, 0) |
| ENSG00000134874(4, 2) | ENSG00000082512(2, 0) |
| ENSG00000135045(4, 2) | ENSG00000086300(2, 0) |
| ENSG00000135116(4, 1) | ENSG00000087266(2, 0) |
| ENSG00000135148(4, 2) | ENSG00000087365(2, 0) |
| ENSG00000135363(4, 1) | ENSG00000088726(2, 0) |
| ENSG00000135519(4, 2) | ENSG00000089154(2, 0) |
| ENSG00000135775(4, 1) | ENSG00000089195(2, 0) |
| ENSG00000136099(4, 2) | ENSG00000090889(2, 0) |
| ENSG00000136167(4, 3) | ENSG00000091972(2, 0) |
| ENSG00000136279(4, 1) | ENSG00000095370(2, 0) |
| ENSG00000136352(4, 2) | ENSG00000095397(2, 0) |
| ENSG00000136813(4, 2) | ENSG00000097021(2, 0) |
| ENSG00000136937(4, 1) | ENSG00000100078(2, 0) |
| ENSG00000137076(4, 2) | ENSG00000100095(2, 0) |
| ENSG00000137135(4, 2) | ENSG00000100401(2, 0) |
| ENSG00000137265(4, 1) | ENSG00000100678(2, 0) |
| ENSG00000137502(4, 2) | ENSG00000100726(2, 0) |
| ENSG00000137834(4, 2) | ENSG00000100836(2, 0) |
| ENSG00000138092(4, 2) | ENSG00000101017(2, 0) |
| ENSG00000138162(4, 3) | ENSG00000101191(2, 0) |
| ENSG00000138180(4, 1) | ENSG00000101220(2, 0) |
| ENSG00000138829(4, 3) | ENSG00000102401(2, 0) |
| ENSG00000139209(4, 3) | ENSG00000102760(2, 0) |
| ENSG00000139219(4, 2) | ENSG00000102967(2, 0) |
| ENSG00000139372(4, 2) | ENSG00000103047(2, 0) |
| ENSG00000139433(4, 3) | ENSG00000103351(2, 0) |
| ENSG00000139597(4, 1) | ENSG00000103496(2, 0) |
| ENSG00000139800(4, 1) | ENSG00000103569(2, 0) |
| ENSG00000140153(4, 1) | ENSG00000104321(2, 0) |
| ENSG00000140299(4, 2) | ENSG00000104695(2, 0) |
| ENSG00000140795(4, 1) | ENSG00000104723(2, 0) |
| ENSG00000141013(4, 1) | ENSG00000104859(2, 0) |
| ENSG00000141141(4, 1) | ENSG00000105011(2, 0) |
| ENSG00000141404(4, 1) | ENSG00000105357(2, 0) |
| ENSG00000142065(4, 1) | ENSG00000105409(2, 0) |
| ENSG00000142528(4, 3) | ENSG00000105538(2, 0) |
| ENSG00000142871(4, 1) | ENSG00000105568(2, 0) |
| ENSG00000143033(4, 2) | ENSG00000105647(2, 0) |
| ENSG00000143140(4, 2) | ENSG00000105700(2, 0) |
| ENSG00000143258(4, 3) | ENSG00000105723(2, 0) |
| ENSG00000143333(4, 1) | ENSG00000105849(2, 0) |
| ENSG00000143367(4, 1) | ENSG00000106541(2, 0) |
| ENSG00000143466(4, 1) | ENSG00000106785(2, 0) |
| ENSG00000143553(4, 1) | ENSG00000107404(2, 0) |
| ENSG00000143786(4, 1) | ENSG00000107625(2, 0) |
| ENSG00000144331(4, 1) | ENSG00000107672(2, 0) |
| ENSG00000144644(4, 1) | ENSG00000107859(2, 0) |

| | |
|---|---|
| ENSG00000144645(4, 2) | ENSG00000108021(2, 0) |
| ENSG00000144827(4, 1) | ENSG00000108344(2, 0) |
| ENSG00000144893(4, 2) | ENSG00000108759(2, 0) |
| ENSG00000145194(4, 3) | ENSG00000109016(2, 0) |
| ENSG00000145244(4, 2) | ENSG00000109101(2, 0) |
| ENSG00000145246(4, 1) | ENSG00000109501(2, 0) |
| ENSG00000145375(4, 1) | ENSG00000109881(2, 0) |
| ENSG00000145715(4, 1) | ENSG00000109971(2, 0) |
| ENSG00000145721(4, 3) | ENSG00000110243(2, 0) |
| ENSG00000145833(4, 1) | ENSG00000110448(2, 0) |
| ENSG00000146425(4, 2) | ENSG00000110484(2, 0) |
| ENSG00000146674(4, 2) | ENSG00000110756(2, 0) |
| ENSG00000146700(4, 1) | ENSG00000111011(2, 0) |
| ENSG00000147255(4, 3) | ENSG00000111206(2, 0) |
| ENSG00000147533(4, 3) | ENSG00000111224(2, 0) |
| ENSG00000147606(4, 1) | ENSG00000111254(2, 0) |
| ENSG00000147649(4, 1) | ENSG00000111530(2, 0) |
| ENSG00000147852(4, 2) | ENSG00000111783(2, 0) |
| ENSG00000148123(4, 2) | ENSG00000112041(2, 0) |
| ENSG00000148396(4, 2) | ENSG00000112096(2, 0) |
| ENSG00000148737(4, 1) | ENSG00000112218(2, 0) |
| ENSG00000149091(4, 2) | ENSG00000112378(2, 0) |
| ENSG00000149313(4, 1) | ENSG00000112837(2, 0) |
| ENSG00000149499(4, 2) | ENSG00000113282(2, 0) |
| ENSG00000149798(4, 1) | ENSG00000113520(2, 0) |
| ENSG00000149930(4, 1) | ENSG00000114107(2, 0) |
| ENSG00000150455(4, 1) | ENSG00000114166(2, 0) |
| ENSG00000151148(4, 3) | ENSG00000114346(2, 0) |
| ENSG00000151224(4, 2) | ENSG00000114923(2, 0) |
| ENSG00000151276(4, 3) | ENSG00000115145(2, 0) |
| ENSG00000151461(4, 1) | ENSG00000115282(2, 0) |
| ENSG00000152268(4, 2) | ENSG00000115364(2, 0) |
| ENSG00000152592(4, 3) | ENSG00000115484(2, 0) |
| ENSG00000153071(4, 1) | ENSG00000115592(2, 0) |
| ENSG00000153130(4, 3) | ENSG00000115756(2, 0) |
| ENSG00000153558(4, 2) | ENSG00000116035(2, 0) |
| ENSG00000153714(4, 2) | ENSG00000116157(2, 0) |
| ENSG00000153767(4, 1) | ENSG00000116266(2, 0) |
| ENSG00000154447(4, 3) | ENSG00000116774(2, 0) |
| ENSG00000155066(4, 2) | ENSG00000116786(2, 0) |
| ENSG00000155099(4, 1) | ENSG00000116918(2, 0) |
| ENSG00000155846(4, 2) | ENSG00000117505(2, 0) |
| ENSG00000156140(4, 1) | ENSG00000118193(2, 0) |
| ENSG00000156515(4, 1) | ENSG00000118412(2, 0) |
| ENSG00000156564(4, 3) | ENSG00000118418(2, 0) |
| ENSG00000157193(4, 1) | ENSG00000118514(2, 0) |
| ENSG00000157483(4, 2) | ENSG00000118640(2, 0) |
| ENSG00000157593(4, 1) | ENSG00000118972(2, 0) |
| ENSG00000157693(4, 2) | ENSG00000119125(2, 0) |
| ENSG00000157978(4, 1) | ENSG00000119457(2, 0) |
| ENSG00000158019(4, 2) | ENSG00000119865(2, 0) |
| ENSG00000158290(4, 2) | ENSG00000119953(2, 0) |
| ENSG00000158773(4, 1) | ENSG00000120149(2, 0) |
| ENSG00000158805(4, 1) | ENSG00000120458(2, 0) |

| | |
|---|---|
| ENSG00000158987(4, 2) | ENSG00000120656(2, 0) |
| ENSG00000159263(4, 3) | ENSG00000120889(2, 0) |
| ENSG00000159592(4, 3) | ENSG00000121769(2, 0) |
| ENSG00000159714(4, 2) | ENSG00000122299(2, 0) |
| ENSG00000160325(4, 2) | ENSG00000122643(2, 0) |
| ENSG00000160392(4, 1) | ENSG00000122729(2, 0) |
| ENSG00000160613(4, 2) | ENSG00000122884(2, 0) |
| ENSG00000160633(4, 2) | ENSG00000124074(2, 0) |
| ENSG00000160716(4, 1) | ENSG00000124217(2, 0) |
| ENSG00000161048(4, 3) | ENSG00000124479(2, 0) |
| ENSG00000161558(4, 3) | ENSG00000124635(2, 0) |
| ENSG00000161647(4, 1) | ENSG00000124693(2, 0) |
| ENSG00000161904(4, 3) | ENSG00000124701(2, 0) |
| ENSG00000162650(4, 2) | ENSG00000125510(2, 0) |
| ENSG00000162736(4, 3) | ENSG00000126952(2, 0) |
| ENSG00000162745(4, 1) | ENSG00000128245(2, 0) |
| ENSG00000162772(4, 3) | ENSG00000128285(2, 0) |
| ENSG00000162775(4, 1) | ENSG00000128422(2, 0) |
| ENSG00000162873(4, 3) | ENSG00000128641(2, 0) |
| ENSG00000162889(4, 1) | ENSG00000129187(2, 0) |
| ENSG00000162951(4, 2) | ENSG00000129493(2, 0) |
| ENSG00000162971(4, 2) | ENSG00000130119(2, 0) |
| ENSG00000162989(4, 2) | ENSG00000130309(2, 0) |
| ENSG00000163069(4, 2) | ENSG00000130544(2, 0) |
| ENSG00000163081(4, 2) | ENSG00000131018(2, 0) |
| ENSG00000163291(4, 2) | ENSG00000131069(2, 0) |
| ENSG00000163378(4, 2) | ENSG00000131095(2, 0) |
| ENSG00000163435(4, 2) | ENSG00000131737(2, 0) |
| ENSG00000163508(4, 3) | ENSG00000131738(2, 0) |
| ENSG00000163590(4, 1) | ENSG00000131746(2, 0) |
| ENSG00000163683(4, 1) | ENSG00000131876(2, 0) |
| ENSG00000163704(4, 3) | ENSG00000132635(2, 0) |
| ENSG00000163814(4, 2) | ENSG00000132670(2, 0) |
| ENSG00000163840(4, 1) | ENSG00000132680(2, 0) |
| ENSG00000163898(4, 1) | ENSG00000132749(2, 0) |
| ENSG00000163904(4, 1) | ENSG00000132874(2, 0) |
| ENSG00000163914(4, 1) | ENSG00000133316(2, 0) |
| ENSG00000163930(4, 2) | ENSG00000133466(2, 0) |
| ENSG00000164117(4, 2) | ENSG00000134013(2, 0) |
| ENSG00000164142(4, 2) | ENSG00000134070(2, 0) |
| ENSG00000164181(4, 2) | ENSG00000134453(2, 0) |
| ENSG00000164292(4, 2) | ENSG00000134516(2, 0) |
| ENSG00000164440(4, 3) | ENSG00000134595(2, 0) |
| ENSG00000164442(4, 3) | ENSG00000134744(2, 0) |
| ENSG00000164736(4, 2) | ENSG00000134882(2, 0) |
| ENSG00000164749(4, 2) | ENSG00000134970(2, 0) |
| ENSG00000164754(4, 1) | ENSG00000135100(2, 0) |
| ENSG00000165072(4, 2) | ENSG00000135124(2, 0) |
| ENSG00000165188(4, 2) | ENSG00000135248(2, 0) |
| ENSG00000165197(4, 1) | ENSG00000135404(2, 0) |
| ENSG00000165246(4, 1) | ENSG00000135446(2, 0) |
| ENSG00000165271(4, 2) | ENSG00000135517(2, 0) |
| ENSG00000165406(4, 2) | ENSG00000135604(2, 0) |
| ENSG00000165661(4, 2) | ENSG00000135625(2, 0) |

| | |
|---|---|
| ENSG00000165675(4, 2) | ENSG00000136378(2, 0) |
| ENSG00000165983(4, 2) | ENSG00000136541(2, 0) |
| ENSG00000166002(4, 3) | ENSG00000136636(2, 0) |
| ENSG00000166037(4, 1) | ENSG00000136709(2, 0) |
| ENSG00000166111(4, 3) | ENSG00000136986(2, 0) |
| ENSG00000166387(4, 1) | ENSG00000137070(2, 0) |
| ENSG00000166405(4, 1) | ENSG00000137198(2, 0) |
| ENSG00000166783(4, 2) | ENSG00000137491(2, 0) |
| ENSG00000167004(4, 1) | ENSG00000137714(2, 0) |
| ENSG00000167178(4, 2) | ENSG00000137959(2, 0) |
| ENSG00000167528(4, 1) | ENSG00000138326(2, 0) |
| ENSG00000167578(4, 1) | ENSG00000138347(2, 0) |
| ENSG00000167601(4, 1) | ENSG00000138380(2, 0) |
| ENSG00000167635(4, 1) | ENSG00000138594(2, 0) |
| ENSG00000167703(4, 2) | ENSG00000138615(2, 0) |
| ENSG00000167904(4, 2) | ENSG00000139117(2, 0) |
| ENSG00000168348(4, 2) | ENSG00000139624(2, 0) |
| ENSG00000169031(4, 2) | ENSG00000139734(2, 0) |
| ENSG00000169071(4, 3) | ENSG00000140259(2, 0) |
| ENSG00000169083(4, 3) | ENSG00000140350(2, 0) |
| ENSG00000169105(4, 1) | ENSG00000140479(2, 0) |
| ENSG00000169180(4, 2) | ENSG00000141446(2, 0) |
| ENSG00000169239(4, 1) | ENSG00000141469(2, 0) |
| ENSG00000169375(4, 2) | ENSG00000141736(2, 0) |
| ENSG00000169682(4, 1) | ENSG00000141738(2, 0) |
| ENSG00000169826(4, 2) | ENSG00000142208(2, 0) |
| ENSG00000170049(4, 1) | ENSG00000142541(2, 0) |
| ENSG00000170214(4, 2) | ENSG00000142623(2, 0) |
| ENSG00000170234(4, 1) | ENSG00000142686(2, 0) |
| ENSG00000170260(4, 2) | ENSG00000143167(2, 0) |
| ENSG00000170365(4, 1) | ENSG00000143457(2, 0) |
| ENSG00000170417(4, 1) | ENSG00000143643(2, 0) |
| ENSG00000170577(4, 2) | ENSG00000143768(2, 0) |
| ENSG00000170581(4, 2) | ENSG00000144021(2, 0) |
| ENSG00000170604(4, 2) | ENSG00000144063(2, 0) |
| ENSG00000170759(4, 3) | ENSG00000144229(2, 0) |
| ENSG00000170927(4, 1) | ENSG00000144290(2, 0) |
| ENSG00000171033(4, 2) | ENSG00000144357(2, 0) |
| ENSG00000171150(4, 2) | ENSG00000144671(2, 0) |
| ENSG00000171169(4, 1) | ENSG00000144746(2, 0) |
| ENSG00000171206(4, 1) | ENSG00000144824(2, 0) |
| ENSG00000171219(4, 3) | ENSG00000144843(2, 0) |
| ENSG00000171777(4, 2) | ENSG00000146112(2, 0) |
| ENSG00000171864(4, 1) | ENSG00000146242(2, 0) |
| ENSG00000171954(4, 1) | ENSG00000146247(2, 0) |
| ENSG00000172379(4, 2) | ENSG00000146574(2, 0) |
| ENSG00000172531(4, 3) | ENSG00000146904(2, 0) |
| ENSG00000172572(4, 3) | ENSG00000147145(2, 0) |
| ENSG00000172575(4, 3) | ENSG00000147316(2, 0) |
| ENSG00000172830(4, 2) | ENSG00000147535(2, 0) |
| ENSG00000172936(4, 2) | ENSG00000147548(2, 0) |
| ENSG00000173698(4, 1) | ENSG00000147813(2, 0) |
| ENSG00000173705(4, 1) | ENSG00000148925(2, 0) |
| ENSG00000173821(4, 3) | ENSG00000149150(2, 0) |

| | |
|---|---|
| ENSG00000173848(4, 3) | ENSG00000149380(2, 0) |
| ENSG00000173905(4, 1) | ENSG00000149636(2, 0) |
| ENSG00000174106(4, 1) | ENSG00000149926(2, 0) |
| ENSG00000174111(4, 2) | ENSG00000150459(2, 0) |
| ENSG00000175606(4, 1) | ENSG00000150477(2, 0) |
| ENSG00000176049(4, 2) | ENSG00000151062(2, 0) |
| ENSG00000176390(4, 1) | ENSG00000151468(2, 0) |
| ENSG00000176971(4, 1) | ENSG00000151514(2, 0) |
| ENSG00000178053(4, 1) | ENSG00000151615(2, 0) |
| ENSG00000179242(4, 1) | ENSG00000152266(2, 0) |
| ENSG00000179335(4, 2) | ENSG00000152495(2, 0) |
| ENSG00000180660(4, 1) | ENSG00000152904(2, 0) |
| ENSG00000180834(4, 1) | ENSG00000153162(2, 0) |
| ENSG00000180979(4, 1) | ENSG00000153956(2, 0) |
| ENSG00000181039(4, 2) | ENSG00000154832(2, 0) |
| ENSG00000181061(4, 1) | ENSG00000155100(2, 0) |
| ENSG00000181072(4, 3) | ENSG00000155380(2, 0) |
| ENSG00000181220(4, 2) | ENSG00000155729(2, 0) |
| ENSG00000181315(4, 2) | ENSG00000155850(2, 0) |
| ENSG00000181333(4, 1) | ENSG00000155975(2, 0) |
| ENSG00000181418(4, 2) | ENSG00000156097(2, 0) |
| ENSG00000181495(4, 1) | ENSG00000158042(2, 0) |
| ENSG00000181555(4, 1) | ENSG00000158106(2, 0) |
| ENSG00000181904(4, 1) | ENSG00000158161(2, 0) |
| ENSG00000181965(4, 2) | ENSG00000158246(2, 0) |
| ENSG00000182087(4, 2) | ENSG00000158296(2, 0) |
| ENSG00000182108(4, 1) | ENSG00000158445(2, 0) |
| ENSG00000182197(4, 1) | ENSG00000159147(2, 0) |
| ENSG00000182400(4, 2) | ENSG00000159374(2, 0) |
| ENSG00000182578(4, 2) | ENSG00000159479(2, 0) |
| ENSG00000182601(4, 1) | ENSG00000159713(2, 0) |
| ENSG00000182704(4, 3) | ENSG00000159733(2, 0) |
| ENSG00000183114(4, 2) | ENSG00000160051(2, 0) |
| ENSG00000183155(4, 3) | ENSG00000160602(2, 0) |
| ENSG00000183337(4, 1) | ENSG00000160867(2, 0) |
| ENSG00000183386(4, 2) | ENSG00000161267(2, 0) |
| ENSG00000183578(4, 2) | ENSG00000161405(2, 0) |
| ENSG00000183688(4, 1) | ENSG00000162139(2, 0) |
| ENSG00000183908(4, 3) | ENSG00000162174(2, 0) |
| ENSG00000184007(4, 2) | ENSG00000162456(2, 0) |
| ENSG00000184277(4, 2) | ENSG00000162769(2, 0) |
| ENSG00000184349(4, 3) | ENSG00000162927(2, 0) |
| ENSG00000184486(4, 1) | ENSG00000162946(2, 0) |
| ENSG00000184811(4, 1) | ENSG00000163067(2, 0) |
| ENSG00000185033(4, 3) | ENSG00000163131(2, 0) |
| ENSG00000185250(4, 3) | ENSG00000163288(2, 0) |
| ENSG00000185262(4, 1) | ENSG00000163444(2, 0) |
| ENSG00000185652(4, 1) | ENSG00000163599(2, 0) |
| ENSG00000185800(4, 1) | ENSG00000163626(2, 0) |
| ENSG00000186288(4, 2) | ENSG00000163660(2, 0) |
| ENSG00000186417(4, 2) | ENSG00000163686(2, 0) |
| ENSG00000186532(4, 1) | ENSG00000163755(2, 0) |
| ENSG00000186564(4, 3) | ENSG00000163815(2, 0) |
| ENSG00000186716(4, 2) | ENSG00000163818(2, 0) |

| | |
|---|---|
| ENSG00000187664(4, 1) | ENSG00000163995(2, 0) |
| ENSG00000187764(4, 1) | ENSG00000164010(2, 0) |
| ENSG00000188026(4, 2) | ENSG00000164099(2, 0) |
| ENSG00000188315(4, 2) | ENSG00000164104(2, 0) |
| ENSG00000188419(4, 2) | ENSG00000164116(2, 0) |
| ENSG00000188612(4, 2) | ENSG00000164307(2, 0) |
| ENSG00000188760(4, 3) | ENSG00000164542(2, 0) |
| ENSG00000188761(4, 1) | ENSG00000164609(2, 0) |
| ENSG00000188763(4, 1) | ENSG00000164695(2, 0) |
| ENSG00000196230(4, 1) | ENSG00000164713(2, 0) |
| ENSG00000196367(4, 3) | ENSG00000165131(2, 0) |
| ENSG00000196422(4, 1) | ENSG00000165288(2, 0) |
| ENSG00000196576(4, 2) | ENSG00000165494(2, 0) |
| ENSG00000196917(4, 2) | ENSG00000165985(2, 0) |
| ENSG00000197406(4, 2) | ENSG00000165996(2, 0) |
| ENSG00000197457(4, 1) | ENSG00000166170(2, 0) |
| ENSG00000197565(4, 3) | ENSG00000166265(2, 0) |
| ENSG00000197587(4, 2) | ENSG00000166333(2, 0) |
| ENSG00000197818(4, 1) | ENSG00000166471(2, 0) |
| ENSG00000197930(4, 2) | ENSG00000166509(2, 0) |
| ENSG00000197971(4, 1) | ENSG00000166689(2, 0) |
| ENSG00000198089(4, 2) | ENSG00000166845(2, 0) |
| ENSG00000198231(4, 2) | ENSG00000166863(2, 0) |
| ENSG00000198408(4, 2) | ENSG00000166920(2, 0) |
| ENSG00000198431(4, 1) | ENSG00000167165(2, 0) |
| ENSG00000198477(4, 2) | ENSG00000167766(2, 0) |
| ENSG00000198626(4, 1) | ENSG00000167768(2, 0) |
| ENSG00000198650(4, 1) | ENSG00000167775(2, 0) |
| ENSG00000198682(4, 2) | ENSG00000167994(2, 0) |
| ENSG00000198704(4, 1) | ENSG00000168081(2, 0) |
| ENSG00000198797(4, 2) | ENSG00000168148(2, 0) |
| ENSG00000198799(4, 2) | ENSG00000168243(2, 0) |
| ENSG00000198814(4, 1) | ENSG00000168274(2, 0) |
| ENSG00000198826(4, 1) | ENSG00000168286(2, 0) |
| ENSG00000198890(4, 1) | ENSG00000168398(2, 0) |
| ENSG00000198944(4, 3) | ENSG00000168481(2, 0) |
| ENSG00000204291(4, 2) | ENSG00000168907(2, 0) |
| ENSG00000204688(4, 2) | ENSG00000169139(2, 0) |
| ENSG00000205302(4, 3) | ENSG00000169193(2, 0) |
| ENSG00000205531(4, 1) | ENSG00000169245(2, 0) |
| ENSG00000205808(4, 2) | ENSG00000169429(2, 0) |
| ENSG00000205927(4, 3) | ENSG00000169564(2, 0) |
| ENSG00000211445(4, 2) | ENSG00000169594(2, 0) |
| ENSG00000213066(4, 3) | ENSG00000169718(2, 0) |
| ENSG00000213071(4, 2) | ENSG00000169884(2, 0) |
| ENSG00000213190(4, 2) | ENSG00000169908(2, 0) |
| ENSG00000213658(4, 1) | ENSG00000170382(2, 0) |
| ENSG00000214193(4, 1) | ENSG00000170681(2, 0) |
| ENSG00000232810(4, 2) | ENSG00000170899(2, 0) |
| ENSG00000241852(4, 2) | ENSG00000170962(2, 0) |
| ENSG00000243335(4, 2) | ENSG00000171320(2, 0) |
| ENSG00000243364(4, 1) | ENSG00000171443(2, 0) |
| ENSG00000243978(4, 2) | ENSG00000172137(2, 0) |
| ENSG00000248857(4, 1) | ENSG00000172159(2, 0) |

| | |
|---|---|
| ENSG00000254147(4, 1) | ENSG00000172399(2, 0) |
| ENSG00000001617(5, 3) | ENSG00000172728(2, 0) |
| ENSG00000002587(5, 3) | ENSG00000173376(2, 0) |
| ENSG00000002746(5, 4) | ENSG00000173540(2, 0) |
| ENSG00000003393(5, 2) | ENSG00000173614(2, 0) |
| ENSG00000004799(5, 2) | ENSG00000173918(2, 0) |
| ENSG00000005249(5, 2) | ENSG00000173950(2, 0) |
| ENSG00000006210(5, 3) | ENSG00000174206(2, 0) |
| ENSG00000007202(5, 3) | ENSG00000174332(2, 0) |
| ENSG00000007923(5, 1) | ENSG00000174370(2, 0) |
| ENSG00000009307(5, 2) | ENSG00000174482(2, 0) |
| ENSG00000010278(5, 1) | ENSG00000174483(2, 0) |
| ENSG00000010539(5, 2) | ENSG00000174567(2, 0) |
| ENSG00000011028(5, 4) | ENSG00000175048(2, 0) |
| ENSG00000011485(5, 2) | ENSG00000175166(2, 0) |
| ENSG00000014216(5, 3) | ENSG00000175262(2, 0) |
| ENSG00000015676(5, 2) | ENSG00000175416(2, 0) |
| ENSG00000021762(5, 1) | ENSG00000175470(2, 0) |
| ENSG00000022355(5, 4) | ENSG00000175482(2, 0) |
| ENSG00000026652(5, 1) | ENSG00000175518(2, 0) |
| ENSG00000030110(5, 2) | ENSG00000175591(2, 0) |
| ENSG00000033178(5, 3) | ENSG00000175895(2, 0) |
| ENSG00000034677(5, 2) | ENSG00000176148(2, 0) |
| ENSG00000035499(5, 2) | ENSG00000176244(2, 0) |
| ENSG00000047578(5, 3) | ENSG00000176273(2, 0) |
| ENSG00000049656(5, 2) | ENSG00000176387(2, 0) |
| ENSG00000050165(5, 1) | ENSG00000176438(2, 0) |
| ENSG00000051341(5, 1) | ENSG00000176444(2, 0) |
| ENSG00000052344(5, 3) | ENSG00000176454(2, 0) |
| ENSG00000056345(5, 4) | ENSG00000176463(2, 0) |
| ENSG00000058063(5, 1) | ENSG00000176532(2, 0) |
| ENSG00000058673(5, 1) | ENSG00000176595(2, 0) |
| ENSG00000064300(5, 3) | ENSG00000176623(2, 0) |
| ENSG00000066468(5, 2) | ENSG00000176678(2, 0) |
| ENSG00000067167(5, 3) | ENSG00000176753(2, 0) |
| ENSG00000067560(5, 3) | ENSG00000177370(2, 0) |
| ENSG00000070778(5, 1) | ENSG00000177508(2, 0) |
| ENSG00000072682(5, 4) | ENSG00000177685(2, 0) |
| ENSG00000073417(5, 3) | ENSG00000178104(2, 0) |
| ENSG00000073584(5, 2) | ENSG00000178449(2, 0) |
| ENSG00000073756(5, 1) | ENSG00000178685(2, 0) |
| ENSG00000074047(5, 3) | ENSG00000178718(2, 0) |
| ENSG00000074219(5, 2) | ENSG00000178772(2, 0) |
| ENSG00000074696(5, 1) | ENSG00000178971(2, 0) |
| ENSG00000075413(5, 3) | ENSG00000179029(2, 0) |
| ENSG00000076826(5, 4) | ENSG00000179455(2, 0) |
| ENSG00000077254(5, 2) | ENSG00000179588(2, 0) |
| ENSG00000079482(5, 2) | ENSG00000180155(2, 0) |
| ENSG00000081014(5, 1) | ENSG00000180259(2, 0) |
| ENSG00000081853(5, 3) | ENSG00000180318(2, 0) |
| ENSG00000082556(5, 2) | ENSG00000180573(2, 0) |
| ENSG00000083642(5, 4) | ENSG00000180596(2, 0) |
| ENSG00000084070(5, 3) | ENSG00000180818(2, 0) |
| ENSG00000084090(5, 3) | ENSG00000181104(2, 0) |

| | |
|---|---|
| ENSG00000084733(5, 2) | ENSG00000181754(2, 0) |
| ENSG00000086062(5, 2) | ENSG00000182168(2, 0) |
| ENSG00000086712(5, 2) | ENSG00000182534(2, 0) |
| ENSG00000087303(5, 2) | ENSG00000182572(2, 0) |
| ENSG00000089558(5, 2) | ENSG00000182782(2, 0) |
| ENSG00000089597(5, 3) | ENSG00000182890(2, 0) |
| ENSG00000090020(5, 2) | ENSG00000183087(2, 0) |
| ENSG00000090054(5, 2) | ENSG00000183137(2, 0) |
| ENSG00000090565(5, 2) | ENSG00000183255(2, 0) |
| ENSG00000091129(5, 3) | ENSG00000183682(2, 0) |
| ENSG00000091409(5, 3) | ENSG00000183718(2, 0) |
| ENSG00000092445(5, 3) | ENSG00000183779(2, 0) |
| ENSG00000092529(5, 3) | ENSG00000183831(2, 0) |
| ENSG00000094880(5, 2) | ENSG00000183833(2, 0) |
| ENSG00000095739(5, 4) | ENSG00000183856(2, 0) |
| ENSG00000099219(5, 1) | ENSG00000184260(2, 0) |
| ENSG00000099365(5, 2) | ENSG00000184270(2, 0) |
| ENSG00000099381(5, 2) | ENSG00000184838(2, 0) |
| ENSG00000099622(5, 2) | ENSG00000184897(2, 0) |
| ENSG00000099953(5, 3) | ENSG00000185015(2, 0) |
| ENSG00000099968(5, 2) | ENSG00000185332(2, 0) |
| ENSG00000100003(5, 2) | ENSG00000185515(2, 0) |
| ENSG00000100056(5, 3) | ENSG00000186075(2, 0) |
| ENSG00000100068(5, 2) | ENSG00000186212(2, 0) |
| ENSG00000100075(5, 2) | ENSG00000186231(2, 0) |
| ENSG00000100100(5, 2) | ENSG00000186326(2, 0) |
| ENSG00000100216(5, 3) | ENSG00000186714(2, 0) |
| ENSG00000100314(5, 3) | ENSG00000187091(2, 0) |
| ENSG00000100485(5, 1) | ENSG00000187800(2, 0) |
| ENSG00000100744(5, 1) | ENSG00000187823(2, 0) |
| ENSG00000100813(5, 3) | ENSG00000187969(2, 0) |
| ENSG00000100842(5, 2) | ENSG00000188133(2, 0) |
| ENSG00000100852(5, 2) | ENSG00000188906(2, 0) |
| ENSG00000100991(5, 3) | ENSG00000189056(2, 0) |
| ENSG00000101082(5, 1) | ENSG00000189221(2, 0) |
| ENSG00000101152(5, 3) | ENSG00000189334(2, 0) |
| ENSG00000101180(5, 2) | ENSG00000196262(2, 0) |
| ENSG00000101856(5, 1) | ENSG00000196371(2, 0) |
| ENSG00000101986(5, 3) | ENSG00000196374(2, 0) |
| ENSG00000102100(5, 3) | ENSG00000196453(2, 0) |
| ENSG00000103035(5, 1) | ENSG00000196505(2, 0) |
| ENSG00000103495(5, 1) | ENSG00000196562(2, 0) |
| ENSG00000103653(5, 1) | ENSG00000196743(2, 0) |
| ENSG00000104164(5, 3) | ENSG00000196747(2, 0) |
| ENSG00000104290(5, 2) | ENSG00000196866(2, 0) |
| ENSG00000104327(5, 1) | ENSG00000196876(2, 0) |
| ENSG00000104365(5, 2) | ENSG00000196998(2, 0) |
| ENSG00000104969(5, 4) | ENSG00000197136(2, 0) |
| ENSG00000105216(5, 3) | ENSG00000197177(2, 0) |
| ENSG00000105866(5, 2) | ENSG00000197245(2, 0) |
| ENSG00000105971(5, 2) | ENSG00000197430(2, 0) |
| ENSG00000105974(5, 3) | ENSG00000197614(2, 0) |
| ENSG00000106415(5, 3) | ENSG00000197694(2, 0) |
| ENSG00000106688(5, 2) | ENSG00000197846(2, 0) |

| | |
|---|---|
| ENSG00000107021(5, 3) | ENSG00000197977(2, 0) |
| ENSG00000107104(5, 2) | ENSG00000198131(2, 0) |
| ENSG00000107187(5, 3) | ENSG00000198242(2, 0) |
| ENSG00000107485(5, 3) | ENSG00000198301(2, 0) |
| ENSG00000107719(5, 4) | ENSG00000198604(2, 0) |
| ENSG00000108231(5, 3) | ENSG00000198643(2, 0) |
| ENSG00000108389(5, 1) | ENSG00000198825(2, 0) |
| ENSG00000108518(5, 2) | ENSG00000198858(2, 0) |
| ENSG00000108797(5, 2) | ENSG00000198887(2, 0) |
| ENSG00000108854(5, 2) | ENSG00000198945(2, 0) |
| ENSG00000109118(5, 2) | ENSG00000203710(2, 0) |
| ENSG00000109220(5, 1) | ENSG00000204065(2, 0) |
| ENSG00000109320(5, 2) | ENSG00000204186(2, 0) |
| ENSG00000109436(5, 2) | ENSG00000204256(2, 0) |
| ENSG00000109814(5, 2) | ENSG00000204469(2, 0) |
| ENSG00000110047(5, 1) | ENSG00000204539(2, 0) |
| ENSG00000110429(5, 1) | ENSG00000204946(2, 0) |
| ENSG00000110786(5, 1) | ENSG00000204977(2, 0) |
| ENSG00000111652(5, 1) | ENSG00000204991(2, 0) |
| ENSG00000111676(5, 2) | ENSG00000206384(2, 0) |
| ENSG00000111696(5, 2) | ENSG00000211452(2, 0) |
| ENSG00000111707(5, 3) | ENSG00000211460(2, 0) |
| ENSG00000111725(5, 2) | ENSG00000213853(2, 0) |
| ENSG00000111728(5, 2) | ENSG00000213928(2, 0) |
| ENSG00000111790(5, 2) | ENSG00000214046(2, 0) |
| ENSG00000111799(5, 1) | ENSG00000214655(2, 0) |
| ENSG00000112290(5, 3) | ENSG00000215421(2, 0) |
| ENSG00000112305(5, 2) | ENSG00000221949(2, 0) |
| ENSG00000112333(5, 2) | ENSG00000221988(2, 0) |
| ENSG00000112893(5, 4) | ENSG00000227500(2, 0) |
| ENSG00000113384(5, 3) | ENSG00000240224(2, 0) |
| ENSG00000113387(5, 3) | ENSG00000240694(2, 0) |
| ENSG00000114270(5, 1) | ENSG00000241119(2, 0) |
| ENSG00000115008(5, 1) | ENSG00000241322(2, 0) |
| ENSG00000115194(5, 2) | ENSG00000241635(2, 0) |
| ENSG00000115295(5, 1) | ENSG00000241697(2, 0) |
| ENSG00000115307(5, 2) | ENSG00000242366(2, 0) |
| ENSG00000115365(5, 1) | ENSG00000242372(2, 0) |
| ENSG00000115520(5, 1) | ENSG00000242515(2, 0) |
| ENSG00000115561(5, 2) | ENSG00000243135(2, 0) |
| ENSG00000115738(5, 1) | ENSG00000243709(2, 0) |
| ENSG00000115896(5, 1) | ENSG00000243943(2, 0) |
| ENSG00000116679(5, 1) | ENSG00000244122(2, 0) |
| ENSG00000117000(5, 1) | ENSG00000244474(2, 0) |
| ENSG00000117318(5, 1) | ENSG00000244617(2, 0) |
| ENSG00000117620(5, 3) | ENSG00000247596(2, 0) |
| ENSG00000118200(5, 1) | ENSG00000253148(2, 0) |
| ENSG00000118242(5, 2) | ENSG00000007062(3, 0) |
| ENSG00000119471(5, 2) | ENSG00000009765(3, 0) |
| ENSG00000119487(5, 1) | ENSG00000011201(3, 0) |
| ENSG00000119537(5, 1) | ENSG00000030066(3, 0) |
| ENSG00000119630(5, 3) | ENSG00000034152(3, 0) |
| ENSG00000119919(5, 1) | ENSG00000048028(3, 0) |
| ENSG00000120075(5, 3) | ENSG00000058085(3, 0) |

| | |
|---|---|
| ENSG00000120088(5, 4) | ENSG00000065361(3, 0) |
| ENSG00000120616(5, 2) | ENSG00000065978(3, 0) |
| ENSG00000120727(5, 2) | ENSG00000067113(3, 0) |
| ENSG00000121060(5, 1) | ENSG00000068137(3, 0) |
| ENSG00000121207(5, 2) | ENSG00000072858(3, 0) |
| ENSG00000121281(5, 2) | ENSG00000074370(3, 0) |
| ENSG00000121774(5, 1) | ENSG00000079313(3, 0) |
| ENSG00000122176(5, 1) | ENSG00000087338(3, 0) |
| ENSG00000123159(5, 4) | ENSG00000088543(3, 0) |
| ENSG00000123342(5, 1) | ENSG00000090530(3, 0) |
| ENSG00000123908(5, 4) | ENSG00000090539(3, 0) |
| ENSG00000124216(5, 4) | ENSG00000091879(3, 0) |
| ENSG00000124772(5, 2) | ENSG00000092096(3, 0) |
| ENSG00000124942(5, 4) | ENSG00000096696(3, 0) |
| ENSG00000125968(5, 1) | ENSG00000100325(3, 0) |
| ENSG00000125970(5, 1) | ENSG00000100348(3, 0) |
| ENSG00000126062(5, 4) | ENSG00000100722(3, 0) |
| ENSG00000126217(5, 3) | ENSG00000100987(3, 0) |
| ENSG00000126581(5, 4) | ENSG00000101294(3, 0) |
| ENSG00000127220(5, 3) | ENSG00000101407(3, 0) |
| ENSG00000127328(5, 2) | ENSG00000101542(3, 0) |
| ENSG00000127603(5, 3) | ENSG00000101638(3, 0) |
| ENSG00000128510(5, 4) | ENSG00000102038(3, 0) |
| ENSG00000128710(5, 3) | ENSG00000102804(3, 0) |
| ENSG00000129152(5, 1) | ENSG00000102931(3, 0) |
| ENSG00000129255(5, 1) | ENSG00000103042(3, 0) |
| ENSG00000129353(5, 4) | ENSG00000105173(3, 0) |
| ENSG00000129521(5, 3) | ENSG00000105229(3, 0) |
| ENSG00000129654(5, 2) | ENSG00000106355(3, 0) |
| ENSG00000130165(5, 2) | ENSG00000107362(3, 0) |
| ENSG00000130559(5, 3) | ENSG00000107679(3, 0) |
| ENSG00000130638(5, 2) | ENSG00000111615(3, 0) |
| ENSG00000130695(5, 2) | ENSG00000111653(3, 0) |
| ENSG00000130821(5, 3) | ENSG00000112200(3, 0) |
| ENSG00000130881(5, 1) | ENSG00000112599(3, 0) |
| ENSG00000131051(5, 1) | ENSG00000112655(3, 0) |
| ENSG00000131370(5, 4) | ENSG00000112697(3, 0) |
| ENSG00000131375(5, 2) | ENSG00000112769(3, 0) |
| ENSG00000131378(5, 1) | ENSG00000113240(3, 0) |
| ENSG00000131725(5, 3) | ENSG00000113263(3, 0) |
| ENSG00000131941(5, 2) | ENSG00000113722(3, 0) |
| ENSG00000132424(5, 3) | ENSG00000114904(3, 0) |
| ENSG00000132694(5, 2) | ENSG00000116783(3, 0) |
| ENSG00000132849(5, 1) | ENSG00000116962(3, 0) |
| ENSG00000133069(5, 2) | ENSG00000117385(3, 0) |
| ENSG00000133393(5, 3) | ENSG00000117697(3, 0) |
| ENSG00000133812(5, 1) | ENSG00000118267(3, 0) |
| ENSG00000134884(5, 1) | ENSG00000118762(3, 0) |
| ENSG00000134897(5, 1) | ENSG00000119699(3, 0) |
| ENSG00000134917(5, 3) | ENSG00000119720(3, 0) |
| ENSG00000135093(5, 1) | ENSG00000120756(3, 0) |
| ENSG00000135272(5, 1) | ENSG00000121905(3, 0) |
| ENSG00000135334(5, 2) | ENSG00000121940(3, 0) |
| ENSG00000135423(5, 1) | ENSG00000123178(3, 0) |

| | |
|---|---|
| ENSG00000135447(5, 2) | ENSG00000123268(3, 0) |
| ENSG00000135537(5, 1) | ENSG00000124882(3, 0) |
| ENSG00000135597(5, 4) | ENSG00000125878(3, 0) |
| ENSG00000135709(5, 2) | ENSG00000126216(3, 0) |
| ENSG00000135776(5, 3) | ENSG00000126461(3, 0) |
| ENSG00000136002(5, 4) | ENSG00000128602(3, 0) |
| ENSG00000136100(5, 2) | ENSG00000130827(3, 0) |
| ENSG00000136634(5, 3) | ENSG00000131043(3, 0) |
| ENSG00000136827(5, 2) | ENSG00000131171(3, 0) |
| ENSG00000137101(5, 4) | ENSG00000131503(3, 0) |
| ENSG00000137558(5, 2) | ENSG00000131668(3, 0) |
| ENSG00000137709(5, 3) | ENSG00000131771(3, 0) |
| ENSG00000137801(5, 2) | ENSG00000132507(3, 0) |
| ENSG00000137842(5, 3) | ENSG00000132591(3, 0) |
| ENSG00000137878(5, 1) | ENSG00000132603(3, 0) |
| ENSG00000138031(5, 2) | ENSG00000132780(3, 0) |
| ENSG00000138071(5, 2) | ENSG00000133789(3, 0) |
| ENSG00000138101(5, 1) | ENSG00000134042(3, 0) |
| ENSG00000138138(5, 1) | ENSG00000134075(3, 0) |
| ENSG00000138246(5, 2) | ENSG00000134452(3, 0) |
| ENSG00000138316(5, 3) | ENSG00000134533(3, 0) |
| ENSG00000138617(5, 1) | ENSG00000135269(3, 0) |
| ENSG00000138698(5, 2) | ENSG00000135443(3, 0) |
| ENSG00000138780(5, 1) | ENSG00000135469(3, 0) |
| ENSG00000139197(5, 1) | ENSG00000135547(3, 0) |
| ENSG00000139318(5, 1) | ENSG00000135842(3, 0) |
| ENSG00000139517(5, 2) | ENSG00000136144(3, 0) |
| ENSG00000139546(5, 3) | ENSG00000136536(3, 0) |
| ENSG00000139567(5, 1) | ENSG00000136816(3, 0) |
| ENSG00000139625(5, 3) | ENSG00000137033(3, 0) |
| ENSG00000139668(5, 1) | ENSG00000137145(3, 0) |
| ENSG00000139687(5, 2) | ENSG00000137285(3, 0) |
| ENSG00000139726(5, 2) | ENSG00000137474(3, 0) |
| ENSG00000139874(5, 1) | ENSG00000137507(3, 0) |
| ENSG00000139890(5, 2) | ENSG00000137726(3, 0) |
| ENSG00000140157(5, 3) | ENSG00000138160(3, 0) |
| ENSG00000140396(5, 3) | ENSG00000138311(3, 0) |
| ENSG00000140807(5, 1) | ENSG00000138448(3, 0) |
| ENSG00000141258(5, 4) | ENSG00000138823(3, 0) |
| ENSG00000141433(5, 4) | ENSG00000140750(3, 0) |
| ENSG00000141441(5, 2) | ENSG00000141127(3, 0) |
| ENSG00000141448(5, 2) | ENSG00000141179(3, 0) |
| ENSG00000141542(5, 2) | ENSG00000142279(3, 0) |
| ENSG00000141644(5, 2) | ENSG00000143248(3, 0) |
| ENSG00000141753(5, 4) | ENSG00000143520(3, 0) |
| ENSG00000141956(5, 2) | ENSG00000143669(3, 0) |
| ENSG00000141985(5, 3) | ENSG00000144119(3, 0) |
| ENSG00000142235(5, 4) | ENSG00000144452(3, 0) |
| ENSG00000143006(5, 2) | ENSG00000145743(3, 0) |
| ENSG00000143157(5, 2) | ENSG00000145860(3, 0) |
| ENSG00000143373(5, 3) | ENSG00000145861(3, 0) |
| ENSG00000143375(5, 2) | ENSG00000147382(3, 0) |
| ENSG00000143420(5, 1) | ENSG00000147421(3, 0) |
| ENSG00000143570(5, 3) | ENSG00000147883(3, 0) |

| | |
|---|---|
| ENSG00000143630(5, 1) | ENSG00000148120(3, 0) |
| ENSG00000143878(5, 1) | ENSG00000149231(3, 0) |
| ENSG00000144118(5, 2) | ENSG00000149596(3, 0) |
| ENSG00000144278(5, 3) | ENSG00000150471(3, 0) |
| ENSG00000144455(5, 2) | ENSG00000151413(3, 0) |
| ENSG00000144741(5, 3) | ENSG00000151576(3, 0) |
| ENSG00000144815(5, 2) | ENSG00000152683(3, 0) |
| ENSG00000144959(5, 2) | ENSG00000152766(3, 0) |
| ENSG00000146469(5, 3) | ENSG00000152804(3, 0) |
| ENSG00000147065(5, 2) | ENSG00000153790(3, 0) |
| ENSG00000147416(5, 4) | ENSG00000153993(3, 0) |
| ENSG00000147471(5, 4) | ENSG00000154175(3, 0) |
| ENSG00000147650(5, 1) | ENSG00000154237(3, 0) |
| ENSG00000148426(5, 2) | ENSG00000154764(3, 0) |
| ENSG00000148468(5, 3) | ENSG00000155090(3, 0) |
| ENSG00000148700(5, 1) | ENSG00000155324(3, 0) |
| ENSG00000148798(5, 3) | ENSG00000155876(3, 0) |
| ENSG00000148926(5, 3) | ENSG00000155984(3, 0) |
| ENSG00000148948(5, 2) | ENSG00000158006(3, 0) |
| ENSG00000149927(5, 2) | ENSG00000158109(3, 0) |
| ENSG00000150051(5, 4) | ENSG00000160963(3, 0) |
| ENSG00000150086(5, 2) | ENSG00000162337(3, 0) |
| ENSG00000150401(5, 2) | ENSG00000162702(3, 0) |
| ENSG00000151014(5, 3) | ENSG00000163071(3, 0) |
| ENSG00000151135(5, 1) | ENSG00000163352(3, 0) |
| ENSG00000151229(5, 2) | ENSG00000163661(3, 0) |
| ENSG00000152049(5, 1) | ENSG00000163703(3, 0) |
| ENSG00000152193(5, 1) | ENSG00000164112(3, 0) |
| ENSG00000152332(5, 2) | ENSG00000164663(3, 0) |
| ENSG00000152518(5, 2) | ENSG00000165409(3, 0) |
| ENSG00000152779(5, 2) | ENSG00000165655(3, 0) |
| ENSG00000152894(5, 2) | ENSG00000166446(3, 0) |
| ENSG00000152954(5, 2) | ENSG00000167552(3, 0) |
| ENSG00000153012(5, 1) | ENSG00000167995(3, 0) |
| ENSG00000153291(5, 2) | ENSG00000168439(3, 0) |
| ENSG00000154274(5, 1) | ENSG00000169155(3, 0) |
| ENSG00000154518(5, 2) | ENSG00000170464(3, 0) |
| ENSG00000154845(5, 3) | ENSG00000170561(3, 0) |
| ENSG00000154864(5, 2) | ENSG00000170925(3, 0) |
| ENSG00000155893(5, 2) | ENSG00000171560(3, 0) |
| ENSG00000156284(5, 1) | ENSG00000171812(3, 0) |
| ENSG00000157077(5, 2) | ENSG00000172349(3, 0) |
| ENSG00000157554(5, 4) | ENSG00000172404(3, 0) |
| ENSG00000157613(5, 2) | ENSG00000172789(3, 0) |
| ENSG00000158301(5, 3) | ENSG00000173275(3, 0) |
| ENSG00000158457(5, 1) | ENSG00000174437(3, 0) |
| ENSG00000158717(5, 4) | ENSG00000174514(3, 0) |
| ENSG00000158941(5, 3) | ENSG00000175084(3, 0) |
| ENSG00000159140(5, 2) | ENSG00000175175(3, 0) |
| ENSG00000159167(5, 1) | ENSG00000175203(3, 0) |
| ENSG00000159251(5, 1) | ENSG00000175274(3, 0) |
| ENSG00000159335(5, 1) | ENSG00000175548(3, 0) |
| ENSG00000159556(5, 2) | ENSG00000175592(3, 0) |
| ENSG00000159921(5, 2) | ENSG00000176381(3, 0) |

| | |
|---|---|
| ENSG00000160050(5, 2) | ENSG00000177300(3, 0) |
| ENSG00000160305(5, 3) | ENSG00000177733(3, 0) |
| ENSG00000160710(5, 2) | ENSG00000179023(3, 0) |
| ENSG00000160789(5, 3) | ENSG00000179886(3, 0) |
| ENSG00000160796(5, 1) | ENSG00000179918(3, 0) |
| ENSG00000161381(5, 1) | ENSG00000180964(3, 0) |
| ENSG00000162188(5, 1) | ENSG00000181652(3, 0) |
| ENSG00000162374(5, 2) | ENSG00000182107(3, 0) |
| ENSG00000162434(5, 1) | ENSG00000182533(3, 0) |
| ENSG00000162551(5, 2) | ENSG00000183354(3, 0) |
| ENSG00000162735(5, 2) | ENSG00000183763(3, 0) |
| ENSG00000162739(5, 2) | ENSG00000184678(3, 0) |
| ENSG00000162959(5, 3) | ENSG00000184900(3, 0) |
| ENSG00000162999(5, 2) | ENSG00000185666(3, 0) |
| ENSG00000163320(5, 3) | ENSG00000186638(3, 0) |
| ENSG00000163347(5, 1) | ENSG00000187778(3, 0) |
| ENSG00000163359(5, 2) | ENSG00000188488(3, 0) |
| ENSG00000163462(5, 3) | ENSG00000188807(3, 0) |
| ENSG00000163659(5, 2) | ENSG00000189091(3, 0) |
| ENSG00000163874(5, 1) | ENSG00000196296(3, 0) |
| ENSG00000163877(5, 2) | ENSG00000196458(3, 0) |
| ENSG00000164118(5, 2) | ENSG00000197779(3, 0) |
| ENSG00000164168(5, 2) | ENSG00000198356(3, 0) |
| ENSG00000164171(5, 2) | ENSG00000198420(3, 0) |
| ENSG00000164626(5, 3) | ENSG00000198576(3, 0) |
| ENSG00000164659(5, 4) | ENSG00000198589(3, 0) |
| ENSG00000164692(5, 3) | ENSG00000198680(3, 0) |
| ENSG00000164815(5, 1) | ENSG00000198822(3, 0) |
| ENSG00000164896(5, 2) | ENSG00000198863(3, 0) |
| ENSG00000165152(5, 1) | ENSG00000198911(3, 0) |
| ENSG00000165300(5, 2) | ENSG00000203791(3, 0) |
| ENSG00000165410(5, 3) | ENSG00000204618(3, 0) |
| ENSG00000165443(5, 3) | ENSG00000205089(3, 0) |
| ENSG00000165556(5, 3) | ENSG00000206527(3, 0) |
| ENSG00000165724(5, 3) | ENSG00000213341(3, 0) |
| ENSG00000165802(5, 3) | ENSG00000213859(3, 0) |
| ENSG00000165813(5, 1) | ENSG00000213977(3, 0) |
| ENSG00000166086(5, 2) | ENSG00000215271(3, 0) |
| ENSG00000166317(5, 1) | ENSG00000241994(3, 0) |
| ENSG00000166326(5, 1) | ENSG00000005893(4, 0) |
| ENSG00000166747(5, 2) | ENSG00000021645(4, 0) |
| ENSG00000166780(5, 1) | ENSG00000054356(4, 0) |
| ENSG00000166913(5, 3) | ENSG00000055130(4, 0) |
| ENSG00000167244(5, 1) | ENSG00000055813(4, 0) |
| ENSG00000167461(5, 1) | ENSG00000067221(4, 0) |
| ENSG00000167654(5, 3) | ENSG00000070718(4, 0) |
| ENSG00000167670(5, 2) | ENSG00000072182(4, 0) |
| ENSG00000167695(5, 3) | ENSG00000073910(4, 0) |
| ENSG00000168066(5, 2) | ENSG00000075975(4, 0) |
| ENSG00000168137(5, 2) | ENSG00000084774(4, 0) |
| ENSG00000168309(5, 2) | ENSG00000085382(4, 0) |
| ENSG00000168385(5, 1) | ENSG00000085449(4, 0) |
| ENSG00000168453(5, 2) | ENSG00000086589(4, 0) |
| ENSG00000168487(5, 2) | ENSG00000091317(4, 0) |

| | |
|---|---|
| ENSG00000168615(5, 3) | ENSG00000094963(4, 0) |
| ENSG00000168702(5, 1) | ENSG00000097096(4, 0) |
| ENSG00000168876(5, 3) | ENSG00000099889(4, 0) |
| ENSG00000168994(5, 2) | ENSG00000100425(4, 0) |
| ENSG00000169218(5, 2) | ENSG00000100968(4, 0) |
| ENSG00000169223(5, 4) | ENSG00000101057(4, 0) |
| ENSG00000169228(5, 3) | ENSG00000101327(4, 0) |
| ENSG00000169252(5, 3) | ENSG00000103018(4, 0) |
| ENSG00000169398(5, 1) | ENSG00000103089(4, 0) |
| ENSG00000169925(5, 3) | ENSG00000104853(4, 0) |
| ENSG00000170006(5, 1) | ENSG00000107623(4, 0) |
| ENSG00000170035(5, 1) | ENSG00000108578(4, 0) |
| ENSG00000170325(5, 2) | ENSG00000109771(4, 0) |
| ENSG00000170473(5, 2) | ENSG00000111186(4, 0) |
| ENSG00000170734(5, 2) | ENSG00000112164(4, 0) |
| ENSG00000170941(5, 1) | ENSG00000114279(4, 0) |
| ENSG00000170961(5, 2) | ENSG00000115363(4, 0) |
| ENSG00000171056(5, 2) | ENSG00000119681(4, 0) |
| ENSG00000171160(5, 1) | ENSG00000122592(4, 0) |
| ENSG00000171396(5, 1) | ENSG00000123096(4, 0) |
| ENSG00000171570(5, 4) | ENSG00000123374(4, 0) |
| ENSG00000172061(5, 1) | ENSG00000123689(4, 0) |
| ENSG00000172667(5, 4) | ENSG00000127561(4, 0) |
| ENSG00000172731(5, 2) | ENSG00000127837(4, 0) |
| ENSG00000172794(5, 1) | ENSG00000130725(4, 0) |
| ENSG00000173482(5, 3) | ENSG00000130939(4, 0) |
| ENSG00000173674(5, 2) | ENSG00000131188(4, 0) |
| ENSG00000173681(5, 4) | ENSG00000135535(4, 0) |
| ENSG00000173991(5, 1) | ENSG00000136940(4, 0) |
| ENSG00000174516(5, 2) | ENSG00000137642(4, 0) |
| ENSG00000174697(5, 2) | ENSG00000143379(4, 0) |
| ENSG00000175093(5, 1) | ENSG00000143443(4, 0) |
| ENSG00000175130(5, 2) | ENSG00000150540(4, 0) |
| ENSG00000175137(5, 3) | ENSG00000151846(4, 0) |
| ENSG00000175354(5, 1) | ENSG00000152229(4, 0) |
| ENSG00000175376(5, 3) | ENSG00000152784(4, 0) |
| ENSG00000175414(5, 1) | ENSG00000153531(4, 0) |
| ENSG00000176018(5, 3) | ENSG00000153827(4, 0) |
| ENSG00000176907(5, 2) | ENSG00000156486(4, 0) |
| ENSG00000177045(5, 1) | ENSG00000161800(4, 0) |
| ENSG00000177352(5, 1) | ENSG00000162928(4, 0) |
| ENSG00000177963(5, 2) | ENSG00000163029(4, 0) |
| ENSG00000178184(5, 2) | ENSG00000163155(4, 0) |
| ENSG00000178202(5, 2) | ENSG00000165240(4, 0) |
| ENSG00000178761(5, 3) | ENSG00000165804(4, 0) |
| ENSG00000179218(5, 3) | ENSG00000166348(4, 0) |
| ENSG00000179362(5, 1) | ENSG00000167614(4, 0) |
| ENSG00000179364(5, 3) | ENSG00000167702(4, 0) |
| ENSG00000179476(5, 3) | ENSG00000168067(4, 0) |
| ENSG00000179837(5, 4) | ENSG00000169181(4, 0) |
| ENSG00000179915(5, 3) | ENSG00000169242(4, 0) |
| ENSG00000179933(5, 1) | ENSG00000173545(4, 0) |
| ENSG00000180198(5, 3) | ENSG00000175182(4, 0) |
| ENSG00000180332(5, 4) | ENSG00000175329(4, 0) |

| | |
|---|---|
| ENSG00000180611(5, 1) | ENSG00000175564(4, 0) |
| ENSG00000180694(5, 3) | ENSG00000175746(4, 0) |
| ENSG00000181274(5, 1) | ENSG00000176597(4, 0) |
| ENSG00000181472(5, 4) | ENSG00000176871(4, 0) |
| ENSG00000181656(5, 1) | ENSG00000179057(4, 0) |
| ENSG00000181744(5, 3) | ENSG00000180228(4, 0) |
| ENSG00000181830(5, 3) | ENSG00000180929(4, 0) |
| ENSG00000181929(5, 2) | ENSG00000181163(4, 0) |
| ENSG00000182040(5, 3) | ENSG00000182606(4, 0) |
| ENSG00000182149(5, 2) | ENSG00000182923(4, 0) |
| ENSG00000182389(5, 1) | ENSG00000182979(4, 0) |
| ENSG00000182481(5, 1) | ENSG00000183643(4, 0) |
| ENSG00000182621(5, 4) | ENSG00000184634(4, 0) |
| ENSG00000182718(5, 2) | ENSG00000185760(4, 0) |
| ENSG00000183049(5, 1) | ENSG00000187486(4, 0) |
| ENSG00000183092(5, 2) | ENSG00000187838(4, 0) |
| ENSG00000183943(5, 1) | ENSG00000188554(4, 0) |
| ENSG00000184005(5, 2) | ENSG00000189184(4, 0) |
| ENSG00000184216(5, 3) | ENSG00000189337(4, 0) |
| ENSG00000184271(5, 1) | ENSG00000197892(4, 0) |
| ENSG00000184388(5, 2) | ENSG00000198353(4, 0) |
| ENSG00000184508(5, 1) | ENSG00000198597(4, 0) |
| ENSG00000184672(5, 1) | ENSG00000213588(4, 0) |
| ENSG00000184916(5, 2) | ENSG00000214827(4, 0) |
| ENSG00000185019(5, 3) | ENSG00000215021(4, 0) |
| ENSG00000185127(5, 4) | ENSG00000251380(4, 0) |
| ENSG00000185219(5, 1) | ENSG00000048649(5, 0) |
| ENSG00000185269(5, 2) | ENSG00000070476(5, 0) |
| ENSG00000185274(5, 1) | ENSG00000074695(5, 0) |
| ENSG00000185624(5, 1) | ENSG00000077585(5, 0) |
| ENSG00000185985(5, 1) | ENSG00000108379(5, 0) |
| ENSG00000186130(5, 2) | ENSG00000109929(5, 0) |
| ENSG00000186310(5, 4) | ENSG00000114349(5, 0) |
| ENSG00000186908(5, 1) | ENSG00000115649(5, 0) |
| ENSG00000187653(5, 2) | ENSG00000115762(5, 0) |
| ENSG00000187840(5, 3) | ENSG00000117152(5, 0) |
| ENSG00000188153(5, 4) | ENSG00000120992(5, 0) |
| ENSG00000188486(5, 3) | ENSG00000122180(5, 0) |
| ENSG00000188580(5, 1) | ENSG00000127418(5, 0) |
| ENSG00000188778(5, 3) | ENSG00000131748(5, 0) |
| ENSG00000189067(5, 3) | ENSG00000132356(5, 0) |
| ENSG00000189369(5, 2) | ENSG00000132612(5, 0) |
| ENSG00000196284(5, 1) | ENSG00000133104(5, 0) |
| ENSG00000196353(5, 1) | ENSG00000135316(5, 0) |
| ENSG00000196639(5, 3) | ENSG00000136267(5, 0) |
| ENSG00000196730(5, 2) | ENSG00000137216(5, 0) |
| ENSG00000196937(5, 3) | ENSG00000137807(5, 0) |
| ENSG00000197006(5, 2) | ENSG00000143409(5, 0) |
| ENSG00000197037(5, 2) | ENSG00000143847(5, 0) |
| ENSG00000197111(5, 2) | ENSG00000144445(5, 0) |
| ENSG00000197226(5, 4) | ENSG00000145687(5, 0) |
| ENSG00000197343(5, 3) | ENSG00000145741(5, 0) |
| ENSG00000197601(5, 1) | ENSG00000145901(5, 0) |
| ENSG00000198081(5, 2) | ENSG00000149792(5, 0) |

| | |
|---|---|
| ENSG00000198355(5, 2) | ENSG00000151657(5, 0) |
| ENSG00000198492(5, 1) | ENSG00000151952(5, 0) |
| ENSG00000198756(5, 1) | ENSG00000152661(5, 0) |
| ENSG00000198894(5, 4) | ENSG00000155657(5, 0) |
| ENSG00000198900(5, 1) | ENSG00000158270(5, 0) |
| ENSG00000198901(5, 3) | ENSG00000158985(5, 0) |
| ENSG00000204435(5, 2) | ENSG00000161960(5, 0) |
| ENSG00000204956(5, 3) | ENSG00000165572(5, 0) |
| ENSG00000205250(5, 2) | ENSG00000165669(5, 0) |
| ENSG00000205542(5, 2) | ENSG00000166803(5, 0) |
| ENSG00000213281(5, 1) | ENSG00000168411(5, 0) |
| ENSG00000213672(5, 3) | ENSG00000170579(5, 0) |
| ENSG00000214140(5, 1) | ENSG00000170653(5, 0) |
| ENSG00000214517(5, 4) | ENSG00000172175(5, 0) |
| ENSG00000221869(5, 3) | ENSG00000179603(5, 0) |
| ENSG00000221899(5, 2) | ENSG00000183853(5, 0) |
| ENSG00000240184(5, 3) | ENSG00000189266(5, 0) |
| ENSG00000240764(5, 3) | ENSG00000197429(5, 0) |
| ENSG00000242419(5, 3) | ENSG00000197713(5, 0) |
| ENSG00000253159(5, 3) | ENSG00000249992(5, 0) |
| ENSG00000253305(5, 3) | ENSG00000253950(5, 0) |
| ENSG00000253485(5, 3) | ENSG00000080815(6, 0) |
| ENSG00000253537(5, 3) | ENSG00000081059(6, 0) |
| ENSG00000253731(5, 3) | ENSG00000091640(6, 0) |
| ENSG00000253767(5, 3) | ENSG00000115380(6, 0) |
| ENSG00000253797(5, 1) | ENSG00000117751(6, 0) |
| ENSG00000253846(5, 3) | ENSG00000117758(6, 0) |
| ENSG00000253873(5, 3) | ENSG00000122025(6, 0) |
| ENSG00000253910(5, 3) | ENSG00000123560(6, 0) |
| ENSG00000253953(5, 3) | ENSG00000131899(6, 0) |
| ENSG00000254122(5, 3) | ENSG00000132155(6, 0) |
| ENSG00000254221(5, 3) | ENSG00000151923(6, 0) |
| ENSG00000254245(5, 3) | ENSG00000156162(6, 0) |
| ENSG00000004478(6, 2) | ENSG00000158636(6, 0) |
| ENSG00000004660(6, 2) | ENSG00000159461(6, 0) |
| ENSG00000005469(6, 2) | ENSG00000161544(6, 0) |
| ENSG00000006025(6, 3) | ENSG00000162461(6, 0) |
| ENSG00000006576(6, 3) | ENSG00000164076(6, 0) |
| ENSG00000010270(6, 2) | ENSG00000164438(6, 0) |
| ENSG00000012171(6, 3) | ENSG00000164976(6, 0) |
| ENSG00000015133(6, 5) | ENSG00000167987(6, 0) |
| ENSG00000018189(6, 1) | ENSG00000168140(6, 0) |
| ENSG00000023909(6, 3) | ENSG00000168255(6, 0) |
| ENSG00000026025(6, 5) | ENSG00000170142(6, 0) |
| ENSG00000027869(6, 2) | ENSG00000172071(6, 0) |
| ENSG00000028137(6, 3) | ENSG00000173898(6, 0) |
| ENSG00000035664(6, 3) | ENSG00000178974(6, 0) |
| ENSG00000038532(6, 4) | ENSG00000183691(6, 0) |
| ENSG00000043093(6, 2) | ENSG00000184203(6, 0) |
| ENSG00000046653(6, 2) | ENSG00000198176(6, 0) |
| ENSG00000049245(6, 5) | ENSG00000010803(7, 0) |
| ENSG00000049246(6, 2) | ENSG00000063244(7, 0) |
| ENSG00000052802(6, 2) | ENSG00000082258(7, 0) |
| ENSG00000054267(6, 3) | ENSG00000090372(7, 0) |

ENSG00000061337(6, 1)
ENSG00000061938(6, 4)
ENSG00000062282(6, 1)
ENSG00000063015(6, 1)
ENSG00000065882(6, 3)
ENSG00000066136(6, 1)
ENSG00000066777(6, 2)
ENSG00000068323(6, 3)
ENSG00000068366(6, 1)
ENSG00000069399(6, 3)
ENSG00000070756(6, 1)
ENSG00000072062(6, 1)
ENSG00000072195(6, 5)
ENSG00000072310(6, 4)
ENSG00000072415(6, 4)
ENSG00000072609(6, 2)
ENSG00000072786(6, 2)
ENSG00000073150(6, 2)
ENSG00000076716(6, 3)
ENSG00000078018(6, 1)
ENSG00000078399(6, 3)
ENSG00000079277(6, 1)
ENSG00000080371(6, 3)
ENSG00000080709(6, 1)
ENSG00000081923(6, 2)
ENSG00000082684(6, 1)
ENSG00000084731(6, 3)
ENSG00000086475(6, 4)
ENSG00000087245(6, 2)
ENSG00000087448(6, 2)
ENSG00000088038(6, 2)
ENSG00000088356(6, 2)
ENSG00000088833(6, 1)
ENSG00000088881(6, 4)
ENSG00000089177(6, 2)
ENSG00000090316(6, 1)
ENSG00000091009(6, 1)
ENSG00000091140(6, 3)
ENSG00000095951(6, 1)
ENSG00000099875(6, 3)
ENSG00000099917(6, 5)
ENSG00000099954(6, 3)
ENSG00000099985(6, 2)
ENSG00000100302(6, 2)
ENSG00000100380(6, 2)
ENSG00000100592(6, 2)
ENSG00000101040(6, 1)
ENSG00000101230(6, 3)
ENSG00000101349(6, 3)
ENSG00000101438(6, 3)
ENSG00000101460(6, 3)
ENSG00000101888(6, 5)
ENSG00000102221(6, 1)
ENSG00000102858(6, 3)
ENSG00000122126(7, 0)
ENSG00000125520(7, 0)
ENSG00000126746(7, 0)
ENSG00000141664(7, 0)
ENSG00000143549(7, 0)
ENSG00000154305(7, 0)
ENSG00000155034(7, 0)
ENSG00000162599(7, 0)
ENSG00000163132(7, 0)
ENSG00000168488(7, 0)
ENSG00000173757(7, 0)
ENSG00000186354(7, 0)
ENSG00000196338(7, 0)
ENSG00000073464(8, 0)
ENSG00000083099(8, 0)
ENSG00000100505(8, 0)
ENSG00000113140(8, 0)
ENSG00000167257(8, 0)
ENSG00000181722(8, 0)
ENSG00000113721(9, 0)
ENSG00000120049(9, 0)
ENSG00000145794(9, 0)
ENSG00000173805(9, 0)
ENSG00000171603(10, 0)
ENSG00000131467(17, 0)

ENSG00000103126(6, 2)
ENSG00000103647(6, 1)
ENSG00000103710(6, 1)
ENSG00000104332(6, 4)
ENSG00000104381(6, 4)
ENSG00000105186(6, 4)
ENSG00000105552(6, 3)
ENSG00000106025(6, 4)
ENSG00000106617(6, 1)
ENSG00000106689(6, 2)
ENSG00000106772(6, 2)
ENSG00000107262(6, 1)
ENSG00000108094(6, 1)
ENSG00000108179(6, 5)
ENSG00000108509(6, 3)
ENSG00000108684(6, 2)
ENSG00000108753(6, 4)
ENSG00000109099(6, 2)
ENSG00000110344(6, 3)
ENSG00000110721(6, 2)
ENSG00000111142(6, 2)
ENSG00000111837(6, 1)
ENSG00000111875(6, 3)
ENSG00000112078(6, 3)
ENSG00000112079(6, 3)
ENSG00000112146(6, 3)
ENSG00000112576(6, 1)
ENSG00000112592(6, 4)
ENSG00000112773(6, 4)
ENSG00000113430(6, 1)
ENSG00000113916(6, 3)
ENSG00000114268(6, 2)
ENSG00000114315(6, 3)
ENSG00000114520(6, 3)
ENSG00000114742(6, 3)
ENSG00000115207(6, 4)
ENSG00000115226(6, 4)
ENSG00000115232(6, 3)
ENSG00000115414(6, 3)
ENSG00000116141(6, 1)
ENSG00000116678(6, 1)
ENSG00000116754(6, 3)
ENSG00000117519(6, 3)
ENSG00000117533(6, 2)
ENSG00000117625(6, 2)
ENSG00000118523(6, 3)
ENSG00000119121(6, 3)
ENSG00000119280(6, 4)
ENSG00000119383(6, 3)
ENSG00000119508(6, 2)
ENSG00000119541(6, 5)
ENSG00000119596(6, 1)
ENSG00000119979(6, 3)
ENSG00000120068(6, 5)

ENSG00000120519(6, 3)
ENSG00000120868(6, 2)
ENSG00000122545(6, 4)
ENSG00000122691(6, 1)
ENSG00000122877(6, 1)
ENSG00000123700(6, 4)
ENSG00000124160(6, 2)
ENSG00000124177(6, 1)
ENSG00000124208(6, 4)
ENSG00000124226(6, 2)
ENSG00000124493(6, 2)
ENSG00000124818(6, 4)
ENSG00000125166(6, 2)
ENSG00000125249(6, 5)
ENSG00000125355(6, 2)
ENSG00000125430(6, 1)
ENSG00000125459(6, 4)
ENSG00000125845(6, 3)
ENSG00000128714(6, 3)
ENSG00000128849(6, 2)
ENSG00000129474(6, 2)
ENSG00000129657(6, 4)
ENSG00000130176(6, 4)
ENSG00000130508(6, 5)
ENSG00000130540(6, 4)
ENSG00000130590(6, 5)
ENSG00000130703(6, 2)
ENSG00000131067(6, 4)
ENSG00000132128(6, 3)
ENSG00000132205(6, 3)
ENSG00000132563(6, 2)
ENSG00000132692(6, 2)
ENSG00000132819(6, 3)
ENSG00000132825(6, 3)
ENSG00000132932(6, 2)
ENSG00000133275(6, 2)
ENSG00000134317(6, 2)
ENSG00000134444(6, 2)
ENSG00000134709(6, 4)
ENSG00000134817(6, 4)
ENSG00000135164(6, 4)
ENSG00000135374(6, 1)
ENSG00000135392(6, 2)
ENSG00000135720(6, 4)
ENSG00000136051(6, 3)
ENSG00000136160(6, 3)
ENSG00000136238(6, 2)
ENSG00000136240(6, 5)
ENSG00000136280(6, 4)
ENSG00000136425(6, 4)
ENSG00000137218(6, 3)
ENSG00000137221(6, 1)
ENSG00000137273(6, 1)
ENSG00000137310(6, 2)

ENSG00000137497(6, 4)
ENSG00000137571(6, 5)
ENSG00000138641(6, 4)
ENSG00000138670(6, 1)
ENSG00000138771(6, 1)
ENSG00000138792(6, 4)
ENSG00000138795(6, 2)
ENSG00000139112(6, 1)
ENSG00000139428(6, 1)
ENSG00000139926(6, 3)
ENSG00000140090(6, 1)
ENSG00000140470(6, 1)
ENSG00000140548(6, 5)
ENSG00000141314(6, 3)
ENSG00000141580(6, 1)
ENSG00000142459(6, 2)
ENSG00000142669(6, 3)
ENSG00000143365(6, 2)
ENSG00000143569(6, 1)
ENSG00000143622(6, 1)
ENSG00000144583(6, 2)
ENSG00000145335(6, 3)
ENSG00000146250(6, 3)
ENSG00000146360(6, 3)
ENSG00000146411(6, 4)
ENSG00000146433(6, 2)
ENSG00000147419(6, 3)
ENSG00000147854(6, 4)
ENSG00000148204(6, 2)
ENSG00000148343(6, 4)
ENSG00000148516(6, 4)
ENSG00000149115(6, 2)
ENSG00000149269(6, 2)
ENSG00000149295(6, 3)
ENSG00000149480(6, 2)
ENSG00000149639(6, 2)
ENSG00000149657(6, 1)
ENSG00000150457(6, 2)
ENSG00000150967(6, 5)
ENSG00000151208(6, 3)
ENSG00000151239(6, 2)
ENSG00000151327(6, 3)
ENSG00000151490(6, 4)
ENSG00000151617(6, 4)
ENSG00000152402(6, 2)
ENSG00000152795(6, 1)
ENSG00000154059(6, 1)
ENSG00000154342(6, 3)
ENSG00000154645(6, 1)
ENSG00000154710(6, 1)
ENSG00000154822(6, 2)
ENSG00000155011(6, 3)
ENSG00000155304(6, 3)
ENSG00000155858(6, 2)

ENSG00000156504(6, 3)
ENSG00000156521(6, 4)
ENSG00000157240(6, 1)
ENSG00000157388(6, 2)
ENSG00000157502(6, 3)
ENSG00000158050(6, 1)
ENSG00000158055(6, 3)
ENSG00000158321(6, 3)
ENSG00000158715(6, 2)
ENSG00000158769(6, 2)
ENSG00000159399(6, 4)
ENSG00000160208(6, 2)
ENSG00000160271(6, 3)
ENSG00000160785(6, 4)
ENSG00000161835(6, 4)
ENSG00000161956(6, 1)
ENSG00000162493(6, 2)
ENSG00000162704(6, 4)
ENSG00000162849(6, 3)
ENSG00000163053(6, 3)
ENSG00000163145(6, 2)
ENSG00000163235(6, 3)
ENSG00000163376(6, 4)
ENSG00000163600(6, 5)
ENSG00000163618(6, 2)
ENSG00000163635(6, 1)
ENSG00000163884(6, 1)
ENSG00000163909(6, 3)
ENSG00000164303(6, 2)
ENSG00000164318(6, 3)
ENSG00000164600(6, 2)
ENSG00000164733(6, 2)
ENSG00000165168(6, 1)
ENSG00000165376(6, 2)
ENSG00000165449(6, 3)
ENSG00000165458(6, 4)
ENSG00000165548(6, 3)
ENSG00000165566(6, 2)
ENSG00000165886(6, 1)
ENSG00000165915(6, 4)
ENSG00000165997(6, 3)
ENSG00000166847(6, 2)
ENSG00000166923(6, 4)
ENSG00000166924(6, 1)
ENSG00000167037(6, 2)
ENSG00000167118(6, 1)
ENSG00000167323(6, 3)
ENSG00000167716(6, 3)
ENSG00000167778(6, 1)
ENSG00000167977(6, 4)
ENSG00000168228(6, 1)
ENSG00000168229(6, 3)
ENSG00000168477(6, 1)
ENSG00000168490(6, 2)

ENSG00000168993(6, 4)
ENSG00000169194(6, 3)
ENSG00000169220(6, 2)
ENSG00000170044(6, 2)
ENSG00000170153(6, 2)
ENSG00000170185(6, 4)
ENSG00000170289(6, 1)
ENSG00000170390(6, 2)
ENSG00000170485(6, 3)
ENSG00000170500(6, 2)
ENSG00000170779(6, 2)
ENSG00000170801(6, 2)
ENSG00000171044(6, 2)
ENSG00000171303(6, 2)
ENSG00000171314(6, 4)
ENSG00000171357(6, 2)
ENSG00000171502(6, 4)
ENSG00000171522(6, 4)
ENSG00000171587(6, 1)
ENSG00000171786(6, 2)
ENSG00000172239(6, 4)
ENSG00000172346(6, 4)
ENSG00000172375(6, 4)
ENSG00000172530(6, 1)
ENSG00000172780(6, 2)
ENSG00000172818(6, 3)
ENSG00000173114(6, 2)
ENSG00000173276(6, 2)
ENSG00000173436(6, 3)
ENSG00000173452(6, 3)
ENSG00000173456(6, 2)
ENSG00000173868(6, 3)
ENSG00000174348(6, 1)
ENSG00000174780(6, 4)
ENSG00000174950(6, 2)
ENSG00000175040(6, 3)
ENSG00000175505(6, 1)
ENSG00000175931(6, 3)
ENSG00000175970(6, 2)
ENSG00000176076(6, 1)
ENSG00000176142(6, 2)
ENSG00000177169(6, 3)
ENSG00000177479(6, 3)
ENSG00000178188(6, 5)
ENSG00000180138(6, 2)
ENSG00000180801(6, 4)
ENSG00000180891(6, 3)
ENSG00000180901(6, 4)
ENSG00000181192(6, 2)
ENSG00000182199(6, 3)
ENSG00000182287(6, 3)
ENSG00000182324(6, 3)
ENSG00000182636(6, 3)
ENSG00000182909(6, 1)

ENSG00000182985(6, 1)
ENSG00000183431(6, 2)
ENSG00000183722(6, 4)
ENSG00000183900(6, 1)
ENSG00000184232(6, 5)
ENSG00000184304(6, 2)
ENSG00000184787(6, 2)
ENSG00000184840(6, 2)
ENSG00000185344(6, 3)
ENSG00000185477(6, 3)
ENSG00000185633(6, 2)
ENSG00000185818(6, 3)
ENSG00000185909(6, 3)
ENSG00000186063(6, 3)
ENSG00000186340(6, 2)
ENSG00000186530(6, 2)
ENSG00000187257(6, 2)
ENSG00000188157(6, 2)
ENSG00000188428(6, 5)
ENSG00000189410(6, 3)
ENSG00000196199(6, 2)
ENSG00000196547(6, 3)
ENSG00000196652(6, 1)
ENSG00000196872(6, 1)
ENSG00000197256(6, 4)
ENSG00000197375(6, 3)
ENSG00000197380(6, 2)
ENSG00000197557(6, 1)
ENSG00000197562(6, 3)
ENSG00000197594(6, 2)
ENSG00000197771(6, 1)
ENSG00000197798(6, 4)
ENSG00000197921(6, 1)
ENSG00000197965(6, 2)
ENSG00000198182(6, 2)
ENSG00000198373(6, 1)
ENSG00000198399(6, 4)
ENSG00000198563(6, 2)
ENSG00000198642(6, 2)
ENSG00000198729(6, 1)
ENSG00000198795(6, 1)
ENSG00000198836(6, 3)
ENSG00000198837(6, 2)
ENSG00000198910(6, 2)
ENSG00000198929(6, 2)
ENSG00000204308(6, 4)
ENSG00000204406(6, 3)
ENSG00000204634(6, 3)
ENSG00000204681(6, 3)
ENSG00000205213(6, 1)
ENSG00000205423(6, 2)
ENSG00000206561(6, 2)
ENSG00000206579(6, 4)
ENSG00000215012(6, 1)

ENSG00000221818(6, 3)
ENSG00000221870(6, 4)
ENSG00000240583(6, 1)
ENSG00000241553(6, 1)
ENSG00000244687(6, 4)
ENSG00000245848(6, 4)
ENSG00000005483(7, 2)
ENSG00000006007(7, 1)
ENSG00000006377(7, 3)
ENSG00000007314(7, 4)
ENSG00000008282(7, 4)
ENSG00000009335(7, 4)
ENSG00000009844(7, 2)
ENSG00000011258(7, 2)
ENSG00000013016(7, 3)
ENSG00000013297(7, 5)
ENSG00000017621(7, 2)
ENSG00000019505(7, 3)
ENSG00000021574(7, 4)
ENSG00000023516(7, 3)
ENSG00000033122(7, 5)
ENSG00000038274(7, 3)
ENSG00000041515(7, 1)
ENSG00000041982(7, 1)
ENSG00000043039(7, 3)
ENSG00000047932(7, 3)
ENSG00000050426(7, 3)
ENSG00000052795(7, 2)
ENSG00000053438(7, 4)
ENSG00000054690(7, 4)
ENSG00000066422(7, 2)
ENSG00000067900(7, 4)
ENSG00000068078(7, 4)
ENSG00000069702(7, 3)
ENSG00000070610(7, 2)
ENSG00000070759(7, 4)
ENSG00000072110(7, 1)
ENSG00000073803(7, 2)
ENSG00000074181(7, 3)
ENSG00000075618(7, 3)
ENSG00000076321(7, 2)
ENSG00000078369(7, 2)
ENSG00000079335(7, 2)
ENSG00000080573(7, 4)
ENSG00000081026(7, 3)
ENSG00000081087(7, 2)
ENSG00000081479(7, 2)
ENSG00000081803(7, 2)
ENSG00000082175(7, 4)
ENSG00000082805(7, 2)
ENSG00000085978(7, 5)
ENSG00000086200(7, 4)
ENSG00000088756(7, 3)
ENSG00000090097(7, 1)

ENSG00000090686(7, 4)
ENSG00000090857(7, 3)
ENSG00000094975(7, 5)
ENSG00000099308(7, 2)
ENSG00000100151(7, 2)
ENSG00000100201(7, 3)
ENSG00000100379(7, 5)
ENSG00000100522(7, 3)
ENSG00000100626(7, 3)
ENSG00000100941(7, 1)
ENSG00000101290(7, 1)
ENSG00000101751(7, 3)
ENSG00000102057(7, 4)
ENSG00000102096(7, 2)
ENSG00000102290(7, 4)
ENSG00000102317(7, 3)
ENSG00000102882(7, 3)
ENSG00000102974(7, 1)
ENSG00000102996(7, 2)
ENSG00000103160(7, 2)
ENSG00000103275(7, 3)
ENSG00000103942(7, 3)
ENSG00000105137(7, 1)
ENSG00000105270(7, 3)
ENSG00000105429(7, 1)
ENSG00000105967(7, 1)
ENSG00000106236(7, 3)
ENSG00000106261(7, 1)
ENSG00000106484(7, 3)
ENSG00000106546(7, 1)
ENSG00000106683(7, 4)
ENSG00000106992(7, 3)
ENSG00000107562(7, 2)
ENSG00000107738(7, 4)
ENSG00000107897(7, 3)
ENSG00000108557(7, 2)
ENSG00000108604(7, 2)
ENSG00000108799(7, 4)
ENSG00000108861(7, 4)
ENSG00000109180(7, 2)
ENSG00000109381(7, 3)
ENSG00000109705(7, 3)
ENSG00000110066(7, 4)
ENSG00000110911(7, 2)
ENSG00000111670(7, 4)
ENSG00000111711(7, 3)
ENSG00000111885(7, 3)
ENSG00000112062(7, 5)
ENSG00000112308(7, 4)
ENSG00000112379(7, 3)
ENSG00000112964(7, 2)
ENSG00000113273(7, 2)
ENSG00000113441(7, 3)
ENSG00000113578(7, 2)

ENSG00000113805(7, 2)
ENSG00000114120(7, 5)
ENSG00000114374(7, 3)
ENSG00000114757(7, 2)
ENSG00000115041(7, 3)
ENSG00000115084(7, 3)
ENSG00000115806(7, 4)
ENSG00000116017(7, 4)
ENSG00000116128(7, 5)
ENSG00000116199(7, 3)
ENSG00000116698(7, 2)
ENSG00000116985(7, 1)
ENSG00000117266(7, 6)
ENSG00000117479(7, 2)
ENSG00000117481(7, 3)
ENSG00000117525(7, 3)
ENSG00000118046(7, 4)
ENSG00000118276(7, 3)
ENSG00000118513(7, 3)
ENSG00000118655(7, 3)
ENSG00000119408(7, 4)
ENSG00000119522(7, 1)
ENSG00000119703(7, 3)
ENSG00000119772(7, 1)
ENSG00000119899(7, 3)
ENSG00000120063(7, 4)
ENSG00000120738(7, 5)
ENSG00000120833(7, 4)
ENSG00000121005(7, 2)
ENSG00000121766(7, 2)
ENSG00000121892(7, 3)
ENSG00000122515(7, 2)
ENSG00000122557(7, 3)
ENSG00000122824(7, 4)
ENSG00000123124(7, 3)
ENSG00000123472(7, 4)
ENSG00000123552(7, 5)
ENSG00000124098(7, 5)
ENSG00000124145(7, 2)
ENSG00000124491(7, 2)
ENSG00000124593(7, 2)
ENSG00000124762(7, 4)
ENSG00000124785(7, 3)
ENSG00000124795(7, 4)
ENSG00000125285(7, 3)
ENSG00000125354(7, 4)
ENSG00000125633(7, 2)
ENSG00000125810(7, 4)
ENSG00000125812(7, 3)
ENSG00000125814(7, 4)
ENSG00000126351(7, 2)
ENSG00000127526(7, 3)
ENSG00000127688(7, 4)
ENSG00000127838(7, 4)

ENSG00000127863(7, 3)
ENSG00000128191(7, 6)
ENSG00000128512(7, 5)
ENSG00000128965(7, 1)
ENSG00000129116(7, 2)
ENSG00000129250(7, 4)
ENSG00000129757(7, 6)
ENSG00000130202(7, 5)
ENSG00000130226(7, 3)
ENSG00000130287(7, 4)
ENSG00000130522(7, 3)
ENSG00000130675(7, 4)
ENSG00000130711(7, 2)
ENSG00000131148(7, 3)
ENSG00000131437(7, 3)
ENSG00000131475(7, 2)
ENSG00000131482(7, 3)
ENSG00000131508(7, 1)
ENSG00000131773(7, 3)
ENSG00000131788(7, 3)
ENSG00000132024(7, 1)
ENSG00000132405(7, 4)
ENSG00000132434(7, 2)
ENSG00000132471(7, 1)
ENSG00000133884(7, 5)
ENSG00000134698(7, 3)
ENSG00000134717(7, 3)
ENSG00000135119(7, 2)
ENSG00000135250(7, 3)
ENSG00000136153(7, 2)
ENSG00000137309(7, 3)
ENSG00000137727(7, 2)
ENSG00000137766(7, 2)
ENSG00000137845(7, 2)
ENSG00000138131(7, 3)
ENSG00000138867(7, 3)
ENSG00000138942(7, 3)
ENSG00000139116(7, 1)
ENSG00000139190(7, 3)
ENSG00000139218(7, 1)
ENSG00000139722(7, 3)
ENSG00000141140(7, 5)
ENSG00000141510(7, 3)
ENSG00000141522(7, 4)
ENSG00000142002(7, 2)
ENSG00000142303(7, 3)
ENSG00000143028(7, 2)
ENSG00000143363(7, 1)
ENSG00000143842(7, 2)
ENSG00000143952(7, 2)
ENSG00000144460(7, 1)
ENSG00000144681(7, 3)
ENSG00000144802(7, 2)
ENSG00000145390(7, 2)

ENSG00000145423(7, 2)
ENSG00000145730(7, 2)
ENSG00000146151(7, 4)
ENSG00000146278(7, 4)
ENSG00000146374(7, 2)
ENSG00000146834(7, 4)
ENSG00000147044(7, 1)
ENSG00000147655(7, 1)
ENSG00000148429(7, 4)
ENSG00000148572(7, 2)
ENSG00000149260(7, 4)
ENSG00000150527(7, 2)
ENSG00000150893(7, 5)
ENSG00000151012(7, 3)
ENSG00000151025(7, 3)
ENSG00000151079(7, 5)
ENSG00000151322(7, 4)
ENSG00000151694(7, 2)
ENSG00000152785(7, 4)
ENSG00000153207(7, 4)
ENSG00000153253(7, 5)
ENSG00000153823(7, 5)
ENSG00000154124(7, 4)
ENSG00000154146(7, 4)
ENSG00000154174(7, 5)
ENSG00000154478(7, 1)
ENSG00000154655(7, 3)
ENSG00000154945(7, 2)
ENSG00000155252(7, 3)
ENSG00000155508(7, 3)
ENSG00000155629(7, 2)
ENSG00000155849(7, 3)
ENSG00000155926(7, 2)
ENSG00000156427(7, 1)
ENSG00000156531(7, 3)
ENSG00000156603(7, 3)
ENSG00000156976(7, 1)
ENSG00000157766(7, 3)
ENSG00000157827(7, 4)
ENSG00000157851(7, 1)
ENSG00000158555(7, 2)
ENSG00000159173(7, 4)
ENSG00000159459(7, 6)
ENSG00000160145(7, 6)
ENSG00000160469(7, 3)
ENSG00000160741(7, 2)
ENSG00000161203(7, 1)
ENSG00000161940(7, 3)
ENSG00000162521(7, 4)
ENSG00000163126(7, 3)
ENSG00000163389(7, 4)
ENSG00000163412(7, 5)
ENSG00000163545(7, 3)
ENSG00000163625(7, 3)

ENSG00000163638(7, 4)
ENSG00000163743(7, 1)
ENSG00000163820(7, 3)
ENSG00000163888(7, 3)
ENSG00000163946(7, 3)
ENSG00000164093(7, 3)
ENSG00000164107(7, 4)
ENSG00000164151(7, 3)
ENSG00000164270(7, 4)
ENSG00000164379(7, 5)
ENSG00000164649(7, 2)
ENSG00000165006(7, 1)
ENSG00000165119(7, 2)
ENSG00000165124(7, 1)
ENSG00000165186(7, 2)
ENSG00000165244(7, 1)
ENSG00000165476(7, 3)
ENSG00000166068(7, 2)
ENSG00000166123(7, 1)
ENSG00000166128(7, 4)
ENSG00000166888(7, 1)
ENSG00000166900(7, 3)
ENSG00000167005(7, 2)
ENSG00000167085(7, 2)
ENSG00000167460(7, 2)
ENSG00000167711(7, 1)
ENSG00000167767(7, 4)
ENSG00000168546(7, 4)
ENSG00000168785(7, 2)
ENSG00000168795(7, 4)
ENSG00000169016(7, 6)
ENSG00000169432(7, 5)
ENSG00000169499(7, 4)
ENSG00000170004(7, 1)
ENSG00000170370(7, 1)
ENSG00000170915(7, 2)
ENSG00000170989(7, 3)
ENSG00000171885(7, 3)
ENSG00000172007(7, 2)
ENSG00000172020(7, 3)
ENSG00000172264(7, 3)
ENSG00000173281(7, 3)
ENSG00000173334(7, 3)
ENSG00000173451(7, 5)
ENSG00000173559(7, 2)
ENSG00000173653(7, 3)
ENSG00000173852(7, 3)
ENSG00000173926(7, 3)
ENSG00000173933(7, 3)
ENSG00000174307(7, 2)
ENSG00000175229(7, 3)
ENSG00000175264(7, 5)
ENSG00000175426(7, 3)
ENSG00000175662(7, 4)

ENSG00000176087(7, 3)
ENSG00000176422(7, 4)
ENSG00000176490(7, 4)
ENSG00000176974(7, 2)
ENSG00000177108(7, 3)
ENSG00000177485(7, 2)
ENSG00000177551(7, 2)
ENSG00000177807(7, 1)
ENSG00000178163(7, 1)
ENSG00000178403(7, 2)
ENSG00000178567(7, 1)
ENSG00000179431(7, 4)
ENSG00000179454(7, 3)
ENSG00000179632(7, 2)
ENSG00000179776(7, 3)
ENSG00000179922(7, 3)
ENSG00000180304(7, 1)
ENSG00000180488(7, 2)
ENSG00000180592(7, 5)
ENSG00000180861(7, 2)
ENSG00000181090(7, 1)
ENSG00000181788(7, 4)
ENSG00000182472(7, 2)
ENSG00000183317(7, 3)
ENSG00000184009(7, 2)
ENSG00000184205(7, 2)
ENSG00000184408(7, 2)
ENSG00000185352(7, 2)
ENSG00000185518(7, 5)
ENSG00000185896(7, 2)
ENSG00000186298(7, 5)
ENSG00000186575(7, 1)
ENSG00000187189(7, 2)
ENSG00000187323(7, 3)
ENSG00000187720(7, 3)
ENSG00000188001(7, 2)
ENSG00000196363(7, 4)
ENSG00000196517(7, 3)
ENSG00000196549(7, 2)
ENSG00000196591(7, 5)
ENSG00000196850(7, 4)
ENSG00000196961(7, 1)
ENSG00000197323(7, 1)
ENSG00000197451(7, 1)
ENSG00000197584(7, 5)
ENSG00000197705(7, 4)
ENSG00000198689(7, 4)
ENSG00000198898(7, 2)
ENSG00000198933(7, 3)
ENSG00000204070(7, 6)
ENSG00000204252(7, 1)
ENSG00000204271(7, 2)
ENSG00000204439(7, 4)
ENSG00000204580(7, 1)

ENSG00000204682(7, 2)
ENSG00000205189(7, 5)
ENSG00000213079(7, 2)
ENSG00000213463(7, 2)
ENSG00000213923(7, 4)
ENSG00000214882(7, 1)
ENSG00000215440(7, 2)
ENSG00000230989(7, 4)
ENSG00000001167(8, 3)
ENSG00000003056(8, 4)
ENSG00000006432(8, 3)
ENSG00000007545(8, 3)
ENSG00000010319(8, 4)
ENSG00000011021(8, 3)
ENSG00000013523(8, 3)
ENSG00000015592(8, 5)
ENSG00000018280(8, 4)
ENSG00000032444(8, 3)
ENSG00000047849(8, 2)
ENSG00000050628(8, 4)
ENSG00000054118(8, 3)
ENSG00000058262(8, 3)
ENSG00000059915(8, 2)
ENSG00000060069(8, 3)
ENSG00000063176(8, 4)
ENSG00000064042(8, 2)
ENSG00000065526(8, 3)
ENSG00000065609(8, 4)
ENSG00000067840(8, 5)
ENSG00000069011(8, 2)
ENSG00000069956(8, 3)
ENSG00000070366(8, 2)
ENSG00000071127(8, 5)
ENSG00000071189(8, 2)
ENSG00000073350(8, 3)
ENSG00000074317(8, 3)
ENSG00000075391(8, 1)
ENSG00000075785(8, 4)
ENSG00000078304(8, 2)
ENSG00000078403(8, 4)
ENSG00000079805(8, 3)
ENSG00000080503(8, 4)
ENSG00000084628(8, 2)
ENSG00000084764(8, 3)
ENSG00000089818(8, 2)
ENSG00000090061(8, 3)
ENSG00000090447(8, 1)
ENSG00000095485(8, 3)
ENSG00000095587(8, 4)
ENSG00000096717(8, 5)
ENSG00000100077(8, 4)
ENSG00000100084(8, 2)
ENSG00000100167(8, 4)
ENSG00000100345(8, 6)

ENSG00000100393(8, 4)
ENSG00000100644(8, 4)
ENSG00000101079(8, 5)
ENSG00000101306(8, 3)
ENSG00000101323(8, 3)
ENSG00000101782(8, 5)
ENSG00000101928(8, 3)
ENSG00000101974(8, 3)
ENSG00000102098(8, 4)
ENSG00000103196(8, 1)
ENSG00000103855(8, 2)
ENSG00000104067(8, 7)
ENSG00000104219(8, 5)
ENSG00000104419(8, 3)
ENSG00000105219(8, 6)
ENSG00000105438(8, 2)
ENSG00000105856(8, 5)
ENSG00000105976(8, 5)
ENSG00000105983(8, 1)
ENSG00000106299(8, 2)
ENSG00000106615(8, 3)
ENSG00000106635(8, 2)
ENSG00000107164(8, 4)
ENSG00000107854(8, 5)
ENSG00000107984(8, 4)
ENSG00000108654(8, 4)
ENSG00000108960(8, 5)
ENSG00000109084(8, 3)
ENSG00000109113(8, 5)
ENSG00000109158(8, 2)
ENSG00000109606(8, 3)
ENSG00000110693(8, 2)
ENSG00000110841(8, 1)
ENSG00000111424(8, 5)
ENSG00000111961(8, 3)
ENSG00000112232(8, 5)
ENSG00000112511(8, 3)
ENSG00000112562(8, 4)
ENSG00000112640(8, 3)
ENSG00000113369(8, 5)
ENSG00000113763(8, 5)
ENSG00000114209(8, 2)
ENSG00000114383(8, 3)
ENSG00000114850(8, 4)
ENSG00000115107(8, 4)
ENSG00000115112(8, 2)
ENSG00000115318(8, 3)
ENSG00000115902(8, 5)
ENSG00000115942(8, 2)
ENSG00000116133(8, 4)
ENSG00000118495(8, 4)
ENSG00000119314(8, 3)
ENSG00000119414(8, 3)
ENSG00000119614(8, 4)

ENSG00000119689(8, 3)
ENSG00000120805(8, 4)
ENSG00000121057(8, 2)
ENSG00000121481(8, 3)
ENSG00000123353(8, 2)
ENSG00000123643(8, 3)
ENSG00000123836(8, 2)
ENSG00000124104(8, 3)
ENSG00000124193(8, 2)
ENSG00000124201(8, 3)
ENSG00000124374(8, 4)
ENSG00000124743(8, 5)
ENSG00000125675(8, 4)
ENSG00000125676(8, 4)
ENSG00000128052(8, 3)
ENSG00000130150(8, 4)
ENSG00000130164(8, 2)
ENSG00000130294(8, 4)
ENSG00000130856(8, 4)
ENSG00000131196(8, 3)
ENSG00000132639(8, 5)
ENSG00000132821(8, 3)
ENSG00000133874(8, 3)
ENSG00000134324(8, 3)
ENSG00000134531(8, 2)
ENSG00000134644(8, 2)
ENSG00000135048(8, 4)
ENSG00000135341(8, 4)
ENSG00000135454(8, 2)
ENSG00000135622(8, 4)
ENSG00000135631(8, 2)
ENSG00000135821(8, 2)
ENSG00000135916(8, 3)
ENSG00000135932(8, 1)
ENSG00000136152(8, 4)
ENSG00000136231(8, 7)
ENSG00000136381(8, 1)
ENSG00000136720(8, 5)
ENSG00000136758(8, 5)
ENSG00000136842(8, 4)
ENSG00000136854(8, 1)
ENSG00000136935(8, 5)
ENSG00000137713(8, 4)
ENSG00000137819(8, 2)
ENSG00000137944(8, 2)
ENSG00000138185(8, 3)
ENSG00000138293(8, 3)
ENSG00000138675(8, 2)
ENSG00000138741(8, 2)
ENSG00000139496(8, 4)
ENSG00000139746(8, 2)
ENSG00000140332(8, 3)
ENSG00000140450(8, 2)
ENSG00000141232(8, 5)

ENSG00000141384(8, 2)
ENSG00000141639(8, 6)
ENSG00000142039(8, 3)
ENSG00000142961(8, 7)
ENSG00000143079(8, 5)
ENSG00000143252(8, 5)
ENSG00000143344(8, 2)
ENSG00000143390(8, 4)
ENSG00000143867(8, 6)
ENSG00000143870(8, 4)
ENSG00000143882(8, 3)
ENSG00000144642(8, 3)
ENSG00000144674(8, 3)
ENSG00000144791(8, 4)
ENSG00000145495(8, 2)
ENSG00000145703(8, 6)
ENSG00000145949(8, 1)
ENSG00000146072(8, 2)
ENSG00000146197(8, 4)
ENSG00000146535(8, 2)
ENSG00000146802(8, 5)
ENSG00000146963(8, 2)
ENSG00000147027(8, 1)
ENSG00000147246(8, 7)
ENSG00000147488(8, 3)
ENSG00000148400(8, 2)
ENSG00000148484(8, 4)
ENSG00000149182(8, 1)
ENSG00000149308(8, 4)
ENSG00000149577(8, 2)
ENSG00000149679(8, 4)
ENSG00000151491(8, 2)
ENSG00000152430(8, 5)
ENSG00000152760(8, 1)
ENSG00000152822(8, 4)
ENSG00000153879(8, 3)
ENSG00000153982(8, 5)
ENSG00000155744(8, 4)
ENSG00000155980(8, 4)
ENSG00000156875(8, 5)
ENSG00000156925(8, 5)
ENSG00000157216(8, 4)
ENSG00000158747(8, 3)
ENSG00000159082(8, 7)
ENSG00000159267(8, 2)
ENSG00000159346(8, 3)
ENSG00000160408(8, 3)
ENSG00000160460(8, 2)
ENSG00000161526(8, 3)
ENSG00000161847(8, 4)
ENSG00000162368(8, 4)
ENSG00000162706(8, 3)
ENSG00000162992(8, 3)
ENSG00000163348(8, 3)

ENSG00000163430(8, 4)
ENSG00000163512(8, 2)
ENSG00000163681(8, 1)
ENSG00000163719(8, 3)
ENSG00000164056(8, 3)
ENSG00000164197(8, 3)
ENSG00000164284(8, 2)
ENSG00000164574(8, 1)
ENSG00000165175(8, 4)
ENSG00000165195(8, 2)
ENSG00000165209(8, 5)
ENSG00000165322(8, 2)
ENSG00000165617(8, 5)
ENSG00000165731(8, 4)
ENSG00000165879(8, 3)
ENSG00000166260(8, 3)
ENSG00000166349(8, 3)
ENSG00000166575(8, 5)
ENSG00000166793(8, 5)
ENSG00000167182(8, 6)
ENSG00000167202(8, 4)
ENSG00000167371(8, 3)
ENSG00000167470(8, 2)
ENSG00000167770(8, 1)
ENSG00000167978(8, 3)
ENSG00000168539(8, 6)
ENSG00000168906(8, 4)
ENSG00000169032(8, 1)
ENSG00000170017(8, 2)
ENSG00000170345(8, 4)
ENSG00000170689(8, 4)
ENSG00000170873(8, 4)
ENSG00000171843(8, 3)
ENSG00000172059(8, 3)
ENSG00000173327(8, 5)
ENSG00000173402(8, 2)
ENSG00000174628(8, 4)
ENSG00000174996(8, 3)
ENSG00000175066(8, 4)
ENSG00000175727(8, 1)
ENSG00000177051(8, 4)
ENSG00000177098(8, 3)
ENSG00000177103(8, 1)
ENSG00000177374(8, 5)
ENSG00000177432(8, 5)
ENSG00000177542(8, 5)
ENSG00000177606(8, 5)
ENSG00000177614(8, 2)
ENSG00000177694(8, 4)
ENSG00000178691(8, 5)
ENSG00000179820(8, 5)
ENSG00000179841(8, 3)
ENSG00000180398(8, 7)
ENSG00000181449(8, 3)

ENSG00000184144(8, 4)
ENSG00000184254(8, 3)
ENSG00000184371(8, 3)
ENSG00000184454(8, 3)
ENSG00000184611(8, 6)
ENSG00000184792(8, 3)
ENSG00000185112(8, 4)
ENSG00000185305(8, 2)
ENSG00000185697(8, 1)
ENSG00000186111(8, 4)
ENSG00000187098(8, 4)
ENSG00000187140(8, 3)
ENSG00000187164(8, 5)
ENSG00000187325(8, 6)
ENSG00000187498(8, 3)
ENSG00000187595(8, 3)
ENSG00000187866(8, 3)
ENSG00000188783(8, 5)
ENSG00000189120(8, 6)
ENSG00000196368(8, 1)
ENSG00000196411(8, 3)
ENSG00000196588(8, 3)
ENSG00000197651(8, 1)
ENSG00000197724(8, 3)
ENSG00000197860(8, 6)
ENSG00000198060(8, 3)
ENSG00000198146(8, 2)
ENSG00000198162(8, 3)
ENSG00000198435(8, 2)
ENSG00000198791(8, 4)
ENSG00000198844(8, 3)
ENSG00000204160(8, 5)
ENSG00000204599(8, 4)
ENSG00000204673(8, 6)
ENSG00000204764(8, 1)
ENSG00000213240(8, 1)
ENSG00000213533(8, 7)
ENSG00000214753(8, 2)
ENSG00000218823(8, 5)
ENSG00000234545(8, 3)
ENSG00000242108(8, 3)
ENSG00000244462(8, 5)
ENSG00000253293(8, 4)
ENSG00000001084(9, 4)
ENSG00000005810(9, 2)
ENSG00000009830(9, 1)
ENSG00000033867(9, 3)
ENSG00000049540(9, 3)
ENSG00000049618(9, 3)
ENSG00000054967(9, 4)
ENSG00000055163(9, 3)
ENSG00000056487(9, 5)
ENSG00000056661(9, 6)
ENSG00000063978(9, 1)

ENSG00000064419(9, 4)
ENSG00000065883(9, 6)
ENSG00000068354(9, 3)
ENSG00000074755(9, 2)
ENSG00000080854(9, 5)
ENSG00000083168(9, 4)
ENSG00000087258(9, 3)
ENSG00000087274(9, 4)
ENSG00000088808(9, 2)
ENSG00000088832(9, 4)
ENSG00000092201(9, 4)
ENSG00000092203(9, 1)
ENSG00000097033(9, 3)
ENSG00000100036(9, 6)
ENSG00000100239(9, 4)
ENSG00000100311(9, 3)
ENSG00000101193(9, 4)
ENSG00000101216(9, 1)
ENSG00000101265(9, 3)
ENSG00000101331(9, 3)
ENSG00000101412(9, 3)
ENSG00000101938(9, 4)
ENSG00000102144(9, 1)
ENSG00000103034(9, 4)
ENSG00000103222(9, 5)
ENSG00000104154(9, 5)
ENSG00000104635(9, 4)
ENSG00000104722(9, 4)
ENSG00000104888(9, 4)
ENSG00000105711(9, 4)
ENSG00000106003(9, 3)
ENSG00000106004(9, 5)
ENSG00000106348(9, 2)
ENSG00000106460(9, 4)
ENSG00000106571(9, 3)
ENSG00000106665(9, 4)
ENSG00000106682(9, 3)
ENSG00000106948(9, 3)
ENSG00000107560(9, 7)
ENSG00000108433(9, 4)
ENSG00000108443(9, 8)
ENSG00000108511(9, 3)
ENSG00000109079(9, 2)
ENSG00000109919(9, 3)
ENSG00000110076(9, 4)
ENSG00000110107(9, 4)
ENSG00000110171(9, 4)
ENSG00000110675(9, 5)
ENSG00000110888(9, 3)
ENSG00000111432(9, 1)
ENSG00000111846(9, 4)
ENSG00000112033(9, 2)
ENSG00000112130(9, 5)
ENSG00000113356(9, 2)

ENSG00000114999(9, 5)
ENSG00000115159(9, 5)
ENSG00000115317(9, 3)
ENSG00000115825(9, 2)
ENSG00000115993(9, 2)
ENSG00000116584(9, 6)
ENSG00000116833(9, 4)
ENSG00000116984(9, 3)
ENSG00000117115(9, 5)
ENSG00000117222(9, 5)
ENSG00000117500(9, 5)
ENSG00000117676(9, 4)
ENSG00000118473(9, 3)
ENSG00000118496(9, 4)
ENSG00000118503(9, 3)
ENSG00000120029(9, 1)
ENSG00000120705(9, 3)
ENSG00000120913(9, 2)
ENSG00000121039(9, 4)
ENSG00000121104(9, 5)
ENSG00000122707(9, 3)
ENSG00000123104(9, 2)
ENSG00000123119(9, 4)
ENSG00000124126(9, 5)
ENSG00000124198(9, 4)
ENSG00000124203(9, 2)
ENSG00000124813(9, 4)
ENSG00000125084(9, 2)
ENSG00000125744(9, 2)
ENSG00000125851(9, 4)
ENSG00000126777(9, 3)
ENSG00000126822(9, 5)
ENSG00000127359(9, 7)
ENSG00000129675(9, 5)
ENSG00000130147(9, 6)
ENSG00000130669(9, 5)
ENSG00000130766(9, 5)
ENSG00000130962(9, 5)
ENSG00000132182(9, 7)
ENSG00000132485(9, 1)
ENSG00000132872(9, 4)
ENSG00000132964(9, 4)
ENSG00000132970(9, 5)
ENSG00000133318(9, 4)
ENSG00000133704(9, 4)
ENSG00000134121(9, 4)
ENSG00000134198(9, 2)
ENSG00000134287(9, 5)
ENSG00000134308(9, 4)
ENSG00000134508(9, 6)
ENSG00000135525(9, 4)
ENSG00000135632(9, 4)
ENSG00000135678(9, 6)
ENSG00000136193(9, 3)

ENSG00000136205(9, 3)
ENSG00000136535(9, 4)
ENSG00000136802(9, 5)
ENSG00000137393(9, 2)
ENSG00000137478(9, 4)
ENSG00000137872(9, 2)
ENSG00000137942(9, 5)
ENSG00000138036(9, 5)
ENSG00000138622(9, 4)
ENSG00000139505(9, 3)
ENSG00000140382(9, 2)
ENSG00000140403(9, 1)
ENSG00000143393(9, 2)
ENSG00000143761(9, 4)
ENSG00000144040(9, 6)
ENSG00000144597(9, 4)
ENSG00000144724(9, 4)
ENSG00000144730(9, 3)
ENSG00000145817(9, 4)
ENSG00000147036(9, 3)
ENSG00000147676(9, 3)
ENSG00000147912(9, 4)
ENSG00000148358(9, 4)
ENSG00000148672(9, 3)
ENSG00000148848(9, 4)
ENSG00000149547(9, 5)
ENSG00000149582(9, 2)
ENSG00000149658(9, 2)
ENSG00000150347(9, 5)
ENSG00000151623(9, 2)
ENSG00000152270(9, 4)
ENSG00000153786(9, 4)
ENSG00000153904(9, 3)
ENSG00000154640(9, 2)
ENSG00000155313(9, 6)
ENSG00000155592(9, 4)
ENSG00000155755(9, 4)
ENSG00000156467(9, 2)
ENSG00000156983(9, 3)
ENSG00000158966(9, 3)
ENSG00000160683(9, 2)
ENSG00000160691(9, 4)
ENSG00000161958(9, 3)
ENSG00000162437(9, 6)
ENSG00000162783(9, 1)
ENSG00000162980(9, 7)
ENSG00000163431(9, 2)
ENSG00000163596(9, 1)
ENSG00000163697(9, 6)
ENSG00000164045(9, 3)
ENSG00000164219(9, 4)
ENSG00000164796(9, 3)
ENSG00000164855(9, 3)
ENSG00000165660(9, 3)

ENSG00000165757(9, 5)
ENSG00000165782(9, 3)
ENSG00000165995(9, 5)
ENSG00000166016(9, 1)
ENSG00000166025(9, 3)
ENSG00000166257(9, 4)
ENSG00000166263(9, 6)
ENSG00000166483(9, 2)
ENSG00000167306(9, 4)
ENSG00000167566(9, 5)
ENSG00000167615(9, 5)
ENSG00000167693(9, 2)
ENSG00000167941(9, 7)
ENSG00000168172(9, 3)
ENSG00000168256(9, 3)
ENSG00000168300(9, 5)
ENSG00000168310(9, 4)
ENSG00000168395(9, 5)
ENSG00000169306(9, 1)
ENSG00000170647(9, 2)
ENSG00000170881(9, 5)
ENSG00000171533(9, 2)
ENSG00000171608(9, 5)
ENSG00000172348(9, 3)
ENSG00000172380(9, 5)
ENSG00000173801(9, 2)
ENSG00000174151(9, 3)
ENSG00000174672(9, 3)
ENSG00000174748(9, 3)
ENSG00000175348(9, 7)
ENSG00000175898(9, 4)
ENSG00000176204(9, 3)
ENSG00000176624(9, 3)
ENSG00000176769(9, 3)
ENSG00000176853(9, 6)
ENSG00000176884(9, 3)
ENSG00000178235(9, 4)
ENSG00000178878(9, 2)
ENSG00000179094(9, 4)
ENSG00000179270(9, 5)
ENSG00000179314(9, 4)
ENSG00000179813(9, 3)
ENSG00000179981(9, 4)
ENSG00000180447(9, 1)
ENSG00000181409(9, 2)
ENSG00000182150(9, 2)
ENSG00000182247(9, 4)
ENSG00000182446(9, 2)
ENSG00000182541(9, 3)
ENSG00000182580(9, 1)
ENSG00000182934(9, 3)
ENSG00000183576(9, 3)
ENSG00000183723(9, 2)
ENSG00000184307(9, 4)

ENSG00000185338(9, 1)
ENSG00000185634(9, 4)
ENSG00000185668(9, 3)
ENSG00000186834(9, 4)
ENSG00000188158(9, 4)
ENSG00000188706(9, 3)
ENSG00000188997(9, 4)
ENSG00000196177(9, 3)
ENSG00000196208(9, 2)
ENSG00000196277(9, 4)
ENSG00000196313(9, 5)
ENSG00000196470(9, 3)
ENSG00000197381(9, 4)
ENSG00000197757(9, 3)
ENSG00000197948(9, 2)
ENSG00000198363(9, 3)
ENSG00000198586(9, 5)
ENSG00000203879(9, 4)
ENSG00000205937(9, 1)
ENSG00000206053(9, 3)
ENSG00000223380(9, 3)
ENSG00000239264(9, 4)
ENSG00000239306(9, 1)
ENSG00000243156(9, 3)
ENSG00000003989(10, 4)
ENSG00000005020(10, 3)
ENSG00000007866(10, 2)
ENSG00000011009(10, 6)
ENSG00000022840(10, 4)
ENSG00000029364(10, 3)
ENSG00000034053(10, 4)
ENSG00000040531(10, 3)
ENSG00000048471(10, 3)
ENSG00000048707(10, 4)
ENSG00000050438(10, 4)
ENSG00000056277(10, 4)
ENSG00000058866(10, 4)
ENSG00000060656(10, 1)
ENSG00000062725(10, 4)
ENSG00000063587(10, 4)
ENSG00000066382(10, 5)
ENSG00000067191(10, 6)
ENSG00000068305(10, 2)
ENSG00000071242(10, 6)
ENSG00000071246(10, 5)
ENSG00000072657(10, 8)
ENSG00000074416(10, 6)
ENSG00000076513(10, 1)
ENSG00000076554(10, 5)
ENSG00000077458(10, 6)
ENSG00000085831(10, 4)
ENSG00000086570(10, 5)
ENSG00000088247(10, 8)
ENSG00000091039(10, 6)

ENSG00000092607(10, 4)
ENSG00000093000(10, 4)
ENSG00000100027(10, 6)
ENSG00000100106(10, 2)
ENSG00000100461(10, 7)
ENSG00000100605(10, 3)
ENSG00000101557(10, 4)
ENSG00000101945(10, 3)
ENSG00000102302(10, 4)
ENSG00000102962(10, 2)
ENSG00000103326(10, 8)
ENSG00000103460(10, 5)
ENSG00000104756(10, 5)
ENSG00000105767(10, 5)
ENSG00000106459(10, 5)
ENSG00000107863(10, 5)
ENSG00000108406(10, 1)
ENSG00000109066(10, 3)
ENSG00000109861(10, 4)
ENSG00000111371(10, 5)
ENSG00000111913(10, 3)
ENSG00000111962(10, 1)
ENSG00000113300(10, 4)
ENSG00000113580(10, 5)
ENSG00000113739(10, 5)
ENSG00000114450(10, 6)
ENSG00000115183(10, 5)
ENSG00000115540(10, 3)
ENSG00000115652(10, 4)
ENSG00000115875(10, 5)
ENSG00000115935(10, 5)
ENSG00000116106(10, 3)
ENSG00000116396(10, 5)
ENSG00000117713(10, 3)
ENSG00000120549(10, 5)
ENSG00000122482(10, 1)
ENSG00000123728(10, 3)
ENSG00000124120(10, 4)
ENSG00000125170(10, 2)
ENSG00000125503(10, 3)
ENSG00000125820(10, 3)
ENSG00000125952(10, 3)
ENSG00000126950(10, 3)
ENSG00000127947(10, 3)
ENSG00000131236(10, 8)
ENSG00000131795(10, 4)
ENSG00000132535(10, 5)
ENSG00000133056(10, 3)
ENSG00000134109(10, 3)
ENSG00000134294(10, 5)
ENSG00000135090(10, 6)
ENSG00000135472(10, 1)
ENSG00000136383(10, 4)
ENSG00000136451(10, 4)

ENSG00000136859(10, 7)
ENSG00000137269(10, 4)
ENSG00000138041(10, 2)
ENSG00000138411(10, 3)
ENSG00000138468(10, 2)
ENSG00000138735(10, 3)
ENSG00000140577(10, 3)
ENSG00000140873(10, 3)
ENSG00000140945(10, 4)
ENSG00000141750(10, 2)
ENSG00000141867(10, 6)
ENSG00000142453(10, 4)
ENSG00000142634(10, 3)
ENSG00000142798(10, 3)
ENSG00000143153(10, 2)
ENSG00000143418(10, 4)
ENSG00000143702(10, 4)
ENSG00000143772(10, 4)
ENSG00000144233(10, 3)
ENSG00000144320(10, 6)
ENSG00000144355(10, 5)
ENSG00000145284(10, 3)
ENSG00000145808(10, 5)
ENSG00000145824(10, 6)
ENSG00000146285(10, 6)
ENSG00000147140(10, 4)
ENSG00000148218(10, 3)
ENSG00000150594(10, 3)
ENSG00000150625(10, 4)
ENSG00000151458(10, 3)
ENSG00000151502(10, 8)
ENSG00000151553(10, 3)
ENSG00000151726(10, 4)
ENSG00000152527(10, 3)
ENSG00000152642(10, 6)
ENSG00000152782(10, 5)
ENSG00000153046(10, 9)
ENSG00000153560(10, 4)
ENSG00000153914(10, 3)
ENSG00000155189(10, 4)
ENSG00000155961(10, 2)
ENSG00000156453(10, 3)
ENSG00000157107(10, 3)
ENSG00000157152(10, 4)
ENSG00000157782(10, 8)
ENSG00000158186(10, 4)
ENSG00000158480(10, 4)
ENSG00000158615(10, 6)
ENSG00000161533(10, 2)
ENSG00000162236(10, 8)
ENSG00000162302(10, 6)
ENSG00000162415(10, 5)
ENSG00000162613(10, 3)
ENSG00000162695(10, 6)

ENSG00000163104(10, 4)
ENSG00000163428(10, 1)
ENSG00000163513(10, 3)
ENSG00000163788(10, 4)
ENSG00000163939(10, 4)
ENSG00000164588(10, 4)
ENSG00000164823(10, 3)
ENSG00000165219(10, 5)
ENSG00000166181(10, 2)
ENSG00000166435(10, 2)
ENSG00000166579(10, 4)
ENSG00000166974(10, 4)
ENSG00000167191(10, 3)
ENSG00000167680(10, 4)
ENSG00000168283(10, 6)
ENSG00000168575(10, 4)
ENSG00000168591(10, 3)
ENSG00000168743(10, 3)
ENSG00000168916(10, 3)
ENSG00000168936(10, 2)
ENSG00000169439(10, 4)
ENSG00000169813(10, 2)
ENSG00000170091(10, 5)
ENSG00000170248(10, 4)
ENSG00000170525(10, 5)
ENSG00000170616(10, 5)
ENSG00000170802(10, 2)
ENSG00000171444(10, 5)
ENSG00000172057(10, 3)
ENSG00000172613(10, 4)
ENSG00000172660(10, 4)
ENSG00000172757(10, 3)
ENSG00000172869(10, 4)
ENSG00000172985(10, 3)
ENSG00000173210(10, 3)
ENSG00000174903(10, 2)
ENSG00000175220(10, 3)
ENSG00000175497(10, 4)
ENSG00000176619(10, 5)
ENSG00000176986(10, 6)
ENSG00000177000(10, 2)
ENSG00000177034(10, 3)
ENSG00000177469(10, 2)
ENSG00000178074(10, 3)
ENSG00000179889(10, 6)
ENSG00000180543(10, 5)
ENSG00000180957(10, 4)
ENSG00000180998(10, 4)
ENSG00000181773(10, 4)
ENSG00000183454(10, 4)
ENSG00000183873(10, 6)
ENSG00000184545(10, 4)
ENSG00000184640(10, 4)
ENSG00000185739(10, 7)

ENSG00000186629(10, 3)
ENSG00000186868(10, 4)
ENSG00000187672(10, 5)
ENSG00000188549(10, 3)
ENSG00000188582(10, 5)
ENSG00000188613(10, 2)
ENSG00000196083(10, 2)
ENSG00000196233(10, 5)
ENSG00000196428(10, 7)
ENSG00000196586(10, 5)
ENSG00000198648(10, 7)
ENSG00000198719(10, 3)
ENSG00000198925(10, 4)
ENSG00000204231(10, 2)
ENSG00000204442(10, 7)
ENSG00000204842(10, 3)
ENSG00000205726(10, 4)
ENSG00000213626(10, 5)
ENSG00000221890(10, 3)
ENSG00000001629(11, 3)
ENSG00000001631(11, 4)
ENSG00000004142(11, 1)
ENSG00000004399(11, 7)
ENSG00000005073(11, 2)
ENSG00000006125(11, 7)
ENSG00000008300(11, 6)
ENSG00000009413(11, 1)
ENSG00000010244(11, 5)
ENSG00000012048(11, 3)
ENSG00000012963(11, 7)
ENSG00000044574(11, 4)
ENSG00000054793(11, 6)
ENSG00000055917(11, 1)
ENSG00000059804(11, 1)
ENSG00000065989(11, 4)
ENSG00000067842(11, 5)
ENSG00000070159(11, 4)
ENSG00000070961(11, 4)
ENSG00000071967(11, 3)
ENSG00000073282(11, 4)
ENSG00000073670(11, 5)
ENSG00000075426(11, 7)
ENSG00000077092(11, 5)
ENSG00000077454(11, 5)
ENSG00000077809(11, 3)
ENSG00000078114(11, 6)
ENSG00000087053(11, 5)
ENSG00000091542(11, 3)
ENSG00000092051(11, 3)
ENSG00000092148(11, 3)
ENSG00000095015(11, 5)
ENSG00000100034(11, 7)
ENSG00000100105(11, 5)
ENSG00000100376(11, 6)

ENSG00000101019(11, 3)
ENSG00000101126(11, 2)
ENSG00000101236(11, 5)
ENSG00000101337(11, 5)
ENSG00000101892(11, 1)
ENSG00000102287(11, 3)
ENSG00000102606(11, 3)
ENSG00000103365(11, 4)
ENSG00000103888(11, 4)
ENSG00000104517(11, 5)
ENSG00000105738(11, 6)
ENSG00000106366(11, 4)
ENSG00000107518(11, 2)
ENSG00000108219(11, 5)
ENSG00000108375(11, 3)
ENSG00000108828(11, 6)
ENSG00000108840(11, 3)
ENSG00000108946(11, 3)
ENSG00000109956(11, 3)
ENSG00000110048(11, 2)
ENSG00000110851(11, 3)
ENSG00000110881(11, 4)
ENSG00000110906(11, 5)
ENSG00000111912(11, 7)
ENSG00000112081(11, 3)
ENSG00000112149(11, 2)
ENSG00000113070(11, 4)
ENSG00000115844(11, 6)
ENSG00000116285(11, 3)
ENSG00000117597(11, 6)
ENSG00000118733(11, 5)
ENSG00000120685(11, 2)
ENSG00000122367(11, 4)
ENSG00000122644(11, 4)
ENSG00000122756(11, 3)
ENSG00000123505(11, 4)
ENSG00000124222(11, 4)
ENSG00000124440(11, 4)
ENSG00000125107(11, 4)
ENSG00000125753(11, 6)
ENSG00000126070(11, 7)
ENSG00000126464(11, 1)
ENSG00000127329(11, 7)
ENSG00000128266(11, 4)
ENSG00000128268(11, 4)
ENSG00000129422(11, 5)
ENSG00000130340(11, 2)
ENSG00000130402(11, 2)
ENSG00000131459(11, 8)
ENSG00000131626(11, 4)
ENSG00000131759(11, 5)
ENSG00000132466(11, 2)
ENSG00000133606(11, 3)
ENSG00000133935(11, 4)

ENSG00000134152(11, 7)
ENSG00000135144(11, 6)
ENSG00000135213(11, 7)
ENSG00000136111(11, 4)
ENSG00000136158(11, 5)
ENSG00000137094(11, 4)
ENSG00000137414(11, 3)
ENSG00000137460(11, 2)
ENSG00000139826(11, 6)
ENSG00000140406(11, 4)
ENSG00000141540(11, 4)
ENSG00000142188(11, 6)
ENSG00000143067(11, 6)
ENSG00000143217(11, 5)
ENSG00000143401(11, 4)
ENSG00000143845(11, 5)
ENSG00000143970(11, 4)
ENSG00000144285(11, 4)
ENSG00000144560(11, 5)
ENSG00000144749(11, 7)
ENSG00000144909(11, 5)
ENSG00000146005(11, 3)
ENSG00000146826(11, 5)
ENSG00000147144(11, 4)
ENSG00000148110(11, 3)
ENSG00000148842(11, 5)
ENSG00000149428(11, 6)
ENSG00000150776(11, 8)
ENSG00000151240(11, 4)
ENSG00000152223(11, 2)
ENSG00000153113(11, 1)
ENSG00000153310(11, 5)
ENSG00000153561(11, 3)
ENSG00000153944(11, 3)
ENSG00000154734(11, 4)
ENSG00000155511(11, 2)
ENSG00000156218(11, 4)
ENSG00000156642(11, 6)
ENSG00000156650(11, 5)
ENSG00000157227(11, 4)
ENSG00000157680(11, 6)
ENSG00000157916(11, 5)
ENSG00000158352(11, 5)
ENSG00000158467(11, 5)
ENSG00000158796(11, 2)
ENSG00000160293(11, 3)
ENSG00000162409(11, 7)
ENSG00000162545(11, 3)
ENSG00000163501(11, 3)
ENSG00000164024(11, 6)
ENSG00000164983(11, 5)
ENSG00000165861(11, 2)
ENSG00000166831(11, 5)
ENSG00000166881(11, 6)

ENSG00000166908(11, 6)
ENSG00000167522(11, 1)
ENSG00000167588(11, 5)
ENSG00000168763(11, 5)
ENSG00000169047(11, 4)
ENSG00000169760(11, 4)
ENSG00000169855(11, 3)
ENSG00000169967(11, 2)
ENSG00000171282(11, 3)
ENSG00000171368(11, 5)
ENSG00000171992(11, 7)
ENSG00000172534(11, 2)
ENSG00000172578(11, 5)
ENSG00000173726(11, 2)
ENSG00000174576(11, 4)
ENSG00000175785(11, 5)
ENSG00000175938(11, 4)
ENSG00000176788(11, 5)
ENSG00000177764(11, 6)
ENSG00000177981(11, 7)
ENSG00000178562(11, 5)
ENSG00000179151(11, 5)
ENSG00000179195(11, 5)
ENSG00000181291(11, 5)
ENSG00000181588(11, 5)
ENSG00000181856(11, 2)
ENSG00000182010(11, 2)
ENSG00000182968(11, 4)
ENSG00000183580(11, 1)
ENSG00000184557(11, 7)
ENSG00000185924(11, 5)
ENSG00000186197(11, 4)
ENSG00000188177(11, 3)
ENSG00000188636(11, 5)
ENSG00000188647(11, 5)
ENSG00000188687(11, 1)
ENSG00000189159(11, 5)
ENSG00000196396(11, 5)
ENSG00000196935(11, 2)
ENSG00000197081(11, 4)
ENSG00000197461(11, 4)
ENSG00000198517(11, 4)
ENSG00000198663(11, 3)
ENSG00000204262(11, 2)
ENSG00000221914(11, 7)
ENSG00000222040(11, 4)
ENSG00000240849(11, 7)
ENSG00000006062(12, 5)
ENSG00000009954(12, 5)
ENSG00000011275(12, 3)
ENSG00000033050(12, 5)
ENSG00000049130(12, 5)
ENSG00000049769(12, 4)
ENSG00000062598(12, 3)

ENSG00000066629(12, 6)
ENSG00000067048(12, 4)
ENSG00000068308(12, 5)
ENSG00000070182(12, 5)
ENSG00000070413(12, 6)
ENSG00000072121(12, 4)
ENSG00000075275(12, 6)
ENSG00000078747(12, 4)
ENSG00000084234(12, 5)
ENSG00000085365(12, 6)
ENSG00000088305(12, 6)
ENSG00000096746(12, 2)
ENSG00000099904(12, 1)
ENSG00000100321(12, 3)
ENSG00000100426(12, 2)
ENSG00000100439(12, 5)
ENSG00000101384(12, 6)
ENSG00000101417(12, 5)
ENSG00000101695(12, 3)
ENSG00000101746(12, 7)
ENSG00000103241(12, 2)
ENSG00000104313(12, 5)
ENSG00000104442(12, 5)
ENSG00000104643(12, 5)
ENSG00000105656(12, 6)
ENSG00000105662(12, 7)
ENSG00000105698(12, 2)
ENSG00000107263(12, 5)
ENSG00000107779(12, 4)
ENSG00000108587(12, 3)
ENSG00000108821(12, 7)
ENSG00000110315(12, 8)
ENSG00000112972(12, 8)
ENSG00000113391(12, 6)
ENSG00000113658(12, 2)
ENSG00000114796(12, 6)
ENSG00000115616(12, 4)
ENSG00000119042(12, 7)
ENSG00000119682(12, 8)
ENSG00000119778(12, 5)
ENSG00000120137(12, 2)
ENSG00000124181(12, 7)
ENSG00000124571(12, 6)
ENSG00000125398(12, 4)
ENSG00000125629(12, 6)
ENSG00000125817(12, 8)
ENSG00000126091(12, 3)
ENSG00000127022(12, 3)
ENSG00000128011(12, 5)
ENSG00000128016(12, 5)
ENSG00000128590(12, 6)
ENSG00000128645(12, 6)
ENSG00000129244(12, 1)
ENSG00000130584(12, 3)

ENSG00000131873(12, 6)
ENSG00000132002(12, 5)
ENSG00000132003(12, 7)
ENSG00000133216(12, 7)
ENSG00000133639(12, 4)
ENSG00000134001(12, 6)
ENSG00000134250(12, 3)
ENSG00000135083(12, 4)
ENSG00000135960(12, 5)
ENSG00000136295(12, 7)
ENSG00000136574(12, 8)
ENSG00000136754(12, 3)
ENSG00000137815(12, 4)
ENSG00000137962(12, 4)
ENSG00000138107(12, 6)
ENSG00000138336(12, 4)
ENSG00000138834(12, 7)
ENSG00000139289(12, 2)
ENSG00000139364(12, 3)
ENSG00000140044(12, 4)
ENSG00000141380(12, 8)
ENSG00000141837(12, 8)
ENSG00000142700(12, 2)
ENSG00000144567(12, 5)
ENSG00000144668(12, 3)
ENSG00000144711(12, 4)
ENSG00000145685(12, 3)
ENSG00000145934(12, 2)
ENSG00000146006(12, 2)
ENSG00000146457(12, 6)
ENSG00000146463(12, 6)
ENSG00000146477(12, 5)
ENSG00000150938(12, 6)
ENSG00000151150(12, 7)
ENSG00000151247(12, 3)
ENSG00000151967(12, 5)
ENSG00000152284(12, 3)
ENSG00000152558(12, 8)
ENSG00000152749(12, 3)
ENSG00000152767(12, 5)
ENSG00000153006(12, 4)
ENSG00000153250(12, 8)
ENSG00000153814(12, 4)
ENSG00000154222(12, 3)
ENSG00000155096(12, 3)
ENSG00000155097(12, 4)
ENSG00000156395(12, 3)
ENSG00000157933(12, 5)
ENSG00000158528(12, 4)
ENSG00000158859(12, 4)
ENSG00000160967(12, 2)
ENSG00000162378(12, 6)
ENSG00000163041(12, 1)
ENSG00000163171(12, 2)

ENSG00000163328(12, 5)
ENSG00000163812(12, 5)
ENSG00000164985(12, 4)
ENSG00000165868(12, 6)
ENSG00000165891(12, 10)
ENSG00000166159(12, 4)
ENSG00000168209(12, 7)
ENSG00000168610(12, 5)
ENSG00000168646(12, 2)
ENSG00000168724(12, 2)
ENSG00000168758(12, 9)
ENSG00000168874(12, 4)
ENSG00000169282(12, 4)
ENSG00000169758(12, 5)
ENSG00000169762(12, 6)
ENSG00000169933(12, 4)
ENSG00000170011(12, 5)
ENSG00000170832(12, 5)
ENSG00000171388(12, 4)
ENSG00000171914(12, 3)
ENSG00000172201(12, 4)
ENSG00000173894(12, 4)
ENSG00000174705(12, 5)
ENSG00000176406(12, 6)
ENSG00000177283(12, 5)
ENSG00000177468(12, 4)
ENSG00000177853(12, 4)
ENSG00000178695(12, 2)
ENSG00000179241(12, 2)
ENSG00000181751(12, 4)
ENSG00000185008(12, 3)
ENSG00000185532(12, 3)
ENSG00000185585(12, 6)
ENSG00000185650(12, 3)
ENSG00000185811(12, 5)
ENSG00000187772(12, 7)
ENSG00000188483(12, 6)
ENSG00000197535(12, 5)
ENSG00000198252(12, 3)
ENSG00000198952(12, 4)
ENSG00000198964(12, 7)
ENSG00000204084(12, 3)
ENSG00000211448(12, 5)
ENSG00000213390(12, 8)
ENSG00000218336(12, 3)
ENSG00000238227(12, 3)
ENSG00000253304(12, 7)
ENSG00000004848(13, 2)
ENSG00000011405(13, 5)
ENSG00000011523(13, 4)
ENSG00000016082(13, 7)
ENSG00000019144(13, 6)
ENSG00000023287(13, 5)
ENSG00000039560(13, 2)

ENSG00000047644(13, 5)
ENSG00000049759(13, 7)
ENSG00000057657(13, 5)
ENSG00000058272(13, 6)
ENSG00000061273(13, 4)
ENSG00000064999(13, 5)
ENSG00000065060(13, 7)
ENSG00000065135(13, 8)
ENSG00000065809(13, 7)
ENSG00000066084(13, 6)
ENSG00000067533(13, 6)
ENSG00000068650(13, 3)
ENSG00000071564(13, 2)
ENSG00000075035(13, 8)
ENSG00000075240(13, 5)
ENSG00000082293(13, 4)
ENSG00000087299(13, 7)
ENSG00000089159(13, 7)
ENSG00000090060(13, 5)
ENSG00000092871(13, 8)
ENSG00000100811(13, 4)
ENSG00000101189(13, 3)
ENSG00000102003(13, 6)
ENSG00000102870(13, 2)
ENSG00000104331(13, 3)
ENSG00000104490(13, 4)
ENSG00000105085(13, 2)
ENSG00000105221(13, 7)
ENSG00000105464(13, 5)
ENSG00000105556(13, 8)
ENSG00000106089(13, 8)
ENSG00000107742(13, 8)
ENSG00000108349(13, 3)
ENSG00000108424(13, 4)
ENSG00000108774(13, 4)
ENSG00000109089(13, 8)
ENSG00000109189(13, 8)
ENSG00000110237(13, 5)
ENSG00000110422(13, 7)
ENSG00000111110(13, 5)
ENSG00000111269(13, 7)
ENSG00000112144(13, 8)
ENSG00000113575(13, 9)
ENSG00000113712(13, 3)
ENSG00000114857(13, 2)
ENSG00000115170(13, 3)
ENSG00000115677(13, 8)
ENSG00000116237(13, 7)
ENSG00000117394(13, 4)
ENSG00000119335(13, 3)
ENSG00000119927(13, 4)
ENSG00000120742(13, 4)
ENSG00000121388(13, 6)
ENSG00000122565(13, 3)

ENSG00000122786(13, 4)
ENSG00000123066(13, 4)
ENSG00000123684(13, 5)
ENSG00000124782(13, 5)
ENSG00000124920(13, 3)
ENSG00000125447(13, 4)
ENSG00000125740(13, 3)
ENSG00000125818(13, 4)
ENSG00000126814(13, 4)
ENSG00000127946(13, 7)
ENSG00000130829(13, 5)
ENSG00000131653(13, 6)
ENSG00000131791(13, 7)
ENSG00000132142(13, 3)
ENSG00000133193(13, 5)
ENSG00000133401(13, 3)
ENSG00000134278(13, 8)
ENSG00000135111(13, 6)
ENSG00000135373(13, 6)
ENSG00000135482(13, 6)
ENSG00000135655(13, 6)
ENSG00000135956(13, 4)
ENSG00000136653(13, 5)
ENSG00000137776(13, 4)
ENSG00000138166(13, 7)
ENSG00000138386(13, 5)
ENSG00000140199(13, 4)
ENSG00000141699(13, 4)
ENSG00000143127(13, 4)
ENSG00000143321(13, 2)
ENSG00000143442(13, 6)
ENSG00000143797(13, 3)
ENSG00000144579(13, 4)
ENSG00000145362(13, 5)
ENSG00000146122(13, 7)
ENSG00000147100(13, 4)
ENSG00000152056(13, 8)
ENSG00000153989(13, 5)
ENSG00000156687(13, 6)
ENSG00000157450(13, 5)
ENSG00000157470(13, 6)
ENSG00000157500(13, 5)
ENSG00000160199(13, 5)
ENSG00000160216(13, 7)
ENSG00000161202(13, 6)
ENSG00000162231(13, 8)
ENSG00000162981(13, 4)
ENSG00000163694(13, 2)
ENSG00000164756(13, 4)
ENSG00000166889(13, 3)
ENSG00000166949(13, 4)
ENSG00000167130(13, 6)
ENSG00000167491(13, 7)
ENSG00000168246(13, 5)

ENSG00000168280(13, 3)
ENSG00000169291(13, 5)
ENSG00000169410(13, 6)
ENSG00000169783(13, 7)
ENSG00000171723(13, 7)
ENSG00000172432(13, 5)
ENSG00000172461(13, 6)
ENSG00000173020(13, 4)
ENSG00000174282(13, 4)
ENSG00000174839(13, 8)
ENSG00000178764(13, 5)
ENSG00000179134(13, 2)
ENSG00000179833(13, 5)
ENSG00000180263(13, 7)
ENSG00000181852(13, 4)
ENSG00000181915(13, 4)
ENSG00000182158(13, 3)
ENSG00000183044(13, 6)
ENSG00000184588(13, 8)
ENSG00000187123(13, 1)
ENSG00000187147(13, 4)
ENSG00000188322(13, 6)
ENSG00000189319(13, 5)
ENSG00000196376(13, 6)
ENSG00000196498(13, 5)
ENSG00000196526(13, 5)
ENSG00000196792(13, 2)
ENSG00000197386(13, 6)
ENSG00000197622(13, 5)
ENSG00000197702(13, 5)
ENSG00000198055(13, 9)
ENSG00000198087(13, 6)
ENSG00000198142(13, 2)
ENSG00000198963(13, 8)
ENSG00000204569(13, 6)
ENSG00000241839(13, 5)
ENSG00000004455(14, 9)
ENSG00000006047(14, 8)
ENSG00000007372(14, 4)
ENSG00000020129(14, 7)
ENSG00000034063(14, 7)
ENSG00000035403(14, 7)
ENSG00000054965(14, 8)
ENSG00000062485(14, 2)
ENSG00000062650(14, 7)
ENSG00000064195(14, 4)
ENSG00000065970(14, 2)
ENSG00000073921(14, 7)
ENSG00000074054(14, 4)
ENSG00000075213(14, 4)
ENSG00000075539(14, 5)
ENSG00000077147(14, 7)
ENSG00000078295(14, 5)
ENSG00000080845(14, 7)

ENSG00000081377(14, 7)
ENSG00000085185(14, 4)
ENSG00000089486(14, 7)
ENSG00000095203(14, 7)
ENSG00000100364(14, 6)
ENSG00000100596(14, 5)
ENSG00000102081(14, 4)
ENSG00000102531(14, 7)
ENSG00000102678(14, 6)
ENSG00000105576(14, 5)
ENSG00000108510(14, 4)
ENSG00000108819(14, 6)
ENSG00000109046(14, 5)
ENSG00000109265(14, 6)
ENSG00000110497(14, 4)
ENSG00000111785(14, 6)
ENSG00000112782(14, 7)
ENSG00000113558(14, 3)
ENSG00000113758(14, 3)
ENSG00000114098(14, 5)
ENSG00000116273(14, 8)
ENSG00000116473(14, 7)
ENSG00000116731(14, 7)
ENSG00000117155(14, 7)
ENSG00000118363(14, 5)
ENSG00000118515(14, 5)
ENSG00000118946(14, 4)
ENSG00000119669(14, 5)
ENSG00000121741(14, 3)
ENSG00000123388(14, 7)
ENSG00000124164(14, 6)
ENSG00000125386(14, 3)
ENSG00000125875(14, 4)
ENSG00000125977(14, 5)
ENSG00000126016(14, 5)
ENSG00000128585(14, 3)
ENSG00000128923(14, 2)
ENSG00000129515(14, 7)
ENSG00000130224(14, 7)
ENSG00000130699(14, 7)
ENSG00000131504(14, 2)
ENSG00000131711(14, 5)
ENSG00000132361(14, 4)
ENSG00000134046(14, 5)
ENSG00000134323(14, 2)
ENSG00000134758(14, 6)
ENSG00000135968(14, 3)
ENSG00000137992(14, 2)
ENSG00000138668(14, 5)
ENSG00000138696(14, 6)
ENSG00000138760(14, 7)
ENSG00000139200(14, 4)
ENSG00000139266(14, 3)
ENSG00000139514(14, 4)

ENSG00000139793(14, 4)
ENSG00000141068(14, 2)
ENSG00000141582(14, 1)
ENSG00000143384(14, 6)
ENSG00000143862(14, 5)
ENSG00000144043(14, 6)
ENSG00000144524(14, 5)
ENSG00000147457(14, 4)
ENSG00000147475(14, 2)
ENSG00000151117(14, 8)
ENSG00000151338(14, 3)
ENSG00000152092(14, 6)
ENSG00000152291(14, 8)
ENSG00000154122(14, 5)
ENSG00000155760(14, 6)
ENSG00000156508(14, 2)
ENSG00000156599(14, 4)
ENSG00000157306(14, 5)
ENSG00000157617(14, 7)
ENSG00000158158(14, 5)
ENSG00000158813(14, 8)
ENSG00000159873(14, 7)
ENSG00000162670(14, 1)
ENSG00000162923(14, 6)
ENSG00000165588(14, 9)
ENSG00000166716(14, 7)
ENSG00000167193(14, 9)
ENSG00000167580(14, 6)
ENSG00000167971(14, 8)
ENSG00000168036(14, 3)
ENSG00000168710(14, 6)
ENSG00000169330(14, 5)
ENSG00000171634(14, 3)
ENSG00000172197(14, 6)
ENSG00000173786(14, 7)
ENSG00000174502(14, 10)
ENSG00000177380(14, 5)
ENSG00000178031(14, 5)
ENSG00000178177(14, 6)
ENSG00000180182(14, 2)
ENSG00000180370(14, 2)
ENSG00000182175(14, 4)
ENSG00000183283(14, 7)
ENSG00000183775(14, 7)
ENSG00000183878(14, 6)
ENSG00000183960(14, 6)
ENSG00000186472(14, 8)
ENSG00000186480(14, 6)
ENSG00000187068(14, 7)
ENSG00000187416(14, 4)
ENSG00000188312(14, 5)
ENSG00000196581(14, 3)
ENSG00000196932(14, 8)
ENSG00000197122(14, 8)

ENSG00000198315(14, 5)
ENSG00000198732(14, 3)
ENSG00000198794(14, 6)
ENSG00000198830(14, 5)
ENSG00000198961(14, 4)
ENSG00000204138(14, 7)
ENSG00000204619(14, 7)
ENSG00000205056(14, 4)
ENSG00000206026(14, 6)
ENSG00000219438(14, 3)
ENSG00000241878(14, 4)
ENSG00000004897(15, 5)
ENSG00000006831(15, 4)
ENSG00000007944(15, 7)
ENSG00000037965(15, 4)
ENSG00000043591(15, 6)
ENSG00000053108(15, 6)
ENSG00000071051(15, 8)
ENSG00000072952(15, 6)
ENSG00000079332(15, 6)
ENSG00000081019(15, 7)
ENSG00000081052(15, 4)
ENSG00000082153(15, 5)
ENSG00000083307(15, 5)
ENSG00000090905(15, 6)
ENSG00000095139(15, 7)
ENSG00000095787(15, 4)
ENSG00000096060(15, 6)
ENSG00000099250(15, 7)
ENSG00000100234(15, 5)
ENSG00000100346(15, 6)
ENSG00000101298(15, 6)
ENSG00000102385(15, 6)
ENSG00000104205(15, 7)
ENSG00000106244(15, 5)
ENSG00000114062(15, 3)
ENSG00000115760(15, 6)
ENSG00000116675(15, 5)
ENSG00000118160(15, 6)
ENSG00000119396(15, 8)
ENSG00000119403(15, 6)
ENSG00000124212(15, 4)
ENSG00000125149(15, 9)
ENSG00000126882(15, 7)
ENSG00000128567(15, 6)
ENSG00000128595(15, 9)
ENSG00000129993(15, 6)
ENSG00000131381(15, 10)
ENSG00000132478(15, 6)
ENSG00000133065(15, 7)
ENSG00000133121(15, 8)
ENSG00000133657(15, 8)
ENSG00000135127(15, 4)
ENSG00000135823(15, 7)

ENSG00000135945(15, 5)
ENSG00000136531(15, 5)
ENSG00000136874(15, 6)
ENSG00000137843(15, 8)
ENSG00000138604(15, 6)
ENSG00000140538(15, 7)
ENSG00000141279(15, 5)
ENSG00000141668(15, 5)
ENSG00000143398(15, 3)
ENSG00000143437(15, 8)
ENSG00000143494(15, 6)
ENSG00000145016(15, 5)
ENSG00000145349(15, 7)
ENSG00000146216(15, 8)
ENSG00000147130(15, 4)
ENSG00000147526(15, 6)
ENSG00000148704(15, 3)
ENSG00000151893(15, 5)
ENSG00000152377(15, 6)
ENSG00000154118(15, 5)
ENSG00000156273(15, 7)
ENSG00000156675(15, 6)
ENSG00000156959(15, 4)
ENSG00000157350(15, 9)
ENSG00000157557(15, 8)
ENSG00000158195(15, 7)
ENSG00000158887(15, 5)
ENSG00000159202(15, 7)
ENSG00000160551(15, 9)
ENSG00000161638(15, 5)
ENSG00000161791(15, 6)
ENSG00000162298(15, 5)
ENSG00000162631(15, 1)
ENSG00000163297(15, 6)
ENSG00000164040(15, 6)
ENSG00000164603(15, 6)
ENSG00000164916(15, 5)
ENSG00000165023(15, 4)
ENSG00000165280(15, 5)
ENSG00000166224(15, 4)
ENSG00000166963(15, 4)
ENSG00000167074(15, 7)
ENSG00000167081(15, 8)
ENSG00000168748(15, 7)
ENSG00000170027(15, 10)
ENSG00000171621(15, 6)
ENSG00000171862(15, 7)
ENSG00000171988(15, 12)
ENSG00000172493(15, 8)
ENSG00000172819(15, 6)
ENSG00000174469(15, 7)
ENSG00000174574(15, 3)
ENSG00000174611(15, 4)
ENSG00000175105(15, 5)

ENSG00000179912(15, 5)
ENSG00000180354(15, 9)
ENSG00000182732(15, 7)
ENSG00000183955(15, 3)
ENSG00000184402(15, 9)
ENSG00000186073(15, 6)
ENSG00000186660(15, 6)
ENSG00000186951(15, 7)
ENSG00000187239(15, 4)
ENSG00000189007(15, 6)
ENSG00000196132(15, 4)
ENSG00000196787(15, 5)
ENSG00000196814(15, 11)
ENSG00000197959(15, 6)
ENSG00000198720(15, 5)
ENSG00000198785(15, 5)
ENSG00000198915(15, 4)
ENSG00000206538(15, 8)
ENSG00000215397(15, 10)
ENSG00000004948(16, 7)
ENSG00000005007(16, 5)
ENSG00000005889(16, 3)
ENSG00000007341(16, 3)
ENSG00000009694(16, 5)
ENSG00000012232(16, 4)
ENSG00000020633(16, 5)
ENSG00000029725(16, 2)
ENSG00000031823(16, 6)
ENSG00000032219(16, 8)
ENSG00000048828(16, 4)
ENSG00000063322(16, 5)
ENSG00000064651(16, 7)
ENSG00000075711(16, 9)
ENSG00000076604(16, 6)
ENSG00000076641(16, 4)
ENSG00000085832(16, 9)
ENSG00000088538(16, 7)
ENSG00000090615(16, 4)
ENSG00000092068(16, 9)
ENSG00000100335(16, 7)
ENSG00000101577(16, 8)
ENSG00000101752(16, 3)
ENSG00000102069(16, 5)
ENSG00000102230(16, 7)
ENSG00000104885(16, 10)
ENSG00000105722(16, 5)
ENSG00000105997(16, 1)
ENSG00000107566(16, 6)
ENSG00000107882(16, 4)
ENSG00000108582(16, 8)
ENSG00000111737(16, 7)
ENSG00000112902(16, 8)
ENSG00000113657(16, 7)
ENSG00000114127(16, 6)

ENSG00000116016(16, 3)
ENSG00000117569(16, 5)
ENSG00000119048(16, 3)
ENSG00000119402(16, 6)
ENSG00000120963(16, 5)
ENSG00000123983(16, 4)
ENSG00000125618(16, 4)
ENSG00000126603(16, 6)
ENSG00000128917(16, 6)
ENSG00000129204(16, 7)
ENSG00000129351(16, 5)
ENSG00000130382(16, 7)
ENSG00000132341(16, 8)
ENSG00000132640(16, 4)
ENSG00000132854(16, 4)
ENSG00000134215(16, 8)
ENSG00000135333(16, 6)
ENSG00000137203(16, 9)
ENSG00000137343(16, 6)
ENSG00000137710(16, 7)
ENSG00000139946(16, 6)
ENSG00000140937(16, 5)
ENSG00000142949(16, 5)
ENSG00000143013(16, 5)
ENSG00000143515(16, 7)
ENSG00000143776(16, 4)
ENSG00000143924(16, 6)
ENSG00000148339(16, 6)
ENSG00000148719(16, 2)
ENSG00000152192(16, 7)
ENSG00000153094(16, 6)
ENSG00000153922(16, 11)
ENSG00000154127(16, 9)
ENSG00000154727(16, 6)
ENSG00000157087(16, 7)
ENSG00000157103(16, 6)
ENSG00000157890(16, 5)
ENSG00000159658(16, 4)
ENSG00000159784(16, 6)
ENSG00000162430(16, 7)
ENSG00000163714(16, 5)
ENSG00000164684(16, 6)
ENSG00000166266(16, 6)
ENSG00000168813(16, 8)
ENSG00000169604(16, 3)
ENSG00000170113(16, 6)
ENSG00000170348(16, 3)
ENSG00000170743(16, 6)
ENSG00000171456(16, 7)
ENSG00000173064(16, 4)
ENSG00000173166(16, 6)
ENSG00000173473(16, 10)
ENSG00000173542(16, 8)
ENSG00000174871(16, 8)

ENSG00000175073(16, 7)
ENSG00000175582(16, 3)
ENSG00000175866(16, 3)
ENSG00000175893(16, 5)
ENSG00000176274(16, 6)
ENSG00000177511(16, 5)
ENSG00000178951(16, 6)
ENSG00000179348(16, 5)
ENSG00000180667(16, 9)
ENSG00000181826(16, 7)
ENSG00000183023(16, 5)
ENSG00000183242(16, 2)
ENSG00000183496(16, 2)
ENSG00000185551(16, 3)
ENSG00000185963(16, 6)
ENSG00000186469(16, 7)
ENSG00000186479(16, 6)
ENSG00000186591(16, 2)
ENSG00000187391(16, 5)
ENSG00000196700(16, 7)
ENSG00000198382(16, 9)
ENSG00000198792(16, 6)
ENSG00000203727(16, 4)
ENSG00000204310(16, 6)
ENSG00000214575(16, 8)
ENSG00000244405(16, 7)
ENSG00000005379(17, 9)
ENSG00000011105(17, 6)
ENSG00000057019(17, 7)
ENSG00000069345(17, 4)
ENSG00000070808(17, 6)
ENSG00000071537(17, 7)
ENSG00000074211(17, 4)
ENSG00000076984(17, 7)
ENSG00000082898(17, 8)
ENSG00000092199(17, 5)
ENSG00000101871(17, 7)
ENSG00000102189(17, 9)
ENSG00000102786(17, 6)
ENSG00000103449(17, 2)
ENSG00000105327(17, 5)
ENSG00000106771(17, 9)
ENSG00000108830(17, 5)
ENSG00000109654(17, 7)
ENSG00000110931(17, 5)
ENSG00000111752(17, 6)
ENSG00000112419(17, 4)
ENSG00000112531(17, 7)
ENSG00000112715(17, 9)
ENSG00000114251(17, 5)
ENSG00000114573(17, 7)
ENSG00000114770(17, 10)
ENSG00000115419(17, 7)
ENSG00000115464(17, 8)

ENSG00000115884(17, 4)
ENSG00000115904(17, 5)
ENSG00000119661(17, 6)
ENSG00000122863(17, 7)
ENSG00000123572(17, 8)
ENSG00000124507(17, 6)
ENSG00000130338(17, 7)
ENSG00000130477(17, 4)
ENSG00000131507(17, 5)
ENSG00000132005(17, 7)
ENSG00000132254(17, 8)
ENSG00000134986(17, 9)
ENSG00000135486(17, 4)
ENSG00000138443(17, 4)
ENSG00000139352(17, 7)
ENSG00000139436(17, 8)
ENSG00000139915(17, 8)
ENSG00000139998(17, 8)
ENSG00000142149(17, 6)
ENSG00000143061(17, 7)
ENSG00000145555(17, 5)
ENSG00000151612(17, 8)
ENSG00000153234(17, 7)
ENSG00000153339(17, 6)
ENSG00000156860(17, 5)
ENSG00000159164(17, 5)
ENSG00000159692(17, 3)
ENSG00000159792(17, 6)
ENSG00000160685(17, 12)
ENSG00000162407(17, 9)
ENSG00000163083(17, 5)
ENSG00000163113(17, 10)
ENSG00000163486(17, 6)
ENSG00000163684(17, 6)
ENSG00000164741(17, 5)
ENSG00000165434(17, 9)
ENSG00000166147(17, 5)
ENSG00000166619(17, 12)
ENSG00000168779(17, 8)
ENSG00000169184(17, 6)
ENSG00000171105(17, 7)
ENSG00000171552(17, 7)
ENSG00000174136(17, 4)
ENSG00000175161(17, 5)
ENSG00000178217(17, 6)
ENSG00000178996(17, 8)
ENSG00000179361(17, 8)
ENSG00000180008(17, 8)
ENSG00000183741(17, 8)
ENSG00000184185(17, 6)
ENSG00000184226(17, 6)
ENSG00000185950(17, 8)
ENSG00000187109(17, 7)
ENSG00000187172(17, 5)

ENSG00000187555(17, 4)
ENSG00000196632(17, 5)
ENSG00000196712(17, 9)
ENSG00000196821(17, 8)
ENSG00000198160(17, 7)
ENSG00000198216(17, 9)
ENSG00000198478(17, 6)
ENSG00000206560(17, 4)
ENSG00000005238(18, 6)
ENSG00000007237(18, 8)
ENSG00000010818(18, 6)
ENSG00000011454(18, 6)
ENSG00000017260(18, 4)
ENSG00000033327(18, 6)
ENSG00000047056(18, 10)
ENSG00000070087(18, 11)
ENSG00000070882(18, 8)
ENSG00000073711(18, 9)
ENSG00000074657(18, 7)
ENSG00000075891(18, 6)
ENSG00000084652(18, 6)
ENSG00000091527(18, 8)
ENSG00000100207(18, 7)
ENSG00000100664(18, 5)
ENSG00000100796(18, 5)
ENSG00000102572(18, 7)
ENSG00000102935(18, 7)
ENSG00000103319(18, 11)
ENSG00000104081(18, 7)
ENSG00000106610(18, 7)
ENSG00000106780(18, 4)
ENSG00000108468(18, 8)
ENSG00000108947(18, 5)
ENSG00000108953(18, 7)
ENSG00000109132(18, 6)
ENSG00000110436(18, 5)
ENSG00000111249(18, 7)
ENSG00000113594(18, 7)
ENSG00000116138(18, 5)
ENSG00000116254(18, 9)
ENSG00000118900(18, 6)
ENSG00000121671(18, 10)
ENSG00000121871(18, 6)
ENSG00000122733(18, 5)
ENSG00000123360(18, 4)
ENSG00000123364(18, 4)
ENSG00000124406(18, 5)
ENSG00000127080(18, 6)
ENSG00000127483(18, 8)
ENSG00000130054(18, 8)
ENSG00000130749(18, 7)
ENSG00000131089(18, 3)
ENSG00000131368(18, 8)
ENSG00000132953(18, 5)

ENSG00000134030(18, 4)
ENSG00000135108(18, 6)
ENSG00000135913(18, 10)
ENSG00000136490(18, 8)
ENSG00000136944(18, 7)
ENSG00000137573(18, 6)
ENSG00000137802(18, 7)
ENSG00000138175(18, 10)
ENSG00000138639(18, 6)
ENSG00000139438(18, 9)
ENSG00000140262(18, 10)
ENSG00000140264(18, 10)
ENSG00000143590(18, 6)
ENSG00000144619(18, 4)
ENSG00000147724(18, 9)
ENSG00000148158(18, 8)
ENSG00000150764(18, 4)
ENSG00000154642(18, 6)
ENSG00000155545(18, 9)
ENSG00000156466(18, 8)
ENSG00000157514(18, 7)
ENSG00000161013(18, 6)
ENSG00000162402(18, 8)
ENSG00000163349(18, 5)
ENSG00000163539(18, 6)
ENSG00000163947(18, 11)
ENSG00000166402(18, 10)
ENSG00000169247(18, 4)
ENSG00000169714(18, 4)
ENSG00000172292(18, 10)
ENSG00000172845(18, 7)
ENSG00000173575(18, 8)
ENSG00000174306(18, 4)
ENSG00000176105(18, 7)
ENSG00000176165(18, 6)
ENSG00000176658(18, 6)
ENSG00000177888(18, 10)
ENSG00000177889(18, 7)
ENSG00000180628(18, 7)
ENSG00000180875(18, 7)
ENSG00000182095(18, 4)
ENSG00000183715(18, 5)
ENSG00000183864(18, 6)
ENSG00000184677(18, 7)
ENSG00000185070(18, 9)
ENSG00000186918(18, 7)
ENSG00000189403(18, 4)
ENSG00000196504(18, 9)
ENSG00000196776(18, 9)
ENSG00000197045(18, 10)
ENSG00000197121(18, 7)
ENSG00000198053(18, 7)
ENSG00000198265(18, 7)
ENSG00000198739(18, 5)

ENSG00000211455(18, 8)
ENSG00000002834(19, 7)
ENSG00000003137(19, 8)
ENSG00000005884(19, 7)
ENSG00000011304(19, 8)
ENSG00000011451(19, 7)
ENSG00000017797(19, 7)
ENSG00000025293(19, 7)
ENSG00000029363(19, 4)
ENSG00000030419(19, 9)
ENSG00000035862(19, 4)
ENSG00000061987(19, 10)
ENSG00000066739(19, 6)
ENSG00000068615(19, 10)
ENSG00000070831(19, 7)
ENSG00000070886(19, 8)
ENSG00000071575(19, 6)
ENSG00000074590(19, 6)
ENSG00000079432(19, 10)
ENSG00000081842(19, 5)
ENSG00000100226(19, 6)
ENSG00000100625(19, 8)
ENSG00000105245(19, 9)
ENSG00000106829(19, 7)
ENSG00000106852(19, 7)
ENSG00000108352(19, 8)
ENSG00000116132(19, 6)
ENSG00000116205(19, 4)
ENSG00000116983(19, 7)
ENSG00000116991(19, 6)
ENSG00000119946(19, 10)
ENSG00000120071(19, 4)
ENSG00000120948(19, 8)
ENSG00000122042(19, 7)
ENSG00000123933(19, 10)
ENSG00000124151(19, 4)
ENSG00000124313(19, 10)
ENSG00000124789(19, 8)
ENSG00000126767(19, 7)
ENSG00000129007(19, 5)
ENSG00000129245(19, 9)
ENSG00000133083(19, 3)
ENSG00000134207(19, 8)
ENSG00000134569(19, 8)
ENSG00000134686(19, 10)
ENSG00000135298(19, 7)
ENSG00000135677(19, 8)
ENSG00000136237(19, 7)
ENSG00000136826(19, 7)
ENSG00000138757(19, 7)
ENSG00000139083(19, 3)
ENSG00000143641(19, 4)
ENSG00000145012(19, 7)
ENSG00000148248(19, 8)

ENSG00000149177(19, 6)
ENSG00000151692(19, 12)
ENSG00000157064(19, 6)
ENSG00000157110(19, 3)
ENSG00000158470(19, 7)
ENSG00000159842(19, 5)
ENSG00000160014(19, 12)
ENSG00000160097(19, 9)
ENSG00000162104(19, 9)
ENSG00000162552(19, 7)
ENSG00000164619(19, 6)
ENSG00000166272(19, 10)
ENSG00000168264(19, 8)
ENSG00000169851(19, 6)
ENSG00000169891(19, 7)
ENSG00000170275(19, 6)
ENSG00000172765(19, 8)
ENSG00000173598(19, 10)
ENSG00000174233(19, 6)
ENSG00000178585(19, 7)
ENSG00000184602(19, 8)
ENSG00000185129(19, 7)
ENSG00000186350(19, 8)
ENSG00000196182(19, 10)
ENSG00000198700(19, 6)
ENSG00000198740(19, 10)
ENSG00000198948(19, 7)
ENSG00000204962(19, 5)
ENSG00000204963(19, 5)
ENSG00000204965(19, 5)
ENSG00000204967(19, 5)
ENSG00000204969(19, 5)
ENSG00000204970(19, 5)
ENSG00000217128(19, 7)
ENSG00000239389(19, 5)
ENSG00000243232(19, 5)
ENSG00000248383(19, 5)
ENSG00000249158(19, 5)
ENSG00000250120(19, 5)
ENSG00000251664(19, 5)
ENSG00000254108(19, 5)
ENSG00000007968(20, 9)
ENSG00000019995(20, 7)
ENSG00000028528(20, 6)
ENSG00000063046(20, 3)
ENSG00000064309(20, 10)
ENSG00000065308(20, 6)
ENSG00000067798(20, 9)
ENSG00000074356(20, 6)
ENSG00000077274(20, 6)
ENSG00000079308(20, 6)
ENSG00000081189(20, 6)
ENSG00000083896(20, 4)
ENSG00000096070(20, 6)

ENSG00000102053(20, 11)
ENSG00000102921(20, 9)
ENSG00000106609(20, 10)
ENSG00000107341(20, 9)
ENSG00000108239(20, 7)
ENSG00000108829(20, 5)
ENSG00000109332(20, 8)
ENSG00000110042(20, 9)
ENSG00000112139(20, 9)
ENSG00000112183(20, 6)
ENSG00000112319(20, 10)
ENSG00000114019(20, 9)
ENSG00000117298(20, 8)
ENSG00000120251(20, 7)
ENSG00000122966(20, 6)
ENSG00000123612(20, 7)
ENSG00000125966(20, 6)
ENSG00000128342(20, 5)
ENSG00000128594(20, 9)
ENSG00000130635(20, 11)
ENSG00000132463(20, 9)
ENSG00000132718(20, 5)
ENSG00000134780(20, 11)
ENSG00000135862(20, 7)
ENSG00000136504(20, 11)
ENSG00000137522(20, 7)
ENSG00000138650(20, 5)
ENSG00000138759(20, 6)
ENSG00000141429(20, 4)
ENSG00000143126(20, 6)
ENSG00000145780(20, 13)
ENSG00000146938(20, 7)
ENSG00000147324(20, 10)
ENSG00000149948(20, 4)
ENSG00000150712(20, 9)
ENSG00000151320(20, 8)
ENSG00000152520(20, 8)
ENSG00000154736(20, 10)
ENSG00000155111(20, 8)
ENSG00000161021(20, 9)
ENSG00000163110(20, 7)
ENSG00000164938(20, 8)
ENSG00000166960(20, 4)
ENSG00000169914(20, 11)
ENSG00000169992(20, 7)
ENSG00000170374(20, 8)
ENSG00000170776(20, 7)
ENSG00000171532(20, 7)
ENSG00000171540(20, 5)
ENSG00000172943(20, 7)
ENSG00000173153(20, 3)
ENSG00000173706(20, 7)
ENSG00000175087(20, 9)
ENSG00000185052(20, 11)

ENSG00000186174(20, 7)
ENSG00000188994(20, 7)
ENSG00000196782(20, 8)
ENSG00000196950(20, 6)
ENSG00000197283(20, 8)
ENSG00000198846(20, 9)
ENSG00000204116(20, 9)
ENSG00000214114(20, 12)
ENSG00000215712(20, 5)
ENSG00000008196(21, 6)
ENSG00000029993(21, 7)
ENSG00000048540(21, 7)
ENSG00000065150(21, 6)
ENSG00000068383(21, 7)
ENSG00000071073(21, 12)
ENSG00000072501(21, 5)
ENSG00000075420(21, 6)
ENSG00000077157(21, 9)
ENSG00000078699(21, 7)
ENSG00000079841(21, 13)
ENSG00000083799(21, 9)
ENSG00000090975(21, 10)
ENSG00000095637(21, 8)
ENSG00000100221(21, 11)
ENSG00000100731(21, 10)
ENSG00000103064(21, 10)
ENSG00000106070(21, 8)
ENSG00000107443(21, 11)
ENSG00000108312(21, 5)
ENSG00000110880(21, 14)
ENSG00000115355(21, 7)
ENSG00000118432(21, 8)
ENSG00000119283(21, 8)
ENSG00000120093(21, 8)
ENSG00000120709(21, 11)
ENSG00000124171(21, 11)
ENSG00000124486(21, 12)
ENSG00000125834(21, 4)
ENSG00000127585(21, 7)
ENSG00000135999(21, 4)
ENSG00000140285(21, 6)
ENSG00000142784(21, 6)
ENSG00000143324(21, 9)
ENSG00000145864(21, 6)
ENSG00000146267(21, 9)
ENSG00000147162(21, 10)
ENSG00000158863(21, 9)
ENSG00000163577(21, 8)
ENSG00000164463(21, 8)
ENSG00000164970(21, 5)
ENSG00000165029(21, 13)
ENSG00000165659(21, 11)
ENSG00000166398(21, 6)
ENSG00000166439(21, 8)

ENSG00000166860(21, 10)
ENSG00000167106(21, 10)
ENSG00000169122(21, 9)
ENSG00000170558(21, 7)
ENSG00000170871(21, 8)
ENSG00000171617(21, 5)
ENSG00000173273(21, 7)
ENSG00000177200(21, 6)
ENSG00000180530(21, 6)
ENSG00000183117(21, 6)
ENSG00000183307(21, 10)
ENSG00000183508(21, 10)
ENSG00000184863(21, 7)
ENSG00000225190(21, 15)
ENSG00000251322(21, 9)
ENSG00000007402(22, 8)
ENSG00000015171(22, 6)
ENSG00000028277(22, 9)
ENSG00000058668(22, 10)
ENSG00000060982(22, 11)
ENSG00000077684(22, 11)
ENSG00000078140(22, 7)
ENSG00000101665(22, 8)
ENSG00000101972(22, 6)
ENSG00000103056(22, 9)
ENSG00000103494(22, 6)
ENSG00000105855(22, 9)
ENSG00000106723(22, 7)
ENSG00000109171(22, 9)
ENSG00000110400(22, 9)
ENSG00000111605(22, 8)
ENSG00000113532(22, 9)
ENSG00000114554(22, 7)
ENSG00000114648(22, 12)
ENSG00000116406(22, 11)
ENSG00000116990(22, 9)
ENSG00000118707(22, 6)
ENSG00000121904(22, 10)
ENSG00000125848(22, 13)
ENSG00000130940(22, 7)
ENSG00000132334(22, 10)
ENSG00000135074(22, 10)
ENSG00000137075(22, 10)
ENSG00000139146(22, 8)
ENSG00000146021(22, 13)
ENSG00000147202(22, 9)
ENSG00000151718(22, 8)
ENSG00000156299(22, 8)
ENSG00000157404(22, 7)
ENSG00000157954(22, 7)
ENSG00000161813(22, 9)
ENSG00000162367(22, 8)
ENSG00000162522(22, 9)
ENSG00000165323(22, 7)

ENSG00000165495(22, 8)
ENSG00000166206(22, 7)
ENSG00000168672(22, 8)
ENSG00000169641(22, 8)
ENSG00000171940(22, 11)
ENSG00000172915(22, 8)
ENSG00000173889(22, 5)
ENSG00000174099(22, 7)
ENSG00000174238(22, 8)
ENSG00000175029(22, 9)
ENSG00000179051(22, 10)
ENSG00000182963(22, 8)
ENSG00000196090(22, 13)
ENSG00000196361(22, 8)
ENSG00000197879(22, 12)
ENSG00000198369(22, 7)
ENSG00000198743(22, 5)
ENSG00000198947(22, 9)
ENSG00000010017(23, 10)
ENSG00000011347(23, 12)
ENSG00000020181(23, 8)
ENSG00000029534(23, 11)
ENSG00000060237(23, 10)
ENSG00000065320(23, 7)
ENSG00000065491(23, 11)
ENSG00000067208(23, 9)
ENSG00000067715(23, 10)
ENSG00000077782(23, 9)
ENSG00000082641(23, 7)
ENSG00000095539(23, 15)
ENSG00000099194(23, 11)
ENSG00000105887(23, 9)
ENSG00000106799(23, 10)
ENSG00000109184(23, 12)
ENSG00000109586(23, 7)
ENSG00000112624(23, 11)
ENSG00000112739(23, 6)
ENSG00000114853(23, 10)
ENSG00000117523(23, 9)
ENSG00000120837(23, 3)
ENSG00000121297(23, 9)
ENSG00000134243(23, 7)
ENSG00000134852(23, 8)
ENSG00000139163(23, 10)
ENSG00000141034(23, 8)
ENSG00000143469(23, 6)
ENSG00000143850(23, 13)
ENSG00000143933(23, 6)
ENSG00000144228(23, 10)
ENSG00000145391(23, 10)
ENSG00000148053(23, 4)
ENSG00000150593(23, 7)
ENSG00000152465(23, 6)
ENSG00000153201(23, 8)

ENSG00000153885(23, 9)
ENSG00000154001(23, 7)
ENSG00000157654(23, 8)
ENSG00000160714(23, 7)
ENSG00000164330(23, 11)
ENSG00000164494(23, 8)
ENSG00000165389(23, 5)
ENSG00000169862(23, 8)
ENSG00000170624(23, 9)
ENSG00000172795(23, 12)
ENSG00000174963(23, 5)
ENSG00000176749(23, 9)
ENSG00000183762(23, 6)
ENSG00000183826(23, 11)
ENSG00000184675(23, 5)
ENSG00000185722(23, 13)
ENSG00000189308(23, 5)
ENSG00000198668(23, 7)
ENSG00000204217(23, 14)
ENSG00000241978(23, 8)
ENSG00000008952(24, 8)
ENSG00000070367(24, 11)
ENSG00000071054(24, 13)
ENSG00000072134(24, 9)
ENSG00000073792(24, 10)
ENSG00000079102(24, 8)
ENSG00000101966(24, 13)
ENSG00000112320(24, 8)
ENSG00000117682(24, 7)
ENSG00000120162(24, 14)
ENSG00000121989(24, 13)
ENSG00000124225(24, 8)
ENSG00000134954(24, 9)
ENSG00000135387(24, 7)
ENSG00000138069(24, 6)
ENSG00000138081(24, 8)
ENSG00000139651(24, 10)
ENSG00000140564(24, 13)
ENSG00000140992(24, 13)
ENSG00000141150(24, 12)
ENSG00000141568(24, 10)
ENSG00000142599(24, 12)
ENSG00000145087(24, 10)
ENSG00000162734(24, 13)
ENSG00000162761(24, 8)
ENSG00000164418(24, 9)
ENSG00000166200(24, 9)
ENSG00000170677(24, 9)
ENSG00000177311(24, 8)
ENSG00000177885(24, 9)
ENSG00000182568(24, 6)
ENSG00000182836(24, 13)
ENSG00000185651(24, 9)
ENSG00000185728(24, 7)

ENSG00000186260(24, 8)
ENSG00000198561(24, 12)
ENSG00000204576(24, 14)
ENSG00000007168(25, 11)
ENSG00000036549(25, 11)
ENSG00000054523(25, 8)
ENSG00000072422(25, 9)
ENSG00000082458(25, 10)
ENSG00000099364(25, 11)
ENSG00000101367(25, 10)
ENSG00000101445(25, 12)
ENSG00000108262(25, 12)
ENSG00000110321(25, 6)
ENSG00000110987(25, 10)
ENSG00000115461(25, 6)
ENSG00000116852(25, 11)
ENSG00000119906(25, 13)
ENSG00000122566(25, 9)
ENSG00000123411(25, 8)
ENSG00000133985(25, 12)
ENSG00000138430(25, 8)
ENSG00000159388(25, 9)
ENSG00000160058(25, 8)
ENSG00000160445(25, 10)
ENSG00000164742(25, 9)
ENSG00000168807(25, 10)
ENSG00000171408(25, 10)
ENSG00000172350(25, 6)
ENSG00000172939(25, 10)
ENSG00000174013(25, 14)
ENSG00000175215(25, 13)
ENSG00000181467(25, 9)
ENSG00000182771(25, 8)
ENSG00000182831(25, 6)
ENSG00000184481(25, 11)
ENSG00000197106(25, 10)
ENSG00000198815(25, 8)
ENSG00000204103(25, 4)
ENSG00000204120(25, 7)
ENSG00000204304(25, 9)
ENSG00000205269(25, 13)
ENSG00000060749(26, 9)
ENSG00000072274(26, 10)
ENSG00000072803(26, 13)
ENSG00000100307(26, 8)
ENSG00000100647(26, 15)
ENSG00000101109(26, 9)
ENSG00000101849(26, 12)
ENSG00000103044(26, 11)
ENSG00000107249(26, 7)
ENSG00000107338(26, 6)
ENSG00000109685(26, 13)
ENSG00000109819(26, 6)
ENSG00000117114(26, 6)

ENSG00000118454(26, 10)
ENSG00000124783(26, 7)
ENSG00000127314(26, 8)
ENSG00000136868(26, 8)
ENSG00000137770(26, 8)
ENSG00000141503(26, 7)
ENSG00000149289(26, 9)
ENSG00000154917(26, 6)
ENSG00000159216(26, 10)
ENSG00000160007(26, 8)
ENSG00000161654(26, 9)
ENSG00000162738(26, 13)
ENSG00000163251(26, 6)
ENSG00000164091(26, 11)
ENSG00000165194(26, 9)
ENSG00000166167(26, 8)
ENSG00000166833(26, 12)
ENSG00000168175(26, 11)
ENSG00000171735(26, 8)
ENSG00000173218(26, 10)
ENSG00000175155(26, 8)
ENSG00000176994(26, 12)
ENSG00000179456(26, 9)
ENSG00000187678(26, 12)
ENSG00000220205(26, 12)
ENSG00000007047(27, 12)
ENSG00000008083(27, 12)
ENSG00000065243(27, 12)
ENSG00000088812(27, 10)
ENSG00000088854(27, 10)
ENSG00000089902(27, 11)
ENSG00000091831(27, 5)
ENSG00000092964(27, 13)
ENSG00000108018(27, 9)
ENSG00000112245(27, 9)
ENSG00000112658(27, 10)
ENSG00000124209(27, 12)
ENSG00000129292(27, 10)
ENSG00000133026(27, 8)
ENSG00000133703(27, 9)
ENSG00000134247(27, 12)
ENSG00000134982(27, 15)
ENSG00000136848(27, 11)
ENSG00000145819(27, 10)
ENSG00000150907(27, 8)
ENSG00000154229(27, 14)
ENSG00000156011(27, 8)
ENSG00000157106(27, 7)
ENSG00000163637(27, 7)
ENSG00000164778(27, 10)
ENSG00000168769(27, 6)
ENSG00000171435(27, 6)
ENSG00000174197(27, 6)
ENSG00000186566(27, 9)

ENSG00000221978(27, 10)
ENSG00000011114(28, 8)
ENSG00000015153(28, 9)
ENSG00000060709(28, 8)
ENSG00000067141(28, 13)
ENSG00000070614(28, 9)
ENSG00000078804(28, 12)
ENSG00000079387(28, 11)
ENSG00000099942(28, 7)
ENSG00000101558(28, 5)
ENSG00000101935(28, 10)
ENSG00000102753(28, 7)
ENSG00000108001(28, 8)
ENSG00000128655(28, 10)
ENSG00000131242(28, 10)
ENSG00000134532(28, 5)
ENSG00000134853(28, 16)
ENSG00000140557(28, 9)
ENSG00000144218(28, 7)
ENSG00000144677(28, 9)
ENSG00000148660(28, 7)
ENSG00000152578(28, 11)
ENSG00000152977(28, 12)
ENSG00000156639(28, 12)
ENSG00000159023(28, 14)
ENSG00000163873(28, 13)
ENSG00000169946(28, 8)
ENSG00000175387(28, 12)
ENSG00000185009(28, 11)
ENSG00000186318(28, 12)
ENSG00000059728(29, 14)
ENSG00000072364(29, 7)
ENSG00000101745(29, 11)
ENSG00000110427(29, 7)
ENSG00000111540(29, 10)
ENSG00000114353(29, 12)
ENSG00000117461(29, 9)
ENSG00000123636(29, 14)
ENSG00000125266(29, 11)
ENSG00000136448(29, 14)
ENSG00000156103(29, 10)
ENSG00000164924(29, 9)
ENSG00000165699(29, 10)
ENSG00000170242(29, 13)
ENSG00000171246(29, 14)
ENSG00000171451(29, 10)
ENSG00000173065(29, 12)
ENSG00000177463(29, 11)
ENSG00000177565(29, 12)
ENSG00000179295(29, 8)
ENSG00000197063(29, 9)
ENSG00000198909(29, 13)
ENSG00000221823(29, 13)
ENSG00000005339(30, 5)

ENSG00000050030(30, 12)
ENSG00000105810(30, 14)
ENSG00000110092(30, 12)
ENSG00000116539(30, 12)
ENSG00000119231(30, 13)
ENSG00000125686(30, 14)
ENSG00000127334(30, 13)
ENSG00000138758(30, 14)
ENSG00000149256(30, 13)
ENSG00000151532(30, 11)
ENSG00000152102(30, 11)
ENSG00000153936(30, 10)
ENSG00000163602(30, 13)
ENSG00000164164(30, 7)
ENSG00000171132(30, 10)
ENSG00000172260(30, 12)
ENSG00000182263(30, 11)
ENSG00000188215(30, 11)
ENSG00000036257(31, 11)
ENSG00000055609(31, 17)
ENSG00000070269(31, 11)
ENSG00000070444(31, 14)
ENSG00000117016(31, 17)
ENSG00000119318(31, 16)
ENSG00000124766(31, 13)
ENSG00000124788(31, 8)
ENSG00000135097(31, 13)
ENSG00000135837(31, 13)
ENSG00000141298(31, 9)
ENSG00000145920(31, 12)
ENSG00000152242(31, 12)
ENSG00000164327(31, 18)
ENSG00000164604(31, 12)
ENSG00000171475(31, 19)
ENSG00000176697(31, 7)
ENSG00000179119(31, 17)
ENSG00000180011(31, 13)
ENSG00000186432(31, 16)
ENSG00000188042(31, 14)
ENSG00000198742(31, 13)
ENSG00000066117(32, 14)
ENSG00000070018(32, 13)
ENSG00000083312(32, 12)
ENSG00000084676(32, 17)
ENSG00000088367(32, 7)
ENSG00000092421(32, 14)
ENSG00000101350(32, 12)
ENSG00000103404(32, 13)
ENSG00000104343(32, 13)
ENSG00000111252(32, 10)
ENSG00000118058(32, 11)
ENSG00000118971(32, 10)
ENSG00000124140(32, 9)
ENSG00000135503(32, 15)

ENSG00000136928(32, 15)
ENSG00000141084(32, 13)
ENSG00000146830(32, 14)
ENSG00000148943(32, 12)
ENSG00000149575(32, 13)
ENSG00000155506(32, 12)
ENSG00000165424(32, 13)
ENSG00000166225(32, 14)
ENSG00000168939(32, 9)
ENSG00000176542(32, 13)
ENSG00000186187(32, 12)
ENSG00000196323(32, 11)
ENSG00000006468(33, 9)
ENSG00000066044(33, 10)
ENSG00000080493(33, 15)
ENSG00000100697(33, 10)
ENSG00000114416(33, 14)
ENSG00000121964(33, 10)
ENSG00000122778(33, 15)
ENSG00000141367(33, 11)
ENSG00000146776(33, 13)
ENSG00000161642(33, 14)
ENSG00000164190(33, 11)
ENSG00000166897(33, 14)
ENSG00000171791(33, 10)
ENSG00000181690(33, 15)
ENSG00000182752(33, 13)
ENSG00000185658(33, 10)
ENSG00000242265(33, 13)
ENSG00000043143(34, 10)
ENSG00000049192(34, 16)
ENSG00000065559(34, 9)
ENSG00000090776(34, 11)
ENSG00000111262(34, 13)
ENSG00000116667(34, 12)
ENSG00000137166(34, 14)
ENSG00000138814(34, 13)
ENSG00000166135(34, 10)
ENSG00000171316(34, 14)
ENSG00000177570(34, 18)
ENSG00000184083(34, 15)
ENSG00000055070(35, 14)
ENSG00000076356(35, 17)
ENSG00000087095(35, 6)
ENSG00000100320(35, 8)
ENSG00000118985(35, 17)
ENSG00000132388(35, 16)
ENSG00000134138(35, 16)
ENSG00000136828(35, 15)
ENSG00000143376(35, 13)
ENSG00000149294(35, 16)
ENSG00000150672(35, 17)
ENSG00000153707(35, 9)
ENSG00000160094(35, 10)

ENSG00000017427(36, 15)
ENSG00000107864(36, 14)
ENSG00000108175(36, 14)
ENSG00000109756(36, 15)
ENSG00000140948(36, 14)
ENSG00000146872(36, 17)
ENSG00000162290(36, 12)
ENSG00000165355(36, 14)
ENSG00000177125(36, 14)
ENSG00000183166(36, 13)
ENSG00000101493(37, 17)
ENSG00000107105(37, 19)
ENSG00000109670(37, 13)
ENSG00000114861(37, 14)
ENSG00000118689(37, 10)
ENSG00000123600(37, 16)
ENSG00000140320(37, 16)
ENSG00000143322(37, 17)
ENSG00000143995(37, 10)
ENSG00000145675(37, 14)
ENSG00000155966(37, 12)
ENSG00000163013(37, 17)
ENSG00000163531(37, 17)
ENSG00000164651(37, 10)
ENSG00000168675(37, 13)
ENSG00000185920(37, 11)
ENSG00000189079(37, 20)
ENSG00000072071(38, 16)
ENSG00000085224(38, 15)
ENSG00000100403(38, 13)
ENSG00000108292(38, 9)
ENSG00000121274(38, 16)
ENSG00000128573(38, 16)
ENSG00000140526(38, 11)
ENSG00000156030(38, 13)
ENSG00000156113(38, 11)
ENSG00000188786(38, 16)
ENSG00000196482(38, 7)
ENSG00000221866(38, 15)
ENSG00000108091(39, 10)
ENSG00000108924(39, 18)
ENSG00000112242(39, 19)
ENSG00000129473(39, 17)
ENSG00000131149(39, 15)
ENSG00000131389(39, 20)
ENSG00000139154(39, 12)
ENSG00000243444(39, 14)
ENSG00000068024(40, 16)
ENSG00000100030(40, 14)
ENSG00000103353(40, 17)
ENSG00000110395(40, 13)
ENSG00000142864(40, 15)
ENSG00000151474(40, 18)
ENSG00000166987(40, 11)

ENSG00000169554(40, 9)
ENSG00000179388(40, 18)
ENSG00000185619(40, 15)
ENSG00000196628(40, 16)
ENSG00000213639(40, 16)
ENSG00000109787(41, 16)
ENSG00000115306(41, 16)
ENSG00000146592(41, 10)
ENSG00000178568(41, 14)
ENSG00000198010(41, 18)
ENSG00000113448(42, 14)
ENSG00000126003(42, 19)
ENSG00000129159(42, 20)
ENSG00000152601(42, 6)
ENSG00000183579(42, 15)
ENSG00000101098(43, 24)
ENSG00000135870(43, 16)
ENSG00000169926(43, 17)
ENSG00000180357(43, 15)
ENSG00000103489(44, 19)
ENSG00000134369(44, 13)
ENSG00000169118(44, 20)
ENSG00000177732(44, 16)
ENSG00000178573(44, 14)
ENSG00000006459(45, 20)
ENSG00000076108(45, 18)
ENSG00000087152(45, 22)
ENSG00000089057(45, 18)
ENSG00000114030(45, 10)
ENSG00000142611(45, 15)
ENSG00000153187(45, 10)
ENSG00000171365(45, 22)
ENSG00000008441(46, 19)
ENSG00000141646(46, 18)
ENSG00000151090(46, 17)
ENSG00000155130(46, 14)
ENSG00000170144(46, 19)
ENSG00000159217(47, 16)
ENSG00000163848(47, 21)
ENSG00000132475(48, 14)
ENSG00000138767(48, 20)
ENSG00000139910(48, 14)
ENSG00000174804(48, 19)
ENSG00000116604(49, 20)
ENSG00000151067(49, 19)
ENSG00000154380(49, 19)
ENSG00000100330(50, 22)
ENSG00000107957(50, 18)
ENSG00000110367(50, 19)
ENSG00000113742(50, 18)
ENSG00000115977(50, 17)
ENSG00000164061(50, 19)
ENSG00000053254(51, 18)
ENSG00000162105(51, 13)

ENSG00000196220(51, 22)
ENSG00000107372(52, 21)
ENSG00000092847(53, 19)
ENSG00000111596(53, 16)
ENSG00000157540(53, 16)
ENSG00000177189(53, 19)
ENSG00000196914(53, 20)
ENSG00000077279(54, 19)
ENSG00000141622(54, 16)
ENSG00000146083(54, 17)
ENSG00000157657(55, 17)
ENSG00000186487(55, 15)
ENSG00000203759(55, 20)
ENSG00000025800(56, 18)
ENSG00000169635(56, 24)
ENSG00000135365(57, 23)
ENSG00000176887(57, 20)
ENSG00000187079(58, 22)
ENSG00000118922(59, 16)
ENSG00000127152(59, 20)
ENSG00000215301(59, 20)
ENSG00000119547(61, 19)
ENSG00000140443(61, 15)
ENSG00000143614(61, 24)
ENSG00000119866(62, 22)
ENSG00000137449(63, 22)
ENSG00000137504(63, 24)
ENSG00000187605(65, 23)
ENSG00000112182(66, 22)
ENSG00000102908(67, 26)
ENSG00000114739(67, 23)
ENSG00000170921(67, 23)
ENSG00000173068(67, 22)
ENSG00000108256(71, 24)
ENSG00000146676(71, 22)
ENSG00000147862(74, 23)
ENSG00000185591(78, 35)
ENSG00000064393(79, 28)
ENSG00000104447(80, 32)
ENSG00000176407(80, 28)
ENSG00000167548(81, 31)
ENSG00000091656(85, 28)
ENSG00000100354(95, 34)
ENSG00000169057(98, 34)
ENSG00000139645(99, 41)
