# Supplementary material for: Insights into the regulation of human CNV-miRNAs from the view of their target genes
Source: BMC Genomics. 2012 Dec 18;13:707. doi: 10.1186/1471-2164-13-707 (PMC3582595; doi:10.1186/1471-2164-13-707)
Supplement: Additional file 4 — Coefficient of variation (CV) of human protein-coding genes in four HapMap ethnic populations. [file 1471-2164-13-707-S4.pdf]

Ensembl Gene ID	CV in YRI	CV in CEU	CV in CHB	CV in JPT
**ENSG00000127720**	*0.039466239*	*0.039092832*	*0.03300057*	*0.035316145*
**ENSG00000109819**	*0.028701726*	*0.025725772*	*0.025247146*	*0.025925952*
**ENSG00000161057**	*0.021643122*	*0.027666774*	*0.026906086*	*0.026938763*
**ENSG00000237787**	*0.014669792*	*0.024676023*	*0.024522119*	*0.015752464*
**ENSG00000051596**	*0.048830556*	*0.037215416*	*0.037659393*	*0.035402659*
**ENSG00000172244**	*0.039442334*	*0.042517218*	*0.031144247*	*0.030413094*
**ENSG00000182511**	*0.048378495*	*0.050503897*	*0.056066256*	*0.048065326*
**ENSG00000100796**	*0.015641039*	*0.025521288*	*0.025380635*	*0.015151878*
**ENSG00000135541**	*0.035027212*	*0.040353311*	*0.033084856*	*0.0331495*
**ENSG00000143376**	*0.034273641*	*0.041679729*	*0.035684194*	*0.026831788*
**ENSG00000165240**	*0.035009162*	*0.038441215*	*0.031838486*	*0.035109638*
**ENSG00000120853**	*0.016414995*	*0.025336277*	*0.025030259*	*0.015952448*
**ENSG00000180739**	*0.016031752*	*0.025104646*	*0.024430713*	*0.015267624*
**ENSG00000172172**	*0.027703771*	*0.030692062*	*0.028981341*	*0.023712781*
**ENSG00000172020**	*0.091461122*	*0.066614445*	*0.033272598*	*0.040482513*
**ENSG00000100218**	*0.024146741*	*0.027552035*	*0.025843513*	*0.024115346*
**ENSG00000029639**	*0.038675005*	*0.036747937*	*0.035843206*	*0.03277403*
**ENSG00000132604**	*0.026124052*	*0.034190371*	*0.029156799*	*0.024053984*
**ENSG00000111218**	*0.014966223*	*0.025430698*	*0.025451699*	*0.015647152*
**ENSG00000142185**	*0.017311823*	*0.026644012*	*0.025570314*	*0.017983217*
**ENSG00000162676**	*0.064259592*	*0.045997887*	*0.052618582*	*0.044429683*
**ENSG00000131724**	*0.104308819*	*0.106366054*	*0.048931752*	*0.074294809*
**ENSG00000213417**	*0.017683838*	*0.025330408*	*0.025657138*	*0.017221358*
**ENSG00000101194**	*0.032625569*	*0.031790866*	*0.031690121*	*0.027916704*
**ENSG00000149483**	*0.046475079*	*0.037837665*	*0.041341746*	*0.039176966*
**ENSG00000112812**	*0.054007245*	*0.045388894*	*0.040567174*	*0.043535054*
**ENSG00000005884**	*0.018022031*	*0.026483557*	*0.025475909*	*0.017427774*
**ENSG00000128595**	*0.030608438*	*0.033473019*	*0.036372331*	*0.032182175*
**ENSG00000136531**	*0.021429785*	*0.026808071*	*0.025286133*	*0.017375345*
**ENSG00000100211**	*0.037961311*	*0.044050213*	*0.051844912*	*0.040735287*
**ENSG00000183617**	*0.032537436*	*0.034951333*	*0.034886777*	*0.030440223*
**ENSG00000175220**	*0.030247616*	*0.035058367*	*0.031435432*	*0.033291036*
**ENSG00000156282**	*0.014620332*	*0.025504744*	*0.024064327*	*0.01452672*
**ENSG00000186479**	*0.013772709*	*0.023819527*	*0.023912648*	*0.013868162*
**ENSG00000112981**	*0.031432028*	*0.033335973*	*0.027874745*	*0.025647969*
**ENSG00000168283**	*0.033920682*	*0.044744559*	*0.039004927*	*0.030984621*
**ENSG00000204165**	*0.05896926*	*0.037101286*	*0.036231119*	*0.043511691*
**ENSG00000034510**	*0.01901018*	*0.024943298*	*0.025732529*	*0.017050132*
**ENSG00000187608**	*0.04343567*	*0.043842146*	*0.039509875*	*0.038629871*
**ENSG00000100652**	*0.01511544*	*0.025043679*	*0.024021672*	*0.016342473*
**ENSG00000167536**	*0.061097257*	*0.051940171*	*0.044355776*	*0.050632535*
**ENSG00000145103**	*0.050634062*	*0.044809553*	*0.043693434*	*0.039561961*
**ENSG00000131652**	*0.036588086*	*0.033637507*	*0.035044653*	*0.029107271*
**ENSG00000102805**	*0.029729285*	*0.034801485*	*0.034093603*	*0.029573071*
**ENSG00000107185**	*0.016646062*	*0.02592485*	*0.025509873*	*0.015589633*
**ENSG00000119314**	*0.032868947*	*0.037297572*	*0.036865814*	*0.026059832*
**ENSG00000159259**	*0.041257229*	*0.037205427*	*0.036559292*	*0.029695166*
**ENSG00000196652**	*0.016910538*	*0.025501239*	*0.025549531*	*0.017210014*
**ENSG00000100568**	*0.025347053*	*0.03130486*	*0.033060354*	*0.027211497*
**ENSG00000008283**	*0.034006451*	*0.036562016*	*0.03621847*	*0.028129983*
**ENSG00000108468**	*0.032308152*	*0.036794706*	*0.033629081*	*0.032823238*

**ENSG00000176566**	*0.016668496*	*0.025745947*	*0.02467226*	*0.014842794*
**ENSG00000158109**	*0.030930442*	*0.036428438*	*0.034151272*	*0.029253692*
**ENSG00000088970**	*0.042246013*	*0.041316017*	*0.036980894*	*0.03191229*
**ENSG00000169313**	*0.018149737*	*0.025639219*	*0.024894773*	*0.015504274*
**ENSG00000136044**	*0.028832057*	*0.033644085*	*0.031757476*	*0.028988703*
**ENSG00000163898**	*0.029065443*	*0.031332111*	*0.033422426*	*0.025840946*
**ENSG00000102057**	*0.027147842*	*0.030495158*	*0.027401179*	*0.023684604*
**ENSG00000189132**	*0.018341332*	*0.025729657*	*0.025952775*	*0.017447232*
**ENSG00000130349**	*0.033262335*	*0.035516179*	*0.030112125*	*0.024468795*
**ENSG00000113163**	*0.0357667*	*0.033425287*	*0.032241842*	*0.02880923*
**ENSG00000141968**	*0.02878118*	*0.031948768*	*0.031055562*	*0.026327145*
**ENSG00000182333**	*0.016123559*	*0.025569481*	*0.025141848*	*0.016188039*
**ENSG00000179776**	*0.017459664*	*0.026544396*	*0.026638798*	*0.015376307*
**ENSG00000155760**	*0.071120798*	*0.061028161*	*0.026515603*	*0.024041587*
**ENSG00000125266**	*0.083567094*	*0.066883578*	*0.048414756*	*0.058673842*
**ENSG00000132669**	*0.085055173*	*0.049050703*	*0.029919867*	*0.034114967*
**ENSG00000184220**	*0.037113856*	*0.038749377*	*0.035112581*	*0.028136141*
**ENSG00000107404**	*0.018630604*	*0.026065005*	*0.025555988*	*0.01740607*
**ENSG00000105829**	*0.032985579*	*0.037333696*	*0.03261776*	*0.030500927*
**ENSG00000244537**	*0.016029823*	*0.025674106*	*0.025606896*	*0.015713672*
**ENSG00000169026**	*0.056148558*	*0.053665551*	*0.044660003*	*0.059638467*
**ENSG00000131238**	*0.025542992*	*0.030279235*	*0.028400417*	*0.025747219*
**ENSG00000139211**	*0.016011932*	*0.025201156*	*0.025531213*	*0.017046866*
**ENSG00000196220**	*0.036099556*	*0.039217143*	*0.032716134*	*0.037659778*
**ENSG00000178804**	*0.01544479*	*0.024739042*	*0.024507982*	*0.015231929*
**ENSG00000088451**	*0.02989545*	*0.032820865*	*0.033641106*	*0.024200728*
**ENSG00000156049**	*0.016050356*	*0.024104211*	*0.025002067*	*0.015048459*
**ENSG00000099330**	*0.031886287*	*0.029001257*	*0.031273818*	*0.028732763*
**ENSG00000221855**	*0.014962168*	*0.025574573*	*0.025537335*	*0.015191534*
**ENSG00000155792**	*0.033913126*	*0.037385754*	*0.029411869*	*0.026895111*
**ENSG00000178567**	*0.03573633*	*0.03643392*	*0.034884052*	*0.03169986*
**ENSG00000131153**	*0.080064809*	*0.061061021*	*0.059754003*	*0.067556257*
**ENSG00000101146**	*0.02691619*	*0.030647577*	*0.033022517*	*0.025503233*
**ENSG00000221834**	*0.016768097*	*0.025346406*	*0.027340916*	*0.018321687*
**ENSG00000164330**	*0.054809427*	*0.050412743*	*0.042561971*	*0.045886592*
**ENSG00000105053**	*0.023345991*	*0.03396513*	*0.031977922*	*0.024859192*
**ENSG00000160285**	*0.033462551*	*0.042226889*	*0.035869632*	*0.035182939*
**ENSG00000121671**	*0.022675745*	*0.029498781*	*0.034759711*	*0.023531942*
**ENSG00000154822**	*0.054526643*	*0.039006983*	*0.040171354*	*0.043970464*
**ENSG00000131686**	*0.01810588*	*0.026298508*	*0.026659761*	*0.020360307*
**ENSG00000183840**	*0.015368686*	*0.025531936*	*0.025641238*	*0.015831733*
**ENSG00000100577**	*0.024929267*	*0.02854222*	*0.033181651*	*0.021862982*
**ENSG00000136026**	*0.071573233*	*0.039068956*	*0.04557701*	*0.061284812*
**ENSG00000133665**	*0.016867032*	*0.025369529*	*0.0247198*	*0.016301346*
**ENSG00000243649**	*0.019955079*	*0.028772929*	*0.025210252*	*0.018787371*
**ENSG00000078114**	*0.026516014*	*0.025446466*	*0.025762264*	*0.017667495*
**ENSG00000169291**	*0.020586036*	*0.02715692*	*0.024590365*	*0.023238425*
**ENSG00000177045**	*0.034265538*	*0.035265848*	*0.036308243*	*0.036762269*
**ENSG00000181852**	*0.028026286*	*0.033966029*	*0.036044231*	*0.025385071*
**ENSG00000167553**	*0.032517495*	*0.032474271*	*0.031914615*	*0.029560567*
**ENSG00000165905**	*0.044404894*	*0.037384834*	*0.034354184*	*0.044289638*
**ENSG00000132139**	*0.015691942*	*0.025454561*	*0.024999631*	*0.016226916*
**ENSG00000181938**	*0.044839133*	*0.037662195*	*0.043308328*	*0.042467267*
**ENSG00000082258**	*0.02642285*	*0.026918835*	*0.026488599*	*0.023920198*

**ENSG00000116062**	*0.034554997*	*0.035692469*	*0.031015546*	*0.027277446*
**ENSG00000076344**	*0.016215263*	*0.025153198*	*0.024955538*	*0.016717196*
**ENSG00000142279**	*0.016618426*	*0.026254247*	*0.026952359*	*0.01603654*
**ENSG00000008710**	*0.023819369*	*0.029200998*	*0.032268739*	*0.028125042*
**ENSG00000170632**	*0.031010528*	*0.035487502*	*0.030074029*	*0.029673041*
**ENSG00000186577**	*0.028744227*	*0.034148514*	*0.032289829*	*0.026204212*
**ENSG00000197943**	*0.032653992*	*0.034211295*	*0.034928342*	*0.035694989*
**ENSG00000082556**	*0.015993472*	*0.025521259*	*0.025331758*	*0.016266499*
**ENSG00000154222**	*0.023857471*	*0.035117272*	*0.033383428*	*0.033464731*
**ENSG00000213341**	*0.033028731*	*0.038878371*	*0.033268424*	*0.037630832*
**ENSG00000184156**	*0.015954788*	*0.02546455*	*0.025301064*	*0.014241604*
**ENSG00000047315**	*0.024647696*	*0.031474829*	*0.030867631*	*0.022922483*
**ENSG00000171824**	*0.028562023*	*0.03496767*	*0.029923296*	*0.024431936*
**ENSG00000051341**	*0.029914345*	*0.031714333*	*0.042778899*	*0.025767556*
**ENSG00000126785**	*0.016761828*	*0.025780779*	*0.025706028*	*0.015376975*
**ENSG00000143217**	*0.021817333*	*0.02442412*	*0.025374115*	*0.015251383*
**ENSG00000106714**	*0.016873884*	*0.024852613*	*0.024979265*	*0.014478518*
**ENSG00000115159**	*0.047448598*	*0.048694868*	*0.041135762*	*0.039009699*
**ENSG00000162636**	*0.017198525*	*0.025164431*	*0.024860262*	*0.016295337*
**ENSG00000044115**	*0.03368339*	*0.038450634*	*0.031081326*	*0.029287229*
**ENSG00000088766**	*0.032045175*	*0.033170019*	*0.030497518*	*0.022261413*
**ENSG00000130700**	*0.015349218*	*0.025020655*	*0.025978776*	*0.015644959*
**ENSG00000100196**	*0.052919127*	*0.047799481*	*0.055791487*	*0.061256392*
**ENSG00000143921**	*0.014884522*	*0.025221017*	*0.024776767*	*0.014183699*
**ENSG00000114480**	*0.049980778*	*0.052469171*	*0.037829955*	*0.049137323*
**ENSG00000110987**	*0.057501699*	*0.052700864*	*0.046546642*	*0.045021395*
**ENSG00000198211**	*0.08174089*	*0.073286493*	*0.043981658*	*0.065560169*
**ENSG00000143457**	*0.031067215*	*0.038684382*	*0.036449627*	*0.031195378*
**ENSG00000130270**	*0.016630806*	*0.025211157*	*0.024929034*	*0.017226858*
**ENSG00000132612**	*0.02136346*	*0.030118477*	*0.035945737*	*0.02205853*
**ENSG00000185269**	*0.019483616*	*0.02912673*	*0.026334865*	*0.024145317*
**ENSG00000163481**	*0.02266052*	*0.033246055*	*0.037309061*	*0.025980402*
**ENSG00000132185**	*0.041381051*	*0.039405637*	*0.0423939*	*0.059287748*
**ENSG00000214226**	*0.020074749*	*0.028452971*	*0.025952935*	*0.019119632*
**ENSG00000170214**	*0.014704145*	*0.024849454*	*0.025332937*	*0.014589973*
**ENSG00000145868**	*0.018428518*	*0.027202556*	*0.024975785*	*0.017100536*
**ENSG00000070669**	*0.016952394*	*0.024499044*	*0.024968277*	*0.016139362*
**ENSG00000125633**	*0.018674795*	*0.026479339*	*0.02542063*	*0.018020928*
**ENSG00000178585**	*0.036212374*	*0.033739731*	*0.035595101*	*0.034542477*
**ENSG00000182359**	*0.038477043*	*0.039121651*	*0.034931431*	*0.036247224*
**ENSG00000162931**	*0.016185903*	*0.025856043*	*0.027262946*	*0.016978057*
**ENSG00000164483**	*0.015193414*	*0.026026571*	*0.025169845*	*0.01693734*
**ENSG00000077312**	*0.027942698*	*0.027444916*	*0.027865074*	*0.022960283*
**ENSG00000196169**	*0.017241526*	*0.025457375*	*0.02525219*	*0.015512844*
**ENSG00000145022**	*0.032343401*	*0.030498408*	*0.033436685*	*0.02896974*
**ENSG00000132275**	*0.028784429*	*0.032856116*	*0.035992537*	*0.022844229*
**ENSG00000179841**	*0.017919346*	*0.025297117*	*0.024965822*	*0.016369922*
**ENSG00000204160**	*0.028148763*	*0.031564758*	*0.033520875*	*0.028865319*
**ENSG00000115310**	*0.01420456*	*0.024709264*	*0.025472602*	*0.015614865*
**ENSG00000119669**	*0.056218331*	*0.047286528*	*0.044686393*	*0.045794144*
**ENSG00000170876**	*0.027033139*	*0.030910587*	*0.029401647*	*0.020477881*
**ENSG00000186063**	*0.040507908*	*0.052836806*	*0.038998216*	*0.043568315*
**ENSG00000171840**	*0.07266543*	*0.047332787*	*0.050597308*	*0.045582279*
**ENSG00000180376**	*0.036059411*	*0.040801561*	*0.036021872*	*0.029887823*

**ENSG00000010319**	*0.015441711*	*0.024650107*	*0.024838365*	*0.015125556*
**ENSG00000074356**	*0.032593983*	*0.035092807*	*0.031711147*	*0.025135104*
**ENSG00000241484**	*0.015235797*	*0.025449612*	*0.024718105*	*0.016300132*
**ENSG00000145861**	*0.015979814*	*0.023707718*	*0.025385514*	*0.015121654*
**ENSG00000007384**	*0.048067884*	*0.039941899*	*0.039016703*	*0.027948322*
**ENSG00000242515**	*0.016627193*	*0.025948346*	*0.025925015*	*0.015531129*
**ENSG00000102595**	*0.050244426*	*0.050241838*	*0.038830808*	*0.039953615*
**ENSG00000101336**	*0.130785096*	*0.094340074*	*0.105813249*	*0.117850408*
**ENSG00000129559**	*0.020038811*	*0.02675768*	*0.026315654*	*0.016796111*
**ENSG00000144026**	*0.029975645*	*0.034580549*	*0.034039278*	*0.025632255*
**ENSG00000186051**	*0.016599235*	*0.025058243*	*0.025058109*	*0.015661358*
**ENSG00000175518**	*0.01968924*	*0.027264622*	*0.027714473*	*0.022205487*
**ENSG00000137501**	*0.017293062*	*0.025197534*	*0.026158356*	*0.01548732*
**ENSG00000041353**	*0.015991438*	*0.025354634*	*0.023938074*	*0.016173254*
**ENSG00000006715**	*0.027109083*	*0.034762017*	*0.032489761*	*0.024621456*
**ENSG00000143799**	*0.025525643*	*0.030756488*	*0.026984225*	*0.021468603*
**ENSG00000137699**	*0.025541551*	*0.027783521*	*0.025738424*	*0.019051885*
**ENSG00000081985**	*0.082068655*	*0.071258377*	*0.054034831*	*0.08072356*
**ENSG00000137842**	*0.033849986*	*0.040883423*	*0.045910008*	*0.030499558*
**ENSG00000188811**	*0.03170523*	*0.03573239*	*0.030865872*	*0.030517978*
**ENSG00000115484**	*0.026309442*	*0.029848602*	*0.028697872*	*0.018592744*
**ENSG00000148229**	*0.036428724*	*0.03707289*	*0.03955742*	*0.036200304*
**ENSG00000129152**	*0.016931105*	*0.025318973*	*0.0247775*	*0.016627729*
**ENSG00000110719**	*0.018246143*	*0.025046547*	*0.024432509*	*0.017102807*
**ENSG00000176219**	*0.016274449*	*0.026051839*	*0.02722631*	*0.016613875*
**ENSG00000185264**	*0.016306231*	*0.025542443*	*0.025805479*	*0.014567465*
**ENSG00000143545**	*0.07519607*	*0.04531145*	*0.052052447*	*0.051448892*
**ENSG00000092470**	*0.034415844*	*0.035315077*	*0.03075373*	*0.024705202*
**ENSG00000100814**	*0.025944928*	*0.029320946*	*0.030884036*	*0.022729845*
**ENSG00000149328**	*0.075954749*	*0.077579082*	*0.070660166*	*0.069809349*
**ENSG00000204291**	*0.01850684*	*0.026223398*	*0.026817209*	*0.018396043*
**ENSG00000163823**	*0.141445455*	*0.097784768*	*0.099055544*	*0.110791681*
**ENSG00000170989**	*0.03658356*	*0.040627353*	*0.035903957*	*0.041667085*
**ENSG00000154710**	*0.024305638*	*0.03645001*	*0.04199657*	*0.024724327*
**ENSG00000108381**	*0.086532942*	*0.065546469*	*0.030753464*	*0.039800029*
**ENSG00000132470**	*0.019055405*	*0.025895519*	*0.026166534*	*0.016763169*
**ENSG00000182118**	*0.044951434*	*0.077540884*	*0.053300472*	*0.039788282*
**ENSG00000162739**	*0.041016146*	*0.036290416*	*0.038051553*	*0.041886125*
**ENSG00000139168**	*0.03263072*	*0.036107355*	*0.034671235*	*0.030406514*
**ENSG00000065054**	*0.016627418*	*0.025318763*	*0.025224732*	*0.015566739*
**ENSG00000164733**	*0.048902518*	*0.046412377*	*0.043025831*	*0.043304887*
**ENSG00000212864**	*0.034811027*	*0.039724253*	*0.033475013*	*0.035073144*
**ENSG00000124164**	*0.021149571*	*0.029638853*	*0.034047973*	*0.021241765*
**ENSG00000198939**	*0.016139839*	*0.0244077*	*0.024464946*	*0.014364376*
**ENSG00000136834**	*0.01583817*	*0.024974952*	*0.025974923*	*0.01567464*
**ENSG00000169951**	*0.023645292*	*0.033975827*	*0.034324084*	*0.024510681*
**ENSG00000187003**	*0.016199188*	*0.0259187*	*0.02614598*	*0.016646208*
**ENSG00000198887**	*0.034148258*	*0.040989754*	*0.035649543*	*0.028558675*
**ENSG00000175164**	*0.019625255*	*0.026913494*	*0.026664049*	*0.018412971*
**ENSG00000164600**	*0.017772423*	*0.024784576*	*0.026261081*	*0.016007054*
**ENSG00000124406**	*0.05873852*	*0.055088235*	*0.041768693*	*0.042084135*
**ENSG00000165959**	*0.080721641*	*0.079338348*	*0.066104385*	*0.066893477*
**ENSG00000166974**	*0.037030606*	*0.041845817*	*0.036951799*	*0.033572841*
**ENSG00000140650**	*0.040487839*	*0.044751603*	*0.061821768*	*0.047214383*

**ENSG00000184787**	*0.019080426*	*0.02508923*	*0.025603871*	*0.016051393*
**ENSG00000130856**	*0.026413711*	*0.037589996*	*0.028320589*	*0.023420847*
**ENSG00000132475**	*0.040846217*	*0.048258735*	*0.046179658*	*0.032780336*
**ENSG00000167104**	*0.015768055*	*0.026328558*	*0.025422352*	*0.0159977*
**ENSG00000162704**	*0.021846285*	*0.02914972*	*0.026777712*	*0.021649601*
**ENSG00000180694**	*0.022919363*	*0.032104948*	*0.02860306*	*0.018523035*
**ENSG00000096006**	*0.018623649*	*0.025537135*	*0.026020134*	*0.015526667*
**ENSG00000115474**	*0.015408847*	*0.026391018*	*0.024912533*	*0.015274742*
**ENSG00000173826**	*0.015994738*	*0.025112791*	*0.025214159*	*0.01548079*
**ENSG00000171396**	*0.015304847*	*0.025048978*	*0.02455014*	*0.015832416*
**ENSG00000204764**	*0.016241094*	*0.026385765*	*0.024855442*	*0.01462829*
**ENSG00000107566**	*0.033697279*	*0.037743177*	*0.034223043*	*0.027951613*
**ENSG00000134588**	*0.016152878*	*0.025296264*	*0.025543195*	*0.015103264*
**ENSG00000136982**	*0.047966737*	*0.044878297*	*0.035315833*	*0.04036234*
**ENSG00000145919**	*0.026339157*	*0.028993716*	*0.028519985*	*0.024927296*
**ENSG00000184792**	*0.019115581*	*0.025744435*	*0.02638085*	*0.017193102*
**ENSG00000167994**	*0.015889486*	*0.024881601*	*0.024190538*	*0.014265839*
**ENSG00000151746**	*0.029379065*	*0.038423539*	*0.032800998*	*0.031700328*
**ENSG00000163125**	*0.028110475*	*0.031897946*	*0.044472394*	*0.025436058*
**ENSG00000065613**	*0.039669393*	*0.041138888*	*0.037127646*	*0.036547755*
**ENSG00000064042**	*0.015379562*	*0.024552563*	*0.024066528*	*0.014892815*
**ENSG00000104320**	*0.033658353*	*0.042105344*	*0.038142282*	*0.040145474*
**ENSG00000147138**	*0.033439995*	*0.038437869*	*0.034793279*	*0.030672166*
**ENSG00000198113**	*0.03247826*	*0.04150894*	*0.051203813*	*0.033466257*
**ENSG00000128683**	*0.058839203*	*0.038705053*	*0.042270474*	*0.053957051*
**ENSG00000116030**	*0.025824147*	*0.029163594*	*0.02700542*	*0.028384906*
**ENSG00000128573**	*0.015639946*	*0.024848796*	*0.024747725*	*0.016451845*
**ENSG00000187187**	*0.033969765*	*0.044557469*	*0.035086437*	*0.031727418*
**ENSG00000141084**	*0.02814523*	*0.031660523*	*0.030774429*	*0.024313622*
**ENSG00000143199**	*0.01541927*	*0.024506103*	*0.02480115*	*0.016166829*
**ENSG00000178665**	*0.017983415*	*0.027322483*	*0.026695753*	*0.022203108*
**ENSG00000168646**	*0.01744237*	*0.026863661*	*0.0257311*	*0.016228417*
**ENSG00000106278**	*0.015974159*	*0.024864612*	*0.024687461*	*0.016221243*
**ENSG00000141371**	*0.016018929*	*0.02602744*	*0.026165862*	*0.015084484*
**ENSG00000014914**	*0.016474597*	*0.025713502*	*0.025429192*	*0.014932134*
**ENSG00000197756**	*0.015312443*	*0.030032155*	*0.031164126*	*0.020009906*
**ENSG00000185527**	*0.073388351*	*0.067564441*	*0.049613227*	*0.064915668*
**ENSG00000144045**	*0.017056462*	*0.026732396*	*0.026187204*	*0.019159836*
**ENSG00000197406**	*0.016339221*	*0.026152451*	*0.025444375*	*0.015747272*
**ENSG00000113391**	*0.035257269*	*0.042148411*	*0.034933196*	*0.030232287*
**ENSG00000104442**	*0.024624324*	*0.031638664*	*0.028257178*	*0.022711314*
**ENSG00000101680**	*0.020421343*	*0.029089863*	*0.025214441*	*0.017592227*
**ENSG00000146223**	*0.037528703*	*0.036171007*	*0.033206243*	*0.027827724*
**ENSG00000184280**	*0.049278985*	*0.042197477*	*0.032540785*	*0.036318786*
**ENSG00000073754**	*0.018616901*	*0.026616772*	*0.025747333*	*0.017842122*
**ENSG00000076321**	*0.023974061*	*0.030259597*	*0.032936858*	*0.021322301*
**ENSG00000066135**	*0.021521101*	*0.028933007*	*0.029105013*	*0.019297375*
**ENSG00000128713**	*0.015983455*	*0.024674548*	*0.025447917*	*0.015770622*
**ENSG00000152078**	*0.022540983*	*0.02948973*	*0.026636665*	*0.019899579*
**ENSG00000170175**	*0.029164966*	*0.028860104*	*0.030487869*	*0.028559631*
**ENSG00000151718**	*0.020950895*	*0.02726602*	*0.026207501*	*0.018050909*
**ENSG00000163331**	*0.017808339*	*0.025850188*	*0.024748481*	*0.015569632*
**ENSG00000108771**	*0.051745999*	*0.04048799*	*0.041457952*	*0.037145976*
**ENSG00000139908**	*0.016688251*	*0.024848141*	*0.024877138*	*0.017056942*

**ENSG00000186153**	*0.017646284*	*0.026762926*	*0.027342911*	*0.018466501*
**ENSG00000069849**	*0.026512556*	*0.030523749*	*0.029596929*	*0.021990934*
**ENSG00000110841**	*0.020329586*	*0.030364244*	*0.025341471*	*0.018751575*
**ENSG00000135476**	*0.039775165*	*0.030772574*	*0.032677041*	*0.027908578*
**ENSG00000141569**	*0.020260407*	*0.025186668*	*0.026502329*	*0.015689365*
**ENSG00000198380**	*0.04331659*	*0.046475327*	*0.041616366*	*0.034543592*
**ENSG00000011143**	*0.030362306*	*0.032879206*	*0.030359078*	*0.023091061*
**ENSG00000152904**	*0.023422837*	*0.032934439*	*0.03075232*	*0.024828023*
**ENSG00000115762**	*0.029564227*	*0.031620191*	*0.035627918*	*0.024394403*
**ENSG00000249709**	*0.022505145*	*0.028923368*	*0.027850953*	*0.020574439*
**ENSG00000164035**	*0.01525089*	*0.025671117*	*0.025451047*	*0.014218886*
**ENSG00000196600**	*0.016184193*	*0.02569002*	*0.025705244*	*0.016749992*
**ENSG00000122966**	*0.045484068*	*0.037161366*	*0.034902866*	*0.028973607*
**ENSG00000244165**	*0.050444121*	*0.044666227*	*0.052112163*	*0.045448912*
**ENSG00000173890**	*0.069822324*	*0.065017429*	*0.06117055*	*0.055713835*
**ENSG00000129595**	*0.05182437*	*0.057329034*	*0.05117826*	*0.056035279*
**ENSG00000151876**	*0.015672314*	*0.024121864*	*0.024722747*	*0.014223023*
**ENSG00000168918**	*0.023374525*	*0.033367451*	*0.030993722*	*0.028302824*
**ENSG00000125355**	*0.048150001*	*0.049095219*	*0.047988316*	*0.042988134*
**ENSG00000112319**	*0.017430816*	*0.024394144*	*0.02718954*	*0.016221737*
**ENSG00000188986**	*0.023814065*	*0.030563404*	*0.033865445*	*0.0237725*
**ENSG00000197181**	*0.017227152*	*0.025515035*	*0.025335675*	*0.016482725*
**ENSG00000136098**	*0.015310814*	*0.024512697*	*0.025014743*	*0.016154857*
**ENSG00000134897**	*0.040254291*	*0.04354315*	*0.032760507*	*0.029594107*
**ENSG00000180305**	*0.015678496*	*0.024980838*	*0.025443713*	*0.015622729*
**ENSG00000240403**	*0.015219039*	*0.026380163*	*0.024201462*	*0.015092998*
**ENSG00000040933**	*0.048311141*	*0.048081419*	*0.048709569*	*0.041130104*
**ENSG00000152763**	*0.016258889*	*0.024345216*	*0.025816852*	*0.015619567*
**ENSG00000235863**	*0.035578266*	*0.031488895*	*0.034963724*	*0.034526774*
**ENSG00000116957**	*0.026669421*	*0.03096551*	*0.031011498*	*0.022683706*
**ENSG00000132703**	*0.017565018*	*0.025700815*	*0.024637113*	*0.015550864*
**ENSG00000131381**	*0.015887946*	*0.025113241*	*0.025209579*	*0.015433705*
**ENSG00000153237**	*0.015222643*	*0.024167933*	*0.025124747*	*0.015175703*
**ENSG00000148426**	*0.028107284*	*0.026978807*	*0.028359816*	*0.020425088*
**ENSG00000121851**	*0.029314919*	*0.034224664*	*0.030345981*	*0.021168925*
**ENSG00000166664**	*0.015760153*	*0.024877355*	*0.0258497*	*0.015019497*
**ENSG00000115944**	*0.021871052*	*0.031052825*	*0.028686573*	*0.020744002*
**ENSG00000137054**	*0.02876727*	*0.03607324*	*0.033791961*	*0.025732348*
**ENSG00000132026**	*0.016774301*	*0.026126815*	*0.024740678*	*0.015273843*
**ENSG00000155886**	*0.016892087*	*0.026113647*	*0.024648702*	*0.016541447*
**ENSG00000179002**	*0.018380641*	*0.024941811*	*0.0273747*	*0.018785018*
**ENSG00000138802**	*0.026697183*	*0.032937128*	*0.029556611*	*0.026681482*
**ENSG00000115266**	*0.020264622*	*0.02717766*	*0.027719788*	*0.018293435*
**ENSG00000164896**	*0.026170229*	*0.03076228*	*0.030163337*	*0.026689377*
**ENSG00000158022**	*0.022103666*	*0.025278276*	*0.024351354*	*0.021489507*
**ENSG00000004864**	*0.044791339*	*0.039399631*	*0.034181092*	*0.040892693*
**ENSG00000151846**	*0.024677864*	*0.025561427*	*0.026444198*	*0.016648276*
**ENSG00000176402**	*0.018949774*	*0.024651652*	*0.025157363*	*0.018102705*
**ENSG00000139613**	*0.017371707*	*0.026075181*	*0.026368114*	*0.01680433*
**ENSG00000181396**	*0.025505881*	*0.029991205*	*0.027620093*	*0.022799162*
**ENSG00000206075**	*0.017201031*	*0.024980288*	*0.025856772*	*0.016826058*
**ENSG00000100425**	*0.03080718*	*0.036392865*	*0.03418745*	*0.026063372*
**ENSG00000047188**	*0.033344509*	*0.038521797*	*0.031890112*	*0.034044682*
**ENSG00000198797**	*0.01585901*	*0.024927873*	*0.025030322*	*0.014738809*

**ENSG00000092421**	*0.023164943*	*0.031114554*	*0.029204493*	*0.020474875*
**ENSG00000187266**	*0.033201489*	*0.044669289*	*0.034120959*	*0.035149524*
**ENSG00000113580**	*0.039434226*	*0.039651749*	*0.03817672*	*0.035103191*
**ENSG00000104853**	*0.024873544*	*0.029320832*	*0.036855702*	*0.029288307*
**ENSG00000169122**	*0.066329002*	*0.051836216*	*0.045831992*	*0.060715614*
**ENSG00000111652**	*0.026502454*	*0.029126708*	*0.029156608*	*0.026258387*
**ENSG00000158864**	*0.025130727*	*0.029435791*	*0.029477917*	*0.020845907*
**ENSG00000168268**	*0.049333254*	*0.047534487*	*0.053143615*	*0.042587004*
**ENSG00000166710**	*0.014677383*	*0.024180389*	*0.0243546*	*0.014277573*
**ENSG00000051825**	*0.041396971*	*0.041213413*	*0.032792912*	*0.025475668*
**ENSG00000078142**	*0.033767682*	*0.040045589*	*0.031682517*	*0.024332203*
**ENSG00000144031**	*0.014821262*	*0.023960176*	*0.025438546*	*0.015427302*
**ENSG00000183783**	*0.015886918*	*0.026572069*	*0.024392767*	*0.014937157*
**ENSG00000146826**	*0.029479187*	*0.03384737*	*0.041637485*	*0.032154608*
**ENSG00000048740**	*0.040243399*	*0.049115368*	*0.033825262*	*0.033555187*
**ENSG00000137857**	*0.016368172*	*0.026502883*	*0.026211074*	*0.016246762*
**ENSG00000186297**	*0.016359803*	*0.024774363*	*0.025844587*	*0.017075079*
**ENSG00000164066**	*0.024521398*	*0.03350417*	*0.026036584*	*0.020141186*
**ENSG00000011566**	*0.03850825*	*0.036953154*	*0.033015903*	*0.037633415*
**ENSG00000014216**	*0.029703597*	*0.034175034*	*0.030664221*	*0.023360808*
**ENSG00000063761**	*0.021366681*	*0.027542388*	*0.02512281*	*0.019567432*
**ENSG00000139874**	*0.015625202*	*0.024983708*	*0.024281637*	*0.01473987*
**ENSG00000152049**	*0.015131786*	*0.025315589*	*0.025068359*	*0.015273801*
**ENSG00000156113**	*0.12960319*	*0.088558302*	*0.064526358*	*0.10902697*
**ENSG00000080493**	*0.051814972*	*0.028016592*	*0.027126397*	*0.0199489*
**ENSG00000079689**	*0.015525013*	*0.025062998*	*0.024437611*	*0.015590498*
**ENSG00000154188**	*0.015099477*	*0.025441748*	*0.024911751*	*0.015768009*
**ENSG00000231925**	*0.037256292*	*0.036635489*	*0.033283319*	*0.032058896*
**ENSG00000253950**	*0.036842174*	*0.035218488*	*0.037003555*	*0.030064287*
**ENSG00000131378**	*0.024378875*	*0.031073224*	*0.029298497*	*0.023977538*
**ENSG00000119714**	*0.014617581*	*0.024057889*	*0.025414623*	*0.015762308*
**ENSG00000133740**	*0.032984101*	*0.039819114*	*0.034772012*	*0.031176311*
**ENSG00000196453**	*0.033048946*	*0.043459033*	*0.041894125*	*0.033436819*
**ENSG00000092096**	*0.01503065*	*0.024873764*	*0.024854616*	*0.015475664*
**ENSG00000131187**	*0.067366932*	*0.05789567*	*0.058753597*	*0.072466815*
**ENSG00000136100**	*0.030038634*	*0.035721345*	*0.032263576*	*0.02392525*
**ENSG00000165655**	*0.140026644*	*0.090006266*	*0.081832483*	*0.091370274*
**ENSG00000163104**	*0.030821724*	*0.035368074*	*0.030726972*	*0.027878004*
**ENSG00000048392**	*0.040661284*	*0.045096584*	*0.035394911*	*0.037786835*
**ENSG00000070601**	*0.016290671*	*0.025739672*	*0.024469482*	*0.014979817*
**ENSG00000121716**	*0.017740014*	*0.02688829*	*0.027442756*	*0.016457273*
**ENSG00000125875**	*0.02725272*	*0.031178366*	*0.031532464*	*0.021933422*
**ENSG00000148795**	*0.017460782*	*0.025046338*	*0.024924348*	*0.01691382*
**ENSG00000107165**	*0.017460084*	*0.025389975*	*0.025064601*	*0.015556617*
**ENSG00000101439**	*0.065219743*	*0.066528436*	*0.054297436*	*0.057174596*
**ENSG00000116525**	*0.016742998*	*0.02522844*	*0.027936708*	*0.016065593*
**ENSG00000163521**	*0.028681487*	*0.032261539*	*0.034561548*	*0.030280726*
**ENSG00000135090**	*0.015106956*	*0.025413267*	*0.024287008*	*0.014015214*
**ENSG00000198049**	*0.015676615*	*0.025201355*	*0.025451929*	*0.015289592*
**ENSG00000091831**	*0.018753267*	*0.030187439*	*0.025425341*	*0.021388208*
**ENSG00000186310**	*0.024602053*	*0.025021794*	*0.024080516*	*0.014952996*
**ENSG00000116151**	*0.0161813*	*0.026471879*	*0.025280975*	*0.025104108*
**ENSG00000181781**	*0.017012511*	*0.025239038*	*0.025216359*	*0.018163845*
**ENSG00000164932**	*0.026437631*	*0.027108827*	*0.025090629*	*0.022898019*

**ENSG00000075945**	*0.042795751*	*0.051182862*	*0.043279014*	*0.037846248*
**ENSG00000158352**	*0.015719451*	*0.025658135*	*0.024250719*	*0.014399983*
**ENSG00000197079**	*0.018856293*	*0.026658458*	*0.024915384*	*0.017623058*
**ENSG00000138434**	*0.05135125*	*0.046441429*	*0.043731088*	*0.043413156*
**ENSG00000162191**	*0.020186447*	*0.02733209*	*0.027724609*	*0.021151679*
**ENSG00000102900**	*0.025493562*	*0.028145457*	*0.030652683*	*0.02436468*
**ENSG00000050438**	*0.021920085*	*0.024901009*	*0.02749368*	*0.019786638*
**ENSG00000124356**	*0.025775305*	*0.031321559*	*0.029042237*	*0.021321728*
**ENSG00000172995**	*0.015155817*	*0.024553239*	*0.024183909*	*0.016218803*
**ENSG00000198597**	*0.01723926*	*0.025161317*	*0.025828869*	*0.017030561*
**ENSG00000213934**	*0.061224933*	*0.026942187*	*0.025689513*	*0.02075979*
**ENSG00000150753**	*0.033211161*	*0.034730196*	*0.031936369*	*0.03012165*
**ENSG00000179195**	*0.029323936*	*0.037872132*	*0.032655164*	*0.02221579*
**ENSG00000172531**	*0.024433706*	*0.027935581*	*0.029475077*	*0.021574757*
**ENSG00000071054**	*0.043937678*	*0.029888064*	*0.027088996*	*0.022018101*
**ENSG00000166321**	*0.024127415*	*0.033858209*	*0.028670692*	*0.023336973*
**ENSG00000124588**	*0.061981204*	*0.070511811*	*0.068506332*	*0.049978037*
**ENSG00000165891**	*0.048403343*	*0.050815205*	*0.044367931*	*0.054625222*
**ENSG00000076258**	*0.034831261*	*0.034699769*	*0.031392096*	*0.028969739*
**ENSG00000188316**	*0.017503454*	*0.025339448*	*0.025915742*	*0.016916152*
**ENSG00000242247**	*0.0264956*	*0.03290134*	*0.035597653*	*0.02805393*
**ENSG00000006128**	*0.026748686*	*0.028499663*	*0.027260338*	*0.030828419*
**ENSG00000151952**	*0.020001806*	*0.028065174*	*0.027371884*	*0.01880825*
**ENSG00000172288**	*0.015920685*	*0.024191749*	*0.024783556*	*0.015489438*
**ENSG00000137880**	*0.047732126*	*0.04776401*	*0.048974266*	*0.045815252*
**ENSG00000112312**	*0.036612819*	*0.031543294*	*0.032145773*	*0.033543131*
**ENSG00000121653**	*0.020632291*	*0.026242147*	*0.026880016*	*0.017330545*
**ENSG00000103855**	*0.017658238*	*0.0259387*	*0.026163029*	*0.017850895*
**ENSG00000157350**	*0.043107687*	*0.041500736*	*0.039412846*	*0.032296454*
**ENSG00000176986**	*0.021395623*	*0.027414926*	*0.031162958*	*0.024224056*
**ENSG00000197329**	*0.036546128*	*0.039077893*	*0.034981355*	*0.032888112*
**ENSG00000144824**	*0.043972873*	*0.033004425*	*0.02668128*	*0.023456598*
**ENSG00000155629**	*0.062105262*	*0.060906629*	*0.058648935*	*0.07717362*
**ENSG00000126107**	*0.02553932*	*0.031047042*	*0.032018286*	*0.028020685*
**ENSG00000197312**	*0.016423085*	*0.025803228*	*0.024925218*	*0.015136507*
**ENSG00000172575**	*0.062236258*	*0.057081073*	*0.050223273*	*0.063479218*
**ENSG00000059915**	*0.017654617*	*0.024821724*	*0.027357994*	*0.015724642*
**ENSG00000198498**	*0.039142799*	*0.03441128*	*0.032086982*	*0.039482781*
**ENSG00000086288**	*0.084983624*	*0.03694198*	*0.036930182*	*0.068238967*
**ENSG00000106665**	*0.026933608*	*0.038048152*	*0.029013307*	*0.026495681*
**ENSG00000198075**	*0.015839505*	*0.025829829*	*0.026705985*	*0.014813489*
**ENSG00000185559**	*0.016115333*	*0.025509638*	*0.025093602*	*0.016111561*
**ENSG00000114854**	*0.020499532*	*0.027146509*	*0.027323166*	*0.02248604*
**ENSG00000185792**	*0.017003225*	*0.02478335*	*0.024493365*	*0.015720905*
**ENSG00000180611**	*0.084756328*	*0.071094534*	*0.094134553*	*0.11257369*
**ENSG00000172732**	*0.022513299*	*0.029667238*	*0.030290186*	*0.024454159*
**ENSG00000129472**	*0.02617192*	*0.030480275*	*0.031600088*	*0.022322173*
**ENSG00000091140**	*0.022762644*	*0.030116382*	*0.029606588*	*0.025421745*
**ENSG00000140675**	*0.019346066*	*0.028343328*	*0.026816383*	*0.01799944*
**ENSG00000084110**	*0.016032318*	*0.025594732*	*0.025006223*	*0.016571131*
**ENSG00000135845**	*0.030127256*	*0.03252799*	*0.033190854*	*0.025733509*
**ENSG00000125319**	*0.03685451*	*0.033094544*	*0.035491219*	*0.029689533*
**ENSG00000076555**	*0.016499607*	*0.025628362*	*0.026759337*	*0.016067866*
**ENSG00000123472**	*0.026160473*	*0.031768509*	*0.032008481*	*0.019394935*

**ENSG00000161526**	*0.025685872*	*0.028346172*	*0.029220548*	*0.022242517*
**ENSG00000185168**	*0.015355331*	*0.024857753*	*0.024454499*	*0.014972408*
**ENSG00000165209**	*0.029636675*	*0.036018956*	*0.035581453*	*0.02963361*
**ENSG00000173699**	*0.026380201*	*0.030172447*	*0.034755074*	*0.022142913*
**ENSG00000187531**	*0.029584087*	*0.034954441*	*0.036740816*	*0.025472418*
**ENSG00000096088**	*0.0163762*	*0.02572883*	*0.02567419*	*0.016314156*
**ENSG00000127743**	*0.017202187*	*0.024426384*	*0.024313465*	*0.015238269*
**ENSG00000132872**	*0.017111241*	*0.025440655*	*0.024995631*	*0.017431669*
**ENSG00000101255**	*0.058930342*	*0.050633146*	*0.041684475*	*0.038380063*
**ENSG00000116120**	*0.020099921*	*0.026749543*	*0.027674202*	*0.018542442*
**ENSG00000168772**	*0.068276949*	*0.07469191*	*0.07078072*	*0.076885625*
**ENSG00000130734**	*0.018277644*	*0.028304998*	*0.027208675*	*0.017434914*
**ENSG00000171735**	*0.019866544*	*0.027887642*	*0.026041117*	*0.020044961*
**ENSG00000197168**	*0.016360591*	*0.024428197*	*0.024728528*	*0.014084931*
**ENSG00000103546**	*0.017134639*	*0.026706373*	*0.025849426*	*0.017399899*
**ENSG00000071242**	*0.042162459*	*0.03718736*	*0.03161013*	*0.035169278*
**ENSG00000078618**	*0.028989*	*0.031520396*	*0.028947028*	*0.025077607*
**ENSG00000172292**	*0.04251036*	*0.041401772*	*0.041320242*	*0.032705377*
**ENSG00000179817**	*0.016912525*	*0.025609287*	*0.025876626*	*0.014498882*
**ENSG00000138115**	*0.018808537*	*0.027273873*	*0.025417374*	*0.018851994*
**ENSG00000105376**	*0.060812534*	*0.052941316*	*0.049538909*	*0.054149596*
**ENSG00000170289**	*0.019186561*	*0.026158054*	*0.025195837*	*0.017860481*
**ENSG00000145040**	*0.015732701*	*0.026326188*	*0.025976263*	*0.016572705*
**ENSG00000122584**	*0.013592563*	*0.024470102*	*0.024945691*	*0.014420553*
**ENSG00000183960**	*0.017656006*	*0.026835495*	*0.026744674*	*0.017106785*
**ENSG00000107262**	*0.024413955*	*0.030838908*	*0.032221658*	*0.025350124*
**ENSG00000125531**	*0.018307403*	*0.026177943*	*0.026269003*	*0.019481742*
**ENSG00000089060**	*0.021745291*	*0.037560465*	*0.035513014*	*0.023655508*
**ENSG00000015676**	*0.026467778*	*0.032552394*	*0.034362617*	*0.024784522*
**ENSG00000112996**	*0.023102324*	*0.028914863*	*0.028150198*	*0.019612528*
**ENSG00000155085**	*0.017995575*	*0.02462937*	*0.025829889*	*0.018009631*
**ENSG00000164296**	*0.01927453*	*0.025935231*	*0.030216508*	*0.017616248*
**ENSG00000125746**	*0.030741861*	*0.030470751*	*0.028781432*	*0.022527766*
**ENSG00000154342**	*0.037014105*	*0.048436663*	*0.02701941*	*0.029933435*
**ENSG00000148136**	*0.015536416*	*0.02511255*	*0.025859891*	*0.015227283*
**ENSG00000196611**	*0.018073257*	*0.026165026*	*0.024779766*	*0.016105722*
**ENSG00000137124**	*0.046116427*	*0.03388285*	*0.034059405*	*0.033507629*
**ENSG00000198715**	*0.034461116*	*0.033601802*	*0.030956062*	*0.028221334*
**ENSG00000005471**	*0.048711023*	*0.054104202*	*0.045792729*	*0.051054953*
**ENSG00000152219**	*0.026809446*	*0.033287507*	*0.035304906*	*0.028449332*
**ENSG00000109466**	*0.031518148*	*0.036239325*	*0.032487095*	*0.030912722*
**ENSG00000185272**	*0.025021718*	*0.026386201*	*0.025947423*	*0.018767864*
**ENSG00000088782**	*0.017044195*	*0.025506455*	*0.025255299*	*0.017502221*
**ENSG00000154451**	*0.095683651*	*0.065096654*	*0.072692807*	*0.076321665*
**ENSG00000180011**	*0.038614885*	*0.039038902*	*0.034825624*	*0.029626959*
**ENSG00000164438**	*0.016597588*	*0.025915167*	*0.02493369*	*0.015214874*
**ENSG00000163882**	*0.029747871*	*0.029977553*	*0.02834275*	*0.02134849*
**ENSG00000081026**	*0.01612674*	*0.024901716*	*0.024125515*	*0.014234356*
**ENSG00000143315**	*0.043105964*	*0.040506408*	*0.034619099*	*0.029335536*
**ENSG00000176797**	*0.015489347*	*0.025728592*	*0.024657316*	*0.014422103*
**ENSG00000163497**	*0.015146302*	*0.024603592*	*0.025333801*	*0.014764417*
**ENSG00000129353**	*0.050700823*	*0.040826333*	*0.041612833*	*0.040206097*
**ENSG00000135776**	*0.032704376*	*0.038354091*	*0.03537036*	*0.028897244*
**ENSG00000104823**	*0.02338359*	*0.031113481*	*0.032055892*	*0.025202939*

**ENSG00000198860**	*0.039534771*	*0.036632537*	*0.035141475*	*0.041200936*
**ENSG00000101883**	*0.029078465*	*0.029778341*	*0.033849435*	*0.029635991*
**ENSG00000125744**	*0.041097631*	*0.03676429*	*0.035104965*	*0.034412043*
**ENSG00000165810**	*0.030620508*	*0.025101182*	*0.026951495*	*0.025381979*
**ENSG00000165792**	*0.027701956*	*0.030025926*	*0.027665275*	*0.023195406*
**ENSG00000187546**	*0.017341997*	*0.024122963*	*0.024908524*	*0.014487144*
**ENSG00000196917**	*0.025025399*	*0.026467223*	*0.02744255*	*0.016982532*
**ENSG00000076356**	*0.016373846*	*0.0258785*	*0.02577594*	*0.014707055*
**ENSG00000117154**	*0.018068438*	*0.026728558*	*0.024774046*	*0.017482038*
**ENSG00000117519**	*0.125323706*	*0.100048281*	*0.077683333*	*0.091067028*
**ENSG00000162482**	*0.016354773*	*0.025430146*	*0.024803446*	*0.018243929*
**ENSG00000139697**	*0.036198521*	*0.045804396*	*0.037941505*	*0.03692376*
**ENSG00000204120**	*0.024839233*	*0.034300314*	*0.029307992*	*0.022681448*
**ENSG00000140511**	*0.019657127*	*0.02772904*	*0.029497517*	*0.021880507*
**ENSG00000115524**	*0.024474153*	*0.032215733*	*0.028962947*	*0.020316749*
**ENSG00000160888**	*0.031936871*	*0.033753295*	*0.036585335*	*0.032386687*
**ENSG00000178057**	*0.037639652*	*0.031114175*	*0.032177486*	*0.039687599*
**ENSG00000143333**	*0.050865203*	*0.045783255*	*0.048367194*	*0.050649433*
**ENSG00000115129**	*0.055838492*	*0.047175232*	*0.04449771*	*0.043425543*
**ENSG00000007541**	*0.028200312*	*0.037912541*	*0.039468711*	*0.034991521*
**ENSG00000184857**	*0.023200492*	*0.028655278*	*0.031692505*	*0.019067301*
**ENSG00000010610**	*0.048094933*	*0.03369937*	*0.030623246*	*0.056501955*
**ENSG00000138207**	*0.015594484*	*0.026145621*	*0.024669871*	*0.018488449*
**ENSG00000072401**	*0.027228419*	*0.035363727*	*0.030804196*	*0.027221712*
**ENSG00000151881**	*0.031975967*	*0.033491765*	*0.034670711*	*0.03263385*
**ENSG00000161807**	*0.016247314*	*0.025096852*	*0.024330367*	*0.015080942*
**ENSG00000205356**	*0.027381904*	*0.033364501*	*0.035028605*	*0.032336044*
**ENSG00000167992**	*0.05111792*	*0.050452698*	*0.036579137*	*0.043082035*
**ENSG00000136522**	*0.028863684*	*0.033608538*	*0.031514386*	*0.037073181*
**ENSG00000108523**	*0.041986212*	*0.044657748*	*0.050435255*	*0.031475764*
**ENSG00000129317**	*0.039309764*	*0.038441677*	*0.032198647*	*0.030190042*
**ENSG00000110148**	*0.016196423*	*0.025388467*	*0.026117887*	*0.015777751*
**ENSG00000105679**	*0.018569421*	*0.026614685*	*0.026434728*	*0.017039572*
**ENSG00000150527**	*0.016159488*	*0.025665257*	*0.024754987*	*0.015947916*
**ENSG00000122696**	*0.018684138*	*0.026391368*	*0.026091065*	*0.020537338*
**ENSG00000132383**	*0.026233984*	*0.033312053*	*0.031301246*	*0.027460058*
**ENSG00000129003**	*0.029505149*	*0.036577078*	*0.0330865*	*0.026580091*
**ENSG00000153779**	*0.016031027*	*0.023764757*	*0.024797075*	*0.014650895*
**ENSG00000002586**	*0.047742453*	*0.053359298*	*0.048873791*	*0.041069227*
**ENSG00000197822**	*0.064354599*	*0.046103195*	*0.038960241*	*0.048617142*
**ENSG00000118271**	*0.016471894*	*0.025787503*	*0.027415919*	*0.015369736*
**ENSG00000186844**	*0.02710905*	*0.025286963*	*0.026551535*	*0.017949645*
**ENSG00000165219**	*0.024833714*	*0.031319968*	*0.033308336*	*0.025668746*
**ENSG00000166896**	*0.046588261*	*0.037370786*	*0.040304051*	*0.027734836*
**ENSG00000091844**	*0.090968872*	*0.049933727*	*0.03637203*	*0.066575096*
**ENSG00000153064**	*0.127120073*	*0.104740705*	*0.09276584*	*0.114480226*
**ENSG00000139546**	*0.029234484*	*0.03346521*	*0.034372864*	*0.025711887*
**ENSG00000119866**	*0.042049093*	*0.043355697*	*0.039366486*	*0.045030254*
**ENSG00000189252**	*0.018043948*	*0.026718961*	*0.025811372*	*0.018235561*
**ENSG00000170482**	*0.016232993*	*0.027021653*	*0.027505831*	*0.015617738*
**ENSG00000139572**	*0.035157948*	*0.0361733*	*0.040631868*	*0.043103037*
**ENSG00000111845**	*0.033122975*	*0.035399068*	*0.036704661*	*0.033500663*
**ENSG00000186446**	*0.015706391*	*0.024694124*	*0.024538584*	*0.015282387*
**ENSG00000101464**	*0.031160283*	*0.031961456*	*0.031896367*	*0.024549356*

**ENSG00000198657**	*0.016869552*	*0.025213765*	*0.024382013*	*0.014566589*
**ENSG00000164972**	*0.02513638*	*0.029478941*	*0.03303895*	*0.020110038*
**ENSG00000077092**	*0.015769277*	*0.026145139*	*0.025375802*	*0.014691264*
**ENSG00000171067**	*0.032452168*	*0.034263591*	*0.035108615*	*0.027765539*
**ENSG00000205277**	*0.024462198*	*0.037727278*	*0.038431383*	*0.030935595*
**ENSG00000182255**	*0.01621551*	*0.026101079*	*0.026259027*	*0.01617408*
**ENSG00000242441**	*0.015447073*	*0.025382507*	*0.024434863*	*0.014973322*
**ENSG00000132744**	*0.120101864*	*0.106959321*	*0.101768987*	*0.11579597*
**ENSG00000135951**	*0.022709551*	*0.028257945*	*0.028669758*	*0.019623195*
**ENSG00000178828**	*0.018964847*	*0.026662393*	*0.026226742*	*0.016456739*
**ENSG00000214819**	*0.015309803*	*0.025190935*	*0.024412733*	*0.015048198*
**ENSG00000066455**	*0.027345932*	*0.034282547*	*0.035914007*	*0.026643645*
**ENSG00000228278**	*0.017818325*	*0.025722899*	*0.027029253*	*0.016003866*
**ENSG00000197530**	*0.036913864*	*0.033348351*	*0.039754263*	*0.030929023*
**ENSG00000171055**	*0.037114835*	*0.043629154*	*0.037111926*	*0.032197282*
**ENSG00000248746**	*0.03689418*	*0.031575451*	*0.027488155*	*0.030296469*
**ENSG00000163221**	*0.016469691*	*0.024927133*	*0.025557091*	*0.015774771*
**ENSG00000198563**	*0.023064149*	*0.027910428*	*0.032978152*	*0.022447351*
**ENSG00000182583**	*0.066708948*	*0.03447031*	*0.037128072*	*0.045082376*
**ENSG00000118217**	*0.023474832*	*0.032026516*	*0.033183278*	*0.026304762*
**ENSG00000184056**	*0.02336888*	*0.031514922*	*0.03020697*	*0.020390873*
**ENSG00000138757**	*0.027260031*	*0.032451627*	*0.032202089*	*0.022241379*
**ENSG00000142867**	*0.016336867*	*0.025964959*	*0.025371156*	*0.016212827*
**ENSG00000187288**	*0.019952905*	*0.027664909*	*0.029508896*	*0.019393672*
**ENSG00000011304**	*0.021877943*	*0.027624811*	*0.026878393*	*0.019760616*
**ENSG00000154975**	*0.016455402*	*0.026193321*	*0.025398643*	*0.015742082*
**ENSG00000147679**	*0.030015995*	*0.037110181*	*0.037125674*	*0.031363331*
**ENSG00000156471**	*0.025618056*	*0.030952781*	*0.03287058*	*0.029312639*
**ENSG00000162458**	*0.015285249*	*0.023987744*	*0.025202911*	*0.014063605*
**ENSG00000135698**	*0.037283761*	*0.038676052*	*0.038443734*	*0.02857532*
**ENSG00000182545**	*0.018935145*	*0.027795445*	*0.028737414*	*0.019744382*
**ENSG00000198650**	*0.015560663*	*0.025333981*	*0.025277029*	*0.015671423*
**ENSG00000102054**	*0.02553676*	*0.029785374*	*0.027479896*	*0.022703899*
**ENSG00000173113**	*0.021647627*	*0.027119054*	*0.027526546*	*0.016881691*
**ENSG00000112096**	*0.03811613*	*0.036308033*	*0.035850663*	*0.025962008*
**ENSG00000134121**	*0.063212473*	*0.053685111*	*0.042366462*	*0.051142546*
**ENSG00000121542**	*0.027457669*	*0.031248425*	*0.030667561*	*0.022210005*
**ENSG00000171291**	*0.068613829*	*0.06282407*	*0.043295104*	*0.055056687*
**ENSG00000141642**	*0.030760136*	*0.038366849*	*0.040665413*	*0.035504021*
**ENSG00000164898**	*0.036053531*	*0.037248092*	*0.035210018*	*0.031973793*
**ENSG00000214575**	*0.051007779*	*0.028709761*	*0.031969078*	*0.037910026*
**ENSG00000173262**	*0.057463994*	*0.055644864*	*0.065982083*	*0.059537264*
**ENSG00000197496**	*0.021372297*	*0.027974181*	*0.028444983*	*0.028270255*
**ENSG00000006282**	*0.064062965*	*0.049352819*	*0.037356823*	*0.062033968*
**ENSG00000171116**	*0.017292949*	*0.027260828*	*0.026601958*	*0.018472315*
**ENSG00000135318**	*0.104735344*	*0.088387264*	*0.084586937*	*0.085860841*
**ENSG00000181036**	*0.016312341*	*0.025156626*	*0.02441298*	*0.015231268*
**ENSG00000146070**	*0.021083701*	*0.025946725*	*0.025483509*	*0.015509107*
**ENSG00000147140**	*0.01931992*	*0.026350125*	*0.02695619*	*0.016656077*
**ENSG00000151239**	*0.028681792*	*0.037809435*	*0.041522894*	*0.031290948*
**ENSG00000172724**	*0.050340491*	*0.072915462*	*0.033687195*	*0.024705853*
**ENSG00000174407**	*0.018478808*	*0.025151143*	*0.026052193*	*0.016753074*
**ENSG00000163736**	*0.014959361*	*0.025073636*	*0.025312933*	*0.015057815*
**ENSG00000161243**	*0.085445351*	*0.068522076*	*0.052479462*	*0.057706241*

**ENSG00000138050**	*0.033788081*	*0.040180738*	*0.035051963*	*0.024434519*
**ENSG00000147475**	*0.019373765*	*0.027646581*	*0.025620878*	*0.016898347*
**ENSG00000179057**	*0.02049801*	*0.025968581*	*0.025370194*	*0.016161098*
**ENSG00000135114**	*0.042057485*	*0.050109428*	*0.047324048*	*0.040419862*
**ENSG00000182318**	*0.021118249*	*0.027942473*	*0.028944332*	*0.019149788*
**ENSG00000204531**	*0.017113171*	*0.025496414*	*0.026069332*	*0.016313518*
**ENSG00000164619**	*0.015083632*	*0.025122746*	*0.026399595*	*0.016107865*
**ENSG00000087087**	*0.033923676*	*0.032594564*	*0.032315824*	*0.031685881*
**ENSG00000147488**	*0.016264069*	*0.026986078*	*0.024141306*	*0.015741192*
**ENSG00000185100**	*0.016296898*	*0.027100627*	*0.025271112*	*0.016706666*
**ENSG00000187601**	*0.030325091*	*0.036313035*	*0.037889246*	*0.026332272*
**ENSG00000086548**	*0.016860398*	*0.02612706*	*0.024838334*	*0.015471906*
**ENSG00000135763**	*0.041922377*	*0.041028353*	*0.046887002*	*0.032742829*
**ENSG00000182272**	*0.015355336*	*0.025698309*	*0.02564701*	*0.015402278*
**ENSG00000099999**	*0.020745037*	*0.027018542*	*0.027205077*	*0.02180431*
**ENSG00000162399**	*0.017052157*	*0.027083219*	*0.026675437*	*0.016309679*
**ENSG00000161395**	*0.036951082*	*0.038496319*	*0.038824516*	*0.032561928*
**ENSG00000085563**	*0.045574703*	*0.043265127*	*0.036608795*	*0.049932982*
**ENSG00000161091**	*0.029074945*	*0.035187227*	*0.033669922*	*0.028440752*
**ENSG00000105467**	*0.017700018*	*0.025341897*	*0.025692187*	*0.01634468*
**ENSG00000108518**	*0.015463075*	*0.024497497*	*0.023874351*	*0.016099392*
**ENSG00000112029**	*0.048629945*	*0.038094745*	*0.03805334*	*0.035576415*
**ENSG00000222040**	*0.021620213*	*0.026044349*	*0.028114462*	*0.016652095*
**ENSG00000184481**	*0.019579811*	*0.028180418*	*0.026685531*	*0.02265524*
**ENSG00000213619**	*0.024695862*	*0.028245604*	*0.027566655*	*0.019828149*
**ENSG00000165935**	*0.016573095*	*0.027282545*	*0.025278207*	*0.019167109*
**ENSG00000172967**	*0.015586141*	*0.024745428*	*0.024950941*	*0.015639568*
**ENSG00000163467**	*0.018349106*	*0.025998584*	*0.024152015*	*0.016476257*
**ENSG00000158220**	*0.015223206*	*0.025122004*	*0.024714768*	*0.016645574*
**ENSG00000179097**	*0.017160582*	*0.026804339*	*0.025012326*	*0.017464065*
**ENSG00000100836**	*0.029402417*	*0.034685375*	*0.031204229*	*0.022048897*
**ENSG00000126756**	*0.027571883*	*0.031659627*	*0.028829938*	*0.028814279*
**ENSG00000135454**	*0.024482659*	*0.026887998*	*0.027382411*	*0.021117282*
**ENSG00000134453**	*0.028148038*	*0.033633353*	*0.031231586*	*0.026631616*
**ENSG00000136274**	*0.015776745*	*0.026585256*	*0.02528356*	*0.014817567*
**ENSG00000004455**	*0.039590697*	*0.030550823*	*0.031988716*	*0.028446037*
**ENSG00000173947**	*0.030087202*	*0.032009771*	*0.027109841*	*0.023353355*
**ENSG00000125462**	*0.066542146*	*0.050609391*	*0.047920062*	*0.058542464*
**ENSG00000213420**	*0.030555129*	*0.03163964*	*0.027472376*	*0.023127877*
**ENSG00000138964**	*0.03502325*	*0.037213203*	*0.040158201*	*0.04635853*
**ENSG00000105136**	*0.023226759*	*0.035364628*	*0.032932086*	*0.022654427*
**ENSG00000167632**	*0.029043367*	*0.036027888*	*0.034283847*	*0.028148579*
**ENSG00000151466**	*0.040189046*	*0.04640645*	*0.034856343*	*0.03177256*
**ENSG00000129480**	*0.029948502*	*0.030232938*	*0.032120663*	*0.026782731*
**ENSG00000188993**	*0.016348753*	*0.024855718*	*0.02466578*	*0.014788723*
**ENSG00000160883**	*0.019299185*	*0.026410534*	*0.026784328*	*0.019681843*
**ENSG00000164076**	*0.018150768*	*0.025958209*	*0.027458295*	*0.017493581*
**ENSG00000187461**	*0.057271177*	*0.040029417*	*0.045554618*	*0.057655638*
**ENSG00000170209**	*0.016209691*	*0.024775496*	*0.025548041*	*0.015836366*
**ENSG00000168079**	*0.015781954*	*0.024452294*	*0.025604845*	*0.014219252*
**ENSG00000185950**	*0.017628651*	*0.02485247*	*0.024811474*	*0.015990403*
**ENSG00000170091**	*0.016094979*	*0.025246594*	*0.024586787*	*0.014877118*
**ENSG00000129071**	*0.025808415*	*0.031824287*	*0.030114487*	*0.022796132*
**ENSG00000206549**	*0.020480593*	*0.02607564*	*0.024830098*	*0.028346763*

**ENSG00000164329**	*0.024412564*	*0.032752098*	*0.029481938*	*0.022688475*
**ENSG00000156983**	*0.030567763*	*0.044373473*	*0.053417738*	*0.03242282*
**ENSG00000047578**	*0.023581991*	*0.031294332*	*0.028740727*	*0.022958808*
**ENSG00000214046**	*0.028385826*	*0.034580486*	*0.032746352*	*0.024786677*
**ENSG00000108669**	*0.023866362*	*0.031616952*	*0.029636227*	*0.022030702*
**ENSG00000119912**	*0.035778442*	*0.041568551*	*0.034844671*	*0.032584228*
**ENSG00000172053**	*0.020918995*	*0.027123031*	*0.026023562*	*0.020413003*
**ENSG00000163132**	*0.123143186*	*0.09562806*	*0.106592934*	*0.112444942*
**ENSG00000228083**	*0.020179796*	*0.025841095*	*0.026794674*	*0.01794086*
**ENSG00000108759**	*0.016337694*	*0.024801402*	*0.024716218*	*0.014692563*
**ENSG00000070371**	*0.015057207*	*0.02534428*	*0.025895588*	*0.015668256*
**ENSG00000107014**	*0.049448244*	*0.041744018*	*0.041494536*	*0.04038023*
**ENSG00000167393**	*0.045667386*	*0.049514128*	*0.04232489*	*0.04101576*
**ENSG00000181562**	*0.017104014*	*0.025158084*	*0.02412428*	*0.016396906*
**ENSG00000006047**	*0.019838959*	*0.028700428*	*0.027510121*	*0.017785034*
**ENSG00000038945**	*0.016863845*	*0.024343664*	*0.026231779*	*0.015338598*
**ENSG00000168813**	*0.016379939*	*0.025260309*	*0.025118044*	*0.015226431*
**ENSG00000158079**	*0.015916618*	*0.025526267*	*0.024147415*	*0.015536826*
**ENSG00000177239**	*0.014267269*	*0.024865634*	*0.025498271*	*0.014043178*
**ENSG00000184012**	*0.018586403*	*0.024853921*	*0.024517914*	*0.015216529*
**ENSG00000053372**	*0.025260984*	*0.029521237*	*0.033070119*	*0.021448972*
**ENSG00000077063**	*0.017596465*	*0.026595945*	*0.026542542*	*0.016194361*
**ENSG00000183813**	*0.016794174*	*0.025653872*	*0.02427687*	*0.014684903*
**ENSG00000134443**	*0.019368114*	*0.025619037*	*0.024781084*	*0.017064988*
**ENSG00000108691**	*0.019372151*	*0.026089809*	*0.025547823*	*0.018911945*
**ENSG00000107147**	*0.014897623*	*0.024882764*	*0.025270615*	*0.015697444*
**ENSG00000170619**	*0.01849838*	*0.02860483*	*0.025757482*	*0.020192668*
**ENSG00000145990**	*0.048801686*	*0.043162422*	*0.042862278*	*0.038687536*
**ENSG00000151632**	*0.06041463*	*0.027409985*	*0.025427508*	*0.015969395*
**ENSG00000121057**	*0.026579149*	*0.028376052*	*0.027382864*	*0.022807409*
**ENSG00000131368**	*0.036203603*	*0.037377876*	*0.033856478*	*0.029205243*
**ENSG00000013573**	*0.038755773*	*0.041565319*	*0.036430614*	*0.029215267*
**ENSG00000126259**	*0.017390402*	*0.026046724*	*0.025459173*	*0.016734384*
**ENSG00000158578**	*0.015718227*	*0.024657079*	*0.025198745*	*0.014410628*
**ENSG00000155890**	*0.01536823*	*0.025351006*	*0.023988696*	*0.014841031*
**ENSG00000165194**	*0.016191236*	*0.025083735*	*0.026427123*	*0.015217039*
**ENSG00000120963**	*0.020948601*	*0.027804866*	*0.028035101*	*0.018074646*
**ENSG00000124226**	*0.020597845*	*0.028707814*	*0.028831221*	*0.018623525*
**ENSG00000159527**	*0.015344686*	*0.024154079*	*0.025157845*	*0.014995039*
**ENSG00000182400**	*0.028976589*	*0.033492333*	*0.033174118*	*0.02403581*
**ENSG00000182162**	*0.036843417*	*0.036794571*	*0.033649367*	*0.026691224*
**ENSG00000184677**	*0.034900141*	*0.040932126*	*0.035172616*	*0.029135382*
**ENSG00000175928**	*0.016979647*	*0.026353656*	*0.024907712*	*0.016200967*
**ENSG00000139197**	*0.029346438*	*0.032937603*	*0.031980515*	*0.021237488*
**ENSG00000183814**	*0.047905192*	*0.048301016*	*0.036078692*	*0.042269672*
**ENSG00000005238**	*0.02614988*	*0.034036257*	*0.030787147*	*0.025013172*
**ENSG00000237110**	*0.016331794*	*0.026585627*	*0.025569253*	*0.01675613*
**ENSG00000134107**	*0.05856395*	*0.035816194*	*0.044895629*	*0.047293261*
**ENSG00000136824**	*0.050514208*	*0.051635987*	*0.036439904*	*0.05496954*
**ENSG00000100122**	*0.015353455*	*0.025723194*	*0.026303743*	*0.015795746*
**ENSG00000164603**	*0.043116253*	*0.050554021*	*0.039713056*	*0.041573342*
**ENSG00000157600**	*0.039184802*	*0.039552711*	*0.037149863*	*0.034589828*
**ENSG00000160746**	*0.081537798*	*0.049269754*	*0.039042526*	*0.06471909*
**ENSG00000175544**	*0.014860196*	*0.024798781*	*0.024568625*	*0.015010194*

**ENSG00000171791**	*0.058810916*	*0.061368754*	*0.052925442*	*0.043955552*
**ENSG00000121691**	*0.041198464*	*0.044234405*	*0.037166797*	*0.026827577*
**ENSG00000121101**	*0.015124507*	*0.025787856*	*0.024901811*	*0.013803444*
**ENSG00000170540**	*0.023262291*	*0.033275257*	*0.030421318*	*0.02293433*
**ENSG00000184983**	*0.031297677*	*0.034202367*	*0.035693992*	*0.032949181*
**ENSG00000146918**	*0.047684242*	*0.052368757*	*0.036972945*	*0.036198284*
**ENSG00000196526**	*0.018342523*	*0.026985881*	*0.026200035*	*0.015751361*
**ENSG00000130522**	*0.029259673*	*0.038250139*	*0.04455492*	*0.028072387*
**ENSG00000111605**	*0.03755756*	*0.037619946*	*0.038424052*	*0.039647176*
**ENSG00000169851**	*0.016424924*	*0.024471125*	*0.025089949*	*0.014785433*
**ENSG00000037280**	*0.049133364*	*0.042632032*	*0.037693132*	*0.047609869*
**ENSG00000173918**	*0.016374491*	*0.025100741*	*0.02494401*	*0.016323088*
**ENSG00000179361**	*0.030312973*	*0.032401942*	*0.03221859*	*0.025351279*
**ENSG00000044524**	*0.018799937*	*0.026323477*	*0.025091387*	*0.016733138*
**ENSG00000066279**	*0.047659524*	*0.040559468*	*0.03733531*	*0.036707396*
**ENSG00000197142**	*0.017476738*	*0.028183632*	*0.027251054*	*0.015890974*
**ENSG00000197965**	*0.038615148*	*0.043514737*	*0.039027843*	*0.032702934*
**ENSG00000189077**	*0.060676791*	*0.051930548*	*0.07214227*	*0.059808045*
**ENSG00000062725**	*0.035920075*	*0.038023204*	*0.034212557*	*0.040077793*
**ENSG00000239306**	*0.031869577*	*0.03767049*	*0.033021871*	*0.025763273*
**ENSG00000137142**	*0.017353606*	*0.025390941*	*0.025357055*	*0.01637759*
**ENSG00000117697**	*0.028036944*	*0.034615635*	*0.033640604*	*0.027176942*
**ENSG00000125815**	*0.015920302*	*0.025310709*	*0.025087393*	*0.015285441*
**ENSG00000072163**	*0.052493449*	*0.042972286*	*0.044259171*	*0.047181735*
**ENSG00000163064**	*0.015561305*	*0.024644369*	*0.024609568*	*0.016512587*
**ENSG00000143702**	*0.033961951*	*0.047792764*	*0.043284593*	*0.046284297*
**ENSG00000189221**	*0.017017813*	*0.024686227*	*0.025390681*	*0.01501046*
**ENSG00000160785**	*0.030539347*	*0.038297813*	*0.03994811*	*0.035484754*
**ENSG00000129988**	*0.016955097*	*0.025542213*	*0.025700037*	*0.016331278*
**ENSG00000106560**	*0.042838435*	*0.044255052*	*0.041558736*	*0.03780565*
**ENSG00000172215**	*0.014668674*	*0.025338238*	*0.024330279*	*0.015057167*
**ENSG00000198728**	*0.017000884*	*0.02717066*	*0.028863611*	*0.015969553*
**ENSG00000214113**	*0.039574323*	*0.035411315*	*0.037461635*	*0.033643429*
**ENSG00000196236**	*0.022315574*	*0.027433998*	*0.029190522*	*0.019150983*
**ENSG00000164535**	*0.034708498*	*0.038255436*	*0.036789256*	*0.028762406*
**ENSG00000115616**	*0.023275892*	*0.030946775*	*0.025581792*	*0.0193344*
**ENSG00000160209**	*0.0363009*	*0.034090556*	*0.038491888*	*0.032183887*
**ENSG00000142405**	*0.016563778*	*0.024622767*	*0.024898013*	*0.015319213*
**ENSG00000173153**	*0.029536642*	*0.037079644*	*0.034224499*	*0.028378961*
**ENSG00000185686**	*0.024308705*	*0.029539565*	*0.026132928*	*0.062744217*
**ENSG00000168958**	*0.027953972*	*0.034588773*	*0.030088117*	*0.025055106*
**ENSG00000104723**	*0.030615226*	*0.025764448*	*0.026233219*	*0.019459699*
**ENSG00000167779**	*0.040996039*	*0.032067869*	*0.029151029*	*0.021050527*
**ENSG00000111667**	*0.017304852*	*0.026472095*	*0.025058469*	*0.01614028*
**ENSG00000177697**	*0.070081245*	*0.050044381*	*0.049493426*	*0.050184005*
**ENSG00000163362**	*0.116876937*	*0.082001457*	*0.080159205*	*0.085973469*
**ENSG00000176692**	*0.014956025*	*0.024729914*	*0.025104336*	*0.014603039*
**ENSG00000137656**	*0.033600966*	*0.035186416*	*0.035133779*	*0.031638852*
**ENSG00000123500**	*0.017753252*	*0.026689361*	*0.025237019*	*0.016151209*
**ENSG00000125482**	*0.027535076*	*0.033720382*	*0.029617975*	*0.020414362*
**ENSG00000105339**	*0.052516013*	*0.04316853*	*0.03995572*	*0.045168231*
**ENSG00000150526**	*0.014752573*	*0.025174566*	*0.025729424*	*0.015701385*
**ENSG00000106617**	*0.036428865*	*0.033618261*	*0.032702074*	*0.027914054*
**ENSG00000104848**	*0.015726314*	*0.025345192*	*0.025475426*	*0.015782807*

**ENSG00000104691**	*0.03305515*	*0.036230056*	*0.034490246*	*0.027024871*
**ENSG00000253313**	*0.015199562*	*0.025571772*	*0.025296867*	*0.016062981*
**ENSG00000204681**	*0.015180408*	*0.026048489*	*0.024541641*	*0.015707918*
**ENSG00000175274**	*0.017501144*	*0.025274304*	*0.024854547*	*0.01531514*
**ENSG00000006634**	*0.036235679*	*0.03541478*	*0.037345609*	*0.036734813*
**ENSG00000118017**	*0.015458532*	*0.024985746*	*0.025019776*	*0.015256606*
**ENSG00000211454**	*0.017581715*	*0.024764846*	*0.026239393*	*0.018543464*
**ENSG00000155304**	*0.03774019*	*0.036593377*	*0.036767194*	*0.042217384*
**ENSG00000221983**	*0.043816496*	*0.046709334*	*0.048331838*	*0.020346347*
**ENSG00000198478**	*0.026435027*	*0.026645102*	*0.026048249*	*0.019611392*
**ENSG00000179454**	*0.041930238*	*0.038127192*	*0.038156003*	*0.044263832*
**ENSG00000130475**	*0.033483945*	*0.034405776*	*0.034724828*	*0.036608775*
**ENSG00000116981**	*0.016641857*	*0.02430243*	*0.025191788*	*0.016124613*
**ENSG00000109390**	*0.036294562*	*0.034217212*	*0.035321637*	*0.035225999*
**ENSG00000143450**	*0.019185308*	*0.028889254*	*0.026167486*	*0.018123592*
**ENSG00000180016**	*0.015340883*	*0.02552255*	*0.025376695*	*0.015659683*
**ENSG00000221878**	*0.018917118*	*0.026018755*	*0.028006493*	*0.020028744*
**ENSG00000145536**	*0.015349903*	*0.024685633*	*0.024241745*	*0.016444108*
**ENSG00000123454**	*0.019248085*	*0.029358359*	*0.025639763*	*0.018012637*
**ENSG00000109063**	*0.029812889*	*0.031784444*	*0.029371584*	*0.020215909*
**ENSG00000145349**	*0.050339594*	*0.051903949*	*0.044704535*	*0.050923674*
**ENSG00000165023**	*0.0172583*	*0.027075094*	*0.025922069*	*0.017048597*
**ENSG00000130962**	*0.01973625*	*0.031579435*	*0.027455172*	*0.018951995*
**ENSG00000137573**	*0.070005029*	*0.024388606*	*0.025154103*	*0.015987507*
**ENSG00000130054**	*0.016719115*	*0.025248564*	*0.024903805*	*0.016032804*
**ENSG00000185567**	*0.019749963*	*0.026523282*	*0.026712268*	*0.019329069*
**ENSG00000165071**	*0.020106588*	*0.044225864*	*0.040231581*	*0.027984338*
**ENSG00000087253**	*0.015169616*	*0.025292929*	*0.024789838*	*0.015826407*
**ENSG00000123091**	*0.021272021*	*0.0355405*	*0.027261406*	*0.036061801*
**ENSG00000196873**	*0.030422071*	*0.032536875*	*0.029046195*	*0.024964645*
**ENSG00000175325**	*0.015876337*	*0.025531257*	*0.025062274*	*0.015531255*
**ENSG00000091527**	*0.025036469*	*0.03459503*	*0.033369197*	*0.021988042*
**ENSG00000160961**	*0.017033809*	*0.025608585*	*0.026042503*	*0.016202411*
**ENSG00000010626**	*0.051585932*	*0.044231459*	*0.056503698*	*0.044423568*
**ENSG00000141655**	*0.061384894*	*0.054638736*	*0.054101459*	*0.053410983*
**ENSG00000120256**	*0.017337957*	*0.025060313*	*0.025310552*	*0.015106662*
**ENSG00000181617**	*0.015225127*	*0.024284931*	*0.02551961*	*0.014650292*
**ENSG00000150760**	*0.014844282*	*0.026653267*	*0.024888887*	*0.015509677*
**ENSG00000234545**	*0.03124592*	*0.035901932*	*0.029988155*	*0.030157805*
**ENSG00000198805**	*0.045126529*	*0.040230006*	*0.039761181*	*0.0428102*
**ENSG00000167377**	*0.029424901*	*0.035239297*	*0.030939526*	*0.021711379*
**ENSG00000108055**	*0.029615504*	*0.035081398*	*0.030051195*	*0.024380599*
**ENSG00000101444**	*0.025218174*	*0.029797843*	*0.028188847*	*0.024352572*
**ENSG00000179564**	*0.017913305*	*0.024699324*	*0.024564453*	*0.015161304*
**ENSG00000174343**	*0.015827734*	*0.025100935*	*0.02484061*	*0.016243496*
**ENSG00000111671**	*0.050920063*	*0.055060392*	*0.045671631*	*0.048387174*
**ENSG00000185038**	*0.014536448*	*0.025189771*	*0.024967511*	*0.014576305*
**ENSG00000141295**	*0.037605029*	*0.03924612*	*0.036524972*	*0.03432528*
**ENSG00000163380**	*0.015612998*	*0.02503065*	*0.024722762*	*0.016362599*
**ENSG00000174792**	*0.019562799*	*0.024474781*	*0.025009775*	*0.015473441*
**ENSG00000157764**	*0.031561745*	*0.036180984*	*0.033981934*	*0.027135156*
**ENSG00000093072**	*0.014768609*	*0.024814311*	*0.025057226*	*0.014575828*
**ENSG00000115641**	*0.085752577*	*0.049100711*	*0.041011118*	*0.054584803*
**ENSG00000005955**	*0.025956186*	*0.037746501*	*0.035767759*	*0.025940986*

**ENSG00000056097**	*0.021012625*	*0.028183575*	*0.030465797*	*0.021148356*
**ENSG00000063244**	*0.026394337*	*0.033883028*	*0.028109534*	*0.02330691*
**ENSG00000106853**	*0.089427792*	*0.090167372*	*0.073168154*	*0.083695249*
**ENSG00000132376**	*0.01706966*	*0.024753833*	*0.024566508*	*0.015282252*
**ENSG00000113273**	*0.026835097*	*0.0314328*	*0.026848609*	*0.022597908*
**ENSG00000147168**	*0.030263873*	*0.032460271*	*0.034759318*	*0.031516081*
**ENSG00000186231**	*0.018474808*	*0.026228433*	*0.025147561*	*0.016872812*
**ENSG00000100012**	*0.015842428*	*0.02501869*	*0.02439156*	*0.014710214*
**ENSG00000197172**	*0.018102484*	*0.025352579*	*0.026620137*	*0.016577709*
**ENSG00000111276**	*0.035427234*	*0.04186352*	*0.039867363*	*0.039614858*
**ENSG00000100604**	*0.048766903*	*0.033314611*	*0.03406062*	*0.034408339*
**ENSG00000235750**	*0.043065814*	*0.046459325*	*0.037313133*	*0.041292047*
**ENSG00000105383**	*0.060406721*	*0.042881992*	*0.054670171*	*0.061541928*
**ENSG00000092067**	*0.015813816*	*0.026121969*	*0.025167128*	*0.017534702*
**ENSG00000091513**	*0.015626571*	*0.026077934*	*0.024877094*	*0.017864134*
**ENSG00000182793**	*0.015248008*	*0.024849966*	*0.024505492*	*0.015388905*
**ENSG00000171819**	*0.015741369*	*0.024440085*	*0.024455278*	*0.015204727*
**ENSG00000106688**	*0.043534071*	*0.051785071*	*0.04241181*	*0.041496247*
**ENSG00000109220**	*0.025521635*	*0.034413712*	*0.034554133*	*0.027578237*
**ENSG00000117174**	*0.031858277*	*0.035712177*	*0.034747973*	*0.031896829*
**ENSG00000147588**	*0.016806424*	*0.025363908*	*0.025205018*	*0.016630856*
**ENSG00000170264**	*0.049462627*	*0.05209385*	*0.04041988*	*0.042720289*
**ENSG00000165102**	*0.02002407*	*0.025802457*	*0.025156675*	*0.016318499*
**ENSG00000166133**	*0.029446284*	*0.034925268*	*0.037020739*	*0.028039891*
**ENSG00000093009**	*0.051011815*	*0.045202568*	*0.037019168*	*0.039446976*
**ENSG00000147234**	*0.015628481*	*0.025071346*	*0.024921626*	*0.014388282*
**ENSG00000166741**	*0.016079745*	*0.0258461*	*0.025453726*	*0.015291487*
**ENSG00000186792**	*0.016738026*	*0.0259094*	*0.025962592*	*0.016769235*
**ENSG00000141279**	*0.027776486*	*0.036218603*	*0.028640663*	*0.023232393*
**ENSG00000101365**	*0.016967256*	*0.026117741*	*0.025954319*	*0.015400514*
**ENSG00000125207**	*0.018209098*	*0.028418565*	*0.025456908*	*0.017214068*
**ENSG00000167695**	*0.045560869*	*0.040292645*	*0.036852005*	*0.031475624*
**ENSG00000134245**	*0.017119376*	*0.024764327*	*0.025384424*	*0.015578701*
**ENSG00000136205**	*0.07669059*	*0.071192411*	*0.058261464*	*0.061508566*
**ENSG00000134256**	*0.020159226*	*0.026238107*	*0.025378112*	*0.017742597*
**ENSG00000186980**	*0.017671124*	*0.025188642*	*0.027141988*	*0.017120735*
**ENSG00000143190**	*0.035069675*	*0.033478049*	*0.030155862*	*0.026852557*
**ENSG00000145604**	*0.026302946*	*0.03037454*	*0.028082884*	*0.019827101*
**ENSG00000197557**	*0.03843107*	*0.051027644*	*0.034229473*	*0.028067939*
**ENSG00000182902**	*0.016396227*	*0.025044882*	*0.025203394*	*0.014338172*
**ENSG00000167081**	*0.055597348*	*0.046034866*	*0.037255362*	*0.052903061*
**ENSG00000146006**	*0.016053238*	*0.024395794*	*0.024229912*	*0.014566875*
**ENSG00000085831**	*0.06352222*	*0.053411725*	*0.044119333*	*0.050068958*
**ENSG00000164825**	*0.015525133*	*0.025332049*	*0.025916441*	*0.015253805*
**ENSG00000143401**	*0.041484546*	*0.045154923*	*0.036180656*	*0.057226732*
**ENSG00000198818**	*0.024052844*	*0.031058758*	*0.027505989*	*0.022136478*
**ENSG00000102898**	*0.027420033*	*0.031842193*	*0.030509905*	*0.025933921*
**ENSG00000182993**	*0.036252504*	*0.041516114*	*0.036145892*	*0.031760569*
**ENSG00000120658**	*0.025369669*	*0.028369168*	*0.02645145*	*0.017535047*
**ENSG00000153531**	*0.021266863*	*0.025834426*	*0.025026864*	*0.017897468*
**ENSG00000119673**	*0.038916768*	*0.041165644*	*0.031833083*	*0.038292302*
**ENSG00000188343**	*0.049455869*	*0.0425078*	*0.045357755*	*0.055393271*
**ENSG00000087077**	*0.040988862*	*0.031353336*	*0.031301371*	*0.028033322*
**ENSG00000187730**	*0.01630377*	*0.025545782*	*0.025141396*	*0.015760379*

**ENSG00000170929**	*0.014931616*	*0.024387468*	*0.024490207*	*0.015147903*
**ENSG00000163026**	*0.027280141*	*0.029610739*	*0.030022182*	*0.024490768*
**ENSG00000242108**	*0.016075589*	*0.026174619*	*0.024989711*	*0.016719292*
**ENSG00000240344**	*0.040304674*	*0.030187946*	*0.029076975*	*0.045760627*
**ENSG00000093144**	*0.029970167*	*0.034136622*	*0.030093009*	*0.027671777*
**ENSG00000083844**	*0.03573845*	*0.039632082*	*0.039689941*	*0.029843783*
**ENSG00000108270**	*0.02124142*	*0.027144485*	*0.028901064*	*0.017731618*
**ENSG00000125841**	*0.042315657*	*0.035964596*	*0.035213874*	*0.037810003*
**ENSG00000135436**	*0.025841082*	*0.026049577*	*0.026715967*	*0.016720051*
**ENSG00000172037**	*0.015855863*	*0.023776439*	*0.025396525*	*0.015076332*
**ENSG00000092850**	*0.014551495*	*0.023867577*	*0.024341009*	*0.01469708*
**ENSG00000136052**	*0.032286052*	*0.038282846*	*0.039586436*	*0.031453742*
**ENSG00000120087**	*0.056446545*	*0.043664832*	*0.041058278*	*0.042207649*
**ENSG00000139719**	*0.029944832*	*0.036671379*	*0.03403055*	*0.033060484*
**ENSG00000213927**	*0.015945008*	*0.025337496*	*0.025545662*	*0.01533503*
**ENSG00000215305**	*0.02582593*	*0.03621522*	*0.031368072*	*0.029692146*
**ENSG00000144583**	*0.016266017*	*0.024641748*	*0.025307232*	*0.0155573*
**ENSG00000162542**	*0.040648684*	*0.039567744*	*0.037969281*	*0.035404033*
**ENSG00000121318**	*0.016959285*	*0.02405266*	*0.025569414*	*0.016186354*
**ENSG00000174628**	*0.048876442*	*0.039516281*	*0.033031148*	*0.036923321*
**ENSG00000117500**	*0.02292788*	*0.030349553*	*0.033611909*	*0.028740867*
**ENSG00000178691**	*0.0265108*	*0.033451225*	*0.028667012*	*0.026368863*
**ENSG00000154065**	*0.105074262*	*0.073323148*	*0.064950276*	*0.076049267*
**ENSG00000005194**	*0.032651464*	*0.037120834*	*0.036078501*	*0.034194841*
**ENSG00000204439**	*0.029536891*	*0.037596661*	*0.036809004*	*0.026796436*
**ENSG00000154127**	*0.032295535*	*0.026103635*	*0.031514778*	*0.028413155*
**ENSG00000155906**	*0.027333018*	*0.036153014*	*0.028714993*	*0.022817519*
**ENSG00000185842**	*0.016744718*	*0.025598643*	*0.024559512*	*0.014871005*
**ENSG00000095139**	*0.021146792*	*0.029768437*	*0.029807234*	*0.022203502*
**ENSG00000075891**	*0.015230198*	*0.025021819*	*0.025657218*	*0.015185801*
**ENSG00000169710**	*0.029139806*	*0.032004167*	*0.026992042*	*0.024585379*
**ENSG00000249115**	*0.021481592*	*0.027900298*	*0.028151917*	*0.01861074*
**ENSG00000182405**	*0.028359482*	*0.036468372*	*0.030917342*	*0.027753738*
**ENSG00000139626**	*0.055325154*	*0.043289206*	*0.041262777*	*0.058016987*
**ENSG00000167964**	*0.02469604*	*0.026608468*	*0.028950835*	*0.026723035*
**ENSG00000160218**	*0.032395025*	*0.035624207*	*0.038185957*	*0.032297591*
**ENSG00000159593**	*0.029246742*	*0.033813318*	*0.031337331*	*0.022199608*
**ENSG00000124215**	*0.016042848*	*0.025149445*	*0.024727039*	*0.015943404*
**ENSG00000112304**	*0.027963614*	*0.030938128*	*0.028051012*	*0.027403138*
**ENSG00000172179**	*0.119104161*	*0.026232163*	*0.025167779*	*0.0372419*
**ENSG00000164761**	*0.125635612*	*0.079920418*	*0.042395251*	*0.05257761*
**ENSG00000146122**	*0.1453242*	*0.081384099*	*0.052190222*	*0.073564381*
**ENSG00000183682**	*0.016622237*	*0.024736954*	*0.024738845*	*0.014510686*
**ENSG00000147119**	*0.039943055*	*0.042684966*	*0.034563151*	*0.033953998*
**ENSG00000118729**	*0.016508411*	*0.024581873*	*0.024973393*	*0.015531783*
**ENSG00000079482**	*0.01610798*	*0.025423569*	*0.02492372*	*0.015762781*
**ENSG00000138175**	*0.035388447*	*0.037597823*	*0.033331721*	*0.025641096*
**ENSG00000119917**	*0.061205304*	*0.069578599*	*0.06440174*	*0.075003803*
**ENSG00000162975**	*0.015857528*	*0.024855646*	*0.025306093*	*0.014803753*
**ENSG00000132823**	*0.02469027*	*0.033188573*	*0.033330965*	*0.025416728*
**ENSG00000173482**	*0.03605037*	*0.027170031*	*0.026847952*	*0.022766168*
**ENSG00000204019**	*0.015507562*	*0.025556068*	*0.025330159*	*0.042468565*
**ENSG00000213030**	*0.016180542*	*0.025377692*	*0.025606406*	*0.016250405*
**ENSG00000169045**	*0.031174817*	*0.040645265*	*0.035195813*	*0.027975221*

**ENSG00000117682**	*0.03002504*	*0.035891265*	*0.039474106*	*0.030053481*
**ENSG00000015568**	*0.03197301*	*0.035111956*	*0.029574077*	*0.023651426*
**ENSG00000132207**	*0.037935086*	*0.036952132*	*0.036466069*	*0.027001344*
**ENSG00000185254**	*0.01885058*	*0.026055393*	*0.025014365*	*0.016827769*
**ENSG00000138193**	*0.017185475*	*0.025151616*	*0.024872142*	*0.016234814*
**ENSG00000119950**	*0.015999235*	*0.024028115*	*0.025103369*	*0.01511471*
**ENSG00000100994**	*0.031426133*	*0.030796143*	*0.032484468*	*0.027338458*
**ENSG00000143633**	*0.026772175*	*0.030710946*	*0.031600046*	*0.025043782*
**ENSG00000065882**	*0.04026856*	*0.038126897*	*0.042049301*	*0.036551585*
**ENSG00000198131**	*0.034976787*	*0.040821257*	*0.049256998*	*0.043653431*
**ENSG00000244486**	*0.018696275*	*0.025233133*	*0.026025266*	*0.017755878*
**ENSG00000205221**	*0.018996856*	*0.03086208*	*0.028744934*	*0.019399913*
**ENSG00000105173**	*0.035835998*	*0.034497147*	*0.037910476*	*0.030394136*
**ENSG00000170180**	*0.016908208*	*0.025597923*	*0.02514049*	*0.016897049*
**ENSG00000171136**	*0.014297255*	*0.024756101*	*0.02524771*	*0.014806785*
**ENSG00000134198**	*0.01632417*	*0.025008474*	*0.02505928*	*0.015748327*
**ENSG00000125398**	*0.081316219*	*0.06292248*	*0.056202448*	*0.07567329*
**ENSG00000183317**	*0.016470434*	*0.025998293*	*0.026119865*	*0.016511584*
**ENSG00000107020**	*0.033128474*	*0.035400202*	*0.032510061*	*0.029675826*
**ENSG00000100156**	*0.016386317*	*0.024710056*	*0.025858951*	*0.014240101*
**ENSG00000112619**	*0.01592259*	*0.024470259*	*0.02490534*	*0.016087213*
**ENSG00000094804**	*0.02077775*	*0.027148798*	*0.025632688*	*0.018504936*
**ENSG00000168615**	*0.028784973*	*0.032562907*	*0.031232133*	*0.020463729*
**ENSG00000109101**	*0.016121194*	*0.025388379*	*0.025787043*	*0.015710044*
**ENSG00000174516**	*0.018032288*	*0.0287927*	*0.025421875*	*0.018788715*
**ENSG00000171103**	*0.031915348*	*0.033792082*	*0.028846648*	*0.023608648*
**ENSG00000196935**	*0.027553842*	*0.031241525*	*0.029471561*	*0.021504315*
**ENSG00000163359**	*0.015550557*	*0.02464161*	*0.024904264*	*0.015733705*
**ENSG00000089154**	*0.027722492*	*0.033140399*	*0.033610106*	*0.022584801*
**ENSG00000124610**	*0.016814718*	*0.024470611*	*0.024717536*	*0.015557599*
**ENSG00000162944**	*0.016628195*	*0.025203209*	*0.024351107*	*0.016489966*
**ENSG00000153802**	*0.016482507*	*0.024496958*	*0.02529453*	*0.014013792*
**ENSG00000121552**	*0.099087878*	*0.104245036*	*0.059202934*	*0.055070494*
**ENSG00000197448**	*0.02465364*	*0.029056111*	*0.028496623*	*0.023224587*
**ENSG00000095370**	*0.031817803*	*0.030152409*	*0.038848481*	*0.038780071*
**ENSG00000197601**	*0.036806024*	*0.038000501*	*0.039489082*	*0.0346715*
**ENSG00000150244**	*0.036903559*	*0.026544263*	*0.035817794*	*0.020314378*
**ENSG00000075624**	*0.013414368*	*0.023587025*	*0.023548045*	*0.013292624*
**ENSG00000139291**	*0.042575312*	*0.03864438*	*0.044634015*	*0.042906991*
**ENSG00000034152**	*0.018029942*	*0.029369488*	*0.026261257*	*0.021345484*
**ENSG00000101670**	*0.090856294*	*0.069880199*	*0.068305321*	*0.071071979*
**ENSG00000174640**	*0.030074024*	*0.026185304*	*0.029957074*	*0.018226859*
**ENSG00000168026**	*0.027708058*	*0.034485398*	*0.030771795*	*0.033321302*
**ENSG00000152784**	*0.017026776*	*0.025352569*	*0.025357551*	*0.014999491*
**ENSG00000157450**	*0.022268791*	*0.031547493*	*0.029152557*	*0.021171092*
**ENSG00000164122**	*0.016348728*	*0.025204168*	*0.024268657*	*0.015750842*
**ENSG00000182871**	*0.022305508*	*0.031552904*	*0.028511392*	*0.023376615*
**ENSG00000136732**	*0.033513541*	*0.029549462*	*0.031229559*	*0.024109176*
**ENSG00000226979**	*0.048775287*	*0.041280514*	*0.0345472*	*0.043544197*
**ENSG00000113648**	*0.030411477*	*0.031986825*	*0.038458664*	*0.033667358*
**ENSG00000152402**	*0.016452531*	*0.023835754*	*0.025243177*	*0.014629843*
**ENSG00000138778**	*0.048953494*	*0.043995894*	*0.037595313*	*0.03747261*
**ENSG00000017373**	*0.015313285*	*0.025136451*	*0.025423078*	*0.013860926*
**ENSG00000241973**	*0.016956866*	*0.024382554*	*0.024678824*	*0.015510547*

**ENSG00000117475**	*0.034643475*	*0.035015222*	*0.037642522*	*0.04095229*
**ENSG00000240583**	*0.016476886*	*0.026930512*	*0.025487817*	*0.015410932*
**ENSG00000012124**	*0.031464471*	*0.038439348*	*0.034298795*	*0.03085004*
**ENSG00000159082**	*0.016148514*	*0.024506041*	*0.024598776*	*0.014895885*
**ENSG00000137393**	*0.025500886*	*0.025340656*	*0.024958505*	*0.017325395*
**ENSG00000181234**	*0.017076794*	*0.026178217*	*0.025836875*	*0.017360444*
**ENSG00000166592**	*0.059407405*	*0.052059924*	*0.048355006*	*0.054097349*
**ENSG00000162390**	*0.024971472*	*0.027498389*	*0.026145456*	*0.023935003*
**ENSG00000125726**	*0.027245132*	*0.02959473*	*0.033182629*	*0.028344084*
**ENSG00000164543**	*0.021773895*	*0.031181241*	*0.027526103*	*0.017241622*
**ENSG00000146276**	*0.017431576*	*0.024498698*	*0.024139301*	*0.016774393*
**ENSG00000186818**	*0.014807381*	*0.024615116*	*0.024435954*	*0.015854049*
**ENSG00000158792**	*0.036162588*	*0.044387593*	*0.046326761*	*0.029462243*
**ENSG00000166246**	*0.018863355*	*0.025754432*	*0.025665033*	*0.017962825*
**ENSG00000173269**	*0.020606534*	*0.026442363*	*0.025212832*	*0.016749539*
**ENSG00000165272**	*0.023763578*	*0.025410145*	*0.027980879*	*0.017949011*
**ENSG00000072506**	*0.033675304*	*0.0351155*	*0.030892655*	*0.02866245*
**ENSG00000131941**	*0.076334185*	*0.050017762*	*0.049501774*	*0.057932094*
**ENSG00000113328**	*0.033773251*	*0.045059991*	*0.036687444*	*0.039351926*
**ENSG00000172640**	*0.015594813*	*0.024519584*	*0.024869198*	*0.016480037*
**ENSG00000198924**	*0.032792595*	*0.040278395*	*0.03321111*	*0.028026017*
**ENSG00000181830**	*0.028108852*	*0.031513094*	*0.040732498*	*0.025543115*
**ENSG00000165807**	*0.015711393*	*0.026656346*	*0.025362168*	*0.015963236*
**ENSG00000178467**	*0.044594794*	*0.039286752*	*0.037575534*	*0.037534339*
**ENSG00000170941**	*0.017915038*	*0.027117017*	*0.027963442*	*0.01765083*
**ENSG00000126550**	*0.01544969*	*0.024476585*	*0.024557468*	*0.014946308*
**ENSG00000144597**	*0.020798616*	*0.02742921*	*0.026174033*	*0.017320228*
**ENSG00000167601**	*0.019366949*	*0.028070204*	*0.026163858*	*0.016435805*
**ENSG00000161513**	*0.04395149*	*0.043158114*	*0.042433063*	*0.034965471*
**ENSG00000221829**	*0.041755659*	*0.039730461*	*0.030441882*	*0.030844159*
**ENSG00000154265**	*0.037153531*	*0.035106229*	*0.034224556*	*0.031063464*
**ENSG00000198718**	*0.036231468*	*0.040911894*	*0.035001573*	*0.035742075*
**ENSG00000155052**	*0.016345869*	*0.025184783*	*0.025171876*	*0.014620515*
**ENSG00000145428**	*0.018904613*	*0.024852152*	*0.025708828*	*0.015224598*
**ENSG00000176083**	*0.016565555*	*0.025142588*	*0.025127717*	*0.016223159*
**ENSG00000125170**	*0.06986115*	*0.056433741*	*0.045591222*	*0.058500282*
**ENSG00000047644**	*0.068214474*	*0.060355914*	*0.049474582*	*0.094069168*
**ENSG00000137074**	*0.033111527*	*0.036904812*	*0.030442696*	*0.023144043*
**ENSG00000174697**	*0.015486463*	*0.024753978*	*0.024396432*	*0.015598733*
**ENSG00000183111**	*0.019179825*	*0.028007893*	*0.025070008*	*0.017357648*
**ENSG00000020577**	*0.027880005*	*0.031263265*	*0.030357581*	*0.026614663*
**ENSG00000115183**	*0.088705304*	*0.064630963*	*0.048957822*	*0.064830484*
**ENSG00000183648**	*0.018387095*	*0.027469863*	*0.025692681*	*0.018509025*
**ENSG00000136856**	*0.041711103*	*0.040413195*	*0.042971366*	*0.035983858*
**ENSG00000152578**	*0.016250282*	*0.024620971*	*0.025811027*	*0.016579903*
**ENSG00000137288**	*0.03229323*	*0.034350252*	*0.03285568*	*0.030613312*
**ENSG00000015133**	*0.031471627*	*0.035782825*	*0.03273827*	*0.023702653*
**ENSG00000167085**	*0.036088569*	*0.0343032*	*0.036430731*	*0.030708337*
**ENSG00000108256**	*0.032110986*	*0.039954876*	*0.043855348*	*0.032822211*
**ENSG00000137285**	*0.129387626*	*0.126244903*	*0.110387245*	*0.112526948*
**ENSG00000186049**	*0.01533067*	*0.024064942*	*0.024964375*	*0.016104994*
**ENSG00000166734**	*0.027451493*	*0.034480143*	*0.03029696*	*0.026780283*
**ENSG00000163281**	*0.028219392*	*0.035383527*	*0.032098514*	*0.029302007*
**ENSG00000176894**	*0.035022704*	*0.035235038*	*0.039165357*	*0.032385968*

**ENSG00000114107**	*0.049416525*	*0.041038666*	*0.044295881*	*0.033475595*
**ENSG00000101452**	*0.03357839*	*0.037144646*	*0.033557809*	*0.029580755*
**ENSG00000101951**	*0.016654172*	*0.025943081*	*0.02492009*	*0.016110433*
**ENSG00000172935**	*0.017674384*	*0.025034462*	*0.024614369*	*0.015602754*
**ENSG00000162078**	*0.089476749*	*0.06972619*	*0.068255871*	*0.082913582*
**ENSG00000110079**	*0.017103319*	*0.02607824*	*0.024927237*	*0.016982976*
**ENSG00000151065**	*0.025508393*	*0.031401129*	*0.027980695*	*0.02180051*
**ENSG00000100129**	*0.017104425*	*0.026183759*	*0.026456674*	*0.018457005*
**ENSG00000130177**	*0.028422337*	*0.033749277*	*0.033907539*	*0.020617549*
**ENSG00000100314**	*0.017530619*	*0.026020779*	*0.025383472*	*0.017539924*
**ENSG00000166507**	*0.032021841*	*0.036642859*	*0.036037876*	*0.027395185*
**ENSG00000126254**	*0.020894435*	*0.02905537*	*0.032010086*	*0.024547387*
**ENSG00000167654**	*0.017873407*	*0.025808321*	*0.025775894*	*0.015699341*
**ENSG00000111371**	*0.02485206*	*0.03032531*	*0.031283513*	*0.022384917*
**ENSG00000156313**	*0.058371602*	*0.059406527*	*0.060856816*	*0.062261368*
**ENSG00000143157**	*0.028217682*	*0.033502147*	*0.034065434*	*0.025202234*
**ENSG00000205531**	*0.024880232*	*0.030243121*	*0.029200144*	*0.021005*
**ENSG00000168872**	*0.025600947*	*0.030482128*	*0.033472516*	*0.025595798*
**ENSG00000108684**	*0.015920495*	*0.025882269*	*0.024700164*	*0.015867336*
**ENSG00000169689**	*0.040544797*	*0.03608169*	*0.032130197*	*0.027898623*
**ENSG00000134996**	*0.030803947*	*0.041343426*	*0.03668516*	*0.024626245*
**ENSG00000111241**	*0.017039487*	*0.024549372*	*0.025697288*	*0.015202951*
**ENSG00000159214**	*0.025224179*	*0.030027084*	*0.031390682*	*0.024052495*
**ENSG00000135968**	*0.020563529*	*0.026818222*	*0.027641978*	*0.021000084*
**ENSG00000020633**	*0.028551034*	*0.032034255*	*0.032645571*	*0.031614941*
**ENSG00000114904**	*0.015396857*	*0.025773801*	*0.025509478*	*0.017165603*
**ENSG00000104133**	*0.033934172*	*0.039984663*	*0.035265612*	*0.030192124*
**ENSG00000038295**	*0.018520761*	*0.025891062*	*0.026017443*	*0.016764142*
**ENSG00000168944**	*0.030240665*	*0.040680499*	*0.033053743*	*0.030221904*
**ENSG00000186827**	*0.076057928*	*0.056885741*	*0.061882917*	*0.071041457*
**ENSG00000165914**	*0.01795398*	*0.026696985*	*0.024665842*	*0.017232471*
**ENSG00000106991**	*0.048744628*	*0.047069828*	*0.040646238*	*0.046233092*
**ENSG00000196531**	*0.014407216*	*0.024680412*	*0.024563393*	*0.014709756*
**ENSG00000167791**	*0.016381528*	*0.025233885*	*0.025701928*	*0.016266395*
**ENSG00000163295**	*0.018435623*	*0.026416554*	*0.026386925*	*0.018575486*
**ENSG00000014123**	*0.036320476*	*0.045241688*	*0.036007197*	*0.036139262*
**ENSG00000204640**	*0.016009786*	*0.024520339*	*0.024733804*	*0.015173128*
**ENSG00000101017**	*0.04419183*	*0.038059845*	*0.04231078*	*0.041053601*
**ENSG00000111344**	*0.109499812*	*0.092210264*	*0.069238567*	*0.089223098*
**ENSG00000166272**	*0.019569711*	*0.028928168*	*0.025944412*	*0.020505332*
**ENSG00000177150**	*0.04010235*	*0.045069415*	*0.042318885*	*0.030750864*
**ENSG00000164400**	*0.039742428*	*0.026499121*	*0.028626128*	*0.018785161*
**ENSG00000204525**	*0.028926441*	*0.029361965*	*0.031097438*	*0.023265863*
**ENSG00000204882**	*0.014863039*	*0.025053243*	*0.024670721*	*0.016421898*
**ENSG00000172742**	*0.016469762*	*0.024916093*	*0.025126063*	*0.014376281*
**ENSG00000078900**	*0.035146058*	*0.039463598*	*0.041066684*	*0.038489338*
**ENSG00000137462**	*0.071982538*	*0.052323082*	*0.036921038*	*0.050557803*
**ENSG00000126231**	*0.016670061*	*0.024818532*	*0.024403681*	*0.015847598*
**ENSG00000187145**	*0.022714826*	*0.025723075*	*0.025676072*	*0.016762161*
**ENSG00000130255**	*0.014411694*	*0.023802043*	*0.023979343*	*0.015934793*
**ENSG00000158715**	*0.064606667*	*0.068123929*	*0.051938385*	*0.068571249*
**ENSG00000112578**	*0.034714189*	*0.032036292*	*0.039152476*	*0.0311467*
**ENSG00000175482**	*0.03718181*	*0.039642323*	*0.038882839*	*0.037100762*
**ENSG00000100345**	*0.023133862*	*0.032973622*	*0.029257724*	*0.022860782*

**ENSG00000092531**	*0.026407713*	*0.031682626*	*0.029315222*	*0.025035005*
**ENSG00000180440**	*0.019351251*	*0.025556933*	*0.026154895*	*0.017558244*
**ENSG00000004779**	*0.022305842*	*0.028162407*	*0.026677097*	*0.020174326*
**ENSG00000109756**	*0.066041645*	*0.055581272*	*0.05418979*	*0.038943343*
**ENSG00000105856**	*0.032011801*	*0.033714329*	*0.040873339*	*0.035482261*
**ENSG00000063015**	*0.017164218*	*0.026628427*	*0.027250037*	*0.015680484*
**ENSG00000198890**	*0.04138729*	*0.04108277*	*0.041337075*	*0.040251483*
**ENSG00000170962**	*0.096158861*	*0.028935113*	*0.027817726*	*0.045840417*
**ENSG00000185823**	*0.014072698*	*0.024135899*	*0.024045357*	*0.014690499*
**ENSG00000069431**	*0.022220035*	*0.02558355*	*0.025834402*	*0.018644549*
**ENSG00000121022**	*0.021862206*	*0.028949987*	*0.02751139*	*0.018895055*
**ENSG00000120875**	*0.095198814*	*0.097415954*	*0.075942624*	*0.110611007*
**ENSG00000134697**	*0.024860979*	*0.027251761*	*0.027914382*	*0.02429251*
**ENSG00000156042**	*0.019747088*	*0.026394567*	*0.024850083*	*0.015322899*
**ENSG00000167110**	*0.028050363*	*0.030383153*	*0.030769028*	*0.026842947*
**ENSG00000154310**	*0.054073515*	*0.052053179*	*0.046846232*	*0.044633818*
**ENSG00000178127**	*0.02088256*	*0.027876598*	*0.027086513*	*0.016138505*
**ENSG00000119321**	*0.040260387*	*0.040815233*	*0.036815295*	*0.035195704*
**ENSG00000139200**	*0.016734714*	*0.024676583*	*0.02444556*	*0.01668702*
**ENSG00000165646**	*0.018629951*	*0.02889804*	*0.026678863*	*0.017792444*
**ENSG00000135870**	*0.040831558*	*0.035349195*	*0.030374926*	*0.034225826*
**ENSG00000167721**	*0.028198522*	*0.036545339*	*0.033950166*	*0.028708227*
**ENSG00000164707**	*0.01724204*	*0.024593963*	*0.026103435*	*0.016443365*
**ENSG00000134183**	*0.021817144*	*0.027625457*	*0.024950304*	*0.018404546*
**ENSG00000081760**	*0.018145385*	*0.025752111*	*0.027523068*	*0.018445088*
**ENSG00000204227**	*0.02478711*	*0.030429855*	*0.03143582*	*0.021222766*
**ENSG00000186807**	*0.088052267*	*0.034338586*	*0.036133961*	*0.074815648*
**ENSG00000071677**	*0.015756171*	*0.024666523*	*0.024863854*	*0.015847917*
**ENSG00000172340**	*0.038224689*	*0.042524637*	*0.041496941*	*0.040841221*
**ENSG00000164853**	*0.015187172*	*0.025335121*	*0.024816701*	*0.014662537*
**ENSG00000206072**	*0.017534213*	*0.02650025*	*0.025663565*	*0.017592703*
**ENSG00000204498**	*0.024200096*	*0.030448022*	*0.031427207*	*0.032594412*
**ENSG00000130066**	*0.047598152*	*0.046493821*	*0.043611062*	*0.041891718*
**ENSG00000156858**	*0.022961826*	*0.028746356*	*0.033654026*	*0.024214332*
**ENSG00000101310**	*0.023794597*	*0.02572209*	*0.026774084*	*0.017656107*
**ENSG00000103978**	*0.031389778*	*0.034596711*	*0.041179484*	*0.032176661*
**ENSG00000175556**	*0.022108358*	*0.027234017*	*0.026145751*	*0.017596003*
**ENSG00000172031**	*0.015184687*	*0.024407747*	*0.025325047*	*0.014631405*
**ENSG00000126803**	*0.02549756*	*0.030248922*	*0.025611248*	*0.023252088*
**ENSG00000171295**	*0.033258709*	*0.032262672*	*0.035590162*	*0.033565195*
**ENSG00000040199**	*0.033319608*	*0.041947493*	*0.036514369*	*0.030239965*
**ENSG00000133059**	*0.019763021*	*0.027464622*	*0.025991661*	*0.018479337*
**ENSG00000114942**	*0.034517837*	*0.030684129*	*0.030017829*	*0.032750013*
**ENSG00000040633**	*0.029154796*	*0.033330234*	*0.036903179*	*0.028954745*
**ENSG00000105640**	*0.013807346*	*0.023874962*	*0.024378858*	*0.014673273*
**ENSG00000174948**	*0.015733544*	*0.024827716*	*0.024697229*	*0.015271703*
**ENSG00000187037**	*0.017024906*	*0.026119402*	*0.024616384*	*0.01453906*
**ENSG00000100373**	*0.016551757*	*0.024326745*	*0.025437358*	*0.016407022*
**ENSG00000147570**	*0.035774357*	*0.076823011*	*0.042919622*	*0.068252798*
**ENSG00000109475**	*0.044615598*	*0.042596*	*0.03566812*	*0.031522747*
**ENSG00000106123**	*0.015793805*	*0.026232867*	*0.024967165*	*0.014923789*
**ENSG00000204856**	*0.032497457*	*0.036003423*	*0.030121355*	*0.026072305*
**ENSG00000168118**	*0.045010908*	*0.042424529*	*0.037459093*	*0.033078961*
**ENSG00000106809**	*0.015296552*	*0.024655219*	*0.024563146*	*0.015169172*

**ENSG00000071189**	*0.030986999*	*0.041824244*	*0.038256392*	*0.024383129*
**ENSG00000134574**	*0.030266356*	*0.03600755*	*0.03604185*	*0.03112244*
**ENSG00000136518**	*0.035981884*	*0.038587797*	*0.031179835*	*0.030313764*
**ENSG00000175567**	*0.043397171*	*0.034279329*	*0.037297978*	*0.031017648*
**ENSG00000162664**	*0.023433203*	*0.026749036*	*0.027912056*	*0.022103301*
**ENSG00000116014**	*0.076973703*	*0.071856944*	*0.065606033*	*0.069193949*
**ENSG00000162896**	*0.120681176*	*0.091613934*	*0.10705028*	*0.120737965*
**ENSG00000147255**	*0.043210542*	*0.02695756*	*0.026598063*	*0.019206013*
**ENSG00000104725**	*0.01711789*	*0.024948626*	*0.02529034*	*0.01481817*
**ENSG00000108950**	*0.015426339*	*0.024313046*	*0.024256029*	*0.013861592*
**ENSG00000169126**	*0.014792366*	*0.024570643*	*0.024645013*	*0.01480068*
**ENSG00000197380**	*0.023090723*	*0.028194032*	*0.025633088*	*0.022608641*
**ENSG00000188092**	*0.028923514*	*0.031245856*	*0.036676488*	*0.023021223*
**ENSG00000122705**	*0.016347184*	*0.025828403*	*0.025317771*	*0.014322305*
**ENSG00000112655**	*0.040308618*	*0.03867999*	*0.026766478*	*0.022818909*
**ENSG00000249437**	*0.021729983*	*0.02990583*	*0.028902567*	*0.025090588*
**ENSG00000198252**	*0.025745226*	*0.036672013*	*0.028961004*	*0.019869997*
**ENSG00000213085**	*0.029929114*	*0.032797899*	*0.032500971*	*0.056925843*
**ENSG00000174903**	*0.025202042*	*0.02964941*	*0.033530547*	*0.024432437*
**ENSG00000128656**	*0.017095185*	*0.024868164*	*0.025771315*	*0.01639486*
**ENSG00000065325**	*0.01625462*	*0.025353357*	*0.024361252*	*0.015153227*
**ENSG00000205436**	*0.017296399*	*0.025553934*	*0.02459302*	*0.013818406*
**ENSG00000113494**	*0.034375238*	*0.034979069*	*0.027679418*	*0.02746519*
**ENSG00000135297**	*0.028757086*	*0.033863902*	*0.031983334*	*0.021824954*
**ENSG00000076604**	*0.04023609*	*0.05537892*	*0.05721221*	*0.045371568*
**ENSG00000137601**	*0.035095185*	*0.041231448*	*0.032608776*	*0.030032838*
**ENSG00000172270**	*0.047196937*	*0.037404736*	*0.037665554*	*0.037878699*
**ENSG00000212128**	*0.016075467*	*0.024172658*	*0.024640559*	*0.015318591*
**ENSG00000101150**	*0.02349557*	*0.030385984*	*0.030760993*	*0.023208157*
**ENSG00000173614**	*0.035138631*	*0.033813172*	*0.034322319*	*0.040462593*
**ENSG00000134548**	*0.021391666*	*0.038412469*	*0.036899668*	*0.023173879*
**ENSG00000109956**	*0.017459178*	*0.02818334*	*0.026268532*	*0.024036324*
**ENSG00000140287**	*0.017278282*	*0.026734625*	*0.025356245*	*0.017148368*
**ENSG00000154330**	*0.016462708*	*0.025296703*	*0.025490948*	*0.016129709*
**ENSG00000071051**	*0.024046125*	*0.032146141*	*0.030300884*	*0.02274763*
**ENSG00000122852**	*0.017188899*	*0.024880058*	*0.025771269*	*0.015027927*
**ENSG00000101955**	*0.023110353*	*0.033780947*	*0.028312603*	*0.021568805*
**ENSG00000176953**	*0.028696072*	*0.030823063*	*0.032824508*	*0.029035765*
**ENSG00000185000**	*0.023821268*	*0.027280746*	*0.027500529*	*0.022365111*
**ENSG00000183726**	*0.029645257*	*0.03505842*	*0.033929875*	*0.033356731*
**ENSG00000170776**	*0.036326193*	*0.037891608*	*0.034610873*	*0.033856738*
**ENSG00000004897**	*0.025105096*	*0.034482937*	*0.032410229*	*0.024787219*
**ENSG00000183186**	*0.017587929*	*0.024796596*	*0.025044538*	*0.016585994*
**ENSG00000025800**	*0.026432599*	*0.02942535*	*0.029842047*	*0.021614206*
**ENSG00000165609**	*0.023771965*	*0.031041521*	*0.027730682*	*0.020639207*
**ENSG00000141873**	*0.029269108*	*0.033562883*	*0.034029129*	*0.032559442*
**ENSG00000147592**	*0.038180639*	*0.036891727*	*0.031959329*	*0.028932506*
**ENSG00000167065**	*0.026835443*	*0.030212513*	*0.027564856*	*0.023836379*
**ENSG00000113532**	*0.05581066*	*0.059272655*	*0.048101594*	*0.044755163*
**ENSG00000168634**	*0.015759302*	*0.024925242*	*0.025272696*	*0.015100826*
**ENSG00000169330**	*0.03309761*	*0.029164069*	*0.029662493*	*0.033138774*
**ENSG00000158683**	*0.015918875*	*0.026382028*	*0.024862887*	*0.015439815*
**ENSG00000205937**	*0.017804178*	*0.025162108*	*0.025009926*	*0.016883189*
**ENSG00000117868**	*0.020454435*	*0.026047078*	*0.026163195*	*0.01655395*

**ENSG00000105409**	*0.060165411*	*0.047415107*	*0.039733645*	*0.040972441*
**ENSG00000184992**	*0.037824052*	*0.044711215*	*0.04073733*	*0.033353571*
**ENSG00000146242**	*0.137308397*	*0.080478837*	*0.063873871*	*0.105425984*
**ENSG00000021762**	*0.015110333*	*0.025630674*	*0.025091351*	*0.016467773*
**ENSG00000196199**	*0.030044053*	*0.03687236*	*0.033349817*	*0.022968552*
**ENSG00000117010**	*0.033254714*	*0.035927942*	*0.036844246*	*0.029064893*
**ENSG00000183979**	*0.021032912*	*0.026252545*	*0.024762113*	*0.018536505*
**ENSG00000229544**	*0.016461838*	*0.02511454*	*0.024857814*	*0.015847916*
**ENSG00000122299**	*0.026162097*	*0.031090861*	*0.039912258*	*0.026583027*
**ENSG00000183791**	*0.015334363*	*0.025055047*	*0.024495039*	*0.014751732*
**ENSG00000104164**	*0.043976317*	*0.041252028*	*0.038244967*	*0.057201464*
**ENSG00000172188**	*0.01888453*	*0.025329495*	*0.026518197*	*0.018094811*
**ENSG00000167920**	*0.033437874*	*0.035936514*	*0.033594936*	*0.029409637*
**ENSG00000143320**	*0.017736568*	*0.025160657*	*0.025078325*	*0.015820766*
**ENSG00000143951**	*0.027781452*	*0.035089359*	*0.027606231*	*0.028267413*
**ENSG00000101493**	*0.052487782*	*0.047829315*	*0.043794833*	*0.045375087*
**ENSG00000139684**	*0.021146797*	*0.028088957*	*0.02775705*	*0.020596717*
**ENSG00000114353**	*0.023150491*	*0.027679204*	*0.028147998*	*0.018949517*
**ENSG00000182348**	*0.016071344*	*0.024812281*	*0.024910143*	*0.016356004*
**ENSG00000139579**	*0.030938722*	*0.034396366*	*0.028586551*	*0.025113373*
**ENSG00000187391**	*0.017270116*	*0.026825429*	*0.027339437*	*0.017792315*
**ENSG00000148803**	*0.032032992*	*0.034376564*	*0.030492417*	*0.034233379*
**ENSG00000113205**	*0.0171874*	*0.026403586*	*0.025345006*	*0.016086729*
**ENSG00000184203**	*0.031417563*	*0.036922797*	*0.036987466*	*0.02451318*
**ENSG00000117479**	*0.05709633*	*0.041562436*	*0.039724816*	*0.037595156*
**ENSG00000184363**	*0.016396317*	*0.026509149*	*0.027545206*	*0.015949262*
**ENSG00000153086**	*0.015552871*	*0.025134968*	*0.02479555*	*0.014080468*
**ENSG00000172500**	*0.02956175*	*0.033357365*	*0.029260515*	*0.027642411*
**ENSG00000159322**	*0.024497029*	*0.031134611*	*0.030828312*	*0.022374093*
**ENSG00000168899**	*0.04369771*	*0.039995895*	*0.041001279*	*0.036797702*
**ENSG00000128510**	*0.064370905*	*0.066930707*	*0.054714939*	*0.064411795*
**ENSG00000182187**	*0.017276045*	*0.023994856*	*0.024566357*	*0.015108048*
**ENSG00000145384**	*0.017204642*	*0.026148815*	*0.027563908*	*0.017187124*
**ENSG00000111704**	*0.016787781*	*0.025408926*	*0.025753515*	*0.018916255*
**ENSG00000091129**	*0.153218751*	*0.07099608*	*0.03394775*	*0.056833789*
**ENSG00000099785**	*0.031140717*	*0.036071756*	*0.035514047*	*0.030331756*
**ENSG00000147432**	*0.018187327*	*0.026746815*	*0.026496303*	*0.017835591*
**ENSG00000132005**	*0.025884835*	*0.036633942*	*0.032971894*	*0.028526887*
**ENSG00000128254**	*0.015288537*	*0.024024515*	*0.025288688*	*0.01499516*
**ENSG00000196793**	*0.054045652*	*0.042182138*	*0.037011371*	*0.034365459*
**ENSG00000129518**	*0.025390448*	*0.038467229*	*0.032562098*	*0.02603*
**ENSG00000145982**	*0.044976194*	*0.033238215*	*0.034167768*	*0.037047597*
**ENSG00000140400**	*0.02492764*	*0.033275381*	*0.035332656*	*0.029586061*
**ENSG00000004838**	*0.024663822*	*0.028793649*	*0.032168952*	*0.024824581*
**ENSG00000164796**	*0.017378904*	*0.027668434*	*0.027444466*	*0.016700265*
**ENSG00000146674**	*0.147332306*	*0.084254346*	*0.029901061*	*0.063040238*
**ENSG00000138356**	*0.018266787*	*0.026953591*	*0.025255956*	*0.017990047*
**ENSG00000121879**	*0.030381096*	*0.03703118*	*0.037893547*	*0.028262186*
**ENSG00000141753**	*0.088691018*	*0.060079232*	*0.060506915*	*0.066017005*
**ENSG00000151414**	*0.016411731*	*0.026029813*	*0.025825531*	*0.016382783*
**ENSG00000065970**	*0.02705656*	*0.03054541*	*0.031727189*	*0.022089299*
**ENSG00000135185**	*0.026807029*	*0.031356381*	*0.032732885*	*0.021600425*
**ENSG00000177842**	*0.037301126*	*0.038730844*	*0.038019157*	*0.043895089*
**ENSG00000185414**	*0.029791625*	*0.040097607*	*0.03225259*	*0.026314907*

**ENSG00000170011**	*0.085225336*	*0.044375977*	*0.042081528*	*0.042920862*
**ENSG00000187033**	*0.037566539*	*0.041033486*	*0.031946448*	*0.036457797*
**ENSG00000168000**	*0.040716875*	*0.035868785*	*0.034422003*	*0.034242014*
**ENSG00000166920**	*0.015183689*	*0.024687728*	*0.023910888*	*0.015153865*
**ENSG00000079337**	*0.019549382*	*0.02680357*	*0.026376146*	*0.016076933*
**ENSG00000185437**	*0.031743521*	*0.032054252*	*0.029159528*	*0.026732918*
**ENSG00000108242**	*0.016663105*	*0.02463333*	*0.02464324*	*0.014475885*
**ENSG00000125753**	*0.020839936*	*0.030877461*	*0.029745605*	*0.022783019*
**ENSG00000167588**	*0.018195003*	*0.026873056*	*0.025860382*	*0.01667778*
**ENSG00000173950**	*0.043189082*	*0.041163474*	*0.036162429*	*0.042945719*
**ENSG00000106927**	*0.016612102*	*0.024842149*	*0.025283946*	*0.015566591*
**ENSG00000141391**	*0.019079429*	*0.026282669*	*0.025597229*	*0.017397979*
**ENSG00000114279**	*0.017498488*	*0.026544962*	*0.025890612*	*0.014780967*
**ENSG00000157851**	*0.015954412*	*0.025325527*	*0.024645059*	*0.016409083*
**ENSG00000081014**	*0.021555932*	*0.027577602*	*0.026783339*	*0.020323654*
**ENSG00000100473**	*0.063153267*	*0.060119847*	*0.052582419*	*0.053488144*
**ENSG00000006788**	*0.016531167*	*0.02498554*	*0.025471834*	*0.015548446*
**ENSG00000108510**	*0.029626666*	*0.033086297*	*0.03712386*	*0.024337354*
**ENSG00000163428**	*0.03961528*	*0.035479287*	*0.03719962*	*0.029939673*
**ENSG00000167286**	*0.044387579*	*0.026606859*	*0.026652808*	*0.095866741*
**ENSG00000152580**	*0.014730908*	*0.025167896*	*0.024709953*	*0.014676961*
**ENSG00000153944**	*0.051440626*	*0.03577443*	*0.033034618*	*0.037185977*
**ENSG00000130304**	*0.022948372*	*0.028368113*	*0.028203571*	*0.021819044*
**ENSG00000170222**	*0.025701841*	*0.032039738*	*0.030555709*	*0.021792577*
**ENSG00000161664**	*0.060245063*	*0.049476865*	*0.047780226*	*0.087116769*
**ENSG00000182230**	*0.015076104*	*0.025732709*	*0.025950711*	*0.019661526*
**ENSG00000178449**	*0.021795501*	*0.027823945*	*0.029723248*	*0.024344387*
**ENSG00000162753**	*0.018194906*	*0.030392395*	*0.027891858*	*0.021253313*
**ENSG00000134871**	*0.0365897*	*0.035983025*	*0.026401752*	*0.022031835*
**ENSG00000132305**	*0.028136667*	*0.033504577*	*0.031454938*	*0.020649129*
**ENSG00000187801**	*0.039876033*	*0.040012865*	*0.04526264*	*0.037705071*
**ENSG00000241468**	*0.030286383*	*0.032505064*	*0.028646058*	*0.026140092*
**ENSG00000161649**	*0.019679209*	*0.028186471*	*0.025562555*	*0.020785997*
**ENSG00000144451**	*0.031353922*	*0.02852727*	*0.027903154*	*0.021230573*
**ENSG00000244122**	*0.016657472*	*0.02545122*	*0.025045022*	*0.016312798*
**ENSG00000204628**	*0.015343904*	*0.024280498*	*0.025463017*	*0.017400085*
**ENSG00000171643**	*0.023123278*	*0.026184308*	*0.024712343*	*0.015668395*
**ENSG00000033867**	*0.050009454*	*0.03983724*	*0.034290344*	*0.033103387*
**ENSG00000185385**	*0.01530777*	*0.024689881*	*0.02412485*	*0.015148724*
**ENSG00000184368**	*0.120793993*	*0.092538412*	*0.065173299*	*0.108195531*
**ENSG00000204518**	*0.015226483*	*0.024571063*	*0.025200204*	*0.01492736*
**ENSG00000167236**	*0.027879401*	*0.025284013*	*0.026448371*	*0.022664449*
**ENSG00000089685**	*0.045584609*	*0.034022539*	*0.04068601*	*0.036447518*
**ENSG00000141933**	*0.031529199*	*0.035261297*	*0.035171958*	*0.029305852*
**ENSG00000023572**	*0.023208555*	*0.029508686*	*0.02512762*	*0.019125476*
**ENSG00000175137**	*0.026315901*	*0.027898809*	*0.029701425*	*0.022806194*
**ENSG00000151500**	*0.034255019*	*0.037180816*	*0.031135338*	*0.027731656*
**ENSG00000144161**	*0.031713884*	*0.032146277*	*0.038411684*	*0.028787282*
**ENSG00000164181**	*0.047739545*	*0.051565529*	*0.054911306*	*0.046894676*
**ENSG00000100908**	*0.030042215*	*0.030676248*	*0.033369818*	*0.027163677*
**ENSG00000119685**	*0.018620358*	*0.026151425*	*0.026349476*	*0.017676263*
**ENSG00000147364**	*0.017585683*	*0.027098645*	*0.026327381*	*0.017120702*
**ENSG00000204560**	*0.022160695*	*0.03177968*	*0.029264573*	*0.020627538*
**ENSG00000174586**	*0.017776143*	*0.025414547*	*0.025848264*	*0.017078241*

**ENSG00000150459**	*0.036820551*	*0.037932506*	*0.032102286*	*0.026654058*
**ENSG00000213722**	*0.028268813*	*0.035539816*	*0.034006351*	*0.028342002*
**ENSG00000105486**	*0.036165121*	*0.032772568*	*0.033370792*	*0.026125412*
**ENSG00000148926**	*0.144476754*	*0.119087782*	*0.139406768*	*0.138717717*
**ENSG00000248098**	*0.035310665*	*0.034675881*	*0.033794155*	*0.02879948*
**ENSG00000015285**	*0.021437444*	*0.027335862*	*0.02831236*	*0.021797507*
**ENSG00000114125**	*0.022488236*	*0.029644287*	*0.028235907*	*0.020415947*
**ENSG00000118596**	*0.024709735*	*0.033112539*	*0.029795039*	*0.020983695*
**ENSG00000115902**	*0.04763009*	*0.045317152*	*0.052462871*	*0.050869515*
**ENSG00000135604**	*0.035820695*	*0.042393901*	*0.044925465*	*0.035052432*
**ENSG00000119471**	*0.028920821*	*0.036497497*	*0.036828565*	*0.035302742*
**ENSG00000182606**	*0.036823062*	*0.040987692*	*0.036823674*	*0.032000282*
**ENSG00000114487**	*0.019093919*	*0.025784093*	*0.027803388*	*0.019941316*
**ENSG00000167565**	*0.016258093*	*0.027507342*	*0.025518304*	*0.017004032*
**ENSG00000186075**	*0.016204703*	*0.024597757*	*0.024898051*	*0.014565947*
**ENSG00000124596**	*0.031182059*	*0.032413263*	*0.028596276*	*0.020009282*
**ENSG00000174206**	*0.03028537*	*0.031264143*	*0.034845243*	*0.019173361*
**ENSG00000204386**	*0.030018048*	*0.031923601*	*0.036240685*	*0.028054931*
**ENSG00000136868**	*0.041765605*	*0.041035769*	*0.036599434*	*0.034789255*
**ENSG00000146733**	*0.040192523*	*0.036529635*	*0.033866605*	*0.040142537*
**ENSG00000166783**	*0.01926933*	*0.028873032*	*0.026694882*	*0.019248872*
**ENSG00000107651**	*0.031783002*	*0.037677312*	*0.031592082*	*0.027989134*
**ENSG00000100249**	*0.016424071*	*0.025609878*	*0.027248913*	*0.017568555*
**ENSG00000172977**	*0.014822159*	*0.024639142*	*0.024474095*	*0.015319705*
**ENSG00000022355**	*0.016563782*	*0.024975851*	*0.02471782*	*0.015553505*
**ENSG00000184374**	*0.016324959*	*0.026084231*	*0.025416429*	*0.015203516*
**ENSG00000055130**	*0.024784356*	*0.031463358*	*0.030928415*	*0.019448367*
**ENSG00000169551**	*0.015116484*	*0.024206711*	*0.024361678*	*0.014781261*
**ENSG00000119383**	*0.038642384*	*0.035181143*	*0.03445255*	*0.033872914*
**ENSG00000131848**	*0.030536087*	*0.034894652*	*0.042034297*	*0.03602496*
**ENSG00000188818**	*0.016811724*	*0.02545345*	*0.024931106*	*0.017217456*
**ENSG00000187244**	*0.015742197*	*0.02475329*	*0.023890719*	*0.016297*
**ENSG00000172297**	*0.013439364*	*0.023893802*	*0.023547794*	*0.01298002*
**ENSG00000148396**	*0.019631219*	*0.029349658*	*0.036356778*	*0.022453848*
**ENSG00000182271**	*0.015625963*	*0.026524951*	*0.02521593*	*0.015593123*
**ENSG00000162290**	*0.030334456*	*0.037150017*	*0.034963188*	*0.029476493*
**ENSG00000119280**	*0.037856889*	*0.032715296*	*0.03524716*	*0.029604846*
**ENSG00000175899**	*0.044602836*	*0.06292713*	*0.036627087*	*0.052888546*
**ENSG00000101161**	*0.048648994*	*0.037845592*	*0.037300859*	*0.045605194*
**ENSG00000126950**	*0.016328284*	*0.025325869*	*0.02555663*	*0.015025185*
**ENSG00000144642**	*0.061087541*	*0.050945413*	*0.037988174*	*0.037044306*
**ENSG00000160271**	*0.025713699*	*0.03393126*	*0.032728145*	*0.029976316*
**ENSG00000107623**	*0.015686695*	*0.02538447*	*0.025644244*	*0.015043396*
**ENSG00000161800**	*0.037551413*	*0.034098016*	*0.031623747*	*0.026925512*
**ENSG00000125900**	*0.019998584*	*0.026330274*	*0.024879636*	*0.020383789*
**ENSG00000188011**	*0.016318468*	*0.025145676*	*0.024926627*	*0.016860541*
**ENSG00000244405**	*0.079518492*	*0.053402503*	*0.056603177*	*0.060523371*
**ENSG00000012817**	*0.225890988*	*0.229641431*	*0.214402944*	*0.213501042*
**ENSG00000163444**	*0.025203167*	*0.031502474*	*0.029549921*	*0.019155785*
**ENSG00000187566**	*0.016075276*	*0.024928424*	*0.024651092*	*0.014636129*
**ENSG00000151117**	*0.017245357*	*0.025472764*	*0.025124385*	*0.015277651*
**ENSG00000110799**	*0.018210149*	*0.027090568*	*0.025986507*	*0.01805171*
**ENSG00000122641**	*0.025185026*	*0.029492189*	*0.029031777*	*0.021491617*
**ENSG00000215251**	*0.033813442*	*0.038740095*	*0.046791699*	*0.034352398*

**ENSG00000103326**	*0.018562656*	*0.027302016*	*0.026620789*	*0.020761607*
**ENSG00000197915**	*0.013509338*	*0.023970626*	*0.023697108*	*0.013577247*
**ENSG00000150269**	*0.017611984*	*0.025601671*	*0.024942343*	*0.016091942*
**ENSG00000137414**	*0.029982845*	*0.038692392*	*0.03781155*	*0.025199463*
**ENSG00000130340**	*0.014955221*	*0.024301245*	*0.024666423*	*0.014543308*
**ENSG00000115461**	*0.01894934*	*0.026192014*	*0.026354904*	*0.017505079*
**ENSG00000106436**	*0.016843288*	*0.025852716*	*0.026005209*	*0.015972567*
**ENSG00000198754**	*0.038105101*	*0.035414305*	*0.038553173*	*0.030062587*
**ENSG00000169247**	*0.01656509*	*0.02589761*	*0.024378156*	*0.014971488*
**ENSG00000223638**	*0.02539009*	*0.041748936*	*0.029762783*	*0.016646319*
**ENSG00000196620**	*0.01667781*	*0.02404549*	*0.025068031*	*0.014323233*
**ENSG00000127603**	*0.017394084*	*0.028586102*	*0.026444378*	*0.018916959*
**ENSG00000100319**	*0.023892606*	*0.027670395*	*0.02706028*	*0.021870528*
**ENSG00000115657**	*0.043175791*	*0.043739737*	*0.034635424*	*0.036439117*
**ENSG00000213760**	*0.038172197*	*0.042521825*	*0.036723835*	*0.032340825*
**ENSG00000221866**	*0.019692654*	*0.026795762*	*0.027027499*	*0.017551092*
**ENSG00000182979**	*0.026930591*	*0.029312172*	*0.03047272*	*0.022384313*
**ENSG00000063322**	*0.026850426*	*0.028552923*	*0.030078107*	*0.022695311*
**ENSG00000013392**	*0.03285572*	*0.037744071*	*0.03922887*	*0.033445691*
**ENSG00000135720**	*0.031073789*	*0.029913791*	*0.032598145*	*0.024592197*
**ENSG00000149100**	*0.019516172*	*0.027839546*	*0.026732448*	*0.017990457*
**ENSG00000177839**	*0.047448776*	*0.043532228*	*0.037509324*	*0.048275805*
**ENSG00000180432**	*0.015454888*	*0.024720734*	*0.024476903*	*0.015720825*
**ENSG00000050820**	*0.052240747*	*0.056000306*	*0.050380006*	*0.055753217*
**ENSG00000070778**	*0.016168219*	*0.025280259*	*0.025292855*	*0.015439104*
**ENSG00000185338**	*0.059640365*	*0.051219076*	*0.051565619*	*0.053243089*
**ENSG00000011105**	*0.017261774*	*0.025451733*	*0.025271205*	*0.017078804*
**ENSG00000114737**	*0.023736907*	*0.032162191*	*0.027517174*	*0.023286739*
**ENSG00000112164**	*0.015188517*	*0.024889011*	*0.025424035*	*0.01475405*
**ENSG00000114503**	*0.018848269*	*0.028528681*	*0.029302463*	*0.019084685*
**ENSG00000121417**	*0.027375732*	*0.03711608*	*0.038600682*	*0.025956452*
**ENSG00000187513**	*0.017722769*	*0.025995076*	*0.025965875*	*0.016984926*
**ENSG00000128050**	*0.028181765*	*0.030478047*	*0.027302604*	*0.022542095*
**ENSG00000128709**	*0.016363817*	*0.025901804*	*0.024662009*	*0.015765585*
**ENSG00000126773**	*0.030467788*	*0.03550027*	*0.032376514*	*0.02596694*
**ENSG00000183307**	*0.016569032*	*0.02558349*	*0.025419609*	*0.014518565*
**ENSG00000162063**	*0.054604363*	*0.037651269*	*0.039528095*	*0.041236234*
**ENSG00000165782**	*0.033751435*	*0.041980423*	*0.039840219*	*0.032909974*
**ENSG00000160050**	*0.036720477*	*0.033605803*	*0.03244242*	*0.035215429*
**ENSG00000106511**	*0.015707941*	*0.024707137*	*0.024601738*	*0.015615208*
**ENSG00000007062**	*0.019603185*	*0.02706087*	*0.029405363*	*0.020978595*
**ENSG00000120738**	*0.089487957*	*0.054260181*	*0.077075597*	*0.073465181*
**ENSG00000119614**	*0.018025223*	*0.026187155*	*0.025182121*	*0.014187194*
**ENSG00000121749**	*0.025872261*	*0.031959039*	*0.0328717*	*0.022465595*
**ENSG00000130772**	*0.018469897*	*0.026479316*	*0.028031272*	*0.019799261*
**ENSG00000204178**	*0.034845182*	*0.036840652*	*0.040790928*	*0.029688222*
**ENSG00000186564**	*0.016675506*	*0.024915998*	*0.025133595*	*0.016259864*
**ENSG00000164687**	*0.038015996*	*0.034591709*	*0.030339064*	*0.038733667*
**ENSG00000152620**	*0.03720497*	*0.035988836*	*0.032285493*	*0.028187394*
**ENSG00000166922**	*0.040016381*	*0.040941913*	*0.027003781*	*0.022511475*
**ENSG00000133142**	*0.030479378*	*0.033181832*	*0.034425873*	*0.023500551*
**ENSG00000116977**	*0.018549822*	*0.026169428*	*0.02749028*	*0.020015387*
**ENSG00000119121**	*0.022142435*	*0.028438522*	*0.028228405*	*0.020683039*
**ENSG00000184575**	*0.044004758*	*0.049580567*	*0.037849825*	*0.04434935*

**ENSG00000198626**	*0.015806164*	*0.025774841*	*0.02483659*	*0.015653881*
**ENSG00000140522**	*0.015316469*	*0.025251894*	*0.023998676*	*0.015886865*
**ENSG00000197324**	*0.027374041*	*0.031372659*	*0.040259194*	*0.026626542*
**ENSG00000171180**	*0.015047253*	*0.024728159*	*0.025384689*	*0.015683716*
**ENSG00000172782**	*0.014750654*	*0.024781638*	*0.024195501*	*0.014516087*
**ENSG00000119333**	*0.038794168*	*0.042308536*	*0.036578096*	*0.031024866*
**ENSG00000168807**	*0.028557245*	*0.028837566*	*0.02676879*	*0.021714282*
**ENSG00000214706**	*0.034294145*	*0.032858576*	*0.032724188*	*0.034225989*
**ENSG00000101544**	*0.043826954*	*0.055993182*	*0.062338714*	*0.053931503*
**ENSG00000117676**	*0.032823333*	*0.032116604*	*0.035216157*	*0.030653995*
**ENSG00000170345**	*0.085830324*	*0.058467941*	*0.0489592*	*0.06323457*
**ENSG00000152503**	*0.017297787*	*0.025175434*	*0.025425988*	*0.01571865*
**ENSG00000016391**	*0.046974208*	*0.047759132*	*0.040867887*	*0.049110521*
**ENSG00000160097**	*0.016897209*	*0.026478109*	*0.025412746*	*0.01639097*
**ENSG00000166682**	*0.015934431*	*0.025392383*	*0.024586223*	*0.016016752*
**ENSG00000139324**	*0.02028426*	*0.026147631*	*0.026916611*	*0.021609863*
**ENSG00000120279**	*0.02567283*	*0.02763863*	*0.0286056*	*0.028637645*
**ENSG00000139055**	*0.048664037*	*0.040654017*	*0.039252388*	*0.037544526*
**ENSG00000164163**	*0.028056213*	*0.032012051*	*0.031992857*	*0.031326942*
**ENSG00000128294**	*0.037094259*	*0.037145785*	*0.035914777*	*0.02871396*
**ENSG00000148120**	*0.021962917*	*0.026980355*	*0.027664877*	*0.019827757*
**ENSG00000120586**	*0.01529763*	*0.025101092*	*0.024447094*	*0.014981845*
**ENSG00000139263**	*0.089842202*	*0.073784317*	*0.074978209*	*0.072811542*
**ENSG00000107959**	*0.059162455*	*0.058746798*	*0.04689001*	*0.054901298*
**ENSG00000165996**	*0.103179287*	*0.093766062*	*0.078400437*	*0.096465162*
**ENSG00000115935**	*0.031066728*	*0.034800888*	*0.030417572*	*0.024600346*
**ENSG00000161265**	*0.029637336*	*0.030385861*	*0.033353385*	*0.027227746*
**ENSG00000125149**	*0.022325778*	*0.028358793*	*0.026163114*	*0.017850611*
**ENSG00000132879**	*0.016100999*	*0.02558613*	*0.023933946*	*0.015496946*
**ENSG00000173114**	*0.023419301*	*0.029617395*	*0.028613813*	*0.020823187*
**ENSG00000073282**	*0.07630944*	*0.071201173*	*0.059247489*	*0.068446144*
**ENSG00000167461**	*0.025624931*	*0.029878976*	*0.033101267*	*0.024078869*
**ENSG00000133103**	*0.036796886*	*0.036228523*	*0.037359081*	*0.029803829*
**ENSG00000234745**	*0.02201478*	*0.029003279*	*0.029898518*	*0.020999417*
**ENSG00000140320**	*0.024456425*	*0.034962812*	*0.035470032*	*0.022339592*
**ENSG00000163110**	*0.030176395*	*0.030503112*	*0.03120132*	*0.02667715*
**ENSG00000182752**	*0.022668432*	*0.027868212*	*0.03201886*	*0.019028964*
**ENSG00000105464**	*0.014681991*	*0.025679947*	*0.024688242*	*0.015363075*
**ENSG00000144476**	*0.097987134*	*0.086924784*	*0.089326566*	*0.098152693*
**ENSG00000169554**	*0.057559049*	*0.045553128*	*0.037860621*	*0.044457124*
**ENSG00000058063**	*0.033554359*	*0.038610114*	*0.033225271*	*0.031281528*
**ENSG00000180155**	*0.01482722*	*0.02467769*	*0.024575178*	*0.016250549*
**ENSG00000204392**	*0.026056781*	*0.026604944*	*0.030208574*	*0.020728388*
**ENSG00000133063**	*0.017556762*	*0.024541994*	*0.026790633*	*0.016225804*
**ENSG00000168763**	*0.036765888*	*0.035048157*	*0.035893121*	*0.038581228*
**ENSG00000129932**	*0.034447098*	*0.040145353*	*0.035505912*	*0.031140187*
**ENSG00000092036**	*0.037750803*	*0.033625122*	*0.032115221*	*0.027675204*
**ENSG00000181885**	*0.020269569*	*0.027320712*	*0.026120643*	*0.018811165*
**ENSG00000212900**	*0.014612987*	*0.024230519*	*0.023794275*	*0.014584181*
**ENSG00000154920**	*0.032160406*	*0.031607548*	*0.030203312*	*0.024655385*
**ENSG00000167311**	*0.016531398*	*0.025677226*	*0.025234855*	*0.01642643*
**ENSG00000110367**	*0.027868123*	*0.028363169*	*0.02971331*	*0.024735428*
**ENSG00000112539**	*0.017366654*	*0.024971266*	*0.025248143*	*0.015972815*
**ENSG00000073921**	*0.04032821*	*0.054468586*	*0.04303777*	*0.042674331*

**ENSG00000187867**	*0.015780416*	*0.02559628*	*0.024296812*	*0.015253132*
**ENSG00000110713**	*0.060471592*	*0.051598717*	*0.051254519*	*0.053156951*
**ENSG00000204138**	*0.025282413*	*0.032063364*	*0.030453912*	*0.02604204*
**ENSG00000164307**	*0.041482571*	*0.044505476*	*0.032651713*	*0.036018975*
**ENSG00000104549**	*0.02967016*	*0.035641676*	*0.030364945*	*0.024231651*
**ENSG00000177398**	*0.043889407*	*0.038019663*	*0.041477905*	*0.032720117*
**ENSG00000156374**	*0.033009936*	*0.04036168*	*0.039461107*	*0.03147529*
**ENSG00000152556**	*0.033847688*	*0.036099897*	*0.031028455*	*0.026904333*
**ENSG00000100644**	*0.042027682*	*0.049875882*	*0.047484101*	*0.06016154*
**ENSG00000104901**	*0.016389091*	*0.024912307*	*0.025674917*	*0.016215966*
**ENSG00000160606**	*0.028428689*	*0.03171273*	*0.030651868*	*0.024513393*
**ENSG00000196071**	*0.016081437*	*0.024511163*	*0.025759325*	*0.015538495*
**ENSG00000172667**	*0.025895923*	*0.028803148*	*0.031364531*	*0.022443144*
**ENSG00000008516**	*0.042597104*	*0.039524768*	*0.036949285*	*0.049791893*
**ENSG00000172459**	*0.015636541*	*0.024379*	*0.024911479*	*0.016458008*
**ENSG00000007080**	*0.026369423*	*0.029599639*	*0.027377908*	*0.023746988*
**ENSG00000148483**	*0.01306356*	*0.023532593*	*0.023527311*	*0.012981818*
**ENSG00000146618**	*0.015929539*	*0.024883434*	*0.025845057*	*0.016463346*
**ENSG00000100387**	*0.017872003*	*0.027276938*	*0.026154718*	*0.017132877*
**ENSG00000008083**	*0.051985164*	*0.048903442*	*0.043007895*	*0.051139331*
**ENSG00000143641**	*0.043916363*	*0.04724623*	*0.038897908*	*0.033604563*
**ENSG00000169129**	*0.103959551*	*0.072107332*	*0.053800143*	*0.093153993*
**ENSG00000166398**	*0.032399457*	*0.037378788*	*0.035240558*	*0.027345693*
**ENSG00000233827**	*0.015614643*	*0.025928246*	*0.024204588*	*0.015411442*
**ENSG00000091073**	*0.040772508*	*0.039156719*	*0.045505806*	*0.036701424*
**ENSG00000170500**	*0.016601326*	*0.024954763*	*0.025440531*	*0.015729139*
**ENSG00000115896**	*0.028753295*	*0.029668507*	*0.024660478*	*0.03991838*
**ENSG00000149346**	*0.040045182*	*0.038142365*	*0.033982207*	*0.038285111*
**ENSG00000145555**	*0.044880952*	*0.043303928*	*0.035245016*	*0.027168982*
**ENSG00000171208**	*0.045860307*	*0.040149611*	*0.035335407*	*0.041322261*
**ENSG00000143363**	*0.026458648*	*0.031389531*	*0.02989427*	*0.025696785*
**ENSG00000135451**	*0.051188778*	*0.036760784*	*0.042317047*	*0.037652464*
**ENSG00000144868**	*0.015727596*	*0.024882794*	*0.024638325*	*0.015481476*
**ENSG00000198961**	*0.022618544*	*0.028475516*	*0.028532371*	*0.022127398*
**ENSG00000143184**	*0.134400204*	*0.119828945*	*0.100873647*	*0.134757472*
**ENSG00000097096**	*0.024652323*	*0.029663071*	*0.028048066*	*0.027165495*
**ENSG00000017797**	*0.027081952*	*0.0332366*	*0.031171087*	*0.031147852*
**ENSG00000115317**	*0.029188434*	*0.030816571*	*0.034500319*	*0.033970668*
**ENSG00000240021**	*0.014938335*	*0.024709632*	*0.025155828*	*0.014452332*
**ENSG00000179542**	*0.01879064*	*0.025238014*	*0.025664965*	*0.017683523*
**ENSG00000071564**	*0.043958467*	*0.036403626*	*0.046439307*	*0.034435579*
**ENSG00000163060**	*0.019775171*	*0.02503598*	*0.025992344*	*0.034484636*
**ENSG00000204569**	*0.037941111*	*0.033333761*	*0.045574605*	*0.030949094*
**ENSG00000138660**	*0.034503126*	*0.037817135*	*0.036139938*	*0.035386055*
**ENSG00000177485**	*0.016412334*	*0.025493971*	*0.025416126*	*0.015098654*
**ENSG00000173588**	*0.034252941*	*0.046483875*	*0.038553761*	*0.02829328*
**ENSG00000176974**	*0.054908816*	*0.043117607*	*0.040614759*	*0.033136915*
**ENSG00000131944**	*0.024542789*	*0.027956187*	*0.028927166*	*0.021064283*
**ENSG00000168594**	*0.024144247*	*0.025530966*	*0.024976895*	*0.01602042*
**ENSG00000118412**	*0.036058621*	*0.040954144*	*0.035431106*	*0.032870692*
**ENSG00000177202**	*0.016781268*	*0.026353604*	*0.025468329*	*0.015833883*
**ENSG00000136881**	*0.015278175*	*0.024442135*	*0.025760909*	*0.014786173*
**ENSG00000167483**	*0.038821328*	*0.033610795*	*0.047946659*	*0.034053184*
**ENSG00000103168**	*0.015584254*	*0.02595113*	*0.025806109*	*0.017728826*

**ENSG00000213424**	*0.014529598*	*0.02481527*	*0.024985683*	*0.014908343*
**ENSG00000131914**	*0.016030523*	*0.024539819*	*0.024773365*	*0.015546361*
**ENSG00000179271**	*0.03182221*	*0.030266989*	*0.034999525*	*0.031408632*
**ENSG00000120500**	*0.018263027*	*0.025840787*	*0.025484081*	*0.017275245*
**ENSG00000094841**	*0.030997137*	*0.036270261*	*0.03236043*	*0.023401502*
**ENSG00000001629**	*0.025651581*	*0.032392413*	*0.02967637*	*0.02490417*
**ENSG00000140941**	*0.030398509*	*0.03971951*	*0.03811879*	*0.026820293*
**ENSG00000176605**	*0.01791848*	*0.026552046*	*0.025513637*	*0.015257458*
**ENSG00000047457**	*0.01641015*	*0.025133414*	*0.024521749*	*0.015944914*
**ENSG00000145414**	*0.030158101*	*0.038002588*	*0.038414841*	*0.041864662*
**ENSG00000112394**	*0.039868156*	*0.032844408*	*0.032353951*	*0.032671029*
**ENSG00000106066**	*0.019957208*	*0.024708996*	*0.025427026*	*0.016057073*
**ENSG00000188603**	*0.026875675*	*0.034952104*	*0.034832851*	*0.03173401*
**ENSG00000099399**	*0.023029289*	*0.024553049*	*0.027343023*	*0.021388906*
**ENSG00000121957**	*0.058846229*	*0.045692025*	*0.038273121*	*0.037367447*
**ENSG00000215421**	*0.01687796*	*0.025425412*	*0.02546964*	*0.016084935*
**ENSG00000171421**	*0.026083374*	*0.028388417*	*0.027552399*	*0.021582351*
**ENSG00000185664**	*0.034292296*	*0.03919379*	*0.029952492*	*0.021443222*
**ENSG00000148488**	*0.016698476*	*0.025051806*	*0.024516306*	*0.017031299*
**ENSG00000196396**	*0.027423497*	*0.029148431*	*0.030832246*	*0.020936719*
**ENSG00000090487**	*0.022309055*	*0.029094972*	*0.028584736*	*0.018269697*
**ENSG00000196850**	*0.033546733*	*0.031858612*	*0.033859347*	*0.037320513*
**ENSG00000157554**	*0.015851661*	*0.025254321*	*0.026658661*	*0.016552835*
**ENSG00000198814**	*0.049429846*	*0.044638968*	*0.039110896*	*0.043141391*
**ENSG00000198265**	*0.03014237*	*0.036874059*	*0.035933766*	*0.033382247*
**ENSG00000174307**	*0.047920054*	*0.043835961*	*0.036780645*	*0.039326154*
**ENSG00000171763**	*0.032098448*	*0.032745515*	*0.031925633*	*0.025670468*
**ENSG00000230657**	*0.015804075*	*0.024679376*	*0.024139189*	*0.014194365*
**ENSG00000179598**	*0.056615657*	*0.060699086*	*0.058970795*	*0.046728295*
**ENSG00000103067**	*0.023413057*	*0.03839737*	*0.041234829*	*0.02217709*
**ENSG00000205329**	*0.01840106*	*0.026794389*	*0.026049197*	*0.01720816*
**ENSG00000187653**	*0.013662781*	*0.023599381*	*0.024010119*	*0.013717197*
**ENSG00000146281**	*0.031558367*	*0.030794553*	*0.029965504*	*0.023481305*
**ENSG00000010704**	*0.016266984*	*0.026507089*	*0.02564474*	*0.016436091*
**ENSG00000141985**	*0.027351033*	*0.032893937*	*0.030689282*	*0.029144725*
**ENSG00000136040**	*0.028085982*	*0.032051192*	*0.033220679*	*0.032600449*
**ENSG00000171847**	*0.025104281*	*0.031437195*	*0.027007921*	*0.021578553*
**ENSG00000148908**	*0.034244531*	*0.034915549*	*0.034966717*	*0.030949811*
**ENSG00000172366**	*0.029695593*	*0.031528262*	*0.031750156*	*0.026683939*
**ENSG00000171444**	*0.035796915*	*0.035164689*	*0.031215821*	*0.027834557*
**ENSG00000184194**	*0.01467641*	*0.025019307*	*0.024504519*	*0.014450434*
**ENSG00000057935**	*0.039273581*	*0.037533118*	*0.032704435*	*0.026920916*
**ENSG00000150627**	*0.041119599*	*0.033889014*	*0.028075799*	*0.019734269*
**ENSG00000108559**	*0.032292477*	*0.033626872*	*0.031009046*	*0.02286038*
**ENSG00000124257**	*0.050671825*	*0.051358483*	*0.040538416*	*0.040711241*
**ENSG00000139436**	*0.030355933*	*0.031186921*	*0.031757102*	*0.02647313*
**ENSG00000244414**	*0.014851213*	*0.024837162*	*0.024848774*	*0.014733584*
**ENSG00000110887**	*0.014686659*	*0.024158982*	*0.024462861*	*0.015181327*
**ENSG00000124693**	*0.063743111*	*0.04699643*	*0.051623796*	*0.062891919*
**ENSG00000108474**	*0.034656458*	*0.036467046*	*0.032559176*	*0.033233802*
**ENSG00000133477**	*0.017286945*	*0.026389625*	*0.025870673*	*0.018662674*
**ENSG00000067221**	*0.037248681*	*0.038481861*	*0.03603854*	*0.036610889*
**ENSG00000167825**	*0.015598192*	*0.024676542*	*0.025284531*	*0.016427677*
**ENSG00000185122**	*0.018458068*	*0.028026801*	*0.033509976*	*0.024190657*

**ENSG00000100028**	*0.032479077*	*0.035070082*	*0.037512712*	*0.034367466*
**ENSG00000177830**	*0.034069487*	*0.033640279*	*0.03192419*	*0.02909418*
**ENSG00000050344**	*0.048489614*	*0.038247585*	*0.033684968*	*0.040202215*
**ENSG00000171004**	*0.015639844*	*0.025213124*	*0.024231854*	*0.015976292*
**ENSG00000166037**	*0.033682122*	*0.032976971*	*0.029564711*	*0.023611049*
**ENSG00000049246**	*0.096383654*	*0.081332782*	*0.08118285*	*0.077100464*
**ENSG00000117593**	*0.040210621*	*0.041807388*	*0.0315829*	*0.029203932*
**ENSG00000023228**	*0.029480357*	*0.035850756*	*0.030057694*	*0.034360115*
**ENSG00000175489**	*0.017284552*	*0.026667023*	*0.024819492*	*0.016144109*
**ENSG00000067992**	*0.038744474*	*0.039037984*	*0.039191877*	*0.028681208*
**ENSG00000110422**	*0.022585015*	*0.027212799*	*0.032303944*	*0.01829717*
**ENSG00000105928**	*0.035659935*	*0.057005248*	*0.060624717*	*0.058200733*
**ENSG00000143845**	*0.014542643*	*0.024443872*	*0.024171396*	*0.014790558*
**ENSG00000064393**	*0.036173607*	*0.038732374*	*0.039096152*	*0.035575746*
**ENSG00000102854**	*0.025034917*	*0.025393599*	*0.030395863*	*0.021901276*
**ENSG00000137177**	*0.019667964*	*0.024777164*	*0.025226309*	*0.015827097*
**ENSG00000205174**	*0.013245672*	*0.023941474*	*0.024113843*	*0.013405844*
**ENSG00000136653**	*0.026000052*	*0.02820842*	*0.028857693*	*0.022968741*
**ENSG00000213689**	*0.031761514*	*0.034748954*	*0.031591337*	*0.025463078*
**ENSG00000137216**	*0.030404813*	*0.03271804*	*0.033136627*	*0.029831603*
**ENSG00000154146**	*0.082814196*	*0.059493046*	*0.062452721*	*0.061251221*
**ENSG00000183347**	*0.020336903*	*0.030261245*	*0.024747761*	*0.026006185*
**ENSG00000105519**	*0.018049877*	*0.027270752*	*0.026411644*	*0.017932515*
**ENSG00000112242**	*0.030010312*	*0.035939079*	*0.034770535*	*0.026844985*
**ENSG00000150054**	*0.014467695*	*0.026077305*	*0.024833477*	*0.016163793*
**ENSG00000113300**	*0.032918607*	*0.034839394*	*0.034647332*	*0.029079393*
**ENSG00000153147**	*0.02523303*	*0.032256855*	*0.028229897*	*0.023186594*
**ENSG00000120341**	*0.023705682*	*0.026146685*	*0.025507752*	*0.015653752*
**ENSG00000087191**	*0.023589915*	*0.029194884*	*0.027357387*	*0.022016461*
**ENSG00000136754**	*0.031419521*	*0.04041061*	*0.032360916*	*0.071669455*
**ENSG00000179335**	*0.031958541*	*0.044119749*	*0.042335536*	*0.032270774*
**ENSG00000198839**	*0.02497531*	*0.03110766*	*0.030256542*	*0.025084154*
**ENSG00000116783**	*0.018868252*	*0.024277749*	*0.026112823*	*0.014605399*
**ENSG00000176293**	*0.021637656*	*0.027325327*	*0.024983918*	*0.019087659*
**ENSG00000143442**	*0.024598886*	*0.032944846*	*0.031306389*	*0.025373566*
**ENSG00000181904**	*0.037543376*	*0.035807885*	*0.038258757*	*0.036094536*
**ENSG00000101246**	*0.029102263*	*0.03248196*	*0.029801529*	*0.027954608*
**ENSG00000160801**	*0.019433083*	*0.027029002*	*0.026736843*	*0.046033319*
**ENSG00000128346**	*0.016576221*	*0.025352765*	*0.024190145*	*0.016387777*
**ENSG00000084070**	*0.025076375*	*0.029297871*	*0.03133735*	*0.023580622*
**ENSG00000134640**	*0.015545323*	*0.025454641*	*0.025211766*	*0.015578828*
**ENSG00000175591**	*0.016186795*	*0.024722268*	*0.024638437*	*0.014331472*
**ENSG00000083814**	*0.027360064*	*0.03266856*	*0.033442314*	*0.023183542*
**ENSG00000147380**	*0.017071335*	*0.025088167*	*0.026206867*	*0.016933327*
**ENSG00000166887**	*0.021646853*	*0.028325461*	*0.031427527*	*0.020154336*
**ENSG00000111581**	*0.032854318*	*0.03542507*	*0.032362521*	*0.024893932*
**ENSG00000105613**	*0.018006089*	*0.026404049*	*0.02740831*	*0.018810504*
**ENSG00000172139**	*0.01621381*	*0.026610759*	*0.025576102*	*0.017023149*
**ENSG00000112486**	*0.022806732*	*0.029183762*	*0.030031603*	*0.022636648*
**ENSG00000152154**	*0.016765441*	*0.025033432*	*0.024806897*	*0.018316603*
**ENSG00000130311**	*0.022678223*	*0.03133812*	*0.037214425*	*0.027312115*
**ENSG00000117650**	*0.04976788*	*0.045239682*	*0.038420836*	*0.036322942*
**ENSG00000197977**	*0.015177775*	*0.02500461*	*0.024347459*	*0.015842445*
**ENSG00000057019**	*0.061285086*	*0.046725791*	*0.040568975*	*0.042316678*

**ENSG00000130203**	*0.020410299*	*0.026201823*	*0.024965613*	*0.016633345*
**ENSG00000117362**	*0.026864027*	*0.028665821*	*0.033468811*	*0.021559919*
**ENSG00000025423**	*0.026669133*	*0.032509749*	*0.030595871*	*0.02063048*
**ENSG00000162594**	*0.06481749*	*0.078765482*	*0.073883148*	*0.062178533*
**ENSG00000197723**	*0.015158607*	*0.024877581*	*0.024873059*	*0.014832754*
**ENSG00000166145**	*0.047265133*	*0.047582591*	*0.046076346*	*0.046932288*
**ENSG00000082293**	*0.030721809*	*0.034794036*	*0.029650544*	*0.02606289*
**ENSG00000127054**	*0.027092233*	*0.027696976*	*0.029144972*	*0.022153565*
**ENSG00000184022**	*0.021780674*	*0.026773429*	*0.026386877*	*0.018635001*
**ENSG00000090060**	*0.030963735*	*0.035700031*	*0.03210884*	*0.028727249*
**ENSG00000151208**	*0.077184617*	*0.064347587*	*0.05704952*	*0.067959618*
**ENSG00000205755**	*0.018015523*	*0.025633258*	*0.024570787*	*0.014719774*
**ENSG00000175879**	*0.015178032*	*0.024782286*	*0.024854981*	*0.014628142*
**ENSG00000119414**	*0.021030539*	*0.029635288*	*0.029625314*	*0.018723547*
**ENSG00000130707**	*0.104445412*	*0.083850712*	*0.073430245*	*0.08077936*
**ENSG00000158164**	*0.03903779*	*0.033645815*	*0.029581351*	*0.022628109*
**ENSG00000096395**	*0.015940774*	*0.025134976*	*0.024850822*	*0.015073998*
**ENSG00000170807**	*0.013371648*	*0.023799761*	*0.023895825*	*0.012856848*
**ENSG00000123933**	*0.051156812*	*0.051366842*	*0.052477194*	*0.049267263*
**ENSG00000151849**	*0.03442357*	*0.033740221*	*0.032247604*	*0.025533682*
**ENSG00000183044**	*0.02036964*	*0.026157486*	*0.025737097*	*0.019122693*
**ENSG00000223865**	*0.022257229*	*0.033164124*	*0.03064108*	*0.028905603*
**ENSG00000154252**	*0.016931284*	*0.025783061*	*0.025415136*	*0.016552958*
**ENSG00000082482**	*0.016353289*	*0.025690115*	*0.02643564*	*0.017129331*
**ENSG00000163701**	*0.018220136*	*0.025613481*	*0.025252858*	*0.01639502*
**ENSG00000122042**	*0.026909136*	*0.037388454*	*0.034686597*	*0.030857118*
**ENSG00000183638**	*0.016291246*	*0.025290688*	*0.024673075*	*0.015517208*
**ENSG00000122194**	*0.042151726*	*0.026598598*	*0.029536371*	*0.023742074*
**ENSG00000175455**	*0.032895662*	*0.03444668*	*0.033272486*	*0.02419876*
**ENSG00000075651**	*0.104196301*	*0.074753925*	*0.066639962*	*0.088529155*
**ENSG00000136114**	*0.02480063*	*0.031893337*	*0.031590082*	*0.019227826*
**ENSG00000123892**	*0.086052768*	*0.076040446*	*0.075783217*	*0.094571198*
**ENSG00000182224**	*0.037580326*	*0.040083616*	*0.040372184*	*0.031817532*
**ENSG00000127995**	*0.034979507*	*0.039843159*	*0.034619757*	*0.037444504*
**ENSG00000100744**	*0.038959779*	*0.044973056*	*0.040971387*	*0.044106303*
**ENSG00000243989**	*0.030034323*	*0.034852667*	*0.034653914*	*0.031461445*
**ENSG00000145945**	*0.042343919*	*0.042520287*	*0.040901052*	*0.033186255*
**ENSG00000162572**	*0.028231728*	*0.038888949*	*0.030420837*	*0.031504822*
**ENSG00000074527**	*0.016093224*	*0.024401076*	*0.024511212*	*0.016111028*
**ENSG00000181368**	*0.062175021*	*0.054754264*	*0.049987331*	*0.055382189*
**ENSG00000162641**	*0.014882551*	*0.025186622*	*0.02536555*	*0.015338248*
**ENSG00000103264**	*0.025874409*	*0.029807879*	*0.034255943*	*0.023554197*
**ENSG00000196517**	*0.015176427*	*0.025183184*	*0.025140242*	*0.014384314*
**ENSG00000171914**	*0.074779561*	*0.031733637*	*0.025460767*	*0.023313937*
**ENSG00000120727**	*0.026662237*	*0.032796842*	*0.028172714*	*0.022966645*
**ENSG00000020129**	*0.026900446*	*0.032200169*	*0.037168987*	*0.032137308*
**ENSG00000065371**	*0.016717995*	*0.025304991*	*0.025417213*	*0.015613388*
**ENSG00000174370**	*0.015939913*	*0.026082403*	*0.025991136*	*0.015587031*
**ENSG00000151498**	*0.033880239*	*0.034770907*	*0.032428874*	*0.027201337*
**ENSG00000166359**	*0.015454124*	*0.02498491*	*0.025241502*	*0.013910649*
**ENSG00000105246**	*0.053447553*	*0.0373408*	*0.042941581*	*0.048610388*
**ENSG00000155097**	*0.033712758*	*0.040686738*	*0.038983627*	*0.028747623*
**ENSG00000157349**	*0.025806319*	*0.030439091*	*0.032798828*	*0.019989706*
**ENSG00000100554**	*0.03035107*	*0.034792934*	*0.031317899*	*0.021880433*

**ENSG00000197134**	*0.032820383*	*0.036166041*	*0.032905435*	*0.024942582*
**ENSG00000109016**	*0.025658242*	*0.029891466*	*0.028618961*	*0.022974099*
**ENSG00000125388**	*0.017040551*	*0.025473357*	*0.024893189*	*0.018201932*
**ENSG00000141627**	*0.025391241*	*0.029727443*	*0.028276188*	*0.021997606*
**ENSG00000204290**	*0.01536777*	*0.02488539*	*0.024809426*	*0.014908015*
**ENSG00000132932**	*0.015204908*	*0.025171504*	*0.024866576*	*0.015435772*
**ENSG00000198574**	*0.018433241*	*0.028100327*	*0.027355872*	*0.017485043*
**ENSG00000186407**	*0.016092594*	*0.025401767*	*0.024982605*	*0.015026589*
**ENSG00000055147**	*0.021163799*	*0.034217896*	*0.030467832*	*0.018451423*
**ENSG00000173281**	*0.02742695*	*0.035132875*	*0.033389384*	*0.02729663*
**ENSG00000204520**	*0.040061443*	*0.050582957*	*0.042574309*	*0.03525455*
**ENSG00000175279**	*0.035151815*	*0.035486681*	*0.03155821*	*0.029515836*
**ENSG00000170748**	*0.016626421*	*0.025303532*	*0.024186092*	*0.014923159*
**ENSG00000150201**	*0.015089287*	*0.024711339*	*0.024695988*	*0.014637375*
**ENSG00000103591**	*0.019585762*	*0.026409559*	*0.028641957*	*0.017030733*
**ENSG00000129910**	*0.021554792*	*0.027193639*	*0.027349107*	*0.023348104*
**ENSG00000122711**	*0.017412942*	*0.025613624*	*0.025163503*	*0.015066576*
**ENSG00000154678**	*0.01976766*	*0.027655856*	*0.02469219*	*0.019825565*
**ENSG00000235987**	*0.019019351*	*0.046747808*	*0.045831507*	*0.035136049*
**ENSG00000206172**	*0.025127864*	*0.024643799*	*0.024404912*	*0.051776769*
**ENSG00000178084**	*0.015871937*	*0.02503614*	*0.024558184*	*0.015373958*
**ENSG00000170923**	*0.012899691*	*0.02344604*	*0.024076084*	*0.013561976*
**ENSG00000075290**	*0.015151523*	*0.025154076*	*0.025371329*	*0.013821588*
**ENSG00000075388**	*0.016966708*	*0.026478974*	*0.025885915*	*0.017344026*
**ENSG00000082929**	*0.020923139*	*0.026912207*	*0.027825131*	*0.017080771*
**ENSG00000162618**	*0.013858699*	*0.023618772*	*0.023523243*	*0.013438397*
**ENSG00000099282**	*0.026587022*	*0.029581578*	*0.027892096*	*0.027040355*
**ENSG00000110200**	*0.030956165*	*0.02921791*	*0.031218625*	*0.030458011*
**ENSG00000111530**	*0.031910247*	*0.037922576*	*0.031627486*	*0.026734871*
**ENSG00000178234**	*0.061419486*	*0.042705495*	*0.04476039*	*0.043424639*
**ENSG00000169213**	*0.019989704*	*0.025866148*	*0.024922451*	*0.0256996*
**ENSG00000175352**	*0.014098185*	*0.025105517*	*0.025072827*	*0.01388615*
**ENSG00000149596**	*0.015742082*	*0.024607949*	*0.024562613*	*0.014320935*
**ENSG00000121764**	*0.0162749*	*0.024635156*	*0.026303486*	*0.014977735*
**ENSG00000143553**	*0.027573642*	*0.032281601*	*0.029307545*	*0.027016634*
**ENSG00000107938**	*0.055764016*	*0.058687761*	*0.043378497*	*0.040699327*
**ENSG00000092929**	*0.030141063*	*0.034360828*	*0.038138718*	*0.028433687*
**ENSG00000151092**	*0.034678093*	*0.035309904*	*0.034597954*	*0.028546305*
**ENSG00000171408**	*0.017873507*	*0.026564626*	*0.026386*	*0.016743491*
**ENSG00000213185**	*0.047190521*	*0.041508107*	*0.037277589*	*0.033392069*
**ENSG00000124939**	*0.016285651*	*0.024920907*	*0.024747148*	*0.015236561*
**ENSG00000146383**	*0.01584404*	*0.025215996*	*0.024823644*	*0.017663149*
**ENSG00000197561**	*0.02287833*	*0.02757511*	*0.029394354*	*0.019357839*
**ENSG00000140543**	*0.020879515*	*0.026372399*	*0.02956145*	*0.019312999*
**ENSG00000140563**	*0.11790507*	*0.076782258*	*0.078091667*	*0.088576241*
**ENSG00000138061**	*0.099480908*	*0.110522667*	*0.078132653*	*0.084726091*
**ENSG00000182931**	*0.01680459*	*0.024308268*	*0.024830903*	*0.015131592*
**ENSG00000222028**	*0.016730884*	*0.02558808*	*0.026637165*	*0.016452105*
**ENSG00000171475**	*0.020242468*	*0.028772547*	*0.033654775*	*0.023771076*
**ENSG00000131100**	*0.023576467*	*0.028674176*	*0.033504715*	*0.023847617*
**ENSG00000181785**	*0.016117172*	*0.026323893*	*0.024803031*	*0.014416659*
**ENSG00000071082**	*0.013483864*	*0.023784298*	*0.023579082*	*0.015443832*
**ENSG00000038219**	*0.019088495*	*0.025568824*	*0.026638595*	*0.017564319*
**ENSG00000187193**	*0.074673917*	*0.078144563*	*0.083052622*	*0.078574806*

**ENSG00000085760**	*0.02639982*	*0.030750338*	*0.027237289*	*0.02431613*
**ENSG00000112799**	*0.039577291*	*0.037515506*	*0.040703569*	*0.043219806*
**ENSG00000099194**	*0.027083446*	*0.027494157*	*0.02595263*	*0.022093861*
**ENSG00000198342**	*0.027161469*	*0.031674847*	*0.02803677*	*0.021955142*
**ENSG00000142973**	*0.015541047*	*0.024556867*	*0.024465677*	*0.014595998*
**ENSG00000085415**	*0.033192457*	*0.034679068*	*0.033161912*	*0.027165218*
**ENSG00000151835**	*0.043930225*	*0.043441495*	*0.037300006*	*0.049267217*
**ENSG00000188976**	*0.030499139*	*0.031185954*	*0.031340754*	*0.024984356*
**ENSG00000158445**	*0.015866019*	*0.025360616*	*0.025779448*	*0.015212373*
**ENSG00000179134**	*0.024115926*	*0.030088049*	*0.037337407*	*0.026737407*
**ENSG00000168806**	*0.035041106*	*0.04010981*	*0.039336689*	*0.033888016*
**ENSG00000175087**	*0.035484507*	*0.037094787*	*0.037783232*	*0.036207116*
**ENSG00000157617**	*0.03965104*	*0.033641882*	*0.033957155*	*0.033878313*
**ENSG00000121742**	*0.01644616*	*0.026307535*	*0.024753873*	*0.015079965*
**ENSG00000197932**	*0.024933572*	*0.028734784*	*0.030438874*	*0.021976776*
**ENSG00000104341**	*0.144942491*	*0.09915858*	*0.09650415*	*0.105910368*
**ENSG00000105325**	*0.022895241*	*0.027864964*	*0.027547736*	*0.019562546*
**ENSG00000060762**	*0.031129402*	*0.039625349*	*0.032794413*	*0.035149617*
**ENSG00000115297**	*0.017367927*	*0.024955457*	*0.025354928*	*0.015845845*
**ENSG00000140577**	*0.028848614*	*0.031278775*	*0.037522346*	*0.025691249*
**ENSG00000157613**	*0.043591114*	*0.040119936*	*0.029863537*	*0.033713924*
**ENSG00000185963**	*0.014839982*	*0.026131192*	*0.024655804*	*0.016256329*
**ENSG00000160654**	*0.015238823*	*0.025472526*	*0.02494804*	*0.01481941*
**ENSG00000065491**	*0.020724213*	*0.031117048*	*0.030994515*	*0.024241578*
**ENSG00000180855**	*0.043982262*	*0.038650356*	*0.046351387*	*0.033382024*
**ENSG00000038002**	*0.049544214*	*0.042811697*	*0.039053308*	*0.030474536*
**ENSG00000133895**	*0.015505045*	*0.025687307*	*0.025138038*	*0.01530802*
**ENSG00000104763**	*0.015487116*	*0.026050081*	*0.024791567*	*0.01578467*
**ENSG00000116221**	*0.025693821*	*0.030262869*	*0.030540281*	*0.018846377*
**ENSG00000152413**	*0.060912234*	*0.056167787*	*0.052994893*	*0.048420101*
**ENSG00000082701**	*0.024185508*	*0.029320406*	*0.034679507*	*0.024567123*
**ENSG00000000457**	*0.021280124*	*0.026781681*	*0.025458215*	*0.020888247*
**ENSG00000087258**	*0.016429595*	*0.024779598*	*0.024374814*	*0.014798816*
**ENSG00000186523**	*0.02513179*	*0.029787408*	*0.0288434*	*0.023011924*
**ENSG00000148541**	*0.015841488*	*0.025950886*	*0.024738774*	*0.016495749*
**ENSG00000155975**	*0.031795577*	*0.038657705*	*0.032099058*	*0.044200794*
**ENSG00000123485**	*0.05137989*	*0.039020357*	*0.040490177*	*0.038896092*
**ENSG00000109184**	*0.037969234*	*0.03694994*	*0.033532458*	*0.033664357*
**ENSG00000077809**	*0.035057477*	*0.037308439*	*0.03254752*	*0.024349083*
**ENSG00000155893**	*0.021408459*	*0.02928617*	*0.027156106*	*0.02211257*
**ENSG00000196476**	*0.017302594*	*0.025144975*	*0.024837962*	*0.016176912*
**ENSG00000188868**	*0.019029488*	*0.026525077*	*0.024675428*	*0.017871563*
**ENSG00000166126**	*0.01626539*	*0.025122445*	*0.024728196*	*0.014991632*
**ENSG00000092964**	*0.050701261*	*0.073101185*	*0.058560708*	*0.06010773*
**ENSG00000091664**	*0.015361695*	*0.0249986*	*0.02501125*	*0.014837142*
**ENSG00000159346**	*0.021212249*	*0.028233713*	*0.028425548*	*0.019750597*
**ENSG00000146083**	*0.025115995*	*0.028348584*	*0.031015595*	*0.026160592*
**ENSG00000167702**	*0.03526414*	*0.038237438*	*0.039744992*	*0.035444864*
**ENSG00000165283**	*0.028568129*	*0.033171683*	*0.029939649*	*0.022431793*
**ENSG00000105993**	*0.032752115*	*0.045875942*	*0.035268814*	*0.03363995*
**ENSG00000234906**	*0.036930979*	*0.030181427*	*0.031028149*	*0.025249859*
**ENSG00000109790**	*0.041854458*	*0.041423622*	*0.038851268*	*0.035264369*
**ENSG00000080618**	*0.017719053*	*0.026150896*	*0.024675507*	*0.016791619*
**ENSG00000026950**	*0.044141218*	*0.037310664*	*0.035823378*	*0.037046626*

**ENSG00000165288**	*0.033634327*	*0.036085718*	*0.031675279*	*0.032872849*
**ENSG00000162437**	*0.053586897*	*0.040438752*	*0.024714714*	*0.015648915*
**ENSG00000175806**	*0.046596457*	*0.037167765*	*0.037118178*	*0.03812944*
**ENSG00000132792**	*0.02532567*	*0.029300974*	*0.028580208*	*0.024650654*
**ENSG00000123908**	*0.039572592*	*0.04105996*	*0.039273649*	*0.03477687*
**ENSG00000221944**	*0.027784674*	*0.032061183*	*0.030176419*	*0.021171635*
**ENSG00000064703**	*0.039287416*	*0.044718229*	*0.046737937*	*0.046055037*
**ENSG00000111199**	*0.015177925*	*0.025323464*	*0.025174113*	*0.014301141*
**ENSG00000106638**	*0.027572967*	*0.031329642*	*0.031781144*	*0.026239769*
**ENSG00000133687**	*0.019909893*	*0.025745923*	*0.024683626*	*0.015915799*
**ENSG00000198523**	*0.014985023*	*0.024162203*	*0.02484124*	*0.014693141*
**ENSG00000166199**	*0.026641133*	*0.032227121*	*0.035033858*	*0.023635825*
**ENSG00000167770**	*0.026049967*	*0.030950686*	*0.039074422*	*0.031176509*
**ENSG00000130725**	*0.023158238*	*0.026640886*	*0.030008655*	*0.024092002*
**ENSG00000166946**	*0.022645617*	*0.034630094*	*0.030377323*	*0.027665741*
**ENSG00000198642**	*0.038067088*	*0.038823574*	*0.036501108*	*0.030787173*
**ENSG00000187583**	*0.016389619*	*0.027585435*	*0.026580059*	*0.019635719*
**ENSG00000100297**	*0.033645321*	*0.031013396*	*0.036977652*	*0.033092265*
**ENSG00000156239**	*0.034081858*	*0.035708431*	*0.031982865*	*0.024058633*
**ENSG00000135372**	*0.027775156*	*0.03452657*	*0.030494879*	*0.023433756*
**ENSG00000175318**	*0.01421073*	*0.024093774*	*0.025199609*	*0.01437479*
**ENSG00000144579**	*0.020788675*	*0.039950653*	*0.028418044*	*0.030001201*
**ENSG00000117395**	*0.027076746*	*0.031893119*	*0.028793721*	*0.028238443*
**ENSG00000177721**	*0.047699839*	*0.051333673*	*0.051424892*	*0.054715457*
**ENSG00000042980**	*0.015769845*	*0.025574654*	*0.025693731*	*0.017030003*
**ENSG00000221938**	*0.015932187*	*0.023922578*	*0.025148332*	*0.015135288*
**ENSG00000112282**	*0.048512634*	*0.049245932*	*0.045740034*	*0.083081549*
**ENSG00000175093**	*0.015672368*	*0.025437324*	*0.026004721*	*0.015955935*
**ENSG00000137106**	*0.035286665*	*0.034950275*	*0.035551074*	*0.034171396*
**ENSG00000172828**	*0.021478618*	*0.029571423*	*0.026727227*	*0.025541154*
**ENSG00000171094**	*0.015510628*	*0.025582093*	*0.026143753*	*0.016187078*
**ENSG00000042813**	*0.018328125*	*0.024949437*	*0.025314378*	*0.016467829*
**ENSG00000166171**	*0.036025781*	*0.038914108*	*0.034676913*	*0.029836177*
**ENSG00000109084**	*0.034789766*	*0.034769558*	*0.035556398*	*0.030096802*
**ENSG00000154639**	*0.01792732*	*0.025192263*	*0.025019151*	*0.023602098*
**ENSG00000189013**	*0.014867796*	*0.023608636*	*0.025417329*	*0.015413273*
**ENSG00000139921**	*0.028838084*	*0.033397606*	*0.031295319*	*0.020898345*
**ENSG00000177047**	*0.016556242*	*0.027148932*	*0.025392062*	*0.015030185*
**ENSG00000176907**	*0.015944319*	*0.024564275*	*0.025457903*	*0.016634517*
**ENSG00000186117**	*0.017349548*	*0.027405699*	*0.024548542*	*0.016875638*
**ENSG00000196387**	*0.031437695*	*0.041060951*	*0.045164428*	*0.034189253*
**ENSG00000101977**	*0.017200697*	*0.025653428*	*0.025483423*	*0.018434162*
**ENSG00000127903**	*0.015565849*	*0.024964022*	*0.024488297*	*0.015567886*
**ENSG00000103522**	*0.027470463*	*0.029985433*	*0.027616394*	*0.018452422*
**ENSG00000141252**	*0.017676216*	*0.028403186*	*0.026181505*	*0.017104343*
**ENSG00000155363**	*0.028594642*	*0.038573452*	*0.039266452*	*0.038179835*
**ENSG00000127780**	*0.018219626*	*0.02577914*	*0.025452828*	*0.018268512*
**ENSG00000184029**	*0.015627511*	*0.024707255*	*0.025177905*	*0.016199478*
**ENSG00000131508**	*0.038560631*	*0.046917881*	*0.037235733*	*0.029875345*
**ENSG00000197734**	*0.019658837*	*0.028678645*	*0.026412128*	*0.018482986*
**ENSG00000140557**	*0.020589618*	*0.024695716*	*0.026229632*	*0.02034394*
**ENSG00000127364**	*0.015417212*	*0.024978888*	*0.025058486*	*0.014343474*
**ENSG00000101003**	*0.038796089*	*0.035072727*	*0.035762456*	*0.035416938*
**ENSG00000227057**	*0.032141612*	*0.029643514*	*0.033289631*	*0.03309129*

**ENSG00000198060**	*0.041092568*	*0.045365232*	*0.054382507*	*0.043284196*
**ENSG00000104808**	*0.030864255*	*0.032406986*	*0.029880215*	*0.057645883*
**ENSG00000186335**	*0.015449108*	*0.024921558*	*0.027124398*	*0.015545532*
**ENSG00000184324**	*0.037789013*	*0.027339994*	*0.029172072*	*0.02698578*
**ENSG00000143627**	*0.015750849*	*0.025077495*	*0.02520277*	*0.014892526*
**ENSG00000136718**	*0.025854599*	*0.029942913*	*0.028834379*	*0.021590563*
**ENSG00000163814**	*0.044661638*	*0.035277149*	*0.034663635*	*0.050177608*
**ENSG00000164188**	*0.016136892*	*0.025346223*	*0.025348551*	*0.017965518*
**ENSG00000146267**	*0.015732005*	*0.026423428*	*0.025308167*	*0.014891923*
**ENSG00000079308**	*0.017075922*	*0.024928362*	*0.0249751*	*0.018828652*
**ENSG00000167508**	*0.02748289*	*0.028389816*	*0.035894151*	*0.031951717*
**ENSG00000175077**	*0.015108086*	*0.025187623*	*0.025239912*	*0.015437313*
**ENSG00000052850**	*0.015944878*	*0.025276422*	*0.024302015*	*0.017023289*
**ENSG00000205038**	*0.057250115*	*0.079530561*	*0.068095198*	*0.059600832*
**ENSG00000118434**	*0.015220483*	*0.024740691*	*0.024687964*	*0.015574502*
**ENSG00000176366**	*0.038715199*	*0.042950199*	*0.033634624*	*0.032798672*
**ENSG00000183475**	*0.019655744*	*0.027437548*	*0.027125718*	*0.01962227*
**ENSG00000120903**	*0.016490664*	*0.025397195*	*0.025041807*	*0.017257898*
**ENSG00000030110**	*0.032940151*	*0.039643584*	*0.043355818*	*0.030248404*
**ENSG00000176920**	*0.015293779*	*0.024697371*	*0.025470779*	*0.015404342*
**ENSG00000181751**	*0.051474055*	*0.042639635*	*0.037077838*	*0.033772326*
**ENSG00000213096**	*0.038313139*	*0.038828676*	*0.032307349*	*0.031146132*
**ENSG00000176927**	*0.015256651*	*0.024621827*	*0.024510344*	*0.014753245*
**ENSG00000119707**	*0.03057143*	*0.034652477*	*0.030852762*	*0.024871229*
**ENSG00000152234**	*0.019698567*	*0.027202987*	*0.025630319*	*0.01760753*
**ENSG00000112218**	*0.045509475*	*0.042120724*	*0.039113705*	*0.038929848*
**ENSG00000135916**	*0.058483657*	*0.04186114*	*0.053596094*	*0.050007758*
**ENSG00000162727**	*0.016262191*	*0.024542531*	*0.024903976*	*0.015578792*
**ENSG00000139278**	*0.03443639*	*0.038843229*	*0.04173135*	*0.036520863*
**ENSG00000135144**	*0.039009721*	*0.033539791*	*0.030611001*	*0.033569567*
**ENSG00000132388**	*0.037854652*	*0.047882804*	*0.038107123*	*0.040741097*
**ENSG00000124766**	*0.049443945*	*0.045550319*	*0.055484597*	*0.054949047*
**ENSG00000197620**	*0.022415379*	*0.03480742*	*0.033259083*	*0.02442681*
**ENSG00000167861**	*0.026571583*	*0.026569929*	*0.028111861*	*0.020197969*
**ENSG00000187122**	*0.042721155*	*0.029846713*	*0.026264251*	*0.020814856*
**ENSG00000136546**	*0.018955554*	*0.024875804*	*0.025914734*	*0.015871141*
**ENSG00000149262**	*0.03817758*	*0.050452858*	*0.035070288*	*0.038941986*
**ENSG00000090857**	*0.036268075*	*0.042509752*	*0.044072443*	*0.035176893*
**ENSG00000047365**	*0.026074537*	*0.02943185*	*0.030047999*	*0.027521776*
**ENSG00000148672**	*0.023264322*	*0.030437825*	*0.030839373*	*0.021397134*
**ENSG00000104388**	*0.027267527*	*0.031736688*	*0.035073479*	*0.02624582*
**ENSG00000169439**	*0.017598398*	*0.025987915*	*0.025765493*	*0.016132561*
**ENSG00000089486**	*0.06413579*	*0.042183754*	*0.040634707*	*0.045950177*
**ENSG00000145217**	*0.014976857*	*0.02559895*	*0.024687637*	*0.016006931*
**ENSG00000096093**	*0.046099583*	*0.044113987*	*0.039031574*	*0.03624329*
**ENSG00000140265**	*0.035899564*	*0.034452629*	*0.037470728*	*0.023326715*
**ENSG00000139370**	*0.028801815*	*0.032161829*	*0.035553683*	*0.031851718*
**ENSG00000253873**	*0.02134135*	*0.026410593*	*0.027104797*	*0.018575858*
**ENSG00000196505**	*0.028937924*	*0.034063638*	*0.031012736*	*0.025196867*
**ENSG00000158113**	*0.018436804*	*0.027233154*	*0.025605672*	*0.017860115*
**ENSG00000186470**	*0.061167303*	*0.045461359*	*0.055306416*	*0.037434524*
**ENSG00000122644**	*0.061227796*	*0.062460881*	*0.061332386*	*0.048785221*
**ENSG00000162552**	*0.024329401*	*0.032846123*	*0.027717859*	*0.031066245*
**ENSG00000136527**	*0.029812391*	*0.033156961*	*0.035657488*	*0.031521044*

**ENSG00000136938**	*0.021627796*	*0.027159527*	*0.025318095*	*0.016260137*
**ENSG00000106477**	*0.035359903*	*0.039828767*	*0.034277093*	*0.030451875*
**ENSG00000125835**	*0.02768816*	*0.029870913*	*0.028745964*	*0.026031947*
**ENSG00000171864**	*0.016392226*	*0.025777547*	*0.024440347*	*0.015923819*
**ENSG00000196787**	*0.01644766*	*0.025557193*	*0.025709323*	*0.016239865*
**ENSG00000149968**	*0.016145193*	*0.024969054*	*0.024644673*	*0.015683399*
**ENSG00000163157**	*0.015699946*	*0.025333883*	*0.026044678*	*0.016273599*
**ENSG00000174990**	*0.016139371*	*0.025723779*	*0.025226688*	*0.017859871*
**ENSG00000155530**	*0.01629544*	*0.025292211*	*0.025098481*	*0.01501614*
**ENSG00000006704**	*0.015412368*	*0.025104399*	*0.024372776*	*0.015287083*
**ENSG00000072364**	*0.022974429*	*0.02716232*	*0.030316811*	*0.018753134*
**ENSG00000101811**	*0.028669807*	*0.033528115*	*0.02892205*	*0.031133706*
**ENSG00000130159**	*0.027195878*	*0.032063766*	*0.028570749*	*0.023360815*
**ENSG00000138750**	*0.029743281*	*0.033254841*	*0.030833964*	*0.029350142*
**ENSG00000005812**	*0.030775822*	*0.038916091*	*0.04072717*	*0.033413669*
**ENSG00000143125**	*0.015037339*	*0.025445297*	*0.024788726*	*0.014875725*
**ENSG00000148053**	*0.01544085*	*0.024920066*	*0.025349836*	*0.015711862*
**ENSG00000177462**	*0.014425452*	*0.024401602*	*0.024633124*	*0.014443155*
**ENSG00000213445**	*0.015118771*	*0.02549322*	*0.02561742*	*0.016823449*
**ENSG00000111269**	*0.030038872*	*0.037411236*	*0.033415934*	*0.032938431*
**ENSG00000104177**	*0.016772201*	*0.026293522*	*0.025975874*	*0.016634382*
**ENSG00000172985**	*0.015692415*	*0.025672479*	*0.025038575*	*0.015201252*
**ENSG00000126368**	*0.020906918*	*0.029028668*	*0.030410777*	*0.025265707*
**ENSG00000185928**	*0.047538815*	*0.044129787*	*0.033473893*	*0.031259163*
**ENSG00000145088**	*0.061192802*	*0.044771487*	*0.047237271*	*0.035839665*
**ENSG00000103018**	*0.024084858*	*0.029299268*	*0.029324167*	*0.024271688*
**ENSG00000181965**	*0.01579432*	*0.025448109*	*0.025100937*	*0.015366441*
**ENSG00000172869**	*0.033582319*	*0.043496206*	*0.033123219*	*0.036778563*
**ENSG00000140025**	*0.015572244*	*0.026126882*	*0.025167954*	*0.014739378*
**ENSG00000110660**	*0.032571383*	*0.034785596*	*0.038179049*	*0.028165982*
**ENSG00000100109**	*0.02818225*	*0.037195936*	*0.038956779*	*0.025585118*
**ENSG00000198758**	*0.016123414*	*0.026573494*	*0.025162424*	*0.014966119*
**ENSG00000178980**	*0.030897987*	*0.035032642*	*0.03466678*	*0.02617313*
**ENSG00000091536**	*0.017453466*	*0.025837025*	*0.025575089*	*0.015901679*
**ENSG00000134759**	*0.02603553*	*0.028873008*	*0.028277396*	*0.025290273*
**ENSG00000100365**	*0.066640488*	*0.055380756*	*0.053698687*	*0.057744881*
**ENSG00000145029**	*0.015841912*	*0.024241175*	*0.024588948*	*0.015293321*
**ENSG00000100097**	*0.037212278*	*0.032634405*	*0.034186286*	*0.036723685*
**ENSG00000157456**	*0.037774539*	*0.033108411*	*0.033359245*	*0.027014094*
**ENSG00000062650**	*0.023990073*	*0.031256651*	*0.031970756*	*0.023027995*
**ENSG00000184840**	*0.030377766*	*0.032282244*	*0.037958595*	*0.030253159*
**ENSG00000177076**	*0.017248967*	*0.025454552*	*0.026956093*	*0.015327361*
**ENSG00000167670**	*0.037098281*	*0.0412147*	*0.036569085*	*0.028341309*
**ENSG00000129657**	*0.032038896*	*0.036047457*	*0.040457007*	*0.031056624*
**ENSG00000116663**	*0.036632085*	*0.041183466*	*0.041551467*	*0.035643956*
**ENSG00000132466**	*0.026468609*	*0.036285782*	*0.030115246*	*0.027006697*
**ENSG00000142619**	*0.019103131*	*0.028872812*	*0.027894758*	*0.017454724*
**ENSG00000171401**	*0.017390336*	*0.02617186*	*0.02434574*	*0.015069543*
**ENSG00000107551**	*0.041427674*	*0.042198428*	*0.042323316*	*0.04331706*
**ENSG00000103415**	*0.016643134*	*0.026099542*	*0.02528744*	*0.015714669*
**ENSG00000090924**	*0.026734055*	*0.031726802*	*0.037883941*	*0.030818896*
**ENSG00000101972**	*0.028358932*	*0.035537552*	*0.030443057*	*0.027615535*
**ENSG00000139648**	*0.015475388*	*0.025035936*	*0.024526063*	*0.015284463*
**ENSG00000152463**	*0.032902733*	*0.033567163*	*0.03477685*	*0.02924344*

**ENSG00000159199**	*0.028966189*	*0.030524078*	*0.029864687*	*0.023208039*
**ENSG00000138081**	*0.031159341*	*0.036479923*	*0.032535123*	*0.027835419*
**ENSG00000158486**	*0.01640535*	*0.024610864*	*0.025218682*	*0.016128012*
**ENSG00000101096**	*0.015611038*	*0.025131219*	*0.024760033*	*0.014687053*
**ENSG00000107643**	*0.021028635*	*0.02720609*	*0.032167211*	*0.020773363*
**ENSG00000196420**	*0.015304318*	*0.026627352*	*0.026797757*	*0.017323779*
**ENSG00000196911**	*0.031993928*	*0.034545612*	*0.046503673*	*0.036202678*
**ENSG00000168484**	*0.017173309*	*0.025077185*	*0.024850705*	*0.015995491*
**ENSG00000110344**	*0.024856146*	*0.031329979*	*0.028953756*	*0.017674034*
**ENSG00000187170**	*0.042533138*	*0.028383691*	*0.026371916*	*0.0251108*
**ENSG00000105127**	*0.033527397*	*0.039442596*	*0.042682685*	*0.032281776*
**ENSG00000050426**	*0.02559958*	*0.028297312*	*0.029460672*	*0.026023178*
**ENSG00000183010**	*0.035042535*	*0.035260736*	*0.040185199*	*0.036939369*
**ENSG00000138684**	*0.017423305*	*0.025041881*	*0.026748792*	*0.015651626*
**ENSG00000196616**	*0.014501444*	*0.024741887*	*0.024497736*	*0.01389029*
**ENSG00000172322**	*0.014975005*	*0.025055919*	*0.024214709*	*0.014589334*
**ENSG00000196131**	*0.01485094*	*0.025976639*	*0.024404161*	*0.016746098*
**ENSG00000117069**	*0.01686376*	*0.026622656*	*0.025085371*	*0.015530519*
**ENSG00000120729**	*0.020985194*	*0.027838865*	*0.029313441*	*0.018117996*
**ENSG00000221988**	*0.015562118*	*0.026434331*	*0.024264603*	*0.015822388*
**ENSG00000134146**	*0.027085241*	*0.032577454*	*0.031104378*	*0.022308503*
**ENSG00000221900**	*0.018131663*	*0.025055874*	*0.025126175*	*0.016775722*
**ENSG00000126953**	*0.034162519*	*0.034473033*	*0.034183476*	*0.032633357*
**ENSG00000011590**	*0.059319557*	*0.04063662*	*0.042287981*	*0.048510679*
**ENSG00000143228**	*0.035355243*	*0.031688655*	*0.031825675*	*0.028502876*
**ENSG00000130770**	*0.053770106*	*0.040235938*	*0.045268733*	*0.049545901*
**ENSG00000187559**	*0.015320224*	*0.024818814*	*0.024487335*	*0.015060179*
**ENSG00000172236**	*0.030356382*	*0.027911154*	*0.025322221*	*0.016458767*
**ENSG00000171033**	*0.020799139*	*0.027385168*	*0.027225414*	*0.018300582*
**ENSG00000135940**	*0.019808769*	*0.026914538*	*0.027079735*	*0.016942734*
**ENSG00000179476**	*0.030012638*	*0.032922795*	*0.031513323*	*0.026792535*
**ENSG00000118733**	*0.020130807*	*0.025131481*	*0.024420656*	*0.01553004*
**ENSG00000108375**	*0.073375181*	*0.063526338*	*0.057567986*	*0.064037406*
**ENSG00000152223**	*0.044976395*	*0.043691079*	*0.040308189*	*0.033209637*
**ENSG00000103154**	*0.016444335*	*0.026452222*	*0.025193724*	*0.016426307*
**ENSG00000171505**	*0.018730321*	*0.026304715*	*0.025712991*	*0.017950527*
**ENSG00000044446**	*0.032113904*	*0.038640843*	*0.037385733*	*0.026510034*
**ENSG00000141570**	*0.016722502*	*0.027586897*	*0.025425985*	*0.015990842*
**ENSG00000089012**	*0.048529134*	*0.04886807*	*0.02543708*	*0.030761582*
**ENSG00000063515**	*0.01634389*	*0.025891168*	*0.024406685*	*0.01670626*
**ENSG00000214681**	*0.013478339*	*0.023637243*	*0.023699304*	*0.014368353*
**ENSG00000138346**	*0.049499341*	*0.049495953*	*0.038539465*	*0.038621921*
**ENSG00000134297**	*0.035483777*	*0.04597964*	*0.036186298*	*0.03053045*
**ENSG00000113070**	*0.029319587*	*0.041276393*	*0.044461242*	*0.036478231*
**ENSG00000133247**	*0.042287514*	*0.039101999*	*0.035017838*	*0.036225552*
**ENSG00000138463**	*0.034593734*	*0.034584814*	*0.032214396*	*0.02661456*
**ENSG00000012174**	*0.021011945*	*0.030437117*	*0.029527423*	*0.018484284*
**ENSG00000100092**	*0.026480107*	*0.03223778*	*0.030960493*	*0.02805188*
**ENSG00000112941**	*0.043123632*	*0.045413255*	*0.04009763*	*0.032314063*
**ENSG00000145757**	*0.015332896*	*0.024637881*	*0.02489159*	*0.014044877*
**ENSG00000087206**	*0.024209214*	*0.03206834*	*0.030571689*	*0.022677902*
**ENSG00000102871**	*0.028113101*	*0.054343138*	*0.037833907*	*0.040417327*
**ENSG00000110074**	*0.034387598*	*0.033604024*	*0.028343711*	*0.027919847*
**ENSG00000120800**	*0.044250841*	*0.035668229*	*0.033520513*	*0.034623077*

**ENSG00000136213**	*0.060067034*	*0.047803616*	*0.047003092*	*0.049940174*
**ENSG00000154229**	*0.016248553*	*0.025018082*	*0.024587878*	*0.015467378*
**ENSG00000187630**	*0.035402617*	*0.039842041*	*0.039233046*	*0.044625651*
**ENSG00000186439**	*0.018676128*	*0.025340834*	*0.028104344*	*0.015502935*
**ENSG00000108702**	*0.106054303*	*0.051818228*	*0.087048159*	*0.032293036*
**ENSG00000127515**	*0.015437918*	*0.024349193*	*0.024880633*	*0.014771995*
**ENSG00000115085**	*0.044157009*	*0.037522742*	*0.03670002*	*0.057430072*
**ENSG00000162631**	*0.0170356*	*0.025568455*	*0.025402216*	*0.017279422*
**ENSG00000167842**	*0.032111348*	*0.037575481*	*0.039946664*	*0.030941699*
**ENSG00000154035**	*0.017170002*	*0.025941143*	*0.025716021*	*0.016509205*
**ENSG00000109911**	*0.04081963*	*0.03866057*	*0.034470517*	*0.029297141*
**ENSG00000197629**	*0.099324391*	*0.09634083*	*0.075764432*	*0.100634465*
**ENSG00000198483**	*0.017439439*	*0.026706884*	*0.027431349*	*0.015266438*
**ENSG00000095777**	*0.015046183*	*0.024585218*	*0.024234815*	*0.015400823*
**ENSG00000139974**	*0.031620904*	*0.037634716*	*0.029873905*	*0.025171576*
**ENSG00000128915**	*0.028892094*	*0.037877485*	*0.02995461*	*0.023574855*
**ENSG00000154099**	*0.015651145*	*0.024755109*	*0.024514879*	*0.014307371*
**ENSG00000130558**	*0.017239141*	*0.025371075*	*0.025423057*	*0.014998332*
**ENSG00000253731**	*0.01623771*	*0.0254693*	*0.024766718*	*0.015695099*
**ENSG00000147419**	*0.028787304*	*0.032459591*	*0.032230439*	*0.020981443*
**ENSG00000179151**	*0.021185147*	*0.028331928*	*0.030658763*	*0.01965187*
**ENSG00000188191**	*0.018897325*	*0.026470359*	*0.031607527*	*0.020066397*
**ENSG00000117586**	*0.0872805*	*0.063607118*	*0.068219923*	*0.081511516*
**ENSG00000165555**	*0.015203156*	*0.024498851*	*0.024805805*	*0.016069313*
**ENSG00000177174**	*0.014802902*	*0.025773764*	*0.024509198*	*0.014852406*
**ENSG00000183729**	*0.015540025*	*0.02486336*	*0.025254227*	*0.014751696*
**ENSG00000178460**	*0.01419993*	*0.025507314*	*0.024819489*	*0.014898945*
**ENSG00000157985**	*0.056848781*	*0.039173588*	*0.038082533*	*0.038900698*
**ENSG00000170545**	*0.032387295*	*0.030986212*	*0.038158897*	*0.033342991*
**ENSG00000067182**	*0.065658755*	*0.048356601*	*0.042316303*	*0.059858819*
**ENSG00000235173**	*0.036879807*	*0.035687296*	*0.032152599*	*0.035835284*
**ENSG00000170364**	*0.033536815*	*0.034467678*	*0.041980054*	*0.044239579*
**ENSG00000180999**	*0.014442104*	*0.02561879*	*0.025251314*	*0.015037918*
**ENSG00000139641**	*0.031784403*	*0.035751693*	*0.029683274*	*0.027118512*
**ENSG00000143772**	*0.070942709*	*0.052597061*	*0.050831976*	*0.050272321*
**ENSG00000089847**	*0.063265245*	*0.047847393*	*0.036454358*	*0.049272458*
**ENSG00000159189**	*0.016931031*	*0.025055323*	*0.024398525*	*0.015415019*
**ENSG00000103353**	*0.025950304*	*0.03236545*	*0.029650224*	*0.024119486*
**ENSG00000186185**	*0.023908162*	*0.026294848*	*0.026967153*	*0.018170571*
**ENSG00000186265**	*0.054099922*	*0.05091084*	*0.037567505*	*0.041218332*
**ENSG00000103479**	*0.028826834*	*0.038221115*	*0.033500817*	*0.027671092*
**ENSG00000101343**	*0.035033576*	*0.030930473*	*0.02946408*	*0.023839767*
**ENSG00000116194**	*0.017042726*	*0.025229676*	*0.025178183*	*0.017838519*
**ENSG00000185973**	*0.020765955*	*0.028459552*	*0.025500281*	*0.021142494*
**ENSG00000185104**	*0.038954894*	*0.041794961*	*0.038400224*	*0.0355598*
**ENSG00000115919**	*0.041895168*	*0.037012109*	*0.035469545*	*0.034213425*
**ENSG00000196083**	*0.01681609*	*0.026353268*	*0.025155586*	*0.016802167*
**ENSG00000125457**	*0.026660864*	*0.033596943*	*0.031510996*	*0.023010578*
**ENSG00000135315**	*0.020057267*	*0.031416655*	*0.027790865*	*0.019504427*
**ENSG00000147613**	*0.015537305*	*0.025911536*	*0.02455604*	*0.014799047*
**ENSG00000178301**	*0.027024166*	*0.030919455*	*0.02818318*	*0.02367248*
**ENSG00000169035**	*0.015172018*	*0.025038275*	*0.024119517*	*0.014855371*
**ENSG00000187054**	*0.018805361*	*0.026072834*	*0.026613066*	*0.016473003*
**ENSG00000136144**	*0.021767663*	*0.033485962*	*0.030949479*	*0.020455311*

**ENSG00000153006**	*0.025161039*	*0.030270181*	*0.029826742*	*0.023193771*
**ENSG00000214014**	*0.025792092*	*0.032083275*	*0.034802152*	*0.027366434*
**ENSG00000111331**	*0.075619284*	*0.050913206*	*0.045361021*	*0.047136705*
**ENSG00000107447**	*0.019280755*	*0.028078444*	*0.028027612*	*0.049082047*
**ENSG00000168300**	*0.04578383*	*0.044102879*	*0.036410759*	*0.036683777*
**ENSG00000182890**	*0.030747174*	*0.03913026*	*0.033227736*	*0.027929562*
**ENSG00000137707**	*0.014889439*	*0.026528687*	*0.025001331*	*0.015920094*
**ENSG00000124593**	*0.022338667*	*0.027042003*	*0.028290887*	*0.024810891*
**ENSG00000151806**	*0.04381603*	*0.047631581*	*0.035513934*	*0.041537792*
**ENSG00000037749**	*0.02920162*	*0.03551619*	*0.036506497*	*0.02334684*
**ENSG00000175104**	*0.017376188*	*0.025884076*	*0.025479481*	*0.016771619*
**ENSG00000100504**	*0.087544898*	*0.081451382*	*0.052597452*	*0.066873904*
**ENSG00000134910**	*0.037649601*	*0.037567203*	*0.037256243*	*0.03406784*
**ENSG00000241476**	*0.024720315*	*0.024482742*	*0.024699323*	*0.015360466*
**ENSG00000100483**	*0.031192231*	*0.038175795*	*0.04081727*	*0.033516898*
**ENSG00000182809**	*0.021355754*	*0.032068098*	*0.027158915*	*0.040697311*
**ENSG00000127337**	*0.029258507*	*0.031801546*	*0.030463578*	*0.034427624*
**ENSG00000182180**	*0.027615795*	*0.029358821*	*0.027536564*	*0.02324426*
**ENSG00000106608**	*0.029288894*	*0.03121801*	*0.039129454*	*0.022984952*
**ENSG00000004139**	*0.036227104*	*0.037565203*	*0.034069219*	*0.030164787*
**ENSG00000163932**	*0.026409082*	*0.03303654*	*0.034586706*	*0.021571018*
**ENSG00000143578**	*0.037806664*	*0.03880253*	*0.030803598*	*0.029834837*
**ENSG00000135045**	*0.041552972*	*0.047875289*	*0.039449147*	*0.031672249*
**ENSG00000087586**	*0.044057133*	*0.035474459*	*0.034840532*	*0.031833148*
**ENSG00000186866**	*0.017753963*	*0.027192531*	*0.025640224*	*0.01792739*
**ENSG00000131263**	*0.018064379*	*0.025024906*	*0.026000985*	*0.016617508*
**ENSG00000154080**	*0.014996618*	*0.024658879*	*0.02396722*	*0.014848525*
**ENSG00000142208**	*0.027280668*	*0.031591894*	*0.033987597*	*0.026423455*
**ENSG00000145979**	*0.032978054*	*0.035481779*	*0.031369118*	*0.028035411*
**ENSG00000114062**	*0.018113057*	*0.024821924*	*0.025570446*	*0.019649576*
**ENSG00000133961**	*0.029646126*	*0.032674612*	*0.033414984*	*0.021092287*
**ENSG00000173013**	*0.024458774*	*0.030550432*	*0.030304896*	*0.021863989*
**ENSG00000146872**	*0.025197171*	*0.033298504*	*0.031990107*	*0.024715573*
**ENSG00000159228**	*0.035484542*	*0.030108852*	*0.02872675*	*0.033871064*
**ENSG00000134352**	*0.022611492*	*0.027414892*	*0.028033335*	*0.020231037*
**ENSG00000127948**	*0.026088543*	*0.033201961*	*0.03678798*	*0.028836198*
**ENSG00000175894**	*0.018648708*	*0.026247633*	*0.025488317*	*0.019059833*
**ENSG00000021461**	*0.016026479*	*0.025530049*	*0.02495045*	*0.015843409*
**ENSG00000177733**	*0.026567385*	*0.031913943*	*0.036265456*	*0.031047766*
**ENSG00000162585**	*0.019526708*	*0.025819084*	*0.026105179*	*0.020667737*
**ENSG00000198785**	*0.015700324*	*0.025379637*	*0.02442461*	*0.01511563*
**ENSG00000149922**	*0.015326557*	*0.02481068*	*0.024477956*	*0.017236283*
**ENSG00000175229**	*0.016998826*	*0.025729289*	*0.025171748*	*0.015376066*
**ENSG00000143476**	*0.041019308*	*0.039101471*	*0.032786064*	*0.031657157*
**ENSG00000164651**	*0.016751156*	*0.0258233*	*0.024485827*	*0.014913188*
**ENSG00000106367**	*0.030757737*	*0.036196104*	*0.034849854*	*0.037463969*
**ENSG00000149380**	*0.015688956*	*0.024766517*	*0.025153645*	*0.014312116*
**ENSG00000125863**	*0.031480731*	*0.035458287*	*0.030960023*	*0.023452723*
**ENSG00000182636**	*0.101545992*	*0.065362088*	*0.063746693*	*0.085295691*
**ENSG00000141867**	*0.021913407*	*0.034603258*	*0.029116681*	*0.023783212*
**ENSG00000187017**	*0.016459216*	*0.023902872*	*0.025551022*	*0.015789113*
**ENSG00000198270**	*0.037723288*	*0.04090227*	*0.036974876*	*0.033294532*
**ENSG00000165066**	*0.015350983*	*0.025175553*	*0.024994749*	*0.014841417*
**ENSG00000104972**	*0.042651707*	*0.044572293*	*0.0373103*	*0.03817703*

**ENSG00000177963**	*0.022853055*	*0.030209996*	*0.038482851*	*0.023543323*
**ENSG00000119787**	*0.021602702*	*0.026401453*	*0.026706893*	*0.017256839*
**ENSG00000166396**	*0.020898042*	*0.025788454*	*0.025025527*	*0.016108054*
**ENSG00000176194**	*0.016742668*	*0.026240694*	*0.025503694*	*0.015777933*
**ENSG00000144619**	*0.01631425*	*0.024562538*	*0.025372397*	*0.015215339*
**ENSG00000181896**	*0.032098762*	*0.039011552*	*0.042983372*	*0.02969687*
**ENSG00000168427**	*0.015329114*	*0.024961333*	*0.024141197*	*0.015072359*
**ENSG00000059588**	*0.036870379*	*0.037604275*	*0.040136896*	*0.0316823*
**ENSG00000236287**	*0.030031916*	*0.03470767*	*0.030302546*	*0.023432911*
**ENSG00000122482**	*0.016722586*	*0.024473974*	*0.024981731*	*0.016448443*
**ENSG00000168556**	*0.038596347*	*0.034361729*	*0.033010898*	*0.024679164*
**ENSG00000203705**	*0.027378507*	*0.033401808*	*0.035883607*	*0.041692033*
**ENSG00000189306**	*0.04978002*	*0.039569163*	*0.039139343*	*0.041637908*
**ENSG00000166997**	*0.029792293*	*0.034682283*	*0.027983551*	*0.024622339*
**ENSG00000155833**	*0.016550098*	*0.025060342*	*0.025116601*	*0.015259478*
**ENSG00000204300**	*0.015914271*	*0.025082077*	*0.024699658*	*0.01549995*
**ENSG00000174944**	*0.018506583*	*0.02665846*	*0.026364683*	*0.019316894*
**ENSG00000188931**	*0.017060553*	*0.025888365*	*0.024985486*	*0.016491984*
**ENSG00000144589**	*0.030170211*	*0.037863011*	*0.033025537*	*0.025550802*
**ENSG00000057663**	*0.039708389*	*0.037394223*	*0.032612089*	*0.030483698*
**ENSG00000171815**	*0.016878073*	*0.025592598*	*0.024247654*	*0.015524102*
**ENSG00000198753**	*0.016257894*	*0.026711668*	*0.025064441*	*0.017647293*
**ENSG00000241399**	*0.014495886*	*0.025173153*	*0.024813573*	*0.014783775*
**ENSG00000083307**	*0.015531195*	*0.024471966*	*0.024716278*	*0.015941524*
**ENSG00000172123**	*0.075210711*	*0.043344156*	*0.049148765*	*0.039393776*
**ENSG00000178860**	*0.029735282*	*0.03115204*	*0.031077856*	*0.031348356*
**ENSG00000186684**	*0.017516859*	*0.029144809*	*0.026918091*	*0.019360156*
**ENSG00000111405**	*0.025775306*	*0.028961227*	*0.027943072*	*0.022516091*
**ENSG00000196597**	*0.020589162*	*0.027453262*	*0.024879845*	*0.020431259*
**ENSG00000241465**	*0.058744969*	*0.024888254*	*0.025964336*	*0.015843267*
**ENSG00000152767**	*0.045707586*	*0.035425119*	*0.035510336*	*0.029876781*
**ENSG00000144580**	*0.032979872*	*0.038027454*	*0.032049904*	*0.033315907*
**ENSG00000162407**	*0.017031066*	*0.024900546*	*0.024890934*	*0.015507842*
**ENSG00000164385**	*0.015459426*	*0.025896563*	*0.025798866*	*0.014876686*
**ENSG00000138653**	*0.015584371*	*0.025759255*	*0.025540924*	*0.015976836*
**ENSG00000184381**	*0.029754936*	*0.033672882*	*0.030525097*	*0.027175729*
**ENSG00000130958**	*0.052670754*	*0.040320187*	*0.044169551*	*0.046236868*
**ENSG00000119630**	*0.038633663*	*0.031290746*	*0.028593093*	*0.027755162*
**ENSG00000196700**	*0.03630682*	*0.037131636*	*0.046552808*	*0.034538592*
**ENSG00000187775**	*0.017473343*	*0.026501668*	*0.025635591*	*0.017857905*
**ENSG00000005001**	*0.015204513*	*0.025890774*	*0.025782601*	*0.015608897*
**ENSG00000089639**	*0.028324416*	*0.031568223*	*0.03911082*	*0.028632406*
**ENSG00000102837**	*0.014833247*	*0.026865052*	*0.024409141*	*0.014960678*
**ENSG00000152433**	*0.016822914*	*0.02534887*	*0.026369819*	*0.017143122*
**ENSG00000036828**	*0.022418349*	*0.027102601*	*0.026989707*	*0.02141747*
**ENSG00000188243**	*0.029204748*	*0.031195948*	*0.030130839*	*0.031471779*
**ENSG00000100403**	*0.019242248*	*0.025842526*	*0.025983741*	*0.019838116*
**ENSG00000150672**	*0.015724147*	*0.024942865*	*0.024250229*	*0.014402491*
**ENSG00000188677**	*0.034919291*	*0.030874298*	*0.037352944*	*0.095118462*
**ENSG00000187260**	*0.024746304*	*0.035978021*	*0.035142998*	*0.023097362*
**ENSG00000135220**	*0.041103424*	*0.024636443*	*0.024445103*	*0.020028766*
**ENSG00000170581**	*0.033853121*	*0.03756006*	*0.033643167*	*0.031480855*
**ENSG00000101049**	*0.015801706*	*0.024386392*	*0.025116256*	*0.013925602*
**ENSG00000111641**	*0.027976631*	*0.031973045*	*0.034990388*	*0.031693604*

**ENSG00000171435**	*0.015970322*	*0.024956733*	*0.025216512*	*0.01431134*
**ENSG00000174292**	*0.015893148*	*0.026439172*	*0.026117615*	*0.016973927*
**ENSG00000163960**	*0.040742209*	*0.040391796*	*0.039258549*	*0.039159191*
**ENSG00000197724**	*0.019150029*	*0.02717968*	*0.025765435*	*0.016617698*
**ENSG00000138623**	*0.042557756*	*0.049000451*	*0.043607534*	*0.045199707*
**ENSG00000183549**	*0.017193474*	*0.026213295*	*0.024618451*	*0.016587876*
**ENSG00000163607**	*0.027670448*	*0.033525392*	*0.033737363*	*0.034169688*
**ENSG00000009307**	*0.019687992*	*0.027714808*	*0.027860801*	*0.021577482*
**ENSG00000162494**	*0.016312372*	*0.025658117*	*0.025056358*	*0.015444683*
**ENSG00000138294**	*0.015472358*	*0.025645534*	*0.025636813*	*0.014621418*
**ENSG00000124608**	*0.037928028*	*0.041547089*	*0.037429193*	*0.028253394*
**ENSG00000205978**	*0.015567149*	*0.024557881*	*0.0248238*	*0.01593537*
**ENSG00000161798**	*0.015127721*	*0.025813313*	*0.024601567*	*0.01612748*
**ENSG00000073417**	*0.031825584*	*0.032808395*	*0.032999926*	*0.031247984*
**ENSG00000167130**	*0.020809786*	*0.027329534*	*0.028825276*	*0.020987538*
**ENSG00000234438**	*0.019335505*	*0.026167668*	*0.026794671*	*0.018676898*
**ENSG00000168264**	*0.032296686*	*0.036800746*	*0.039069905*	*0.034346565*
**ENSG00000130764**	*0.021481885*	*0.028323402*	*0.02939598*	*0.019233971*
**ENSG00000163746**	*0.014490555*	*0.025580995*	*0.024237905*	*0.01480559*
**ENSG00000185652**	*0.014585389*	*0.024532506*	*0.023813075*	*0.014870476*
**ENSG00000243251**	*0.044472835*	*0.0389052*	*0.039821153*	*0.039204404*
**ENSG00000148143**	*0.0805799*	*0.040870093*	*0.043310081*	*0.031157569*
**ENSG00000052841**	*0.026314324*	*0.030053793*	*0.034397399*	*0.028598818*
**ENSG00000186666**	*0.036120068*	*0.037923754*	*0.036055806*	*0.030731159*
**ENSG00000115556**	*0.017186574*	*0.024859991*	*0.024225181*	*0.014439163*
**ENSG00000125510**	*0.028348541*	*0.036211419*	*0.030971743*	*0.044061715*
**ENSG00000186205**	*0.038262681*	*0.038813802*	*0.038761832*	*0.030736236*
**ENSG00000110811**	*0.0439132*	*0.039052933*	*0.036718925*	*0.032913379*
**ENSG00000027644**	*0.016781696*	*0.025141359*	*0.026361216*	*0.01608122*
**ENSG00000156172**	*0.016855879*	*0.026448009*	*0.024941708*	*0.01672732*
**ENSG00000042445**	*0.02493914*	*0.032372713*	*0.029810473*	*0.020534139*
**ENSG00000126777**	*0.02810653*	*0.033014451*	*0.029282391*	*0.023218031*
**ENSG00000155465**	*0.074214814*	*0.093892824*	*0.097614634*	*0.081666112*
**ENSG00000115598**	*0.015305423*	*0.024563165*	*0.024713232*	*0.01533571*
**ENSG00000152147**	*0.030786259*	*0.034846319*	*0.030790219*	*0.022763112*
**ENSG00000149305**	*0.016268929*	*0.025802431*	*0.025418898*	*0.01750611*
**ENSG00000173915**	*0.115591868*	*0.116630705*	*0.110911216*	*0.058560127*
**ENSG00000109851**	*0.016136027*	*0.025062121*	*0.02436983*	*0.015359381*
**ENSG00000204842**	*0.030991095*	*0.043127834*	*0.036537304*	*0.031492298*
**ENSG00000104957**	*0.027936808*	*0.032468538*	*0.032128929*	*0.026508147*
**ENSG00000103126**	*0.015988485*	*0.024467968*	*0.024146149*	*0.013552323*
**ENSG00000143158**	*0.034133112*	*0.033996158*	*0.031655873*	*0.027301687*
**ENSG00000181924**	*0.026685001*	*0.028625877*	*0.032159895*	*0.02303194*
**ENSG00000183283**	*0.028705407*	*0.037922235*	*0.035302868*	*0.028807119*
**ENSG00000157152**	*0.015590363*	*0.025823562*	*0.024662206*	*0.014951857*
**ENSG00000165097**	*0.015184691*	*0.024850899*	*0.02470248*	*0.015894847*
**ENSG00000169862**	*0.020893662*	*0.025440152*	*0.025676228*	*0.017362609*
**ENSG00000100142**	*0.024003144*	*0.027642543*	*0.027693747*	*0.022755605*
**ENSG00000158863**	*0.024652578*	*0.032985503*	*0.034391524*	*0.026999137*
**ENSG00000113100**	*0.016435721*	*0.025151211*	*0.026868384*	*0.016838714*
**ENSG00000144035**	*0.017273414*	*0.02613247*	*0.027302307*	*0.016400092*
**ENSG00000003393**	*0.031899899*	*0.042694466*	*0.033203565*	*0.032101877*
**ENSG00000127564**	*0.028277921*	*0.028205877*	*0.031562677*	*0.026362217*
**ENSG00000171450**	*0.014233916*	*0.025254482*	*0.024616968*	*0.014306097*

**ENSG00000140990**	*0.024641136*	*0.026418365*	*0.027228087*	*0.020497039*
**ENSG00000148719**	*0.020530538*	*0.027504044*	*0.02815366*	*0.019096378*
**ENSG00000172578**	*0.094948429*	*0.081134876*	*0.079587677*	*0.073850332*
**ENSG00000204267**	*0.029222395*	*0.038479654*	*0.04321666*	*0.021431878*
**ENSG00000173852**	*0.085941723*	*0.05838393*	*0.05543569*	*0.057946652*
**ENSG00000149735**	*0.017825473*	*0.026725455*	*0.025716284*	*0.015243528*
**ENSG00000186895**	*0.02033035*	*0.028285687*	*0.027184367*	*0.025100989*
**ENSG00000008441**	*0.016932874*	*0.025229515*	*0.024786056*	*0.015436018*
**ENSG00000186314**	*0.026268428*	*0.029902763*	*0.031753731*	*0.021233343*
**ENSG00000112679**	*0.031477659*	*0.03482743*	*0.030515377*	*0.027635415*
**ENSG00000007237**	*0.073746525*	*0.059947923*	*0.06880455*	*0.070759809*
**ENSG00000165188**	*0.03606185*	*0.098043769*	*0.072958668*	*0.094598737*
**ENSG00000197993**	*0.02034549*	*0.025737512*	*0.024322799*	*0.017398929*
**ENSG00000196268**	*0.029723018*	*0.031034624*	*0.029680176*	*0.025368615*
**ENSG00000171195**	*0.014878355*	*0.025601419*	*0.024536582*	*0.016892498*
**ENSG00000205111**	*0.016903527*	*0.025419798*	*0.024804969*	*0.015340207*
**ENSG00000182685**	*0.01915117*	*0.030517369*	*0.026548276*	*0.017272657*
**ENSG00000198829**	*0.059247965*	*0.046336436*	*0.045000207*	*0.038462354*
**ENSG00000140854**	*0.033418424*	*0.037611453*	*0.029127972*	*0.029833042*
**ENSG00000144034**	*0.03167775*	*0.031165709*	*0.033409687*	*0.024531362*
**ENSG00000136807**	*0.02691915*	*0.034476133*	*0.033343366*	*0.023903321*
**ENSG00000142765**	*0.058804524*	*0.051402535*	*0.062694602*	*0.046982655*
**ENSG00000100206**	*0.037869436*	*0.035952563*	*0.036793137*	*0.041798524*
**ENSG00000179772**	*0.015055292*	*0.024046255*	*0.024684828*	*0.015094749*
**ENSG00000168418**	*0.017247642*	*0.025176643*	*0.024739058*	*0.016332091*
**ENSG00000122592**	*0.031430627*	*0.02691429*	*0.033396277*	*0.043554033*
**ENSG00000117834**	*0.01553597*	*0.024783635*	*0.025163098*	*0.014616378*
**ENSG00000125999**	*0.01602738*	*0.024460833*	*0.024043272*	*0.015242139*
**ENSG00000142751**	*0.023456546*	*0.032192269*	*0.033142341*	*0.022666031*
**ENSG00000143858**	*0.014730738*	*0.024936058*	*0.024951038*	*0.014682889*
**ENSG00000163319**	*0.026051748*	*0.030823605*	*0.032807085*	*0.024214755*
**ENSG00000064547**	*0.030648452*	*0.036528382*	*0.034928817*	*0.02787937*
**ENSG00000125814**	*0.035528364*	*0.037358723*	*0.031767069*	*0.030485491*
**ENSG00000148841**	*0.026071492*	*0.032458552*	*0.033795194*	*0.027386068*
**ENSG00000070413**	*0.030429471*	*0.032473922*	*0.03545899*	*0.032507163*
**ENSG00000138107**	*0.029407385*	*0.036704234*	*0.036119076*	*0.030552403*
**ENSG00000123080**	*0.074255987*	*0.052122441*	*0.043255429*	*0.054884068*
**ENSG00000198952**	*0.024789901*	*0.032392245*	*0.037861854*	*0.029416478*
**ENSG00000153060**	*0.0461677*	*0.064246856*	*0.05243712*	*0.046755324*
**ENSG00000102934**	*0.044339308*	*0.042432157*	*0.035221111*	*0.035114268*
**ENSG00000179546**	*0.017887728*	*0.025838383*	*0.025034485*	*0.014734703*
**ENSG00000173145**	*0.030852559*	*0.035443394*	*0.031781573*	*0.023404035*
**ENSG00000138111**	*0.023967661*	*0.028750341*	*0.026354404*	*0.019591426*
**ENSG00000055955**	*0.030534378*	*0.042964716*	*0.031945791*	*0.030459485*
**ENSG00000086589**	*0.026797372*	*0.032098631*	*0.032283511*	*0.021686925*
**ENSG00000205464**	*0.022792483*	*0.029428675*	*0.02738734*	*0.022669055*
**ENSG00000128590**	*0.042262892*	*0.039204968*	*0.047381308*	*0.048991611*
**ENSG00000149527**	*0.064550165*	*0.06887106*	*0.057857984*	*0.067299351*
**ENSG00000213809**	*0.032064963*	*0.041949256*	*0.036380983*	*0.054434718*
**ENSG00000160460**	*0.016349735*	*0.025970381*	*0.024848145*	*0.014946289*
**ENSG00000170890**	*0.016650159*	*0.026029161*	*0.025643015*	*0.016743923*
**ENSG00000143622**	*0.056166158*	*0.050110038*	*0.047535507*	*0.055505238*
**ENSG00000163681**	*0.022418851*	*0.028773481*	*0.025846371*	*0.020437921*
**ENSG00000173369**	*0.015737376*	*0.024218273*	*0.024260492*	*0.01438225*

**ENSG00000079385**	*0.067769423*	*0.07171265*	*0.054435207*	*0.065437949*
**ENSG00000105538**	*0.036909667*	*0.059434979*	*0.092245247*	*0.102055187*
**ENSG00000213171**	*0.015851406*	*0.024777836*	*0.025046208*	*0.014892306*
**ENSG00000153558**	*0.017444556*	*0.026261734*	*0.024524276*	*0.015723833*
**ENSG00000114021**	*0.032918522*	*0.03364578*	*0.033028811*	*0.025875785*
**ENSG00000006016**	*0.014955547*	*0.025012642*	*0.025327652*	*0.015572187*
**ENSG00000173210**	*0.015714786*	*0.02517937*	*0.024102845*	*0.015100009*
**ENSG00000128731**	*0.035468134*	*0.047778863*	*0.035062147*	*0.051489051*
**ENSG00000197362**	*0.039424474*	*0.036579877*	*0.038284511*	*0.044817006*
**ENSG00000019186**	*0.016494904*	*0.024320396*	*0.025584931*	*0.014341596*
**ENSG00000179528**	*0.016071598*	*0.024957042*	*0.024598623*	*0.014956294*
**ENSG00000116521**	*0.022180812*	*0.026471583*	*0.027277236*	*0.022788914*
**ENSG00000134363**	*0.017613648*	*0.026391851*	*0.025389925*	*0.01628536*
**ENSG00000165699**	*0.034628406*	*0.03702523*	*0.031753136*	*0.029906234*
**ENSG00000189376**	*0.023283393*	*0.030350536*	*0.032783563*	*0.022008008*
**ENSG00000120253**	*0.034500811*	*0.032333419*	*0.035911627*	*0.032083772*
**ENSG00000112761**	*0.017777646*	*0.025641558*	*0.025764959*	*0.015412276*
**ENSG00000140807**	*0.016088738*	*0.024646337*	*0.024197814*	*0.016331964*
**ENSG00000076641**	*0.058339926*	*0.048231617*	*0.042948206*	*0.059445025*
**ENSG00000107679**	*0.040748898*	*0.043756602*	*0.044741238*	*0.038476157*
**ENSG00000165799**	*0.015955917*	*0.026505347*	*0.025705863*	*0.014113506*
**ENSG00000070159**	*0.024934854*	*0.026103874*	*0.026269752*	*0.018368425*
**ENSG00000165443**	*0.017829975*	*0.024585427*	*0.024372183*	*0.015605542*
**ENSG00000187116**	*0.02898878*	*0.026591383*	*0.024822896*	*0.016087551*
**ENSG00000132581**	*0.022303937*	*0.031373807*	*0.032626674*	*0.021143403*
**ENSG00000168970**	*0.039058267*	*0.040440538*	*0.038037421*	*0.035220907*
**ENSG00000154493**	*0.017825989*	*0.026310172*	*0.02561119*	*0.018550154*
**ENSG00000128609**	*0.028368517*	*0.030093599*	*0.028109463*	*0.034630476*
**ENSG00000211584**	*0.030328169*	*0.03244724*	*0.037050581*	*0.034000213*
**ENSG00000108813**	*0.016653913*	*0.02559945*	*0.025162292*	*0.016654084*
**ENSG00000072080**	*0.016320611*	*0.02585285*	*0.026074671*	*0.016300111*
**ENSG00000089692**	*0.102433392*	*0.066265525*	*0.078156204*	*0.095791786*
**ENSG00000119986**	*0.047017781*	*0.040589328*	*0.041256482*	*0.040991635*
**ENSG00000172381**	*0.017916684*	*0.028574001*	*0.027096336*	*0.017476071*
**ENSG00000125740**	*0.060859594*	*0.072600896*	*0.068876771*	*0.040621885*
**ENSG00000176136**	*0.018527337*	*0.026539629*	*0.02714809*	*0.016790367*
**ENSG00000021488**	*0.016681714*	*0.025016098*	*0.025721378*	*0.016221281*
**ENSG00000119403**	*0.044881*	*0.04192561*	*0.035420274*	*0.033606851*
**ENSG00000197594**	*0.016371336*	*0.024793839*	*0.024771908*	*0.01653191*
**ENSG00000102096**	*0.043601047*	*0.033119337*	*0.039089421*	*0.042356347*
**ENSG00000132911**	*0.018202198*	*0.029608799*	*0.027632574*	*0.017776591*
**ENSG00000113712**	*0.023067613*	*0.030168794*	*0.032867679*	*0.025707762*
**ENSG00000120885**	*0.053722679*	*0.028089386*	*0.030810142*	*0.038449198*
**ENSG00000118849**	*0.018865879*	*0.027255328*	*0.026140378*	*0.017629053*
**ENSG00000162613**	*0.028200578*	*0.031628531*	*0.030660809*	*0.023605685*
**ENSG00000160957**	*0.034965898*	*0.032478001*	*0.034942319*	*0.029668946*
**ENSG00000161277**	*0.057821366*	*0.054110824*	*0.039844164*	*0.041085833*
**ENSG00000129810**	*0.0467479*	*0.043541305*	*0.033681327*	*0.030565054*
**ENSG00000185420**	*0.032996791*	*0.035230433*	*0.029716577*	*0.031588813*
**ENSG00000064218**	*0.01555116*	*0.02480291*	*0.024147201*	*0.015364457*
**ENSG00000100603**	*0.022263901*	*0.03065129*	*0.029594493*	*0.017907752*
**ENSG00000196839**	*0.045669544*	*0.044894322*	*0.039595758*	*0.042206178*
**ENSG00000140497**	*0.038426988*	*0.039237181*	*0.039737822*	*0.031224455*
**ENSG00000213054**	*0.087723229*	*0.080663901*	*0.070083963*	*0.070330166*

**ENSG00000170430**	*0.084547305*	*0.039113502*	*0.034352207*	*0.032659789*
**ENSG00000167106**	*0.048844864*	*0.056831582*	*0.049417193*	*0.053273031*
**ENSG00000104755**	*0.015325848*	*0.024949622*	*0.025022545*	*0.015682143*
**ENSG00000088808**	*0.045247735*	*0.042430359*	*0.041397206*	*0.038102455*
**ENSG00000179468**	*0.016107329*	*0.02543158*	*0.02455638*	*0.015581019*
**ENSG00000188107**	*0.015593027*	*0.02467535*	*0.026138778*	*0.015072093*
**ENSG00000103042**	*0.02590672*	*0.03386246*	*0.033475815*	*0.026933477*
**ENSG00000068400**	*0.020717878*	*0.028824401*	*0.03012753*	*0.029357121*
**ENSG00000139266**	*0.036305875*	*0.032574186*	*0.032761763*	*0.034517484*
**ENSG00000203811**	*0.056193763*	*0.054774331*	*0.071236983*	*0.068707807*
**ENSG00000112130**	*0.01868253*	*0.027452996*	*0.026933537*	*0.017207681*
**ENSG00000164463**	*0.03895997*	*0.036520872*	*0.041598113*	*0.038904197*
**ENSG00000144802**	*0.048038351*	*0.053589098*	*0.051382593*	*0.049355782*
**ENSG00000137876**	*0.020475396*	*0.027873497*	*0.027465099*	*0.019290732*
**ENSG00000184945**	*0.016462675*	*0.02487015*	*0.025320933*	*0.015861891*
**ENSG00000138795**	*0.110610545*	*0.084124199*	*0.069643027*	*0.091145865*
**ENSG00000183258**	*0.023862013*	*0.029186396*	*0.031215816*	*0.019685972*
**ENSG00000110060**	*0.023959934*	*0.034182372*	*0.035374834*	*0.021376154*
**ENSG00000163312**	*0.028843425*	*0.035734482*	*0.036842576*	*0.027562306*
**ENSG00000125966**	*0.015550492*	*0.0251318*	*0.026964914*	*0.014710985*
**ENSG00000153292**	*0.014229096*	*0.024504009*	*0.024707203*	*0.013945766*
**ENSG00000160791**	*0.021195164*	*0.027007889*	*0.02551986*	*0.018383739*
**ENSG00000167874**	*0.017467356*	*0.02960958*	*0.026702505*	*0.017526564*
**ENSG00000186442**	*0.017047758*	*0.026077161*	*0.026441042*	*0.017113167*
**ENSG00000135638**	*0.087893115*	*0.061738886*	*0.04887799*	*0.064653434*
**ENSG00000243710**	*0.022242889*	*0.024934203*	*0.025520874*	*0.017056609*
**ENSG00000124003**	*0.017424386*	*0.025024982*	*0.024951108*	*0.016951526*
**ENSG00000122126**	*0.03407536*	*0.037590002*	*0.033461158*	*0.02982469*
**ENSG00000064655**	*0.078016175*	*0.059114086*	*0.072490311*	*0.049495966*
**ENSG00000081181**	*0.02223337*	*0.032734836*	*0.033292752*	*0.024648299*
**ENSG00000140297**	*0.096303092*	*0.070633328*	*0.089518209*	*0.079000985*
**ENSG00000135569**	*0.015875591*	*0.025455217*	*0.025499176*	*0.014948761*
**ENSG00000162511**	*0.028586759*	*0.034085668*	*0.031541438*	*0.027516052*
**ENSG00000185697**	*0.055222186*	*0.053699891*	*0.045653433*	*0.043570193*
**ENSG00000106772**	*0.081117284*	*0.04710195*	*0.027461289*	*0.043648587*
**ENSG00000204315**	*0.028160871*	*0.028407538*	*0.029007126*	*0.020036614*
**ENSG00000168530**	*0.015159125*	*0.025734833*	*0.024790667*	*0.014745585*
**ENSG00000172315**	*0.030991103*	*0.033834521*	*0.031292009*	*0.028363514*
**ENSG00000163320**	*0.034134691*	*0.043105188*	*0.032523642*	*0.02790433*
**ENSG00000058056**	*0.043038411*	*0.041506427*	*0.03867026*	*0.039669273*
**ENSG00000184076**	*0.018705336*	*0.025818112*	*0.02569995*	*0.016592879*
**ENSG00000001561**	*0.052031476*	*0.041937145*	*0.044679482*	*0.045990129*
**ENSG00000180398**	*0.031979366*	*0.037833549*	*0.032215071*	*0.031410639*
**ENSG00000162692**	*0.129645471*	*0.107943858*	*0.070446202*	*0.083023962*
**ENSG00000169612**	*0.026024872*	*0.03484085*	*0.036908953*	*0.02440772*
**ENSG00000124721**	*0.017052553*	*0.025482049*	*0.026005531*	*0.018322642*
**ENSG00000071539**	*0.048532927*	*0.044384404*	*0.037973411*	*0.047215015*
**ENSG00000101474**	*0.026410014*	*0.031813373*	*0.035712683*	*0.026440595*
**ENSG00000174145**	*0.015858044*	*0.02519685*	*0.024633788*	*0.016219075*
**ENSG00000086504**	*0.034574466*	*0.037154383*	*0.037797641*	*0.023765708*
**ENSG00000112379**	*0.016910181*	*0.024631219*	*0.026579031*	*0.014892728*
**ENSG00000111052**	*0.104183013*	*0.075154431*	*0.076222705*	*0.082692943*
**ENSG00000118058**	*0.017926177*	*0.028422627*	*0.024585631*	*0.015596416*
**ENSG00000073605**	*0.041079622*	*0.04495521*	*0.039975218*	*0.054743394*

**ENSG00000176895**	*0.015996346*	*0.025104917*	*0.025631689*	*0.015846488*
**ENSG00000067836**	*0.051363399*	*0.041818901*	*0.037878611*	*0.043640154*
**ENSG00000011523**	*0.026833762*	*0.037388852*	*0.031241679*	*0.030206623*
**ENSG00000139438**	*0.015280647*	*0.024592404*	*0.024946528*	*0.014822334*
**ENSG00000076826**	*0.069731653*	*0.055112394*	*0.048564895*	*0.056860872*
**ENSG00000137133**	*0.025760894*	*0.029792354*	*0.029976088*	*0.025579057*
**ENSG00000101752**	*0.032791078*	*0.03428706*	*0.035150868*	*0.031208581*
**ENSG00000197879**	*0.026444783*	*0.03198151*	*0.031652989*	*0.034108011*
**ENSG00000179766**	*0.013425576*	*0.023890392*	*0.023677688*	*0.013691714*
**ENSG00000135469**	*0.033810736*	*0.035744358*	*0.032816149*	*0.023991404*
**ENSG00000196660**	*0.016843949*	*0.024841495*	*0.024541853*	*0.015910675*
**ENSG00000171428**	*0.029562768*	*0.036095077*	*0.037638162*	*0.031091644*
**ENSG00000172819**	*0.030838649*	*0.032433906*	*0.028869676*	*0.026436498*
**ENSG00000042286**	*0.028212161*	*0.033136684*	*0.031914055*	*0.024415351*
**ENSG00000084731**	*0.016482593*	*0.026543613*	*0.025445435*	*0.017218072*
**ENSG00000095485**	*0.028325193*	*0.032539845*	*0.032419915*	*0.022130222*
**ENSG00000140153**	*0.02772168*	*0.038617658*	*0.036364425*	*0.028229628*
**ENSG00000159339**	*0.061724337*	*0.044139909*	*0.053517965*	*0.055455346*
**ENSG00000019485**	*0.015635291*	*0.02502727*	*0.024745547*	*0.01468841*
**ENSG00000100226**	*0.022422952*	*0.030530302*	*0.03026102*	*0.021794894*
**ENSG00000167550**	*0.052933817*	*0.042609754*	*0.041571505*	*0.041403035*
**ENSG00000185156**	*0.0311151*	*0.033159343*	*0.029383267*	*0.032304714*
**ENSG00000100564**	*0.027817576*	*0.033435686*	*0.03603677*	*0.027458997*
**ENSG00000196366**	*0.015382935*	*0.024329352*	*0.024383332*	*0.015883953*
**ENSG00000161905**	*0.016106307*	*0.02554501*	*0.025183235*	*0.016436582*
**ENSG00000137343**	*0.019453444*	*0.027315703*	*0.027008263*	*0.017511293*
**ENSG00000151012**	*0.022353507*	*0.027300142*	*0.026540402*	*0.023743044*
**ENSG00000171747**	*0.01531442*	*0.024681642*	*0.024689663*	*0.032744626*
**ENSG00000118705**	*0.026222343*	*0.030134168*	*0.033770463*	*0.02554413*
**ENSG00000185015**	*0.036973747*	*0.043418042*	*0.036473557*	*0.034134945*
**ENSG00000124831**	*0.028413039*	*0.028426457*	*0.032306153*	*0.024218923*
**ENSG00000221972**	*0.017063162*	*0.026476611*	*0.027185985*	*0.019213487*
**ENSG00000168887**	*0.030741361*	*0.034026289*	*0.033732261*	*0.027347324*
**ENSG00000113368**	*0.049180914*	*0.04250449*	*0.036829039*	*0.042532335*
**ENSG00000126461**	*0.021654676*	*0.03485086*	*0.030318081*	*0.028859522*
**ENSG00000101337**	*0.018954574*	*0.02584281*	*0.037575119*	*0.024181848*
**ENSG00000079257**	*0.04668788*	*0.039721642*	*0.036759823*	*0.03273667*
**ENSG00000142733**	*0.035893176*	*0.032337322*	*0.028107532*	*0.023301913*
**ENSG00000175535**	*0.015982504*	*0.02539685*	*0.024455307*	*0.014243155*
**ENSG00000171159**	*0.026213989*	*0.032145072*	*0.032856705*	*0.035150367*
**ENSG00000134765**	*0.055459339*	*0.054876086*	*0.053575976*	*0.068195889*
**ENSG00000129673**	*0.015780759*	*0.025203947*	*0.025254577*	*0.014984123*
**ENSG00000196126**	*0.169617663*	*0.17389295*	*0.198304651*	*0.246757131*
**ENSG00000111707**	*0.026969622*	*0.036615752*	*0.031859182*	*0.022932775*
**ENSG00000060558**	*0.071184208*	*0.05056657*	*0.045892161*	*0.06404303*
**ENSG00000166595**	*0.024986921*	*0.027975474*	*0.029287014*	*0.021959153*
**ENSG00000107821**	*0.025481266*	*0.031746133*	*0.029932605*	*0.024934404*
**ENSG00000159685**	*0.072321927*	*0.049697003*	*0.041450857*	*0.064521268*
**ENSG00000151491**	*0.167271324*	*0.10550878*	*0.101510308*	*0.123482287*
**ENSG00000171236**	*0.070859487*	*0.050017403*	*0.054174805*	*0.046167634*
**ENSG00000102032**	*0.044977544*	*0.038002076*	*0.040903327*	*0.037388161*
**ENSG00000186795**	*0.015535385*	*0.024836196*	*0.025074125*	*0.015720586*
**ENSG00000154370**	*0.025235308*	*0.030296924*	*0.043017014*	*0.029339134*
**ENSG00000171126**	*0.015793414*	*0.026042501*	*0.025103276*	*0.014808605*

**ENSG00000189182**	*0.020609557*	*0.026269922*	*0.026865745*	*0.018392736*
**ENSG00000053501**	*0.023501095*	*0.029570564*	*0.031278051*	*0.022824928*
**ENSG00000138246**	*0.032683297*	*0.040222509*	*0.031698003*	*0.026073511*
**ENSG00000092529**	*0.039163527*	*0.039397516*	*0.04257064*	*0.040161177*
**ENSG00000101052**	*0.026654297*	*0.030287083*	*0.028558639*	*0.022827564*
**ENSG00000091164**	*0.022778692*	*0.028153959*	*0.027122571*	*0.02136723*
**ENSG00000198720**	*0.019119875*	*0.026994475*	*0.026750644*	*0.017168769*
**ENSG00000197548**	*0.032726677*	*0.044747374*	*0.03397576*	*0.027164868*
**ENSG00000106952**	*0.022417914*	*0.024744825*	*0.025573001*	*0.017315404*
**ENSG00000128606**	*0.021986329*	*0.030936285*	*0.026829397*	*0.024279591*
**ENSG00000196734**	*0.074769623*	*0.035377927*	*0.030697454*	*0.043180987*
**ENSG00000089335**	*0.023260395*	*0.026901864*	*0.027855762*	*0.019990398*
**ENSG00000117448**	*0.026208633*	*0.029334756*	*0.029515177*	*0.020347285*
**ENSG00000091106**	*0.029638426*	*0.032609959*	*0.032382021*	*0.027872862*
**ENSG00000121486**	*0.038967007*	*0.055516502*	*0.037744895*	*0.036639779*
**ENSG00000102935**	*0.017132858*	*0.026179955*	*0.026248255*	*0.017457723*
**ENSG00000145506**	*0.103592416*	*0.091763052*	*0.087799891*	*0.115023141*
**ENSG00000221914**	*0.02304532*	*0.029995702*	*0.029650788*	*0.019677667*
**ENSG00000138614**	*0.028245815*	*0.036295566*	*0.034611238*	*0.022790441*
**ENSG00000164318**	*0.017671697*	*0.025125941*	*0.024668814*	*0.016064289*
**ENSG00000172296**	*0.015396151*	*0.025183068*	*0.024365152*	*0.017943168*
**ENSG00000197465**	*0.064480893*	*0.05728648*	*0.042203477*	*0.043312916*
**ENSG00000136451**	*0.035355993*	*0.042645489*	*0.04367759*	*0.026671761*
**ENSG00000106483**	*0.015482101*	*0.026932185*	*0.024861648*	*0.015992795*
**ENSG00000125246**	*0.066240569*	*0.070143415*	*0.055628222*	*0.058703706*
**ENSG00000133661**	*0.017521478*	*0.024988003*	*0.025442539*	*0.016460764*
**ENSG00000127511**	*0.029513882*	*0.031094934*	*0.030258228*	*0.02517518*
**ENSG00000197565**	*0.018396165*	*0.02623658*	*0.024322495*	*0.015976714*
**ENSG00000112139**	*0.0182074*	*0.028171377*	*0.026183339*	*0.017864417*
**ENSG00000161939**	*0.027333032*	*0.027006568*	*0.030525071*	*0.031015155*
**ENSG00000172247**	*0.017531752*	*0.028763843*	*0.025629113*	*0.017507616*
**ENSG00000214290**	*0.072829851*	*0.069169885*	*0.058986583*	*0.066664343*
**ENSG00000134058**	*0.024612042*	*0.031125672*	*0.03073442*	*0.023239176*
**ENSG00000116260**	*0.014877083*	*0.025299515*	*0.025678415*	*0.015098919*
**ENSG00000183172**	*0.03580485*	*0.043043644*	*0.035416821*	*0.029458796*
**ENSG00000101236**	*0.034796406*	*0.032899956*	*0.037112542*	*0.03969559*
**ENSG00000164509**	*0.015342618*	*0.024737245*	*0.024773481*	*0.014648516*
**ENSG00000072422**	*0.015858239*	*0.025065259*	*0.025223034*	*0.014922189*
**ENSG00000150893**	*0.015239421*	*0.026588734*	*0.025888645*	*0.015081431*
**ENSG00000136770**	*0.035089993*	*0.035901362*	*0.035090762*	*0.030950036*
**ENSG00000173598**	*0.029345288*	*0.037827548*	*0.032466818*	*0.023358178*
**ENSG00000176988**	*0.014990665*	*0.025403918*	*0.026156654*	*0.016377505*
**ENSG00000180053**	*0.015582756*	*0.024937268*	*0.024671372*	*0.015819906*
**ENSG00000151461**	*0.024949505*	*0.032197785*	*0.030325852*	*0.02239038*
**ENSG00000168495**	*0.036290216*	*0.038967673*	*0.036176297*	*0.036575036*
**ENSG00000176209**	*0.024558189*	*0.031826258*	*0.029727664*	*0.023978976*
**ENSG00000123095**	*0.033090539*	*0.042256693*	*0.032216857*	*0.050246694*
**ENSG00000100285**	*0.113632739*	*0.091544031*	*0.087742055*	*0.095055895*
**ENSG00000170233**	*0.024083179*	*0.029266424*	*0.030924935*	*0.025649335*
**ENSG00000116604**	*0.032033683*	*0.040983098*	*0.035582822*	*0.035939243*
**ENSG00000183035**	*0.017852923*	*0.024408353*	*0.024130935*	*0.015463768*
**ENSG00000141428**	*0.023403146*	*0.029054472*	*0.033149015*	*0.019964703*
**ENSG00000171617**	*0.072243712*	*0.063955922*	*0.047566473*	*0.069611453*
**ENSG00000101846**	*0.082796225*	*0.060719431*	*0.057077313*	*0.068656599*

**ENSG00000221870**	*0.021811277*	*0.025410732*	*0.02529101*	*0.017531023*
**ENSG00000152240**	*0.041076745*	*0.042727759*	*0.033124691*	*0.029560446*
**ENSG00000125652**	*0.024511463*	*0.028036358*	*0.03013165*	*0.023039122*
**ENSG00000147081**	*0.014907352*	*0.025265439*	*0.024452297*	*0.015007167*
**ENSG00000173230**	*0.029295927*	*0.039066379*	*0.034674142*	*0.026191674*
**ENSG00000170473**	*0.029195529*	*0.031452712*	*0.036008304*	*0.027518686*
**ENSG00000162869**	*0.034819124*	*0.04485121*	*0.034374119*	*0.028509883*
**ENSG00000136487**	*0.015761603*	*0.025800318*	*0.024652089*	*0.015250733*
**ENSG00000112759**	*0.030350412*	*0.030764439*	*0.034791054*	*0.030541694*
**ENSG00000147883**	*0.016729911*	*0.025146849*	*0.025215777*	*0.016190902*
**ENSG00000115165**	*0.029768886*	*0.033036863*	*0.033751457*	*0.028796796*
**ENSG00000161573**	*0.015141602*	*0.025758513*	*0.025125516*	*0.014207457*
**ENSG00000156299**	*0.017304962*	*0.026829106*	*0.02487266*	*0.016547215*
**ENSG00000120440**	*0.016515413*	*0.025502721*	*0.02616388*	*0.016296132*
**ENSG00000171962**	*0.01649664*	*0.024987995*	*0.025324455*	*0.015398716*
**ENSG00000090238**	*0.042823033*	*0.035578761*	*0.040879991*	*0.043074617*
**ENSG00000183155**	*0.028442474*	*0.031637291*	*0.030260303*	*0.034531026*
**ENSG00000130377**	*0.016415326*	*0.025028039*	*0.026224135*	*0.016053215*
**ENSG00000104643**	*0.035533231*	*0.044925191*	*0.044289796*	*0.033573824*
**ENSG00000136828**	*0.038009152*	*0.032360476*	*0.033048003*	*0.030161167*
**ENSG00000133606**	*0.018930771*	*0.026569151*	*0.028866492*	*0.018733945*
**ENSG00000141556**	*0.035238203*	*0.035756586*	*0.034603189*	*0.027556905*
**ENSG00000162139**	*0.019200837*	*0.028844679*	*0.026958009*	*0.018154689*
**ENSG00000128833**	*0.045534878*	*0.040756705*	*0.036156832*	*0.036219543*
**ENSG00000161847**	*0.023616295*	*0.028853647*	*0.035332292*	*0.024639779*
**ENSG00000169704**	*0.01808632*	*0.025542178*	*0.026088001*	*0.015963241*
**ENSG00000168393**	*0.039509387*	*0.036664432*	*0.035443583*	*0.031062118*
**ENSG00000164104**	*0.032973585*	*0.035415863*	*0.030986723*	*0.024661599*
**ENSG00000205856**	*0.01654692*	*0.02663234*	*0.024784775*	*0.015854715*
**ENSG00000142408**	*0.017554232*	*0.024923659*	*0.025394979*	*0.017356325*
**ENSG00000142182**	*0.017508437*	*0.026245035*	*0.02493005*	*0.016868881*
**ENSG00000136010**	*0.039421656*	*0.035473147*	*0.034721149*	*0.030637631*
**ENSG00000175374**	*0.054203641*	*0.04446563*	*0.038893933*	*0.03488569*
**ENSG00000006747**	*0.026398743*	*0.029231205*	*0.027663667*	*0.028725464*
**ENSG00000102221**	*0.098424374*	*0.08295069*	*0.06123366*	*0.074464204*
**ENSG00000162961**	*0.023422356*	*0.028941776*	*0.028154867*	*0.019015623*
**ENSG00000108306**	*0.030597137*	*0.040131356*	*0.033800282*	*0.025587504*
**ENSG00000167476**	*0.151338797*	*0.109751952*	*0.121918832*	*0.140561025*
**ENSG00000144746**	*0.028915342*	*0.033006649*	*0.030136441*	*0.022204517*
**ENSG00000095739**	*0.10357208*	*0.061622042*	*0.057687423*	*0.063903196*
**ENSG00000095585**	*0.046438526*	*0.040401504*	*0.032908646*	*0.030388632*
**ENSG00000166603**	*0.01635773*	*0.02616284*	*0.025432574*	*0.015779129*
**ENSG00000134313**	*0.026480228*	*0.034982741*	*0.033111473*	*0.028907132*
**ENSG00000064787**	*0.099858394*	*0.085227704*	*0.083913115*	*0.090381698*
**ENSG00000135902**	*0.016040011*	*0.025253666*	*0.025063032*	*0.015412153*
**ENSG00000181374**	*0.014960397*	*0.025662056*	*0.024537153*	*0.016414369*
**ENSG00000042088**	*0.031857946*	*0.035200998*	*0.02863878*	*0.022412072*
**ENSG00000137269**	*0.106857426*	*0.079001375*	*0.077300416*	*0.093544157*
**ENSG00000111962**	*0.120788677*	*0.102281459*	*0.079732309*	*0.101994602*
**ENSG00000064961**	*0.018953131*	*0.027273443*	*0.029350264*	*0.018312512*
**ENSG00000182372**	*0.032727988*	*0.040916184*	*0.034727581*	*0.028345402*
**ENSG00000126216**	*0.037992164*	*0.037004631*	*0.034514568*	*0.037149306*
**ENSG00000113441**	*0.038816033*	*0.045213597*	*0.043210672*	*0.033043817*
**ENSG00000144792**	*0.01732219*	*0.027179*	*0.026205818*	*0.016852036*

**ENSG00000183655**	*0.035421668*	*0.033305993*	*0.035289564*	*0.029219742*
**ENSG00000118298**	*0.016586762*	*0.025682807*	*0.026304969*	*0.016191359*
**ENSG00000121644**	*0.039996015*	*0.034956395*	*0.034375613*	*0.033390499*
**ENSG00000161202**	*0.02427691*	*0.032455784*	*0.033045869*	*0.022154888*
**ENSG00000115204**	*0.023565878*	*0.028163587*	*0.030685204*	*0.025066215*
**ENSG00000245848**	*0.021773727*	*0.02681222*	*0.02943632*	*0.020265672*
**ENSG00000176444**	*0.024302087*	*0.039294988*	*0.032492583*	*0.025686*
**ENSG00000107562**	*0.151776564*	*0.10383979*	*0.088137682*	*0.110854206*
**ENSG00000138735**	*0.016001561*	*0.024753205*	*0.026567497*	*0.015507453*
**ENSG00000166821**	*0.056022449*	*0.059981337*	*0.051401026*	*0.052938267*
**ENSG00000181104**	*0.074863975*	*0.052650439*	*0.058112713*	*0.076233222*
**ENSG00000166189**	*0.028248871*	*0.033929198*	*0.032867879*	*0.026390202*
**ENSG00000221888**	*0.016274986*	*0.02484684*	*0.024688433*	*0.014166323*
**ENSG00000105085**	*0.02707438*	*0.033462366*	*0.038503831*	*0.026985907*
**ENSG00000100442**	*0.024549784*	*0.030918898*	*0.026963544*	*0.025275097*
**ENSG00000114742**	*0.031755011*	*0.034239381*	*0.03876192*	*0.02693222*
**ENSG00000107854**	*0.016918861*	*0.026750341*	*0.027954394*	*0.017376487*
**ENSG00000159788**	*0.016196777*	*0.025617517*	*0.024939565*	*0.016968151*
**ENSG00000172845**	*0.02609758*	*0.032533009*	*0.029075462*	*0.025826436*
**ENSG00000248383**	*0.015941494*	*0.024910387*	*0.024660879*	*0.015180575*
**ENSG00000086205**	*0.017622816*	*0.026027836*	*0.025249228*	*0.016207647*
**ENSG00000116649**	*0.031798916*	*0.033597429*	*0.032100447*	*0.031853509*
**ENSG00000163563**	*0.057021065*	*0.069581664*	*0.056733836*	*0.079217314*
**ENSG00000073969**	*0.030481836*	*0.039246084*	*0.034791801*	*0.029731201*
**ENSG00000144218**	*0.015556125*	*0.024739053*	*0.025403135*	*0.016655308*
**ENSG00000135413**	*0.018300662*	*0.026727662*	*0.025494115*	*0.01679087*
**ENSG00000105610**	*0.017087233*	*0.025956377*	*0.026733125*	*0.016383028*
**ENSG00000105370**	*0.016944042*	*0.025455247*	*0.026406072*	*0.01581761*
**ENSG00000132646**	*0.044415492*	*0.059021603*	*0.038796413*	*0.071663523*
**ENSG00000164941**	*0.029649048*	*0.036349109*	*0.031222734*	*0.022954662*
**ENSG00000034239**	*0.016151831*	*0.025382829*	*0.025447185*	*0.015602127*
**ENSG00000125637**	*0.028154558*	*0.030914246*	*0.032052608*	*0.027135685*
**ENSG00000224051**	*0.028418894*	*0.033565413*	*0.036538141*	*0.028449422*
**ENSG00000155511**	*0.016629641*	*0.026276391*	*0.025938291*	*0.016429822*
**ENSG00000154174**	*0.02685469*	*0.031218914*	*0.029168186*	*0.031944882*
**ENSG00000082153**	*0.022904525*	*0.029150047*	*0.028175469*	*0.024058545*
**ENSG00000113140**	*0.061694765*	*0.057807414*	*0.044156107*	*0.052052395*
**ENSG00000080224**	*0.015200285*	*0.025631867*	*0.02412197*	*0.015015217*
**ENSG00000135355**	*0.014874648*	*0.024832768*	*0.025637916*	*0.015070444*
**ENSG00000143569**	*0.029725159*	*0.031735746*	*0.035065676*	*0.028085385*
**ENSG00000205571**	*0.101644956*	*0.074321694*	*0.074300783*	*0.114584841*
**ENSG00000214097**	*0.01569735*	*0.026664363*	*0.024281098*	*0.014475235*
**ENSG00000101198**	*0.015548851*	*0.025940814*	*0.025927212*	*0.014978903*
**ENSG00000254147**	*0.016024824*	*0.024664909*	*0.025198433*	*0.016135135*
**ENSG00000104324**	*0.021691579*	*0.02668314*	*0.025930831*	*0.031502052*
**ENSG00000179913**	*0.01649971*	*0.024825385*	*0.024953527*	*0.016850672*
**ENSG00000135801**	*0.029032541*	*0.044294449*	*0.043223152*	*0.030824318*
**ENSG00000110107**	*0.030781603*	*0.03085822*	*0.032511508*	*0.028939488*
**ENSG00000010318**	*0.016756142*	*0.025504332*	*0.025420554*	*0.016184918*
**ENSG00000148154**	*0.038435242*	*0.047465324*	*0.046003851*	*0.043815569*
**ENSG00000173762**	*0.020977544*	*0.025889945*	*0.024654588*	*0.015997429*
**ENSG00000156603**	*0.025990936*	*0.032936075*	*0.037404825*	*0.026569788*
**ENSG00000136275**	*0.014929669*	*0.024775878*	*0.024609069*	*0.015511317*
**ENSG00000175898**	*0.016916069*	*0.025058062*	*0.023676031*	*0.015077903*

**ENSG00000178401**	*0.015292374*	*0.025119382*	*0.02491096*	*0.014713835*
**ENSG00000175581**	*0.030961743*	*0.035122128*	*0.033207801*	*0.026187543*
**ENSG00000155897**	*0.015718059*	*0.024597897*	*0.02525686*	*0.014504511*
**ENSG00000204859**	*0.02618714*	*0.03000248*	*0.032641877*	*0.021511681*
**ENSG00000124214**	*0.025766349*	*0.031338571*	*0.029023138*	*0.018546416*
**ENSG00000141699**	*0.024086333*	*0.032384365*	*0.030256823*	*0.026264915*
**ENSG00000100462**	*0.028140129*	*0.030436634*	*0.028824702*	*0.025476461*
**ENSG00000173542**	*0.04550115*	*0.050999968*	*0.052044238*	*0.052351306*
**ENSG00000156026**	*0.033263897*	*0.035609752*	*0.037869655*	*0.030534696*
**ENSG00000171431**	*0.015707951*	*0.024168983*	*0.024139014*	*0.015109895*
**ENSG00000130988**	*0.016455078*	*0.02588605*	*0.025001976*	*0.015301403*
**ENSG00000185753**	*0.033017438*	*0.032464286*	*0.036550956*	*0.030548972*
**ENSG00000197860**	*0.016813899*	*0.024891427*	*0.024624016*	*0.015872434*
**ENSG00000108018**	*0.018543527*	*0.026577368*	*0.025239916*	*0.016446737*
**ENSG00000099985**	*0.04453552*	*0.040050587*	*0.041188399*	*0.03663713*
**ENSG00000144152**	*0.020079094*	*0.026565477*	*0.027613157*	*0.015641777*
**ENSG00000114026**	*0.039872258*	*0.039686881*	*0.032307388*	*0.030152791*
**ENSG00000178537**	*0.031457032*	*0.033853495*	*0.032286875*	*0.026315287*
**ENSG00000161653**	*0.016413677*	*0.026057484*	*0.025014683*	*0.016830695*
**ENSG00000137815**	*0.02419345*	*0.031520082*	*0.032627169*	*0.02255319*
**ENSG00000079332**	*0.031401259*	*0.036898013*	*0.041893632*	*0.031162964*
**ENSG00000169953**	*0.023182677*	*0.025966675*	*0.024987315*	*0.020453267*
**ENSG00000164885**	*0.043070965*	*0.04641362*	*0.038207094*	*0.031661502*
**ENSG00000108379**	*0.018160027*	*0.027476086*	*0.025691713*	*0.017693636*
**ENSG00000132256**	*0.029458157*	*0.04119168*	*0.031286002*	*0.031311539*
**ENSG00000131264**	*0.016265907*	*0.02434468*	*0.02519055*	*0.014089051*
**ENSG00000180644**	*0.037948279*	*0.056747582*	*0.041985129*	*0.036319422*
**ENSG00000175143**	*0.015629121*	*0.024945919*	*0.024524116*	*0.014944557*
**ENSG00000165376**	*0.017664055*	*0.024726252*	*0.024343194*	*0.015599343*
**ENSG00000108852**	*0.017444231*	*0.027939705*	*0.025285769*	*0.017627957*
**ENSG00000166582**	*0.039403101*	*0.032211289*	*0.034461648*	*0.026874755*
**ENSG00000164741**	*0.015185776*	*0.024414224*	*0.024768001*	*0.01540568*
**ENSG00000186451**	*0.016138412*	*0.025025571*	*0.024623498*	*0.01641007*
**ENSG00000042832**	*0.016891614*	*0.029314144*	*0.024461727*	*0.015326896*
**ENSG00000229117**	*0.013015292*	*0.02353833*	*0.023427344*	*0.013192505*
**ENSG00000145241**	*0.028467851*	*0.035539504*	*0.035539122*	*0.022202323*
**ENSG00000150093**	*0.017981323*	*0.026658287*	*0.02610188*	*0.015582605*
**ENSG00000175084**	*0.015617415*	*0.025211284*	*0.024945421*	*0.01550734*
**ENSG00000171060**	*0.01515416*	*0.025207475*	*0.024478674*	*0.014824423*
**ENSG00000187191**	*0.01608235*	*0.024915605*	*0.024257061*	*0.015462743*
**ENSG00000141040**	*0.019835205*	*0.026733418*	*0.024586412*	*0.016522364*
**ENSG00000114982**	*0.026327265*	*0.031568772*	*0.030017936*	*0.021304904*
**ENSG00000168610**	*0.035961993*	*0.035699312*	*0.037278202*	*0.035558039*
**ENSG00000149571**	*0.016018192*	*0.026626777*	*0.02579277*	*0.01548676*
**ENSG00000172058**	*0.023834001*	*0.029676044*	*0.027334353*	*0.02856865*
**ENSG00000168439**	*0.031426694*	*0.036454759*	*0.031984729*	*0.03473012*
**ENSG00000183269**	*0.016905723*	*0.026190169*	*0.026000159*	*0.017549906*
**ENSG00000133027**	*0.018593832*	*0.025551178*	*0.026109133*	*0.017234734*
**ENSG00000132109**	*0.027218878*	*0.035484464*	*0.037263756*	*0.035312466*
**ENSG00000149591**	*0.026473508*	*0.029003284*	*0.028645248*	*0.020774846*
**ENSG00000214215**	*0.017208452*	*0.02518538*	*0.025702283*	*0.016189546*
**ENSG00000150455**	*0.027642728*	*0.029626654*	*0.029261376*	*0.023796587*
**ENSG00000141552**	*0.022792241*	*0.028776455*	*0.026657236*	*0.01865948*
**ENSG00000167699**	*0.025707521*	*0.030731749*	*0.030298501*	*0.023215043*

**ENSG00000155130**	*0.043107323*	*0.046941985*	*0.041652626*	*0.051772982*
**ENSG00000111725**	*0.032358608*	*0.040804368*	*0.042782405*	*0.032000745*
**ENSG00000149273**	*0.018635403*	*0.025884117*	*0.026252582*	*0.018531512*
**ENSG00000149743**	*0.034683454*	*0.031048232*	*0.030892464*	*0.034850423*
**ENSG00000110906**	*0.026930032*	*0.032751813*	*0.03451203*	*0.026496231*
**ENSG00000179403**	*0.103235292*	*0.076735735*	*0.066120062*	*0.084542964*
**ENSG00000071205**	*0.042128933*	*0.043335949*	*0.037335263*	*0.040892356*
**ENSG00000176597**	*0.016594296*	*0.026310227*	*0.025509323*	*0.016015624*
**ENSG00000167333**	*0.031686819*	*0.03296522*	*0.033964954*	*0.022502055*
**ENSG00000254221**	*0.020180131*	*0.025143781*	*0.025770876*	*0.018857874*
**ENSG00000102145**	*0.015923336*	*0.024872249*	*0.025765178*	*0.018015182*
**ENSG00000213079**	*0.029233328*	*0.03766339*	*0.038259594*	*0.027533743*
**ENSG00000099331**	*0.024636291*	*0.028520419*	*0.035475149*	*0.0276083*
**ENSG00000138071**	*0.039465646*	*0.046908259*	*0.043070227*	*0.041533249*
**ENSG00000128655**	*0.017108446*	*0.025694551*	*0.02483639*	*0.016477188*
**ENSG00000184588**	*0.054126874*	*0.054233144*	*0.048095711*	*0.046122893*
**ENSG00000175311**	*0.019717968*	*0.026755076*	*0.027324361*	*0.015322051*
**ENSG00000132357**	*0.027384186*	*0.032555407*	*0.034593675*	*0.024909328*
**ENSG00000104415**	*0.015227902*	*0.025375402*	*0.024985734*	*0.015147074*
**ENSG00000077514**	*0.034575844*	*0.036002503*	*0.032677816*	*0.027896661*
**ENSG00000122012**	*0.017425775*	*0.024630773*	*0.02465401*	*0.016311151*
**ENSG00000158941**	*0.025882208*	*0.033893855*	*0.036228757*	*0.029130226*
**ENSG00000104689**	*0.064008166*	*0.060730167*	*0.044055317*	*0.03891652*
**ENSG00000100949**	*0.034373438*	*0.035855603*	*0.034914876*	*0.028950332*
**ENSG00000178053**	*0.076725317*	*0.050437693*	*0.03909881*	*0.038410716*
**ENSG00000126746**	*0.022258912*	*0.029003894*	*0.034406531*	*0.020026355*
**ENSG00000133019**	*0.015950777*	*0.024846357*	*0.025320202*	*0.016894079*
**ENSG00000111711**	*0.034279832*	*0.041476363*	*0.045051953*	*0.029048158*
**ENSG00000198692**	*0.35483954*	*0.372940687*	*0.351787251*	*0.323761069*
**ENSG00000181689**	*0.018076826*	*0.025479641*	*0.025888975*	*0.016501824*
**ENSG00000143153**	*0.098668609*	*0.061466877*	*0.06921208*	*0.08810799*
**ENSG00000136628**	*0.027166761*	*0.030163166*	*0.028554797*	*0.018579672*
**ENSG00000071553**	*0.023512966*	*0.03008772*	*0.032323084*	*0.028590766*
**ENSG00000163286**	*0.016365695*	*0.025560415*	*0.02626835*	*0.015436119*
**ENSG00000078487**	*0.02858218*	*0.033859682*	*0.029737774*	*0.025256568*
**ENSG00000118007**	*0.038025784*	*0.043586573*	*0.033709556*	*0.03036637*
**ENSG00000101251**	*0.015916446*	*0.025763176*	*0.025048002*	*0.016195966*
**ENSG00000114948**	*0.140527731*	*0.040281708*	*0.029448443*	*0.090173551*
**ENSG00000105472**	*0.036902588*	*0.035067711*	*0.03705277*	*0.029132522*
**ENSG00000117122**	*0.017475544*	*0.026336686*	*0.0249376*	*0.015913621*
**ENSG00000164331**	*0.04645576*	*0.041316305*	*0.041060292*	*0.037334512*
**ENSG00000176204**	*0.027560236*	*0.024609152*	*0.026209628*	*0.021107975*
**ENSG00000158023**	*0.03805449*	*0.033492149*	*0.028312418*	*0.026995095*
**ENSG00000189144**	*0.03725129*	*0.040401005*	*0.031848633*	*0.028306596*
**ENSG00000151338**	*0.01779534*	*0.024837514*	*0.024646878*	*0.015737884*
**ENSG00000099769**	*0.018031467*	*0.026203565*	*0.026513431*	*0.017096606*
**ENSG00000086570**	*0.017191577*	*0.025467111*	*0.025056303*	*0.015146436*
**ENSG00000183675**	*0.0267782*	*0.030768869*	*0.030548295*	*0.028752162*
**ENSG00000110002**	*0.06823233*	*0.056019971*	*0.044046087*	*0.048316397*
**ENSG00000188659**	*0.029228778*	*0.026243283*	*0.028128487*	*0.027818241*
**ENSG00000183378**	*0.026000853*	*0.028938104*	*0.029742946*	*0.022238115*
**ENSG00000139618**	*0.038896221*	*0.044452324*	*0.034635974*	*0.039536162*
**ENSG00000179165**	*0.040001825*	*0.029302791*	*0.030407865*	*0.026902622*
**ENSG00000092201**	*0.023873699*	*0.032030355*	*0.031331997*	*0.022831412*

**ENSG00000054356**	*0.016763982*	*0.025120246*	*0.024713264*	*0.015886049*
**ENSG00000160870**	*0.018366217*	*0.025725731*	*0.025517161*	*0.019458496*
**ENSG00000204397**	*0.048496989*	*0.043095944*	*0.041437233*	*0.043063392*
**ENSG00000130816**	*0.033016323*	*0.03855293*	*0.033592182*	*0.025332309*
**ENSG00000156395**	*0.028529008*	*0.02674337*	*0.027472724*	*0.019319918*
**ENSG00000105176**	*0.022060475*	*0.02934369*	*0.029628088*	*0.019408762*
**ENSG00000163914**	*0.015951509*	*0.025105899*	*0.024762389*	*0.01626551*
**ENSG00000122756**	*0.017751207*	*0.025140744*	*0.025167948*	*0.016547876*
**ENSG00000152822**	*0.016384489*	*0.0252385*	*0.025444627*	*0.017068339*
**ENSG00000196547**	*0.036482333*	*0.035624454*	*0.040020962*	*0.031111284*
**ENSG00000169903**	*0.016886551*	*0.026618497*	*0.025889718*	*0.015479424*
**ENSG00000242866**	*0.018450889*	*0.026539467*	*0.027577189*	*0.019318818*
**ENSG00000132205**	*0.062633257*	*0.050017038*	*0.045960222*	*0.055914863*
**ENSG00000100266**	*0.035741919*	*0.033263793*	*0.033842734*	*0.033322536*
**ENSG00000205639**	*0.013458905*	*0.02408737*	*0.024283446*	*0.013136268*
**ENSG00000132824**	*0.034771332*	*0.035318816*	*0.046719173*	*0.038047928*
**ENSG00000108599**	*0.025310724*	*0.036514143*	*0.031818518*	*0.026223005*
**ENSG00000079616**	*0.037895781*	*0.029239824*	*0.033728487*	*0.027044139*
**ENSG00000143971**	*0.029665597*	*0.03451918*	*0.030833778*	*0.044831881*
**ENSG00000112893**	*0.076801276*	*0.06033728*	*0.054420109*	*0.056232741*
**ENSG00000197857**	*0.019921617*	*0.02649804*	*0.025628372*	*0.0169986*
**ENSG00000160445**	*0.028221822*	*0.038055505*	*0.031509475*	*0.02899515*
**ENSG00000114656**	*0.01574449*	*0.024709201*	*0.025283551*	*0.015935069*
**ENSG00000143149**	*0.028469524*	*0.031997574*	*0.028841432*	*0.029751007*
**ENSG00000089916**	*0.03178502*	*0.032392526*	*0.02999634*	*0.027530898*
**ENSG00000131943**	*0.024719536*	*0.032485494*	*0.034702215*	*0.028130288*
**ENSG00000137868**	*0.074522696*	*0.053958246*	*0.040739945*	*0.057708939*
**ENSG00000053254**	*0.029580738*	*0.032608573*	*0.031811297*	*0.024912344*
**ENSG00000134253**	*0.0189902*	*0.028820388*	*0.027658407*	*0.018725696*
**ENSG00000120952**	*0.015484957*	*0.024821598*	*0.02561195*	*0.015550035*
**ENSG00000035499**	*0.048747555*	*0.046991158*	*0.038046699*	*0.036503384*
**ENSG00000162337**	*0.054181114*	*0.045032923*	*0.03654873*	*0.046685989*
**ENSG00000146670**	*0.044502324*	*0.031698772*	*0.036937056*	*0.03322297*
**ENSG00000133124**	*0.017401918*	*0.026788152*	*0.024323108*	*0.015161195*
**ENSG00000184881**	*0.01635338*	*0.024978473*	*0.024819511*	*0.0146571*
**ENSG00000170276**	*0.016076701*	*0.024396785*	*0.023979532*	*0.014517781*
**ENSG00000196184**	*0.016998883*	*0.02539087*	*0.025298897*	*0.017596671*
**ENSG00000087128**	*0.01614297*	*0.026827415*	*0.026066636*	*0.015390566*
**ENSG00000158427**	*0.058692704*	*0.054034745*	*0.047730464*	*0.058088662*
**ENSG00000068831**	*0.022005905*	*0.036180135*	*0.036138273*	*0.035181882*
**ENSG00000087074**	*0.039370869*	*0.045034057*	*0.039452993*	*0.035301992*
**ENSG00000150990**	*0.032528314*	*0.03408829*	*0.033599953*	*0.025688357*
**ENSG00000170260**	*0.022375853*	*0.031936855*	*0.033638247*	*0.020670278*
**ENSG00000182631**	*0.015611453*	*0.025278348*	*0.025034379*	*0.013978539*
**ENSG00000198099**	*0.019298831*	*0.027613748*	*0.027137747*	*0.018784227*
**ENSG00000172264**	*0.016139921*	*0.024916865*	*0.024868326*	*0.014892693*
**ENSG00000146166**	*0.018048902*	*0.026353257*	*0.025407571*	*0.015144255*
**ENSG00000197818**	*0.017676546*	*0.025891757*	*0.026464101*	*0.016575191*
**ENSG00000171560**	*0.016629549*	*0.025070089*	*0.025568641*	*0.015435031*
**ENSG00000112977**	*0.028257682*	*0.03581107*	*0.033833446*	*0.029656557*
**ENSG00000100884**	*0.020525071*	*0.027258458*	*0.024669383*	*0.018372506*
**ENSG00000146926**	*0.016686909*	*0.026632611*	*0.024065594*	*0.016588918*
**ENSG00000064115**	*0.039789011*	*0.038978326*	*0.034070949*	*0.032872557*
**ENSG00000187747**	*0.017454879*	*0.026810799*	*0.026761164*	*0.019705716*

**ENSG00000104918**	*0.015246367*	*0.025634907*	*0.025091456*	*0.015220795*
**ENSG00000197124**	*0.028280747*	*0.031523394*	*0.028267751*	*0.025643622*
**ENSG00000196663**	*0.032835273*	*0.037071265*	*0.032317953*	*0.027960378*
**ENSG00000115539**	*0.040133426*	*0.038392613*	*0.038592969*	*0.033468815*
**ENSG00000070182**	*0.017521551*	*0.027126622*	*0.025195107*	*0.016634683*
**ENSG00000186925**	*0.01637945*	*0.026061201*	*0.02495967*	*0.015914068*
**ENSG00000166166**	*0.031018223*	*0.046061091*	*0.049061182*	*0.034300973*
**ENSG00000164199**	*0.043779435*	*0.026576667*	*0.02540766*	*0.024257914*
**ENSG00000120327**	*0.017020606*	*0.024997628*	*0.025268484*	*0.015739247*
**ENSG00000179583**	*0.043645606*	*0.046154528*	*0.046416454*	*0.047004639*
**ENSG00000172175**	*0.039093732*	*0.039360152*	*0.037171112*	*0.034131554*
**ENSG00000188783**	*0.015165903*	*0.024591068*	*0.02487855*	*0.0144168*
**ENSG00000144285**	*0.016131688*	*0.025230038*	*0.024329294*	*0.01468778*
**ENSG00000145975**	*0.016380131*	*0.024389437*	*0.025095106*	*0.015456004*
**ENSG00000164659**	*0.112575805*	*0.095182739*	*0.080336021*	*0.092098263*
**ENSG00000126890**	*0.015555075*	*0.02576161*	*0.024638949*	*0.016007151*
**ENSG00000123570**	*0.032536439*	*0.035264619*	*0.035267167*	*0.030309845*
**ENSG00000167968**	*0.017161699*	*0.024024009*	*0.025311396*	*0.016002657*
**ENSG00000000005**	*0.016002577*	*0.024958023*	*0.024507024*	*0.014396774*
**ENSG00000171557**	*0.014950371*	*0.024968138*	*0.025542437*	*0.014936687*
**ENSG00000166596**	*0.014848888*	*0.023704226*	*0.024857722*	*0.014340649*
**ENSG00000166349**	*0.019207568*	*0.026089637*	*0.02534506*	*0.018905977*
**ENSG00000253293**	*0.015756154*	*0.024464641*	*0.02533653*	*0.015730856*
**ENSG00000102069**	*0.026860697*	*0.033654487*	*0.029408254*	*0.023159915*
**ENSG00000197891**	*0.019441819*	*0.027895177*	*0.02978561*	*0.023294405*
**ENSG00000109519**	*0.030540315*	*0.032708262*	*0.032582368*	*0.025435919*
**ENSG00000169252**	*0.042383502*	*0.049459018*	*0.032869561*	*0.033246058*
**ENSG00000105227**	*0.018986048*	*0.026782883*	*0.02648874*	*0.0167316*
**ENSG00000153879**	*0.0352069*	*0.041746817*	*0.039944574*	*0.033129913*
**ENSG00000112699**	*0.040549346*	*0.045334387*	*0.036580675*	*0.027635188*
**ENSG00000129925**	*0.034303166*	*0.033486488*	*0.036556292*	*0.030736193*
**ENSG00000147183**	*0.016689633*	*0.026555697*	*0.0262606*	*0.016653091*
**ENSG00000140598**	*0.028212519*	*0.035686107*	*0.030184676*	*0.024547334*
**ENSG00000112640**	*0.025962131*	*0.032983674*	*0.029016736*	*0.026943018*
**ENSG00000159596**	*0.023374843*	*0.028127992*	*0.029830038*	*0.021675882*
**ENSG00000123165**	*0.018201524*	*0.026407263*	*0.027342821*	*0.015138195*
**ENSG00000154096**	*0.043418969*	*0.03991936*	*0.027665561*	*0.029003466*
**ENSG00000204644**	*0.026156549*	*0.030341975*	*0.028597093*	*0.021009034*
**ENSG00000117984**	*0.03130569*	*0.029920875*	*0.032787289*	*0.030762606*
**ENSG00000188938**	*0.02710134*	*0.032871305*	*0.031236665*	*0.022657491*
**ENSG00000205220**	*0.023078557*	*0.028890388*	*0.028042748*	*0.017749491*
**ENSG00000179532**	*0.015488618*	*0.024539892*	*0.025561505*	*0.016973453*
**ENSG00000181649**	*0.089976762*	*0.06660561*	*0.062680358*	*0.080792092*
**ENSG00000186976**	*0.017503692*	*0.025255088*	*0.025299447*	*0.016201244*
**ENSG00000108296**	*0.037470322*	*0.040585737*	*0.038336268*	*0.035496538*
**ENSG00000143079**	*0.03426683*	*0.041614097*	*0.035555117*	*0.030247418*
**ENSG00000015413**	*0.016732602*	*0.025298594*	*0.024736684*	*0.014889873*
**ENSG00000166913**	*0.022441992*	*0.029449841*	*0.028122895*	*0.021754952*
**ENSG00000128335**	*0.016762936*	*0.024771215*	*0.025452999*	*0.017460968*
**ENSG00000174446**	*0.032303307*	*0.032958778*	*0.033959625*	*0.031071268*
**ENSG00000174915**	*0.024993571*	*0.02874702*	*0.034616926*	*0.024495118*
**ENSG00000164008**	*0.024427275*	*0.026706409*	*0.0321961*	*0.022939311*
**ENSG00000122367**	*0.016092654*	*0.025028332*	*0.024579456*	*0.015081768*
**ENSG00000143473**	*0.018200396*	*0.025886426*	*0.025192423*	*0.016809548*

**ENSG00000129437**	*0.016320849*	*0.025291851*	*0.024773375*	*0.016103161*
**ENSG00000154059**	*0.019929086*	*0.025885605*	*0.026280788*	*0.019292926*
**ENSG00000128710**	*0.015036434*	*0.025170166*	*0.024963003*	*0.015148589*
**ENSG00000136997**	*0.036797941*	*0.030740017*	*0.029090636*	*0.031921556*
**ENSG00000032219**	*0.027890754*	*0.034037977*	*0.03375318*	*0.026980723*
**ENSG00000062822**	*0.040819343*	*0.035710803*	*0.032766479*	*0.029407594*
**ENSG00000167904**	*0.03689054*	*0.04398271*	*0.040937718*	*0.033366988*
**ENSG00000122483**	*0.042093678*	*0.04296524*	*0.033916289*	*0.030523979*
**ENSG00000100124**	*0.032248345*	*0.033933769*	*0.030259532*	*0.025464403*
**ENSG00000214717**	*0.023554427*	*0.035028975*	*0.032277427*	*0.032451296*
**ENSG00000129484**	*0.038637519*	*0.040505642*	*0.036821216*	*0.026113712*
**ENSG00000176531**	*0.016158333*	*0.027584599*	*0.026090077*	*0.01741735*
**ENSG00000102409**	*0.13342339*	*0.083958362*	*0.068404141*	*0.09030517*
**ENSG00000118160**	*0.016567964*	*0.02520211*	*0.024280642*	*0.015433096*
**ENSG00000112851**	*0.025862373*	*0.035021141*	*0.033669204*	*0.032145788*
**ENSG00000145916**	*0.027339325*	*0.032079857*	*0.028730962*	*0.026834855*
**ENSG00000205420**	*0.015089444*	*0.024409444*	*0.025134173*	*0.015610418*
**ENSG00000170584**	*0.030141162*	*0.032106433*	*0.029080265*	*0.020714491*
**ENSG00000105220**	*0.028264993*	*0.030605246*	*0.02845351*	*0.022399111*
**ENSG00000198951**	*0.029837843*	*0.033333398*	*0.03612506*	*0.027683485*
**ENSG00000110075**	*0.031207647*	*0.034236305*	*0.035230832*	*0.027270241*
**ENSG00000104731**	*0.040381519*	*0.037056984*	*0.033806157*	*0.03292173*
**ENSG00000096063**	*0.032360667*	*0.035894607*	*0.031010519*	*0.02743772*
**ENSG00000124614**	*0.014790924*	*0.023983842*	*0.024409748*	*0.015579267*
**ENSG00000160336**	*0.036123952*	*0.039602711*	*0.036442043*	*0.042840221*
**ENSG00000185448**	*0.015257597*	*0.024503258*	*0.024745169*	*0.015343225*
**ENSG00000112531**	*0.015633971*	*0.025541857*	*0.02437415*	*0.015328332*
**ENSG00000151287**	*0.039025246*	*0.036095535*	*0.034156656*	*0.035842082*
**ENSG00000171951**	*0.017050103*	*0.025042276*	*0.024831425*	*0.016026327*
**ENSG00000163634**	*0.020463326*	*0.027174993*	*0.026950704*	*0.018025058*
**ENSG00000012963**	*0.016410636*	*0.026990948*	*0.025529578*	*0.016564097*
**ENSG00000116254**	*0.015137236*	*0.025195694*	*0.025345929*	*0.01457512*
**ENSG00000221838**	*0.027212752*	*0.027980701*	*0.030330167*	*0.021986922*
**ENSG00000162728**	*0.018020311*	*0.026246398*	*0.0252215*	*0.014563883*
**ENSG00000115756**	*0.016465736*	*0.028659897*	*0.027426741*	*0.01764838*
**ENSG00000170956**	*0.01635385*	*0.02450651*	*0.024852621*	*0.015653292*
**ENSG00000128578**	*0.023579889*	*0.026733375*	*0.029581838*	*0.0206405*
**ENSG00000068308**	*0.027011789*	*0.035592253*	*0.036279741*	*0.026069344*
**ENSG00000125656**	*0.023301943*	*0.027348463*	*0.026747099*	*0.018031273*
**ENSG00000083635**	*0.025576947*	*0.030281277*	*0.029043551*	*0.023213973*
**ENSG00000181588**	*0.024175795*	*0.031900502*	*0.030090191*	*0.019674636*
**ENSG00000166340**	*0.031466473*	*0.03368854*	*0.034765752*	*0.026844018*
**ENSG00000213029**	*0.051745608*	*0.043383053*	*0.037941165*	*0.034384924*
**ENSG00000188729**	*0.016004583*	*0.025358245*	*0.02538152*	*0.014912714*
**ENSG00000128802**	*0.014704317*	*0.025697103*	*0.024525341*	*0.015003168*
**ENSG00000171222**	*0.029977729*	*0.032273759*	*0.035544505*	*0.023500945*
**ENSG00000106004**	*0.03167566*	*0.030414574*	*0.028900344*	*0.034787124*
**ENSG00000197888**	*0.146435848*	*0.23035304*	*0.216147924*	*0.113393406*
**ENSG00000136929**	*0.016695495*	*0.025097341*	*0.025252336*	*0.015870434*
**ENSG00000146373**	*0.037665736*	*0.027759286*	*0.027451644*	*0.020275528*
**ENSG00000103534**	*0.015571437*	*0.024854702*	*0.025110529*	*0.015314795*
**ENSG00000227234**	*0.04836841*	*0.031739102*	*0.027433995*	*0.020836476*
**ENSG00000205126**	*0.018351015*	*0.027836*	*0.02483369*	*0.015135621*
**ENSG00000083312**	*0.022134375*	*0.026197804*	*0.026698592*	*0.015841498*

**ENSG00000139187**	*0.027925881*	*0.030908303*	*0.028707467*	*0.01799867*
**ENSG00000137161**	*0.034070527*	*0.030995945*	*0.040296968*	*0.037404882*
**ENSG00000121621**	*0.045748362*	*0.037607109*	*0.035680744*	*0.036651882*
**ENSG00000118900**	*0.029299875*	*0.035486351*	*0.033896633*	*0.025774666*
**ENSG00000143434**	*0.016948885*	*0.025057189*	*0.025485114*	*0.015089111*
**ENSG00000106683**	*0.015525838*	*0.025009383*	*0.024587296*	*0.016483106*
**ENSG00000002834**	*0.025868596*	*0.032135155*	*0.031960017*	*0.021955413*
**ENSG00000128645**	*0.018345393*	*0.027807366*	*0.026831921*	*0.01886043*
**ENSG00000005187**	*0.061118629*	*0.044149442*	*0.043925559*	*0.044256673*
**ENSG00000118894**	*0.026629351*	*0.028247942*	*0.028631986*	*0.024516526*
**ENSG00000119139**	*0.043534923*	*0.04447811*	*0.039476784*	*0.046088652*
**ENSG00000158796**	*0.024251367*	*0.031836739*	*0.034122135*	*0.021082521*
**ENSG00000136169**	*0.03647463*	*0.038985222*	*0.034318491*	*0.024571664*
**ENSG00000164338**	*0.021423389*	*0.027604289*	*0.024477542*	*0.025100453*
**ENSG00000123106**	*0.034595523*	*0.042820067*	*0.036661884*	*0.031062307*
**ENSG00000146842**	*0.028514513*	*0.033143582*	*0.035691811*	*0.0297292*
**ENSG00000254093**	*0.030249609*	*0.031436504*	*0.033571675*	*0.02775743*
**ENSG00000114541**	*0.01879732*	*0.025700329*	*0.025182512*	*0.017169782*
**ENSG00000173838**	*0.016126445*	*0.026416811*	*0.026148805*	*0.016347641*
**ENSG00000181634**	*0.035177943*	*0.034356762*	*0.036621271*	*0.034512463*
**ENSG00000134853**	*0.019854284*	*0.026429711*	*0.025881805*	*0.016384458*
**ENSG00000137070**	*0.016771945*	*0.025267183*	*0.025034764*	*0.016759427*
**ENSG00000033327**	*0.016726583*	*0.024769024*	*0.024575714*	*0.015504154*
**ENSG00000092208**	*0.038906542*	*0.040549836*	*0.032102117*	*0.027490183*
**ENSG00000178764**	*0.050191378*	*0.042011775*	*0.040199007*	*0.038599097*
**ENSG00000152894**	*0.128891204*	*0.105956062*	*0.088682451*	*0.109269887*
**ENSG00000000419**	*0.023308714*	*0.029989698*	*0.03144476*	*0.02324104*
**ENSG00000117472**	*0.071439332*	*0.051544962*	*0.051425376*	*0.051791525*
**ENSG00000117000**	*0.04429443*	*0.039359326*	*0.038599484*	*0.037838651*
**ENSG00000183091**	*0.021404018*	*0.029113263*	*0.026542337*	*0.020653262*
**ENSG00000176563**	*0.015921311*	*0.024555765*	*0.025869545*	*0.014936304*
**ENSG00000005020**	*0.042512167*	*0.044352733*	*0.040924243*	*0.032096221*
**ENSG00000023041**	*0.031569393*	*0.036088835*	*0.033858511*	*0.024798596*
**ENSG00000172728**	*0.020323513*	*0.026983442*	*0.027267751*	*0.019015504*
**ENSG00000159873**	*0.034703552*	*0.040176478*	*0.032973671*	*0.031451469*
**ENSG00000170906**	*0.025261417*	*0.029568492*	*0.029102003*	*0.024419937*
**ENSG00000172362**	*0.016260347*	*0.025047048*	*0.026308847*	*0.015070654*
**ENSG00000093183**	*0.027657674*	*0.032392263*	*0.031464721*	*0.024921346*
**ENSG00000187954**	*0.042529483*	*0.041115876*	*0.036185097*	*0.038148382*
**ENSG00000071991**	*0.01602606*	*0.025786059*	*0.02619105*	*0.015601689*
**ENSG00000164715**	*0.041484332*	*0.041219786*	*0.051933312*	*0.050151066*
**ENSG00000159658**	*0.021388293*	*0.030643226*	*0.03049508*	*0.022117094*
**ENSG00000085276**	*0.015619697*	*0.025687959*	*0.025142128*	*0.014676138*
**ENSG00000120686**	*0.033922494*	*0.040066096*	*0.0420809*	*0.030919163*
**ENSG00000118960**	*0.014958809*	*0.026333555*	*0.025166289*	*0.016608357*
**ENSG00000135040**	*0.027215453*	*0.031041427*	*0.03524382*	*0.022104746*
**ENSG00000143061**	*0.019182339*	*0.02731227*	*0.026273341*	*0.015457882*
**ENSG00000198883**	*0.016895083*	*0.025966818*	*0.025785507*	*0.016062505*
**ENSG00000118689**	*0.015663711*	*0.025564782*	*0.024690579*	*0.014442657*
**ENSG00000147689**	*0.01543411*	*0.025383177*	*0.024220394*	*0.01502601*
**ENSG00000137959**	*0.054055375*	*0.041948757*	*0.035656581*	*0.043221761*
**ENSG00000188994**	*0.037823597*	*0.045282839*	*0.034297959*	*0.055409298*
**ENSG00000101916**	*0.016334624*	*0.025150236*	*0.024674206*	*0.015227822*
**ENSG00000118503**	*0.035520205*	*0.033868285*	*0.036950794*	*0.035417286*

**ENSG00000158169**	*0.048855642*	*0.04756642*	*0.041658517*	*0.043473408*
**ENSG00000100726**	*0.036378124*	*0.036585859*	*0.036985592*	*0.03215754*
**ENSG00000185247**	*0.015746717*	*0.025230949*	*0.025091252*	*0.016520119*
**ENSG00000077150**	*0.037319338*	*0.033320607*	*0.036824468*	*0.038374082*
**ENSG00000182541**	*0.01701375*	*0.026022089*	*0.025317026*	*0.015292495*
**ENSG00000146197**	*0.015664175*	*0.025616826*	*0.025649619*	*0.014591366*
**ENSG00000204669**	*0.018037925*	*0.026849439*	*0.025474374*	*0.015879161*
**ENSG00000162413**	*0.036811696*	*0.041138973*	*0.041094644*	*0.029991711*
**ENSG00000108231**	*0.016307239*	*0.024970812*	*0.025131682*	*0.01528891*
**ENSG00000176485**	*0.085582482*	*0.068212205*	*0.059943193*	*0.072106856*
**ENSG00000120647**	*0.036446269*	*0.046279066*	*0.033726675*	*0.022819576*
**ENSG00000137198**	*0.083192737*	*0.06777542*	*0.057872111*	*0.069723561*
**ENSG00000132953**	*0.029023672*	*0.032212927*	*0.030077027*	*0.023201144*
**ENSG00000099995**	*0.023670891*	*0.030421613*	*0.029241113*	*0.023116704*
**ENSG00000134884**	*0.024239233*	*0.031015898*	*0.030061179*	*0.019106011*
**ENSG00000170291**	*0.033154903*	*0.044523079*	*0.053271264*	*0.032948174*
**ENSG00000182324**	*0.015366018*	*0.025672914*	*0.024908548*	*0.016918496*
**ENSG00000160305**	*0.017116673*	*0.02588747*	*0.025727337*	*0.017342486*
**ENSG00000129562**	*0.033226221*	*0.031660912*	*0.033297452*	*0.027852549*
**ENSG00000005022**	*0.023034261*	*0.027239118*	*0.028050178*	*0.02125774*
**ENSG00000174429**	*0.016538253*	*0.025214902*	*0.026105111*	*0.015324452*
**ENSG00000119650**	*0.033384754*	*0.036185195*	*0.032535573*	*0.028624821*
**ENSG00000180233**	*0.03266899*	*0.039569659*	*0.04160111*	*0.024868544*
**ENSG00000165730**	*0.034696108*	*0.02809405*	*0.028649103*	*0.016091981*
**ENSG00000084710**	*0.016435071*	*0.025294241*	*0.025467642*	*0.014873727*
**ENSG00000108344**	*0.027572624*	*0.031665621*	*0.034875009*	*0.029563366*
**ENSG00000203837**	*0.015127564*	*0.026479877*	*0.024842984*	*0.014658955*
**ENSG00000065675**	*0.016955201*	*0.034114334*	*0.026160269*	*0.017596234*
**ENSG00000127080**	*0.032259354*	*0.035065621*	*0.033168035*	*0.026536335*
**ENSG00000074855**	*0.021109429*	*0.026998692*	*0.027767725*	*0.018594026*
**ENSG00000122692**	*0.031840053*	*0.040862266*	*0.037312644*	*0.038069892*
**ENSG00000168310**	*0.035225968*	*0.051812411*	*0.05004658*	*0.056368106*
**ENSG00000213397**	*0.042214681*	*0.039853199*	*0.037151706*	*0.033287311*
**ENSG00000094631**	*0.026261435*	*0.033042552*	*0.033532631*	*0.029476309*
**ENSG00000075884**	*0.024121835*	*0.028133915*	*0.028483659*	*0.021806135*
**ENSG00000159202**	*0.022052491*	*0.027952185*	*0.027745728*	*0.019664158*
**ENSG00000100417**	*0.02982263*	*0.029511044*	*0.030925508*	*0.022458761*
**ENSG00000182557**	*0.027974239*	*0.029106173*	*0.02764229*	*0.026809973*
**ENSG00000140988**	*0.01355623*	*0.02325386*	*0.023818271*	*0.014368619*
**ENSG00000090402**	*0.017629736*	*0.025113524*	*0.028371988*	*0.015780911*
**ENSG00000077522**	*0.014788753*	*0.024732853*	*0.023996109*	*0.014679379*
**ENSG00000188293**	*0.016525153*	*0.025231509*	*0.02435816*	*0.016068521*
**ENSG00000135253**	*0.015823786*	*0.02456639*	*0.024693612*	*0.015827852*
**ENSG00000138101**	*0.047250679*	*0.053892508*	*0.047105054*	*0.045209293*
**ENSG00000166902**	*0.023470185*	*0.02844648*	*0.027960185*	*0.021878907*
**ENSG00000240563**	*0.137944642*	*0.146492635*	*0.149933565*	*0.106708967*
**ENSG00000112116**	*0.01655184*	*0.025230822*	*0.025266346*	*0.01545606*
**ENSG00000141431**	*0.01676029*	*0.024934475*	*0.024275012*	*0.015396315*
**ENSG00000071859**	*0.032028975*	*0.030230158*	*0.033165068*	*0.023524674*
**ENSG00000116774**	*0.016570174*	*0.027331647*	*0.025637753*	*0.018684684*
**ENSG00000214042**	*0.016500724*	*0.024753651*	*0.02537993*	*0.01514807*
**ENSG00000007933**	*0.017663237*	*0.02528813*	*0.025429412*	*0.016863199*
**ENSG00000132570**	*0.036885827*	*0.037099686*	*0.032694131*	*0.025692777*
**ENSG00000170791**	*0.032726812*	*0.038815847*	*0.043556541*	*0.031210304*

**ENSG00000090863**	*0.033118344*	*0.031692884*	*0.033404018*	*0.029908546*
**ENSG00000211445**	*0.015416083*	*0.024991809*	*0.025886729*	*0.015842399*
**ENSG00000104427**	*0.050280079*	*0.04178521*	*0.038307972*	*0.048136603*
**ENSG00000126752**	*0.022696549*	*0.02643232*	*0.02445089*	*0.014885761*
**ENSG00000176809**	*0.016457523*	*0.026076219*	*0.023945421*	*0.014506713*
**ENSG00000171928**	*0.015439028*	*0.025739919*	*0.024634264*	*0.016304866*
**ENSG00000056661**	*0.082936977*	*0.062020236*	*0.062787063*	*0.0709379*
**ENSG00000188710**	*0.016127714*	*0.025223021*	*0.024747016*	*0.014042595*
**ENSG00000152527**	*0.017095694*	*0.026020747*	*0.024614118*	*0.01891928*
**ENSG00000154269**	*0.016798414*	*0.024951613*	*0.025131156*	*0.016696337*
**ENSG00000140740**	*0.019581284*	*0.026437248*	*0.026839322*	*0.016147621*
**ENSG00000100324**	*0.025771951*	*0.028316936*	*0.028262478*	*0.019462292*
**ENSG00000126351**	*0.015640598*	*0.025909847*	*0.025480273*	*0.015419097*
**ENSG00000164638**	*0.045404391*	*0.04775371*	*0.041107716*	*0.053802203*
**ENSG00000149201**	*0.01692778*	*0.026558004*	*0.025105708*	*0.015379476*
**ENSG00000183844**	*0.025549749*	*0.02877223*	*0.037150961*	*0.027579845*
**ENSG00000122679**	*0.017764777*	*0.025860577*	*0.024826442*	*0.016113115*
**ENSG00000164265**	*0.014206857*	*0.024367929*	*0.024853825*	*0.013695742*
**ENSG00000092203**	*0.022704251*	*0.032125024*	*0.031875707*	*0.026449948*
**ENSG00000197935**	*0.018913394*	*0.027512298*	*0.025435403*	*0.02153073*
**ENSG00000180532**	*0.033114241*	*0.024508477*	*0.027252741*	*0.016770201*
**ENSG00000162639**	*0.061639313*	*0.054064922*	*0.056752748*	*0.063003499*
**ENSG00000151692**	*0.062199798*	*0.065255092*	*0.062817885*	*0.062728387*
**ENSG00000143575**	*0.023059166*	*0.027017397*	*0.02949369*	*0.022196467*
**ENSG00000167987**	*0.032101258*	*0.035896541*	*0.031792425*	*0.028792053*
**ENSG00000196967**	*0.032789667*	*0.036659051*	*0.031845869*	*0.02871866*
**ENSG00000102076**	*0.016311238*	*0.024152257*	*0.024197645*	*0.015539354*
**ENSG00000142173**	*0.017678129*	*0.024559931*	*0.024855213*	*0.016117035*
**ENSG00000099910**	*0.040852078*	*0.042677898*	*0.042094944*	*0.036353185*
**ENSG00000163939**	*0.015478522*	*0.024651973*	*0.025542759*	*0.015281887*
**ENSG00000196337**	*0.01620731*	*0.025081805*	*0.025051847*	*0.015937117*
**ENSG00000102890**	*0.021566949*	*0.028299227*	*0.029078966*	*0.019591039*
**ENSG00000196188**	*0.014949379*	*0.025636162*	*0.025062222*	*0.015561242*
**ENSG00000125780**	*0.016810018*	*0.026192054*	*0.025000808*	*0.014153156*
**ENSG00000204580**	*0.017549231*	*0.025900921*	*0.026363402*	*0.018479973*
**ENSG00000134389**	*0.01670202*	*0.025314555*	*0.025693306*	*0.016635046*
**ENSG00000178685**	*0.0337113*	*0.03917419*	*0.031457039*	*0.025805383*
**ENSG00000178075**	*0.049487049*	*0.032436178*	*0.036628578*	*0.049279001*
**ENSG00000170915**	*0.079862624*	*0.075325613*	*0.068837146*	*0.047409862*
**ENSG00000050130**	*0.030343046*	*0.037371748*	*0.035796294*	*0.024271458*
**ENSG00000198466**	*0.041476131*	*0.046999855*	*0.042514777*	*0.035408649*
**ENSG00000138834**	*0.023349434*	*0.041115017*	*0.027225791*	*0.033498064*
**ENSG00000117751**	*0.038284471*	*0.034686463*	*0.033405003*	*0.031071899*
**ENSG00000131584**	*0.028087567*	*0.047722356*	*0.033927311*	*0.034628492*
**ENSG00000151552**	*0.059936695*	*0.039768322*	*0.039388889*	*0.054815862*
**ENSG00000140092**	*0.026689393*	*0.027560796*	*0.025445008*	*0.019485086*
**ENSG00000122386**	*0.033915439*	*0.034989585*	*0.035656629*	*0.028680455*
**ENSG00000146707**	*0.096217838*	*0.084467701*	*0.041211381*	*0.06705406*
**ENSG00000070770**	*0.025748217*	*0.032801594*	*0.030930322*	*0.027930161*
**ENSG00000130711**	*0.021620745*	*0.029416247*	*0.026427048*	*0.019317392*
**ENSG00000126432**	*0.021854474*	*0.026652489*	*0.025815127*	*0.019975282*
**ENSG00000128536**	*0.01749768*	*0.02552238*	*0.024689035*	*0.015425644*
**ENSG00000107742**	*0.050836506*	*0.049931903*	*0.029935539*	*0.035100782*
**ENSG00000152082**	*0.043934314*	*0.041607517*	*0.038020772*	*0.030170083*

**ENSG00000121690**	*0.075444691*	*0.059799825*	*0.059648125*	*0.061628371*
**ENSG00000145388**	*0.032332998*	*0.03713948*	*0.03453713*	*0.025395644*
**ENSG00000105722**	*0.0239528*	*0.030038349*	*0.031442285*	*0.026380316*
**ENSG00000143393**	*0.032821853*	*0.034754224*	*0.044528303*	*0.035198576*
**ENSG00000066777**	*0.025672496*	*0.034824886*	*0.030528859*	*0.02232207*
**ENSG00000091181**	*0.017739466*	*0.026203721*	*0.026323656*	*0.015438533*
**ENSG00000125351**	*0.029488151*	*0.035230741*	*0.033086442*	*0.029261982*
**ENSG00000157014**	*0.031902696*	*0.034174726*	*0.035113282*	*0.028905679*
**ENSG00000168397**	*0.038007207*	*0.039530174*	*0.038404886*	*0.036534104*
**ENSG00000167644**	*0.015964037*	*0.024826801*	*0.024506935*	*0.014466187*
**ENSG00000158747**	*0.015816208*	*0.026130806*	*0.025396393*	*0.015224272*
**ENSG00000167757**	*0.016902219*	*0.025280776*	*0.024691644*	*0.015533303*
**ENSG00000159261**	*0.029705582*	*0.031875641*	*0.029924201*	*0.02507893*
**ENSG00000183395**	*0.037783812*	*0.034140423*	*0.034366468*	*0.03330244*
**ENSG00000121210**	*0.025259995*	*0.033669311*	*0.030526544*	*0.026760383*
**ENSG00000101327**	*0.016741797*	*0.026621769*	*0.024796138*	*0.017414716*
**ENSG00000133789**	*0.031819828*	*0.056508254*	*0.033842978*	*0.0313275*
**ENSG00000115386**	*0.014965783*	*0.024024468*	*0.024662362*	*0.014793087*
**ENSG00000126581**	*0.022417575*	*0.029149472*	*0.031185685*	*0.02383131*
**ENSG00000135829**	*0.04666047*	*0.078509121*	*0.045986566*	*0.075533057*
**ENSG00000176014**	*0.094338192*	*0.055899157*	*0.052800945*	*0.066535912*
**ENSG00000205744**	*0.025804669*	*0.029618737*	*0.029328605*	*0.021968327*
**ENSG00000144290**	*0.017044244*	*0.025744698*	*0.024746574*	*0.015163601*
**ENSG00000080371**	*0.028891122*	*0.031337315*	*0.030029306*	*0.026653426*
**ENSG00000103121**	*0.027699436*	*0.031345664*	*0.029498371*	*0.020127711*
**ENSG00000179059**	*0.017638376*	*0.026692866*	*0.026340682*	*0.017085156*
**ENSG00000169085**	*0.017415958*	*0.026730943*	*0.025865537*	*0.016592579*
**ENSG00000141837**	*0.017603227*	*0.031810498*	*0.029203333*	*0.021844589*
**ENSG00000114331**	*0.038515799*	*0.038233739*	*0.042930511*	*0.026701399*
**ENSG00000118985**	*0.043777692*	*0.045161527*	*0.052004596*	*0.048712508*
**ENSG00000168993**	*0.020457513*	*0.028119708*	*0.027025976*	*0.021159645*
**ENSG00000124523**	*0.03301097*	*0.04097002*	*0.03412129*	*0.029550565*
**ENSG00000180921**	*0.04354317*	*0.033310518*	*0.035702643*	*0.033004601*
**ENSG00000009335**	*0.029776504*	*0.036059941*	*0.032297058*	*0.026677711*
**ENSG00000188629**	*0.028241809*	*0.03129868*	*0.029241543*	*0.024847759*
**ENSG00000173467**	*0.017524781*	*0.024012772*	*0.025460149*	*0.016955451*
**ENSG00000106100**	*0.02639785*	*0.031679847*	*0.034259864*	*0.030847768*
**ENSG00000177463**	*0.02621221*	*0.032998293*	*0.029834258*	*0.022150432*
**ENSG00000161681**	*0.016386787*	*0.025389388*	*0.025059307*	*0.015131843*
**ENSG00000206560**	*0.028712935*	*0.034651125*	*0.031221505*	*0.024457988*
**ENSG00000155868**	*0.029226648*	*0.033503613*	*0.031839714*	*0.020009075*
**ENSG00000145882**	*0.035070038*	*0.039052773*	*0.033493681*	*0.028331383*
**ENSG00000124574**	*0.040283881*	*0.040410755*	*0.032849598*	*0.034812648*
**ENSG00000104147**	*0.045567361*	*0.041369066*	*0.034099332*	*0.03546517*
**ENSG00000115523**	*0.016368531*	*0.025398371*	*0.024885322*	*0.015213976*
**ENSG00000163114**	*0.016888237*	*0.025832234*	*0.024651684*	*0.016426861*
**ENSG00000039537**	*0.017080171*	*0.025005397*	*0.024448705*	*0.014706645*
**ENSG00000198160**	*0.030687044*	*0.037610816*	*0.034103825*	*0.02720719*
**ENSG00000103319**	*0.0412959*	*0.037530641*	*0.032512761*	*0.030739249*
**ENSG00000099204**	*0.034039826*	*0.031532048*	*0.031483812*	*0.034322766*
**ENSG00000002587**	*0.086754148*	*0.09137417*	*0.077706133*	*0.090875061*
**ENSG00000165185**	*0.015493484*	*0.025451118*	*0.025409987*	*0.015835073*
**ENSG00000196581**	*0.015481358*	*0.025326267*	*0.024315199*	*0.015248707*
**ENSG00000132671**	*0.017501809*	*0.026965055*	*0.02555939*	*0.014747729*

**ENSG00000171703**	*0.030706516*	*0.027893303*	*0.028334096*	*0.029303278*
**ENSG00000007255**	*0.027184922*	*0.029564824*	*0.03431393*	*0.028613791*
**ENSG00000136986**	*0.031298852*	*0.036334377*	*0.036911245*	*0.026960181*
**ENSG00000166401**	*0.047951972*	*0.064133473*	*0.059474232*	*0.058610176*
**ENSG00000091732**	*0.020488718*	*0.028728969*	*0.028099665*	*0.02087262*
**ENSG00000120937**	*0.016356994*	*0.025341498*	*0.025025705*	*0.015600344*
**ENSG00000164746**	*0.015408759*	*0.024231686*	*0.025402271*	*0.015517267*
**ENSG00000111537**	*0.070263234*	*0.061617718*	*0.085003303*	*0.083526528*
**ENSG00000164530**	*0.019690342*	*0.025146538*	*0.02553628*	*0.019103122*
**ENSG00000205669**	*0.016047756*	*0.024010867*	*0.025480608*	*0.015764098*
**ENSG00000183864**	*0.020299816*	*0.025539886*	*0.0255031*	*0.017594321*
**ENSG00000166483**	*0.037975749*	*0.041857252*	*0.038618603*	*0.035525888*
**ENSG00000066697**	*0.03775867*	*0.038987988*	*0.03341247*	*0.030958816*
**ENSG00000165046**	*0.016180799*	*0.025811202*	*0.025164906*	*0.019508928*
**ENSG00000188158**	*0.018034285*	*0.028159347*	*0.026124026*	*0.017643389*
**ENSG00000163206**	*0.020304091*	*0.026702878*	*0.030515521*	*0.0170548*
**ENSG00000002745**	*0.014463963*	*0.025249323*	*0.024966274*	*0.014160133*
**ENSG00000134955**	*0.049237846*	*0.05271475*	*0.049782566*	*0.056900179*
**ENSG00000163918**	*0.033623714*	*0.034313306*	*0.028731034*	*0.028738511*
**ENSG00000167196**	*0.04017253*	*0.036646297*	*0.031013971*	*0.029410167*
**ENSG00000170312**	*0.038953955*	*0.036950033*	*0.031752367*	*0.030209097*
**ENSG00000154978**	*0.021095356*	*0.029567639*	*0.02882174*	*0.021897137*
**ENSG00000182168**	*0.016147492*	*0.024485472*	*0.024970286*	*0.0141762*
**ENSG00000157884**	*0.015840601*	*0.024580855*	*0.024803501*	*0.016132707*
**ENSG00000156453**	*0.015744031*	*0.025194078*	*0.024620963*	*0.015839883*
**ENSG00000105258**	*0.023510094*	*0.028025729*	*0.027524843*	*0.018693056*
**ENSG00000116580**	*0.025205107*	*0.035503282*	*0.040172921*	*0.027264772*
**ENSG00000090889**	*0.044861235*	*0.038793166*	*0.034913575*	*0.032155371*
**ENSG00000163950**	*0.033764765*	*0.032282826*	*0.033168454*	*0.030432773*
**ENSG00000197702**	*0.018746947*	*0.027321068*	*0.02704217*	*0.020390088*
**ENSG00000170558**	*0.133397115*	*0.082586775*	*0.042432267*	*0.064972204*
**ENSG00000117724**	*0.053104618*	*0.045252429*	*0.03795297*	*0.041500705*
**ENSG00000130540**	*0.015857413*	*0.024935775*	*0.023946826*	*0.014221132*
**ENSG00000095574**	*0.027630243*	*0.03813544*	*0.029760156*	*0.042741747*
**ENSG00000197711**	*0.01550623*	*0.024480521*	*0.024695489*	*0.014845522*
**ENSG00000166529**	*0.031951494*	*0.044204553*	*0.042974303*	*0.026104845*
**ENSG00000138942**	*0.02389873*	*0.03138314*	*0.035239157*	*0.020515618*
**ENSG00000159140**	*0.03102963*	*0.038023892*	*0.038437662*	*0.029095695*
**ENSG00000150756**	*0.034819412*	*0.035275512*	*0.031394845*	*0.029087978*
**ENSG00000206530**	*0.017548507*	*0.025686954*	*0.025542486*	*0.016907452*
**ENSG00000025770**	*0.022829989*	*0.030852786*	*0.027495432*	*0.020687447*
**ENSG00000112425**	*0.034458697*	*0.031432984*	*0.030454358*	*0.028748173*
**ENSG00000182223**	*0.017801313*	*0.025926165*	*0.024538325*	*0.015786202*
**ENSG00000160789**	*0.055156001*	*0.047020346*	*0.047024054*	*0.042026367*
**ENSG00000222014**	*0.027059272*	*0.030013939*	*0.032959415*	*0.026582388*
**ENSG00000058804**	*0.044611764*	*0.041648725*	*0.036166514*	*0.029289214*
**ENSG00000168014**	*0.016669835*	*0.027789811*	*0.025864188*	*0.016489269*
**ENSG00000104980**	*0.025765771*	*0.028848746*	*0.033904067*	*0.022786855*
**ENSG00000150086**	*0.01727783*	*0.024672081*	*0.026084694*	*0.016224266*
**ENSG00000131748**	*0.020724586*	*0.02809556*	*0.034768534*	*0.024668495*
**ENSG00000003989**	*0.015586105*	*0.025105191*	*0.025013974*	*0.015364779*
**ENSG00000159905**	*0.029060602*	*0.035188445*	*0.028599567*	*0.024465067*
**ENSG00000108591**	*0.024118329*	*0.028320424*	*0.03356492*	*0.024997389*
**ENSG00000143341**	*0.016442964*	*0.025695637*	*0.024978714*	*0.015998658*

**ENSG00000131042**	*0.048843064*	*0.030954985*	*0.025379357*	*0.016975378*
**ENSG00000163251**	*0.028110345*	*0.033535306*	*0.031183538*	*0.025531778*
**ENSG00000203667**	*0.035173632*	*0.036290316*	*0.030100376*	*0.030411708*
**ENSG00000203805**	*0.018310089*	*0.027745048*	*0.026557913*	*0.017141708*
**ENSG00000145907**	*0.021774842*	*0.027858594*	*0.026908135*	*0.019042395*
**ENSG00000135407**	*0.029849283*	*0.02683479*	*0.027696705*	*0.019193214*
**ENSG00000139132**	*0.017855969*	*0.025525078*	*0.025388719*	*0.016218688*
**ENSG00000244474**	*0.018446831*	*0.024942987*	*0.024684463*	*0.016940563*
**ENSG00000100294**	*0.031813798*	*0.033690852*	*0.033721894*	*0.02916515*
**ENSG00000106565**	*0.022432245*	*0.024120063*	*0.02746517*	*0.014737903*
**ENSG00000204950**	*0.013535252*	*0.02369377*	*0.023418145*	*0.013160018*
**ENSG00000171302**	*0.021673252*	*0.031921883*	*0.038706942*	*0.023132064*
**ENSG00000148090**	*0.041626781*	*0.04737797*	*0.036883723*	*0.040628791*
**ENSG00000163040**	*0.106177445*	*0.077448878*	*0.078356042*	*0.084960325*
**ENSG00000105698**	*0.02443556*	*0.0325103*	*0.034933563*	*0.026154786*
**ENSG00000171711**	*0.09918622*	*0.050600527*	*0.052521663*	*0.054679744*
**ENSG00000153303**	*0.016029632*	*0.024284757*	*0.025676686*	*0.014701237*
**ENSG00000164850**	*0.097856833*	*0.072897123*	*0.059648812*	*0.07401732*
**ENSG00000130338**	*0.037286401*	*0.035769376*	*0.034649151*	*0.036485329*
**ENSG00000054179**	*0.083370799*	*0.045114766*	*0.036611757*	*0.06122421*
**ENSG00000173641**	*0.015479217*	*0.024992926*	*0.02425692*	*0.015029777*
**ENSG00000101412**	*0.038082534*	*0.032492216*	*0.042720086*	*0.031945187*
**ENSG00000100739**	*0.018137784*	*0.024979716*	*0.026332699*	*0.015161266*
**ENSG00000172534**	*0.031965078*	*0.0327226*	*0.038038862*	*0.029269487*
**ENSG00000134020**	*0.019021854*	*0.026639334*	*0.026726474*	*0.021948562*
**ENSG00000160917**	*0.026220702*	*0.029407273*	*0.030512369*	*0.024268057*
**ENSG00000132688**	*0.016987655*	*0.024816564*	*0.025272565*	*0.016805432*
**ENSG00000186832**	*0.016482486*	*0.02567582*	*0.025959658*	*0.016869866*
**ENSG00000146282**	*0.027377803*	*0.030309932*	*0.032811313*	*0.01841812*
**ENSG00000104081**	*0.020527942*	*0.030874918*	*0.027022299*	*0.020059322*
**ENSG00000189433**	*0.014943347*	*0.024809986*	*0.024965098*	*0.0138683*
**ENSG00000047621**	*0.03212917*	*0.042685024*	*0.034231924*	*0.029840784*
**ENSG00000115414**	*0.016428402*	*0.025275607*	*0.024847338*	*0.015865736*
**ENSG00000112297**	*0.049473334*	*0.040584647*	*0.04316362*	*0.043321931*
**ENSG00000128298**	*0.023604908*	*0.028851077*	*0.029182064*	*0.024861713*
**ENSG00000067057**	*0.035505568*	*0.030609492*	*0.027523772*	*0.025452157*
**ENSG00000105726**	*0.038071343*	*0.039911313*	*0.034970791*	*0.029140989*
**ENSG00000166925**	*0.022438395*	*0.030115155*	*0.028557979*	*0.023801693*
**ENSG00000204389**	*0.060202499*	*0.044211113*	*0.04729918*	*0.047138926*
**ENSG00000136480**	*0.041468415*	*0.044732583*	*0.032709865*	*0.030680805*
**ENSG00000175857**	*0.054995601*	*0.065299271*	*0.050843864*	*0.07826177*
**ENSG00000107831**	*0.016177901*	*0.027518783*	*0.026480212*	*0.017049708*
**ENSG00000171843**	*0.038144041*	*0.035263139*	*0.035198729*	*0.028231763*
**ENSG00000184348**	*0.027503513*	*0.027989495*	*0.038669451*	*0.029135483*
**ENSG00000035141**	*0.034316356*	*0.035784148*	*0.036088863*	*0.031836758*
**ENSG00000162745**	*0.014989147*	*0.024642753*	*0.024253248*	*0.0157531*
**ENSG00000177700**	*0.026664772*	*0.029785359*	*0.028714823*	*0.029543971*
**ENSG00000213064**	*0.019776716*	*0.025666579*	*0.028256662*	*0.017510568*
**ENSG00000169181**	*0.01588068*	*0.026193948*	*0.025697659*	*0.015786084*
**ENSG00000134262**	*0.030712312*	*0.036409835*	*0.03128216*	*0.026458292*
**ENSG00000135392**	*0.027424953*	*0.033563834*	*0.03381903*	*0.024061078*
**ENSG00000204245**	*0.018801222*	*0.024801684*	*0.024571198*	*0.017796067*
**ENSG00000188283**	*0.033529263*	*0.03952812*	*0.035613047*	*0.025705932*
**ENSG00000092199**	*0.020046379*	*0.027801607*	*0.027969894*	*0.016509328*

**ENSG00000171931**	*0.016273181*	*0.025389282*	*0.024985519*	*0.015847539*
**ENSG00000131634**	*0.015038701*	*0.02591063*	*0.026299386*	*0.018761171*
**ENSG00000003137**	*0.01566006*	*0.025282994*	*0.02568616*	*0.016392196*
**ENSG00000118873**	*0.028386569*	*0.035152506*	*0.031825303*	*0.024306201*
**ENSG00000071462**	*0.02457367*	*0.029343628*	*0.029899617*	*0.0220487*
**ENSG00000151726**	*0.039126235*	*0.042666772*	*0.036224106*	*0.033172354*
**ENSG00000187824**	*0.040620279*	*0.038460483*	*0.038290387*	*0.042796818*
**ENSG00000173908**	*0.015951355*	*0.024610654*	*0.024652004*	*0.015560448*
**ENSG00000084090**	*0.024947403*	*0.026250656*	*0.027334169*	*0.022679177*
**ENSG00000176623**	*0.030225267*	*0.030768962*	*0.029931445*	*0.019732395*
**ENSG00000196576**	*0.034544487*	*0.039756691*	*0.040952864*	*0.031578287*
**ENSG00000146950**	*0.022749759*	*0.02807799*	*0.024819305*	*0.018257193*
**ENSG00000131089**	*0.047508133*	*0.046282389*	*0.044866957*	*0.03692219*
**ENSG00000075292**	*0.03224106*	*0.040131297*	*0.035019401*	*0.027166115*
**ENSG00000113790**	*0.047144883*	*0.0402437*	*0.036621816*	*0.035711628*
**ENSG00000242110**	*0.039043194*	*0.03540035*	*0.033049774*	*0.033086762*
**ENSG00000169548**	*0.014602881*	*0.024590126*	*0.025726569*	*0.014994176*
**ENSG00000162772**	*0.018449185*	*0.027882845*	*0.026594075*	*0.018396162*
**ENSG00000010295**	*0.016293626*	*0.025374179*	*0.025612083*	*0.015937719*
**ENSG00000143590**	*0.017078435*	*0.024816311*	*0.02489661*	*0.015473409*
**ENSG00000197136**	*0.027313973*	*0.033463355*	*0.037432773*	*0.033624441*
**ENSG00000186468**	*0.013729473*	*0.023643715*	*0.024163639*	*0.014867874*
**ENSG00000123689**	*0.168349923*	*0.089792488*	*0.099889473*	*0.129237771*
**ENSG00000163516**	*0.049234237*	*0.043215965*	*0.049362136*	*0.043650533*
**ENSG00000139780**	*0.016417795*	*0.026026227*	*0.025546198*	*0.017105404*
**ENSG00000110171**	*0.016191119*	*0.025360971*	*0.025591468*	*0.014749051*
**ENSG00000143851**	*0.015496419*	*0.024148558*	*0.024187244*	*0.014205346*
**ENSG00000175445**	*0.016379077*	*0.02485049*	*0.025438006*	*0.01418836*
**ENSG00000145777**	*0.017490732*	*0.025756885*	*0.025910087*	*0.015805938*
**ENSG00000116761**	*0.062796042*	*0.056819055*	*0.046801342*	*0.049033818*
**ENSG00000181191**	*0.031040309*	*0.038384687*	*0.032168535*	*0.029751823*
**ENSG00000083457**	*0.041405639*	*0.040006755*	*0.039045389*	*0.033672483*
**ENSG00000087365**	*0.020666066*	*0.027585907*	*0.030335297*	*0.017744754*
**ENSG00000086991**	*0.019237163*	*0.025599311*	*0.027838173*	*0.017140701*
**ENSG00000145703**	*0.054166931*	*0.040284148*	*0.036120661*	*0.029880407*
**ENSG00000169840**	*0.014561464*	*0.024773237*	*0.025231604*	*0.014011574*
**ENSG00000185737**	*0.016912915*	*0.024232179*	*0.026045797*	*0.015433855*
**ENSG00000243477**	*0.038725023*	*0.033657846*	*0.033110796*	*0.030267787*
**ENSG00000109452**	*0.109221385*	*0.045816278*	*0.039807974*	*0.04226725*
**ENSG00000121413**	*0.106621979*	*0.070807226*	*0.089898483*	*0.092268877*
**ENSG00000158813**	*0.019041334*	*0.026493057*	*0.027297897*	*0.019078578*
**ENSG00000152954**	*0.020993386*	*0.027467279*	*0.025883879*	*0.024206947*
**ENSG00000167617**	*0.117824525*	*0.089368842*	*0.077971939*	*0.081089281*
**ENSG00000167157**	*0.017629525*	*0.025136976*	*0.025515672*	*0.016523006*
**ENSG00000165898**	*0.026971331*	*0.031967957*	*0.028828858*	*0.019869567*
**ENSG00000119508**	*0.040592463*	*0.046623471*	*0.046985184*	*0.033129577*
**ENSG00000155816**	*0.016046985*	*0.024905439*	*0.025168528*	*0.01405759*
**ENSG00000180198**	*0.040692595*	*0.033946201*	*0.033555271*	*0.03387404*
**ENSG00000188039**	*0.025349063*	*0.02835129*	*0.029663328*	*0.021557023*
**ENSG00000150593**	*0.016038374*	*0.025821057*	*0.024674715*	*0.017710677*
**ENSG00000117335**	*0.033517773*	*0.037231727*	*0.033003855*	*0.028195039*
**ENSG00000101448**	*0.015773592*	*0.024909009*	*0.024282702*	*0.015773093*
**ENSG00000176547**	*0.015957378*	*0.024414722*	*0.024970405*	*0.015243302*
**ENSG00000176401**	*0.040248203*	*0.041579232*	*0.035139789*	*0.029041361*

**ENSG00000134323**	*0.01694557*	*0.024727863*	*0.023632794*	*0.016631817*
**ENSG00000197635**	*0.083911524*	*0.066313385*	*0.056535353*	*0.075853779*
**ENSG00000049089**	*0.036385932*	*0.031258522*	*0.038038853*	*0.037273876*
**ENSG00000205076**	*0.015080198*	*0.024814563*	*0.025213834*	*0.015502909*
**ENSG00000101203**	*0.017847307*	*0.025816334*	*0.027011982*	*0.0165198*
**ENSG00000102316**	*0.023008702*	*0.029811425*	*0.03256865*	*0.022219297*
**ENSG00000235109**	*0.032722795*	*0.035932303*	*0.03597612*	*0.030553111*
**ENSG00000168385**	*0.020140475*	*0.029259773*	*0.027911627*	*0.020287503*
**ENSG00000101282**	*0.015061225*	*0.025550263*	*0.025891655*	*0.015671418*
**ENSG00000107341**	*0.028060458*	*0.031714277*	*0.030008229*	*0.024043948*
**ENSG00000050327**	*0.048618876*	*0.037008134*	*0.044401077*	*0.034463631*
**ENSG00000101138**	*0.02896633*	*0.033240786*	*0.034331444*	*0.023513257*
**ENSG00000147124**	*0.015418345*	*0.025059464*	*0.024730651*	*0.016749074*
**ENSG00000102445**	*0.102590702*	*0.065324643*	*0.080728426*	*0.090829987*
**ENSG00000164867**	*0.016592002*	*0.024969799*	*0.025492367*	*0.017158832*
**ENSG00000171827**	*0.031082293*	*0.032935524*	*0.033726582*	*0.028465363*
**ENSG00000197050**	*0.038698318*	*0.038860349*	*0.031911021*	*0.043280785*
**ENSG00000162194**	*0.027574747*	*0.030884559*	*0.03251694*	*0.038788634*
**ENSG00000158987**	*0.024245129*	*0.032845108*	*0.0310757*	*0.021151261*
**ENSG00000102981**	*0.043019683*	*0.043816562*	*0.038978453*	*0.033226645*
**ENSG00000131966**	*0.029633421*	*0.034812964*	*0.030626431*	*0.030054077*
**ENSG00000118482**	*0.024868125*	*0.030813174*	*0.030296023*	*0.023082041*
**ENSG00000078081**	*0.051641969*	*0.03675075*	*0.043185613*	*0.041499566*
**ENSG00000085449**	*0.034785701*	*0.038052475*	*0.037922627*	*0.024731095*
**ENSG00000135926**	*0.039000852*	*0.037285533*	*0.037244807*	*0.039021524*
**ENSG00000204296**	*0.015865699*	*0.02438501*	*0.024246871*	*0.01432435*
**ENSG00000112664**	*0.029601816*	*0.033318017*	*0.036526545*	*0.028260049*
**ENSG00000123561**	*0.015605951*	*0.025224237*	*0.025145495*	*0.014649523*
**ENSG00000156140**	*0.014714475*	*0.02488536*	*0.024706396*	*0.014692145*
**ENSG00000163071**	*0.078386429*	*0.075112082*	*0.046065909*	*0.059805032*
**ENSG00000120539**	*0.040046249*	*0.040843324*	*0.041722661*	*0.044235035*
**ENSG00000175215**	*0.02927978*	*0.03571145*	*0.032045447*	*0.025868424*
**ENSG00000169836**	*0.016125276*	*0.026442033*	*0.024763826*	*0.016084421*
**ENSG00000096264**	*0.025862721*	*0.029830525*	*0.025448099*	*0.018040246*
**ENSG00000198382**	*0.025777573*	*0.031094177*	*0.033809398*	*0.025469594*
**ENSG00000144355**	*0.019214751*	*0.024952513*	*0.026816729*	*0.018096429*
**ENSG00000170448**	*0.032836171*	*0.032601129*	*0.031401404*	*0.026501556*
**ENSG00000197037**	*0.0172593*	*0.027221638*	*0.026226102*	*0.017250717*
**ENSG00000130695**	*0.020913662*	*0.026063245*	*0.029662593*	*0.015679723*
**ENSG00000135002**	*0.027163656*	*0.032815093*	*0.032636436*	*0.025373404*
**ENSG00000151779**	*0.032544214*	*0.035212245*	*0.032465754*	*0.027688106*
**ENSG00000204466**	*0.015516629*	*0.024710215*	*0.024102012*	*0.014925528*
**ENSG00000125967**	*0.033641794*	*0.034459879*	*0.034235069*	*0.034468963*
**ENSG00000161677**	*0.033677641*	*0.034467881*	*0.03397512*	*0.034129777*
**ENSG00000237441**	*0.029636525*	*0.03482543*	*0.035018119*	*0.031837686*
**ENSG00000157741**	*0.022274107*	*0.027736332*	*0.030726605*	*0.023711675*
**ENSG00000166452**	*0.014997226*	*0.024673368*	*0.024489264*	*0.015257825*
**ENSG00000105321**	*0.033328278*	*0.036330615*	*0.040960041*	*0.028417557*
**ENSG00000168096**	*0.017226362*	*0.027253111*	*0.02677851*	*0.016961765*
**ENSG00000198400**	*0.021141175*	*0.026971817*	*0.028337463*	*0.021531815*
**ENSG00000133067**	*0.01487951*	*0.025630736*	*0.024397819*	*0.015823622*
**ENSG00000030066**	*0.029050843*	*0.036220852*	*0.033242004*	*0.026880498*
**ENSG00000204174**	*0.014410219*	*0.024419194*	*0.024257896*	*0.015028162*
**ENSG00000152611**	*0.022658613*	*0.026054928*	*0.026075249*	*0.016212829*

**ENSG00000173744**	*0.026114617*	*0.033714173*	*0.029350865*	*0.022351556*
**ENSG00000188171**	*0.078342208*	*0.060928231*	*0.048559214*	*0.065094617*
**ENSG00000155229**	*0.03386075*	*0.036438211*	*0.034483633*	*0.03104949*
**ENSG00000157426**	*0.031576111*	*0.038975442*	*0.034110427*	*0.022342135*
**ENSG00000152128**	*0.096034727*	*0.094393723*	*0.088370913*	*0.103871607*
**ENSG00000166926**	*0.050286384*	*0.034074476*	*0.030700592*	*0.040028252*
**ENSG00000185332**	*0.015714629*	*0.025349155*	*0.025950667*	*0.01450596*
**ENSG00000072041**	*0.015001525*	*0.024682196*	*0.024612176*	*0.016254889*
**ENSG00000160325**	*0.018894495*	*0.029623364*	*0.028373015*	*0.022233353*
**ENSG00000133454**	*0.112008205*	*0.109222406*	*0.066889966*	*0.071806261*
**ENSG00000136099**	*0.017426411*	*0.025120445*	*0.024824164*	*0.015636535*
**ENSG00000160323**	*0.016133949*	*0.025783653*	*0.024670083*	*0.015762933*
**ENSG00000137700**	*0.035989963*	*0.033595349*	*0.030481505*	*0.029535915*
**ENSG00000198453**	*0.034372604*	*0.03599676*	*0.037513747*	*0.042644487*
**ENSG00000170835**	*0.025241054*	*0.031095032*	*0.02785248*	*0.025898773*
**ENSG00000133704**	*0.029681896*	*0.033116334*	*0.03401796*	*0.021550811*
**ENSG00000001167**	*0.025766273*	*0.032627072*	*0.031886312*	*0.023250867*
**ENSG00000198920**	*0.032194761*	*0.041115587*	*0.030181925*	*0.029942501*
**ENSG00000110429**	*0.018518017*	*0.026100863*	*0.026510098*	*0.017052769*
**ENSG00000158517**	*0.035541411*	*0.036661222*	*0.033578634*	*0.029228234*
**ENSG00000089902**	*0.018891823*	*0.026660596*	*0.026490501*	*0.017833216*
**ENSG00000140379**	*0.036421737*	*0.043780623*	*0.041099814*	*0.039226382*
**ENSG00000177981**	*0.022567739*	*0.031332221*	*0.034809129*	*0.019448429*
**ENSG00000061918**	*0.141704676*	*0.117172291*	*0.111720392*	*0.107642689*
**ENSG00000166828**	*0.016107697*	*0.024863821*	*0.024281532*	*0.014851784*
**ENSG00000171467**	*0.036153803*	*0.044533449*	*0.037404818*	*0.035523714*
**ENSG00000109205**	*0.014714109*	*0.02461763*	*0.024572366*	*0.015541992*
**ENSG00000143367**	*0.045791953*	*0.05149498*	*0.045241216*	*0.040935739*
**ENSG00000163624**	*0.018195782*	*0.026374255*	*0.026281398*	*0.017405116*
**ENSG00000174939**	*0.021853572*	*0.028226346*	*0.026232604*	*0.017409386*
**ENSG00000164304**	*0.015165597*	*0.024606569*	*0.024311547*	*0.015564029*
**ENSG00000171241**	*0.021170783*	*0.027143574*	*0.026081825*	*0.020898179*
**ENSG00000239590**	*0.017671022*	*0.025322203*	*0.025986922*	*0.01631025*
**ENSG00000164089**	*0.014909141*	*0.025076877*	*0.024213648*	*0.014400263*
**ENSG00000155749**	*0.014998894*	*0.025368193*	*0.024618203*	*0.016262817*
**ENSG00000170075**	*0.014870826*	*0.024698666*	*0.025006125*	*0.015538139*
**ENSG00000143614**	*0.027543921*	*0.030440012*	*0.037797173*	*0.032575208*
**ENSG00000243130**	*0.016587611*	*0.025384128*	*0.025680088*	*0.015186789*
**ENSG00000108292**	*0.017720656*	*0.029759895*	*0.025946527*	*0.022652362*
**ENSG00000182601**	*0.017626955*	*0.027727841*	*0.028520405*	*0.018947895*
**ENSG00000173163**	*0.029035583*	*0.030651727*	*0.031385608*	*0.028940029*
**ENSG00000185291**	*0.068215316*	*0.065891032*	*0.049132511*	*0.067530557*
**ENSG00000157483**	*0.025146776*	*0.030040337*	*0.02850085*	*0.025720165*
**ENSG00000132361**	*0.031792119*	*0.030392814*	*0.029973911*	*0.033606259*
**ENSG00000026297**	*0.045640144*	*0.032559263*	*0.030137073*	*0.025578582*
**ENSG00000171566**	*0.02308499*	*0.031075273*	*0.028487271*	*0.017930471*
**ENSG00000166800**	*0.01957487*	*0.026481608*	*0.025469844*	*0.018799665*
**ENSG00000107021**	*0.022542934*	*0.032182085*	*0.035526001*	*0.02179587*
**ENSG00000118363**	*0.03186854*	*0.036719471*	*0.030228323*	*0.025716346*
**ENSG00000181929**	*0.028353823*	*0.031209308*	*0.0312424*	*0.02785137*
**ENSG00000126705**	*0.02847293*	*0.036564987*	*0.031907172*	*0.032794383*
**ENSG00000174083**	*0.051516936*	*0.039303232*	*0.040004665*	*0.046954021*
**ENSG00000007866**	*0.019165197*	*0.024835583*	*0.026901042*	*0.016358604*
**ENSG00000176788**	*0.043089601*	*0.032068603*	*0.034464593*	*0.02780444*

**ENSG00000180638**	*0.015759453*	*0.024612321*	*0.024601902*	*0.015555846*
**ENSG00000162852**	*0.031455845*	*0.035091201*	*0.032995287*	*0.025965859*
**ENSG00000130368**	*0.016792199*	*0.026656635*	*0.027385936*	*0.017074981*
**ENSG00000106799**	*0.01968963*	*0.026770272*	*0.029745654*	*0.019517325*
**ENSG00000116791**	*0.046230469*	*0.045775603*	*0.040959053*	*0.040037101*
**ENSG00000185022**	*0.017503519*	*0.024989503*	*0.02527249*	*0.016686596*
**ENSG00000239886**	*0.015404123*	*0.024798755*	*0.024819533*	*0.016148566*
**ENSG00000140945**	*0.015668934*	*0.024343818*	*0.025435919*	*0.015131954*
**ENSG00000169908**	*0.016044777*	*0.024919365*	*0.024551853*	*0.014441053*
**ENSG00000010438**	*0.018812039*	*0.02605348*	*0.025246079*	*0.017394121*
**ENSG00000154319**	*0.08439645*	*0.08877955*	*0.093099405*	*0.089785388*
**ENSG00000102271**	*0.017308408*	*0.027944527*	*0.026110884*	*0.015412192*
**ENSG00000129226**	*0.019849405*	*0.029049982*	*0.026814581*	*0.019707426*
**ENSG00000136696**	*0.015355211*	*0.026028151*	*0.024273612*	*0.015573541*
**ENSG00000113722**	*0.016129432*	*0.024585191*	*0.024287793*	*0.016066382*
**ENSG00000242259**	*0.024333022*	*0.031687674*	*0.029121618*	*0.024442698*
**ENSG00000101447**	*0.056710623*	*0.040110829*	*0.045745725*	*0.043255069*
**ENSG00000104969**	*0.024098303*	*0.030413108*	*0.034570129*	*0.023071973*
**ENSG00000148602**	*0.015568508*	*0.025431309*	*0.025238782*	*0.016231692*
**ENSG00000198547**	*0.017801574*	*0.027227386*	*0.026054351*	*0.016714484*
**ENSG00000171448**	*0.031123182*	*0.034279405*	*0.031119492*	*0.027118466*
**ENSG00000110108**	*0.039404226*	*0.032928371*	*0.036296735*	*0.032250948*
**ENSG00000187013**	*0.01575147*	*0.025240995*	*0.024648495*	*0.016419285*
**ENSG00000158201**	*0.047330478*	*0.048522406*	*0.036532701*	*0.032055026*
**ENSG00000070047**	*0.027599686*	*0.034377391*	*0.03244698*	*0.025918748*
**ENSG00000176024**	*0.029809147*	*0.038594176*	*0.041297444*	*0.025478434*
**ENSG00000103707**	*0.032048086*	*0.036053908*	*0.032363747*	*0.025577995*
**ENSG00000183742**	*0.064984625*	*0.055715028*	*0.056996263*	*0.069490556*
**ENSG00000107281**	*0.065725838*	*0.040240844*	*0.033434842*	*0.046126723*
**ENSG00000007216**	*0.016208264*	*0.025055065*	*0.024632738*	*0.015428633*
**ENSG00000183434**	*0.015056827*	*0.02488176*	*0.02427183*	*0.014654492*
**ENSG00000111843**	*0.024318307*	*0.031680708*	*0.028510197*	*0.024278952*
**ENSG00000103502**	*0.018124692*	*0.026539795*	*0.027685326*	*0.023533145*
**ENSG00000130385**	*0.01896688*	*0.024734804*	*0.02484826*	*0.015866746*
**ENSG00000117419**	*0.024007087*	*0.030659458*	*0.027995723*	*0.020362185*
**ENSG00000179104**	*0.039206378*	*0.038487059*	*0.035201447*	*0.028270379*
**ENSG00000122359**	*0.019934831*	*0.025980808*	*0.026516668*	*0.021981049*
**ENSG00000123191**	*0.018027017*	*0.032257951*	*0.027527846*	*0.022938451*
**ENSG00000102974**	*0.031461518*	*0.03246234*	*0.031505384*	*0.027055387*
**ENSG00000140505**	*0.021819631*	*0.025456865*	*0.02564312*	*0.01726246*
**ENSG00000128928**	*0.040092915*	*0.044858641*	*0.038874843*	*0.034274779*
**ENSG00000180035**	*0.016373659*	*0.025624388*	*0.025514105*	*0.016080747*
**ENSG00000198203**	*0.016294431*	*0.025192495*	*0.024814889*	*0.015755166*
**ENSG00000127125**	*0.025623988*	*0.031433852*	*0.029677213*	*0.035515588*
**ENSG00000176490**	*0.044192541*	*0.047774781*	*0.040029909*	*0.043006734*
**ENSG00000072134**	*0.027963597*	*0.031162529*	*0.033367215*	*0.026313721*
**ENSG00000176383**	*0.017830031*	*0.027179276*	*0.026241799*	*0.018099006*
**ENSG00000099812**	*0.016403714*	*0.023886618*	*0.024904743*	*0.015334963*
**ENSG00000176225**	*0.043232723*	*0.040273417*	*0.034766979*	*0.039519937*
**ENSG00000157445**	*0.016650214*	*0.024605877*	*0.024253529*	*0.014571803*
**ENSG00000008513**	*0.018591115*	*0.028183568*	*0.026400677*	*0.022135296*
**ENSG00000116586**	*0.028208919*	*0.032493119*	*0.028433546*	*0.025656574*
**ENSG00000185730**	*0.031815675*	*0.035063659*	*0.032410006*	*0.03036404*
**ENSG00000145050**	*0.034317453*	*0.03435671*	*0.037389701*	*0.036484759*

**ENSG00000101470**	*0.016831071*	*0.024860655*	*0.024582388*	*0.018384203*
**ENSG00000181458**	*0.08391894*	*0.06443536*	*0.063206352*	*0.064417933*
**ENSG00000106327**	*0.02233974*	*0.030752677*	*0.027256912*	*0.019299604*
**ENSG00000108578**	*0.028493328*	*0.033394064*	*0.029510537*	*0.024241332*
**ENSG00000167768**	*0.015790825*	*0.024226392*	*0.02466552*	*0.015182135*
**ENSG00000138075**	*0.031473902*	*0.031291571*	*0.029793672*	*0.017579131*
**ENSG00000151892**	*0.01557343*	*0.025915667*	*0.024882841*	*0.015694274*
**ENSG00000072657**	*0.016963872*	*0.024224048*	*0.025019165*	*0.01514854*
**ENSG00000147459**	*0.01902047*	*0.024891738*	*0.025629042*	*0.018668398*
**ENSG00000115687**	*0.055714187*	*0.050646547*	*0.053631495*	*0.053825928*
**ENSG00000106034**	*0.015804978*	*0.025135711*	*0.024933703*	*0.015230922*
**ENSG00000135097**	*0.018902136*	*0.028414767*	*0.027611695*	*0.017482443*
**ENSG00000145375**	*0.015567444*	*0.02495243*	*0.024397912*	*0.015409929*
**ENSG00000104814**	*0.027836443*	*0.031689823*	*0.029473475*	*0.025338559*
**ENSG00000128585**	*0.03018806*	*0.035301344*	*0.033911149*	*0.024755568*
**ENSG00000010361**	*0.019298168*	*0.02790186*	*0.025466427*	*0.018794819*
**ENSG00000135517**	*0.015965796*	*0.024944279*	*0.024733781*	*0.014164138*
**ENSG00000156475**	*0.014788432*	*0.025486242*	*0.025432368*	*0.014427058*
**ENSG00000100003**	*0.042042943*	*0.03682297*	*0.032240516*	*0.029998544*
**ENSG00000181847**	*0.01870349*	*0.026556948*	*0.027840075*	*0.015670313*
**ENSG00000115507**	*0.016158557*	*0.026497033*	*0.025118892*	*0.016305526*
**ENSG00000167562**	*0.03349866*	*0.037226715*	*0.040596964*	*0.043999171*
**ENSG00000185787**	*0.020831188*	*0.026828968*	*0.026333401*	*0.018547616*
**ENSG00000064309**	*0.015290889*	*0.025650172*	*0.025267009*	*0.016131294*
**ENSG00000128276**	*0.015578616*	*0.024943454*	*0.02476774*	*0.015853171*
**ENSG00000102524**	*0.064215589*	*0.048920159*	*0.049150967*	*0.057434422*
**ENSG00000175826**	*0.026045945*	*0.033025389*	*0.041834049*	*0.03789774*
**ENSG00000176102**	*0.0430993*	*0.050003161*	*0.040287313*	*0.034739696*
**ENSG00000166961**	*0.018105523*	*0.02598244*	*0.027483294*	*0.016363622*
**ENSG00000158859**	*0.016489711*	*0.025561685*	*0.025037072*	*0.015221335*
**ENSG00000103150**	*0.030591692*	*0.039899389*	*0.034773215*	*0.036178201*
**ENSG00000137673**	*0.204689587*	*0.146915561*	*0.131663118*	*0.16001956*
**ENSG00000164100**	*0.016082047*	*0.024709189*	*0.024129475*	*0.014807151*
**ENSG00000127527**	*0.024550272*	*0.032747543*	*0.028892924*	*0.023948291*
**ENSG00000204852**	*0.054638724*	*0.043474298*	*0.033333723*	*0.045144278*
**ENSG00000052749**	*0.036969965*	*0.036698804*	*0.037192373*	*0.036705114*
**ENSG00000174579**	*0.03079814*	*0.039542926*	*0.03623733*	*0.025595441*
**ENSG00000176742**	*0.017190324*	*0.025567028*	*0.024766567*	*0.014970855*
**ENSG00000125363**	*0.015157201*	*0.024675391*	*0.025272501*	*0.015814286*
**ENSG00000198431**	*0.047421836*	*0.042187227*	*0.039605817*	*0.035155788*
**ENSG00000170803**	*0.019024623*	*0.025924049*	*0.027751014*	*0.017745403*
**ENSG00000150750**	*0.016717018*	*0.02574251*	*0.025815692*	*0.016760765*
**ENSG00000130508**	*0.109354792*	*0.130305392*	*0.099686832*	*0.125314177*
**ENSG00000196767**	*0.015772086*	*0.024810616*	*0.024486627*	*0.014836826*
**ENSG00000166816**	*0.01749105*	*0.025717799*	*0.0256635*	*0.017522394*
**ENSG00000072682**	*0.097839278*	*0.063698435*	*0.06213783*	*0.074422912*
**ENSG00000187840**	*0.042208722*	*0.031434232*	*0.031984412*	*0.029689528*
**ENSG00000159455**	*0.015713913*	*0.025029335*	*0.024818963*	*0.015693856*
**ENSG00000007314**	*0.016100123*	*0.02603141*	*0.024562531*	*0.014984013*
**ENSG00000142515**	*0.015979204*	*0.025508617*	*0.025650536*	*0.014349552*
**ENSG00000114554**	*0.043141158*	*0.03690123*	*0.036242823*	*0.039150739*
**ENSG00000160202**	*0.017266376*	*0.025639366*	*0.025874296*	*0.015281448*
**ENSG00000004660**	*0.0149606*	*0.023911478*	*0.024582647*	*0.0143303*
**ENSG00000134184**	*0.202087581*	*0.184818758*	*0.165499833*	*0.16917334*

**ENSG00000072694**	*0.057900079*	*0.051840145*	*0.048107508*	*0.060102158*
**ENSG00000178996**	*0.016992864*	*0.02480386*	*0.024536804*	*0.015286803*
**ENSG00000165496**	*0.015388739*	*0.025551574*	*0.025678692*	*0.016381917*
**ENSG00000162782**	*0.019887334*	*0.025736849*	*0.024891909*	*0.017942569*
**ENSG00000165389**	*0.034782482*	*0.036905461*	*0.034034961*	*0.03059118*
**ENSG00000135972**	*0.02672556*	*0.031468888*	*0.028848055*	*0.023739071*
**ENSG00000140443**	*0.015472364*	*0.024684436*	*0.025266222*	*0.015109258*
**ENSG00000124209**	*0.034676897*	*0.040926684*	*0.03662952*	*0.031357596*
**ENSG00000204131**	*0.016551906*	*0.024892362*	*0.024338659*	*0.015690231*
**ENSG00000137845**	*0.034269093*	*0.039733482*	*0.037108257*	*0.026461625*
**ENSG00000100106**	*0.033087019*	*0.032184854*	*0.031403915*	*0.027278093*
**ENSG00000211456**	*0.032428031*	*0.033689528*	*0.030928137*	*0.041981838*
**ENSG00000114446**	*0.040974959*	*0.04082242*	*0.0370246*	*0.035703322*
**ENSG00000134779**	*0.051814857*	*0.046960885*	*0.044742622*	*0.034426241*
**ENSG00000086189**	*0.02397562*	*0.033040695*	*0.028823673*	*0.023497854*
**ENSG00000145632**	*0.055783814*	*0.047395675*	*0.040639052*	*0.041493759*
**ENSG00000103254**	*0.035502928*	*0.032948127*	*0.03264287*	*0.030963868*
**ENSG00000185418**	*0.038256215*	*0.040476786*	*0.040375246*	*0.036013079*
**ENSG00000154736**	*0.016634238*	*0.025476702*	*0.026958831*	*0.016020325*
**ENSG00000225697**	*0.03213253*	*0.035530782*	*0.029880791*	*0.037297668*
**ENSG00000109686**	*0.016772119*	*0.024734522*	*0.024683726*	*0.015940852*
**ENSG00000183067**	*0.015626737*	*0.025481867*	*0.024345915*	*0.0156227*
**ENSG00000148346**	*0.015786776*	*0.025569249*	*0.025226965*	*0.014871201*
**ENSG00000147457**	*0.036360118*	*0.035286932*	*0.033880027*	*0.02512192*
**ENSG00000180772**	*0.01574225*	*0.024431941*	*0.02570387*	*0.016115955*
**ENSG00000182004**	*0.019967656*	*0.026106855*	*0.025891993*	*0.018282951*
**ENSG00000116690**	*0.017010306*	*0.02581257*	*0.026570653*	*0.017542768*
**ENSG00000136014**	*0.015304436*	*0.025051908*	*0.024758382*	*0.014590092*
**ENSG00000197870**	*0.016149347*	*0.024508443*	*0.024925459*	*0.015447279*
**ENSG00000100348**	*0.024290794*	*0.026356805*	*0.031418695*	*0.025131006*
**ENSG00000172404**	*0.015490362*	*0.025481229*	*0.024438729*	*0.016419586*
**ENSG00000068976**	*0.017035565*	*0.024767558*	*0.02504247*	*0.015010038*
**ENSG00000086061**	*0.031077608*	*0.033112157*	*0.029571148*	*0.024958912*
**ENSG00000137100**	*0.020898115*	*0.027442501*	*0.02879966*	*0.017029116*
**ENSG00000126226**	*0.029538369*	*0.032224501*	*0.030687255*	*0.025514021*
**ENSG00000178921**	*0.03807592*	*0.03680176*	*0.033811021*	*0.032802198*
**ENSG00000174721**	*0.015585115*	*0.025889921*	*0.025053905*	*0.016653079*
**ENSG00000170647**	*0.034217902*	*0.033329202*	*0.029105575*	*0.031632143*
**ENSG00000154359**	*0.042809817*	*0.042694481*	*0.038314517*	*0.036744083*
**ENSG00000135535**	*0.023896607*	*0.031259855*	*0.029521457*	*0.022238273*
**ENSG00000173610**	*0.04616538*	*0.035041808*	*0.025302888*	*0.028705797*
**ENSG00000140368**	*0.068522601*	*0.061670336*	*0.044238355*	*0.061962694*
**ENSG00000183048**	*0.051526914*	*0.039457819*	*0.046625499*	*0.039814768*
**ENSG00000148735**	*0.015326707*	*0.02491033*	*0.024620316*	*0.016640232*
**ENSG00000162909**	*0.099251968*	*0.07152148*	*0.068814359*	*0.075547629*
**ENSG00000161921**	*0.037799464*	*0.033532241*	*0.031360645*	*0.040890108*
**ENSG00000141429**	*0.032065019*	*0.037235987*	*0.032079443*	*0.033914438*
**ENSG00000186625**	*0.026214137*	*0.036640739*	*0.033734388*	*0.026256328*
**ENSG00000138468**	*0.020883997*	*0.030809705*	*0.028120886*	*0.022159499*
**ENSG00000231389**	*0.017171188*	*0.027463257*	*0.025796099*	*0.020870041*
**ENSG00000133116**	*0.09514386*	*0.07542626*	*0.046067662*	*0.07495192*
**ENSG00000164080**	*0.023342713*	*0.032089654*	*0.035227146*	*0.025877227*
**ENSG00000103507**	*0.032414994*	*0.029441006*	*0.033114526*	*0.028614466*
**ENSG00000145949**	*0.015484725*	*0.024260941*	*0.023830807*	*0.015396316*

**ENSG00000136783**	*0.043501724*	*0.04986632*	*0.036654277*	*0.074141181*
**ENSG00000113303**	*0.015288899*	*0.024669276*	*0.024586095*	*0.014663926*
**ENSG00000100614**	*0.017450729*	*0.02588578*	*0.02757722*	*0.016046414*
**ENSG00000134531**	*0.036753578*	*0.036350245*	*0.033074619*	*0.023949475*
**ENSG00000165525**	*0.025654616*	*0.03402358*	*0.029672744*	*0.023838864*
**ENSG00000198366**	*0.039922139*	*0.032333224*	*0.041364704*	*0.046782175*
**ENSG00000065243**	*0.028284619*	*0.034697195*	*0.035833536*	*0.022963009*
**ENSG00000182093**	*0.031193211*	*0.038381433*	*0.032879133*	*0.029830367*
**ENSG00000070882**	*0.037153952*	*0.038174339*	*0.038077134*	*0.037665016*
**ENSG00000166971**	*0.030616262*	*0.035480353*	*0.037516734*	*0.029598953*
**ENSG00000171204**	*0.026350685*	*0.033683716*	*0.029272598*	*0.020666881*
**ENSG00000138617**	*0.02794627*	*0.028981754*	*0.029236787*	*0.022077674*
**ENSG00000143494**	*0.07007774*	*0.05606889*	*0.050912097*	*0.070439292*
**ENSG00000025434**	*0.01742553*	*0.029623065*	*0.027548184*	*0.021576095*
**ENSG00000187555**	*0.027061253*	*0.033007583*	*0.032911897*	*0.023214874*
**ENSG00000128519**	*0.016387231*	*0.02548386*	*0.024330429*	*0.016161704*
**ENSG00000162892**	*0.018252278*	*0.025211721*	*0.027889166*	*0.016222876*
**ENSG00000141736**	*0.017041025*	*0.025097011*	*0.026437494*	*0.017520054*
**ENSG00000141503**	*0.014209612*	*0.024819445*	*0.02492035*	*0.014765453*
**ENSG00000125730**	*0.020838438*	*0.035355005*	*0.026958101*	*0.029304377*
**ENSG00000106078**	*0.017853897*	*0.025919084*	*0.02768896*	*0.017360273*
**ENSG00000155008**	*0.045166103*	*0.050588338*	*0.045088115*	*0.042068148*
**ENSG00000204503**	*0.0204188*	*0.027487624*	*0.030256421*	*0.016685121*
**ENSG00000143196**	*0.01581329*	*0.025843113*	*0.02594572*	*0.016269387*
**ENSG00000066933**	*0.037568154*	*0.039405612*	*0.037740529*	*0.034212539*
**ENSG00000169962**	*0.013586174*	*0.023764781*	*0.024376185*	*0.013633511*
**ENSG00000081377**	*0.044939701*	*0.02728367*	*0.024995762*	*0.026118618*
**ENSG00000135069**	*0.058989371*	*0.047523822*	*0.041310915*	*0.050995441*
**ENSG00000136872**	*0.058856215*	*0.02512173*	*0.024713434*	*0.017677542*
**ENSG00000196372**	*0.018320943*	*0.02739352*	*0.030243429*	*0.017896614*
**ENSG00000139233**	*0.02485901*	*0.03305313*	*0.033082589*	*0.024614829*
**ENSG00000242498**	*0.017496575*	*0.026197645*	*0.024835905*	*0.023702336*
**ENSG00000157601**	*0.037276895*	*0.033373031*	*0.037533209*	*0.037722882*
**ENSG00000187764**	*0.028426391*	*0.03372784*	*0.030549657*	*0.023327192*
**ENSG00000146857**	*0.015459651*	*0.024294186*	*0.02469189*	*0.014974343*
**ENSG00000015479**	*0.022028766*	*0.029526702*	*0.02698206*	*0.021510146*
**ENSG00000133056**	*0.020670079*	*0.030199182*	*0.028747277*	*0.021365608*
**ENSG00000071127**	*0.02744147*	*0.03259549*	*0.032149885*	*0.031961255*
**ENSG00000100341**	*0.017439274*	*0.025390314*	*0.025607989*	*0.016740558*
**ENSG00000011007**	*0.027213261*	*0.034001238*	*0.037796584*	*0.027618302*
**ENSG00000133243**	*0.024566694*	*0.029410023*	*0.029813978*	*0.024217175*
**ENSG00000156804**	*0.016363387*	*0.025171458*	*0.024854307*	*0.016340701*
**ENSG00000168124**	*0.018011111*	*0.025003752*	*0.025235331*	*0.015370606*
**ENSG00000173406**	*0.015695715*	*0.025209317*	*0.025969879*	*0.015472739*
**ENSG00000136867**	*0.028059698*	*0.035446714*	*0.036561528*	*0.021894772*
**ENSG00000179142**	*0.017234746*	*0.026169727*	*0.025538436*	*0.016925354*
**ENSG00000007129**	*0.031099315*	*0.037627231*	*0.041412279*	*0.036323549*
**ENSG00000154768**	*0.016876054*	*0.025743234*	*0.024328986*	*0.01613117*
**ENSG00000121931**	*0.033747252*	*0.040380212*	*0.037877236*	*0.032229147*
**ENSG00000100351**	*0.094999276*	*0.073994621*	*0.060142086*	*0.074155422*
**ENSG00000186166**	*0.032291678*	*0.03483645*	*0.034725096*	*0.027405384*
**ENSG00000136950**	*0.027983373*	*0.031916018*	*0.035704577*	*0.031709389*
**ENSG00000041982**	*0.022340175*	*0.026562706*	*0.026397575*	*0.020366966*
**ENSG00000186105**	*0.04555381*	*0.036294154*	*0.035868063*	*0.034563627*

**ENSG00000239474**	*0.015526003*	*0.026352663*	*0.027018654*	*0.015979561*
**ENSG00000182330**	*0.027867856*	*0.024405189*	*0.026364794*	*0.015278867*
**ENSG00000169372**	*0.034227132*	*0.036584914*	*0.034004782*	*0.023832343*
**ENSG00000188921**	*0.031567516*	*0.033801738*	*0.032281157*	*0.026493036*
**ENSG00000185340**	*0.015446024*	*0.025182988*	*0.025436598*	*0.015459809*
**ENSG00000169855**	*0.020714048*	*0.025464502*	*0.025805521*	*0.015355198*
**ENSG00000152661**	*0.016702932*	*0.024865684*	*0.024862977*	*0.016445924*
**ENSG00000116815**	*0.024380543*	*0.029989285*	*0.028554048*	*0.02577007*
**ENSG00000056998**	*0.015650743*	*0.024265079*	*0.02441574*	*0.015120364*
**ENSG00000176340**	*0.018665207*	*0.025390584*	*0.02609897*	*0.018466661*
**ENSG00000080986**	*0.044955665*	*0.03787861*	*0.036462901*	*0.037714273*
**ENSG00000169914**	*0.018430406*	*0.026763365*	*0.027015054*	*0.017544206*
**ENSG00000182611**	*0.018916177*	*0.027068721*	*0.025439129*	*0.016190981*
**ENSG00000128250**	*0.018319656*	*0.027196302*	*0.02608076*	*0.01780557*
**ENSG00000100823**	*0.022282309*	*0.027750545*	*0.026260033*	*0.019429444*
**ENSG00000197927**	*0.039538444*	*0.041811057*	*0.045443982*	*0.045317106*
**ENSG00000198934**	*0.020617446*	*0.02660439*	*0.026001112*	*0.017985922*
**ENSG00000006625**	*0.031676773*	*0.034171261*	*0.029401085*	*0.027453981*
**ENSG00000187664**	*0.016774719*	*0.02616415*	*0.025293721*	*0.016083242*
**ENSG00000031691**	*0.040038487*	*0.037000447*	*0.032593807*	*0.035275725*
**ENSG00000143947**	*0.017611757*	*0.024821725*	*0.024790453*	*0.020578445*
**ENSG00000145365**	*0.039186676*	*0.043961849*	*0.038568507*	*0.034721094*
**ENSG00000116688**	*0.026941266*	*0.033133002*	*0.031589191*	*0.025372637*
**ENSG00000119878**	*0.034113727*	*0.03897659*	*0.03473349*	*0.024402341*
**ENSG00000099624**	*0.021408078*	*0.026836169*	*0.028497735*	*0.021724143*
**ENSG00000121858**	*0.071042481*	*0.066745672*	*0.073794963*	*0.065301039*
**ENSG00000013523**	*0.027723179*	*0.028026993*	*0.028639344*	*0.022002765*
**ENSG00000107897**	*0.030571047*	*0.037304441*	*0.032265912*	*0.033430633*
**ENSG00000122863**	*0.019878012*	*0.029205587*	*0.025635743*	*0.022994381*
**ENSG00000123405**	*0.018866115*	*0.02549702*	*0.025547035*	*0.014596715*
**ENSG00000160994**	*0.016508516*	*0.025568904*	*0.02582758*	*0.017826037*
**ENSG00000102081**	*0.034281084*	*0.040387892*	*0.032281699*	*0.030681901*
**ENSG00000157214**	*0.075196108*	*0.049214813*	*0.041208011*	*0.049016683*
**ENSG00000188086**	*0.016866518*	*0.025368532*	*0.025456593*	*0.022403957*
**ENSG00000107593**	*0.016236643*	*0.024300201*	*0.024754798*	*0.016796178*
**ENSG00000144908**	*0.029433907*	*0.025216942*	*0.027064558*	*0.020848466*
**ENSG00000078898**	*0.015609247*	*0.024875266*	*0.024499621*	*0.01510654*
**ENSG00000243364**	*0.038432718*	*0.036017587*	*0.035838891*	*0.035706648*
**ENSG00000070388**	*0.016024969*	*0.024598649*	*0.024283984*	*0.016036953*
**ENSG00000188690**	*0.034311382*	*0.03827538*	*0.039301417*	*0.032108611*
**ENSG00000185808**	*0.028298154*	*0.030292427*	*0.032137166*	*0.026280389*
**ENSG00000090372**	*0.026416526*	*0.030825253*	*0.040244656*	*0.029736266*
**ENSG00000076513**	*0.034120518*	*0.036183133*	*0.032941083*	*0.02902812*
**ENSG00000184148**	*0.016101244*	*0.024853084*	*0.025034452*	*0.016309823*
**ENSG00000127588**	*0.017851757*	*0.026173012*	*0.026755969*	*0.016489988*
**ENSG00000165548**	*0.016194267*	*0.025095796*	*0.024863038*	*0.014320006*
**ENSG00000140961**	*0.016613757*	*0.025773384*	*0.024349996*	*0.016032387*
**ENSG00000023839**	*0.018189654*	*0.027265598*	*0.026239621*	*0.017379225*
**ENSG00000184281**	*0.031891576*	*0.036508874*	*0.04018149*	*0.034370021*
**ENSG00000100201**	*0.043130299*	*0.04896487*	*0.060666694*	*0.072792305*
**ENSG00000136152**	*0.029465328*	*0.0356921*	*0.034267926*	*0.026203538*
**ENSG00000087995**	*0.02906815*	*0.033772165*	*0.032332259*	*0.029036377*
**ENSG00000100228**	*0.025369777*	*0.026937184*	*0.032839861*	*0.019410287*
**ENSG00000112739**	*0.040325962*	*0.03884961*	*0.031668605*	*0.032123562*

**ENSG00000206418**	*0.048885253*	*0.06155834*	*0.04367876*	*0.064204653*
**ENSG00000027001**	*0.035641902*	*0.039625662*	*0.036248123*	*0.028172571*
**ENSG00000156413**	*0.029795559*	*0.030172766*	*0.032822474*	*0.025967647*
**ENSG00000159899**	*0.025662777*	*0.028417857*	*0.030586534*	*0.021696948*
**ENSG00000151623**	*0.01582568*	*0.024696924*	*0.025826561*	*0.016008685*
**ENSG00000196177**	*0.039415365*	*0.041772507*	*0.038426253*	*0.043006715*
**ENSG00000111832**	*0.019892955*	*0.027716991*	*0.026864685*	*0.019239905*
**ENSG00000188682**	*0.016501401*	*0.026122987*	*0.024501685*	*0.014622718*
**ENSG00000198488**	*0.016925483*	*0.025922235*	*0.02620925*	*0.017400351*
**ENSG00000196431**	*0.01634601*	*0.025566636*	*0.026977006*	*0.015587402*
**ENSG00000131097**	*0.015881586*	*0.025957134*	*0.025237957*	*0.016634154*
**ENSG00000198171**	*0.030071853*	*0.031677598*	*0.034198999*	*0.024861939*
**ENSG00000159885**	*0.027466204*	*0.036848569*	*0.03615764*	*0.021698858*
**ENSG00000087916**	*0.018169738*	*0.024643114*	*0.026129813*	*0.015561641*
**ENSG00000198551**	*0.032053602*	*0.037196669*	*0.034153337*	*0.027685826*
**ENSG00000136840**	*0.039424247*	*0.038744059*	*0.041139787*	*0.035359133*
**ENSG00000143319**	*0.03060584*	*0.033607104*	*0.039581764*	*0.030558036*
**ENSG00000204427**	*0.030051358*	*0.030207711*	*0.034143202*	*0.027910688*
**ENSG00000100433**	*0.015332686*	*0.024650143*	*0.025380722*	*0.014453153*
**ENSG00000178199**	*0.01623689*	*0.024544363*	*0.025181113*	*0.015219009*
**ENSG00000134755**	*0.113779297*	*0.055258228*	*0.029879957*	*0.053414904*
**ENSG00000240505**	*0.046481349*	*0.038555253*	*0.033389063*	*0.032360983*
**ENSG00000065548**	*0.021784186*	*0.029404521*	*0.030129192*	*0.024423831*
**ENSG00000131016**	*0.015768717*	*0.024543202*	*0.024325983*	*0.015685424*
**ENSG00000184898**	*0.016603368*	*0.025357724*	*0.024397496*	*0.014978073*
**ENSG00000205495**	*0.017065606*	*0.025172082*	*0.025305092*	*0.017174951*
**ENSG00000166578**	*0.016496587*	*0.025608179*	*0.025073023*	*0.014332436*
**ENSG00000162344**	*0.01712521*	*0.026003095*	*0.026732836*	*0.016027307*
**ENSG00000103642**	*0.035199929*	*0.037429988*	*0.037055809*	*0.030060454*
**ENSG00000145358**	*0.099481698*	*0.074099322*	*0.082696351*	*0.079141037*
**ENSG00000105676**	*0.029374218*	*0.030069777*	*0.03368158*	*0.033402605*
**ENSG00000108515**	*0.045275767*	*0.047068403*	*0.03927339*	*0.039795184*
**ENSG00000176567**	*0.024244865*	*0.029390649*	*0.030552811*	*0.021104115*
**ENSG00000112796**	*0.068663034*	*0.052887837*	*0.04290396*	*0.061863259*
**ENSG00000070010**	*0.023838972*	*0.031951083*	*0.029270913*	*0.028085143*
**ENSG00000197614**	*0.027690424*	*0.029887996*	*0.030805612*	*0.033145715*
**ENSG00000187522**	*0.029154269*	*0.033502692*	*0.035884545*	*0.03029932*
**ENSG00000104518**	*0.035008832*	*0.037830636*	*0.035235432*	*0.030272748*
**ENSG00000164393**	*0.016194438*	*0.024678595*	*0.024803097*	*0.016028195*
**ENSG00000159079**	*0.015772834*	*0.024418781*	*0.024982781*	*0.014832026*
**ENSG00000090621**	*0.036703708*	*0.035182428*	*0.035799696*	*0.037940131*
**ENSG00000141030**	*0.030070062*	*0.032778146*	*0.033801207*	*0.027314077*
**ENSG00000180537**	*0.014605261*	*0.024405334*	*0.024480543*	*0.014299416*
**ENSG00000148948**	*0.070521606*	*0.049143234*	*0.050861751*	*0.043488884*
**ENSG00000141665**	*0.021535368*	*0.028794986*	*0.026077138*	*0.018306442*
**ENSG00000180616**	*0.070353439*	*0.05050777*	*0.043736687*	*0.053880379*
**ENSG00000131844**	*0.035002299*	*0.044348952*	*0.033033577*	*0.032558358*
**ENSG00000179218**	*0.035475943*	*0.035980369*	*0.03633577*	*0.039677255*
**ENSG00000186881**	*0.015662762*	*0.024878754*	*0.025718493*	*0.015453121*
**ENSG00000110048**	*0.021340409*	*0.028648639*	*0.034093378*	*0.021252486*
**ENSG00000154721**	*0.081658787*	*0.051667711*	*0.057478556*	*0.055929923*
**ENSG00000169230**	*0.032793475*	*0.033071283*	*0.03019261*	*0.031676933*
**ENSG00000183098**	*0.016416694*	*0.0264163*	*0.027282469*	*0.019095883*
**ENSG00000130165**	*0.021683749*	*0.028204471*	*0.029757346*	*0.018761071*

**ENSG00000142409**	*0.054002935*	*0.049942685*	*0.053047455*	*0.035694151*
**ENSG00000065427**	*0.026079876*	*0.029810949*	*0.02864386*	*0.01870709*
**ENSG00000102383**	*0.017624079*	*0.025457061*	*0.025740637*	*0.017422415*
**ENSG00000140259**	*0.024597568*	*0.032782409*	*0.031251258*	*0.020599333*
**ENSG00000147437**	*0.018906149*	*0.028901611*	*0.027249039*	*0.02065134*
**ENSG00000169435**	*0.116721999*	*0.08676646*	*0.079006654*	*0.09404154*
**ENSG00000196605**	*0.015431388*	*0.026660838*	*0.024532337*	*0.017856698*
**ENSG00000204421**	*0.015892254*	*0.023693295*	*0.024083368*	*0.01448074*
**ENSG00000147251**	*0.03039445*	*0.037449344*	*0.036428769*	*0.032779188*
**ENSG00000133216**	*0.02855451*	*0.029879527*	*0.027808011*	*0.036076857*
**ENSG00000142632**	*0.028047718*	*0.029596148*	*0.032374729*	*0.025712367*
**ENSG00000100302**	*0.016656603*	*0.02639359*	*0.02595308*	*0.019147171*
**ENSG00000213937**	*0.017009658*	*0.025703386*	*0.024633409*	*0.016243817*
**ENSG00000145451**	*0.015378291*	*0.025234278*	*0.025359048*	*0.01519635*
**ENSG00000179958**	*0.037071982*	*0.029638029*	*0.032291607*	*0.032326677*
**ENSG00000138326**	*0.013700438*	*0.023401509*	*0.023558501*	*0.015092526*
**ENSG00000111817**	*0.029862413*	*0.032195676*	*0.030940748*	*0.028596514*
**ENSG00000189319**	*0.045134939*	*0.037795222*	*0.037850808*	*0.040803842*
**ENSG00000131738**	*0.015746981*	*0.023932291*	*0.024517763*	*0.015429224*
**ENSG00000157823**	*0.01951272*	*0.028870979*	*0.026863108*	*0.018674796*
**ENSG00000136160**	*0.018753286*	*0.025266377*	*0.025149286*	*0.015809833*
**ENSG00000132640**	*0.016246026*	*0.025127018*	*0.025017017*	*0.017784185*
**ENSG00000128789**	*0.019646994*	*0.02605785*	*0.027873093*	*0.019207057*
**ENSG00000176165**	*0.123209291*	*0.025806611*	*0.061591804*	*0.033910776*
**ENSG00000109132**	*0.015794687*	*0.024466627*	*0.024952709*	*0.014588428*
**ENSG00000176945**	*0.035885451*	*0.03956692*	*0.037731205*	*0.040324882*
**ENSG00000161992**	*0.014315422*	*0.025058327*	*0.024279316*	*0.015513678*
**ENSG00000148331**	*0.016345221*	*0.026130011*	*0.026253355*	*0.017656804*
**ENSG00000075151**	*0.032212314*	*0.037367386*	*0.035400186*	*0.02956546*
**ENSG00000112697**	*0.032299181*	*0.035330334*	*0.03516809*	*0.026583408*
**ENSG00000165804**	*0.029356459*	*0.032539966*	*0.026413832*	*0.024843169*
**ENSG00000176236**	*0.015847992*	*0.025029167*	*0.025277106*	*0.015749596*
**ENSG00000197905**	*0.146407078*	*0.119212344*	*0.10250148*	*0.112924044*
**ENSG00000132561**	*0.019347912*	*0.024314831*	*0.025105084*	*0.017161711*
**ENSG00000101745**	*0.033549945*	*0.034694594*	*0.034612438*	*0.032444487*
**ENSG00000056050**	*0.038178583*	*0.036880133*	*0.032312938*	*0.032726234*
**ENSG00000103995**	*0.045851183*	*0.047469837*	*0.032438432*	*0.038260869*
**ENSG00000075142**	*0.032625304*	*0.036230549*	*0.030644286*	*0.030710877*
**ENSG00000148219**	*0.016055441*	*0.025414212*	*0.024970134*	*0.016030154*
**ENSG00000228716**	*0.053303224*	*0.045683905*	*0.036564817*	*0.047216107*
**ENSG00000176177**	*0.047719027*	*0.085865094*	*0.029161436*	*0.047445523*
**ENSG00000104660**	*0.032671218*	*0.035312032*	*0.034067993*	*0.03013433*
**ENSG00000100307**	*0.039901724*	*0.037178259*	*0.04350922*	*0.038150559*
**ENSG00000028203**	*0.029857196*	*0.031830261*	*0.040833603*	*0.0242766*
**ENSG00000179262**	*0.022776285*	*0.026225222*	*0.028334382*	*0.022082887*
**ENSG00000204463**	*0.02097008*	*0.026672872*	*0.032756664*	*0.022194599*
**ENSG00000081148**	*0.015283914*	*0.025911141*	*0.024391591*	*0.014824243*
**ENSG00000146648**	*0.018117391*	*0.027272126*	*0.025683284*	*0.020227918*
**ENSG00000127530**	*0.015700139*	*0.025690969*	*0.025123159*	*0.015904424*
**ENSG00000161999**	*0.022601897*	*0.033446023*	*0.032598867*	*0.027113426*
**ENSG00000176393**	*0.034103265*	*0.040265102*	*0.031119234*	*0.028592905*
**ENSG00000100867**	*0.028155838*	*0.027244648*	*0.027473853*	*0.018414917*
**ENSG00000164404**	*0.016669229*	*0.025928581*	*0.025356071*	*0.015597635*
**ENSG00000145879**	*0.014897292*	*0.024514376*	*0.02461941*	*0.014445779*

**ENSG00000113851**	*0.029547971*	*0.038009751*	*0.034583297*	*0.025972933*
**ENSG00000204195**	*0.016859822*	*0.025340504*	*0.025227335*	*0.015764172*
**ENSG00000119946**	*0.01938112*	*0.026726909*	*0.026137423*	*0.017973648*
**ENSG00000132386**	*0.034778765*	*0.037411487*	*0.040594949*	*0.025160245*
**ENSG00000163479**	*0.021993923*	*0.028490958*	*0.030763622*	*0.020098164*
**ENSG00000142694**	*0.053398008*	*0.05300575*	*0.043869466*	*0.054915407*
**ENSG00000142748**	*0.016566819*	*0.025510122*	*0.024792555*	*0.015960272*
**ENSG00000183808**	*0.029818696*	*0.034559032*	*0.036514541*	*0.026205522*
**ENSG00000113212**	*0.018906823*	*0.027489563*	*0.028079404*	*0.01923142*
**ENSG00000120054**	*0.018269286*	*0.025810452*	*0.026145563*	*0.017975327*
**ENSG00000035720**	*0.050773793*	*0.051425691*	*0.041361329*	*0.038800265*
**ENSG00000181991**	*0.027475239*	*0.031839752*	*0.030294353*	*0.026096593*
**ENSG00000141385**	*0.02487716*	*0.030942699*	*0.031994895*	*0.023688381*
**ENSG00000177272**	*0.027027322*	*0.030194588*	*0.030936209*	*0.029212726*
**ENSG00000171532**	*0.030494043*	*0.028263217*	*0.026976809*	*0.018302944*
**ENSG00000063601**	*0.025849114*	*0.034036495*	*0.026681324*	*0.035771092*
**ENSG00000124762**	*0.032635214*	*0.032223151*	*0.033055058*	*0.026138576*
**ENSG00000144591**	*0.032056298*	*0.046354318*	*0.041823511*	*0.034225141*
**ENSG00000196781**	*0.050084669*	*0.037122636*	*0.039928793*	*0.057672588*
**ENSG00000135439**	*0.017813786*	*0.026782946*	*0.024900603*	*0.019022815*
**ENSG00000188176**	*0.015540491*	*0.026229759*	*0.025936551*	*0.016856151*
**ENSG00000151229**	*0.034656822*	*0.039300316*	*0.032542645*	*0.0340526*
**ENSG00000156502**	*0.03000155*	*0.035813442*	*0.03194088*	*0.027453454*
**ENSG00000109320**	*0.023416212*	*0.030148157*	*0.029584451*	*0.022336034*
**ENSG00000173930**	*0.018212671*	*0.025818401*	*0.025648146*	*0.017946889*
**ENSG00000105672**	*0.030811405*	*0.03234028*	*0.031197236*	*0.022729782*
**ENSG00000143178**	*0.018826212*	*0.029428473*	*0.027284671*	*0.02307622*
**ENSG00000180767**	*0.016804573*	*0.025680267*	*0.024418985*	*0.015065181*
**ENSG00000185053**	*0.016002288*	*0.02577771*	*0.02457005*	*0.016077272*
**ENSG00000011376**	*0.030304395*	*0.035360357*	*0.032114399*	*0.026938988*
**ENSG00000120471**	*0.016072933*	*0.026023256*	*0.025659748*	*0.015225004*
**ENSG00000241839**	*0.0282452*	*0.033743426*	*0.040047982*	*0.031250859*
**ENSG00000198464**	*0.046786626*	*0.045730101*	*0.04368048*	*0.048071557*
**ENSG00000146094**	*0.038011269*	*0.036836574*	*0.037614465*	*0.036921537*
**ENSG00000089356**	*0.01549802*	*0.025071491*	*0.025349886*	*0.015643148*
**ENSG00000159640**	*0.016231969*	*0.026668616*	*0.025202735*	*0.016547646*
**ENSG00000103174**	*0.022686967*	*0.029449134*	*0.029785876*	*0.028569626*
**ENSG00000100890**	*0.016784167*	*0.024758082*	*0.025978396*	*0.015994855*
**ENSG00000182909**	*0.023798045*	*0.030216835*	*0.028083481*	*0.026878618*
**ENSG00000178462**	*0.017542847*	*0.027522942*	*0.025835049*	*0.017642436*
**ENSG00000152467**	*0.016181085*	*0.024287097*	*0.025538793*	*0.016311563*
**ENSG00000080854**	*0.017690898*	*0.025718576*	*0.027035684*	*0.017427402*
**ENSG00000158006**	*0.029766838*	*0.03236671*	*0.033343352*	*0.032726291*
**ENSG00000148290**	*0.029465221*	*0.033008822*	*0.034779709*	*0.025857509*
**ENSG00000128203**	*0.037215166*	*0.04438978*	*0.043020675*	*0.030731907*
**ENSG00000163082**	*0.046016663*	*0.042466772*	*0.043377378*	*0.042732473*
**ENSG00000102904**	*0.016823949*	*0.025181774*	*0.02454173*	*0.015126384*
**ENSG00000171016**	*0.020040371*	*0.025481845*	*0.025079605*	*0.018557888*
**ENSG00000142675**	*0.085302806*	*0.077151197*	*0.075012555*	*0.080046496*
**ENSG00000101306**	*0.01622831*	*0.025250588*	*0.025164861*	*0.016224072*
**ENSG00000029363**	*0.030154507*	*0.032003162*	*0.036195788*	*0.037413733*
**ENSG00000154001**	*0.027171747*	*0.032878784*	*0.031289891*	*0.020688055*
**ENSG00000130544**	*0.029230267*	*0.037353494*	*0.035922211*	*0.02622375*
**ENSG00000100632**	*0.022606018*	*0.029114376*	*0.026987681*	*0.019422341*

**ENSG00000083093**	*0.023720565*	*0.031373186*	*0.030682047*	*0.019741912*
**ENSG00000151365**	*0.016512252*	*0.024503432*	*0.024506581*	*0.015023185*
**ENSG00000183801**	*0.015655669*	*0.02522007*	*0.024801141*	*0.016642734*
**ENSG00000186493**	*0.017001322*	*0.025170228*	*0.026339204*	*0.016000197*
**ENSG00000212933**	*0.018230869*	*0.026995178*	*0.027529283*	*0.018898601*
**ENSG00000112877**	*0.02859956*	*0.032607073*	*0.028673187*	*0.023184401*
**ENSG00000024526**	*0.046071232*	*0.035432236*	*0.035547628*	*0.031047565*
**ENSG00000144040**	*0.03873443*	*0.03114851*	*0.030438711*	*0.028047898*
**ENSG00000155754**	*0.015696743*	*0.02413029*	*0.02433742*	*0.015809421*
**ENSG00000113396**	*0.017116877*	*0.025761966*	*0.025824497*	*0.016931303*
**ENSG00000146476**	*0.033579483*	*0.042946876*	*0.033502916*	*0.024502662*
**ENSG00000133460**	*0.019989133*	*0.026027779*	*0.024456008*	*0.018160312*
**ENSG00000038427**	*0.098230407*	*0.077881367*	*0.071957756*	*0.084460917*
**ENSG00000110011**	*0.024029362*	*0.029848496*	*0.032306213*	*0.02849339*
**ENSG00000173389**	*0.018312884*	*0.02871523*	*0.026237101*	*0.018130738*
**ENSG00000165862**	*0.016174118*	*0.02476999*	*0.027218441*	*0.01566825*
**ENSG00000146757**	*0.048157849*	*0.053243891*	*0.042708035*	*0.064186143*
**ENSG00000185523**	*0.021357882*	*0.026273493*	*0.024876373*	*0.017619572*
**ENSG00000100290**	*0.082861299*	*0.070065252*	*0.074636469*	*0.093461868*
**ENSG00000108826**	*0.049405016*	*0.037294483*	*0.04760093*	*0.037583305*
**ENSG00000089472**	*0.015554526*	*0.02472605*	*0.025006095*	*0.014699642*
**ENSG00000243232**	*0.019406089*	*0.027277003*	*0.029585658*	*0.016615688*
**ENSG00000102007**	*0.049885794*	*0.035029037*	*0.038521808*	*0.040551722*
**ENSG00000180090**	*0.017378437*	*0.025315568*	*0.02611322*	*0.014869705*
**ENSG00000103494**	*0.020831109*	*0.029337782*	*0.0270652*	*0.017601323*
**ENSG00000130254**	*0.026088595*	*0.030443615*	*0.035931689*	*0.023406304*
**ENSG00000108830**	*0.014507616*	*0.025525388*	*0.024833235*	*0.014684912*
**ENSG00000095970**	*0.015305932*	*0.025068235*	*0.02497988*	*0.014747819*
**ENSG00000140279**	*0.016025052*	*0.026211569*	*0.025569453*	*0.016772984*
**ENSG00000138039**	*0.022075936*	*0.031095544*	*0.025546215*	*0.020632393*
**ENSG00000197837**	*0.0345969*	*0.030906092*	*0.034224699*	*0.036289248*
**ENSG00000169282**	*0.014900705*	*0.02485227*	*0.024385786*	*0.016020782*
**ENSG00000130244**	*0.040139838*	*0.031209039*	*0.033860825*	*0.031290967*
**ENSG00000116668**	*0.034516337*	*0.034290371*	*0.031361074*	*0.030335985*
**ENSG00000203784**	*0.015894418*	*0.024351778*	*0.024585754*	*0.01518423*
**ENSG00000112333**	*0.018376493*	*0.026540792*	*0.029015358*	*0.01582858*
**ENSG00000141577**	*0.020042379*	*0.025878998*	*0.025275047*	*0.01881297*
**ENSG00000159496**	*0.071256843*	*0.040632141*	*0.051227858*	*0.065904681*
**ENSG00000075131**	*0.039913454*	*0.039011231*	*0.036292912*	*0.04110648*
**ENSG00000012822**	*0.032185865*	*0.03152621*	*0.035579872*	*0.036774851*
**ENSG00000174374**	*0.028356797*	*0.032164844*	*0.033412903*	*0.026140313*
**ENSG00000178952**	*0.024749628*	*0.029598538*	*0.026662772*	*0.023376521*
**ENSG00000111049**	*0.01516249*	*0.025201438*	*0.025962338*	*0.015208444*
**ENSG00000120910**	*0.032783926*	*0.033411742*	*0.034582075*	*0.025466104*
**ENSG00000137409**	*0.0209724*	*0.029107148*	*0.028602407*	*0.019230409*
**ENSG00000008869**	*0.038433636*	*0.043976446*	*0.034284975*	*0.033629472*
**ENSG00000105427**	*0.023391147*	*0.030075186*	*0.026639909*	*0.031527697*
**ENSG00000068878**	*0.020862527*	*0.030241964*	*0.029789528*	*0.020777398*
**ENSG00000136371**	*0.037765275*	*0.045419315*	*0.036439847*	*0.038673435*
**ENSG00000160767**	*0.026984396*	*0.028132506*	*0.030011097*	*0.025180131*
**ENSG00000146700**	*0.019049625*	*0.028527084*	*0.031373108*	*0.017831423*
**ENSG00000185624**	*0.031103708*	*0.028466761*	*0.0357226*	*0.029589405*
**ENSG00000006210**	*0.041021329*	*0.032238*	*0.038428507*	*0.01890812*
**ENSG00000138030**	*0.022430508*	*0.028538174*	*0.031833287*	*0.018408749*

**ENSG00000110934**	*0.038824024*	*0.039712441*	*0.042344324*	*0.03889189*
**ENSG00000124641**	*0.042353094*	*0.040555976*	*0.032849657*	*0.02779126*
**ENSG00000109189**	*0.033063615*	*0.038974936*	*0.034888685*	*0.025673352*
**ENSG00000109046**	*0.030492392*	*0.030275447*	*0.029291618*	*0.028550227*
**ENSG00000169032**	*0.025229128*	*0.030691957*	*0.030184531*	*0.024826315*
**ENSG00000137265**	*0.023406221*	*0.027736274*	*0.028923506*	*0.020542028*
**ENSG00000070985**	*0.015902456*	*0.026724633*	*0.024386463*	*0.016053357*
**ENSG00000100982**	*0.027428334*	*0.028834834*	*0.03388983*	*0.025070991*
**ENSG00000110435**	*0.02724122*	*0.030623648*	*0.031366107*	*0.025877194*
**ENSG00000115758**	*0.037855695*	*0.033420199*	*0.031502077*	*0.031010789*
**ENSG00000243279**	*0.036529966*	*0.03141467*	*0.029306242*	*0.031079087*
**ENSG00000182197**	*0.042677143*	*0.040434873*	*0.043967225*	*0.035924644*
**ENSG00000117632**	*0.015742195*	*0.025125918*	*0.025943221*	*0.016650856*
**ENSG00000138641**	*0.030783823*	*0.038187372*	*0.032770791*	*0.030743962*
**ENSG00000105479**	*0.016382523*	*0.024058015*	*0.024343121*	*0.016798072*
**ENSG00000179172**	*0.027606307*	*0.035632536*	*0.034083719*	*0.036035508*
**ENSG00000198822**	*0.058343854*	*0.04298435*	*0.04107565*	*0.037522026*
**ENSG00000135250**	*0.022263611*	*0.03145936*	*0.030183264*	*0.023753173*
**ENSG00000110245**	*0.016011477*	*0.025174918*	*0.024649286*	*0.016118821*
**ENSG00000122729**	*0.041951381*	*0.048463572*	*0.040173029*	*0.036817569*
**ENSG00000184032**	*0.015104254*	*0.023634969*	*0.024078144*	*0.01439412*
**ENSG00000169018**	*0.021950174*	*0.027044773*	*0.028109075*	*0.01818562*
**ENSG00000152332**	*0.029288458*	*0.036764245*	*0.052880411*	*0.032083878*
**ENSG00000163394**	*0.015671879*	*0.0250803*	*0.024685562*	*0.015301065*
**ENSG00000160310**	*0.031683539*	*0.038124688*	*0.035518402*	*0.0333975*
**ENSG00000129244**	*0.040322004*	*0.029522452*	*0.033026091*	*0.030947288*
**ENSG00000121297**	*0.016609893*	*0.024878789*	*0.024746795*	*0.015166249*
**ENSG00000176476**	*0.025788191*	*0.029379046*	*0.027394921*	*0.024385518*
**ENSG00000080644**	*0.018223185*	*0.026151839*	*0.027984744*	*0.015227419*
**ENSG00000176679**	*0.015716195*	*0.026356841*	*0.026358911*	*0.016798854*
**ENSG00000101210**	*0.153478512*	*0.127188423*	*0.099423895*	*0.131062527*
**ENSG00000089101**	*0.015712825*	*0.025133922*	*0.024877829*	*0.014411088*
**ENSG00000169764**	*0.024829497*	*0.033087065*	*0.030419243*	*0.019719767*
**ENSG00000129465**	*0.024628378*	*0.028645805*	*0.028968915*	*0.023722556*
**ENSG00000198331**	*0.04424222*	*0.044401412*	*0.047055478*	*0.040983741*
**ENSG00000124787**	*0.044278597*	*0.034747601*	*0.038681358*	*0.043473002*
**ENSG00000163710**	*0.033174643*	*0.030029721*	*0.02706776*	*0.026408857*
**ENSG00000160213**	*0.034773732*	*0.036528861*	*0.03890627*	*0.031572364*
**ENSG00000016602**	*0.015623597*	*0.025737914*	*0.025587363*	*0.01606847*
**ENSG00000130598**	*0.016214675*	*0.026374513*	*0.025019465*	*0.016207391*
**ENSG00000175782**	*0.03142141*	*0.036924669*	*0.037384073*	*0.032191166*
**ENSG00000176198**	*0.016829104*	*0.02461686*	*0.024879992*	*0.015851535*
**ENSG00000011052**	*0.020837447*	*0.02734305*	*0.027053578*	*0.019964799*
**ENSG00000166762**	*0.029929801*	*0.030251352*	*0.03359215*	*0.026515133*
**ENSG00000164938**	*0.069849411*	*0.059472613*	*0.055759435*	*0.055917991*
**ENSG00000181856**	*0.0152801*	*0.023776641*	*0.024781052*	*0.015118889*
**ENSG00000183722**	*0.080600738*	*0.041759571*	*0.04928359*	*0.056503751*
**ENSG00000180861**	*0.015828421*	*0.024386044*	*0.024898075*	*0.01488225*
**ENSG00000157193**	*0.015508568*	*0.024420742*	*0.025124125*	*0.015117777*
**ENSG00000070018**	*0.016460157*	*0.026311435*	*0.026162052*	*0.01766893*
**ENSG00000198914**	*0.015337063*	*0.025369962*	*0.025491785*	*0.016716725*
**ENSG00000167283**	*0.03166661*	*0.031664459*	*0.033627711*	*0.030253967*
**ENSG00000175224**	*0.026184755*	*0.030385783*	*0.039814711*	*0.022397624*
**ENSG00000172469**	*0.045797212*	*0.043141751*	*0.037990373*	*0.040018103*

**ENSG00000104904**	*0.016433508*	*0.025174292*	*0.025245218*	*0.014669779*
**ENSG00000102924**	*0.018169908*	*0.027000472*	*0.026234923*	*0.017779314*
**ENSG00000132017**	*0.030720656*	*0.030227553*	*0.03454125*	*0.030308437*
**ENSG00000145192**	*0.014505357*	*0.024690595*	*0.025159165*	*0.016457821*
**ENSG00000109881**	*0.018708258*	*0.025768735*	*0.026214183*	*0.017804262*
**ENSG00000165457**	*0.045251107*	*0.040035243*	*0.040038666*	*0.03971136*
**ENSG00000188487**	*0.016707441*	*0.025187111*	*0.025812308*	*0.016248453*
**ENSG00000160439**	*0.031572202*	*0.02940023*	*0.036889287*	*0.023010852*
**ENSG00000023445**	*0.043122222*	*0.037396148*	*0.03598301*	*0.041238381*
**ENSG00000134597**	*0.017274496*	*0.027857235*	*0.025282343*	*0.017352558*
**ENSG00000213218**	*0.017584273*	*0.025111909*	*0.025300459*	*0.016380047*
**ENSG00000185069**	*0.015945721*	*0.025351907*	*0.024583442*	*0.014587995*
**ENSG00000185888**	*0.015375043*	*0.025753509*	*0.024342006*	*0.015405894*
**ENSG00000198081**	*0.032607226*	*0.039925298*	*0.037155451*	*0.028332104*
**ENSG00000064933**	*0.045847774*	*0.050031029*	*0.041229073*	*0.030619594*
**ENSG00000126562**	*0.015367847*	*0.024915922*	*0.024255252*	*0.014631104*
**ENSG00000182481**	*0.030914561*	*0.031840598*	*0.033126224*	*0.032972867*
**ENSG00000139725**	*0.030236123*	*0.035072158*	*0.034012544*	*0.032112382*
**ENSG00000107736**	*0.015809831*	*0.025647615*	*0.024405557*	*0.014670599*
**ENSG00000148677**	*0.017091524*	*0.025150179*	*0.024373807*	*0.016083127*
**ENSG00000133636**	*0.042932015*	*0.029890355*	*0.030107135*	*0.027024814*
**ENSG00000181467**	*0.017195485*	*0.025019567*	*0.024653214*	*0.015887674*
**ENSG00000159840**	*0.029869119*	*0.034105687*	*0.038602817*	*0.036995673*
**ENSG00000083535**	*0.020116294*	*0.030180692*	*0.026605416*	*0.03322132*
**ENSG00000116017**	*0.028932323*	*0.034150655*	*0.035911403*	*0.023363973*
**ENSG00000187806**	*0.015322514*	*0.02523222*	*0.024585724*	*0.015057969*
**ENSG00000105877**	*0.017181198*	*0.026613658*	*0.025743518*	*0.016693407*
**ENSG00000196074**	*0.032124995*	*0.034935542*	*0.036157382*	*0.031215099*
**ENSG00000158497**	*0.078251157*	*0.066265381*	*0.066491497*	*0.067637458*
**ENSG00000125629**	*0.054557243*	*0.05228055*	*0.055398452*	*0.047973773*
**ENSG00000099917**	*0.028367833*	*0.038057943*	*0.039204958*	*0.037601367*
**ENSG00000181982**	*0.018791781*	*0.025420877*	*0.026412565*	*0.01707498*
**ENSG00000168619**	*0.023167649*	*0.031522224*	*0.02896661*	*0.026619195*
**ENSG00000115257**	*0.02533544*	*0.029947425*	*0.028361872*	*0.024845509*
**ENSG00000144118**	*0.052378658*	*0.076515831*	*0.059396742*	*0.062128976*
**ENSG00000101346**	*0.020008011*	*0.027501807*	*0.026179623*	*0.01566467*
**ENSG00000145782**	*0.025879761*	*0.031125091*	*0.031548818*	*0.020663687*
**ENSG00000132141**	*0.039852585*	*0.042737106*	*0.039416706*	*0.044496322*
**ENSG00000101958**	*0.016227254*	*0.025065482*	*0.025458259*	*0.014709753*
**ENSG00000129873**	*0.015110067*	*0.025184046*	*0.025418435*	*0.015517434*
**ENSG00000175390**	*0.01648993*	*0.025537569*	*0.025136006*	*0.017131568*
**ENSG00000134698**	*0.0221992*	*0.028468255*	*0.031494071*	*0.023127053*
**ENSG00000186862**	*0.015330322*	*0.024417264*	*0.025244382*	*0.015379607*
**ENSG00000164251**	*0.024521899*	*0.025797276*	*0.02472624*	*0.017242448*
**ENSG00000090273**	*0.030025086*	*0.030914999*	*0.031383525*	*0.026269463*
**ENSG00000060656**	*0.014255676*	*0.024837949*	*0.024848364*	*0.014819167*
**ENSG00000136830**	*0.059750375*	*0.094949292*	*0.076253887*	*0.059621579*
**ENSG00000088882**	*0.12879588*	*0.074092282*	*0.068613714*	*0.09592604*
**ENSG00000057704**	*0.037581038*	*0.045396019*	*0.042782037*	*0.041477846*
**ENSG00000188483**	*0.016341234*	*0.026434161*	*0.02607364*	*0.017738635*
**ENSG00000161048**	*0.036189864*	*0.041437119*	*0.033174526*	*0.029098406*
**ENSG00000156030**	*0.027727109*	*0.038551156*	*0.036237056*	*0.020843743*
**ENSG00000002919**	*0.026242066*	*0.031540238*	*0.030730329*	*0.029218019*
**ENSG00000146457**	*0.015505867*	*0.025416255*	*0.025737085*	*0.015635058*

**ENSG00000188643**	*0.015296837*	*0.024724201*	*0.02452712*	*0.016460263*
**ENSG00000162645**	*0.029094575*	*0.037741582*	*0.03414568*	*0.031217757*
**ENSG00000188076**	*0.015383524*	*0.024981651*	*0.025761733*	*0.014194173*
**ENSG00000197409**	*0.058998326*	*0.04922568*	*0.049271857*	*0.053140735*
**ENSG00000086200**	*0.029912781*	*0.035985012*	*0.033986465*	*0.025110354*
**ENSG00000145734**	*0.01592845*	*0.026278309*	*0.025762246*	*0.017043327*
**ENSG00000116717**	*0.038902059*	*0.032533254*	*0.040116241*	*0.040189356*
**ENSG00000171115**	*0.073903063*	*0.066672404*	*0.067280392*	*0.075235178*
**ENSG00000196678**	*0.02213608*	*0.030326288*	*0.029342299*	*0.018591911*
**ENSG00000175175**	*0.03407066*	*0.025083691*	*0.025796947*	*0.019484551*
**ENSG00000174600**	*0.056125012*	*0.04433185*	*0.034665622*	*0.04557791*
**ENSG00000113141**	*0.024658209*	*0.032405376*	*0.030327649*	*0.019186042*
**ENSG00000118263**	*0.02025316*	*0.0282514*	*0.027589708*	*0.017858933*
**ENSG00000067208**	*0.038505962*	*0.039229584*	*0.036014808*	*0.045848821*
**ENSG00000151792**	*0.043733856*	*0.046590192*	*0.040862015*	*0.038773672*
**ENSG00000118762**	*0.040443363*	*0.041941969*	*0.042631794*	*0.036852907*
**ENSG00000072501**	*0.022545985*	*0.028928576*	*0.030007716*	*0.019058044*
**ENSG00000167526**	*0.032357544*	*0.036754204*	*0.032083825*	*0.031112226*
**ENSG00000107443**	*0.021489976*	*0.027973637*	*0.028307386*	*0.022499756*
**ENSG00000164610**	*0.028149079*	*0.030801433*	*0.030238763*	*0.023558001*
**ENSG00000116898**	*0.019264739*	*0.0268091*	*0.025895065*	*0.017196589*
**ENSG00000196358**	*0.018023994*	*0.027118394*	*0.027571634*	*0.021080756*
**ENSG00000177302**	*0.021124918*	*0.027918198*	*0.031366198*	*0.021902764*
**ENSG00000155980**	*0.016221498*	*0.024561462*	*0.025198624*	*0.015702868*
**ENSG00000186113**	*0.016694074*	*0.025730556*	*0.025592919*	*0.015333953*
**ENSG00000105976**	*0.015860227*	*0.024401259*	*0.025259642*	*0.015125915*
**ENSG00000065135**	*0.035913526*	*0.037836088*	*0.033115566*	*0.03587597*
**ENSG00000102109**	*0.02408058*	*0.025890038*	*0.025066429*	*0.017055823*
**ENSG00000105609**	*0.017371565*	*0.025574804*	*0.027311836*	*0.015797722*
**ENSG00000145425**	*0.01332499*	*0.023713021*	*0.023619595*	*0.014522328*
**ENSG00000108688**	*0.017788759*	*0.025523472*	*0.027500983*	*0.017524371*
**ENSG00000113732**	*0.017610031*	*0.028065817*	*0.027737179*	*0.016460818*
**ENSG00000131389**	*0.016363565*	*0.02615194*	*0.024687383*	*0.016057355*
**ENSG00000170871**	*0.03258974*	*0.029804489*	*0.034518877*	*0.032840797*
**ENSG00000171863**	*0.014536994*	*0.023769201*	*0.024030865*	*0.01575267*
**ENSG00000187939**	*0.017752695*	*0.024819875*	*0.02637407*	*0.015802026*
**ENSG00000144214**	*0.020537165*	*0.026856875*	*0.029427154*	*0.019950787*
**ENSG00000134070**	*0.018951705*	*0.027730902*	*0.026400121*	*0.02082864*
**ENSG00000124302**	*0.019830365*	*0.032603567*	*0.031416201*	*0.021121285*
**ENSG00000144381**	*0.015401835*	*0.025346575*	*0.024493758*	*0.015212446*
**ENSG00000104679**	*0.027182098*	*0.033876487*	*0.041347459*	*0.023393106*
**ENSG00000088325**	*0.04627831*	*0.036985206*	*0.037111884*	*0.031321899*
**ENSG00000178928**	*0.016043243*	*0.026294344*	*0.024723315*	*0.015427787*
**ENSG00000134987**	*0.029970542*	*0.031596036*	*0.029849588*	*0.035392763*
**ENSG00000054654**	*0.014378781*	*0.025119656*	*0.025286333*	*0.015828721*
**ENSG00000141564**	*0.025917063*	*0.035109615*	*0.037917759*	*0.029874064*
**ENSG00000137563**	*0.039817424*	*0.033336129*	*0.031657496*	*0.031172697*
**ENSG00000185829**	*0.030704373*	*0.027629718*	*0.02706115*	*0.018978557*
**ENSG00000180287**	*0.016652204*	*0.025405222*	*0.025523651*	*0.014831107*
**ENSG00000125454**	*0.032988844*	*0.034020543*	*0.036038591*	*0.027918622*
**ENSG00000120160**	*0.015509247*	*0.02501599*	*0.024940223*	*0.015370133*
**ENSG00000141447**	*0.014502917*	*0.025073957*	*0.024925985*	*0.015521729*
**ENSG00000035115**	*0.037650344*	*0.040960135*	*0.038845228*	*0.030776783*
**ENSG00000160233**	*0.015189557*	*0.024943021*	*0.02482851*	*0.014032766*

**ENSG00000119927**	*0.0261136*	*0.028738162*	*0.031638325*	*0.020246684*
**ENSG00000118193**	*0.049509764*	*0.040734783*	*0.037111647*	*0.037715001*
**ENSG00000157895**	*0.025276708*	*0.029691284*	*0.032465525*	*0.024069279*
**ENSG00000164889**	*0.025797233*	*0.032413589*	*0.032959953*	*0.028741501*
**ENSG00000170498**	*0.015547486*	*0.025214345*	*0.025107057*	*0.016016263*
**ENSG00000156697**	*0.028624964*	*0.032118647*	*0.036346485*	*0.032122379*
**ENSG00000133195**	*0.035022776*	*0.035577828*	*0.030803291*	*0.028990548*
**ENSG00000173171**	*0.023145963*	*0.027079474*	*0.030992866*	*0.022365226*
**ENSG00000113645**	*0.057037852*	*0.055010857*	*0.057552957*	*0.049923891*
**ENSG00000196562**	*0.087606513*	*0.062487213*	*0.063384183*	*0.061427276*
**ENSG00000213658**	*0.059406028*	*0.050336368*	*0.050109901*	*0.050613284*
**ENSG00000205560**	*0.041477887*	*0.050222998*	*0.042678687*	*0.039173697*
**ENSG00000188582**	*0.016184864*	*0.026082857*	*0.024998041*	*0.015743874*
**ENSG00000143515**	*0.021049527*	*0.02823156*	*0.027744803*	*0.020362449*
**ENSG00000143761**	*0.021145808*	*0.02694497*	*0.02824696*	*0.02003463*
**ENSG00000070404**	*0.054413923*	*0.040782573*	*0.038848873*	*0.056260138*
**ENSG00000179021**	*0.029233502*	*0.035300858*	*0.039042693*	*0.024876193*
**ENSG00000113966**	*0.016846412*	*0.024707666*	*0.026333633*	*0.016259446*
**ENSG00000118855**	*0.035234064*	*0.035368858*	*0.030722207*	*0.030144757*
**ENSG00000105369**	*0.037695371*	*0.035483554*	*0.038586426*	*0.035922172*
**ENSG00000107099**	*0.035937511*	*0.044006085*	*0.031699125*	*0.02889716*
**ENSG00000152443**	*0.029857958*	*0.03954695*	*0.037844561*	*0.032696808*
**ENSG00000104154**	*0.015187913*	*0.025067219*	*0.025105826*	*0.014950284*
**ENSG00000239732**	*0.014976589*	*0.025141294*	*0.024804915*	*0.015188309*
**ENSG00000015592**	*0.019448935*	*0.02492333*	*0.025514553*	*0.015178747*
**ENSG00000116747**	*0.027235824*	*0.030433794*	*0.031597392*	*0.021786049*
**ENSG00000144224**	*0.020688617*	*0.027719314*	*0.027500687*	*0.020791087*
**ENSG00000166046**	*0.01856019*	*0.029035845*	*0.026420358*	*0.019511696*
**ENSG00000108264**	*0.029661933*	*0.029330398*	*0.036067536*	*0.033191919*
**ENSG00000188269**	*0.017404021*	*0.024683422*	*0.02577883*	*0.015830279*
**ENSG00000134825**	*0.024234297*	*0.030289741*	*0.032699943*	*0.025950575*
**ENSG00000066422**	*0.030507567*	*0.035377467*	*0.030673618*	*0.036140095*
**ENSG00000198681**	*0.024351864*	*0.026245321*	*0.025674985*	*0.016243258*
**ENSG00000126903**	*0.029422549*	*0.032456141*	*0.040029797*	*0.023743257*
**ENSG00000154143**	*0.015970044*	*0.02576693*	*0.025424724*	*0.014858275*
**ENSG00000168056**	*0.027211478*	*0.030645662*	*0.039375282*	*0.022862924*
**ENSG00000105793**	*0.034862829*	*0.032289671*	*0.040846267*	*0.037203874*
**ENSG00000196329**	*0.131698218*	*0.103643774*	*0.095284756*	*0.117860348*
**ENSG00000169131**	*0.043104582*	*0.042414431*	*0.03963625*	*0.032393877*
**ENSG00000089682**	*0.029796378*	*0.032230468*	*0.030439717*	*0.022276133*
**ENSG00000087589**	*0.01582483*	*0.025298541*	*0.024695854*	*0.015320419*
**ENSG00000174132**	*0.028015883*	*0.033865709*	*0.031385078*	*0.024430289*
**ENSG00000172794**	*0.082026823*	*0.065543327*	*0.059014916*	*0.07322027*
**ENSG00000069998**	*0.042521323*	*0.038603198*	*0.035851422*	*0.034205616*
**ENSG00000187821**	*0.01455395*	*0.024791168*	*0.024855988*	*0.014373975*
**ENSG00000171509**	*0.015269937*	*0.024643067*	*0.024772538*	*0.015358165*
**ENSG00000112290**	*0.062161829*	*0.070656918*	*0.058360559*	*0.077115988*
**ENSG00000134202**	*0.03475589*	*0.035395506*	*0.031183368*	*0.035721326*
**ENSG00000164379**	*0.015515106*	*0.025830382*	*0.024651381*	*0.014653795*
**ENSG00000100985**	*0.040719713*	*0.033103428*	*0.033989196*	*0.059535638*
**ENSG00000149575**	*0.01466978*	*0.025140564*	*0.024904381*	*0.014415304*
**ENSG00000179331**	*0.015545546*	*0.024923925*	*0.024281776*	*0.016823654*
**ENSG00000009765**	*0.015792444*	*0.025268161*	*0.025650852*	*0.015959076*
**ENSG00000122140**	*0.025157018*	*0.028331371*	*0.034249113*	*0.0238001*

**ENSG00000141469**	*0.024319967*	*0.030750294*	*0.028955277*	*0.023746834*
**ENSG00000078018**	*0.016027538*	*0.025019312*	*0.023936281*	*0.0146636*
**ENSG00000092853**	*0.031259954*	*0.032993458*	*0.026708623*	*0.021115801*
**ENSG00000100558**	*0.01589597*	*0.025557054*	*0.025319239*	*0.01590476*
**ENSG00000164073**	*0.03249762*	*0.034937682*	*0.039429477*	*0.026172883*
**ENSG00000161956**	*0.023142663*	*0.030791959*	*0.031378503*	*0.024488001*
**ENSG00000188778**	*0.016429897*	*0.02574303*	*0.024813275*	*0.01554273*
**ENSG00000178761**	*0.026323191*	*0.036106939*	*0.035988223*	*0.026496111*
**ENSG00000178732**	*0.014833756*	*0.024327382*	*0.024293564*	*0.014686145*
**ENSG00000198589**	*0.026014107*	*0.033340325*	*0.031788729*	*0.022031527*
**ENSG00000106028**	*0.020589034*	*0.026422441*	*0.027533025*	*0.02134622*
**ENSG00000138821**	*0.035224154*	*0.042939557*	*0.036715406*	*0.030017413*
**ENSG00000036448**	*0.062331115*	*0.067178817*	*0.051059382*	*0.061693054*
**ENSG00000111886**	*0.015756658*	*0.025117283*	*0.025359247*	*0.015075429*
**ENSG00000178295**	*0.032589452*	*0.032313513*	*0.030420828*	*0.032049658*
**ENSG00000163536**	*0.04411412*	*0.041702635*	*0.0362893*	*0.034345611*
**ENSG00000172943**	*0.019570744*	*0.026394065*	*0.027186898*	*0.016591124*
**ENSG00000197272**	*0.016736424*	*0.023939672*	*0.024641985*	*0.01607997*
**ENSG00000114786**	*0.032810905*	*0.031706591*	*0.030712012*	*0.025367733*
**ENSG00000138594**	*0.024636989*	*0.031314139*	*0.030204988*	*0.021464652*
**ENSG00000174485**	*0.02996501*	*0.034229531*	*0.031822694*	*0.026202096*
**ENSG00000116213**	*0.033078929*	*0.033199523*	*0.034704686*	*0.031161603*
**ENSG00000095380**	*0.037292296*	*0.03216849*	*0.032767134*	*0.028832013*
**ENSG00000119915**	*0.014723492*	*0.024198004*	*0.025715048*	*0.01495461*
**ENSG00000174963**	*0.018470244*	*0.024525496*	*0.028220579*	*0.018403859*
**ENSG00000119684**	*0.016729381*	*0.02621197*	*0.026123269*	*0.017070284*
**ENSG00000151575**	*0.062200458*	*0.085780988*	*0.056462113*	*0.064544033*
**ENSG00000128245**	*0.02890858*	*0.031883245*	*0.030588347*	*0.026517186*
**ENSG00000170367**	*0.014099057*	*0.024837851*	*0.025149407*	*0.014051224*
**ENSG00000170903**	*0.030858123*	*0.036257615*	*0.03568956*	*0.030363844*
**ENSG00000159182**	*0.02662446*	*0.024534193*	*0.024558337*	*0.015174344*
**ENSG00000185046**	*0.016365257*	*0.024782242*	*0.025241533*	*0.01584637*
**ENSG00000079435**	*0.032039131*	*0.035954756*	*0.030596365*	*0.030810884*
**ENSG00000130487**	*0.017918375*	*0.026573556*	*0.025868776*	*0.017431563*
**ENSG00000131148**	*0.032543739*	*0.044027317*	*0.044162335*	*0.035249895*
**ENSG00000102302**	*0.016792555*	*0.025783462*	*0.024883633*	*0.016445115*
**ENSG00000166091**	*0.016899833*	*0.024200509*	*0.02689904*	*0.015734777*
**ENSG00000167930**	*0.029115206*	*0.041086773*	*0.039018541*	*0.034334159*
**ENSG00000234616**	*0.021088917*	*0.028775523*	*0.029382242*	*0.021087001*
**ENSG00000122420**	*0.015557629*	*0.024977533*	*0.025176023*	*0.014854736*
**ENSG00000243024**	*0.033500238*	*0.033186441*	*0.031700667*	*0.024149718*
**ENSG00000078596**	*0.051495477*	*0.039951973*	*0.045357078*	*0.046172417*
**ENSG00000120049**	*0.016944343*	*0.02531699*	*0.027248781*	*0.015982431*
**ENSG00000253910**	*0.016930969*	*0.025427093*	*0.025587397*	*0.016667756*
**ENSG00000013588**	*0.056492386*	*0.065190363*	*0.045179909*	*0.059148627*
**ENSG00000129990**	*0.01633265*	*0.024881672*	*0.025488842*	*0.016857416*
**ENSG00000244754**	*0.044493331*	*0.04550025*	*0.045964857*	*0.036523102*
**ENSG00000105738**	*0.018261506*	*0.026615191*	*0.026981561*	*0.021747381*
**ENSG00000215612**	*0.015489102*	*0.025009386*	*0.024980427*	*0.015212966*
**ENSG00000170927**	*0.015998118*	*0.024653621*	*0.025261287*	*0.015438616*
**ENSG00000174547**	*0.022571548*	*0.02726551*	*0.026789414*	*0.020151257*
**ENSG00000123338**	*0.025015782*	*0.029230924*	*0.029820358*	*0.021373701*
**ENSG00000129993**	*0.016068201*	*0.024919613*	*0.025990289*	*0.015233611*
**ENSG00000125843**	*0.031033757*	*0.03136254*	*0.037151451*	*0.034864186*

**ENSG00000054219**	*0.039137217*	*0.037297998*	*0.037176863*	*0.051449393*
**ENSG00000184905**	*0.158502044*	*0.132381762*	*0.122764044*	*0.130919481*
**ENSG00000158477**	*0.016041397*	*0.025011808*	*0.024766657*	*0.016811267*
**ENSG00000137726**	*0.035543272*	*0.03047923*	*0.028304752*	*0.021473583*
**ENSG00000152457**	*0.041959628*	*0.038480813*	*0.04270616*	*0.035130034*
**ENSG00000124145**	*0.055181938*	*0.048923197*	*0.043781011*	*0.052699826*
**ENSG00000149557**	*0.119851791*	*0.089890786*	*0.070022367*	*0.095149347*
**ENSG00000214595**	*0.01289763*	*0.023437364*	*0.023898508*	*0.012858465*
**ENSG00000112210**	*0.015722844*	*0.024942962*	*0.024773246*	*0.014817756*
**ENSG00000122224**	*0.089271784*	*0.081402645*	*0.061806964*	*0.067964799*
**ENSG00000028528**	*0.029921127*	*0.036667243*	*0.029981105*	*0.028367462*
**ENSG00000142552**	*0.035877844*	*0.040503218*	*0.049329183*	*0.035989263*
**ENSG00000125384**	*0.027608267*	*0.033717609*	*0.039282056*	*0.043862782*
**ENSG00000163818**	*0.050863941*	*0.044867513*	*0.034598972*	*0.049218746*
**ENSG00000241644**	*0.015861567*	*0.025267636*	*0.025671535*	*0.015748521*
**ENSG00000113594**	*0.014363587*	*0.024686574*	*0.025102642*	*0.014361653*
**ENSG00000189233**	*0.025331702*	*0.029345268*	*0.031202204*	*0.029531124*
**ENSG00000144554**	*0.021458728*	*0.02584403*	*0.027973337*	*0.017186596*
**ENSG00000203909**	*0.015527016*	*0.024623242*	*0.025405042*	*0.015016789*
**ENSG00000124104**	*0.019400452*	*0.028448553*	*0.025805208*	*0.016861092*
**ENSG00000140043**	*0.01759875*	*0.026206596*	*0.026462311*	*0.015679658*
**ENSG00000181023**	*0.017693561*	*0.023944016*	*0.023976609*	*0.016468253*
**ENSG00000135362**	*0.018481095*	*0.026012718*	*0.026758178*	*0.016008074*
**ENSG00000109180**	*0.025848909*	*0.029985164*	*0.029625056*	*0.034550116*
**ENSG00000168961**	*0.031051186*	*0.036206403*	*0.038603734*	*0.032765131*
**ENSG00000142700**	*0.015259161*	*0.025161146*	*0.024618793*	*0.015847971*
**ENSG00000100253**	*0.029719013*	*0.02749265*	*0.026098415*	*0.019741284*
**ENSG00000137513**	*0.030356892*	*0.035049287*	*0.030931123*	*0.024183298*
**ENSG00000186197**	*0.024155678*	*0.026114155*	*0.026724975*	*0.022031574*
**ENSG00000099958**	*0.076969476*	*0.047603268*	*0.053478183*	*0.060854888*
**ENSG00000134940**	*0.016399884*	*0.025282253*	*0.024186214*	*0.015943996*
**ENSG00000133107**	*0.015676281*	*0.025109243*	*0.024896069*	*0.014694895*
**ENSG00000163171**	*0.040050485*	*0.028579559*	*0.042455917*	*0.026043531*
**ENSG00000196655**	*0.03322302*	*0.03311469*	*0.03043936*	*0.02400882*
**ENSG00000187726**	*0.019869129*	*0.027039107*	*0.025664037*	*0.019038053*
**ENSG00000149541**	*0.022474498*	*0.029809131*	*0.034261991*	*0.024573942*
**ENSG00000197265**	*0.02627881*	*0.031192462*	*0.029813075*	*0.020932355*
**ENSG00000163798**	*0.02634648*	*0.030962616*	*0.03163995*	*0.021147859*
**ENSG00000156875**	*0.025670464*	*0.032970921*	*0.031363008*	*0.024678169*
**ENSG00000132321**	*0.016862762*	*0.025174188*	*0.02521805*	*0.016637434*
**ENSG00000172766**	*0.028763473*	*0.042566696*	*0.045679961*	*0.034919097*
**ENSG00000101986**	*0.017133291*	*0.027265571*	*0.025814391*	*0.017752901*
**ENSG00000172146**	*0.015804858*	*0.024788147*	*0.02678034*	*0.018423343*
**ENSG00000127249**	*0.015650388*	*0.025169763*	*0.024619282*	*0.014974648*
**ENSG00000182518**	*0.024810999*	*0.029158093*	*0.027771008*	*0.020139108*
**ENSG00000006327**	*0.033048024*	*0.029566369*	*0.027786736*	*0.026791362*
**ENSG00000213626**	*0.073435349*	*0.062294197*	*0.054278383*	*0.056644078*
**ENSG00000143006**	*0.016265474*	*0.024933241*	*0.025670443*	*0.014807287*
**ENSG00000125630**	*0.044286221*	*0.041076429*	*0.035537556*	*0.033413908*
**ENSG00000163006**	*0.036657899*	*0.038157527*	*0.033818924*	*0.037503987*
**ENSG00000007376**	*0.035215205*	*0.031548875*	*0.044180819*	*0.040782042*
**ENSG00000166123**	*0.071468264*	*0.044006839*	*0.043457032*	*0.046962533*
**ENSG00000136897**	*0.027050374*	*0.033914578*	*0.035067075*	*0.021124138*
**ENSG00000160877**	*0.017224721*	*0.025635015*	*0.025702801*	*0.016148087*

**ENSG00000133935**	*0.017273696*	*0.026804784*	*0.027120141*	*0.015395832*
**ENSG00000213759**	*0.128478695*	*0.191652615*	*0.179222249*	*0.105697396*
**ENSG00000185220**	*0.038448292*	*0.036489456*	*0.034926732*	*0.032314984*
**ENSG00000088387**	*0.042587798*	*0.038553619*	*0.032328511*	*0.027132846*
**ENSG00000106635**	*0.029872811*	*0.04196074*	*0.045996393*	*0.029365418*
**ENSG00000120948**	*0.031345979*	*0.03469331*	*0.032594271*	*0.023369522*
**ENSG00000105997**	*0.016338378*	*0.025174282*	*0.024931527*	*0.015547113*
**ENSG00000198039**	*0.018053909*	*0.025044508*	*0.02532244*	*0.015802543*
**ENSG00000090905**	*0.026293287*	*0.032531627*	*0.034385728*	*0.021666101*
**ENSG00000112159**	*0.019342811*	*0.028435343*	*0.026103639*	*0.017435074*
**ENSG00000143742**	*0.020155138*	*0.028066555*	*0.026465192*	*0.019578722*
**ENSG00000105352**	*0.015489436*	*0.02518774*	*0.025383778*	*0.015467068*
**ENSG00000160471**	*0.015415337*	*0.025871874*	*0.02445192*	*0.016338865*
**ENSG00000064225**	*0.113677836*	*0.100841214*	*0.056992832*	*0.098026917*
**ENSG00000129195**	*0.083524267*	*0.054395769*	*0.051651875*	*0.065810078*
**ENSG00000166619**	*0.027924486*	*0.033647072*	*0.0337373*	*0.02826826*
**ENSG00000113387**	*0.034100317*	*0.03716729*	*0.03371568*	*0.03643354*
**ENSG00000120699**	*0.02928361*	*0.031003252*	*0.028276173*	*0.021831416*
**ENSG00000155957**	*0.028269407*	*0.029611666*	*0.030876826*	*0.024671047*
**ENSG00000132692**	*0.029850703*	*0.045908705*	*0.033275948*	*0.023511484*
**ENSG00000131398**	*0.015604258*	*0.024537602*	*0.024497698*	*0.014894658*
**ENSG00000170370**	*0.014755973*	*0.025857236*	*0.024559769*	*0.015046*
**ENSG00000158435**	*0.0288615*	*0.03766567*	*0.03151151*	*0.030101844*
**ENSG00000170043**	*0.022463214*	*0.031372849*	*0.03001353*	*0.026108178*
**ENSG00000078804**	*0.038851618*	*0.057516552*	*0.046303776*	*0.036941741*
**ENSG00000198768**	*0.016213245*	*0.024925778*	*0.024777473*	*0.015407008*
**ENSG00000187736**	*0.016295521*	*0.025453495*	*0.025458517*	*0.015900464*
**ENSG00000149257**	*0.040097417*	*0.03735564*	*0.035622535*	*0.038873395*
**ENSG00000131504**	*0.023817291*	*0.030023173*	*0.031167378*	*0.024116295*
**ENSG00000221813**	*0.018028328*	*0.026301197*	*0.026211002*	*0.019373691*
**ENSG00000144810**	*0.020956552*	*0.024445621*	*0.024888826*	*0.01714138*
**ENSG00000160013**	*0.029801863*	*0.033954145*	*0.033516082*	*0.030752367*
**ENSG00000081307**	*0.018020572*	*0.025937672*	*0.025902748*	*0.017531114*
**ENSG00000177096**	*0.029025374*	*0.030558773*	*0.031603183*	*0.025505911*
**ENSG00000167380**	*0.042919133*	*0.037742595*	*0.041236184*	*0.036336556*
**ENSG00000101849**	*0.052751993*	*0.060726569*	*0.045266831*	*0.047179887*
**ENSG00000128602**	*0.047136695*	*0.046578039*	*0.036471356*	*0.039632125*
**ENSG00000136925**	*0.023629572*	*0.029382712*	*0.026933243*	*0.019986036*
**ENSG00000006283**	*0.015517635*	*0.024850234*	*0.024471558*	*0.014258555*
**ENSG00000135919**	*0.05033154*	*0.058919674*	*0.056159678*	*0.06057798*
**ENSG00000106780**	*0.042211401*	*0.056037678*	*0.038184501*	*0.042242339*
**ENSG00000120925**	*0.025640477*	*0.032266091*	*0.028782442*	*0.026878289*
**ENSG00000204962**	*0.014954851*	*0.025448943*	*0.025101832*	*0.014884887*
**ENSG00000159403**	*0.017741372*	*0.026189987*	*0.028048532*	*0.018792935*
**ENSG00000141367**	*0.024957546*	*0.031953068*	*0.027007273*	*0.028654536*
**ENSG00000182450**	*0.014805413*	*0.024911686*	*0.024831178*	*0.015747466*
**ENSG00000134452**	*0.032319185*	*0.033702817*	*0.031641205*	*0.027903382*
**ENSG00000111404**	*0.016819302*	*0.025124181*	*0.024259211*	*0.015245454*
**ENSG00000167633**	*0.015136723*	*0.025227287*	*0.024683507*	*0.015488441*
**ENSG00000186432**	*0.03083622*	*0.032862421*	*0.032812187*	*0.030801579*
**ENSG00000107954**	*0.015374522*	*0.025578166*	*0.024719876*	*0.015301793*
**ENSG00000167244**	*0.015514512*	*0.024883549*	*0.025490088*	*0.015657401*
**ENSG00000131015**	*0.021567894*	*0.03076143*	*0.029869145*	*0.015103334*
**ENSG00000166947**	*0.016105769*	*0.025379058*	*0.02451265*	*0.015157897*

**ENSG00000213901**	*0.030883765*	*0.031466561*	*0.026634685*	*0.026738205*
**ENSG00000154274**	*0.02311675*	*0.029306825*	*0.029224513*	*0.028530859*
**ENSG00000184497**	*0.021186323*	*0.026575702*	*0.027651013*	*0.016992153*
**ENSG00000130748**	*0.028779385*	*0.029155784*	*0.028199101*	*0.023107144*
**ENSG00000129128**	*0.041551408*	*0.040009956*	*0.045746476*	*0.039133635*
**ENSG00000183196**	*0.026615306*	*0.03263635*	*0.029963119*	*0.022668957*
**ENSG00000159267**	*0.01850803*	*0.027407612*	*0.025938129*	*0.017511071*
**ENSG00000117394**	*0.044241232*	*0.05261246*	*0.043249089*	*0.048458795*
**ENSG00000124333**	*0.022017406*	*0.037466486*	*0.030759691*	*0.026208404*
**ENSG00000016402**	*0.017074316*	*0.025384462*	*0.025133263*	*0.015991404*
**ENSG00000231322**	*0.015430632*	*0.025330607*	*0.024508159*	*0.014381236*
**ENSG00000008197**	*0.015121263*	*0.025193747*	*0.025727397*	*0.015731641*
**ENSG00000205362**	*0.015441772*	*0.025255602*	*0.025318063*	*0.016116437*
**ENSG00000169967**	*0.031922614*	*0.035532664*	*0.033733796*	*0.030939323*
**ENSG00000163131**	*0.04087321*	*0.032669185*	*0.037395252*	*0.029990473*
**ENSG00000132429**	*0.016588417*	*0.024870951*	*0.024781726*	*0.015521121*
**ENSG00000141298**	*0.035779584*	*0.037880024*	*0.031709318*	*0.031324358*
**ENSG00000139151**	*0.016088074*	*0.02488095*	*0.025044128*	*0.01689191*
**ENSG00000165702**	*0.018239207*	*0.026254598*	*0.024992859*	*0.017581481*
**ENSG00000182872**	*0.023321536*	*0.027985536*	*0.030194549*	*0.022319605*
**ENSG00000168259**	*0.02299336*	*0.029134335*	*0.029357098*	*0.021562259*
**ENSG00000099864**	*0.062788751*	*0.031779122*	*0.027913149*	*0.026488539*
**ENSG00000152464**	*0.025770509*	*0.033913461*	*0.037027026*	*0.02510541*
**ENSG00000213015**	*0.030504041*	*0.036640787*	*0.043011634*	*0.032618854*
**ENSG00000049245**	*0.031027988*	*0.037139489*	*0.034327266*	*0.033945437*
**ENSG00000124688**	*0.023577781*	*0.033867696*	*0.036223317*	*0.021617071*
**ENSG00000183624**	*0.048907201*	*0.041266959*	*0.038064115*	*0.028924316*
**ENSG00000109743**	*0.01763999*	*0.025483811*	*0.02751463*	*0.018497267*
**ENSG00000165527**	*0.03901999*	*0.047225774*	*0.045962234*	*0.055285536*
**ENSG00000126500**	*0.015503744*	*0.025801643*	*0.024703386*	*0.01442639*
**ENSG00000071282**	*0.109094375*	*0.089146443*	*0.075833832*	*0.092483663*
**ENSG00000142089**	*0.060987034*	*0.051144535*	*0.057870623*	*0.053093079*
**ENSG00000031544**	*0.015442623*	*0.024942992*	*0.024474776*	*0.0149384*
**ENSG00000250834**	*0.022651587*	*0.026429002*	*0.028990045*	*0.020589575*
**ENSG00000221937**	*0.018192203*	*0.026217569*	*0.027821869*	*0.01781491*
**ENSG00000243709**	*0.019619472*	*0.026809527*	*0.026038394*	*0.016180035*
**ENSG00000134250**	*0.033377796*	*0.028045145*	*0.029790547*	*0.02822442*
**ENSG00000166111**	*0.01405063*	*0.025045128*	*0.024966106*	*0.014517541*
**ENSG00000116824**	*0.059182091*	*0.063209306*	*0.026774475*	*0.027442766*
**ENSG00000159579**	*0.02903393*	*0.035619557*	*0.04076421*	*0.023757082*
**ENSG00000107338**	*0.034020298*	*0.03797472*	*0.033917842*	*0.028241699*
**ENSG00000153560**	*0.028022956*	*0.035239841*	*0.036272052*	*0.024763191*
**ENSG00000135314**	*0.016221021*	*0.025001433*	*0.025889878*	*0.016985951*
**ENSG00000011260**	*0.032423127*	*0.038957061*	*0.03126044*	*0.030564125*
**ENSG00000161011**	*0.077492004*	*0.067665975*	*0.054928729*	*0.052263029*
**ENSG00000106070**	*0.01697243*	*0.02665599*	*0.026942173*	*0.014815497*
**ENSG00000135697**	*0.062198662*	*0.078711283*	*0.072910931*	*0.085392676*
**ENSG00000145390**	*0.017349758*	*0.027069762*	*0.025962263*	*0.017751869*
**ENSG00000241370**	*0.024704999*	*0.028406654*	*0.030524138*	*0.023299878*
**ENSG00000123607**	*0.016087648*	*0.024883057*	*0.024209726*	*0.015353037*
**ENSG00000167965**	*0.025812319*	*0.028431857*	*0.032654167*	*0.028589803*
**ENSG00000167191**	*0.025030146*	*0.027358028*	*0.028477473*	*0.024970277*
**ENSG00000139182**	*0.016922818*	*0.027686136*	*0.025826917*	*0.020184668*
**ENSG00000089169**	*0.016975905*	*0.027394951*	*0.02649864*	*0.016302475*

**ENSG00000187796**	*0.119011261*	*0.07837556*	*0.075407995*	*0.080095032*
**ENSG00000179477**	*0.016497784*	*0.025648055*	*0.025185398*	*0.016199464*
**ENSG00000053371**	*0.032522381*	*0.036973627*	*0.036298222*	*0.032792912*
**ENSG00000166278**	*0.016540483*	*0.02630238*	*0.025732123*	*0.01698829*
**ENSG00000175322**	*0.040394476*	*0.041474426*	*0.036408947*	*0.041765271*
**ENSG00000170296**	*0.018536417*	*0.026109582*	*0.028401915*	*0.018833539*
**ENSG00000164663**	*0.015212608*	*0.025187287*	*0.025565992*	*0.015659722*
**ENSG00000170128**	*0.030674228*	*0.028530061*	*0.026877683*	*0.016843346*
**ENSG00000106541**	*0.015533565*	*0.024414021*	*0.025267309*	*0.014057696*
**ENSG00000158186**	*0.019832454*	*0.026226301*	*0.024993477*	*0.017805211*
**ENSG00000184007**	*0.032507101*	*0.034648947*	*0.033454006*	*0.032566622*
**ENSG00000122378**	*0.027766084*	*0.033535535*	*0.033968954*	*0.029792801*
**ENSG00000212901**	*0.015957823*	*0.025707054*	*0.024644837*	*0.014587194*
**ENSG00000154642**	*0.038896796*	*0.046428001*	*0.060953636*	*0.042174976*
**ENSG00000122824**	*0.019232519*	*0.025336052*	*0.025517162*	*0.016789197*
**ENSG00000104332**	*0.071087748*	*0.040243536*	*0.039865846*	*0.056530407*
**ENSG00000242221**	*0.015798976*	*0.025675244*	*0.025079283*	*0.015669967*
**ENSG00000184586**	*0.016650534*	*0.026048936*	*0.025850572*	*0.016881264*
**ENSG00000127084**	*0.037967385*	*0.035853149*	*0.035509698*	*0.03477696*
**ENSG00000164597**	*0.029389184*	*0.031549205*	*0.030550006*	*0.02746588*
**ENSG00000122335**	*0.019602302*	*0.027093068*	*0.026452977*	*0.017466417*
**ENSG00000005381**	*0.018416363*	*0.027169311*	*0.026512768*	*0.017361721*
**ENSG00000125449**	*0.034275721*	*0.037670411*	*0.045680365*	*0.032387817*
**ENSG00000223380**	*0.023014823*	*0.029771734*	*0.031669979*	*0.023210422*
**ENSG00000124570**	*0.091992312*	*0.079412142*	*0.055331334*	*0.092592096*
**ENSG00000116922**	*0.033745059*	*0.036755076*	*0.034267291*	*0.033879726*
**ENSG00000198729**	*0.018021092*	*0.027027755*	*0.026696918*	*0.016813646*
**ENSG00000188313**	*0.061742678*	*0.054381767*	*0.055334566*	*0.051088486*
**ENSG00000101323**	*0.017759191*	*0.026600511*	*0.026290943*	*0.01623809*
**ENSG00000175348**	*0.027171659*	*0.037364844*	*0.039354202*	*0.028233768*
**ENSG00000184611**	*0.016831718*	*0.024996438*	*0.02519532*	*0.016673999*
**ENSG00000106571**	*0.015520162*	*0.024557484*	*0.025683239*	*0.014673068*
**ENSG00000156261**	*0.020919281*	*0.027933521*	*0.027034192*	*0.021177013*
**ENSG00000179083**	*0.022302401*	*0.026083783*	*0.026568328*	*0.017301784*
**ENSG00000236613**	*0.016213428*	*0.026020531*	*0.025104942*	*0.015022331*
**ENSG00000107581**	*0.025323484*	*0.031047914*	*0.029242189*	*0.023895232*
**ENSG00000176381**	*0.104669209*	*0.108171904*	*0.093613349*	*0.102178036*
**ENSG00000168509**	*0.037089198*	*0.038731734*	*0.028870718*	*0.023147394*
**ENSG00000111666**	*0.066336202*	*0.063885857*	*0.05806083*	*0.049423231*
**ENSG00000204611**	*0.031251847*	*0.039196826*	*0.031537805*	*0.031342345*
**ENSG00000100296**	*0.015012932*	*0.025349677*	*0.025262662*	*0.017329152*
**ENSG00000110619**	*0.032810808*	*0.030619199*	*0.035344752*	*0.029066242*
**ENSG00000159335**	*0.043914201*	*0.042812131*	*0.047648925*	*0.048410648*
**ENSG00000096996**	*0.018180578*	*0.027714632*	*0.026504176*	*0.018482814*
**ENSG00000085872**	*0.027675847*	*0.033557132*	*0.039425913*	*0.028744372*
**ENSG00000154864**	*0.142268912*	*0.132347972*	*0.106725611*	*0.11456401*
**ENSG00000013503**	*0.03924195*	*0.039185724*	*0.03571196*	*0.043683514*
**ENSG00000100147**	*0.023622745*	*0.02898508*	*0.026302575*	*0.019567101*
**ENSG00000117226**	*0.068123209*	*0.062504667*	*0.062408705*	*0.04526541*
**ENSG00000118508**	*0.07845356*	*0.032135531*	*0.030119968*	*0.033666896*
**ENSG00000139323**	*0.03661468*	*0.034643965*	*0.032562973*	*0.029878016*
**ENSG00000196963**	*0.017899955*	*0.025320506*	*0.025531997*	*0.016488241*
**ENSG00000109158**	*0.015348574*	*0.024780487*	*0.024612801*	*0.014988103*
**ENSG00000164742**	*0.112309621*	*0.049809477*	*0.042502676*	*0.060922548*

**ENSG00000177508**	*0.016597255*	*0.025021857*	*0.02506307*	*0.015469068*
**ENSG00000102962**	*0.06543253*	*0.044098851*	*0.042214974*	*0.052849672*
**ENSG00000155287**	*0.024360756*	*0.030219757*	*0.033604409*	*0.02317996*
**ENSG00000196966**	*0.031202309*	*0.037449338*	*0.038680565*	*0.039538567*
**ENSG00000125148**	*0.067425349*	*0.063500302*	*0.06463028*	*0.068188705*
**ENSG00000172208**	*0.017080567*	*0.025211737*	*0.024277238*	*0.015082789*
**ENSG00000122965**	*0.036928628*	*0.034955083*	*0.035703254*	*0.036545785*
**ENSG00000184060**	*0.073844608*	*0.068523777*	*0.067030186*	*0.054515643*
**ENSG00000106025**	*0.074863327*	*0.055374495*	*0.055937752*	*0.053526197*
**ENSG00000130724**	*0.019047117*	*0.026701722*	*0.027937666*	*0.017056911*
**ENSG00000186193**	*0.079793829*	*0.055365927*	*0.0509357*	*0.061113093*
**ENSG00000167900**	*0.049088684*	*0.042362625*	*0.034862022*	*0.03338484*
**ENSG00000152601**	*0.033214784*	*0.035589954*	*0.034443747*	*0.024202369*
**ENSG00000156968**	*0.015899236*	*0.025378431*	*0.024836223*	*0.015053068*
**ENSG00000158805**	*0.022350694*	*0.028640521*	*0.027815435*	*0.021339681*
**ENSG00000185149**	*0.016651807*	*0.026070955*	*0.024597278*	*0.016092475*
**ENSG00000136247**	*0.017050575*	*0.02596043*	*0.02526757*	*0.016891832*
**ENSG00000135740**	*0.060938814*	*0.048554141*	*0.042409521*	*0.034691062*
**ENSG00000198901**	*0.037741313*	*0.030607115*	*0.032991424*	*0.027192426*
**ENSG00000137968**	*0.017631725*	*0.024551522*	*0.024347492*	*0.015695778*
**ENSG00000108671**	*0.025122161*	*0.030246194*	*0.028816399*	*0.020722655*
**ENSG00000105374**	*0.029391084*	*0.038124811*	*0.030698511*	*0.043919475*
**ENSG00000025039**	*0.101588668*	*0.0663478*	*0.055849213*	*0.07686704*
**ENSG00000183765**	*0.02865857*	*0.035414376*	*0.029011201*	*0.03821018*
**ENSG00000016082**	*0.01779934*	*0.025513646*	*0.024784764*	*0.016041802*
**ENSG00000118513**	*0.04956336*	*0.056698576*	*0.047584257*	*0.049813364*
**ENSG00000132437**	*0.021781556*	*0.025650426*	*0.02595374*	*0.016494006*
**ENSG00000007312**	*0.056951742*	*0.053968701*	*0.050444377*	*0.041575338*
**ENSG00000184330**	*0.015502607*	*0.02516881*	*0.025822618*	*0.016132215*
**ENSG00000102230**	*0.018386527*	*0.026734954*	*0.025339867*	*0.019827334*
**ENSG00000185070**	*0.01538586*	*0.024552495*	*0.02568977*	*0.015595237*
**ENSG00000163466**	*0.021430018*	*0.030345465*	*0.028755*	*0.02312261*
**ENSG00000124449**	*0.014600623*	*0.024782129*	*0.024619374*	*0.013787355*
**ENSG00000116147**	*0.014456133*	*0.024410877*	*0.024717911*	*0.013827689*
**ENSG00000160193**	*0.046835352*	*0.044241007*	*0.036539905*	*0.040605288*
**ENSG00000124103**	*0.016875412*	*0.027883273*	*0.025156552*	*0.015519771*
**ENSG00000136436**	*0.031689652*	*0.034278416*	*0.032210834*	*0.034917*
**ENSG00000100711**	*0.032022028*	*0.039260086*	*0.033048767*	*0.031128374*
**ENSG00000180138**	*0.014785388*	*0.024961762*	*0.025742702*	*0.015991187*
**ENSG00000107807**	*0.016890186*	*0.026384993*	*0.024937746*	*0.016830071*
**ENSG00000123989**	*0.0465446*	*0.04257507*	*0.046114259*	*0.046835017*
**ENSG00000167778**	*0.028190775*	*0.031230527*	*0.03515972*	*0.031446114*
**ENSG00000109272**	*0.016484124*	*0.026679775*	*0.025948374*	*0.015972986*
**ENSG00000179636**	*0.017029405*	*0.025085641*	*0.025171907*	*0.015683583*
**ENSG00000082126**	*0.016511154*	*0.025016812*	*0.025319869*	*0.015772867*
**ENSG00000214900**	*0.022670206*	*0.029527604*	*0.027975761*	*0.019488929*
**ENSG00000143466**	*0.029564871*	*0.036493447*	*0.029583672*	*0.028745651*
**ENSG00000186834**	*0.042086396*	*0.047577919*	*0.053098492*	*0.041534101*
**ENSG00000142920**	*0.017322693*	*0.025635167*	*0.024682891*	*0.017393149*
**ENSG00000085552**	*0.018619223*	*0.026191897*	*0.025692966*	*0.01502194*
**ENSG00000130768**	*0.018073201*	*0.026630945*	*0.02623604*	*0.020459351*
**ENSG00000179455**	*0.017596147*	*0.024711417*	*0.024837217*	*0.016140142*
**ENSG00000103343**	*0.019181985*	*0.027358695*	*0.027782271*	*0.0176485*
**ENSG00000174004**	*0.044445127*	*0.052529904*	*0.056851406*	*0.044721508*

**ENSG00000188807**	*0.024678474*	*0.032835919*	*0.031988301*	*0.026684855*
**ENSG00000108753**	*0.129571205*	*0.065543745*	*0.071577233*	*0.096812323*
**ENSG00000115738**	*0.069361744*	*0.06667176*	*0.062153326*	*0.067885624*
**ENSG00000071626**	*0.017446934*	*0.026072893*	*0.026369966*	*0.018833795*
**ENSG00000122971**	*0.029517348*	*0.036800142*	*0.031926985*	*0.027029847*
**ENSG00000088827**	*0.015262805*	*0.025623837*	*0.025063691*	*0.015671153*
**ENSG00000123353**	*0.024210834*	*0.029578352*	*0.027570299*	*0.022079617*
**ENSG00000048405**	*0.033255139*	*0.047538945*	*0.05240847*	*0.03804811*
**ENSG00000237172**	*0.039766199*	*0.041175381*	*0.04671397*	*0.044121996*
**ENSG00000101076**	*0.016145789*	*0.025135214*	*0.02616549*	*0.016739128*
**ENSG00000139190**	*0.018028548*	*0.025924418*	*0.025758626*	*0.016012685*
**ENSG00000121406**	*0.030109289*	*0.031466648*	*0.031850782*	*0.028118909*
**ENSG00000115419**	*0.040180699*	*0.044702631*	*0.036366074*	*0.034619961*
**ENSG00000100565**	*0.018294526*	*0.026554933*	*0.025561893*	*0.015569802*
**ENSG00000185485**	*0.024030055*	*0.03432241*	*0.031020729*	*0.028984324*
**ENSG00000130175**	*0.024451041*	*0.032916071*	*0.035839355*	*0.026564179*
**ENSG00000106397**	*0.031337301*	*0.034634985*	*0.032029185*	*0.031372893*
**ENSG00000244462**	*0.037741464*	*0.039759816*	*0.037191077*	*0.029190219*
**ENSG00000170191**	*0.015051984*	*0.024914534*	*0.025312531*	*0.015595992*
**ENSG00000101084**	*0.026068557*	*0.030513725*	*0.031732663*	*0.02664332*
**ENSG00000099365**	*0.016309672*	*0.024017031*	*0.024948293*	*0.015055325*
**ENSG00000105364**	*0.019145026*	*0.029646956*	*0.027007057*	*0.018197907*
**ENSG00000173992**	*0.029502248*	*0.030211796*	*0.033317104*	*0.031645671*
**ENSG00000136122**	*0.037383049*	*0.03605911*	*0.037079803*	*0.031019681*
**ENSG00000185962**	*0.020147365*	*0.027616438*	*0.030559411*	*0.018843616*
**ENSG00000115295**	*0.029255001*	*0.034924289*	*0.030866318*	*0.024661311*
**ENSG00000132199**	*0.043382178*	*0.050587484*	*0.04712897*	*0.036342342*
**ENSG00000103653**	*0.031314529*	*0.031572691*	*0.031584655*	*0.02599557*
**ENSG00000070614**	*0.01584431*	*0.025319181*	*0.025644383*	*0.015424039*
**ENSG00000226372**	*0.018871079*	*0.026635888*	*0.025930497*	*0.017555486*
**ENSG00000164604**	*0.015808704*	*0.024405657*	*0.024411964*	*0.013740736*
**ENSG00000187815**	*0.030531144*	*0.038531132*	*0.039029183*	*0.030867886*
**ENSG00000151116**	*0.033997659*	*0.044653424*	*0.037887603*	*0.039333743*
**ENSG00000169057**	*0.020667738*	*0.030064894*	*0.02991229*	*0.021431178*
**ENSG00000162599**	*0.024558592*	*0.024874127*	*0.025666745*	*0.016109917*
**ENSG00000173175**	*0.016996741*	*0.026335752*	*0.025353857*	*0.028607786*
**ENSG00000205730**	*0.018980316*	*0.028156956*	*0.028492274*	*0.018350776*
**ENSG00000163209**	*0.015812253*	*0.024537403*	*0.025071985*	*0.015877264*
**ENSG00000088320**	*0.015462545*	*0.025282282*	*0.02422588*	*0.015539827*
**ENSG00000167513**	*0.04496506*	*0.045272202*	*0.040545089*	*0.041633224*
**ENSG00000102218**	*0.015457142*	*0.024670208*	*0.024800113*	*0.015813291*
**ENSG00000130299**	*0.015989173*	*0.024802792*	*0.024356761*	*0.014285545*
**ENSG00000144648**	*0.015397908*	*0.025773672*	*0.024501874*	*0.014877911*
**ENSG00000169896**	*0.081707003*	*0.066650528*	*0.061163119*	*0.063734015*
**ENSG00000066336**	*0.033223792*	*0.037838479*	*0.041322006*	*0.041535021*
**ENSG00000189056**	*0.01654397*	*0.024678661*	*0.024796338*	*0.015434224*
**ENSG00000139767**	*0.016066115*	*0.025167745*	*0.025006317*	*0.015720585*
**ENSG00000126251**	*0.015920708*	*0.025615265*	*0.024549746*	*0.014886755*
**ENSG00000160882**	*0.014818905*	*0.025626247*	*0.024725115*	*0.015672696*
**ENSG00000172201**	*0.016455448*	*0.025079035*	*0.026040275*	*0.015766074*
**ENSG00000196975**	*0.039786215*	*0.043117146*	*0.039409406*	*0.040219122*
**ENSG00000035403**	*0.065292497*	*0.051370245*	*0.058421116*	*0.059512272*
**ENSG00000168329**	*0.016996254*	*0.025424844*	*0.026524511*	*0.015728544*
**ENSG00000175065**	*0.017041812*	*0.02738195*	*0.027082725*	*0.017886463*

**ENSG00000063438**	*0.026983202*	*0.034102635*	*0.032377009*	*0.030031884*
**ENSG00000055813**	*0.044342552*	*0.0247405*	*0.024904319*	*0.016183201*
**ENSG00000110700**	*0.015921497*	*0.024054427*	*0.024348545*	*0.016357486*
**ENSG00000173221**	*0.036132527*	*0.04621873*	*0.037658654*	*0.035611405*
**ENSG00000163935**	*0.029054001*	*0.033384136*	*0.030759033*	*0.026570571*
**ENSG00000111335**	*0.052892187*	*0.054386637*	*0.042373165*	*0.04104099*
**ENSG00000213585**	*0.02090388*	*0.027649194*	*0.026233513*	*0.020143082*
**ENSG00000164347**	*0.015285586*	*0.025755602*	*0.025514321*	*0.014998237*
**ENSG00000159792**	*0.035103452*	*0.033100858*	*0.032251373*	*0.028675211*
**ENSG00000084764**	*0.027531379*	*0.02774944*	*0.025799864*	*0.024487287*
**ENSG00000185650**	*0.024454581*	*0.031793523*	*0.032858426*	*0.024456761*
**ENSG00000143458**	*0.027626443*	*0.030459816*	*0.029221944*	*0.028788364*
**ENSG00000197405**	*0.144033685*	*0.098376467*	*0.062942529*	*0.08103761*
**ENSG00000231924**	*0.017905657*	*0.025220195*	*0.024390624*	*0.016588461*
**ENSG00000170949**	*0.023934106*	*0.034131495*	*0.031821959*	*0.021526645*
**ENSG00000061987**	*0.018115193*	*0.027075285*	*0.026546292*	*0.01712984*
**ENSG00000130635**	*0.161358965*	*0.099772473*	*0.086906324*	*0.121332948*
**ENSG00000179889**	*0.0209707*	*0.025017913*	*0.026600265*	*0.018049594*
**ENSG00000136295**	*0.014333823*	*0.023537401*	*0.023677569*	*0.013647539*
**ENSG00000131495**	*0.020945809*	*0.02795925*	*0.027181908*	*0.018079158*
**ENSG00000136279**	*0.032741325*	*0.03512513*	*0.035066991*	*0.028364379*
**ENSG00000145740**	*0.032091472*	*0.035039754*	*0.031349318*	*0.035669644*
**ENSG00000140403**	*0.070404383*	*0.05704668*	*0.060237905*	*0.049432487*
**ENSG00000129292**	*0.025051019*	*0.026847504*	*0.034279212*	*0.022669196*
**ENSG00000163138**	*0.016650544*	*0.025662197*	*0.02641763*	*0.01727699*
**ENSG00000128284**	*0.014667306*	*0.024869125*	*0.024788156*	*0.015882047*
**ENSG00000166589**	*0.016631223*	*0.024833612*	*0.02420331*	*0.016517917*
**ENSG00000160408**	*0.037549063*	*0.037268695*	*0.036210803*	*0.035124244*
**ENSG00000160683**	*0.016545711*	*0.025099584*	*0.025315469*	*0.017230418*
**ENSG00000091656**	*0.035116217*	*0.032829415*	*0.025114742*	*0.018941703*
**ENSG00000149403**	*0.021593752*	*0.026131561*	*0.026103314*	*0.019114885*
**ENSG00000171804**	*0.014480695*	*0.025465486*	*0.025081521*	*0.015695137*
**ENSG00000164764**	*0.016979461*	*0.025091656*	*0.024652744*	*0.015084831*
**ENSG00000130699**	*0.028435076*	*0.035405718*	*0.032937618*	*0.0240749*
**ENSG00000182393**	*0.017187168*	*0.024969972*	*0.025035908*	*0.015223261*
**ENSG00000173409**	*0.028880717*	*0.03137057*	*0.033593468*	*0.029738019*
**ENSG00000108406**	*0.024093711*	*0.031836898*	*0.032540622*	*0.02085447*
**ENSG00000149923**	*0.019099946*	*0.028265477*	*0.031269164*	*0.020810583*
**ENSG00000034971**	*0.053969504*	*0.024605521*	*0.025473498*	*0.014511897*
**ENSG00000105707**	*0.016097905*	*0.024744011*	*0.024596064*	*0.015665941*
**ENSG00000145819**	*0.031648948*	*0.031032789*	*0.028938624*	*0.029421036*
**ENSG00000154438**	*0.015885792*	*0.024127411*	*0.024566711*	*0.014937065*
**ENSG00000163067**	*0.02449292*	*0.029050189*	*0.025930332*	*0.020312121*
**ENSG00000213401**	*0.018510966*	*0.024598901*	*0.027411787*	*0.015263466*
**ENSG00000173546**	*0.021110715*	*0.02950256*	*0.028782222*	*0.024075103*
**ENSG00000083845**	*0.016850498*	*0.025043325*	*0.025422*	*0.017043176*
**ENSG00000112592**	*0.025412695*	*0.031525159*	*0.032317928*	*0.02065085*
**ENSG00000125124**	*0.052023051*	*0.045936196*	*0.034467848*	*0.047027608*
**ENSG00000157796**	*0.038842314*	*0.044517167*	*0.036860099*	*0.030857113*
**ENSG00000170178**	*0.01681672*	*0.024962025*	*0.025638258*	*0.014680069*
**ENSG00000198601**	*0.0134951*	*0.023964759*	*0.024263967*	*0.013221488*
**ENSG00000177119**	*0.029507531*	*0.036702661*	*0.036820584*	*0.028445509*
**ENSG00000106153**	*0.017225676*	*0.025113578*	*0.024983262*	*0.017026635*
**ENSG00000087338**	*0.045504271*	*0.047926406*	*0.037404151*	*0.037492042*

**ENSG00000105695**	*0.016232537*	*0.025605172*	*0.025038914*	*0.015808448*
**ENSG00000170242**	*0.026225041*	*0.03521415*	*0.035510731*	*0.022349511*
**ENSG00000146192**	*0.067371193*	*0.050147639*	*0.044330428*	*0.051790526*
**ENSG00000132382**	*0.03757976*	*0.034647941*	*0.034181157*	*0.034406455*
**ENSG00000105967**	*0.023702683*	*0.026020248*	*0.026940296*	*0.036094025*
**ENSG00000139668**	*0.032360305*	*0.036728007*	*0.036108571*	*0.032316241*
**ENSG00000163406**	*0.029738942*	*0.033834126*	*0.029199898*	*0.033583425*
**ENSG00000069509**	*0.032833695*	*0.032963484*	*0.036693605*	*0.028121649*
**ENSG00000116095**	*0.025881547*	*0.035785977*	*0.036242815*	*0.025958581*
**ENSG00000172468**	*0.016536075*	*0.026385656*	*0.02492682*	*0.016875937*
**ENSG00000184260**	*0.062489251*	*0.062112676*	*0.05436015*	*0.050793549*
**ENSG00000163762**	*0.017008575*	*0.025591748*	*0.025263899*	*0.017612282*
**ENSG00000143409**	*0.027133014*	*0.030963324*	*0.029733049*	*0.025314478*
**ENSG00000100997**	*0.077241048*	*0.056004135*	*0.067425345*	*0.060317767*
**ENSG00000169688**	*0.015302493*	*0.024089521*	*0.024874368*	*0.015320851*
**ENSG00000175893**	*0.032334708*	*0.041287186*	*0.036449657*	*0.033453881*
**ENSG00000162543**	*0.023323199*	*0.026843991*	*0.034077039*	*0.028844123*
**ENSG00000156831**	*0.028871681*	*0.031111328*	*0.034543134*	*0.022741036*
**ENSG00000112305**	*0.034661594*	*0.040023936*	*0.031250323*	*0.028180732*
**ENSG00000108294**	*0.022506711*	*0.027821979*	*0.027079028*	*0.020486338*
**ENSG00000100505**	*0.037423905*	*0.047170592*	*0.034336931*	*0.023974556*
**ENSG00000144674**	*0.03349664*	*0.038804304*	*0.038383682*	*0.041216773*
**ENSG00000142949**	*0.026424928*	*0.031680823*	*0.028985374*	*0.024079007*
**ENSG00000082438**	*0.017296585*	*0.025470451*	*0.025486231*	*0.017865593*
**ENSG00000159592**	*0.021724495*	*0.032256423*	*0.03170155*	*0.017484705*
**ENSG00000239264**	*0.044925372*	*0.044865557*	*0.043949188*	*0.04386954*
**ENSG00000125962**	*0.05693032*	*0.037446718*	*0.036446912*	*0.047615901*
**ENSG00000164877**	*0.022591236*	*0.029994646*	*0.032641093*	*0.019664615*
**ENSG00000164649**	*0.048276679*	*0.036073052*	*0.032678475*	*0.029668535*
**ENSG00000145244**	*0.015829207*	*0.025579619*	*0.025570344*	*0.015591584*
**ENSG00000109787**	*0.018345283*	*0.02636787*	*0.026276097*	*0.018219662*
**ENSG00000147224**	*0.030665982*	*0.03248661*	*0.030530686*	*0.027225967*
**ENSG00000106804**	*0.039231874*	*0.044049718*	*0.033919448*	*0.032515921*
**ENSG00000065717**	*0.020951476*	*0.026961928*	*0.026906173*	*0.02068224*
**ENSG00000181541**	*0.014988776*	*0.026600142*	*0.025013292*	*0.015020643*
**ENSG00000049323**	*0.040265932*	*0.027120025*	*0.025240624*	*0.01979808*
**ENSG00000058668**	*0.016018516*	*0.025306328*	*0.025241073*	*0.014902639*
**ENSG00000121377**	*0.014756531*	*0.023999901*	*0.02433758*	*0.016450826*
**ENSG00000178035**	*0.025107802*	*0.028058838*	*0.027881712*	*0.021808688*
**ENSG00000116721**	*0.025990612*	*0.024755368*	*0.025069087*	*0.016966312*
**ENSG00000104907**	*0.027280673*	*0.030713879*	*0.029131173*	*0.024190934*
**ENSG00000109861**	*0.049641999*	*0.053883033*	*0.051177386*	*0.056043371*
**ENSG00000104213**	*0.035552678*	*0.038887567*	*0.029608182*	*0.02716735*
**ENSG00000087263**	*0.024646731*	*0.032612569*	*0.03303974*	*0.02613434*
**ENSG00000100445**	*0.026501686*	*0.034653502*	*0.035112214*	*0.032159565*
**ENSG00000152292**	*0.015013313*	*0.025636534*	*0.024506154*	*0.014630407*
**ENSG00000164830**	*0.038650557*	*0.045409706*	*0.037404105*	*0.033302439*
**ENSG00000169740**	*0.033805299*	*0.037334805*	*0.035241137*	*0.027007391*
**ENSG00000157765**	*0.014694895*	*0.025227492*	*0.024862832*	*0.013957338*
**ENSG00000123240**	*0.036664333*	*0.034930257*	*0.03560397*	*0.02946831*
**ENSG00000111305**	*0.017092425*	*0.025599363*	*0.025060765*	*0.014029636*
**ENSG00000019995**	*0.042708303*	*0.039280325*	*0.042170554*	*0.041555069*
**ENSG00000005421**	*0.014902258*	*0.024741173*	*0.024176994*	*0.015332501*
**ENSG00000174373**	*0.029625255*	*0.035484579*	*0.032612811*	*0.03022583*

**ENSG00000099617**	*0.016474055*	*0.025745006*	*0.025718517*	*0.016929039*
**ENSG00000124788**	*0.055883813*	*0.048421451*	*0.049429682*	*0.040415202*
**ENSG00000078369**	*0.018346758*	*0.026311056*	*0.026999989*	*0.017916345*
**ENSG00000086967**	*0.090611167*	*0.078407533*	*0.076308334*	*0.060926098*
**ENSG00000165072**	*0.014850492*	*0.025385847*	*0.024773124*	*0.015545591*
**ENSG00000126249**	*0.02987611*	*0.029987214*	*0.031443105*	*0.027908706*
**ENSG00000138587**	*0.046004928*	*0.050794953*	*0.037418447*	*0.044604368*
**ENSG00000172059**	*0.019613173*	*0.025288574*	*0.025504074*	*0.016819924*
**ENSG00000134057**	*0.037679429*	*0.034309319*	*0.034846414*	*0.033466441*
**ENSG00000172199**	*0.016068096*	*0.024723057*	*0.025059628*	*0.016515369*
**ENSG00000115761**	*0.01667737*	*0.025478732*	*0.025037763*	*0.01478924*
**ENSG00000123307**	*0.016726705*	*0.025596742*	*0.023942556*	*0.014933809*
**ENSG00000117713**	*0.016874792*	*0.026330865*	*0.026319067*	*0.016193916*
**ENSG00000185899**	*0.016271329*	*0.025712185*	*0.024624649*	*0.015288844*
**ENSG00000130985**	*0.016358159*	*0.025476179*	*0.025551137*	*0.016946409*
**ENSG00000152520**	*0.027186986*	*0.032678415*	*0.032895626*	*0.02709534*
**ENSG00000166780**	*0.018982717*	*0.026928887*	*0.024988183*	*0.01817466*
**ENSG00000136827**	*0.030592435*	*0.035487245*	*0.042757997*	*0.031423248*
**ENSG00000125779**	*0.015044037*	*0.02592399*	*0.024154773*	*0.015216578*
**ENSG00000171606**	*0.022696958*	*0.030481611*	*0.032706639*	*0.025917777*
**ENSG00000130649**	*0.03309595*	*0.03221001*	*0.02944686*	*0.0304395*
**ENSG00000150625**	*0.07533226*	*0.032887974*	*0.025563444*	*0.018449549*
**ENSG00000138670**	*0.017275465*	*0.027521028*	*0.025641807*	*0.017156384*
**ENSG00000178184**	*0.015575157*	*0.025704133*	*0.024799139*	*0.015613008*
**ENSG00000023734**	*0.022653031*	*0.027783254*	*0.02961863*	*0.026635749*
**ENSG00000179152**	*0.021087313*	*0.030400698*	*0.030109489*	*0.020506629*
**ENSG00000068383**	*0.051836681*	*0.04696515*	*0.048570002*	*0.05132136*
**ENSG00000251664**	*0.016465984*	*0.026105563*	*0.025631678*	*0.016503504*
**ENSG00000078579**	*0.016340452*	*0.025323885*	*0.025240091*	*0.016897377*
**ENSG00000172508**	*0.016408515*	*0.024659284*	*0.024208678*	*0.016586015*
**ENSG00000137473**	*0.016718522*	*0.025059249*	*0.024770087*	*0.015467211*
**ENSG00000204086**	*0.016795246*	*0.024741811*	*0.026077802*	*0.015796884*
**ENSG00000127318**	*0.016175203*	*0.024596259*	*0.024892602*	*0.015281927*
**ENSG00000164123**	*0.018565521*	*0.025556622*	*0.026040986*	*0.017689601*
**ENSG00000105254**	*0.025951642*	*0.032563979*	*0.031265853*	*0.027737241*
**ENSG00000125944**	*0.025261331*	*0.03272932*	*0.029309291*	*0.027181097*
**ENSG00000134480**	*0.027738893*	*0.030385344*	*0.029547615*	*0.025257648*
**ENSG00000178306**	*0.015284573*	*0.024897912*	*0.024977194*	*0.015256643*
**ENSG00000079277**	*0.023174242*	*0.031796254*	*0.035643808*	*0.022546124*
**ENSG00000126016**	*0.101078276*	*0.102123102*	*0.101075375*	*0.082073036*
**ENSG00000146007**	*0.024550125*	*0.030566272*	*0.032711295*	*0.022414266*
**ENSG00000101558**	*0.032990319*	*0.035445567*	*0.035891675*	*0.030312922*
**ENSG00000205423**	*0.024803416*	*0.031082292*	*0.031535881*	*0.026412255*
**ENSG00000178935**	*0.022288213*	*0.033050304*	*0.028779781*	*0.022615413*
**ENSG00000153310**	*0.031633242*	*0.036963017*	*0.035925023*	*0.029727806*
**ENSG00000186943**	*0.015432413*	*0.024810771*	*0.026134723*	*0.015147665*
**ENSG00000013619**	*0.016272134*	*0.025339494*	*0.025262512*	*0.016188818*
**ENSG00000102390**	*0.035807369*	*0.03286147*	*0.036243645*	*0.030106031*
**ENSG00000188958**	*0.086096316*	*0.122524282*	*0.111064579*	*0.100580614*
**ENSG00000104728**	*0.036782637*	*0.030000279*	*0.034701697*	*0.024927073*
**ENSG00000137507**	*0.081446342*	*0.064017943*	*0.067036567*	*0.060941223*
**ENSG00000111249**	*0.015409007*	*0.024498673*	*0.024980341*	*0.014087046*
**ENSG00000074219**	*0.033656463*	*0.035695445*	*0.0380475*	*0.033278596*
**ENSG00000114999**	*0.015451579*	*0.025302168*	*0.024748058*	*0.015766189*

**ENSG00000204444**	*0.035456787*	*0.034041074*	*0.033819896*	*0.022601078*
**ENSG00000095906**	*0.032798517*	*0.036091022*	*0.03189814*	*0.022164022*
**ENSG00000134061**	*0.031645718*	*0.042254635*	*0.043630623*	*0.035460231*
**ENSG00000113083**	*0.018915663*	*0.028202208*	*0.026231509*	*0.01593719*
**ENSG00000150773**	*0.022590658*	*0.02945509*	*0.027542337*	*0.022936244*
**ENSG00000186652**	*0.055326628*	*0.056126898*	*0.044822426*	*0.051066637*
**ENSG00000144357**	*0.02965787*	*0.031830766*	*0.036664416*	*0.02893884*
**ENSG00000180801**	*0.018076446*	*0.026054092*	*0.02655611*	*0.017600836*
**ENSG00000100364**	*0.046337899*	*0.049150368*	*0.047939744*	*0.039387679*
**ENSG00000120662**	*0.0269689*	*0.032701592*	*0.030207276*	*0.019691112*
**ENSG00000173261**	*0.016747781*	*0.025994362*	*0.025477575*	*0.016719085*
**ENSG00000181031**	*0.072088608*	*0.045146014*	*0.043949978*	*0.037061613*
**ENSG00000165349**	*0.016113625*	*0.027362768*	*0.024193402*	*0.016664185*
**ENSG00000108272**	*0.029660792*	*0.034112302*	*0.032084569*	*0.022429521*
**ENSG00000085832**	*0.028584046*	*0.032198752*	*0.030640569*	*0.024650578*
**ENSG00000198846**	*0.07177868*	*0.072365972*	*0.058835085*	*0.07624414*
**ENSG00000076706**	*0.017023093*	*0.026331249*	*0.026113297*	*0.017781461*
**ENSG00000173093**	*0.015713265*	*0.025366603*	*0.025635946*	*0.015745488*
**ENSG00000105223**	*0.028901353*	*0.029245063*	*0.035673428*	*0.033740412*
**ENSG00000100802**	*0.036382573*	*0.040549269*	*0.036970699*	*0.029813099*
**ENSG00000125864**	*0.019331326*	*0.027331334*	*0.02643153*	*0.017356563*
**ENSG00000149231**	*0.048814545*	*0.047424852*	*0.038950342*	*0.066518571*
**ENSG00000168243**	*0.016550813*	*0.024672225*	*0.025056383*	*0.015788584*
**ENSG00000186376**	*0.023779826*	*0.032421692*	*0.026906762*	*0.022762178*
**ENSG00000147905**	*0.038056111*	*0.037584194*	*0.034809959*	*0.038130016*
**ENSG00000102125**	*0.024630938*	*0.028711057*	*0.031082273*	*0.023924361*
**ENSG00000072832**	*0.019274132*	*0.026547215*	*0.026752955*	*0.016748545*
**ENSG00000105656**	*0.020472654*	*0.03092817*	*0.029095344*	*0.025051343*
**ENSG00000170515**	*0.022593207*	*0.02650688*	*0.026570117*	*0.023024833*
**ENSG00000162366**	*0.014878642*	*0.025642964*	*0.025302907*	*0.015289397*
**ENSG00000204590**	*0.019796306*	*0.028346872*	*0.02871535*	*0.018659277*
**ENSG00000164283**	*0.015245824*	*0.024784427*	*0.024753283*	*0.014721367*
**ENSG00000188037**	*0.016428543*	*0.025842387*	*0.025868814*	*0.016257082*
**ENSG00000183032**	*0.018479263*	*0.025512207*	*0.025978887*	*0.015524974*
**ENSG00000167910**	*0.015390404*	*0.024462773*	*0.024587402*	*0.015950331*
**ENSG00000143753**	*0.039956145*	*0.038269619*	*0.037637579*	*0.027798429*
**ENSG00000136631**	*0.031945882*	*0.038104739*	*0.032045658*	*0.028974155*
**ENSG00000169105**	*0.022923717*	*0.029860341*	*0.027354404*	*0.022495396*
**ENSG00000110328**	*0.05601145*	*0.051165187*	*0.046238567*	*0.045789003*
**ENSG00000137872**	*0.016839649*	*0.025706979*	*0.026064792*	*0.016152714*
**ENSG00000166860**	*0.02445106*	*0.034593481*	*0.031727312*	*0.019208742*
**ENSG00000225830**	*0.021477596*	*0.027967437*	*0.026699843*	*0.017043956*
**ENSG00000164011**	*0.027753719*	*0.036837062*	*0.029418443*	*0.025431794*
**ENSG00000184602**	*0.065247548*	*0.071908332*	*0.05957631*	*0.068191744*
**ENSG00000173214**	*0.021565365*	*0.02948256*	*0.026872362*	*0.022177725*
**ENSG00000196408**	*0.021377462*	*0.034322305*	*0.030043555*	*0.020129604*
**ENSG00000114491**	*0.028550033*	*0.032476299*	*0.028417944*	*0.02241656*
**ENSG00000080822**	*0.021400007*	*0.028736473*	*0.028895008*	*0.020844003*
**ENSG00000170456**	*0.055367636*	*0.045139683*	*0.053059567*	*0.051082891*
**ENSG00000121236**	*0.073188688*	*0.055617166*	*0.046252506*	*0.0625385*
**ENSG00000198908**	*0.05682207*	*0.041267352*	*0.035523794*	*0.038175531*
**ENSG00000072756**	*0.032416967*	*0.039053377*	*0.030148624*	*0.034282125*
**ENSG00000145808**	*0.016877559*	*0.024675208*	*0.024854111*	*0.018238561*
**ENSG00000110347**	*0.018054654*	*0.026800323*	*0.026316643*	*0.017761829*

**ENSG00000103710**	*0.01501547*	*0.023798264*	*0.024343849*	*0.015632171*
**ENSG00000122735**	*0.015596947*	*0.025418635*	*0.024308597*	*0.017020281*
**ENSG00000088386**	*0.016077189*	*0.025084339*	*0.024352565*	*0.013831703*
**ENSG00000186009**	*0.033557337*	*0.033656368*	*0.028652643*	*0.019246194*
**ENSG00000091986**	*0.01557377*	*0.025239307*	*0.025259322*	*0.014731598*
**ENSG00000140691**	*0.038112615*	*0.039222199*	*0.056865949*	*0.040745948*
**ENSG00000176435**	*0.014873759*	*0.023772976*	*0.025601973*	*0.015463119*
**ENSG00000213145**	*0.049999862*	*0.042196742*	*0.03912911*	*0.040224362*
**ENSG00000177602**	*0.050798757*	*0.043121879*	*0.045859019*	*0.040963499*
**ENSG00000158828**	*0.04420453*	*0.03992408*	*0.038466192*	*0.038167435*
**ENSG00000108848**	*0.05002422*	*0.047013231*	*0.03775017*	*0.053734689*
**ENSG00000107789**	*0.036821011*	*0.040581732*	*0.030628052*	*0.02895661*
**ENSG00000104760**	*0.015730037*	*0.025089692*	*0.026165649*	*0.016338051*
**ENSG00000101361**	*0.041414659*	*0.038290363*	*0.038386141*	*0.038225416*
**ENSG00000179344**	*0.032479429*	*0.041649824*	*0.04263354*	*0.040339939*
**ENSG00000116574**	*0.091435707*	*0.078762766*	*0.070921005*	*0.084641142*
**ENSG00000169251**	*0.044649856*	*0.048476716*	*0.041182755*	*0.038786507*
**ENSG00000136235**	*0.057977132*	*0.054361323*	*0.053476396*	*0.050177271*
**ENSG00000107968**	*0.042769814*	*0.042872246*	*0.047819146*	*0.049305631*
**ENSG00000085511**	*0.035796591*	*0.040762576*	*0.036076828*	*0.031550602*
**ENSG00000167397**	*0.028853094*	*0.027342708*	*0.030254006*	*0.023776199*
**ENSG00000173193**	*0.035186041*	*0.036162463*	*0.038036723*	*0.03545583*
**ENSG00000164645**	*0.017694623*	*0.027137337*	*0.025436076*	*0.018704345*
**ENSG00000160856**	*0.018911395*	*0.02768298*	*0.028649209*	*0.02670794*
**ENSG00000018510**	*0.035545166*	*0.037734904*	*0.032112992*	*0.024921252*
**ENSG00000075391**	*0.020963417*	*0.025197381*	*0.025969863*	*0.015474459*
**ENSG00000143862**	*0.020551126*	*0.027444427*	*0.027989962*	*0.021780385*
**ENSG00000117450**	*0.021458078*	*0.026419322*	*0.027215165*	*0.019048721*
**ENSG00000143771**	*0.02547637*	*0.028528731*	*0.028613576*	*0.021583168*
**ENSG00000187514**	*0.022626496*	*0.027602494*	*0.026285204*	*0.017754692*
**ENSG00000126545**	*0.017973313*	*0.025547607*	*0.027518448*	*0.017488904*
**ENSG00000109079**	*0.023101559*	*0.031406089*	*0.030854078*	*0.023421455*
**ENSG00000138347**	*0.015001957*	*0.024914338*	*0.024681593*	*0.014412754*
**ENSG00000153066**	*0.048004957*	*0.038572253*	*0.048624495*	*0.045624082*
**ENSG00000153162**	*0.056651448*	*0.062741016*	*0.05344526*	*0.063977371*
**ENSG00000171102**	*0.016547649*	*0.024961089*	*0.02645885*	*0.015711412*
**ENSG00000139116**	*0.044054714*	*0.076305913*	*0.072509568*	*0.067690738*
**ENSG00000115825**	*0.048743852*	*0.044729004*	*0.043296034*	*0.042400816*
**ENSG00000116874**	*0.036481531*	*0.040075089*	*0.034566909*	*0.0292589*
**ENSG00000151948**	*0.144280788*	*0.110986295*	*0.096545868*	*0.105615534*
**ENSG00000129187**	*0.029941578*	*0.039227149*	*0.036131819*	*0.02933436*
**ENSG00000119888**	*0.041751279*	*0.039321774*	*0.035214362*	*0.028520008*
**ENSG00000180089**	*0.021255085*	*0.028306466*	*0.02759357*	*0.021284869*
**ENSG00000174791**	*0.01529419*	*0.025618355*	*0.02398392*	*0.015786313*
**ENSG00000120055**	*0.015134131*	*0.024337768*	*0.025108919*	*0.01459952*
**ENSG00000070729**	*0.014982741*	*0.025508051*	*0.025839554*	*0.015731546*
**ENSG00000102879**	*0.034428228*	*0.030687744*	*0.031737927*	*0.030826624*
**ENSG00000183640**	*0.017388837*	*0.027623506*	*0.026875243*	*0.016991029*
**ENSG00000240857**	*0.029899653*	*0.036181324*	*0.03406073*	*0.0268481*
**ENSG00000121481**	*0.015532444*	*0.024682317*	*0.026333564*	*0.015731587*
**ENSG00000154309**	*0.019341105*	*0.027209783*	*0.025416244*	*0.018470335*
**ENSG00000144452**	*0.025405453*	*0.026562824*	*0.027413572*	*0.017649702*
**ENSG00000104381**	*0.021449487*	*0.025515057*	*0.026445921*	*0.017770692*
**ENSG00000122145**	*0.016886752*	*0.025481346*	*0.024840249*	*0.015355767*

**ENSG00000129295**	*0.016346521*	*0.0256046*	*0.024784404*	*0.014878552*
**ENSG00000133328**	*0.053697306*	*0.049160753*	*0.045069867*	*0.051819284*
**ENSG00000125834**	*0.02493561*	*0.030656101*	*0.031063918*	*0.024105906*
**ENSG00000174442**	*0.045491772*	*0.045976937*	*0.036783462*	*0.039950819*
**ENSG00000130226**	*0.01591404*	*0.024781019*	*0.025852906*	*0.014391719*
**ENSG00000153404**	*0.016738961*	*0.025746538*	*0.025776747*	*0.015296108*
**ENSG00000171100**	*0.037323912*	*0.03631559*	*0.037054607*	*0.037040021*
**ENSG00000095015**	*0.048936486*	*0.044889846*	*0.036117884*	*0.033495506*
**ENSG00000162630**	*0.016731151*	*0.02572855*	*0.025018771*	*0.015258266*
**ENSG00000172399**	*0.016632052*	*0.026003109*	*0.025235061*	*0.016099172*
**ENSG00000180806**	*0.017646751*	*0.024476153*	*0.025711925*	*0.016172069*
**ENSG00000151704**	*0.018184874*	*0.024811462*	*0.026615755*	*0.018584762*
**ENSG00000244355**	*0.015113242*	*0.025280297*	*0.025598023*	*0.015136529*
**ENSG00000172269**	*0.040350589*	*0.042430752*	*0.033966379*	*0.03326849*
**ENSG00000160401**	*0.016106464*	*0.024795893*	*0.026667124*	*0.015271953*
**ENSG00000140660**	*0.038341974*	*0.039082784*	*0.039957724*	*0.033930784*
**ENSG00000163735**	*0.017523756*	*0.026166218*	*0.025415914*	*0.015727829*
**ENSG00000213024**	*0.024520183*	*0.027456999*	*0.030178248*	*0.028037825*
**ENSG00000125037**	*0.02344318*	*0.029063253*	*0.03240636*	*0.020630996*
**ENSG00000147647**	*0.035793857*	*0.027332637*	*0.025453929*	*0.032663988*
**ENSG00000092068**	*0.015508515*	*0.024652951*	*0.024998719*	*0.015861943*
**ENSG00000164978**	*0.051800806*	*0.072810461*	*0.089024753*	*0.05154294*
**ENSG00000159459**	*0.02381761*	*0.031302302*	*0.034002234*	*0.023669746*
**ENSG00000197043**	*0.028731678*	*0.033924877*	*0.033281678*	*0.02699326*
**ENSG00000171777**	*0.017249453*	*0.025552414*	*0.025188049*	*0.015221325*
**ENSG00000022567**	*0.020568192*	*0.027359746*	*0.026779928*	*0.019929192*
**ENSG00000165474**	*0.07385993*	*0.046055186*	*0.043157974*	*0.063158664*
**ENSG00000162391**	*0.017140125*	*0.02593036*	*0.025627218*	*0.017292229*
**ENSG00000182816**	*0.016052598*	*0.024783183*	*0.024117047*	*0.015486682*
**ENSG00000117281**	*0.017951153*	*0.028386649*	*0.026191415*	*0.018769601*
**ENSG00000180008**	*0.019503856*	*0.025266921*	*0.026648014*	*0.016096602*
**ENSG00000196743**	*0.046597259*	*0.037313842*	*0.043735726*	*0.052908152*
**ENSG00000111445**	*0.038701364*	*0.033261895*	*0.031861059*	*0.029732037*
**ENSG00000132915**	*0.015927685*	*0.025676787*	*0.024765355*	*0.015818576*
**ENSG00000072315**	*0.017045947*	*0.025642766*	*0.02802715*	*0.016901095*
**ENSG00000153012**	*0.015672678*	*0.02477682*	*0.024027284*	*0.014501211*
**ENSG00000104863**	*0.020855354*	*0.027127218*	*0.028794046*	*0.024482928*
**ENSG00000198925**	*0.023759108*	*0.031646782*	*0.031635321*	*0.031072282*
**ENSG00000178718**	*0.047858401*	*0.045034638*	*0.04602024*	*0.045366533*
**ENSG00000109919**	*0.029380611*	*0.035596445*	*0.029725251*	*0.025430618*
**ENSG00000154832**	*0.041190614*	*0.03794023*	*0.042536039*	*0.042640074*
**ENSG00000170522**	*0.059545527*	*0.051110429*	*0.0445529*	*0.03937013*
**ENSG00000139549**	*0.015794616*	*0.025120469*	*0.0252854*	*0.01529958*
**ENSG00000105568**	*0.019064509*	*0.026767334*	*0.030299261*	*0.019403314*
**ENSG00000204873**	*0.017467124*	*0.02699434*	*0.026736344*	*0.016547316*
**ENSG00000123066**	*0.063171614*	*0.072869979*	*0.071884893*	*0.069257429*
**ENSG00000172830**	*0.016180824*	*0.024853426*	*0.024282717*	*0.014533799*
**ENSG00000100575**	*0.022123222*	*0.030019751*	*0.030284135*	*0.023293217*
**ENSG00000196865**	*0.026367053*	*0.033328063*	*0.026842654*	*0.052643247*
**ENSG00000099338**	*0.016331953*	*0.027045767*	*0.023953992*	*0.014458506*
**ENSG00000125901**	*0.028835346*	*0.027637257*	*0.029284804*	*0.021209998*
**ENSG00000171056**	*0.015122415*	*0.025760042*	*0.026081128*	*0.014570135*
**ENSG00000169750**	*0.030584385*	*0.0315838*	*0.031624954*	*0.02669716*
**ENSG00000171794**	*0.015457062*	*0.025290473*	*0.024379826*	*0.020864711*

**ENSG00000154764**	*0.017285017*	*0.026311959*	*0.026419935*	*0.01698993*
**ENSG00000123411**	*0.021258316*	*0.027228646*	*0.02770619*	*0.020039485*
**ENSG00000145687**	*0.027340559*	*0.037822933*	*0.040509952*	*0.029768029*
**ENSG00000123154**	*0.024077704*	*0.029973549*	*0.03013066*	*0.022863782*
**ENSG00000131899**	*0.015030881*	*0.025077402*	*0.02544104*	*0.015835204*
**ENSG00000095981**	*0.014980074*	*0.025753184*	*0.025098073*	*0.015438689*
**ENSG00000167971**	*0.015982674*	*0.025033902*	*0.024452838*	*0.01535671*
**ENSG00000132517**	*0.017358814*	*0.026111723*	*0.025444072*	*0.016879646*
**ENSG00000113812**	*0.033224187*	*0.039585363*	*0.033204777*	*0.032430827*
**ENSG00000218891**	*0.029865204*	*0.029018252*	*0.032577152*	*0.02738417*
**ENSG00000101997**	*0.020722681*	*0.02898196*	*0.029788324*	*0.025612494*
**ENSG00000182195**	*0.024787728*	*0.029484361*	*0.029935344*	*0.024820484*
**ENSG00000107159**	*0.037508277*	*0.043754454*	*0.049699338*	*0.033110208*
**ENSG00000185519**	*0.01855591*	*0.026997307*	*0.02532878*	*0.018085347*
**ENSG00000147481**	*0.015546312*	*0.025763264*	*0.024843863*	*0.015584008*
**ENSG00000198824**	*0.036641276*	*0.035842654*	*0.043181145*	*0.031856224*
**ENSG00000100060**	*0.027379023*	*0.033673506*	*0.035343542*	*0.026426423*
**ENSG00000165724**	*0.036595019*	*0.035916801*	*0.039502802*	*0.031397393*
**ENSG00000136869**	*0.018440425*	*0.025399976*	*0.02658146*	*0.01759618*
**ENSG00000183303**	*0.015420348*	*0.025264419*	*0.0246053*	*0.015999351*
**ENSG00000127586**	*0.04983563*	*0.051852805*	*0.043915011*	*0.033103018*
**ENSG00000149084**	*0.026891073*	*0.030390811*	*0.029466012*	*0.026590073*
**ENSG00000157358**	*0.026531009*	*0.026396712*	*0.028077851*	*0.015661079*
**ENSG00000135900**	*0.027617575*	*0.035748107*	*0.033158177*	*0.02397848*
**ENSG00000170961**	*0.016650107*	*0.026886581*	*0.024887674*	*0.016960524*
**ENSG00000170100**	*0.016826238*	*0.026492072*	*0.026688529*	*0.017244845*
**ENSG00000086015**	*0.031291877*	*0.034769414*	*0.030790367*	*0.027221489*
**ENSG00000114779**	*0.029213024*	*0.028893672*	*0.032667384*	*0.032376671*
**ENSG00000151617**	*0.059888089*	*0.032225288*	*0.026109359*	*0.033549286*
**ENSG00000203747**	*0.016062666*	*0.024695366*	*0.025797945*	*0.015426417*
**ENSG00000182858**	*0.023766457*	*0.027658682*	*0.033887108*	*0.025618956*
**ENSG00000100578**	*0.03704759*	*0.03919291*	*0.03096319*	*0.024796773*
**ENSG00000179456**	*0.017261348*	*0.025668114*	*0.024733819*	*0.015232167*
**ENSG00000182378**	*0.063623215*	*0.053200847*	*0.046188117*	*0.04465017*
**ENSG00000105619**	*0.023297545*	*0.028144675*	*0.029646412*	*0.025666678*
**ENSG00000237649**	*0.047695294*	*0.036491824*	*0.03527599*	*0.035150224*
**ENSG00000214107**	*0.014820969*	*0.025827506*	*0.024363502*	*0.015614691*
**ENSG00000100024**	*0.063962154*	*0.05501916*	*0.051831498*	*0.059178224*
**ENSG00000184155**	*0.016194082*	*0.025246172*	*0.024544748*	*0.015219571*
**ENSG00000158773**	*0.03624152*	*0.037445782*	*0.039367163*	*0.035711648*
**ENSG00000167554**	*0.021518847*	*0.035837681*	*0.026127755*	*0.019138553*
**ENSG00000117425**	*0.015193624*	*0.025444196*	*0.024880176*	*0.015608046*
**ENSG00000104231**	*0.026874291*	*0.033560241*	*0.031697719*	*0.022890845*
**ENSG00000204897**	*0.016203389*	*0.025597413*	*0.024420171*	*0.016335082*
**ENSG00000188095**	*0.017253232*	*0.026650247*	*0.027726496*	*0.015525524*
**ENSG00000124198**	*0.035333366*	*0.036894479*	*0.039440399*	*0.034664198*
**ENSG00000115841**	*0.017646318*	*0.027499868*	*0.027514339*	*0.017452676*
**ENSG00000147571**	*0.016723068*	*0.026189288*	*0.026340815*	*0.017310625*
**ENSG00000159714**	*0.018708606*	*0.026132287*	*0.026562396*	*0.018009004*
**ENSG00000116128**	*0.02592784*	*0.033919104*	*0.033327933*	*0.024142376*
**ENSG00000122591**	*0.018810105*	*0.025689374*	*0.029746416*	*0.017405583*
**ENSG00000167977**	*0.023497076*	*0.027797505*	*0.03073278*	*0.023879086*
**ENSG00000035862**	*0.018274505*	*0.030294459*	*0.035037854*	*0.02147578*
**ENSG00000179111**	*0.029889465*	*0.038615184*	*0.040117844*	*0.037856174*

**ENSG00000104218**	*0.034448593*	*0.033697356*	*0.0302111*	*0.031798794*
**ENSG00000111077**	*0.015471045*	*0.024700816*	*0.024859217*	*0.015395874*
**ENSG00000159884**	*0.033622564*	*0.038806729*	*0.035128464*	*0.032894273*
**ENSG00000130701**	*0.020305715*	*0.02731711*	*0.02751799*	*0.020322314*
**ENSG00000081853**	*0.016147717*	*0.02655432*	*0.025225469*	*0.015329684*
**ENSG00000134470**	*0.015729721*	*0.025486914*	*0.027845809*	*0.015466093*
**ENSG00000088035**	*0.03696295*	*0.03848984*	*0.034805495*	*0.026659837*
**ENSG00000100219**	*0.045830491*	*0.042611797*	*0.042930652*	*0.041839884*
**ENSG00000161180**	*0.017416521*	*0.026828748*	*0.026850161*	*0.016982672*
**ENSG00000180190**	*0.016323966*	*0.025608846*	*0.02499296*	*0.014830348*
**ENSG00000118520**	*0.015814731*	*0.025086652*	*0.024480538*	*0.015535084*
**ENSG00000172458**	*0.029772912*	*0.029743992*	*0.028884026*	*0.025750978*
**ENSG00000120705**	*0.031301776*	*0.034980585*	*0.032367708*	*0.030685526*
**ENSG00000101182**	*0.019164406*	*0.026496115*	*0.025057091*	*0.01984892*
**ENSG00000093010**	*0.025470625*	*0.032231417*	*0.032232489*	*0.033099121*
**ENSG00000129515**	*0.026684819*	*0.031535835*	*0.033043806*	*0.023711963*
**ENSG00000127533**	*0.016130016*	*0.025379419*	*0.026385074*	*0.016947584*
**ENSG00000119929**	*0.028439288*	*0.030997752*	*0.034920917*	*0.02299016*
**ENSG00000168883**	*0.027046569*	*0.031828131*	*0.032101372*	*0.023817196*
**ENSG00000125977**	*0.020166344*	*0.027983293*	*0.027001636*	*0.018891332*
**ENSG00000175315**	*0.017472204*	*0.026729231*	*0.026073978*	*0.016087619*
**ENSG00000186352**	*0.087022946*	*0.066395841*	*0.079007068*	*0.072078359*
**ENSG00000141668**	*0.023475814*	*0.024776486*	*0.02658666*	*0.016547486*
**ENSG00000078304**	*0.01814214*	*0.026953114*	*0.024791347*	*0.015522663*
**ENSG00000178381**	*0.037754716*	*0.03589921*	*0.037822132*	*0.029028704*
**ENSG00000154114**	*0.037087175*	*0.038678426*	*0.035169699*	*0.033063016*
**ENSG00000197081**	*0.032544778*	*0.03948857*	*0.039053345*	*0.034400553*
**ENSG00000112599**	*0.015806941*	*0.026343386*	*0.025886029*	*0.018665398*
**ENSG00000167769**	*0.014969477*	*0.025547586*	*0.025593591*	*0.01523014*
**ENSG00000101901**	*0.03986304*	*0.044647637*	*0.051147374*	*0.037899533*
**ENSG00000179178**	*0.016227673*	*0.025922762*	*0.026532075*	*0.01655444*
**ENSG00000068615**	*0.033034954*	*0.02816078*	*0.025020891*	*0.022843182*
**ENSG00000122877**	*0.072985541*	*0.053319986*	*0.054095609*	*0.060073974*
**ENSG00000163486**	*0.043963174*	*0.037415374*	*0.038597238*	*0.038892465*
**ENSG00000078246**	*0.016576462*	*0.025381933*	*0.025770493*	*0.015163322*
**ENSG00000166169**	*0.02360263*	*0.027757232*	*0.031011229*	*0.027834507*
**ENSG00000133561**	*0.101638319*	*0.075834299*	*0.08164734*	*0.090417972*
**ENSG00000167685**	*0.023480953*	*0.029972657*	*0.028405372*	*0.022329961*
**ENSG00000027075**	*0.137717885*	*0.107238396*	*0.117988824*	*0.133605871*
**ENSG00000180817**	*0.018523617*	*0.026781466*	*0.026476126*	*0.018620997*
**ENSG00000188026**	*0.017682883*	*0.026608167*	*0.02545192*	*0.016825058*
**ENSG00000111737**	*0.033046353*	*0.03934554*	*0.039282495*	*0.034566366*
**ENSG00000167720**	*0.038663878*	*0.040361229*	*0.032808689*	*0.029845835*
**ENSG00000169564**	*0.020450229*	*0.027597315*	*0.025376927*	*0.016104102*
**ENSG00000196653**	*0.075381639*	*0.066476922*	*0.071559938*	*0.048480363*
**ENSG00000125954**	*0.034145283*	*0.03862297*	*0.033817132*	*0.023832811*
**ENSG00000185010**	*0.019764136*	*0.026938132*	*0.02564801*	*0.018073056*
**ENSG00000121005**	*0.015444504*	*0.025972853*	*0.024172049*	*0.014646013*
**ENSG00000188001**	*0.093128849*	*0.072657738*	*0.05345026*	*0.075254844*
**ENSG00000097021**	*0.060833949*	*0.043188078*	*0.045230379*	*0.060585189*
**ENSG00000169174**	*0.015035691*	*0.024597862*	*0.025078324*	*0.014982319*
**ENSG00000102287**	*0.018040648*	*0.02425222*	*0.025216354*	*0.016852788*
**ENSG00000111181**	*0.021715112*	*0.02715735*	*0.025188506*	*0.016485947*
**ENSG00000132164**	*0.016152222*	*0.026110449*	*0.025560972*	*0.014687767*

**ENSG00000134758**	*0.034960551*	*0.040887215*	*0.038663937*	*0.035590793*
**ENSG00000140367**	*0.032179705*	*0.035247432*	*0.035278964*	*0.028140219*
**ENSG00000165197**	*0.016509412*	*0.02524501*	*0.026609065*	*0.016034603*
**ENSG00000106263**	*0.034624825*	*0.026978463*	*0.030389457*	*0.028196468*
**ENSG00000120451**	*0.026955629*	*0.035963351*	*0.028359331*	*0.025986425*
**ENSG00000055163**	*0.026790051*	*0.031346597*	*0.031208997*	*0.035031648*
**ENSG00000100911**	*0.026511491*	*0.033300641*	*0.03036438*	*0.024227119*
**ENSG00000134684**	*0.023391408*	*0.028853522*	*0.029719727*	*0.021883785*
**ENSG00000172748**	*0.020810603*	*0.029302277*	*0.028478946*	*0.019119135*
**ENSG00000108187**	*0.038821141*	*0.037837266*	*0.034454496*	*0.036609487*
**ENSG00000117133**	*0.02823265*	*0.032035266*	*0.033632895*	*0.029549398*
**ENSG00000183020**	*0.024709546*	*0.033993141*	*0.031704702*	*0.023681345*
**ENSG00000171459**	*0.013128741*	*0.023511326*	*0.02384858*	*0.012890179*
**ENSG00000136156**	*0.062432475*	*0.055571255*	*0.052035147*	*0.040912056*
**ENSG00000167080**	*0.020492264*	*0.025585003*	*0.025469634*	*0.018174112*
**ENSG00000176160**	*0.040356418*	*0.033425178*	*0.030065738*	*0.029288757*
**ENSG00000171044**	*0.017923935*	*0.025773159*	*0.024447467*	*0.015611225*
**ENSG00000104419**	*0.109936616*	*0.103773912*	*0.080361109*	*0.108566903*
**ENSG00000198363**	*0.015786163*	*0.025398318*	*0.025516862*	*0.016036377*
**ENSG00000165501**	*0.04178768*	*0.034927468*	*0.035257378*	*0.033476718*
**ENSG00000127311**	*0.022392381*	*0.031949504*	*0.028123146*	*0.025478145*
**ENSG00000095397**	*0.030502045*	*0.039366629*	*0.026946015*	*0.022166194*
**ENSG00000132405**	*0.028319099*	*0.03561406*	*0.032807159*	*0.023308253*
**ENSG00000205090**	*0.015981151*	*0.02495132*	*0.02484918*	*0.015580049*
**ENSG00000102038**	*0.074287851*	*0.060127364*	*0.034989733*	*0.046250535*
**ENSG00000105146**	*0.022202486*	*0.029656352*	*0.033722732*	*0.023167165*
**ENSG00000180263**	*0.060696168*	*0.057720934*	*0.058647474*	*0.049400895*
**ENSG00000079432**	*0.023184144*	*0.031508381*	*0.033188008*	*0.02536399*
**ENSG00000144771**	*0.016077443*	*0.024590525*	*0.024597546*	*0.016171485*
**ENSG00000206073**	*0.017675371*	*0.025385738*	*0.026563607*	*0.017071202*
**ENSG00000136839**	*0.018129031*	*0.027038938*	*0.025759043*	*0.015956527*
**ENSG00000222004**	*0.015445409*	*0.025621455*	*0.025396764*	*0.014984261*
**ENSG00000113600**	*0.01603131*	*0.024593071*	*0.024563625*	*0.015754967*
**ENSG00000196811**	*0.016025873*	*0.024930967*	*0.024378753*	*0.015909434*
**ENSG00000095002**	*0.040931373*	*0.040780892*	*0.032530921*	*0.037305256*
**ENSG00000156150**	*0.015715822*	*0.026226371*	*0.024362233*	*0.015394175*
**ENSG00000151148**	*0.031109909*	*0.037950558*	*0.030599375*	*0.026107419*
**ENSG00000110442**	*0.024849825*	*0.030316852*	*0.033910154*	*0.029581931*
**ENSG00000143119**	*0.022569418*	*0.033629726*	*0.032169899*	*0.025274549*
**ENSG00000175920**	*0.016903586*	*0.025232291*	*0.02501253*	*0.016553309*
**ENSG00000104435**	*0.016450372*	*0.025049761*	*0.025078772*	*0.01647338*
**ENSG00000169918**	*0.030898027*	*0.033302583*	*0.031281343*	*0.028206365*
**ENSG00000141433**	*0.016119464*	*0.027201562*	*0.025213284*	*0.015136571*
**ENSG00000062096**	*0.015604095*	*0.026429735*	*0.024429667*	*0.016159272*
**ENSG00000146425**	*0.034908939*	*0.038360226*	*0.041307255*	*0.032987593*
**ENSG00000131849**	*0.042420288*	*0.033275719*	*0.033073804*	*0.025669182*
**ENSG00000121905**	*0.015330647*	*0.025653294*	*0.024053564*	*0.014615147*
**ENSG00000176182**	*0.028715559*	*0.035424637*	*0.030389946*	*0.024621579*
**ENSG00000197841**	*0.023762716*	*0.028253298*	*0.026080315*	*0.019209576*
**ENSG00000161405**	*0.033930005*	*0.041869139*	*0.038092958*	*0.035880354*
**ENSG00000148935**	*0.105194883*	*0.074277212*	*0.083716067*	*0.093052119*
**ENSG00000165802**	*0.01456605*	*0.025996533*	*0.025288586*	*0.014972778*
**ENSG00000019505**	*0.015111088*	*0.024737502*	*0.024124859*	*0.014602049*
**ENSG00000230989**	*0.029413851*	*0.030887488*	*0.030483156*	*0.021546*

**ENSG00000204252**	*0.042170721*	*0.048249746*	*0.036410767*	*0.065030623*
**ENSG00000124216**	*0.015546082*	*0.024885098*	*0.025082332*	*0.016472772*
**ENSG00000181027**	*0.032002832*	*0.038530177*	*0.04315124*	*0.035034324*
**ENSG00000198851**	*0.066998058*	*0.044743009*	*0.032803721*	*0.055092847*
**ENSG00000067560**	*0.018054421*	*0.027160602*	*0.026587944*	*0.017845231*
**ENSG00000103111**	*0.031764112*	*0.037916682*	*0.03780385*	*0.033251407*
**ENSG00000118322**	*0.015958268*	*0.024831083*	*0.024746356*	*0.016248063*
**ENSG00000172000**	*0.016955871*	*0.025198018*	*0.024945424*	*0.015825082*
**ENSG00000176732**	*0.029847445*	*0.030780339*	*0.029879592*	*0.020198946*
**ENSG00000196632**	*0.017418913*	*0.025653973*	*0.026384229*	*0.015494289*
**ENSG00000155016**	*0.043917558*	*0.04072831*	*0.038641257*	*0.039227785*
**ENSG00000224201**	*0.019772162*	*0.025807651*	*0.025993083*	*0.016816759*
**ENSG00000079393**	*0.022750571*	*0.027969634*	*0.029472172*	*0.018701287*
**ENSG00000156206**	*0.015452579*	*0.024668782*	*0.025201067*	*0.015022628*
**ENSG00000137494**	*0.019255701*	*0.025774088*	*0.025480984*	*0.018955301*
**ENSG00000162885**	*0.021595819*	*0.027230043*	*0.027897618*	*0.019777442*
**ENSG00000186190**	*0.015999107*	*0.02416372*	*0.024590287*	*0.014919207*
**ENSG00000142453**	*0.024188959*	*0.028103291*	*0.030244461*	*0.022799724*
**ENSG00000107104**	*0.014061763*	*0.02508967*	*0.024434388*	*0.016138904*
**ENSG00000227345**	*0.030887699*	*0.040435598*	*0.03017451*	*0.022906668*
**ENSG00000129151**	*0.046461126*	*0.027447326*	*0.02553978*	*0.019834175*
**ENSG00000137500**	*0.027813315*	*0.031663979*	*0.027923942*	*0.018655491*
**ENSG00000085719**	*0.025862699*	*0.032401314*	*0.031636505*	*0.023479124*
**ENSG00000154813**	*0.030453707*	*0.034337517*	*0.037804301*	*0.030747218*
**ENSG00000132661**	*0.0309391*	*0.039617804*	*0.041523233*	*0.027901655*
**ENSG00000165006**	*0.022823377*	*0.032280504*	*0.03705005*	*0.018096948*
**ENSG00000160182**	*0.018233573*	*0.026687121*	*0.026233822*	*0.015971747*
**ENSG00000168303**	*0.0232223*	*0.028964968*	*0.029549058*	*0.019500899*
**ENSG00000169297**	*0.016750637*	*0.025884189*	*0.024586044*	*0.016141087*
**ENSG00000155659**	*0.017379558*	*0.025572332*	*0.026431839*	*0.017122745*
**ENSG00000105700**	*0.021199539*	*0.027379887*	*0.030252036*	*0.021387156*
**ENSG00000155380**	*0.042027513*	*0.052505012*	*0.04425277*	*0.076327804*
**ENSG00000163586**	*0.018422958*	*0.025285971*	*0.02650427*	*0.01593942*
**ENSG00000158301**	*0.033570389*	*0.033371759*	*0.032580824*	*0.026216572*
**ENSG00000164117**	*0.027285884*	*0.035054023*	*0.031433923*	*0.025905789*
**ENSG00000101335**	*0.04771224*	*0.052985732*	*0.032686077*	*0.038016896*
**ENSG00000138092**	*0.037871949*	*0.032811263*	*0.034360053*	*0.02609292*
**ENSG00000142186**	*0.037958632*	*0.042684096*	*0.044128197*	*0.035588581*
**ENSG00000126856**	*0.054875142*	*0.043564057*	*0.037517325*	*0.042880069*
**ENSG00000186074**	*0.018555419*	*0.028478129*	*0.027666979*	*0.019540709*
**ENSG00000132975**	*0.015481657*	*0.024585259*	*0.024934648*	*0.013861216*
**ENSG00000163806**	*0.015242431*	*0.024678606*	*0.024714332*	*0.014258415*
**ENSG00000196141**	*0.100314617*	*0.068820551*	*0.075279377*	*0.091904611*
**ENSG00000117151**	*0.031506412*	*0.036573354*	*0.041670329*	*0.032113065*
**ENSG00000136197**	*0.034859048*	*0.03618874*	*0.030408238*	*0.026372455*
**ENSG00000170616**	*0.016158753*	*0.025492799*	*0.024962885*	*0.016277693*
**ENSG00000112041**	*0.015557892*	*0.025380767*	*0.024089633*	*0.015475473*
**ENSG00000186150**	*0.016829782*	*0.024684675*	*0.025047987*	*0.016089192*
**ENSG00000198015**	*0.031872065*	*0.033487941*	*0.030737657*	*0.025785681*
**ENSG00000186184**	*0.022893936*	*0.03254945*	*0.029097252*	*0.026760137*
**ENSG00000156976**	*0.029388999*	*0.03029247*	*0.03082121*	*0.022999017*
**ENSG00000147813**	*0.070205427*	*0.137092771*	*0.125493041*	*0.089715414*
**ENSG00000215203**	*0.014323335*	*0.024349271*	*0.023645397*	*0.013763507*
**ENSG00000173320**	*0.041064536*	*0.02587478*	*0.025366512*	*0.016951051*

**ENSG00000148730**	*0.0268788*	*0.033072348*	*0.030781708*	*0.022411641*
**ENSG00000161654**	*0.022378005*	*0.027889258*	*0.02957378*	*0.019514825*
**ENSG00000163126**	*0.039362965*	*0.047246856*	*0.037747768*	*0.038193251*
**ENSG00000134962**	*0.016150115*	*0.025358719*	*0.024643572*	*0.016350759*
**ENSG00000138738**	*0.015357709*	*0.025844097*	*0.025162537*	*0.015907408*
**ENSG00000118564**	*0.017600875*	*0.026916688*	*0.0266854*	*0.018180707*
**ENSG00000083123**	*0.046254642*	*0.04984258*	*0.03918009*	*0.033056219*
**ENSG00000161031**	*0.026186294*	*0.029795259*	*0.030996369*	*0.028647518*
**ENSG00000163902**	*0.027809094*	*0.030093324*	*0.033525113*	*0.028527917*
**ENSG00000131459**	*0.04203586*	*0.031308527*	*0.031237957*	*0.022077011*
**ENSG00000096746**	*0.029836386*	*0.030981138*	*0.02969813*	*0.025578661*
**ENSG00000144134**	*0.036698652*	*0.041111264*	*0.038337739*	*0.0350109*
**ENSG00000166855**	*0.026694443*	*0.032219175*	*0.031005377*	*0.025857007*
**ENSG00000131480**	*0.016437496*	*0.024448781*	*0.02474128*	*0.016666914*
**ENSG00000165621**	*0.016584129*	*0.025480508*	*0.024842126*	*0.015508537*
**ENSG00000100055**	*0.046913779*	*0.04005679*	*0.040561648*	*0.040864662*
**ENSG00000198390**	*0.015793817*	*0.02476322*	*0.025040865*	*0.016006024*
**ENSG00000151348**	*0.038901786*	*0.041754926*	*0.036944883*	*0.031441835*
**ENSG00000128191**	*0.035347457*	*0.033900857*	*0.032102096*	*0.036040023*
**ENSG00000145287**	*0.07782621*	*0.050620416*	*0.053115091*	*0.051652459*
**ENSG00000161610**	*0.015436379*	*0.027517261*	*0.02794715*	*0.015940763*
**ENSG00000181408**	*0.015981851*	*0.025754598*	*0.024627213*	*0.014875888*
**ENSG00000198324**	*0.016394911*	*0.025479889*	*0.025266176*	*0.014228833*
**ENSG00000177685**	*0.060353413*	*0.044774997*	*0.040817949*	*0.041906327*
**ENSG00000105887**	*0.034550639*	*0.040256552*	*0.0340323*	*0.030658427*
**ENSG00000136874**	*0.020748185*	*0.029990249*	*0.029504543*	*0.019585455*
**ENSG00000143543**	*0.020754971*	*0.028409833*	*0.026078003*	*0.018370421*
**ENSG00000115339**	*0.033955482*	*0.03612598*	*0.037101158*	*0.041154841*
**ENSG00000120306**	*0.047230891*	*0.038361076*	*0.040759874*	*0.046281687*
**ENSG00000135678**	*0.018742174*	*0.025774893*	*0.025809453*	*0.017081207*
**ENSG00000197483**	*0.0165773*	*0.029615739*	*0.030388894*	*0.018289759*
**ENSG00000132970**	*0.015862097*	*0.025399058*	*0.025736953*	*0.018031127*
**ENSG00000175121**	*0.016183781*	*0.024812787*	*0.02435028*	*0.015993188*
**ENSG00000105379**	*0.03252978*	*0.033434528*	*0.035462477*	*0.028125469*
**ENSG00000142611**	*0.016490985*	*0.025084289*	*0.024178642*	*0.01510016*
**ENSG00000243147**	*0.019942565*	*0.028060051*	*0.027816334*	*0.017652772*
**ENSG00000137819**	*0.017084072*	*0.026642273*	*0.025664994*	*0.017086494*
**ENSG00000169957**	*0.034073853*	*0.035697341*	*0.038463582*	*0.029337889*
**ENSG00000148798**	*0.059040699*	*0.031511447*	*0.035960168*	*0.052361537*
**ENSG00000143493**	*0.035506868*	*0.036585554*	*0.038135664*	*0.040393201*
**ENSG00000127824**	*0.044064529*	*0.033090909*	*0.031836147*	*0.031021005*
**ENSG00000175727**	*0.05091619*	*0.047977943*	*0.047162734*	*0.042614346*
**ENSG00000168597**	*0.022030938*	*0.02913511*	*0.026958493*	*0.021458409*
**ENSG00000177335**	*0.022988122*	*0.028016372*	*0.027924158*	*0.020232366*
**ENSG00000111058**	*0.016337149*	*0.024965414*	*0.024038182*	*0.014581469*
**ENSG00000152061**	*0.043099278*	*0.042291911*	*0.038532234*	*0.046873848*
**ENSG00000140859**	*0.030043847*	*0.030724553*	*0.028553589*	*0.021309823*
**ENSG00000146066**	*0.021496551*	*0.027939305*	*0.029545156*	*0.028670974*
**ENSG00000177613**	*0.028431998*	*0.040564927*	*0.040073074*	*0.026581818*
**ENSG00000188800**	*0.014370996*	*0.025165747*	*0.025521661*	*0.014470049*
**ENSG00000117620**	*0.032933*	*0.036071276*	*0.032659487*	*0.023367784*
**ENSG00000146535**	*0.027533575*	*0.029104991*	*0.026293498*	*0.02339336*
**ENSG00000152455**	*0.027030798*	*0.029707417*	*0.029308066*	*0.024011489*
**ENSG00000140398**	*0.085115181*	*0.062309579*	*0.059404123*	*0.081524073*

**ENSG00000174562**	*0.01430295*	*0.024877638*	*0.025019761*	*0.015180825*
**ENSG00000176208**	*0.042212902*	*0.04154257*	*0.033746335*	*0.040144318*
**ENSG00000133800**	*0.01512985*	*0.024611696*	*0.025181823*	*0.01477264*
**ENSG00000111653**	*0.016173933*	*0.024845899*	*0.024781777*	*0.015488759*
**ENSG00000102362**	*0.055149432*	*0.025331855*	*0.025858087*	*0.026782639*
**ENSG00000179750**	*0.070676991*	*0.127929457*	*0.12701674*	*0.060184376*
**ENSG00000059691**	*0.028919148*	*0.033648329*	*0.032391841*	*0.024926924*
**ENSG00000138650**	*0.015368956*	*0.024567173*	*0.025317702*	*0.016123486*
**ENSG00000120509**	*0.024396797*	*0.030756233*	*0.027717068*	*0.020396327*
**ENSG00000175497**	*0.016508298*	*0.025468115*	*0.025365446*	*0.015654181*
**ENSG00000140519**	*0.014935795*	*0.025216891*	*0.024879622*	*0.014131129*
**ENSG00000197714**	*0.024721418*	*0.031934193*	*0.029434217*	*0.021604153*
**ENSG00000138162**	*0.026455162*	*0.028874578*	*0.025031055*	*0.01510334*
**ENSG00000155876**	*0.022980419*	*0.030313528*	*0.030732144*	*0.020116483*
**ENSG00000109089**	*0.019203161*	*0.025636973*	*0.026648658*	*0.017722918*
**ENSG00000101160**	*0.058922337*	*0.050734384*	*0.05233446*	*0.066848908*
**ENSG00000175182**	*0.051242584*	*0.046442342*	*0.038667393*	*0.042990346*
**ENSG00000182504**	*0.016535172*	*0.024972682*	*0.025845423*	*0.015973683*
**ENSG00000198077**	*0.01687137*	*0.024966053*	*0.024511055*	*0.014747795*
**ENSG00000100146**	*0.015576909*	*0.024802039*	*0.024955954*	*0.015318407*
**ENSG00000174502**	*0.014896583*	*0.024949717*	*0.024434416*	*0.015935904*
**ENSG00000177575**	*0.015340386*	*0.025009463*	*0.025579834*	*0.015084528*
**ENSG00000198858**	*0.016833548*	*0.026345674*	*0.026309101*	*0.016193729*
**ENSG00000137267**	*0.078534028*	*0.087192199*	*0.083935592*	*0.082672406*
**ENSG00000145780**	*0.033085079*	*0.04256348*	*0.036505934*	*0.028049192*
**ENSG00000171150**	*0.015806831*	*0.024906613*	*0.025272938*	*0.015679006*
**ENSG00000244045**	*0.029031711*	*0.036398329*	*0.035336916*	*0.034742895*
**ENSG00000171453**	*0.031243266*	*0.034834853*	*0.03832904*	*0.028181895*
**ENSG00000100842**	*0.016804038*	*0.025737645*	*0.024746736*	*0.014425101*
**ENSG00000166152**	*0.015457803*	*0.024858009*	*0.024876497*	*0.016055196*
**ENSG00000095261**	*0.029911289*	*0.033900963*	*0.031929435*	*0.022862355*
**ENSG00000171443**	*0.033096734*	*0.03565059*	*0.032619602*	*0.028653949*
**ENSG00000186648**	*0.016351348*	*0.027909797*	*0.02781237*	*0.016425419*
**ENSG00000099901**	*0.030938802*	*0.033015065*	*0.030812936*	*0.026929141*
**ENSG00000141971**	*0.03106119*	*0.031962557*	*0.032190557*	*0.024649139*
**ENSG00000136603**	*0.043998744*	*0.039705417*	*0.041749381*	*0.030209877*
**ENSG00000068079**	*0.03360724*	*0.034734695*	*0.033566859*	*0.029648774*
**ENSG00000171848**	*0.072089868*	*0.062327984*	*0.047065886*	*0.055115542*
**ENSG00000058262**	*0.029580862*	*0.033228472*	*0.043939853*	*0.036838855*
**ENSG00000173898**	*0.028664053*	*0.036547197*	*0.027446896*	*0.038795164*
**ENSG00000072274**	*0.027106651*	*0.029604369*	*0.0283862*	*0.024764473*
**ENSG00000116218**	*0.015148876*	*0.024617961*	*0.024611091*	*0.014960873*
**ENSG00000108175**	*0.056127761*	*0.039022897*	*0.036276804*	*0.041729425*
**ENSG00000155324**	*0.051727201*	*0.055390436*	*0.048686201*	*0.058590197*
**ENSG00000116698**	*0.01631847*	*0.025251138*	*0.026079071*	*0.01556465*
**ENSG00000147526**	*0.023785112*	*0.034654981*	*0.03329879*	*0.027072973*
**ENSG00000144560**	*0.047249477*	*0.041799313*	*0.048909355*	*0.038962434*
**ENSG00000134508**	*0.05341447*	*0.035937727*	*0.035585193*	*0.038354645*
**ENSG00000233224**	*0.031371108*	*0.031326231*	*0.034777142*	*0.033713846*
**ENSG00000172530**	*0.030696026*	*0.032526405*	*0.040088743*	*0.034313537*
**ENSG00000175471**	*0.047310197*	*0.040673501*	*0.041328381*	*0.044283029*
**ENSG00000115760**	*0.025986535*	*0.037654856*	*0.029388557*	*0.025573634*
**ENSG00000152705**	*0.018489276*	*0.028723892*	*0.02649135*	*0.017522128*
**ENSG00000116701**	*0.054674451*	*0.051265305*	*0.043425105*	*0.047291257*

**ENSG00000188452**	*0.031513309*	*0.027499075*	*0.026592062*	*0.022914324*
**ENSG00000051523**	*0.02690749*	*0.031314835*	*0.029108598*	*0.023588994*
**ENSG00000196345**	*0.026394273*	*0.031575745*	*0.026879962*	*0.02120584*
**ENSG00000205809**	*0.016808769*	*0.024653088*	*0.025105933*	*0.017089988*
**ENSG00000010165**	*0.034281519*	*0.041282829*	*0.034875303*	*0.029795272*
**ENSG00000048540**	*0.142422309*	*0.099611166*	*0.089717418*	*0.09541886*
**ENSG00000176294**	*0.016755601*	*0.025400879*	*0.028233529*	*0.014417903*
**ENSG00000174951**	*0.017055389*	*0.025664231*	*0.024762778*	*0.018166269*
**ENSG00000084774**	*0.03485247*	*0.039946471*	*0.035008554*	*0.026391109*
**ENSG00000107372**	*0.026588733*	*0.037354101*	*0.035762266*	*0.024877784*
**ENSG00000084628**	*0.034131442*	*0.029421868*	*0.02484013*	*0.017960954*
**ENSG00000144749**	*0.051960687*	*0.055063693*	*0.044945855*	*0.046943652*
**ENSG00000073803**	*0.024718899*	*0.030192155*	*0.032885564*	*0.027660222*
**ENSG00000070061**	*0.029634225*	*0.035654125*	*0.031261969*	*0.019874107*
**ENSG00000001626**	*0.016267878*	*0.02685182*	*0.026407607*	*0.016458894*
**ENSG00000074416**	*0.036805959*	*0.03788355*	*0.034690162*	*0.037069874*
**ENSG00000206053**	*0.030142199*	*0.033761033*	*0.031879475*	*0.025190439*
**ENSG00000129277**	*0.074254686*	*0.050752006*	*0.053359377*	*0.06156874*
**ENSG00000092054**	*0.024516323*	*0.025357589*	*0.02604863*	*0.018539681*
**ENSG00000164946**	*0.021377187*	*0.025347594*	*0.024374395*	*0.023277707*
**ENSG00000113068**	*0.020140494*	*0.028781308*	*0.030716148*	*0.021849889*
**ENSG00000125648**	*0.044726504*	*0.043714706*	*0.036706917*	*0.036460222*
**ENSG00000125798**	*0.016965345*	*0.02524879*	*0.025841808*	*0.016854168*
**ENSG00000144840**	*0.029943727*	*0.034123876*	*0.03534576*	*0.026806675*
**ENSG00000164587**	*0.012998215*	*0.023582113*	*0.023388337*	*0.014560897*
**ENSG00000168234**	*0.052261572*	*0.061492756*	*0.060091292*	*0.060118835*
**ENSG00000243056**	*0.048325602*	*0.053848005*	*0.04517061*	*0.05202232*
**ENSG00000198755**	*0.016400273*	*0.02508864*	*0.025795425*	*0.017130672*
**ENSG00000168917**	*0.083817065*	*0.072595688*	*0.044995369*	*0.052724872*
**ENSG00000152689**	*0.03487496*	*0.046842858*	*0.037358235*	*0.030730096*
**ENSG00000076770**	*0.017938306*	*0.02660067*	*0.026827505*	*0.022363596*
**ENSG00000151893**	*0.022375726*	*0.028548933*	*0.029027405*	*0.018442543*
**ENSG00000130518**	*0.014791957*	*0.024550846*	*0.024101022*	*0.014403915*
**ENSG00000132702**	*0.014845624*	*0.024589184*	*0.02404918*	*0.013698753*
**ENSG00000090013**	*0.049874983*	*0.038661804*	*0.039528908*	*0.040640425*
**ENSG00000115009**	*0.155882674*	*0.108535503*	*0.047663986*	*0.104359733*
**ENSG00000067048**	*0.253568148*	*0.283227114*	*0.251468315*	*0.248287655*
**ENSG00000168010**	*0.044748828*	*0.039552024*	*0.03965922*	*0.036456216*
**ENSG00000184984**	*0.01552645*	*0.025516721*	*0.025032485*	*0.015295694*
**ENSG00000137693**	*0.018616246*	*0.025095873*	*0.024612252*	*0.015467747*
**ENSG00000187068**	*0.017372052*	*0.025161113*	*0.02512231*	*0.015944714*
**ENSG00000114113**	*0.016760765*	*0.025241307*	*0.026273021*	*0.015684445*
**ENSG00000135749**	*0.02156049*	*0.028520456*	*0.026017634*	*0.022623351*
**ENSG00000157927**	*0.015667742*	*0.025390858*	*0.024445856*	*0.014920082*
**ENSG00000039600**	*0.017504404*	*0.025514656*	*0.025433174*	*0.024936745*
**ENSG00000168066**	*0.053354307*	*0.069744954*	*0.06889615*	*0.080363798*
**ENSG00000072954**	*0.030883737*	*0.031919749*	*0.030963447*	*0.03731589*
**ENSG00000136068**	*0.065121737*	*0.058543157*	*0.050667773*	*0.051993538*
**ENSG00000178342**	*0.013981092*	*0.025387429*	*0.024978275*	*0.015295038*
**ENSG00000180061**	*0.014912183*	*0.02523392*	*0.025728084*	*0.016100602*
**ENSG00000164758**	*0.038310142*	*0.037027834*	*0.033287842*	*0.029347093*
**ENSG00000165714**	*0.026826404*	*0.029766126*	*0.027584821*	*0.019415786*
**ENSG00000196950**	*0.058174931*	*0.045977319*	*0.039341091*	*0.038228681*
**ENSG00000163683**	*0.044072939*	*0.041623104*	*0.036323589*	*0.041617946*

**ENSG00000136108**	*0.040400665*	*0.043130328*	*0.037721799*	*0.03352914*
**ENSG00000100426**	*0.028124526*	*0.040874442*	*0.038228539*	*0.027434361*
**ENSG00000147889**	*0.014651339*	*0.024785469*	*0.024475682*	*0.015175559*
**ENSG00000026025**	*0.078316916*	*0.050983415*	*0.046364638*	*0.066813634*
**ENSG00000173020**	*0.016104037*	*0.025019569*	*0.025444589*	*0.015560293*
**ENSG00000142224**	*0.040658814*	*0.038525554*	*0.034150506*	*0.029191107*
**ENSG00000072840**	*0.089363397*	*0.049000473*	*0.037778567*	*0.058774252*
**ENSG00000184502**	*0.017895976*	*0.027698857*	*0.025418378*	*0.017176545*
**ENSG00000029993**	*0.040410739*	*0.038350586*	*0.034221778*	*0.034102703*
**ENSG00000149636**	*0.03044862*	*0.030996755*	*0.03224965*	*0.02395805*
**ENSG00000167346**	*0.015504365*	*0.025895583*	*0.024824386*	*0.015620026*
**ENSG00000168412**	*0.017881631*	*0.02602907*	*0.025908116*	*0.016322947*
**ENSG00000169413**	*0.070867683*	*0.057878072*	*0.055288578*	*0.066162267*
**ENSG00000184564**	*0.019626335*	*0.026579523*	*0.026647428*	*0.017369705*
**ENSG00000148848**	*0.021480616*	*0.024028791*	*0.024472152*	*0.01629631*
**ENSG00000171564**	*0.015748964*	*0.024750305*	*0.025220736*	*0.016143325*
**ENSG00000167625**	*0.032072041*	*0.03269629*	*0.034119022*	*0.025706123*
**ENSG00000182010**	*0.016812939*	*0.025422196*	*0.026358211*	*0.016351534*
**ENSG00000161640**	*0.017020082*	*0.026134822*	*0.024116117*	*0.014982001*
**ENSG00000143420**	*0.02675069*	*0.035779239*	*0.033857517*	*0.028634759*
**ENSG00000198739**	*0.015206826*	*0.024974035*	*0.024739154*	*0.015954079*
**ENSG00000104964**	*0.020110463*	*0.030366087*	*0.028186928*	*0.020720315*
**ENSG00000171522**	*0.024484058*	*0.026783647*	*0.028072115*	*0.018881691*
**ENSG00000116991**	*0.015742091*	*0.025462956*	*0.025480511*	*0.017739605*
**ENSG00000160226**	*0.016671318*	*0.025272534*	*0.026439474*	*0.017871066*
**ENSG00000189190**	*0.024682013*	*0.029009346*	*0.028920295*	*0.023481442*
**ENSG00000137947**	*0.024626627*	*0.032332627*	*0.036633519*	*0.021093673*
**ENSG00000067900**	*0.035068161*	*0.04574274*	*0.037256281*	*0.03231753*
**ENSG00000118707**	*0.026377709*	*0.034142533*	*0.031895846*	*0.022787608*
**ENSG00000141977**	*0.019704706*	*0.025754185*	*0.02565446*	*0.018102459*
**ENSG00000213416**	*0.01893877*	*0.026709688*	*0.028494553*	*0.017591944*
**ENSG00000188396**	*0.016618694*	*0.025272229*	*0.025928878*	*0.015855224*
**ENSG00000100335**	*0.027865455*	*0.033250498*	*0.038418695*	*0.029039404*
**ENSG00000105245**	*0.019437554*	*0.03163805*	*0.033502972*	*0.028120683*
**ENSG00000149212**	*0.053609042*	*0.052315205*	*0.037122079*	*0.041222751*
**ENSG00000135709**	*0.081746378*	*0.051547674*	*0.05653588*	*0.05527522*
**ENSG00000196730**	*0.080270868*	*0.071331207*	*0.067628551*	*0.061242903*
**ENSG00000186513**	*0.016748392*	*0.0258212*	*0.02488086*	*0.015457993*
**ENSG00000161270**	*0.017312262*	*0.026154226*	*0.027277289*	*0.016944174*
**ENSG00000196226**	*0.050774945*	*0.049039723*	*0.053024784*	*0.051561448*
**ENSG00000150076**	*0.022364687*	*0.026382874*	*0.028272598*	*0.018420071*
**ENSG00000082458**	*0.037768721*	*0.034276598*	*0.030230793*	*0.02787038*
**ENSG00000136933**	*0.032506104*	*0.034187476*	*0.029716705*	*0.022032113*
**ENSG00000122218**	*0.019805501*	*0.028370393*	*0.030308452*	*0.020547979*
**ENSG00000165660**	*0.025657068*	*0.034225718*	*0.032388133*	*0.020884317*
**ENSG00000179284**	*0.017852545*	*0.026046623*	*0.02613511*	*0.017523659*
**ENSG00000155158**	*0.047618168*	*0.048876186*	*0.048761403*	*0.041377026*
**ENSG00000141540**	*0.05410078*	*0.050422085*	*0.049214959*	*0.050382793*
**ENSG00000180902**	*0.046710295*	*0.043447103*	*0.039862395*	*0.036178662*
**ENSG00000125743**	*0.022515804*	*0.027442382*	*0.027635599*	*0.020356281*
**ENSG00000130332**	*0.023402057*	*0.027233926*	*0.029695054*	*0.020466426*
**ENSG00000130164**	*0.043411405*	*0.042743271*	*0.043391558*	*0.033852021*
**ENSG00000179914**	*0.016526504*	*0.025347666*	*0.024428366*	*0.015139262*
**ENSG00000228314**	*0.046478302*	*0.040810174*	*0.037917323*	*0.052023863*

**ENSG00000143248**	*0.017438109*	*0.027193823*	*0.026161446*	*0.019986781*
**ENSG00000144406**	*0.019123276*	*0.026762373*	*0.026320138*	*0.017949287*
**ENSG00000197641**	*0.017373105*	*0.025334809*	*0.0258893*	*0.015768631*
**ENSG00000188827**	*0.015491945*	*0.025852243*	*0.025491209*	*0.015086016*
**ENSG00000082512**	*0.018266522*	*0.02585689*	*0.025091573*	*0.01654075*
**ENSG00000070081**	*0.05747625*	*0.050099401*	*0.048762761*	*0.054525106*
**ENSG00000182718**	*0.040941734*	*0.039345719*	*0.035256383*	*0.039344119*
**ENSG00000171316**	*0.027841308*	*0.033247624*	*0.033570443*	*0.026670753*
**ENSG00000117625**	*0.027992392*	*0.034966018*	*0.031906463*	*0.025367193*
**ENSG00000198796**	*0.094890637*	*0.076073242*	*0.065302976*	*0.065447927*
**ENSG00000117153**	*0.024855164*	*0.034529059*	*0.03074075*	*0.024435527*
**ENSG00000178363**	*0.014807731*	*0.025755447*	*0.025136191*	*0.014150189*
**ENSG00000109854**	*0.031600438*	*0.035934036*	*0.033493679*	*0.024678507*
**ENSG00000131969**	*0.016351592*	*0.02506849*	*0.024420891*	*0.014589321*
**ENSG00000077157**	*0.015463806*	*0.024770744*	*0.024596466*	*0.015495836*
**ENSG00000138756**	*0.038859477*	*0.031044495*	*0.030211269*	*0.025050132*
**ENSG00000188573**	*0.017729195*	*0.025144093*	*0.024687506*	*0.014871472*
**ENSG00000163661**	*0.015828047*	*0.025007252*	*0.025524677*	*0.014222997*
**ENSG00000183145**	*0.070913514*	*0.059125793*	*0.059507919*	*0.074152424*
**ENSG00000196465**	*0.022066946*	*0.030382935*	*0.028643493*	*0.026521552*
**ENSG00000121753**	*0.020652384*	*0.027752309*	*0.026250423*	*0.023084605*
**ENSG00000165795**	*0.015905564*	*0.025340444*	*0.025017943*	*0.01544737*
**ENSG00000163424**	*0.016278464*	*0.024882468*	*0.025129302*	*0.01497469*
**ENSG00000134490**	*0.043968465*	*0.04096061*	*0.042370889*	*0.038164227*
**ENSG00000102471**	*0.034220825*	*0.038916179*	*0.040556949*	*0.037153946*
**ENSG00000138796**	*0.044289489*	*0.042288049*	*0.035945308*	*0.030243177*
**ENSG00000227826**	*0.107741841*	*0.148766627*	*0.134662087*	*0.109525912*
**ENSG00000100439**	*0.044261229*	*0.040436947*	*0.046474324*	*0.042855249*
**ENSG00000169228**	*0.028134994*	*0.037142103*	*0.032519402*	*0.024059254*
**ENSG00000075073**	*0.016338451*	*0.025234981*	*0.024730715*	*0.015405537*
**ENSG00000166681**	*0.127278052*	*0.132542685*	*0.094318283*	*0.128707887*
**ENSG00000119705**	*0.021139615*	*0.027548307*	*0.026291701*	*0.020145469*
**ENSG00000183426**	*0.020944444*	*0.026564141*	*0.033224365*	*0.019375349*
**ENSG00000143479**	*0.052183281*	*0.041651966*	*0.042489031*	*0.037376688*
**ENSG00000097033**	*0.026364614*	*0.033653729*	*0.033709283*	*0.020673107*
**ENSG00000125787**	*0.015871791*	*0.025257243*	*0.024881542*	*0.015526703*
**ENSG00000121381**	*0.016408759*	*0.025208853*	*0.024534104*	*0.014501749*
**ENSG00000083857**	*0.099617583*	*0.050399749*	*0.028847072*	*0.050436222*
**ENSG00000170633**	*0.017186265*	*0.026245453*	*0.026110477*	*0.016802404*
**ENSG00000182533**	*0.016829932*	*0.025075648*	*0.026228467*	*0.016611005*
**ENSG00000144057**	*0.015878474*	*0.026182584*	*0.024262351*	*0.015447453*
**ENSG00000119865**	*0.018171405*	*0.026846859*	*0.026242908*	*0.015775644*
**ENSG00000157379**	*0.040402723*	*0.039959288*	*0.041570197*	*0.044882789*
**ENSG00000184650**	*0.021861855*	*0.029207447*	*0.027433424*	*0.020021362*
**ENSG00000079459**	*0.022211542*	*0.028858096*	*0.027362221*	*0.020586124*
**ENSG00000068781**	*0.015230156*	*0.024905408*	*0.025057991*	*0.015464092*
**ENSG00000172260**	*0.015845005*	*0.02451496*	*0.024462382*	*0.014955697*
**ENSG00000119408**	*0.034864232*	*0.043479985*	*0.042792318*	*0.039477417*
**ENSG00000129596**	*0.035736654*	*0.029221085*	*0.031277483*	*0.021020951*
**ENSG00000115155**	*0.035910876*	*0.035712291*	*0.041609503*	*0.034690845*
**ENSG00000146151**	*0.017011006*	*0.025028412*	*0.025314777*	*0.015580148*
**ENSG00000165775**	*0.027354354*	*0.029719627*	*0.032486113*	*0.027826207*
**ENSG00000171209**	*0.015515506*	*0.024648471*	*0.024228573*	*0.014138952*
**ENSG00000092295**	*0.032498741*	*0.035720019*	*0.035685887*	*0.029055251*

**ENSG00000148688**	*0.020044565*	*0.027432572*	*0.026247927*	*0.017203464*
**ENSG00000109929**	*0.030527509*	*0.034728613*	*0.038416766*	*0.02913874*
**ENSG00000179058**	*0.016338209*	*0.025619327*	*0.024580121*	*0.015278468*
**ENSG00000122958**	*0.023917271*	*0.03036276*	*0.028838436*	*0.023118555*
**ENSG00000186567**	*0.014218219*	*0.025170638*	*0.024470071*	*0.015341423*
**ENSG00000176248**	*0.02490966*	*0.028673098*	*0.028815468*	*0.025156464*
**ENSG00000106244**	*0.024777204*	*0.030071765*	*0.030876162*	*0.024244356*
**ENSG00000124813**	*0.01661014*	*0.025208824*	*0.025102282*	*0.014569248*
**ENSG00000179869**	*0.021537912*	*0.0280001*	*0.026136732*	*0.018264706*
**ENSG00000198246**	*0.015831004*	*0.024604926*	*0.024442686*	*0.014363767*
**ENSG00000198074**	*0.015361751*	*0.024589056*	*0.024723918*	*0.01566041*
**ENSG00000154124**	*0.035985175*	*0.039997949*	*0.041583807*	*0.032047614*
**ENSG00000088543**	*0.017913398*	*0.028179143*	*0.025085964*	*0.017814714*
**ENSG00000168078**	*0.048655446*	*0.037824941*	*0.035095162*	*0.037670097*
**ENSG00000223730**	*0.015867731*	*0.024675945*	*0.02496338*	*0.016456929*
**ENSG00000183340**	*0.025136738*	*0.034310633*	*0.028712939*	*0.021613779*
**ENSG00000103723**	*0.016539722*	*0.025238369*	*0.024814714*	*0.016326596*
**ENSG00000169248**	*0.01977118*	*0.026564856*	*0.026330058*	*0.015560784*
**ENSG00000156219**	*0.015387666*	*0.024948439*	*0.025298291*	*0.015332075*
**ENSG00000166823**	*0.052575478*	*0.049889508*	*0.046273043*	*0.044409802*
**ENSG00000162654**	*0.104295726*	*0.100500946*	*0.095493115*	*0.112277505*
**ENSG00000107223**	*0.016184748*	*0.024257109*	*0.024445773*	*0.01531503*
**ENSG00000221955**	*0.065760186*	*0.05070852*	*0.042758157*	*0.063599972*
**ENSG00000049239**	*0.01674936*	*0.024902024*	*0.023788423*	*0.015153609*
**ENSG00000144837**	*0.088297711*	*0.051954734*	*0.055388065*	*0.06714615*
**ENSG00000133627**	*0.03559944*	*0.035462872*	*0.032143262*	*0.030520161*
**ENSG00000100038**	*0.025382473*	*0.032359428*	*0.033729589*	*0.024445647*
**ENSG00000104067**	*0.08006845*	*0.031056064*	*0.027536544*	*0.040470152*
**ENSG00000187173**	*0.015635115*	*0.024164422*	*0.025463498*	*0.014941641*
**ENSG00000129235**	*0.028100335*	*0.03140443*	*0.026892941*	*0.025655136*
**ENSG00000115318**	*0.075318779*	*0.057654705*	*0.05109257*	*0.064507764*
**ENSG00000008294**	*0.032874724*	*0.033539159*	*0.034826107*	*0.024740193*
**ENSG00000106299**	*0.027850918*	*0.035418023*	*0.035111661*	*0.024714281*
**ENSG00000149582**	*0.018529236*	*0.028011462*	*0.026423325*	*0.018053946*
**ENSG00000197885**	*0.033971361*	*0.035208192*	*0.034422868*	*0.030516542*
**ENSG00000100523**	*0.034304677*	*0.039824649*	*0.032327399*	*0.031267605*
**ENSG00000148399**	*0.028979625*	*0.032793804*	*0.032775768*	*0.023900778*
**ENSG00000130427**	*0.016989304*	*0.025007889*	*0.026074655*	*0.018012762*
**ENSG00000185668**	*0.027004656*	*0.030422804*	*0.027636648*	*0.029158225*
**ENSG00000109111**	*0.020460812*	*0.028928294*	*0.030515592*	*0.022555215*
**ENSG00000135973**	*0.017614831*	*0.026577491*	*0.026606659*	*0.016697808*
**ENSG00000127928**	*0.01722319*	*0.025651418*	*0.02542835*	*0.016856237*
**ENSG00000186395**	*0.026859923*	*0.038981719*	*0.039935363*	*0.026658773*
**ENSG00000076554**	*0.020309128*	*0.026605539*	*0.027039747*	*0.016364153*
**ENSG00000114738**	*0.046730433*	*0.043940163*	*0.047336487*	*0.046670891*
**ENSG00000170348**	*0.032257278*	*0.035117829*	*0.037489904*	*0.029897156*
**ENSG00000105193**	*0.020606796*	*0.02737089*	*0.026267721*	*0.021310714*
**ENSG00000104883**	*0.018859589*	*0.027369431*	*0.024573796*	*0.016468467*
**ENSG00000115594**	*0.115406661*	*0.098671061*	*0.056684816*	*0.089605947*
**ENSG00000123473**	*0.016929191*	*0.026749585*	*0.025746678*	*0.017040754*
**ENSG00000122566**	*0.025877215*	*0.03145163*	*0.02783204*	*0.023503812*
**ENSG00000162627**	*0.025209923*	*0.025396366*	*0.025040802*	*0.016581618*
**ENSG00000180483**	*0.017095844*	*0.025461011*	*0.024923719*	*0.017687971*
**ENSG00000111300**	*0.03337373*	*0.032734993*	*0.033318842*	*0.029355792*

**ENSG00000086232**	*0.022768059*	*0.030236511*	*0.035657375*	*0.023672566*
**ENSG00000140678**	*0.062853714*	*0.053322396*	*0.049557818*	*0.082745547*
**ENSG00000173120**	*0.022093456*	*0.031581701*	*0.037018064*	*0.023599174*
**ENSG00000166432**	*0.025987313*	*0.029504238*	*0.027356546*	*0.026402101*
**ENSG00000129514**	*0.050466078*	*0.033936786*	*0.030457691*	*0.019678023*
**ENSG00000170035**	*0.014926281*	*0.024846278*	*0.025476184*	*0.014538397*
**ENSG00000142273**	*0.019468872*	*0.027881335*	*0.025996138*	*0.020659161*
**ENSG00000167749**	*0.049809311*	*0.05416763*	*0.045599851*	*0.057150976*
**ENSG00000163702**	*0.015262804*	*0.025703224*	*0.024442885*	*0.014968705*
**ENSG00000186081**	*0.028093747*	*0.024655569*	*0.025966027*	*0.019392446*
**ENSG00000013375**	*0.035822092*	*0.037174323*	*0.041259803*	*0.037064138*
**ENSG00000221986**	*0.017023242*	*0.026176736*	*0.024435074*	*0.015804418*
**ENSG00000133863**	*0.033453014*	*0.035824572*	*0.027159189*	*0.03173027*
**ENSG00000122884**	*0.061546758*	*0.038008041*	*0.035746485*	*0.040396563*
**ENSG00000176749**	*0.073791856*	*0.049850626*	*0.044908038*	*0.066003093*
**ENSG00000017621**	*0.016047284*	*0.025897924*	*0.024936005*	*0.015944318*
**ENSG00000248144**	*0.020403188*	*0.024336142*	*0.026123472*	*0.016789047*
**ENSG00000109445**	*0.027236066*	*0.031319703*	*0.031029985*	*0.020405668*
**ENSG00000188352**	*0.044322497*	*0.039630076*	*0.036974369*	*0.031656904*
**ENSG00000108561**	*0.025634467*	*0.027670555*	*0.026889078*	*0.020577716*
**ENSG00000153107**	*0.028616811*	*0.033269543*	*0.029877931*	*0.020203628*
**ENSG00000132541**	*0.053530818*	*0.048349407*	*0.038367852*	*0.040489648*
**ENSG00000223802**	*0.017932047*	*0.024901281*	*0.025599986*	*0.014990541*
**ENSG00000042429**	*0.023600183*	*0.030760325*	*0.027007888*	*0.019237292*
**ENSG00000125551**	*0.028879132*	*0.030557943*	*0.034607939*	*0.024129398*
**ENSG00000177082**	*0.031706248*	*0.035111474*	*0.042090098*	*0.027443172*
**ENSG00000134201**	*0.015962494*	*0.024653327*	*0.024558575*	*0.015650112*
**ENSG00000054116**	*0.028320605*	*0.033401713*	*0.032352615*	*0.02375788*
**ENSG00000198876**	*0.026511338*	*0.032219348*	*0.029467964*	*0.020894866*
**ENSG00000119778**	*0.023201281*	*0.032950446*	*0.029500745*	*0.020763045*
**ENSG00000169594**	*0.014848157*	*0.024394851*	*0.024768983*	*0.015692434*
**ENSG00000109099**	*0.015051004*	*0.025053714*	*0.024401794*	*0.026780195*
**ENSG00000140992**	*0.02549078*	*0.031938842*	*0.031573722*	*0.022675792*
**ENSG00000172888**	*0.028273905*	*0.031288793*	*0.030982269*	*0.023824914*
**ENSG00000149294**	*0.015613427*	*0.024469095*	*0.025078584*	*0.01620213*
**ENSG00000175376**	*0.03192916*	*0.035688875*	*0.03691153*	*0.024095842*
**ENSG00000140521**	*0.020775822*	*0.030281035*	*0.028757991*	*0.0200509*
**ENSG00000072786**	*0.029463244*	*0.033304377*	*0.034659596*	*0.0248956*
**ENSG00000006116**	*0.032759594*	*0.026436869*	*0.025210236*	*0.038742331*
**ENSG00000198719**	*0.036755412*	*0.051089887*	*0.042457149*	*0.050253191*
**ENSG00000186300**	*0.017945362*	*0.026987865*	*0.024663975*	*0.016625931*
**ENSG00000243667**	*0.036595095*	*0.04473641*	*0.036111621*	*0.031216614*
**ENSG00000196323**	*0.036929073*	*0.036978784*	*0.038387888*	*0.034951709*
**ENSG00000007306**	*0.015068531*	*0.024856297*	*0.024469923*	*0.015077528*
**ENSG00000196532**	*0.067619846*	*0.050237343*	*0.066201593*	*0.073517507*
**ENSG00000107937**	*0.028260208*	*0.034396131*	*0.033607291*	*0.030219119*
**ENSG00000183955**	*0.033165052*	*0.035584933*	*0.037039394*	*0.040025964*
**ENSG00000183431**	*0.026436365*	*0.030426563*	*0.033520855*	*0.026138545*
**ENSG00000145335**	*0.048266391*	*0.028709453*	*0.027149104*	*0.040569302*
**ENSG00000213347**	*0.043705752*	*0.032294837*	*0.034338313*	*0.029841097*
**ENSG00000150991**	*0.014074979*	*0.02377619*	*0.024638588*	*0.013515026*
**ENSG00000182108**	*0.03382288*	*0.036872078*	*0.036043233*	*0.033771092*
**ENSG00000125821**	*0.029133572*	*0.032201426*	*0.03341834*	*0.023511746*
**ENSG00000109182**	*0.016704662*	*0.025514029*	*0.02500827*	*0.016381879*

**ENSG00000169116**	*0.023321252*	*0.028749397*	*0.027225706*	*0.024325342*
**ENSG00000196943**	*0.023224617*	*0.027537615*	*0.027215227*	*0.017926357*
**ENSG00000142511**	*0.014956617*	*0.024018489*	*0.024287215*	*0.01440346*
**ENSG00000158555**	*0.068971562*	*0.084019337*	*0.063433251*	*0.052496597*
**ENSG00000196376**	*0.016586026*	*0.02469643*	*0.024628169*	*0.015089233*
**ENSG00000124380**	*0.03333763*	*0.035884397*	*0.037050256*	*0.027192694*
**ENSG00000196565**	*0.067220104*	*0.026413282*	*0.025102266*	*0.021358345*
**ENSG00000114030**	*0.027933648*	*0.032127735*	*0.034731025*	*0.028644263*
**ENSG00000176853**	*0.039617047*	*0.040272536*	*0.046602455*	*0.033152434*
**ENSG00000182810**	*0.030676119*	*0.037726202*	*0.046649824*	*0.031595446*
**ENSG00000139610**	*0.01972326*	*0.025460446*	*0.025474285*	*0.017449417*
**ENSG00000087903**	*0.022074775*	*0.028665506*	*0.027247726*	*0.021396309*
**ENSG00000119723**	*0.017604347*	*0.025273216*	*0.025525617*	*0.016055503*
**ENSG00000124783**	*0.028056754*	*0.032666623*	*0.03305931*	*0.028010072*
**ENSG00000168438**	*0.034169806*	*0.040778757*	*0.034743538*	*0.02801558*
**ENSG00000151923**	*0.015400012*	*0.025918437*	*0.025274877*	*0.014124095*
**ENSG00000142528**	*0.024251559*	*0.02746832*	*0.028600697*	*0.030544991*
**ENSG00000165409**	*0.016335178*	*0.025578171*	*0.026744072*	*0.016559357*
**ENSG00000123453**	*0.032141293*	*0.040916271*	*0.037017905*	*0.04449024*
**ENSG00000061656**	*0.076001435*	*0.04647411*	*0.047846201*	*0.049464651*
**ENSG00000156136**	*0.046564262*	*0.052542091*	*0.039460105*	*0.033424238*
**ENSG00000081386**	*0.026438743*	*0.034688843*	*0.031646736*	*0.025640428*
**ENSG00000140830**	*0.0302002*	*0.02871868*	*0.035425491*	*0.026808846*
**ENSG00000163600**	*0.061788495*	*0.094602463*	*0.086661045*	*0.084955344*
**ENSG00000162174**	*0.098149246*	*0.049921756*	*0.042729534*	*0.065340412*
**ENSG00000198585**	*0.022044369*	*0.028138294*	*0.028772048*	*0.022383304*
**ENSG00000109381**	*0.02988408*	*0.04038693*	*0.040036071*	*0.026782651*
**ENSG00000123836**	*0.014392864*	*0.024925724*	*0.024357849*	*0.01403394*
**ENSG00000183734**	*0.025200529*	*0.028618767*	*0.034447049*	*0.021698112*
**ENSG00000185860**	*0.01731295*	*0.024813396*	*0.025633708*	*0.015403454*
**ENSG00000111186**	*0.016830683*	*0.024969604*	*0.02556782*	*0.016133881*
**ENSG00000134278**	*0.020142219*	*0.023932363*	*0.025273932*	*0.015422684*
**ENSG00000126091**	*0.02686868*	*0.030251361*	*0.03186969*	*0.021098229*
**ENSG00000177752**	*0.016724962*	*0.025893511*	*0.025883604*	*0.016687183*
**ENSG00000094796**	*0.014994925*	*0.024586607*	*0.024341751*	*0.014862905*
**ENSG00000119535**	*0.018455597*	*0.026411487*	*0.025693433*	*0.017254582*
**ENSG00000188321**	*0.040128366*	*0.045568136*	*0.051539518*	*0.033756051*
**ENSG00000138744**	*0.063161793*	*0.04521069*	*0.045995171*	*0.056402574*
**ENSG00000181577**	*0.085944995*	*0.070420216*	*0.061848707*	*0.073638244*
**ENSG00000157500**	*0.033237481*	*0.044376642*	*0.038937939*	*0.035587343*
**ENSG00000099977**	*0.018604435*	*0.026885934*	*0.027535838*	*0.022320009*
**ENSG00000173681**	*0.038169766*	*0.036376663*	*0.033771022*	*0.036134023*
**ENSG00000131171**	*0.021239204*	*0.031756835*	*0.028189075*	*0.022893735*
**ENSG00000141150**	*0.01909183*	*0.025616436*	*0.026079631*	*0.017074593*
**ENSG00000162924**	*0.050610365*	*0.054336766*	*0.053010395*	*0.041154604*
**ENSG00000143612**	*0.018205842*	*0.026713407*	*0.026991563*	*0.018800548*
**ENSG00000173917**	*0.106656017*	*0.079936004*	*0.067234339*	*0.074961039*
**ENSG00000090659**	*0.014849904*	*0.025593427*	*0.025192937*	*0.016004837*
**ENSG00000175334**	*0.030249229*	*0.033161225*	*0.033152091*	*0.031690058*
**ENSG00000213654**	*0.021392066*	*0.028397566*	*0.033942793*	*0.022672622*
**ENSG00000163719**	*0.036966765*	*0.036854151*	*0.047264109*	*0.036048599*
**ENSG00000117592**	*0.030855064*	*0.036793644*	*0.033688679*	*0.024018243*
**ENSG00000188611**	*0.020654058*	*0.027333727*	*0.028689686*	*0.019187436*
**ENSG00000065609**	*0.018487489*	*0.027335765*	*0.024851447*	*0.015631227*

**ENSG00000152785**	*0.015867326*	*0.024775093*	*0.024523507*	*0.015428787*
**ENSG00000138135**	*0.015937151*	*0.02688434*	*0.025624767*	*0.015855178*
**ENSG00000129221**	*0.017014896*	*0.025334808*	*0.024217595*	*0.015692699*
**ENSG00000173728**	*0.01674246*	*0.024779612*	*0.025292761*	*0.014733528*
**ENSG00000197497**	*0.029104196*	*0.02993024*	*0.031413731*	*0.025577156*
**ENSG00000105612**	*0.046521193*	*0.039108029*	*0.038491791*	*0.029933374*
**ENSG00000132623**	*0.020218555*	*0.026942358*	*0.025442818*	*0.015905635*
**ENSG00000171487**	*0.016650464*	*0.025330664*	*0.024834247*	*0.015619724*
**ENSG00000213380**	*0.019177607*	*0.027402436*	*0.031165976*	*0.020015407*
**ENSG00000118094**	*0.01681347*	*0.024542912*	*0.025220966*	*0.015431892*
**ENSG00000141425**	*0.034132239*	*0.036971004*	*0.032117776*	*0.028813746*
**ENSG00000173846**	*0.028302054*	*0.033990733*	*0.038281587*	*0.025451646*
**ENSG00000163565**	*0.025780532*	*0.029801768*	*0.029222006*	*0.021928227*
**ENSG00000162992**	*0.017908966*	*0.026866608*	*0.027423837*	*0.015829761*
**ENSG00000197976**	*0.023817975*	*0.0315203*	*0.029166243*	*0.024837092*
**ENSG00000132326**	*0.036804053*	*0.038139563*	*0.040215605*	*0.033324288*
**ENSG00000181035**	*0.035646181*	*0.039988696*	*0.042165436*	*0.042433997*
**ENSG00000203326**	*0.030251719*	*0.036236204*	*0.036354712*	*0.032798462*
**ENSG00000175115**	*0.03331427*	*0.036589675*	*0.037310502*	*0.033671822*
**ENSG00000135686**	*0.030758359*	*0.034807382*	*0.036221357*	*0.024963597*
**ENSG00000068323**	*0.018673826*	*0.028664903*	*0.031342704*	*0.019617228*
**ENSG00000106823**	*0.022329277*	*0.028982831*	*0.028660574*	*0.021323663*
**ENSG00000100116**	*0.045108716*	*0.036449955*	*0.036876616*	*0.033460821*
**ENSG00000092841**	*0.021854117*	*0.028812606*	*0.02679955*	*0.021211225*
**ENSG00000146360**	*0.017213844*	*0.026816048*	*0.024367453*	*0.015762991*
**ENSG00000216937**	*0.01836482*	*0.026958418*	*0.026822162*	*0.015853058*
**ENSG00000047617**	*0.014968292*	*0.024334387*	*0.024641722*	*0.015293016*
**ENSG00000111110**	*0.014742373*	*0.025051531*	*0.024179469*	*0.014511326*
**ENSG00000184307**	*0.048903436*	*0.040824055*	*0.040719991*	*0.038182331*
**ENSG00000174886**	*0.020397983*	*0.02724423*	*0.02781318*	*0.017821034*
**ENSG00000027869**	*0.080168391*	*0.051198107*	*0.045607796*	*0.054619029*
**ENSG00000126391**	*0.031514517*	*0.034889867*	*0.036777961*	*0.031591382*
**ENSG00000131379**	*0.017842863*	*0.026274069*	*0.025911888*	*0.017383351*
**ENSG00000172725**	*0.030474938*	*0.035537612*	*0.028501072*	*0.031371066*
**ENSG00000013306**	*0.028778364*	*0.029737869*	*0.028238058*	*0.02245035*
**ENSG00000113595**	*0.017186837*	*0.025653381*	*0.026172092*	*0.016621349*
**ENSG00000171766**	*0.06249615*	*0.054204322*	*0.046046967*	*0.048891656*
**ENSG00000133104**	*0.092886331*	*0.068071152*	*0.054158162*	*0.067907852*
**ENSG00000148516**	*0.04423714*	*0.048600945*	*0.041813447*	*0.051041212*
**ENSG00000213215**	*0.016081543*	*0.024453374*	*0.026365635*	*0.014848964*
**ENSG00000089063**	*0.02428784*	*0.030218735*	*0.026550031*	*0.020646684*
**ENSG00000053524**	*0.017209478*	*0.025584084*	*0.026475187*	*0.016230062*
**ENSG00000110244**	*0.017954254*	*0.027121276*	*0.026109534*	*0.016945888*
**ENSG00000110076**	*0.016963008*	*0.025432257*	*0.02704474*	*0.01701892*
**ENSG00000107890**	*0.032341362*	*0.03765741*	*0.032615298*	*0.030373272*
**ENSG00000091972**	*0.057976325*	*0.065924842*	*0.050759955*	*0.056354084*
**ENSG00000150636**	*0.017839795*	*0.025071261*	*0.024796923*	*0.017940141*
**ENSG00000236637**	*0.016421029*	*0.02607165*	*0.025689064*	*0.017057371*
**ENSG00000070019**	*0.021613596*	*0.026861448*	*0.029272828*	*0.024028585*
**ENSG00000119599**	*0.039839372*	*0.046997226*	*0.054922513*	*0.038558677*
**ENSG00000134216**	*0.016279267*	*0.024353894*	*0.024738908*	*0.015302602*
**ENSG00000237651**	*0.051709105*	*0.052516783*	*0.052526744*	*0.045815646*
**ENSG00000165181**	*0.017539746*	*0.027010354*	*0.025709752*	*0.016840193*
**ENSG00000175793**	*0.037416189*	*0.042145264*	*0.032616666*	*0.027274835*

**ENSG00000206384**	*0.015666404*	*0.024911097*	*0.025393015*	*0.015320336*
**ENSG00000129749**	*0.020774606*	*0.027972508*	*0.028804288*	*0.016485233*
**ENSG00000152242**	*0.024885567*	*0.030632295*	*0.032815438*	*0.021135955*
**ENSG00000124469**	*0.020742548*	*0.026292209*	*0.026701478*	*0.018597476*
**ENSG00000124370**	*0.035461168*	*0.034524337*	*0.035910961*	*0.029878655*
**ENSG00000167230**	*0.016174844*	*0.025630605*	*0.02530964*	*0.0163408*
**ENSG00000113569**	*0.01694987*	*0.025386496*	*0.025101116*	*0.015569285*
**ENSG00000074266**	*0.033253205*	*0.039095486*	*0.033460411*	*0.025813573*
**ENSG00000124564**	*0.015794334*	*0.024669061*	*0.025261959*	*0.016831648*
**ENSG00000164256**	*0.01808022*	*0.027605204*	*0.028397738*	*0.016568742*
**ENSG00000131043**	*0.026254609*	*0.038357814*	*0.043970699*	*0.027713042*
**ENSG00000158639**	*0.01804644*	*0.027003887*	*0.025985548*	*0.016553053*
**ENSG00000167139**	*0.016185712*	*0.025063067*	*0.024330561*	*0.014808785*
**ENSG00000167895**	*0.041197761*	*0.054232387*	*0.043481305*	*0.037627944*
**ENSG00000043355**	*0.018023095*	*0.024865771*	*0.02610207*	*0.017450828*
**ENSG00000108588**	*0.026704596*	*0.032569237*	*0.029029282*	*0.027262829*
**ENSG00000177108**	*0.015481524*	*0.024492429*	*0.024838662*	*0.015016651*
**ENSG00000142961**	*0.015506983*	*0.025975983*	*0.025814151*	*0.014918395*
**ENSG00000221926**	*0.032485669*	*0.038800901*	*0.039103972*	*0.032182952*
**ENSG00000130204**	*0.031815018*	*0.032843096*	*0.035775896*	*0.036082778*
**ENSG00000056277**	*0.035204911*	*0.033861465*	*0.031270807*	*0.026225091*
**ENSG00000187048**	*0.017054023*	*0.026085089*	*0.024583807*	*0.017615423*
**ENSG00000137193**	*0.065207849*	*0.05492716*	*0.046997216*	*0.053828195*
**ENSG00000085491**	*0.037681943*	*0.029100929*	*0.027974467*	*0.019390559*
**ENSG00000198087**	*0.053688023*	*0.059326204*	*0.041845842*	*0.090617669*
**ENSG00000178882**	*0.018419896*	*0.025578967*	*0.02784341*	*0.015699691*
**ENSG00000171160**	*0.042841793*	*0.047184635*	*0.037920603*	*0.042950045*
**ENSG00000119640**	*0.036988204*	*0.034083664*	*0.036275749*	*0.040482926*
**ENSG00000156515**	*0.017615306*	*0.027879652*	*0.026148019*	*0.017885937*
**ENSG00000163626**	*0.028814511*	*0.031470532*	*0.028954561*	*0.021859381*
**ENSG00000215009**	*0.015640715*	*0.02523398*	*0.024712952*	*0.014605429*
**ENSG00000121988**	*0.017741925*	*0.025805771*	*0.024616446*	*0.015698476*
**ENSG00000066427**	*0.037172182*	*0.041566279*	*0.03783536*	*0.034583802*
**ENSG00000133112**	*0.0161849*	*0.024815678*	*0.025194637*	*0.017633061*
**ENSG00000163217**	*0.016526787*	*0.026716225*	*0.026348048*	*0.016518445*
**ENSG00000186583**	*0.029822292*	*0.032710713*	*0.031451826*	*0.024928964*
**ENSG00000172209**	*0.015887494*	*0.025208777*	*0.025181989*	*0.014184632*
**ENSG00000008405**	*0.078821085*	*0.065070976*	*0.053091874*	*0.065448666*
**ENSG00000105971**	*0.067016748*	*0.057687315*	*0.051218589*	*0.019251075*
**ENSG00000180834**	*0.036198821*	*0.035255195*	*0.0431736*	*0.034967173*
**ENSG00000204815**	*0.025880338*	*0.030037955*	*0.02682994*	*0.025523079*
**ENSG00000151773**	*0.018157352*	*0.025708209*	*0.025607493*	*0.016915767*
**ENSG00000127580**	*0.019194317*	*0.028181033*	*0.028494654*	*0.022266831*
**ENSG00000169154**	*0.015681965*	*0.024907316*	*0.024986594*	*0.015276965*
**ENSG00000166292**	*0.039486296*	*0.032689545*	*0.037445626*	*0.051764015*
**ENSG00000116176**	*0.016962234*	*0.02526699*	*0.024359475*	*0.016508358*
**ENSG00000196189**	*0.041882331*	*0.048766691*	*0.048230141*	*0.040743233*
**ENSG00000138069**	*0.021522574*	*0.029459388*	*0.031202387*	*0.022177537*
**ENSG00000136280**	*0.029665054*	*0.034990603*	*0.036945315*	*0.026088808*
**ENSG00000056736**	*0.110614721*	*0.080326494*	*0.081287674*	*0.101137443*
**ENSG00000162695**	*0.030083505*	*0.034765498*	*0.038882819*	*0.029239669*
**ENSG00000166326**	*0.025790741*	*0.032980775*	*0.029620944*	*0.023325715*
**ENSG00000182853**	*0.01749477*	*0.025109562*	*0.026686759*	*0.016310867*
**ENSG00000164107**	*0.01546831*	*0.024525586*	*0.024556842*	*0.014998059*

**ENSG00000157107**	*0.021296607*	*0.028492809*	*0.026507819*	*0.022330783*
**ENSG00000182934**	*0.025963117*	*0.031042956*	*0.036758068*	*0.029660031*
**ENSG00000159398**	*0.017521663*	*0.026002698*	*0.024739177*	*0.014669675*
**ENSG00000213588**	*0.020087255*	*0.029197402*	*0.027708573*	*0.017559305*
**ENSG00000122787**	*0.016437518*	*0.024984319*	*0.025181447*	*0.015264615*
**ENSG00000021574**	*0.023059763*	*0.028844008*	*0.027398583*	*0.021630115*
**ENSG00000083168**	*0.02698163*	*0.031059633*	*0.032610914*	*0.02412522*
**ENSG00000253846**	*0.017054366*	*0.026763455*	*0.025276228*	*0.016647426*
**ENSG00000135837**	*0.028304695*	*0.035467061*	*0.032326978*	*0.028866063*
**ENSG00000073598**	*0.015869292*	*0.024672287*	*0.023790159*	*0.014872444*
**ENSG00000143185**	*0.148617157*	*0.136802821*	*0.130465001*	*0.144641035*
**ENSG00000171163**	*0.042651321*	*0.046292733*	*0.037918754*	*0.03299579*
**ENSG00000100311**	*0.01524148*	*0.025454884*	*0.025389339*	*0.015827367*
**ENSG00000056291**	*0.014733451*	*0.024686991*	*0.024768208*	*0.015672894*
**ENSG00000105507**	*0.015998755*	*0.024387068*	*0.02443824*	*0.014280803*
**ENSG00000109083**	*0.025188447*	*0.03110809*	*0.034875363*	*0.021788606*
**ENSG00000173258**	*0.017746832*	*0.024832315*	*0.026374932*	*0.015754898*
**ENSG00000148498**	*0.036380064*	*0.035948889*	*0.035554549*	*0.035708931*
**ENSG00000138640**	*0.075606725*	*0.061480958*	*0.049827283*	*0.06681908*
**ENSG00000249915**	*0.019562593*	*0.028949755*	*0.027809197*	*0.021485894*
**ENSG00000134440**	*0.027681594*	*0.030522161*	*0.029141874*	*0.02200279*
**ENSG00000136286**	*0.030263202*	*0.030832696*	*0.036463192*	*0.028361649*
**ENSG00000144713**	*0.014375577*	*0.024274996*	*0.024253927*	*0.015718597*
**ENSG00000102078**	*0.027232251*	*0.033408661*	*0.03113814*	*0.025512449*
**ENSG00000136877**	*0.03711762*	*0.041617344*	*0.037245247*	*0.0354217*
**ENSG00000196139**	*0.02754551*	*0.026530949*	*0.024762525*	*0.018240901*
**ENSG00000139926**	*0.091551293*	*0.054725704*	*0.047233389*	*0.084014519*
**ENSG00000197208**	*0.022494969*	*0.026929881*	*0.025963644*	*0.019247866*
**ENSG00000115977**	*0.015086182*	*0.026332078*	*0.024473829*	*0.015310073*
**ENSG00000099795**	*0.024469089*	*0.028572326*	*0.02740563*	*0.019423193*
**ENSG00000137275**	*0.032798972*	*0.039339083*	*0.044349014*	*0.030415462*
**ENSG00000197084**	*0.084762865*	*0.047765979*	*0.038141449*	*0.056638617*
**ENSG00000172660**	*0.02803902*	*0.033577875*	*0.031460881*	*0.025458559*
**ENSG00000168781**	*0.028036221*	*0.039371981*	*0.037777715*	*0.02950674*
**ENSG00000033030**	*0.031968456*	*0.035573957*	*0.032925374*	*0.024712497*
**ENSG00000164961**	*0.029393433*	*0.03429584*	*0.028337642*	*0.026764587*
**ENSG00000108479**	*0.042490397*	*0.036253779*	*0.030930366*	*0.025897761*
**ENSG00000165059**	*0.016928812*	*0.026350658*	*0.024609564*	*0.016632501*
**ENSG00000136003**	*0.026305321*	*0.030094159*	*0.028973449*	*0.022591802*
**ENSG00000161888**	*0.050189121*	*0.036167156*	*0.036381477*	*0.032519221*
**ENSG00000147874**	*0.039052305*	*0.039370771*	*0.034043966*	*0.046430782*
**ENSG00000124140**	*0.016174803*	*0.024876815*	*0.02470898*	*0.014903803*
**ENSG00000085998**	*0.030680253*	*0.0314541*	*0.029746183*	*0.025619288*
**ENSG00000083720**	*0.039533939*	*0.039588928*	*0.033359039*	*0.033648675*
**ENSG00000107984**	*0.017889843*	*0.025944058*	*0.025591106*	*0.017263787*
**ENSG00000130881**	*0.035053164*	*0.031008031*	*0.037495586*	*0.019017057*
**ENSG00000205403**	*0.018799175*	*0.025966121*	*0.025739069*	*0.01518561*
**ENSG00000126804**	*0.023849391*	*0.033363135*	*0.032215691*	*0.026756547*
**ENSG00000172320**	*0.018340381*	*0.025750009*	*0.025025189*	*0.015522724*
**ENSG00000111729**	*0.071473998*	*0.070131732*	*0.050169833*	*0.108463487*
**ENSG00000101290**	*0.033290027*	*0.036194758*	*0.033668464*	*0.029081284*
**ENSG00000175040**	*0.05196421*	*0.045095014*	*0.044302135*	*0.057614323*
**ENSG00000171649**	*0.050507438*	*0.048589569*	*0.054778534*	*0.042869213*
**ENSG00000198848**	*0.017166542*	*0.026346072*	*0.028207603*	*0.015845138*

**ENSG00000079101**	*0.017082454*	*0.025453163*	*0.024685261*	*0.015829057*
**ENSG00000144596**	*0.017310179*	*0.027728844*	*0.024954074*	*0.016326333*
**ENSG00000175868**	*0.017238724*	*0.025446627*	*0.025375987*	*0.015846132*
**ENSG00000112874**	*0.079708844*	*0.062606879*	*0.039735441*	*0.065847997*
**ENSG00000174606**	*0.031639694*	*0.035074728*	*0.032652824*	*0.029707669*
**ENSG00000178175**	*0.016927958*	*0.025613206*	*0.025491191*	*0.016806119*
**ENSG00000134046**	*0.014735696*	*0.024887612*	*0.025145873*	*0.016230427*
**ENSG00000197381**	*0.032153174*	*0.030431082*	*0.025451937*	*0.01469685*
**ENSG00000213949**	*0.052307591*	*0.051672797*	*0.039190371*	*0.050502561*
**ENSG00000145087**	*0.015815821*	*0.025567746*	*0.025322607*	*0.0157773*
**ENSG00000030304**	*0.015363031*	*0.023959376*	*0.024227586*	*0.014663111*
**ENSG00000065361**	*0.016072111*	*0.025913253*	*0.023940233*	*0.013905802*
**ENSG00000248099**	*0.034100442*	*0.033344136*	*0.031396569*	*0.030736533*
**ENSG00000022267**	*0.102797137*	*0.074423006*	*0.081980241*	*0.100198062*
**ENSG00000151657**	*0.025315589*	*0.030464911*	*0.034659704*	*0.029235473*
**ENSG00000244509**	*0.030553007*	*0.033995638*	*0.032367286*	*0.028721181*
**ENSG00000165757**	*0.019478279*	*0.024850575*	*0.025005118*	*0.015394181*
**ENSG00000108961**	*0.026768387*	*0.029467108*	*0.030081758*	*0.019208302*
**ENSG00000159387**	*0.022210956*	*0.028956181*	*0.027134242*	*0.01912015*
**ENSG00000101391**	*0.026354621*	*0.030826676*	*0.029629935*	*0.021839912*
**ENSG00000167671**	*0.031635174*	*0.037072105*	*0.036602024*	*0.029135713*
**ENSG00000156795**	*0.034226475*	*0.033345921*	*0.03457617*	*0.026407964*
**ENSG00000079156**	*0.016333615*	*0.026373997*	*0.02559204*	*0.016355937*
**ENSG00000162959**	*0.023106614*	*0.031235121*	*0.0288582*	*0.021143443*
**ENSG00000184434**	*0.017749228*	*0.026210185*	*0.024543088*	*0.016838296*
**ENSG00000155542**	*0.046181007*	*0.057886466*	*0.047327578*	*0.052074596*
**ENSG00000054598**	*0.021793612*	*0.027448721*	*0.027815935*	*0.018100815*
**ENSG00000135164**	*0.025613826*	*0.035503912*	*0.032076108*	*0.021504398*
**ENSG00000177535**	*0.015309*	*0.024892917*	*0.025548547*	*0.016160023*
**ENSG00000198223**	*0.02753292*	*0.032124677*	*0.033117579*	*0.019510831*
**ENSG00000129007**	*0.042844792*	*0.041427548*	*0.046376867*	*0.03885195*
**ENSG00000171772**	*0.034941712*	*0.024761434*	*0.025685429*	*0.036892508*
**ENSG00000132465**	*0.061793153*	*0.046386102*	*0.045897919*	*0.04025927*
**ENSG00000089234**	*0.021688574*	*0.030092775*	*0.031869877*	*0.020604659*
**ENSG00000189001**	*0.02355021*	*0.025809973*	*0.025157368*	*0.018287358*
**ENSG00000166265**	*0.015573689*	*0.024842928*	*0.025642161*	*0.016578792*
**ENSG00000197746**	*0.03394462*	*0.035152482*	*0.040134566*	*0.042030289*
**ENSG00000204314**	*0.016104092*	*0.02558126*	*0.024437171*	*0.014950326*
**ENSG00000029153**	*0.037008768*	*0.03736895*	*0.038138089*	*0.042243002*
**ENSG00000085224**	*0.025414681*	*0.035994767*	*0.031002635*	*0.028507974*
**ENSG00000108821**	*0.017151785*	*0.026531483*	*0.025076111*	*0.014036578*
**ENSG00000060566**	*0.01653784*	*0.024639504*	*0.023847937*	*0.015166117*
**ENSG00000126870**	*0.033737851*	*0.039657574*	*0.033075456*	*0.040347714*
**ENSG00000138032**	*0.035698994*	*0.039257892*	*0.040051844*	*0.040830893*
**ENSG00000167703**	*0.066611358*	*0.055769012*	*0.043756938*	*0.053960924*
**ENSG00000165813**	*0.025393561*	*0.033838574*	*0.033298354*	*0.028664695*
**ENSG00000007372**	*0.019646785*	*0.026187438*	*0.028045351*	*0.019545017*
**ENSG00000185917**	*0.027209155*	*0.033928508*	*0.035144983*	*0.023935489*
**ENSG00000023843**	*0.018224481*	*0.02526462*	*0.028572531*	*0.018792967*
**ENSG00000072110**	*0.128311224*	*0.092451295*	*0.1000383*	*0.109845898*
**ENSG00000177947**	*0.01745527*	*0.026024365*	*0.025579382*	*0.015883711*
**ENSG00000101868**	*0.023835362*	*0.03221342*	*0.025563757*	*0.026239215*
**ENSG00000082515**	*0.023495969*	*0.028422605*	*0.029993387*	*0.018868888*
**ENSG00000133275**	*0.025874433*	*0.031355298*	*0.034496391*	*0.025574555*

**ENSG00000174175**	*0.017688587*	*0.030393647*	*0.027413026*	*0.023264839*
**ENSG00000100139**	*0.014932646*	*0.024826597*	*0.024651263*	*0.014164507*
**ENSG00000070785**	*0.040671363*	*0.037655895*	*0.03088003*	*0.029987885*
**ENSG00000163009**	*0.027157189*	*0.026122629*	*0.026862378*	*0.024433118*
**ENSG00000124532**	*0.034420705*	*0.037094685*	*0.031962198*	*0.034479713*
**ENSG00000177992**	*0.015303698*	*0.024591134*	*0.025295314*	*0.016093103*
**ENSG00000160948**	*0.018558427*	*0.025289959*	*0.025400026*	*0.016938589*
**ENSG00000233927**	*0.017951294*	*0.024608429*	*0.02674496*	*0.01967061*
**ENSG00000151651**	*0.034754898*	*0.038411665*	*0.038610596*	*0.041651991*
**ENSG00000113211**	*0.017505355*	*0.027907591*	*0.026936559*	*0.016510828*
**ENSG00000105576**	*0.024306216*	*0.034820279*	*0.027457149*	*0.02842014*
**ENSG00000146350**	*0.035421234*	*0.033935644*	*0.035262445*	*0.042717248*
**ENSG00000165917**	*0.016212145*	*0.023842243*	*0.02511594*	*0.015648076*
**ENSG00000162496**	*0.059311174*	*0.053347094*	*0.05028978*	*0.061284416*
**ENSG00000001084**	*0.044185727*	*0.041016573*	*0.039167616*	*0.036406588*
**ENSG00000174282**	*0.030417008*	*0.034751555*	*0.037180836*	*0.03032257*
**ENSG00000106976**	*0.016590337*	*0.024927297*	*0.025570681*	*0.015151456*
**ENSG00000159871**	*0.018526452*	*0.025797753*	*0.02626263*	*0.016570867*
**ENSG00000164951**	*0.01628745*	*0.025337808*	*0.025151047*	*0.015326101*
**ENSG00000112337**	*0.016820215*	*0.024944762*	*0.024506138*	*0.015983681*
**ENSG00000166200**	*0.024553506*	*0.03099348*	*0.032821369*	*0.02141411*
**ENSG00000159256**	*0.042619108*	*0.046435483*	*0.039425211*	*0.046446061*
**ENSG00000158716**	*0.078722353*	*0.053297115*	*0.041138375*	*0.041650688*
**ENSG00000159761**	*0.032143736*	*0.031301607*	*0.028193884*	*0.030143037*
**ENSG00000115970**	*0.030168385*	*0.044413913*	*0.031921675*	*0.028079218*
**ENSG00000135052**	*0.017852194*	*0.025878423*	*0.024765249*	*0.016843065*
**ENSG00000109775**	*0.032424895*	*0.03019625*	*0.032085452*	*0.024739815*
**ENSG00000109654**	*0.1007494*	*0.079262268*	*0.055966794*	*0.069673749*
**ENSG00000100485**	*0.026251079*	*0.028658142*	*0.028543785*	*0.021554195*
**ENSG00000127152**	*0.016249009*	*0.024836252*	*0.024413709*	*0.015287221*
**ENSG00000163328**	*0.030687038*	*0.032093926*	*0.035644866*	*0.027959474*
**ENSG00000184937**	*0.044185047*	*0.03291294*	*0.030718133*	*0.039792474*
**ENSG00000111144**	*0.025406293*	*0.030529063*	*0.027257244*	*0.021954738*
**ENSG00000177494**	*0.082828399*	*0.066865045*	*0.078745307*	*0.088178522*
**ENSG00000073067**	*0.015826861*	*0.024852204*	*0.025059285*	*0.015239441*
**ENSG00000160753**	*0.034937826*	*0.037266552*	*0.033749118*	*0.032489729*
**ENSG00000147160**	*0.015648011*	*0.02505467*	*0.02570682*	*0.015228237*
**ENSG00000132718**	*0.040183711*	*0.03634904*	*0.045902141*	*0.041524245*
**ENSG00000124767**	*0.023506835*	*0.032754913*	*0.031059143*	*0.023612655*
**ENSG00000065413**	*0.027267648*	*0.03050767*	*0.028058784*	*0.028970127*
**ENSG00000072062**	*0.019786981*	*0.028829842*	*0.027225995*	*0.018885486*
**ENSG00000164342**	*0.026649732*	*0.029304033*	*0.027191594*	*0.020564036*
**ENSG00000123388**	*0.014936716*	*0.025067531*	*0.024095043*	*0.015063175*
**ENSG00000221996**	*0.016644496*	*0.026292379*	*0.025869905*	*0.015321744*
**ENSG00000169635**	*0.028741289*	*0.041602472*	*0.044118212*	*0.027182233*
**ENSG00000094661**	*0.015590278*	*0.024198396*	*0.025158426*	*0.014200315*
**ENSG00000151612**	*0.034914005*	*0.040048938*	*0.03560934*	*0.032613449*
**ENSG00000161542**	*0.032527928*	*0.035875412*	*0.032538657*	*0.021854467*
**ENSG00000135443**	*0.014292785*	*0.024523564*	*0.02593281*	*0.014611119*
**ENSG00000185475**	*0.022700831*	*0.029362302*	*0.031946679*	*0.024642937*
**ENSG00000138411**	*0.132187797*	*0.116380125*	*0.116973188*	*0.112432397*
**ENSG00000140987**	*0.02353085*	*0.036385113*	*0.04172033*	*0.024767196*
**ENSG00000133398**	*0.030489411*	*0.039218643*	*0.041030588*	*0.028230251*
**ENSG00000175003**	*0.016859146*	*0.026105687*	*0.026014906*	*0.015144195*

**ENSG00000108733**	*0.022765682*	*0.031803209*	*0.029356263*	*0.023172452*
**ENSG00000023287**	*0.025894597*	*0.033449149*	*0.032954729*	*0.025487473*
**ENSG00000099817**	*0.04987473*	*0.050879134*	*0.049184507*	*0.039570321*
**ENSG00000116675**	*0.017062134*	*0.025329459*	*0.02493335*	*0.017830565*
**ENSG00000162714**	*0.02215864*	*0.027114357*	*0.027186272*	*0.018241801*
**ENSG00000148704**	*0.016578839*	*0.025373417*	*0.02535053*	*0.014818927*
**ENSG00000165280**	*0.028863743*	*0.033831217*	*0.034902397*	*0.035484027*
**ENSG00000198283**	*0.017645345*	*0.025824123*	*0.024852509*	*0.016575057*
**ENSG00000140968**	*0.045009806*	*0.046754407*	*0.048463304*	*0.054478931*
**ENSG00000073861**	*0.021974195*	*0.027797999*	*0.028928771*	*0.026491588*
**ENSG00000213297**	*0.017351624*	*0.026114128*	*0.02612582*	*0.017111771*
**ENSG00000087053**	*0.016676062*	*0.024931853*	*0.024952732*	*0.017131601*
**ENSG00000198000**	*0.026041112*	*0.030330908*	*0.029756339*	*0.02098674*
**ENSG00000183741**	*0.031496171*	*0.035838657*	*0.032455849*	*0.025491374*
**ENSG00000135338**	*0.02383183*	*0.026378691*	*0.027122143*	*0.024435495*
**ENSG00000145216**	*0.022938771*	*0.031813782*	*0.031269724*	*0.023887669*
**ENSG00000165948**	*0.039230508*	*0.034895414*	*0.035502783*	*0.028942777*
**ENSG00000115806**	*0.019792324*	*0.027852974*	*0.032084384*	*0.021670332*
**ENSG00000101577**	*0.030352977*	*0.032800272*	*0.03350034*	*0.028343914*
**ENSG00000162687**	*0.015596136*	*0.02507234*	*0.024603875*	*0.015289304*
**ENSG00000250254**	*0.036741259*	*0.031863571*	*0.032872344*	*0.026931354*
**ENSG00000112972**	*0.04032646*	*0.040381802*	*0.040885506*	*0.032437394*
**ENSG00000133393**	*0.024879286*	*0.030136786*	*0.031801386*	*0.022948301*
**ENSG00000139354**	*0.020938487*	*0.026398163*	*0.02572191*	*0.019658499*
**ENSG00000011021**	*0.018979913*	*0.026349645*	*0.025604733*	*0.015553682*
**ENSG00000122512**	*0.033603912*	*0.034190813*	*0.032245409*	*0.027621091*
**ENSG00000143919**	*0.015786539*	*0.024999122*	*0.02446034*	*0.016476444*
**ENSG00000244607**	*0.01518935*	*0.024931083*	*0.025243264*	*0.015198392*
**ENSG00000100101**	*0.030926699*	*0.031296827*	*0.037519959*	*0.031392207*
**ENSG00000177663**	*0.03062826*	*0.032755472*	*0.036819467*	*0.033369856*
**ENSG00000127947**	*0.035373096*	*0.037783342*	*0.036662634*	*0.031894364*
**ENSG00000106302**	*0.024080745*	*0.02819735*	*0.027652205*	*0.02234149*
**ENSG00000046774**	*0.01663409*	*0.026070623*	*0.025748275*	*0.017119376*
**ENSG00000165325**	*0.015456759*	*0.029260772*	*0.026407963*	*0.015031082*
**ENSG00000178395**	*0.017617843*	*0.024679415*	*0.026490407*	*0.016632576*
**ENSG00000177283**	*0.018496128*	*0.026496711*	*0.026613133*	*0.018027118*
**ENSG00000167011**	*0.020566507*	*0.026818067*	*0.028013745*	*0.017719426*
**ENSG00000163297**	*0.068688079*	*0.062383394*	*0.05752253*	*0.050605128*
**ENSG00000151014**	*0.037704301*	*0.038390181*	*0.043063815*	*0.030516483*
**ENSG00000105750**	*0.068681695*	*0.04965402*	*0.039942821*	*0.054927865*
**ENSG00000168309**	*0.016232637*	*0.027275684*	*0.027700384*	*0.018240044*
**ENSG00000104312**	*0.041486788*	*0.041047943*	*0.040594897*	*0.037225973*
**ENSG00000161996**	*0.042119055*	*0.044355688*	*0.037172767*	*0.043059746*
**ENSG00000175520**	*0.038900498*	*0.036423259*	*0.034245117*	*0.0358735*
**ENSG00000068366**	*0.041881084*	*0.049096342*	*0.04125598*	*0.038797349*
**ENSG00000108602**	*0.018720891*	*0.030126968*	*0.027851576*	*0.033502508*
**ENSG00000083097**	*0.03266903*	*0.042943091*	*0.035526675*	*0.029591921*
**ENSG00000085978**	*0.022422972*	*0.025147793*	*0.031888432*	*0.019197859*
**ENSG00000104973**	*0.02977084*	*0.033872762*	*0.033664069*	*0.020821886*
**ENSG00000144677**	*0.034810864*	*0.031633414*	*0.028104921*	*0.024984157*
**ENSG00000153246**	*0.016003046*	*0.024914841*	*0.025222442*	*0.014883503*
**ENSG00000006025**	*0.018780473*	*0.025215008*	*0.027624854*	*0.017340563*
**ENSG00000101400**	*0.037326521*	*0.031631421*	*0.032976829*	*0.033627446*
**ENSG00000173674**	*0.049337738*	*0.046894881*	*0.046352946*	*0.03410953*

**ENSG00000119632**	*0.025647185*	*0.032767897*	*0.030213664*	*0.025601237*
**ENSG00000125872**	*0.015875078*	*0.025906641*	*0.024716222*	*0.015228798*
**ENSG00000163527**	*0.039762636*	*0.043650539*	*0.041910191*	*0.039844026*
**ENSG00000196507**	*0.038800574*	*0.039542743*	*0.038167821*	*0.027694681*
**ENSG00000198919**	*0.036930846*	*0.038900104*	*0.031591929*	*0.029743546*
**ENSG00000153187**	*0.016046778*	*0.025186305*	*0.024480028*	*0.01464308*
**ENSG00000197121**	*0.022823615*	*0.028580208*	*0.026902463*	*0.018957706*
**ENSG00000140459**	*0.02174869*	*0.033708643*	*0.031755501*	*0.02178584*
**ENSG00000146090**	*0.015478667*	*0.025207567*	*0.024956064*	*0.016224154*
**ENSG00000028310**	*0.026700568*	*0.031252312*	*0.035509617*	*0.027134197*
**ENSG00000213494**	*0.014864846*	*0.024749587*	*0.024629094*	*0.015687413*
**ENSG00000178358**	*0.015794264*	*0.025188212*	*0.024464871*	*0.01588756*
**ENSG00000165416**	*0.024100468*	*0.029194489*	*0.028231271*	*0.023747134*
**ENSG00000166033**	*0.015841745*	*0.025338523*	*0.025922555*	*0.015390707*
**ENSG00000126603**	*0.015415977*	*0.024945828*	*0.024086798*	*0.014631969*
**ENSG00000178403**	*0.102758179*	*0.076010125*	*0.086209752*	*0.0899817*
**ENSG00000150456**	*0.037758429*	*0.038088202*	*0.032475802*	*0.033376232*
**ENSG00000163239**	*0.016772879*	*0.025782303*	*0.025376708*	*0.019162181*
**ENSG00000120675**	*0.038379065*	*0.037364728*	*0.031621747*	*0.02295015*
**ENSG00000125450**	*0.030625536*	*0.033442831*	*0.032634291*	*0.023940059*
**ENSG00000185480**	*0.037840048*	*0.036718723*	*0.036988656*	*0.035004481*
**ENSG00000079215**	*0.027852736*	*0.028378474*	*0.02560258*	*0.018168635*
**ENSG00000128617**	*0.018583383*	*0.026291265*	*0.025485858*	*0.015754967*
**ENSG00000104765**	*0.048916752*	*0.036203145*	*0.033873187*	*0.032005246*
**ENSG00000174236**	*0.016647893*	*0.02568693*	*0.02447488*	*0.015727815*
**ENSG00000186280**	*0.017152998*	*0.026167639*	*0.024935327*	*0.016382022*
**ENSG00000166351**	*0.016687592*	*0.025606685*	*0.024946298*	*0.014630228*
**ENSG00000160447**	*0.030199559*	*0.039867013*	*0.035131724*	*0.027042003*
**ENSG00000244115**	*0.02884324*	*0.035528744*	*0.035098669*	*0.02625914*
**ENSG00000180626**	*0.047454437*	*0.04663559*	*0.033210286*	*0.039507318*
**ENSG00000157181**	*0.023944449*	*0.028679189*	*0.028798235*	*0.021104167*
**ENSG00000110497**	*0.028604325*	*0.030818894*	*0.033531004*	*0.035937611*
**ENSG00000203857**	*0.015394231*	*0.025659973*	*0.025317265*	*0.015643798*
**ENSG00000166148**	*0.016445003*	*0.025188679*	*0.026720447*	*0.015832287*
**ENSG00000055208**	*0.027246649*	*0.032675904*	*0.030650099*	*0.030083909*
**ENSG00000168067**	*0.028515948*	*0.033320744*	*0.032490155*	*0.023282523*
**ENSG00000172382**	*0.017424027*	*0.025406837*	*0.024966471*	*0.014374134*
**ENSG00000157578**	*0.015225281*	*0.02480516*	*0.025164065*	*0.015783461*
**ENSG00000198910**	*0.015724073*	*0.025485587*	*0.024977818*	*0.016350106*
**ENSG00000172164**	*0.113628785*	*0.085400656*	*0.083112782*	*0.082596287*
**ENSG00000189269**	*0.015926808*	*0.025990971*	*0.02497489*	*0.015824688*
**ENSG00000163666**	*0.03472954*	*0.038583775*	*0.040250202*	*0.030472044*
**ENSG00000223496**	*0.034928479*	*0.040207501*	*0.04784686*	*0.036059027*
**ENSG00000182070**	*0.016221683*	*0.02616862*	*0.025285159*	*0.016449563*
**ENSG00000112077**	*0.015905589*	*0.024635969*	*0.025298741*	*0.014447646*
**ENSG00000132763**	*0.018122318*	*0.026854614*	*0.025437201*	*0.015361212*
**ENSG00000077942**	*0.016891455*	*0.025230263*	*0.026298307*	*0.016718247*
**ENSG00000196569**	*0.016386637*	*0.025848851*	*0.024859593*	*0.015491996*
**ENSG00000160299**	*0.029830214*	*0.037747653*	*0.032506425*	*0.025659071*
**ENSG00000136383**	*0.020988004*	*0.027335642*	*0.029333609*	*0.019619588*
**ENSG00000186765**	*0.018089074*	*0.02631822*	*0.024852083*	*0.021609934*
**ENSG00000162735**	*0.029329654*	*0.031556332*	*0.032833692*	*0.022501546*
**ENSG00000142606**	*0.018794708*	*0.026552858*	*0.024854633*	*0.01725244*
**ENSG00000113525**	*0.017669332*	*0.02759857*	*0.027887669*	*0.018014949*

**ENSG00000170860**	*0.024407979*	*0.028023704*	*0.027324063*	*0.02024594*
**ENSG00000138376**	*0.039590158*	*0.037739761*	*0.038414902*	*0.031617847*
**ENSG00000096060**	*0.048919813*	*0.041943251*	*0.036785093*	*0.041901412*
**ENSG00000185974**	*0.016555485*	*0.025541344*	*0.026035954*	*0.01608609*
**ENSG00000149313**	*0.031762703*	*0.040393104*	*0.030165463*	*0.02725711*
**ENSG00000146378**	*0.017182851*	*0.025863735*	*0.026959488*	*0.01674054*
**ENSG00000144445**	*0.017081587*	*0.024740377*	*0.024846212*	*0.016035059*
**ENSG00000133020**	*0.016582092*	*0.025527265*	*0.02484834*	*0.016299019*
**ENSG00000152439**	*0.02005717*	*0.027735628*	*0.032490914*	*0.018482218*
**ENSG00000148826**	*0.015151865*	*0.024384949*	*0.02533148*	*0.014451025*
**ENSG00000120280**	*0.06528578*	*0.054778779*	*0.044894956*	*0.051194559*
**ENSG00000138606**	*0.033069006*	*0.03319433*	*0.034190805*	*0.027120724*
**ENSG00000155368**	*0.018857763*	*0.0251252*	*0.025198699*	*0.01611885*
**ENSG00000182185**	*0.030621053*	*0.031869175*	*0.030649424*	*0.031206238*
**ENSG00000085433**	*0.028048791*	*0.040062277*	*0.033994763*	*0.032207845*
**ENSG00000144063**	*0.015237892*	*0.024406344*	*0.024238157*	*0.014032436*
**ENSG00000089280**	*0.031079295*	*0.030322839*	*0.033743317*	*0.033164615*
**ENSG00000160256**	*0.028245519*	*0.029774901*	*0.029069801*	*0.025986157*
**ENSG00000130193**	*0.052704653*	*0.046786307*	*0.043535341*	*0.041379728*
**ENSG00000125458**	*0.030345139*	*0.02931284*	*0.031580411*	*0.025590188*
**ENSG00000184162**	*0.029321846*	*0.028678994*	*0.030713098*	*0.021065007*
**ENSG00000239382**	*0.027174143*	*0.032883209*	*0.031686546*	*0.020590402*
**ENSG00000105989**	*0.046958183*	*0.030181302*	*0.029862131*	*0.050311167*
**ENSG00000215440**	*0.04398518*	*0.040615342*	*0.043522152*	*0.041306504*
**ENSG00000137310**	*0.030217261*	*0.035841684*	*0.029646593*	*0.031143709*
**ENSG00000165060**	*0.03070667*	*0.030367557*	*0.034027182*	*0.025945209*
**ENSG00000111875**	*0.045149755*	*0.041728214*	*0.036478611*	*0.032766682*
**ENSG00000153283**	*0.107683297*	*0.077647699*	*0.084676715*	*0.11508813*
**ENSG00000170579**	*0.015825137*	*0.02499246*	*0.02520502*	*0.015067569*
**ENSG00000204376**	*0.0180217*	*0.026966435*	*0.027964135*	*0.018919645*
**ENSG00000155115**	*0.02957547*	*0.032624975*	*0.030499659*	*0.031180183*
**ENSG00000115232**	*0.064405154*	*0.065417384*	*0.045264399*	*0.046671452*
**ENSG00000183337**	*0.035079748*	*0.036831316*	*0.037327664*	*0.028894474*
**ENSG00000169660**	*0.04389002*	*0.047211192*	*0.039206352*	*0.040206235*
**ENSG00000182670**	*0.031615162*	*0.03386015*	*0.034857828*	*0.027960812*
**ENSG00000197056**	*0.035594646*	*0.039338937*	*0.036885316*	*0.038658872*
**ENSG00000065833**	*0.084462073*	*0.074135637*	*0.071618962*	*0.08828417*
**ENSG00000120333**	*0.029267974*	*0.030844081*	*0.028431669*	*0.021162713*
**ENSG00000079785**	*0.026637588*	*0.029345886*	*0.029699683*	*0.024138892*
**ENSG00000143162**	*0.044957394*	*0.039845109*	*0.044879879*	*0.030879653*
**ENSG00000166311**	*0.029152888*	*0.036428199*	*0.041666443*	*0.035071821*
**ENSG00000176635**	*0.017598855*	*0.02495485*	*0.024904417*	*0.016092024*
**ENSG00000075043**	*0.045855766*	*0.028763149*	*0.035939143*	*0.041644133*
**ENSG00000204371**	*0.0309288*	*0.03536395*	*0.030580553*	*0.026830873*
**ENSG00000137731**	*0.211043196*	*0.132501562*	*0.122784243*	*0.171088875*
**ENSG00000181061**	*0.026414135*	*0.032526069*	*0.029306304*	*0.035056694*
**ENSG00000174796**	*0.041289492*	*0.04452421*	*0.049096176*	*0.039136015*
**ENSG00000119973**	*0.01681578*	*0.024426329*	*0.025290053*	*0.01673363*
**ENSG00000168216**	*0.029301525*	*0.03659766*	*0.034007261*	*0.02411401*
**ENSG00000243772**	*0.016014477*	*0.024549968*	*0.025225505*	*0.015176039*
**ENSG00000136937**	*0.038121632*	*0.04016148*	*0.031403223*	*0.029274387*
**ENSG00000059573**	*0.025082411*	*0.030797433*	*0.03008965*	*0.021307729*
**ENSG00000172680**	*0.01740168*	*0.025414621*	*0.026977874*	*0.017339534*
**ENSG00000168538**	*0.027566807*	*0.033859055*	*0.032176752*	*0.020894522*

**ENSG00000120289**	*0.015762433*	*0.025160835*	*0.024915754*	*0.015042162*
**ENSG00000137154**	*0.014939997*	*0.023958453*	*0.024304319*	*0.016204956*
**ENSG00000006075**	*0.044501635*	*0.045536674*	*0.049597723*	*0.042792246*
**ENSG00000184924**	*0.035758727*	*0.03756119*	*0.032189037*	*0.025965156*
**ENSG00000136143**	*0.033643315*	*0.040808939*	*0.031638896*	*0.027458758*
**ENSG00000111339**	*0.016239208*	*0.025476216*	*0.025220247*	*0.014900532*
**ENSG00000077800**	*0.014991957*	*0.025457045*	*0.02419346*	*0.014315645*
**ENSG00000136842**	*0.06074902*	*0.058643061*	*0.055244143*	*0.052043494*
**ENSG00000147408**	*0.031592798*	*0.033794984*	*0.039334662*	*0.040710402*
**ENSG00000136636**	*0.043946723*	*0.036659884*	*0.038890248*	*0.032313116*
**ENSG00000050405**	*0.044930938*	*0.035804529*	*0.033777001*	*0.044040877*
**ENSG00000105492**	*0.017473134*	*0.025987376*	*0.024899832*	*0.020678475*
**ENSG00000188706**	*0.016606886*	*0.024923577*	*0.024703498*	*0.014396979*
**ENSG00000174576**	*0.015994463*	*0.025741642*	*0.026932194*	*0.015935517*
**ENSG00000100258**	*0.02681489*	*0.032391668*	*0.030812898*	*0.023440283*
**ENSG00000148341**	*0.030075893*	*0.038224178*	*0.036267637*	*0.027574603*
**ENSG00000167785**	*0.071838531*	*0.041106759*	*0.042778391*	*0.053235794*
**ENSG00000243509**	*0.018633888*	*0.026976923*	*0.025941366*	*0.018319322*
**ENSG00000164039**	*0.032242987*	*0.041620851*	*0.036949489*	*0.029541073*
**ENSG00000168653**	*0.024554519*	*0.030851302*	*0.032321351*	*0.020580347*
**ENSG00000067191**	*0.015124329*	*0.024354745*	*0.025348826*	*0.01476311*
**ENSG00000197959**	*0.064618319*	*0.064887235*	*0.040482712*	*0.04137803*
**ENSG00000010671**	*0.025802942*	*0.029534748*	*0.030775969*	*0.023429264*
**ENSG00000010219**	*0.027535024*	*0.03067231*	*0.030644052*	*0.020854814*
**ENSG00000143630**	*0.028394503*	*0.035133166*	*0.02782359*	*0.025376632*
**ENSG00000181903**	*0.017697552*	*0.025224856*	*0.025042864*	*0.014927025*
**ENSG00000122136**	*0.019291669*	*0.025978523*	*0.027735374*	*0.017092213*
**ENSG00000146243**	*0.035884159*	*0.039725816*	*0.034279062*	*0.028665755*
**ENSG00000178209**	*0.039850045*	*0.044313265*	*0.036436252*	*0.037430061*
**ENSG00000083799**	*0.035820475*	*0.036230994*	*0.037433222*	*0.03503744*
**ENSG00000164694**	*0.016881069*	*0.026277235*	*0.025037181*	*0.015286861*
**ENSG00000103160**	*0.022871696*	*0.028003833*	*0.028852047*	*0.02206652*
**ENSG00000167522**	*0.029127349*	*0.033083831*	*0.03756111*	*0.027833104*
**ENSG00000162105**	*0.016091812*	*0.026248845*	*0.026354536*	*0.017099106*
**ENSG00000184831**	*0.035542693*	*0.037222036*	*0.031489258*	*0.027195*
**ENSG00000198208**	*0.05666256*	*0.03898369*	*0.043698286*	*0.037058692*
**ENSG00000196235**	*0.022320609*	*0.031428507*	*0.040609336*	*0.02479211*
**ENSG00000092758**	*0.081335877*	*0.089640359*	*0.092812358*	*0.101402962*
**ENSG00000088682**	*0.02586667*	*0.031244619*	*0.030008509*	*0.022237669*
**ENSG00000105855**	*0.029619132*	*0.040862017*	*0.033750597*	*0.028185289*
**ENSG00000101158**	*0.029342357*	*0.030123467*	*0.032568668*	*0.026648507*
**ENSG00000178591**	*0.015515026*	*0.025133581*	*0.025520318*	*0.016171551*
**ENSG00000174123**	*0.042200713*	*0.051671198*	*0.058634069*	*0.042039906*
**ENSG00000176903**	*0.042782707*	*0.038937926*	*0.036896215*	*0.035241114*
**ENSG00000132498**	*0.015175858*	*0.02548729*	*0.025079701*	*0.017700823*
**ENSG00000172243**	*0.015219167*	*0.02473021*	*0.024669598*	*0.015947256*
**ENSG00000112167**	*0.033206031*	*0.034375435*	*0.035046833*	*0.02884873*
**ENSG00000138231**	*0.032327876*	*0.034755958*	*0.038241791*	*0.029749671*
**ENSG00000140479**	*0.029842018*	*0.033791363*	*0.031242982*	*0.035177916*
**ENSG00000114346**	*0.043735658*	*0.034818378*	*0.035087194*	*0.036850854*
**ENSG00000174327**	*0.018194221*	*0.026190014*	*0.025347733*	*0.018819968*
**ENSG00000082175**	*0.016618689*	*0.025312205*	*0.025096097*	*0.015191634*
**ENSG00000148482**	*0.029129583*	*0.02568323*	*0.025513197*	*0.015822662*
**ENSG00000175161**	*0.019971036*	*0.024871837*	*0.024742973*	*0.015658181*

**ENSG00000006071**	*0.016381013*	*0.026611228*	*0.025756398*	*0.01642093*
**ENSG00000198369**	*0.05124871*	*0.053930404*	*0.049248299*	*0.055100164*
**ENSG00000131981**	*0.053270749*	*0.046800778*	*0.048078286*	*0.058358364*
**ENSG00000179399**	*0.046208115*	*0.033843217*	*0.026850179*	*0.021342796*
**ENSG00000171303**	*0.017584562*	*0.026204432*	*0.02530869*	*0.017204343*
**ENSG00000067715**	*0.017196273*	*0.025315344*	*0.024774116*	*0.017435914*
**ENSG00000135241**	*0.018084524*	*0.027966026*	*0.024457464*	*0.018999282*
**ENSG00000137463**	*0.015976877*	*0.025221094*	*0.025764657*	*0.015945742*
**ENSG00000113649**	*0.025794078*	*0.029282492*	*0.028862346*	*0.021664664*
**ENSG00000135452**	*0.029980854*	*0.03460027*	*0.034349672*	*0.025826194*
**ENSG00000099810**	*0.03468355*	*0.034602048*	*0.02939473*	*0.04731647*
**ENSG00000161609**	*0.021228046*	*0.027210365*	*0.027610972*	*0.023990718*
**ENSG00000178741**	*0.017641195*	*0.026160894*	*0.026441628*	*0.019310206*
**ENSG00000168461**	*0.094764144*	*0.08720413*	*0.088378106*	*0.085564009*
**ENSG00000243696**	*0.01907804*	*0.026238437*	*0.026544164*	*0.017195354*
**ENSG00000120694**	*0.045338661*	*0.050660925*	*0.040863301*	*0.037492541*
**ENSG00000166006**	*0.021231195*	*0.027926803*	*0.027008594*	*0.019167389*
**ENSG00000129675**	*0.051044501*	*0.042522071*	*0.039647333*	*0.037209651*
**ENSG00000197619**	*0.036623963*	*0.047504818*	*0.049196678*	*0.028132176*
**ENSG00000058866**	*0.036059745*	*0.031621934*	*0.031551868*	*0.026568163*
**ENSG00000152766**	*0.030082956*	*0.029421533*	*0.02854329*	*0.023006494*
**ENSG00000134668**	*0.016847318*	*0.025198565*	*0.025583861*	*0.016523429*
**ENSG00000012983**	*0.034351849*	*0.037826518*	*0.036624465*	*0.035513228*
**ENSG00000152670**	*0.016316313*	*0.024785286*	*0.024831114*	*0.015137073*
**ENSG00000119715**	*0.016963828*	*0.026471696*	*0.026404058*	*0.015249427*
**ENSG00000008735**	*0.016071929*	*0.02507406*	*0.025276975*	*0.015334243*
**ENSG00000182264**	*0.015388245*	*0.02440428*	*0.024734199*	*0.01568072*
**ENSG00000114388**	*0.030397659*	*0.03458903*	*0.033078776*	*0.0232897*
**ENSG00000124935**	*0.015613806*	*0.025390963*	*0.024931316*	*0.015241039*
**ENSG00000141441**	*0.041395829*	*0.02597401*	*0.024609313*	*0.01639624*
**ENSG00000169393**	*0.016093058*	*0.025045374*	*0.025344346*	*0.015272998*
**ENSG00000105641**	*0.015625557*	*0.024932547*	*0.026562114*	*0.014985866*
**ENSG00000158055**	*0.068404322*	*0.039151133*	*0.029583339*	*0.039819212*
**ENSG00000155744**	*0.033702524*	*0.038380921*	*0.036388933*	*0.030374178*
**ENSG00000010030**	*0.041022058*	*0.04822527*	*0.037334985*	*0.047491116*
**ENSG00000099984**	*0.015885354*	*0.025672734*	*0.025728982*	*0.015856442*
**ENSG00000116205**	*0.026453257*	*0.043638323*	*0.032959468*	*0.035397229*
**ENSG00000198765**	*0.016907693*	*0.024970791*	*0.025052282*	*0.015410978*
**ENSG00000172007**	*0.020079142*	*0.02710892*	*0.02986789*	*0.020125749*
**ENSG00000151665**	*0.027803496*	*0.033705146*	*0.031199329*	*0.02283934*
**ENSG00000141258**	*0.031083111*	*0.034642134*	*0.041734854*	*0.030901825*
**ENSG00000140718**	*0.027638936*	*0.032332708*	*0.034765234*	*0.02343982*
**ENSG00000100372**	*0.044630869*	*0.043217983*	*0.039291056*	*0.041636042*
**ENSG00000170234**	*0.020103484*	*0.030957291*	*0.026888526*	*0.02169343*
**ENSG00000174156**	*0.016348501*	*0.025550577*	*0.024666804*	*0.014415585*
**ENSG00000162738**	*0.044904336*	*0.045499192*	*0.027399747*	*0.029887008*
**ENSG00000242173**	*0.014953806*	*0.025187724*	*0.024770369*	*0.015034935*
**ENSG00000116117**	*0.014568118*	*0.024365374*	*0.026402096*	*0.014934558*
**ENSG00000181638**	*0.017072676*	*0.025106224*	*0.02474936*	*0.015710699*
**ENSG00000105875**	*0.047325252*	*0.039847738*	*0.036437053*	*0.039255981*
**ENSG00000183093**	*0.026253016*	*0.028556539*	*0.027719056*	*0.02175146*
**ENSG00000172348**	*0.015509576*	*0.024320119*	*0.025132091*	*0.016056396*
**ENSG00000163577**	*0.05533973*	*0.046072601*	*0.037967341*	*0.033859178*
**ENSG00000124256**	*0.069501768*	*0.064870853*	*0.064210647*	*0.067150085*

**ENSG00000164292**	*0.088650382*	*0.063994093*	*0.064287705*	*0.070389246*
**ENSG00000183778**	*0.017898082*	*0.025625359*	*0.024755216*	*0.016373925*
**ENSG00000161911**	*0.017859871*	*0.028406026*	*0.026250761*	*0.018927486*
**ENSG00000123171**	*0.015363872*	*0.024454269*	*0.024817208*	*0.014514418*
**ENSG00000148303**	*0.015839602*	*0.02452774*	*0.0246125*	*0.015516439*
**ENSG00000120093**	*0.040935005*	*0.03913736*	*0.033926998*	*0.034857321*
**ENSG00000147852**	*0.083492475*	*0.07897495*	*0.059333949*	*0.058512968*
**ENSG00000213337**	*0.028757415*	*0.032534727*	*0.032303335*	*0.028103466*
**ENSG00000166402**	*0.015843059*	*0.025209845*	*0.0267215*	*0.015989812*
**ENSG00000121440**	*0.017512287*	*0.032201951*	*0.02536622*	*0.01583481*
**ENSG00000198759**	*0.045436559*	*0.027678372*	*0.031276862*	*0.036069242*
**ENSG00000118816**	*0.019444544*	*0.025972885*	*0.028487309*	*0.019582939*
**ENSG00000250571**	*0.031795927*	*0.031255674*	*0.029114958*	*0.026469086*
**ENSG00000081842**	*0.016437475*	*0.02452375*	*0.024475816*	*0.016512413*
**ENSG00000198315**	*0.015705711*	*0.024586341*	*0.02583606*	*0.016582256*
**ENSG00000114391**	*0.013758482*	*0.023706023*	*0.023918733*	*0.015074674*
**ENSG00000166704**	*0.032401047*	*0.037851637*	*0.030675837*	*0.023882629*
**ENSG00000091157**	*0.031721167*	*0.038762513*	*0.033219587*	*0.026447847*
**ENSG00000143324**	*0.035269823*	*0.03756084*	*0.031842722*	*0.02555559*
**ENSG00000077420**	*0.029628687*	*0.032608321*	*0.032828862*	*0.034490592*
**ENSG00000103257**	*0.056907448*	*0.048994295*	*0.05170032*	*0.063755267*
**ENSG00000121207**	*0.016655368*	*0.024994837*	*0.02684906*	*0.015719611*
**ENSG00000126456**	*0.024480636*	*0.03130301*	*0.033612494*	*0.025227472*
**ENSG00000121895**	*0.055270987*	*0.052175782*	*0.054437086*	*0.049992127*
**ENSG00000186130**	*0.031046435*	*0.038094115*	*0.032066441*	*0.024255001*
**ENSG00000010072**	*0.03826791*	*0.04238601*	*0.046334823*	*0.041871905*
**ENSG00000197646**	*0.049689704*	*0.045329131*	*0.038401345*	*0.040129923*
**ENSG00000036257**	*0.019279641*	*0.026206974*	*0.025926389*	*0.016731079*
**ENSG00000182521**	*0.015155747*	*0.026964022*	*0.024442339*	*0.015691564*
**ENSG00000126733**	*0.015860702*	*0.025293185*	*0.026599321*	*0.016786188*
**ENSG00000183166**	*0.018418796*	*0.025256785*	*0.026461311*	*0.015245286*
**ENSG00000103275**	*0.018563338*	*0.02788605*	*0.025838246*	*0.018810905*
**ENSG00000182492**	*0.014732784*	*0.025113754*	*0.025160115*	*0.014991199*
**ENSG00000179222**	*0.030698957*	*0.032242099*	*0.035485472*	*0.029657662*
**ENSG00000108511**	*0.01740953*	*0.025144803*	*0.026141893*	*0.014588151*
**ENSG00000170465**	*0.018024243*	*0.026418581*	*0.024695417*	*0.016452995*
**ENSG00000077152**	*0.03973838*	*0.034052588*	*0.030971324*	*0.029645084*
**ENSG00000206069**	*0.016651329*	*0.025745173*	*0.025994738*	*0.016790895*
**ENSG00000142556**	*0.034796229*	*0.035676058*	*0.044694692*	*0.028752785*
**ENSG00000183495**	*0.016601658*	*0.026733467*	*0.026046231*	*0.015849348*
**ENSG00000128951**	*0.051949263*	*0.045972515*	*0.038560665*	*0.039243979*
**ENSG00000156709**	*0.031125244*	*0.035229112*	*0.031194226*	*0.028951977*
**ENSG00000102043**	*0.014893844*	*0.024892033*	*0.025243284*	*0.016107067*
**ENSG00000164182**	*0.028590857*	*0.030961777*	*0.031192808*	*0.029446729*
**ENSG00000172062**	*0.034894881*	*0.034475942*	*0.03247658*	*0.031984946*
**ENSG00000197757**	*0.017810843*	*0.025925805*	*0.027142307*	*0.015141262*
**ENSG00000064102**	*0.036493172*	*0.043861362*	*0.033801893*	*0.027070647*
**ENSG00000126266**	*0.019575522*	*0.026328682*	*0.028460875*	*0.033919285*
**ENSG00000127252**	*0.015787526*	*0.025276378*	*0.024331706*	*0.014797984*
**ENSG00000186532**	*0.033947644*	*0.037696139*	*0.037121951*	*0.028859552*
**ENSG00000138722**	*0.037291056*	*0.028428982*	*0.025747661*	*0.019272004*
**ENSG00000149451**	*0.014910157*	*0.024551236*	*0.025230313*	*0.01564407*
**ENSG00000173391**	*0.036195134*	*0.02592584*	*0.026171204*	*0.020720496*
**ENSG00000124479**	*0.017334248*	*0.025516133*	*0.024812892*	*0.016396115*

**ENSG00000168679**	*0.017974169*	*0.031388584*	*0.025729314*	*0.021073751*
**ENSG00000197415**	*0.018161477*	*0.02466792*	*0.025200466*	*0.017595545*
**ENSG00000143340**	*0.016890416*	*0.026565748*	*0.024554369*	*0.017648926*
**ENSG00000130561**	*0.019805651*	*0.026203901*	*0.025308153*	*0.017163367*
**ENSG00000198836**	*0.029266131*	*0.033842529*	*0.03094025*	*0.037726352*
**ENSG00000131876**	*0.026345579*	*0.030124408*	*0.031071054*	*0.024872298*
**ENSG00000138442**	*0.032666748*	*0.033905341*	*0.029569873*	*0.028333426*
**ENSG00000173976**	*0.016455834*	*0.024747113*	*0.026126219*	*0.016196996*
**ENSG00000005156**	*0.016493371*	*0.025835777*	*0.025037147*	*0.01600769*
**ENSG00000166839**	*0.089906051*	*0.048335511*	*0.048657722*	*0.052347459*
**ENSG00000014257**	*0.085833466*	*0.067125771*	*0.058125932*	*0.0637496*
**ENSG00000133703**	*0.017157096*	*0.025946756*	*0.024894486*	*0.015929297*
**ENSG00000151233**	*0.02427575*	*0.031951336*	*0.031699774*	*0.025142256*
**ENSG00000040608**	*0.017584332*	*0.02697498*	*0.027056812*	*0.024556966*
**ENSG00000110881**	*0.015140669*	*0.025440651*	*0.025249295*	*0.015830821*
**ENSG00000178252**	*0.022610029*	*0.027177595*	*0.031360912*	*0.019505092*
**ENSG00000151131**	*0.023105848*	*0.030929459*	*0.032215143*	*0.022224435*
**ENSG00000162383**	*0.01772203*	*0.025749088*	*0.0256758*	*0.018567149*
**ENSG00000198517**	*0.013981161*	*0.025619486*	*0.024675437*	*0.01594043*
**ENSG00000135446**	*0.026735746*	*0.030196387*	*0.030724513*	*0.026660272*
**ENSG00000122873**	*0.04046881*	*0.041258946*	*0.035819431*	*0.030773955*
**ENSG00000027697**	*0.046772502*	*0.054699309*	*0.057076634*	*0.055295841*
**ENSG00000112367**	*0.030158549*	*0.030859214*	*0.031698905*	*0.029871657*
**ENSG00000053747**	*0.014983493*	*0.024393805*	*0.024684191*	*0.015492082*
**ENSG00000174417**	*0.017952511*	*0.024996828*	*0.025404906*	*0.018554501*
**ENSG00000151135**	*0.031458229*	*0.031286042*	*0.033329916*	*0.026089622*
**ENSG00000091136**	*0.059780809*	*0.070573984*	*0.054520278*	*0.057003617*
**ENSG00000152700**	*0.032522352*	*0.038530617*	*0.037695734*	*0.030010436*
**ENSG00000090861**	*0.026612647*	*0.029536409*	*0.030333565*	*0.024201189*
**ENSG00000196821**	*0.031173184*	*0.032072509*	*0.034757013*	*0.028025502*
**ENSG00000101193**	*0.024709421*	*0.029592927*	*0.03490815*	*0.019077057*
**ENSG00000112659**	*0.027828562*	*0.032122899*	*0.030173645*	*0.025604801*
**ENSG00000179813**	*0.015455468*	*0.024980678*	*0.025555737*	*0.015656688*
**ENSG00000123178**	*0.030610746*	*0.035797617*	*0.032507763*	*0.023383976*
**ENSG00000189280**	*0.014775028*	*0.024924833*	*0.02433362*	*0.015050756*
**ENSG00000156076**	*0.014868791*	*0.025141916*	*0.024549179*	*0.015782571*
**ENSG00000166562**	*0.027895782*	*0.03035591*	*0.029703123*	*0.03053857*
**ENSG00000156127**	*0.043520724*	*0.037369302*	*0.036873131*	*0.030172941*
**ENSG00000155066**	*0.01927578*	*0.026745493*	*0.029257427*	*0.017441824*
**ENSG00000081692**	*0.02301431*	*0.026862601*	*0.028487126*	*0.020448762*
**ENSG00000143373**	*0.017274149*	*0.027009931*	*0.026961893*	*0.017289976*
**ENSG00000104872**	*0.025275461*	*0.029354438*	*0.030463249*	*0.018939806*
**ENSG00000065485**	*0.041546848*	*0.033674956*	*0.040501286*	*0.042410023*
**ENSG00000250799**	*0.019473352*	*0.025260969*	*0.025477025*	*0.016448923*
**ENSG00000079819**	*0.063866865*	*0.049653077*	*0.051603333*	*0.050651206*
**ENSG00000158473**	*0.01641089*	*0.026062968*	*0.024878481*	*0.03248995*
**ENSG00000167618**	*0.014854897*	*0.024865677*	*0.024525109*	*0.014202414*
**ENSG00000010278**	*0.118333527*	*0.072473697*	*0.085041444*	*0.086210548*
**ENSG00000164520**	*0.016346749*	*0.025171909*	*0.02537173*	*0.015257039*
**ENSG00000165731**	*0.015699112*	*0.024948634*	*0.025011315*	*0.016404517*
**ENSG00000041515**	*0.016869624*	*0.024766326*	*0.024407941*	*0.016967489*
**ENSG00000044012**	*0.015185889*	*0.024779212*	*0.026032316*	*0.015187309*
**ENSG00000182952**	*0.026476038*	*0.030461387*	*0.030818382*	*0.023639561*
**ENSG00000136463**	*0.028466987*	*0.032803052*	*0.03420136*	*0.026910989*

**ENSG00000141034**	*0.014456082*	*0.024398552*	*0.024406236*	*0.014786594*
**ENSG00000159917**	*0.035588002*	*0.046943471*	*0.052019162*	*0.038196222*
**ENSG00000179604**	*0.069814649*	*0.055253731*	*0.04522177*	*0.059782249*
**ENSG00000110025**	*0.038763798*	*0.042325452*	*0.03836035*	*0.032060187*
**ENSG00000166558**	*0.017565061*	*0.026436125*	*0.024442623*	*0.014740642*
**ENSG00000155495**	*0.015603408*	*0.024742068*	*0.025041642*	*0.015515237*
**ENSG00000143954**	*0.018868239*	*0.027736734*	*0.026778101*	*0.017904865*
**ENSG00000165490**	*0.044210501*	*0.036163508*	*0.037629571*	*0.04035155*
**ENSG00000119335**	*0.022969423*	*0.028129703*	*0.026559139*	*0.021286523*
**ENSG00000112293**	*0.019074389*	*0.027388588*	*0.026291736*	*0.017015245*
**ENSG00000113312**	*0.025069635*	*0.030125884*	*0.031047751*	*0.022344849*
**ENSG00000119013**	*0.031263093*	*0.037296891*	*0.0293262*	*0.024614396*
**ENSG00000100897**	*0.023372184*	*0.035931697*	*0.03128879*	*0.035819867*
**ENSG00000135775**	*0.028782272*	*0.033239172*	*0.033270663*	*0.028067586*
**ENSG00000104824**	*0.030729315*	*0.033246511*	*0.031490412*	*0.02760244*
**ENSG00000160321**	*0.016849791*	*0.025827129*	*0.025732422*	*0.016889424*
**ENSG00000173207**	*0.032601011*	*0.030766594*	*0.029165491*	*0.022051466*
**ENSG00000171490**	*0.024954906*	*0.029613275*	*0.029099022*	*0.021329238*
**ENSG00000171723**	*0.033048282*	*0.040910764*	*0.035072139*	*0.034748618*
**ENSG00000153291**	*0.01795711*	*0.02649985*	*0.025684516*	*0.018233927*
**ENSG00000130433**	*0.017073426*	*0.024988453*	*0.025005305*	*0.016277535*
**ENSG00000155508**	*0.027799136*	*0.035579179*	*0.030934079*	*0.023533165*
**ENSG00000169575**	*0.016214452*	*0.025361968*	*0.025033201*	*0.014592689*
**ENSG00000108622**	*0.035377367*	*0.032408611*	*0.034967364*	*0.034849864*
**ENSG00000102119**	*0.024673092*	*0.030162385*	*0.030358761*	*0.02127809*
**ENSG00000037042**	*0.035733476*	*0.036134624*	*0.034722204*	*0.041853375*
**ENSG00000204130**	*0.021309236*	*0.030215536*	*0.027967914*	*0.020754855*
**ENSG00000046653**	*0.027364108*	*0.029949162*	*0.028408102*	*0.020955422*
**ENSG00000176798**	*0.014799445*	*0.024211123*	*0.025183165*	*0.0151283*
**ENSG00000086598**	*0.029990675*	*0.034641754*	*0.037363156*	*0.027840381*
**ENSG00000102172**	*0.026600766*	*0.036733089*	*0.030784431*	*0.025787365*
**ENSG00000107201**	*0.048625438*	*0.063059049*	*0.050503298*	*0.049732814*
**ENSG00000114745**	*0.027778502*	*0.032455009*	*0.034091955*	*0.027626737*
**ENSG00000106462**	*0.032999147*	*0.032519701*	*0.030317777*	*0.026001469*
**ENSG00000170953**	*0.015834976*	*0.025409098*	*0.024953002*	*0.016171728*
**ENSG00000169241**	*0.027928669*	*0.029891714*	*0.031724231*	*0.023858593*
**ENSG00000130303**	*0.028081915*	*0.041886157*	*0.036976029*	*0.031767361*
**ENSG00000240849**	*0.034210799*	*0.037797585*	*0.034527008*	*0.035834505*
**ENSG00000165138**	*0.018137972*	*0.026831123*	*0.025775774*	*0.016972406*
**ENSG00000125522**	*0.103016382*	*0.084344674*	*0.083526387*	*0.091724894*
**ENSG00000126583**	*0.015339047*	*0.024491322*	*0.024632786*	*0.014047888*
**ENSG00000131732**	*0.025397332*	*0.030377709*	*0.029697385*	*0.01926758*
**ENSG00000131037**	*0.015192109*	*0.025434887*	*0.02527881*	*0.014651522*
**ENSG00000153956**	*0.016470635*	*0.02512613*	*0.025201488*	*0.016702339*
**ENSG00000206527**	*0.030562996*	*0.036571804*	*0.03378799*	*0.028026044*
**ENSG00000177707**	*0.016677878*	*0.025813035*	*0.0239836*	*0.016307891*
**ENSG00000136694**	*0.016826137*	*0.024141193*	*0.024383438*	*0.014771396*
**ENSG00000116273**	*0.026799178*	*0.039158925*	*0.038455425*	*0.025697039*
**ENSG00000138398**	*0.024704791*	*0.031113263*	*0.033368903*	*0.034161456*
**ENSG00000160058**	*0.025756551*	*0.030073614*	*0.032805289*	*0.022845014*
**ENSG00000047346**	*0.050244204*	*0.043109222*	*0.041104063*	*0.035603776*
**ENSG00000165630**	*0.023537691*	*0.031156021*	*0.03405093*	*0.02971349*
**ENSG00000169429**	*0.14514876*	*0.112330592*	*0.104988304*	*0.096369395*
**ENSG00000100029**	*0.032867178*	*0.034137388*	*0.031853112*	*0.030344077*

**ENSG00000134285**	*0.045796345*	*0.043254285*	*0.044322421*	*0.051709964*
**ENSG00000169515**	*0.016374828*	*0.025205572*	*0.025098695*	*0.015950628*
**ENSG00000115275**	*0.022193553*	*0.028931707*	*0.03287581*	*0.024661677*
**ENSG00000171960**	*0.03098451*	*0.031634629*	*0.029931098*	*0.026150919*
**ENSG00000123975**	*0.036166707*	*0.034290398*	*0.031036761*	*0.0240352*
**ENSG00000105810**	*0.016454648*	*0.025344092*	*0.025114171*	*0.016498215*
**ENSG00000111913**	*0.04864511*	*0.048775483*	*0.049931509*	*0.042064251*
**ENSG00000165269**	*0.01473041*	*0.024768846*	*0.024590918*	*0.014363274*
**ENSG00000160404**	*0.0237869*	*0.027653956*	*0.026274227*	*0.019862614*
**ENSG00000082269**	*0.042399242*	*0.042615905*	*0.035451188*	*0.038176908*
**ENSG00000183607**	*0.017698951*	*0.026459236*	*0.027626479*	*0.015721933*
**ENSG00000080293**	*0.017293328*	*0.026106506*	*0.025355355*	*0.01668259*
**ENSG00000125735**	*0.056215231*	*0.040114003*	*0.045045569*	*0.054606412*
**ENSG00000148985**	*0.027744519*	*0.028349782*	*0.031900159*	*0.030417837*
**ENSG00000119457**	*0.014051634*	*0.025076184*	*0.024546617*	*0.015034308*
**ENSG00000166963**	*0.018351334*	*0.025612526*	*0.024610651*	*0.015050364*
**ENSG00000176533**	*0.018060062*	*0.02568538*	*0.025476714*	*0.019200237*
**ENSG00000118491**	*0.028495805*	*0.028297295*	*0.028603932*	*0.026029228*
**ENSG00000172426**	*0.018510526*	*0.026586578*	*0.024465596*	*0.023523032*
**ENSG00000204687**	*0.015285804*	*0.024509455*	*0.0248109*	*0.0153694*
**ENSG00000121577**	*0.026507255*	*0.028648133*	*0.027653787*	*0.019331824*
**ENSG00000166226**	*0.025748481*	*0.029828957*	*0.027734746*	*0.026814962*
**ENSG00000182759**	*0.016559905*	*0.025104833*	*0.024089179*	*0.015006758*
**ENSG00000174437**	*0.032554654*	*0.032660584*	*0.038228058*	*0.03485626*
**ENSG00000107815**	*0.042343821*	*0.043639145*	*0.048486218*	*0.045357254*
**ENSG00000135899**	*0.038699982*	*0.038609809*	*0.038866589*	*0.048212982*
**ENSG00000108786**	*0.016082006*	*0.024443543*	*0.024849317*	*0.015933856*
**ENSG00000115966**	*0.027861289*	*0.033078154*	*0.035439928*	*0.025228606*
**ENSG00000182885**	*0.016567846*	*0.025772101*	*0.026986506*	*0.01912093*
**ENSG00000107625**	*0.027083428*	*0.031394186*	*0.031913086*	*0.021412912*
**ENSG00000243978**	*0.030685482*	*0.030939125*	*0.025947467*	*0.020736894*
**ENSG00000121848**	*0.022210594*	*0.032984371*	*0.037936488*	*0.025844428*
**ENSG00000086289**	*0.111326842*	*0.1003186*	*0.08732898*	*0.106539413*
**ENSG00000067798**	*0.016599576*	*0.025566204*	*0.025305748*	*0.015170534*
**ENSG00000128340**	*0.021913178*	*0.02813611*	*0.030943128*	*0.02641483*
**ENSG00000187969**	*0.015751117*	*0.024475008*	*0.026114642*	*0.015097741*
**ENSG00000140280**	*0.035360479*	*0.040946368*	*0.038922434*	*0.043770741*
**ENSG00000155974**	*0.017430615*	*0.026614367*	*0.025491268*	*0.019267031*
**ENSG00000125812**	*0.023998459*	*0.035305627*	*0.038754239*	*0.024799394*
**ENSG00000164399**	*0.014621211*	*0.024503912*	*0.024762634*	*0.014984199*
**ENSG00000162444**	*0.025124831*	*0.041581138*	*0.034271957*	*0.030238129*
**ENSG00000126215**	*0.03343189*	*0.034694296*	*0.03855656*	*0.032377444*
**ENSG00000173706**	*0.036720573*	*0.040438279*	*0.040558044*	*0.037202547*
**ENSG00000163508**	*0.076031701*	*0.05596503*	*0.05903556*	*0.084795157*
**ENSG00000054803**	*0.023823097*	*0.028821894*	*0.031759526*	*0.018985688*
**ENSG00000128591**	*0.038463582*	*0.037672476*	*0.03195181*	*0.032206441*
**ENSG00000115233**	*0.025517709*	*0.029236197*	*0.02598387*	*0.028289249*
**ENSG00000137822**	*0.033739311*	*0.0334256*	*0.032377053*	*0.03428113*
**ENSG00000135083**	*0.017898356*	*0.024867535*	*0.024683545*	*0.016652421*
**ENSG00000128581**	*0.015635153*	*0.026042935*	*0.024622274*	*0.015563886*
**ENSG00000198242**	*0.014876102*	*0.02426315*	*0.024485891*	*0.015104098*
**ENSG00000188707**	*0.051144797*	*0.061677916*	*0.053926903*	*0.053331213*
**ENSG00000177363**	*0.01497262*	*0.024932848*	*0.024400704*	*0.014809986*
**ENSG00000121741**	*0.025814585*	*0.032386078*	*0.033339435*	*0.026440445*

**ENSG00000137413**	*0.017776741*	*0.027561373*	*0.028196189*	*0.018362758*
**ENSG00000100292**	*0.123476636*	*0.095462746*	*0.118968169*	*0.103715483*
**ENSG00000198742**	*0.01637695*	*0.024626703*	*0.026407855*	*0.014196592*
**ENSG00000152952**	*0.171427341*	*0.085959456*	*0.06059866*	*0.070965702*
**ENSG00000100342**	*0.019454004*	*0.029673669*	*0.027206051*	*0.017268768*
**ENSG00000113905**	*0.016227032*	*0.02541689*	*0.024146579*	*0.015265455*
**ENSG00000120211**	*0.015138527*	*0.024485172*	*0.024460329*	*0.014862565*
**ENSG00000157510**	*0.015966898*	*0.025742458*	*0.024851566*	*0.016956008*
**ENSG00000115850**	*0.032449005*	*0.052038055*	*0.03356133*	*0.029013571*
**ENSG00000197457**	*0.052798201*	*0.05439503*	*0.059764417*	*0.072166493*
**ENSG00000169744**	*0.023736487*	*0.027764014*	*0.025765043*	*0.017710119*
**ENSG00000205336**	*0.066364311*	*0.065604471*	*0.065485519*	*0.080907073*
**ENSG00000105664**	*0.020600753*	*0.030249311*	*0.028751747*	*0.019780322*
**ENSG00000164287**	*0.016970439*	*0.025400942*	*0.024705755*	*0.015979694*
**ENSG00000114374**	*0.108501133*	*0.120339256*	*0.101955712*	*0.100759967*
**ENSG00000130733**	*0.035260448*	*0.034192365*	*0.041741137*	*0.033349381*
**ENSG00000129354**	*0.01639184*	*0.025188572*	*0.025476382*	*0.015308732*
**ENSG00000137807**	*0.039005373*	*0.037967662*	*0.030664442*	*0.033354246*
**ENSG00000144278**	*0.015150097*	*0.024369866*	*0.024986631*	*0.015441577*
**ENSG00000084234**	*0.052444146*	*0.062982014*	*0.03951551*	*0.042519503*
**ENSG00000102755**	*0.04612947*	*0.051553709*	*0.045058553*	*0.031722787*
**ENSG00000167747**	*0.0334566*	*0.03324617*	*0.036175194*	*0.037045366*
**ENSG00000133835**	*0.034151148*	*0.036663484*	*0.030159955*	*0.0259613*
**ENSG00000083817**	*0.0278553*	*0.047800473*	*0.049757169*	*0.027843554*
**ENSG00000117877**	*0.045595198*	*0.041659675*	*0.04001103*	*0.046847254*
**ENSG00000164548**	*0.024776838*	*0.030319525*	*0.029001165*	*0.021835496*
**ENSG00000197111**	*0.017243313*	*0.025815658*	*0.027348445*	*0.017157815*
**ENSG00000174469**	*0.030620905*	*0.026709356*	*0.025900041*	*0.018257969*
**ENSG00000134049**	*0.027167139*	*0.030051548*	*0.033925727*	*0.031125754*
**ENSG00000107263**	*0.018402376*	*0.0253395*	*0.025830898*	*0.016927203*
**ENSG00000136960**	*0.098910255*	*0.084727662*	*0.07326167*	*0.086531504*
**ENSG00000103423**	*0.027293325*	*0.033052111*	*0.030310101*	*0.021766059*
**ENSG00000123159**	*0.05286504*	*0.048561703*	*0.041493797*	*0.046539136*
**ENSG00000169306**	*0.015871531*	*0.024613691*	*0.024438448*	*0.015698358*
**ENSG00000179846**	*0.015897253*	*0.027683509*	*0.025850975*	*0.015226875*
**ENSG00000135093**	*0.029053324*	*0.033880555*	*0.035569377*	*0.023935877*
**ENSG00000091010**	*0.017680963*	*0.026353012*	*0.026292412*	*0.016837565*
**ENSG00000174498**	*0.017512395*	*0.025962372*	*0.025201023*	*0.015074329*
**ENSG00000006453**	*0.105841246*	*0.079082838*	*0.090430653*	*0.102293366*
**ENSG00000204301**	*0.026950024*	*0.037004128*	*0.035163311*	*0.027209663*
**ENSG00000136888**	*0.020771761*	*0.030264682*	*0.03039102*	*0.022774994*
**ENSG00000006432**	*0.034238146*	*0.033817397*	*0.031212125*	*0.044659205*
**ENSG00000117318**	*0.071591964*	*0.056844751*	*0.048642681*	*0.06060457*
**ENSG00000158158**	*0.035954295*	*0.033960847*	*0.034603183*	*0.031352077*
**ENSG00000167113**	*0.033476257*	*0.033113763*	*0.030462947*	*0.028190377*
**ENSG00000188404**	*0.090980729*	*0.079134976*	*0.077009272*	*0.091717571*
**ENSG00000095713**	*0.015357996*	*0.025644922*	*0.02445838*	*0.015535083*
**ENSG00000183753**	*0.01520479*	*0.025309344*	*0.024910394*	*0.014499365*
**ENSG00000160588**	*0.021051378*	*0.026553583*	*0.028593328*	*0.020602917*
**ENSG00000069275**	*0.024148136*	*0.028206438*	*0.029076718*	*0.01913807*
**ENSG00000121388**	*0.032771789*	*0.036299758*	*0.0327278*	*0.03500293*
**ENSG00000159423**	*0.038730988*	*0.043273557*	*0.035572679*	*0.045374661*
**ENSG00000058085**	*0.018489214*	*0.026312224*	*0.025080466*	*0.015259377*
**ENSG00000132746**	*0.019610639*	*0.026860887*	*0.027634501*	*0.021893374*

**ENSG00000187151**	*0.017057185*	*0.024676765*	*0.025102719*	*0.0161944*
**ENSG00000186897**	*0.01506841*	*0.02548483*	*0.024901387*	*0.014873349*
**ENSG00000116199**	*0.029836824*	*0.033535912*	*0.033530469*	*0.023987919*
**ENSG00000170689**	*0.036126725*	*0.037989095*	*0.032176963*	*0.047095039*
**ENSG00000165995**	*0.015370925*	*0.025389311*	*0.024674323*	*0.015776062*
**ENSG00000183773**	*0.016454557*	*0.026652607*	*0.024554094*	*0.014510772*
**ENSG00000165816**	*0.015625877*	*0.025473308*	*0.024262778*	*0.014814999*
**ENSG00000123094**	*0.016081796*	*0.025218234*	*0.025415862*	*0.016961447*
**ENSG00000127129**	*0.021178148*	*0.027519098*	*0.02571883*	*0.016576627*
**ENSG00000114013**	*0.075252089*	*0.056368342*	*0.065210044*	*0.073770683*
**ENSG00000123297**	*0.028521187*	*0.030296712*	*0.034629011*	*0.026999743*
**ENSG00000078401**	*0.094243706*	*0.074125259*	*0.072682951*	*0.07652464*
**ENSG00000105404**	*0.039013393*	*0.037171146*	*0.042515366*	*0.043730113*
**ENSG00000077585**	*0.040043159*	*0.040984953*	*0.041498494*	*0.039718859*
**ENSG00000042317**	*0.047471649*	*0.043545783*	*0.040909063*	*0.048030377*
**ENSG00000066382**	*0.019039792*	*0.026727421*	*0.026028753*	*0.019263582*
**ENSG00000106615**	*0.025440908*	*0.035624513*	*0.033600805*	*0.023967803*
**ENSG00000178055**	*0.021809596*	*0.028698774*	*0.026604439*	*0.021278545*
**ENSG00000103091**	*0.021957917*	*0.028359491*	*0.030497269*	*0.024607122*
**ENSG00000007516**	*0.020467382*	*0.030540657*	*0.029058263*	*0.024996896*
**ENSG00000178015**	*0.017680907*	*0.02444924*	*0.025194259*	*0.016058621*
**ENSG00000108387**	*0.014622308*	*0.025498956*	*0.025044718*	*0.015279636*
**ENSG00000180771**	*0.029425967*	*0.037808781*	*0.042998238*	*0.026253983*
**ENSG00000115415**	*0.026797746*	*0.030833205*	*0.028484062*	*0.020545219*
**ENSG00000110911**	*0.040357953*	*0.048818973*	*0.042796314*	*0.035138477*
**ENSG00000108556**	*0.024699515*	*0.026610447*	*0.028025731*	*0.019878297*
**ENSG00000163349**	*0.018469904*	*0.026955509*	*0.025949414*	*0.018562507*
**ENSG00000181481**	*0.039594448*	*0.034324035*	*0.03356477*	*0.045016869*
**ENSG00000120533**	*0.021528021*	*0.026681915*	*0.027649572*	*0.018426788*
**ENSG00000124942**	*0.023302908*	*0.029374293*	*0.026482204*	*0.021906294*
**ENSG00000184343**	*0.019609311*	*0.027373857*	*0.027119232*	*0.017064586*
**ENSG00000241595**	*0.017117572*	*0.025016442*	*0.025728283*	*0.014769085*
**ENSG00000125354**	*0.033969723*	*0.039089833*	*0.035528598*	*0.035652707*
**ENSG00000175189**	*0.016778954*	*0.025773332*	*0.024649211*	*0.015670172*
**ENSG00000117016**	*0.079890611*	*0.062243477*	*0.060171209*	*0.073264816*
**ENSG00000116031**	*0.015398663*	*0.025846578*	*0.025452931*	*0.015603264*
**ENSG00000196890**	*0.029741795*	*0.030532942*	*0.033718355*	*0.0239889*
**ENSG00000110484**	*0.015119829*	*0.024912442*	*0.024116665*	*0.014836928*
**ENSG00000152137**	*0.01706546*	*0.026777513*	*0.025772436*	*0.015754768*
**ENSG00000142444**	*0.022173129*	*0.027861116*	*0.032479217*	*0.01905533*
**ENSG00000108849**	*0.021167986*	*0.025897045*	*0.02542825*	*0.020465717*
**ENSG00000181784**	*0.016179618*	*0.024440629*	*0.024827892*	*0.015565456*
**ENSG00000198373**	*0.015463786*	*0.024598152*	*0.024197773*	*0.01484033*
**ENSG00000197299**	*0.049747903*	*0.040987921*	*0.039633987*	*0.033020764*
**ENSG00000137145**	*0.031275065*	*0.031272654*	*0.033398869*	*0.029433459*
**ENSG00000102978**	*0.027926726*	*0.032727826*	*0.03862718*	*0.027745838*
**ENSG00000137203**	*0.017568727*	*0.026060974*	*0.024552395*	*0.015941285*
**ENSG00000138293**	*0.020571944*	*0.028675242*	*0.027666823*	*0.019349494*
**ENSG00000092439**	*0.038852902*	*0.042719701*	*0.037012574*	*0.027559938*
**ENSG00000187144**	*0.015679558*	*0.024734473*	*0.024752362*	*0.015944669*
**ENSG00000127220**	*0.026417877*	*0.032890544*	*0.036660469*	*0.030264643*
**ENSG00000072609**	*0.024174844*	*0.033396417*	*0.033988625*	*0.027267063*
**ENSG00000010322**	*0.025460386*	*0.028395769*	*0.034031567*	*0.023499016*
**ENSG00000150471**	*0.015841543*	*0.024817228*	*0.02441786*	*0.014353516*

**ENSG00000154783**	*0.036517823*	*0.025708871*	*0.026108508*	*0.016480364*
**ENSG00000160194**	*0.034187388*	*0.035215396*	*0.043893773*	*0.019725058*
**ENSG00000167315**	*0.030874631*	*0.031806579*	*0.031047004*	*0.021891187*
**ENSG00000087460**	*0.014685449*	*0.024414306*	*0.025073518*	*0.014391344*
**ENSG00000086065**	*0.025141869*	*0.031311687*	*0.029200441*	*0.021036732*
**ENSG00000122741**	*0.027383535*	*0.034817246*	*0.033165662*	*0.021990238*
**ENSG00000176105**	*0.050081444*	*0.051229792*	*0.048371645*	*0.045447074*
**ENSG00000158195**	*0.027219792*	*0.026824268*	*0.029915226*	*0.031744519*
**ENSG00000116514**	*0.036496906*	*0.039196379*	*0.045897597*	*0.042150663*
**ENSG00000169641**	*0.01977561*	*0.025181263*	*0.026079985*	*0.01834222*
**ENSG00000173928**	*0.038013378*	*0.035124691*	*0.035571564*	*0.036322641*
**ENSG00000164024**	*0.034254381*	*0.036227618*	*0.030920271*	*0.027904233*
**ENSG00000165675**	*0.020808112*	*0.026639662*	*0.025957164*	*0.017708445*
**ENSG00000198685**	*0.015979791*	*0.024746547*	*0.024541275*	*0.014480827*
**ENSG00000185236**	*0.021908946*	*0.031751181*	*0.027687905*	*0.025630687*
**ENSG00000077264**	*0.017005318*	*0.027468612*	*0.025115554*	*0.017980823*
**ENSG00000180530**	*0.098515835*	*0.067723407*	*0.064897615*	*0.065880484*
**ENSG00000138079**	*0.016627925*	*0.0251388*	*0.025557103*	*0.017087661*
**ENSG00000143183**	*0.026773684*	*0.03200429*	*0.032952665*	*0.023354986*
**ENSG00000110786**	*0.018359001*	*0.026966711*	*0.028192678*	*0.017118174*
**ENSG00000088726**	*0.016521256*	*0.025402512*	*0.024392483*	*0.014773323*
**ENSG00000197694**	*0.027534483*	*0.038381396*	*0.036596322*	*0.03229993*
**ENSG00000171984**	*0.035928803*	*0.042891977*	*0.034286593*	*0.034845392*
**ENSG00000101890**	*0.015870645*	*0.025283538*	*0.024478201*	*0.015568143*
**ENSG00000162415**	*0.028397809*	*0.027235127*	*0.025334595*	*0.017468433*
**ENSG00000145824**	*0.135541979*	*0.060336782*	*0.059325172*	*0.074128693*
**ENSG00000185728**	*0.038673911*	*0.045063255*	*0.054442079*	*0.038767352*
**ENSG00000213088**	*0.048661531*	*0.036295243*	*0.026721825*	*0.025634517*
**ENSG00000203710**	*0.014731834*	*0.025151933*	*0.024425782*	*0.014539442*
**ENSG00000129521**	*0.114762849*	*0.081030593*	*0.073638944*	*0.087016615*
**ENSG00000006007**	*0.02441601*	*0.03263725*	*0.032719136*	*0.024034285*
**ENSG00000197256**	*0.054801474*	*0.045695738*	*0.040320206*	*0.042094135*
**ENSG00000151715**	*0.016063606*	*0.026192502*	*0.024508663*	*0.016244217*
**ENSG00000156787**	*0.0412491*	*0.041635574*	*0.034456536*	*0.028264412*
**ENSG00000243566**	*0.016644695*	*0.025307485*	*0.024814534*	*0.018161517*
**ENSG00000168542**	*0.017599381*	*0.026263247*	*0.02675309*	*0.017121797*
**ENSG00000008517**	*0.091417562*	*0.055594832*	*0.055417488*	*0.067621627*
**ENSG00000197479**	*0.019346598*	*0.026409374*	*0.029169986*	*0.017865618*
**ENSG00000184277**	*0.04207822*	*0.037143949*	*0.037116223*	*0.040268559*
**ENSG00000072042**	*0.024219863*	*0.028704129*	*0.026188924*	*0.018791948*
**ENSG00000113492**	*0.017134849*	*0.025983836*	*0.024898207*	*0.016183664*
**ENSG00000168267**	*0.019912015*	*0.029151174*	*0.027241984*	*0.019008152*
**ENSG00000142910**	*0.019271111*	*0.026145986*	*0.025501822*	*0.019457815*
**ENSG00000124157**	*0.019352583*	*0.024901687*	*0.025333288*	*0.016095058*
**ENSG00000069206**	*0.015263419*	*0.024904879*	*0.025181856*	*0.014780763*
**ENSG00000151690**	*0.029807163*	*0.032475361*	*0.031377953*	*0.029205346*
**ENSG00000111412**	*0.035491852*	*0.040765268*	*0.040086922*	*0.034267469*
**ENSG00000139722**	*0.066014604*	*0.054256442*	*0.056871654*	*0.047172531*
**ENSG00000008394**	*0.114292931*	*0.074925543*	*0.066920717*	*0.094474625*
**ENSG00000184574**	*0.036173796*	*0.034154527*	*0.036864309*	*0.036962982*
**ENSG00000163655**	*0.027431477*	*0.032733534*	*0.029358847*	*0.024693066*
**ENSG00000188620**	*0.129710955*	*0.105811608*	*0.100302255*	*0.119242712*
**ENSG00000163539**	*0.03775639*	*0.053822772*	*0.036557952*	*0.043419429*
**ENSG00000159723**	*0.020913375*	*0.028386699*	*0.025859319*	*0.017139473*

**ENSG00000253729**	*0.038934573*	*0.050330839*	*0.036372089*	*0.033777014*
**ENSG00000135249**	*0.015404759*	*0.025137111*	*0.024794602*	*0.01496746*
**ENSG00000203836**	*0.021113437*	*0.029109092*	*0.027122589*	*0.020559014*
**ENSG00000175393**	*0.016009336*	*0.024148842*	*0.024665699*	*0.015435412*
**ENSG00000108352**	*0.036608811*	*0.029894434*	*0.028736273*	*0.041061179*
**ENSG00000188290**	*0.115973436*	*0.077773466*	*0.071580143*	*0.10544063*
**ENSG00000101782**	*0.025344242*	*0.030240125*	*0.030191852*	*0.025890844*
**ENSG00000169714**	*0.024093473*	*0.031815073*	*0.029545655*	*0.021718739*
**ENSG00000103942**	*0.041692656*	*0.034866168*	*0.036468831*	*0.029624973*
**ENSG00000185024**	*0.015606127*	*0.025413219*	*0.024725108*	*0.0145261*
**ENSG00000146678**	*0.016336137*	*0.024335191*	*0.025361487*	*0.015687288*
**ENSG00000171657**	*0.03275816*	*0.029795432*	*0.028490202*	*0.024712401*
**ENSG00000111961**	*0.057612011*	*0.045261072*	*0.040700163*	*0.048920606*
**ENSG00000145284**	*0.016361921*	*0.026718856*	*0.026118002*	*0.015956636*
**ENSG00000005981**	*0.015574868*	*0.024958775*	*0.024448559*	*0.015376914*
**ENSG00000226761**	*0.015744176*	*0.025179365*	*0.024656166*	*0.014929587*
**ENSG00000077616**	*0.030298467*	*0.038187128*	*0.032852111*	*0.046178235*
**ENSG00000156925**	*0.018024698*	*0.026442974*	*0.025183867*	*0.016860829*
**ENSG00000188342**	*0.029433255*	*0.031321235*	*0.028639844*	*0.02318612*
**ENSG00000178795**	*0.014941204*	*0.024367629*	*0.025784755*	*0.01555327*
**ENSG00000126561**	*0.040111681*	*0.036413019*	*0.037221631*	*0.033678442*
**ENSG00000075914**	*0.030489335*	*0.02815929*	*0.030875121*	*0.02631761*
**ENSG00000102195**	*0.016441006*	*0.024568965*	*0.025168299*	*0.015274445*
**ENSG00000178188**	*0.023006243*	*0.027818501*	*0.035250149*	*0.023193745*
**ENSG00000124575**	*0.060597265*	*0.039742459*	*0.049275026*	*0.063074762*
**ENSG00000165076**	*0.017960649*	*0.025532023*	*0.026526567*	*0.015307226*
**ENSG00000000003**	*0.022314088*	*0.025220688*	*0.024583592*	*0.021506493*
**ENSG00000171867**	*0.059992152*	*0.048975528*	*0.042674756*	*0.045163014*
**ENSG00000213265**	*0.015711222*	*0.025186953*	*0.024446041*	*0.015078584*
**ENSG00000077044**	*0.022439142*	*0.027328637*	*0.029541402*	*0.021831421*
**ENSG00000065600**	*0.016243785*	*0.024316143*	*0.024451634*	*0.016070626*
**ENSG00000196305**	*0.023137686*	*0.02888723*	*0.026409957*	*0.020704776*
**ENSG00000232112**	*0.023944021*	*0.027469604*	*0.030426193*	*0.020519052*
**ENSG00000096384**	*0.020235967*	*0.025549199*	*0.025337671*	*0.01989584*
**ENSG00000092009**	*0.072543563*	*0.046407399*	*0.04303195*	*0.043675238*
**ENSG00000160145**	*0.015330769*	*0.025515895*	*0.024385981*	*0.014755945*
**ENSG00000178409**	*0.014657847*	*0.024037982*	*0.025215462*	*0.013884533*
**ENSG00000140416**	*0.019642145*	*0.025180744*	*0.026923551*	*0.016371097*
**ENSG00000075303**	*0.016586349*	*0.025923654*	*0.025352177*	*0.016031901*
**ENSG00000105894**	*0.019711377*	*0.024867422*	*0.024787525*	*0.01712741*
**ENSG00000138085**	*0.020838418*	*0.027228416*	*0.027874755*	*0.020059893*
**ENSG00000157077**	*0.01963688*	*0.026281154*	*0.026634298*	*0.018606523*
**ENSG00000105699**	*0.014853555*	*0.025097541*	*0.025706164*	*0.015159687*
**ENSG00000119953**	*0.027015706*	*0.032099872*	*0.030122026*	*0.025281209*
**ENSG00000215695**	*0.026624417*	*0.036088041*	*0.041720645*	*0.026880249*
**ENSG00000139835**	*0.016525088*	*0.026649422*	*0.024628361*	*0.016237936*
**ENSG00000179600**	*0.016318586*	*0.027361352*	*0.025265293*	*0.016093677*
**ENSG00000166930**	*0.015646399*	*0.025862023*	*0.025071124*	*0.01502431*
**ENSG00000198010**	*0.015507073*	*0.025007561*	*0.026276765*	*0.015759733*
**ENSG00000100600**	*0.05248797*	*0.042926182*	*0.037467824*	*0.043363565*
**ENSG00000116667**	*0.023670441*	*0.027205837*	*0.025674312*	*0.021995052*
**ENSG00000109323**	*0.041977012*	*0.043516979*	*0.043148108*	*0.041447967*
**ENSG00000149179**	*0.030255709*	*0.037035003*	*0.031827434*	*0.026434737*
**ENSG00000137270**	*0.016259632*	*0.027273884*	*0.025688824*	*0.016175394*

**ENSG00000170819**	*0.077223753*	*0.071452333*	*0.05868572*	*0.066593364*
**ENSG00000170802**	*0.019418299*	*0.026764139*	*0.027290963*	*0.020692055*
**ENSG00000164325**	*0.014894757*	*0.025010109*	*0.025300185*	*0.014528602*
**ENSG00000170323**	*0.021052088*	*0.026334924*	*0.028443889*	*0.019064672*
**ENSG00000171484**	*0.017247219*	*0.027301022*	*0.027968883*	*0.01644353*
**ENSG00000168703**	*0.017395682*	*0.026125553*	*0.026038757*	*0.017596895*
**ENSG00000171773**	*0.015400258*	*0.024781974*	*0.025334602*	*0.014216034*
**ENSG00000187105**	*0.024206632*	*0.024153697*	*0.025424032*	*0.021091476*
**ENSG00000157131**	*0.017449454*	*0.025798774*	*0.028797417*	*0.018065791*
**ENSG00000185250**	*0.016341118*	*0.02480572*	*0.024846131*	*0.016501317*
**ENSG00000205496**	*0.018539511*	*0.025906168*	*0.02666403*	*0.018069197*
**ENSG00000143390**	*0.019380531*	*0.029137812*	*0.029267778*	*0.022099756*
**ENSG00000182968**	*0.015575538*	*0.024963572*	*0.025105914*	*0.015114134*
**ENSG00000129170**	*0.015367887*	*0.025378135*	*0.024921446*	*0.014758365*
**ENSG00000178695**	*0.057765587*	*0.043295406*	*0.042966344*	*0.043164491*
**ENSG00000010810**	*0.154965042*	*0.090414646*	*0.09866739*	*0.136573513*
**ENSG00000042062**	*0.01859666*	*0.02719396*	*0.026345238*	*0.016925684*
**ENSG00000109066**	*0.021971574*	*0.028511645*	*0.026574061*	*0.018202042*
**ENSG00000169019**	*0.030100208*	*0.037407653*	*0.03143788*	*0.032227274*
**ENSG00000198271**	*0.015689505*	*0.024669331*	*0.025462072*	*0.015151771*
**ENSG00000100906**	*0.028743398*	*0.031970629*	*0.029083422*	*0.025550197*
**ENSG00000186188**	*0.015746122*	*0.024600163*	*0.02438532*	*0.015103786*
**ENSG00000166024**	*0.031641304*	*0.033919038*	*0.033464288*	*0.024925824*
**ENSG00000153885**	*0.016489574*	*0.025247715*	*0.024488315*	*0.014829872*
**ENSG00000143344**	*0.044308753*	*0.047397807*	*0.041271924*	*0.058972159*
**ENSG00000168795**	*0.023804789*	*0.034722062*	*0.031420647*	*0.023381156*
**ENSG00000141522**	*0.026050363*	*0.030956074*	*0.035642909*	*0.027617138*
**ENSG00000092200**	*0.015452846*	*0.025085618*	*0.024395329*	*0.015335859*
**ENSG00000164458**	*0.065112696*	*0.055278441*	*0.035622777*	*0.0509268*
**ENSG00000124126**	*0.027029862*	*0.031647949*	*0.036584501*	*0.034489067*
**ENSG00000111676**	*0.022447273*	*0.031572149*	*0.039622454*	*0.03010309*
**ENSG00000174914**	*0.013187615*	*0.02393331*	*0.024213887*	*0.013443484*
**ENSG00000134882**	*0.044904069*	*0.045067634*	*0.043428668*	*0.041120676*
**ENSG00000147912**	*0.033346904*	*0.031400215*	*0.031803765*	*0.027697371*
**ENSG00000166747**	*0.028855235*	*0.033878674*	*0.036329917*	*0.02652654*
**ENSG00000198558**	*0.04088249*	*0.036748894*	*0.04070753*	*0.042968844*
**ENSG00000132485**	*0.029478537*	*0.028927459*	*0.02764296*	*0.018527891*
**ENSG00000213240**	*0.069479627*	*0.061004781*	*0.06692451*	*0.07303826*
**ENSG00000240303**	*0.045721877*	*0.052523575*	*0.039194686*	*0.04839082*
**ENSG00000174667**	*0.016246914*	*0.026571168*	*0.024820327*	*0.017240649*
**ENSG00000020426**	*0.030799159*	*0.037310791*	*0.031681882*	*0.023325227*
**ENSG00000178028**	*0.027342456*	*0.031450243*	*0.031818549*	*0.024664772*
**ENSG00000138771**	*0.121288674*	*0.103146067*	*0.12475618*	*0.104033045*
**ENSG00000135547**	*0.021877041*	*0.027152681*	*0.025460371*	*0.021625786*
**ENSG00000205339**	*0.042293863*	*0.04746428*	*0.039445455*	*0.043138704*
**ENSG00000127554**	*0.015146685*	*0.025618441*	*0.025453901*	*0.016274256*
**ENSG00000197535**	*0.036131181*	*0.040263837*	*0.036811266*	*0.033870828*
**ENSG00000115607**	*0.078109827*	*0.083696835*	*0.051141937*	*0.059122538*
**ENSG00000144668**	*0.023205555*	*0.025578049*	*0.024991319*	*0.015452464*
**ENSG00000164631**	*0.029570861*	*0.035599375*	*0.032641056*	*0.029169311*
**ENSG00000079134**	*0.030740392*	*0.033267926*	*0.03182181*	*0.021917543*
**ENSG00000173548**	*0.021035321*	*0.029244098*	*0.027632764*	*0.01924319*
**ENSG00000138430**	*0.023978447*	*0.027309088*	*0.026116968*	*0.019278988*
**ENSG00000144369**	*0.018483742*	*0.024402419*	*0.024339912*	*0.01673213*

**ENSG00000130720**	*0.016435172*	*0.025349565*	*0.025578975*	*0.017702789*
**ENSG00000089157**	*0.019608273*	*0.027617931*	*0.02842399*	*0.020992082*
**ENSG00000107611**	*0.016816629*	*0.024947116*	*0.024375603*	*0.015418016*
**ENSG00000173757**	*0.033411746*	*0.040125957*	*0.041285316*	*0.031558685*
**ENSG00000181019**	*0.069711051*	*0.04829446*	*0.047927867*	*0.06131601*
**ENSG00000132471**	*0.027741398*	*0.035741151*	*0.037056224*	*0.036443009*
**ENSG00000070540**	*0.034620822*	*0.035179324*	*0.038653774*	*0.03539413*
**ENSG00000119242**	*0.040940265*	*0.041110307*	*0.035508379*	*0.036304512*
**ENSG00000198561**	*0.063450681*	*0.057854216*	*0.037266677*	*0.060825592*
**ENSG00000117691**	*0.030704934*	*0.033970538*	*0.032110105*	*0.023655777*
**ENSG00000131233**	*0.018230735*	*0.025695254*	*0.026251796*	*0.018580836*
**ENSG00000117322**	*0.0851468*	*0.0861642*	*0.079809082*	*0.087754026*
**ENSG00000101665**	*0.042553973*	*0.0477045*	*0.047562507*	*0.039878788*
**ENSG00000122545**	*0.026283577*	*0.029093294*	*0.028887509*	*0.032163544*
**ENSG00000179348**	*0.016566494*	*0.024272409*	*0.025498195*	*0.016368926*
**ENSG00000110696**	*0.019706808*	*0.028607707*	*0.027167985*	*0.018265995*
**ENSG00000006074**	*0.01818914*	*0.026413236*	*0.025843555*	*0.019303077*
**ENSG00000126012**	*0.040151571*	*0.041647866*	*0.050162298*	*0.040396224*
**ENSG00000168092**	*0.026944496*	*0.030579858*	*0.034993522*	*0.026685223*
**ENSG00000091651**	*0.042856307*	*0.040026985*	*0.034902678*	*0.033660777*
**ENSG00000104960**	*0.026127338*	*0.03015922*	*0.031306897*	*0.026430763*
**ENSG00000163098**	*0.016929682*	*0.025177045*	*0.024895829*	*0.014086996*
**ENSG00000187955**	*0.015979028*	*0.024717831*	*0.025514543*	*0.015334613*
**ENSG00000134294**	*0.026432532*	*0.032477103*	*0.035296934*	*0.027488298*
**ENSG00000173040**	*0.033705797*	*0.034861276*	*0.028602859*	*0.026372838*
**ENSG00000075089**	*0.032538175*	*0.035184023*	*0.03034521*	*0.027518714*
**ENSG00000141574**	*0.016564311*	*0.024400477*	*0.026398319*	*0.017624072*
**ENSG00000134830**	*0.018628403*	*0.028017997*	*0.025897115*	*0.017238399*
**ENSG00000102466**	*0.018553565*	*0.027996976*	*0.027611642*	*0.019899996*
**ENSG00000215301**	*0.035656238*	*0.045423388*	*0.051877611*	*0.03146208*
**ENSG00000214114**	*0.042145159*	*0.045415875*	*0.034069147*	*0.030355861*
**ENSG00000113522**	*0.017344814*	*0.024522978*	*0.024949105*	*0.01646261*
**ENSG00000160781**	*0.031542223*	*0.036183047*	*0.030660149*	*0.024298865*
**ENSG00000124557**	*0.07292317*	*0.058212421*	*0.043778255*	*0.043124158*
**ENSG00000115520**	*0.029359736*	*0.044543012*	*0.048607416*	*0.033912717*
**ENSG00000163285**	*0.017001774*	*0.025982724*	*0.025447615*	*0.015790805*
**ENSG00000198604**	*0.02701728*	*0.035283741*	*0.032514896*	*0.028051321*
**ENSG00000137814**	*0.032934683*	*0.033697516*	*0.032309101*	*0.041093251*
**ENSG00000198915**	*0.031129786*	*0.042190104*	*0.040183637*	*0.029246382*
**ENSG00000085382**	*0.020690884*	*0.026177472*	*0.029405825*	*0.02104012*
**ENSG00000162729**	*0.023960699*	*0.030062009*	*0.030924755*	*0.025871735*
**ENSG00000109758**	*0.017037142*	*0.025186706*	*0.025208954*	*0.015034656*
**ENSG00000110583**	*0.031636472*	*0.032052118*	*0.032051045*	*0.027596557*
**ENSG00000137745**	*0.015136904*	*0.025799712*	*0.02487667*	*0.01583112*
**ENSG00000105649**	*0.069502933*	*0.074377049*	*0.066840815*	*0.071213371*
**ENSG00000064763**	*0.095987491*	*0.065389966*	*0.063003655*	*0.07461855*
**ENSG00000063046**	*0.023662785*	*0.030315009*	*0.030967993*	*0.028461952*
**ENSG00000114988**	*0.034661329*	*0.040223171*	*0.037541239*	*0.026062819*
**ENSG00000168779**	*0.015969491*	*0.025620957*	*0.025098139*	*0.016425053*
**ENSG00000139567**	*0.015236884*	*0.025936404*	*0.02633747*	*0.015734015*
**ENSG00000129250**	*0.029052057*	*0.033045798*	*0.033043876*	*0.030644797*
**ENSG00000143870**	*0.037369814*	*0.036354126*	*0.034851866*	*0.036815196*
**ENSG00000141027**	*0.019976418*	*0.029749959*	*0.030044051*	*0.018223061*
**ENSG00000138496**	*0.048352574*	*0.041656786*	*0.04088179*	*0.039602932*

**ENSG00000112530**	*0.015188307*	*0.025284282*	*0.024737998*	*0.015506652*
**ENSG00000175600**	*0.018435564*	*0.025580589*	*0.024888773*	*0.018164257*
**ENSG00000167962**	*0.015709814*	*0.026744329*	*0.024534676*	*0.015982017*
**ENSG00000186930**	*0.015317706*	*0.025193665*	*0.025059248*	*0.014689141*
**ENSG00000139679**	*0.148119443*	*0.103162724*	*0.099881538*	*0.101874122*
**ENSG00000153391**	*0.022132096*	*0.030524187*	*0.029422577*	*0.026999697*
**ENSG00000187416**	*0.017967883*	*0.025992403*	*0.025159712*	*0.017183101*
**ENSG00000184486**	*0.015859779*	*0.025227333*	*0.025752003*	*0.015084018*
**ENSG00000162927**	*0.025668691*	*0.031950009*	*0.031378583*	*0.020102905*
**ENSG00000159217**	*0.014729561*	*0.024952147*	*0.024268775*	*0.015095685*
**ENSG00000188234**	*0.027114888*	*0.030159683*	*0.030648365*	*0.025305136*
**ENSG00000005249**	*0.0175771*	*0.024382216*	*0.024252418*	*0.016166659*
**ENSG00000170608**	*0.060718056*	*0.081493375*	*0.053600234*	*0.066407884*
**ENSG00000101307**	*0.02679495*	*0.03341033*	*0.02523724*	*0.017299021*
**ENSG00000214530**	*0.038505699*	*0.03659964*	*0.034718311*	*0.04101496*
**ENSG00000146701**	*0.019888674*	*0.025624587*	*0.026076729*	*0.016243764*
**ENSG00000068028**	*0.032078683*	*0.044647696*	*0.043483802*	*0.037017096*
**ENSG00000116299**	*0.087317174*	*0.069144436*	*0.053232107*	*0.098555238*
**ENSG00000104313**	*0.018650375*	*0.026467069*	*0.025504993*	*0.016091752*
**ENSG00000168878**	*0.018709903*	*0.026370063*	*0.026997471*	*0.020495938*
**ENSG00000205250**	*0.026617742*	*0.03416864*	*0.038457183*	*0.027742008*
**ENSG00000160688**	*0.029710411*	*0.032846564*	*0.034200708*	*0.029415654*
**ENSG00000153975**	*0.028054186*	*0.03191822*	*0.031082864*	*0.02277132*
**ENSG00000140694**	*0.033516036*	*0.037042199*	*0.037687294*	*0.029323031*
**ENSG00000181555**	*0.034794006*	*0.039803553*	*0.035943952*	*0.030724759*
**ENSG00000144815**	*0.036617406*	*0.03469911*	*0.037338008*	*0.034134886*
**ENSG00000078070**	*0.034221515*	*0.034468108*	*0.032520501*	*0.027352125*
**ENSG00000108506**	*0.024696415*	*0.032457358*	*0.029732593*	*0.025302485*
**ENSG00000162396**	*0.027238506*	*0.031041598*	*0.027341687*	*0.019579126*
**ENSG00000024422**	*0.016043762*	*0.025324998*	*0.024763763*	*0.014694671*
**ENSG00000129968**	*0.025248437*	*0.031938502*	*0.033671959*	*0.027855504*
**ENSG00000090530**	*0.167799476*	*0.105820363*	*0.102492116*	*0.122625253*
**ENSG00000124243**	*0.0379245*	*0.036337748*	*0.036644224*	*0.033513793*
**ENSG00000148291**	*0.027457529*	*0.029732907*	*0.030594936*	*0.023466568*
**ENSG00000161509**	*0.016426922*	*0.02496315*	*0.02540625*	*0.016070022*
**ENSG00000183092**	*0.035943457*	*0.036624101*	*0.033589656*	*0.023059143*
**ENSG00000169583**	*0.018237699*	*0.025320298*	*0.025291945*	*0.015360713*
**ENSG00000131471**	*0.022240785*	*0.026742668*	*0.027180208*	*0.017322535*
**ENSG00000178531**	*0.049994285*	*0.038145871*	*0.034498255*	*0.044931951*
**ENSG00000005102**	*0.016180673*	*0.025584765*	*0.026059648*	*0.016694042*
**ENSG00000170325**	*0.032847222*	*0.046089945*	*0.03324678*	*0.032536566*
**ENSG00000167302**	*0.029029597*	*0.031723004*	*0.034495706*	*0.030767068*
**ENSG00000179213**	*0.016842812*	*0.025575508*	*0.025298573*	*0.015864169*
**ENSG00000115274**	*0.023787039*	*0.028594061*	*0.03105952*	*0.023474021*
**ENSG00000197892**	*0.03453865*	*0.037487105*	*0.041401454*	*0.026889125*
**ENSG00000173464**	*0.015236762*	*0.024883943*	*0.024880081*	*0.015688095*
**ENSG00000221852**	*0.020063909*	*0.026841319*	*0.025257525*	*0.017098804*
**ENSG00000159314**	*0.059896468*	*0.060206353*	*0.050918801*	*0.056982104*
**ENSG00000139112**	*0.07101167*	*0.062305726*	*0.047868878*	*0.056770241*
**ENSG00000160838**	*0.017351566*	*0.024543488*	*0.02563552*	*0.015478434*
**ENSG00000196544**	*0.029461101*	*0.036825965*	*0.033269898*	*0.03405993*
**ENSG00000177374**	*0.042310869*	*0.059766194*	*0.043964978*	*0.061628956*
**ENSG00000119318**	*0.029662044*	*0.032298604*	*0.029498006*	*0.023369179*
**ENSG00000166348**	*0.028957612*	*0.037021669*	*0.032916298*	*0.03103939*

**ENSG00000087274**	*0.022773555*	*0.03151517*	*0.034047416*	*0.020420787*
**ENSG00000079691**	*0.090701075*	*0.084057731*	*0.079722542*	*0.069386619*
**ENSG00000168710**	*0.015237767*	*0.024331381*	*0.024921381*	*0.014239127*
**ENSG00000127920**	*0.113874617*	*0.089746633*	*0.087282281*	*0.087476105*
**ENSG00000161040**	*0.019816894*	*0.02512015*	*0.02464816*	*0.015890012*
**ENSG00000148337**	*0.017853281*	*0.026353264*	*0.028069861*	*0.017006418*
**ENSG00000136270**	*0.025780319*	*0.030767329*	*0.029975045*	*0.023353834*
**ENSG00000167088**	*0.039248032*	*0.038015246*	*0.033946599*	*0.038577931*
**ENSG00000034677**	*0.028259143*	*0.033991938*	*0.034738565*	*0.03332925*
**ENSG00000006744**	*0.028973426*	*0.03192495*	*0.03509274*	*0.031763426*
**ENSG00000120549**	*0.023468805*	*0.036049556*	*0.028398032*	*0.024366966*
**ENSG00000172586**	*0.024993493*	*0.030549689*	*0.028370587*	*0.022378441*
**ENSG00000103485**	*0.041269794*	*0.033416503*	*0.036346939*	*0.042585892*
**ENSG00000243729**	*0.016362166*	*0.025477378*	*0.025416409*	*0.015104409*
**ENSG00000175387**	*0.023533004*	*0.03209564*	*0.030638577*	*0.02163163*
**ENSG00000116678**	*0.021510139*	*0.0275369*	*0.027079475*	*0.026038796*
**ENSG00000172482**	*0.016383756*	*0.025561338*	*0.024374196*	*0.015359893*
**ENSG00000242372**	*0.016469727*	*0.024705187*	*0.025026599*	*0.015094321*
**ENSG00000231738**	*0.015683904*	*0.025622883*	*0.024744659*	*0.01671965*
**ENSG00000101405**	*0.016797327*	*0.025563544*	*0.027108071*	*0.017543443*
**ENSG00000176087**	*0.026895089*	*0.031128881*	*0.034589478*	*0.028051565*
**ENSG00000111245**	*0.03977527*	*0.038142277*	*0.034641021*	*0.034257861*
**ENSG00000126549**	*0.015546407*	*0.024716438*	*0.023891905*	*0.014285632*
**ENSG00000129083**	*0.019884262*	*0.02853526*	*0.029821578*	*0.019409701*
**ENSG00000176302**	*0.016924444*	*0.025098555*	*0.027060556*	*0.016458713*
**ENSG00000124784**	*0.018740183*	*0.027605071*	*0.025861618*	*0.02202342*
**ENSG00000182674**	*0.016421196*	*0.024645288*	*0.024926702*	*0.014781005*
**ENSG00000167914**	*0.015294838*	*0.024568779*	*0.025266156*	*0.015095382*
**ENSG00000198721**	*0.015647865*	*0.025639695*	*0.024633214*	*0.017443654*
**ENSG00000107957**	*0.022575526*	*0.028150323*	*0.025428895*	*0.01933188*
**ENSG00000160345**	*0.035124552*	*0.032304081*	*0.032173407*	*0.030540138*
**ENSG00000159958**	*0.045965152*	*0.036792696*	*0.040903229*	*0.043207003*
**ENSG00000138399**	*0.033634218*	*0.034869532*	*0.028328011*	*0.023253032*
**ENSG00000176171**	*0.058722955*	*0.039337783*	*0.035815359*	*0.03650427*
**ENSG00000104976**	*0.022521494*	*0.026709154*	*0.031640209*	*0.02140628*
**ENSG00000112276**	*0.016701132*	*0.025310882*	*0.025420976*	*0.015392304*
**ENSG00000165388**	*0.014709051*	*0.024466349*	*0.024040045*	*0.014682732*
**ENSG00000157916**	*0.030400551*	*0.036434934*	*0.031438446*	*0.024591858*
**ENSG00000178031**	*0.016385826*	*0.024833979*	*0.024800999*	*0.014921694*
**ENSG00000137078**	*0.090629892*	*0.058651396*	*0.052744064*	*0.046768182*
**ENSG00000196747**	*0.029778729*	*0.02850569*	*0.034697352*	*0.030902315*
**ENSG00000171148**	*0.040114136*	*0.037943875*	*0.037283762*	*0.045799511*
**ENSG00000180878**	*0.021444943*	*0.025162269*	*0.027999679*	*0.019318255*
**ENSG00000163191**	*0.060343941*	*0.047808885*	*0.040722923*	*0.056107529*
**ENSG00000163739**	*0.0177185*	*0.02591185*	*0.026318177*	*0.017590504*
**ENSG00000205581**	*0.024343087*	*0.029617758*	*0.027593326*	*0.022673289*
**ENSG00000023902**	*0.03026795*	*0.035207687*	*0.036085707*	*0.040294497*
**ENSG00000125817**	*0.032998099*	*0.03790674*	*0.04444718*	*0.038110195*
**ENSG00000149054**	*0.05771079*	*0.057000329*	*0.047778144*	*0.064012243*
**ENSG00000188175**	*0.016933078*	*0.026147084*	*0.02605417*	*0.015613598*
**ENSG00000150556**	*0.089873303*	*0.081509635*	*0.053072579*	*0.089742788*
**ENSG00000133597**	*0.039730109*	*0.040551073*	*0.034006862*	*0.030364869*
**ENSG00000075702**	*0.053127192*	*0.043780794*	*0.040926733*	*0.044610259*
**ENSG00000245680**	*0.030020068*	*0.035089777*	*0.030303958*	*0.022915839*

**ENSG00000168505**	*0.015264063*	*0.024805752*	*0.025113332*	*0.014400717*
**ENSG00000160886**	*0.01659117*	*0.025407975*	*0.026077856*	*0.014991351*
**ENSG00000128011**	*0.018578513*	*0.026406365*	*0.02611377*	*0.02084368*
**ENSG00000143514**	*0.061378764*	*0.053400829*	*0.048088413*	*0.048940106*
**ENSG00000118407**	*0.073332311*	*0.036386189*	*0.02775382*	*0.034409958*
**ENSG00000173875**	*0.036934541*	*0.038107519*	*0.046318059*	*0.031365614*
**ENSG00000125657**	*0.03053323*	*0.032732921*	*0.034790453*	*0.034522445*
**ENSG00000061794**	*0.026082042*	*0.030160014*	*0.030063897*	*0.025505631*
**ENSG00000011485**	*0.027988083*	*0.031139963*	*0.030131958*	*0.02417742*
**ENSG00000188223**	*0.025974873*	*0.033192928*	*0.035919815*	*0.025615936*
**ENSG00000184304**	*0.026527653*	*0.03344728*	*0.028255895*	*0.028745951*
**ENSG00000169684**	*0.031719427*	*0.031008205*	*0.031483651*	*0.03019788*
**ENSG00000167986**	*0.023088519*	*0.031503974*	*0.028826371*	*0.020827446*
**ENSG00000139155**	*0.015425755*	*0.024816784*	*0.02506*	*0.014141282*
**ENSG00000163682**	*0.018319228*	*0.025738531*	*0.02420282*	*0.021846029*
**ENSG00000165949**	*0.130171844*	*0.161957556*	*0.123774588*	*0.137818144*
**ENSG00000148082**	*0.021011326*	*0.02487068*	*0.024156682*	*0.014477852*
**ENSG00000172115**	*0.050488562*	*0.057327875*	*0.055648165*	*0.051156355*
**ENSG00000198682**	*0.039829849*	*0.046080465*	*0.042129121*	*0.035381592*
**ENSG00000167384**	*0.035822015*	*0.040138385*	*0.043270505*	*0.03701907*
**ENSG00000021852**	*0.017827048*	*0.026272479*	*0.029240479*	*0.016346553*
**ENSG00000105329**	*0.016758722*	*0.025555316*	*0.024999438*	*0.016969436*
**ENSG00000145833**	*0.030800512*	*0.034373532*	*0.029736706*	*0.029666917*
**ENSG00000229314**	*0.015272927*	*0.024529741*	*0.024720784*	*0.01489674*
**ENSG00000186675**	*0.015682585*	*0.024195678*	*0.02441685*	*0.015154664*
**ENSG00000081189**	*0.024003183*	*0.030201973*	*0.029744729*	*0.022443721*
**ENSG00000163001**	*0.041996791*	*0.041345934*	*0.035835047*	*0.04195148*
**ENSG00000103260**	*0.041874646*	*0.032503953*	*0.033954828*	*0.032430989*
**ENSG00000111897**	*0.025888302*	*0.031098427*	*0.039526233*	*0.025402198*
**ENSG00000182512**	*0.023591937*	*0.030165914*	*0.028693075*	*0.022542843*
**ENSG00000144182**	*0.031516738*	*0.039921279*	*0.034006562*	*0.02254728*
**ENSG00000054690**	*0.018694587*	*0.029068704*	*0.025398941*	*0.018904153*
**ENSG00000197020**	*0.016565088*	*0.025607172*	*0.025211401*	*0.015269626*
**ENSG00000163430**	*0.0170386*	*0.025258766*	*0.025345573*	*0.015739994*
**ENSG00000138180**	*0.042324415*	*0.03898754*	*0.033590826*	*0.031574886*
**ENSG00000102891**	*0.042361042*	*0.034384594*	*0.030086181*	*0.026232226*
**ENSG00000154655**	*0.017056555*	*0.025294591*	*0.024264688*	*0.015295817*
**ENSG00000160472**	*0.016797108*	*0.02567601*	*0.025903096*	*0.015541539*
**ENSG00000197863**	*0.045726112*	*0.042008302*	*0.039313093*	*0.050588255*
**ENSG00000163145**	*0.016352746*	*0.026025963*	*0.024993605*	*0.015840983*
**ENSG00000213551**	*0.037961359*	*0.032806455*	*0.038441866*	*0.036799098*
**ENSG00000250120**	*0.017560101*	*0.026108737*	*0.025779498*	*0.015950366*
**ENSG00000180185**	*0.038756741*	*0.041550088*	*0.040923183*	*0.035074785*
**ENSG00000134444**	*0.029872018*	*0.034685186*	*0.030594634*	*0.025445576*
**ENSG00000131507**	*0.034563203*	*0.035594244*	*0.030267789*	*0.025519715*
**ENSG00000148513**	*0.017861669*	*0.027315596*	*0.027310754*	*0.015780897*
**ENSG00000104343**	*0.027444846*	*0.035056121*	*0.034477857*	*0.024448292*
**ENSG00000166128**	*0.031135898*	*0.038395768*	*0.03934506*	*0.033155529*
**ENSG00000137834**	*0.016071274*	*0.02534584*	*0.026468955*	*0.017908021*
**ENSG00000148842**	*0.022869362*	*0.029388672*	*0.032891669*	*0.024724537*
**ENSG00000165684**	*0.039062318*	*0.04011467*	*0.043235097*	*0.033088138*
**ENSG00000179071**	*0.01743132*	*0.025038494*	*0.026134044*	*0.01685038*
**ENSG00000164411**	*0.017904202*	*0.025249028*	*0.025798603*	*0.016611376*
**ENSG00000163795**	*0.021690285*	*0.029012047*	*0.033725394*	*0.023618061*

**ENSG00000105072**	*0.032854825*	*0.03611995*	*0.031777524*	*0.026573633*
**ENSG00000062716**	*0.038532506*	*0.037200265*	*0.035003568*	*0.033267065*
**ENSG00000197150**	*0.022858822*	*0.02919577*	*0.026553751*	*0.021998858*
**ENSG00000081277**	*0.017894379*	*0.025486385*	*0.025809209*	*0.017731406*
**ENSG00000139946**	*0.023779836*	*0.03048776*	*0.029679998*	*0.020705128*
**ENSG00000135931**	*0.058176476*	*0.055999742*	*0.044213145*	*0.043531421*
**ENSG00000100721**	*0.079651879*	*0.040893421*	*0.041042156*	*0.054698269*
**ENSG00000126522**	*0.043587933*	*0.037781949*	*0.032113348*	*0.03456467*
**ENSG00000158882**	*0.034332623*	*0.035110031*	*0.034425043*	*0.03573147*
**ENSG00000180938**	*0.026095584*	*0.03168818*	*0.03081973*	*0.024856361*
**ENSG00000181333**	*0.016306922*	*0.024952538*	*0.02498409*	*0.016022496*
**ENSG00000104783**	*0.041567345*	*0.040950526*	*0.043119259*	*0.044729844*
**ENSG00000115286**	*0.023523035*	*0.027049175*	*0.026855843*	*0.019940401*
**ENSG00000163866**	*0.020936978*	*0.027493007*	*0.026381618*	*0.017079715*
**ENSG00000146839**	*0.017765307*	*0.025565793*	*0.026037867*	*0.015107179*
**ENSG00000139194**	*0.015354106*	*0.025193546*	*0.025697185*	*0.015875049*
**ENSG00000171596**	*0.015628999*	*0.02452493*	*0.024087787*	*0.014749161*
**ENSG00000143156**	*0.039188285*	*0.040962425*	*0.035154841*	*0.037477864*
**ENSG00000170421**	*0.017511597*	*0.027221986*	*0.026044658*	*0.016197515*
**ENSG00000187792**	*0.017806589*	*0.026423869*	*0.027091136*	*0.01735942*
**ENSG00000234829**	*0.015946786*	*0.024604807*	*0.025758249*	*0.015321896*
**ENSG00000120159**	*0.026353234*	*0.033093599*	*0.03022007*	*0.023968784*
**ENSG00000130559**	*0.028753634*	*0.038739469*	*0.036253707*	*0.028865085*
**ENSG00000139734**	*0.042204736*	*0.042509212*	*0.032439775*	*0.037868853*
**ENSG00000114867**	*0.017944998*	*0.026787427*	*0.025833159*	*0.017626354*
**ENSG00000171320**	*0.046823661*	*0.047772597*	*0.033860781*	*0.03698581*
**ENSG00000169783**	*0.025581525*	*0.027630005*	*0.026489233*	*0.017814584*
**ENSG00000111087**	*0.015176267*	*0.025324399*	*0.026137309*	*0.014981182*
**ENSG00000156097**	*0.015417916*	*0.025748249*	*0.024471794*	*0.014525784*
**ENSG00000141480**	*0.025549965*	*0.027001064*	*0.028144484*	*0.023948826*
**ENSG00000172071**	*0.039058137*	*0.038241814*	*0.037076712*	*0.036598483*
**ENSG00000107331**	*0.016387426*	*0.025292835*	*0.025169963*	*0.015408574*
**ENSG00000127585**	*0.025743546*	*0.036493008*	*0.035474235*	*0.033135251*
**ENSG00000177504**	*0.058738222*	*0.031431379*	*0.032982883*	*0.038956077*
**ENSG00000075336**	*0.032117564*	*0.035748539*	*0.03060623*	*0.023859569*
**ENSG00000111879**	*0.032330587*	*0.030873124*	*0.028824296*	*0.021418175*
**ENSG00000103769**	*0.02980671*	*0.035861435*	*0.032992891*	*0.027170446*
**ENSG00000128563**	*0.032000274*	*0.037193258*	*0.037946418*	*0.031983433*
**ENSG00000120659**	*0.016604313*	*0.025192787*	*0.025656195*	*0.01650995*
**ENSG00000118972**	*0.01882159*	*0.026903421*	*0.028135123*	*0.01718778*
**ENSG00000163815**	*0.01782074*	*0.025683658*	*0.024672263*	*0.015553726*
**ENSG00000116127**	*0.054265963*	*0.058290537*	*0.041817382*	*0.043441835*
**ENSG00000063854**	*0.037036086*	*0.038228736*	*0.03763789*	*0.031619782*
**ENSG00000075413**	*0.028956609*	*0.033998*	*0.030456424*	*0.023619508*
**ENSG00000166317**	*0.104699223*	*0.100425861*	*0.076488395*	*0.099699012*
**ENSG00000009790**	*0.03742763*	*0.041243189*	*0.040175272*	*0.037566534*
**ENSG00000154305**	*0.01600775*	*0.02648681*	*0.025250287*	*0.014772014*
**ENSG00000137218**	*0.035585078*	*0.041340953*	*0.031426266*	*0.027009023*
**ENSG00000162755**	*0.028607867*	*0.029645902*	*0.029394522*	*0.026906293*
**ENSG00000157637**	*0.019671862*	*0.026444152*	*0.0260544*	*0.017788701*
**ENSG00000165182**	*0.014753432*	*0.024824378*	*0.024676087*	*0.01478037*
**ENSG00000148153**	*0.024022426*	*0.027789928*	*0.027873108*	*0.02306824*
**ENSG00000153574**	*0.033024946*	*0.035163016*	*0.032728875*	*0.033480672*
**ENSG00000132963**	*0.019825875*	*0.027347176*	*0.028458472*	*0.02221775*

**ENSG00000131910**	*0.016507861*	*0.025244725*	*0.025478308*	*0.016194324*
**ENSG00000011198**	*0.045074408*	*0.039812903*	*0.03813485*	*0.038042558*
**ENSG00000184470**	*0.017292655*	*0.025584825*	*0.024582202*	*0.016259438*
**ENSG00000173156**	*0.036376466*	*0.032001324*	*0.029373721*	*0.025229208*
**ENSG00000140015**	*0.017317549*	*0.02607388*	*0.025424923*	*0.014576413*
**ENSG00000106144**	*0.038071944*	*0.038587011*	*0.036626897*	*0.032085396*
**ENSG00000117222**	*0.02628305*	*0.030126572*	*0.029087336*	*0.021454276*
**ENSG00000155313**	*0.033052865*	*0.037480602*	*0.029947083*	*0.027727062*
**ENSG00000104044**	*0.028466601*	*0.024351567*	*0.026446554*	*0.014787989*
**ENSG00000165029**	*0.0361244*	*0.053261336*	*0.037971948*	*0.054004174*
**ENSG00000135914**	*0.073415913*	*0.054507899*	*0.051607209*	*0.042392471*
**ENSG00000189052**	*0.01538951*	*0.02571182*	*0.026851742*	*0.01454325*
**ENSG00000158552**	*0.032635602*	*0.033078511*	*0.034986166*	*0.02663239*
**ENSG00000140157**	*0.024795834*	*0.03500173*	*0.036786259*	*0.031774727*
**ENSG00000204435**	*0.019918043*	*0.026748932*	*0.028174181*	*0.017081368*
**ENSG00000135480**	*0.053955961*	*0.036267575*	*0.031671126*	*0.031088069*
**ENSG00000179409**	*0.032838244*	*0.0349903*	*0.034074995*	*0.027608953*
**ENSG00000135956**	*0.023502096*	*0.036350851*	*0.040876794*	*0.022254925*
**ENSG00000110446**	*0.048138792*	*0.038882838*	*0.042758275*	*0.032969472*
**ENSG00000154277**	*0.128347032*	*0.140355757*	*0.119879585*	*0.146236602*
**ENSG00000168038**	*0.017723154*	*0.026051295*	*0.025660856*	*0.016992741*
**ENSG00000158887**	*0.02338185*	*0.029490109*	*0.032581346*	*0.018877174*
**ENSG00000103657**	*0.040211746*	*0.036772777*	*0.041263589*	*0.038950669*
**ENSG00000158545**	*0.024804213*	*0.030069543*	*0.031061715*	*0.027304024*
**ENSG00000104884**	*0.028424806*	*0.029425395*	*0.032866657*	*0.022854579*
**ENSG00000189139**	*0.015115511*	*0.025883386*	*0.024604791*	*0.014664719*
**ENSG00000213020**	*0.031237493*	*0.035524982*	*0.037747428*	*0.028306347*
**ENSG00000213465**	*0.032513738*	*0.036734402*	*0.034362242*	*0.026205201*
**ENSG00000114204**	*0.014820319*	*0.024864249*	*0.025087131*	*0.013574359*
**ENSG00000116962**	*0.065247865*	*0.053057743*	*0.045164793*	*0.056785258*
**ENSG00000109511**	*0.016031721*	*0.025810936*	*0.025440201*	*0.014856571*
**ENSG00000138658**	*0.017892546*	*0.025321518*	*0.025188897*	*0.01612991*
**ENSG00000185222**	*0.082631492*	*0.062789339*	*0.045503955*	*0.062746084*
**ENSG00000132341**	*0.037399013*	*0.037654369*	*0.034480929*	*0.037136426*
**ENSG00000182257**	*0.016947684*	*0.024962597*	*0.024378545*	*0.015909608*
**ENSG00000204599**	*0.02561068*	*0.032292074*	*0.036605011*	*0.019761356*
**ENSG00000111332**	*0.053653469*	*0.042377097*	*0.040609529*	*0.041171041*
**ENSG00000162946**	*0.037603547*	*0.031641038*	*0.027439124*	*0.027242737*
**ENSG00000106105**	*0.023608986*	*0.029504943*	*0.029157671*	*0.022054259*
**ENSG00000162595**	*0.01656121*	*0.02531199*	*0.024651586*	*0.015059913*
**ENSG00000135447**	*0.015388094*	*0.025117971*	*0.025016754*	*0.015967279*
**ENSG00000139193**	*0.033864383*	*0.043228677*	*0.036647082*	*0.053881731*
**ENSG00000162598**	*0.016491698*	*0.025342664*	*0.025686252*	*0.016320658*
**ENSG00000215475**	*0.014562459*	*0.024312788*	*0.024267848*	*0.014471202*
**ENSG00000019991**	*0.025649162*	*0.02529797*	*0.026386141*	*0.025077331*
**ENSG00000106392**	*0.054044828*	*0.049616623*	*0.046390147*	*0.048456052*
**ENSG00000160951**	*0.015708195*	*0.025642544*	*0.024996919*	*0.014468698*
**ENSG00000167792**	*0.023993518*	*0.029240479*	*0.029822022*	*0.019990646*
**ENSG00000149021**	*0.019644792*	*0.026255471*	*0.025807079*	*0.016967303*
**ENSG00000118579**	*0.024090482*	*0.033035339*	*0.031215784*	*0.026333426*
**ENSG00000154930**	*0.047252872*	*0.044598615*	*0.035490471*	*0.039877528*
**ENSG00000126001**	*0.026569096*	*0.02788037*	*0.031067051*	*0.020319304*
**ENSG00000099875**	*0.065655217*	*0.056072484*	*0.050359557*	*0.064974376*
**ENSG00000185681**	*0.016664292*	*0.026708638*	*0.024749231*	*0.016409826*

**ENSG00000129255**	*0.032628177*	*0.030964752*	*0.032973595*	*0.032190887*
**ENSG00000103888**	*0.017314366*	*0.02458554*	*0.024697859*	*0.015339723*
**ENSG00000174946**	*0.058614901*	*0.074129314*	*0.079374062*	*0.085556575*
**ENSG00000134222**	*0.063671885*	*0.041211674*	*0.037769311*	*0.04644056*
**ENSG00000173914**	*0.030823761*	*0.032191841*	*0.034643087*	*0.02810943*
**ENSG00000163931**	*0.023990892*	*0.027014521*	*0.02854379*	*0.021553302*
**ENSG00000204748**	*0.020449291*	*0.025385034*	*0.026112917*	*0.018027207*
**ENSG00000204256**	*0.024551087*	*0.032262039*	*0.03904121*	*0.021676466*
**ENSG00000105767**	*0.017984256*	*0.025296519*	*0.025752633*	*0.016470366*
**ENSG00000110395**	*0.018505799*	*0.025603936*	*0.025096191*	*0.016216632*
**ENSG00000158901**	*0.015562248*	*0.024918849*	*0.024865369*	*0.01688624*
**ENSG00000103037**	*0.038070964*	*0.035078126*	*0.031454014*	*0.03124126*
**ENSG00000187741**	*0.022583629*	*0.029311149*	*0.029454125*	*0.019214498*
**ENSG00000204536**	*0.03951127*	*0.046505026*	*0.040265719*	*0.035950066*
**ENSG00000055483**	*0.061857516*	*0.077483749*	*0.060171845*	*0.044551533*
**ENSG00000124568**	*0.016823803*	*0.025063656*	*0.024934524*	*0.016718152*
**ENSG00000196814**	*0.016141696*	*0.026765698*	*0.026057325*	*0.017190134*
**ENSG00000130182**	*0.015437162*	*0.02426946*	*0.026037585*	*0.015214522*
**ENSG00000185187**	*0.027661648*	*0.029856491*	*0.030094018*	*0.026532138*
**ENSG00000160972**	*0.041168925*	*0.046400356*	*0.03993135*	*0.032460356*
**ENSG00000176601**	*0.019427384*	*0.026354611*	*0.029908651*	*0.01921868*
**ENSG00000114784**	*0.02167357*	*0.029839224*	*0.030908045*	*0.021948675*
**ENSG00000219073**	*0.015737841*	*0.02392381*	*0.025083791*	*0.015218762*
**ENSG00000157335**	*0.015063344*	*0.024849973*	*0.025719155*	*0.01487507*
**ENSG00000090554**	*0.025687762*	*0.028668749*	*0.033274641*	*0.027876667*
**ENSG00000127990**	*0.058691283*	*0.055700697*	*0.055104102*	*0.05163737*
**ENSG00000176697**	*0.016975567*	*0.024738927*	*0.024360237*	*0.015179125*
**ENSG00000007038**	*0.089658792*	*0.082548985*	*0.061631097*	*0.043333674*
**ENSG00000144468**	*0.037132551*	*0.039897298*	*0.035506945*	*0.034098398*
**ENSG00000133302**	*0.042599913*	*0.049885582*	*0.034478839*	*0.031009409*
**ENSG00000183826**	*0.01513165*	*0.024971551*	*0.025153837*	*0.014885114*
**ENSG00000163093**	*0.034134023*	*0.029379864*	*0.033653853*	*0.031735712*
**ENSG00000107175**	*0.030263069*	*0.032274582*	*0.037204356*	*0.027511386*
**ENSG00000136738**	*0.034988975*	*0.040836471*	*0.039915846*	*0.035493634*
**ENSG00000203666**	*0.083030207*	*0.070191842*	*0.084857252*	*0.070257766*
**ENSG00000182199**	*0.029438621*	*0.029446889*	*0.028095391*	*0.022277291*
**ENSG00000170185**	*0.026810992*	*0.035708678*	*0.032571908*	*0.027071442*
**ENSG00000137434**	*0.033936415*	*0.029387059*	*0.029746091*	*0.037092918*
**ENSG00000168994**	*0.085438541*	*0.082458785*	*0.056758244*	*0.073103508*
**ENSG00000196954**	*0.030907173*	*0.030399974*	*0.034206582*	*0.021584896*
**ENSG00000047249**	*0.022918275*	*0.032259816*	*0.029399078*	*0.022428362*
**ENSG00000139800**	*0.015790684*	*0.025033457*	*0.02476246*	*0.014974091*
**ENSG00000116830**	*0.032550467*	*0.031314365*	*0.03282633*	*0.025916901*
**ENSG00000107862**	*0.025452392*	*0.032788424*	*0.033311177*	*0.029252539*
**ENSG00000118655**	*0.052407801*	*0.055516137*	*0.04313417*	*0.058403452*
**ENSG00000183628**	*0.019182082*	*0.027190269*	*0.025626949*	*0.017927913*
**ENSG00000122557**	*0.030141036*	*0.037665893*	*0.031460411*	*0.039591866*
**ENSG00000198931**	*0.024382976*	*0.030364255*	*0.03064704*	*0.031890715*
**ENSG00000123200**	*0.020621158*	*0.026944427*	*0.028705431*	*0.019897794*
**ENSG00000242485**	*0.022291441*	*0.027181126*	*0.027714309*	*0.022648616*
**ENSG00000166888**	*0.031588477*	*0.040243953*	*0.040556987*	*0.038669732*
**ENSG00000132881**	*0.015885699*	*0.024971894*	*0.024145235*	*0.015063317*
**ENSG00000154582**	*0.020520741*	*0.027295426*	*0.026708171*	*0.018231295*
**ENSG00000158161**	*0.017187552*	*0.027156441*	*0.025761034*	*0.017008496*

**ENSG00000004809**	*0.020234954*	*0.025613241*	*0.025586124*	*0.014496686*
**ENSG00000154845**	*0.030624854*	*0.035590949*	*0.039473352*	*0.033095032*
**ENSG00000104835**	*0.02914848*	*0.031084974*	*0.030744035*	*0.025892694*
**ENSG00000018869**	*0.028374127*	*0.035142499*	*0.035900318*	*0.025865694*
**ENSG00000108590**	*0.017913125*	*0.026618458*	*0.025250862*	*0.016093677*
**ENSG00000122970**	*0.015640247*	*0.025283449*	*0.025872525*	*0.015464463*
**ENSG00000135828**	*0.035238989*	*0.042450713*	*0.036034337*	*0.028595012*
**ENSG00000133983**	*0.025553671*	*0.032720339*	*0.030057509*	*0.02076335*
**ENSG00000197780**	*0.021707904*	*0.027151406*	*0.028964585*	*0.020798257*
**ENSG00000182307**	*0.034651759*	*0.034039824*	*0.034790101*	*0.027727416*
**ENSG00000136870**	*0.032407855*	*0.03414727*	*0.031662002*	*0.026307289*
**ENSG00000248483**	*0.015386306*	*0.025202705*	*0.025695521*	*0.014242717*
**ENSG00000107105**	*0.016819366*	*0.026562167*	*0.02515713*	*0.016861452*
**ENSG00000010327**	*0.016514364*	*0.025916749*	*0.024474108*	*0.01639355*
**ENSG00000164047**	*0.097338829*	*0.065935228*	*0.070022396*	*0.083806938*
**ENSG00000014164**	*0.023569745*	*0.0303335*	*0.037882212*	*0.024949928*
**ENSG00000166153**	*0.015871584*	*0.025209225*	*0.025042081*	*0.015506199*
**ENSG00000205268**	*0.050855626*	*0.057427129*	*0.050875082*	*0.048530861*
**ENSG00000110880**	*0.026352445*	*0.03243344*	*0.030832821*	*0.020824142*
**ENSG00000165028**	*0.031765914*	*0.043000987*	*0.040102486*	*0.031961755*
**ENSG00000243444**	*0.014872612*	*0.024519337*	*0.02448185*	*0.014982428*
**ENSG00000164054**	*0.042406932*	*0.037329139*	*0.037327156*	*0.0337727*
**ENSG00000182132**	*0.016478889*	*0.023997404*	*0.024266035*	*0.015081953*
**ENSG00000176170**	*0.01450618*	*0.024402745*	*0.024030802*	*0.014719267*
**ENSG00000130950**	*0.015870483*	*0.025587147*	*0.025599777*	*0.016106247*
**ENSG00000244624**	*0.015308779*	*0.025044397*	*0.024955226*	*0.014445963*
**ENSG00000108384**	*0.043204129*	*0.059003692*	*0.047497257*	*0.033374548*
**ENSG00000166851**	*0.050905437*	*0.032686339*	*0.04739466*	*0.041526139*
**ENSG00000095627**	*0.070619331*	*0.049806618*	*0.036776276*	*0.023147373*
**ENSG00000141965**	*0.026082421*	*0.040300036*	*0.047479894*	*0.03083272*
**ENSG00000103313**	*0.015778064*	*0.025060574*	*0.025190969*	*0.01594449*
**ENSG00000101442**	*0.034889651*	*0.036846544*	*0.034969993*	*0.026927381*
**ENSG00000100714**	*0.037023654*	*0.036988785*	*0.033076426*	*0.027771427*
**ENSG00000165244**	*0.01574607*	*0.025686751*	*0.026937244*	*0.01612367*
**ENSG00000128654**	*0.028710584*	*0.032597927*	*0.028642202*	*0.022178124*
**ENSG00000140545**	*0.095365355*	*0.064854591*	*0.064841572*	*0.075792395*
**ENSG00000111615**	*0.029544083*	*0.039985364*	*0.029018629*	*0.025459399*
**ENSG00000099246**	*0.024002953*	*0.031768287*	*0.031628356*	*0.031254628*
**ENSG00000138166**	*0.045321295*	*0.048482455*	*0.056646141*	*0.046910052*
**ENSG00000079263**	*0.051723238*	*0.042718197*	*0.049879027*	*0.046255574*
**ENSG00000155875**	*0.016166406*	*0.025167534*	*0.025006011*	*0.016367397*
**ENSG00000125931**	*0.015260608*	*0.025386368*	*0.025042656*	*0.014245216*
**ENSG00000131778**	*0.031263724*	*0.037493645*	*0.029950961*	*0.027619659*
**ENSG00000174448**	*0.016731582*	*0.025561649*	*0.025381868*	*0.016123881*
**ENSG00000003056**	*0.021249815*	*0.030745907*	*0.029282547*	*0.018303721*
**ENSG00000153214**	*0.026098807*	*0.034983484*	*0.029454528*	*0.020720204*
**ENSG00000135437**	*0.047701608*	*0.036444467*	*0.033247692*	*0.036911701*
**ENSG00000173327**	*0.022508861*	*0.035606819*	*0.045244007*	*0.028361781*
**ENSG00000138315**	*0.015228436*	*0.025587249*	*0.024112356*	*0.014437762*
**ENSG00000111252**	*0.033828558*	*0.051585528*	*0.051611873*	*0.066380319*
**ENSG00000185201**	*0.070022219*	*0.049256212*	*0.053294509*	*0.058037003*
**ENSG00000104953**	*0.033349525*	*0.035841639*	*0.034290931*	*0.030975424*
**ENSG00000069399**	*0.03364882*	*0.042480237*	*0.051740437*	*0.043303812*
**ENSG00000242550**	*0.128461196*	*0.106860691*	*0.092398654*	*0.097334954*

**ENSG00000169490**	*0.026547491*	*0.032961144*	*0.033891063*	*0.026016558*
**ENSG00000166508**	*0.014911784*	*0.02532238*	*0.025025603*	*0.016948509*
**ENSG00000108179**	*0.040826899*	*0.033992652*	*0.038958331*	*0.036809984*
**ENSG00000206203**	*0.018019566*	*0.026772238*	*0.029185855*	*0.016281239*
**ENSG00000131050**	*0.015405282*	*0.024206337*	*0.025160239*	*0.014749084*
**ENSG00000162512**	*0.055938692*	*0.048283262*	*0.044788043*	*0.033833098*
**ENSG00000151748**	*0.037541402*	*0.042861091*	*0.038755344*	*0.030724071*
**ENSG00000138303**	*0.032625421*	*0.033627574*	*0.032268151*	*0.025587718*
**ENSG00000204700**	*0.017284937*	*0.026854675*	*0.026423267*	*0.016138643*
**ENSG00000186665**	*0.018529443*	*0.02710937*	*0.026819331*	*0.018377286*
**ENSG00000168062**	*0.016696077*	*0.025330686*	*0.024881639*	*0.015919102*
**ENSG00000104613**	*0.034026859*	*0.036589477*	*0.033404319*	*0.022779724*
**ENSG00000124116**	*0.018839355*	*0.027619019*	*0.026866943*	*0.017871241*
**ENSG00000141096**	*0.019579518*	*0.029801824*	*0.038173533*	*0.017323642*
**ENSG00000136193**	*0.03322884*	*0.0344836*	*0.030704158*	*0.031949714*
**ENSG00000165449**	*0.07321246*	*0.065373412*	*0.050557786*	*0.063095084*
**ENSG00000130429**	*0.022065828*	*0.027827866*	*0.028047426*	*0.02180616*
**ENSG00000174839**	*0.036076206*	*0.034746013*	*0.035732701*	*0.02890957*
**ENSG00000127226**	*0.031656002*	*0.035303755*	*0.037387584*	*0.032901595*
**ENSG00000157404**	*0.021917936*	*0.031542157*	*0.029080123*	*0.038163985*
**ENSG00000204614**	*0.01562457*	*0.024742465*	*0.024228888*	*0.015174663*
**ENSG00000143793**	*0.029600165*	*0.030616822*	*0.03147476*	*0.021395741*
**ENSG00000173338**	*0.015319955*	*0.024492976*	*0.024750519*	*0.015118563*
**ENSG00000147164**	*0.024259504*	*0.030500234*	*0.028707911*	*0.025861456*
**ENSG00000203797**	*0.018482401*	*0.025192236*	*0.025983183*	*0.015066172*
**ENSG00000128422**	*0.108209552*	*0.085609622*	*0.064350913*	*0.082868867*
**ENSG00000104903**	*0.060421482*	*0.056354582*	*0.046709619*	*0.049199835*
**ENSG00000130024**	*0.045043114*	*0.046403503*	*0.039674318*	*0.030259379*
**ENSG00000160932**	*0.047379718*	*0.036831665*	*0.041009477*	*0.035047191*
**ENSG00000071246**	*0.029986081*	*0.038330313*	*0.029676682*	*0.027735616*
**ENSG00000170777**	*0.017415334*	*0.026400247*	*0.026381828*	*0.017390975*
**ENSG00000178662**	*0.028569413*	*0.02902966*	*0.024440254*	*0.015578471*
**ENSG00000135486**	*0.017970929*	*0.025464004*	*0.024758106*	*0.016858951*
**ENSG00000057608**	*0.02280525*	*0.029320773*	*0.026528437*	*0.019219976*
**ENSG00000100697**	*0.016489098*	*0.024795658*	*0.025067721*	*0.016431639*
**ENSG00000163520**	*0.016722998*	*0.025127692*	*0.024541917*	*0.015369237*
**ENSG00000158869**	*0.025227628*	*0.053314413*	*0.045862954*	*0.051489906*
**ENSG00000149418**	*0.017612414*	*0.026803272*	*0.025471953*	*0.017014189*
**ENSG00000105202**	*0.022788569*	*0.026033758*	*0.026229162*	*0.02062011*
**ENSG00000101871**	*0.014967537*	*0.02528541*	*0.024301465*	*0.014879449*
**ENSG00000077684**	*0.02401949*	*0.033836939*	*0.028911047*	*0.020300297*
**ENSG00000198730**	*0.030754069*	*0.04113492*	*0.03388525*	*0.030377664*
**ENSG00000158258**	*0.016865269*	*0.025119983*	*0.025410136*	*0.017595218*
**ENSG00000138459**	*0.023571236*	*0.036164377*	*0.032108494*	*0.024071475*
**ENSG00000213995**	*0.029707802*	*0.035780613*	*0.036066123*	*0.02463013*
**ENSG00000120438**	*0.026451467*	*0.031233249*	*0.027686613*	*0.020817359*
**ENSG00000124205**	*0.016903006*	*0.025160952*	*0.025043226*	*0.016447001*
**ENSG00000136942**	*0.01320482*	*0.023754781*	*0.024064533*	*0.014288458*
**ENSG00000158955**	*0.016002882*	*0.024708143*	*0.024410051*	*0.016100703*
**ENSG00000165115**	*0.014811405*	*0.02484244*	*0.025537559*	*0.015743714*
**ENSG00000138435**	*0.020110865*	*0.027324789*	*0.026473188*	*0.015769435*
**ENSG00000184508**	*0.028689027*	*0.032383133*	*0.03255743*	*0.028273497*
**ENSG00000186038**	*0.015906429*	*0.024431698*	*0.025400263*	*0.015176426*
**ENSG00000198793**	*0.030616042*	*0.040698349*	*0.032879739*	*0.02916835*

**ENSG00000196367**	*0.032571887*	*0.03731573*	*0.03349451*	*0.025937035*
**ENSG00000102243**	*0.015626045*	*0.02447208*	*0.025714931*	*0.01549993*
**ENSG00000143643**	*0.035104452*	*0.037442808*	*0.036097404*	*0.026764428*
**ENSG00000198826**	*0.040650182*	*0.035209725*	*0.033032931*	*0.031764271*
**ENSG00000181499**	*0.01660055*	*0.026160869*	*0.025775159*	*0.016385713*
**ENSG00000197753**	*0.016867201*	*0.02588373*	*0.025741746*	*0.014431109*
**ENSG00000211460**	*0.039698893*	*0.036943641*	*0.037602803*	*0.048687963*
**ENSG00000172497**	*0.017198085*	*0.026451292*	*0.024975654*	*0.015438751*
**ENSG00000100084**	*0.031418849*	*0.032546642*	*0.034678421*	*0.030759988*
**ENSG00000171853**	*0.022966014*	*0.031158929*	*0.034405565*	*0.026025204*
**ENSG00000162408**	*0.022098682*	*0.026640883*	*0.02711335*	*0.024203529*
**ENSG00000073734**	*0.015736332*	*0.025748566*	*0.024582382*	*0.016939255*
**ENSG00000124391**	*0.016684281*	*0.026738169*	*0.024179028*	*0.015886756*
**ENSG00000142676**	*0.014445745*	*0.024227066*	*0.024239845*	*0.015256792*
**ENSG00000151474**	*0.101482634*	*0.085796455*	*0.076494348*	*0.081280939*
**ENSG00000161533**	*0.016278375*	*0.024781549*	*0.024802504*	*0.015498212*
**ENSG00000164346**	*0.020969072*	*0.026373463*	*0.026266787*	*0.023352286*
**ENSG00000100422**	*0.016458478*	*0.025082801*	*0.025021862*	*0.015957952*
**ENSG00000204822**	*0.018626424*	*0.029533956*	*0.032137063*	*0.024332733*
**ENSG00000105983**	*0.029628119*	*0.036023084*	*0.031650206*	*0.022546799*
**ENSG00000204616**	*0.016416328*	*0.024540787*	*0.025333759*	*0.015003112*
**ENSG00000080839**	*0.029390225*	*0.034706974*	*0.027843569*	*0.020576087*
**ENSG00000072958**	*0.022620613*	*0.028997996*	*0.028880384*	*0.026280133*
**ENSG00000061492**	*0.015628718*	*0.025158732*	*0.024520498*	*0.016418457*
**ENSG00000167634**	*0.119278735*	*0.087567963*	*0.093822008*	*0.094384445*
**ENSG00000125409**	*0.014925825*	*0.024324976*	*0.02533252*	*0.016309209*
**ENSG00000152430**	*0.016574206*	*0.026426192*	*0.025374882*	*0.017281369*
**ENSG00000197808**	*0.031816629*	*0.0387319*	*0.040025949*	*0.03022711*
**ENSG00000148834**	*0.027263273*	*0.029715757*	*0.029437032*	*0.030038057*
**ENSG00000172818**	*0.021876728*	*0.028311349*	*0.027893433*	*0.02215918*
**ENSG00000182261**	*0.016069692*	*0.025829053*	*0.025444903*	*0.016071028*
**ENSG00000170917**	*0.034990034*	*0.034949099*	*0.032270931*	*0.028721135*
**ENSG00000104812**	*0.035704587*	*0.034401735*	*0.034685773*	*0.032715365*
**ENSG00000088832**	*0.024826938*	*0.030457823*	*0.029206501*	*0.025179227*
**ENSG00000166069**	*0.015166207*	*0.024730892*	*0.025207468*	*0.015386756*
**ENSG00000241553**	*0.025775468*	*0.030802958*	*0.032006241*	*0.028895631*
**ENSG00000205352**	*0.01986995*	*0.028285255*	*0.027426765*	*0.018917991*
**ENSG00000184719**	*0.016292985*	*0.025205298*	*0.025545859*	*0.016127872*
**ENSG00000131373**	*0.025636185*	*0.032084053*	*0.031761998*	*0.022591428*
**ENSG00000185386**	*0.024241058*	*0.030904144*	*0.02881379*	*0.026155043*
**ENSG00000165509**	*0.016334295*	*0.025504096*	*0.025513117*	*0.015911636*
**ENSG00000244187**	*0.033453902*	*0.031370528*	*0.028583378*	*0.029801145*
**ENSG00000162493**	*0.016084403*	*0.027185761*	*0.025915412*	*0.017093651*
**ENSG00000203933**	*0.015663633*	*0.025765233*	*0.025878428*	*0.014702078*
**ENSG00000114473**	*0.076374682*	*0.106804685*	*0.068273836*	*0.088745036*
**ENSG00000196209**	*0.017353335*	*0.027274357*	*0.024944372*	*0.016042897*
**ENSG00000011638**	*0.055902196*	*0.043797391*	*0.042949983*	*0.052880589*
**ENSG00000143933**	*0.019049162*	*0.027189253*	*0.026907256*	*0.018970534*
**ENSG00000000460**	*0.051072343*	*0.053065555*	*0.05010576*	*0.035864924*
**ENSG00000128408**	*0.018263894*	*0.025427962*	*0.025286635*	*0.017087636*
**ENSG00000171132**	*0.018407461*	*0.028011576*	*0.028241424*	*0.020196449*
**ENSG00000130511**	*0.03683141*	*0.030474013*	*0.037138751*	*0.030400811*
**ENSG00000147036**	*0.015217745*	*0.024815209*	*0.024887596*	*0.014429895*
**ENSG00000124635**	*0.071316909*	*0.060965451*	*0.064444825*	*0.061457664*

**ENSG00000133315**	*0.033477453*	*0.03649404*	*0.0358453*	*0.027002237*
**ENSG00000167107**	*0.052951377*	*0.037795532*	*0.041779246*	*0.05222762*
**ENSG00000015532**	*0.028353216*	*0.029316923*	*0.029705014*	*0.025579041*
**ENSG00000120733**	*0.024915259*	*0.033832253*	*0.031096775*	*0.021453634*
**ENSG00000163156**	*0.023506338*	*0.032380563*	*0.033987382*	*0.022014136*
**ENSG00000186908**	*0.028331902*	*0.033302501*	*0.031921979*	*0.026002254*
**ENSG00000108465**	*0.028990825*	*0.031299723*	*0.032635736*	*0.024586401*
**ENSG00000152256**	*0.051897*	*0.051460606*	*0.039770542*	*0.051495292*
**ENSG00000131475**	*0.02209975*	*0.029107112*	*0.030680877*	*0.022627685*
**ENSG00000171988**	*0.032945713*	*0.036541486*	*0.034200107*	*0.031864698*
**ENSG00000168913**	*0.014872824*	*0.024752319*	*0.024900469*	*0.014427863*
**ENSG00000127412**	*0.015242172*	*0.024803838*	*0.024248595*	*0.01550751*
**ENSG00000168350**	*0.017889944*	*0.0274438*	*0.025995101*	*0.018423198*
**ENSG00000165637**	*0.021737406*	*0.027485888*	*0.027111523*	*0.019938875*
**ENSG00000168903**	*0.01885588*	*0.02532*	*0.027199192*	*0.017296156*
**ENSG00000163376**	*0.049605987*	*0.071440371*	*0.064022169*	*0.066132552*
**ENSG00000164418**	*0.015646447*	*0.024702218*	*0.024868443*	*0.015791898*
**ENSG00000111642**	*0.027355783*	*0.032203474*	*0.033793433*	*0.025037363*
**ENSG00000221857**	*0.015649101*	*0.025004872*	*0.025727677*	*0.015869737*
**ENSG00000152518**	*0.036596334*	*0.047061013*	*0.042994904*	*0.038893069*
**ENSG00000081041**	*0.029385701*	*0.02840402*	*0.027360618*	*0.018931629*
**ENSG00000170727**	*0.034772206*	*0.033267353*	*0.035276369*	*0.031929044*
**ENSG00000197622**	*0.035791567*	*0.042365757*	*0.048723644*	*0.03561906*
**ENSG00000197183**	*0.023247664*	*0.02702504*	*0.025869106*	*0.019901714*
**ENSG00000135655**	*0.029225227*	*0.036589177*	*0.032771058*	*0.026767219*
**ENSG00000173894**	*0.015716026*	*0.024736807*	*0.025434988*	*0.01510802*
**ENSG00000214265**	*0.041913188*	*0.029579787*	*0.031629225*	*0.027192639*
**ENSG00000132749**	*0.01820519*	*0.026683896*	*0.026938727*	*0.017904907*
**ENSG00000196341**	*0.016239046*	*0.025065105*	*0.02415687*	*0.014871491*
**ENSG00000088930**	*0.022923171*	*0.029739475*	*0.030747197*	*0.021112028*
**ENSG00000152969**	*0.137597309*	*0.10033782*	*0.090643132*	*0.115704037*
**ENSG00000150630**	*0.016148349*	*0.024527263*	*0.024734037*	*0.016394427*
**ENSG00000088538**	*0.015154818*	*0.024708621*	*0.024955763*	*0.014768074*
**ENSG00000112246**	*0.018929276*	*0.02625695*	*0.026532639*	*0.018527433*
**ENSG00000119522**	*0.049437865*	*0.04119878*	*0.039488672*	*0.039260851*
**ENSG00000253506**	*0.031502962*	*0.04257552*	*0.034575083*	*0.064985461*
**ENSG00000215021**	*0.023157549*	*0.030020225*	*0.02941145*	*0.019221837*
**ENSG00000117308**	*0.033695296*	*0.031436195*	*0.038215941*	*0.031520674*
**ENSG00000148429**	*0.051575952*	*0.043419464*	*0.048351888*	*0.039958701*
**ENSG00000178386**	*0.024899544*	*0.029320853*	*0.025245002*	*0.019452044*
**ENSG00000162670**	*0.016641807*	*0.024658595*	*0.024115865*	*0.016123877*
**ENSG00000182831**	*0.028557038*	*0.036368339*	*0.035362777*	*0.027188784*
**ENSG00000189320**	*0.015035705*	*0.024194329*	*0.024363985*	*0.014904706*
**ENSG00000163689**	*0.026399018*	*0.028828083*	*0.025849589*	*0.028116257*
**ENSG00000148660**	*0.036465661*	*0.035509655*	*0.030944508*	*0.029362839*
**ENSG00000115590**	*0.128696264*	*0.073136487*	*0.05435888*	*0.092311328*
**ENSG00000136819**	*0.029142496*	*0.034002469*	*0.032507196*	*0.02462958*
**ENSG00000185721**	*0.023231506*	*0.029417494*	*0.029506921*	*0.017810809*
**ENSG00000079805**	*0.023437899*	*0.030351973*	*0.035810343*	*0.025768362*
**ENSG00000085377**	*0.034416722*	*0.039877507*	*0.03478144*	*0.025367236*
**ENSG00000066117**	*0.017912911*	*0.028027838*	*0.027354936*	*0.019880141*
**ENSG00000111348**	*0.023422642*	*0.028196273*	*0.026202174*	*0.021094602*
**ENSG00000215186**	*0.016074356*	*0.025095862*	*0.025323641*	*0.015907514*
**ENSG00000105355**	*0.030602924*	*0.030925481*	*0.033903303*	*0.029905377*

**ENSG00000140931**	*0.017707483*	*0.024508159*	*0.026050637*	*0.015710666*
**ENSG00000115884**	*0.137696539*	*0.1193151*	*0.108205495*	*0.121850354*
**ENSG00000130829**	*0.02230537*	*0.028281225*	*0.032147498*	*0.019362816*
**ENSG00000183621**	*0.03634033*	*0.036709728*	*0.035180859*	*0.032323352*
**ENSG00000099783**	*0.024127858*	*0.028400143*	*0.028055065*	*0.027115564*
**ENSG00000007968**	*0.079053173*	*0.055363975*	*0.049580633*	*0.057532069*
**ENSG00000136521**	*0.022729675*	*0.030300095*	*0.027210327*	*0.020559174*
**ENSG00000161634**	*0.015541045*	*0.02653573*	*0.024374409*	*0.015928484*
**ENSG00000091039**	*0.026446051*	*0.032082719*	*0.034249068*	*0.033731878*
**ENSG00000204655**	*0.016418449*	*0.024437407*	*0.025163399*	*0.01636488*
**ENSG00000102606**	*0.020225589*	*0.031081747*	*0.027563361*	*0.018647849*
**ENSG00000150776**	*0.025267988*	*0.03771951*	*0.039523953*	*0.030270814*
**ENSG00000213865**	*0.017985613*	*0.02578682*	*0.024621237*	*0.016950003*
**ENSG00000211450**	*0.019360349*	*0.026971433*	*0.026682152*	*0.01756045*
**ENSG00000120708**	*0.091227827*	*0.061279918*	*0.044472298*	*0.100913323*
**ENSG00000167754**	*0.016531229*	*0.025478302*	*0.024953814*	*0.014552175*
**ENSG00000089693**	*0.02070369*	*0.029035429*	*0.035116644*	*0.023740171*
**ENSG00000163344**	*0.028931637*	*0.034600087*	*0.032716723*	*0.026089221*
**ENSG00000103335**	*0.031315661*	*0.034630735*	*0.036158084*	*0.032656613*
**ENSG00000167858**	*0.019251623*	*0.025918701*	*0.026018243*	*0.019751837*
**ENSG00000149474**	*0.030947955*	*0.03349045*	*0.033639402*	*0.026863203*
**ENSG00000156253**	*0.0482064*	*0.040727076*	*0.03238743*	*0.037747792*
**ENSG00000184925**	*0.015715809*	*0.026059857*	*0.025129197*	*0.016318382*
**ENSG00000111481**	*0.021778121*	*0.030037941*	*0.031423067*	*0.021952535*
**ENSG00000165495**	*0.016456404*	*0.025417893*	*0.025818414*	*0.015962789*
**ENSG00000101654**	*0.02971262*	*0.031637939*	*0.032259744*	*0.026624055*
**ENSG00000141644**	*0.031077287*	*0.031812919*	*0.038128763*	*0.036747695*
**ENSG00000150768**	*0.029234257*	*0.034767479*	*0.029563329*	*0.034577975*
**ENSG00000107882**	*0.018084889*	*0.026293634*	*0.026271466*	*0.018553253*
**ENSG00000135821**	*0.084242834*	*0.067060874*	*0.046364383*	*0.055435862*
**ENSG00000165806**	*0.018258168*	*0.026722144*	*0.024652791*	*0.019780728*
**ENSG00000135631**	*0.015525393*	*0.024979046*	*0.02526708*	*0.016189749*
**ENSG00000184988**	*0.030590081*	*0.035803893*	*0.032019288*	*0.032167773*
**ENSG00000115241**	*0.030692698*	*0.031410284*	*0.028936375*	*0.024758684*
**ENSG00000164778**	*0.015994168*	*0.024314684*	*0.025306417*	*0.01621964*
**ENSG00000089558**	*0.016758894*	*0.025539251*	*0.025318603*	*0.016342005*
**ENSG00000134539**	*0.016139075*	*0.024612552*	*0.024617838*	*0.01594799*
**ENSG00000180884**	*0.031563789*	*0.036889046*	*0.032656335*	*0.022761476*
**ENSG00000139287**	*0.016511218*	*0.025551412*	*0.024968663*	*0.01534696*
**ENSG00000105373**	*0.021278774*	*0.029262117*	*0.027308213*	*0.022515269*
**ENSG00000076650**	*0.034392376*	*0.032882157*	*0.039116426*	*0.024573732*
**ENSG00000104290**	*0.066887167*	*0.048755234*	*0.050009756*	*0.057284898*
**ENSG00000054282**	*0.033446996*	*0.035769393*	*0.033489714*	*0.031188362*
**ENSG00000111713**	*0.015556258*	*0.0249812*	*0.025227929*	*0.015501166*
**ENSG00000133818**	*0.034940073*	*0.035882265*	*0.033363779*	*0.027474803*
**ENSG00000253159**	*0.017834147*	*0.024666*	*0.024884684*	*0.01584175*
**ENSG00000084207**	*0.030929611*	*0.033486649*	*0.034584807*	*0.029269745*
**ENSG00000108582**	*0.038309373*	*0.040509973*	*0.034876317*	*0.029709449*
**ENSG00000173511**	*0.035712999*	*0.032561669*	*0.036037894*	*0.0345407*
**ENSG00000100234**	*0.016054716*	*0.024338125*	*0.024852409*	*0.014690578*
**ENSG00000115361**	*0.016578477*	*0.024789211*	*0.024905343*	*0.015894789*
**ENSG00000166446**	*0.017018253*	*0.024900868*	*0.024598683*	*0.016783022*
**ENSG00000124253**	*0.015507525*	*0.025196056*	*0.02433966*	*0.015133192*
**ENSG00000162650**	*0.030491929*	*0.031981603*	*0.040067308*	*0.033224215*

**ENSG00000158528**	*0.015847095*	*0.025122273*	*0.025769676*	*0.014797913*
**ENSG00000228198**	*0.014372038*	*0.024524942*	*0.024689629*	*0.016472732*
**ENSG00000169224**	*0.01589128*	*0.024910679*	*0.024968909*	*0.015396518*
**ENSG00000161036**	*0.040242478*	*0.040920226*	*0.039758389*	*0.040227852*
**ENSG00000111907**	*0.039819989*	*0.02486291*	*0.02537188*	*0.015812581*
**ENSG00000187239**	*0.019128663*	*0.027479339*	*0.02573012*	*0.018536846*
**ENSG00000106336**	*0.021015843*	*0.026051122*	*0.026038193*	*0.017695857*
**ENSG00000160679**	*0.026462044*	*0.030778668*	*0.027995186*	*0.021824904*
**ENSG00000163515**	*0.018967506*	*0.025907324*	*0.026097355*	*0.022077342*
**ENSG00000177888**	*0.016692775*	*0.024911751*	*0.025373642*	*0.017537151*
**ENSG00000180697**	*0.015172629*	*0.02503389*	*0.024582552*	*0.015620802*
**ENSG00000167680**	*0.015925707*	*0.026731205*	*0.025550006*	*0.017139083*
**ENSG00000205085**	*0.016976013*	*0.024863779*	*0.024975069*	*0.014652925*
**ENSG00000198053**	*0.107844516*	*0.068761856*	*0.078912272*	*0.078370877*
**ENSG00000204713**	*0.030938372*	*0.030068811*	*0.032143967*	*0.026199428*
**ENSG00000049449**	*0.036047873*	*0.0354458*	*0.033964645*	*0.037259035*
**ENSG00000170484**	*0.015457713*	*0.024327138*	*0.024777017*	*0.016148935*
**ENSG00000143850**	*0.018785579*	*0.025859008*	*0.025308349*	*0.016131698*
**ENSG00000178999**	*0.042380627*	*0.03225696*	*0.033756428*	*0.029602726*
**ENSG00000240764**	*0.015518284*	*0.02437871*	*0.024682014*	*0.014325406*
**ENSG00000156738**	*0.053071709*	*0.059582155*	*0.053405366*	*0.052682947*
**ENSG00000166526**	*0.018301488*	*0.024858064*	*0.024576431*	*0.015639038*
**ENSG00000139746**	*0.029442452*	*0.034159808*	*0.02954704*	*0.024925099*
**ENSG00000104497**	*0.043628177*	*0.044125158*	*0.043272308*	*0.03171177*
**ENSG00000092445**	*0.015584946*	*0.02640505*	*0.024677019*	*0.015329973*
**ENSG00000122121**	*0.025673624*	*0.028527658*	*0.029300345*	*0.023377503*
**ENSG00000121966**	*0.082594271*	*0.0582108*	*0.054204836*	*0.071448093*
**ENSG00000077009**	*0.021703861*	*0.027724985*	*0.026063072*	*0.019442757*
**ENSG00000135108**	*0.031348436*	*0.037325455*	*0.036568074*	*0.030604005*
**ENSG00000116786**	*0.027767007*	*0.032260194*	*0.03816723*	*0.026190378*
**ENSG00000183696**	*0.016388968*	*0.026333691*	*0.026155493*	*0.015901367*
**ENSG00000164976**	*0.019782174*	*0.025267885*	*0.025101526*	*0.0147995*
**ENSG00000162614**	*0.075580672*	*0.067707456*	*0.050958925*	*0.046067162*
**ENSG00000181135**	*0.02004349*	*0.028611985*	*0.029272452*	*0.021973946*
**ENSG00000214078**	*0.042038613*	*0.043728245*	*0.038200286*	*0.037282009*
**ENSG00000113658**	*0.042535663*	*0.037699739*	*0.03822712*	*0.038593778*
**ENSG00000214309**	*0.020723976*	*0.02584017*	*0.027758286*	*0.018642238*
**ENSG00000154473**	*0.025144301*	*0.032198563*	*0.030726234*	*0.026247255*
**ENSG00000123415**	*0.02591381*	*0.031648344*	*0.027765912*	*0.018624175*
**ENSG00000112763**	*0.030924749*	*0.036865209*	*0.033531229*	*0.025115873*
**ENSG00000147896**	*0.016539038*	*0.025530319*	*0.025676038*	*0.016616718*
**ENSG00000005379**	*0.053671672*	*0.046686382*	*0.046253021*	*0.054834786*
**ENSG00000028137**	*0.046518678*	*0.040441956*	*0.034917677*	*0.041045373*
**ENSG00000205189**	*0.02928216*	*0.033675875*	*0.03448439*	*0.028637118*
**ENSG00000004848**	*0.018970124*	*0.026091647*	*0.024457658*	*0.019682593*
**ENSG00000102226**	*0.024953993*	*0.027888942*	*0.030067355*	*0.02161817*
**ENSG00000117560**	*0.01527278*	*0.025119658*	*0.024413342*	*0.015458237*
**ENSG00000136449**	*0.015360079*	*0.025000246*	*0.024727555*	*0.0147283*
**ENSG00000204967**	*0.015378176*	*0.024923284*	*0.025673471*	*0.014585213*
**ENSG00000139350**	*0.048911886*	*0.048996605*	*0.04238374*	*0.045316693*
**ENSG00000145730**	*0.049429607*	*0.041306687*	*0.042104279*	*0.040152154*
**ENSG00000183023**	*0.016628581*	*0.024636107*	*0.024585215*	*0.016152567*
**ENSG00000069020**	*0.041918747*	*0.028565262*	*0.034444926*	*0.030205671*
**ENSG00000163633**	*0.020461804*	*0.027545349*	*0.025446336*	*0.017795567*

**ENSG00000177459**	*0.122108632*	*0.077140257*	*0.080737783*	*0.08451474*
**ENSG00000161298**	*0.01843226*	*0.025859565*	*0.026621714*	*0.018350947*
**ENSG00000146729**	*0.027013241*	*0.031633341*	*0.031030281*	*0.032187024*
**ENSG00000166573**	*0.015121311*	*0.02572269*	*0.024142435*	*0.014752382*
**ENSG00000184357**	*0.056478788*	*0.03785126*	*0.048001841*	*0.056060768*
**ENSG00000131845**	*0.025201866*	*0.035572597*	*0.034224532*	*0.021900996*
**ENSG00000145864**	*0.016907057*	*0.0255887*	*0.02552668*	*0.01606047*
**ENSG00000100410**	*0.033025104*	*0.034946053*	*0.03765651*	*0.030724348*
**ENSG00000205981**	*0.032166648*	*0.030192909*	*0.03056864*	*0.021936244*
**ENSG00000167797**	*0.038699556*	*0.035010939*	*0.033891274*	*0.036500224*
**ENSG00000132846**	*0.04617484*	*0.040761805*	*0.037339118*	*0.038371134*
**ENSG00000105058**	*0.021573832*	*0.028889514*	*0.033064565*	*0.02173181*
**ENSG00000136155**	*0.019529968*	*0.027479241*	*0.026962532*	*0.018799227*
**ENSG00000163121**	*0.133736789*	*0.08589833*	*0.094848622*	*0.114613634*
**ENSG00000243335**	*0.018310449*	*0.025905056*	*0.024789398*	*0.01576285*
**ENSG00000161671**	*0.026629655*	*0.032414426*	*0.031609616*	*0.027680074*
**ENSG00000169093**	*0.033657894*	*0.034107653*	*0.042900303*	*0.044681262*
**ENSG00000158786**	*0.015428609*	*0.025640859*	*0.02465825*	*0.015526099*
**ENSG00000152495**	*0.039650361*	*0.046883258*	*0.037645985*	*0.039932968*
**ENSG00000115604**	*0.135415444*	*0.112256187*	*0.103474972*	*0.119414198*
**ENSG00000162813**	*0.030708161*	*0.036510276*	*0.031065647*	*0.026266512*
**ENSG00000100350**	*0.037989338*	*0.03362529*	*0.031260065*	*0.028681778*
**ENSG00000231852**	*0.016076159*	*0.025640539*	*0.025949225*	*0.015693847*
**ENSG00000198440**	*0.020363051*	*0.02591716*	*0.028484777*	*0.023038917*
**ENSG00000151093**	*0.029193758*	*0.033857772*	*0.028653219*	*0.021112382*
**ENSG00000118600**	*0.033977386*	*0.032041636*	*0.033758742*	*0.030425264*
**ENSG00000109133**	*0.037812675*	*0.037101436*	*0.03129955*	*0.033606508*
**ENSG00000189129**	*0.015108425*	*0.024972269*	*0.025009169*	*0.014632021*
**ENSG00000023318**	*0.023740617*	*0.028964414*	*0.027711848*	*0.019155667*
**ENSG00000141934**	*0.01558649*	*0.025175162*	*0.02582433*	*0.015935973*
**ENSG00000019169**	*0.031080031*	*0.029540217*	*0.027407462*	*0.021791996*
**ENSG00000183036**	*0.016739785*	*0.025386756*	*0.024791611*	*0.015026906*
**ENSG00000169314**	*0.015981935*	*0.025450617*	*0.024211108*	*0.015058176*
**ENSG00000156958**	*0.037710065*	*0.037199012*	*0.03269758*	*0.029819257*
**ENSG00000046889**	*0.023394115*	*0.030413238*	*0.028442111*	*0.023583297*
**ENSG00000101190**	*0.06938211*	*0.063159365*	*0.049845124*	*0.056877644*
**ENSG00000187990**	*0.055007166*	*0.044150273*	*0.045860148*	*0.043087192*
**ENSG00000186073**	*0.041595374*	*0.042405594*	*0.032209223*	*0.034163969*
**ENSG00000101751**	*0.034911489*	*0.037247971*	*0.031290981*	*0.033357132*
**ENSG00000173531**	*0.047551791*	*0.049509563*	*0.042821141*	*0.051148286*
**ENSG00000179562**	*0.025733549*	*0.039867335*	*0.041793225*	*0.025584531*
**ENSG00000184788**	*0.0204708*	*0.024717608*	*0.032758293*	*0.016611584*
**ENSG00000164451**	*0.017038455*	*0.024671629*	*0.025236521*	*0.016030274*
**ENSG00000125898**	*0.045746241*	*0.050272573*	*0.039017568*	*0.038642624*
**ENSG00000164935**	*0.01641673*	*0.025368573*	*0.024499237*	*0.014883826*
**ENSG00000132780**	*0.022631477*	*0.027880209*	*0.027643325*	*0.020937905*
**ENSG00000149929**	*0.036157066*	*0.034015626*	*0.031244426*	*0.027579093*
**ENSG00000105011**	*0.047265916*	*0.040652459*	*0.041307792*	*0.037055633*
**ENSG00000040341**	*0.03756061*	*0.045717059*	*0.033711432*	*0.033213774*
**ENSG00000147065**	*0.022212362*	*0.029819429*	*0.028357256*	*0.019984677*
**ENSG00000017483**	*0.038047537*	*0.031472583*	*0.033544054*	*0.0368018*
**ENSG00000101435**	*0.030084867*	*0.03545362*	*0.032517461*	*0.029596186*
**ENSG00000116685**	*0.026665254*	*0.030976556*	*0.037130389*	*0.025205659*
**ENSG00000152669**	*0.015595785*	*0.025009637*	*0.025480658*	*0.015438816*

**ENSG00000148356**	*0.026797035*	*0.030528462*	*0.027757611*	*0.021653319*
**ENSG00000196242**	*0.01557781*	*0.024601931*	*0.024907994*	*0.01540431*
**ENSG00000143140**	*0.015009688*	*0.025497477*	*0.024651849*	*0.014858771*
**ENSG00000173349**	*0.03031873*	*0.037489427*	*0.03496327*	*0.026008088*
**ENSG00000108774**	*0.030737074*	*0.037270901*	*0.032523991*	*0.033757367*
**ENSG00000179115**	*0.024133737*	*0.028249871*	*0.029379684*	*0.023766039*
**ENSG00000065029**	*0.033524862*	*0.036771624*	*0.033765927*	*0.02870081*
**ENSG00000131771**	*0.01542869*	*0.025105293*	*0.024261521*	*0.014022842*
**ENSG00000179364**	*0.014959148*	*0.024703295*	*0.02452265*	*0.014197984*
**ENSG00000106829**	*0.058269774*	*0.049958539*	*0.044467237*	*0.04916383*
**ENSG00000140464**	*0.018575938*	*0.027125131*	*0.02569266*	*0.016287633*
**ENSG00000172478**	*0.017489763*	*0.02660803*	*0.025536748*	*0.017377917*
**ENSG00000103994**	*0.032941081*	*0.0382093*	*0.03195011*	*0.025039316*
**ENSG00000105497**	*0.035740482*	*0.037903142*	*0.038312917*	*0.026198351*
**ENSG00000140254**	*0.015196966*	*0.02502865*	*0.024118585*	*0.015782052*
**ENSG00000178257**	*0.015600981*	*0.025730554*	*0.024631775*	*0.01630571*
**ENSG00000084444**	*0.022591176*	*0.034787096*	*0.031012337*	*0.023639252*
**ENSG00000181085**	*0.014958829*	*0.025887372*	*0.023879849*	*0.01450203*
**ENSG00000174840**	*0.02349148*	*0.031433872*	*0.030706181*	*0.02539161*
**ENSG00000142959**	*0.025878908*	*0.028912259*	*0.026861385*	*0.020435156*
**ENSG00000141664**	*0.016343605*	*0.024972505*	*0.025533542*	*0.015235809*
**ENSG00000187091**	*0.027711829*	*0.033020461*	*0.027211752*	*0.028773703*
**ENSG00000241233**	*0.018467964*	*0.026172977*	*0.026347973*	*0.017763735*
**ENSG00000197375**	*0.031346269*	*0.033177664*	*0.028897874*	*0.021667688*
**ENSG00000171365**	*0.016927446*	*0.025978853*	*0.025170327*	*0.017223176*
**ENSG00000163877**	*0.034638914*	*0.049901502*	*0.047850721*	*0.035768632*
**ENSG00000125492**	*0.016068114*	*0.027585268*	*0.026197708*	*0.016197673*
**ENSG00000181284**	*0.020146054*	*0.02789312*	*0.027948573*	*0.022978091*
**ENSG00000183876**	*0.015320644*	*0.02553416*	*0.025417167*	*0.016120543*
**ENSG00000187242**	*0.01504733*	*0.024562791*	*0.024962038*	*0.014862273*
**ENSG00000095464**	*0.016467964*	*0.025731855*	*0.024529056*	*0.015603114*
**ENSG00000157800**	*0.030303432*	*0.033954065*	*0.038848976*	*0.025733259*
**ENSG00000204843**	*0.016276098*	*0.024533117*	*0.025397648*	*0.015487261*
**ENSG00000099960**	*0.015130077*	*0.024730036*	*0.024633452*	*0.014858126*
**ENSG00000082781**	*0.104252683*	*0.068265693*	*0.079068751*	*0.100903404*
**ENSG00000159461**	*0.043990117*	*0.053460296*	*0.034716948*	*0.058066532*
**ENSG00000061676**	*0.082766568*	*0.078732069*	*0.066343415*	*0.07525707*
**ENSG00000103671**	*0.026802938*	*0.034045008*	*0.0334919*	*0.034443441*
**ENSG00000139515**	*0.019560524*	*0.02699277*	*0.026352841*	*0.017248018*
**ENSG00000131668**	*0.025287174*	*0.029524863*	*0.025468253*	*0.016985493*
**ENSG00000161547**	*0.028364534*	*0.033318259*	*0.035623354*	*0.029322092*
**ENSG00000162881**	*0.017242962*	*0.027498104*	*0.026287369*	*0.020348848*
**ENSG00000080189**	*0.018926547*	*0.026123124*	*0.027047555*	*0.018962914*
**ENSG00000080561**	*0.02128261*	*0.028269106*	*0.026739566*	*0.017801305*
**ENSG00000107242**	*0.062784759*	*0.06976943*	*0.050163162*	*0.052285851*
**ENSG00000110057**	*0.037504875*	*0.066327882*	*0.050844841*	*0.051723203*
**ENSG00000151503**	*0.039264936*	*0.038047432*	*0.032565984*	*0.026078685*
**ENSG00000013441**	*0.028937843*	*0.035108376*	*0.038281081*	*0.037704012*
**ENSG00000176390**	*0.027346061*	*0.033649178*	*0.032466614*	*0.02608051*
**ENSG00000137033**	*0.017665336*	*0.025405674*	*0.02454199*	*0.016535331*
**ENSG00000065618**	*0.01923173*	*0.029362618*	*0.027283589*	*0.019200049*
**ENSG00000132681**	*0.015904713*	*0.024779692*	*0.023870909*	*0.01465435*
**ENSG00000143549**	*0.021758092*	*0.028876589*	*0.027562798*	*0.020546291*
**ENSG00000008988**	*0.016122666*	*0.024129398*	*0.025164387*	*0.016598583*

**ENSG00000104979**	*0.01798016*	*0.026868495*	*0.027707301*	*0.019881374*
**ENSG00000184524**	*0.099194613*	*0.072488273*	*0.066978952*	*0.083425511*
**ENSG00000148450**	*0.035327963*	*0.042306619*	*0.040063525*	*0.029419962*
**ENSG00000160181**	*0.083832278*	*0.029486308*	*0.029071899*	*0.042965067*
**ENSG00000212127**	*0.019061609*	*0.027136188*	*0.028511064*	*0.018969577*
**ENSG00000197008**	*0.0395475*	*0.041901143*	*0.036438426*	*0.034191854*
**ENSG00000086696**	*0.016470284*	*0.02552616*	*0.024696164*	*0.014807641*
**ENSG00000160844**	*0.037618704*	*0.033420772*	*0.034079523*	*0.022771016*
**ENSG00000116266**	*0.034695079*	*0.038654811*	*0.035040056*	*0.038655255*
**ENSG00000148734**	*0.017777154*	*0.027541323*	*0.025232252*	*0.016927458*
**ENSG00000123562**	*0.033611945*	*0.034559836*	*0.033593444*	*0.023486263*
**ENSG00000179796**	*0.017273938*	*0.025880648*	*0.026660259*	*0.017052757*
**ENSG00000090534**	*0.014547409*	*0.024679093*	*0.02419535*	*0.013851722*
**ENSG00000101331**	*0.01553207*	*0.02425229*	*0.024391152*	*0.015631554*
**ENSG00000075426**	*0.069544962*	*0.067973253*	*0.059149285*	*0.080543175*
**ENSG00000112742**	*0.043090309*	*0.036929978*	*0.03553822*	*0.036715201*
**ENSG00000127837**	*0.024489014*	*0.027850705*	*0.030925649*	*0.021332694*
**ENSG00000018699**	*0.035727559*	*0.040324447*	*0.033103373*	*0.030106678*
**ENSG00000162923**	*0.032198388*	*0.033785191*	*0.035292032*	*0.030992348*
**ENSG00000103852**	*0.024144605*	*0.034704051*	*0.02700779*	*0.024497679*
**ENSG00000116857**	*0.042294454*	*0.038529822*	*0.038935648*	*0.03141369*
**ENSG00000156711**	*0.081259283*	*0.045199125*	*0.059389053*	*0.053334931*
**ENSG00000196639**	*0.016459832*	*0.026801609*	*0.025996359*	*0.015888461*
**ENSG00000115255**	*0.016969024*	*0.025422858*	*0.02615667*	*0.016086041*
**ENSG00000058600**	*0.026441421*	*0.031842102*	*0.038658625*	*0.024700919*
**ENSG00000117569**	*0.035317778*	*0.037144827*	*0.040008921*	*0.029951583*
**ENSG00000115488**	*0.017394183*	*0.025265893*	*0.025557284*	*0.015378094*
**ENSG00000127191**	*0.02175663*	*0.029258467*	*0.030017614*	*0.022352293*
**ENSG00000122085**	*0.034091692*	*0.03583493*	*0.034980308*	*0.030831383*
**ENSG00000099341**	*0.033320781*	*0.035242226*	*0.034759237*	*0.025946405*
**ENSG00000164032**	*0.026846835*	*0.028904578*	*0.028100871*	*0.02092373*
**ENSG00000144567**	*0.023017915*	*0.029968226*	*0.032359419*	*0.026132754*
**ENSG00000065308**	*0.06822187*	*0.051427646*	*0.049430039*	*0.059652801*
**ENSG00000012061**	*0.024722619*	*0.030144654*	*0.031459252*	*0.023598998*
**ENSG00000114349**	*0.017276978*	*0.025866569*	*0.025000755*	*0.017864274*
**ENSG00000176428**	*0.05209132*	*0.04721033*	*0.037862326*	*0.045701805*
**ENSG00000205649**	*0.017211855*	*0.024910245*	*0.025401301*	*0.016324705*
**ENSG00000099822**	*0.015210253*	*0.024939703*	*0.024795287*	*0.014593414*
**ENSG00000114023**	*0.016349242*	*0.024810479*	*0.025150903*	*0.015033389*
**ENSG00000127884**	*0.023055058*	*0.031159742*	*0.031703498*	*0.023451409*
**ENSG00000041357**	*0.026216146*	*0.033048801*	*0.030378531*	*0.025995759*
**ENSG00000136141**	*0.016343387*	*0.025085256*	*0.024401714*	*0.017089646*
**ENSG00000196247**	*0.033255054*	*0.041434962*	*0.041372009*	*0.031668009*
**ENSG00000152127**	*0.017974626*	*0.028401761*	*0.027600296*	*0.018936533*
**ENSG00000107796**	*0.044748801*	*0.052354542*	*0.048926795*	*0.047791535*
**ENSG00000136243**	*0.02807779*	*0.034043473*	*0.035218257*	*0.028838934*
**ENSG00000169021**	*0.0192488*	*0.026289631*	*0.027935701*	*0.018605593*
**ENSG00000142546**	*0.025983834*	*0.030155202*	*0.029733189*	*0.020820443*
**ENSG00000169442**	*0.030991564*	*0.032852611*	*0.02671545*	*0.027804653*
**ENSG00000129911**	*0.025738256*	*0.037579579*	*0.043521044*	*0.027631085*
**ENSG00000124194**	*0.015348655*	*0.025758366*	*0.024162099*	*0.015241383*
**ENSG00000146858**	*0.016808234*	*0.02633397*	*0.025124992*	*0.015539582*
**ENSG00000196924**	*0.03312732*	*0.041919901*	*0.034008473*	*0.038390735*
**ENSG00000120616**	*0.034521861*	*0.042948629*	*0.040406111*	*0.030214181*

**ENSG00000117614**	*0.026220367*	*0.03247688*	*0.031620409*	*0.024388718*
**ENSG00000186104**	*0.019267383*	*0.027817411*	*0.026909357*	*0.018738056*
**ENSG00000180758**	*0.019098081*	*0.028050462*	*0.027109316*	*0.017745685*
**ENSG00000175832**	*0.052262828*	*0.052188727*	*0.044000882*	*0.052386216*
**ENSG00000158106**	*0.025162181*	*0.041436172*	*0.031797339*	*0.038322552*
**ENSG00000124795**	*0.030977689*	*0.033148851*	*0.034829021*	*0.036345658*
**ENSG00000177103**	*0.040004693*	*0.033777253*	*0.028893241*	*0.031107753*
**ENSG00000143195**	*0.016301575*	*0.025255927*	*0.024095753*	*0.015869311*
**ENSG00000161570**	*0.053425085*	*0.049632918*	*0.044309428*	*0.058903612*
**ENSG00000172183**	*0.042190037*	*0.042879666*	*0.037191882*	*0.036568758*
**ENSG00000156110**	*0.041157919*	*0.043201709*	*0.035908097*	*0.03826636*
**ENSG00000241404**	*0.015202885*	*0.025274612*	*0.02443488*	*0.014444687*
**ENSG00000221874**	*0.016875651*	*0.024343385*	*0.024615114*	*0.014344993*
**ENSG00000147082**	*0.015590172*	*0.025797567*	*0.023990462*	*0.015814571*
**ENSG00000177556**	*0.033300183*	*0.033107746*	*0.029398928*	*0.030917776*
**ENSG00000100441**	*0.020701777*	*0.029990938*	*0.027138827*	*0.025718298*
**ENSG00000103199**	*0.017256366*	*0.02517307*	*0.024961636*	*0.016796206*
**ENSG00000111700**	*0.019167263*	*0.0259398*	*0.025873224*	*0.016634472*
**ENSG00000205765**	*0.030960142*	*0.03292472*	*0.029136766*	*0.021890173*
**ENSG00000156298**	*0.037335272*	*0.028487509*	*0.025024891*	*0.024861145*
**ENSG00000066136**	*0.025302802*	*0.033096132*	*0.031521681*	*0.023631485*
**ENSG00000160392**	*0.03435726*	*0.029893279*	*0.044990979*	*0.042622083*
**ENSG00000167100**	*0.027486215*	*0.030572203*	*0.034330837*	*0.027992608*
**ENSG00000108106**	*0.041046743*	*0.031525293*	*0.042666247*	*0.037810725*
**ENSG00000176371**	*0.021541326*	*0.027179591*	*0.027433324*	*0.019617902*
**ENSG00000004766**	*0.024144513*	*0.031468467*	*0.02702184*	*0.023217454*
**ENSG00000205426**	*0.016320918*	*0.024694929*	*0.025171765*	*0.014931303*
**ENSG00000008323**	*0.041378433*	*0.034374266*	*0.02895613*	*0.031211914*
**ENSG00000197063**	*0.035798303*	*0.043295427*	*0.03675055*	*0.033555067*
**ENSG00000078699**	*0.032204921*	*0.037912821*	*0.029920333*	*0.032478301*
**ENSG00000151422**	*0.031645387*	*0.036411132*	*0.031951513*	*0.025792628*
**ENSG00000122133**	*0.01764408*	*0.027854143*	*0.025137743*	*0.023981942*
**ENSG00000115844**	*0.049558412*	*0.040668021*	*0.042940694*	*0.046458767*
**ENSG00000160216**	*0.038774724*	*0.042361952*	*0.036578623*	*0.042330269*
**ENSG00000198146**	*0.015649489*	*0.026815794*	*0.025922663*	*0.016802684*
**ENSG00000170965**	*0.019057292*	*0.02754042*	*0.025990057*	*0.02307926*
**ENSG00000125618**	*0.017265414*	*0.026915535*	*0.026035889*	*0.01661758*
**ENSG00000144535**	*0.021563135*	*0.028176057*	*0.027412481*	*0.021050718*
**ENSG00000139531**	*0.020518468*	*0.028363855*	*0.029365364*	*0.020350719*
**ENSG00000071575**	*0.073297433*	*0.045222877*	*0.064315228*	*0.069152016*
**ENSG00000109805**	*0.04104086*	*0.036155855*	*0.036740408*	*0.033695022*
**ENSG00000114770**	*0.03179579*	*0.034270629*	*0.032589664*	*0.024851013*
**ENSG00000168685**	*0.038779169*	*0.038391424*	*0.030538725*	*0.022265948*
**ENSG00000166181**	*0.023090081*	*0.032251103*	*0.028535872*	*0.021082343*
**ENSG00000104321**	*0.016810886*	*0.025729285*	*0.025073113*	*0.016628937*
**ENSG00000120594**	*0.121694802*	*0.071958066*	*0.043596887*	*0.078322241*
**ENSG00000196878**	*0.07765909*	*0.060342583*	*0.052918003*	*0.05745339*
**ENSG00000160714**	*0.030477251*	*0.038668979*	*0.037036532*	*0.033776946*
**ENSG00000124160**	*0.034348878*	*0.033057929*	*0.035611041*	*0.034019234*
**ENSG00000129055**	*0.044446188*	*0.051812924*	*0.044281958*	*0.073169276*
**ENSG00000181963**	*0.015375816*	*0.025957443*	*0.025575902*	*0.01483925*
**ENSG00000198216**	*0.017340147*	*0.025788889*	*0.024358634*	*0.01738791*
**ENSG00000197323**	*0.016250102*	*0.025382847*	*0.026419872*	*0.015987318*
**ENSG00000141452**	*0.029324024*	*0.033919298*	*0.031420061*	*0.025321533*

**ENSG00000250361**	*0.016439639*	*0.025132925*	*0.024966754*	*0.015827429*
**ENSG00000171790**	*0.016677604*	*0.024900159*	*0.024303967*	*0.015114195*
**ENSG00000124659**	*0.026311272*	*0.033060794*	*0.033053017*	*0.021399436*
**ENSG00000169509**	*0.015126616*	*0.024493086*	*0.024637161*	*0.015527144*
**ENSG00000117118**	*0.024807725*	*0.030809171*	*0.027305615*	*0.020691176*
**ENSG00000124839**	*0.016843584*	*0.025314377*	*0.025262616*	*0.015470702*
**ENSG00000168081**	*0.046920964*	*0.038527065*	*0.034701664*	*0.03970935*
**ENSG00000076924**	*0.028803636*	*0.02952063*	*0.034215359*	*0.027357682*
**ENSG00000115866**	*0.02922995*	*0.030660692*	*0.027708429*	*0.020873967*
**ENSG00000090565**	*0.028788118*	*0.03666448*	*0.038107913*	*0.023121959*
**ENSG00000186416**	*0.02406838*	*0.031295219*	*0.030763227*	*0.021249686*
**ENSG00000100593**	*0.016808462*	*0.027362654*	*0.025158281*	*0.01842167*
**ENSG00000167555**	*0.025875655*	*0.028172773*	*0.028158129*	*0.02011621*
**ENSG00000013275**	*0.026775618*	*0.031064962*	*0.030138209*	*0.030309756*
**ENSG00000162241**	*0.043249267*	*0.038230309*	*0.034312846*	*0.038224913*
**ENSG00000177169**	*0.036539135*	*0.039099226*	*0.0398047*	*0.034798011*
**ENSG00000182612**	*0.021839965*	*0.029326651*	*0.028098963*	*0.027160055*
**ENSG00000139083**	*0.029190235*	*0.036508273*	*0.032538974*	*0.038752008*
**ENSG00000121281**	*0.038209021*	*0.042748328*	*0.039029303*	*0.029396212*
**ENSG00000164007**	*0.015660701*	*0.026935822*	*0.024998228*	*0.015803192*
**ENSG00000129048**	*0.017820002*	*0.026513347*	*0.025836908*	*0.018776526*
**ENSG00000111328**	*0.032955073*	*0.033888329*	*0.027965343*	*0.035783622*
**ENSG00000163263**	*0.013725373*	*0.024013036*	*0.02422063*	*0.013720633*
**ENSG00000102158**	*0.028699362*	*0.03246445*	*0.033565017*	*0.027394867*
**ENSG00000171942**	*0.014991004*	*0.025143268*	*0.024732823*	*0.015002505*
**ENSG00000105552**	*0.025630804*	*0.027920608*	*0.029712189*	*0.023874924*
**ENSG00000068650**	*0.03269478*	*0.028024712*	*0.027545423*	*0.025742263*
**ENSG00000206262**	*0.014874758*	*0.023838076*	*0.0247229*	*0.013903233*
**ENSG00000150316**	*0.026609947*	*0.03109339*	*0.030098525*	*0.020641196*
**ENSG00000172602**	*0.031085312*	*0.031179606*	*0.034380852*	*0.032554121*
**ENSG00000012660**	*0.032017827*	*0.036280165*	*0.031810151*	*0.024447122*
**ENSG00000072310**	*0.051254684*	*0.038402967*	*0.043227793*	*0.035445526*
**ENSG00000173698**	*0.053551784*	*0.028264278*	*0.025732658*	*0.021389073*
**ENSG00000169446**	*0.022664353*	*0.028602917*	*0.03143625*	*0.02289938*
**ENSG00000151366**	*0.033619318*	*0.033114492*	*0.035022351*	*0.037091238*
**ENSG00000182218**	*0.015509007*	*0.026458557*	*0.024865926*	*0.015284669*
**ENSG00000102901**	*0.022498653*	*0.03113291*	*0.030595661*	*0.021802344*
**ENSG00000148700**	*0.056897784*	*0.04502997*	*0.04289536*	*0.038418196*
**ENSG00000172197**	*0.035808209*	*0.033914737*	*0.036357144*	*0.028536864*
**ENSG00000141200**	*0.016636949*	*0.024947505*	*0.025220225*	*0.016934413*
**ENSG00000213281**	*0.030724474*	*0.035261703*	*0.039726817*	*0.034882024*
**ENSG00000136490**	*0.029565439*	*0.028276786*	*0.029462312*	*0.025437178*
**ENSG00000178602**	*0.017386725*	*0.024241857*	*0.024906057*	*0.016290943*
**ENSG00000233822**	*0.018916734*	*0.026862342*	*0.026343803*	*0.017045479*
**ENSG00000148110**	*0.044387022*	*0.062732182*	*0.04752766*	*0.107305569*
**ENSG00000241258**	*0.022334924*	*0.027396414*	*0.028873681*	*0.02173572*
**ENSG00000004799**	*0.015850267*	*0.025563955*	*0.025459847*	*0.01637481*
**ENSG00000157259**	*0.027016481*	*0.03357057*	*0.032531231*	*0.025829832*
**ENSG00000105287**	*0.029898328*	*0.029569387*	*0.036776122*	*0.024699147*
**ENSG00000169885**	*0.016096826*	*0.024588551*	*0.024291563*	*0.014448103*
**ENSG00000157540**	*0.027880573*	*0.033761174*	*0.035712414*	*0.028952538*
**ENSG00000125257**	*0.041046175*	*0.045587819*	*0.039692641*	*0.041296249*
**ENSG00000178171**	*0.015877607*	*0.024853068*	*0.024729523*	*0.014538716*
**ENSG00000112214**	*0.015777992*	*0.024800505*	*0.025174964*	*0.015295856*

**ENSG00000186977**	*0.018208182*	*0.02549086*	*0.027126658*	*0.016831594*
**ENSG00000155329**	*0.030941055*	*0.038257558*	*0.037856349*	*0.030147962*
**ENSG00000180066**	*0.020174373*	*0.032324185*	*0.025923566*	*0.017568512*
**ENSG00000188784**	*0.015357684*	*0.026201306*	*0.024843742*	*0.014927849*
**ENSG00000074935**	*0.042593252*	*0.045286599*	*0.036852881*	*0.038290214*
**ENSG00000160563**	*0.028631091*	*0.035198917*	*0.035239827*	*0.030351729*
**ENSG00000010256**	*0.025924061*	*0.028837874*	*0.027402993*	*0.021049077*
**ENSG00000102580**	*0.043472997*	*0.040939407*	*0.056287946*	*0.04593887*
**ENSG00000060069**	*0.032724006*	*0.039438444*	*0.037917289*	*0.031584864*
**ENSG00000197125**	*0.017478899*	*0.026284972*	*0.025752912*	*0.016958192*
**ENSG00000206026**	*0.017028467*	*0.025132469*	*0.025518365*	*0.015885072*
**ENSG00000171501**	*0.015926497*	*0.025940701*	*0.024133708*	*0.015863312*
**ENSG00000133321**	*0.04965819*	*0.052840466*	*0.043316179*	*0.047966254*
**ENSG00000184750**	*0.015909622*	*0.023972302*	*0.024187729*	*0.015332488*
**ENSG00000101844**	*0.031587019*	*0.037735685*	*0.032828283*	*0.029474566*
**ENSG00000068001**	*0.017544593*	*0.027196436*	*0.027310158*	*0.018467378*
**ENSG00000091317**	*0.037797262*	*0.045620852*	*0.039262204*	*0.045115295*
**ENSG00000025156**	*0.029484099*	*0.03301761*	*0.031891165*	*0.025003116*
**ENSG00000163012**	*0.01585571*	*0.025947977*	*0.025872593*	*0.01515838*
**ENSG00000155428**	*0.037601145*	*0.0375138*	*0.039316661*	*0.038959253*
**ENSG00000100077**	*0.03517225*	*0.035356474*	*0.038265407*	*0.031674506*
**ENSG00000113088**	*0.024625713*	*0.031938294*	*0.032080325*	*0.036759971*
**ENSG00000103061**	*0.022295066*	*0.028523411*	*0.029226572*	*0.022471951*
**ENSG00000205022**	*0.015609733*	*0.024202527*	*0.024194284*	*0.013579206*
**ENSG00000084072**	*0.049661641*	*0.044521475*	*0.037533834*	*0.040228898*
**ENSG00000215560**	*0.017388765*	*0.025919045*	*0.026112732*	*0.016378278*
**ENSG00000157343**	*0.023176705*	*0.027239115*	*0.026031287*	*0.017376315*
**ENSG00000142615**	*0.017212032*	*0.025501208*	*0.025554537*	*0.016166541*
**ENSG00000112852**	*0.017284764*	*0.025984343*	*0.024303378*	*0.01439481*
**ENSG00000186212**	*0.016646175*	*0.025157125*	*0.026394633*	*0.015654733*
**ENSG00000113889**	*0.019394071*	*0.025641046*	*0.025564292*	*0.017764186*
**ENSG00000181935**	*0.015632976*	*0.024860501*	*0.024322891*	*0.015135587*
**ENSG00000068078**	*0.025354756*	*0.027042335*	*0.026829047*	*0.019491393*
**ENSG00000151576**	*0.023105197*	*0.025770558*	*0.028750261*	*0.020333317*
**ENSG00000177303**	*0.017728201*	*0.027698731*	*0.025619075*	*0.018324141*
**ENSG00000110237**	*0.056885178*	*0.058586902*	*0.053254265*	*0.070514552*
**ENSG00000175711**	*0.022654122*	*0.030576847*	*0.030413899*	*0.022140619*
**ENSG00000158764**	*0.01632577*	*0.025652694*	*0.02416449*	*0.015920762*
**ENSG00000127870**	*0.017666349*	*0.026218513*	*0.025996507*	*0.017159085*
**ENSG00000205323**	*0.019221635*	*0.028137008*	*0.028226635*	*0.017112871*
**ENSG00000152291**	*0.020903202*	*0.027381382*	*0.030037198*	*0.024806555*
**ENSG00000175592**	*0.018151065*	*0.027036433*	*0.025425134*	*0.01862935*
**ENSG00000115145**	*0.037898253*	*0.037545473*	*0.032987054*	*0.042426949*
**ENSG00000166912**	*0.014072096*	*0.024406843*	*0.024862923*	*0.014767982*
**ENSG00000136560**	*0.054506437*	*0.045616284*	*0.055232829*	*0.055392434*
**ENSG00000164134**	*0.034566432*	*0.035559842*	*0.032121317*	*0.02982457*
**ENSG00000177646**	*0.03574871*	*0.035649562*	*0.032723928*	*0.029467933*
**ENSG00000174231**	*0.022261657*	*0.029936697*	*0.029707666*	*0.019922608*
**ENSG00000150045**	*0.051286497*	*0.045802677*	*0.035077457*	*0.039149114*
**ENSG00000073792**	*0.040107285*	*0.02717032*	*0.026162878*	*0.020453071*
**ENSG00000184956**	*0.018477607*	*0.025598261*	*0.026839325*	*0.015963384*
**ENSG00000033170**	*0.041591938*	*0.039625244*	*0.046444015*	*0.045924009*
**ENSG00000181315**	*0.030101204*	*0.037653612*	*0.034483952*	*0.03125591*
**ENSG00000163755**	*0.017548956*	*0.025738925*	*0.027608056*	*0.018172604*

**ENSG00000109762**	*0.032621987*	*0.042064724*	*0.032632711*	*0.034679448*
**ENSG00000198590**	*0.015983098*	*0.026646522*	*0.025284059*	*0.015750015*
**ENSG00000108960**	*0.038074862*	*0.041424*	*0.036057786*	*0.034857737*
**ENSG00000132434**	*0.055018571*	*0.045893534*	*0.053963917*	*0.056013806*
**ENSG00000136731**	*0.04624348*	*0.041038141*	*0.044809942*	*0.039170882*
**ENSG00000105549**	*0.016366019*	*0.024892546*	*0.02432072*	*0.016080927*
**ENSG00000148444**	*0.027808868*	*0.032084254*	*0.030499368*	*0.0218084*
**ENSG00000165480**	*0.045503967*	*0.04671728*	*0.036161234*	*0.036514534*
**ENSG00000175606**	*0.057164115*	*0.04905953*	*0.046028161*	*0.053868629*
**ENSG00000157954**	*0.015655696*	*0.025787245*	*0.02482755*	*0.015514472*
**ENSG00000162236**	*0.025698729*	*0.030500321*	*0.043619408*	*0.023878978*
**ENSG00000114796**	*0.040138593*	*0.043802918*	*0.046714807*	*0.042957055*
**ENSG00000113597**	*0.031046266*	*0.031152982*	*0.031678756*	*0.028056434*
**ENSG00000149970**	*0.015890216*	*0.025874195*	*0.025006281*	*0.016093365*
**ENSG00000127472**	*0.015524566*	*0.024934118*	*0.025653413*	*0.016224371*
**ENSG00000137757**	*0.021577739*	*0.027617626*	*0.033848103*	*0.018869155*
**ENSG00000136689**	*0.017644121*	*0.025598402*	*0.026325149*	*0.01755491*
**ENSG00000188486**	*0.04222295*	*0.040154823*	*0.04080108*	*0.035504115*
**ENSG00000073756**	*0.052883442*	*0.030768923*	*0.029908851*	*0.024294509*
**ENSG00000154589**	*0.033025335*	*0.040806641*	*0.034832213*	*0.02833643*
**ENSG00000152229**	*0.101227012*	*0.071140005*	*0.05828755*	*0.068098812*
**ENSG00000179837**	*0.033264856*	*0.0316088*	*0.031810268*	*0.029522428*
**ENSG00000165970**	*0.018948213*	*0.025333137*	*0.025577458*	*0.015483628*
**ENSG00000176463**	*0.017588478*	*0.025964512*	*0.02555551*	*0.018629224*
**ENSG00000185479**	*0.017131845*	*0.024645976*	*0.026864372*	*0.017165196*
**ENSG00000204316**	*0.025980336*	*0.026789777*	*0.030431733*	*0.027177258*
**ENSG00000143224**	*0.034409637*	*0.039909403*	*0.029996366*	*0.02655289*
**ENSG00000232119**	*0.025736966*	*0.029612128*	*0.028307398*	*0.018997253*
**ENSG00000175874**	*0.016717369*	*0.025185868*	*0.02469906*	*0.016879388*
**ENSG00000029364**	*0.048379446*	*0.051128192*	*0.042549833*	*0.031582823*
**ENSG00000198862**	*0.029194373*	*0.037334935*	*0.031540628*	*0.029201311*
**ENSG00000005075**	*0.027776985*	*0.036367629*	*0.03092659*	*0.023892187*
**ENSG00000158483**	*0.01751387*	*0.026581565*	*0.026617995*	*0.019713639*
**ENSG00000132600**	*0.032732347*	*0.033845903*	*0.035218764*	*0.027067317*
**ENSG00000214087**	*0.038448339*	*0.033489875*	*0.038862284*	*0.034703543*
**ENSG00000004059**	*0.024591947*	*0.032429551*	*0.030052064*	*0.025062703*
**ENSG00000162817**	*0.126407906*	*0.112239004*	*0.118611515*	*0.122101147*
**ENSG00000106305**	*0.032409387*	*0.033456598*	*0.032609585*	*0.033879541*
**ENSG00000039523**	*0.031668837*	*0.03809457*	*0.038895295*	*0.045128603*
**ENSG00000149925**	*0.026538437*	*0.035969121*	*0.030668676*	*0.029239882*
**ENSG00000137770**	*0.031616658*	*0.035855808*	*0.033327422*	*0.031963642*
**ENSG00000149187**	*0.036618742*	*0.041591745*	*0.03558688*	*0.024715984*
**ENSG00000183723**	*0.015475104*	*0.024565451*	*0.024856567*	*0.015420191*
**ENSG00000109472**	*0.015848351*	*0.024733732*	*0.025142137*	*0.015116476*
**ENSG00000185379**	*0.029589251*	*0.038635066*	*0.031392723*	*0.026023913*
**ENSG00000183690**	*0.017128227*	*0.027642203*	*0.026218731*	*0.032928965*
**ENSG00000130813**	*0.032375527*	*0.038669463*	*0.033497168*	*0.02818388*
**ENSG00000187753**	*0.018132859*	*0.025785297*	*0.029489772*	*0.018662059*
**ENSG00000169397**	*0.017130826*	*0.024938464*	*0.026105351*	*0.016890989*
**ENSG00000135702**	*0.014818513*	*0.024828493*	*0.025050348*	*0.015832172*
**ENSG00000087494**	*0.016273148*	*0.025738856*	*0.024527175*	*0.01537834*
**ENSG00000137936**	*0.093433893*	*0.085832383*	*0.094328284*	*0.114705023*
**ENSG00000168421**	*0.031955447*	*0.037435425*	*0.034249314*	*0.026462015*
**ENSG00000139726**	*0.023707914*	*0.032843918*	*0.029249609*	*0.025201755*

**ENSG00000147246**	*0.016074644*	*0.025498936*	*0.025420789*	*0.014899184*
**ENSG00000054148**	*0.023169024*	*0.028376627*	*0.028000485*	*0.021371241*
**ENSG00000157184**	*0.020984227*	*0.027472682*	*0.037164419*	*0.028239598*
**ENSG00000166130**	*0.015572697*	*0.025801711*	*0.024430605*	*0.015945704*
**ENSG00000005007**	*0.028421828*	*0.030880519*	*0.037406735*	*0.02725749*
**ENSG00000124493**	*0.015602005*	*0.026156611*	*0.025735533*	*0.016834477*
**ENSG00000121316**	*0.066900445*	*0.054302788*	*0.069269791*	*0.078775499*
**ENSG00000213625**	*0.035948797*	*0.034193958*	*0.030641975*	*0.026770436*
**ENSG00000072818**	*0.031709665*	*0.029369742*	*0.031548392*	*0.028181155*
**ENSG00000128487**	*0.043524488*	*0.037358283*	*0.037827499*	*0.032050997*
**ENSG00000117114**	*0.037570013*	*0.025810582*	*0.024993406*	*0.017246512*
**ENSG00000179588**	*0.029663402*	*0.030844401*	*0.03686632*	*0.026935973*
**ENSG00000155093**	*0.072404613*	*0.05921292*	*0.057656508*	*0.065362834*
**ENSG00000083642**	*0.035306306*	*0.035397406*	*0.033564044*	*0.028547641*
**ENSG00000177938**	*0.016161353*	*0.024907797*	*0.025111835*	*0.015265977*
**ENSG00000160584**	*0.037092016*	*0.036839541*	*0.037288865*	*0.038430726*
**ENSG00000135049**	*0.024857202*	*0.028863263*	*0.031931913*	*0.02636569*
**ENSG00000122034**	*0.028622931*	*0.032418009*	*0.029003872*	*0.024564953*
**ENSG00000137200**	*0.02144803*	*0.029194833*	*0.028764514*	*0.018789382*
**ENSG00000100427**	*0.017165013*	*0.024787973*	*0.02614523*	*0.015680931*
**ENSG00000189134**	*0.023221086*	*0.028489039*	*0.027502993*	*0.021015492*
**ENSG00000196503**	*0.017402906*	*0.030330584*	*0.028407888*	*0.015672786*
**ENSG00000051620**	*0.055681028*	*0.076513795*	*0.073454974*	*0.135468504*
**ENSG00000065615**	*0.031619934*	*0.038871206*	*0.033725708*	*0.028774986*
**ENSG00000161692**	*0.022760225*	*0.02730784*	*0.0280537*	*0.0194358*
**ENSG00000109771**	*0.015415899*	*0.024123646*	*0.025531607*	*0.015479885*
**ENSG00000162409**	*0.015057605*	*0.025342718*	*0.02568512*	*0.015029967*
**ENSG00000164252**	*0.032501451*	*0.044555044*	*0.030703715*	*0.027744962*
**ENSG00000174738**	*0.030599346*	*0.036345089*	*0.034966827*	*0.031010173*
**ENSG00000163535**	*0.021174977*	*0.030809381*	*0.026947132*	*0.017253494*
**ENSG00000104941**	*0.034458448*	*0.033520039*	*0.035833376*	*0.040541017*
**ENSG00000174871**	*0.040570403*	*0.038181218*	*0.039936058*	*0.033776954*
**ENSG00000159961**	*0.017644949*	*0.02592487*	*0.026290895*	*0.019858206*
**ENSG00000188517**	*0.018366307*	*0.026105616*	*0.025607937*	*0.017731245*
**ENSG00000213928**	*0.025186065*	*0.031218152*	*0.029936032*	*0.021084037*
**ENSG00000178038**	*0.017344972*	*0.025228147*	*0.026009435*	*0.016774641*
**ENSG00000137869**	*0.016241546*	*0.026280009*	*0.024899296*	*0.015456657*
**ENSG00000117597**	*0.027179171*	*0.030022012*	*0.028338375*	*0.020990881*
**ENSG00000157224**	*0.042859861*	*0.039777287*	*0.040334252*	*0.036015101*
**ENSG00000121351**	*0.016577221*	*0.025234214*	*0.02478572*	*0.01543501*
**ENSG00000197838**	*0.015246019*	*0.02477436*	*0.024990356*	*0.014977771*
**ENSG00000042493**	*0.070757062*	*0.061698002*	*0.055781541*	*0.070767448*
**ENSG00000134086**	*0.034300714*	*0.034936282*	*0.034120421*	*0.030050927*
**ENSG00000205678**	*0.015941284*	*0.026151695*	*0.026081607*	*0.016502432*
**ENSG00000099800**	*0.033787304*	*0.03954925*	*0.040387455*	*0.035619173*
**ENSG00000152348**	*0.023240684*	*0.028039626*	*0.027661143*	*0.024167602*
**ENSG00000137440**	*0.02147663*	*0.024828505*	*0.025266004*	*0.015143922*
**ENSG00000141580**	*0.032764427*	*0.037572437*	*0.035599979*	*0.034375632*
**ENSG00000111885**	*0.060018408*	*0.049823861*	*0.046153541*	*0.048247789*
**ENSG00000130363**	*0.032343317*	*0.039451511*	*0.033126643*	*0.026610015*
**ENSG00000206013**	*0.013477379*	*0.023768372*	*0.023495009*	*0.013146153*
**ENSG00000132142**	*0.017186946*	*0.026402678*	*0.025792545*	*0.017399239*
**ENSG00000112406**	*0.051018933*	*0.04722956*	*0.048578104*	*0.045293859*
**ENSG00000186868**	*0.016816635*	*0.025348041*	*0.026379277*	*0.015826731*

**ENSG00000102743**	*0.047452548*	*0.040429782*	*0.037583094*	*0.038027252*
**ENSG00000131469**	*0.015078922*	*0.024316674*	*0.024714438*	*0.015470162*
**ENSG00000138136**	*0.018875054*	*0.026017143*	*0.025558589*	*0.018444911*
**ENSG00000139343**	*0.023129609*	*0.027757173*	*0.02791482*	*0.018620348*
**ENSG00000152192**	*0.037117093*	*0.030987658*	*0.029446934*	*0.028801894*
**ENSG00000135597**	*0.030313851*	*0.032268451*	*0.03574117*	*0.029461317*
**ENSG00000067533**	*0.035107851*	*0.037678532*	*0.040445113*	*0.040900357*
**ENSG00000140688**	*0.028559344*	*0.032240615*	*0.031380301*	*0.025961486*
**ENSG00000163705**	*0.01656313*	*0.025047422*	*0.025845486*	*0.016788919*
**ENSG00000183580**	*0.019323955*	*0.025413804*	*0.024553164*	*0.015239725*
**ENSG00000158488**	*0.013371238*	*0.023792743*	*0.02401923*	*0.014275723*
**ENSG00000117481**	*0.032045119*	*0.030319941*	*0.030686652*	*0.027422752*
**ENSG00000168517**	*0.035115325*	*0.043020004*	*0.036759183*	*0.033178656*
**ENSG00000083828**	*0.027227532*	*0.034301967*	*0.035993481*	*0.023239663*
**ENSG00000126602**	*0.031384523*	*0.030388966*	*0.031838278*	*0.025983967*
**ENSG00000185499**	*0.020231056*	*0.030122604*	*0.030138731*	*0.02577398*
**ENSG00000170425**	*0.051809608*	*0.034983126*	*0.026953577*	*0.037765343*
**ENSG00000153132**	*0.072282541*	*0.037325675*	*0.026681713*	*0.023544066*
**ENSG00000120526**	*0.044390011*	*0.051534919*	*0.038076202*	*0.039982837*
**ENSG00000010932**	*0.014109828*	*0.025082037*	*0.024777361*	*0.014800333*
**ENSG00000092607**	*0.14169471*	*0.107919369*	*0.085438383*	*0.107393979*
**ENSG00000065060**	*0.016893303*	*0.025384997*	*0.025157826*	*0.016704576*
**ENSG00000102554**	*0.064286672*	*0.037899978*	*0.033924012*	*0.040372546*
**ENSG00000166261**	*0.01552373*	*0.02437804*	*0.024826473*	*0.01504438*
**ENSG00000205060**	*0.029797397*	*0.034846202*	*0.035246358*	*0.033058567*
**ENSG00000166479**	*0.039421779*	*0.040866104*	*0.034371894*	*0.025498652*
**ENSG00000149742**	*0.01988762*	*0.025699579*	*0.0262255*	*0.015672578*
**ENSG00000115446**	*0.026249557*	*0.032308051*	*0.032540049*	*0.022993865*
**ENSG00000101421**	*0.022852259*	*0.032218705*	*0.032474457*	*0.022983009*
**ENSG00000091542**	*0.042809003*	*0.033832012*	*0.03838289*	*0.034656034*
**ENSG00000127989**	*0.03836799*	*0.038916494*	*0.044059961*	*0.033330143*
**ENSG00000197506**	*0.015324941*	*0.024821741*	*0.025020334*	*0.0159598*
**ENSG00000183421**	*0.074207404*	*0.045626212*	*0.044226972*	*0.052965152*
**ENSG00000171989**	*0.017107253*	*0.024660756*	*0.023824658*	*0.015532186*
**ENSG00000157303**	*0.051872277*	*0.038496832*	*0.041058932*	*0.042401475*
**ENSG00000130684**	*0.032051914*	*0.036254118*	*0.035805759*	*0.035769293*
**ENSG00000187134**	*0.088295252*	*0.038215447*	*0.025218468*	*0.033464444*
**ENSG00000197019**	*0.037318825*	*0.042355637*	*0.055283111*	*0.032044665*
**ENSG00000134242**	*0.016329406*	*0.024708537*	*0.024957726*	*0.017564607*
**ENSG00000116560**	*0.035293039*	*0.040102269*	*0.041852565*	*0.042884968*
**ENSG00000142192**	*0.134567762*	*0.077814181*	*0.074680601*	*0.092732093*
**ENSG00000168476**	*0.038742632*	*0.030968*	*0.037568547*	*0.028111216*
**ENSG00000204624**	*0.023843675*	*0.024989136*	*0.024860304*	*0.019528351*
**ENSG00000068784**	*0.039794717*	*0.039921824*	*0.031100887*	*0.029146175*
**ENSG00000106868**	*0.049358127*	*0.045607299*	*0.054053463*	*0.051691401*
**ENSG00000147606**	*0.01934912*	*0.02500592*	*0.025619694*	*0.021555543*
**ENSG00000187838**	*0.033300907*	*0.036808889*	*0.032671984*	*0.029223169*
**ENSG00000141456**	*0.027841996*	*0.027863819*	*0.035121791*	*0.027598078*
**ENSG00000197928**	*0.017055532*	*0.02667467*	*0.026104167*	*0.016034621*
**ENSG00000159224**	*0.016035396*	*0.024766334*	*0.024521312*	*0.014627512*
**ENSG00000114200**	*0.120982889*	*0.089605868*	*0.029626565*	*0.02689986*
**ENSG00000196090**	*0.015678488*	*0.025241778*	*0.024472619*	*0.016786054*
**ENSG00000196502**	*0.016855006*	*0.026119974*	*0.025099656*	*0.022128401*
**ENSG00000147231**	*0.062643111*	*0.053053995*	*0.042183807*	*0.041515848*

**ENSG00000133874**	*0.043730618*	*0.038234858*	*0.043688919*	*0.036707387*
**ENSG00000185896**	*0.029418149*	*0.034564232*	*0.032128702*	*0.030080222*
**ENSG00000143776**	*0.026870303*	*0.029467948*	*0.02689546*	*0.022525174*
**ENSG00000146374**	*0.015503007*	*0.025369568*	*0.02481034*	*0.014913953*
**ENSG00000115750**	*0.042113644*	*0.039911275*	*0.040967714*	*0.036968067*
**ENSG00000143603**	*0.082203519*	*0.07658081*	*0.063747966*	*0.055969696*
**ENSG00000177479**	*0.028765491*	*0.032884386*	*0.033767997*	*0.027119344*
**ENSG00000119977**	*0.030554528*	*0.033972321*	*0.035970962*	*0.027203686*
**ENSG00000022556**	*0.171478017*	*0.145839275*	*0.142843609*	*0.135590104*
**ENSG00000184117**	*0.028371656*	*0.029720106*	*0.034391799*	*0.032845973*
**ENSG00000115363**	*0.01551645*	*0.025315475*	*0.024159679*	*0.015240679*
**ENSG00000100105**	*0.035476177*	*0.036467283*	*0.037713406*	*0.040816152*
**ENSG00000175984**	*0.016914081*	*0.025252063*	*0.02471537*	*0.015644605*
**ENSG00000186377**	*0.01625746*	*0.025226314*	*0.02487708*	*0.014719019*
**ENSG00000155827**	*0.034448823*	*0.037851961*	*0.032855815*	*0.025304975*
**ENSG00000111728**	*0.014649976*	*0.024399436*	*0.024633191*	*0.02681669*
**ENSG00000184672**	*0.015154557*	*0.024851162*	*0.025718841*	*0.013703952*
**ENSG00000244025**	*0.017865793*	*0.02648054*	*0.025623517*	*0.015076406*
**ENSG00000104325**	*0.02573888*	*0.031395934*	*0.029366781*	*0.019997662*
**ENSG00000158089**	*0.091417194*	*0.044016368*	*0.044931527*	*0.084721818*
**ENSG00000168434**	*0.018424612*	*0.027342561*	*0.02830848*	*0.01655756*
**ENSG00000114646**	*0.016130545*	*0.025327944*	*0.024293971*	*0.015729789*
**ENSG00000116016**	*0.016003968*	*0.02462852*	*0.024408478*	*0.014195242*
**ENSG00000144893**	*0.016348644*	*0.025081275*	*0.024516885*	*0.015987903*
**ENSG00000103599**	*0.016851331*	*0.025517109*	*0.024692535*	*0.015641693*
**ENSG00000175538**	*0.020010576*	*0.025753889*	*0.024127356*	*0.014650994*
**ENSG00000156304**	*0.027428881*	*0.032478801*	*0.035331853*	*0.028691401*
**ENSG00000173402**	*0.02578237*	*0.034472881*	*0.033885973*	*0.02500267*
**ENSG00000116903**	*0.03544268*	*0.038649788*	*0.039127737*	*0.038912768*
**ENSG00000198353**	*0.018416307*	*0.025544687*	*0.02560997*	*0.017365343*
**ENSG00000029725**	*0.040713663*	*0.030366325*	*0.032746872*	*0.028475261*
**ENSG00000114923**	*0.018248385*	*0.025759978*	*0.026689493*	*0.016873007*
**ENSG00000106538**	*0.135672322*	*0.097116431*	*0.0732806*	*0.094929344*
**ENSG00000145014**	*0.051290741*	*0.046802565*	*0.053296091*	*0.052768631*
**ENSG00000170893**	*0.015712264*	*0.025227216*	*0.025090408*	*0.015248005*
**ENSG00000137824**	*0.023269929*	*0.03018522*	*0.029639104*	*0.020712521*
**ENSG00000178096**	*0.038817294*	*0.040610249*	*0.03752214*	*0.030309304*
**ENSG00000104915**	*0.024602036*	*0.030032795*	*0.031384452*	*0.03181753*
**ENSG00000038274**	*0.029660246*	*0.034483374*	*0.038714915*	*0.026860663*
**ENSG00000102001**	*0.021722561*	*0.032528448*	*0.028996225*	*0.020163175*
**ENSG00000127377**	*0.01645105*	*0.024841819*	*0.02502151*	*0.016459643*
**ENSG00000176153**	*0.014797971*	*0.024651038*	*0.024756579*	*0.014317732*
**ENSG00000099949**	*0.026018796*	*0.037807761*	*0.031370456*	*0.029312913*
**ENSG00000111880**	*0.04854555*	*0.046256581*	*0.042280988*	*0.040817915*
**ENSG00000075461**	*0.017067819*	*0.024749755*	*0.025279597*	*0.01582189*
**ENSG00000054983**	*0.045346105*	*0.041059728*	*0.036179259*	*0.034379419*
**ENSG00000198356**	*0.026824533*	*0.032569742*	*0.029951493*	*0.027294798*
**ENSG00000198689**	*0.034843277*	*0.041245294*	*0.035904989*	*0.028851421*
**ENSG00000130844**	*0.043485539*	*0.063345634*	*0.051094084*	*0.031948598*
**ENSG00000104897**	*0.023409865*	*0.028510779*	*0.029471008*	*0.023002376*
**ENSG00000137875**	*0.074774997*	*0.05316748*	*0.048279925*	*0.052752767*
**ENSG00000183831**	*0.017990942*	*0.025413346*	*0.026877716*	*0.017563351*
**ENSG00000185352**	*0.014944334*	*0.024379556*	*0.025716738*	*0.015385414*
**ENSG00000109576**	*0.016697062*	*0.024293056*	*0.024418063*	*0.016081584*

**ENSG00000129824**	*0.368804182*	*0.384301412*	*0.368915922*	*0.340430059*
**ENSG00000169900**	*0.015143021*	*0.025427679*	*0.024957966*	*0.014574296*
**ENSG00000143556**	*0.020951905*	*0.028578342*	*0.027544856*	*0.019249874*
**ENSG00000183762**	*0.01753937*	*0.026127531*	*0.026521057*	*0.015974338*
**ENSG00000136536**	*0.035343288*	*0.045746504*	*0.03939003*	*0.04286444*
**ENSG00000176239**	*0.0165799*	*0.025125996*	*0.025905691*	*0.015536243*
**ENSG00000158050**	*0.051312922*	*0.037478883*	*0.039966071*	*0.042189709*
**ENSG00000186496**	*0.016836171*	*0.025282862*	*0.025372046*	*0.016866176*
**ENSG00000140396**	*0.020111055*	*0.027817196*	*0.027718137*	*0.01921957*
**ENSG00000169403**	*0.089234978*	*0.072241684*	*0.055301288*	*0.07141188*
**ENSG00000176095**	*0.025400233*	*0.033502318*	*0.03376588*	*0.026291233*
**ENSG00000131067**	*0.017215731*	*0.025822359*	*0.024958233*	*0.016854358*
**ENSG00000180098**	*0.02232647*	*0.028466202*	*0.026981229*	*0.017132016*
**ENSG00000229361**	*0.015991899*	*0.026433855*	*0.0248865*	*0.016056791*
**ENSG00000132768**	*0.031065747*	*0.032377056*	*0.031974089*	*0.033025098*
**ENSG00000198842**	*0.01683254*	*0.025079181*	*0.025052992*	*0.015593169*
**ENSG00000108039**	*0.02714617*	*0.030536683*	*0.030262642*	*0.020817574*
**ENSG00000198885**	*0.01945732*	*0.025183778*	*0.02860192*	*0.018613971*
**ENSG00000144120**	*0.025196985*	*0.028167612*	*0.027678954*	*0.021685041*
**ENSG00000110274**	*0.02832081*	*0.034600692*	*0.03026587*	*0.024839815*
**ENSG00000099942**	*0.023576469*	*0.030785107*	*0.030855391*	*0.019329612*
**ENSG00000158406**	*0.059614787*	*0.052248211*	*0.042448711*	*0.04516011*
**ENSG00000177432**	*0.032826381*	*0.033398521*	*0.034213563*	*0.033696891*
**ENSG00000169598**	*0.031307295*	*0.034275431*	*0.034200246*	*0.026375088*
**ENSG00000163214**	*0.015979067*	*0.025738029*	*0.02400864*	*0.015098257*
**ENSG00000143815**	*0.015278556*	*0.025161279*	*0.024297448*	*0.01568271*
**ENSG00000175216**	*0.02806412*	*0.035402264*	*0.02878568*	*0.022043995*
**ENSG00000131462**	*0.035977729*	*0.037306643*	*0.032233845*	*0.031839021*
**ENSG00000171403**	*0.015545443*	*0.024737264*	*0.025536484*	*0.015188597*
**ENSG00000135074**	*0.099470894*	*0.090578582*	*0.070692506*	*0.094987774*
**ENSG00000167272**	*0.036788307*	*0.036396629*	*0.035718464*	*0.032841458*
**ENSG00000140983**	*0.024493497*	*0.030503011*	*0.029556484*	*0.020199223*
**ENSG00000176407**	*0.021764396*	*0.028245129*	*0.026466015*	*0.017533454*
**ENSG00000109680**	*0.029367078*	*0.038090455*	*0.029291863*	*0.025244562*
**ENSG00000104299**	*0.02542154*	*0.033702805*	*0.033677148*	*0.030217583*
**ENSG00000140835**	*0.076117356*	*0.067857194*	*0.049916483*	*0.04914639*
**ENSG00000006194**	*0.029012119*	*0.041237106*	*0.044570042*	*0.024136306*
**ENSG00000019102**	*0.019440075*	*0.026941261*	*0.026301676*	*0.017266731*
**ENSG00000173660**	*0.027256706*	*0.027786927*	*0.027809817*	*0.020316333*
**ENSG00000105954**	*0.014320091*	*0.024554095*	*0.025463433*	*0.016212197*
**ENSG00000158014**	*0.015208723*	*0.025184967*	*0.025048919*	*0.01521277*
**ENSG00000130640**	*0.026726474*	*0.031091863*	*0.032379998*	*0.022343375*
**ENSG00000131697**	*0.043166084*	*0.04598499*	*0.041357021*	*0.031470479*
**ENSG00000147010**	*0.029288503*	*0.028339251*	*0.03170193*	*0.0233631*
**ENSG00000139133**	*0.026725599*	*0.033391624*	*0.031965652*	*0.022523657*
**ENSG00000158042**	*0.030018816*	*0.029430934*	*0.034445927*	*0.021677985*
**ENSG00000178773**	*0.089523556*	*0.058661559*	*0.047924713*	*0.059481502*
**ENSG00000160551**	*0.026674559*	*0.034211992*	*0.033656098*	*0.036287846*
**ENSG00000177917**	*0.04499026*	*0.043426003*	*0.035613184*	*0.030042618*
**ENSG00000135363**	*0.104379918*	*0.094099747*	*0.101564015*	*0.111392024*
**ENSG00000185087**	*0.017224064*	*0.025032414*	*0.025803151*	*0.018219786*
**ENSG00000187837**	*0.066367814*	*0.07313054*	*0.068866781*	*0.0642724*
**ENSG00000136750**	*0.020285125*	*0.025868532*	*0.027576773*	*0.019426568*
**ENSG00000174844**	*0.01470137*	*0.024758763*	*0.025295525*	*0.014971218*

**ENSG00000170145**	*0.016218168*	*0.025537024*	*0.024690884*	*0.01527739*
**ENSG00000079462**	*0.039260215*	*0.037041859*	*0.033701935*	*0.029492928*
**ENSG00000197408**	*0.016827172*	*0.025581093*	*0.024512522*	*0.018041164*
**ENSG00000159348**	*0.030471446*	*0.031337287*	*0.037285779*	*0.024032633*
**ENSG00000126947**	*0.099520655*	*0.058026857*	*0.058992266*	*0.054162502*
**ENSG00000120458**	*0.03182928*	*0.040462555*	*0.035212016*	*0.029820585*
**ENSG00000011132**	*0.027654394*	*0.028103934*	*0.033678383*	*0.030037126*
**ENSG00000109943**	*0.024223019*	*0.025225691*	*0.026346615*	*0.016483375*
**ENSG00000072133**	*0.022846311*	*0.027000913*	*0.026265409*	*0.019925646*
**ENSG00000111490**	*0.093158775*	*0.050692971*	*0.026838693*	*0.045845966*
**ENSG00000162591**	*0.067567242*	*0.058415122*	*0.059315753*	*0.060834492*
**ENSG00000134986**	*0.062251969*	*0.049254882*	*0.050113496*	*0.051982778*
**ENSG00000109832**	*0.018814945*	*0.025449317*	*0.024350572*	*0.016544676*
**ENSG00000166819**	*0.01488791*	*0.02533628*	*0.024042815*	*0.014763062*
**ENSG00000111536**	*0.020423438*	*0.0262282*	*0.027977404*	*0.017865983*
**ENSG00000023330**	*0.033471184*	*0.034849409*	*0.031861448*	*0.026374279*
**ENSG00000156096**	*0.015877783*	*0.024730743*	*0.026160375*	*0.01656492*
**ENSG00000130208**	*0.084542293*	*0.059039656*	*0.054854448*	*0.061985142*
**ENSG00000111783**	*0.024045514*	*0.028237193*	*0.028258485*	*0.027637019*
**ENSG00000159197**	*0.017611779*	*0.025609633*	*0.025444216*	*0.016559887*
**ENSG00000102100**	*0.015604529*	*0.025031586*	*0.025239404*	*0.016722759*
**ENSG00000118194**	*0.01583933*	*0.02542114*	*0.024723134*	*0.015298538*
**ENSG00000136715**	*0.028330085*	*0.035348665*	*0.03210648*	*0.021584903*
**ENSG00000158373**	*0.065578993*	*0.066402533*	*0.054722253*	*0.062831911*
**ENSG00000113249**	*0.016635196*	*0.027400066*	*0.026648481*	*0.016995364*
**ENSG00000142669**	*0.025565649*	*0.031948651*	*0.027720763*	*0.023599354*
**ENSG00000120820**	*0.016224719*	*0.024839255*	*0.025778888*	*0.014376783*
**ENSG00000172774**	*0.01534113*	*0.025466113*	*0.02469061*	*0.014742756*
**ENSG00000166881**	*0.019424149*	*0.027232471*	*0.026181081*	*0.017480545*
**ENSG00000196586**	*0.0905593*	*0.063647269*	*0.063793755*	*0.057420277*
**ENSG00000100271**	*0.031500803*	*0.030339069*	*0.031718313*	*0.028724926*
**ENSG00000173275**	*0.028292163*	*0.039280013*	*0.029565059*	*0.025718589*
**ENSG00000170743**	*0.018547091*	*0.026691958*	*0.027429556*	*0.018180919*
**ENSG00000070756**	*0.014976136*	*0.024331382*	*0.025127443*	*0.014698083*
**ENSG00000164171**	*0.017114839*	*0.025566173*	*0.02418892*	*0.015740794*
**ENSG00000003436**	*0.028053552*	*0.026645013*	*0.024819573*	*0.016110312*
**ENSG00000150995**	*0.04667243*	*0.042634048*	*0.036175816*	*0.040706867*
**ENSG00000175110**	*0.031183075*	*0.030255094*	*0.030488563*	*0.021782515*
**ENSG00000161682**	*0.017839225*	*0.024087126*	*0.024910966*	*0.015327457*
**ENSG00000214897**	*0.035324584*	*0.04121915*	*0.03309336*	*0.034782399*
**ENSG00000101079**	*0.034355464*	*0.038483823*	*0.034975899*	*0.031154719*
**ENSG00000182901**	*0.016273721*	*0.024756735*	*0.024210715*	*0.015575778*
**ENSG00000141446**	*0.027176783*	*0.032973514*	*0.032802427*	*0.027810556*
**ENSG00000130758**	*0.016875371*	*0.025012378*	*0.024846344*	*0.014765557*
**ENSG00000180425**	*0.040602911*	*0.03812812*	*0.031519273*	*0.026101453*
**ENSG00000131435**	*0.015694421*	*0.026239755*	*0.024476997*	*0.015207661*
**ENSG00000150594**	*0.017898258*	*0.026223364*	*0.024851126*	*0.015860997*
**ENSG00000136810**	*0.022447291*	*0.027433487*	*0.026294032*	*0.017841177*
**ENSG00000082014**	*0.016262908*	*0.024576573*	*0.025215748*	*0.015507281*
**ENSG00000139372**	*0.02507238*	*0.033504724*	*0.035480761*	*0.029337579*
**ENSG00000089163**	*0.030142059*	*0.037030669*	*0.035825485*	*0.025725476*
**ENSG00000087502**	*0.02972973*	*0.033082033*	*0.031839083*	*0.024143989*
**ENSG00000134438**	*0.017537175*	*0.027782431*	*0.025912937*	*0.016941459*
**ENSG00000198873**	*0.046900929*	*0.042254384*	*0.045259053*	*0.046914199*

**ENSG00000146039**	*0.015922867*	*0.025086259*	*0.024210638*	*0.014760235*
**ENSG00000153029**	*0.032672764*	*0.035911777*	*0.036480008*	*0.032124891*
**ENSG00000157881**	*0.025813616*	*0.03188177*	*0.031390276*	*0.022320736*
**ENSG00000239713**	*0.032959501*	*0.03408833*	*0.034006856*	*0.028095835*
**ENSG00000149485**	*0.043882866*	*0.037147564*	*0.032008623*	*0.033636436*
**ENSG00000103034**	*0.016312696*	*0.027053109*	*0.026059343*	*0.01622349*
**ENSG00000153046**	*0.0162478*	*0.024376284*	*0.026560728*	*0.015467776*
**ENSG00000076201**	*0.026292407*	*0.029105792*	*0.043649539*	*0.030866505*
**ENSG00000183891**	*0.040498603*	*0.051030684*	*0.049368072*	*0.035242687*
**ENSG00000160991**	*0.043213463*	*0.041008844*	*0.053555783*	*0.041894191*
**ENSG00000009694**	*0.01600888*	*0.025241916*	*0.025596228*	*0.015799815*
**ENSG00000197771**	*0.039005403*	*0.033274231*	*0.030610475*	*0.033958531*
**ENSG00000150977**	*0.034268714*	*0.034677464*	*0.034210977*	*0.033411243*
**ENSG00000168488**	*0.022986408*	*0.041613094*	*0.042552107*	*0.042776477*
**ENSG00000165819**	*0.03615418*	*0.036430598*	*0.031482229*	*0.023516667*
**ENSG00000132693**	*0.015995444*	*0.025540898*	*0.02552797*	*0.015602074*
**ENSG00000101413**	*0.01754737*	*0.024433247*	*0.025102407*	*0.015784107*
**ENSG00000162433**	*0.02312745*	*0.031766355*	*0.030990035*	*0.024925848*
**ENSG00000185811**	*0.068294395*	*0.068902077*	*0.089576286*	*0.071363582*
**ENSG00000185818**	*0.016069547*	*0.025316584*	*0.025915272*	*0.014570289*
**ENSG00000099998**	*0.017366292*	*0.026305188*	*0.027389799*	*0.016668142*
**ENSG00000178473**	*0.014847398*	*0.02509529*	*0.024987283*	*0.014741529*
**ENSG00000114124**	*0.015823936*	*0.025582987*	*0.024704331*	*0.01678269*
**ENSG00000076242**	*0.035891629*	*0.036667932*	*0.030028918*	*0.025594082*
**ENSG00000215114**	*0.019935167*	*0.027852255*	*0.027026262*	*0.018834701*
**ENSG00000178726**	*0.019865813*	*0.025189235*	*0.024941527*	*0.015572032*
**ENSG00000197444**	*0.07023563*	*0.04370421*	*0.048387271*	*0.033628697*
**ENSG00000214274**	*0.054174439*	*0.041565628*	*0.050588972*	*0.047964487*
**ENSG00000237190**	*0.017421371*	*0.026395673*	*0.024935597*	*0.018220587*
**ENSG00000165591**	*0.036102725*	*0.032887626*	*0.032227544*	*0.030155439*
**ENSG00000160868**	*0.030731507*	*0.030677107*	*0.032254673*	*0.028195058*
**ENSG00000213390**	*0.017980235*	*0.025934202*	*0.025115686*	*0.016489374*
**ENSG00000116750**	*0.028624238*	*0.034138515*	*0.028863407*	*0.027430819*
**ENSG00000085982**	*0.01601893*	*0.029851527*	*0.025310073*	*0.017030664*
**ENSG00000204564**	*0.02662035*	*0.031344945*	*0.028898983*	*0.026830792*
**ENSG00000127483**	*0.031584306*	*0.037827248*	*0.031219174*	*0.021789282*
**ENSG00000105996**	*0.027560629*	*0.031586724*	*0.040592435*	*0.022762303*
**ENSG00000189159**	*0.027208869*	*0.03064209*	*0.02878275*	*0.024739042*
**ENSG00000156876**	*0.036814151*	*0.034066258*	*0.033457218*	*0.037589306*
**ENSG00000137642**	*0.039273834*	*0.050560952*	*0.042858307*	*0.057002403*
**ENSG00000106992**	*0.043391297*	*0.040106502*	*0.039045622*	*0.051834086*
**ENSG00000232382**	*0.016143854*	*0.025868014*	*0.02428446*	*0.015393454*
**ENSG00000114978**	*0.035943975*	*0.042127746*	*0.036523065*	*0.052108028*
**ENSG00000170837**	*0.016544205*	*0.02558181*	*0.025271599*	*0.016828025*
**ENSG00000198477**	*0.067556043*	*0.050897984*	*0.054426723*	*0.048102337*
**ENSG00000149547**	*0.030695883*	*0.03205208*	*0.032214241*	*0.029642281*
**ENSG00000130479**	*0.02420887*	*0.035082023*	*0.044574246*	*0.026167066*
**ENSG00000205208**	*0.035375945*	*0.03883809*	*0.035448218*	*0.026094218*
**ENSG00000170412**	*0.10583769*	*0.066405646*	*0.058906339*	*0.075429992*
**ENSG00000177932**	*0.017042822*	*0.02711748*	*0.025884641*	*0.016722529*
**ENSG00000204388**	*0.067235338*	*0.040138727*	*0.050859649*	*0.052437886*
**ENSG00000166840**	*0.018097204*	*0.026201227*	*0.025220209*	*0.016810588*
**ENSG00000103449**	*0.016257612*	*0.026275524*	*0.025187138*	*0.017216133*
**ENSG00000144230**	*0.015695878*	*0.025290898*	*0.025103788*	*0.015558695*

**ENSG00000155961**	*0.038065649*	*0.036355786*	*0.032746404*	*0.038242621*
**ENSG00000005513**	*0.016882646*	*0.026419826*	*0.026327771*	*0.014474932*
**ENSG00000151914**	*0.030516611*	*0.033283868*	*0.033426825*	*0.032229302*
**ENSG00000116641**	*0.037318444*	*0.044278118*	*0.032905644*	*0.034063589*
**ENSG00000183671**	*0.029808062*	*0.031672934*	*0.031098998*	*0.029201643*
**ENSG00000197859**	*0.044553145*	*0.03470295*	*0.029669291*	*0.027810313*
**ENSG00000171659**	*0.076633249*	*0.039041161*	*0.031780259*	*0.043119664*
**ENSG00000171169**	*0.022058025*	*0.026428959*	*0.026897065*	*0.017966125*
**ENSG00000065911**	*0.029873618*	*0.03135657*	*0.028560875*	*0.02769211*
**ENSG00000115107**	*0.018139033*	*0.025369599*	*0.025275706*	*0.016917428*
**ENSG00000128039**	*0.032984801*	*0.033274819*	*0.03316112*	*0.038727184*
**ENSG00000197273**	*0.019386767*	*0.025618454*	*0.025003202*	*0.017181915*
**ENSG00000146047**	*0.014983043*	*0.02531766*	*0.025057334*	*0.015051905*
**ENSG00000165410**	*0.019146336*	*0.027043249*	*0.02496234*	*0.017068711*
**ENSG00000012223**	*0.017480837*	*0.024991132*	*0.025324862*	*0.026954628*
**ENSG00000083223**	*0.029046898*	*0.037599915*	*0.035716466*	*0.029294469*
**ENSG00000145936**	*0.086557254*	*0.103411122*	*0.090205509*	*0.098758933*
**ENSG00000169519**	*0.05192666*	*0.077164455*	*0.070311445*	*0.07209653*
**ENSG00000110851**	*0.032453617*	*0.043280099*	*0.041474887*	*0.029856834*
**ENSG00000116489**	*0.024736975*	*0.032042298*	*0.030360003*	*0.02930262*
**ENSG00000183354**	*0.028835507*	*0.034342133*	*0.035443333*	*0.026710728*
**ENSG00000085063**	*0.03240507*	*0.032259678*	*0.031670631*	*0.027359374*
**ENSG00000146285**	*0.01672057*	*0.025017221*	*0.024397953*	*0.016232011*
**ENSG00000196670**	*0.031407079*	*0.035196787*	*0.032308489*	*0.025810179*
**ENSG00000170946**	*0.031271602*	*0.036661516*	*0.031515722*	*0.026302102*
**ENSG00000138308**	*0.015641081*	*0.024918486*	*0.024777176*	*0.015843153*
**ENSG00000175106**	*0.018397955*	*0.0254816*	*0.026090418*	*0.017841856*
**ENSG00000127074**	*0.167543334*	*0.086822829*	*0.066400066*	*0.135156929*
**ENSG00000238023**	*0.017722099*	*0.025115611*	*0.024381269*	*0.016593818*
**ENSG00000075239**	*0.030956983*	*0.034513253*	*0.031298293*	*0.022603973*
**ENSG00000183632**	*0.028098252*	*0.056384083*	*0.04004436*	*0.031271284*
**ENSG00000177138**	*0.016175223*	*0.026976334*	*0.025009585*	*0.015050046*
**ENSG00000090920**	*0.046260281*	*0.056499951*	*0.054565163*	*0.048233319*
**ENSG00000147548**	*0.033481096*	*0.036408362*	*0.035546311*	*0.028592549*
**ENSG00000123358**	*0.01620504*	*0.026477753*	*0.025568909*	*0.016067256*
**ENSG00000100312**	*0.015914389*	*0.026385289*	*0.025517303*	*0.015215826*
**ENSG00000106554**	*0.02627771*	*0.030021929*	*0.028150953*	*0.024510332*
**ENSG00000084674**	*0.015237926*	*0.024214729*	*0.024863996*	*0.015041339*
**ENSG00000119689**	*0.023100674*	*0.031821335*	*0.036105284*	*0.02656267*
**ENSG00000146834**	*0.037475353*	*0.034268305*	*0.041823957*	*0.030839995*
**ENSG00000129991**	*0.017745748*	*0.024331582*	*0.025539713*	*0.019166335*
**ENSG00000163751**	*0.01687079*	*0.025511943*	*0.025387783*	*0.01564725*
**ENSG00000072778**	*0.025452714*	*0.030105133*	*0.028635912*	*0.02232063*
**ENSG00000144455**	*0.034708296*	*0.034249867*	*0.031855431*	*0.035885783*
**ENSG00000162722**	*0.027921411*	*0.029168262*	*0.030063443*	*0.024242378*
**ENSG00000158793**	*0.034148915*	*0.033755293*	*0.029924168*	*0.023820468*
**ENSG00000138131**	*0.042490376*	*0.036576976*	*0.034311561*	*0.030055728*
**ENSG00000144741**	*0.02368893*	*0.030426505*	*0.02958091*	*0.02305515*
**ENSG00000106331**	*0.014729258*	*0.025723006*	*0.024694816*	*0.014961879*
**ENSG00000165584**	*0.017180569*	*0.025814462*	*0.025370486*	*0.017981781*
**ENSG00000105732**	*0.027459775*	*0.030998212*	*0.03639603*	*0.030013691*
**ENSG00000168785**	*0.036541497*	*0.034862503*	*0.028699107*	*0.028851885*
**ENSG00000122691**	*0.015435834*	*0.024063864*	*0.024269828*	*0.014725779*
**ENSG00000137675**	*0.01460821*	*0.025888004*	*0.024487318*	*0.015811002*

**ENSG00000101928**	*0.026132938*	*0.033391844*	*0.033260452*	*0.021415999*
**ENSG00000124602**	*0.020498252*	*0.02662319*	*0.027257685*	*0.024082621*
**ENSG00000163694**	*0.038222491*	*0.035195578*	*0.03560638*	*0.033576021*
**ENSG00000231500**	*0.015249804*	*0.0238596*	*0.023789199*	*0.015581297*
**ENSG00000179133**	*0.01574662*	*0.025153395*	*0.024290295*	*0.015037806*
**ENSG00000125459**	*0.040149843*	*0.036472879*	*0.039386672*	*0.037132693*
**ENSG00000134815**	*0.033039683*	*0.038305853*	*0.029132639*	*0.026517582*
**ENSG00000168286**	*0.024694773*	*0.031258529*	*0.02805423*	*0.020158666*
**ENSG00000164113**	*0.015040074*	*0.025510284*	*0.024770633*	*0.014287592*
**ENSG00000134077**	*0.030092027*	*0.034380591*	*0.028657566*	*0.022915945*
**ENSG00000090316**	*0.025382448*	*0.032299751*	*0.030536082*	*0.027463925*
**ENSG00000169340**	*0.017012215*	*0.024875777*	*0.024412419*	*0.015585007*
**ENSG00000189409**	*0.05540893*	*0.060343224*	*0.060809666*	*0.04434409*
**ENSG00000169258**	*0.027900059*	*0.026067455*	*0.028259559*	*0.027385361*
**ENSG00000072071**	*0.017911106*	*0.025560475*	*0.026410023*	*0.017821339*
**ENSG00000188676**	*0.019947253*	*0.027530683*	*0.025819827*	*0.023515647*
**ENSG00000116679**	*0.056129567*	*0.04627864*	*0.038738246*	*0.03988234*
**ENSG00000163220**	*0.031099665*	*0.025602143*	*0.027099547*	*0.030585973*
**ENSG00000187094**	*0.03739009*	*0.040977503*	*0.025814859*	*0.017344762*
**ENSG00000160505**	*0.080286526*	*0.050573821*	*0.075220184*	*0.080280565*
**ENSG00000186143**	*0.015984982*	*0.024160275*	*0.024804254*	*0.015043487*
**ENSG00000143382**	*0.017795787*	*0.02580404*	*0.025878131*	*0.016008622*
**ENSG00000103528**	*0.058421554*	*0.04970631*	*0.044954194*	*0.04643822*
**ENSG00000214946**	*0.016646884*	*0.024092588*	*0.025279088*	*0.014761911*
**ENSG00000049249**	*0.063448193*	*0.055837487*	*0.046817552*	*0.058608486*
**ENSG00000146587**	*0.022638634*	*0.02807008*	*0.028567695*	*0.024494827*
**ENSG00000144233**	*0.031276106*	*0.041567444*	*0.038087602*	*0.026870021*
**ENSG00000004777**	*0.023179619*	*0.02851574*	*0.027766223*	*0.022461584*
**ENSG00000057149**	*0.015420564*	*0.024984317*	*0.025190776*	*0.014997665*
**ENSG00000126267**	*0.018279117*	*0.025459397*	*0.024924747*	*0.015186694*
**ENSG00000163884**	*0.016291105*	*0.024706605*	*0.024766969*	*0.015294826*
**ENSG00000163072**	*0.015436374*	*0.024803465*	*0.025832214*	*0.015460476*
**ENSG00000120899**	*0.03777326*	*0.034096763*	*0.035086042*	*0.029611716*
**ENSG00000124313**	*0.018015369*	*0.026576838*	*0.02547686*	*0.018729479*
**ENSG00000123569**	*0.016392*	*0.024972447*	*0.024485975*	*0.015247421*
**ENSG00000067596**	*0.020802109*	*0.029490044*	*0.031873752*	*0.023123225*
**ENSG00000163945**	*0.028361339*	*0.038961221*	*0.032714543*	*0.023333035*
**ENSG00000080802**	*0.026106658*	*0.032125435*	*0.03125865*	*0.023007131*
**ENSG00000103351**	*0.030156281*	*0.028211929*	*0.027072599*	*0.020643865*
**ENSG00000182880**	*0.016899545*	*0.025769189*	*0.024379909*	*0.015679045*
**ENSG00000175073**	*0.033628542*	*0.0407002*	*0.033063117*	*0.030815131*
**ENSG00000144354**	*0.055541873*	*0.054591987*	*0.048789382*	*0.054496675*
**ENSG00000147655**	*0.016342196*	*0.025561789*	*0.025486861*	*0.015340841*
**ENSG00000138759**	*0.018248701*	*0.025274262*	*0.026098405*	*0.017161924*
**ENSG00000181472**	*0.032676223*	*0.035050667*	*0.039361642*	*0.026980961*
**ENSG00000172889**	*0.033026343*	*0.032993705*	*0.030687969*	*0.030491748*
**ENSG00000157429**	*0.016250344*	*0.026720419*	*0.025367105*	*0.017121129*
**ENSG00000164683**	*0.104301353*	*0.086048319*	*0.095320901*	*0.104783007*
**ENSG00000180104**	*0.024609758*	*0.02862215*	*0.029937257*	*0.020987955*
**ENSG00000134709**	*0.083391472*	*0.062312513*	*0.057235645*	*0.070830018*
**ENSG00000144834**	*0.022620547*	*0.033651971*	*0.026476412*	*0.019002955*
**ENSG00000101109**	*0.021557328*	*0.026551841*	*0.029348376*	*0.020651219*
**ENSG00000117407**	*0.018258043*	*0.024454789*	*0.026838711*	*0.015922906*
**ENSG00000164983**	*0.068195323*	*0.06225246*	*0.048429025*	*0.053018724*

**ENSG00000153933**	*0.023596695*	*0.031966664*	*0.02751954*	*0.023159582*
**ENSG00000205572**	*0.024011983*	*0.027664352*	*0.029881193*	*0.02159379*
**ENSG00000100478**	*0.01511918*	*0.024024539*	*0.024886305*	*0.014517219*
**ENSG00000161914**	*0.02951972*	*0.036369038*	*0.034700006*	*0.026171384*
**ENSG00000141316**	*0.039434164*	*0.029446013*	*0.026337618*	*0.028018058*
**ENSG00000162521**	*0.025976214*	*0.033645248*	*0.030232161*	*0.02425743*
**ENSG00000183049**	*0.025249437*	*0.03005613*	*0.032107743*	*0.024206895*
**ENSG00000164128**	*0.018075082*	*0.025452262*	*0.024720899*	*0.015687401*
**ENSG00000104371**	*0.034386786*	*0.031589232*	*0.027522114*	*0.026080694*
**ENSG00000140526**	*0.021076359*	*0.028826777*	*0.026569253*	*0.019177324*
**ENSG00000169856**	*0.016518861*	*0.024974401*	*0.025564284*	*0.016109723*
**ENSG00000109193**	*0.017604698*	*0.026490251*	*0.024894619*	*0.015559978*
**ENSG00000119574**	*0.028725926*	*0.032835702*	*0.036471229*	*0.029301333*
**ENSG00000185532**	*0.017494503*	*0.027051305*	*0.026023216*	*0.016965573*
**ENSG00000176101**	*0.021578751*	*0.02764653*	*0.026844945*	*0.022570199*
**ENSG00000184108**	*0.015913239*	*0.025282432*	*0.025922701*	*0.016634546*
**ENSG00000124702**	*0.029932143*	*0.035744535*	*0.029587352*	*0.026363553*
**ENSG00000125968**	*0.020012125*	*0.026141165*	*0.027022174*	*0.019177502*
**ENSG00000099260**	*0.015215605*	*0.025107264*	*0.025610807*	*0.014496639*
**ENSG00000120210**	*0.015715664*	*0.025857591*	*0.024895692*	*0.015347732*
**ENSG00000183853**	*0.020524337*	*0.026403186*	*0.026787083*	*0.021357074*
**ENSG00000175573**	*0.02830433*	*0.030416655*	*0.030189238*	*0.027002557*
**ENSG00000165061**	*0.015505447*	*0.025596816*	*0.025647535*	*0.014723121*
**ENSG00000204022**	*0.018455699*	*0.025408081*	*0.025781305*	*0.018035075*
**ENSG00000066379**	*0.025352682*	*0.032672308*	*0.036331509*	*0.029262943*
**ENSG00000184349**	*0.018513787*	*0.026161487*	*0.024928869*	*0.015768665*
**ENSG00000189043**	*0.017380952*	*0.025690491*	*0.02486755*	*0.018008091*
**ENSG00000243137**	*0.0153471*	*0.025819606*	*0.024756614*	*0.016060735*
**ENSG00000166803**	*0.043242269*	*0.036681913*	*0.030262736*	*0.026507493*
**ENSG00000167889**	*0.016069113*	*0.026316159*	*0.024506658*	*0.016636493*
**ENSG00000114315**	*0.040641876*	*0.033629495*	*0.034921863*	*0.039599713*
**ENSG00000009844**	*0.026996999*	*0.034613126*	*0.031791304*	*0.028574443*
**ENSG00000181322**	*0.016211994*	*0.024669122*	*0.024592337*	*0.014745531*
**ENSG00000136327**	*0.016050551*	*0.025694946*	*0.025497736*	*0.015399626*
**ENSG00000013563**	*0.039037073*	*0.033730039*	*0.034220593*	*0.03195059*
**ENSG00000125304**	*0.026268195*	*0.033735489*	*0.031784474*	*0.026565805*
**ENSG00000166262**	*0.017199509*	*0.026254068*	*0.025224654*	*0.016572937*
**ENSG00000153951**	*0.017222804*	*0.02539582*	*0.025321091*	*0.014502892*
**ENSG00000126934**	*0.024627305*	*0.026931998*	*0.027389111*	*0.025690528*
**ENSG00000130300**	*0.026080756*	*0.036854118*	*0.028424619*	*0.029393418*
**ENSG00000115008**	*0.162379424*	*0.11551867*	*0.09411282*	*0.113112351*
**ENSG00000181773**	*0.027761475*	*0.028830949*	*0.027393368*	*0.020354404*
**ENSG00000123427**	*0.051396652*	*0.041232859*	*0.044445355*	*0.040029196*
**ENSG00000132958**	*0.021136697*	*0.025518147*	*0.026268813*	*0.019015123*
**ENSG00000104142**	*0.023973111*	*0.044106707*	*0.045552825*	*0.031932921*
**ENSG00000147536**	*0.046934927*	*0.045745227*	*0.035027638*	*0.036719236*
**ENSG00000176884**	*0.077524211*	*0.065165002*	*0.057350759*	*0.076766071*
**ENSG00000138336**	*0.016963382*	*0.024532083*	*0.025337574*	*0.015409749*
**ENSG00000034053**	*0.015544061*	*0.025509518*	*0.024460821*	*0.015582733*
**ENSG00000118495**	*0.059066312*	*0.041838766*	*0.042886425*	*0.06381995*
**ENSG00000122642**	*0.024464011*	*0.034816051*	*0.030130649*	*0.027850976*
**ENSG00000159111**	*0.028137941*	*0.033889253*	*0.032723465*	*0.022356992*
**ENSG00000176020**	*0.023247283*	*0.027513119*	*0.028514162*	*0.01705136*
**ENSG00000143839**	*0.017664558*	*0.026253893*	*0.024120824*	*0.015835647*

**ENSG00000008952**	*0.028274624*	*0.037391369*	*0.037148588*	*0.0271561*
**ENSG00000164167**	*0.026222341*	*0.029100024*	*0.029157842*	*0.025228884*
**ENSG00000187010**	*0.015265824*	*0.024838692*	*0.025366596*	*0.015285755*
**ENSG00000168874**	*0.02661134*	*0.026000909*	*0.024389781*	*0.020778628*
**ENSG00000142512**	*0.016845257*	*0.027434894*	*0.02444711*	*0.017418977*
**ENSG00000197540**	*0.016446811*	*0.026350574*	*0.026236452*	*0.02582009*
**ENSG00000116985**	*0.028214946*	*0.030113864*	*0.029548423*	*0.020430379*
**ENSG00000186136**	*0.015775648*	*0.024831689*	*0.025018447*	*0.017301073*
**ENSG00000067842**	*0.015241442*	*0.024919381*	*0.024753636*	*0.014866603*
**ENSG00000127022**	*0.021246709*	*0.027696387*	*0.026242048*	*0.020276964*
**ENSG00000186815**	*0.029173723*	*0.029918402*	*0.031759899*	*0.026190557*
**ENSG00000095066**	*0.021765511*	*0.028265135*	*0.026509393*	*0.02499106*
**ENSG00000180340**	*0.042402398*	*0.044402334*	*0.037411958*	*0.047098277*
**ENSG00000130935**	*0.028692535*	*0.032181022*	*0.030206409*	*0.028298497*
**ENSG00000075340**	*0.015955444*	*0.025525418*	*0.026733368*	*0.016669227*
**ENSG00000152953**	*0.025843825*	*0.027986374*	*0.025313323*	*0.030749697*
**ENSG00000112038**	*0.017434439*	*0.024603039*	*0.026312519*	*0.016335185*
**ENSG00000130307**	*0.016209365*	*0.025858349*	*0.025537201*	*0.016563969*
**ENSG00000177868**	*0.033620268*	*0.033668459*	*0.034775931*	*0.027887583*
**ENSG00000140743**	*0.035084304*	*0.040821118*	*0.041202475*	*0.031329253*
**ENSG00000113296**	*0.016522325*	*0.025412925*	*0.025354249*	*0.014787658*
**ENSG00000184347**	*0.015465252*	*0.025351682*	*0.024229626*	*0.014534325*
**ENSG00000161328**	*0.030872295*	*0.032396229*	*0.033502687*	*0.024622893*
**ENSG00000162779**	*0.016376369*	*0.025024862*	*0.024133526*	*0.017248356*
**ENSG00000140284**	*0.04115485*	*0.039215206*	*0.038772828*	*0.032802244*
**ENSG00000168140**	*0.021554869*	*0.030563764*	*0.027745139*	*0.024097081*
**ENSG00000175264**	*0.016161086*	*0.02533498*	*0.026426618*	*0.014656919*
**ENSG00000240386**	*0.066937828*	*0.036796013*	*0.030789203*	*0.041723983*
**ENSG00000144021**	*0.025995288*	*0.029953078*	*0.030334942*	*0.019559556*
**ENSG00000182508**	*0.021494727*	*0.029994892*	*0.028262513*	*0.02126922*
**ENSG00000091409**	*0.016081111*	*0.024855411*	*0.02534083*	*0.015400404*
**ENSG00000117525**	*0.016924248*	*0.02549516*	*0.025679925*	*0.016584645*
**ENSG00000182253**	*0.06666748*	*0.075045164*	*0.067368691*	*0.060514461*
**ENSG00000145247**	*0.041593364*	*0.03997925*	*0.036837288*	*0.040431903*
**ENSG00000067646**	*0.070227235*	*0.065465019*	*0.062442952*	*0.052315394*
**ENSG00000176782**	*0.016128886*	*0.026183246*	*0.026308951*	*0.016188546*
**ENSG00000185774**	*0.015356052*	*0.024645824*	*0.025558058*	*0.015381301*
**ENSG00000141098**	*0.032242528*	*0.035728957*	*0.033037794*	*0.035018257*
**ENSG00000111424**	*0.085915616*	*0.069423929*	*0.071987099*	*0.085724437*
**ENSG00000120334**	*0.058119425*	*0.078389139*	*0.071156943*	*0.050766578*
**ENSG00000128886**	*0.05771737*	*0.061434678*	*0.061636082*	*0.059163175*
**ENSG00000147316**	*0.024915701*	*0.032113969*	*0.030956668*	*0.030820909*
**ENSG00000103742**	*0.042239854*	*0.038366587*	*0.028883008*	*0.028033806*
**ENSG00000157087**	*0.01793203*	*0.02468593*	*0.025900327*	*0.016304732*
**ENSG00000134324**	*0.048018201*	*0.051882941*	*0.050063255*	*0.050219487*
**ENSG00000138363**	*0.027549214*	*0.030549416*	*0.029088398*	*0.02548412*
**ENSG00000120215**	*0.015082028*	*0.025477897*	*0.02568971*	*0.015435114*
**ENSG00000167447**	*0.024283323*	*0.030518844*	*0.031902795*	*0.020283492*
**ENSG00000135218**	*0.054082955*	*0.025621242*	*0.024371168*	*0.016214151*
**ENSG00000120645**	*0.016462877*	*0.024647785*	*0.025132762*	*0.015690185*
**ENSG00000159164**	*0.044851868*	*0.041620493*	*0.037498452*	*0.041745123*
**ENSG00000164209**	*0.025599069*	*0.032429268*	*0.036802515*	*0.031763571*
**ENSG00000152931**	*0.015918805*	*0.026052347*	*0.025129444*	*0.015037416*
**ENSG00000173200**	*0.049122636*	*0.057117047*	*0.05049084*	*0.048293556*

**ENSG00000170549**	*0.016952863*	*0.025180271*	*0.025064473*	*0.015150236*
**ENSG00000106723**	*0.031384737*	*0.032555496*	*0.035171872*	*0.032278955*
**ENSG00000099899**	*0.017616381*	*0.026603671*	*0.026784589*	*0.016287285*
**ENSG00000155903**	*0.038040725*	*0.035653283*	*0.040340441*	*0.03513864*
**ENSG00000073711**	*0.01667942*	*0.025607348*	*0.026943679*	*0.015516242*
**ENSG00000115194**	*0.015517199*	*0.025618459*	*0.02587141*	*0.016423647*
**ENSG00000136695**	*0.015945878*	*0.026387529*	*0.024947001*	*0.015767143*
**ENSG00000176971**	*0.01625593*	*0.025435531*	*0.025522012*	*0.014604051*
**ENSG00000132530**	*0.072970304*	*0.059922513*	*0.060311144*	*0.074089098*
**ENSG00000187650**	*0.013076892*	*0.023329564*	*0.023787117*	*0.013394331*
**ENSG00000125970**	*0.022621652*	*0.026029321*	*0.030747652*	*0.022057843*
**ENSG00000075826**	*0.01761787*	*0.025823149*	*0.027484512*	*0.017800215*
**ENSG00000196456**	*0.037168466*	*0.043221692*	*0.038028314*	*0.037781056*
**ENSG00000141294**	*0.023091686*	*0.027602873*	*0.027641384*	*0.020858858*
**ENSG00000197584**	*0.039345829*	*0.029427763*	*0.030544802*	*0.022093424*
**ENSG00000149089**	*0.037325131*	*0.045992567*	*0.039693528*	*0.030055432*
**ENSG00000178445**	*0.087052074*	*0.075535738*	*0.05912588*	*0.057106981*
**ENSG00000156574**	*0.016467632*	*0.02533991*	*0.02543163*	*0.014698666*
**ENSG00000165572**	*0.022702524*	*0.026894656*	*0.028928219*	*0.019001604*
**ENSG00000204246**	*0.015635701*	*0.024370652*	*0.025360882*	*0.014650682*
**ENSG00000145675**	*0.021328036*	*0.025636038*	*0.024720342*	*0.02026979*
**ENSG00000070761**	*0.032921221*	*0.033811898*	*0.035147057*	*0.032007085*
**ENSG00000110218**	*0.033190057*	*0.039427213*	*0.042906708*	*0.031182457*
**ENSG00000196418**	*0.038458848*	*0.044804849*	*0.053208966*	*0.041999589*
**ENSG00000152382**	*0.030727302*	*0.036057287*	*0.030127139*	*0.027664521*
**ENSG00000157093**	*0.01630103*	*0.025585558*	*0.025569033*	*0.015336561*
**ENSG00000135406**	*0.024023414*	*0.026384527*	*0.027321352*	*0.01664132*
**ENSG00000233816**	*0.016689083*	*0.025444079*	*0.025947153*	*0.019038118*
**ENSG00000104888**	*0.016402476*	*0.025383107*	*0.025113525*	*0.016337288*
**ENSG00000166924**	*0.017929976*	*0.024971102*	*0.025247643*	*0.017460799*
**ENSG00000126337**	*0.016369274*	*0.024668666*	*0.024482236*	*0.014991951*
**ENSG00000160094**	*0.062183123*	*0.060345907*	*0.053453634*	*0.0643956*
**ENSG00000181092**	*0.015377516*	*0.025608132*	*0.025514942*	*0.0161869*
**ENSG00000123983**	*0.049098471*	*0.042465911*	*0.036521616*	*0.034462482*
**ENSG00000164902**	*0.027880029*	*0.030876554*	*0.032746184*	*0.028453337*
**ENSG00000172663**	*0.029216605*	*0.031609968*	*0.035204425*	*0.03066879*
**ENSG00000168792**	*0.045116165*	*0.045449877*	*0.037927737*	*0.038648832*
**ENSG00000177354**	*0.015553724*	*0.025346498*	*0.024752368*	*0.014664613*
**ENSG00000103064**	*0.033379096*	*0.034754947*	*0.036060335*	*0.031129536*
**ENSG00000198933**	*0.025797655*	*0.031890595*	*0.031399662*	*0.035258121*
**ENSG00000126821**	*0.052361033*	*0.055113843*	*0.051193193*	*0.06108286*
**ENSG00000171135**	*0.027048082*	*0.042779495*	*0.042398613*	*0.030057346*
**ENSG00000155636**	*0.034854394*	*0.039379833*	*0.035659835*	*0.033422217*
**ENSG00000174740**	*0.017257138*	*0.025551747*	*0.025308544*	*0.015754702*
**ENSG00000142002**	*0.023877703*	*0.031251708*	*0.032450663*	*0.027359244*
**ENSG00000125879**	*0.015373629*	*0.024548444*	*0.024300959*	*0.015038959*
**ENSG00000221882**	*0.021299036*	*0.024373401*	*0.025876183*	*0.016034423*
**ENSG00000164244**	*0.026190711*	*0.035721657*	*0.033734233*	*0.029290228*
**ENSG00000163623**	*0.016902908*	*0.026389292*	*0.027054554*	*0.017257428*
**ENSG00000168263**	*0.018761002*	*0.026154451*	*0.025997231*	*0.01651485*
**ENSG00000130818**	*0.03054145*	*0.039529213*	*0.039621687*	*0.027055068*
**ENSG00000163659**	*0.041317435*	*0.040289107*	*0.034527992*	*0.032181666*
**ENSG00000170786**	*0.015550851*	*0.024741118*	*0.025027765*	*0.015210132*
**ENSG00000181610**	*0.022782642*	*0.028643171*	*0.02744901*	*0.02105902*

**ENSG00000147121**	*0.024900396*	*0.031720356*	*0.02820607*	*0.023697508*
**ENSG00000092140**	*0.025560909*	*0.027883061*	*0.029110582*	*0.021733927*
**ENSG00000183486**	*0.044739289*	*0.044349617*	*0.047811946*	*0.059667233*
**ENSG00000142197**	*0.03485508*	*0.039531263*	*0.037161236*	*0.031722512*
**ENSG00000108953**	*0.022362032*	*0.027496541*	*0.033567597*	*0.023994334*
**ENSG00000139970**	*0.017738414*	*0.025538608*	*0.025402686*	*0.015392084*
**ENSG00000168314**	*0.015749001*	*0.024718937*	*0.024914982*	*0.014771633*
**ENSG00000180228**	*0.026697065*	*0.030408905*	*0.034339996*	*0.03042511*
**ENSG00000183718**	*0.015640804*	*0.025280131*	*0.02543117*	*0.016734138*
**ENSG00000147099**	*0.025400024*	*0.033584418*	*0.029551268*	*0.0293339*
**ENSG00000123737**	*0.028821335*	*0.032648122*	*0.033257049*	*0.02535332*
**ENSG00000171720**	*0.026884038*	*0.029243325*	*0.028567465*	*0.022178662*
**ENSG00000165650**	*0.04290875*	*0.040530951*	*0.040591743*	*0.033463907*
**ENSG00000186019**	*0.016646672*	*0.025459409*	*0.025325983*	*0.01712135*
**ENSG00000122550**	*0.036418096*	*0.045227918*	*0.035318564*	*0.035236334*
**ENSG00000157514**	*0.01945941*	*0.027967563*	*0.025990674*	*0.021645424*
**ENSG00000160326**	*0.039262013*	*0.038644722*	*0.036994975*	*0.048343609*
**ENSG00000188987**	*0.059006871*	*0.042055183*	*0.035380839*	*0.056872812*
**ENSG00000164303**	*0.02251827*	*0.030968544*	*0.026182856*	*0.026768141*
**ENSG00000004468**	*0.124077986*	*0.074646816*	*0.07263905*	*0.07652104*
**ENSG00000067141**	*0.022510594*	*0.035540788*	*0.025632463*	*0.020159965*
**ENSG00000180745**	*0.049365287*	*0.025749271*	*0.024636534*	*0.014953467*
**ENSG00000160072**	*0.028972992*	*0.027801954*	*0.030089034*	*0.026147073*
**ENSG00000099377**	*0.022659959*	*0.028322587*	*0.026159736*	*0.022294473*
**ENSG00000100813**	*0.023868989*	*0.030648275*	*0.03424216*	*0.023693771*
**ENSG00000004142**	*0.028668317*	*0.034238073*	*0.031619956*	*0.027180252*
**ENSG00000203880**	*0.038247568*	*0.041865917*	*0.036042926*	*0.027109591*
**ENSG00000214193**	*0.016508982*	*0.02555815*	*0.025901809*	*0.016870966*
**ENSG00000166801**	*0.031651276*	*0.036323844*	*0.03370765*	*0.027132241*
**ENSG00000132000**	*0.017145409*	*0.024913389*	*0.025066491*	*0.016149205*
**ENSG00000047662**	*0.016331651*	*0.025374243*	*0.024664172*	*0.016418942*
**ENSG00000163399**	*0.028099856*	*0.031105077*	*0.028982394*	*0.025872751*
**ENSG00000102401**	*0.01500451*	*0.025775985*	*0.024694159*	*0.015466504*
**ENSG00000178809**	*0.050211237*	*0.040618035*	*0.037302617*	*0.039260504*
**ENSG00000116251**	*0.022337163*	*0.030562225*	*0.027439639*	*0.021507294*
**ENSG00000243910**	*0.020381338*	*0.024858849*	*0.024533727*	*0.016107683*
**ENSG00000197919**	*0.017683862*	*0.029205675*	*0.025596813*	*0.018935549*
**ENSG00000138380**	*0.018747304*	*0.029722405*	*0.027161869*	*0.01707064*
**ENSG00000164088**	*0.040443622*	*0.033781282*	*0.034846737*	*0.033032718*
**ENSG00000165669**	*0.020128007*	*0.027999015*	*0.027067535*	*0.017725877*
**ENSG00000135333**	*0.017651437*	*0.02501656*	*0.024591038*	*0.016697014*
**ENSG00000122859**	*0.016285023*	*0.026352778*	*0.026290943*	*0.01579866*
**ENSG00000100207**	*0.018155045*	*0.025398811*	*0.027622167*	*0.015283659*
**ENSG00000144306**	*0.01834383*	*0.026288153*	*0.025291203*	*0.017759809*
**ENSG00000242019**	*0.018495943*	*0.027629921*	*0.026690692*	*0.017010574*
**ENSG00000157005**	*0.017033162*	*0.024216432*	*0.025420683*	*0.01578743*
**ENSG00000130348**	*0.04219094*	*0.039850041*	*0.034309581*	*0.028071787*
**ENSG00000251322**	*0.016542058*	*0.025250713*	*0.025423635*	*0.015516679*
**ENSG00000121594**	*0.047749728*	*0.042857626*	*0.039791725*	*0.047095664*
**ENSG00000187079**	*0.016481943*	*0.024939954*	*0.024774714*	*0.017072008*
**ENSG00000019144**	*0.015495194*	*0.025849941*	*0.025073685*	*0.016486472*
**ENSG00000250510**	*0.016238053*	*0.026008668*	*0.025796441*	*0.015354027*
**ENSG00000154760**	*0.019410516*	*0.02594928*	*0.02657898*	*0.018093647*
**ENSG00000181220**	*0.023665032*	*0.037966356*	*0.040366459*	*0.026978383*

**ENSG00000139687**	*0.07526374*	*0.085538503*	*0.06526947*	*0.064935821*
**ENSG00000052723**	*0.020028765*	*0.028763788*	*0.02797129*	*0.018110516*
**ENSG00000169067**	*0.022836261*	*0.032704388*	*0.028858458*	*0.027475539*
**ENSG00000167614**	*0.014975053*	*0.025559294*	*0.024611215*	*0.014978454*
**ENSG00000108370**	*0.016752037*	*0.025387383*	*0.025707544*	*0.014627514*
**ENSG00000176919**	*0.015721764*	*0.025474313*	*0.025143543*	*0.015620045*
**ENSG00000189266**	*0.023114451*	*0.031207278*	*0.030843614*	*0.021611553*
**ENSG00000150938**	*0.06656242*	*0.047795191*	*0.043157304*	*0.045348693*
**ENSG00000168297**	*0.038281794*	*0.041667817*	*0.04018422*	*0.033592223*
**ENSG00000177692**	*0.015580826*	*0.026326074*	*0.025787387*	*0.016087645*
**ENSG00000143222**	*0.021755089*	*0.02853916*	*0.027849191*	*0.017759265*
**ENSG00000165458**	*0.029873451*	*0.031182686*	*0.032667799*	*0.031632079*
**ENSG00000128694**	*0.040526064*	*0.049913191*	*0.036001939*	*0.036421262*
**ENSG00000168939**	*0.015024533*	*0.025859893*	*0.025227159*	*0.015982673*
**ENSG00000177468**	*0.016479491*	*0.037430175*	*0.025134465*	*0.021050598*
**ENSG00000162891**	*0.01646731*	*0.0263735*	*0.026249222*	*0.017328619*
**ENSG00000153015**	*0.032255047*	*0.035690863*	*0.030235359*	*0.024451146*
**ENSG00000119711**	*0.033340513*	*0.038639809*	*0.038116376*	*0.030635136*
**ENSG00000165698**	*0.026838841*	*0.029540646*	*0.030862848*	*0.024556637*
**ENSG00000239697**	*0.060707419*	*0.061426544*	*0.046262136*	*0.054750983*
**ENSG00000136021**	*0.037753332*	*0.039375139*	*0.040776732*	*0.04802203*
**ENSG00000166634**	*0.018383509*	*0.028551087*	*0.026920453*	*0.018125374*
**ENSG00000134594**	*0.048916671*	*0.040044362*	*0.036914414*	*0.030821117*
**ENSG00000079313**	*0.023232709*	*0.036212506*	*0.045265888*	*0.029393883*
**ENSG00000101940**	*0.021552455*	*0.028823195*	*0.031794336*	*0.020537248*
**ENSG00000141504**	*0.02634916*	*0.030305141*	*0.034208592*	*0.024759804*
**ENSG00000196371**	*0.071127948*	*0.064202973*	*0.060574984*	*0.067337533*
**ENSG00000187862**	*0.027079866*	*0.028034318*	*0.027776455*	*0.024449515*
**ENSG00000168757**	*0.01637858*	*0.024170984*	*0.024639478*	*0.015986263*
**ENSG00000149809**	*0.046128461*	*0.040139721*	*0.031062563*	*0.036609992*
**ENSG00000101695**	*0.019945754*	*0.029875389*	*0.027831105*	*0.019547914*
**ENSG00000007392**	*0.044223889*	*0.043519374*	*0.044629821*	*0.03464995*
**ENSG00000142327**	*0.025591221*	*0.028236453*	*0.03262023*	*0.024118172*
**ENSG00000095209**	*0.041738855*	*0.037969294*	*0.042323966*	*0.034019611*
**ENSG00000163964**	*0.023921553*	*0.030703908*	*0.030044183*	*0.027146964*
**ENSG00000141959**	*0.026972812*	*0.033153796*	*0.031534243*	*0.026032086*
**ENSG00000113460**	*0.026941616*	*0.029932354*	*0.032342783*	*0.029091907*
**ENSG00000147027**	*0.015139367*	*0.025136292*	*0.025597264*	*0.015997993*
**ENSG00000105852**	*0.031838138*	*0.027050213*	*0.025312216*	*0.016066055*
**ENSG00000131730**	*0.016115711*	*0.025467368*	*0.024395341*	*0.016593516*
**ENSG00000165192**	*0.016409491*	*0.025470099*	*0.024946482*	*0.015375521*
**ENSG00000177191**	*0.019648693*	*0.029843332*	*0.026581277*	*0.017838712*
**ENSG00000159720**	*0.030850868*	*0.035189521*	*0.036168953*	*0.035417253*
**ENSG00000133055**	*0.042237134*	*0.051741092*	*0.042364077*	*0.031240738*
**ENSG00000134812**	*0.01707218*	*0.025676829*	*0.024745556*	*0.01584369*
**ENSG00000168496**	*0.038664319*	*0.031895551*	*0.035253126*	*0.036805898*
**ENSG00000134551**	*0.014639019*	*0.025308714*	*0.024362518*	*0.015589668*
**ENSG00000159210**	*0.027209178*	*0.032433403*	*0.030036341*	*0.025199878*
**ENSG00000094975**	*0.0163841*	*0.024178276*	*0.025302727*	*0.015584736*
**ENSG00000158711**	*0.063997408*	*0.064770735*	*0.066528199*	*0.065599495*
**ENSG00000169752**	*0.088004458*	*0.040180133*	*0.031049403*	*0.044526203*
**ENSG00000147596**	*0.018030617*	*0.026616215*	*0.02874135*	*0.015560022*
**ENSG00000138785**	*0.023739899*	*0.032434817*	*0.033582217*	*0.021212122*
**ENSG00000166569**	*0.015320802*	*0.02525036*	*0.02536123*	*0.015488153*

**ENSG00000144048**	*0.029268294*	*0.031721255*	*0.035502846*	*0.024271431*
**ENSG00000107249**	*0.017514596*	*0.02528888*	*0.02435975*	*0.015916224*
**ENSG00000175294**	*0.018756183*	*0.027264284*	*0.026845403*	*0.019927956*
**ENSG00000176472**	*0.01560423*	*0.024495266*	*0.025068472*	*0.015452459*
**ENSG00000006831**	*0.024321213*	*0.031346568*	*0.029446872*	*0.01953893*
**ENSG00000078295**	*0.015439072*	*0.025511465*	*0.024563398*	*0.015157507*
**ENSG00000101347**	*0.019290559*	*0.028774934*	*0.027153444*	*0.017504742*
**ENSG00000163599**	*0.016799366*	*0.027647372*	*0.026348919*	*0.017800772*
**ENSG00000244687**	*0.022853405*	*0.030657118*	*0.029697192*	*0.019928843*
**ENSG00000188069**	*0.01675212*	*0.026311704*	*0.025583388*	*0.01776294*
**ENSG00000116209**	*0.029310137*	*0.031983598*	*0.032142367*	*0.020184336*
**ENSG00000152583**	*0.016361153*	*0.024335431*	*0.024770426*	*0.015255626*
**ENSG00000162222**	*0.027937441*	*0.034994744*	*0.032319926*	*0.023729128*
**ENSG00000153902**	*0.015828056*	*0.025948278*	*0.02488074*	*0.016274379*
**ENSG00000143256**	*0.022751228*	*0.028162164*	*0.028131699*	*0.024769194*
**ENSG00000171488**	*0.079974237*	*0.065161719*	*0.072335629*	*0.068949808*
**ENSG00000106603**	*0.037471342*	*0.032908671*	*0.037023363*	*0.035521499*
**ENSG00000133422**	*0.036368469*	*0.041059947*	*0.034650872*	*0.029663046*
**ENSG00000119048**	*0.025572412*	*0.032037329*	*0.032395072*	*0.024876234*
**ENSG00000049656**	*0.035675565*	*0.038697547*	*0.038822487*	*0.041645955*
**ENSG00000115268**	*0.0356334*	*0.036966007*	*0.031423553*	*0.03540348*
**ENSG00000186965**	*0.01500698*	*0.024486507*	*0.024672186*	*0.015341859*
**ENSG00000105523**	*0.016669755*	*0.024874428*	*0.024444485*	*0.016350051*
**ENSG00000181026**	*0.030494847*	*0.043340398*	*0.045842437*	*0.036810978*
**ENSG00000187140**	*0.016812134*	*0.026056192*	*0.025459239*	*0.01671178*
**ENSG00000123496**	*0.015102604*	*0.02476649*	*0.025819216*	*0.01514155*
**ENSG00000139146**	*0.027525488*	*0.03174131*	*0.030924804*	*0.026771719*
**ENSG00000135387**	*0.015813586*	*0.025561752*	*0.025335377*	*0.016467833*
**ENSG00000137101**	*0.045258188*	*0.036856502*	*0.039459746*	*0.045820569*
**ENSG00000160201**	*0.023628658*	*0.028945339*	*0.028554583*	*0.023125018*
**ENSG00000170419**	*0.015921064*	*0.024795896*	*0.024856737*	*0.015967606*
**ENSG00000164949**	*0.019030067*	*0.02522333*	*0.024912949*	*0.017969302*
**ENSG00000182022**	*0.047806002*	*0.051937565*	*0.057124012*	*0.060372655*
**ENSG00000169777**	*0.014869968*	*0.024182462*	*0.025689363*	*0.014896924*
**ENSG00000112031**	*0.026272238*	*0.033348864*	*0.035316054*	*0.028810308*
**ENSG00000168003**	*0.047473556*	*0.043931202*	*0.052037635*	*0.037356204*
**ENSG00000079335**	*0.018132365*	*0.026752783*	*0.027340595*	*0.018711569*
**ENSG00000141698**	*0.048604173*	*0.046723721*	*0.049881434*	*0.038946243*
**ENSG00000142494**	*0.110877961*	*0.080944525*	*0.082046174*	*0.079554115*
**ENSG00000141497**	*0.015692416*	*0.025512016*	*0.0241707*	*0.015236804*
**ENSG00000142484**	*0.01664552*	*0.026028582*	*0.025026715*	*0.016044232*
**ENSG00000113209**	*0.015673703*	*0.024169355*	*0.024601305*	*0.014829289*
**ENSG00000239789**	*0.027848406*	*0.030246073*	*0.030164384*	*0.024414578*
**ENSG00000162783**	*0.025161118*	*0.030824935*	*0.03285943*	*0.025983479*
**ENSG00000114771**	*0.014601696*	*0.024371889*	*0.025768134*	*0.015216904*
**ENSG00000101132**	*0.031942078*	*0.033873033*	*0.028717601*	*0.030726931*
**ENSG00000103187**	*0.04703748*	*0.036187983*	*0.035728611*	*0.040228052*
**ENSG00000198026**	*0.02379985*	*0.029319509*	*0.028529659*	*0.020085102*
**ENSG00000078061**	*0.022786909*	*0.029508108*	*0.036048162*	*0.022752226*
**ENSG00000158315**	*0.016735189*	*0.025874102*	*0.025138092*	*0.016459523*
**ENSG00000163820**	*0.038949047*	*0.03636021*	*0.031569697*	*0.029396758*
**ENSG00000241106**	*0.062471469*	*0.05868827*	*0.053908854*	*0.067536937*
**ENSG00000075790**	*0.035103649*	*0.039138934*	*0.039380847*	*0.034320162*
**ENSG00000152818**	*0.016743342*	*0.026270512*	*0.026192273*	*0.016173911*

**ENSG00000015475**	*0.039112633*	*0.043895418*	*0.047054956*	*0.048738715*
**ENSG00000117411**	*0.037295962*	*0.033582371*	*0.039160389*	*0.028601297*
**ENSG00000119638**	*0.025235601*	*0.032077691*	*0.028304365*	*0.027529565*
**ENSG00000128610**	*0.014849261*	*0.023964613*	*0.023960934*	*0.014631061*
**ENSG00000138813**	*0.017851428*	*0.0257161*	*0.026663707*	*0.017997323*
**ENSG00000065559**	*0.029693125*	*0.033739513*	*0.034762889*	*0.022976664*
**ENSG00000089248**	*0.03350145*	*0.031795708*	*0.034424381*	*0.029842413*
**ENSG00000101197**	*0.108576649*	*0.094573457*	*0.068724201*	*0.054424919*
**ENSG00000123144**	*0.018967723*	*0.026109579*	*0.031298527*	*0.020654428*
**ENSG00000170523**	*0.01987916*	*0.027318084*	*0.025420579*	*0.017238027*
**ENSG00000153914**	*0.036279549*	*0.03918227*	*0.033451746*	*0.032375987*
**ENSG00000169504**	*0.037646322*	*0.036409696*	*0.033317713*	*0.041185112*
**ENSG00000162069**	*0.025551303*	*0.038502606*	*0.033182534*	*0.033066052*
**ENSG00000076108**	*0.016564324*	*0.025430648*	*0.025989861*	*0.015794649*
**ENSG00000204304**	*0.023875728*	*0.029465253*	*0.030822485*	*0.024703673*
**ENSG00000197070**	*0.028232215*	*0.037002905*	*0.040076145*	*0.031239294*
**ENSG00000127526**	*0.024460375*	*0.028999273*	*0.030492672*	*0.028042517*
**ENSG00000143353**	*0.032146354*	*0.034978503*	*0.030158782*	*0.028549003*
**ENSG00000186960**	*0.025850569*	*0.023655092*	*0.023639175*	*0.01307655*
**ENSG00000103253**	*0.062299945*	*0.048213324*	*0.064649794*	*0.064128688*
**ENSG00000138767**	*0.017571721*	*0.025737221*	*0.025484236*	*0.016466538*
**ENSG00000184292**	*0.019103175*	*0.027749238*	*0.025735447*	*0.017193991*
**ENSG00000108798**	*0.037434053*	*0.037152156*	*0.035737978*	*0.036284461*
**ENSG00000100242**	*0.020631933*	*0.027039207*	*0.026078313*	*0.017774377*
**ENSG00000182568**	*0.023642585*	*0.084260324*	*0.068297095*	*0.074523672*
**ENSG00000204516**	*0.037425596*	*0.042996543*	*0.041199981*	*0.028346904*
**ENSG00000221867**	*0.016451107*	*0.025693322*	*0.025132263*	*0.014691635*
**ENSG00000178105**	*0.026652221*	*0.034920217*	*0.032995425*	*0.021476129*
**ENSG00000203756**	*0.072864364*	*0.029946193*	*0.027257185*	*0.037800326*
**ENSG00000037757**	*0.032263148*	*0.032562951*	*0.03238341*	*0.025696573*
**ENSG00000172377**	*0.015305045*	*0.02424744*	*0.025309613*	*0.015412295*
**ENSG00000215193**	*0.027944234*	*0.031329394*	*0.029934171*	*0.028194402*
**ENSG00000165118**	*0.038452434*	*0.041959411*	*0.039763081*	*0.03115713*
**ENSG00000169062**	*0.031585166*	*0.037693784*	*0.036301094*	*0.024013071*
**ENSG00000117640**	*0.021908722*	*0.028585302*	*0.031670409*	*0.021889996*
**ENSG00000161929**	*0.057112296*	*0.058326195*	*0.058795183*	*0.061590039*
**ENSG00000181790**	*0.016605559*	*0.026670706*	*0.026288546*	*0.01799919*
**ENSG00000166387**	*0.036881956*	*0.038634247*	*0.034475893*	*0.031273389*
**ENSG00000148308**	*0.0260606*	*0.032809397*	*0.032090496*	*0.021447258*
**ENSG00000161850**	*0.018722447*	*0.026339664*	*0.026982014*	*0.016825016*
**ENSG00000166670**	*0.016334503*	*0.025223057*	*0.024737319*	*0.014566375*
**ENSG00000168386**	*0.069603873*	*0.073536196*	*0.053608528*	*0.048685856*
**ENSG00000172367**	*0.02457269*	*0.032678365*	*0.027764764*	*0.023808901*
**ENSG00000186767**	*0.015975875*	*0.025111924*	*0.024293223*	*0.014536343*
**ENSG00000154175**	*0.016214166*	*0.027670865*	*0.026080094*	*0.017479506*
**ENSG00000175309**	*0.023168387*	*0.030752481*	*0.031621557*	*0.035513292*
**ENSG00000124374**	*0.034495866*	*0.037183758*	*0.030702124*	*0.031276253*
**ENSG00000214435**	*0.082839969*	*0.079438033*	*0.057469276*	*0.07453483*
**ENSG00000173473**	*0.0227267*	*0.031869108*	*0.029260793*	*0.020876906*
**ENSG00000140307**	*0.020978045*	*0.02937284*	*0.030285995*	*0.02150825*
**ENSG00000102683**	*0.017904904*	*0.026719734*	*0.026119886*	*0.01800842*
**ENSG00000196792**	*0.029874102*	*0.036817124*	*0.031621408*	*0.031844015*
**ENSG00000138688**	*0.034046211*	*0.037918209*	*0.033591901*	*0.03249639*
**ENSG00000198513**	*0.019248163*	*0.025573129*	*0.025385261*	*0.019065801*

**ENSG00000144867**	*0.032632967*	*0.040256613*	*0.046304428*	*0.038532562*
**ENSG00000089006**	*0.040920574*	*0.047417427*	*0.037205852*	*0.048518103*
**ENSG00000176774**	*0.021063819*	*0.025612358*	*0.024973933*	*0.019829093*
**ENSG00000157933**	*0.017231179*	*0.025633705*	*0.02559284*	*0.015119153*
**ENSG00000175787**	*0.015201237*	*0.026704602*	*0.024621276*	*0.014608607*
**ENSG00000164508**	*0.014910126*	*0.025199277*	*0.026452885*	*0.015225247*
**ENSG00000091137**	*0.033072656*	*0.032023593*	*0.035926254*	*0.026723114*
**ENSG00000136488**	*0.015127923*	*0.025432404*	*0.025448481*	*0.014630174*
**ENSG00000119397**	*0.035843923*	*0.040543897*	*0.03241629*	*0.027863025*
**ENSG00000156469**	*0.039222701*	*0.043052026*	*0.038695613*	*0.027167842*
**ENSG00000154767**	*0.032198175*	*0.036469151*	*0.035184872*	*0.025200418*
**ENSG00000008130**	*0.025999844*	*0.032946055*	*0.030023535*	*0.0281803*
**ENSG00000105186**	*0.028486625*	*0.034522777*	*0.030973307*	*0.02602995*
**ENSG00000153993**	*0.014908395*	*0.024885363*	*0.024697981*	*0.014605108*
**ENSG00000205309**	*0.045856725*	*0.040887108*	*0.041500504*	*0.042394028*
**ENSG00000170950**	*0.015031142*	*0.024740063*	*0.025188426*	*0.015205613*
**ENSG00000198612**	*0.019901358*	*0.025716938*	*0.025126492*	*0.017811057*
**ENSG00000184185**	*0.017606217*	*0.027676653*	*0.027224706*	*0.017710213*
**ENSG00000110047**	*0.035397339*	*0.032463122*	*0.035654886*	*0.030276395*
**ENSG00000154237**	*0.054076012*	*0.042999377*	*0.035338922*	*0.078410195*
**ENSG00000140451**	*0.021737779*	*0.025959687*	*0.025938658*	*0.019280879*
**ENSG00000243811**	*0.017028971*	*0.025667564*	*0.026528861*	*0.01590795*
**ENSG00000126262**	*0.026031775*	*0.030426515*	*0.029989139*	*0.032259123*
**ENSG00000115170**	*0.096703611*	*0.067315496*	*0.054167245*	*0.058902657*
**ENSG00000175467**	*0.022443793*	*0.029030966*	*0.030787578*	*0.0250496*
**ENSG00000204420**	*0.015499614*	*0.024757147*	*0.025966994*	*0.016133027*
**ENSG00000166167**	*0.016963491*	*0.026008436*	*0.026620643*	*0.016860961*
**ENSG00000137331**	*0.060402176*	*0.046332484*	*0.061202848*	*0.050559733*
**ENSG00000232810**	*0.049677113*	*0.049099698*	*0.047341651*	*0.047281714*
**ENSG00000100983**	*0.030375937*	*0.03330346*	*0.029852061*	*0.021735314*
**ENSG00000124357**	*0.040532853*	*0.039211662*	*0.034212128*	*0.039331928*
**ENSG00000186562**	*0.016450944*	*0.025488063*	*0.025819926*	*0.015454727*
**ENSG00000146263**	*0.03758874*	*0.043972585*	*0.035566556*	*0.030029734*
**ENSG00000062524**	*0.026158082*	*0.0425642*	*0.030678289*	*0.019940545*
**ENSG00000198853**	*0.016121237*	*0.026321512*	*0.024944541*	*0.016360132*
**ENSG00000169550**	*0.017480973*	*0.026650081*	*0.024714229*	*0.015515208*
**ENSG00000177426**	*0.017834751*	*0.025192794*	*0.025556074*	*0.016709852*
**ENSG00000162877**	*0.01560693*	*0.026048194*	*0.025716571*	*0.017054391*
**ENSG00000110931**	*0.059402035*	*0.061125571*	*0.054319957*	*0.052763923*
**ENSG00000181827**	*0.034398293*	*0.039216662*	*0.029999449*	*0.028407754*
**ENSG00000167751**	*0.017994706*	*0.027726858*	*0.026540029*	*0.021021979*
**ENSG00000181722**	*0.06202068*	*0.058541322*	*0.038099878*	*0.04559534*
**ENSG00000101266**	*0.022226332*	*0.030712435*	*0.029518771*	*0.02096595*
**ENSG00000188859**	*0.03214969*	*0.032073511*	*0.033105261*	*0.030291461*
**ENSG00000132589**	*0.059479763*	*0.051710569*	*0.047158841*	*0.049680696*
**ENSG00000140623**	*0.017079796*	*0.024360387*	*0.02481814*	*0.015240346*
**ENSG00000151320**	*0.028895333*	*0.026002469*	*0.024400484*	*0.020387649*
**ENSG00000040487**	*0.015283242*	*0.025602633*	*0.025551477*	*0.015134735*
**ENSG00000077713**	*0.038795046*	*0.039544064*	*0.031694815*	*0.038739809*
**ENSG00000115540**	*0.016326276*	*0.025277028*	*0.024946249*	*0.015507763*
**ENSG00000164690**	*0.01643028*	*0.024424558*	*0.024278861*	*0.01522449*
**ENSG00000054796**	*0.014862134*	*0.02441954*	*0.025437197*	*0.015335595*
**ENSG00000164405**	*0.022918996*	*0.029631151*	*0.027153505*	*0.021519753*
**ENSG00000151967**	*0.098735654*	*0.073356249*	*0.069729083*	*0.066502948*

**ENSG00000146411**	*0.018439118*	*0.029014048*	*0.02576275*	*0.02113715*
**ENSG00000153347**	*0.017014622*	*0.025599618*	*0.026005467*	*0.016428043*
**ENSG00000142538**	*0.019229848*	*0.03007159*	*0.024275183*	*0.015640175*
**ENSG00000162302**	*0.037363198*	*0.041315798*	*0.03307274*	*0.034425007*
**ENSG00000162757**	*0.032786629*	*0.0356479*	*0.033240664*	*0.023545616*
**ENSG00000064199**	*0.037883304*	*0.043394271*	*0.045371756*	*0.044832992*
**ENSG00000166337**	*0.019477031*	*0.026048063*	*0.027438774*	*0.022309289*
**ENSG00000063180**	*0.026257774*	*0.029774434*	*0.033015238*	*0.030877539*
**ENSG00000187609**	*0.024681855*	*0.028465307*	*0.033231456*	*0.023338035*
**ENSG00000198286**	*0.042377166*	*0.043582021*	*0.039811041*	*0.036987272*
**ENSG00000003402**	*0.036586222*	*0.034768565*	*0.034686556*	*0.032050565*
**ENSG00000103175**	*0.016817897*	*0.024606181*	*0.025093286*	*0.016921493*
**ENSG00000182346**	*0.016431097*	*0.024700383*	*0.025082519*	*0.0169375*
**ENSG00000173421**	*0.015576861*	*0.026365226*	*0.025774018*	*0.015474496*
**ENSG00000163590**	*0.017522094*	*0.024709145*	*0.025187527*	*0.015676542*
**ENSG00000010270**	*0.032973632*	*0.036565074*	*0.03382627*	*0.033943393*
**ENSG00000139209**	*0.020176909*	*0.026660882*	*0.025475792*	*0.016155598*
**ENSG00000182742**	*0.061776787*	*0.051982872*	*0.044863793*	*0.057049691*
**ENSG00000172016**	*0.013990595*	*0.024746831*	*0.024685956*	*0.014859666*
**ENSG00000169271**	*0.018825276*	*0.026319197*	*0.026323055*	*0.015815775*
**ENSG00000111602**	*0.042662145*	*0.043360872*	*0.033856785*	*0.035724239*
**ENSG00000153446**	*0.016232482*	*0.025504814*	*0.024660348*	*0.015618975*
**ENSG00000198646**	*0.025232589*	*0.035285497*	*0.028025865*	*0.026335048*
**ENSG00000100376**	*0.071592125*	*0.086008339*	*0.065150247*	*0.063713026*
**ENSG00000204576**	*0.03354959*	*0.035970435*	*0.035503557*	*0.031366853*
**ENSG00000196159**	*0.015997973*	*0.026406177*	*0.025537143*	*0.01511671*
**ENSG00000168675**	*0.051298109*	*0.054532565*	*0.049549473*	*0.052276215*
**ENSG00000183609**	*0.015950885*	*0.025123285*	*0.025197904*	*0.015961131*
**ENSG00000065357**	*0.016023717*	*0.025134094*	*0.02454543*	*0.014472473*
**ENSG00000171603**	*0.031858488*	*0.031418172*	*0.033682318*	*0.025544667*
**ENSG00000105865**	*0.03151304*	*0.033239953*	*0.031850096*	*0.02461972*
**ENSG00000079246**	*0.023697406*	*0.029925564*	*0.029206779*	*0.021548298*
**ENSG00000132259**	*0.018892842*	*0.026612395*	*0.027247858*	*0.015928217*
**ENSG00000179431**	*0.060883871*	*0.048242201*	*0.056556866*	*0.061765478*
**ENSG00000205944**	*0.015616279*	*0.024335954*	*0.025277799*	*0.014329836*
**ENSG00000156521**	*0.037362326*	*0.038454381*	*0.039986725*	*0.027883514*
**ENSG00000231887**	*0.01889594*	*0.029199018*	*0.030849008*	*0.018309654*
**ENSG00000177879**	*0.033739944*	*0.031946347*	*0.02978895*	*0.023561706*
**ENSG00000005483**	*0.034895318*	*0.040675877*	*0.034856097*	*0.03023119*
**ENSG00000123685**	*0.050239784*	*0.054208843*	*0.045839124*	*0.060931134*
**ENSG00000116748**	*0.029177365*	*0.02628476*	*0.024809833*	*0.020560204*
**ENSG00000162601**	*0.019093308*	*0.027691895*	*0.02604197*	*0.018556025*
**ENSG00000179674**	*0.036101535*	*0.030456627*	*0.027144969*	*0.018025362*
**ENSG00000241635**	*0.017842921*	*0.0257985*	*0.025141667*	*0.019016736*
**ENSG00000169194**	*0.052294899*	*0.03322102*	*0.028476769*	*0.027250552*
**ENSG00000052802**	*0.024078392*	*0.033232757*	*0.031831936*	*0.021695705*
**ENSG00000129691**	*0.028889752*	*0.033836606*	*0.03290349*	*0.022672614*
**ENSG00000243244**	*0.039143118*	*0.035978377*	*0.039072358*	*0.044982568*
**ENSG00000050165**	*0.014566691*	*0.025128786*	*0.02538281*	*0.01446972*
**ENSG00000087884**	*0.039630972*	*0.039133508*	*0.042017288*	*0.02992133*
**ENSG00000175203**	*0.02225244*	*0.027998448*	*0.028481194*	*0.021152914*
**ENSG00000159399**	*0.064290337*	*0.072507875*	*0.066525986*	*0.060243054*
**ENSG00000170852**	*0.039733713*	*0.044056577*	*0.036733749*	*0.034337677*
**ENSG00000239961**	*0.080749444*	*0.07136538*	*0.038844279*	*0.043497228*

**ENSG00000152778**	*0.045424223*	*0.051152216*	*0.045801358*	*0.039297215*
**ENSG00000197969**	*0.034120417*	*0.041724804*	*0.034659113*	*0.033910192*
**ENSG00000182575**	*0.019492494*	*0.028570694*	*0.02525471*	*0.018177138*
**ENSG00000196409**	*0.042732809*	*0.043338482*	*0.041585519*	*0.036212367*
**ENSG00000138443**	*0.028154149*	*0.032152238*	*0.031931667*	*0.023326654*
**ENSG00000173769**	*0.014232171*	*0.023516211*	*0.024002102*	*0.012855021*
**ENSG00000124151**	*0.020717926*	*0.030261534*	*0.028048775*	*0.024179567*
**ENSG00000188906**	*0.022830125*	*0.026825134*	*0.027330519*	*0.018060003*
**ENSG00000127329**	*0.018552762*	*0.02691023*	*0.025118063*	*0.017657839*
**ENSG00000095637**	*0.057439107*	*0.064930677*	*0.030513451*	*0.034417224*
**ENSG00000163449**	*0.028830844*	*0.031159049*	*0.026916079*	*0.022981847*
**ENSG00000109103**	*0.043740434*	*0.044752299*	*0.043129782*	*0.042209741*
**ENSG00000184361**	*0.0162232*	*0.025462345*	*0.025759369*	*0.015732898*
**ENSG00000100450**	*0.030959031*	*0.031309494*	*0.029350886*	*0.057257717*
**ENSG00000105701**	*0.027837105*	*0.037528291*	*0.042340335*	*0.036341904*
**ENSG00000249948**	*0.044275921*	*0.027296346*	*0.025788025*	*0.016327452*
**ENSG00000163602**	*0.041230979*	*0.041766579*	*0.042980136*	*0.065842473*
**ENSG00000048052**	*0.072651665*	*0.066795014*	*0.055131345*	*0.057216228*
**ENSG00000157216**	*0.051574125*	*0.049461166*	*0.048730536*	*0.057570345*
**ENSG00000062282**	*0.039357717*	*0.034342883*	*0.03529534*	*0.033858461*
**ENSG00000196155**	*0.017490351*	*0.027905092*	*0.027295383*	*0.020485602*
**ENSG00000142871**	*0.015625444*	*0.024713662*	*0.024812453*	*0.016048332*
**ENSG00000006468**	*0.02076132*	*0.025835943*	*0.028554566*	*0.017658623*
**ENSG00000102174**	*0.020399254*	*0.027161381*	*0.028262217*	*0.01805601*
**ENSG00000175130**	*0.042992221*	*0.047612393*	*0.051489119*	*0.05107237*
**ENSG00000142539**	*0.047380704*	*0.039967043*	*0.041893777*	*0.060444566*
**ENSG00000114654**	*0.017449434*	*0.028124411*	*0.026377148*	*0.015789766*
**ENSG00000115486**	*0.021308129*	*0.028688707*	*0.027866072*	*0.01928293*
**ENSG00000100479**	*0.050681292*	*0.045635536*	*0.037703416*	*0.038170754*
**ENSG00000196458**	*0.027961005*	*0.031523987*	*0.02939803*	*0.025149053*
**ENSG00000249124**	*0.027805432*	*0.028566031*	*0.028602765*	*0.023670958*
**ENSG00000117477**	*0.017789308*	*0.025512491*	*0.024825266*	*0.015875099*
**ENSG00000133135**	*0.016435953*	*0.024939333*	*0.025009123*	*0.014826369*
**ENSG00000178372**	*0.018096121*	*0.024456128*	*0.025868586*	*0.016516261*
**ENSG00000171496**	*0.016240973*	*0.024692834*	*0.025779627*	*0.016076224*
**ENSG00000183117**	*0.016258072*	*0.024098851*	*0.023806738*	*0.015829944*
**ENSG00000164794**	*0.064001435*	*0.030801301*	*0.030302107*	*0.035489753*
**ENSG00000168564**	*0.036156991*	*0.044105081*	*0.041827906*	*0.046886797*
**ENSG00000127529**	*0.01631563*	*0.024338551*	*0.024702134*	*0.015591809*
**ENSG00000086102**	*0.025864464*	*0.03021361*	*0.030922075*	*0.0218995*
**ENSG00000140455**	*0.031830023*	*0.03032434*	*0.0344939*	*0.024077941*
**ENSG00000136159**	*0.027671454*	*0.031382788*	*0.032074473*	*0.027052352*
**ENSG00000188089**	*0.014464689*	*0.024096279*	*0.024162966*	*0.013176876*
**ENSG00000123594**	*0.016558329*	*0.026034392*	*0.025043228*	*0.01708503*
**ENSG00000120029**	*0.034275314*	*0.033976478*	*0.03037124*	*0.025441835*
**ENSG00000136161**	*0.056809086*	*0.059403719*	*0.049057028*	*0.056298662*
**ENSG00000141956**	*0.070789187*	*0.057835666*	*0.058009036*	*0.070360636*
**ENSG00000153922**	*0.027624326*	*0.039266716*	*0.042149901*	*0.031216729*
**ENSG00000120332**	*0.03333198*	*0.02732936*	*0.026625139*	*0.024250144*
**ENSG00000170561**	*0.014513756*	*0.024386913*	*0.024591151*	*0.015702394*
**ENSG00000075673**	*0.01597935*	*0.024126426*	*0.025034792*	*0.016047981*
**ENSG00000114405**	*0.161724225*	*0.097689612*	*0.057241137*	*0.090934993*
**ENSG00000137628**	*0.078257156*	*0.065422812*	*0.050056999*	*0.065214664*
**ENSG00000184661**	*0.050311472*	*0.036716011*	*0.044234503*	*0.039719101*

**ENSG00000189420**	*0.0162363*	*0.02473039*	*0.024504286*	*0.014144249*
**ENSG00000085265**	*0.041736595*	*0.030254841*	*0.035097317*	*0.029635526*
**ENSG00000081052**	*0.066202399*	*0.077906007*	*0.057988012*	*0.076368312*
**ENSG00000166211**	*0.019582386*	*0.02552221*	*0.026383523*	*0.015446517*
**ENSG00000134757**	*0.018426241*	*0.025220041*	*0.02565095*	*0.01534033*
**ENSG00000100379**	*0.018154338*	*0.02789622*	*0.026197829*	*0.017071241*
**ENSG00000183625**	*0.01688182*	*0.024532757*	*0.025593985*	*0.015822317*
**ENSG00000241127**	*0.027446197*	*0.033597101*	*0.034781375*	*0.024248495*
**ENSG00000050628**	*0.018209767*	*0.025721036*	*0.025613092*	*0.020048039*
**ENSG00000054523**	*0.017520839*	*0.02531172*	*0.025235065*	*0.018473584*
**ENSG00000185615**	*0.015431811*	*0.024677571*	*0.024725258*	*0.015570765*
**ENSG00000173572**	*0.015663719*	*0.026023546*	*0.024395352*	*0.015545928*
**ENSG00000130714**	*0.028668973*	*0.035503206*	*0.0339526*	*0.024910017*
**ENSG00000155846**	*0.033162355*	*0.037278094*	*0.043219877*	*0.035824939*
**ENSG00000241563**	*0.017495818*	*0.02576971*	*0.02450342*	*0.0157146*
**ENSG00000186628**	*0.020994052*	*0.027305761*	*0.025822795*	*0.021475116*
**ENSG00000134516**	*0.023458821*	*0.032598436*	*0.028987698*	*0.019260976*
**ENSG00000168274**	*0.049480293*	*0.047904327*	*0.04721569*	*0.042735974*
**ENSG00000110721**	*0.045294275*	*0.036474626*	*0.042404717*	*0.033934903*
**ENSG00000065154**	*0.10792969*	*0.067426306*	*0.055842575*	*0.083959723*
**ENSG00000178826**	*0.016030866*	*0.024820217*	*0.024594774*	*0.01630419*
**ENSG00000177098**	*0.028363202*	*0.030551724*	*0.033411624*	*0.031940212*
**ENSG00000080709**	*0.036488873*	*0.033495622*	*0.034048657*	*0.032469741*
**ENSG00000173039**	*0.02357679*	*0.035362078*	*0.042763192*	*0.024300474*
**ENSG00000140829**	*0.025083921*	*0.031626226*	*0.031974357*	*0.023767606*
**ENSG00000125755**	*0.030723786*	*0.04567073*	*0.045565654*	*0.040911959*
**ENSG00000138109**	*0.015568343*	*0.024458721*	*0.024872101*	*0.015791411*
**ENSG00000081019**	*0.030232108*	*0.040088298*	*0.037833092*	*0.03136802*
**ENSG00000165512**	*0.034744299*	*0.036405229*	*0.036461303*	*0.026694563*
**ENSG00000115904**	*0.029869215*	*0.036837695*	*0.035246542*	*0.039921516*
**ENSG00000176046**	*0.018062526*	*0.026032882*	*0.025940876*	*0.016260063*
**ENSG00000043462**	*0.053096952*	*0.048636037*	*0.049002261*	*0.056217062*
**ENSG00000115963**	*0.104515045*	*0.074328818*	*0.034315388*	*0.045700782*
**ENSG00000160199**	*0.015585945*	*0.024785863*	*0.024860933*	*0.014882474*
**ENSG00000081138**	*0.015855027*	*0.024296482*	*0.024250079*	*0.015274154*
**ENSG00000165516**	*0.0284152*	*0.032268901*	*0.0327798*	*0.025169833*
**ENSG00000141378**	*0.022838303*	*0.032294762*	*0.03530333*	*0.023798674*
**ENSG00000121898**	*0.036338031*	*0.025804097*	*0.025207494*	*0.022056613*
**ENSG00000099337**	*0.057019552*	*0.050608202*	*0.053638686*	*0.060721129*
**ENSG00000090376**	*0.090622613*	*0.06653202*	*0.063585667*	*0.06827838*
**ENSG00000106993**	*0.025145864*	*0.034529655*	*0.036250708*	*0.026605264*
**ENSG00000005073**	*0.016531524*	*0.024992272*	*0.025926318*	*0.016942607*
**ENSG00000197689**	*0.014885574*	*0.024750244*	*0.025100115*	*0.015449773*
**ENSG00000120242**	*0.020537925*	*0.027696666*	*0.029334406*	*0.017752813*
**ENSG00000174130**	*0.048057823*	*0.058344712*	*0.054432728*	*0.035224215*
**ENSG00000100056**	*0.02254463*	*0.028145004*	*0.033534614*	*0.022680817*
**ENSG00000184779**	*0.018587926*	*0.025214564*	*0.025641349*	*0.017108213*
**ENSG00000100300**	*0.058789848*	*0.043188137*	*0.039170465*	*0.0470449*
**ENSG00000134873**	*0.015632947*	*0.024657349*	*0.024500049*	*0.016102453*
**ENSG00000145439**	*0.044270377*	*0.039903407*	*0.034129365*	*0.040249987*
**ENSG00000134215**	*0.046965634*	*0.025749402*	*0.024564102*	*0.016527645*
**ENSG00000196363**	*0.03854463*	*0.039093511*	*0.035355779*	*0.030604148*
**ENSG00000116863**	*0.029324498*	*0.032598278*	*0.031719756*	*0.021678619*
**ENSG00000205707**	*0.027226096*	*0.029744992*	*0.03529887*	*0.02837006*

**ENSG00000073009**	*0.027386272*	*0.036646011*	*0.034006063*	*0.025652148*
**ENSG00000117533**	*0.038890832*	*0.036313748*	*0.040788409*	*0.036485854*
**ENSG00000163283**	*0.042399692*	*0.039979389*	*0.040474684*	*0.045182459*
**ENSG00000096433**	*0.044752785*	*0.037182195*	*0.033437861*	*0.029681214*
**ENSG00000104327**	*0.018256648*	*0.027010541*	*0.026361728*	*0.015922256*
**ENSG00000157388**	*0.016436001*	*0.024646202*	*0.02440631*	*0.01488639*
**ENSG00000148300**	*0.024553176*	*0.027994337*	*0.029580015*	*0.026991655*
**ENSG00000214367**	*0.036243893*	*0.036919249*	*0.029328057*	*0.031736864*
**ENSG00000156050**	*0.014571422*	*0.024937547*	*0.024647497*	*0.015905534*
**ENSG00000188419**	*0.018197508*	*0.03302964*	*0.026178861*	*0.017707878*
**ENSG00000006114**	*0.032016273*	*0.034266935*	*0.034086091*	*0.026852856*
**ENSG00000142784**	*0.026613449*	*0.031785342*	*0.036670159*	*0.030169353*
**ENSG00000078124**	*0.027085504*	*0.032507284*	*0.032147094*	*0.026462066*
**ENSG00000150403**	*0.031087934*	*0.041344272*	*0.034474164*	*0.034163282*
**ENSG00000128881**	*0.015627712*	*0.026006779*	*0.02568749*	*0.016387989*
**ENSG00000142235**	*0.04938564*	*0.062977526*	*0.062100418*	*0.053264434*
**ENSG00000164291**	*0.020420052*	*0.026552002*	*0.026113025*	*0.019558059*
**ENSG00000156675**	*0.055021283*	*0.048221426*	*0.05075909*	*0.049461339*
**ENSG00000196176**	*0.028259105*	*0.027961519*	*0.032669958*	*0.029964446*
**ENSG00000078725**	*0.017615753*	*0.02520836*	*0.026728947*	*0.015751357*
**ENSG00000146109**	*0.019678114*	*0.025573571*	*0.025480187*	*0.016799435*
**ENSG00000169946**	*0.037238814*	*0.033637075*	*0.030171992*	*0.030809548*
**ENSG00000118245**	*0.016548253*	*0.025501248*	*0.024823761*	*0.015119769*
**ENSG00000173545**	*0.022539309*	*0.031080594*	*0.03463313*	*0.020413931*
**ENSG00000135966**	*0.032125085*	*0.035876229*	*0.038479386*	*0.033815795*
**ENSG00000138944**	*0.017255869*	*0.025139848*	*0.025819391*	*0.016415598*
**ENSG00000213599**	*0.017153293*	*0.025178494*	*0.024400493*	*0.017066635*
**ENSG00000169006**	*0.016895087*	*0.024344855*	*0.025213983*	*0.015949293*
**ENSG00000089041**	*0.022748784*	*0.02779483*	*0.02936071*	*0.020904993*
**ENSG00000144791**	*0.040510731*	*0.045575606*	*0.037629967*	*0.036010117*
**ENSG00000234511**	*0.015760346*	*0.02632022*	*0.024481788*	*0.014959628*
**ENSG00000155189**	*0.032385655*	*0.034720385*	*0.031097076*	*0.035934437*
**ENSG00000080573**	*0.047281826*	*0.038313639*	*0.028359374*	*0.037060529*
**ENSG00000130751**	*0.023957037*	*0.026814659*	*0.027584084*	*0.021736328*
**ENSG00000145362**	*0.019569842*	*0.02524124*	*0.026788245*	*0.015957985*
**ENSG00000151079**	*0.017871185*	*0.025061346*	*0.024964348*	*0.014726625*
**ENSG00000135898**	*0.067261173*	*0.042255755*	*0.046618351*	*0.062309453*
**ENSG00000183060**	*0.042850611*	*0.042124241*	*0.038852083*	*0.041519968*
**ENSG00000117595**	*0.023549622*	*0.027252299*	*0.026612233*	*0.016997653*
**ENSG00000132938**	*0.016721069*	*0.025340805*	*0.024747875*	*0.014856479*
**ENSG00000174332**	*0.014855405*	*0.025606561*	*0.024602734*	*0.015944542*
**ENSG00000100557**	*0.120732921*	*0.081936098*	*0.081420899*	*0.102459354*
**ENSG00000176142**	*0.034153521*	*0.035823511*	*0.035633183*	*0.036849856*
**ENSG00000141759**	*0.019182334*	*0.025783667*	*0.026812074*	*0.017355603*
**ENSG00000180979**	*0.025284047*	*0.034221922*	*0.034551739*	*0.023796406*
**ENSG00000198892**	*0.015458452*	*0.024899748*	*0.024784263*	*0.014753007*
**ENSG00000116785**	*0.016629686*	*0.024439573*	*0.024395322*	*0.015141196*
**ENSG00000145012**	*0.018537849*	*0.027851219*	*0.026603786*	*0.020206301*
**ENSG00000168671**	*0.016023419*	*0.025334937*	*0.025079276*	*0.015073383*
**ENSG00000115112**	*0.034798086*	*0.030701349*	*0.034887882*	*0.020566062*
**ENSG00000187238**	*0.01475296*	*0.024736767*	*0.024919794*	*0.015551248*
**ENSG00000186191**	*0.018508804*	*0.026068525*	*0.027290147*	*0.016697514*
**ENSG00000105639**	*0.033102542*	*0.046712894*	*0.037709508*	*0.039904366*
**ENSG00000117862**	*0.028402808*	*0.030498523*	*0.030337887*	*0.021177439*

**ENSG00000131408**	*0.022029809*	*0.028117609*	*0.033450246*	*0.024233619*
**ENSG00000119919**	*0.016549737*	*0.024787221*	*0.024763282*	*0.015612995*
**ENSG00000173480**	*0.03332916*	*0.032813453*	*0.032877383*	*0.030591897*
**ENSG00000105851**	*0.065337051*	*0.068952273*	*0.063750473*	*0.053812347*
**ENSG00000136908**	*0.025975937*	*0.030993239*	*0.034400402*	*0.02754123*
**ENSG00000158296**	*0.01526837*	*0.025597279*	*0.024958473*	*0.015397873*
**ENSG00000179087**	*0.016037665*	*0.024690253*	*0.025021497*	*0.015490843*
**ENSG00000101181**	*0.025211012*	*0.027061376*	*0.027355678*	*0.018805666*
**ENSG00000008086**	*0.015337817*	*0.025036509*	*0.024640788*	*0.014618996*
**ENSG00000143207**	*0.024567846*	*0.034531865*	*0.032260138*	*0.026223874*
**ENSG00000165861**	*0.031357017*	*0.031756936*	*0.032390736*	*0.027880933*
**ENSG00000184384**	*0.032442381*	*0.037086753*	*0.033407269*	*0.032096527*
**ENSG00000159200**	*0.043381074*	*0.0509169*	*0.050255813*	*0.050146663*
**ENSG00000074803**	*0.015955965*	*0.025740017*	*0.026319322*	*0.016328197*
**ENSG00000149657**	*0.018405588*	*0.029957175*	*0.027085588*	*0.019250094*
**ENSG00000114378**	*0.017300448*	*0.025750136*	*0.027129758*	*0.017075581*
**ENSG00000112499**	*0.015911632*	*0.024220008*	*0.024337883*	*0.016060509*
**ENSG00000073146**	*0.017665278*	*0.024714586*	*0.025232105*	*0.018901147*
**ENSG00000116459**	*0.018770625*	*0.026421805*	*0.025802291*	*0.017276819*
**ENSG00000107669**	*0.019866034*	*0.027495258*	*0.025776103*	*0.018102291*
**ENSG00000162894**	*0.040148944*	*0.036445725*	*0.039015323*	*0.046653761*
**ENSG00000126233**	*0.017080384*	*0.024397194*	*0.024820964*	*0.015581486*
**ENSG00000204604**	*0.022877537*	*0.03210897*	*0.034638803*	*0.021368578*
**ENSG00000176386**	*0.025266745*	*0.030828104*	*0.030935446*	*0.019837066*
**ENSG00000196152**	*0.040285351*	*0.036124464*	*0.039499345*	*0.033717294*
**ENSG00000167619**	*0.040290069*	*0.037697146*	*0.033136427*	*0.056074791*
**ENSG00000167264**	*0.028093376*	*0.031470678*	*0.030984155*	*0.025127723*
**ENSG00000069345**	*0.021855713*	*0.029344839*	*0.03013815*	*0.021072395*
**ENSG00000101773**	*0.016563814*	*0.026442009*	*0.02491389*	*0.014755284*
**ENSG00000103184**	*0.016324501*	*0.025164443*	*0.024386494*	*0.015276088*
**ENSG00000221978**	*0.027573104*	*0.035026271*	*0.034413702*	*0.027900228*
**ENSG00000112299**	*0.042574647*	*0.028310218*	*0.025355903*	*0.021683302*
**ENSG00000250722**	*0.100139503*	*0.04290266*	*0.025480203*	*0.031473278*
**ENSG00000125885**	*0.029110497*	*0.031866655*	*0.030332243*	*0.031890788*
**ENSG00000147256**	*0.01511329*	*0.024868867*	*0.024680714*	*0.015004024*
**ENSG00000141255**	*0.03827411*	*0.045143811*	*0.042995699*	*0.022073136*
**ENSG00000166143**	*0.016610116*	*0.025305089*	*0.025010398*	*0.016526543*
**ENSG00000104880**	*0.028569181*	*0.033139704*	*0.034261518*	*0.023678506*
**ENSG00000175906**	*0.033056135*	*0.033717567*	*0.032099941*	*0.02965399*
**ENSG00000183134**	*0.016940588*	*0.025739628*	*0.025960657*	*0.014997913*
**ENSG00000183251**	*0.050683926*	*0.024339939*	*0.023851296*	*0.016387589*
**ENSG00000188257**	*0.016842986*	*0.026428721*	*0.025479948*	*0.015867442*
**ENSG00000110680**	*0.017320389*	*0.025669695*	*0.02529297*	*0.015639721*
**ENSG00000122406**	*0.015128393*	*0.024368325*	*0.024243991*	*0.016011026*
**ENSG00000105642**	*0.049185624*	*0.04592218*	*0.04696675*	*0.048213566*
**ENSG00000168228**	*0.030487616*	*0.041772942*	*0.03816159*	*0.023865628*
**ENSG00000105514**	*0.02465364*	*0.030219007*	*0.0263115*	*0.019760177*
**ENSG00000145386**	*0.050927878*	*0.042777671*	*0.037666353*	*0.034750529*
**ENSG00000204683**	*0.014086385*	*0.023935736*	*0.023938071*	*0.01361177*
**ENSG00000135677**	*0.027395519*	*0.03574855*	*0.029369794*	*0.025185559*
**ENSG00000151332**	*0.026382876*	*0.038235325*	*0.034608874*	*0.020183497*
**ENSG00000204308**	*0.031630177*	*0.031627507*	*0.030403007*	*0.020213283*
**ENSG00000119616**	*0.036171382*	*0.043202646*	*0.044844271*	*0.038437637*
**ENSG00000134186**	*0.039007081*	*0.037794057*	*0.032269372*	*0.034374588*

**ENSG00000166435**	*0.03320708*	*0.036486197*	*0.037749545*	*0.025968086*
**ENSG00000167094**	*0.017903621*	*0.025009586*	*0.025383488*	*0.016108663*
**ENSG00000186466**	*0.016218315*	*0.025105475*	*0.0263047*	*0.016259074*
**ENSG00000168148**	*0.016336126*	*0.024288707*	*0.024662445*	*0.015042236*
**ENSG00000069493**	*0.086493722*	*0.048417133*	*0.046610629*	*0.040573763*
**ENSG00000123146**	*0.039046672*	*0.025051567*	*0.032529375*	*0.028452863*
**ENSG00000189430**	*0.015241251*	*0.025374004*	*0.024877193*	*0.015556297*
**ENSG00000184752**	*0.022187331*	*0.030074643*	*0.02874187*	*0.019987597*
**ENSG00000177301**	*0.01770446*	*0.025414553*	*0.027493336*	*0.017865037*
**ENSG00000180316**	*0.018312273*	*0.026829712*	*0.029011706*	*0.020884332*
**ENSG00000196374**	*0.083493017*	*0.055019979*	*0.073835237*	*0.075398287*
**ENSG00000114859**	*0.016630971*	*0.026155487*	*0.026043555*	*0.016042732*
**ENSG00000162873**	*0.015252261*	*0.024591699*	*0.024569081*	*0.014740136*
**ENSG00000132406**	*0.02673217*	*0.036162141*	*0.031070222*	*0.026973284*
**ENSG00000111452**	*0.016446902*	*0.025521845*	*0.024848276*	*0.016862915*
**ENSG00000175486**	*0.016117701*	*0.024736281*	*0.024468929*	*0.015509272*
**ENSG00000078053**	*0.028723122*	*0.026978586*	*0.026984048*	*0.017520479*
**ENSG00000185926**	*0.016591398*	*0.025018206*	*0.024588361*	*0.014430309*
**ENSG00000170873**	*0.0414558*	*0.049381446*	*0.052950023*	*0.034136154*
**ENSG00000150540**	*0.083121288*	*0.045943903*	*0.052720018*	*0.06547923*
**ENSG00000167656**	*0.01600545*	*0.024794869*	*0.02542257*	*0.017747585*
**ENSG00000167693**	*0.028332278*	*0.028362736*	*0.029844682*	*0.021378716*
**ENSG00000013297**	*0.048006085*	*0.040746037*	*0.028839872*	*0.072390715*
**ENSG00000182896**	*0.015448777*	*0.025124737*	*0.02451654*	*0.01482561*
**ENSG00000198205**	*0.023938475*	*0.030686883*	*0.029659221*	*0.023413979*
**ENSG00000162066**	*0.031336616*	*0.030118487*	*0.030265939*	*0.026595143*
**ENSG00000115282**	*0.021436369*	*0.027508691*	*0.029725448*	*0.021170032*
**ENSG00000138768**	*0.025423344*	*0.032686672*	*0.027862403*	*0.022335493*
**ENSG00000171680**	*0.015217825*	*0.024658669*	*0.024677795*	*0.014867133*
**ENSG00000198707**	*0.033754445*	*0.037991021*	*0.031964575*	*0.026758865*
**ENSG00000184478**	*0.013519231*	*0.024227115*	*0.023726115*	*0.013884397*
**ENSG00000253304**	*0.018342228*	*0.026839457*	*0.025674301*	*0.01808786*
**ENSG00000106610**	*0.027419305*	*0.031785064*	*0.029493912*	*0.020703609*
**ENSG00000137692**	*0.027788784*	*0.030819774*	*0.030223632*	*0.022417283*
**ENSG00000178982**	*0.019289181*	*0.025510157*	*0.025234359*	*0.01712485*
**ENSG00000117505**	*0.026797938*	*0.034670699*	*0.039117464*	*0.030450803*
**ENSG00000172785**	*0.054791749*	*0.055414254*	*0.050114851*	*0.066160078*
**ENSG00000080823**	*0.099842698*	*0.09167952*	*0.071322545*	*0.078583485*
**ENSG00000105402**	*0.026537198*	*0.033468385*	*0.033530434*	*0.027913528*
**ENSG00000137449**	*0.019567307*	*0.029537937*	*0.028088038*	*0.026246251*
**ENSG00000133065**	*0.014992949*	*0.024313926*	*0.02546328*	*0.01471741*
**ENSG00000141505**	*0.020114772*	*0.027067496*	*0.027225944*	*0.018632378*
**ENSG00000132446**	*0.015166628*	*0.025293051*	*0.025939469*	*0.014611094*
**ENSG00000091490**	*0.098200345*	*0.059615379*	*0.051155571*	*0.058220445*
**ENSG00000153721**	*0.118831826*	*0.082835476*	*0.069953356*	*0.093167297*
**ENSG00000197471**	*0.033030185*	*0.034604479*	*0.040993007*	*0.049717971*
**ENSG00000197912**	*0.028936807*	*0.031325415*	*0.029234983*	*0.025705765*
**ENSG00000239672**	*0.041614165*	*0.043324908*	*0.039644964*	*0.030216209*
**ENSG00000024048**	*0.031445805*	*0.036768665*	*0.0315183*	*0.025898765*
**ENSG00000177728**	*0.029840433*	*0.029366768*	*0.037454193*	*0.033679839*
**ENSG00000159110**	*0.023408816*	*0.029097634*	*0.027720126*	*0.020606087*
**ENSG00000148942**	*0.015880067*	*0.025575563*	*0.024612963*	*0.015528688*
**ENSG00000177212**	*0.016290263*	*0.025623503*	*0.024831738*	*0.016381847*
**ENSG00000204231**	*0.022077407*	*0.027124809*	*0.030205572*	*0.021173164*

**ENSG00000126882**	*0.026076279*	*0.029602597*	*0.029338308*	*0.025388098*
**ENSG00000206052**	*0.016170593*	*0.024077113*	*0.025447756*	*0.015096812*
**ENSG00000152558**	*0.036636235*	*0.045321508*	*0.041809333*	*0.047683887*
**ENSG00000145850**	*0.134146609*	*0.076173138*	*0.078933858*	*0.103757817*
**ENSG00000063176**	*0.026570196*	*0.032699743*	*0.02993937*	*0.031071784*
**ENSG00000148481**	*0.035382281*	*0.034190655*	*0.031411169*	*0.022725503*
**ENSG00000020181**	*0.016360971*	*0.024801008*	*0.024233713*	*0.015737841*
**ENSG00000113838**	*0.024870034*	*0.033203329*	*0.029794636*	*0.022431427*
**ENSG00000170340**	*0.050205719*	*0.04936986*	*0.048157305*	*0.035835775*
**ENSG00000112237**	*0.029060773*	*0.041153365*	*0.031235164*	*0.028026512*
**ENSG00000162526**	*0.015814645*	*0.029599345*	*0.027103609*	*0.01742113*
**ENSG00000135624**	*0.022848324*	*0.028336911*	*0.026165696*	*0.019468667*
**ENSG00000178966**	*0.046540542*	*0.045920533*	*0.034894866*	*0.033270221*
**ENSG00000146830**	*0.025527448*	*0.026879127*	*0.028381785*	*0.02723462*
**ENSG00000116212**	*0.028966997*	*0.032934932*	*0.031484714*	*0.02773618*
**ENSG00000070961**	*0.031555165*	*0.034731074*	*0.032365144*	*0.036219882*
**ENSG00000189057**	*0.053106405*	*0.045329177*	*0.045261495*	*0.052958738*
**ENSG00000138668**	*0.023042942*	*0.027976362*	*0.026792479*	*0.020426308*
**ENSG00000069667**	*0.017088689*	*0.024737095*	*0.024697434*	*0.016717357*
**ENSG00000113558**	*0.018766032*	*0.026685361*	*0.026357554*	*0.018323539*
**ENSG00000178222**	*0.050314499*	*0.038060435*	*0.038064611*	*0.046898963*
**ENSG00000143536**	*0.016355075*	*0.026162281*	*0.026035058*	*0.016557686*
**ENSG00000152253**	*0.033278534*	*0.038647628*	*0.035293695*	*0.03513669*
**ENSG00000168411**	*0.035389719*	*0.036965983*	*0.038812903*	*0.037191263*
**ENSG00000236398**	*0.015090064*	*0.024478552*	*0.024974816*	*0.014743636*
**ENSG00000174080**	*0.016702903*	*0.025208368*	*0.025127508*	*0.017200123*
**ENSG00000145191**	*0.022656563*	*0.029112713*	*0.030592703*	*0.021525469*
**ENSG00000146005**	*0.016288595*	*0.024572667*	*0.025366939*	*0.015230553*
**ENSG00000131165**	*0.025895998*	*0.032512083*	*0.04557209*	*0.028159035*
**ENSG00000135824**	*0.01463593*	*0.025944816*	*0.02578589*	*0.015590847*
**ENSG00000167182**	*0.033589203*	*0.034719491*	*0.041920692*	*0.031214875*
**ENSG00000011426**	*0.054947754*	*0.04552909*	*0.035833054*	*0.041683871*
**ENSG00000133612**	*0.033060537*	*0.035173622*	*0.033522136*	*0.03828424*
**ENSG00000197191**	*0.024279115*	*0.02950683*	*0.026178653*	*0.020590067*
**ENSG00000073150**	*0.016775959*	*0.027535095*	*0.026391365*	*0.017941321*
**ENSG00000172238**	*0.017791056*	*0.026042175*	*0.026350214*	*0.017438148*
**ENSG00000157657**	*0.016726234*	*0.024608481*	*0.025217776*	*0.015273505*
**ENSG00000078814**	*0.016177211*	*0.025002356*	*0.025205903*	*0.015831009*
**ENSG00000188672**	*0.015728022*	*0.025942815*	*0.025056532*	*0.01485573*
**ENSG00000095794**	*0.046533962*	*0.047953844*	*0.04559819*	*0.047929691*
**ENSG00000119943**	*0.102877007*	*0.05442209*	*0.04346781*	*0.059710735*
**ENSG00000134595**	*0.016073418*	*0.024802604*	*0.025135779*	*0.01601337*
**ENSG00000149600**	*0.033050693*	*0.031979439*	*0.032539012*	*0.02572152*
**ENSG00000128512**	*0.018815396*	*0.02552754*	*0.026594992*	*0.016798701*
**ENSG00000148604**	*0.016134537*	*0.024747696*	*0.024221551*	*0.015445864*
**ENSG00000081923**	*0.017383101*	*0.024958672*	*0.024481169*	*0.017268801*
**ENSG00000174151**	*0.019387756*	*0.026835041*	*0.028994696*	*0.019986031*
**ENSG00000189037**	*0.017308104*	*0.024681799*	*0.025303333*	*0.016143133*
**ENSG00000064726**	*0.025151154*	*0.033351697*	*0.030289118*	*0.02191745*
**ENSG00000166928**	*0.051176873*	*0.040702341*	*0.040321152*	*0.048589851*
**ENSG00000165417**	*0.016670812*	*0.02614129*	*0.025158776*	*0.019336877*
**ENSG00000005059**	*0.036195948*	*0.034673189*	*0.031425695*	*0.028321995*
**ENSG00000170921**	*0.026562058*	*0.027594991*	*0.027001413*	*0.021387292*
**ENSG00000164985**	*0.028731118*	*0.033376392*	*0.034149356*	*0.033452866*

**ENSG00000134308**	*0.021854262*	*0.029159429*	*0.027585857*	*0.023944936*
**ENSG00000174276**	*0.02944918*	*0.030823349*	*0.035019963*	*0.030151432*
**ENSG00000170439**	*0.021274192*	*0.025962916*	*0.025482517*	*0.019262426*
**ENSG00000166797**	*0.025412275*	*0.029190846*	*0.02642234*	*0.019510669*
**ENSG00000175809**	*0.017538333*	*0.026143875*	*0.027013834*	*0.015941184*
**ENSG00000076685**	*0.027871588*	*0.030178749*	*0.033294612*	*0.026232159*
**ENSG00000047579**	*0.029200726*	*0.034320762*	*0.032709054*	*0.024529947*
**ENSG00000139915**	*0.019679823*	*0.02473872*	*0.02457973*	*0.015649298*
**ENSG00000112183**	*0.01649934*	*0.024962528*	*0.024967378*	*0.015264906*
**ENSG00000074657**	*0.043373217*	*0.036461005*	*0.035174617*	*0.037980346*
**ENSG00000165140**	*0.038820611*	*0.03298421*	*0.034008764*	*0.019890253*
**ENSG00000143365**	*0.015359526*	*0.025340294*	*0.023961761*	*0.015481193*
**ENSG00000168528**	*0.119060986*	*0.085857981*	*0.09296589*	*0.101602092*
**ENSG00000081059**	*0.056214561*	*0.049149019*	*0.052372651*	*0.05684796*
**ENSG00000118181**	*0.015790568*	*0.024650452*	*0.024470245*	*0.01904765*
**ENSG00000175691**	*0.024734926*	*0.035136079*	*0.035341733*	*0.026290797*
**ENSG00000143882**	*0.016190734*	*0.025438034*	*0.024928909*	*0.015054376*
**ENSG00000140943**	*0.030356972*	*0.032768904*	*0.03482246*	*0.031580888*
**ENSG00000169508**	*0.084453348*	*0.050230992*	*0.054634221*	*0.05939913*
**ENSG00000111785**	*0.034278597*	*0.044237174*	*0.031930409*	*0.030515469*
**ENSG00000145741**	*0.016070166*	*0.024880178*	*0.025403289*	*0.015090851*
**ENSG00000066032**	*0.094255766*	*0.037239214*	*0.031265607*	*0.034561089*
**ENSG00000082898**	*0.027138207*	*0.031490613*	*0.027804293*	*0.021280885*
**ENSG00000138413**	*0.041895585*	*0.044133743*	*0.035757307*	*0.030445811*
**ENSG00000212122**	*0.015281356*	*0.025157921*	*0.024704924*	*0.014748468*
**ENSG00000166689**	*0.061586964*	*0.05616825*	*0.054219779*	*0.056521617*
**ENSG00000165671**	*0.019560348*	*0.027165025*	*0.030172883*	*0.020198039*
**ENSG00000197951**	*0.023221557*	*0.029435188*	*0.031419077*	*0.020930747*
**ENSG00000120051**	*0.016060688*	*0.025329239*	*0.025724619*	*0.016358871*
**ENSG00000188801**	*0.045038265*	*0.042348973*	*0.050754648*	*0.042166336*
**ENSG00000121634**	*0.01521701*	*0.024889721*	*0.02481547*	*0.014808606*
**ENSG00000169344**	*0.015125009*	*0.024493433*	*0.024686226*	*0.015313658*
**ENSG00000145248**	*0.016448759*	*0.02559306*	*0.025393214*	*0.016139005*
**ENSG00000116106**	*0.020387525*	*0.025363505*	*0.025721012*	*0.016740817*
**ENSG00000206557**	*0.028861829*	*0.032731378*	*0.027849169*	*0.022635789*
**ENSG00000166035**	*0.017872593*	*0.029064134*	*0.024756347*	*0.017695778*
**ENSG00000171310**	*0.051443093*	*0.046168223*	*0.051897985*	*0.043983375*
**ENSG00000154217**	*0.09813124*	*0.060786326*	*0.058176242*	*0.082512383*
**ENSG00000172594**	*0.040658108*	*0.039167493*	*0.029773558*	*0.0320331*
**ENSG00000177951**	*0.023797287*	*0.035159735*	*0.039617407*	*0.023333306*
**ENSG00000139637**	*0.02199921*	*0.027095477*	*0.026898317*	*0.021826171*
**ENSG00000106355**	*0.022964129*	*0.027720295*	*0.027907399*	*0.022340813*
**ENSG00000165644**	*0.03510998*	*0.032296843*	*0.031558597*	*0.031700126*
**ENSG00000125844**	*0.04859949*	*0.046941181*	*0.052876938*	*0.049684845*
**ENSG00000134460**	*0.057140558*	*0.051874921*	*0.053492897*	*0.046408479*
**ENSG00000110697**	*0.038668293*	*0.032689213*	*0.042463505*	*0.035056619*
**ENSG00000171724**	*0.022882395*	*0.02905573*	*0.03175005*	*0.018598491*
**ENSG00000139330**	*0.01542452*	*0.024173379*	*0.025760091*	*0.015204554*
**ENSG00000168490**	*0.017921347*	*0.025129986*	*0.026780041*	*0.01694066*
**ENSG00000137338**	*0.042208183*	*0.04233864*	*0.044523708*	*0.037425044*
**ENSG00000242612**	*0.042604918*	*0.039778202*	*0.041242389*	*0.038003578*
**ENSG00000093167**	*0.061355183*	*0.04622356*	*0.048961515*	*0.048058774*
**ENSG00000167766**	*0.036519074*	*0.041828505*	*0.037198956*	*0.039303966*
**ENSG00000196284**	*0.059361974*	*0.057382709*	*0.050807736*	*0.044875979*

**ENSG00000203734**	*0.014990597*	*0.025170729*	*0.025097611*	*0.01434043*
**ENSG00000163644**	*0.033651566*	*0.039250065*	*0.036945122*	*0.03074129*
**ENSG00000137274**	*0.03329227*	*0.040790653*	*0.035881636*	*0.037251097*
**ENSG00000109047**	*0.016369301*	*0.025238808*	*0.025336199*	*0.015340147*
**ENSG00000131370**	*0.031136411*	*0.030783695*	*0.034834139*	*0.027865204*
**ENSG00000149300**	*0.014760353*	*0.024948885*	*0.026348513*	*0.015048*
**ENSG00000078549**	*0.015581847*	*0.025553695*	*0.024134092*	*0.015000438*
**ENSG00000198722**	*0.052716914*	*0.064658847*	*0.042441432*	*0.044352623*
**ENSG00000146802**	*0.029323744*	*0.03477045*	*0.033486344*	*0.023522956*
**ENSG00000176022**	*0.041148536*	*0.0481714*	*0.049363414*	*0.047123986*
**ENSG00000117906**	*0.033155911*	*0.039184215*	*0.033447254*	*0.028679173*
**ENSG00000145781**	*0.032684296*	*0.039876405*	*0.034191509*	*0.027517985*
**ENSG00000206047**	*0.016263544*	*0.025920487*	*0.02534266*	*0.016356382*
**ENSG00000166822**	*0.033403276*	*0.034959276*	*0.036012307*	*0.029667685*
**ENSG00000150764**	*0.027380606*	*0.032167508*	*0.031333525*	*0.024423238*
**ENSG00000071203**	*0.014674934*	*0.024047173*	*0.023658679*	*0.01463859*
**ENSG00000160055**	*0.02571023*	*0.033378552*	*0.026627517*	*0.021183513*
**ENSG00000175866**	*0.016449214*	*0.026326122*	*0.024232725*	*0.019444306*
**ENSG00000137948**	*0.015890076*	*0.02543098*	*0.02467548*	*0.016194242*
**ENSG00000138483**	*0.017278236*	*0.027416306*	*0.025842269*	*0.017271694*
**ENSG00000127241**	*0.015451487*	*0.026487962*	*0.025851138*	*0.015557147*
**ENSG00000136883**	*0.021579338*	*0.028022237*	*0.028803181*	*0.017275522*
**ENSG00000123136**	*0.033566685*	*0.031827394*	*0.03349523*	*0.032499395*
**ENSG00000108557**	*0.034911754*	*0.045350304*	*0.035352876*	*0.045790633*
**ENSG00000164106**	*0.015163521*	*0.024643918*	*0.024170196*	*0.014767707*
**ENSG00000009724**	*0.017291028*	*0.024861167*	*0.02463603*	*0.015939602*
**ENSG00000137266**	*0.080643048*	*0.098823845*	*0.034128709*	*0.033386855*
**ENSG00000062194**	*0.02714987*	*0.035408521*	*0.030979319*	*0.022377374*
**ENSG00000168884**	*0.026562279*	*0.033668823*	*0.037376215*	*0.026001821*
**ENSG00000170502**	*0.031691502*	*0.033164098*	*0.029690305*	*0.02425743*
**ENSG00000007908**	*0.016070513*	*0.024966667*	*0.024597074*	*0.015766897*
**ENSG00000164168**	*0.026223565*	*0.037154468*	*0.038595974*	*0.027552367*
**ENSG00000139714**	*0.018916369*	*0.025165821*	*0.025397159*	*0.017852908*
**ENSG00000070367**	*0.025943157*	*0.031993822*	*0.030474357*	*0.033565797*
**ENSG00000146247**	*0.027638434*	*0.033004652*	*0.029148837*	*0.022323402*
**ENSG00000198033**	*0.039638547*	*0.038091752*	*0.039733637*	*0.059755327*
**ENSG00000092931**	*0.027959397*	*0.03356792*	*0.039696795*	*0.02512151*
**ENSG00000204704**	*0.014495603*	*0.024028215*	*0.024220059*	*0.01511373*
**ENSG00000146731**	*0.031416071*	*0.035884427*	*0.031631765*	*0.030587058*
**ENSG00000108839**	*0.019691019*	*0.025792957*	*0.028001247*	*0.018993734*
**ENSG00000136305**	*0.054693065*	*0.050106444*	*0.03978441*	*0.03682303*
**ENSG00000148468**	*0.104930597*	*0.093178193*	*0.05752117*	*0.089370706*
**ENSG00000211452**	*0.017276152*	*0.026138469*	*0.025156284*	*0.017531829*
**ENSG00000101265**	*0.018787485*	*0.028944578*	*0.028297255*	*0.018590155*
**ENSG00000106086**	*0.015650402*	*0.025353228*	*0.025523861*	*0.014511274*
**ENSG00000198837**	*0.024894516*	*0.030188889*	*0.031084296*	*0.021816097*
**ENSG00000102678**	*0.028524221*	*0.029722827*	*0.02910096*	*0.020965281*
**ENSG00000187147**	*0.028768871*	*0.03541215*	*0.04032711*	*0.027092409*
**ENSG00000182379**	*0.017578544*	*0.028891137*	*0.028203692*	*0.018919967*
**ENSG00000169302**	*0.022301143*	*0.025031299*	*0.024631851*	*0.014977075*
**ENSG00000055950**	*0.03064162*	*0.04393088*	*0.037869209*	*0.036160202*
**ENSG00000153140**	*0.03974472*	*0.039791967*	*0.031672461*	*0.034454819*
**ENSG00000198756**	*0.078740433*	*0.055356744*	*0.027702991*	*0.026964361*
**ENSG00000160963**	*0.017550562*	*0.025577146*	*0.026500194*	*0.015244427*

**ENSG00000179295**	*0.033863796*	*0.037480474*	*0.032107903*	*0.026851343*
**ENSG00000180543**	*0.114622931*	*0.065691291*	*0.067054113*	*0.086288406*
**ENSG00000168077**	*0.014939468*	*0.025202387*	*0.025827089*	*0.015106821*
**ENSG00000139631**	*0.034221236*	*0.037283731*	*0.034685166*	*0.041300757*
**ENSG00000188266**	*0.057591674*	*0.039859555*	*0.036234132*	*0.03928034*
**ENSG00000198967**	*0.014640189*	*0.024243335*	*0.025070977*	*0.01481584*
**ENSG00000162441**	*0.042865374*	*0.060775402*	*0.044080346*	*0.032792209*
**ENSG00000006837**	*0.033166851*	*0.034843661*	*0.036633056*	*0.032305138*
**ENSG00000085514**	*0.071707004*	*0.056637358*	*0.04154912*	*0.05192633*
**ENSG00000188056**	*0.017279918*	*0.025631599*	*0.025080381*	*0.017570088*
**ENSG00000196923**	*0.046426733*	*0.039417175*	*0.045996564*	*0.047125263*
**ENSG00000152266**	*0.015897396*	*0.025271559*	*0.024852959*	*0.017018592*
**ENSG00000185359**	*0.02469602*	*0.030001066*	*0.03264901*	*0.019427381*
**ENSG00000165355**	*0.031849221*	*0.033793772*	*0.03499839*	*0.027892235*
**ENSG00000176681**	*0.018853162*	*0.026175768*	*0.026517655*	*0.016555685*
**ENSG00000165152**	*0.019268826*	*0.0255214*	*0.026038474*	*0.01636893*
**ENSG00000179055**	*0.015564599*	*0.025857821*	*0.025311847*	*0.016031879*
**ENSG00000111319**	*0.01621233*	*0.024467959*	*0.025266062*	*0.014916128*
**ENSG00000167759**	*0.016527708*	*0.02478122*	*0.02566435*	*0.015939597*
**ENSG00000106245**	*0.020949088*	*0.028548979*	*0.02947953*	*0.020487618*
**ENSG00000186566**	*0.015755404*	*0.024696871*	*0.024149246*	*0.015300779*
**ENSG00000168952**	*0.147477451*	*0.096192603*	*0.073966329*	*0.066071945*
**ENSG00000164124**	*0.025314949*	*0.029071463*	*0.028423977*	*0.023272627*
**ENSG00000126353**	*0.050737087*	*0.052576306*	*0.051365349*	*0.06075798*
**ENSG00000169249**	*0.037016229*	*0.042136935*	*0.040123314*	*0.040331608*
**ENSG00000166444**	*0.017882256*	*0.027016474*	*0.025606022*	*0.019344096*
**ENSG00000138073**	*0.033433453*	*0.030767514*	*0.033967116*	*0.03106554*
**ENSG00000069122**	*0.01614816*	*0.02486275*	*0.025219404*	*0.015959296*
**ENSG00000147206**	*0.015435489*	*0.023883195*	*0.024357799*	*0.014082028*
**ENSG00000115053**	*0.043012709*	*0.029628503*	*0.033181096*	*0.026947098*
**ENSG00000166598**	*0.029510093*	*0.033016347*	*0.034395833*	*0.03006179*
**ENSG00000116670**	*0.029114149*	*0.028538247*	*0.029718531*	*0.022156348*
**ENSG00000241685**	*0.025911893*	*0.031446836*	*0.029080586*	*0.021714216*
**ENSG00000061337**	*0.018415861*	*0.025773072*	*0.025343827*	*0.016161995*
**ENSG00000175213**	*0.021515001*	*0.028351388*	*0.038133481*	*0.021095301*
**ENSG00000187172**	*0.019168224*	*0.028665713*	*0.028328289*	*0.022399875*
**ENSG00000111011**	*0.026932339*	*0.033799458*	*0.029987358*	*0.01968027*
**ENSG00000167468**	*0.026944595*	*0.030754618*	*0.032025766*	*0.024554504*
**ENSG00000198624**	*0.038603254*	*0.036118912*	*0.036184884*	*0.027454563*
**ENSG00000112715**	*0.027680643*	*0.033647027*	*0.028594696*	*0.023160651*
**ENSG00000170044**	*0.017744366*	*0.025055492*	*0.024303455*	*0.015683511*
**ENSG00000175634**	*0.034419659*	*0.037187366*	*0.036485363*	*0.033003497*
**ENSG00000153395**	*0.03627385*	*0.035457829*	*0.036865984*	*0.03656992*
**ENSG00000110955**	*0.016602628*	*0.025274413*	*0.025156744*	*0.016102545*
**ENSG00000130383**	*0.019106972*	*0.024688455*	*0.02490428*	*0.01808444*
**ENSG00000090269**	*0.015575571*	*0.024920676*	*0.024095284*	*0.014536041*
**ENSG00000183305**	*0.016763204*	*0.024606553*	*0.026944662*	*0.015124167*
**ENSG00000158985**	*0.032529545*	*0.041257642*	*0.040447156*	*0.027271057*
**ENSG00000159248**	*0.018054996*	*0.025511428*	*0.024779202*	*0.016400876*
**ENSG00000136895**	*0.044385973*	*0.041056145*	*0.03554142*	*0.041386239*
**ENSG00000121410**	*0.021830552*	*0.029839139*	*0.026782644*	*0.021137657*
**ENSG00000135974**	*0.036527634*	*0.033461148*	*0.03413208*	*0.031199237*
**ENSG00000167748**	*0.05119244*	*0.038552398*	*0.039148026*	*0.051455361*
**ENSG00000128271**	*0.033156964*	*0.041943315*	*0.039287108*	*0.037673229*

**ENSG00000173705**	*0.017377525*	*0.025008243*	*0.027087256*	*0.017321855*
**ENSG00000120314**	*0.025651675*	*0.033509866*	*0.031726709*	*0.023563878*
**ENSG00000086666**	*0.042432402*	*0.057425303*	*0.044393106*	*0.053719283*
**ENSG00000164087**	*0.045245599*	*0.045999392*	*0.039806288*	*0.03230437*
**ENSG00000180346**	*0.052957274*	*0.048039158*	*0.039869663*	*0.04048317*
**ENSG00000180245**	*0.01513542*	*0.025163613*	*0.025828685*	*0.014049807*
**ENSG00000143294**	*0.026131888*	*0.034046792*	*0.035851834*	*0.02492369*
**ENSG00000006607**	*0.029943101*	*0.031524359*	*0.030906781*	*0.029362127*
**ENSG00000178307**	*0.026801401*	*0.030634231*	*0.033952535*	*0.02405696*
**ENSG00000107864**	*0.054932909*	*0.050117566*	*0.043076252*	*0.043879118*
**ENSG00000205777**	*0.018782885*	*0.026537338*	*0.025587368*	*0.017989908*
**ENSG00000116793**	*0.045763294*	*0.053318251*	*0.03979925*	*0.037585874*
**ENSG00000170085**	*0.032981601*	*0.037543748*	*0.03383798*	*0.021861163*
**ENSG00000128159**	*0.021315555*	*0.030760521*	*0.035129*	*0.026232834*
**ENSG00000150477**	*0.022846925*	*0.031204535*	*0.027165187*	*0.019738457*
**ENSG00000101000**	*0.043218321*	*0.033089366*	*0.032789965*	*0.035750318*
**ENSG00000134265**	*0.02945091*	*0.035042536*	*0.037118713*	*0.025856581*
**ENSG00000185674**	*0.014397685*	*0.025188151*	*0.024346032*	*0.013822785*
**ENSG00000099284**	*0.047914593*	*0.033405775*	*0.027165941*	*0.03433831*
**ENSG00000179915**	*0.014883595*	*0.02583291*	*0.024238423*	*0.015532162*
**ENSG00000241119**	*0.015800377*	*0.025434886*	*0.025431304*	*0.016031498*
**ENSG00000144407**	*0.032228835*	*0.047594068*	*0.042486723*	*0.029280862*
**ENSG00000203782**	*0.017749399*	*0.032905941*	*0.025524368*	*0.015243397*
**ENSG00000143226**	*0.053046191*	*0.035348527*	*0.031816359*	*0.02659194*
**ENSG00000168894**	*0.022647179*	*0.027290314*	*0.028904747*	*0.018758411*
**ENSG00000171936**	*0.016571192*	*0.02526155*	*0.024706818*	*0.015451025*
**ENSG00000184492**	*0.017664427*	*0.024616434*	*0.026283523*	*0.017581959*
**ENSG00000177383**	*0.041784175*	*0.040383933*	*0.032035226*	*0.033394416*
**ENSG00000102030**	*0.028111978*	*0.030606443*	*0.02776622*	*0.026555593*
**ENSG00000172955**	*0.016399601*	*0.025592017*	*0.025635046*	*0.015185827*
**ENSG00000120262**	*0.016244433*	*0.025625505*	*0.02418956*	*0.015733858*
**ENSG00000166049**	*0.01671733*	*0.026076866*	*0.025076893*	*0.015964958*
**ENSG00000168936**	*0.01885311*	*0.028714771*	*0.029293042*	*0.020838753*
**ENSG00000185361**	*0.036575604*	*0.038395721*	*0.036821956*	*0.038604676*
**ENSG00000169919**	*0.030816504*	*0.029951771*	*0.030814712*	*0.030562298*
**ENSG00000168765**	*0.020584121*	*0.028647011*	*0.027540666*	*0.028029055*
**ENSG00000204176**	*0.05795889*	*0.036660287*	*0.037463883*	*0.034098914*
**ENSG00000138386**	*0.04254917*	*0.041646262*	*0.038185907*	*0.038430657*
**ENSG00000118113**	*0.015623764*	*0.025117678*	*0.025222409*	*0.014850957*
**ENSG00000143179**	*0.034424524*	*0.034076166*	*0.034043426*	*0.034972145*
**ENSG00000112273**	*0.017311338*	*0.024896053*	*0.024257056*	*0.014866529*
**ENSG00000174243**	*0.023311201*	*0.030582821*	*0.032561483*	*0.021744515*
**ENSG00000171862**	*0.034157234*	*0.038585178*	*0.03784461*	*0.031195455*
**ENSG00000101542**	*0.015234126*	*0.025203402*	*0.025228594*	*0.014789528*
**ENSG00000137818**	*0.014310435*	*0.02340775*	*0.024200901*	*0.016141185*
**ENSG00000127804**	*0.021154666*	*0.026318275*	*0.026137293*	*0.018326104*
**ENSG00000125998**	*0.015606291*	*0.024392482*	*0.025860786*	*0.014943831*
**ENSG00000121578**	*0.03004818*	*0.036249931*	*0.03329582*	*0.027977355*
**ENSG00000136630**	*0.068159921*	*0.06446153*	*0.030453614*	*0.024554045*
**ENSG00000165494**	*0.03355923*	*0.041150951*	*0.03719043*	*0.030876816*
**ENSG00000132716**	*0.030960935*	*0.034537253*	*0.034185504*	*0.024837106*
**ENSG00000011600**	*0.029641255*	*0.040385001*	*0.038053904*	*0.03345*
**ENSG00000118496**	*0.036456797*	*0.040018417*	*0.043765082*	*0.038730953*
**ENSG00000132522**	*0.015481787*	*0.025468909*	*0.025084263*	*0.015333671*

**ENSG00000168065**	*0.016409735*	*0.024218109*	*0.026111846*	*0.015783759*
**ENSG00000035681**	*0.031445848*	*0.035421397*	*0.033961385*	*0.032450788*
**ENSG00000125485**	*0.016355073*	*0.024973377*	*0.024407008*	*0.014661504*
**ENSG00000049541**	*0.029059228*	*0.033301368*	*0.032256043*	*0.024026051*
**ENSG00000215644**	*0.031250233*	*0.049323288*	*0.025833444*	*0.028572397*
**ENSG00000168454**	*0.018213926*	*0.02565289*	*0.025784713*	*0.01524102*
**ENSG00000184863**	*0.020725432*	*0.030774049*	*0.031964431*	*0.022065821*
**ENSG00000116809**	*0.026872149*	*0.02763581*	*0.037250441*	*0.022720129*
**ENSG00000181733**	*0.018133808*	*0.027097622*	*0.026172148*	*0.016062228*
**ENSG00000130997**	*0.018256325*	*0.02772961*	*0.026157028*	*0.020147409*
**ENSG00000180209**	*0.016637428*	*0.024977071*	*0.024003786*	*0.015577229*
**ENSG00000165661**	*0.032746044*	*0.037481962*	*0.035338518*	*0.028057326*
**ENSG00000183386**	*0.070120018*	*0.049403226*	*0.058161909*	*0.057735292*
**ENSG00000121775**	*0.024448823*	*0.027986514*	*0.03512463*	*0.023524843*
**ENSG00000121068**	*0.037994995*	*0.037648343*	*0.036863124*	*0.041851458*
**ENSG00000186603**	*0.077854019*	*0.04737762*	*0.047520487*	*0.049119452*
**ENSG00000164904**	*0.098898906*	*0.068154154*	*0.063116659*	*0.075231456*
**ENSG00000105143**	*0.018670874*	*0.026374098*	*0.025870622*	*0.019290786*
**ENSG00000123560**	*0.015897902*	*0.024325838*	*0.02432004*	*0.01615102*
**ENSG00000173065**	*0.030862134*	*0.037595972*	*0.035780376*	*0.027144575*
**ENSG00000042753**	*0.0230549*	*0.029598843*	*0.027055397*	*0.021176462*
**ENSG00000198336**	*0.024325646*	*0.026744348*	*0.029260336*	*0.025871393*
**ENSG00000134686**	*0.017596547*	*0.026334826*	*0.025918521*	*0.01760017*
**ENSG00000167578**	*0.028937976*	*0.033430711*	*0.031715912*	*0.031174846*
**ENSG00000240694**	*0.016607615*	*0.029894298*	*0.025316631*	*0.01482035*
**ENSG00000232258**	*0.05345262*	*0.057169882*	*0.077071682*	*0.047443621*
**ENSG00000158571**	*0.016880928*	*0.026017266*	*0.025315953*	*0.016567838*
**ENSG00000188782**	*0.019296651*	*0.029239188*	*0.030272241*	*0.018280779*
**ENSG00000167004**	*0.023257432*	*0.030067456*	*0.030281353*	*0.023753043*
**ENSG00000105677**	*0.029389957*	*0.029691523*	*0.028192533*	*0.026775564*
**ENSG00000134817**	*0.016611971*	*0.026792509*	*0.025446113*	*0.016898685*
**ENSG00000131143**	*0.016900768*	*0.025069454*	*0.025844153*	*0.016363059*
**ENSG00000101974**	*0.034897233*	*0.04031091*	*0.034449571*	*0.040190071*
**ENSG00000140548**	*0.045995939*	*0.026732005*	*0.025751528*	*0.018279429*
**ENSG00000084073**	*0.031994561*	*0.036300773*	*0.035439237*	*0.039910613*
**ENSG00000141013**	*0.063996645*	*0.059490551*	*0.061488411*	*0.071968459*
**ENSG00000243725**	*0.028109104*	*0.034652687*	*0.03833253*	*0.026654223*
**ENSG00000128185**	*0.036629334*	*0.034113587*	*0.034302807*	*0.024523509*
**ENSG00000160633**	*0.02828726*	*0.031317196*	*0.034025455*	*0.026102784*
**ENSG00000164924**	*0.042972692*	*0.053368771*	*0.05154608*	*0.049727693*
**ENSG00000178821**	*0.036687219*	*0.039029688*	*0.041930662*	*0.029964709*
**ENSG00000198740**	*0.027907556*	*0.030603057*	*0.030696275*	*0.02434987*
**ENSG00000135625**	*0.022481206*	*0.026913561*	*0.025277176*	*0.017300677*
**ENSG00000047932**	*0.028699413*	*0.032474705*	*0.036373018*	*0.025145979*
**ENSG00000140326**	*0.03197221*	*0.037946374*	*0.034170891*	*0.025788129*
**ENSG00000229035**	*0.069206771*	*0.038341954*	*0.026170213*	*0.041739906*
**ENSG00000146085**	*0.025659499*	*0.031946131*	*0.03164932*	*0.021736645*
**ENSG00000013374**	*0.039425184*	*0.043309196*	*0.034526642*	*0.032106937*
**ENSG00000181433**	*0.015545953*	*0.025964206*	*0.025520143*	*0.014820442*
**ENSG00000105991**	*0.016610059*	*0.025326094*	*0.024045169*	*0.015529476*
**ENSG00000187720**	*0.018721067*	*0.026892358*	*0.02641007*	*0.018193882*
**ENSG00000127578**	*0.016790342*	*0.026750153*	*0.025888478*	*0.015979965*
**ENSG00000168005**	*0.026110261*	*0.031848625*	*0.034278837*	*0.026408172*
**ENSG00000197930**	*0.048736995*	*0.038577989*	*0.042450168*	*0.034375523*

**ENSG00000072736**	*0.032958102*	*0.032764982*	*0.032239611*	*0.033113705*
**ENSG00000174851**	*0.026859994*	*0.029424505*	*0.031896255*	*0.024974267*
**ENSG00000129194**	*0.016313075*	*0.024057268*	*0.025413204*	*0.016775775*
**ENSG00000143469**	*0.020021107*	*0.027297669*	*0.026299425*	*0.019100132*
**ENSG00000105392**	*0.014930888*	*0.02561597*	*0.024877651*	*0.016388747*
**ENSG00000121871**	*0.015484829*	*0.026248964*	*0.024460696*	*0.016252899*
**ENSG00000169696**	*0.018249404*	*0.026980481*	*0.028598315*	*0.019042472*
**ENSG00000148680**	*0.056205418*	*0.025367298*	*0.026519143*	*0.014256785*
**ENSG00000175792**	*0.036219775*	*0.037579437*	*0.03178466*	*0.031211414*
**ENSG00000163697**	*0.027067857*	*0.024380973*	*0.026071841*	*0.017881628*
**ENSG00000198417**	*0.05959506*	*0.054247915*	*0.065990155*	*0.046478925*
**ENSG00000149554**	*0.042740883*	*0.040237891*	*0.033955531*	*0.039884961*
**ENSG00000179673**	*0.014848864*	*0.024397665*	*0.025113046*	*0.015580202*
**ENSG00000104361**	*0.016858353*	*0.025231479*	*0.025046994*	*0.017363442*
**ENSG00000132010**	*0.03948256*	*0.033746523*	*0.036205487*	*0.02975761*
**ENSG00000167202**	*0.03595884*	*0.042285661*	*0.033828207*	*0.031383935*
**ENSG00000180917**	*0.041876102*	*0.040224867*	*0.039297181*	*0.039589889*
**ENSG00000145194**	*0.015624472*	*0.023911988*	*0.024261348*	*0.014977759*
**ENSG00000106636**	*0.024808899*	*0.032926723*	*0.037691617*	*0.029424626*
**ENSG00000138663**	*0.026022871*	*0.031068845*	*0.030193018*	*0.02246301*
**ENSG00000135317**	*0.023464889*	*0.027635066*	*0.031084312*	*0.021306594*
**ENSG00000118507**	*0.10345725*	*0.078776022*	*0.097584162*	*0.08859851*
**ENSG00000196510**	*0.025631833*	*0.031733666*	*0.031010312*	*0.0254283*
**ENSG00000150681**	*0.016967729*	*0.027237106*	*0.025564519*	*0.016119681*
**ENSG00000187808**	*0.025082201*	*0.032652219*	*0.029037549*	*0.025646701*
**ENSG00000124733**	*0.027927033*	*0.0308695*	*0.02843291*	*0.02348597*
**ENSG00000143127**	*0.016295281*	*0.025241838*	*0.026140108*	*0.015226356*
**ENSG00000160908**	*0.032195121*	*0.046238341*	*0.057784408*	*0.035045158*
**ENSG00000130749**	*0.018312598*	*0.028513523*	*0.02560699*	*0.017678073*
**ENSG00000238243**	*0.015576489*	*0.025356353*	*0.025335863*	*0.017223579*
**ENSG00000125375**	*0.030469588*	*0.03536329*	*0.032354107*	*0.029176472*
**ENSG00000204963**	*0.015620293*	*0.025286946*	*0.024767664*	*0.014542309*
**ENSG00000163812**	*0.027803789*	*0.03438784*	*0.03008612*	*0.028781257*
**ENSG00000122783**	*0.036896592*	*0.040985966*	*0.033573584*	*0.038092089*
**ENSG00000171530**	*0.022196138*	*0.026683455*	*0.026314946*	*0.018508009*
**ENSG00000011295**	*0.031393157*	*0.031308626*	*0.032985476*	*0.029182602*
**ENSG00000182156**	*0.01682513*	*0.026293654*	*0.025636328*	*0.016528573*
**ENSG00000215704**	*0.015842509*	*0.024782416*	*0.025527191*	*0.015108124*
**ENSG00000100095**	*0.015648424*	*0.026437193*	*0.025542278*	*0.015798858*
**ENSG00000172156**	*0.016099386*	*0.025861297*	*0.025969957*	*0.016142469*
**ENSG00000123360**	*0.021006456*	*0.02675754*	*0.028120017*	*0.019618665*
**ENSG00000160014**	*0.034464111*	*0.040017913*	*0.03470795*	*0.031083389*
**ENSG00000143033**	*0.026521967*	*0.031287752*	*0.03294945*	*0.021342443*
**ENSG00000204577**	*0.112020271*	*0.072534537*	*0.063434411*	*0.049034996*
**ENSG00000076248**	*0.035376773*	*0.0396469*	*0.030454613*	*0.031499417*
**ENSG00000127362**	*0.014606594*	*0.024659101*	*0.024733924*	*0.015505016*
**ENSG00000131503**	*0.039689323*	*0.042935551*	*0.040088063*	*0.035233368*
**ENSG00000154611**	*0.17555734*	*0.105514857*	*0.094227941*	*0.134518227*
**ENSG00000107874**	*0.022638148*	*0.029185714*	*0.028710048*	*0.019365003*
**ENSG00000163141**	*0.018708902*	*0.025665424*	*0.025447215*	*0.0172816*
**ENSG00000112280**	*0.015268245*	*0.024094459*	*0.026289776*	*0.014088395*
**ENSG00000174599**	*0.028201232*	*0.024650922*	*0.024982336*	*0.018821772*
**ENSG00000138029**	*0.021629512*	*0.027818113*	*0.028408845*	*0.020934791*
**ENSG00000185803**	*0.03162137*	*0.03641137*	*0.044612055*	*0.033384703*

**ENSG00000077782**	*0.017202039*	*0.025578642*	*0.025241616*	*0.016634725*
**ENSG00000073670**	*0.016789995*	*0.026557811*	*0.024971379*	*0.015037072*
**ENSG00000165688**	*0.023009914*	*0.028502987*	*0.033363422*	*0.024563777*
**ENSG00000080166**	*0.016491641*	*0.025625658*	*0.024986499*	*0.015943557*
**ENSG00000119760**	*0.016696598*	*0.02609605*	*0.024756387*	*0.0158917*
**ENSG00000120756**	*0.049321333*	*0.035825133*	*0.032968184*	*0.035278885*
**ENSG00000157330**	*0.014764739*	*0.024920976*	*0.025022768*	*0.014169922*
**ENSG00000166847**	*0.027204656*	*0.032075647*	*0.035823563*	*0.025154575*
**ENSG00000182591**	*0.018598807*	*0.027385892*	*0.026806705*	*0.020895188*
**ENSG00000048028**	*0.047055772*	*0.054307724*	*0.039787391*	*0.034092536*
**ENSG00000138036**	*0.025037547*	*0.028200288*	*0.027101005*	*0.027061643*
**ENSG00000188229**	*0.029862299*	*0.027675382*	*0.033179048*	*0.029942521*
**ENSG00000159674**	*0.080256956*	*0.070713382*	*0.064027902*	*0.074266556*
**ENSG00000166106**	*0.015660743*	*0.024413287*	*0.024581924*	*0.014963322*
**ENSG00000184708**	*0.035190352*	*0.039005845*	*0.039661576*	*0.032734774*
**ENSG00000134864**	*0.051105726*	*0.077754941*	*0.079966213*	*0.052275653*
**ENSG00000127399**	*0.024041831*	*0.031176186*	*0.030626018*	*0.023040365*
**ENSG00000182944**	*0.030932327*	*0.033602467*	*0.035519034*	*0.030284095*
**ENSG00000106415**	*0.052131133*	*0.056975934*	*0.043848591*	*0.042063304*
**ENSG00000073060**	*0.032141069*	*0.034937817*	*0.03456805*	*0.027172321*
**ENSG00000111266**	*0.038410211*	*0.038086978*	*0.041095119*	*0.048279052*
**ENSG00000196591**	*0.024681047*	*0.030829991*	*0.028409833*	*0.024798154*
**ENSG00000099953**	*0.017659738*	*0.026324517*	*0.026603839*	*0.016520502*
**ENSG00000145736**	*0.035054715*	*0.041564726*	*0.040169023*	*0.033966683*
**ENSG00000198668**	*0.031612434*	*0.036488672*	*0.034106088*	*0.031802492*
**ENSG00000145495**	*0.03817244*	*0.040698234*	*0.035469579*	*0.037191838*
**ENSG00000176909**	*0.01807038*	*0.024704292*	*0.024144844*	*0.017684603*
**ENSG00000067167**	*0.025828655*	*0.031746484*	*0.034071231*	*0.025998696*
**ENSG00000100628**	*0.116295001*	*0.095722425*	*0.085660323*	*0.119740684*
**ENSG00000100395**	*0.033210028*	*0.034359789*	*0.035979216*	*0.030754601*
**ENSG00000125285**	*0.015325236*	*0.024604153*	*0.024997757*	*0.014579281*
**ENSG00000170681**	*0.015482481*	*0.024692064*	*0.025194453*	*0.01442265*
**ENSG00000121989**	*0.041114143*	*0.030776124*	*0.026546972*	*0.025304776*
**ENSG00000101888**	*0.032595604*	*0.034820989*	*0.032165696*	*0.02297181*
**ENSG00000149489**	*0.017012536*	*0.025759683*	*0.026295566*	*0.015958486*
**ENSG00000146463**	*0.036819606*	*0.043699271*	*0.032321966*	*0.02984753*
**ENSG00000166090**	*0.017460097*	*0.025523213*	*0.025554841*	*0.015769258*
**ENSG00000154165**	*0.05504959*	*0.053227364*	*0.045711448*	*0.048808483*
**ENSG00000163840**	*0.045729697*	*0.050981809*	*0.049759042*	*0.041392833*
**ENSG00000132819**	*0.037905433*	*0.043899886*	*0.036266734*	*0.038592821*
**ENSG00000072195**	*0.016854747*	*0.025542265*	*0.025687255*	*0.016037466*
**ENSG00000132591**	*0.025887555*	*0.032505079*	*0.036463769*	*0.029843933*
**ENSG00000205358**	*0.055379313*	*0.060908842*	*0.065766204*	*0.06383946*
**ENSG00000135374**	*0.015553195*	*0.025373053*	*0.024964288*	*0.015661439*
**ENSG00000170017**	*0.039294821*	*0.043608481*	*0.040930609*	*0.043075664*
**ENSG00000132323**	*0.027301397*	*0.032156252*	*0.032710241*	*0.023969415*
**ENSG00000099250**	*0.020109635*	*0.027537952*	*0.027582947*	*0.016313496*
**ENSG00000168143**	*0.01599816*	*0.024093952*	*0.025252515*	*0.014816545*
**ENSG00000188747**	*0.017232985*	*0.026451921*	*0.026734586*	*0.019030577*
**ENSG00000141526**	*0.080097525*	*0.049901896*	*0.044785381*	*0.049855164*
**ENSG00000101489**	*0.020124409*	*0.028433662*	*0.026982482*	*0.018315806*
**ENSG00000149292**	*0.028975216*	*0.028775235*	*0.027184343*	*0.034971279*
**ENSG00000118137**	*0.017253085*	*0.025367026*	*0.025518612*	*0.018899483*
**ENSG00000155090**	*0.0456647*	*0.047020941*	*0.042502666*	*0.035070868*

**ENSG00000160752**	*0.023744017*	*0.02830442*	*0.02650764*	*0.019859463*
**ENSG00000101298**	*0.016327215*	*0.024394658*	*0.025828375*	*0.014831002*
**ENSG00000147669**	*0.02324956*	*0.029197606*	*0.027426806*	*0.021385208*
**ENSG00000144891**	*0.015950347*	*0.024293056*	*0.024987993*	*0.014431238*
**ENSG00000104671**	*0.027580391*	*0.032479756*	*0.032171405*	*0.022230486*
**ENSG00000111046**	*0.024013746*	*0.026695242*	*0.025539299*	*0.018909825*
**ENSG00000110756**	*0.029042924*	*0.032732698*	*0.029603837*	*0.024860575*
**ENSG00000188322**	*0.012709858*	*0.023716982*	*0.023823376*	*0.013691245*
**ENSG00000163703**	*0.052836674*	*0.045963037*	*0.036638244*	*0.044430304*
**ENSG00000114520**	*0.02901546*	*0.029953968*	*0.029537812*	*0.030147178*
**ENSG00000203618**	*0.045107351*	*0.037599805*	*0.035394955*	*0.025693437*
**ENSG00000196944**	*0.016490322*	*0.025852586*	*0.025027866*	*0.016256163*
**ENSG00000129116**	*0.099054911*	*0.041396884*	*0.040540436*	*0.037777006*
**ENSG00000076928**	*0.024469798*	*0.034915379*	*0.044884557*	*0.036861583*
**ENSG00000185313**	*0.017117378*	*0.025201817*	*0.025002795*	*0.014338806*
**ENSG00000185651**	*0.034380551*	*0.038012425*	*0.03704272*	*0.033281442*
**ENSG00000103496**	*0.025512773*	*0.029324553*	*0.030602548*	*0.026149574*
**ENSG00000185627**	*0.023435631*	*0.029757205*	*0.029150451*	*0.024067386*
**ENSG00000099814**	*0.039669938*	*0.035828707*	*0.034764386*	*0.030235222*
**ENSG00000092871**	*0.025662976*	*0.029908789*	*0.024868789*	*0.021145738*
**ENSG00000176714**	*0.018635402*	*0.027076805*	*0.025996008*	*0.020064787*
**ENSG00000127993**	*0.019053323*	*0.027328036*	*0.026359488*	*0.016800526*
**ENSG00000171517**	*0.014819036*	*0.024381422*	*0.025068519*	*0.014417699*
**ENSG00000155269**	*0.016254371*	*0.025546506*	*0.025300529*	*0.015964017*
**ENSG00000071894**	*0.031670386*	*0.041490153*	*0.03553148*	*0.026739951*
**ENSG00000100075**	*0.028315914*	*0.029516933*	*0.034064329*	*0.029295714*
**ENSG00000165506**	*0.030782511*	*0.033395178*	*0.030676214*	*0.029756068*
**ENSG00000180210**	*0.017024575*	*0.026578057*	*0.026534852*	*0.015934558*
**ENSG00000129696**	*0.026221075*	*0.03174163*	*0.031877727*	*0.023936122*
**ENSG00000198576**	*0.015951004*	*0.024979747*	*0.024344168*	*0.016251055*
**ENSG00000103355**	*0.014777531*	*0.025835949*	*0.024704833*	*0.016312046*
**ENSG00000156398**	*0.063527081*	*0.069808637*	*0.056248131*	*0.049521799*
**ENSG00000204450**	*0.01538022*	*0.024642589*	*0.02474729*	*0.015605375*
**ENSG00000105926**	*0.038454521*	*0.035094093*	*0.03470398*	*0.033222732*
**ENSG00000205517**	*0.020982153*	*0.028086303*	*0.030041559*	*0.030658521*
**ENSG00000114302**	*0.026678364*	*0.030958661*	*0.029504165*	*0.025822617*
**ENSG00000064692**	*0.09588746*	*0.03540795*	*0.037433026*	*0.024968663*
**ENSG00000083812**	*0.019503183*	*0.03188108*	*0.033238465*	*0.019928546*
**ENSG00000151151**	*0.017348636*	*0.026585856*	*0.027001984*	*0.018556315*
**ENSG00000213920**	*0.026132572*	*0.029792906*	*0.029254253*	*0.026220157*
**ENSG00000151790**	*0.063334275*	*0.03079933*	*0.029354751*	*0.029756835*
**ENSG00000065809**	*0.052343008*	*0.044047984*	*0.041599654*	*0.049558306*
**ENSG00000170369**	*0.015583846*	*0.024837204*	*0.025003509*	*0.015144941*
**ENSG00000143727**	*0.022838809*	*0.029832467*	*0.031092064*	*0.024294356*
**ENSG00000196628**	*0.034760339*	*0.038695577*	*0.033845426*	*0.032434831*
**ENSG00000172733**	*0.015753371*	*0.024849177*	*0.025739963*	*0.015190767*
**ENSG00000196666**	*0.016407943*	*0.024354851*	*0.024613922*	*0.016010086*
**ENSG00000073737**	*0.102171202*	*0.098095628*	*0.095974686*	*0.128178738*
**ENSG00000183943**	*0.045700649*	*0.041016729*	*0.044425927*	*0.032827257*
**ENSG00000196482**	*0.017242917*	*0.025789514*	*0.025108583*	*0.017464238*
**ENSG00000170144**	*0.027308711*	*0.031897107*	*0.029289705*	*0.021066568*
**ENSG00000173041**	*0.03381051*	*0.036759305*	*0.030102793*	*0.026913013*
**ENSG00000099849**	*0.044995467*	*0.048858272*	*0.03813773*	*0.039582315*
**ENSG00000161082**	*0.018441103*	*0.025082533*	*0.025942162*	*0.015939449*

**ENSG00000137976**	*0.016477956*	*0.024864676*	*0.025484544*	*0.016344357*
**ENSG00000100625**	*0.017034437*	*0.026090063*	*0.027908764*	*0.020075144*
**ENSG00000185946**	*0.029739065*	*0.034265711*	*0.035755025*	*0.032673319*
**ENSG00000139173**	*0.038410859*	*0.039305907*	*0.04432615*	*0.030491187*
**ENSG00000133101**	*0.07413242*	*0.068278293*	*0.070277516*	*0.063145079*
**ENSG00000120725**	*0.03615064*	*0.035141854*	*0.039203349*	*0.036855452*
**ENSG00000188732**	*0.048058546*	*0.047212525*	*0.047804831*	*0.044641096*
**ENSG00000152454**	*0.043936205*	*0.049949923*	*0.055683099*	*0.042289408*
**ENSG00000171970**	*0.028971025*	*0.03667926*	*0.035577249*	*0.027435919*
**ENSG00000128218**	*0.084457402*	*0.078663266*	*0.081393835*	*0.086830485*
**ENSG00000121807**	*0.017742128*	*0.026075282*	*0.026440348*	*0.017404724*
**ENSG00000114120**	*0.039391028*	*0.037315966*	*0.043144254*	*0.037312679*
**ENSG00000164010**	*0.044766778*	*0.043618528*	*0.031432469*	*0.036412422*
**ENSG00000137474**	*0.01562761*	*0.023925095*	*0.024791965*	*0.016176461*
**ENSG00000044090**	*0.031133331*	*0.031210964*	*0.034829665*	*0.031956839*
**ENSG00000103249**	*0.030484654*	*0.037464972*	*0.033449755*	*0.029964689*
**ENSG00000112514**	*0.022695324*	*0.028073853*	*0.030525252*	*0.021310156*
**ENSG00000072415**	*0.031251222*	*0.033450233*	*0.035118351*	*0.031343128*
**ENSG00000139977**	*0.040386712*	*0.046384754*	*0.03838565*	*0.031598453*
**ENSG00000162804**	*0.018246711*	*0.024956357*	*0.025702805*	*0.016242563*
**ENSG00000162616**	*0.040060185*	*0.038618849*	*0.035802035*	*0.031800737*
**ENSG00000219481**	*0.028150969*	*0.034083296*	*0.030443656*	*0.025084143*
**ENSG00000170381**	*0.032868674*	*0.033565932*	*0.034626625*	*0.031562888*
**ENSG00000124102**	*0.03157304*	*0.038179*	*0.026677815*	*0.021651117*
**ENSG00000137804**	*0.038088447*	*0.036588264*	*0.032035181*	*0.024913721*
**ENSG00000071909**	*0.047335167*	*0.039569613*	*0.038791887*	*0.046433999*
**ENSG00000124789**	*0.032173537*	*0.033773866*	*0.031206459*	*0.030221442*
**ENSG00000174807**	*0.018868088*	*0.026948133*	*0.024680249*	*0.015948101*
**ENSG00000182107**	*0.0166272*	*0.025597023*	*0.024830946*	*0.016885458*
**ENSG00000104205**	*0.035421037*	*0.030873292*	*0.037261784*	*0.027646295*
**ENSG00000105556**	*0.042030856*	*0.041642755*	*0.039962827*	*0.026703797*
**ENSG00000116096**	*0.054341359*	*0.057588748*	*0.045073871*	*0.050437099*
**ENSG00000104892**	*0.024180031*	*0.028018694*	*0.027947816*	*0.017901263*
**ENSG00000135778**	*0.042378813*	*0.039398874*	*0.038449495*	*0.029971834*
**ENSG00000105419**	*0.019619236*	*0.028758442*	*0.028520834*	*0.017957255*
**ENSG00000145332**	*0.032390292*	*0.039139185*	*0.040309355*	*0.027401455*
**ENSG00000174125**	*0.052144207*	*0.07169487*	*0.069349032*	*0.064797515*
**ENSG00000140030**	*0.047147396*	*0.054487052*	*0.045954825*	*0.038122627*
**ENSG00000080298**	*0.023958862*	*0.02696602*	*0.027244441*	*0.019844655*
**ENSG00000183473**	*0.0169462*	*0.024896472*	*0.02511719*	*0.01717149*
**ENSG00000108679**	*0.040961258*	*0.079534437*	*0.07603617*	*0.090752439*
**ENSG00000142661**	*0.015252948*	*0.02471924*	*0.024541315*	*0.015577645*
**ENSG00000145708**	*0.015585572*	*0.02504623*	*0.026026501*	*0.015627464*
**ENSG00000130045**	*0.016549548*	*0.024984922*	*0.024909255*	*0.015988055*
**ENSG00000127324**	*0.01526887*	*0.025396146*	*0.024709351*	*0.015853797*
**ENSG00000184701**	*0.038421427*	*0.037890769*	*0.03494573*	*0.038116832*
**ENSG00000177889**	*0.028441883*	*0.040192843*	*0.033792602*	*0.024877782*
**ENSG00000179163**	*0.066711388*	*0.043049645*	*0.050461855*	*0.057341902*
**ENSG00000116171**	*0.025426663*	*0.030454272*	*0.028271359*	*0.021241425*
**ENSG00000165929**	*0.070154532*	*0.06837468*	*0.054421961*	*0.063433314*
**ENSG00000179010**	*0.028118147*	*0.033233505*	*0.030871901*	*0.023419413*
**ENSG00000099721**	*0.017334723*	*0.024982708*	*0.024675891*	*0.01593064*
**ENSG00000087299**	*0.028635726*	*0.03261943*	*0.030636487*	*0.02273631*
**ENSG00000171405**	*0.018229242*	*0.024521644*	*0.025316644*	*0.017466728*

**ENSG00000198643**	*0.0330844*	*0.027154286*	*0.024981718*	*0.020181791*
**ENSG00000206337**	*0.026334818*	*0.038340874*	*0.031870092*	*0.026230916*
**ENSG00000184811**	*0.015157624*	*0.025591871*	*0.026002306*	*0.015513138*
**ENSG00000119737**	*0.017779726*	*0.025194483*	*0.024955687*	*0.016125293*
**ENSG00000140470**	*0.016851707*	*0.025034879*	*0.025430562*	*0.015305256*
**ENSG00000160803**	*0.022794678*	*0.030331672*	*0.027491838*	*0.019086443*
**ENSG00000182687**	*0.023452486*	*0.026887782*	*0.030490845*	*0.021663356*
**ENSG00000132801**	*0.032063714*	*0.03237553*	*0.03020045*	*0.022712612*
**ENSG00000174788**	*0.031741003*	*0.043780591*	*0.038275905*	*0.043055625*
**ENSG00000153551**	*0.135491397*	*0.122634331*	*0.09189791*	*0.129472804*
**ENSG00000134917**	*0.01575311*	*0.024795593*	*0.026240484*	*0.015561043*
**ENSG00000108700**	*0.015357374*	*0.024737343*	*0.024897312*	*0.016029665*
**ENSG00000076043**	*0.067939347*	*0.062901497*	*0.045688553*	*0.052692212*
**ENSG00000141579**	*0.020317499*	*0.026027957*	*0.025527958*	*0.01816807*
**ENSG00000050030**	*0.065106846*	*0.056732865*	*0.045834073*	*0.058803384*
**ENSG00000154227**	*0.015436945*	*0.027121834*	*0.026015223*	*0.015464718*
**ENSG00000124786**	*0.03705662*	*0.042093044*	*0.033038963*	*0.033468255*
**ENSG00000138311**	*0.016385787*	*0.024981588*	*0.025453423*	*0.016902672*
**ENSG00000123843**	*0.101342435*	*0.064327866*	*0.0441929*	*0.06830588*
**ENSG00000119147**	*0.016960001*	*0.025021895*	*0.025090586*	*0.014576166*
**ENSG00000175197**	*0.052343924*	*0.051014205*	*0.048125537*	*0.042615162*
**ENSG00000175602**	*0.033399183*	*0.031364215*	*0.032788474*	*0.034755518*
**ENSG00000244005**	*0.02346783*	*0.030010964*	*0.027013237*	*0.020151908*
**ENSG00000105429**	*0.017503756*	*0.026193886*	*0.026300404*	*0.017103185*
**ENSG00000151532**	*0.020910636*	*0.028672355*	*0.027873715*	*0.018151124*
**ENSG00000129682**	*0.015429743*	*0.024519555*	*0.024215752*	*0.014265329*
**ENSG00000164081**	*0.025324094*	*0.030112305*	*0.030412002*	*0.022450494*
**ENSG00000115306**	*0.030083266*	*0.035955388*	*0.029488285*	*0.033791462*
**ENSG00000153563**	*0.017814806*	*0.026995213*	*0.026248273*	*0.025150452*
**ENSG00000038210**	*0.030324925*	*0.039278796*	*0.032722007*	*0.02347164*
**ENSG00000168787**	*0.015961792*	*0.025210265*	*0.025661098*	*0.015880642*
**ENSG00000104140**	*0.05495316*	*0.049304323*	*0.047563773*	*0.050572542*
**ENSG00000164849**	*0.092129325*	*0.072938499*	*0.067394225*	*0.080820845*
**ENSG00000110925**	*0.032924391*	*0.035007284*	*0.035061506*	*0.026480947*
**ENSG00000116285**	*0.063630336*	*0.055646137*	*0.043932067*	*0.048816261*
**ENSG00000186971**	*0.017292344*	*0.025493501*	*0.024845108*	*0.015617294*
**ENSG00000068654**	*0.037035268*	*0.051160509*	*0.054828007*	*0.057890421*
**ENSG00000154856**	*0.018496441*	*0.024727412*	*0.024390898*	*0.015808615*
**ENSG00000177599**	*0.015511991*	*0.024534933*	*0.024379459*	*0.014581699*
**ENSG00000198909**	*0.01593556*	*0.024900675*	*0.024962691*	*0.013836119*
**ENSG00000184903**	*0.043876281*	*0.043799063*	*0.042845235*	*0.040496806*
**ENSG00000124177**	*0.029486143*	*0.040750232*	*0.033584677*	*0.034143011*
**ENSG00000099956**	*0.039729148*	*0.039190899*	*0.044449179*	*0.038253542*
**ENSG00000235718**	*0.015811487*	*0.02614338*	*0.024870665*	*0.015012445*
**ENSG00000166987**	*0.017562984*	*0.027081628*	*0.025060842*	*0.014528915*
**ENSG00000188766**	*0.018674046*	*0.025720999*	*0.026579003*	*0.015743083*
**ENSG00000130811**	*0.017928957*	*0.025891687*	*0.026599376*	*0.018512963*
**ENSG00000203879**	*0.022746518*	*0.028979281*	*0.035024428*	*0.031598644*
**ENSG00000214513**	*0.01505341*	*0.024618992*	*0.024977908*	*0.014994707*
**ENSG00000128311**	*0.061912187*	*0.045543896*	*0.044076255*	*0.057912744*
**ENSG00000167700**	*0.050827322*	*0.040867057*	*0.044582314*	*0.037761228*
**ENSG00000178149**	*0.026008988*	*0.029953413*	*0.029738689*	*0.026302567*
**ENSG00000100138**	*0.028247668*	*0.030463882*	*0.032615802*	*0.031212684*
**ENSG00000189298**	*0.034067305*	*0.042650142*	*0.031690185*	*0.02979671*

**ENSG00000124201**	*0.030382087*	*0.044434329*	*0.048095836*	*0.031124124*
**ENSG00000091704**	*0.014983577*	*0.025822158*	*0.02480803*	*0.015899553*
**ENSG00000177058**	*0.034258609*	*0.036937121*	*0.033708284*	*0.029589114*
**ENSG00000166960**	*0.017563078*	*0.02674987*	*0.02528432*	*0.017298808*
**ENSG00000100448**	*0.016590502*	*0.025012838*	*0.024283432*	*0.015379185*
**ENSG00000213983**	*0.028936869*	*0.040959565*	*0.037525458*	*0.031309107*
**ENSG00000186318**	*0.030932928*	*0.030154896*	*0.028913384*	*0.024887555*
**ENSG00000112855**	*0.02967598*	*0.033590258*	*0.029416228*	*0.022884905*
**ENSG00000127946**	*0.026871871*	*0.029267606*	*0.028035015*	*0.026939103*
**ENSG00000166716**	*0.019798542*	*0.028090177*	*0.027536739*	*0.018399086*
**ENSG00000157570**	*0.020948765*	*0.032225813*	*0.035659678*	*0.034550493*
**ENSG00000183160**	*0.068344007*	*0.04034485*	*0.031445343*	*0.027564444*
**ENSG00000156414**	*0.026413445*	*0.026404111*	*0.031361988*	*0.020474576*
**ENSG00000105388**	*0.015854146*	*0.024807049*	*0.025100888*	*0.014797272*
**ENSG00000182634**	*0.01635088*	*0.025908835*	*0.025903659*	*0.017761585*
**ENSG00000171124**	*0.015313067*	*0.024581475*	*0.025025839*	*0.014652979*
**ENSG00000147601**	*0.032367688*	*0.034624624*	*0.036189494*	*0.029514657*
**ENSG00000162998**	*0.030784264*	*0.025778442*	*0.026494255*	*0.015878686*
**ENSG00000160211**	*0.034246473*	*0.036560987*	*0.03613913*	*0.033572393*
**ENSG00000120833**	*0.086274193*	*0.054713161*	*0.058333699*	*0.062419674*
**ENSG00000156920**	*0.017795034*	*0.025890609*	*0.026497512*	*0.015673726*
**ENSG00000081913**	*0.06408583*	*0.047071165*	*0.052598857*	*0.041981265*
**ENSG00000158402**	*0.047214039*	*0.043143937*	*0.040985663*	*0.047568751*
**ENSG00000167173**	*0.034821002*	*0.040729872*	*0.055063654*	*0.034387646*
**ENSG00000174677**	*0.017480723*	*0.025579567*	*0.027282022*	*0.017087762*
**ENSG00000163352**	*0.017226054*	*0.024630674*	*0.026604122*	*0.015021775*
**ENSG00000180613**	*0.015374289*	*0.02562546*	*0.025432291*	*0.015519476*
**ENSG00000012779**	*0.06986243*	*0.038170647*	*0.036046935*	*0.054982609*
**ENSG00000137672**	*0.016277587*	*0.024915282*	*0.024925226*	*0.014948554*
**ENSG00000162688**	*0.016133315*	*0.024471108*	*0.024498418*	*0.015051596*
**ENSG00000171956**	*0.024600238*	*0.030001479*	*0.026500891*	*0.024624571*
**ENSG00000183691**	*0.017522438*	*0.02583725*	*0.026131543*	*0.016587129*
**ENSG00000157191**	*0.03160969*	*0.034232417*	*0.032578336*	*0.034334114*
**ENSG00000188305**	*0.015618632*	*0.025068056*	*0.024625652*	*0.014081425*
**ENSG00000089094**	*0.035229656*	*0.034349245*	*0.030158644*	*0.028509175*
**ENSG00000148377**	*0.018835837*	*0.024513392*	*0.026483886*	*0.016546891*
**ENSG00000135596**	*0.036901088*	*0.041246169*	*0.0353266*	*0.030967265*
**ENSG00000149781**	*0.022592659*	*0.037323148*	*0.031193984*	*0.024258461*
**ENSG00000179965**	*0.016668544*	*0.024072224*	*0.024685944*	*0.015388339*
**ENSG00000184743**	*0.030625826*	*0.031547104*	*0.03364625*	*0.02916938*
**ENSG00000162980**	*0.033638715*	*0.041219109*	*0.039290882*	*0.032335951*
**ENSG00000139357**	*0.042903823*	*0.04211223*	*0.041087024*	*0.052173253*
**ENSG00000152484**	*0.042735758*	*0.047521161*	*0.047569429*	*0.03183389*
**ENSG00000135047**	*0.018574703*	*0.02465003*	*0.025438554*	*0.01653152*
**ENSG00000126067**	*0.024320287*	*0.029188279*	*0.029241416*	*0.022872799*
**ENSG00000106400**	*0.022986691*	*0.026495277*	*0.027019674*	*0.019771148*
**ENSG00000142227**	*0.030433631*	*0.029909276*	*0.033498264*	*0.028305312*
**ENSG00000120860**	*0.061074606*	*0.045965128*	*0.036725022*	*0.035897871*
**ENSG00000165694**	*0.014934241*	*0.02524879*	*0.024124734*	*0.01506577*
**ENSG00000197616**	*0.044202509*	*0.03214063*	*0.028445596*	*0.025766441*
**ENSG00000108405**	*0.065973771*	*0.054694359*	*0.054470555*	*0.056315622*
**ENSG00000157045**	*0.02330837*	*0.03059712*	*0.03151208*	*0.020605487*
**ENSG00000189308**	*0.032174281*	*0.032970039*	*0.034558872*	*0.029761382*
**ENSG00000186480**	*0.037983374*	*0.036526791*	*0.034635087*	*0.030488597*

**ENSG00000136720**	*0.016410937*	*0.024790696*	*0.024986311*	*0.016127785*
**ENSG00000130638**	*0.029467762*	*0.03218065*	*0.031708574*	*0.02595734*
**ENSG00000144671**	*0.016989633*	*0.026154678*	*0.026162476*	*0.016633747*
**ENSG00000197766**	*0.023282432*	*0.027412854*	*0.028169224*	*0.023128277*
**ENSG00000128482**	*0.018547015*	*0.025453948*	*0.029172768*	*0.016592553*
**ENSG00000110768**	*0.032242484*	*0.040138907*	*0.030813192*	*0.027300926*
**ENSG00000114757**	*0.017419024*	*0.025578481*	*0.02495366*	*0.01694022*
**ENSG00000066027**	*0.024403849*	*0.030335885*	*0.030936669*	*0.023862156*
**ENSG00000163083**	*0.016482067*	*0.025379997*	*0.024856851*	*0.01537182*
**ENSG00000185554**	*0.015096238*	*0.025716528*	*0.024667772*	*0.016258294*
**ENSG00000100532**	*0.027078969*	*0.039316633*	*0.04383131*	*0.030180172*
**ENSG00000118432**	*0.09008295*	*0.080559969*	*0.049986942*	*0.08013879*
**ENSG00000132254**	*0.023260336*	*0.030399468*	*0.040189075*	*0.028127599*
**ENSG00000133193**	*0.024407169*	*0.031505963*	*0.031557494*	*0.021570573*
**ENSG00000127666**	*0.034082168*	*0.034709547*	*0.031606975*	*0.030333899*
**ENSG00000118292**	*0.093124919*	*0.068446089*	*0.058755107*	*0.068326047*
**ENSG00000196422**	*0.089399331*	*0.07124822*	*0.066183769*	*0.076530381*
**ENSG00000172086**	*0.035675159*	*0.053361374*	*0.046160555*	*0.042968525*
**ENSG00000117791**	*0.145732708*	*0.092192912*	*0.104885781*	*0.118592136*
**ENSG00000103202**	*0.043374442*	*0.042881787*	*0.03803881*	*0.039169501*
**ENSG00000121060**	*0.027185908*	*0.030031508*	*0.030738856*	*0.024326962*
**ENSG00000249158**	*0.014811701*	*0.025133134*	*0.025106689*	*0.015828614*
**ENSG00000160593**	*0.086355337*	*0.080388766*	*0.071083986*	*0.092917995*
**ENSG00000173452**	*0.021070903*	*0.026218911*	*0.028681489*	*0.01894124*
**ENSG00000178338**	*0.036472726*	*0.031922304*	*0.032540636*	*0.03270119*
**ENSG00000186472**	*0.015517495*	*0.023591816*	*0.024217284*	*0.013916958*
**ENSG00000182352**	*0.015658161*	*0.02587418*	*0.024194606*	*0.01750665*
**ENSG00000090104**	*0.051082677*	*0.046004968*	*0.048282838*	*0.040958281*
**ENSG00000068745**	*0.027750609*	*0.03091003*	*0.036982391*	*0.026522222*
**ENSG00000160049**	*0.030609154*	*0.032090062*	*0.030995126*	*0.025464727*
**ENSG00000102878**	*0.073437058*	*0.061129107*	*0.045286393*	*0.065836106*
**ENSG00000099992**	*0.024809443*	*0.033945367*	*0.031269686*	*0.02572202*
**ENSG00000135365**	*0.035075902*	*0.034959401*	*0.033982094*	*0.0267959*
**ENSG00000167716**	*0.033456678*	*0.036656347*	*0.042759512*	*0.034879565*
**ENSG00000100784**	*0.016627818*	*0.025571128*	*0.025268366*	*0.014400198*
**ENSG00000167323**	*0.026705277*	*0.031159877*	*0.033880396*	*0.027769912*
**ENSG00000079557**	*0.015771526*	*0.024952124*	*0.025494058*	*0.016195187*
**ENSG00000135070**	*0.029494466*	*0.034034203*	*0.032221715*	*0.027557625*
**ENSG00000196352**	*0.049538689*	*0.051173953*	*0.04443434*	*0.050344933*
**ENSG00000197498**	*0.024752712*	*0.02874998*	*0.027620726*	*0.0252224*
**ENSG00000113248**	*0.015606818*	*0.026696231*	*0.025265659*	*0.015807224*
**ENSG00000106330**	*0.037819782*	*0.039525297*	*0.034208989*	*0.029652085*
**ENSG00000149480**	*0.026568998*	*0.029774911*	*0.038000341*	*0.029604797*
**ENSG00000186115**	*0.035271074*	*0.031724086*	*0.027185231*	*0.024847744*
**ENSG00000159231**	*0.064819137*	*0.054580353*	*0.051095932*	*0.02608449*
**ENSG00000133466**	*0.023415143*	*0.030006106*	*0.030040018*	*0.02782965*
**ENSG00000075399**	*0.024464617*	*0.027885852*	*0.030427812*	*0.020829602*
**ENSG00000167363**	*0.046193227*	*0.049390085*	*0.039378761*	*0.05790517*
**ENSG00000101417**	*0.02166164*	*0.028676367*	*0.027606321*	*0.022033308*
**ENSG00000129535**	*0.016784464*	*0.025011228*	*0.024866579*	*0.015373035*
**ENSG00000151470**	*0.036971745*	*0.039671894*	*0.032984839*	*0.039191568*
**ENSG00000167549**	*0.026895638*	*0.030020199*	*0.029587435*	*0.028076877*
**ENSG00000184160**	*0.017351279*	*0.026066436*	*0.024646022*	*0.015449476*
**ENSG00000135502**	*0.01529218*	*0.024877893*	*0.024789039*	*0.014552021*

**ENSG00000174672**	*0.015311238*	*0.024861375*	*0.024372867*	*0.015258028*
**ENSG00000182747**	*0.018512914*	*0.02649789*	*0.025520374*	*0.016670158*
**ENSG00000132514**	*0.023438918*	*0.026511616*	*0.025394742*	*0.020326483*
**ENSG00000170471**	*0.023858363*	*0.032115917*	*0.032540998*	*0.02199838*
**ENSG00000113262**	*0.014846895*	*0.024236213*	*0.024393387*	*0.014305603*
**ENSG00000168758**	*0.022873168*	*0.031546573*	*0.030548027*	*0.022536499*
**ENSG00000108587**	*0.022050618*	*0.033316405*	*0.034763224*	*0.020338221*
**ENSG00000198420**	*0.024521799*	*0.038318313*	*0.030893579*	*0.029524555*
**ENSG00000136425**	*0.0195871*	*0.025801525*	*0.025217905*	*0.01728626*
**ENSG00000243468**	*0.017298964*	*0.026754268*	*0.026930528*	*0.015405547*
**ENSG00000149016**	*0.024950691*	*0.029986428*	*0.030421142*	*0.022787855*
**ENSG00000149972**	*0.018156877*	*0.027096617*	*0.024657207*	*0.017318331*
**ENSG00000170889**	*0.017512069*	*0.025217207*	*0.026055356*	*0.018319394*
**ENSG00000155099**	*0.035257308*	*0.033553899*	*0.033745357*	*0.034020973*
**ENSG00000164266**	*0.016105711*	*0.024756462*	*0.026013901*	*0.01613679*
**ENSG00000167123**	*0.032887351*	*0.046388482*	*0.038571154*	*0.046739858*
**ENSG00000149269**	*0.040524959*	*0.038983182*	*0.033423874*	*0.026823672*
**ENSG00000162073**	*0.054730303*	*0.048589822*	*0.041806137*	*0.043393725*
**ENSG00000116711**	*0.091978556*	*0.080227528*	*0.078139116*	*0.073792938*
**ENSG00000185875**	*0.05830762*	*0.055094483*	*0.045855581*	*0.04038087*
**ENSG00000116819**	*0.01581956*	*0.025319288*	*0.024576246*	*0.015544187*
**ENSG00000146216**	*0.017303289*	*0.025028424*	*0.026724822*	*0.0171918*
**ENSG00000159184**	*0.015472879*	*0.025682722*	*0.025889066*	*0.01399348*
**ENSG00000178607**	*0.016864149*	*0.025670842*	*0.026492198*	*0.015516301*
**ENSG00000196588**	*0.034859205*	*0.040929428*	*0.040583274*	*0.040155584*
**ENSG00000116741**	*0.119139332*	*0.087045384*	*0.090011187*	*0.095879634*
**ENSG00000213699**	*0.015612799*	*0.024145723*	*0.02422251*	*0.015441593*
**ENSG00000067955**	*0.031163947*	*0.032722595*	*0.031657328*	*0.025006056*
**ENSG00000180828**	*0.020297462*	*0.025964375*	*0.025864555*	*0.020213874*
**ENSG00000135617**	*0.031847391*	*0.033736096*	*0.032236898*	*0.029893807*
**ENSG00000240432**	*0.015265883*	*0.025166329*	*0.024073804*	*0.014377799*
**ENSG00000131979**	*0.038772146*	*0.04690149*	*0.046846102*	*0.038304449*
**ENSG00000136059**	*0.028758702*	*0.034766199*	*0.030520895*	*0.025548013*
**ENSG00000158008**	*0.016043868*	*0.025576314*	*0.025972313*	*0.014811079*
**ENSG00000147324**	*0.058399889*	*0.048897332*	*0.046636779*	*0.053745992*
**ENSG00000109705**	*0.015269292*	*0.025011691*	*0.024700268*	*0.014639552*
**ENSG00000197223**	*0.081944121*	*0.077274762*	*0.09452856*	*0.067754622*
**ENSG00000140105**	*0.037695332*	*0.038842589*	*0.03622328*	*0.033989658*
**ENSG00000174992**	*0.016099827*	*0.0264735*	*0.024834439*	*0.015500484*
**ENSG00000150907**	*0.053961578*	*0.051022214*	*0.045733799*	*0.042467529*
**ENSG00000141499**	*0.027150881*	*0.029757338*	*0.030095456*	*0.024497006*
**ENSG00000173786**	*0.024303635*	*0.031328146*	*0.034640344*	*0.027339236*
**ENSG00000162367**	*0.017018394*	*0.026978244*	*0.026542247*	*0.01697651*
**ENSG00000051009**	*0.036518116*	*0.034473196*	*0.035912652*	*0.033545576*
**ENSG00000185955**	*0.029406079*	*0.032219666*	*0.031877991*	*0.026942174*
**ENSG00000166856**	*0.014883814*	*0.02428354*	*0.024470452*	*0.013656576*
**ENSG00000132464**	*0.021608714*	*0.028238062*	*0.025908878*	*0.01878238*
**ENSG00000105939**	*0.030586048*	*0.032798547*	*0.040774634*	*0.029709478*
**ENSG00000068097**	*0.040718385*	*0.038494663*	*0.035536361*	*0.035900463*
**ENSG00000170516**	*0.017458862*	*0.027149401*	*0.025658765*	*0.015397591*
**ENSG00000134970**	*0.035369379*	*0.039048188*	*0.041273547*	*0.039948567*
**ENSG00000167863**	*0.024365771*	*0.028936413*	*0.026784804*	*0.018351203*
**ENSG00000100413**	*0.03345532*	*0.035377528*	*0.037323291*	*0.038523028*
**ENSG00000175414**	*0.017264866*	*0.02657894*	*0.025088928*	*0.015344577*

**ENSG00000034713**	*0.025500414*	*0.029531465*	*0.02987199*	*0.021584643*
**ENSG00000152465**	*0.060089804*	*0.049592082*	*0.048832008*	*0.055785742*
**ENSG00000141384**	*0.044311634*	*0.039971189*	*0.040116369*	*0.037955207*
**ENSG00000101199**	*0.025440555*	*0.037883482*	*0.047048599*	*0.027999819*
**ENSG00000063241**	*0.049984391*	*0.031184141*	*0.035440703*	*0.03874005*
**ENSG00000157470**	*0.07010108*	*0.0680379*	*0.069186122*	*0.06780515*
**ENSG00000167112**	*0.025865643*	*0.031441211*	*0.033544963*	*0.025907364*
**ENSG00000117054**	*0.022046357*	*0.027340707*	*0.028449922*	*0.021708175*
**ENSG00000137634**	*0.016572982*	*0.025366578*	*0.024571199*	*0.01564914*
**ENSG00000179008**	*0.019680104*	*0.025908758*	*0.024587788*	*0.018241206*
**ENSG00000138083**	*0.057928182*	*0.07652153*	*0.055414901*	*0.071009924*
**ENSG00000216490**	*0.033506899*	*0.036803077*	*0.033296607*	*0.032305069*
**ENSG00000131737**	*0.015954375*	*0.025488974*	*0.025077219*	*0.014929851*
**ENSG00000096654**	*0.035398426*	*0.035361959*	*0.037386355*	*0.024643948*
**ENSG00000163166**	*0.029593018*	*0.035878114*	*0.033643253*	*0.024646047*
**ENSG00000196782**	*0.017743555*	*0.026318488*	*0.026391644*	*0.015559187*
**ENSG00000187049**	*0.032972681*	*0.037648602*	*0.038637391*	*0.033146612*
**ENSG00000172867**	*0.016267239*	*0.02542288*	*0.025294435*	*0.015882547*
**ENSG00000165912**	*0.018941661*	*0.028726151*	*0.02598106*	*0.016748486*
**ENSG00000127952**	*0.037420401*	*0.038024646*	*0.035831523*	*0.035177802*
**ENSG00000203872**	*0.018945368*	*0.026926655*	*0.027266183*	*0.016143284*
**ENSG00000139842**	*0.027609611*	*0.034984347*	*0.029265276*	*0.022476794*
**ENSG00000180479**	*0.03021509*	*0.034343186*	*0.033729673*	*0.025219321*
**ENSG00000181192**	*0.032817313*	*0.03506762*	*0.035491992*	*0.03401253*
**ENSG00000225190**	*0.029004756*	*0.036004632*	*0.036006053*	*0.025116715*
**ENSG00000173253**	*0.029595187*	*0.025433498*	*0.024668269*	*0.022956157*
**ENSG00000137225**	*0.01571174*	*0.024784454*	*0.02503222*	*0.016510528*
**ENSG00000156006**	*0.01798553*	*0.024974599*	*0.025286748*	*0.015885696*
**ENSG00000184113**	*0.017778027*	*0.025791071*	*0.025153746*	*0.014974285*
**ENSG00000092020**	*0.029482286*	*0.036193857*	*0.03358193*	*0.025034852*
**ENSG00000126467**	*0.021890807*	*0.036509465*	*0.027066263*	*0.026193739*
**ENSG00000128965**	*0.069868922*	*0.066404271*	*0.072925845*	*0.069124278*
**ENSG00000183775**	*0.017310062*	*0.026935119*	*0.026504277*	*0.017921341*
**ENSG00000100865**	*0.026617515*	*0.028812545*	*0.033514469*	*0.031944106*
**ENSG00000132436**	*0.042527566*	*0.046689188*	*0.037555365*	*0.040001381*
**ENSG00000106009**	*0.033267005*	*0.036491774*	*0.038703371*	*0.028086251*
**ENSG00000123728**	*0.026710541*	*0.033045008*	*0.033677679*	*0.023249538*
**ENSG00000152217**	*0.107160934*	*0.076650167*	*0.058334234*	*0.084321099*
**ENSG00000141048**	*0.017492281*	*0.02537347*	*0.025860448*	*0.015341145*
**ENSG00000189171**	*0.069214779*	*0.07663343*	*0.066011398*	*0.06501992*
**ENSG00000155034**	*0.015717258*	*0.024453621*	*0.024525394*	*0.0169296*
**ENSG00000108773**	*0.03005563*	*0.035058657*	*0.035666367*	*0.025752951*
**ENSG00000132840**	*0.016162566*	*0.024711709*	*0.024799743*	*0.015522621*
**ENSG00000197603**	*0.033498062*	*0.041902618*	*0.03113448*	*0.026375601*
**ENSG00000120693**	*0.017542737*	*0.024662903*	*0.025510142*	*0.014910047*
**ENSG00000100280**	*0.029084926*	*0.035526014*	*0.031997006*	*0.034546135*
**ENSG00000196381**	*0.015692846*	*0.02423655*	*0.024607759*	*0.015522996*
**ENSG00000101115**	*0.015220691*	*0.024435194*	*0.024365411*	*0.015811156*
**ENSG00000174567**	*0.033788306*	*0.042440708*	*0.035477346*	*0.034475259*
**ENSG00000141510**	*0.033989888*	*0.03509195*	*0.03596134*	*0.035258793*
**ENSG00000213719**	*0.026055467*	*0.027998427*	*0.030082588*	*0.018506088*
**ENSG00000159713**	*0.015826497*	*0.024641063*	*0.024911415*	*0.014995676*
**ENSG00000119185**	*0.03111898*	*0.036113058*	*0.033930869*	*0.026319356*
**ENSG00000156735**	*0.025257241*	*0.027695053*	*0.03520555*	*0.024387376*

**ENSG00000130702**	*0.085569572*	*0.076170231*	*0.064117216*	*0.079822617*
**ENSG00000107960**	*0.031786332*	*0.030648565*	*0.034562264*	*0.026796196*
**ENSG00000183643**	*0.018916209*	*0.027316903*	*0.026776681*	*0.016131727*
**ENSG00000170866**	*0.047916639*	*0.033475428*	*0.027700924*	*0.020027546*
**ENSG00000137090**	*0.02970318*	*0.027219184*	*0.026869622*	*0.018191439*
**ENSG00000172061**	*0.015978548*	*0.024874135*	*0.024116216*	*0.016402975*
**ENSG00000157693**	*0.050234695*	*0.044403478*	*0.04605653*	*0.053651468*
**ENSG00000096070**	*0.037702629*	*0.040698929*	*0.047083514*	*0.034344847*
**ENSG00000167220**	*0.04169124*	*0.041825027*	*0.041506668*	*0.032108561*
**ENSG00000184216**	*0.032525064*	*0.03574066*	*0.036671056*	*0.03173756*
**ENSG00000187624**	*0.119731746*	*0.115914597*	*0.113840688*	*0.090366082*
**ENSG00000104129**	*0.027540225*	*0.032483925*	*0.029692648*	*0.022464588*
**ENSG00000152591**	*0.014396873*	*0.02412345*	*0.024557628*	*0.014201751*
**ENSG00000145863**	*0.016078912*	*0.025998287*	*0.024491544*	*0.016341374*
**ENSG00000183684**	*0.03281071*	*0.029555822*	*0.029942478*	*0.029884652*
**ENSG00000164398**	*0.015805012*	*0.025067304*	*0.024940533*	*0.015504189*
**ENSG00000176787**	*0.018122105*	*0.027097189*	*0.02684766*	*0.018143944*
**ENSG00000144847**	*0.015482713*	*0.025085801*	*0.025155563*	*0.015042448*
**ENSG00000214128**	*0.017700653*	*0.024929137*	*0.025528065*	*0.015774949*
**ENSG00000128059**	*0.031978596*	*0.035571862*	*0.031623704*	*0.021939223*
**ENSG00000168487**	*0.022952385*	*0.031423624*	*0.029811878*	*0.024303393*
**ENSG00000136936**	*0.027098895*	*0.037883418*	*0.038982127*	*0.027549015*
**ENSG00000188636**	*0.11170547*	*0.081236943*	*0.06487301*	*0.08026463*
**ENSG00000214655**	*0.039613569*	*0.040312362*	*0.040046919*	*0.036368634*
**ENSG00000213316**	*0.05814501*	*0.052294874*	*0.042490491*	*0.060727665*
**ENSG00000185518**	*0.11391624*	*0.065060177*	*0.067645868*	*0.093450133*
**ENSG00000086506**	*0.028065734*	*0.029709192*	*0.027974986*	*0.028683543*
**ENSG00000170683**	*0.015498681*	*0.024869983*	*0.024441748*	*0.014088235*
**ENSG00000120802**	*0.052943604*	*0.060274816*	*0.043107981*	*0.060346697*
**ENSG00000172167**	*0.034665381*	*0.033996155*	*0.029307443*	*0.0293445*
**ENSG00000185262**	*0.042443595*	*0.043247139*	*0.047895091*	*0.032378518*
**ENSG00000066629**	*0.028898248*	*0.028204142*	*0.02559382*	*0.023536584*
**ENSG00000144736**	*0.029519811*	*0.036221782*	*0.03629609*	*0.031963351*
**ENSG00000110871**	*0.029401064*	*0.034380623*	*0.031642281*	*0.022487241*
**ENSG00000182866**	*0.053311345*	*0.045397631*	*0.041983457*	*0.065414127*
**ENSG00000061938**	*0.043059003*	*0.048058586*	*0.038695933*	*0.039070201*
**ENSG00000115073**	*0.031647286*	*0.033205303*	*0.030583724*	*0.028808899*
**ENSG00000167978**	*0.024861472*	*0.03247382*	*0.03695222*	*0.027476036*
**ENSG00000154040**	*0.077184468*	*0.060122332*	*0.057440452*	*0.062072524*
**ENSG00000168356**	*0.032371662*	*0.035760717*	*0.037434606*	*0.033789812*
**ENSG00000100354**	*0.020420135*	*0.028637711*	*0.032392953*	*0.022209331*
**ENSG00000109991**	*0.016609653*	*0.026091856*	*0.02513059*	*0.016239305*
**ENSG00000189050**	*0.035699866*	*0.042900568*	*0.037493458*	*0.038219147*
**ENSG00000171433**	*0.014576352*	*0.024455444*	*0.024291241*	*0.013282949*
**ENSG00000167291**	*0.021436468*	*0.02662444*	*0.027550564*	*0.019479879*
**ENSG00000187097**	*0.022429178*	*0.026218325*	*0.026362785*	*0.017474038*
**ENSG00000171201**	*0.015723125*	*0.025160213*	*0.024893678*	*0.015377294*
**ENSG00000198001**	*0.028323208*	*0.034049213*	*0.032826849*	*0.021698525*
**ENSG00000168152**	*0.021682206*	*0.02642388*	*0.028959306*	*0.022425036*
**ENSG00000141905**	*0.021060017*	*0.027947548*	*0.026878899*	*0.023085657*
**ENSG00000126778**	*0.01653449*	*0.026003272*	*0.024857607*	*0.015024883*
**ENSG00000161055**	*0.062763421*	*0.052326597*	*0.030973134*	*0.04873854*
**ENSG00000134982**	*0.02276813*	*0.030760167*	*0.027435804*	*0.020504511*
**ENSG00000221886**	*0.041108767*	*0.062140055*	*0.045575256*	*0.043592576*

**ENSG00000178110**	*0.015774213*	*0.025415083*	*0.024720979*	*0.015103582*
**ENSG00000125850**	*0.028795743*	*0.030291653*	*0.029414852*	*0.028382638*
**ENSG00000188162**	*0.018667055*	*0.025953466*	*0.029236065*	*0.020987546*
**ENSG00000100592**	*0.065123607*	*0.057769665*	*0.047751341*	*0.047425552*
**ENSG00000162032**	*0.02252823*	*0.028851035*	*0.033662292*	*0.025209135*
**ENSG00000184402**	*0.016490421*	*0.025713605*	*0.026825652*	*0.01608492*
**ENSG00000156411**	*0.026141576*	*0.032093056*	*0.029304768*	*0.023177767*
**ENSG00000146904**	*0.017382888*	*0.02471835*	*0.025573466*	*0.016458469*
**ENSG00000142330**	*0.018993671*	*0.026560374*	*0.025650647*	*0.019710463*
**ENSG00000126858**	*0.035561371*	*0.05224956*	*0.041000728*	*0.031264317*
**ENSG00000088367**	*0.014493256*	*0.024877595*	*0.024553327*	*0.01546901*
**ENSG00000166813**	*0.063313422*	*0.060326098*	*0.038264801*	*0.064617162*
**ENSG00000144724**	*0.10287471*	*0.060499061*	*0.033828333*	*0.047727494*
**ENSG00000085721**	*0.03190515*	*0.035560971*	*0.036454947*	*0.030309358*
**ENSG00000165502**	*0.020423044*	*0.034284717*	*0.027532332*	*0.022605239*
**ENSG00000198937**	*0.025094047*	*0.031324709*	*0.03231205*	*0.026605196*
**ENSG00000181449**	*0.018012778*	*0.028090647*	*0.027728944*	*0.019272116*
**ENSG00000102967**	*0.036577823*	*0.034841129*	*0.030393585*	*0.022705832*
**ENSG00000120094**	*0.018930474*	*0.025578255*	*0.025951253*	*0.016074435*
**ENSG00000179119**	*0.040534099*	*0.038483903*	*0.048114558*	*0.036442541*
**ENSG00000110717**	*0.021712696*	*0.027006215*	*0.027399014*	*0.020529957*
**ENSG00000142731**	*0.044099817*	*0.038222838*	*0.034515129*	*0.030679066*
**ENSG00000182578**	*0.059160706*	*0.051179529*	*0.045187899*	*0.065514899*
**ENSG00000141434**	*0.015410056*	*0.024301683*	*0.024935195*	*0.015201366*
**ENSG00000128872**	*0.027313767*	*0.034448427*	*0.031160595*	*0.027929233*
**ENSG00000143595**	*0.015786814*	*0.024362349*	*0.024924661*	*0.014450509*
**ENSG00000158125**	*0.017665453*	*0.025207292*	*0.023872521*	*0.014987132*
**ENSG00000136861**	*0.030720042*	*0.043534782*	*0.038456036*	*0.038807873*
**ENSG00000160185**	*0.035980277*	*0.033683733*	*0.030066231*	*0.030460485*
**ENSG00000117152**	*0.016229564*	*0.025161151*	*0.024257208*	*0.016171677*
**ENSG00000125895**	*0.020532904*	*0.026626184*	*0.028525926*	*0.029570214*
**ENSG00000134153**	*0.023967221*	*0.02900101*	*0.033010725*	*0.023481386*
**ENSG00000074370**	*0.047225844*	*0.040523202*	*0.044785447*	*0.035481851*
**ENSG00000125888**	*0.015519514*	*0.024423698*	*0.025432954*	*0.015974414*
**ENSG00000214517**	*0.045372819*	*0.037446631*	*0.035990264*	*0.03648298*
**ENSG00000151789**	*0.019854492*	*0.02832769*	*0.027908642*	*0.019478535*
**ENSG00000153896**	*0.01625303*	*0.025848606*	*0.025016831*	*0.018300955*
**ENSG00000131558**	*0.026126553*	*0.030078203*	*0.032102904*	*0.023585133*
**ENSG00000167552**	*0.045342333*	*0.048534735*	*0.033610153*	*0.044359306*
**ENSG00000073536**	*0.041222225*	*0.045872825*	*0.049970199*	*0.034300131*
**ENSG00000181804**	*0.029445487*	*0.032548944*	*0.030611941*	*0.025780026*
**ENSG00000185594**	*0.016357946*	*0.025541367*	*0.026254732*	*0.015193217*
**ENSG00000165246**	*0.107362889*	*0.113391932*	*0.084380631*	*0.101251084*
**ENSG00000221946**	*0.015700491*	*0.02453891*	*0.024813815*	*0.015526688*
**ENSG00000182836**	*0.015829692*	*0.024311531*	*0.025193772*	*0.014491256*
**ENSG00000179988**	*0.023253858*	*0.028040931*	*0.027794772*	*0.021564588*
**ENSG00000166831**	*0.166290929*	*0.113349064*	*0.114368238*	*0.137246964*
**ENSG00000198799**	*0.028052814*	*0.034685596*	*0.033367516*	*0.026665069*
**ENSG00000167741**	*0.017952154*	*0.026128913*	*0.026582231*	*0.017540427*
**ENSG00000196468**	*0.016768081*	*0.025562119*	*0.02525782*	*0.014802103*
**ENSG00000164038**	*0.032621606*	*0.041530441*	*0.031255532*	*0.023957559*
**ENSG00000160959**	*0.038215738*	*0.041768866*	*0.039267202*	*0.031707474*
**ENSG00000125871**	*0.032433848*	*0.036922183*	*0.032013256*	*0.025134187*
**ENSG00000169020**	*0.025426341*	*0.0308089*	*0.029400084*	*0.02469468*

**ENSG00000070950**	*0.024279192*	*0.02938014*	*0.029418777*	*0.023004111*
**ENSG00000171462**	*0.016888449*	*0.025318545*	*0.025243391*	*0.01517161*
**ENSG00000127688**	*0.016015605*	*0.025365827*	*0.026392201*	*0.01543472*
**ENSG00000075415**	*0.034249392*	*0.039994635*	*0.039098172*	*0.036327837*
**ENSG00000144043**	*0.027023731*	*0.031030503*	*0.03791996*	*0.0321264*
**ENSG00000052126**	*0.036603016*	*0.025830244*	*0.025288809*	*0.01617427*
**ENSG00000112983**	*0.01493414*	*0.025125405*	*0.025739959*	*0.01560545*
**ENSG00000198538**	*0.013943713*	*0.023268802*	*0.023869161*	*0.01350916*
**ENSG00000131236**	*0.018630783*	*0.027432992*	*0.026630805*	*0.01858277*
**ENSG00000126787**	*0.04403211*	*0.043720308*	*0.036949109*	*0.035192448*
**ENSG00000237077**	*0.014450626*	*0.024088517*	*0.024035573*	*0.014655891*
**ENSG00000146859**	*0.067169282*	*0.05348084*	*0.056438274*	*0.060485404*
**ENSG00000123179**	*0.033653855*	*0.032894964*	*0.030908461*	*0.032273229*
**ENSG00000007264**	*0.015810304*	*0.02546367*	*0.025489045*	*0.016321675*
**ENSG00000113161**	*0.035152297*	*0.042788553*	*0.033530236*	*0.034741128*
**ENSG00000166377**	*0.025301258*	*0.02873675*	*0.028540566*	*0.022825699*
**ENSG00000241935**	*0.046941873*	*0.031681487*	*0.03118416*	*0.043937302*
**ENSG00000186118**	*0.014381236*	*0.023549413*	*0.024133212*	*0.014119496*
**ENSG00000162604**	*0.029916105*	*0.0364698*	*0.032921881*	*0.028873911*
**ENSG00000129159**	*0.015989175*	*0.0249253*	*0.025199456*	*0.014980624*
**ENSG00000187325**	*0.036775531*	*0.042701647*	*0.036970233*	*0.040541509*
**ENSG00000179046**	*0.016425042*	*0.024909529*	*0.025618816*	*0.015829936*
**ENSG00000082516**	*0.038205238*	*0.042914532*	*0.035642145*	*0.029099077*
**ENSG00000125650**	*0.017949915*	*0.025665199*	*0.024428275*	*0.015812384*
**ENSG00000122068**	*0.030103703*	*0.036704983*	*0.034693673*	*0.030177721*
**ENSG00000101188**	*0.015427077*	*0.025406463*	*0.025827421*	*0.016566306*
**ENSG00000176922**	*0.016687144*	*0.026820727*	*0.025754343*	*0.017756129*
**ENSG00000158669**	*0.027268607*	*0.033239106*	*0.037055289*	*0.025961787*
**ENSG00000171817**	*0.019662401*	*0.027151587*	*0.026752118*	*0.023845418*
**ENSG00000176887**	*0.018799552*	*0.026823861*	*0.027071486*	*0.016863454*
**ENSG00000175048**	*0.111998754*	*0.100111557*	*0.083003457*	*0.076611209*
**ENSG00000165092**	*0.017281943*	*0.026244432*	*0.024732491*	*0.0152863*
**ENSG00000169410**	*0.015679395*	*0.025417281*	*0.024662546*	*0.016689785*
**ENSG00000181761**	*0.016109448*	*0.025139034*	*0.025062975*	*0.014977717*
**ENSG00000185013**	*0.016471412*	*0.025933295*	*0.025478236*	*0.016489965*
**ENSG00000153443**	*0.032805967*	*0.044545984*	*0.048751724*	*0.038410405*
**ENSG00000151388**	*0.015706414*	*0.024751399*	*0.024935992*	*0.014855249*
**ENSG00000090054**	*0.035286282*	*0.044323849*	*0.03882586*	*0.059192073*
**ENSG00000127418**	*0.054395515*	*0.055461916*	*0.05459183*	*0.051986438*
**ENSG00000183615**	*0.016124176*	*0.025335802*	*0.026189703*	*0.016035727*
**ENSG00000215864**	*0.015677102*	*0.025431238*	*0.025148314*	*0.015721711*
**ENSG00000100216**	*0.026009753*	*0.030372873*	*0.029001387*	*0.020832608*
**ENSG00000101144**	*0.016721078*	*0.02423714*	*0.02624343*	*0.01625903*
**ENSG00000131650**	*0.034953586*	*0.041155731*	*0.031240268*	*0.037204458*
**ENSG00000104826**	*0.016128209*	*0.024592467*	*0.025498144*	*0.016398788*
**ENSG00000167193**	*0.036163907*	*0.034612485*	*0.035273829*	*0.030722238*
**ENSG00000103932**	*0.032612197*	*0.036418424*	*0.030154653*	*0.029258255*
**ENSG00000136574**	*0.02362816*	*0.029764622*	*0.028981564*	*0.021076849*
**ENSG00000186222**	*0.024512881*	*0.034329242*	*0.032264344*	*0.020385576*
**ENSG00000177464**	*0.014911401*	*0.025164464*	*0.025290003*	*0.015655383*
**ENSG00000179603**	*0.110377633*	*0.074422756*	*0.059048626*	*0.078815572*
**ENSG00000188536**	*0.016741517*	*0.025428625*	*0.024789454*	*0.025269971*
**ENSG00000156885**	*0.014369077*	*0.024227151*	*0.024450575*	*0.014264064*
**ENSG00000147400**	*0.029849704*	*0.035776843*	*0.029674865*	*0.030340637*

**ENSG00000107618**	*0.020435422*	*0.028134611*	*0.026241779*	*0.01735654*
**ENSG00000124490**	*0.015784543*	*0.025066537*	*0.024620042*	*0.015250915*
**ENSG00000069764**	*0.015306864*	*0.025020822*	*0.024765609*	*0.014904211*
**ENSG00000169255**	*0.019199195*	*0.028138318*	*0.027441728*	*0.017874548*
**ENSG00000143374**	*0.02404796*	*0.028131245*	*0.031197319*	*0.022869888*
**ENSG00000162373**	*0.056330421*	*0.10200357*	*0.107114067*	*0.108518999*
**ENSG00000198881**	*0.015722236*	*0.027165487*	*0.025495235*	*0.016097657*
**ENSG00000164494**	*0.032600573*	*0.034603832*	*0.031327052*	*0.026266508*
**ENSG00000176842**	*0.016707519*	*0.025050111*	*0.025785014*	*0.015669872*
**ENSG00000140263**	*0.033664364*	*0.035594405*	*0.030679076*	*0.029644615*
**ENSG00000008277**	*0.015959972*	*0.024671544*	*0.024674384*	*0.016071532*
**ENSG00000138792**	*0.024100497*	*0.031204338*	*0.025808807*	*0.016674727*
**ENSG00000130560**	*0.024523933*	*0.030994692*	*0.028724622*	*0.022841034*
**ENSG00000148655**	*0.116848985*	*0.091965277*	*0.059723422*	*0.068335934*
**ENSG00000135587**	*0.022915348*	*0.026561112*	*0.029522541*	*0.02045022*
**ENSG00000112294**	*0.051039577*	*0.05236248*	*0.055739408*	*0.056830083*
**ENSG00000174010**	*0.021064137*	*0.027468962*	*0.030803705*	*0.017743166*
**ENSG00000103381**	*0.051220436*	*0.039340642*	*0.036058883*	*0.037549321*
**ENSG00000242185**	*0.211503785*	*0.180000038*	*0.193335496*	*0.203610925*
**ENSG00000116833**	*0.018724262*	*0.027278175*	*0.025880309*	*0.01817133*
**ENSG00000198056**	*0.047584521*	*0.042474303*	*0.03484635*	*0.029295524*
**ENSG00000173821**	*0.059719308*	*0.078506675*	*0.055460609*	*0.057956913*
**ENSG00000163576**	*0.01714842*	*0.026549696*	*0.025271126*	*0.019721741*
**ENSG00000130762**	*0.015800774*	*0.024638688*	*0.024612946*	*0.014358894*
**ENSG00000100883**	*0.026209582*	*0.031814159*	*0.030741347*	*0.02408938*
**ENSG00000138161**	*0.028553615*	*0.028228665*	*0.025957175*	*0.018612543*
**ENSG00000244617**	*0.018137791*	*0.026900368*	*0.024957492*	*0.016662517*
**ENSG00000141519**	*0.016986255*	*0.026422789*	*0.026571458*	*0.016854281*
**ENSG00000119777**	*0.030224821*	*0.030157834*	*0.038531795*	*0.028371241*
**ENSG00000127184**	*0.014443672*	*0.024113433*	*0.024590498*	*0.016354111*
**ENSG00000169084**	*0.024287711*	*0.027599819*	*0.02610804*	*0.027768926*
**ENSG00000132855**	*0.014695019*	*0.023821097*	*0.024074681*	*0.014454512*
**ENSG00000135847**	*0.026539043*	*0.029210845*	*0.030085101*	*0.023157098*
**ENSG00000163638**	*0.016732992*	*0.025118021*	*0.024747581*	*0.015233761*
**ENSG00000106351**	*0.031793137*	*0.03366418*	*0.029365832*	*0.02945117*
**ENSG00000167972**	*0.044642037*	*0.045479971*	*0.031599612*	*0.048027482*
**ENSG00000161618**	*0.033733582*	*0.035906102*	*0.03612934*	*0.034459405*
**ENSG00000128165**	*0.016332571*	*0.02641867*	*0.026748174*	*0.017377101*
**ENSG00000100220**	*0.024550267*	*0.033469671*	*0.033935856*	*0.025021067*
**ENSG00000187164**	*0.06895365*	*0.04903965*	*0.041966155*	*0.061684977*
**ENSG00000177225**	*0.033834287*	*0.035612087*	*0.032059241*	*0.029025464*
**ENSG00000163378**	*0.046470305*	*0.045491438*	*0.046206365*	*0.051852825*
**ENSG00000104408**	*0.018245347*	*0.026765069*	*0.025896999*	*0.018096795*
**ENSG00000089195**	*0.033704174*	*0.043008768*	*0.040655479*	*0.03693131*
**ENSG00000048707**	*0.018425073*	*0.029461469*	*0.02760378*	*0.022464446*
**ENSG00000114416**	*0.020605665*	*0.028832189*	*0.027308661*	*0.021185793*
**ENSG00000183963**	*0.040252845*	*0.033366535*	*0.037433074*	*0.032119878*
**ENSG00000198792**	*0.036073998*	*0.045171211*	*0.051718129*	*0.045104738*
**ENSG00000127463**	*0.031027186*	*0.033667633*	*0.034758516*	*0.029012473*
**ENSG00000174776**	*0.016121028*	*0.025541819*	*0.024391767*	*0.01498648*
**ENSG00000134056**	*0.021418754*	*0.027112021*	*0.027458425*	*0.019159724*
**ENSG00000184154**	*0.022029152*	*0.028741938*	*0.025485028*	*0.018078327*
**ENSG00000121361**	*0.016200869*	*0.02533734*	*0.024799632*	*0.015572553*
**ENSG00000177030**	*0.029248715*	*0.030001065*	*0.033614508*	*0.027488094*

**ENSG00000132635**	*0.028740305*	*0.033410703*	*0.033391888*	*0.031967527*
**ENSG00000137821**	*0.045208885*	*0.032688397*	*0.02781206*	*0.027948666*
**ENSG00000171793**	*0.036114963*	*0.03568692*	*0.034797968*	*0.038548321*
**ENSG00000108924**	*0.029019091*	*0.026435802*	*0.025802369*	*0.017756275*
**ENSG00000114439**	*0.02786416*	*0.030375038*	*0.031254547*	*0.02232427*
**ENSG00000203485**	*0.056218123*	*0.053304884*	*0.041501435*	*0.038912156*
**ENSG00000100902**	*0.01977529*	*0.0277436*	*0.025899373*	*0.020711977*
**ENSG00000156466**	*0.016970679*	*0.026350369*	*0.028346083*	*0.017468564*
**ENSG00000100033**	*0.026451561*	*0.025534488*	*0.025582473*	*0.020464861*
**ENSG00000090932**	*0.019438606*	*0.028975127*	*0.026796276*	*0.017130596*
**ENSG00000204220**	*0.027277255*	*0.029714702*	*0.029604031*	*0.023824102*
**ENSG00000135932**	*0.022248587*	*0.032305233*	*0.033010808*	*0.024153416*
**ENSG00000160539**	*0.032243196*	*0.031373794*	*0.027403256*	*0.030826931*
**ENSG00000109670**	*0.036979542*	*0.048464155*	*0.048635802*	*0.047245498*
**ENSG00000176055**	*0.034218995*	*0.038502254*	*0.034829905*	*0.027734824*
**ENSG00000163879**	*0.015981256*	*0.02475519*	*0.024436481*	*0.015922039*
**ENSG00000146399**	*0.015503786*	*0.024253691*	*0.02541597*	*0.015005451*
**ENSG00000014824**	*0.028229763*	*0.031731519*	*0.030198076*	*0.024368319*
**ENSG00000164002**	*0.035138799*	*0.034041247*	*0.034283982*	*0.036088733*
**ENSG00000145623**	*0.016410361*	*0.025166439*	*0.02467508*	*0.016013889*
**ENSG00000163993**	*0.020336895*	*0.025176475*	*0.02648381*	*0.017450142*
**ENSG00000124217**	*0.018945136*	*0.026984915*	*0.027066785*	*0.018975909*
**ENSG00000115840**	*0.029270247*	*0.034528271*	*0.037268472*	*0.034501923*
**ENSG00000149452**	*0.015051139*	*0.025222831*	*0.024999686*	*0.015463537*
**ENSG00000124657**	*0.015096467*	*0.024555689*	*0.024272432*	*0.015728457*
**ENSG00000067248**	*0.031108222*	*0.035875043*	*0.033819163*	*0.031013053*
**ENSG00000165588**	*0.015871443*	*0.025418985*	*0.024350696*	*0.014573041*
**ENSG00000221859**	*0.017566346*	*0.030084083*	*0.027894565*	*0.018928492*
**ENSG00000171631**	*0.015535933*	*0.024917797*	*0.024406923*	*0.015281771*
**ENSG00000164116**	*0.134284451*	*0.100672127*	*0.101039818*	*0.100496738*
**ENSG00000187098**	*0.015100434*	*0.024553714*	*0.025933763*	*0.015870228*
**ENSG00000092377**	*0.015894444*	*0.024996741*	*0.025320768*	*0.014891348*
**ENSG00000158104**	*0.015447456*	*0.025414468*	*0.024565418*	*0.015309522*
**ENSG00000140463**	*0.030272066*	*0.037191688*	*0.034878316*	*0.025904271*
**ENSG00000204688**	*0.016876042*	*0.026825211*	*0.026527718*	*0.017555042*
**ENSG00000214063**	*0.081961904*	*0.071078951*	*0.059299984*	*0.0736286*
**ENSG00000166669**	*0.043644234*	*0.046729009*	*0.036583398*	*0.039415405*
**ENSG00000058091**	*0.04517485*	*0.04750042*	*0.042778682*	*0.042684045*
**ENSG00000148335**	*0.029363096*	*0.029172116*	*0.03017272*	*0.023704726*
**ENSG00000173530**	*0.036122601*	*0.035937422*	*0.032687061*	*0.02403242*
**ENSG00000188215**	*0.020378576*	*0.027942038*	*0.030903422*	*0.01699885*
**ENSG00000157399**	*0.015222*	*0.024872576*	*0.024761965*	*0.014907451*
**ENSG00000204264**	*0.026654244*	*0.031636244*	*0.029757911*	*0.026696166*
**ENSG00000144401**	*0.029855228*	*0.030913471*	*0.029965078*	*0.029363573*
**ENSG00000164729**	*0.015977759*	*0.02448009*	*0.024520725*	*0.014248892*
**ENSG00000174106**	*0.030204235*	*0.034228088*	*0.033344658*	*0.026868823*
**ENSG00000189067**	*0.020260015*	*0.02783079*	*0.032231025*	*0.023056012*
**ENSG00000179886**	*0.04365618*	*0.044182818*	*0.042278652*	*0.041328894*
**ENSG00000213780**	*0.03023948*	*0.03647124*	*0.032082279*	*0.028813951*
**ENSG00000101460**	*0.017474985*	*0.026956685*	*0.02620672*	*0.016086209*
**ENSG00000129355**	*0.016028403*	*0.024702799*	*0.025213946*	*0.015816429*
**ENSG00000074590**	*0.014915389*	*0.025149689*	*0.024539732*	*0.015374653*
**ENSG00000163257**	*0.026397574*	*0.03282942*	*0.032759015*	*0.027633729*
**ENSG00000134317**	*0.020056022*	*0.025207965*	*0.025470715*	*0.016800487*

**ENSG00000166510**	*0.018631456*	*0.030713574*	*0.028112873*	*0.01962518*
**ENSG00000105248**	*0.034014005*	*0.038279638*	*0.045086134*	*0.029855145*
**ENSG00000129028**	*0.057698254*	*0.056823135*	*0.04071462*	*0.043462863*
**ENSG00000206562**	*0.030416658*	*0.035597314*	*0.033916696*	*0.026750643*
**ENSG00000249853**	*0.018088668*	*0.024884044*	*0.026288638*	*0.016732665*
**ENSG00000187713**	*0.02304005*	*0.030557615*	*0.032085339*	*0.020519977*
**ENSG00000178201**	*0.018393429*	*0.027139415*	*0.026436648*	*0.017245363*
**ENSG00000148835**	*0.034546647*	*0.041438501*	*0.033803817*	*0.025552342*
**ENSG00000156687**	*0.019114029*	*0.025575851*	*0.028198567*	*0.017065767*
**ENSG00000105825**	*0.125706804*	*0.086600902*	*0.060376573*	*0.066429535*
**ENSG00000155966**	*0.017630621*	*0.027155867*	*0.026523214*	*0.015260484*
**ENSG00000131013**	*0.024068942*	*0.028427218*	*0.028945964*	*0.023365368*
**ENSG00000084112**	*0.027868604*	*0.03347291*	*0.036020942*	*0.028520075*
**ENSG00000145020**	*0.02854162*	*0.032442777*	*0.031526954*	*0.034037066*
**ENSG00000133149**	*0.017469613*	*0.029811353*	*0.027487191*	*0.020094393*
**ENSG00000113361**	*0.015671416*	*0.025057228*	*0.024424872*	*0.014512363*
**ENSG00000182870**	*0.032518153*	*0.034650029*	*0.029816639*	*0.027030921*
**ENSG00000107485**	*0.091602974*	*0.064615731*	*0.055762688*	*0.069438196*
**ENSG00000197162**	*0.020818058*	*0.027619148*	*0.026726921*	*0.022283101*
**ENSG00000163545**	*0.023564425*	*0.029297072*	*0.027483042*	*0.026784061*
**ENSG00000160953**	*0.04416269*	*0.043513757*	*0.042270739*	*0.044171712*
**ENSG00000184500**	*0.018844893*	*0.02847975*	*0.025295607*	*0.017372038*
**ENSG00000113448**	*0.019517705*	*0.026774633*	*0.028694346*	*0.029950538*
**ENSG00000169223**	*0.029151559*	*0.028220631*	*0.028952838*	*0.024054576*
**ENSG00000105808**	*0.041008187*	*0.039285719*	*0.040814374*	*0.034780573*
**ENSG00000163938**	*0.02486641*	*0.029571116*	*0.030368498*	*0.024007372*
**ENSG00000161204**	*0.020445256*	*0.027300011*	*0.03175768*	*0.022355821*
**ENSG00000130037**	*0.016933105*	*0.028962933*	*0.02537475*	*0.016870174*
**ENSG00000104825**	*0.025596504*	*0.028758945*	*0.034685291*	*0.031806275*
**ENSG00000116005**	*0.036850417*	*0.034598641*	*0.036543857*	*0.038095785*
**ENSG00000167098**	*0.016164329*	*0.024882244*	*0.025970569*	*0.016523276*
**ENSG00000198298**	*0.026185432*	*0.029015805*	*0.031054526*	*0.023113903*
**ENSG00000131779**	*0.027700767*	*0.031233819*	*0.032671501*	*0.022840959*
**ENSG00000048545**	*0.015296415*	*0.025836104*	*0.024553587*	*0.015304713*
**ENSG00000187714**	*0.015321559*	*0.025276803*	*0.025092208*	*0.014353092*
**ENSG00000161940**	*0.020707322*	*0.034611766*	*0.034392015*	*0.028857902*
**ENSG00000204619**	*0.024773274*	*0.032226159*	*0.032213749*	*0.02388225*
**ENSG00000152804**	*0.121615389*	*0.083808655*	*0.078409192*	*0.10560598*
**ENSG00000163743**	*0.03105033*	*0.036563112*	*0.037539692*	*0.029843916*
**ENSG00000139880**	*0.016030972*	*0.027000549*	*0.026575368*	*0.016347448*
**ENSG00000108395**	*0.035943389*	*0.038842529*	*0.033449346*	*0.037623193*
**ENSG00000175643**	*0.056459103*	*0.060236954*	*0.05171736*	*0.040259212*
**ENSG00000112624**	*0.024714041*	*0.031167358*	*0.030900453*	*0.021862713*
**ENSG00000138755**	*0.121802443*	*0.093333464*	*0.031205425*	*0.114317489*
**ENSG00000172757**	*0.025844798*	*0.029943007*	*0.030160323*	*0.029251104*
**ENSG00000198830**	*0.025832229*	*0.028400079*	*0.02627557*	*0.02303981*
**ENSG00000165732**	*0.033914049*	*0.03852396*	*0.037615938*	*0.033271368*
**ENSG00000115526**	*0.038781004*	*0.038113798*	*0.047281394*	*0.052201524*
**ENSG00000100263**	*0.028380743*	*0.032675599*	*0.037844042*	*0.030116426*
**ENSG00000070476**	*0.027109035*	*0.03080511*	*0.033078278*	*0.024756038*
**ENSG00000108551**	*0.022954982*	*0.0264171*	*0.025290011*	*0.017788286*
**ENSG00000124212**	*0.015871125*	*0.025003344*	*0.024836844*	*0.0147031*
**ENSG00000102098**	*0.016219924*	*0.025793437*	*0.027207761*	*0.017841435*
**ENSG00000156531**	*0.042447829*	*0.054899037*	*0.039993955*	*0.038025811*

**ENSG00000152942**	*0.014271253*	*0.02546565*	*0.025346475*	*0.016048391*
**ENSG00000119906**	*0.03967196*	*0.048369916*	*0.040339724*	*0.03153602*
**ENSG00000186448**	*0.024080977*	*0.031637448*	*0.029578355*	*0.023241799*
**ENSG00000182473**	*0.03014428*	*0.032389075*	*0.034457806*	*0.028005101*
**ENSG00000103544**	*0.025598073*	*0.03225558*	*0.02760569*	*0.026963708*
**ENSG00000175183**	*0.112158225*	*0.091521249*	*0.067732005*	*0.098006295*
**ENSG00000108984**	*0.040259097*	*0.045193751*	*0.038099297*	*0.033828685*
**ENSG00000133657**	*0.026135833*	*0.026338873*	*0.029463693*	*0.02112417*
**ENSG00000198189**	*0.040417855*	*0.036064139*	*0.037362751*	*0.030109644*
**ENSG00000154917**	*0.031107215*	*0.033462243*	*0.038651372*	*0.0251198*
**ENSG00000204610**	*0.016142709*	*0.025055148*	*0.024732599*	*0.01470156*
**ENSG00000151883**	*0.032355963*	*0.038365662*	*0.037052207*	*0.02650072*
**ENSG00000128309**	*0.038116901*	*0.031266328*	*0.034140414*	*0.041098676*
**ENSG00000149218**	*0.070070374*	*0.051374811*	*0.048505462*	*0.051051015*
**ENSG00000197903**	*0.044120463*	*0.054231839*	*0.048367458*	*0.042747181*
**ENSG00000230522**	*0.017613265*	*0.027033696*	*0.024276604*	*0.014722609*
**ENSG00000122718**	*0.015253597*	*0.025956209*	*0.026053078*	*0.016876179*
**ENSG00000197044**	*0.022212627*	*0.025408366*	*0.025772618*	*0.020662096*
**ENSG00000111799**	*0.016234118*	*0.024368927*	*0.024521014*	*0.015261252*
**ENSG00000182447**	*0.015584634*	*0.024567523*	*0.025737021*	*0.015970399*
**ENSG00000169756**	*0.034621299*	*0.034105499*	*0.033148591*	*0.027564372*
**ENSG00000104756**	*0.039071993*	*0.042398728*	*0.037101306*	*0.035017979*
**ENSG00000197849**	*0.016989946*	*0.025795426*	*0.025251813*	*0.016494936*
**ENSG00000168748**	*0.015668807*	*0.02457584*	*0.025069935*	*0.015770248*
**ENSG00000170315**	*0.014624089*	*0.024647619*	*0.025014036*	*0.015665376*
**ENSG00000115956**	*0.025287463*	*0.031899716*	*0.033092844*	*0.030843985*
**ENSG00000079387**	*0.04373737*	*0.045673607*	*0.037393289*	*0.039599184*
**ENSG00000156508**	*0.013152077*	*0.023545511*	*0.023844292*	*0.014093267*
**ENSG00000167800**	*0.0155988*	*0.026020168*	*0.025228811*	*0.017416103*
**ENSG00000104517**	*0.029727592*	*0.036767254*	*0.033074677*	*0.024567256*
**ENSG00000155666**	*0.017787712*	*0.026623976*	*0.026247129*	*0.016945522*
**ENSG00000137392**	*0.015715671*	*0.02431059*	*0.024935027*	*0.014586222*
**ENSG00000136244**	*0.052260007*	*0.031213926*	*0.032323722*	*0.036203065*
**ENSG00000155850**	*0.038421729*	*0.036529363*	*0.033602399*	*0.029827544*
**ENSG00000138621**	*0.017387894*	*0.025119054*	*0.026367839*	*0.01505795*
**ENSG00000077935**	*0.026975794*	*0.026457734*	*0.028295653*	*0.017976079*
**ENSG00000100979**	*0.025404667*	*0.035386054*	*0.033792106*	*0.025083647*
**ENSG00000002746**	*0.014451969*	*0.024547142*	*0.024057363*	*0.014233102*
**ENSG00000137491**	*0.075335782*	*0.074401934*	*0.062454291*	*0.066142722*
**ENSG00000111752**	*0.046116178*	*0.054103637*	*0.047557585*	*0.051297415*
**ENSG00000122779**	*0.038006447*	*0.036230601*	*0.038140606*	*0.029139769*
**ENSG00000183856**	*0.038873188*	*0.038564704*	*0.03827959*	*0.033798118*
**ENSG00000165794**	*0.015696896*	*0.024959342*	*0.025437147*	*0.01518853*
**ENSG00000095110**	*0.016068527*	*0.024755516*	*0.025242129*	*0.015041064*
**ENSG00000107371**	*0.025714109*	*0.031769357*	*0.029209221*	*0.026194657*
**ENSG00000131626**	*0.038797704*	*0.035404544*	*0.03783513*	*0.034221889*
**ENSG00000115942**	*0.037772985*	*0.037731256*	*0.03281249*	*0.023218493*
**ENSG00000134287**	*0.027472617*	*0.033377492*	*0.030652878*	*0.029487027*
**ENSG00000130921**	*0.033789016*	*0.035725526*	*0.033015785*	*0.031293049*
**ENSG00000186207**	*0.014307843*	*0.02521258*	*0.024925932*	*0.015202511*
**ENSG00000104938**	*0.017195156*	*0.025639085*	*0.026270717*	*0.017195845*
**ENSG00000132024**	*0.025364691*	*0.028181242*	*0.031276657*	*0.024189885*
**ENSG00000127831**	*0.028258837*	*0.025799835*	*0.0253129*	*0.016477031*
**ENSG00000162706**	*0.017058635*	*0.025603463*	*0.025035119*	*0.01565428*

**ENSG00000183735**	*0.022021368*	*0.031537997*	*0.033840728*	*0.019957715*
**ENSG00000069974**	*0.048656877*	*0.045264767*	*0.046854564*	*0.052475981*
**ENSG00000198374**	*0.046539223*	*0.041281225*	*0.052144981*	*0.04922617*
**ENSG00000185633**	*0.035552374*	*0.059293516*	*0.034241418*	*0.032825744*
**ENSG00000153814**	*0.056311476*	*0.040410554*	*0.036421221*	*0.053060381*
**ENSG00000160282**	*0.017510013*	*0.028040284*	*0.027903207*	*0.018314431*
**ENSG00000123352**	*0.030559698*	*0.035076115*	*0.033267534*	*0.026119472*
**ENSG00000111647**	*0.029889568*	*0.032749728*	*0.034873036*	*0.029785703*
**ENSG00000182087**	*0.026076464*	*0.035338569*	*0.034699449*	*0.031590564*
**ENSG00000186642**	*0.01838516*	*0.024485818*	*0.02516412*	*0.017899858*
**ENSG00000132664**	*0.026955128*	*0.034835849*	*0.036621081*	*0.034083425*
**ENSG00000179314**	*0.018585805*	*0.026053428*	*0.026903952*	*0.017385618*
**ENSG00000160813**	*0.032604131*	*0.033786994*	*0.033905486*	*0.026884374*
**ENSG00000110315**	*0.031173376*	*0.030629559*	*0.029100929*	*0.02031521*
**ENSG00000187240**	*0.015907138*	*0.026082575*	*0.02558945*	*0.016196738*
**ENSG00000163686**	*0.074315559*	*0.062547481*	*0.061181827*	*0.062419016*
**ENSG00000147113**	*0.016157707*	*0.024826194*	*0.025154957*	*0.015442316*
**ENSG00000198734**	*0.061887229*	*0.02612024*	*0.026255737*	*0.022565202*
**ENSG00000219438**	*0.038888361*	*0.037706296*	*0.027848784*	*0.021341218*
**ENSG00000100522**	*0.02404236*	*0.027788801*	*0.028863823*	*0.02089631*
**ENSG00000006756**	*0.020772474*	*0.02706534*	*0.025943162*	*0.01936599*
**ENSG00000164574**	*0.015721373*	*0.024527799*	*0.024026817*	*0.015501992*
**ENSG00000182179**	*0.028182798*	*0.034381465*	*0.03304126*	*0.029841352*
**ENSG00000173334**	*0.064687236*	*0.051485245*	*0.059301725*	*0.059480217*
**ENSG00000169807**	*0.015803755*	*0.02420156*	*0.024320627*	*0.015199472*
**ENSG00000179751**	*0.021157639*	*0.027909874*	*0.028872422*	*0.021204014*
**ENSG00000179933**	*0.015637257*	*0.024687758*	*0.024463144*	*0.015267477*
**ENSG00000183024**	*0.015765452*	*0.025488615*	*0.024438746*	*0.01500933*
**ENSG00000126653**	*0.028401398*	*0.035555698*	*0.036122624*	*0.02357217*
**ENSG00000128283**	*0.016659649*	*0.025620548*	*0.026080713*	*0.015552835*
**ENSG00000109667**	*0.016419967*	*0.025616191*	*0.024750137*	*0.016353886*
**ENSG00000016864**	*0.038189858*	*0.044230585*	*0.041135769*	*0.046390847*
**ENSG00000232434**	*0.014558171*	*0.023784664*	*0.024343284*	*0.015181033*
**ENSG00000188000**	*0.027370458*	*0.034433687*	*0.034635466*	*0.035206755*
**ENSG00000128604**	*0.064445189*	*0.060795056*	*0.064323904*	*0.049555845*
**ENSG00000161594**	*0.019445918*	*0.026558982*	*0.025912855*	*0.019222986*
**ENSG00000170509**	*0.020935784*	*0.031987155*	*0.027021365*	*0.022903077*
**ENSG00000088833**	*0.033801444*	*0.03954862*	*0.037690878*	*0.030231827*
**ENSG00000163684**	*0.030046745*	*0.037500794*	*0.040500411*	*0.028029675*
**ENSG00000123124**	*0.028559547*	*0.031565944*	*0.031250579*	*0.024267408*
**ENSG00000148204**	*0.01748322*	*0.026038622*	*0.027995374*	*0.019933178*
**ENSG00000099821**	*0.026522477*	*0.031690329*	*0.031567772*	*0.02593455*
**ENSG00000160223**	*0.025812893*	*0.03336381*	*0.032868771*	*0.027157316*
**ENSG00000167165**	*0.01613579*	*0.02476449*	*0.024349429*	*0.015282284*
**ENSG00000160691**	*0.016976184*	*0.025652974*	*0.025336082*	*0.01634752*
**ENSG00000183798**	*0.016080336*	*0.023718495*	*0.024569235*	*0.014990497*
**ENSG00000146469**	*0.016691676*	*0.026806556*	*0.02493766*	*0.015421663*
**ENSG00000064651**	*0.043665056*	*0.051408014*	*0.054005995*	*0.054110311*
**ENSG00000197563**	*0.017346115*	*0.025690172*	*0.025618215*	*0.016132012*
**ENSG00000163041**	*0.039315051*	*0.034785432*	*0.033441726*	*0.03936927*
**ENSG00000186891**	*0.115968942*	*0.075234174*	*0.075623701*	*0.08242764*
**ENSG00000127445**	*0.023672851*	*0.028961737*	*0.027459626*	*0.021370581*
**ENSG00000114248**	*0.065227216*	*0.075565052*	*0.044837953*	*0.040716973*
**ENSG00000164068**	*0.020604098*	*0.031000681*	*0.027284251*	*0.022654336*

**ENSG00000137760**	*0.031775045*	*0.031880754*	*0.032016736*	*0.028757164*
**ENSG00000156170**	*0.037337511*	*0.03775281*	*0.036740849*	*0.044241583*
**ENSG00000119844**	*0.024694456*	*0.034354223*	*0.032048105*	*0.027932666*
**ENSG00000170855**	*0.026987577*	*0.030064746*	*0.029457846*	*0.023203632*
**ENSG00000180354**	*0.05446796*	*0.065455145*	*0.044908528*	*0.057420355*
**ENSG00000197006**	*0.03678573*	*0.037006101*	*0.036382757*	*0.029658536*
**ENSG00000162039**	*0.015904733*	*0.024645712*	*0.024738829*	*0.014773639*
**ENSG00000163106**	*0.023220009*	*0.025787821*	*0.026597622*	*0.017693965*
**ENSG00000011478**	*0.020620029*	*0.028235503*	*0.026351333*	*0.0190629*
**ENSG00000143669**	*0.043955934*	*0.046847338*	*0.042816748*	*0.042230046*
**ENSG00000076944**	*0.027586914*	*0.029742386*	*0.033211683*	*0.023365839*
**ENSG00000146453**	*0.016272188*	*0.024658933*	*0.026539346*	*0.058412549*
**ENSG00000163512**	*0.03069467*	*0.034869182*	*0.035334684*	*0.028588038*
**ENSG00000142627**	*0.02133993*	*0.028319612*	*0.027091039*	*0.021095129*
**ENSG00000112964**	*0.136098226*	*0.037346284*	*0.025843084*	*0.0741961*
**ENSG00000116198**	*0.023875231*	*0.031684949*	*0.031488782*	*0.025436081*
**ENSG00000099804**	*0.024044846*	*0.031643681*	*0.031893097*	*0.025499745*
**ENSG00000137808**	*0.01821761*	*0.025660995*	*0.025458154*	*0.017055688*
**ENSG00000001617**	*0.014877451*	*0.024623375*	*0.024769341*	*0.014448891*
**ENSG00000125691**	*0.01848926*	*0.026111924*	*0.025020656*	*0.023562537*
**ENSG00000082996**	*0.018164851*	*0.024659454*	*0.02607166*	*0.017726565*
**ENSG00000060642**	*0.037620601*	*0.039862028*	*0.032920386*	*0.029096425*
**ENSG00000186393**	*0.015261053*	*0.026022185*	*0.024151669*	*0.015651632*
**ENSG00000168826**	*0.017369456*	*0.025481758*	*0.025422648*	*0.014873702*
**ENSG00000065665**	*0.028684369*	*0.031941667*	*0.031263036*	*0.027509187*
**ENSG00000178150**	*0.061543007*	*0.057581169*	*0.050522807*	*0.051463041*
**ENSG00000115593**	*0.019362449*	*0.02674563*	*0.025121293*	*0.018246847*
**ENSG00000124772**	*0.049707876*	*0.0424604*	*0.053267185*	*0.052119652*
**ENSG00000145545**	*0.030165311*	*0.036084864*	*0.033325742*	*0.026314361*
**ENSG00000114098**	*0.035562772*	*0.035606956*	*0.038321918*	*0.037164965*
**ENSG00000115368**	*0.043278006*	*0.035492412*	*0.034691364*	*0.043794527*
**ENSG00000174885**	*0.015354147*	*0.025574607*	*0.025520053*	*0.015674602*
**ENSG00000162843**	*0.016775302*	*0.026267631*	*0.024687315*	*0.015977763*
**ENSG00000165175**	*0.057285732*	*0.053605445*	*0.04802007*	*0.059915156*
**ENSG00000108654**	*0.024403626*	*0.032186319*	*0.033972325*	*0.026379714*
**ENSG00000068024**	*0.055706811*	*0.055760289*	*0.044603312*	*0.041262707*
**ENSG00000110169**	*0.020511606*	*0.027683658*	*0.026560754*	*0.016575549*
**ENSG00000163519**	*0.060023681*	*0.026505669*	*0.02457805*	*0.031856684*
**ENSG00000164414**	*0.042210896*	*0.044855528*	*0.034962439*	*0.029944257*
**ENSG00000183273**	*0.015584824*	*0.024154988*	*0.025682978*	*0.015283795*
**ENSG00000087470**	*0.035992877*	*0.046525782*	*0.031771529*	*0.029615757*
**ENSG00000232040**	*0.018128531*	*0.02557928*	*0.025525736*	*0.018072724*
**ENSG00000115289**	*0.021378614*	*0.026991232*	*0.033547157*	*0.023400238*
**ENSG00000100030**	*0.037866481*	*0.036674153*	*0.039129547*	*0.037244065*
**ENSG00000141376**	*0.087423375*	*0.058058188*	*0.056988382*	*0.045869925*
**ENSG00000204482**	*0.022676938*	*0.025976675*	*0.026329829*	*0.015939327*
**ENSG00000241322**	*0.01651918*	*0.025990148*	*0.024938889*	*0.016023668*
**ENSG00000163975**	*0.018664479*	*0.026872556*	*0.026026907*	*0.017147793*
**ENSG00000204410**	*0.03367217*	*0.03292244*	*0.031475978*	*0.024517723*
**ENSG00000164934**	*0.032259552*	*0.034841732*	*0.032696972*	*0.030483885*
**ENSG00000112110**	*0.026912385*	*0.030627408*	*0.027778124*	*0.020989262*
**ENSG00000180881**	*0.01802139*	*0.025947211*	*0.025344076*	*0.01550536*
**ENSG00000102313**	*0.015580058*	*0.025340517*	*0.025386836*	*0.015286216*
**ENSG00000164111**	*0.03908082*	*0.032678886*	*0.033779939*	*0.036452628*

**ENSG00000140450**	*0.023501223*	*0.064827639*	*0.048230476*	*0.043269025*
**ENSG00000067840**	*0.015180735*	*0.025427557*	*0.024873099*	*0.015220849*
**ENSG00000160113**	*0.048870014*	*0.043224462*	*0.041574581*	*0.041647189*
**ENSG00000164327**	*0.024324285*	*0.028791356*	*0.033784*	*0.021211663*
**ENSG00000204956**	*0.01681238*	*0.025170065*	*0.026475824*	*0.015329759*
**ENSG00000135248**	*0.0168181*	*0.026454896*	*0.026319128*	*0.015942741*
**ENSG00000121380**	*0.020224082*	*0.026902902*	*0.026430396*	*0.017732114*
**ENSG00000244731**	*0.024570731*	*0.027617073*	*0.02705748*	*0.029540365*
**ENSG00000182827**	*0.0306492*	*0.033701956*	*0.033589353*	*0.028845933*
**ENSG00000132912**	*0.031581652*	*0.037120353*	*0.034933231*	*0.029452923*
**ENSG00000164221**	*0.038647651*	*0.144014226*	*0.140043487*	*0.033520016*
**ENSG00000112249**	*0.033848036*	*0.040066466*	*0.029056641*	*0.030291137*
**ENSG00000125245**	*0.075212238*	*0.055133936*	*0.065776733*	*0.080426684*
**ENSG00000236249**	*0.049035513*	*0.024855888*	*0.026094453*	*0.015260968*
**ENSG00000143819**	*0.017618233*	*0.024921131*	*0.026574288*	*0.015877643*
**ENSG00000175416**	*0.016295168*	*0.024095281*	*0.025091025*	*0.015418211*
**ENSG00000149357**	*0.021278674*	*0.026548541*	*0.02959082*	*0.025727852*
**ENSG00000185306**	*0.015132717*	*0.024703024*	*0.024814842*	*0.015109266*
**ENSG00000110921**	*0.030101768*	*0.032856759*	*0.031500746*	*0.027014478*
**ENSG00000130032**	*0.016609278*	*0.024797049*	*0.02497241*	*0.016307784*
**ENSG00000177971**	*0.021213964*	*0.027504062*	*0.028338902*	*0.017001076*
**ENSG00000178078**	*0.060244322*	*0.050220805*	*0.05119139*	*0.043366988*
**ENSG00000103310**	*0.015669753*	*0.025143614*	*0.025185645*	*0.014969077*
**ENSG00000116539**	*0.022639951*	*0.028444505*	*0.025792896*	*0.01867247*
**ENSG00000144199**	*0.028988465*	*0.030623967*	*0.029319862*	*0.025161291*
**ENSG00000158417**	*0.024067617*	*0.03027347*	*0.028575886*	*0.019618034*
**ENSG00000240263**	*0.015398936*	*0.024901138*	*0.024039222*	*0.015111409*
**ENSG00000124019**	*0.016126564*	*0.02501213*	*0.026046572*	*0.01623091*
**ENSG00000124780**	*0.015398089*	*0.025027149*	*0.024296366*	*0.015633426*
**ENSG00000188612**	*0.029953943*	*0.034490476*	*0.027998268*	*0.023937496*
**ENSG00000114054**	*0.02591*	*0.028309786*	*0.030868519*	*0.025997488*
**ENSG00000118231**	*0.015638497*	*0.025216448*	*0.024634513*	*0.014941233*
**ENSG00000071655**	*0.033342955*	*0.035692592*	*0.036725726*	*0.028933137*
**ENSG00000011347**	*0.017543528*	*0.026727857*	*0.026008154*	*0.017046865*
**ENSG00000138185**	*0.037224487*	*0.039522652*	*0.043939931*	*0.034670695*
**ENSG00000173621**	*0.043688668*	*0.040129011*	*0.04055129*	*0.037177337*
**ENSG00000139624**	*0.038440359*	*0.035102569*	*0.034346738*	*0.037295384*
**ENSG00000096717**	*0.034628336*	*0.039912277*	*0.044299027*	*0.035823833*
**ENSG00000157326**	*0.033762506*	*0.041186071*	*0.039871327*	*0.044610243*
**ENSG00000087116**	*0.015439959*	*0.024478134*	*0.024410317*	*0.01508983*
**ENSG00000161813**	*0.04047903*	*0.041775485*	*0.038100418*	*0.039389379*
**ENSG00000004939**	*0.017849387*	*0.026918896*	*0.026328202*	*0.016462997*
**ENSG00000112782**	*0.018482831*	*0.025578135*	*0.025676699*	*0.019867*
**ENSG00000152404**	*0.02798186*	*0.036198638*	*0.03079983*	*0.027575141*
**ENSG00000155087**	*0.024575458*	*0.034170307*	*0.027042467*	*0.022405855*
**ENSG00000105737**	*0.016517773*	*0.026251342*	*0.025011388*	*0.01623873*
**ENSG00000162623**	*0.048056666*	*0.037897507*	*0.035450263*	*0.047444336*
**ENSG00000146278**	*0.020796059*	*0.030622268*	*0.029090543*	*0.028575063*
**ENSG00000105171**	*0.022971337*	*0.027996118*	*0.029144674*	*0.021123519*
**ENSG00000142178**	*0.064511565*	*0.05799182*	*0.051659658*	*0.055141748*
**ENSG00000160967**	*0.034626784*	*0.035422879*	*0.034555113*	*0.029733452*
**ENSG00000181004**	*0.015306401*	*0.025044573*	*0.024606263*	*0.015295993*
**ENSG00000180914**	*0.066325851*	*0.046986192*	*0.052001062*	*0.06401852*
**ENSG00000142082**	*0.033491689*	*0.045804333*	*0.039953722*	*0.033072421*

**ENSG00000175354**	*0.031924612*	*0.040703793*	*0.036091503*	*0.027560039*
**ENSG00000123407**	*0.015858791*	*0.026117282*	*0.025639591*	*0.016121632*
**ENSG00000164190**	*0.028332708*	*0.03430503*	*0.032318267*	*0.029029431*
**ENSG00000132622**	*0.016558103*	*0.023699034*	*0.024771569*	*0.015198933*
**ENSG00000168131**	*0.028313821*	*0.028870333*	*0.026451955*	*0.023472838*
**ENSG00000139053**	*0.015596662*	*0.025713385*	*0.025055134*	*0.016448367*
**ENSG00000184675**	*0.026231455*	*0.033028083*	*0.032491113*	*0.027204004*
**ENSG00000120068**	*0.021619642*	*0.030071207*	*0.028429415*	*0.02231372*
**ENSG00000176273**	*0.040006443*	*0.038394348*	*0.048307727*	*0.040995678*
**ENSG00000173805**	*0.01591345*	*0.025751845*	*0.024361977*	*0.015753286*
**ENSG00000157782**	*0.090445269*	*0.076250832*	*0.062236659*	*0.073377078*
**ENSG00000170909**	*0.017621209*	*0.027241321*	*0.027226866*	*0.018725961*
**ENSG00000115998**	*0.03443373*	*0.037120838*	*0.032832849*	*0.030820369*
**ENSG00000135341**	*0.022447745*	*0.031940823*	*0.031898631*	*0.021232947*
**ENSG00000138674**	*0.020662554*	*0.028179934*	*0.030980161*	*0.021534772*
**ENSG00000132740**	*0.031294942*	*0.036362395*	*0.033968683*	*0.026554687*
**ENSG00000170310**	*0.030214961*	*0.029508032*	*0.030673456*	*0.024695085*
**ENSG00000102531**	*0.034604941*	*0.034442052*	*0.031619777*	*0.033226241*
**ENSG00000134207**	*0.016739269*	*0.026573056*	*0.025965533*	*0.016028892*
**ENSG00000013016**	*0.03228537*	*0.034793157*	*0.028018827*	*0.022375971*
**ENSG00000163092**	*0.01722862*	*0.025029991*	*0.024862246*	*0.017877496*
**ENSG00000240972**	*0.019970954*	*0.02486369*	*0.025715898*	*0.017610947*
**ENSG00000198133**	*0.016852814*	*0.024845488*	*0.0255371*	*0.015901488*
**ENSG00000164713**	*0.036019599*	*0.037332973*	*0.032809698*	*0.029213544*
**ENSG00000196754**	*0.028951047*	*0.038076249*	*0.034657862*	*0.030282264*
**ENSG00000134258**	*0.017113795*	*0.025435513*	*0.026356761*	*0.017057875*
**ENSG00000077097**	*0.027369234*	*0.03469105*	*0.034446538*	*0.027863819*
**ENSG00000130449**	*0.0224411*	*0.027466308*	*0.027319367*	*0.021647282*
**ENSG00000180182**	*0.017291802*	*0.027789788*	*0.027272376*	*0.016739296*
**ENSG00000139537**	*0.016570378*	*0.024300701*	*0.024947912*	*0.014710767*
**ENSG00000179388**	*0.056227172*	*0.042846278*	*0.048923608*	*0.047614922*
**ENSG00000137575**	*0.03883748*	*0.038356404*	*0.038101781*	*0.030631677*
**ENSG00000121211**	*0.051270314*	*0.038290188*	*0.03609787*	*0.035122038*
**ENSG00000130234**	*0.01841537*	*0.025451627*	*0.027482991*	*0.015890376*
**ENSG00000102104**	*0.018788974*	*0.028573286*	*0.026728974*	*0.02111508*
**ENSG00000109171**	*0.028509523*	*0.040406134*	*0.036800325*	*0.031074205*
**ENSG00000137801**	*0.042053903*	*0.030936421*	*0.036144003*	*0.024157263*
**ENSG00000148175**	*0.039463495*	*0.049424333*	*0.049762659*	*0.038021189*
**ENSG00000140986**	*0.016502617*	*0.024934496*	*0.024846092*	*0.0158534*
**ENSG00000123213**	*0.042394151*	*0.040732643*	*0.033660811*	*0.036735344*
**ENSG00000145526**	*0.015552176*	*0.025043222*	*0.024565986*	*0.014001544*
**ENSG00000197140**	*0.017347722*	*0.027557393*	*0.026769428*	*0.017527827*
**ENSG00000134200**	*0.016131826*	*0.027499526*	*0.025736205*	*0.01593123*
**ENSG00000133731**	*0.030022042*	*0.035870709*	*0.033185152*	*0.025177869*
**ENSG00000036054**	*0.031474079*	*0.037621655*	*0.043909703*	*0.038709414*
**ENSG00000182240**	*0.082283202*	*0.072559708*	*0.096344168*	*0.100689934*
**ENSG00000149177**	*0.020129531*	*0.027130576*	*0.026340894*	*0.016665725*
**ENSG00000106404**	*0.037868074*	*0.039021921*	*0.042691736*	*0.038691873*
**ENSG00000173264**	*0.017031888*	*0.026612181*	*0.025661229*	*0.017582996*
**ENSG00000166938**	*0.049587184*	*0.046292813*	*0.040373263*	*0.037236777*
**ENSG00000083444**	*0.040503964*	*0.041893275*	*0.040226985*	*0.030500993*
**ENSG00000159216**	*0.061566201*	*0.03814275*	*0.040802578*	*0.054298463*
**ENSG00000163564**	*0.027740499*	*0.033629484*	*0.035309978*	*0.038500486*
**ENSG00000140993**	*0.037775404*	*0.039391117*	*0.035262367*	*0.035607538*

**ENSG00000174225**	*0.01291491*	*0.023383165*	*0.023328863*	*0.012931429*
**ENSG00000171298**	*0.034210799*	*0.036938341*	*0.034483006*	*0.033647293*
**ENSG00000129084**	*0.033434558*	*0.035771067*	*0.035630031*	*0.030953624*
**ENSG00000117245**	*0.015482448*	*0.025110822*	*0.024998304*	*0.015852659*
**ENSG00000185565**	*0.080874562*	*0.060463841*	*0.061132076*	*0.074661746*
**ENSG00000124749**	*0.016259772*	*0.024903027*	*0.026137202*	*0.015626073*
**ENSG00000122694**	*0.048785714*	*0.060058819*	*0.056649023*	*0.057906484*
**ENSG00000067334**	*0.031331413*	*0.034908496*	*0.038698098*	*0.029789322*
**ENSG00000075035**	*0.018549906*	*0.026229385*	*0.028897953*	*0.016886562*
**ENSG00000124578**	*0.016242455*	*0.025690684*	*0.02630969*	*0.016519196*
**ENSG00000151650**	*0.015811567*	*0.025067315*	*0.025550169*	*0.01568386*
**ENSG00000100316**	*0.017480591*	*0.024858636*	*0.025839191*	*0.018025267*
**ENSG00000174695**	*0.02941385*	*0.033666424*	*0.03251141*	*0.023112822*
**ENSG00000163608**	*0.022218601*	*0.031347572*	*0.032187224*	*0.022318562*
**ENSG00000198700**	*0.030672493*	*0.035463551*	*0.029641723*	*0.023860859*
**ENSG00000164970**	*0.028098725*	*0.032414995*	*0.029634551*	*0.0282315*
**ENSG00000205176**	*0.016510097*	*0.025024354*	*0.024616596*	*0.014953366*
**ENSG00000198807**	*0.016329762*	*0.025503498*	*0.024599949*	*0.015005377*
**ENSG00000169359**	*0.035381802*	*0.037701218*	*0.042042083*	*0.036749897*
**ENSG00000189377**	*0.017104595*	*0.025517917*	*0.025184744*	*0.017629188*
**ENSG00000120137**	*0.024987795*	*0.030063232*	*0.028658092*	*0.020118058*
**ENSG00000109065**	*0.023821548*	*0.030446828*	*0.032694649*	*0.025459936*
**ENSG00000049130**	*0.01741814*	*0.025932357*	*0.026439116*	*0.016174314*
**ENSG00000130876**	*0.016552044*	*0.024742709*	*0.025144613*	*0.015386283*
**ENSG00000168071**	*0.037160103*	*0.040253758*	*0.040462148*	*0.028821212*
**ENSG00000183527**	*0.029379978*	*0.029943022*	*0.029138917*	*0.029576795*
**ENSG00000146072**	*0.118517012*	*0.083957837*	*0.068882302*	*0.084945032*
**ENSG00000170606**	*0.022941677*	*0.029194391*	*0.028429107*	*0.023035997*
**ENSG00000169621**	*0.016288204*	*0.026402361*	*0.025160539*	*0.016446086*
**ENSG00000182326**	*0.019109043*	*0.025491992*	*0.025031826*	*0.019972903*
**ENSG00000160613**	*0.028250145*	*0.030532943*	*0.028702532*	*0.025504007*
**ENSG00000168631**	*0.016180459*	*0.02508626*	*0.02451508*	*0.015298728*
**ENSG00000166086**	*0.069266969*	*0.061430008*	*0.047654788*	*0.065467876*
**ENSG00000176595**	*0.018101396*	*0.025680382*	*0.028035744*	*0.017244335*
**ENSG00000114127**	*0.033700861*	*0.044249627*	*0.041070359*	*0.032583653*
**ENSG00000198795**	*0.030480959*	*0.02615982*	*0.025258159*	*0.017615361*
**ENSG00000146205**	*0.038153743*	*0.035964899*	*0.035078252*	*0.034161696*
**ENSG00000169155**	*0.030093904*	*0.046888144*	*0.044624324*	*0.028374163*
**ENSG00000130675**	*0.118589109*	*0.080317578*	*0.075283293*	*0.097969133*
**ENSG00000100722**	*0.024984903*	*0.031074036*	*0.032643317*	*0.02392289*
**ENSG00000189212**	*0.032395566*	*0.028969059*	*0.026482491*	*0.023491487*
**ENSG00000173991**	*0.01551324*	*0.025679413*	*0.025032757*	*0.015874376*
**ENSG00000204257**	*0.028956982*	*0.034405161*	*0.035011436*	*0.038466685*
**ENSG00000145794**	*0.015160849*	*0.023671437*	*0.024387212*	*0.014044908*
**ENSG00000149136**	*0.027125316*	*0.027680326*	*0.02953811*	*0.02417209*
**ENSG00000148288**	*0.058795382*	*0.053280053*	*0.052591837*	*0.063673655*
**ENSG00000147873**	*0.01679428*	*0.025424573*	*0.02510894*	*0.01579541*
**ENSG00000197576**	*0.017365671*	*0.025299548*	*0.025471685*	*0.014929302*
**ENSG00000053438**	*0.045492894*	*0.029977653*	*0.031353546*	*0.027048756*
**ENSG00000143155**	*0.044211608*	*0.045063471*	*0.050432974*	*0.055322355*
**ENSG00000144647**	*0.020503715*	*0.026497483*	*0.025691006*	*0.02000956*
**ENSG00000171053**	*0.016740999*	*0.025518885*	*0.024342778*	*0.015874793*
**ENSG00000125538**	*0.104471005*	*0.111365919*	*0.094954808*	*0.093325509*
**ENSG00000144785**	*0.035670099*	*0.042752976*	*0.036206638*	*0.029440313*

**ENSG00000165983**	*0.064381884*	*0.071816054*	*0.060761396*	*0.054250608*
**ENSG00000139496**	*0.019398417*	*0.026791799*	*0.025969912*	*0.019971416*
**ENSG00000074317**	*0.018106769*	*0.024964643*	*0.025641584*	*0.016446836*
**ENSG00000119446**	*0.030184377*	*0.036230325*	*0.030300609*	*0.022604409*
**ENSG00000174007**	*0.030562348*	*0.029839883*	*0.033290107*	*0.02579777*
**ENSG00000115290**	*0.018457321*	*0.02825238*	*0.027377432*	*0.0182528*
**ENSG00000172938**	*0.015352513*	*0.024609248*	*0.025132398*	*0.014138033*
**ENSG00000147677**	*0.019383163*	*0.02661768*	*0.026473887*	*0.019353373*
**ENSG00000172940**	*0.017348066*	*0.024900607*	*0.025300701*	*0.016112961*
**ENSG00000157593**	*0.024772624*	*0.036404768*	*0.038184256*	*0.031758641*
**ENSG00000115514**	*0.035728834*	*0.040184134*	*0.036836726*	*0.05575011*
**ENSG00000215790**	*0.032105558*	*0.035984999*	*0.034661658*	*0.031930541*
**ENSG00000035664**	*0.03298857*	*0.033141654*	*0.033701657*	*0.046921423*
**ENSG00000085999**	*0.05005148*	*0.04413127*	*0.034559538*	*0.028928618*
**ENSG00000131018**	*0.017577178*	*0.028687521*	*0.026169683*	*0.01710895*
**ENSG00000102359**	*0.017967623*	*0.026125721*	*0.02627761*	*0.017748019*
**ENSG00000125571**	*0.016092914*	*0.024140945*	*0.024980252*	*0.01650406*
**ENSG00000131351**	*0.042004501*	*0.033743006*	*0.034841249*	*0.029691088*
**ENSG00000181218**	*0.069434923*	*0.058744395*	*0.050049424*	*0.053288921*
**ENSG00000181656**	*0.014222854*	*0.025036394*	*0.025828308*	*0.01531254*
**ENSG00000164743**	*0.023015452*	*0.02633817*	*0.028689253*	*0.019045006*
**ENSG00000100330**	*0.024569402*	*0.031139364*	*0.036939828*	*0.022669296*
**ENSG00000172476**	*0.017755285*	*0.026048692*	*0.025423343*	*0.025184619*
**ENSG00000172070**	*0.063106017*	*0.042291448*	*0.054281149*	*0.039887385*
**ENSG00000165359**	*0.02898342*	*0.035000562*	*0.032276286*	*0.025306633*
**ENSG00000141401**	*0.077972183*	*0.060187007*	*0.050818101*	*0.054428794*
**ENSG00000134748**	*0.034967438*	*0.036201931*	*0.037047798*	*0.032792645*
**ENSG00000134014**	*0.02499076*	*0.033494675*	*0.031023558*	*0.02225908*
**ENSG00000125430**	*0.019596032*	*0.026384775*	*0.026277586*	*0.019996898*
**ENSG00000204248**	*0.020919144*	*0.025627148*	*0.025720766*	*0.018521721*
**ENSG00000125823**	*0.015829156*	*0.024966292*	*0.025101626*	*0.015243666*
**ENSG00000205810**	*0.01809156*	*0.027586204*	*0.025669665*	*0.01634929*
**ENSG00000186635**	*0.016525704*	*0.025200029*	*0.027371965*	*0.016433816*
**ENSG00000186994**	*0.024687908*	*0.029069902*	*0.026861424*	*0.023653499*
**ENSG00000141026**	*0.031046357*	*0.037203886*	*0.036231469*	*0.030912061*
**ENSG00000104221**	*0.027725188*	*0.033199939*	*0.039960011*	*0.02662117*
**ENSG00000130943**	*0.015238411*	*0.02581235*	*0.025194108*	*0.016360099*
**ENSG00000148584**	*0.015779711*	*0.024655399*	*0.025567209*	*0.015849029*
**ENSG00000170954**	*0.060929553*	*0.051305392*	*0.0560297*	*0.06335567*
**ENSG00000115380**	*0.015468439*	*0.025745188*	*0.02524202*	*0.016027538*
**ENSG00000122257**	*0.039620789*	*0.036969546*	*0.029197807*	*0.032247796*
**ENSG00000119801**	*0.02942241*	*0.036375674*	*0.037778792*	*0.026584647*
**ENSG00000132128**	*0.031235024*	*0.038073057*	*0.033461633*	*0.02668294*
**ENSG00000003987**	*0.020864078*	*0.026817254*	*0.029793701*	*0.016247052*
**ENSG00000158077**	*0.018770894*	*0.025239509*	*0.025012227*	*0.018262094*
**ENSG00000163541**	*0.024788028*	*0.029791965*	*0.031420699*	*0.020323701*
**ENSG00000204007**	*0.016761266*	*0.024984802*	*0.024729169*	*0.017243928*
**ENSG00000163961**	*0.023293599*	*0.02815074*	*0.027943524*	*0.019886651*
**ENSG00000134333**	*0.024158393*	*0.026603622*	*0.024989515*	*0.018393194*
**ENSG00000204394**	*0.04374855*	*0.04113133*	*0.03933508*	*0.039067739*
**ENSG00000126460**	*0.047384017*	*0.048244988*	*0.04115174*	*0.061162143*
**ENSG00000137076**	*0.032029536*	*0.047201564*	*0.040303804*	*0.038633342*
**ENSG00000166788**	*0.031426267*	*0.030672067*	*0.028747457*	*0.02691766*
**ENSG00000040531**	*0.031315011*	*0.03325788*	*0.034503202*	*0.027256488*

**ENSG00000149131**	*0.018164566*	*0.025253659*	*0.024832245*	*0.01660493*
**ENSG00000133773**	*0.025994734*	*0.030749649*	*0.033639204*	*0.024242208*
**ENSG00000186094**	*0.016168715*	*0.02456321*	*0.024775953*	*0.015127065*
**ENSG00000155561**	*0.027014352*	*0.034058352*	*0.028193811*	*0.022141692*
**ENSG00000139505**	*0.032868508*	*0.039138849*	*0.04183634*	*0.039539782*
**ENSG00000179051**	*0.024705106*	*0.030490144*	*0.03190584*	*0.027250294*
**ENSG00000106211**	*0.054511869*	*0.041986111*	*0.048569151*	*0.050581929*
**ENSG00000149948**	*0.058520935*	*0.027991488*	*0.033449693*	*0.016787037*
**ENSG00000179630**	*0.017201946*	*0.027000953*	*0.02585751*	*0.017299269*
**ENSG00000149532**	*0.015766287*	*0.026173898*	*0.025611862*	*0.017524022*
**ENSG00000134109**	*0.033921071*	*0.034858641*	*0.035477767*	*0.026741785*
**ENSG00000100150**	*0.021910609*	*0.02904496*	*0.028057056*	*0.019609338*
**ENSG00000205929**	*0.015615363*	*0.0246893*	*0.027328165*	*0.016344453*
**ENSG00000173218**	*0.021857098*	*0.024405589*	*0.025416866*	*0.020631974*
**ENSG00000156931**	*0.027768154*	*0.030210246*	*0.030329581*	*0.027411123*
**ENSG00000185432**	*0.090155767*	*0.074203184*	*0.087868253*	*0.107761742*
**ENSG00000125084**	*0.016070783*	*0.025267751*	*0.024637959*	*0.014666119*
**ENSG00000196116**	*0.031900955*	*0.038011866*	*0.033964101*	*0.029030732*
**ENSG00000136450**	*0.024153574*	*0.028820347*	*0.027480431*	*0.023611195*
**ENSG00000142599**	*0.04468405*	*0.042934102*	*0.03959438*	*0.0385542*
**ENSG00000164309**	*0.027625343*	*0.026625204*	*0.027442728*	*0.021309088*
**ENSG00000196683**	*0.019058836*	*0.027364978*	*0.026373849*	*0.022666804*
**ENSG00000113578**	*0.015516751*	*0.024576901*	*0.026045824*	*0.016277433*
**ENSG00000177469**	*0.016911017*	*0.025577513*	*0.027762657*	*0.015800827*
**ENSG00000169047**	*0.03233685*	*0.03743855*	*0.035508761*	*0.025831757*
**ENSG00000172116**	*0.018034524*	*0.026396233*	*0.02537018*	*0.015628111*
**ENSG00000066294**	*0.058304591*	*0.046627294*	*0.046951207*	*0.047819646*
**ENSG00000106399**	*0.02968309*	*0.030102604*	*0.028825687*	*0.020534101*
**ENSG00000100162**	*0.048239151*	*0.03803154*	*0.030495223*	*0.03450353*
**ENSG00000188338**	*0.01578546*	*0.024705689*	*0.024622818*	*0.015733097*
**ENSG00000185252**	*0.019054836*	*0.027665873*	*0.025708505*	*0.017883682*
**ENSG00000169905**	*0.023117244*	*0.031183389*	*0.027429837*	*0.020162542*
**ENSG00000074211**	*0.017938531*	*0.025968763*	*0.02496045*	*0.015029828*
**ENSG00000161010**	*0.018787794*	*0.025042135*	*0.027077019*	*0.018834509*
**ENSG00000163625**	*0.015686288*	*0.02500443*	*0.024173544*	*0.015487662*
**ENSG00000085274**	*0.0267701*	*0.032866864*	*0.034181224*	*0.021871742*
**ENSG00000146809**	*0.018431*	*0.025809577*	*0.025565432*	*0.017493038*
**ENSG00000134075**	*0.024183667*	*0.034515893*	*0.031637081*	*0.023636093*
**ENSG00000198088**	*0.052337071*	*0.047145938*	*0.043824948*	*0.035425656*
**ENSG00000039139**	*0.015677686*	*0.025267741*	*0.025153462*	*0.014224439*
**ENSG00000007171**	*0.018482003*	*0.025863056*	*0.028034846*	*0.016079047*
**ENSG00000135175**	*0.016380334*	*0.02436459*	*0.024453775*	*0.014737359*
**ENSG00000100014**	*0.015801538*	*0.025564793*	*0.024175472*	*0.015727238*
**ENSG00000163218**	*0.021440787*	*0.025740308*	*0.026100401*	*0.017597269*
**ENSG00000107816**	*0.031619898*	*0.0323438*	*0.030174919*	*0.026844737*
**ENSG00000091947**	*0.026249411*	*0.030773388*	*0.031635364*	*0.021978077*
**ENSG00000112414**	*0.015625375*	*0.024295596*	*0.025166268*	*0.015019885*
**ENSG00000167523**	*0.035163726*	*0.034404392*	*0.035732953*	*0.033555923*
**ENSG00000138604**	*0.059278457*	*0.050919634*	*0.032658614*	*0.036825283*
**ENSG00000196937**	*0.042376939*	*0.043484779*	*0.04036711*	*0.036368151*
**ENSG00000112146**	*0.017724873*	*0.026296936*	*0.027185793*	*0.018536364*
**ENSG00000122375**	*0.018502731*	*0.024972475*	*0.025214377*	*0.018162738*
**ENSG00000102760**	*0.081407933*	*0.057562186*	*0.050530622*	*0.062101242*
**ENSG00000088926**	*0.018380066*	*0.026618079*	*0.026593053*	*0.018012493*

**ENSG00000185158**	*0.026072868*	*0.030560946*	*0.031053596*	*0.031324398*
**ENSG00000197901**	*0.015884482*	*0.024620341*	*0.025433599*	*0.015156428*
**ENSG00000146143**	*0.037379657*	*0.040757462*	*0.032403615*	*0.026190536*
**ENSG00000205544**	*0.021111248*	*0.027418982*	*0.027570941*	*0.026284887*
**ENSG00000174514**	*0.018518685*	*0.025452752*	*0.024846852*	*0.016755919*
**ENSG00000157036**	*0.041105104*	*0.034708283*	*0.03893491*	*0.043603488*
**ENSG00000136238**	*0.020428085*	*0.028189984*	*0.028987124*	*0.019425833*
**ENSG00000008438**	*0.015162546*	*0.024332031*	*0.024280171*	*0.014762611*
**ENSG00000164816**	*0.015199461*	*0.024416969*	*0.02437207*	*0.014259464*
**ENSG00000099860**	*0.044541726*	*0.045413479*	*0.03923332*	*0.043230977*
**ENSG00000166025**	*0.016483432*	*0.024931546*	*0.024586861*	*0.014972034*
**ENSG00000176438**	*0.01599375*	*0.025450359*	*0.025052027*	*0.015781305*
**ENSG00000105255**	*0.019494736*	*0.029856891*	*0.036383774*	*0.019646667*
**ENSG00000243156**	*0.085250367*	*0.074355007*	*0.065073435*	*0.063102249*
**ENSG00000100023**	*0.018228241*	*0.029237718*	*0.028655683*	*0.019566184*
**ENSG00000171224**	*0.038637248*	*0.044994765*	*0.042519634*	*0.038044642*
**ENSG00000136931**	*0.018126321*	*0.025846107*	*0.027031164*	*0.016993202*
**ENSG00000153989**	*0.03107694*	*0.037536002*	*0.035509626*	*0.027494015*
**ENSG00000148296**	*0.032921626*	*0.032098537*	*0.032249038*	*0.034507515*
**ENSG00000165475**	*0.044045129*	*0.041746472*	*0.037806057*	*0.044836658*
**ENSG00000151224**	*0.017072712*	*0.025415864*	*0.026299589*	*0.017334041*
**ENSG00000105270**	*0.081788394*	*0.04961773*	*0.046448234*	*0.059117992*
**ENSG00000109625**	*0.017202581*	*0.025339906*	*0.024802894*	*0.015095012*
**ENSG00000197956**	*0.079404976*	*0.058359698*	*0.05273146*	*0.064157531*
**ENSG00000001460**	*0.023425622*	*0.026929436*	*0.02688357*	*0.020515244*
**ENSG00000179902**	*0.013982048*	*0.023991724*	*0.023868765*	*0.015020682*
**ENSG00000142798**	*0.014678119*	*0.02531863*	*0.02505711*	*0.013933305*
**ENSG00000125484**	*0.032091665*	*0.03686599*	*0.042220827*	*0.032775457*
**ENSG00000128000**	*0.017163664*	*0.026742531*	*0.025255277*	*0.015890853*
**ENSG00000088356**	*0.025337039*	*0.035001605*	*0.040228291*	*0.028547689*
**ENSG00000146676**	*0.018174854*	*0.027256863*	*0.027501206*	*0.018709992*
**ENSG00000155367**	*0.018712778*	*0.028567339*	*0.027918307*	*0.019645526*
**ENSG00000143412**	*0.018134477*	*0.02526551*	*0.026235254*	*0.018197299*
**ENSG00000178719**	*0.026804249*	*0.048819222*	*0.052529776*	*0.048076204*
**ENSG00000196659**	*0.016552197*	*0.025999254*	*0.024806581*	*0.016077193*
**ENSG00000087510**	*0.020589092*	*0.026493961*	*0.027772856*	*0.017788455*
**ENSG00000179855**	*0.016276919*	*0.025338514*	*0.024376826*	*0.016056305*
**ENSG00000204140**	*0.014767554*	*0.025552435*	*0.025209068*	*0.01505371*
**ENSG00000121904**	*0.018747027*	*0.026395615*	*0.026487522*	*0.017311694*
**ENSG00000180785**	*0.01942855*	*0.025493235*	*0.025735453*	*0.017589044*
**ENSG00000142039**	*0.028071177*	*0.032991381*	*0.037070995*	*0.032815268*
**ENSG00000102893**	*0.037164146*	*0.035586135*	*0.03199267*	*0.026540491*
**ENSG00000171105**	*0.056725728*	*0.048593824*	*0.045735451*	*0.060171354*
**ENSG00000118257**	*0.020757646*	*0.027844022*	*0.029323901*	*0.028183571*
**ENSG00000131094**	*0.016108956*	*0.024599247*	*0.024927535*	*0.016039658*
**ENSG00000183597**	*0.02696827*	*0.032658334*	*0.028905654*	*0.021917367*
**ENSG00000106006**	*0.016289991*	*0.026247348*	*0.025212867*	*0.017513793*
**ENSG00000106013**	*0.028080109*	*0.028510164*	*0.026236263*	*0.021427903*
**ENSG00000111247**	*0.042916993*	*0.037457757*	*0.034836925*	*0.038363751*
**ENSG00000166736**	*0.048243276*	*0.034755432*	*0.037257345*	*0.027495787*
**ENSG00000178502**	*0.020528927*	*0.028069508*	*0.028741633*	*0.018905691*
**ENSG00000143252**	*0.021354488*	*0.026925*	*0.027445539*	*0.018646606*
**ENSG00000167083**	*0.033214561*	*0.038514575*	*0.033476917*	*0.035693319*
**ENSG00000078589**	*0.037549896*	*0.040485566*	*0.043221482*	*0.030783617*

**ENSG00000164120**	*0.034925367*	*0.035718483*	*0.030008776*	*0.048184067*
**ENSG00000083842**	*0.017394113*	*0.027925469*	*0.026697131*	*0.016286671*
**ENSG00000126010**	*0.018058163*	*0.025517374*	*0.026187622*	*0.016551347*
**ENSG00000138395**	*0.015843807*	*0.024610126*	*0.02420455*	*0.014820267*
**ENSG00000131069**	*0.035093663*	*0.034095555*	*0.029617569*	*0.028239967*
**ENSG00000105696**	*0.015521189*	*0.025627542*	*0.024670906*	*0.016194736*
**ENSG00000170373**	*0.016244395*	*0.024864675*	*0.024577778*	*0.015143766*
**ENSG00000124383**	*0.027015161*	*0.031702596*	*0.034886012*	*0.029731051*
**ENSG00000083807**	*0.039893145*	*0.046614172*	*0.040786143*	*0.035028687*
**ENSG00000133083**	*0.015047034*	*0.024746403*	*0.024991621*	*0.014869942*
**ENSG00000168040**	*0.028210504*	*0.031946207*	*0.035885826*	*0.02719002*
**ENSG00000188996**	*0.025976106*	*0.031875441*	*0.032194525*	*0.021297561*
**ENSG00000204941**	*0.015669896*	*0.02479069*	*0.024490772*	*0.016279689*
**ENSG00000123119**	*0.031902907*	*0.034430888*	*0.036474451*	*0.035755517*
**ENSG00000132359**	*0.030181528*	*0.027338444*	*0.030204606*	*0.02648375*
**ENSG00000130755**	*0.031397464*	*0.031070621*	*0.030803793*	*0.024258386*
**ENSG00000205302**	*0.027268203*	*0.036633737*	*0.033668596*	*0.027540917*
**ENSG00000143398**	*0.024388369*	*0.032266202*	*0.036088215*	*0.021386752*
**ENSG00000113583**	*0.029978431*	*0.032736241*	*0.032715005*	*0.023264274*
**ENSG00000204642**	*0.029413454*	*0.034570406*	*0.033901092*	*0.033723778*
**ENSG00000138095**	*0.034827156*	*0.04006464*	*0.033385145*	*0.032183225*
**ENSG00000132549**	*0.016712799*	*0.02686809*	*0.02574305*	*0.017227232*
**ENSG00000180573**	*0.056078279*	*0.051495375*	*0.049850198*	*0.043313161*
**ENSG00000141127**	*0.025190667*	*0.030385672*	*0.027893968*	*0.021261535*
**ENSG00000132313**	*0.032225938*	*0.032205886*	*0.032722751*	*0.035430607*
**ENSG00000154263**	*0.020719434*	*0.026433121*	*0.026315167*	*0.016537752*
**ENSG00000154727**	*0.034511426*	*0.046425658*	*0.037347304*	*0.036595812*
**ENSG00000177084**	*0.026314824*	*0.028485441*	*0.030603318*	*0.021418695*
**ENSG00000112419**	*0.051914002*	*0.042996467*	*0.036348246*	*0.036093126*
**ENSG00000123104**	*0.051499516*	*0.052497224*	*0.040761686*	*0.046924321*
**ENSG00000168246**	*0.049072952*	*0.045071049*	*0.041599533*	*0.0377982*
**ENSG00000128944**	*0.035913389*	*0.034511954*	*0.03212162*	*0.026839328*
**ENSG00000101935**	*0.021477754*	*0.027914214*	*0.031186254*	*0.016735029*
**ENSG00000077943**	*0.01693527*	*0.025759499*	*0.024655338*	*0.016251864*
**ENSG00000167531**	*0.016710762*	*0.024860237*	*0.024669757*	*0.017629434*
**ENSG00000112658**	*0.037750667*	*0.040712493*	*0.048310063*	*0.047057456*
**ENSG00000126895**	*0.015278381*	*0.025944384*	*0.025436317*	*0.014827474*
**ENSG00000069956**	*0.031494294*	*0.033548554*	*0.033870194*	*0.028591036*
**ENSG00000196405**	*0.046660867*	*0.040167652*	*0.03894074*	*0.038611516*
**ENSG00000105204**	*0.020102958*	*0.028973587*	*0.025460846*	*0.021357026*
**ENSG00000102181**	*0.02972785*	*0.030688623*	*0.032025826*	*0.03131603*
**ENSG00000106266**	*0.02994993*	*0.035026356*	*0.037955274*	*0.02979814*
**ENSG00000136167**	*0.019245776*	*0.026573367*	*0.02620969*	*0.020334378*
**ENSG00000197747**	*0.087318674*	*0.066164494*	*0.049114578*	*0.095067721*
**ENSG00000177476**	*0.015221646*	*0.024643096*	*0.025404567*	*0.014687132*
**ENSG00000051382**	*0.036099243*	*0.041783001*	*0.037831225*	*0.042283165*
**ENSG00000184083**	*0.018401002*	*0.025188574*	*0.024770149*	*0.016182303*
**ENSG00000104131**	*0.037281556*	*0.043253513*	*0.043590758*	*0.042113415*
**ENSG00000108666**	*0.036370175*	*0.042540482*	*0.033482328*	*0.029944884*
**ENSG00000089327**	*0.039632218*	*0.029706378*	*0.031825907*	*0.02736791*
**ENSG00000167733**	*0.017913576*	*0.02680959*	*0.026812726*	*0.017628122*
**ENSG00000173599**	*0.016219489*	*0.024788769*	*0.025676518*	*0.015159836*
**ENSG00000196290**	*0.030739906*	*0.035496688*	*0.031734253*	*0.02044741*
**ENSG00000116329**	*0.016995077*	*0.025171009*	*0.026615362*	*0.015650941*

**ENSG00000165617**	*0.059883645*	*0.0435756*	*0.042339072*	*0.039312361*
**ENSG00000215788**	*0.053848371*	*0.038161002*	*0.026626751*	*0.024840683*
**ENSG00000150457**	*0.024078796*	*0.032497648*	*0.03023165*	*0.022754415*
**ENSG00000187678**	*0.032110582*	*0.034393791*	*0.033139978*	*0.030701925*
**ENSG00000235860**	*0.015636689*	*0.025248471*	*0.025178585*	*0.016199159*
**ENSG00000163734**	*0.015099099*	*0.025995412*	*0.024857491*	*0.016526434*
**ENSG00000165805**	*0.015686833*	*0.02485153*	*0.025222701*	*0.014668899*
**ENSG00000106628**	*0.032033149*	*0.031933924*	*0.033728004*	*0.021575796*
**ENSG00000157227**	*0.023824666*	*0.026200412*	*0.025784363*	*0.017603264*
**ENSG00000157856**	*0.016347062*	*0.026128901*	*0.025669766*	*0.016095949*
**ENSG00000104472**	*0.031457187*	*0.036796638*	*0.036730606*	*0.026889729*
**ENSG00000238269**	*0.013678382*	*0.023568474*	*0.023812175*	*0.012903844*
**ENSG00000116035**	*0.016717596*	*0.026215921*	*0.025152784*	*0.015885959*
**ENSG00000066405**	*0.020039615*	*0.026648534*	*0.026404338*	*0.017766896*
**ENSG00000124678**	*0.018498228*	*0.024878774*	*0.025523117*	*0.01765432*
**ENSG00000112308**	*0.022011479*	*0.031057581*	*0.02849659*	*0.017051043*
**ENSG00000101224**	*0.050005396*	*0.041645945*	*0.041874967*	*0.041662*
**ENSG00000188785**	*0.03221093*	*0.041281253*	*0.034053514*	*0.02425675*
**ENSG00000139517**	*0.030550544*	*0.036467932*	*0.033899856*	*0.02452211*
**ENSG00000127083**	*0.016576511*	*0.025055629*	*0.026077723*	*0.016031532*
**ENSG00000138772**	*0.114437597*	*0.08086981*	*0.053564226*	*0.059520515*
**ENSG00000102393**	*0.047231967*	*0.041362301*	*0.036416788*	*0.035227602*
**ENSG00000153071**	*0.028946161*	*0.026157512*	*0.024731249*	*0.025366492*
**ENSG00000197785**	*0.035438001*	*0.032182712*	*0.039890403*	*0.036351864*
**ENSG00000171130**	*0.037201675*	*0.045884506*	*0.039058027*	*0.040547386*
**ENSG00000010818**	*0.044711164*	*0.03788552*	*0.034977648*	*0.039654575*
**ENSG00000198911**	*0.025578699*	*0.029908424*	*0.033722057*	*0.02432551*
**ENSG00000156284**	*0.017017588*	*0.025354165*	*0.024705326*	*0.016176831*
**ENSG00000139410**	*0.071273883*	*0.057704293*	*0.055070262*	*0.052308928*
**ENSG00000198816**	*0.075943353*	*0.063861636*	*0.058821748*	*0.064399549*
**ENSG00000214212**	*0.021860311*	*0.029150025*	*0.027589787*	*0.023623734*
**ENSG00000116954**	*0.032759332*	*0.041617165*	*0.040287863*	*0.031138166*
**ENSG00000137831**	*0.042763658*	*0.038590237*	*0.032424238*	*0.035515413*
**ENSG00000189186**	*0.014654556*	*0.024641814*	*0.024237513*	*0.014780833*
**ENSG00000122547**	*0.035350978*	*0.031453808*	*0.025954474*	*0.024028062*
**ENSG00000161835**	*0.035847403*	*0.037109581*	*0.03323215*	*0.028269362*
**ENSG00000141002**	*0.026379942*	*0.031742056*	*0.03195192*	*0.023347548*
**ENSG00000253797**	*0.037634754*	*0.0446879*	*0.035675401*	*0.03505205*
**ENSG00000197122**	*0.015038759*	*0.025074446*	*0.025301414*	*0.015822815*
**ENSG00000187080**	*0.016340382*	*0.024589839*	*0.025049789*	*0.015782961*
**ENSG00000124237**	*0.016401953*	*0.02550239*	*0.024970565*	*0.015066176*
**ENSG00000174348**	*0.022819501*	*0.027877905*	*0.030272229*	*0.021333397*
**ENSG00000070814**	*0.033149994*	*0.033952039*	*0.033781339*	*0.030544766*
**ENSG00000170638**	*0.033816805*	*0.033261066*	*0.044482401*	*0.027042354*
**ENSG00000143436**	*0.026479524*	*0.034265107*	*0.030392673*	*0.021777335*
**ENSG00000251297**	*0.015937466*	*0.026018003*	*0.024647806*	*0.015139248*
**ENSG00000179144**	*0.108376664*	*0.082309666*	*0.088637089*	*0.093950468*
**ENSG00000121289**	*0.025983272*	*0.036167399*	*0.031204052*	*0.026488968*
**ENSG00000124827**	*0.017591807*	*0.026233622*	*0.025094474*	*0.017019449*
**ENSG00000043514**	*0.032083244*	*0.034086224*	*0.031998385*	*0.028640383*
**ENSG00000213931**	*0.092714643*	*0.025508305*	*0.025845227*	*0.032798721*
**ENSG00000173157**	*0.017416608*	*0.024972856*	*0.025005347*	*0.01546046*
**ENSG00000085662**	*0.028900736*	*0.035204621*	*0.030198419*	*0.025594837*
**ENSG00000101367**	*0.023082114*	*0.029883857*	*0.030942511*	*0.023726509*

**ENSG00000239900**	*0.028659694*	*0.032270121*	*0.030350843*	*0.021835987*
**ENSG00000187688**	*0.032187112*	*0.040774834*	*0.041593952*	*0.037894184*
**ENSG00000133997**	*0.036021602*	*0.041494793*	*0.039777101*	*0.045420613*
**ENSG00000124074**	*0.041726807*	*0.042467603*	*0.035048324*	*0.033545334*
**ENSG00000213160**	*0.036021294*	*0.036665777*	*0.032811196*	*0.030500588*
**ENSG00000186803**	*0.015763084*	*0.025278158*	*0.024862517*	*0.014975237*
**ENSG00000132481**	*0.046378951*	*0.042183194*	*0.044202055*	*0.05414898*
**ENSG00000166352**	*0.089486173*	*0.086113754*	*0.061504719*	*0.08588635*
**ENSG00000239779**	*0.023649338*	*0.029710419*	*0.031068776*	*0.02349157*
**ENSG00000130413**	*0.041069685*	*0.037519105*	*0.029532181*	*0.028572092*
**ENSG00000061273**	*0.017475283*	*0.025607411*	*0.025010309*	*0.015447702*
**ENSG00000015171**	*0.036950862*	*0.043067055*	*0.03400198*	*0.036133455*
**ENSG00000134247**	*0.055319469*	*0.045670201*	*0.02532334*	*0.017860943*
**ENSG00000115042**	*0.032877116*	*0.033302757*	*0.035182826*	*0.029065774*
**ENSG00000213246**	*0.030779972*	*0.035170389*	*0.039326441*	*0.031992141*
**ENSG00000166743**	*0.021541332*	*0.026922815*	*0.027998544*	*0.018613152*
**ENSG00000144460**	*0.017006738*	*0.025122123*	*0.024820148*	*0.016071833*
**ENSG00000106125**	*0.036793102*	*0.036196199*	*0.030949096*	*0.028169333*
**ENSG00000160446**	*0.027400803*	*0.030283574*	*0.030609314*	*0.026262463*
**ENSG00000160255**	*0.046409816*	*0.038523398*	*0.040096042*	*0.036486566*
**ENSG00000166484**	*0.021219054*	*0.030531265*	*0.032064663*	*0.023425936*
**ENSG00000196866**	*0.045719238*	*0.038996095*	*0.038632297*	*0.034621249*
**ENSG00000077327**	*0.039056899*	*0.034347695*	*0.029878034*	*0.027585059*
**ENSG00000168811**	*0.06215239*	*0.052748883*	*0.051650508*	*0.053799373*
**ENSG00000239605**	*0.016029801*	*0.026694408*	*0.02588845*	*0.015501663*
**ENSG00000133392**	*0.032519811*	*0.028659698*	*0.037987962*	*0.032071383*
**ENSG00000103274**	*0.024828629*	*0.03130151*	*0.031773241*	*0.025331644*
**ENSG00000005339**	*0.029616641*	*0.037075475*	*0.035148124*	*0.030007253*
**ENSG00000120656**	*0.028938933*	*0.03456839*	*0.031421839*	*0.023646317*
**ENSG00000130021**	*0.054584562*	*0.051695904*	*0.057221712*	*0.04731959*
**ENSG00000196661**	*0.015555824*	*0.024606447*	*0.025740694*	*0.015473324*
**ENSG00000118454**	*0.034861688*	*0.039981795*	*0.032653527*	*0.03644078*
**ENSG00000100650**	*0.022442804*	*0.034037482*	*0.031738395*	*0.024494762*
**ENSG00000178896**	*0.025259304*	*0.028787864*	*0.031472762*	*0.025617015*
**ENSG00000105447**	*0.038144314*	*0.036798169*	*0.04562937*	*0.040161617*
**ENSG00000213402**	*0.03825903*	*0.037529644*	*0.03765021*	*0.036174218*
**ENSG00000166863**	*0.016140297*	*0.024971478*	*0.025881366*	*0.016140494*
**ENSG00000221909**	*0.03787729*	*0.03663676*	*0.038407419*	*0.035990327*
**ENSG00000138378**	*0.046018964*	*0.038231908*	*0.037444904*	*0.034113897*
**ENSG00000215012**	*0.037801579*	*0.031689109*	*0.03262577*	*0.02701714*
**ENSG00000132821**	*0.02975786*	*0.027718975*	*0.025653406*	*0.020829056*
**ENSG00000166135**	*0.022888575*	*0.031743587*	*0.027996482*	*0.023048729*
**ENSG00000170571**	*0.056015401*	*0.063965702*	*0.060269538*	*0.055552248*
**ENSG00000072803**	*0.023769493*	*0.030366354*	*0.030794853*	*0.023092435*
**ENSG00000203993**	*0.031110687*	*0.034640023*	*0.03404458*	*0.02414376*
**ENSG00000187957**	*0.017608102*	*0.025199845*	*0.025912271*	*0.015535317*
**ENSG00000100401**	*0.030368792*	*0.035541078*	*0.032162257*	*0.031616756*
**ENSG00000011201**	*0.049327284*	*0.024981251*	*0.025407479*	*0.021664622*
**ENSG00000175206**	*0.015605384*	*0.024692868*	*0.02623245*	*0.015145499*
**ENSG00000145293**	*0.033443059*	*0.035210792*	*0.031199605*	*0.023374089*
**ENSG00000177548**	*0.032908372*	*0.03534609*	*0.031650202*	*0.029500084*
**ENSG00000150361**	*0.016209772*	*0.024661897*	*0.025150801*	*0.014744924*
**ENSG00000164197**	*0.053459983*	*0.035381012*	*0.026153047*	*0.022280966*
**ENSG00000137648**	*0.016980057*	*0.025407906*	*0.026948706*	*0.016187836*

**ENSG00000170270**	*0.040426519*	*0.039953247*	*0.041116438*	*0.036593632*
**ENSG00000184634**	*0.027877438*	*0.039821366*	*0.032382752*	*0.027353944*
**ENSG00000184261**	*0.078062054*	*0.05907718*	*0.054625398*	*0.067595485*
**ENSG00000135747**	*0.018561446*	*0.027260859*	*0.027709552*	*0.017082254*
**ENSG00000196335**	*0.015673342*	*0.025418171*	*0.024333475*	*0.014769259*
**ENSG00000167491**	*0.025524244*	*0.031193455*	*0.036057487*	*0.027345523*
**ENSG00000175193**	*0.021834362*	*0.027677*	*0.030717355*	*0.021841743*
**ENSG00000133812**	*0.016832484*	*0.02539875*	*0.025142954*	*0.017283453*
**ENSG00000124208**	*0.017932673*	*0.02887969*	*0.026082079*	*0.017672293*
**ENSG00000242413**	*0.01657074*	*0.026059377*	*0.02508953*	*0.016136573*
**ENSG00000143631**	*0.015758165*	*0.026148397*	*0.024717634*	*0.015836031*
**ENSG00000081248**	*0.014913635*	*0.025748619*	*0.025084276*	*0.015661795*
**ENSG00000147117**	*0.016374103*	*0.025736824*	*0.025129581*	*0.014503616*
**ENSG00000232427**	*0.029120187*	*0.034024891*	*0.038154667*	*0.024708915*
**ENSG00000172465**	*0.028106488*	*0.034861351*	*0.033748711*	*0.024981764*
**ENSG00000179921**	*0.016648985*	*0.025508502*	*0.024941219*	*0.015352618*
**ENSG00000205213**	*0.086367647*	*0.057437782*	*0.052330939*	*0.060218584*
**ENSG00000115648**	*0.026306467*	*0.025884114*	*0.025305038*	*0.023019875*
**ENSG00000137713**	*0.014235222*	*0.024938212*	*0.024392427*	*0.014947627*
**ENSG00000204540**	*0.09112392*	*0.069680626*	*0.072939169*	*0.111037799*
**ENSG00000155366**	*0.027888154*	*0.033296822*	*0.030849862*	*0.031049136*
**ENSG00000168522**	*0.018510357*	*0.028563213*	*0.028108278*	*0.020239003*
**ENSG00000178226**	*0.016213892*	*0.025081558*	*0.026420799*	*0.016938403*
**ENSG00000185947**	*0.036985769*	*0.039288673*	*0.037512376*	*0.04568451*
**ENSG00000144331**	*0.016874864*	*0.026258801*	*0.024934809*	*0.016115367*
**ENSG00000112701**	*0.027745462*	*0.036685922*	*0.030627618*	*0.031130071*
**ENSG00000065268**	*0.04459628*	*0.04150228*	*0.037670263*	*0.033910831*
**ENSG00000164164**	*0.035015982*	*0.044938692*	*0.043073409*	*0.033132142*
**ENSG00000152760**	*0.014802827*	*0.025029142*	*0.025343608*	*0.016305745*
**ENSG00000166548**	*0.067080299*	*0.074493598*	*0.080217769*	*0.081196116*
**ENSG00000142937**	*0.01542602*	*0.024323264*	*0.025110411*	*0.016033522*
**ENSG00000179091**	*0.021976*	*0.026024016*	*0.025927298*	*0.020477947*
**ENSG00000105501**	*0.026763309*	*0.033562493*	*0.030209556*	*0.023167389*
**ENSG00000160298**	*0.016053757*	*0.02499437*	*0.025495982*	*0.014868693*
**ENSG00000167755**	*0.016206146*	*0.025192594*	*0.024670591*	*0.016909965*
**ENSG00000187773**	*0.018557904*	*0.028130841*	*0.026751579*	*0.02028737*
**ENSG00000122180**	*0.015608618*	*0.024803052*	*0.025001327*	*0.014363665*
**ENSG00000054277**	*0.046885866*	*0.061335673*	*0.044144631*	*0.043888987*
**ENSG00000147485**	*0.054693584*	*0.070406522*	*0.026223521*	*0.016384754*
**ENSG00000203883**	*0.181874847*	*0.124408477*	*0.104862399*	*0.138580591*
**ENSG00000142677**	*0.015255097*	*0.024883218*	*0.025294976*	*0.015411789*
**ENSG00000184545**	*0.029728767*	*0.042068279*	*0.039544326*	*0.030059238*
**ENSG00000049860**	*0.026858379*	*0.028489404*	*0.02815704*	*0.020036226*
**ENSG00000008853**	*0.031515471*	*0.039203371*	*0.041635487*	*0.045645742*
**ENSG00000100968**	*0.019422285*	*0.02640471*	*0.025185873*	*0.017975636*
**ENSG00000171469**	*0.021551503*	*0.027449851*	*0.027993096*	*0.016565524*
**ENSG00000174279**	*0.016035959*	*0.024913682*	*0.024550603*	*0.01496062*
**ENSG00000198945**	*0.039938835*	*0.037321658*	*0.038794217*	*0.030202919*
**ENSG00000176978**	*0.030789704*	*0.031317542*	*0.032372598*	*0.028950051*
**ENSG00000159374**	*0.026274171*	*0.02941871*	*0.036318639*	*0.020929106*
**ENSG00000132003**	*0.037645231*	*0.042060483*	*0.033407969*	*0.033473113*
**ENSG00000167850**	*0.041944561*	*0.041493464*	*0.037022603*	*0.022343424*
**ENSG00000151468**	*0.016666611*	*0.02544532*	*0.02621751*	*0.023407222*
**ENSG00000113971**	*0.038012298*	*0.04428964*	*0.038749166*	*0.039881845*

**ENSG00000177200**	*0.028284581*	*0.033731296*	*0.02951725*	*0.023798353*
**ENSG00000187231**	*0.04820782*	*0.049263515*	*0.049247285*	*0.049807812*
**ENSG00000174529**	*0.014465153*	*0.024280222*	*0.024777756*	*0.014508823*
**ENSG00000113739**	*0.025269475*	*0.02733863*	*0.027432479*	*0.020368594*
**ENSG00000167635**	*0.024975478*	*0.030857907*	*0.029481581*	*0.026754395*
**ENSG00000147535**	*0.036101381*	*0.034226229*	*0.037500944*	*0.036938269*
**ENSG00000131196**	*0.061126358*	*0.049655252*	*0.047011353*	*0.041536245*
**ENSG00000137171**	*0.018641893*	*0.026531521*	*0.025391429*	*0.017462125*
**ENSG00000101247**	*0.018434697*	*0.02555168*	*0.029180036*	*0.016410184*
**ENSG00000198930**	*0.016924698*	*0.025390681*	*0.025605631*	*0.0169178*
**ENSG00000185920**	*0.030127228*	*0.034849269*	*0.030842313*	*0.024192972*
**ENSG00000136104**	*0.033324377*	*0.034437678*	*0.032537192*	*0.027799617*
**ENSG00000164082**	*0.016155087*	*0.024313423*	*0.024726717*	*0.015875898*
**ENSG00000125144**	*0.03025373*	*0.035917175*	*0.065606671*	*0.038540949*
**ENSG00000161944**	*0.015432015*	*0.02567285*	*0.025897537*	*0.015929328*
**ENSG00000011422**	*0.077675608*	*0.066327689*	*0.064776375*	*0.053952502*
**ENSG00000068903**	*0.024722686*	*0.027756931*	*0.029822069*	*0.023973864*
**ENSG00000198917**	*0.023915072*	*0.031103116*	*0.029160821*	*0.025508566*
**ENSG00000168002**	*0.024947634*	*0.027685681*	*0.027059268*	*0.020483244*
**ENSG00000122515**	*0.046503607*	*0.042177373*	*0.033538395*	*0.037142788*
**ENSG00000204103**	*0.022295728*	*0.032613951*	*0.03292509*	*0.029299587*
**ENSG00000187823**	*0.015844756*	*0.024740041*	*0.024602966*	*0.015081112*
**ENSG00000165672**	*0.026592903*	*0.032504354*	*0.029564471*	*0.019891817*
**ENSG00000115325**	*0.039332861*	*0.031683115*	*0.033519574*	*0.032829382*
**ENSG00000196208**	*0.018632655*	*0.028369386*	*0.027030515*	*0.019113832*
**ENSG00000185231**	*0.016384826*	*0.024817506*	*0.025333585*	*0.015519316*
**ENSG00000108576**	*0.092702057*	*0.072221262*	*0.04273587*	*0.076443857*
**ENSG00000093217**	*0.035074499*	*0.037584649*	*0.028333129*	*0.023736543*
**ENSG00000182150**	*0.017012006*	*0.025763613*	*0.025195301*	*0.017887727*
**ENSG00000102886**	*0.029586225*	*0.035942866*	*0.034288409*	*0.035474801*
**ENSG00000143322**	*0.015270878*	*0.025338936*	*0.025882578*	*0.014819493*
**ENSG00000240184**	*0.017144905*	*0.026370486*	*0.024761601*	*0.015094244*
**ENSG00000101892**	*0.01568629*	*0.024803004*	*0.0249784*	*0.015630579*
**ENSG00000134899**	*0.026646614*	*0.032029571*	*0.030636993*	*0.020455953*
**ENSG00000164220**	*0.015269751*	*0.02553816*	*0.025200564*	*0.014858945*
**ENSG00000123609**	*0.030631008*	*0.032108584*	*0.030551457*	*0.029663591*
**ENSG00000143278**	*0.017095385*	*0.027584683*	*0.025308024*	*0.016240499*
**ENSG00000197128**	*0.032663613*	*0.033796521*	*0.028910843*	*0.031788373*
**ENSG00000115685**	*0.030568989*	*0.033878198*	*0.033518417*	*0.025015135*
**ENSG00000141527**	*0.016836244*	*0.024952448*	*0.02513728*	*0.015173248*
**ENSG00000136698**	*0.016592309*	*0.024748484*	*0.024542079*	*0.016417879*
**ENSG00000140285**	*0.015248015*	*0.025668846*	*0.025597967*	*0.01530509*
**ENSG00000124429**	*0.094941618*	*0.07064005*	*0.069197736*	*0.055386954*
**ENSG00000128052**	*0.014977503*	*0.025670972*	*0.024285714*	*0.014729313*
**ENSG00000176495**	*0.016540435*	*0.026582936*	*0.026101804*	*0.015011248*
**ENSG00000162771**	*0.014747215*	*0.025218819*	*0.02581218*	*0.01589909*
**ENSG00000135472**	*0.017840098*	*0.02563299*	*0.025179845*	*0.01789227*
**ENSG00000171987**	*0.017354171*	*0.027880462*	*0.026881841*	*0.015957136*
**ENSG00000164237**	*0.061568866*	*0.066085395*	*0.050916418*	*0.059090956*
**ENSG00000141293**	*0.037118705*	*0.039307114*	*0.036819597*	*0.03307259*
**ENSG00000133119**	*0.03638518*	*0.03711902*	*0.03542287*	*0.035347804*
**ENSG00000102882**	*0.032041203*	*0.034873439*	*0.03486486*	*0.026884199*
**ENSG00000131080**	*0.021671221*	*0.028843481*	*0.029699379*	*0.017664745*
**ENSG00000163528**	*0.039056705*	*0.037560417*	*0.037784857*	*0.026155073*

**ENSG00000090989**	*0.030035531*	*0.033698884*	*0.032697183*	*0.025699669*
**ENSG00000143811**	*0.027288159*	*0.031958264*	*0.029878871*	*0.023542587*
**ENSG00000004776**	*0.017118905*	*0.025968816*	*0.026086556*	*0.017258488*
**ENSG00000126070**	*0.032348217*	*0.036248804*	*0.033925041*	*0.023785946*
**ENSG00000132356**	*0.029272904*	*0.033126639*	*0.039032064*	*0.028417638*
**ENSG00000166509**	*0.015939936*	*0.025765873*	*0.025627072*	*0.014900941*
**ENSG00000126457**	*0.023560136*	*0.025919675*	*0.02643307*	*0.022616614*
**ENSG00000187474**	*0.01826318*	*0.027687907*	*0.026804046*	*0.017329626*
**ENSG00000021776**	*0.024001109*	*0.031385187*	*0.031718744*	*0.02179346*
**ENSG00000084636**	*0.070717359*	*0.074050675*	*0.057290883*	*0.061779136*
**ENSG00000183423**	*0.015704624*	*0.025456317*	*0.024806776*	*0.01569187*
**ENSG00000136943**	*0.028016987*	*0.03274382*	*0.037773617*	*0.029405906*
**ENSG00000184887**	*0.043529261*	*0.043974461*	*0.039705058*	*0.033459711*
**ENSG00000113924**	*0.015350068*	*0.025610957*	*0.024014483*	*0.015944732*
**ENSG00000117859**	*0.015742868*	*0.024882924*	*0.025518208*	*0.014684132*
**ENSG00000111801**	*0.039330635*	*0.037755575*	*0.032397694*	*0.03749339*
**ENSG00000113758**	*0.015825371*	*0.025385558*	*0.02528095*	*0.015902215*
**ENSG00000006712**	*0.024439765*	*0.027881499*	*0.030452863*	*0.027307602*
**ENSG00000177807**	*0.017520971*	*0.026117007*	*0.026704579*	*0.014923718*
**ENSG00000110693**	*0.016793701*	*0.024799206*	*0.025351014*	*0.017103659*
**ENSG00000163793**	*0.017429588*	*0.025686903*	*0.026052598*	*0.016440626*
**ENSG00000102996**	*0.062838817*	*0.069143604*	*0.057284406*	*0.085750526*
**ENSG00000143369**	*0.021496567*	*0.026620888*	*0.027076323*	*0.020337577*
**ENSG00000128274**	*0.097389657*	*0.074378094*	*0.06486461*	*0.0725772*
**ENSG00000104967**	*0.014992052*	*0.024652231*	*0.024290546*	*0.015408119*
**ENSG00000125775**	*0.022932648*	*0.029096122*	*0.0306958*	*0.020050072*
**ENSG00000182974**	*0.016272463*	*0.025714818*	*0.024309428*	*0.014731789*
**ENSG00000032742**	*0.03513459*	*0.033790553*	*0.033070635*	*0.03500403*
**ENSG00000184371**	*0.021423812*	*0.02784904*	*0.026873075*	*0.020588422*
**ENSG00000184164**	*0.047886266*	*0.043023345*	*0.049879983*	*0.055944821*
**ENSG00000105982**	*0.018735568*	*0.028085525*	*0.026667058*	*0.023233293*
**ENSG00000100678**	*0.016498164*	*0.025437327*	*0.02457237*	*0.016209572*
**ENSG00000131323**	*0.031961599*	*0.034242844*	*0.033178604*	*0.032156823*
**ENSG00000145491**	*0.015442587*	*0.025502024*	*0.02642411*	*0.016337495*
**ENSG00000173083**	*0.029884642*	*0.031788104*	*0.034451199*	*0.028870376*
**ENSG00000124535**	*0.028326*	*0.036924676*	*0.030495887*	*0.022728708*
**ENSG00000169245**	*0.152224868*	*0.172931103*	*0.107133358*	*0.144717473*
**ENSG00000057657**	*0.044186459*	*0.03726856*	*0.037712376*	*0.037884045*
**ENSG00000186509**	*0.016492446*	*0.025389586*	*0.024561596*	*0.015616868*
**ENSG00000181619**	*0.0165115*	*0.02443386*	*0.025845616*	*0.015358996*
**ENSG00000174021**	*0.02436534*	*0.029968973*	*0.029240325*	*0.024708945*
**ENSG00000077254**	*0.016235907*	*0.025615849*	*0.025643397*	*0.016128616*
**ENSG00000006652**	*0.034426114*	*0.038772835*	*0.03650593*	*0.026537583*
**ENSG00000172155**	*0.037234698*	*0.027711236*	*0.026112772*	*0.021319637*
**ENSG00000163431**	*0.016565103*	*0.02557905*	*0.024900867*	*0.014823037*
**ENSG00000189060**	*0.082492264*	*0.064413849*	*0.04842888*	*0.059478494*
**ENSG00000198963**	*0.015643321*	*0.0256016*	*0.024739647*	*0.014824808*
**ENSG00000156959**	*0.015903062*	*0.025389086*	*0.025712476*	*0.015424566*
**ENSG00000112186**	*0.016248352*	*0.027064806*	*0.024851482*	*0.01453492*
**ENSG00000120709**	*0.029959787*	*0.039435767*	*0.043511324*	*0.027677995*
**ENSG00000184895**	*0.016079471*	*0.026616348*	*0.024815071*	*0.014560506*
**ENSG00000189283**	*0.043934468*	*0.041120997*	*0.042920573*	*0.042503887*
**ENSG00000166441**	*0.015899977*	*0.024412297*	*0.023928904*	*0.020492045*
**ENSG00000076662**	*0.02433363*	*0.033760662*	*0.032110286*	*0.028623817*

**ENSG00000115084**	*0.033895819*	*0.03786914*	*0.039104285*	*0.031946075*
**ENSG00000140749**	*0.016730897*	*0.024752815*	*0.025102604*	*0.016216129*
**ENSG00000250565**	*0.037511596*	*0.036676108*	*0.032214768*	*0.03001075*
**ENSG00000176358**	*0.017279257*	*0.025386292*	*0.025812018*	*0.016829768*
**ENSG00000101019**	*0.025994648*	*0.031852921*	*0.031650636*	*0.024816692*
**ENSG00000157368**	*0.016222673*	*0.025707051*	*0.024544254*	*0.015011915*
**ENSG00000169800**	*0.017791484*	*0.026439952*	*0.026172999*	*0.017188031*
**ENSG00000149435**	*0.016807515*	*0.025561002*	*0.024841831*	*0.017169588*
**ENSG00000183454**	*0.034418424*	*0.027910867*	*0.025248028*	*0.01601415*
**ENSG00000070031**	*0.026307759*	*0.043185225*	*0.03764324*	*0.051294511*
**ENSG00000172638**	*0.039272669*	*0.041895943*	*0.036345269*	*0.048612304*
**ENSG00000026036**	*0.018978505*	*0.027512129*	*0.025393667*	*0.017119034*
**ENSG00000096872**	*0.034167971*	*0.038211442*	*0.03333206*	*0.03366383*
**ENSG00000221864**	*0.015444654*	*0.024921992*	*0.024654246*	*0.015003221*
**ENSG00000198042**	*0.031218469*	*0.038035622*	*0.039401226*	*0.034179409*
**ENSG00000110711**	*0.02412175*	*0.027762278*	*0.031548019*	*0.024010999*
**ENSG00000122707**	*0.044395677*	*0.053571864*	*0.040010563*	*0.050192939*
**ENSG00000165168**	*0.058527988*	*0.050211814*	*0.044563974*	*0.04770577*
**ENSG00000173960**	*0.025131539*	*0.031342743*	*0.03604132*	*0.023414798*
**ENSG00000165156**	*0.026291723*	*0.031718681*	*0.03723927*	*0.025784617*
**ENSG00000118307**	*0.029707645*	*0.029665143*	*0.033377684*	*0.02038269*
**ENSG00000105668**	*0.051562682*	*0.055519638*	*0.047686754*	*0.062844276*
**ENSG00000127838**	*0.015736575*	*0.025265631*	*0.024419632*	*0.014797889*
**ENSG00000224916**	*0.014505197*	*0.024537744*	*0.02472937*	*0.014904781*
**ENSG00000198964**	*0.056815163*	*0.047172441*	*0.039188174*	*0.042812234*
**ENSG00000085733**	*0.058569059*	*0.055849705*	*0.049225963*	*0.049733211*
**ENSG00000051180**	*0.023673385*	*0.0277675*	*0.028950312*	*0.021136269*
**ENSG00000113318**	*0.029092907*	*0.030532384*	*0.032338435*	*0.025432301*
**ENSG00000010017**	*0.03055565*	*0.041970421*	*0.035727377*	*0.031717456*
**ENSG00000106443**	*0.029181375*	*0.033941566*	*0.035401933*	*0.026496514*
**ENSG00000107859**	*0.016916299*	*0.025382079*	*0.025485557*	*0.014868578*
**ENSG00000143106**	*0.021836246*	*0.028650214*	*0.026443989*	*0.021204941*
**ENSG00000160051**	*0.040028145*	*0.039750815*	*0.038804523*	*0.032051941*
**ENSG00000169989**	*0.016668807*	*0.0246871*	*0.02524302*	*0.015174178*
**ENSG00000177683**	*0.04850705*	*0.032635673*	*0.036205725*	*0.046637088*
**ENSG00000186111**	*0.055824947*	*0.039963767*	*0.041694707*	*0.040922275*
**ENSG00000213614**	*0.017693222*	*0.025215376*	*0.026608239*	*0.016912157*
**ENSG00000164465**	*0.037510283*	*0.031502495*	*0.033878603*	*0.028457264*
**ENSG00000118680**	*0.017887153*	*0.027299804*	*0.026508356*	*0.018824328*
**ENSG00000103222**	*0.027531298*	*0.035057611*	*0.02881651*	*0.024492807*
**ENSG00000129455**	*0.017484314*	*0.025767201*	*0.024788652*	*0.0171534*
**ENSG00000135423**	*0.024439001*	*0.032661107*	*0.031082151*	*0.022451122*
**ENSG00000132698**	*0.016600416*	*0.025383171*	*0.02569076*	*0.015878407*
**ENSG00000171446**	*0.01670211*	*0.023942524*	*0.024647231*	*0.015427103*
**ENSG00000006659**	*0.023014641*	*0.027509911*	*0.028954754*	*0.020530085*
**ENSG00000122122**	*0.032816403*	*0.037073793*	*0.038281161*	*0.032200924*
**ENSG00000005302**	*0.027302063*	*0.031752733*	*0.02959624*	*0.027135841*
**ENSG00000164306**	*0.042423534*	*0.047143633*	*0.034577284*	*0.030205786*
**ENSG00000163017**	*0.017117959*	*0.026541003*	*0.025053961*	*0.015931914*
**ENSG00000144820**	*0.015797973*	*0.02538636*	*0.025684509*	*0.015506954*
**ENSG00000104064**	*0.033522401*	*0.0381049*	*0.039235204*	*0.036209643*
**ENSG00000150967**	*0.017482627*	*0.024570325*	*0.024709305*	*0.015055966*
**ENSG00000168061**	*0.042731819*	*0.038394246*	*0.039447111*	*0.041102785*
**ENSG00000184344**	*0.016225217*	*0.024977039*	*0.025345865*	*0.016299668*

**ENSG00000090581**	*0.021884071*	*0.029979525*	*0.029379989*	*0.026824499*
**ENSG00000119326**	*0.049623073*	*0.047311399*	*0.038265101*	*0.035604913*
**ENSG00000163923**	*0.05144443*	*0.039476159*	*0.035732347*	*0.031955255*
**ENSG00000134716**	*0.072665964*	*0.056951257*	*0.05314609*	*0.063813231*
**ENSG00000166233**	*0.029134333*	*0.0349006*	*0.033967217*	*0.025827564*
**ENSG00000145113**	*0.024902649*	*0.026490266*	*0.026226357*	*0.017886829*
**ENSG00000125734**	*0.025689082*	*0.028533166*	*0.032950221*	*0.030310517*
**ENSG00000117707**	*0.018943957*	*0.025146237*	*0.025218993*	*0.015949855*
**ENSG00000117385**	*0.032316972*	*0.0363143*	*0.036535599*	*0.027688208*
**ENSG00000136634**	*0.071979487*	*0.064682343*	*0.057519803*	*0.062709617*
**ENSG00000164754**	*0.025236415*	*0.031580122*	*0.027893554*	*0.022801666*
**ENSG00000186522**	*0.078697687*	*0.052990474*	*0.04824913*	*0.063817445*
**ENSG00000117036**	*0.028430909*	*0.031478475*	*0.029100192*	*0.020373733*
**ENSG00000185722**	*0.024079642*	*0.029943126*	*0.029869562*	*0.021270738*
**ENSG00000185989**	*0.058222533*	*0.047543565*	*0.047153577*	*0.045743219*
**ENSG00000126261**	*0.044233835*	*0.051817536*	*0.04700009*	*0.043389246*
**ENSG00000116133**	*0.031048439*	*0.033634273*	*0.038123655*	*0.036841551*
**ENSG00000173612**	*0.0176229*	*0.024872913*	*0.024464231*	*0.015262098*
**ENSG00000177511**	*0.013886285*	*0.025124088*	*0.025783157*	*0.014799761*
**ENSG00000173208**	*0.029378744*	*0.03147552*	*0.027460898*	*0.028518243*
**ENSG00000140600**	*0.016937814*	*0.02430904*	*0.025423252*	*0.016064697*
**ENSG00000138472**	*0.015218198*	*0.025006957*	*0.024892688*	*0.015189493*
**ENSG00000177875**	*0.01905105*	*0.027160904*	*0.026397351*	*0.020692547*
**ENSG00000112039**	*0.04244499*	*0.040107629*	*0.033066208*	*0.032278639*
**ENSG00000158815**	*0.014991215*	*0.024554284*	*0.025596363*	*0.014108742*
**ENSG00000183862**	*0.016549433*	*0.025459229*	*0.026754776*	*0.016219853*
**ENSG00000168769**	*0.030064375*	*0.033556678*	*0.034595257*	*0.027075876*
**ENSG00000150281**	*0.015309758*	*0.024267391*	*0.025821625*	*0.016056476*
**ENSG00000110324**	*0.051148399*	*0.061318659*	*0.062802319*	*0.038005343*
**ENSG00000160124**	*0.044062655*	*0.051338335*	*0.048536466*	*0.037078778*
**ENSG00000123999**	*0.016312802*	*0.026503328*	*0.025283197*	*0.015020938*
**ENSG00000187045**	*0.032785068*	*0.042582356*	*0.039790021*	*0.033922432*
**ENSG00000171051**	*0.017798272*	*0.026846171*	*0.029315627*	*0.017601402*
**ENSG00000118418**	*0.025419146*	*0.030163825*	*0.028399077*	*0.021564696*
**ENSG00000151005**	*0.015807931*	*0.026147835*	*0.025115742*	*0.017096435*
**ENSG00000176473**	*0.021854715*	*0.028243617*	*0.026279996*	*0.019570921*
**ENSG00000137309**	*0.017294066*	*0.025198489*	*0.025747029*	*0.014132935*
**ENSG00000205409**	*0.013249167*	*0.023405671*	*0.023504969*	*0.013211756*
**ENSG00000221931**	*0.013346544*	*0.02365346*	*0.024396195*	*0.014086034*
**ENSG00000162747**	*0.017131732*	*0.025581964*	*0.024868514*	*0.014844089*
**ENSG00000118162**	*0.031299653*	*0.035749995*	*0.032789438*	*0.029774513*
**ENSG00000103647**	*0.015482059*	*0.024750793*	*0.025334355*	*0.016613035*
**ENSG00000067704**	*0.025479957*	*0.033006186*	*0.028567977*	*0.023543833*
**ENSG00000225968**	*0.017367002*	*0.024962583*	*0.026014286*	*0.016012024*
**ENSG00000111912**	*0.033089218*	*0.039913742*	*0.038594032*	*0.036432874*
**ENSG00000123268**	*0.034924638*	*0.03663957*	*0.034472665*	*0.02986508*
**ENSG00000073584**	*0.023129734*	*0.030913796*	*0.033841927*	*0.019490297*
**ENSG00000115267**	*0.038249325*	*0.032898872*	*0.033146767*	*0.031696262*
**ENSG00000104529**	*0.01685128*	*0.024835564*	*0.024826151*	*0.016444886*
**ENSG00000047230**	*0.020838666*	*0.025501115*	*0.02773372*	*0.019931271*
**ENSG00000212126**	*0.018262082*	*0.028959086*	*0.0270872*	*0.017593564*
**ENSG00000172893**	*0.03097189*	*0.033323869*	*0.038706098*	*0.031549966*
**ENSG00000163946**	*0.01608958*	*0.025958098*	*0.025947784*	*0.01999814*
**ENSG00000100321**	*0.101474775*	*0.083529914*	*0.062175812*	*0.068764307*

**ENSG00000168546**	*0.019314908*	*0.026617804*	*0.028351926*	*0.01571722*
**ENSG00000164920**	*0.090770432*	*0.081856946*	*0.066241329*	*0.081382032*
**ENSG00000112245**	*0.027022919*	*0.034923115*	*0.038082676*	*0.028984245*
**ENSG00000147202**	*0.036684428*	*0.040176049*	*0.037131532*	*0.029289653*
**ENSG00000198522**	*0.0244088*	*0.030139158*	*0.030904198*	*0.020566189*
**ENSG00000077274**	*0.018076503*	*0.02535749*	*0.028204898*	*0.018753743*
**ENSG00000166923**	*0.017729824*	*0.02514774*	*0.025686918*	*0.017097741*
**ENSG00000165626**	*0.016069074*	*0.02594954*	*0.024576613*	*0.015104784*
**ENSG00000079841**	*0.016336143*	*0.027109355*	*0.026565914*	*0.016985046*
**ENSG00000161970**	*0.016235297*	*0.024585478*	*0.024212904*	*0.017915422*
**ENSG00000162129**	*0.016436424*	*0.025792465*	*0.025706109*	*0.017094642*
**ENSG00000109436**	*0.102317843*	*0.089555074*	*0.077825601*	*0.096702696*
**ENSG00000122986**	*0.035349986*	*0.033318666*	*0.03454812*	*0.038644162*
**ENSG00000187994**	*0.029624471*	*0.031185519*	*0.029735041*	*0.027480673*
**ENSG00000198003**	*0.119625551*	*0.08055236*	*0.053935759*	*0.088113347*
**ENSG00000100353**	*0.022038867*	*0.026602166*	*0.032237298*	*0.022923157*
**ENSG00000185002**	*0.014901238*	*0.024971084*	*0.024689202*	*0.015536225*
**ENSG00000254108**	*0.016365238*	*0.025443065*	*0.025075338*	*0.016295443*
**ENSG00000182613**	*0.054419552*	*0.027691079*	*0.025602847*	*0.04638813*
**ENSG00000137259**	*0.045621204*	*0.035041754*	*0.044541605*	*0.048622935*
**ENSG00000106366**	*0.026764452*	*0.028851977*	*0.029392154*	*0.023749814*
**ENSG00000072571**	*0.053330817*	*0.060169399*	*0.043346056*	*0.04121073*
**ENSG00000198301**	*0.032551141*	*0.034560634*	*0.047438604*	*0.039941966*
**ENSG00000254087**	*0.021411386*	*0.02832483*	*0.030119876*	*0.019584879*
**ENSG00000120324**	*0.016134413*	*0.025329107*	*0.025370522*	*0.015327036*
**ENSG00000183137**	*0.049027503*	*0.042642778*	*0.034723153*	*0.033185923*
**ENSG00000090339**	*0.039257185*	*0.038962188*	*0.04176699*	*0.039774509*
**ENSG00000139620**	*0.024421393*	*0.029628416*	*0.031868964*	*0.021114664*
**ENSG00000102385**	*0.017998435*	*0.025500279*	*0.025155324*	*0.014907867*
**ENSG00000105185**	*0.025036774*	*0.031143137*	*0.026663702*	*0.023579647*
**ENSG00000160703**	*0.028802634*	*0.033196956*	*0.035598152*	*0.024474168*
**ENSG00000130830**	*0.04965668*	*0.044475447*	*0.038860648*	*0.042513623*
**ENSG00000145839**	*0.016950126*	*0.026379978*	*0.024707138*	*0.016389917*
**ENSG00000214013**	*0.023064647*	*0.032333741*	*0.027920587*	*0.020068406*
**ENSG00000171858**	*0.013763228*	*0.023945225*	*0.023975774*	*0.01527621*
**ENSG00000134802**	*0.032652295*	*0.029525538*	*0.033330874*	*0.029563251*
**ENSG00000197263**	*0.014993543*	*0.025556758*	*0.024849759*	*0.014296389*
**ENSG00000130669**	*0.022590793*	*0.029817271*	*0.036790723*	*0.021280109*
**ENSG00000184979**	*0.051829421*	*0.054355318*	*0.054435761*	*0.060298395*
**ENSG00000185619**	*0.016137321*	*0.025537071*	*0.024947335*	*0.015121538*
**ENSG00000183668**	*0.017657091*	*0.025861903*	*0.025828398*	*0.017543282*
**ENSG00000157322**	*0.021607461*	*0.035264659*	*0.029781704*	*0.017169339*
**ENSG00000189180**	*0.028799332*	*0.03137296*	*0.030003241*	*0.032567048*
**ENSG00000165202**	*0.016963656*	*0.025952843*	*0.025629637*	*0.015835466*
**ENSG00000133884**	*0.024340079*	*0.033785666*	*0.031814927*	*0.025788147*
**ENSG00000184209**	*0.022651257*	*0.029938811*	*0.033487138*	*0.023654723*
**ENSG00000168256**	*0.021919411*	*0.029618304*	*0.031696302*	*0.024100049*
**ENSG00000167261**	*0.032737734*	*0.034985301*	*0.039351001*	*0.039277927*
**ENSG00000125686**	*0.025747272*	*0.032200473*	*0.032659075*	*0.023835868*
**ENSG00000138449**	*0.02755796*	*0.031058926*	*0.036200558*	*0.02182156*
**ENSG00000196433**	*0.042626092*	*0.047342582*	*0.05038125*	*0.034119562*
**ENSG00000135378**	*0.042450885*	*0.048096682*	*0.048898193*	*0.042420823*
**ENSG00000198518**	*0.036139078*	*0.030681957*	*0.03717504*	*0.038184691*
**ENSG00000131095**	*0.017571163*	*0.025262092*	*0.025251514*	*0.016669074*

**ENSG00000069329**	*0.023157415*	*0.03225689*	*0.027442953*	*0.01866328*
**ENSG00000130592**	*0.034979634*	*0.036120369*	*0.039862504*	*0.034408375*
**ENSG00000171798**	*0.015704522*	*0.027848643*	*0.025373643*	*0.015520399*
**ENSG00000122176**	*0.030761377*	*0.024754225*	*0.024814043*	*0.01422433*
**ENSG00000136002**	*0.019627402*	*0.029115112*	*0.029978637*	*0.022699262*
**ENSG00000104413**	*0.03223833*	*0.030737173*	*0.030605354*	*0.017569445*
**ENSG00000089220**	*0.0243953*	*0.030727173*	*0.032431294*	*0.026840393*
**ENSG00000142303**	*0.015481257*	*0.025246635*	*0.0253413*	*0.016100677*
**ENSG00000131669**	*0.055944305*	*0.048455875*	*0.040557527*	*0.044266562*
**ENSG00000006059**	*0.017602121*	*0.024906648*	*0.024963734*	*0.015675015*
**ENSG00000196172**	*0.055677386*	*0.056142122*	*0.038237793*	*0.069396858*
**ENSG00000168904**	*0.039043777*	*0.037173229*	*0.036474506*	*0.035916447*
**ENSG00000134533**	*0.017203125*	*0.025186422*	*0.026627723*	*0.019509479*
**ENSG00000113805**	*0.01693474*	*0.025385833*	*0.025080467*	*0.015789694*
**ENSG00000178573**	*0.015722263*	*0.024961461*	*0.025417918*	*0.014636779*
**ENSG00000205155**	*0.022376094*	*0.028270548*	*0.030466309*	*0.022176319*
**ENSG00000197253**	*0.020805537*	*0.028546604*	*0.028427888*	*0.031104268*
**ENSG00000138760**	*0.06559425*	*0.06533791*	*0.067233656*	*0.072084483*
**ENSG00000174652**	*0.018458281*	*0.024786787*	*0.025017143*	*0.016509828*
**ENSG00000133131**	*0.059504266*	*0.040651659*	*0.031258043*	*0.034415284*
**ENSG00000147434**	*0.049136165*	*0.060258597*	*0.04494526*	*0.052423963*
**ENSG00000103549**	*0.022886243*	*0.028661122*	*0.035872792*	*0.022750823*
**ENSG00000185009**	*0.022987659*	*0.031259991*	*0.026648333*	*0.020632671*
**ENSG00000145283**	*0.016194901*	*0.024575863*	*0.024923216*	*0.014850947*
**ENSG00000163162**	*0.050440575*	*0.044381948*	*0.040321652*	*0.03901557*
**ENSG00000172345**	*0.026468872*	*0.028956425*	*0.029549223*	*0.023042443*
**ENSG00000159842**	*0.015962755*	*0.024473234*	*0.025469174*	*0.016953136*
**ENSG00000167723**	*0.016661247*	*0.02499848*	*0.025236702*	*0.015387081*
**ENSG00000111790**	*0.030683525*	*0.039453452*	*0.036755653*	*0.029607767*
**ENSG00000148343**	*0.026056287*	*0.034867142*	*0.036923663*	*0.023068441*
**ENSG00000144061**	*0.01534043*	*0.025340469*	*0.02469266*	*0.015717176*
**ENSG00000180447**	*0.016727772*	*0.024849344*	*0.024760142*	*0.016910423*
**ENSG00000197114**	*0.025571286*	*0.032358548*	*0.030993268*	*0.027440381*
**ENSG00000166197**	*0.036636284*	*0.035625264*	*0.036692515*	*0.035804072*
**ENSG00000177674**	*0.055444096*	*0.042689155*	*0.040097093*	*0.04252114*
**ENSG00000164616**	*0.016230514*	*0.02413705*	*0.024960866*	*0.017309098*
**ENSG00000149090**	*0.014833043*	*0.02638367*	*0.026322775*	*0.015541082*
**ENSG00000229937**	*0.015626873*	*0.02457747*	*0.024922597*	*0.016089421*
**ENSG00000117899**	*0.01674845*	*0.025221893*	*0.024795164*	*0.015744303*
**ENSG00000196151**	*0.035067043*	*0.036165055*	*0.042978788*	*0.026388914*
**ENSG00000144366**	*0.020298359*	*0.024454208*	*0.024245353*	*0.015236941*
**ENSG00000166002**	*0.054763715*	*0.049808264*	*0.051468322*	*0.04508318*
**ENSG00000157212**	*0.037793879*	*0.040847579*	*0.035460785*	*0.026717849*
**ENSG00000103051**	*0.032424156*	*0.038521785*	*0.03750008*	*0.027696609*
**ENSG00000106348**	*0.015585665*	*0.026139927*	*0.024179339*	*0.016206273*
**ENSG00000138685**	*0.027225918*	*0.027720959*	*0.026183666*	*0.019920616*
**ENSG00000104687**	*0.033524573*	*0.04335251*	*0.03497035*	*0.035776628*
**ENSG00000075856**	*0.030901472*	*0.036178061*	*0.030001527*	*0.023319548*
**ENSG00000174749**	*0.044438742*	*0.046493405*	*0.048181813*	*0.048222285*
**ENSG00000109738**	*0.015429571*	*0.024705569*	*0.024675181*	*0.014802426*
**ENSG00000105048**	*0.01595667*	*0.026822652*	*0.026772611*	*0.018650798*
**ENSG00000155592**	*0.018766475*	*0.027632704*	*0.030101248*	*0.019668627*
**ENSG00000122861**	*0.104476887*	*0.067931733*	*0.066248725*	*0.075555748*
**ENSG00000102048**	*0.119356674*	*0.085845796*	*0.079123523*	*0.096975472*

**ENSG00000186020**	*0.034301134*	*0.039336993*	*0.037506281*	*0.03028023*
**ENSG00000108840**	*0.029851575*	*0.034555483*	*0.038458498*	*0.032325193*
**ENSG00000110077**	*0.020777944*	*0.025346496*	*0.023965303*	*0.016058658*
**ENSG00000178623**	*0.025648233*	*0.029968*	*0.032111427*	*0.020223656*
**ENSG00000144857**	*0.015387653*	*0.024849726*	*0.024464899*	*0.015113797*
**ENSG00000181240**	*0.01885048*	*0.025296928*	*0.025967081*	*0.017071472*
**ENSG00000010244**	*0.030351269*	*0.03390502*	*0.03113526*	*0.031890576*
**ENSG00000108061**	*0.02345461*	*0.033918663*	*0.034237439*	*0.023168781*
**ENSG00000135632**	*0.019525444*	*0.02633918*	*0.029247338*	*0.020014333*
**ENSG00000130810**	*0.02817599*	*0.028854147*	*0.031063586*	*0.026711581*
**ENSG00000101280**	*0.019724177*	*0.030394538*	*0.028135827*	*0.019978132*
**ENSG00000198399**	*0.014990375*	*0.0254028*	*0.025194285*	*0.015407367*
**ENSG00000099622**	*0.029604128*	*0.034989318*	*0.032760935*	*0.030667857*
**ENSG00000115137**	*0.026376304*	*0.031437213*	*0.029321132*	*0.02001194*
**ENSG00000055609**	*0.026318948*	*0.032956131*	*0.033141395*	*0.035771661*
**ENSG00000126088**	*0.032863806*	*0.03218924*	*0.029953015*	*0.028153108*
**ENSG00000144659**	*0.026025578*	*0.032906379*	*0.03727139*	*0.025379769*
**ENSG00000116032**	*0.017229646*	*0.024922097*	*0.024290371*	*0.014368485*
**ENSG00000131203**	*0.015628622*	*0.025173942*	*0.025550919*	*0.020168712*
**ENSG00000102225**	*0.014766128*	*0.024977211*	*0.024997986*	*0.014503033*
**ENSG00000168135**	*0.015968106*	*0.025420679*	*0.025051804*	*0.015549379*
**ENSG00000164818**	*0.031592448*	*0.035699744*	*0.033609868*	*0.031376864*
**ENSG00000110046**	*0.024727586*	*0.029449128*	*0.034592225*	*0.025888691*
**ENSG00000186910**	*0.026170396*	*0.026375184*	*0.025503379*	*0.032526137*
**ENSG00000100304**	*0.040198946*	*0.03812073*	*0.041903499*	*0.03827492*
**ENSG00000114573**	*0.030473359*	*0.031052473*	*0.029646395*	*0.025088948*
**ENSG00000125508**	*0.01457017*	*0.024622505*	*0.024461809*	*0.015239187*
**ENSG00000111215**	*0.039400929*	*0.050167943*	*0.042944176*	*0.036137399*
**ENSG00000189108**	*0.01806901*	*0.025206425*	*0.025129855*	*0.01523309*
**ENSG00000198648**	*0.035594034*	*0.026652449*	*0.026095112*	*0.028282033*
**ENSG00000119772**	*0.030352587*	*0.037852228*	*0.039051432*	*0.031433072*
**ENSG00000078902**	*0.02646919*	*0.030390571*	*0.029902903*	*0.021967965*
**ENSG00000070831**	*0.055795659*	*0.037939983*	*0.037224967*	*0.048924081*
**ENSG00000198835**	*0.018926316*	*0.025714636*	*0.02549259*	*0.015857306*
**ENSG00000182446**	*0.030806886*	*0.048572376*	*0.044270199*	*0.038376886*
**ENSG00000213066**	*0.052153369*	*0.046898455*	*0.049086948*	*0.0349725*
**ENSG00000139364**	*0.021990418*	*0.025402345*	*0.025029092*	*0.015130638*
**ENSG00000178125**	*0.015818575*	*0.025027163*	*0.024482107*	*0.014861731*
**ENSG00000171388**	*0.01566368*	*0.026406757*	*0.025589892*	*0.014966474*
**ENSG00000035928**	*0.033629003*	*0.036220448*	*0.032493188*	*0.025627835*
**ENSG00000175877**	*0.015405636*	*0.025043586*	*0.024552941*	*0.015598233*
**ENSG00000116455**	*0.038718107*	*0.035814046*	*0.036601286*	*0.037655545*
**ENSG00000108094**	*0.025383347*	*0.033661638*	*0.031208171*	*0.020095016*
**ENSG00000142166**	*0.020245465*	*0.032820559*	*0.029140303*	*0.021320874*
**ENSG00000060971**	*0.028278185*	*0.035985095*	*0.030568633*	*0.028048809*
**ENSG00000146410**	*0.044901099*	*0.035974283*	*0.040788328*	*0.041626817*
**ENSG00000151475**	*0.01675114*	*0.025407473*	*0.028103461*	*0.016728837*
**ENSG00000121454**	*0.015360272*	*0.025609315*	*0.025299196*	*0.014538337*
**ENSG00000028839**	*0.021971149*	*0.029582569*	*0.03034478*	*0.023591815*
**ENSG00000109572**	*0.035615197*	*0.041346455*	*0.032884886*	*0.026668443*
**ENSG00000100083**	*0.031661593*	*0.035184228*	*0.040931394*	*0.03759485*
**ENSG00000152270**	*0.023813423*	*0.03840165*	*0.032418259*	*0.024734298*
**ENSG00000118526**	*0.015918468*	*0.026251137*	*0.025682632*	*0.015062897*
**ENSG00000071967**	*0.065350074*	*0.07205642*	*0.063777894*	*0.061999551*

**ENSG00000134780**	*0.018701867*	*0.024761394*	*0.025294324*	*0.015603582*
**ENSG00000164406**	*0.014534704*	*0.025803207*	*0.025169825*	*0.015444847*
**ENSG00000125337**	*0.016758491*	*0.025960212*	*0.025714242*	*0.0163486*
**ENSG00000161267**	*0.016453169*	*0.025005201*	*0.024978447*	*0.014720004*
**ENSG00000005175**	*0.032324468*	*0.037924962*	*0.030875786*	*0.027209377*
**ENSG00000197487**	*0.01469022*	*0.024085967*	*0.024558273*	*0.016385617*
**ENSG00000113231**	*0.017087482*	*0.025085241*	*0.02463931*	*0.01576902*
**ENSG00000160200**	*0.026946326*	*0.035334024*	*0.030522656*	*0.022706388*
**ENSG00000105819**	*0.023898191*	*0.033250173*	*0.029758551*	*0.020771405*
**ENSG00000167414**	*0.099924567*	*0.073935307*	*0.066100034*	*0.082286801*
**ENSG00000141738**	*0.015059559*	*0.025262093*	*0.024567733*	*0.015903337*
**ENSG00000165119**	*0.02239854*	*0.027246908*	*0.026997874*	*0.030065803*
**ENSG00000188408**	*0.015435497*	*0.024380695*	*0.024474561*	*0.015127492*
**ENSG00000147059**	*0.015196381*	*0.024421466*	*0.025590027*	*0.015440465*
**ENSG00000105963**	*0.062278052*	*0.043033986*	*0.047992536*	*0.04633033*
**ENSG00000167548**	*0.017241585*	*0.026073808*	*0.024313129*	*0.019022257*
**ENSG00000166908**	*0.027270552*	*0.038618585*	*0.041459869*	*0.027811482*
**ENSG00000094755**	*0.016765118*	*0.024789772*	*0.024032485*	*0.015078095*
**ENSG00000177885**	*0.023629162*	*0.03068036*	*0.033289133*	*0.021675554*
**ENSG00000162430**	*0.136099086*	*0.120416555*	*0.124079807*	*0.117964164*
**ENSG00000177324**	*0.015867332*	*0.025238451*	*0.024936864*	*0.014493661*
**ENSG00000186417**	*0.014813036*	*0.024712817*	*0.024206438*	*0.014465331*
**ENSG00000154734**	*0.015937749*	*0.024792283*	*0.024821479*	*0.015556286*
**ENSG00000172789**	*0.034505606*	*0.027449719*	*0.026042631*	*0.019989164*
**ENSG00000071073**	*0.073808558*	*0.053652382*	*0.046572432*	*0.056118373*
**ENSG00000153827**	*0.020802877*	*0.032237947*	*0.028540856*	*0.019653224*
**ENSG00000173080**	*0.015273406*	*0.02403179*	*0.024802633*	*0.014825232*
**ENSG00000149716**	*0.017646964*	*0.026747442*	*0.026877663*	*0.017944197*
**ENSG00000151379**	*0.018676803*	*0.026066017*	*0.025041668*	*0.019405548*
**ENSG00000177192**	*0.036506428*	*0.035747976*	*0.036719219*	*0.03630128*
**ENSG00000167615**	*0.016025332*	*0.026593318*	*0.025345212*	*0.0187809*
**ENSG00000174326**	*0.022219468*	*0.041504637*	*0.032705893*	*0.024944368*
**ENSG00000132554**	*0.034485526*	*0.030642219*	*0.026639421*	*0.027646383*
**ENSG00000100243**	*0.01663541*	*0.025650968*	*0.025586464*	*0.014651573*
**ENSG00000197872**	*0.076061847*	*0.059271727*	*0.039802424*	*0.07818037*
**ENSG00000137460**	*0.015582105*	*0.024700288*	*0.025211945*	*0.015777775*
**ENSG00000206288**	*0.016304005*	*0.025039719*	*0.026443174*	*0.015822835*
**ENSG00000060982**	*0.061047672*	*0.076374925*	*0.074574265*	*0.06772247*
**ENSG00000122643**	*0.032937732*	*0.037545356*	*0.033336572*	*0.024230125*
**ENSG00000107833**	*0.033953848*	*0.031566909*	*0.031835452*	*0.027100488*
**ENSG00000151502**	*0.029699604*	*0.032099163*	*0.032309435*	*0.02975383*
**ENSG00000112984**	*0.04819516*	*0.037694385*	*0.036241062*	*0.037550565*
**ENSG00000162104**	*0.023298379*	*0.029453171*	*0.028990461*	*0.039829413*
**ENSG00000179270**	*0.015721702*	*0.024772867*	*0.025179497*	*0.014369326*
**ENSG00000204428**	*0.021277438*	*0.026848934*	*0.026024989*	*0.019164679*
**ENSG00000162761**	*0.015204197*	*0.025273446*	*0.025530394*	*0.015935158*
**ENSG00000135722**	*0.026409224*	*0.03338622*	*0.028714721*	*0.027398628*
**ENSG00000120337**	*0.016569973*	*0.025433389*	*0.025304096*	*0.014818839*
**ENSG00000168672**	*0.016501378*	*0.024990483*	*0.025218291*	*0.015834029*
**ENSG00000103569**	*0.080073891*	*0.059852812*	*0.041357861*	*0.053730831*
**ENSG00000064989**	*0.049250423*	*0.029947344*	*0.032134057*	*0.024037849*
**ENSG00000180370**	*0.03742094*	*0.043045514*	*0.030857819*	*0.032816416*
**ENSG00000205143**	*0.022924473*	*0.028454057*	*0.03170863*	*0.024146732*
**ENSG00000052344**	*0.017002173*	*0.024919491*	*0.026061942*	*0.014825575*

**ENSG00000122481**	*0.032479423*	*0.041594584*	*0.032931636*	*0.031004051*
**ENSG00000105711**	*0.019115447*	*0.03113448*	*0.029912232*	*0.021639331*
**ENSG00000184557**	*0.0168583*	*0.025784889*	*0.025740611*	*0.015269594*
**ENSG00000120370**	*0.033322991*	*0.036340721*	*0.036655804*	*0.031195773*
**ENSG00000166454**	*0.032447801*	*0.038626605*	*0.039649652*	*0.031734561*
**ENSG00000118804**	*0.025812569*	*0.027250133*	*0.028119165*	*0.019662562*
**ENSG00000145996**	*0.015123245*	*0.024929236*	*0.024388234*	*0.014995271*
**ENSG00000143486**	*0.026052769*	*0.034009983*	*0.03160962*	*0.02402315*
**ENSG00000100346**	*0.018079074*	*0.02585074*	*0.026052148*	*0.018387144*
**ENSG00000100612**	*0.031311896*	*0.03028759*	*0.030815605*	*0.025955435*
**ENSG00000162384**	*0.031128715*	*0.033852736*	*0.03233742*	*0.021755765*
**ENSG00000004700**	*0.035564166*	*0.038619828*	*0.031553951*	*0.027741081*
**ENSG00000083290**	*0.046834885*	*0.049017539*	*0.03823088*	*0.0354187*
**ENSG00000203759**	*0.038166881*	*0.036704193*	*0.032081634*	*0.030109002*
**ENSG00000133805**	*0.046282458*	*0.048046041*	*0.051597698*	*0.044141222*
**ENSG00000111850**	*0.035696253*	*0.038212576*	*0.029853126*	*0.026375882*
**ENSG00000138764**	*0.05534088*	*0.067973973*	*0.065403269*	*0.060359883*
**ENSG00000171206**	*0.027491716*	*0.033833673*	*0.03454238*	*0.028110122*
**ENSG00000096092**	*0.041042873*	*0.04769489*	*0.038923961*	*0.028013332*
**ENSG00000168028**	*0.015649431*	*0.024329668*	*0.024163063*	*0.014924088*
**ENSG00000162910**	*0.024526104*	*0.027456697*	*0.029547929*	*0.020405645*
**ENSG00000153498**	*0.021266494*	*0.026897349*	*0.025281021*	*0.016325743*
**ENSG00000075618**	*0.043702606*	*0.047356334*	*0.050667295*	*0.051139309*
**ENSG00000143167**	*0.018148534*	*0.026352522*	*0.026969695*	*0.016885437*
**ENSG00000100519**	*0.024626034*	*0.03191352*	*0.027529442*	*0.023370069*
**ENSG00000119969**	*0.036098392*	*0.042150423*	*0.032051695*	*0.034782296*
**ENSG00000117215**	*0.017508929*	*0.0251873*	*0.026281822*	*0.014855879*
**ENSG00000177706**	*0.01805113*	*0.02545287*	*0.024862669*	*0.016225845*
**ENSG00000187210**	*0.097530098*	*0.063086032*	*0.07031565*	*0.073569944*
**ENSG00000171219**	*0.016916141*	*0.025477922*	*0.027388289*	*0.016309577*
**ENSG00000186047**	*0.016042886*	*0.02556252*	*0.025435388*	*0.016339877*
**ENSG00000215217**	*0.015151895*	*0.024558916*	*0.024976073*	*0.014598448*
**ENSG00000181072**	*0.015006806*	*0.024601054*	*0.024700103*	*0.014788655*
**ENSG00000175105**	*0.018981325*	*0.028864231*	*0.027682823*	*0.019870657*
**ENSG00000185942**	*0.017871226*	*0.025024488*	*0.025526341*	*0.015307979*
**ENSG00000133114**	*0.041079097*	*0.050850177*	*0.038622559*	*0.055180161*
**ENSG00000143194**	*0.033663257*	*0.031007274*	*0.026594495*	*0.023279445*
**ENSG00000156802**	*0.043624942*	*0.042154243*	*0.03380203*	*0.028867432*
**ENSG00000004478**	*0.045494885*	*0.042590398*	*0.039912357*	*0.033661537*
**ENSG00000137843**	*0.02397883*	*0.02752525*	*0.027682317*	*0.019318995*
**ENSG00000143452**	*0.017409422*	*0.025526717*	*0.026353944*	*0.016845109*
**ENSG00000030582**	*0.028764193*	*0.033346201*	*0.030345415*	*0.034170485*
**ENSG00000121933**	*0.024609256*	*0.028931474*	*0.027790006*	*0.018964374*
**ENSG00000152977**	*0.017616705*	*0.024871137*	*0.025956335*	*0.016005379*
**ENSG00000197376**	*0.017351973*	*0.024802392*	*0.024615378*	*0.015091277*
**ENSG00000241945**	*0.027794435*	*0.033002811*	*0.033568828*	*0.025338561*
**ENSG00000126861**	*0.017324051*	*0.024712592*	*0.024509642*	*0.01606966*
**ENSG00000197555**	*0.023963323*	*0.033749261*	*0.032778758*	*0.023380084*
**ENSG00000240251**	*0.02502964*	*0.029047417*	*0.029830952*	*0.018996739*
**ENSG00000144935**	*0.053840901*	*0.049643304*	*0.032442098*	*0.045242936*
**ENSG00000168938**	*0.074941043*	*0.071325651*	*0.053022069*	*0.081464422*
**ENSG00000115234**	*0.025693351*	*0.029500416*	*0.029890327*	*0.02175705*
**ENSG00000145016**	*0.030909158*	*0.034325338*	*0.035248352*	*0.028285812*
**ENSG00000159167**	*0.019856427*	*0.02549752*	*0.027399452*	*0.019483737*

**ENSG00000135299**	*0.058857845*	*0.041072969*	*0.0451222*	*0.044660479*
**ENSG00000011243**	*0.023868874*	*0.032428459*	*0.039159191*	*0.021785072*
**ENSG00000129534**	*0.040012784*	*0.039848579*	*0.033860923*	*0.034485397*
**ENSG00000090615**	*0.026321029*	*0.033941896*	*0.036177066*	*0.028893184*
**ENSG00000164611**	*0.033402167*	*0.032082428*	*0.030645141*	*0.024423439*
**ENSG00000173404**	*0.019942454*	*0.026467523*	*0.025703655*	*0.017617141*
**ENSG00000173585**	*0.016047863*	*0.02640138*	*0.024772083*	*0.016835813*
**ENSG00000137478**	*0.027963422*	*0.034419876*	*0.030356427*	*0.023324971*
**ENSG00000145220**	*0.036258896*	*0.030864564*	*0.034953685*	*0.036659943*
**ENSG00000132196**	*0.029363784*	*0.030872786*	*0.032693186*	*0.02920945*
**ENSG00000155096**	*0.032139338*	*0.039696997*	*0.037250239*	*0.02854971*
**ENSG00000116771**	*0.041183478*	*0.037762001*	*0.033880302*	*0.033950271*
**ENSG00000161281**	*0.016672572*	*0.024698418*	*0.026623006*	*0.023256345*
**ENSG00000106609**	*0.024014653*	*0.034106483*	*0.034319223*	*0.01959161*
**ENSG00000178950**	*0.032903083*	*0.035168416*	*0.032025052*	*0.028219349*
**ENSG00000173566**	*0.054221178*	*0.045512852*	*0.037471032*	*0.040547014*
**ENSG00000101098**	*0.017913193*	*0.02583941*	*0.025534406*	*0.017033542*
**ENSG00000186187**	*0.015723909*	*0.025101288*	*0.025027508*	*0.014946615*
**ENSG00000089199**	*0.017212808*	*0.025146062*	*0.024928807*	*0.016137901*
**ENSG00000163558**	*0.042404476*	*0.040486711*	*0.037546631*	*0.031944562*
**ENSG00000166016**	*0.061398014*	*0.060234013*	*0.064186445*	*0.062285049*
**ENSG00000129467**	*0.035940346*	*0.034496426*	*0.031262686*	*0.02923366*
**ENSG00000185751**	*0.018146672*	*0.027229512*	*0.025912162*	*0.015985408*
**ENSG00000139433**	*0.03178065*	*0.03650575*	*0.034528436*	*0.024853865*
**ENSG00000189350**	*0.015198011*	*0.024751119*	*0.02526279*	*0.015495058*
**ENSG00000147044**	*0.065494723*	*0.049872623*	*0.040990644*	*0.043576437*
**ENSG00000139344**	*0.019432144*	*0.026330998*	*0.025684892*	*0.017953538*
**ENSG00000242366**	*0.022590896*	*0.029280466*	*0.025208622*	*0.025093841*
**ENSG00000116191**	*0.057951038*	*0.053478832*	*0.057432204*	*0.052572184*
**ENSG00000075218**	*0.025899323*	*0.028098553*	*0.025722265*	*0.02084557*
**ENSG00000092330**	*0.040100146*	*0.035932953*	*0.038236165*	*0.026673028*
**ENSG00000133639**	*0.019753094*	*0.028270057*	*0.027380235*	*0.020112227*
**ENSG00000176049**	*0.01655992*	*0.025615053*	*0.024754366*	*0.015215236*
**ENSG00000123505**	*0.027551584*	*0.034238952*	*0.034182384*	*0.03130187*
**ENSG00000179300**	*0.022513217*	*0.030481927*	*0.027909097*	*0.018566547*
**ENSG00000048544**	*0.032437245*	*0.036673466*	*0.037776891*	*0.038190908*
**ENSG00000177542**	*0.026094742*	*0.033517865*	*0.038816438*	*0.027956278*
**ENSG00000212734**	*0.026453853*	*0.03360947*	*0.03549893*	*0.026402636*
**ENSG00000198040**	*0.02983576*	*0.034844113*	*0.031847075*	*0.024744118*
**ENSG00000109846**	*0.026291324*	*0.032366266*	*0.031314395*	*0.035161815*
**ENSG00000183918**	*0.086365889*	*0.099001301*	*0.10857905*	*0.086519914*
**ENSG00000008324**	*0.019152175*	*0.028528234*	*0.031205809*	*0.018497661*
**ENSG00000091428**	*0.016336574*	*0.024932399*	*0.025287084*	*0.015143165*
**ENSG00000173638**	*0.037044258*	*0.03172482*	*0.03544475*	*0.033568864*
**ENSG00000105216**	*0.025358632*	*0.030542221*	*0.029541895*	*0.021264864*
**ENSG00000143443**	*0.023688676*	*0.02716173*	*0.029627856*	*0.021217222*
**ENSG00000160293**	*0.020558565*	*0.030068697*	*0.02561175*	*0.021924826*
**ENSG00000181666**	*0.043797808*	*0.039415092*	*0.037270587*	*0.03614952*
**ENSG00000125818**	*0.014923398*	*0.02451506*	*0.024932232*	*0.015201994*
**ENSG00000173715**	*0.057041252*	*0.050712104*	*0.047265737*	*0.039914877*
**ENSG00000088876**	*0.027535949*	*0.02909489*	*0.029572427*	*0.019450746*
**ENSG00000004534**	*0.030176719*	*0.035186851*	*0.031306067*	*0.023369877*
**ENSG00000133110**	*0.015854402*	*0.024397103*	*0.024405563*	*0.015595799*
**ENSG00000169762**	*0.034930777*	*0.038673076*	*0.035968788*	*0.031459549*

**ENSG00000108417**	*0.016163605*	*0.024899194*	*0.024396571*	*0.015364782*
**ENSG00000158636**	*0.031577108*	*0.033116617*	*0.031716449*	*0.027361906*
**ENSG00000169418**	*0.047322768*	*0.053566436*	*0.053547183*	*0.050659193*
**ENSG00000143499**	*0.038581806*	*0.041242807*	*0.034289174*	*0.033003066*
**ENSG00000124785**	*0.12701717*	*0.126537087*	*0.09133679*	*0.135792354*
**ENSG00000106803**	*0.023663408*	*0.028945715*	*0.031804281*	*0.026428316*
**ENSG00000177595**	*0.050811118*	*0.050523448*	*0.037801133*	*0.037069105*
**ENSG00000149150**	*0.041303309*	*0.036219341*	*0.036277101*	*0.029772773*
**ENSG00000149573**	*0.021779039*	*0.026321135*	*0.025209741*	*0.019668459*
**ENSG00000099139**	*0.016288644*	*0.025826596*	*0.025611375*	*0.015370519*
**ENSG00000088298**	*0.03201069*	*0.032602578*	*0.034880652*	*0.030517358*
**ENSG00000145214**	*0.016694084*	*0.024836482*	*0.024762082*	*0.016097307*
**ENSG00000117480**	*0.033253903*	*0.030031171*	*0.026888246*	*0.021983896*
**ENSG00000119686**	*0.058775714*	*0.054028307*	*0.049189306*	*0.051694988*
**ENSG00000071794**	*0.042915752*	*0.039167239*	*0.034339012*	*0.029542709*
**ENSG00000140506**	*0.015422967*	*0.025151982*	*0.025104479*	*0.01524318*
**ENSG00000176979**	*0.016272864*	*0.025427281*	*0.025017958*	*0.015689634*
**ENSG00000102575**	*0.081083899*	*0.054290149*	*0.045248174*	*0.052163411*
**ENSG00000122507**	*0.021079838*	*0.026425768*	*0.028382881*	*0.021237207*
**ENSG00000186334**	*0.016523233*	*0.026138814*	*0.025694439*	*0.015583767*
**ENSG00000154781**	*0.02281052*	*0.032582216*	*0.032671131*	*0.02491835*
**ENSG00000148671**	*0.081235995*	*0.065384515*	*0.060510245*	*0.074777964*
**ENSG00000171121**	*0.015892906*	*0.025618103*	*0.025449753*	*0.014644318*
**ENSG00000137860**	*0.015629798*	*0.024718931*	*0.024788831*	*0.014781338*
**ENSG00000168702**	*0.01637809*	*0.024892964*	*0.025250851*	*0.016643596*
**ENSG00000147454**	*0.041621678*	*0.03725004*	*0.03750013*	*0.039708502*
**ENSG00000205726**	*0.016329548*	*0.024226492*	*0.024824972*	*0.01614814*
**ENSG00000179761**	*0.06126441*	*0.050587549*	*0.046134483*	*0.061412252*
**ENSG00000134138**	*0.016354822*	*0.025291067*	*0.024504857*	*0.015912047*
**ENSG00000153790**	*0.020415048*	*0.027425178*	*0.025937812*	*0.020529445*
**ENSG00000070731**	*0.028019355*	*0.026886614*	*0.026756911*	*0.030877266*
**ENSG00000135334**	*0.024877338*	*0.033438413*	*0.032144413*	*0.021634288*
**ENSG00000184226**	*0.01560583*	*0.024482607*	*0.025638989*	*0.016372144*
**ENSG00000127334**	*0.018761138*	*0.029435426*	*0.029616094*	*0.019474231*
**ENSG00000181418**	*0.038558235*	*0.034454688*	*0.039182877*	*0.031620428*
**ENSG00000146966**	*0.015679412*	*0.024501038*	*0.024260662*	*0.014365723*
**ENSG00000243480**	*0.031512257*	*0.038345516*	*0.031062851*	*0.031139877*
**ENSG00000104859**	*0.020832757*	*0.032933855*	*0.038333941*	*0.027655979*
**ENSG00000113013**	*0.024540242*	*0.029353102*	*0.027664928*	*0.019273865*
**ENSG00000119913**	*0.015632722*	*0.024582312*	*0.024648948*	*0.015137887*
**ENSG00000059377**	*0.078469623*	*0.070331467*	*0.06464926*	*0.066409284*
**ENSG00000168306**	*0.015897628*	*0.02488665*	*0.024719649*	*0.015098854*
**ENSG00000087301**	*0.016437885*	*0.024664628*	*0.026283334*	*0.014873764*
**ENSG00000105968**	*0.027619266*	*0.029633993*	*0.029367118*	*0.021278323*
**ENSG00000239389**	*0.016169729*	*0.024497432*	*0.024639202*	*0.016020921*
**ENSG00000179241**	*0.032736284*	*0.026228555*	*0.024459136*	*0.017810036*
**ENSG00000203910**	*0.016500078*	*0.024789559*	*0.02496124*	*0.016265968*
**ENSG00000120837**	*0.039007313*	*0.0445748*	*0.036362659*	*0.028372843*
**ENSG00000170836**	*0.035167575*	*0.038014141*	*0.036007188*	*0.025394846*
**ENSG00000169484**	*0.017622693*	*0.026339925*	*0.025183597*	*0.016247219*
**ENSG00000178694**	*0.023096144*	*0.030177263*	*0.028080709*	*0.020294226*
**ENSG00000112462**	*0.015283563*	*0.024204832*	*0.02490725*	*0.015475393*
**ENSG00000148814**	*0.033763076*	*0.036888179*	*0.036285515*	*0.026620624*
**ENSG00000104356**	*0.039416774*	*0.048264796*	*0.036976545*	*0.040111752*

**ENSG00000186860**	*0.114847272*	*0.077420239*	*0.042920148*	*0.09067478*
**ENSG00000203730**	*0.015261525*	*0.025512715*	*0.024519929*	*0.015096861*
**ENSG00000047410**	*0.021736713*	*0.031816807*	*0.02942829*	*0.019520844*
**ENSG00000124422**	*0.028334751*	*0.027998578*	*0.03406073*	*0.027685206*
**ENSG00000237763**	*0.026554403*	*0.033107972*	*0.029756907*	*0.023461327*
**ENSG00000184544**	*0.015952205*	*0.026230731*	*0.025243363*	*0.01486154*
**ENSG00000151655**	*0.015598523*	*0.024810097*	*0.025072472*	*0.014780234*
**ENSG00000008056**	*0.01517368*	*0.025245655*	*0.02543726*	*0.01449779*
**ENSG00000137709**	*0.027639313*	*0.05646628*	*0.038710371*	*0.031684593*
**ENSG00000102452**	*0.015179709*	*0.026097063*	*0.025314753*	*0.016725635*
**ENSG00000165323**	*0.016775585*	*0.025639304*	*0.025078037*	*0.016259614*
**ENSG00000100601**	*0.027657207*	*0.033755784*	*0.034074741*	*0.025159851*
**ENSG00000140848**	*0.045767788*	*0.049031837*	*0.039873729*	*0.049804776*
**ENSG00000102781**	*0.040923857*	*0.047025693*	*0.036141574*	*0.040019854*
**ENSG00000169884**	*0.017412569*	*0.025863623*	*0.024571448*	*0.017555206*
**ENSG00000254218**	*0.018416229*	*0.024489027*	*0.024824193*	*0.01644483*
**ENSG00000147050**	*0.048341915*	*0.049795392*	*0.053754503*	*0.038672889*
**ENSG00000143847**	*0.028835482*	*0.031542333*	*0.030520713*	*0.030779847*
**ENSG00000170801**	*0.035462184*	*0.029604342*	*0.032981617*	*0.027219573*
**ENSG00000152193**	*0.02927439*	*0.036859123*	*0.037639997*	*0.029048356*
**ENSG00000130803**	*0.025114333*	*0.034927145*	*0.040274535*	*0.025309096*
**ENSG00000166173**	*0.106661436*	*0.062713234*	*0.035007445*	*0.05952565*
**ENSG00000178498**	*0.032238062*	*0.036391531*	*0.040274146*	*0.032488497*
**ENSG00000076382**	*0.033159099*	*0.032020726*	*0.030371922*	*0.022015048*
**ENSG00000076984**	*0.01926279*	*0.030874405*	*0.028617986*	*0.022824394*
**ENSG00000204920**	*0.046714585*	*0.039837769*	*0.04022534*	*0.027130402*
**ENSG00000144962**	*0.017922272*	*0.028395553*	*0.024850395*	*0.018903632*
**ENSG00000151353**	*0.040996826*	*0.047658263*	*0.035859779*	*0.032032416*
**ENSG00000136478**	*0.041721091*	*0.041028309*	*0.037229377*	*0.030864688*
**ENSG00000182103**	*0.016052556*	*0.025494129*	*0.025380048*	*0.014782518*
**ENSG00000135525**	*0.019217042*	*0.025049444*	*0.025689864*	*0.018162789*
**ENSG00000130305**	*0.026267174*	*0.032079003*	*0.029969802*	*0.025367596*
**ENSG00000054967**	*0.015255673*	*0.024970233*	*0.026006475*	*0.01492502*
**ENSG00000166794**	*0.033059406*	*0.031844057*	*0.036838419*	*0.036716431*
**ENSG00000183908**	*0.017376136*	*0.027016557*	*0.025094939*	*0.018054343*
**ENSG00000087269**	*0.026359615*	*0.029481252*	*0.029741774*	*0.024809907*
**ENSG00000150048**	*0.016227465*	*0.024802037*	*0.024034932*	*0.015478622*
**ENSG00000141349**	*0.033554103*	*0.029331829*	*0.029006471*	*0.030989903*
**ENSG00000156671**	*0.015287759*	*0.025673091*	*0.024291252*	*0.014721556*
**ENSG00000130202**	*0.016417254*	*0.025531403*	*0.024754282*	*0.015108119*
**ENSG00000131242**	*0.045392156*	*0.041506146*	*0.035370658*	*0.03944555*
**ENSG00000153575**	*0.037704463*	*0.035898142*	*0.038137315*	*0.046131125*
**ENSG00000145723**	*0.0395526*	*0.041644495*	*0.033169565*	*0.026489433*
**ENSG00000153406**	*0.034669228*	*0.032466189*	*0.030786139*	*0.038962617*
**ENSG00000136485**	*0.029209086*	*0.032400974*	*0.040849245*	*0.025677883*
**ENSG00000177106**	*0.059470514*	*0.05872893*	*0.045988315*	*0.051585864*
**ENSG00000078140**	*0.026621032*	*0.034310443*	*0.029764379*	*0.0267726*
**ENSG00000241343**	*0.015338522*	*0.024160869*	*0.023903728*	*0.018547865*
**ENSG00000179833**	*0.033956144*	*0.036986541*	*0.036928368*	*0.032592545*
**ENSG00000117280**	*0.055102721*	*0.041705202*	*0.038985281*	*0.042695398*
**ENSG00000099385**	*0.028096945*	*0.034786365*	*0.030196364*	*0.030619329*
**ENSG00000104375**	*0.055468912*	*0.053700163*	*0.045304259*	*0.052207663*
**ENSG00000167772**	*0.014668827*	*0.025090892*	*0.025414864*	*0.015232487*
**ENSG00000000938**	*0.077558017*	*0.059044042*	*0.048682091*	*0.065532833*

**ENSG00000178033**	*0.016371143*	*0.025788661*	*0.026326443*	*0.015983542*
**ENSG00000225921**	*0.026377906*	*0.029319977*	*0.029891379*	*0.022722631*
**ENSG00000125861**	*0.016455993*	*0.025168181*	*0.026573353*	*0.014970586*
**ENSG00000172081**	*0.024829372*	*0.031627239*	*0.030275291*	*0.027516683*
**ENSG00000153037**	*0.026016953*	*0.03471891*	*0.038601231*	*0.029589987*
**ENSG00000100241**	*0.032993403*	*0.036932582*	*0.036478266*	*0.026711187*
**ENSG00000154262**	*0.050740742*	*0.048288154*	*0.035846088*	*0.042192263*
**ENSG00000033122**	*0.015145608*	*0.025141981*	*0.024894071*	*0.015536891*
**ENSG00000134072**	*0.049048374*	*0.046861916*	*0.035486979*	*0.042251516*
**ENSG00000198598**	*0.083357765*	*0.084242656*	*0.083323598*	*0.086732814*
**ENSG00000166136**	*0.022504957*	*0.028561473*	*0.027653428*	*0.019756918*
**ENSG00000111291**	*0.038821679*	*0.031094831*	*0.032467524*	*0.02344288*
**ENSG00000100100**	*0.058463079*	*0.053853207*	*0.054989422*	*0.061871487*
**ENSG00000080007**	*0.096864966*	*0.077489304*	*0.084133159*	*0.079774256*
**ENSG00000170577**	*0.019481982*	*0.0269483*	*0.028464833*	*0.017859725*
**ENSG00000103266**	*0.024411538*	*0.026700088*	*0.028965374*	*0.020175471*
**ENSG00000181518**	*0.015980513*	*0.025492251*	*0.025630049*	*0.014623132*
**ENSG00000204351**	*0.030472684*	*0.033870639*	*0.031579083*	*0.023067012*
**ENSG00000187243**	*0.029246974*	*0.024590123*	*0.02574703*	*0.017265165*
**ENSG00000135744**	*0.016862175*	*0.025324445*	*0.025825859*	*0.015929664*
**ENSG00000137077**	*0.015451213*	*0.024759819*	*0.025318843*	*0.014940644*
**ENSG00000104921**	*0.042114134*	*0.049173913*	*0.050382693*	*0.062063951*
**ENSG00000115211**	*0.015750645*	*0.024675565*	*0.024359268*	*0.016832355*
**ENSG00000133872**	*0.025185437*	*0.029826713*	*0.031771153*	*0.019891106*
**ENSG00000173250**	*0.016294926*	*0.024811361*	*0.02519823*	*0.016787756*
**ENSG00000156234**	*0.051786151*	*0.049887669*	*0.050536087*	*0.038578562*
**ENSG00000125848**	*0.01949977*	*0.025651377*	*0.025411493*	*0.01622811*
**ENSG00000166260**	*0.043088464*	*0.046031154*	*0.034227659*	*0.034744112*
**ENSG00000183260**	*0.017065084*	*0.026009054*	*0.024966146*	*0.01642668*
**ENSG00000112137**	*0.056747397*	*0.062616064*	*0.052072891*	*0.07236883*
**ENSG00000093000**	*0.015533298*	*0.024418391*	*0.02486549*	*0.014032699*
**ENSG00000137185**	*0.026225162*	*0.034051067*	*0.031679208*	*0.025247276*
**ENSG00000170255**	*0.014632132*	*0.025248348*	*0.025764009*	*0.01591843*
**ENSG00000168477**	*0.01648748*	*0.024580771*	*0.024209251*	*0.014842013*
**ENSG00000164989**	*0.025918195*	*0.029206617*	*0.030011956*	*0.025233531*
**ENSG00000173451**	*0.031919214*	*0.045358386*	*0.042180406*	*0.029232005*
**ENSG00000105443**	*0.02456214*	*0.029329234*	*0.033329283*	*0.02772691*
**ENSG00000173456**	*0.032400438*	*0.029978126*	*0.03747505*	*0.027882977*
**ENSG00000172780**	*0.024695668*	*0.030610371*	*0.032855074*	*0.024044116*
**ENSG00000147885**	*0.016671539*	*0.027070474*	*0.025282022*	*0.017271833*
**ENSG00000112335**	*0.027291466*	*0.030711933*	*0.027301817*	*0.021956738*
**ENSG00000149782**	*0.023264108*	*0.030491621*	*0.029613285*	*0.024024116*
**ENSG00000178172**	*0.017538501*	*0.026549137*	*0.026220702*	*0.017742206*
**ENSG00000254122**	*0.018529062*	*0.025876022*	*0.027119412*	*0.016653067*
**ENSG00000131931**	*0.029783644*	*0.035134139*	*0.034623012*	*0.02925933*
**ENSG00000229809**	*0.022865238*	*0.027469127*	*0.029756469*	*0.023441395*
**ENSG00000186340**	*0.016395269*	*0.02554968*	*0.025346336*	*0.014179527*
**ENSG00000116745**	*0.016340246*	*0.025080244*	*0.02590134*	*0.016256823*
**ENSG00000174607**	*0.032159968*	*0.039223194*	*0.029106674*	*0.038204656*
**ENSG00000100299**	*0.038184925*	*0.038389696*	*0.03605128*	*0.033772137*
**ENSG00000174173**	*0.032934868*	*0.039654136*	*0.044743555*	*0.032942657*
**ENSG00000185298**	*0.027912966*	*0.034124082*	*0.038336214*	*0.029122283*
**ENSG00000104537**	*0.017196079*	*0.025518014*	*0.024955685*	*0.016609697*
**ENSG00000163754**	*0.0380115*	*0.039820982*	*0.036393338*	*0.033400113*

**ENSG00000188580**	*0.039578259*	*0.026526952*	*0.025865393*	*0.016537036*
**ENSG00000183087**	*0.048877072*	*0.051793045*	*0.048997219*	*0.049158154*
**ENSG00000111325**	*0.025373995*	*0.030385154*	*0.032363742*	*0.027751911*
**ENSG00000099326**	*0.034374885*	*0.037899123*	*0.035413868*	*0.029414961*
**ENSG00000131126**	*0.016904341*	*0.026599458*	*0.026019081*	*0.016227149*
**ENSG00000181873**	*0.03328481*	*0.032663113*	*0.038614206*	*0.034220047*
**ENSG00000080845**	*0.016820167*	*0.02552189*	*0.025227227*	*0.014940883*
**ENSG00000166323**	*0.027414227*	*0.029680202*	*0.028513654*	*0.026974503*
**ENSG00000164253**	*0.071032032*	*0.039942822*	*0.037253367*	*0.029667856*
**ENSG00000144481**	*0.016539646*	*0.02506112*	*0.026317573*	*0.015657366*
**ENSG00000158480**	*0.037225106*	*0.036926909*	*0.034481136*	*0.027109351*
**ENSG00000163534**	*0.017519847*	*0.028653003*	*0.026259365*	*0.018063879*
**ENSG00000168778**	*0.014963441*	*0.025947787*	*0.025109188*	*0.015063397*
**ENSG00000108828**	*0.027599358*	*0.032181477*	*0.03085231*	*0.029894039*
**ENSG00000179918**	*0.036677334*	*0.035544362*	*0.036536037*	*0.030694245*
**ENSG00000185345**	*0.015694996*	*0.025052756*	*0.024827238*	*0.014768825*
**ENSG00000132286**	*0.031395082*	*0.037407874*	*0.043357762*	*0.032552624*
**ENSG00000005436**	*0.031815688*	*0.036791761*	*0.032542434*	*0.026512702*
**ENSG00000181552**	*0.016146831*	*0.025163254*	*0.025140187*	*0.015494013*
**ENSG00000186501**	*0.025111192*	*0.034900312*	*0.038455824*	*0.024451122*
**ENSG00000106460**	*0.035675929*	*0.044894255*	*0.038724646*	*0.029735835*
**ENSG00000158457**	*0.030885443*	*0.036284628*	*0.034063575*	*0.034741047*
**ENSG00000129422**	*0.034399601*	*0.028457797*	*0.026078172*	*0.033018856*
**ENSG00000132510**	*0.034015729*	*0.038610434*	*0.040854847*	*0.034273297*
**ENSG00000165841**	*0.015195424*	*0.025318026*	*0.025192153*	*0.015401415*
**ENSG00000111261**	*0.017915595*	*0.024990338*	*0.025849966*	*0.017834457*
**ENSG00000169100**	*0.022912855*	*0.02815356*	*0.028141189*	*0.022617071*
**ENSG00000090006**	*0.033954895*	*0.029748465*	*0.038396034*	*0.034531172*
**ENSG00000147576**	*0.046918331*	*0.039820593*	*0.037162154*	*0.040274424*
**ENSG00000085185**	*0.052241148*	*0.045492741*	*0.038214511*	*0.038051408*
**ENSG00000185504**	*0.031341841*	*0.038219245*	*0.036966089*	*0.027853913*
**ENSG00000172324**	*0.01671574*	*0.025770197*	*0.025907617*	*0.015121045*
**ENSG00000083544**	*0.032607305*	*0.036569268*	*0.030262755*	*0.023464603*
**ENSG00000040731**	*0.017353326*	*0.025499929*	*0.025002703*	*0.017090183*
**ENSG00000170627**	*0.247734321*	*0.156626613*	*0.13124397*	*0.177745352*
**ENSG00000084093**	*0.017960755*	*0.026534132*	*0.025498135*	*0.016589776*
**ENSG00000134343**	*0.08327361*	*0.038213392*	*0.025808665*	*0.029404688*
**ENSG00000130313**	*0.023881031*	*0.030695752*	*0.030547526*	*0.027775205*
**ENSG00000182580**	*0.014760424*	*0.024753045*	*0.02507466*	*0.014469115*
**ENSG00000104899**	*0.02245244*	*0.028148074*	*0.026155718*	*0.017778096*
**ENSG00000086730**	*0.035008058*	*0.044940658*	*0.040701958*	*0.059215228*
**ENSG00000132964**	*0.032942118*	*0.038833266*	*0.031557516*	*0.026315952*
**ENSG00000244038**	*0.02271916*	*0.027078056*	*0.027154089*	*0.022276955*
**ENSG00000103152**	*0.024119857*	*0.027026873*	*0.028909027*	*0.022633396*
**ENSG00000104365**	*0.032001254*	*0.036277277*	*0.03534737*	*0.031082232*
**ENSG00000178297**	*0.017493052*	*0.025360058*	*0.025396969*	*0.016781924*
**ENSG00000213398**	*0.032601409*	*0.034973518*	*0.028826867*	*0.032931013*
**ENSG00000157680**	*0.031621785*	*0.029523736*	*0.031768496*	*0.028477484*
**ENSG00000139428**	*0.025980918*	*0.034494921*	*0.031897606*	*0.022465829*
**ENSG00000070610**	*0.025746247*	*0.033276954*	*0.032079611*	*0.030107173*
**ENSG00000139160**	*0.015907316*	*0.024164723*	*0.025388731*	*0.014919221*
**ENSG00000183072**	*0.01612534*	*0.024255106*	*0.025641349*	*0.014323036*
**ENSG00000133105**	*0.017388142*	*0.024944729*	*0.024773709*	*0.016974077*
**ENSG00000131019**	*0.015909137*	*0.026064343*	*0.025412344*	*0.014948108*

**ENSG00000125945**	*0.027101627*	*0.034349254*	*0.028450333*	*0.024005537*
**ENSG00000155254**	*0.048141674*	*0.04229426*	*0.036755317*	*0.034221092*
**ENSG00000106633**	*0.018167468*	*0.025623234*	*0.025633135*	*0.015669068*
**ENSG00000077279**	*0.015830913*	*0.024840966*	*0.024326435*	*0.016010098*
**ENSG00000152936**	*0.01577842*	*0.026504228*	*0.024933753*	*0.015918836*
**ENSG00000074319**	*0.025462869*	*0.032625224*	*0.029731964*	*0.024018857*
**ENSG00000172425**	*0.016520875*	*0.026363342*	*0.024847494*	*0.016488698*
**ENSG00000196091**	*0.020452509*	*0.026086784*	*0.027270623*	*0.017485424*
**ENSG00000226929**	*0.016699191*	*0.024952739*	*0.025430445*	*0.016126594*
**ENSG00000244752**	*0.071350211*	*0.095569022*	*0.082207731*	*0.074804017*
**ENSG00000162734**	*0.030460694*	*0.031962189*	*0.029557879*	*0.026571534*
**ENSG00000120896**	*0.035824297*	*0.035057905*	*0.029627022*	*0.028989433*
**ENSG00000111145**	*0.017751237*	*0.025588167*	*0.02657967*	*0.019157726*
**ENSG00000141380**	*0.037630811*	*0.038398059*	*0.034620883*	*0.036662726*
**ENSG00000213906**	*0.016325107*	*0.026359619*	*0.025080107*	*0.016344976*
**ENSG00000108349**	*0.023467907*	*0.030094355*	*0.032223876*	*0.022628115*
**ENSG00000112212**	*0.017133324*	*0.024105912*	*0.024600174*	*0.015354769*
**ENSG00000057294**	*0.0296516*	*0.027844672*	*0.027506467*	*0.030917202*
**ENSG00000117143**	*0.035595701*	*0.033450059*	*0.037504234*	*0.034025894*
**ENSG00000197971**	*0.016937402*	*0.026480593*	*0.026138315*	*0.015032563*
**ENSG00000110888**	*0.016452013*	*0.026359808*	*0.025430538*	*0.01697878*
**ENSG00000171161**	*0.023903937*	*0.030341499*	*0.030999977*	*0.024828878*
**ENSG00000065150**	*0.043791858*	*0.050030437*	*0.034892916*	*0.039559078*
**ENSG00000066248**	*0.016548329*	*0.024607335*	*0.02440422*	*0.015088978*
**ENSG00000151062**	*0.036516229*	*0.040001909*	*0.032413047*	*0.035626177*
**ENSG00000161647**	*0.015240008*	*0.024902271*	*0.024529949*	*0.014909*
**ENSG00000157064**	*0.016324458*	*0.025574693*	*0.024898344*	*0.015451095*
**ENSG00000110243**	*0.014868848*	*0.025481797*	*0.024947621*	*0.015821409*
**ENSG00000004846**	*0.015973079*	*0.025476493*	*0.024327915*	*0.016331585*
**ENSG00000128917**	*0.016883581*	*0.027984397*	*0.025619621*	*0.017755732*
**ENSG00000171402**	*0.016593462*	*0.025448275*	*0.026399808*	*0.01597561*
**ENSG00000142156**	*0.022039232*	*0.025852719*	*0.024526644*	*0.017891437*
**ENSG00000196497**	*0.035405901*	*0.037962488*	*0.037004313*	*0.034536217*
**ENSG00000196689**	*0.016245322*	*0.025356576*	*0.025418644*	*0.016623593*
**ENSG00000111716**	*0.017203906*	*0.025293042*	*0.024947901*	*0.01823831*
**ENSG00000164308**	*0.026759334*	*0.029479953*	*0.032388893*	*0.022556412*
**ENSG00000156500**	*0.032486393*	*0.036763729*	*0.031548835*	*0.028599088*
**ENSG00000080546**	*0.046177283*	*0.048901748*	*0.041177227*	*0.04082755*
**ENSG00000166750**	*0.061432403*	*0.061764843*	*0.069621554*	*0.056367793*
**ENSG00000064195**	*0.01709525*	*0.026612274*	*0.025149017*	*0.016749002*
**ENSG00000113575**	*0.019305951*	*0.027487025*	*0.028282035*	*0.019495793*
**ENSG00000198573**	*0.037817578*	*0.025059499*	*0.025443914*	*0.015155805*
**ENSG00000145391**	*0.016992931*	*0.024727359*	*0.025960782*	*0.016174988*
**ENSG00000174016**	*0.016263252*	*0.026462277*	*0.026696101*	*0.01715988*
**ENSG00000164048**	*0.037396706*	*0.037140753*	*0.042040432*	*0.035945467*
**ENSG00000173401**	*0.025558818*	*0.027333112*	*0.026949501*	*0.01636651*
**ENSG00000164022**	*0.029315889*	*0.033086493*	*0.030816501*	*0.022081401*
**ENSG00000155252**	*0.028739636*	*0.030331472*	*0.032142985*	*0.026922348*
**ENSG00000130528**	*0.015662307*	*0.024669079*	*0.025037317*	*0.015125267*
**ENSG00000153250**	*0.074228743*	*0.064726818*	*0.054169482*	*0.076175551*
**ENSG00000133256**	*0.078852576*	*0.054630293*	*0.049880735*	*0.059910339*
**ENSG00000198788**	*0.018545183*	*0.025694809*	*0.027791957*	*0.016284942*
**ENSG00000126878**	*0.018062306*	*0.024945262*	*0.02546386*	*0.066756836*
**ENSG00000142230**	*0.026019496*	*0.030136205*	*0.028465718*	*0.022257317*

**ENSG00000198874**	*0.02731784*	*0.032470337*	*0.028173224*	*0.02402898*
**ENSG00000025796**	*0.027212831*	*0.030968654*	*0.029956743*	*0.027157358*
**ENSG00000157734**	*0.032346982*	*0.034387312*	*0.030142238*	*0.026669188*
**ENSG00000143740**	*0.030253317*	*0.035268924*	*0.03256237*	*0.024738755*
**ENSG00000057757**	*0.024304975*	*0.029974266*	*0.031907283*	*0.019953667*
**ENSG00000011083**	*0.015530767*	*0.02471772*	*0.024954183*	*0.014468907*
**ENSG00000101082**	*0.0183458*	*0.031481052*	*0.030503093*	*0.023131193*
**ENSG00000164363**	*0.016455411*	*0.025108895*	*0.025588339*	*0.01502335*
**ENSG00000131759**	*0.03490069*	*0.037826353*	*0.034882353*	*0.038897688*
**ENSG00000100749**	*0.035212803*	*0.036049333*	*0.03389034*	*0.033910365*
**ENSG00000162694**	*0.048647788*	*0.044407959*	*0.046439044*	*0.045218369*
**ENSG00000177731**	*0.025331207*	*0.031362445*	*0.027248946*	*0.023157746*
**ENSG00000140950**	*0.017288716*	*0.025652756*	*0.024648213*	*0.01838469*
**ENSG00000196470**	*0.033587699*	*0.046176923*	*0.039844768*	*0.030170876*
**ENSG00000151572**	*0.014854423*	*0.02512116*	*0.024590666*	*0.013913374*
**ENSG00000184205**	*0.034689954*	*0.034517988*	*0.037679217*	*0.033300871*
**ENSG00000162723**	*0.014606913*	*0.024608443*	*0.025460164*	*0.014754084*
**ENSG00000119782**	*0.030705105*	*0.032774823*	*0.031970939*	*0.030875292*
**ENSG00000081870**	*0.028455566*	*0.030381772*	*0.029394844*	*0.025545873*
**ENSG00000106012**	*0.022377969*	*0.025908964*	*0.027480879*	*0.019874141*
**ENSG00000167711**	*0.016547898*	*0.025546622*	*0.025689547*	*0.015533176*
**ENSG00000165553**	*0.016568576*	*0.025021437*	*0.025202168*	*0.015757587*
**ENSG00000008256**	*0.059854758*	*0.059873459*	*0.054701024*	*0.072478237*
**ENSG00000176619**	*0.029796066*	*0.031126059*	*0.033118532*	*0.026023782*
**ENSG00000112695**	*0.022029572*	*0.026896079*	*0.028085979*	*0.017077134*
**ENSG00000074201**	*0.024409872*	*0.030072918*	*0.028433146*	*0.018368375*
**ENSG00000188033**	*0.030048989*	*0.037548144*	*0.030594689*	*0.027349952*
**ENSG00000110628**	*0.018863004*	*0.027447048*	*0.026358762*	*0.020446277*
**ENSG00000131808**	*0.015454945*	*0.024418657*	*0.024824884*	*0.014758124*
**ENSG00000135838**	*0.070828967*	*0.056376065*	*0.039073284*	*0.055807907*
**ENSG00000115816**	*0.024843096*	*0.02943628*	*0.029932677*	*0.023699106*
**ENSG00000123329**	*0.036078886*	*0.036981622*	*0.035691578*	*0.035316173*
**ENSG00000170365**	*0.025609283*	*0.031508543*	*0.028033397*	*0.026772947*
**ENSG00000134612**	*0.015467029*	*0.024551421*	*0.024849249*	*0.014895329*
**ENSG00000163930**	*0.019938505*	*0.029175825*	*0.032170272*	*0.025257861*
**ENSG00000130939**	*0.029267572*	*0.034253013*	*0.029009899*	*0.023735468*
**ENSG00000155959**	*0.022890263*	*0.029514429*	*0.027955915*	*0.025708058*
**ENSG00000134900**	*0.035061661*	*0.036299426*	*0.032665179*	*0.029470127*
**ENSG00000134030**	*0.02077169*	*0.030220315*	*0.029671*	*0.023312854*
**ENSG00000014138**	*0.04070312*	*0.038498476*	*0.033854104*	*0.029337586*
**ENSG00000146592**	*0.01569976*	*0.025118203*	*0.024466722*	*0.016340657*
**ENSG00000084733**	*0.030738182*	*0.035521832*	*0.029663289*	*0.023534738*
**ENSG00000104805**	*0.028432292*	*0.029466526*	*0.034014853*	*0.028033666*
**ENSG00000163825**	*0.016808824*	*0.025183589*	*0.025045554*	*0.01567675*
**ENSG00000147383**	*0.024495171*	*0.030078769*	*0.03040281*	*0.022998429*
**ENSG00000047597**	*0.022659413*	*0.027591479*	*0.02561074*	*0.016142759*
**ENSG00000163389**	*0.038849883*	*0.038566389*	*0.039640178*	*0.03408696*
**ENSG00000101222**	*0.016827807*	*0.025742984*	*0.024249969*	*0.015729533*
**ENSG00000020256**	*0.039819384*	*0.042602448*	*0.034754826*	*0.030190584*
**ENSG00000183878**	*0.152333082*	*0.169655655*	*0.14721303*	*0.146050598*
**ENSG00000132563**	*0.053843314*	*0.046906459*	*0.045729036*	*0.051378656*
**ENSG00000181274**	*0.043506134*	*0.037612452*	*0.037496418*	*0.035941408*
**ENSG00000144681**	*0.016416828*	*0.02602207*	*0.026178072*	*0.016181822*
**ENSG00000154719**	*0.027340576*	*0.031719883*	*0.028806352*	*0.022398815*

**ENSG00000151164**	*0.018065245*	*0.027145268*	*0.025188055*	*0.015263671*
**ENSG00000184033**	*0.015564992*	*0.025649274*	*0.026295092*	*0.015017344*
**ENSG00000153832**	*0.025359664*	*0.026885426*	*0.027136685*	*0.018992105*
**ENSG00000136110**	*0.022593183*	*0.037233048*	*0.024854835*	*0.032686828*
**ENSG00000163867**	*0.018008295*	*0.025621548*	*0.0242867*	*0.015047185*
**ENSG00000144730**	*0.01681815*	*0.02502639*	*0.024558803*	*0.014813048*
**ENSG00000186907**	*0.015164201*	*0.024482934*	*0.024427334*	*0.015191406*
**ENSG00000005448**	*0.034028134*	*0.032122306*	*0.032196539*	*0.026944317*
**ENSG00000136146**	*0.024079032*	*0.028915625*	*0.030155351*	*0.022377254*
**ENSG00000105607**	*0.03903511*	*0.035142079*	*0.03171247*	*0.032217617*
**ENSG00000213859**	*0.01580686*	*0.026370952*	*0.025008764*	*0.016393006*
**ENSG00000170248**	*0.022975268*	*0.03132462*	*0.028239928*	*0.021429477*
**ENSG00000196119**	*0.017169227*	*0.025038893*	*0.024370679*	*0.016189261*
**ENSG00000175283**	*0.035798452*	*0.047954245*	*0.049598433*	*0.040371102*
**ENSG00000137547**	*0.023345322*	*0.029024381*	*0.027894996*	*0.023418506*
**ENSG00000101441**	*0.01581247*	*0.024815825*	*0.024347469*	*0.015365458*
**ENSG00000180176**	*0.019781656*	*0.025157568*	*0.02582605*	*0.017058398*
**ENSG00000023608**	*0.048536908*	*0.043842947*	*0.044297917*	*0.055549245*
**ENSG00000105974**	*0.034732554*	*0.029536427*	*0.03314522*	*0.023601152*
**ENSG00000185885**	*0.041578515*	*0.035806849*	*0.038088298*	*0.035634934*
**ENSG00000101825**	*0.0169422*	*0.025240757*	*0.024392638*	*0.015334524*
**ENSG00000161326**	*0.045820996*	*0.052481568*	*0.044038978*	*0.046719747*
**ENSG00000221963**	*0.031037478*	*0.034415667*	*0.037918068*	*0.026747348*
**ENSG00000132906**	*0.031755271*	*0.036892256*	*0.040833892*	*0.027419959*
**ENSG00000160075**	*0.025672732*	*0.031143295*	*0.029889171*	*0.022203556*
**ENSG00000149823**	*0.020743983*	*0.027423491*	*0.030184207*	*0.020664605*
**ENSG00000134827**	*0.016346545*	*0.024652814*	*0.026063536*	*0.018131124*
**ENSG00000136404**	*0.064910868*	*0.063448615*	*0.050425595*	*0.073600837*
**ENSG00000163605**	*0.029761445*	*0.033236861*	*0.034754935*	*0.031474249*
**ENSG00000163785**	*0.038244198*	*0.043542434*	*0.035311736*	*0.036019613*
**ENSG00000240038**	*0.031370225*	*0.033841167*	*0.032493197*	*0.027908801*
**ENSG00000125878**	*0.023953924*	*0.030863742*	*0.028670873*	*0.021804004*
**ENSG00000154447**	*0.050295045*	*0.040810369*	*0.036939403*	*0.042477507*
**ENSG00000164175**	*0.016979794*	*0.025638932*	*0.024234627*	*0.018313654*
**ENSG00000163518**	*0.094026254*	*0.089927901*	*0.066465315*	*0.090425331*
**ENSG00000133937**	*0.014714111*	*0.025083432*	*0.024743626*	*0.01492261*
**ENSG00000204475**	*0.07176846*	*0.075615461*	*0.093164193*	*0.072276041*
**ENSG00000181264**	*0.04470009*	*0.038496063*	*0.033094703*	*0.037001995*
**ENSG00000145920**	*0.016634152*	*0.024847354*	*0.025184609*	*0.015372365*
**ENSG00000105141**	*0.014949974*	*0.024714983*	*0.025510843*	*0.014356657*
**ENSG00000149260**	*0.017761342*	*0.026000358*	*0.026402797*	*0.016098759*
**ENSG00000058729**	*0.028633515*	*0.033685302*	*0.035405751*	*0.02393019*
**ENSG00000144485**	*0.018252497*	*0.025507982*	*0.025089075*	*0.015266513*
**ENSG00000141140**	*0.035817144*	*0.041537871*	*0.033033236*	*0.026265198*
**ENSG00000166220**	*0.023115562*	*0.026685021*	*0.030444496*	*0.018322901*
**ENSG00000139574**	*0.016932401*	*0.026581747*	*0.025344334*	*0.015732585*
**ENSG00000196378**	*0.026817251*	*0.030568474*	*0.036509687*	*0.02794329*
**ENSG00000175344**	*0.017026525*	*0.026370944*	*0.025951064*	*0.017027328*
**ENSG00000100629**	*0.059998794*	*0.059219468*	*0.04842248*	*0.042635193*
**ENSG00000123364**	*0.015557403*	*0.02525114*	*0.024917023*	*0.01701932*
**ENSG00000187581**	*0.01952695*	*0.030404387*	*0.025888551*	*0.020814837*
**ENSG00000078674**	*0.023689951*	*0.032882546*	*0.029180156*	*0.024225477*
**ENSG00000168172**	*0.029699809*	*0.039946378*	*0.03277661*	*0.027437962*
**ENSG00000135390**	*0.019579278*	*0.025105801*	*0.02610856*	*0.01673815*

**ENSG00000184995**	*0.017242453*	*0.026038138*	*0.025426658*	*0.01744632*
**ENSG00000182950**	*0.01682306*	*0.025049116*	*0.025308563*	*0.014583336*
**ENSG00000095539**	*0.0165681*	*0.024943784*	*0.024569319*	*0.016520254*
**ENSG00000129473**	*0.037459622*	*0.034139434*	*0.028443728*	*0.025320617*
**ENSG00000154429**	*0.03045335*	*0.027582236*	*0.030106396*	*0.024336263*
**ENSG00000077458**	*0.040065364*	*0.04369602*	*0.038223442*	*0.037087199*
**ENSG00000166889**	*0.026159079*	*0.031215259*	*0.031317924*	*0.022616691*
**ENSG00000105755**	*0.0382973*	*0.035005195*	*0.040764041*	*0.039005075*
**ENSG00000187866**	*0.028420405*	*0.033782241*	*0.03158013*	*0.020824619*
**ENSG00000198838**	*0.026870359*	*0.026090325*	*0.026591245*	*0.01787237*
**ENSG00000188641**	*0.112522687*	*0.075730975*	*0.069922883*	*0.068397809*
**ENSG00000117360**	*0.025554967*	*0.030150877*	*0.029064498*	*0.023660674*
**ENSG00000150782**	*0.032154818*	*0.032222821*	*0.032269524*	*0.033131106*
**ENSG00000120251**	*0.018114213*	*0.026201827*	*0.026073861*	*0.017261563*
**ENSG00000168818**	*0.024616773*	*0.033914673*	*0.033544206*	*0.024626757*
**ENSG00000155393**	*0.033842972*	*0.036599594*	*0.034013614*	*0.026718583*
**ENSG00000100344**	*0.015531428*	*0.024771979*	*0.024868137*	*0.014461612*
**ENSG00000185274**	*0.015812052*	*0.025310028*	*0.025714507*	*0.016364155*
**ENSG00000185112**	*0.078368669*	*0.054536849*	*0.063211335*	*0.059845358*
**ENSG00000170925**	*0.016561652*	*0.025550703*	*0.024842748*	*0.015962255*
**ENSG00000165887**	*0.026143263*	*0.028590772*	*0.026102615*	*0.024859438*
**ENSG00000121064**	*0.033467076*	*0.038146905*	*0.035923992*	*0.036405501*
**ENSG00000197147**	*0.026312789*	*0.029393668*	*0.027506267*	*0.022242994*
**ENSG00000163646**	*0.015835548*	*0.025290483*	*0.024497427*	*0.015730871*
**ENSG00000178522**	*0.016139764*	*0.02520175*	*0.024806178*	*0.014885817*
**ENSG00000186106**	*0.041726439*	*0.041213184*	*0.035889856*	*0.029121846*
**ENSG00000109536**	*0.023475197*	*0.029049424*	*0.027388294*	*0.027730767*
**ENSG00000139985**	*0.015865115*	*0.025346223*	*0.024793801*	*0.015696741*
**ENSG00000138316**	*0.016122271*	*0.027298505*	*0.025874459*	*0.01601599*
**ENSG00000078967**	*0.036666561*	*0.040081917*	*0.03133514*	*0.032216514*
**ENSG00000171014**	*0.016781617*	*0.025096055*	*0.02494487*	*0.016194552*
**ENSG00000169871**	*0.030298864*	*0.034251792*	*0.02979683*	*0.028000905*
**ENSG00000198198**	*0.018811167*	*0.02685486*	*0.027448214*	*0.023293443*
**ENSG00000105198**	*0.019223972*	*0.025009853*	*0.025342492*	*0.016004387*
**ENSG00000132676**	*0.020966*	*0.026502109*	*0.026622752*	*0.01734102*
**ENSG00000119729**	*0.048787375*	*0.043252222*	*0.046934511*	*0.042553785*
**ENSG00000108819**	*0.01714085*	*0.026739968*	*0.024938327*	*0.01793339*
**ENSG00000137561**	*0.015687404*	*0.024939831*	*0.024543655*	*0.015299292*
**ENSG00000129625**	*0.037155104*	*0.042494663*	*0.036066011*	*0.028745843*
**ENSG00000135312**	*0.017729466*	*0.025772223*	*0.027274998*	*0.015766549*
**ENSG00000183888**	*0.017536259*	*0.027221039*	*0.025002164*	*0.020490594*
**ENSG00000121766**	*0.028902553*	*0.035545411*	*0.032776788*	*0.02147593*
**ENSG00000135913**	*0.033730885*	*0.04700652*	*0.036653142*	*0.035862051*
**ENSG00000069696**	*0.020819441*	*0.027225986*	*0.027112576*	*0.019518774*
**ENSG00000130643**	*0.014565541*	*0.025016205*	*0.02489278*	*0.014731794*
**ENSG00000113621**	*0.031979162*	*0.034584369*	*0.035446886*	*0.033145382*
**ENSG00000110852**	*0.049199621*	*0.062177342*	*0.055939711*	*0.066867613*
**ENSG00000145423**	*0.014597296*	*0.024971281*	*0.024629392*	*0.014752397*
**ENSG00000182256**	*0.016441024*	*0.025730353*	*0.025083917*	*0.017213004*
**ENSG00000123349**	*0.016790108*	*0.025208886*	*0.02602729*	*0.018206311*
**ENSG00000137312**	*0.026023208*	*0.031936469*	*0.03611066*	*0.027397611*
**ENSG00000117758**	*0.03167775*	*0.037205558*	*0.035497004*	*0.02920175*
**ENSG00000111361**	*0.024234492*	*0.029804858*	*0.030670704*	*0.02130459*
**ENSG00000127616**	*0.033282411*	*0.035228641*	*0.033586325*	*0.028441752*

**ENSG00000070808**	*0.015822132*	*0.025478183*	*0.025608114*	*0.014937551*
**ENSG00000119041**	*0.029718195*	*0.033742216*	*0.030950355*	*0.028375339*
**ENSG00000150275**	*0.017012112*	*0.025092788*	*0.025100391*	*0.01634324*
**ENSG00000151445**	*0.02888455*	*0.034669472*	*0.030400665*	*0.025227085*
**ENSG00000169826**	*0.048359555*	*0.049085819*	*0.041648094*	*0.041556564*
**ENSG00000147003**	*0.019339797*	*0.028079385*	*0.026812923*	*0.019931975*
**ENSG00000124171**	*0.016641396*	*0.025248044*	*0.02542389*	*0.017118988*
**ENSG00000170892**	*0.024900062*	*0.030293921*	*0.030974583*	*0.027163068*
**ENSG00000155265**	*0.017578427*	*0.026269468*	*0.026405169*	*0.016594337*
**ENSG00000166183**	*0.015966982*	*0.026460607*	*0.024451412*	*0.014784015*
**ENSG00000162951**	*0.017189524*	*0.024952771*	*0.024804278*	*0.016331479*
**ENSG00000180929**	*0.016338833*	*0.025234985*	*0.024827981*	*0.014540865*
**ENSG00000130766**	*0.032199332*	*0.03282531*	*0.034225977*	*0.032076905*
**ENSG00000174384**	*0.015736659*	*0.025136597*	*0.02540097*	*0.015005792*
**ENSG00000161958**	*0.019727276*	*0.031061083*	*0.026603527*	*0.018892517*
**ENSG00000133069**	*0.015682983*	*0.025692535*	*0.024768683*	*0.015800585*
**ENSG00000166415**	*0.016404228*	*0.025616544*	*0.0251674*	*0.01616252*
**ENSG00000162643**	*0.054988105*	*0.04227094*	*0.038703021*	*0.040042606*
**ENSG00000205758**	*0.026339126*	*0.03522896*	*0.032059483*	*0.023281544*
**ENSG00000161714**	*0.018446372*	*0.025720206*	*0.025606281*	*0.01706755*
**ENSG00000156873**	*0.020856908*	*0.02956581*	*0.02984699*	*0.020824285*
**ENSG00000147145**	*0.016436679*	*0.027172337*	*0.026526232*	*0.016911975*
**ENSG00000162062**	*0.050172673*	*0.049829597*	*0.03753342*	*0.037001291*
**ENSG00000141562**	*0.038224491*	*0.035997768*	*0.035804393*	*0.038863865*
**ENSG00000206422**	*0.01498921*	*0.025230554*	*0.024135574*	*0.015354163*
**ENSG00000204370**	*0.02159454*	*0.02823717*	*0.026862914*	*0.021979927*
**ENSG00000142864**	*0.01927952*	*0.025934157*	*0.027848308*	*0.01674218*
**ENSG00000047936**	*0.015928909*	*0.024928064*	*0.025212809*	*0.016215856*
**ENSG00000108309**	*0.017485769*	*0.024645277*	*0.02461709*	*0.015697959*
**ENSG00000131374**	*0.032665541*	*0.041701631*	*0.034503773*	*0.02946621*
**ENSG00000103056**	*0.019449661*	*0.026502278*	*0.025946735*	*0.018616718*
**ENSG00000129315**	*0.015337154*	*0.025643163*	*0.02803896*	*0.016536094*
**ENSG00000124508**	*0.019544101*	*0.028487787*	*0.028463758*	*0.022066595*
**ENSG00000154814**	*0.040073031*	*0.038263357*	*0.040598904*	*0.045599747*
**ENSG00000081818**	*0.015649065*	*0.024656795*	*0.02518582*	*0.014811057*
**ENSG00000213886**	*0.138559471*	*0.106363508*	*0.107347078*	*0.120090777*
**ENSG00000135636**	*0.015711608*	*0.025017758*	*0.02381977*	*0.014357504*
**ENSG00000223501**	*0.02799454*	*0.033569376*	*0.032230677*	*0.024086966*
**ENSG00000152133**	*0.035693309*	*0.033061752*	*0.033426153*	*0.033473795*
**ENSG00000146063**	*0.023036443*	*0.02872466*	*0.03598414*	*0.020034212*
**ENSG00000075275**	*0.017267009*	*0.025406782*	*0.025168235*	*0.014534106*
**ENSG00000142623**	*0.017416913*	*0.025389438*	*0.025799008*	*0.016478435*
**ENSG00000101624**	*0.034409182*	*0.03800752*	*0.033380973*	*0.032538558*
**ENSG00000168291**	*0.026435849*	*0.030029601*	*0.029364976*	*0.021842827*
**ENSG00000164897**	*0.024264635*	*0.027072309*	*0.037421627*	*0.024458858*
**ENSG00000128849**	*0.079206769*	*0.040130417*	*0.033105543*	*0.045402736*
**ENSG00000097046**	*0.048455962*	*0.038354694*	*0.036891241*	*0.031827436*
**ENSG00000179862**	*0.072592755*	*0.05766785*	*0.058593692*	*0.049782101*
**ENSG00000236981**	*0.016221744*	*0.026595373*	*0.024733532*	*0.016843367*
**ENSG00000164129**	*0.015251543*	*0.024222579*	*0.024705263*	*0.013862638*
**ENSG00000188064**	*0.018442841*	*0.026731025*	*0.027013659*	*0.018004537*
**ENSG00000143751**	*0.020104251*	*0.032687911*	*0.039991017*	*0.020991709*
**ENSG00000103426**	*0.0264302*	*0.033846229*	*0.046564762*	*0.035414818*
**ENSG00000153294**	*0.017706262*	*0.026128766*	*0.024682776*	*0.016900803*

**ENSG00000183709**	*0.018955845*	*0.026495223*	*0.026606767*	*0.018521862*
**ENSG00000171495**	*0.016463702*	*0.025624065*	*0.024594068*	*0.014613516*
**ENSG00000178021**	*0.016216827*	*0.025831088*	*0.025629512*	*0.017180784*
**ENSG00000083750**	*0.036257828*	*0.036740286*	*0.039486911*	*0.029033504*
**ENSG00000171314**	*0.03000313*	*0.032226811*	*0.030985829*	*0.023980364*
**ENSG00000066557**	*0.030854858*	*0.038518229*	*0.030239522*	*0.027910506*
**ENSG00000206190**	*0.02562544*	*0.028490671*	*0.027296213*	*0.023172721*
**ENSG00000140090**	*0.016333402*	*0.026098019*	*0.025158413*	*0.016807209*
**ENSG00000182645**	*0.017164444*	*0.025577894*	*0.025196334*	*0.015123616*
**ENSG00000185483**	*0.061076418*	*0.045229158*	*0.031732694*	*0.038574321*
**ENSG00000126218**	*0.015340014*	*0.024781093*	*0.024307062*	*0.014992008*
**ENSG00000172543**	*0.024275316*	*0.036614701*	*0.029925406*	*0.046822127*
**ENSG00000169857**	*0.038472331*	*0.042380008*	*0.035359231*	*0.034482547*
**ENSG00000165583**	*0.019479756*	*0.0275022*	*0.028797743*	*0.022624567*
**ENSG00000164099**	*0.018534291*	*0.02763224*	*0.026837349*	*0.01711706*
**ENSG00000156535**	*0.045543904*	*0.038234776*	*0.034483381*	*0.037381447*
**ENSG00000170485**	*0.015871004*	*0.024889866*	*0.024641104*	*0.015452352*
**ENSG00000182795**	*0.01960718*	*0.027051257*	*0.025378609*	*0.01904612*
**ENSG00000158470**	*0.036776093*	*0.042945108*	*0.040973234*	*0.036019633*
**ENSG00000065057**	*0.043986287*	*0.035912979*	*0.03626937*	*0.032877973*
**ENSG00000189042**	*0.02783242*	*0.037041398*	*0.03451024*	*0.028933348*
**ENSG00000171861**	*0.028260204*	*0.031214628*	*0.034075545*	*0.023471985*
**ENSG00000108528**	*0.035956825*	*0.034501476*	*0.029900767*	*0.03204883*
**ENSG00000018625**	*0.015340776*	*0.024552661*	*0.024234434*	*0.01373202*
**ENSG00000106588**	*0.019808761*	*0.027674986*	*0.026379399*	*0.016595758*
**ENSG00000113552**	*0.049627059*	*0.042098101*	*0.034404382*	*0.036698515*
**ENSG00000135842**	*0.044401176*	*0.045814992*	*0.044844366*	*0.052704982*
**ENSG00000099968**	*0.028114603*	*0.034517039*	*0.032369853*	*0.02354787*
**ENSG00000115541**	*0.022947394*	*0.029366688*	*0.026890989*	*0.019581239*
**ENSG00000146233**	*0.018954735*	*0.027176675*	*0.026783719*	*0.01695007*
**ENSG00000139117**	*0.057397837*	*0.07977396*	*0.051935083*	*0.058617763*
**ENSG00000071537**	*0.035376059*	*0.037933763*	*0.036937404*	*0.032474182*
**ENSG00000121073**	*0.031950668*	*0.033884267*	*0.035887296*	*0.035186561*
**ENSG00000188042**	*0.039299515*	*0.067158226*	*0.053780121*	*0.057489098*
**ENSG00000154945**	*0.03228328*	*0.031738062*	*0.036975568*	*0.030671359*
**ENSG00000179774**	*0.015078479*	*0.024516325*	*0.024732871*	*0.014947563*
**ENSG00000137571**	*0.058560877*	*0.051937282*	*0.056296052*	*0.048523464*
**ENSG00000172716**	*0.054890701*	*0.040849942*	*0.042051116*	*0.038003529*
**ENSG00000179593**	*0.046344683*	*0.047787719*	*0.038464286*	*0.038788953*
**ENSG00000112706**	*0.01570291*	*0.025226862*	*0.024999916*	*0.014578456*
**ENSG00000205583**	*0.01713319*	*0.026356993*	*0.026402687*	*0.016616463*
**ENSG00000187475**	*0.015718338*	*0.024537147*	*0.024672833*	*0.01562946*
**ENSG00000144115**	*0.063472342*	*0.054156815*	*0.054776363*	*0.067372792*
**ENSG00000126246**	*0.056251672*	*0.041271376*	*0.036205044*	*0.038745795*
**ENSG00000197217**	*0.03395798*	*0.032873842*	*0.037114836*	*0.033159691*
**ENSG00000250486**	*0.038673811*	*0.035908416*	*0.028927961*	*0.029749686*
**ENSG00000171612**	*0.038798341*	*0.042561326*	*0.045092591*	*0.037436108*
**ENSG00000169221**	*0.020093565*	*0.028954252*	*0.0338022*	*0.023035282*
**ENSG00000185745**	*0.09477877*	*0.086805875*	*0.078430716*	*0.076877184*
**ENSG00000119401**	*0.03437881*	*0.035786595*	*0.030745953*	*0.023131689*
**ENSG00000138698**	*0.045575628*	*0.045110295*	*0.03636613*	*0.038542929*
**ENSG00000157353**	*0.025008468*	*0.03284269*	*0.028818393*	*0.025311806*
**ENSG00000196705**	*0.015876106*	*0.024795673*	*0.025118942*	*0.016226184*
**ENSG00000125534**	*0.024824706*	*0.031647352*	*0.033189332*	*0.02662508*

**ENSG00000125046**	*0.018316014*	*0.027482947*	*0.027708075*	*0.018585764*
**ENSG00000107554**	*0.047030055*	*0.039530021*	*0.032926355*	*0.040404717*
**ENSG00000184825**	*0.041259858*	*0.038017064*	*0.048320136*	*0.035405751*
**ENSG00000117408**	*0.035166145*	*0.039219118*	*0.028545304*	*0.028354352*
**ENSG00000001497**	*0.02816482*	*0.030345058*	*0.030783267*	*0.025846973*
**ENSG00000168016**	*0.053944962*	*0.060751256*	*0.045616412*	*0.04907559*
**ENSG00000173535**	*0.017024535*	*0.024279436*	*0.025474597*	*0.014995127*
**ENSG00000188394**	*0.014203004*	*0.024885201*	*0.025170606*	*0.016485718*
**ENSG00000163322**	*0.044841893*	*0.047901598*	*0.036773503*	*0.032042309*
**ENSG00000117410**	*0.025617301*	*0.031847697*	*0.032651826*	*0.025673368*
**ENSG00000089050**	*0.039607277*	*0.044231188*	*0.036257795*	*0.039949728*
**ENSG00000158525**	*0.025355954*	*0.026669431*	*0.023768243*	*0.018470462*
**ENSG00000159147**	*0.044140843*	*0.05625176*	*0.038626767*	*0.053772284*
**ENSG00000069482**	*0.014741058*	*0.025833643*	*0.024985687*	*0.015450333*
**ENSG00000105655**	*0.064056468*	*0.042643781*	*0.049924723*	*0.063355265*
**ENSG00000188738**	*0.028347687*	*0.032655146*	*0.035932632*	*0.046590269*
**ENSG00000157890**	*0.015133659*	*0.024758976*	*0.02466222*	*0.014761572*
**ENSG00000196344**	*0.020931473*	*0.028679788*	*0.027645782*	*0.020283803*
**ENSG00000163491**	*0.01647119*	*0.024967929*	*0.024931446*	*0.016387059*
**ENSG00000178279**	*0.018283566*	*0.026425773*	*0.024790013*	*0.018907821*
**ENSG00000198663**	*0.025362814*	*0.031040824*	*0.038836943*	*0.025560499*
**ENSG00000145246**	*0.043952506*	*0.059200843*	*0.046637091*	*0.039480909*
**ENSG00000108799**	*0.025342464*	*0.028325259*	*0.028933385*	*0.028365949*
**ENSG00000066185**	*0.028729468*	*0.029904864*	*0.02861243*	*0.025344556*
**ENSG00000134538**	*0.015806815*	*0.025746767*	*0.025087725*	*0.016303845*
**ENSG00000156504**	*0.044406073*	*0.039768303*	*0.036915536*	*0.033221035*
**ENSG00000164070**	*0.042622946*	*0.045089203*	*0.038782344*	*0.039191847*
**ENSG00000156427**	*0.021555656*	*0.027013062*	*0.027560748*	*0.017271018*
**ENSG00000136816**	*0.025341996*	*0.03338525*	*0.033791114*	*0.023198348*
**ENSG00000197057**	*0.055608758*	*0.037969159*	*0.03563588*	*0.04324361*
**ENSG00000183530**	*0.015487692*	*0.024599664*	*0.024299303*	*0.015114427*
**ENSG00000203813**	*0.034842291*	*0.036806577*	*0.036456955*	*0.033798759*
**ENSG00000135346**	*0.018151317*	*0.025755694*	*0.024933796*	*0.017604594*
**ENSG00000147654**	*0.01700745*	*0.025296777*	*0.026212599*	*0.017069936*
**ENSG00000038382**	*0.042580413*	*0.038337185*	*0.039611*	*0.036722472*
**ENSG00000009780**	*0.030837169*	*0.036331359*	*0.031897455*	*0.023376426*
**ENSG00000125910**	*0.049134165*	*0.051841809*	*0.046036534*	*0.045336652*
**ENSG00000139329**	*0.020556044*	*0.024952266*	*0.024980106*	*0.015006731*
**ENSG00000213516**	*0.041981015*	*0.040732216*	*0.048669676*	*0.044687268*
**ENSG00000122565**	*0.025173235*	*0.033287915*	*0.033733973*	*0.027650949*
**ENSG00000160867**	*0.01992963*	*0.030049033*	*0.027242524*	*0.023579272*
**ENSG00000173627**	*0.015632862*	*0.02531402*	*0.02478185*	*0.016764626*
**ENSG00000163159**	*0.028782557*	*0.035718235*	*0.031352174*	*0.023397715*
**ENSG00000073910**	*0.052722772*	*0.05238155*	*0.051747073*	*0.058129887*
**ENSG00000074621**	*0.049422755*	*0.043788238*	*0.033471603*	*0.033360123*
**ENSG00000161981**	*0.036136146*	*0.030795725*	*0.031121244*	*0.033210976*
**ENSG00000186517**	*0.024685642*	*0.030664526*	*0.03032508*	*0.027448503*
**ENSG00000101938**	*0.016581908*	*0.024501431*	*0.024903513*	*0.016038775*
**ENSG00000182389**	*0.072405562*	*0.070299515*	*0.058590304*	*0.075868929*
**ENSG00000089057**	*0.016470788*	*0.02663257*	*0.024287392*	*0.015585317*
**ENSG00000104889**	*0.03988137*	*0.036764438*	*0.032232988*	*0.029311682*
**ENSG00000164893**	*0.017123532*	*0.02445427*	*0.024843805*	*0.016380707*
**ENSG00000113742**	*0.056444741*	*0.046081624*	*0.048709036*	*0.032679318*
**ENSG00000125845**	*0.0816796*	*0.056115992*	*0.059624315*	*0.061935275*

**ENSG00000004975**	*0.025357657*	*0.027640789*	*0.029071214*	*0.024259635*
**ENSG00000124875**	*0.016780488*	*0.025055069*	*0.025731439*	*0.015775396*
**ENSG00000171759**	*0.044251278*	*0.036626546*	*0.024903524*	*0.030585362*
**ENSG00000140527**	*0.016886823*	*0.024804326*	*0.026012014*	*0.014939946*
**ENSG00000129270**	*0.016113185*	*0.024738239*	*0.024936125*	*0.015320255*
**ENSG00000121853**	*0.017926589*	*0.027300941*	*0.026538461*	*0.015597065*
**ENSG00000153982**	*0.020794816*	*0.028725486*	*0.02711535*	*0.022815255*
**ENSG00000110944**	*0.060953683*	*0.05749162*	*0.056429935*	*0.051066121*
**ENSG00000182500**	*0.024945367*	*0.030651151*	*0.034429999*	*0.032852664*
**ENSG00000182175**	*0.020535182*	*0.027116783*	*0.02560244*	*0.018630065*
**ENSG00000163382**	*0.042244519*	*0.038976638*	*0.03704989*	*0.040055641*
**ENSG00000103227**	*0.016311892*	*0.026153666*	*0.024735096*	*0.015466752*
**ENSG00000133028**	*0.028877789*	*0.03208138*	*0.032769141*	*0.026547846*
**ENSG00000033100**	*0.036243666*	*0.047077979*	*0.049139749*	*0.033019039*
**ENSG00000178690**	*0.018604371*	*0.026087019*	*0.026924235*	*0.018269066*
**ENSG00000134108**	*0.025974605*	*0.03123618*	*0.033522843*	*0.028625135*
**ENSG00000185883**	*0.020552714*	*0.027403136*	*0.02943716*	*0.019813113*
**ENSG00000113119**	*0.038363957*	*0.039508683*	*0.032594994*	*0.031124058*
**ENSG00000126790**	*0.045714978*	*0.040642678*	*0.038771977*	*0.043615137*
**ENSG00000165633**	*0.016605539*	*0.025408931*	*0.025882459*	*0.016727108*
**ENSG00000196449**	*0.032437809*	*0.038762862*	*0.04784175*	*0.037086506*
**ENSG00000092148**	*0.024829147*	*0.032503701*	*0.029527309*	*0.023079367*
**ENSG00000214882**	*0.058792379*	*0.044523024*	*0.050273798*	*0.045954722*
**ENSG00000167664**	*0.016863723*	*0.025160284*	*0.026470331*	*0.015744877*
**ENSG00000169618**	*0.015098154*	*0.024547994*	*0.024990489*	*0.015342042*
**ENSG00000189403**	*0.034539709*	*0.034417949*	*0.031308889*	*0.030421897*
**ENSG00000188315**	*0.044127254*	*0.045964264*	*0.044515177*	*0.037039703*
**ENSG00000160695**	*0.021613844*	*0.028931433*	*0.027652048*	*0.022666328*
**ENSG00000172137**	*0.017321809*	*0.026144166*	*0.024951919*	*0.015702374*
**ENSG00000119636**	*0.02819813*	*0.03013041*	*0.026223719*	*0.03632955*
**ENSG00000138381**	*0.025247152*	*0.032267449*	*0.033797759*	*0.027538973*
**ENSG00000010282**	*0.01749758*	*0.02625967*	*0.024334562*	*0.015247577*
**ENSG00000214160**	*0.033312878*	*0.034342671*	*0.030074224*	*0.026422356*
**ENSG00000184560**	*0.015332901*	*0.024550567*	*0.025753516*	*0.015562458*
**ENSG00000185404**	*0.036566698*	*0.036848382*	*0.035782807*	*0.036158984*
**ENSG00000205916**	*0.017783748*	*0.027525462*	*0.025961473*	*0.017806041*
**ENSG00000138297**	*0.02538603*	*0.031733558*	*0.03494512*	*0.030907788*
**ENSG00000130956**	*0.058394882*	*0.04016029*	*0.040153546*	*0.048163691*
**ENSG00000111859**	*0.0473238*	*0.053243714*	*0.052240052*	*0.04603981*
**ENSG00000116703**	*0.015619793*	*0.024724894*	*0.024742822*	*0.015043239*
**ENSG00000235568**	*0.01625181*	*0.025342259*	*0.025173162*	*0.015797968*
**ENSG00000166448**	*0.018241996*	*0.025747033*	*0.026125593*	*0.01663767*
**ENSG00000099381**	*0.019967173*	*0.02785935*	*0.031061775*	*0.02385093*
**ENSG00000127423**	*0.047304788*	*0.041630347*	*0.043199385*	*0.042937306*
**ENSG00000109920**	*0.023135641*	*0.028793161*	*0.028394829*	*0.023893002*
**ENSG00000116396**	*0.030378036*	*0.02960433*	*0.029030689*	*0.024574457*
**ENSG00000186141**	*0.026081486*	*0.030624713*	*0.0322968*	*0.02112306*
**ENSG00000160396**	*0.016319169*	*0.025213679*	*0.024302132*	*0.015448634*
**ENSG00000106236**	*0.018829722*	*0.025008362*	*0.025379836*	*0.014796183*
**ENSG00000171155**	*0.036286854*	*0.038387248*	*0.033468865*	*0.028055012*
**ENSG00000140939**	*0.03580048*	*0.038019583*	*0.036202991*	*0.036053479*
**ENSG00000074964**	*0.01970928*	*0.02979497*	*0.029324795*	*0.020338681*
**ENSG00000130347**	*0.041372048*	*0.04235074*	*0.035577206*	*0.032821163*
**ENSG00000111640**	*0.019930527*	*0.024688071*	*0.026032629*	*0.018431893*

**ENSG00000158604**	*0.03187917*	*0.043707915*	*0.042553405*	*0.035237758*
**ENSG00000174099**	*0.027685258*	*0.030767529*	*0.029733202*	*0.026911963*
**ENSG00000165309**	*0.018919154*	*0.026437963*	*0.027739301*	*0.017968715*
**ENSG00000169926**	*0.023657734*	*0.036073009*	*0.033715414*	*0.021570314*
**ENSG00000236699**	*0.016042437*	*0.02488277*	*0.025396511*	*0.014886473*
**ENSG00000174942**	*0.015955474*	*0.025601877*	*0.026300242*	*0.015312713*
**ENSG00000083520**	*0.018855631*	*0.02602633*	*0.025242301*	*0.016637059*
**ENSG00000147118**	*0.026123841*	*0.032789607*	*0.03093425*	*0.029647082*
**ENSG00000185798**	*0.027656188*	*0.035176743*	*0.037255101*	*0.024930808*
**ENSG00000168589**	*0.028134707*	*0.027646531*	*0.026338313*	*0.024084111*
**ENSG00000123643**	*0.016436601*	*0.025505177*	*0.02441509*	*0.014910152*
**ENSG00000184985**	*0.015318612*	*0.025006548*	*0.025091746*	*0.01566457*
**ENSG00000127314**	*0.022140428*	*0.032289959*	*0.030296684*	*0.024774662*
**ENSG00000158092**	*0.029615379*	*0.034258287*	*0.032922329*	*0.027161265*
**ENSG00000187634**	*0.019489237*	*0.026284373*	*0.029437702*	*0.016007208*
**ENSG00000197826**	*0.029213083*	*0.032308076*	*0.027172101*	*0.023375237*
**ENSG00000164675**	*0.01655661*	*0.025449234*	*0.025015663*	*0.014322023*
**ENSG00000126106**	*0.027288795*	*0.03131333*	*0.031966374*	*0.023989706*
**ENSG00000137497**	*0.021479704*	*0.029165414*	*0.031066533*	*0.023935848*
**ENSG00000204356**	*0.028559252*	*0.030827634*	*0.032021965*	*0.021424966*
**ENSG00000149308**	*0.027410953*	*0.03669774*	*0.031418963*	*0.032335344*
**ENSG00000165632**	*0.01619289*	*0.024494629*	*0.024151677*	*0.016096069*
**ENSG00000133742**	*0.015930394*	*0.024624283*	*0.025091215*	*0.016073746*
**ENSG00000204344**	*0.021691677*	*0.02960645*	*0.036408701*	*0.022079414*
**ENSG00000149792**	*0.02245149*	*0.027941608*	*0.033132145*	*0.021201231*
**ENSG00000169692**	*0.05812852*	*0.040659225*	*0.039383808*	*0.034930727*
**ENSG00000160179**	*0.085951615*	*0.059924653*	*0.062735327*	*0.077381515*
**ENSG00000204262**	*0.045999881*	*0.02811372*	*0.026030741*	*0.029911854*
**ENSG00000072182**	*0.015397916*	*0.025175814*	*0.024926224*	*0.015314146*
**ENSG00000111906**	*0.024772351*	*0.030143144*	*0.028717199*	*0.023945714*
**ENSG00000182040**	*0.020513472*	*0.027744999*	*0.027023217*	*0.026739438*
**ENSG00000088038**	*0.026357716*	*0.029757041*	*0.042208249*	*0.02650259*
**ENSG00000170266**	*0.028454509*	*0.033571552*	*0.032855994*	*0.023962217*
**ENSG00000130287**	*0.022680102*	*0.026201478*	*0.027862676*	*0.017509347*
**ENSG00000105708**	*0.035718905*	*0.034237699*	*0.035988481*	*0.034221536*
**ENSG00000036565**	*0.015834705*	*0.024116341*	*0.024168814*	*0.014149675*
**ENSG00000221858**	*0.014204531*	*0.023865669*	*0.024665915*	*0.015577475*
**ENSG00000143624**	*0.026647313*	*0.030963606*	*0.029642407*	*0.025982986*
**ENSG00000204983**	*0.016696178*	*0.024597596*	*0.025090647*	*0.015449365*
**ENSG00000115946**	*0.043402142*	*0.056078193*	*0.058889829*	*0.053030547*
**ENSG00000067082**	*0.044294808*	*0.044983724*	*0.046419374*	*0.040791056*
**ENSG00000164744**	*0.016469371*	*0.026289343*	*0.026491765*	*0.01646232*
**ENSG00000120868**	*0.047642758*	*0.055203033*	*0.047614659*	*0.038881167*
**ENSG00000168374**	*0.023106167*	*0.031945018*	*0.037095678*	*0.027130617*
**ENSG00000089116**	*0.040742492*	*0.039589787*	*0.036143127*	*0.032367018*
**ENSG00000107317**	*0.01516441*	*0.0250432*	*0.024444102*	*0.015571694*
**ENSG00000147465**	*0.046687335*	*0.042744321*	*0.039254436*	*0.031917052*
**ENSG00000116478**	*0.024155316*	*0.032106861*	*0.029268054*	*0.02068823*
**ENSG00000138160**	*0.045264359*	*0.038925177*	*0.040060092*	*0.038668663*
**ENSG00000163472**	*0.032665023*	*0.039801731*	*0.037890498*	*0.027555156*
**ENSG00000142655**	*0.024313131*	*0.028425215*	*0.036152561*	*0.024110178*
**ENSG00000147262**	*0.017162661*	*0.024753241*	*0.025029855*	*0.015341271*
**ENSG00000179934**	*0.033489365*	*0.032768606*	*0.039566851*	*0.024022478*
**ENSG00000171345**	*0.015527362*	*0.024500491*	*0.024650121*	*0.014746655*

**ENSG00000109610**	*0.017096633*	*0.025438931*	*0.025283313*	*0.016764037*
**ENSG00000180535**	*0.016720172*	*0.024730323*	*0.024807945*	*0.016190137*
**ENSG00000110675**	*0.016708506*	*0.025750151*	*0.024929813*	*0.015548815*
**ENSG00000105583**	*0.019247502*	*0.026937254*	*0.027753805*	*0.018278581*
**ENSG00000158865**	*0.016212764*	*0.025300235*	*0.026166377*	*0.01407249*
**ENSG00000157654**	*0.039247435*	*0.04167764*	*0.037315394*	*0.038304428*
**ENSG00000062485**	*0.021320472*	*0.027363344*	*0.02806065*	*0.02183061*
**ENSG00000114268**	*0.105940787*	*0.052542863*	*0.050908546*	*0.057798044*
**ENSG00000177455**	*0.02840635*	*0.032917413*	*0.034045752*	*0.031760329*
**ENSG00000120942**	*0.038311386*	*0.03962886*	*0.044108706*	*0.042125475*
**ENSG00000106819**	*0.021377087*	*0.024443264*	*0.025991267*	*0.021417371*
**ENSG00000233838**	*0.022411446*	*0.028425114*	*0.026690963*	*0.019414368*
**ENSG00000012232**	*0.024491648*	*0.031825829*	*0.032465739*	*0.025706873*
**ENSG00000057468**	*0.021992954*	*0.027022656*	*0.030960563*	*0.019348962*
**ENSG00000135736**	*0.047718693*	*0.048328806*	*0.056172869*	*0.05147274*
**ENSG00000157150**	*0.016072862*	*0.025555136*	*0.025053593*	*0.015698175*
**ENSG00000081237**	*0.017002195*	*0.025674866*	*0.025721249*	*0.015252334*
**ENSG00000204613**	*0.015352276*	*0.025726297*	*0.024929821*	*0.015303721*
**ENSG00000168916**	*0.083385279*	*0.080623212*	*0.064439905*	*0.0737595*
**ENSG00000184507**	*0.013463018*	*0.024172767*	*0.023663267*	*0.013547695*
**ENSG00000124444**	*0.028556891*	*0.030754861*	*0.035128134*	*0.031153899*
**ENSG00000160710**	*0.020923721*	*0.028254932*	*0.028268818*	*0.018133483*
**ENSG00000221836**	*0.01319677*	*0.023872236*	*0.023665055*	*0.013274551*
**ENSG00000138823**	*0.109097439*	*0.079372875*	*0.069785376*	*0.075720602*
**ENSG00000106701**	*0.038422006*	*0.027438865*	*0.034230035*	*0.0298217*
**ENSG00000056345**	*0.038974841*	*0.04040497*	*0.040585018*	*0.043519836*
**ENSG00000140319**	*0.016926738*	*0.026383578*	*0.025727133*	*0.018270374*
**ENSG00000071243**	*0.059041944*	*0.063164512*	*0.070626347*	*0.05769572*
**ENSG00000115596**	*0.017537335*	*0.026523368*	*0.025701605*	*0.017525789*
**ENSG00000165953**	*0.017435325*	*0.025927306*	*0.024700487*	*0.015729122*
**ENSG00000107186**	*0.018273715*	*0.026278299*	*0.027809712*	*0.015935432*
**ENSG00000164691**	*0.066503138*	*0.05336021*	*0.05365503*	*0.042744013*
**ENSG00000113520**	*0.030089254*	*0.038936153*	*0.037127306*	*0.036341265*
**ENSG00000183579**	*0.014824831*	*0.0248771*	*0.024077796*	*0.015286094*
**ENSG00000103196**	*0.029003131*	*0.025450483*	*0.024307215*	*0.015236736*
**ENSG00000173692**	*0.029120922*	*0.036345452*	*0.029770081*	*0.035679224*
**ENSG00000152213**	*0.015217474*	*0.026710451*	*0.025478009*	*0.01629457*
**ENSG00000139540**	*0.016691963*	*0.025985707*	*0.025261212*	*0.014697676*
**ENSG00000184436**	*0.024705207*	*0.028872872*	*0.036730694*	*0.023406187*
**ENSG00000213977**	*0.039888152*	*0.033993859*	*0.035674635*	*0.039843204*
**ENSG00000114450**	*0.040081807*	*0.03855042*	*0.03732765*	*0.05192253*
**ENSG00000213930**	*0.039118563*	*0.040456237*	*0.033223742*	*0.03469516*
**ENSG00000136206**	*0.015989663*	*0.025477081*	*0.024737136*	*0.01732226*
**ENSG00000090971**	*0.036601131*	*0.034454294*	*0.033912722*	*0.031644092*
**ENSG00000196406**	*0.033879243*	*0.026897952*	*0.025389705*	*0.017121397*
**ENSG00000137040**	*0.041198945*	*0.043654017*	*0.043771556*	*0.041987611*
**ENSG00000164841**	*0.018336705*	*0.026441695*	*0.026536303*	*0.017423163*
**ENSG00000137364**	*0.037883113*	*0.029399762*	*0.034623408*	*0.031096849*
**ENSG00000140488**	*0.0243659*	*0.02927819*	*0.026988962*	*0.022604868*
**ENSG00000196104**	*0.01664948*	*0.024974718*	*0.026039515*	*0.017075248*
**ENSG00000164442**	*0.060473719*	*0.056477895*	*0.048520763*	*0.052844284*
**ENSG00000198178**	*0.046517367*	*0.042459639*	*0.040911313*	*0.058109908*
**ENSG00000120889**	*0.032539907*	*0.035571646*	*0.03398235*	*0.028697746*
**ENSG00000236104**	*0.024915722*	*0.031508786*	*0.035510273*	*0.024146372*

**ENSG00000179041**	*0.042481067*	*0.041611588*	*0.046654321*	*0.046736888*
**ENSG00000155984**	*0.027825965*	*0.040355373*	*0.036686636*	*0.036213074*
**ENSG00000103404**	*0.016891558*	*0.025337867*	*0.025352651*	*0.015525379*
**ENSG00000164822**	*0.015378373*	*0.025231485*	*0.02539988*	*0.01516099*
**ENSG00000204977**	*0.026564276*	*0.031957069*	*0.031309113*	*0.026619716*
**ENSG00000169992**	*0.017402606*	*0.027084009*	*0.028121783*	*0.017937835*
**ENSG00000132874**	*0.015214506*	*0.02534517*	*0.026156389*	*0.01630103*
**ENSG00000168591**	*0.018510193*	*0.029172189*	*0.031691837*	*0.018550802*
**ENSG00000108433**	*0.03607718*	*0.040193329*	*0.036222753*	*0.032448368*
**ENSG00000162068**	*0.014890305*	*0.026148485*	*0.025557813*	*0.015685051*
**ENSG00000158411**	*0.031875053*	*0.032806477*	*0.031090948*	*0.022008493*
**ENSG00000101166**	*0.026879311*	*0.03157267*	*0.030576854*	*0.022178583*
**ENSG00000116918**	*0.029817307*	*0.038839339*	*0.034619589*	*0.031622802*
**ENSG00000085871**	*0.075276408*	*0.068743433*	*0.067695225*	*0.058927342*
**ENSG00000197701**	*0.018025979*	*0.024400081*	*0.02581886*	*0.017318913*
**ENSG00000122435**	*0.034704695*	*0.038791971*	*0.030736967*	*0.024043644*
**ENSG00000146221**	*0.015454134*	*0.02551214*	*0.02692033*	*0.016304064*
**ENSG00000183309**	*0.029363443*	*0.032271414*	*0.029662985*	*0.030736447*
**ENSG00000011332**	*0.022787612*	*0.025965326*	*0.027003837*	*0.018797167*
**ENSG00000105438**	*0.02903634*	*0.031624127*	*0.035829388*	*0.028225075*
**ENSG00000176261**	*0.023315425*	*0.030543167*	*0.03137128*	*0.021065609*
**ENSG00000171307**	*0.017207011*	*0.025272956*	*0.025990966*	*0.017470393*
**ENSG00000105372**	*0.014417147*	*0.023949402*	*0.023938248*	*0.015485454*
**ENSG00000100596**	*0.037163654*	*0.04185952*	*0.034567504*	*0.041096952*
**ENSG00000179023**	*0.020036227*	*0.02869548*	*0.028716914*	*0.018739607*
**ENSG00000140873**	*0.019310205*	*0.027108686*	*0.026349631*	*0.018763924*
**ENSG00000107537**	*0.049736068*	*0.045799704*	*0.048673035*	*0.060213738*
**ENSG00000134193**	*0.015431676*	*0.02439215*	*0.024885222*	*0.01424776*
**ENSG00000099889**	*0.017793762*	*0.02639914*	*0.027143491*	*0.01694463*
**ENSG00000161849**	*0.018037073*	*0.028385174*	*0.026484164*	*0.018477339*
**ENSG00000129460**	*0.026995301*	*0.034917836*	*0.034157366*	*0.022030996*
**ENSG00000072952**	*0.016562256*	*0.024081622*	*0.02427254*	*0.015022096*
**ENSG00000198954**	*0.036250926*	*0.040154878*	*0.035220142*	*0.037344936*
**ENSG00000154079**	*0.034539511*	*0.031025445*	*0.034244591*	*0.029502976*
**ENSG00000144029**	*0.027876985*	*0.031548158*	*0.028684423*	*0.020644238*
**ENSG00000149761**	*0.029547618*	*0.031110647*	*0.030697929*	*0.031321555*
**ENSG00000101057**	*0.044532772*	*0.04194003*	*0.044024202*	*0.042688939*
**ENSG00000114744**	*0.029818744*	*0.038274374*	*0.03034626*	*0.042390523*
**ENSG00000139629**	*0.041741726*	*0.044197243*	*0.047546282*	*0.042101263*
**ENSG00000113657**	*0.080081961*	*0.061264774*	*0.041912514*	*0.095395438*
**ENSG00000007545**	*0.020311189*	*0.02932081*	*0.028600195*	*0.017796921*
**ENSG00000163485**	*0.017629533*	*0.024933857*	*0.025123846*	*0.016404156*
**ENSG00000176783**	*0.026523773*	*0.034549461*	*0.03344586*	*0.024164933*
**ENSG00000022840**	*0.03772159*	*0.039464096*	*0.045562673*	*0.032548246*
**ENSG00000106733**	*0.050677692*	*0.050435614*	*0.052696984*	*0.03641857*
**ENSG00000161558**	*0.028938818*	*0.035605587*	*0.035977929*	*0.025601751*
**ENSG00000135506**	*0.026431104*	*0.029153854*	*0.036930204*	*0.027141169*
**ENSG00000144524**	*0.023704417*	*0.028174777*	*0.032891131*	*0.022113378*
**ENSG00000196227**	*0.032952824*	*0.032461232*	*0.033306432*	*0.02924042*
**ENSG00000166948**	*0.01595133*	*0.025876921*	*0.025694608*	*0.015277382*
**ENSG00000172493**	*0.031541446*	*0.033634636*	*0.030255108*	*0.027079043*
**ENSG00000164919**	*0.01724412*	*0.026564738*	*0.025000278*	*0.018005196*
**ENSG00000124900**	*0.017280339*	*0.024688653*	*0.024792095*	*0.015845598*
**ENSG00000171813**	*0.056607653*	*0.066675375*	*0.056511977*	*0.06266469*

**ENSG00000177764**	*0.025458644*	*0.033522299*	*0.034352341*	*0.023946355*
**ENSG00000076003**	*0.047844861*	*0.039697782*	*0.039422281*	*0.0483257*
**ENSG00000177000**	*0.019138189*	*0.027958922*	*0.024867725*	*0.016355019*
**ENSG00000113327**	*0.01828939*	*0.028312146*	*0.02711072*	*0.016722677*
**ENSG00000204539**	*0.015415583*	*0.024482106*	*0.025200014*	*0.015032822*
**ENSG00000160180**	*0.015802399*	*0.025689195*	*0.025132595*	*0.015904494*
**ENSG00000152944**	*0.032476308*	*0.036937535*	*0.032614063*	*0.033344959*
**ENSG00000130294**	*0.016083222*	*0.025392876*	*0.025150631*	*0.016139203*
**ENSG00000115252**	*0.01634811*	*0.026053644*	*0.025576623*	*0.016455332*
**ENSG00000134762**	*0.110542482*	*0.046781707*	*0.027659207*	*0.072725519*
**ENSG00000168395**	*0.040397858*	*0.042716989*	*0.04625103*	*0.036182599*
**ENSG00000154380**	*0.015461626*	*0.024933228*	*0.024661021*	*0.015159032*
**ENSG00000187135**	*0.017411562*	*0.025094507*	*0.024220762*	*0.016400646*
**ENSG00000111732**	*0.044903337*	*0.04652518*	*0.041552437*	*0.049300609*
**ENSG00000169071**	*0.031761574*	*0.029323526*	*0.025961863*	*0.054329479*
**ENSG00000172992**	*0.030860768*	*0.030186408*	*0.030036881*	*0.026709937*
**ENSG00000116001**	*0.030159087*	*0.039404311*	*0.034134369*	*0.02719716*
**ENSG00000106948**	*0.032710583*	*0.031158749*	*0.033422849*	*0.030823968*
**ENSG00000124228**	*0.029246688*	*0.028369126*	*0.03177501*	*0.031663913*
**ENSG00000173540**	*0.038210972*	*0.03586005*	*0.03177245*	*0.035616267*
**ENSG00000135245**	*0.125618646*	*0.102135458*	*0.129898033*	*0.122455584*
**ENSG00000090661**	*0.026623726*	*0.034616877*	*0.029439304*	*0.031653003*
**ENSG00000169955**	*0.019449607*	*0.025405032*	*0.025716462*	*0.018270572*
**ENSG00000100605**	*0.046345144*	*0.044700296*	*0.040325301*	*0.047954131*
**ENSG00000165424**	*0.026791242*	*0.031838228*	*0.033191731*	*0.027106233*
**ENSG00000128313**	*0.016047139*	*0.025157956*	*0.024811295*	*0.015375084*
**ENSG00000170820**	*0.015604901*	*0.025032287*	*0.024600621*	*0.015139944*
**ENSG00000227500**	*0.031305501*	*0.035629696*	*0.043937051*	*0.034054938*
**ENSG00000130035**	*0.019199498*	*0.02613976*	*0.025782648*	*0.016651657*
**ENSG00000163507**	*0.047536475*	*0.047431235*	*0.035457715*	*0.04312769*
**ENSG00000248871**	*0.047094264*	*0.045364419*	*0.036837502*	*0.045317571*
**ENSG00000106692**	*0.029327037*	*0.033228892*	*0.02895591*	*0.023468947*
**ENSG00000011405**	*0.03540636*	*0.04670212*	*0.039853454*	*0.037819998*
**ENSG00000162419**	*0.029394336*	*0.030589124*	*0.035965666*	*0.027209716*
**ENSG00000091262**	*0.014953118*	*0.023962047*	*0.024074382*	*0.014428525*
**ENSG00000100065**	*0.017120662*	*0.024381343*	*0.024329676*	*0.01534773*
**ENSG00000154118**	*0.015906132*	*0.025445736*	*0.023846065*	*0.014617675*
**ENSG00000163874**	*0.027421273*	*0.040303561*	*0.041287961*	*0.031655304*
**ENSG00000197798**	*0.018856763*	*0.027208857*	*0.02788596*	*0.021279161*
**ENSG00000148737**	*0.018264546*	*0.026233625*	*0.026198752*	*0.015869633*
**ENSG00000139832**	*0.091465551*	*0.071643988*	*0.056991886*	*0.071356561*
**ENSG00000158467**	*0.038478488*	*0.037743145*	*0.032200378*	*0.032558564*
**ENSG00000101608**	*0.018958968*	*0.028002676*	*0.026728496*	*0.019813185*
**ENSG00000198900**	*0.027913547*	*0.036388821*	*0.031055852*	*0.030317171*
**ENSG00000108107**	*0.035240134*	*0.03648889*	*0.03374285*	*0.043895366*
**ENSG00000168447**	*0.038426006*	*0.045906272*	*0.035326784*	*0.04826442*
**ENSG00000140932**	*0.014700784*	*0.025452576*	*0.024488192*	*0.014987096*
**ENSG00000168175**	*0.027102395*	*0.036676015*	*0.032599644*	*0.026632974*
**ENSG00000167996**	*0.020179353*	*0.02719613*	*0.025868071*	*0.02035705*
**ENSG00000124743**	*0.015990024*	*0.025314853*	*0.024795477*	*0.015621277*
**ENSG00000126003**	*0.034365973*	*0.038020253*	*0.043860088*	*0.033093727*
**ENSG00000064419**	*0.026870903*	*0.032272342*	*0.032224248*	*0.022378881*
**ENSG00000198894**	*0.035628697*	*0.040997327*	*0.040264997*	*0.033832382*
**ENSG00000198929**	*0.016137252*	*0.024618664*	*0.024891665*	*0.01566407*

**ENSG00000114455**	*0.038539973*	*0.032402617*	*0.030247408*	*0.02058488*
**ENSG00000106178**	*0.017052895*	*0.025712213*	*0.024501679*	*0.016830556*
**ENSG00000005882**	*0.019124179*	*0.027740151*	*0.02590448*	*0.018033691*
**ENSG00000242689**	*0.01615246*	*0.024818858*	*0.023790509*	*0.015639062*
**ENSG00000050767**	*0.021426759*	*0.028877072*	*0.028550519*	*0.018825766*
**ENSG00000113555**	*0.015890344*	*0.025118215*	*0.025182995*	*0.014263231*
**ENSG00000121039**	*0.017083774*	*0.02516812*	*0.025625848*	*0.016308963*
**ENSG00000196248**	*0.014421471*	*0.02449045*	*0.024481098*	*0.016005458*
**ENSG00000159915**	*0.04746289*	*0.039026211*	*0.03173871*	*0.028637724*
**ENSG00000243646**	*0.027124625*	*0.031967103*	*0.03169179*	*0.024821959*
**ENSG00000068697**	*0.024420607*	*0.031705195*	*0.033880543*	*0.025442942*
**ENSG00000146909**	*0.03042815*	*0.030301436*	*0.033145187*	*0.022154018*
**ENSG00000142102**	*0.050393393*	*0.058476939*	*0.056537076*	*0.056693211*
**ENSG00000172331**	*0.039838727*	*0.047097823*	*0.038897736*	*0.038721774*
**ENSG00000148248**	*0.026808051*	*0.03125831*	*0.037738839*	*0.028344275*
**ENSG00000156103**	*0.016461642*	*0.025399832*	*0.025459972*	*0.016171165*
**ENSG00000089876**	*0.048834061*	*0.037319238*	*0.037695416*	*0.0321184*
**ENSG00000196277**	*0.017180128*	*0.025399949*	*0.024985188*	*0.01730888*
**ENSG00000114739**	*0.048885428*	*0.046696221*	*0.041140657*	*0.04053965*
**ENSG00000148156**	*0.015492223*	*0.024990349*	*0.024925521*	*0.014710141*
**ENSG00000142634**	*0.030268077*	*0.034665157*	*0.032352922*	*0.032035733*
**ENSG00000173198**	*0.046411886*	*0.04376784*	*0.045865882*	*0.049095613*
**ENSG00000243943**	*0.031418502*	*0.037782153*	*0.031945298*	*0.027499115*
**ENSG00000116406**	*0.04396973*	*0.041582632*	*0.038046715*	*0.046909039*
**ENSG00000163069**	*0.055538528*	*0.056130395*	*0.049323877*	*0.050339679*
**ENSG00000173575**	*0.01860427*	*0.026081874*	*0.025826843*	*0.018518427*
**ENSG00000112195**	*0.052481692*	*0.039314469*	*0.033504511*	*0.04463224*
**ENSG00000163872**	*0.048037459*	*0.051693921*	*0.051672593*	*0.045908426*
**ENSG00000089127**	*0.077197293*	*0.07735364*	*0.051659084*	*0.107739648*
**ENSG00000172590**	*0.047238944*	*0.047662791*	*0.040793948*	*0.035603625*
**ENSG00000112773**	*0.121961001*	*0.078919378*	*0.066682937*	*0.090832616*
**ENSG00000239704**	*0.058574272*	*0.047732478*	*0.057297376*	*0.065076825*
**ENSG00000123416**	*0.020419238*	*0.026139161*	*0.025128688*	*0.021286139*
**ENSG00000100490**	*0.019779551*	*0.027250942*	*0.027469551*	*0.017475059*
**ENSG00000160685**	*0.023543257*	*0.03244025*	*0.028964997*	*0.025426072*
**ENSG00000088448**	*0.037925382*	*0.03807028*	*0.035731386*	*0.032365236*
**ENSG00000053108**	*0.017355448*	*0.025506426*	*0.025897787*	*0.016082437*
**ENSG00000053918**	*0.014924454*	*0.025769288*	*0.024822565*	*0.014457943*
**ENSG00000166557**	*0.028547174*	*0.03340721*	*0.034210517*	*0.028578736*
**ENSG00000115350**	*0.033875379*	*0.030102848*	*0.035824174*	*0.030690186*
**ENSG00000117602**	*0.033135788*	*0.030668651*	*0.028027021*	*0.024094879*
**ENSG00000118785**	*0.026118316*	*0.03337916*	*0.033889817*	*0.022848431*
**ENSG00000189184**	*0.027740389*	*0.02515829*	*0.025153023*	*0.017887325*
**ENSG00000089775**	*0.045572663*	*0.042679612*	*0.044455069*	*0.047629226*
**ENSG00000120697**	*0.027313843*	*0.031472134*	*0.033422619*	*0.027033866*
**ENSG00000188425**	*0.017677652*	*0.024632644*	*0.02607292*	*0.01489523*
**ENSG00000140481**	*0.019268197*	*0.025736661*	*0.025945166*	*0.019022138*
**ENSG00000165752**	*0.0478231*	*0.052342237*	*0.038406153*	*0.040906434*
**ENSG00000168843**	*0.034395039*	*0.028758008*	*0.037302106*	*0.019695209*
**ENSG00000114686**	*0.024345658*	*0.028172193*	*0.027154825*	*0.025414726*
**ENSG00000007923**	*0.031129136*	*0.032318797*	*0.033500925*	*0.030039141*
**ENSG00000162600**	*0.037391469*	*0.041057095*	*0.032873727*	*0.032152493*
**ENSG00000125903**	*0.017798719*	*0.02821322*	*0.027321454*	*0.018543828*
**ENSG00000110801**	*0.033235594*	*0.040527741*	*0.036136054*	*0.034385737*

**ENSG00000169193**	*0.047723913*	*0.037715953*	*0.03586144*	*0.035251539*
**ENSG00000186272**	*0.029270325*	*0.03893259*	*0.041042166*	*0.025630948*
**ENSG00000110723**	*0.049158964*	*0.051579856*	*0.047646393*	*0.053368932*
**ENSG00000124098**	*0.030505364*	*0.033806944*	*0.031142969*	*0.026130484*
**ENSG00000137135**	*0.029120528*	*0.027535249*	*0.028939844*	*0.025134567*
**ENSG00000100918**	*0.047512683*	*0.045214741*	*0.047096639*	*0.050441595*
**ENSG00000132972**	*0.038719828*	*0.045125252*	*0.024613361*	*0.039296391*
**ENSG00000134874**	*0.049052512*	*0.035742519*	*0.027642195*	*0.026097383*
**ENSG00000253953**	*0.017741688*	*0.025149929*	*0.024123249*	*0.014834955*
**ENSG00000134321**	*0.03610397*	*0.041206496*	*0.038959715*	*0.035744325*
**ENSG00000166426**	*0.031583241*	*0.042759749*	*0.026026064*	*0.02343265*
**ENSG00000133265**	*0.04135742*	*0.033703266*	*0.037072115*	*0.039337428*
**ENSG00000130713**	*0.025659666*	*0.029611075*	*0.029175379*	*0.02410947*
**ENSG00000008118**	*0.017004355*	*0.026636255*	*0.024631452*	*0.016702725*
**ENSG00000163636**	*0.022417355*	*0.029338328*	*0.029055147*	*0.023973338*
**ENSG00000112561**	*0.032945813*	*0.040171307*	*0.037097832*	*0.03509325*
**ENSG00000140905**	*0.045054585*	*0.047538899*	*0.038879755*	*0.045463027*
**ENSG00000197697**	*0.056992398*	*0.06140401*	*0.046080109*	*0.048728549*
**ENSG00000204186**	*0.034269854*	*0.032244207*	*0.033222865*	*0.025865682*
**ENSG00000118690**	*0.02012597*	*0.024462579*	*0.026281588*	*0.018444676*
**ENSG00000177854**	*0.033845754*	*0.034785691*	*0.032721403*	*0.034642758*
**ENSG00000178802**	*0.039403618*	*0.034767871*	*0.036471157*	*0.034772078*
**ENSG00000081803**	*0.097921461*	*0.05438271*	*0.043485999*	*0.06706311*
**ENSG00000101191**	*0.033748254*	*0.037978066*	*0.037382284*	*0.024006334*
**ENSG00000187268**	*0.016349382*	*0.026040419*	*0.025199518*	*0.015264887*
**ENSG00000113761**	*0.021011464*	*0.033553179*	*0.03032983*	*0.026111162*
**ENSG00000068985**	*0.014825568*	*0.024808291*	*0.024138867*	*0.015786525*
**ENSG00000120688**	*0.030552386*	*0.035725003*	*0.031461464*	*0.028335797*
**ENSG00000059728**	*0.017983245*	*0.025655423*	*0.026439208*	*0.019080991*
**ENSG00000133316**	*0.03327396*	*0.032073639*	*0.034835545*	*0.026353751*
**ENSG00000171234**	*0.115810629*	*0.168429834*	*0.158797566*	*0.098574721*
**ENSG00000168497**	*0.020298973*	*0.025940174*	*0.025304076*	*0.014787094*
**ENSG00000150051**	*0.015731385*	*0.025284159*	*0.025331532*	*0.015543477*
**ENSG00000110031**	*0.025787273*	*0.031656211*	*0.028486879*	*0.022242727*
**ENSG00000088836**	*0.020716023*	*0.028514357*	*0.025415339*	*0.017094292*
**ENSG00000133958**	*0.016047155*	*0.025470126*	*0.025319385*	*0.015183154*
**ENSG00000109501**	*0.036035356*	*0.030873345*	*0.03123718*	*0.029637214*
**ENSG00000157542**	*0.014277913*	*0.024927692*	*0.024815051*	*0.016137877*
**ENSG00000169933**	*0.016975101*	*0.024789824*	*0.024626794*	*0.015630307*
**ENSG00000162882**	*0.030099386*	*0.030404843*	*0.027584501*	*0.025406904*
**ENSG00000178163**	*0.039462852*	*0.045889367*	*0.042051006*	*0.036334478*
**ENSG00000177425**	*0.050934286*	*0.043244007*	*0.033642884*	*0.040218712*
**ENSG00000177034**	*0.020627519*	*0.025307744*	*0.026595274*	*0.016082714*
**ENSG00000136928**	*0.015515106*	*0.026071214*	*0.024831729*	*0.015122165*
**ENSG00000164326**	*0.018271659*	*0.024960382*	*0.024610665*	*0.01762437*
**ENSG00000106591**	*0.028236699*	*0.032876313*	*0.031511043*	*0.023067277*
**ENSG00000138814**	*0.03659925*	*0.048279378*	*0.037791175*	*0.033646473*
**ENSG00000075240**	*0.051090139*	*0.041226663*	*0.034908934*	*0.03686221*
**ENSG00000167780**	*0.031635745*	*0.032191128*	*0.031797006*	*0.024591568*
**ENSG00000198327**	*0.023674988*	*0.028034382*	*0.029455215*	*0.020763479*
**ENSG00000159409**	*0.015215914*	*0.025036869*	*0.025095803*	*0.015911135*
**ENSG00000102034**	*0.024497403*	*0.031079488*	*0.037251457*	*0.02383874*
**ENSG00000002933**	*0.050777544*	*0.02616946*	*0.025022348*	*0.016930221*
**ENSG00000163440**	*0.015768032*	*0.026264377*	*0.02496036*	*0.015174649*

**ENSG00000172554**	*0.025512624*	*0.026564929*	*0.025261331*	*0.024297967*
**ENSG00000196455**	*0.026029741*	*0.03526257*	*0.030685973*	*0.022502622*
**ENSG00000186575**	*0.016659106*	*0.028991643*	*0.024911436*	*0.017065754*
**ENSG00000132849**	*0.019766683*	*0.029265048*	*0.029744292*	*0.019432856*
**ENSG00000110651**	*0.026124334*	*0.030200571*	*0.030311426*	*0.021225305*
**ENSG00000162378**	*0.034457683*	*0.038239185*	*0.03281636*	*0.027476424*
**ENSG00000149798**	*0.041889878*	*0.04189714*	*0.044574582*	*0.044008802*
**ENSG00000155463**	*0.021376518*	*0.028225563*	*0.028211576*	*0.019144053*
**ENSG00000166407**	*0.016890507*	*0.025495224*	*0.026150068*	*0.016625579*
**ENSG00000144566**	*0.021821073*	*0.032016*	*0.034885577*	*0.022625671*
**ENSG00000116984**	*0.02608131*	*0.03154511*	*0.031598327*	*0.028563958*
**ENSG00000145626**	*0.016386326*	*0.024339698*	*0.024802144*	*0.015681355*
**ENSG00000101189**	*0.032993647*	*0.039221409*	*0.033667041*	*0.023322653*
**ENSG00000002549**	*0.027083382*	*0.032087557*	*0.029420228*	*0.021733335*
**ENSG00000183153**	*0.017574608*	*0.026305113*	*0.026659966*	*0.016173554*
**ENSG00000162878**	*0.030362611*	*0.025287979*	*0.02553385*	*0.018314916*
**ENSG00000099308**	*0.033713438*	*0.035476297*	*0.036324586*	*0.034967738*
**ENSG00000117528**	*0.038173904*	*0.044618799*	*0.035055957*	*0.029103131*
**ENSG00000196368**	*0.014781059*	*0.024975817*	*0.02445334*	*0.015690799*
**ENSG00000127863**	*0.164508146*	*0.143665963*	*0.122629797*	*0.155995293*
**ENSG00000059122**	*0.024300919*	*0.032362123*	*0.033664807*	*0.027228115*
**ENSG00000173825**	*0.014862909*	*0.024824227*	*0.025603582*	*0.015670997*
**ENSG00000169239**	*0.046334295*	*0.037672088*	*0.041369715*	*0.039695464*
**ENSG00000186431**	*0.032709106*	*0.032902046*	*0.038741067*	*0.031090741*
**ENSG00000196981**	*0.01968064*	*0.028381826*	*0.026015997*	*0.017240049*
**ENSG00000170634**	*0.01890644*	*0.025412196*	*0.02538746*	*0.016938779*
**ENSG00000172831**	*0.027894218*	*0.028753365*	*0.032748412*	*0.022358403*
**ENSG00000184697**	*0.017561725*	*0.026850878*	*0.027096974*	*0.019220308*
**ENSG00000203690**	*0.015271733*	*0.024512271*	*0.025512573*	*0.014243129*
**ENSG00000173110**	*0.071807263*	*0.080257054*	*0.061033895*	*0.076151226*
**ENSG00000133943**	*0.032520617*	*0.039809574*	*0.034702377*	*0.036853274*
**ENSG00000154839**	*0.047748623*	*0.038032114*	*0.037053791*	*0.036106135*
**ENSG00000170469**	*0.028982728*	*0.033352371*	*0.0293757*	*0.035059136*
**ENSG00000170231**	*0.077142349*	*0.061986451*	*0.046493211*	*0.05718678*
**ENSG00000109674**	*0.033813678*	*0.033495521*	*0.029140683*	*0.02405288*
**ENSG00000170615**	*0.018792488*	*0.026399953*	*0.025553913*	*0.017490111*
**ENSG00000163581**	*0.036226307*	*0.025527513*	*0.025855196*	*0.015122594*
**ENSG00000196693**	*0.043158116*	*0.040210279*	*0.033266501*	*0.038667453*
**ENSG00000162889**	*0.029378797*	*0.039386479*	*0.048445099*	*0.032695612*
**ENSG00000188566**	*0.0410583*	*0.039665335*	*0.031747534*	*0.031246125*
**ENSG00000242520**	*0.01477592*	*0.025045044*	*0.024141937*	*0.014887498*
**ENSG00000165716**	*0.102475185*	*0.105091992*	*0.068585548*	*0.071034811*
**ENSG00000108298**	*0.013382362*	*0.023441205*	*0.023728891*	*0.014118253*
**ENSG00000177710**	*0.014986838*	*0.024885331*	*0.024448981*	*0.014390068*
**ENSG00000172840**	*0.020593404*	*0.027834139*	*0.026865214*	*0.019466983*
**ENSG00000142686**	*0.028855129*	*0.034149264*	*0.034552894*	*0.028170282*
**ENSG00000105221**	*0.025904159*	*0.047996113*	*0.034260615*	*0.040071441*
**ENSG00000205808**	*0.037495838*	*0.036299676*	*0.031523904*	*0.027595516*
**ENSG00000165471**	*0.01681822*	*0.025664943*	*0.026811611*	*0.016279206*
**ENSG00000101882**	*0.026116874*	*0.031774045*	*0.030701022*	*0.021583831*
**ENSG00000234560**	*0.019819999*	*0.026008543*	*0.025604069*	*0.01761636*
**ENSG00000151176**	*0.036209821*	*0.034129751*	*0.030985827*	*0.042459611*
**ENSG00000117289**	*0.06333762*	*0.082617193*	*0.065887458*	*0.06671446*
**ENSG00000242852**	*0.02048182*	*0.027499403*	*0.026346229*	*0.017435132*

**ENSG00000206579**	*0.058926949*	*0.045911027*	*0.027292788*	*0.033089902*
**ENSG00000184351**	*0.0158714*	*0.026042079*	*0.024552094*	*0.017796895*
**ENSG00000166073**	*0.016013485*	*0.02521459*	*0.025969512*	*0.015006044*
**ENSG00000174917**	*0.022982388*	*0.02733807*	*0.02758085*	*0.020275649*
**ENSG00000197893**	*0.015554096*	*0.025736565*	*0.024938686*	*0.015076002*
**ENSG00000106631**	*0.016244264*	*0.024533083*	*0.025152594*	*0.016343753*
**ENSG00000124107**	*0.042139659*	*0.046470304*	*0.024855812*	*0.025917877*
**ENSG00000102786**	*0.032976116*	*0.03748861*	*0.036514164*	*0.036697589*
**ENSG00000174444**	*0.0141917*	*0.0242539*	*0.024109554*	*0.015031322*
**ENSG00000113946**	*0.047124583*	*0.027058275*	*0.026230002*	*0.019735213*
**ENSG00000170653**	*0.015168873*	*0.025154794*	*0.025223185*	*0.01519726*
**ENSG00000090097**	*0.031214414*	*0.037893966*	*0.034932729*	*0.032449014*
**ENSG00000115828**	*0.078718988*	*0.046038984*	*0.056802392*	*0.063216041*
**ENSG00000106785**	*0.01969245*	*0.026724138*	*0.026989074*	*0.018364596*
**ENSG00000139797**	*0.016581243*	*0.025021376*	*0.025306291*	*0.015472471*
**ENSG00000165259**	*0.041704517*	*0.043339874*	*0.034764044*	*0.030416901*
**ENSG00000105251**	*0.016978739*	*0.025442384*	*0.025445575*	*0.016954824*
**ENSG00000171953**	*0.020608379*	*0.029659083*	*0.033526077*	*0.026489223*
**ENSG00000166748**	*0.015491761*	*0.024832985*	*0.024031402*	*0.013917448*
**ENSG00000176769**	*0.015334641*	*0.024962216*	*0.025288593*	*0.026279089*
**ENSG00000145416**	*0.046449452*	*0.060090559*	*0.045813807*	*0.047906147*
**ENSG00000176834**	*0.017731538*	*0.026784128*	*0.025030978*	*0.016783194*
**ENSG00000102984**	*0.02998677*	*0.027887625*	*0.031356036*	*0.032119983*
**ENSG00000162607**	*0.0438659*	*0.037768011*	*0.036253472*	*0.037548534*
**ENSG00000125820**	*0.016746245*	*0.025993786*	*0.025339623*	*0.01557703*
**ENSG00000102897**	*0.029242605*	*0.035237789*	*0.032771233*	*0.023829359*
**ENSG00000158825**	*0.071959923*	*0.083687474*	*0.102732354*	*0.079934443*
**ENSG00000135077**	*0.126704407*	*0.069897791*	*0.074291057*	*0.092940323*
**ENSG00000125868**	*0.040627705*	*0.042280251*	*0.036491451*	*0.037347373*
**ENSG00000031823**	*0.021430093*	*0.027229186*	*0.032323296*	*0.0222114*
**ENSG00000169715**	*0.015485964*	*0.026601162*	*0.025334463*	*0.014309416*
**ENSG00000160207**	*0.019150865*	*0.028073601*	*0.026539824*	*0.020981389*
**ENSG00000146147**	*0.015226943*	*0.025280303*	*0.024287391*	*0.015328932*
**ENSG00000239857**	*0.026455948*	*0.034234371*	*0.036420525*	*0.026478977*
**ENSG00000184967**	*0.028196895*	*0.029553484*	*0.032366191*	*0.03219529*
**ENSG00000137878**	*0.035744353*	*0.042192121*	*0.036399913*	*0.02810896*
**ENSG00000156162**	*0.036262866*	*0.039795996*	*0.034352975*	*0.037915619*
**ENSG00000186306**	*0.016518523*	*0.024788163*	*0.024500814*	*0.015731779*
**ENSG00000141750**	*0.019754563*	*0.02906026*	*0.025110824*	*0.018344896*
**ENSG00000134248**	*0.021203307*	*0.027006721*	*0.027083855*	*0.018583912*
**ENSG00000169760**	*0.015692621*	*0.024129754*	*0.025290751*	*0.01613615*
**ENSG00000136999**	*0.062447238*	*0.03264418*	*0.026255278*	*0.027795456*
**ENSG00000173465**	*0.026058587*	*0.030419428*	*0.030651394*	*0.027588525*
**ENSG00000171574**	*0.020170419*	*0.026337174*	*0.02845379*	*0.016528602*
**ENSG00000149926**	*0.017712606*	*0.02620543*	*0.025622737*	*0.017662272*
**ENSG00000104267**	*0.114960039*	*0.086601298*	*0.087092147*	*0.091621455*
**ENSG00000142794**	*0.035134611*	*0.038863571*	*0.033544634*	*0.032601761*
**ENSG00000204310**	*0.014729816*	*0.024924048*	*0.024033336*	*0.01435344*
**ENSG00000167775**	*0.032946886*	*0.03410387*	*0.031884973*	*0.031928666*
**ENSG00000135503**	*0.043818276*	*0.030377284*	*0.032129673*	*0.029703561*
**ENSG00000065621**	*0.027593924*	*0.029044127*	*0.029920573*	*0.027869251*
**ENSG00000198018**	*0.023468619*	*0.029089252*	*0.02622441*	*0.019705442*
**ENSG00000147416**	*0.022692308*	*0.030052762*	*0.030194449*	*0.023849152*
**ENSG00000204694**	*0.016989642*	*0.025119984*	*0.024172332*	*0.015576275*

**ENSG00000198553**	*0.01719695*	*0.026402231*	*0.026549765*	*0.018998043*
**ENSG00000124006**	*0.017861128*	*0.026770764*	*0.025114252*	*0.017821756*
**ENSG00000136688**	*0.01864131*	*0.027082578*	*0.025555573*	*0.01785663*
**ENSG00000155275**	*0.018691296*	*0.026751962*	*0.026130762*	*0.018312222*
**ENSG00000178093**	*0.02905173*	*0.031147554*	*0.032817044*	*0.024934685*
**ENSG00000198863**	*0.032191722*	*0.034180903*	*0.031433098*	*0.027072379*
**ENSG00000106344**	*0.036928733*	*0.037539469*	*0.034834518*	*0.03138189*
**ENSG00000150394**	*0.019603852*	*0.028886011*	*0.028813522*	*0.018984291*
**ENSG00000221823**	*0.025377646*	*0.036347656*	*0.031510929*	*0.022123972*
**ENSG00000120563**	*0.017241924*	*0.025172194*	*0.025904348*	*0.015870485*
**ENSG00000136854**	*0.019745049*	*0.026139935*	*0.026266*	*0.018068165*
**ENSG00000151611**	*0.051501404*	*0.039716024*	*0.039224441*	*0.039430342*
**ENSG00000180628**	*0.041756202*	*0.042048346*	*0.038250077*	*0.037147671*
**ENSG00000033178**	*0.029567994*	*0.035836519*	*0.032316403*	*0.029318445*
**ENSG00000007341**	*0.037986733*	*0.051324433*	*0.037359809*	*0.032635547*
**ENSG00000188021**	*0.026020008*	*0.029157404*	*0.028181609*	*0.027378993*
**ENSG00000197372**	*0.040713685*	*0.038697346*	*0.038021911*	*0.052179417*
**ENSG00000168875**	*0.016046483*	*0.024588987*	*0.024431686*	*0.014906173*
**ENSG00000163249**	*0.037189868*	*0.035763068*	*0.037350819*	*0.032238618*
**ENSG00000132423**	*0.033995275*	*0.036012399*	*0.032753019*	*0.03669373*
**ENSG00000170881**	*0.01904756*	*0.025243442*	*0.026209652*	*0.016037713*
**ENSG00000184445**	*0.044423174*	*0.041805865*	*0.034591216*	*0.031597694*
**ENSG00000172738**	*0.026758979*	*0.029507841*	*0.029972433*	*0.023404915*
**ENSG00000151067**	*0.014798318*	*0.024250198*	*0.02436598*	*0.015718799*
**ENSG00000143147**	*0.015261285*	*0.025203687*	*0.025068916*	*0.01553793*
**ENSG00000086544**	*0.021449886*	*0.027584727*	*0.026827001*	*0.017360113*
**ENSG00000153044**	*0.041699831*	*0.039716373*	*0.0339409*	*0.034208561*
**ENSG00000143171**	*0.016226804*	*0.02494992*	*0.024794081*	*0.015263893*
**ENSG00000160999**	*0.053143215*	*0.054517478*	*0.059323644*	*0.064540536*
**ENSG00000189334**	*0.023339597*	*0.02801095*	*0.027683361*	*0.018112361*
**ENSG00000166206**	*0.019688778*	*0.026305046*	*0.025801381*	*0.019092666*
**ENSG00000085365**	*0.024985154*	*0.030687977*	*0.029419756*	*0.023567046*
**ENSG00000214253**	*0.030921328*	*0.028732045*	*0.028825232*	*0.026597594*
**ENSG00000131795**	*0.025501817*	*0.027494487*	*0.027993574*	*0.021404788*
**ENSG00000091127**	*0.045417498*	*0.043747462*	*0.032391343*	*0.031111234*
**ENSG00000180549**	*0.133125261*	*0.107764608*	*0.085145797*	*0.108275436*
**ENSG00000124249**	*0.015289485*	*0.025366833*	*0.024322172*	*0.016031483*
**ENSG00000139514**	*0.019094696*	*0.027035278*	*0.025925011*	*0.018138513*
**ENSG00000198105**	*0.044389876*	*0.046768284*	*0.042249227*	*0.037779531*
**ENSG00000123575**	*0.041360969*	*0.045310729*	*0.035535948*	*0.029487095*
**ENSG00000164151**	*0.028264823*	*0.034415614*	*0.035451391*	*0.026045098*
**ENSG00000111554**	*0.019759479*	*0.027131646*	*0.026634502*	*0.016261665*
**ENSG00000206344**	*0.039786646*	*0.041471237*	*0.0354039*	*0.04250027*
**ENSG00000150873**	*0.01541416*	*0.025643686*	*0.025094577*	*0.015517117*
**ENSG00000137166**	*0.044219654*	*0.0412778*	*0.037838154*	*0.039570666*
**ENSG00000163833**	*0.018120184*	*0.027298395*	*0.026121847*	*0.018147387*
**ENSG00000147687**	*0.021133275*	*0.028326634*	*0.029781574*	*0.023496243*
**ENSG00000163617**	*0.021447671*	*0.027566644*	*0.027467235*	*0.018788486*
**ENSG00000136378**	*0.016961001*	*0.025886973*	*0.024164437*	*0.015140979*
**ENSG00000121900**	*0.032952286*	*0.031418693*	*0.026823205*	*0.023179257*
**ENSG00000189162**	*0.016457113*	*0.024620209*	*0.024862068*	*0.015899569*
**ENSG00000243414**	*0.017583074*	*0.026021319*	*0.027465508*	*0.01701507*
**ENSG00000004866**	*0.051788979*	*0.042020513*	*0.045349147*	*0.056586181*
**ENSG00000176723**	*0.017818764*	*0.027129489*	*0.0255941*	*0.018323871*

**ENSG00000158874**	*0.015678436*	*0.025265899*	*0.024928963*	*0.015742701*
**ENSG00000133794**	*0.047063781*	*0.041880281*	*0.035213164*	*0.032315024*
**ENSG00000169599**	*0.029875231*	*0.032231975*	*0.028530529*	*0.021873621*
**ENSG00000169604**	*0.015024173*	*0.024734765*	*0.025304292*	*0.01483973*
**ENSG00000204970**	*0.016526476*	*0.025762555*	*0.026224512*	*0.016776598*
**ENSG00000179526**	*0.024909392*	*0.030529704*	*0.030288688*	*0.023839708*
**ENSG00000102804**	*0.016775308*	*0.025890673*	*0.025817625*	*0.017211633*
**ENSG00000134851**	*0.031219281*	*0.03559881*	*0.031918621*	*0.031567656*
**ENSG00000150551**	*0.015507854*	*0.024503455*	*0.024607949*	*0.015549232*
**ENSG00000173077**	*0.016955361*	*0.024657693*	*0.025170412*	*0.017608812*
**ENSG00000112232**	*0.016407439*	*0.02450947*	*0.025070871*	*0.015588371*
**ENSG00000177414**	*0.016987566*	*0.024965951*	*0.0255273*	*0.016111948*
**ENSG00000204740**	*0.01584718*	*0.025095788*	*0.025561985*	*0.014711671*
**ENSG00000196428**	*0.032436482*	*0.042738078*	*0.039945441*	*0.034047758*
**ENSG00000198841**	*0.028326491*	*0.035234285*	*0.041721798*	*0.028703468*
**ENSG00000165685**	*0.043168161*	*0.028864774*	*0.026384771*	*0.031639846*
**ENSG00000083067**	*0.01502851*	*0.02466362*	*0.026047713*	*0.015134958*
**ENSG00000163808**	*0.046301658*	*0.042767455*	*0.035083877*	*0.035650285*
**ENSG00000183019**	*0.01607004*	*0.025152566*	*0.024556319*	*0.014736132*
**ENSG00000163728**	*0.036746735*	*0.040342766*	*0.038181772*	*0.032543467*
**ENSG00000104522**	*0.031623492*	*0.032855213*	*0.032870282*	*0.034483651*
**ENSG00000130653**	*0.042585178*	*0.039065289*	*0.038643054*	*0.048306208*
**ENSG00000184258**	*0.017463345*	*0.025847481*	*0.025483056*	*0.016090733*
**ENSG00000173933**	*0.025542388*	*0.032369431*	*0.033705285*	*0.028261635*
**ENSG00000165120**	*0.014706524*	*0.024908722*	*0.025423481*	*0.014624714*
**ENSG00000135605**	*0.033880593*	*0.034996298*	*0.035699448*	*0.032185447*
**ENSG00000130167**	*0.018036709*	*0.027138379*	*0.024621339*	*0.017971469*
**ENSG00000163348**	*0.022768924*	*0.029787466*	*0.028646915*	*0.026061757*
**ENSG00000174255**	*0.014905314*	*0.02668052*	*0.024751397*	*0.015585696*
**ENSG00000143502**	*0.025813668*	*0.026525564*	*0.024652827*	*0.014635193*
**ENSG00000114735**	*0.032889897*	*0.037691926*	*0.03329125*	*0.03908989*
**ENSG00000128739**	*0.040074087*	*0.028631411*	*0.030639374*	*0.024913128*
**ENSG00000142945**	*0.04092991*	*0.03633596*	*0.036230077*	*0.029224399*
**ENSG00000115364**	*0.031458527*	*0.036979174*	*0.032430212*	*0.027925709*
**ENSG00000154723**	*0.018972183*	*0.025817647*	*0.025391744*	*0.017523564*
**ENSG00000137941**	*0.041891075*	*0.025971669*	*0.025545295*	*0.022495739*
**ENSG00000150656**	*0.017100379*	*0.025971137*	*0.024714734*	*0.016368997*
**ENSG00000183185**	*0.016291196*	*0.024635379*	*0.024678803*	*0.015369804*
**ENSG00000100034**	*0.033869869*	*0.035860113*	*0.042957632*	*0.030134773*
**ENSG00000158122**	*0.021295836*	*0.027596064*	*0.025115332*	*0.019253303*
**ENSG00000104998**	*0.052417553*	*0.040127062*	*0.044248714*	*0.04139685*
**ENSG00000087076**	*0.049029003*	*0.054094418*	*0.04427568*	*0.05199784*
**ENSG00000196182**	*0.029796212*	*0.032235568*	*0.046875785*	*0.02950362*
**ENSG00000183207**	*0.03047993*	*0.031007231*	*0.029269032*	*0.026484419*
**ENSG00000198276**	*0.029616691*	*0.03167786*	*0.0317362*	*0.024736316*
**ENSG00000164050**	*0.032581703*	*0.032439479*	*0.030295934*	*0.030707378*
**ENSG00000163288**	*0.015801289*	*0.024836645*	*0.024751396*	*0.014916199*
**ENSG00000001631**	*0.016646514*	*0.025582097*	*0.025613968*	*0.015891987*
**ENSG00000136940**	*0.015355266*	*0.024830588*	*0.024412852*	*0.016101834*
**ENSG00000136802**	*0.027313653*	*0.031173367*	*0.035794145*	*0.035959127*
**ENSG00000120907**	*0.015691944*	*0.025652931*	*0.024201542*	*0.015660378*
**ENSG00000131746**	*0.016940814*	*0.024812109*	*0.024889648*	*0.016835755*
**ENSG00000196976**	*0.030900073*	*0.032323308*	*0.035186744*	*0.034017389*
**ENSG00000111727**	*0.028435632*	*0.035213774*	*0.03072885*	*0.028449116*

**ENSG00000184451**	*0.029662105*	*0.029462478*	*0.028332368*	*0.023834466*
**ENSG00000146828**	*0.038900492*	*0.044512103*	*0.042596973*	*0.035983851*
**ENSG00000196646**	*0.035109759*	*0.03459968*	*0.037853394*	*0.029583902*
**ENSG00000100368**	*0.023140566*	*0.030089531*	*0.027931563*	*0.02208072*
**ENSG00000166342**	*0.032900291*	*0.029395067*	*0.032719767*	*0.024290858*
**ENSG00000062370**	*0.023060017*	*0.030564556*	*0.028199807*	*0.019500713*
**ENSG00000153930**	*0.021625214*	*0.027826096*	*0.024873439*	*0.016504077*
**ENSG00000128596**	*0.027405659*	*0.032328604*	*0.031912123*	*0.027507559*
**ENSG00000198812**	*0.017031415*	*0.024466097*	*0.024592611*	*0.016722856*
**ENSG00000136271**	*0.028941378*	*0.030238748*	*0.029327969*	*0.025047258*
**ENSG00000171476**	*0.09392748*	*0.074214364*	*0.060628738*	*0.082179696*
**ENSG00000197016**	*0.015585514*	*0.026323285*	*0.025578673*	*0.016122908*
**ENSG00000113504**	*0.114824756*	*0.07395718*	*0.072141856*	*0.095678218*
**ENSG00000129646**	*0.034380906*	*0.035855117*	*0.02975226*	*0.023883696*
**ENSG00000204305**	*0.02554503*	*0.032718789*	*0.03078865*	*0.027774558*
**ENSG00000185924**	*0.014341481*	*0.024867804*	*0.024363559*	*0.013443606*
**ENSG00000136231**	*0.060942987*	*0.057136454*	*0.051442057*	*0.058449004*
**ENSG00000204406**	*0.0319912*	*0.036259492*	*0.031551865*	*0.028510193*
**ENSG00000142789**	*0.020320976*	*0.026736861*	*0.02503094*	*0.01783113*
**ENSG00000049319**	*0.015638907*	*0.024819408*	*0.024981497*	*0.015255726*
**ENSG00000135018**	*0.032333103*	*0.037266154*	*0.034470524*	*0.03150425*
**ENSG00000146038**	*0.021721278*	*0.025806451*	*0.025851765*	*0.015537531*
**ENSG00000142252**	*0.015727963*	*0.024208377*	*0.025859595*	*0.015402565*
**ENSG00000108651**	*0.024826165*	*0.029361902*	*0.02824961*	*0.020388794*
**ENSG00000171199**	*0.016044696*	*0.02554931*	*0.02626697*	*0.018159477*
**ENSG00000186790**	*0.016259482*	*0.025369663*	*0.024387188*	*0.016590339*
**ENSG00000164879**	*0.017895265*	*0.025398871*	*0.024939927*	*0.01524856*
**ENSG00000116039**	*0.017010482*	*0.02584903*	*0.024311902*	*0.015502855*
**ENSG00000163462**	*0.016027447*	*0.026432556*	*0.024510089*	*0.015378108*
**ENSG00000169860**	*0.020377633*	*0.026229362*	*0.027314154*	*0.018686317*
**ENSG00000064545**	*0.035908351*	*0.032184353*	*0.036035033*	*0.024608778*
**ENSG00000183506**	*0.022485291*	*0.029093559*	*0.029976062*	*0.031049923*
**ENSG00000066739**	*0.035402421*	*0.043517921*	*0.035904269*	*0.026733818*
**ENSG00000170322**	*0.018619399*	*0.02672486*	*0.028449625*	*0.018547305*
**ENSG00000130717**	*0.028784849*	*0.033463398*	*0.035662897*	*0.024534038*
**ENSG00000148200**	*0.015440143*	*0.024581783*	*0.024991694*	*0.015328947*
**ENSG00000109471**	*0.015746541*	*0.024242453*	*0.024998275*	*0.015440297*
**ENSG00000066044**	*0.042637001*	*0.040316733*	*0.041523404*	*0.038513724*
**ENSG00000087495**	*0.039556738*	*0.025908989*	*0.027088994*	*0.028276952*
**ENSG00000159128**	*0.028601432*	*0.032894972*	*0.029733698*	*0.028959874*
**ENSG00000132031**	*0.019040324*	*0.027433972*	*0.025325058*	*0.0222878*
**ENSG00000113749**	*0.015884126*	*0.024358526*	*0.025326927*	*0.015469189*
**ENSG00000153823**	*0.016345378*	*0.023914439*	*0.02583319*	*0.014868506*
**ENSG00000120329**	*0.01536889*	*0.023991022*	*0.024476945*	*0.014020577*
**ENSG00000181789**	*0.024272617*	*0.029649037*	*0.035450703*	*0.026668672*
**ENSG00000108424**	*0.021355945*	*0.028154907*	*0.025960492*	*0.022929358*
**ENSG00000139826**	*0.025047608*	*0.038580842*	*0.034597674*	*0.026341165*
**ENSG00000130948**	*0.014896693*	*0.025136389*	*0.024820148*	*0.014346535*
**ENSG00000114251**	*0.067667935*	*0.045162908*	*0.037071565*	*0.03736683*
**ENSG00000100647**	*0.032010698*	*0.041075059*	*0.034568276*	*0.033596926*
**ENSG00000121053**	*0.015500087*	*0.025022812*	*0.024804667*	*0.015430928*
**ENSG00000196549**	*0.015662929*	*0.024791664*	*0.024397168*	*0.016895461*
**ENSG00000159692**	*0.021154559*	*0.029172684*	*0.025596574*	*0.032791275*
**ENSG00000143878**	*0.022061612*	*0.029375501*	*0.034626058*	*0.027967275*

**ENSG00000141639**	*0.015719529*	*0.02609713*	*0.025055591*	*0.01566703*
**ENSG00000198183**	*0.018288844*	*0.027458998*	*0.025622664*	*0.017199097*
**ENSG00000160190**	*0.038353866*	*0.041268334*	*0.041063054*	*0.031693076*
**ENSG00000198162**	*0.039673169*	*0.030001053*	*0.028795861*	*0.029551078*
**ENSG00000134255**	*0.036898506*	*0.037320841*	*0.033520397*	*0.028637845*
**ENSG00000095564**	*0.03465975*	*0.038898427*	*0.03433661*	*0.034600407*
**ENSG00000076351**	*0.015664048*	*0.024365752*	*0.024353033*	*0.014832286*
**ENSG00000185760**	*0.016209182*	*0.024472654*	*0.024600475*	*0.014332521*
**ENSG00000176658**	*0.035599024*	*0.031096394*	*0.033103078*	*0.02641224*
**ENSG00000101463**	*0.014635588*	*0.024247874*	*0.025468423*	*0.014611778*
**ENSG00000089177**	*0.018984984*	*0.026320239*	*0.02697984*	*0.018095603*
**ENSG00000130222**	*0.099711207*	*0.071198757*	*0.07782125*	*0.072577742*
**ENSG00000063127**	*0.026644626*	*0.032310116*	*0.029051817*	*0.026142667*
**ENSG00000122035**	*0.015121296*	*0.025031767*	*0.025236628*	*0.015087462*
**ENSG00000168754**	*0.014430386*	*0.024716477*	*0.024377491*	*0.015181736*
**ENSG00000115705**	*0.018279452*	*0.024863383*	*0.024446053*	*0.016324521*
**ENSG00000100664**	*0.024152059*	*0.027631482*	*0.026942252*	*0.021043227*
**ENSG00000162814**	*0.017480172*	*0.025022935*	*0.024528449*	*0.015108703*
**ENSG00000171551**	*0.017840496*	*0.025252808*	*0.02439715*	*0.015129396*
**ENSG00000067177**	*0.023482417*	*0.044182336*	*0.038671501*	*0.035599315*
**ENSG00000124920**	*0.088432251*	*0.088153152*	*0.052718326*	*0.075593777*
**ENSG00000180083**	*0.016131857*	*0.026104414*	*0.024622192*	*0.014815567*
**ENSG00000228300**	*0.028943043*	*0.033697439*	*0.029618685*	*0.027215345*
**ENSG00000109107**	*0.051979315*	*0.042251625*	*0.03349299*	*0.042228086*
**ENSG00000122026**	*0.015739881*	*0.0241802*	*0.023401279*	*0.017433485*
**ENSG00000212899**	*0.016351681*	*0.024996349*	*0.025921515*	*0.015374919*
**ENSG00000135048**	*0.059814505*	*0.060216089*	*0.05785394*	*0.06514082*
**ENSG00000159363**	*0.028310737*	*0.033346491*	*0.033822376*	*0.039415282*
**ENSG00000053702**	*0.015641693*	*0.024151529*	*0.024520271*	*0.015783727*
**ENSG00000168661**	*0.052190231*	*0.0438243*	*0.043778826*	*0.034606749*
**ENSG00000011009**	*0.02336281*	*0.031653192*	*0.034732066*	*0.027359723*
**ENSG00000125122**	*0.020085718*	*0.027287234*	*0.025191087*	*0.019944634*
**ENSG00000163050**	*0.039134572*	*0.035026501*	*0.034472133*	*0.031866017*
**ENSG00000213672**	*0.064841525*	*0.052498278*	*0.042688466*	*0.046509948*
**ENSG00000100526**	*0.039503669*	*0.032061234*	*0.033465027*	*0.028350511*
**ENSG00000169607**	*0.051116932*	*0.04004399*	*0.039834326*	*0.038343192*
**ENSG00000100078**	*0.015169173*	*0.024496754*	*0.02522668*	*0.015758914*
**ENSG00000026652**	*0.02350237*	*0.026472809*	*0.026599402*	*0.019935618*
**ENSG00000159263**	*0.018327927*	*0.027240187*	*0.024968779*	*0.016781021*
**ENSG00000001036**	*0.033743553*	*0.032973499*	*0.030840359*	*0.031719577*
**ENSG00000171873**	*0.014730927*	*0.025179353*	*0.024995221*	*0.014291679*
**ENSG00000120742**	*0.028915256*	*0.034874261*	*0.034409668*	*0.029583514*
**ENSG00000160007**	*0.038348531*	*0.04339877*	*0.035220807*	*0.035165661*
**ENSG00000169398**	*0.130317302*	*0.09011789*	*0.087303612*	*0.109845643*
**ENSG00000108176**	*0.06201443*	*0.048693444*	*0.035951994*	*0.032252218*
**ENSG00000026751**	*0.027531022*	*0.032902892*	*0.033548003*	*0.025595796*
**ENSG00000059804**	*0.050015946*	*0.04965571*	*0.071959386*	*0.063436383*
**ENSG00000185658**	*0.034902468*	*0.051261296*	*0.050021444*	*0.03496537*
**ENSG00000221819**	*0.016337437*	*0.024863795*	*0.024421675*	*0.014635942*
**ENSG00000164654**	*0.031275801*	*0.031647924*	*0.03043221*	*0.024998099*
**ENSG00000117155**	*0.020539796*	*0.02757368*	*0.026429404*	*0.016199002*
**ENSG00000181704**	*0.029121669*	*0.034331195*	*0.032497306*	*0.022686835*
**ENSG00000115525**	*0.074112548*	*0.071092828*	*0.048653972*	*0.056132967*
**ENSG00000189079**	*0.033082101*	*0.032815848*	*0.03210668*	*0.024965468*

**ENSG00000174032**	*0.04336525*	*0.045635685*	*0.037604188*	*0.03221862*
**ENSG00000103021**	*0.035183469*	*0.033812108*	*0.034468143*	*0.033932453*
**ENSG00000163235**	*0.014979305*	*0.024412309*	*0.025649439*	*0.015083653*
**ENSG00000060688**	*0.025863966*	*0.030393417*	*0.032971467*	*0.026205059*
**ENSG00000172653**	*0.01637212*	*0.024315407*	*0.024793798*	*0.015073187*
**ENSG00000127325**	*0.015628475*	*0.026589427*	*0.025848304*	*0.017313715*
**ENSG00000003096**	*0.019098719*	*0.025576573*	*0.025876672*	*0.022229595*
**ENSG00000106624**	*0.079027575*	*0.066459221*	*0.05964046*	*0.059850274*
**ENSG00000178974**	*0.026638612*	*0.037030629*	*0.033838821*	*0.019622261*
**ENSG00000169474**	*0.015295454*	*0.024498808*	*0.024407685*	*0.015527992*
**ENSG00000088280**	*0.019510396*	*0.024954055*	*0.024407391*	*0.014317835*
**ENSG00000094916**	*0.033518313*	*0.035137243*	*0.033867987*	*0.030044063*
**ENSG00000177465**	*0.071077528*	*0.056088253*	*0.040666438*	*0.044053954*
**ENSG00000156017**	*0.016198065*	*0.02446275*	*0.025221498*	*0.014765774*
**ENSG00000136826**	*0.064235316*	*0.038975706*	*0.031425605*	*0.03708229*
**ENSG00000126838**	*0.016553311*	*0.025604315*	*0.024518261*	*0.014624862*
**ENSG00000082213**	*0.033840092*	*0.041246373*	*0.041195918*	*0.037529552*
**ENSG00000158806**	*0.045555207*	*0.039780568*	*0.042040557*	*0.042220531*
**ENSG00000164211**	*0.036100473*	*0.037282957*	*0.038187752*	*0.037035143*
**ENSG00000165025**	*0.059553944*	*0.049428212*	*0.046018974*	*0.042900956*
**ENSG00000221995**	*0.03652178*	*0.033747153*	*0.032768889*	*0.034369077*
**ENSG00000124120**	*0.039172129*	*0.038544073*	*0.034842373*	*0.040011243*
**ENSG00000120457**	*0.01462346*	*0.025136728*	*0.024864333*	*0.014370925*
**ENSG00000165533**	*0.036173687*	*0.039555892*	*0.035778106*	*0.05235629*
**ENSG00000173276**	*0.01834317*	*0.028705026*	*0.02658787*	*0.015187706*
**ENSG00000162592**	*0.01720805*	*0.02528591*	*0.025183545*	*0.018286553*
**ENSG00000179873**	*0.082065145*	*0.033630224*	*0.050621824*	*0.049860597*
**ENSG00000165507**	*0.097540477*	*0.09898511*	*0.097091527*	*0.092466993*
**ENSG00000173267**	*0.018831506*	*0.024277408*	*0.024109391*	*0.014264391*
**ENSG00000204390**	*0.032385553*	*0.030357014*	*0.030263826*	*0.024918735*
**ENSG00000115718**	*0.015638588*	*0.02485108*	*0.024472157*	*0.01548491*
**ENSG00000119541**	*0.025440784*	*0.030957564*	*0.0313818*	*0.027241532*
**ENSG00000143198**	*0.037239329*	*0.038049062*	*0.039593384*	*0.03062077*
**ENSG00000109944**	*0.019177271*	*0.026673265*	*0.025722772*	*0.018263365*
**ENSG00000162989**	*0.016467682*	*0.025484508*	*0.024713905*	*0.015727605*
**ENSG00000213023**	*0.016557806*	*0.024937921*	*0.026794447*	*0.015398157*
**ENSG00000204472**	*0.016365043*	*0.02518547*	*0.024787598*	*0.016170085*
**ENSG00000197713**	*0.045317979*	*0.048193547*	*0.039576117*	*0.030553616*
**ENSG00000143842**	*0.036287294*	*0.026492506*	*0.02607799*	*0.018858136*
**ENSG00000139351**	*0.01898251*	*0.02788297*	*0.028447118*	*0.014503626*
**ENSG00000183763**	*0.039653672*	*0.031248358*	*0.033918243*	*0.031918156*
**ENSG00000109606**	*0.028510496*	*0.035211358*	*0.028317399*	*0.029618352*
**ENSG00000175324**	*0.022620437*	*0.030851437*	*0.032955097*	*0.022967601*
**ENSG00000007402**	*0.017532233*	*0.024702648*	*0.02460512*	*0.015324679*
**ENSG00000173273**	*0.023120136*	*0.029986282*	*0.028514621*	*0.019788104*
**ENSG00000172613**	*0.033572588*	*0.031103635*	*0.031599706*	*0.024114693*
**ENSG00000103512**	*0.025472635*	*0.030319948*	*0.03147734*	*0.02692767*
**ENSG00000139910**	*0.026239879*	*0.028225792*	*0.031881912*	*0.026306614*
**ENSG00000073464**	*0.025988977*	*0.026109667*	*0.025181384*	*0.019925429*
**ENSG00000165916**	*0.027569352*	*0.030285739*	*0.030187857*	*0.027700971*
**ENSG00000166527**	*0.018672709*	*0.025995986*	*0.026662629*	*0.025295715*
**ENSG00000176246**	*0.0187651*	*0.027439513*	*0.02648575*	*0.017463859*
**ENSG00000006125**	*0.026766567*	*0.033147348*	*0.032715267*	*0.025986158*
**ENSG00000109062**	*0.048675872*	*0.044228821*	*0.040333168*	*0.039768384*

**ENSG00000108312**	*0.032727565*	*0.03393951*	*0.031805883*	*0.030757245*
**ENSG00000128266**	*0.066313162*	*0.047095217*	*0.03093469*	*0.026764469*
**ENSG00000125445**	*0.0277688*	*0.032512025*	*0.031651975*	*0.025878204*
**ENSG00000120156**	*0.016583483*	*0.0247091*	*0.024474808*	*0.015768062*
**ENSG00000164161**	*0.021594226*	*0.028059014*	*0.026324598*	*0.019547599*
**ENSG00000168582**	*0.020463595*	*0.027459359*	*0.028538009*	*0.018962451*
**ENSG00000185621**	*0.014556463*	*0.025027492*	*0.024635847*	*0.015913486*
**ENSG00000103316**	*0.138613781*	*0.083985195*	*0.091932412*	*0.114254697*
**ENSG00000075711**	*0.045785677*	*0.049461525*	*0.044130412*	*0.046136703*
**ENSG00000173801**	*0.033148707*	*0.029846618*	*0.031753597*	*0.0247811*
**ENSG00000134243**	*0.018595122*	*0.025922859*	*0.028480464*	*0.016341806*
**ENSG00000178202**	*0.051631945*	*0.049433767*	*0.038166769*	*0.042825473*
**ENSG00000128714**	*0.014734096*	*0.025228577*	*0.02595694*	*0.015470021*
**ENSG00000163629**	*0.116521214*	*0.05015581*	*0.028495569*	*0.052547833*
**ENSG00000088881**	*0.016635392*	*0.025613567*	*0.02613835*	*0.017088524*
**ENSG00000067064**	*0.025105348*	*0.031010908*	*0.028527861*	*0.022052599*
**ENSG00000205683**	*0.016925828*	*0.025763724*	*0.02417146*	*0.01482482*
**ENSG00000156575**	*0.016934923*	*0.024524989*	*0.024581034*	*0.015088355*
**ENSG00000130656**	*0.015817994*	*0.02531148*	*0.025298878*	*0.016378913*
**ENSG00000025772**	*0.036825932*	*0.05216023*	*0.044707483*	*0.034843156*
**ENSG00000127561**	*0.040085288*	*0.040658878*	*0.035137208*	*0.034015945*
**ENSG00000128567**	*0.020141865*	*0.027733247*	*0.027299631*	*0.017119345*
**ENSG00000167612**	*0.026718954*	*0.028103006*	*0.027667938*	*0.030589624*
**ENSG00000221916**	*0.017355287*	*0.025917413*	*0.026269628*	*0.01803438*
**ENSG00000154025**	*0.018153833*	*0.026413102*	*0.02845549*	*0.017000065*
**ENSG00000115355**	*0.016417735*	*0.024714456*	*0.02504479*	*0.016237555*
**ENSG00000146576**	*0.030662411*	*0.038916936*	*0.046566388*	*0.032974612*
**ENSG00000124802**	*0.030913613*	*0.032259139*	*0.032310658*	*0.034828815*
**ENSG00000196331**	*0.017639977*	*0.026099238*	*0.026330466*	*0.015136164*
**ENSG00000163219**	*0.097115474*	*0.089849251*	*0.080481474*	*0.086171752*
**ENSG00000205629**	*0.024695946*	*0.03313662*	*0.030995249*	*0.020657859*
**ENSG00000161896**	*0.015958247*	*0.026214915*	*0.026009323*	*0.016706622*
**ENSG00000100191**	*0.017160214*	*0.024378739*	*0.024490049*	*0.016521518*
**ENSG00000092621**	*0.091324349*	*0.053261826*	*0.044355667*	*0.059217501*
**ENSG00000117399**	*0.042033993*	*0.038878427*	*0.035778217*	*0.037770832*
**ENSG00000117305**	*0.036951927*	*0.034732467*	*0.035510011*	*0.033426661*
**ENSG00000139174**	*0.04327855*	*0.044256888*	*0.049122159*	*0.045857556*
**ENSG00000108854**	*0.037584403*	*0.036372104*	*0.040558521*	*0.026584987*
**ENSG00000137210**	*0.026030311*	*0.028737279*	*0.027851442*	*0.028898399*
**ENSG00000239305**	*0.0389452*	*0.043072488*	*0.047786536*	*0.038212413*
**ENSG00000112208**	*0.033193061*	*0.033781576*	*0.032578345*	*0.029440065*
**ENSG00000181163**	*0.017675986*	*0.02592497*	*0.024900945*	*0.015636043*
**ENSG00000160469**	*0.018077503*	*0.027074774*	*0.027251472*	*0.015411075*
**ENSG00000119698**	*0.074712223*	*0.040841884*	*0.035252233*	*0.102793874*
**ENSG00000126759**	*0.048237592*	*0.057791802*	*0.046541558*	*0.05763986*
**ENSG00000197937**	*0.026307045*	*0.032671269*	*0.027687094*	*0.025825674*
**ENSG00000048649**	*0.022213129*	*0.029800774*	*0.0315326*	*0.02003033*
**ENSG00000122490**	*0.028500549*	*0.032769604*	*0.031326179*	*0.02175351*
**ENSG00000064313**	*0.027729369*	*0.035347321*	*0.031819261*	*0.024762081*
**ENSG00000089250**	*0.016541134*	*0.025296365*	*0.024165564*	*0.014831681*
**ENSG00000089723**	*0.018690406*	*0.026874399*	*0.027689634*	*0.01928935*
**ENSG00000116691**	*0.066260577*	*0.045335472*	*0.047319936*	*0.048287034*
**ENSG00000116882**	*0.01498325*	*0.026148847*	*0.025218647*	*0.015225552*
**ENSG00000145348**	*0.067432558*	*0.062871969*	*0.047113427*	*0.044675118*

**ENSG00000156970**	*0.042480061*	*0.038345954*	*0.034422426*	*0.034748498*
**ENSG00000217555**	*0.085161078*	*0.083464348*	*0.091985536*	*0.089997759*
**ENSG00000143786**	*0.05525231*	*0.036854752*	*0.031316032*	*0.034714212*
**ENSG00000022277**	*0.019742048*	*0.028106367*	*0.027871487*	*0.018274563*
**ENSG00000113269**	*0.068653607*	*0.077142418*	*0.054021336*	*0.067947446*
**ENSG00000183785**	*0.015697525*	*0.02517463*	*0.024859258*	*0.015608266*
**ENSG00000225932**	*0.048714375*	*0.048184359*	*0.043937762*	*0.050621344*
**ENSG00000165832**	*0.030753974*	*0.042406061*	*0.032173077*	*0.029616332*
**ENSG00000099937**	*0.031810177*	*0.059704173*	*0.045514206*	*0.039981901*
**ENSG00000055044**	*0.025383953*	*0.029446071*	*0.033750525*	*0.029500317*
**ENSG00000125249**	*0.03500425*	*0.038364143*	*0.033229089*	*0.029812844*
**ENSG00000028116**	*0.03553591*	*0.042310475*	*0.033301942*	*0.029335669*
**ENSG00000103043**	*0.025409116*	*0.031365942*	*0.033358809*	*0.033388101*
**ENSG00000114861**	*0.106246383*	*0.103078869*	*0.086380887*	*0.104627391*
**ENSG00000103024**	*0.033418689*	*0.032078452*	*0.032846323*	*0.034808495*
**ENSG00000114316**	*0.025144403*	*0.032315705*	*0.029994988*	*0.024490118*
**ENSG00000115459**	*0.022606372*	*0.031587501*	*0.027850379*	*0.041571332*
**ENSG00000073050**	*0.031093751*	*0.033789271*	*0.028741673*	*0.023646306*
**ENSG00000064666**	*0.048404059*	*0.050468297*	*0.060510623*	*0.050666604*
**ENSG00000213853**	*0.033028093*	*0.035878906*	*0.029471392*	*0.02973352*
**ENSG00000186226**	*0.040843016*	*0.03385098*	*0.028933738*	*0.027384659*
**ENSG00000185324**	*0.020302891*	*0.027818922*	*0.026591813*	*0.019003755*
**ENSG00000129173**	*0.057712124*	*0.036648712*	*0.035898048*	*0.03687085*
**ENSG00000139737**	*0.055541136*	*0.054032767*	*0.045947562*	*0.044793941*
**ENSG00000179094**	*0.034398676*	*0.048503143*	*0.049723332*	*0.052849199*
**ENSG00000056558**	*0.03737994*	*0.029316092*	*0.032135193*	*0.031319765*
**ENSG00000119514**	*0.030507613*	*0.043472474*	*0.03510636*	*0.030114267*
**ENSG00000159208**	*0.031093847*	*0.032347825*	*0.029856699*	*0.028051718*
**ENSG00000204003**	*0.017897473*	*0.026963272*	*0.02527559*	*0.01670561*
**ENSG00000070915**	*0.022503023*	*0.026174535*	*0.027649508*	*0.025529001*
**ENSG00000109163**	*0.016535412*	*0.024710807*	*0.02663227*	*0.014626322*
**ENSG00000100888**	*0.02468467*	*0.03330271*	*0.030513019*	*0.021707914*
**ENSG00000114115**	*0.044927253*	*0.054238054*	*0.042998748*	*0.052580595*
**ENSG00000144559**	*0.042514744*	*0.039392792*	*0.035278814*	*0.026650066*
**ENSG00000197858**	*0.037548997*	*0.035726615*	*0.036746768*	*0.036307335*
**ENSG00000179168**	*0.016789656*	*0.025578133*	*0.026227162*	*0.016336109*
**ENSG00000119720**	*0.019096949*	*0.026675255*	*0.027162218*	*0.019659666*
**ENSG00000151292**	*0.030113747*	*0.036705546*	*0.037024024*	*0.031513651*
**ENSG00000188992**	*0.018551758*	*0.02643893*	*0.025916839*	*0.016914516*
**ENSG00000197170**	*0.039467063*	*0.044959966*	*0.037057183*	*0.033889662*
**ENSG00000149311**	*0.015179837*	*0.025258341*	*0.025128805*	*0.014827408*
**ENSG00000174808**	*0.015766796*	*0.02627156*	*0.024791145*	*0.018036064*
**ENSG00000087095**	*0.027939875*	*0.04010816*	*0.03775955*	*0.029631927*
**ENSG00000126453**	*0.029832793*	*0.030162872*	*0.028111564*	*0.024480491*
**ENSG00000152409**	*0.04651155*	*0.057438923*	*0.05491614*	*0.050351405*
**ENSG00000185129**	*0.024710648*	*0.031005954*	*0.029141133*	*0.024578995*
**ENSG00000149639**	*0.021073317*	*0.027547875*	*0.026352199*	*0.020926008*
**ENSG00000137955**	*0.029594519*	*0.030226075*	*0.02727326*	*0.021253777*
**ENSG00000142149**	*0.016724206*	*0.025469258*	*0.02649469*	*0.015810072*
**ENSG00000157017**	*0.03700909*	*0.032004278*	*0.031759819*	*0.02955568*
**ENSG00000168612**	*0.016305107*	*0.026941745*	*0.02685601*	*0.016266171*
**ENSG00000033800**	*0.022001049*	*0.028884505*	*0.030732866*	*0.022673893*
**ENSG00000230463**	*0.227990217*	*0.245892985*	*0.24195951*	*0.143518506*
**ENSG00000077549**	*0.022713961*	*0.030230126*	*0.028489414*	*0.023114371*

**ENSG00000250423**	*0.0475919*	*0.03009931*	*0.024953953*	*0.01476162*
**ENSG00000161249**	*0.103718138*	*0.090916479*	*0.074412097*	*0.086277186*
**ENSG00000102543**	*0.017901472*	*0.026315074*	*0.026169986*	*0.017093754*
**ENSG00000128463**	*0.025833504*	*0.030865027*	*0.030100934*	*0.020979186*
**ENSG00000180660**	*0.022630508*	*0.026089852*	*0.02713868*	*0.020509134*
**ENSG00000108342**	*0.017470193*	*0.027829192*	*0.027492241*	*0.01905982*
**ENSG00000104043**	*0.053919449*	*0.034756658*	*0.032567026*	*0.027516674*
**ENSG00000182534**	*0.068595167*	*0.089366884*	*0.086905797*	*0.108203663*
**ENSG00000171368**	*0.018572096*	*0.02690532*	*0.025290473*	*0.016429181*
**ENSG00000142856**	*0.030141559*	*0.034436504*	*0.029765958*	*0.024298146*
**ENSG00000164112**	*0.015907581*	*0.025562043*	*0.025222651*	*0.016219053*
**ENSG00000213638**	*0.037188628*	*0.037878464*	*0.043932175*	*0.035272331*
**ENSG00000177565**	*0.034582794*	*0.03433191*	*0.033315822*	*0.025850742*
**ENSG00000149050**	*0.024902894*	*0.028898179*	*0.027069017*	*0.019271314*
**ENSG00000196961**	*0.02735018*	*0.035169989*	*0.02893173*	*0.02688306*
**ENSG00000180424**	*0.018061816*	*0.025359211*	*0.024894443*	*0.01711995*
**ENSG00000240654**	*0.014462409*	*0.024904713*	*0.024184868*	*0.014988073*
**ENSG00000050555**	*0.017490876*	*0.025939233*	*0.02563221*	*0.016070832*
**ENSG00000174989**	*0.014870765*	*0.025473662*	*0.024302791*	*0.014428205*
**ENSG00000186510**	*0.04990005*	*0.05113753*	*0.048637386*	*0.046661658*
**ENSG00000136878**	*0.023729087*	*0.031592311*	*0.033223593*	*0.026389997*
**ENSG00000152492**	*0.017849089*	*0.026680522*	*0.02630307*	*0.020629904*
**ENSG00000184640**	*0.03112829*	*0.031797681*	*0.03139953*	*0.025850551*
**ENSG00000187026**	*0.018886048*	*0.0265769*	*0.026106074*	*0.017979714*
**ENSG00000146833**	*0.037145169*	*0.04041359*	*0.041500173*	*0.028787248*
**ENSG00000188153**	*0.053974501*	*0.026062813*	*0.025288543*	*0.021258635*
**ENSG00000039123**	*0.027569338*	*0.032862914*	*0.029320684*	*0.020866869*
**ENSG00000087111**	*0.02722953*	*0.032948491*	*0.031204587*	*0.026785909*
**ENSG00000130826**	*0.029312063*	*0.032711918*	*0.033418274*	*0.027992177*
**ENSG00000169683**	*0.059517616*	*0.0530124*	*0.045673914*	*0.03831195*
**ENSG00000172817**	*0.117900205*	*0.079424422*	*0.078807507*	*0.105571111*
**ENSG00000180423**	*0.020208949*	*0.025878621*	*0.027314601*	*0.019804909*
**ENSG00000188959**	*0.01358332*	*0.024670207*	*0.024039882*	*0.013267592*
**ENSG00000164062**	*0.033003827*	*0.033923686*	*0.030450846*	*0.026142724*
**ENSG00000165264**	*0.030363274*	*0.030911101*	*0.028266177*	*0.026755424*
**ENSG00000163154**	*0.05222055*	*0.056734511*	*0.044985*	*0.045180991*
**ENSG00000159337**	*0.015099091*	*0.024787162*	*0.025327711*	*0.014976927*
**ENSG00000086619**	*0.035231646*	*0.035251442*	*0.039723573*	*0.03476654*
**ENSG00000008196**	*0.045915249*	*0.025035296*	*0.025508793*	*0.02805607*
**ENSG00000047056**	*0.022853063*	*0.029119731*	*0.028501941*	*0.020640551*
**ENSG00000239998**	*0.046160043*	*0.047479047*	*0.051804501*	*0.064693431*
**ENSG00000141337**	*0.02388426*	*0.027908055*	*0.029783624*	*0.020467211*
**ENSG00000050730**	*0.049084851*	*0.041558316*	*0.035625736*	*0.050105468*
**ENSG00000249760**	*0.041111258*	*0.030683079*	*0.035226771*	*0.027251037*
**ENSG00000213918**	*0.023362578*	*0.034504078*	*0.029742975*	*0.023964726*
**ENSG00000196684**	*0.033942199*	*0.033148267*	*0.04396656*	*0.035122842*
**ENSG00000183103**	*0.01707995*	*0.025278367*	*0.026107162*	*0.015264867*
**ENSG00000003509**	*0.039271338*	*0.045249874*	*0.035841893*	*0.031247059*
**ENSG00000115271**	*0.034018349*	*0.034891018*	*0.02977684*	*0.02745647*
**ENSG00000181544**	*0.0366865*	*0.045006081*	*0.030461324*	*0.027166879*
**ENSG00000160410**	*0.027917762*	*0.032016286*	*0.035347034*	*0.029856272*
**ENSG00000241690**	*0.01595131*	*0.02476713*	*0.025057084*	*0.014920671*
**ENSG00000136811**	*0.025394986*	*0.029644983*	*0.028091678*	*0.018977676*
**ENSG00000177600**	*0.016941177*	*0.02542295*	*0.026016969*	*0.017124491*

**ENSG00000066230**	*0.016532433*	*0.024918848*	*0.024967823*	*0.016162513*
**ENSG00000143768**	*0.015978379*	*0.024938887*	*0.026085043*	*0.016125179*
**ENSG00000124701**	*0.018973023*	*0.0271673*	*0.028649347*	*0.015973957*
**ENSG00000197153**	*0.062813842*	*0.046367586*	*0.063325329*	*0.066025973*
**ENSG00000125503**	*0.033879361*	*0.039969638*	*0.031773566*	*0.031824437*
**ENSG00000100764**	*0.022018797*	*0.02775502*	*0.027681978*	*0.020016226*
**ENSG00000107290**	*0.026745069*	*0.030393315*	*0.034422431*	*0.035604586*
**ENSG00000130227**	*0.033245755*	*0.042757111*	*0.033585981*	*0.025916254*
**ENSG00000062038**	*0.015980601*	*0.024529059*	*0.024358217*	*0.016319693*
**ENSG00000101843**	*0.026669763*	*0.032913375*	*0.029309161*	*0.020435*
**ENSG00000088826**	*0.015625099*	*0.025508242*	*0.025263476*	*0.015346569*
**ENSG00000151917**	*0.034587074*	*0.029148471*	*0.029282351*	*0.025668437*
**ENSG00000132639**	*0.01622415*	*0.025765284*	*0.025894585*	*0.021124354*
**ENSG00000063587**	*0.032406851*	*0.032806958*	*0.03446908*	*0.032645459*
**ENSG00000133138**	*0.021045099*	*0.02795177*	*0.028355728*	*0.019826858*
**ENSG00000186526**	*0.015174265*	*0.024819827*	*0.024972204*	*0.015896457*
**ENSG00000182585**	*0.0158317*	*0.025233169*	*0.02486135*	*0.016381266*
**ENSG00000145721**	*0.016873627*	*0.026577154*	*0.02556085*	*0.016685113*
**ENSG00000074054**	*0.027043583*	*0.035108538*	*0.033457262*	*0.024167177*
**ENSG00000069011**	*0.018805841*	*0.026961101*	*0.027009669*	*0.018088779*
**ENSG00000172538**	*0.01823585*	*0.025431418*	*0.027708213*	*0.016383881*
**ENSG00000012504**	*0.022277439*	*0.029353746*	*0.025331999*	*0.020385111*
**ENSG00000135750**	*0.103878712*	*0.1104387*	*0.083605864*	*0.094562235*
**ENSG00000167394**	*0.024206482*	*0.029601213*	*0.034889495*	*0.026486986*
**ENSG00000240891**	*0.017529625*	*0.025517404*	*0.025145669*	*0.016369226*
**ENSG00000113845**	*0.032589147*	*0.036847607*	*0.029646238*	*0.024666443*
**ENSG00000120328**	*0.014972711*	*0.024846976*	*0.025349017*	*0.015257656*
**ENSG00000169087**	*0.040331382*	*0.041780441*	*0.038494157*	*0.037742848*
**ENSG00000204006**	*0.01611981*	*0.025511849*	*0.025727836*	*0.014787612*
**ENSG00000176845**	*0.046114235*	*0.057573052*	*0.047741358*	*0.039014744*
**ENSG00000143507**	*0.051494914*	*0.043395945*	*0.049129771*	*0.044156107*
**ENSG00000124562**	*0.025493463*	*0.026696951*	*0.027708217*	*0.023291951*
**ENSG00000120669**	*0.017281484*	*0.025838351*	*0.026023285*	*0.016568305*
**ENSG00000136542**	*0.033432951*	*0.025480471*	*0.025395354*	*0.015678204*
**ENSG00000122786**	*0.029283941*	*0.025567417*	*0.026224211*	*0.018625003*
**ENSG00000223609**	*0.085935894*	*0.033199073*	*0.042799869*	*0.083043489*
**ENSG00000156009**	*0.023890928*	*0.024394397*	*0.025545271*	*0.016007832*
**ENSG00000166405**	*0.030182286*	*0.030912882*	*0.027329053*	*0.023643346*
**ENSG00000131437**	*0.046315985*	*0.041044963*	*0.043320929*	*0.03966687*
**ENSG00000177370**	*0.05269897*	*0.052399014*	*0.057888441*	*0.054099979*
**ENSG00000122390**	*0.018186339*	*0.028579792*	*0.03185809*	*0.025033509*
**ENSG00000070495**	*0.016843569*	*0.025478967*	*0.025162102*	*0.01598017*
**ENSG00000158710**	*0.025969898*	*0.029777191*	*0.031415356*	*0.032058968*
**ENSG00000103005**	*0.035706924*	*0.04136199*	*0.033775049*	*0.034093723*
**ENSG00000184408**	*0.074876262*	*0.062663521*	*0.047459687*	*0.065238911*
**ENSG00000058673**	*0.020934865*	*0.028940313*	*0.030259411*	*0.018406434*
**ENSG00000091128**	*0.01719517*	*0.027652472*	*0.026702296*	*0.017423585*
**ENSG00000167232**	*0.019384111*	*0.026351775*	*0.02806328*	*0.017139584*
**ENSG00000251380**	*0.016088486*	*0.025440098*	*0.025577058*	*0.015858176*
**ENSG00000101126**	*0.026887175*	*0.03179545*	*0.029097994*	*0.022012707*
**ENSG00000114650**	*0.026020642*	*0.036247715*	*0.032505642*	*0.02484927*
**ENSG00000085741**	*0.053122879*	*0.049118564*	*0.043227272*	*0.050456182*
**ENSG00000077080**	*0.017560749*	*0.025495536*	*0.025294511*	*0.018882761*
**ENSG00000152932**	*0.058455844*	*0.041433895*	*0.051119721*	*0.060204541*

**ENSG00000109971**	*0.027304622*	*0.03296588*	*0.033943442*	*0.024281984*
**ENSG00000064490**	*0.022786097*	*0.028875126*	*0.028385754*	*0.02243642*
**ENSG00000171872**	*0.014524868*	*0.026069684*	*0.025131616*	*0.015094534*
**ENSG00000144119**	*0.018009734*	*0.026567762*	*0.026697606*	*0.016175371*
**ENSG00000165566**	*0.032842388*	*0.027576174*	*0.025712959*	*0.019791663*
**ENSG00000143032**	*0.017782824*	*0.025170633*	*0.025115091*	*0.016166701*
**ENSG00000129566**	*0.030826934*	*0.031511708*	*0.03395909*	*0.028113429*
**ENSG00000205359**	*0.015979051*	*0.025444067*	*0.024709427*	*0.016054412*
**ENSG00000046647**	*0.040626095*	*0.036191515*	*0.031275215*	*0.035787514*
**ENSG00000188770**	*0.014061745*	*0.024438328*	*0.025786839*	*0.015277622*
**ENSG00000185088**	*0.020169608*	*0.027830362*	*0.026691522*	*0.017583972*
**ENSG00000182851**	*0.015348698*	*0.024953924*	*0.025293415*	*0.01471782*
**ENSG00000164077**	*0.034481575*	*0.034472595*	*0.034498511*	*0.033621622*
**ENSG00000175513**	*0.015399746*	*0.025274855*	*0.025659641*	*0.015281766*
**ENSG00000169495**	*0.015508462*	*0.025471148*	*0.026598314*	*0.014016519*
**ENSG00000120235**	*0.014846828*	*0.024723102*	*0.024898027*	*0.014950554*
**ENSG00000169906**	*0.018315305*	*0.025723194*	*0.024943803*	*0.017044787*
**ENSG00000101842**	*0.028528561*	*0.030807638*	*0.029262581*	*0.032815459*
**ENSG00000196664**	*0.049362312*	*0.050537991*	*0.049266214*	*0.047248789*
**ENSG00000166959**	*0.020791503*	*0.026664502*	*0.025402783*	*0.020140987*
**ENSG00000184709**	*0.097674489*	*0.083079347*	*0.073743059*	*0.086865571*
**ENSG00000135148**	*0.0292402*	*0.037315544*	*0.03786352*	*0.03803886*
**ENSG00000001630**	*0.029637246*	*0.030932077*	*0.032152334*	*0.026625167*
**ENSG00000121067**	*0.022913987*	*0.031712014*	*0.030083749*	*0.025090106*
**ENSG00000115425**	*0.035219444*	*0.037601125*	*0.032519104*	*0.029534341*
**ENSG00000221957**	*0.016569989*	*0.024961194*	*0.024814797*	*0.015558753*
**ENSG00000197978**	*0.056894551*	*0.047535104*	*0.045679028*	*0.070723592*
**ENSG00000088888**	*0.026133477*	*0.029948175*	*0.029725987*	*0.0225729*
**ENSG00000181323**	*0.016303789*	*0.026637716*	*0.025159827*	*0.015432639*
**ENSG00000128253**	*0.01587144*	*0.024412492*	*0.026084665*	*0.015478904*
**ENSG00000164109**	*0.039476245*	*0.033055747*	*0.03046293*	*0.030347419*
**ENSG00000148334**	*0.032651907*	*0.034927982*	*0.031756888*	*0.030567196*
**ENSG00000087086**	*0.027554069*	*0.028693181*	*0.027528287*	*0.023762912*
**ENSG00000106113**	*0.015749011*	*0.025258806*	*0.025418507*	*0.017277188*
**ENSG00000134809**	*0.048302406*	*0.040925418*	*0.051987186*	*0.042521381*
**ENSG00000062582**	*0.025172026*	*0.027263029*	*0.028865162*	*0.022015912*
**ENSG00000215712**	*0.021211605*	*0.03019963*	*0.028240799*	*0.020606203*
**ENSG00000140564**	*0.05055241*	*0.050505074*	*0.05362728*	*0.044690065*
**ENSG00000003249**	*0.101591275*	*0.075531396*	*0.081757977*	*0.104295643*
**ENSG00000123636**	*0.043260855*	*0.051711296*	*0.046125379*	*0.045525462*
**ENSG00000154556**	*0.015550962*	*0.025552633*	*0.025307618*	*0.014776113*
**ENSG00000171054**	*0.014994343*	*0.024825666*	*0.024472717*	*0.014847769*
**ENSG00000162836**	*0.054330194*	*0.050032767*	*0.038991606*	*0.040451009*
**ENSG00000172954**	*0.032516294*	*0.030345457*	*0.032028188*	*0.036844522*
**ENSG00000213533**	*0.023345146*	*0.030915713*	*0.027091295*	*0.021291235*
**ENSG00000182149**	*0.024910002*	*0.031398666*	*0.033696674*	*0.021090028*
**ENSG00000049540**	*0.016263588*	*0.024850708*	*0.024731735*	*0.016034797*
**ENSG00000107798**	*0.031376809*	*0.033397745*	*0.029457726*	*0.03803298*
**ENSG00000197279**	*0.054664359*	*0.080651911*	*0.078423615*	*0.053834629*
**ENSG00000080815**	*0.015967945*	*0.024409557*	*0.024757598*	*0.014925137*
**ENSG00000196712**	*0.018508103*	*0.026394069*	*0.026689473*	*0.018596365*
**ENSG00000168630**	*0.087648922*	*0.074639007*	*0.078668719*	*0.080603102*
**ENSG00000138758**	*0.049342334*	*0.062363976*	*0.052163495*	*0.058784955*
**ENSG00000204175**	*0.015726641*	*0.025287065*	*0.025519489*	*0.01548411*

**ENSG00000198157**	*0.017532576*	*0.027295613*	*0.024539573*	*0.01698538*
**ENSG00000136352**	*0.017680619*	*0.025454238*	*0.02492636*	*0.017470324*
**ENSG00000168924**	*0.033146027*	*0.03259715*	*0.03178332*	*0.03344891*
**ENSG00000137877**	*0.015072975*	*0.025296307*	*0.02494654*	*0.015123526*
**ENSG00000135269**	*0.033416483*	*0.039216388*	*0.033797665*	*0.029767365*
**ENSG00000186919**	*0.01604428*	*0.024288317*	*0.024317277*	*0.015623423*
**ENSG00000226288**	*0.015292381*	*0.025544104*	*0.024824415*	*0.014887236*
**ENSG00000165338**	*0.031102231*	*0.03399872*	*0.028059582*	*0.023267505*
**ENSG00000196419**	*0.021704356*	*0.027375938*	*0.027922529*	*0.01826701*
**ENSG00000125505**	*0.025178489*	*0.029711491*	*0.033367638*	*0.031541024*
**ENSG00000109424**	*0.016808051*	*0.026081355*	*0.026096817*	*0.015539434*
**ENSG00000120129**	*0.062321385*	*0.047740024*	*0.047742713*	*0.055634705*
**ENSG00000144711**	*0.038120108*	*0.038178392*	*0.034607104*	*0.033228772*
**ENSG00000111639**	*0.028595718*	*0.030770688*	*0.029776726*	*0.025281405*
**ENSG00000198046**	*0.040273225*	*0.037817635*	*0.035684646*	*0.044977019*
**ENSG00000196511**	*0.055146776*	*0.044869136*	*0.034462364*	*0.036801671*
**ENSG00000158234**	*0.073031502*	*0.046529666*	*0.051521568*	*0.059608583*
**ENSG00000127540**	*0.02305819*	*0.028898802*	*0.027374294*	*0.02021377*
**ENSG00000170037**	*0.029375614*	*0.028427539*	*0.034632212*	*0.024914655*
**ENSG00000175745**	*0.016682451*	*0.026057274*	*0.024529356*	*0.015733108*
**ENSG00000180096**	*0.02382097*	*0.029465221*	*0.030519356*	*0.025027504*
**ENSG00000174953**	*0.028404368*	*0.032984628*	*0.03496496*	*0.027270825*
**ENSG00000165282**	*0.025974489*	*0.029461988*	*0.028932342*	*0.021236138*
**ENSG00000125695**	*0.027904753*	*0.033857469*	*0.033457564*	*0.024759946*
**ENSG00000161381**	*0.015206703*	*0.025635082*	*0.02466705*	*0.015464941*
**ENSG00000119725**	*0.024187034*	*0.035730849*	*0.037628385*	*0.021493706*
**ENSG00000155926**	*0.016975954*	*0.027102662*	*0.027142644*	*0.018581089*
**ENSG00000189046**	*0.029761907*	*0.031262833*	*0.032879651*	*0.026139214*
**ENSG00000147697**	*0.016085735*	*0.02499743*	*0.025124566*	*0.016168069*
**ENSG00000182263**	*0.01667663*	*0.026034789*	*0.025789945*	*0.016175015*
**ENSG00000104369**	*0.015408209*	*0.024174094*	*0.025145408*	*0.015248435*
**ENSG00000145337**	*0.022847054*	*0.030760957*	*0.029868517*	*0.022934447*
**ENSG00000183837**	*0.017131446*	*0.026897279*	*0.025927341*	*0.016797663*
**ENSG00000105135**	*0.031492855*	*0.029480209*	*0.035599825*	*0.021267611*
**ENSG00000187175**	*0.015208431*	*0.024758966*	*0.024272745*	*0.014913446*
**ENSG00000142347**	*0.03537187*	*0.035703675*	*0.031089116*	*0.036243769*
**ENSG00000064999**	*0.033282397*	*0.033662183*	*0.029137974*	*0.025975128*
**ENSG00000172216**	*0.050480002*	*0.059064321*	*0.056485*	*0.049239401*
**ENSG00000104894**	*0.024159331*	*0.028099772*	*0.032182716*	*0.028773121*
**ENSG00000096080**	*0.026747577*	*0.030691763*	*0.031333124*	*0.022948131*
**ENSG00000188050**	*0.016260395*	*0.024308503*	*0.025437253*	*0.015456291*
**ENSG00000141448**	*0.032226637*	*0.031848307*	*0.030700617*	*0.020265937*
**ENSG00000186687**	*0.039985756*	*0.036262747*	*0.041347971*	*0.041468939*
**ENSG00000108100**	*0.041536768*	*0.041718657*	*0.03947135*	*0.039201547*
**ENSG00000149932**	*0.024617437*	*0.029906617*	*0.030086267*	*0.029996309*
**ENSG00000166157**	*0.028297369*	*0.027263925*	*0.02451994*	*0.024709216*
**ENSG00000077380**	*0.027597417*	*0.034636292*	*0.029253176*	*0.027016483*
**ENSG00000213762**	*0.026799807*	*0.047427641*	*0.052289374*	*0.032700641*
**ENSG00000110203**	*0.016390385*	*0.025531365*	*0.025040476*	*0.015516343*
**ENSG00000146414**	*0.028425184*	*0.035293809*	*0.030259037*	*0.023459813*
**ENSG00000140832**	*0.016413449*	*0.025552567*	*0.025148418*	*0.015839942*
**ENSG00000140534**	*0.054416827*	*0.059345334*	*0.041939124*	*0.03861331*
**ENSG00000148702**	*0.015952484*	*0.024853901*	*0.02453214*	*0.015016662*
**ENSG00000130741**	*0.026302583*	*0.029489842*	*0.030238295*	*0.023906434*

**ENSG00000205927**	*0.01650043*	*0.025157595*	*0.025663216*	*0.016601394*
**ENSG00000188306**	*0.015832235*	*0.025505506*	*0.024488139*	*0.016441996*
**ENSG00000144228**	*0.037615954*	*0.036605438*	*0.035639141*	*0.039295375*
**ENSG00000211455**	*0.059227558*	*0.063588483*	*0.057576633*	*0.050398805*
**ENSG00000129538**	*0.016193659*	*0.025668706*	*0.025163625*	*0.015980684*
**ENSG00000155256**	*0.017023837*	*0.026732273*	*0.026252528*	*0.021457408*
**ENSG00000145331**	*0.036587105*	*0.039076091*	*0.03042761*	*0.024915811*
**ENSG00000166411**	*0.032881844*	*0.03494016*	*0.030722168*	*0.029371796*
**ENSG00000078808**	*0.017042519*	*0.025767771*	*0.025882691*	*0.018170375*
**ENSG00000103510**	*0.029278256*	*0.029061882*	*0.031253909*	*0.028005398*
**ENSG00000157060**	*0.015709733*	*0.024614172*	*0.024556691*	*0.014908781*
**ENSG00000196757**	*0.029377608*	*0.032942049*	*0.035969365*	*0.024082766*
**ENSG00000181786**	*0.016007862*	*0.025586801*	*0.024659533*	*0.01601116*
**ENSG00000109618**	*0.032089724*	*0.034428423*	*0.031177315*	*0.024325054*
**ENSG00000041880**	*0.037580605*	*0.035539828*	*0.032581514*	*0.037933932*
**ENSG00000185344**	*0.037473991*	*0.03663827*	*0.037731332*	*0.032455517*
**ENSG00000007168**	*0.031201269*	*0.039312327*	*0.036111374*	*0.031662369*
**ENSG00000125255**	*0.045265789*	*0.03040695*	*0.027977262*	*0.021721666*
**ENSG00000118620**	*0.031141353*	*0.031992779*	*0.034410828*	*0.027885726*
**ENSG00000134760**	*0.039279633*	*0.024814078*	*0.02500118*	*0.015416154*
**ENSG00000166363**	*0.017330401*	*0.025908179*	*0.025796851*	*0.016625683*
**ENSG00000126768**	*0.029472563*	*0.032152994*	*0.031701029*	*0.025331807*
**ENSG00000176715**	*0.02135407*	*0.139754951*	*0.149019386*	*0.037395516*
**ENSG00000060491**	*0.027611634*	*0.035756966*	*0.043360639*	*0.026093934*
**ENSG00000089289**	*0.023289728*	*0.029763613*	*0.029681835*	*0.022485686*
**ENSG00000138592**	*0.027826537*	*0.035656155*	*0.032321551*	*0.022210701*
**ENSG00000213047**	*0.016495742*	*0.025344714*	*0.025393414*	*0.015989983*
**ENSG00000109534**	*0.035507668*	*0.03511993*	*0.033848585*	*0.032643982*
**ENSG00000185278**	*0.015561647*	*0.02453401*	*0.024454994*	*0.015034286*
**ENSG00000170426**	*0.014195117*	*0.025625415*	*0.025091758*	*0.01364394*
**ENSG00000172350**	*0.015666537*	*0.025201209*	*0.025003915*	*0.015372373*
**ENSG00000108439**	*0.025619502*	*0.030840879*	*0.031045915*	*0.022830945*
**ENSG00000164609**	*0.025419824*	*0.028587677*	*0.033337129*	*0.024905263*
**ENSG00000163781**	*0.03444388*	*0.040641181*	*0.032421982*	*0.034159644*
**ENSG00000088256**	*0.038477184*	*0.038243216*	*0.037235672*	*0.03084552*
**ENSG00000137273**	*0.015280614*	*0.024811115*	*0.025142609*	*0.014242778*
**ENSG00000119411**	*0.075948611*	*0.057171015*	*0.061949304*	*0.082992686*
**ENSG00000184678**	*0.055751603*	*0.051749696*	*0.042952836*	*0.0371419*
**ENSG00000120784**	*0.015290489*	*0.024854622*	*0.025324793*	*0.016087757*
**ENSG00000170477**	*0.015972437*	*0.025623666*	*0.024419885*	*0.016402371*
**ENSG00000139998**	*0.050451647*	*0.060667225*	*0.050904014*	*0.054061582*
**ENSG00000115808**	*0.017758267*	*0.026969551*	*0.026085263*	*0.019837607*
**ENSG00000168453**	*0.014378271*	*0.024590855*	*0.024561396*	*0.015804107*
**ENSG00000088812**	*0.028568508*	*0.036265189*	*0.028966744*	*0.025562237*
**ENSG00000164136**	*0.01870919*	*0.026215021*	*0.025364317*	*0.018034657*
**ENSG00000087250**	*0.018808501*	*0.028372045*	*0.025497098*	*0.035706215*
**ENSG00000165195**	*0.015991711*	*0.024531929*	*0.025285175*	*0.015548609*
**ENSG00000166118**	*0.024404452*	*0.028553401*	*0.025749318*	*0.02157758*
**ENSG00000066583**	*0.037451617*	*0.033933148*	*0.033936161*	*0.030974674*
**ENSG00000164440**	*0.063048361*	*0.045221728*	*0.054818626*	*0.062340464*
**ENSG00000176555**	*0.016218082*	*0.024902324*	*0.02445684*	*0.014168161*
**ENSG00000055332**	*0.036652097*	*0.040351368*	*0.035833432*	*0.045815118*
**ENSG00000073350**	*0.076387596*	*0.063028308*	*0.053070712*	*0.05933898*
**ENSG00000163885**	*0.016907368*	*0.025506581*	*0.025090717*	*0.015479631*

**ENSG00000004487**	*0.032545527*	*0.029676354*	*0.028848969*	*0.024294685*
**ENSG00000106605**	*0.034514046*	*0.033466196*	*0.029725309*	*0.028612743*
**ENSG00000185825**	*0.024200132*	*0.030369061*	*0.02846248*	*0.02535619*
**ENSG00000170442**	*0.050264787*	*0.03673347*	*0.03160261*	*0.031269894*
**ENSG00000147162**	*0.032838593*	*0.034305767*	*0.032442146*	*0.027510939*
**ENSG00000121769**	*0.113557783*	*0.092609342*	*0.056427038*	*0.102612406*
**ENSG00000155858**	*0.01583348*	*0.024404881*	*0.025020702*	*0.016287813*
**ENSG00000187848**	*0.015942897*	*0.024320251*	*0.025232248*	*0.015496146*
**ENSG00000139988**	*0.014916386*	*0.024187291*	*0.024092899*	*0.014086303*
**ENSG00000169814**	*0.018721838*	*0.025862591*	*0.0267583*	*0.019714828*
**ENSG00000105605**	*0.015363369*	*0.025081115*	*0.024943668*	*0.015466932*
**ENSG00000104722**	*0.018039454*	*0.02576892*	*0.027999612*	*0.015688326*
**ENSG00000010404**	*0.034862359*	*0.040018712*	*0.038688791*	*0.031114346*
**ENSG00000148358**	*0.030112418*	*0.031056009*	*0.034286835*	*0.025423026*
**ENSG00000172375**	*0.025922424*	*0.030149911*	*0.028762619*	*0.020680147*
**ENSG00000100987**	*0.014543317*	*0.025199822*	*0.025870218*	*0.014487249*
**ENSG00000204001**	*0.015667008*	*0.025098701*	*0.02652186*	*0.016126601*
**ENSG00000105122**	*0.034808602*	*0.038736646*	*0.033092425*	*0.02541124*
**ENSG00000110514**	*0.021153418*	*0.028927347*	*0.028088598*	*0.022630442*
**ENSG00000149634**	*0.017352912*	*0.026410914*	*0.024802984*	*0.017129338*
**ENSG00000117091**	*0.021888725*	*0.026417944*	*0.026878996*	*0.019658286*
**ENSG00000094914**	*0.029048141*	*0.030097965*	*0.031786867*	*0.025456521*
**ENSG00000184647**	*0.019195943*	*0.025651937*	*0.027797279*	*0.015697828*
**ENSG00000162148**	*0.024989281*	*0.033984896*	*0.032881997*	*0.029774964*
**ENSG00000177732**	*0.017069464*	*0.025727243*	*0.025968945*	*0.016928131*
**ENSG00000149428**	*0.041146308*	*0.040122846*	*0.045482545*	*0.050087277*
**ENSG00000168004**	*0.015121731*	*0.024315212*	*0.02496818*	*0.014816095*
**ENSG00000138379**	*0.016120446*	*0.024996862*	*0.026271473*	*0.016542913*
**ENSG00000113263**	*0.028952963*	*0.040239401*	*0.029591051*	*0.026145247*
**ENSG00000249992**	*0.052742161*	*0.028949556*	*0.029544561*	*0.048216502*
**ENSG00000204361**	*0.03559828*	*0.036188616*	*0.033590186*	*0.036087589*
**ENSG00000118242**	*0.046458948*	*0.045733236*	*0.043480921*	*0.048344827*
**ENSG00000143093**	*0.016870405*	*0.024913192*	*0.024784775*	*0.015384535*
**ENSG00000175595**	*0.018603933*	*0.025769185*	*0.026073674*	*0.016961772*
**ENSG00000105643**	*0.027199792*	*0.031515956*	*0.031419625*	*0.026214225*
**ENSG00000211448**	*0.015017352*	*0.025057695*	*0.024947584*	*0.014578478*
**ENSG00000172461**	*0.016980169*	*0.026067062*	*0.025553208*	*0.017924396*
**ENSG00000021355**	*0.120637366*	*0.077203078*	*0.078506823*	*0.075159895*
**ENSG00000084754**	*0.028613225*	*0.033742264*	*0.035636218*	*0.025807443*
**ENSG00000136381**	*0.032987746*	*0.040169272*	*0.034040594*	*0.032116554*
**ENSG00000138035**	*0.048567115*	*0.04662042*	*0.050784182*	*0.05486405*
**ENSG00000213203**	*0.050992197*	*0.040311801*	*0.04748033*	*0.055993252*
**ENSG00000166862**	*0.018791214*	*0.02638295*	*0.026757177*	*0.018699209*
**ENSG00000157240**	*0.053748691*	*0.053219885*	*0.042761772*	*0.04944155*
**ENSG00000140382**	*0.027221102*	*0.034158768*	*0.029660706*	*0.022139741*
**ENSG00000119537**	*0.02344984*	*0.029866595*	*0.029908784*	*0.022495003*
**ENSG00000105948**	*0.03388391*	*0.034503651*	*0.035605587*	*0.029879722*
**ENSG00000158485**	*0.015520638*	*0.024886871*	*0.024355863*	*0.014257587*
**ENSG00000170374**	*0.017178721*	*0.025384967*	*0.02469156*	*0.017047817*
**ENSG00000204219**	*0.016658671*	*0.025566543*	*0.026937797*	*0.015215037*
**ENSG00000156928**	*0.021035457*	*0.02731816*	*0.028233336*	*0.01903319*
**ENSG00000121797**	*0.065542455*	*0.057581677*	*0.047730838*	*0.058352182*
**ENSG00000164736**	*0.016886145*	*0.024643814*	*0.024380456*	*0.014836381*
**ENSG00000115207**	*0.021034428*	*0.028279852*	*0.029362084*	*0.022731891*

**ENSG00000171243**	*0.017529231*	*0.026981429*	*0.026422817*	*0.018752687*
**ENSG00000138271**	*0.019268008*	*0.025328926*	*0.024447549*	*0.019158661*
**ENSG00000120088**	*0.01702678*	*0.025236703*	*0.025287343*	*0.015613502*
**ENSG00000169217**	*0.030160161*	*0.03292443*	*0.036636192*	*0.02643696*
**ENSG00000143379**	*0.020560658*	*0.030610701*	*0.027280833*	*0.022413137*
**ENSG00000134419**	*0.026147479*	*0.028229032*	*0.027799612*	*0.026528554*
**ENSG00000115694**	*0.035660937*	*0.041214341*	*0.040954622*	*0.024755124*
**ENSG00000196132**	*0.018971644*	*0.027566911*	*0.027572945*	*0.020619877*
**ENSG00000148925**	*0.026802843*	*0.035817985*	*0.031394102*	*0.023617166*
**ENSG00000007202**	*0.033199289*	*0.032667058*	*0.029933114*	*0.030253756*
**ENSG00000113384**	*0.02450509*	*0.035258379*	*0.034639395*	*0.021768012*
**ENSG00000125870**	*0.025792614*	*0.030995313*	*0.028213919*	*0.026270287*
**ENSG00000197429**	*0.034821591*	*0.038149003*	*0.038454638*	*0.024135798*
**ENSG00000136261**	*0.078272903*	*0.078887097*	*0.067473353*	*0.076369357*
**ENSG00000179922**	*0.026300899*	*0.033004631*	*0.031910436*	*0.025835069*
**ENSG00000143970**	*0.028121307*	*0.035614486*	*0.029535472*	*0.029804829*
**ENSG00000141101**	*0.024558377*	*0.029830874*	*0.032611952*	*0.023160701*
**ENSG00000176641**	*0.017957602*	*0.026411074*	*0.027595424*	*0.016186823*
**ENSG00000134571**	*0.015654395*	*0.025330621*	*0.026191695*	*0.015492762*
**ENSG00000169727**	*0.02843617*	*0.031296837*	*0.030117687*	*0.025095912*
**ENSG00000123143**	*0.023491125*	*0.031047744*	*0.031958307*	*0.031348566*
**ENSG00000184232**	*0.045247691*	*0.057099998*	*0.068181459*	*0.073110911*
**ENSG00000066056**	*0.022424777*	*0.0256813*	*0.025393438*	*0.026584224*
**ENSG00000163554**	*0.016743302*	*0.026497853*	*0.0259451*	*0.015933707*
**ENSG00000204438**	*0.027145911*	*0.029966432*	*0.029463452*	*0.022433603*
**ENSG00000142892**	*0.036310674*	*0.039974402*	*0.032630964*	*0.028811726*
**ENSG00000111224**	*0.027533722*	*0.037644784*	*0.035218194*	*0.021450453*
**ENSG00000135929**	*0.082199206*	*0.059441825*	*0.042037808*	*0.075686808*
**ENSG00000164403**	*0.022519257*	*0.031827067*	*0.028735519*	*0.02182502*
**ENSG00000203859**	*0.015316703*	*0.024351424*	*0.024248131*	*0.014910716*
**ENSG00000170396**	*0.039056997*	*0.033750256*	*0.029574642*	*0.024605584*
**ENSG00000184613**	*0.016428327*	*0.026492143*	*0.025635274*	*0.016264191*
**ENSG00000171360**	*0.014498046*	*0.025826385*	*0.025167482*	*0.01593599*
**ENSG00000146755**	*0.015708676*	*0.025346374*	*0.02397241*	*0.014722461*
**ENSG00000105426**	*0.019421216*	*0.02640431*	*0.026354309*	*0.015894844*
**ENSG00000174238**	*0.038121171*	*0.033782378*	*0.033168957*	*0.033857155*
**ENSG00000181631**	*0.019155794*	*0.024907026*	*0.025946342*	*0.015428602*
**ENSG00000138593**	*0.030980141*	*0.033957921*	*0.036574747*	*0.027754516*
**ENSG00000139163**	*0.036766158*	*0.043622666*	*0.036651555*	*0.029516202*
**ENSG00000204711**	*0.015296058*	*0.02427431*	*0.025025556*	*0.014998111*
**ENSG00000138138**	*0.028444648*	*0.03467128*	*0.033083835*	*0.033437076*
**ENSG00000169379**	*0.017359113*	*0.026936872*	*0.02493451*	*0.01528066*
**ENSG00000143632**	*0.015956694*	*0.026823324*	*0.026582737*	*0.01724348*
**ENSG00000242265**	*0.07439448*	*0.068637519*	*0.060130774*	*0.067418472*
**ENSG00000105401**	*0.024155055*	*0.028487331*	*0.030520338*	*0.022881529*
**ENSG00000139292**	*0.016842804*	*0.025400787*	*0.025906499*	*0.017001958*
**ENSG00000135521**	*0.033001975*	*0.039105507*	*0.042049604*	*0.038501739*
**ENSG00000112936**	*0.015493444*	*0.025022096*	*0.024238056*	*0.015530967*
**ENSG00000120992**	*0.032873903*	*0.045309129*	*0.038906743*	*0.032343844*
**ENSG00000122728**	*0.035040416*	*0.042695427*	*0.038737405*	*0.035070084*
**ENSG00000037241**	*0.024550564*	*0.029065723*	*0.026605547*	*0.02439379*
**ENSG00000182782**	*0.018300914*	*0.025511009*	*0.025850783*	*0.016341786*
**ENSG00000168298**	*0.050521798*	*0.034163713*	*0.037624451*	*0.045264404*
**ENSG00000198570**	*0.017275338*	*0.025454396*	*0.028094137*	*0.016935186*

**ENSG00000101445**	*0.031796932*	*0.035045851*	*0.033977359*	*0.026383137*
**ENSG00000147789**	*0.02302032*	*0.034759283*	*0.038241247*	*0.022436834*
**ENSG00000106852**	*0.015963177*	*0.024260703*	*0.024529276*	*0.013985368*
**ENSG00000253767**	*0.017125178*	*0.025173991*	*0.024402287*	*0.015162843*
**ENSG00000149564**	*0.084313037*	*0.066364998*	*0.051538323*	*0.057369559*
**ENSG00000185972**	*0.015837974*	*0.025419984*	*0.02504116*	*0.015494691*
**ENSG00000131482**	*0.015533067*	*0.024735463*	*0.025647075*	*0.015455733*
**ENSG00000108423**	*0.031518708*	*0.036906851*	*0.030929081*	*0.029957905*
**ENSG00000114648**	*0.017094394*	*0.025561647*	*0.025589031*	*0.016222383*
**ENSG00000079950**	*0.038092065*	*0.041028892*	*0.034701894*	*0.03613024*
**ENSG00000105219**	*0.015278299*	*0.025537599*	*0.024518523*	*0.016007131*
**ENSG00000120162**	*0.082303272*	*0.059983534*	*0.051053657*	*0.064755751*
**ENSG00000083782**	*0.014758318*	*0.024399746*	*0.024882475*	*0.015053482*
**ENSG00000071994**	*0.033568483*	*0.036089924*	*0.041745804*	*0.036212882*
**ENSG00000124491**	*0.144034104*	*0.116946196*	*0.120419281*	*0.137389944*
**ENSG00000163513**	*0.016304888*	*0.025814781*	*0.02608555*	*0.016213072*
**ENSG00000168734**	*0.0711144*	*0.072864187*	*0.054697434*	*0.068329358*
**ENSG00000110917**	*0.034741423*	*0.035370019*	*0.030222482*	*0.024569156*
**ENSG00000090382**	*0.016833033*	*0.025690361*	*0.025451857*	*0.015586176*
**ENSG00000115365**	*0.035144571*	*0.037442958*	*0.032971785*	*0.028911631*
**ENSG00000198948**	*0.013842977*	*0.023658472*	*0.023673363*	*0.013052373*
**ENSG00000146376**	*0.060294666*	*0.047108178*	*0.037135618*	*0.039692136*
**ENSG00000114767**	*0.034804942*	*0.032075733*	*0.037001128*	*0.034075872*
**ENSG00000164434**	*0.018280347*	*0.025049926*	*0.024527222*	*0.01507224*
**ENSG00000118473**	*0.015356492*	*0.02451021*	*0.024208859*	*0.016061737*
**ENSG00000175707**	*0.01977688*	*0.025515253*	*0.027750693*	*0.014759624*
**ENSG00000117013**	*0.016390187*	*0.026111745*	*0.024998379*	*0.016096039*
**ENSG00000221949**	*0.022058818*	*0.029407515*	*0.030464005*	*0.024065054*
**ENSG00000198932**	*0.03289385*	*0.032058363*	*0.035508379*	*0.031272334*
**ENSG00000122025**	*0.020022074*	*0.025588803*	*0.025543091*	*0.016985388*
**ENSG00000230666**	*0.018870453*	*0.024448939*	*0.025909132*	*0.018251394*
**ENSG00000105281**	*0.032919511*	*0.032849537*	*0.036280542*	*0.033127524*
**ENSG00000147174**	*0.030962826*	*0.044957148*	*0.038286904*	*0.039827207*
**ENSG00000110013**	*0.038895108*	*0.041316782*	*0.035118005*	*0.029839969*
**ENSG00000104833**	*0.021614101*	*0.028935096*	*0.02676727*	*0.022248002*
**ENSG00000166263**	*0.0365254*	*0.032250136*	*0.031523838*	*0.029657858*
**ENSG00000103429**	*0.026968172*	*0.032577911*	*0.028839239*	*0.020914317*
**ENSG00000130309**	*0.032706305*	*0.029119098*	*0.034993001*	*0.028665638*
**ENSG00000120915**	*0.068119425*	*0.055793556*	*0.046923554*	*0.06845532*
**ENSG00000068305**	*0.032304685*	*0.033083877*	*0.043235478*	*0.031608323*
**ENSG00000240682**	*0.025287565*	*0.033293792*	*0.032171509*	*0.019346775*
**ENSG00000103876**	*0.043849192*	*0.046596358*	*0.046898052*	*0.03830277*
**ENSG00000186001**	*0.027254016*	*0.035186906*	*0.028433083*	*0.025129047*
**ENSG00000179449**	*0.027930675*	*0.032186907*	*0.037432603*	*0.020971857*
**ENSG00000170054**	*0.033937864*	*0.028236674*	*0.025581291*	*0.026015165*
**ENSG00000204099**	*0.026012139*	*0.035365467*	*0.027557616*	*0.022638122*
**ENSG00000241878**	*0.024000104*	*0.03334109*	*0.034126924*	*0.02703881*
**ENSG00000176256**	*0.017283946*	*0.02442365*	*0.025009707*	*0.015640396*
**ENSG00000204193**	*0.015666491*	*0.024669262*	*0.024367473*	*0.014458665*
**ENSG00000167701**	*0.019378316*	*0.026602502*	*0.025568258*	*0.017193329*
**ENSG00000134259**	*0.017924471*	*0.025984865*	*0.026328833*	*0.016496934*
**ENSG00000167925**	*0.029550711*	*0.032372717*	*0.039447668*	*0.028220329*
**ENSG00000158062**	*0.015495454*	*0.025706472*	*0.02455301*	*0.01574918*
**ENSG00000167600**	*0.019005335*	*0.031502253*	*0.027358338*	*0.017933155*

**ENSG00000105289**	*0.017265907*	*0.026604457*	*0.027498765*	*0.017390774*
**ENSG00000180891**	*0.05172373*	*0.058994957*	*0.036857953*	*0.036698511*
**ENSG00000196475**	*0.015856498*	*0.025776906*	*0.024293765*	*0.014856293*
**ENSG00000140104**	*0.027499549*	*0.027861929*	*0.028989461*	*0.029503697*
**ENSG00000112053**	*0.015288036*	*0.025316813*	*0.024861946*	*0.014409338*
**ENSG00000132463**	*0.032721091*	*0.03473502*	*0.030821964*	*0.027959923*
**ENSG00000165923**	*0.034561413*	*0.03600557*	*0.029781351*	*0.032081178*
**ENSG00000178177**	*0.02384155*	*0.033212773*	*0.029858378*	*0.024090991*
**ENSG00000164953**	*0.015146628*	*0.024567467*	*0.025195683*	*0.014448556*
**ENSG00000196876**	*0.018117472*	*0.027394244*	*0.026266991*	*0.016173094*
**ENSG00000175097**	*0.016738245*	*0.024977949*	*0.025320234*	*0.015518351*
**ENSG00000204673**	*0.026582788*	*0.029245537*	*0.031499484*	*0.025164194*
**ENSG00000156587**	*0.015956413*	*0.025915144*	*0.025294462*	*0.014514325*
**ENSG00000177628**	*0.036967615*	*0.033638944*	*0.029639342*	*0.031016657*
**ENSG00000205209**	*0.016245763*	*0.024536151*	*0.02570876*	*0.01520485*
**ENSG00000127507**	*0.062943646*	*0.058060094*	*0.061649267*	*0.072718488*
**ENSG00000155438**	*0.031875101*	*0.036623588*	*0.03847574*	*0.03391175*
**ENSG00000072210**	*0.053786649*	*0.041413332*	*0.042988207*	*0.034248479*
**ENSG00000149609**	*0.016633505*	*0.02478318*	*0.024450001*	*0.016451929*
**ENSG00000090674**	*0.024248935*	*0.031124833*	*0.031180508*	*0.022225117*
**ENSG00000184432**	*0.028211125*	*0.035046214*	*0.03285936*	*0.023327618*
**ENSG00000174059**	*0.015579128*	*0.025452809*	*0.024679797*	*0.015873234*
**ENSG00000108797**	*0.059949978*	*0.050915247*	*0.04637542*	*0.047996898*
**ENSG00000111669**	*0.026804794*	*0.030657484*	*0.030229781*	*0.022164141*
**ENSG00000197024**	*0.031234202*	*0.039879553*	*0.042669811*	*0.029809465*
**ENSG00000169976**	*0.023165245*	*0.027037856*	*0.027047925*	*0.019295783*
**ENSG00000113734**	*0.025003475*	*0.033614446*	*0.036266961*	*0.024492508*
**ENSG00000181894**	*0.040136921*	*0.049629568*	*0.051131937*	*0.033818426*
**ENSG00000168515**	*0.015825193*	*0.024845199*	*0.024302591*	*0.015482133*
**ENSG00000108823**	*0.018955415*	*0.026585278*	*0.025976371*	*0.019347041*
**ENSG00000127951**	*0.056332093*	*0.067540963*	*0.052457466*	*0.054071891*
**ENSG00000173141**	*0.026197634*	*0.028496006*	*0.031562384*	*0.022731121*
**ENSG00000082196**	*0.017257712*	*0.026045045*	*0.024280596*	*0.017557685*
**ENSG00000141542**	*0.047919425*	*0.048316742*	*0.047861828*	*0.04632723*
**ENSG00000134291**	*0.041167029*	*0.040072502*	*0.03350589*	*0.027694832*
**ENSG00000178700**	*0.029367122*	*0.030634422*	*0.025798518*	*0.028807093*
**ENSG00000176956**	*0.015038732*	*0.025143689*	*0.025006609*	*0.015266815*
**ENSG00000103495**	*0.033785907*	*0.051190526*	*0.042033486*	*0.037308695*
**ENSG00000167840**	*0.041929202*	*0.039637344*	*0.032736508*	*0.027980208*
**ENSG00000186812**	*0.020576896*	*0.029159385*	*0.026835575*	*0.018500977*
**ENSG00000134001**	*0.038670815*	*0.046824704*	*0.056903728*	*0.064371562*
**ENSG00000054938**	*0.01560218*	*0.025382279*	*0.025091825*	*0.016626842*
**ENSG00000204969**	*0.018349313*	*0.025258501*	*0.025635106*	*0.017376362*
**ENSG00000116161**	*0.024129067*	*0.028742344*	*0.028750566*	*0.01866341*
**ENSG00000112339**	*0.032782059*	*0.030864739*	*0.032977338*	*0.028443415*
**ENSG00000166228**	*0.039359399*	*0.034860133*	*0.03308395*	*0.030137229*
**ENSG00000185477**	*0.028148207*	*0.032051353*	*0.034160122*	*0.035022365*
**ENSG00000133980**	*0.015285929*	*0.024981946*	*0.026366891*	*0.014963104*
**ENSG00000167981**	*0.022030816*	*0.033368322*	*0.038604816*	*0.022362742*
**ENSG00000123572**	*0.013776462*	*0.02480477*	*0.024298611*	*0.014844685*
**ENSG00000164627**	*0.022271584*	*0.025810908*	*0.025822256*	*0.015956742*
**ENSG00000158717**	*0.02929815*	*0.037139702*	*0.034147665*	*0.024716826*
**ENSG00000173812**	*0.029011197*	*0.036732237*	*0.0354866*	*0.024436293*
**ENSG00000171757**	*0.074290476*	*0.0569148*	*0.050673729*	*0.057248669*

**ENSG00000197852**	*0.026974981*	*0.031311924*	*0.027357769*	*0.02114165*
**ENSG00000169609**	*0.031585556*	*0.036465851*	*0.033251505*	*0.02596209*
**ENSG00000172171**	*0.028748762*	*0.03635786*	*0.031689997*	*0.024144972*
**ENSG00000175395**	*0.041629743*	*0.04028358*	*0.040261229*	*0.034119842*
**ENSG00000143570**	*0.027916371*	*0.031371163*	*0.032332479*	*0.027820501*
**ENSG00000075234**	*0.03116708*	*0.030771489*	*0.034605308*	*0.043268164*
**ENSG00000138639**	*0.028035824*	*0.030122111*	*0.025598206*	*0.031701086*
**ENSG00000177519**	*0.039287553*	*0.026058299*	*0.024168199*	*0.028620011*
**ENSG00000115239**	*0.037141429*	*0.057762424*	*0.039818591*	*0.048178667*
**ENSG00000119922**	*0.058907196*	*0.061160924*	*0.049774684*	*0.064509839*
**ENSG00000067066**	*0.026774346*	*0.027687314*	*0.028061264*	*0.021106603*
**ENSG00000137776**	*0.030392757*	*0.036664271*	*0.031695581*	*0.025594626*
**ENSG00000171954**	*0.015417928*	*0.02645534*	*0.025048834*	*0.015218499*
**ENSG00000164258**	*0.01875174*	*0.027024295*	*0.028659752*	*0.019390253*
**ENSG00000176871**	*0.029316186*	*0.031221024*	*0.030421698*	*0.026683725*
**ENSG00000187109**	*0.027646022*	*0.037350505*	*0.02981843*	*0.025387632*
**ENSG00000009709**	*0.015669492*	*0.025365372*	*0.026222499*	*0.015924676*
**ENSG00000167325**	*0.032032465*	*0.033646529*	*0.028770866*	*0.024000606*
**ENSG00000125831**	*0.014927859*	*0.024676037*	*0.02467249*	*0.015464323*
**ENSG00000241794**	*0.020190562*	*0.028199799*	*0.024933084*	*0.015220738*
**ENSG00000114529**	*0.048784805*	*0.042147292*	*0.039686976*	*0.058936971*
**ENSG00000005243**	*0.03213799*	*0.026863335*	*0.032417239*	*0.026621531*
**ENSG00000174100**	*0.02411375*	*0.029466273*	*0.027633239*	*0.024453927*
**ENSG00000085788**	*0.031606458*	*0.035088549*	*0.031624975*	*0.024124802*
**ENSG00000116337**	*0.015820369*	*0.024731033*	*0.025143475*	*0.014475745*
**ENSG00000135441**	*0.028965397*	*0.027597664*	*0.029640239*	*0.023304931*
**ENSG00000147724**	*0.014872072*	*0.025469149*	*0.02467842*	*0.014620805*
**ENSG00000109906**	*0.017876805*	*0.02621694*	*0.026261061*	*0.016287296*
**ENSG00000131477**	*0.015916236*	*0.026057603*	*0.024044212*	*0.016374961*
**ENSG00000111321**	*0.063563177*	*0.051064003*	*0.054717246*	*0.037853588*
**ENSG00000164172**	*0.033665216*	*0.036800236*	*0.03242382*	*0.026899679*
**ENSG00000146013**	*0.016478865*	*0.025476165*	*0.02521889*	*0.015390908*
**ENSG00000162551**	*0.044403279*	*0.045235002*	*0.029264904*	*0.020961943*
**ENSG00000159650**	*0.015419581*	*0.023936959*	*0.024369002*	*0.014378023*
**ENSG00000130414**	*0.023967994*	*0.028639519*	*0.028340119*	*0.024159865*
**ENSG00000144852**	*0.013936821*	*0.024746234*	*0.025061609*	*0.014686615*
**ENSG00000254245**	*0.018438312*	*0.025678497*	*0.024766023*	*0.017367242*
**ENSG00000106089**	*0.033519727*	*0.04221575*	*0.03701291*	*0.03992404*
**ENSG00000140632**	*0.02658055*	*0.035087044*	*0.030352518*	*0.023357225*
**ENSG00000160949**	*0.024989977*	*0.029720609*	*0.02769312*	*0.022998425*
**ENSG00000162976**	*0.053772854*	*0.038847157*	*0.04496969*	*0.040555982*
**ENSG00000171489**	*0.015988596*	*0.025147977*	*0.024523299*	*0.015266932*
**ENSG00000188488**	*0.016338968*	*0.025053538*	*0.025546415*	*0.015213974*
**ENSG00000101276**	*0.015322929*	*0.024477392*	*0.024817132*	*0.015288384*
**ENSG00000102349**	*0.052088863*	*0.047704246*	*0.044555716*	*0.049012833*
**ENSG00000015153**	*0.029075437*	*0.035466418*	*0.033438556*	*0.023096217*
**ENSG00000023909**	*0.040810736*	*0.03632372*	*0.035620653*	*0.035331025*
**ENSG00000088992**	*0.116377925*	*0.068635571*	*0.063723498*	*0.067698054*
**ENSG00000138696**	*0.016634553*	*0.024794458*	*0.024818276*	*0.016861752*
**ENSG00000127663**	*0.027964005*	*0.034152887*	*0.033544741*	*0.02505293*
**ENSG00000184221**	*0.01860519*	*0.028308505*	*0.026003376*	*0.018759065*
**ENSG00000182985**	*0.148513451*	*0.097139258*	*0.08037484*	*0.125903436*
**ENSG00000172922**	*0.029913429*	*0.031605382*	*0.030320777*	*0.026549386*
**ENSG00000144231**	*0.029305971*	*0.033796144*	*0.040504159*	*0.026079264*

**ENSG00000173581**	*0.033289427*	*0.040121112*	*0.030823851*	*0.031525623*
**ENSG00000198028**	*0.015801478*	*0.025118023*	*0.02560399*	*0.014067557*
**ENSG00000198300**	*0.015209402*	*0.024897623*	*0.024429149*	*0.014613367*
**ENSG00000166451**	*0.041491261*	*0.042808061*	*0.047652981*	*0.041160265*
**ENSG00000170854**	*0.016376617*	*0.025606235*	*0.025056087*	*0.015155096*
**ENSG00000172009**	*0.025476114*	*0.030900419*	*0.032474386*	*0.028935666*
**ENSG00000197417**	*0.035384855*	*0.033218612*	*0.035984992*	*0.026978124*
**ENSG00000013725**	*0.018180326*	*0.026417495*	*0.025048637*	*0.018300602*
**ENSG00000127124**	*0.020056364*	*0.027313107*	*0.026069167*	*0.018173319*
**ENSG00000127528**	*0.06324398*	*0.056554361*	*0.057900723*	*0.074763604*
**ENSG00000198142**	*0.07424847*	*0.054596328*	*0.050243187*	*0.065417229*
**ENSG00000073008**	*0.019925683*	*0.026276974*	*0.025434676*	*0.020056203*
**ENSG00000099797**	*0.036100469*	*0.032624284*	*0.032083247*	*0.025829877*
**ENSG00000159131**	*0.036056597*	*0.038332253*	*0.031925536*	*0.030839926*
**ENSG00000075429**	*0.016178555*	*0.0256213*	*0.025045043*	*0.015041226*
**ENSG00000121897**	*0.030898272*	*0.034075855*	*0.033126531*	*0.024957758*
**ENSG00000175550**	*0.027982767*	*0.031722193*	*0.031549563*	*0.024907029*
**ENSG00000114126**	*0.037688481*	*0.037297043*	*0.035599727*	*0.039681599*
**ENSG00000111364**	*0.028262159*	*0.029383101*	*0.031578063*	*0.02657793*
**ENSG00000132300**	*0.028998747*	*0.033670057*	*0.032751624*	*0.023812621*
**ENSG00000153201**	*0.038524525*	*0.036813121*	*0.040766074*	*0.03354417*
**ENSG00000105647**	*0.016919803*	*0.025642457*	*0.025611669*	*0.015763083*
**ENSG00000166478**	*0.025189691*	*0.029194618*	*0.029968786*	*0.020909143*
**ENSG00000241962**	*0.029372901*	*0.030840648*	*0.033153168*	*0.028667459*
**ENSG00000166289**	*0.04206034*	*0.040274537*	*0.033956116*	*0.033932786*
**ENSG00000046604**	*0.064039951*	*0.044527388*	*0.037697999*	*0.044056744*
**ENSG00000101134**	*0.017188432*	*0.024494795*	*0.025297588*	*0.015711368*
**ENSG00000159763**	*0.014502767*	*0.023838463*	*0.024882652*	*0.015471821*
**ENSG00000138675**	*0.018617576*	*0.024163241*	*0.024470138*	*0.014369031*
**ENSG00000082146**	*0.052016432*	*0.056796066*	*0.055615852*	*0.045846974*
**ENSG00000198521**	*0.045076126*	*0.041744539*	*0.032539646*	*0.047293708*
**ENSG00000253485**	*0.016651377*	*0.02680351*	*0.025177802*	*0.017280259*
**ENSG00000139547**	*0.017672939*	*0.024775329*	*0.026532516*	*0.018780531*
**ENSG00000164512**	*0.022264119*	*0.026193408*	*0.025959864*	*0.016014894*
**ENSG00000180448**	*0.030657414*	*0.034462028*	*0.035661474*	*0.032886456*
**ENSG00000102858**	*0.024316451*	*0.035140833*	*0.045663734*	*0.032611037*
**ENSG00000197386**	*0.02736149*	*0.030223524*	*0.031394382*	*0.025792909*
**ENSG00000084734**	*0.015441709*	*0.02441837*	*0.025288815*	*0.014945051*
**ENSG00000198443**	*0.017259854*	*0.024831795*	*0.026209173*	*0.017247253*
**ENSG00000187553**	*0.01629922*	*0.02567229*	*0.024969834*	*0.015397616*
**ENSG00000128394**	*0.029832979*	*0.038652623*	*0.029068996*	*0.028818364*
**ENSG00000114423**	*0.057028252*	*0.040252146*	*0.049354923*	*0.046909183*
**ENSG00000095587**	*0.016997554*	*0.025842766*	*0.024863172*	*0.015161102*
**ENSG00000169738**	*0.030778358*	*0.032197487*	*0.032479916*	*0.031571976*
**ENSG00000110066**	*0.03836887*	*0.044760659*	*0.045798648*	*0.044429071*
**ENSG00000155962**	*0.106961183*	*0.081378577*	*0.073172556*	*0.099163026*
**ENSG00000157911**	*0.014471514*	*0.025638444*	*0.024887059*	*0.016584102*
**ENSG00000169184**	*0.015209092*	*0.025093449*	*0.02594372*	*0.015854289*
**ENSG00000134769**	*0.028455916*	*0.026163757*	*0.026908771*	*0.017357814*
**ENSG00000143375**	*0.083943193*	*0.077838653*	*0.062956714*	*0.082271337*
**ENSG00000137812**	*0.015463124*	*0.024650363*	*0.02635019*	*0.014343745*
**ENSG00000165164**	*0.015426449*	*0.025352547*	*0.024284948*	*0.016092468*
**ENSG00000101311**	*0.083182091*	*0.062421343*	*0.031929806*	*0.050021627*
**ENSG00000196353**	*0.030822232*	*0.026342984*	*0.027257036*	*0.019432174*

**ENSG00000081721**	*0.028400018*	*0.032376253*	*0.034826596*	*0.029567955*
**ENSG00000119396**	*0.019159606*	*0.025122821*	*0.025048918*	*0.016417811*
**ENSG00000102931**	*0.030508735*	*0.036055042*	*0.035755541*	*0.028877275*
**ENSG00000170236**	*0.01907992*	*0.025323988*	*0.025289096*	*0.015753574*
**ENSG00000162300**	*0.0293286*	*0.031789168*	*0.033653942*	*0.022209348*
**ENSG00000205838**	*0.015699614*	*0.025515416*	*0.024402381*	*0.014598003*
**ENSG00000137825**	*0.046781344*	*0.052803248*	*0.038721559*	*0.035768158*
**ENSG00000111911**	*0.031348365*	*0.038250222*	*0.031580656*	*0.024717132*
**ENSG00000125347**	*0.037368451*	*0.036267368*	*0.0415854*	*0.030554926*
**ENSG00000123096**	*0.040153033*	*0.048328445*	*0.036766507*	*0.035041181*
**ENSG00000175221**	*0.028817616*	*0.030633393*	*0.039374824*	*0.025991239*
**ENSG00000091138**	*0.016484157*	*0.025599397*	*0.024745853*	*0.016332081*
**ENSG00000151413**	*0.032472588*	*0.034352305*	*0.032546959*	*0.033726893*
**ENSG00000119125**	*0.022597424*	*0.035916102*	*0.034109766*	*0.034403799*
**ENSG00000101187**	*0.054748918*	*0.04600905*	*0.039396968*	*0.036870926*
**ENSG00000128594**	*0.015671184*	*0.026608823*	*0.025291269*	*0.014908126*
**ENSG00000156463**	*0.016075003*	*0.025312397*	*0.025725423*	*0.015645087*
**ENSG00000163354**	*0.015448704*	*0.024274266*	*0.02439179*	*0.015390039*
**ENSG00000173868**	*0.016293056*	*0.025310741*	*0.024697791*	*0.015229859*
**ENSG00000108846**	*0.024732883*	*0.025861098*	*0.025989689*	*0.02014302*
**ENSG00000114354**	*0.024832697*	*0.029928845*	*0.031132894*	*0.024033191*
**ENSG00000101213**	*0.016661171*	*0.024632709*	*0.025590229*	*0.016068837*
**ENSG00000133424**	*0.098416105*	*0.072921533*	*0.075311373*	*0.072203563*
**ENSG00000143653**	*0.038652029*	*0.054376673*	*0.045844069*	*0.051891418*
**ENSG00000124159**	*0.017900512*	*0.025361621*	*0.026334489*	*0.017975003*
**ENSG00000181803**	*0.016720095*	*0.025205556*	*0.024550774*	*0.016289563*
**ENSG00000168481**	*0.014832441*	*0.025774123*	*0.025033244*	*0.014316569*
**ENSG00000127415**	*0.031189423*	*0.036037951*	*0.034146828*	*0.032887138*
**ENSG00000172775**	*0.033333058*	*0.037953355*	*0.035976985*	*0.030815807*
**ENSG00000091640**	*0.024364413*	*0.028638929*	*0.032370598*	*0.024097911*
**ENSG00000106049**	*0.04009489*	*0.038912803*	*0.034125957*	*0.032746073*
**ENSG00000169469**	*0.017354087*	*0.025929959*	*0.026267081*	*0.018351308*
**ENSG00000174720**	*0.025278902*	*0.033355364*	*0.030568937*	*0.02154075*
**ENSG00000198515**	*0.015343368*	*0.025379153*	*0.024573336*	*0.015710625*
**ENSG00000123901**	*0.015293092*	*0.025495518*	*0.02518862*	*0.01534628*
**ENSG00000152990**	*0.090869641*	*0.075173614*	*0.035394387*	*0.038456484*
**ENSG00000123595**	*0.0352567*	*0.036031905*	*0.033857118*	*0.030247291*
**ENSG00000136045**	*0.02296633*	*0.02860276*	*0.030323895*	*0.020074817*
**ENSG00000164366**	*0.026198115*	*0.027492337*	*0.030166311*	*0.022106846*
**ENSG00000196154**	*0.106808346*	*0.070369602*	*0.055627671*	*0.096317242*
**ENSG00000139220**	*0.015703792*	*0.025347099*	*0.025231623*	*0.015183138*
**ENSG00000010539**	*0.016611621*	*0.02534948*	*0.025857506*	*0.015145649*
**ENSG00000167460**	*0.027011594*	*0.028440929*	*0.037089039*	*0.037563918*
**ENSG00000251247**	*0.020167526*	*0.030012146*	*0.027992492*	*0.023570333*
**ENSG00000196914**	*0.028732354*	*0.031006412*	*0.028632309*	*0.023094278*
**ENSG00000012048**	*0.048776909*	*0.055460085*	*0.04259675*	*0.036771638*
**ENSG00000164815**	*0.019225922*	*0.026689436*	*0.025179891*	*0.020135138*
**ENSG00000089225**	*0.016618779*	*0.025848877*	*0.025063943*	*0.015527008*
**ENSG00000196275**	*0.034018996*	*0.028445276*	*0.029913068*	*0.029430483*
**ENSG00000165521**	*0.016417991*	*0.026115115*	*0.025709172*	*0.016364312*
**ENSG00000184517**	*0.029586102*	*0.035720874*	*0.033262811*	*0.030562527*
**ENSG00000100811**	*0.023595955*	*0.031541505*	*0.03017174*	*0.019041048*
**ENSG00000171940**	*0.034417829*	*0.039791732*	*0.036238574*	*0.030591224*
**ENSG00000161021**	*0.032004235*	*0.037807555*	*0.039591353*	*0.026706063*

**ENSG00000151702**	*0.046773237*	*0.045980666*	*0.035953171*	*0.032242359*
**ENSG00000138448**	*0.015872329*	*0.024276479*	*0.024980461*	*0.016150008*
**ENSG00000113048**	*0.027937479*	*0.031789723*	*0.032259507*	*0.02735072*
**ENSG00000205021**	*0.058309509*	*0.053854867*	*0.05963652*	*0.051236331*
**ENSG00000108788**	*0.022055809*	*0.028835671*	*0.032542716*	*0.021835449*
**ENSG00000161980**	*0.032034703*	*0.036772437*	*0.035715149*	*0.030352767*
**ENSG00000151240**	*0.024109797*	*0.026914696*	*0.025563228*	*0.016084327*
**ENSG00000108932**	*0.018461822*	*0.026230388*	*0.026786865*	*0.019596493*
**ENSG00000173702**	*0.109167011*	*0.075060633*	*0.056854208*	*0.048356222*
**ENSG00000185614**	*0.059176894*	*0.036451002*	*0.048424001*	*0.048134943*
**ENSG00000135298**	*0.01743165*	*0.025446267*	*0.024797097*	*0.017478297*
**ENSG00000155918**	*0.015693906*	*0.024724388*	*0.025520382*	*0.014664337*
**ENSG00000189058**	*0.038257568*	*0.036810627*	*0.032720089*	*0.018932598*
**ENSG00000167528**	*0.017524488*	*0.025748049*	*0.02495394*	*0.018463775*
**ENSG00000187682**	*0.016431922*	*0.025815591*	*0.025305932*	*0.0163932*
**ENSG00000197617**	*0.015170836*	*0.025368982*	*0.025326444*	*0.014555947*
**ENSG00000204385**	*0.02596554*	*0.029930433*	*0.027889373*	*0.025466698*
**ENSG00000203943**	*0.073533932*	*0.057385887*	*0.054714449*	*0.055379468*
**ENSG00000171202**	*0.024153741*	*0.028001024*	*0.029372651*	*0.026113387*
**ENSG00000171502**	*0.068000999*	*0.061133886*	*0.053055614*	*0.062755947*
**ENSG00000151023**	*0.019922457*	*0.02739662*	*0.030490097*	*0.02061691*
**ENSG00000166224**	*0.031487348*	*0.034477704*	*0.033705688*	*0.03302228*
**ENSG00000141506**	*0.045094407*	*0.041338274*	*0.038192576*	*0.03858825*
**ENSG00000143869**	*0.01577634*	*0.025547308*	*0.024707159*	*0.01581677*
**ENSG00000133878**	*0.017584861*	*0.02547668*	*0.024560918*	*0.017215564*
**ENSG00000171621**	*0.045720385*	*0.051271589*	*0.03788235*	*0.029170033*
**ENSG00000167657**	*0.02616186*	*0.037886362*	*0.045930863*	*0.02632589*
**ENSG00000084693**	*0.027091503*	*0.030648176*	*0.028183731*	*0.024616424*
**ENSG00000163377**	*0.016592264*	*0.025025976*	*0.025107184*	*0.015794224*
**ENSG00000159445**	*0.030855145*	*0.032466317*	*0.035343756*	*0.030561144*
**ENSG00000162711**	*0.016670775*	*0.029328529*	*0.02527252*	*0.014899588*
**ENSG00000135960**	*0.015323411*	*0.025010715*	*0.024558856*	*0.016150068*
**ENSG00000198176**	*0.028926756*	*0.034241202*	*0.028606263*	*0.026454141*
**ENSG00000183542**	*0.015970422*	*0.025126844*	*0.025351569*	*0.015598658*
**ENSG00000147533**	*0.029064373*	*0.033243769*	*0.03164362*	*0.025536323*
**ENSG00000164484**	*0.085957748*	*0.086538376*	*0.12970416*	*0.097227235*
**ENSG00000107929**	*0.022924354*	*0.034148186*	*0.028481225*	*0.023793047*
**ENSG00000105854**	*0.096869834*	*0.090615938*	*0.080789695*	*0.084514155*
**ENSG00000100221**	*0.02914708*	*0.040715655*	*0.032418713*	*0.023400783*
**ENSG00000171604**	*0.037345187*	*0.033843635*	*0.03178831*	*0.032173873*
**ENSG00000156639**	*0.03043621*	*0.037908946*	*0.034493073*	*0.030785613*
**ENSG00000132773**	*0.034973236*	*0.033932538*	*0.0517139*	*0.043875996*
**ENSG00000147257**	*0.016967172*	*0.02646791*	*0.025000118*	*0.017502032*
**ENSG00000174780**	*0.024802577*	*0.030659*	*0.031132184*	*0.026261868*
**ENSG00000152268**	*0.016272*	*0.025292182*	*0.025785363*	*0.015936436*
**ENSG00000175485**	*0.01630115*	*0.02551835*	*0.025666848*	*0.0158539*
**ENSG00000117090**	*0.034640045*	*0.030356153*	*0.030707526*	*0.031144612*
**ENSG00000159166**	*0.022510981*	*0.027222218*	*0.028200728*	*0.020142836*
**ENSG00000095951**	*0.036177427*	*0.038048042*	*0.040084491*	*0.030804106*
**ENSG00000198746**	*0.031652398*	*0.041630023*	*0.040651545*	*0.032681297*
**ENSG00000127774**	*0.023030278*	*0.030700633*	*0.030554451*	*0.0253376*
**ENSG00000100385**	*0.036484342*	*0.038269961*	*0.038746584*	*0.029531669*
**ENSG00000182154**	*0.02758716*	*0.031170234*	*0.030080216*	*0.031455571*
**ENSG00000070886**	*0.016219676*	*0.024811018*	*0.024437105*	*0.015851156*

**ENSG00000101200**	*0.015716974*	*0.024447858*	*0.024440738*	*0.014870969*
**ENSG00000186452**	*0.016496132*	*0.025267521*	*0.025650103*	*0.01461281*
**ENSG00000171992**	*0.01722807*	*0.026603113*	*0.024978047*	*0.016509486*
**ENSG00000184922**	*0.031840793*	*0.036570313*	*0.040154956*	*0.027077274*
**ENSG00000103194**	*0.023128979*	*0.028933569*	*0.030750112*	*0.019838328*
**ENSG00000164037**	*0.022537528*	*0.02735792*	*0.028928122*	*0.023325466*
**ENSG00000043591**	*0.01774413*	*0.025095584*	*0.024721478*	*0.015805504*
**ENSG00000167543**	*0.032375644*	*0.032256336*	*0.035745885*	*0.029657229*
**ENSG00000144015**	*0.069787546*	*0.025334317*	*0.04791658*	*0.027843205*
**ENSG00000163216**	*0.024992884*	*0.034125547*	*0.024751563*	*0.017284452*
**ENSG00000185591**	*0.042293721*	*0.034019679*	*0.033049733*	*0.02640456*
**ENSG00000100197**	*0.017776986*	*0.028938707*	*0.025629025*	*0.018127734*
**ENSG00000176387**	*0.01564358*	*0.025227322*	*0.024956555*	*0.01541481*
**ENSG00000037637**	*0.02602854*	*0.032159121*	*0.032063635*	*0.02557035*
**ENSG00000167487**	*0.018992307*	*0.026874576*	*0.030387076*	*0.018986047*
**ENSG00000161013**	*0.014980934*	*0.025152346*	*0.025341817*	*0.014712199*
**ENSG00000148297**	*0.032591967*	*0.038442789*	*0.03362389*	*0.03035523*
**ENSG00000132613**	*0.017729186*	*0.0254698*	*0.026267489*	*0.016065806*
**ENSG00000135211**	*0.023302354*	*0.031839218*	*0.028802411*	*0.019511456*
**ENSG00000149476**	*0.028602166*	*0.028047812*	*0.031186449*	*0.031565212*
**ENSG00000110400**	*0.017326778*	*0.025479407*	*0.025883133*	*0.016857705*
**ENSG00000169676**	*0.018777135*	*0.025696379*	*0.025295015*	*0.017115631*
**ENSG00000180992**	*0.024884853*	*0.029813316*	*0.030409181*	*0.022718075*
**ENSG00000140395**	*0.028620838*	*0.030822649*	*0.03031286*	*0.023828265*
**ENSG00000138769**	*0.016829004*	*0.026381448*	*0.025745287*	*0.017605961*
**ENSG00000182566**	*0.049297181*	*0.044339921*	*0.030879423*	*0.031076746*
**ENSG00000178586**	*0.01607369*	*0.024837519*	*0.024455642*	*0.016083354*
**ENSG00000119965**	*0.034432845*	*0.037836924*	*0.03973058*	*0.034667297*
**ENSG00000183873**	*0.016146737*	*0.025247583*	*0.024620046*	*0.015147565*
**ENSG00000184293**	*0.08423958*	*0.043838975*	*0.042283162*	*0.039962239*
**ENSG00000033011**	*0.031978543*	*0.035556955*	*0.033177811*	*0.03743893*
**ENSG00000136848**	*0.015349292*	*0.025301336*	*0.025166038*	*0.015626098*
**ENSG00000124279**	*0.029430366*	*0.04343544*	*0.039861841*	*0.032854444*
**ENSG00000113643**	*0.023053903*	*0.028689694*	*0.028456099*	*0.022245702*
**ENSG00000167183**	*0.015948058*	*0.025107057*	*0.02544483*	*0.01790023*
**ENSG00000110042**	*0.017634013*	*0.027251956*	*0.026218489*	*0.016677621*
**ENSG00000197245**	*0.015793378*	*0.025795188*	*0.024573186*	*0.015628102*
**ENSG00000135924**	*0.030738914*	*0.033211289*	*0.036719686*	*0.034030322*
**ENSG00000141052**	*0.015448553*	*0.024860923*	*0.024985294*	*0.015410126*
**ENSG00000213139**	*0.028566264*	*0.031164155*	*0.032272967*	*0.022805836*
**ENSG00000055070**	*0.023391256*	*0.03126104*	*0.028376413*	*0.022792768*
**ENSG00000151090**	*0.016545555*	*0.025984062*	*0.024865536*	*0.01544957*
**ENSG00000132274**	*0.02437928*	*0.029638679*	*0.029188766*	*0.027107577*
**ENSG00000016490**	*0.016084868*	*0.024861517*	*0.025275515*	*0.015626272*
**ENSG00000132952**	*0.028782671*	*0.036393124*	*0.038433533*	*0.026788386*
**ENSG00000112033**	*0.017035854*	*0.026925585*	*0.02663556*	*0.016323718*
**ENSG00000085465**	*0.057294578*	*0.041196235*	*0.044747013*	*0.04810483*
**ENSG00000165478**	*0.016708064*	*0.025936768*	*0.024971516*	*0.016335167*
**ENSG00000165868**	*0.03341016*	*0.035574721*	*0.029345772*	*0.030787256*
**ENSG00000078098**	*0.015434508*	*0.025975244*	*0.024731429*	*0.014993528*
**ENSG00000137441**	*0.073923203*	*0.076071545*	*0.051351018*	*0.074545943*
**ENSG00000069535**	*0.018588719*	*0.025406103*	*0.026141725*	*0.016527544*
**ENSG00000143164**	*0.023724058*	*0.034591439*	*0.028988824*	*0.020499084*
**ENSG00000150347**	*0.022556616*	*0.02875302*	*0.030058862*	*0.021227594*

**ENSG00000110375**	*0.048138716*	*0.05147966*	*0.044807565*	*0.052869568*
**ENSG00000074660**	*0.061058637*	*0.062424411*	*0.069075978*	*0.066872876*
**ENSG00000174748**	*0.018833978*	*0.027944942*	*0.026413949*	*0.020175231*
**ENSG00000113194**	*0.023226663*	*0.031348134*	*0.033818696*	*0.023448397*
**ENSG00000169064**	*0.018766212*	*0.02679213*	*0.026824787*	*0.018427027*
**ENSG00000172349**	*0.058037868*	*0.067583809*	*0.066973821*	*0.051576905*
**ENSG00000196218**	*0.01832313*	*0.026266557*	*0.026770762*	*0.022979369*
**ENSG00000108641**	*0.037766569*	*0.040067177*	*0.035253436*	*0.03806467*
**ENSG00000240428**	*0.017012779*	*0.02445228*	*0.025543297*	*0.01614562*
**ENSG00000204228**	*0.034946875*	*0.032070441*	*0.040080944*	*0.03465463*
**ENSG00000177551**	*0.015057606*	*0.025695276*	*0.024594763*	*0.014451232*
**ENSG00000135482**	*0.037781539*	*0.040142751*	*0.031284439*	*0.02933319*
**ENSG00000179397**	*0.015187319*	*0.025390485*	*0.024757952*	*0.013803385*
**ENSG00000144028**	*0.022676265*	*0.029041133*	*0.031548064*	*0.019475524*
**ENSG00000121350**	*0.034990477*	*0.043566921*	*0.038105208*	*0.030963414*
**ENSG00000131653**	*0.028363405*	*0.032394434*	*0.029037743*	*0.02660241*
**ENSG00000125447**	*0.015968524*	*0.02550715*	*0.024764676*	*0.015587993*
**ENSG00000158850**	*0.034540615*	*0.034984268*	*0.040339062*	*0.034491293*
**ENSG00000242574**	*0.034414783*	*0.039270458*	*0.0407222*	*0.045795488*
**ENSG00000153714**	*0.021912623*	*0.026201056*	*0.02515535*	*0.01490901*
**ENSG00000176454**	*0.03988396*	*0.035687847*	*0.032487994*	*0.029837172*
**ENSG00000172159**	*0.026180094*	*0.047010189*	*0.02986482*	*0.025812267*
**ENSG00000163116**	*0.015797246*	*0.024695474*	*0.024087525*	*0.014869742*
**ENSG00000107140**	*0.027649571*	*0.030575152*	*0.03078556*	*0.026899556*
**ENSG00000244362**	*0.016221136*	*0.025077257*	*0.0257067*	*0.014895266*
**ENSG00000149658**	*0.026686858*	*0.038539956*	*0.046642557*	*0.027169084*
**ENSG00000123838**	*0.016194201*	*0.024973241*	*0.024797547*	*0.014952858*
**ENSG00000073111**	*0.029025734*	*0.032773622*	*0.030628376*	*0.025673102*
**ENSG00000054611**	*0.030831945*	*0.035397303*	*0.032294871*	*0.032851579*
**ENSG00000164086**	*0.018608772*	*0.030460148*	*0.026879039*	*0.021921305*
**ENSG00000104412**	*0.028346559*	*0.031871576*	*0.029924285*	*0.027370038*
**ENSG00000064270**	*0.015135171*	*0.024388871*	*0.024942653*	*0.015206252*
**ENSG00000177984**	*0.029817346*	*0.029344463*	*0.027674028*	*0.024607904*
**ENSG00000163032**	*0.018267929*	*0.025288026*	*0.02676917*	*0.016450953*
**ENSG00000168090**	*0.030132778*	*0.033941476*	*0.030047521*	*0.024037865*
**ENSG00000111796**	*0.014960847*	*0.023663875*	*0.024797167*	*0.015900888*
**ENSG00000055957**	*0.033758503*	*0.036670806*	*0.028425885*	*0.049079203*
**ENSG00000197580**	*0.018521532*	*0.02708352*	*0.026203417*	*0.019520428*
**ENSG00000163530**	*0.018556077*	*0.029225685*	*0.028000963*	*0.018080735*
**ENSG00000106128**	*0.02284364*	*0.031412825*	*0.029989272*	*0.020762612*
**ENSG00000139636**	*0.032648032*	*0.031465818*	*0.035499655*	*0.034822518*
**ENSG00000133115**	*0.020857871*	*0.028490904*	*0.02805874*	*0.021410555*
**ENSG00000183508**	*0.074070865*	*0.075036343*	*0.081289574*	*0.07608551*
**ENSG00000175336**	*0.017673821*	*0.025433147*	*0.026418167*	*0.018320365*
**ENSG00000103066**	*0.024797605*	*0.030689157*	*0.032816122*	*0.023334566*
**ENSG00000163347**	*0.087592311*	*0.026683657*	*0.024984725*	*0.017662415*
**ENSG00000164169**	*0.02934431*	*0.03360248*	*0.032632919*	*0.024967696*
**ENSG00000070269**	*0.027719573*	*0.035723999*	*0.030360641*	*0.025278051*
**ENSG00000104974**	*0.016001781*	*0.026435604*	*0.025957638*	*0.01660384*
**ENSG00000145779**	*0.026860919*	*0.030078556*	*0.029630091*	*0.022858965*
**ENSG00000197632**	*0.075838668*	*0.031634007*	*0.025003763*	*0.043097034*
**ENSG00000120805**	*0.02427706*	*0.030890759*	*0.032895103*	*0.026614794*
**ENSG00000176009**	*0.015982152*	*0.026253851*	*0.02508888*	*0.014866994*
**ENSG00000135519**	*0.047119323*	*0.046484898*	*0.041917724*	*0.037991203*

**ENSG00000178922**	*0.05618428*	*0.048453576*	*0.037849849*	*0.047788794*
**ENSG00000049759**	*0.053156972*	*0.049322393*	*0.036410818*	*0.035320966*
**ENSG00000185238**	*0.032888838*	*0.033217428*	*0.033365973*	*0.027204427*
**ENSG00000003756**	*0.02068453*	*0.028256178*	*0.031175596*	*0.020064793*
**ENSG00000149503**	*0.016593987*	*0.024656984*	*0.025651245*	*0.015100629*
**ENSG00000179088**	*0.049208812*	*0.040697107*	*0.03703343*	*0.045470108*
**ENSG00000170027**	*0.021185961*	*0.029505217*	*0.028818863*	*0.022465978*
**ENSG00000137674**	*0.0495637*	*0.046885192*	*0.027848231*	*0.02814156*
**ENSG00000131269**	*0.024898772*	*0.030510002*	*0.030864573*	*0.02345767*
**ENSG00000115504**	*0.03581126*	*0.043175526*	*0.0396209*	*0.044041077*
**ENSG00000156510**	*0.014228929*	*0.025295025*	*0.025063932*	*0.017196155*
**ENSG00000176200**	*0.01609905*	*0.025409599*	*0.024160151*	*0.014941847*
**ENSG00000102312**	*0.022251239*	*0.031329679*	*0.028263985*	*0.026419049*
**ENSG00000149124**	*0.017914434*	*0.026883302*	*0.026187435*	*0.01621775*
**ENSG00000188389**	*0.037919332*	*0.043087635*	*0.045805703*	*0.042020681*
**ENSG00000069943**	*0.027511548*	*0.032958827*	*0.03177292*	*0.0219276*
**ENSG00000124587**	*0.065570682*	*0.074958755*	*0.057552161*	*0.046866396*
**ENSG00000070366**	*0.021027189*	*0.031182062*	*0.030325938*	*0.023994127*
**ENSG00000162231**	*0.033230327*	*0.031894618*	*0.035656782*	*0.028730569*
**ENSG00000164871**	*0.015037185*	*0.023885327*	*0.024382097*	*0.015211076*
**ENSG00000133401**	*0.016012885*	*0.024982294*	*0.02605872*	*0.015759775*
**ENSG00000110958**	*0.026553235*	*0.03169231*	*0.0273263*	*0.01985424*
**ENSG00000185513**	*0.016233816*	*0.026802718*	*0.027162302*	*0.015462036*
**ENSG00000138622**	*0.017275945*	*0.025641887*	*0.02515336*	*0.015366182*
**ENSG00000118515**	*0.090329362*	*0.07727976*	*0.077278128*	*0.079152796*
**ENSG00000117594**	*0.01577396*	*0.024943346*	*0.0248412*	*0.015390382*
**ENSG00000188833**	*0.017198141*	*0.025574033*	*0.026535858*	*0.019356133*
**ENSG00000144827**	*0.037587688*	*0.035418434*	*0.033799418*	*0.023078677*
**ENSG00000131188**	*0.046839482*	*0.039309379*	*0.039857125*	*0.040822827*
**ENSG00000066923**	*0.067098635*	*0.056529396*	*0.061708168*	*0.06846349*
**ENSG00000114853**	*0.016198346*	*0.025185397*	*0.025882432*	*0.016898598*
**ENSG00000185909**	*0.018142378*	*0.026702407*	*0.027154691*	*0.017474028*
**ENSG00000129219**	*0.02329322*	*0.028333714*	*0.027701091*	*0.021658101*
**ENSG00000179950**	*0.032849576*	*0.032036825*	*0.035587621*	*0.031060198*
**ENSG00000102387**	*0.015443296*	*0.025675167*	*0.024375092*	*0.015466087*
**ENSG00000081051**	*0.01701186*	*0.025753017*	*0.02456846*	*0.015657933*
**ENSG00000101638**	*0.069967577*	*0.051200801*	*0.039193948*	*0.054616928*
**ENSG00000187118**	*0.038289895*	*0.037546984*	*0.034954329*	*0.029044054*
**ENSG00000106537**	*0.015892236*	*0.024775118*	*0.024932723*	*0.015672618*
**ENSG00000109917**	*0.028364061*	*0.030773985*	*0.031459891*	*0.01965433*
**ENSG00000015520**	*0.017368948*	*0.026623675*	*0.026211228*	*0.015105765*
**ENSG00000124089**	*0.015284434*	*0.024507517*	*0.024635949*	*0.014375014*
**ENSG00000162520**	*0.0155536*	*0.0254199*	*0.025815204*	*0.017839124*
**ENSG00000102239**	*0.014011336*	*0.024734286*	*0.024894036*	*0.014166574*
**ENSG00000162460**	*0.013194896*	*0.023800981*	*0.023812836*	*0.01375836*
**ENSG00000242252**	*0.017752224*	*0.026635157*	*0.025503267*	*0.016068559*
**ENSG00000197721**	*0.017119514*	*0.02734508*	*0.026012836*	*0.016986786*
**ENSG00000187778**	*0.026474585*	*0.030292971*	*0.034672545*	*0.028529322*
**ENSG00000167945**	*0.016016673*	*0.024325402*	*0.024885339*	*0.014511573*
**ENSG00000164692**	*0.017733513*	*0.026689707*	*0.026503418*	*0.015596945*
**ENSG00000101384**	*0.04850996*	*0.036241122*	*0.02708916*	*0.028955116*
**ENSG00000100934**	*0.040153826*	*0.045823937*	*0.039485325*	*0.037966161*
**ENSG00000178965**	*0.015478793*	*0.025321564*	*0.0251894*	*0.015491602*
**ENSG00000131174**	*0.020448527*	*0.027254571*	*0.025601402*	*0.01861729*

**ENSG00000173926**	*0.055584577*	*0.060243941*	*0.047838748*	*0.053511297*
**ENSG00000198130**	*0.038493575*	*0.038974325*	*0.03413477*	*0.032121184*
**ENSG00000197430**	*0.015010758*	*0.024233524*	*0.025320168*	*0.015779596*
**ENSG00000117115**	*0.019050097*	*0.024939403*	*0.025148778*	*0.020269255*
**ENSG00000125356**	*0.017674625*	*0.026334698*	*0.026714192*	*0.018131693*
**ENSG00000142449**	*0.015692827*	*0.025501139*	*0.025260134*	*0.015262858*
**ENSG00000197683**	*0.016058085*	*0.024753859*	*0.023757191*	*0.014845753*
**ENSG00000140374**	*0.02045299*	*0.029015552*	*0.028364588*	*0.019045907*
**ENSG00000079112**	*0.1264057*	*0.030419532*	*0.055668118*	*0.087723575*
**ENSG00000145826**	*0.017380298*	*0.025411622*	*0.024712739*	*0.01613881*
**ENSG00000187024**	*0.032582485*	*0.031171584*	*0.031071984*	*0.027112749*
**ENSG00000172409**	*0.027241553*	*0.032513898*	*0.039635099*	*0.025948913*
**ENSG00000181195**	*0.017018331*	*0.026015719*	*0.025155597*	*0.017756377*
**ENSG00000156222**	*0.019416313*	*0.02582542*	*0.025831017*	*0.017117915*
**ENSG00000122033**	*0.027313103*	*0.029857271*	*0.031168781*	*0.029589005*
**ENSG00000100852**	*0.014959735*	*0.025459035*	*0.025090893*	*0.017501158*
**ENSG00000064201**	*0.017399115*	*0.025733728*	*0.0253254*	*0.016404949*
**ENSG00000145041**	*0.032046517*	*0.034991082*	*0.030280185*	*0.025537989*
**ENSG00000149548**	*0.029844669*	*0.033171297*	*0.03267556*	*0.026807176*
**ENSG00000119812**	*0.026267103*	*0.030121137*	*0.027064856*	*0.024546178*
**ENSG00000104852**	*0.023803178*	*0.02860174*	*0.032060979*	*0.024293694*
**ENSG00000143797**	*0.106331135*	*0.078070253*	*0.058545992*	*0.099117785*
**ENSG00000078237**	*0.030914519*	*0.034467595*	*0.03539368*	*0.039710079*
**ENSG00000196437**	*0.022367385*	*0.029150107*	*0.027621405*	*0.018237369*
**ENSG00000183011**	*0.03235149*	*0.030785603*	*0.029688004*	*0.02680457*
**ENSG00000130775**	*0.050116262*	*0.046156594*	*0.049490432*	*0.046754552*
**ENSG00000173166**	*0.015476682*	*0.025522567*	*0.024332937*	*0.014785066*
**ENSG00000158156**	*0.030348394*	*0.043345224*	*0.043207919*	*0.027254308*
**ENSG00000163435**	*0.038298181*	*0.033879235*	*0.03258544*	*0.028964279*
**ENSG00000119203**	*0.030233405*	*0.034955221*	*0.031133152*	*0.024053533*
**ENSG00000170788**	*0.016842729*	*0.02523146*	*0.025595728*	*0.014874026*
**ENSG00000006757**	*0.039551724*	*0.040420098*	*0.035820373*	*0.029133801*
**ENSG00000162736**	*0.022226816*	*0.029250364*	*0.032739471*	*0.024705177*
**ENSG00000108861**	*0.047619663*	*0.038039435*	*0.037699526*	*0.038158818*
**ENSG00000126883**	*0.021605662*	*0.031221093*	*0.034811218*	*0.025219872*
**ENSG00000186298**	*0.021980946*	*0.028705277*	*0.026446316*	*0.019375862*
**ENSG00000167208**	*0.026571429*	*0.031690146*	*0.028625033*	*0.02547476*
**ENSG00000143384**	*0.046608221*	*0.057746913*	*0.052968291*	*0.0473077*
**ENSG00000151150**	*0.015515371*	*0.024221312*	*0.023914343*	*0.014718779*
**ENSG00000116473**	*0.026631626*	*0.03161809*	*0.029129613*	*0.023192284*
**ENSG00000125498**	*0.016565112*	*0.025773555*	*0.025501135*	*0.016423383*
**ENSG00000133574**	*0.137567689*	*0.123360136*	*0.114054556*	*0.136320332*
**ENSG00000068120**	*0.025893585*	*0.029141209*	*0.030211271*	*0.018811987*
**ENSG00000083454**	*0.017562317*	*0.026780327*	*0.02436757*	*0.016447922*
**ENSG00000151376**	*0.035710055*	*0.039262196*	*0.02660892*	*0.02815261*
**ENSG00000180353**	*0.024417611*	*0.028554246*	*0.028867609*	*0.026939464*
**ENSG00000197459**	*0.054669369*	*0.057109328*	*0.048628755*	*0.050534006*
**ENSG00000088179**	*0.033141722*	*0.037450033*	*0.034580644*	*0.030647582*
**ENSG00000168214**	*0.035806126*	*0.033113176*	*0.036201735*	*0.032864007*
**ENSG00000005469**	*0.046824865*	*0.043936216*	*0.035300704*	*0.030737927*
**ENSG00000173418**	*0.026208357*	*0.031159715*	*0.029393425*	*0.021229579*
**ENSG00000167740**	*0.038865877*	*0.036578721*	*0.032591621*	*0.03301577*
**ENSG00000139318**	*0.052972499*	*0.041476593*	*0.047919222*	*0.052342001*
**ENSG00000161642**	*0.031152001*	*0.033281692*	*0.027692341*	*0.032074976*

**ENSG00000163630**	*0.016302799*	*0.025532123*	*0.025587095*	*0.014502153*
**ENSG00000156011**	*0.054528132*	*0.032395865*	*0.028757204*	*0.028750753*
**ENSG00000112149**	*0.063468914*	*0.064906345*	*0.059573657*	*0.061320169*
**ENSG00000174521**	*0.015199375*	*0.025510095*	*0.024112357*	*0.014717092*
**ENSG00000007047**	*0.017256784*	*0.026016266*	*0.025974131*	*0.016049487*
**ENSG00000145715**	*0.017802681*	*0.025476579*	*0.027258718*	*0.016859097*
**ENSG00000182919**	*0.030491274*	*0.039528583*	*0.033788865*	*0.030593116*
**ENSG00000165733**	*0.024674453*	*0.035241396*	*0.028266246*	*0.024649456*
**ENSG00000180596**	*0.056594044*	*0.056074806*	*0.04929082*	*0.04469135*
**ENSG00000176406**	*0.098002161*	*0.036906079*	*0.031391491*	*0.051004184*
**ENSG00000151689**	*0.031634266*	*0.037202239*	*0.032118002*	*0.029911741*
**ENSG00000124818**	*0.015833241*	*0.024766944*	*0.025243335*	*0.015584385*
**ENSG00000154358**	*0.015219655*	*0.025147083*	*0.025070006*	*0.016143877*
**ENSG00000130147**	*0.017211033*	*0.025561452*	*0.025247476*	*0.01549557*
**ENSG00000031003**	*0.034390532*	*0.039555496*	*0.039071121*	*0.032186461*
**ENSG00000121579**	*0.041151352*	*0.0421048*	*0.035679244*	*0.030457543*
**ENSG00000157870**	*0.06167558*	*0.048312968*	*0.041384467*	*0.051037767*
**ENSG00000128342**	*0.023772101*	*0.030684965*	*0.027551*	*0.024090181*
**ENSG00000081791**	*0.027831557*	*0.03145417*	*0.0283789*	*0.020427714*
**ENSG00000150779**	*0.023770255*	*0.030354974*	*0.028782751*	*0.025302834*
**ENSG00000181718**	*0.015450977*	*0.02428278*	*0.024841827*	*0.014988104*
**ENSG00000175063**	*0.045562695*	*0.035763877*	*0.038840905*	*0.035104216*
**ENSG00000100209**	*0.023430933*	*0.028567459*	*0.028854775*	*0.021286511*
**ENSG00000080031**	*0.017508651*	*0.025642969*	*0.024976917*	*0.017530199*
**ENSG00000155506**	*0.024664038*	*0.030347712*	*0.035167835*	*0.025274979*
**ENSG00000090612**	*0.03121414*	*0.033141567*	*0.033530691*	*0.02455925*
**ENSG00000107295**	*0.017469701*	*0.024755876*	*0.025073973*	*0.019461655*
**ENSG00000221968**	*0.042570657*	*0.041201375*	*0.034430335*	*0.036640302*
**ENSG00000107863**	*0.043882845*	*0.03629518*	*0.037890921*	*0.031761414*
**ENSG00000130055**	*0.015765947*	*0.024200865*	*0.024511313*	*0.015227936*
**ENSG00000111846**	*0.015026017*	*0.02412091*	*0.024886743*	*0.014320888*
**ENSG00000131055**	*0.024197137*	*0.028063823*	*0.029746633*	*0.01949553*
**ENSG00000036530**	*0.018603497*	*0.025994888*	*0.028588654*	*0.018104688*
**ENSG00000102753**	*0.026433595*	*0.032123523*	*0.029553458*	*0.030713958*
**ENSG00000087448**	*0.042231717*	*0.041028396*	*0.034806592*	*0.03037176*
**ENSG00000121774**	*0.020284185*	*0.026946519*	*0.02653511*	*0.020544237*
**ENSG00000166897**	*0.017329157*	*0.024894123*	*0.023935094*	*0.015616696*
**ENSG00000067606**	*0.045206556*	*0.045376489*	*0.043400772*	*0.047652301*
**ENSG00000106246**	*0.025340788*	*0.031104273*	*0.035389847*	*0.025811992*
**ENSG00000151882**	*0.078309354*	*0.050718478*	*0.056990779*	*0.063920249*
**ENSG00000099625**	*0.018442099*	*0.025155173*	*0.027388916*	*0.015977533*
**ENSG00000163421**	*0.018544807*	*0.027306521*	*0.024950421*	*0.017303469*
**ENSG00000165084**	*0.019644359*	*0.025562877*	*0.027061442*	*0.016952296*
**ENSG00000091483**	*0.036389364*	*0.045533189*	*0.034222799*	*0.041696632*
**ENSG00000205667**	*0.015569719*	*0.024717379*	*0.025158879*	*0.015201955*
**ENSG00000149043**	*0.015264331*	*0.024577269*	*0.024371025*	*0.015489503*
**ENSG00000129451**	*0.016366457*	*0.024150735*	*0.024770298*	*0.01528394*
**ENSG00000043143**	*0.024215266*	*0.029839351*	*0.028937805*	*0.022482016*
**ENSG00000196932**	*0.015904193*	*0.025526974*	*0.025244266*	*0.014926735*
**ENSG00000002330**	*0.019747455*	*0.029211839*	*0.026654994*	*0.021760804*
**ENSG00000185271**	*0.017910666*	*0.025286203*	*0.025372894*	*0.015925398*
**ENSG00000172403**	*0.016845941*	*0.02526176*	*0.025218943*	*0.015028401*
**ENSG00000177352**	*0.028470331*	*0.03305186*	*0.038839872*	*0.034308718*
**ENSG00000188120**	*0.014765525*	*0.024887748*	*0.024444002*	*0.015869401*

**ENSG00000008311**	*0.036933442*	*0.032563899*	*0.032376762*	*0.02782391*
**ENSG00000106648**	*0.024205024*	*0.02689661*	*0.02759129*	*0.022740428*
**ENSG00000249481**	*0.021254089*	*0.024691112*	*0.024834179*	*0.016082166*
**ENSG00000135537**	*0.036350789*	*0.036346048*	*0.030073801*	*0.022358081*
**ENSG00000137337**	*0.025236983*	*0.030699792*	*0.032035046*	*0.02199299*
**ENSG00000182544**	*0.028527129*	*0.033473538*	*0.043252854*	*0.028901104*
**ENSG00000021826**	*0.041240528*	*0.042001148*	*0.031848161*	*0.032876375*
**ENSG00000150787**	*0.038734809*	*0.033734557*	*0.032227641*	*0.032619247*
**ENSG00000181523**	*0.031860654*	*0.04565957*	*0.050765203*	*0.034584417*
**ENSG00000099715**	*0.020990732*	*0.031199314*	*0.027203472*	*0.021723882*
**ENSG00000132694**	*0.015529551*	*0.025489806*	*0.0251276*	*0.01519767*
**ENSG00000174891**	*0.040621967*	*0.041235364*	*0.034091735*	*0.031588185*
**ENSG00000167851**	*0.04548745*	*0.039329519*	*0.044070284*	*0.042718699*
**ENSG00000111877**	*0.033924606*	*0.035138845*	*0.031151595*	*0.027471409*
**ENSG00000131068**	*0.016948048*	*0.025407552*	*0.025030176*	*0.016516884*
**ENSG00000181744**	*0.036567548*	*0.042365542*	*0.036067966*	*0.033365462*
**ENSG00000198625**	*0.045470707*	*0.05663974*	*0.043474396*	*0.035122581*
**ENSG00000120278**	*0.054756525*	*0.038330727*	*0.031400528*	*0.043900698*
**ENSG00000133706**	*0.036514322*	*0.037189965*	*0.029073914*	*0.023718018*
**ENSG00000162775**	*0.036598577*	*0.041053031*	*0.047437118*	*0.031127374*
**ENSG00000143387**	*0.036625884*	*0.0324381*	*0.038059447*	*0.027119967*
**ENSG00000176076**	*0.019862417*	*0.027114637*	*0.028963079*	*0.016936882*
**ENSG00000002822**	*0.051422985*	*0.035264966*	*0.04127232*	*0.043561628*
**ENSG00000013288**	*0.015291638*	*0.025347326*	*0.024933029*	*0.014433399*
**ENSG00000185339**	*0.080674524*	*0.073506738*	*0.063290069*	*0.070689284*
**ENSG00000176125**	*0.019374109*	*0.024765518*	*0.026204081*	*0.015987059*
**ENSG00000123131**	*0.040252615*	*0.035319*	*0.036904979*	*0.031557133*
**ENSG00000162434**	*0.053780448*	*0.07318848*	*0.043773672*	*0.062052031*
**ENSG00000166927**	*0.078782643*	*0.061840616*	*0.058131413*	*0.067500745*
**ENSG00000143355**	*0.014852471*	*0.024760284*	*0.025113265*	*0.016340311*
**ENSG00000125810**	*0.134278832*	*0.059661052*	*0.045062867*	*0.096748191*
**ENSG00000110090**	*0.01902116*	*0.026509311*	*0.026109084*	*0.0178682*
**ENSG00000130382**	*0.017112746*	*0.027986814*	*0.027438622*	*0.02500257*
**ENSG00000224586**	*0.015291118*	*0.024670626*	*0.024713626*	*0.014894776*
**ENSG00000006530**	*0.025868497*	*0.031927917*	*0.03135486*	*0.025758117*
**ENSG00000173372**	*0.017739665*	*0.0253481*	*0.028207159*	*0.016182452*
**ENSG00000182054**	*0.034513295*	*0.034556319*	*0.029911141*	*0.030824181*
**ENSG00000120498**	*0.015487397*	*0.02516745*	*0.024660372*	*0.0161153*
**ENSG00000172731**	*0.016303696*	*0.025503521*	*0.024428522*	*0.015324987*
**ENSG00000125520**	*0.04138628*	*0.035781652*	*0.032803083*	*0.033698372*
**ENSG00000056487**	*0.020136598*	*0.027775603*	*0.027266484*	*0.021002644*
**ENSG00000009830**	*0.020641894*	*0.028146331*	*0.029103292*	*0.018669488*
**ENSG00000174371**	*0.040994288*	*0.03880308*	*0.033846529*	*0.033393036*
**ENSG00000100626**	*0.015819683*	*0.026476667*	*0.025036338*	*0.015667704*
**ENSG00000157168**	*0.015435249*	*0.026057453*	*0.025263494*	*0.015498159*
**ENSG00000180318**	*0.015135605*	*0.024935497*	*0.025256577*	*0.01490713*
**ENSG00000176746**	*0.014491357*	*0.02484534*	*0.025812196*	*0.014796126*
**ENSG00000137996**	*0.030676884*	*0.033650712*	*0.029578742*	*0.022461652*
**ENSG00000118965**	*0.027911274*	*0.035456256*	*0.031862571*	*0.022706276*
**ENSG00000102103**	*0.021983285*	*0.027218382*	*0.027961945*	*0.021218414*
**ENSG00000137574**	*0.02025229*	*0.02887169*	*0.029295951*	*0.023837752*
**ENSG00000180776**	*0.043687923*	*0.063324317*	*0.047999522*	*0.054696893*
**ENSG00000181867**	*0.014854056*	*0.024487901*	*0.024731403*	*0.014424192*
**ENSG00000117748**	*0.024408437*	*0.028919389*	*0.031404434*	*0.020066905*

**ENSG00000171227**	*0.020714169*	*0.031897425*	*0.028616298*	*0.026033659*
**ENSG00000164620**	*0.038574577*	*0.037699706*	*0.035957381*	*0.035943335*
**ENSG00000152380**	*0.016296926*	*0.025298627*	*0.024445129*	*0.016339231*
**ENSG00000008838**	*0.0243633*	*0.031208292*	*0.030918469*	*0.021976754*
**ENSG00000163464**	*0.015438906*	*0.0264113*	*0.024195508*	*0.015657637*
**ENSG00000163933**	*0.019364701*	*0.025901062*	*0.026134906*	*0.016886642*
**ENSG00000204592**	*0.030356175*	*0.032236251*	*0.03235679*	*0.024991042*
**ENSG00000081320**	*0.043471136*	*0.052520184*	*0.04586868*	*0.048409345*
**ENSG00000160862**	*0.01925144*	*0.027194183*	*0.026543359*	*0.01737697*
**ENSG00000006042**	*0.047451733*	*0.031340179*	*0.034783211*	*0.040985645*
**ENSG00000244734**	*0.017257171*	*0.024827644*	*0.025089243*	*0.015356075*
**ENSG00000136273**	*0.017440319*	*0.025819994*	*0.025291069*	*0.015954127*
**ENSG00000079739**	*0.03220638*	*0.032504319*	*0.030828837*	*0.023909338*
**ENSG00000110104**	*0.039443639*	*0.042631772*	*0.040756194*	*0.04006722*
**ENSG00000134339**	*0.039000896*	*0.047566917*	*0.048054538*	*0.050170065*
**ENSG00000168959**	*0.016942236*	*0.025487964*	*0.025596579*	*0.015954043*
**ENSG00000118518**	*0.030536856*	*0.039788404*	*0.03628725*	*0.029167129*
**ENSG00000075188**	*0.03464445*	*0.032713165*	*0.031003142*	*0.02443914*
**ENSG00000159625**	*0.016266993*	*0.026609601*	*0.02502415*	*0.016931982*
**ENSG00000185033**	*0.015006957*	*0.024701841*	*0.024424568*	*0.014692202*
**ENSG00000128228**	*0.048004537*	*0.044883618*	*0.044890818*	*0.051846344*
**ENSG00000134627**	*0.036543923*	*0.041099671*	*0.033118887*	*0.042337165*
**ENSG00000008018**	*0.021447215*	*0.027010312*	*0.027040603*	*0.017008028*
**ENSG00000130402**	*0.024219928*	*0.02878239*	*0.02848394*	*0.022765692*
**ENSG00000119900**	*0.045082596*	*0.036809349*	*0.036554468*	*0.030673209*
**ENSG00000111540**	*0.030874596*	*0.031538077*	*0.031295371*	*0.032875811*
**ENSG00000172915**	*0.068748835*	*0.045013673*	*0.042285748*	*0.049555527*
**ENSG00000198542**	*0.026765676*	*0.027031474*	*0.025462185*	*0.015801431*
**ENSG00000185043**	*0.038005159*	*0.032718841*	*0.033324257*	*0.032565422*
**ENSG00000157978**	*0.034133802*	*0.030971165*	*0.033270159*	*0.032865965*
**ENSG00000147133**	*0.026275979*	*0.031333177*	*0.029774885*	*0.027391651*
**ENSG00000134463**	*0.053635983*	*0.04996047*	*0.052696743*	*0.058030509*
**ENSG00000174483**	*0.029678515*	*0.03567196*	*0.028361216*	*0.028569617*
**ENSG00000139192**	*0.047262172*	*0.043500805*	*0.049819586*	*0.039807626*
**ENSG00000129951**	*0.015706006*	*0.025610021*	*0.025600554*	*0.014666217*
**ENSG00000136710**	*0.024516814*	*0.034466665*	*0.032187668*	*0.023009845*
**ENSG00000167641**	*0.017903564*	*0.026544301*	*0.02599902*	*0.019718203*
**ENSG00000167646**	*0.018211962*	*0.026631959*	*0.025423245*	*0.020403273*
**ENSG00000134028**	*0.045488088*	*0.059826032*	*0.026726205*	*0.038948161*
**ENSG00000033050**	*0.032581786*	*0.039957275*	*0.033305515*	*0.035095355*
**ENSG00000168070**	*0.016318733*	*0.02659444*	*0.025751181*	*0.018093458*
**ENSG00000171540**	*0.110149831*	*0.057956126*	*0.037498389*	*0.043351302*
**ENSG00000099991**	*0.028845392*	*0.035573805*	*0.031871401*	*0.02734725*
**ENSG00000114850**	*0.042757873*	*0.041550176*	*0.037378397*	*0.039263316*
**ENSG00000111275**	*0.080710717*	*0.057571894*	*0.055496343*	*0.057391907*
**ENSG00000164270**	*0.014711614*	*0.024983874*	*0.025843834*	*0.015012358*
**ENSG00000183751**	*0.031158955*	*0.034436107*	*0.028704077*	*0.028756141*
**ENSG00000140006**	*0.024002378*	*0.029892339*	*0.030377282*	*0.019540486*
**ENSG00000165837**	*0.01551119*	*0.025031577*	*0.025546151*	*0.015662278*
**ENSG00000163956**	*0.026777625*	*0.032999806*	*0.036672842*	*0.029821184*
**ENSG00000182334**	*0.01778126*	*0.024845848*	*0.027136321*	*0.017662244*
**ENSG00000101321**	*0.017504181*	*0.027340361*	*0.025791936*	*0.018097837*
**ENSG00000153789**	*0.020575307*	*0.025723105*	*0.028596806*	*0.015721864*
**ENSG00000089048**	*0.031559598*	*0.033202494*	*0.03482595*	*0.029295028*

**ENSG00000140995**	*0.049214583*	*0.047254817*	*0.046357717*	*0.042835181*
**ENSG00000112062**	*0.019808107*	*0.028969393*	*0.027430768*	*0.016576149*
**ENSG00000167515**	*0.027994318*	*0.031218084*	*0.033352926*	*0.027008955*
**ENSG00000129636**	*0.045797858*	*0.04682217*	*0.038911601*	*0.069355994*
**ENSG00000002726**	*0.047108817*	*0.024881095*	*0.02552917*	*0.041108249*
**ENSG00000196072**	*0.046335614*	*0.032516721*	*0.042210639*	*0.082211096*
**ENSG00000113240**	*0.030431351*	*0.035063026*	*0.037553538*	*0.032912505*
**ENSG00000123374**	*0.03219074*	*0.037709599*	*0.031645076*	*0.028300182*
**ENSG00000174827**	*0.025133025*	*0.026981811*	*0.028715786*	*0.020971002*
**ENSG00000198034**	*0.01827803*	*0.026407125*	*0.02656396*	*0.018014678*
**ENSG00000197914**	*0.032967052*	*0.03203287*	*0.035467232*	*0.033866235*
**ENSG00000101911**	*0.030340273*	*0.034390072*	*0.031056839*	*0.024990638*
**ENSG00000105929**	*0.015371525*	*0.02514034*	*0.02454021*	*0.015549069*
**ENSG00000179632**	*0.023274083*	*0.027028496*	*0.039043199*	*0.028905429*
**ENSG00000037897**	*0.036023854*	*0.0335428*	*0.034231254*	*0.035052281*
**ENSG00000120322**	*0.015644998*	*0.024875285*	*0.02522749*	*0.01606837*
**ENSG00000162545**	*0.093892943*	*0.065242709*	*0.057316186*	*0.062389168*
**ENSG00000167604**	*0.018891654*	*0.02551667*	*0.025619833*	*0.016864339*
**ENSG00000149654**	*0.016118503*	*0.025881649*	*0.026037567*	*0.015179352*
**ENSG00000182667**	*0.015168444*	*0.02544225*	*0.025908727*	*0.015658848*
**ENSG00000105723**	*0.016222006*	*0.024695765*	*0.02491559*	*0.015652632*
**ENSG00000164749**	*0.022957342*	*0.026898632*	*0.024927825*	*0.026554068*
**ENSG00000165462**	*0.014761346*	*0.024924684*	*0.024279885*	*0.014831553*
**ENSG00000111450**	*0.03911067*	*0.035800319*	*0.037279134*	*0.0372683*
**ENSG00000131788**	*0.025444196*	*0.032669777*	*0.035079601*	*0.034715092*
**ENSG00000123610**	*0.092527702*	*0.08437447*	*0.081629183*	*0.084277376*
**ENSG00000042781**	*0.01630496*	*0.025874931*	*0.025229142*	*0.015114386*
**ENSG00000189227**	*0.027949852*	*0.030880281*	*0.031875599*	*0.02593835*
**ENSG00000154654**	*0.041156075*	*0.041422858*	*0.055850298*	*0.036991732*
**ENSG00000149599**	*0.016511869*	*0.025765172*	*0.024763371*	*0.014862368*
**ENSG00000122203**	*0.026026465*	*0.032926233*	*0.03323951*	*0.025477043*
**ENSG00000182621**	*0.046430315*	*0.026489834*	*0.024432457*	*0.026088973*
**ENSG00000166575**	*0.036667847*	*0.035040557*	*0.030639996*	*0.029030479*
**ENSG00000132330**	*0.051103332*	*0.049117555*	*0.050751557*	*0.051864033*
**ENSG00000146416**	*0.047946738*	*0.049256083*	*0.042973832*	*0.04188561*
**ENSG00000196636**	*0.037029039*	*0.037983762*	*0.036752042*	*0.03417161*
**ENSG00000113916**	*0.015221378*	*0.024567101*	*0.025743539*	*0.015665699*
**ENSG00000166313**	*0.019592667*	*0.028314698*	*0.029229858*	*0.019950859*
**ENSG00000165215**	*0.015941947*	*0.025552304*	*0.024850844*	*0.016392132*
**ENSG00000169118**	*0.015905561*	*0.024975322*	*0.026024115*	*0.015011418*
**ENSG00000128652**	*0.0196101*	*0.027798649*	*0.030288886*	*0.017703149*
**ENSG00000184599**	*0.015168237*	*0.025480328*	*0.024464096*	*0.01559306*
**ENSG00000129214**	*0.015343832*	*0.025013644*	*0.024340042*	*0.015797101*
**ENSG00000135404**	*0.031746667*	*0.034207393*	*0.033460815*	*0.027407206*
**ENSG00000142319**	*0.018130637*	*0.025817948*	*0.02477975*	*0.01612307*
**ENSG00000120063**	*0.030374069*	*0.035020065*	*0.033169224*	*0.030241907*
**ENSG00000069248**	*0.03114023*	*0.034567253*	*0.029464615*	*0.02681684*
**ENSG00000156599**	*0.031963204*	*0.039362989*	*0.040382104*	*0.029256587*
**ENSG00000182551**	*0.039180465*	*0.039581966*	*0.037547489*	*0.033617243*
**ENSG00000125166**	*0.026524229*	*0.033182928*	*0.031638805*	*0.029049238*
**ENSG00000118971**	*0.022249967*	*0.028563055*	*0.0266324*	*0.022173432*
**ENSG00000186889**	*0.034576783*	*0.032898798*	*0.034423241*	*0.031978676*
**ENSG00000105559**	*0.049578904*	*0.060396101*	*0.048578291*	*0.058302197*
**ENSG00000139914**	*0.016731342*	*0.0253551*	*0.024747804*	*0.017380166*

**ENSG00000023516**	*0.035015115*	*0.04267308*	*0.03367696*	*0.030475538*
**ENSG00000075785**	*0.024382589*	*0.031260157*	*0.031289518*	*0.022709158*
**ENSG00000137802**	*0.024557764*	*0.040917441*	*0.0314007*	*0.031173458*
**ENSG00000168301**	*0.031022027*	*0.032285244*	*0.033548104*	*0.023622154*
**ENSG00000183304**	*0.015018885*	*0.025166372*	*0.024090249*	*0.014470666*
**ENSG00000162571**	*0.01732529*	*0.024560061*	*0.024925307*	*0.015708592*
**ENSG00000144191**	*0.016684683*	*0.027746889*	*0.025389472*	*0.017554209*
**ENSG00000111203**	*0.03234199*	*0.035426047*	*0.034991448*	*0.028873657*
**ENSG00000023892**	*0.034287853*	*0.033266153*	*0.03223394*	*0.023455225*
**ENSG00000153233**	*0.018311326*	*0.026258411*	*0.026834095*	*0.017260163*
**ENSG00000158163**	*0.015036861*	*0.024852341*	*0.024623914*	*0.014718211*
**ENSG00000104047**	*0.0360742*	*0.040353428*	*0.032332931*	*0.028613849*
**ENSG00000081087**	*0.034433317*	*0.039938429*	*0.033987287*	*0.030622251*
**ENSG00000179639**	*0.036150086*	*0.024670499*	*0.025603043*	*0.022084378*
**ENSG00000123612**	*0.021252784*	*0.030400547*	*0.025133861*	*0.019446454*
**ENSG00000101363**	*0.026253855*	*0.027667155*	*0.029200176*	*0.020770528*
**ENSG00000166886**	*0.025273963*	*0.032914363*	*0.035014174*	*0.031522883*
**ENSG00000130706**	*0.024151911*	*0.02885899*	*0.035910054*	*0.030533749*
**ENSG00000247746**	*0.019038466*	*0.02918837*	*0.028197399*	*0.017748119*
**ENSG00000143126**	*0.018198237*	*0.024683004*	*0.025460936*	*0.018192966*
**ENSG00000101350**	*0.036712581*	*0.035722344*	*0.039589491*	*0.032352702*
**ENSG00000162949**	*0.016048954*	*0.024978446*	*0.025859896*	*0.015184995*
**ENSG00000145908**	*0.133473946*	*0.074664818*	*0.081325293*	*0.076080964*
**ENSG00000248541**	*0.075821926*	*0.051992526*	*0.072717156*	*0.073446576*
**ENSG00000185115**	*0.041689755*	*0.040708222*	*0.041041117*	*0.037897804*
**ENSG00000141560**	*0.032776303*	*0.03692007*	*0.037119636*	*0.030986464*
**ENSG00000173431**	*0.016971714*	*0.02644387*	*0.025470419*	*0.017059092*
**ENSG00000175166**	*0.031677295*	*0.038982688*	*0.033586533*	*0.031534675*
**ENSG00000186723**	*0.018228219*	*0.025488667*	*0.025348489*	*0.014629677*
**ENSG00000170382**	*0.017326081*	*0.025745437*	*0.024619769*	*0.015772006*
**ENSG00000145901**	*0.023198907*	*0.029506428*	*0.029190374*	*0.021160024*
**ENSG00000116871**	*0.020774219*	*0.031889568*	*0.035648268*	*0.023862432*
**ENSG00000149196**	*0.025291568*	*0.029314203*	*0.029556626*	*0.019921264*
**ENSG00000143067**	*0.013700971*	*0.024186052*	*0.02413549*	*0.0138548*
**ENSG00000065457**	*0.024935223*	*0.030852983*	*0.030349126*	*0.02244968*
**ENSG00000106459**	*0.026039515*	*0.029476624*	*0.029344602*	*0.019680945*
**ENSG00000170955**	*0.075256305*	*0.047536523*	*0.047935236*	*0.05106536*
**ENSG00000187676**	*0.059967678*	*0.04432417*	*0.042606441*	*0.041861843*
**ENSG00000167767**	*0.017913785*	*0.02880603*	*0.024948429*	*0.02595196*
**ENSG00000111196**	*0.037429086*	*0.035018363*	*0.03762798*	*0.038247724*
**ENSG00000187123**	*0.015080817*	*0.024575591*	*0.025117695*	*0.014667252*
**ENSG00000135046**	*0.114979351*	*0.109896464*	*0.10333301*	*0.119110673*
**ENSG00000168237**	*0.028871544*	*0.029621509*	*0.02886997*	*0.025493973*
**ENSG00000025708**	*0.041350111*	*0.041204428*	*0.038089962*	*0.034700535*
**ENSG00000100665**	*0.01808377*	*0.026642282*	*0.027091208*	*0.016544604*
**ENSG00000142065**	*0.015738839*	*0.026112566*	*0.025463982*	*0.014010782*
**ENSG00000187742**	*0.023933787*	*0.03050853*	*0.027955193*	*0.023990655*
**ENSG00000171865**	*0.027496107*	*0.032965664*	*0.033257334*	*0.028077804*
**ENSG00000142507**	*0.02393636*	*0.028268814*	*0.028369827*	*0.020905671*
**ENSG00000120318**	*0.02269449*	*0.025383682*	*0.024688758*	*0.016549021*
**ENSG00000141424**	*0.038491381*	*0.043547131*	*0.040638294*	*0.04686115*
**ENSG00000139508**	*0.038969744*	*0.033486112*	*0.038646866*	*0.03485369*
**ENSG00000145494**	*0.026041786*	*0.02949942*	*0.028884378*	*0.023474663*
**ENSG00000155011**	*0.016252666*	*0.025524784*	*0.024993072*	*0.014627411*

**ENSG00000135373**	*0.016391913*	*0.026719689*	*0.025380512*	*0.016059553*
**ENSG00000120437**	*0.030896175*	*0.031610727*	*0.031809389*	*0.026255702*
**ENSG00000127452**	*0.028801466*	*0.036626277*	*0.040408283*	*0.028146451*
**ENSG00000137221**	*0.030436955*	*0.035817809*	*0.031595869*	*0.02659456*
**ENSG00000130312**	*0.026069973*	*0.028548544*	*0.029240176*	*0.02476277*
**ENSG00000198723**	*0.015484347*	*0.025008396*	*0.025136603*	*0.015073412*
**ENSG00000135679**	*0.029522779*	*0.037470725*	*0.038841146*	*0.034536944*
**ENSG00000171133**	*0.01718764*	*0.030464396*	*0.02529533*	*0.017864821*
**ENSG00000078269**	*0.041584081*	*0.040813485*	*0.044306377*	*0.036229562*
**ENSG00000135917**	*0.018008202*	*0.025632358*	*0.026436983*	*0.016284689*
**ENSG00000105397**	*0.027661211*	*0.031254967*	*0.030770135*	*0.028729369*
**ENSG00000087088**	*0.017160067*	*0.027995548*	*0.025770155*	*0.019632872*
**ENSG00000101438**	*0.061086116*	*0.056614662*	*0.043312864*	*0.042636574*
**ENSG00000147381**	*0.015767791*	*0.024195011*	*0.025455569*	*0.014599024*
**ENSG00000154162**	*0.018274127*	*0.025141739*	*0.024948048*	*0.016311452*
**ENSG00000154122**	*0.053413065*	*0.047132394*	*0.044536092*	*0.043088176*
**ENSG00000163611**	*0.043033325*	*0.053215699*	*0.040544817*	*0.033393583*
**ENSG00000126247**	*0.021296149*	*0.027724901*	*0.027728869*	*0.023474966*
**ENSG00000186967**	*0.019078372*	*0.025315981*	*0.025725763*	*0.01944365*
**ENSG00000122376**	*0.033409054*	*0.035589033*	*0.030741537*	*0.028161197*
**ENSG00000171174**	*0.031396648*	*0.033348479*	*0.032259191*	*0.033832375*
**ENSG00000100027**	*0.026140058*	*0.029776701*	*0.029189853*	*0.028242145*
**ENSG00000183695**	*0.016314548*	*0.025701286*	*0.025578635*	*0.01608455*
**ENSG00000118402**	*0.02895821*	*0.027960286*	*0.026621793*	*0.022949105*
**ENSG00000149488**	*0.01630175*	*0.02469788*	*0.02497986*	*0.015284118*
**ENSG00000213186**	*0.023676866*	*0.027768132*	*0.027240157*	*0.021665153*
**ENSG00000033627**	*0.026603614*	*0.035862824*	*0.030853228*	*0.023575893*
**ENSG00000184058**	*0.017217028*	*0.025103625*	*0.024809283*	*0.014279639*
**ENSG00000100151**	*0.024658068*	*0.031356278*	*0.035496542*	*0.024125643*
**ENSG00000013561**	*0.017403385*	*0.026775064*	*0.025605171*	*0.016255706*
**ENSG00000039319**	*0.034931631*	*0.035603613*	*0.032696292*	*0.040637597*
**ENSG00000121743**	*0.016023588*	*0.02464665*	*0.025117376*	*0.015573796*
**ENSG00000170417**	*0.026871388*	*0.031425983*	*0.029442842*	*0.028536844*
**ENSG00000167862**	*0.0278845*	*0.029117158*	*0.029050415*	*0.025636614*
**ENSG00000174482**	*0.016438797*	*0.025185894*	*0.024506087*	*0.016046683*
**ENSG00000135111**	*0.01608753*	*0.024531871*	*0.024692605*	*0.017474075*
**ENSG00000112175**	*0.023728395*	*0.027350203*	*0.025990296*	*0.017448863*
**ENSG00000068394**	*0.029427866*	*0.032303784*	*0.031876772*	*0.025326127*
**ENSG00000134717**	*0.033149018*	*0.037263352*	*0.036747438*	*0.047779454*
**ENSG00000114638**	*0.014947492*	*0.024947769*	*0.025127421*	*0.015127021*
**ENSG00000143303**	*0.022038731*	*0.029725908*	*0.031145181*	*0.034250215*
**ENSG00000090520**	*0.030994541*	*0.038004392*	*0.036309003*	*0.03538298*
**ENSG00000142684**	*0.03089473*	*0.035138385*	*0.038740619*	*0.035173295*
**ENSG00000121075**	*0.015051985*	*0.024479613*	*0.025444793*	*0.016193955*
**ENSG00000135100**	*0.016147378*	*0.025782413*	*0.02524635*	*0.014934664*
**ENSG00000165434**	*0.01951702*	*0.025957239*	*0.02526369*	*0.015222174*
**ENSG00000128040**	*0.039799467*	*0.035087049*	*0.038100257*	*0.043472109*
**ENSG00000060339**	*0.029918952*	*0.033134687*	*0.029036302*	*0.031666243*
**ENSG00000198554**	*0.039899134*	*0.040874089*	*0.034552328*	*0.031959314*
**ENSG00000167005**	*0.026831015*	*0.029305539*	*0.029937694*	*0.019795293*
**ENSG00000070423**	*0.027619887*	*0.034608597*	*0.045269049*	*0.026091959*
**ENSG00000196968**	*0.075340983*	*0.059511995*	*0.059608358*	*0.055678165*
**ENSG00000142657**	*0.029941029*	*0.031430022*	*0.033594356*	*0.027224651*
**ENSG00000055732**	*0.023067077*	*0.027264147*	*0.025187339*	*0.019492732*

**ENSG00000121864**	*0.016257657*	*0.024666183*	*0.024620322*	*0.014514071*
**ENSG00000073331**	*0.043976596*	*0.038350196*	*0.035462426*	*0.036356316*
**ENSG00000103248**	*0.038121025*	*0.036494327*	*0.03173067*	*0.033464667*
**ENSG00000118898**	*0.016888612*	*0.025539233*	*0.025303345*	*0.015551567*
**ENSG00000137509**	*0.018305737*	*0.024616997*	*0.025387445*	*0.017375825*
**ENSG00000126767**	*0.024827516*	*0.031653901*	*0.03176224*	*0.027782761*
**ENSG00000186912**	*0.014928346*	*0.024282026*	*0.025030135*	*0.01497453*
**ENSG00000165125**	*0.016735354*	*0.025086998*	*0.025696908*	*0.0158597*
**ENSG00000123219**	*0.050172523*	*0.050564537*	*0.037741855*	*0.037309848*
**ENSG00000152208**	*0.015768718*	*0.025803483*	*0.024461443*	*0.01458241*
**ENSG00000196739**	*0.016206356*	*0.027224375*	*0.024880393*	*0.015349929*
**ENSG00000174226**	*0.016322458*	*0.025336952*	*0.025491533*	*0.01645832*
**ENSG00000159352**	*0.027388766*	*0.031824945*	*0.033147543*	*0.02404308*
**ENSG00000128310**	*0.016487447*	*0.024194084*	*0.024752195*	*0.015247818*
**ENSG00000179363**	*0.015633888*	*0.024852696*	*0.024562735*	*0.01608964*
**ENSG00000125971**	*0.018370378*	*0.027296279*	*0.027400893*	*0.021454894*
**ENSG00000004399**	*0.017236637*	*0.026869592*	*0.027375846*	*0.016454787*
**ENSG00000155714**	*0.015322107*	*0.025746801*	*0.025223342*	*0.016173659*
**ENSG00000132965**	*0.070024386*	*0.062226904*	*0.07991446*	*0.08227877*
**ENSG00000149930**	*0.023131976*	*0.031069599*	*0.027299148*	*0.022601697*
**ENSG00000125089**	*0.044652208*	*0.045898783*	*0.044629851*	*0.048698203*
**ENSG00000100298**	*0.04802617*	*0.050967512*	*0.049539014*	*0.044054039*
**ENSG00000130812**	*0.036459496*	*0.033483585*	*0.039640224*	*0.041844162*
**ENSG00000099904**	*0.034341297*	*0.03720384*	*0.033265825*	*0.024164818*
**ENSG00000147676**	*0.015718437*	*0.025472867*	*0.024628948*	*0.01426469*
**ENSG00000074842**	*0.031048077*	*0.032565297*	*0.035626292*	*0.033200674*
**ENSG00000129282**	*0.035721998*	*0.043030126*	*0.032648131*	*0.028415963*
**ENSG00000168621**	*0.01569193*	*0.025740342*	*0.024751714*	*0.01515042*
**ENSG00000173714**	*0.016877703*	*0.0244172*	*0.025054546*	*0.017899707*
**ENSG00000089737**	*0.020825737*	*0.028475384*	*0.032377201*	*0.018241255*
**ENSG00000181016**	*0.021710852*	*0.029155642*	*0.025658991*	*0.017530767*
**ENSG00000117228**	*0.058889647*	*0.060556879*	*0.058205909*	*0.056818141*
**ENSG00000162438**	*0.016610665*	*0.025780372*	*0.024466194*	*0.017684448*
**ENSG00000159176**	*0.037440017*	*0.037794219*	*0.037030271*	*0.028320404*
**ENSG00000163002**	*0.036324094*	*0.034798083*	*0.037366328*	*0.037870507*
**ENSG00000196365**	*0.031140001*	*0.030711869*	*0.034369662*	*0.027556527*
**ENSG00000173085**	*0.075026106*	*0.064243279*	*0.054325498*	*0.058365398*
**ENSG00000136319**	*0.031677378*	*0.036316605*	*0.036555348*	*0.028251356*
**ENSG00000227507**	*0.086212141*	*0.067495068*	*0.072976135*	*0.08217916*
**ENSG00000060140**	*0.019801385*	*0.024796242*	*0.025121406*	*0.017560066*
**ENSG00000100804**	*0.028208411*	*0.031564037*	*0.029575219*	*0.023006228*
**ENSG00000106789**	*0.068000467*	*0.117652057*	*0.094008284*	*0.099619668*
**ENSG00000080511**	*0.017262045*	*0.025459081*	*0.026183763*	*0.015530294*
**ENSG00000108255**	*0.015328759*	*0.024757108*	*0.026034665*	*0.016117239*
**ENSG00000113407**	*0.022669872*	*0.030720812*	*0.032031734*	*0.02429204*
**ENSG00000147854**	*0.032765404*	*0.038479344*	*0.035526144*	*0.026719051*
**ENSG00000102144**	*0.033706103*	*0.031682242*	*0.036622952*	*0.026986757*
**ENSG00000108829**	*0.034421566*	*0.036699782*	*0.042098028*	*0.036660161*
**ENSG00000159377**	*0.025602916*	*0.030646771*	*0.034273203*	*0.020230845*
**ENSG00000108001**	*0.016531154*	*0.024989268*	*0.025018861*	*0.017715394*
**ENSG00000147471**	*0.027700655*	*0.038781236*	*0.035136598*	*0.028541926*
**ENSG00000101457**	*0.025799245*	*0.030684874*	*0.031945902*	*0.024718194*
**ENSG00000171570**	*0.027528611*	*0.041250554*	*0.043448577*	*0.033897909*
**ENSG00000204446**	*0.018729206*	*0.02598547*	*0.025336739*	*0.016946763*

**ENSG00000131781**	*0.016747627*	*0.02680267*	*0.02514922*	*0.017012718*
**ENSG00000107731**	*0.016045282*	*0.024757708*	*0.026189594*	*0.016169717*
**ENSG00000197110**	*0.016834642*	*0.02886113*	*0.025966097*	*0.017268365*
**ENSG00000184867**	*0.081566064*	*0.048138054*	*0.050799637*	*0.05932807*
**ENSG00000184009**	*0.018156633*	*0.025183902*	*0.025707659*	*0.017314176*
**ENSG00000166501**	*0.044659448*	*0.040981196*	*0.039654681*	*0.03670241*
**ENSG00000166170**	*0.033680368*	*0.040766607*	*0.042956035*	*0.031506206*
**ENSG00000011677**	*0.017177956*	*0.027664721*	*0.024531044*	*0.017640205*
**ENSG00000162368**	*0.026447047*	*0.033236933*	*0.029244051*	*0.027295428*
**ENSG00000165186**	*0.015040473*	*0.025481865*	*0.024555423*	*0.015749406*
**ENSG00000179387**	*0.034548577*	*0.038433075*	*0.033707265*	*0.028623399*
**ENSG00000182050**	*0.015968265*	*0.025093544*	*0.025194896*	*0.016416491*
**ENSG00000119547**	*0.01621957*	*0.025247363*	*0.024976899*	*0.015143575*
**ENSG00000010379**	*0.017924473*	*0.025743776*	*0.026072073*	*0.015645864*
**ENSG00000167434**	*0.064035202*	*0.047849364*	*0.040018019*	*0.052037255*
**ENSG00000072849**	*0.031663276*	*0.031996192*	*0.040608575*	*0.030483988*
**ENSG00000148180**	*0.022314688*	*0.025659537*	*0.028264936*	*0.018184665*
**ENSG00000172432**	*0.028565464*	*0.032464535*	*0.030365594*	*0.023516989*
**ENSG00000080824**	*0.030673129*	*0.037622286*	*0.032285945*	*0.036446618*
**ENSG00000100889**	*0.029102585*	*0.029574307*	*0.030786142*	*0.022585977*
**ENSG00000179715**	*0.074258737*	*0.057268468*	*0.067485755*	*0.05932664*
**ENSG00000137168**	*0.034439462*	*0.035367249*	*0.032956626*	*0.034312228*
**ENSG00000118369**	*0.039109042*	*0.03614835*	*0.042476604*	*0.03299201*
**ENSG00000171700**	*0.042376706*	*0.046412384*	*0.040968405*	*0.042133142*
**ENSG00000182791**	*0.014724438*	*0.023885811*	*0.02455389*	*0.014272423*
**ENSG00000172551**	*0.018281779*	*0.024780566*	*0.024854254*	*0.01623226*
**ENSG00000126214**	*0.027193604*	*0.031646712*	*0.03318313*	*0.025252104*
**ENSG00000140391**	*0.034352062*	*0.035427169*	*0.033507706*	*0.039203667*
**ENSG00000143816**	*0.015315955*	*0.025437718*	*0.02520331*	*0.01548521*
**ENSG00000019582**	*0.02210617*	*0.030711649*	*0.028322266*	*0.0274537*
**ENSG00000128791**	*0.054287859*	*0.041356204*	*0.037285191*	*0.041177863*
**ENSG00000237289**	*0.029325927*	*0.03461811*	*0.028491617*	*0.017374908*
**ENSG00000163492**	*0.015705128*	*0.025375485*	*0.024069748*	*0.015099098*
**ENSG00000184254**	*0.027754998*	*0.027998674*	*0.026279188*	*0.051648697*
**ENSG00000111206**	*0.016590405*	*0.026070452*	*0.025672116*	*0.015622543*
**ENSG00000160973**	*0.01641393*	*0.025762318*	*0.024083166*	*0.014805676*
**ENSG00000148606**	*0.018897733*	*0.02905348*	*0.025856104*	*0.017970502*
**ENSG00000090512**	*0.018903734*	*0.026236395*	*0.02554833*	*0.016624587*
**ENSG00000176571**	*0.017762973*	*0.027101519*	*0.02718019*	*0.018128637*
**ENSG00000134371**	*0.028793897*	*0.036069389*	*0.032921705*	*0.028722731*
**ENSG00000185640**	*0.016733858*	*0.024970611*	*0.024863081*	*0.014891926*
**ENSG00000204936**	*0.015464055*	*0.024964067*	*0.025310319*	*0.015603703*
**ENSG00000172803**	*0.015892669*	*0.027302778*	*0.026431778*	*0.016158968*
**ENSG00000176928**	*0.015704136*	*0.025106923*	*0.024683328*	*0.015278037*
**ENSG00000212916**	*0.040365895*	*0.035872821*	*0.037060455*	*0.032644397*
**ENSG00000253958**	*0.058684348*	*0.06243211*	*0.060093698*	*0.057334461*
**ENSG00000064300**	*0.019180093*	*0.02619135*	*0.026164366*	*0.018375384*
**ENSG00000170279**	*0.014867239*	*0.024621793*	*0.025229411*	*0.014438071*
**ENSG00000136237**	*0.085420443*	*0.077491314*	*0.066963308*	*0.073797298*
**ENSG00000180879**	*0.029183883*	*0.029794388*	*0.033900922*	*0.031002802*
**ENSG00000178789**	*0.020580069*	*0.035339095*	*0.029171859*	*0.02587161*
**ENSG00000142583**	*0.095085113*	*0.065656629*	*0.065899753*	*0.07669893*
**ENSG00000152683**	*0.02103429*	*0.029156666*	*0.02878007*	*0.024937277*
**ENSG00000180787**	*0.041128461*	*0.03962049*	*0.039241448*	*0.034674156*

**ENSG00000143891**	*0.073985718*	*0.058918896*	*0.056386558*	*0.06605052*
**ENSG00000115993**	*0.029468156*	*0.03614608*	*0.033032227*	*0.025286096*
**ENSG00000177025**	*0.0183527*	*0.026231247*	*0.025376737*	*0.019954328*
**ENSG00000131446**	*0.026509873*	*0.028471432*	*0.037749983*	*0.02855365*
**ENSG00000186654**	*0.017116864*	*0.02615642*	*0.026708707*	*0.01712645*
**ENSG00000163982**	*0.016190452*	*0.024899031*	*0.024704255*	*0.014994476*
**ENSG00000157703**	*0.016213541*	*0.025974151*	*0.024994645*	*0.014802967*
**ENSG00000171823**	*0.026013156*	*0.032367227*	*0.029098805*	*0.02370926*
**ENSG00000166523**	*0.016412256*	*0.024313017*	*0.024763965*	*0.015016143*
**ENSG00000127922**	*0.019819652*	*0.027263401*	*0.027595295*	*0.017167857*
**ENSG00000122733**	*0.016568443*	*0.025257408*	*0.024682864*	*0.018276007*
**ENSG00000135905**	*0.047582906*	*0.051518405*	*0.042891181*	*0.03731006*
**ENSG00000100416**	*0.024315753*	*0.029629967*	*0.027761586*	*0.024161307*
**ENSG00000171049**	*0.015768704*	*0.025300179*	*0.02496074*	*0.014479878*
**ENSG00000101425**	*0.015256743*	*0.02496275*	*0.024785301*	*0.015152997*
**ENSG00000140323**	*0.015195368*	*0.024023802*	*0.024192319*	*0.016185764*
**ENSG00000080503**	*0.043306296*	*0.044213974*	*0.042142887*	*0.039169372*
**ENSG00000035687**	*0.030382631*	*0.03459223*	*0.033049595*	*0.028662288*
**ENSG00000143314**	*0.035633442*	*0.035845328*	*0.034000223*	*0.023608328*
**ENSG00000120057**	*0.015417022*	*0.024901618*	*0.025114429*	*0.015424317*
**ENSG00000164647**	*0.155759857*	*0.088568517*	*0.09314466*	*0.101663866*
**ENSG00000185130**	*0.037056524*	*0.030744504*	*0.040703926*	*0.032344465*
**ENSG00000104055**	*0.101147466*	*0.091527404*	*0.078336146*	*0.082290023*
**ENSG00000157110**	*0.125649976*	*0.105399123*	*0.082076075*	*0.118765845*
**ENSG00000139223**	*0.015691756*	*0.024896661*	*0.024306985*	*0.014535023*
**ENSG00000138777**	*0.036647022*	*0.050161611*	*0.049676484*	*0.056270192*
**ENSG00000197651**	*0.023038143*	*0.032105562*	*0.030155038*	*0.020391652*
**ENSG00000156486**	*0.017324131*	*0.025291758*	*0.026661835*	*0.01713829*
**ENSG00000130158**	*0.038452913*	*0.039119975*	*0.04165965*	*0.039123751*
**ENSG00000163737**	*0.018596549*	*0.025394123*	*0.025977337*	*0.015563669*
**ENSG00000157557**	*0.041213333*	*0.044481842*	*0.04334482*	*0.035657165*
**ENSG00000244057**	*0.015484781*	*0.024541704*	*0.025061998*	*0.015623439*
**ENSG00000166535**	*0.01637484*	*0.024388141*	*0.024802791*	*0.015568355*
**ENSG00000185219**	*0.016199977*	*0.026753761*	*0.025138391*	*0.01596716*
**ENSG00000188559**	*0.016951989*	*0.025342279*	*0.023975673*	*0.015090001*
**ENSG00000164056**	*0.069140102*	*0.079617778*	*0.080294867*	*0.088649179*
**ENSG00000136859**	*0.057904034*	*0.079138872*	*0.073062949*	*0.075056975*
**ENSG00000154485**	*0.017995366*	*0.025504727*	*0.024573504*	*0.017242432*
**ENSG00000186471**	*0.016803776*	*0.026941469*	*0.026785413*	*0.017027529*
**ENSG00000157992**	*0.018796369*	*0.025409567*	*0.026540721*	*0.017022442*
**ENSG00000128805**	*0.022750073*	*0.027502112*	*0.02873857*	*0.021806344*
**ENSG00000094963**	*0.016622873*	*0.025750509*	*0.024801668*	*0.01560127*
**ENSG00000173905**	*0.037299362*	*0.038527679*	*0.035506301*	*0.036092556*
**ENSG00000147649**	*0.025874991*	*0.030575773*	*0.030578643*	*0.025322461*
**ENSG00000174574**	*0.029168493*	*0.038293847*	*0.039466746*	*0.028942226*
**ENSG00000183161**	*0.045225408*	*0.046050462*	*0.032274791*	*0.032844615*
**ENSG00000074706**	*0.122626715*	*0.084725467*	*0.077061854*	*0.084317958*
**ENSG00000180998**	*0.022381192*	*0.026183839*	*0.025807345*	*0.018057068*
**ENSG00000181817**	*0.024752411*	*0.031381953*	*0.033600102*	*0.029955303*
**ENSG00000188467**	*0.015621321*	*0.024436365*	*0.023645854*	*0.015060436*
**ENSG00000173239**	*0.01448977*	*0.02498006*	*0.025007993*	*0.016196316*
**ENSG00000153253**	*0.021972668*	*0.025182893*	*0.024531312*	*0.016128033*
**ENSG00000186806**	*0.034104595*	*0.041887399*	*0.041600481*	*0.03790183*
**ENSG00000011454**	*0.029622783*	*0.035157295*	*0.033244204*	*0.027689746*

**ENSG00000138669**	*0.016474972*	*0.025264854*	*0.026142777*	*0.016151118*
**ENSG00000137204**	*0.015881891*	*0.025818649*	*0.025047739*	*0.015150968*
**ENSG00000108592**	*0.029314735*	*0.033168062*	*0.035374535*	*0.026186351*
**ENSG00000118939**	*0.030716393*	*0.03368994*	*0.03267346*	*0.026348963*
**ENSG00000169169**	*0.015407269*	*0.026075553*	*0.024304476*	*0.024466993*
**ENSG00000144655**	*0.041915326*	*0.041696639*	*0.04298312*	*0.038568978*
**ENSG00000132837**	*0.015539545*	*0.025228815*	*0.027523496*	*0.014498391*
**ENSG00000198947**	*0.016349607*	*0.025256635*	*0.025250402*	*0.01641806*
**ENSG00000254004**	*0.017827083*	*0.026307284*	*0.02637485*	*0.019874187*
**ENSG00000005844**	*0.064899747*	*0.046084521*	*0.048837207*	*0.042576347*
**ENSG00000160741**	*0.027439182*	*0.031427172*	*0.036153702*	*0.028904411*
**ENSG00000075643**	*0.099617529*	*0.081265268*	*0.068391823*	*0.074465955*
**ENSG00000156642**	*0.026042872*	*0.030741406*	*0.030223254*	*0.023049097*
**ENSG00000112303**	*0.040978926*	*0.053662981*	*0.040845367*	*0.052466111*
**ENSG00000186260**	*0.016550752*	*0.025486544*	*0.024829759*	*0.016662289*
**ENSG00000184166**	*0.018925017*	*0.026596136*	*0.025931859*	*0.01862991*
**ENSG00000183287**	*0.022598464*	*0.027630204*	*0.032640597*	*0.019412354*
**ENSG00000082068**	*0.031668909*	*0.032805931*	*0.031572256*	*0.024496767*
**ENSG00000094880**	*0.025315532*	*0.031692339*	*0.031499715*	*0.021200826*
**ENSG00000147041**	*0.014134836*	*0.024225889*	*0.025321342*	*0.015309954*
**ENSG00000138190**	*0.053534878*	*0.049509529*	*0.039455445*	*0.047096208*
**ENSG00000171855**	*0.029218305*	*0.02870226*	*0.027221457*	*0.015448243*
**ENSG00000134489**	*0.014514762*	*0.025887232*	*0.025006024*	*0.01582125*
**ENSG00000109814**	*0.035938352*	*0.041082545*	*0.031763432*	*0.02908131*
**ENSG00000123810**	*0.033771074*	*0.043774335*	*0.035939237*	*0.037115663*
**ENSG00000196296**	*0.015791185*	*0.024324029*	*0.025165606*	*0.014840253*
**ENSG00000180219**	*0.015059226*	*0.024219889*	*0.025001322*	*0.015015314*
**ENSG00000113389**	*0.01579418*	*0.026692534*	*0.026305254*	*0.015897714*
**ENSG00000105697**	*0.017215516*	*0.025599162*	*0.025197776*	*0.017201467*
**ENSG00000124207**	*0.035327511*	*0.035891799*	*0.033581428*	*0.026287284*
**ENSG00000166428**	*0.018221627*	*0.027096558*	*0.02938537*	*0.016137162*
**ENSG00000099866**	*0.016754916*	*0.024622601*	*0.024837939*	*0.016492196*
**ENSG00000145912**	*0.024501732*	*0.028411357*	*0.028904167*	*0.019730614*
**ENSG00000168906**	*0.045702529*	*0.045501268*	*0.052322203*	*0.044178588*
**ENSG00000119042**	*0.016644591*	*0.02498726*	*0.024459068*	*0.018527635*
**ENSG00000125703**	*0.045758096*	*0.037145937*	*0.032669452*	*0.026722596*
**ENSG00000087303**	*0.019445969*	*0.028828652*	*0.026307165*	*0.019927576*
**ENSG00000070190**	*0.033091538*	*0.036752324*	*0.037142332*	*0.034146613*
**ENSG00000137714**	*0.026847733*	*0.036568267*	*0.03470361*	*0.034013419*
**ENSG00000241837**	*0.016461378*	*0.025191101*	*0.025169742*	*0.015798839*
**ENSG00000105784**	*0.020894112*	*0.027155572*	*0.025291119*	*0.040051053*
**ENSG00000100503**	*0.036101914*	*0.039544833*	*0.032032707*	*0.028984407*
**ENSG00000156381**	*0.054289274*	*0.038353593*	*0.037823527*	*0.038692754*
**ENSG00000188674**	*0.013528402*	*0.023444434*	*0.023518746*	*0.013005628*
**ENSG00000123684**	*0.045853532*	*0.044381211*	*0.046759264*	*0.045581085*
**ENSG00000079150**	*0.022540009*	*0.030122827*	*0.027137642*	*0.025310798*
**ENSG00000074696**	*0.034620461*	*0.036738784*	*0.033298316*	*0.034149095*
**ENSG00000018236**	*0.015564821*	*0.026710287*	*0.025229897*	*0.015997296*
**ENSG00000175198**	*0.036952458*	*0.035777702*	*0.031256303*	*0.032619603*
**ENSG00000165695**	*0.032758975*	*0.035848978*	*0.033866913*	*0.028704496*
**ENSG00000151514**	*0.022512545*	*0.025546025*	*0.026085071*	*0.015738216*
**ENSG00000158423**	*0.017254701*	*0.024566833*	*0.024650013*	*0.015326917*
**ENSG00000139625**	*0.025391907*	*0.032485298*	*0.027709486*	*0.022804176*
**ENSG00000112200**	*0.039689524*	*0.034149903*	*0.033887789*	*0.02905784*

**ENSG00000151834**	*0.016010414*	*0.024950528*	*0.025878684*	*0.015831573*
**ENSG00000184459**	*0.01520824*	*0.024839588*	*0.024466077*	*0.015708244*
**ENSG00000068489**	*0.041539058*	*0.035537057*	*0.034755015*	*0.028780402*
**ENSG00000128524**	*0.019894133*	*0.027970437*	*0.027510539*	*0.020722177*
**ENSG00000169718**	*0.022974674*	*0.027475662*	*0.02692133*	*0.026955807*
**ENSG00000159450**	*0.018117197*	*0.025432969*	*0.026603172*	*0.019401169*
**ENSG00000164334**	*0.017511239*	*0.026474479*	*0.025159246*	*0.015477642*
**ENSG00000100991**	*0.020738908*	*0.026039478*	*0.031615042*	*0.021255804*
**ENSG00000111674**	*0.067353999*	*0.04861233*	*0.044907088*	*0.05833817*
**ENSG00000144635**	*0.029047436*	*0.036249828*	*0.035065912*	*0.037549927*
**ENSG00000111670**	*0.034362841*	*0.035732492*	*0.04320069*	*0.032854496*
**ENSG00000115041**	*0.015909822*	*0.025220753*	*0.024883139*	*0.015407981*
**ENSG00000074582**	*0.032787511*	*0.032309877*	*0.029580554*	*0.02374932*
**ENSG00000116141**	*0.039227914*	*0.030157992*	*0.03502987*	*0.021888507*
**ENSG00000133808**	*0.016480755*	*0.025733629*	*0.024804916*	*0.015001833*
**ENSG00000102870**	*0.015069113*	*0.024754988*	*0.024877743*	*0.015216223*
**ENSG00000102189**	*0.02262195*	*0.030785461*	*0.026990612*	*0.023090502*
**ENSG00000213199**	*0.017347993*	*0.025842732*	*0.02618707*	*0.01534516*
**ENSG00000185880**	*0.034506396*	*0.044336874*	*0.031622265*	*0.025485879*
**ENSG00000007174**	*0.016341627*	*0.023945435*	*0.024716821*	*0.015931237*
**ENSG00000102710**	*0.025537755*	*0.031653746*	*0.029169787*	*0.026039425*
**ENSG00000188295**	*0.040978108*	*0.037498078*	*0.03850051*	*0.0399744*
**ENSG00000165973**	*0.018856563*	*0.025117255*	*0.025426106*	*0.015920154*
**ENSG00000157119**	*0.015731494*	*0.024818252*	*0.024456991*	*0.015672343*
**ENSG00000067113**	*0.016134626*	*0.026245231*	*0.026270835*	*0.015874866*
**ENSG00000101180**	*0.016223313*	*0.025186752*	*0.025365246*	*0.015386452*
**ENSG00000102539**	*0.018031651*	*0.025422988*	*0.024053546*	*0.017070136*
**ENSG00000152795**	*0.040462259*	*0.037737852*	*0.039273304*	*0.037350211*
**ENSG00000089159**	*0.038213621*	*0.03574414*	*0.034571901*	*0.033725199*
**ENSG00000100528**	*0.028411456*	*0.037481672*	*0.030694678*	*0.022655078*
**ENSG00000186638**	*0.026869543*	*0.03284934*	*0.036213881*	*0.02186154*
**ENSG00000131116**	*0.031838333*	*0.035645395*	*0.032520523*	*0.03084677*
**ENSG00000119655**	*0.033503184*	*0.031759839*	*0.032312319*	*0.029239924*
**ENSG00000197705**	*0.034846419*	*0.064762967*	*0.063281475*	*0.052754077*
**ENSG00000188763**	*0.07081437*	*0.067792585*	*0.055767436*	*0.049689182*
**ENSG00000166986**	*0.02723214*	*0.033796811*	*0.030598624*	*0.028324242*
**ENSG00000120071**	*0.021545244*	*0.029392157*	*0.033557135*	*0.023284839*
**ENSG00000147439**	*0.030168754*	*0.034436625*	*0.037347928*	*0.026534446*
**ENSG00000172936**	*0.034611409*	*0.041110746*	*0.0454327*	*0.037503876*
**ENSG00000006576**	*0.035352624*	*0.03889348*	*0.032572202*	*0.031214093*
**ENSG00000170537**	*0.015747951*	*0.025682962*	*0.024344347*	*0.01548655*
**ENSG00000170162**	*0.01823607*	*0.027216012*	*0.026397197*	*0.018514855*
**ENSG00000131142**	*0.027220271*	*0.029185131*	*0.032494174*	*0.028677226*
**ENSG00000133048**	*0.049731177*	*0.070888377*	*0.057118674*	*0.057780019*
**ENSG00000170271**	*0.016158421*	*0.02587627*	*0.02661521*	*0.016704839*
**ENSG00000120832**	*0.017028186*	*0.026704208*	*0.02546786*	*0.016924016*
**ENSG00000175582**	*0.033558564*	*0.041679892*	*0.034156626*	*0.031534644*
**ENSG00000131061**	*0.017691282*	*0.024511439*	*0.025987373*	*0.014991284*
**ENSG00000125952**	*0.021749703*	*0.029133512*	*0.029581361*	*0.024541925*
**ENSG00000152034**	*0.016906944*	*0.02481452*	*0.024107387*	*0.015796434*
**ENSG00000070526**	*0.01666587*	*0.024935165*	*0.025086248*	*0.014439549*
**ENSG00000113272**	*0.020209009*	*0.029150741*	*0.028995627*	*0.020586013*
**ENSG00000186970**	*0.017345364*	*0.024591138*	*0.025888618*	*0.015446616*
**ENSG00000242950**	*0.01479616*	*0.024464352*	*0.025145004*	*0.015477808*

**ENSG00000234024**	*0.045503828*	*0.02990147*	*0.027048816*	*0.036768147*
**ENSG00000121903**	*0.014509429*	*0.02620092*	*0.0248803*	*0.016087273*
**ENSG00000135549**	*0.019224066*	*0.026200371*	*0.024782645*	*0.020137465*
**ENSG00000156564**	*0.015920899*	*0.025678493*	*0.025440524*	*0.016008249*
**ENSG00000102921**	*0.048888156*	*0.041640152*	*0.042704117*	*0.039833726*
**ENSG00000170265**	*0.01966611*	*0.029100679*	*0.034191533*	*0.022312136*
**ENSG00000180329**	*0.02496946*	*0.030770705*	*0.030200374*	*0.023141934*
**ENSG00000176108**	*0.021550935*	*0.027106357*	*0.030743668*	*0.020325246*
**ENSG00000078399**	*0.015427817*	*0.024494427*	*0.025444655*	*0.014963299*
**ENSG00000112309**	*0.017305946*	*0.026858931*	*0.026949926*	*0.018865394*
**ENSG00000047634**	*0.112154765*	*0.090702714*	*0.067244932*	*0.090533306*
**ENSG00000163909**	*0.029047289*	*0.02945186*	*0.031091295*	*0.028055345*
**ENSG00000106080**	*0.038578362*	*0.034984217*	*0.037409145*	*0.040667027*
**ENSG00000169562**	*0.016203309*	*0.025266657*	*0.025678016*	*0.014786082*
**ENSG00000126698**	*0.026307894*	*0.031723458*	*0.031454607*	*0.02272842*
**ENSG00000092108**	*0.020013046*	*0.028267906*	*0.02979595*	*0.022660484*
**ENSG00000243955**	*0.016832284*	*0.024698751*	*0.025352711*	*0.016431448*
**ENSG00000128845**	*0.015335933*	*0.025279018*	*0.024373854*	*0.016287486*
**ENSG00000072864**	*0.029595042*	*0.0373206*	*0.032545933*	*0.028564469*
**ENSG00000077147**	*0.024296555*	*0.029936787*	*0.030611056*	*0.02175547*
**ENSG00000088205**	*0.0287293*	*0.034364961*	*0.032234943*	*0.026555841*
**ENSG00000140525**	*0.043243912*	*0.042927487*	*0.034147302*	*0.033513967*
**ENSG00000133169**	*0.028730023*	*0.02738518*	*0.026919867*	*0.018013496*
**ENSG00000142544**	*0.026120045*	*0.031081303*	*0.029251789*	*0.031107947*
**ENSG00000154328**	*0.031412175*	*0.034869542*	*0.033157688*	*0.026297851*
**ENSG00000189068**	*0.015167436*	*0.026050182*	*0.02408134*	*0.015603625*
**ENSG00000168229**	*0.016828749*	*0.025704778*	*0.024113695*	*0.015501233*
**ENSG00000189369**	*0.027318081*	*0.036586423*	*0.033732329*	*0.025587175*
**ENSG00000197846**	*0.079633301*	*0.056249278*	*0.07666212*	*0.07408853*
**ENSG00000104818**	*0.019041357*	*0.029780231*	*0.026301443*	*0.017325414*
**ENSG00000141682**	*0.03171686*	*0.032356099*	*0.036303809*	*0.030545019*
**ENSG00000140332**	*0.042888203*	*0.038835422*	*0.038031968*	*0.036980412*
**ENSG00000152592**	*0.015737463*	*0.025468696*	*0.025285269*	*0.01499645*
**ENSG00000183034**	*0.017214956*	*0.026212842*	*0.024791375*	*0.01723533*
**ENSG00000065328**	*0.047627705*	*0.043586116*	*0.035131466*	*0.04001927*
**ENSG00000181378**	*0.014172115*	*0.025140462*	*0.024826657*	*0.014628143*
**ENSG00000097007**	*0.035789452*	*0.036229893*	*0.03845273*	*0.028715733*
**ENSG00000032444**	*0.028540811*	*0.031348959*	*0.027123217*	*0.026963285*
**ENSG00000141325**	*0.029529519*	*0.035133157*	*0.033755157*	*0.022634595*
**ENSG00000174500**	*0.054991005*	*0.067609206*	*0.060421349*	*0.063197118*
**ENSG00000048140**	*0.025124613*	*0.033423091*	*0.032799839*	*0.025591431*
**ENSG00000138798**	*0.016925226*	*0.024167052*	*0.02498375*	*0.015176756*
**ENSG00000163645**	*0.016496616*	*0.0247461*	*0.024685194*	*0.015477786*
**ENSG00000132329**	*0.118489067*	*0.106225747*	*0.105592641*	*0.102949832*
**ENSG00000136051**	*0.034756575*	*0.038831244*	*0.034905631*	*0.032905914*
**ENSG00000150628**	*0.016878195*	*0.025005838*	*0.025426624*	*0.015370531*
**ENSG00000173726**	*0.025543967*	*0.032672946*	*0.028982323*	*0.02575044*
**ENSG00000140955**	*0.026899938*	*0.029157309*	*0.043286408*	*0.018419957*
**ENSG00000133488**	*0.01543281*	*0.025972941*	*0.02598216*	*0.016387266*
**ENSG00000160716**	*0.01635548*	*0.024148027*	*0.024108886*	*0.015194838*
**ENSG00000147443**	*0.03378707*	*0.044330517*	*0.04151049*	*0.03891837*
**ENSG00000160284**	*0.06658035*	*0.044490973*	*0.03771814*	*0.135364676*
**ENSG00000048162**	*0.031211614*	*0.033119601*	*0.035373718*	*0.033325649*
**ENSG00000004948**	*0.016139416*	*0.024149075*	*0.024832288*	*0.015899082*

**ENSG00000168995**	*0.016587331*	*0.026291064*	*0.027667863*	*0.015798864*
**ENSG00000145860**	*0.037465422*	*0.043327334*	*0.042649601*	*0.037453483*
**ENSG00000172336**	*0.026796821*	*0.027368162*	*0.032388251*	*0.027921043*
**ENSG00000036672**	*0.017062393*	*0.026162432*	*0.025419877*	*0.018875074*
**ENSG00000041802**	*0.029729487*	*0.031292056*	*0.031691514*	*0.027386012*
**ENSG00000164430**	*0.045289132*	*0.041184144*	*0.04252722*	*0.037296622*
**ENSG00000123444**	*0.014654444*	*0.025696849*	*0.024814234*	*0.014827518*
**ENSG00000065000**	*0.029859487*	*0.035486906*	*0.034397502*	*0.028162804*
**ENSG00000180964**	*0.02747872*	*0.0300815*	*0.02755073*	*0.019780816*
**ENSG00000101333**	*0.03029719*	*0.029195539*	*0.028295912*	*0.023759083*
**ENSG00000111596**	*0.019077915*	*0.02805485*	*0.029618119*	*0.018246255*
**ENSG00000166347**	*0.041272537*	*0.035907733*	*0.034085052*	*0.032777953*
**ENSG00000182704**	*0.016309095*	*0.025198568*	*0.025213062*	*0.015844986*
**ENSG00000179520**	*0.018184603*	*0.025095282*	*0.025350689*	*0.014342084*
**ENSG00000080910**	*0.01631619*	*0.025557611*	*0.025905649*	*0.017053885*
**ENSG00000113638**	*0.037592879*	*0.043079177*	*0.033802833*	*0.030074147*
**ENSG00000130703**	*0.024342199*	*0.030300194*	*0.030435119*	*0.021506469*
**ENSG00000130827**	*0.021261646*	*0.030572432*	*0.03112097*	*0.019001921*
**ENSG00000152254**	*0.015637796*	*0.024433785*	*0.024798492*	*0.01595826*
**ENSG00000164821**	*0.016443978*	*0.026922937*	*0.027303276*	*0.016882244*
**ENSG00000214026**	*0.022313103*	*0.027648482*	*0.027608612*	*0.018755877*
**ENSG00000155657**	*0.020367857*	*0.027622545*	*0.029253805*	*0.018449652*
**ENSG00000125851**	*0.015383884*	*0.025124772*	*0.024789688*	*0.016951887*
**ENSG00000140350**	*0.036620328*	*0.031402022*	*0.028274358*	*0.028134103*
**ENSG00000181752**	*0.017556764*	*0.027490177*	*0.02672084*	*0.018886269*
**ENSG00000006062**	*0.038196065*	*0.044614322*	*0.043255492*	*0.03271385*
**ENSG00000137992**	*0.03210633*	*0.037377416*	*0.032238939*	*0.025174233*
**ENSG00000108639**	*0.035813292*	*0.038600404*	*0.031255224*	*0.035096164*
**ENSG00000118246**	*0.0266301*	*0.031963434*	*0.030028135*	*0.027506841*
**ENSG00000153179**	*0.018806374*	*0.026577797*	*0.025593747*	*0.016481506*
**ENSG00000139352**	*0.120560526*	*0.098155164*	*0.063091418*	*0.091849825*
**ENSG00000176034**	*0.015425495*	*0.025107531*	*0.025280456*	*0.01545639*
**ENSG00000135226**	*0.06948393*	*0.049131774*	*0.029414432*	*0.046112281*
**ENSG00000125965**	*0.016528754*	*0.025188771*	*0.025873395*	*0.015400581*
**ENSG00000139269**	*0.095832071*	*0.064210873*	*0.055269849*	*0.067126429*
**ENSG00000188428**	*0.032788603*	*0.03356919*	*0.031415555*	*0.022769652*
**ENSG00000161574**	*0.017011116*	*0.026036198*	*0.025590936*	*0.016209313*
**ENSG00000138615**	*0.021665786*	*0.026876836*	*0.025997832*	*0.015779883*
**ENSG00000180332**	*0.015775512*	*0.024768315*	*0.025427869*	*0.01417047*
**ENSG00000205030**	*0.015413598*	*0.025972143*	*0.024763166*	*0.016109171*
**ENSG00000130477**	*0.01516768*	*0.024950358*	*0.024668882*	*0.015148912*
**ENSG00000090020**	*0.038209412*	*0.039701399*	*0.038758206*	*0.039083227*
**ENSG00000138495**	*0.025086899*	*0.028768353*	*0.031679821*	*0.022657394*
**ENSG00000169385**	*0.015495269*	*0.025948796*	*0.024487721*	*0.015463449*
**ENSG00000119820**	*0.024045557*	*0.030955295*	*0.028749239*	*0.023427847*
**ENSG00000198856**	*0.029630539*	*0.033368121*	*0.032851734*	*0.028152293*
**ENSG00000174564**	*0.022408179*	*0.030081027*	*0.032428732*	*0.020340734*
**ENSG00000105866**	*0.01546038*	*0.024808764*	*0.025021351*	*0.015462108*
**ENSG00000168032**	*0.015034409*	*0.024879854*	*0.024182765*	*0.014766271*
**ENSG00000056586**	*0.0256528*	*0.034621248*	*0.03240452*	*0.020182212*
**ENSG00000068971**	*0.051796048*	*0.039465558*	*0.040791207*	*0.047481957*
**ENSG00000187189**	*0.040478395*	*0.036806918*	*0.034315898*	*0.035914439*
**ENSG00000039068**	*0.131207618*	*0.109332643*	*0.108601569*	*0.115540444*
**ENSG00000153487**	*0.019550544*	*0.02869203*	*0.026186521*	*0.020442013*

**ENSG00000185261**	*0.0180295*	*0.026924848*	*0.02551276*	*0.015960442*
**ENSG00000116752**	*0.025673258*	*0.034580415*	*0.033495548*	*0.034232004*
**ENSG00000166257**	*0.016322908*	*0.025185191*	*0.025414251*	*0.014513931*
**ENSG00000074771**	*0.015475143*	*0.025338882*	*0.024501731*	*0.015702325*
**ENSG00000137817**	*0.025809943*	*0.033538847*	*0.032079255*	*0.028166421*
**ENSG00000166473**	*0.016033142*	*0.024577789*	*0.024357581*	*0.014927797*
**ENSG00000169592**	*0.028077813*	*0.030736571*	*0.033183839*	*0.025601434*
**ENSG00000112234**	*0.022823364*	*0.03385945*	*0.031480782*	*0.020707207*
**ENSG00000053770**	*0.029444619*	*0.036496274*	*0.031386505*	*0.029199895*
**ENSG00000107560**	*0.032876529*	*0.038448353*	*0.039993341*	*0.032864224*
**ENSG00000178852**	*0.015062213*	*0.025404616*	*0.024774057*	*0.015301264*
**ENSG00000085840**	*0.052420595*	*0.042003193*	*0.035130042*	*0.036793995*
**ENSG00000187180**	*0.016429268*	*0.025021457*	*0.026107286*	*0.016317767*
**ENSG00000197748**	*0.015063351*	*0.025749227*	*0.025037014*	*0.014507957*
**ENSG00000060138**	*0.032252652*	*0.031564199*	*0.029191006*	*0.028728876*
**ENSG00000162733**	*0.059323801*	*0.047878276*	*0.039075461*	*0.046057628*
**ENSG00000137841**	*0.063315699*	*0.054429845*	*0.043899679*	*0.043015021*
**ENSG00000107745**	*0.030831924*	*0.032036323*	*0.03145583*	*0.022634014*
**ENSG00000153208**	*0.045925899*	*0.037910674*	*0.033590285*	*0.031008653*
**ENSG00000162298**	*0.03427238*	*0.036180031*	*0.045338615*	*0.033571705*
**ENSG00000175564**	*0.015735473*	*0.025147406*	*0.025077205*	*0.01525043*
**ENSG00000144821**	*0.016887286*	*0.025660451*	*0.025430666*	*0.016717949*
**ENSG00000153976**	*0.019962502*	*0.026831541*	*0.024730905*	*0.018274282*
**ENSG00000134775**	*0.126444703*	*0.116335721*	*0.055903698*	*0.090297396*
**ENSG00000100591**	*0.028844626*	*0.034805807*	*0.031427123*	*0.028593164*
**ENSG00000153774**	*0.083306722*	*0.075318239*	*0.065682508*	*0.086025304*
**ENSG00000185736**	*0.039100755*	*0.033810974*	*0.030932172*	*0.036677634*
**ENSG00000163596**	*0.016138563*	*0.025348506*	*0.025033049*	*0.015985756*
**ENSG00000187516**	*0.015254107*	*0.024110918*	*0.024762707*	*0.0151314*
**ENSG00000160087**	*0.016951649*	*0.026166152*	*0.027289132*	*0.018105641*
**ENSG00000181693**	*0.018898302*	*0.025011577*	*0.026330591*	*0.01768504*
**ENSG00000132677**	*0.015575752*	*0.025803924*	*0.025529612*	*0.015870905*
**ENSG00000112343**	*0.034404309*	*0.038900154*	*0.045487395*	*0.028639192*
**ENSG00000158321**	*0.108837563*	*0.099185129*	*0.079481827*	*0.090271278*
**ENSG00000188910**	*0.019324164*	*0.027915365*	*0.028085017*	*0.018566619*
**ENSG00000175646**	*0.014992994*	*0.02392901*	*0.02483546*	*0.01446405*
**ENSG00000149115**	*0.045260183*	*0.036704848*	*0.040348935*	*0.03493354*
**ENSG00000187021**	*0.019348374*	*0.026043685*	*0.029239084*	*0.016873894*
**ENSG00000159251**	*0.021227408*	*0.025810974*	*0.025505551*	*0.015990424*
**ENSG00000205364**	*0.054225195*	*0.057497798*	*0.064509628*	*0.059428487*
**ENSG00000188846**	*0.050814898*	*0.046962005*	*0.059507259*	*0.039017917*
**ENSG00000144320**	*0.035228966*	*0.038538715*	*0.030559162*	*0.02421144*
**ENSG00000115109**	*0.016942639*	*0.026354733*	*0.025530662*	*0.017842195*
**ENSG00000177143**	*0.017208266*	*0.025959203*	*0.026320518*	*0.016908441*
**ENSG00000132182**	*0.031770488*	*0.03440515*	*0.032107836*	*0.023527218*
**ENSG00000146555**	*0.014529434*	*0.024844732*	*0.024961684*	*0.014372642*
**ENSG00000170113**	*0.02881147*	*0.031919815*	*0.032587019*	*0.028901647*
**ENSG00000089123**	*0.026252824*	*0.035851467*	*0.029733407*	*0.020879238*
**ENSG00000171262**	*0.019139773*	*0.025611669*	*0.025627652*	*0.017050012*
**ENSG00000147614**	*0.016249218*	*0.025886482*	*0.026364667*	*0.017382097*
**ENSG00000070501**	*0.030447308*	*0.033817863*	*0.032480237*	*0.024726392*
**ENSG00000154415**	*0.017672061*	*0.025634888*	*0.02531956*	*0.015349441*
**ENSG00000154743**	*0.035168185*	*0.034458355*	*0.037268158*	*0.031218337*
**ENSG00000122882**	*0.022083715*	*0.028613067*	*0.030378945*	*0.023389669*

**ENSG00000170775**	*0.01589616*	*0.024772412*	*0.024799652*	*0.015355038*
**ENSG00000079999**	*0.017073162*	*0.026267287*	*0.024547928*	*0.015102123*
**ENSG00000177791**	*0.031606519*	*0.045199023*	*0.032951429*	*0.027637717*
**ENSG00000095303**	*0.088745404*	*0.076421758*	*0.054103686*	*0.069350542*
**ENSG00000123243**	*0.01793784*	*0.026477533*	*0.026589524*	*0.017248369*
**ENSG00000124196**	*0.016083858*	*0.024566645*	*0.024275851*	*0.017794207*
**ENSG00000175938**	*0.038915967*	*0.033631761*	*0.033479709*	*0.035548407*
**ENSG00000177427**	*0.020480585*	*0.029564151*	*0.028508711*	*0.021847661*
**ENSG00000196642**	*0.03344841*	*0.029847424*	*0.035041392*	*0.031363825*
**ENSG00000074047**	*0.016355929*	*0.024716223*	*0.025877026*	*0.014198033*
**ENSG00000107902**	*0.061716976*	*0.06110329*	*0.063500948*	*0.062198011*
**ENSG00000170703**	*0.014930325*	*0.025164903*	*0.024580303*	*0.01432463*
**ENSG00000140199**	*0.040048894*	*0.039100115*	*0.041107761*	*0.040419757*
**ENSG00000162374**	*0.021241262*	*0.024869935*	*0.026251809*	*0.015484821*
**ENSG00000164933**	*0.033193016*	*0.037456877*	*0.030098544*	*0.034818665*
**ENSG00000102547**	*0.018120131*	*0.026484686*	*0.024770932*	*0.015600002*
**ENSG00000068724**	*0.016853101*	*0.026019585*	*0.025939057*	*0.015615219*
**ENSG00000168158**	*0.014969314*	*0.024718651*	*0.025786904*	*0.015300928*
**ENSG00000161544**	*0.017887877*	*0.02453507*	*0.025029388*	*0.015846341*
**ENSG00000132854**	*0.014913033*	*0.024526743*	*0.025253541*	*0.014393743*
**ENSG00000115163**	*0.053335913*	*0.042406843*	*0.042284923*	*0.043144326*
**ENSG00000105617**	*0.026491311*	*0.035703298*	*0.040216052*	*0.025394763*
**ENSG00000204568**	*0.020450543*	*0.02586878*	*0.02889983*	*0.020450875*
**ENSG00000036549**	*0.030204043*	*0.032933226*	*0.034934622*	*0.025642178*
**ENSG00000065534**	*0.015965773*	*0.025993219*	*0.025815768*	*0.015844944*
**ENSG00000132130**	*0.023651176*	*0.026660846*	*0.025974946*	*0.02231945*
**ENSG00000152377**	*0.023653717*	*0.025519186*	*0.027369769*	*0.019000031*
**ENSG00000135094**	*0.020512133*	*0.026302226*	*0.026350687*	*0.017654247*
**ENSG00000091622**	*0.016107696*	*0.025048279*	*0.024610943*	*0.015330721*
**ENSG00000138433**	*0.020250477*	*0.027184759*	*0.029462164*	*0.018784414*
**ENSG00000148158**	*0.035748888*	*0.044858725*	*0.042367739*	*0.040192131*
**ENSG00000214022**	*0.017085092*	*0.025746441*	*0.025249924*	*0.015109616*
**ENSG00000115307**	*0.022836938*	*0.027051018*	*0.02851476*	*0.023434081*
**ENSG00000167580**	*0.017536337*	*0.026006489*	*0.02661342*	*0.01643952*
**ENSG00000171595**	*0.017523499*	*0.025050612*	*0.024902734*	*0.016748268*
**ENSG00000175931**	*0.025194602*	*0.030543635*	*0.033293818*	*0.024028602*
**ENSG00000174038**	*0.016613195*	*0.025901009*	*0.026409018*	*0.015599515*
**ENSG00000140682**	*0.064645778*	*0.064115262*	*0.054907323*	*0.075363508*
**ENSG00000147894**	*0.044167368*	*0.042451559*	*0.043632519*	*0.04467567*
**ENSG00000177675**	*0.159146695*	*0.118271728*	*0.100510099*	*0.12105643*
**ENSG00000074071**	*0.027140133*	*0.028659791*	*0.030232695*	*0.024683325*
**ENSG00000139219**	*0.021515388*	*0.027623127*	*0.02601112*	*0.017366493*
**ENSG00000184014**	*0.074595405*	*0.0603203*	*0.067365459*	*0.065290057*
**ENSG00000100288**	*0.037669104*	*0.046374593*	*0.043044162*	*0.039849354*
**ENSG00000183570**	*0.061372876*	*0.056886808*	*0.062040434*	*0.053444976*
**ENSG00000112079**	*0.034776761*	*0.042488803*	*0.033090287*	*0.033383404*
**ENSG00000133710**	*0.015028504*	*0.024890081*	*0.024758209*	*0.014914983*
**ENSG00000131828**	*0.022602283*	*0.027268906*	*0.026475108*	*0.019788884*
**ENSG00000167395**	*0.018587974*	*0.029301648*	*0.02806004*	*0.019207397*
**ENSG00000178913**	*0.02710763*	*0.035098079*	*0.037539546*	*0.02581727*
**ENSG00000148965**	*0.016510543*	*0.027406034*	*0.026043588*	*0.01633298*
**ENSG00000112902**	*0.030428036*	*0.028311916*	*0.025110703*	*0.021052244*
**ENSG00000174804**	*0.016935703*	*0.025746173*	*0.026022505*	*0.016418634*
**ENSG00000137691**	*0.017777903*	*0.026086661*	*0.025814174*	*0.017393471*

**ENSG00000101162**	*0.016806914*	*0.02654282*	*0.025803356*	*0.015819666*
**ENSG00000168830**	*0.015820686*	*0.025516089*	*0.025383822*	*0.01424009*
**ENSG00000150337**	*0.01884406*	*0.027350384*	*0.027378569*	*0.020624744*
**ENSG00000136930**	*0.020133339*	*0.027713959*	*0.027846616*	*0.02019988*
**ENSG00000161960**	*0.022415278*	*0.026491065*	*0.026215376*	*0.018035768*
**ENSG00000164944**	*0.028746087*	*0.034733921*	*0.033711375*	*0.022932729*
**ENSG00000168101**	*0.025284831*	*0.030930629*	*0.032329283*	*0.022902381*
**ENSG00000185436**	*0.087082565*	*0.060096415*	*0.05954729*	*0.077243807*
**ENSG00000126970**	*0.026155235*	*0.030196781*	*0.029198498*	*0.025377557*
**ENSG00000203896**	*0.073718619*	*0.065154338*	*0.063020016*	*0.065638627*
**ENSG00000137962**	*0.016177229*	*0.025902775*	*0.025183895*	*0.016915368*
**ENSG00000125651**	*0.023286217*	*0.032595245*	*0.035741874*	*0.021688764*
**ENSG00000198889**	*0.01636072*	*0.025186809*	*0.024947743*	*0.01480117*
**ENSG00000204618**	*0.015327965*	*0.025098323*	*0.025461526*	*0.01548387*
**ENSG00000165091**	*0.015061037*	*0.02491229*	*0.024384116*	*0.014734313*
**ENSG00000163482**	*0.042342584*	*0.047980284*	*0.036450949*	*0.031292999*
**ENSG00000156650**	*0.028629238*	*0.030263781*	*0.033163719*	*0.02772414*
**ENSG00000125378**	*0.016274814*	*0.024924527*	*0.025305194*	*0.014799641*
**ENSG00000143952**	*0.032328204*	*0.03871059*	*0.032818444*	*0.031558559*
**ENSG00000103966**	*0.025181053*	*0.030939256*	*0.036045276*	*0.029205351*
**ENSG00000170631**	*0.028184051*	*0.040863787*	*0.043204646*	*0.032269612*
**ENSG00000173212**	*0.057757387*	*0.050950444*	*0.038485965*	*0.039652185*
**ENSG00000108389**	*0.025667936*	*0.030737211*	*0.030150975*	*0.022796296*
**ENSG00000005961**	*0.03403885*	*0.029323148*	*0.03041595*	*0.023274244*
**ENSG00000167799**	*0.048706247*	*0.040511365*	*0.043105257*	*0.050066201*
**ENSG00000065978**	*0.017016981*	*0.025135747*	*0.024703919*	*0.017666114*
**ENSG00000189195**	*0.017133434*	*0.025959854*	*0.024139338*	*0.015701232*
**ENSG00000166164**	*0.02686516*	*0.034567992*	*0.029676921*	*0.019277829*
**ENSG00000110448**	*0.01827876*	*0.024712299*	*0.024452147*	*0.016238841*
**ENSG00000005801**	*0.028015761*	*0.034199292*	*0.036828311*	*0.030547127*
**ENSG00000113369**	*0.063041216*	*0.059457086*	*0.05259218*	*0.058987587*
**ENSG00000110455**	*0.056249565*	*0.075051708*	*0.064959307*	*0.029191718*
**ENSG00000110318**	*0.015743753*	*0.024758093*	*0.025063725*	*0.014879899*
**ENSG00000066926**	*0.037400175*	*0.044961354*	*0.044334905*	*0.033189316*
**ENSG00000162227**	*0.031012784*	*0.036738982*	*0.042732375*	*0.032250717*
**ENSG00000120265**	*0.02872953*	*0.030925876*	*0.028830605*	*0.02272588*
**ENSG00000105516**	*0.037762315*	*0.033752387*	*0.035019666*	*0.036028541*
**ENSG00000184838**	*0.016233161*	*0.025172669*	*0.025694194*	*0.016294085*
**ENSG00000090975**	*0.015369747*	*0.024965768*	*0.024344057*	*0.015014122*
**ENSG00000135999**	*0.031225613*	*0.042065688*	*0.038685207*	*0.035944904*
**ENSG00000186119**	*0.017378638*	*0.02624292*	*0.024195883*	*0.015602183*
**ENSG00000198569**	*0.017001908*	*0.025074597*	*0.024878788*	*0.015455454*
**ENSG00000119487**	*0.030146174*	*0.032766973*	*0.030526798*	*0.027096066*
**ENSG00000131721**	*0.015639701*	*0.02517677*	*0.024768369*	*0.016867167*
**ENSG00000170454**	*0.017842996*	*0.02799471*	*0.026000069*	*0.019151955*
**ENSG00000203780**	*0.079797744*	*0.038704516*	*0.026171366*	*0.034017524*
**ENSG00000148384**	*0.028427183*	*0.033173595*	*0.037368246*	*0.029074048*
**ENSG00000178568**	*0.013894301*	*0.02419*	*0.024188275*	*0.013884115*
**ENSG00000203740**	*0.01588983*	*0.02491781*	*0.024427785*	*0.016704301*
**ENSG00000157103**	*0.017069921*	*0.027120106*	*0.027613769*	*0.01681192*
**ENSG00000156194**	*0.015545743*	*0.024821857*	*0.024054771*	*0.015891534*
**ENSG00000112541**	*0.019684264*	*0.027950216*	*0.027444728*	*0.018313982*
**ENSG00000167103**	*0.016550042*	*0.025716315*	*0.024667921*	*0.01624241*
**ENSG00000141485**	*0.016899937*	*0.025297136*	*0.025395437*	*0.015870356*

**ENSG00000034063**	*0.052160168*	*0.046051787*	*0.038948565*	*0.04837451*
**ENSG00000185085**	*0.024533988*	*0.030960416*	*0.039517952*	*0.027589147*
**ENSG00000075213**	*0.052111968*	*0.025940078*	*0.026441319*	*0.016399041*
**ENSG00000030419**	*0.054144186*	*0.046299274*	*0.053404257*	*0.049029886*
**ENSG00000113810**	*0.039402606*	*0.040664778*	*0.03475864*	*0.031722644*
**ENSG00000110777**	*0.030062573*	*0.030023055*	*0.031139316*	*0.025332307*
**ENSG00000100567**	*0.025630471*	*0.031562686*	*0.028561111*	*0.024392754*
**ENSG00000151304**	*0.032529871*	*0.034285109*	*0.034837632*	*0.028484958*
**ENSG00000144909**	*0.03243851*	*0.039042123*	*0.032874467*	*0.040507352*
**ENSG00000172410**	*0.015377733*	*0.026425216*	*0.0255041*	*0.015571089*
**ENSG00000143321**	*0.023696525*	*0.027290416*	*0.028424238*	*0.021971064*
**ENSG00000005889**	*0.024043131*	*0.027486503*	*0.029284828*	*0.021032636*
**ENSG00000143995**	*0.015993231*	*0.024526369*	*0.024649392*	*0.015367613*
**ENSG00000117009**	*0.058016588*	*0.054810839*	*0.049540833*	*0.052684317*
**ENSG00000169813**	*0.043272671*	*0.036326021*	*0.036188865*	*0.03678132*
**ENSG00000196715**	*0.03602286*	*0.038103357*	*0.035986751*	*0.027872665*
**ENSG00000119718**	*0.027348481*	*0.034081124*	*0.03331091*	*0.024612382*
**ENSG00000102738**	*0.033992882*	*0.033788944*	*0.031718848*	*0.024174768*
**ENSG00000132603**	*0.034952213*	*0.042565336*	*0.047274985*	*0.042810833*
**ENSG00000077238**	*0.040002682*	*0.047062788*	*0.044852585*	*0.038715116*
**ENSG00000164756**	*0.015790684*	*0.025654536*	*0.026930409*	*0.019530378*
**ENSG00000064652**	*0.044273832*	*0.040860127*	*0.031806325*	*0.034518958*
**ENSG00000155621**	*0.028889538*	*0.033042649*	*0.035897916*	*0.034533772*
**ENSG00000163501**	*0.015988532*	*0.024676639*	*0.024633922*	*0.016454322*
**ENSG00000087008**	*0.037420542*	*0.039505284*	*0.044350981*	*0.027892639*
**ENSG00000013810**	*0.042177865*	*0.033797968*	*0.044734147*	*0.038791012*
**ENSG00000036473**	*0.014149385*	*0.024033667*	*0.0247401*	*0.015632082*
**ENSG00000160224**	*0.021468561*	*0.027275637*	*0.026096623*	*0.021636217*
**ENSG00000104228**	*0.017477158*	*0.024819206*	*0.026489791*	*0.016819382*
**ENSG00000105137**	*0.016613949*	*0.025001252*	*0.025449975*	*0.015061064*
**ENSG00000101856**	*0.041215096*	*0.038900329*	*0.033962933*	*0.034083925*
**ENSG00000064886**	*0.157930161*	*0.096659094*	*0.08225214*	*0.127999829*
**ENSG00000089053**	*0.022008742*	*0.027204106*	*0.027744882*	*0.020825102*
**ENSG00000082074**	*0.069047028*	*0.04039197*	*0.037619675*	*0.029403911*
**ENSG00000186579**	*0.016359781*	*0.025415374*	*0.025632207*	*0.01657449*
**ENSG00000179941**	*0.033263271*	*0.036961837*	*0.03131281*	*0.025163379*
**ENSG00000166848**	*0.018718833*	*0.027104836*	*0.028822717*	*0.018503297*
**ENSG00000145242**	*0.016410846*	*0.024880734*	*0.024852584*	*0.017040101*
**ENSG00000162244**	*0.016568667*	*0.024236873*	*0.024877615*	*0.016536757*
**ENSG00000168209**	*0.053868233*	*0.051831567*	*0.069845632*	*0.051181925*
**ENSG00000132781**	*0.039253877*	*0.039186194*	*0.036741517*	*0.033266223*
**ENSG00000198960**	*0.022865786*	*0.028059648*	*0.030309576*	*0.020861157*
**ENSG00000168930**	*0.01634173*	*0.024405561*	*0.025027548*	*0.015780143*
**ENSG00000124440**	*0.016467785*	*0.02477827*	*0.025590519*	*0.013850498*
**ENSG00000166012**	*0.043955836*	*0.037714883*	*0.043311713*	*0.035362215*
**ENSG00000139180**	*0.025067303*	*0.030577227*	*0.029440279*	*0.021845461*
**ENSG00000121310**	*0.057022196*	*0.06524464*	*0.056447812*	*0.036859998*
**ENSG00000172150**	*0.017366784*	*0.026530452*	*0.024903701*	*0.016249808*
**ENSG00000168827**	*0.033197248*	*0.034083569*	*0.032050197*	*0.031463515*
**ENSG00000124067**	*0.048003112*	*0.043155129*	*0.040197287*	*0.038004827*
**ENSG00000170486**	*0.022014481*	*0.029830993*	*0.029650709*	*0.018933087*
**ENSG00000164466**	*0.03354338*	*0.032759851*	*0.030488857*	*0.026526667*
**ENSG00000161217**	*0.029119849*	*0.034770748*	*0.029325109*	*0.027549025*
**ENSG00000197728**	*0.034094271*	*0.039686774*	*0.039861068*	*0.023926125*

**ENSG00000105357**	*0.015585597*	*0.025988163*	*0.025588997*	*0.015192599*
**ENSG00000165392**	*0.031369176*	*0.034745972*	*0.033747384*	*0.030762663*
**ENSG00000146574**	*0.022403383*	*0.030681788*	*0.030005481*	*0.024161572*
**ENSG00000172023**	*0.014894814*	*0.024209515*	*0.024824917*	*0.014965863*
**ENSG00000113763**	*0.015267931*	*0.025938036*	*0.024250855*	*0.015493675*
**ENSG00000165899**	*0.017088399*	*0.024529088*	*0.025945638*	*0.016099734*
**ENSG00000130038**	*0.016383445*	*0.025199502*	*0.026431544*	*0.016056164*
**ENSG00000174775**	*0.035339134*	*0.031020774*	*0.034692661*	*0.029260049*
**ENSG00000170613**	*0.016168559*	*0.026654784*	*0.02516107*	*0.016799851*
**ENSG00000139793**	*0.053521893*	*0.043587999*	*0.041941879*	*0.03855583*
**ENSG00000146856**	*0.018068834*	*0.025342826*	*0.025456919*	*0.018104292*
**ENSG00000127954**	*0.014772683*	*0.025310476*	*0.024419456*	*0.014983249*
**ENSG00000169891**	*0.022614529*	*0.032979903*	*0.024153316*	*0.01793531*
**ENSG00000185164**	*0.025290224*	*0.030610208*	*0.031612774*	*0.026978361*
**ENSG00000160678**	*0.016552376*	*0.026491885*	*0.028078503*	*0.017785366*
**ENSG00000080572**	*0.027301538*	*0.030332875*	*0.031734969*	*0.02501476*
**ENSG00000058335**	*0.016835798*	*0.025028793*	*0.024860559*	*0.015791784*
**ENSG00000131470**	*0.039751167*	*0.038250326*	*0.030830288*	*0.026828366*
**ENSG00000138385**	*0.026407488*	*0.02926502*	*0.027685957*	*0.022782923*
**ENSG00000112378**	*0.130314328*	*0.092065286*	*0.065319681*	*0.084674577*
**ENSG00000129933**	*0.024699158*	*0.032929129*	*0.031623924*	*0.023651883*
**ENSG00000136448**	*0.032546482*	*0.034430992*	*0.033448166*	*0.033734278*
**ENSG00000174460**	*0.015532114*	*0.025648741*	*0.026695836*	*0.016787991*
**ENSG00000197045**	*0.026753116*	*0.032049241*	*0.035560122*	*0.03284758*
**ENSG00000168260**	*0.015658262*	*0.025447583*	*0.024659265*	*0.015594071*
**ENSG00000110063**	*0.027859114*	*0.029634314*	*0.029156921*	*0.02405941*
**ENSG00000077348**	*0.033169707*	*0.033170346*	*0.030752776*	*0.027933359*
**ENSG00000147394**	*0.074209478*	*0.057314836*	*0.075350328*	*0.055527668*
**ENSG00000135823**	*0.026305206*	*0.031420659*	*0.030612025*	*0.027685693*
**ENSG00000163904**	*0.028289911*	*0.035253642*	*0.034727922*	*0.026966608*
**ENSG00000063177**	*0.014648653*	*0.023691445*	*0.024181838*	*0.016281137*
**ENSG00000181090**	*0.044302084*	*0.048564541*	*0.04304183*	*0.038749931*
**ENSG00000184860**	*0.032411676*	*0.029589201*	*0.031890215*	*0.023870633*
**ENSG00000078795**	*0.017642115*	*0.024397656*	*0.025256048*	*0.014956014*
**ENSG00000143621**	*0.020496222*	*0.027829513*	*0.028841157*	*0.021496862*
**ENSG00000196415**	*0.016109322*	*0.024734033*	*0.025383057*	*0.016662431*
**ENSG00000113319**	*0.018916809*	*0.025549291*	*0.027912034*	*0.018195348*
**ENSG00000198794**	*0.045119716*	*0.040305188*	*0.042325753*	*0.06620909*
**ENSG00000183246**	*0.058996983*	*0.075151245*	*0.052504188*	*0.065098357*
**ENSG00000170293**	*0.016507084*	*0.024972216*	*0.024992651*	*0.023414196*
**ENSG00000167178**	*0.026273132*	*0.027339424*	*0.02651436*	*0.018450838*
**ENSG00000186847**	*0.021847655*	*0.028050798*	*0.026617298*	*0.017351983*
**ENSG00000155660**	*0.045191712*	*0.043512219*	*0.042972005*	*0.048064188*
**ENSG00000147509**	*0.025041268*	*0.024720219*	*0.024238967*	*0.023774628*
**ENSG00000242732**	*0.015605396*	*0.024736021*	*0.025794044*	*0.014880744*
**ENSG00000112818**	*0.016774427*	*0.024989841*	*0.024588431*	*0.01570852*
**ENSG00000163714**	*0.039627303*	*0.039969446*	*0.039355817*	*0.034111658*
**ENSG00000160307**	*0.01574176*	*0.024154064*	*0.024793596*	*0.015685344*
**ENSG00000107672**	*0.028048311*	*0.030988511*	*0.028625002*	*0.024372796*
**ENSG00000198492**	*0.024070542*	*0.034950139*	*0.03873997*	*0.026228558*
**ENSG00000124225**	*0.018094503*	*0.02528774*	*0.025307356*	*0.016736877*
**ENSG00000133313**	*0.024276972*	*0.038211258*	*0.036357332*	*0.027101785*
**ENSG00000143110**	*0.104212552*	*0.056235308*	*0.054565158*	*0.07863519*
**ENSG00000166266**	*0.019671582*	*0.027294077*	*0.027263232*	*0.018112098*

**ENSG00000136011**	*0.016700131*	*0.025722081*	*0.02492418*	*0.017285193*
**ENSG00000101945**	*0.034297722*	*0.028451722*	*0.040628061*	*0.034693872*
**ENSG00000100031**	*0.01531603*	*0.02614035*	*0.024226113*	*0.01484349*
**ENSG00000007952**	*0.015765775*	*0.025524096*	*0.024599769*	*0.014448997*
**ENSG00000104885**	*0.029813361*	*0.037339626*	*0.042603068*	*0.031973809*
**ENSG00000119523**	*0.024613995*	*0.034255722*	*0.037030053*	*0.02351581*
**ENSG00000106258**	*0.017410528*	*0.025016791*	*0.025915181*	*0.014906924*
**ENSG00000108947**	*0.015245883*	*0.02473995*	*0.024817593*	*0.01452777*
**ENSG00000130595**	*0.050984857*	*0.059153482*	*0.057080934*	*0.072092028*
**ENSG00000126814**	*0.030948743*	*0.034110081*	*0.03330513*	*0.025643059*
**ENSG00000051108**	*0.045868946*	*0.046903162*	*0.048820531*	*0.042409264*
**ENSG00000138119**	*0.027640722*	*0.025510359*	*0.024984473*	*0.015569243*
**ENSG00000132507**	*0.03593735*	*0.039054636*	*0.039073766*	*0.040380358*
**ENSG00000144136**	*0.030321377*	*0.039126693*	*0.034297694*	*0.029679656*
**ENSG00000134318**	*0.039365316*	*0.03453776*	*0.035590538*	*0.029040454*
**ENSG00000102021**	*0.021147229*	*0.025892484*	*0.025904868*	*0.016342025*
**ENSG00000076864**	*0.058874109*	*0.055151376*	*0.063532894*	*0.057471373*
**ENSG00000106018**	*0.016745359*	*0.025839844*	*0.025710442*	*0.016020388*
**ENSG00000250067**	*0.019019915*	*0.026126723*	*0.024927235*	*0.017329098*
**ENSG00000175449**	*0.039720893*	*0.037233493*	*0.032905083*	*0.031129817*
**ENSG00000069966**	*0.01717973*	*0.025155512*	*0.02454596*	*0.016665393*
**ENSG00000179029**	*0.029196691*	*0.045715291*	*0.032629447*	*0.039179231*
**ENSG00000129009**	*0.016148673*	*0.02624698*	*0.025563592*	*0.016263818*
**ENSG00000168242**	*0.057859004*	*0.045788969*	*0.064763663*	*0.061074947*
**ENSG00000131791**	*0.017403357*	*0.026488482*	*0.026703322*	*0.015858733*
**ENSG00000169507**	*0.018077479*	*0.024447045*	*0.02554986*	*0.017145461*
**ENSG00000119328**	*0.027722526*	*0.036659766*	*0.032905158*	*0.024022924*
**ENSG00000105063**	*0.029147697*	*0.032551991*	*0.032245347*	*0.02807426*
**ENSG00000128708**	*0.027683339*	*0.03171721*	*0.028079334*	*0.031220426*
**ENSG00000090447**	*0.018211482*	*0.025744538*	*0.025816763*	*0.015552557*
**ENSG00000115221**	*0.016054097*	*0.02617347*	*0.025300124*	*0.016125285*
**ENSG00000135953**	*0.016709027*	*0.024689685*	*0.025800675*	*0.016594864*
**ENSG00000006606**	*0.01575391*	*0.025154198*	*0.024734069*	*0.015158617*
**ENSG00000121058**	*0.027142828*	*0.032029471*	*0.03173263*	*0.022574537*
**ENSG00000157778**	*0.02760788*	*0.031111369*	*0.033732899*	*0.028027711*
**ENSG00000167771**	*0.015318032*	*0.024963817*	*0.024125568*	*0.015069687*
**ENSG00000159648**	*0.014876639*	*0.024611837*	*0.024869274*	*0.014630786*
**ENSG00000152284**	*0.016613183*	*0.024519129*	*0.025096373*	*0.016195733*
**ENSG00000163029**	*0.036335469*	*0.04061581*	*0.03552898*	*0.029426094*
**ENSG00000105371**	*0.016903122*	*0.025912667*	*0.026518825*	*0.016183667*
**ENSG00000121964**	*0.01683849*	*0.025834274*	*0.024192228*	*0.015740834*
**ENSG00000137261**	*0.018837103*	*0.025813345*	*0.026960336*	*0.017445069*
**ENSG00000197321**	*0.017944087*	*0.026302732*	*0.025072715*	*0.016930091*
**ENSG00000009954**	*0.025903122*	*0.032641393*	*0.031930961*	*0.025282703*
**ENSG00000083937**	*0.033404109*	*0.039206543*	*0.034872834*	*0.031916458*
**ENSG00000185666**	*0.01604756*	*0.024913997*	*0.024451472*	*0.016572841*
**ENSG00000183346**	*0.015834883*	*0.025397268*	*0.025138201*	*0.014967463*
**ENSG00000018610**	*0.032941461*	*0.032805621*	*0.03422191*	*0.029496611*
**ENSG00000168491**	*0.056450931*	*0.05496603*	*0.048477379*	*0.050911227*
**ENSG00000138629**	*0.034958705*	*0.036691009*	*0.03287826*	*0.033615324*
**ENSG00000131051**	*0.040898969*	*0.044941994*	*0.039244119*	*0.064263004*
**ENSG00000107951**	*0.036893936*	*0.031577483*	*0.038275799*	*0.02885434*
**ENSG00000128989**	*0.028043563*	*0.033304444*	*0.030421037*	*0.022750513*
**ENSG00000119421**	*0.031618792*	*0.033893054*	*0.032870554*	*0.024824584*

**ENSG00000140093**	*0.01552153*	*0.025182862*	*0.024500631*	*0.014818213*
**ENSG00000171587**	*0.016305057*	*0.025701455*	*0.025268804*	*0.015589366*
**ENSG00000184465**	*0.020339337*	*0.031378389*	*0.027494968*	*0.022226683*
**ENSG00000159733**	*0.016769059*	*0.025101465*	*0.023984972*	*0.016419889*
**ENSG00000165304**	*0.043124299*	*0.042359528*	*0.032532498*	*0.036840134*
**ENSG00000115020**	*0.01627574*	*0.025472435*	*0.024165077*	*0.017685363*
**ENSG00000218823**	*0.015663933*	*0.024822971*	*0.025060303*	*0.015263498*
**ENSG00000168398**	*0.016723594*	*0.025029809*	*0.025294966*	*0.014777019*
**ENSG00000151465**	*0.028760639*	*0.033027497*	*0.031544993*	*0.026524059*
**ENSG00000162928**	*0.026725238*	*0.033913148*	*0.040299703*	*0.020733951*
**ENSG00000054267**	*0.027102836*	*0.033770806*	*0.03442225*	*0.026787437*
**ENSG00000169299**	*0.033170834*	*0.041715874*	*0.033783347*	*0.028798036*
**ENSG00000077498**	*0.015029583*	*0.025070301*	*0.024480046*	*0.015853625*
**ENSG00000204519**	*0.028121793*	*0.044431875*	*0.04704824*	*0.030673199*
**ENSG00000058272**	*0.032804525*	*0.04492765*	*0.03845482*	*0.049583503*
**ENSG00000135862**	*0.06123733*	*0.05347829*	*0.046959685*	*0.043467182*
**ENSG00000163637**	*0.059900556*	*0.035848611*	*0.028960546*	*0.031765353*
**ENSG00000135414**	*0.020006349*	*0.028565168*	*0.030682744*	*0.019668313*
**ENSG00000068793**	*0.031769184*	*0.040491115*	*0.036545382*	*0.033717857*
**ENSG00000008382**	*0.028952543*	*0.029315118*	*0.031309137*	*0.025337217*
**ENSG00000119938**	*0.033128846*	*0.033719975*	*0.039415523*	*0.02768806*
**ENSG00000160349**	*0.016020997*	*0.025137173*	*0.024723367*	*0.014909901*
**ENSG00000204632**	*0.039399771*	*0.048496043*	*0.042184265*	*0.053462199*
**ENSG00000148187**	*0.034541321*	*0.037755344*	*0.034568769*	*0.030464046*
**ENSG00000102241**	*0.034548874*	*0.036728269*	*0.032764145*	*0.026524365*
**ENSG00000108469**	*0.022395117*	*0.02963015*	*0.029110155*	*0.026579506*
**ENSG00000165689**	*0.039199799*	*0.040402414*	*0.036204925*	*0.030112063*
**ENSG00000118777**	*0.015810939*	*0.025345298*	*0.024337665*	*0.014911788*
**ENSG00000172572**	*0.018074263*	*0.025564293*	*0.024878143*	*0.017333029*
**ENSG00000177156**	*0.023893844*	*0.030799244*	*0.027451056*	*0.021337983*
**ENSG00000115592**	*0.014176829*	*0.025837674*	*0.026526073*	*0.014496346*
**ENSG00000164708**	*0.016705034*	*0.024931788*	*0.025430633*	*0.017975321*
**ENSG00000129158**	*0.028242627*	*0.029207954*	*0.030492947*	*0.029925613*
**ENSG00000128564**	*0.016185764*	*0.025544443*	*0.024672278*	*0.01504669*
**ENSG00000103241**	*0.015583459*	*0.025767775*	*0.024682471*	*0.01560344*
**ENSG00000162402**	*0.017487839*	*0.025284172*	*0.02760847*	*0.016486697*
**ENSG00000183780**	*0.077192931*	*0.070041547*	*0.040067691*	*0.046685684*
**ENSG00000112118**	*0.032329224*	*0.032456204*	*0.030177581*	*0.026149363*
**ENSG00000177380**	*0.017500761*	*0.02604714*	*0.025541543*	*0.016764038*
**ENSG00000120158**	*0.035747214*	*0.036709501*	*0.041722593*	*0.037263021*
**ENSG00000134905**	*0.033032567*	*0.036467922*	*0.03496244*	*0.02947855*
**ENSG00000105499**	*0.100792232*	*0.095002079*	*0.081746414*	*0.072830079*
**ENSG00000198258**	*0.018224142*	*0.027455013*	*0.025334453*	*0.016874952*
**ENSG00000070759**	*0.040168309*	*0.035928704*	*0.038005134*	*0.035382158*
**ENSG00000134152**	*0.037579362*	*0.036880666*	*0.038999484*	*0.031552153*
**ENSG00000206181**	*0.019115068*	*0.028692951*	*0.026638875*	*0.017961086*
**ENSG00000198798**	*0.016823971*	*0.027162738*	*0.026274874*	*0.01703367*
**ENSG00000166825**	*0.017862561*	*0.029939278*	*0.025495555*	*0.034541597*
**ENSG00000183323**	*0.024540455*	*0.033800631*	*0.028864896*	*0.023177609*
**ENSG00000197587**	*0.016034609*	*0.025409176*	*0.024318708*	*0.015508036*
**ENSG00000105483**	*0.044901873*	*0.048369497*	*0.037388143*	*0.032120784*
**ENSG00000139154**	*0.037487425*	*0.039893849*	*0.03308241*	*0.028396891*
**ENSG00000095932**	*0.023736522*	*0.031133827*	*0.02723522*	*0.025030551*
**ENSG00000039560**	*0.096180221*	*0.110599276*	*0.04038661*	*0.068640008*

**ENSG00000102572**	*0.024160408*	*0.031089103*	*0.029285308*	*0.020719097*
**ENSG00000197562**	*0.032192381*	*0.037384507*	*0.033668476*	*0.027774646*
**ENSG00000011258**	*0.026547297*	*0.02944552*	*0.032183606*	*0.026571154*
**ENSG00000165131**	*0.015373818*	*0.025191323*	*0.024700805*	*0.015977869*
**ENSG00000136944**	*0.016666199*	*0.025776149*	*0.025317133*	*0.016507546*
**ENSG00000121570**	*0.017819275*	*0.02578158*	*0.025199059*	*0.01691238*
**ENSG00000156384**	*0.023978612*	*0.02975406*	*0.026126191*	*0.023526787*
**ENSG00000170262**	*0.015684839*	*0.025446355*	*0.025368618*	*0.014775824*
**ENSG00000188060**	*0.0788725*	*0.073333148*	*0.050714444*	*0.067700939*
**ENSG00000163848**	*0.024667952*	*0.029819611*	*0.031480663*	*0.022548512*
**ENSG00000187855**	*0.015944817*	*0.025842682*	*0.024849999*	*0.016833631*
**ENSG00000174982**	*0.015778996*	*0.025184948*	*0.024893816*	*0.014561481*
**ENSG00000204217**	*0.031424256*	*0.033820164*	*0.036841894*	*0.031823176*
**ENSG00000178645**	*0.016178509*	*0.024990428*	*0.024312976*	*0.015280113*
**ENSG00000125995**	*0.022353605*	*0.025838857*	*0.025697215*	*0.01918263*
**ENSG00000141979**	*0.015159527*	*0.025169807*	*0.024838608*	*0.015160721*
**ENSG00000177189**	*0.033724111*	*0.044713427*	*0.034653347*	*0.031051827*
**ENSG00000125414**	*0.019532055*	*0.030219114*	*0.027163105*	*0.018682708*
**ENSG00000174898**	*0.015070283*	*0.025022302*	*0.025089037*	*0.016107216*
**ENSG00000154478**	*0.015471199*	*0.025688927*	*0.024775943*	*0.015888179*
**ENSG00000106038**	*0.01560964*	*0.02562541*	*0.026085814*	*0.016139063*
**ENSG00000139405**	*0.022240137*	*0.028999882*	*0.02935127*	*0.02414476*
**ENSG00000113360**	*0.028834946*	*0.034094214*	*0.029182456*	*0.020464135*
**ENSG00000248405**	*0.016334497*	*0.024926138*	*0.025015984*	*0.014706482*
**ENSG00000138074**	*0.043663684*	*0.04237461*	*0.038943304*	*0.046542703*
**ENSG00000223953**	*0.016719797*	*0.025903644*	*0.024557308*	*0.016224805*
**ENSG00000196459**	*0.027157416*	*0.03217189*	*0.034447753*	*0.026447063*
**ENSG00000135336**	*0.029841426*	*0.035153221*	*0.029569879*	*0.026498258*
**ENSG00000156509**	*0.053001589*	*0.044099879*	*0.039254664*	*0.041456655*
**ENSG00000150510**	*0.016373432*	*0.027667612*	*0.026614521*	*0.0154512*
**ENSG00000039650**	*0.029050243*	*0.031393778*	*0.028024277*	*0.026895213*
**ENSG00000172456**	*0.050248954*	*0.043632417*	*0.033188466*	*0.037564118*
**ENSG00000180304**	*0.030566328*	*0.03330924*	*0.031475616*	*0.02495932*
**ENSG00000134369**	*0.030070386*	*0.028845921*	*0.031620306*	*0.026742071*
**ENSG00000185608**	*0.024981822*	*0.031323927*	*0.033163979*	*0.020462607*
**ENSG00000123700**	*0.102790097*	*0.058578504*	*0.026925895*	*0.016128557*
**ENSG00000138801**	*0.038035403*	*0.040568189*	*0.036305155*	*0.027704985*
**ENSG00000178425**	*0.035854672*	*0.033099569*	*0.033793177*	*0.024792959*
**ENSG00000107829**	*0.022104794*	*0.029309305*	*0.029877159*	*0.021684437*
**ENSG00000213741**	*0.017571708*	*0.025050283*	*0.0246774*	*0.018014004*
**ENSG00000130283**	*0.016862691*	*0.02548497*	*0.024838033*	*0.016605065*
**ENSG00000115263**	*0.015999096*	*0.027708866*	*0.025735789*	*0.017461626*
**ENSG00000128891**	*0.030679573*	*0.031858898*	*0.034431414*	*0.025404744*
**ENSG00000116996**	*0.022886694*	*0.02499801*	*0.02477605*	*0.015593821*
**ENSG00000184659**	*0.014740415*	*0.026028296*	*0.024562796*	*0.014683658*
**ENSG00000082641**	*0.026244878*	*0.030486636*	*0.033161583*	*0.029130161*
**ENSG00000111981**	*0.016730533*	*0.02573291*	*0.025222261*	*0.015364396*
**ENSG00000186732**	*0.015855177*	*0.025687908*	*0.024848324*	*0.015977171*
**ENSG00000166796**	*0.024996788*	*0.025828729*	*0.026889205*	*0.020756655*
**ENSG00000069869**	*0.016533579*	*0.024404434*	*0.024339124*	*0.018190388*
**ENSG00000166068**	*0.091053439*	*0.076280059*	*0.072507352*	*0.087960268*
**ENSG00000172678**	*0.016216242*	*0.024697592*	*0.025395508*	*0.015920606*
**ENSG00000174718**	*0.036456897*	*0.040750477*	*0.037516112*	*0.034312281*
**ENSG00000177614**	*0.017441761*	*0.027197021*	*0.025646493*	*0.017256296*

**ENSG00000090266**	*0.024531416*	*0.032940917*	*0.029618098*	*0.030241682*
**ENSG00000112473**	*0.032092434*	*0.036778732*	*0.043520588*	*0.036772538*
**ENSG00000131773**	*0.076831065*	*0.027779281*	*0.025393847*	*0.024897012*
**ENSG00000167371**	*0.021090275*	*0.02704985*	*0.028561227*	*0.018682657*
**ENSG00000204348**	*0.035691123*	*0.027808188*	*0.028471552*	*0.023059377*
**ENSG00000128607**	*0.037695351*	*0.049072606*	*0.046094657*	*0.039601546*
**ENSG00000104093**	*0.035990674*	*0.03476969*	*0.032715636*	*0.025713487*
**ENSG00000115649**	*0.02892723*	*0.035109214*	*0.034172014*	*0.031070444*
**ENSG00000197343**	*0.025616327*	*0.036772183*	*0.035244724*	*0.02947197*
**ENSG00000077984**	*0.021017305*	*0.038628623*	*0.035606318*	*0.056566567*
**ENSG00000087245**	*0.017757825*	*0.025798278*	*0.025112079*	*0.015339764*
**ENSG00000133318**	*0.027662035*	*0.030395012*	*0.029772845*	*0.023025205*
**ENSG00000198815**	*0.023400114*	*0.03043612*	*0.032442269*	*0.024623259*
**ENSG00000114857**	*0.036313629*	*0.038457923*	*0.034175508*	*0.031438753*
**ENSG00000132424**	*0.02427086*	*0.031957816*	*0.030049538*	*0.022107694*
**ENSG00000136541**	*0.027339638*	*0.035856644*	*0.034846625*	*0.023587785*
**ENSG00000101216**	*0.025613156*	*0.037323867*	*0.038019025*	*0.023776691*
**ENSG00000129862**	*0.026671835*	*0.02500639*	*0.025839766*	*0.018889434*
**ENSG00000182899**	*0.014288266*	*0.023892859*	*0.024339323*	*0.014752049*
**ENSG00000139644**	*0.019679534*	*0.02847038*	*0.03024008*	*0.018710725*
**ENSG00000152684**	*0.032438209*	*0.03096755*	*0.033807503*	*0.026066862*
**ENSG00000164093**	*0.016535135*	*0.025864178*	*0.02441266*	*0.016023796*
**ENSG00000157423**	*0.017732305*	*0.025159645*	*0.025458531*	*0.015720705*
**ENSG00000134007**	*0.018406845*	*0.026287031*	*0.025017148*	*0.015603023*
**ENSG00000163081**	*0.017267659*	*0.025503702*	*0.02574849*	*0.016301705*
**ENSG00000204965**	*0.01832874*	*0.025247504*	*0.026246389*	*0.01884406*
**ENSG00000203877**	*0.015708559*	*0.024667893*	*0.024412859*	*0.014550974*
**ENSG00000163810**	*0.017244662*	*0.025944812*	*0.025361383*	*0.018363311*
**ENSG00000108244**	*0.019990242*	*0.027557385*	*0.025756192*	*0.018942981*
**ENSG00000123545**	*0.033452888*	*0.035277841*	*0.033344294*	*0.028671886*
**ENSG00000148824**	*0.019575425*	*0.027841556*	*0.02618915*	*0.018032215*
**ENSG00000122304**	*0.016373323*	*0.025105571*	*0.025480454*	*0.015298637*
**ENSG00000167941**	*0.020662275*	*0.030112815*	*0.027269202*	*0.022959945*
**ENSG00000087302**	*0.018680554*	*0.026521353*	*0.026094319*	*0.019197346*
**ENSG00000132016**	*0.015453829*	*0.024761552*	*0.025232312*	*0.014681023*
**ENSG00000082805**	*0.016986543*	*0.025799527*	*0.024368426*	*0.015982793*
**ENSG00000187833**	*0.021837385*	*0.025454894*	*0.024756419*	*0.015580808*
**ENSG00000107077**	*0.029837619*	*0.037175598*	*0.030602933*	*0.025672965*
**ENSG00000213190**	*0.062138605*	*0.042903822*	*0.046686563*	*0.056898316*
**ENSG00000165322**	*0.033294989*	*0.036060163*	*0.03695222*	*0.033904645*
**ENSG00000123064**	*0.033402633*	*0.030888414*	*0.031060909*	*0.029139478*
**ENSG00000158856**	*0.018141951*	*0.025402955*	*0.026248369*	*0.016438116*
**ENSG00000141576**	*0.057440895*	*0.052605726*	*0.050955589*	*0.051553178*
**ENSG00000170848**	*0.015576446*	*0.025552742*	*0.025452069*	*0.015641393*
**ENSG00000038358**	*0.03872517*	*0.041844163*	*0.041305383*	*0.02786464*
**ENSG00000124713**	*0.022967348*	*0.025927353*	*0.026236762*	*0.018243588*
**ENSG00000168348**	*0.015833607*	*0.02457395*	*0.024421052*	*0.014564029*
**ENSG00000184515**	*0.058581219*	*0.041965378*	*0.042188239*	*0.048774226*
**ENSG00000082212**	*0.037387304*	*0.045411426*	*0.035732135*	*0.034861051*
**ENSG00000148600**	*0.020263193*	*0.028140737*	*0.025655233*	*0.025195193*
**ENSG00000172671**	*0.042735267*	*0.044357489*	*0.035122931*	*0.033745415*
**ENSG00000133250**	*0.035356143*	*0.039519071*	*0.044335912*	*0.030762792*
**ENSG00000182628**	*0.043976045*	*0.043266046*	*0.037569245*	*0.032502443*
**ENSG00000167014**	*0.016554032*	*0.02594052*	*0.024521273*	*0.016145181*

**ENSG00000160219**	*0.023671963*	*0.02704627*	*0.026746452*	*0.020334406*
**ENSG00000116906**	*0.027179485*	*0.033264336*	*0.027789519*	*0.023077258*
**ENSG00000137103**	*0.037353225*	*0.034045941*	*0.038429941*	*0.034456967*
**ENSG00000013364**	*0.032499154*	*0.038044025*	*0.034215243*	*0.03213362*
**ENSG00000231256**	*0.018293298*	*0.025102032*	*0.026380226*	*0.016521893*
**ENSG00000155100**	*0.025366818*	*0.030905909*	*0.029223124*	*0.020181052*
**ENSG00000183255**	*0.027825221*	*0.034819505*	*0.035208223*	*0.026419956*
**ENSG00000198089**	*0.03798031*	*0.042558246*	*0.043757496*	*0.039078599*
**ENSG00000104450**	*0.029947071*	*0.031738862*	*0.033261121*	*0.033179452*
**ENSG00000124172**	*0.018031365*	*0.02590002*	*0.025130736*	*0.017486558*
**ENSG00000133026**	*0.089267161*	*0.068698868*	*0.070079822*	*0.093093553*
**ENSG00000124134**	*0.016442631*	*0.02499005*	*0.025555593*	*0.015878167*
**ENSG00000163660**	*0.032807205*	*0.039736047*	*0.041027389*	*0.025094565*
**ENSG00000110436**	*0.016250424*	*0.02595631*	*0.025113547*	*0.015136195*
**ENSG00000183941**	*0.058556484*	*0.044699744*	*0.055883903*	*0.058369677*
**ENSG00000165556**	*0.017251089*	*0.028435612*	*0.027312669*	*0.0170786*
**ENSG00000197238**	*0.049389473*	*0.05073224*	*0.053055625*	*0.042018477*
**ENSG00000187492**	*0.015442946*	*0.025154907*	*0.024838188*	*0.014592795*
**ENSG00000162769**	*0.038387222*	*0.042130777*	*0.032097395*	*0.035426833*
**ENSG00000160570**	*0.01872799*	*0.026899659*	*0.029333245*	*0.025452963*
**ENSG00000148773**	*0.048996877*	*0.044849333*	*0.038872862*	*0.036815125*
**ENSG00000170423**	*0.014775922*	*0.024730372*	*0.024726213*	*0.015150563*
**ENSG00000140386**	*0.044211275*	*0.04377753*	*0.039462845*	*0.031723505*
**ENSG00000118702**	*0.017504451*	*0.025849641*	*0.023990512*	*0.01602289*
**ENSG00000108219**	*0.034308887*	*0.037787243*	*0.032787744*	*0.038374204*
**ENSG00000176937**	*0.018367407*	*0.026143092*	*0.027065258*	*0.017898127*
**ENSG00000170464**	*0.021991478*	*0.029469441*	*0.028853754*	*0.022073628*
**ENSG00000162994**	*0.041156493*	*0.043997137*	*0.038922147*	*0.030168477*
**ENSG00000111726**	*0.028709921*	*0.036548081*	*0.03128425*	*0.021308907*
**ENSG00000131864**	*0.016812064*	*0.024955269*	*0.024883363*	*0.01508094*
**ENSG00000163517**	*0.02019685*	*0.026175552*	*0.026484828*	*0.0175413*
**ENSG00000178878**	*0.057627179*	*0.056716482*	*0.059470171*	*0.066585975*
**ENSG00000111229**	*0.018238925*	*0.02673265*	*0.026408092*	*0.024139427*
**ENSG00000135409**	*0.017071221*	*0.025457219*	*0.024543368*	*0.016188757*
**ENSG00000198783**	*0.027240478*	*0.032713813*	*0.032730122*	*0.024739646*
**ENSG00000146049**	*0.017299288*	*0.024587542*	*0.024540107*	*0.013984278*
**ENSG00000244482**	*0.018210897*	*0.025727245*	*0.025698067*	*0.01773869*
**ENSG00000141551**	*0.023950609*	*0.031498466*	*0.035769857*	*0.031086025*
**ENSG00000138375**	*0.028839439*	*0.03541664*	*0.033514889*	*0.026699722*
**ENSG00000130201**	*0.016566247*	*0.025180298*	*0.025070949*	*0.01538234*
**ENSG00000163393**	*0.021834321*	*0.024633994*	*0.0255356*	*0.017919919*
**ENSG00000143337**	*0.025360945*	*0.034821951*	*0.035572938*	*0.022579727*
**ENSG00000121314**	*0.018786688*	*0.027946992*	*0.025945746*	*0.016095602*
**ENSG00000106341**	*0.01662578*	*0.024475584*	*0.028064303*	*0.017151282*
**ENSG00000186591**	*0.022463301*	*0.029891628*	*0.032422634*	*0.022057178*
**ENSG00000213366**	*0.071682778*	*0.066322245*	*0.068796064*	*0.064641918*
**ENSG00000143498**	*0.030373495*	*0.036452266*	*0.028568741*	*0.023713731*
**ENSG00000145321**	*0.016093538*	*0.024981588*	*0.024704399*	*0.015791087*
**ENSG00000163510**	*0.027625125*	*0.031339033*	*0.034631772*	*0.029652057*
**ENSG00000153130**	*0.029256808*	*0.035519756*	*0.030176783*	*0.025569931*
**ENSG00000129245**	*0.02247856*	*0.028046334*	*0.028152442*	*0.027201521*
**ENSG00000198743**	*0.028256672*	*0.033069686*	*0.032852149*	*0.036147552*
**ENSG00000183770**	*0.025902731*	*0.027181249*	*0.029581713*	*0.025025147*
**ENSG00000109689**	*0.026925047*	*0.030486768*	*0.029812879*	*0.021833032*

**ENSG00000196136**	*0.015076248*	*0.025804371*	*0.025633566*	*0.015360553*
**ENSG00000163995**	*0.016583696*	*0.026128098*	*0.025709023*	*0.016163402*
**ENSG00000054118**	*0.030857318*	*0.034688883*	*0.031840802*	*0.030774718*
**ENSG00000128272**	*0.037166662*	*0.037891591*	*0.036000313*	*0.04130073*
**ENSG00000063245**	*0.025780129*	*0.037010504*	*0.042428188*	*0.032259344*
**ENSG00000134954**	*0.038304372*	*0.046213193*	*0.047427182*	*0.045726108*
**ENSG00000017427**	*0.079695589*	*0.077454711*	*0.049365913*	*0.065761878*
**ENSG00000187008**	*0.018428769*	*0.025947417*	*0.025904217*	*0.016588178*
**ENSG00000074695**	*0.046451529*	*0.046854424*	*0.044299466*	*0.044759868*
**ENSG00000180871**	*0.038794214*	*0.030564519*	*0.031415144*	*0.032099105*
**ENSG00000055211**	*0.02471604*	*0.031285696*	*0.030367853*	*0.027725464*
**ENSG00000104237**	*0.016201655*	*0.025063456*	*0.025742503*	*0.015632761*
**ENSG00000153822**	*0.018632663*	*0.027418371*	*0.026029435*	*0.016981719*
**ENSG00000197958**	*0.016177409*	*0.025332121*	*0.025641275*	*0.016325734*
**ENSG00000049192**	*0.031965329*	*0.030396781*	*0.02627459*	*0.025271313*
**ENSG00000213799**	*0.027037245*	*0.032780107*	*0.032044186*	*0.031785647*
**ENSG00000132432**	*0.021922678*	*0.027051049*	*0.027375785*	*0.020378266*
**ENSG00000086827**	*0.035710741*	*0.038798155*	*0.034013136*	*0.029264192*
**ENSG00000184897**	*0.040664333*	*0.042168714*	*0.040976098*	*0.034481204*
**ENSG00000115216**	*0.025066335*	*0.031250074*	*0.037490609*	*0.022570341*
**ENSG00000134824**	*0.039498789*	*0.034802578*	*0.030461747*	*0.033725029*
**ENSG00000170624**	*0.020029236*	*0.026006727*	*0.025636218*	*0.017380257*
**ENSG00000166192**	*0.035852117*	*0.038989408*	*0.03279486*	*0.028056654*
**ENSG00000157211**	*0.015876829*	*0.024267195*	*0.024699639*	*0.016663335*
**ENSG00000114544**	*0.033684822*	*0.039054813*	*0.037698592*	*0.030659805*
**ENSG00000244067**	*0.016663923*	*0.027425954*	*0.027802501*	*0.01754468*
**ENSG00000167705**	*0.02055186*	*0.02855069*	*0.027033863*	*0.020681394*
**ENSG00000114166**	*0.019589856*	*0.028231436*	*0.025233194*	*0.017294427*
**ENSG00000112992**	*0.018471239*	*0.027028276*	*0.026784117*	*0.018513339*
**ENSG00000119138**	*0.062239223*	*0.041204918*	*0.041402774*	*0.044324875*
**ENSG00000158481**	*0.044476782*	*0.041450463*	*0.055132594*	*0.066978977*
**ENSG00000167332**	*0.016327994*	*0.024622601*	*0.025522547*	*0.016624941*
**ENSG00000139597**	*0.040198483*	*0.036685318*	*0.043627866*	*0.04170415*
**ENSG00000196357**	*0.019237891*	*0.026923353*	*0.027615296*	*0.01543807*
**ENSG00000138080**	*0.105111906*	*0.099944593*	*0.092999601*	*0.11662332*
**ENSG00000106333**	*0.030627008*	*0.036879815*	*0.028399743*	*0.027666112*
**ENSG00000151364**	*0.112662008*	*0.10987413*	*0.118040367*	*0.077781621*
**ENSG00000159086**	*0.02841636*	*0.034762418*	*0.037950397*	*0.025956496*
**ENSG00000131127**	*0.038382689*	*0.039557159*	*0.033602119*	*0.034761239*
**ENSG00000174996**	*0.036147336*	*0.035144656*	*0.0352395*	*0.036536972*
**ENSG00000163631**	*0.018116383*	*0.024734517*	*0.025193038*	*0.015949857*
**ENSG00000126822**	*0.015774766*	*0.025462912*	*0.025691272*	*0.015578166*
**ENSG00000198125**	*0.018740456*	*0.024962853*	*0.025766643*	*0.017364832*
**ENSG00000171533**	*0.01662516*	*0.026174833*	*0.024394991*	*0.015280814*
**ENSG00000239402**	*0.018256222*	*0.025653517*	*0.025649719*	*0.018108092*
**ENSG00000177946**	*0.032695399*	*0.038060428*	*0.037674697*	*0.030625894*
**ENSG00000174928**	*0.02084072*	*0.026919344*	*0.026459391*	*0.01668919*
**ENSG00000101294**	*0.032722615*	*0.03845706*	*0.041983692*	*0.038474021*
**ENSG00000164187**	*0.025401028*	*0.032425888*	*0.030398308*	*0.025091864*
**ENSG00000154553**	*0.057230661*	*0.044797181*	*0.03378614*	*0.024443292*
**ENSG00000136492**	*0.026563177*	*0.033579829*	*0.02947952*	*0.034059582*
**ENSG00000069702**	*0.116374857*	*0.083140983*	*0.07580353*	*0.088641497*
**ENSG00000169139**	*0.023880442*	*0.03144424*	*0.029035834*	*0.022065659*
**ENSG00000171017**	*0.01829224*	*0.02927509*	*0.027313259*	*0.017253681*

**ENSG00000154518**	*0.018959545*	*0.025784889*	*0.025410815*	*0.016843867*
**ENSG00000110321**	*0.019439088*	*0.02883852*	*0.028529645*	*0.021945607*
**ENSG00000178562**	*0.059233491*	*0.067528801*	*0.064214643*	*0.060505796*
**ENSG00000074603**	*0.025356958*	*0.034537409*	*0.034419477*	*0.024336773*
**ENSG00000100276**	*0.019237897*	*0.026398672*	*0.026018848*	*0.015420345*
**ENSG00000132518**	*0.016982429*	*0.025550346*	*0.025215896*	*0.015071487*
**ENSG00000006118**	*0.038469879*	*0.045634008*	*0.040851717*	*0.043613739*
**ENSG00000134852**	*0.028477552*	*0.034809321*	*0.031361452*	*0.024164139*
**ENSG00000189091**	*0.031362459*	*0.036341953*	*0.030727421*	*0.023928309*
**ENSG00000137502**	*0.066111945*	*0.059521439*	*0.061430694*	*0.048705477*
**ENSG00000106268**	*0.033818568*	*0.03017867*	*0.034189483*	*0.034338626*
**ENSG00000129757**	*0.029441705*	*0.037408875*	*0.029970644*	*0.0325932*
**ENSG00000184702**	*0.062867456*	*0.049208074*	*0.05054683*	*0.048597527*
**ENSG00000143297**	*0.099426355*	*0.065729059*	*0.077216659*	*0.100605213*
**ENSG00000137960**	*0.018670614*	*0.026910469*	*0.024948823*	*0.018953617*
**ENSG00000087266**	*0.033066178*	*0.036118245*	*0.034255254*	*0.03036999*
**ENSG00000152104**	*0.028056236*	*0.026269906*	*0.024692319*	*0.016922231*
**ENSG00000136267**	*0.01478573*	*0.025310306*	*0.0243714*	*0.015017305*
**ENSG00000176890**	*0.030542191*	*0.032580648*	*0.028019278*	*0.024907648*
**ENSG00000196867**	*0.021987225*	*0.025754962*	*0.026299052*	*0.017788083*
**ENSG00000183779**	*0.024837552*	*0.02945697*	*0.026334899*	*0.021076457*
**ENSG00000099203**	*0.02610971*	*0.029483042*	*0.033821456*	*0.021539577*
**ENSG00000044459**	*0.025153428*	*0.031326607*	*0.030318154*	*0.02021485*
**ENSG00000169605**	*0.016362377*	*0.024707873*	*0.025567052*	*0.014972359*
**ENSG00000124232**	*0.018507508*	*0.026384138*	*0.027046829*	*0.016954044*
**ENSG00000120696**	*0.040121362*	*0.042917462*	*0.030929518*	*0.0264013*
**ENSG00000079102**	*0.016817732*	*0.024717017*	*0.025232869*	*0.017722835*
**ENSG00000237541**	*0.282420222*	*0.264067524*	*0.265088455*	*0.200045966*
**ENSG00000197102**	*0.027515143*	*0.041694036*	*0.035026443*	*0.029420868*
**ENSG00000170899**	*0.069695868*	*0.04609917*	*0.046916151*	*0.061916596*
**ENSG00000123576**	*0.014995411*	*0.024625695*	*0.024093271*	*0.014208627*
**ENSG00000177105**	*0.025380064*	*0.030902091*	*0.033552083*	*0.027369351*
**ENSG00000169136**	*0.061555534*	*0.056194775*	*0.05131311*	*0.062038518*
**ENSG00000166793**	*0.038650805*	*0.045948833*	*0.041082207*	*0.050380296*
**ENSG00000092098**	*0.023568928*	*0.031260411*	*0.03745544*	*0.02321251*
**ENSG00000134901**	*0.038991963*	*0.035294345*	*0.034538226*	*0.033833742*
**ENSG00000118997**	*0.026512722*	*0.024831616*	*0.024744136*	*0.020823352*
**ENSG00000150961**	*0.036658052*	*0.037486335*	*0.036754745*	*0.037558784*
**ENSG00000186924**	*0.016010094*	*0.025926387*	*0.025304878*	*0.015269742*
**ENSG00000153767**	*0.033359637*	*0.037675152*	*0.031065729*	*0.029192591*
**ENSG00000234776**	*0.015633028*	*0.024765851*	*0.025528359*	*0.01636624*
**ENSG00000165828**	*0.015283376*	*0.025051962*	*0.024043149*	*0.015686368*
**ENSG00000138031**	*0.038455641*	*0.035896284*	*0.032084535*	*0.02796523*
**ENSG00000102053**	*0.016908498*	*0.024918561*	*0.025477744*	*0.015063582*
**ENSG00000166275**	*0.032679528*	*0.0402959*	*0.040791795*	*0.031562025*
**ENSG00000165972**	*0.016714227*	*0.025057263*	*0.024544792*	*0.014634214*
**ENSG00000121104**	*0.029638156*	*0.034714524*	*0.038138194*	*0.029274919*
**ENSG00000149927**	*0.017232822*	*0.027216751*	*0.024942909*	*0.015913393*
**ENSG00000108963**	*0.022185144*	*0.029461401*	*0.031088218*	*0.018697326*
**ENSG00000137558**	*0.015547116*	*0.024663377*	*0.024954343*	*0.014873304*
**ENSG00000184182**	*0.02746138*	*0.036276184*	*0.034438755*	*0.026081023*
**ENSG00000003147**	*0.070182177*	*0.064011116*	*0.057734692*	*0.102101374*
**ENSG00000205020**	*0.064307898*	*0.083275906*	*0.073437946*	*0.087197365*
**ENSG00000136891**	*0.026380289*	*0.030563921*	*0.028154703*	*0.021974131*

**ENSG00000181585**	*0.015482899*	*0.024683552*	*0.025087954*	*0.013814759*
**ENSG00000136643**	*0.027239122*	*0.033270385*	*0.032717288*	*0.026073202*
**ENSG00000106069**	*0.017391276*	*0.024649895*	*0.026015518*	*0.016063908*
**ENSG00000117298**	*0.049474193*	*0.042498508*	*0.043766194*	*0.041887182*
**ENSG00000152022**	*0.036390144*	*0.043872928*	*0.03849706*	*0.028530548*
**ENSG00000169188**	*0.028387735*	*0.034162312*	*0.032492496*	*0.026232162*
**ENSG00000138892**	*0.018136764*	*0.025073874*	*0.025219957*	*0.016390281*
**ENSG00000114670**	*0.016171092*	*0.025540875*	*0.024373955*	*0.01537446*
**ENSG00000153936**	*0.029799099*	*0.032491936*	*0.034445045*	*0.028443064*
**ENSG00000183389**	*0.016844281*	*0.025946159*	*0.025250957*	*0.017498056*
**ENSG00000176532**	*0.015325331*	*0.025197742*	*0.025442923*	*0.014391915*
**ENSG00000196584**	*0.034492762*	*0.033372656*	*0.036000654*	*0.034005457*
**ENSG00000166866**	*0.020910824*	*0.028053951*	*0.026259609*	*0.018334653*
**ENSG00000134249**	*0.017405878*	*0.026706989*	*0.026581708*	*0.016430897*
**ENSG00000095917**	*0.015957973*	*0.025865651*	*0.026434215*	*0.016747175*
**ENSG00000101546**	*0.028175941*	*0.029360778*	*0.03337836*	*0.025629742*
**ENSG00000110844**	*0.024565853*	*0.030995155*	*0.025777698*	*0.020535775*
**ENSG00000140750**	*0.041209152*	*0.043180655*	*0.040969374*	*0.037821405*
**ENSG00000162999**	*0.028823017*	*0.031846636*	*0.03229995*	*0.026662457*
**ENSG00000070718**	*0.052014166*	*0.050409068*	*0.04327088*	*0.044653705*
**ENSG00000183313**	*0.014910223*	*0.024069025*	*0.024635277*	*0.015339536*
**ENSG00000112576**	*0.054676694*	*0.050478299*	*0.04224659*	*0.050541763*
**ENSG00000125869**	*0.15897682*	*0.142694502*	*0.129409108*	*0.135118063*
**ENSG00000173567**	*0.017970536*	*0.025101859*	*0.026146455*	*0.015607098*
**ENSG00000108878**	*0.016040446*	*0.023961362*	*0.02401639*	*0.015926872*
**ENSG00000184724**	*0.01603841*	*0.024974701*	*0.024551529*	*0.015325455*
**ENSG00000053900**	*0.034889018*	*0.040553741*	*0.033708958*	*0.025106348*
**ENSG00000188997**	*0.037414391*	*0.028605*	*0.031074864*	*0.023392261*
**ENSG00000127366**	*0.015000605*	*0.02439243*	*0.025936426*	*0.015759194*
**ENSG00000188581**	*0.014685881*	*0.025074252*	*0.02525357*	*0.014109484*
**ENSG00000122678**	*0.024276268*	*0.028638977*	*0.030913244*	*0.023694806*
**ENSG00000185049**	*0.024710621*	*0.032297886*	*0.033825929*	*0.023662548*
**ENSG00000005893**	*0.031175157*	*0.032984563*	*0.030679844*	*0.028965832*
**ENSG00000143001**	*0.015801717*	*0.024805264*	*0.02436075*	*0.015103953*
**ENSG00000124459**	*0.02757646*	*0.038123148*	*0.038594376*	*0.027221147*
**ENSG00000092847**	*0.03180559*	*0.034057106*	*0.02913507*	*0.027800301*
**ENSG00000159479**	*0.023681904*	*0.02961002*	*0.030186667*	*0.02428472*
**ENSG00000102977**	*0.033828701*	*0.039299369*	*0.033937997*	*0.028783634*
**ENSG00000118640**	*0.023506997*	*0.028024207*	*0.031175036*	*0.023102786*
**ENSG00000178235**	*0.017127244*	*0.026134783*	*0.025580048*	*0.017972238*
**ENSG00000092969**	*0.015899802*	*0.025342338*	*0.026000571*	*0.015385065*
**ENSG00000159708**	*0.020640344*	*0.025156736*	*0.025669637*	*0.021357751*
**ENSG00000108946**	*0.020007436*	*0.030251933*	*0.028487322*	*0.017757888*
**ENSG00000043039**	*0.01843634*	*0.024167908*	*0.02427548*	*0.016771276*
**ENSG00000183900**	*0.031771427*	*0.032258387*	*0.028891946*	*0.032330926*
**ENSG00000111837**	*0.018314908*	*0.029252392*	*0.025743415*	*0.018617794*
**ENSG00000128534**	*0.033935836*	*0.040525511*	*0.034418445*	*0.029864947*
**ENSG00000126264**	*0.049581218*	*0.04930171*	*0.04415664*	*0.040680794*
**ENSG00000171097**	*0.032702343*	*0.036551845*	*0.035505923*	*0.03123195*
**ENSG00000139973**	*0.015613511*	*0.024942298*	*0.023881811*	*0.015737063*
**ENSG00000176678**	*0.015742742*	*0.024734914*	*0.02423733*	*0.015404481*
**ENSG00000104804**	*0.051902306*	*0.046228103*	*0.038728516*	*0.043892865*
**ENSG00000186462**	*0.04531214*	*0.03446968*	*0.040290661*	*0.025102631*
**ENSG00000162517**	*0.023889251*	*0.028836841*	*0.028596747*	*0.023617437*

**ENSG00000130176**	*0.015592929*	*0.026268416*	*0.025383393*	*0.016431083*
**ENSG00000008282**	*0.026177241*	*0.029511743*	*0.029936753*	*0.023051119*
**ENSG00000100325**	*0.023883994*	*0.03298599*	*0.030167498*	*0.021685571*
**ENSG00000198670**	*0.03009087*	*0.026321531*	*0.025366706*	*0.018667133*
**ENSG00000089818**	*0.024951345*	*0.032881353*	*0.037356602*	*0.024530649*
**ENSG00000070748**	*0.015599715*	*0.024969182*	*0.024082456*	*0.015537127*
**ENSG00000137727**	*0.039765416*	*0.048028921*	*0.032994381*	*0.049849871*
**ENSG00000162188**	*0.017278822*	*0.024709078*	*0.02486785*	*0.017801816*
**ENSG00000119004**	*0.037686328*	*0.037773927*	*0.043822094*	*0.04998138*
**ENSG00000160131**	*0.029185924*	*0.033206267*	*0.03219782*	*0.028510369*
**ENSG00000206561**	*0.016359333*	*0.025773812*	*0.025340618*	*0.015028861*
**ENSG00000213694**	*0.015427802*	*0.026627171*	*0.02562898*	*0.016222714*
**ENSG00000185813**	*0.036892016*	*0.043342941*	*0.033378921*	*0.030825172*
**ENSG00000124496**	*0.015875958*	*0.025614264*	*0.02430632*	*0.014916729*
**ENSG00000176396**	*0.029704988*	*0.036659105*	*0.038290277*	*0.023716414*
**ENSG00000184900**	*0.032545407*	*0.033544268*	*0.032642817*	*0.029188816*
**ENSG00000122223**	*0.088889458*	*0.087834521*	*0.097683191*	*0.104586674*
**ENSG00000099256**	*0.034004284*	*0.030259556*	*0.026059087*	*0.023083365*
**ENSG00000102010**	*0.01588868*	*0.02477336*	*0.025293239*	*0.015669617*
**ENSG00000173653**	*0.030101336*	*0.034343483*	*0.045858753*	*0.031855865*
**ENSG00000205542**	*0.015695813*	*0.024116616*	*0.023681363*	*0.016607755*
**ENSG00000133858**	*0.023616619*	*0.032049838*	*0.029223324*	*0.021330341*
**ENSG00000131183**	*0.01467679*	*0.024144848*	*0.024471961*	*0.014670151*
**ENSG00000154144**	*0.031843806*	*0.030018753*	*0.033703036*	*0.030923825*
**ENSG00000185551**	*0.207136143*	*0.173838398*	*0.139732355*	*0.161551406*
**ENSG00000169288**	*0.029507978*	*0.034491126*	*0.035051994*	*0.028690133*
**ENSG00000101292**	*0.015946135*	*0.024941616*	*0.024502927*	*0.01496218*
**ENSG00000156273**	*0.045222273*	*0.050522432*	*0.043681828*	*0.036308137*
**ENSG00000197157**	*0.023413184*	*0.026677388*	*0.029344196*	*0.021025154*
**ENSG00000151553**	*0.032833052*	*0.034630617*	*0.033266161*	*0.030699619*
**ENSG00000167207**	*0.047696782*	*0.039277865*	*0.038450355*	*0.04174355*
**ENSG00000101040**	*0.027806291*	*0.036203176*	*0.030972158*	*0.022511202*
**ENSG00000197442**	*0.037379325*	*0.042063184*	*0.0347054*	*0.031305537*
**ENSG00000170006**	*0.056722113*	*0.048297432*	*0.041531347*	*0.037726023*
**ENSG00000188822**	*0.029206295*	*0.02910163*	*0.027645659*	*0.023163355*
**ENSG00000130520**	*0.031487613*	*0.032157564*	*0.031861595*	*0.026559289*
**ENSG00000185090**	*0.104061115*	*0.076164916*	*0.070163555*	*0.060665935*
**ENSG00000147130**	*0.019608654*	*0.026686157*	*0.029220402*	*0.020075926*
**ENSG00000198736**	*0.046182666*	*0.046593852*	*0.034752014*	*0.038515869*
**ENSG00000126458**	*0.040193271*	*0.035818313*	*0.034444557*	*0.042369168*
**ENSG00000136111**	*0.090481323*	*0.097636879*	*0.098935827*	*0.081924132*
**ENSG00000147402**	*0.018571355*	*0.024985813*	*0.02526812*	*0.016157542*
**ENSG00000188735**	*0.022454087*	*0.029432923*	*0.03045131*	*0.02881825*
**ENSG00000155970**	*0.017305081*	*0.025360976*	*0.025363787*	*0.016136823*
**ENSG00000118096**	*0.03024829*	*0.032936671*	*0.030143286*	*0.031917912*
**ENSG00000155307**	*0.065501917*	*0.051046528*	*0.057767021*	*0.054248061*
**ENSG00000182287**	*0.035862391*	*0.035076898*	*0.033459357*	*0.031261286*
**ENSG00000137965**	*0.049757581*	*0.04111067*	*0.036126251*	*0.038126389*
**ENSG00000165934**	*0.027023145*	*0.031990012*	*0.031937539*	*0.027484547*
**ENSG00000102970**	*0.12644928*	*0.102716229*	*0.100241686*	*0.114426458*
**ENSG00000128045**	*0.016248116*	*0.02475442*	*0.025771659*	*0.015041527*
**ENSG00000125912**	*0.026871923*	*0.032350452*	*0.034564817*	*0.027081592*
**ENSG00000050748**	*0.029307389*	*0.035088582*	*0.032144323*	*0.022929104*
**ENSG00000169877**	*0.019610383*	*0.025123437*	*0.025411968*	*0.076192946*

**ENSG00000198586**	*0.027846706*	*0.030631652*	*0.033070514*	*0.027084533*
**ENSG00000160318**	*0.033657992*	*0.035341278*	*0.033469556*	*0.032591631*
**ENSG00000196187**	*0.05218025*	*0.052344874*	*0.043854951*	*0.041952746*
**ENSG00000137252**	*0.015728168*	*0.024764165*	*0.024516078*	*0.015662061*
**ENSG00000168658**	*0.01478133*	*0.024788538*	*0.02464435*	*0.015059777*
**ENSG00000131059**	*0.016976612*	*0.024689326*	*0.02424048*	*0.016178816*
**ENSG00000164929**	*0.016616349*	*0.024818387*	*0.025209308*	*0.016802341*
**ENSG00000153048**	*0.046163349*	*0.038886842*	*0.033964481*	*0.026396752*
**ENSG00000116726**	*0.03074638*	*0.027411476*	*0.031320899*	*0.019393379*
**ENSG00000170290**	*0.017585035*	*0.026293863*	*0.0246057*	*0.018372985*
**ENSG00000172361**	*0.016311983*	*0.025317772*	*0.024720488*	*0.016808943*
**ENSG00000162576**	*0.016521132*	*0.027261707*	*0.025595443*	*0.016364185*
**ENSG00000181609**	*0.017821377*	*0.025864337*	*0.025498608*	*0.01828588*
**ENSG00000135773**	*0.01651326*	*0.025066844*	*0.025316427*	*0.015999005*
**ENSG00000154016**	*0.026337705*	*0.029199734*	*0.032722818*	*0.027532144*
**ENSG00000122254**	*0.016092962*	*0.025392612*	*0.024141331*	*0.015769851*
**ENSG00000150637**	*0.0456058*	*0.043287439*	*0.035108282*	*0.046886751*
**ENSG00000151812**	*0.016875626*	*0.025045101*	*0.024267762*	*0.014664284*
**ENSG00000105880**	*0.014964899*	*0.024452981*	*0.025025991*	*0.015162924*
**ENSG00000177042**	*0.028974605*	*0.034255453*	*0.032527307*	*0.031755963*
**ENSG00000143774**	*0.020887272*	*0.027227235*	*0.028420703*	*0.016922241*
**ENSG00000170759**	*0.037634264*	*0.040105847*	*0.039792074*	*0.032572199*
**ENSG00000188124**	*0.022638557*	*0.02744736*	*0.031257334*	*0.019106937*
**ENSG00000173432**	*0.017890432*	*0.029398588*	*0.02595043*	*0.019589807*
**ENSG00000185245**	*0.037505355*	*0.031472014*	*0.031272075*	*0.026477772*
**ENSG00000179820**	*0.066455299*	*0.048067501*	*0.048531886*	*0.047702739*
**ENSG00000188786**	*0.023563981*	*0.038881539*	*0.036788278*	*0.026779727*
**ENSG00000221818**	*0.019384952*	*0.025415002*	*0.027468219*	*0.016716874*
**ENSG00000182325**	*0.030892193*	*0.032379341*	*0.035385736*	*0.030258342*
**ENSG00000189292**	*0.016060989*	*0.025783685*	*0.025923083*	*0.015240696*
**ENSG00000137767**	*0.026426034*	*0.031601681*	*0.032328463*	*0.021175411*
**ENSG00000171786**	*0.018094212*	*0.024964864*	*0.025313078*	*0.016181985*
**ENSG00000158714**	*0.025990988*	*0.025212718*	*0.026361379*	*0.030549065*
**ENSG00000133106**	*0.042677505*	*0.034724816*	*0.035204882*	*0.031022182*
**ENSG00000131467**	*0.035241025*	*0.032396777*	*0.03889539*	*0.043907299*
**ENSG00000196092**	*0.01474455*	*0.025430114*	*0.024419404*	*0.015487561*
**ENSG00000124275**	*0.016749146*	*0.025016036*	*0.025967146*	*0.016903572*
**ENSG00000100931**	*0.027463989*	*0.030639996*	*0.031854792*	*0.024765708*
**ENSG00000158966**	*0.014759095*	*0.025510552*	*0.024215106*	*0.014579066*
**ENSG00000149133**	*0.014796091*	*0.023992812*	*0.024500975*	*0.014776549*
**ENSG00000105953**	*0.030346833*	*0.032774384*	*0.030505089*	*0.029593712*
**ENSG00000165055**	*0.0315001*	*0.031703501*	*0.032307757*	*0.027098176*
**ENSG00000084676**	*0.038541311*	*0.037920334*	*0.036934336*	*0.036436463*
**ENSG00000169972**	*0.027561*	*0.030007939*	*0.033935764*	*0.024227296*
**ENSG00000049883**	*0.031440111*	*0.033727645*	*0.030862078*	*0.022759969*
**ENSG00000197982**	*0.028980489*	*0.033349814*	*0.031799419*	*0.028558752*
**ENSG00000166477**	*0.030200352*	*0.035641542*	*0.030710298*	*0.023247618*
**ENSG00000145198**	*0.016168114*	*0.025166803*	*0.025047847*	*0.016294333*
**ENSG00000181513**	*0.018926712*	*0.026697452*	*0.027798763*	*0.020849351*
**ENSG00000101349**	*0.017048782*	*0.025424734*	*0.025193301*	*0.01496905*
**ENSG00000176754**	*0.015845182*	*0.025125321*	*0.02467699*	*0.014615063*
**ENSG00000067225**	*0.016305557*	*0.024721657*	*0.025275945*	*0.015328198*
**ENSG00000102178**	*0.02616562*	*0.042348598*	*0.03164485*	*0.038821772*
**ENSG00000120685**	*0.028616301*	*0.033910781*	*0.038284161*	*0.033947238*

**ENSG00000138028**	*0.018814476*	*0.026000019*	*0.025682738*	*0.016556855*
**ENSG00000166685**	*0.020826794*	*0.02709082*	*0.030955997*	*0.019559135*
**ENSG00000164023**	*0.031790432*	*0.034516352*	*0.036412868*	*0.030066014*
**ENSG00000101353**	*0.033242916*	*0.032377184*	*0.033715184*	*0.023674317*
**ENSG00000149646**	*0.024103398*	*0.02739008*	*0.027702168*	*0.016279523*
**ENSG00000175764**	*0.016395768*	*0.025630749*	*0.025554762*	*0.016097517*
**ENSG00000165300**	*0.023371926*	*0.025297586*	*0.026466678*	*0.01567747*
**ENSG00000129744**	*0.015359318*	*0.024210174*	*0.025028475*	*0.014831962*
**ENSG00000103269**	*0.029853161*	*0.036190004*	*0.037455034*	*0.024263283*
**ENSG00000167744**	*0.016421332*	*0.023814036*	*0.024233097*	*0.015366319*
**ENSG00000141404**	*0.014856705*	*0.024476266*	*0.024083376*	*0.016304371*
**ENSG00000118260**	*0.029594059*	*0.031790515*	*0.032706183*	*0.025807799*
**ENSG00000171492**	*0.040422408*	*0.039160923*	*0.036282445*	*0.031785937*
**ENSG00000037965**	*0.015506693*	*0.02494213*	*0.024506198*	*0.014582065*
**ENSG00000164576**	*0.029414999*	*0.032064263*	*0.03145821*	*0.026344245*
**ENSG00000221826**	*0.018553175*	*0.025884147*	*0.025373784*	*0.017995763*
**ENSG00000203737**	*0.017127493*	*0.026435633*	*0.026012337*	*0.017570386*
**ENSG00000114631**	*0.042889289*	*0.040752097*	*0.034341881*	*0.035155746*
**ENSG00000147872**	*0.048828647*	*0.038538827*	*0.039576672*	*0.043395736*
**ENSG00000113356**	*0.034703496*	*0.034853743*	*0.033739063*	*0.031967295*
**ENSG00000188883**	*0.019453615*	*0.024828296*	*0.024753376*	*0.017596228*
**ENSG00000112357**	*0.033080494*	*0.038785667*	*0.030511477*	*0.029355232*
**ENSG00000164967**	*0.019414621*	*0.028485048*	*0.026675235*	*0.023113936*
**ENSG00000143036**	*0.017110171*	*0.024731307*	*0.025803581*	*0.0161906*
**ENSG00000172380**	*0.041393449*	*0.04154168*	*0.026794585*	*0.032266178*
**ENSG00000105131**	*0.016063576*	*0.025015188*	*0.024458623*	*0.014615338*
**ENSG00000185246**	*0.028440754*	*0.032265632*	*0.029821775*	*0.025177832*
**ENSG00000167359**	*0.017342563*	*0.026023774*	*0.025587885*	*0.015818297*
**ENSG00000099957**	*0.015830162*	*0.025906529*	*0.024975395*	*0.014779972*
**ENSG00000138152**	*0.017915805*	*0.031350253*	*0.029831581*	*0.02054282*
**ENSG00000105717**	*0.064339185*	*0.060800154*	*0.046653044*	*0.055117373*
**ENSG00000120798**	*0.03202922*	*0.033231891*	*0.03051581*	*0.031146934*
**ENSG00000203908**	*0.027876507*	*0.028360073*	*0.028503528*	*0.022230517*
**ENSG00000144895**	*0.022530329*	*0.02835733*	*0.029232321*	*0.025557583*
**ENSG00000103365**	*0.02746063*	*0.041125833*	*0.031494671*	*0.02738309*
**ENSG00000121270**	*0.018217655*	*0.026533264*	*0.029379688*	*0.017811526*
**ENSG00000091583**	*0.016217328*	*0.025442055*	*0.0255961*	*0.015812073*
**ENSG00000084092**	*0.021361658*	*0.028633255*	*0.028108813*	*0.020747037*
**ENSG00000213923**	*0.017125058*	*0.025344941*	*0.026960362*	*0.01631692*
**ENSG00000183833**	*0.019239206*	*0.024787067*	*0.025317154*	*0.015538277*
**ENSG00000141458**	*0.040699785*	*0.041425296*	*0.037446868*	*0.036287857*
**ENSG00000186716**	*0.017948916*	*0.027130869*	*0.026540515*	*0.018088151*
**ENSG00000165915**	*0.022649217*	*0.029977486*	*0.031673963*	*0.023907617*
**ENSG00000185821**	*0.016384465*	*0.025377004*	*0.024447033*	*0.015297354*
**ENSG00000150783**	*0.015708836*	*0.025333425*	*0.02552056*	*0.014925241*
**ENSG00000085117**	*0.040169951*	*0.044052523*	*0.044665584*	*0.047528506*
**ENSG00000172987**	*0.018727488*	*0.026477877*	*0.025090848*	*0.018510829*
**ENSG00000064995**	*0.022634019*	*0.030241793*	*0.031700211*	*0.018747662*
**ENSG00000108883**	*0.016740516*	*0.025689179*	*0.026740409*	*0.015988045*
**ENSG00000086758**	*0.038639295*	*0.051389796*	*0.035295259*	*0.03431607*
**ENSG00000175329**	*0.12619317*	*0.068121518*	*0.083309238*	*0.111268674*
**ENSG00000155026**	*0.017644746*	*0.027650372*	*0.026768016*	*0.017034502*
**ENSG00000140538**	*0.015482025*	*0.025108946*	*0.025016875*	*0.01579047*
**ENSG00000140262**	*0.035573685*	*0.047821161*	*0.038705047*	*0.038418113*

**ENSG00000171944**	*0.015512699*	*0.024751961*	*0.024903222*	*0.015438349*
**ENSG00000157869**	*0.033568168*	*0.034764108*	*0.031765546*	*0.025767427*
**ENSG00000110080**	*0.027729496*	*0.036888574*	*0.030214808*	*0.024624378*
**ENSG00000188687**	*0.015441278*	*0.024425817*	*0.024647161*	*0.015074358*
**ENSG00000123395**	*0.03272891*	*0.040945491*	*0.050753689*	*0.034750353*
**ENSG00000171877**	*0.014944934*	*0.025918898*	*0.02486895*	*0.015077038*
**ENSG00000170049**	*0.018478711*	*0.028117741*	*0.025960597*	*0.019018067*
**ENSG00000162819**	*0.020503354*	*0.030906974*	*0.027770008*	*0.021352726*
**ENSG00000250589**	*0.015339111*	*0.024897224*	*0.025135285*	*0.014495935*
**ENSG00000206538**	*0.017343204*	*0.024734593*	*0.026513352*	*0.016552422*
**ENSG00000047849**	*0.031706001*	*0.058599708*	*0.034961663*	*0.056128575*
**ENSG00000169758**	*0.024434895*	*0.024909965*	*0.024164663*	*0.017561951*
**ENSG00000132004**	*0.032019589*	*0.034378694*	*0.034364347*	*0.025942387*
**ENSG00000145107**	*0.017620426*	*0.024901231*	*0.027275121*	*0.023977091*
**ENSG00000166333**	*0.029225528*	*0.033355141*	*0.031268761*	*0.022421377*
**ENSG00000143554**	*0.05296639*	*0.057241308*	*0.046708496*	*0.051959977*
**ENSG00000147166**	*0.019505285*	*0.029052288*	*0.026072605*	*0.018555186*
**ENSG00000184674**	*0.137923293*	*0.181139182*	*0.14981277*	*0.159648661*
**ENSG00000143013**	*0.051578855*	*0.047283222*	*0.040826632*	*0.041419737*
**ENSG00000101204**	*0.016258255*	*0.025167383*	*0.025865806*	*0.016060177*
**ENSG00000129103**	*0.032945549*	*0.033066825*	*0.034322606*	*0.033353421*
**ENSG00000022976**	*0.034269845*	*0.03861817*	*0.035822439*	*0.029082415*
**ENSG00000143368**	*0.030335362*	*0.039867287*	*0.044261547*	*0.046996417*
**ENSG00000170891**	*0.022873396*	*0.024418895*	*0.024004377*	*0.01553168*
**ENSG00000206503**	*0.109155518*	*0.096839957*	*0.175658572*	*0.119251699*
**ENSG00000187166**	*0.015132072*	*0.025078973*	*0.024648156*	*0.014043441*
**ENSG00000079931**	*0.15976078*	*0.095504837*	*0.065737688*	*0.129224907*
**ENSG00000012211**	*0.017937357*	*0.027150622*	*0.025260177*	*0.01753003*
**ENSG00000198467**	*0.080924166*	*0.069601286*	*0.061395308*	*0.070183634*
**ENSG00000057593**	*0.016375653*	*0.02518151*	*0.024722183*	*0.016696986*
**ENSG00000166008**	*0.01561619*	*0.026112401*	*0.025574301*	*0.015294547*
**ENSG00000110195**	*0.016036637*	*0.024646988*	*0.024538455*	*0.015115765*
**ENSG00000184908**	*0.015489212*	*0.025132867*	*0.026056565*	*0.01564281*
**ENSG00000148832**	*0.031263772*	*0.034343525*	*0.029747985*	*0.031308406*
**ENSG00000158748**	*0.018208409*	*0.025696341*	*0.027649208*	*0.017682802*
**ENSG00000167114**	*0.016889691*	*0.025740103*	*0.024889665*	*0.01602357*
**ENSG00000108091**	*0.03608415*	*0.038575592*	*0.038228472*	*0.03265682*
**ENSG00000167645**	*0.035165985*	*0.034691455*	*0.032031591*	*0.029600903*
**ENSG00000162144**	*0.062673394*	*0.04890003*	*0.045007807*	*0.050178902*
**ENSG00000148225**	*0.018149583*	*0.025569344*	*0.025788117*	*0.017974886*
**ENSG00000171503**	*0.028989715*	*0.036032056*	*0.0295256*	*0.023710439*
**ENSG00000134160**	*0.015160273*	*0.024742572*	*0.024842956*	*0.014857588*
**ENSG00000149397**	*0.038092056*	*0.036838562*	*0.036993028*	*0.031134387*
**ENSG00000180720**	*0.015105422*	*0.025877635*	*0.024551583*	*0.015669543*
**ENSG00000107018**	*0.040922623*	*0.039105518*	*0.030024771*	*0.029810702*
**ENSG00000185873**	*0.022483949*	*0.025559162*	*0.024713572*	*0.016219921*
**ENSG00000136717**	*0.073117345*	*0.058363458*	*0.04961558*	*0.055826321*
**ENSG00000166225**	*0.017026276*	*0.025040906*	*0.025323819*	*0.016907891*
**ENSG00000172379**	*0.020716672*	*0.02766876*	*0.025935478*	*0.016878355*
**ENSG00000163606**	*0.021322213*	*0.027300643*	*0.026228225*	*0.017181062*
**ENSG00000149295**	*0.015395782*	*0.024810875*	*0.025410142*	*0.016082117*
**ENSG00000138741**	*0.014488817*	*0.024286107*	*0.024454826*	*0.013989463*
**ENSG00000132680**	*0.03197066*	*0.035103622*	*0.032110107*	*0.028209542*
**ENSG00000109339**	*0.016243821*	*0.025447317*	*0.024304286*	*0.014436324*

**ENSG00000100393**	*0.029758665*	*0.033613354*	*0.042874519*	*0.026505259*
**ENSG00000166833**	*0.019499072*	*0.025955927*	*0.026084809*	*0.018984697*
**ENSG00000175356**	*0.014845249*	*0.025379025*	*0.024496715*	*0.014763094*
**ENSG00000145147**	*0.014940996*	*0.024719195*	*0.024763416*	*0.014162137*
**ENSG00000174842**	*0.019743741*	*0.028202247*	*0.025777774*	*0.031184334*
**ENSG00000196313**	*0.02296863*	*0.030047222*	*0.029503832*	*0.02094145*
**ENSG00000140009**	*0.020982166*	*0.028046415*	*0.026132626*	*0.018540145*
**ENSG00000141582**	*0.017886852*	*0.027395606*	*0.026401848*	*0.018850016*
**ENSG00000182973**	*0.024498029*	*0.031720872*	*0.030617506*	*0.023085346*
**ENSG00000152422**	*0.039007797*	*0.036177761*	*0.0329199*	*0.026401093*
**ENSG00000172301**	*0.043031958*	*0.039721342*	*0.04105628*	*0.033655038*
**ENSG00000146054**	*0.015821337*	*0.025458418*	*0.024483515*	*0.014927639*
**ENSG00000066735**	*0.015934913*	*0.025122322*	*0.024544775*	*0.014532632*
**ENSG00000164344**	*0.01820679*	*0.026426734*	*0.027945085*	*0.016581249*
**ENSG00000136697**	*0.015256947*	*0.026209037*	*0.023950069*	*0.015684412*
**ENSG00000154133**	*0.014583448*	*0.024328817*	*0.024242412*	*0.014091496*
**ENSG00000180475**	*0.015911147*	*0.024813199*	*0.024247591*	*0.013886544*
**ENSG00000156265**	*0.104272421*	*0.078924847*	*0.059181634*	*0.078627335*
**ENSG00000122477**	*0.016884903*	*0.025632703*	*0.024710021*	*0.017676072*
**ENSG00000106536**	*0.01475986*	*0.02476627*	*0.024160321*	*0.01462552*
**ENSG00000137752**	*0.075161531*	*0.062432545*	*0.053391021*	*0.061752688*
**ENSG00000101342**	*0.021052401*	*0.028040812*	*0.030234653*	*0.021711382*
**ENSG00000172354**	*0.028541843*	*0.034663252*	*0.030185375*	*0.022634028*
**ENSG00000102003**	*0.018061164*	*0.027517243*	*0.026744547*	*0.017487447*
**ENSG00000167470**	*0.03300406*	*0.039842904*	*0.038351383*	*0.032903803*
**ENSG00000185905**	*0.034632127*	*0.035642416*	*0.036955548*	*0.030979558*
**ENSG00000183678**	*0.017771069*	*0.044872288*	*0.025372017*	*0.024135802*
**ENSG00000163788**	*0.033696042*	*0.03717912*	*0.03636423*	*0.033587498*
**ENSG00000164828**	*0.03043946*	*0.036436841*	*0.033979132*	*0.026346794*
**ENSG00000124181**	*0.045565239*	*0.04431741*	*0.043204965*	*0.034865451*
**ENSG00000109181**	*0.08491492*	*0.074337274*	*0.051422267*	*0.08303128*
**ENSG00000186660**	*0.027392641*	*0.034809983*	*0.033900755*	*0.026060289*
**ENSG00000113593**	*0.030778083*	*0.03538299*	*0.032132529*	*0.022323235*
**ENSG00000141141**	*0.030616192*	*0.035478046*	*0.033651444*	*0.030825813*
**ENSG00000135473**	*0.043582191*	*0.042576816*	*0.040992488*	*0.047633533*
**ENSG00000100731**	*0.028901478*	*0.040481014*	*0.037200514*	*0.030968344*
**ENSG00000184990**	*0.030987717*	*0.030208961*	*0.03155695*	*0.027425564*
**ENSG00000162086**	*0.034624032*	*0.037975364*	*0.043774353*	*0.038034295*
**ENSG00000198677**	*0.025290946*	*0.029585121*	*0.030071738*	*0.022327248*
**ENSG00000185522**	*0.026159718*	*0.037869431*	*0.037244475*	*0.038385476*
**ENSG00000148572**	*0.025612379*	*0.035078854*	*0.040615179*	*0.023902617*
**ENSG00000185978**	*0.015482106*	*0.025497561*	*0.023698011*	*0.015611022*
**ENSG00000146938**	*0.015638907*	*0.024862789*	*0.024615397*	*0.015481379*
**ENSG00000129474**	*0.016682558*	*0.024214884*	*0.025112406*	*0.015698862*
**ENSG00000148408**	*0.019076149*	*0.028947975*	*0.029205434*	*0.021717297*
**ENSG00000114019**	*0.016731102*	*0.024535865*	*0.024916612*	*0.017304026*
**ENSG00000183715**	*0.014965853*	*0.025440816*	*0.024474749*	*0.014366594*
**ENSG00000158019**	*0.025321077*	*0.030181961*	*0.030823568*	*0.024873062*
**ENSG00000184270**	*0.027714246*	*0.024889201*	*0.027795374*	*0.021201878*
**ENSG00000178971**	*0.033139748*	*0.03564792*	*0.035494205*	*0.029194919*
**ENSG00000186867**	*0.016108674*	*0.025363059*	*0.024511304*	*0.016793804*
**ENSG00000170745**	*0.019116234*	*0.025854717*	*0.02660372*	*0.016122722*
**ENSG00000160808**	*0.017342543*	*0.024402328*	*0.024815479*	*0.016019839*
**ENSG00000174306**	*0.028303097*	*0.031555588*	*0.029006127*	*0.026696114*

**ENSG00000157106**	*0.032160864*	*0.037368012*	*0.035647828*	*0.031899158*
**ENSG00000116731**	*0.030982824*	*0.041209661*	*0.036281992*	*0.032111557*
**ENSG00000187961**	*0.038103005*	*0.038832191*	*0.039474409*	*0.030751727*
**ENSG00000087842**	*0.062524708*	*0.042954551*	*0.039630655*	*0.04543245*
**ENSG00000153786**	*0.024162791*	*0.030022071*	*0.031897946*	*0.030269831*
**ENSG00000141179**	*0.044650582*	*0.042263436*	*0.048183961*	*0.044954296*
**ENSG00000104856**	*0.035572499*	*0.043069849*	*0.041441328*	*0.039634525*
**ENSG00000132535**	*0.019048837*	*0.029450547*	*0.027518744*	*0.021035171*
**ENSG00000182117**	*0.019534147*	*0.027574684*	*0.028317668*	*0.020572138*
**ENSG00000125611**	*0.022876348*	*0.028744316*	*0.02863483*	*0.021258714*
**ENSG00000135424**	*0.017491236*	*0.0248556*	*0.025243275*	*0.015022639*
**ENSG00000242802**	*0.045679736*	*0.036291777*	*0.033783925*	*0.038133648*
**ENSG00000139144**	*0.019004013*	*0.025383493*	*0.027456413*	*0.020810719*
**ENSG00000023191**	*0.030912048*	*0.030507988*	*0.028779196*	*0.024896742*
**ENSG00000151322**	*0.01573722*	*0.025583838*	*0.024533186*	*0.014741625*
**ENSG00000158516**	*0.043340963*	*0.027119499*	*0.025312522*	*0.022171841*
**ENSG00000175155**	*0.015386514*	*0.026868559*	*0.02536195*	*0.016963928*
**ENSG00000105327**	*0.024995817*	*0.035711157*	*0.033011691*	*0.030705443*
**ENSG00000188596**	*0.016408489*	*0.025046406*	*0.024278867*	*0.015965863*
**ENSG00000120217**	*0.040841377*	*0.040832826*	*0.046336838*	*0.041915396*
**ENSG00000171722**	*0.017402926*	*0.025696321*	*0.025321709*	*0.015910287*
**ENSG00000112320**	*0.073903333*	*0.051804818*	*0.054485012*	*0.062112614*
**ENSG00000185985**	*0.018968457*	*0.024892758*	*0.0255355*	*0.016745214*
**ENSG00000004961**	*0.028762303*	*0.034532992*	*0.033318003*	*0.028893521*
**ENSG00000137098**	*0.017872063*	*0.026280766*	*0.026671243*	*0.017440024*
**ENSG00000184886**	*0.044306014*	*0.040952247*	*0.041102575*	*0.048936944*
**ENSG00000157653**	*0.016200483*	*0.025399744*	*0.024219363*	*0.015954593*
**ENSG00000156171**	*0.033818745*	*0.039093033*	*0.038509221*	*0.03331666*
**ENSG00000174358**	*0.015332701*	*0.02557141*	*0.025790573*	*0.014789758*
**ENSG00000056972**	*0.018826079*	*0.028628761*	*0.028041544*	*0.018762433*
**ENSG00000153904**	*0.119689811*	*0.052673476*	*0.029941409*	*0.041943281*
**ENSG00000115677**	*0.016198547*	*0.026211043*	*0.026348933*	*0.016552719*
**ENSG00000144036**	*0.059739892*	*0.057571355*	*0.038642054*	*0.044264946*
**ENSG00000221880**	*0.017235952*	*0.024597332*	*0.026145132*	*0.01527371*
**ENSG00000120519**	*0.015282963*	*0.025076577*	*0.024792183*	*0.016612893*
**ENSG00000186088**	*0.058210009*	*0.050372026*	*0.042263865*	*0.040389862*
**ENSG00000141622**	*0.01561155*	*0.024681699*	*0.025595649*	*0.01494477*
**ENSG00000150676**	*0.017229149*	*0.026623056*	*0.025257129*	*0.017173511*
**ENSG00000125877**	*0.030453281*	*0.032090123*	*0.032570396*	*0.026570349*
**ENSG00000105879**	*0.030448779*	*0.045200728*	*0.050650539*	*0.031044332*
**ENSG00000104870**	*0.076119256*	*0.049968459*	*0.037439788*	*0.0514294*
**ENSG00000162642**	*0.034627026*	*0.034782687*	*0.031967966*	*0.025980999*
**ENSG00000101440**	*0.017978797*	*0.025378633*	*0.026445648*	*0.015466455*
**ENSG00000130119**	*0.031993996*	*0.03265705*	*0.034843541*	*0.03017093*
**ENSG00000198898**	*0.028630387*	*0.039306633*	*0.031399274*	*0.035431033*
**ENSG00000160161**	*0.01615238*	*0.025488467*	*0.024887934*	*0.016598547*
**ENSG00000082397**	*0.08847762*	*0.058582183*	*0.048487478*	*0.128984914*
**ENSG00000076053**	*0.031675537*	*0.038401625*	*0.035068252*	*0.029206372*
**ENSG00000078747**	*0.035857849*	*0.035239882*	*0.039380063*	*0.034411332*
**ENSG00000186174**	*0.018872241*	*0.028681581*	*0.027151789*	*0.020486057*
**ENSG00000204209**	*0.024553998*	*0.029944529*	*0.034010704*	*0.02578751*
**ENSG00000100938**	*0.025150495*	*0.027837311*	*0.028113462*	*0.021177694*
**ENSG00000186854**	*0.036096722*	*0.041822244*	*0.033265194*	*0.026065283*
**ENSG00000128641**	*0.066555877*	*0.070350995*	*0.045719094*	*0.05873353*

**ENSG00000151640**	*0.097300902*	*0.080838244*	*0.094211719*	*0.101962583*
**ENSG00000112511**	*0.04158871*	*0.042667988*	*0.046517544*	*0.047877706*
**ENSG00000116885**	*0.01598141*	*0.024919406*	*0.025351023*	*0.016092717*
**ENSG00000109208**	*0.014440718*	*0.024757911*	*0.024367319*	*0.01498757*
**ENSG00000141198**	*0.067297577*	*0.047207653*	*0.046758043*	*0.048684531*
**ENSG00000181626**	*0.016642072*	*0.024660976*	*0.024447824*	*0.015344398*
**ENSG00000166900**	*0.04615051*	*0.054593208*	*0.049200876*	*0.045580533*
**ENSG00000171189**	*0.016339316*	*0.023897401*	*0.024998731*	*0.015719502*
**ENSG00000104827**	*0.016877468*	*0.024774986*	*0.025054285*	*0.01513201*
**ENSG00000105771**	*0.024929575*	*0.032094498*	*0.031601354*	*0.028142802*
**ENSG00000163673**	*0.015224176*	*0.025331598*	*0.025211615*	*0.013895025*
**ENSG00000101981**	*0.01726497*	*0.02401622*	*0.024355797*	*0.016195619*
**ENSG00000113196**	*0.02314356*	*0.026470828*	*0.024889644*	*0.02218443*
**ENSG00000118922**	*0.062684148*	*0.053428565*	*0.05413084*	*0.047462109*
**ENSG00000132670**	*0.015806335*	*0.024563044*	*0.025465722*	*0.016452279*
**ENSG00000198821**	*0.030556798*	*0.032291206*	*0.027867266*	*0.029900115*
**ENSG00000177627**	*0.087125761*	*0.035044934*	*0.038176282*	*0.062171438*
**ENSG00000064205**	*0.015084515*	*0.025465877*	*0.024688068*	*0.01400589*
**ENSG00000148943**	*0.032120185*	*0.036333537*	*0.036628118*	*0.02660163*
**ENSG00000144504**	*0.014415334*	*0.024583718*	*0.024285541*	*0.014577067*
**ENSG00000187051**	*0.023521971*	*0.028550406*	*0.02809898*	*0.020606545*
**ENSG00000143318**	*0.048308081*	*0.031519432*	*0.029319205*	*0.026220767*
**ENSG00000185128**	*0.030949852*	*0.031444532*	*0.03251471*	*0.048649781*
**ENSG00000182646**	*0.029565412*	*0.031939245*	*0.031030052*	*0.024005956*
**ENSG00000187223**	*0.015246335*	*0.025183195*	*0.024432332*	*0.015504479*
**ENSG00000156256**	*0.027580354*	*0.033935532*	*0.034942111*	*0.029136957*
**ENSG00000119392**	*0.020993616*	*0.029455111*	*0.027148263*	*0.017988227*
**ENSG00000144339**	*0.019254378*	*0.02774011*	*0.027414688*	*0.020578165*
**ENSG00000145681**	*0.01850902*	*0.024583689*	*0.024271259*	*0.01513394*
**ENSG00000198339**	*0.024080632*	*0.032646676*	*0.039736395*	*0.030359914*
**ENSG00000142233**	*0.017150942*	*0.024933294*	*0.0264027*	*0.016591261*
**ENSG00000153495**	*0.017498565*	*0.02752296*	*0.024933672*	*0.01851231*
**ENSG00000138134**	*0.048414288*	*0.05921667*	*0.048547192*	*0.043339211*
**ENSG00000133111**	*0.016714337*	*0.02527569*	*0.024421329*	*0.015199446*
**ENSG00000171885**	*0.015678342*	*0.024056216*	*0.02532605*	*0.015813691*
**ENSG00000112667**	*0.016150318*	*0.02456248*	*0.02523315*	*0.016024624*
**ENSG00000204574**	*0.030770194*	*0.03377057*	*0.033896427*	*0.02746297*
**ENSG00000184916**	*0.016794394*	*0.026265502*	*0.026613956*	*0.017896785*
**ENSG00000163113**	*0.019700095*	*0.028081721*	*0.027627555*	*0.020863766*
**ENSG00000239839**	*0.016391498*	*0.025419396*	*0.024267247*	*0.015109176*
**ENSG00000116132**	*0.018969794*	*0.025778847*	*0.025909543*	*0.017924494*
**ENSG00000141946**	*0.01644005*	*0.025374657*	*0.02641859*	*0.015049479*
**ENSG00000165678**	*0.02470089*	*0.030072316*	*0.026400006*	*0.026760986*
**ENSG00000189120**	*0.016062759*	*0.025672288*	*0.027331103*	*0.015635121*
**ENSG00000109458**	*0.024532009*	*0.027234681*	*0.028867644*	*0.019212877*
**ENSG00000169375**	*0.026605782*	*0.030526685*	*0.032221753*	*0.025001764*
**ENSG00000139437**	*0.033836834*	*0.043011696*	*0.037534552*	*0.031342149*
**ENSG00000125733**	*0.032576007*	*0.033284617*	*0.031354504*	*0.031859631*
**ENSG00000154928**	*0.111338918*	*0.077802779*	*0.071039282*	*0.088577765*
**ENSG00000243135**	*0.015920949*	*0.024891431*	*0.02466351*	*0.0145085*
**ENSG00000197948**	*0.016255015*	*0.024896828*	*0.0252212*	*0.015034437*
**ENSG00000088247**	*0.0234799*	*0.027764401*	*0.029669741*	*0.028352772*
**ENSG00000102024**	*0.217922055*	*0.13821585*	*0.120053156*	*0.156266995*
**ENSG00000049247**	*0.015791289*	*0.061363171*	*0.045936634*	*0.030329052*

**ENSG00000164300**	*0.015713027*	*0.025766727*	*0.025272339*	*0.016542666*
**ENSG00000143416**	*0.015939368*	*0.026112613*	*0.026280923*	*0.015062612*
**ENSG00000197451**	*0.034463868*	*0.034057368*	*0.032783093*	*0.0338388*
**ENSG00000127955**	*0.018151717*	*0.026069243*	*0.024926103*	*0.015821285*
**ENSG00000078403**	*0.021149554*	*0.028086435*	*0.026659884*	*0.02414396*
**ENSG00000159063**	*0.033147006*	*0.035217569*	*0.030919365*	*0.028604117*
**ENSG00000173209**	*0.030635935*	*0.042037583*	*0.034245302*	*0.032385062*
**ENSG00000130779**	*0.024625204*	*0.031806802*	*0.028546866*	*0.018854437*
**ENSG00000196998**	*0.033414257*	*0.0325093*	*0.037397961*	*0.031743674*
**ENSG00000167658**	*0.018842483*	*0.025690445*	*0.025225929*	*0.019416328*
**ENSG00000111142**	*0.023922595*	*0.030109357*	*0.029926574*	*0.02045641*
**ENSG00000011465**	*0.016842707*	*0.026364409*	*0.025065656*	*0.016961446*
**ENSG00000154640**	*0.03141868*	*0.032219554*	*0.031632517*	*0.025718317*
**ENSG00000086159**	*0.016481378*	*0.024919051*	*0.024407009*	*0.014953399*
**ENSG00000187323**	*0.018326044*	*0.027278507*	*0.026290577*	*0.017164979*
**ENSG00000167916**	*0.017887806*	*0.027178247*	*0.028050453*	*0.017224936*
**ENSG00000169427**	*0.016698291*	*0.025688252*	*0.026334092*	*0.018385859*
**ENSG00000161904**	*0.029555026*	*0.032167093*	*0.033224908*	*0.02752278*
**ENSG00000164299**	*0.014802942*	*0.025139703*	*0.025006987*	*0.014881366*
**ENSG00000160602**	*0.035691722*	*0.038797211*	*0.039087685*	*0.032630601*
**ENSG00000088053**	*0.016613789*	*0.02450749*	*0.024819181*	*0.015207609*
**ENSG00000106524**	*0.038093605*	*0.038557467*	*0.036379922*	*0.03115214*
**ENSG00000169083**	*0.030397623*	*0.025526622*	*0.02545124*	*0.017804923*
**ENSG00000104879**	*0.015690996*	*0.024582027*	*0.025265146*	*0.014955702*
**ENSG00000134283**	*0.02919334*	*0.040294411*	*0.031485244*	*0.025420502*
**ENSG00000149507**	*0.015057059*	*0.024228447*	*0.026206228*	*0.014849699*
**ENSG00000113721**	*0.064716056*	*0.066684417*	*0.057182793*	*0.051829794*
**ENSG00000136250**	*0.019469941*	*0.025260528*	*0.025707539*	*0.016795361*
**ENSG00000128626**	*0.015955375*	*0.025430532*	*0.025131159*	*0.017645216*
**ENSG00000197275**	*0.015528955*	*0.024634682*	*0.024820817*	*0.016544439*
**ENSG00000185634**	*0.03329468*	*0.033684929*	*0.02990229*	*0.025001737*
**ENSG00000102309**	*0.031523752*	*0.026860747*	*0.026492006*	*0.021751719*
**ENSG00000105509**	*0.016252998*	*0.026139475*	*0.025041719*	*0.016345194*
**ENSG00000163947**	*0.067570757*	*0.058942239*	*0.062358926*	*0.062777076*
**ENSG00000169981**	*0.036161248*	*0.065227625*	*0.067407879*	*0.051504544*
**ENSG00000164040**	*0.034070269*	*0.034163196*	*0.03515596*	*0.025820289*
**ENSG00000187908**	*0.017821811*	*0.026604063*	*0.0257071*	*0.017321923*
**ENSG00000132170**	*0.125191558*	*0.092222357*	*0.073986445*	*0.093286763*
**ENSG00000106771**	*0.030493629*	*0.035298449*	*0.02912682*	*0.028833322*
**ENSG00000187535**	*0.015571266*	*0.026667244*	*0.025801367*	*0.017638843*
**ENSG00000117643**	*0.029295106*	*0.036793549*	*0.034815226*	*0.040428376*
**ENSG00000164506**	*0.039447071*	*0.045361569*	*0.043919387*	*0.045014677*
**ENSG00000100320**	*0.01647093*	*0.025084023*	*0.024618234*	*0.01526643*
**ENSG00000157551**	*0.037243565*	*0.026039641*	*0.025647419*	*0.015707905*
**ENSG00000148400**	*0.046281811*	*0.048648677*	*0.041684185*	*0.043587849*
**ENSG00000101850**	*0.016892439*	*0.024569175*	*0.024941897*	*0.0157943*
**ENSG00000186487**	*0.016690771*	*0.025263911*	*0.024582195*	*0.015856354*
**ENSG00000135643**	*0.044067085*	*0.042711084*	*0.041184872*	*0.043068895*
**ENSG00000135637**	*0.019395503*	*0.029184404*	*0.025452055*	*0.023168194*
**ENSG00000143924**	*0.039775443*	*0.036886731*	*0.037847441*	*0.035054753*
**ENSG00000156853**	*0.03637793*	*0.043604068*	*0.04459203*	*0.035525988*
**ENSG00000068796**	*0.025752652*	*0.037341898*	*0.032653789*	*0.025005345*
**ENSG00000100380**	*0.026409435*	*0.028456361*	*0.027467907*	*0.0296406*
**ENSG00000072135**	*0.030109439*	*0.032697716*	*0.033074598*	*0.02917269*

**ENSG00000163794**	*0.023916737*	*0.028304782*	*0.028414714*	*0.026983998*
**ENSG00000028277**	*0.035367835*	*0.034303586*	*0.037196398*	*0.03547272*
**ENSG00000139289**	*0.024684425*	*0.033768988*	*0.027072589*	*0.023519539*
**ENSG00000138709**	*0.047278299*	*0.04453627*	*0.047725082*	*0.04636924*
**ENSG00000175575**	*0.030536543*	*0.038807928*	*0.035295873*	*0.022933403*
**ENSG00000154027**	*0.019023687*	*0.026510832*	*0.029379557*	*0.017598038*
**ENSG00000106031**	*0.017802591*	*0.025652983*	*0.024209012*	*0.016907397*
**ENSG00000185915**	*0.024448196*	*0.030000258*	*0.032415155*	*0.027371422*
**ENSG00000151729**	*0.033945876*	*0.032216777*	*0.034140298*	*0.030464698*
**ENSG00000084463**	*0.023138093*	*0.02884468*	*0.029407042*	*0.023217245*
**ENSG00000198408**	*0.022665179*	*0.030676162*	*0.029994089*	*0.020613637*
**ENSG00000241186**	*0.015496988*	*0.025366938*	*0.025417712*	*0.01661967*
**ENSG00000108443**	*0.019251559*	*0.026069779*	*0.027577484*	*0.020820274*
**ENSG00000110975**	*0.018602008*	*0.025456436*	*0.025980586*	*0.016459612*
**ENSG00000162341**	*0.050594895*	*0.049069381*	*0.043678873*	*0.042995141*
**ENSG00000132394**	*0.028350487*	*0.028785077*	*0.03001438*	*0.027596297*
**ENSG00000171311**	*0.018087468*	*0.026063744*	*0.029106739*	*0.017661911*
**ENSG00000185670**	*0.027866896*	*0.036186721*	*0.036884625*	*0.024998548*
**ENSG00000099219**	*0.044360182*	*0.041464234*	*0.037355273*	*0.038663117*
**ENSG00000148484**	*0.044515979*	*0.052036964*	*0.043929937*	*0.044689529*
**ENSG00000197361**	*0.024941611*	*0.028982395*	*0.029614264*	*0.025222475*
**ENSG00000065518**	*0.017732369*	*0.026807179*	*0.025757834*	*0.015983379*
**ENSG00000046651**	*0.035539935*	*0.040714875*	*0.039571228*	*0.034593966*
**ENSG00000179148**	*0.01851583*	*0.025198832*	*0.025149247*	*0.015075878*
**ENSG00000165997**	*0.038382195*	*0.043660136*	*0.045717241*	*0.038254822*
**ENSG00000162777**	*0.054706209*	*0.060087212*	*0.058771555*	*0.048000917*
**ENSG00000182472**	*0.047678223*	*0.05185399*	*0.042845233*	*0.053319412*
**ENSG00000241058**	*0.038552151*	*0.040185427*	*0.037417607*	*0.029474097*
**ENSG00000117971**	*0.017429712*	*0.025640595*	*0.025137239*	*0.015816647*
**ENSG00000110172**	*0.049936569*	*0.060844803*	*0.050693868*	*0.0402766*
**ENSG00000168701**	*0.030753948*	*0.033920913*	*0.038936343*	*0.031955538*
**ENSG00000091482**	*0.016377866*	*0.025576151*	*0.024490749*	*0.015335487*
**ENSG00000221843**	*0.016109762*	*0.024831944*	*0.024532139*	*0.015217589*
**ENSG00000128918**	*0.027025928*	*0.024892137*	*0.025378356*	*0.017531947*
**ENSG00000072858**	*0.037182252*	*0.044541898*	*0.035502478*	*0.035048982*
**ENSG00000143105**	*0.016888868*	*0.02609741*	*0.025401729*	*0.015592891*
**ENSG00000133030**	*0.030967279*	*0.035906799*	*0.032227477*	*0.02728501*
**ENSG00000175785**	*0.017058856*	*0.025033961*	*0.02628611*	*0.016407497*
**ENSG00000156689**	*0.04197554*	*0.036829007*	*0.030259204*	*0.023165845*
**ENSG00000183914**	*0.015775504*	*0.025966194*	*0.026077989*	*0.015295117*
**ENSG00000072201**	*0.036341481*	*0.041134429*	*0.026259869*	*0.016453974*
**ENSG00000136573**	*0.06130535*	*0.072314539*	*0.070715427*	*0.058312793*
**ENSG00000172005**	*0.105210679*	*0.084767058*	*0.079267797*	*0.090332721*
**ENSG00000170684**	*0.035274304*	*0.030712419*	*0.03031917*	*0.031900496*
**ENSG00000100170**	*0.016690807*	*0.026271939*	*0.025418281*	*0.016410428*
**ENSG00000140948**	*0.051475122*	*0.042331972*	*0.034922838*	*0.045374533*
**ENSG00000229676**	*0.030343727*	*0.032305183*	*0.03130773*	*0.027790999*
**ENSG00000100362**	*0.015402939*	*0.025790068*	*0.025020091*	*0.015601135*
**ENSG00000157827**	*0.09328674*	*0.068749902*	*0.065499946*	*0.071911109*
**ENSG00000129450**	*0.015062531*	*0.025012837*	*0.024628912*	*0.015232755*
**ENSG00000213339**	*0.032951055*	*0.033892467*	*0.030037744*	*0.026197654*
**ENSG00000160799**	*0.026538367*	*0.03013756*	*0.033310976*	*0.026725418*
**ENSG00000170166**	*0.016420761*	*0.024600907*	*0.024408824*	*0.015120506*
**ENSG00000100429**	*0.017992198*	*0.02866778*	*0.028137595*	*0.016734718*

**ENSG00000089597**	*0.030129579*	*0.029022022*	*0.035336073*	*0.027471863*
**ENSG00000144550**	*0.019084357*	*0.026369964*	*0.024958014*	*0.017147592*
**ENSG00000175463**	*0.032029943*	*0.030389571*	*0.033724995*	*0.03089836*
**ENSG00000149499**	*0.024720536*	*0.028969087*	*0.030785718*	*0.026185453*
**ENSG00000095203**	*0.016416461*	*0.026042629*	*0.025798433*	*0.01669305*
**ENSG00000185008**	*0.032883323*	*0.025671283*	*0.024628427*	*0.015910083*
**ENSG00000148344**	*0.015170888*	*0.02513278*	*0.025066724*	*0.014702885*
**ENSG00000141744**	*0.019828081*	*0.026955595*	*0.025979509*	*0.019188108*
**ENSG00000075975**	*0.025186863*	*0.03248365*	*0.035997878*	*0.0262369*
**ENSG00000183066**	*0.017136498*	*0.026261525*	*0.025919575*	*0.02330951*
**ENSG00000116983**	*0.020817332*	*0.02606958*	*0.025944572*	*0.017559406*
**ENSG00000168925**	*0.015138014*	*0.024777351*	*0.024789537*	*0.014853935*
**ENSG00000064012**	*0.039780955*	*0.037941154*	*0.033164514*	*0.032510351*
**ENSG00000184345**	*0.015902363*	*0.02554277*	*0.024995188*	*0.014683617*
**ENSG00000138646**	*0.050783771*	*0.047254837*	*0.038530112*	*0.038343973*
**ENSG00000169432**	*0.016189609*	*0.024759328*	*0.024619863*	*0.01495561*
**ENSG00000164144**	*0.026522253*	*0.033815939*	*0.030934642*	*0.025941343*
**ENSG00000180205**	*0.017378852*	*0.025472644*	*0.024527991*	*0.016758393*
**ENSG00000087157**	*0.027804117*	*0.032957007*	*0.033949165*	*0.02988805*
**ENSG00000069424**	*0.022408345*	*0.026756916*	*0.030535375*	*0.021426168*
**ENSG00000127328**	*0.034305078*	*0.034799816*	*0.03458402*	*0.031516253*
**ENSG00000198610**	*0.060612105*	*0.028476554*	*0.025104491*	*0.019524167*
**ENSG00000164615**	*0.027939046*	*0.031453082*	*0.031088016*	*0.023825594*
**ENSG00000100068**	*0.056342785*	*0.049509842*	*0.04205413*	*0.050328579*
**ENSG00000124155**	*0.032208658*	*0.033889649*	*0.034812231*	*0.025671488*
**ENSG00000196150**	*0.025936874*	*0.028212705*	*0.030155786*	*0.019822691*
**ENSG00000196535**	*0.016833066*	*0.026216477*	*0.025182141*	*0.014786207*
**ENSG00000176973**	*0.037793459*	*0.043242138*	*0.045842017*	*0.034441038*
**ENSG00000172232**	*0.016106942*	*0.025487339*	*0.02507858*	*0.017527053*
**ENSG00000134013**	*0.066150903*	*0.055503165*	*0.069629675*	*0.062237357*
**ENSG00000137486**	*0.020608878*	*0.028513142*	*0.026883211*	*0.01937581*
**ENSG00000012171**	*0.016187897*	*0.025554458*	*0.025101906*	*0.015490844*
**ENSG00000138686**	*0.031148705*	*0.040338192*	*0.034804677*	*0.031387748*
**ENSG00000115464**	*0.029154634*	*0.039187249*	*0.033200058*	*0.02589162*
**ENSG00000174595**	*0.023722734*	*0.026735798*	*0.026029227*	*0.021897191*
**ENSG00000124664**	*0.01601871*	*0.025372478*	*0.025884682*	*0.014545026*
**ENSG00000101004**	*0.061037269*	*0.056843017*	*0.066148249*	*0.063478724*
**ENSG00000013293**	*0.018721041*	*0.025899922*	*0.027348163*	*0.016729511*
**ENSG00000196735**	*0.331149452*	*0.391001966*	*0.283226671*	*0.32766191*
**ENSG00000159921**	*0.032556958*	*0.036567147*	*0.036398263*	*0.028584975*
**ENSG00000180071**	*0.017766105*	*0.025799981*	*0.02527947*	*0.018621952*
**ENSG00000158270**	*0.100317346*	*0.05610344*	*0.054078573*	*0.079201056*
**ENSG00000105671**	*0.036246027*	*0.038092196*	*0.033295201*	*0.035337023*
**ENSG00000197403**	*0.016076232*	*0.025274545*	*0.024530857*	*0.016801052*
**ENSG00000077235**	*0.024164751*	*0.030165955*	*0.035374859*	*0.026707221*
**ENSG00000011028**	*0.032391836*	*0.031608852*	*0.029877367*	*0.026134001*
**ENSG00000164180**	*0.023125031*	*0.029863878*	*0.028916239*	*0.020125799*
**ENSG00000162897**	*0.019436959*	*0.027060124*	*0.026618587*	*0.018295992*
**ENSG00000174611**	*0.017672743*	*0.025043188*	*0.025860347*	*0.016653046*
**ENSG00000126860**	*0.04654253*	*0.049369308*	*0.039683119*	*0.041083793*
**ENSG00000135324**	*0.015671605*	*0.025608129*	*0.024446148*	*0.016695659*
**ENSG00000176720**	*0.015689464*	*0.025805778*	*0.0254361*	*0.015448536*
**ENSG00000159784**	*0.015300761*	*0.025029153*	*0.024800376*	*0.016865097*
**ENSG00000068912**	*0.032350827*	*0.036538131*	*0.041602261*	*0.03386383*

**ENSG00000181222**	*0.02508174*	*0.029858315*	*0.039867825*	*0.026266079*
**ENSG00000090686**	*0.031997762*	*0.035415892*	*0.031764654*	*0.02489152*
**ENSG00000198393**	*0.034395772*	*0.035058061*	*0.033588509*	*0.027527352*
**ENSG00000103740**	*0.03897952*	*0.038163115*	*0.028947119*	*0.024690133*
**ENSG00000133226**	*0.02214481*	*0.028891778*	*0.02779246*	*0.016886524*
**ENSG00000153707**	*0.019130821*	*0.027065037*	*0.026030467*	*0.019053685*
**ENSG00000135457**	*0.041841148*	*0.048477559*	*0.036103091*	*0.036408646*
**ENSG00000183665**	*0.035189746*	*0.033032311*	*0.047472596*	*0.037100288*
**ENSG00000152910**	*0.017268737*	*0.024623357*	*0.025965339*	*0.016508328*
**ENSG00000100453**	*0.028209158*	*0.036104623*	*0.039449296*	*0.025263818*
**ENSG00000188613**	*0.019841589*	*0.028083705*	*0.027104683*	*0.020005317*
**ENSG00000160221**	*0.027553486*	*0.033858234*	*0.029066183*	*0.02979036*
**ENSG00000183439**	*0.05227931*	*0.040470731*	*0.03456834*	*0.036444025*
**ENSG00000134575**	*0.026710099*	*0.033141173*	*0.033405893*	*0.029351094*
**ENSG00000132002**	*0.042924962*	*0.033511989*	*0.031384992*	*0.031410931*
**ENSG00000214050**	*0.033396407*	*0.033135814*	*0.033631506*	*0.029735774*
**ENSG00000166394**	*0.102829957*	*0.077897594*	*0.071475001*	*0.085649647*
**ENSG00000102384**	*0.043391622*	*0.048250837*	*0.033664618*	*0.029165202*
**ENSG00000204889**	*0.018591355*	*0.026172696*	*0.026430464*	*0.018379504*
**ENSG00000130513**	*0.095438545*	*0.068142492*	*0.073133741*	*0.086714193*
**ENSG00000186017**	*0.026554004*	*0.030306608*	*0.027395373*	*0.024682684*
**ENSG00000135903**	*0.014956949*	*0.0246308*	*0.024683454*	*0.014624391*
**ENSG00000143537**	*0.04206206*	*0.041871018*	*0.037723231*	*0.036754428*
**ENSG00000115468**	*0.016467786*	*0.024559856*	*0.025715995*	*0.016295683*
**ENSG00000127419**	*0.037248351*	*0.049358989*	*0.040999566*	*0.035227903*
**ENSG00000112144**	*0.022901965*	*0.026989773*	*0.028422886*	*0.021524603*
**ENSG00000116584**	*0.025471491*	*0.031901332*	*0.031605379*	*0.025475343*
**ENSG00000162851**	*0.031183702*	*0.035968778*	*0.040248841*	*0.035101038*
**ENSG00000198108**	*0.017069519*	*0.025803622*	*0.024895809*	*0.015603572*
**ENSG00000170445**	*0.019235882*	*0.028517641*	*0.028151096*	*0.022328914*
**ENSG00000074181**	*0.014935958*	*0.024806016*	*0.024580006*	*0.014235546*
**ENSG00000241697**	*0.017204314*	*0.027696386*	*0.025227005*	*0.017271561*
**ENSG00000159388**	*0.033523905*	*0.032963483*	*0.03134753*	*0.027824405*
**ENSG00000140406**	*0.038965977*	*0.04660064*	*0.05346841*	*0.043641682*
**ENSG00000178394**	*0.015335896*	*0.024797888*	*0.024856545*	*0.015426726*
**ENSG00000180658**	*0.017952935*	*0.025274854*	*0.02563217*	*0.015326317*
**ENSG00000070214**	*0.06361619*	*0.051302078*	*0.043356673*	*0.046135524*
**ENSG00000142698**	*0.016081239*	*0.024182574*	*0.024691689*	*0.015924132*
**ENSG00000117148**	*0.01749336*	*0.026061257*	*0.025343873*	*0.016647177*
**ENSG00000169964**	*0.032821648*	*0.03335983*	*0.033244457*	*0.028157186*
**ENSG00000154620**	*0.16768477*	*0.151314*	*0.138781254*	*0.144325397*
**ENSG00000118200**	*0.036648672*	*0.042808512*	*0.040917621*	*0.042590977*
**ENSG00000136514**	*0.048497962*	*0.049798493*	*0.044674719*	*0.041614139*
**ENSG00000253537**	*0.017454879*	*0.025328646*	*0.024579783*	*0.01596127*
**ENSG00000164305**	*0.017640405*	*0.025542625*	*0.025079247*	*0.015673704*
**ENSG00000146385**	*0.015499549*	*0.024426024*	*0.025091811*	*0.016699445*
**ENSG00000173889**	*0.032029917*	*0.037176762*	*0.037384167*	*0.025831949*
**ENSG00000065989**	*0.062875682*	*0.055638865*	*0.067252355*	*0.045510524*
**ENSG00000189366**	*0.022312848*	*0.028645247*	*0.027257381*	*0.022850227*
**ENSG00000104112**	*0.021242587*	*0.028531891*	*0.026358927*	*0.022392941*
**ENSG00000026508**	*0.047665576*	*0.046279538*	*0.051361509*	*0.055993413*
**ENSG00000174943**	*0.026236009*	*0.031728941*	*0.031545151*	*0.02811746*
**ENSG00000161203**	*0.028596671*	*0.035439132*	*0.031005858*	*0.024429073*
**ENSG00000163453**	*0.015769884*	*0.025154026*	*0.024712303*	*0.016179*

**ENSG00000243452**	*0.028075871*	*0.03357365*	*0.030063376*	*0.025071964*
**ENSG00000181778**	*0.015852575*	*0.025109208*	*0.025176475*	*0.015508155*
**ENSG00000031698**	*0.021344228*	*0.026641233*	*0.03062093*	*0.018411032*
**ENSG00000212938**	*0.015853432*	*0.024866889*	*0.025032054*	*0.014838711*
**ENSG00000112365**	*0.033734381*	*0.035970546*	*0.035837092*	*0.029105958*
**ENSG00000122484**	*0.029933229*	*0.036415966*	*0.030090139*	*0.023886147*
**ENSG00000119661**	*0.019622306*	*0.02689981*	*0.026500073*	*0.017865272*
**ENSG00000080608**	*0.032131064*	*0.032529403*	*0.031226106*	*0.031085134*
**ENSG00000169679**	*0.035421465*	*0.042170814*	*0.034934856*	*0.028968148*
**ENSG00000130489**	*0.031033508*	*0.039777104*	*0.044782828*	*0.028734758*
**ENSG00000003400**	*0.016056454*	*0.025067597*	*0.025658154*	*0.018270594*
**ENSG00000107438**	*0.034151284*	*0.03356279*	*0.034973099*	*0.036118633*
**ENSG00000075407**	*0.034808591*	*0.035466301*	*0.034915857*	*0.035404714*
**ENSG00000108064**	*0.030148491*	*0.044201901*	*0.047642648*	*0.024924736*
**ENSG00000128699**	*0.056048905*	*0.061357819*	*0.059150114*	*0.049060717*
**ENSG00000149182**	*0.024809688*	*0.029226445*	*0.035551529*	*0.027095325*
**ENSG00000186918**	*0.080917183*	*0.047431831*	*0.049527861*	*0.05148218*
**ENSG00000162385**	*0.028956448*	*0.03374509*	*0.032214834*	*0.024921179*
**ENSG00000197696**	*0.036157513*	*0.043333356*	*0.037035449*	*0.031729826*
**ENSG00000165629**	*0.0204377*	*0.027066676*	*0.025013729*	*0.016477455*
**ENSG00000128513**	*0.034063902*	*0.040564945*	*0.033289995*	*0.037292441*
**ENSG00000254014**	*0.02700847*	*0.030483416*	*0.030781471*	*0.026013134*
**ENSG00000142875**	*0.016032197*	*0.024690629*	*0.026510685*	*0.015429753*
**ENSG00000100347**	*0.024648257*	*0.029253057*	*0.030199027*	*0.024137976*
**ENSG00000132467**	*0.025525503*	*0.03427502*	*0.037180552*	*0.026795008*
**ENSG00000162624**	*0.015482816*	*0.025154424*	*0.024880936*	*0.015246502*
**ENSG00000068137**	*0.046493744*	*0.037665133*	*0.041905593*	*0.041492168*
**ENSG00000129167**	*0.016672176*	*0.025534784*	*0.02560252*	*0.015198995*
**ENSG00000204542**	*0.015956323*	*0.025213014*	*0.024676829*	*0.017279649*
**ENSG00000157219**	*0.014695021*	*0.025361718*	*0.025484545*	*0.015602541*
**ENSG00000175697**	*0.017116738*	*0.025692256*	*0.027329771*	*0.017048237*
**ENSG00000179292**	*0.016970031*	*0.02522455*	*0.024918826*	*0.015426013*
**ENSG00000197472**	*0.06870288*	*0.040229815*	*0.044102909*	*0.050534699*
**ENSG00000104946**	*0.040294594*	*0.046388686*	*0.038702553*	*0.039676725*
**ENSG00000043093**	*0.030276757*	*0.036843816*	*0.034656603*	*0.024925407*
**ENSG00000115652**	*0.040784227*	*0.046089926*	*0.039189441*	*0.03961619*
**ENSG00000166147**	*0.059899927*	*0.063037587*	*0.056849933*	*0.069581123*
**ENSG00000135925**	*0.089151122*	*0.064906536*	*0.079263342*	*0.07761203*
**ENSG00000119681**	*0.017666869*	*0.025637698*	*0.024882688*	*0.015072945*
**ENSG00000167186**	*0.033506832*	*0.032398454*	*0.032206133*	*0.029335629*
**ENSG00000197123**	*0.016158999*	*0.024635561*	*0.024394164*	*0.015170297*
**ENSG00000167419**	*0.017575998*	*0.026579066*	*0.025121897*	*0.015686811*
**ENSG00000141524**	*0.033198987*	*0.031258695*	*0.035354462*	*0.030802323*
**ENSG00000204381**	*0.068612646*	*0.041460589*	*0.032617261*	*0.055680884*
**ENSG00000113302**	*0.051642451*	*0.039436468*	*0.050945636*	*0.044235615*
**ENSG00000184144**	*0.022645098*	*0.029605735*	*0.027849238*	*0.01971271*
**ENSG00000146776**	*0.015804494*	*0.024539591*	*0.025446167*	*0.014377249*
**ENSG00000112078**	*0.022356418*	*0.02857413*	*0.028566585*	*0.018769449*
**ENSG00000100079**	*0.055411051*	*0.025227524*	*0.027446656*	*0.016351959*
**ENSG00000188386**	*0.017971823*	*0.027761814*	*0.026684132*	*0.017036982*
**ENSG00000101966**	*0.03291145*	*0.030358459*	*0.034389284*	*0.024362821*
**ENSG00000130150**	*0.034925816*	*0.037722605*	*0.033277112*	*0.028141603*
**ENSG00000148459**	*0.03699216*	*0.035165129*	*0.032119221*	*0.038700241*
**ENSG00000171246**	*0.015966722*	*0.025148717*	*0.025769899*	*0.02816293*

**ENSG00000163161**	*0.022067273*	*0.031115379*	*0.034564415*	*0.02485458*
**ENSG00000168724**	*0.025185576*	*0.030362761*	*0.032333213*	*0.023540738*
**ENSG00000125247**	*0.044108855*	*0.040233079*	*0.034334179*	*0.033562958*
**ENSG00000197249**	*0.076297039*	*0.039001619*	*0.029153441*	*0.059581192*
**ENSG00000066084**	*0.036921007*	*0.036179821*	*0.037551268*	*0.036828292*
**ENSG00000130940**	*0.052869393*	*0.045571427*	*0.040069061*	*0.046703166*
**ENSG00000187626**	*0.030255706*	*0.035057889*	*0.032054389*	*0.023392814*
**ENSG00000139178**	*0.029492243*	*0.036214157*	*0.030848467*	*0.026327446*
**ENSG00000178538**	*0.022650287*	*0.025492181*	*0.025001721*	*0.020488665*
**ENSG00000213231**	*0.016763338*	*0.025151201*	*0.024722455*	*0.016020855*
**ENSG00000172689**	*0.01607832*	*0.02453622*	*0.025968044*	*0.016585663*
**ENSG00000198855**	*0.039780788*	*0.039036109*	*0.053372158*	*0.045419843*
**ENSG00000215018**	*0.018588404*	*0.026971826*	*0.027405802*	*0.020105152*
**ENSG00000135766**	*0.053995114*	*0.043475717*	*0.042699355*	*0.039945441*
**ENSG00000102796**	*0.033694772*	*0.033498996*	*0.036762031*	*0.028855091*
**ENSG00000179299**	*0.03538459*	*0.040238299*	*0.030087219*	*0.023131359*
**ENSG00000131873**	*0.032293112*	*0.034341709*	*0.032149652*	*0.025691006*
**ENSG00000074800**	*0.018138178*	*0.024816755*	*0.024687919*	*0.016464194*
**ENSG00000175054**	*0.033593266*	*0.039732448*	*0.03123267*	*0.029638827*
**ENSG00000166965**	*0.030754713*	*0.0310613*	*0.028300561*	*0.023758411*
**ENSG00000131711**	*0.059033006*	*0.034294442*	*0.026118006*	*0.024754547*
**ENSG00000172428**	*0.014976216*	*0.025359167*	*0.024764678*	*0.014272547*
**ENSG00000151458**	*0.054890945*	*0.044782745*	*0.039998559*	*0.042237137*
**ENSG00000189410**	*0.026339364*	*0.03478876*	*0.028839405*	*0.022912659*
**ENSG00000118557**	*0.017135683*	*0.026790704*	*0.025008644*	*0.017427048*
**ENSG00000184428**	*0.032988364*	*0.03565335*	*0.036260412*	*0.047139349*
**ENSG00000125772**	*0.038896682*	*0.039887666*	*0.043916571*	*0.040042877*
**ENSG00000140365**	*0.020125786*	*0.026695277*	*0.025588401*	*0.017779284*
**ENSG00000154803**	*0.038965083*	*0.040363204*	*0.038386191*	*0.026574764*
**ENSG00000156467**	*0.033701023*	*0.035542787*	*0.033167561*	*0.025210628*
**ENSG00000103089**	*0.051913871*	*0.062401904*	*0.051106222*	*0.051054912*
**ENSG00000166979**	*0.018443488*	*0.026029513*	*0.026087777*	*0.016274487*
**ENSG00000130590**	*0.020463708*	*0.026604324*	*0.026081628*	*0.020120905*
**ENSG00000162009**	*0.016685538*	*0.025619288*	*0.024532514*	*0.015692798*
**ENSG00000159023**	*0.018297675*	*0.025499701*	*0.026098736*	*0.016011825*
**ENSG00000196498**	*0.021876561*	*0.031435749*	*0.033460696*	*0.025207234*
**ENSG00000164051**	*0.029495941*	*0.032441354*	*0.028839887*	*0.021471643*
**ENSG00000225485**	*0.016042404*	*0.024942463*	*0.025270265*	*0.01582117*
**ENSG00000006638**	*0.014952535*	*0.024444316*	*0.025177067*	*0.014301003*
**ENSG00000166165**	*0.096875857*	*0.06535707*	*0.06179984*	*0.079716247*
**ENSG00000145649**	*0.01438675*	*0.02589983*	*0.025413965*	*0.024733709*
**ENSG00000138835**	*0.015996422*	*0.02585325*	*0.024827012*	*0.015466788*
**ENSG00000105428**	*0.01482555*	*0.024201701*	*0.024436796*	*0.014523308*
**ENSG00000100225**	*0.019952154*	*0.028868882*	*0.032422811*	*0.021979651*
**ENSG00000101746**	*0.073636704*	*0.030422622*	*0.025805304*	*0.025854292*
**ENSG00000091879**	*0.015295929*	*0.025189136*	*0.025237803*	*0.014198943*
**ENSG00000111358**	*0.036531835*	*0.044439463*	*0.035880226*	*0.042275477*
**ENSG00000112559**	*0.016393302*	*0.024870685*	*0.025731415*	*0.016173101*
**ENSG00000163053**	*0.043214068*	*0.032032235*	*0.032273327*	*0.029091415*
**ENSG00000171729**	*0.143554414*	*0.095664814*	*0.096107969*	*0.111462022*
**ENSG00000108784**	*0.029475181*	*0.031234772*	*0.031562486*	*0.024364861*
**ENSG00000129204**	*0.036246609*	*0.040129029*	*0.035915261*	*0.041497724*
**ENSG00000196233**	*0.034356814*	*0.039838125*	*0.045206288*	*0.030293374*
**ENSG00000125731**	*0.020812994*	*0.026948408*	*0.028825775*	*0.021327317*

**ENSG00000196776**	*0.030332558*	*0.041153388*	*0.045025017*	*0.035753754*
**ENSG00000111262**	*0.033601332*	*0.03119579*	*0.027715269*	*0.026816443*
**ENSG00000186160**	*0.015321155*	*0.024620806*	*0.025470661*	*0.014547293*
**ENSG00000103197**	*0.015433511*	*0.024965543*	*0.024914583*	*0.015981873*
**ENSG00000138613**	*0.032225995*	*0.04670985*	*0.043981876*	*0.028039167*
**ENSG00000136048**	*0.037263571*	*0.046746323*	*0.047896974*	*0.035598178*
**ENSG00000176155**	*0.015794481*	*0.025688755*	*0.025386471*	*0.017869246*
**ENSG00000134690**	*0.043213863*	*0.03761903*	*0.038026465*	*0.036421433*
**ENSG00000176148**	*0.017510423*	*0.026422574*	*0.026604441*	*0.017819249*
**ENSG00000185305**	*0.03013984*	*0.031927063*	*0.02897159*	*0.022132208*
**ENSG00000076716**	*0.17265957*	*0.11641444*	*0.102407962*	*0.129722524*
**ENSG00000180974**	*0.022459043*	*0.024770732*	*0.02433629*	*0.019947485*
**ENSG00000171608**	*0.026866504*	*0.030840391*	*0.02911911*	*0.028268943*
**ENSG00000187372**	*0.019849936*	*0.026668611*	*0.026782568*	*0.018863604*
**ENSG00000172890**	*0.027667773*	*0.033079894*	*0.031247645*	*0.028210648*
**ENSG00000144747**	*0.038788143*	*0.04130449*	*0.042302315*	*0.036242942*
**ENSG00000180433**	*0.016273109*	*0.025441547*	*0.023969181*	*0.016096253*
**ENSG00000172927**	*0.082384506*	*0.073065661*	*0.069260268*	*0.098450902*
**ENSG00000149091**	*0.017087519*	*0.026267073*	*0.025308643*	*0.015649245*
**ENSG00000118523**	*0.015301299*	*0.025038947*	*0.026152922*	*0.016599738*
**ENSG00000164105**	*0.039493824*	*0.038930485*	*0.036465498*	*0.03254776*
**ENSG00000174937**	*0.017012592*	*0.025696351*	*0.024363258*	*0.015483004*
**ENSG00000176029**	*0.01793703*	*0.026774188*	*0.026996595*	*0.015157995*
**ENSG00000179912**	*0.0354549*	*0.035407485*	*0.031107671*	*0.034009147*
**ENSG00000064601**	*0.032818085*	*0.037212093*	*0.033900065*	*0.030128402*
**ENSG00000126217**	*0.016383232*	*0.024800753*	*0.024849309*	*0.014921755*
**ENSG00000114270**	*0.047925625*	*0.044845004*	*0.035400644*	*0.045020666*
**ENSG00000174325**	*0.015650534*	*0.025493143*	*0.024917564*	*0.014556622*
**ENSG00000198918**	*0.014042003*	*0.023838595*	*0.023849453*	*0.015839742*
**ENSG00000179930**	*0.015284634*	*0.025117438*	*0.024575305*	*0.015494018*
**ENSG00000086062**	*0.03665265*	*0.037743495*	*0.047080688*	*0.046274961*
**ENSG00000143257**	*0.015853234*	*0.024304766*	*0.025367649*	*0.016097255*
**ENSG00000067369**	*0.026810515*	*0.03435654*	*0.029703782*	*0.021085081*
**ENSG00000196407**	*0.015800116*	*0.024527816*	*0.024891769*	*0.016743572*
**ENSG00000196557**	*0.023113067*	*0.030720972*	*0.030654419*	*0.02373514*
**ENSG00000182732**	*0.083503497*	*0.038380157*	*0.030688761*	*0.047862464*
**ENSG00000100099**	*0.016706434*	*0.025140931*	*0.025571438*	*0.016135688*
**ENSG00000185862**	*0.040237697*	*0.040913068*	*0.040992033*	*0.034574612*
**ENSG00000121152**	*0.04271697*	*0.034425771*	*0.036941785*	*0.03462744*
**ENSG00000049167**	*0.033816072*	*0.039669038*	*0.031619954*	*0.029241406*
**ENSG00000124222**	*0.032262471*	*0.044573489*	*0.036932702*	*0.031139464*
**ENSG00000143801**	*0.039621344*	*0.037391623*	*0.037614861*	*0.034345696*
**ENSG00000175756**	*0.022201854*	*0.026616461*	*0.02659324*	*0.01933684*
**ENSG00000138382**	*0.023833321*	*0.029677869*	*0.02781799*	*0.019218639*
**ENSG00000104626**	*0.037196557*	*0.036563871*	*0.031352313*	*0.038451529*
**ENSG00000126709**	*0.05373567*	*0.046788744*	*0.040181029*	*0.037838816*
**ENSG00000106052**	*0.026177532*	*0.03371801*	*0.031351886*	*0.02866746*
**ENSG00000204538**	*0.017458699*	*0.024457576*	*0.025169939*	*0.016418583*
**ENSG00000134532**	*0.016812182*	*0.025715278*	*0.025404463*	*0.016087558*
**ENSG00000156052**	*0.016295326*	*0.02412283*	*0.025584595*	*0.015128205*
**ENSG00000172346**	*0.016028181*	*0.024522228*	*0.025377317*	*0.015060294*
**ENSG00000104738**	*0.015898402*	*0.024368843*	*0.024742582*	*0.01530812*
**ENSG00000006451**	*0.031229963*	*0.03616*	*0.032761346*	*0.031815841*
**ENSG00000221869**	*0.070992676*	*0.063730944*	*0.072098243*	*0.071021682*

**ENSG00000155545**	*0.015122148*	*0.025352087*	*0.025317643*	*0.015233495*
**ENSG00000204396**	*0.015848641*	*0.025609473*	*0.025486911*	*0.015536932*
**ENSG00000136682**	*0.020873614*	*0.026865419*	*0.027672159*	*0.020474305*
**ENSG00000108010**	*0.015405635*	*0.025546076*	*0.024399753*	*0.016234303*
**ENSG00000159495**	*0.015629294*	*0.025378825*	*0.024642922*	*0.015214019*
**ENSG00000119559**	*0.028249938*	*0.031516365*	*0.026424271*	*0.030749484*
**ENSG00000106682**	*0.021583158*	*0.027672531*	*0.028453*	*0.017714886*
**ENSG00000221954**	*0.015838589*	*0.024787475*	*0.024644031*	*0.014929188*
**ENSG00000111775**	*0.020959239*	*0.026630401*	*0.026459409*	*0.017600014*
**ENSG00000165487**	*0.037704386*	*0.048632198*	*0.037000882*	*0.036787437*
**ENSG00000168876**	*0.026562549*	*0.035758164*	*0.031170675*	*0.019420479*
**ENSG00000187456**	*0.029226904*	*0.03299121*	*0.030687862*	*0.023453487*
**ENSG00000179826**	*0.015652927*	*0.025551326*	*0.024716908*	*0.014902275*
**ENSG00000152642**	*0.043688062*	*0.036363206*	*0.03709466*	*0.03171328*
**ENSG00000162398**	*0.014775067*	*0.024894186*	*0.025292811*	*0.014405126*
**ENSG00000186007**	*0.013231618*	*0.023739884*	*0.023896568*	*0.014628989*
**ENSG00000155755**	*0.037766864*	*0.037359602*	*0.038910549*	*0.044464008*
**ENSG00000156973**	*0.026164047*	*0.029255507*	*0.030667613*	*0.023948794*
**ENSG00000109113**	*0.124194536*	*0.073614938*	*0.067391669*	*0.068894894*
**ENSG00000107949**	*0.039698398*	*0.041051928*	*0.034258335*	*0.034893336*
**ENSG00000162076**	*0.033464771*	*0.035820115*	*0.032489121*	*0.041032979*
**ENSG00000064932**	*0.028143138*	*0.035120748*	*0.041519125*	*0.032841365*
**ENSG00000169717**	*0.018622949*	*0.026684005*	*0.029079231*	*0.016065961*
**ENSG00000186090**	*0.016662777*	*0.026204563*	*0.02520687*	*0.016954084*
**ENSG00000109255**	*0.01759871*	*0.02460317*	*0.025489531*	*0.014988495*
**ENSG00000100941**	*0.032325193*	*0.04542945*	*0.051350837*	*0.025657765*
**ENSG00000170458**	*0.015696379*	*0.024640911*	*0.024531805*	*0.015605476*
**ENSG00000180488**	*0.042251525*	*0.038104343*	*0.039604477*	*0.040005333*
**ENSG00000130545**	*0.028998289*	*0.036373172*	*0.033152823*	*0.02597714*
**ENSG00000184005**	*0.019214255*	*0.024931215*	*0.025154051*	*0.017844819*
**ENSG00000131115**	*0.029477139*	*0.039220806*	*0.041942164*	*0.024094842*
**ENSG00000108825**	*0.028015983*	*0.029982327*	*0.029378433*	*0.023757561*
**ENSG00000147869**	*0.020207218*	*0.03139903*	*0.027868331*	*0.021397633*
**ENSG00000100461**	*0.027363538*	*0.033065161*	*0.031226508*	*0.027648304*
**ENSG00000181045**	*0.057863465*	*0.051659585*	*0.053257474*	*0.053413935*
**ENSG00000110848**	*0.076341778*	*0.061147329*	*0.057807704*	*0.058220146*
**ENSG00000183576**	*0.018725057*	*0.026996122*	*0.026651894*	*0.018220666*
**ENSG00000163618**	*0.015845935*	*0.024330135*	*0.024690291*	*0.015579995*
**ENSG00000135912**	*0.015714204*	*0.02422803*	*0.024981767*	*0.016110345*
**ENSG00000185739**	*0.016718*	*0.026691546*	*0.025155215*	*0.016614043*
**ENSG00000114209**	*0.031373562*	*0.035220925*	*0.033779961*	*0.025665229*
**ENSG00000156990**	*0.036093336*	*0.033844302*	*0.029094797*	*0.023668732*
**ENSG00000178997**	*0.022200261*	*0.030994154*	*0.028447807*	*0.026157586*
**ENSG00000170153**	*0.013124972*	*0.0237269*	*0.023720956*	*0.012948918*
**ENSG00000179085**	*0.017468256*	*0.025132197*	*0.024423131*	*0.016091528*
**ENSG00000156482**	*0.014827397*	*0.024850738*	*0.024853087*	*0.01661744*
**ENSG00000116661**	*0.019119506*	*0.028256122*	*0.027483605*	*0.023396505*
**ENSG00000198774**	*0.017279437*	*0.024830739*	*0.026150817*	*0.01616711*
**ENSG00000167034**	*0.027491526*	*0.034105437*	*0.028963652*	*0.02381507*
**ENSG00000065320**	*0.017253865*	*0.026170758*	*0.026099167*	*0.016959231*
**ENSG00000165863**	*0.01656958*	*0.024364799*	*0.024193845*	*0.01629551*
**ENSG00000166482**	*0.018690757*	*0.028189887*	*0.025880916*	*0.018807144*
**ENSG00000133816**	*0.025673418*	*0.03268634*	*0.028654172*	*0.026351804*
**ENSG00000059758**	*0.029441251*	*0.032239376*	*0.033265922*	*0.030083311*

**ENSG00000077232**	*0.031338568*	*0.034549127*	*0.03349179*	*0.032784845*
**ENSG00000154007**	*0.016839463*	*0.026226168*	*0.025714738*	*0.015945294*
**ENSG00000148339**	*0.035472394*	*0.037748535*	*0.03512625*	*0.033524851*
**ENSG00000215271**	*0.029687083*	*0.032185622*	*0.029996764*	*0.020503151*
**ENSG00000107521**	*0.027074199*	*0.031270622*	*0.033685438*	*0.027453639*
**ENSG00000109270**	*0.024559179*	*0.030709811*	*0.033410688*	*0.025009719*
**ENSG00000095752**	*0.027949281*	*0.029580559*	*0.027780476*	*0.017454846*
**ENSG00000115827**	*0.031592913*	*0.033735472*	*0.028394583*	*0.02526804*
**ENSG00000188379**	*0.027606135*	*0.038564759*	*0.028887545*	*0.025457556*
**ENSG00000172057**	*0.027808832*	*0.035481454*	*0.035628461*	*0.029762846*
**ENSG00000102468**	*0.024359287*	*0.031474439*	*0.02657257*	*0.032258936*
**ENSG00000180875**	*0.016937801*	*0.026743528*	*0.02641633*	*0.017749244*
**ENSG00000178951**	*0.033356962*	*0.043937628*	*0.059398934*	*0.030561908*
**ENSG00000181690**	*0.056934163*	*0.05382977*	*0.046274982*	*0.059921012*
**ENSG00000182749**	*0.016765682*	*0.025975468*	*0.026729853*	*0.016214048*
**ENSG00000136153**	*0.054116922*	*0.048892755*	*0.039320687*	*0.033593217*
**ENSG00000188372**	*0.018101293*	*0.02661556*	*0.026338008*	*0.015961983*
**ENSG00000085231**	*0.02794892*	*0.032323139*	*0.030228607*	*0.026217775*
**ENSG00000102763**	*0.037317759*	*0.048727189*	*0.03319513*	*0.029930518*
**ENSG00000187950**	*0.015923083*	*0.025488012*	*0.024284914*	*0.01466219*
**ENSG00000188760**	*0.026239805*	*0.032737721*	*0.028019852*	*0.026331249*
**ENSG00000165271**	*0.029503903*	*0.031794913*	*0.031694986*	*0.029902536*
**ENSG00000160214**	*0.016046402*	*0.02487102*	*0.025241509*	*0.014462648*
**ENSG00000157502**	*0.038846388*	*0.026030505*	*0.025863693*	*0.018383918*
**ENSG00000160339**	*0.017794639*	*0.025953641*	*0.026539102*	*0.019493034*
**ENSG00000135116**	*0.021240329*	*0.025675614*	*0.026074266*	*0.016336066*
**ENSG00000233276**	*0.021131575*	*0.029807507*	*0.026545427*	*0.0210385*
**ENSG00000088986**	*0.022797435*	*0.029291734*	*0.027410318*	*0.021718621*
**ENSG00000138172**	*0.070536071*	*0.056032266*	*0.045080494*	*0.056398896*
**ENSG00000116497**	*0.041619326*	*0.045686245*	*0.03576122*	*0.038039165*
**ENSG00000220205**	*0.031876873*	*0.044019315*	*0.045052286*	*0.029270804*
**ENSG00000177294**	*0.01537865*	*0.025011185*	*0.025065738*	*0.01846759*
**ENSG00000109321**	*0.07992815*	*0.07218331*	*0.066015772*	*0.076108612*
**ENSG00000114812**	*0.017389705*	*0.026029938*	*0.024728876*	*0.018790678*
**ENSG00000131473**	*0.02326372*	*0.03132731*	*0.02727884*	*0.021379379*
**ENSG00000197894**	*0.038041431*	*0.038831371*	*0.031961584*	*0.032003441*
**ENSG00000079974**	*0.036700133*	*0.040146974*	*0.037409461*	*0.033605409*
**ENSG00000146540**	*0.032325732*	*0.036366389*	*0.031750427*	*0.024486394*
**ENSG00000120053**	*0.030408071*	*0.032001691*	*0.029539369*	*0.032160442*
**ENSG00000055917**	*0.02808847*	*0.034451542*	*0.030323689*	*0.024884351*
**ENSG00000141994**	*0.041193861*	*0.040339427*	*0.043464295*	*0.03479723*
**ENSG00000188130**	*0.031530602*	*0.034965863*	*0.034280952*	*0.030503166*
**ENSG00000197782**	*0.020555197*	*0.026285248*	*0.026788288*	*0.022656721*
**ENSG00000169059**	*0.047914493*	*0.029097582*	*0.030579193*	*0.029801352*
**ENSG00000031081**	*0.032129776*	*0.039489983*	*0.035589377*	*0.036559683*
**ENSG00000176946**	*0.025274739*	*0.031500096*	*0.029506436*	*0.020041301*
**ENSG00000130821**	*0.020641345*	*0.029508913*	*0.026965899*	*0.020760348*
**ENSG00000188177**	*0.03126108*	*0.039335465*	*0.039514519*	*0.036650193*
**ENSG00000167774**	*0.029363427*	*0.028569631*	*0.031751994*	*0.023448916*
**ENSG00000111012**	*0.036946063*	*0.033973208*	*0.035222356*	*0.026253874*
**ENSG00000175548**	*0.015212837*	*0.025130828*	*0.024625897*	*0.01530139*
**ENSG00000100121**	*0.017243087*	*0.025212277*	*0.025873687*	*0.015687673*
**ENSG00000142687**	*0.030108632*	*0.029952129*	*0.03035012*	*0.027719254*
**ENSG00000204335**	*0.013471233*	*0.024088832*	*0.023862435*	*0.013326197*

**ENSG00000177606**	*0.049216371*	*0.044820995*	*0.040550956*	*0.03851965*
**ENSG00000147862**	*0.097010405*	*0.050409504*	*0.049956644*	*0.059356116*
**ENSG00000014641**	*0.024156177*	*0.031573914*	*0.029545647*	*0.022270489*
**ENSG00000164162**	*0.030259708*	*0.03364997*	*0.031583117*	*0.026159837*
**ENSG00000197353**	*0.01469406*	*0.025798697*	*0.024433542*	*0.016328606*
**ENSG00000184828**	*0.015504772*	*0.025522079*	*0.025270521*	*0.015675298*
**ENSG00000147180**	*0.043789647*	*0.032823951*	*0.029734484*	*0.025944856*
**ENSG00000171109**	*0.038970467*	*0.034670929*	*0.040432956*	*0.05078068*
**ENSG00000120436**	*0.016647051*	*0.02468213*	*0.024426309*	*0.016521156*
**ENSG00000117501**	*0.016274619*	*0.025349404*	*0.024937156*	*0.015171685*
**ENSG00000118004**	*0.017874401*	*0.025004918*	*0.026463974*	*0.016808799*
**ENSG00000066654**	*0.023253503*	*0.031250513*	*0.031511941*	*0.019887319*
**ENSG00000154240**	*0.018713293*	*0.026596504*	*0.024761324*	*0.016256652*
**ENSG00000117461**	*0.04219742*	*0.050964857*	*0.042649929*	*0.042270581*
**ENSG00000149806**	*0.014217446*	*0.0241849*	*0.024106451*	*0.014559488*
**ENSG00000108509**	*0.01711578*	*0.026725406*	*0.025443051*	*0.017245658*
**ENSG00000125788**	*0.017257669*	*0.025209063*	*0.025369886*	*0.015730275*
**ENSG00000081800**	*0.017480654*	*0.027336607*	*0.025169729*	*0.018806982*
**ENSG00000106546**	*0.024312215*	*0.028214827*	*0.028323905*	*0.023376274*
**ENSG00000160294**	*0.02316472*	*0.029995303*	*0.031013012*	*0.020796368*
**ENSG00000118046**	*0.029559977*	*0.037057717*	*0.03732508*	*0.026399122*
**ENSG00000165275**	*0.018978457*	*0.02575619*	*0.027982767*	*0.018993204*
**ENSG00000205502**	*0.02165499*	*0.025790975*	*0.026807486*	*0.019251362*
**ENSG00000105229**	*0.026762264*	*0.033666685*	*0.034363669*	*0.02484568*
**ENSG00000135945**	*0.033845989*	*0.036171408*	*0.033281731*	*0.028481304*
**ENSG00000018280**	*0.016889666*	*0.026569176*	*0.026114835*	*0.017031397*
**ENSG00000124299**	*0.03381411*	*0.036784483*	*0.033872792*	*0.02725556*
**ENSG00000104219**	*0.056413384*	*0.044012408*	*0.046426637*	*0.042375466*
**ENSG00000143889**	*0.080964567*	*0.064727517*	*0.057001654*	*0.072217934*
**ENSG00000164588**	*0.016284752*	*0.026306433*	*0.02500222*	*0.015979082*
**ENSG00000164930**	*0.064364552*	*0.062373845*	*0.060703291*	*0.063731019*
**ENSG00000152749**	*0.035163722*	*0.036184055*	*0.030582693*	*0.025996881*
**ENSG00000009413**	*0.033698377*	*0.038362207*	*0.032059931*	*0.036505281*
**ENSG00000070193**	*0.016415731*	*0.024731066*	*0.024849692*	*0.015470263*
**ENSG00000100412**	*0.024129195*	*0.030736807*	*0.030966163*	*0.027763567*
**ENSG00000069018**	*0.015673067*	*0.025677845*	*0.025584706*	*0.015268325*
**ENSG00000115421**	*0.017348039*	*0.024737736*	*0.025608365*	*0.016419156*
**ENSG00000163888**	*0.0170005*	*0.02520412*	*0.026940922*	*0.016399921*
**ENSG00000029559**	*0.016175689*	*0.024998904*	*0.025901971*	*0.015652969*
**ENSG00000158428**	*0.01730047*	*0.026477709*	*0.026004499*	*0.017200649*
**ENSG00000136457**	*0.019670642*	*0.024760042*	*0.025707236*	*0.015308228*
**ENSG00000172466**	*0.043596865*	*0.052447674*	*0.054773362*	*0.053926638*
**ENSG00000145569**	*0.033518229*	*0.032152391*	*0.028044345*	*0.026405819*
**ENSG00000253598**	*0.015724829*	*0.024602255*	*0.024811983*	*0.015133063*
**ENSG00000103489**	*0.034977301*	*0.030452332*	*0.028211984*	*0.02599413*
**ENSG00000166341**	*0.015846765*	*0.025015847*	*0.025285042*	*0.016116355*
**ENSG00000184350**	*0.015495552*	*0.025694977*	*0.02439687*	*0.014438581*
**ENSG00000057252**	*0.041722535*	*0.040785884*	*0.037958261*	*0.037412468*
**ENSG00000047648**	*0.017074857*	*0.02533985*	*0.026697411*	*0.015024659*
**ENSG00000130517**	*0.02170442*	*0.027427796*	*0.027289899*	*0.024266683*
**ENSG00000005100**	*0.038096668*	*0.03632622*	*0.035587114*	*0.028911065*
**ENSG00000089820**	*0.030821056*	*0.032122282*	*0.036041556*	*0.028786417*
**ENSG00000167653**	*0.015020223*	*0.025544944*	*0.024472551*	*0.015625823*
**ENSG00000112685**	*0.026557942*	*0.03184864*	*0.031776413*	*0.025486988*

**ENSG00000181652**	*0.016401176*	*0.025903687*	*0.025640717*	*0.016282762*
**ENSG00000121083**	*0.037453064*	*0.043194773*	*0.043851669*	*0.032758953*
**ENSG00000198218**	*0.025257953*	*0.031214186*	*0.033008532*	*0.020340839*
**ENSG00000168269**	*0.016042469*	*0.024507243*	*0.025744333*	*0.015294275*
**ENSG00000059378**	*0.061550244*	*0.056450663*	*0.050099434*	*0.064698023*
**ENSG00000143512**	*0.014939119*	*0.02542937*	*0.024590175*	*0.014483675*
**ENSG00000132825**	*0.038135292*	*0.038533339*	*0.031982356*	*0.028151536*
**ENSG00000152214**	*0.014756017*	*0.024709405*	*0.024686561*	*0.016037645*
**ENSG00000124260**	*0.015100798*	*0.024574208*	*0.024844634*	*0.014520948*
**ENSG00000157766**	*0.016512534*	*0.024921262*	*0.024823799*	*0.014039391*
**ENSG00000165078**	*0.018886001*	*0.026265107*	*0.028572888*	*0.01571568*
**ENSG00000161179**	*0.030627687*	*0.030528131*	*0.034574792*	*0.027329081*
**ENSG00000138780**	*0.017448154*	*0.025374991*	*0.025656487*	*0.015117935*
**ENSG00000112306**	*0.020887945*	*0.029575353*	*0.029078863*	*0.020360731*
**ENSG00000141219**	*0.037240564*	*0.043220989*	*0.0326339*	*0.030507553*
**ENSG00000163202**	*0.017240328*	*0.025644279*	*0.027920637*	*0.01858823*
**ENSG00000186474**	*0.01470035*	*0.024650915*	*0.024881916*	*0.014854951*
**ENSG00000170379**	*0.019356013*	*0.02887523*	*0.028248307*	*0.021757596*
**ENSG00000184933**	*0.015460534*	*0.024564908*	*0.024566164*	*0.01436118*
**ENSG00000071889**	*0.022859533*	*0.030303545*	*0.03499064*	*0.029155942*
**ENSG00000137710**	*0.031554654*	*0.039471239*	*0.031962555*	*0.033615078*
**ENSG00000183150**	*0.025496595*	*0.030464625*	*0.027687112*	*0.019109438*
**ENSG00000178187**	*0.039639392*	*0.034212104*	*0.039082595*	*0.03576105*
**ENSG00000205413**	*0.043896408*	*0.050949904*	*0.049199773*	*0.044871921*
**ENSG00000171806**	*0.036917414*	*0.034091063*	*0.033626594*	*0.025939712*
**ENSG00000162972**	*0.025261194*	*0.030058063*	*0.029687922*	*0.02369151*
**ENSG00000132704**	*0.042712008*	*0.028766681*	*0.029333267*	*0.027141911*
**ENSG00000143977**	*0.030943668*	*0.034513857*	*0.030536776*	*0.03032903*
**ENSG00000179242**	*0.015340802*	*0.024929179*	*0.024899216*	*0.014783015*
**ENSG00000104611**	*0.018123857*	*0.035618293*	*0.03096999*	*0.023868161*
**ENSG00000174669**	*0.026115534*	*0.031902474*	*0.030430626*	*0.027562316*
**ENSG00000147382**	*0.030677111*	*0.032929819*	*0.034438445*	*0.034835363*
**ENSG00000110876**	*0.053548634*	*0.047666761*	*0.054835218*	*0.057892945*
**ENSG00000158623**	*0.036740787*	*0.042113379*	*0.039568116*	*0.038113493*
**ENSG00000154153**	*0.097435964*	*0.092903801*	*0.082127845*	*0.108040228*
**ENSG00000136297**	*0.015964859*	*0.025447514*	*0.02423782*	*0.015769891*
**ENSG00000171552**	*0.036864548*	*0.049031727*	*0.041263405*	*0.047782811*
**ENSG00000181788**	*0.036489111*	*0.032353902*	*0.038771124*	*0.035535211*
**ENSG00000111863**	*0.107733655*	*0.079441657*	*0.090872869*	*0.090565354*
**ENSG00000163913**	*0.020604294*	*0.027341655*	*0.025762291*	*0.019831786*
**ENSG00000141314**	*0.017832358*	*0.02990567*	*0.026656382*	*0.017119415*
**ENSG00000136699**	*0.016562834*	*0.024960602*	*0.024482471*	*0.01636934*
**ENSG00000147642**	*0.082070047*	*0.076573536*	*0.060636342*	*0.061157083*
**ENSG00000196862**	*0.029762973*	*0.030258631*	*0.034489673*	*0.028413617*
**ENSG00000105849**	*0.028365349*	*0.039872372*	*0.0396803*	*0.029653317*
**ENSG00000187699**	*0.105859236*	*0.078641426*	*0.065748205*	*0.073823965*
**ENSG00000167969**	*0.029946937*	*0.033018583*	*0.03036504*	*0.027097758*
**ENSG00000117601**	*0.017407498*	*0.026619967*	*0.028525319*	*0.017411796*
**ENSG00000224389**	*0.017322696*	*0.025055106*	*0.025606796*	*0.018632022*
**ENSG00000197061**	*0.025621873*	*0.025926337*	*0.025552943*	*0.017069625*
**ENSG00000162490**	*0.016957194*	*0.026034378*	*0.0254252*	*0.01551938*
**ENSG00000087237**	*0.084383783*	*0.066848313*	*0.06527483*	*0.083917135*
**ENSG00000176410**	*0.029970341*	*0.030176694*	*0.030591692*	*0.02015153*
**ENSG00000167967**	*0.028911827*	*0.030523465*	*0.041585222*	*0.026187575*

**ENSG00000141232**	*0.028659854*	*0.034252309*	*0.035472888*	*0.027883397*
**ENSG00000171045**	*0.017268536*	*0.024683903*	*0.025613826*	*0.01699593*
**ENSG00000168803**	*0.016953923*	*0.025415104*	*0.025135441*	*0.017043662*
**ENSG00000187498**	*0.042608425*	*0.036075913*	*0.024972779*	*0.021555292*
**ENSG00000113073**	*0.018559145*	*0.025128481*	*0.026531454*	*0.016417861*
**ENSG00000102780**	*0.015809621*	*0.024525672*	*0.025109337*	*0.014366925*
**ENSG00000103375**	*0.016228299*	*0.025050671*	*0.026022909*	*0.016881686*
**ENSG00000186230**	*0.020683966*	*0.028663125*	*0.028295934*	*0.019653702*
**ENSG00000164332**	*0.026474093*	*0.034481277*	*0.03246653*	*0.030925125*
**ENSG00000180357**	*0.027010205*	*0.037003267*	*0.031699227*	*0.030120953*
**ENSG00000128016**	*0.034189794*	*0.034896255*	*0.032356885*	*0.028592853*
**ENSG00000126749**	*0.017591255*	*0.025116466*	*0.024726416*	*0.015698397*
**ENSG00000165105**	*0.042474209*	*0.040428895*	*0.04072736*	*0.046114128*
**ENSG00000122778**	*0.038760206*	*0.031401635*	*0.030423091*	*0.028704109*
**ENSG00000204645**	*0.01926725*	*0.025988654*	*0.025985191*	*0.018713869*
**ENSG00000163807**	*0.029565028*	*0.032084913*	*0.033338408*	*0.025361324*
**ENSG00000187735**	*0.024508185*	*0.030758548*	*0.029099496*	*0.021414259*
**ENSG00000111678**	*0.026014234*	*0.029310062*	*0.03004202*	*0.033438224*
**ENSG00000119431**	*0.030541679*	*0.041572935*	*0.035384218*	*0.024809722*
**ENSG00000182196**	*0.014899055*	*0.026165972*	*0.025155403*	*0.015171925*
**ENSG00000170275**	*0.056536863*	*0.046755514*	*0.04787315*	*0.039482857*
**ENSG00000151327**	*0.040877295*	*0.040016149*	*0.039988102*	*0.041213469*
**ENSG00000239388**	*0.016087648*	*0.024704089*	*0.024388877*	*0.01513408*
**ENSG00000170525**	*0.025537588*	*0.031644061*	*0.030640025*	*0.024775323*
**ENSG00000067365**	*0.030432055*	*0.033064035*	*0.035087513*	*0.025625576*
**ENSG00000198825**	*0.103242938*	*0.074510803*	*0.057660211*	*0.070476488*
**ENSG00000213965**	*0.031203651*	*0.037743452*	*0.030830991*	*0.034911044*
**ENSG00000117020**	*0.017664515*	*0.025221231*	*0.025445565*	*0.016653038*
**ENSG00000189007**	*0.023031815*	*0.028088092*	*0.026066983*	*0.019844668*
**ENSG00000136875**	*0.025917323*	*0.030562499*	*0.033391343*	*0.02208469*
**ENSG00000114698**	*0.0153008*	*0.02448929*	*0.024983238*	*0.01440057*
**ENSG00000213463**	*0.033125974*	*0.040159633*	*0.038376219*	*0.029106253*
**ENSG00000223443**	*0.057706307*	*0.039216191*	*0.049577353*	*0.03089989*
**ENSG00000158615**	*0.039298392*	*0.040263356*	*0.044862884*	*0.033513859*
**ENSG00000106689**	*0.139453939*	*0.095306033*	*0.05611377*	*0.110295462*
**ENSG00000187257**	*0.029168306*	*0.039543005*	*0.033089556*	*0.0234326*
**ENSG00000152926**	*0.017707996*	*0.025671085*	*0.024957843*	*0.018084214*
**ENSG00000172273**	*0.023694834*	*0.032348263*	*0.030531244*	*0.021122895*
**ENSG00000131002**	*0.199109213*	*0.181368706*	*0.170067472*	*0.178917334*
**ENSG00000129654**	*0.022011789*	*0.028203036*	*0.028460295*	*0.020581181*
**ENSG00000154645**	*0.023080617*	*0.029689428*	*0.028096978*	*0.024189549*
**ENSG00000198182**	*0.034224803*	*0.034132421*	*0.033663684*	*0.02370848*
**ENSG00000142541**	*0.018987728*	*0.0249854*	*0.024805839*	*0.018331343*
**ENSG00000121390**	*0.030058205*	*0.032254782*	*0.036759705*	*0.031037899*
**ENSG00000115353**	*0.01627528*	*0.024573044*	*0.026353935*	*0.015680806*
**ENSG00000084453**	*0.016661631*	*0.025228383*	*0.025191153*	*0.015957156*
**ENSG00000182632**	*0.016903902*	*0.025229688*	*0.026861682*	*0.017197594*
**ENSG00000111679**	*0.017262263*	*0.025635589*	*0.02587679*	*0.015763425*
**ENSG00000134042**	*0.015633896*	*0.024918579*	*0.026737703*	*0.015063389*
**ENSG00000124529**	*0.09748596*	*0.073620508*	*0.083060163*	*0.092312355*
**ENSG00000189143**	*0.01500743*	*0.025192982*	*0.024984239*	*0.014337963*
**ENSG00000152207**	*0.016660394*	*0.02721898*	*0.025825225*	*0.016675466*
**ENSG00000076067**	*0.024039521*	*0.031356602*	*0.030663288*	*0.022471917*
**ENSG00000197586**	*0.024171262*	*0.029071059*	*0.032883431*	*0.025505512*

**ENSG00000023697**	*0.029493818*	*0.030606534*	*0.029391338*	*0.02592949*
**ENSG00000083838**	*0.022616338*	*0.02632006*	*0.030337286*	*0.024980478*
**ENSG00000134240**	*0.016273668*	*0.024386355*	*0.024865353*	*0.015685702*
**ENSG00000155729**	*0.027037374*	*0.036846793*	*0.032442346*	*0.025166635*
**ENSG00000110492**	*0.058595813*	*0.056667853*	*0.039713299*	*0.055777834*
**ENSG00000175029**	*0.059857241*	*0.046817333*	*0.043166244*	*0.050478602*
**ENSG00000182247**	*0.031523824*	*0.032243988*	*0.030888264*	*0.026409767*
**ENSG00000109061**	*0.016294647*	*0.024172244*	*0.024621737*	*0.015914663*
**ENSG00000040275**	*0.039146754*	*0.041975301*	*0.032234361*	*0.032939765*
**ENSG00000120913**	*0.016901264*	*0.026731309*	*0.026111477*	*0.018217213*
**ENSG00000130052**	*0.051728248*	*0.041011939*	*0.039323177*	*0.036016957*
**ENSG00000146477**	*0.016026422*	*0.026085255*	*0.024834914*	*0.015781003*
**ENSG00000136709**	*0.028256533*	*0.030179164*	*0.036365871*	*0.025807968*
**ENSG00000203668**	*0.019220542*	*0.026181587*	*0.027271563*	*0.017612792*
**ENSG00000183558**	*0.063610711*	*0.066647503*	*0.056950237*	*0.050559617*
**ENSG00000183688**	*0.094790117*	*0.08111698*	*0.071946596*	*0.087660651*
**ENSG00000176399**	*0.015546992*	*0.026044063*	*0.025250578*	*0.016649009*
**ENSG00000198546**	*0.030893317*	*0.02991931*	*0.028568997*	*0.025640674*
**ENSG00000234127**	*0.022994193*	*0.032367263*	*0.038280823*	*0.026255138*
**ENSG00000132122**	*0.015056908*	*0.025039325*	*0.025225575*	*0.015570347*
**ENSG00000054392**	*0.052149913*	*0.045784677*	*0.040404688*	*0.043344466*
**ENSG00000171346**	*0.015531253*	*0.025058749*	*0.024057595*	*0.019113554*
**ENSG00000160191**	*0.057596831*	*0.056253057*	*0.046322442*	*0.05425764*
**ENSG00000182898**	*0.018631745*	*0.025498376*	*0.024615537*	*0.01697731*
**ENSG00000170385**	*0.063057744*	*0.070345552*	*0.081155707*	*0.059654764*
**ENSG00000104886**	*0.025891842*	*0.031891579*	*0.030848404*	*0.024761178*
**ENSG00000105197**	*0.023013617*	*0.027950892*	*0.029778853*	*0.021042613*
**ENSG00000165113**	*0.044529137*	*0.04297645*	*0.035852028*	*0.038459884*
**ENSG00000213022**	*0.016106576*	*0.025701152*	*0.025875995*	*0.015353534*
**ENSG00000095787**	*0.019311938*	*0.027529692*	*0.02807459*	*0.020443314*
**ENSG00000144645**	*0.024299416*	*0.028022859*	*0.03405746*	*0.030690135*
**ENSG00000127481**	*0.027566278*	*0.032000774*	*0.036535009*	*0.02758831*
**ENSG00000141741**	*0.021756618*	*0.030608237*	*0.033247223*	*0.021547092*
**ENSG00000145692**	*0.01583359*	*0.025457514*	*0.024621869*	*0.016919494*
**ENSG00000182552**	*0.034132032*	*0.035981434*	*0.032143357*	*0.03179072*
**ENSG00000215717**	*0.022837445*	*0.032616049*	*0.032381218*	*0.022316765*
**ENSG00000187005**	*0.015744146*	*0.025122476*	*0.025633431*	*0.014954349*
**ENSG00000175664**	*0.017233257*	*0.026314074*	*0.02448318*	*0.015826581*
**ENSG00000107362**	*0.038954056*	*0.048274328*	*0.045266239*	*0.045379878*
**ENSG00000144229**	*0.018657512*	*0.024779378*	*0.025506073*	*0.015131149*
**ENSG00000163468**	*0.025743843*	*0.031367155*	*0.028235762*	*0.024580298*
**ENSG00000106261**	*0.021332155*	*0.028189724*	*0.027616843*	*0.022932693*
**ENSG00000183828**	*0.044519692*	*0.042107463*	*0.042166864*	*0.042315844*
**ENSG00000143756**	*0.035249657*	*0.041872252*	*0.04198248*	*0.036015103*
**ENSG00000172795**	*0.038147934*	*0.038927285*	*0.031035607*	*0.029908761*
**ENSG00000102290**	*0.016935506*	*0.024675447*	*0.024997967*	*0.015229916*
**ENSG00000196338**	*0.016528628*	*0.024862561*	*0.024713245*	*0.016337086*
**ENSG00000124334**	*0.024335972*	*0.027807753*	*0.026597485*	*0.020514464*
**ENSG00000101074**	*0.015526157*	*0.025500084*	*0.025217797*	*0.016607624*
**ENSG00000250506**	*0.019394132*	*0.025556256*	*0.026744161*	*0.019247818*
**ENSG00000112727**	*0.075478684*	*0.055714189*	*0.059065272*	*0.077978685*
**ENSG00000138829**	*0.045193579*	*0.025951499*	*0.025465377*	*0.019636619*
**ENSG00000177689**	*0.031310962*	*0.029944719*	*0.037037186*	*0.02954932*
**ENSG00000119231**	*0.031678667*	*0.037528368*	*0.038603973*	*0.027165821*

**ENSG00000140575**	*0.022721123*	*0.030553582*	*0.028952362*	*0.020102518*
**ENSG00000140553**	*0.023310302*	*0.029956032*	*0.029824924*	*0.029815161*
**ENSG00000115392**	*0.043808666*	*0.051463603*	*0.042952013*	*0.034174386*
**ENSG00000127081**	*0.031935924*	*0.033504322*	*0.032178604*	*0.025701054*
**ENSG00000165886**	*0.021198441*	*0.029015776*	*0.026283439*	*0.027169274*
**ENSG00000204366**	*0.015756806*	*0.025085081*	*0.024818473*	*0.015234636*
**ENSG00000244682**	*0.046185636*	*0.04735826*	*0.039381341*	*0.045473129*
**ENSG00000182220**	*0.022710311*	*0.029664428*	*0.026712982*	*0.022460795*
**ENSG00000113282**	*0.045806639*	*0.041722131*	*0.034107397*	*0.036454314*
**ENSG00000088305**	*0.016499385*	*0.024334119*	*0.025785892*	*0.015966319*
**ENSG00000173597**	*0.02028786*	*0.026413911*	*0.02412283*	*0.015699772*
**ENSG00000095596**	*0.017904931*	*0.025082126*	*0.02511547*	*0.016936298*
**ENSG00000138182**	*0.038078916*	*0.036478356*	*0.035776214*	*0.025034521*
**ENSG00000174109**	*0.025641051*	*0.034314663*	*0.037137226*	*0.023892907*
**ENSG00000215375**	*0.038854111*	*0.043217628*	*0.035560131*	*0.038448776*
**ENSG00000103035**	*0.025747198*	*0.030302938*	*0.031918625*	*0.025404336*
**ENSG00000007001**	*0.014245173*	*0.024464119*	*0.025349156*	*0.015706876*
**ENSG00000183647**	*0.026098116*	*0.034960262*	*0.042535744*	*0.029898556*
**ENSG00000243927**	*0.025727676*	*0.030343405*	*0.028819966*	*0.020507602*
**ENSG00000185189**	*0.030257953*	*0.035517581*	*0.031610206*	*0.032436018*
**ENSG00000157020**	*0.024788404*	*0.029555825*	*0.032657964*	*0.026326517*
**ENSG00000173227**	*0.032394403*	*0.037930617*	*0.031709397*	*0.029182315*
**ENSG00000092010**	*0.021540986*	*0.028132087*	*0.028536978*	*0.017418556*
**ENSG00000179407**	*0.017426077*	*0.025242635*	*0.024625861*	*0.016660946*
**ENSG00000178814**	*0.020586139*	*0.029857172*	*0.026647677*	*0.020294448*
**ENSG00000185585**	*0.065748104*	*0.059683262*	*0.067441286*	*0.045516751*
**ENSG00000186998**	*0.070143496*	*0.058058511*	*0.057197098*	*0.062626964*
**ENSG00000185960**	*0.016191302*	*0.026830197*	*0.026232254*	*0.017500428*
**ENSG00000006555**	*0.015419514*	*0.024780963*	*0.025977595*	*0.015459951*
**ENSG00000111271**	*0.021552735*	*0.02808303*	*0.029922375*	*0.025031945*
**ENSG00000164219**	*0.038694029*	*0.044825157*	*0.038351665*	*0.038660259*
**ENSG00000148411**	*0.025228322*	*0.029046187*	*0.029561947*	*0.020685026*
**ENSG00000196724**	*0.023366017*	*0.02892108*	*0.026469322*	*0.020416392*
**ENSG00000002016**	*0.025044557*	*0.034370406*	*0.027609979*	*0.020900459*
**ENSG00000234734**	*0.014435425*	*0.025079016*	*0.02413197*	*0.014414474*
**ENSG00000100815**	*0.030671903*	*0.042244158*	*0.036999749*	*0.028646372*
**ENSG00000167525**	*0.019132753*	*0.028885847*	*0.027529902*	*0.019843564*
**ENSG00000167136**	*0.034544089*	*0.036886898*	*0.042328188*	*0.03402222*
**ENSG00000183484**	*0.031700591*	*0.062725966*	*0.059321968*	*0.045835488*
**ENSG00000134909**	*0.028527973*	*0.031194473*	*0.031109877*	*0.028104927*
**ENSG00000124571**	*0.030383784*	*0.034391027*	*0.030347433*	*0.031757831*
**ENSG00000123977**	*0.015197889*	*0.026697494*	*0.025056397*	*0.016725762*
**ENSG00000171456**	*0.037175288*	*0.038016977*	*0.037116012*	*0.033443925*
**ENSG00000120254**	*0.036751507*	*0.054622636*	*0.044415047*	*0.046417885*
**ENSG00000132334**	*0.015775675*	*0.025154977*	*0.02465014*	*0.015718227*
**ENSG00000174405**	*0.039901566*	*0.047698319*	*0.046866022*	*0.038377795*
**ENSG00000141338**	*0.021510806*	*0.027602303*	*0.028728655*	*0.018896919*
**ENSG00000108278**	*0.021243002*	*0.027840163*	*0.031111975*	*0.018827169*
**ENSG00000099940**	*0.042865613*	*0.044734499*	*0.035532232*	*0.038809217*
**ENSG00000008300**	*0.066471546*	*0.054299865*	*0.057579252*	*0.054215814*
**ENSG00000130224**	*0.056985689*	*0.025149733*	*0.025613832*	*0.015918273*
**ENSG00000176659**	*0.038725008*	*0.038106766*	*0.036243162*	*0.032731983*
**ENSG00000184845**	*0.016617456*	*0.024316842*	*0.024624722*	*0.015927213*
**ENSG00000137080**	*0.017364174*	*0.026308052*	*0.025143029*	*0.015178816*

**ENSG00000116044**	*0.031550014*	*0.041398579*	*0.039172205*	*0.030814892*
**ENSG00000182134**	*0.025271342*	*0.027784688*	*0.026413989*	*0.020204852*
**ENSG00000168955**	*0.017602873*	*0.026980775*	*0.026047361*	*0.019396778*
**ENSG00000147684**	*0.018998589*	*0.025241547*	*0.026043366*	*0.018469176*
**ENSG00000063169**	*0.019428303*	*0.028006901*	*0.029153202*	*0.019256441*
**ENSG00000060237**	*0.033192421*	*0.033968381*	*0.036088496*	*0.033185952*
**ENSG00000169895**	*0.031418341*	*0.032526359*	*0.033013824*	*0.028206775*
**ENSG00000196704**	*0.02737591*	*0.032708583*	*0.030721682*	*0.02594545*
**ENSG00000173867**	*0.026820799*	*0.030614596*	*0.034774235*	*0.027039891*
**ENSG00000159173**	*0.017317105*	*0.025726037*	*0.024434566*	*0.016324135*
**ENSG00000213903**	*0.016947847*	*0.027935068*	*0.024900537*	*0.017164403*
**ENSG00000213123**	*0.033520892*	*0.037638304*	*0.03473102*	*0.031025146*
**ENSG00000137975**	*0.015309981*	*0.024512169*	*0.023864544*	*0.015602673*
**ENSG00000064687**	*0.015128761*	*0.024320665*	*0.024043834*	*0.015588159*
**ENSG00000063978**	*0.02462759*	*0.035902924*	*0.034124319*	*0.026912821*
**ENSG00000247596**	*0.030458688*	*0.031683494*	*0.031134693*	*0.03121545*
**ENSG00000164045**	*0.021208247*	*0.025122465*	*0.026444984*	*0.018546714*
**ENSG00000141756**	*0.021056229*	*0.025951497*	*0.024051434*	*0.016613788*
**ENSG00000133985**	*0.027403401*	*0.033698504*	*0.028188369*	*0.030412069*
**ENSG00000125434**	*0.020765711*	*0.028401173*	*0.026439211*	*0.018893805*
**ENSG00000164061**	*0.014864514*	*0.025831375*	*0.025131338*	*0.015939996*
**ENSG00000197712**	*0.023967868*	*0.029869692*	*0.030125799*	*0.021624844*
**ENSG00000172113**	*0.024336585*	*0.029166691*	*0.031841633*	*0.021520642*
**ENSG00000196361**	*0.016437333*	*0.024422628*	*0.02648945*	*0.016388743*
**ENSG00000169220**	*0.034403725*	*0.042696588*	*0.03601222*	*0.035650821*
**ENSG00000185052**	*0.015236807*	*0.027640883*	*0.024692733*	*0.015348847*
**ENSG00000070444**	*0.03137743*	*0.048654044*	*0.056982635*	*0.029849601*
**ENSG00000018189**	*0.036721282*	*0.042211058*	*0.037582619*	*0.0326895*
**ENSG00000149534**	*0.015862226*	*0.025397931*	*0.024264988*	*0.014970706*
**ENSG00000213639**	*0.027310934*	*0.032828151*	*0.029456092*	*0.025467217*
**ENSG00000198093**	*0.047025042*	*0.043868357*	*0.038760355*	*0.033691697*
**ENSG00000099290**	*0.027113066*	*0.033173501*	*0.030669336*	*0.021624287*
**ENSG00000178074**	*0.039119819*	*0.041080421*	*0.035630908*	*0.047492369*
**ENSG00000140299**	*0.027219002*	*0.034969426*	*0.039666295*	*0.030374798*
**ENSG00000169682**	*0.030460258*	*0.033396023*	*0.031817809*	*0.028719979*
**ENSG00000147100**	*0.017472547*	*0.026898408*	*0.025845403*	*0.016792076*
**ENSG00000123992**	*0.030531738*	*0.02896061*	*0.030586702*	*0.023905584*
**ENSG00000107130**	*0.016869051*	*0.025424636*	*0.026034577*	*0.01679869*
**ENSG00000105366**	*0.028685088*	*0.029400976*	*0.028176501*	*0.030554917*
**ENSG00000151033**	*0.016444629*	*0.024966825*	*0.026634638*	*0.015156702*
**ENSG00000146021**	*0.056880783*	*0.053483874*	*0.042737903*	*0.046799979*
**ENSG00000126524**	*0.029963857*	*0.036810093*	*0.035983494*	*0.026617673*
**ENSG00000170734**	*0.042223531*	*0.040802262*	*0.0418385*	*0.050233676*
**ENSG00000122952**	*0.048913996*	*0.043662447*	*0.038573097*	*0.038401083*
**ENSG00000122188**	*0.085382439*	*0.068512552*	*0.066516547*	*0.072236707*
**ENSG00000170604**	*0.019783983*	*0.02698477*	*0.028965951*	*0.017436152*
**ENSG00000197165**	*0.016259538*	*0.025613408*	*0.025240476*	*0.018105707*
**ENSG00000180957**	*0.023678598*	*0.029319174*	*0.03012353*	*0.022421216*
**ENSG00000179709**	*0.017150762*	*0.024655507*	*0.025044776*	*0.015976268*
**ENSG00000124882**	*0.019585362*	*0.026353479*	*0.029974584*	*0.015529069*
**ENSG00000100167**	*0.015072884*	*0.025863077*	*0.025020807*	*0.015866222*
**ENSG00000067829**	*0.017800387*	*0.026939184*	*0.026328695*	*0.017201241*
**ENSG00000141012**	*0.025300219*	*0.031820222*	*0.031340925*	*0.033014875*
**ENSG00000187790**	*0.016533545*	*0.026202507*	*0.026060797*	*0.01931799*

**ENSG00000147144**	*0.048053455*	*0.037537279*	*0.038457531*	*0.041290223*
**ENSG00000084652**	*0.03014212*	*0.027693575*	*0.027567666*	*0.021575168*
**ENSG00000163568**	*0.069767584*	*0.067935152*	*0.059732039*	*0.050302011*
**ENSG00000172073**	*0.015644755*	*0.024942733*	*0.025689459*	*0.014746511*
**ENSG00000133962**	*0.018250972*	*0.026040814*	*0.025639269*	*0.016859076*
**ENSG00000163864**	*0.07708714*	*0.054918643*	*0.064325842*	*0.081195344*
**ENSG00000173486**	*0.01835741*	*0.027476408*	*0.027338924*	*0.016854167*
**ENSG00000128973**	*0.035153785*	*0.037819563*	*0.033847033*	*0.030607924*
**ENSG00000116990**	*0.019130064*	*0.028167557*	*0.026729156*	*0.018117002*
**ENSG00000159618**	*0.084897053*	*0.071025283*	*0.089589992*	*0.094341616*
**ENSG00000166295**	*0.020924766*	*0.028291379*	*0.027939517*	*0.018638259*
**ENSG00000090432**	*0.025966985*	*0.03724444*	*0.042275295*	*0.02986083*
**ENSG00000121680**	*0.027038616*	*0.033492036*	*0.031674948*	*0.022324635*
**ENSG00000120949**	*0.04496632*	*0.041707208*	*0.03704147*	*0.037674183*
**ENSG00000167037**	*0.015594469*	*0.025105044*	*0.024747036*	*0.014757478*
**ENSG00000103145**	*0.039055066*	*0.033445788*	*0.034738812*	*0.033252425*
**ENSG00000147573**	*0.048374256*	*0.02905515*	*0.027887573*	*0.027622329*
**ENSG00000026559**	*0.015380846*	*0.024978709*	*0.025261182*	*0.014654368*
**ENSG00000105398**	*0.015159193*	*0.024635503*	*0.024362781*	*0.014389535*
**ENSG00000126243**	*0.071914034*	*0.062595295*	*0.054130763*	*0.058393634*
**ENSG00000140022**	*0.019085524*	*0.027038312*	*0.027700928*	*0.018814695*
**ENSG00000186810**	*0.117075214*	*0.066626564*	*0.071799498*	*0.077468418*
**ENSG00000182463**	*0.016465446*	*0.025499779*	*0.025152991*	*0.017415658*
**ENSG00000108239**	*0.024481439*	*0.027263135*	*0.028484626*	*0.018787781*
**ENSG00000151962**	*0.016671592*	*0.025088174*	*0.024375965*	*0.015306715*
**ENSG00000078668**	*0.021014005*	*0.028271816*	*0.026689713*	*0.017668895*
**ENSG00000090470**	*0.022167943*	*0.029785098*	*0.030085597*	*0.023711237*
**ENSG00000165682**	*0.017148125*	*0.026587956*	*0.025416423*	*0.0157786*
**ENSG00000171119**	*0.016953773*	*0.024855768*	*0.025686925*	*0.014659442*
**ENSG00000103044**	*0.015033027*	*0.024406135*	*0.024329534*	*0.014705047*
**ENSG00000175426**	*0.0156513*	*0.024801818*	*0.025871092*	*0.01525914*
**ENSG00000197776**	*0.034888338*	*0.035621417*	*0.03201554*	*0.032149294*
**ENSG00000111664**	*0.016764806*	*0.027640724*	*0.02558568*	*0.017332796*
**ENSG00000095059**	*0.026090427*	*0.028521*	*0.030705695*	*0.028385289*
**ENSG00000188493**	*0.025263485*	*0.031797472*	*0.0295931*	*0.025302575*
**ENSG00000092094**	*0.033212919*	*0.034288883*	*0.032434409*	*0.027762003*
**ENSG00000159307**	*0.015635529*	*0.023925548*	*0.024304825*	*0.015758192*
**ENSG00000184584**	*0.042898201*	*0.041890065*	*0.040216642*	*0.037758448*
**ENSG00000152315**	*0.01583511*	*0.026250212*	*0.027301376*	*0.016227516*
**ENSG00000175895**	*0.041207323*	*0.044767004*	*0.044626054*	*0.04416799*
**ENSG00000140057**	*0.01847924*	*0.0260108*	*0.027404849*	*0.01729368*
**ENSG00000140067**	*0.015223654*	*0.024819595*	*0.025360813*	*0.015976394*
**ENSG00000078043**	*0.025557373*	*0.031489597*	*0.031545995*	*0.02269542*
**ENSG00000148218**	*0.022151579*	*0.027666121*	*0.029021657*	*0.022419941*
**ENSG00000131149**	*0.029258713*	*0.031775048*	*0.036612971*	*0.028980452*
**ENSG00000118961**	*0.036371567*	*0.040980108*	*0.03561025*	*0.026549059*
**ENSG00000068438**	*0.017041238*	*0.026481665*	*0.02659883*	*0.017538833*
**ENSG00000204065**	*0.017216751*	*0.026695823*	*0.024608227*	*0.027004666*
**ENSG00000116288**	*0.018278648*	*0.026112825*	*0.025605254*	*0.01672842*
**ENSG00000158246**	*0.016862255*	*0.027436988*	*0.027193053*	*0.016174054*
**ENSG00000157538**	*0.027559027*	*0.034809687*	*0.033513898*	*0.03093662*
**ENSG00000111432**	*0.015000067*	*0.023901727*	*0.024984395*	*0.01492326*
**ENSG00000119699**	*0.018563693*	*0.025828754*	*0.025149722*	*0.015618154*
**ENSG00000092978**	*0.035296043*	*0.04181077*	*0.03367707*	*0.04001172*

**ENSG00000109072**	*0.015589356*	*0.025156756*	*0.024720687*	*0.016696341*
**ENSG00000243543**	*0.015080267*	*0.025259832*	*0.024835965*	*0.014603424*
**ENSG00000164591**	*0.017963055*	*0.0246422*	*0.024810418*	*0.014893313*
**ENSG00000167881**	*0.024889469*	*0.028124681*	*0.030836189*	*0.020139472*
**ENSG00000113108**	*0.034024462*	*0.03431438*	*0.032748834*	*0.038600852*
**ENSG00000102265**	*0.045630094*	*0.043023208*	*0.042700005*	*0.03816963*
**ENSG00000187758**	*0.017022901*	*0.024487438*	*0.024828501*	*0.016746599*
**ENSG00000060709**	*0.152375573*	*0.110059917*	*0.082614657*	*0.114266587*
**ENSG00000130726**	*0.029461823*	*0.029893081*	*0.042165729*	*0.031754206*
**ENSG00000104490**	*0.086061682*	*0.070770828*	*0.075429142*	*0.070075419*
**ENSG00000105393**	*0.026203059*	*0.02910937*	*0.028034078*	*0.0205666*
**ENSG00000120075**	*0.016769181*	*0.027434623*	*0.026497074*	*0.021597043*
**ENSG00000198691**	*0.039499251*	*0.024761427*	*0.024558762*	*0.021773273*
**ENSG00000170099**	*0.0505505*	*0.026960597*	*0.024556651*	*0.037323996*
**ENSG00000020922**	*0.033340646*	*0.039719961*	*0.029998371*	*0.023409957*
**ENSG00000135577**	*0.01562687*	*0.025570762*	*0.024770817*	*0.014115754*
**ENSG00000163817**	*0.015825792*	*0.025031973*	*0.024379321*	*0.016751866*
**ENSG00000155719**	*0.02846199*	*0.026200334*	*0.024756103*	*0.016320992*
**ENSG00000129667**	*0.032793294*	*0.038257027*	*0.03598878*	*0.039414766*
**ENSG00000159556**	*0.082697987*	*0.061765526*	*0.061424333*	*0.066555289*
**ENSG00000175265**	*0.0575396*	*0.048704336*	*0.043439794*	*0.054881257*
**ENSG00000164674**	*0.069182541*	*0.061135415*	*0.063928614*	*0.057441541*
**ENSG00000140044**	*0.050453613*	*0.042136*	*0.041538691*	*0.045146774*
**ENSG00000131831**	*0.016489719*	*0.024839165*	*0.025157131*	*0.015530194*
**ENSG00000148840**	*0.035483687*	*0.044389808*	*0.051169629*	*0.0422396*
**ENSG00000132631**	*0.016884075*	*0.02583066*	*0.025462281*	*0.017498446*
**ENSG00000137094**	*0.049031271*	*0.043467882*	*0.042482289*	*0.044632061*
**ENSG00000196263**	*0.017317545*	*0.027732684*	*0.028513662*	*0.017440811*
**ENSG00000189299**	*0.016154131*	*0.025676047*	*0.025051848*	*0.014468272*
**ENSG00000120149**	*0.018508089*	*0.025841942*	*0.028961233*	*0.015504672*
**ENSG00000235631**	*0.034340737*	*0.025817473*	*0.025538295*	*0.017862539*
**ENSG00000160783**	*0.034939319*	*0.031516965*	*0.035104699*	*0.025822316*
**ENSG00000172824**	*0.057194932*	*0.041312715*	*0.037171377*	*0.041413891*
**ENSG00000114993**	*0.0753506*	*0.055227766*	*0.045489777*	*0.0636157*
**ENSG00000213714**	*0.016996055*	*0.032363729*	*0.027113501*	*0.017657389*
**ENSG00000010292**	*0.04193658*	*0.045696984*	*0.044619342*	*0.034271033*
**ENSG00000096401**	*0.024282446*	*0.030770201*	*0.030343861*	*0.021894315*
**ENSG00000108021**	*0.030196528*	*0.036990627*	*0.040462786*	*0.033688612*
**ENSG00000106484**	*0.026540706*	*0.033368572*	*0.025848104*	*0.023808455*
**ENSG00000111237**	*0.025038375*	*0.031913534*	*0.027289806*	*0.020116275*
**ENSG00000134602**	*0.031399942*	*0.036982844*	*0.032723164*	*0.033808134*
**ENSG00000174015**	*0.022758462*	*0.025655251*	*0.026185809*	*0.017714528*
**ENSG00000127980**	*0.03269289*	*0.040889966*	*0.034776318*	*0.027750357*
**ENSG00000135119**	*0.016922441*	*0.025084911*	*0.024603891*	*0.016938488*
**ENSG00000131747**	*0.038142625*	*0.034408389*	*0.032662914*	*0.028507161*
**ENSG00000130529**	*0.019496098*	*0.030238406*	*0.029538516*	*0.021944727*
**ENSG00000135316**	*0.033144351*	*0.035359709*	*0.037464339*	*0.038386164*
**ENSG00000168575**	*0.02256073*	*0.029175949*	*0.029409562*	*0.022743778*
**ENSG00000151929**	*0.092913447*	*0.0605449*	*0.051877247*	*0.058295208*
**ENSG00000025293**	*0.017622018*	*0.02544883*	*0.025698495*	*0.015839772*
**ENSG00000179044**	*0.039480223*	*0.044426263*	*0.034517772*	*0.035101512*
**ENSG00000119402**	*0.027272722*	*0.034177829*	*0.03309994*	*0.029864648*
**ENSG00000163827**	*0.016416351*	*0.024904019*	*0.025107959*	*0.017033369*
**ENSG00000197446**	*0.015312545*	*0.024440115*	*0.024643421*	*0.01575181*

**ENSG00000180919**	*0.016345691*	*0.026345164*	*0.024307091*	*0.016230142*
**ENSG00000185163**	*0.043133213*	*0.04154002*	*0.044262569*	*0.032301047*
**ENSG00000165030**	*0.065047637*	*0.068789404*	*0.066742377*	*0.073662955*
**ENSG00000007350**	*0.018595777*	*0.024920039*	*0.024249024*	*0.015773796*
**ENSG00000185630**	*0.015623171*	*0.024464895*	*0.025029667*	*0.01634429*
**ENSG00000171681**	*0.031861757*	*0.038573613*	*0.045488465*	*0.036839911*
**ENSG00000133317**	*0.016335742*	*0.025008761*	*0.024481315*	*0.015534071*
**ENSG00000134375**	*0.033996531*	*0.03605299*	*0.030538392*	*0.030321536*
**ENSG00000212125**	*0.015573191*	*0.025647377*	*0.024891173*	*0.016546753*
**ENSG00000115457**	*0.053166087*	*0.082260288*	*0.063840088*	*0.063560135*
**ENSG00000169925**	*0.037454559*	*0.039257046*	*0.039263841*	*0.03679601*
**ENSG00000111644**	*0.02315653*	*0.027317723*	*0.026487131*	*0.022916759*
**ENSG00000136935**	*0.029015101*	*0.03476491*	*0.03085935*	*0.028842387*
**ENSG00000253305**	*0.01912575*	*0.026382831*	*0.02575263*	*0.015835183*
**ENSG00000145431**	*0.062016591*	*0.025052651*	*0.025046166*	*0.014896925*
**ENSG00000110092**	*0.086448285*	*0.069117514*	*0.062231974*	*0.071007075*
**ENSG00000104331**	*0.0355779*	*0.037630267*	*0.039371811*	*0.032149764*
**ENSG00000104774**	*0.029202344*	*0.029877312*	*0.031851045*	*0.033037683*
**ENSG00000106304**	*0.019079088*	*0.025393289*	*0.025614625*	*0.017675894*
**ENSG00000102699**	*0.023833827*	*0.029569753*	*0.028472128*	*0.022039844*
**ENSG00000166455**	*0.017745102*	*0.025164166*	*0.025977809*	*0.017283796*
**ENSG00000198844**	*0.017983767*	*0.02550673*	*0.024428567*	*0.016976708*
**ENSG00000137404**	*0.050724199*	*0.044483073*	*0.04012114*	*0.034613157*
**ENSG00000081177**	*0.030171425*	*0.039496429*	*0.030939805*	*0.032752059*
**ENSG00000179979**	*0.037379149*	*0.032309968*	*0.036657305*	*0.024830266*
**ENSG00000118276**	*0.04283443*	*0.051910093*	*0.044660733*	*0.041622563*
**ENSG00000163346**	*0.049904853*	*0.049342748*	*0.039309844*	*0.035070688*
**ENSG00000115665**	*0.016275876*	*0.024933566*	*0.024469349*	*0.015497975*
**ENSG00000105669**	*0.022366816*	*0.026124058*	*0.029163948*	*0.020105366*
**ENSG00000118420**	*0.022348742*	*0.028209348*	*0.025141848*	*0.017506465*
**ENSG00000006534**	*0.02060781*	*0.024581199*	*0.025536099*	*0.017963989*
**ENSG00000109927**	*0.015936318*	*0.024756799*	*0.024965886*	*0.015759324*
**ENSG00000102802**	*0.015657083*	*0.025244413*	*0.024963604*	*0.014958957*
**ENSG00000110330**	*0.031191673*	*0.034640796*	*0.034841502*	*0.028073313*
**ENSG00000186350**	*0.043859514*	*0.050335238*	*0.040073879*	*0.046202645*
**ENSG00000143748**	*0.029414379*	*0.034421588*	*0.029787557*	*0.02558104*
**ENSG00000161551**	*0.029666728*	*0.031380229*	*0.026803543*	*0.029981519*
**ENSG00000177181**	*0.092155798*	*0.064031204*	*0.058249765*	*0.056297999*
**ENSG00000156232**	*0.03598567*	*0.047349905*	*0.046727907*	*0.030422178*
**ENSG00000055118**	*0.019449102*	*0.026444415*	*0.025496602*	*0.01666146*
**ENSG00000166845**	*0.035089817*	*0.037830212*	*0.029991324*	*0.025743304*
**ENSG00000041988**	*0.015869352*	*0.026147169*	*0.026249281*	*0.019129018*
**ENSG00000063660**	*0.015281592*	*0.024747462*	*0.024799728*	*0.014621698*
**ENSG00000240871**	*0.016287842*	*0.024476995*	*0.024636132*	*0.01457301*
**ENSG00000136444**	*0.032796425*	*0.031016881*	*0.029297267*	*0.021248527*
**ENSG00000096968**	*0.037305338*	*0.04420649*	*0.036371522*	*0.03088478*
**ENSG00000085644**	*0.025621505*	*0.030668346*	*0.033339467*	*0.027109068*
**ENSG00000116183**	*0.014986771*	*0.025281548*	*0.024875415*	*0.015075278*
**ENSG00000196391**	*0.015228614*	*0.025282431*	*0.024424353*	*0.01554916*
**ENSG00000042304**	*0.015606821*	*0.024448672*	*0.024930172*	*0.016424372*
**ENSG00000184786**	*0.015442402*	*0.025185625*	*0.024602917*	*0.014810828*
**ENSG00000131620**	*0.016960683*	*0.025528407*	*0.02471711*	*0.015109319*
**ENSG00000134569**	*0.016640654*	*0.025936731*	*0.02523356*	*0.016017065*
**ENSG00000164284**	*0.036992733*	*0.040784949*	*0.040531572*	*0.035458808*

**ENSG00000161973**	*0.016032794*	*0.025367566*	*0.024702334*	*0.016261524*
**ENSG00000105662**	*0.015966367*	*0.025879147*	*0.02604245*	*0.017247511*
**ENSG00000112115**	*0.015660075*	*0.02437928*	*0.024921375*	*0.015728514*
**ENSG00000075223**	*0.020802802*	*0.025867825*	*0.025156919*	*0.018287041*
**ENSG00000129351**	*0.043302226*	*0.055551111*	*0.040538799*	*0.044965912*
**ENSG00000198673**	*0.017069224*	*0.025240768*	*0.024765767*	*0.015836429*
**ENSG00000186204**	*0.019437765*	*0.025460305*	*0.026640186*	*0.01528987*
**ENSG00000188038**	*0.015690931*	*0.024442925*	*0.024541689*	*0.016363895*
**ENSG00000174111**	*0.016350428*	*0.026477164*	*0.026763937*	*0.016806926*
**ENSG00000078177**	*0.031326274*	*0.03243222*	*0.033040955*	*0.031624855*
**ENSG00000188554**	*0.027857597*	*0.032059492*	*0.03117239*	*0.028341725*
**ENSG00000184911**	*0.017804976*	*0.025169371*	*0.024566299*	*0.015302858*
**ENSG00000198959**	*0.055840737*	*0.055387024*	*0.048038269*	*0.04010671*
**ENSG00000151693**	*0.032190494*	*0.038336071*	*0.025546502*	*0.015419102*
**ENSG00000125846**	*0.036669214*	*0.04166411*	*0.040474774*	*0.034091593*
**ENSG00000145592**	*0.016192364*	*0.024870115*	*0.025156587*	*0.020050429*
**ENSG00000188282**	*0.021472147*	*0.032822608*	*0.027735993*	*0.027133852*
**ENSG00000124812**	*0.014955642*	*0.025230332*	*0.025667234*	*0.01405087*
**ENSG00000185215**	*0.062572952*	*0.054266283*	*0.051683855*	*0.072524759*
**ENSG00000142188**	*0.040111695*	*0.040842676*	*0.047481655*	*0.031512107*
**ENSG00000066855**	*0.034892015*	*0.034457486*	*0.034671864*	*0.030822084*
**ENSG00000182572**	*0.033481642*	*0.030031081*	*0.03374177*	*0.032393944*
**ENSG00000177954**	*0.013377519*	*0.023560006*	*0.023341876*	*0.014750626*
**ENSG00000081154**	*0.025833386*	*0.031538996*	*0.027646669*	*0.020417854*
**ENSG00000113430**	*0.019088389*	*0.024777524*	*0.025232918*	*0.015795557*
**ENSG00000104432**	*0.061887082*	*0.059007034*	*0.05883271*	*0.063832027*
**ENSG00000140795**	*0.016290655*	*0.024573912*	*0.024545688*	*0.015135396*
**ENSG00000034533**	*0.030561813*	*0.032986183*	*0.034511887*	*0.027911309*
**ENSG00000204657**	*0.018154844*	*0.027651009*	*0.029058696*	*0.022607495*
**ENSG00000136158**	*0.120442654*	*0.08991662*	*0.090147028*	*0.096454463*
**ENSG00000171497**	*0.033316608*	*0.03749305*	*0.0385186*	*0.030240022*
**ENSG00000065183**	*0.028987524*	*0.030235175*	*0.028911431*	*0.032134936*
**ENSG00000159433**	*0.03061701*	*0.03276231*	*0.032661052*	*0.031713771*
**ENSG00000034693**	*0.035083294*	*0.036715133*	*0.032183995*	*0.028791201*
**ENSG00000032389**	*0.028933324*	*0.033191247*	*0.03462808*	*0.029098387*
**ENSG00000170948**	*0.015229354*	*0.024928153*	*0.024592761*	*0.014824008*
**ENSG00000118308**	*0.044462717*	*0.04205501*	*0.05227346*	*0.041268514*
**ENSG00000130957**	*0.015830359*	*0.025506628*	*0.024798355*	*0.015404782*
**ENSG00000184986**	*0.031041468*	*0.033807334*	*0.035848454*	*0.025839648*
**ENSG00000147604**	*0.017388842*	*0.024792222*	*0.023902737*	*0.017569335*
**ENSG00000128383**	*0.025754505*	*0.029230465*	*0.029213016*	*0.023367321*
**ENSG00000153266**	*0.020698602*	*0.026781885*	*0.026945162*	*0.018042698*
**ENSG00000204414**	*0.015336957*	*0.024078476*	*0.024553665*	*0.015281881*
**ENSG00000100767**	*0.015859145*	*0.025126614*	*0.024940598*	*0.016210025*
**ENSG00000111254**	*0.021071062*	*0.02946399*	*0.027195298*	*0.02166751*
**ENSG00000176624**	*0.02807613*	*0.032968581*	*0.032983981*	*0.028235973*
**ENSG00000103245**	*0.024325165*	*0.031483863*	*0.034599848*	*0.027736428*
**ENSG00000124251**	*0.015169454*	*0.024855419*	*0.024931233*	*0.014708416*
**ENSG00000125352**	*0.033162979*	*0.032258473*	*0.032681992*	*0.024720518*
**ENSG00000039987**	*0.019106719*	*0.02537032*	*0.0254977*	*0.016944231*
**ENSG00000165169**	*0.030861883*	*0.032413044*	*0.033511577*	*0.031529378*
**ENSG00000198771**	*0.035242798*	*0.038862105*	*0.037243083*	*0.030073654*
**ENSG00000173578**	*0.01813673*	*0.027625289*	*0.027271657*	*0.017984686*
**ENSG00000070087**	*0.113681133*	*0.094735441*	*0.069934364*	*0.08898886*

**ENSG00000198965**	*0.018159065*	*0.026831222*	*0.028961097*	*0.016868833*
**ENSG00000072121**	*0.039192718*	*0.043893999*	*0.034710384*	*0.030467765*
**ENSG00000058404**	*0.020754044*	*0.028726049*	*0.027290622*	*0.018446044*
**ENSG00000213782**	*0.027819495*	*0.034008899*	*0.030920321*	*0.028323054*
**ENSG00000158526**	*0.023143115*	*0.030102389*	*0.029875513*	*0.022936665*
**ENSG00000167074**	*0.036853434*	*0.040511081*	*0.041028103*	*0.030496066*
**ENSG00000171860**	*0.01479047*	*0.024329447*	*0.024973276*	*0.015502528*
**ENSG00000188015**	*0.027259974*	*0.03071398*	*0.032177407*	*0.021679834*
**ENSG00000167105**	*0.015549884*	*0.025835093*	*0.024859578*	*0.015182705*
**ENSG00000165171**	*0.02717211*	*0.040786652*	*0.031689557*	*0.029367143*
**ENSG00000167535**	*0.017520006*	*0.025836502*	*0.025012296*	*0.015670746*
**ENSG00000185482**	*0.025376085*	*0.034237825*	*0.032381887*	*0.023307318*
**ENSG00000157111**	*0.016408318*	*0.024453943*	*0.024810144*	*0.015264954*
**ENSG00000125741**	*0.022060013*	*0.028030367*	*0.028198971*	*0.021548504*
**ENSG00000168539**	*0.01656382*	*0.024905599*	*0.025815236*	*0.014969846*
**ENSG00000187569**	*0.016327373*	*0.025056182*	*0.026204672*	*0.017660655*
**ENSG00000198231**	*0.02337534*	*0.030764599*	*0.031924714*	*0.02231113*
**ENSG00000197093**	*0.055801679*	*0.04903808*	*0.038803179*	*0.045283247*
**ENSG00000103363**	*0.019566998*	*0.025724412*	*0.026890075*	*0.01818065*
**ENSG00000137720**	*0.037833916*	*0.034629069*	*0.037064516*	*0.035923857*
**ENSG00000165704**	*0.02971255*	*0.034885985*	*0.030528832*	*0.024385722*
**ENSG00000124507**	*0.030618811*	*0.05657609*	*0.038991891*	*0.033406663*
**ENSG00000165659**	*0.018626037*	*0.027747622*	*0.025926105*	*0.021691523*
**ENSG00000104881**	*0.033761715*	*0.040272579*	*0.037241016*	*0.04497369*
**ENSG00000164751**	*0.038175624*	*0.031445722*	*0.039256741*	*0.033074585*
**ENSG00000049768**	*0.016179602*	*0.024666269*	*0.025025999*	*0.015255434*
**ENSG00000145685**	*0.045830451*	*0.04151948*	*0.036408918*	*0.039764499*
**ENSG00000163254**	*0.015768274*	*0.024943013*	*0.024126719*	*0.015020276*
**ENSG00000087085**	*0.015411209*	*0.025923919*	*0.025017415*	*0.016435865*
**ENSG00000122585**	*0.074009837*	*0.035322262*	*0.03016924*	*0.035133729*
**ENSG00000198055**	*0.01908759*	*0.025264256*	*0.026506925*	*0.017825415*
**ENSG00000169347**	*0.016286571*	*0.025151523*	*0.025313107*	*0.015777938*
**ENSG00000167118**	*0.02266807*	*0.028784905*	*0.035662165*	*0.024721708*
**ENSG00000126062**	*0.028797312*	*0.035271689*	*0.038090366*	*0.027157339*
**ENSG00000135622**	*0.028141971*	*0.033932531*	*0.029777037*	*0.024819642*
**ENSG00000173137**	*0.030108261*	*0.035452523*	*0.03091679*	*0.025606255*
**ENSG00000171425**	*0.024767411*	*0.029798731*	*0.033249972*	*0.030121145*
**ENSG00000115548**	*0.056727587*	*0.05205795*	*0.061529981*	*0.050029139*
**ENSG00000021300**	*0.019056722*	*0.027048159*	*0.027367317*	*0.030332693*
**ENSG00000092051**	*0.047686743*	*0.038522702*	*0.03004428*	*0.031700162*
**ENSG00000153113**	*0.036930943*	*0.038204382*	*0.032539633*	*0.038093136*
**ENSG00000138100**	*0.019331724*	*0.027620843*	*0.027374127*	*0.017101951*
**ENSG00000147274**	*0.021088669*	*0.026877391*	*0.027344673*	*0.021920137*
**ENSG00000180347**	*0.015892899*	*0.025243818*	*0.025877367*	*0.015686094*
**ENSG00000186951**	*0.020198338*	*0.026885342*	*0.026711083*	*0.019963947*
**ENSG00000167815**	*0.01698839*	*0.024849198*	*0.025751825*	*0.015690188*
**ENSG00000138439**	*0.018663546*	*0.027219042*	*0.029144149*	*0.019810509*
**ENSG00000188937**	*0.014998583*	*0.024204829*	*0.024855189*	*0.014831815*
**ENSG00000160117**	*0.032429773*	*0.032262056*	*0.034497093*	*0.025895756*
**ENSG00000167984**	*0.015717414*	*0.024811563*	*0.02500673*	*0.016588167*
**ENSG00000163900**	*0.032919083*	*0.032663137*	*0.035048256*	*0.027170389*
**ENSG00000212710**	*0.016572864*	*0.02608626*	*0.025876472*	*0.014996171*
**ENSG00000139718**	*0.033351641*	*0.03437538*	*0.03806162*	*0.028672524*
**ENSG00000177182**	*0.020481306*	*0.027734577*	*0.025886368*	*0.020486938*

**ENSG00000060718**	*0.016776988*	*0.02526856*	*0.025182301*	*0.015553374*
**ENSG00000074410**	*0.015743876*	*0.025391188*	*0.025090994*	*0.016345473*
**ENSG00000164091**	*0.031159223*	*0.03216575*	*0.034685313*	*0.026907416*
**ENSG00000182963**	*0.025037479*	*0.028751449*	*0.029116819*	*0.01891523*
**ENSG00000181495**	*0.017138208*	*0.026396234*	*0.024958362*	*0.019014227*
**ENSG00000168394**	*0.022203422*	*0.030220135*	*0.032514774*	*0.020736709*
**ENSG00000153339**	*0.025459032*	*0.033633529*	*0.031378966*	*0.028391248*
**ENSG00000168060**	*0.040539389*	*0.037817615*	*0.032590751*	*0.033036897*
**ENSG00000115947**	*0.027636237*	*0.030018348*	*0.028467361*	*0.023719267*
**ENSG00000107282**	*0.014729724*	*0.02504696*	*0.02365675*	*0.014943291*
**ENSG00000212124**	*0.01521115*	*0.024821999*	*0.024815622*	*0.014399025*
**ENSG00000106328**	*0.017961352*	*0.025625868*	*0.026106263*	*0.016806111*
**ENSG00000111341**	*0.016808133*	*0.025322995*	*0.025787442*	*0.015794197*
**ENSG00000083896**	*0.022475028*	*0.028927404*	*0.028656698*	*0.017372975*
**ENSG00000162510**	*0.023641018*	*0.029584681*	*0.026677961*	*0.018356056*
**ENSG00000049618**	*0.014660422*	*0.025219964*	*0.024704307*	*0.014621626*
**ENSG00000131375**	*0.035649135*	*0.044213244*	*0.035501246*	*0.040023003*
**ENSG00000147155**	*0.035694536*	*0.031594951*	*0.030954424*	*0.031077954*
**ENSG00000130005**	*0.016266819*	*0.02526652*	*0.025344261*	*0.015538727*
**ENSG00000117543**	*0.031666207*	*0.032709778*	*0.03244199*	*0.026719276*
**ENSG00000141646**	*0.025457624*	*0.031982781*	*0.029236631*	*0.023685405*
**ENSG00000145817**	*0.031786812*	*0.03217956*	*0.038915199*	*0.030482419*
**ENSG00000170190**	*0.054823325*	*0.046893641*	*0.041441137*	*0.048261253*
**ENSG00000221994**	*0.03653664*	*0.037225435*	*0.035860118*	*0.037070506*
**ENSG00000010803**	*0.024757064*	*0.0291482*	*0.031625944*	*0.023295332*
**ENSG00000140853**	*0.034445966*	*0.036946645*	*0.033979092*	*0.035466732*
**ENSG00000006611**	*0.01691125*	*0.025773101*	*0.025888012*	*0.015610182*
**ENSG00000105675**	*0.041173092*	*0.041262707*	*0.046588857*	*0.04495732*
**ENSG00000107779**	*0.067230955*	*0.044009435*	*0.044329807*	*0.030761457*
**ENSG00000186283**	*0.038530054*	*0.037033861*	*0.044218602*	*0.040837655*
**ENSG00000102245**	*0.01693001*	*0.025425213*	*0.025169092*	*0.016277704*
**ENSG00000182957**	*0.026283723*	*0.033993265*	*0.030014051*	*0.034804012*
**ENSG00000104951**	*0.016323839*	*0.026113449*	*0.025746702*	*0.01602727*
**ENSG00000182916**	*0.029982325*	*0.029182301*	*0.026763211*	*0.01963475*
**ENSG00000084623**	*0.021512661*	*0.028085065*	*0.027336063*	*0.017448033*
**ENSG00000090263**	*0.024249021*	*0.028841711*	*0.027413169*	*0.018501857*
**ENSG00000169902**	*0.059606549*	*0.056902098*	*0.059070517*	*0.058431832*
**ENSG00000138041**	*0.025643368*	*0.033893021*	*0.029217899*	*0.023291352*
**ENSG00000073578**	*0.025321556*	*0.027893811*	*0.029362887*	*0.018432257*
**ENSG00000139044**	*0.016486641*	*0.026377842*	*0.024212613*	*0.016407846*
**ENSG00000165879**	*0.021698113*	*0.026328724*	*0.025632703*	*0.02016349*
**ENSG00000081479**	*0.016263598*	*0.026114915*	*0.024644227*	*0.015234259*
**ENSG00000019549**	*0.015512862*	*0.025644455*	*0.025684218*	*0.015224398*
**ENSG00000134365**	*0.016457813*	*0.025251384*	*0.024479027*	*0.015212203*
**ENSG00000242419**	*0.014767263*	*0.02487402*	*0.024263441*	*0.015046753*
**ENSG00000101574**	*0.033725962*	*0.03438596*	*0.033875697*	*0.024078198*
**ENSG00000196411**	*0.017351665*	*0.027245256*	*0.02770455*	*0.016487544*
**ENSG00000167378**	*0.015843425*	*0.024900241*	*0.024797354*	*0.015104796*
**ENSG00000170832**	*0.035930085*	*0.036268365*	*0.035855801*	*0.028849321*
**ENSG00000119703**	*0.046831962*	*0.047503479*	*0.037568076*	*0.034066512*
**ENSG00000125813**	*0.014771491*	*0.025158022*	*0.024463755*	*0.015260798*
**ENSG00000180818**	*0.015176722*	*0.024203914*	*0.024150863*	*0.015720788*
**ENSG00000111231**	*0.03287994*	*0.035861554*	*0.032905336*	*0.02523442*
**ENSG00000174938**	*0.016639573*	*0.025416885*	*0.024347808*	*0.015511283*

**ENSG00000185900**	*0.01557225*	*0.024471083*	*0.024783622*	*0.01556761*
**ENSG00000240053**	*0.019153517*	*0.026493244*	*0.025952231*	*0.017100894*
**ENSG00000154957**	*0.026113567*	*0.028197646*	*0.028803224*	*0.019939467*
**ENSG00000183784**	*0.02003005*	*0.025740596*	*0.02716186*	*0.020051698*
**ENSG00000172239**	*0.021786356*	*0.029187051*	*0.029748526*	*0.026287397*
**ENSG00000072518**	*0.025760364*	*0.032510787*	*0.02999005*	*0.023983661*
**ENSG00000125991**	*0.020660945*	*0.026066647*	*0.028448586*	*0.02498125*
**ENSG00000213413**	*0.068640876*	*0.043327575*	*0.043585015*	*0.063805898*
**ENSG00000197558**	*0.01810098*	*0.027267297*	*0.025105814*	*0.022702991*
**ENSG00000171466**	*0.027900026*	*0.04113202*	*0.040515865*	*0.030510888*
**ENSG00000131096**	*0.016026006*	*0.024358791*	*0.025142041*	*0.01606513*
**ENSG00000163207**	*0.043097316*	*0.033730677*	*0.027013374*	*0.023046925*
**ENSG00000044574**	*0.049000277*	*0.044145619*	*0.049672338*	*0.049230736*
**ENSG00000242885**	*0.015154067*	*0.024748266*	*0.025843837*	*0.015602905*
**ENSG00000080603**	*0.020318254*	*0.035807375*	*0.040866638*	*0.031377161*
**ENSG00000048462**	*0.040555255*	*0.038835965*	*0.038374722*	*0.043293877*
**ENSG00000131409**	*0.016039157*	*0.02603561*	*0.024986098*	*0.0153255*
**ENSG00000151276**	*0.015659855*	*0.025023743*	*0.02448769*	*0.015227548*
**ENSG00000234414**	*0.016574036*	*0.024288756*	*0.02594644*	*0.015785231*
**ENSG00000073849**	*0.061259886*	*0.055986267*	*0.057058327*	*0.059938244*
**ENSG00000116704**	*0.035633597*	*0.034663806*	*0.037360796*	*0.028907082*
**ENSG00000173442**	*0.034086107*	*0.035504773*	*0.038527822*	*0.029623219*
**ENSG00000125107**	*0.025397639*	*0.033829954*	*0.028521483*	*0.019901007*
**ENSG00000136535**	*0.018820269*	*0.027240713*	*0.025559358*	*0.018867371*
**ENSG00000184601**	*0.017416922*	*0.026472445*	*0.025862542*	*0.017856512*
**ENSG00000165379**	*0.015844938*	*0.02515214*	*0.024261844*	*0.014912554*
**ENSG00000119899**	*0.035369306*	*0.040095281*	*0.03829488*	*0.034671907*
**ENSG00000118514**	*0.020590765*	*0.027822795*	*0.029160763*	*0.023407734*
**ENSG00000048991**	*0.023879023*	*0.034312717*	*0.028262153*	*0.022328707*
**ENSG00000174837**	*0.091564178*	*0.036250658*	*0.045079271*	*0.082238304*
**ENSG00000096696**	*0.017526861*	*0.024724982*	*0.025097709*	*0.017504884*
**ENSG00000161791**	*0.029970886*	*0.033236823*	*0.028865165*	*0.027281939*
**ENSG00000102317**	*0.023336892*	*0.0280492*	*0.026915339*	*0.02834031*
**ENSG00000162702**	*0.031397847*	*0.037631914*	*0.041317277*	*0.029978421*
**ENSG00000165966**	*0.016346512*	*0.024921747*	*0.024481672*	*0.014653914*
**ENSG00000180509**	*0.015186944*	*0.024539025*	*0.024549196*	*0.015458189*
**ENSG00000184178**	*0.037955372*	*0.041202718*	*0.033819165*	*0.032970103*
**ENSG00000204287**	*0.024260103*	*0.032227535*	*0.033561349*	*0.033968567*
**ENSG00000148950**	*0.031431679*	*0.034571346*	*0.033456541*	*0.027369191*
**ENSG00000074966**	*0.030960273*	*0.02963791*	*0.030045546*	*0.028921163*
**ENSG00000088002**	*0.01584185*	*0.024518223*	*0.025887819*	*0.01558142*
**ENSG00000204104**	*0.020390619*	*0.02794876*	*0.025940538*	*0.018909597*
**ENSG00000090776**	*0.019579059*	*0.025421155*	*0.025151099*	*0.019378523*
**ENSG00000075420**	*0.05641715*	*0.046117598*	*0.047234692*	*0.045562845*
**ENSG00000184489**	*0.052033395*	*0.044805754*	*0.047442952*	*0.054547068*
**ENSG00000171611**	*0.019097192*	*0.029427377*	*0.027615637*	*0.017686366*
**ENSG00000133246**	*0.01734505*	*0.02703106*	*0.025781073*	*0.019944557*
**ENSG00000112494**	*0.01697879*	*0.024625738*	*0.027722049*	*0.015195202*
**ENSG00000180900**	*0.016873687*	*0.025114613*	*0.024892218*	*0.015224575*
**ENSG00000147403**	*0.015195385*	*0.024158288*	*0.025495025*	*0.015627298*
**ENSG00000086475**	*0.037759865*	*0.036714643*	*0.0324573*	*0.033003478*
**ENSG00000104447**	*0.018800865*	*0.026479449*	*0.026391795*	*0.016149261*
**ENSG00000155849**	*0.016447731*	*0.026052022*	*0.024877061*	*0.016679903*
**ENSG00000144848**	*0.024653662*	*0.030291015*	*0.029410466*	*0.020023493*

**ENSG00000123815**	*0.026155577*	*0.032412392*	*0.034113282*	*0.025404168*
**ENSG00000164176**	*0.019742571*	*0.026778177*	*0.024910844*	*0.017895006*
**ENSG00000161638**	*0.030274354*	*0.036729367*	*0.02842531*	*0.021678003*
**ENSG00000144843**	*0.028915639*	*0.038701666*	*0.032209334*	*0.028408728*
**ENSG00000122008**	*0.031880662*	*0.034035872*	*0.032205233*	*0.032037056*
**ENSG00000100053**	*0.016601171*	*0.025659755*	*0.025138406*	*0.016122814*
**ENSG00000171812**	*0.016442352*	*0.025440737*	*0.02512215*	*0.016440361*
**ENSG00000161920**	*0.026855735*	*0.032197457*	*0.029834113*	*0.02758988*
**ENSG00000010310**	*0.01910974*	*0.027138586*	*0.025860385*	*0.017600914*
**ENSG00000183090**	*0.01865444*	*0.026822933*	*0.025922903*	*0.016980941*
**ENSG00000161016**	*0.043081942*	*0.039963311*	*0.038952955*	*0.039524274*
**ENSG00000174799**	*0.023423203*	*0.027909805*	*0.02923873*	*0.020782851*
**ENSG00000165623**	*0.022746799*	*0.025206064*	*0.025694956*	*0.015628871*
**ENSG00000109332**	*0.022077761*	*0.029366372*	*0.028771106*	*0.019146652*
**ENSG00000114933**	*0.016406557*	*0.024696105*	*0.02488136*	*0.014491266*
**ENSG00000115138**	*0.150433371*	*0.095029701*	*0.070833142*	*0.103683275*
**ENSG00000150667**	*0.020202347*	*0.025607445*	*0.024474876*	*0.016180909*
**ENSG00000111731**	*0.035680536*	*0.037890243*	*0.032307645*	*0.026355457*
**ENSG00000184210**	*0.020616984*	*0.03521657*	*0.030574796*	*0.038180901*
**ENSG00000187554**	*0.020305332*	*0.027472184*	*0.02522849*	*0.015953044*
**ENSG00000101557**	*0.035692802*	*0.03538548*	*0.037340045*	*0.034173572*
**ENSG00000138794**	*0.036323997*	*0.042616467*	*0.041641552*	*0.049933929*
**ENSG00000152782**	*0.022176547*	*0.02769119*	*0.028637449*	*0.022276433*
**ENSG00000134115**	*0.044081143*	*0.024629593*	*0.02429247*	*0.01573161*
**ENSG00000160188**	*0.079041712*	*0.096006933*	*0.073060106*	*0.076219119*
**ENSG00000171223**	*0.017323354*	*0.025522612*	*0.025935403*	*0.019520425*
**ENSG00000140612**	*0.023449031*	*0.028971657*	*0.028677006*	*0.020093029*
**ENSG00000149516**	*0.01661774*	*0.026297721*	*0.026058402*	*0.017066254*
**ENSG00000132155**	*0.022700992*	*0.02879748*	*0.033305969*	*0.021733936*
**ENSG00000129965**	*0.018786298*	*0.026203598*	*0.024689822*	*0.017285587*
**ENSG00000124613**	*0.020086185*	*0.028365842*	*0.028676068*	*0.018412641*
**ENSG00000163875**	*0.029382272*	*0.032228248*	*0.03188932*	*0.026208361*
**ENSG00000148634**	*0.039062837*	*0.051507261*	*0.038085012*	*0.033012448*
**ENSG00000136866**	*0.069033791*	*0.065299178*	*0.051658765*	*0.048271863*
**ENSG00000116237**	*0.031231353*	*0.039366547*	*0.033131762*	*0.03511259*
**ENSG00000167608**	*0.0165074*	*0.026747492*	*0.026295713*	*0.017908441*
**ENSG00000170542**	*0.05943417*	*0.047005283*	*0.046433892*	*0.045449007*
**ENSG00000188739**	*0.05350106*	*0.054618028*	*0.041285405*	*0.088762827*
**ENSG00000130783**	*0.017412674*	*0.025278436*	*0.024483847*	*0.015839456*
**ENSG00000145888**	*0.01653341*	*0.025384638*	*0.025218525*	*0.015912445*
**ENSG00000213593**	*0.026515196*	*0.031197357*	*0.031880522*	*0.024851425*
**ENSG00000204052**	*0.016576875*	*0.025613298*	*0.025268098*	*0.01661239*
**ENSG00000074755**	*0.023944708*	*0.029467528*	*0.031925998*	*0.02332889*
**ENSG00000160360**	*0.016453202*	*0.025297372*	*0.025772652*	*0.017152763*
**ENSG00000215910**	*0.015135123*	*0.026095371*	*0.024774984*	*0.014769477*
**ENSG00000108262**	*0.018641279*	*0.026150444*	*0.027679083*	*0.019069715*
**ENSG00000180269**	*0.016229076*	*0.02446695*	*0.024972454*	*0.015347116*
**ENSG00000104524**	*0.041295968*	*0.037166516*	*0.03668682*	*0.04140013*
**ENSG00000115226**	*0.015764329*	*0.025991775*	*0.024647246*	*0.015846901*
**ENSG00000116353**	*0.027977778*	*0.029495848*	*0.031609322*	*0.024955604*
**ENSG00000169371**	*0.024461914*	*0.030349043*	*0.030674819*	*0.023226847*
**ENSG00000147127**	*0.0166735*	*0.025108602*	*0.024525189*	*0.016054153*
**ENSG00000163357**	*0.017204095*	*0.02477403*	*0.024971049*	*0.015466799*
**ENSG00000106077**	*0.015974621*	*0.025387978*	*0.024777533*	*0.015278437*

**ENSG00000159753**	*0.026923608*	*0.030389639*	*0.032004145*	*0.025903667*
**ENSG00000140937**	*0.029606731*	*0.027464285*	*0.024901551*	*0.014040524*
**ENSG00000158290**	*0.037950577*	*0.033785555*	*0.039708208*	*0.032631119*
**ENSG00000160183**	*0.190544108*	*0.140837082*	*0.123842189*	*0.143582024*
**ENSG00000168116**	*0.03120319*	*0.038188157*	*0.03426361*	*0.027988255*
**ENSG00000134545**	*0.020153009*	*0.027087745*	*0.025037436*	*0.019205382*
**ENSG00000136240**	*0.037889564*	*0.041195242*	*0.045789396*	*0.040754015*
**ENSG00000122912**	*0.031527655*	*0.038442791*	*0.03357583*	*0.029388425*
**ENSG00000151247**	*0.031502811*	*0.038716757*	*0.032017787*	*0.027072196*
**ENSG00000188730**	*0.038198329*	*0.02663175*	*0.030309242*	*0.026057996*
**ENSG00000167995**	*0.017104729*	*0.026084086*	*0.026911762*	*0.016639923*
**ENSG00000184698**	*0.015031509*	*0.024527933*	*0.025110736*	*0.015643262*
**ENSG00000021645**	*0.093189952*	*0.052176069*	*0.02905144*	*0.074128342*
**ENSG00000155640**	*0.020492746*	*0.027645122*	*0.026804189*	*0.017942804*
**ENSG00000181915**	*0.033389273*	*0.034940825*	*0.037187803*	*0.03140322*
**ENSG00000109685**	*0.019580959*	*0.027387156*	*0.025299332*	*0.016995194*
**ENSG00000165238**	*0.015287386*	*0.025118671*	*0.025410994*	*0.015253019*
**ENSG00000152595**	*0.016260891*	*0.025269261*	*0.024828576*	*0.01600601*
**ENSG00000133121**	*0.016538378*	*0.026041248*	*0.026076057*	*0.015639704*
**ENSG00000137942**	*0.022792548*	*0.030387556*	*0.034120065*	*0.020766241*
**ENSG00000215041**	*0.025922269*	*0.03166736*	*0.030325343*	*0.023310223*
**ENSG00000105550**	*0.014695709*	*0.024428546*	*0.024472435*	*0.015574071*
**ENSG00000165370**	*0.01517923*	*0.024911432*	*0.023946368*	*0.014713743*
**ENSG00000159055**	*0.0394595*	*0.033533514*	*0.033400478*	*0.029254872*
**ENSG00000119227**	*0.029327691*	*0.035515539*	*0.030357183*	*0.039467977*
**ENSG00000107187**	*0.020181867*	*0.025740416*	*0.02537801*	*0.018822572*
**ENSG00000168036**	*0.025358451*	*0.037952906*	*0.033753419*	*0.033793097*
**ENSG00000144488**	*0.087016879*	*0.068918639*	*0.055891456*	*0.063457263*
**ENSG00000066827**	*0.036362962*	*0.036062586*	*0.031847012*	*0.029129331*
**ENSG00000124203**	*0.021517898*	*0.027754433*	*0.027288482*	*0.022766023*
**ENSG00000169567**	*0.015596502*	*0.024708934*	*0.024595735*	*0.016914874*
**ENSG00000184302**	*0.016773092*	*0.02448622*	*0.025381422*	*0.014569316*
**ENSG00000123739**	*0.033682018*	*0.039620883*	*0.032514063*	*0.025211929*
**ENSG00000157873**	*0.039136198*	*0.039590813*	*0.032580561*	*0.035106413*
**ENSG00000147378**	*0.016463595*	*0.025365919*	*0.025120711*	*0.01529147*
**ENSG00000112238**	*0.027533313*	*0.028781838*	*0.029927141*	*0.021533176*
**ENSG00000164823**	*0.031279567*	*0.036110494*	*0.03279629*	*0.031101512*
**ENSG00000185155**	*0.020383688*	*0.029380053*	*0.028251392*	*0.019771373*
**ENSG00000183230**	*0.015989867*	*0.024121696*	*0.025021057*	*0.015910096*
**ENSG00000198502**	*0.104196322*	*0.232441204*	*0.210916685*	*0.290062123*
**ENSG00000177558**	*0.01823952*	*0.025938245*	*0.025545736*	*0.017789318*
**ENSG00000014919**	*0.032961649*	*0.036606349*	*0.032838693*	*0.024228654*
**ENSG00000160712**	*0.022609403*	*0.026214613*	*0.026327768*	*0.022432142*
**ENSG00000146386**	*0.02858332*	*0.030300698*	*0.030366209*	*0.026747683*
**ENSG00000170142**	*0.030972645*	*0.038120237*	*0.036269099*	*0.036872425*
**ENSG00000169231**	*0.030000939*	*0.032970752*	*0.02920569*	*0.035672371*
**ENSG00000140465**	*0.09099832*	*0.056630203*	*0.054360104*	*0.06061427*
**ENSG00000124343**	*0.015084476*	*0.0267858*	*0.025321173*	*0.015138087*
**ENSG00000168334**	*0.033868342*	*0.032816261*	*0.038733907*	*0.02388902*
**ENSG00000128908**	*0.026633722*	*0.030243011*	*0.032019612*	*0.023988061*
**ENSG00000198732**	*0.017577154*	*0.026381342*	*0.025530518*	*0.017230136*
**ENSG00000166188**	*0.02504852*	*0.031386178*	*0.033864029*	*0.022696047*
**ENSG00000182013**	*0.128557866*	*0.105524708*	*0.081576545*	*0.104223998*
**ENSG00000135723**	*0.03516283*	*0.037634154*	*0.037975142*	*0.035242619*

**ENSG00000205439**	*0.01661303*	*0.024907852*	*0.025791774*	*0.01531362*
**ENSG00000027847**	*0.034968967*	*0.038373772*	*0.03619521*	*0.030272571*
**ENSG00000159212**	*0.099137386*	*0.08406913*	*0.097165256*	*0.094978254*
**ENSG00000147421**	*0.049556294*	*0.034449118*	*0.04507739*	*0.065846044*
**ENSG00000136504**	*0.020459357*	*0.028841868*	*0.030981628*	*0.021029152*
**ENSG00000163273**	*0.016788999*	*0.02568177*	*0.026816437*	*0.017074102*
**ENSG00000129038**	*0.015353282*	*0.024069996*	*0.02429412*	*0.014978128*
**ENSG00000204524**	*0.020627755*	*0.032498732*	*0.038178103*	*0.021364648*
**ENSG00000186010**	*0.020958959*	*0.027276061*	*0.026217627*	*0.020053717*
**ENSG00000077454**	*0.037148783*	*0.039134186*	*0.032463831*	*0.031266844*
**ENSG00000178919**	*0.016963129*	*0.026482939*	*0.027272019*	*0.018479042*
**ENSG00000117139**	*0.093195217*	*0.05431836*	*0.052110556*	*0.057495749*
**ENSG00000132478**	*0.030288469*	*0.036100862*	*0.035138956*	*0.024722363*
**ENSG00000109118**	*0.015127911*	*0.024785993*	*0.025292061*	*0.01503171*
**ENSG00000185019**	*0.016034862*	*0.02548526*	*0.025535066*	*0.014542729*
**ENSG00000166949**	*0.046978391*	*0.045099687*	*0.051973616*	*0.051260672*
**ENSG00000147586**	*0.030428607*	*0.034349475*	*0.029897695*	*0.026160682*
**ENSG00000128242**	*0.033873782*	*0.024866157*	*0.026152748*	*0.019838381*
**ENSG00000143546**	*0.055265354*	*0.036961187*	*0.02452885*	*0.043136367*
**ENSG00000182938**	*0.01502288*	*0.025279031*	*0.02470298*	*0.014878259*
**ENSG00000180481**	*0.015743561*	*0.024906023*	*0.024320322*	*0.016660885*
**ENSG00000180773**	*0.03723731*	*0.035269922*	*0.032995193*	*0.034020937*
**ENSG00000135222**	*0.018754329*	*0.026927131*	*0.028176267*	*0.019008684*
**ENSG00000100246**	*0.024007312*	*0.031334238*	*0.029141969*	*0.02685557*
**ENSG00000104499**	*0.016128902*	*0.025417302*	*0.025390672*	*0.016431787*
**ENSG00000165233**	*0.04187052*	*0.042567*	*0.038967093*	*0.037991587*
**ENSG00000132842**	*0.035105569*	*0.04205681*	*0.029896366*	*0.032418828*
**ENSG00000148362**	*0.034870363*	*0.032092532*	*0.034780817*	*0.035420999*
**ENSG00000103342**	*0.023005686*	*0.028071308*	*0.028955004*	*0.021073435*
**ENSG00000114395**	*0.030360234*	*0.033231728*	*0.039925515*	*0.030613124*
**ENSG00000141086**	*0.022961797*	*0.031662291*	*0.032665169*	*0.02328658*
**ENSG00000170779**	*0.040227295*	*0.037946554*	*0.040963798*	*0.034610703*
**ENSG00000107719**	*0.085705056*	*0.07735479*	*0.069911541*	*0.082326839*
**ENSG00000137806**	*0.034696699*	*0.033616895*	*0.035959855*	*0.023848275*
**ENSG00000176925**	*0.015733767*	*0.025081727*	*0.024254476*	*0.014686993*
**ENSG00000114902**	*0.020354853*	*0.026587021*	*0.029002597*	*0.022445724*
**ENSG00000101605**	*0.022767149*	*0.031792*	*0.02634459*	*0.025278337*
**ENSG00000145309**	*0.016913924*	*0.024798371*	*0.024461737*	*0.015617204*
**ENSG00000163463**	*0.026908165*	*0.026926105*	*0.030249391*	*0.026063741*
**ENSG00000105650**	*0.034714817*	*0.034671773*	*0.033889956*	*0.035127858*
**ENSG00000169570**	*0.022021899*	*0.028340074*	*0.028139959*	*0.027282499*
**ENSG00000204866**	*0.027991936*	*0.025426127*	*0.024933053*	*0.015286666*
**ENSG00000086300**	*0.045336209*	*0.04642465*	*0.04105881*	*0.037097888*
**ENSG00000196260**	*0.015413199*	*0.025348411*	*0.025048001*	*0.015040367*
**ENSG00000092345**	*0.041293657*	*0.034098268*	*0.025507799*	*0.030175037*
**ENSG00000198185**	*0.01569851*	*0.026111631*	*0.025068083*	*0.015312899*
**ENSG00000169436**	*0.024596938*	*0.026208215*	*0.025134199*	*0.022534823*
**ENSG00000162267**	*0.027033384*	*0.028872814*	*0.024998313*	*0.017337556*
**ENSG00000049283**	*0.020774952*	*0.028726652*	*0.029997923*	*0.021648179*
**ENSG00000132294**	*0.02828766*	*0.041778702*	*0.034748856*	*0.028169403*
**ENSG00000168566**	*0.030214002*	*0.030725024*	*0.030358119*	*0.032305531*
**ENSG00000163499**	*0.015929837*	*0.024384919*	*0.027234062*	*0.016333239*
**ENSG00000161572**	*0.017050717*	*0.027955458*	*0.025214711*	*0.01547851*
**ENSG00000100284**	*0.047568238*	*0.041850918*	*0.044623097*	*0.044627619*

**ENSG00000017260**	*0.029606733*	*0.037887298*	*0.032121401*	*0.024459402*
**ENSG00000197461**	*0.078122622*	*0.052684228*	*0.061299648*	*0.070765983*
**ENSG00000198791**	*0.028154457*	*0.036217479*	*0.030261854*	*0.02643716*
**ENSG00000156966**	*0.015427717*	*0.025047121*	*0.025808048*	*0.015704916*
**ENSG00000135930**	*0.021224114*	*0.028594792*	*0.029002661*	*0.020526994*
**ENSG00000124731**	*0.059398313*	*0.03322715*	*0.030084278*	*0.025378528*
**ENSG00000146232**	*0.040952091*	*0.034890699*	*0.034007235*	*0.03474935*
**ENSG00000203795**	*0.015431775*	*0.024324035*	*0.024724823*	*0.014837321*
**ENSG00000138678**	*0.11176019*	*0.11151635*	*0.093872726*	*0.093400824*
**ENSG00000077721**	*0.021131184*	*0.024935143*	*0.02801482*	*0.021315788*
**ENSG00000112769**	*0.01806791*	*0.026250767*	*0.025309206*	*0.016819083*
**ENSG00000164485**	*0.016288814*	*0.024673148*	*0.025613254*	*0.015742845*
**ENSG00000166579**	*0.029368178*	*0.031393468*	*0.033736041*	*0.025683134*
**ENSG00000168288**	*0.020384775*	*0.027741026*	*0.029456527*	*0.023686731*
**ENSG00000128322**	*0.017498967*	*0.026212194*	*0.025857284*	*0.016015105*
**ENSG00000141161**	*0.017789851*	*0.026188194*	*0.026482493*	*0.01749482*
**ENSG00000115561**	*0.029754107*	*0.036248105*	*0.030969883*	*0.027616129*
**ENSG00000181450**	*0.015611055*	*0.025358266*	*0.0248409*	*0.016532365*
**ENSG00000105290**	*0.023795491*	*0.024756454*	*0.025040514*	*0.018410468*
**ENSG00000172339**	*0.035662045*	*0.034627262*	*0.036968417*	*0.038781159*
**ENSG00000124092**	*0.016069575*	*0.025296525*	*0.025024045*	*0.016602506*
**ENSG00000100227**	*0.022131191*	*0.027014086*	*0.029306653*	*0.019977784*
**ENSG00000167880**	*0.016318806*	*0.024117105*	*0.025750936*	*0.015994541*
**ENSG00000164695**	*0.053223056*	*0.052797206*	*0.055525736*	*0.052781781*
**ENSG00000151615**	*0.018548276*	*0.024760693*	*0.025134866*	*0.014671059*
**ENSG00000137492**	*0.037168302*	*0.042884037*	*0.041320303*	*0.032563404*
**ENSG00000102910**	*0.028910422*	*0.032622338*	*0.029743685*	*0.019788944*
**ENSG00000162377**	*0.039072949*	*0.038838389*	*0.040516736*	*0.037957432*
**ENSG00000174233**	*0.04285394*	*0.039264947*	*0.035853666*	*0.036448489*
**ENSG00000198944**	*0.060466858*	*0.056006334*	*0.052456595*	*0.05375361*
**ENSG00000170468**	*0.047866015*	*0.049912428*	*0.050349332*	*0.042537469*
**ENSG00000177054**	*0.034953991*	*0.040561565*	*0.033135855*	*0.027852929*
**ENSG00000006695**	*0.024937847*	*0.031168401*	*0.029604166*	*0.02350126*
**ENSG00000171385**	*0.01745451*	*0.025783322*	*0.025025748*	*0.018147177*
**ENSG00000184735**	*0.01573481*	*0.024678039*	*0.024853722*	*0.016226824*
**ENSG00000189127**	*0.014600195*	*0.025986132*	*0.025261742*	*0.015421819*
**ENSG00000111816**	*0.054571373*	*0.046589061*	*0.038139439*	*0.038161562*
**ENSG00000134644**	*0.020913179*	*0.030048262*	*0.028802513*	*0.023107108*
**ENSG00000176994**	*0.016880001*	*0.02506579*	*0.025007125*	*0.014680217*
**ENSG00000183520**	*0.029626218*	*0.031742064*	*0.032306351*	*0.027865926*
**ENSG00000115602**	*0.014871428*	*0.025155351*	*0.024561936*	*0.015820544*
**ENSG00000115091**	*0.023140107*	*0.03080592*	*0.028096755*	*0.022553535*
**ENSG00000197579**	*0.028142386*	*0.035672149*	*0.040866214*	*0.028210531*
**ENSG00000187486**	*0.016478477*	*0.026333205*	*0.02571558*	*0.016431496*
**ENSG00000117400**	*0.01754354*	*0.024452799*	*0.024847497*	*0.014944807*
**ENSG00000126945**	*0.033990338*	*0.037647645*	*0.03792913*	*0.027619835*
**ENSG00000111665**	*0.047796775*	*0.032630912*	*0.035915532*	*0.039867*
**ENSG00000171451**	*0.014517598*	*0.024798929*	*0.025030629*	*0.014476431*
**ENSG00000242220**	*0.021090692*	*0.026855028*	*0.028015367*	*0.01716342*
**ENSG00000169242**	*0.042013434*	*0.031632717*	*0.028114842*	*0.026467878*
**ENSG00000142168**	*0.021178701*	*0.028705783*	*0.027016819*	*0.019295196*
**ENSG00000114383**	*0.025427526*	*0.036513317*	*0.039355507*	*0.024914873*
**ENSG00000141076**	*0.029117783*	*0.028622702*	*0.031738589*	*0.028060068*
**ENSG00000024862**	*0.02884675*	*0.0348698*	*0.037046905*	*0.029504179*

**ENSG00000186400**	*0.016255469*	*0.025005231*	*0.026058182*	*0.015127318*
**ENSG00000185838**	*0.047424229*	*0.043572082*	*0.037337174*	*0.03092747*
**ENSG00000163873**	*0.017237476*	*0.025296172*	*0.024669151*	*0.017264591*
**ENSG00000198121**	*0.062175437*	*0.050144141*	*0.027335744*	*0.02786723*
**ENSG00000107758**	*0.022454175*	*0.029707841*	*0.027785128*	*0.019319759*
**ENSG00000103460**	*0.074528688*	*0.055094024*	*0.026159846*	*0.017852998*
**ENSG00000160993**	*0.021144801*	*0.030921464*	*0.030319492*	*0.019920508*
**ENSG00000157315**	*0.019342408*	*0.028301867*	*0.027272622*	*0.018037504*
**ENSG00000120690**	*0.023110975*	*0.030292951*	*0.029574912*	*0.018694747*
**ENSG00000066322**	*0.026442136*	*0.029547999*	*0.033486989*	*0.026277358*
**ENSG00000037474**	*0.024264703*	*0.033187882*	*0.033314861*	*0.02725881*
**ENSG00000112584**	*0.028556444*	*0.035605512*	*0.032115518*	*0.023318737*
**ENSG00000188917**	*0.025651705*	*0.02962492*	*0.027965232*	*0.021433384*
**ENSG00000164776**	*0.016436391*	*0.025174116*	*0.024704371*	*0.015439529*
**ENSG00000139890**	*0.029460512*	*0.032108662*	*0.0306222*	*0.027760563*
**ENSG00000121940**	*0.02969646*	*0.02988724*	*0.033187283*	*0.026754924*
**ENSG00000180815**	*0.014294964*	*0.024677018*	*0.023830366*	*0.015366048*
**ENSG00000100926**	*0.029234036*	*0.031690598*	*0.03469138*	*0.029792592*
**ENSG00000164626**	*0.086511137*	*0.071145729*	*0.050655773*	*0.071110768*
**ENSG00000171634**	*0.015944753*	*0.025169511*	*0.02485935*	*0.016630297*
**ENSG00000112651**	*0.029897277*	*0.041709681*	*0.037964206*	*0.043111546*
**ENSG00000064835**	*0.015006711*	*0.025326842*	*0.025002932*	*0.015235015*
**ENSG00000000971**	*0.017560382*	*0.026407*	*0.025670019*	*0.015393324*
**ENSG00000162065**	*0.037418183*	*0.034817208*	*0.031354045*	*0.03118528*
**ENSG00000065923**	*0.018956255*	*0.02759048*	*0.028053929*	*0.021494763*
**ENSG00000151490**	*0.062096204*	*0.055060897*	*0.044560673*	*0.04758907*
**ENSG00000104936**	*0.036877361*	*0.036625016*	*0.041237409*	*0.029536775*
**ENSG00000158560**	*0.017192293*	*0.026170368*	*0.024401286*	*0.015900746*
**ENSG00000138279**	*0.019377033*	*0.026017703*	*0.026621053*	*0.018516307*
**ENSG00000163687**	*0.105297497*	*0.1136747*	*0.103738314*	*0.095369786*
**ENSG00000151418**	*0.016667414*	*0.024987956*	*0.024622246*	*0.017610692*
**ENSG00000172318**	*0.015887811*	*0.024228657*	*0.025330351*	*0.014316754*
**ENSG00000182158**	*0.017656856*	*0.024892563*	*0.025547165*	*0.016103323*
**ENSG00000184047**	*0.026991241*	*0.029983608*	*0.029056301*	*0.031456607*
**ENSG00000161652**	*0.01575747*	*0.025135401*	*0.024712543*	*0.015234788*
**ENSG00000165526**	*0.024362335*	*0.02959934*	*0.031665274*	*0.026194059*
**ENSG00000163635**	*0.016492384*	*0.024653766*	*0.025444403*	*0.015018722*
**ENSG00000153561**	*0.018482028*	*0.027495212*	*0.026077416*	*0.015745511*
**ENSG00000153002**	*0.016439916*	*0.024670125*	*0.02604774*	*0.015419343*
**ENSG00000065526**	*0.027917432*	*0.032750481*	*0.033906078*	*0.022804972*
**ENSG00000173848**	*0.033275512*	*0.034769452*	*0.03741598*	*0.034651715*
**ENSG00000051128**	*0.025219906*	*0.031121784*	*0.032362108*	*0.023932224*
**ENSG00000179981**	*0.039025249*	*0.042324297*	*0.037398782*	*0.030955374*
**ENSG00000089022**	*0.02506148*	*0.031181618*	*0.030114274*	*0.021729093*
**ENSG00000197410**	*0.021951451*	*0.029880319*	*0.026494728*	*0.018856572*
**ENSG00000146250**	*0.018305843*	*0.024667901*	*0.025489524*	*0.014975779*
**ENSG00000112182**	*0.015309845*	*0.024741876*	*0.025224575*	*0.014804861*
**ENSG00000204961**	*0.015638583*	*0.024715121*	*0.024667399*	*0.015081245*
**ENSG00000137251**	*0.054769158*	*0.050423256*	*0.05454716*	*0.059428859*
**ENSG00000128285**	*0.019409801*	*0.027165495*	*0.024982687*	*0.015770922*
**ENSG00000112562**	*0.017650582*	*0.025109015*	*0.026425087*	*0.015012522*
**ENSG00000166368**	*0.014811952*	*0.024627992*	*0.02478545*	*0.014283236*
**ENSG00000146963**	*0.028183657*	*0.029394199*	*0.029503202*	*0.03190401*
**ENSG00000164078**	*0.022763446*	*0.026824983*	*0.027801989*	*0.019080108*

**ENSG00000182923**	*0.022664691*	*0.027079061*	*0.029796434*	*0.021494136*
**ENSG00000131725**	*0.033833498*	*0.039670757*	*0.03377578*	*0.028639978*
**ENSG00000119509**	*0.019397777*	*0.029536052*	*0.029120629*	*0.019157248*
