# Supplementary material for: Insights into the regulation of human CNV-miRNAs from the view of their target genes
Source: BMC Genomics. 2012 Dec 18;13:707. doi: 10.1186/1471-2164-13-707 (PMC3582595; doi:10.1186/1471-2164-13-707)
Supplement: Additional file 5 — Minor allele frequencies (MAFs) of 5′UTR- and 3′UTR-SNPs in four HapMap ethnic populations. [file 1471-2164-13-707-S5.pdf]

Ensembl Gene ID	MAF of 5'UTR in YRI	MAF of 3'UTR in YRI	MAF of 5'UTR in CEU	MAF of 3'UTR in CEU	MAF of 5'UTR in CHB	MAF of 3'UTR in CHB	MAF of 5'UTR in JPT	MAF of 3'UTR in JPT
**ENSG00000256574**		*0.16666667*		*0.166666667*				
**ENSG00000109819**		*0.46969697*		*0.46969697*				*0.4709302*
**ENSG00000152778**		*0.49085366*		*0.490853659*				*0.4593023*
**ENSG00000161057**		*0.1030303*		*0.103030303*				*0.2732558*
**ENSG00000197969**	*0.06886228*	*0.47272727*	*0.06886228*	*0.472727273*	*0.071428571*			*0.3823529*
**ENSG00000182575**		*0.40555556*		*0.405555556*				*0.0909091*
**ENSG00000138443**		*0.42682927*		*0.426829268*				*0.4352941*
**ENSG00000188906**		*0.42378049*		*0.423780488*				*0.4761905*
**ENSG00000051596**		*0.25*		*0.25*				*0.2352941*
**ENSG00000124151**	*0.09659091*	*0.32121212*	*0.09659091*	*0.321212121*				*0.2159091*
**ENSG00000127329**		*0.44785276*		*0.447852761*				*0.4886364*
**ENSG00000095637**		*0.45426829*		*0.454268293*				*0.3953488*
**ENSG00000172244**	*0.32335329*		*0.32335329*		*0.095238095*		*0.1746988*	
**ENSG00000189241**		*0.32424242*		*0.324242424*				*0.4651163*
**ENSG00000182511**	*0.23952096*		*0.23952096*					
**ENSG00000109103**		*0.16666667*		*0.166666667*				
**ENSG00000184361**	*0.5*		*0.5*		*0.455555556*		*0.3666667*	
**ENSG00000100450**								*0.0581395*
**ENSG00000100796**		*0.44252874*		*0.442528736*				*0.4940476*
**ENSG00000135541**	*0.17777778*	*0.46363636*	*0.17777778*	*0.463636364*	*0.272727273*		*0.2073171*	*0.2848837*
**ENSG00000258588**	*0.4491018*	*0.05757576*	*0.4491018*	*0.057575758*	*0.452380952*		*0.4825581*	
**ENSG00000143376**		*0.35454545*		*0.354545455*				*0.3953488*
**ENSG00000163602**		*0.23636364*		*0.236363636*				*0.2209302*
**ENSG00000048052**		*0.48484848*		*0.484848485*				*0.4360465*
**ENSG00000247270**		*0.09090909*		*0.090909091*				*0.1547619*
**ENSG00000214814**	*0.31437126*	*0.24545455*	*0.31437126*	*0.245454545*	*0.148809524*		*0.0988372*	*0.4418605*
**ENSG00000196155**	*0.2754491*	*0.10909091*	*0.2754491*	*0.109090909*				
**ENSG00000062282**	*0.45555556*	*0.10606061*	*0.45555556*	*0.106060606*	*0.233333333*		*0.2727273*	*0.3139535*
**ENSG00000006468**	*0.14772727*	*0.4*	*0.14772727*	*0.4*				*0.3197674*
**ENSG00000102174**	*0.33233533*	*0.40243902*	*0.33233533*	*0.402439024*	*0.297619048*		*0.3255814*	*0.2325581*
**ENSG00000165240**		*0.26380368*		*0.263803681*				*0.3430233*
**ENSG00000142539**		*0.16111111*		*0.161111111*				*0.0795455*
**ENSG00000180739**					*0.19047619*		*0.1627907*	
**ENSG00000185068**		*0.278125*		*0.278125*				*0.4534884*
**ENSG00000172172**		*0.4047619*		*0.404761905*				*0.4709302*
**ENSG00000172020**		*0.30909091*		*0.309090909*				*0.1104651*
**ENSG00000100218**	*0.31111111*		*0.31111111*		*0.377777778*		*0.3222222*	
**ENSG00000115486**		*0.38888889*		*0.388888889*				*0.3111111*
**ENSG00000100479**	*0.06927711*	*0.22865854*	*0.06927711*	*0.228658537*				
**ENSG00000196458**	*0.22155689*	*0.4969697*	*0.22155689*	*0.496969697*	*0.327380952*		*0.2732558*	*0.3705882*
**ENSG00000029639**		*0.31976744*		*0.319767442*				
**ENSG00000139218**	*0.14670659*		*0.14670659*		*0.455555556*		*0.3988095*	
**ENSG00000111218**	*0.15963855*	*0.12121212*	*0.15963855*	*0.121212121*				*0.1046512*
**ENSG00000117477**	*0.10778443*		*0.10778443*		*0.101190476*		*0.1046512*	
**ENSG00000142185**	*0.5*		*0.5*					
**ENSG00000162676**		*0.32777778*		*0.327777778*				*0.1860465*
**ENSG00000131724**	*0.48333333*	*0.13719512*	*0.48333333*	*0.137195122*	*0.446428571*		*0.4886364*	*0.4825581*
**ENSG00000183117**		*0.1*		*0.1*				
**ENSG00000164794**		*0.09090909*		*0.090909091*				*0.0872093*
**ENSG00000204823**	*0.43712575*		*0.43712575*		*0.322222222*		*0.2674419*	

**ENSG00000101194**		*0.48787879*		*0.487878788*			*0.2732558*
**ENSG00000112812**							*0.0639535*
**ENSG00000149483**	*0.11077844*	*0.14848485*	*0.11077844*	*0.148484848*			
**ENSG00000005884**		*0.29393939*		*0.293939394*			*0.372093*
**ENSG00000128595**		*0.34848485*		*0.348484848*			*0.0555556*
**ENSG00000136531**		*0.43888889*		*0.438888889*			*0.3977273*
**ENSG00000086102**	*0.16467066*	*0.10606061*	*0.16467066*	*0.106060606*	*0.136904762*	*0.2235294*	
**ENSG00000140455**		*0.39393939*		*0.393939394*			*0.1022727*
**ENSG00000100211**	*0.31666667*	*0.28181818*	*0.31666667*	*0.281818182*	*0.397727273*	*0.3522727*	*0.3117647*
**ENSG00000175220**		*0.08484848*		*0.084848485*			
**ENSG00000183617**	*0.49401198*		*0.49401198*		*0.321428571*	*0.2616279*	
**ENSG00000186479**		*0.31818182*		*0.318181818*			*0.4360465*
**ENSG00000188089**	*0.24251497*	*0.30606061*	*0.24251497*	*0.306060606*	*0.226190476*	*0.3255814*	*0.377907*
**ENSG00000120029**		*0.36363636*		*0.363636364*			*0.1918605*
**ENSG00000136161**	*0.0508982*		*0.0508982*				
**ENSG00000141956**		*0.44886364*		*0.448863636*			*0.4555556*
**ENSG00000187608**	*0.29341317*		*0.29341317*				
**ENSG00000170561**		*0.23636364*		*0.236363636*			*0.4302326*
**ENSG00000235935**							*0.122093*
**ENSG00000075673**		*0.15757576*		*0.157575758*			*0.127907*
**ENSG00000240510**		*0.44242424*		*0.442424242*			*0.3372093*
**ENSG00000174684**	*0.23952096*		*0.23952096*		*0.154761905*	*0.1511628*	
**ENSG00000184661**	*0.48780488*	*0.33333333*	*0.48780488*	*0.333333333*	*0.375*	*0.3488372*	*0.2613636*
**ENSG00000137628**	*0.21686747*		*0.21686747*		*0.12195122*	*0.122093*	
**ENSG00000213822**	*0.25568182*		*0.25568182*		*0.102272727*	*0.0909091*	
**ENSG00000167536**		*0.18181818*		*0.181818182*			*0.2093023*
**ENSG00000085265**		*0.43597561*		*0.43597561*			*0.4534884*
**ENSG00000090539**		*0.17575758*		*0.175757576*			*0.3294118*
**ENSG00000107185**		*0.36666667*		*0.366666667*			*0.377907*
**ENSG00000102805**	*0.4491018*	*0.0969697*	*0.4491018*	*0.096969697*	*0.31547619*	*0.2383721*	*0.3333333*
**ENSG00000081052**	*0.4251497*	*0.45092025*	*0.4251497*	*0.450920245*			*0.3604651*
**ENSG00000119314**		*0.43939394*		*0.439393939*			*0.4127907*
**ENSG00000134757**	*0.47727273*	*0.40490798*	*0.47727273*	*0.404907975*	*0.122222222*	*0.1363636*	*0.2142857*
**ENSG00000159259**		*0.10365854*		*0.103658537*			
**ENSG00000196652**	*0.26969697*	*0.15*	*0.26969697*	*0.15*	*0.355555556*	*0.2954545*	*0.2840909*
**ENSG00000100568**		*0.32919255*		*0.329192547*			*0.3953488*
**ENSG00000008283**		*0.49393939*		*0.493939394*			*0.2325581*
**ENSG00000241127**		*0.18965517*		*0.189655172*			*0.1363636*
**ENSG00000108468**		*0.25280899*		*0.252808989*			*0.2386364*
**ENSG00000176566**		*0.06666667*		*0.066666667*			
**ENSG00000164114**	*0.14071856*	*0.45555556*	*0.14071856*	*0.455555556*	*0.214285714*	*0.127907*	*0.4659091*
**ENSG00000050628**		*0.45555556*		*0.455555556*			*0.4127907*
**ENSG00000158109**		*0.21646341*		*0.216463415*			*0.3430233*
**ENSG00000169313**	*0.16766467*		*0.16766467*		*0.232142857*	*0.1511628*	
**ENSG00000054523**		*0.49393939*		*0.493939394*			*0.372093*
**ENSG00000151136**	*0.10479042*	*0.29393939*	*0.10479042*	*0.293939394*	*0.255952381*	*0.244186*	*0.4534884*
**ENSG00000152582**	*0.16467066*	*0.49090909*	*0.16467066*	*0.490909091*			*0.2674419*
**ENSG00000136044**	*0.39221557*	*0.43333333*	*0.39221557*	*0.433333333*	*0.428571429*	*0.4883721*	*0.4352941*
**ENSG00000163898**		*0.47878788*		*0.478787879*			*0.3255814*
**ENSG00000102057**					*0.397727273*	*0.3837209*	
**ENSG00000189132**		*0.22392638*		*0.22392638*			*0.3095238*
**ENSG00000130714**		*0.07878788*		*0.078787879*			*0.1058824*
**ENSG00000155846**		*0.28484848*		*0.284848485*			*0.2151163*
**ENSG00000113163**		*0.23030303*		*0.23030303*			*0.3837209*

**ENSG00000130349**	*0.14071856*		*0.14071856*				
**ENSG00000179776**	*0.23493976*	*0.41111111*	*0.23493976*	*0.411111111*	*0.154761905*	*0.1918605*	*0.1590909*
**ENSG00000186628**		*0.18181818*		*0.181818182*			*0.4069767*
**ENSG00000155760**		*0.28787879*		*0.287878788*			*0.2906977*
**ENSG00000125266**		*0.4030303*		*0.403030303*			*0.4767442*
**ENSG00000134516**	*0.31437126*	*0.33636364*	*0.31437126*	*0.336363636*	*0.148809524*	*0.2267442*	*0.4593023*
**ENSG00000110721**		*0.06707317*		*0.067073171*			*0.1162791*
**ENSG00000065154**		*0.13888889*		*0.138888889*			*0.0639535*
**ENSG00000132669**		*0.23030303*		*0.23030303*			*0.2383721*
**ENSG00000184220**		*0.1509434*		*0.150943396*			*0.0722892*
**ENSG00000177098**		*0.37878788*		*0.378787879*			*0.3023256*
**ENSG00000105829**		*0.3969697*		*0.396969697*			*0.4709302*
**ENSG00000080709**		*0.44444444*		*0.444444444*	*0.147727273*	*0.0909091*	*0.2840909*
**ENSG00000244537**		*0.46341463*		*0.463414634*	*0.083333333*		*0.3604651*
**ENSG00000173039**	*0.48502994*	*0.15517241*	*0.48502994*	*0.155172414*	*0.398809524*	*0.4302326*	*0.4545455*
**ENSG00000140829**		*0.48888889*		*0.488888889*			*0.3863636*
**ENSG00000165985**		*0.45402299*		*0.454022989*			*0.3977273*
**ENSG00000160111**		*0.49393939*		*0.493939394*			*0.2383721*
**ENSG00000169026**	*0.12874251*		*0.12874251*				
**ENSG00000139211**		*0.19817073*		*0.198170732*			*0.3*
**ENSG00000196220**		*0.33636364*		*0.336363636*			*0.452381*
**ENSG00000131238**	*0.17261905*	*0.32012195*	*0.17261905*	*0.320121951*	*0.43452381*	*0.4476744*	*0.4186047*
**ENSG00000125755**		*0.05151515*		*0.051515152*			*0.4476744*
**ENSG00000077463**		*0.15555556*		*0.155555556*			*0.3181818*
**ENSG00000178804**	*0.5*		*0.5*		*0.44047619*	*0.4244186*	
**ENSG00000138109**		*0.1554878*		*0.155487805*			
**ENSG00000165512**	*0.22777778*	*0.25460123*	*0.22777778*	*0.254601227*	*0.306818182*	*0.4090909*	*0.3614458*
**ENSG00000188816**		*0.11515152*		*0.115151515*			*0.3313953*
**ENSG00000156049**	*0.28143713*		*0.28143713*		*0.208333333*	*0.2176471*	*0.2151163*
**ENSG00000115904**	*0.10778443*	*0.4*	*0.10778443*	*0.4*		*0.0581395*	*0.3197674*
**ENSG00000043462**	*0.13888889*	*0.47222222*	*0.13888889*	*0.472222222*	*0.477777778*	*0.4659091*	*0.4111111*
**ENSG00000099330**		*0.07575758*		*0.075757576*			
**ENSG00000115963**		*0.16358025*		*0.163580247*			*0.4642857*
**ENSG00000155792**		*0.42727273*		*0.427272727*			*0.4011628*
**ENSG00000160199**		*0.43636364*		*0.436363636*			*0.4534884*
**ENSG00000081138**	*0.43113772*	*0.26666667*	*0.43113772*	*0.266666667*	*0.297619048*	*0.3197674*	*0.0568182*
**ENSG00000178567**		*0.48780488*		*0.487804878*			*0.2034884*
**ENSG00000165516**	*0.29341317*		*0.29341317*				
**ENSG00000141378**	*0.39772727*		*0.39772727*		*0.470238095*	*0.4593023*	
**ENSG00000099337**		*0.19090909*		*0.190909091*			*0.1764706*
**ENSG00000121898**	*0.14371257*	*0.47272727*	*0.14371257*	*0.472727273*	*0.345238095*	*0.4186047*	*0.3837209*
**ENSG00000131153**		*0.32926829*		*0.329268293*			*0.4127907*
**ENSG00000101146**		*0.46590909*		*0.465909091*			*0.4360465*
**ENSG00000090376**		*0.47256098*		*0.472560976*			*0.377907*
**ENSG00000106993**		*0.39444444*		*0.394444444*			*0.4659091*
**ENSG00000211973**	*0.20833333*		*0.20833333*		*0.422222222*	*0.4318182*	
**ENSG00000231213**	*0.21666667*	*0.08484848*	*0.21666667*	*0.084848485*			*0.1162791*
**ENSG00000120242**		*0.05555556*		*0.055555556*			*0.1*
**ENSG00000174130**							*0.2386364*
**ENSG00000100056**		*0.21212121*		*0.212121212*			*0.2383721*
**ENSG00000160285**		*0.36969697*		*0.36969697*			*0.2559524*
**ENSG00000105053**	*0.42222222*	*0.21084337*	*0.42222222*	*0.210843373*	*0.204545455*	*0.2840909*	*0.3636364*
**ENSG00000121671**		*0.32121212*		*0.321212121*			*0.4244186*
**ENSG00000154822**	*0.35843373*	*0.22727273*	*0.35843373*	*0.227272727*	*0.464285714*	*0.4882353*	

**ENSG00000100577**	*0.31460674*	*0.4969697*	*0.31460674*	*0.496969697*	*0.482142857*		*0.4767442*	*0.4069767*
**ENSG00000136026**	*0.12048193*		*0.12048193*					
**ENSG00000133665**		*0.32777778*		*0.327777778*				*0.0681818*
**ENSG00000100300**	*0.35628743*	*0.28220859*	*0.35628743*	*0.282208589*				*0.2093023*
**ENSG00000134873**	*0.08682635*	*0.32121212*	*0.08682635*	*0.321212121*	*0.142857143*		*0.2034884*	*0.4588235*
**ENSG00000145439**		*0.24444444*		*0.244444444*				*0.0930233*
**ENSG00000134215**		*0.36060606*		*0.360606061*				*0.4825581*
**ENSG00000243649**		*0.3902439*		*0.390243902*				*0.4127907*
**ENSG00000258847**		*0.1*		*0.1*				
**ENSG00000078114**		*0.40909091*		*0.409090909*				*0.4352941*
**ENSG00000169291**		*0.42424242*		*0.424242424*				*0.255814*
**ENSG00000177045**		*0.46111111*		*0.461111111*				*0.2954545*
**ENSG00000167553**		*0.34482759*		*0.344827586*				*0.2297297*
**ENSG00000132139**								*0.2383721*
**ENSG00000205707**	*0.43902439*	*0.49695122*	*0.43902439*	*0.49695122*	*0.160714286*		*0.0697674*	*0.1882353*
**ENSG00000116863**	*0.05389222*		*0.05389222*		*0.220238095*		*0.1627907*	*0.0705882*
**ENSG00000117533**		*0.32121212*		*0.321212121*				*0.2790698*
**ENSG00000180592**	*0.23353293*		*0.23353293*					
**ENSG00000082258**		*0.38484848*		*0.384848485*	*0.095238095*		*0.1511628*	*0.2383721*
**ENSG00000116062**	*0.14371257*	*0.29573171*	*0.14371257*	*0.295731707*				*0.2151163*
**ENSG00000076344**		*0.09444444*		*0.094444444*				*0.4090909*
**ENSG00000142279**		*0.26470588*		*0.264705882*				*0.5*
**ENSG00000157388**	*0.38888889*	*0.10670732*	*0.38888889*	*0.106707317*	*0.06547619*			*0.1941176*
**ENSG00000148300**		*0.05*		*0.05*				
**ENSG00000170632**		*0.11666667*		*0.116666667*				*0.3841463*
**ENSG00000008710**		*0.13068182*		*0.130681818*				
**ENSG00000186577**	*0.45508982*		*0.45508982*		*0.130952381*		*0.2267442*	
**ENSG00000105618**	*0.09580838*		*0.09580838*		*0.25*		*0.2151163*	
**ENSG00000197943**		*0.06060606*		*0.060606061*				
**ENSG00000214367**		*0.1097561*		*0.109756098*				*0.3081395*
**ENSG00000082556**	*0.06321839*	*0.14545455*	*0.06321839*	*0.145454545*				*0.0697674*
**ENSG00000156050**		*0.12727273*		*0.127272727*				*0.4666667*
**ENSG00000154222**		*0.46646341*		*0.466463415*				*0.1162791*
**ENSG00000006114**		*0.36060606*		*0.360606061*				*0.4*
**ENSG00000188419**		*0.2484472*		*0.248447205*				*0.1785714*
**ENSG00000184156**		*0.49393939*		*0.493939394*				*0.3372093*
**ENSG00000047315**	*0.13772455*		*0.13772455*					
**ENSG00000078124**	*0.40963855*	*0.41818182*	*0.40963855*	*0.418181818*	*0.347560976*		*0.2941176*	*0.4069767*
**ENSG00000150403**		*0.18012422*		*0.180124224*				*0.1829268*
**ENSG00000171824**		*0.24242424*		*0.242424242*				*0.1395349*
**ENSG00000128881**					*0.422619048*		*0.4360465*	
**ENSG00000164291**		*0.14939024*		*0.149390244*				*0.1918605*
**ENSG00000126785**	*0.44311377*	*0.3117284*	*0.44311377*	*0.311728395*	*0.494047619*		*0.4352941*	*0.2380952*
**ENSG00000143217**		*0.27777778*		*0.277777778*				*0.0872093*
**ENSG00000106714**		*0.28333333*		*0.283333333*				*0.3522727*
**ENSG00000115159**		*0.28333333*		*0.283333333*				*0.2613636*
**ENSG00000162636**		*0.05*		*0.05*				*0.1818182*
**ENSG00000156675**		*0.26666667*		*0.266666667*				*0.0639535*
**ENSG00000196176**		*0.10909091*		*0.109090909*				
**ENSG00000044115**		*0.29090909*		*0.290909091*				
**ENSG00000146109**		*0.11515152*		*0.115151515*				*0.0697674*
**ENSG00000215570**		*0.28888889*		*0.288888889*				
**ENSG00000169946**	*0.08982036*		*0.08982036*		*0.130952381*		*0.1470588*	
**ENSG00000135966**		*0.2969697*		*0.296969697*	*0.125*		*0.1686047*	*0.4666667*

**ENSG00000138944**	*0.16666667*	*0.31609195*	*0.16666667*	*0.316091954*				*0.4888889*
**ENSG00000186714**	*0.28742515*	*0.27878788*	*0.28742515*	*0.278787879*	*0.240963855*		*0.2034884*	*0.0823529*
**ENSG00000088766**		*0.49090909*		*0.490909091*				*0.452381*
**ENSG00000130700**		*0.23312883*		*0.233128834*				*0.0755814*
**ENSG00000169006**		*0.15*		*0.15*				*0.0568182*
**ENSG00000100196**		*0.31818182*		*0.318181818*				*0.3837209*
**ENSG00000143921**					*0.155555556*		*0.2222222*	
**ENSG00000110987**		*0.23333333*		*0.233333333*	*0.226190476*		*0.1802326*	*0.1860465*
**ENSG00000251201**		*0.49393939*		*0.493939394*				*0.4709302*
**ENSG00000114480**	*0.28353659*	*0.45402299*	*0.28353659*	*0.454022989*	*0.444444444*		*0.375*	*0.127907*
**ENSG00000089041**	*0.3*	*0.45757576*	*0.3*	*0.457575758*	*0.333333333*		*0.4418605*	*0.4418605*
**ENSG00000144791**	*0.30113636*	*0.43209877*	*0.30113636*	*0.432098765*	*0.344444444*		*0.3636364*	*0.2325581*
**ENSG00000155189**		*0.26086957*		*0.260869565*				*0.3197674*
**ENSG00000234511**	*0.29341317*	*0.46969697*	*0.29341317*	*0.46969697*	*0.130952381*		*0.1453488*	*0.4825581*
**ENSG00000080573**		*0.33128834*		*0.331288344*				*0.1744186*
**ENSG00000151079**	*0.4011976*	*0.38787879*	*0.4011976*	*0.387878788*	*0.482142857*		*0.4647059*	*0.4709302*
**ENSG00000145362**	*0.07303371*	*0.13333333*	*0.07303371*	*0.133333333*				
**ENSG00000198211**		*0.07878788*		*0.078787879*				
**ENSG00000183060**		*0.42941176*		*0.429411765*				*0.4186047*
**ENSG00000135898**		*0.31515152*		*0.315151515*				*0.3255814*
**ENSG00000143457**		*0.4054878*		*0.405487805*				*0.2383721*
**ENSG00000130270**	*0.35542169*	*0.48787879*	*0.35542169*	*0.487878788*	*0.386904762*		*0.4642857*	*0.4767442*
**ENSG00000117595**	*0.47590361*	*0.26060606*	*0.47590361*	*0.260606061*	*0.363095238*		*0.4069767*	*0.4825581*
**ENSG00000132612**		*0.06363636*		*0.063636364*				
**ENSG00000174332**		*0.05*		*0.05*				*0.1136364*
**ENSG00000100557**	*0.18373494*	*0.3*	*0.18373494*	*0.3*				*0.054878*
**ENSG00000163481**	*0.43413174*		*0.43413174*		*0.142857143*		*0.1569767*	*0.1363636*
**ENSG00000132185**	*0.4760479*		*0.4760479*		*0.19047619*		*0.2325581*	
**ENSG00000214226**		*0.17987805*		*0.179878049*	*0.077380952*			
**ENSG00000176142**	*0.0748503*	*0.13636364*	*0.0748503*	*0.136363636*	*0.083333333*		*0.1046512*	*0.2647059*
**ENSG00000073614**		*0.20909091*		*0.209090909*				*0.5*
**ENSG00000180979**	*0.15868263*	*0.07012195*	*0.15868263*	*0.070121951*				
**ENSG00000141759**	*0.40229885*	*0.3030303*	*0.40229885*	*0.303030303*	*0.088888889*		*0.0681818*	*0.4651163*
**ENSG00000164402**		*0.125*		*0.125*				*0.3953488*
**ENSG00000198892**		*0.36627907*		*0.36627907*				*0.4404762*
**ENSG00000116785**	*0.15168539*	*0.21428571*	*0.15168539*	*0.214285714*	*0.477777778*		*0.4886364*	*0.4702381*
**ENSG00000125633**		*0.38181818*		*0.381818182*				*0.2848837*
**ENSG00000145012**	*0.31460674*	*0.49090909*	*0.31460674*	*0.490909091*	*0.433333333*		*0.3409091*	*0.4939759*
**ENSG00000178585**		*0.25280899*		*0.252808989*				*0.1046512*
**ENSG00000182359**	*0.18263473*	*0.33647799*	*0.18263473*	*0.336477987*			*0.0523256*	*0.4127907*
**ENSG00000205702**							*0.05*	
**ENSG00000162931**	*0.09756098*		*0.09756098*		*0.303571429*		*0.1860465*	
**ENSG00000253138**		*0.07777778*		*0.077777778*				
**ENSG00000164483**	*0.06586826*	*0.13939394*	*0.06586826*	*0.139393939*	*0.410714286*		*0.4545455*	*0.0697674*
**ENSG00000077312**		*0.10606061*		*0.106060606*	*0.196428571*		*0.2848837*	*0.2848837*
**ENSG00000168671**	*0.20625*		*0.20625*					*0.0647059*
**ENSG00000145022**		*0.27575758*		*0.275757576*				*0.1162791*
**ENSG00000132275**		*0.46363636*		*0.463636364*				*0.3255814*
**ENSG00000204160**		*0.3597561*		*0.359756098*				
**ENSG00000115112**		*0.42682927*		*0.426829268*				*0.4545455*
**ENSG00000115310**	*0.34730539*		*0.34730539*		*0.113095238*		*0.0523256*	
**ENSG00000186191**		*0.33333333*		*0.333333333*				*0.2470588*
**ENSG00000105639**	*0.32222222*	*0.42424242*	*0.32222222*	*0.424242424*	*0.244444444*		*0.2333333*	*0.494186*
**ENSG00000170876**		*0.39090909*		*0.390909091*				*0.4186047*

**ENSG00000117862**	*0.32335329*		*0.32335329*		*0.130952381*		*0.0813953*	
**ENSG00000119919**		*0.27575758*		*0.275757576*				*0.372093*
**ENSG00000173480**		*0.48888889*		*0.488888889*				*0.494186*
**ENSG00000105851**		*0.46969697*		*0.46969697*	*0.071428571*			*0.4883721*
**ENSG00000141696**		*0.09937888*		*0.099378882*	*0.089285714*		*0.0872093*	
**ENSG00000136908**	*0.30838323*		*0.30838323*		*0.375*		*0.4069767*	
**ENSG00000171840**	*0.28143713*	*0.09090909*	*0.28143713*	*0.090909091*	*0.403614458*		*0.4058824*	*0.1860465*
**ENSG00000158296**		*0.2969697*		*0.296969697*				*0.3058824*
**ENSG00000180376**	*0.05*	*0.43333333*	*0.05*	*0.433333333*				*0.4886364*
**ENSG00000010319**		*0.45757576*		*0.457575758*				*0.3953488*
**ENSG00000101181**		*0.47575758*		*0.475757576*				*0.3139535*
**ENSG00000241484**	*0.43888889*	*0.26060606*	*0.43888889*	*0.260606061*	*0.133333333*		*0.0777778*	*0.3662791*
**ENSG00000074356**	*0.18862275*		*0.18862275*					
**ENSG00000007384**		*0.17241379*		*0.172413793*				*0.5*
**ENSG00000145861**		*0.42528736*		*0.425287356*				*0.4545455*
**ENSG00000165861**	*0.05487805*	*0.34090909*	*0.05487805*	*0.340909091*	*0.195121951*		*0.1764706*	*0.1333333*
**ENSG00000242515**		*0.22777778*		*0.227777778*				*0.1590909*
**ENSG00000184384**	*0.22159091*	*0.36890244*	*0.22159091*	*0.368902439*	*0.444444444*		*0.4767442*	*0.372093*
**ENSG00000159200**	*0.4939759*	*0.07012195*	*0.4939759*	*0.070121951*	*0.391566265*		*0.372093*	
**ENSG00000102595**		*0.45151515*		*0.451515152*				*0.2790698*
**ENSG00000074803**	*0.34730539*		*0.34730539*		*0.311111111*		*0.5*	*0.4823529*
**ENSG00000114378**		*0.13636364*		*0.136363636*	*0.36746988*		*0.4825581*	
**ENSG00000112499**	*0.12275449*	*0.22424242*	*0.12275449*	*0.224242424*	*0.083333333*		*0.0697674*	*0.1395349*
**ENSG00000073146**	*0.19886364*	*0.33030303*	*0.19886364*	*0.33030303*	*0.377777778*		*0.5*	*0.2840909*
**ENSG00000175518**	*0.23952096*	*0.24242424*	*0.23952096*	*0.242424242*	*0.380952381*		*0.3953488*	*0.3430233*
**ENSG00000137501**	*0.43373494*		*0.43373494*		*0.398809524*		*0.4302326*	
**ENSG00000256683**		*0.13333333*		*0.133333333*				*0.1802326*
**ENSG00000116459**	*0.44311377*	*0.35*	*0.44311377*	*0.35*	*0.255952381*		*0.255814*	*0.2674419*
**ENSG00000107669**	*0.12777778*	*0.14545455*	*0.12777778*	*0.145454545*	*0.344444444*		*0.3068182*	*0.4529412*
**ENSG00000259119**	*0.21856287*	*0.32822086*	*0.21856287*	*0.328220859*	*0.25*		*0.2732558*	*0.1802326*
**ENSG00000204604**	*0.09195402*	*0.42727273*	*0.09195402*	*0.427272727*			*0.0568182*	*0.4011628*
**ENSG00000176386**	*0.32183908*		*0.32183908*		*0.360465116*		*0.4090909*	
**ENSG00000006715**		*0.37878788*		*0.378787879*				*0.3895349*
**ENSG00000196152**					*0.25*		*0.25*	
**ENSG00000143799**		*0.38181818*		*0.381818182*				*0.2151163*
**ENSG00000139656**								*0.125*
**ENSG00000137699**	*0.15868263*	*0.14848485*	*0.15868263*	*0.148484848*	*0.5*		*0.4418605*	
**ENSG00000081985**	*0.38333333*	*0.35151515*	*0.38333333*	*0.351515152*	*0.25*		*0.2732558*	*0.4360465*
**ENSG00000101773**	*0.3742515*		*0.3742515*					
**ENSG00000105835**		*0.3902439*		*0.390243902*				*0.0813953*
**ENSG00000115484**		*0.06969697*		*0.06969697*				
**ENSG00000148229**		*0.36280488*		*0.362804878*				
**ENSG00000188811**		*0.0969697*		*0.096969697*				*0.1627907*
**ENSG00000204850**		*0.07222222*		*0.072222222*				*0.1477273*
**ENSG00000175066**		*0.38787879*		*0.387878788*				*0.2383721*
**ENSG00000103184**		*0.48787879*		*0.487878788*				*0.3411765*
**ENSG00000185264**	*0.19578313*		*0.19578313*		*0.482142857*		*0.3023256*	
**ENSG00000092470**		*0.06134969*		*0.061349693*				*0.2235294*
**ENSG00000112299**		*0.31818182*		*0.318181818*				*0.2941176*
**ENSG00000250722**	*0.47222222*	*0.3030303*	*0.47222222*	*0.303030303*	*0.388888889*		*0.4666667*	*0.3392857*
**ENSG00000147256**		*0.17878788*		*0.178787879*				*0.4882353*
**ENSG00000125885**	*0.39221557*		*0.39221557*		*0.083333333*		*0.0813953*	
**ENSG00000141255**		*0.16111111*		*0.161111111*				*0.3555556*
**ENSG00000100814**	*0.24550898*		*0.24550898*		*0.44047619*		*0.4825581*	

**ENSG00000245017**		*0.375*		*0.375*				
**ENSG00000119688**	*0.15340909*	*0.2969697*	*0.15340909*	*0.296969697*	*0.255952381*		*0.244186*	*0.244186*
**ENSG00000104880**	*0.24157303*	*0.31212121*	*0.24157303*	*0.312121212*	*0.344444444*		*0.3488372*	*0.3953488*
**ENSG00000175906**		*0.30606061*		*0.306060606*				*0.372093*
**ENSG00000149328**		*0.43636364*		*0.436363636*				*0.1453488*
**ENSG00000204291**		*0.22699387*		*0.226993865*			*0.0681818*	*0.3176471*
**ENSG00000163823**		*0.08484848*		*0.084848485*				
**ENSG00000188257**	*0.14204545*	*0.19444444*	*0.14204545*	*0.194444444*				*0.2176471*
**ENSG00000170989**	*0.08083832*		*0.08083832*		*0.297619048*		*0.2*	
**ENSG00000154710**		*0.23030303*		*0.23030303*				*0.4*
**ENSG00000132470**		*0.42944785*		*0.429447853*				*0.4069767*
**ENSG00000108381**		*0.35757576*		*0.357575758*				*0.3863636*
**ENSG00000258405**		*0.43939394*		*0.439393939*				*0.4127907*
**ENSG00000161955**		*0.19090909*		*0.190909091*				*0.0823529*
**ENSG00000139168**	*0.38202247*		*0.38202247*		*0.455555556*		*0.4090909*	
**ENSG00000065054**		*0.15151515*		*0.151515152*				
**ENSG00000122406**	*0.33532934*		*0.33532934*					
**ENSG00000164733**	*0.32335329*	*0.35757576*	*0.32335329*	*0.357575758*	*0.470238095*		*0.4709302*	*0.4705882*
**ENSG00000258873**		*0.48484848*		*0.484848485*				*0.3666667*
**ENSG00000105642**		*0.48484848*		*0.484848485*				*0.4764706*
**ENSG00000168228**		*0.42424242*		*0.424242424*				*0.4880952*
**ENSG00000124164**		*0.41818182*		*0.418181818*				*0.3977273*
**ENSG00000105514**		*0.09090909*		*0.090909091*				*0.2325581*
**ENSG00000198939**	*0.28888889*	*0.08181818*	*0.28888889*	*0.081818182*	*0.244444444*		*0.3555556*	*0.1686047*
**ENSG00000204683**		*0.33030303*		*0.33030303*				*0.3588235*
**ENSG00000151332**	*0.37777778*	*0.06748466*	*0.37777778*	*0.067484663*	*0.055555556*			
**ENSG00000135677**	*0.33832335*	*0.40697674*	*0.33832335*	*0.406976744*	*0.107142857*		*0.1744186*	*0.2325581*
**ENSG00000145386**	*0.0505618*		*0.0505618*		*0.219512195*		*0.2619048*	*0.0872093*
**ENSG00000066813**		*0.1552795*		*0.155279503*				*0.4529412*
**ENSG00000204308**		*0.2030303*		*0.203030303*				*0.2674419*
**ENSG00000166435**	*0.09090909*	*0.44512195*	*0.09090909*	*0.445121951*	*0.476190476*		*0.4411765*	*0.4476744*
**ENSG00000167094**		*0.45757576*		*0.457575758*				*0.3837209*
**ENSG00000187003**		*0.16363636*		*0.163636364*				*0.1511628*
**ENSG00000198887**		*0.32777778*		*0.327777778*				*0.4090909*
**ENSG00000222005**		*0.46060606*		*0.460606061*				*0.372093*
**ENSG00000164600**		*0.28658537*		*0.286585366*				*0.1802326*
**ENSG00000124406**		*0.37037037*		*0.37037037*				*0.4625*
**ENSG00000166974**		*0.33333333*		*0.333333333*				*0.4352941*
**ENSG00000165959**	*0.18862275*		*0.18862275*		*0.321428571*		*0.4*	
**ENSG00000140650**		*0.1969697*		*0.196969697*				*0.3235294*
**ENSG00000069493**	*0.20555556*	*0.42727273*	*0.20555556*	*0.427272727*	*0.3*		*0.375*	*0.3604651*
**ENSG00000123146**	*0.47468354*		*0.47468354*		*0.146341463*		*0.1125*	
**ENSG00000184787**		*0.3969697*		*0.396969697*				*0.4772727*
**ENSG00000130856**		*0.47560976*		*0.475609756*				*0.4244186*
**ENSG00000167104**		*0.48780488*		*0.487804878*				*0.2882353*
**ENSG00000132475**		*0.44848485*		*0.448484848*				*0.4011628*
**ENSG00000162704**		*0.49090909*		*0.490909091*				*0.1627907*
**ENSG00000184752**		*0.23170732*		*0.231707317*				*0.0722892*
**ENSG00000180694**		*0.36060606*		*0.360606061*				*0.3555556*
**ENSG00000175305**		*0.15757576*		*0.157575758*				*0.0988372*
**ENSG00000177301**	*0.49700599*	*0.16363636*	*0.49700599*	*0.163636364*	*0.392857143*		*0.3988095*	*0.4464286*
**ENSG00000115474**		*0.29090909*		*0.290909091*				*0.2848837*
**ENSG00000096006**		*0.0969697*		*0.096969697*				
**ENSG00000204764**		*0.35393258*		*0.353932584*				*0.2613636*

**ENSG00000136982**		0.43333333		0.433333333				0.4090909
**ENSG00000134588**	0.43888889		0.43888889					
**ENSG00000114859**	0.49700599		0.49700599		0.410714286		0.4069767	
**ENSG00000107566**	0.38622754		0.38622754		0.095238095		0.1860465	0.4759036
**ENSG00000145919**		0.13333333		0.133333333				0.0888889
**ENSG00000162873**	0.49375	0.30113636	0.49375	0.301136364	0.487804878		0.4594595	
**ENSG00000111452**		0.40606061		0.406060606	0.220930233		0.3222222	0.3953488
**ENSG00000132406**	0.5	0.17878788	0.5	0.178787879	0.452380952		0.4011628	0.2616279
**ENSG00000184792**	0.2826087	0.32727273	0.2826087	0.327272727	0.160493827		0.2159091	0.4069767
**ENSG00000078053**	0.06111111	0.37116564	0.06111111	0.371165644				0.4329268
**ENSG00000167994**		0.43030303		0.43030303	0.255952381		0.2965116	0.0595238
**ENSG00000186787**					0.333333333		0.2616279	
**ENSG00000151746**		0.49050633		0.490506329				0.4593023
**ENSG00000170873**	0.45808383	0.18484848	0.45808383	0.184848485	0.31547619		0.3823529	
**ENSG00000163125**		0.43333333		0.433333333				0.3662791
**ENSG00000065613**		0.18539326		0.185393258				0.4186047
**ENSG00000064042**	0.1	0.15757576	0.1	0.157575758				0.1511628
**ENSG00000104320**	0.29041916	0.34848485	0.29041916	0.348484848	0.452380952		0.4360465	0.4360465
**ENSG00000150540**	0.46706587	0.2202381	0.46706587	0.220238095	0.327380952		0.3372093	0.2666667
**ENSG00000167693**		0.47878788		0.478787879				0.3
**ENSG00000147138**	0.46407186		0.46407186		0.44047619		0.4767442	
**ENSG00000198113**		0.2607362		0.260736196				0.4939759
**ENSG00000128683**		0.26993865		0.26993865	0.232142857		0.2647059	0.3081395
**ENSG00000146426**	0.40718563	0.31976744	0.40718563	0.319767442	0.488636364		0.4817073	
**ENSG00000128573**		0.48876404		0.488764045				0.3313953
**ENSG00000255986**								0.0595238
**ENSG00000187187**	0.47305389		0.47305389		0.160714286		0.127907	0.0777778
**ENSG00000198205**								0.1931818
**ENSG00000143199**		0.41212121		0.412121212				0.1976744
**ENSG00000141084**		0.1030303		0.103030303				0.0523256
**ENSG00000178665**		0.48333333		0.483333333				0.4333333
**ENSG00000168646**		0.43333333		0.433333333				0.377907
**ENSG00000014914**	0.28742515		0.28742515		0.255952381		0.4418605	
**ENSG00000138768**					0.069767442			
**ENSG00000115282**		0.11515152		0.115151515	0.154761905		0.1802326	0.1860465
**ENSG00000197756**	0.43413174	0.14242424	0.43413174	0.142424242	0.410714286		0.4244186	0.1511628
**ENSG00000185527**		0.39090909		0.390909091				0.2906977
**ENSG00000144045**		0.12424242		0.124242424				0.1918605
**ENSG00000171680**	0.45783133	0.12121212	0.45783133	0.121212121	0.267857143		0.25	
**ENSG00000197406**		0.11515152		0.115151515				
**ENSG00000198707**	0.31736527		0.31736527		0.201219512		0.3588235	0.3588235
**ENSG00000104442**	0.22159091	0.35454545	0.22159091	0.354545455	0.465909091		0.4444444	0.4318182
**ENSG00000113391**	0.1	0.14285714	0.1	0.142857143	0.088888889		0.1	0.1025641
**ENSG00000101680**	0.08383234		0.08383234					
**ENSG00000253304**		0.14634146		0.146341463				0.4767442
**ENSG00000073754**		0.17777778		0.177777778	0.136904762		0.2383721	0.3444444
**ENSG00000106610**		0.49425287		0.494252874				0.4418605
**ENSG00000137692**					0.181818182		0.1818182	
**ENSG00000128713**		0.33841463		0.338414634				0.4651163
**ENSG00000152078**		0.30909091		0.309090909				0.3614458
**ENSG00000117505**	0.26204819	0.13333333	0.26204819	0.133333333	0.31547619		0.3294118	0.0523256
**ENSG00000151718**		0.24719101		0.247191011				0.2272727
**ENSG00000170175**		0.17878788		0.178787879				0.3372093
**ENSG00000163331**		0.35151515		0.351515152				0.1337209

**ENSG00000108771**	0.10227273		0.10227273		0.088888889	0.1111111	
**ENSG00000214106**	0.44311377	0.42045455	0.44311377	0.420454545	0.244047619	0.2383721	0.4593023
**ENSG00000080823**	0.38922156	0.19393939	0.38922156	0.193939394	0.071428571	0.0930233	0.1176471
**ENSG00000105402**		0.12424242		0.124242424			0.2267442
**ENSG00000186153**	0.1497006		0.1497006		0.178571429	0.1918605	
**ENSG00000137449**							0.3409091
**ENSG00000110841**		0.43678161		0.436781609		0.0568182	0.4666667
**ENSG00000133065**		0.46987952		0.469879518			0.4011628
**ENSG00000069849**		0.24367089		0.243670886			0.0705882
**ENSG00000141505**	0.07185629		0.07185629				
**ENSG00000091490**	0.1	0.4689441	0.1	0.468944099			0.2906977
**ENSG00000153721**	0.31515152	0.19393939	0.31515152	0.193939394	0.297619048	0.1686047	0.1627907
**ENSG00000141569**		0.23333333		0.233333333			0.1802326
**ENSG00000135476**	0.17065868	0.30606061	0.17065868	0.306060606			
**ENSG00000197912**		0.44512195		0.445121951			0.2176471
**ENSG00000198380**		0.41111111		0.411111111			0.4476744
**ENSG00000011143**		0.0797546		0.079754601			0.0755814
**ENSG00000152904**	0.38622754	0.49444444	0.38622754	0.494444444	0.210843373	0.3255814	0.3863636
**ENSG00000115762**		0.10555556		0.105555556			0.1333333
**ENSG00000164035**		0.27044025		0.270440252			0.1046512
**ENSG00000239672**	0.35329341	0.48787879	0.35329341	0.487878788	0.273809524	0.2674419	0.5
**ENSG00000196600**		0.48148148		0.481481481			0.4
**ENSG00000024048**		0.36969697		0.36969697			0.4470588
**ENSG00000122966**	0.48502994	0.34969325	0.48502994	0.349693252	0.464285714	0.3941176	0.1046512
**ENSG00000244165**		0.44827586		0.448275862			0.4431818
**ENSG00000173890**		0.31212121		0.312121212			0.4534884
**ENSG00000159110**		0.33860759		0.338607595			0.4882353
**ENSG00000129595**		0.33939394		0.339393939			0.377907
**ENSG00000148942**	0.3125	0.48181818	0.3125	0.481818182	0.111111111	0.1363636	0.4888889
**ENSG00000204231**		0.31547619		0.31547619			0.2159091
**ENSG00000126882**	0.21556886		0.21556886		0.476190476	0.494186	
**ENSG00000151876**		0.06741573		0.06741573			
**ENSG00000168918**		0.18787879		0.187878788			0.3588235
**ENSG00000112319**		0.41463415		0.414634146			0.25
**ENSG00000206052**		0.46629213		0.466292135			0.3977273
**ENSG00000152558**	0.19886364	0.16111111	0.19886364	0.161111111	0.060240964	0.0714286	
**ENSG00000188986**		0.05590062		0.055900621			
**ENSG00000197181**	0.10479042		0.10479042		0.172619048	0.1337209	
**ENSG00000063176**	0.22754491	0.13483146	0.22754491	0.134831461			0.0681818
**ENSG00000251192**		0.42331288		0.423312883			0.0588235
**ENSG00000136098**	0.22727273	0.46111111	0.22727273	0.461111111	0.464285714	0.494186	0.4204545
**ENSG00000134897**		0.35555556		0.355555556			0.3604651
**ENSG00000258436**		0.18181818		0.181818182			0.2093023
**ENSG00000148481**	0.44277108		0.44277108		0.166666667	0.1569767	
**ENSG00000180305**	0.21556886		0.21556886		0.363095238	0.3139535	
**ENSG00000240403**							0.1627907
**ENSG00000203970**					0.081395349	0.0792683	
**ENSG00000040933**		0.23030303		0.23030303			0.2906977
**ENSG00000235863**		0.15454545		0.154545455			0.0872093
**ENSG00000152763**	0.08333333	0.48159509	0.08333333	0.481595092		0.1477273	0.377907
**ENSG00000116957**		0.07222222		0.072222222			
**ENSG00000113838**	0.48203593		0.48203593		0.125	0.1744186	
**ENSG00000110427**		0.31818182		0.318181818			0.255814
**ENSG00000170340**		0.13333333		0.133333333			0.377907

**ENSG00000112237**		0.4030303		0.403030303				0.377907
**ENSG00000131381**	0.33832335	0.32424242	0.33832335	0.324242424				0.1511628
**ENSG00000153237**		0.22121212		0.221212121			0.0523256	0.1918605
**ENSG00000148426**		0.44478528		0.444785276				0.1190476
**ENSG00000135624**	0.08682635		0.08682635		0.476190476		0.3662791	
**ENSG00000172765**		0.08787879		0.087878788				0.127907
**ENSG00000146830**	0.15269461	0.37931034	0.15269461	0.379310345	0.487951807		0.4825581	0.1136364
**ENSG00000178966**	0.42222222		0.42222222		0.233333333		0.1931818	
**ENSG00000137054**	0.40718563	0.23030303	0.40718563	0.23030303				0.1569767
**ENSG00000115944**	0.0748503	0.46969697	0.0748503	0.46969697				0.4302326
**ENSG00000116212**	0.38622754		0.38622754		0.083333333		0.0581395	
**ENSG00000144712**		0.17816092		0.17816092				0.1125
**ENSG00000172568**		0.32424242		0.324242424				0.2732558
**ENSG00000233863**	0.25		0.25		0.125		0.0639535	
**ENSG00000132026**	0.11144578	0.17575758	0.11144578	0.175757576				0.1337209
**ENSG00000155886**		0.27222222		0.272222222				0.4418605
**ENSG00000138668**		0.26060606		0.260606061				0.3882353
**ENSG00000115266**	0.49401198		0.49401198		0.244444444		0.2613636	
**ENSG00000164896**								0.2202381
**ENSG00000069667**		0.44207317		0.442073171				0.0639535
**ENSG00000158022**		0.34848485		0.348484848				0.1569767
**ENSG00000113558**	0.39520958		0.39520958		0.462962963		0.4767442	
**ENSG00000178222**		0.34545455		0.345454545				0.4476744
**ENSG00000143536**		0.12804878		0.12804878				0.4476744
**ENSG00000152253**	0.14444444		0.14444444					
**ENSG00000168411**		0.43678161		0.436781609				0.2045455
**ENSG00000174080**		0.27878788		0.278787879				0.3139535
**ENSG00000151846**		0.31666667		0.316666667				0.2777778
**ENSG00000181396**		0.32621951		0.326219512				0.4302326
**ENSG00000145191**		0.31818182		0.318181818				0.4709302
**ENSG00000206075**	0.1497006	0.43939394	0.1497006	0.439393939	0.452380952		0.4883721	0.4825581
**ENSG00000146005**		0.11818182		0.118181818				0.1162791
**ENSG00000131165**		0.42727273		0.427272727				
**ENSG00000135824**	0.44610778		0.44610778		0.204819277		0.1511628	0.1569767
**ENSG00000100425**	0.42215569		0.42215569		0.148809524		0.1569767	
**ENSG00000133612**								0.0697674
**ENSG00000073150**		0.1		0.1				0.2674419
**ENSG00000172238**	0.1257485		0.1257485		0.178571429		0.3235294	
**ENSG00000011426**	0.16167665	0.125	0.16167665	0.125	0.446428571		0.4011628	0.1144578
**ENSG00000157657**		0.42727273		0.427272727				0.3662791
**ENSG00000078814**		0.36969697		0.36969697				0.1802326
**ENSG00000047188**	0.38068182	0.45	0.38068182	0.45	0.188888889		0.1477273	0.2705882
**ENSG00000184949**		0.34831461		0.348314607				0.4476744
**ENSG00000092421**		0.07575758		0.075757576	0.196428571		0.2	0.1337209
**ENSG00000198797**	0.26946108	0.23333333	0.26946108	0.233333333				
**ENSG00000095794**	0.39820359	0.18787879	0.39820359	0.187878788	0.357142857		0.255814	0.2267442
**ENSG00000113580**		0.1969697		0.196969697				
**ENSG00000169122**		0.33030303		0.33030303				0.4186047
**ENSG00000104853**	0.21666667	0.1030303	0.21666667	0.103030303			0.1022727	0.0813953
**ENSG00000189157**		0.13030303		0.13030303				0.2093023
**ENSG00000111652**		0.38787879		0.387878788	0.280487805		0.2209302	0.3023256
**ENSG00000158864**		0.14110429		0.141104294	0.347560976		0.375	0.2965116
**ENSG00000166710**								0.1363636
**ENSG00000095321**		0.23333333		0.233333333				0.375

**ENSG00000051825**	*0.08383234*	*0.3902439*	*0.08383234*	*0.390243902*			*0.2732558*
**ENSG00000128512**		*0.3*		*0.3*			*0.4302326*
**ENSG00000078142**		*0.5*		*0.5*			*0.3977273*
**ENSG00000144031**		*0.24712644*		*0.247126437*			*0.1453488*
**ENSG00000148604**		*0.25609756*		*0.256097561*			*0.0697674*
**ENSG00000081923**		*0.25555556*		*0.255555556*			*0.4879518*
**ENSG00000174151**		*0.44848485*		*0.448484848*			*0.3352941*
**ENSG00000064726**		*0.44545455*		*0.445454545*			*0.1162791*
**ENSG00000165417**		*0.23939394*		*0.239393939*			*0.3139535*
**ENSG00000166928**		*0.38787879*		*0.387878788*			*0.4883721*
**ENSG00000005059**		*0.38719512*		*0.387195122*			*0.4647059*
**ENSG00000164985**		*0.08333333*		*0.083333333*			*0.3295455*
**ENSG00000048740**		*0.38787879*		*0.387878788*			*0.2093023*
**ENSG00000170439**		*0.48181818*		*0.481818182*			*0.0755814*
**ENSG00000204343**		*0.11801242*		*0.118012422*			*0.4302326*
**ENSG00000076685**		*0.39393939*		*0.393939394*	*0.113095238*	*0.0529412*	*0.3895349*
**ENSG00000047579**	*0.33832335*	*0.36363636*	*0.33832335*	*0.363636364*	*0.288888889*	*0.2790698*	*0.4534884*
**ENSG00000198738**	*0.17878788*		*0.17878788*		*0.166666667*	*0.1802326*	
**ENSG00000139915**	*0.16111111*	*0.42424242*	*0.16111111*	*0.424242424*	*0.133333333*	*0.0888889*	*0.3181818*
**ENSG00000137857**		*0.43939394*		*0.439393939*			*0.4772727*
**ENSG00000227115**	*0.25149701*		*0.25149701*		*0.232142857*	*0.255814*	*0.2034884*
**ENSG00000168528**							*0.3333333*
**ENSG00000143365**		*0.4030303*		*0.403030303*			*0.4476744*
**ENSG00000164066**		*0.47878788*		*0.478787879*			*0.494186*
**ENSG00000165140**	*0.14117647*	*0.48181818*	*0.14117647*	*0.481818182*	*0.355555556*	*0.0795455*	*0.4011628*
**ENSG00000081059**	*0.45930233*		*0.45930233*		*0.066666667*		
**ENSG00000175691**		*0.10909091*		*0.109090909*			*0.3372093*
**ENSG00000118181**	*0.47904192*		*0.47904192*			*0.0988372*	
**ENSG00000011566**	*0.26347305*	*0.15432099*	*0.26347305*	*0.154320988*	*0.154761905*	*0.2058824*	*0.2771084*
**ENSG00000250264**	*0.42777778*		*0.42777778*		*0.4*	*0.4431818*	
**ENSG00000014216**	*0.07777778*		*0.07777778*		*0.233333333*	*0.3409091*	
**ENSG00000143882**		*0.41212121*		*0.412121212*			*0.3604651*
**ENSG00000063761**		*0.43939394*		*0.439393939*			*0.3882353*
**ENSG00000093134**		*0.26363636*		*0.263636364*			*0.3430233*
**ENSG00000140943**	*0.34730539*	*0.32317073*	*0.34730539*	*0.323170732*	*0.306818182*	*0.3192771*	*0.4294118*
**ENSG00000169508**		*0.2191358*		*0.219135802*			*0.3604651*
**ENSG00000139874**	*0.11377246*	*0.37078652*	*0.11377246*	*0.370786517*			*0.4127907*
**ENSG00000111785**	*0.37724551*	*0.23333333*	*0.37724551*	*0.233333333*	*0.30952381*	*0.2619048*	*0.4294118*
**ENSG00000152049**		*0.06666667*		*0.066666667*			*0.0681818*
**ENSG00000082898**		*0.12222222*		*0.122222222*			*0.347561*
**ENSG00000236624**	*0.1*	*0.3*	*0.1*	*0.3*	*0.077777778*		
**ENSG00000066032**	*0.28143713*	*0.3902439*	*0.28143713*	*0.390243902*	*0.333333333*	*0.3372093*	*0.422619*
**ENSG00000156113**	*0.28089888*	*0.46666667*	*0.28089888*	*0.466666667*	*0.068181818*	*0.1222222*	*0.4659091*
**ENSG00000138413**					*0.464285714*	*0.4069767*	
**ENSG00000080493**		*0.26111111*		*0.261111111*			*0.4*
**ENSG00000177483**		*0.11666667*		*0.116666667*			*0.3977273*
**ENSG00000212122**		*0.16860465*		*0.168604651*			*0.1363636*
**ENSG00000079689**		*0.28484848*		*0.284848485*			*0.2848837*
**ENSG00000154188**	*0.30681818*	*0.36627907*	*0.30681818*	*0.36627907*	*0.277777778*	*0.2613636*	*0.3604651*
**ENSG00000166689**		*0.46625767*		*0.466257669*			*0.4476744*
**ENSG00000231925**	*0.41317365*	*0.4969697*	*0.41317365*	*0.496969697*	*0.31547619*	*0.3255814*	*0.3546512*
**ENSG00000165671**		*0.18181818*		*0.181818182*			*0.4767442*
**ENSG00000197951**		*0.34545455*		*0.345454545*	*0.155555556*		*0.3604651*
**ENSG00000120051**							*0.122093*

**ENSG00000131378**		*0.30555556*		*0.305555556*			*0.4767442*
**ENSG00000119714**							*0.0872093*
**ENSG00000145248**		*0.21646341*		*0.216463415*			*0.0813953*
**ENSG00000133740**	*0.20555556*		*0.20555556*				
**ENSG00000198912**		*0.1030303*		*0.103030303*			*0.3630952*
**ENSG00000116106**		*0.37878788*		*0.378787879*			*0.2840909*
**ENSG00000166035**	*0.21666667*	*0.48295455*	*0.21666667*	*0.482954545*	*0.133333333*	*0.0888889*	*0.4767442*
**ENSG00000171310**	*0.12121212*	*0.31515152*	*0.12121212*	*0.315151515*	*0.071428571*		*0.4709302*
**ENSG00000154217**					*0.280487805*	*0.4090909*	
**ENSG00000188511**	*0.16566265*	*0.46111111*	*0.16566265*	*0.461111111*	*0.398809524*	*0.3690476*	*0.4011628*
**ENSG00000131187**	*0.40419162*		*0.40419162*		*0.30952381*	*0.3023256*	
**ENSG00000136100**		*0.40606061*		*0.406060606*			*0.3470588*
**ENSG00000165655**	*0.38922156*		*0.38922156*				
**ENSG00000163104**		*0.44654088*		*0.446540881*			*0.4235294*
**ENSG00000172594**		*0.30434783*		*0.304347826*			*0.4823529*
**ENSG00000048392**		*0.13109756*		*0.131097561*			
**ENSG00000136932**	*0.17365269*	*0.31818182*	*0.17365269*	*0.318181818*	*0.226190476*	*0.1511628*	*0.1647059*
**ENSG00000070601**	*0.48802395*		*0.48802395*		*0.148809524*	*0.127907*	
**ENSG00000121716**	*0.2005988*	*0.15757576*	*0.2005988*	*0.157575758*	*0.151162791*	*0.1976744*	
**ENSG00000125875**		*0.44848485*		*0.448484848*			*0.4127907*
**ENSG00000177951**		*0.32121212*		*0.321212121*			*0.4360465*
**ENSG00000139637**	*0.26347305*		*0.26347305*				
**ENSG00000106355**	*0.39221557*	*0.45454545*	*0.39221557*	*0.454545455*	*0.255555556*	*0.0988372*	*0.2882353*
**ENSG00000125844**		*0.2*		*0.2*			*0.4651163*
**ENSG00000148795**	*0.28443114*		*0.28443114*		*0.410714286*	*0.4593023*	
**ENSG00000134460**		*0.4030303*		*0.403030303*			*0.2428571*
**ENSG00000171724**		*0.44545455*		*0.445454545*			*0.4090909*
**ENSG00000101439**		*0.35955056*		*0.359550562*			*0.3111111*
**ENSG00000107165**	*0.18862275*	*0.35454545*	*0.18862275*	*0.354545455*			
**ENSG00000139330**		*0.05182927*		*0.051829268*			*0.1222222*
**ENSG00000116525**		*0.23636364*		*0.236363636*		*0.1022727*	*0.377907*
**ENSG00000163521**	*0.2994012*		*0.2994012*		*0.160714286*	*0.0988372*	
**ENSG00000135090**	*0.28313253*		*0.28313253*		*0.255952381*	*0.4069767*	
**ENSG00000137338**		*0.0969697*		*0.096969697*			*0.1882353*
**ENSG00000178796**	*0.13473054*	*0.23333333*	*0.13473054*	*0.233333333*	*0.232142857*	*0.244186*	*0.3372093*
**ENSG00000198049**	*0.13173653*		*0.13173653*				
**ENSG00000093167**	*0.16167665*	*0.48484848*	*0.16167665*	*0.484848485*			*0.2117647*
**ENSG00000167766**	*0.36826347*	*0.46625767*	*0.36826347*	*0.466257669*	*0.477777778*	*0.4883721*	*0.3255814*
**ENSG00000196284**		*0.46646341*		*0.466463415*	*0.266666667*	*0.2666667*	*0.2790698*
**ENSG00000091831**	*0.49700599*	*0.44848485*	*0.49700599*	*0.448484848*	*0.398809524*	*0.3488372*	*0.3604651*
**ENSG00000203734**	*0.26704545*	*0.47777778*	*0.26704545*	*0.477777778*	*0.261363636*	*0.3*	*0.3809524*
**ENSG00000116151**		*0.34848485*		*0.348484848*			*0.4069767*
**ENSG00000137274**	*0.15269461*		*0.15269461*		*0.078313253*	*0.0595238*	
**ENSG00000181781**		*0.22121212*		*0.221212121*			*0.3255814*
**ENSG00000164932**	*0.21666667*		*0.21666667*		*0.433333333*	*0.3636364*	
**ENSG00000109047**	*0.17664671*	*0.27325581*	*0.17664671*	*0.273255814*	*0.351190476*	*0.3647059*	*0.1024096*
**ENSG00000131370**	*0.4030303*	*0.29651163*	*0.4030303*	*0.296511628*	*0.30952381*	*0.2125*	
**ENSG00000198722**		*0.25454545*		*0.254545455*			
**ENSG00000078549**		*0.2969697*		*0.296969697*			*0.2666667*
**ENSG00000146802**		*0.27044025*		*0.270440252*			*0.0681818*
**ENSG00000177186**		*0.30337079*		*0.303370787*			*0.1860465*
**ENSG00000075945**	*0.18862275*	*0.12727273*	*0.18862275*	*0.127272727*	*0.130952381*	*0.1162791*	*0.1511628*
**ENSG00000158352**		*0.31111111*		*0.311111111*			*0.1363636*
**ENSG00000166822**		*0.4*		*0.4*			*0.5*

**ENSG00000203722**		*0.15151515*		*0.151515152*				*0.4476744*
**ENSG00000150764**		*0.29573171*		*0.295731707*				*0.3953488*
**ENSG00000071203**		*0.42424242*		*0.424242424*				*0.3081395*
**ENSG00000160055**		*0.05*		*0.05*				
**ENSG00000175866**	*0.0988024*	*0.26060606*	*0.0988024*	*0.260606061*	*0.228915663*		*0.1744186*	*0.3837209*
**ENSG00000138434**	*0.15662651*	*0.14242424*	*0.15662651*	*0.142424242*	*0.05952381*		*0.0639535*	*0.1590909*
**ENSG00000137948**	*0.44578313*		*0.44578313*		*0.416666667*		*0.4069767*	
**ENSG00000138483**					*0.172619048*		*0.1162791*	
**ENSG00000127241**	*0.24850299*	*0.48484848*	*0.24850299*	*0.484848485*	*0.244047619*		*0.1337209*	*0.5*
**ENSG00000050438**	*0.06111111*	*0.45757576*	*0.06111111*	*0.457575758*				*0.4651163*
**ENSG00000124356**	*0.45555556*	*0.2969697*	*0.45555556*	*0.296969697*	*0.363095238*		*0.4593023*	*0.4777778*
**ENSG00000206531**	*0.21111111*	*0.39444444*	*0.21111111*	*0.394444444*				
**ENSG00000118946**		*0.27878788*		*0.278787879*				*0.4823529*
**ENSG00000172995**	*0.38323353*	*0.36363636*	*0.38323353*	*0.363636364*	*0.101190476*		*0.2034884*	*0.4476744*
**ENSG00000213934**		*0.4137931*		*0.413793103*				*0.3068182*
**ENSG00000198597**		*0.17378049*		*0.173780488*				*0.3662791*
**ENSG00000164106**	*0.28089888*		*0.28089888*					
**ENSG00000009724**		*0.14044944*		*0.140449438*				*0.1136364*
**ENSG00000137266**	*0.25287356*	*0.12222222*	*0.25287356*	*0.122222222*	*0.31547619*		*0.2674419*	*0.3977273*
**ENSG00000062194**		*0.41818182*		*0.418181818*	*0.058139535*			*0.255814*
**ENSG00000168884**		*0.12121212*		*0.121212121*				*0.0581395*
**ENSG00000150753**	*0.2994012*	*0.1969697*	*0.2994012*	*0.196969697*	*0.083333333*		*0.1104651*	*0.1686047*
**ENSG00000179195**	*0.39221557*	*0.21022727*	*0.39221557*	*0.210227273*	*0.101190476*		*0.127907*	
**ENSG00000164168**								*0.1744186*
**ENSG00000007908**	*0.07228916*	*0.24233129*	*0.07228916*	*0.242331288*				*0.373494*
**ENSG00000071054**	*0.121875*	*0.4030303*	*0.121875*	*0.403030303*			*0.246988*	*0.4529412*
**ENSG00000166321**		*0.05*		*0.05*				*0.2954545*
**ENSG00000165891**		*0.46111111*		*0.461111111*				*0.3181818*
**ENSG00000124588**	*0.35928144*	*0.13333333*	*0.35928144*	*0.133333333*	*0.307228916*		*0.3255814*	*0.1627907*
**ENSG00000188316**		*0.20186335*		*0.201863354*				*0.1941176*
**ENSG00000117266**	*0.15868263*	*0.39880952*	*0.15868263*	*0.398809524*	*0.071428571*		*0.0813953*	*0.3170732*
**ENSG00000070367**		*0.17295597*		*0.172955975*				*0.0892857*
**ENSG00000139714**		*0.07055215*		*0.070552147*				
**ENSG00000242247**		*0.48181818*		*0.481818182*				*0.3488372*
**ENSG00000255103**		*0.18333333*		*0.183333333*				*0.2093023*
**ENSG00000006128**		*0.18484848*		*0.184848485*				*0.2616279*
**ENSG00000250151**		*0.11890244*		*0.118902439*				*0.0731707*
**ENSG00000151952**	*0.36526946*	*0.26969697*	*0.36526946*	*0.26969697*	*0.369047619*		*0.372093*	*0.1686047*
**ENSG00000198033**		*0.05194805*		*0.051948052*				
**ENSG00000092931**		*0.07716049*		*0.077160494*				*0.4634146*
**ENSG00000137880**	*0.22222222*		*0.22222222*					
**ENSG00000136305**	*0.15361446*		*0.15361446*		*0.154761905*		*0.1823529*	*0.2*
**ENSG00000112312**	*0.28888889*		*0.28888889*		*0.113636364*			
**ENSG00000148468**		*0.16666667*		*0.166666667*				*0.1453488*
**ENSG00000103855**		*0.49090909*		*0.490909091*				*0.4127907*
**ENSG00000101265**	*0.07777778*	*0.39393939*	*0.07777778*	*0.393939394*	*0.1*		*0.0681818*	*0.4069767*
**ENSG00000211452**	*0.25*	*0.37272727*	*0.25*	*0.372727273*	*0.119047619*		*0.1046512*	*0.1511628*
**ENSG00000106086**		*0.23563218*		*0.235632184*				*0.1860465*
**ENSG00000187147**		*0.35151515*		*0.351515152*				*0.0930233*
**ENSG00000102678**		*0.13888889*		*0.138888889*				
**ENSG00000176986**	*0.15568862*	*0.11515152*	*0.15568862*	*0.115151515*	*0.172619048*		*0.1569767*	*0.127907*
**ENSG00000055950**		*0.47727273*		*0.477272727*				
**ENSG00000169302**		*0.21212121*		*0.212121212*				*0.1569767*
**ENSG00000197329**	*0.14444444*	*0.12727273*	*0.14444444*	*0.127272727*	*0.155555556*		*0.1022727*	*0.0823529*

**ENSG00000160963**		*0.21818182*		*0.218181818*			*0.2732558*
**ENSG00000198756**	*0.08982036*	*0.46319018*	*0.08982036*	*0.463190184*	*0.273809524*	*0.2674419*	*0.4883721*
**ENSG00000144824**	*0.25149701*	*0.37804878*	*0.25149701*	*0.37804878*	*0.214285714*	*0.2267442*	*0.3555556*
**ENSG00000155629**		*0.3030303*		*0.303030303*			*0.1337209*
**ENSG00000126107**		*0.23636364*		*0.236363636*			*0.2823529*
**ENSG00000197312**		*0.41666667*		*0.416666667*			*0.377907*
**ENSG00000059915**		*0.36363636*		*0.363636364*			*0.2034884*
**ENSG00000168077**		*0.26744186*		*0.26744186*			*0.5*
**ENSG00000180543**		*0.39393939*		*0.393939394*			*0.4593023*
**ENSG00000198498**	*0.27844311*	*0.5*	*0.27844311*	*0.5*	*0.451807229*	*0.4058824*	*0.3666667*
**ENSG00000086288**	*0.05*		*0.05*		*0.113636364*	*0.1627907*	
**ENSG00000139631**	*0.16666667*	*0.21341463*	*0.16666667*	*0.213414634*	*0.111111111*	*0.255814*	*0.375*
**ENSG00000106665**		*0.33333333*		*0.333333333*			*0.1704545*
**ENSG00000162441**	*0.17272727*		*0.17272727*		*0.398809524*	*0.3197674*	
**ENSG00000188056**	*0.22754491*	*0.48484848*	*0.22754491*	*0.484848485*	*0.101190476*		*0.4534884*
**ENSG00000083099**		*0.47777778*		*0.477777778*			*0.2045455*
**ENSG00000196923**		*0.45705521*		*0.457055215*			*0.3081395*
**ENSG00000165355**		*0.23636364*		*0.236363636*			*0.494186*
**ENSG00000185359**		*0.12777778*		*0.127777778*			*0.2941176*
**ENSG00000165152**	*0.06666667*	*0.44545455*	*0.06666667*	*0.445454545*	*0.077777778*		*0.4651163*
**ENSG00000179055**		*0.27575758*		*0.275757576*			*0.4476744*
**ENSG00000111319**		*0.25555556*		*0.255555556*			*0.3111111*
**ENSG00000167759**	*0.43413174*	*0.45151515*	*0.43413174*	*0.451515152*	*0.369047619*	*0.3023256*	*0.372093*
**ENSG00000185792**		*0.14634146*		*0.146341463*			*0.1511628*
**ENSG00000180611**		*0.4695122*		*0.469512195*			*0.4352941*
**ENSG00000168952**	*0.5*	*0.42727273*	*0.5*	*0.427272727*	*0.166666667*	*0.255814*	*0.4883721*
**ENSG00000129472**		*0.16363636*		*0.163636364*			*0.0639535*
**ENSG00000172732**	*0.17065868*		*0.17065868*		*0.271084337*	*0.4*	
**ENSG00000091140**		*0.43030303*		*0.43030303*			*0.4825581*
**ENSG00000164124**	*0.16111111*	*0.43030303*	*0.16111111*	*0.43030303*	*0.068181818*	*0.0697674*	*0.2117647*
**ENSG00000084110**	*0.12275449*	*0.39506173*	*0.12275449*	*0.395061728*			*0.4360465*
**ENSG00000166444**	*0.18888889*		*0.18888889*		*0.088888889*		
**ENSG00000138073**		*0.16363636*		*0.163636364*			*0.1744186*
**ENSG00000135845**	*0.10479042*		*0.10479042*		*0.327380952*	*0.3546512*	
**ENSG00000069122**	*0.22155689*	*0.3006135*	*0.22155689*	*0.300613497*	*0.18452381*	*0.1686047*	*0.4117647*
**ENSG00000125319**		*0.33231707*		*0.332317073*			*0.4176471*
**ENSG00000115053**							*0.1744186*
**ENSG00000076555**	*0.42134831*	*0.17777778*	*0.42134831*	*0.177777778*	*0.322222222*	*0.3662791*	*0.2906977*
**ENSG00000123472**		*0.26111111*		*0.261111111*			*0.4166667*
**ENSG00000166598**	*0.47222222*		*0.47222222*		*0.444444444*	*0.4886364*	
**ENSG00000116670**		*0.2*		*0.2*	*0.422222222*	*0.4431818*	*0.1860465*
**ENSG00000244694**		*0.49090909*		*0.490909091*			*0.2383721*
**ENSG00000165209**		*0.15454545*		*0.154545455*			*0.1453488*
**ENSG00000110900**		*0.49090909*		*0.490909091*			*0.4767442*
**ENSG00000173699**		*0.44848485*		*0.448484848*			*0.4767442*
**ENSG00000236882**		*0.27439024*		*0.274390244*			*0.0697674*
**ENSG00000241685**		*0.06363636*		*0.063636364*			
**ENSG00000061337**		*0.44705882*		*0.447058824*			*0.4069767*
**ENSG00000096088**		*0.06976744*		*0.069767442*			
**ENSG00000132872**		*0.27439024*		*0.274390244*			*0.4235294*
**ENSG00000101255**							*0.122093*
**ENSG00000253457**		*0.34662577*		*0.346625767*			*0.375*
**ENSG00000116120**	*0.23952096*	*0.36969697*	*0.23952096*	*0.36969697*	*0.488888889*	*0.4156627*	*0.3977273*
**ENSG00000168772**		*0.38953488*		*0.389534884*			*0.3522727*

**ENSG00000171735**		*0.18404908*		*0.18404908*				*0.3294118*
**ENSG00000111011**		*0.23619632*		*0.236196319*				*0.0941176*
**ENSG00000197168**	*0.27272727*	*0.3*	*0.27272727*	*0.3*	*0.422222222*		*0.4651163*	*0.4651163*
**ENSG00000167468**		*0.44242424*		*0.442424242*				*0.3941176*
**ENSG00000103546**	*0.25449102*	*0.13030303*	*0.25449102*	*0.13030303*	*0.297619048*		*0.3662791*	*0.1802326*
**ENSG00000071242**	*0.48888889*	*0.35151515*	*0.48888889*	*0.351515152*	*0.3*		*0.2777778*	*0.1918605*
**ENSG00000198624**	*0.38333333*	*0.45757576*	*0.38333333*	*0.457575758*	*0.344444444*		*0.4333333*	*0.4333333*
**ENSG00000112715**	*0.06287425*	*0.43030303*	*0.06287425*	*0.43030303*	*0.095238095*		*0.1529412*	*0.1511628*
**ENSG00000078618**	*0.22777778*		*0.22777778*		*0.159090909*		*0.1927711*	
**ENSG00000172292**		*0.32012195*		*0.320121951*				*0.4411765*
**ENSG00000175634**		*0.47272727*		*0.472727273*				*0.2727273*
**ENSG00000170044**	*0.07777778*	*0.38636364*	*0.07777778*	*0.386363636*				*0.1627907*
**ENSG00000138115**		*0.18012422*		*0.180124224*				*0.3941176*
**ENSG00000153395**		*0.3*		*0.3*				*0.4821429*
**ENSG00000170289**	*0.17777778*	*0.26666667*	*0.17777778*	*0.266666667*	*0.133333333*			*0.4244186*
**ENSG00000145040**								*0.1104651*
**ENSG00000122584**		*0.33333333*		*0.333333333*				*0.1511628*
**ENSG00000204572**		*0.42727273*		*0.427272727*				*0.3546512*
**ENSG00000130383**	*0.43888889*	*0.18333333*	*0.43888889*	*0.183333333*	*0.122222222*		*0.255814*	*0.1333333*
**ENSG00000183960**								*0.2151163*
**ENSG00000107262**		*0.11818182*		*0.118181818*				*0.2209302*
**ENSG00000158985**	*0.45481928*		*0.45481928*					
**ENSG00000089060**		*0.1402439*		*0.140243902*				*0.2616279*
**ENSG00000015676**		*0.23529412*		*0.235294118*	*0.066666667*			
**ENSG00000155085**	*0.31437126*	*0.38484848*	*0.31437126*	*0.384848485*	*0.329545455*		*0.2777778*	*0.372093*
**ENSG00000164296**	*0.34730539*	*0.14242424*	*0.34730539*	*0.142424242*	*0.22972973*		*0.2195122*	*0.1022727*
**ENSG00000135974**	*0.18975904*	*0.05617978*	*0.18975904*	*0.056179775*	*0.138554217*		*0.2093023*	
**ENSG00000167748**	*0.20786517*		*0.20786517*		*0.2*		*0.3111111*	
**ENSG00000154342**		*0.2962963*		*0.296296296*				*0.2034884*
**ENSG00000120314**	*0.43413174*	*0.41818182*	*0.43413174*	*0.418181818*	*0.445121951*		*0.4651163*	*0.4886364*
**ENSG00000196611**		*0.34545455*		*0.345454545*			*0.0872093*	*0.4111111*
**ENSG00000164087**		*0.15454545*		*0.154545455*				*0.494186*
**ENSG00000137124**		*0.36363636*		*0.363636364*				*0.494186*
**ENSG00000198715**		*0.26666667*		*0.266666667*				*0.2209302*
**ENSG00000214338**	*0.27245509*	*0.44444444*	*0.27245509*	*0.444444444*	*0.095238095*		*0.1511628*	*0.3372093*
**ENSG00000005471**	*0.31437126*		*0.31437126*					
**ENSG00000180245**		*0.18292683*		*0.182926829*				*0.4176471*
**ENSG00000180346**	*0.20359281*	*0.22222222*	*0.20359281*	*0.222222222*	*0.19047619*		*0.1802326*	*0.2159091*
**ENSG00000255310**	*0.20114943*	*0.40909091*	*0.20114943*	*0.409090909*			*0.0769231*	
**ENSG00000152219**	*0.48630137*	*0.13636364*	*0.48630137*	*0.136363636*	*0.342105263*		*0.3780488*	*0.4189189*
**ENSG00000185272**		*0.39240506*		*0.392405063*				*0.4705882*
**ENSG00000006607**		*0.23030303*		*0.23030303*				*0.4011628*
**ENSG00000088782**					*0.464285714*		*0.4883721*	
**ENSG00000107864**		*0.3030303*		*0.303030303*				*0.4360465*
**ENSG00000205777**		*0.42424242*		*0.424242424*				*0.1627907*
**ENSG00000154451**		*0.44382022*		*0.443820225*	*0.277108434*		*0.3095238*	*0.2386364*
**ENSG00000180011**		*0.49079755*		*0.490797546*	*0.452380952*		*0.4883721*	*0.4588235*
**ENSG00000116793**		*0.23636364*		*0.236363636*				*0.4294118*
**ENSG00000170085**		*0.18181818*		*0.181818182*				*0.1807229*
**ENSG00000081026**	*0.13333333*	*0.29393939*	*0.13333333*	*0.293939394*	*0.311111111*		*0.4886364*	*0.1411765*
**ENSG00000150477**		*0.24848485*		*0.248484848*				*0.4534884*
**ENSG00000101000**		*0.44252874*		*0.442528736*				*0.2727273*
**ENSG00000143315**	*0.12275449*	*0.06666667*	*0.12275449*	*0.066666667*				*0.3352941*
**ENSG00000134265**		*0.38888889*		*0.388888889*				*0.4634146*

**ENSG00000224361**	*0.26646707*		*0.26646707*					
**ENSG00000179915**	*0.06896552*	*0.27222222*	*0.06896552*	*0.272222222*	*0.318181818*		*0.2674419*	*0.3888889*
**ENSG00000241119**		*0.22777778*		*0.227777778*				*0.1590909*
**ENSG00000144407**		*0.20909091*		*0.209090909*				*0.4117647*
**ENSG00000129353**		*0.39393939*		*0.393939394*				*0.3529412*
**ENSG00000167077**	*0.30838323*	*0.20909091*	*0.30838323*	*0.209090909*	*0.136904762*		*0.3255814*	
**ENSG00000143226**		*0.49390244*		*0.493902439*				*0.2710843*
**ENSG00000135776**		*0.17948718*		*0.179487179*				*0.0609756*
**ENSG00000168894**		*0.23125*		*0.23125*	*0.05*			*0.3546512*
**ENSG00000198860**		*0.07575758*		*0.075757576*				
**ENSG00000165810**		*0.4847561*		*0.484756098*				*0.4302326*
**ENSG00000196979**	*0.40419162*	*0.08333333*	*0.40419162*	*0.083333333*	*0.386904762*		*0.2383721*	*0.2666667*
**ENSG00000185507**	*0.11666667*		*0.11666667*		*0.055555556*			
**ENSG00000165792**	*0.13173653*	*0.22121212*	*0.13173653*	*0.221212121*	*0.18452381*		*0.2267442*	*0.2674419*
**ENSG00000196917**		*0.38181818*		*0.381818182*				*0.494186*
**ENSG00000102030**		*0.21296296*		*0.212962963*				*0.3546512*
**ENSG00000076356**		*0.37931034*		*0.379310345*				*0.3895349*
**ENSG00000117154**		*0.42727273*		*0.427272727*				*0.3081395*
**ENSG00000117519**		*0.08181818*		*0.081818182*				
**ENSG00000172955**		*0.47272727*		*0.472727273*				*0.0988372*
**ENSG00000139697**		*0.13030303*		*0.13030303*				*0.2674419*
**ENSG00000204120**	*0.25149701*	*0.29444444*	*0.25149701*	*0.294444444*	*0.31547619*		*0.2848837*	*0.3295455*
**ENSG00000120262**		*0.44848485*		*0.448484848*				*0.377907*
**ENSG00000140511**		*0.09090909*		*0.090909091*				*0.1470588*
**ENSG00000166049**	*0.12222222*	*0.26829268*	*0.12222222*	*0.268292683*				*0.0697674*
**ENSG00000168936**		*0.36363636*		*0.363636364*				*0.3313953*
**ENSG00000204311**		*0.1030303*		*0.103030303*				
**ENSG00000115524**		*0.30674847*		*0.306748466*				*0.2831325*
**ENSG00000143333**		*0.4127907*		*0.412790698*				*0.372093*
**ENSG00000180901**		*0.1954023*		*0.195402299*	*0.18452381*		*0.2321429*	*0.4204545*
**ENSG00000185361**		*0.49431818*		*0.494318182*				*0.2647059*
**ENSG00000169919**		*0.45757576*		*0.457575758*				*0.1785714*
**ENSG00000168765**	*0.25280899*		*0.25280899*		*0.363636364*		*0.375*	
**ENSG00000258850**	*0.20359281*		*0.20359281*		*0.290697674*		*0.2738095*	
**ENSG00000138386**	*0.20180723*		*0.20180723*		*0.351190476*		*0.3372093*	
**ENSG00000118113**	*0.15868263*	*0.49085366*	*0.15868263*	*0.490853659*	*0.422619048*		*0.3953488*	*0.3953488*
**ENSG00000143179**		*0.42727273*		*0.427272727*				*0.25*
**ENSG00000007541**		*0.47272727*		*0.472727273*				*0.3243243*
**ENSG00000112273**	*0.07865169*	*0.34482759*	*0.07865169*	*0.344827586*				*0.2325581*
**ENSG00000184857**		*0.46060606*		*0.460606061*				*0.5*
**ENSG00000010610**		*0.45705521*		*0.457055215*				*0.4883721*
**ENSG00000174243**		*0.45151515*		*0.451515152*				*0.3662791*
**ENSG00000171862**		*0.36196319*		*0.36196319*				*0.4058824*
**ENSG00000101542**	*0.42215569*	*0.08045977*	*0.42215569*	*0.08045977*	*0.19047619*		*0.1395349*	*0.4431818*
**ENSG00000137818**	*0.05172414*		*0.05172414*					
**ENSG00000127804**	*0.13473054*	*0.33333333*	*0.13473054*	*0.333333333*				*0.3352941*
**ENSG00000151881**	*0.32335329*		*0.32335329*		*0.083333333*		*0.1627907*	*0.0666667*
**ENSG00000136630**	*0.30681818*	*0.15454545*	*0.30681818*	*0.154545455*	*0.415662651*		*0.3764706*	
**ENSG00000121578**	*0.27777778*	*0.16292135*	*0.27777778*	*0.162921348*	*0.4*		*0.2954545*	*0.2222222*
**ENSG00000205356**		*0.3445122*		*0.344512195*				*0.4825581*
**ENSG00000132716**								*0.0523256*
**ENSG00000011600**								*0.122093*
**ENSG00000136522**	*0.14457831*		*0.14457831*		*0.148809524*		*0.122093*	*0.122093*
**ENSG00000129317**		*0.08787879*		*0.087878788*				

**ENSG00000108523**		*0.18965517*		*0.189655172*				*0.5*
**ENSG00000110148**	*0.38095238*	*0.38787879*	*0.38095238*	*0.387878788*				*0.4*
**ENSG00000132522**	*0.35628743*	*0.37575758*	*0.35628743*	*0.375757576*	*0.054216867*		*0.0755814*	
**ENSG00000150527**							*0.060241*	
**ENSG00000122696**								*0.1022727*
**ENSG00000035681**		*0.33435583*		*0.334355828*				*0.2093023*
**ENSG00000125485**		*0.24848485*		*0.248484848*	*0.339285714*		*0.5*	*0.4186047*
**ENSG00000049541**		*0.05757576*		*0.057575758*				*0.0930233*
**ENSG00000184863**	*0.33233533*	*0.42727273*	*0.33233533*	*0.427272727*	*0.337349398*		*0.3235294*	*0.3313953*
**ENSG00000116809**	*0.05120482*		*0.05120482*					
**ENSG00000130997**		*0.12195122*		*0.12195122*				*0.2267442*
**ENSG00000168743**		*0.39506173*		*0.395061728*				*0.172619*
**ENSG00000132383**		*0.35454545*		*0.354545455*				*0.4767442*
**ENSG00000105298**	*0.20786517*	*0.43636364*	*0.20786517*	*0.436363636*				*0.3372093*
**ENSG00000129003**		*0.39570552*		*0.395705521*				*0.1411765*
**ENSG00000229450**		*0.23636364*		*0.236363636*				*0.4011628*
**ENSG00000197822**	*0.06626506*	*0.05974843*	*0.06626506*	*0.059748428*	*0.136904762*		*0.2383721*	
**ENSG00000165661**		*0.48787879*		*0.487878788*				*0.5*
**ENSG00000248713**		*0.32515337*		*0.325153374*				*0.3023256*
**ENSG00000166896**		*0.39939024*		*0.399390244*				*0.1802326*
**ENSG00000165219**	*0.43712575*	*0.49695122*	*0.43712575*	*0.49695122*	*0.422619048*		*0.4411765*	*0.5*
**ENSG00000214248**	*0.37724551*		*0.37724551*		*0.273809524*		*0.4186047*	
**ENSG00000153064**	*0.48203593*	*0.18787879*	*0.48203593*	*0.187878788*	*0.494047619*		*0.3953488*	*0.2383721*
**ENSG00000139546**		*0.13690476*		*0.136904762*				*0.1046512*
**ENSG00000119866**		*0.43636364*		*0.436363636*				
**ENSG00000121775**		*0.06402439*		*0.06402439*				*0.1705882*
**ENSG00000078328**		*0.48787879*		*0.487878788*				*0.2906977*
**ENSG00000164904**	*0.38719512*	*0.46629213*	*0.38719512*	*0.466292135*				*0.5*
**ENSG00000105143**	*0.28443114*	*0.3045977*	*0.28443114*	*0.304597701*	*0.06547619*			*0.3255814*
**ENSG00000173065**								*0.1190476*
**ENSG00000123560**	*0.29041916*		*0.29041916*		*0.351190476*		*0.3837209*	
**ENSG00000186446**	*0.39520958*	*0.35454545*	*0.39520958*	*0.354545455*	*0.113095238*		*0.1046512*	*0.1136364*
**ENSG00000111845**	*0.30555556*		*0.30555556*					
**ENSG00000042753**		*0.18181818*		*0.181818182*				
**ENSG00000007944**		*0.21515152*		*0.215151515*				*0.3023256*
**ENSG00000134686**	*0.33939394*	*0.37777778*	*0.33939394*	*0.377777778*	*0.273809524*		*0.2325581*	*0.3511905*
**ENSG00000077092**		*0.08484848*		*0.084848485*				*0.2034884*
**ENSG00000240694**	*0.16766467*	*0.44545455*	*0.16766467*	*0.445454545*				*0.3372093*
**ENSG00000167578**	*0.12424242*	*0.43292683*	*0.12424242*	*0.432926829*	*0.220238095*		*0.2906977*	*0.2790698*
**ENSG00000132744**	*0.26646707*		*0.26646707*		*0.220238095*		*0.2732558*	
**ENSG00000232258**		*0.28651685*		*0.286516854*				
**ENSG00000188782**		*0.25280899*		*0.252808989*				*0.1453488*
**ENSG00000135951**	*0.24698795*	*0.28484848*	*0.24698795*	*0.284848485*	*0.31547619*		*0.372093*	*0.1162791*
**ENSG00000178828**								*0.2383721*
**ENSG00000167004**		*0.29444444*		*0.294444444*				*0.4659091*
**ENSG00000174899**		*0.35454545*		*0.354545455*				*0.2142857*
**ENSG00000105677**		*0.36666667*		*0.366666667*				*0.4360465*
**ENSG00000134817**	*0.05688623*	*0.33030303*	*0.05688623*	*0.33030303*	*0.19047619*		*0.3081395*	*0.4651163*
**ENSG00000101974**								*0.3197674*
**ENSG00000140548**		*0.35882353*		*0.358823529*				*0.3522727*
**ENSG00000084073**		*0.06969697*		*0.06969697*				*0.2151163*
**ENSG00000171055**	*0.42771084*	*0.35454545*	*0.42771084*	*0.354545455*	*0.458333333*		*0.3604651*	*0.2325581*
**ENSG00000248746**								*0.1918605*
**ENSG00000163221**		*0.09090909*		*0.090909091*				*0.0639535*

**ENSG00000198563**	*0.48235294*	*0.21646341*	*0.48235294*	*0.216463415*	*0.433333333*		*0.4204545*	*0.0988372*
**ENSG00000141013**		*0.40853659*		*0.408536585*				*0.3604651*
**ENSG00000243725**		*0.48787879*		*0.487878788*				*0.125*
**ENSG00000118217**		*0.46363636*		*0.463636364*				*0.4772727*
**ENSG00000184056**	*0.11445783*	*0.13888889*	*0.11445783*	*0.138888889*			*0.0568182*	*0.4659091*
**ENSG00000178821**	*0.46604938*		*0.46604938*		*0.225*			
**ENSG00000198740**		*0.48484848*		*0.484848485*				*0.4011628*
**ENSG00000135625**		*0.05031447*		*0.050314465*				*0.4883721*
**ENSG00000138757**		*0.18181818*		*0.181818182*				*0.1162791*
**ENSG00000226180**		*0.14545455*		*0.145454545*				*0.1627907*
**ENSG00000215883**	*0.16566265*	*0.09333333*	*0.16566265*	*0.093333333*	*0.202380952*		*0.2906977*	*0.2965116*
**ENSG00000047932**	*0.24251497*	*0.47575758*	*0.24251497*	*0.475757576*	*0.196428571*		*0.2294118*	*0.3488372*
**ENSG00000140326**		*0.2208589*		*0.220858896*				*0.2674419*
**ENSG00000142867**	*0.21385542*	*0.49386503*	*0.21385542*	*0.493865031*	*0.306818182*		*0.2954545*	*0.4244186*
**ENSG00000187288**		*0.19090909*		*0.190909091*				*0.3895349*
**ENSG00000149633**	*0.15662651*	*0.47222222*	*0.15662651*	*0.472222222*	*0.214285714*		*0.3023256*	*0.494186*
**ENSG00000147679**		*0.15151515*		*0.151515152*				*0.3409091*
**ENSG00000154975**		*0.48888889*		*0.488888889*				*0.3636364*
**ENSG00000156471**		*0.40606061*		*0.406060606*				*0.3546512*
**ENSG00000146085**		*0.36060606*		*0.360606061*				*0.4011628*
**ENSG00000162458**		*0.39751553*		*0.397515528*				*0.2906977*
**ENSG00000013374**		*0.21515152*		*0.215151515*				*0.4127907*
**ENSG00000135698**		*0.37222222*		*0.372222222*				*0.3888889*
**ENSG00000187720**		*0.36363636*		*0.363636364*				*0.4244186*
**ENSG00000198650**		*0.35555556*		*0.355555556*				*0.2954545*
**ENSG00000168005**		*0.48484848*		*0.484848485*				*0.3081395*
**ENSG00000197930**	*0.26666667*	*0.10606061*	*0.26666667*	*0.106060606*	*0.162790698*		*0.2159091*	*0.1918605*
**ENSG00000072736**	*0.18263473*	*0.11585366*	*0.18263473*	*0.115853659*				*0.1395349*
**ENSG00000112096**	*0.38622754*	*0.43939394*	*0.38622754*	*0.439393939*	*0.154761905*		*0.1802326*	*0.4651163*
**ENSG00000173113**	*0.23493976*		*0.23493976*		*0.130952381*		*0.122093*	
**ENSG00000129194**	*0.12874251*		*0.12874251*					
**ENSG00000143469**		*0.35714286*		*0.357142857*				*0.4659091*
**ENSG00000105392**	*0.31736527*	*0.42424242*	*0.31736527*	*0.424242424*				*0.1453488*
**ENSG00000134121**		*0.23312883*		*0.233128834*				*0.2619048*
**ENSG00000119596**		*0.23780488*		*0.237804878*				*0.3035714*
**ENSG00000121871**		*0.07575758*		*0.075757576*				
**ENSG00000121542**	*0.48203593*	*0.18888889*	*0.48203593*	*0.188888889*	*0.255952381*		*0.25*	*0.1222222*
**ENSG00000148680**		*0.2*		*0.2*				*0.3081395*
**ENSG00000175792**	*0.2439759*	*0.24712644*	*0.2439759*	*0.247126437*	*0.111111111*		*0.1704545*	*0.0872093*
**ENSG00000141642**		*0.38181818*		*0.381818182*				*0.3837209*
**ENSG00000214575**					*0.244047619*		*0.2034884*	
**ENSG00000173262**	*0.29651163*	*0.42121212*	*0.29651163*	*0.421212121*	*0.271604938*		*0.3571429*	*0.255814*
**ENSG00000197496**		*0.39877301*		*0.398773006*				*0.4698795*
**ENSG00000163697**	*0.13772455*	*0.36890244*	*0.13772455*	*0.368902439*			*0.0523256*	*0.127907*
**ENSG00000006282**	*0.24137931*	*0.31666667*	*0.24137931*	*0.316666667*	*0.095238095*		*0.1*	*0.1*
**ENSG00000135318**	*0.05688623*	*0.34545455*	*0.05688623*	*0.345454545*				*0.3470588*
**ENSG00000188051**		*0.44545455*		*0.445454545*				*0.3837209*
**ENSG00000149554**	*0.35227273*	*0.49438202*	*0.35227273*	*0.494382022*	*0.428571429*		*0.3452381*	*0.3571429*
**ENSG00000181036**		*0.16969697*		*0.16969697*				*0.127907*
**ENSG00000146070**	*0.15909091*		*0.15909091*		*0.144444444*		*0.2386364*	
**ENSG00000172724**								*0.1104651*
**ENSG00000104361**		*0.49390244*		*0.493902439*				*0.3895349*
**ENSG00000161243**		*0.32727273*		*0.327272727*				*0.5*
**ENSG00000132010**		*0.12424242*		*0.124242424*				*0.2383721*

**ENSG00000138050**		0.46666667		0.466666667				0.2383721
**ENSG00000167202**		0.26219512		0.262195122				0.3895349
**ENSG00000179057**		0.40243902		0.402439024				0.1104651
**ENSG00000080819**		0.32424242		0.324242424				0.377907
**ENSG00000135114**		0.19207317		0.192073171				0.0872093
**ENSG00000182318**								0.0930233
**ENSG00000180917**		0.11515152		0.115151515				0.4404762
**ENSG00000145194**		0.28125		0.28125				0.4825581
**ENSG00000204531**	0.24850299	0.47272727	0.24850299	0.472727273	0.386904762		0.4767442	0.5
**ENSG00000106636**		0.06666667		0.066666667				
**ENSG00000164619**		0.32926829		0.329268293				0.2823529
**ENSG00000087087**	0.49700599	0.48787879	0.49700599	0.487878788	0.428571429		0.4476744	0.2
**ENSG00000118507**								0.2790698
**ENSG00000147488**	0.0508982	0.12424242	0.0508982	0.124242424				0.0872093
**ENSG00000185100**		0.40909091		0.409090909	0.363095238		0.4418605	0.4418605
**ENSG00000150681**	0.17771084		0.17771084		0.317073171		0.3023256	
**ENSG00000143127**		0.15555556		0.155555556				0.125
**ENSG00000130749**		0.34756098		0.347560976				0.2710843
**ENSG00000005810**	0.32934132		0.32934132		0.455555556		0.4709302	
**ENSG00000125375**	0.46706587	0.44512195	0.46706587	0.445121951	0.404761905		0.3430233	0.3430233
**ENSG00000238243**	0.09337349	0.30182927	0.09337349	0.301829268	0.166666667		0.1046512	0.4883721
**ENSG00000254999**		0.13333333		0.133333333				
**ENSG00000204963**		0.05151515		0.051515152				0.1162791
**ENSG00000086548**		0.43636364		0.436363636				0.494186
**ENSG00000163812**		0.47865854		0.478658537				0.1104651
**ENSG00000135763**		0.48773006		0.487730061				0.4058824
**ENSG00000122783**		0.46875		0.46875				0.3882353
**ENSG00000171530**		0.44444444		0.444444444				0.4888889
**ENSG00000099999**		0.22222222		0.222222222				
**ENSG00000162399**	0.47727273	0.41975309	0.47727273	0.419753086	0.409090909		0.4534884	0.2045455
**ENSG00000161395**		0.29874214		0.298742138				0.4705882
**ENSG00000167131**	0.06287425	0.33030303	0.06287425	0.33030303	0.05952381		0.127907	0.2034884
**ENSG00000215704**		0.21515152		0.215151515				0.4883721
**ENSG00000085563**	0.05688623	0.47878788	0.05688623	0.478787879	0.071428571		0.0581395	0.4593023
**ENSG00000100095**	0.28742515	0.48757764	0.28742515	0.48757764				0.0666667
**ENSG00000259518**		0.38719512		0.387195122				0.2151163
**ENSG00000112029**		0.17777778		0.177777778				0.1818182
**ENSG00000172156**		0.23030303		0.23030303				0.0595238
**ENSG00000160014**		0.49390244		0.493902439				0.4647059
**ENSG00000143033**		0.18333333		0.183333333				
**ENSG00000123360**	0.39820359	0.48484848	0.39820359	0.484848485	0.30952381		0.3488372	0.4886364
**ENSG00000222040**		0.31818182		0.318181818				0.3895349
**ENSG00000204577**		0.30379747		0.303797468				0.2840909
**ENSG00000132793**	0.28143713	0.16969697	0.28143713	0.16969697				
**ENSG00000076248**	0.23888889		0.23888889		0.107142857		0.175	
**ENSG00000127362**					0.279761905		0.2267442	
**ENSG00000104714**	0.2875	0.42222222	0.2875	0.422222222	0.104651163		0.1511628	0.4767442
**ENSG00000184481**								0.1235294
**ENSG00000154611**								0.2093023
**ENSG00000158220**		0.42378049		0.423780488				0.4418605
**ENSG00000179097**		0.39393939		0.393939394				0.3546512
**ENSG00000163141**	0.33532934	0.2	0.33532934	0.2				0.0930233
**ENSG00000205846**	0.37724551		0.37724551					
**ENSG00000138029**		0.08333333		0.083333333				0.1704545

**ENSG00000112280**	*0.23333333*	*0.44848485*	*0.23333333*	*0.448484848*			*0.3895349*
**ENSG00000135454**	*0.38023952*	*0.25914634*	*0.38023952*	*0.259146341*	*0.172619048*	*0.1046512*	*0.1453488*
**ENSG00000134453**	*0.10542169*	*0.13030303*	*0.10542169*	*0.13030303*			*0.077381*
**ENSG00000004455**		*0.28484848*		*0.284848485*			*0.3837209*
**ENSG00000077782**		*0.26666667*		*0.266666667*			*0.3895349*
**ENSG00000073670**		*0.3969697*		*0.396969697*			*0.1976744*
**ENSG00000173947**		*0.43902439*		*0.43902439*			*0.4651163*
**ENSG00000165688**		*0.34756098*		*0.347560976*	*0.125*	*0.1104651*	*0.1686047*
**ENSG00000119760**	*0.1257485*	*0.40184049*	*0.1257485*	*0.401840491*			*0.0892857*
**ENSG00000080166**	*0.11676647*		*0.11676647*				
**ENSG00000125462**		*0.20552147*		*0.205521472*			*0.3139535*
**ENSG00000213420**		*0.44242424*		*0.442424242*			*0.2647059*
**ENSG00000138964**		*0.32317073*		*0.323170732*			*0.4883721*
**ENSG00000120756**	*0.19161677*		*0.19161677*		*0.410714286*	*0.4*	*0.0697674*
**ENSG00000215472**	*0.26347305*		*0.26347305*		*0.119047619*		
**ENSG00000157330**	*0.36666667*	*0.31212121*	*0.36666667*	*0.312121212*	*0.06547619*	*0.0988372*	*0.3*
**ENSG00000105136**	*0.30838323*	*0.2969697*	*0.30838323*	*0.296969697*	*0.36746988*	*0.3604651*	*0.255814*
**ENSG00000166847**		*0.20121951*		*0.201219512*			*0.4011628*
**ENSG00000167632**		*0.13836478*		*0.13836478*			*0.1488095*
**ENSG00000151466**		*0.19090909*		*0.190909091*			*0.2093023*
**ENSG00000129480**		*0.42528736*		*0.425287356*			*0.3953488*
**ENSG00000048028**		*0.20909091*		*0.209090909*			*0.1860465*
**ENSG00000188993**		*0.27743902*		*0.277439024*			*0.3546512*
**ENSG00000138036**		*0.3*		*0.3*	*0.191358025*	*0.1294118*	*0.127907*
**ENSG00000159674**		*0.07222222*		*0.072222222*			*0.1111111*
**ENSG00000164076**	*0.30538922*	*0.4695122*	*0.30538922*	*0.469512195*	*0.113095238*	*0.1802326*	*0.1918605*
**ENSG00000256870**		*0.38650307*		*0.386503067*			*0.2*
**ENSG00000170209**		*0.16060606*		*0.160606061*			*0.4011628*
**ENSG00000166106**		*0.22777778*		*0.227777778*			*0.3295455*
**ENSG00000184708**		*0.41818182*		*0.418181818*			
**ENSG00000127399**	*0.4702381*		*0.4702381*		*0.26744186*	*0.4268293*	*0.1125*
**ENSG00000106415**		*0.47272727*		*0.472727273*			*0.4709302*
**ENSG00000073060**		*0.28658537*		*0.286585366*			*0.4756098*
**ENSG00000168079**	*0.38253012*	*0.43251534*	*0.38253012*	*0.432515337*	*0.403614458*	*0.3333333*	*0.4941176*
**ENSG00000111266**		*0.05194805*		*0.051948052*			*0.1931818*
**ENSG00000185950**		*0.29885057*		*0.298850575*			*0.4772727*
**ENSG00000196591**	*0.11666667*	*0.46202532*	*0.11666667*	*0.462025316*	*0.268292683*	*0.3658537*	*0.3392857*
**ENSG00000170091**	*0.28742515*	*0.29447853*	*0.28742515*	*0.294478528*			*0.125*
**ENSG00000129071**		*0.05757576*		*0.057575758*			
**ENSG00000206549**	*0.49700599*		*0.49700599*		*0.15*	*0.1428571*	
**ENSG00000198668**		*0.39655172*		*0.396551724*			*0.4940476*
**ENSG00000204147**	*0.15*	*0.15555556*	*0.15*	*0.155555556*	*0.088888889*	*0.1*	*0.1*
**ENSG00000145495**		*0.10606061*		*0.106060606*			*0.3430233*
**ENSG00000164329**		*0.18787879*		*0.187878788*			*0.2267442*
**ENSG00000047578**		*0.14942529*		*0.149425287*			
**ENSG00000067167**		*0.17222222*		*0.172222222*			*0.2621951*
**ENSG00000214046**		*0.10606061*		*0.106060606*			*0.2790698*
**ENSG00000100628**	*0.2994012*	*0.15757576*	*0.2994012*	*0.157575758*	*0.392857143*	*0.3488372*	*0.4709302*
**ENSG00000100395**		*0.33333333*		*0.333333333*			*0.4418605*
**ENSG00000170681**	*0.26666667*	*0.05*	*0.26666667*	*0.05*	*0.111111111*	*0.0568182*	*0.1666667*
**ENSG00000121989**		*0.43888889*		*0.438888889*			*0.1162791*
**ENSG00000108669**		*0.4244186*		*0.424418605*			*0.3409091*
**ENSG00000119912**	*0.39939024*		*0.39939024*		*0.125*	*0.2771084*	
**ENSG00000172053**		*0.11890244*		*0.118902439*			*0.0872093*

**ENSG00000163132**		*0.25151515*		*0.251515152*				*0.4476744*
**ENSG00000101888**		*0.34848485*		*0.348484848*				*0.1395349*
**ENSG00000108759**	*0.17065868*		*0.17065868*		*0.34939759*		*0.3035714*	
**ENSG00000149489**	*0.25625*		*0.25625*					
**ENSG00000154165**					*0.154761905*		*0.2267442*	
**ENSG00000070371**		*0.48787879*		*0.487878788*				*0.2267442*
**ENSG00000166090**	*0.13173653*	*0.28963415*	*0.13173653*	*0.289634146*	*0.30952381*		*0.1860465*	*0.1802326*
**ENSG00000163840**		*0.31818182*		*0.318181818*				*0.5*
**ENSG00000107014**	*0.18333333*		*0.18333333*					
**ENSG00000181562**	*0.12275449*		*0.12275449*					
**ENSG00000006047**		*0.35454545*		*0.354545455*				*0.3139535*
**ENSG00000132819**		*0.22424242*		*0.224242424*				
**ENSG00000072195**	*0.11676647*	*0.48787879*	*0.11676647*	*0.487878788*	*0.30952381*		*0.377907*	*0.4069767*
**ENSG00000132591**		*0.20606061*		*0.206060606*				*0.4244186*
**ENSG00000038945**	*0.44011976*	*0.23636364*	*0.44011976*	*0.236363636*	*0.130952381*		*0.0872093*	*0.5*
**ENSG00000135374**		*0.5*		*0.5*				*0.4883721*
**ENSG00000168813**	*0.49438202*	*0.46969697*	*0.49438202*	*0.46969697*	*0.466666667*		*0.3977273*	*0.5*
**ENSG00000184012**		*0.16969697*		*0.16969697*				*0.4767442*
**ENSG00000177239**		*0.46666667*		*0.466666667*				*0.3139535*
**ENSG00000170017**	*0.15568862*	*0.17878788*	*0.15568862*	*0.178787879*	*0.148809524*		*0.1569767*	*0.1627907*
**ENSG00000053372**	*0.40419162*	*0.23333333*	*0.40419162*	*0.233333333*	*0.178571429*		*0.1627907*	*0.1590909*
**ENSG00000077063**		*0.06896552*		*0.068965517*				
**ENSG00000099250**	*0.36144578*	*0.49444444*	*0.36144578*	*0.494444444*	*0.488095238*		*0.452381*	*0.5*
**ENSG00000183813**		*0.1030303*		*0.103030303*				*0.372093*
**ENSG00000108691**		*0.31212121*		*0.312121212*				*0.372093*
**ENSG00000107147**		*0.32424242*		*0.324242424*				*0.4418605*
**ENSG00000211829**		*0.17378049*		*0.173780488*				
**ENSG00000170619**	*0.31034483*	*0.37272727*	*0.31034483*	*0.372727273*	*0.267857143*		*0.372093*	*0.4302326*
**ENSG00000155090**		*0.47878788*		*0.478787879*				*0.494186*
**ENSG00000149292**	*0.28143713*	*0.48170732*	*0.28143713*	*0.481707317*	*0.482142857*		*0.4186047*	*0.0823529*
**ENSG00000145990**		*0.48181818*		*0.481818182*				*0.3222222*
**ENSG00000101298**		*0.44047619*		*0.44047619*				*0.4647059*
**ENSG00000147669**		*0.19444444*		*0.194444444*				*0.0795455*
**ENSG00000250584**	*0.13772455*		*0.13772455*		*0.215909091*		*0.0988372*	
**ENSG00000151632**	*0.14772727*	*0.24242424*	*0.14772727*	*0.242424242*				
**ENSG00000144891**	*0.06886228*		*0.06886228*		*0.226190476*		*0.1744186*	
**ENSG00000111046**	*0.20958084*	*0.39393939*	*0.20958084*	*0.393939394*				*0.255814*
**ENSG00000121057**		*0.4969697*		*0.496969697*				*0.4302326*
**ENSG00000131368**		*0.13803681*		*0.13803681*				*0.0988372*
**ENSG00000110756**	*0.21686747*	*0.28787879*	*0.21686747*	*0.287878788*	*0.107142857*		*0.1764706*	*0.3941176*
**ENSG00000013573**	*0.23595506*	*0.44753086*	*0.23595506*	*0.447530864*				*0.225*
**ENSG00000188322**		*0.3*		*0.3*				*0.0588235*
**ENSG00000220201**	*0.37777778*	*0.1030303*	*0.37777778*	*0.103030303*	*0.088888889*		*0.0568182*	
**ENSG00000163703**	*0.11976048*		*0.11976048*					
**ENSG00000165194**	*0.1626506*	*0.34848485*	*0.1626506*	*0.348484848*				*0.2142857*
**ENSG00000114520**		*0.11046512*		*0.110465116*				*0.1764706*
**ENSG00000124226**		*0.46363636*		*0.463636364*				*0.4285714*
**ENSG00000120963**	*0.14444444*		*0.14444444*					
**ENSG00000159527**	*0.31626506*	*0.29411765*	*0.31626506*	*0.294117647*	*0.208333333*		*0.1569767*	*0.2045455*
**ENSG00000182400**		*0.24848485*		*0.248484848*				*0.3895349*
**ENSG00000182162**		*0.14906832*		*0.149068323*				
**ENSG00000184677**	*0.48802395*	*0.36666667*	*0.48802395*	*0.366666667*	*0.392857143*		*0.4476744*	*0.4476744*
**ENSG00000175928**	*0.46407186*		*0.46407186*		*0.363095238*		*0.3895349*	*0.1395349*
**ENSG00000185313**		*0.46060606*		*0.460606061*				*0.1860465*

**ENSG00000139197**		*0.425*		*0.425*	*0.196428571*		*0.2093023*	*0.3780488*
**ENSG00000129116**	*0.2754491*	*0.34848485*	*0.2754491*	*0.348484848*	*0.369047619*		*0.4825581*	*0.1395349*
**ENSG00000185651**		*0.18597561*		*0.18597561*				*0.4825581*
**ENSG00000103496**	*0.3045977*		*0.3045977*				*0.0952381*	
**ENSG00000183814**		*0.38343558*		*0.383435583*				*0.4534884*
**ENSG00000005238**	*0.20958084*		*0.20958084*		*0.380952381*		*0.3139535*	
**ENSG00000099814**	*0.33888889*		*0.33888889*		*0.166666667*		*0.127907*	*0.1627907*
**ENSG00000185627**	*0.08682635*	*0.25151515*	*0.08682635*	*0.251515152*	*0.166666667*		*0.1860465*	*0.4772727*
**ENSG00000176714**		*0.42405063*		*0.424050633*				*0.0662651*
**ENSG00000092871**		*0.35454545*		*0.354545455*				*0.1931818*
**ENSG00000204954**	*0.39444444*	*0.49393939*	*0.39444444*	*0.493939394*	*0.388888889*		*0.5*	*0.5*
**ENSG00000127993**		*0.14242424*		*0.142424242*				*0.2411765*
**ENSG00000171517**		*0.23030303*		*0.23030303*				*0.25*
**ENSG00000134107**		*0.15757576*		*0.157575758*				*0.1569767*
**ENSG00000154134**	*0.26807229*		*0.26807229*		*0.083333333*			
**ENSG00000155269**	*0.18562874*	*0.5*	*0.18562874*	*0.5*	*0.458333333*		*0.4825581*	*0.4642857*
**ENSG00000136824**	*0.46407186*	*0.49393939*	*0.46407186*	*0.493939394*	*0.266666667*		*0.1363636*	*0.1511628*
**ENSG00000255604**	*0.2005988*		*0.2005988*		*0.232142857*		*0.1686047*	
**ENSG00000164603**	*0.21111111*	*0.11666667*	*0.21111111*	*0.116666667*				
**ENSG00000180210**	*0.10555556*		*0.10555556*					
**ENSG00000171791**	*0.1*	*0.25153374*	*0.1*	*0.251533742*	*0.433333333*		*0.4318182*	*0.2906977*
**ENSG00000170540**	*0.20180723*	*0.08181818*	*0.20180723*	*0.081818182*				*0.0755814*
**ENSG00000121691**	*0.45*		*0.45*		*0.255555556*		*0.2666667*	
**ENSG00000129696**	*0.46111111*		*0.46111111*		*0.443181818*		*0.4940476*	
**ENSG00000198576**		*0.49691358*		*0.49691358*				*0.1705882*
**ENSG00000184983**		*0.47878788*		*0.478787879*				*0.4593023*
**ENSG00000146918**		*0.39393939*		*0.393939394*				*0.4825581*
**ENSG00000196526**		*0.43939394*		*0.439393939*				*0.4360465*
**ENSG00000130522**		*0.27272727*		*0.272727273*				*0.494186*
**ENSG00000111605**		*0.40555556*		*0.405555556*				*0.3295455*
**ENSG00000205740**		*0.43209877*		*0.432098765*				*0.3154762*
**ENSG00000037280**		*0.09937888*		*0.099378882*	*0.154761905*		*0.122093*	*0.3928571*
**ENSG00000177688**								*0.2692308*
**ENSG00000178597**		*0.36969697*		*0.36969697*				*0.4825581*
**ENSG00000103355**	*0.40060241*		*0.40060241*					
**ENSG00000173918**	*0.31736527*	*0.3*	*0.31736527*	*0.3*	*0.457831325*		*0.4529412*	*0.4759036*
**ENSG00000179361**		*0.30909091*		*0.309090909*				*0.4593023*
**ENSG00000205147**	*0.40449438*		*0.40449438*		*0.066666667*			
**ENSG00000105926**		*0.36666667*		*0.366666667*				*0.1627907*
**ENSG00000044524**		*0.44242424*		*0.442424242*				*0.1337209*
**ENSG00000205517**		*0.06748466*		*0.067484663*				*0.2650602*
**ENSG00000114302**		*0.37272727*		*0.372727273*				*0.0872093*
**ENSG00000064692**	*0.12777778*	*0.10606061*	*0.12777778*	*0.106060606*	*0.147727273*		*0.1111111*	*0.0872093*
**ENSG00000083812**	*0.3*		*0.3*		*0.494047619*		*0.4593023*	
**ENSG00000066279**		*0.09393939*		*0.093939394*				
**ENSG00000197142**		*0.3030303*		*0.303030303*				*0.2941176*
**ENSG00000151151**		*0.17222222*		*0.172222222*				
**ENSG00000197965**		*0.46987952*		*0.469879518*				*0.3546512*
**ENSG00000198879**		*0.30909091*		*0.309090909*				*0.3662791*
**ENSG00000211795**	*0.07777778*		*0.07777778*		*0.433333333*		*0.4444444*	
**ENSG00000239306**		*0.2*		*0.2*				
**ENSG00000137142**		*0.33333333*		*0.333333333*				*0.3604651*
**ENSG00000117697**		*0.26666667*		*0.266666667*				*0.4302326*
**ENSG00000065809**	*0.37724551*	*0.2*	*0.37724551*	*0.2*	*0.455555556*		*0.4216867*	*0.1625*

**ENSG00000072163**	*0.46875*		*0.46875*		*0.351190476*	*0.3023256*	
**ENSG00000143727**		*0.2969697*		*0.296969697*			*0.1686047*
**ENSG00000196628**	*0.13473054*	*0.375*	*0.13473054*	*0.375*			*0.2383721*
**ENSG00000138286**		*0.13636364*		*0.136363636*			*0.3555556*
**ENSG00000180539**	*0.23888889*	*0.36890244*	*0.23888889*	*0.368902439*	*0.177777778*	*0.2613636*	*0.2093023*
**ENSG00000172733**		*0.30909091*		*0.309090909*			
**ENSG00000131591**		*0.13030303*		*0.13030303*			*0.1352941*
**ENSG00000073737**	*0.08982036*	*0.17222222*	*0.08982036*	*0.172222222*	*0.119047619*	*0.1860465*	*0.2727273*
**ENSG00000189221**	*0.48493976*	*0.23333333*	*0.48493976*	*0.233333333*			*0.3068182*
**ENSG00000143702**	*0.43636364*		*0.43636364*		*0.303571429*	*0.2267442*	
**ENSG00000160785**		*0.36309524*		*0.363095238*			*0.3295455*
**ENSG00000106560**		*0.39939024*		*0.399390244*			*0.4235294*
**ENSG00000183943**		*0.33231707*		*0.332317073*			*0.1941176*
**ENSG00000196482**	*0.46706587*	*0.06666667*	*0.46706587*	*0.066666667*	*0.476190476*	*0.4709302*	*0.1802326*
**ENSG00000172215**	*0.14371257*	*0.49090909*	*0.14371257*	*0.490909091*	*0.361445783*	*0.3764706*	*0.4709302*
**ENSG00000170144**		*0.20555556*		*0.205555556*			*0.2613636*
**ENSG00000173041**	*0.33333333*	*0.27878788*	*0.33333333*	*0.278787879*	*0.488095238*	*0.4825581*	*0.3875*
**ENSG00000099849**							*0.1744186*
**ENSG00000161082**		*0.44242424*		*0.442424242*			*0.2383721*
**ENSG00000214113**		*0.44520548*		*0.445205479*			*0.2666667*
**ENSG00000100625**		*0.26363636*		*0.263636364*			*0.3662791*
**ENSG00000196236**		*0.47575758*		*0.475757576*			*0.0813953*
**ENSG00000185946**		*0.35454545*		*0.354545455*			*0.3139535*
**ENSG00000164535**		*0.46969697*		*0.46969697*			*0.3895349*
**ENSG00000115616**		*0.27987421*		*0.279874214*			*0.1569767*
**ENSG00000160209**		*0.49090909*		*0.490909091*			*0.4588235*
**ENSG00000205670**		*0.46604938*		*0.466049383*			*0.4011628*
**ENSG00000197591**	*0.4*		*0.4*				
**ENSG00000142405**	*0.27844311*	*0.11280488*	*0.27844311*	*0.112804878*	*0.386904762*	*0.3662791*	*0.0755814*
**ENSG00000133101**					*0.144444444*	*0.1136364*	
**ENSG00000173153**					*0.147727273*		
**ENSG00000188732**		*0.30674847*		*0.306748466*			*0.327381*
**ENSG00000185686**	*0.40697674*		*0.40697674*		*0.211111111*	*0.3068182*	*0.3373494*
**ENSG00000104723**		*0.28181818*		*0.281818182*			*0.2965116*
**ENSG00000168958**	*0.48850575*		*0.48850575*		*0.3*	*0.255814*	*0.4886364*
**ENSG00000171970**	*0.43258427*		*0.43258427*		*0.255555556*	*0.1477273*	
**ENSG00000121807**		*0.23636364*		*0.236363636*			*0.2616279*
**ENSG00000114120**	*0.17964072*	*0.08333333*	*0.17964072*	*0.083333333*	*0.214285714*	*0.2151163*	*0.2727273*
**ENSG00000164010**	*0.46706587*	*0.17272727*	*0.46706587*	*0.172727273*	*0.380952381*	*0.4186047*	*0.1744186*
**ENSG00000137474**	*0.15361446*		*0.15361446*		*0.494047619*	*0.494186*	
**ENSG00000111667**							*0.3255814*
**ENSG00000177697**	*0.33233533*	*0.29878049*	*0.33233533*	*0.298780488*	*0.125*	*0.1627907*	*0.4127907*
**ENSG00000176692**							*0.1022727*
**ENSG00000163362**	*0.16111111*		*0.16111111*		*0.144444444*	*0.2045455*	
**ENSG00000137656**		*0.07777778*		*0.077777778*			*0.1627907*
**ENSG00000103249**		*0.5*		*0.5*			*0.2093023*
**ENSG00000125482**	*0.48802395*		*0.48802395*		*0.463855422*	*0.4702381*	
**ENSG00000112514**	*0.23353293*		*0.23353293*		*0.053571429*	*0.0523256*	
**ENSG00000150526**		*0.41477273*		*0.414772727*			*0.4883721*
**ENSG00000105339**	*0.4760479*	*0.43939394*	*0.4760479*	*0.439393939*	*0.43452381*	*0.4186047*	*0.4244186*
**ENSG00000072415**	*0.39457831*	*0.07777778*	*0.39457831*	*0.077777778*	*0.363095238*	*0.4941176*	*0.4772727*
**ENSG00000106617**		*0.35454545*		*0.354545455*			*0.2529412*
**ENSG00000139977**		*0.11818182*		*0.118181818*			*0.0930233*
**ENSG00000162804**		*0.37272727*		*0.372727273*			*0.4772727*

**ENSG00000178734**	*0.07185629*	*0.47852761*	*0.07185629*	*0.478527607*				*0.3777778*
**ENSG00000104848**		*0.4969697*		*0.496969697*				*0.2790698*
**ENSG00000162616**								*0.1309524*
**ENSG00000204978**		*0.12727273*		*0.127272727*				*0.3372093*
**ENSG00000253313**		*0.47878788*		*0.478787879*				*0.3488372*
**ENSG00000170381**		*0.14444444*		*0.144444444*				*0.125*
**ENSG00000258231**					*0.31547619*		*0.1802326*	
**ENSG00000204681**		*0.46103896*		*0.461038961*				*0.3023256*
**ENSG00000124789**								*0.2848837*
**ENSG00000071909**		*0.17682927*		*0.176829268*				
**ENSG00000182107**		*0.32727273*		*0.327272727*				*0.4883721*
**ENSG00000175287**		*0.43030303*		*0.43030303*				*0.2151163*
**ENSG00000104205**	*0.42168675*		*0.42168675*		*0.226190476*		*0.25*	
**ENSG00000175274**	*0.16666667*	*0.17272727*	*0.16666667*	*0.172727273*	*0.464285714*		*0.4011628*	*0.122093*
**ENSG00000169991**		*0.05*		*0.05*				*0.0909091*
**ENSG00000105419**		*0.1011236*		*0.101123596*				
**ENSG00000006634**	*0.1257485*	*0.1030303*	*0.1257485*	*0.103030303*	*0.06547619*		*0.0988372*	*0.2732558*
**ENSG00000145332**		*0.42073171*		*0.420731707*				*0.4767442*
**ENSG00000174125**	*0.16467066*		*0.16467066*		*0.464285714*		*0.4825581*	
**ENSG00000118017**	*0.4186747*		*0.4186747*		*0.404761905*		*0.4651163*	
**ENSG00000109684**	*0.31137725*	*0.46067416*	*0.31137725*	*0.460674157*	*0.363095238*		*0.4127907*	*0.4647059*
**ENSG00000140030**	*0.06886228*		*0.06886228*					
**ENSG00000182141**		*0.31481481*		*0.314814815*				*0.3139535*
**ENSG00000080298**	*0.20958084*	*0.26363636*	*0.20958084*	*0.263636364*				*0.4534884*
**ENSG00000198478**		*0.34545455*		*0.345454545*				*0.494186*
**ENSG00000221983**		*0.39090909*		*0.390909091*				*0.3430233*
**ENSG00000155304**		*0.33939394*		*0.339393939*				*0.5*
**ENSG00000186409**	*0.05555556*	*0.23636364*	*0.05555556*	*0.236363636*	*0.233333333*		*0.1363636*	*0.3662791*
**ENSG00000183473**	*0.33333333*	*0.32822086*	*0.33333333*	*0.328220859*	*0.222222222*		*0.1428571*	*0.4487179*
**ENSG00000211454**	*0.11494253*	*0.17987805*	*0.11494253*	*0.179878049*	*0.136363636*		*0.1162791*	*0.1235294*
**ENSG00000145708**		*0.43251534*		*0.432515337*				*0.4651163*
**ENSG00000142661**	*0.18862275*	*0.33030303*	*0.18862275*	*0.33030303*	*0.19047619*		*0.1453488*	
**ENSG00000179454**	*0.4494382*		*0.4494382*		*0.119047619*		*0.127907*	
**ENSG00000204038**	*0.10778443*		*0.10778443*		*0.494047619*		*0.494186*	
**ENSG00000109390**	*0.24712644*		*0.24712644*		*0.113636364*		*0.0714286*	
**ENSG00000143450**	*0.17065868*		*0.17065868*		*0.398809524*		*0.2588235*	
**ENSG00000123454**		*0.49090909*		*0.490909091*				*0.3*
**ENSG00000130045**		*0.07575758*		*0.075757576*				*0.3197674*
**ENSG00000145536**		*0.48837209*		*0.488372093*				*0.3589744*
**ENSG00000127324**	*0.11077844*	*0.39444444*	*0.11077844*	*0.394444444*				*0.3409091*
**ENSG00000165023**		*0.0862069*		*0.086206897*				*0.25*
**ENSG00000145349**	*0.15030675*	*0.26969697*	*0.15030675*	*0.26969697*	*0.488095238*		*0.4767442*	*0.4302326*
**ENSG00000177889**		*0.0969697*		*0.096969697*				
**ENSG00000182208**	*0.21666667*		*0.21666667*		*0.277777778*		*0.4777778*	
**ENSG00000130962**		*0.07575758*		*0.075757576*				*0.0639535*
**ENSG00000137573**	*0.30538922*	*0.26751592*	*0.30538922*	*0.267515924*	*0.261904762*		*0.2732558*	*0.4069767*
**ENSG00000255633**		*0.39325843*		*0.393258427*				*0.4431818*
**ENSG00000130054**		*0.29573171*		*0.295731707*				*0.1823529*
**ENSG00000185567**		*0.47575758*		*0.475757576*				*0.377907*
**ENSG00000165071**		*0.46969697*		*0.46969697*				*0.3975904*
**ENSG00000087253**	*0.07222222*	*0.5*	*0.07222222*	*0.5*	*0.222222222*		*0.2666667*	*0.3333333*
**ENSG00000116171**		*0.10606061*		*0.106060606*				*0.4111111*
**ENSG00000165929**	*0.19161677*	*0.08484848*	*0.19161677*	*0.084848485*	*0.470238095*		*0.4186047*	*0.0813953*
**ENSG00000179010**		*0.16666667*		*0.166666667*				*0.0681818*

**ENSG00000087299**	*0.46706587*	*0.45454545*	*0.46706587*	*0.454545455*	*0.404761905*	*0.3430233*	*0.3294118*
**ENSG00000198643**	*0.14204545*		*0.14204545*		*0.466666667*	*0.375*	
**ENSG00000175325**		*0.30606061*		*0.306060606*			*0.2732558*
**ENSG00000160961**		*0.41818182*		*0.418181818*			*0.2840909*
**ENSG00000091527**		*0.11585366*		*0.115853659*			
**ENSG00000141655**		*0.27439024*		*0.274390244*			*0.25*
**ENSG00000010626**	*0.4*	*0.32727273*	*0.4*	*0.327272727*	*0.4*	*0.4886364*	*0.4767442*
**ENSG00000184811**		*0.31460674*		*0.314606742*			*0.2616279*
**ENSG00000181617**		*0.15030675*		*0.150306748*			
**ENSG00000120256**		*0.34545455*		*0.345454545*			*0.2267442*
**ENSG00000140470**		*0.47272727*		*0.472727273*			*0.494186*
**ENSG00000166250**		*0.2030303*		*0.203030303*			*0.4235294*
**ENSG00000150760**		*0.49090909*		*0.490909091*			*0.3139535*
**ENSG00000198805**	*0.24251497*	*0.06666667*	*0.24251497*	*0.066666667*	*0.208333333*	*0.2616279*	
**ENSG00000167377**	*0.37222222*		*0.37222222*		*0.25*	*0.3139535*	
**ENSG00000179564**		*0.15151515*		*0.151515152*			*0.4127907*
**ENSG00000132801**		*0.05487805*		*0.054878049*			*0.1569767*
**ENSG00000125386**		*0.06666667*		*0.066666667*			*0.1104651*
**ENSG00000153551**		*0.19886364*		*0.198863636*			*0.4318182*
**ENSG00000134917**		*0.07272727*		*0.072727273*			*0.1744186*
**ENSG00000111671**					*0.226190476*	*0.1686047*	*0.1590909*
**ENSG00000076043**	*0.25*		*0.25*		*0.380952381*	*0.4759036*	
**ENSG00000141579**	*0.3404908*		*0.3404908*		*0.083333333*	*0.0697674*	
**ENSG00000050030**		*0.07668712*		*0.076687117*			
**ENSG00000154227**		*0.36666667*		*0.366666667*			*0.4204545*
**ENSG00000124786**		*0.45505618*		*0.45505618*			*0.4285714*
**ENSG00000185038**	*0.1257485*		*0.1257485*				
**ENSG00000138311**		*0.23333333*		*0.233333333*			*0.1453488*
**ENSG00000141295**		*0.14848485*		*0.148484848*			*0.0833333*
**ENSG00000163380**							*0.377907*
**ENSG00000175197**	*0.32934132*		*0.32934132*		*0.392857143*	*0.1395349*	
**ENSG00000174792**		*0.22424242*		*0.224242424*			*0.2732558*
**ENSG00000116852**		*0.32022472*		*0.320224719*			*0.2272727*
**ENSG00000244005**		*0.05757576*		*0.057575758*			
**ENSG00000157764**		*0.4375*		*0.4375*			*0.1136364*
**ENSG00000093072**	*0.33433735*	*0.41666667*	*0.33433735*	*0.416666667*	*0.285714286*	*0.2034884*	*0.3068182*
**ENSG00000151532**		*0.24242424*		*0.242424242*			*0.4244186*
**ENSG00000115641**	*0.23053892*	*0.47878788*	*0.23053892*	*0.478787879*	*0.351190476*	*0.2325581*	*0.2616279*
**ENSG00000164081**	*0.15555556*	*0.13719512*	*0.15555556*	*0.137195122*	*0.079545455*	*0.0813953*	*0.0639535*
**ENSG00000129682**	*0.2*	*0.08895706*	*0.2*	*0.088957055*	*0.273809524*	*0.255814*	
**ENSG00000115306**		*0.32424242*		*0.324242424*			*0.4476744*
**ENSG00000153563**	*0.48502994*	*0.14848485*	*0.48502994*	*0.148484848*	*0.119047619*	*0.1860465*	*0.1860465*
**ENSG00000106853**		*0.08282209*		*0.082822086*			*0.2965116*
**ENSG00000038210**		*0.42901235*		*0.429012346*			*0.3081395*
**ENSG00000063244**	*0.23493976*	*0.24712644*	*0.23493976*	*0.247126437*	*0.470238095*	*0.4011628*	*0.4069767*
**ENSG00000168787**		*0.5*		*0.5*			*0.3604651*
**ENSG00000198843**		*0.35060976*		*0.350609756*			*0.439759*
**ENSG00000164849**							*0.1918605*
**ENSG00000204277**		*0.48314607*		*0.483146067*			*0.3372093*
**ENSG00000110925**		*0.21590909*		*0.215909091*			*0.3636364*
**ENSG00000116285**		*0.44545455*		*0.445454545*			
**ENSG00000146433**		*0.18333333*		*0.183333333*			*0.1333333*
**ENSG00000132376**		*0.19393939*		*0.193939394*			
**ENSG00000113273**	*0.1626506*	*0.5*	*0.1626506*	*0.5*	*0.130952381*	*0.0963855*	*0.4411765*

**ENSG00000186971**		*0.43636364*		*0.436363636*			*0.3941176*
**ENSG00000186231**	*0.12874251*		*0.12874251*		*0.095238095*	*0.0581395*	
**ENSG00000100012**		*0.41515152*		*0.415151515*	*0.122222222*	*0.125*	*0.4127907*
**ENSG00000068654**	*0.13173653*	*0.28181818*	*0.13173653*	*0.281818182*	*0.172619048*	*0.2034884*	*0.4666667*
**ENSG00000154856**		*0.42727273*		*0.427272727*			*0.3662791*
**ENSG00000111276**	*0.40718563*	*0.35151515*	*0.40718563*	*0.351515152*	*0.470238095*	*0.4883721*	*0.1802326*
**ENSG00000100604**		*0.27272727*		*0.272727273*			*0.2954545*
**ENSG00000177599**		*0.31790123*		*0.317901235*			*0.3409091*
**ENSG00000198909**		*0.31818182*		*0.318181818*			
**ENSG00000184903**	*0.1257485*	*0.33536585*	*0.1257485*	*0.335365854*	*0.5*	*0.4709302*	*0.2411765*
**ENSG00000105323**	*0.39090909*	*0.15730337*	*0.39090909*	*0.157303371*	*0.363095238*	*0.255814*	
**ENSG00000124177**		*0.31818182*		*0.318181818*			*0.372093*
**ENSG00000105383**		*0.46060606*		*0.460606061*			*0.2209302*
**ENSG00000092067**	*0.17065868*		*0.17065868*		*0.232142857*	*0.4360465*	
**ENSG00000091513**	*0.28409091*	*0.12424242*	*0.28409091*	*0.124242424*	*0.277777778*	*0.244186*	*0.2267442*
**ENSG00000235718**	*0.4760479*	*0.18888889*	*0.4760479*	*0.188888889*	*0.244047619*	*0.2352941*	*0.0681818*
**ENSG00000171819**							*0.0872093*
**ENSG00000106688**		*0.32777778*		*0.327777778*			*0.1590909*
**ENSG00000166987**	*0.33233533*		*0.33233533*				
**ENSG00000258961**		*0.14242424*		*0.142424242*			*0.122093*
**ENSG00000130811**		*0.44827586*		*0.448275862*			*0.4431818*
**ENSG00000253309**		*0.12804878*		*0.12804878*	*0.088888889*	*0.1333333*	*0.2650602*
**ENSG00000117174**		*0.36060606*		*0.360606061*			*0.4883721*
**ENSG00000230797**	*0.31736527*	*0.23595506*	*0.31736527*	*0.235955056*	*0.351190476*	*0.4*	*0.4659091*
**ENSG00000183569**		*0.06818182*		*0.068181818*			*0.3928571*
**ENSG00000198471**		*0.38181818*		*0.381818182*			*0.3255814*
**ENSG00000170264**		*0.32727273*		*0.327272727*			*0.2987805*
**ENSG00000147588**		*0.30909091*		*0.309090909*			*0.372093*
**ENSG00000227706**		*0.42424242*		*0.424242424*			*0.1686047*
**ENSG00000093009**						*0.0705882*	
**ENSG00000166741**	*0.49096386*	*0.19393939*	*0.49096386*	*0.193939394*	*0.416666667*	*0.4825581*	*0.0988372*
**ENSG00000100138**		*0.3969697*		*0.396969697*			*0.2790698*
**ENSG00000186792**	*0.19186047*		*0.19186047*		*0.386363636*	*0.4886364*	
**ENSG00000141279**							*0.1022727*
**ENSG00000258838**	*0.12275449*		*0.12275449*		*0.306818182*	*0.3181818*	
**ENSG00000189298**		*0.3125*		*0.3125*			*0.2972973*
**ENSG00000167695**		*0.32424242*		*0.324242424*			*0.1705882*
**ENSG00000134245**	*0.12874251*	*0.27272727*	*0.12874251*	*0.272727273*			*0.4360465*
**ENSG00000125207**	*0.14670659*	*0.42424242*	*0.14670659*	*0.424242424*	*0.077380952*	*0.1104651*	*0.372093*
**ENSG00000166770**	*0.11676647*		*0.11676647*				
**ENSG00000124201**		*0.30898876*		*0.308988764*			*0.2848837*
**ENSG00000187959**		*0.23030303*		*0.23030303*			*0.1860465*
**ENSG00000136205**	*0.49444444*	*0.43333333*	*0.49444444*	*0.433333333*	*0.466666667*	*0.3488372*	*0.4709302*
**ENSG00000177058**	*0.375*	*0.36666667*	*0.375*	*0.366666667*	*0.433333333*	*0.5*	*0.5*
**ENSG00000143190**	*0.37222222*	*0.49390244*	*0.37222222*	*0.493902439*			*0.2906977*
**ENSG00000145604**							*0.377907*
**ENSG00000197557**	*0.18263473*	*0.25*	*0.18263473*	*0.25*			*0.3222222*
**ENSG00000254997**	*0.23030303*	*0.41954023*	*0.23030303*	*0.41954023*	*0.136904762*	*0.0988372*	*0.4476744*
**ENSG00000182902**		*0.18965517*		*0.189655172*			*0.2954545*
**ENSG00000167081**		*0.20909091*		*0.209090909*			*0.3647059*
**ENSG00000156869**					*0.363095238*	*0.3662791*	
**ENSG00000146006**	*0.13855422*		*0.13855422*				*0.0755814*
**ENSG00000085831**	*0.21556886*	*0.12777778*	*0.21556886*	*0.127777778*			*0.3068182*
**ENSG00000164825**	*0.39520958*	*0.12424242*	*0.39520958*	*0.124242424*	*0.43452381*	*0.4764706*	*0.3197674*

**ENSG00000143401**		*0.06818182*		*0.068181818*				
**ENSG00000186318**	*0.11042945*	*0.44817073*	*0.11042945*	*0.448170732*	*0.311111111*		*0.3409091*	*0.1162791*
**ENSG00000198818**	*0.23888889*		*0.23888889*					
**ENSG00000102898**		*0.15454545*		*0.154545455*				*0.0523256*
**ENSG00000182993**		*0.18181818*		*0.181818182*				
**ENSG00000187258**	*0.15432099*	*0.36666667*	*0.15432099*	*0.366666667*	*0.122222222*		*0.2*	*0.2823529*
**ENSG00000224453**		*0.33536585*		*0.335365854*				*0.4651163*
**ENSG00000120658**	*0.22754491*	*0.41515152*	*0.22754491*	*0.415151515*	*0.19047619*		*0.1860465*	*0.4069767*
**ENSG00000166716**		*0.24545455*		*0.245454545*				*0.4360465*
**ENSG00000127946**		*0.43030303*		*0.43030303*				*0.0523256*
**ENSG00000188343**		*0.31595092*		*0.31595092*				*0.4117647*
**ENSG00000087077**		*0.48275862*		*0.482758621*				*0.4476744*
**ENSG00000183571**	*0.5*		*0.5*				*0.0588235*	
**ENSG00000234719**	*0.09444444*		*0.09444444*					
**ENSG00000163026**		*0.47777778*		*0.477777778*				*0.1444444*
**ENSG00000157570**	*0.17365269*	*0.49418605*	*0.17365269*	*0.494186047*	*0.119047619*		*0.1395349*	*0.4545455*
**ENSG00000183160**		*0.11818182*		*0.118181818*	*0.158536585*		*0.1309524*	*0.2965116*
**ENSG00000240344**		*0.13636364*		*0.136363636*				
**ENSG00000156414**		*0.45886076*		*0.458860759*				*0.4216867*
**ENSG00000105388**		*0.42134831*		*0.421348315*				*0.2727273*
**ENSG00000083844**		*0.37647059*		*0.376470588*				*0.3139535*
**ENSG00000093144**	*0.18862275*	*0.39444444*	*0.18862275*	*0.394444444*	*0.077380952*		*0.1363636*	
**ENSG00000125841**		*0.1*		*0.1*				
**ENSG00000171124**	*0.08383234*	*0.39570552*	*0.08383234*	*0.395705521*				*0.3588235*
**ENSG00000092850**		*0.0969697*		*0.096969697*				
**ENSG00000162998**		*0.44545455*		*0.445454545*				*0.1395349*
**ENSG00000136052**		*0.15243902*		*0.152439024*				*0.244186*
**ENSG00000120087**		*0.23939394*		*0.239393939*	*0.273809524*		*0.2383721*	*0.2616279*
**ENSG00000139719**		*0.29938272*		*0.299382716*				*0.372093*
**ENSG00000120833**	*0.38622754*	*0.23033708*	*0.38622754*	*0.230337079*	*0.220238095*		*0.1453488*	*0.4418605*
**ENSG00000162542**		*0.18787879*		*0.187878788*				*0.4534884*
**ENSG00000114805**	*0.07784431*	*0.11818182*	*0.07784431*	*0.118181818*	*0.464285714*		*0.4588235*	
**ENSG00000117500**		*0.28484848*		*0.284848485*				*0.372093*
**ENSG00000158402**								*0.0697674*
**ENSG00000178691**		*0.18333333*		*0.183333333*				
**ENSG00000154065**	*0.11666667*	*0.46111111*	*0.11666667*	*0.461111111*				*0.2267442*
**ENSG00000204439**	*0.13772455*		*0.13772455*		*0.202380952*		*0.0988372*	
**ENSG00000154127**	*0.32335329*	*0.32941176*	*0.32335329*	*0.329411765*	*0.154761905*		*0.1569767*	*0.2823529*
**ENSG00000155906**		*0.21511628*		*0.215116279*	*0.198795181*		*0.1470588*	*0.4659091*
**ENSG00000137672**		*0.27272727*		*0.272727273*	*0.43902439*		*0.4625*	*0.2093023*
**ENSG00000185842**		*0.38484848*		*0.384848485*				*0.2613636*
**ENSG00000162688**	*0.48333333*	*0.484375*	*0.48333333*	*0.484375*	*0.386904762*		*0.4593023*	*0.4244186*
**ENSG00000095139**		*0.16666667*		*0.166666667*				*0.4634146*
**ENSG00000157191**		*0.11818182*		*0.118181818*				
**ENSG00000145911**		*0.26829268*		*0.268292683*				*0.2954545*
**ENSG00000075891**	*0.26347305*		*0.26347305*					*0.1162791*
**ENSG00000188305**		*0.33030303*		*0.33030303*				*0.4545455*
**ENSG00000089094**		*0.08484848*		*0.084848485*				*0.1104651*
**ENSG00000148377**		*0.20909091*		*0.209090909*				*0.2777778*
**ENSG00000175262**		*0.32317073*		*0.323170732*				*0.2470588*
**ENSG00000169710**	*0.45209581*	*0.44242424*	*0.45209581*	*0.442424242*	*0.192771084*		*0.1860465*	*0.2906977*
**ENSG00000227392**		*0.45555556*		*0.455555556*				*0.3636364*
**ENSG00000205476**	*0.44011976*	*0.39444444*	*0.44011976*	*0.394444444*				*0.4659091*
**ENSG00000160218**	*0.25748503*	*0.18484848*	*0.25748503*	*0.184848485*	*0.226190476*		*0.3255814*	*0.3255814*

**ENSG00000159593**					*0.339285714*	*0.3882353*	
**ENSG00000135596**	*0.23493976*		*0.23493976*		*0.488095238*	*0.4588235*	
**ENSG00000124215**	*0.32934132*	*0.33333333*	*0.32934132*	*0.333333333*			*0.4767442*
**ENSG00000149781**	*0.27840909*		*0.27840909*		*0.295454545*	*0.2272727*	
**ENSG00000184743**	*0.2245509*	*0.14545455*	*0.2245509*	*0.145454545*			
**ENSG00000188878**	*0.24719101*		*0.24719101*			*0.0777778*	
**ENSG00000112304**	*0.06586826*	*0.31402439*	*0.06586826*	*0.31402439*	*0.154761905*	*0.127907*	*0.3313953*
**ENSG00000162980**		*0.1969697*		*0.196969697*			*0.494186*
**ENSG00000152484**		*0.42121212*		*0.421212121*			*0.3823529*
**ENSG00000135047**	*0.46706587*		*0.46706587*		*0.321428571*	*0.2848837*	
**ENSG00000164761**	*0.20786517*		*0.20786517*		*0.444444444*	*0.3181818*	
**ENSG00000186453**		*0.37272727*		*0.372727273*			*0.2325581*
**ENSG00000146122**	*0.08083832*	*0.5*	*0.08083832*	*0.5*	*0.321428571*	*0.4534884*	*0.2790698*
**ENSG00000147119**		*0.42121212*		*0.421212121*			*0.2325581*
**ENSG00000183682**		*0.36060606*		*0.360606061*			*0.3546512*
**ENSG00000106400**	*0.21556886*	*0.09444444*	*0.21556886*	*0.094444444*	*0.210843373*	*0.1453488*	
**ENSG00000118729**	*0.20987654*	*0.38050314*	*0.20987654*	*0.380503145*			*0.2470588*
**ENSG00000079482**		*0.36060606*		*0.360606061*			*0.255814*
**ENSG00000142227**		*0.42727273*		*0.427272727*			*0.2352941*
**ENSG00000138175**		*0.12222222*		*0.122222222*			*0.4555556*
**ENSG00000119917**		*0.48888889*		*0.488888889*			*0.1444444*
**ENSG00000132823**	*0.33231707*	*0.28787879*	*0.33231707*	*0.287878788*	*0.488095238*	*0.4360465*	
**ENSG00000173482**	*0.40662651*		*0.40662651*		*0.144578313*	*0.1104651*	
**ENSG00000117682**	*0.48493976*	*0.2969697*	*0.48493976*	*0.296969697*	*0.321428571*	*0.2034884*	*0.2034884*
**ENSG00000169045**	*0.34131737*		*0.34131737*		*0.261904762*	*0.3941176*	
**ENSG00000165694**		*0.37037037*		*0.37037037*			*0.0639535*
**ENSG00000108405**		*0.37575758*		*0.375757576*			*0.1860465*
**ENSG00000138193**	*0.49401198*	*0.11235955*	*0.49401198*	*0.112359551*	*0.494047619*	*0.4823529*	*0.1860465*
**ENSG00000119950**		*0.46666667*		*0.466666667*			*0.4772727*
**ENSG00000100994**		*0.48181818*		*0.481818182*			*0.0988372*
**ENSG00000143633**		*0.22121212*		*0.221212121*			*0.3882353*
**ENSG00000065882**	*0.40740741*	*0.35*	*0.40740741*	*0.35*	*0.398809524*	*0.4204545*	*0.3837209*
**ENSG00000215014**		*0.34375*		*0.34375*			*0.1511628*
**ENSG00000205221**	*0.2005988*		*0.2005988*			*0.0523256*	*0.1162791*
**ENSG00000189308**		*0.27743902*		*0.277439024*			*0.4705882*
**ENSG00000105173**		*0.25304878*		*0.25304878*			*0.4360465*
**ENSG00000226800**							*0.1802326*
**ENSG00000170180**	*0.45555556*	*0.47222222*	*0.45555556*	*0.472222222*	*0.488636364*	*0.4431818*	*0.4431818*
**ENSG00000171136**					*0.134146341*	*0.202381*	
**ENSG00000136720**		*0.23148148*		*0.231481481*			*0.2159091*
**ENSG00000134198**		*0.47575758*		*0.475757576*			*0.494186*
**ENSG00000130638**		*0.12222222*		*0.122222222*			*0.1*
**ENSG00000250641**							*0.0833333*
**ENSG00000144671**	*0.07185629*		*0.07185629*		*0.136904762*	*0.1162791*	
**ENSG00000125398**		*0.37195122*		*0.37195122*			*0.4360465*
**ENSG00000110768**	*0.05722892*	*0.35*	*0.05722892*	*0.35*	*0.452380952*	*0.4470588*	*0.3636364*
**ENSG00000114757**	*0.23837209*	*0.37272727*	*0.23837209*	*0.372727273*	*0.160714286*	*0.1686047*	*0.255814*
**ENSG00000206199**	*0.21084337*	*0.44848485*	*0.21084337*	*0.448484848*	*0.154761905*	*0.1104651*	*0.4767442*
**ENSG00000066027**		*0.35632184*		*0.356321839*			*0.375*
**ENSG00000183317**	*0.08333333*	*0.27222222*	*0.08333333*	*0.272222222*	*0.085714286*	*0.0625*	
**ENSG00000205014**	*0.29885057*	*0.07738095*	*0.29885057*	*0.077380952*	*0.055555556*		*0.1625*
**ENSG00000100532**							*0.1511628*
**ENSG00000112619**		*0.29878049*		*0.298780488*			*0.3372093*
**ENSG00000188373**		*0.3*		*0.3*			*0.125*

**ENSG00000118432**	*0.47222222*	*0.25757576*	*0.47222222*	*0.257575758*	*0.375*	*0.3372093*	*0.494186*
**ENSG00000132254**	*0.25555556*		*0.25555556*			*0.0555556*	
**ENSG00000168615**		*0.14117647*		*0.141176471*			*0.0909091*
**ENSG00000133193**		*0.07272727*		*0.072727273*			*0.1744186*
**ENSG00000109101**		*0.31111111*		*0.311111111*			*0.375*
**ENSG00000127666**		*0.25757576*		*0.257575758*			*0.4593023*
**ENSG00000174516**		*0.27575758*		*0.275757576*			*0.3081395*
**ENSG00000181877**		*0.07926829*		*0.079268293*			*0.1104651*
**ENSG00000172086**		*0.32022472*		*0.320224719*			*0.2383721*
**ENSG00000196422**	*0.35628743*		*0.35628743*		*0.369047619*	*0.377907*	*0.4318182*
**ENSG00000117791**							*0.0639535*
**ENSG00000196935**	*0.06287425*	*0.43030303*	*0.06287425*	*0.43030303*			*0.2616279*
**ENSG00000163359**		*0.11666667*		*0.116666667*			*0.1136364*
**ENSG00000089154**		*0.43902439*		*0.43902439*			*0.4825581*
**ENSG00000103202**	*0.32634731*	*0.3908046*	*0.32634731*	*0.390804598*		*0.1744186*	*0.4772727*
**ENSG00000121060**		*0.4030303*		*0.403030303*			*0.2117647*
**ENSG00000249158**		*0.05151515*		*0.051515152*			*0.1162791*
**ENSG00000160593**	*0.25748503*	*0.39263804*	*0.25748503*	*0.392638037*	*0.279761905*	*0.2325581*	*0.3604651*
**ENSG00000162944**		*0.3*		*0.3*			*0.4186047*
**ENSG00000173452**		*0.22392638*		*0.22392638*			*0.5*
**ENSG00000153802**		*0.1*		*0.1*			*0.2682927*
**ENSG00000178338**		*0.12804878*		*0.12804878*			
**ENSG00000121552**		*0.15757576*		*0.157575758*			*0.3953488*
**ENSG00000186472**		*0.49444444*		*0.494444444*			*0.4825581*
**ENSG00000182352**	*0.48888889*	*0.48773006*	*0.48888889*	*0.487730061*	*0.1*	*0.3222222*	*0.3953488*
**ENSG00000095370**	*0.25862069*		*0.25862069*		*0.066666667*		
**ENSG00000150244**							*0.1744186*
**ENSG00000197601**	*0.18333333*	*0.14649682*	*0.18333333*	*0.146496815*			*0.3433735*
**ENSG00000090104**		*0.08484848*		*0.084848485*			
**ENSG00000139291**		*0.14939024*		*0.149390244*	*0.470238095*	*0.4058824*	*0.4302326*
**ENSG00000068745**	*0.28333333*	*0.11818182*	*0.28333333*	*0.118181818*			
**ENSG00000160049**							*0.2727273*
**ENSG00000101670**		*0.06666667*		*0.066666667*			*0.4883721*
**ENSG00000174640**		*0.22121212*		*0.221212121*			*0.4069767*
**ENSG00000168026**		*0.3030303*		*0.303030303*			*0.2848837*
**ENSG00000164122**		*0.20606061*		*0.206060606*			*0.1395349*
**ENSG00000099992**	*0.08982036*	*0.13333333*	*0.08982036*	*0.133333333*	*0.125*	*0.0930233*	*0.3139535*
**ENSG00000135365**		*0.27777778*		*0.277777778*			
**ENSG00000164916**	*0.30538922*	*0.0969697*	*0.30538922*	*0.096969697*	*0.119047619*	*0.0813953*	*0.0813953*
**ENSG00000167716**		*0.25914634*		*0.259146341*	*0.133333333*	*0.1444444*	*0.4464286*
**ENSG00000182871**		*0.39329268*		*0.393292683*			*0.4360465*
**ENSG00000136732**	*0.47005988*	*0.26060606*	*0.47005988*	*0.260606061*	*0.428571429*	*0.2906977*	*0.3313953*
**ENSG00000167323**	*0.4011976*	*0.11818182*	*0.4011976*	*0.118181818*	*0.476190476*	*0.494186*	*0.2325581*
**ENSG00000233527**		*0.20909091*		*0.209090909*			*0.1569767*
**ENSG00000226979**	*0.28443114*		*0.28443114*		*0.351190476*	*0.4476744*	
**ENSG00000135070**		*0.23295455*		*0.232954545*			*0.4431818*
**ENSG00000113648**		*0.07575758*		*0.075757576*			*0.0523256*
**ENSG00000171101**		*0.06969697*		*0.06969697*			*0.4767442*
**ENSG00000152402**		*0.10909091*		*0.109090909*			*0.0795455*
**ENSG00000017373**		*0.22121212*		*0.221212121*			*0.1162791*
**ENSG00000241973**		*0.06134969*		*0.061349693*			*0.0523256*
**ENSG00000117475**	*0.29819277*	*0.36060606*	*0.29819277*	*0.360606061*	*0.133333333*	*0.1569767*	*0.0639535*
**ENSG00000240583**		*0.37575758*		*0.375757576*			*0.3895349*
**ENSG00000012124**		*0.07575758*		*0.075757576*			*0.1569767*

**ENSG00000181409**		*0.17241379*		*0.172413793*			*0.1704545*
**ENSG00000159082**		*0.49689441*		*0.49689441*			*0.4588235*
**ENSG00000106330**					*0.101190476*	*0.0988372*	
**ENSG00000149480**		*0.15757576*		*0.157575758*			*0.2674419*
**ENSG00000137393**	*0.18263473*	*0.18181818*	*0.18263473*	*0.181818182*	*0.422619048*	*0.4166667*	*0.2383721*
**ENSG00000181234**		*0.11212121*		*0.112121212*			*0.2616279*
**ENSG00000186115**		*0.19333333*		*0.193333333*	*0.083333333*		
**ENSG00000133466**		*0.24242424*		*0.242424242*			*0.3941176*
**ENSG00000162390**		*0.47647059*		*0.476470588*			*0.4186047*
**ENSG00000075399**							*0.1104651*
**ENSG00000101417**		*0.45731707*		*0.457317073*			*0.2383721*
**ENSG00000167363**		*0.43636364*		*0.436363636*	*0.3*	*0.3863636*	*0.4302326*
**ENSG00000129535**	*0.08522727*	*0.2804878*	*0.08522727*	*0.280487805*			*0.4767442*
**ENSG00000151470**	*0.34269663*	*0.37878788*	*0.34269663*	*0.378787879*	*0.1*	*0.1888889*	*0.2790698*
**ENSG00000125726**	*0.15568862*	*0.07954545*	*0.15568862*	*0.079545455*			
**ENSG00000167549**		*0.4969697*		*0.496969697*			*0.255814*
**ENSG00000164543**	*0.48502994*	*0.43333333*	*0.48502994*	*0.433333333*	*0.333333333*	*0.2906977*	*0.4360465*
**ENSG00000146276**	*0.49700599*	*0.4030303*	*0.49700599*	*0.403030303*	*0.095238095*		*0.2965116*
**ENSG00000174672**		*0.31666667*		*0.316666667*			
**ENSG00000186818**	*0.49698795*		*0.49698795*		*0.493975904*	*0.4360465*	
**ENSG00000158792**		*0.38888889*		*0.388888889*			*0.3974359*
**ENSG00000166246**	*0.12111801*	*0.32926829*	*0.12111801*	*0.329268293*			*0.2965116*
**ENSG00000132514**	*0.09281437*	*0.07222222*	*0.09281437*	*0.072222222*	*0.071428571*	*0.1162791*	*0.125*
**ENSG00000173269**		*0.39634146*		*0.396341463*			*0.0813953*
**ENSG00000113262**		*0.34242424*		*0.342424242*			*0.3235294*
**ENSG00000165272**		*0.09090909*		*0.090909091*			*0.2134146*
**ENSG00000222012**		*0.39877301*		*0.398773006*			*0.3869048*
**ENSG00000108587**		*0.43030303*		*0.43030303*			*0.2732558*
**ENSG00000198420**	*0.15269461*		*0.15269461*		*0.386904762*	*0.4302326*	
**ENSG00000131941**	*0.49096386*	*0.1554878*	*0.49096386*	*0.155487805*	*0.222891566*	*0.2093023*	*0.0523256*
**ENSG00000113328**	*0.15*		*0.15*				
**ENSG00000136425**		*0.3117284*		*0.311728395*			*0.2093023*
**ENSG00000165807**	*0.29938272*	*0.46666667*	*0.29938272*	*0.466666667*	*0.398809524*	*0.3197674*	*0.3197674*
**ENSG00000181830**	*0.4251497*	*0.2969697*	*0.4251497*	*0.296969697*	*0.130952381*	*0.1627907*	*0.3470588*
**ENSG00000198924**	*0.34431138*		*0.34431138*		*0.107142857*	*0.2267442*	
**ENSG00000166984**	*0.15555556*		*0.15555556*		*0.159090909*	*0.0777778*	
**ENSG00000170941**		*0.36666667*		*0.366666667*			*0.2267442*
**ENSG00000149972**		*0.47297297*		*0.472972973*			*0.4880952*
**ENSG00000117507**		*0.19393939*		*0.193939394*	*0.166666667*	*0.1569767*	*0.2117647*
**ENSG00000170889**	*0.48802395*	*0.42682927*	*0.48802395*	*0.426829268*	*0.488095238*	*0.4534884*	*0.4360465*
**ENSG00000178404**	*0.3742515*		*0.3742515*		*0.289156627*	*0.255814*	
**ENSG00000126550**		*0.29444444*		*0.294444444*			*0.1111111*
**ENSG00000131023**	*0.49431818*	*0.34242424*	*0.49431818*	*0.342424242*	*0.211111111*	*0.2954545*	*0.4285714*
**ENSG00000164266**							*0.1294118*
**ENSG00000144597**		*0.44827586*		*0.448275862*			*0.4204545*
**ENSG00000167601**							*0.0930233*
**ENSG00000170677**		*0.05757576*		*0.057575758*			*0.0882353*
**ENSG00000161513**					*0.357142857*	*0.4011628*	
**ENSG00000167123**	*0.2*	*0.20909091*	*0.2*	*0.209090909*	*0.366666667*	*0.3636364*	*0.3588235*
**ENSG00000149269**	*0.48809524*	*0.35151515*	*0.48809524*	*0.351515152*	*0.306818182*	*0.2727273*	*0.4709302*
**ENSG00000162073**		*0.42073171*		*0.420731707*			*0.125*
**ENSG00000221829**							*0.1337209*
**ENSG00000154265**		*0.45555556*		*0.455555556*			*0.3977273*
**ENSG00000198718**							*0.1337209*

**ENSG00000185875**		*0.27878788*		*0.278787879*			*0.494186*
**ENSG00000125170**		*0.28484848*		*0.284848485*			*0.5*
**ENSG00000145428**		*0.39090909*		*0.390909091*			*0.2616279*
**ENSG00000116819**	*0.0988024*		*0.0988024*		*0.232142857*	*0.1744186*	
**ENSG00000176083**	*0.42168675*	*0.39090909*	*0.42168675*	*0.390909091*	*0.398809524*	*0.3662791*	*0.25*
**ENSG00000137074**	*0.21111111*		*0.21111111*		*0.458333333*	*0.4011628*	
**ENSG00000047644**	*0.48333333*	*0.14848485*	*0.48333333*	*0.148484848*			*0.1046512*
**ENSG00000174697**	*0.46407186*	*0.43333333*	*0.46407186*	*0.433333333*	*0.19047619*	*0.1918605*	*0.2616279*
**ENSG00000020577**		*0.42727273*		*0.427272727*			*0.4235294*
**ENSG00000183111**		*0.48787879*		*0.487878788*			*0.3081395*
**ENSG00000178607**		*0.13068182*		*0.130681818*			*0.1818182*
**ENSG00000115183**	*0.15568862*	*0.31515152*	*0.15568862*	*0.315151515*	*0.119047619*	*0.1627907*	*0.3529412*
**ENSG00000180667**	*0.12048193*	*0.46067416*	*0.12048193*	*0.460674157*			*0.2272727*
**ENSG00000150722**		*0.21348315*		*0.213483146*			*0.3111111*
**ENSG00000183648**	*0.36826347*	*0.35151515*	*0.36826347*	*0.351515152*	*0.369047619*	*0.3546512*	*0.122093*
**ENSG00000213699**		*0.33333333*		*0.333333333*			*0.3352941*
**ENSG00000180828**		*0.08484848*		*0.084848485*			
**ENSG00000136856**		*0.42528736*		*0.425287356*			*0.0909091*
**ENSG00000152578**		*0.48333333*		*0.483333333*			*0.4555556*
**ENSG00000015133**		*0.19631902*		*0.196319018*			*0.3111111*
**ENSG00000167085**	*0.27844311*	*0.17272727*	*0.27844311*	*0.172727273*	*0.119047619*	*0.2674419*	*0.4127907*
**ENSG00000136059**		*0.15454545*		*0.154545455*			
**ENSG00000131979**	*0.1497006*	*0.26086957*	*0.1497006*	*0.260869565*		*0.0523256*	*0.4329268*
**ENSG00000108256**		*0.20245399*		*0.202453988*			*0.4360465*
**ENSG00000158008**	*0.06321839*	*0.35534591*	*0.06321839*	*0.355345912*	*0.085365854*	*0.0609756*	*0.1011905*
**ENSG00000147324**		*0.43030303*		*0.43030303*			*0.1395349*
**ENSG00000174227**	*0.45209581*	*0.24085366*	*0.45209581*	*0.240853659*	*0.244047619*	*0.3197674*	*0.3197674*
**ENSG00000166734**							*0.127907*
**ENSG00000109705**		*0.16060606*		*0.160606061*			*0.0639535*
**ENSG00000197223**		*0.13333333*		*0.133333333*			*0.3181818*
**ENSG00000163281**		*0.47222222*		*0.472222222*			*0.4333333*
**ENSG00000140105**	*0.48502994*	*0.2247191*	*0.48502994*	*0.224719101*	*0.397590361*	*0.4476744*	*0.3488372*
**ENSG00000150907**							*0.372093*
**ENSG00000176894**		*0.42424242*		*0.424242424*			*0.2559524*
**ENSG00000141499**		*0.23809524*		*0.238095238*		*0.0588235*	*0.3636364*
**ENSG00000114107**		*0.30606061*		*0.306060606*			*0.4418605*
**ENSG00000173786**		*0.23333333*		*0.233333333*			*0.4011628*
**ENSG00000101452**	*0.10542169*	*0.18181818*	*0.10542169*	*0.181818182*	*0.107142857*	*0.1162791*	*0.122093*
**ENSG00000162367**		*0.43030303*		*0.43030303*	*0.055555556*		*0.4534884*
**ENSG00000051009**	*0.17222222*	*0.05757576*	*0.17222222*	*0.057575758*			
**ENSG00000162078**		*0.29393939*		*0.293939394*			*0.4011628*
**ENSG00000172935**		*0.24842767*		*0.248427673*			*0.2738095*
**ENSG00000110079**	*0.31402439*	*0.29393939*	*0.31402439*	*0.293939394*	*0.446428571*	*0.2764706*	*0.3139535*
**ENSG00000151065**		*0.41111111*		*0.411111111*			*0.25*
**ENSG00000105939**	*0.19662921*	*0.48787879*	*0.19662921*	*0.487878788*	*0.355555556*	*0.4090909*	*0.3295455*
**ENSG00000068097**	*0.17613636*		*0.17613636*		*0.055555556*	*0.0681818*	
**ENSG00000250305**	*0.19760479*	*0.48181818*	*0.19760479*	*0.481818182*	*0.416666667*	*0.5*	*0.4011628*
**ENSG00000137871**	*0.19578313*	*0.34545455*	*0.19578313*	*0.345454545*	*0.381578947*	*0.3882353*	*0.4883721*
**ENSG00000134970**		*0.42331288*		*0.423312883*			*0.3977273*
**ENSG00000170516**	*0.18562874*	*0.21111111*	*0.18562874*	*0.211111111*	*0.273809524*	*0.2848837*	*0.3068182*
**ENSG00000100314**		*0.07575758*		*0.075757576*			*0.3895349*
**ENSG00000167863**	*0.34431138*		*0.34431138*		*0.428571429*	*0.4127907*	
**ENSG00000175746**	*0.17664671*	*0.14848485*	*0.17664671*	*0.148484848*	*0.055555556*	*0.0555556*	*0.3197674*
**ENSG00000100413**		*0.23619632*		*0.236196319*			*0.3430233*

**ENSG00000166507**		*0.37931034*		*0.379310345*			*0.0555556*	*0.4651163*
**ENSG00000175414**		*0.45454545*		*0.454545455*				*0.4302326*
**ENSG00000152465**	*0.33888889*		*0.33888889*		*0.055555556*		*0.1190476*	
**ENSG00000141384**		*0.44545455*		*0.445454545*				*0.1686047*
**ENSG00000205246**	*0.09090909*		*0.09090909*					
**ENSG00000149577**	*0.26666667*	*0.19817073*	*0.26666667*	*0.198170732*	*0.05952381*		*0.1363636*	*0.377907*
**ENSG00000167654**		*0.31111111*		*0.311111111*				*0.372093*
**ENSG00000111371**	*0.24846626*		*0.24846626*		*0.457317073*		*0.4302326*	
**ENSG00000063241**	*0.26646707*		*0.26646707*		*0.44047619*		*0.377907*	
**ENSG00000157470**	*0.23353293*		*0.23353293*		*0.494047619*		*0.2965116*	
**ENSG00000167112**		*0.17777778*		*0.177777778*				
**ENSG00000137634**		*0.12576687*		*0.125766871*				
**ENSG00000117054**		*0.3030303*		*0.303030303*				
**ENSG00000156313**								*0.0666667*
**ENSG00000143157**		*0.47575758*		*0.475757576*				*0.494186*
**ENSG00000138083**		*0.11515152*		*0.115151515*				
**ENSG00000179008**	*0.49382716*	*0.29320988*	*0.49382716*	*0.293209877*	*0.166666667*		*0.1363636*	*0.4767442*
**ENSG00000205531**	*0.19760479*		*0.19760479*					
**ENSG00000151725**		*0.12727273*		*0.127272727*				*0.3953488*
**ENSG00000216490**	*0.30239521*	*0.23939394*	*0.30239521*	*0.239393939*	*0.101190476*		*0.1395349*	*0.1511628*
**ENSG00000131737**		*0.12424242*		*0.124242424*				*0.0639535*
**ENSG00000108684**		*0.36666667*		*0.366666667*				*0.4882353*
**ENSG00000222019**		*0.27586207*		*0.275862069*				*0.3197674*
**ENSG00000096654**		*0.4556962*		*0.455696203*				*0.3977273*
**ENSG00000163166**		*0.31818182*		*0.318181818*				*0.3255814*
**ENSG00000169689**	*0.4251497*		*0.4251497*		*0.25*		*0.3023256*	
**ENSG00000134996**		*0.3597561*		*0.359756098*				*0.3941176*
**ENSG00000111241**		*0.30232558*		*0.302325581*				*0.2325581*
**ENSG00000159214**	*0.09281437*		*0.09281437*		*0.386904762*		*0.4360465*	
**ENSG00000135968**	*0.40588235*	*0.35849057*	*0.40588235*	*0.358490566*	*0.053571429*		*0.2034884*	*0.1931818*
**ENSG00000114904**		*0.47647059*		*0.476470588*				*0.5*
**ENSG00000020633**		*0.06060606*		*0.060606061*				
**ENSG00000187049**	*0.31437126*	*0.17272727*	*0.31437126*	*0.172727273*				
**ENSG00000038295**	*0.4011976*	*0.45426829*	*0.4011976*	*0.454268293*	*0.482142857*		*0.4767442*	*0.4940476*
**ENSG00000172867**								*0.127907*
**ENSG00000168944**		*0.41515152*		*0.415151515*				*0.4883721*
**ENSG00000186827**								*0.1818182*
**ENSG00000165912**	*0.3961039*		*0.3961039*		*0.147727273*		*0.0813953*	
**ENSG00000127952**	*0.15730337*	*0.30909091*	*0.15730337*	*0.309090909*	*0.477777778*		*0.3255814*	*0.4886364*
**ENSG00000214401**		*0.16292135*		*0.162921348*				
**ENSG00000203872**	*0.16167665*	*0.08588957*	*0.16167665*	*0.085889571*				*0.126506*
**ENSG00000165914**	*0.46385542*	*0.26666667*	*0.46385542*	*0.266666667*	*0.380952381*		*0.3154762*	*0.4709302*
**ENSG00000139842**	*0.10479042*		*0.10479042*					*0.2383721*
**ENSG00000106991**	*0.22155689*		*0.22155689*		*0.053571429*		*0.0813953*	
**ENSG00000196531**	*0.47005988*		*0.47005988*		*0.214285714*		*0.3604651*	
**ENSG00000181192**		*0.08333333*		*0.083333333*				*0.0568182*
**ENSG00000225190**		*0.16666667*		*0.166666667*				
**ENSG00000167791**		*0.47272727*		*0.472727273*				*0.2848837*
**ENSG00000163295**	*0.1*	*0.11515152*	*0.1*	*0.115151515*	*0.077777778*		*0.1022727*	*0.2674419*
**ENSG00000173253**	*0.14044944*	*0.3969697*	*0.14044944*	*0.396969697*			*0.0568182*	*0.4886364*
**ENSG00000156006**	*0.28333333*		*0.28333333*					
**ENSG00000014123**	*0.07185629*		*0.07185629*				*0.122093*	
**ENSG00000256892**	*0.39444444*	*0.32222222*	*0.39444444*	*0.322222222*	*0.466666667*		*0.4189189*	*0.4111111*
**ENSG00000184113**		*0.11111111*		*0.111111111*	*0.283950617*		*0.2616279*	*0.2840909*

**ENSG00000092020**	*0.46706587*	*0.06111111*	*0.46706587*	*0.061111111*	*0.422619048*		*0.4127907*	
**ENSG00000101017**	*0.35928144*	*0.05172414*	*0.35928144*	*0.051724138*	*0.307228916*		*0.5*	
**ENSG00000111344**		*0.07386364*		*0.073863636*				*0.25*
**ENSG00000183775**		*0.41975309*		*0.419753086*				*0.2926829*
**ENSG00000100865**		*0.31707317*		*0.317073171*				*0.4411765*
**ENSG00000166272**		*0.43820225*		*0.438202247*				*0.494186*
**ENSG00000132436**	*0.20359281*	*0.2654321*	*0.20359281*	*0.265432099*	*0.386904762*		*0.4011628*	*0.4705882*
**ENSG00000106009**		*0.14606742*		*0.146067416*				
**ENSG00000258366**		*0.24242424*		*0.242424242*	*0.095238095*		*0.0882353*	*0.4204545*
**ENSG00000259645**		*0.07894737*		*0.078947368*				*0.4125*
**ENSG00000177150**		*0.37195122*		*0.37195122*				*0.127907*
**ENSG00000152217**	*0.0748503*	*0.12777778*	*0.0748503*	*0.127777778*				*0.1590909*
**ENSG00000189171**	*0.47005988*		*0.47005988*		*0.351190476*		*0.3081395*	
**ENSG00000204525**	*0.22159091*	*0.33018868*	*0.22159091*	*0.330188679*	*0.307692308*		*0.2125*	*0.2941176*
**ENSG00000155034**		*0.43939394*		*0.439393939*				*0.2647059*
**ENSG00000078900**		*0.21341463*		*0.213414634*				*0.4651163*
**ENSG00000132840**	*0.20481928*		*0.20481928*		*0.398809524*		*0.494186*	
**ENSG00000120693**		*0.08333333*		*0.083333333*				*0.244186*
**ENSG00000130255**					*0.055555556*			
**ENSG00000187145**	*0.3742515*	*0.31666667*	*0.3742515*	*0.316666667*				*0.244186*
**ENSG00000196381**	*0.28742515*	*0.15151515*	*0.28742515*	*0.151515152*	*0.119047619*		*0.1511628*	*0.3352941*
**ENSG00000158715**		*0.08333333*		*0.083333333*				*0.1802326*
**ENSG00000112578**		*0.06666667*		*0.066666667*				*0.2034884*
**ENSG00000174567**		*0.06666667*		*0.066666667*				
**ENSG00000141510**	*0.33146067*		*0.33146067*		*0.488888889*		*0.4090909*	*0.1162791*
**ENSG00000100345**	*0.0748503*	*0.12727273*	*0.0748503*	*0.127272727*	*0.155555556*		*0.0777778*	*0.3536585*
**ENSG00000159713**		*0.07575758*		*0.075757576*				
**ENSG00000119185**	*0.25*	*0.48484848*	*0.25*	*0.484848485*				*0.1395349*
**ENSG00000092531**		*0.25*		*0.25*				
**ENSG00000156735**	*0.11111111*	*0.25555556*	*0.11111111*	*0.255555556*				*0.3409091*
**ENSG00000107960**	*0.18333333*		*0.18333333*		*0.122222222*		*0.2045455*	
**ENSG00000180440**		*0.45454545*		*0.454545455*				*0.494186*
**ENSG00000183643**	*0.46107784*	*0.20909091*	*0.46107784*	*0.209090909*	*0.458333333*		*0.4767442*	*0.1931818*
**ENSG00000170866**					*0.077380952*		*0.122093*	
**ENSG00000137090**		*0.16158537*		*0.161585366*				*0.2470588*
**ENSG00000162929**	*0.41477273*	*0.44242424*	*0.41477273*	*0.442424242*	*0.275*		*0.3372093*	*0.3895349*
**ENSG00000172061**		*0.3030303*		*0.303030303*				*0.1477273*
**ENSG00000063015**		*0.15454545*		*0.154545455*				*0.1046512*
**ENSG00000157693**		*0.15151515*		*0.151515152*				*0.2559524*
**ENSG00000198890**		*0.20909091*		*0.209090909*				*0.1337209*
**ENSG00000170962**		*0.46666667*		*0.466666667*				*0.4825581*
**ENSG00000096070**	*0.2994012*	*0.38764045*	*0.2994012*	*0.387640449*	*0.30952381*		*0.3488372*	*0.375*
**ENSG00000167220**		*0.16666667*		*0.166666667*				*0.1941176*
**ENSG00000069431**		*0.36666667*		*0.366666667*				*0.2325581*
**ENSG00000185823**		*0.30898876*		*0.308988764*				*0.2790698*
**ENSG00000184216**		*0.20555556*		*0.205555556*				*0.2045455*
**ENSG00000121022**	*0.09580838*		*0.09580838*					
**ENSG00000120875**	*0.39520958*	*0.07272727*	*0.39520958*	*0.072727273*	*0.428571429*		*0.4107143*	
**ENSG00000156042**	*0.14444444*		*0.14444444*					
**ENSG00000167110**		*0.19393939*		*0.193939394*				*0.2906977*
**ENSG00000154310**		*0.26060606*		*0.260606061*				*0.3139535*
**ENSG00000119321**	*0.12048193*	*0.28333333*	*0.12048193*	*0.283333333*	*0.375*		*0.3430233*	*0.4360465*
**ENSG00000256223**	*0.10778443*	*0.27439024*	*0.10778443*	*0.274390244*	*0.077380952*		*0.0872093*	*0.0647059*
**ENSG00000135870**		*0.11235955*		*0.112359551*				*0.2613636*

**ENSG00000152591**		*0.12121212*		*0.121212121*			*0.1337209*
**ENSG00000165646**		*0.49390244*		*0.493902439*			*0.4642857*
**ENSG00000145863**	*0.3502994*	*0.35151515*	*0.3502994*	*0.351515152*	*0.375*	*0.3333333*	*0.3313953*
**ENSG00000167721**		*0.26111111*		*0.261111111*			*0.1555556*
**ENSG00000164398**	*0.15972222*	*0.23030303*	*0.15972222*	*0.23030303*	*0.5*	*0.4090909*	*0.4235294*
**ENSG00000134183**	*0.06586826*		*0.06586826*		*0.101190476*	*0.1046512*	
**ENSG00000164707**	*0.48484848*		*0.48484848*		*0.43373494*	*0.4352941*	
**ENSG00000081760**	*0.49700599*	*0.22121212*	*0.49700599*	*0.221212121*	*0.470238095*	*0.375*	*0.3294118*
**ENSG00000186807**							*0.3863636*
**ENSG00000144847**	*0.48802395*		*0.48802395*		*0.470238095*	*0.3953488*	
**ENSG00000143942**		*0.16969697*		*0.16969697*			*0.0813953*
**ENSG00000172340**		*0.46363636*		*0.463636364*			*0.3953488*
**ENSG00000214128**		*0.46060606*		*0.460606061*			*0.25*
**ENSG00000128059**		*0.43939394*		*0.439393939*	*0.1*	*0.2333333*	*0.2325581*
**ENSG00000206072**	*0.4760479*	*0.28181818*	*0.4760479*	*0.281818182*	*0.488888889*	*0.4318182*	*0.1744186*
**ENSG00000204498**		*0.12424242*		*0.124242424*			*0.2906977*
**ENSG00000184774**							*0.1818182*
**ENSG00000168487**		*0.24848485*		*0.248484848*			*0.2093023*
**ENSG00000136936**	*0.22754491*		*0.22754491*		*0.476190476*	*0.4880952*	*0.0872093*
**ENSG00000101310**	*0.36931818*		*0.36931818*		*0.266666667*	*0.4090909*	
**ENSG00000103978**		*0.09302326*		*0.093023256*	*0.345238095*	*0.2790698*	*0.1022727*
**ENSG00000214655**		*0.41515152*		*0.415151515*			*0.2848837*
**ENSG00000188636**		*0.49079755*		*0.490797546*			*0.4204545*
**ENSG00000175556**		*0.33636364*		*0.336363636*			*0.2176471*
**ENSG00000248167**		*0.12727273*		*0.127272727*			*0.372093*
**ENSG00000185518**	*0.08383234*	*0.44444444*	*0.08383234*	*0.444444444*	*0.172619048*	*0.0988372*	*0.4545455*
**ENSG00000171295**	*0.47126437*	*0.32515337*	*0.47126437*	*0.325153374*	*0.166666667*	*0.0853659*	*0.3139535*
**ENSG00000040199**		*0.34545455*		*0.345454545*			*0.3181818*
**ENSG00000219159**	*0.34730539*		*0.34730539*		*0.392857143*	*0.4294118*	
**ENSG00000170683**	*0.26946108*		*0.26946108*		*0.357142857*	*0.255814*	
**ENSG00000120802**		*0.33333333*		*0.333333333*			
**ENSG00000185262**		*0.37575758*		*0.375757576*			*0.3529412*
**ENSG00000066629**	*0.23333333*	*0.32317073*	*0.23333333*	*0.323170732*			*0.4476744*
**ENSG00000110871**	*0.0748503*	*0.30606061*	*0.0748503*	*0.306060606*	*0.488095238*	*0.4476744*	*0.4476744*
**ENSG00000133059**		*0.45426829*		*0.454268293*			*0.4883721*
**ENSG00000105640**		*0.31707317*		*0.317073171*			*0.4883721*
**ENSG00000061938**							*0.1117647*
**ENSG00000115073**		*0.29192547*		*0.291925466*			*0.3588235*
**ENSG00000187037**	*0.32634731*		*0.32634731*		*0.232142857*	*0.1976744*	
**ENSG00000147570**					*0.058139535*	*0.0731707*	
**ENSG00000259220**		*0.10606061*		*0.106060606*			*0.0581395*
**ENSG00000167978**		*0.2*		*0.2*			*0.4011628*
**ENSG00000154040**	*0.10588235*	*0.4*	*0.10588235*	*0.4*	*0.077380952*	*0.1976744*	*0.5*
**ENSG00000179627**		*0.11235955*		*0.112359551*			
**ENSG00000106123**	*0.0748503*		*0.0748503*				
**ENSG00000204856**	*0.17878788*		*0.17878788*		*0.054216867*	*0.0705882*	
**ENSG00000168356**		*0.49393939*		*0.493939394*			*0.4360465*
**ENSG00000109991**		*0.41666667*		*0.416666667*			*0.4186047*
**ENSG00000100354**	*0.17065868*	*0.48780488*	*0.17065868*	*0.487804878*			*0.4823529*
**ENSG00000106809**		*0.39534884*		*0.395348837*			*0.3409091*
**ENSG00000168118**		*0.48170732*		*0.481707317*			*0.4647059*
**ENSG00000167291**		*0.41515152*		*0.415151515*			*0.3488372*
**ENSG00000198001**		*0.46052632*		*0.460526316*			*0.3717949*
**ENSG00000187097**		*0.44545455*		*0.445454545*	*0.303571429*	*0.3081395*	*0.3546512*

**ENSG00000171201**	*0.12790698*	*0.38333333*	*0.12790698*	*0.383333333*				*0.3863636*
**ENSG00000071189**		*0.49090909*		*0.490909091*				*0.4767442*
**ENSG00000134574**	*0.42134831*		*0.42134831*					
**ENSG00000175567**	*0.2245509*		*0.2245509*					
**ENSG00000168152**		*0.44242424*		*0.442424242*				*0.255814*
**ENSG00000162664**		*0.49393939*		*0.493939394*				*0.0813953*
**ENSG00000162896**								*0.3068182*
**ENSG00000126778**	*0.36227545*	*0.06666667*	*0.36227545*	*0.066666667*	*0.166666667*		*0.2151163*	*0.1686047*
**ENSG00000182986**		*0.29090909*		*0.290909091*			*0.2621951*	*0.3604651*
**ENSG00000213057**	*0.0988024*	*0.42727273*	*0.0988024*	*0.427272727*	*0.398809524*		*0.3895349*	*0.4534884*
**ENSG00000180264**	*0.18263473*		*0.18263473*		*0.144578313*		*0.1607143*	
**ENSG00000108950**	*0.13173653*	*0.2030303*	*0.13173653*	*0.203030303*	*0.355555556*		*0.3777778*	*0.2738095*
**ENSG00000169126**	*0.16467066*	*0.47727273*	*0.16467066*	*0.477272727*				*0.4333333*
**ENSG00000197380**		*0.16969697*		*0.16969697*				*0.2034884*
**ENSG00000168159**		*0.46060606*		*0.460606061*				*0.4476744*
**ENSG00000134982**		*0.37195122*		*0.37195122*				*0.2732558*
**ENSG00000221886**	*0.44277108*		*0.44277108*		*0.380952381*		*0.3895349*	
**ENSG00000178110**	*0.246875*		*0.246875*		*0.214285714*		*0.244186*	
**ENSG00000125850**		*0.41158537*		*0.411585366*				*0.2790698*
**ENSG00000122705**	*0.20658683*	*0.1*	*0.20658683*	*0.1*	*0.154761905*		*0.127907*	*0.1882353*
**ENSG00000253161**		*0.38636364*		*0.386363636*				*0.1111111*
**ENSG00000112655**		*0.41158537*		*0.411585366*				*0.3470588*
**ENSG00000188162**	*0.16111111*	*0.26363636*	*0.16111111*	*0.263636364*	*0.174418605*		*0.1818182*	*0.1162791*
**ENSG00000213085**					*0.25*		*0.2325581*	
**ENSG00000100592**					*0.166666667*		*0.1511628*	
**ENSG00000184402**								*0.1117647*
**ENSG00000128656**	*0.31736527*		*0.31736527*		*0.291666667*		*0.2906977*	
**ENSG00000156411**		*0.25151515*		*0.251515152*				*0.3662791*
**ENSG00000065325**		*0.42424242*		*0.424242424*				*0.3255814*
**ENSG00000146904**		*0.05757576*		*0.057575758*				
**ENSG00000088367**		*0.17272727*		*0.172727273*				*0.1964286*
**ENSG00000142330**		*0.42222222*		*0.422222222*				*0.375*
**ENSG00000166813**		*0.31402439*		*0.31402439*				*0.3647059*
**ENSG00000135297**	*0.19444444*	*0.16770186*	*0.19444444*	*0.167701863*			*0.1136364*	*0.2117647*
**ENSG00000113494**	*0.40449438*	*0.17777778*	*0.40449438*	*0.177777778*	*0.193181818*		*0.25*	*0.3181818*
**ENSG00000085721**		*0.11212121*		*0.112121212*				*0.1627907*
**ENSG00000144724**		*0.34242424*		*0.342424242*				*0.2616279*
**ENSG00000165502**	*0.42222222*	*0.43030303*	*0.42222222*	*0.43030303*	*0.215909091*		*0.125*	*0.0813953*
**ENSG00000076604**		*0.1993865*		*0.199386503*				*0.3139535*
**ENSG00000137601**	*0.08083832*	*0.36060606*	*0.08083832*	*0.360606061*	*0.18452381*		*0.1569767*	*0.1647059*
**ENSG00000198937**		*0.30606061*		*0.306060606*				*0.5*
**ENSG00000181449**		*0.21666667*		*0.216666667*				
**ENSG00000184208**		*0.2030303*		*0.203030303*				
**ENSG00000102967**		*0.49444444*		*0.494444444*				*0.3636364*
**ENSG00000204511**	*0.10227273*	*0.25*	*0.10227273*	*0.25*	*0.125*		*0.1363636*	*0.1111111*
**ENSG00000256235**	*0.28742515*		*0.28742515*		*0.295180723*		*0.2159091*	
**ENSG00000188385**		*0.48484848*		*0.484848485*				*0.0755814*
**ENSG00000172270**		*0.37647059*		*0.376470588*				*0.1704545*
**ENSG00000179119**		*0.4695122*		*0.469512195*				*0.2202381*
**ENSG00000212128**		*0.43081761*		*0.43081761*				*0.2045455*
**ENSG00000110717**	*0.23888889*	*0.16363636*	*0.23888889*	*0.163636364*	*0.238095238*		*0.2333333*	*0.2093023*
**ENSG00000101150**		*0.5*		*0.5*				*0.1046512*
**ENSG00000134548**		*0.34146341*		*0.341463415*				*0.2142857*
**ENSG00000109956**	*0.39240506*	*0.2804878*	*0.39240506*	*0.280487805*	*0.119047619*		*0.1104651*	

**ENSG00000140287**							*0.0568182*
**ENSG00000182578**	*0.28614458*	*0.1954023*	*0.28614458*	*0.195402299*	*0.220238095*	*0.1918605*	*0.3977273*
**ENSG00000128872**		*0.49085366*		*0.490853659*			*0.5*
**ENSG00000071051**		*0.31212121*		*0.312121212*			*0.3139535*
**ENSG00000198520**	*0.18562874*		*0.18562874*				
**ENSG00000101955**		*0.11235955*		*0.112359551*			*0.0714286*
**ENSG00000158125**		*0.46060606*		*0.460606061*			*0.3953488*
**ENSG00000136861**		*0.25555556*		*0.255555556*			*0.3430233*
**ENSG00000176953**		*0.29393939*		*0.293939394*			*0.0697674*
**ENSG00000183726**		*0.46060606*		*0.460606061*			*0.3139535*
**ENSG00000160185**		*0.42073171*		*0.420731707*			*0.3255814*
**ENSG00000170776**	*0.45808383*	*0.5*	*0.45808383*	*0.5*	*0.5*	*0.4767442*	*0.4767442*
**ENSG00000004897**		*0.40243902*		*0.402439024*	*0.077777778*	*0.0697674*	*0.2176471*
**ENSG00000117152**	*0.44311377*	*0.2791411*	*0.44311377*	*0.279141104*	*0.416666667*	*0.4767442*	*0.3705882*
**ENSG00000183186**		*0.2652439*		*0.265243902*			*0.4767442*
**ENSG00000165609**		*0.47575758*		*0.475757576*			*0.4772727*
**ENSG00000125895**	*0.17065868*		*0.17065868*				
**ENSG00000169330**		*0.43030303*		*0.43030303*			*0.3662791*
**ENSG00000147592**		*0.08888889*		*0.088888889*			
**ENSG00000168634**		*0.41818182*		*0.418181818*			*0.2674419*
**ENSG00000113532**	*0.4011976*	*0.39939024*	*0.4011976*	*0.399390244*	*0.259036145*	*0.3546512*	*0.3705882*
**ENSG00000167065**	*0.41111111*	*0.43030303*	*0.41111111*	*0.43030303*	*0.416666667*	*0.4825581*	*0.4883721*
**ENSG00000214517**		*0.15454545*		*0.154545455*	*0.464285714*	*0.4588235*	*0.2674419*
**ENSG00000125888**	*0.16467066*		*0.16467066*				
**ENSG00000074370**	*0.35928144*	*0.46060606*	*0.35928144*	*0.460606061*	*0.089285714*	*0.0813953*	*0.4146341*
**ENSG00000151789**	*0.19461078*	*0.49074074*	*0.19461078*	*0.490740741*		*0.1104651*	*0.5*
**ENSG00000250588**					*0.244444444*	*0.2444444*	
**ENSG00000158683**		*0.11235955*		*0.112359551*			
**ENSG00000131558**	*0.40361446*	*0.42727273*	*0.40361446*	*0.427272727*	*0.244047619*	*0.2093023*	*0.2790698*
**ENSG00000205937**	*0.34431138*	*0.21818182*	*0.34431138*	*0.218181818*	*0.416666667*	*0.4302326*	
**ENSG00000117868**	*0.0505618*	*0.11818182*	*0.0505618*	*0.118181818*		*0.0666667*	*0.4879518*
**ENSG00000167552**	*0.16666667*		*0.16666667*		*0.295180723*	*0.3255814*	
**ENSG00000105409**		*0.35465116*		*0.354651163*			*0.2386364*
**ENSG00000184992**		*0.3969697*		*0.396969697*			*0.3255814*
**ENSG00000146242**	*0.4251497*	*0.32424242*	*0.4251497*	*0.324242424*	*0.4625*	*0.4529412*	*0.3604651*
**ENSG00000021762**	*0.42168675*	*0.24695122*	*0.42168675*	*0.24695122*	*0.166666667*	*0.2616279*	*0.1022727*
**ENSG00000073536**	*0.15568862*		*0.15568862*		*0.05952381*	*0.0639535*	*0.1395349*
**ENSG00000185594**	*0.35628743*		*0.35628743*		*0.5*	*0.4709302*	
**ENSG00000181804**	*0.11077844*	*0.35757576*	*0.11077844*	*0.357575758*			*0.2857143*
**ENSG00000183470**	*0.36746988*	*0.48787879*	*0.36746988*	*0.487878788*	*0.202380952*	*0.2209302*	*0.4883721*
**ENSG00000165246**							*0.4347826*
**ENSG00000196199**	*0.24550898*		*0.24550898*				
**ENSG00000182836**		*0.26666667*		*0.266666667*			*0.4090909*
**ENSG00000179988**	*0.38922156*	*0.36585366*	*0.38922156*	*0.365853659*	*0.355421687*	*0.4476744*	*0.4411765*
**ENSG00000117010**		*0.38125*		*0.38125*			*0.3588235*
**ENSG00000176222**		*0.428125*		*0.428125*			*0.1705882*
**ENSG00000180458**		*0.15151515*		*0.151515152*			*0.3313953*
**ENSG00000167741**		*0.20606061*		*0.206060606*	*0.05952381*	*0.075*	*0.1453488*
**ENSG00000164038**							*0.1022727*
**ENSG00000160959**		*0.49090909*		*0.490909091*			*0.3895349*
**ENSG00000125871**		*0.3452381*		*0.345238095*			*0.5*
**ENSG00000258311**		*0.20786517*		*0.207865169*			*0.1860465*
**ENSG00000171462**	*0.13772455*	*0.30487805*	*0.13772455*	*0.304878049*	*0.053571429*	*0.1046512*	*0.1117647*
**ENSG00000070950**	*0.30838323*	*0.30909091*	*0.30838323*	*0.309090909*	*0.446428571*	*0.4534884*	*0.4418605*

**ENSG00000104164**		*0.19090909*		*0.190909091*			*0.1162791*
**ENSG00000149506**		*0.26666667*		*0.266666667*	*0.375*	*0.4011628*	*0.4011628*
**ENSG00000143320**							*0.1860465*
**ENSG00000127688**		*0.45757576*		*0.457575758*			*0.3313953*
**ENSG00000143951**		*0.41304348*		*0.413043478*	*0.166666667*	*0.2045455*	*0.2965116*
**ENSG00000144043**		*0.36206897*		*0.362068966*			*0.4772727*
**ENSG00000052126**	*0.24444444*	*0.20909091*	*0.24444444*	*0.209090909*	*0.077777778*	*0.0666667*	*0.3589744*
**ENSG00000112983**	*0.3372093*	*0.36890244*	*0.3372093*	*0.368902439*	*0.397727273*	*0.4204545*	*0.4360465*
**ENSG00000235865**	*0.3974359*		*0.3974359*		*0.380952381*	*0.377907*	
**ENSG00000101493**	*0.05389222*	*0.21212121*	*0.05389222*	*0.212121212*	*0.172619048*	*0.1627907*	*0.1686047*
**ENSG00000253258**					*0.178571429*	*0.2325581*	
**ENSG00000184785**		*0.40909091*		*0.409090909*			*0.4651163*
**ENSG00000198538**		*0.31111111*		*0.311111111*			*0.2666667*
**ENSG00000131236**	*0.44277108*	*0.10869565*	*0.44277108*	*0.108695652*	*0.414634146*	*0.5*	*0.3414634*
**ENSG00000139579**	*0.2005988*		*0.2005988*				
**ENSG00000126787**	*0.2994012*		*0.2994012*				
**ENSG00000146859**							*0.0681818*
**ENSG00000187391**	*0.06666667*	*0.29878049*	*0.06666667*	*0.298780488*			*0.4886364*
**ENSG00000113161**		*0.39393939*		*0.393939394*			*0.4647059*
**ENSG00000196355**	*0.26946108*		*0.26946108*		*0.18452381*	*0.1511628*	
**ENSG00000166377**	*0.34444444*	*0.47222222*	*0.34444444*	*0.472222222*	*0.422222222*	*0.375*	*0.375*
**ENSG00000241935**		*0.36060606*		*0.360606061*			*0.255814*
**ENSG00000113205**		*0.28888889*		*0.288888889*			*0.4302326*
**ENSG00000162604**	*0.18562874*		*0.18562874*				
**ENSG00000129159**		*0.2030303*		*0.203030303*			*0.1744186*
**ENSG00000187325**		*0.22611465*		*0.22611465*			*0.4666667*
**ENSG00000184203**		*0.33908046*		*0.33908046*			*0.4605263*
**ENSG00000179046**		*0.36842105*		*0.368421053*			*0.4883721*
**ENSG00000117479**	*0.36805556*	*0.22121212*	*0.36805556*	*0.221212121*	*0.109756098*	*0.1323529*	*0.1162791*
**ENSG00000168890**		*0.41515152*		*0.415151515*			*0.4360465*
**ENSG00000204930**		*0.44545455*		*0.445454545*			*0.3372093*
**ENSG00000082516**		*0.43292683*		*0.432926829*			
**ENSG00000101188**		*0.12420382*		*0.124203822*			*0.1022727*
**ENSG00000122068**	*0.40419162*	*0.13030303*	*0.40419162*	*0.13030303*	*0.088888889*	*0.0681818*	*0.0639535*
**ENSG00000125650**	*0.14465409*		*0.14465409*				
**ENSG00000153086**	*0.0508982*		*0.0508982*				
**ENSG00000172500**		*0.22121212*		*0.221212121*			*0.3255814*
**ENSG00000159322**		*0.31707317*		*0.317073171*			*0.2034884*
**ENSG00000158669**	*0.47305389*	*0.48484848*	*0.47305389*	*0.484848485*	*0.439759036*	*0.4883721*	*0.4941176*
**ENSG00000168899**							*0.3647059*
**ENSG00000128510**		*0.41515152*		*0.415151515*			*0.494186*
**ENSG00000182187**	*0.07575758*		*0.07575758*		*0.125*	*0.127907*	
**ENSG00000176887**		*0.34848485*		*0.348484848*			*0.494186*
**ENSG00000175048**		*0.37804878*		*0.37804878*			*0.4883721*
**ENSG00000257230**	*0.42215569*		*0.42215569*				
**ENSG00000145384**		*0.43939394*		*0.439393939*	*0.06547619*	*0.0639535*	*0.4534884*
**ENSG00000111704**	*0.08333333*		*0.08333333*			*0.1022727*	
**ENSG00000091129**	*0.11377246*	*0.11212121*	*0.11377246*	*0.112121212*	*0.182926829*	*0.255814*	*0.25*
**ENSG00000132005**		*0.26969697*		*0.26969697*			*0.244186*
**ENSG00000147432**	*0.15269461*	*0.06363636*	*0.15269461*	*0.063636364*	*0.25*	*0.1337209*	
**ENSG00000256530**		*0.29545455*		*0.295454545*			*0.1463415*
**ENSG00000198680**		*0.49375*		*0.49375*			*0.3674699*
**ENSG00000128254**	*0.46111111*		*0.46111111*		*0.233333333*	*0.1818182*	
**ENSG00000203791**		*0.46341463*		*0.463414634*			*0.4470588*

**ENSG00000185013**		*0.17777778*		*0.177777778*			*0.1136364*
**ENSG00000196793**	*0.11676647*	*0.43678161*	*0.11676647*	*0.436781609*	*0.410714286*	*0.4360465*	*0.4360465*
**ENSG00000151388**		*0.43939394*		*0.439393939*			*0.2732558*
**ENSG00000145982**	*0.36227545*		*0.36227545*		*0.285714286*	*0.2613636*	
**ENSG00000127418**	*0.15269461*	*0.45454545*	*0.15269461*	*0.454545455*		*0.0581395*	*0.2848837*
**ENSG00000004838**					*0.375*	*0.4886364*	*0.4825581*
**ENSG00000164796**	*0.44382022*	*0.09195402*	*0.44382022*	*0.091954023*	*0.181818182*	*0.1111111*	*0.2613636*
**ENSG00000146674**		*0.2*		*0.2*			
**ENSG00000121879**		*0.19393939*		*0.193939394*			*0.0930233*
**ENSG00000151414**	*0.35057471*	*0.35670732*	*0.35057471*	*0.356707317*	*0.222222222*	*0.2727273*	*0.25*
**ENSG00000100216**		*0.35393258*		*0.353932584*			*0.4651163*
**ENSG00000065970**	*0.16566265*	*0.34545455*	*0.16566265*	*0.345454545*	*0.05952381*		*0.2848837*
**ENSG00000101144**		*0.48333333*		*0.483333333*			*0.4222222*
**ENSG00000135185**		*0.11764706*		*0.117647059*			*0.425*
**ENSG00000131650**	*0.44827586*		*0.44827586*				
**ENSG00000167193**		*0.08181818*		*0.081818182*			*0.1860465*
**ENSG00000177842**		*0.36060606*		*0.360606061*			*0.372093*
**ENSG00000185414**	*0.10179641*	*0.375*	*0.10179641*	*0.375*	*0.266666667*	*0.2386364*	*0.1162791*
**ENSG00000186222**		*0.37575758*		*0.375757576*			*0.3882353*
**ENSG00000136574**		*0.44827586*		*0.448275862*			*0.4772727*
**ENSG00000163291**		*0.22891566*		*0.228915663*			*0.3409091*
**ENSG00000170011**	*0.17964072*	*0.37272727*	*0.17964072*	*0.372727273*	*0.071428571*	*0.1352941*	*0.304878*
**ENSG00000177464**	*0.3742515*	*0.40606061*	*0.3742515*	*0.406060606*	*0.403614458*	*0.3895349*	*0.3895349*
**ENSG00000179603**		*0.38484848*		*0.384848485*			*0.377907*
**ENSG00000187033**		*0.08787879*		*0.087878788*		*0.1104651*	*0.2058824*
**ENSG00000147400**		*0.2030303*		*0.203030303*			*0.1569767*
**ENSG00000124490**		*0.4695122*		*0.469512195*			*0.1395349*
**ENSG00000197467**	*0.24850299*	*0.3*	*0.24850299*	*0.3*	*0.113095238*	*0.1511628*	*0.4318182*
**ENSG00000138002**		*0.40123457*		*0.401234568*			*0.0988372*
**ENSG00000079337**	*0.0988024*	*0.3030303*	*0.0988024*	*0.303030303*	*0.073170732*	*0.0705882*	*0.3372093*
**ENSG00000185437**		*0.06707317*		*0.067073171*			
**ENSG00000108242**		*0.375*		*0.375*			*0.2906977*
**ENSG00000169255**	*0.08682635*	*0.24233129*	*0.08682635*	*0.242331288*			*0.347561*
**ENSG00000143374**	*0.21111111*		*0.21111111*		*0.1*	*0.1444444*	
**ENSG00000162373**	*0.14071856*	*0.16111111*	*0.14071856*	*0.161111111*	*0.220238095*	*0.4244186*	*0.2142857*
**ENSG00000164494**					*0.090909091*	*0.0581395*	*0.0697674*
**ENSG00000176842**		*0.21428571*		*0.214285714*			*0.0764706*
**ENSG00000125753**		*0.32121212*		*0.321212121*			*0.1569767*
**ENSG00000173950**		*0.33333333*		*0.333333333*			*0.3953488*
**ENSG00000141391**		*0.06666667*		*0.066666667*			*0.0777778*
**ENSG00000114279**	*0.17365269*	*0.40449438*	*0.17365269*	*0.404494382*	*0.18452381*	*0.3058824*	*0.4878049*
**ENSG00000157851**		*0.39393939*		*0.393939394*			*0.3139535*
**ENSG00000138792**		*0.39393939*		*0.393939394*			*0.4069767*
**ENSG00000130560**		*0.45757576*		*0.457575758*			*0.4593023*
**ENSG00000008277**	*0.4246988*	*0.3445122*	*0.4246988*	*0.344512195*	*0.160714286*	*0.1627907*	*0.4883721*
**ENSG00000148655**	*0.14371257*		*0.14371257*		*0.303571429*	*0.4534884*	
**ENSG00000081014**		*0.42424242*		*0.424242424*			*0.4244186*
**ENSG00000006788**		*0.07878788*		*0.078787879*			*0.3372093*
**ENSG00000100473**	*0.10778443*	*0.34444444*	*0.10778443*	*0.344444444*	*0.095238095*	*0.1046512*	
**ENSG00000163428**		*0.45757576*		*0.457575758*			*0.3255814*
**ENSG00000180869**		*0.07777778*		*0.077777778*			*0.1777778*
**ENSG00000135587**	*0.25301205*		*0.25301205*		*0.375*	*0.4411765*	
**ENSG00000174010**		*0.21341463*		*0.213414634*			*0.3139535*
**ENSG00000112294**		*0.33536585*		*0.335365854*			*0.0764706*

**ENSG00000103381**		*0.06060606*		*0.060606061*			*0.372093*
**ENSG00000152580**		*0.42727273*		*0.427272727*			*0.4883721*
**ENSG00000153944**		*0.23333333*		*0.233333333*			*0.3604651*
**ENSG00000161664**		*0.25914634*		*0.259146341*			*0.4767442*
**ENSG00000170222**		*0.4030303*		*0.403030303*			*0.3372093*
**ENSG00000130304**		*0.48255814*		*0.48255814*			*0.3604651*
**ENSG00000116833**		*0.43333333*		*0.433333333*			*0.4825581*
**ENSG00000218336**		*0.36666667*		*0.366666667*			*0.1744186*
**ENSG00000162753**		*0.2030303*		*0.203030303*			*0.3953488*
**ENSG00000198056**		*0.3597561*		*0.359756098*			*0.3604651*
**ENSG00000134871**		*0.4695122*		*0.469512195*			*0.2034884*
**ENSG00000173821**		*0.49393939*		*0.493939394*			*0.1511628*
**ENSG00000163576**	*0.40419162*		*0.40419162*		*0.361445783*	*0.3081395*	
**ENSG00000132305**	*0.47005988*		*0.47005988*		*0.208333333*	*0.3869048*	
**ENSG00000187801**					*0.160714286*	*0.1511628*	*0.0963855*
**ENSG00000144451**		*0.41515152*		*0.415151515*			*0.4534884*
**ENSG00000244122**		*0.37222222*		*0.372222222*			*0.1627907*
**ENSG00000204628**	*0.38372093*		*0.38372093*		*0.273809524*	*0.3837209*	*0.3197674*
**ENSG00000256895**	*0.45508982*	*0.0969697*	*0.45508982*	*0.096969697*	*0.345238095*	*0.3313953*	*0.2732558*
**ENSG00000171643**	*0.47904192*	*0.34444444*	*0.47904192*	*0.344444444*	*0.398809524*	*0.4476744*	*0.4545455*
**ENSG00000033867**	*0.22155689*	*0.42352941*	*0.22155689*	*0.423529412*		*0.1046512*	*0.1891892*
**ENSG00000100883**	*0.29341317*		*0.29341317*		*0.4*	*0.4166667*	
**ENSG00000138161**	*0.49401198*		*0.49401198*		*0.5*	*0.4709302*	
**ENSG00000244617**		*0.16969697*		*0.16969697*			*0.3837209*
**ENSG00000089685**	*0.38922156*	*0.43636364*	*0.38922156*	*0.436363636*	*0.494047619*	*0.4127907*	*0.4651163*
**ENSG00000141519**		*0.40909091*		*0.409090909*			*0.4825581*
**ENSG00000127184**	*0.1091954*		*0.1091954*				*0.1882353*
**ENSG00000248905**	*0.07777778*	*0.48876404*	*0.07777778*	*0.488764045*	*0.079545455*	*0.0666667*	*0.4767442*
**ENSG00000023572**	*0.26807229*		*0.26807229*		*0.397590361*	*0.4638554*	
**ENSG00000175137**	*0.2994012*	*0.14242424*	*0.2994012*	*0.142424242*	*0.464285714*	*0.4767442*	*0.4294118*
**ENSG00000132855**	*0.1257485*		*0.1257485*		*0.06547619*	*0.0523256*	
**ENSG00000196230**		*0.18787879*		*0.187878788*			*0.2325581*
**ENSG00000135847**	*0.19879518*		*0.19879518*		*0.285714286*	*0.4244186*	
**ENSG00000151500**	*0.07142857*		*0.07142857*				
**ENSG00000144161**		*0.15454545*		*0.154545455*			
**ENSG00000119685**		*0.38823529*		*0.388235294*	*0.321428571*	*0.4302326*	*0.4534884*
**ENSG00000164181**	*0.27575758*	*0.11666667*	*0.27575758*	*0.116666667*	*0.05952381*	*0.1104651*	*0.1918605*
**ENSG00000163638**		*0.28181818*		*0.281818182*			*0.4534884*
**ENSG00000147364**		*0.28235294*		*0.282352941*			*0.2159091*
**ENSG00000204560**	*0.24719101*		*0.24719101*		*0.420454545*	*0.3604651*	
**ENSG00000106351**		*0.16666667*		*0.166666667*			
**ENSG00000150459**		*0.49085366*		*0.490853659*			*0.3081395*
**ENSG00000174586**		*0.46060606*		*0.460606061*			*0.3068182*
**ENSG00000167972**	*0.25149701*		*0.25149701*				
**ENSG00000213204**		*0.26060606*		*0.260606061*	*0.188888889*	*0.1777778*	*0.2352941*
**ENSG00000161618**		*0.30909091*		*0.309090909*			*0.1860465*
**ENSG00000213722**	*0.13529412*		*0.13529412*		*0.428571429*	*0.4659091*	
**ENSG00000128165**		*0.34242424*		*0.342424242*			*0.3139535*
**ENSG00000105486**	*0.30681818*	*0.44848485*	*0.30681818*	*0.448484848*	*0.088888889*		*0.2151163*
**ENSG00000100220**		*0.16969697*		*0.16969697*			*0.4418605*
**ENSG00000148926**	*0.11363636*		*0.11363636*				
**ENSG00000248098**	*0.29640719*		*0.29640719*		*0.458333333*	*0.4883721*	
**ENSG00000163378**		*0.1969697*		*0.196969697*			*0.4651163*
**ENSG00000177225**		*0.471875*		*0.471875*			*0.4593023*

**ENSG00000187164**		*0.12121212*		*0.121212121*				
**ENSG00000114125**		*0.16969697*		*0.16969697*				*0.1744186*
**ENSG00000118596**	*0.1875*	*0.375*	*0.1875*	*0.375*	*0.126506024*		*0.1802326*	*0.3546512*
**ENSG00000115902**		*0.10135135*		*0.101351351*				
**ENSG00000205300**	*0.18263473*		*0.18263473*		*0.267857143*		*0.2093023*	
**ENSG00000048707**								*0.125*
**ENSG00000089195**		*0.17878788*		*0.178787879*				*0.4069767*
**ENSG00000114416**	*0.16467066*		*0.16467066*					
**ENSG00000135604**								*0.297619*
**ENSG00000119471**		*0.35757576*		*0.357575758*				*0.4069767*
**ENSG00000182606**		*0.39393939*		*0.393939394*				*0.475*
**ENSG00000183963**	*0.30722892*	*0.0969697*	*0.30722892*	*0.096969697*	*0.433333333*		*0.4886364*	*0.4244186*
**ENSG00000198792**		*0.25757576*		*0.257575758*				*0.3255814*
**ENSG00000127463**		*0.14417178*		*0.144171779*				
**ENSG00000167565**	*0.13068182*	*0.14814815*	*0.13068182*	*0.148148148*	*0.111111111*		*0.0909091*	
**ENSG00000134056**	*0.175*		*0.175*		*0.390243902*		*0.2159091*	
**ENSG00000186075**		*0.49444444*		*0.494444444*				*0.2727273*
**ENSG00000184154**		*0.06060606*		*0.060606061*				*0.2202381*
**ENSG00000124596**		*0.1969697*		*0.196969697*				*0.244186*
**ENSG00000175766**	*0.18562874*		*0.18562874*		*0.053571429*			
**ENSG00000137821**	*0.44886364*		*0.44886364*		*0.322222222*		*0.4659091*	
**ENSG00000132635**	*0.45508982*		*0.45508982*		*0.144578313*		*0.1627907*	
**ENSG00000174206**		*0.46060606*		*0.460606061*				*0.4302326*
**ENSG00000171793**		*0.06666667*		*0.066666667*				*0.1569767*
**ENSG00000214021**	*0.16766467*	*0.40909091*	*0.16766467*	*0.409090909*	*0.210843373*		*0.3197674*	*0.4883721*
**ENSG00000108924**		*0.31707317*		*0.317073171*				*0.3837209*
**ENSG00000253207**	*0.48447205*		*0.48447205*		*0.303571429*		*0.3058824*	
**ENSG00000204386**								*0.0872093*
**ENSG00000187533**		*0.14848485*		*0.148484848*				
**ENSG00000136868**		*0.43902439*		*0.43902439*				*0.4777778*
**ENSG00000114439**		*0.19186047*		*0.191860465*			*0.1352941*	*0.0853659*
**ENSG00000171282**		*0.20909091*		*0.209090909*				*0.3023256*
**ENSG00000146733**	*0.08383234*	*0.23595506*	*0.08383234*	*0.235955056*			*0.0882353*	*0.3571429*
**ENSG00000166783**		*0.08181818*		*0.081818182*				*0.1976744*
**ENSG00000107651**		*0.48787879*		*0.487878788*				*0.4176471*
**ENSG00000203485**		*0.42993631*		*0.429936306*				*0.1369048*
**ENSG00000022355**		*0.34662577*		*0.346625767*				*0.4705882*
**ENSG00000184374**								*0.2790698*
**ENSG00000156466**		*0.05151515*		*0.051515152*				
**ENSG00000100902**	*0.37125749*		*0.37125749*		*0.377777778*		*0.4090909*	
**ENSG00000055130**	*0.16111111*		*0.16111111*		*0.375*		*0.4545455*	
**ENSG00000100033**	*0.49444444*		*0.49444444*		*0.288888889*		*0.2840909*	
**ENSG00000188818**	*0.4011976*		*0.4011976*		*0.19047619*		*0.2616279*	*0.1410256*
**ENSG00000119383**	*0.23888889*	*0.26436782*	*0.23888889*	*0.264367816*	*0.4*		*0.3863636*	*0.3895349*
**ENSG00000187244**		*0.33333333*		*0.333333333*				*0.4127907*
**ENSG00000204220**	*0.19277108*		*0.19277108*		*0.355421687*		*0.3255814*	
**ENSG00000135932**		*0.21515152*		*0.215151515*				*0.3139535*
**ENSG00000148396**	*0.15116279*	*0.47878788*	*0.15116279*	*0.478787879*				*0.2093023*
**ENSG00000259024**		*0.07878788*		*0.078787879*				*0.4069767*
**ENSG00000160539**	*0.22754491*	*0.3030303*	*0.22754491*	*0.303030303*	*0.470238095*		*0.4709302*	*0.2470588*
**ENSG00000109670**	*0.08682635*		*0.08682635*		*0.476190476*		*0.4883721*	
**ENSG00000119280**		*0.17575758*		*0.175757576*				*0.2965116*
**ENSG00000176055**		*0.40909091*		*0.409090909*				*0.3372093*
**ENSG00000175899**	*0.39520958*		*0.39520958*		*0.119047619*		*0.1104651*	

**ENSG00000163879**	*0.08982036*	*0.49393939*	*0.08982036*	*0.493939394*	*0.380952381*	*0.2267442*	*0.372093*
**ENSG00000144642**		*0.33529412*		*0.335294118*			*0.4512195*
**ENSG00000160271**	*0.24850299*	*0.40606061*	*0.24850299*	*0.406060606*	*0.488095238*	*0.4764706*	*0.4302326*
**ENSG00000014824**	*0.16060606*	*0.26060606*	*0.16060606*	*0.260606061*			
**ENSG00000107623**		*0.18390805*		*0.183908046*			*0.2613636*
**ENSG00000164002**	*0.25748503*		*0.25748503*		*0.482142857*	*0.3604651*	
**ENSG00000145623**		*0.41717791*		*0.417177914*			*0.4318182*
**ENSG00000180525**							*0.0903614*
**ENSG00000161800**	*0.29640719*		*0.29640719*				
**ENSG00000115840**	*0.40963855*	*0.30555556*	*0.40963855*	*0.305555556*	*0.115853659*	*0.0872093*	*0.2045455*
**ENSG00000149452**	*0.05688623*		*0.05688623*		*0.261904762*	*0.2764706*	
**ENSG00000125900**	*0.41916168*		*0.41916168*		*0.255952381*	*0.2965116*	
**ENSG00000012817**		*0.29545455*		*0.295454545*			
**ENSG00000165588**		*0.0969697*		*0.096969697*			
**ENSG00000163444**		*0.39393939*		*0.393939394*			*0.1744186*
**ENSG00000171631**	*0.18125*	*0.11419753*	*0.18125*	*0.114197531*			*0.0795455*
**ENSG00000151117**		*0.16060606*		*0.160606061*			*0.3470588*
**ENSG00000103326**		*0.24848485*		*0.248484848*			*0.255814*
**ENSG00000164116**	*0.3258427*	*0.3902439*	*0.3258427*	*0.390243902*	*0.226190476*	*0.2616279*	*0.1882353*
**ENSG00000197915**		*0.16363636*		*0.163636364*			*0.3430233*
**ENSG00000187098**	*0.05688623*	*0.28484848*	*0.05688623*	*0.284848485*	*0.482142857*	*0.4360465*	*0.3255814*
**ENSG00000130340**		*0.23939394*		*0.239393939*			*0.1686047*
**ENSG00000137414**	*0.30555556*	*0.24848485*	*0.30555556*	*0.248484848*		*0.0795455*	*0.0581395*
**ENSG00000115461**		*0.05*		*0.05*			*0.0568182*
**ENSG00000158104**	*0.28443114*		*0.28443114*		*0.178571429*	*0.2151163*	
**ENSG00000140463**		*0.08787879*		*0.087878788*			*0.2151163*
**ENSG00000169247**	*0.43113772*	*0.49090909*	*0.43113772*	*0.490909091*	*0.25*	*0.2848837*	*0.3255814*
**ENSG00000196466**		*0.49418605*		*0.494186047*			*0.4204545*
**ENSG00000127603**	*0.46625767*	*0.44848485*	*0.46625767*	*0.448484848*	*0.186746988*	*0.1918605*	*0.3197674*
**ENSG00000204688**	*0.07777778*	*0.4*	*0.07777778*	*0.4*	*0.177777778*	*0.1511628*	*0.4545455*
**ENSG00000214063**	*0.41317365*	*0.48888889*	*0.41317365*	*0.488888889*	*0.44047619*	*0.4418605*	*0.3181818*
**ENSG00000213760**		*0.34545455*		*0.345454545*			*0.4069767*
**ENSG00000115657**	*0.40804598*		*0.40804598*		*0.182926829*	*0.1463415*	
**ENSG00000104866**	*0.13772455*	*0.07575758*	*0.13772455*	*0.075757576*			*0.0764706*
**ENSG00000221866**		*0.25757576*		*0.257575758*			*0.4476744*
**ENSG00000166669**	*0.478125*	*0.38787879*	*0.478125*	*0.387878788*		*0.347561*	*0.4302326*
**ENSG00000182979**	*0.0797546*		*0.0797546*		*0.333333333*	*0.3895349*	
**ENSG00000063322**		*0.41049383*		*0.410493827*			*0.3690476*
**ENSG00000058091**	*0.15568862*	*0.21515152*	*0.15568862*	*0.215151515*	*0.077380952*		*0.25*
**ENSG00000149100**	*0.07228916*	*0.3045977*	*0.07228916*	*0.304597701*			*0.0909091*
**ENSG00000233198**					*0.19047619*	*0.1453488*	
**ENSG00000180432**		*0.45454545*		*0.454545455*			*0.2093023*
**ENSG00000173530**		*0.22424242*		*0.224242424*			*0.3604651*
**ENSG00000178229**	*0.28742515*		*0.28742515*		*0.458333333*	*0.4360465*	
**ENSG00000050820**	*0.36526946*	*0.49444444*	*0.36526946*	*0.494444444*	*0.428571429*	*0.4244186*	*0.5*
**ENSG00000070778**		*0.31818182*		*0.318181818*			*0.377907*
**ENSG00000011105**		*0.45151515*		*0.451515152*			*0.3023256*
**ENSG00000112164**		*0.33333333*		*0.333333333*			*0.3522727*
**ENSG00000122417**		*0.38414634*		*0.384146341*	*0.089285714*	*0.1511628*	*0.3928571*
**ENSG00000114737**	*0.16467066*	*0.16768293*	*0.16467066*	*0.167682927*	*0.337349398*	*0.2674419*	*0.3255814*
**ENSG00000144401**	*0.4251497*	*0.38333333*	*0.4251497*	*0.383333333*	*0.214285714*	*0.1162791*	*0.25*
**ENSG00000114503**	*0.11976048*	*0.31212121*	*0.11976048*	*0.312121212*	*0.083333333*	*0.0930233*	*0.2732558*
**ENSG00000164729**		*0.38109756*		*0.381097561*			
**ENSG00000156218**		*0.24848485*		*0.248484848*			

**ENSG00000121417**	*0.23493976*	*0.14329268*	*0.23493976*	*0.143292683*	*0.329545455*	*0.2682927*	*0.2916667*
**ENSG00000189067**		*0.33333333*		*0.333333333*			*0.4534884*
**ENSG00000174106**		*0.31288344*		*0.312883436*			*0.2034884*
**ENSG00000179886**		*0.39534884*		*0.395348837*			*0.3295455*
**ENSG00000213780**	*0.25149701*	*0.11818182*	*0.25149701*	*0.118181818*	*0.452380952*	*0.3953488*	
**ENSG00000187513**		*0.34545455*		*0.345454545*			*0.3313953*
**ENSG00000128050**		*0.246875*		*0.246875*			*0.4534884*
**ENSG00000203630**	*0.10795455*		*0.10795455*				
**ENSG00000126773**		*0.10606061*		*0.106060606*	*0.144444444*	*0.1666667*	*0.3953488*
**ENSG00000128709**	*0.12727273*	*0.32424242*	*0.12727273*	*0.324242424*	*0.422619048*	*0.4244186*	*0.4176471*
**ENSG00000074590**		*0.38787879*		*0.387878788*			*0.0755814*
**ENSG00000183307**	*0.47305389*	*0.33939394*	*0.47305389*	*0.339393939*	*0.488095238*	*0.4764706*	*0.3941176*
**ENSG00000160050**		*0.26993865*		*0.26993865*			*0.1744186*
**ENSG00000134317**	*0.4491018*	*0.41158537*	*0.4491018*	*0.411585366*			*0.3795181*
**ENSG00000166510**		*0.29268293*		*0.292682927*			*0.3988095*
**ENSG00000106511**	*0.2754491*	*0.3258427*	*0.2754491*	*0.325842697*	*0.273809524*	*0.1802326*	*0.4825581*
**ENSG00000129028**	*0.05389222*	*0.30487805*	*0.05389222*	*0.304878049*	*0.178571429*	*0.127907*	*0.3139535*
**ENSG00000007062**	*0.48802395*	*0.48484848*	*0.48802395*	*0.484848485*	*0.345238095*	*0.2179487*	*0.4186047*
**ENSG00000206562**		*0.3969697*		*0.396969697*			*0.4825581*
**ENSG00000249853**		*0.12121212*		*0.121212121*	*0.193181818*	*0.2*	*0.2777778*
**ENSG00000156687**							*0.0639535*
**ENSG00000119614**		*0.17791411*		*0.17791411*			*0.2941176*
**ENSG00000105825**	*0.15789474*		*0.15789474*				*0.2209302*
**ENSG00000155966**		*0.1030303*		*0.103030303*			*0.1058824*
**ENSG00000121749**		*0.26363636*		*0.263636364*			*0.1802326*
**ENSG00000084112**		*0.27439024*		*0.274390244*			*0.3255814*
**ENSG00000145020**		*0.27575758*		*0.275757576*			*0.1190476*
**ENSG00000186564**	*0.14371257*		*0.14371257*		*0.202380952*	*0.2034884*	
**ENSG00000152620**	*0.18862275*	*0.31111111*	*0.18862275*	*0.311111111*	*0.327380952*	*0.2951807*	*0.1111111*
**ENSG00000113361**		*0.48787879*		*0.487878788*			*0.4476744*
**ENSG00000182870**					*0.208333333*	*0.244186*	
**ENSG00000107485**		*0.22424242*		*0.224242424*			*0.3197674*
**ENSG00000133142**	*0.39820359*		*0.39820359*		*0.476190476*	*0.4352941*	
**ENSG00000197162**		*0.15454545*		*0.154545455*			
**ENSG00000163545**		*0.13333333*		*0.133333333*			*0.1569767*
**ENSG00000116977**	*0.38554217*	*0.36666667*	*0.38554217*	*0.366666667*	*0.457831325*	*0.4883721*	*0.4404762*
**ENSG00000251283**	*0.21987952*	*0.3258427*	*0.21987952*	*0.325842697*			*0.4268293*
**ENSG00000160953**	*0.29041916*	*0.25454545*	*0.29041916*	*0.254545455*	*0.409638554*	*0.3313953*	*0.4411765*
**ENSG00000119121**		*0.46666667*		*0.466666667*			*0.2383721*
**ENSG00000215428**		*0.12121212*		*0.121212121*			*0.4593023*
**ENSG00000184575**	*0.38764045*		*0.38764045*		*0.222222222*	*0.1477273*	
**ENSG00000198626**		*0.34848485*		*0.348484848*			*0.2848837*
**ENSG00000113448**	*0.21856287*	*0.49425287*	*0.21856287*	*0.494252874*	*0.392857143*	*0.377907*	*0.4156627*
**ENSG00000197324**		*0.09815951*		*0.098159509*			*0.1369048*
**ENSG00000140522**	*0.10240964*	*0.38109756*	*0.10240964*	*0.381097561*			*0.202381*
**ENSG00000164556**	*0.25449102*	*0.09393939*	*0.25449102*	*0.093939394*	*0.470238095*	*0.4651163*	
**ENSG00000188828**		*0.37037037*		*0.37037037*			*0.3430233*
**ENSG00000172782**		*0.2183908*		*0.218390805*			*0.3837209*
**ENSG00000119333**		*0.13939394*		*0.139393939*			*0.4767442*
**ENSG00000163938**	*0.48203593*		*0.48203593*		*0.494047619*	*0.4767442*	
**ENSG00000168807**		*0.29878049*		*0.298780488*			*0.4709302*
**ENSG00000130037**	*0.43712575*	*0.33536585*	*0.43712575*	*0.335365854*	*0.337349398*	*0.452381*	*0.4534884*
**ENSG00000104825**		*0.26687117*		*0.266871166*			*0.372093*
**ENSG00000255317**	*0.25748503*		*0.25748503*		*0.398809524*	*0.3313953*	

**ENSG00000116005**	*0.12643678*	*0.2816092*	*0.12643678*	*0.281609195*	*0.088888889*		*0.2386364*	*0.3636364*
**ENSG00000214706**					*0.232142857*		*0.3333333*	
**ENSG00000101544**		*0.38888889*		*0.388888889*				*0.125*
**ENSG00000198298**	*0.09444444*		*0.09444444*					
**ENSG00000117676**	*0.29518072*	*0.44545455*	*0.29518072*	*0.445454545*	*0.232142857*		*0.25*	*0.2732558*
**ENSG00000170345**					*0.392857143*		*0.4825581*	
**ENSG00000152503**	*0.19375*		*0.19375*					*0.3977273*
**ENSG00000048545**	*0.42814371*		*0.42814371*		*0.138554217*		*0.1352941*	
**ENSG00000016391**	*0.21341463*	*0.08333333*	*0.21341463*	*0.083333333*	*0.05*		*0.1590909*	*0.0731707*
**ENSG00000187714**	*0.06586826*		*0.06586826*		*0.267857143*		*0.2093023*	
**ENSG00000160097**		*0.39329268*		*0.393292683*				*0.2294118*
**ENSG00000161940**	*0.1257485*	*0.08333333*	*0.1257485*	*0.083333333*				
**ENSG00000204619**	*0.05688623*	*0.10555556*	*0.05688623*	*0.105555556*	*0.05952381*		*0.1104651*	*0.3488372*
**ENSG00000166682**	*0.13483146*	*0.34848485*	*0.13483146*	*0.348484848*	*0.297619048*		*0.4186047*	*0.1860465*
**ENSG00000163743**		*0.4*		*0.4*				*0.3488372*
**ENSG00000139880**		*0.40243902*		*0.402439024*				*0.1395349*
**ENSG00000175643**		*0.26666667*		*0.266666667*				*0.3214286*
**ENSG00000138755**		*0.49393939*		*0.493939394*				*0.0714286*
**ENSG00000112624**		*0.06441718*		*0.064417178*				*0.1130952*
**ENSG00000108395**	*0.15361446*		*0.15361446*		*0.5*		*0.4294118*	
**ENSG00000120279**		*0.45757576*		*0.457575758*				*0.4418605*
**ENSG00000139324**		*0.21515152*		*0.215151515*				
**ENSG00000257482**	*0.40662651*		*0.40662651*		*0.234939759*		*0.3488372*	
**ENSG00000172757**	*0.38922156*		*0.38922156*		*0.06547619*		*0.0639535*	
**ENSG00000139055**	*0.43712575*		*0.43712575*		*0.130952381*		*0.1744186*	
**ENSG00000128294**		*0.45757576*		*0.457575758*				*0.372093*
**ENSG00000165732**		*0.37931034*		*0.379310345*				*0.125*
**ENSG00000115526**	*0.35928144*	*0.47878788*	*0.35928144*	*0.478787879*	*0.375*		*0.3430233*	*0.3081395*
**ENSG00000070476**		*0.4969697*		*0.496969697*				*0.4709302*
**ENSG00000148120**		*0.22699387*		*0.226993865*	*0.077380952*		*0.0930233*	*0.4186047*
**ENSG00000139263**								*0.1022727*
**ENSG00000108551**		*0.18235294*		*0.182352941*				*0.3333333*
**ENSG00000107959**	*0.45454545*	*0.325*	*0.45454545*	*0.325*	*0.38372093*		*0.3333333*	*0.255814*
**ENSG00000156531**		*0.20253165*		*0.202531646*				*0.4235294*
**ENSG00000152942**	*0.45783133*		*0.45783133*		*0.103658537*		*0.1506024*	
**ENSG00000119906**		*0.17222222*		*0.172222222*				*0.4431818*
**ENSG00000117523**		*0.2*		*0.2*				*0.1686747*
**ENSG00000103544**		*0.34545455*		*0.345454545*				*0.4470588*
**ENSG00000182473**	*0.34269663*	*0.5*	*0.34269663*	*0.5*	*0.077777778*		*0.1477273*	*0.4476744*
**ENSG00000115935**		*0.48466258*		*0.484662577*				*0.127907*
**ENSG00000198189**					*0.136904762*		*0.1104651*	
**ENSG00000133657**		*0.20606061*		*0.206060606*	*0.144444444*		*0.1022727*	*0.2235294*
**ENSG00000154917**		*0.23333333*		*0.233333333*				*0.4302326*
**ENSG00000204610**	*0.2994012*	*0.0969697*	*0.2994012*	*0.096969697*	*0.095238095*		*0.1235294*	*0.1744186*
**ENSG00000151883**		*0.38535032*		*0.385350318*				*0.3470588*
**ENSG00000125149**		*0.44817073*		*0.448170732*				*0.4244186*
**ENSG00000149218**		*0.47701149*		*0.477011494*				*0.377907*
**ENSG00000132879**		*0.43333333*		*0.433333333*				*0.3809524*
**ENSG00000173114**	*0.21556886*		*0.21556886*		*0.31547619*		*0.2325581*	
**ENSG00000215595**		*0.46363636*		*0.463636364*				*0.2965116*
**ENSG00000167461**		*0.31666667*		*0.316666667*				*0.3313953*
**ENSG00000133103**		*0.38050314*		*0.380503145*				*0.4767442*
**ENSG00000171903**	*0.25748503*	*0.45454545*	*0.25748503*	*0.454545455*	*0.355263158*		*0.4125*	*0.3181818*
**ENSG00000197044**		*0.36363636*		*0.363636364*				*0.4127907*

**ENSG00000132297**		*0.32777778*		*0.327777778*			*0.5*
**ENSG00000213221**		*0.45426829*		*0.454268293*			*0.3289474*
**ENSG00000234745**		*0.45*		*0.45*			*0.2906977*
**ENSG00000169756**		*0.47222222*		*0.472222222*			*0.1363636*
**ENSG00000081665**		*0.34242424*		*0.342424242*			*0.3430233*
**ENSG00000061936**	*0.32121212*	*0.24848485*	*0.32121212*	*0.248484848*			
**ENSG00000140320**	*0.07784431*		*0.07784431*				
**ENSG00000163110**		*0.49444444*		*0.494444444*			*0.4642857*
**ENSG00000182752**	*0.49431818*	*0.43333333*	*0.49431818*	*0.433333333*	*0.433333333*	*0.4318182*	*0.4069767*
**ENSG00000137747**	*0.38181818*	*0.35060976*	*0.38181818*	*0.350609756*	*0.353658537*	*0.3470588*	*0.4204545*
**ENSG00000079387**		*0.18787879*		*0.187878788*	*0.238095238*	*0.1931818*	*0.1976744*
**ENSG00000115956**	*0.34431138*	*0.42987805*	*0.34431138*	*0.429878049*	*0.244047619*	*0.3023256*	*0.2111111*
**ENSG00000156508**	*0.43712575*	*0.24444444*	*0.43712575*	*0.244444444*	*0.452380952*	*0.3837209*	*0.3181818*
**ENSG00000058063**	*0.37724551*	*0.16470588*	*0.37724551*	*0.164705882*		*0.0697674*	*0.4418605*
**ENSG00000167800**		*0.45555556*		*0.455555556*			*0.3863636*
**ENSG00000180155**	*0.1746988*	*0.22777778*	*0.1746988*	*0.227777778*	*0.470238095*	*0.3764706*	*0.373494*
**ENSG00000233488**		*0.22988506*		*0.229885057*			*0.4883721*
**ENSG00000168763**		*0.07777778*		*0.077777778*			
**ENSG00000133063**		*0.09770115*		*0.097701149*			*0.1022727*
**ENSG00000129932**	*0.35227273*	*0.25454545*	*0.35227273*	*0.254545455*	*0.3*	*0.3295455*	*0.4127907*
**ENSG00000143674**		*0.46969697*		*0.46969697*			*0.3197674*
**ENSG00000155666**	*0.26047904*	*0.42682927*	*0.26047904*	*0.426829268*			*0.4651163*
**ENSG00000181885**		*0.45*		*0.45*			*0.2616279*
**ENSG00000155850**		*0.2030303*		*0.203030303*			*0.4047619*
**ENSG00000077935**		*0.46363636*		*0.463636364*			*0.4941176*
**ENSG00000167311**	*0.2994012*	*0.22424242*	*0.2994012*	*0.224242424*	*0.458333333*	*0.4651163*	*0.2529412*
**ENSG00000100979**	*0.39520958*		*0.39520958*		*0.446428571*	*0.372093*	
**ENSG00000110367**		*0.40229885*		*0.402298851*			*0.4772727*
**ENSG00000213512**		*0.38372093*		*0.38372093*			*0.195122*
**ENSG00000178385**		*0.35757576*		*0.357575758*			
**ENSG00000112539**	*0.38787879*		*0.38787879*				
**ENSG00000073921**		*0.20606061*		*0.206060606*			*0.255814*
**ENSG00000137491**	*0.43712575*	*0.19393939*	*0.43712575*	*0.193939394*	*0.404761905*	*0.4476744*	*0.4011628*
**ENSG00000110713**		*0.47575758*		*0.475757576*			*0.4127907*
**ENSG00000111752**	*0.48863636*	*0.48447205*	*0.48863636*	*0.48447205*	*0.255555556*	*0.3409091*	*0.4883721*
**ENSG00000204138**		*0.29754601*		*0.297546012*			*0.0930233*
**ENSG00000164307**	*0.47752809*	*0.35757576*	*0.47752809*	*0.357575758*	*0.466666667*	*0.5*	*0.372093*
**ENSG00000122779**	*0.40718563*	*0.28658537*	*0.40718563*	*0.286585366*			*0.4360465*
**ENSG00000165794**		*0.2969697*		*0.296969697*			*0.4186047*
**ENSG00000104549**	*0.29041916*		*0.29041916*				*0.0909091*
**ENSG00000177398**	*0.19161677*	*0.42987805*	*0.19161677*	*0.429878049*			*0.494186*
**ENSG00000107371**		*0.16158537*		*0.161585366*			*0.127907*
**ENSG00000095110**	*0.4494382*		*0.4494382*		*0.410714286*	*0.4709302*	
**ENSG00000131626**	*0.13173653*	*0.37423313*	*0.13173653*	*0.374233129*	*0.125*	*0.1860465*	*0.2093023*
**ENSG00000152556**	*0.36227545*	*0.39634146*	*0.36227545*	*0.396341463*	*0.267857143*	*0.1931818*	*0.2325581*
**ENSG00000100644**		*0.1030303*		*0.103030303*			*0.2383721*
**ENSG00000248727**		*0.21511628*		*0.215116279*			*0.2954545*
**ENSG00000115942**	*0.48333333*		*0.48333333*				*0.0581395*
**ENSG00000227975**		*0.49386503*		*0.493865031*			*0.402439*
**ENSG00000134287**	*0.45151515*	*0.34146341*	*0.45151515*	*0.341463415*			*0.4767442*
**ENSG00000196071**		*0.18787879*		*0.187878788*			*0.4*
**ENSG00000130921**	*0.33832335*	*0.23030303*	*0.33832335*	*0.23030303*	*0.416666667*	*0.2616279*	
**ENSG00000172667**		*0.39939024*		*0.399390244*	*0.083333333*		*0.0568182*
**ENSG00000008516**		*0.10493827*		*0.104938272*			

**ENSG00000104938**		*0.31212121*		*0.312121212*			*0.3430233*
**ENSG00000127831**		*0.38181818*		*0.381818182*			*0.1235294*
**ENSG00000132024**	*0.31736527*	*0.28181818*	*0.31736527*	*0.281818182*			*0.2882353*
**ENSG00000100387**		*0.42727273*		*0.427272727*			*0.2209302*
**ENSG00000008083**		*0.14666667*		*0.146666667*			
**ENSG00000143641**		*0.45757576*		*0.457575758*			*0.4772727*
**ENSG00000169129**		*0.18787879*		*0.187878788*			*0.3895349*
**ENSG00000162706**		*0.12643678*		*0.126436782*			*0.4545455*
**ENSG00000069974**		*0.22413793*		*0.224137931*			*0.125*
**ENSG00000166436**		*0.33636364*		*0.336363636*			*0.4360465*
**ENSG00000166398**	*0.19161677*	*0.3597561*	*0.19161677*	*0.359756098*			*0.1802326*
**ENSG00000232774**		*0.20552147*		*0.205521472*			*0.4879518*
**ENSG00000153814**		*0.25842697*		*0.258426966*			*0.3636364*
**ENSG00000160282**					*0.380952381*	*0.4411765*	*0.5*
**ENSG00000091073**		*0.35882353*		*0.358823529*			*0.4204545*
**ENSG00000123352**					*0.125*	*0.127907*	
**ENSG00000170500**		*0.5*		*0.5*			*0.4767442*
**ENSG00000111647**	*0.3742515*	*0.22424242*	*0.3742515*	*0.224242424*	*0.071428571*		
**ENSG00000115896**		*0.5*		*0.5*			*0.4204545*
**ENSG00000182087**		*0.10606061*		*0.106060606*			
**ENSG00000145555**	*0.2245509*		*0.2245509*		*0.111111111*	*0.1590909*	
**ENSG00000171208**		*0.05454545*		*0.054545455*			
**ENSG00000186642**	*0.13473054*	*0.31402439*	*0.13473054*	*0.31402439*			*0.2616279*
**ENSG00000179314**		*0.43478261*		*0.434782609*			*0.4883721*
**ENSG00000132664**	*0.28488372*	*0.18902439*	*0.28488372*	*0.18902439*			*0.4404762*
**ENSG00000110315**		*0.48876404*		*0.488764045*			*0.4204545*
**ENSG00000198961**		*0.46932515*		*0.469325153*		*0.0813953*	*0.3409091*
**ENSG00000144868**	*0.23913043*	*0.46969697*	*0.23913043*	*0.46969697*	*0.452380952*	*0.4593023*	*0.4593023*
**ENSG00000143184**		*0.28787879*		*0.287878788*			*0.2409639*
**ENSG00000187240**	*0.44011976*	*0.32621951*	*0.44011976*	*0.326219512*	*0.321428571*	*0.3023256*	*0.4767442*
**ENSG00000163686**		*0.43030303*		*0.43030303*			*0.2965116*
**ENSG00000097096**		*0.0625*		*0.0625*			*0.0609756*
**ENSG00000017797**	*0.30421687*	*0.30681818*	*0.30421687*	*0.306818182*		*0.0647059*	*0.4*
**ENSG00000147113**		*0.48170732*		*0.481707317*			*0.4777778*
**ENSG00000198734**	*0.4491018*		*0.4491018*		*0.345238095*	*0.4709302*	*0.313253*
**ENSG00000100522**		*0.38095238*		*0.380952381*			*0.4090909*
**ENSG00000219438**		*0.43030303*		*0.43030303*			*0.2619048*
**ENSG00000006756**		*0.06134969*		*0.061349693*			*0.3529412*
**ENSG00000164574**		*0.31288344*		*0.312883436*			*0.4709302*
**ENSG00000240021**	*0.12275449*	*0.36060606*	*0.12275449*	*0.360606061*			*0.4651163*
**ENSG00000179542**	*0.20555556*	*0.36280488*	*0.20555556*	*0.362804878*	*0.465909091*	*0.4772727*	*0.4883721*
**ENSG00000173334**	*0.11976048*	*0.30487805*	*0.11976048*	*0.304878049*	*0.215909091*	*0.1818182*	*0.127907*
**ENSG00000071564**		*0.23333333*		*0.233333333*			*0.4127907*
**ENSG00000179933**		*0.47619048*		*0.476190476*			*0.4333333*
**ENSG00000197953**		*0.29878049*		*0.298780488*			*0.4352941*
**ENSG00000204569**							*0.0872093*
**ENSG00000016864**	*0.10778443*	*0.33636364*	*0.10778443*	*0.336363636*	*0.476190476*	*0.5*	*0.5*
**ENSG00000177485**		*0.4*		*0.4*			*0.5*
**ENSG00000188000**		*0.31481481*		*0.314814815*			*0.4882353*
**ENSG00000128604**					*0.06547619*	*0.0872093*	
**ENSG00000176974**	*0.13333333*	*0.3372093*	*0.13333333*	*0.337209302*	*0.222222222*	*0.2272727*	*0.0697674*
**ENSG00000168594**	*0.20359281*		*0.20359281*		*0.078313253*		
**ENSG00000131944**	*0.28977273*	*0.12941176*	*0.28977273*	*0.129411765*	*0.369047619*	*0.2906977*	*0.0681818*
**ENSG00000161594**	*0.24850299*		*0.24850299*		*0.222891566*	*0.122093*	

**ENSG00000136881**		*0.19393939*		*0.193939394*				*0.2906977*
**ENSG00000170509**		*0.44817073*		*0.448170732*				*0.372093*
**ENSG00000103168**	*0.21686747*	*0.48484848*	*0.21686747*	*0.484848485*	*0.482142857*		*0.4777778*	*0.4825581*
**ENSG00000167483**	*0.2739726*	*0.26969697*	*0.2739726*	*0.26969697*	*0.313253012*		*0.3176471*	*0.122093*
**ENSG00000163684**	*0.41666667*	*0.40490798*	*0.41666667*	*0.404907975*	*0.186046512*		*0.1904762*	*0.2325581*
**ENSG00000088833**	*0.34659091*	*0.48787879*	*0.34659091*	*0.487878788*	*0.255555556*		*0.3522727*	*0.3139535*
**ENSG00000131914**		*0.48780488*		*0.487804878*				*0.1022727*
**ENSG00000123124**								*0.0731707*
**ENSG00000120500**	*0.12777778*		*0.12777778*		*0.444444444*		*0.3863636*	
**ENSG00000180259**	*0.5*		*0.5*		*0.30952381*		*0.2848837*	
**ENSG00000103811**		*0.30337079*		*0.303370787*				*0.1882353*
**ENSG00000001629**		*0.45454545*		*0.454545455*				*0.5*
**ENSG00000140941**	*0.37650602*	*0.14848485*	*0.37650602*	*0.148484848*				*0.4244186*
**ENSG00000160223**		*0.47752809*		*0.47752809*				*0.4207317*
**ENSG00000145414**	*0.21666667*	*0.25454545*	*0.21666667*	*0.254545455*	*0.233333333*		*0.2386364*	
**ENSG00000249160**		*0.44047619*		*0.44047619*				*0.1627907*
**ENSG00000112394**		*0.44444444*		*0.444444444*				*0.3895349*
**ENSG00000226757**					*0.469135802*		*0.4418605*	
**ENSG00000106066**	*0.31137725*		*0.31137725*					
**ENSG00000188603**	*0.19760479*		*0.19760479*					
**ENSG00000167165**	*0.43333333*	*0.22777778*	*0.43333333*	*0.227777778*	*0.233333333*		*0.1477273*	*0.1590909*
**ENSG00000099399**		*0.34242424*		*0.342424242*				*0.0523256*
**ENSG00000121957**	*0.13636364*	*0.36060606*	*0.13636364*	*0.360606061*				*0.494186*
**ENSG00000160691**					*0.078313253*			*0.0697674*
**ENSG00000215421**		*0.21515152*		*0.215151515*				
**ENSG00000146067**		*0.3030303*		*0.303030303*				*0.3488372*
**ENSG00000171421**	*0.25748503*		*0.25748503*		*0.398809524*		*0.4709302*	
**ENSG00000183798**		*0.17575758*		*0.175757576*				*0.4476744*
**ENSG00000146469**		*0.43030303*		*0.43030303*				*0.2176471*
**ENSG00000064651**		*0.25555556*		*0.255555556*				*0.4*
**ENSG00000148488**		*0.37640449*		*0.376404494*				*0.1777778*
**ENSG00000251369**		*0.26666667*		*0.266666667*				*0.1162791*
**ENSG00000185664**	*0.16666667*		*0.16666667*					
**ENSG00000197563**	*0.13173653*		*0.13173653*		*0.18452381*		*0.2034884*	
**ENSG00000196850**		*0.33333333*		*0.333333333*				*0.494186*
**ENSG00000090487**		*0.46060606*		*0.460606061*				*0.0813953*
**ENSG00000157554**		*0.42424242*		*0.424242424*	*0.244444444*		*0.1590909*	*0.4011628*
**ENSG00000198814**		*0.22077922*		*0.220779221*				*0.1444444*
**ENSG00000163041**		*0.31097561*		*0.31097561*				*0.25*
**ENSG00000198265**		*0.4601227*		*0.460122699*				*0.3197674*
**ENSG00000186891**								*0.1918605*
**ENSG00000174307**		*0.37345679*		*0.37345679*				*0.3863636*
**ENSG00000250493**	*0.11976048*	*0.3969697*	*0.11976048*	*0.396969697*				*0.4244186*
**ENSG00000171763**	*0.24550898*		*0.24550898*		*0.15060241*		*0.1411765*	
**ENSG00000230657**		*0.16463415*		*0.164634146*				*0.1860465*
**ENSG00000127445**	*0.17222222*	*0.45*	*0.17222222*	*0.45*				*0.3863636*
**ENSG00000114248**		*0.37272727*		*0.372727273*				*0.2034884*
**ENSG00000179598**		*0.40449438*		*0.404494382*				*0.3295455*
**ENSG00000103067**		*0.10670732*		*0.106707317*				*0.1428571*
**ENSG00000164068**		*0.46666667*		*0.466666667*				*0.2383721*
**ENSG00000137760**		*0.27878788*		*0.278787879*				*0.3023256*
**ENSG00000156170**	*0.36890244*	*0.23888889*	*0.36890244*	*0.238888889*	*0.244047619*		*0.2616279*	*0.2529412*
**ENSG00000180354**	*0.49700599*	*0.43636364*	*0.49700599*	*0.436363636*	*0.398809524*		*0.3372093*	*0.5*
**ENSG00000146281**		*0.3030303*		*0.303030303*				*0.2034884*

**ENSG00000010704**		*0.37272727*		*0.372727273*	*0.125*			*0.1511628*
**ENSG00000141985**		*0.27607362*		*0.27607362*				
**ENSG00000197006**	*0.22754491*		*0.22754491*		*0.44047619*		*0.4588235*	
**ENSG00000011478**		*0.12359551*		*0.123595506*				*0.1555556*
**ENSG00000136040**		*0.21818182*		*0.218181818*				*0.1860465*
**ENSG00000188984**	*0.44827586*	*0.29090909*	*0.44827586*	*0.290909091*	*0.375*		*0.4625*	*0.255814*
**ENSG00000143669**		*0.42121212*		*0.421212121*				*0.1104651*
**ENSG00000171847**	*0.17045455*	*0.21428571*	*0.17045455*	*0.214285714*	*0.133333333*		*0.0909091*	*0.3409091*
**ENSG00000163512**	*0.44207317*	*0.29090909*	*0.44207317*	*0.290909091*	*0.168674699*		*0.202381*	*0.2529412*
**ENSG00000171444**		*0.47272727*		*0.472727273*				*0.377907*
**ENSG00000142627**								*0.0755814*
**ENSG00000184194**		*0.24390244*		*0.243902439*				*0.2529412*
**ENSG00000057935**		*0.47126437*		*0.471264368*				*0.25*
**ENSG00000116198**		*0.4969697*		*0.496969697*				*0.4767442*
**ENSG00000112964**	*0.40419162*	*0.45454545*	*0.40419162*	*0.454545455*	*0.146341463*		*0.1190476*	*0.4186047*
**ENSG00000150627**		*0.24848485*		*0.248484848*				*0.3117647*
**ENSG00000099804**		*0.37974684*		*0.379746835*				*0.4634146*
**ENSG00000137808**								*0.2034884*
**ENSG00000001617**					*0.345238095*		*0.494186*	
**ENSG00000259030**		*0.32222222*		*0.322222222*				*0.3197674*
**ENSG00000124257**	*0.14371257*	*0.41158537*	*0.14371257*	*0.411585366*	*0.482142857*		*0.4534884*	*0.2764706*
**ENSG00000139436**		*0.26666667*		*0.266666667*				*0.3081395*
**ENSG00000177596**	*0.17964072*	*0.33636364*	*0.17964072*	*0.336363636*	*0.148809524*		*0.2034884*	*0.4011628*
**ENSG00000125691**		*0.47878788*		*0.478787879*				*0.4825581*
**ENSG00000082996**		*0.27878788*		*0.278787879*	*0.398809524*		*0.3488372*	*0.1686047*
**ENSG00000110887**		*0.26060606*		*0.260606061*				*0.494186*
**ENSG00000135426**	*0.43975904*	*0.32121212*	*0.43975904*	*0.321212121*	*0.089285714*		*0.1162791*	*0.3837209*
**ENSG00000124693**		*0.35582822*		*0.355828221*				*0.1104651*
**ENSG00000108474**		*0.47272727*		*0.472727273*				*0.0930233*
**ENSG00000168826**	*0.19879518*	*0.22727273*	*0.19879518*	*0.227272727*	*0.345238095*		*0.3588235*	*0.3588235*
**ENSG00000138400**	*0.2994012*	*0.1097561*	*0.2994012*	*0.109756098*			*0.0581395*	*0.4825581*
**ENSG00000065665**	*0.13580247*	*0.47575758*	*0.13580247*	*0.475757576*			*0.4880952*	*0.4659091*
**ENSG00000067221**		*0.2347561*		*0.234756098*				*0.3604651*
**ENSG00000177830**		*0.49695122*		*0.49695122*				*0.2678571*
**ENSG00000050344**	*0.17045455*		*0.17045455*					*0.0872093*
**ENSG00000115593**	*0.24545455*	*0.29393939*	*0.24545455*	*0.293939394*	*0.077777778*		*0.0777778*	*0.4709302*
**ENSG00000171004**		*0.27300613*		*0.273006135*				*0.3117647*
**ENSG00000124772**		*0.46036585*		*0.460365854*				*0.4821429*
**ENSG00000145545**		*0.46060606*		*0.460606061*				*0.3139535*
**ENSG00000166037**		*0.49079755*		*0.490797546*				*0.4285714*
**ENSG00000114098**		*0.09393939*		*0.093939394*				
**ENSG00000115368**		*0.33333333*		*0.333333333*				*0.3662791*
**ENSG00000049246**	*0.09202454*		*0.09202454*		*0.291666667*		*0.2352941*	
**ENSG00000117593**	*0.13772455*		*0.13772455*					
**ENSG00000165175**					*0.375*		*0.4883721*	
**ENSG00000162843**		*0.42777778*		*0.427777778*				*0.4204545*
**ENSG00000254986**	*0.41017964*	*0.24444444*	*0.41017964*	*0.244444444*	*0.285714286*		*0.4939024*	*0.1931818*
**ENSG00000023228**		*0.39090909*		*0.390909091*				*0.2159091*
**ENSG00000108654**		*0.06111111*		*0.061111111*	*0.339285714*		*0.3139535*	
**ENSG00000068024**	*0.33433735*	*0.43333333*	*0.33433735*	*0.433333333*	*0.410714286*		*0.3430233*	*0.4294118*
**ENSG00000163519**	*0.09580838*	*0.48787879*	*0.09580838*	*0.487878788*				*0.122093*
**ENSG00000164414**	*0.17777778*	*0.47222222*	*0.17777778*	*0.472222222*	*0.466666667*		*0.4888889*	*0.4940476*
**ENSG00000183273**	*0.2005988*	*0.33030303*	*0.2005988*	*0.33030303*	*0.398809524*		*0.4360465*	*0.1918605*
**ENSG00000110422**		*0.37878788*		*0.378787879*				*0.3988095*

**ENSG00000087470**	0.16766467	0.16060606	0.16766467	0.160606061	0.053571429			0.0731707
**ENSG00000105928**	0.2	0.14848485	0.2	0.148484848	0.476190476		0.4651163	0.2209302
**ENSG00000115289**					0.154761905		0.1764706	
**ENSG00000143845**	0.11627907		0.11627907					
**ENSG00000100030**		0.5		0.5				0.377907
**ENSG00000064393**		0.33333333		0.333333333				0.2093023
**ENSG00000141376**		0.07575758		0.075757576				
**ENSG00000102854**		0.19767442		0.197674419				0.2954545
**ENSG00000137177**		0.45555556		0.455555556				0.2848837
**ENSG00000174501**		0.3030303		0.303030303				0.3837209
**ENSG00000136653**		0.46551724		0.465517241				0.4545455
**ENSG00000204482**		0.26060606		0.260606061				0.2674419
**ENSG00000213689**					0.06626506			
**ENSG00000137216**		0.08787879		0.087878788				0.2674419
**ENSG00000163975**		0.31288344		0.312883436				0.1117647
**ENSG00000088727**	0.23652695		0.23652695		0.464285714		0.4244186	0.1337209
**ENSG00000164934**		0.21818182		0.218181818				0.3488372
**ENSG00000204410**	0.26946108	0.27575758	0.26946108	0.275757576	0.172619048		0.3430233	0.4470588
**ENSG00000105519**		0.3		0.3				0.2093023
**ENSG00000159588**		0.32317073		0.323170732				0.3604651
**ENSG00000112110**		0.47272727		0.472727273				0.1802326
**ENSG00000189023**	0.26666667	0.43865031	0.26666667	0.438650307				0.2267442
**ENSG00000180881**	0.18263473	0.27743902	0.18263473	0.277439024	0.19047619		0.1647059	0.1647059
**ENSG00000112242**		0.25		0.25				0.2195122
**ENSG00000113300**		0.19207317		0.192073171				0.0872093
**ENSG00000153147**		0.48787879		0.487878788				0.4777778
**ENSG00000120341**	0.40718563	0.35454545	0.40718563	0.354545455	0.404761905		0.4011628	0.4
**ENSG00000150054**	0.11875	0.06097561	0.11875	0.06097561	0.420454545		0.4302326	0.4476744
**ENSG00000238227**		0.08888889		0.088888889				
**ENSG00000136754**		0.17777778		0.177777778				
**ENSG00000140450**		0.36060606		0.360606061				0.2383721
**ENSG00000198839**		0.0625		0.0625				
**ENSG00000067840**		0.08333333		0.083333333				0.1931818
**ENSG00000143442**								0.2882353
**ENSG00000176293**	0.36227545	0.4	0.36227545	0.4	0.452380952		0.4186047	0.3777778
**ENSG00000164327**		0.39939024		0.399390244				0.5
**ENSG00000204956**		0.20224719		0.202247191				0.1477273
**ENSG00000100580**		0.47752809		0.47752809				0.452381
**ENSG00000205659**	0.39520958	0.2	0.39520958	0.2	0.222222222		0.2954545	0.3197674
**ENSG00000135248**		0.47222222		0.472222222	0.297619048		0.3081395	0.4545455
**ENSG00000121380**	0.11746988	0.26060606	0.11746988	0.260606061	0.375		0.3111111	0.3662791
**ENSG00000160801**		0.36666667		0.366666667				0.4476744
**ENSG00000182827**		0.20606061		0.206060606				0.0823529
**ENSG00000132912**	0.31137725		0.31137725		0.291666667		0.2034884	0.0988372
**ENSG00000084070**					0.061728395			
**ENSG00000134640**		0.48181818		0.481818182				0.3372093
**ENSG00000112249**	0.3988764	0.48181818	0.3988764	0.481818182	0.25		0.2840909	0.4534884
**ENSG00000125245**	0.0508982		0.0508982					
**ENSG00000099994**		0.2030303		0.203030303				0.4593023
**ENSG00000143819**		0.08841463		0.088414634				0.0697674
**ENSG00000175416**		0.30434783		0.304347826				0.3176471
**ENSG00000175591**	0.2754491		0.2754491					
**ENSG00000083814**		0.43030303		0.43030303				0.25
**ENSG00000110921**	0.49700599	0.20606061	0.49700599	0.206060606	0.166666667		0.1686047	0.127907

**ENSG00000130032**	*0.26111111*	*0.3969697*	*0.26111111*	*0.396969697*	*0.177777778*	*0.125*	*0.4476744*
**ENSG00000177971**	*0.44409938*		*0.44409938*		*0.233333333*	*0.4767442*	
**ENSG00000166887**		*0.1*		*0.1*			*0.1744186*
**ENSG00000111581**	*0.26047904*	*0.25316456*	*0.26047904*	*0.253164557*	*0.322222222*	*0.2888889*	
**ENSG00000172139**		*0.33939394*		*0.339393939*			*0.4825581*
**ENSG00000112486**	*0.41017964*	*0.15454545*	*0.41017964*	*0.154545455*	*0.4*	*0.4659091*	
**ENSG00000124224**	*0.23939394*	*0.48484848*	*0.23939394*	*0.484848485*	*0.494047619*	*0.377907*	*0.4767442*
**ENSG00000258256**		*0.34269663*		*0.342696629*			*0.3888889*
**ENSG00000130311**		*0.31402439*		*0.31402439*			*0.2613636*
**ENSG00000158417**		*0.4*		*0.4*			*0.2840909*
**ENSG00000117650**		*0.21341463*		*0.213414634*			*0.5*
**ENSG00000057019**		*0.42424242*		*0.424242424*			*0.2906977*
**ENSG00000197977**		*0.48181818*		*0.481818182*			*0.255814*
**ENSG00000124019**	*0.18562874*	*0.18181818*	*0.18562874*	*0.181818182*			*0.2848837*
**ENSG00000114054**		*0.26666667*		*0.266666667*			*0.1931818*
**ENSG00000117362**		*0.17272727*		*0.172727273*	*0.297619048*	*0.3255814*	
**ENSG00000118231**							*0.4186047*
**ENSG00000071655**		*0.34090909*		*0.340909091*			*0.4666667*
**ENSG00000025423**	*0.19760479*		*0.19760479*		*0.267857143*	*0.2209302*	
**ENSG00000162594**	*0.47904192*	*0.30606061*	*0.47904192*	*0.306060606*	*0.369047619*	*0.377907*	*0.3255814*
**ENSG00000138185**		*0.47878788*		*0.478787879*			*0.3452381*
**ENSG00000166145**		*0.16666667*		*0.166666667*			*0.2529412*
**ENSG00000139624**	*0.30722892*	*0.42424242*	*0.30722892*	*0.424242424*	*0.297619048*	*0.244186*	*0.2439024*
**ENSG00000082293**		*0.49068323*		*0.49068323*			*0.3895349*
**ENSG00000096717**		*0.09872611*		*0.098726115*			*0.3604651*
**ENSG00000087116**		*0.41818182*		*0.418181818*			*0.4709302*
**ENSG00000157326**	*0.27011494*		*0.27011494*				
**ENSG00000161813**		*0.0617284*		*0.061728395*			*0.4418605*
**ENSG00000004939**		*0.27272727*		*0.272727273*			*0.4127907*
**ENSG00000127054**		*0.18484848*		*0.184848485*			
**ENSG00000112782**		*0.40449438*		*0.404494382*			*0.4333333*
**ENSG00000152404**		*0.47575758*		*0.475757576*	*0.154761905*	*0.0872093*	*0.4069767*
**ENSG00000090060**		*0.21515152*		*0.215151515*			*0.1235294*
**ENSG00000151208**		*0.37878788*		*0.378787879*			*0.4883721*
**ENSG00000155087**	*0.43113772*		*0.43113772*		*0.363095238*	*0.3255814*	
**ENSG00000105737**		*0.06666667*		*0.066666667*			*0.0755814*
**ENSG00000162623**		*0.24242424*		*0.242424242*			*0.3554217*
**ENSG00000175879**					*0.089285714*	*0.1104651*	
**ENSG00000131044**		*0.43939394*		*0.439393939*			*0.1162791*
**ENSG00000105171**		*0.41818182*		*0.418181818*			*0.4222222*
**ENSG00000119414**	*0.48255814*	*0.48888889*	*0.48255814*	*0.488888889*	*0.43902439*	*0.5*	*0.5*
**ENSG00000142178**		*0.34482759*		*0.344827586*			*0.3522727*
**ENSG00000181143**		*0.11111111*		*0.111111111*			*0.1022727*
**ENSG00000181004**		*0.36666667*		*0.366666667*			*0.3522727*
**ENSG00000180914**		*0.375*		*0.375*			*0.0930233*
**ENSG00000096395**	*0.20958084*		*0.20958084*		*0.083333333*	*0.1511628*	
**ENSG00000142082**	*0.07185629*	*0.27575758*	*0.07185629*	*0.275757576*	*0.142857143*	*0.1705882*	*0.2888889*
**ENSG00000170807**	*0.14071856*	*0.48837209*	*0.14071856*	*0.488372093*	*0.166666667*	*0.1569767*	*0.4127907*
**ENSG00000123933**		*0.17777778*		*0.177777778*			*0.3081395*
**ENSG00000163584**		*0.16111111*		*0.161111111*			*0.3295455*
**ENSG00000132622**		*0.3375*		*0.3375*			*0.1976744*
**ENSG00000223865**		*0.32386364*		*0.323863636*			*0.4476744*
**ENSG00000183044**	*0.41616766*	*0.5*	*0.41616766*	*0.5*	*0.455555556*	*0.3554217*	*0.494186*
**ENSG00000139053**	*0.14071856*		*0.14071856*		*0.083333333*	*0.127907*	*0.0639535*

**ENSG00000172046**	*0.46107784*	*0.36666667*	*0.46107784*	*0.366666667*	*0.06547619*		*0.0872093*	*0.0882353*
**ENSG00000153234**		*0.48484848*		*0.484848485*	*0.144444444*		*0.1363636*	*0.2093023*
**ENSG00000082482**		*0.21022727*		*0.210227273*				*0.2727273*
**ENSG00000176273**		*0.46363636*		*0.463636364*				*0.122093*
**ENSG00000163701**		*0.46969697*		*0.46969697*				
**ENSG00000122042**	*0.30538922*	*0.26363636*	*0.30538922*	*0.263636364*			*0.1235294*	*0.3647059*
**ENSG00000183638**		*0.38787879*		*0.387878788*	*0.155555556*		*0.1022727*	*0.4825581*
**ENSG00000122194**	*0.46666667*	*0.26060606*	*0.46666667*	*0.260606061*	*0.4*		*0.4318182*	*0.4476744*
**ENSG00000173805**		*0.11212121*		*0.112121212*				*0.122093*
**ENSG00000157782**	*0.49101796*		*0.49101796*		*0.267857143*		*0.1941176*	
**ENSG00000170909**					*0.462962963*		*0.4772727*	
**ENSG00000175455**	*0.32934132*	*0.35454545*	*0.32934132*	*0.354545455*	*0.5*		*0.4764706*	*0.1395349*
**ENSG00000075651**		*0.38484848*		*0.384848485*				*0.3488372*
**ENSG00000115998**	*0.31736527*		*0.31736527*		*0.130952381*		*0.1627907*	
**ENSG00000123892**	*0.16564417*	*0.43030303*	*0.16564417*	*0.43030303*	*0.156626506*		*0.2034884*	*0.3882353*
**ENSG00000127995**		*0.40909091*		*0.409090909*				*0.2034884*
**ENSG00000170310**		*0.36666667*		*0.366666667*				*0.126506*
**ENSG00000138674**	*0.31818182*	*0.24545455*	*0.31818182*	*0.245454545*	*0.422222222*		*0.4431818*	*0.4709302*
**ENSG00000135341**	*0.31097561*	*0.38764045*	*0.31097561*	*0.387640449*	*0.384146341*		*0.4186047*	*0.3783784*
**ENSG00000100744**	*0.05988024*	*0.46363636*	*0.05988024*	*0.463636364*	*0.416666667*		*0.4534884*	*0.1860465*
**ENSG00000132740**	*0.07185629*	*0.33116883*	*0.07185629*	*0.331168831*				
**ENSG00000102531**		*0.06363636*		*0.063636364*				*0.0523256*
**ENSG00000134207**		*0.47575758*		*0.475757576*				*0.4941176*
**ENSG00000013016**		*0.22352941*		*0.223529412*				*0.1395349*
**ENSG00000163092**		*0.12068966*		*0.120689655*	*0.071428571*			*0.2678571*
**ENSG00000145945**		*0.47222222*		*0.472222222*				*0.2738095*
**ENSG00000162572**								*0.1931818*
**ENSG00000074527**		*0.46363636*		*0.463636364*				*0.494186*
**ENSG00000103264**		*0.44512195*		*0.445121951*				*0.25*
**ENSG00000198133**		*0.49090909*		*0.490909091*				*0.3058824*
**ENSG00000162641**	*0.46706587*	*0.35151515*	*0.46706587*	*0.351515152*			*0.0581395*	*0.3647059*
**ENSG00000196517**		*0.09444444*		*0.094444444*				*0.2727273*
**ENSG00000171914**	*0.05681818*	*0.16463415*	*0.05681818*	*0.164634146*				*0.2294118*
**ENSG00000142609**		*0.12222222*		*0.122222222*				*0.1136364*
**ENSG00000020129**								*0.1802326*
**ENSG00000196754**	*0.36526946*	*0.07272727*	*0.36526946*	*0.072727273*				
**ENSG00000174370**		*0.44135802*		*0.441358025*				*0.3941176*
**ENSG00000151498**		*0.08235294*		*0.082352941*				
**ENSG00000204839**		*0.26708075*		*0.267080745*				
**ENSG00000134258**		*0.30555556*		*0.305555556*	*0.259036145*		*0.2906977*	*0.1104651*
**ENSG00000077097**	*0.4939759*		*0.4939759*		*0.303571429*		*0.3953488*	
**ENSG00000166359**		*0.1011236*		*0.101123596*				*0.3444444*
**ENSG00000105246**		*0.28488372*		*0.284883721*				*0.3604651*
**ENSG00000155097**	*0.32934132*	*0.28484848*	*0.32934132*	*0.284848485*				*0.4204545*
**ENSG00000139537**	*0.07185629*	*0.33636364*	*0.07185629*	*0.336363636*	*0.44047619*		*0.4651163*	*0.4651163*
**ENSG00000179388**		*0.46296296*		*0.462962963*				*0.452381*
**ENSG00000157349**	*0.27245509*	*0.10909091*	*0.27245509*	*0.109090909*				*0.127907*
**ENSG00000137575**	*0.33333333*		*0.33333333*		*0.433333333*		*0.375*	*0.3953488*
**ENSG00000100554**	*0.41017964*		*0.41017964*		*0.357142857*		*0.5*	*0.4940476*
**ENSG00000255767**		*0.4*		*0.4*				*0.3111111*
**ENSG00000197134**	*0.4494382*	*0.40797546*	*0.4494382*	*0.40797546*	*0.344444444*		*0.4761905*	*0.3235294*
**ENSG00000125388**		*0.37222222*		*0.372222222*				*0.0795455*
**ENSG00000181350**		*0.36060606*		*0.360606061*				*0.3023256*
**ENSG00000109171**		*0.34337349*		*0.343373494*				*0.4545455*

**ENSG00000204290**	*0.14670659*	*0.47272727*	*0.14670659*	*0.472727273*	*0.202380952*		*0.1802326*	*0.4880952*
**ENSG00000137801**	*0.2005988*	*0.14545455*	*0.2005988*	*0.145454545*	*0.331325301*		*0.4476744*	*0.4011628*
**ENSG00000132932**	*0.36227545*	*0.49393939*	*0.36227545*	*0.493939394*	*0.077380952*		*0.1744186*	*0.4764706*
**ENSG00000148175**		*0.3*		*0.3*				*0.3068182*
**ENSG00000198574**		*0.34242424*		*0.342424242*				*0.4352941*
**ENSG00000123213**		*0.27743902*		*0.277439024*				*0.4879518*
**ENSG00000145526**	*0.21556886*		*0.21556886*					
**ENSG00000186407**	*0.3908046*	*0.3006135*	*0.3908046*	*0.300613497*	*0.25*		*0.3222222*	*0.125*
**ENSG00000055147**	*0.44311377*	*0.38333333*	*0.44311377*	*0.383333333*	*0.148809524*		*0.1511628*	*0.1555556*
**ENSG00000173281**		*0.28888889*		*0.288888889*				*0.3313953*
**ENSG00000175279**		*0.42675159*		*0.426751592*				*0.4117647*
**ENSG00000204520**	*0.48863636*	*0.40740741*	*0.48863636*	*0.407407407*	*0.295454545*		*0.3222222*	*0.2804878*
**ENSG00000150201**	*0.3742515*		*0.3742515*		*0.415662651*		*0.4294118*	
**ENSG00000103591**	*0.33532934*	*0.49090909*	*0.33532934*	*0.490909091*	*0.119047619*		*0.1802326*	*0.4651163*
**ENSG00000174136**		*0.29090909*		*0.290909091*				*0.2906977*
**ENSG00000197140**		*0.2652439*		*0.265243902*				*0.2732558*
**ENSG00000133731**	*0.45757576*	*0.40606061*	*0.45757576*	*0.406060606*	*0.296296296*		*0.2470588*	*0.3823529*
**ENSG00000154678**		*0.40909091*		*0.409090909*				*0.4186047*
**ENSG00000178084**		*0.43636364*		*0.436363636*				*0.2058824*
**ENSG00000036054**		*0.36969697*		*0.36969697*				*0.0523256*
**ENSG00000182240**		*0.47878788*		*0.478787879*				*0.3372093*
**ENSG00000149177**		*0.44848485*		*0.448484848*				*0.4360465*
**ENSG00000106404**	*0.4011976*	*0.25304878*	*0.4011976*	*0.25304878*	*0.196428571*		*0.1453488*	*0.1453488*
**ENSG00000075290**	*0.16167665*	*0.21818182*	*0.16167665*	*0.218181818*	*0.142857143*		*0.1918605*	*0.1918605*
**ENSG00000166938**		*0.36060606*		*0.360606061*				*0.246988*
**ENSG00000173264**	*0.15151515*		*0.15151515*		*0.166666667*		*0.2151163*	
**ENSG00000075388**		*0.26060606*		*0.260606061*				*0.2848837*
**ENSG00000082929**	*0.27777778*	*0.25757576*	*0.27777778*	*0.257575758*	*0.322222222*		*0.3666667*	*0.127907*
**ENSG00000259471**		*0.17791411*		*0.17791411*				*0.2093023*
**ENSG00000162618**		*0.31034483*		*0.310344828*				*0.2840909*
**ENSG00000159216**		*0.06969697*		*0.06969697*				
**ENSG00000099282**		*0.38484848*		*0.384848485*				*0.1428571*
**ENSG00000254901**		*0.44545455*		*0.445454545*				*0.4244186*
**ENSG00000174225**		*0.16060606*		*0.160606061*				
**ENSG00000110200**	*0.19161677*		*0.19161677*		*0.303571429*		*0.2*	
**ENSG00000171298**		*0.35151515*		*0.351515152*				*0.1627907*
**ENSG00000117245**		*0.22327044*		*0.22327044*	*0.261904762*		*0.3975904*	*0.0588235*
**ENSG00000129084**	*0.34545455*		*0.34545455*		*0.341463415*		*0.3546512*	
**ENSG00000111530**	*0.06626506*	*0.41515152*	*0.06626506*	*0.415151515*	*0.488095238*		*0.3255814*	*0.3444444*
**ENSG00000178234**	*0.47891566*		*0.47891566*		*0.154761905*		*0.1686047*	*0.1802326*
**ENSG00000169213**		*0.37804878*		*0.37804878*				*0.1046512*
**ENSG00000175352**		*0.48181818*		*0.481818182*				*0.4069767*
**ENSG00000185565**		*0.41358025*		*0.413580247*				*0.3662791*
**ENSG00000149596**	*0.48295455*	*0.48888889*	*0.48295455*	*0.488888889*	*0.422222222*		*0.375*	*0.4825581*
**ENSG00000124749**	*0.35555556*	*0.40606061*	*0.35555556*	*0.406060606*	*0.066666667*		*0.1555556*	*0.2267442*
**ENSG00000121764**		*0.33146067*		*0.331460674*				*0.3111111*
**ENSG00000143553**		*0.48181818*		*0.481818182*				*0.3081395*
**ENSG00000107938**		*0.11349693*		*0.113496933*				*0.2235294*
**ENSG00000237353**		*0.13636364*		*0.136363636*				*0.2888889*
**ENSG00000092929**	*0.13483146*	*0.06666667*	*0.13483146*	*0.066666667*			*0.0795455*	
**ENSG00000206535**	*0.47727273*	*0.42424242*	*0.47727273*	*0.424242424*	*0.433333333*		*0.3977273*	*0.3604651*
**ENSG00000171408**								*0.2267442*
**ENSG00000122694**		*0.30606061*		*0.306060606*				*0.3863636*
**ENSG00000067334**		*0.34848485*		*0.348484848*	*0.398809524*		*0.4418605*	*0.4418605*

**ENSG00000075035**	*0.28614458*	*0.46666667*	*0.28614458*	*0.466666667*	*0.089285714*	*0.1046512*	*0.4709302*
**ENSG00000140543**		*0.41818182*		*0.418181818*			*0.4651163*
**ENSG00000151650**		*0.0969697*		*0.096969697*			*0.0813953*
**ENSG00000174695**		*0.48181818*		*0.481818182*			*0.2732558*
**ENSG00000140563**	*0.35555556*	*0.48876404*	*0.35555556*	*0.488764045*	*0.277777778*	*0.3409091*	*0.3409091*
**ENSG00000163608**		*0.17777778*		*0.177777778*			*0.4090909*
**ENSG00000138061**	*0.16467066*	*0.46969697*	*0.16467066*	*0.46969697*			*0.1162791*
**ENSG00000222028**		*0.40909091*		*0.409090909*			*0.1627907*
**ENSG00000131100**	*0.26204819*		*0.26204819*		*0.196428571*	*0.25*	
**ENSG00000071082**	*0.42222222*	*0.3*	*0.42222222*	*0.3*	*0.181818182*	*0.1666667*	*0.1888889*
**ENSG00000198700**		*0.35454545*		*0.354545455*			*0.202381*
**ENSG00000038219**		*0.34848485*		*0.348484848*			*0.0581395*
**ENSG00000164970**		*0.4695122*		*0.469512195*			*0.4825581*
**ENSG00000198807**	*0.41916168*	*0.47777778*	*0.41916168*	*0.477777778*	*0.345238095*	*0.2848837*	*0.4318182*
**ENSG00000169359**	*0.45783133*		*0.45783133*		*0.481927711*	*0.3764706*	*0.0872093*
**ENSG00000189377**	*0.15662651*		*0.15662651*		*0.06547619*	*0.0581395*	
**ENSG00000120137**		*0.34848485*		*0.348484848*			*0.4375*
**ENSG00000110665**		*0.40909091*		*0.409090909*			*0.2797619*
**ENSG00000198326**		*0.08181818*		*0.081818182*	*0.285714286*	*0.372093*	*0.1569767*
**ENSG00000109065**		*0.49425287*		*0.494252874*			*0.2325581*
**ENSG00000085760**	*0.43413174*		*0.43413174*		*0.291666667*	*0.2965116*	
**ENSG00000049130**	*0.11235955*	*0.18787879*	*0.11235955*	*0.187878788*			*0.4772727*
**ENSG00000099194**		*0.35151515*		*0.351515152*			*0.3837209*
**ENSG00000112799**	*0.18333333*		*0.18333333*		*0.1*	*0.2111111*	
**ENSG00000168071**	*0.31137725*	*0.47865854*	*0.31137725*	*0.478658537*	*0.386904762*	*0.4767442*	*0.2209302*
**ENSG00000183527**		*0.43820225*		*0.438202247*			*0.4767442*
**ENSG00000085415**		*0.45151515*		*0.451515152*			*0.2151163*
**ENSG00000142973**	*0.1257485*	*0.15757576*	*0.1257485*	*0.157575758*	*0.210843373*	*0.3294118*	*0.377907*
**ENSG00000151835**	*0.12576687*	*0.44242424*	*0.12576687*	*0.442424242*	*0.445783133*	*0.4186047*	*0.4534884*
**ENSG00000173876**		*0.39655172*		*0.396551724*			*0.2*
**ENSG00000170606**		*0.26136364*		*0.261363636*			*0.0697674*
**ENSG00000146072**	*0.23353293*	*0.1*	*0.23353293*	*0.1*	*0.386904762*	*0.4302326*	
**ENSG00000165124**		*0.49444444*		*0.494444444*			*0.4204545*
**ENSG00000169621**		*0.084375*		*0.084375*			
**ENSG00000182326**	*0.1497006*	*0.07272727*	*0.1497006*	*0.072727273*	*0.101190476*	*0.0697674*	*0.0705882*
**ENSG00000160613**	*0.17365269*	*0.375*	*0.17365269*	*0.375*	*0.273809524*	*0.2965116*	*0.3837209*
**ENSG00000158445**		*0.27272727*		*0.272727273*			*0.3255814*
**ENSG00000168631**	*0.11212121*	*0.32424242*	*0.11212121*	*0.324242424*	*0.266666667*	*0.25*	*0.494186*
**ENSG00000166086**		*0.5*		*0.5*			*0.4767442*
**ENSG00000176595**	*0.47005988*	*0.30909091*	*0.47005988*	*0.309090909*	*0.279761905*	*0.2616279*	
**ENSG00000179134**		*0.15151515*		*0.151515152*			*0.1511628*
**ENSG00000114127**		*0.39393939*		*0.393939394*			*0.3372093*
**ENSG00000168806**		*0.2347561*		*0.234756098*			
**ENSG00000175087**		*0.21818182*		*0.218181818*			
**ENSG00000157617**		*0.38414634*		*0.384146341*			*0.4418605*
**ENSG00000146205**		*0.26969697*		*0.26969697*			*0.2267442*
**ENSG00000198711**		*0.43030303*		*0.43030303*			*0.2325581*
**ENSG00000169155**	*0.44252874*		*0.44252874*		*0.051282051*		*0.3430233*
**ENSG00000149635**		*0.08484848*		*0.084848485*			*0.0813953*
**ENSG00000121742**	*0.12275449*	*0.19393939*	*0.12275449*	*0.193939394*			*0.297619*
**ENSG00000104341**		*0.38109756*		*0.381097561*			*0.4534884*
**ENSG00000244274**		*0.23939394*		*0.239393939*			*0.4107143*
**ENSG00000130675**	*0.33832335*		*0.33832335*				
**ENSG00000105325**		*0.27134146*		*0.271341463*			*0.1744186*

**ENSG00000140577**		*0.21818182*		*0.218181818*			*0.3139535*
**ENSG00000060762**	*0.14545455*	*0.44545455*	*0.14545455*	*0.445454545*			*0.4127907*
**ENSG00000157613**		*0.33030303*		*0.33030303*			*0.2093023*
**ENSG00000173991**	*0.1257485*		*0.1257485*		*0.5*	*0.4709302*	
**ENSG00000185963**		*0.27575758*		*0.275757576*			*0.4666667*
**ENSG00000204257**		*0.08181818*		*0.081818182*			
**ENSG00000160654**		*0.2804878*		*0.280487805*			*0.4318182*
**ENSG00000065491**		*0.35670732*		*0.356707317*			*0.0581395*
**ENSG00000180855**	*0.35227273*		*0.35227273*		*0.344444444*	*0.3977273*	
**ENSG00000148288**	*0.24850299*		*0.24850299*		*0.488095238*	*0.4764706*	
**ENSG00000145794**	*0.14670659*	*0.2183908*	*0.14670659*	*0.218390805*	*0.343373494*	*0.3895349*	*0.4285714*
**ENSG00000149136**	*0.39520958*		*0.39520958*				
**ENSG00000197576**		*0.12121212*		*0.121212121*			*0.1569767*
**ENSG00000143155**		*0.30909091*		*0.309090909*			*0.3953488*
**ENSG00000144647**	*0.24251497*	*0.14110429*	*0.24251497*	*0.141104294*	*0.410714286*	*0.377907*	
**ENSG00000171053**		*0.5*		*0.5*			*0.4127907*
**ENSG00000104763**		*0.36060606*		*0.360606061*			*0.4736842*
**ENSG00000125538**	*0.34131737*		*0.34131737*		*0.463855422*	*0.4825581*	
**ENSG00000184313**	*0.2994012*	*0.48787879*	*0.2994012*	*0.487878788*	*0.168674699*	*0.2034884*	*0.1976744*
**ENSG00000152413**		*0.44848485*		*0.448484848*			*0.2732558*
**ENSG00000165983**	*0.11627907*	*0.45*	*0.11627907*	*0.45*	*0.388888889*	*0.3571429*	*0.3977273*
**ENSG00000082701**		*0.32121212*		*0.321212121*			*0.5*
**ENSG00000000457**		*0.11666667*		*0.116666667*			
**ENSG00000139496**		*0.37575758*		*0.375757576*			*0.4244186*
**ENSG00000074317**							*0.4529412*
**ENSG00000158691**		*0.36363636*		*0.363636364*			*0.4709302*
**ENSG00000119446**		*0.48787879*		*0.487878788*			*0.1882353*
**ENSG00000148541**	*0.35795455*	*0.38888889*	*0.35795455*	*0.388888889*	*0.465116279*	*0.4404762*	*0.3977273*
**ENSG00000257860**	*0.34883721*		*0.34883721*		*0.452380952*	*0.475*	
**ENSG00000188991**		*0.49375*		*0.49375*			*0.2682927*
**ENSG00000155975**		*0.48484848*		*0.484848485*			*0.3823529*
**ENSG00000178722**		*0.24545455*		*0.245454545*			*0.2888889*
**ENSG00000174007**	*0.2245509*	*0.37575758*	*0.2245509*	*0.375757576*	*0.43452381*	*0.4476744*	*0.0697674*
**ENSG00000118197**	*0.17664671*	*0.42352941*	*0.17664671*	*0.423529412*	*0.357142857*	*0.3662791*	*0.4767442*
**ENSG00000115290**	*0.13772455*	*0.39444444*	*0.13772455*	*0.394444444*	*0.279761905*	*0.494186*	*0.375*
**ENSG00000129347**	*0.22155689*	*0.49390244*	*0.22155689*	*0.493902439*	*0.386904762*	*0.2764706*	*0.1337209*
**ENSG00000123485**		*0.47878788*		*0.478787879*			*0.377907*
**ENSG00000147677**		*0.11515152*		*0.115151515*			*0.4588235*
**ENSG00000109184**	*0.2245509*	*0.33139535*	*0.2245509*	*0.331395349*	*0.375*	*0.3372093*	*0.3444444*
**ENSG00000157593**		*0.17575758*		*0.175757576*			*0.4651163*
**ENSG00000077809**	*0.23053892*		*0.23053892*		*0.071428571*	*0.0813953*	
**ENSG00000115514**		*0.49444444*		*0.494444444*			*0.3181818*
**ENSG00000215790**		*0.48314607*		*0.483146067*			*0.3777778*
**ENSG00000189167**	*0.47005988*	*0.14634146*	*0.47005988*	*0.146341463*	*0.477272727*	*0.4404762*	*0.1352941*
**ENSG00000035664**	*0.28742515*		*0.28742515*				*0.2*
**ENSG00000085999**	*0.08982036*		*0.08982036*				
**ENSG00000131018**		*0.20909091*		*0.209090909*			*0.4186047*
**ENSG00000155893**	*0.48809524*	*0.22727273*	*0.48809524*	*0.227272727*	*0.172619048*	*0.1666667*	*0.4390244*
**ENSG00000102359**	*0.15*		*0.15*		*0.177777778*	*0.1931818*	
**ENSG00000196476**		*0.45454545*		*0.454545455*			*0.4825581*
**ENSG00000166126**	*0.43113772*	*0.31818182*	*0.43113772*	*0.318181818*	*0.238095238*	*0.2906977*	*0.125*
**ENSG00000092964**		*0.44817073*		*0.448170732*			*0.4883721*
**ENSG00000091664**		*0.41111111*		*0.411111111*			*0.3588235*
**ENSG00000164743**	*0.0952381*		*0.0952381*				

**ENSG00000181656**	*0.14670659*	*0.37267081*	*0.14670659*	*0.372670807*	*0.303571429*	*0.25*	*0.3837209*
**ENSG00000159346**		*0.28651685*		*0.286516854*			*0.1860465*
**ENSG00000100330**	*0.1*	*0.42777778*	*0.1*	*0.427777778*	*0.144444444*		*0.4431818*
**ENSG00000172476**	*0.29341317*		*0.29341317*				
**ENSG00000172070**		*0.3030303*		*0.303030303*			*0.4302326*
**ENSG00000165359**	*0.08383234*		*0.08383234*			*0.1046512*	
**ENSG00000146083**							*0.0681818*
**ENSG00000135272**		*0.20555556*		*0.205555556*			*0.2738095*
**ENSG00000141401**		*0.05454545*		*0.054545455*			
**ENSG00000134748**		*0.23595506*		*0.235955056*			*0.1395349*
**ENSG00000105993**		*0.39090909*		*0.390909091*			*0.4470588*
**ENSG00000234906**	*0.2439759*	*0.31609195*	*0.2439759*	*0.316091954*	*0.297619048*	*0.3058824*	*0.4756098*
**ENSG00000109790**	*0.40340909*	*0.44444444*	*0.40340909*	*0.444444444*	*0.155555556*	*0.1477273*	*0.3604651*
**ENSG00000080618**		*0.33742331*		*0.337423313*			*0.2906977*
**ENSG00000134014**	*0.11805556*	*0.44382022*	*0.11805556*	*0.443820225*	*0.473684211*	*0.3235294*	
**ENSG00000026950**	*0.19461078*	*0.17878788*	*0.19461078*	*0.178787879*	*0.172619048*	*0.1802326*	*0.0526316*
**ENSG00000165288**							*0.4244186*
**ENSG00000175806**		*0.36969697*		*0.36969697*			*0.4705882*
**ENSG00000162437**		*0.40123457*		*0.401234568*			*0.35*
**ENSG00000125430**	*0.23053892*	*0.42134831*	*0.23053892*	*0.421348315*	*0.303571429*	*0.3023256*	*0.4709302*
**ENSG00000123908**		*0.42121212*		*0.421212121*			*0.5*
**ENSG00000221944**	*0.15568862*		*0.15568862*		*0.142857143*	*0.0941176*	
**ENSG00000064703**					*0.188888889*	*0.2159091*	
**ENSG00000111199**		*0.15151515*		*0.151515152*			*0.1918605*
**ENSG00000106638**		*0.30588235*		*0.305882353*			*0.1104651*
**ENSG00000133687**		*0.49378882*		*0.49378882*			*0.494186*
**ENSG00000198523**	*0.0508982*	*0.29444444*	*0.0508982*	*0.294444444*			*0.2954545*
**ENSG00000258818**	*0.47904192*	*0.25914634*	*0.47904192*	*0.259146341*			*0.4186047*
**ENSG00000186635**	*0.48888889*		*0.48888889*		*0.344444444*	*0.3068182*	
**ENSG00000166199**	*0.05421687*	*0.35757576*	*0.05421687*	*0.357575758*			*0.1395349*
**ENSG00000197322**		*0.43636364*		*0.436363636*			*0.494186*
**ENSG00000166946**		*0.08484848*		*0.084848485*			*0.0666667*
**ENSG00000130943**		*0.1554878*		*0.155487805*			
**ENSG00000148584**	*0.19760479*		*0.19760479*		*0.05952381*	*0.0755814*	
**ENSG00000170954**	*0.36526946*	*0.45121951*	*0.36526946*	*0.451219512*	*0.339285714*	*0.2790698*	*0.3764706*
**ENSG00000122257**	*0.05405405*		*0.05405405*				
**ENSG00000115380**	*0.06111111*		*0.06111111*				
**ENSG00000198642**		*0.19393939*		*0.193939394*			*0.5*
**ENSG00000119801**		*0.15*		*0.15*			*0.375*
**ENSG00000003987**		*0.30606061*		*0.306060606*			*0.3953488*
**ENSG00000156239**		*0.33908046*		*0.33908046*			*0.4534884*
**ENSG00000135372**		*0.14848485*		*0.148484848*			*0.4651163*
**ENSG00000158077**	*0.07303371*		*0.07303371*		*0.155555556*	*0.1704545*	
**ENSG00000163541**		*0.06060606*		*0.060606061*			*0.0523256*
**ENSG00000187772**		*0.29213483*		*0.292134831*			*0.5*
**ENSG00000204007**		*0.15757576*		*0.157575758*			*0.2209302*
**ENSG00000042980**	*0.18263473*	*0.45454545*	*0.18263473*	*0.454545455*	*0.404761905*	*0.3882353*	*0.4186047*
**ENSG00000177721**	*0.38323353*		*0.38323353*				
**ENSG00000163961**	*0.375*	*0.41212121*	*0.375*	*0.412121212*	*0.377777778*	*0.3295455*	*0.1411765*
**ENSG00000134333**	*0.05681818*	*0.41212121*	*0.05681818*	*0.412121212*			*0.4360465*
**ENSG00000112282**		*0.31515152*		*0.315151515*			*0.3855422*
**ENSG00000175093**	*0.43113772*	*0.10559006*	*0.43113772*	*0.105590062*			*0.1569767*
**ENSG00000204394**	*0.09638554*		*0.09638554*				
**ENSG00000137106**	*0.27844311*		*0.27844311*		*0.160714286*	*0.1453488*	*0.1477273*

**ENSG00000126460**	*0.40419162*		*0.40419162*		*0.232142857*	*0.1569767*	
**ENSG00000137076**							*0.0529412*
**ENSG00000040531**		*0.22121212*		*0.221212121*	*0.240963855*	*0.2790698*	*0.2616279*
**ENSG00000149131**		*0.2*		*0.2*			
**ENSG00000178776**		*0.0505618*		*0.050561798*			*0.1222222*
**ENSG00000133773**		*0.15151515*		*0.151515152*			
**ENSG00000109084**		*0.05454545*		*0.054545455*			*0.1627907*
**ENSG00000248485**		*0.18023256*		*0.180232558*			*0.2564103*
**ENSG00000186094**		*0.40909091*		*0.409090909*			*0.4127907*
**ENSG00000154639**		*0.37222222*		*0.372222222*			*0.4545455*
**ENSG00000139505**	*0.0748503*	*0.16666667*	*0.0748503*	*0.166666667*	*0.409638554*	*0.4709302*	*0.4888889*
**ENSG00000179051**		*0.33030303*		*0.33030303*			*0.4883721*
**ENSG00000139921**		*0.31609195*		*0.316091954*			*0.255814*
**ENSG00000106211**	*0.06060606*		*0.06060606*				
**ENSG00000177047**		*0.3969697*		*0.396969697*			*0.1453488*
**ENSG00000175911**		*0.16666667*		*0.166666667*			*0.0813953*
**ENSG00000176907**		*0.35555556*		*0.355555556*			*0.4431818*
**ENSG00000196387**	*0.43712575*	*0.4*	*0.43712575*	*0.4*	*0.214285714*	*0.1529412*	*0.4659091*
**ENSG00000101977**	*0.06363636*		*0.06363636*		*0.113095238*	*0.0764706*	
**ENSG00000149948**		*0.49090909*		*0.490909091*			*0.3081395*
**ENSG00000259486**		*0.24545455*		*0.245454545*			*0.4352941*
**ENSG00000134109**		*0.46060606*		*0.460606061*			*0.4360465*
**ENSG00000179630**		*0.4689441*		*0.468944099*	*0.101190476*	*0.0872093*	*0.3072289*
**ENSG00000149532**	*0.45508982*		*0.45508982*				
**ENSG00000100150**		*0.14329268*		*0.143292683*			*0.1352941*
**ENSG00000103522**		*0.24848485*		*0.248484848*			*0.1453488*
**ENSG00000141252**		*0.45890411*		*0.45890411*			*0.3295455*
**ENSG00000127903**	*0.38922156*		*0.38922156*		*0.30952381*	*0.3895349*	
**ENSG00000155363**					*0.077777778*	*0.0795455*	
**ENSG00000205929**		*0.25555556*		*0.255555556*			
**ENSG00000173218**		*0.38484848*		*0.384848485*			*0.4651163*
**ENSG00000156931**	*0.28089888*	*0.36809816*	*0.28089888*	*0.36809816*	*0.344444444*	*0.4333333*	*0.4529412*
**ENSG00000211660**	*0.11585366*		*0.11585366*				
**ENSG00000184029**		*0.38343558*		*0.383435583*			*0.1*
**ENSG00000254979**		*0.31707317*		*0.317073171*			*0.3176471*
**ENSG00000136450**		*0.16666667*		*0.166666667*			
**ENSG00000140557**		*0.36060606*		*0.360606061*			*0.4444444*
**ENSG00000164309**		*0.13333333*		*0.133333333*			*0.0639535*
**ENSG00000142599**	*0.39263804*	*0.46666667*	*0.39263804*	*0.466666667*		*0.0697674*	*0.2176471*
**ENSG00000101003**		*0.46646341*		*0.466463415*			*0.1046512*
**ENSG00000197816**	*0.17365269*	*0.45031056*	*0.17365269*	*0.450310559*	*0.119047619*	*0.0988372*	*0.4593023*
**ENSG00000177469**		*0.13496933*		*0.134969325*			*0.1395349*
**ENSG00000113578**		*0.40243902*		*0.402439024*			*0.3846154*
**ENSG00000198060**		*0.44545455*		*0.445454545*	*0.233333333*	*0.0909091*	*0.4593023*
**ENSG00000227057**	*0.19277108*		*0.19277108*		*0.355421687*	*0.3255814*	
**ENSG00000196683**	*0.24251497*	*0.40555556*	*0.24251497*	*0.405555556*	*0.386904762*	*0.372093*	*0.3837209*
**ENSG00000116729**		*0.35454545*		*0.354545455*			*0.4651163*
**ENSG00000104808**		*0.08181818*		*0.081818182*			*0.0872093*
**ENSG00000066294**		*0.39090909*		*0.390909091*			*0.4011628*
**ENSG00000172116**		*0.26060606*		*0.260606061*			*0.0523256*
**ENSG00000100162**					*0.154761905*	*0.1337209*	
**ENSG00000106399**	*0.33233533*	*0.18209877*	*0.33233533*	*0.182098765*	*0.148809524*	*0.0813953*	*0.0882353*
**ENSG00000186335**		*0.49444444*		*0.494444444*			*0.1590909*
**ENSG00000185252**		*0.4030303*		*0.403030303*			*0.377907*

**ENSG00000169905**		*0.46969697*		*0.46969697*			*0.2906977*
**ENSG00000074211**	*0.34242424*	*0.5*	*0.34242424*	*0.5*	*0.226190476*	*0.2882353*	*0.4709302*
**ENSG00000161010**	*0.13473054*	*0.5*	*0.13473054*	*0.5*	*0.214285714*	*0.1976744*	*0.4883721*
**ENSG00000143627**		*0.29393939*		*0.293939394*			*0.2616279*
**ENSG00000136718**	*0.1257485*		*0.1257485*				
**ENSG00000163814**		*0.42424242*		*0.424242424*			*0.2965116*
**ENSG00000164188**							*0.3928571*
**ENSG00000205041**		*0.36969697*		*0.36969697*			*0.4117647*
**ENSG00000085274**		*0.23333333*		*0.233333333*			*0.2261905*
**ENSG00000146809**	*0.15555556*		*0.15555556*		*0.196428571*	*0.2674419*	
**ENSG00000198088**	*0.16766467*	*0.34848485*	*0.16766467*	*0.348484848*	*0.482142857*	*0.4764706*	*0.4767442*
**ENSG00000174945**	*0.38888889*	*0.49090909*	*0.38888889*	*0.490909091*	*0.311111111*	*0.2272727*	*0.2151163*
**ENSG00000039139**		*0.4969697*		*0.496969697*			*0.4244186*
**ENSG00000079308**	*0.32335329*	*0.47575758*	*0.32335329*	*0.475757576*	*0.416666667*	*0.4651163*	*0.4651163*
**ENSG00000007171**	*0.25*		*0.25*		*0.488888889*	*0.4090909*	
**ENSG00000135175**		*0.44512195*		*0.445121951*			*0.3636364*
**ENSG00000253976**		*0.44848485*		*0.448484848*	*0.321428571*	*0.3895349*	*0.3255814*
**ENSG00000100014**		*0.49090909*		*0.490909091*			*0.3313953*
**ENSG00000107816**	*0.43712575*	*0.2030303*	*0.43712575*	*0.203030303*	*0.493975904*	*0.4534884*	*0.4418605*
**ENSG00000163218**	*0.28527607*	*0.4969697*	*0.28527607*	*0.496969697*	*0.092592593*	*0.0654762*	*0.244186*
**ENSG00000175077**		*0.14848485*		*0.148484848*			*0.3255814*
**ENSG00000167508**		*0.36666667*		*0.366666667*			*0.4767442*
**ENSG00000052850**		*0.35365854*		*0.353658537*			*0.3809524*
**ENSG00000112414**		*0.18902439*		*0.18902439*			*0.2333333*
**ENSG00000167523**		*0.26111111*		*0.261111111*			*0.4222222*
**ENSG00000174705**		*0.43939394*		*0.439393939*			*0.494186*
**ENSG00000138604**		*0.34242424*		*0.342424242*			*0.494186*
**ENSG00000196937**		*0.15243902*		*0.152439024*			
**ENSG00000235931**	*0.22754491*	*0.49393939*	*0.22754491*	*0.493939394*	*0.196428571*	*0.1337209*	*0.4659091*
**ENSG00000173226**		*0.33939394*		*0.339393939*			*0.3430233*
**ENSG00000112146**	*0.25748503*	*0.21818182*	*0.25748503*	*0.218181818*			*0.1627907*
**ENSG00000196542**	*0.24698795*	*0.4969697*	*0.24698795*	*0.496969697*	*0.482142857*	*0.4709302*	*0.4*
**ENSG00000122375**		*0.16969697*		*0.16969697*			*0.4302326*
**ENSG00000183475**		*0.24390244*		*0.243902439*			*0.1162791*
**ENSG00000253327**		*0.05757576*		*0.057575758*			*0.25*
**ENSG00000030110**		*0.31111111*		*0.311111111*			*0.2045455*
**ENSG00000120903**	*0.30239521*	*0.47126437*	*0.30239521*	*0.471264368*	*0.202380952*	*0.3081395*	*0.4772727*
**ENSG00000001461**	*0.46686747*	*0.49079755*	*0.46686747*	*0.490797546*	*0.321428571*	*0.2732558*	*0.3411765*
**ENSG00000088926**	*0.37790698*	*0.35151515*	*0.37790698*	*0.351515152*	*0.344444444*	*0.2272727*	*0.2093023*
**ENSG00000176920**		*0.43636364*		*0.436363636*			
**ENSG00000048828**		*0.38235294*		*0.382352941*			*0.4886364*
**ENSG00000185158**		*0.14545455*		*0.145454545*			
**ENSG00000197901**	*0.41573034*		*0.41573034*		*0.272727273*	*0.2777778*	
**ENSG00000181751**		*0.43181818*		*0.431818182*			*0.3095238*
**ENSG00000211810**	*0.30239521*		*0.30239521*		*0.422619048*	*0.4534884*	
**ENSG00000178932**							*0.3333333*
**ENSG00000119707**		*0.0969697*		*0.096969697*			*0.1453488*
**ENSG00000152234**	*0.28571429*	*0.41358025*	*0.28571429*	*0.413580247*	*0.426829268*	*0.4294118*	*0.0529412*
**ENSG00000176927**	*0.40662651*		*0.40662651*		*0.304878049*	*0.3095238*	
**ENSG00000112218**	*0.17777778*		*0.17777778*				
**ENSG00000135916**	*0.23888889*		*0.23888889*		*0.107142857*	*0.2848837*	
**ENSG00000139278**		*0.42331288*		*0.423312883*			*0.494186*
**ENSG00000174514**		*0.15757576*		*0.157575758*			*0.4090909*
**ENSG00000157036**	*0.4760479*	*0.4*	*0.4760479*	*0.4*	*0.369047619*	*0.4418605*	*0.4651163*

**ENSG00000136238**		*0.22121212*		*0.221212121*			*0.1569767*
**ENSG00000135144**	*0.46385542*		*0.46385542*		*0.360465116*	*0.325*	
**ENSG00000099860**							*0.1111111*
**ENSG00000132388**		*0.32621951*		*0.326219512*			*0.3081395*
**ENSG00000166025**		*0.16871166*		*0.168711656*			*0.0833333*
**ENSG00000197620**	*0.0753012*		*0.0753012*		*0.095238095*	*0.1511628*	
**ENSG00000176438**	*0.29444444*	*0.43030303*	*0.29444444*	*0.43030303*			*0.3488372*
**ENSG00000233889**	*0.2826087*		*0.2826087*				
**ENSG00000167861**		*0.46969697*		*0.46969697*			*0.4204545*
**ENSG00000187122**		*0.23030303*		*0.23030303*			*0.127907*
**ENSG00000243156**		*0.42424242*		*0.424242424*			*0.2906977*
**ENSG00000136546**		*0.32424242*		*0.324242424*			*0.2616279*
**ENSG00000149262**		*0.29393939*		*0.293939394*			*0.1860465*
**ENSG00000090857**		*0.34659091*		*0.346590909*			*0.4302326*
**ENSG00000047365**	*0.17272727*	*0.49090909*	*0.17272727*	*0.490909091*	*0.259259259*	*0.1363636*	*0.2764706*
**ENSG00000100023**		*0.39444444*		*0.394444444*			*0.4244186*
**ENSG00000171224**		*0.35454545*		*0.354545455*			*0.1569767*
**ENSG00000136931**		*0.4382716*		*0.438271605*			*0.1976744*
**ENSG00000104388**		*0.40909091*		*0.409090909*			*0.4360465*
**ENSG00000148296**		*0.43636364*		*0.436363636*			*0.2616279*
**ENSG00000165475**		*0.28963415*		*0.289634146*			*0.4235294*
**ENSG00000151224**		*0.27575758*		*0.275757576*			*0.0988372*
**ENSG00000169439**	*0.39772727*	*0.23888889*	*0.39772727*	*0.238888889*	*0.411111111*	*0.4659091*	*0.1333333*
**ENSG00000105270**							*0.1511628*
**ENSG00000183653**		*0.42638037*		*0.426380368*			*0.2797619*
**ENSG00000089486**		*0.38764045*		*0.387640449*			*0.3139535*
**ENSG00000109625**	*0.45808383*	*0.5*	*0.45808383*	*0.5*	*0.386904762*	*0.3117647*	*0.4642857*
**ENSG00000145217**		*0.12424242*		*0.124242424*			*0.122093*
**ENSG00000140265**		*0.23333333*		*0.233333333*			*0.2272727*
**ENSG00000096093**		*0.20909091*		*0.209090909*			*0.2151163*
**ENSG00000001460**	*0.23053892*	*0.14242424*	*0.23053892*	*0.142424242*	*0.451219512*	*0.4709302*	
**ENSG00000139370**	*0.43975904*	*0.48787879*	*0.43975904*	*0.487878788*	*0.277777778*	*0.2176471*	*0.4318182*
**ENSG00000253873**		*0.20224719*		*0.202247191*			*0.1477273*
**ENSG00000196505**		*0.45*		*0.45*			*0.125*
**ENSG00000186470**		*0.30909091*		*0.309090909*			*0.1888889*
**ENSG00000122644**	*0.06886228*	*0.08484848*	*0.06886228*	*0.084848485*	*0.351190476*	*0.3953488*	*0.2151163*
**ENSG00000162552**							*0.1704545*
**ENSG00000125484**		*0.37272727*		*0.372727273*			*0.4659091*
**ENSG00000106477**	*0.30239521*	*0.37222222*	*0.30239521*	*0.372222222*	*0.31547619*	*0.2848837*	*0.1744186*
**ENSG00000128000**							*0.127907*
**ENSG00000146676**		*0.4494382*		*0.449438202*			*0.244186*
**ENSG00000125835**	*0.06586826*	*0.08988764*	*0.06586826*	*0.08988764*	*0.196428571*	*0.3255814*	*0.3111111*
**ENSG00000171864**		*0.4847561*		*0.484756098*			*0.4819277*
**ENSG00000111860**		*0.45348837*		*0.453488372*			*0.3295455*
**ENSG00000149968**		*0.43333333*		*0.433333333*			*0.2727273*
**ENSG00000178719**		*0.41515152*		*0.415151515*			*0.1453488*
**ENSG00000179909**		*0.48765432*		*0.487654321*			*0.4642857*
**ENSG00000179855**		*0.48888889*		*0.488888889*			*0.3081395*
**ENSG00000204140**							*0.0769231*
**ENSG00000121904**	*0.2754491*	*0.39130435*	*0.2754491*	*0.391304348*	*0.119047619*	*0.1882353*	*0.4235294*
**ENSG00000155530**							*0.1162791*
**ENSG00000180785**	*0.28143713*	*0.49090909*	*0.28143713*	*0.490909091*	*0.369047619*	*0.4127907*	*0.2235294*
**ENSG00000142039**		*0.12883436*		*0.128834356*			*0.255814*
**ENSG00000006704**	*0.30239521*		*0.30239521*		*0.3*	*0.4886364*	

**ENSG00000072364**		*0.44545455*		*0.445454545*			*0.4941176*
**ENSG00000171105**		*0.42121212*		*0.421212121*			*0.4651163*
**ENSG00000176542**		*0.3030303*		*0.303030303*			*0.2965116*
**ENSG00000118257**	*0.4491018*	*0.45454545*	*0.4491018*	*0.454545455*	*0.445783133*	*0.4588235*	*0.4825581*
**ENSG00000131094**		*0.49444444*		*0.494444444*			
**ENSG00000183597**	*0.33832335*	*0.40184049*	*0.33832335*	*0.401840491*	*0.446428571*	*0.4360465*	*0.5*
**ENSG00000237896**							*0.1511628*
**ENSG00000106006**							*0.2261905*
**ENSG00000106013**	*0.05769231*		*0.05769231*		*0.097560976*	*0.0512821*	
**ENSG00000166736**	*0.48203593*	*0.24545455*	*0.48203593*	*0.245454545*	*0.246987952*	*0.2790698*	*0.2267442*
**ENSG00000178502**		*0.06666667*		*0.066666667*			
**ENSG00000130159**		*0.05521472*		*0.055214724*			
**ENSG00000138750**		*0.37195122*		*0.37195122*			*0.3414634*
**ENSG00000167083**	*0.08982036*	*0.36666667*	*0.08982036*	*0.366666667*	*0.053571429*		*0.0823529*
**ENSG00000005812**							*0.3023256*
**ENSG00000168367**	*0.32335329*		*0.32335329*		*0.208333333*	*0.2352941*	
**ENSG00000258804**	*0.20359281*		*0.20359281*		*0.291666667*	*0.2965116*	
**ENSG00000164120**	*0.18562874*	*0.41515152*	*0.18562874*	*0.415151515*	*0.452380952*	*0.4418605*	*0.494186*
**ENSG00000126010**	*0.36585366*	*0.25153374*	*0.36585366*	*0.251533742*	*0.089285714*	*0.2093023*	*0.2209302*
**ENSG00000083842**	*0.13473054*	*0.36280488*	*0.13473054*	*0.362804878*			*0.4360465*
**ENSG00000138395**	*0.47777778*	*0.33030303*	*0.47777778*	*0.33030303*	*0.388888889*	*0.4545455*	*0.3604651*
**ENSG00000143125**		*0.1097561*		*0.109756098*			
**ENSG00000105696**		*0.41818182*		*0.418181818*			*0.3941176*
**ENSG00000131069**	*0.48502994*	*0.38484848*	*0.48502994*	*0.384848485*	*0.409638554*	*0.3430233*	*0.3430233*
**ENSG00000148053**		*0.47272727*		*0.472727273*			*0.4545455*
**ENSG00000005700**		*0.24213836*		*0.242138365*			*0.2840909*
**ENSG00000111269**		*0.44545455*		*0.445454545*			*0.494186*
**ENSG00000213445**	*0.14071856*		*0.14071856*		*0.244047619*	*0.2151163*	
**ENSG00000163738**		*0.24342105*		*0.243421053*			*0.1941176*
**ENSG00000124383**	*0.2247191*		*0.2247191*		*0.369047619*	*0.3837209*	
**ENSG00000104177**							*0.4534884*
**ENSG00000111696**		*0.47272727*		*0.472727273*			*0.3511905*
**ENSG00000133083**	*0.10119048*	*0.46646341*	*0.10119048*	*0.466463415*			*0.4534884*
**ENSG00000172985**		*0.15151515*		*0.151515152*			*0.3023256*
**ENSG00000168040**		*0.4695122*		*0.469512195*			*0.3255814*
**ENSG00000126368**	*0.36227545*		*0.36227545*		*0.464285714*	*0.4476744*	
**ENSG00000204941**		*0.171875*		*0.171875*			*0.4702381*
**ENSG00000123119**		*0.32317073*		*0.323170732*			*0.2882353*
**ENSG00000132359**		*0.41666667*		*0.416666667*			*0.3139535*
**ENSG00000103018**		*0.41515152*		*0.415151515*			*0.4651163*
**ENSG00000145088**		*0.12727273*		*0.127272727*			*0.3430233*
**ENSG00000181965**					*0.214285714*	*0.2151163*	
**ENSG00000138095**		*0.09444444*		*0.094444444*			
**ENSG00000113583**		*0.08841463*		*0.088414634*			*0.0639535*
**ENSG00000204642**	*0.27840909*	*0.47619048*	*0.27840909*	*0.476190476*	*0.411111111*	*0.375*	*0.494186*
**ENSG00000140025**	*0.1497006*	*0.49386503*	*0.1497006*	*0.493865031*			*0.4772727*
**ENSG00000110660**	*0.2245509*	*0.48333333*	*0.2245509*	*0.483333333*	*0.404761905*	*0.4302326*	*0.3176471*
**ENSG00000100109**		*0.07222222*		*0.072222222*	*0.277108434*	*0.2380952*	*0.125*
**ENSG00000132549**		*0.20606061*		*0.206060606*			*0.1*
**ENSG00000198758**		*0.40555556*		*0.405555556*			*0.3181818*
**ENSG00000123552**	*0.14444444*	*0.47272727*	*0.14444444*	*0.472727273*	*0.494047619*	*0.4069767*	*0.3837209*
**ENSG00000178980**							*0.0909091*
**ENSG00000091536**	*0.23030303*	*0.22121212*	*0.23030303*	*0.221212121*			*0.3546512*
**ENSG00000180573**	*0.25449102*		*0.25449102*		*0.267857143*	*0.1686047*	

**ENSG00000204620**		*0.06666667*		*0.066666667*			*0.0523256*
**ENSG00000100365**		*0.05454545*		*0.054545455*			
**ENSG00000145029**		*0.2826087*		*0.282608696*			
**ENSG00000134759**	*0.36445783*	*0.32621951*	*0.36445783*	*0.326219512*	*0.18452381*	*0.2058824*	*0.2228916*
**ENSG00000141127**	*0.39221557*	*0.17575758*	*0.39221557*	*0.175757576*	*0.333333333*	*0.3372093*	*0.0588235*
**ENSG00000132313**		*0.48333333*		*0.483333333*			*0.4883721*
**ENSG00000157456**	*0.07185629*		*0.07185629*				
**ENSG00000154727**							*0.0714286*
**ENSG00000154263**		*0.41515152*		*0.415151515*	*0.108433735*	*0.0639535*	*0.4127907*
**ENSG00000184840**		*0.46666667*		*0.466666667*			*0.4659091*
**ENSG00000177084**	*0.38323353*	*0.43678161*	*0.38323353*	*0.436781609*	*0.386904762*	*0.2965116*	*0.25*
**ENSG00000177076**		*0.45454545*		*0.454545455*			*0.1111111*
**ENSG00000112419**		*0.23333333*		*0.233333333*			*0.3953488*
**ENSG00000167670**		*0.23939394*		*0.239393939*			*0.3255814*
**ENSG00000129657**	*0.3502994*	*0.30909091*	*0.3502994*	*0.309090909*	*0.433333333*	*0.3488372*	*0.25*
**ENSG00000153898**		*0.33841463*		*0.338414634*			*0.3546512*
**ENSG00000132466**		*0.18944099*		*0.189440994*			*0.3095238*
**ENSG00000123104**		*0.33939394*		*0.339393939*			*0.3895349*
**ENSG00000171401**		*0.44968553*		*0.449685535*			*0.4573171*
**ENSG00000142619**		*0.31410256*		*0.314102564*			*0.0581395*
**ENSG00000107551**	*0.43413174*		*0.43413174*		*0.464285714*	*0.4186047*	*0.1744186*
**ENSG00000168246**		*0.29878049*		*0.298780488*			*0.4702381*
**ENSG00000128944**		*0.14939024*		*0.149390244*			
**ENSG00000103415**		*0.4030303*		*0.403030303*			*0.3411765*
**ENSG00000167531**		*0.09444444*		*0.094444444*			*0.125*
**ENSG00000126895**		*0.37195122*		*0.37195122*	*0.066666667*	*0.0568182*	*0.255814*
**ENSG00000090924**		*0.45*		*0.45*			*0.375*
**ENSG00000112658**		*0.05454545*		*0.054545455*			*0.3488372*
**ENSG00000077943**	*0.0748503*	*0.32121212*	*0.0748503*	*0.321212121*	*0.119047619*	*0.1802326*	*0.4756098*
**ENSG00000139648**	*0.28742515*	*0.48850575*	*0.28742515*	*0.488505747*			*0.4772727*
**ENSG00000204283**		*0.10795455*		*0.107954545*			*0.2159091*
**ENSG00000196405**	*0.31137725*	*0.23939394*	*0.31137725*	*0.239393939*			*0.4360465*
**ENSG00000152463**		*0.42682927*		*0.426829268*			*0.3488372*
**ENSG00000102181**		*0.41515152*		*0.415151515*			*0.2411765*
**ENSG00000136167**	*0.2994012*	*0.42424242*	*0.2994012*	*0.424242424*	*0.321428571*	*0.3023256*	*0.4825581*
**ENSG00000106266**	*0.44578313*		*0.44578313*		*0.125*	*0.1627907*	
**ENSG00000197747**	*0.0508982*		*0.0508982*				
**ENSG00000051382**		*0.17977528*		*0.179775281*			
**ENSG00000159199**	*0.49700599*		*0.49700599*		*0.119047619*	*0.1802326*	
**ENSG00000205334**		*0.2195122*		*0.219512195*			*0.4360465*
**ENSG00000138081**		*0.05757576*		*0.057575758*			
**ENSG00000158486**		*0.44242424*		*0.442424242*			*0.4244186*
**ENSG00000101096**		*0.48333333*		*0.483333333*			*0.4011628*
**ENSG00000107643**		*0.48888889*		*0.488888889*			*0.3095238*
**ENSG00000089327**		*0.39263804*		*0.392638037*			*0.2616279*
**ENSG00000116329**		*0.44817073*		*0.448170732*			*0.1860465*
**ENSG00000196911**		*0.23006135*		*0.23006135*			*0.4069767*
**ENSG00000168484**		*0.28787879*		*0.287878788*			*0.4318182*
**ENSG00000105127**		*0.26190476*		*0.261904762*			
**ENSG00000110344**		*0.14939024*		*0.149390244*		*0.0813953*	*0.4534884*
**ENSG00000187170**	*0.08333333*		*0.08333333*		*0.122222222*		
**ENSG00000050426**	*0.45808383*	*0.44848485*	*0.45808383*	*0.448484848*	*0.363095238*	*0.2674419*	*0.3197674*
**ENSG00000188375**		*0.17977528*		*0.179775281*			*0.2034884*
**ENSG00000165617**		*0.2*		*0.2*			*0.1590909*

**ENSG00000196616**		*0.496875*		*0.496875*				*0.2613636*
**ENSG00000172322**		*0.3757764*		*0.375776398*				*0.2151163*
**ENSG00000197647**	*0.1257485*		*0.1257485*					
**ENSG00000117069**		*0.07012195*		*0.070121951*				*0.3895349*
**ENSG00000150457**	*0.37777778*	*0.30909091*	*0.37777778*	*0.309090909*	*0.433333333*		*0.4886364*	*0.2034884*
**ENSG00000120729**		*0.17272727*		*0.172727273*				*0.3255814*
**ENSG00000187678**		*0.36111111*		*0.361111111*	*0.30952381*		*0.377907*	*0.4090909*
**ENSG00000221988**	*0.05988024*		*0.05988024*					
**ENSG00000134146**		*0.26829268*		*0.268292683*				*0.3863636*
**ENSG00000165805**		*0.10555556*		*0.105555556*				
**ENSG00000106628**	*0.15963855*		*0.15963855*		*0.232142857*		*0.2034884*	
**ENSG00000170959**	*0.13473054*	*0.36666667*	*0.13473054*	*0.366666667*	*0.125*		*0.0872093*	*0.4302326*
**ENSG00000104472**		*0.20555556*		*0.205555556*				*0.1666667*
**ENSG00000157856**		*0.15151515*		*0.151515152*			*0.122093*	*0.1395349*
**ENSG00000157227**		*0.48780488*		*0.487804878*				*0.494186*
**ENSG00000203907**	*0.48850575*		*0.48850575*		*0.211111111*		*0.2954545*	
**ENSG00000143228**	*0.17222222*	*0.20496894*	*0.17222222*	*0.204968944*	*0.088888889*		*0.0888889*	
**ENSG00000130770**		*0.34848485*		*0.348484848*				*0.4186047*
**ENSG00000066405**		*0.45*		*0.45*	*0.232142857*		*0.3313953*	*0.3295455*
**ENSG00000187559**	*0.16352201*		*0.16352201*					
**ENSG00000124678**	*0.26946108*	*0.14848485*	*0.26946108*	*0.148484848*	*0.166666667*		*0.255814*	*0.1046512*
**ENSG00000171033**	*0.05151515*	*0.16060606*	*0.05151515*	*0.160606061*				*0.127907*
**ENSG00000152223**		*0.4030303*		*0.403030303*				*0.4825581*
**ENSG00000108375**		*0.40606061*		*0.406060606*				*0.2906977*
**ENSG00000103154**		*0.42682927*		*0.426829268*				*0.4882353*
**ENSG00000112308**		*0.37025316*		*0.370253165*	*0.166666667*		*0.122093*	*0.1744186*
**ENSG00000213676**		*0.27575758*		*0.275757576*				*0.2906977*
**ENSG00000101224**	*0.09580838*	*0.10493827*	*0.09580838*	*0.104938272*	*0.311111111*		*0.2272727*	
**ENSG00000188785**	*0.37724551*	*0.19753086*	*0.37724551*	*0.197530864*	*0.05952381*		*0.0697674*	*0.0641026*
**ENSG00000139517**		*0.14634146*		*0.146341463*	*0.088888889*		*0.1*	*0.2333333*
**ENSG00000186871**		*0.28527607*		*0.285276074*				*0.4883721*
**ENSG00000138772**	*0.0748503*	*0.31402439*	*0.0748503*	*0.31402439*				*0.3895349*
**ENSG00000141570**		*0.28484848*		*0.284848485*				*0.1802326*
**ENSG00000089012**		*0.27906977*		*0.279069767*				*0.3081395*
**ENSG00000063515**								*0.4651163*
**ENSG00000138346**	*0.37724551*	*0.33636364*	*0.37724551*	*0.336363636*	*0.136904762*		*0.1104651*	*0.2093023*
**ENSG00000113070**		*0.47272727*		*0.472727273*				*0.4360465*
**ENSG00000228594**		*0.20606061*		*0.206060606*				*0.2325581*
**ENSG00000182768**		*0.09202454*		*0.09202454*				
**ENSG00000133247**		*0.42424242*		*0.424242424*				*0.2616279*
**ENSG00000138463**	*0.18333333*	*0.23030303*	*0.18333333*	*0.23030303*				*0.2647059*
**ENSG00000012174**		*0.33231707*		*0.332317073*				*0.4186047*
**ENSG00000153071**	*0.32634731*	*0.45151515*	*0.32634731*	*0.451515152*	*0.311111111*		*0.2965116*	*0.2732558*
**ENSG00000171130**		*0.4030303*		*0.403030303*	*0.222222222*		*0.2926829*	*0.375*
**ENSG00000100092**	*0.45731707*	*0.1030303*	*0.45731707*	*0.103030303*	*0.234939759*		*0.313253*	
**ENSG00000112941**		*0.32727273*		*0.327272727*				*0.3837209*
**ENSG00000156284**		*0.15151515*		*0.151515152*				*0.3953488*
**ENSG00000198911**	*0.23652695*	*0.1030303*	*0.23652695*	*0.103030303*	*0.148809524*		*0.3117647*	*0.0697674*
**ENSG00000010818**	*0.27777778*	*0.34969325*	*0.27777778*	*0.349693252*				*0.2034884*
**ENSG00000145757**		*0.35151515*		*0.351515152*				*0.3333333*
**ENSG00000139410**	*0.21176471*		*0.21176471*					
**ENSG00000204710**	*0.0988024*		*0.0988024*		*0.119047619*		*0.0988372*	
**ENSG00000087206**	*0.12727273*	*0.48780488*	*0.12727273*	*0.487804878*	*0.481481481*		*0.494186*	*0.4709302*
**ENSG00000110074**	*0.48192771*	*0.22988506*	*0.48192771*	*0.229885057*				*0.3863636*

**ENSG00000116954**								*0.3081395*
**ENSG00000120800**		*0.22360248*		*0.223602484*				
**ENSG00000226807**	*0.09939759*	*0.48295455*	*0.09939759*	*0.482954545*				*0.4709302*
**ENSG00000154229**		*0.30909091*		*0.309090909*				*0.377907*
**ENSG00000137831**	*0.49401198*	*0.27575758*	*0.49401198*	*0.275757576*	*0.089285714*			*0.4360465*
**ENSG00000253831**		*0.39634146*		*0.396341463*				*0.2674419*
**ENSG00000186439**		*0.44848485*		*0.448484848*				*0.4534884*
**ENSG00000122547**		*0.4969697*		*0.496969697*				*0.452381*
**ENSG00000197851**	*0.18862275*	*0.36503067*	*0.18862275*	*0.365030675*	*0.136904762*		*0.0755814*	*0.0523256*
**ENSG00000243284**		*0.29754601*		*0.297546012*				*0.077381*
**ENSG00000141002**		*0.10606061*		*0.106060606*				
**ENSG00000197122**	*0.34131737*	*0.08484848*	*0.34131737*	*0.084848485*	*0.160714286*		*0.2176471*	
**ENSG00000253797**	*0.08383234*	*0.36206897*	*0.08383234*	*0.362068966*	*0.482142857*		*0.4821429*	*0.4709302*
**ENSG00000115085**		*0.30113636*		*0.301136364*	*0.358024691*		*0.1818182*	*0.1022727*
**ENSG00000174348**								*0.2588235*
**ENSG00000162631**	*0.23333333*	*0.47575758*	*0.23333333*	*0.475757576*	*0.155555556*			*0.3895349*
**ENSG00000167842**	*0.39221557*	*0.23939394*	*0.39221557*	*0.239393939*	*0.31547619*		*0.3139535*	*0.3117647*
**ENSG00000070814**		*0.12962963*		*0.12962963*				*0.3197674*
**ENSG00000197629**		*0.46111111*		*0.461111111*				*0.4127907*
**ENSG00000170638**		*0.24233129*		*0.242331288*				*0.0892857*
**ENSG00000095777**	*0.17222222*	*0.44207317*	*0.17222222*	*0.442073171*				*0.4186047*
**ENSG00000139974**	*0.17365269*		*0.17365269*		*0.202380952*		*0.2941176*	*0.3*
**ENSG00000128915**	*0.39221557*	*0.49393939*	*0.39221557*	*0.493939394*	*0.096385542*			
**ENSG00000154099**		*0.48484848*		*0.484848485*				*0.4825581*
**ENSG00000130558**	*0.11111111*	*0.23939394*	*0.11111111*	*0.239393939*	*0.292682927*		*0.445122*	*0.1976744*
**ENSG00000253731**		*0.20224719*		*0.202247191*				*0.1477273*
**ENSG00000147419**	*0.39156627*	*0.46666667*	*0.39156627*	*0.466666667*	*0.136904762*		*0.2151163*	*0.4186047*
**ENSG00000179151**		*0.07386364*		*0.073863636*				*0.3333333*
**ENSG00000124827**	*0.48502994*		*0.48502994*		*0.05952381*		*0.0697674*	
**ENSG00000043514**	*0.24550898*	*0.44545455*	*0.24550898*	*0.445454545*	*0.273809524*		*0.3546512*	*0.372093*
**ENSG00000117586**		*0.30909091*		*0.309090909*				*0.0755814*
**ENSG00000173157**		*0.3969697*		*0.396969697*				*0.0662651*
**ENSG00000085662**	*0.46*	*0.3030303*	*0.46*	*0.303030303*	*0.214285714*		*0.2702703*	*0.0755814*
**ENSG00000165555**		*0.49090909*		*0.490909091*				*0.0755814*
**ENSG00000101367**		*0.42767296*		*0.427672956*				
**ENSG00000183729**	*0.32035928*		*0.32035928*		*0.482142857*		*0.494186*	
**ENSG00000178460**		*0.05681818*		*0.056818182*				
**ENSG00000187688**	*0.46060606*	*0.31818182*	*0.46060606*	*0.318181818*	*0.246987952*		*0.1162791*	*0.244186*
**ENSG00000133997**		*0.22121212*		*0.221212121*				*0.4534884*
**ENSG00000157985**		*0.37575758*		*0.375757576*				*0.1823529*
**ENSG00000213160**		*0.3969697*		*0.396969697*				*0.4886364*
**ENSG00000186803**		*0.17045455*		*0.170454545*				*0.4047619*
**ENSG00000166352**	*0.25555556*	*0.08181818*	*0.25555556*	*0.081818182*	*0.090909091*			
**ENSG00000130413**	*0.38953488*	*0.47222222*	*0.38953488*	*0.472222222*	*0.44047619*		*0.494186*	*0.3837209*
**ENSG00000061273**		*0.22121212*		*0.221212121*				*0.0909091*
**ENSG00000180999**		*0.48787879*		*0.487878788*				*0.3197674*
**ENSG00000170364**	*0.4*		*0.4*		*0.188888889*		*0.1477273*	
**ENSG00000143772**		*0.27575758*		*0.275757576*				*0.4011628*
**ENSG00000134247**		*0.46646341*		*0.466463415*				*0.4518072*
**ENSG00000089847**	*0.22754491*		*0.22754491*		*0.422619048*		*0.4476744*	
**ENSG00000159189**	*0.14044944*	*0.33146067*	*0.14044944*	*0.331460674*				*0.4111111*
**ENSG00000186185**		*0.26363636*		*0.263636364*				*0.0872093*
**ENSG00000103353**	*0.20958084*	*0.09756098*	*0.20958084*	*0.097560976*				*0.2529412*
**ENSG00000186265**								*0.2034884*

**ENSG00000103479**		*0.38787879*		*0.387878788*				*0.1176471*
**ENSG00000213246**		*0.48787879*		*0.487878788*				*0.255814*
**ENSG00000101343**	*0.40718563*		*0.40718563*		*0.279761905*		*0.4418605*	*0.4651163*
**ENSG00000116194**		*0.38414634*		*0.384146341*				*0.4294118*
**ENSG00000185104**	*0.11377246*	*0.1*	*0.11377246*	*0.1*				*0.0697674*
**ENSG00000196083**		*0.2804878*		*0.280487805*				*0.4888889*
**ENSG00000115919**	*0.11235955*	*0.09509202*	*0.11235955*	*0.095092025*				*0.2619048*
**ENSG00000166743**		*0.16666667*		*0.166666667*				*0.3662791*
**ENSG00000144460**		*0.41158537*		*0.411585366*				*0.4534884*
**ENSG00000160255**		*0.28787879*		*0.287878788*	*0.285714286*		*0.2965116*	*0.2906977*
**ENSG00000186973**	*0.2183908*		*0.2183908*		*0.273809524*		*0.3023256*	
**ENSG00000135315**	*0.20555556*		*0.20555556*					
**ENSG00000147613**		*0.34969325*		*0.349693252*				*0.4302326*
**ENSG00000169035**	*0.47590361*		*0.47590361*		*0.432926829*		*0.4882353*	
**ENSG00000168811**		*0.1402439*		*0.140243902*				*0.1506024*
**ENSG00000187054**	*0.47904192*	*0.42727273*	*0.47904192*	*0.427272727*	*0.5*		*0.4186047*	*0.375*
**ENSG00000255394**		*0.43333333*		*0.433333333*				*0.4883721*
**ENSG00000136144**		*0.45833333*		*0.458333333*				*0.4352941*
**ENSG00000188133**		*0.29393939*		*0.293939394*				*0.0697674*
**ENSG00000153006**	*0.19318182*	*0.44444444*	*0.19318182*	*0.444444444*	*0.488888889*		*0.4545455*	*0.4431818*
**ENSG00000103274**		*0.19393939*		*0.193939394*				*0.3313953*
**ENSG00000107447**		*0.13333333*		*0.133333333*				*0.0639535*
**ENSG00000111331**	*0.48181818*	*0.3803681*	*0.48181818*	*0.380368098*	*0.325301205*		*0.1511628*	*0.4069767*
**ENSG00000168300**		*0.30113636*		*0.301136364*				*0.3846154*
**ENSG00000005339**	*0.28443114*	*0.27272727*	*0.28443114*	*0.272727273*	*0.220238095*		*0.0813953*	*0.3255814*
**ENSG00000120656**	*0.21257485*		*0.21257485*					
**ENSG00000130021**		*0.42682927*		*0.426829268*				*0.3953488*
**ENSG00000255730**	*0.18862275*		*0.18862275*		*0.470238095*		*0.4825581*	
**ENSG00000151806**		*0.3902439*		*0.390243902*				*0.4176471*
**ENSG00000124593**		*0.47878788*		*0.478787879*	*0.273809524*		*0.1337209*	*0.3647059*
**ENSG00000118454**	*0.07777778*		*0.07777778*					
**ENSG00000100650**					*0.160714286*		*0.1337209*	
**ENSG00000059769**		*0.12424242*		*0.124242424*				*0.4823529*
**ENSG00000037749**	*0.48502994*	*0.37777778*	*0.48502994*	*0.377777778*	*0.30952381*		*0.2831325*	*0.1590909*
**ENSG00000175104**		*0.14367816*		*0.143678161*				*0.4534884*
**ENSG00000100504**		*0.33636364*		*0.336363636*				*0.4302326*
**ENSG00000134910**	*0.37125749*		*0.37125749*		*0.102409639*		*0.1235294*	
**ENSG00000214140**		*0.19318182*		*0.193181818*				*0.1477273*
**ENSG00000100483**		*0.40555556*		*0.405555556*				*0.2682927*
**ENSG00000166863**					*0.122222222*		*0.1022727*	
**ENSG00000127337**					*0.307228916*		*0.3023256*	
**ENSG00000213402**	*0.08682635*		*0.08682635*		*0.31547619*		*0.2616279*	
**ENSG00000182180**		*0.05*		*0.05*				*0.3181818*
**ENSG00000004139**		*0.45757576*		*0.457575758*				*0.2267442*
**ENSG00000106608**	*0.24850299*	*0.33636364*	*0.24850299*	*0.336363636*	*0.289156627*		*0.3546512*	*0.3430233*
**ENSG00000143578**	*0.38622754*		*0.38622754*		*0.05952381*		*0.0697674*	
**ENSG00000138378**		*0.4030303*		*0.403030303*				*0.2209302*
**ENSG00000087586**	*0.3502994*	*0.22699387*	*0.3502994*	*0.226993865*				*0.3705882*
**ENSG00000215012**		*0.3*		*0.3*				*0.4244186*
**ENSG00000170571**		*0.47752809*		*0.47752809*				*0.3488372*
**ENSG00000166135**	*0.13473054*	*0.2030303*	*0.13473054*	*0.203030303*	*0.148809524*		*0.2034884*	*0.1453488*
**ENSG00000259141**		*0.14545455*		*0.145454545*				*0.3895349*
**ENSG00000186866**		*0.38650307*		*0.386503067*				*0.4011628*
**ENSG00000203993**	*0.34131737*		*0.34131737*		*0.077380952*		*0.1162791*	

**ENSG00000131263**		*0.08588957*		*0.085889571*			
**ENSG00000142208**	*0.38343558*		*0.38343558*		*0.084337349*	*0.126506*	
**ENSG00000145979**		*0.11111111*		*0.111111111*			*0.5*
**ENSG00000011201**		*0.46111111*		*0.461111111*			*0.3333333*
**ENSG00000100401**	*0.34730539*	*0.40909091*	*0.34730539*	*0.409090909*	*0.244047619*	*0.2267442*	
**ENSG00000175206**		*0.13030303*		*0.13030303*			
**ENSG00000145293**	*0.26946108*		*0.26946108*		*0.083333333*	*0.0813953*	*0.127907*
**ENSG00000133961**	*0.19444444*	*0.25757576*	*0.19444444*	*0.257575758*			*0.2333333*
**ENSG00000159228**		*0.41818182*		*0.418181818*			*0.3555556*
**ENSG00000146872**		*0.31111111*		*0.311111111*			*0.2674419*
**ENSG00000134352**		*0.10555556*		*0.105555556*			
**ENSG00000127948**	*0.33333333*	*0.35151515*	*0.33333333*	*0.351515152*	*0.465116279*	*0.4418605*	*0.3604651*
**ENSG00000150361**		*0.15740741*		*0.157407407*			
**ENSG00000164197**		*0.30898876*		*0.308988764*			*0.1627907*
**ENSG00000137648**		*0.39090909*		*0.390909091*			*0.3546512*
**ENSG00000021461**		*0.12727273*		*0.127272727*			
**ENSG00000106686**	*0.06024096*	*0.22916667*	*0.06024096*	*0.229166667*			*0.494186*
**ENSG00000177733**		*0.2030303*		*0.203030303*			*0.0523256*
**ENSG00000175892**		*0.30606061*		*0.306060606*			*0.4882353*
**ENSG00000162585**	*0.44011976*	*0.41212121*	*0.44011976*	*0.412121212*	*0.31547619*	*0.1931818*	*0.1931818*
**ENSG00000198785**		*0.46666667*		*0.466666667*			*0.2965116*
**ENSG00000149922**					*0.450617284*	*0.4111111*	
**ENSG00000135747**	*0.21987952*	*0.44252874*	*0.21987952*	*0.442528736*	*0.101190476*	*0.1511628*	*0.5*
**ENSG00000196335**	*0.49401198*		*0.49401198*		*0.398809524*	*0.3430233*	
**ENSG00000175229**		*0.18333333*		*0.183333333*			
**ENSG00000175193**		*0.49444444*		*0.494444444*			*0.4772727*
**ENSG00000167491**		*0.37272727*		*0.372727273*			*0.2732558*
**ENSG00000143476**		*0.41463415*		*0.414634146*	*0.05952381*		*0.3430233*
**ENSG00000106367**		*0.43333333*		*0.433333333*			*0.4404762*
**ENSG00000164651**	*0.06287425*		*0.06287425*		*0.363095238*	*0.3953488*	
**ENSG00000149380**		*0.17272727*		*0.172727273*			*0.2848837*
**ENSG00000133812**		*0.39393939*		*0.393939394*			*0.3647059*
**ENSG00000141867**		*0.48787879*		*0.487878788*			*0.3764706*
**ENSG00000187017**		*0.10909091*		*0.109090909*			
**ENSG00000124208**		*0.33146067*		*0.331460674*			*0.2613636*
**ENSG00000198270**	*0.31024096*	*0.19090909*	*0.31024096*	*0.190909091*	*0.304878049*	*0.2235294*	*0.2235294*
**ENSG00000205084**		*0.23333333*		*0.233333333*			*0.244186*
**ENSG00000165066**		*0.41463415*		*0.414634146*			*0.3139535*
**ENSG00000104972**		*0.15243902*		*0.152439024*			
**ENSG00000177963**		*0.16091954*		*0.16091954*			*0.4651163*
**ENSG00000179921**	*0.2005988*		*0.2005988*		*0.421686747*	*0.4011628*	
**ENSG00000119787**		*0.32777778*		*0.327777778*			*0.1860465*
**ENSG00000166396**	*0.33333333*	*0.49438202*	*0.33333333*	*0.494382022*	*0.4*	*0.3295455*	*0.4642857*
**ENSG00000197991**	*0.40662651*	*0.32716049*	*0.40662651*	*0.327160494*	*0.470238095*	*0.4360465*	*0.3941176*
**ENSG00000181896**					*0.233333333*	*0.0909091*	
**ENSG00000205213**		*0.38181818*		*0.381818182*			*0.3546512*
**ENSG00000144619**	*0.26047904*	*0.48295455*	*0.26047904*	*0.482954545*			*0.4518072*
**ENSG00000168427**		*0.44478528*		*0.444785276*			*0.3117647*
**ENSG00000236287**	*0.06287425*		*0.06287425*				
**ENSG00000115648**	*0.27222222*	*0.13888889*	*0.27222222*	*0.138888889*	*0.244444444*	*0.2093023*	*0.5*
**ENSG00000122482**		*0.12727273*		*0.127272727*			*0.0523256*
**ENSG00000137713**		*0.38414634*		*0.384146341*			*0.1686047*
**ENSG00000189306**		*0.19318182*		*0.193181818*			*0.3181818*
**ENSG00000204540**	*0.30538922*	*0.21515152*	*0.30538922*	*0.215151515*	*0.369047619*	*0.4593023*	*0.3023256*

**ENSG00000155366**	*0.4251497*		*0.4251497*		*0.327380952*		*0.3604651*	
**ENSG00000187902**		*0.5*		*0.5*				*0.2058824*
**ENSG00000255408**		*0.05151515*		*0.051515152*				*0.1162791*
**ENSG00000155833**		*0.37272727*		*0.372727273*				*0.1453488*
**ENSG00000144331**		*0.1*		*0.1*				*0.4651163*
**ENSG00000204300**		*0.46604938*		*0.466049383*				*0.4823529*
**ENSG00000188931**		*0.06666667*		*0.066666667*				*0.3058824*
**ENSG00000112701**	*0.26666667*	*0.33888889*	*0.26666667*	*0.338888889*	*0.188888889*		*0.1818182*	*0.313253*
**ENSG00000198455**		*0.18787879*		*0.187878788*				*0.2588235*
**ENSG00000144589**	*0.38023952*		*0.38023952*					
**ENSG00000164164**		*0.45402299*		*0.454022989*				*0.3888889*
**ENSG00000152760**		*0.34756098*		*0.347560976*				*0.0813953*
**ENSG00000166548**	*0.45454545*		*0.45454545*		*0.355555556*		*0.1931818*	*0.1569767*
**ENSG00000142937**	*0.34730539*		*0.34730539*		*0.470238095*		*0.494186*	
**ENSG00000198753**	*0.0873494*		*0.0873494*					
**ENSG00000241399**		*0.41768293*		*0.417682927*				*0.4011628*
**ENSG00000105501**		*0.05454545*		*0.054545455*				*0.1*
**ENSG00000083307**		*0.41463415*		*0.414634146*			*0.0795455*	*0.4069767*
**ENSG00000160298**		*0.42592593*		*0.425925926*				*0.3588235*
**ENSG00000172123**	*0.30113636*		*0.30113636*		*0.303571429*		*0.1976744*	
**ENSG00000178860**		*0.28333333*		*0.283333333*				*0.255814*
**ENSG00000187773**		*0.40606061*		*0.406060606*				*0.5*
**ENSG00000122180**	*0.47839506*		*0.47839506*		*0.481707317*		*0.4647059*	
**ENSG00000186684**		*0.46060606*		*0.460606061*				*0.2965116*
**ENSG00000147485**		*0.25151515*		*0.251515152*				*0.1162791*
**ENSG00000142677**		*0.43081761*		*0.43081761*				*0.2325581*
**ENSG00000184545**		*0.43030303*		*0.43030303*				*0.4431818*
**ENSG00000049860**	*0.24550898*		*0.24550898*		*0.196428571*		*0.3058824*	
**ENSG00000008853**	*0.41616766*	*0.4*	*0.41616766*	*0.4*	*0.101190476*		*0.2093023*	*0.494186*
**ENSG00000196597**	*0.45555556*		*0.45555556*		*0.311111111*		*0.3409091*	*0.3197674*
**ENSG00000100968**	*0.43712575*	*0.33939394*	*0.43712575*	*0.339393939*	*0.25*		*0.3081395*	*0.4534884*
**ENSG00000205593**		*0.05345912*		*0.053459119*				
**ENSG00000198945**		*0.11515152*		*0.115151515*				
**ENSG00000171469**	*0.17365269*	*0.17272727*	*0.17365269*	*0.172727273*				
**ENSG00000152767**	*0.05747126*	*0.36666667*	*0.05747126*	*0.366666667*				*0.2848837*
**ENSG00000144580**	*0.21341463*		*0.21341463*		*0.25*		*0.2764706*	*0.125*
**ENSG00000132003**		*0.2969697*		*0.296969697*				*0.2790698*
**ENSG00000167850**					*0.446428571*		*0.4186047*	
**ENSG00000151468**	*0.06586826*	*0.33636364*	*0.06586826*	*0.336363636*	*0.375*		*0.2848837*	*0.2790698*
**ENSG00000177200**		*0.26687117*		*0.266871166*				*0.3012048*
**ENSG00000162407**		*0.48170732*		*0.481707317*				*0.4883721*
**ENSG00000113971**	*0.13888889*	*0.13939394*	*0.13888889*	*0.139393939*	*0.088888889*			*0.3255814*
**ENSG00000177800**		*0.28963415*		*0.289634146*	*0.226190476*		*0.1627907*	*0.3430233*
**ENSG00000187231**		*0.30909091*		*0.309090909*				*0.2619048*
**ENSG00000113739**								*0.0988372*
**ENSG00000138653**		*0.12777778*		*0.127777778*				*0.2325581*
**ENSG00000164385**	*0.15568862*	*0.20606061*	*0.15568862*	*0.206060606*	*0.214285714*		*0.2034884*	*0.255814*
**ENSG00000167635**	*0.19760479*		*0.19760479*					
**ENSG00000147535**	*0.12777778*		*0.12777778*		*0.333333333*		*0.3372093*	
**ENSG00000184381**	*0.10909091*	*0.41666667*	*0.10909091*	*0.416666667*	*0.130952381*		*0.122093*	*0.4709302*
**ENSG00000131196**		*0.17272727*		*0.172727273*				*0.1453488*
**ENSG00000137171**	*0.41017964*	*0.12121212*	*0.41017964*	*0.121212121*	*0.208333333*		*0.2383721*	*0.244186*
**ENSG00000119630**		*0.17073171*		*0.170731707*				*0.2*
**ENSG00000101247**		*0.32727273*		*0.327272727*				*0.4767442*

**ENSG00000196700**		*0.11111111*		*0.111111111*				
**ENSG00000198930**		*0.43209877*		*0.432098765*				*0.2857143*
**ENSG00000233539**		*0.25757576*		*0.257575758*				*0.4302326*
**ENSG00000185920**	*0.17241379*	*0.26704545*	*0.17241379*	*0.267045455*	*0.136363636*		*0.1395349*	*0.3837209*
**ENSG00000225683**		*0.38636364*		*0.386363636*				*0.4767442*
**ENSG00000005001**		*0.22988506*		*0.229885057*				*0.0681818*
**ENSG00000089639**		*0.27878788*		*0.278787879*				*0.1337209*
**ENSG00000102837**	*0.15361446*	*0.5*	*0.15361446*	*0.5*	*0.178571429*		*0.2209302*	*0.4709302*
**ENSG00000152433**	*0.26111111*	*0.46060606*	*0.26111111*	*0.460606061*	*0.266666667*		*0.3409091*	*0.3255814*
**ENSG00000036828**		*0.36060606*		*0.360606061*				*0.4411765*
**ENSG00000188243**		*0.28235294*		*0.282352941*	*0.077777778*		*0.0777778*	*0.1590909*
**ENSG00000100403**		*0.26060606*		*0.260606061*				*0.375*
**ENSG00000236301**	*0.31818182*	*0.3969697*	*0.31818182*	*0.396969697*	*0.44047619*		*0.3488372*	*0.297619*
**ENSG00000150672**	*0.28742515*	*0.36060606*	*0.28742515*	*0.360606061*	*0.066666667*		*0.1888889*	*0.2616279*
**ENSG00000188677**	*0.45205479*	*0.38787879*	*0.45205479*	*0.387878788*	*0.182926829*		*0.1686747*	*0.2616279*
**ENSG00000187260**								*0.0581395*
**ENSG00000135220**		*0.35057471*		*0.350574713*				*0.3977273*
**ENSG00000101049**	*0.25149701*	*0.1554878*	*0.25149701*	*0.155487805*	*0.344444444*		*0.2222222*	*0.2619048*
**ENSG00000171435**		*0.42073171*		*0.420731707*				*0.1686047*
**ENSG00000174292**		*0.12121212*		*0.121212121*				*0.3372093*
**ENSG00000197933**		*0.47204969*		*0.472049689*				*0.3823529*
**ENSG00000229619**		*0.07272727*		*0.072727273*				
**ENSG00000068903**		*0.35365854*		*0.353658537*				*0.4011628*
**ENSG00000168002**		*0.23636364*		*0.236363636*				*0.1802326*
**ENSG00000198917**		*0.18787879*		*0.187878788*				*0.2732558*
**ENSG00000197724**		*0.38787879*		*0.387878788*				*0.2965116*
**ENSG00000163960**		*0.48181818*		*0.481818182*				*0.1222222*
**ENSG00000122515**		*0.49393939*		*0.493939394*				*0.1860465*
**ENSG00000138623**		*0.06179775*		*0.061797753*				*0.127907*
**ENSG00000204103**		*0.11212121*		*0.112121212*				
**ENSG00000183549**	*0.49401198*	*0.24390244*	*0.49401198*	*0.243902439*	*0.083333333*		*0.0872093*	*0.1569767*
**ENSG00000163607**	*0.12275449*		*0.12275449*		*0.347560976*		*0.3863636*	
**ENSG00000009307**	*0.12921348*	*0.19090909*	*0.12921348*	*0.190909091*				*0.0930233*
**ENSG00000132153**	*0.49401198*		*0.49401198*		*0.240963855*		*0.2529412*	
**ENSG00000162494**		*0.38181818*		*0.381818182*				*0.4186047*
**ENSG00000138294**	*0.20426829*		*0.20426829*					
**ENSG00000165672**		*0.41818182*		*0.418181818*				*0.3977273*
**ENSG00000205978**		*0.28181818*		*0.281818182*				*0.4883721*
**ENSG00000196208**	*0.19161677*	*0.49090909*	*0.19161677*	*0.490909091*	*0.476190476*		*0.4940476*	*0.4360465*
**ENSG00000206043**	*0.46407186*	*0.40506329*	*0.46407186*	*0.405063291*	*0.43452381*		*0.4593023*	*0.5*
**ENSG00000167130**					*0.19047619*		*0.255814*	*0.1627907*
**ENSG00000234438**		*0.46060606*		*0.460606061*				*0.1976744*
**ENSG00000185231**		*0.48484848*		*0.484848485*				*0.4883721*
**ENSG00000168264**		*0.31707317*		*0.317073171*				*0.3072289*
**ENSG00000108576**	*0.30722892*	*0.40606061*	*0.30722892*	*0.406060606*	*0.136904762*		*0.1162791*	*0.1860465*
**ENSG00000130764**		*0.46646341*		*0.466463415*				*0.1104651*
**ENSG00000093217**	*0.28143713*	*0.4*	*0.28143713*	*0.4*				*0.5*
**ENSG00000182150**	*0.45808383*	*0.31402439*	*0.45808383*	*0.31402439*	*0.333333333*		*0.422619*	*0.377907*
**ENSG00000182177**	*0.32934132*	*0.11515152*	*0.32934132*	*0.115151515*	*0.232142857*		*0.122093*	
**ENSG00000143322**		*0.46666667*		*0.466666667*				*0.4555556*
**ENSG00000163746**	*0.18862275*	*0.33333333*	*0.18862275*	*0.333333333*	*0.351190476*		*0.4534884*	*0.2267442*
**ENSG00000240184**		*0.20224719*		*0.202247191*				*0.1477273*
**ENSG00000101892**		*0.39393939*		*0.393939394*				*0.3430233*
**ENSG00000134899**	*0.3502994*	*0.42424242*	*0.3502994*	*0.424242424*	*0.380952381*		*0.3647059*	*0.4764706*

**ENSG00000164220**		*0.49695122*		*0.49695122*			*0.3953488*
**ENSG00000052841**							*0.0666667*
**ENSG00000186666**		*0.11818182*		*0.118181818*			
**ENSG00000115556**		*0.40606061*		*0.406060606*	*0.119047619*	*0.127907*	*0.2352941*
**ENSG00000123609**		*0.43030303*		*0.43030303*			*0.3511905*
**ENSG00000143278**		*0.38484848*		*0.384848485*			*0.1764706*
**ENSG00000125510**	*0.46407186*	*0.08181818*	*0.46407186*	*0.081818182*	*0.410714286*	*0.372093*	
**ENSG00000255212**		*0.21515152*		*0.215151515*			*0.3430233*
**ENSG00000197128**		*0.38787879*		*0.387878788*			*0.3630952*
**ENSG00000115685**		*0.20909091*		*0.209090909*	*0.226190476*	*0.2093023*	*0.3571429*
**ENSG00000141527**	*0.47005988*	*0.37878788*	*0.47005988*	*0.378787879*	*0.172619048*	*0.244186*	*0.3255814*
**ENSG00000186205**		*0.13888889*		*0.138888889*			*0.2732558*
**ENSG00000110811**	*0.13173653*	*0.47839506*	*0.13173653*	*0.478395062*	*0.44047619*	*0.4651163*	*0.4146341*
**ENSG00000140285**		*0.44545455*		*0.445454545*			*0.472973*
**ENSG00000027644**		*0.12068966*		*0.120689655*			*0.1477273*
**ENSG00000124429**		*0.11212121*		*0.112121212*			*0.4069767*
**ENSG00000128052**	*0.39102564*		*0.39102564*		*0.284090909*	*0.2790698*	
**ENSG00000162771**	*0.29041916*		*0.29041916*		*0.44047619*	*0.4709302*	
**ENSG00000152092**		*0.36363636*		*0.363636364*			*0.3313953*
**ENSG00000042445**		*0.4127907*		*0.412790698*			*0.4431818*
**ENSG00000177453**	*0.34831461*		*0.34831461*		*0.186746988*	*0.2666667*	
**ENSG00000135472**	*0.19461078*	*0.36363636*	*0.19461078*	*0.363636364*	*0.297619048*	*0.3235294*	*0.372093*
**ENSG00000126777**	*0.27325581*		*0.27325581*		*0.395348837*	*0.3888889*	
**ENSG00000155465**	*0.49700599*	*0.13030303*	*0.49700599*	*0.13030303*	*0.44047619*	*0.4651163*	*0.1104651*
**ENSG00000152147**	*0.31437126*	*0.05757576*	*0.31437126*	*0.057575758*	*0.154761905*	*0.122093*	*0.3255814*
**ENSG00000149305**		*0.20909091*		*0.209090909*			*0.1627907*
**ENSG00000173915**	*0.23652695*		*0.23652695*		*0.398809524*	*0.4767442*	
**ENSG00000204842**	*0.28333333*	*0.19393939*	*0.28333333*	*0.193939394*	*0.452380952*	*0.4418605*	*0.4431818*
**ENSG00000133119**		*0.07575758*		*0.075757576*			*0.3430233*
**ENSG00000131080**		*0.15757576*		*0.157575758*			
**ENSG00000102882**		*0.11666667*		*0.116666667*			
**ENSG00000090989**		*0.15644172*		*0.156441718*			*0.4883721*
**ENSG00000259075**		*0.35582822*		*0.355828221*			*0.1802326*
**ENSG00000143811**		*0.14545455*		*0.145454545*			*0.0930233*
**ENSG00000103126**	*0.17964072*	*0.21818182*	*0.17964072*	*0.218181818*	*0.113095238*	*0.1046512*	*0.1162791*
**ENSG00000143158**		*0.23888889*		*0.238888889*			*0.0909091*
**ENSG00000183283**	*0.47005988*	*0.18125*	*0.47005988*	*0.18125*	*0.327380952*	*0.3430233*	*0.4352941*
**ENSG00000181924**	*0.26966292*		*0.26966292*		*0.193181818*	*0.125*	
**ENSG00000165097**		*0.21779141*		*0.217791411*			*0.1686747*
**ENSG00000166509**		*0.46666667*		*0.466666667*			*0.4404762*
**ENSG00000188167**		*0.22865854*		*0.228658537*			*0.0581395*
**ENSG00000169862**	*0.38323353*	*0.38484848*	*0.38323353*	*0.384848485*	*0.398809524*	*0.4244186*	*0.494186*
**ENSG00000100142**		*0.34242424*		*0.342424242*			*0.3372093*
**ENSG00000187474**	*0.2994012*	*0.33333333*	*0.2994012*	*0.333333333*	*0.267857143*	*0.122093*	*0.4772727*
**ENSG00000021776**	*0.44879518*	*0.46666667*	*0.44879518*	*0.466666667*	*0.053571429*		*0.2888889*
**ENSG00000183423**	*0.45209581*	*0.16768293*	*0.45209581*	*0.167682927*	*0.494047619*	*0.4709302*	*0.3433735*
**ENSG00000158863**		*0.45*		*0.45*			*0.3977273*
**ENSG00000136943**		*0.13414634*		*0.134146341*			*0.4235294*
**ENSG00000177669**		*0.37222222*		*0.372222222*			*0.2*
**ENSG00000144426**							*0.0755814*
**ENSG00000003393**		*0.1*		*0.1*			*0.2352941*
**ENSG00000127564**	*0.44886364*		*0.44886364*		*0.202380952*	*0.2267442*	
**ENSG00000184887**		*0.26060606*		*0.260606061*			*0.1511628*
**ENSG00000171450**		*0.05757576*		*0.057575758*			

**ENSG00000148719**								*0.0697674*
**ENSG00000172578**		*0.47191011*		*0.471910112*				*0.2325581*
**ENSG00000006459**		*0.37222222*		*0.372222222*				*0.3444444*
**ENSG00000117859**	*0.21856287*	*0.16363636*	*0.21856287*	*0.163636364*	*0.261904762*		*0.2383721*	*0.0941176*
**ENSG00000204267**	*0.42777778*	*0.3*	*0.42777778*	*0.3*	*0.4*		*0.4431818*	*0.4186047*
**ENSG00000149735**		*0.2969697*		*0.296969697*				*0.4418605*
**ENSG00000173852**		*0.3006135*		*0.300613497*				*0.1158537*
**ENSG00000111801**							*0.0705882*	*0.127907*
**ENSG00000186314**		*0.15151515*		*0.151515152*				*0.25*
**ENSG00000112679**		*0.41666667*		*0.416666667*				*0.2222222*
**ENSG00000007237**		*0.4969697*		*0.496969697*	*0.11875*		*0.1666667*	*0.4939759*
**ENSG00000130023**					*0.43452381*		*0.4534884*	
**ENSG00000232196**	*0.11111111*	*0.33522727*	*0.11111111*	*0.335227273*				*0.1931818*
**ENSG00000165188**	*0.5*	*0.3258427*	*0.5*	*0.325842697*	*0.458333333*		*0.4186047*	*0.0595238*
**ENSG00000177807**		*0.36666667*		*0.366666667*				*0.3372093*
**ENSG00000196268**		*0.30606061*		*0.306060606*				*0.2613636*
**ENSG00000110693**	*0.17065868*	*0.44848485*	*0.17065868*	*0.448484848*				*0.4302326*
**ENSG00000171195**	*0.33832335*	*0.48787879*	*0.33832335*	*0.487878788*	*0.154761905*		*0.0930233*	*0.3375*
**ENSG00000163793**		*0.20606061*		*0.206060606*				*0.0697674*
**ENSG00000102996**		*0.38484848*		*0.384848485*				*0.4709302*
**ENSG00000205111**								*0.1744186*
**ENSG00000128274**		*0.34242424*		*0.342424242*				*0.4476744*
**ENSG00000143369**	*0.11676647*		*0.11676647*					
**ENSG00000198829**		*0.48136646*		*0.48136646*				*0.313253*
**ENSG00000149679**		*0.43636364*		*0.436363636*				*0.0930233*
**ENSG00000140854**	*0.21385542*	*0.22121212*	*0.21385542*	*0.221212121*				*0.0813953*
**ENSG00000125775**	*0.29213483*	*0.38888889*	*0.29213483*	*0.388888889*			*0.0813953*	*0.4882353*
**ENSG00000181007**		*0.2969697*		*0.296969697*				*0.3604651*
**ENSG00000165606**		*0.09202454*		*0.09202454*				
**ENSG00000032742**	*0.22777778*	*0.17378049*	*0.22777778*	*0.173780488*	*0.411111111*		*0.2790698*	
**ENSG00000136807**		*0.35060976*		*0.350609756*				*0.4011628*
**ENSG00000125676**		*0.35632184*		*0.356321839*				*0.2272727*
**ENSG00000184371**		*0.43103448*		*0.431034483*				*0.4204545*
**ENSG00000142765**	*0.33233533*	*0.3136646*	*0.33233533*	*0.313664596*				*0.255814*
**ENSG00000105982**		*0.5*		*0.5*				*0.5*
**ENSG00000235978**	*0.36526946*		*0.36526946*		*0.133333333*		*0.0930233*	
**ENSG00000259133**	*0.08083832*		*0.08083832*		*0.142857143*		*0.1453488*	
**ENSG00000100678**	*0.25149701*	*0.29090909*	*0.25149701*	*0.290909091*	*0.333333333*		*0.3372093*	*0.4709302*
**ENSG00000131323**		*0.14848485*		*0.148484848*				*0.3529412*
**ENSG00000179772**	*0.46629213*		*0.46629213*					
**ENSG00000173083**		*0.3*		*0.3*				*0.1802326*
**ENSG00000117834**		*0.17575758*		*0.175757576*				*0.2151163*
**ENSG00000125999**		*0.47222222*		*0.472222222*				*0.1704545*
**ENSG00000169245**		*0.5*		*0.5*				*0.0588235*
**ENSG00000124535**		*0.33939394*		*0.339393939*				*0.4*
**ENSG00000143858**		*0.44242424*		*0.442424242*				*0.5*
**ENSG00000057657**		*0.05555556*		*0.055555556*				*0.0813953*
**ENSG00000186509**	*0.2125*	*0.35454545*	*0.2125*	*0.354545455*				*0.1627907*
**ENSG00000142751**	*0.07185629*	*0.4068323*	*0.07185629*	*0.406832298*				
**ENSG00000181619**		*0.32317073*		*0.323170732*				*0.1511628*
**ENSG00000174021**	*0.48295455*		*0.48295455*		*0.18452381*		*0.1976744*	
**ENSG00000064547**		*0.38181818*		*0.381818182*				*0.4360465*
**ENSG00000006652**	*0.49404762*	*0.46036585*	*0.49404762*	*0.460365854*	*0.2*		*0.1511628*	*0.2209302*
**ENSG00000163319**	*0.48203593*	*0.45121951*	*0.48203593*	*0.451219512*				*0.375*

**ENSG00000125814**		*0.33939394*		*0.339393939*				*0.3255814*
**ENSG00000163431**	*0.35329341*	*0.35454545*	*0.35329341*	*0.354545455*				*0.0697674*
**ENSG00000148841**	*0.29341317*	*0.36363636*	*0.29341317*	*0.363636364*	*0.214285714*		*0.2588235*	*0.255814*
**ENSG00000198963**		*0.07954545*		*0.079545455*				*0.3488372*
**ENSG00000189060**		*0.26969697*		*0.26969697*				*0.494186*
**ENSG00000070413**	*0.27222222*	*0.41463415*	*0.27222222*	*0.414634146*	*0.397727273*		*0.3295455*	*0.4047619*
**ENSG00000120709**		*0.34545455*		*0.345454545*				*0.5*
**ENSG00000138107**		*0.43939394*		*0.439393939*				*0.3809524*
**ENSG00000112186**	*0.14371257*	*0.40909091*	*0.14371257*	*0.409090909*	*0.125*		*0.1337209*	*0.25*
**ENSG00000156959**	*0.33636364*		*0.33636364*		*0.494047619*		*0.4302326*	
**ENSG00000123080**	*0.08682635*	*0.0969697*	*0.08682635*	*0.096969697*	*0.133333333*		*0.1136364*	*0.0813953*
**ENSG00000198952**		*0.46666667*		*0.466666667*				*0.4058824*
**ENSG00000189283**		*0.39090909*		*0.390909091*				*0.1976744*
**ENSG00000166441**	*0.18562874*	*0.45454545*	*0.18562874*	*0.454545455*			*0.0647059*	*0.3255814*
**ENSG00000140749**		*0.12727273*		*0.127272727*				*0.2176471*
**ENSG00000115084**	*0.48484848*	*0.42121212*	*0.48484848*	*0.421212121*	*0.455555556*		*0.3181818*	*0.3111111*
**ENSG00000102934**		*0.1*		*0.1*				*0.3139535*
**ENSG00000179546**		*0.33333333*		*0.333333333*				*0.244186*
**ENSG00000250565**	*0.45180723*	*0.33030303*	*0.45180723*	*0.33030303*	*0.160714286*		*0.1860465*	*0.372093*
**ENSG00000173145**	*0.10778443*	*0.11666667*	*0.10778443*	*0.116666667*				
**ENSG00000101019**		*0.33333333*		*0.333333333*				*0.2674419*
**ENSG00000138111**	*0.32335329*	*0.45426829*	*0.32335329*	*0.454268293*	*0.345238095*		*0.3902439*	*0.4588235*
**ENSG00000055955**	*0.24850299*		*0.24850299*		*0.494047619*		*0.3888889*	
**ENSG00000157368**	*0.24157303*		*0.24157303*		*0.488888889*		*0.4090909*	*0.2613636*
**ENSG00000258735**		*0.43167702*		*0.431677019*				*0.4642857*
**ENSG00000183454**		*0.46060606*		*0.460606061*				*0.5*
**ENSG00000172638**		*0.45454545*		*0.454545455*				*0.25*
**ENSG00000205464**					*0.056818182*			
**ENSG00000149527**		*0.3*		*0.3*				
**ENSG00000213809**		*0.28735632*		*0.287356322*				*0.3522727*
**ENSG00000198042**	*0.32098765*	*0.37777778*	*0.32098765*	*0.377777778*	*0.430232558*		*0.4558824*	*0.4767442*
**ENSG00000165168**		*0.09393939*		*0.093939394*				
**ENSG00000143622**	*0.34659091*	*0.27575758*	*0.34659091*	*0.275757576*	*0.088888889*		*0.0795455*	*0.2616279*
**ENSG00000173960**		*0.24242424*		*0.242424242*				*0.0930233*
**ENSG00000163681**	*0.16766467*	*0.39090909*	*0.16766467*	*0.390909091*	*0.488095238*		*0.4593023*	*0.4940476*
**ENSG00000173369**	*0.3030303*		*0.3030303*		*0.348837209*		*0.2613636*	
**ENSG00000118307**	*0.13772455*	*0.39329268*	*0.13772455*	*0.393292683*	*0.279761905*		*0.1941176*	*0.1941176*
**ENSG00000165156**	*0.17777778*		*0.17777778*					
**ENSG00000079385**	*0.37724551*	*0.08695652*	*0.37724551*	*0.086956522*	*0.06547619*		*0.0581395*	*0.2151163*
**ENSG00000105668**		*0.44444444*		*0.444444444*				*0.4534884*
**ENSG00000213171**		*0.3969697*		*0.396969697*				*0.4476744*
**ENSG00000153558**	*0.25449102*	*0.41139241*	*0.25449102*	*0.411392405*	*0.089285714*		*0.0930233*	*0.3941176*
**ENSG00000127838**		*0.43636364*		*0.436363636*				*0.3197674*
**ENSG00000224916**		*0.48181818*		*0.481818182*				*0.4756098*
**ENSG00000198964**		*0.47878788*		*0.478787879*	*0.079268293*		*0.1529412*	*0.255814*
**ENSG00000085733**		*0.29573171*		*0.295731707*				*0.0581395*
**ENSG00000051180**	*0.26436782*	*0.45757576*	*0.26436782*	*0.457575758*	*0.133333333*		*0.1463415*	*0.1704545*
**ENSG00000173210**	*0.43413174*	*0.44545455*	*0.43413174*	*0.445454545*	*0.404761905*		*0.4825581*	*0.4767442*
**ENSG00000106443**								*0.1190476*
**ENSG00000019186**		*0.18787879*		*0.187878788*				*0.4156627*
**ENSG00000179528**	*0.17088608*	*0.1097561*	*0.17088608*	*0.109756098*				*0.1764706*
**ENSG00000177994**		*0.27439024*		*0.274390244*				*0.3372093*
**ENSG00000116521**	*0.5*		*0.5*		*0.244047619*		*0.255814*	
**ENSG00000177683**	*0.08383234*	*0.46969697*	*0.08383234*	*0.46969697*	*0.494047619*		*0.5*	*0.3953488*

**ENSG00000165699**		*0.42727273*		*0.427272727*				*0.4709302*
**ENSG00000186111**		*0.40909091*		*0.409090909*				*0.4825581*
**ENSG00000213614**	*0.42814371*		*0.42814371*		*0.095238095*		*0.1704545*	*0.3662791*
**ENSG00000120253**	*0.43113772*	*0.34242424*	*0.43113772*	*0.342424242*	*0.238095238*		*0.2383721*	*0.2202381*
**ENSG00000112761**	*0.27586207*	*0.23939394*	*0.27586207*	*0.239393939*	*0.377777778*		*0.2727273*	*0.2616279*
**ENSG00000140807**		*0.35151515*		*0.351515152*				*0.3372093*
**ENSG00000164465**	*0.46407186*	*0.45151515*	*0.46407186*	*0.451515152*	*0.142857143*		*0.0523256*	*0.4534884*
**ENSG00000076641**	*0.2245509*	*0.07575758*	*0.2245509*	*0.075757576*	*0.463855422*		*0.4882353*	*0.1511628*
**ENSG00000107679**	*0.13333333*	*0.43902439*	*0.13333333*	*0.43902439*	*0.177777778*		*0.2256098*	*0.1918605*
**ENSG00000165799**	*0.24251497*	*0.32121212*	*0.24251497*	*0.321212121*	*0.345238095*		*0.4069767*	*0.3372093*
**ENSG00000070159**	*0.48502994*		*0.48502994*		*0.277777778*		*0.3333333*	
**ENSG00000165443**	*0.17777778*	*0.18333333*	*0.17777778*	*0.183333333*				*0.2045455*
**ENSG00000165821**		*0.28484848*		*0.284848485*				*0.2267442*
**ENSG00000132581**		*0.25151515*		*0.251515152*				*0.1395349*
**ENSG00000168970**		*0.48295455*		*0.482954545*				*0.3139535*
**ENSG00000154493**	*0.34730539*	*0.29393939*	*0.34730539*	*0.293939394*	*0.363095238*		*0.4418605*	*0.3*
**ENSG00000103222**	*0.24251497*	*0.38888889*	*0.24251497*	*0.388888889*	*0.452380952*		*0.4302326*	*0.2965116*
**ENSG00000128609**	*0.05988024*	*0.28409091*	*0.05988024*	*0.284090909*				*0.4069767*
**ENSG00000254528**		*0.40909091*		*0.409090909*				*0.25*
**ENSG00000135423**		*0.19207317*		*0.192073171*				*0.1*
**ENSG00000108813**		*0.14848485*		*0.148484848*				*0.3546512*
**ENSG00000211584**	*0.20783133*	*0.16363636*	*0.20783133*	*0.163636364*	*0.355421687*		*0.372093*	*0.3488372*
**ENSG00000006659**		*0.24444444*		*0.244444444*				*0.125*
**ENSG00000072080**		*0.05151515*		*0.051515152*				
**ENSG00000089692**	*0.23053892*		*0.23053892*					
**ENSG00000119986**	*0.23006135*		*0.23006135*		*0.43452381*		*0.4069767*	
**ENSG00000125740**		*0.37575758*		*0.375757576*				*0.2151163*
**ENSG00000122122**		*0.39444444*		*0.394444444*				*0.1136364*
**ENSG00000176136**	*0.20958084*	*0.16060606*	*0.20958084*	*0.160606061*	*0.25*		*0.2209302*	*0.4186047*
**ENSG00000005302**								*0.1704545*
**ENSG00000184909**		*0.42727273*		*0.427272727*				*0.4534884*
**ENSG00000164306**	*0.20359281*	*0.19090909*	*0.20359281*	*0.190909091*				
**ENSG00000119403**	*0.19760479*	*0.32121212*	*0.19760479*	*0.321212121*	*0.177777778*		*0.125*	*0.4186047*
**ENSG00000021488**	*0.39772727*	*0.15730337*	*0.39772727*	*0.157303371*	*0.422222222*		*0.4090909*	*0.2727273*
**ENSG00000187398**		*0.49386503*		*0.493865031*				*0.2045455*
**ENSG00000163017**	*0.49700599*	*0.46590909*	*0.49700599*	*0.465909091*	*0.398809524*		*0.4767442*	*0.4588235*
**ENSG00000197594**		*0.24545455*		*0.245454545*				*0.4767442*
**ENSG00000144820**	*0.10479042*		*0.10479042*					
**ENSG00000104064**	*0.44*	*0.11818182*	*0.44*	*0.118181818*	*0.348837209*		*0.3378378*	
**ENSG00000168824**	*0.46407186*	*0.0945122*	*0.46407186*	*0.094512195*	*0.398809524*		*0.4058824*	*0.0581395*
**ENSG00000132911**		*0.20606061*		*0.206060606*				
**ENSG00000150967**	*0.4054878*	*0.11515152*	*0.4054878*	*0.115151515*	*0.428571429*		*0.3863636*	*0.1918605*
**ENSG00000113712**		*0.18493151*		*0.184931507*				*0.2235294*
**ENSG00000120885**	*0.15568862*	*0.17378049*	*0.15568862*	*0.173780488*	*0.411111111*		*0.4431818*	*0.3255814*
**ENSG00000090581**		*0.06363636*		*0.063636364*				
**ENSG00000119326**		*0.05487805*		*0.054878049*				
**ENSG00000180257**		*0.37078652*		*0.370786517*				*0.3409091*
**ENSG00000118849**		*0.18888889*		*0.188888889*				*0.2209302*
**ENSG00000163923**	*0.1*		*0.1*					
**ENSG00000134716**								*0.1046512*
**ENSG00000161277**		*0.33125*		*0.33125*	*0.255555556*		*0.1590909*	*0.4404762*
**ENSG00000166233**		*0.07575758*		*0.075757576*				*0.1666667*
**ENSG00000129810**		*0.35151515*		*0.351515152*				*0.0625*
**ENSG00000185420**	*0.40555556*	*0.25153374*	*0.40555556*	*0.251533742*	*0.077777778*		*0.0909091*	*0.4529412*

**ENSG00000064218**	*0.24850299*	*0.46060606*	*0.24850299*	*0.460606061*	*0.267857143*		*0.3*	*0.4651163*
**ENSG00000100603**		*0.46666667*		*0.466666667*				*0.3255814*
**ENSG00000162522**		*0.16111111*		*0.161111111*				*0.2045455*
**ENSG00000145113**		*0.5*		*0.5*				*0.3255814*
**ENSG00000125734**	*0.12222222*		*0.12222222*		*0.122222222*			
**ENSG00000140497**		*0.39090909*		*0.390909091*				*0.4883721*
**ENSG00000117707**	*0.23353293*		*0.23353293*		*0.416666667*		*0.4709302*	*0.0795455*
**ENSG00000117385**		*0.35454545*		*0.354545455*				*0.4*
**ENSG00000167106**		*0.08787879*		*0.087878788*				*0.4069767*
**ENSG00000136634**		*0.4847561*		*0.484756098*				
**ENSG00000164754**	*0.36666667*	*0.46629213*	*0.36666667*	*0.466292135*	*0.202380952*		*0.1818182*	*0.1818182*
**ENSG00000167985**		*0.15454545*		*0.154545455*				
**ENSG00000186522**	*0.3502994*		*0.3502994*				*0.0930233*	*0.25*
**ENSG00000117036**		*0.1030303*		*0.103030303*				*0.4534884*
**ENSG00000185989**		*0.46363636*		*0.463636364*				*0.2848837*
**ENSG00000185722**		*0.13333333*		*0.133333333*				*0.3313953*
**ENSG00000151694**		*0.47272727*		*0.472727273*				
**ENSG00000126261**	*0.48502994*		*0.48502994*		*0.19047619*		*0.127907*	
**ENSG00000177511**		*0.4695122*		*0.469512195*				*0.5*
**ENSG00000116133**		*0.30606061*		*0.306060606*				*0.4659091*
**ENSG00000173064**	*0.10240964*	*0.10909091*	*0.10240964*	*0.109090909*	*0.077380952*		*0.0988372*	*0.4090909*
**ENSG00000173208**		*0.23030303*		*0.23030303*				*0.3488372*
**ENSG00000188107**	*0.38787879*	*0.41573034*	*0.38787879*	*0.415730337*	*0.107142857*		*0.0882353*	*0.2777778*
**ENSG00000214999**		*0.31818182*		*0.318181818*				*0.4111111*
**ENSG00000103042**	*0.0508982*	*0.3*	*0.0508982*	*0.3*				*0.0909091*
**ENSG00000139266**		*0.25304878*		*0.25304878*				*0.1511628*
**ENSG00000177875**		*0.39090909*		*0.390909091*				*0.4235294*
**ENSG00000112130**	*0.35227273*	*0.34242424*	*0.35227273*	*0.342424242*	*0.244444444*		*0.2840909*	*0.1918605*
**ENSG00000164463**		*0.29573171*		*0.295731707*				*0.2195122*
**ENSG00000137876**	*0.44242424*	*0.22560976*	*0.44242424*	*0.225609756*	*0.192771084*		*0.0872093*	*0.0882353*
**ENSG00000138795**		*0.15757576*		*0.157575758*				
**ENSG00000183862**		*0.12068966*		*0.120689655*				*0.1470588*
**ENSG00000110060**	*0.44886364*		*0.44886364*		*0.066666667*		*0.1818182*	
**ENSG00000258744**		*0.20909091*		*0.209090909*				*0.1627907*
**ENSG00000163312**	*0.39820359*		*0.39820359*					
**ENSG00000125966**		*0.38109756*		*0.381097561*				*0.2470588*
**ENSG00000153292**	*0.46666667*	*0.25454545*	*0.46666667*	*0.254545455*	*0.266666667*		*0.1590909*	*0.1976744*
**ENSG00000168769**		*0.40909091*		*0.409090909*				*0.1976744*
**ENSG00000178243**		*0.49411765*		*0.494117647*				*0.2368421*
**ENSG00000122043**	*0.31437126*		*0.31437126*		*0.214285714*		*0.1453488*	
**ENSG00000150281**		*0.34782609*		*0.347826087*				*0.0988372*
**ENSG00000160791**	*0.25149701*		*0.25149701*		*0.416666667*		*0.4883721*	
**ENSG00000110324**	*0.20958084*	*0.4969697*	*0.20958084*	*0.496969697*	*0.053571429*		*0.0697674*	*0.494186*
**ENSG00000187045**	*0.13580247*		*0.13580247*		*0.284090909*		*0.4222222*	
**ENSG00000118418**		*0.10909091*		*0.109090909*				
**ENSG00000135638**		*0.246875*		*0.246875*				*0.1470588*
**ENSG00000243710**	*0.125*	*0.41515152*	*0.125*	*0.415151515*				*0.3421053*
**ENSG00000176473**	*0.46407186*	*0.45757576*	*0.46407186*	*0.457575758*	*0.397590361*		*0.4476744*	*0.3372093*
**ENSG00000122126**		*0.27272727*		*0.272727273*				*0.3895349*
**ENSG00000064655**		*0.5*		*0.5*				*0.494186*
**ENSG00000081181**								*0.2045455*
**ENSG00000140297**								*0.3313953*
**ENSG00000162747**		*0.26404494*		*0.264044944*				
**ENSG00000118162**		*0.11890244*		*0.118902439*				*0.2267442*

**ENSG00000162511**		*0.47865854*		*0.478658537*				*0.327381*
**ENSG00000067704**		*0.14723926*		*0.147239264*				*0.1904762*
**ENSG00000103647**	*0.31437126*	*0.3969697*	*0.31437126*	*0.396969697*	*0.120481928*		*0.0755814*	*0.4659091*
**ENSG00000106772**	*0.32228916*	*0.11585366*	*0.32228916*	*0.115853659*	*0.43452381*		*0.5*	*0.1918605*
**ENSG00000204315**	*0.23353293*		*0.23353293*		*0.089285714*		*0.1176471*	
**ENSG00000168530**	*0.34939759*		*0.34939759*		*0.428571429*		*0.4767442*	
**ENSG00000163320**	*0.48333333*	*0.19886364*	*0.48333333*	*0.198863636*	*0.193181818*		*0.0681818*	*0.0813953*
**ENSG00000172315**	*0.25287356*	*0.11212121*	*0.25287356*	*0.112121212*	*0.255555556*		*0.2613636*	
**ENSG00000058056**		*0.29444444*		*0.294444444*				*0.1931818*
**ENSG00000111912**	*0.35632184*	*0.43333333*	*0.35632184*	*0.433333333*	*0.069767442*		*0.0595238*	*0.4011628*
**ENSG00000123268**		*0.42424242*		*0.424242424*				*0.3882353*
**ENSG00000001561**	*0.18*	*0.45092025*	*0.18*	*0.450920245*	*0.238095238*		*0.2209302*	*0.3895349*
**ENSG00000073584**	*0.1497006*	*0.13291139*	*0.1497006*	*0.132911392*	*0.279761905*		*0.244186*	*0.2906977*
**ENSG00000180398**		*0.3*		*0.3*				*0.3555556*
**ENSG00000162692**	*0.11377246*		*0.11377246*					*0.0595238*
**ENSG00000104529**	*0.44610778*	*0.33939394*	*0.44610778*	*0.339393939*	*0.458333333*		*0.4883721*	*0.373494*
**ENSG00000124721**								*0.2777778*
**ENSG00000071539**		*0.07878788*		*0.078787879*				*0.127907*
**ENSG00000256087**					*0.327380952*		*0.3488372*	
**ENSG00000174145**		*0.49393939*		*0.493939394*				*0.4593023*
**ENSG00000101474**	*0.19444444*	*0.24848485*	*0.19444444*	*0.248484848*	*0.066666667*		*0.0777778*	*0.127907*
**ENSG00000197302**	*0.45808383*	*0.34269663*	*0.45808383*	*0.342696629*	*0.321428571*		*0.3255814*	*0.4360465*
**ENSG00000186458**		*0.47272727*		*0.472727273*				*0.4767442*
**ENSG00000164684**		*0.36363636*		*0.363636364*				*0.4825581*
**ENSG00000112379**		*0.07272727*		*0.072727273*				*0.1802326*
**ENSG00000172893**	*0.2754491*	*0.2804878*	*0.2754491*	*0.280487805*	*0.43452381*		*0.4011628*	*0.4651163*
**ENSG00000219545**		*0.46363636*		*0.463636364*				*0.4593023*
**ENSG00000228032**		*0.42073171*		*0.420731707*				*0.4235294*
**ENSG00000111052**		*0.11235955*		*0.112359551*				*0.125*
**ENSG00000100321**		*0.32515337*		*0.325153374*				*0.0714286*
**ENSG00000163946**	*0.23684211*	*0.23333333*	*0.23684211*	*0.233333333*	*0.410714286*		*0.3194444*	*0.4888889*
**ENSG00000168546**		*0.46363636*		*0.463636364*				*0.4709302*
**ENSG00000118058**		*0.21212121*		*0.212121212*			*0.0581395*	*0.4767442*
**ENSG00000073605**		*0.48484848*		*0.484848485*				*0.3139535*
**ENSG00000164920**	*0.21666667*		*0.21666667*		*0.111111111*			
**ENSG00000067836**		*0.46932515*		*0.469325153*				*0.3352941*
**ENSG00000091009**		*0.2804878*		*0.280487805*				*0.3181818*
**ENSG00000172006**		*0.30909091*		*0.309090909*				
**ENSG00000144395**		*0.22413793*		*0.224137931*				*0.1818182*
**ENSG00000112245**		*0.07575758*		*0.075757576*				*0.2202381*
**ENSG00000147202**		*0.11515152*		*0.115151515*				
**ENSG00000166923**		*0.42121212*		*0.421212121*				*0.4593023*
**ENSG00000198522**	*0.35928144*	*0.41515152*	*0.35928144*	*0.415151515*	*0.277777778*		*0.4318182*	*0.4886364*
**ENSG00000077274**	*0.26807229*	*0.05151515*	*0.26807229*	*0.051515152*				
**ENSG00000011523**	*0.26347305*	*0.23333333*	*0.26347305*	*0.233333333*	*0.283950617*			*0.3488372*
**ENSG00000165626**	*0.26946108*	*0.38484848*	*0.26946108*	*0.384848485*	*0.259036145*		*0.1352941*	*0.3255814*
**ENSG00000229689**		*0.35151515*		*0.351515152*				*0.2034884*
**ENSG00000101752**		*0.3*		*0.3*				*0.4825581*
**ENSG00000079841**		*0.47575758*		*0.475757576*				*0.1686047*
**ENSG00000223572**		*0.3373494*		*0.337349398*				*0.4090909*
**ENSG00000162129**		*0.40555556*		*0.405555556*				*0.3863636*
**ENSG00000196660**		*0.19512195*		*0.195121951*				*0.127907*
**ENSG00000109436**		*0.17777778*		*0.177777778*				*0.0681818*
**ENSG00000259426**								*0.2666667*

**ENSG00000187994**	*0.33832335*		*0.33832335*		*0.428571429*	*0.3255814*	
**ENSG00000196440**	*0.34638554*	*0.26363636*	*0.34638554*	*0.263636364*	*0.095238095*	*0.0988372*	*0.4659091*
**ENSG00000172819**	*0.44886364*	*0.11818182*	*0.44886364*	*0.118181818*	*0.188888889*	*0.2613636*	*0.0988372*
**ENSG00000171428**	*0.06666667*	*0.25471698*	*0.06666667*	*0.254716981*	*0.363095238*	*0.4709302*	*0.4529412*
**ENSG00000042286**		*0.15454545*		*0.154545455*			*0.4883721*
**ENSG00000140153**		*0.20555556*		*0.205555556*			*0.1022727*
**ENSG00000084731**		*0.26060606*		*0.260606061*			*0.0930233*
**ENSG00000095485**	*0.20359281*		*0.20359281*		*0.107142857*	*0.0872093*	
**ENSG00000100353**	*0.06111111*		*0.06111111*				
**ENSG00000185002**		*0.19393939*		*0.193939394*			*0.4186047*
**ENSG00000019485**		*0.29573171*		*0.295731707*			*0.3313953*
**ENSG00000100226**		*0.11212121*		*0.112121212*			*0.127907*
**ENSG00000106366**		*0.43333333*		*0.433333333*			*0.4360465*
**ENSG00000198301**		*0.41111111*		*0.411111111*			*0.2906977*
**ENSG00000072571**	*0.07575758*		*0.07575758*		*0.279761905*	*0.244186*	
**ENSG00000100564**		*0.20121951*		*0.201219512*			*0.2093023*
**ENSG00000258659**	*0.4491018*	*0.05757576*	*0.4491018*	*0.057575758*	*0.452380952*	*0.4825581*	
**ENSG00000196366**		*0.1993865*		*0.199386503*			
**ENSG00000213523**	*0.25748503*		*0.25748503*		*0.404761905*	*0.5*	
**ENSG00000254087**	*0.08282209*		*0.08282209*			*0.0639535*	
**ENSG00000137343**		*0.47575758*		*0.475757576*			*0.4069767*
**ENSG00000161905**	*0.33532934*	*0.31212121*	*0.33532934*	*0.312121212*			*0.4069767*
**ENSG00000120324**		*0.43939394*		*0.439393939*			*0.2732558*
**ENSG00000151012**		*0.43636364*		*0.436363636*			*0.4593023*
**ENSG00000183137**	*0.33832335*		*0.33832335*		*0.06547619*	*0.0639535*	
**ENSG00000090339**	*0.24137931*	*0.31515152*	*0.24137931*	*0.315151515*			*0.0639535*
**ENSG00000139620**	*0.26047904*	*0.38484848*	*0.26047904*	*0.384848485*	*0.253012048*	*0.2790698*	*0.2790698*
**ENSG00000102385**	*0.11280488*	*0.41358025*	*0.11280488*	*0.413580247*	*0.125*		*0.2674419*
**ENSG00000185015**		*0.37272727*		*0.372727273*			*0.3823529*
**ENSG00000124831**		*0.17575758*		*0.175757576*			*0.4186047*
**ENSG00000105185**		*0.34444444*		*0.344444444*			*0.1022727*
**ENSG00000221972**	*0.23353293*		*0.23353293*				
**ENSG00000168887**		*0.08484848*		*0.084848485*			
**ENSG00000160703**	*0.25449102*	*0.43939394*	*0.25449102*	*0.439393939*	*0.138554217*	*0.1764706*	*0.3197674*
**ENSG00000136813**	*0.21987952*		*0.21987952*		*0.160714286*	*0.2117647*	*0.0909091*
**ENSG00000113368**		*0.46969697*		*0.46969697*			*0.377907*
**ENSG00000130830**		*0.27300613*		*0.273006135*			*0.1337209*
**ENSG00000183977**	*0.46407186*		*0.46407186*		*0.19047619*	*0.1764706*	
**ENSG00000126461**		*0.26404494*		*0.264044944*			
**ENSG00000101337**		*0.44242424*		*0.442424242*			
**ENSG00000214013**		*0.11515152*		*0.115151515*			*0.1162791*
**ENSG00000129946**		*0.26666667*		*0.266666667*			*0.4651163*
**ENSG00000171858**		*0.19393939*		*0.193939394*			*0.377907*
**ENSG00000134802**	*0.21856287*	*0.13636364*	*0.21856287*	*0.136363636*	*0.119047619*	*0.1162791*	
**ENSG00000197263**	*0.38855422*		*0.38855422*		*0.196428571*	*0.2891566*	
**ENSG00000130669**		*0.31818182*		*0.318181818*	*0.25*	*0.244186*	*0.3863636*
**ENSG00000173517**		*0.37878788*		*0.378787879*			*0.4555556*
**ENSG00000184979**	*0.18333333*		*0.18333333*			*0.1162791*	
**ENSG00000134765**		*0.05151515*		*0.051515152*			
**ENSG00000185619**		*0.34242424*		*0.342424242*			*0.2093023*
**ENSG00000111707**		*0.44242424*		*0.442424242*			*0.3081395*
**ENSG00000189180**		*0.16060606*		*0.160606061*			*0.1686047*
**ENSG00000258890**	*0.17575758*		*0.17575758*				
**ENSG00000165202**		*0.41818182*		*0.418181818*			*0.4418605*

**ENSG00000107821**		*0.13636364*		*0.136363636*			
**ENSG00000168256**	*0.41111111*		*0.41111111*		*0.155555556*	*0.1477273*	
**ENSG00000138449**	*0.37222222*	*0.38787879*	*0.37222222*	*0.387878788*	*0.133333333*	*0.1477273*	*0.1395349*
**ENSG00000167261**	*0.47305389*		*0.47305389*		*0.107142857*	*0.1395349*	
**ENSG00000151491**	*0.23053892*	*0.15151515*	*0.23053892*	*0.151515152*	*0.054878049*		*0.0523256*
**ENSG00000135378**		*0.49693252*		*0.496932515*			*0.4883721*
**ENSG00000131095**		*0.16363636*		*0.163636364*			*0.2034884*
**ENSG00000198518**		*0.45555556*		*0.455555556*			*0.1046512*
**ENSG00000069329**					*0.077777778*		
**ENSG00000130592**	*0.36969697*	*0.08484848*	*0.36969697*	*0.084848485*	*0.25*	*0.2647059*	
**ENSG00000122176**	*0.35625*	*0.46969697*	*0.35625*	*0.46969697*	*0.433333333*	*0.4222222*	*0.4176471*
**ENSG00000136002**	*0.34444444*		*0.34444444*		*0.19047619*	*0.1337209*	
**ENSG00000175202**	*0.23619632*		*0.23619632*		*0.228395062*	*0.1976744*	
**ENSG00000104413**		*0.36280488*		*0.362804878*			*0.4767442*
**ENSG00000171126**		*0.44545455*		*0.445454545*			*0.3941176*
**ENSG00000232859**		*0.22424242*		*0.224242424*			*0.4882353*
**ENSG00000189182**	*0.46107784*	*0.42424242*	*0.46107784*	*0.424242424*	*0.267857143*	*0.1511628*	*0.3488372*
**ENSG00000089220**	*0.11746988*	*0.39329268*	*0.11746988*	*0.393292683*	*0.077380952*	*0.0581395*	*0.4127907*
**ENSG00000131669**		*0.13888889*		*0.138888889*			*0.1590909*
**ENSG00000196172**		*0.05*		*0.05*			
**ENSG00000168904**		*0.27134146*		*0.271341463*			*0.3953488*
**ENSG00000138246**		*0.24848485*		*0.248484848*			*0.1627907*
**ENSG00000092529**	*0.2754491*		*0.2754491*		*0.113095238*	*0.1071429*	
**ENSG00000134533**	*0.47878788*	*0.12777778*	*0.47878788*	*0.127777778*	*0.246987952*	*0.3197674*	*0.1477273*
**ENSG00000091164**	*0.40963855*	*0.43888889*	*0.40963855*	*0.438888889*	*0.303571429*	*0.3546512*	*0.4222222*
**ENSG00000197548**		*0.42727273*		*0.427272727*			*0.3372093*
**ENSG00000253200**	*0.43888889*	*0.31818182*	*0.43888889*	*0.318181818*	*0.366666667*	*0.3333333*	*0.3430233*
**ENSG00000113805**		*0.25454545*		*0.254545455*			*0.2732558*
**ENSG00000178573**		*0.33024691*		*0.330246914*			*0.3895349*
**ENSG00000174744**	*0.09281437*		*0.09281437*				
**ENSG00000106952**		*0.4030303*		*0.403030303*			*0.4821429*
**ENSG00000138760**		*0.46060606*		*0.460606061*			*0.3414634*
**ENSG00000174652**	*0.48863636*	*0.43333333*	*0.48863636*	*0.433333333*	*0.466666667*	*0.4090909*	*0.4294118*
**ENSG00000148123**	*0.48888889*		*0.48888889*		*0.277777778*	*0.3636364*	
**ENSG00000128606**					*0.125*	*0.1337209*	
**ENSG00000156345**	*0.2245509*	*0.08536585*	*0.2245509*	*0.085365854*	*0.321428571*	*0.3197674*	*0.2705882*
**ENSG00000147434**		*0.26363636*		*0.263636364*			*0.1511628*
**ENSG00000103549**	*0.23652695*	*0.24085366*	*0.23652695*	*0.240853659*	*0.077380952*	*0.0988372*	*0.0872093*
**ENSG00000089335**	*0.06586826*	*0.36969697*	*0.06586826*	*0.36969697*			*0.3953488*
**ENSG00000185009**		*0.24545455*		*0.245454545*	*0.077777778*		*0.4464286*
**ENSG00000205269**		*0.39090909*		*0.390909091*			*0.494186*
**ENSG00000091106**	*0.24550898*		*0.24550898*		*0.428571429*	*0.4360465*	
**ENSG00000172345**		*0.4030303*		*0.403030303*			*0.4941176*
**ENSG00000159842**	*0.08888889*	*0.3*	*0.08888889*	*0.3*	*0.355555556*	*0.3636364*	*0.4457831*
**ENSG00000121486**		*0.38650307*		*0.386503067*			*0.4772727*
**ENSG00000167723**		*0.45454545*		*0.454545455*			*0.2965116*
**ENSG00000111790**	*0.06886228*	*0.17878788*	*0.06886228*	*0.178787879*			*0.1744186*
**ENSG00000144061**		*0.31515152*		*0.315151515*			*0.3855422*
**ENSG00000148343**	*0.35329341*		*0.35329341*		*0.142857143*	*0.1918605*	
**ENSG00000180447**	*0.0988024*		*0.0988024*				
**ENSG00000197114**		*0.1030303*		*0.103030303*			
**ENSG00000166197**	*0.38888889*	*0.48484848*	*0.38888889*	*0.484848485*			*0.3604651*
**ENSG00000177674**		*0.32317073*		*0.323170732*			*0.2848837*
**ENSG00000149090**	*0.44382022*	*0.44848485*	*0.44382022*	*0.448484848*	*0.5*	*0.4545455*	*0.3837209*

**ENSG00000221914**		*0.09393939*		*0.093939394*			
**ENSG00000186529**		*0.48765432*		*0.487654321*			*0.4777778*
**ENSG00000117899**		*0.2*		*0.2*			*0.2674419*
**ENSG00000138614**	*0.0748503*		*0.0748503*		*0.166666667*	*0.2882353*	
**ENSG00000164318**	*0.36227545*	*0.12195122*	*0.36227545*	*0.12195122*	*0.255952381*	*0.1823529*	*0.1626506*
**ENSG00000172296**	*0.39221557*	*0.22699387*	*0.39221557*	*0.226993865*	*0.297619048*	*0.3895349*	*0.255814*
**ENSG00000197465**		*0.30909091*		*0.309090909*			*0.372093*
**ENSG00000185105**		*0.30909091*		*0.309090909*			*0.377907*
**ENSG00000144366**	*0.41477273*	*0.15030675*	*0.41477273*	*0.150306748*	*0.155555556*	*0.1931818*	*0.1590909*
**ENSG00000106483**		*0.28181818*		*0.281818182*			*0.3235294*
**ENSG00000186710**		*0.06402439*		*0.06402439*			*0.0930233*
**ENSG00000125246**		*0.30337079*		*0.303370787*			*0.3823529*
**ENSG00000180389**	*0.47904192*		*0.47904192*		*0.196428571*	*0.1511628*	
**ENSG00000166002**	*0.07228916*	*0.48255814*	*0.07228916*	*0.48255814*			*0.0568182*
**ENSG00000157212**		*0.26666667*		*0.266666667*			*0.25*
**ENSG00000127511**		*0.42528736*		*0.425287356*			*0.4318182*
**ENSG00000197565**		*0.4054878*		*0.405487805*			*0.0647059*
**ENSG00000103051**		*0.3969697*		*0.396969697*			*0.494186*
**ENSG00000112139**		*0.47575758*		*0.475757576*			*0.4761905*
**ENSG00000106348**		*0.25151515*		*0.251515152*			*0.3895349*
**ENSG00000138685**		*0.49695122*		*0.49695122*			*0.4767442*
**ENSG00000104687**		*0.40123457*		*0.401234568*			*0.2045455*
**ENSG00000168333**		*0.48876404*		*0.488764045*			*0.3895349*
**ENSG00000075856**	*0.2247191*	*0.13636364*	*0.2247191*	*0.136363636*	*0.277777778*	*0.2727273*	*0.1162791*
**ENSG00000119862**		*0.30487805*		*0.304878049*			*0.2764706*
**ENSG00000174749**		*0.43030303*		*0.43030303*			*0.1341463*
**ENSG00000214290**	*0.07222222*	*0.41818182*	*0.07222222*	*0.418181818*			*0.4186047*
**ENSG00000134058**					*0.226190476*	*0.1395349*	
**ENSG00000109738**		*0.44444444*		*0.444444444*			*0.4204545*
**ENSG00000249647**		*0.46666667*		*0.466666667*			*0.3255814*
**ENSG00000116260**		*0.28484848*		*0.284848485*			*0.5*
**ENSG00000183172**	*0.4137931*		*0.4137931*		*0.2*	*0.1818182*	
**ENSG00000155592**		*0.43939394*		*0.439393939*			*0.4470588*
**ENSG00000122861**		*0.37777778*		*0.377777778*			*0.125*
**ENSG00000101236**		*0.49444444*		*0.494444444*			*0.3555556*
**ENSG00000102048**	*0.45783133*	*0.21348315*	*0.45783133*	*0.213483146*	*0.277108434*	*0.3139535*	*0.2616279*
**ENSG00000186020**	*0.2183908*	*0.16363636*	*0.2183908*	*0.163636364*	*0.222222222*	*0.1428571*	
**ENSG00000072422**	*0.39221557*	*0.12727273*	*0.39221557*	*0.127272727*	*0.196428571*	*0.2674419*	*0.0639535*
**ENSG00000164509**	*0.47289157*	*0.26666667*	*0.47289157*	*0.266666667*	*0.43452381*	*0.4883721*	*0.1395349*
**ENSG00000108840**		*0.5*		*0.5*			*0.1918605*
**ENSG00000110077**	*0.32934132*	*0.46969697*	*0.32934132*	*0.46969697*	*0.30952381*	*0.3255814*	*0.3430233*
**ENSG00000178623**	*0.40419162*	*0.27300613*	*0.40419162*	*0.273006135*	*0.366666667*	*0.4360465*	
**ENSG00000100916**		*0.10674157*		*0.106741573*			*0.2888889*
**ENSG00000144857**	*0.22777778*	*0.20909091*	*0.22777778*	*0.209090909*	*0.322222222*	*0.3333333*	*0.5*
**ENSG00000150893**		*0.49085366*		*0.490853659*			*0.4476744*
**ENSG00000185610**		*0.44848485*		*0.448484848*			*0.2034884*
**ENSG00000010244**		*0.48888889*		*0.488888889*			*0.1222222*
**ENSG00000173598**		*0.22121212*		*0.221212121*			*0.3139535*
**ENSG00000181240**		*0.34848485*		*0.348484848*			*0.4883721*
**ENSG00000176988**							*0.1158537*
**ENSG00000108061**							*0.2916667*
**ENSG00000151461**		*0.07878788*		*0.078787879*			
**ENSG00000123095**		*0.4244186*		*0.424418605*			*0.1707317*
**ENSG00000168495**		*0.43888889*		*0.438888889*			*0.4593023*

**ENSG00000136367**		*0.40909091*		*0.409090909*	*0.25*	*0.255814*	*0.3529412*
**ENSG00000100285**		*0.38181818*		*0.381818182*			*0.1395349*
**ENSG00000135632**	*0.16049383*		*0.16049383*				
**ENSG00000130810**	*0.0748503*		*0.0748503*				
**ENSG00000198399**		*0.33333333*		*0.333333333*			*0.1860465*
**ENSG00000101280**		*0.11656442*		*0.116564417*			*0.127907*
**ENSG00000230000**	*0.06097561*		*0.06097561*		*0.476190476*	*0.3837209*	
**ENSG00000116604**		*0.34269663*		*0.342696629*			*0.2840909*
**ENSG00000141428**	*0.35928144*		*0.35928144*		*0.273809524*	*0.2674419*	
**ENSG00000171617**		*0.06060606*		*0.060606061*			
**ENSG00000101846**		*0.32389937*		*0.323899371*			*0.3529412*
**ENSG00000221870**		*0.31707317*		*0.317073171*			*0.4695122*
**ENSG00000115137**	*0.44011976*	*0.47865854*	*0.44011976*	*0.478658537*			*0.4882353*
**ENSG00000055609**		*0.13803681*		*0.13803681*			*0.3117647*
**ENSG00000173230**		*0.31818182*		*0.318181818*			*0.3953488*
**ENSG00000144659**		*0.12359551*		*0.123595506*			
**ENSG00000168135**		*0.23636364*		*0.236363636*			*0.2790698*
**ENSG00000102225**	*0.30120482*		*0.30120482*		*0.244047619*	*0.2705882*	
**ENSG00000164818**		*0.18292683*		*0.182926829*			*0.4647059*
**ENSG00000100304**		*0.3969697*		*0.396969697*			*0.2613636*
**ENSG00000162869**	*0.44886364*		*0.44886364*		*0.220238095*	*0.2616279*	
**ENSG00000114573**		*0.3030303*		*0.303030303*			*0.3139535*
**ENSG00000147883**		*0.43292683*		*0.432926829*			*0.4176471*
**ENSG00000115165**	*0.44444444*		*0.44444444*				*0.1136364*
**ENSG00000161573**		*0.27575758*		*0.275757576*			*0.4825581*
**ENSG00000156299**	*0.47305389*	*0.47852761*	*0.47305389*	*0.478527607*	*0.422222222*	*0.4545455*	*0.2882353*
**ENSG00000120440**	*0.20958084*		*0.20958084*			*0.0882353*	*0.1*
**ENSG00000078902**		*0.49386503*		*0.493865031*			*0.2674419*
**ENSG00000183155**		*0.36363636*		*0.363636364*			*0.3095238*
**ENSG00000198835**							*0.2034884*
**ENSG00000104643**		*0.46666667*		*0.466666667*			*0.3197674*
**ENSG00000182446**	*0.39820359*	*0.46551724*	*0.39820359*	*0.465517241*	*0.220238095*	*0.3023256*	*0.5*
**ENSG00000139364**		*0.49393939*		*0.493939394*			*0.3197674*
**ENSG00000213066**		*0.43888889*		*0.438888889*			*0.4418605*
**ENSG00000136828**		*0.45555556*		*0.455555556*			*0.3522727*
**ENSG00000171388**		*0.07926829*		*0.079268293*			*0.3430233*
**ENSG00000035928**	*0.11077844*	*0.47222222*	*0.11077844*	*0.472222222*			*0.3095238*
**ENSG00000175877**	*0.38622754*	*0.39393939*	*0.38622754*	*0.393939394*	*0.428571429*	*0.3882353*	*0.4302326*
**ENSG00000133606**	*0.27245509*	*0.17857143*	*0.27245509*	*0.178571429*	*0.154761905*	*0.1976744*	*0.1931818*
**ENSG00000141556**	*0.24850299*	*0.40606061*	*0.24850299*	*0.406060606*	*0.261904762*	*0.2209302*	*0.4302326*
**ENSG00000162139**	*0.14156627*		*0.14156627*		*0.202380952*	*0.1987952*	
**ENSG00000128833**		*0.27011494*		*0.270114943*			*0.4825581*
**ENSG00000103472**		*0.33888889*		*0.338888889*			
**ENSG00000116455**	*0.44311377*	*0.27777778*	*0.44311377*	*0.277777778*	*0.253012048*	*0.255814*	*0.1363636*
**ENSG00000108094**		*0.2*		*0.2*			*0.1931818*
**ENSG00000142166**		*0.25454545*		*0.254545455*			
**ENSG00000161847**		*0.21515152*		*0.215151515*			*0.0595238*
**ENSG00000060971**		*0.15*		*0.15*			*0.2386364*
**ENSG00000205856**		*0.35*		*0.35*			*0.1022727*
**ENSG00000142408**		*0.34662577*		*0.346625767*			*0.2045455*
**ENSG00000111684**		*0.07878788*		*0.078787879*			*0.0755814*
**ENSG00000121454**	*0.11077844*	*0.30909091*	*0.11077844*	*0.309090909*			*0.4545455*
**ENSG00000028839**		*0.07926829*		*0.079268293*			
**ENSG00000109572**	*0.14670659*	*0.47575758*	*0.14670659*	*0.475757576*			*0.2142857*

**ENSG00000100083**	*0.11976048*	*0.08333333*	*0.11976048*	*0.083333333*	*0.439759036*	*0.4705882*	*0.2848837*
**ENSG00000118526**		*0.37575758*		*0.375757576*			*0.4593023*
**ENSG00000136010**		*0.26219512*		*0.262195122*			*0.2888889*
**ENSG00000071967**		*0.41818182*		*0.418181818*			*0.3953488*
**ENSG00000006747**		*0.38109756*		*0.381097561*			*0.4518072*
**ENSG00000134780**		*0.4030303*		*0.403030303*			*0.3411765*
**ENSG00000102221**		*0.41818182*		*0.418181818*			*0.255814*
**ENSG00000108306**		*0.26060606*		*0.260606061*			*0.1976744*
**ENSG00000164406**	*0.47305389*	*0.29878049*	*0.47305389*	*0.298780488*	*0.476190476*	*0.4767442*	*0.4593023*
**ENSG00000125337**	*0.38323353*	*0.06707317*	*0.38323353*	*0.067073171*	*0.255952381*	*0.2674419*	*0.2151163*
**ENSG00000256980**	*0.38333333*	*0.47878788*	*0.38333333*	*0.478787879*	*0.3*	*0.2613636*	*0.372093*
**ENSG00000167476**					*0.1*	*0.1363636*	
**ENSG00000161267**		*0.34242424*		*0.342424242*			*0.1337209*
**ENSG00000144746**		*0.46666667*		*0.466666667*			*0.4360465*
**ENSG00000113231**		*0.49693252*		*0.496932515*			*0.4302326*
**ENSG00000197487**		*0.48333333*		*0.483333333*			*0.3546512*
**ENSG00000160200**	*0.13333333*	*0.40909091*	*0.13333333*	*0.409090909*	*0.232142857*	*0.2906977*	*0.4709302*
**ENSG00000105819**		*0.15243902*		*0.152439024*	*0.166666667*	*0.2083333*	*0.0813953*
**ENSG00000095585**	*0.08682635*		*0.08682635*				
**ENSG00000134313**		*0.30909091*		*0.309090909*			*0.1860465*
**ENSG00000165119**	*0.42222222*		*0.42222222*		*0.285714286*	*0.2588235*	
**ENSG00000064787**	*0.42814371*	*0.46060606*	*0.42814371*	*0.460606061*	*0.380952381*	*0.3430233*	*0.4709302*
**ENSG00000135902**	*0.28977273*	*0.32121212*	*0.28977273*	*0.321212121*	*0.3*	*0.3977273*	*0.4659091*
**ENSG00000042088**	*0.39520958*		*0.39520958*		*0.422222222*	*0.4090909*	*0.1*
**ENSG00000147059**	*0.4760479*		*0.4760479*				
**ENSG00000137269**		*0.4969697*		*0.496969697*			*0.3081395*
**ENSG00000167548**		*0.05454545*		*0.054545455*			
**ENSG00000064961**		*0.11212121*		*0.112121212*			*0.0581395*
**ENSG00000182372**		*0.35632184*		*0.356321839*			*0.3068182*
**ENSG00000094755**	*0.4494382*	*0.44545455*	*0.4494382*	*0.445454545*	*0.295454545*	*0.2272727*	*0.2790698*
**ENSG00000177885**		*0.23333333*		*0.233333333*			*0.0523256*
**ENSG00000162430**		*0.23030303*		*0.23030303*			*0.3313953*
**ENSG00000126216**		*0.2030303*		*0.203030303*			*0.494186*
**ENSG00000240230**		*0.41212121*		*0.412121212*			*0.372093*
**ENSG00000186417**		*0.43888889*		*0.438888889*			*0.5*
**ENSG00000113441**	*0.07784431*	*0.49444444*	*0.07784431*	*0.494444444*	*0.06547619*	*0.0988372*	*0.4431818*
**ENSG00000172789**		*0.06666667*		*0.066666667*			*0.0581395*
**ENSG00000144792**		*0.2375*		*0.2375*			
**ENSG00000183655**	*0.28143713*	*0.26060606*	*0.28143713*	*0.260606061*	*0.166666667*	*0.127907*	*0.4825581*
**ENSG00000154734**	*0.05421687*	*0.12424242*	*0.05421687*	*0.124242424*	*0.403614458*	*0.4117647*	*0.4767442*
**ENSG00000071073**		*0.23030303*		*0.23030303*			*0.2965116*
**ENSG00000005206**		*0.40606061*		*0.406060606*			*0.3588235*
**ENSG00000153827**		*0.13333333*		*0.133333333*			*0.0930233*
**ENSG00000121644**		*0.20426829*		*0.204268293*			*0.3941176*
**ENSG00000149716**		*0.30606061*		*0.306060606*			*0.1337209*
**ENSG00000161202**		*0.22727273*		*0.227272727*			
**ENSG00000245848**		*0.19631902*		*0.196319018*			
**ENSG00000167615**	*0.32228916*	*0.1*	*0.32228916*	*0.1*	*0.344444444*	*0.3915663*	*0.3837209*
**ENSG00000132554**	*0.10542169*		*0.10542169*		*0.280487805*	*0.244186*	
**ENSG00000100243**	*0.09281437*	*0.13888889*	*0.09281437*	*0.138888889*	*0.452380952*	*0.4302326*	*0.3953488*
**ENSG00000138735**		*0.23636364*		*0.236363636*			*0.4090909*
**ENSG00000107562**		*0.37222222*		*0.372222222*			*0.4767442*
**ENSG00000197872**	*0.18373494*	*0.35093168*	*0.18373494*	*0.350931677*			*0.4883721*
**ENSG00000137460**		*0.10060976*		*0.100609756*			

**ENSG00000166821**	*0.2005988*	*0.32317073*	*0.2005988*	*0.323170732*	*0.30952381*	*0.3176471*	*0.3197674*
**ENSG00000181104**		*0.24848485*		*0.248484848*			*0.1235294*
**ENSG00000060982**	*0.05988024*	*0.41515152*	*0.05988024*	*0.415151515*	*0.188888889*	*0.1363636*	*0.4940476*
**ENSG00000233670**	*0.21111111*	*0.26969697*	*0.21111111*	*0.26969697*			*0.1704545*
**ENSG00000166189**		*0.17575758*		*0.175757576*			*0.4069767*
**ENSG00000122643**	*0.05113636*	*0.23493976*	*0.05113636*	*0.234939759*			*0.4767442*
**ENSG00000151502**		*0.30792683*		*0.307926829*			*0.2325581*
**ENSG00000123130**	*0.45508982*	*0.22560976*	*0.45508982*	*0.225609756*	*0.321428571*	*0.3197674*	
**ENSG00000221888**	*0.44578313*		*0.44578313*			*0.0523256*	
**ENSG00000162104**		*0.37575758*		*0.375757576*			*0.3837209*
**ENSG00000179270**		*0.46604938*		*0.466049383*			*0.4705882*
**ENSG00000114742**	*0.25*	*0.28484848*	*0.25*	*0.284848485*	*0.398809524*	*0.3023256*	*0.4011628*
**ENSG00000107854**		*0.10909091*		*0.109090909*			*0.3181818*
**ENSG00000162761**		*0.25609756*		*0.256097561*			*0.0930233*
**ENSG00000159788**	*0.06886228*	*0.25555556*	*0.06886228*	*0.255555556*			*0.4545455*
**ENSG00000167281**	*0.42727273*		*0.42727273*		*0.102409639*	*0.1744186*	
**ENSG00000172845**		*0.38888889*		*0.388888889*			*0.3902439*
**ENSG00000173295**	*0.3*		*0.3*		*0.209876543*	*0.1363636*	*0.0639535*
**ENSG00000120337**		*0.26219512*		*0.262195122*			*0.2151163*
**ENSG00000168672**					*0.101190476*	*0.0930233*	*0.0523256*
**ENSG00000248383**		*0.42378049*		*0.423780488*			*0.4705882*
**ENSG00000103569**		*0.14596273*		*0.145962733*			*0.1823529*
**ENSG00000064989**	*0.36111111*	*0.28353659*	*0.36111111*	*0.283536585*	*0.111111111*	*0.0909091*	*0.0588235*
**ENSG00000086205**	*0.36144578*	*0.32777778*	*0.36144578*	*0.327777778*	*0.285714286*	*0.4244186*	*0.4244186*
**ENSG00000180370**		*0.44848485*		*0.448484848*			*0.1162791*
**ENSG00000073969**		*0.18181818*		*0.181818182*			
**ENSG00000239887**	*0.08982036*	*0.41818182*	*0.08982036*	*0.418181818*	*0.19047619*	*0.122093*	*0.4534884*
**ENSG00000005486**		*0.27272727*		*0.272727273*			*0.3604651*
**ENSG00000105705**		*0.19444444*		*0.194444444*			
**ENSG00000122481**		*0.1*		*0.1*			*0.1176471*
**ENSG00000144218**	*0.19161677*	*0.42134831*	*0.19161677*	*0.421348315*			
**ENSG00000184557**		*0.14848485*		*0.148484848*			*0.4069767*
**ENSG00000120370**		*0.3969697*		*0.396969697*			*0.0581395*
**ENSG00000166454**		*0.13333333*		*0.133333333*			*0.3023256*
**ENSG00000132646**		*0.10555556*		*0.105555556*			
**ENSG00000164941**		*0.48417722*		*0.484177215*			*0.3430233*
**ENSG00000145996**		*0.39655172*		*0.396551724*			*0.2613636*
**ENSG00000118804**	*0.26347305*	*0.13030303*	*0.26347305*	*0.13030303*	*0.363095238*	*0.4011628*	*0.2093023*
**ENSG00000214708**		*0.17073171*		*0.170731707*			*0.2857143*
**ENSG00000034239**		*0.21341463*		*0.213414634*			*0.3081395*
**ENSG00000100346**		*0.20909091*		*0.209090909*			
**ENSG00000100612**	*0.26347305*	*0.11280488*	*0.26347305*	*0.112804878*	*0.06547619*		*0.3941176*
**ENSG00000162384**		*0.36969697*		*0.36969697*			*0.4764706*
**ENSG00000004700**	*0.4760479*	*0.5*	*0.4760479*	*0.5*			*0.372093*
**ENSG00000154174**	*0.47727273*	*0.32121212*	*0.47727273*	*0.321212121*	*0.433333333*	*0.3977273*	*0.2209302*
**ENSG00000196224**	*0.23493976*	*0.16768293*	*0.23493976*	*0.167682927*		*0.0523256*	*0.4647059*
**ENSG00000083290**		*0.06111111*		*0.061111111*			*0.3255814*
**ENSG00000133805**	*0.47289157*	*0.38181818*	*0.47289157*	*0.381818182*	*0.178571429*	*0.2616279*	*0.3837209*
**ENSG00000218357**	*0.13473054*	*0.35802469*	*0.13473054*	*0.358024691*	*0.148809524*	*0.1918605*	*0.1802326*
**ENSG00000082153**	*0.30239521*		*0.30239521*		*0.311111111*	*0.1569767*	
**ENSG00000135355**		*0.36585366*		*0.365853659*			
**ENSG00000113140**		*0.48275862*		*0.482758621*			*0.4659091*
**ENSG00000143569**		*0.47272727*		*0.472727273*			*0.4476744*
**ENSG00000214097**		*0.37878788*		*0.378787879*			*0.0588235*

**ENSG00000145700**		*0.36363636*		*0.363636364*			*0.4767442*
**ENSG00000164031**		*0.23563218*		*0.235632184*			*0.1704545*
**ENSG00000111850**	*0.09337349*	*0.40797546*	*0.09337349*	*0.40797546*	*0.113095238*	*0.1162791*	*0.3975904*
**ENSG00000138764**	*0.07185629*	*0.46060606*	*0.07185629*	*0.460606061*			*0.4883721*
**ENSG00000101198**	*0.34730539*	*0.13803681*	*0.34730539*	*0.13803681*	*0.113095238*	*0.1453488*	*0.2965116*
**ENSG00000168028**	*0.15868263*		*0.15868263*		*0.279761905*	*0.3176471*	
**ENSG00000162910**	*0.23652695*		*0.23652695*		*0.21686747*	*0.2034884*	
**ENSG00000153498**	*0.05389222*		*0.05389222*		*0.301204819*	*0.3372093*	
**ENSG00000179913**		*0.22256098*		*0.222560976*			*0.4186047*
**ENSG00000075618**	*0.33532934*		*0.33532934*		*0.113095238*	*0.1337209*	
**ENSG00000135801**		*0.43636364*		*0.436363636*			*0.494186*
**ENSG00000143167**	*0.43113772*	*0.46646341*	*0.43113772*	*0.466463415*	*0.196428571*	*0.1802326*	*0.4651163*
**ENSG00000100519**		*0.4054878*		*0.405487805*			*0.4244186*
**ENSG00000148154**		*0.30606061*		*0.306060606*			*0.25*
**ENSG00000119969**		*0.43333333*		*0.433333333*			*0.3255814*
**ENSG00000136275**	*0.35632184*	*0.5*	*0.35632184*	*0.5*	*0.420454545*	*0.2906977*	*0.2840909*
**ENSG00000117215**		*0.47777778*		*0.477777778*			*0.3522727*
**ENSG00000175898**		*0.31515152*		*0.315151515*			*0.3953488*
**ENSG00000178401**	*0.28443114*		*0.28443114*				*0.1022727*
**ENSG00000205108**		*0.4969697*		*0.496969697*			*0.4534884*
**ENSG00000187210**	*0.2994012*	*0.45977011*	*0.2994012*	*0.459770115*	*0.156626506*		*0.255814*
**ENSG00000175581**	*0.17664671*	*0.07222222*	*0.17664671*	*0.072222222*	*0.44047619*	*0.4883721*	*0.0666667*
**ENSG00000155897**	*0.28313253*		*0.28313253*		*0.28313253*	*0.3470588*	
**ENSG00000171219**		*0.22777778*		*0.227777778*			*0.4222222*
**ENSG00000204859**	*0.0988024*		*0.0988024*				
**ENSG00000215217**		*0.43636364*		*0.436363636*			*0.3662791*
**ENSG00000186047**		*0.0969697*		*0.096969697*			*0.2093023*
**ENSG00000181072**		*0.47777778*		*0.477777778*			*0.3977273*
**ENSG00000175105**	*0.47005988*	*0.16463415*	*0.47005988*	*0.164634146*	*0.172619048*	*0.0813953*	*0.0813953*
**ENSG00000205441**		*0.0872093*		*0.087209302*			*0.1395349*
**ENSG00000124214**		*0.35555556*		*0.355555556*			*0.2888889*
**ENSG00000185942**	*0.43888889*	*0.41011236*	*0.43888889*	*0.41011236*	*0.433333333*	*0.3863636*	*0.2383721*
**ENSG00000205922**		*0.42528736*		*0.425287356*	*0.366666667*	*0.1704545*	*0.5*
**ENSG00000133114**							*0.1666667*
**ENSG00000141699**		*0.37575758*		*0.375757576*			*0.4593023*
**ENSG00000156802**		*0.34545455*		*0.345454545*			*0.4825581*
**ENSG00000173542**		*0.21036585*		*0.210365854*			*0.2764706*
**ENSG00000171431**		*0.41011236*		*0.41011236*			*0.4127907*
**ENSG00000030582**		*0.24242424*		*0.242424242*			*0.2267442*
**ENSG00000137843**		*0.35060976*		*0.350609756*	*0.077380952*		*0.2267442*
**ENSG00000130988**		*0.1*		*0.1*	*0.178571429*	*0.2325581*	
**ENSG00000185753**	*0.36445783*		*0.36445783*		*0.130952381*	*0.1860465*	
**ENSG00000121933**		*0.44545455*		*0.445454545*			*0.4069767*
**ENSG00000152977**		*0.32424242*		*0.324242424*			*0.2906977*
**ENSG00000197860**		*0.31111111*		*0.311111111*			*0.3869048*
**ENSG00000197555**					*0.077380952*	*0.0639535*	
**ENSG00000144152**		*0.22777778*		*0.227777778*			
**ENSG00000114026**		*0.2183908*		*0.218390805*			*0.4772727*
**ENSG00000168938**		*0.4068323*		*0.406832298*			
**ENSG00000144935**	*0.19760479*	*0.18209877*	*0.19760479*	*0.182098765*	*0.37195122*	*0.3614458*	*0.2916667*
**ENSG00000099985**	*0.13888889*	*0.16363636*	*0.13888889*	*0.163636364*			*0.2209302*
**ENSG00000159167**		*0.14939024*		*0.149390244*			*0.1470588*
**ENSG00000145016**		*0.06666667*		*0.066666667*			*0.125*
**ENSG00000161653**		*0.34545455*		*0.345454545*			*0.2209302*

**ENSG00000135299**		0.26875		0.26875			0.2727273
**ENSG00000137815**		0.47272727		0.472727273			0.2732558
**ENSG00000090615**		0.22121212		0.221212121			0.4244186
**ENSG00000164611**	0.15454545		0.15454545		0.160714286	0.1860465	
**ENSG00000079332**		0.42236025		0.422360248	0.090909091	0.0581395	0.0666667
**ENSG00000173585**		0.10795455		0.107954545			
**ENSG00000253350**		0.23333333		0.233333333			0.3470588
**ENSG00000145220**	0.39820359		0.39820359		0.487804878	0.4651163	
**ENSG00000155096**	0.07386364	0.32777778	0.07386364	0.327777778	0.222222222	0.1704545	0.2386364
**ENSG00000164885**	0.41566265		0.41566265		0.297619048	0.2906977	
**ENSG00000132256**	0.38068182	0.11515152	0.38068182	0.115151515	0.5	0.4825581	0.4941176
**ENSG00000161281**	0.26646707		0.26646707		0.321428571	0.3837209	
**ENSG00000106609**		0.25786164		0.257861635	0.069767442	0.127907	0.25
**ENSG00000198854**		0.35757576		0.357575758			0.1453488
**ENSG00000108852**	0.37724551	0.20909091	0.37724551	0.209090909	0.375	0.377907	
**ENSG00000165376**	0.37222222		0.37222222				
**ENSG00000054793**		0.35454545		0.354545455			0.4825581
**ENSG00000178950**		0.32424242		0.324242424			0.2235294
**ENSG00000166582**		0.48484848		0.484848485			
**ENSG00000173566**		0.46969697		0.46969697			0.4117647
**ENSG00000164741**	0.46407186	0.33636364	0.46407186	0.336363636	0.416666667	0.4302326	0.4011628
**ENSG00000101098**		0.46969697		0.46969697			0.4651163
**ENSG00000117598**		0.31515152		0.315151515			0.3546512
**ENSG00000186187**	0.0988024	0.49695122	0.0988024	0.49695122	0.226190476	0.1104651	0.1686047
**ENSG00000042832**	0.38253012	0.4969697	0.38253012	0.496969697	0.345238095	0.3546512	0.3604651
**ENSG00000229117**					0.095238095	0.0697674	
**ENSG00000188523**		0.41111111		0.411111111			0.1136364
**ENSG00000089199**		0.42592593		0.425925926			0.494186
**ENSG00000163558**		0.17878788		0.178787879			
**ENSG00000138867**		0.14444444		0.144444444			0.2674419
**ENSG00000129467**		0.12209302		0.122093023			
**ENSG00000166016**	0.28443114		0.28443114		0.333333333	0.3953488	
**ENSG00000145241**		0.37575758		0.375757576			0.2383721
**ENSG00000150093**	0.16060606	0.08426966	0.16060606	0.084269663	0.244047619	0.255814	
**ENSG00000171060**							0.25
**ENSG00000141040**		0.06060606		0.060606061			
**ENSG00000168610**		0.4		0.4			0.4390244
**ENSG00000149571**	0.16766467	0.48333333	0.16766467	0.483333333	0.422619048	0.3255814	0.4302326
**ENSG00000254647**		0.23636364		0.236363636			
**ENSG00000147044**		0.11515152		0.115151515			0.0823529
**ENSG00000139344**	0.0988024		0.0988024		0.408536585	0.3941176	
**ENSG00000163412**		0.15243902		0.152439024			0.3977273
**ENSG00000168439**	0.18787879		0.18787879		0.409638554	0.4941176	
**ENSG00000116191**		0.41212121		0.412121212			0.3081395
**ENSG00000242366**	0.10555556	0.22777778	0.10555556	0.227777778			0.1590909
**ENSG00000133027**					0.077777778	0.0568182	
**ENSG00000183269**		0.49090909		0.490909091			0.3197674
**ENSG00000176049**							0.1627907
**ENSG00000133639**	0.46107784	0.16969697	0.46107784	0.16969697	0.416666667	0.3255814	0.3372093
**ENSG00000132109**	0.2245509	0.0862069	0.2245509	0.086206897	0.226190476	0.2732558	0.4941176
**ENSG00000149591**	0.18562874		0.18562874				
**ENSG00000150455**	0.05988024	0.24085366	0.05988024	0.240853659			0.172619
**ENSG00000123505**							0.3705882
**ENSG00000155130**		0.43939394		0.439393939			0.2965116

**ENSG00000144649**	*0.11666667*	*0.2*	*0.11666667*	*0.2*			*0.1588235*
**ENSG00000048544**		*0.3404908*		*0.340490798*			*0.1802326*
**ENSG00000111725**	*0.125*	*0.35060976*	*0.125*	*0.350609756*	*0.477777778*	*0.4318182*	
**ENSG00000149273**		*0.2*		*0.2*	*0.208333333*	*0.1104651*	*0.3081395*
**ENSG00000177542**	*0.17365269*	*0.46604938*	*0.17365269*	*0.466049383*			*0.4764706*
**ENSG00000149743**	*0.12352941*		*0.12352941*				
**ENSG00000212734**		*0.14848485*		*0.148484848*			*0.4476744*
**ENSG00000198040**	*0.43113772*	*0.35060976*	*0.43113772*	*0.350609756*	*0.208333333*	*0.1511628*	*0.127907*
**ENSG00000110906**	*0.31736527*	*0.25925926*	*0.31736527*	*0.259259259*	*0.339285714*	*0.2674419*	*0.1235294*
**ENSG00000254221**		*0.20224719*		*0.202247191*			*0.1477273*
**ENSG00000176597**	*0.07784431*		*0.07784431*		*0.208333333*	*0.244186*	
**ENSG00000167333**	*0.48493976*	*0.35151515*	*0.48493976*	*0.351515152*			*0.3411765*
**ENSG00000183918**		*0.14545455*		*0.145454545*			*0.3352941*
**ENSG00000109846**	*0.18263473*	*0.32121212*	*0.18263473*	*0.321212121*	*0.148809524*	*0.1976744*	*0.1976744*
**ENSG00000215346**	*0.4760479*		*0.4760479*		*0.053571429*		
**ENSG00000008324**		*0.33333333*		*0.333333333*			*0.4764706*
**ENSG00000213079**		*0.42777778*		*0.427777778*			
**ENSG00000091428**	*0.48802395*		*0.48802395*		*0.345238095*	*0.4476744*	
**ENSG00000099331**	*0.33832335*		*0.33832335*		*0.261904762*	*0.2906977*	
**ENSG00000138071**		*0.3*		*0.3*			*0.2906977*
**ENSG00000128655**	*0.30120482*	*0.27878788*	*0.30120482*	*0.278787879*	*0.19047619*	*0.2383721*	*0.3488372*
**ENSG00000173638**	*0.2994012*	*0.42696629*	*0.2994012*	*0.426966292*	*0.5*	*0.4418605*	*0.4360465*
**ENSG00000132357**		*0.42901235*		*0.429012346*			*0.4695122*
**ENSG00000104415**		*0.37272727*		*0.372727273*			*0.0755814*
**ENSG00000175311**		*0.27878788*		*0.278787879*			
**ENSG00000184588**		*0.26060606*		*0.260606061*			
**ENSG00000255062**	*0.0748503*	*0.23333333*	*0.0748503*	*0.233333333*	*0.331325301*	*0.4176471*	
**ENSG00000077514**	*0.36227545*	*0.17878788*	*0.36227545*	*0.178787879*	*0.297619048*	*0.372093*	*0.2727273*
**ENSG00000122012**	*0.1497006*	*0.49393939*	*0.1497006*	*0.493939394*	*0.30952381*	*0.3081395*	*0.4772727*
**ENSG00000158941**	*0.49700599*	*0.34545455*	*0.49700599*	*0.345454545*	*0.291666667*	*0.3430233*	*0.4709302*
**ENSG00000181666**					*0.058139535*	*0.0697674*	
**ENSG00000173715**		*0.27586207*		*0.275862069*			*0.1022727*
**ENSG00000104689**	*0.20359281*		*0.20359281*				
**ENSG00000125818**	*0.20454545*	*0.40909091*	*0.20454545*	*0.409090909*	*0.410714286*	*0.4642857*	*0.4069767*
**ENSG00000088876**	*0.41017964*	*0.23939394*	*0.41017964*	*0.239393939*	*0.238095238*	*0.3058824*	*0.3255814*
**ENSG00000004534**	*0.30538922*	*0.47272727*	*0.30538922*	*0.472727273*	*0.148809524*	*0.1918605*	*0.1802326*
**ENSG00000100949**		*0.29878049*		*0.298780488*			*0.3953488*
**ENSG00000133110**		*0.13125*		*0.13125*	*0.333333333*	*0.2777778*	*0.0714286*
**ENSG00000169762**		*0.42528736*		*0.425287356*			*0.2965116*
**ENSG00000178053**	*0.33333333*	*0.42424242*	*0.33333333*	*0.424242424*		*0.1111111*	*0.4102564*
**ENSG00000126746**		*0.47126437*		*0.471264368*			
**ENSG00000133019**	*0.14670659*	*0.38484848*	*0.14670659*	*0.384848485*			*0.4534884*
**ENSG00000111711**	*0.26946108*	*0.4847561*	*0.26946108*	*0.484756098*			*0.4883721*
**ENSG00000158636**		*0.5*		*0.5*			*0.5*
**ENSG00000143153**							*0.2375*
**ENSG00000163286**		*0.1*		*0.1*			*0.2272727*
**ENSG00000071553**		*0.07453416*		*0.074534161*			*0.1046512*
**ENSG00000136628**		*0.45977011*		*0.459770115*			*0.4318182*
**ENSG00000143499**							*0.2906977*
**ENSG00000078487**	*0.15269461*	*0.16969697*	*0.15269461*	*0.16969697*	*0.154761905*	*0.244186*	
**ENSG00000118007**		*0.47204969*		*0.472049689*			*0.2045455*
**ENSG00000101251**	*0.13473054*	*0.26666667*	*0.13473054*	*0.266666667*	*0.255952381*	*0.3139535*	*0.122093*
**ENSG00000114948**		*0.09090909*		*0.090909091*			
**ENSG00000164331**	*0.41616766*	*0.31818182*	*0.41616766*	*0.318181818*	*0.232142857*	*0.0930233*	*0.1744186*

**ENSG00000176204**		*0.44242424*		*0.442424242*			*0.4418605*
**ENSG00000177595**	*0.05*	*0.49691358*	*0.05*	*0.49691358*	*0.077777778*		*0.4709302*
**ENSG00000149150**		*0.23333333*		*0.233333333*			*0.1860465*
**ENSG00000158023**	*0.45679012*		*0.45679012*		*0.475903614*	*0.4651163*	
**ENSG00000149573**		*0.43902439*		*0.43902439*			*0.3470588*
**ENSG00000189144**		*0.15757576*		*0.157575758*	*0.107142857*	*0.0581395*	*0.3313953*
**ENSG00000099139**	*0.48502994*	*0.43939394*	*0.48502994*	*0.439393939*			*0.4476744*
**ENSG00000151338**	*0.21856287*	*0.37116564*	*0.21856287*	*0.371165644*	*0.392857143*	*0.4011628*	*0.2045455*
**ENSG00000088298**		*0.38484848*		*0.384848485*			*0.2117647*
**ENSG00000086570**		*0.22727273*		*0.227272727*			*0.1511628*
**ENSG00000110002**		*0.47701149*		*0.477011494*			*0.4360465*
**ENSG00000188659**	*0.11746988*	*0.5*	*0.11746988*	*0.5*	*0.182926829*	*0.2117647*	*0.2840909*
**ENSG00000145214**		*0.49393939*		*0.493939394*			*0.4244186*
**ENSG00000117480**	*0.43072289*		*0.43072289*		*0.142857143*	*0.1860465*	
**ENSG00000139618**	*0.08888889*	*0.29090909*	*0.08888889*	*0.290909091*	*0.297619048*	*0.4651163*	*0.1802326*
**ENSG00000140506**							*0.1511628*
**ENSG00000071794**		*0.35*		*0.35*			*0.377907*
**ENSG00000092201**		*0.07222222*		*0.072222222*			*0.122093*
**ENSG00000160870**		*0.08024691*		*0.080246914*			*0.2647059*
**ENSG00000102575**	*0.27245509*		*0.27245509*		*0.439759036*	*0.4476744*	
**ENSG00000205863**		*0.1875*		*0.1875*			
**ENSG00000122507**	*0.06927711*	*0.27272727*	*0.06927711*	*0.272727273*	*0.136904762*	*0.122093*	*0.3181818*
**ENSG00000186334**	*0.32222222*	*0.22121212*	*0.32222222*	*0.221212121*	*0.464285714*	*0.4705882*	*0.2034884*
**ENSG00000107738**		*0.45151515*		*0.451515152*			*0.3941176*
**ENSG00000156395**		*0.22727273*		*0.227272727*			*0.2674419*
**ENSG00000154781**		*0.38719512*		*0.387195122*			*0.5*
**ENSG00000148671**		*0.45151515*		*0.451515152*			*0.3255814*
**ENSG00000163914**	*0.24850299*		*0.24850299*		*0.411111111*	*0.4418605*	*0.4886364*
**ENSG00000171121**	*0.46111111*	*0.18888889*	*0.46111111*	*0.188888889*	*0.113095238*	*0.127907*	*0.0777778*
**ENSG00000119608**		*0.05151515*		*0.051515152*			
**ENSG00000152822**		*0.46646341*		*0.466463415*			*0.4772727*
**ENSG00000169903**	*0.28313253*	*0.1*	*0.28313253*	*0.1*	*0.13253012*	*0.1453488*	*0.3372093*
**ENSG00000196547**	*0.30838323*	*0.32727273*	*0.30838323*	*0.327272727*			
**ENSG00000168702**	*0.22754491*	*0.33030303*	*0.22754491*	*0.33030303*	*0.351190476*	*0.2764706*	*0.4767442*
**ENSG00000147454**		*0.43636364*		*0.436363636*			*0.4470588*
**ENSG00000242866**		*0.07777778*		*0.077777778*			*0.3975904*
**ENSG00000205726**		*0.5*		*0.5*			*0.4418605*
**ENSG00000132205**		*0.40555556*		*0.405555556*			*0.3430233*
**ENSG00000100266**		*0.47272727*		*0.472727273*			*0.3953488*
**ENSG00000205639**		*0.1*		*0.1*			
**ENSG00000108599**		*0.29090909*		*0.290909091*			
**ENSG00000132824**		*0.16363636*		*0.163636364*			*0.0581395*
**ENSG00000226742**		*0.43209877*		*0.432098765*			*0.4819277*
**ENSG00000153790**		*0.22727273*		*0.227272727*			*0.25*
**ENSG00000169218**		*0.45757576*		*0.457575758*			*0.2848837*
**ENSG00000256618**	*0.35955056*		*0.35955056*		*0.411111111*	*0.3863636*	
**ENSG00000070731**		*0.13939394*		*0.139393939*			*0.0697674*
**ENSG00000112893**		*0.07878788*		*0.078787879*			
**ENSG00000135334**	*0.30838323*		*0.30838323*		*0.327380952*	*0.2965116*	
**ENSG00000160445**		*0.38484848*		*0.384848485*			*0.2529412*
**ENSG00000197857**	*0.23652695*		*0.23652695*		*0.351190476*	*0.2823529*	
**ENSG00000114656**		*0.41818182*		*0.418181818*	*0.148809524*	*0.1046512*	*0.5*
**ENSG00000184226**	*0.14071856*	*0.41515152*	*0.14071856*	*0.415151515*	*0.428571429*	*0.4767442*	*0.494186*
**ENSG00000127334**		*0.40909091*		*0.409090909*			*0.3546512*

**ENSG00000143149**	*0.12874251*	*0.10606061*	*0.12874251*	*0.106060606*	*0.392857143*	*0.3197674*	*0.1746988*
**ENSG00000214117**	*0.12275449*		*0.12275449*		*0.196428571*	*0.2093023*	
**ENSG00000089916**	*0.45180723*	*0.4*	*0.45180723*	*0.4*	*0.377777778*	*0.3604651*	*0.4360465*
**ENSG00000137868**		*0.45731707*		*0.457317073*			*0.4244186*
**ENSG00000131943**		*0.42424242*		*0.424242424*			*0.4647059*
**ENSG00000053254**		*0.37575758*		*0.375757576*			*0.4764706*
**ENSG00000181418**		*0.35454545*		*0.354545455*			*0.4011628*
**ENSG00000134253**	*0.20658683*	*0.41212121*	*0.20658683*	*0.412121212*			*0.1104651*
**ENSG00000146966**		*0.49393939*		*0.493939394*			*0.1104651*
**ENSG00000162337**		*0.25757576*		*0.257575758*			*0.3352941*
**ENSG00000062598**	*0.34431138*	*0.09146341*	*0.34431138*	*0.091463415*	*0.259036145*	*0.3647059*	*0.2444444*
**ENSG00000146670**		*0.38181818*		*0.381818182*			*0.1627907*
**ENSG00000184682**						*0.0512821*	
**ENSG00000184881**		*0.12424242*		*0.124242424*			*0.4767442*
**ENSG00000104859**	*0.0508982*		*0.0508982*				
**ENSG00000113013**	*0.15168539*	*0.46969697*	*0.15168539*	*0.46969697*			*0.1744186*
**ENSG00000170276**	*0.16167665*		*0.16167665*		*0.232142857*	*0.1860465*	
**ENSG00000196184**	*0.33233533*		*0.33233533*				
**ENSG00000059377**		*0.17272727*		*0.172727273*			*0.0988372*
**ENSG00000168306**	*0.32035928*	*0.08787879*	*0.32035928*	*0.087878788*			*0.2111111*
**ENSG00000087301**		*0.19817073*		*0.198170732*			*0.2616279*
**ENSG00000068831**	*0.40419162*	*0.12121212*	*0.40419162*	*0.121212121*	*0.470238095*	*0.3546512*	*0.3488372*
**ENSG00000087074**	*0.24850299*		*0.24850299*		*0.119047619*	*0.1627907*	
**ENSG00000150990**		*0.31666667*		*0.316666667*			*0.2093023*
**ENSG00000239389**		*0.05151515*		*0.051515152*			*0.1162791*
**ENSG00000179241**		*0.27272727*		*0.272727273*			*0.1802326*
**ENSG00000257390**		*0.32222222*		*0.322222222*			*0.3181818*
**ENSG00000105968**		*0.41666667*		*0.416666667*			*0.2176471*
**ENSG00000120837**		*0.32022472*		*0.320224719*			*0.4545455*
**ENSG00000170260**		*0.49390244*		*0.493902439*			*0.4941176*
**ENSG00000182631**		*0.39325843*		*0.393258427*			*0.1022727*
**ENSG00000198099**		*0.27575758*		*0.275757576*			
**ENSG00000164542**		*0.4054878*		*0.405487805*			*0.4011628*
**ENSG00000172264**	*0.39506173*	*0.44767442*	*0.39506173*	*0.447674419*	*0.068181818*	*0.0888889*	*0.4360465*
**ENSG00000146166**		*0.46060606*		*0.460606061*			*0.5*
**ENSG00000112462**		*0.46583851*		*0.465838509*	*0.148809524*	*0.1046512*	*0.2678571*
**ENSG00000197818**	*0.40718563*	*0.1402439*	*0.40718563*	*0.140243902*			*0.3139535*
**ENSG00000148814**	*0.12874251*	*0.5*	*0.12874251*	*0.5*	*0.375*	*0.3662791*	*0.1918605*
**ENSG00000104356**		*0.28651685*		*0.286516854*			*0.1976744*
**ENSG00000170324**	*0.41317365*		*0.41317365*				
**ENSG00000203730**		*0.49090909*		*0.490909091*	*0.18452381*	*0.2151163*	*0.255814*
**ENSG00000186860**	*0.17065868*		*0.17065868*		*0.297619048*	*0.3313953*	
**ENSG00000171560**	*0.16467066*	*0.15151515*	*0.16467066*	*0.151515152*	*0.43452381*	*0.4883721*	*0.1352941*
**ENSG00000112977**		*0.24085366*		*0.240853659*			*0.4186047*
**ENSG00000100884**		*0.19631902*		*0.196319018*			*0.3953488*
**ENSG00000124422**		*0.29444444*		*0.294444444*			*0.4883721*
**ENSG00000146926**		*0.3908046*		*0.390804598*			*0.4431818*
**ENSG00000064115**	*0.07185629*	*0.07236842*	*0.07185629*	*0.072368421*			
**ENSG00000124782**		*0.45151515*		*0.451515152*			*0.4244186*
**ENSG00000082684**	*0.2245509*		*0.2245509*		*0.380952381*	*0.3953488*	
**ENSG00000197124**	*0.22155689*		*0.22155689*		*0.44047619*	*0.4886364*	*0.1860465*
**ENSG00000196663**		*0.33939394*		*0.339393939*		*0.0697674*	*0.4411765*
**ENSG00000070182**	*0.3988764*	*0.15757576*	*0.3988764*	*0.157575758*	*0.327380952*	*0.3255814*	*0.0755814*
**ENSG00000115539**	*0.07185629*		*0.07185629*				

**ENSG00000256222**	*0.11111111*	*0.44382022*	*0.11111111*	*0.443820225*				*0.2555556*
**ENSG00000166166**		*0.39329268*		*0.393292683*				*0.2790698*
**ENSG00000164199**	*0.13772455*	*0.46969697*	*0.13772455*	*0.46969697*				*0.5*
**ENSG00000137709**	*0.09580838*		*0.09580838*					
**ENSG00000102452**		*0.40606061*		*0.406060606*				*0.4418605*
**ENSG00000215099**		*0.18787879*		*0.187878788*				*0.2267442*
**ENSG00000100601**		*0.37878788*		*0.378787879*				*0.4302326*
**ENSG00000179583**		*0.42424242*		*0.424242424*				*0.4512195*
**ENSG00000165323**		*0.37575758*		*0.375757576*				*0.4593023*
**ENSG00000172175**		*0.46111111*		*0.461111111*				*0.4318182*
**ENSG00000102781**		*0.45757576*		*0.457575758*				*0.4176471*
**ENSG00000169884**	*0.16467066*	*0.33636364*	*0.16467066*	*0.336363636*	*0.44047619*		*0.4069767*	*0.4651163*
**ENSG00000134330**		*0.47272727*		*0.472727273*				
**ENSG00000188783**		*0.30487805*		*0.304878049*				*0.3081395*
**ENSG00000144285**		*0.35454545*		*0.354545455*				*0.372093*
**ENSG00000145975**		*0.30745342*		*0.307453416*				*0.2965116*
**ENSG00000147050**		*0.23333333*		*0.233333333*				*0.4767442*
**ENSG00000164659**		*0.09393939*		*0.093939394*				
**ENSG00000123570**		*0.17378049*		*0.173780488*				*0.5*
**ENSG00000143847**	*0.30239521*	*0.41768293*	*0.30239521*	*0.417682927*	*0.420454545*		*0.4418605*	*0.3882353*
**ENSG00000171557**		*0.2969697*		*0.296969697*				*0.2058824*
**ENSG00000152193**		*0.37037037*		*0.37037037*				*0.3690476*
**ENSG00000166173**		*0.46646341*		*0.466463415*				*0.4593023*
**ENSG00000166596**		*0.23780488*		*0.237804878*				*0.0882353*
**ENSG00000130803**	*0.37724551*	*0.43333333*	*0.37724551*	*0.433333333*	*0.113095238*		*0.0988372*	*0.3430233*
**ENSG00000178498**	*0.29518072*		*0.29518072*		*0.244047619*		*0.2151163*	
**ENSG00000130723**	*0.43103448*	*0.20909091*	*0.43103448*	*0.209090909*	*0.077380952*		*0.1084337*	*0.1046512*
**ENSG00000198092**		*0.21212121*		*0.212121212*				*0.1569767*
**ENSG00000166349**	*0.46067416*	*0.36516854*	*0.46067416*	*0.365168539*	*0.072289157*			*0.25*
**ENSG00000102069**		*0.29444444*		*0.294444444*				*0.4545455*
**ENSG00000076984**		*0.35*		*0.35*				*0.4318182*
**ENSG00000204920**	*0.4251497*	*0.27272727*	*0.4251497*	*0.272727273*	*0.428571429*		*0.4767442*	*0.4360465*
**ENSG00000197891**	*0.23952096*	*0.11515152*	*0.23952096*	*0.115151515*	*0.261363636*		*0.1818182*	*0.2906977*
**ENSG00000151353**		*0.42727273*		*0.427272727*				*0.4360465*
**ENSG00000109519**	*0.41818182*	*0.23863636*	*0.41818182*	*0.238636364*	*0.279761905*		*0.3953488*	*0.4011628*
**ENSG00000169252**	*0.14071856*		*0.14071856*		*0.107142857*		*0.1162791*	
**ENSG00000174453**		*0.27011494*		*0.270114943*				*0.2209302*
**ENSG00000182103**		*0.4030303*		*0.403030303*				*0.3488372*
**ENSG00000105227**		*0.5*		*0.5*				*0.1627907*
**ENSG00000153879**		*0.23939394*		*0.239393939*				
**ENSG00000129925**	*0.32608696*	*0.42727273*	*0.32608696*	*0.427272727*	*0.313253012*		*0.2732558*	*0.2906977*
**ENSG00000135525**		*0.11212121*		*0.112121212*				*0.4939024*
**ENSG00000249860**	*0.47904192*		*0.47904192*		*0.166666667*		*0.1627907*	
**ENSG00000054967**		*0.22777778*		*0.227777778*				
**ENSG00000147183**		*0.26687117*		*0.266871166*				*0.4767442*
**ENSG00000183908**		*0.46666667*		*0.466666667*				*0.4011628*
**ENSG00000150048**		*0.13939394*		*0.139393939*	*0.215909091*		*0.1931818*	*0.4186047*
**ENSG00000159596**	*0.47891566*		*0.47891566*		*0.427710843*		*0.3372093*	
**ENSG00000123165**		*0.05*		*0.05*				
**ENSG00000183605**	*0.48203593*		*0.48203593*		*0.273809524*		*0.3255814*	*0.2209302*
**ENSG00000141349**					*0.188888889*		*0.2159091*	
**ENSG00000156671**		*0.25*		*0.25*				*0.4235294*
**ENSG00000130202**		*0.43333333*		*0.433333333*				*0.3604651*
**ENSG00000154096**	*0.33146067*	*0.42567568*	*0.33146067*	*0.425675676*	*0.088888889*		*0.1976744*	*0.2272727*

**ENSG00000131242**		*0.38484848*		*0.384848485*			*0.4709302*
**ENSG00000204644**	*0.28888889*		*0.28888889*		*0.333333333*	*0.3837209*	
**ENSG00000145723**		*0.30606061*		*0.306060606*			*0.4825581*
**ENSG00000153575**	*0.19578313*	*0.08231707*	*0.19578313*	*0.082317073*	*0.072289157*	*0.0588235*	*0.4360465*
**ENSG00000153406**	*0.33707865*		*0.33707865*				
**ENSG00000177106**	*0.18862275*	*0.44817073*	*0.18862275*	*0.448170732*	*0.210843373*	*0.2267442*	*0.3430233*
**ENSG00000078140**		*0.24242424*		*0.242424242*			*0.1046512*
**ENSG00000179532**	*0.20481928*	*0.49090909*	*0.20481928*	*0.490909091*	*0.19047619*	*0.244186*	*0.372093*
**ENSG00000205220**	*0.21556886*		*0.21556886*				
**ENSG00000188938**	*0.48765432*	*0.33030303*	*0.48765432*	*0.33030303*	*0.178571429*	*0.1395349*	*0.4470588*
**ENSG00000179833**		*0.42727273*		*0.427272727*			*0.4127907*
**ENSG00000181649**		*0.15454545*		*0.154545455*			*0.1*
**ENSG00000117280**	*0.38023952*	*0.47575758*	*0.38023952*	*0.475757576*	*0.482142857*	*0.4825581*	*0.4244186*
**ENSG00000099385**		*0.38719512*		*0.387195122*			*0.1097561*
**ENSG00000108296**		*0.43333333*		*0.433333333*			*0.2176471*
**ENSG00000186976**	*0.12359551*		*0.12359551*				
**ENSG00000104375**	*0.38922156*	*0.39937107*	*0.38922156*	*0.399371069*	*0.494047619*	*0.4464286*	*0.4821429*
**ENSG00000143079**		*0.20426829*		*0.204268293*			*0.4821429*
**ENSG00000166913**	*0.45*	*0.08181818*	*0.45*	*0.081818182*	*0.072289157*	*0.1337209*	
**ENSG00000128335**	*0.31736527*	*0.45398773*	*0.31736527*	*0.45398773*	*0.172619048*	*0.1569767*	*0.0609756*
**ENSG00000178033**		*0.41515152*		*0.415151515*			*0.4772727*
**ENSG00000174446**		*0.21666667*		*0.216666667*			*0.2411765*
**ENSG00000174915**		*0.0969697*		*0.096969697*			
**ENSG00000122367**							*0.0581395*
**ENSG00000125861**		*0.34242424*		*0.342424242*			*0.494186*
**ENSG00000172081**		*0.36627907*		*0.36627907*			*0.2272727*
**ENSG00000143473**		*0.31976744*		*0.319767442*			*0.4166667*
**ENSG00000154059**		*0.33333333*		*0.333333333*			*0.2906977*
**ENSG00000153037**	*0.12275449*	*0.43636364*	*0.12275449*	*0.436363636*	*0.30952381*	*0.2470588*	*0.2840909*
**ENSG00000154262**		*0.42777778*		*0.427777778*			*0.244186*
**ENSG00000128710**	*0.35628743*		*0.35628743*				
**ENSG00000136997**	*0.06886228*		*0.06886228*		*0.130952381*	*0.127907*	*0.1136364*
**ENSG00000033122**					*0.107142857*	*0.1860465*	
**ENSG00000032219**		*0.08282209*		*0.082822086*			
**ENSG00000134072**		*0.1402439*		*0.140243902*			
**ENSG00000166136**	*0.13888889*		*0.13888889*		*0.133333333*	*0.1777778*	
**ENSG00000198598**	*0.18862275*		*0.18862275*		*0.30952381*	*0.4244186*	*0.2948718*
**ENSG00000100100**		*0.24137931*		*0.24137931*			*0.3895349*
**ENSG00000080007**	*0.48850575*	*0.42424242*	*0.48850575*	*0.424242424*	*0.211111111*	*0.2954545*	*0.4705882*
**ENSG00000170577**		*0.12424242*		*0.124242424*			
**ENSG00000167904**	*0.48333333*	*0.37804878*	*0.48333333*	*0.37804878*	*0.311111111*	*0.3409091*	*0.3837209*
**ENSG00000122483**	*0.25568182*	*0.17777778*	*0.25568182*	*0.177777778*	*0.273809524*	*0.3081395*	*0.2613636*
**ENSG00000181518**		*0.31097561*		*0.31097561*			*0.2588235*
**ENSG00000100124**		*0.17222222*		*0.172222222*			
**ENSG00000124143**	*0.12359551*	*0.4030303*	*0.12359551*	*0.403030303*			*0.4090909*
**ENSG00000204351**	*0.12275449*	*0.29393939*	*0.12275449*	*0.293939394*		*0.0755814*	*0.377907*
**ENSG00000214717**		*0.46969697*		*0.46969697*			*0.425*
**ENSG00000135744**	*0.11890244*	*0.33939394*	*0.11890244*	*0.339393939*	*0.214285714*	*0.1686047*	*0.122093*
**ENSG00000137077**					*0.068181818*		
**ENSG00000133872**		*0.12727273*		*0.127272727*			*0.1860465*
**ENSG00000173250**		*0.36280488*		*0.362804878*			*0.2529412*
**ENSG00000156234**		*0.13939394*		*0.139393939*			*0.0581395*
**ENSG00000125848**		*0.25555556*		*0.255555556*	*0.05952381*	*0.0988372*	*0.3295455*
**ENSG00000129484**		*0.24848485*		*0.248484848*			*0.244186*

**ENSG00000166260**		*0.38181818*		*0.381818182*			*0.1918605*
**ENSG00000189275**	*0.4246988*	*0.48101266*	*0.4246988*	*0.481012658*	*0.458333333*	*0.4709302*	*0.4878049*
**ENSG00000176531**		*0.29393939*		*0.293939394*			*0.1511628*
**ENSG00000102409**		*0.34567901*		*0.345679012*	*0.05952381*	*0.1511628*	*0.25*
**ENSG00000178217**		*0.19090909*		*0.190909091*			*0.2674419*
**ENSG00000118160**		*0.22392638*		*0.22392638*	*0.060240964*		*0.2840909*
**ENSG00000112851**		*0.09659091*		*0.096590909*		*0.0681818*	*0.4651163*
**ENSG00000145916**		*0.43333333*		*0.433333333*			*0.1104651*
**ENSG00000205420**		*0.31609195*		*0.316091954*			*0.25*
**ENSG00000105220**		*0.42424242*		*0.424242424*			*0.4593023*
**ENSG00000198951**	*0.49431818*	*0.49393939*	*0.49431818*	*0.493939394*	*0.2*	*0.1818182*	*0.1802326*
**ENSG00000112137**		*0.29090909*		*0.290909091*			*0.4127907*
**ENSG00000093000**	*0.33832335*	*0.48170732*	*0.33832335*	*0.481707317*	*0.494047619*	*0.4709302*	*0.4764706*
**ENSG00000110075**		*0.29393939*		*0.293939394*			*0.25*
**ENSG00000170255**	*0.26704545*		*0.26704545*		*0.2375*	*0.2738095*	
**ENSG00000137185**	*0.20555556*	*0.34848485*	*0.20555556*	*0.348484848*	*0.255555556*	*0.3181818*	*0.3837209*
**ENSG00000104731**		*0.17987805*		*0.179878049*		*0.1511628*	*0.127907*
**ENSG00000164989**	*0.43712575*	*0.47777778*	*0.43712575*	*0.477777778*	*0.392857143*	*0.4651163*	*0.4318182*
**ENSG00000173451**	*0.11976048*	*0.26363636*	*0.11976048*	*0.263636364*			*0.1802326*
**ENSG00000203876**		*0.08181818*		*0.081818182*			*0.3197674*
**ENSG00000105443**	*0.41916168*		*0.41916168*		*0.452380952*	*0.4767442*	
**ENSG00000173456**	*0.23053892*	*0.18944099*	*0.23053892*	*0.189440994*	*0.160714286*	*0.2383721*	*0.0588235*
**ENSG00000204544**		*0.25151515*		*0.251515152*			*0.1511628*
**ENSG00000257242**	*0.16111111*		*0.16111111*				
**ENSG00000112531**	*0.46107784*	*0.46341463*	*0.46107784*	*0.463414634*			*0.3604651*
**ENSG00000151287**		*0.17878788*		*0.178787879*			*0.4127907*
**ENSG00000147885**		*0.05454545*		*0.054545455*			*0.1046512*
**ENSG00000112335**							*0.1046512*
**ENSG00000204161**		*0.47272727*		*0.472727273*			*0.3081395*
**ENSG00000254122**		*0.20224719*		*0.202247191*			*0.1477273*
**ENSG00000131931**		*0.06666667*		*0.066666667*			
**ENSG00000012963**	*0.17701863*	*0.47272727*	*0.17701863*	*0.472727273*	*0.15*	*0.1011905*	*0.3430233*
**ENSG00000116254**	*0.48314607*	*0.35454545*	*0.48314607*	*0.354545455*	*0.404761905*	*0.4302326*	*0.4534884*
**ENSG00000221838**		*0.37878788*		*0.378787879*			*0.1337209*
**ENSG00000162728**		*0.35151515*		*0.351515152*			*0.1860465*
**ENSG00000186340**		*0.48787879*		*0.487878788*			*0.4767442*
**ENSG00000100299**		*0.10606061*		*0.106060606*			
**ENSG00000115756**	*0.47159091*	*0.44207317*	*0.47159091*	*0.442073171*	*0.322222222*	*0.3571429*	*0.372093*
**ENSG00000100336**	*0.45808383*	*0.49438202*	*0.45808383*	*0.494382022*	*0.178571429*	*0.2151163*	*0.2650602*
**ENSG00000170956**		*0.44171779*		*0.441717791*			*0.4886364*
**ENSG00000185298**		*0.40184049*		*0.401840491*			*0.3546512*
**ENSG00000174173**	*0.48502994*	*0.06666667*	*0.48502994*	*0.066666667*	*0.077777778*		*0.2159091*
**ENSG00000104537**	*0.09580838*	*0.34337349*	*0.09580838*	*0.343373494*			*0.3837209*
**ENSG00000128578**		*0.25757576*		*0.257575758*			*0.2965116*
**ENSG00000163754**		*0.29444444*		*0.294444444*			*0.3295455*
**ENSG00000188580**		*0.125*		*0.125*			*0.1058824*
**ENSG00000111325**		*0.33333333*		*0.333333333*			*0.3313953*
**ENSG00000166391**	*0.25149701*		*0.25149701*		*0.119047619*	*0.1686047*	*0.0523256*
**ENSG00000166340**	*0.13173653*	*0.24390244*	*0.13173653*	*0.243902439*			*0.0813953*
**ENSG00000188729**		*0.5*		*0.5*			*0.1569767*
**ENSG00000166676**		*0.2969697*		*0.296969697*			*0.3837209*
**ENSG00000213029**	*0.21556886*		*0.21556886*		*0.386904762*	*0.4647059*	
**ENSG00000128802**							*0.1686047*
**ENSG00000181873**		*0.3908046*		*0.390804598*			*0.2840909*

**ENSG00000171222**							*0.0523256*	
**ENSG00000080845**	*0.14071856*	*0.25151515*	*0.14071856*	*0.251515152*				*0.4*
**ENSG00000166323**		*0.44545455*		*0.445454545*				*0.4360465*
**ENSG00000164253**		*0.42727273*		*0.427272727*				*0.5*
**ENSG00000144481**	*0.05*	*0.41111111*	*0.05*	*0.411111111*	*0.3*		*0.1704545*	*0.4117647*
**ENSG00000146373**	*0.13473054*	*0.43030303*	*0.13473054*	*0.43030303*				*0.2613636*
**ENSG00000189164**		*0.14545455*		*0.145454545*				*0.3081395*
**ENSG00000103534**	*0.30555556*	*0.14545455*	*0.30555556*	*0.145454545*				*0.2209302*
**ENSG00000083312**		*0.36969697*		*0.36969697*	*0.337209302*		*0.3222222*	*0.3604651*
**ENSG00000205126**		*0.22424242*		*0.224242424*				*0.1529412*
**ENSG00000168778**	*0.13772455*		*0.13772455*					
**ENSG00000108828**		*0.31515152*		*0.315151515*				*0.0639535*
**ENSG00000139187**		*0.34444444*		*0.344444444*				*0.4777778*
**ENSG00000176040**		*0.07012195*		*0.070121951*				
**ENSG00000179918**								*0.0755814*
**ENSG00000137161**		*0.26666667*		*0.266666667*	*0.475609756*		*0.4418605*	
**ENSG00000185345**	*0.08982036*	*0.13636364*	*0.08982036*	*0.136363636*	*0.404761905*		*0.3941176*	*0.0755814*
**ENSG00000118900**	*0.24096386*	*0.42948718*	*0.24096386*	*0.429487179*				*0.3488372*
**ENSG00000204752**	*0.31137725*		*0.31137725*		*0.113095238*		*0.1046512*	
**ENSG00000132286**	*0.25*	*0.46363636*	*0.25*	*0.463636364*			*0.0568182*	*0.244186*
**ENSG00000143434**	*0.28977273*	*0.19393939*	*0.28977273*	*0.193939394*	*0.066666667*		*0.1104651*	*0.1046512*
**ENSG00000005436**		*0.45402299*		*0.454022989*				*0.3295455*
**ENSG00000002834**		*0.37575758*		*0.375757576*				*0.4764706*
**ENSG00000173011**	*0.25595238*	*0.23333333*	*0.25595238*	*0.233333333*	*0.291666667*		*0.4011628*	*0.3636364*
**ENSG00000005187**	*0.06586826*		*0.06586826*					
**ENSG00000181552**		*0.42721519*		*0.42721519*				*0.2383721*
**ENSG00000186501**		*0.44817073*		*0.448170732*				*0.3488372*
**ENSG00000106460**	*0.21590909*	*0.44545455*	*0.21590909*	*0.445454545*	*0.311111111*		*0.4204545*	*0.4529412*
**ENSG00000119139**	*0.17065868*	*0.42073171*	*0.17065868*	*0.420731707*				*0.3764706*
**ENSG00000158457**		*0.20555556*		*0.205555556*				*0.1453488*
**ENSG00000136169**		*0.40909091*		*0.409090909*				*0.4883721*
**ENSG00000197226**		*0.39772727*		*0.397727273*				*0.3546512*
**ENSG00000129422**	*0.21556886*	*0.42121212*	*0.21556886*	*0.421212121*	*0.428571429*		*0.4883721*	*0.4127907*
**ENSG00000164338**	*0.41616766*	*0.2*	*0.41616766*	*0.2*	*0.232142857*		*0.0930233*	*0.0930233*
**ENSG00000111261**	*0.47005988*	*0.23939394*	*0.47005988*	*0.239393939*	*0.363095238*		*0.4767442*	*0.1802326*
**ENSG00000123106**		*0.33030303*		*0.33030303*	*0.088888889*		*0.1333333*	*0.3662791*
**ENSG00000090006**	*0.35795455*	*0.36969697*	*0.35795455*	*0.36969697*	*0.339285714*		*0.3488372*	*0.2882353*
**ENSG00000243440**		*0.41818182*		*0.418181818*				*0.4302326*
**ENSG00000147576**		*0.46341463*		*0.463414634*				*0.4940476*
**ENSG00000146842**		*0.2030303*		*0.203030303*				*0.3863636*
**ENSG00000254093**		*0.29012346*		*0.290123457*				*0.0813953*
**ENSG00000114541**	*0.48192771*		*0.48192771*		*0.331325301*		*0.3837209*	*0.372093*
**ENSG00000185504**	*0.49101796*	*0.26060606*	*0.49101796*	*0.260606061*	*0.398809524*		*0.4883721*	*0.3604651*
**ENSG00000173838**	*0.4494382*	*0.46666667*	*0.4494382*	*0.466666667*	*0.333333333*		*0.3222222*	*0.2383721*
**ENSG00000198865**		*0.3030303*		*0.303030303*	*0.244444444*		*0.3555556*	*0.3392857*
**ENSG00000134853**		*0.16060606*		*0.160606061*				*0.2151163*
**ENSG00000187689**	*0.14759036*		*0.14759036*					
**ENSG00000165511**		*0.11212121*		*0.112121212*				*0.2209302*
**ENSG00000033327**	*0.38922156*	*0.15757576*	*0.38922156*	*0.157575758*	*0.357142857*		*0.4825581*	*0.4705882*
**ENSG00000092208**	*0.15868263*		*0.15868263*		*0.375*		*0.3662791*	
**ENSG00000178764**	*0.12874251*	*0.46969697*	*0.12874251*	*0.46969697*	*0.136904762*		*0.127907*	*0.3255814*
**ENSG00000170627**	*0.22590361*	*0.0969697*	*0.22590361*	*0.096969697*	*0.05952381*		*0.0988372*	*0.0988372*
**ENSG00000152894**		*0.38484848*		*0.384848485*				*0.3488372*
**ENSG00000084093**		*0.27777778*		*0.277777778*				

**ENSG00000134343**	*0.23192771*	*0.45151515*	*0.23192771*	*0.451515152*	*0.466666667*	*0.5*	*0.3953488*
**ENSG00000182580**		*0.22327044*		*0.22327044*			*0.2083333*
**ENSG00000167765**	*0.26047904*	*0.20909091*	*0.26047904*	*0.209090909*			*0.2732558*
**ENSG00000183091**	*0.43072289*		*0.43072289*		*0.477777778*	*0.4593023*	
**ENSG00000005020**		*0.40490798*		*0.404907975*	*0.119047619*	*0.122093*	*0.1785714*
**ENSG00000023041**	*0.45209581*	*0.0872093*	*0.45209581*	*0.087209302*	*0.452380952*	*0.4360465*	*0.2093023*
**ENSG00000103152**	*0.1497006*		*0.1497006*		*0.119047619*	*0.122093*	
**ENSG00000104365**	*0.07222222*		*0.07222222*		*0.444444444*	*0.4545455*	*0.0568182*
**ENSG00000159873**		*0.33841463*		*0.338414634*	*0.122222222*	*0.125*	*0.3809524*
**ENSG00000172728**	*0.11377246*		*0.11377246*		*0.054878049*		*0.1337209*
**ENSG00000093183**	*0.36144578*	*0.32727273*	*0.36144578*	*0.327272727*	*0.476190476*	*0.4825581*	*0.4825581*
**ENSG00000187954**	*0.32222222*	*0.48170732*	*0.32222222*	*0.481707317*	*0.470238095*	*0.4534884*	*0.3522727*
**ENSG00000071991**		*0.33636364*		*0.336363636*			*0.3809524*
**ENSG00000189362**	*0.29640719*	*0.43597561*	*0.29640719*	*0.43597561*	*0.476190476*	*0.3372093*	*0.4464286*
**ENSG00000139428**		*0.46363636*		*0.463636364*			*0.2840909*
**ENSG00000157680**		*0.46969697*		*0.46969697*			*0.4709302*
**ENSG00000164715**		*0.15454545*		*0.154545455*			*0.3023256*
**ENSG00000159658**	*0.39520958*	*0.28963415*	*0.39520958*	*0.289634146*	*0.267857143*	*0.2117647*	*0.4011628*
**ENSG00000070610**	*0.41111111*		*0.41111111*		*0.386363636*	*0.3662791*	
**ENSG00000139160**		*0.47575758*		*0.475757576*			*0.2151163*
**ENSG00000183072**		*0.35757576*		*0.357575758*			*0.1046512*
**ENSG00000085276**	*0.38372093*	*0.39939024*	*0.38372093*	*0.399390244*	*0.494047619*	*0.4883721*	*0.2440476*
**ENSG00000181908**		*0.34545455*		*0.345454545*			*0.25*
**ENSG00000109586**							*0.1511628*
**ENSG00000246477**		*0.43030303*		*0.43030303*			*0.3352941*
**ENSG00000167216**	*0.4251497*		*0.4251497*		*0.458333333*	*0.494186*	
**ENSG00000118960**		*0.38787879*		*0.387878788*			*0.4476744*
**ENSG00000131019**		*0.43636364*		*0.436363636*			*0.372093*
**ENSG00000133105**		*0.43333333*		*0.433333333*			*0.2176471*
**ENSG00000164500**		*0.27575758*		*0.275757576*			
**ENSG00000125945**		*0.22530864*		*0.225308642*			*0.2848837*
**ENSG00000152936**	*0.46686747*		*0.46686747*		*0.273809524*	*0.2151163*	
**ENSG00000074319**					*0.211111111*	*0.3977273*	*0.0666667*
**ENSG00000135040**		*0.21666667*		*0.216666667*			*0.4318182*
**ENSG00000196091**		*0.44242424*		*0.442424242*			*0.372093*
**ENSG00000198883**	*0.20555556*		*0.20555556*				
**ENSG00000118689**	*0.15555556*	*0.28181818*	*0.15555556*	*0.281818182*	*0.2*	*0.2045455*	*0.1918605*
**ENSG00000147689**	*0.33333333*		*0.33333333*		*0.186746988*	*0.2325581*	
**ENSG00000137959**	*0.12650602*		*0.12650602*		*0.476190476*	*0.4651163*	
**ENSG00000188994**		*0.47777778*		*0.477777778*			*0.4651163*
**ENSG00000101916**	*0.26807229*	*0.19444444*	*0.26807229*	*0.194444444*	*0.208333333*	*0.2209302*	
**ENSG00000111145**							*0.1511628*
**ENSG00000120896**		*0.46060606*		*0.460606061*			*0.2965116*
**ENSG00000205307**					*0.261904762*	*0.2965116*	
**ENSG00000158169**	*0.11797753*	*0.43939394*	*0.11797753*	*0.439393939*	*0.122222222*	*0.0909091*	*0.4578313*
**ENSG00000141380**	*0.17613636*	*0.18888889*	*0.17613636*	*0.188888889*	*0.144444444*	*0.0555556*	
**ENSG00000100726**	*0.23353293*	*0.21111111*	*0.23353293*	*0.211111111*	*0.488095238*	*0.4705882*	*0.3977273*
**ENSG00000112212**	*0.27245509*		*0.27245509*		*0.259036145*	*0.2325581*	
**ENSG00000213906**	*0.15361446*		*0.15361446*		*0.154761905*	*0.1764706*	
**ENSG00000057294**		*0.34242424*		*0.342424242*			*0.1860465*
**ENSG00000117143**					*0.337349398*	*0.255814*	
**ENSG00000185247**		*0.42424242*		*0.424242424*			
**ENSG00000197971**		*0.41666667*		*0.416666667*			*0.4*
**ENSG00000146197**		*0.16666667*		*0.166666667*			*0.2093023*

**ENSG00000182541**	*0.48203593*	*0.29393939*	*0.48203593*	*0.293939394*				*0.4767442*
**ENSG00000162413**	*0.1*	*0.33231707*	*0.1*	*0.332317073*	*0.4*		*0.4111111*	*0.4457831*
**ENSG00000110888**		*0.43888889*		*0.438888889*	*0.28313253*		*0.3117647*	*0.4659091*
**ENSG00000112787**		*0.18484848*		*0.184848485*				*0.1802326*
**ENSG00000120647**	*0.37125749*	*0.23939394*	*0.37125749*	*0.239393939*	*0.327380952*		*0.3197674*	*0.3837209*
**ENSG00000171161**	*0.0754717*	*0.06363636*	*0.0754717*	*0.063636364*				*0.1627907*
**ENSG00000137198**		*0.27777778*		*0.277777778*				*0.0888889*
**ENSG00000065150**	*0.18390805*	*0.48780488*	*0.18390805*	*0.487804878*	*0.2*		*0.2045455*	*0.25*
**ENSG00000066248**		*0.34242424*		*0.342424242*				*0.4545455*
**ENSG00000099995**		*0.20909091*		*0.209090909*				*0.3139535*
**ENSG00000132953**		*0.43333333*		*0.433333333*				*0.1918605*
**ENSG00000151062**		*0.42727273*		*0.427272727*				*0.494186*
**ENSG00000256060**	*0.26111111*		*0.26111111*		*0.266666667*		*0.3409091*	
**ENSG00000255087**		*0.4969697*		*0.496969697*				*0.1686047*
**ENSG00000170291**		*0.4*		*0.4*				*0.372093*
**ENSG00000157064**		*0.11212121*		*0.112121212*				*0.4360465*
**ENSG00000160305**		*0.1882716*		*0.188271605*				*0.3662791*
**ENSG00000129562**	*0.23053892*	*0.09393939*	*0.23053892*	*0.093939394*	*0.178571429*		*0.1802326*	*0.0872093*
**ENSG00000004846**	*0.41317365*	*0.34302326*	*0.41317365*	*0.343023256*	*0.095238095*		*0.1046512*	*0.127907*
**ENSG00000110243**	*0.16467066*	*0.21111111*	*0.16467066*	*0.211111111*	*0.261904762*		*0.372093*	*0.3255814*
**ENSG00000119650**		*0.33636364*		*0.336363636*				*0.3536585*
**ENSG00000174429**		*0.24444444*		*0.244444444*				*0.2272727*
**ENSG00000171402**		*0.37078652*		*0.370786517*				*0.4302326*
**ENSG00000142156**		*0.13109756*		*0.131097561*				*0.0813953*
**ENSG00000224383**		*0.42546584*		*0.425465839*				*0.3529412*
**ENSG00000196497**		*0.49090909*		*0.490909091*				*0.4651163*
**ENSG00000108344**		*0.15116279*		*0.151162791*				
**ENSG00000084710**		*0.23170732*		*0.231707317*				*0.4127907*
**ENSG00000203837**	*0.44242424*	*0.05757576*	*0.44242424*	*0.057575758*	*0.410714286*		*0.4939759*	*0.25*
**ENSG00000196689**	*0.32934132*	*0.44545455*	*0.32934132*	*0.445454545*				*0.25*
**ENSG00000143994**		*0.16969697*		*0.16969697*				*0.1744186*
**ENSG00000111716**	*0.24444444*		*0.24444444*					
**ENSG00000065675**		*0.48181818*		*0.481818182*				*0.2383721*
**ENSG00000164308**	*0.43113772*	*0.49090909*	*0.43113772*	*0.490909091*	*0.375*		*0.494186*	*0.4761905*
**ENSG00000156500**		*0.08484848*		*0.084848485*				
**ENSG00000080546**		*0.0969697*		*0.096969697*				
**ENSG00000122692**		*0.46666667*		*0.466666667*				*0.3409091*
**ENSG00000213397**	*0.20783133*	*0.31097561*	*0.20783133*	*0.31097561*				*0.3488372*
**ENSG00000166750**		*0.42987805*		*0.429878049*				*0.3235294*
**ENSG00000064195**	*0.28143713*	*0.13636364*	*0.28143713*	*0.136363636*	*0.397590361*		*0.3289474*	*0.4011628*
**ENSG00000113575**		*0.12424242*		*0.124242424*				*0.4941176*
**ENSG00000145391**		*0.19393939*		*0.193939394*				
**ENSG00000159202**		*0.49393939*		*0.493939394*				*0.2732558*
**ENSG00000075884**	*0.25449102*		*0.25449102*		*0.291666667*		*0.3023256*	
**ENSG00000164048**	*0.15*	*0.34848485*	*0.15*	*0.348484848*	*0.144444444*		*0.1590909*	*0.2325581*
**ENSG00000182557**	*0.42613636*	*0.30909091*	*0.42613636*	*0.309090909*	*0.144444444*		*0.1136364*	
**ENSG00000188010**	*0.0748503*	*0.47777778*	*0.0748503*	*0.477777778*	*0.077380952*		*0.0755814*	*0.1931818*
**ENSG00000164022**	*0.17771084*	*0.21515152*	*0.17771084*	*0.215151515*	*0.136904762*		*0.2764706*	*0.2732558*
**ENSG00000155252**		*0.35151515*		*0.351515152*				*0.4529412*
**ENSG00000077522**		*0.29393939*		*0.293939394*				*0.3068182*
**ENSG00000153250**	*0.31626506*		*0.31626506*		*0.154761905*		*0.1511628*	
**ENSG00000188293**		*0.46363636*		*0.463636364*				*0.4651163*
**ENSG00000133256**		*0.33333333*		*0.333333333*				*0.3941176*
**ENSG00000138101**	*0.13772455*	*0.37575758*	*0.13772455*	*0.375757576*	*0.452380952*		*0.4244186*	

**ENSG00000198788**		*0.07058824*		*0.070588235*				*0.0526316*
**ENSG00000166902**	*0.08988764*		*0.08988764*				*0.0666667*	
**ENSG00000126878**	*0.13888889*	*0.32317073*	*0.13888889*	*0.323170732*				*0.3977273*
**ENSG00000240563**		*0.45151515*		*0.451515152*				*0.1162791*
**ENSG00000215455**		*0.31460674*		*0.314606742*				*0.4886364*
**ENSG00000141431**		*0.12424242*		*0.124242424*				*0.2848837*
**ENSG00000228835**		*0.36666667*		*0.366666667*				*0.3430233*
**ENSG00000025796**		*0.21518987*		*0.215189873*				*0.45*
**ENSG00000116774**		*0.2*		*0.2*			*0.0697674*	*0.1976744*
**ENSG00000157734**		*0.1554878*		*0.155487805*				*0.0529412*
**ENSG00000132570**		*0.14848485*		*0.148484848*				*0.1976744*
**ENSG00000143740**	*0.41111111*	*0.47222222*	*0.41111111*	*0.472222222*	*0.411111111*		*0.3488372*	*0.2386364*
**ENSG00000057757**		*0.3969697*		*0.396969697*				*0.3604651*
**ENSG00000011083**		*0.49090909*		*0.490909091*				*0.3313953*
**ENSG00000090863**		*0.44545455*		*0.445454545*				*0.3662791*
**ENSG00000211445**		*0.13030303*		*0.13030303*				*0.4418605*
**ENSG00000164363**	*0.19318182*		*0.19318182*		*0.482142857*		*0.3977273*	
**ENSG00000126752**		*0.5*		*0.5*				*0.4058824*
**ENSG00000196832**		*0.41358025*		*0.413580247*				*0.3081395*
**ENSG00000196470**		*0.15853659*		*0.158536585*				
**ENSG00000140950**	*0.39520958*	*0.49393939*	*0.39520958*	*0.493939394*	*0.458333333*		*0.4883721*	*0.2790698*
**ENSG00000162694**	*0.05688623*	*0.36363636*	*0.05688623*	*0.363636364*	*0.470238095*		*0.3197674*	*0.4011628*
**ENSG00000112837**		*0.35454545*		*0.354545455*				*0.1046512*
**ENSG00000151572**	*0.06325301*	*0.15730337*	*0.06325301*	*0.157303371*				*0.2093023*
**ENSG00000056661**		*0.42121212*		*0.421212121*				*0.3081395*
**ENSG00000073712**		*0.08787879*		*0.087878788*				*0.2325581*
**ENSG00000152527**		*0.28787879*		*0.287878788*				*0.122093*
**ENSG00000154269**		*0.25304878*		*0.25304878*				*0.3647059*
**ENSG00000231171**		*0.1433121*		*0.143312102*				*0.2058824*
**ENSG00000081870**		*0.12121212*		*0.121212121*				*0.060241*
**ENSG00000106012**		*0.4847561*		*0.484756098*				*0.5*
**ENSG00000100324**		*0.3030303*		*0.303030303*				
**ENSG00000126351**		*0.12777778*		*0.127777778*				*0.4207317*
**ENSG00000164638**		*0.20588235*		*0.205882353*				*0.0813953*
**ENSG00000149201**	*0.20783133*	*0.25609756*	*0.20783133*	*0.256097561*	*0.279761905*		*0.2616279*	*0.2848837*
**ENSG00000167711**		*0.44242424*		*0.442424242*				*0.4593023*
**ENSG00000107771**		*0.47777778*		*0.477777778*				*0.4695122*
**ENSG00000122679**		*0.31212121*		*0.312121212*				*0.1046512*
**ENSG00000248050**		*0.09393939*		*0.093939394*				
**ENSG00000197935**		*0.17222222*		*0.172222222*				*0.125*
**ENSG00000092203**	*0.49401198*	*0.25454545*	*0.49401198*	*0.254545455*	*0.267857143*		*0.2209302*	*0.1976744*
**ENSG00000008256**	*0.19760479*	*0.18484848*	*0.19760479*	*0.184848485*	*0.297619048*		*0.2378049*	*0.2613636*
**ENSG00000180532**	*0.27586207*		*0.27586207*		*0.255555556*		*0.1931818*	
**ENSG00000151692**		*0.47575758*		*0.475757576*				*0.1686047*
**ENSG00000176619**		*0.48809524*		*0.488095238*				*0.4883721*
**ENSG00000237521**		*0.18484848*		*0.184848485*				*0.4360465*
**ENSG00000112695**	*0.15868263*		*0.15868263*		*0.220238095*		*0.1744186*	
**ENSG00000188033**	*0.19631902*	*0.34444444*	*0.19631902*	*0.344444444*	*0.136904762*		*0.2352941*	*0.3181818*
**ENSG00000110628**	*0.14371257*		*0.14371257*		*0.238095238*		*0.2790698*	
**ENSG00000182111**		*0.41666667*		*0.416666667*				*0.4883721*
**ENSG00000143575**					*0.445783133*		*0.4156627*	
**ENSG00000131808**		*0.35757576*		*0.357575758*				*0.3139535*
**ENSG00000167987**		*0.43636364*		*0.436363636*				
**ENSG00000135838**		*0.07272727*		*0.072727273*				*0.1860465*

**ENSG00000196967**		*0.36666667*		*0.366666667*			*0.1162791*
**ENSG00000120664**	*0.4251497*		*0.4251497*		*0.220238095*	*0.2093023*	
**ENSG00000115816**	*0.1497006*		*0.1497006*		*0.404761905*	*0.4709302*	
**ENSG00000236032**	*0.39102564*		*0.39102564*		*0.452380952*	*0.3409091*	
**ENSG00000142173**	*0.30239521*	*0.42528736*	*0.30239521*	*0.425287356*	*0.089285714*		*0.5*
**ENSG00000099910**		*0.35151515*		*0.351515152*			*0.4772727*
**ENSG00000082497**		*0.10736196*		*0.107361963*			*0.122093*
**ENSG00000196337**	*0.49438202*		*0.49438202*		*0.355555556*	*0.3181818*	
**ENSG00000163939**	*0.05120482*	*0.33231707*	*0.05120482*	*0.332317073*			*0.494186*
**ENSG00000130939**	*0.0988024*		*0.0988024*		*0.380952381*	*0.2965116*	*0.0666667*
**ENSG00000155959**		*0.25776398*		*0.257763975*			*0.1395349*
**ENSG00000134030**		*0.2*		*0.2*			*0.1647059*
**ENSG00000134900**		*0.48787879*		*0.487878788*			*0.4651163*
**ENSG00000014138**		*0.08333333*		*0.083333333*			
**ENSG00000146592**		*0.19512195*		*0.195121951*			*0.3255814*
**ENSG00000084733**		*0.23888889*		*0.238888889*			*0.1363636*
**ENSG00000104805**	*0.49438202*	*0.29393939*	*0.49438202*	*0.293939394*		*0.1590909*	*0.372093*
**ENSG00000163825**					*0.05952381*		
**ENSG00000147383**		*0.2030303*		*0.203030303*			*0.0529412*
**ENSG00000182459**		*0.36196319*		*0.36196319*		*0.0666667*	*0.4470588*
**ENSG00000236669**		*0.45402299*		*0.454022989*			*0.375*
**ENSG00000047597**		*0.14044944*		*0.140449438*			
**ENSG00000125780**		*0.4*		*0.4*			*0.1162791*
**ENSG00000204580**	*0.3258427*	*0.4030303*	*0.3258427*	*0.403030303*	*0.452380952*	*0.3977273*	*0.4534884*
**ENSG00000134389**	*0.48502994*	*0.47515528*	*0.48502994*	*0.47515528*			*0.1627907*
**ENSG00000163389**		*0.28333333*		*0.283333333*			*0.2954545*
**ENSG00000172986**		*0.39090909*		*0.390909091*			*0.4825581*
**ENSG00000101222**	*0.17964072*		*0.17964072*		*0.261904762*	*0.255814*	*0.122093*
**ENSG00000020256**		*0.17261905*		*0.172619048*			
**ENSG00000178685**	*0.32934132*		*0.32934132*		*0.166666667*	*0.1453488*	
**ENSG00000178075**					*0.357142857*	*0.3081395*	
**ENSG00000132563**		*0.47484277*		*0.474842767*			*0.2261905*
**ENSG00000144681**		*0.17272727*		*0.172727273*			*0.244186*
**ENSG00000170915**	*0.36666667*	*0.25757576*	*0.36666667*	*0.257575758*	*0.255555556*	*0.2555556*	*0.4651163*
**ENSG00000050130**	*0.41916168*	*0.21212121*	*0.41916168*	*0.212121212*	*0.130952381*	*0.1511628*	*0.1511628*
**ENSG00000198466**	*0.10479042*	*0.39329268*	*0.10479042*	*0.393292683*	*0.214285714*	*0.1337209*	*0.4886364*
**ENSG00000138834**		*0.46319018*		*0.463190184*			*0.327381*
**ENSG00000117751**	*0.42814371*		*0.42814371*		*0.267857143*	*0.2093023*	
**ENSG00000131584**							*0.4244186*
**ENSG00000151164**							*0.0755814*
**ENSG00000151552**		*0.35757576*		*0.357575758*			*0.4767442*
**ENSG00000153832**		*0.41212121*		*0.412121212*	*0.148809524*	*0.2267442*	*0.2848837*
**ENSG00000136110**		*0.07272727*		*0.072727273*			
**ENSG00000163867**							*0.3895349*
**ENSG00000233701**		*0.05757576*		*0.057575758*			
**ENSG00000144730**	*0.44886364*	*0.21515152*	*0.44886364*	*0.215151515*	*0.066666667*	*0.1136364*	*0.5*
**ENSG00000146707**							*0.3977273*
**ENSG00000130711**		*0.09090909*		*0.090909091*			*0.4593023*
**ENSG00000128536**	*0.26047904*	*0.42121212*	*0.26047904*	*0.421212121*	*0.5*	*0.4767442*	*0.2790698*
**ENSG00000136146**							*0.0523256*
**ENSG00000107742**		*0.48295455*		*0.482954545*			*0.4593023*
**ENSG00000145388**					*0.101190476*	*0.0523256*	
**ENSG00000169876**		*0.48484848*		*0.484848485*			*0.3139535*
**ENSG00000105607**		*0.42236025*		*0.422360248*			*0.1686047*

**ENSG00000213859**		*0.16564417*		*0.165644172*				*0.1488095*
**ENSG00000170248**		*0.44705882*		*0.447058824*				*0.3888889*
**ENSG00000125351**		*0.06603774*		*0.066037736*				
**ENSG00000023608**	*0.28089888*		*0.28089888*		*0.233333333*		*0.2777778*	
**ENSG00000091181**	*0.37650602*	*0.49695122*	*0.37650602*	*0.49695122*	*0.452380952*		*0.2674419*	*0.2325581*
**ENSG00000136758**	*0.28915663*	*0.17272727*	*0.28915663*	*0.172727273*	*0.403614458*		*0.3604651*	*0.2848837*
**ENSG00000168397**		*0.28658537*		*0.286585366*				*0.4883721*
**ENSG00000157014**	*0.07185629*	*0.44242424*	*0.07185629*	*0.442424242*				*0.3647059*
**ENSG00000167644**	*0.14071856*		*0.14071856*		*0.422619048*		*0.4593023*	
**ENSG00000158747**		*0.28787879*		*0.287878788*				*0.4476744*
**ENSG00000105974**		*0.25*		*0.25*				*0.5*
**ENSG00000101825**		*0.06321839*		*0.063218391*				
**ENSG00000167757**		*0.18484848*		*0.184848485*			*0.0529412*	*0.3546512*
**ENSG00000159261**	*0.11976048*		*0.11976048*					
**ENSG00000161326**	*0.14670659*	*0.12424242*	*0.14670659*	*0.124242424*	*0.488095238*		*0.3895349*	*0.1569767*
**ENSG00000221963**								*0.1705882*
**ENSG00000253251**	*0.41916168*		*0.41916168*		*0.396341463*		*0.3571429*	
**ENSG00000101327**		*0.24528302*		*0.245283019*				*0.2529412*
**ENSG00000132906**	*0.3875*	*0.48787879*	*0.3875*	*0.487878788*	*0.375*		*0.3662791*	*0.3888889*
**ENSG00000133789**		*0.43939394*		*0.439393939*				*0.3197674*
**ENSG00000115386**		*0.43030303*		*0.43030303*				*0.2732558*
**ENSG00000250317**	*0.10778443*	*0.24848485*	*0.10778443*	*0.248484848*	*0.369047619*		*0.3372093*	*0.3372093*
**ENSG00000160075**		*0.34848485*		*0.348484848*	*0.43902439*		*0.4230769*	*0.3837209*
**ENSG00000176014**	*0.49691358*		*0.49691358*		*0.39375*		*0.3597561*	
**ENSG00000163605**		*0.48255814*		*0.48255814*				*0.4709302*
**ENSG00000080371**		*0.21515152*		*0.215151515*				*0.5*
**ENSG00000103121**	*0.1*		*0.1*		*0.344444444*		*0.375*	*0.0930233*
**ENSG00000169085**								*0.2045455*
**ENSG00000179059**		*0.17272727*		*0.172727273*				*0.297619*
**ENSG00000145832**	*0.28443114*	*0.39393939*	*0.28443114*	*0.393939394*	*0.339285714*		*0.2906977*	*0.2674419*
**ENSG00000215298**	*0.10778443*	*0.38181818*	*0.10778443*	*0.381818182*	*0.452380952*		*0.4470588*	*0.2209302*
**ENSG00000163785**	*0.14071856*		*0.14071856*		*0.44047619*		*0.4825581*	*0.25*
**ENSG00000240038**	*0.46875*		*0.46875*		*0.404761905*		*0.3139535*	
**ENSG00000141837**		*0.45151515*		*0.451515152*				*0.3690476*
**ENSG00000167637**		*0.35227273*		*0.352272727*				*0.4555556*
**ENSG00000118985**		*0.27272727*		*0.272727273*				*0.2613636*
**ENSG00000114331**		*0.31097561*		*0.31097561*				*0.2848837*
**ENSG00000125878**								*0.2674419*
**ENSG00000163959**		*0.43103448*		*0.431034483*				*0.1818182*
**ENSG00000228075**		*0.32121212*		*0.321212121*				*0.4318182*
**ENSG00000168993**		*0.42528736*		*0.425287356*				*0.3068182*
**ENSG00000259176**								*0.1428571*
**ENSG00000154447**	*0.38202247*	*0.17987805*	*0.38202247*	*0.179878049*				*0.0647059*
**ENSG00000164175**								*0.4302326*
**ENSG00000124523**	*0.2754491*		*0.2754491*		*0.130952381*		*0.1627907*	
**ENSG00000180921**		*0.14367816*		*0.143678161*				*0.4886364*
**ENSG00000009335**		*0.38484848*		*0.384848485*				*0.4825581*
**ENSG00000173467**		*0.35151515*		*0.351515152*				*0.2965116*
**ENSG00000188629**	*0.48314607*	*0.45151515*	*0.48314607*	*0.451515152*	*0.477777778*		*0.4090909*	*0.4127907*
**ENSG00000163518**		*0.38888889*		*0.388888889*				*0.4302326*
**ENSG00000106100**	*0.36526946*	*0.09393939*	*0.36526946*	*0.093939394*	*0.297619048*		*0.4767442*	*0.0755814*
**ENSG00000204475**		*0.05757576*		*0.057575758*				*0.1860465*
**ENSG00000155868**	*0.13888889*	*0.38181818*	*0.13888889*	*0.381818182*				*0.2616279*
**ENSG00000181264**		*0.3969697*		*0.396969697*				*0.2965116*

**ENSG00000145882**		*0.4847561*		*0.484756098*				*0.3546512*
**ENSG00000124574**	*0.11676647*		*0.11676647*				*0.0523256*	
**ENSG00000115523**	*0.48802395*	*0.43820225*	*0.48802395*	*0.438202247*	*0.357142857*		*0.3953488*	*0.4555556*
**ENSG00000198160**		*0.28888889*		*0.288888889*				*0.1444444*
**ENSG00000039537**	*0.17065868*	*0.39329268*	*0.17065868*	*0.393292683*	*0.083333333*		*0.1569767*	*0.1976744*
**ENSG00000145920**	*0.20359281*	*0.49695122*	*0.20359281*	*0.49695122*	*0.077777778*		*0.1022727*	*0.4244186*
**ENSG00000103319**		*0.10909091*		*0.109090909*				*0.1860465*
**ENSG00000058729**		*0.46666667*		*0.466666667*				*0.494186*
**ENSG00000149260**		*0.47575758*		*0.475757576*				*0.4534884*
**ENSG00000099204**	*0.10795455*	*0.46666667*	*0.10795455*	*0.466666667*	*0.422619048*		*0.4651163*	*0.4651163*
**ENSG00000144485**								*0.1402439*
**ENSG00000165185**		*0.30909091*		*0.309090909*				*0.494186*
**ENSG00000002587**		*0.37575758*		*0.375757576*				*0.2965116*
**ENSG00000196581**		*0.38505747*		*0.385057471*				*0.3411765*
**ENSG00000223474**		*0.25460123*		*0.254601227*				*0.3294118*
**ENSG00000141140**	*0.21084337*	*0.43258427*	*0.21084337*	*0.43258427*	*0.202380952*		*0.0705882*	
**ENSG00000198083**		*0.33333333*		*0.333333333*				*0.3977273*
**ENSG00000136986**		*0.39308176*		*0.393081761*				*0.4244186*
**ENSG00000091732**		*0.42727273*		*0.427272727*				*0.2848837*
**ENSG00000166401**	*0.46706587*	*0.46060606*	*0.46706587*	*0.460606061*	*0.231707317*		*0.2411765*	*0.4186047*
**ENSG00000100629**	*0.39156627*	*0.43518519*	*0.39156627*	*0.435185185*	*0.30952381*		*0.3546512*	*0.3546512*
**ENSG00000164746**	*0.17365269*	*0.39329268*	*0.17365269*	*0.393292683*	*0.452380952*		*0.3546512*	*0.2325581*
**ENSG00000123364**		*0.27575758*		*0.275757576*				*0.1686047*
**ENSG00000185689**	*0.1835443*	*0.46363636*	*0.1835443*	*0.463636364*	*0.3*		*0.3154762*	*0.3139535*
**ENSG00000164530**	*0.4375*	*0.3969697*	*0.4375*	*0.396969697*	*0.466666667*		*0.4476744*	*0.494186*
**ENSG00000183864**								*0.3372093*
**ENSG00000205669**	*0.25149701*		*0.25149701*		*0.357142857*		*0.2732558*	
**ENSG00000166483**		*0.14329268*		*0.143292683*				*0.244186*
**ENSG00000078674**		*0.41212121*		*0.412121212*				*0.4444444*
**ENSG00000205036**	*0.47289157*		*0.47289157*		*0.458333333*		*0.4883721*	
**ENSG00000135390**	*0.43820225*		*0.43820225*		*0.433333333*		*0.3777778*	
**ENSG00000165046**	*0.16766467*		*0.16766467*		*0.375*		*0.3546512*	
**ENSG00000095539**		*0.48314607*		*0.483146067*				*0.4534884*
**ENSG00000188158**	*0.38333333*		*0.38333333*					
**ENSG00000129473**		*0.07575758*		*0.075757576*				*0.0930233*
**ENSG00000154429**		*0.4494382*		*0.449438202*				*0.4555556*
**ENSG00000077458**	*0.2245509*	*0.49056604*	*0.2245509*	*0.490566038*	*0.404761905*		*0.4476744*	*0.422619*
**ENSG00000163206**	*0.1257485*		*0.1257485*		*0.422619048*		*0.4418605*	
**ENSG00000187866**		*0.3969697*		*0.396969697*				*0.3953488*
**ENSG00000002745**		*0.41212121*		*0.412121212*				*0.1569767*
**ENSG00000134955**		*0.43333333*		*0.433333333*				*0.4588235*
**ENSG00000188641**		*0.4969697*		*0.496969697*				*0.4593023*
**ENSG00000198838**		*0.24242424*		*0.242424242*				*0.4411765*
**ENSG00000163918**	*0.4760479*	*0.17575758*	*0.4760479*	*0.175757576*	*0.410714286*		*0.4476744*	*0.0872093*
**ENSG00000170312**		*0.25617284*		*0.25617284*				*0.5*
**ENSG00000167196**		*0.13939394*		*0.139393939*				*0.1453488*
**ENSG00000163597**	*0.32222222*	*0.32222222*	*0.32222222*	*0.322222222*	*0.455555556*		*0.4204545*	*0.4593023*
**ENSG00000176753**		*0.38787879*		*0.387878788*				*0.1453488*
**ENSG00000185966**		*0.21818182*		*0.218181818*				*0.2906977*
**ENSG00000154978**	*0.36826347*	*0.18902439*	*0.36826347*	*0.18902439*	*0.05952381*		*0.0930233*	*0.0697674*
**ENSG00000120251**	*0.44848485*	*0.44817073*	*0.44848485*	*0.448170732*	*0.160714286*		*0.2142857*	*0.372093*
**ENSG00000150782**	*0.23053892*		*0.23053892*		*0.071428571*		*0.1627907*	
**ENSG00000168818**	*0.22155689*	*0.15*	*0.22155689*	*0.15*				*0.0694444*
**ENSG00000182168**		*0.47752809*		*0.47752809*				*0.4111111*

**ENSG00000157884**		*0.48787879*		*0.487878788*			*0.3430233*
**ENSG00000100344**		*0.29090909*		*0.290909091*			*0.4418605*
**ENSG00000156453**		*0.32727273*		*0.327272727*			*0.4476744*
**ENSG00000185274**	*0.18888889*	*0.14545455*	*0.18888889*	*0.145454545*			
**ENSG00000105258**	*0.23053892*		*0.23053892*		*0.168674699*	*0.2034884*	
**ENSG00000185112**	*0.18562874*		*0.18562874*		*0.428571429*	*0.4883721*	
**ENSG00000116580**		*0.36060606*		*0.360606061*			*0.2209302*
**ENSG00000165887**	*0.28143713*		*0.28143713*		*0.488888889*	*0.4294118*	
**ENSG00000163950**		*0.35582822*		*0.355828221*			*0.3214286*
**ENSG00000123342**		*0.2030303*		*0.203030303*			*0.1190476*
**ENSG00000090889**	*0.38323353*		*0.38323353*				*0.0813953*
**ENSG00000197702**		*0.36111111*		*0.361111111*			*0.3181818*
**ENSG00000197147**		*0.25151515*		*0.251515152*			*0.2151163*
**ENSG00000170558**		*0.08841463*		*0.088414634*			*0.3409091*
**ENSG00000163646**	*0.26347305*	*0.44242424*	*0.26347305*	*0.442424242*	*0.458333333*	*0.5*	*0.2705882*
**ENSG00000149651**		*0.40909091*		*0.409090909*			*0.4476744*
**ENSG00000250699**	*0.10479042*		*0.10479042*				
**ENSG00000109536**	*0.4939759*		*0.4939759*		*0.398809524*	*0.3658537*	
**ENSG00000186106**	*0.38323353*	*0.30792683*	*0.38323353*	*0.307926829*	*0.142857143*	*0.122093*	*0.2732558*
**ENSG00000117724**	*0.16666667*		*0.16666667*				
**ENSG00000130540**		*0.19207317*		*0.192073171*			*0.1104651*
**ENSG00000095574**	*0.40909091*	*0.16363636*	*0.40909091*	*0.163636364*	*0.133333333*	*0.2159091*	*0.4418605*
**ENSG00000171014**		*0.16363636*		*0.163636364*			*0.2209302*
**ENSG00000138316**		*0.48787879*		*0.487878788*			*0.4555556*
**ENSG00000078967**		*0.14242424*		*0.142424242*			*0.0654762*
**ENSG00000169871**		*0.06060606*		*0.060606061*			
**ENSG00000198198**		*0.44848485*		*0.448484848*			*0.3895349*
**ENSG00000169629**	*0.06666667*		*0.06666667*				
**ENSG00000132676**	*0.12424242*		*0.12424242*		*0.280487805*	*0.2616279*	
**ENSG00000119729**		*0.07777778*		*0.077777778*			*0.0697674*
**ENSG00000171695**		*0.28735632*		*0.287356322*			*0.2613636*
**ENSG00000166529**	*0.06287425*		*0.06287425*		*0.470238095*	*0.494186*	
**ENSG00000138942**		*0.29090909*		*0.290909091*			*0.0930233*
**ENSG00000129625**		*0.44545455*		*0.445454545*			*0.3333333*
**ENSG00000135312**	*0.18888889*		*0.18888889*		*0.088888889*	*0.0666667*	
**ENSG00000183888**		*0.15030675*		*0.150306748*			
**ENSG00000121766**		*0.409375*		*0.409375*			*0.1802326*
**ENSG00000135913**	*0.40340909*	*0.38181818*	*0.40340909*	*0.381818182*	*0.253012048*	*0.2790698*	*0.1235294*
**ENSG00000150756**	*0.27607362*	*0.17177914*	*0.27607362*	*0.171779141*	*0.19047619*	*0.1453488*	*0.0581395*
**ENSG00000206530**	*0.11077844*	*0.48484848*	*0.11077844*	*0.484848485*	*0.433333333*	*0.2888889*	*0.494186*
**ENSG00000025770**		*0.41212121*		*0.412121212*			*0.3197674*
**ENSG00000112425**	*0.1863354*	*0.41818182*	*0.1863354*	*0.418181818*	*0.182926829*	*0.297619*	*0.3511905*
**ENSG00000130643**		*0.1097561*		*0.109756098*			*0.1104651*
**ENSG00000113621**		*0.3969697*		*0.396969697*			*0.3604651*
**ENSG00000254081**	*0.3502994*		*0.3502994*		*0.397590361*	*0.4011628*	
**ENSG00000170390**	*0.18181818*		*0.18181818*				
**ENSG00000110852**	*0.28443114*	*0.4127907*	*0.28443114*	*0.412790698*	*0.380952381*	*0.2906977*	*0.4545455*
**ENSG00000198169**							*0.4302326*
**ENSG00000099834**	*0.42814371*	*0.36280488*	*0.42814371*	*0.362804878*			*0.2529412*
**ENSG00000145423**							*0.2111111*
**ENSG00000182223**		*0.17222222*		*0.172222222*			*0.0777778*
**ENSG00000182256**		*0.45731707*		*0.457317073*			*0.255814*
**ENSG00000222014**	*0.44848485*		*0.44848485*		*0.148809524*	*0.1686047*	
**ENSG00000123349**	*0.12777778*		*0.12777778*				

**ENSG00000117758**							*0.1818182*
**ENSG00000168014**		*0.17878788*		*0.178787879*			*0.2882353*
**ENSG00000111361**		*0.34848485*		*0.348484848*			*0.0523256*
**ENSG00000104980**		*0.32022472*		*0.320224719*			*0.4545455*
**ENSG00000150086**		*0.44512195*		*0.445121951*			*0.2209302*
**ENSG00000070808**	*0.15963855*	*0.07777778*	*0.15963855*	*0.077777778*	*0.222222222*	*0.25*	
**ENSG00000003989**	*0.16666667*	*0.18484848*	*0.16666667*	*0.184848485*			*0.1395349*
**ENSG00000220891**							*0.3837209*
**ENSG00000119041**		*0.0969697*		*0.096969697*			*0.0930233*
**ENSG00000163251**		*0.40555556*		*0.405555556*			*0.2727273*
**ENSG00000150275**	*0.45508982*	*0.41455696*	*0.45508982*	*0.414556962*	*0.178571429*	*0.0930233*	*0.4651163*
**ENSG00000008128**					*0.175*	*0.1222222*	
**ENSG00000169826**		*0.278125*		*0.278125*			*0.4767442*
**ENSG00000151445**		*0.49393939*		*0.493939394*			*0.0755814*
**ENSG00000205882**		*0.48888889*		*0.488888889*			*0.4651163*
**ENSG00000203805**		*0.19817073*		*0.198170732*			*0.0588235*
**ENSG00000124171**		*0.3445122*		*0.344512195*			*0.3176471*
**ENSG00000145907**		*0.43030303*		*0.43030303*	*0.232142857*	*0.2470588*	*0.2732558*
**ENSG00000205791**		*0.4847561*		*0.484756098*			*0.3058824*
**ENSG00000139132**	*0.25149701*	*0.45*	*0.25149701*	*0.45*	*0.255952381*	*0.1686047*	*0.1705882*
**ENSG00000244474**		*0.22777778*		*0.227777778*			*0.1590909*
**ENSG00000100294**		*0.3030303*		*0.303030303*			*0.377907*
**ENSG00000170892**	*0.26875*		*0.26875*		*0.174698795*	*0.1707317*	*0.1764706*
**ENSG00000106565**	*0.48295455*	*0.49393939*	*0.48295455*	*0.493939394*	*0.366666667*	*0.2790698*	*0.3662791*
**ENSG00000155265**		*0.45757576*		*0.457575758*			*0.2151163*
**ENSG00000204950**		*0.13803681*		*0.13803681*			*0.4411765*
**ENSG00000162951**							*0.1*
**ENSG00000171302**	*0.19760479*	*0.13888889*	*0.19760479*	*0.138888889*	*0.28313253*	*0.3081395*	*0.3081395*
**ENSG00000180929**		*0.40449438*		*0.404494382*			*0.1931818*
**ENSG00000161958**		*0.44848485*		*0.448484848*			*0.25*
**ENSG00000163040**		*0.18888889*		*0.188888889*			*0.2857143*
**ENSG00000133069**	*0.10179641*	*0.11656442*	*0.10179641*	*0.116564417*		*0.0755814*	
**ENSG00000166415**		*0.41515152*		*0.415151515*			*0.4418605*
**ENSG00000162643**	*0.22754491*	*0.14556962*	*0.22754491*	*0.14556962*	*0.476190476*	*0.4360465*	*0.2317073*
**ENSG00000153303**	*0.11392405*	*0.4591195*	*0.11392405*	*0.459119497*	*0.05952381*	*0.1162791*	*0.4702381*
**ENSG00000205758**		*0.41011236*		*0.41011236*	*0.148809524*	*0.1976744*	*0.5*
**ENSG00000130338**	*0.24545455*	*0.49693252*	*0.24545455*	*0.496932515*	*0.452380952*	*0.4709302*	*0.5*
**ENSG00000164850**	*0.21856287*	*0.35057471*	*0.21856287*	*0.350574713*	*0.196428571*	*0.2058824*	*0.4431818*
**ENSG00000161714**		*0.20606061*		*0.206060606*			*0.0755814*
**ENSG00000173641**	*0.36526946*	*0.39939024*	*0.36526946*	*0.399390244*	*0.321428571*	*0.3452381*	*0.2823529*
**ENSG00000147145**		*0.24242424*		*0.242424242*			
**ENSG00000100739**					*0.130952381*	*0.1337209*	
**ENSG00000134020**	*0.19461078*		*0.19461078*		*0.386904762*	*0.3255814*	
**ENSG00000213002**	*0.47865854*	*0.17222222*	*0.47865854*	*0.172222222*	*0.3*	*0.2621951*	*0.25*
**ENSG00000248874**		*0.19444444*		*0.194444444*			*0.0777778*
**ENSG00000142864**		*0.42424242*		*0.424242424*			*0.4518072*
**ENSG00000215372**							*0.2625*
**ENSG00000047936**		*0.38181818*		*0.381818182*			*0.372093*
**ENSG00000160917**		*0.15*		*0.15*			*0.3255814*
**ENSG00000131374**	*0.47777778*	*0.30606061*	*0.47777778*	*0.306060606*			*0.0639535*
**ENSG00000103056**		*0.42613636*		*0.426136364*	*0.155555556*	*0.1136364*	*0.1818182*
**ENSG00000214787**		*0.10429448*		*0.104294479*			*0.1428571*
**ENSG00000129315**	*0.20555556*	*0.38484848*	*0.20555556*	*0.384848485*	*0.277777778*	*0.2954545*	*0.2790698*
**ENSG00000132688**		*0.29393939*		*0.293939394*			*0.2848837*

**ENSG00000204033**	*0.19461078*	*0.40909091*	*0.19461078*	*0.409090909*	*0.083333333*			*0.3430233*
**ENSG00000124508**	*0.49700599*	*0.48484848*	*0.49700599*	*0.484848485*	*0.089285714*		*0.1162791*	*0.3197674*
**ENSG00000154814**		*0.43333333*		*0.433333333*				*0.4709302*
**ENSG00000081818**		*0.26969697*		*0.26969697*				*0.4096386*
**ENSG00000146282**		*0.1030303*		*0.103030303*				*0.1411765*
**ENSG00000213886**	*0.48802395*	*0.46103896*	*0.48802395*	*0.461038961*	*0.148809524*		*0.2151163*	*0.3023256*
**ENSG00000165383**		*0.18711656*		*0.187116564*				
**ENSG00000104081**		*0.46666667*		*0.466666667*				*0.5*
**ENSG00000189433**								*0.1569767*
**ENSG00000047621**		*0.17391304*		*0.173913043*				*0.1569767*
**ENSG00000135636**	*0.1746988*		*0.1746988*					
**ENSG00000197106**		*0.48787879*		*0.487878788*				*0.4318182*
**ENSG00000223501**	*0.07831325*	*0.33939394*	*0.07831325*	*0.339393939*				*0.3255814*
**ENSG00000115414**		*0.36666667*		*0.366666667*				*0.25*
**ENSG00000152133**		*0.36060606*		*0.360606061*				*0.1860465*
**ENSG00000112297**	*0.38333333*	*0.35555556*	*0.38333333*	*0.355555556*	*0.411111111*		*0.4222222*	*0.3139535*
**ENSG00000146063**	*0.14367816*		*0.14367816*		*0.410714286*		*0.4244186*	
**ENSG00000067057**	*0.48802395*	*0.31212121*	*0.48802395*	*0.312121212*	*0.470238095*		*0.4476744*	*0.372093*
**ENSG00000204389**	*0.08888889*		*0.08888889*		*0.311111111*		*0.1976744*	
**ENSG00000166925**	*0.26946108*		*0.26946108*					
**ENSG00000075275**		*0.46666667*		*0.466666667*				*0.2267442*
**ENSG00000175857**	*0.20658683*	*0.3969697*	*0.20658683*	*0.396969697*	*0.120481928*			
**ENSG00000171843**		*0.21604938*		*0.216049383*				*0.3372093*
**ENSG00000186814**	*0.09580838*	*0.36666667*	*0.09580838*	*0.366666667*				*0.5*
**ENSG00000168137**		*0.13636364*		*0.136363636*				*0.0755814*
**ENSG00000035141**		*0.37272727*		*0.372727273*				*0.4404762*
**ENSG00000162745**		*0.41515152*		*0.415151515*				*0.3488372*
**ENSG00000177700**		*0.2969697*		*0.296969697*				*0.2732558*
**ENSG00000168291**		*0.33636364*		*0.336363636*				*0.2209302*
**ENSG00000213064**		*0.29141104*		*0.291411043*				*0.5*
**ENSG00000169181**	*0.45508982*	*0.38484848*	*0.45508982*	*0.384848485*	*0.392857143*		*0.4709302*	*0.4534884*
**ENSG00000134262**	*0.26646707*		*0.26646707*		*0.114457831*		*0.2638889*	
**ENSG00000128849**		*0.44817073*		*0.448170732*				*0.3604651*
**ENSG00000097046**	*0.25748503*	*0.47575758*	*0.25748503*	*0.475757576*	*0.208333333*		*0.2529412*	*0.0755814*
**ENSG00000092199**	*0.05988024*	*0.36969697*	*0.05988024*	*0.36969697*				*0.3313953*
**ENSG00000131634**		*0.30909091*		*0.309090909*				*0.4411765*
**ENSG00000118873**		*0.08333333*		*0.083333333*				
**ENSG00000003137**		*0.20731707*		*0.207317073*				*0.4709302*
**ENSG00000253459**	*0.3125*		*0.3125*		*0.188888889*		*0.1136364*	
**ENSG00000071462**	*0.10674157*	*0.47575758*	*0.10674157*	*0.475757576*	*0.444444444*		*0.4659091*	*0.2906977*
**ENSG00000217702**	*0.05*		*0.05*		*0.5*		*0.4659091*	*0.0639535*
**ENSG00000151726**		*0.35757576*		*0.357575758*	*0.08125*		*0.0795455*	*0.4166667*
**ENSG00000143751**		*0.37078652*		*0.370786517*				*0.25*
**ENSG00000188064**		*0.30606061*		*0.306060606*				*0.2267442*
**ENSG00000146910**	*0.2245509*	*0.39090909*	*0.2245509*	*0.390909091*	*0.303571429*		*0.2848837*	*0.494186*
**ENSG00000187824**		*0.4054878*		*0.405487805*				*0.4767442*
**ENSG00000103426**	*0.08982036*	*0.11515152*	*0.08982036*	*0.115151515*	*0.154761905*		*0.1802326*	*0.0764706*
**ENSG00000173908**		*0.29090909*		*0.290909091*				*0.377907*
**ENSG00000084090**		*0.17878788*		*0.178787879*				*0.1555556*
**ENSG00000176623**		*0.44155844*		*0.441558442*				*0.4825581*
**ENSG00000146950**		*0.19207317*		*0.192073171*				*0.4470588*
**ENSG00000171495**	*0.21818182*		*0.21818182*		*0.329268293*		*0.3953488*	
**ENSG00000178021**		*0.45757576*		*0.457575758*				*0.3837209*
**ENSG00000075292**	*0.14670659*	*0.25*	*0.14670659*	*0.25*	*0.071428571*		*0.2352941*	*0.2848837*

**ENSG00000242110**		0.32121212		0.321212121				0.3636364
**ENSG00000083750**	0.42777778		0.42777778		0.266666667		0.2159091	
**ENSG00000066557**		0.20224719		0.202247191				0.3139535
**ENSG00000206190**		0.43865031		0.438650307				0.4302326
**ENSG00000169548**					0.37804878		0.325	0.4457831
**ENSG00000162772**		0.2030303		0.203030303				0.4709302
**ENSG00000143590**		0.48787879		0.487878788				0.0697674
**ENSG00000140090**	0.4760479	0.4054878	0.4760479	0.405487805				0.4883721
**ENSG00000182645**	0.19631902	0.07954545	0.19631902	0.079545455			0.255814	0.3409091
**ENSG00000185483**	0.17964072	0.23602484	0.17964072	0.236024845	0.488095238		0.4476744	0.0647059
**ENSG00000126218**		0.13030303		0.13030303				0.3837209
**ENSG00000186468**	0.35628743	0.41818182	0.35628743	0.418181818	0.05952381			
**ENSG00000123689**								0.2840909
**ENSG00000169857**								0.2790698
**ENSG00000114956**	0.18674699	0.3	0.18674699	0.3				0.4360465
**ENSG00000139780**		0.3136646		0.313664596				
**ENSG00000143851**	0.32634731	0.32222222	0.32634731	0.322222222	0.19047619		0.372093	0.2117647
**ENSG00000110171**	0.24550898	0.17878788	0.24550898	0.178787879			0.0581395	
**ENSG00000145777**	0.45454545	0.18787879	0.45454545	0.187878788	0.339285714		0.3081395	
**ENSG00000175445**	0.48255814	0.31212121	0.48255814	0.312121212				0.2267442
**ENSG00000116761**	0.10674157		0.10674157					
**ENSG00000181191**		0.11490683		0.114906832				
**ENSG00000165583**	0.15662651		0.15662651					
**ENSG00000083457**					0.238095238		0.2674419	
**ENSG00000086991**	0.22159091		0.22159091		0.475		0.4518072	
**ENSG00000164099**		0.10365854		0.103658537				0.1309524
**ENSG00000145703**	0.36363636	0.45454545	0.36363636	0.454545455	0.244444444		0.2840909	0.4651163
**ENSG00000170485**		0.32926829		0.329268293				0.4709302
**ENSG00000156535**	0.0508982	0.33939394	0.0508982	0.339393939				0.4186047
**ENSG00000185737**	0.24666667	0.4845679	0.24666667	0.484567901	0.304878049		0.3809524	0.3023256
**ENSG00000182795**	0.45508982	0.42424242	0.45508982	0.424242424				0.1976744
**ENSG00000158470**		0.38202247		0.382022472				0.4659091
**ENSG00000109452**		0.18888889		0.188888889				0.372093
**ENSG00000243477**	0.19186047		0.19186047		0.386363636		0.4886364	
**ENSG00000185442**		0.37878788		0.378787879				0.25
**ENSG00000233932**								0.3977273
**ENSG00000121413**	0.45808383	0.17878788	0.45808383	0.178787879	0.077380952		0.1477273	
**ENSG00000158813**		0.35670732		0.356707317				0.4069767
**ENSG00000152954**		0.48484848		0.484848485				0.3604651
**ENSG00000205086**	0.4186747	0.39393939	0.4186747	0.393939394	0.089285714		0.0813953	0.4883721
**ENSG00000196167**	0.33233533	0.30792683	0.33233533	0.307926829	0.428571429		0.4883721	0.4883721
**ENSG00000171861**							0.0581395	
**ENSG00000167617**		0.06481481		0.064814815				0.1
**ENSG00000165898**		0.35454545		0.354545455				0.372093
**ENSG00000108528**		0.05454545		0.054545455				0.1511628
**ENSG00000018625**		0.20606061		0.206060606				0.25
**ENSG00000106588**		0.16292135		0.162921348				0.0888889
**ENSG00000119508**		0.3969697		0.396969697				0.4069767
**ENSG00000113552**		0.45454545		0.454545455				
**ENSG00000135842**		0.44242424		0.442424242				0.4244186
**ENSG00000099968**	0.45808383	0.47256098	0.45808383	0.472560976	0.119047619		0.122093	0.2647059
**ENSG00000155816**	0.32934132	0.36060606	0.32934132	0.360606061	0.475903614		0.494186	0.4883721
**ENSG00000049769**								0.0670732
**ENSG00000146233**		0.21341463		0.213414634				0.1104651

**ENSG00000139117**					*0.220238095*	*0.244186*	
**ENSG00000071537**		*0.44444444*		*0.444444444*			*0.4666667*
**ENSG00000121073**	*0.0748503*	*0.06666667*	*0.0748503*	*0.066666667*			
**ENSG00000180198**	*0.40419162*		*0.40419162*				
**ENSG00000188042**		*0.46363636*		*0.463636364*			*0.3571429*
**ENSG00000188039**	*0.45783133*	*0.48181818*	*0.45783133*	*0.481818182*	*0.44047619*	*0.3837209*	*0.3837209*
**ENSG00000154945**		*0.34848485*		*0.348484848*			*0.3662791*
**ENSG00000179774**	*0.28143713*		*0.28143713*		*0.410714286*	*0.3023256*	
**ENSG00000147650**		*0.06666667*		*0.066666667*			
**ENSG00000150593**		*0.0621118*		*0.062111801*			*0.3546512*
**ENSG00000117335**		*0.40432099*		*0.404320988*			*0.1235294*
**ENSG00000137571**	*0.1741573*	*0.2969697*	*0.1741573*	*0.296969697*	*0.277777778*	*0.3522727*	*0.2267442*
**ENSG00000172716**	*0.17222222*	*0.06969697*	*0.17222222*	*0.06969697*	*0.322222222*	*0.2386364*	*0.122093*
**ENSG00000230055**		*0.42121212*		*0.421212121*			*0.3081395*
**ENSG00000112706**		*0.36111111*		*0.361111111*			*0.4573171*
**ENSG00000101448**	*0.24850299*	*0.14634146*	*0.24850299*	*0.146341463*	*0.469879518*	*0.4244186*	*0.1222222*
**ENSG00000134376**	*0.48314607*	*0.45426829*	*0.48314607*	*0.454268293*	*0.25*	*0.2325581*	*0.3139535*
**ENSG00000176401**		*0.25454545*		*0.254545455*			*0.3023256*
**ENSG00000144115**	*0.20658683*		*0.20658683*		*0.452380952*	*0.4767442*	
**ENSG00000134323**		*0.23333333*		*0.233333333*			*0.3977273*
**ENSG00000197635**		*0.36363636*		*0.363636364*			*0.2093023*
**ENSG00000049089**							*0.0588235*
**ENSG00000151025**		*0.2030303*		*0.203030303*			*0.2151163*
**ENSG00000126246**		*0.14110429*		*0.141104294*			
**ENSG00000101203**		*0.325*		*0.325*			*0.3895349*
**ENSG00000197217**		*0.42987805*		*0.429878049*			*0.5*
**ENSG00000153207**		*0.22222222*		*0.222222222*			*0.1976744*
**ENSG00000235109**	*0.25149701*	*0.334375*	*0.25149701*	*0.334375*	*0.476190476*	*0.4883721*	*0.3809524*
**ENSG00000188981**	*0.06287425*	*0.47222222*	*0.06287425*	*0.472222222*	*0.482142857*	*0.4244186*	*0.4011628*
**ENSG00000107341**		*0.4030303*		*0.403030303*			*0.2732558*
**ENSG00000250486**		*0.33030303*		*0.33030303*			*0.0843373*
**ENSG00000101282**		*0.13636364*		*0.136363636*			*0.2702703*
**ENSG00000168385**	*0.40419162*	*0.2125*	*0.40419162*	*0.2125*	*0.428571429*	*0.3604651*	*0.3953488*
**ENSG00000050327**		*0.42424242*		*0.424242424*			*0.1918605*
**ENSG00000169221**		*0.29444444*		*0.294444444*			
**ENSG00000101138**	*0.26807229*	*0.26969697*	*0.26807229*	*0.26969697*	*0.089285714*	*0.0639535*	*0.3*
**ENSG00000147124**	*0.22121212*	*0.30555556*	*0.22121212*	*0.305555556*	*0.130952381*	*0.2732558*	*0.3863636*
**ENSG00000095383**	*0.08383234*		*0.08383234*				
**ENSG00000102445**	*0.38888889*	*0.31818182*	*0.38888889*	*0.318181818*	*0.455555556*	*0.4659091*	*0.5*
**ENSG00000138698**		*0.05151515*		*0.051515152*			
**ENSG00000119401**		*0.33333333*		*0.333333333*			*0.2954545*
**ENSG00000164867**		*0.13939394*		*0.139393939*			*0.0523256*
**ENSG00000157353**	*0.17065868*		*0.17065868*		*0.355421687*	*0.2848837*	
**ENSG00000125046**	*0.37078652*	*0.38888889*	*0.37078652*	*0.388888889*	*0.202380952*	*0.1511628*	*0.4777778*
**ENSG00000197050**	*0.06024096*	*0.49090909*	*0.06024096*	*0.490909091*			*0.1337209*
**ENSG00000107554**	*0.46666667*	*0.48181818*	*0.46666667*	*0.481818182*	*0.446428571*	*0.3662791*	*0.2383721*
**ENSG00000117408**	*0.19760479*		*0.19760479*		*0.380952381*	*0.4534884*	
**ENSG00000162194**	*0.46590909*	*0.46666667*	*0.46590909*	*0.466666667*	*0.119047619*	*0.075*	*0.2045455*
**ENSG00000184825**	*0.2439759*		*0.2439759*				
**ENSG00000168016**	*0.3742515*	*0.38787879*	*0.3742515*	*0.387878788*	*0.428571429*	*0.4883721*	*0.3430233*
**ENSG00000173535**	*0.20658683*		*0.20658683*		*0.117283951*	*0.1607143*	
**ENSG00000163322**		*0.45121951*		*0.451219512*			*0.375*
**ENSG00000158987**		*0.20606061*		*0.206060606*			*0.4709302*
**ENSG00000117410**		*0.08787879*		*0.087878788*			*0.1104651*

**ENSG00000131966**		0.3988764		0.398876404			0.3522727
**ENSG00000158525**	0.43413174		0.43413174		0.5	0.4772727	
**ENSG00000069482**		0.29393939		0.293939394			0.2383721
**ENSG00000159147**		0.09393939		0.093939394			0.4883721
**ENSG00000085449**		0.4695122		0.469512195			0.4821429
**ENSG00000105655**		0.33636364		0.336363636			0.25
**ENSG00000188738**		0.49079755		0.490797546			0.4642857
**ENSG00000135926**	0.26347305	0.37878788	0.26347305	0.378787879	0.386904762	0.375	0.3863636
**ENSG00000157890**		0.0969697		0.096969697			
**ENSG00000196344**		0.2		0.2			0.2857143
**ENSG00000204296**	0.29573171	0.3969697	0.29573171	0.396969697	0.267857143	0.3372093	0.3647059
**ENSG00000163491**	0.13772455	0.1554878	0.13772455	0.155487805	0.196428571	0.25	0.1395349
**ENSG00000145246**		0.33231707		0.332317073			0.4825581
**ENSG00000198663**		0.19393939		0.193939394			0.3139535
**ENSG00000108799**		0.41463415		0.414634146			0.4360465
**ENSG00000136816**		0.31818182		0.318181818			0.2848837
**ENSG00000163071**		0.14242424		0.142424242			0.3023256
**ENSG00000120539**	0.1497006	0.35757576	0.1497006	0.357575758	0.409090909	0.3076923	0.3988095
**ENSG00000197057**	0.13473054	0.18181818	0.13473054	0.181818182	0.178571429	0.1162791	0.0813953
**ENSG00000197385**	0.33333333		0.33333333		0.311111111	0.375	
**ENSG00000169836**	0.14071856	0.29090909	0.14071856	0.290909091	0.273809524	0.3546512	0.4825581
**ENSG00000096264**	0.48502994		0.48502994		0.138554217	0.122093	
**ENSG00000183530**		0.22727273		0.227272727			0.127907
**ENSG00000215204**		0.33939394		0.339393939			0.3197674
**ENSG00000198382**		0.06363636		0.063636364			0.2674419
**ENSG00000144355**	0.19444444	0.28787879	0.19444444	0.287878788	0.111111111	0.1704545	0.2209302
**ENSG00000170448**		0.35		0.35			
**ENSG00000135346**		0.37078652		0.370786517			0.4772727
**ENSG00000234444**	0.26190476		0.26190476				
**ENSG00000254440**	0.16467066	0.07763975	0.16467066	0.077639752	0.398809524	0.3953488	0.4107143
**ENSG00000197037**							0.2848837
**ENSG00000038382**	0.34269663	0.3597561	0.34269663	0.359756098			0.246988
**ENSG00000147654**	0.10479042	0.35757576	0.10479042	0.357575758	0.345238095	0.3647059	0.4476744
**ENSG00000130695**	0.3313253	0.13333333	0.3313253	0.133333333	0.160714286	0.1511628	
**ENSG00000139329**		0.13719512		0.137195122	0.279761905	0.1802326	0.1802326
**ENSG00000255332**		0.22121212		0.221212121			0.0697674
**ENSG00000160867**	0.21257485	0.24848485	0.21257485	0.248484848			
**ENSG00000151779**		0.38764045		0.387640449			0.2888889
**ENSG00000125967**							0.1463415
**ENSG00000237441**	0.16853933		0.16853933		0.079545455	0.0930233	
**ENSG00000157741**							0.1823529
**ENSG00000173627**	0.29041916	0.37575758	0.29041916	0.375757576	0.077380952		0.0843373
**ENSG00000073910**	0.49401198	0.43333333	0.49401198	0.433333333	0.178571429	0.0872093	0.0872093
**ENSG00000168096**		0.47222222		0.472222222			0.3295455
**ENSG00000074621**					0.166666667	0.3068182	
**ENSG00000198400**	0.35628743	0.21646341	0.35628743	0.216463415	0.130952381	0.1744186	0.3895349
**ENSG00000133067**		0.42405063		0.424050633			0.3214286
**ENSG00000186517**		0.3		0.3			0.2954545
**ENSG00000161981**	0.25149701	0.16969697	0.25149701	0.16969697	0.397590361	0.4529412	0.4593023
**ENSG00000101938**		0.44767442		0.447674419			
**ENSG00000182389**		0.19444444		0.194444444			0.3863636
**ENSG00000030066**		0.45454545		0.454545455	0.148809524	0.0833333	0.3837209
**ENSG00000089057**		0.46363636		0.463636364			0.4244186
**ENSG00000152611**	0.06741573	0.32317073	0.06741573	0.323170732			0.0529412

**ENSG00000164893**		*0.08181818*		*0.081818182*			*0.1569767*
**ENSG00000188171**	*0.3*	*0.35151515*	*0.3*	*0.351515152*	*0.068181818*		*0.2272727*
**ENSG00000125845**		*0.18484848*		*0.184848485*			*0.3895349*
**ENSG00000113742**	*0.45508982*	*0.4*	*0.45508982*	*0.4*	*0.357142857*	*0.3372093*	*0.4578313*
**ENSG00000204653**					*0.19047619*	*0.372093*	
**ENSG00000155229**	*0.2*	*0.46666667*	*0.2*	*0.466666667*			*0.4127907*
**ENSG00000152128**		*0.33939394*		*0.339393939*			*0.3588235*
**ENSG00000124875**		*0.36060606*		*0.360606061*			*0.2906977*
**ENSG00000166926**		*0.24242424*		*0.242424242*			*0.3294118*
**ENSG00000157426**	*0.27844311*	*0.44848485*	*0.27844311*	*0.448484848*	*0.172619048*	*0.2325581*	*0.4709302*
**ENSG00000171759**	*0.34730539*	*0.20909091*	*0.34730539*	*0.209090909*			
**ENSG00000185332**		*0.26506024*		*0.265060241*			*0.2261905*
**ENSG00000140527**		*0.41411043*		*0.414110429*			*0.2352941*
**ENSG00000072041**		*0.24848485*		*0.248484848*			
**ENSG00000160325**		*0.125*		*0.125*	*0.233333333*	*0.25*	*0.1022727*
**ENSG00000205930**	*0.45808383*		*0.45808383*		*0.101190476*	*0.0697674*	
**ENSG00000168502**	*0.23053892*	*0.42777778*	*0.23053892*	*0.427777778*	*0.053571429*		*0.4825581*
**ENSG00000133454**		*0.38181818*		*0.381818182*			*0.25*
**ENSG00000153982**		*0.32727273*		*0.327272727*			*0.4302326*
**ENSG00000160323**	*0.08383234*	*0.46060606*	*0.08383234*	*0.460606061*			*0.2267442*
**ENSG00000198453**	*0.36666667*	*0.41212121*	*0.36666667*	*0.412121212*	*0.34939759*	*0.2965116*	*0.3546512*
**ENSG00000182500**		*0.25454545*		*0.254545455*			*0.3895349*
**ENSG00000182175**	*0.4760479*	*0.28484848*	*0.4760479*	*0.284848485*			*0.2906977*
**ENSG00000133028**		*0.40555556*		*0.405555556*			*0.4545455*
**ENSG00000103227**	*0.44311377*	*0.15454545*	*0.44311377*	*0.154545455*	*0.375*	*0.3430233*	*0.3139535*
**ENSG00000033100**		*0.40963855*		*0.409638554*			*0.4431818*
**ENSG00000133704**	*0.0625*	*0.49090909*	*0.0625*	*0.490909091*	*0.279761905*	*0.3197674*	*0.4772727*
**ENSG00000145743**		*0.26969697*		*0.26969697*			*0.2555556*
**ENSG00000168569**		*0.41717791*		*0.417177914*			*0.4529412*
**ENSG00000198920**	*0.26047904*	*0.46625767*	*0.26047904*	*0.466257669*	*0.345238095*	*0.3705882*	*0.3895349*
**ENSG00000126790**	*0.22754491*		*0.22754491*		*0.136904762*	*0.1511628*	
**ENSG00000110429**	*0.46629213*	*0.37575758*	*0.46629213*	*0.375757576*	*0.444444444*	*0.3636364*	*0.3662791*
**ENSG00000089902**		*0.22121212*		*0.221212121*			*0.4534884*
**ENSG00000177337**		*0.33636364*		*0.336363636*			*0.2848837*
**ENSG00000165633**		*0.41818182*		*0.418181818*			*0.2906977*
**ENSG00000092148**							*0.0697674*
**ENSG00000177981**	*0.1030303*	*0.27878788*	*0.1030303*	*0.278787879*			*0.1976744*
**ENSG00000061918**		*0.21666667*		*0.216666667*			*0.0795455*
**ENSG00000166828**		*0.45454545*		*0.454545455*			*0.1590909*
**ENSG00000171467**		*0.28787879*		*0.287878788*			*0.3411765*
**ENSG00000109205**		*0.24695122*		*0.24695122*			*0.3764706*
**ENSG00000143367**	*0.19161677*	*0.28181818*	*0.19161677*	*0.281818182*			*0.4823529*
**ENSG00000163624**		*0.47575758*		*0.475757576*			*0.4756098*
**ENSG00000218672**		*0.25842697*		*0.258426966*			*0.3181818*
**ENSG00000164304**		*0.15757576*		*0.157575758*	*0.422222222*	*0.4772727*	*0.0813953*
**ENSG00000189403**	*0.05*	*0.35151515*	*0.05*	*0.351515152*	*0.111111111*	*0.1888889*	*0.1918605*
**ENSG00000171241**	*0.07228916*	*0.05487805*	*0.07228916*	*0.054878049*			
**ENSG00000155749**	*0.28742515*	*0.43292683*	*0.28742515*	*0.432926829*	*0.482142857*	*0.4156627*	*0.3869048*
**ENSG00000164089**	*0.36666667*	*0.08484848*	*0.36666667*	*0.084848485*			*0.4709302*
**ENSG00000172137**		*0.24137931*		*0.24137931*			*0.4659091*
**ENSG00000170075**	*0.34848485*		*0.34848485*		*0.457831325*	*0.4545455*	
**ENSG00000119636**	*0.43888889*	*0.18484848*	*0.43888889*	*0.184848485*	*0.088888889*	*0.0681818*	*0.2732558*
**ENSG00000143614**		*0.42121212*		*0.421212121*			*0.1918605*
**ENSG00000010282**		*0.42331288*		*0.423312883*			*0.3313953*

**ENSG00000138381**	*0.31736527*		*0.31736527*		*0.277777778*	*0.255814*	
**ENSG00000248835**	*0.49401198*	*0.08787879*	*0.49401198*	*0.087878788*	*0.295180723*	*0.3023256*	*0.3430233*
**ENSG00000214160**		*0.24848485*		*0.248484848*			*0.1627907*
**ENSG00000182601**		*0.44242424*		*0.442424242*			*0.4186047*
**ENSG00000185404**	*0.11077844*	*0.35889571*	*0.11077844*	*0.358895706*	*0.107142857*	*0.1130952*	*0.1744186*
**ENSG00000173163**							*0.1046512*
**ENSG00000205325**		*0.40606061*		*0.406060606*			*0.3928571*
**ENSG00000185291**	*0.30239521*		*0.30239521*		*0.43452381*	*0.4302326*	
**ENSG00000130956**		*0.17575758*		*0.175757576*			
**ENSG00000163075**	*0.42222222*	*0.36969697*	*0.42222222*	*0.36969697*	*0.3*	*0.2613636*	*0.4069767*
**ENSG00000111859**	*0.06586826*	*0.43333333*	*0.06586826*	*0.433333333*	*0.089285714*	*0.1046512*	*0.4588235*
**ENSG00000116703**	*0.48802395*	*0.13636364*	*0.48802395*	*0.136363636*	*0.053571429*		
**ENSG00000235568**		*0.4969697*		*0.496969697*			*0.5*
**ENSG00000157483**		*0.46363636*		*0.463636364*			*0.4360465*
**ENSG00000166448**		*0.23333333*		*0.233333333*			*0.2325581*
**ENSG00000099381**		*0.35465116*		*0.354651163*			*0.1136364*
**ENSG00000183562**	*0.44318182*		*0.44318182*		*0.144444444*	*0.1818182*	
**ENSG00000026297**		*0.19393939*		*0.193939394*			
**ENSG00000234602**		*0.13888889*		*0.138888889*			*0.2159091*
**ENSG00000116396**							*0.122093*
**ENSG00000106236**		*0.09393939*		*0.093939394*			*0.2093023*
**ENSG00000140939**	*0.41017964*	*0.08484848*	*0.41017964*	*0.084848485*			
**ENSG00000074964**		*0.13924051*		*0.139240506*			*0.0853659*
**ENSG00000171566**		*0.33333333*		*0.333333333*			*0.2034884*
**ENSG00000107021**		*0.20731707*		*0.207317073*			*0.3522727*
**ENSG00000118363**	*0.14371257*	*0.43939394*	*0.14371257*	*0.439393939*	*0.208333333*	*0.1918605*	*0.1918605*
**ENSG00000130347**		*0.14329268*		*0.143292683*			*0.1607143*
**ENSG00000181929**	*0.44444444*		*0.44444444*		*0.3*	*0.4777778*	
**ENSG00000111640**	*0.37878788*		*0.37878788*		*0.476190476*	*0.4360465*	
**ENSG00000065802**		*0.4202454*		*0.420245399*			*0.3488372*
**ENSG00000158604**		*0.37349398*		*0.373493976*			*0.4244186*
**ENSG00000166450**		*0.4969697*		*0.496969697*			*0.4659091*
**ENSG00000180822**	*0.08426966*	*0.28651685*	*0.08426966*	*0.286516854*			*0.5*
**ENSG00000174099**	*0.23888889*	*0.45402299*	*0.23888889*	*0.454022989*			*0.4659091*
**ENSG00000007866**		*0.09393939*		*0.093939394*			*0.3895349*
**ENSG00000165309**		*0.31111111*		*0.311111111*			*0.4090909*
**ENSG00000162852**	*0.21257485*	*0.36206897*	*0.21257485*	*0.362068966*	*0.380952381*	*0.3313953*	*0.2590361*
**ENSG00000236699**		*0.1969697*		*0.196969697*			*0.0755814*
**ENSG00000169926**		*0.42424242*		*0.424242424*			*0.4069767*
**ENSG00000106799**		*0.22560976*		*0.225609756*	*0.455555556*	*0.4204545*	*0.494186*
**ENSG00000116791**		*0.47272727*		*0.472727273*			*0.25*
**ENSG00000185022**	*0.31666667*		*0.31666667*				
**ENSG00000140945**		*0.07777778*		*0.077777778*			*0.0535714*
**ENSG00000176018**		*0.14545455*		*0.145454545*			*0.3895349*
**ENSG00000083520**		*0.36969697*		*0.36969697*			*0.3333333*
**ENSG00000169908**		*0.17222222*		*0.172222222*			*0.5*
**ENSG00000154319**	*0.48203593*	*0.48170732*	*0.48203593*	*0.481707317*	*0.380952381*	*0.3255814*	
**ENSG00000102271**		*0.32022472*		*0.320224719*			*0.4464286*
**ENSG00000168589**	*0.12424242*	*0.27134146*	*0.12424242*	*0.271341463*			
**ENSG00000123643**	*0.46111111*	*0.27878788*	*0.46111111*	*0.278787879*	*0.446428571*	*0.4*	*0.3837209*
**ENSG00000126775**		*0.47777778*		*0.477777778*			*0.3636364*
**ENSG00000184985**		*0.4*		*0.4*			*0.4883721*
**ENSG00000127314**	*0.23795181*		*0.23795181*				
**ENSG00000158092**		*0.10670732*		*0.106707317*			

**ENSG00000136696**	*0.48802395*	*0.49090909*	*0.48802395*	*0.490909091*	*0.339285714*	*0.2848837*	*0.2647059*
**ENSG00000165406**		*0.06363636*		*0.063636364*			*0.1046512*
**ENSG00000242259**		*0.28658537*		*0.286585366*			*0.4011628*
**ENSG00000197826**		*0.10344828*		*0.103448276*			*0.0581395*
**ENSG00000104969**		*0.28378378*		*0.283783784*			*0.4294118*
**ENSG00000164675**	*0.07777778*		*0.07777778*				
**ENSG00000126106**		*0.34848485*		*0.348484848*			*0.1395349*
**ENSG00000137497**	*0.37640449*	*0.06666667*	*0.37640449*	*0.066666667*	*0.322222222*	*0.2272727*	*0.1931818*
**ENSG00000171448**		*0.16463415*		*0.164634146*			*0.1802326*
**ENSG00000149308**							*0.0872093*
**ENSG00000204356**		*0.06363636*		*0.063636364*			*0.0755814*
**ENSG00000204344**		*0.29268293*		*0.292682927*			*0.3023256*
**ENSG00000133742**	*0.30239521*	*0.48181818*	*0.30239521*	*0.481818182*	*0.488095238*	*0.4244186*	*0.4352941*
**ENSG00000204702**	*0.48888889*		*0.48888889*		*0.177777778*	*0.125*	
**ENSG00000187013**		*0.25766871*		*0.257668712*			*0.3604651*
**ENSG00000176024**					*0.255952381*	*0.3023256*	
**ENSG00000158769**		*0.33146067*		*0.331460674*			*0.2555556*
**ENSG00000070047**		*0.25*		*0.25*			
**ENSG00000103707**		*0.5*		*0.5*			*0.0755814*
**ENSG00000169692**		*0.16060606*		*0.160606061*			*0.0988372*
**ENSG00000160179**		*0.4847561*		*0.484756098*			*0.4117647*
**ENSG00000088854**	*0.43712575*	*0.46060606*	*0.43712575*	*0.460606061*	*0.18452381*	*0.244186*	*0.4767442*
**ENSG00000107281**		*0.31707317*		*0.317073171*			*0.2*
**ENSG00000183742**		*0.26363636*		*0.263636364*			*0.2965116*
**ENSG00000251695**		*0.29393939*		*0.293939394*			*0.3953488*
**ENSG00000111843**	*0.41916168*		*0.41916168*		*0.095238095*	*0.0654762*	
**ENSG00000204262**		*0.13030303*		*0.13030303*			*0.1744186*
**ENSG00000103502**	*0.49700599*	*0.45151515*	*0.49700599*	*0.451515152*	*0.107142857*	*0.0930233*	*0.0930233*
**ENSG00000179104**		*0.47272727*		*0.472727273*			*0.3*
**ENSG00000122359**	*0.36526946*	*0.48170732*	*0.36526946*	*0.481707317*	*0.357142857*	*0.3313953*	*0.3604651*
**ENSG00000123191**	*0.19444444*	*0.48787879*	*0.19444444*	*0.487878788*	*0.422222222*	*0.375*	*0.2906977*
**ENSG00000102974**		*0.08181818*		*0.081818182*			
**ENSG00000111906**		*0.43902439*		*0.43902439*			*0.3235294*
**ENSG00000182040**		*0.24375*		*0.24375*			*0.3795181*
**ENSG00000128928**		*0.48795181*		*0.487951807*			*0.3139535*
**ENSG00000088038**	*0.49700599*	*0.42682927*	*0.49700599*	*0.426829268*	*0.267857143*	*0.2151163*	*0.494186*
**ENSG00000180035**		*0.36781609*		*0.367816092*			*0.0909091*
**ENSG00000170266**		*0.30909091*		*0.309090909*	*0.464285714*	*0.4476744*	*0.1686047*
**ENSG00000198203**		*0.44242424*		*0.442424242*			
**ENSG00000130287**		*0.16060606*		*0.160606061*			*0.122093*
**ENSG00000127125**		*0.38181818*		*0.381818182*			*0.4698795*
**ENSG00000105708**		*0.44817073*		*0.448170732*			*0.4476744*
**ENSG00000036565**	*0.11746988*	*0.35454545*	*0.11746988*	*0.354545455*	*0.232142857*	*0.1976744*	*0.3823529*
**ENSG00000176490**		*0.49438202*		*0.494382022*			*0.3181818*
**ENSG00000072134**		*0.22727273*		*0.227272727*			*0.127907*
**ENSG00000143624**	*0.06111111*	*0.0862069*	*0.06111111*	*0.086206897*			
**ENSG00000176383**		*0.43597561*		*0.43597561*			*0.2616279*
**ENSG00000099812**							*0.2383721*
**ENSG00000176225**		*0.22424242*		*0.224242424*			*0.1744186*
**ENSG00000115946**		*0.3836478*		*0.383647799*			*0.4583333*
**ENSG00000067082**		*0.41818182*		*0.418181818*			*0.5*
**ENSG00000214262**		*0.29090909*		*0.290909091*			*0.4883721*
**ENSG00000120868**					*0.214285714*	*0.1411765*	
**ENSG00000157445**	*0.36526946*	*0.44545455*	*0.36526946*	*0.445454545*	*0.43452381*	*0.3488372*	*0.4534884*

**ENSG00000008513**	*0.32934132*	*0.45151515*	*0.32934132*	*0.451515152*	*0.458333333*	*0.3953488*	*0.3888889*
**ENSG00000168374**	*0.14071856*		*0.14071856*		*0.428571429*	*0.4825581*	
**ENSG00000107317**		*0.27272727*		*0.272727273*			*0.2117647*
**ENSG00000185730**		*0.4845679*		*0.484567901*	*0.244444444*	*0.2386364*	*0.2616279*
**ENSG00000172717**		*0.08045977*		*0.08045977*			*0.5*
**ENSG00000185267**		*0.09393939*		*0.093939394*			*0.4534884*
**ENSG00000258474**		*0.43636364*		*0.436363636*			*0.4709302*
**ENSG00000256525**	*0.19760479*		*0.19760479*				
**ENSG00000101470**		*0.37272727*		*0.372727273*			*0.2906977*
**ENSG00000138160**		*0.48787879*		*0.487878788*			*0.3430233*
**ENSG00000181458**	*0.17964072*		*0.17964072*		*0.325301205*	*0.377907*	
**ENSG00000163472**							*0.3068182*
**ENSG00000106327**		*0.18888889*		*0.188888889*			*0.1024096*
**ENSG00000167768**		*0.12121212*		*0.121212121*			
**ENSG00000138075**	*0.36046512*	*0.32424242*	*0.36046512*	*0.324242424*			*0.1337209*
**ENSG00000151892**	*0.49700599*	*0.30909091*	*0.49700599*	*0.309090909*	*0.357142857*	*0.3647059*	*0.4772727*
**ENSG00000180336**	*0.11676647*	*0.09770115*	*0.11676647*	*0.097701149*	*0.148809524*		
**ENSG00000072657**		*0.17272727*		*0.172727273*			*0.3895349*
**ENSG00000147459**		*0.49390244*		*0.493902439*			*0.4651163*
**ENSG00000115687**	*0.22754491*	*0.41212121*	*0.22754491*	*0.412121212*	*0.321428571*	*0.3372093*	*0.494186*
**ENSG00000106034**	*0.26111111*	*0.30606061*	*0.26111111*	*0.306060606*	*0.077777778*	*0.0795455*	*0.3197674*
**ENSG00000135097**		*0.47575758*		*0.475757576*			*0.4476744*
**ENSG00000180535**					*0.311111111*	*0.3488372*	
**ENSG00000110675**							*0.3546512*
**ENSG00000157654**		*0.49691358*		*0.49691358*			*0.4883721*
**ENSG00000188803**		*0.40606061*		*0.406060606*			*0.1453488*
**ENSG00000104814**					*0.273809524*	*0.25*	
**ENSG00000114268**		*0.32012195*		*0.320121951*			*0.2209302*
**ENSG00000128585**	*0.38323353*	*0.47752809*	*0.38323353*	*0.47752809*	*0.307692308*	*0.3676471*	*0.4651163*
**ENSG00000120942**							*0.1818182*
**ENSG00000010361**		*0.11666667*		*0.116666667*			*0.1590909*
**ENSG00000106819**		*0.4969697*		*0.496969697*			*0.3313953*
**ENSG00000012232**		*0.31818182*		*0.318181818*			*0.3372093*
**ENSG00000135517**		*0.43939394*		*0.439393939*			*0.3546512*
**ENSG00000156475**	*0.24550898*		*0.24550898*		*0.470238095*	*0.4651163*	
**ENSG00000135736**		*0.24390244*		*0.243902439*			
**ENSG00000100003**	*0.49698795*	*0.44545455*	*0.49698795*	*0.445454545*	*0.357142857*	*0.4186047*	*0.4036145*
**ENSG00000181847**	*0.30838323*	*0.47272727*	*0.30838323*	*0.472727273*	*0.119047619*	*0.1453488*	*0.1184211*
**ENSG00000157150**		*0.07878788*		*0.078787879*			*0.1395349*
**ENSG00000081237**		*0.18181818*		*0.181818182*	*0.130952381*	*0.0930233*	*0.3493976*
**ENSG00000204613**		*0.35454545*		*0.354545455*			*0.4069767*
**ENSG00000168916**	*0.25149701*		*0.25149701*		*0.220238095*	*0.1352941*	
**ENSG00000184507**	*0.15568862*		*0.15568862*		*0.333333333*	*0.3604651*	
**ENSG00000167562**		*0.1954023*		*0.195402299*			*0.4886364*
**ENSG00000185787**	*0.1497006*	*0.12424242*	*0.1497006*	*0.124242424*		*0.0523256*	*0.2267442*
**ENSG00000203546**		*0.3969697*		*0.396969697*			*0.3895349*
**ENSG00000160710**		*0.46319018*		*0.463190184*			*0.4709302*
**ENSG00000064309**	*0.21348315*	*0.49390244*	*0.21348315*	*0.493902439*	*0.329545455*	*0.3604651*	*0.4882353*
**ENSG00000138823**	*0.20731707*	*0.42727273*	*0.20731707*	*0.427272727*	*0.122222222*	*0.2111111*	*0.2159091*
**ENSG00000128276**							*0.4277108*
**ENSG00000176102**	*0.16467066*	*0.39393939*	*0.16467066*	*0.393939394*	*0.303571429*	*0.3953488*	*0.2559524*
**ENSG00000166961**		*0.46111111*		*0.461111111*			*0.1802326*
**ENSG00000185669**		*0.32121212*		*0.321212121*			*0.3953488*
**ENSG00000106701**	*0.06886228*	*0.41212121*	*0.06886228*	*0.412121212*	*0.464285714*	*0.4302326*	*0.4186047*

**ENSG00000140319**	*0.05988024*	*0.36666667*	*0.05988024*	*0.366666667*	*0.219512195*	*0.2352941*	*0.3470588*
**ENSG00000180846**		*0.45121951*		*0.451219512*			*0.4058824*
**ENSG00000103150**		*0.06969697*		*0.06969697*			*0.1744186*
**ENSG00000137673**		*0.19207317*		*0.192073171*			*0.1976744*
**ENSG00000071243**		*0.19090909*		*0.190909091*			*0.3875*
**ENSG00000165953**	*0.46407186*		*0.46407186*		*0.404761905*	*0.4883721*	
**ENSG00000172262**		*0.39877301*		*0.398773006*			*0.2117647*
**ENSG00000229377**		*0.07471264*		*0.074712644*			
**ENSG00000107186**	*0.13333333*	*0.39393939*	*0.13333333*	*0.393939394*	*0.155555556*	*0.1744186*	*0.4127907*
**ENSG00000164691**	*0.29341317*	*0.10670732*	*0.29341317*	*0.106707317*	*0.331325301*	*0.3139535*	*0.5*
**ENSG00000174579**					*0.154761905*	*0.1744186*	
**ENSG00000064607**		*0.25151515*		*0.251515152*			*0.1860465*
**ENSG00000116350**		*0.22727273*		*0.227272727*			*0.122093*
**ENSG00000198431**	*0.33333333*	*0.1*	*0.33333333*	*0.1*	*0.422222222*	*0.4333333*	*0.2777778*
**ENSG00000170803**		*0.4969697*		*0.496969697*			*0.2906977*
**ENSG00000183579**		*0.48787879*		*0.487878788*			*0.4883721*
**ENSG00000113520**	*0.48203593*		*0.48203593*		*0.261904762*	*0.2790698*	
**ENSG00000150750**		*0.21818182*		*0.218181818*			*0.3081395*
**ENSG00000130508**		*0.434375*		*0.434375*			*0.3571429*
**ENSG00000103196**	*0.21666667*	*0.42121212*	*0.21666667*	*0.421212121*	*0.411111111*	*0.4886364*	*0.3588235*
**ENSG00000121274**		*0.23030303*		*0.23030303*			*0.3604651*
**ENSG00000152213**		*0.46932515*		*0.469325153*			*0.2159091*
**ENSG00000072682**	*0.2754491*		*0.2754491*		*0.208333333*	*0.3488372*	*0.122093*
**ENSG00000213977**		*0.0862069*		*0.086206897*			*0.3181818*
**ENSG00000213930**		*0.10606061*		*0.106060606*			
**ENSG00000114450**	*0.17365269*	*0.25*	*0.17365269*	*0.25*			*0.4418605*
**ENSG00000007314**		*0.35757576*		*0.357575758*			*0.4647059*
**ENSG00000090971**		*0.48888889*		*0.488888889*			*0.25*
**ENSG00000114554**		*0.1097561*		*0.109756098*			*0.4090909*
**ENSG00000142515**		*0.38787879*		*0.387878788*			*0.4941176*
**ENSG00000137040**		*0.2*		*0.2*			*0.1860465*
**ENSG00000004660**		*0.49695122*		*0.49695122*			*0.4825581*
**ENSG00000137364**		*0.2*		*0.2*			*0.2727273*
**ENSG00000153317**	*0.08333333*	*0.49393939*	*0.08333333*	*0.493939394*	*0.111111111*	*0.1777778*	*0.4011628*
**ENSG00000178996**		*0.43597561*		*0.43597561*			*0.4709302*
**ENSG00000140488**	*0.21818182*		*0.21818182*		*0.446428571*	*0.3235294*	*0.377907*
**ENSG00000196104**		*0.39090909*		*0.390909091*			*0.377907*
**ENSG00000163749**		*0.48787879*		*0.487878788*			*0.0813953*
**ENSG00000164442**		*0.14242424*		*0.142424242*			
**ENSG00000198178**	*0.0508982*		*0.0508982*		*0.208333333*	*0.255814*	
**ENSG00000120889**	*0.24157303*	*0.45555556*	*0.24157303*	*0.455555556*			*0.4431818*
**ENSG00000236104**		*0.48787879*		*0.487878788*			*0.3372093*
**ENSG00000103404**	*0.13473054*	*0.34545455*	*0.13473054*	*0.345454545*	*0.148809524*	*0.1395349*	*0.2151163*
**ENSG00000164822**		*0.18484848*		*0.184848485*			*0.0697674*
**ENSG00000140443**	*0.26234568*	*0.48333333*	*0.26234568*	*0.483333333*	*0.077380952*	*0.0714286*	*0.4888889*
**ENSG00000135972**	*0.45508982*	*0.23333333*	*0.45508982*	*0.233333333*	*0.369047619*	*0.2732558*	*0.125*
**ENSG00000204131**		*0.28220859*		*0.282208589*			
**ENSG00000124209**		*0.49393939*		*0.493939394*			*0.4186047*
**ENSG00000204977**		*0.22222222*		*0.222222222*			*0.4204545*
**ENSG00000169992**		*0.36666667*		*0.366666667*			*0.2267442*
**ENSG00000132874**		*0.43939394*		*0.439393939*			*0.3662791*
**ENSG00000137845**	*0.26646707*		*0.26646707*		*0.130952381*	*0.1162791*	
**ENSG00000168591**		*0.33939394*		*0.339393939*			*0.3176471*
**ENSG00000108433**	*0.3125*	*0.48181818*	*0.3125*	*0.481818182*	*0.422222222*	*0.4545455*	*0.4593023*

**ENSG00000100106**		*0.5*		*0.5*				*0.4767442*
**ENSG00000101166**		*0.46036585*		*0.460365854*				*0.1569767*
**ENSG00000085871**	*0.2754491*		*0.2754491*		*0.154761905*		*0.244186*	
**ENSG00000197701**		*0.18888889*		*0.188888889*				*0.2*
**ENSG00000211456**	*0.39820359*	*0.36666667*	*0.39820359*	*0.366666667*	*0.397590361*		*0.4047619*	*0.3139535*
**ENSG00000114446**		*0.12727273*		*0.127272727*				*0.1647059*
**ENSG00000215019**								*0.2616279*
**ENSG00000134779**		*0.18787879*		*0.187878788*				
**ENSG00000122435**		*0.07272727*		*0.072727273*				*0.0755814*
**ENSG00000086189**		*0.29503106*		*0.295031056*				*0.297619*
**ENSG00000145632**		*0.33333333*		*0.333333333*				*0.25*
**ENSG00000146221**	*0.39520958*	*0.18787879*	*0.39520958*	*0.187878788*	*0.44047619*		*0.3023256*	*0.3023256*
**ENSG00000183309**	*0.17777778*	*0.21341463*	*0.17777778*	*0.213414634*	*0.188888889*		*0.2045455*	*0.3546512*
**ENSG00000150433**	*0.29041916*	*0.45454545*	*0.29041916*	*0.454545455*	*0.291666667*		*0.372093*	*0.372093*
**ENSG00000185418**		*0.22121212*		*0.221212121*				*0.2383721*
**ENSG00000011332**	*0.32035928*	*0.30898876*	*0.32035928*	*0.308988764*	*0.422619048*		*0.2674419*	*0.1477273*
**ENSG00000154736**		*0.3969697*		*0.396969697*				*0.4767442*
**ENSG00000105438**		*0.1969697*		*0.196969697*				*0.2732558*
**ENSG00000075539**	*0.16467066*		*0.16467066*		*0.422619048*		*0.4186047*	
**ENSG00000176261**								*0.1104651*
**ENSG00000225697**		*0.0969697*		*0.096969697*				*0.1046512*
**ENSG00000100596**		*0.35534591*		*0.355345912*				*0.3863636*
**ENSG00000109686**	*0.26047904*	*0.05555556*	*0.26047904*	*0.055555556*	*0.297619048*		*0.1860465*	
**ENSG00000140873**		*0.49390244*		*0.493902439*				*0.2848837*
**ENSG00000183067**		*0.33030303*		*0.33030303*				*0.4302326*
**ENSG00000179023**		*0.47560976*		*0.475609756*				*0.3488372*
**ENSG00000147457**	*0.38068182*	*0.48484848*	*0.38068182*	*0.484848485*	*0.291666667*		*0.4764706*	*0.3863636*
**ENSG00000107537**	*0.29166667*	*0.26666667*	*0.29166667*	*0.266666667*	*0.076923077*		*0.0595238*	*0.2383721*
**ENSG00000099889**		*0.32012195*		*0.320121951*				*0.3647059*
**ENSG00000134193**	*0.08682635*	*0.49425287*	*0.08682635*	*0.494252874*				*0.4011628*
**ENSG00000180772**		*0.36363636*		*0.363636364*				*0.2965116*
**ENSG00000161849**	*0.38922156*		*0.38922156*		*0.208333333*		*0.1823529*	
**ENSG00000129460**		*0.20496894*		*0.204968944*	*0.06547619*			*0.2951807*
**ENSG00000130584**		*0.49074074*		*0.490740741*				
**ENSG00000196214**		*0.096875*		*0.096875*				
**ENSG00000198954**		*0.43636364*		*0.436363636*				*0.3837209*
**ENSG00000072952**	*0.4760479*	*0.43636364*	*0.4760479*	*0.436363636*	*0.279761905*		*0.3197674*	*0.3546512*
**ENSG00000182004**		*0.21666667*		*0.216666667*				*0.3409091*
**ENSG00000178104**	*0.38202247*	*0.17857143*	*0.38202247*	*0.178571429*	*0.238095238*		*0.1818182*	*0.3963415*
**ENSG00000136014**					*0.222222222*		*0.3295455*	
**ENSG00000197870**		*0.10909091*		*0.109090909*				*0.0988372*
**ENSG00000100348**	*0.27840909*	*0.08841463*	*0.27840909*	*0.088414634*	*0.3*		*0.3181818*	*0.1162791*
**ENSG00000165568**		*0.11585366*		*0.115853659*	*0.107142857*		*0.0930233*	*0.0930233*
**ENSG00000149761**	*0.12352941*		*0.12352941*					
**ENSG00000068976**	*0.41017964*		*0.41017964*		*0.43452381*		*0.3953488*	
**ENSG00000086061**	*0.44318182*	*0.46428571*	*0.44318182*	*0.464285714*	*0.43902439*		*0.3823529*	*0.1477273*
**ENSG00000137100**		*0.08282209*		*0.082822086*				
**ENSG00000114744**		*0.46666667*		*0.466666667*				*0.2906977*
**ENSG00000166004**	*0.42222222*		*0.42222222*		*0.3*		*0.4886364*	
**ENSG00000126226**	*0.10479042*		*0.10479042*					
**ENSG00000139629**		*0.46363636*		*0.463636364*				*0.4666667*
**ENSG00000113657**		*0.15454545*		*0.154545455*				*0.1802326*
**ENSG00000178921**	*0.39820359*	*0.43209877*	*0.39820359*	*0.432098765*	*0.369047619*		*0.372093*	*0.1705882*
**ENSG00000007545**		*0.21515152*		*0.215151515*				*0.4823529*

**ENSG00000163485**		*0.06666667*		*0.066666667*			*0.3430233*
**ENSG00000022840**	*0.07784431*		*0.07784431*		*0.488095238*	*0.4476744*	*0.3181818*
**ENSG00000176783**	*0.42814371*	*0.36666667*	*0.42814371*	*0.366666667*	*0.232142857*	*0.2647059*	*0.2738095*
**ENSG00000258869**	*0.43712575*	*0.39090909*	*0.43712575*	*0.390909091*	*0.066666667*	*0.1117647*	*0.3023256*
**ENSG00000170647**		*0.33030303*		*0.33030303*			*0.1444444*
**ENSG00000174721**		*0.278125*		*0.278125*			*0.2083333*
**ENSG00000161558**		*0.38109756*		*0.381097561*			*0.2383721*
**ENSG00000106733**	*0.14371257*	*0.48181818*	*0.14371257*	*0.481818182*			*0.3068182*
**ENSG00000154359**	*0.33532934*		*0.33532934*		*0.083333333*	*0.0639535*	
**ENSG00000135535**		*0.38787879*		*0.387878788*			*0.4583333*
**ENSG00000135506**		*0.4*		*0.4*			*0.2727273*
**ENSG00000144524**		*0.32424242*		*0.324242424*			*0.1627907*
**ENSG00000166948**		*0.31111111*		*0.311111111*			*0.4666667*
**ENSG00000172493**	*0.40662651*	*0.43939394*	*0.40662651*	*0.439393939*	*0.464285714*	*0.4534884*	*0.4476744*
**ENSG00000173610**		*0.38650307*		*0.386503067*			*0.3235294*
**ENSG00000164919**	*0.49101796*		*0.49101796*		*0.160714286*	*0.1104651*	
**ENSG00000183048**		*0.43865031*		*0.438650307*			*0.3690476*
**ENSG00000148735**	*0.41317365*	*0.4030303*	*0.41317365*	*0.403030303*	*0.404761905*	*0.4651163*	*0.4302326*
**ENSG00000124900**		*0.47619048*		*0.476190476*			*0.4411765*
**ENSG00000162909**		*0.39329268*		*0.393292683*			*0.4518072*
**ENSG00000076003**							*0.0697674*
**ENSG00000177764**	*0.18862275*	*0.32424242*	*0.18862275*	*0.324242424*	*0.291666667*	*0.2965116*	*0.4302326*
**ENSG00000254685**		*0.46319018*		*0.463190184*			*0.3333333*
**ENSG00000177000**	*0.23652695*	*0.35955056*	*0.23652695*	*0.359550562*	*0.1*	*0.122093*	*0.1777778*
**ENSG00000113327**		*0.41666667*		*0.416666667*			*0.4166667*
**ENSG00000161921**		*0.30606061*		*0.306060606*			*0.4767442*
**ENSG00000141429**							*0.3895349*
**ENSG00000204539**	*0.44117647*	*0.48181818*	*0.44117647*	*0.481818182*	*0.444444444*	*0.4772727*	*0.3953488*
**ENSG00000160180**	*0.09281437*		*0.09281437*				
**ENSG00000152944**	*0.2195122*	*0.37272727*	*0.2195122*	*0.372727273*			
**ENSG00000186625**							*0.2205882*
**ENSG00000130294**		*0.46060606*		*0.460606061*			*0.25*
**ENSG00000138468**		*0.43030303*		*0.43030303*			*0.3823529*
**ENSG00000134762**		*0.49090909*		*0.490909091*			*0.4651163*
**ENSG00000154380**		*0.28181818*		*0.281818182*			*0.3313953*
**ENSG00000168395**		*0.48181818*		*0.481818182*			*0.3081395*
**ENSG00000231389**		*0.16969697*		*0.16969697*			*0.4666667*
**ENSG00000115252**		*0.47191011*		*0.471910112*			*0.3068182*
**ENSG00000133116**		*0.14545455*		*0.145454545*			*0.3081395*
**ENSG00000136783**		*0.2*		*0.2*			*0.125*
**ENSG00000111732**		*0.05454545*		*0.054545455*			*0.0697674*
**ENSG00000145949**	*0.13636364*	*0.48333333*	*0.13636364*	*0.483333333*			*0.4545455*
**ENSG00000113303**	*0.30838323*		*0.30838323*		*0.422619048*	*0.3529412*	
**ENSG00000100614**	*0.44879518*	*0.21646341*	*0.44879518*	*0.216463415*	*0.166666667*	*0.1022727*	*0.1590909*
**ENSG00000134531**	*0.36516854*	*0.46666667*	*0.36516854*	*0.466666667*			*0.4638554*
**ENSG00000169071**	*0.4491018*	*0.08181818*	*0.4491018*	*0.081818182*	*0.291666667*	*0.3081395*	*0.1176471*
**ENSG00000114790**	*0.48295455*	*0.36363636*	*0.48295455*	*0.363636364*	*0.422222222*	*0.4204545*	*0.3430233*
**ENSG00000142046**	*0.26807229*	*0.42378049*	*0.26807229*	*0.423780488*	*0.476190476*	*0.4825581*	*0.494186*
**ENSG00000215277**		*0.47865854*		*0.478658537*			*0.4709302*
**ENSG00000162763**	*0.36363636*	*0.26666667*	*0.36363636*	*0.266666667*	*0.125*	*0.127907*	*0.1686047*
**ENSG00000065243**		*0.43939394*		*0.439393939*	*0.345238095*	*0.3895349*	*0.4883721*
**ENSG00000182093**		*0.35393258*		*0.353932584*			*0.4318182*
**ENSG00000070882**	*0.08441558*	*0.30909091*	*0.08441558*	*0.309090909*			*0.255814*
**ENSG00000176349**		*0.39090909*		*0.390909091*			*0.4176471*

**ENSG00000172992**		0.3969697		0.396969697			0.3546512
**ENSG00000116001**							0.1647059
**ENSG00000171204**		0.16463415		0.164634146			0.4464286
**ENSG00000257103**		0.43030303		0.43030303			
**ENSG00000103356**		0.12121212		0.121212121			0.255814
**ENSG00000106948**	0.18562874	0.43902439	0.18562874	0.43902439			0.4244186
**ENSG00000173540**		0.31515152		0.315151515			0.2383721
**ENSG00000135245**		0.18292683		0.182926829			0.0952381
**ENSG00000143494**		0.34242424		0.342424242			0.4593023
**ENSG00000025434**					0.095238095	0.0930233	
**ENSG00000090661**		0.41573034		0.415730337			0.2333333
**ENSG00000100605**		0.45731707		0.457317073			0.3313953
**ENSG00000187555**	0.42222222		0.42222222		0.066666667		
**ENSG00000165424**		0.44848485		0.448484848			0.4709302
**ENSG00000162892**	0.10179641	0.44545455	0.10179641	0.445454545			0.2732558
**ENSG00000170820**	0.26047904		0.26047904		0.5	0.4825581	
**ENSG00000106078**		0.44444444		0.444444444			0.4111111
**ENSG00000227500**	0.46407186	0.43209877	0.46407186	0.432098765	0.363095238	0.494186	0.494186
**ENSG00000155008**	0.13636364		0.13636364		0.355555556	0.2727273	
**ENSG00000130035**		0.42378049		0.423780488			0.3430233
**ENSG00000143196**		0.05813953		0.058139535			
**ENSG00000205106**	0.18862275		0.18862275		0.470238095	0.4588235	
**ENSG00000163507**		0.08787879		0.087878788			0.4285714
**ENSG00000066933**	0.22727273	0.19886364	0.22727273	0.198863636			0.4069767
**ENSG00000169962**							0.0639535
**ENSG00000214029**		0.32777778		0.327777778	0.136904762	0.0697674	0.0714286
**ENSG00000081377**		0.17575758		0.175757576			
**ENSG00000135069**	0.47777778		0.47777778		0.255555556	0.2954545	
**ENSG00000248871**		0.19090909		0.190909091			0.0823529
**ENSG00000106692**	0.24157303	0.12121212	0.24157303	0.121212121	0.077777778	0.0568182	0.2058824
**ENSG00000011405**		0.46363636		0.463636364			0.3409091
**ENSG00000136872**		0.49444444		0.494444444			0.4555556
**ENSG00000162419**		0.4695122		0.469512195			0.1941176
**ENSG00000091262**		0.24085366		0.240853659			0.25
**ENSG00000255800**	0.30864198		0.30864198		0.404761905	0.3658537	
**ENSG00000196372**		0.46363636		0.463636364			0.4886364
**ENSG00000222011**		0.15		0.15			
**ENSG00000100065**	0.28888889	0.08787879	0.28888889	0.087878788	0.166666667	0.2555556	0.2790698
**ENSG00000139233**	0.29444444		0.29444444		0.155555556	0.1022727	
**ENSG00000242498**		0.45121951		0.451219512	0.146341463	0.125	0.2848837
**ENSG00000148737**							0.1395349
**ENSG00000197798**	0.25149701		0.25149701		0.19047619	0.2209302	
**ENSG00000157601**	0.47777778	0.05617978	0.47777778	0.056179775	0.4	0.3977273	0.2386364
**ENSG00000158467**		0.34545455		0.345454545	0.375	0.4127907	0.4476744
**ENSG00000139832**		0.15116279		0.151162791			0.2840909
**ENSG00000101608**		0.11818182		0.118181818			0.2848837
**ENSG00000187764**	0.28313253	0.31818182	0.28313253	0.318181818	0.398809524	0.3809524	0.3837209
**ENSG00000108107**	0.29041916	0.40449438	0.29041916	0.404494382	0.119047619	0.1337209	0.2272727
**ENSG00000015479**	0.14371257	0.33333333	0.14371257	0.333333333	0.15060241	0.2790698	0.2790698
**ENSG00000071127**		0.23780488		0.237804878			0.1117647
**ENSG00000100341**		0.15454545		0.154545455			
**ENSG00000133056**	0.10778443	0.18888889	0.10778443	0.188888889	0.261904762	0.1976744	0.4593023
**ENSG00000011007**		0.26060606		0.260606061			
**ENSG00000133243**		0.3030303		0.303030303			0.4176471

**ENSG00000156804**		*0.23636364*		*0.236363636*			*0.3604651*
**ENSG00000167996**							*0.2151163*
**ENSG00000124743**	*0.11676647*	*0.36969697*	*0.11676647*	*0.36969697*			*0.3953488*
**ENSG00000255622**		*0.42424242*		*0.424242424*			*0.4058824*
**ENSG00000126003**		*0.45*		*0.45*			*0.3181818*
**ENSG00000173406**		*0.23030303*		*0.23030303*			*0.4651163*
**ENSG00000203684**	*0.11676647*		*0.11676647*				
**ENSG00000136867**		*0.28333333*		*0.283333333*			*0.4360465*
**ENSG00000179142**		*0.4969697*		*0.496969697*			*0.3837209*
**ENSG00000007129**	*0.44512195*	*0.27878788*	*0.44512195*	*0.278787879*	*0.44047619*	*0.494186*	*0.4709302*
**ENSG00000198894**	*0.18888889*	*0.40243902*	*0.18888889*	*0.402439024*	*0.3*	*0.1704545*	*0.4882353*
**ENSG00000121931**		*0.40606061*		*0.406060606*			*0.4709302*
**ENSG00000114455**	*0.19161677*	*0.08333333*	*0.19161677*	*0.083333333*	*0.4*	*0.4772727*	*0.5*
**ENSG00000198929**	*0.43674699*	*0.46666667*	*0.43674699*	*0.466666667*	*0.232142857*	*0.2209302*	*0.4127907*
**ENSG00000100351**	*0.13333333*	*0.17575758*	*0.13333333*	*0.175757576*	*0.284090909*	*0.2159091*	*0.377907*
**ENSG00000186166**		*0.37777778*		*0.377777778*			*0.1136364*
**ENSG00000167257**		*0.44817073*		*0.448170732*			
**ENSG00000136950**		*0.13030303*		*0.13030303*			
**ENSG00000005882**	*0.19318182*	*0.47256098*	*0.19318182*	*0.472560976*	*0.297619048*	*0.375*	*0.3895349*
**ENSG00000141449**		*0.23939394*		*0.239393939*			
**ENSG00000050767**		*0.45151515*		*0.451515152*			*0.3176471*
**ENSG00000242689**		*0.49393939*		*0.493939394*			*0.3470588*
**ENSG00000121039**		*0.1969697*		*0.196969697*			*0.0823529*
**ENSG00000113555**		*0.46932515*		*0.469325153*			*0.122093*
**ENSG00000196248**		*0.4*		*0.4*			*0.3*
**ENSG00000188921**		*0.33636364*		*0.336363636*			*0.0813953*
**ENSG00000243646**		*0.40853659*		*0.408536585*			*0.5*
**ENSG00000146909**		*0.45151515*		*0.451515152*			*0.3139535*
**ENSG00000068697**	*0.29341317*		*0.29341317*		*0.392857143*	*0.4186047*	
**ENSG00000185340**	*0.13888889*		*0.13888889*		*0.233333333*	*0.2954545*	
**ENSG00000169855**	*0.10179641*	*0.1969697*	*0.10179641*	*0.196969697*			*0.3452381*
**ENSG00000142102**		*0.39655172*		*0.396551724*			*0.5*
**ENSG00000152661**		*0.12921348*		*0.129213483*			
**ENSG00000116815**		*0.15168539*		*0.151685393*			*0.3409091*
**ENSG00000172331**	*0.24550898*		*0.24550898*		*0.214285714*	*0.2209302*	
**ENSG00000148248**		*0.46646341*		*0.466463415*	*0.267857143*	*0.2117647*	*0.2840909*
**ENSG00000056998**		*0.06481481*		*0.064814815*			*0.2678571*
**ENSG00000156103**		*0.47878788*		*0.478787879*			*0.3690476*
**ENSG00000169914**		*0.44817073*		*0.448170732*			*0.2093023*
**ENSG00000128250**	*0.05389222*	*0.19444444*	*0.05389222*	*0.194444444*			
**ENSG00000182611**	*0.21686747*		*0.21686747*		*0.103658537*	*0.0813953*	
**ENSG00000100823**							*0.0523256*
**ENSG00000187664**		*0.16363636*		*0.163636364*			
**ENSG00000006625**	*0.45209581*	*0.17878788*	*0.45209581*	*0.178787879*	*0.446428571*	*0.372093*	*0.5*
**ENSG00000196277**		*0.23780488*		*0.237804878*			*0.1918605*
**ENSG00000031691**		*0.39197531*		*0.391975309*			*0.3941176*
**ENSG00000148156**	*0.34730539*		*0.34730539*		*0.446428571*	*0.4825581*	
**ENSG00000173198**		*0.23939394*		*0.239393939*			*0.494186*
**ENSG00000243943**		*0.17878788*		*0.178787879*			*0.2790698*
**ENSG00000198870**		*0.43939394*		*0.439393939*			*0.255814*
**ENSG00000116406**		*0.5*		*0.5*			*0.4411765*
**ENSG00000145365**	*0.17241379*	*0.5*	*0.17241379*	*0.5*	*0.377777778*	*0.3777778*	*0.1882353*
**ENSG00000163069**		*0.43030303*		*0.43030303*			*0.3470588*
**ENSG00000119878**		*0.40606061*		*0.406060606*			*0.1976744*

**ENSG00000116688**	0.25449102		0.25449102		0.410714286	0.3604651	
**ENSG00000121858**		0.41158537		0.411585366			0.4127907
**ENSG00000013523**	0.13173653	0.32317073	0.13173653	0.323170732	0.238095238	0.2647059	0.3546512
**ENSG00000122863**		0.48484848		0.484848485			0.4302326
**ENSG00000173575**	0.47305389	0.49375	0.47305389	0.49375			0.1235294
**ENSG00000173237**		0.05660377		0.056603774			
**ENSG00000089127**		0.41860465		0.418604651			0.1590909
**ENSG00000163872**		0.48181818		0.481818182			0.3895349
**ENSG00000112195**		0.35555556		0.355555556			0.3295455
**ENSG00000172590**	0.44011976	0.19393939	0.44011976	0.193939394	0.482142857	0.4418605	0.2965116
**ENSG00000188916**	0.14371257	0.31790123	0.14371257	0.317901235	0.274390244	0.1941176	0.122093
**ENSG00000112773**		0.33888889		0.338888889			0.2045455
**ENSG00000102081**		0.23602484		0.236024845			0.4418605
**ENSG00000157214**	0.35151515	0.22727273	0.35151515	0.227272727	0.369047619	0.4302326	0.5
**ENSG00000259289**	0.19161677		0.19161677		0.066666667		
**ENSG00000188086**	0.3502994		0.3502994		0.066666667	0.1428571	
**ENSG00000107593**		0.10365854		0.103658537			0.1511628
**ENSG00000144908**		0.17272727		0.172727273			0.2588235
**ENSG00000078898**		0.26436782		0.264367816			0.1375
**ENSG00000123416**		0.36280488		0.362804878			0.3255814
**ENSG00000240747**	0.43636364	0.41818182	0.43636364	0.418181818		0.0755814	0.2906977
**ENSG00000253105**	0.07784431	0.45555556	0.07784431	0.455555556			0.1136364
**ENSG00000185808**	0.24550898		0.24550898		0.327380952	0.377907	
**ENSG00000188690**	0.33888889	0.49090909	0.33888889	0.490909091	0.311111111	0.3295455	0.3837209
**ENSG00000090372**		0.2030303		0.203030303			0.4360465
**ENSG00000076513**	0.33832335	0.06402439	0.33832335	0.06402439	0.05952381	0.1337209	0.1337209
**ENSG00000165548**	0.22222222	0.32121212	0.22222222	0.321212121	0.177777778	0.0568182	0.3895349
**ENSG00000100490**	0.44011976	0.44242424	0.44011976	0.442424242	0.30952381	0.244186	0.3372093
**ENSG00000140961**	0.23652695	0.18181818	0.23652695	0.181818182	0.238095238	0.1976744	0.1918605
**ENSG00000023839**		0.40606061		0.406060606	0.214285714	0.1918605	0.2093023
**ENSG00000100201**		0.32727273		0.327272727			0.3837209
**ENSG00000160685**		0.48181818		0.481818182			0.0581395
**ENSG00000088448**		0.4		0.4			0.2034884
**ENSG00000136152**		0.11666667		0.116666667			0.0568182
**ENSG00000100228**		0.47777778		0.477777778	0.102272727		0.377907
**ENSG00000053918**		0.38181818		0.381818182			0.1104651
**ENSG00000053108**		0.48484848		0.484848485			0.4534884
**ENSG00000117602**		0.46969697		0.46969697			0.2383721
**ENSG00000115350**		0.23333333		0.233333333			0.1976744
**ENSG00000112739**		0.35151515		0.351515152			0.1764706
**ENSG00000206418**		0.13636364		0.136363636			
**ENSG00000118785**		0.19090909		0.190909091			0.4090909
**ENSG00000156413**	0.0873494	0.31176471	0.0873494	0.311764706	0.302469136	0.3452381	0.3444444
**ENSG00000151623**	0.18674699	0.43333333	0.18674699	0.433333333	0.273809524	0.3313953	0.1686047
**ENSG00000189184**		0.375		0.375			0.4529412
**ENSG00000089775**	0.05389222		0.05389222		0.202380952	0.2209302	
**ENSG00000196177**	0.11144578	0.42727273	0.11144578	0.427272727	0.101190476	0.1337209	0.2034884
**ENSG00000188425**		0.16060606		0.160606061			0.2790698
**ENSG00000140481**	0.39520958	0.37272727	0.39520958	0.372727273	0.5	0.3662791	0.3081395
**ENSG00000166546**		0.09090909		0.090909091			0.1569767
**ENSG00000165752**		0.43030303		0.43030303			0.3928571
**ENSG00000111832**		0.30555556		0.305555556			0.375
**ENSG00000007923**		0.33636364		0.336363636			0.4360465
**ENSG00000162600**	0.33928571	0.46363636	0.33928571	0.463636364	0.094594595		0.3411765

**ENSG00000125903**	*0.36516854*		*0.36516854*		*0.209302326*	*0.0888889*	
**ENSG00000110801**	*0.2*	*0.3*	*0.2*	*0.3*			*0.4651163*
**ENSG00000188682**		*0.40909091*		*0.409090909*			*0.3974359*
**ENSG00000167807**		*0.40490798*		*0.404907975*			*0.4534884*
**ENSG00000186272**		*0.06481481*		*0.064814815*	*0.172619048*	*0.2151163*	*0.1744186*
**ENSG00000124098**		*0.22222222*		*0.222222222*			*0.375*
**ENSG00000137135**	*0.43674699*	*0.28787879*	*0.43674699*	*0.287878788*	*0.444444444*	*0.4090909*	*0.4411765*
**ENSG00000110723**	*0.47191011*	*0.13414634*	*0.47191011*	*0.134146341*	*0.166666667*	*0.2222222*	*0.244186*
**ENSG00000100918**	*0.4760479*		*0.4760479*		*0.464285714*	*0.4534884*	
**ENSG00000132972**					*0.285714286*	*0.1976744*	
**ENSG00000134874**		*0.48314607*		*0.483146067*			*0.3*
**ENSG00000198488**		*0.3404908*		*0.340490798*	*0.307228916*	*0.2093023*	*0.2616279*
**ENSG00000253953**		*0.20224719*		*0.202247191*			*0.1477273*
**ENSG00000134321**	*0.35555556*	*0.35795455*	*0.35555556*	*0.357954545*			*0.2666667*
**ENSG00000166426**					*0.363095238*	*0.3941176*	
**ENSG00000008118**		*0.34242424*		*0.342424242*			*0.1046512*
**ENSG00000163636**	*0.15868263*	*0.17575758*	*0.15868263*	*0.175757576*	*0.119047619*	*0.0813953*	*0.1705882*
**ENSG00000140905**		*0.10365854*		*0.103658537*			*0.2272727*
**ENSG00000112561**		*0.3961039*		*0.396103896*			*0.2560976*
**ENSG00000159885**	*0.2994012*		*0.2994012*				
**ENSG00000087916**		*0.46629213*		*0.466292135*			*0.4345238*
**ENSG00000204186**		*0.35151515*		*0.351515152*			*0.4069767*
**ENSG00000118690**		*0.13030303*		*0.13030303*			*0.0639535*
**ENSG00000198551**		*0.41818182*		*0.418181818*			*0.2151163*
**ENSG00000187446**		*0.19325153*		*0.193251534*			
**ENSG00000178802**		*0.40243902*		*0.402439024*			*0.3023256*
**ENSG00000136840**		*0.08181818*		*0.081818182*			*0.2093023*
**ENSG00000205081**							*0.1818182*
**ENSG00000152672**							*0.0888889*
**ENSG00000143319**	*0.13939394*	*0.09444444*	*0.13939394*	*0.094444444*	*0.096385542*	*0.0833333*	
**ENSG00000081803**	*0.36526946*	*0.21818182*	*0.36526946*	*0.218181818*	*0.416666667*	*0.377907*	*0.127907*
**ENSG00000204427**		*0.30909091*		*0.309090909*			*0.4302326*
**ENSG00000101191**		*0.31212121*		*0.312121212*			*0.377907*
**ENSG00000113761**		*0.5*		*0.5*			*0.4244186*
**ENSG00000100433**	*0.45209581*	*0.33333333*	*0.45209581*	*0.333333333*	*0.113095238*	*0.1488095*	*0.494186*
**ENSG00000178199**	*0.07386364*	*0.12424242*	*0.07386364*	*0.124242424*	*0.292682927*	*0.3953488*	*0.3705882*
**ENSG00000120688**		*0.14848485*		*0.148484848*			*0.1046512*
**ENSG00000059728**		*0.49691358*		*0.49691358*			*0.3352941*
**ENSG00000133316**	*0.15*		*0.15*		*0.1*	*0.1363636*	*0.1511628*
**ENSG00000145354**		*0.49444444*		*0.494444444*			*0.4767442*
**ENSG00000131016**		*0.33333333*		*0.333333333*			*0.4476744*
**ENSG00000184898**		*0.45151515*		*0.451515152*			*0.4709302*
**ENSG00000225614**		*0.18333333*		*0.183333333*			*0.0568182*
**ENSG00000166578**	*0.14670659*	*0.13333333*	*0.14670659*	*0.133333333*	*0.067073171*	*0.1104651*	
**ENSG00000150051**		*0.3969697*		*0.396969697*			*0.3888889*
**ENSG00000168497**		*0.46646341*		*0.466463415*			*0.3235294*
**ENSG00000169733**							*0.1627907*
**ENSG00000162344**		*0.21212121*		*0.212121212*			*0.4302326*
**ENSG00000189367**		*0.41818182*		*0.418181818*	*0.369047619*	*0.3313953*	*0.0697674*
**ENSG00000110031**		*0.49431818*		*0.494318182*			*0.3571429*
**ENSG00000103642**	*0.08383234*	*0.3030303*	*0.08383234*	*0.303030303*			
**ENSG00000109501**	*0.09281437*	*0.34242424*	*0.09281437*	*0.342424242*	*0.130952381*	*0.1395349*	*0.0813953*
**ENSG00000145358**		*0.44512195*		*0.445121951*			*0.3941176*
**ENSG00000105676**	*0.37125749*		*0.37125749*		*0.136904762*	*0.1046512*	

**ENSG00000169933**		0.41104294		0.411042945				0.2790698
**ENSG00000162882**	0.12068966	0.07575758	0.12068966	0.075757576	0.277777778		0.1931818	0.1071429
**ENSG00000217442**	0.24251497		0.24251497					
**ENSG00000157837**	0.46084337	0.48780488	0.46084337	0.487804878	0.452380952		0.4709302	0.4702381
**ENSG00000178163**		0.37575758		0.375757576				0.2034884
**ENSG00000108515**	0.21666667	0.41818182	0.21666667	0.418181818	0.244444444		0.1022727	0.4069767
**ENSG00000177425**		0.1741573		0.174157303				0.2325581
**ENSG00000177034**		0.375		0.375				0.4709302
**ENSG00000112796**		0.44545455		0.445454545	0.277777778		0.2444444	0.452381
**ENSG00000070010**	0.46111111	0.49382716	0.46111111	0.49382716	0.344444444		0.375	0.2906977
**ENSG00000136928**		0.29310345		0.293103448				0.3522727
**ENSG00000149443**	0.0988024		0.0988024		0.416666667		0.3081395	
**ENSG00000106591**		0.16111111		0.161111111				0.0897436
**ENSG00000138814**	0.05988024	0.08484848	0.05988024	0.084848485				0.1453488
**ENSG00000197614**		0.12121212		0.121212121				
**ENSG00000075240**	0.16111111	0.47777778	0.16111111	0.477777778				0.4659091
**ENSG00000198327**	0.23939394		0.23939394		0.130952381		0.1162791	
**ENSG00000102034**		0.23780488		0.237804878				0.3295455
**ENSG00000159409**		0.0969697		0.096969697				0.2093023
**ENSG00000002933**		0.06896552		0.068965517				0.0681818
**ENSG00000104518**	0.18888889		0.18888889					
**ENSG00000172554**		0.26969697		0.26969697				0.2965116
**ENSG00000186575**		0.47777778		0.477777778				0.494186
**ENSG00000196455**	0.2754491		0.2754491		0.482142857		0.4294118	
**ENSG00000159079**		0.17378049		0.173780488				0.3313953
**ENSG00000164393**	0.49382716	0.3902439	0.49382716	0.390243902	0.295180723		0.3072289	0.2831325
**ENSG00000090621**		0.23030303		0.23030303				0.1104651
**ENSG00000180537**		0.5		0.5				0.4647059
**ENSG00000132849**	0.5	0.42378049	0.5	0.423780488	0.432926829		0.4011628	0.0764706
**ENSG00000141665**					0.162650602		0.1511628	
**ENSG00000148948**	0.25		0.25		0.077777778		0.0777778	
**ENSG00000180616**		0.21515152		0.215151515				0.372093
**ENSG00000110651**	0.41017964		0.41017964					
**ENSG00000131844**		0.30337079		0.303370787				0.4886364
**ENSG00000149798**	0.0505618		0.0505618					0.2159091
**ENSG00000162378**	0.25149701	0.35555556	0.25149701	0.355555556	0.327380952		0.3023256	0.2840909
**ENSG00000179218**		0.41212121		0.412121212				0.2325581
**ENSG00000110048**		0.06363636		0.063636364				0.0930233
**ENSG00000154721**		0.16860465		0.168604651				0.3636364
**ENSG00000169230**		0.08484848		0.084848485				0.4476744
**ENSG00000155463**	0.43413174		0.43413174		0.196428571		0.1511628	
**ENSG00000089505**	0.18604651		0.18604651					
**ENSG00000166407**	0.3502994		0.3502994		0.226190476		0.2906977	
**ENSG00000183098**					0.071428571		0.0581395	
**ENSG00000130165**		0.17878788		0.178787879				0.0930233
**ENSG00000116984**	0.06666667	0.43251534	0.06666667	0.432515337			0.0666667	0.3253012
**ENSG00000144566**	0.35795455	0.14367816	0.35795455	0.143678161	0.188888889		0.1477273	0.1162791
**ENSG00000145626**		0.47575758		0.475757576				0.3023256
**ENSG00000150401**		0.37272727		0.372727273				0.3662791
**ENSG00000002549**		0.35		0.35				
**ENSG00000101189**		0.44545455		0.445454545				0.4302326
**ENSG00000102383**	0.12	0.20909091	0.12	0.209090909	0.060240964			
**ENSG00000162878**		0.325		0.325				0.4939024
**ENSG00000140259**	0.34730539		0.34730539		0.327380952		0.255814	

**ENSG00000169435**		*0.11656442*		*0.116564417*	*0.363095238*		*0.2732558*	*0.1744186*
**ENSG00000117528**	*0.09393939*	*0.21036585*	*0.09393939*	*0.210365854*	*0.297619048*		*0.1764706*	*0.4244186*
**ENSG00000181826**	*0.33522727*		*0.33522727*		*0.125*		*0.0568182*	*0.3068182*
**ENSG00000127863**		*0.29090909*		*0.290909091*				*0.4011628*
**ENSG00000204421**	*0.16167665*	*0.18484848*	*0.16167665*	*0.184848485*	*0.130952381*		*0.0930233*	*0.3430233*
**ENSG00000147251**		*0.17222222*		*0.172222222*				*0.1453488*
**ENSG00000133216**	*0.38323353*		*0.38323353*		*0.385542169*		*0.4069767*	
**ENSG00000169239**		*0.46932515*		*0.469325153*				*0.4207317*
**ENSG00000142632**		*0.23333333*		*0.233333333*				*0.1337209*
**ENSG00000059122**	*0.49700599*	*0.47575758*	*0.49700599*	*0.475757576*	*0.321428571*		*0.3197674*	*0.439759*
**ENSG00000100302**		*0.35757576*		*0.357575758*				*0.4593023*
**ENSG00000213937**					*0.363095238*		*0.4360465*	
**ENSG00000196981**		*0.43030303*		*0.43030303*				*0.3444444*
**ENSG00000186431**	*0.29041916*	*0.1969697*	*0.29041916*	*0.196969697*	*0.339285714*		*0.3764706*	
**ENSG00000184697**		*0.20606061*		*0.206060606*				*0.2848837*
**ENSG00000170634**		*0.22839506*		*0.228395062*				*0.2926829*
**ENSG00000105821**	*0.30898876*	*0.14444444*	*0.30898876*	*0.144444444*	*0.3*		*0.4090909*	
**ENSG00000145451**		*0.40555556*		*0.405555556*				*0.4772727*
**ENSG00000101152**		*0.06666667*		*0.066666667*				*0.1046512*
**ENSG00000203690**		*0.38181818*		*0.381818182*				*0.3023256*
**ENSG00000173110**		*0.34*		*0.34*				*0.3378378*
**ENSG00000130396**		*0.37272727*		*0.372727273*				*0.3953488*
**ENSG00000133943**	*0.32777778*	*0.38414634*	*0.32777778*	*0.384146341*	*0.1*		*0.1144578*	*0.1453488*
**ENSG00000111817**	*0.08383234*	*0.35403727*	*0.08383234*	*0.354037267*	*0.422222222*		*0.3522727*	*0.122093*
**ENSG00000189319**		*0.47727273*		*0.477272727*				*0.4302326*
**ENSG00000122574**		*0.41818182*		*0.418181818*				*0.3430233*
**ENSG00000131738**								*0.0639535*
**ENSG00000157823**	*0.48493976*	*0.48773006*	*0.48493976*	*0.487730061*	*0.416666667*		*0.3372093*	*0.3764706*
**ENSG00000136160**		*0.11212121*		*0.112121212*				
**ENSG00000109674**		*0.2208589*		*0.220858896*	*0.279761905*		*0.2941176*	*0.4767442*
**ENSG00000170231**		*0.36931818*		*0.369318182*				*0.3837209*
**ENSG00000132640**		*0.47575758*		*0.475757576*				*0.3837209*
**ENSG00000154839**	*0.11666667*	*0.30909091*	*0.11666667*	*0.309090909*	*0.111111111*		*0.1444444*	*0.4186047*
**ENSG00000163581**		*0.29573171*		*0.295731707*				*0.2383721*
**ENSG00000109132**		*0.38484848*		*0.384848485*				*0.2093023*
**ENSG00000249034**		*0.41666667*		*0.416666667*				*0.4431818*
**ENSG00000188761**		*0.31707317*		*0.317073171*				*0.1058824*
**ENSG00000196693**		*0.38764045*		*0.387640449*				*0.4318182*
**ENSG00000177943**		*0.25454545*		*0.254545455*				*0.1046512*
**ENSG00000176945**		*0.44848485*		*0.448484848*				*0.3837209*
**ENSG00000204365**	*0.19461078*	*0.23780488*	*0.19461078*	*0.237804878*	*0.25*		*0.2647059*	*0.3313953*
**ENSG00000165716**	*0.30625*		*0.30625*		*0.192771084*		*0.1046512*	
**ENSG00000177710**	*0.13043478*		*0.13043478*					
**ENSG00000172840**		*0.09393939*		*0.093939394*				*0.1569767*
**ENSG00000148331**		*0.45555556*		*0.455555556*				*0.5*
**ENSG00000205784**		*0.32727273*		*0.327272727*				
**ENSG00000075151**							*0.0523256*	
**ENSG00000142686**	*0.09580838*		*0.09580838*					*0.3488372*
**ENSG00000165804**	*0.28888889*	*0.18965517*	*0.28888889*	*0.189655172*	*0.411111111*		*0.4772727*	*0.3181818*
**ENSG00000112697**	*0.22155689*	*0.05952381*	*0.22155689*	*0.05952381*	*0.452380952*		*0.4069767*	*0.1428571*
**ENSG00000176236**		*0.15757576*		*0.157575758*				
**ENSG00000105221**		*0.5*		*0.5*	*0.301204819*		*0.3470588*	*0.4476744*
**ENSG00000197905**	*0.05722892*		*0.05722892*		*0.125*		*0.0921053*	
**ENSG00000132561**		*0.35151515*		*0.351515152*				*0.3588235*

**ENSG00000205808**	*0.08682635*	*0.28651685*	*0.08682635*	*0.286516854*	*0.261904762*	*0.3023256*	*0.2732558*
**ENSG00000165471**		*0.42424242*		*0.424242424*			*0.3953488*
**ENSG00000101745**	*0.06741573*	*0.39393939*	*0.06741573*	*0.393939394*		*0.1860465*	*0.4883721*
**ENSG00000151176**							*0.0813953*
**ENSG00000103995**	*0.15269461*	*0.18965517*	*0.15269461*	*0.189655172*	*0.321428571*	*0.2790698*	*0.4886364*
**ENSG00000117289**					*0.202380952*	*0.255814*	*0.3235294*
**ENSG00000242852**		*0.19393939*		*0.193939394*			*0.4418605*
**ENSG00000075142**		*0.29573171*		*0.295731707*			*0.0941176*
**ENSG00000148219**	*0.29310345*	*0.47777778*	*0.29310345*	*0.477777778*	*0.476190476*	*0.4302326*	*0.2840909*
**ENSG00000228716**					*0.392857143*	*0.2790698*	
**ENSG00000184351**		*0.43292683*		*0.432926829*			*0.4*
**ENSG00000166073**		*0.36503067*		*0.365030675*			*0.2732558*
**ENSG00000176177**		*0.09090909*		*0.090909091*			*0.0581395*
**ENSG00000104660**							*0.1744186*
**ENSG00000100307**		*0.48850575*		*0.488505747*			*0.4302326*
**ENSG00000130822**	*0.11676647*		*0.11676647*		*0.142857143*	*0.127907*	
**ENSG00000179262**							*0.0909091*
**ENSG00000028203**		*0.490625*		*0.490625*			*0.4886364*
**ENSG00000197893**	*0.49101796*		*0.49101796*		*0.279761905*	*0.2093023*	
**ENSG00000174444**		*0.10060976*		*0.100609756*			*0.4709302*
**ENSG00000170653**		*0.13939394*		*0.139393939*			*0.1235294*
**ENSG00000102786**		*0.40555556*		*0.405555556*			*0.2272727*
**ENSG00000204463**	*0.30838323*		*0.30838323*		*0.398809524*	*0.4333333*	
**ENSG00000203871**	*0.37222222*	*0.29310345*	*0.37222222*	*0.293103448*	*0.188888889*	*0.1931818*	*0.1931818*
**ENSG00000090097**	*0.34431138*		*0.34431138*		*0.095238095*		
**ENSG00000146648**		*0.41573034*		*0.415730337*			*0.4431818*
**ENSG00000054965**		*0.21515152*		*0.215151515*			*0.0930233*
**ENSG00000115828**	*0.23652695*	*0.06321839*	*0.23652695*	*0.063218391*	*0.494047619*	*0.3953488*	*0.4302326*
**ENSG00000161999**		*0.17272727*		*0.172727273*			
**ENSG00000139797**		*0.07222222*		*0.072222222*			*0.1022727*
**ENSG00000106785**	*0.46706587*	*0.27575758*	*0.46706587*	*0.275757576*			*0.0930233*
**ENSG00000176393**	*0.37724551*		*0.37724551*		*0.392857143*	*0.3953488*	
**ENSG00000105251**	*0.30538922*		*0.30538922*		*0.446428571*	*0.3604651*	
**ENSG00000171953**							*0.0523256*
**ENSG00000176769**		*0.4030303*		*0.403030303*			*0.3764706*
**ENSG00000100867**	*0.49401198*		*0.49401198*		*0.214285714*	*0.122093*	
**ENSG00000164404**	*0.47305389*		*0.47305389*		*0.214285714*	*0.2267442*	
**ENSG00000113456**		*0.07621951*		*0.076219512*	*0.055555556*		*0.4294118*
**ENSG00000176834**		*0.44242424*		*0.442424242*			*0.4767442*
**ENSG00000177873**	*0.47005988*	*0.33030303*	*0.47005988*	*0.33030303*	*0.397590361*	*0.4*	*0.4360465*
**ENSG00000145416**	*0.19879518*	*0.5*	*0.19879518*	*0.5*			*0.4593023*
**ENSG00000119946**		*0.22121212*		*0.221212121*			*0.3522727*
**ENSG00000162607**	*0.11676647*	*0.34848485*	*0.11676647*	*0.348484848*			*0.1686047*
**ENSG00000132386**	*0.16470588*		*0.16470588*			*0.1219512*	
**ENSG00000142694**					*0.06547619*	*0.1104651*	
**ENSG00000158825**					*0.06547619*	*0.0523256*	
**ENSG00000135077**	*0.2754491*	*0.16666667*	*0.2754491*	*0.166666667*			
**ENSG00000125868**							*0.0930233*
**ENSG00000166329**		*0.48484848*		*0.484848485*			*0.4647059*
**ENSG00000183808**	*0.45808383*	*0.33125*	*0.45808383*	*0.33125*	*0.493975904*	*0.4294118*	*0.4285714*
**ENSG00000035720**		*0.24242424*		*0.242424242*			*0.4651163*
**ENSG00000181991**	*0.36666667*		*0.36666667*				
**ENSG00000141385**		*0.21666667*		*0.216666667*			*0.3837209*
**ENSG00000169715**		*0.14634146*		*0.146341463*			*0.2151163*

**ENSG00000160207**	*0.47777778*	*0.44242424*	*0.47777778*	*0.442424242*	*0.188888889*	*0.2272727*	*0.1860465*
**ENSG00000146147**		*0.10625*		*0.10625*			*0.1918605*
**ENSG00000177272**		*0.31515152*		*0.315151515*			*0.4047619*
**ENSG00000239857**	*0.05113636*	*0.29393939*	*0.05113636*	*0.293939394*	*0.211111111*	*0.1022727*	*0.2906977*
**ENSG00000124762**							*0.4011628*
**ENSG00000063601**		*0.43209877*		*0.432098765*			*0.2228916*
**ENSG00000137878**		*0.2*		*0.2*	*0.426829268*	*0.494186*	*0.4244186*
**ENSG00000184967**	*0.45808383*		*0.45808383*		*0.488095238*	*0.4069767*	
**ENSG00000156162**		*0.1*		*0.1*			*0.0581395*
**ENSG00000144591**	*0.25449102*		*0.25449102*		*0.18452381*	*0.2093023*	
**ENSG00000141750**		*0.44848485*		*0.448484848*			*0.3313953*
**ENSG00000141194**		*0.24848485*		*0.248484848*			*0.1162791*
**ENSG00000196781**	*0.06111111*	*0.11515152*	*0.06111111*	*0.115151515*	*0.144444444*	*0.1363636*	
**ENSG00000134248**	*0.38622754*		*0.38622754*		*0.455555556*	*0.4886364*	
**ENSG00000169760**	*0.3742515*	*0.47575758*	*0.3742515*	*0.475757576*	*0.386904762*	*0.4476744*	*0.4476744*
**ENSG00000136999**		*0.2*		*0.2*			*0.1860465*
**ENSG00000188176**	*0.26946108*		*0.26946108*		*0.202380952*	*0.244186*	
**ENSG00000140876**							*0.3095238*
**ENSG00000173465**		*0.07303371*		*0.073033708*			*0.0568182*
**ENSG00000171574**	*0.08682635*	*0.47575758*	*0.08682635*	*0.475757576*	*0.06547619*		*0.3555556*
**ENSG00000100239**		*0.27272727*		*0.272727273*			*0.0581395*
**ENSG00000151229**		*0.35*		*0.35*			*0.3837209*
**ENSG00000149926**	*0.22754491*		*0.22754491*		*0.391566265*	*0.2790698*	
**ENSG00000173930**		*0.14329268*		*0.143292683*			*0.1627907*
**ENSG00000204310**		*0.45757576*		*0.457575758*	*0.060240964*	*0.0783133*	*0.4244186*
**ENSG00000142794**	*0.47159091*		*0.47159091*		*0.111111111*	*0.2613636*	
**ENSG00000174171**	*0.25149701*	*0.12883436*	*0.25149701*	*0.128834356*	*0.261904762*	*0.2325581*	
**ENSG00000167775**	*0.38823529*		*0.38823529*				
**ENSG00000135503**		*0.22560976*		*0.225609756*			*0.4302326*
**ENSG00000065621**	*0.39444444*		*0.39444444*		*0.154761905*	*0.1627907*	
**ENSG00000143178**	*0.05688623*	*0.43939394*	*0.05688623*	*0.439393939*			*0.5*
**ENSG00000198018**		*0.48701299*		*0.487012987*			*0.4487179*
**ENSG00000147416**		*0.10909091*		*0.109090909*			*0.0639535*
**ENSG00000185053**		*0.13333333*		*0.133333333*			
**ENSG00000204694**	*0.14670659*	*0.36196319*	*0.14670659*	*0.36196319*	*0.255555556*	*0.2111111*	*0.4360465*
**ENSG00000136688**		*0.32424242*		*0.324242424*			
**ENSG00000172687**	*0.23652695*		*0.23652695*		*0.289156627*	*0.3372093*	*0.3181818*
**ENSG00000120471**	*0.1741573*	*0.29090909*	*0.1741573*	*0.290909091*	*0.477777778*	*0.4302326*	*0.2267442*
**ENSG00000145725**		*0.36111111*		*0.361111111*			*0.4883721*
**ENSG00000241839**		*0.44409938*		*0.444099379*			*0.1569767*
**ENSG00000198464**		*0.3*		*0.3*			*0.3295455*
**ENSG00000198863**		*0.11890244*		*0.118902439*			
**ENSG00000146094**	*0.21604938*	*0.46666667*	*0.21604938*	*0.466666667*	*0.23255814*	*0.1309524*	*0.375*
**ENSG00000178093**	*0.16455696*		*0.16455696*				
**ENSG00000106344**		*0.3597561*		*0.359756098*			*0.4825581*
**ENSG00000089356**		*0.42307692*		*0.423076923*			*0.4244186*
**ENSG00000159640**	*0.43820225*		*0.43820225*		*0.244444444*	*0.4431818*	
**ENSG00000103174**		*0.5*		*0.5*			*0.3953488*
**ENSG00000150394**		*0.21036585*		*0.210365854*			*0.3837209*
**ENSG00000221823**		*0.26219512*		*0.262195122*			*0.1627907*
**ENSG00000100890**	*0.46706587*	*0.15757576*	*0.46706587*	*0.157575758*	*0.422619048*	*0.4318182*	
**ENSG00000182909**							*0.1046512*
**ENSG00000033178**		*0.49431818*		*0.494318182*			*0.4390244*
**ENSG00000180628**		*0.41515152*		*0.415151515*			*0.1190476*

**ENSG00000151611**	*0.35628743*	*0.25151515*	*0.35628743*	*0.251515152*	*0.470238095*	*0.4825581*	*0.494186*
**ENSG00000178462**		*0.24242424*		*0.242424242*			*0.1802326*
**ENSG00000007341**		*0.47256098*		*0.472560976*			*0.3662791*
**ENSG00000080854**		*0.41212121*		*0.412121212*			*0.3139535*
**ENSG00000152467**		*0.30981595*		*0.309815951*			*0.2674419*
**ENSG00000158006**	*0.33888889*	*0.22727273*	*0.33888889*	*0.227272727*	*0.369047619*	*0.255814*	*0.1686047*
**ENSG00000197372**	*0.14444444*		*0.14444444*				
**ENSG00000132423**	*0.11676647*		*0.11676647*		*0.494047619*	*0.3837209*	
**ENSG00000128203**		*0.48888889*		*0.488888889*			*0.3977273*
**ENSG00000148057**	*0.20606061*	*0.17272727*	*0.20606061*	*0.172727273*		*0.0988372*	
**ENSG00000163082**		*0.16666667*		*0.166666667*			
**ENSG00000184445**	*0.16467066*	*0.24848485*	*0.16467066*	*0.248484848*			*0.494186*
**ENSG00000102904**	*0.25748503*		*0.25748503*				
**ENSG00000171016**		*0.40555556*		*0.405555556*			*0.4767442*
**ENSG00000142675**		*0.11818182*		*0.118181818*			*0.1802326*
**ENSG00000172738**		*0.44848485*		*0.448484848*			*0.0988372*
**ENSG00000154001**	*0.34090909*	*0.1*	*0.34090909*	*0.1*	*0.277777778*	*0.3068182*	*0.4431818*
**ENSG00000130544**		*0.38181818*		*0.381818182*			*0.4767442*
**ENSG00000151067**		*0.26993865*		*0.26993865*			*0.5*
**ENSG00000100632**	*0.25842697*		*0.25842697*				
**ENSG00000083093**	*0.15168539*		*0.15168539*				
**ENSG00000183801**		*0.5*		*0.5*			*0.2616279*
**ENSG00000151365**		*0.11111111*		*0.111111111*			*0.375*
**ENSG00000112877**		*0.23333333*		*0.233333333*			*0.3662791*
**ENSG00000086544**		*0.47777778*		*0.477777778*			*0.4090909*
**ENSG00000143147**	*0.31437126*		*0.31437126*		*0.19047619*	*0.1802326*	
**ENSG00000143171**		*0.15882353*		*0.158823529*			*0.0666667*
**ENSG00000024526**		*0.14444444*		*0.144444444*			*0.0813953*
**ENSG00000144040**		*0.24242424*		*0.242424242*			*0.4011628*
**ENSG00000155754**		*0.33939394*		*0.339393939*			
**ENSG00000160999**					*0.219512195*	*0.3846154*	
**ENSG00000113396**	*0.36526946*	*0.29310345*	*0.36526946*	*0.293103448*	*0.101190476*	*0.0952381*	
**ENSG00000189334**		*0.48787879*		*0.487878788*			*0.3546512*
**ENSG00000166206**	*0.25*	*0.3*	*0.25*	*0.3*	*0.26744186*	*0.1888889*	*0.2613636*
**ENSG00000146476**	*0.09281437*		*0.09281437*		*0.267857143*	*0.3372093*	
**ENSG00000133460**		*0.48148148*		*0.481481481*			*0.4411765*
**ENSG00000214253**					*0.321428571*	*0.4418605*	
**ENSG00000179626**	*0.43413174*		*0.43413174*		*0.119047619*	*0.0755814*	
**ENSG00000180549**	*0.23888889*	*0.39393939*	*0.23888889*	*0.393939394*	*0.177777778*	*0.2613636*	*0.2151163*
**ENSG00000038427**		*0.49358974*		*0.493589744*			*0.4264706*
**ENSG00000143258**		*0.12727273*		*0.127272727*			
**ENSG00000139514**		*0.1993865*		*0.199386503*			*0.372093*
**ENSG00000165862**		*0.48787879*		*0.487878788*			*0.2383721*
**ENSG00000146757**		*0.13939394*		*0.139393939*			*0.2906977*
**ENSG00000198105**		*0.14444444*		*0.144444444*			*0.1111111*
**ENSG00000108826**	*0.36666667*	*0.45348837*	*0.36666667*	*0.453488372*			*0.1022727*
**ENSG00000164151**							*0.2045455*
**ENSG00000123575**		*0.33229814*		*0.332298137*			*0.4705882*
**ENSG00000107819**	*0.20359281*		*0.20359281*		*0.238095238*	*0.2325581*	
**ENSG00000111554**	*0.15868263*	*0.29393939*	*0.15868263*	*0.293939394*	*0.410714286*	*0.3837209*	*0.3837209*
**ENSG00000206344**		*0.4*		*0.4*			*0.4883721*
**ENSG00000089472**	*0.41017964*		*0.41017964*				
**ENSG00000243232**		*0.05151515*		*0.051515152*			*0.1162791*
**ENSG00000153093**		*0.47560976*		*0.475609756*			*0.494186*

**ENSG00000150873**							*0.0813953*
**ENSG00000103494**		*0.36666667*		*0.366666667*			*0.4659091*
**ENSG00000108830**		*0.34444444*		*0.344444444*			*0.2840909*
**ENSG00000163833**		*0.46969697*		*0.46969697*			*0.4360465*
**ENSG00000137166**		*0.1969697*		*0.196969697*			*0.2837838*
**ENSG00000140279**	*0.08682635*	*0.05151515*	*0.08682635*	*0.051515152*			*0.0681818*
**ENSG00000138039**		*0.40184049*		*0.401840491*			*0.2325581*
**ENSG00000163617**		*0.1*		*0.1*			*0.1590909*
**ENSG00000147687**	*0.2816092*		*0.2816092*		*0.306818182*	*0.372093*	
**ENSG00000169282**	*0.45180723*	*0.20121951*	*0.45180723*	*0.201219512*	*0.402439024*	*0.4756098*	
**ENSG00000197837**	*0.31437126*	*0.46969697*	*0.31437126*	*0.46969697*			*0.3488372*
**ENSG00000136378**		*0.43258427*		*0.43258427*			
**ENSG00000121900**		*0.05151515*		*0.051515152*			
**ENSG00000130244**		*0.05182927*		*0.051829268*			*0.2093023*
**ENSG00000243414**		*0.49393939*		*0.493939394*			*0.4709302*
**ENSG00000004866**		*0.22222222*		*0.222222222*	*0.466666667*	*0.4659091*	*0.0795455*
**ENSG00000113716**	*0.34730539*	*0.12121212*	*0.34730539*	*0.121212121*	*0.22972973*	*0.2195122*	*0.0813953*
**ENSG00000159496**	*0.40419162*		*0.40419162*		*0.386904762*	*0.3255814*	
**ENSG00000075131**	*0.05722892*		*0.05722892*				
**ENSG00000133794**	*0.42771084*		*0.42771084*		*0.494047619*	*0.4593023*	
**ENSG00000012822**		*0.27439024*		*0.274390244*	*0.285714286*	*0.3372093*	*0.4444444*
**ENSG00000169599**	*0.38333333*	*0.37195122*	*0.38333333*	*0.37195122*	*0.222222222*	*0.1444444*	*0.4411765*
**ENSG00000169604**	*0.20987654*		*0.20987654*				
**ENSG00000204970**		*0.05151515*		*0.051515152*			*0.1162791*
**ENSG00000178952**	*0.36645963*		*0.36645963*		*0.228915663*	*0.2176471*	
**ENSG00000134851**		*0.28888889*		*0.288888889*			*0.2045455*
**ENSG00000179526**	*0.30120482*		*0.30120482*				
**ENSG00000102804**	*0.26646707*		*0.26646707*				
**ENSG00000150551**		*0.48181818*		*0.481818182*			*0.4651163*
**ENSG00000173077**	*0.09444444*	*0.15151515*	*0.09444444*	*0.151515152*			*0.2906977*
**ENSG00000258289**		*0.321875*		*0.321875*			*0.4529412*
**ENSG00000177414**	*0.30357143*	*0.24848485*	*0.30357143*	*0.248484848*	*0.422222222*	*0.452381*	*0.2093023*
**ENSG00000111049**	*0.0988024*	*0.24848485*	*0.0988024*	*0.248484848*	*0.238095238*	*0.255814*	
**ENSG00000120910**		*0.5*		*0.5*			*0.4418605*
**ENSG00000204740**	*0.47005988*		*0.47005988*		*0.357142857*	*0.3837209*	
**ENSG00000244476**		*0.21515152*		*0.215151515*			*0.3837209*
**ENSG00000008869**	*0.08888889*		*0.08888889*				*0.0523256*
**ENSG00000068878**		*0.47865854*		*0.478658537*			*0.4431818*
**ENSG00000160767**							*0.077381*
**ENSG00000165685**		*0.19631902*		*0.196319018*			*0.2616279*
**ENSG00000146700**	*0.17664671*	*0.23939394*	*0.17664671*	*0.239393939*			*0.2674419*
**ENSG00000083067**	*0.33233533*	*0.47256098*	*0.33233533*	*0.472560976*	*0.255952381*	*0.3546512*	*0.4476744*
**ENSG00000104522**		*0.47674419*		*0.476744186*			*0.1744186*
**ENSG00000163808**		*0.46969697*		*0.46969697*			*0.1453488*
**ENSG00000163728**	*0.06886228*	*0.18292683*	*0.06886228*	*0.182926829*			
**ENSG00000173933**		*0.05555556*		*0.055555556*			*0.1333333*
**ENSG00000135605**		*0.41158537*		*0.411585366*			*0.3372093*
**ENSG00000165120**		*0.26969697*		*0.26969697*			
**ENSG00000006210**		*0.31402439*		*0.31402439*			*0.4476744*
**ENSG00000124233**	*0.06111111*		*0.06111111*				
**ENSG00000130167**	*0.16477273*	*0.45121951*	*0.16477273*	*0.451219512*	*0.285714286*	*0.255814*	*0.2906977*
**ENSG00000143502**	*0.19760479*		*0.19760479*		*0.196428571*	*0.2151163*	*0.0872093*
**ENSG00000138030**		*0.403125*		*0.403125*			*0.2142857*
**ENSG00000110934**							*0.3023256*

**ENSG00000182903**		*0.37575758*		*0.375757576*			*0.2882353*
**ENSG00000114735**	*0.05688623*	*0.16463415*	*0.05688623*	*0.164634146*	*0.339285714*	*0.2732558*	*0.3546512*
**ENSG00000124641**	*0.4375*	*0.33888889*	*0.4375*	*0.338888889*	*0.5*	*0.3295455*	*0.2840909*
**ENSG00000128739**	*0.44311377*		*0.44311377*		*0.458333333*	*0.4651163*	
**ENSG00000140107**	*0.47005988*	*0.2183908*	*0.47005988*	*0.218390805*	*0.196428571*	*0.2965116*	*0.1590909*
**ENSG00000142945**	*0.39221557*		*0.39221557*		*0.279761905*	*0.25*	
**ENSG00000115364**		*0.45505618*		*0.45505618*			*0.3295455*
**ENSG00000109189**		*0.16666667*		*0.166666667*			*0.0909091*
**ENSG00000154723**		*0.16860465*		*0.168604651*			*0.2840909*
**ENSG00000137941**		*0.28220859*		*0.282208589*			*0.0714286*
**ENSG00000109046**		*0.36196319*		*0.36196319*			*0.1705882*
**ENSG00000150656**		*0.37272727*		*0.372727273*			*0.3488372*
**ENSG00000169032**		*0.10555556*		*0.105555556*			
**ENSG00000059145**		*0.39939024*		*0.399390244*			*0.2151163*
**ENSG00000137265**		*0.49090909*		*0.490909091*			*0.4883721*
**ENSG00000100034**	*0.36363636*	*0.10122699*	*0.36363636*	*0.101226994*	*0.255555556*	*0.25*	*0.3139535*
**ENSG00000196182**		*0.21515152*		*0.215151515*			*0.1337209*
**ENSG00000183207**	*0.10778443*		*0.10778443*		*0.095238095*		
**ENSG00000100982**		*0.16969697*		*0.16969697*			
**ENSG00000186615**	*0.05688623*		*0.05688623*				
**ENSG00000115758**	*0.22754491*		*0.22754491*		*0.404761905*	*0.4767442*	
**ENSG00000110435**	*0.46107784*	*0.23030303*	*0.46107784*	*0.23030303*	*0.255952381*	*0.3197674*	*0.3081395*
**ENSG00000163288**		*0.26666667*		*0.266666667*			*0.1506024*
**ENSG00000001631**		*0.09090909*		*0.090909091*			*0.3*
**ENSG00000136802**		*0.25454545*		*0.254545455*			*0.3488372*
**ENSG00000136940**		*0.23312883*		*0.233128834*			*0.3823529*
**ENSG00000182197**		*0.45294118*		*0.452941176*			*0.4634146*
**ENSG00000120907**	*0.46686747*	*0.38484848*	*0.46686747*	*0.384848485*	*0.464285714*	*0.4186047*	*0.4767442*
**ENSG00000138641**	*0.08888889*	*0.29393939*	*0.08888889*	*0.293939394*	*0.333333333*	*0.3313953*	*0.3837209*
**ENSG00000131746**		*0.14444444*		*0.144444444*			
**ENSG00000105479**		*0.35454545*		*0.354545455*			*0.4011628*
**ENSG00000179172**					*0.161764706*	*0.1282051*	
**ENSG00000111727**		*0.05769231*		*0.057692308*			*0.2727273*
**ENSG00000198822**						*0.0581395*	
**ENSG00000135250**		*0.21428571*		*0.214285714*			*0.3081395*
**ENSG00000146828**		*0.48787879*		*0.487878788*			*0.2*
**ENSG00000100368**		*0.14848485*		*0.148484848*			*0.1395349*
**ENSG00000110245**		*0.08181818*		*0.081818182*			*0.3823529*
**ENSG00000196646**	*0.09444444*		*0.09444444*				
**ENSG00000122729**		*0.38271605*		*0.382716049*			*0.3604651*
**ENSG00000169018**		*0.31212121*		*0.312121212*			
**ENSG00000152332**		*0.44318182*		*0.443181818*			*0.4882353*
**ENSG00000166342**	*0.20958084*	*0.40243902*	*0.20958084*	*0.402439024*	*0.071428571*		*0.3313953*
**ENSG00000062370**	*0.05617978*	*0.46969697*	*0.05617978*	*0.46969697*		*0.0666667*	*0.4360465*
**ENSG00000163394**	*0.1686747*		*0.1686747*		*0.136904762*	*0.1860465*	
**ENSG00000220032**	*0.08333333*	*0.28787879*	*0.08333333*	*0.287878788*	*0.172619048*	*0.202381*	*0.2674419*
**ENSG00000160310**	*0.48802395*	*0.49438202*	*0.48802395*	*0.494382022*	*0.377777778*	*0.3863636*	*0.4512195*
**ENSG00000129244**		*0.43636364*		*0.436363636*	*0.382716049*	*0.4207317*	*0.4431818*
**ENSG00000101596**		*0.43333333*		*0.433333333*			*0.3392857*
**ENSG00000198812**		*0.32121212*		*0.321212121*			*0.4883721*
**ENSG00000136271**		*0.45151515*		*0.451515152*			*0.372093*
**ENSG00000171476**	*0.30555556*		*0.30555556*		*0.411111111*	*0.3953488*	
**ENSG00000080644**	*0.33532934*	*0.36280488*	*0.33532934*	*0.362804878*	*0.297619048*	*0.3604651*	*0.1627907*
**ENSG00000197016**	*0.16766467*	*0.3445122*	*0.16766467*	*0.344512195*	*0.36746988*	*0.3837209*	*0.3488372*

**ENSG00000113504**		*0.47878788*		*0.478787879*			*0.494186*
**ENSG00000129646**		*0.28787879*		*0.287878788*			*0.3255814*
**ENSG00000185924**		*0.28658537*		*0.286585366*			*0.4418605*
**ENSG00000204305**	*0.1402439*		*0.1402439*		*0.238095238*	*0.127907*	
**ENSG00000204406**		*0.27878788*		*0.278787879*			*0.2674419*
**ENSG00000136231**		*0.4*		*0.4*			*0.3409091*
**ENSG00000089101**	*0.39939024*	*0.48787879*	*0.39939024*	*0.487878788*	*0.295454545*	*0.4698795*	*0.4651163*
**ENSG00000154646**		*0.20858896*		*0.208588957*			
**ENSG00000169764**	*0.14071856*		*0.14071856*		*0.119047619*		
**ENSG00000129465**	*0.17964072*	*0.05792683*	*0.17964072*	*0.057926829*	*0.333333333*	*0.2209302*	*0.077381*
**ENSG00000198331**	*0.19642857*	*0.31212121*	*0.19642857*	*0.312121212*			*0.2151163*
**ENSG00000135018**		*0.35*		*0.35*			*0.0568182*
**ENSG00000124787**		*0.31818182*		*0.318181818*			*0.2297297*
**ENSG00000163710**		*0.24705882*		*0.247058824*			*0.3294118*
**ENSG00000146038**		*0.24117647*		*0.241176471*			*0.4883721*
**ENSG00000142252**		*0.3445122*		*0.344512195*			*0.1104651*
**ENSG00000016602**	*0.14285714*	*0.11212121*	*0.14285714*	*0.112121212*			*0.060241*
**ENSG00000108651**	*0.40555556*	*0.1954023*	*0.40555556*	*0.195402299*	*0.147727273*	*0.1477273*	*0.1511628*
**ENSG00000175782**		*0.23809524*		*0.238095238*			
**ENSG00000186790**		*0.10248447*		*0.102484472*			*0.3197674*
**ENSG00000164879**	*0.22155689*	*0.45151515*	*0.22155689*	*0.451515152*	*0.482142857*	*0.4411765*	*0.4431818*
**ENSG00000176879**					*0.196428571*	*0.2267442*	
**ENSG00000116039**	*0.34131737*	*0.43939394*	*0.34131737*	*0.439393939*	*0.482142857*	*0.4593023*	*0.3430233*
**ENSG00000166762**		*0.22222222*		*0.222222222*			*0.2738095*
**ENSG00000086712**		*0.4030303*		*0.403030303*			*0.3823529*
**ENSG00000169860**							*0.0755814*
**ENSG00000164938**		*0.27575758*		*0.275757576*			*0.244186*
**ENSG00000181856**	*0.21910112*	*0.35454545*	*0.21910112*	*0.354545455*	*0.397435897*	*0.3111111*	*0.3139535*
**ENSG00000064545**	*0.11419753*	*0.15151515*	*0.11419753*	*0.151515152*			
**ENSG00000183722**							*0.2325581*
**ENSG00000066739**		*0.44785276*		*0.447852761*			*0.4883721*
**ENSG00000170322**		*0.28787879*		*0.287878788*			*0.3977273*
**ENSG00000180861**	*0.19461078*	*0.37037037*	*0.19461078*	*0.37037037*	*0.343373494*	*0.3313953*	*0.3372093*
**ENSG00000157193**	*0.19161677*	*0.45757576*	*0.19161677*	*0.457575758*	*0.464285714*	*0.5*	*0.4767442*
**ENSG00000070018**		*0.18787879*		*0.187878788*			*0.0813953*
**ENSG00000130717**		*0.08484848*		*0.084848485*			*0.0952381*
**ENSG00000198914**		*0.40909091*		*0.409090909*			*0.3604651*
**ENSG00000167283**		*0.07926829*		*0.079268293*			*0.0813953*
**ENSG00000171483**	*0.3125*		*0.3125*		*0.114457831*		
**ENSG00000175224**		*0.23780488*		*0.237804878*			*0.3977273*
**ENSG00000172469**	*0.42696629*	*0.42424242*	*0.42696629*	*0.424242424*	*0.375*	*0.4127907*	*0.2771084*
**ENSG00000104904**							*0.0639535*
**ENSG00000148200**		*0.07222222*		*0.072222222*			*0.0639535*
**ENSG00000102924**		*0.22121212*		*0.221212121*			
**ENSG00000227450**		*0.25465839*		*0.254658385*			*0.4470588*
**ENSG00000066044**		*0.31515152*		*0.315151515*			*0.4883721*
**ENSG00000213132**	*0.45092025*	*0.46060606*	*0.45092025*	*0.460606061*	*0.162650602*	*0.1294118*	*0.4207317*
**ENSG00000087495**					*0.05952381*	*0.0529412*	
**ENSG00000177666**		*0.40606061*		*0.406060606*			*0.3662791*
**ENSG00000196758**		*0.49085366*		*0.490853659*			*0.4647059*
**ENSG00000109881**		*0.3*		*0.3*			*0.494186*
**ENSG00000159128**	*0.21257485*	*0.4969697*	*0.21257485*	*0.496969697*	*0.422619048*	*0.4767442*	*0.4767442*
**ENSG00000132031**		*0.3908046*		*0.390804598*			*0.202381*
**ENSG00000187922**		*0.14545455*		*0.145454545*			*0.2093023*

**ENSG00000153823**		*0.48181818*		*0.481818182*			*0.4883721*
**ENSG00000113749**	*0.34131737*		*0.34131737*		*0.244444444*	*0.122093*	*0.1058824*
**ENSG00000188487**		*0.42987805*		*0.429878049*			*0.1927711*
**ENSG00000160439**	*0.22754491*	*0.27950311*	*0.22754491*	*0.279503106*			*0.2616279*
**ENSG00000254833**					*0.06547619*	*0.0755814*	
**ENSG00000196844**		*0.46625767*		*0.466257669*			*0.4011628*
**ENSG00000023445**		*0.3988764*		*0.398876404*			*0.3888889*
**ENSG00000184939**		*0.21212121*		*0.212121212*			*0.1590909*
**ENSG00000139826**		*0.17222222*		*0.172222222*			*0.1363636*
**ENSG00000184224**	*0.20658683*	*0.41212121*	*0.20658683*	*0.412121212*			*0.1162791*
**ENSG00000134597**	*0.17065868*		*0.17065868*				
**ENSG00000185888**							*0.0681818*
**ENSG00000185069**		*0.47777778*		*0.477777778*			*0.3977273*
**ENSG00000198081**		*0.43939394*		*0.439393939*			*0.1647059*
**ENSG00000130948**					*0.208333333*	*0.244186*	
**ENSG00000064933**	*0.26136364*		*0.26136364*		*0.177777778*	*0.2159091*	
**ENSG00000114251**		*0.46363636*		*0.463636364*			*0.2965116*
**ENSG00000100647**		*0.1030303*		*0.103030303*			*0.1666667*
**ENSG00000121053**	*0.38023952*		*0.38023952*		*0.351190476*	*0.2906977*	
**ENSG00000196549**	*0.36227545*	*0.39393939*	*0.36227545*	*0.393939394*	*0.220238095*	*0.2209302*	*0.4880952*
**ENSG00000126562**	*0.24545455*		*0.24545455*				
**ENSG00000159692**	*0.46686747*	*0.43939394*	*0.46686747*	*0.439393939*	*0.226190476*	*0.4069767*	*0.4404762*
**ENSG00000215504**	*0.27409639*		*0.27409639*		*0.071428571*	*0.1453488*	
**ENSG00000107736**		*0.28484848*		*0.284848485*			*0.3777778*
**ENSG00000139725**		*0.16111111*		*0.161111111*			*0.3222222*
**ENSG00000204979**	*0.0988024*	*0.33783784*	*0.0988024*	*0.337837838*			*0.2179487*
**ENSG00000143878**		*0.47878788*		*0.478787879*			*0.1976744*
**ENSG00000141639**	*0.43373494*		*0.43373494*		*0.25*	*0.2151163*	
**ENSG00000198183**		*0.23636364*		*0.236363636*			*0.1352941*
**ENSG00000133636**							*0.2470588*
**ENSG00000148677**	*0.41463415*	*0.45454545*	*0.41463415*	*0.454545455*	*0.365853659*	*0.4*	*0.3197674*
**ENSG00000160190**		*0.45402299*		*0.454022989*	*0.136904762*	*0.1860465*	*0.25*
**ENSG00000181467**		*0.46036585*		*0.460365854*			*0.3023256*
**ENSG00000198162**	*0.06586826*	*0.44025157*	*0.06586826*	*0.440251572*	*0.463855422*	*0.3882353*	*0.4534884*
**ENSG00000134255**	*0.22754491*		*0.22754491*		*0.154761905*	*0.1976744*	
**ENSG00000076351**		*0.45757576*		*0.457575758*			*0.2888889*
**ENSG00000095564**		*0.36890244*		*0.368902439*			*0.1918605*
**ENSG00000185760**		*0.36309524*		*0.363095238*			*0.3430233*
**ENSG00000116017**		*0.26363636*		*0.263636364*			*0.4360465*
**ENSG00000176658**	*0.11515152*	*0.45882353*	*0.11515152*	*0.458823529*			*0.1470588*
**ENSG00000089177**	*0.10060976*	*0.47575758*	*0.10060976*	*0.475757576*	*0.30952381*	*0.3081395*	*0.4216867*
**ENSG00000130222**						*0.0921053*	
**ENSG00000122035**	*0.30838323*		*0.30838323*		*0.463855422*	*0.4058824*	
**ENSG00000168754**	*0.38323353*		*0.38323353*		*0.337349398*	*0.2588235*	
**ENSG00000105877**		*0.34662577*		*0.346625767*			*0.1162791*
**ENSG00000115705**	*0.41463415*	*0.42073171*	*0.41463415*	*0.420731707*	*0.337349398*	*0.372093*	*0.494186*
**ENSG00000100664**	*0.36526946*	*0.26060606*	*0.36526946*	*0.260606061*	*0.291666667*	*0.3953488*	*0.3176471*
**ENSG00000162814**		*0.33333333*		*0.333333333*			*0.1111111*
**ENSG00000171551**		*0.2*		*0.2*			*0.4404762*
**ENSG00000099917**							*0.3953488*
**ENSG00000122026**	*0.3*		*0.3*		*0.055555556*	*0.0639535*	
**ENSG00000181982**		*0.46363636*		*0.463636364*			*0.4767442*
**ENSG00000212899**		*0.38484848*		*0.384848485*			*0.4883721*
**ENSG00000135048**	*0.34131737*	*0.46666667*	*0.34131737*	*0.466666667*	*0.107142857*	*0.0813953*	*0.4058824*

**ENSG00000205414**	*0.39221557*		*0.39221557*		*0.05952381*			
**ENSG00000162971**		*0.44545455*		*0.445454545*				*0.3546512*
**ENSG00000159363**		*0.07222222*		*0.072222222*				
**ENSG00000053702**		*0.13333333*		*0.133333333*				*0.2823529*
**ENSG00000115257**		*0.43558282*		*0.435582822*				*0.3837209*
**ENSG00000168661**	*0.32335329*	*0.16969697*	*0.32335329*	*0.16969697*	*0.380952381*		*0.4069767*	*0.2965116*
**ENSG00000168619**	*0.29341317*		*0.29341317*					
**ENSG00000124193**		*0.37878788*		*0.378787879*				*0.2093023*
**ENSG00000125122**	*0.17777778*	*0.06363636*	*0.17777778*	*0.063636364*				
**ENSG00000166471**		*0.47575758*		*0.475757576*				*0.4529412*
**ENSG00000163050**	*0.46407186*	*0.24085366*	*0.46407186*	*0.240853659*	*0.18452381*		*0.2790698*	*0.3953488*
**ENSG00000144118**	*0.28888889*	*0.35555556*	*0.28888889*	*0.355555556*	*0.388888889*		*0.3409091*	*0.3295455*
**ENSG00000101346**	*0.29341317*		*0.29341317*					
**ENSG00000100526**								*0.0813953*
**ENSG00000145782**		*0.39393939*		*0.393939394*				*0.4642857*
**ENSG00000204279**					*0.31547619*		*0.3176471*	
**ENSG00000101958**	*0.08982036*	*0.39090909*	*0.08982036*	*0.390909091*				
**ENSG00000169607**		*0.5*		*0.5*				*0.2383721*
**ENSG00000100078**		*0.34545455*		*0.345454545*				*0.4534884*
**ENSG00000026652**		*0.41515152*		*0.415151515*				*0.2790698*
**ENSG00000134698**		*0.05757576*		*0.057575758*				
**ENSG00000159263**	*0.0748503*	*0.44545455*	*0.0748503*	*0.445454545*	*0.216049383*		*0.1976744*	*0.3430233*
**ENSG00000175390**	*0.45508982*	*0.46363636*	*0.45508982*	*0.463636364*	*0.451807229*		*0.377907*	*0.1453488*
**ENSG00000001036**	*0.06586826*	*0.32777778*	*0.06586826*	*0.327777778*				*0.3444444*
**ENSG00000164251**	*0.15568862*	*0.1*	*0.15568862*	*0.1*	*0.279761905*		*0.2619048*	*0.0555556*
**ENSG00000090273**	*0.23595506*	*0.07668712*	*0.23595506*	*0.076687117*				
**ENSG00000171873**		*0.46625767*		*0.466257669*				*0.25*
**ENSG00000160007**		*0.31818182*		*0.318181818*				*0.125*
**ENSG00000060656**		*0.08181818*		*0.081818182*				
**ENSG00000169398**	*0.41049383*	*0.46666667*	*0.41049383*	*0.466666667*	*0.345238095*		*0.3953488*	*0.3522727*
**ENSG00000120742**	*0.38636364*	*0.36363636*	*0.38636364*	*0.363636364*	*0.122222222*		*0.0872093*	*0.4302326*
**ENSG00000108176**					*0.422619048*		*0.3662791*	
**ENSG00000136830**		*0.32777778*		*0.327777778*	*0.255555556*		*0.2386364*	*0.1363636*
**ENSG00000026751**		*0.4*		*0.4*				*0.4244186*
**ENSG00000256591**		*0.22121212*		*0.221212121*				
**ENSG00000059804**		*0.13888889*		*0.138888889*				
**ENSG00000185658**	*0.40555556*	*0.42727273*	*0.40555556*	*0.427272727*	*0.5*		*0.3636364*	*0.4941176*
**ENSG00000057704**		*0.42424242*		*0.424242424*				*0.4593023*
**ENSG00000188483**	*0.12777778*	*0.23030303*	*0.12777778*	*0.23030303*				*0.0988372*
**ENSG00000161048**	*0.41616766*	*0.24444444*	*0.41616766*	*0.244444444*				*0.5*
**ENSG00000002919**	*0.18181818*	*0.18390805*	*0.18181818*	*0.183908046*	*0.222222222*		*0.3068182*	*0.1931818*
**ENSG00000156030**	*0.10179641*	*0.24695122*	*0.10179641*	*0.24695122*	*0.130952381*		*0.1453488*	*0.1294118*
**ENSG00000146457**	*0.41616766*		*0.41616766*		*0.43452381*		*0.4476744*	
**ENSG00000117155**		*0.33939394*		*0.339393939*				*0.2411765*
**ENSG00000164654**	*0.10179641*		*0.10179641*		*0.053571429*		*0.0581395*	
**ENSG00000188643**	*0.11976048*		*0.11976048*		*0.327380952*		*0.3488372*	
**ENSG00000162645**		*0.34545455*		*0.345454545*				*0.2209302*
**ENSG00000086200**					*0.055555556*		*0.0888889*	
**ENSG00000197409**	*0.05617978*		*0.05617978*					
**ENSG00000145734**	*0.48493976*	*0.48484848*	*0.48493976*	*0.484848485*	*0.464285714*		*0.5*	*0.5*
**ENSG00000181704**		*0.10736196*		*0.107361963*	*0.083333333*		*0.1046512*	*0.1046512*
**ENSG00000163013**		*0.5*		*0.5*				*0.3764706*
**ENSG00000115525**		*0.2347561*		*0.234756098*				*0.2325581*
**ENSG00000171115**								*0.122093*

**ENSG00000196678**		*0.06969697*		*0.06969697*			*0.1569767*
**ENSG00000174032**	*0.21856287*	*0.44444444*	*0.21856287*	*0.444444444*	*0.148809524*	*0.1395349*	*0.2386364*
**ENSG00000103021**		*0.41463415*		*0.414634146*			*0.4302326*
**ENSG00000175175**		*0.40229885*		*0.402298851*			*0.25*
**ENSG00000060688**		*0.16257669*		*0.162576687*			*0.1686747*
**ENSG00000163235**		*0.41823899*		*0.418238994*			*0.3444444*
**ENSG00000226137**		*0.31818182*		*0.318181818*			*0.1860465*
**ENSG00000174600**		*0.47272727*		*0.472727273*			*0.3546512*
**ENSG00000113141**		*0.14444444*		*0.144444444*			
**ENSG00000108448**	*0.31927711*		*0.31927711*		*0.452380952*	*0.4883721*	
**ENSG00000127325**	*0.27777778*	*0.39090909*	*0.27777778*	*0.390909091*	*0.266666667*	*0.1627907*	*0.4011628*
**ENSG00000118263**	*0.35*	*0.36363636*	*0.35*	*0.363636364*	*0.111111111*	*0.0581395*	*0.1777778*
**ENSG00000003096**	*0.43975904*		*0.43975904*				
**ENSG00000067208**		*0.34662577*		*0.346625767*			*0.0755814*
**ENSG00000140836**							*0.25*
**ENSG00000106624**		*0.4695122*		*0.469512195*			*0.2965116*
**ENSG00000178974**		*0.40449438*		*0.404494382*			*0.3777778*
**ENSG00000169474**		*0.46969697*		*0.46969697*			*0.372093*
**ENSG00000072501**					*0.422619048*	*0.4117647*	
**ENSG00000118762**		*0.3969697*		*0.396969697*			*0.4186047*
**ENSG00000167526**		*0.11212121*		*0.112121212*			*0.0823529*
**ENSG00000107443**		*0.3125*		*0.3125*			*0.4390244*
**ENSG00000094916**	*0.17065868*	*0.43258427*	*0.17065868*	*0.43258427*	*0.470238095*	*0.4418605*	*0.4069767*
**ENSG00000088280**	*0.13772455*	*0.13109756*	*0.13772455*	*0.131097561*			*0.0697674*
**ENSG00000116898**	*0.09580838*	*0.26363636*	*0.09580838*	*0.263636364*	*0.31547619*	*0.244186*	*0.3139535*
**ENSG00000167159**	*0.09580838*		*0.09580838*				
**ENSG00000156017**		*0.36666667*		*0.366666667*			*0.1511628*
**ENSG00000196358**		*0.41666667*		*0.416666667*			*0.4659091*
**ENSG00000177302**	*0.30113636*	*0.2*	*0.30113636*	*0.2*	*0.090909091*		*0.2386364*
**ENSG00000155980**		*0.38181818*		*0.381818182*			*0.4204545*
**ENSG00000105976**		*0.45757576*		*0.457575758*			*0.4418605*
**ENSG00000126838**		*0.3969697*		*0.396969697*			*0.4883721*
**ENSG00000065135**	*0.14371257*	*0.47878788*	*0.14371257*	*0.478787879*	*0.289156627*	*0.3647059*	*0.3666667*
**ENSG00000082213**		*0.39090909*		*0.390909091*	*0.211111111*	*0.2727273*	*0.2848837*
**ENSG00000185869**		*0.41666667*		*0.416666667*			
**ENSG00000254521**							*0.1071429*
**ENSG00000105609**		*0.3006135*		*0.300613497*			*0.127907*
**ENSG00000145425**		*0.07878788*		*0.078787879*	*0.220238095*	*0.1511628*	*0.1860465*
**ENSG00000113732**		*0.13939394*		*0.139393939*			*0.0639535*
**ENSG00000164211**	*0.16666667*	*0.24719101*	*0.16666667*	*0.247191011*	*0.139534884*	*0.1*	*0.2840909*
**ENSG00000165025**		*0.38181818*		*0.381818182*			*0.4709302*
**ENSG00000131389**	*0.07471264*	*0.43292683*	*0.07471264*	*0.432926829*	*0.411111111*	*0.4666667*	*0.494186*
**ENSG00000221995**	*0.11377246*		*0.11377246*		*0.095238095*	*0.127907*	
**ENSG00000170871**		*0.39444444*		*0.394444444*			*0.4418605*
**ENSG00000124120**		*0.48773006*		*0.487730061*			*0.0581395*
**ENSG00000246705**		*0.43888889*		*0.438888889*			*0.2840909*
**ENSG00000120457**	*0.09281437*	*0.27011494*	*0.09281437*	*0.270114943*	*0.410714286*	*0.4651163*	*0.2714286*
**ENSG00000220575**		*0.33939394*		*0.339393939*			*0.4360465*
**ENSG00000144381**		*0.30555556*		*0.305555556*			*0.4186047*
**ENSG00000165533**		*0.24431818*		*0.244318182*			*0.2444444*
**ENSG00000173276**	*0.29640719*	*0.31818182*	*0.29640719*	*0.318181818*		*0.0755814*	*0.3662791*
**ENSG00000104679**	*0.35628743*	*0.4*	*0.35628743*	*0.4*	*0.136904762*	*0.2616279*	*0.2588235*
**ENSG00000179873**	*0.48493976*		*0.48493976*		*0.5*	*0.4883721*	
**ENSG00000178928**		*0.46590909*		*0.465909091*			*0.2790698*

**ENSG00000134987**		0.30792683		0.307926829				0.1333333
**ENSG00000088325**	0.06287425		0.06287425					
**ENSG00000254556**	0.3742515	0.35670732	0.3742515	0.356707317				
**ENSG00000054654**	0.45783133	0.4	0.45783133	0.4	0.327380952		0.372093	0.4011628
**ENSG00000141564**	0.41916168	0.44242424	0.41916168	0.442424242	0.30952381		0.2848837	0.4825581
**ENSG00000173267**	0.4845679	0.08235294	0.4845679	0.082352941	0.345238095		0.3353659	
**ENSG00000115718**	0.35057471		0.35057471		0.2		0.1590909	
**ENSG00000137563**	0.14444444	0.10909091	0.14444444	0.109090909	0.222222222		0.3604651	0.0988372
**ENSG00000180287**	0.38484848	0.34242424	0.38484848	0.342424242	0.13253012		0.125	
**ENSG00000205795**		0.2030303		0.203030303				0.2012195
**ENSG00000125454**	0.30838323	0.32777778	0.30838323	0.327777778				0.0681818
**ENSG00000120160**		0.45555556		0.455555556				0.4318182
**ENSG00000143198**	0.14071856	0.42727273	0.14071856	0.427272727	0.220238095		0.1802326	0.1453488
**ENSG00000109944**	0.07228916	0.43030303	0.07228916	0.43030303	0.076923077		0.1282051	0.2965116
**ENSG00000162989**		0.37654321		0.37654321				
**ENSG00000213023**	0.15		0.15		0.089285714		0.1046512	
**ENSG00000206532**	0.08682635		0.08682635					
**ENSG00000141447**		0.44817073		0.448170732				0.3863636
**ENSG00000035115**	0.15662651	0.3875	0.15662651	0.3875	0.446428571		0.4476744	0.4011628
**ENSG00000204472**					0.470238095		0.4476744	
**ENSG00000197713**		0.35276074		0.352760736				0.2378049
**ENSG00000160233**		0.22121212		0.221212121				0.2732558
**ENSG00000143842**		0.10606061		0.106060606	0.166666667		0.3809524	0.3372093
**ENSG00000119927**		0.43506494		0.435064935				0.4404762
**ENSG00000118193**	0.09659091	0.47878788	0.09659091	0.478787879	0.222222222		0.2727273	0.4047619
**ENSG00000157895**	0.47058824	0.38181818	0.47058824	0.381818182	0.122222222		0.0795455	0.3546512
**ENSG00000183763**		0.46629213		0.466292135				0.1744186
**ENSG00000164889**	0.3742515		0.3742515		0.297619048		0.2848837	
**ENSG00000170498**	0.17168675		0.17168675					
**ENSG00000219626**		0.37272727		0.372727273				0.1744186
**ENSG00000175324**	0.15555556		0.15555556		0.333333333		0.3409091	
**ENSG00000100154**		0.1		0.1				0.3977273
**ENSG00000173273**		0.22699387		0.226993865				0.3888889
**ENSG00000255953**	0.28333333	0.21428571	0.28333333	0.214285714	0.155555556		0.1363636	0.1527778
**ENSG00000133195**		0.48466258		0.484662577				0.3313953
**ENSG00000103512**		0.12269939		0.122699387				0.1627907
**ENSG00000172613**		0.07575758		0.075757576				0.2034884
**ENSG00000139910**	0.31137725	0.2375	0.31137725	0.2375				0.3333333
**ENSG00000113645**		0.15454545		0.154545455				0.2559524
**ENSG00000073464**		0.35670732		0.356707317				0.4360465
**ENSG00000196562**	0.25316456	0.13109756	0.25316456	0.131097561	0.290697674		0.2625	
**ENSG00000205560**	0.42222222	0.12424242	0.42222222	0.124242424	0.404761905		0.3488372	0.4204545
**ENSG00000165916**	0.42045455		0.42045455		0.409090909		0.3068182	
**ENSG00000188582**		0.1969697		0.196969697				0.2209302
**ENSG00000143515**		0.16666667		0.166666667				0.1744186
**ENSG00000070404**		0.21515152		0.215151515				0.4294118
**ENSG00000179021**	0.44610778	0.16363636	0.44610778	0.163636364	0.172619048		0.0714286	0.0813953
**ENSG00000099974**		0.2969697		0.296969697				0.3604651
**ENSG00000113966**		0.10555556		0.105555556				
**ENSG00000006125**		0.25454545		0.254545455				0.4404762
**ENSG00000108312**		0.25304878		0.25304878				0.4069767
**ENSG00000109062**		0.43939394		0.439393939				0.2209302
**ENSG00000118855**		0.35151515		0.351515152				0.3546512
**ENSG00000128266**	0.15068493	0.36969697	0.15068493	0.36969697				0.3392857

**ENSG00000125445**	*0.22839506*	*0.07317073*	*0.22839506*	*0.073170732*				
**ENSG00000120156**		*0.13636364*		*0.136363636*				*0.1511628*
**ENSG00000164161**		*0.49390244*		*0.493902439*				*0.3636364*
**ENSG00000254585**		*0.48787879*		*0.487878788*				*0.1627907*
**ENSG00000152443**		*0.43258427*		*0.43258427*				*0.4418605*
**ENSG00000107099**	*0.48802395*	*0.43939394*	*0.48802395*	*0.439393939*	*0.375*		*0.3546512*	*0.3546512*
**ENSG00000104154**		*0.29393939*		*0.293939394*				*0.1818182*
**ENSG00000015592**		*0.34848485*		*0.348484848*	*0.255952381*		*0.3023256*	*0.4418605*
**ENSG00000185621**		*0.19090909*		*0.190909091*				*0.1162791*
**ENSG00000103316**	*0.06666667*		*0.06666667*		*0.115384615*		*0.0657895*	
**ENSG00000075711**		*0.43209877*		*0.432098765*	*0.102272727*			*0.3780488*
**ENSG00000204952**	*0.12777778*		*0.12777778*				*0.0681818*	
**ENSG00000116747**	*0.07361963*	*0.4*	*0.07361963*	*0.4*				*0.4593023*
**ENSG00000144224**		*0.20496894*		*0.204968944*				*0.4940476*
**ENSG00000166046**	*0.13855422*	*0.46111111*	*0.13855422*	*0.461111111*				*0.2159091*
**ENSG00000108264**	*0.21556886*		*0.21556886*					
**ENSG00000178202**		*0.33939394*		*0.339393939*				*0.494186*
**ENSG00000134243**		*0.46666667*		*0.466666667*				*0.3823529*
**ENSG00000232629**	*0.40361446*	*0.48214286*	*0.40361446*	*0.482142857*	*0.273809524*		*0.3895349*	*0.3294118*
**ENSG00000134825**		*0.11111111*		*0.111111111*	*0.321428571*		*0.3023256*	
**ENSG00000163629**	*0.33888889*	*0.13636364*	*0.33888889*	*0.136363636*	*0.3*		*0.375*	
**ENSG00000088881**		*0.33939394*		*0.339393939*				*0.3313953*
**ENSG00000067064**	*0.19444444*	*0.18670886*	*0.19444444*	*0.186708861*				*0.2256098*
**ENSG00000205683**		*0.49695122*		*0.49695122*				*0.2727273*
**ENSG00000225950**		*0.48314607*		*0.483146067*				*0.3181818*
**ENSG00000254774**	*0.2*		*0.2*					
**ENSG00000025772**								*0.1686047*
**ENSG00000128567**		*0.38888889*		*0.388888889*				*0.4090909*
**ENSG00000168056**	*0.20481928*		*0.20481928*		*0.36746988*		*0.3546512*	
**ENSG00000167612**		*0.27134146*		*0.271341463*				*0.1842105*
**ENSG00000133641**		*0.42777778*		*0.427777778*				*0.4886364*
**ENSG00000105793**	*0.37724551*	*0.25151515*	*0.37724551*	*0.251515152*	*0.208333333*		*0.2325581*	*0.2386364*
**ENSG00000197177**	*0.09281437*		*0.09281437*				*0.1*	
**ENSG00000154025**	*0.13473054*	*0.2*	*0.13473054*	*0.2*	*0.357142857*		*0.4651163*	
**ENSG00000196329**	*0.30538922*	*0.32758621*	*0.30538922*	*0.327586207*				*0.3430233*
**ENSG00000115355**	*0.26704545*	*0.43333333*	*0.26704545*	*0.433333333*	*0.2*		*0.1904762*	*0.4176471*
**ENSG00000163219**	*0.21686747*	*0.46296296*	*0.21686747*	*0.462962963*	*0.44047619*		*0.3837209*	*0.4883721*
**ENSG00000089682**		*0.33939394*		*0.339393939*				*0.4888889*
**ENSG00000092621**	*0.47005988*		*0.47005988*		*0.416666667*		*0.4186047*	
**ENSG00000161896**	*0.13173653*	*0.47272727*	*0.13173653*	*0.472727273*	*0.136904762*		*0.1744186*	*0.2*
**ENSG00000117399**	*0.14071856*		*0.14071856*					
**ENSG00000174132**		*0.49393939*		*0.493939394*				*0.127907*
**ENSG00000087589**	*0.1*	*0.30909091*	*0.1*	*0.309090909*				*0.0872093*
**ENSG00000172794**	*0.30555556*	*0.3969697*	*0.30555556*	*0.396969697*	*0.148809524*		*0.1686047*	*0.1395349*
**ENSG00000117305**		*0.08787879*		*0.087878788*				*0.1395349*
**ENSG00000139174**	*0.1*	*0.42777778*	*0.1*	*0.427777778*	*0.211111111*		*0.1136364*	*0.4111111*
**ENSG00000171509**	*0.37724551*		*0.37724551*		*0.083333333*		*0.0872093*	
**ENSG00000112290**	*0.40432099*		*0.40432099*		*0.213414634*			*0.2848837*
**ENSG00000137210**		*0.3969697*		*0.396969697*	*0.052631579*			*0.4302326*
**ENSG00000211898**								*0.3941176*
**ENSG00000134202**	*0.13173653*	*0.3988764*	*0.13173653*	*0.398876404*	*0.142857143*		*0.1744186*	*0.2790698*
**ENSG00000164379**	*0.19461078*		*0.19461078*		*0.30952381*		*0.3081395*	
**ENSG00000100985**	*0.10465116*		*0.10465116*		*0.073170732*		*0.1666667*	
**ENSG00000149575**		*0.49390244*		*0.493902439*				*0.255814*

**ENSG00000239305**	*0.2005988*		*0.2005988*				*0.0639535*	
**ENSG00000179331**		*0.43333333*		*0.433333333*				*0.2965116*
**ENSG00000009765**		*0.43939394*		*0.439393939*				*0.4777778*
**ENSG00000181163**		*0.36585366*		*0.365853659*				*0.4534884*
**ENSG00000122140**	*0.37724551*	*0.45705521*	*0.37724551*	*0.457055215*	*0.172619048*		*0.1569767*	*0.127907*
**ENSG00000119698**	*0.4251497*	*0.15730337*	*0.4251497*	*0.157303371*	*0.404761905*		*0.3488372*	*0.375*
**ENSG00000197937**	*0.37777778*	*0.23333333*	*0.37777778*	*0.233333333*	*0.188888889*		*0.1477273*	
**ENSG00000048649**	*0.39820359*	*0.13888889*	*0.39820359*	*0.138888889*	*0.446428571*		*0.494186*	*0.1802326*
**ENSG00000141469**	*0.26047904*	*0.49393939*	*0.26047904*	*0.493939394*	*0.463414634*		*0.4302326*	*0.4772727*
**ENSG00000078018**	*0.05988024*	*0.07575758*	*0.05988024*	*0.075757576*				*0.2325581*
**ENSG00000257545**		*0.47878788*		*0.478787879*				*0.2383721*
**ENSG00000064313**	*0.4760479*		*0.4760479*					
**ENSG00000089250**		*0.33841463*		*0.338414634*				*0.452381*
**ENSG00000089723**		*0.38787879*		*0.387878788*	*0.1*		*0.0795455*	*0.5*
**ENSG00000164073**								*0.3255814*
**ENSG00000116882**	*0.13772455*		*0.13772455*					
**ENSG00000161956**		*0.36060606*		*0.360606061*				*0.2790698*
**ENSG00000178761**		*0.4*		*0.4*				*0.3313953*
**ENSG00000145348**	*0.11976048*	*0.33636364*	*0.11976048*	*0.336363636*	*0.13253012*		*0.2764706*	*0.2732558*
**ENSG00000156970**	*0.20454545*	*0.38181818*	*0.20454545*	*0.381818182*	*0.397435897*		*0.4102564*	*0.1918605*
**ENSG00000198589**		*0.4*		*0.4*				*0.4709302*
**ENSG00000144644**		*0.39444444*		*0.394444444*				*0.4761905*
**ENSG00000106028**	*0.08231707*		*0.08231707*				*0.0882353*	
**ENSG00000143786**	*0.06886228*	*0.29393939*	*0.06886228*	*0.293939394*	*0.148809524*		*0.1337209*	*0.0930233*
**ENSG00000022277**		*0.43939394*		*0.439393939*				*0.3095238*
**ENSG00000138821**		*0.48484848*		*0.484848485*				*0.5*
**ENSG00000113269**		*0.37575758*		*0.375757576*				*0.2034884*
**ENSG00000165832**	*0.48295455*	*0.40243902*	*0.48295455*	*0.402439024*				
**ENSG00000036448**	*0.41573034*	*0.28658537*	*0.41573034*	*0.286585366*	*0.244444444*		*0.1395349*	*0.2117647*
**ENSG00000099937**	*0.39457831*	*0.08231707*	*0.39457831*	*0.082317073*	*0.43452381*		*0.4418605*	
**ENSG00000178295**		*0.47272727*		*0.472727273*				*0.1046512*
**ENSG00000055044**		*0.14044944*		*0.140449438*				*0.0777778*
**ENSG00000184619**	*0.25149701*		*0.25149701*		*0.357142857*		*0.3662791*	
**ENSG00000163536**	*0.38888889*		*0.38888889*					
**ENSG00000125249**		*0.41212121*		*0.412121212*				*0.4302326*
**ENSG00000028116**		*0.3445122*		*0.344512195*	*0.397727273*		*0.3*	*0.4011628*
**ENSG00000214357**		*0.46363636*		*0.463636364*				*0.4651163*
**ENSG00000172943**		*0.13030303*		*0.13030303*				
**ENSG00000257028**		*0.26666667*		*0.266666667*				*0.0813953*
**ENSG00000166105**		*0.42121212*		*0.421212121*				*0.3255814*
**ENSG00000114861**	*0.15269461*		*0.15269461*		*0.279761905*		*0.1117647*	*0.1235294*
**ENSG00000197272**		*0.44242424*		*0.442424242*				*0.2965116*
**ENSG00000115459**	*0.11676647*	*0.07055215*	*0.11676647*	*0.070552147*	*0.05952381*		*0.0755814*	
**ENSG00000073050**	*0.0508982*		*0.0508982*					
**ENSG00000064666**	*0.12951807*	*0.10909091*	*0.12951807*	*0.109090909*	*0.333333333*		*0.4882353*	*0.1802326*
**ENSG00000138594**	*0.38888889*	*0.45512821*	*0.38888889*	*0.455128205*	*0.388888889*		*0.4545455*	*0.4772727*
**ENSG00000174485**	*0.14367816*		*0.14367816*		*0.261363636*		*0.3414634*	
**ENSG00000213853**		*0.45757576*		*0.457575758*				*0.4476744*
**ENSG00000185324**	*0.39520958*	*0.10606061*	*0.39520958*	*0.106060606*	*0.172619048*		*0.244186*	*0.2093023*
**ENSG00000095380**		*0.22121212*		*0.221212121*	*0.357142857*		*0.3081395*	*0.0697674*
**ENSG00000129173**		*0.16158537*		*0.161585366*				*0.3588235*
**ENSG00000174963**	*0.09281437*	*0.36363636*	*0.09281437*	*0.363636364*			*0.122093*	*0.4186047*
**ENSG00000139737**	*0.08682635*	*0.2037037*	*0.08682635*	*0.203703704*	*0.119047619*			
**ENSG00000128245**		*0.25*		*0.25*				*0.0681818*

**ENSG00000151575**		*0.47530864*		*0.475308642*			*0.2108434*
**ENSG00000119684**	*0.40229885*	*0.41818182*	*0.40229885*	*0.418181818*	*0.125*	*0.1395349*	*0.1860465*
**ENSG00000170367**		*0.3974359*		*0.397435897*			*0.4268293*
**ENSG00000170903**		*0.39329268*		*0.393292683*	*0.318181818*	*0.3068182*	*0.4127907*
**ENSG00000159182**	*0.36227545*		*0.36227545*		*0.403614458*	*0.3255814*	
**ENSG00000188508**		*0.26060606*		*0.260606061*			
**ENSG00000056558**	*0.41616766*	*0.49090909*	*0.41616766*	*0.490909091*	*0.488095238*	*0.4186047*	*0.4545455*
**ENSG00000130487**		*0.48876404*		*0.488764045*			*0.3977273*
**ENSG00000131148**		*0.17272727*		*0.172727273*			*0.2906977*
**ENSG00000102302**		*0.05487805*		*0.054878049*			*0.4418605*
**ENSG00000119514**							*0.25*
**ENSG00000159208**	*0.25*	*0.30555556*	*0.25*	*0.305555556*	*0.208333333*	*0.2848837*	*0.4*
**ENSG00000204003**	*0.259375*	*0.14545455*	*0.259375*	*0.145454545*	*0.292682927*	*0.186747*	*0.2093023*
**ENSG00000070915**		*0.48333333*		*0.483333333*			*0.4529412*
**ENSG00000167930**	*0.11931818*		*0.11931818*		*0.444444444*	*0.375*	
**ENSG00000109163**		*0.42682927*		*0.426829268*			*0.2678571*
**ENSG00000174473**	*0.32335329*	*0.26969697*	*0.32335329*	*0.26969697*	*0.154761905*	*0.1104651*	*0.0523256*
**ENSG00000122420**		*0.2*		*0.2*			*0.1529412*
**ENSG00000120008**	*0.22289157*	*0.34848485*	*0.22289157*	*0.348484848*	*0.077777778*	*0.0568182*	*0.3470588*
**ENSG00000177990**	*0.12359551*	*0.18404908*	*0.12359551*	*0.18404908*	*0.1*		*0.3409091*
**ENSG00000114115**		*0.25294118*		*0.252941176*			*0.0697674*
**ENSG00000177570**		*0.35955056*		*0.359550562*			*0.4651163*
**ENSG00000144559**	*0.47126437*	*0.44242424*	*0.47126437*	*0.442424242*	*0.266666667*	*0.2380952*	*0.25*
**ENSG00000128815**		*0.44848485*		*0.448484848*			*0.3604651*
**ENSG00000078596**		*0.33333333*		*0.333333333*			*0.4593023*
**ENSG00000120049**		*0.27777778*		*0.277777778*			*0.4244186*
**ENSG00000253910**		*0.20224719*		*0.202247191*			*0.1477273*
**ENSG00000137766**		*0.11818182*		*0.118181818*			*0.1162791*
**ENSG00000119720**		*0.44242424*		*0.442424242*			*0.4764706*
**ENSG00000151292**		*0.42222222*		*0.422222222*			*0.3181818*
**ENSG00000013588**		*0.5*		*0.5*			*0.2790698*
**ENSG00000188992**		*0.5*		*0.5*			*0.4825581*
**ENSG00000176422**		*0.14723926*		*0.147239264*			*0.0988372*
**ENSG00000129990**	*0.15882353*		*0.15882353*		*0.277777778*	*0.2906977*	
**ENSG00000244754**		*0.33707865*		*0.337078652*			*0.1704545*
**ENSG00000105738**		*0.45121951*		*0.451219512*			*0.3081395*
**ENSG00000183631**		*0.20786517*		*0.207865169*			*0.1477273*
**ENSG00000197536**		*0.47575758*		*0.475757576*			*0.3953488*
**ENSG00000174547**		*0.27469136*		*0.274691358*			*0.3081395*
**ENSG00000170927**		*0.45454545*		*0.454545455*			*0.1566265*
**ENSG00000197170**		*0.40797546*		*0.40797546*			*0.3977273*
**ENSG00000123338**		*0.25280899*		*0.252808989*			*0.3255814*
**ENSG00000125843**	*0.10179641*		*0.10179641*				
**ENSG00000174808**		*0.29268293*		*0.292682927*			*0.4825581*
**ENSG00000149311**		*0.47865854*		*0.478658537*			*0.4244186*
**ENSG00000054219**		*0.43030303*		*0.43030303*			*0.3837209*
**ENSG00000152409**		*0.41212121*		*0.412121212*			*0.4825581*
**ENSG00000126453**	*0.30239521*	*0.06969697*	*0.30239521*	*0.06969697*	*0.273809524*	*0.2151163*	
**ENSG00000158477**	*0.41317365*		*0.41317365*		*0.380952381*	*0.3255814*	
**ENSG00000137726**	*0.47305389*	*0.40909091*	*0.47305389*	*0.409090909*	*0.476190476*	*0.4651163*	*0.2616279*
**ENSG00000149639**		*0.32208589*		*0.32208589*			*0.2882353*
**ENSG00000152457**	*0.2994012*	*0.24242424*	*0.2994012*	*0.242424242*	*0.28313253*	*0.3139535*	*0.4761905*
**ENSG00000124145**	*0.11976048*	*0.23636364*	*0.11976048*	*0.236363636*	*0.44047619*	*0.4534884*	*0.4476744*
**ENSG00000149557**	*0.32335329*		*0.32335329*		*0.083333333*	*0.122093*	

**ENSG00000112210**		*0.14848485*		*0.148484848*			
**ENSG00000214595**		*0.4137931*		*0.413793103*			*0.4318182*
**ENSG00000142149**		*0.36890244*		*0.368902439*			*0.372093*
**ENSG00000122224**		*0.49444444*		*0.494444444*			*0.3222222*
**ENSG00000157017**	*0.28742515*		*0.28742515*		*0.344444444*	*0.3444444*	
**ENSG00000028528**		*0.2*		*0.2*			*0.1918605*
**ENSG00000142552**	*0.05688623*		*0.05688623*				
**ENSG00000077549**	*0.42215569*	*0.14705882*	*0.42215569*	*0.147058824*	*0.297619048*	*0.3895349*	*0.3375*
**ENSG00000125384**		*0.13125*		*0.13125*			*0.3837209*
**ENSG00000250423**							*0.4294118*
**ENSG00000241644**		*0.10625*		*0.10625*			*0.2048193*
**ENSG00000163818**		*0.36363636*		*0.363636364*			*0.4651163*
**ENSG00000113594**		*0.39393939*		*0.393939394*			*0.4090909*
**ENSG00000189233**		*0.46629213*		*0.466292135*			*0.1904762*
**ENSG00000144554**		*0.40606061*		*0.406060606*			*0.1744186*
**ENSG00000102543**		*0.42777778*		*0.427777778*			*0.4886364*
**ENSG00000180660**		*0.33939394*		*0.339393939*			*0.4360465*
**ENSG00000108342**	*0.38068182*	*0.37575758*	*0.38068182*	*0.375757576*	*0.388888889*	*0.4418605*	*0.3953488*
**ENSG00000124104**		*0.05172414*		*0.051724138*			
**ENSG00000104043**	*0.38636364*	*0.47777778*	*0.38636364*	*0.477777778*	*0.433333333*	*0.4772727*	*0.4345238*
**ENSG00000169894**		*0.27777778*		*0.277777778*			
**ENSG00000221923**		*0.37272727*		*0.372727273*			*0.3255814*
**ENSG00000140043**	*0.43333333*	*0.39329268*	*0.43333333*	*0.393292683*	*0.422619048*	*0.4204545*	*0.4476744*
**ENSG00000182534**		*0.10060976*		*0.100609756*			*0.4705882*
**ENSG00000135362**	*0.09337349*	*0.41212121*	*0.09337349*	*0.412121212*			*0.2093023*
**ENSG00000171368**		*0.31212121*		*0.312121212*			*0.4069767*
**ENSG00000168961**	*0.13173653*	*0.21910112*	*0.13173653*	*0.219101124*	*0.345238095*	*0.3058824*	*0.25*
**ENSG00000100253**		*0.33908046*		*0.33908046*			*0.4431818*
**ENSG00000257727**		*0.05757576*		*0.057575758*	*0.19047619*	*0.2093023*	*0.3176471*
**ENSG00000164112**	*0.11842105*	*0.17575758*	*0.11842105*	*0.175757576*	*0.5*	*0.4127907*	*0.0930233*
**ENSG00000137513**	*0.47878788*	*0.13333333*	*0.47878788*	*0.133333333*	*0.458333333*	*0.4069767*	*0.4204545*
**ENSG00000186197**	*0.44736842*	*0.39634146*	*0.44736842*	*0.396341463*	*0.4125*	*0.4102564*	*0.25*
**ENSG00000099958**		*0.13636364*		*0.136363636*			*0.3837209*
**ENSG00000134940**		*0.3969697*		*0.396969697*			*0.2325581*
**ENSG00000213888**		*0.14545455*		*0.145454545*			*0.4767442*
**ENSG00000133107**	*0.45508982*	*0.19444444*	*0.45508982*	*0.194444444*	*0.321428571*	*0.3197674*	*0.3604651*
**ENSG00000163171**		*0.27222222*		*0.272222222*		*0.0523256*	*0.4666667*
**ENSG00000177565**		*0.39444444*		*0.394444444*			*0.5*
**ENSG00000213638**	*0.46407186*		*0.46407186*		*0.363095238*	*0.494186*	
**ENSG00000196655**	*0.39520958*	*0.2347561*	*0.39520958*	*0.234756098*		*0.0988372*	*0.1046512*
**ENSG00000214725**	*0.49700599*		*0.49700599*		*0.109756098*	*0.0952381*	
**ENSG00000187726**		*0.2969697*		*0.296969697*			*0.3023256*
**ENSG00000149541**	*0.23952096*		*0.23952096*				
**ENSG00000204084**		*0.25757576*		*0.257575758*			*0.377907*
**ENSG00000174989**		*0.32727273*		*0.327272727*			*0.3372093*
**ENSG00000050555**		*0.45977011*		*0.459770115*			*0.4825581*
**ENSG00000156875**		*0.35795455*		*0.357954545*			*0.3777778*
**ENSG00000259491**	*0.27108434*	*0.2607362*	*0.27108434*	*0.260736196*	*0.220238095*	*0.1860465*	*0.1686047*
**ENSG00000132321**		*0.16969697*		*0.16969697*			
**ENSG00000186510**	*0.2245509*		*0.2245509*				
**ENSG00000172766**		*0.30681818*		*0.306818182*			*0.2954545*
**ENSG00000101986**	*0.07272727*		*0.07272727*				
**ENSG00000152492**		*0.46341463*		*0.463414634*			*0.4886364*
**ENSG00000184640**	*0.35542169*	*0.5*	*0.35542169*	*0.5*	*0.43452381*	*0.4235294*	*0.1802326*

**ENSG00000169189**		*0.39090909*		*0.390909091*			*0.4651163*
**ENSG00000174165**	*0.19161677*	*0.27878788*	*0.19161677*	*0.278787879*	*0.119047619*	*0.1511628*	*0.3214286*
**ENSG00000255112**		*0.20909091*		*0.209090909*	*0.375*	*0.2333333*	
**ENSG00000146833**		*0.49444444*		*0.494444444*			*0.2954545*
**ENSG00000127249**		*0.43636364*		*0.436363636*			*0.4941176*
**ENSG00000006327**		*0.26666667*		*0.266666667*			*0.1136364*
**ENSG00000213626**		*0.25842697*		*0.258426966*			*0.2325581*
**ENSG00000039123**	*0.40718563*	*0.05757576*	*0.40718563*	*0.057575758*	*0.5*	*0.4244186*	*0.244186*
**ENSG00000204128**		*0.15757576*		*0.157575758*			*0.2034884*
**ENSG00000125630**		*0.47222222*		*0.472222222*			*0.3636364*
**ENSG00000130826**		*0.10122699*		*0.101226994*			
**ENSG00000237765**	*0.0505618*		*0.0505618*				
**ENSG00000164975**		*0.16257669*		*0.162576687*			*0.2738095*
**ENSG00000101220**					*0.113095238*	*0.1104651*	
**ENSG00000188959**	*0.41111111*	*0.28963415*	*0.41111111*	*0.289634146*	*0.172619048*	*0.1686047*	*0.1764706*
**ENSG00000178772**		*0.37272727*		*0.372727273*			*0.3529412*
**ENSG00000164062**		*0.25454545*		*0.254545455*			*0.0813953*
**ENSG00000228146**	*0.34131737*		*0.34131737*		*0.160714286*	*0.1744186*	
**ENSG00000163006**		*0.22413793*		*0.224137931*			*0.1627907*
**ENSG00000007376**		*0.22727273*		*0.227272727*			*0.3*
**ENSG00000166123**		*0.08484848*		*0.084848485*			
**ENSG00000136897**		*0.33636364*		*0.336363636*			*0.4772727*
**ENSG00000165264**	*0.0873494*	*0.47191011*	*0.0873494*	*0.471910112*			*0.0909091*
**ENSG00000160877**		*0.32515337*		*0.325153374*			*0.1022727*
**ENSG00000008196**	*0.3502994*	*0.2797619*	*0.3502994*	*0.279761905*	*0.196428571*	*0.2209302*	*0.2325581*
**ENSG00000086619**	*0.15555556*	*0.46969697*	*0.15555556*	*0.46969697*			*0.313253*
**ENSG00000047056**		*0.41573034*		*0.415730337*			*0.4418605*
**ENSG00000141337**					*0.285714286*	*0.3953488*	
**ENSG00000050730**	*0.09281437*	*0.3*	*0.09281437*	*0.3*			
**ENSG00000088387**	*0.24850299*	*0.41954023*	*0.24850299*	*0.41954023*	*0.297619048*	*0.3197674*	*0.4204545*
**ENSG00000196684**		*0.11212121*		*0.112121212*			
**ENSG00000213918**	*0.13190184*		*0.13190184*				
**ENSG00000106635**	*0.36809816*		*0.36809816*				*0.1802326*
**ENSG00000183103**	*0.12275449*		*0.12275449*				
**ENSG00000188971**		*0.45757576*		*0.457575758*			*0.1802326*
**ENSG00000105997**	*0.40909091*		*0.40909091*		*0.133333333*	*0.1590909*	
**ENSG00000003509**	*0.28443114*	*0.26363636*	*0.28443114*	*0.263636364*			*0.4767442*
**ENSG00000198039**		*0.47727273*		*0.477272727*			*0.4886364*
**ENSG00000115271**	*0.42215569*		*0.42215569*		*0.107142857*	*0.1666667*	
**ENSG00000181544**	*0.17777778*		*0.17777778*		*0.211111111*	*0.1704545*	
**ENSG00000090905**		*0.16666667*		*0.166666667*			*0.0639535*
**ENSG00000241690**							*0.1569767*
**ENSG00000160410**					*0.273809524*	*0.1918605*	
**ENSG00000112159**		*0.31111111*		*0.311111111*			
**ENSG00000167306**		*0.49444444*		*0.494444444*			*0.4886364*
**ENSG00000105352**							*0.4545455*
**ENSG00000064225**	*0.35393258*	*0.33333333*	*0.35393258*	*0.333333333*	*0.222222222*	*0.1777778*	*0.2111111*
**ENSG00000124701**		*0.11111111*		*0.111111111*			
**ENSG00000129195**		*0.33333333*		*0.333333333*			*0.2797619*
**ENSG00000166619**	*0.12650602*		*0.12650602*				
**ENSG00000113387**	*0.29775281*	*0.39325843*	*0.29775281*	*0.393258427*	*0.344444444*	*0.375*	*0.4*
**ENSG00000120699**	*0.4011976*		*0.4011976*		*0.279761905*	*0.25*	
**ENSG00000132692**		*0.46363636*		*0.463636364*			*0.4941176*
**ENSG00000155957**	*0.23652695*	*0.37575758*	*0.23652695*	*0.375757576*	*0.4*	*0.4545455*	*0.4659091*

**ENSG00000131398**		0.36060606		0.360606061			0.3705882
**ENSG00000107290**		0.17682927		0.176829268			0.4939759
**ENSG00000130227**		0.48255814		0.48255814			
**ENSG00000062038**	0.2962963	0.10606061	0.2962963	0.106060606			0.2325581
**ENSG00000182983**		0.24375		0.24375			0.0795455
**ENSG00000151917**		0.20245399		0.202453988			0.0952381
**ENSG00000132639**		0.37272727		0.372727273			0.3470588
**ENSG00000063587**	0.41616766	0.4695122	0.41616766	0.469512195	0.451807229	0.4882353	0.3977273
**ENSG00000168273**		0.40243902		0.402439024			0.494186
**ENSG00000188825**	0.32335329	0.08522727	0.32335329	0.085227273	0.345238095	0.3546512	0.2159091
**ENSG00000133138**	0.28143713		0.28143713				
**ENSG00000186526**	0.46590909	0.29310345	0.46590909	0.293103448	0.4	0.4318182	0.2840909
**ENSG00000145721**	0.19760479	0.2969697	0.19760479	0.296969697			0.3588235
**ENSG00000074054**		0.2208589		0.220858896			0.1130952
**ENSG00000187736**		0.24545455		0.245454545			0.4651163
**ENSG00000198768**	0.05	0.42424242	0.05	0.424242424			0.327381
**ENSG00000149257**	0.44610778	0.28353659	0.44610778	0.283536585	0.321428571	0.4302326	0.1686047
**ENSG00000131504**	0.16149068	0.08181818	0.16149068	0.081818182			
**ENSG00000144810**	0.34730539	0.37078652	0.34730539	0.370786517	0.154761905	0.1395349	0.2209302
**ENSG00000154548**		0.45757576		0.457575758			0.2823529
**ENSG00000160013**		0.34545455		0.345454545			
**ENSG00000081307**	0.41515152	0.1402439	0.41515152	0.140243902	0.488095238	0.3197674	0.3235294
**ENSG00000012504**							0.3139535
**ENSG00000135750**	0.39520958		0.39520958		0.345238095	0.4127907	
**ENSG00000167394**	0.20658683		0.20658683		0.053571429	0.1	
**ENSG00000179698**		0.07878788		0.078787879			
**ENSG00000177096**	0.09281437	0.3045977	0.09281437	0.304597701	0.172619048	0.1802326	0.1818182
**ENSG00000167380**	0.0748503		0.0748503		0.470238095	0.4767442	
**ENSG00000101849**		0.47575758		0.475757576			0.3895349
**ENSG00000128602**		0.21818182		0.218181818			0.2616279
**ENSG00000136925**	0.39759036	0.36969697	0.39759036	0.36969697	0.481927711	0.4883721	0.4883721
**ENSG00000006283**		0.10909091		0.109090909			0.0755814
**ENSG00000240891**		0.12804878		0.12804878			
**ENSG00000113845**		0.2030303		0.203030303			0.2965116
**ENSG00000106780**		0.34545455		0.345454545			0.3882353
**ENSG00000259347**		0.05		0.05			
**ENSG00000120925**		0.30606061		0.306060606			0.3313953
**ENSG00000120328**	0.21257485		0.21257485		0.107142857	0.1918605	
**ENSG00000204962**		0.05151515		0.051515152			0.1162791
**ENSG00000169087**	0.49101796	0.1030303	0.49101796	0.103030303	0.369047619	0.4294118	0.127907
**ENSG00000253389**		0.42121212		0.421212121			0.4709302
**ENSG00000159403**		0.16666667		0.166666667			0.4476744
**ENSG00000111404**		0.11212121		0.112121212			
**ENSG00000182450**	0.48502994	0.19135802	0.48502994	0.191358025	0.101190476	0.1046512	0.4375
**ENSG00000167633**							0.1627907
**ENSG00000213213**	0.19461078	0.17575758	0.19461078	0.175757576	0.168674699	0.2705882	0.1162791
**ENSG00000186432**		0.47575758		0.475757576			0.2352941
**ENSG00000104059**		0.20114943		0.201149425			0.4352941
**ENSG00000143507**	0.39204545		0.39204545		0.077777778	0.1363636	
**ENSG00000124562**	0.21856287		0.21856287		0.154761905	0.0988372	
**ENSG00000178233**		0.26060606		0.260606061			0.377907
**ENSG00000120669**		0.39444444		0.394444444			0.1363636
**ENSG00000122786**		0.23333333		0.233333333			0.3546512
**ENSG00000166405**	0.31437126	0.36060606	0.31437126	0.360606061	0.5	0.3662791	0.4880952

**ENSG00000107954**		*0.36060606*		*0.360606061*				*0.2674419*
**ENSG00000167244**	*0.09580838*	*0.33939394*	*0.09580838*	*0.339393939*	*0.379518072*		*0.4651163*	*0.4823529*
**ENSG00000213901**		*0.24545455*		*0.245454545*				*0.4651163*
**ENSG00000166947**	*0.1547619*	*0.08484848*	*0.1547619*	*0.084848485*				*0.0666667*
**ENSG00000131437**		*0.2987013*		*0.298701299*				*0.2777778*
**ENSG00000177370**								*0.255814*
**ENSG00000154274**		*0.28353659*		*0.283536585*				*0.4235294*
**ENSG00000122390**	*0.19161677*	*0.25151515*	*0.19161677*	*0.251515152*	*0.113095238*		*0.0872093*	*0.4534884*
**ENSG00000158710**	*0.19736842*		*0.19736842*					
**ENSG00000070495**		*0.49390244*		*0.493902439*				*0.2732558*
**ENSG00000103005**					*0.113095238*			
**ENSG00000184408**	*0.19760479*	*0.17391304*	*0.19760479*	*0.173913043*				*0.439759*
**ENSG00000058673**	*0.16666667*		*0.16666667*		*0.366666667*		*0.3372093*	
**ENSG00000129128**		*0.15337423*		*0.153374233*				*0.2732558*
**ENSG00000183196**		*0.43636364*		*0.436363636*				*0.4476744*
**ENSG00000091128**		*0.05487805*		*0.054878049*				
**ENSG00000167232**		*0.41111111*		*0.411111111*				*0.4418605*
**ENSG00000159267**	*0.10542169*	*0.41954023*	*0.10542169*	*0.41954023*			*0.0581395*	*0.4659091*
**ENSG00000251380**		*0.26363636*		*0.263636364*				*0.2732558*
**ENSG00000101126**		*0.25914634*		*0.259146341*	*0.095238095*		*0.0813953*	*0.4360465*
**ENSG00000117394**		*0.27011494*		*0.270114943*				*0.3636364*
**ENSG00000114650**	*0.46706587*		*0.46706587*					
**ENSG00000016402**		*0.3969697*		*0.396969697*	*0.096385542*			*0.1918605*
**ENSG00000172519**	*0.14444444*		*0.14444444*		*0.2*		*0.1*	
**ENSG00000085741**		*0.29444444*		*0.294444444*				
**ENSG00000077080**		*0.15454545*		*0.154545455*				
**ENSG00000152932**		*0.48863636*		*0.488636364*				*0.5*
**ENSG00000256061**	*0.40588235*	*0.43636364*	*0.40588235*	*0.436363636*	*0.071428571*			*0.3953488*
**ENSG00000169967**		*0.40606061*		*0.406060606*				*0.3522727*
**ENSG00000163131**	*0.11666667*	*0.43030303*	*0.11666667*	*0.43030303*	*0.388888889*		*0.5*	*0.3941176*
**ENSG00000149256**	*0.09281437*	*0.35454545*	*0.09281437*	*0.354545455*				*0.4186047*
**ENSG00000173436**		*0.2*		*0.2*				*0.3546512*
**ENSG00000064490**	*0.19461078*		*0.19461078*		*0.327380952*		*0.3662791*	
**ENSG00000109971**	*0.49401198*		*0.49401198*		*0.463414634*		*0.4767442*	
**ENSG00000141298**		*0.49090909*		*0.490909091*				*0.2674419*
**ENSG00000139151**	*0.37951807*	*0.08282209*	*0.37951807*	*0.082822086*	*0.353658537*		*0.4302326*	
**ENSG00000144119**		*0.25454545*		*0.254545455*				*0.3662791*
**ENSG00000165566**		*0.20555556*		*0.205555556*				*0.4302326*
**ENSG00000165702**		*0.44252874*		*0.442528736*	*0.357142857*		*0.2916667*	*0.4545455*
**ENSG00000143032**		*0.44409938*		*0.444099379*				*0.4117647*
**ENSG00000183128**		*0.26363636*		*0.263636364*				*0.1453488*
**ENSG00000129566**	*0.45209581*	*0.40606061*	*0.45209581*	*0.406060606*	*0.125*		*0.0581395*	*0.3953488*
**ENSG00000168259**	*0.41111111*		*0.41111111*		*0.155555556*		*0.1477273*	
**ENSG00000099864**	*0.26234568*	*0.31460674*	*0.26234568*	*0.314606742*	*0.482142857*		*0.4638554*	
**ENSG00000046647**	*0.21818182*		*0.21818182*					
**ENSG00000152464**	*0.49700599*		*0.49700599*		*0.054216867*			
**ENSG00000185088**	*0.16666667*	*0.07272727*	*0.16666667*	*0.072727273*	*0.493975904*		*0.4659091*	*0.4651163*
**ENSG00000213015**	*0.23353293*	*0.26969697*	*0.23353293*	*0.26969697*	*0.416666667*		*0.4069767*	*0.1744186*
**ENSG00000049245**		*0.20114943*		*0.201149425*				
**ENSG00000124688**		*0.29393939*		*0.293939394*				
**ENSG00000183624**		*0.1*		*0.1*				
**ENSG00000109743**		*0.23636364*		*0.236363636*				*0.3863636*
**ENSG00000182851**		*0.221875*		*0.221875*				*0.3372093*
**ENSG00000227051**	*0.31481481*	*0.39393939*	*0.31481481*	*0.393939394*			*0.195122*	*0.4883721*

**ENSG00000071282**	*0.35555556*	*0.32727273*	*0.35555556*	*0.327272727*	*0.066666667*			*0.0697674*
**ENSG00000126500**	*0.38505747*	*0.30606061*	*0.38505747*	*0.306060606*	*0.395348837*		*0.4659091*	*0.2678571*
**ENSG00000243709**								*0.255814*
**ENSG00000169495**		*0.47878788*		*0.478787879*				*0.3176471*
**ENSG00000206150**	*0.31437126*	*0.36969697*	*0.31437126*	*0.36969697*	*0.095238095*		*0.0988372*	*0.3352941*
**ENSG00000134250**		*0.11212121*		*0.112121212*			*0.0662651*	
**ENSG00000166111**		*0.36969697*		*0.36969697*	*0.1125*		*0.1341463*	*0.4825581*
**ENSG00000135148**		*0.11890244*		*0.118902439*				*0.2325581*
**ENSG00000166959**	*0.18562874*		*0.18562874*		*0.05952381*		*0.0581395*	
**ENSG00000001630**		*0.4*		*0.4*				*0.1777778*
**ENSG00000159579**	*0.41916168*		*0.41916168*		*0.380952381*		*0.2848837*	
**ENSG00000214279**	*0.06741573*	*0.22727273*	*0.06741573*	*0.227272727*	*0.323170732*		*0.2650602*	*0.4534884*
**ENSG00000121067**		*0.07878788*		*0.078787879*				*0.1666667*
**ENSG00000107338**								*0.122093*
**ENSG00000115425**		*0.43518519*		*0.435185185*				*0.4529412*
**ENSG00000135314**	*0.18888889*		*0.18888889*		*0.3*		*0.2272727*	
**ENSG00000161011**		*0.5*		*0.5*				*0.1976744*
**ENSG00000213971**		*0.35*		*0.35*				*0.3181818*
**ENSG00000106070**	*0.35329341*	*0.18181818*	*0.35329341*	*0.181818182*	*0.386904762*		*0.3117647*	*0.0639535*
**ENSG00000241370**		*0.13888889*		*0.138888889*				*0.4772727*
**ENSG00000088888**		*0.41818182*		*0.418181818*				*0.372093*
**ENSG00000145390**	*0.42814371*	*0.41818182*	*0.42814371*	*0.418181818*	*0.273809524*		*0.2209302*	*0.4593023*
**ENSG00000167965**		*0.48742138*		*0.487421384*				*0.4268293*
**ENSG00000128253**	*0.35*		*0.35*					
**ENSG00000123607**		*0.47878788*		*0.478787879*				*0.4825581*
**ENSG00000167191**		*0.28484848*		*0.284848485*				*0.4534884*
**ENSG00000139182**		*0.07317073*		*0.073170732*				
**ENSG00000089169**	*0.39444444*	*0.48181818*	*0.39444444*	*0.481818182*	*0.166666667*		*0.2111111*	*0.4709302*
**ENSG00000187796**		*0.43888889*		*0.438888889*				*0.0795455*
**ENSG00000179477**	*0.48757764*		*0.48757764*		*0.369047619*		*0.2848837*	
**ENSG00000175322**		*0.36842105*		*0.368421053*				*0.2631579*
**ENSG00000166278**		*0.07361963*		*0.073619632*				*0.0755814*
**ENSG00000170296**		*0.11666667*		*0.116666667*				*0.2616279*
**ENSG00000164663**		*0.45757576*		*0.457575758*				*0.372093*
**ENSG00000106541**	*0.2754491*	*0.22727273*	*0.2754491*	*0.227272727*	*0.416666667*		*0.4244186*	*0.3295455*
**ENSG00000106113**	*0.43125*	*0.07386364*	*0.43125*	*0.073863636*	*0.465909091*		*0.3522727*	*0.2159091*
**ENSG00000158186**	*0.37777778*	*0.16158537*	*0.37777778*	*0.161585366*	*0.166666667*		*0.3977273*	*0.2151163*
**ENSG00000062582**		*0.08333333*		*0.083333333*				*0.1931818*
**ENSG00000215712**		*0.27575758*		*0.275757576*				*0.4117647*
**ENSG00000184007**		*0.12804878*		*0.12804878*				*0.4186047*
**ENSG00000122378**		*0.33636364*		*0.336363636*				*0.1686047*
**ENSG00000140564**		*0.39090909*		*0.390909091*				*0.4825581*
**ENSG00000180438**	*0.23170732*	*0.37804878*	*0.23170732*	*0.37804878*	*0.353658537*		*0.3235294*	*0.3372093*
**ENSG00000003249**								*0.0705882*
**ENSG00000225362**		*0.17977528*		*0.179775281*				*0.2093023*
**ENSG00000135835**	*0.32634731*	*0.43939394*	*0.32634731*	*0.439393939*	*0.377777778*		*0.4634146*	*0.4883721*
**ENSG00000258792**		*0.29090909*		*0.290909091*				*0.3139535*
**ENSG00000154642**		*0.47256098*		*0.472560976*				*0.5*
**ENSG00000166104**		*0.48876404*		*0.488764045*				*0.1363636*
**ENSG00000122824**	*0.43373494*		*0.43373494*		*0.267857143*		*0.2034884*	
**ENSG00000154556**	*0.40419162*	*0.23170732*	*0.40419162*	*0.231707317*	*0.321428571*		*0.2267442*	*0.2848837*
**ENSG00000196559**		*0.37962963*		*0.37962963*				*0.2151163*
**ENSG00000242221**					*0.088888889*			
**ENSG00000104332**		*0.41818182*		*0.418181818*				*0.4186047*

**ENSG00000162836**	*0.35555556*		*0.35555556*		*0.422222222*	*0.25*	
**ENSG00000127084**	*0.46107784*		*0.46107784*		*0.482142857*	*0.377907*	
**ENSG00000164597**		*0.47575758*		*0.475757576*			*0.4534884*
**ENSG00000213533**		*0.33231707*		*0.332317073*			*0.3888889*
**ENSG00000182149**		*0.25454545*		*0.254545455*	*0.144444444*	*0.1444444*	
**ENSG00000172954**		*0.3969697*		*0.396969697*			*0.1918605*
**ENSG00000049540**							*0.2159091*
**ENSG00000122335**		*0.43333333*		*0.433333333*			*0.3068182*
**ENSG00000125449**		*0.36363636*		*0.363636364*			*0.2823529*
**ENSG00000107798**	*0.27844311*	*0.16091954*	*0.27844311*	*0.16091954*	*0.439759036*	*0.3662791*	
**ENSG00000197279**					*0.422619048*	*0.3895349*	
**ENSG00000196712**		*0.38484848*		*0.384848485*			*0.4883721*
**ENSG00000080815**		*0.43333333*		*0.433333333*	*0.4*	*0.4545455*	*0.4767442*
**ENSG00000124570**	*0.23952096*		*0.23952096*		*0.488095238*	*0.5*	
**ENSG00000116922**	*0.19886364*	*0.29192547*	*0.19886364*	*0.291925466*			*0.4878049*
**ENSG00000138758**	*0.28443114*	*0.44047619*	*0.28443114*	*0.44047619*	*0.488095238*	*0.4883721*	*0.3139535*
**ENSG00000257726**		*0.07055215*		*0.070552147*			
**ENSG00000198729**		*0.18484848*		*0.184848485*			*0.2411765*
**ENSG00000204175**	*0.44444444*		*0.44444444*				
**ENSG00000101323**							*0.4651163*
**ENSG00000188313**	*0.38023952*	*0.08888889*	*0.38023952*	*0.088888889*	*0.089285714*	*0.122093*	
**ENSG00000136352**					*0.2*	*0.3068182*	
**ENSG00000175348**	*0.08231707*	*0.17241379*	*0.08231707*	*0.172413793*			*0.4090909*
**ENSG00000137877**		*0.47575758*		*0.475757576*			*0.3081395*
**ENSG00000135269**		*0.3*		*0.3*			*0.4777778*
**ENSG00000106571**		*0.34848485*		*0.348484848*			*0.4593023*
**ENSG00000186919**		*0.11212121*		*0.112121212*			*0.2616279*
**ENSG00000182308**		*0.22727273*		*0.227272727*			
**ENSG00000156261**	*0.29819277*		*0.29819277*		*0.192771084*	*0.2352941*	
**ENSG00000179083**							*0.0697674*
**ENSG00000165338**	*0.35542169*		*0.35542169*		*0.09375*	*0.0941176*	*0.0872093*
**ENSG00000176381**							*0.0872093*
**ENSG00000111666**	*0.06886228*	*0.5*	*0.06886228*	*0.5*	*0.375*	*0.3430233*	*0.4090909*
**ENSG00000196419**	*0.32335329*	*0.39634146*	*0.32335329*	*0.396341463*	*0.095238095*		*0.2209302*
**ENSG00000124486**		*0.08588957*		*0.085889571*			
**ENSG00000125505**	*0.22674419*	*0.5*	*0.22674419*	*0.5*	*0.477777778*	*0.4772727*	*0.1453488*
**ENSG00000100296**		*0.4054878*		*0.405487805*			*0.4767442*
**ENSG00000119285**		*0.31818182*		*0.318181818*			*0.4360465*
**ENSG00000110619**	*0.05120482*	*0.31818182*	*0.05120482*	*0.318181818*	*0.148809524*	*0.1046512*	*0.3*
**ENSG00000144711**		*0.18484848*		*0.184848485*			*0.494186*
**ENSG00000203727**		*0.42424242*		*0.424242424*			*0.3139535*
**ENSG00000096996**	*0.10555556*		*0.10555556*			*0.2613636*	
**ENSG00000255561**		*0.32208589*		*0.32208589*			*0.1976744*
**ENSG00000085872**		*0.34242424*		*0.342424242*			*0.1104651*
**ENSG00000154864**		*0.44805195*		*0.448051948*			*0.4578313*
**ENSG00000100147**					*0.083333333*		
**ENSG00000013503**	*0.31460674*	*0.21341463*	*0.31460674*	*0.213414634*		*0.0581395*	*0.3058824*
**ENSG00000117226**		*0.48787879*		*0.487878788*			*0.4186047*
**ENSG00000176274**		*0.44785276*		*0.447852761*			*0.494186*
**ENSG00000196963**	*0.2994012*	*0.12424242*	*0.2994012*	*0.124242424*	*0.488095238*	*0.4534884*	*0.1337209*
**ENSG00000111639**	*0.42215569*	*0.30606061*	*0.42215569*	*0.306060606*	*0.180722892*	*0.2613636*	*0.25*
**ENSG00000198046**	*0.14071856*		*0.14071856*		*0.101190476*		
**ENSG00000164742**		*0.36969697*		*0.36969697*			*0.4127907*
**ENSG00000109158**	*0.34730539*	*0.4969697*	*0.34730539*	*0.496969697*	*0.4*	*0.3529412*	*0.4111111*

**ENSG00000102962**		*0.14880952*		*0.148809524*				*0.1395349*
**ENSG00000177508**	*0.11377246*		*0.11377246*		*0.416666667*		*0.494186*	
**ENSG00000256660**		*0.4556962*		*0.455696203*				*0.2409639*
**ENSG00000170037**	*0.3742515*	*0.49390244*	*0.3742515*	*0.493902439*	*0.289156627*		*0.3430233*	*0.0697674*
**ENSG00000174953**		*0.39393939*		*0.393939394*				*0.4767442*
**ENSG00000125148**		*0.25*		*0.25*				*0.1931818*
**ENSG00000122965**		*0.4969697*		*0.496969697*	*0.125*		*0.1818182*	*0.4883721*
**ENSG00000225781**	*0.4760479*		*0.4760479*		*0.053571429*			
**ENSG00000106025**	*0.05988024*	*0.22727273*	*0.05988024*	*0.227272727*	*0.30952381*		*0.3197674*	
**ENSG00000125695**		*0.29268293*		*0.292682927*				*0.0523256*
**ENSG00000186193**		*0.21036585*		*0.210365854*				*0.3430233*
**ENSG00000167900**		*0.30981595*		*0.309815951*				*0.4593023*
**ENSG00000161381**	*0.37125749*	*0.4969697*	*0.37125749*	*0.496969697*	*0.172619048*		*0.1162791*	*0.372093*
**ENSG00000119725**	*0.5*	*0.39393939*	*0.5*	*0.393939394*	*0.377777778*		*0.4204545*	*0.3546512*
**ENSG00000152601**		*0.2195122*		*0.219512195*				*0.25*
**ENSG00000158805**		*0.45*		*0.45*				*0.1511628*
**ENSG00000155926**	*0.46706587*	*0.46666667*	*0.46706587*	*0.466666667*	*0.433333333*		*0.3522727*	*0.2093023*
**ENSG00000136247**	*0.39204545*		*0.39204545*		*0.211111111*		*0.1666667*	
**ENSG00000259642**		*0.26666667*		*0.266666667*				*0.2333333*
**ENSG00000135740**		*0.11212121*		*0.112121212*				
**ENSG00000237693**	*0.48295455*		*0.48295455*		*0.422222222*		*0.3863636*	*0.3863636*
**ENSG00000198901**		*0.26666667*		*0.266666667*				
**ENSG00000137968**	*0.39457831*	*0.37777778*	*0.39457831*	*0.377777778*	*0.053571429*			*0.125*
**ENSG00000182263**		*0.29090909*		*0.290909091*				*0.4883721*
**ENSG00000219200**		*0.15454545*		*0.154545455*				*0.1337209*
**ENSG00000025039**		*0.36666667*		*0.366666667*				*0.4222222*
**ENSG00000104369**		*0.24848485*		*0.248484848*				*0.1686047*
**ENSG00000105374**	*0.35329341*	*0.43030303*	*0.35329341*	*0.43030303*				*0.1860465*
**ENSG00000183765**	*0.27586207*		*0.27586207*		*0.388888889*		*0.3444444*	
**ENSG00000016082**	*0.33832335*		*0.33832335*		*0.083333333*		*0.1547619*	
**ENSG00000118513**		*0.44242424*		*0.442424242*				*0.3139535*
**ENSG00000183837**		*0.41158537*		*0.411585366*				*0.4886364*
**ENSG00000132437**	*0.375*	*0.08787879*	*0.375*	*0.087878788*	*0.470238095*		*0.4709302*	
**ENSG00000134461**		*0.15454545*		*0.154545455*				*0.1744186*
**ENSG00000007312**		*0.36419753*		*0.364197531*				*0.4651163*
**ENSG00000123600**	*0.10714286*		*0.10714286*					
**ENSG00000184330**	*0.40419162*	*0.08181818*	*0.40419162*	*0.081818182*	*0.18452381*		*0.0872093*	*0.0988372*
**ENSG00000185070**		*0.30864198*		*0.308641975*				*0.4534884*
**ENSG00000102230**	*0.05688623*	*0.40277778*	*0.05688623*	*0.402777778*	*0.273809524*		*0.2790698*	*0.4882353*
**ENSG00000105135**	*0.42777778*	*0.46060606*	*0.42777778*	*0.460606061*	*0.455555556*		*0.4772727*	*0.3895349*
**ENSG00000163466**	*0.07878788*		*0.07878788*		*0.410714286*		*0.3941176*	
**ENSG00000142347**		*0.22121212*		*0.221212121*				*0.1588235*
**ENSG00000124449**		*0.16666667*		*0.166666667*				*0.1136364*
**ENSG00000099954**		*0.48484848*		*0.484848485*				*0.3647059*
**ENSG00000160193**		*0.45061728*		*0.450617284*				*0.4011628*
**ENSG00000136436**	*0.15*	*0.37222222*	*0.15*	*0.372222222*				*0.1818182*
**ENSG00000185684**	*0.21764706*	*0.11515152*	*0.21764706*	*0.115151515*	*0.175*		*0.25*	*0.1860465*
**ENSG00000124103**	*0.11111111*		*0.11111111*		*0.355555556*		*0.2272727*	
**ENSG00000064999**	*0.35329341*	*0.14848485*	*0.35329341*	*0.148484848*	*0.458333333*		*0.4534884*	*0.2682927*
**ENSG00000100711**		*0.38484848*		*0.384848485*				*0.3255814*
**ENSG00000104894**		*0.22727273*		*0.227272727*	*0.232142857*		*0.1918605*	*0.1918605*
**ENSG00000180138**	*0.41666667*	*0.37116564*	*0.41666667*	*0.371165644*	*0.208333333*		*0.2151163*	*0.2797619*
**ENSG00000107807**	*0.25568182*	*0.35757576*	*0.25568182*	*0.357575758*	*0.30952381*		*0.3522727*	*0.3222222*
**ENSG00000123989**	*0.33233533*	*0.5*	*0.33233533*	*0.5*	*0.380952381*		*0.2588235*	*0.255814*

**ENSG00000255837**		*0.35393258*		*0.353932584*			*0.2045455*
**ENSG00000167778**		*0.08895706*		*0.088957055*			*0.2555556*
**ENSG00000109272**					*0.416666667*	*0.3837209*	
**ENSG00000181291**		*0.37356322*		*0.373563218*			*0.0581395*
**ENSG00000179636**		*0.34848485*		*0.348484848*			*0.4534884*
**ENSG00000082126**		*0.49393939*		*0.493939394*			*0.4352941*
**ENSG00000214900**	*0.39820359*	*0.25757576*	*0.39820359*	*0.257575758*	*0.19047619*	*0.1162791*	
**ENSG00000143466**	*0.2005988*	*0.45151515*	*0.2005988*	*0.451515152*		*0.0697674*	*0.4011628*
**ENSG00000235098**	*0.2875*		*0.2875*		*0.084337349*	*0.1860465*	
**ENSG00000158458**		*0.09090909*		*0.090909091*			
**ENSG00000186834**	*0.26136364*	*0.0969697*	*0.26136364*	*0.096969697*	*0.366666667*	*0.3181818*	*0.3255814*
**ENSG00000187527**	*0.36227545*		*0.36227545*		*0.136904762*	*0.2916667*	
**ENSG00000141448**		*0.41111111*		*0.411111111*			*0.3522727*
**ENSG00000085552**	*0.28443114*	*0.42352941*	*0.28443114*	*0.423529412*	*0.148809524*	*0.122093*	*0.2317073*
**ENSG00000186687**		*0.17575758*		*0.175757576*			*0.1058824*
**ENSG00000175662**		*0.33333333*		*0.333333333*			*0.0697674*
**ENSG00000108100**	*0.36826347*	*0.32424242*	*0.36826347*	*0.324242424*	*0.420731707*	*0.3764706*	*0.3764706*
**ENSG00000166157**		*0.12337662*		*0.123376623*			*0.1511628*
**ENSG00000213762**	*0.11445783*	*0.12804878*	*0.11445783*	*0.12804878*	*0.168674699*	*0.122093*	*0.2325581*
**ENSG00000130768**	*0.31460674*	*0.3*	*0.31460674*	*0.3*			*0.2906977*
**ENSG00000103343**	*0.30838323*		*0.30838323*		*0.125*	*0.1453488*	
**ENSG00000110203**		*0.05757576*		*0.057575758*			
**ENSG00000146414**	*0.43413174*	*0.48333333*	*0.43413174*	*0.483333333*	*0.234939759*	*0.3554217*	*0.3977273*
**ENSG00000128268**		*0.1969697*		*0.196969697*			*0.1627907*
**ENSG00000140832**		*0.26060606*		*0.260606061*			
**ENSG00000140534**		*0.45454545*		*0.454545455*			*0.3809524*
**ENSG00000108753**		*0.44444444*		*0.444444444*			*0.4888889*
**ENSG00000204398**		*0.41212121*		*0.412121212*			*0.4709302*
**ENSG00000148702**	*0.22222222*	*0.1*	*0.22222222*	*0.1*	*0.166666667*	*0.1931818*	*0.0872093*
**ENSG00000205927**	*0.12275449*	*0.08787879*	*0.12275449*	*0.087878788*	*0.345238095*	*0.3953488*	*0.1337209*
**ENSG00000144228**		*0.12777778*		*0.127777778*			*0.3192771*
**ENSG00000211455**		*0.42424242*		*0.424242424*			*0.3546512*
**ENSG00000122971**		*0.3030303*		*0.303030303*			*0.2559524*
**ENSG00000088827**		*0.42424242*		*0.424242424*			*0.4593023*
**ENSG00000048405**	*0.37951807*	*0.10365854*	*0.37951807*	*0.103658537*	*0.166666667*	*0.1477273*	
**ENSG00000163811**	*0.43888889*	*0.29444444*	*0.43888889*	*0.294444444*	*0.220238095*	*0.2352941*	*0.2383721*
**ENSG00000156269**		*0.45555556*		*0.455555556*			*0.1555556*
**ENSG00000129538**							*0.0666667*
**ENSG00000145331**	*0.20359281*	*0.25454545*	*0.20359281*	*0.254545455*	*0.113095238*	*0.1569767*	*0.4302326*
**ENSG00000155256**	*0.2754491*	*0.20689655*	*0.2754491*	*0.206896552*	*0.339285714*	*0.3647059*	*0.4431818*
**ENSG00000101076**		*0.13190184*		*0.13190184*			*0.4069767*
**ENSG00000139190**	*0.46629213*	*0.47575758*	*0.46629213*	*0.475757576*	*0.441860465*	*0.3555556*	*0.3777778*
**ENSG00000166411**	*0.19461078*	*0.42424242*	*0.19461078*	*0.424242424*	*0.273809524*	*0.2790698*	*0.2*
**ENSG00000078808**		*0.1030303*		*0.103030303*			*0.2093023*
**ENSG00000196757**						*0.0833333*	
**ENSG00000186235**	*0.06547619*		*0.06547619*		*0.107142857*	*0.122093*	
**ENSG00000115419**		*0.428125*		*0.428125*			*0.1705882*
**ENSG00000100565**	*0.38622754*	*0.34545455*	*0.38622754*	*0.345454545*			*0.0988372*
**ENSG00000109618**		*0.48484848*		*0.484848485*			*0.2093023*
**ENSG00000185344**	*0.41011236*	*0.42528736*	*0.41011236*	*0.425287356*	*0.083333333*		
**ENSG00000130175**		*0.08536585*		*0.085365854*			*0.0872093*
**ENSG00000007168**		*0.06969697*		*0.06969697*			*0.1627907*
**ENSG00000244462**		*0.18484848*		*0.184848485*			*0.1511628*
**ENSG00000170191**	*0.49425287*	*0.43678161*	*0.49425287*	*0.436781609*		*0.0681818*	*0.4186047*

**ENSG00000125255**	*0.21556886*	*0.46111111*	*0.21556886*	*0.461111111*	*0.113095238*		*0.0581395*	*0.3977273*
**ENSG00000118620**		*0.20786517*		*0.207865169*	*0.111111111*		*0.2386364*	
**ENSG00000091592**	*0.14670659*	*0.44242424*	*0.14670659*	*0.442424242*				*0.1477273*
**ENSG00000204767**							*0.0639535*	
**ENSG00000099365**		*0.36111111*		*0.361111111*				*0.1136364*
**ENSG00000105364**		*0.45987654*		*0.459876543*				*0.4702381*
**ENSG00000173992**					*0.130952381*		*0.0882353*	
**ENSG00000136122**		*0.36969697*		*0.36969697*				*0.1744186*
**ENSG00000115295**	*0.10179641*	*0.36666667*	*0.10179641*	*0.366666667*				*0.3546512*
**ENSG00000134760**		*0.25304878*		*0.25304878*				*0.1104651*
**ENSG00000132199**	*0.11377246*	*0.35889571*	*0.11377246*	*0.358895706*				*0.3470588*
**ENSG00000166363**		*0.3030303*		*0.303030303*				*0.3647059*
**ENSG00000176715**		*0.07575758*		*0.075757576*				*0.3546512*
**ENSG00000060491**	*0.46590909*	*0.39393939*	*0.46590909*	*0.393939394*	*0.411111111*		*0.3409091*	*0.3197674*
**ENSG00000103653**		*0.26363636*		*0.263636364*				*0.4764706*
**ENSG00000138592**					*0.466666667*		*0.2613636*	*0.1627907*
**ENSG00000070614**	*0.12650602*	*0.49393939*	*0.12650602*	*0.493939394*	*0.168674699*		*0.2647059*	*0.3372093*
**ENSG00000164604**	*0.23952096*	*0.48181818*	*0.23952096*	*0.481818182*				*0.0639535*
**ENSG00000213047**		*0.44817073*		*0.448170732*				*0.3058824*
**ENSG00000109534**		*0.35393258*		*0.353932584*				
**ENSG00000187815**	*0.15555556*		*0.15555556*					
**ENSG00000185278**	*0.36227545*	*0.35555556*	*0.36227545*	*0.355555556*				*0.5*
**ENSG00000170426**		*0.14110429*		*0.141104294*				*0.1860465*
**ENSG00000172350**	*0.0508982*	*0.07621951*	*0.0508982*	*0.076219512*				*0.1337209*
**ENSG00000108439**		*0.33636364*		*0.336363636*				*0.4476744*
**ENSG00000164609**	*0.4*	*0.23809524*	*0.4*	*0.238095238*	*0.160714286*		*0.2151163*	*0.1477273*
**ENSG00000217455**	*0.47005988*	*0.1*	*0.47005988*	*0.1*	*0.089285714*		*0.1686047*	
**ENSG00000151116**		*0.14545455*		*0.145454545*	*0.273809524*		*0.3488372*	*0.3352941*
**ENSG00000169057**		*0.2030303*		*0.203030303*				*0.2325581*
**ENSG00000162599**								*0.0639535*
**ENSG00000205730**								*0.1818182*
**ENSG00000119411**		*0.32727273*		*0.327272727*				*0.3372093*
**ENSG00000173175**	*0.2245509*	*0.25151515*	*0.2245509*	*0.251515152*	*0.422619048*		*0.4244186*	
**ENSG00000163209**	*0.46022727*	*0.5*	*0.46022727*	*0.5*	*0.377777778*		*0.3181818*	*0.3181818*
**ENSG00000184678**		*0.12424242*		*0.124242424*				
**ENSG00000120784**		*0.18292683*		*0.182926829*	*0.06547619*			*0.3313953*
**ENSG00000167513**		*0.21590909*		*0.215909091*				*0.4888889*
**ENSG00000139998**		*0.33939394*		*0.339393939*				*0.4244186*
**ENSG00000170477**		*0.16158537*		*0.161585366*				*0.0988372*
**ENSG00000130299**		*0.15337423*		*0.153374233*				*0.1453488*
**ENSG00000144648**		*0.41515152*		*0.415151515*				*0.3662791*
**ENSG00000066336**		*0.35632184*		*0.356321839*				*0.4883721*
**ENSG00000169896**		*0.33333333*		*0.333333333*				*0.2857143*
**ENSG00000088812**		*0.43333333*		*0.433333333*				*0.494186*
**ENSG00000168453**	*0.28614458*	*0.40606061*	*0.28614458*	*0.406060606*	*0.351190476*		*0.4302326*	*0.3953488*
**ENSG00000164136**	*0.44311377*	*0.44242424*	*0.44311377*	*0.442424242*	*0.488372093*		*0.494186*	*0.4883721*
**ENSG00000165195**		*0.35555556*		*0.355555556*				*0.3214286*
**ENSG00000189056**	*0.30337079*		*0.30337079*		*0.166666667*		*0.1705882*	
**ENSG00000139767**	*0.45454545*	*0.46969697*	*0.45454545*	*0.46969697*	*0.233333333*		*0.3295455*	*0.4476744*
**ENSG00000254671**		*0.06363636*		*0.063636364*				
**ENSG00000160882**		*0.4847561*		*0.484756098*				*0.1764706*
**ENSG00000066583**		*0.38484848*		*0.384848485*				*0.1337209*
**ENSG00000172201**		*0.33030303*		*0.33030303*				*0.1686047*
**ENSG00000166118**		*0.28484848*		*0.284848485*				*0.3882353*

**ENSG00000035403**	*0.25748503*	*0.22222222*	*0.25748503*	*0.222222222*	*0.226190476*	*0.2674419*	*0.4886364*
**ENSG00000168329**		*0.26363636*		*0.263636364*			*0.0639535*
**ENSG00000164440**		*0.20689655*		*0.206896552*			*0.1625*
**ENSG00000256349**		*0.41463415*		*0.414634146*			*0.4823529*
**ENSG00000063438**		*0.40804598*		*0.408045977*			*0.4772727*
**ENSG00000055332**	*0.07784431*	*0.3625*	*0.07784431*	*0.3625*	*0.273809524*	*0.1569767*	*0.2529412*
**ENSG00000055813**	*0.31437126*	*0.32727273*	*0.31437126*	*0.327272727*	*0.136904762*	*0.0872093*	*0.1569767*
**ENSG00000173221**		*0.37658228*		*0.376582278*			*0.3636364*
**ENSG00000073350**	*0.48203593*	*0.35757576*	*0.48203593*	*0.357575758*	*0.160714286*	*0.1569767*	*0.1453488*
**ENSG00000163885**		*0.0969697*		*0.096969697*			
**ENSG00000163935**	*0.17333333*	*0.37777778*	*0.17333333*	*0.377777778*	*0.077380952*		*0.4069767*
**ENSG00000111335**		*0.39634146*		*0.396341463*			*0.4767442*
**ENSG00000170442**		*0.43820225*		*0.438202247*			*0.4318182*
**ENSG00000164347**	*0.36666667*		*0.36666667*		*0.170454545*	*0.1666667*	
**ENSG00000213585**		*0.37222222*		*0.372222222*			*0.3409091*
**ENSG00000185650**	*0.36826347*	*0.48484848*	*0.36826347*	*0.484848485*	*0.273809524*	*0.2882353*	*0.2906977*
**ENSG00000143458**		*0.1969697*		*0.196969697*			*0.1058824*
**ENSG00000155858**		*0.41111111*		*0.411111111*			*0.494186*
**ENSG00000231924**	*0.09638554*	*0.12345679*	*0.09638554*	*0.12345679*			*0.3430233*
**ENSG00000170949**	*0.40740741*	*0.3597561*	*0.40740741*	*0.359756098*	*0.2*	*0.2*	*0.3888889*
**ENSG00000124191**	*0.25149701*		*0.25149701*		*0.44047619*	*0.4069767*	
**ENSG00000169814**	*0.08536585*	*0.08231707*	*0.08536585*	*0.082317073*	*0.339285714*	*0.3352941*	*0.3352941*
**ENSG00000061987**		*0.36969697*		*0.36969697*			*0.3780488*
**ENSG00000130635**	*0.25*	*0.4375*	*0.25*	*0.4375*	*0.130952381*	*0.1104651*	*0.4647059*
**ENSG00000179889**		*0.29393939*		*0.293939394*			*0.4761905*
**ENSG00000136295**		*0.16463415*		*0.164634146*			*0.122093*
**ENSG00000131495**	*0.29341317*		*0.29341317*		*0.457831325*	*0.4647059*	
**ENSG00000104722**	*0.0625*	*0.16459627*	*0.0625*	*0.164596273*	*0.433333333*	*0.4090909*	*0.3895349*
**ENSG00000136279**		*0.21779141*		*0.217791411*			*0.1046512*
**ENSG00000010404**	*0.28484848*	*0.47777778*	*0.28484848*	*0.477777778*			*0.1411765*
**ENSG00000148358**		*0.46363636*		*0.463636364*			*0.372093*
**ENSG00000172375**		*0.46060606*		*0.460606061*			*0.3023256*
**ENSG00000145740**		*0.47222222*		*0.472222222*			*0.494186*
**ENSG00000140403**		*0.43636364*		*0.436363636*			*0.3895349*
**ENSG00000197182**	*0.06325301*	*0.31818182*	*0.06325301*	*0.318181818*	*0.148809524*	*0.2034884*	*0.0697674*
**ENSG00000129292**	*0.5*	*0.42777778*	*0.5*	*0.427777778*	*0.357142857*	*0.4302326*	*0.3719512*
**ENSG00000204001**	*0.30838323*		*0.30838323*		*0.297619048*	*0.2034884*	
**ENSG00000163138**	*0.22777778*		*0.22777778*				
**ENSG00000110514**	*0.45808383*		*0.45808383*		*0.369047619*	*0.297619*	
**ENSG00000117091**		*0.18787879*		*0.187878788*			*0.4360465*
**ENSG00000184647**		*0.33939394*		*0.339393939*			*0.4011628*
**ENSG00000177461**	*0.1497006*	*0.2969697*	*0.1497006*	*0.296969697*			*0.3895349*
**ENSG00000162148**	*0.21348315*		*0.21348315*				
**ENSG00000229415**		*0.11212121*		*0.112121212*			
**ENSG00000177732**		*0.1*		*0.1*			*0.1704545*
**ENSG00000149428**	*0.10778443*	*0.49418605*	*0.10778443*	*0.494186047*			*0.3895349*
**ENSG00000128284**	*0.37048193*	*0.48837209*	*0.37048193*	*0.488372093*	*0.351190476*	*0.4418605*	*0.4418605*
**ENSG00000160683**		*0.39329268*		*0.393292683*	*0.125*	*0.1444444*	*0.2325581*
**ENSG00000091656**	*0.35928144*	*0.43820225*	*0.35928144*	*0.438202247*	*0.416666667*	*0.4244186*	*0.3181818*
**ENSG00000149403**		*0.38505747*		*0.385057471*			*0.4777778*
**ENSG00000168004**	*0.39820359*	*0.08181818*	*0.39820359*	*0.081818182*	*0.166666667*	*0.1590909*	*0.1395349*
**ENSG00000164764**		*0.23636364*		*0.236363636*			*0.0930233*
**ENSG00000171804**	*0.4251497*		*0.4251497*		*0.428571429*	*0.4186047*	
**ENSG00000130699**		*0.06289308*		*0.062893082*			

**ENSG00000108406**	*0.09090909*		*0.09090909*				
**ENSG00000113263**		*0.12727273*		*0.127272727*			*0.3837209*
**ENSG00000253190**	*0.08333333*		*0.08333333*				
**ENSG00000249992**		*0.17177914*		*0.171779141*			*0.2228916*
**ENSG00000204361**		*0.37209302*		*0.372093023*			*0.3953488*
**ENSG00000105707**		*0.35151515*		*0.351515152*			*0.2906977*
**ENSG00000034971**	*0.09580838*		*0.09580838*				
**ENSG00000118242**		*0.40909091*		*0.409090909*			*0.2529412*
**ENSG00000145819**		*0.49393939*		*0.493939394*	*0.464285714*	*0.4127907*	*0.3895349*
**ENSG00000154438**		*0.10909091*		*0.109090909*			*0.3604651*
**ENSG00000163067**		*0.26969697*		*0.26969697*			*0.2857143*
**ENSG00000175595**		*0.32515337*		*0.325153374*			*0.3222222*
**ENSG00000173546**		*0.47256098*		*0.472560976*			*0.2848837*
**ENSG00000213401**	*0.07575758*	*0.43902439*	*0.07575758*	*0.43902439*			*0.2674419*
**ENSG00000124466**	*0.23888889*		*0.23888889*		*0.38372093*	*0.3295455*	
**ENSG00000105643**		*0.28484848*		*0.284848485*			*0.1647059*
**ENSG00000083845**		*0.43888889*		*0.438888889*			*0.4880952*
**ENSG00000211448**		*0.40606061*		*0.406060606*	*0.130952381*	*0.1686047*	*0.4418605*
**ENSG00000157796**		*0.33888889*		*0.338888889*			*0.1477273*
**ENSG00000125124**	*0.19444444*		*0.19444444*				
**ENSG00000170178**		*0.34545455*		*0.345454545*			
**ENSG00000177119**		*0.08888889*		*0.088888889*			*0.3522727*
**ENSG00000106153**		*0.17816092*		*0.17816092*	*0.091463415*	*0.0681818*	*0.0681818*
**ENSG00000170242**	*0.17664671*	*0.46666667*	*0.17664671*	*0.466666667*	*0.166666667*	*0.1104651*	*0.3662791*
**ENSG00000087338**		*0.5*		*0.5*			*0.3235294*
**ENSG00000146192**	*0.31818182*	*0.47256098*	*0.31818182*	*0.472560976*	*0.411111111*	*0.4204545*	*0.5*
**ENSG00000084754**		*0.19393939*		*0.193939394*			*0.1104651*
**ENSG00000132382**	*0.14772727*		*0.14772727*				
**ENSG00000021355**		*0.23291925*		*0.232919255*			*0.2831325*
**ENSG00000136381**	*0.13772455*	*0.39090909*	*0.13772455*	*0.390909091*			*0.5*
**ENSG00000138035**		*0.48787879*		*0.487878788*			*0.1818182*
**ENSG00000105967**		*0.17878788*		*0.178787879*			*0.3255814*
**ENSG00000213203**		*0.13333333*		*0.133333333*			*0.2444444*
**ENSG00000157240**		*0.14242424*		*0.142424242*			*0.2383721*
**ENSG00000163406**	*0.06886228*	*0.2347561*	*0.06886228*	*0.234756098*	*0.083333333*	*0.0697674*	*0.0639535*
**ENSG00000069509**	*0.19631902*		*0.19631902*				
**ENSG00000140382**		*0.41717791*		*0.417177914*			*0.1686047*
**ENSG00000119537**		*0.46060606*		*0.460606061*			*0.3235294*
**ENSG00000108604**		*0.37878788*		*0.378787879*			*0.4011628*
**ENSG00000158485**		*0.13030303*		*0.13030303*			*0.3255814*
**ENSG00000116095**		*0.33939394*		*0.339393939*			*0.3882353*
**ENSG00000184566**	*0.31437126*	*0.22727273*	*0.31437126*	*0.227272727*	*0.297619048*	*0.3546512*	*0.1453488*
**ENSG00000143409**	*0.30239521*		*0.30239521*		*0.208333333*	*0.1104651*	
**ENSG00000163762**		*0.48787879*		*0.487878788*			*0.2222222*
**ENSG00000178636**		*0.28484848*		*0.284848485*			*0.5*
**ENSG00000188655**	*0.41515152*	*0.19090909*	*0.41515152*	*0.190909091*	*0.101190476*	*0.0639535*	*0.3372093*
**ENSG00000100997**		*0.45555556*		*0.455555556*			*0.0795455*
**ENSG00000156928**		*0.10060976*		*0.100609756*			*0.0813953*
**ENSG00000175893**		*0.47515528*		*0.47515528*			*0.3953488*
**ENSG00000162543**		*0.21212121*		*0.212121212*			*0.4186047*
**ENSG00000183206**		*0.21111111*		*0.211111111*			*0.4545455*
**ENSG00000112305**		*0.42121212*		*0.421212121*			*0.2616279*
**ENSG00000100505**		*0.42857143*		*0.428571429*			*0.3863636*
**ENSG00000108294**	*0.39520958*		*0.39520958*		*0.113095238*	*0.0639535*	

**ENSG00000115207**	*0.32035928*		*0.32035928*		*0.125*	*0.0930233*	
**ENSG00000171243**	*0.06586826*		*0.06586826*		*0.077380952*	*0.0523256*	
**ENSG00000246223**		*0.44171779*		*0.441717791*			*0.4593023*
**ENSG00000188649**	*0.38554217*	*0.06969697*	*0.38554217*	*0.06969697*	*0.180722892*	*0.25*	
**ENSG00000138271**	*0.44311377*	*0.23636364*	*0.44311377*	*0.236363636*	*0.380952381*	*0.4470588*	*0.1453488*
**ENSG00000120088**		*0.21036585*		*0.210365854*	*0.077380952*	*0.0639535*	
**ENSG00000159592**	*0.21856287*		*0.21856287*		*0.363095238*	*0.2790698*	
**ENSG00000082438**		*0.46969697*		*0.46969697*			*0.0697674*
**ENSG00000164808**	*0.16111111*		*0.16111111*		*0.155555556*	*0.2159091*	*0.1511628*
**ENSG00000239264**		*0.15408805*		*0.15408805*			*0.0639535*
**ENSG00000065883**		*0.16060606*		*0.160606061*			*0.2790698*
**ENSG00000169217**		*0.36363636*		*0.363636364*			*0.127907*
**ENSG00000143379**		*0.37575758*		*0.375757576*			*0.4761905*
**ENSG00000134419**	*0.08682635*		*0.08682635*				
**ENSG00000103540**		*0.07222222*		*0.072222222*			
**ENSG00000204387**	*0.21856287*	*0.14242424*	*0.21856287*	*0.142424242*	*0.233333333*	*0.3139535*	*0.2674419*
**ENSG00000164649**		*0.36969697*		*0.36969697*			*0.1162791*
**ENSG00000145244**		*0.32424242*		*0.324242424*			*0.4761905*
**ENSG00000196132**	*0.0988024*	*0.43597561*	*0.0988024*	*0.43597561*	*0.369047619*	*0.4709302*	*0.0988372*
**ENSG00000115694**	*0.25449102*	*0.37222222*	*0.25449102*	*0.372222222*	*0.255952381*	*0.1976744*	*0.3117647*
**ENSG00000109787**		*0.4*		*0.4*			*0.3470588*
**ENSG00000148925**		*0.18292683*		*0.182926829*			*0.2325581*
**ENSG00000197345**		*0.26380368*		*0.263803681*			*0.3035714*
**ENSG00000088756**	*0.40718563*	*0.3030303*	*0.40718563*	*0.303030303*	*0.2*	*0.0777778*	*0.3511905*
**ENSG00000180081**		*0.08484848*		*0.084848485*			*0.127907*
**ENSG00000125870**	*0.23952096*	*0.34848485*	*0.23952096*	*0.348484848*	*0.238095238*	*0.2823529*	*0.3313953*
**ENSG00000186354**	*0.13772455*	*0.49085366*	*0.13772455*	*0.490853659*			*0.3176471*
**ENSG00000197429**		*0.37777778*		*0.377777778*			*0.3181818*
**ENSG00000065717**		*0.16363636*		*0.163636364*			*0.3255814*
**ENSG00000181541**		*0.07865169*		*0.078651685*			*0.1931818*
**ENSG00000198346**		*0.46969697*		*0.46969697*			*0.3111111*
**ENSG00000203987**	*0.17664671*	*0.43939394*	*0.17664671*	*0.439393939*	*0.380952381*	*0.4476744*	*0.4476744*
**ENSG00000179922**		*0.21818182*		*0.218181818*			*0.2093023*
**ENSG00000143970**		*0.24848485*		*0.248484848*			*0.2*
**ENSG00000173285**	*0.28614458*	*0.32621951*	*0.28614458*	*0.326219512*	*0.375*	*0.4360465*	*0.4352941*
**ENSG00000058668**	*0.42814371*	*0.44207317*	*0.42814371*	*0.442073171*	*0.404761905*	*0.3546512*	*0.4882353*
**ENSG00000049323**		*0.42944785*		*0.429447853*			*0.4883721*
**ENSG00000134571**							*0.0568182*
**ENSG00000176641**		*0.42682927*		*0.426829268*			*0.2732558*
**ENSG00000169727**		*0.33908046*		*0.33908046*			*0.4090909*
**ENSG00000184232**		*0.15517241*		*0.155172414*			*0.1022727*
**ENSG00000116721**		*0.28735632*		*0.287356322*			
**ENSG00000104907**	*0.19461078*		*0.19461078*			*0.0523256*	
**ENSG00000163554**		*0.3969697*		*0.396969697*			*0.4186047*
**ENSG00000204438**	*0.26111111*	*0.22727273*	*0.26111111*	*0.227272727*	*0.156626506*	*0.244186*	*0.4302326*
**ENSG00000109861**	*0.10542169*	*0.21341463*	*0.10542169*	*0.213414634*			*0.4470588*
**ENSG00000142892**		*0.41515152*		*0.415151515*			*0.5*
**ENSG00000087263**		*0.34545455*		*0.345454545*			*0.3809524*
**ENSG00000111224**		*0.38505747*		*0.385057471*			*0.4090909*
**ENSG00000100445**	*0.47005988*	*0.1030303*	*0.47005988*	*0.103030303*	*0.125*	*0.2034884*	*0.2034884*
**ENSG00000203859**		*0.13888889*		*0.138888889*			*0.3837209*
**ENSG00000170396**	*0.21666667*		*0.21666667*		*0.2*	*0.0795455*	
**ENSG00000244242**	*0.4251497*	*0.26966292*	*0.4251497*	*0.269662921*	*0.18452381*	*0.1976744*	*0.2840909*
**ENSG00000152292**	*0.22777778*		*0.22777778*		*0.4*	*0.4166667*	

**ENSG00000139304**	*0.38414634*	*0.40555556*	*0.38414634*	*0.405555556*	*0.255952381*	*0.2732558*	*0.255814*
**ENSG00000184613**	*0.2826087*	*0.22424242*	*0.2826087*	*0.224242424*	*0.44375*	*0.5*	*0.4058824*
**ENSG00000236756**		*0.06060606*		*0.060606061*			*0.3181818*
**ENSG00000171360**		*0.29393939*		*0.293939394*			*0.0581395*
**ENSG00000105426**		*0.31212121*		*0.312121212*			*0.3895349*
**ENSG00000164830**	*0.16766467*	*0.41515152*	*0.16766467*	*0.415151515*	*0.375*	*0.3837209*	*0.3869048*
**ENSG00000174238**		*0.20606061*		*0.206060606*			*0.1395349*
**ENSG00000123240**					*0.172619048*	*0.1511628*	
**ENSG00000157765**		*0.43939394*		*0.439393939*			*0.3176471*
**ENSG00000181631**		*0.30909091*		*0.309090909*			*0.2965116*
**ENSG00000138593**	*0.18862275*	*0.36280488*	*0.18862275*	*0.362804878*	*0.386904762*	*0.3023256*	*0.2616279*
**ENSG00000119979**		*0.24846626*		*0.248466258*			
**ENSG00000019995**		*0.25454545*		*0.254545455*			*0.3139535*
**ENSG00000179240**	*0.16467066*	*0.23333333*	*0.16467066*	*0.233333333*	*0.101190476*	*0.0755814*	*0.4642857*
**ENSG00000005421**	*0.43902439*	*0.32424242*	*0.43902439*	*0.324242424*	*0.133333333*	*0.1222222*	*0.4418605*
**ENSG00000204711**		*0.38333333*		*0.383333333*			*0.3095238*
**ENSG00000124788**		*0.31707317*		*0.317073171*			*0.5*
**ENSG00000122548**		*0.41515152*		*0.415151515*			*0.2848837*
**ENSG00000165072**							*0.4545455*
**ENSG00000138138**	*0.42222222*	*0.29447853*	*0.42222222*	*0.294478528*	*0.122222222*	*0.1777778*	*0.1566265*
**ENSG00000242265**		*0.29090909*		*0.290909091*			*0.2470588*
**ENSG00000138587**	*0.18263473*		*0.18263473*				
**ENSG00000139292**		*0.20121951*		*0.201219512*			*0.4186047*
**ENSG00000172059**		*0.49367089*		*0.493670886*			*0.3*
**ENSG00000112936**		*0.37962963*		*0.37962963*			*0.3058824*
**ENSG00000134057**	*0.48203593*		*0.48203593*		*0.428571429*	*0.3895349*	
**ENSG00000204278**		*0.09146341*		*0.091463415*			*0.2209302*
**ENSG00000120992**	*0.12275449*		*0.12275449*		*0.214285714*	*0.3197674*	
**ENSG00000122728**							*0.2616279*
**ENSG00000115761**		*0.08823529*		*0.088235294*			*0.3690476*
**ENSG00000037241**	*0.19760479*	*0.06818182*	*0.19760479*	*0.068181818*			
**ENSG00000182782**		*0.42777778*		*0.427777778*			*0.4593023*
**ENSG00000123307**		*0.25925926*		*0.259259259*			*0.3809524*
**ENSG00000117713**		*0.07272727*		*0.072727273*			
**ENSG00000130985**	*0.06287425*		*0.06287425*				
**ENSG00000166780**		*0.4*		*0.4*			*0.3295455*
**ENSG00000152520**		*0.35151515*		*0.351515152*			*0.2848837*
**ENSG00000198570**	*0.31137725*	*0.11363636*	*0.31137725*	*0.113636364*	*0.44047619*	*0.372093*	*0.4767442*
**ENSG00000136827**		*0.08333333*		*0.083333333*			
**ENSG00000255154**	*0.2005988*	*0.40490798*	*0.2005988*	*0.404907975*			*0.2325581*
**ENSG00000101445**		*0.27272727*		*0.272727273*			*0.3662791*
**ENSG00000125779**		*0.39329268*		*0.393292683*			*0.2261905*
**ENSG00000171606**	*0.22159091*		*0.22159091*				
**ENSG00000130649**		*0.12727273*		*0.127272727*			*0.4756098*
**ENSG00000147789**		*0.35757576*		*0.357575758*			*0.327381*
**ENSG00000138670**		*0.34756098*		*0.347560976*			*0.2117647*
**ENSG00000150625**	*0.11077844*	*0.09202454*	*0.11077844*	*0.09202454*	*0.43452381*	*0.2906977*	*0.2142857*
**ENSG00000106852**		*0.40909091*		*0.409090909*			*0.4302326*
**ENSG00000253767**		*0.20224719*		*0.202247191*			*0.1477273*
**ENSG00000149564**		*0.23333333*		*0.233333333*			*0.3197674*
**ENSG00000137522**		*0.06111111*		*0.061111111*			*0.2034884*
**ENSG00000178184**		*0.23333333*		*0.233333333*			*0.0639535*
**ENSG00000179152**		*0.06060606*		*0.060606061*			
**ENSG00000185972**	*0.4760479*		*0.4760479*		*0.428571429*	*0.3430233*	

**ENSG00000251664**		0.05151515		0.051515152			0.1162791
**ENSG00000068383**	0.06586826	0.07272727	0.06586826	0.072727273	0.297619048	0.2732558	
**ENSG00000131482**		0.24698795		0.246987952			0.3953488
**ENSG00000078579**		0.29320988		0.293209877			0.4709302
**ENSG00000172508**	0.49101796	0.41212121	0.49101796	0.412121212			0.377907
**ENSG00000114648**		0.44848485		0.448484848			0.4176471
**ENSG00000079950**		0.38719512		0.387195122			0.4709302
**ENSG00000105219**		0.24545455		0.245454545			0.3197674
**ENSG00000120162**		0.41304348		0.413043478			0.4
**ENSG00000071994**		0.48466258		0.484662577			0.25
**ENSG00000124491**		0.4969697		0.496969697			0.4186047
**ENSG00000163513**		0.09393939		0.093939394			0.2325581
**ENSG00000110917**	0.45508982	0.45757576	0.45508982	0.457575758	0.470238095	0.3953488	0.4302326
**ENSG00000090382**	0.16167665	0.48787879	0.16167665	0.487878788			0.3372093
**ENSG00000115365**	0.14156627	0.42592593	0.14156627	0.425925926	0.380952381	0.4318182	0.3372093
**ENSG00000146376**							0.0795455
**ENSG00000198948**	0.13772455	0.2030303	0.13772455	0.203030303	0.18452381	0.1976744	0.2616279
**ENSG00000196504**	0.31666667	0.18333333	0.31666667	0.183333333	0.322222222	0.2840909	0.2840909
**ENSG00000198633**	0.16766467		0.16766467				0.3023256
**ENSG00000164434**		0.41818182		0.418181818			0.4360465
**ENSG00000173862**	0.2245509		0.2245509		0.255952381	0.3313953	
**ENSG00000134480**	0.11077844		0.11077844				
**ENSG00000126016**		0.15340909		0.153409091	0.188888889	0.1888889	0.3181818
**ENSG00000079277**	0.06586826	0.26666667	0.06586826	0.266666667			0.3604651
**ENSG00000118473**	0.46084337	0.45454545	0.46084337	0.454545455	0.446428571	0.4883721	0.2727273
**ENSG00000101558**		0.49090909		0.490909091			0.4709302
**ENSG00000205423**		0.06111111		0.061111111			
**ENSG00000178935**		0.17575758		0.175757576			0.1411765
**ENSG00000013619**		0.43888889		0.438888889			0.3488372
**ENSG00000188958**		0.47222222		0.472222222	0.3	0.1777778	0.125
**ENSG00000104728**		0.36969697		0.36969697			0.4593023
**ENSG00000117013**		0.16091954		0.16091954			0.0795455
**ENSG00000221949**		0.35151515		0.351515152			0.3662791
**ENSG00000198932**							0.1363636
**ENSG00000111249**		0.22121212		0.221212121			0.127907
**ENSG00000122025**		0.48787879		0.487878788			0.4360465
**ENSG00000137507**		0.47272727		0.472727273			0.3869048
**ENSG00000114999**		0.20689655		0.206896552			0.4302326
**ENSG00000146215**		0.39090909		0.390909091			0.3488372
**ENSG00000095906**		0.28888889		0.288888889			0.4444444
**ENSG00000134061**		0.0969697		0.096969697			0.2222222
**ENSG00000105281**	0.37650602		0.37650602		0.488888889	0.4666667	
**ENSG00000113083**		0.15		0.15			
**ENSG00000105778**		0.43636364		0.436363636			0.3604651
**ENSG00000150773**	0.06287425		0.06287425		0.375	0.3837209	
**ENSG00000110013**							0.1385542
**ENSG00000104833**		0.42424242		0.424242424			0.3658537
**ENSG00000166263**		0.4		0.4			0.4545455
**ENSG00000144357**		0.3		0.3			0.494186
**ENSG00000130309**		0.5		0.5			0.1046512
**ENSG00000137073**	0.46107784	0.4030303	0.46107784	0.403030303	0.202380952	0.2771084	0.2848837
**ENSG00000106479**		0.4127907		0.412790698			0.3977273
**ENSG00000180801**	0.29341317	0.25	0.29341317	0.25			0.3837209
**ENSG00000120915**		0.23939394		0.239393939			0.4235294

**ENSG00000068305**	*0.18862275*	*0.48170732*	*0.18862275*	*0.481707317*		*0.0813953*	*0.4882353*
**ENSG00000240682**							*0.0755814*
**ENSG00000100364**	*0.41017964*	*0.44848485*	*0.41017964*	*0.448484848*	*0.388888889*	*0.4555556*	*0.4825581*
**ENSG00000181031**		*0.22222222*		*0.222222222*			
**ENSG00000103876**		*0.47878788*		*0.478787879*	*0.166666667*	*0.1125*	*0.3604651*
**ENSG00000186001**		*0.11111111*		*0.111111111*			*0.0595238*
**ENSG00000085832**		*0.21515152*		*0.215151515*			
**ENSG00000198846**		*0.41818182*		*0.418181818*			*0.4709302*
**ENSG00000170054**	*0.28125*	*0.45061728*	*0.28125*	*0.450617284*			*0.4593023*
**ENSG00000076706**		*0.28963415*		*0.289634146*			*0.4069767*
**ENSG00000173093**	*0.2012987*		*0.2012987*		*0.189189189*	*0.1785714*	
**ENSG00000204099**	*0.48795181*	*0.41515152*	*0.48795181*	*0.415151515*	*0.404761905*	*0.1488095*	*0.3488372*
**ENSG00000105223**	*0.23493976*	*0.30555556*	*0.23493976*	*0.305555556*	*0.125*	*0.1453488*	
**ENSG00000100802**		*0.33030303*		*0.33030303*			*0.1744186*
**ENSG00000241878**	*0.05*	*0.28787879*	*0.05*	*0.287878788*	*0.113095238*	*0.0523256*	*0.377907*
**ENSG00000125864**		*0.41212121*		*0.412121212*			*0.4709302*
**ENSG00000204193**		*0.20606061*		*0.206060606*			*0.3488372*
**ENSG00000168243**		*0.48484848*		*0.484848485*			*0.3197674*
**ENSG00000147905**							*0.2383721*
**ENSG00000186376**	*0.42222222*	*0.32777778*	*0.42222222*	*0.327777778*	*0.366666667*	*0.4659091*	*0.0581395*
**ENSG00000100104**		*0.07222222*		*0.072222222*			*0.125*
**ENSG00000102125**							*0.1477273*
**ENSG00000164488**		*0.16363636*		*0.163636364*			*0.1860465*
**ENSG00000072832**						*0.0681818*	*0.1162791*
**ENSG00000105656**		*0.35126582*		*0.351265823*			*0.2380952*
**ENSG00000170515**	*0.18862275*		*0.18862275*				
**ENSG00000158062**	*0.41011236*	*0.25*	*0.41011236*	*0.25*	*0.43452381*	*0.4476744*	*0.4534884*
**ENSG00000167600**	*0.12222222*	*0.24545455*	*0.12222222*	*0.245454545*	*0.122222222*	*0.1555556*	*0.2325581*
**ENSG00000204590**		*0.32727273*		*0.327272727*			*0.2588235*
**ENSG00000164283**		*0.46666667*		*0.466666667*			*0.3197674*
**ENSG00000105289**	*0.16566265*	*0.41515152*	*0.16566265*	*0.415151515*	*0.369047619*	*0.3941176*	*0.377907*
**ENSG00000180891**	*0.19512195*		*0.19512195*				
**ENSG00000167910**		*0.39197531*		*0.391975309*			*0.255814*
**ENSG00000143753**		*0.31081081*		*0.310810811*			*0.4078947*
**ENSG00000183032**	*0.16666667*	*0.46646341*	*0.16666667*	*0.466463415*			*0.4058824*
**ENSG00000136631**	*0.29640719*		*0.29640719*				
**ENSG00000169105**		*0.07878788*		*0.078787879*			*0.1104651*
**ENSG00000197653**							*0.0697674*
**ENSG00000140104**	*0.38323353*	*0.19393939*	*0.38323353*	*0.193939394*	*0.273809524*	*0.377907*	*0.372093*
**ENSG00000137872**	*0.10778443*	*0.3597561*	*0.10778443*	*0.359756098*	*0.477272727*	*0.4666667*	*0.2333333*
**ENSG00000112053**		*0.16060606*		*0.160606061*			*0.0697674*
**ENSG00000132463**	*0.17613636*	*0.05113636*	*0.17613636*	*0.051136364*	*0.155555556*	*0.2386364*	
**ENSG00000165923**		*0.45151515*		*0.451515152*			*0.2848837*
**ENSG00000164953**		*0.41212121*		*0.412121212*			*0.2034884*
**ENSG00000178177**		*0.49444444*		*0.494444444*			*0.2674419*
**ENSG00000196876**	*0.41566265*	*0.24848485*	*0.41566265*	*0.248484848*			*0.5*
**ENSG00000166860**		*0.07272727*		*0.072727273*			*0.1590909*
**ENSG00000175097**		*0.37341772*		*0.373417722*			*0.2317073*
**ENSG00000204673**	*0.44578313*	*0.15454545*	*0.44578313*	*0.154545455*	*0.476190476*	*0.4069767*	*0.0988372*
**ENSG00000225830**	*0.23053892*	*0.3902439*	*0.23053892*	*0.390243902*	*0.306818182*	*0.3181818*	*0.3313953*
**ENSG00000156587**	*0.3742515*	*0.43333333*	*0.3742515*	*0.433333333*	*0.06547619*	*0.1162791*	*0.3255814*
**ENSG00000177628**	*0.47305389*		*0.47305389*		*0.255952381*	*0.2616279*	
**ENSG00000127507**	*0.10479042*	*0.28787879*	*0.10479042*	*0.287878788*	*0.089285714*	*0.0523256*	*0.2840909*
**ENSG00000164011**		*0.17272727*		*0.172727273*			

**ENSG00000155438**								*0.2325581*
**ENSG00000072210**		*0.46363636*		*0.463636364*				*0.1130952*
**ENSG00000174059**	*0.32335329*	*0.15757576*	*0.32335329*	*0.157575758*	*0.291666667*		*0.3255814*	*0.4186047*
**ENSG00000108797**	*0.05389222*	*0.07012195*	*0.05389222*	*0.070121951*				
**ENSG00000111669**	*0.40718563*		*0.40718563*		*0.397590361*		*0.3372093*	
**ENSG00000184602**		*0.46060606*		*0.460606061*				*0.4709302*
**ENSG00000169976**	*0.17065868*		*0.17065868*					
**ENSG00000197024**	*0.39393939*	*0.35151515*	*0.39393939*	*0.351515152*			*0.0952381*	*0.0872093*
**ENSG00000113734**		*0.14242424*		*0.142424242*				
**ENSG00000257591**	*0.24550898*	*0.45151515*	*0.24550898*	*0.451515152*				*0.4127907*
**ENSG00000173214**	*0.15*	*0.44242424*	*0.15*	*0.442424242*	*0.487951807*		*0.4476744*	*0.4302326*
**ENSG00000151360**	*0.43072289*		*0.43072289*		*0.327380952*		*0.3529412*	
**ENSG00000181894**		*0.16060606*		*0.160606061*				*0.3953488*
**ENSG00000114491**	*0.27469136*	*0.16111111*	*0.27469136*	*0.161111111*	*0.398809524*		*0.4883721*	*0.2727273*
**ENSG00000170456**		*0.46646341*		*0.466463415*				*0.4058824*
**ENSG00000253314**		*0.2969697*		*0.296969697*				*0.1744186*
**ENSG00000229164**		*0.49090909*		*0.490909091*				*0.1627907*
**ENSG00000082196**	*0.26347305*	*0.35454545*	*0.26347305*	*0.354545455*	*0.119047619*		*0.127907*	*0.494186*
**ENSG00000141542**		*0.30487805*		*0.304878049*				*0.3647059*
**ENSG00000121236**	*0.4491018*	*0.12962963*	*0.4491018*	*0.12962963*	*0.452380952*		*0.4825581*	*0.3963415*
**ENSG00000198908**		*0.09006211*		*0.090062112*				*0.1823529*
**ENSG00000134291**		*0.45505618*		*0.45505618*				*0.1022727*
**ENSG00000072756**	*0.24157303*	*0.1030303*	*0.24157303*	*0.103030303*				
**ENSG00000145808**		*0.09444444*		*0.094444444*				*0.0872093*
**ENSG00000103710**		*0.15151515*		*0.151515152*				
**ENSG00000176956**		*0.5*		*0.5*				*0.3488372*
**ENSG00000122735**	*0.38922156*	*0.16969697*	*0.38922156*	*0.16969697*	*0.178571429*		*0.125*	*0.3295455*
**ENSG00000167840**		*0.20606061*		*0.206060606*				
**ENSG00000186812**	*0.09337349*	*0.47272727*	*0.09337349*	*0.472727273*	*0.404761905*		*0.3095238*	*0.3372093*
**ENSG00000091986**	*0.33832335*		*0.33832335*		*0.136904762*		*0.0988372*	
**ENSG00000140691**	*0.19161677*		*0.19161677*		*0.285714286*		*0.2674419*	*0.0795455*
**ENSG00000054938**	*0.18373494*		*0.18373494*		*0.43373494*		*0.4302326*	
**ENSG00000134001**	*0.4491018*		*0.4491018*		*0.404761905*		*0.5*	*0.4659091*
**ENSG00000176435**	*0.30821918*		*0.30821918*		*0.397590361*		*0.3918919*	
**ENSG00000213145**	*0.43113772*		*0.43113772*					
**ENSG00000108848**		*0.45283019*		*0.452830189*				*0.4186047*
**ENSG00000177602**		*0.49444444*		*0.494444444*				*0.4659091*
**ENSG00000158828**		*0.46428571*		*0.464285714*				*0.4659091*
**ENSG00000204969**		*0.44545455*		*0.445454545*				*0.4939759*
**ENSG00000107789**	*0.07831325*		*0.07831325*		*0.220238095*		*0.3372093*	
**ENSG00000101361**		*0.32121212*		*0.321212121*	*0.25*		*0.2209302*	*0.2325581*
**ENSG00000179344**	*0.26047904*	*0.41818182*	*0.26047904*	*0.418181818*	*0.488888889*		*0.4418605*	*0.4767442*
**ENSG00000104760**	*0.49101796*		*0.49101796*		*0.433333333*		*0.4360465*	
**ENSG00000116161**		*0.21212121*		*0.212121212*	*0.333333333*		*0.4651163*	*0.0872093*
**ENSG00000116574**		*0.26666667*		*0.266666667*				*0.255814*
**ENSG00000112339**	*0.3*	*0.46363636*	*0.3*	*0.463636364*	*0.433333333*		*0.5*	*0.494186*
**ENSG00000023171**	*0.29640719*	*0.375*	*0.29640719*	*0.375*	*0.261904762*		*0.2411765*	*0.3139535*
**ENSG00000169251**		*0.48255814*		*0.48255814*			*0.0755814*	
**ENSG00000136235**	*0.11976048*	*0.31818182*	*0.11976048*	*0.318181818*	*0.202380952*		*0.2383721*	*0.1395349*
**ENSG00000107968**	*0.39655172*	*0.12727273*	*0.39655172*	*0.127272727*			*0.0555556*	
**ENSG00000185477**		*0.49404762*		*0.494047619*				*0.4767442*
**ENSG00000167397**		*0.38636364*		*0.386363636*				*0.25*
**ENSG00000085511**		*0.4*		*0.4*				*0.2267442*
**ENSG00000173193**	*0.1257485*	*0.4378882*	*0.1257485*	*0.437888199*	*0.06626506*		*0.152439*	*0.4244186*

**ENSG00000164645**	*0.28484848*		*0.28484848*		*0.077777778*	*0.1445783*	
**ENSG00000160856**	*0.39820359*	*0.41212121*	*0.39820359*	*0.412121212*	*0.366666667*	*0.4886364*	*0.4476744*
**ENSG00000075391**		*0.12121212*		*0.121212121*			*0.0529412*
**ENSG00000204695**		*0.17575758*		*0.175757576*			*0.2034884*
**ENSG00000018510**		*0.23333333*		*0.233333333*			*0.3863636*
**ENSG00000117450**	*0.07185629*		*0.07185629*		*0.142857143*	*0.0930233*	
**ENSG00000133980**	*0.10479042*		*0.10479042*		*0.380952381*	*0.4186047*	
**ENSG00000143771**		*0.3*		*0.3*			
**ENSG00000167981**		*0.27878788*		*0.278787879*			*0.1511628*
**ENSG00000123572**	*0.49401198*	*0.37116564*	*0.49401198*	*0.371165644*			*0.0595238*
**ENSG00000187514**	*0.40718563*		*0.40718563*		*0.172619048*	*0.0697674*	
**ENSG00000109079**		*0.05454545*		*0.054545455*			*0.1686047*
**ENSG00000175470**		*0.33939394*		*0.339393939*			*0.4545455*
**ENSG00000138347**	*0.17964072*	*0.48170732*	*0.17964072*	*0.481707317*	*0.25*	*0.2209302*	*0.4318182*
**ENSG00000153066**		*0.30128205*		*0.301282051*			
**ENSG00000153162**		*0.2652439*		*0.265243902*			*0.2559524*
**ENSG00000171102**		*0.0969697*		*0.096969697*			
**ENSG00000139116**		*0.18181818*		*0.181818182*			*0.3117647*
**ENSG00000164627**		*0.47852761*		*0.478527607*	*0.25*	*0.2267442*	*0.4709302*
**ENSG00000115825**		*0.35493827*		*0.354938272*			*0.4767442*
**ENSG00000116874**		*0.47878788*		*0.478787879*			*0.4593023*
**ENSG00000151948**	*0.47005988*	*0.20606061*	*0.47005988*	*0.206060606*	*0.25*	*0.25*	*0.3313953*
**ENSG00000158717**	*0.28353659*	*0.11042945*	*0.28353659*	*0.110429448*	*0.311111111*	*0.3522727*	
**ENSG00000255247**					*0.077777778*	*0.1363636*	
**ENSG00000129187**		*0.42073171*		*0.420731707*			*0.3529412*
**ENSG00000069188**	*0.47305389*	*0.43333333*	*0.47305389*	*0.433333333*	*0.107142857*	*0.0755814*	*0.3255814*
**ENSG00000197852**		*0.45454545*		*0.454545455*			*0.4534884*
**ENSG00000119888**		*0.17272727*		*0.172727273*			*0.1453488*
**ENSG00000180089**		*0.42283951*		*0.422839506*			*0.2823529*
**ENSG00000174791**		*0.36363636*		*0.363636364*			*0.372093*
**ENSG00000169609**	*0.2439759*		*0.2439759*		*0.323170732*	*0.2235294*	
**ENSG00000172171**		*0.1097561*		*0.109756098*			*0.2857143*
**ENSG00000018408**	*0.32022472*	*0.25*	*0.32022472*	*0.25*	*0.433333333*	*0.4318182*	*0.2045455*
**ENSG00000175395**		*0.48780488*		*0.487804878*			*0.4882353*
**ENSG00000143570**	*0.23053892*	*0.13939394*	*0.23053892*	*0.139393939*	*0.488095238*	*0.4244186*	
**ENSG00000138639**	*0.3502994*	*0.26436782*	*0.3502994*	*0.264367816*	*0.452380952*	*0.4127907*	*0.3863636*
**ENSG00000075234**		*0.14942529*		*0.149425287*			
**ENSG00000177519**		*0.47222222*		*0.472222222*			*0.1860465*
**ENSG00000183640**		*0.48484848*		*0.484848485*			*0.3313953*
**ENSG00000121481**		*0.05454545*		*0.054545455*			*0.3895349*
**ENSG00000115239**	*0.47904192*	*0.20348837*	*0.47904192*	*0.203488372*	*0.464285714*	*0.4534884*	*0.2954545*
**ENSG00000119922**		*0.21515152*		*0.215151515*			
**ENSG00000067066**	*0.37125749*	*0.40490798*	*0.37125749*	*0.404907975*	*0.464285714*	*0.4476744*	*0.2209302*
**ENSG00000137776**		*0.21036585*		*0.210365854*			*0.4470588*
**ENSG00000171954**		*0.48484848*		*0.484848485*			*0.5*
**ENSG00000104381**		*0.32022472*		*0.320224719*			*0.3295455*
**ENSG00000205786**	*0.39520958*		*0.39520958*		*0.345238095*	*0.4352941*	
**ENSG00000129295**		*0.09393939*		*0.093939394*			
**ENSG00000164258**		*0.49375*		*0.49375*			*0.3095238*
**ENSG00000125834**	*0.22727273*	*0.49074074*	*0.22727273*	*0.490740741*	*0.142857143*	*0.2151163*	*0.0930233*
**ENSG00000176871**		*0.28787879*		*0.287878788*			*0.4651163*
**ENSG00000174442**	*0.26807229*	*0.09393939*	*0.26807229*	*0.093939394*	*0.493975904*	*0.4709302*	
**ENSG00000187109**		*0.39634146*		*0.396341463*			*0.4888889*
**ENSG00000009709**		*0.43939394*		*0.439393939*	*0.071428571*	*0.0697674*	*0.4418605*

**ENSG00000130226**		*0.21212121*		*0.212121212*				*0.25*
**ENSG00000172878**	*0.16981132*	*0.48742138*	*0.16981132*	*0.487421384*	*0.18125*		*0.1325301*	*0.4085366*
**ENSG00000167325**		*0.46111111*		*0.461111111*	*0.366666667*		*0.4222222*	*0.4222222*
**ENSG00000153404**		*0.46060606*		*0.460606061*				*0.474359*
**ENSG00000162630**		*0.05454545*		*0.054545455*				
**ENSG00000095015**		*0.38888889*		*0.388888889*				*0.1097561*
**ENSG00000241794**	*0.10179641*		*0.10179641*					*0.0681818*
**ENSG00000172399**		*0.25454545*		*0.254545455*				*0.4883721*
**ENSG00000114529**		*0.45*		*0.45*				*0.5*
**ENSG00000103023**	*0.09281437*	*0.05182927*	*0.09281437*	*0.051829268*	*0.37804878*		*0.4244186*	
**ENSG00000151704**	*0.17964072*	*0.16666667*	*0.17964072*	*0.166666667*	*0.083333333*		*0.0639535*	*0.0755814*
**ENSG00000005243**		*0.38181818*		*0.381818182*				*0.1976744*
**ENSG00000172269**	*0.11377246*	*0.45454545*	*0.11377246*	*0.454545455*	*0.25*		*0.2034884*	*0.4651163*
**ENSG00000085788**		*0.26666667*		*0.266666667*				*0.3488372*
**ENSG00000140660**		*0.28333333*		*0.283333333*				*0.4886364*
**ENSG00000160401**		*0.46969697*		*0.46969697*				*0.3255814*
**ENSG00000116337**		*0.17575758*		*0.175757576*				
**ENSG00000147724**		*0.39634146*		*0.396341463*	*0.066666667*		*0.1136364*	*0.4418605*
**ENSG00000163735**		*0.3*		*0.3*				*0.372093*
**ENSG00000213024**	*0.20359281*	*0.40490798*	*0.20359281*	*0.404907975*	*0.445783133*		*0.4418605*	*0.3837209*
**ENSG00000109906**	*0.33707865*		*0.33707865*		*0.388888889*		*0.4555556*	
**ENSG00000092068**	*0.47159091*	*0.47575758*	*0.47159091*	*0.475757576*	*0.466666667*		*0.4772727*	*0.4534884*
**ENSG00000111321**		*0.25757576*		*0.257575758*				*0.1705882*
**ENSG00000159459**		*0.11818182*		*0.118181818*				*0.2209302*
**ENSG00000164978**	*0.43113772*		*0.43113772*		*0.43452381*		*0.4127907*	
**ENSG00000171777**								*0.2674419*
**ENSG00000197043**	*0.28143713*	*0.06111111*	*0.28143713*	*0.061111111*	*0.410714286*		*0.4941176*	
**ENSG00000214447**	*0.22155689*	*0.24848485*	*0.22155689*	*0.248484848*	*0.357142857*		*0.2034884*	*0.1964286*
**ENSG00000022567**	*0.18562874*	*0.44252874*	*0.18562874*	*0.442528736*	*0.196428571*		*0.1744186*	*0.4294118*
**ENSG00000164172**		*0.25*		*0.25*	*0.172619048*		*0.0813953*	*0.0988372*
**ENSG00000146013**		*0.31666667*		*0.316666667*				*0.375*
**ENSG00000162551**		*0.22121212*		*0.221212121*				*0.3863636*
**ENSG00000165474**		*0.17575758*		*0.175757576*				*0.494186*
**ENSG00000159650**		*0.42121212*		*0.421212121*				*0.4764706*
**ENSG00000130414**		*0.44848485*		*0.448484848*				*0.4883721*
**ENSG00000162391**	*0.05988024*	*0.05*	*0.05988024*	*0.05*				*0.2428571*
**ENSG00000144852**	*0.40718563*	*0.20245399*	*0.40718563*	*0.202453988*	*0.289156627*		*0.2823529*	*0.494186*
**ENSG00000254245**		*0.20224719*		*0.202247191*				*0.1477273*
**ENSG00000182816**	*0.07784431*		*0.07784431*					
**ENSG00000106089**		*0.26666667*		*0.266666667*				*0.2209302*
**ENSG00000140632**		*0.23939394*		*0.239393939*				*0.1976744*
**ENSG00000180008**		*0.44242424*		*0.442424242*				*0.25*
**ENSG00000162976**		*0.1954023*		*0.195402299*				
**ENSG00000139675**	*0.07777778*		*0.07777778*					
**ENSG00000117281**		*0.30606061*		*0.306060606*				*0.1941176*
**ENSG00000137944**		*0.47575758*		*0.475757576*				*0.494186*
**ENSG00000188488**	*0.34431138*	*0.47878788*	*0.34431138*	*0.478787879*	*0.340909091*		*0.4*	*0.1590909*
**ENSG00000196743**	*0.49700599*	*0.33888889*	*0.49700599*	*0.338888889*	*0.422619048*		*0.4534884*	*0.3333333*
**ENSG00000111445**	*0.20658683*		*0.20658683*		*0.178571429*		*0.2034884*	
**ENSG00000205078**		*0.23888889*		*0.238888889*				
**ENSG00000101276**	*0.18263473*	*0.25757576*	*0.18263473*	*0.257575758*	*0.380952381*		*0.4767442*	*0.3662791*
**ENSG00000102349**		*0.09090909*		*0.090909091*				
**ENSG00000175604**	*0.34431138*	*0.33707865*	*0.34431138*	*0.337078652*	*0.204819277*		*0.2848837*	*0.2857143*
**ENSG00000132915**		*0.44242424*		*0.442424242*				*0.4651163*

**ENSG00000072315**	*0.35928144*		*0.35928144*				
**ENSG00000213889**					*0.077380952*		
**ENSG00000015153**		*0.43636364*		*0.436363636*			*0.4352941*
**ENSG00000153012**		*0.4969697*		*0.496969697*			*0.3882353*
**ENSG00000023909**		*0.28571429*		*0.285714286*			*0.2650602*
**ENSG00000198925**		*0.28220859*		*0.282208589*			*0.1463415*
**ENSG00000232103**	*0.17964072*		*0.17964072*		*0.313253012*	*0.4047619*	
**ENSG00000088992**		*0.2*		*0.2*			*0.2325581*
**ENSG00000138696**		*0.46666667*		*0.466666667*			*0.377907*
**ENSG00000127663**		*0.43452381*		*0.43452381*			*0.3197674*
**ENSG00000154832**						*0.0795455*	
**ENSG00000170522**		*0.34090909*		*0.340909091*			*0.4593023*
**ENSG00000105568**	*0.25748503*	*0.32222222*	*0.25748503*	*0.322222222*	*0.089285714*		*0.1918605*
**ENSG00000111701**		*0.24539877*		*0.245398773*			*0.4204545*
**ENSG00000182985**		*0.4969697*		*0.496969697*			*0.1627907*
**ENSG00000172922**		*0.38333333*		*0.383333333*			*0.3522727*
**ENSG00000137496**	*0.43674699*	*0.06179775*	*0.43674699*	*0.061797753*	*0.345238095*	*0.255814*	*0.1931818*
**ENSG00000100575**	*0.44578313*		*0.44578313*				
**ENSG00000196865**		*0.28181818*		*0.281818182*			*0.2294118*
**ENSG00000099338**		*0.49090909*		*0.490909091*			*0.4651163*
**ENSG00000125901**							*0.3488372*
**ENSG00000171056**		*0.28484848*		*0.284848485*			*0.4418605*
**ENSG00000198300**		*0.4969697*		*0.496969697*			*0.4651163*
**ENSG00000166451**	*0.1*		*0.1*		*0.344444444*	*0.375*	
**ENSG00000172009**	*0.22392638*		*0.22392638*		*0.36746988*	*0.3647059*	
**ENSG00000123411**							*0.1136364*
**ENSG00000122870**		*0.05*		*0.05*			*0.0666667*
**ENSG00000176593**	*0.15568862*	*0.41212121*	*0.15568862*	*0.412121212*	*0.083333333*	*0.0581395*	*0.4011628*
**ENSG00000154764**		*0.41515152*		*0.415151515*			*0.4*
**ENSG00000145687**		*0.28787879*		*0.287878788*			*0.1*
**ENSG00000095981**	*0.40419162*	*0.46363636*	*0.40419162*	*0.463636364*	*0.380952381*	*0.2823529*	*0.3662791*
**ENSG00000176771**	*0.1257485*	*0.08181818*	*0.1257485*	*0.081818182*	*0.068181818*		*0.3895349*
**ENSG00000197417**		*0.33939394*		*0.339393939*			*0.3823529*
**ENSG00000167971**		*0.05521472*		*0.055214724*			
**ENSG00000013725**		*0.37804878*		*0.37804878*			*0.1*
**ENSG00000132517**	*0.2*		*0.2*		*0.102409639*	*0.1046512*	
**ENSG00000009950**		*0.18484848*		*0.184848485*			*0.1104651*
**ENSG00000127124**	*0.18072289*	*0.42121212*	*0.18072289*	*0.421212121*	*0.392857143*	*0.4411765*	*0.4545455*
**ENSG00000113812**	*0.12121212*	*0.29393939*	*0.12121212*	*0.293939394*	*0.084337349*	*0.1705882*	*0.3895349*
**ENSG00000127528**		*0.3258427*		*0.325842697*			*0.2840909*
**ENSG00000218891**		*0.08484848*		*0.084848485*			
**ENSG00000198142**		*0.43636364*		*0.436363636*			*0.4302326*
**ENSG00000073008**		*0.19090909*		*0.190909091*			*0.1352941*
**ENSG00000204682**		*0.30606061*		*0.306060606*			
**ENSG00000145476**		*0.48333333*		*0.483333333*			*0.3863636*
**ENSG00000164294**		*0.20606061*		*0.206060606*			*0.2151163*
**ENSG00000182195**	*0.38068182*	*0.35757576*	*0.38068182*	*0.357575758*	*0.266666667*	*0.2159091*	*0.1744186*
**ENSG00000214960**		*0.15822785*		*0.158227848*			*0.1627907*
**ENSG00000159131**		*0.23939394*		*0.239393939*	*0.214285714*	*0.2093023*	*0.1976744*
**ENSG00000107159**	*0.07831325*		*0.07831325*		*0.162650602*	*0.3352941*	
**ENSG00000121897**		*0.36666667*		*0.366666667*			*0.3295455*
**ENSG00000147481**	*0.08083832*		*0.08083832*				
**ENSG00000175550**	*0.12951807*		*0.12951807*		*0.18452381*	*0.0813953*	
**ENSG00000114126**	*0.4011976*	*0.37575758*	*0.4011976*	*0.375757576*	*0.339285714*	*0.3662791*	*0.2674419*

**ENSG00000111364**		*0.34444444*		*0.344444444*				
**ENSG00000155761**		*0.49085366*		*0.490853659*				*0.4583333*
**ENSG00000132300**		*0.47272727*		*0.472727273*				*0.4011628*
**ENSG00000136869**		*0.14444444*		*0.144444444*				*0.25*
**ENSG00000183303**		*0.27272727*		*0.272727273*				*0.122093*
**ENSG00000153201**		*0.22121212*		*0.221212121*				*0.1046512*
**ENSG00000105647**		*0.12068966*		*0.120689655*				
**ENSG00000038532**	*0.29545455*	*0.06179775*	*0.29545455*	*0.061797753*				*0.1555556*
**ENSG00000182771**		*0.45757576*		*0.457575758*				*0.3546512*
**ENSG00000166478**					*0.05952381*		*0.0581395*	
**ENSG00000241962**	*0.10179641*	*0.375*	*0.10179641*	*0.375*	*0.266666667*		*0.2386364*	*0.2764706*
**ENSG00000166289**		*0.06666667*		*0.066666667*				*0.2111111*
**ENSG00000149084**		*0.29447853*		*0.294478528*				*0.1927711*
**ENSG00000186132**	*0.22754491*	*0.38109756*	*0.22754491*	*0.381097561*	*0.101190476*		*0.0755814*	*0.3666667*
**ENSG00000046604**		*0.38888889*		*0.388888889*				*0.2386364*
**ENSG00000101134**		*0.45757576*		*0.457575758*				*0.4534884*
**ENSG00000139131**		*0.16158537*		*0.161585366*				
**ENSG00000135900**		*0.44444444*		*0.444444444*				*0.25*
**ENSG00000138675**	*0.39156627*	*0.35151515*	*0.39156627*	*0.351515152*	*0.053571429*		*0.0823529*	*0.2616279*
**ENSG00000170961**	*0.49700599*	*0.42777778*	*0.49700599*	*0.427777778*	*0.25*		*0.3313953*	
**ENSG00000082146**		*0.44444444*		*0.444444444*				*0.2840909*
**ENSG00000198521**	*0.17365269*		*0.17365269*		*0.077380952*		*0.1686047*	
**ENSG00000253485**		*0.20224719*		*0.202247191*				*0.1477273*
**ENSG00000170100**	*0.29885057*		*0.29885057*		*0.077380952*		*0.0639535*	
**ENSG00000180448**		*0.44545455*		*0.445454545*	*0.392857143*		*0.313253*	*0.1337209*
**ENSG00000188817**		*0.49090909*		*0.490909091*				*0.4888889*
**ENSG00000086015**	*0.36526946*		*0.36526946*		*0.333333333*		*0.2732558*	
**ENSG00000102858**		*0.47126437*		*0.471264368*	*0.166666667*		*0.0795455*	*0.3430233*
**ENSG00000197386**		*0.29503106*		*0.295031056*				*0.3470588*
**ENSG00000198443**		*0.08181818*		*0.081818182*	*0.466666667*		*0.402439*	
**ENSG00000151617**		*0.45505618*		*0.45505618*	*0.343373494*		*0.4302326*	*0.4666667*
**ENSG00000114423**	*0.46969697*	*0.45426829*	*0.46969697*	*0.454268293*	*0.433333333*		*0.3522727*	*0.3197674*
**ENSG00000128394**	*0.10843373*	*0.5*	*0.10843373*	*0.5*				*0.2705882*
**ENSG00000095587**		*0.34545455*		*0.345454545*				*0.4883721*
**ENSG00000203747**		*0.25842697*		*0.258426966*	*0.121621622*		*0.0595238*	
**ENSG00000182858**	*0.15060241*	*0.23030303*	*0.15060241*	*0.23030303*	*0.144578313*		*0.1860465*	*0.1802326*
**ENSG00000100578**					*0.226190476*		*0.2616279*	
**ENSG00000212712**		*0.27878788*		*0.278787879*				*0.3555556*
**ENSG00000110066**		*0.41011236*		*0.41011236*				*0.0795455*
**ENSG00000237649**	*0.09444444*		*0.09444444*		*0.395348837*		*0.2380952*	
**ENSG00000214107**	*0.13333333*		*0.13333333*		*0.422222222*		*0.2840909*	
**ENSG00000182378**		*0.31460674*		*0.314606742*				*0.4772727*
**ENSG00000100024**	*0.34269663*	*0.42771084*	*0.34269663*	*0.427710843*	*0.466666667*		*0.3863636*	*0.4545455*
**ENSG00000155962**		*0.18181818*		*0.181818182*				*0.1569767*
**ENSG00000162040**		*0.18012422*		*0.180124224*				*0.2267442*
**ENSG00000167554**	*0.47560976*	*0.35582822*	*0.47560976*	*0.355828221*	*0.380952381*		*0.3488372*	*0.2790698*
**ENSG00000158773**	*0.16766467*	*0.27878788*	*0.16766467*	*0.278787879*	*0.345238095*		*0.3255814*	*0.2093023*
**ENSG00000117425**								*0.3513514*
**ENSG00000204897**								*0.1976744*
**ENSG00000104231**	*0.14071856*	*0.15757576*	*0.14071856*	*0.157575758*	*0.477777778*		*0.4318182*	*0.3837209*
**ENSG00000183657**	*0.2245509*	*0.37078652*	*0.2245509*	*0.370786517*	*0.277777778*		*0.2325581*	*0.2272727*
**ENSG00000169184**		*0.08181818*		*0.081818182*				*0.1111111*
**ENSG00000203772**		*0.33231707*		*0.332317073*				*0.4529412*
**ENSG00000134769**		*0.25555556*		*0.255555556*				

**ENSG00000124198**		*0.38333333*		*0.383333333*				*0.2916667*
**ENSG00000115841**	*0.37222222*	*0.21515152*	*0.37222222*	*0.215151515*	*0.277777778*		*0.2111111*	*0.4069767*
**ENSG00000147571**	*0.06481481*		*0.06481481*					
**ENSG00000116128**	*0.08083832*	*0.15454545*	*0.08083832*	*0.154545455*	*0.327380952*		*0.3372093*	*0.372093*
**ENSG00000122591**		*0.48214286*		*0.482142857*				*0.472973*
**ENSG00000143375**		*0.14939024*		*0.149390244*				*0.244186*
**ENSG00000179111**		*0.29090909*		*0.290909091*				*0.4011628*
**ENSG00000104218**		*0.10365854*		*0.103658537*				
**ENSG00000111077**		*0.08484848*		*0.084848485*				*0.2267442*
**ENSG00000137812**	*0.23595506*	*0.37575758*	*0.23595506*	*0.375757576*	*0.266666667*		*0.3409091*	*0.3409091*
**ENSG00000133678**		*0.31515152*		*0.315151515*				*0.3488372*
**ENSG00000159884**		*0.28787879*		*0.287878788*				*0.4411765*
**ENSG00000165164**								*0.2209302*
**ENSG00000101311**	*0.40718563*	*0.2247191*	*0.40718563*	*0.224719101*	*0.351190476*		*0.3546512*	*0.3392857*
**ENSG00000145934**	*0.30555556*	*0.33030303*	*0.30555556*	*0.33030303*				*0.0930233*
**ENSG00000130701**		*0.46518987*		*0.465189873*				*0.1104651*
**ENSG00000140474**		*0.26969697*		*0.26969697*				*0.2848837*
**ENSG00000081853**		*0.20224719*		*0.202247191*				*0.1477273*
**ENSG00000134470**	*0.18333333*	*0.49090909*	*0.18333333*	*0.490909091*				*0.3837209*
**ENSG00000196353**	*0.48876404*		*0.48876404*		*0.5*		*0.494186*	
**ENSG00000230891**	*0.14071856*		*0.14071856*				*0.1162791*	
**ENSG00000088035**		*0.43292683*		*0.432926829*				*0.402439*
**ENSG00000119396**		*0.45*		*0.45*				*0.1590909*
**ENSG00000124091**	*0.19879518*		*0.19879518*					
**ENSG00000170236**								*0.1511628*
**ENSG00000180190**		*0.46341463*		*0.463414634*				*0.4360465*
**ENSG00000172458**		*0.42424242*		*0.424242424*				*0.2790698*
**ENSG00000111834**		*0.23939394*		*0.239393939*				*0.372093*
**ENSG00000187812**	*0.23780488*		*0.23780488*		*0.108108108*		*0.2142857*	
**ENSG00000101182**	*0.09444444*	*0.2195122*	*0.09444444*	*0.219512195*	*0.233333333*		*0.2045455*	*0.1704545*
**ENSG00000129515**		*0.43820225*		*0.438202247*				*0.2674419*
**ENSG00000205838**		*0.37272727*		*0.372727273*				*0.4011628*
**ENSG00000093010**	*0.22891566*	*0.29573171*	*0.22891566*	*0.295731707*				*0.377907*
**ENSG00000127533**					*0.121621622*		*0.0641026*	
**ENSG00000111911**		*0.33030303*		*0.33030303*				
**ENSG00000125347**	*0.3258427*	*0.4*	*0.3258427*	*0.4*	*0.406976744*		*0.2674419*	*0.3555556*
**ENSG00000123096**		*0.44242424*		*0.442424242*				*0.3154762*
**ENSG00000091138**		*0.21341463*		*0.213414634*				*0.2674419*
**ENSG00000175221**		*0.26666667*		*0.266666667*				
**ENSG00000119125**		*0.4969697*		*0.496969697*				*0.494186*
**ENSG00000151413**		*0.36060606*		*0.360606061*				*0.4825581*
**ENSG00000101187**	*0.14670659*	*0.45426829*	*0.14670659*	*0.454268293*	*0.172619048*		*0.2151163*	*0.4709302*
**ENSG00000128594**		*0.41818182*		*0.418181818*				*0.1802326*
**ENSG00000119929**	*0.47305389*		*0.47305389*		*0.410714286*		*0.4244186*	
**ENSG00000168883**								*0.3488372*
**ENSG00000187987**	*0.45808383*	*0.16666667*	*0.45808383*	*0.166666667*	*0.464285714*		*0.4709302*	*0.1333333*
**ENSG00000154898**		*0.05757576*		*0.057575758*				
**ENSG00000141668**	*0.21590909*	*0.14545455*	*0.21590909*	*0.145454545*	*0.488888889*		*0.4886364*	*0.4941176*
**ENSG00000156463**		*0.38787879*		*0.387878788*				*0.2727273*
**ENSG00000078304**		*0.14545455*		*0.145454545*				*0.297619*
**ENSG00000152779**	*0.07784431*		*0.07784431*		*0.279761905*		*0.1976744*	
**ENSG00000178381**	*0.46107784*	*0.2030303*	*0.46107784*	*0.203030303*	*0.21686747*		*0.2906977*	
**ENSG00000259272**		*0.08888889*		*0.088888889*				
**ENSG00000154114**	*0.11931818*	*0.30606061*	*0.11931818*	*0.306060606*	*0.261904762*		*0.2954545*	*0.2674419*

**ENSG00000108846**		*0.2*		*0.2*			*0.1976744*
**ENSG00000114354**							*0.2142857*
**ENSG00000197081**		*0.16666667*		*0.166666667*			*0.1444444*
**ENSG00000112599**	*0.06287425*		*0.06287425*		*0.071428571*	*0.0523256*	*0.0988372*
**ENSG00000133424**	*0.20658683*	*0.23030303*	*0.20658683*	*0.23030303*	*0.18452381*	*0.2209302*	*0.2034884*
**ENSG00000179178**	*0.1686747*		*0.1686747*		*0.172619048*	*0.1764706*	
**ENSG00000068615**		*0.46363636*		*0.463636364*			*0.4476744*
**ENSG00000143653**		*0.42727273*		*0.427272727*			*0.2926829*
**ENSG00000163486**	*0.40419162*	*0.43888889*	*0.40419162*	*0.438888889*	*0.261904762*	*0.2906977*	*0.3139535*
**ENSG00000166169**		*0.31515152*		*0.315151515*			
**ENSG00000078246**		*0.17878788*		*0.178787879*			*0.3023256*
**ENSG00000168481**		*0.21212121*		*0.212121212*			*0.0988372*
**ENSG00000133561**	*0.37222222*	*0.3030303*	*0.37222222*	*0.303030303*	*0.458333333*	*0.4222222*	*0.375*
**ENSG00000127415**	*0.26946108*		*0.26946108*		*0.476190476*	*0.2848837*	
**ENSG00000117600**	*0.14071856*		*0.14071856*				
**ENSG00000167685**	*0.17045455*	*0.09393939*	*0.17045455*	*0.093939394*	*0.476190476*	*0.4352941*	*0.3081395*
**ENSG00000027075**	*0.46107784*	*0.07575758*	*0.46107784*	*0.075757576*	*0.453488372*	*0.4111111*	*0.1235294*
**ENSG00000172775**		*0.06666667*		*0.066666667*			
**ENSG00000188026**	*0.41916168*	*0.38484848*	*0.41916168*	*0.384848485*			
**ENSG00000172365**	*0.24719101*		*0.24719101*		*0.144444444*	*0.1818182*	
**ENSG00000144959**	*0.18862275*	*0.30606061*	*0.18862275*	*0.306060606*	*0.25*	*0.2616279*	*0.1764706*
**ENSG00000111737**		*0.19620253*		*0.196202532*			*0.4883721*
**ENSG00000174720**		*0.06321839*		*0.063218391*			*0.3409091*
**ENSG00000169469**	*0.43413174*		*0.43413174*		*0.375*	*0.4418605*	
**ENSG00000167720**		*0.05521472*		*0.055214724*			
**ENSG00000106049**		*0.32777778*		*0.327777778*			*0.1162791*
**ENSG00000196653**	*0.0988024*	*0.07878788*	*0.0988024*	*0.078787879*			
**ENSG00000198515**	*0.31437126*		*0.31437126*		*0.398809524*	*0.4186047*	
**ENSG00000152990**		*0.3*		*0.3*			*0.2613636*
**ENSG00000123901**		*0.45*		*0.45*			*0.3333333*
**ENSG00000185010**		*0.23939394*		*0.239393939*			*0.1104651*
**ENSG00000136045**		*0.37777778*		*0.377777778*			*0.2727273*
**ENSG00000231824**		*0.39655172*		*0.396551724*			*0.4360465*
**ENSG00000164366**		*0.25454545*		*0.254545455*			
**ENSG00000121005**	*0.48314607*	*0.34259259*	*0.48314607*	*0.342592593*	*0.1*	*0.0813953*	*0.3*
**ENSG00000188001**		*0.34269663*		*0.342696629*	*0.428571429*	*0.4358974*	*0.4825581*
**ENSG00000196154**	*0.44311377*		*0.44311377*				
**ENSG00000097021**							*0.2383721*
**ENSG00000139220**	*0.25304878*	*0.18333333*	*0.25304878*	*0.183333333*	*0.136904762*	*0.1294118*	*0.2045455*
**ENSG00000259202**	*0.05988024*	*0.41768293*	*0.05988024*	*0.417682927*			*0.4244186*
**ENSG00000010539**		*0.47701149*		*0.477011494*			*0.3977273*
**ENSG00000167460**	*0.12359551*		*0.12359551*				
**ENSG00000169174**		*0.30232558*		*0.302325581*			*0.1022727*
**ENSG00000102287**	*0.39221557*	*0.375*	*0.39221557*	*0.375*	*0.101190476*	*0.1395349*	*0.1395349*
**ENSG00000188549**	*0.15697674*	*0.28787879*	*0.15697674*	*0.287878788*	*0.125*	*0.0909091*	*0.3522727*
**ENSG00000196914**		*0.2*		*0.2*			
**ENSG00000012048**		*0.33939394*		*0.339393939*		*0.0941176*	*0.2941176*
**ENSG00000164815**	*0.46067416*		*0.46067416*		*0.488636364*	*0.5*	
**ENSG00000111181**		*0.36363636*		*0.363636364*			*0.3372093*
**ENSG00000132164**		*0.15757576*		*0.157575758*			*0.1818182*
**ENSG00000089225**		*0.23333333*		*0.233333333*			*0.3764706*
**ENSG00000140367**		*0.06111111*		*0.061111111*			
**ENSG00000134758**		*0.48181818*		*0.481818182*			*0.3095238*
**ENSG00000168275**	*0.26204819*		*0.26204819*		*0.398809524*	*0.4360465*	

**ENSG00000165521**		*0.07926829*		*0.079268293*			
**ENSG00000165197**	*0.4760479*		*0.4760479*		*0.408536585*	*0.3411765*	
**ENSG00000120451**	*0.46590909*	*0.44848485*	*0.46590909*	*0.448484848*	*0.473684211*	*0.3488372*	*0.5*
**ENSG00000055163**	*0.46107784*	*0.32121212*	*0.46107784*	*0.321212121*	*0.44047619*	*0.494186*	*0.4127907*
**ENSG00000184517**		*0.46932515*		*0.469325153*			*0.2848837*
**ENSG00000100811**		*0.07272727*		*0.072727273*			
**ENSG00000162620**	*0.16666667*	*0.08484848*	*0.16666667*	*0.084848485*	*0.31547619*	*0.3409091*	
**ENSG00000100911**	*0.26047904*		*0.26047904*		*0.369047619*	*0.3895349*	
**ENSG00000172748**	*0.10606061*	*0.37878788*	*0.10606061*	*0.378787879*	*0.422619048*	*0.3430233*	*0.4593023*
**ENSG00000108187**		*0.18944099*		*0.189440994*			*0.2840909*
**ENSG00000151702**		*0.26470588*		*0.264705882*			*0.275*
**ENSG00000171940**	*0.27844311*	*0.17575758*	*0.27844311*	*0.175757576*	*0.244186047*	*0.3111111*	
**ENSG00000161021**		*0.33333333*		*0.333333333*			*0.3068182*
**ENSG00000138448**		*0.49393939*		*0.493939394*			*0.1976744*
**ENSG00000113048**		*0.33333333*		*0.333333333*			*0.3139535*
**ENSG00000183020**	*0.40419162*	*0.43333333*	*0.40419162*	*0.433333333*	*0.273809524*	*0.3604651*	*0.377907*
**ENSG00000108788**		*0.37804878*		*0.37804878*			*0.4418605*
**ENSG00000161980**		*0.17073171*		*0.170731707*			
**ENSG00000136156**							*0.0681818*
**ENSG00000167080**	*0.43113772*		*0.43113772*		*0.160714286*	*0.0988372*	
**ENSG00000151240**		*0.11212121*		*0.112121212*			
**ENSG00000171044**		*0.36206897*		*0.362068966*			*0.1744186*
**ENSG00000108932**		*0.22222222*		*0.222222222*			*0.2272727*
**ENSG00000173702**		*0.20909091*		*0.209090909*			*0.1104651*
**ENSG00000135298**	*0.19461078*		*0.19461078*		*0.220238095*	*0.1511628*	
**ENSG00000143520**		*0.14545455*		*0.145454545*			*0.4651163*
**ENSG00000189401**		*0.3597561*		*0.359756098*			
**ENSG00000104419**	*0.18263473*	*0.38181818*	*0.18263473*	*0.381818182*			*0.2235294*
**ENSG00000167528**	*0.05988024*	*0.41515152*	*0.05988024*	*0.415151515*	*0.255952381*	*0.2093023*	*0.3895349*
**ENSG00000198363**	*0.10555556*	*0.49693252*	*0.10555556*	*0.496932515*	*0.295454545*	*0.25*	*0.4883721*
**ENSG00000203943**		*0.06111111*		*0.061111111*			
**ENSG00000165501**	*0.3125*	*0.43333333*	*0.3125*	*0.433333333*	*0.188888889*	*0.1136364*	*0.1*
**ENSG00000095397**	*0.4*		*0.4*		*0.340909091*	*0.5*	
**ENSG00000127311**		*0.35757576*		*0.357575758*			*0.3604651*
**ENSG00000171202**	*0.05747126*		*0.05747126*				
**ENSG00000171502**	*0.0508982*	*0.5*	*0.0508982*	*0.5*	*0.113095238*	*0.0639535*	*0.4476744*
**ENSG00000151023**		*0.34545455*		*0.345454545*			*0.4883721*
**ENSG00000188848**		*0.36111111*		*0.361111111*			*0.0882353*
**ENSG00000132405**		*0.47575758*		*0.475757576*			*0.4534884*
**ENSG00000166224**		*0.2030303*		*0.203030303*			*0.4302326*
**ENSG00000141506**	*0.15269461*		*0.15269461*				
**ENSG00000203863**	*0.20394737*	*0.2652439*	*0.20394737*	*0.265243902*			*0.1744186*
**ENSG00000105146**					*0.126506024*	*0.1453488*	
**ENSG00000258653**	*0.4375*	*0.48181818*	*0.4375*	*0.481818182*	*0.422619048*	*0.4204545*	*0.4127907*
**ENSG00000133878**	*0.40361446*		*0.40361446*		*0.494047619*	*0.4651163*	
**ENSG00000180263**		*0.32822086*		*0.328220859*			*0.3604651*
**ENSG00000171621**		*0.45454545*		*0.454545455*			*0.3139535*
**ENSG00000167657**		*0.20689655*		*0.206896552*			*0.2840909*
**ENSG00000144771**	*0.16467066*		*0.16467066*		*0.107142857*	*0.1162791*	
**ENSG00000084693**		*0.36890244*		*0.368902439*			*0.1882353*
**ENSG00000163377**		*0.3908046*		*0.390804598*	*0.488095238*	*0.4705882*	*0.1860465*
**ENSG00000162711**		*0.3258427*		*0.325842697*			*0.4555556*
**ENSG00000159445**		*0.42073171*		*0.420731707*			*0.3837209*
**ENSG00000113600**					*0.053571429*		

**ENSG00000198176**		*0.05*		*0.05*	*0.083333333*		*0.0764706*	
**ENSG00000135960**	*0.49438202*	*0.12727273*	*0.49438202*	*0.127272727*	*0.11627907*		*0.1363636*	*0.1704545*
**ENSG00000196811**								*0.1569767*
**ENSG00000165476**		*0.47468354*		*0.474683544*				*0.4880952*
**ENSG00000246922**	*0.10555556*		*0.10555556*					
**ENSG00000095002**	*0.0988024*	*0.38181818*	*0.0988024*	*0.381818182*				*0.4302326*
**ENSG00000151148**	*0.21856287*	*0.46646341*	*0.21856287*	*0.466463415*	*0.107142857*		*0.122093*	*0.25*
**ENSG00000147533**		*0.26666667*		*0.266666667*				*0.2159091*
**ENSG00000175920**		*0.45757576*		*0.457575758*				*0.4625*
**ENSG00000143119**	*0.08682635*	*0.13414634*	*0.08682635*	*0.134146341*	*0.363095238*		*0.3313953*	*0.1686047*
**ENSG00000164484**	*0.3258427*		*0.3258427*		*0.348837209*		*0.4666667*	
**ENSG00000105854**		*0.22222222*		*0.222222222*				*0.2159091*
**ENSG00000107929**		*0.49090909*		*0.490909091*				*0.5*
**ENSG00000169918**	*0.19760479*	*0.4847561*	*0.19760479*	*0.484756098*	*0.246987952*		*0.3214286*	*0.3430233*
**ENSG00000100221**		*0.29393939*		*0.293939394*				*0.4705882*
**ENSG00000156639**		*0.14942529*		*0.149425287*				*0.2333333*
**ENSG00000141433**	*0.41017964*	*0.28353659*	*0.41017964*	*0.283536585*	*0.083333333*		*0.1309524*	*0.2093023*
**ENSG00000146425**	*0.19444444*		*0.19444444*		*0.244444444*		*0.2727273*	
**ENSG00000131849**	*0.36227545*	*0.47575758*	*0.36227545*	*0.475757576*				*0.244186*
**ENSG00000121905**		*0.05151515*		*0.051515152*				
**ENSG00000174780**		*0.31818182*		*0.318181818*				*0.3139535*
**ENSG00000152268**	*0.11676647*	*0.32386364*	*0.11676647*	*0.323863636*				*0.4411765*
**ENSG00000197841**		*0.32183908*		*0.32183908*				*0.4244186*
**ENSG00000175485**	*0.48235294*	*0.06666667*	*0.48235294*	*0.066666667*				*0.1686047*
**ENSG00000161405**		*0.46363636*		*0.463636364*				*0.372093*
**ENSG00000148935**	*0.08383234*		*0.08383234*					
**ENSG00000253379**		*0.33939394*		*0.339393939*				*0.3255814*
**ENSG00000117090**	*0.11676647*	*0.4068323*	*0.11676647*	*0.406832298*	*0.36746988*		*0.2823529*	*0.3666667*
**ENSG00000095951**								*0.3372093*
**ENSG00000159166**		*0.06321839*		*0.063218391*				
**ENSG00000019505**		*0.48181818*		*0.481818182*				*0.3882353*
**ENSG00000230989**		*0.45151515*		*0.451515152*				
**ENSG00000204252**	*0.13772455*	*0.43939394*	*0.13772455*	*0.439393939*	*0.154761905*		*0.2209302*	*0.4127907*
**ENSG00000124216**		*0.28472222*		*0.284722222*				*0.3095238*
**ENSG00000100385**	*0.45*	*0.32758621*	*0.45*	*0.327586207*	*0.279069767*		*0.3809524*	*0.3181818*
**ENSG00000198851**					*0.25625*		*0.2386364*	*0.0568182*
**ENSG00000067560**		*0.28963415*		*0.289634146*				
**ENSG00000070886**		*0.32758621*		*0.327586207*				*0.3372093*
**ENSG00000186452**		*0.19512195*		*0.195121951*				*0.2732558*
**ENSG00000118322**	*0.36526946*	*0.29393939*	*0.36526946*	*0.293939394*	*0.345238095*		*0.3488372*	*0.244186*
**ENSG00000103111**	*0.08682635*	*0.27878788*	*0.08682635*	*0.278787879*	*0.261904762*		*0.2790698*	*0.4418605*
**ENSG00000171992**	*0.08181818*	*0.49695122*	*0.08181818*	*0.49695122*				*0.4883721*
**ENSG00000155016**	*0.39506173*	*0.46470588*	*0.39506173*	*0.464705882*	*0.283783784*		*0.3625*	*0.3588235*
**ENSG00000103194**		*0.32777778*		*0.327777778*	*0.351190476*		*0.377907*	*0.377907*
**ENSG00000224201**								*0.4470588*
**ENSG00000156206**								*0.1395349*
**ENSG00000079393**	*0.45508982*	*0.42121212*	*0.45508982*	*0.421212121*	*0.410714286*		*0.4069767*	*0.4069767*
**ENSG00000164037**	*0.29640719*	*0.47222222*	*0.29640719*	*0.472222222*	*0.452380952*		*0.4709302*	*0.5*
**ENSG00000163216**		*0.49386503*		*0.493865031*	*0.470238095*		*0.4360465*	*0.4821429*
**ENSG00000162885**		*0.46666667*		*0.466666667*	*0.159090909*		*0.0681818*	*0.4418605*
**ENSG00000137494**	*0.33974359*	*0.46067416*	*0.33974359*	*0.460674157*	*0.055555556*			*0.4431818*
**ENSG00000185591**		*0.07317073*		*0.073170732*				
**ENSG00000135063**	*0.28571429*		*0.28571429*		*0.409638554*		*0.4761905*	
**ENSG00000037637**	*0.12349398*	*0.38050314*	*0.12349398*	*0.380503145*				*0.1882353*

**ENSG00000167487**		0.32424242		0.324242424				0.3546512
**ENSG00000107104**	0.34730539	0.3908046	0.34730539	0.390804598	0.432926829		0.4216867	0.494186
**ENSG00000161013**	0.32634731		0.32634731					
**ENSG00000227345**		0.475		0.475				0.4352941
**ENSG00000148297**	0.18562874	0.22727273	0.18562874	0.227272727	0.220238095		0.244186	0.3430233
**ENSG00000129151**	0.3742515	0.1	0.3742515	0.1	0.482142857		0.4534884	
**ENSG00000137500**	0.11976048	0.46666667	0.11976048	0.466666667	0.452380952		0.4825581	0.2674419
**ENSG00000110400**								0.0795455
**ENSG00000135211**		0.31707317		0.317073171				0.2117647
**ENSG00000140395**		0.13333333		0.133333333				0.1046512
**ENSG00000180992**		0.1030303		0.103030303				
**ENSG00000138769**		0.40909091		0.409090909				0.0697674
**ENSG00000085719**	0.42215569	0.36666667	0.42215569	0.366666667	0.398809524		0.4302326	0.4882353
**ENSG00000162669**	0.17065868		0.17065868					
**ENSG00000182566**	0.24251497		0.24251497					
**ENSG00000154813**		0.46666667		0.466666667				0.4777778
**ENSG00000183873**		0.31515152		0.315151515				0.5
**ENSG00000184293**		0.45757576		0.457575758				0.3604651
**ENSG00000136848**	0.11676647	0.09937888	0.11676647	0.099378882				
**ENSG00000165006**		0.31609195		0.316091954				0.3863636
**ENSG00000257198**	0.38323353		0.38323353		0.339285714		0.3197674	
**ENSG00000160182**					0.416666667		0.4825581	
**ENSG00000175779**		0.43820225		0.438202247				0.4352941
**ENSG00000124279**		0.26829268		0.268292683				0.2965116
**ENSG00000169297**		0.1969697		0.196969697				0.3255814
**ENSG00000113643**	0.23952096	0.37575758	0.23952096	0.375757576	0.107142857		0.1104651	0.1566265
**ENSG00000167183**		0.36280488		0.362804878				0.4418605
**ENSG00000155659**		0.1554878		0.155487805				0.1162791
**ENSG00000110042**		0.43636364		0.436363636				0.4127907
**ENSG00000155380**		0.39090909		0.390909091				0.327381
**ENSG00000135924**								0.1590909
**ENSG00000197245**		0.24545455		0.245454545	0.277777778		0.1704545	
**ENSG00000179840**		0.3597561		0.359756098				0.1
**ENSG00000141052**								0.4695122
**ENSG00000163586**		0.33128834		0.331288344				0.4709302
**ENSG00000055070**		0.42727273		0.427272727				0.2732558
**ENSG00000158301**					0.071428571		0.0588235	0.1453488
**ENSG00000151090**		0.43333333		0.433333333				0.5
**ENSG00000164117**	0.08383234	0.06969697	0.08383234	0.06969697				0.1144578
**ENSG00000132274**	0.48192771	0.2	0.48192771	0.2	0.357142857		0.3863636	0.4127907
**ENSG00000138092**		0.42378049		0.423780488				0.3139535
**ENSG00000016490**	0.35889571	0.26060606	0.35889571	0.260606061	0.458333333		0.4825581	0.3953488
**ENSG00000126856**	0.17771084	0.24848485	0.17771084	0.248484848	0.232142857		0.2965116	0.3546512
**ENSG00000186074**		0.18787879		0.187878788				0.4593023
**ENSG00000112033**	0.31137725	0.23939394	0.31137725	0.239393939	0.273809524		0.2267442	0.2325581
**ENSG00000232070**		0.39444444		0.394444444				0.4772727
**ENSG00000132975**		0.29090909		0.290909091				
**ENSG00000196141**	0.21856287		0.21856287		0.244444444		0.2613636	
**ENSG00000085465**	0.26646707		0.26646707		0.259036145		0.255814	
**ENSG00000117151**		0.2969697		0.296969697				0.1860465
**ENSG00000136197**		0.12727273		0.127272727				0.0697674
**ENSG00000165478**		0.35454545		0.354545455				0.0697674
**ENSG00000165868**		0.26666667		0.266666667				0.25
**ENSG00000078098**	0.05182927		0.05182927					0.1136364

**ENSG00000112041**		*0.41818182*		*0.418181818*			*0.4883721*
**ENSG00000198015**		*0.42682927*		*0.426829268*	*0.151162791*	*0.0941176*	*0.1453488*
**ENSG00000186184**		*0.45731707*		*0.457317073*			*0.4883721*
**ENSG00000196166**		*0.19090909*		*0.190909091*			*0.2151163*
**ENSG00000162913**		*0.40116279*		*0.401162791*			*0.3571429*
**ENSG00000147813**		*0.21666667*		*0.216666667*			*0.2727273*
**ENSG00000166159**	*0.17365269*	*0.17272727*	*0.17365269*	*0.172727273*	*0.339285714*	*0.2965116*	*0.0523256*
**ENSG00000215203**		*0.34705882*		*0.347058824*			
**ENSG00000141748**	*0.25449102*		*0.25449102*		*0.279761905*	*0.1918605*	
**ENSG00000137441**	*0.11976048*		*0.11976048*		*0.25*	*0.1976744*	
**ENSG00000069535**							*0.2647059*
**ENSG00000184385**		*0.42987805*		*0.429878049*			*0.4470588*
**ENSG00000148730**		*0.48888889*		*0.488888889*			*0.4659091*
**ENSG00000184716**		*0.06666667*		*0.066666667*			*0.255814*
**ENSG00000150347**					*0.166666667*	*0.1162791*	*0.1511628*
**ENSG00000143164**		*0.35393258*		*0.353932584*			*0.1444444*
**ENSG00000110375**	*0.17964072*		*0.17964072*				
**ENSG00000074660**							*0.0795455*
**ENSG00000134962**		*0.48787879*		*0.487878788*			*0.4069767*
**ENSG00000254790**		*0.38181818*		*0.381818182*			*0.3023256*
**ENSG00000174748**		*0.17222222*		*0.172222222*			*0.0810811*
**ENSG00000118564**		*0.38787879*		*0.387878788*			*0.4883721*
**ENSG00000138738**		*0.27272727*		*0.272727273*			*0.2111111*
**ENSG00000083123**		*0.23888889*		*0.238888889*			*0.2840909*
**ENSG00000163902**	*0.46666667*		*0.46666667*		*0.244047619*	*0.1918605*	
**ENSG00000113194**	*0.11746988*	*0.38787879*	*0.11746988*	*0.387878788*			*0.4418605*
**ENSG00000131459**		*0.23333333*		*0.233333333*			*0.3035714*
**ENSG00000196218**	*0.11377246*		*0.11377246*				
**ENSG00000172349**	*0.45508982*	*0.49438202*	*0.45508982*	*0.494382022*	*0.428571429*	*0.372093*	*0.4941176*
**ENSG00000108641**					*0.077777778*	*0.1136364*	
**ENSG00000096746**	*0.45209581*	*0.09393939*	*0.45209581*	*0.093939394*	*0.226190476*	*0.1162791*	*0.1162791*
**ENSG00000165621**	*0.26704545*		*0.26704545*		*0.355555556*	*0.3636364*	
**ENSG00000100055**	*0.48502994*	*0.35632184*	*0.48502994*	*0.356321839*	*0.5*	*0.5*	*0.494186*
**ENSG00000177551**	*0.5*	*0.43939394*	*0.5*	*0.439393939*	*0.452380952*	*0.4825581*	*0.4534884*
**ENSG00000135482**	*0.49101796*		*0.49101796*				
**ENSG00000128191**		*0.48787879*		*0.487878788*			*0.4476744*
**ENSG00000151348**	*0.39520958*		*0.39520958*		*0.202380952*	*0.2965116*	
**ENSG00000145287**	*0.38023952*	*0.11235955*	*0.38023952*	*0.112359551*	*0.375*	*0.3197674*	
**ENSG00000217825**		*0.20121951*		*0.201219512*			*0.1097561*
**ENSG00000144028**		*0.32121212*		*0.321212121*			*0.3546512*
**ENSG00000121350**	*0.47530864*	*0.5*	*0.47530864*	*0.5*	*0.476190476*	*0.4156627*	*0.372093*
**ENSG00000198324**		*0.34242424*		*0.342424242*			*0.1976744*
**ENSG00000131653**		*0.05521472*		*0.055214724*			
**ENSG00000158850**		*0.21666667*		*0.216666667*			*0.4545455*
**ENSG00000105887**		*0.08181818*		*0.081818182*			*0.0988372*
**ENSG00000136874**	*0.27245509*	*0.33333333*	*0.27245509*	*0.333333333*	*0.285714286*	*0.2325581*	*0.2209302*
**ENSG00000143543**	*0.13173653*		*0.13173653*				
**ENSG00000215131**							*0.0568182*
**ENSG00000242574**		*0.44545455*		*0.445454545*			*0.4767442*
**ENSG00000187607**	*0.07317073*	*0.41818182*	*0.07317073*	*0.418181818*			*0.4127907*
**ENSG00000115339**		*0.47777778*		*0.477777778*			*0.2444444*
**ENSG00000172159**		*0.48407643*		*0.484076433*			*0.4011628*
**ENSG00000120306**	*0.12222222*		*0.12222222*		*0.261363636*	*0.3068182*	
**ENSG00000163116**		*0.3*		*0.3*			*0.2616279*

**ENSG00000107140**	*0.45209581*		*0.45209581*		*0.392857143*	*0.3837209*	
**ENSG00000135678**		*0.40243902*		*0.402439024*			*0.3546512*
**ENSG00000132970**		*0.29090909*		*0.290909091*			*0.0581395*
**ENSG00000175121**		*0.32727273*		*0.327272727*			*0.0872093*
**ENSG00000073111**		*0.21515152*		*0.215151515*			*0.3430233*
**ENSG00000104368**	*0.46706587*	*0.0945122*	*0.46706587*	*0.094512195*	*0.321428571*	*0.3197674*	*0.1588235*
**ENSG00000137819**		*0.06896552*		*0.068965517*			*0.3953488*
**ENSG00000142611**		*0.23636364*		*0.236363636*			*0.4880952*
**ENSG00000054611**		*0.46969697*		*0.46969697*			*0.4534884*
**ENSG00000148798**		*0.46629213*		*0.466292135*			*0.4431818*
**ENSG00000143493**		*0.25151515*		*0.251515152*			*0.4764706*
**ENSG00000151743**	*0.23053892*	*0.4*	*0.23053892*	*0.4*	*0.125*	*0.1104651*	*0.1162791*
**ENSG00000164086**		*0.06060606*		*0.060606061*			*0.377907*
**ENSG00000175727**	*0.44578313*	*0.49393939*	*0.44578313*	*0.493939394*	*0.43452381*	*0.4186047*	*0.4069767*
**ENSG00000104412**	*0.0508982*		*0.0508982*		*0.458333333*	*0.4*	
**ENSG00000168597**		*0.32758621*		*0.327586207*	*0.226190476*	*0.3139535*	*0.2727273*
**ENSG00000064270**		*0.11890244*		*0.118902439*			*0.4244186*
**ENSG00000177335**		*0.28787879*		*0.287878788*			*0.0523256*
**ENSG00000163032**	*0.2754491*	*0.31212121*	*0.2754491*	*0.312121212*	*0.097560976*	*0.1176471*	
**ENSG00000111796**		*0.46111111*		*0.461111111*			*0.5*
**ENSG00000111058**		*0.30674847*		*0.306748466*			*0.4476744*
**ENSG00000152061**	*0.34444444*	*0.30606061*	*0.34444444*	*0.306060606*		*0.0909091*	*0.4593023*
**ENSG00000055957**		*0.2969697*		*0.296969697*			*0.4647059*
**ENSG00000197580**	*0.09281437*	*0.24545455*	*0.09281437*	*0.245454545*	*0.457831325*	*0.4127907*	*0.1918605*
**ENSG00000140859**	*0.20359281*		*0.20359281*		*0.416666667*	*0.3176471*	
**ENSG00000177613**		*0.16363636*		*0.163636364*			*0.3372093*
**ENSG00000106128**	*0.20783133*		*0.20783133*		*0.18452381*	*0.1744186*	
**ENSG00000133115**		*0.25925926*		*0.259259259*			*0.3511905*
**ENSG00000117620**	*0.0508982*	*0.18787879*	*0.0508982*	*0.187878788*	*0.06547619*		*0.0872093*
**ENSG00000146535**	*0.11666667*	*0.48850575*	*0.11666667*	*0.488505747*	*0.3*	*0.2*	*0.3953488*
**ENSG00000152455**	*0.27108434*	*0.09090909*	*0.27108434*	*0.090909091*	*0.410714286*	*0.3895349*	
**ENSG00000183508**		*0.36666667*		*0.366666667*			*0.3023256*
**ENSG00000176208**	*0.10493827*		*0.10493827*		*0.125*	*0.2916667*	
**ENSG00000174562**		*0.25757576*		*0.257575758*			*0.4235294*
**ENSG00000133800**		*0.37575758*		*0.375757576*			*0.3430233*
**ENSG00000103066**	*0.18888889*	*0.27777778*	*0.18888889*	*0.277777778*			*0.0568182*
**ENSG00000111653**							*0.3470588*
**ENSG00000102362**		*0.41666667*		*0.416666667*			*0.494186*
**ENSG00000170160**		*0.1*		*0.1*			
**ENSG00000163347**	*0.20689655*	*0.47169811*	*0.20689655*	*0.471698113*	*0.151162791*	*0.1363636*	*0.1585366*
**ENSG00000138650**		*0.38650307*		*0.386503067*			*0.4821429*
**ENSG00000059691**		*0.47256098*		*0.472560976*			*0.4825581*
**ENSG00000258945**		*0.48787879*		*0.487878788*			*0.3647059*
**ENSG00000175497**	*0.23353293*	*0.47575758*	*0.23353293*	*0.475757576*			*0.4534884*
**ENSG00000070269**	*0.36046512*	*0.31212121*	*0.36046512*	*0.312121212*	*0.458333333*	*0.4186047*	*0.2965116*
**ENSG00000104974**		*0.35151515*		*0.351515152*			*0.3953488*
**ENSG00000140519**		*0.46646341*		*0.466463415*			*0.2965116*
**ENSG00000145779**	*0.28742515*	*0.29444444*	*0.28742515*	*0.294444444*	*0.494047619*	*0.4651163*	*0.1117647*
**ENSG00000197714**		*0.47878788*		*0.478787879*			*0.1802326*
**ENSG00000257923**	*0.49101796*	*0.45426829*	*0.49101796*	*0.454268293*	*0.089285714*		*0.0755814*
**ENSG00000138162**	*0.39655172*	*0.22121212*	*0.39655172*	*0.221212121*	*0.327380952*	*0.4069767*	*0.2093023*
**ENSG00000197632**	*0.06111111*	*0.23333333*	*0.06111111*	*0.233333333*	*0.232142857*	*0.1395349*	*0.3352941*
**ENSG00000120805**	*0.2*	*0.20909091*	*0.2*	*0.209090909*	*0.446428571*	*0.3522727*	*0.3488372*
**ENSG00000101160**		*0.26666667*		*0.266666667*			*0.0813953*

**ENSG00000175182**	*0.41616766*	*0.37272727*	*0.41616766*	*0.372727273*	*0.404761905*	*0.4360465*	*0.25*
**ENSG00000182504**		*0.34146341*		*0.341463415*			*0.3372093*
**ENSG00000198077**	*0.27777778*		*0.27777778*		*0.477777778*	*0.4431818*	
**ENSG00000100146**		*0.33128834*		*0.331288344*			*0.3294118*
**ENSG00000049759**	*0.46107784*	*0.41212121*	*0.46107784*	*0.412121212*	*0.494047619*	*0.3197674*	*0.25*
**ENSG00000185238**							*0.0795455*
**ENSG00000003756**		*0.47272727*		*0.472727273*			*0.1802326*
**ENSG00000174502**		*0.12222222*		*0.122222222*			*0.4545455*
**ENSG00000179088**	*0.26404494*	*0.23888889*	*0.26404494*	*0.238888889*	*0.454545455*	*0.4886364*	*0.4659091*
**ENSG00000170027**		*0.23636364*		*0.236363636*			*0.2906977*
**ENSG00000177575**		*0.48773006*		*0.487730061*			*0.3882353*
**ENSG00000145780**		*0.46363636*		*0.463636364*			*0.4302326*
**ENSG00000244045**		*0.47272727*		*0.472727273*			*0.2267442*
**ENSG00000197706**	*0.0748503*		*0.0748503*		*0.095238095*	*0.0813953*	*0.0813953*
**ENSG00000171150**		*0.34756098*		*0.347560976*			*0.0581395*
**ENSG00000100842**		*0.25304878*		*0.25304878*			*0.2674419*
**ENSG00000095261**		*0.13636364*		*0.136363636*			
**ENSG00000115504**		*0.13030303*		*0.13030303*			*0.122093*
**ENSG00000156510**		*0.45*		*0.45*			*0.2727273*
**ENSG00000099901**		*0.28181818*		*0.281818182*		*0.0909091*	*0.1453488*
**ENSG00000141971**					*0.41025641*	*0.4428571*	
**ENSG00000149124**		*0.17378049*		*0.173780488*			*0.4011628*
**ENSG00000188389**		*0.12121212*		*0.121212121*			*0.4883721*
**ENSG00000068079**		*0.11818182*		*0.118181818*			
**ENSG00000069943**	*0.29640719*	*0.08787879*	*0.29640719*	*0.087878788*	*0.452380952*	*0.4186047*	
**ENSG00000136603**	*0.42215569*	*0.12777778*	*0.42215569*	*0.127777778*	*0.192771084*	*0.1411765*	
**ENSG00000171848**		*0.15853659*		*0.158536585*	*0.366666667*	*0.3522727*	
**ENSG00000058262**	*0.05988024*	*0.25*	*0.05988024*	*0.25*			*0.0843373*
**ENSG00000072274**		*0.38787879*		*0.387878788*			*0.2209302*
**ENSG00000124587**	*0.35628743*	*0.45151515*	*0.35628743*	*0.451515152*	*0.113095238*	*0.1453488*	*0.1453488*
**ENSG00000116218**		*0.26060606*		*0.260606061*			*0.4069767*
**ENSG00000108175**	*0.0988024*	*0.36781609*	*0.0988024*	*0.367816092*	*0.446428571*	*0.3430233*	*0.4888889*
**ENSG00000155324**		*0.18181818*		*0.181818182*			*0.0523256*
**ENSG00000162231**		*0.35151515*		*0.351515152*			*0.3941176*
**ENSG00000070366**		*0.35632184*		*0.356321839*			*0.0755814*
**ENSG00000116698**		*0.36781609*		*0.367816092*			*0.4772727*
**ENSG00000133401**	*0.2245509*	*0.29878049*	*0.2245509*	*0.298780488*	*0.095238095*	*0.0813953*	*0.3372093*
**ENSG00000185513**	*0.48802395*	*0.48275862*	*0.48802395*	*0.482758621*	*0.43452381*	*0.4651163*	*0.4825581*
**ENSG00000144560**					*0.06547619*	*0.1369048*	*0.1369048*
**ENSG00000147526**	*0.34730539*	*0.14634146*	*0.34730539*	*0.146341463*	*0.25*	*0.1941176*	*0.2058824*
**ENSG00000134508**		*0.49090909*		*0.490909091*			*0.494186*
**ENSG00000172530**		*0.25757576*		*0.257575758*		*0.0581395*	*0.2616279*
**ENSG00000138622**		*0.33939394*		*0.339393939*			*0.4821429*
**ENSG00000118515**		*0.42424242*		*0.424242424*	*0.055555556*	*0.0777778*	*0.4764706*
**ENSG00000144827**	*0.17664671*	*0.21666667*	*0.17664671*	*0.216666667*	*0.142857143*	*0.122093*	*0.122093*
**ENSG00000188833**		*0.29573171*		*0.295731707*			*0.2906977*
**ENSG00000186275**		*0.14197531*		*0.141975309*			*0.2162162*
**ENSG00000175471**	*0.36826347*	*0.30813953*	*0.36826347*	*0.308139535*	*0.470238095*	*0.4302326*	*0.3863636*
**ENSG00000116701**	*0.48502994*	*0.1*	*0.48502994*	*0.1*	*0.071428571*	*0.0581395*	
**ENSG00000188452**		*0.31515152*		*0.315151515*			*0.4476744*
**ENSG00000051523**		*0.43333333*		*0.433333333*			*0.4651163*
**ENSG00000196345**	*0.42814371*	*0.28735632*	*0.42814371*	*0.287356322*			
**ENSG00000205809**		*0.16770186*		*0.167701863*			*0.2321429*
**ENSG00000184999**	*0.24096386*	*0.47560976*	*0.24096386*	*0.475609756*	*0.446428571*	*0.3941176*	*0.3953488*

**ENSG00000048540**	*0.05555556*		*0.05555556*					
**ENSG00000010165**	*0.33832335*	*0.26969697*	*0.33832335*	*0.26969697*	*0.369047619*		*0.372093*	*0.4647059*
**ENSG00000066923**	*0.40555556*	*0.45731707*	*0.40555556*	*0.457317073*	*0.422222222*		*0.4090909*	*0.3170732*
**ENSG00000174951**	*0.10479042*	*0.24848485*	*0.10479042*	*0.248484848*				*0.3255814*
**ENSG00000107372**		*0.47852761*		*0.478527607*				*0.5*
**ENSG00000114853**	*0.46226415*	*0.33435583*	*0.46226415*	*0.334355828*	*0.476190476*		*0.3554217*	*0.4146341*
**ENSG00000084628**		*0.27878788*		*0.278787879*				*0.4823529*
**ENSG00000144749**								*0.0666667*
**ENSG00000185909**		*0.24242424*		*0.242424242*				
**ENSG00000073803**	*0.27840909*	*0.46363636*	*0.27840909*	*0.463636364*	*0.122222222*		*0.1477273*	*0.4111111*
**ENSG00000223547**	*0.30606061*	*0.49371069*	*0.30606061*	*0.493710692*	*0.1*		*0.1337209*	*0.4880952*
**ENSG00000070061**	*0.34090909*	*0.30606061*	*0.34090909*	*0.306060606*	*0.5*		*0.4318182*	*0.4529412*
**ENSG00000206053**		*0.05555556*		*0.055555556*				*0.1136364*
**ENSG00000001626**		*0.28333333*		*0.283333333*				
**ENSG00000074416**	*0.23053892*	*0.40490798*	*0.23053892*	*0.404907975*				*0.375*
**ENSG00000081051**		*0.42121212*		*0.421212121*				*0.3372093*
**ENSG00000092054**		*0.05454545*		*0.054545455*				*0.0523256*
**ENSG00000113068**		*0.27272727*		*0.272727273*				*0.372093*
**ENSG00000164946**	*0.4*	*0.3902439*	*0.4*	*0.390243902*	*0.38372093*		*0.4054054*	*0.494186*
**ENSG00000187118**		*0.29090909*		*0.290909091*				*0.2529412*
**ENSG00000125648**		*0.49390244*		*0.493902439*	*0.055555556*			*0.3294118*
**ENSG00000125798**								*0.2267442*
**ENSG00000109917**		*0.11890244*		*0.118902439*				*0.3313953*
**ENSG00000106537**	*0.40963855*	*0.25914634*	*0.40963855*	*0.259146341*	*0.25*		*0.1860465*	*0.3023256*
**ENSG00000144840**	*0.20658683*		*0.20658683*		*0.130952381*		*0.2267442*	
**ENSG00000164587**	*0.26136364*	*0.32424242*	*0.26136364*	*0.324242424*	*0.288888889*		*0.2727273*	*0.244186*
**ENSG00000168234**	*0.44311377*	*0.37222222*	*0.44311377*	*0.372222222*	*0.458333333*		*0.4127907*	*0.4222222*
**ENSG00000015520**		*0.17878788*		*0.178787879*				
**ENSG00000162520**					*0.107142857*		*0.1104651*	*0.1046512*
**ENSG00000124089**	*0.41616766*		*0.41616766*		*0.226190476*		*0.2267442*	
**ENSG00000198755**								*0.0930233*
**ENSG00000187778**		*0.06707317*		*0.067073171*				*0.0777778*
**ENSG00000164692**		*0.10060976*		*0.100609756*				*0.122093*
**ENSG00000101384**	*0.33832335*	*0.41515152*	*0.33832335*	*0.415151515*	*0.155555556*		*0.2666667*	*0.2674419*
**ENSG00000168917**	*0.38922156*		*0.38922156*		*0.148809524*		*0.1395349*	
**ENSG00000100934**	*0.07954545*	*0.12777778*	*0.07954545*	*0.127777778*	*0.344444444*		*0.3333333*	*0.4090909*
**ENSG00000178965**		*0.36666667*		*0.366666667*				*0.4127907*
**ENSG00000173926**		*0.4202454*		*0.420245399*				*0.3963415*
**ENSG00000198130**	*0.29216867*		*0.29216867*		*0.458333333*		*0.4883721*	
**ENSG00000117115**		*0.32941176*		*0.329411765*				*0.4117647*
**ENSG00000197430**		*0.18787879*		*0.187878788*				*0.3988095*
**ENSG00000152689**	*0.46706587*	*0.26363636*	*0.46706587*	*0.263636364*	*0.28313253*		*0.3139535*	*0.0697674*
**ENSG00000076770**		*0.29393939*		*0.293939394*				*0.0581395*
**ENSG00000205636**	*0.36309524*		*0.36309524*				*0.1341463*	
**ENSG00000125356**	*0.16768293*		*0.16768293*		*0.178571429*		*0.125*	
**ENSG00000151893**		*0.14117647*		*0.141176471*				*0.2176471*
**ENSG00000130518**	*0.43413174*		*0.43413174*		*0.488095238*		*0.4883721*	
**ENSG00000142449**	*0.0988024*	*0.14444444*	*0.0988024*	*0.144444444*	*0.172619048*		*0.1918605*	*0.3636364*
**ENSG00000197683**	*0.44311377*	*0.13939394*	*0.44311377*	*0.139393939*	*0.083333333*		*0.0697674*	*0.4302326*
**ENSG00000139865**		*0.46666667*		*0.466666667*				*0.4117647*
**ENSG00000140374**		*0.47159091*		*0.471590909*				*0.3255814*
**ENSG00000079112**		*0.48484848*		*0.484848485*				*0.4825581*
**ENSG00000184984**	*0.21556886*		*0.21556886*		*0.071428571*		*0.1337209*	
**ENSG00000145826**	*0.4760479*	*0.37804878*	*0.4760479*	*0.37804878*	*0.363095238*		*0.3895349*	*0.4186047*

**ENSG00000137693**	*0.17771084*	*0.35057471*	*0.17771084*	*0.350574713*	*0.261904762*	*0.2151163*	*0.5*
**ENSG00000187068**		*0.14723926*		*0.147239264*			*0.1337209*
**ENSG00000187024**	*0.24251497*		*0.24251497*		*0.261904762*	*0.2906977*	
**ENSG00000135749**	*0.41017964*	*0.24848485*	*0.41017964*	*0.248484848*	*0.232142857*	*0.2117647*	*0.2294118*
**ENSG00000157927**	*0.05757576*	*0.32183908*	*0.05757576*	*0.32183908*	*0.469879518*	*0.4709302*	*0.3522727*
**ENSG00000181195**	*0.16666667*	*0.44848485*	*0.16666667*	*0.448484848*			*0.3511905*
**ENSG00000039600**		*0.08950617*		*0.089506173*			
**ENSG00000168066**	*0.14371257*	*0.11515152*	*0.14371257*	*0.115151515*	*0.404761905*	*0.3662791*	*0.3895349*
**ENSG00000156222**	*0.0748503*	*0.12121212*	*0.0748503*	*0.121212121*			*0.0523256*
**ENSG00000122033**					*0.06547619*	*0.0988372*	
**ENSG00000072954**		*0.12921348*		*0.129213483*			*0.0666667*
**ENSG00000069812**		*0.13333333*		*0.133333333*			*0.2616279*
**ENSG00000136068**		*0.48484848*		*0.484848485*			
**ENSG00000165714**		*0.39393939*		*0.393939394*			*0.2209302*
**ENSG00000180061**	*0.42814371*		*0.42814371*		*0.077380952*	*0.0588235*	
**ENSG00000164758**	*0.4251497*		*0.4251497*		*0.255952381*	*0.244186*	
**ENSG00000163683**							*0.1204819*
**ENSG00000100852**		*0.10555556*		*0.105555556*			*0.1477273*
**ENSG00000136108**		*0.39506173*		*0.395061728*			*0.3488372*
**ENSG00000105341**	*0.17777778*		*0.17777778*		*0.443181818*	*0.4545455*	
**ENSG00000145041**		*0.17878788*		*0.178787879*			*0.2117647*
**ENSG00000149548**	*0.1497006*	*0.36363636*	*0.1497006*	*0.363636364*	*0.297619048*	*0.255814*	*0.4352941*
**ENSG00000119812**		*0.27607362*		*0.27607362*			*0.4069767*
**ENSG00000100426**		*0.18888889*		*0.188888889*			*0.2093023*
**ENSG00000104852**	*0.15269461*	*0.13030303*	*0.15269461*	*0.13030303*	*0.125*	*0.0639535*	*0.0639535*
**ENSG00000184271**	*0.0625*	*0.43333333*	*0.0625*	*0.433333333*	*0.155555556*	*0.0568182*	*0.3636364*
**ENSG00000143797**		*0.28181818*		*0.281818182*			*0.0930233*
**ENSG00000147889**		*0.13333333*		*0.133333333*			*0.0639535*
**ENSG00000026025**		*0.36111111*		*0.361111111*			*0.244186*
**ENSG00000183011**					*0.392857143*	*0.4069767*	
**ENSG00000078237**		*0.40606061*		*0.406060606*			*0.4939759*
**ENSG00000142224**	*0.23888889*	*0.24545455*	*0.23888889*	*0.245454545*	*0.255555556*	*0.2272727*	*0.3953488*
**ENSG00000072840**		*0.44545455*		*0.445454545*			*0.2325581*
**ENSG00000129197**	*0.28614458*	*0.21212121*	*0.28614458*	*0.212121212*	*0.054216867*		*0.2235294*
**ENSG00000130775**		*0.38650307*		*0.386503067*			*0.422619*
**ENSG00000173166**		*0.42121212*		*0.421212121*			*0.4418605*
**ENSG00000158156**					*0.25*		
**ENSG00000183747**		*0.21111111*		*0.211111111*			*0.4431818*
**ENSG00000255216**	*0.32954545*		*0.32954545*		*0.488636364*	*0.4333333*	
**ENSG00000163435**	*0.48502994*	*0.32121212*	*0.48502994*	*0.321212121*	*0.5*	*0.4011628*	*0.3546512*
**ENSG00000148848**		*0.4695122*		*0.469512195*			*0.3764706*
**ENSG00000184564**	*0.30239521*	*0.35393258*	*0.30239521*	*0.353932584*	*0.119047619*	*0.2823529*	*0.4375*
**ENSG00000171564**	*0.11976048*	*0.21666667*	*0.11976048*	*0.216666667*	*0.19047619*	*0.1162791*	*0.125*
**ENSG00000170788**		*0.13333333*		*0.133333333*		*0.0666667*	
**ENSG00000232838**		*0.09393939*		*0.093939394*			*0.2151163*
**ENSG00000006757**		*0.45121951*		*0.451219512*			*0.3023256*
**ENSG00000182010**	*0.06886228*	*0.43888889*	*0.06886228*	*0.438888889*			*0.1463415*
**ENSG00000161640**		*0.23030303*		*0.23030303*			*0.2906977*
**ENSG00000198739**	*0.36516854*		*0.36516854*				
**ENSG00000162736**	*0.29041916*		*0.29041916*		*0.060240964*	*0.0581395*	
**ENSG00000143420**		*0.47777778*		*0.477777778*			*0.3546512*
**ENSG00000171522**		*0.13333333*		*0.133333333*			*0.3139535*
**ENSG00000104964**		*0.2969697*		*0.296969697*			*0.1046512*
**ENSG00000108861**		*0.27272727*		*0.272727273*			*0.1569767*

**ENSG00000116991**	*0.25757576*	*0.20909091*	*0.25757576*	*0.209090909*			
**ENSG00000126883**	*0.46666667*		*0.46666667*		*0.433333333*	*0.4545455*	
**ENSG00000189190**	*0.09444444*		*0.09444444*		*0.077777778*	*0.127907*	*0.3068182*
**ENSG00000186298**	*0.39325843*	*0.13719512*	*0.39325843*	*0.137195122*		*0.0523256*	*0.0523256*
**ENSG00000118707**		*0.12424242*		*0.124242424*			*0.2151163*
**ENSG00000067900**	*0.44610778*		*0.44610778*		*0.077380952*	*0.1337209*	
**ENSG00000143384**		*0.42222222*		*0.422222222*			*0.3977273*
**ENSG00000141977**		*0.2030303*		*0.203030303*			*0.0639535*
**ENSG00000151150**	*0.26646707*	*0.44545455*	*0.26646707*	*0.445454545*	*0.244047619*	*0.2034884*	*0.1744186*
**ENSG00000116473**	*0.21257485*	*0.17272727*	*0.21257485*	*0.172727273*			*0.1162791*
**ENSG00000068120**	*0.07185629*	*0.41818182*	*0.07185629*	*0.418181818*			*0.4476744*
**ENSG00000133574**	*0.29341317*		*0.29341317*		*0.05952381*	*0.0523256*	*0.0523256*
**ENSG00000083454**		*0.12121212*		*0.121212121*			*0.2325581*
**ENSG00000151376**		*0.34756098*		*0.347560976*			*0.3662791*
**ENSG00000180353**		*0.30792683*		*0.307926829*			*0.4069767*
**ENSG00000105245**		*0.13636364*		*0.136363636*			
**ENSG00000100335**		*0.32424242*		*0.324242424*			*0.1309524*
**ENSG00000088179**	*0.3742515*	*0.30368098*	*0.3742515*	*0.303680982*	*0.238095238*	*0.1627907*	*0.1627907*
**ENSG00000168214**	*0.11111111*	*0.1097561*	*0.11111111*	*0.109756098*	*0.288888889*	*0.3409091*	
**ENSG00000149212**		*0.39393939*		*0.393939394*			*0.3953488*
**ENSG00000005469**		*0.1030303*		*0.103030303*			
**ENSG00000135709**		*0.4847561*		*0.484756098*			*0.3555556*
**ENSG00000196730**		*0.45151515*		*0.451515152*			*0.4235294*
**ENSG00000186513**		*0.30606061*		*0.306060606*			*0.3255814*
**ENSG00000167740**		*0.28484848*		*0.284848485*			*0.0697674*
**ENSG00000150076**	*0.30606061*	*0.47865854*	*0.30606061*	*0.478658537*	*0.140243902*	*0.172619*	*0.1046512*
**ENSG00000137809**		*0.32608696*		*0.326086957*			
**ENSG00000082458**		*0.40243902*		*0.402439024*			*0.3235294*
**ENSG00000139318**	*0.23333333*	*0.19393939*	*0.23333333*	*0.193939394*			
**ENSG00000136933**	*0.49101796*	*0.05151515*	*0.49101796*	*0.051515152*	*0.357142857*	*0.4011628*	*0.1162791*
**ENSG00000163630**	*0.08888889*	*0.3969697*	*0.08888889*	*0.396969697*	*0.066666667*		*0.2848837*
**ENSG00000156011**	*0.09444444*	*0.41212121*	*0.09444444*	*0.412121212*			*0.2529412*
**ENSG00000112149**		*0.46060606*		*0.460606061*			*0.4647059*
**ENSG00000007047**		*0.3*		*0.3*			*0.2616279*
**ENSG00000155158**	*0.30838323*		*0.30838323*			*0.0639535*	
**ENSG00000141540**	*0.23053892*	*0.2*	*0.23053892*	*0.2*	*0.089285714*	*0.1511628*	*0.0666667*
**ENSG00000185940**		*0.35714286*		*0.357142857*			*0.4011628*
**ENSG00000145715**	*0.17791411*		*0.17791411*		*0.426829268*	*0.3837209*	
**ENSG00000165733**		*0.3030303*		*0.303030303*			*0.4534884*
**ENSG00000182919**	*0.35928144*	*0.24848485*	*0.35928144*	*0.248484848*	*0.071428571*	*0.0988372*	
**ENSG00000130164**		*0.43636364*		*0.436363636*			*0.4709302*
**ENSG00000176406**		*0.32222222*		*0.322222222*			
**ENSG00000180596**		*0.45151515*		*0.451515152*			*0.1686047*
**ENSG00000151689**	*0.26111111*		*0.26111111*		*0.482142857*	*0.5*	
**ENSG00000124818**	*0.28443114*	*0.3030303*	*0.28443114*	*0.303030303*	*0.083333333*		*0.1511628*
**ENSG00000143248**		*0.39090909*		*0.390909091*			*0.4588235*
**ENSG00000144406**		*0.43939394*		*0.439393939*			*0.4882353*
**ENSG00000187510**	*0.32035928*	*0.12222222*	*0.32035928*	*0.122222222*	*0.107142857*	*0.0581395*	*0.054878*
**ENSG00000106290**	*0.43820225*	*0.36516854*	*0.43820225*	*0.365168539*	*0.122222222*	*0.1363636*	*0.1333333*
**ENSG00000130147**					*0.089285714*		
**ENSG00000188827**		*0.13333333*		*0.133333333*			
**ENSG00000154358**	*0.4969697*	*0.3969697*	*0.4969697*	*0.396969697*	*0.476190476*	*0.4222222*	*0.3181818*
**ENSG00000197641**		*0.32121212*		*0.321212121*			*0.4127907*
**ENSG00000031003**		*0.22727273*		*0.227272727*			

**ENSG00000082512**		*0.14444444*		*0.144444444*			
**ENSG00000121579**	*0.29213483*		*0.29213483*		*0.422222222*	*0.3636364*	
**ENSG00000157870**		*0.35757576*		*0.357575758*			*0.3488372*
**ENSG00000070081**		*0.34545455*		*0.345454545*	*0.053571429*	*0.0639535*	*0.3546512*
**ENSG00000182718**	*0.32035928*		*0.32035928*		*0.101190476*		
**ENSG00000128342**		*0.38484848*		*0.384848485*			*0.4186047*
**ENSG00000198796**		*0.43636364*		*0.436363636*	*0.380952381*	*0.2294118*	*0.3139535*
**ENSG00000117625**		*0.16969697*		*0.16969697*			*0.0813953*
**ENSG00000117153**		*0.4054878*		*0.405487805*			*0.3139535*
**ENSG00000178363**		*0.32424242*		*0.324242424*			*0.0588235*
**ENSG00000109854**							*0.4431818*
**ENSG00000131969**		*0.16969697*		*0.16969697*			*0.5*
**ENSG00000081791**		*0.10674157*		*0.106741573*			
**ENSG00000077157**	*0.39444444*	*0.38181818*	*0.39444444*	*0.381818182*	*0.301204819*	*0.2616279*	*0.3023256*
**ENSG00000142459**		*0.11585366*		*0.115853659*			*0.1162791*
**ENSG00000137055**		*0.08787879*		*0.087878788*			
**ENSG00000181718**		*0.115625*		*0.115625*			
**ENSG00000211786**	*0.10625*		*0.10625*		*0.111111111*	*0.1282051*	
**ENSG00000138756**							*0.2882353*
**ENSG00000155506**	*0.23053892*	*0.32777778*	*0.23053892*	*0.327777778*	*0.107142857*	*0.1046512*	*0.0909091*
**ENSG00000090612**		*0.33939394*		*0.339393939*	*0.083333333*	*0.0872093*	*0.0892857*
**ENSG00000107295**		*0.42424242*		*0.424242424*			*0.2352941*
**ENSG00000173068**		*0.43333333*		*0.433333333*			*0.2840909*
**ENSG00000165795**	*0.28742515*	*0.33229814*	*0.28742515*	*0.332298137*	*0.375*	*0.4302326*	*0.3529412*
**ENSG00000134490**		*0.47575758*		*0.475757576*	*0.107142857*	*0.0755814*	*0.3662791*
**ENSG00000102471**		*0.49411765*		*0.494117647*			*0.25*
**ENSG00000215494**		*0.15853659*		*0.158536585*			*0.3430233*
**ENSG00000138796**		*0.071875*		*0.071875*			
**ENSG00000111846**	*0.43113772*	*0.10606061*	*0.43113772*	*0.106060606*	*0.297619048*	*0.3604651*	*0.1453488*
**ENSG00000100439**		*0.28395062*		*0.283950617*			*0.2*
**ENSG00000169228**		*0.17272727*		*0.172727273*			*0.4593023*
**ENSG00000036530**	*0.49700599*	*0.11764706*	*0.49700599*	*0.117647059*	*0.321428571*	*0.244186*	
**ENSG00000101639**	*0.45402299*	*0.47575758*	*0.45402299*	*0.475757576*	*0.443181818*	*0.4659091*	*0.4186047*
**ENSG00000075073**	*0.37125749*		*0.37125749*				
**ENSG00000102753**		*0.30909091*		*0.309090909*			*0.4268293*
**ENSG00000087448**		*0.45138889*		*0.451388889*			*0.3068182*
**ENSG00000166897**	*0.35628743*	*0.36728395*	*0.35628743*	*0.367283951*	*0.30952381*	*0.3139535*	*0.313253*
**ENSG00000067606**	*0.41566265*		*0.41566265*		*0.357142857*	*0.2267442*	
**ENSG00000119705**		*0.42901235*		*0.429012346*			*0.3614458*
**ENSG00000106246**		*0.09090909*		*0.090909091*			
**ENSG00000125787**					*0.363095238*	*0.1860465*	
**ENSG00000151882**		*0.42222222*		*0.422222222*			*0.2333333*
**ENSG00000170633**		*0.08484848*		*0.084848485*	*0.386904762*	*0.4244186*	*0.1104651*
**ENSG00000083857**	*0.2754491*	*0.49090909*	*0.2754491*	*0.490909091*	*0.380952381*	*0.3313953*	*0.494186*
**ENSG00000182533**		*0.44252874*		*0.442528736*			*0.2325581*
**ENSG00000144057**		*0.44242424*		*0.442424242*			*0.1511628*
**ENSG00000119865**		*0.07575758*		*0.075757576*			
**ENSG00000157379**		*0.4494382*		*0.449438202*	*0.088888889*	*0.1333333*	*0.3604651*
**ENSG00000079459**	*0.32634731*	*0.34545455*	*0.32634731*	*0.345454545*	*0.43902439*	*0.4411765*	*0.4583333*
**ENSG00000163421**		*0.07055215*		*0.070552147*			
**ENSG00000068781**		*0.23939394*		*0.239393939*			*0.3823529*
**ENSG00000165084**	*0.44610778*	*0.48181818*	*0.44610778*	*0.481818182*	*0.476190476*	*0.4651163*	*0.4534884*
**ENSG00000119408**		*0.4030303*		*0.403030303*			*0.4058824*
**ENSG00000129596**	*0.43712575*		*0.43712575*				

**ENSG00000149043**		*0.11818182*		*0.118181818*			*0.3953488*
**ENSG00000129451**	*0.27409639*	*0.42378049*	*0.27409639*	*0.423780488*	*0.325301205*	*0.3662791*	*0.4318182*
**ENSG00000043143**	*0.26666667*	*0.09090909*	*0.26666667*	*0.090909091*	*0.111111111*		*0.2764706*
**ENSG00000196932**	*0.30120482*	*0.33024691*	*0.30120482*	*0.330246914*	*0.097222222*		*0.4518072*
**ENSG00000165775**		*0.3404908*		*0.340490798*			*0.2209302*
**ENSG00000146151**		*0.39263804*		*0.392638037*			*0.4825581*
**ENSG00000255366**		*0.38888889*		*0.388888889*			*0.3604651*
**ENSG00000251209**	*0.06818182*	*0.38343558*	*0.06818182*	*0.383435583*	*0.125*	*0.1802326*	*0.1470588*
**ENSG00000092295**	*0.23888889*	*0.18787879*	*0.23888889*	*0.187878788*	*0.466666667*	*0.5*	*0.4705882*
**ENSG00000148688**		*0.33333333*		*0.333333333*			*0.4767442*
**ENSG00000109929**		*0.32621951*		*0.326219512*			*0.3255814*
**ENSG00000122958**	*0.28787879*	*0.36184211*	*0.28787879*	*0.361842105*			*0.122093*
**ENSG00000255552**							*0.0930233*
**ENSG00000186567**	*0.27222222*	*0.26136364*	*0.27222222*	*0.261363636*		*0.0581395*	*0.4294118*
**ENSG00000204946**		*0.46625767*		*0.466257669*			*0.4883721*
**ENSG00000179869**		*0.43939394*		*0.439393939*			*0.4883721*
**ENSG00000185271**	*0.0508982*		*0.0508982*		*0.160714286*	*0.2267442*	
**ENSG00000124813**		*0.1969697*		*0.196969697*			
**ENSG00000198246**		*0.08181818*		*0.081818182*			*0.0523256*
**ENSG00000251503**		*0.42675159*		*0.426751592*			*0.4117647*
**ENSG00000172403**		*0.43030303*		*0.43030303*			*0.4593023*
**ENSG00000198074**	*0.36826347*		*0.36826347*		*0.168674699*	*0.1860465*	
**ENSG00000177352**		*0.15740741*		*0.157407407*			*0.0535714*
**ENSG00000154124**		*0.1*		*0.1*			*0.25*
**ENSG00000088543**		*0.15151515*		*0.151515152*			*0.0872093*
**ENSG00000168078**		*0.39444444*		*0.394444444*			*0.3666667*
**ENSG00000249481**	*0.24550898*	*0.24444444*	*0.24550898*	*0.244444444*	*0.36746988*	*0.2848837*	*0.1363636*
**ENSG00000106648**	*0.36781609*	*0.11280488*	*0.36781609*	*0.112804878*	*0.380952381*	*0.375*	*0.2267442*
**ENSG00000183340**		*0.42727273*		*0.427272727*			*0.2383721*
**ENSG00000135537**	*0.32934132*		*0.32934132*		*0.142857143*	*0.1976744*	
**ENSG00000103723**		*0.37575758*		*0.375757576*			*0.4360465*
**ENSG00000169248**	*0.29341317*	*0.5*	*0.29341317*	*0.5*	*0.053571429*	*0.0581395*	*0.3546512*
**ENSG00000156219**		*0.25*		*0.25*			*0.4883721*
**ENSG00000166823**		*0.38181818*		*0.381818182*			*0.2383721*
**ENSG00000162654**		*0.44242424*		*0.442424242*			*0.3430233*
**ENSG00000221955**		*0.43636364*		*0.436363636*			*0.4302326*
**ENSG00000137337**	*0.45808383*	*0.13030303*	*0.45808383*	*0.13030303*	*0.166666667*	*0.2616279*	*0.1136364*
**ENSG00000049239**		*0.37658228*		*0.376582278*			*0.2965116*
**ENSG00000182544**	*0.16666667*		*0.16666667*		*0.130952381*	*0.3*	
**ENSG00000144837**	*0.4760479*	*0.21818182*	*0.4760479*	*0.218181818*	*0.5*	*0.4825581*	*0.3488372*
**ENSG00000021826**		*0.42727273*		*0.427272727*			*0.1470588*
**ENSG00000150787**		*0.28527607*		*0.285276074*			*0.2625*
**ENSG00000181523**		*0.36060606*		*0.360606061*			*0.3255814*
**ENSG00000132694**	*0.41317365*	*0.20426829*	*0.41317365*	*0.204268293*	*0.477777778*	*0.2386364*	*0.4772727*
**ENSG00000174891**	*0.4491018*		*0.4491018*		*0.452380952*	*0.4593023*	
**ENSG00000182173**	*0.49444444*	*0.31460674*	*0.49444444*	*0.314606742*			*0.4772727*
**ENSG00000100038**	*0.11077844*	*0.14285714*	*0.11077844*	*0.142857143*	*0.5*	*0.4069767*	*0.0769231*
**ENSG00000167851**		*0.32758621*		*0.327586207*			*0.4090909*
**ENSG00000224186**		*0.1969697*		*0.196969697*			*0.1802326*
**ENSG00000187173**		*0.06111111*		*0.061111111*			*0.2386364*
**ENSG00000111877**	*0.4251497*	*0.23888889*	*0.4251497*	*0.238888889*	*0.327380952*	*0.3117647*	*0.244186*
**ENSG00000129235**	*0.26047904*	*0.29775281*	*0.26047904*	*0.297752809*	*0.345238095*	*0.3895349*	*0.4090909*
**ENSG00000181744**	*0.2*	*0.36196319*	*0.2*	*0.36196319*	*0.233333333*	*0.3636364*	*0.3795181*
**ENSG00000008294**		*0.09756098*		*0.097560976*			

**ENSG00000106299**		*0.11818182*		*0.118181818*			*0.0705882*
**ENSG00000198625**		*0.48876404*		*0.488764045*			*0.2906977*
**ENSG00000149582**		*0.29090909*		*0.290909091*			*0.4534884*
**ENSG00000197885**	*0.38068182*	*0.17878788*	*0.38068182*	*0.178787879*	*0.410714286*	*0.4767442*	*0.0755814*
**ENSG00000120278**		*0.25842697*		*0.258426966*	*0.18452381*	*0.1860465*	*0.2034884*
**ENSG00000249016**	*0.2752809*		*0.2752809*				
**ENSG00000148399**		*0.17575758*		*0.175757576*			*0.0988372*
**ENSG00000130427**		*0.11515152*		*0.115151515*			
**ENSG00000162775**	*0.22727273*		*0.22727273*		*0.188888889*	*0.1931818*	
**ENSG00000109111**	*0.31736527*		*0.31736527*		*0.196428571*	*0.1395349*	
**ENSG00000133706**		*0.30487805*		*0.304878049*			*0.2411765*
**ENSG00000127928**		*0.3404908*		*0.340490798*			*0.2882353*
**ENSG00000257040**	*0.29640719*	*0.43333333*	*0.29640719*	*0.433333333*	*0.196428571*	*0.2444444*	*0.4886364*
**ENSG00000114738**	*0.16467066*	*0.18390805*	*0.16467066*	*0.183908046*	*0.4*	*0.3571429*	*0.375*
**ENSG00000176076**		*0.36280488*		*0.362804878*			*0.0872093*
**ENSG00000170348**		*0.49085366*		*0.490853659*			*0.2168675*
**ENSG00000105193**		*0.15*		*0.15*			*0.0909091*
**ENSG00000226397**		*0.07317073*		*0.073170732*			*0.4127907*
**ENSG00000185294**		*0.20909091*		*0.209090909*			
**ENSG00000115594**	*0.07865169*	*0.37575758*	*0.07865169*	*0.375757576*	*0.144444444*	*0.1395349*	*0.4337349*
**ENSG00000013288**		*0.12643678*		*0.126436782*			*0.195122*
**ENSG00000212719**		*0.47575758*		*0.475757576*			*0.172619*
**ENSG00000185339**		*0.49444444*		*0.494444444*	*0.113095238*	*0.1395349*	*0.4659091*
**ENSG00000123473**		*0.46666667*		*0.466666667*			*0.4235294*
**ENSG00000122566**							*0.3255814*
**ENSG00000123131**	*0.34444444*		*0.34444444*		*0.411111111*	*0.375*	
**ENSG00000162434**		*0.07272727*		*0.072727273*			
**ENSG00000162627**		*0.27575758*		*0.275757576*			*0.3430233*
**ENSG00000180483**		*0.47575758*		*0.475757576*			*0.2529412*
**ENSG00000111300**		*0.11656442*		*0.116564417*			*0.4111111*
**ENSG00000091436**		*0.47575758*		*0.475757576*			*0.3068182*
**ENSG00000166927**		*0.3*		*0.3*			*0.2666667*
**ENSG00000173088**		*0.42682927*		*0.426829268*			*0.4709302*
**ENSG00000143355**	*0.1497006*	*0.42073171*	*0.1497006*	*0.420731707*	*0.452380952*	*0.4011628*	*0.3953488*
**ENSG00000086232**		*0.4030303*		*0.403030303*			*0.4360465*
**ENSG00000140678**		*0.45121951*		*0.451219512*			*0.3953488*
**ENSG00000125810**		*0.44848485*		*0.448484848*			*0.2732558*
**ENSG00000173120**		*0.08181818*		*0.081818182*			
**ENSG00000129514**		*0.36969697*		*0.36969697*			*0.1337209*
**ENSG00000166432**		*0.1375*		*0.1375*			
**ENSG00000110090**		*0.4068323*		*0.406832298*			*0.2325581*
**ENSG00000196417**	*0.44444444*	*0.28888889*	*0.44444444*	*0.288888889*	*0.255813953*	*0.1818182*	*0.4529412*
**ENSG00000224586**		*0.31212121*		*0.312121212*			*0.4705882*
**ENSG00000006530**	*0.30681818*		*0.30681818*		*0.055555556*	*0.0568182*	
**ENSG00000163702**		*0.46540881*		*0.465408805*			
**ENSG00000182054**							*0.2093023*
**ENSG00000186081**		*0.08841463*		*0.088414634*	*0.322222222*	*0.2777778*	
**ENSG00000139445**		*0.49393939*		*0.493939394*			*0.377907*
**ENSG00000120498**	*0.06470588*		*0.06470588*				
**ENSG00000172731**		*0.36363636*		*0.363636364*			*0.4244186*
**ENSG00000133863**		*0.16060606*		*0.160606061*			
**ENSG00000013375**	*0.48314607*	*0.25151515*	*0.48314607*	*0.251515152*	*0.284090909*	*0.3488372*	*0.2409639*
**ENSG00000254936**	*0.0748503*	*0.48170732*	*0.0748503*	*0.481707317*	*0.053571429*	*0.0529412*	
**ENSG00000125520**		*0.22424242*		*0.224242424*			*0.4244186*

**ENSG00000122884**		*0.06363636*		*0.063636364*			*0.3863636*
**ENSG00000176749**		*0.44848485*		*0.448484848*			
**ENSG00000056487**		*0.4*		*0.4*			*0.4011628*
**ENSG00000009830**	*0.16981132*	*0.47878788*	*0.16981132*	*0.478787879*	*0.475903614*	*0.4941176*	*0.5*
**ENSG00000017621**		*0.09393939*		*0.093939394*			
**ENSG00000248144**		*0.11515152*		*0.115151515*			*0.0523256*
**ENSG00000174371**	*0.46706587*	*0.4030303*	*0.46706587*	*0.403030303*	*0.295180723*	*0.3823529*	*0.4186047*
**ENSG00000100626**		*0.46969697*		*0.46969697*			*0.375*
**ENSG00000157168**		*0.16060606*		*0.160606061*			
**ENSG00000109445**		*0.271875*		*0.271875*			*0.3529412*
**ENSG00000243207**		*0.10248447*		*0.102484472*			*0.2650602*
**ENSG00000153107**	*0.2439759*	*0.33536585*	*0.2439759*	*0.335365854*	*0.487951807*	*0.4883721*	*0.4418605*
**ENSG00000176746**	*0.43113772*	*0.31666667*	*0.43113772*	*0.316666667*	*0.470238095*	*0.4476744*	*0.4886364*
**ENSG00000132541**		*0.26666667*		*0.266666667*			*0.0523256*
**ENSG00000223802**		*0.08333333*		*0.083333333*			*0.2386364*
**ENSG00000118965**		*0.39090909*		*0.390909091*			*0.3139535*
**ENSG00000042429**	*0.05389222*	*0.13030303*	*0.05389222*	*0.13030303*			*0.2034884*
**ENSG00000212768**	*0.46666667*		*0.46666667*				
**ENSG00000177082**		*0.24242424*		*0.242424242*			*0.372093*
**ENSG00000134201**		*0.43888889*		*0.438888889*	*0.3*	*0.3444444*	*0.3295455*
**ENSG00000137574**	*0.21515152*	*0.12121212*	*0.21515152*	*0.121212121*			
**ENSG00000099840**		*0.32183908*		*0.32183908*			*0.2159091*
**ENSG00000171227**		*0.15555556*		*0.155555556*			*0.0888889*
**ENSG00000117748**		*0.38414634*		*0.384146341*			*0.4058824*
**ENSG00000054116**	*0.07954545*		*0.07954545*				
**ENSG00000198876**		*0.33030303*		*0.33030303*			*0.2034884*
**ENSG00000119778**		*0.43030303*		*0.43030303*			*0.1777778*
**ENSG00000109099**		*0.45454545*		*0.454545455*			*0.3488372*
**ENSG00000008838**		*0.40804598*		*0.408045977*			*0.4431818*
**ENSG00000140992**		*0.05757576*		*0.057575758*			
**ENSG00000149294**	*0.07784431*	*0.38484848*	*0.07784431*	*0.384848485*	*0.31547619*	*0.2790698*	*0.0930233*
**ENSG00000172888**		*0.36060606*		*0.360606061*			*0.2674419*
**ENSG00000175376**	*0.12874251*	*0.1969697*	*0.12874251*	*0.196969697*	*0.178571429*	*0.0872093*	*0.1860465*
**ENSG00000163933**		*0.41212121*		*0.412121212*			*0.1802326*
**ENSG00000140521**		*0.07878788*		*0.078787879*			
**ENSG00000204592**		*0.31111111*		*0.311111111*			*0.3181818*
**ENSG00000081320**		*0.33146067*		*0.331460674*			*0.1842105*
**ENSG00000072786**		*0.18787879*		*0.187878788*			
**ENSG00000006116**	*0.09281437*	*0.23636364*	*0.09281437*	*0.236363636*	*0.18452381*	*0.1337209*	*0.4244186*
**ENSG00000198719**	*0.06287425*	*0.39393939*	*0.06287425*	*0.393939394*	*0.386904762*	*0.4534884*	*0.2906977*
**ENSG00000006042**		*0.33536585*		*0.335365854*			*0.2906977*
**ENSG00000186300**		*0.40490798*		*0.404907975*			*0.3139535*
**ENSG00000244734**					*0.351190476*	*0.4534884*	
**ENSG00000243667**	*0.07784431*		*0.07784431*				
**ENSG00000079739**	*0.2005988*	*0.14545455*	*0.2005988*	*0.145454545*			*0.1511628*
**ENSG00000136273**	*0.25568182*	*0.46111111*	*0.25568182*	*0.461111111*	*0.422222222*	*0.5*	*0.5*
**ENSG00000196323**		*0.42121212*		*0.421212121*			*0.4186047*
**ENSG00000110104**		*0.36904762*		*0.369047619*			*0.3488372*
**ENSG00000135976**		*0.09937888*		*0.099378882*			*0.0823529*
**ENSG00000198833**		*0.15151515*		*0.151515152*			*0.3529412*
**ENSG00000134339**		*0.49444444*		*0.494444444*			*0.1162791*
**ENSG00000168959**		*0.49090909*		*0.490909091*			*0.4294118*
**ENSG00000159625**						*0.0697674*	
**ENSG00000185033**	*0.49700599*	*0.46363636*	*0.49700599*	*0.463636364*	*0.333333333*	*0.3255814*	*0.4302326*

**ENSG00000146530**	*0.47126437*	*0.44968553*	*0.47126437*	*0.449685535*	*0.445783133*	*0.3555556*	*0.4235294*
**ENSG00000184454**		*0.36206897*		*0.362068966*			*0.494186*
**ENSG00000107937**		*0.20909091*		*0.209090909*			*0.2777778*
**ENSG00000234949**		*0.22560976*		*0.225609756*			*0.4593023*
**ENSG00000183955**		*0.08333333*		*0.083333333*			
**ENSG00000008018**	*0.12874251*		*0.12874251*				
**ENSG00000134627**	*0.17664671*	*0.44785276*	*0.17664671*	*0.447852761*		*0.1*	*0.4761905*
**ENSG00000183431**	*0.3502994*	*0.478125*	*0.3502994*	*0.478125*	*0.339285714*	*0.4360465*	*0.4302326*
**ENSG00000213347**		*0.36363636*		*0.363636364*			*0.4476744*
**ENSG00000150991**	*0.41616766*		*0.41616766*		*0.369047619*	*0.4476744*	
**ENSG00000145335**	*0.19318182*	*0.46363636*	*0.19318182*	*0.463636364*	*0.122222222*	*0.0568182*	*0.4186047*
**ENSG00000235194**		*0.28484848*		*0.284848485*			*0.4593023*
**ENSG00000130402**		*0.24719101*		*0.247191011*			*0.2383721*
**ENSG00000182108**		*0.42424242*		*0.424242424*			*0.4294118*
**ENSG00000111540**	*0.06969697*	*0.0969697*	*0.06969697*	*0.096969697*	*0.288888889*	*0.2613636*	
**ENSG00000125821**		*0.20909091*		*0.209090909*			
**ENSG00000172915**	*0.27743902*	*0.42424242*	*0.27743902*	*0.424242424*	*0.369047619*	*0.2616279*	*0.25*
**ENSG00000198542**							*0.3023256*
**ENSG00000109182**	*0.17065868*	*0.28963415*	*0.17065868*	*0.289634146*	*0.279761905*	*0.2848837*	*0.3430233*
**ENSG00000169116**		*0.31212121*		*0.312121212*			*0.3139535*
**ENSG00000196943**		*0.19090909*		*0.190909091*			*0.2272727*
**ENSG00000169994**	*0.29341317*	*0.23333333*	*0.29341317*	*0.233333333*	*0.154761905*	*0.0705882*	*0.1453488*
**ENSG00000157978**		*0.48484848*		*0.484848485*			*0.4127907*
**ENSG00000147133**	*0.28742515*		*0.28742515*				
**ENSG00000174483**		*0.41463415*		*0.414634146*			*0.4823529*
**ENSG00000134463**	*0.4126506*		*0.4126506*				
**ENSG00000139192**	*0.1257485*		*0.1257485*				
**ENSG00000214753**							*0.1744186*
**ENSG00000158555**	*0.34036145*	*0.38484848*	*0.34036145*	*0.384848485*	*0.148809524*	*0.25*	*0.375*
**ENSG00000196376**		*0.14848485*		*0.148484848*			
**ENSG00000196565**	*0.08888889*		*0.08888889*		*0.077777778*	*0.1477273*	
**ENSG00000124380**		*0.45*		*0.45*			*0.4318182*
**ENSG00000114030**		*0.16363636*		*0.163636364*			*0.3430233*
**ENSG00000176853**	*0.07471264*	*0.13372093*	*0.07471264*	*0.13372093*	*0.455555556*	*0.3295455*	*0.2906977*
**ENSG00000167646**		*0.13939394*		*0.139393939*			*0.2647059*
**ENSG00000134028**	*0.18333333*	*0.43333333*	*0.18333333*	*0.433333333*	*0.466666667*	*0.4545455*	*0.2411765*
**ENSG00000182810**							*0.3139535*
**ENSG00000139610**		*0.33030303*		*0.33030303*			*0.3035714*
**ENSG00000033050**		*0.41954023*		*0.41954023*			*0.4285714*
**ENSG00000087903**		*0.05063291*		*0.050632911*			
**ENSG00000168070**	*0.33832335*	*0.32424242*	*0.33832335*	*0.324242424*	*0.172619048*	*0.1511628*	*0.1511628*
**ENSG00000171540**	*0.28313253*		*0.28313253*		*0.339285714*	*0.3352941*	
**ENSG00000119723**	*0.08988764*	*0.44545455*	*0.08988764*	*0.445454545*	*0.205128205*	*0.25*	*0.3522727*
**ENSG00000168438**		*0.06666667*		*0.066666667*	*0.125*	*0.1058824*	*0.122093*
**ENSG00000124783**		*0.36969697*		*0.36969697*			*0.4127907*
**ENSG00000111275**							*0.2383721*
**ENSG00000114850**		*0.28658537*		*0.286585366*			*0.2325581*
**ENSG00000183751**	*0.11676647*		*0.11676647*				
**ENSG00000164270**		*0.40909091*		*0.409090909*			*0.2383721*
**ENSG00000140006**	*0.42215569*		*0.42215569*				
**ENSG00000151923**	*0.33229814*	*0.05151515*	*0.33229814*	*0.051515152*	*0.422619048*	*0.3614458*	
**ENSG00000163956**		*0.49425287*		*0.494252874*			*0.4186047*
**ENSG00000142528**	*0.24550898*		*0.24550898*				
**ENSG00000101321**		*0.29090909*		*0.290909091*			*0.1162791*

**ENSG00000165409**	*0.22891566*	*0.43636364*	*0.22891566*	*0.436363636*	*0.3*	*0.3109756*	*0.4593023*
**ENSG00000153789**							*0.0581395*
**ENSG00000184040**		*0.31212121*		*0.312121212*			*0.4647059*
**ENSG00000123453**	*0.25287356*	*0.35365854*	*0.25287356*	*0.353658537*	*0.333333333*	*0.3255814*	*0.375*
**ENSG00000061656**	*0.32934132*		*0.32934132*		*0.090361446*	*0.0988372*	
**ENSG00000205177**		*0.36363636*		*0.363636364*			*0.4647059*
**ENSG00000156136**		*0.07012195*		*0.070121951*			
**ENSG00000081386**	*0.46706587*		*0.46706587*		*0.345238095*	*0.3882353*	
**ENSG00000163600**		*0.25454545*		*0.254545455*			*0.1976744*
**ENSG00000198585**	*0.48333333*	*0.05454545*	*0.48333333*	*0.054545455*			*0.3181818*
**ENSG00000162174**	*0.33532934*	*0.39090909*	*0.33532934*	*0.390909091*	*0.243902439*	*0.2647059*	*0.2790698*
**ENSG00000089048**	*0.29341317*	*0.1*	*0.29341317*	*0.1*	*0.410714286*	*0.3546512*	
**ENSG00000187905**		*0.41954023*		*0.41954023*			*0.2267442*
**ENSG00000109381**					*0.166666667*	*0.2613636*	
**ENSG00000140995**		*0.15555556*		*0.155555556*			
**ENSG00000123836**	*0.16766467*	*0.44545455*	*0.16766467*	*0.445454545*	*0.154761905*	*0.0764706*	*0.2048193*
**ENSG00000112062**		*0.47191011*		*0.471910112*			*0.4764706*
**ENSG00000167515**		*0.34242424*		*0.342424242*			*0.4360465*
**ENSG00000183734**	*0.29341317*		*0.29341317*		*0.488095238*	*0.4534884*	
**ENSG00000185860**		*0.3969697*		*0.396969697*			*0.4883721*
**ENSG00000002726**	*0.38855422*	*0.17575758*	*0.38855422*	*0.175757576*	*0.476190476*	*0.4651163*	*0.4651163*
**ENSG00000111186**	*0.16566265*	*0.26363636*	*0.16566265*	*0.263636364*	*0.19047619*	*0.3255814*	*0.2674419*
**ENSG00000196072**	*0.18333333*		*0.18333333*				*0.0988372*
**ENSG00000113240**		*0.49390244*		*0.493902439*			*0.3197674*
**ENSG00000134278**	*0.30113636*	*0.2969697*	*0.30113636*	*0.296969697*	*0.366666667*	*0.3863636*	*0.1744186*
**ENSG00000123374**		*0.07471264*		*0.074712644*			*0.122093*
**ENSG00000174827**					*0.148809524*	*0.2267442*	
**ENSG00000126091**		*0.31515152*		*0.315151515*			*0.4360465*
**ENSG00000177752**		*0.4030303*		*0.403030303*			*0.4767442*
**ENSG00000094796**		*0.2012987*		*0.201298701*			*0.1*
**ENSG00000119535**	*0.12048193*		*0.12048193*		*0.071428571*	*0.0988372*	
**ENSG00000105929**		*0.18787879*		*0.187878788*			
**ENSG00000037897**	*0.10493827*	*0.32926829*	*0.10493827*	*0.329268293*	*0.380952381*	*0.375*	*0.2267442*
**ENSG00000188321**		*0.43939394*		*0.439393939*			*0.4244186*
**ENSG00000185742**		*0.37195122*		*0.37195122*			*0.3662791*
**ENSG00000138744**		*0.41954023*		*0.41954023*			*0.0681818*
**ENSG00000149654**	*0.19318182*		*0.19318182*		*0.177777778*	*0.1931818*	
**ENSG00000181577**							*0.2294118*
**ENSG00000119682**	*0.38333333*	*0.4*	*0.38333333*	*0.4*	*0.288888889*	*0.2954545*	*0.4244186*
**ENSG00000182667**		*0.35582822*		*0.355828221*			*0.1802326*
**ENSG00000173681**		*0.14848485*		*0.148484848*			*0.0523256*
**ENSG00000157500**	*0.32424242*	*0.39570552*	*0.32424242*	*0.395705521*			*0.1190476*
**ENSG00000164749**		*0.47619048*		*0.476190476*			*0.4268293*
**ENSG00000141150**							*0.2383721*
**ENSG00000131171**		*0.34444444*		*0.344444444*			*0.1363636*
**ENSG00000143612**		*0.42727273*		*0.427272727*			*0.4476744*
**ENSG00000173917**		*0.36363636*		*0.363636364*			*0.2848837*
**ENSG00000229972**							*0.0764706*
**ENSG00000090659**		*0.40909091*		*0.409090909*			*0.3444444*
**ENSG00000111450**		*0.44444444*		*0.444444444*			*0.1931818*
**ENSG00000123610**		*0.15853659*		*0.158536585*			*0.0705882*
**ENSG00000213654**	*0.11377246*		*0.11377246*				
**ENSG00000117592**		*0.23333333*		*0.233333333*			*0.4127907*
**ENSG00000042781**		*0.19444444*		*0.194444444*			*0.3255814*

**ENSG00000189227**		*0.20858896*		*0.208588957*			*0.494186*
**ENSG00000065609**		*0.26060606*		*0.260606061*			*0.2848837*
**ENSG00000152785**		*0.20552147*		*0.205521472*			*0.1891892*
**ENSG00000154654**		*0.48333333*		*0.483333333*			*0.255814*
**ENSG00000149599**	*0.15568862*	*0.40555556*	*0.15568862*	*0.405555556*	*0.345238095*	*0.3430233*	
**ENSG00000122203**	*0.2*	*0.23030303*	*0.2*	*0.23030303*	*0.333333333*	*0.4888889*	*0.2790698*
**ENSG00000182621**		*0.44117647*		*0.441176471*			*0.4244186*
**ENSG00000132330**	*0.34730539*	*0.46511628*	*0.34730539*	*0.465116279*			*0.4470588*
**ENSG00000166575**		*0.2*		*0.2*			*0.4011628*
**ENSG00000146416**		*0.17878788*		*0.178787879*			*0.452381*
**ENSG00000196636**	*0.2754491*	*0.46666667*	*0.2754491*	*0.466666667*	*0.452380952*	*0.4588235*	*0.4659091*
**ENSG00000129221**		*0.26060606*		*0.260606061*			*0.2674419*
**ENSG00000113916**		*0.36363636*		*0.363636364*	*0.144444444*	*0.3636364*	*0.2034884*
**ENSG00000197497**		*0.43597561*		*0.43597561*			*0.3139535*
**ENSG00000179253**	*0.38023952*	*0.33544304*	*0.38023952*	*0.335443038*	*0.404761905*	*0.5*	*0.2965116*
**ENSG00000132623**		*0.16853933*		*0.168539326*			
**ENSG00000169118**							*0.0581395*
**ENSG00000213380**		*0.38505747*		*0.385057471*			*0.0909091*
**ENSG00000179066**	*0.40718563*		*0.40718563*		*0.452380952*	*0.4186047*	
**ENSG00000128652**	*0.49401198*	*0.35454545*	*0.49401198*	*0.354545455*	*0.071428571*	*0.1453488*	*0.1931818*
**ENSG00000118094**		*0.29090909*		*0.290909091*			*0.494186*
**ENSG00000135404**	*0.21111111*		*0.21111111*				
**ENSG00000198690**		*0.30606061*		*0.306060606*			*0.1823529*
**ENSG00000188334**		*0.5*		*0.5*			*0.2555556*
**ENSG00000141425**		*0.21111111*		*0.211111111*			*0.25*
**ENSG00000142319**		*0.27575758*		*0.275757576*			*0.122093*
**ENSG00000156599**	*0.23952096*	*0.44242424*	*0.23952096*	*0.442424242*			*0.1046512*
**ENSG00000182551**	*0.4*	*0.19047619*	*0.4*	*0.19047619*	*0.311111111*	*0.2380952*	*0.3181818*
**ENSG00000162992**	*0.18263473*		*0.18263473*		*0.095238095*	*0.0755814*	
**ENSG00000163565**	*0.25748503*		*0.25748503*		*0.345238095*	*0.3023256*	
**ENSG00000125166**		*0.37267081*		*0.372670807*			*0.3895349*
**ENSG00000132326**	*0.08383234*	*0.17272727*	*0.08383234*	*0.172727273*	*0.05952381*	*0.0581395*	*0.2732558*
**ENSG00000197976**		*0.43888889*		*0.438888889*			*0.3636364*
**ENSG00000118971**	*0.48802395*	*0.35757576*	*0.48802395*	*0.357575758*	*0.271084337*	*0.3837209*	*0.3235294*
**ENSG00000181035**		*0.14814815*		*0.148148148*			*0.0882353*
**ENSG00000186889**		*0.16768293*		*0.167682927*			*0.4476744*
**ENSG00000203326**	*0.41666667*	*0.32777778*	*0.41666667*	*0.327777778*	*0.433333333*	*0.3444444*	*0.4534884*
**ENSG00000175115**	*0.1497006*	*0.23030303*	*0.1497006*	*0.23030303*	*0.272727273*	*0.2159091*	*0.2209302*
**ENSG00000135686**		*0.3902439*		*0.390243902*			*0.4302326*
**ENSG00000148690**		*0.11818182*		*0.118181818*			
**ENSG00000139914**	*0.26646707*		*0.26646707*		*0.363095238*	*0.3953488*	
**ENSG00000100116**		*0.28181818*		*0.281818182*			*0.2529412*
**ENSG00000106823**		*0.43030303*		*0.43030303*			*0.2823529*
**ENSG00000087152**		*0.33536585*		*0.335365854*			*0.3139535*
**ENSG00000023516**		*0.35151515*		*0.351515152*			*0.25*
**ENSG00000092841**		*0.13333333*		*0.133333333*			
**ENSG00000075785**							*0.0813953*
**ENSG00000146360**		*0.41515152*		*0.415151515*			*0.2616279*
**ENSG00000214711**	*0.27222222*	*0.46666667*	*0.27222222*	*0.466666667*	*0.5*	*0.3977273*	*0.255814*
**ENSG00000168301**	*0.43113772*		*0.43113772*		*0.273809524*	*0.3197674*	
**ENSG00000137802**		*0.38953488*		*0.389534884*			*0.3139535*
**ENSG00000144191**					*0.182926829*	*0.1969697*	
**ENSG00000162571**		*0.13939394*		*0.139393939*			*0.3837209*
**ENSG00000216937**		*0.48181818*		*0.481818182*			*0.1686047*

**ENSG00000111203**		*0.1030303*		*0.103030303*				*0.2151163*
**ENSG00000241978**	*0.34431138*	*0.49691358*	*0.34431138*	*0.49691358*	*0.375*		*0.4534884*	*0.4883721*
**ENSG00000257093**		*0.45757576*		*0.457575758*				*0.4418605*
**ENSG00000023892**	*0.2030303*	*0.07926829*	*0.2030303*	*0.079268293*				
**ENSG00000153233**	*0.21556886*		*0.21556886*		*0.060240964*		*0.0755814*	
**ENSG00000158163**		*0.05813953*		*0.058139535*				*0.4418605*
**ENSG00000111110**		*0.22121212*		*0.221212121*				*0.3895349*
**ENSG00000184307**		*0.4030303*		*0.403030303*				*0.4069767*
**ENSG00000104047**		*0.26969697*		*0.26969697*				*0.4593023*
**ENSG00000126391**		*0.20909091*		*0.209090909*				*0.0523256*
**ENSG00000174886**	*0.38323353*		*0.38323353*		*0.25*		*0.2674419*	
**ENSG00000081087**		*0.27878788*		*0.278787879*				*0.4651163*
**ENSG00000131379**	*0.20658683*		*0.20658683*		*0.244047619*		*0.3023256*	
**ENSG00000179639**		*0.15454545*		*0.154545455*				
**ENSG00000123612**	*0.26946108*		*0.26946108*		*0.303571429*		*0.2151163*	
**ENSG00000101363**		*0.38181818*		*0.381818182*				*0.2674419*
**ENSG00000159882**	*0.45508982*	*0.17272727*	*0.45508982*	*0.172727273*	*0.488888889*		*0.4431818*	*0.2034884*
**ENSG00000113595**	*0.37356322*	*0.36781609*	*0.37356322*	*0.367816092*	*0.453488372*		*0.3111111*	*0.3023256*
**ENSG00000171766**	*0.4491018*	*0.38271605*	*0.4491018*	*0.382716049*	*0.177777778*		*0.1931818*	*0.1931818*
**ENSG00000133104**	*0.20555556*	*0.26969697*	*0.20555556*	*0.26969697*	*0.166666667*		*0.2222222*	*0.4476744*
**ENSG00000247746**								*0.2559524*
**ENSG00000143126**		*0.29885057*		*0.298850575*				*0.0755814*
**ENSG00000101350**		*0.36666667*		*0.366666667*				*0.3197674*
**ENSG00000148516**	*0.22754491*	*0.05757576*	*0.22754491*	*0.057575758*				*0.1744186*
**ENSG00000182698**	*0.05120482*		*0.05120482*		*0.18452381*		*0.1764706*	
**ENSG00000136918**		*0.46666667*		*0.466666667*				*0.372093*
**ENSG00000053524**		*0.20679012*		*0.206790123*				*0.3823529*
**ENSG00000089063**		*0.47272727*		*0.472727273*				*0.1369048*
**ENSG00000162949**	*0.32035928*	*0.36196319*	*0.32035928*	*0.36196319*	*0.238095238*		*0.3023256*	*0.5*
**ENSG00000145908**		*0.06707317*		*0.067073171*				*0.1764706*
**ENSG00000110076**		*0.05590062*		*0.055900621*				*0.2848837*
**ENSG00000107890**		*0.05757576*		*0.057575758*	*0.25*		*0.2790698*	*0.3470588*
**ENSG00000141560**		*0.3*		*0.3*				*0.494186*
**ENSG00000091972**	*0.0748503*	*0.06969697*	*0.0748503*	*0.06969697*	*0.119047619*		*0.1569767*	*0.1976744*
**ENSG00000173431**	*0.38622754*		*0.38622754*		*0.410714286*		*0.3372093*	
**ENSG00000175166**	*0.36526946*	*0.49691358*	*0.36526946*	*0.49691358*	*0.404761905*		*0.4651163*	*0.2235294*
**ENSG00000236637**		*0.16358025*		*0.163580247*				*0.5*
**ENSG00000170382**	*0.37125749*	*0.46060606*	*0.37125749*	*0.460606061*	*0.142857143*		*0.122093*	*0.2674419*
**ENSG00000182584**	*0.246875*		*0.246875*		*0.385542169*		*0.4036145*	
**ENSG00000145901**	*0.28443114*	*0.06363636*	*0.28443114*	*0.063636364*	*0.416666667*		*0.494186*	*0.122093*
**ENSG00000116871**	*0.05555556*		*0.05555556*		*0.077777778*		*0.1477273*	
**ENSG00000119599**	*0.45454545*	*0.28484848*	*0.45454545*	*0.284848485*	*0.392857143*		*0.4825581*	*0.4883721*
**ENSG00000149196**	*0.28143713*	*0.26969697*	*0.28143713*	*0.26969697*	*0.226190476*		*0.25*	*0.2790698*
**ENSG00000143067**		*0.43333333*		*0.433333333*				*0.4583333*
**ENSG00000106459**	*0.2005988*	*0.18484848*	*0.2005988*	*0.184848485*	*0.391566265*		*0.4244186*	*0.2325581*
**ENSG00000170790**	*0.15568862*		*0.15568862*		*0.351190476*		*0.2906977*	
**ENSG00000170955**	*0.36904762*		*0.36904762*		*0.445945946*		*0.4358974*	
**ENSG00000134216**	*0.26404494*	*0.36890244*	*0.26404494*	*0.368902439*	*0.416666667*		*0.3604651*	*0.5*
**ENSG00000237651**	*0.41477273*		*0.41477273*		*0.275*		*0.3372093*	
**ENSG00000187676**		*0.25882353*		*0.258823529*				*0.4090909*
**ENSG00000167767**		*0.39393939*		*0.393939394*				*0.4534884*
**ENSG00000256391**	*0.47878788*	*0.44545455*	*0.47878788*	*0.445454545*	*0.253012048*		*0.3430233*	*0.3430233*
**ENSG00000165181**	*0.24431818*	*0.45454545*	*0.24431818*	*0.454545455*				*0.1845238*
**ENSG00000111196**	*0.47222222*	*0.20786517*	*0.47222222*	*0.207865169*				

**ENSG00000135046**	*0.26946108*		*0.26946108*		*0.166666667*	*0.1704545*	
**ENSG00000187123**		*0.22256098*		*0.222560976*			*0.127907*
**ENSG00000183513**		*0.23030303*		*0.23030303*			*0.2674419*
**ENSG00000168237**		*0.1097561*		*0.109756098*			
**ENSG00000100665**	*0.36666667*		*0.36666667*		*0.452380952*	*0.5*	
**ENSG00000142065**		*0.4030303*		*0.403030303*			*0.4186047*
**ENSG00000206384**		*0.15151515*		*0.151515152*			*0.3546512*
**ENSG00000129749**		*0.08522727*		*0.085227273*			*0.1104651*
**ENSG00000152242**		*0.21515152*		*0.215151515*			*0.4883721*
**ENSG00000171865**		*0.38343558*		*0.383435583*			*0.2674419*
**ENSG00000217128**		*0.24545455*		*0.245454545*			*0.4698795*
**ENSG00000124370**						*0.0833333*	
**ENSG00000113569**	*0.05555556*	*0.16666667*	*0.05555556*	*0.166666667*			*0.0755814*
**ENSG00000141424**	*0.35628743*		*0.35628743*		*0.19047619*	*0.2209302*	
**ENSG00000074266**	*0.26136364*	*0.40606061*	*0.26136364*	*0.406060606*	*0.318181818*	*0.3081395*	*0.2674419*
**ENSG00000139508**		*0.47239264*		*0.472392638*			*0.4069767*
**ENSG00000155011**		*0.33636364*		*0.336363636*	*0.255952381*	*0.1162791*	*0.4186047*
**ENSG00000135373**	*0.31437126*	*0.43604651*	*0.31437126*	*0.436046512*	*0.465909091*	*0.4883721*	*0.4709302*
**ENSG00000164256**	*0.14444444*	*0.2030303*	*0.14444444*	*0.203030303*	*0.2*	*0.1363636*	*0.4302326*
**ENSG00000115568**	*0.15454545*	*0.40243902*	*0.15454545*	*0.402439024*	*0.208333333*	*0.244186*	*0.244186*
**ENSG00000158639**	*0.43333333*		*0.43333333*				
**ENSG00000120437**	*0.1257485*	*0.1969697*	*0.1257485*	*0.196969697*	*0.154761905*	*0.1802326*	*0.4476744*
**ENSG00000167139**		*0.19090909*		*0.190909091*			*0.0523256*
**ENSG00000135124**		*0.49090909*		*0.490909091*			*0.3255814*
**ENSG00000130312**		*0.41515152*		*0.415151515*			*0.122093*
**ENSG00000167895**	*0.11363636*	*0.5*	*0.11363636*	*0.5*	*0.477777778*	*0.4318182*	*0.4659091*
**ENSG00000198723**	*0.2005988*		*0.2005988*		*0.136904762*	*0.3255814*	
**ENSG00000135679**		*0.4969697*		*0.496969697*			*0.2325581*
**ENSG00000043355**		*0.21646341*		*0.216463415*			*0.2965116*
**ENSG00000177108**	*0.08383234*	*0.23939394*	*0.08383234*	*0.239393939*	*0.238095238*	*0.2034884*	*0.3666667*
**ENSG00000108588**		*0.36890244*		*0.368902439*			*0.3941176*
**ENSG00000142961**	*0.49101796*	*0.45333333*	*0.49101796*	*0.453333333*	*0.273809524*	*0.3255814*	*0.1046512*
**ENSG00000221926**	*0.290625*		*0.290625*		*0.494047619*	*0.4534884*	
**ENSG00000214941**		*0.48181818*		*0.481818182*			*0.2613636*
**ENSG00000130204**		*0.29775281*		*0.297752809*			*0.1022727*
**ENSG00000078269**	*0.24251497*	*0.45454545*	*0.24251497*	*0.454545455*	*0.428571429*	*0.4709302*	*0.4545455*
**ENSG00000135917**	*0.19161677*	*0.46969697*	*0.19161677*	*0.46969697*	*0.196428571*	*0.1918605*	
**ENSG00000137193**		*0.31818182*		*0.318181818*			*0.0697674*
**ENSG00000056277**		*0.39393939*		*0.393939394*			*0.3882353*
**ENSG00000187048**		*0.15757576*		*0.157575758*			*0.2034884*
**ENSG00000185761**		*0.47272727*		*0.472727273*	*0.084337349*	*0.0813953*	*0.377907*
**ENSG00000105397**	*0.32934132*	*0.12121212*	*0.32934132*	*0.121212121*	*0.06547619*	*0.0523256*	
**ENSG00000147381**	*0.37222222*		*0.37222222*		*0.322222222*	*0.4431818*	
**ENSG00000126247**		*0.06748466*		*0.067484663*			
**ENSG00000154122**		*0.22424242*		*0.224242424*			*0.255814*
**ENSG00000163611**	*0.06886228*	*0.47272727*	*0.06886228*	*0.472727273*	*0.071428571*	*0.125*	*0.1941176*
**ENSG00000085491**	*0.42777778*	*0.23333333*	*0.42777778*	*0.233333333*			*0.0568182*
**ENSG00000198087**		*0.3447205*		*0.344720497*			*0.4090909*
**ENSG00000178882**		*0.29813665*		*0.298136646*			*0.4825581*
**ENSG00000171174**					*0.077380952*	*0.1918605*	*0.3095238*
**ENSG00000171160**		*0.2125*		*0.2125*			*0.2380952*
**ENSG00000100027**		*0.36060606*		*0.360606061*			*0.3546512*
**ENSG00000119640**		*0.49382716*		*0.49382716*			*0.2294118*
**ENSG00000183695**		*0.2652439*		*0.265243902*			*0.2222222*

**ENSG00000118402**		0.22159091		0.221590909			0.1976744
**ENSG00000185800**		0.46590909		0.465909091			0.2954545
**ENSG00000163626**		0.45151515		0.451515152			0.3647059
**ENSG00000149488**		0.4847561		0.484756098			0.4476744
**ENSG00000162456**		0.25454545		0.254545455			0.3837209
**ENSG00000213186**	0.31818182	0.4969697	0.31818182	0.496969697	0.181818182	0.2666667	0.25
**ENSG00000033627**		0.11818182		0.118181818			0.2034884
**ENSG00000184058**		0.38484848		0.384848485			0.3662791
**ENSG00000100151**	0.2		0.2		0.363636364	0.3522727	
**ENSG00000066427**		0.28888889		0.288888889			0.4777778
**ENSG00000121988**	0.25748503	0.29090909	0.25748503	0.290909091	0.160714286	0.1918605	0.1941176
**ENSG00000133112**	0.26388889	0.39090909	0.26388889	0.390909091	0.36746988	0.3117647	0.2411765
**ENSG00000198090**		0.44827586		0.448275862			0.4294118
**ENSG00000186583**	0.10179641		0.10179641				
**ENSG00000013561**	0.18589744	0.44444444	0.18589744	0.444444444			
**ENSG00000008405**	0.43181818	0.41104294	0.43181818	0.411042945	0.329545455	0.2380952	0.2411765
**ENSG00000039319**		0.07317073		0.073170732			
**ENSG00000180834**		0.48787879		0.487878788			0.4882353
**ENSG00000105971**		0.49393939		0.493939394			0.2732558
**ENSG00000151773**		0.05555556		0.055555556			0.3977273
**ENSG00000121743**		0.30909091		0.309090909			0.2965116
**ENSG00000166292**	0.36826347		0.36826347		0.238095238	0.2674419	
**ENSG00000170417**		0.36728395		0.367283951			0.3176471
**ENSG00000196189**		0.35454545		0.354545455			0.0697674
**ENSG00000174482**						0.0795455	0.1860465
**ENSG00000135111**	0.09883721	0.24242424	0.09883721	0.242424242	0.488095238	0.4047619	0.4404762
**ENSG00000112175**	0.07777778	0.1969697	0.07777778	0.196969697			0.244186
**ENSG00000136280**	0.3313253	0.43023256	0.3313253	0.430232558	0.208333333	0.1411765	0.2619048
**ENSG00000134717**	0.32335329		0.32335329		0.130952381	0.0813953	0.0813953
**ENSG00000114638**					0.244444444	0.1686047	
**ENSG00000056736**		0.33333333		0.333333333			0.4880952
**ENSG00000143303**	0.13939394		0.13939394		0.096385542	0.0833333	
**ENSG00000142684**	0.12777778	0.38953488	0.12777778	0.389534884	0.243902439	0.2621951	0.3430233
**ENSG00000090520**	0.48203593		0.48203593		0.125	0.1744186	
**ENSG00000135100**		0.38181818		0.381818182			0.3604651
**ENSG00000121075**		0.19393939		0.193939394			0.0813953
**ENSG00000162695**		0.28181818		0.281818182			0.0697674
**ENSG00000166326**		0.33888889		0.338888889			0.2272727
**ENSG00000165434**		0.42424242		0.424242424			0.4418605
**ENSG00000060339**	0.05151515	0.12424242	0.05151515	0.124242424	0.331325301	0.3313953	0.3313953
**ENSG00000167005**		0.38787879		0.387878788			0.3352941
**ENSG00000198554**	0.06287425	0.49695122	0.06287425	0.49695122		0.0523256	0.2267442
**ENSG00000221910**	0.23652695	0.36280488	0.23652695	0.362804878	0.5	0.494186	0.4941176
**ENSG00000164107**		0.45757576		0.457575758			0.4127907
**ENSG00000157107**		0.47826087		0.47826087			0.4702381
**ENSG00000182934**	0.06111111	0.24695122	0.06111111	0.24695122			0.1764706
**ENSG00000213588**	0.05681818		0.05681818		0.344444444	0.25	
**ENSG00000159398**	0.42771084		0.42771084		0.369047619	0.4117647	
**ENSG00000164049**	0.25149701		0.25149701		0.321428571	0.4186047	
**ENSG00000122787**		0.2		0.2			0.2209302
**ENSG00000143028**		0.3030303		0.303030303			
**ENSG00000055732**		0.48484848		0.484848485			0.4069767
**ENSG00000073331**		0.49444444		0.494444444			0.3837209
**ENSG00000021574**		0.28658537		0.286585366			0.4352941

**ENSG00000103248**		*0.325*		*0.325*			*0.4244186*
**ENSG00000118898**		*0.48765432*		*0.487654321*			*0.3430233*
**ENSG00000137509**	*0.34848485*	*0.40909091*	*0.34848485*	*0.409090909*			*0.2034884*
**ENSG00000253846**		*0.20224719*		*0.202247191*			*0.1477273*
**ENSG00000083168**	*0.13772455*		*0.13772455*		*0.433333333*	*0.25*	
**ENSG00000135837**		*0.05172414*		*0.051724138*			
**ENSG00000073598**							*0.1395349*
**ENSG00000165125**	*0.36826347*		*0.36826347*				
**ENSG00000171163**							*0.2*
**ENSG00000123219**							*0.0813953*
**ENSG00000258227**		*0.2969697*		*0.296969697*			*0.4011628*
**ENSG00000244291**							*0.3255814*
**ENSG00000196739**		*0.43636364*		*0.436363636*			*0.4941176*
**ENSG00000216895**	*0.45209581*		*0.45209581*		*0.327380952*	*0.3372093*	
**ENSG00000152102**		*0.1*		*0.1*			*0.1046512*
**ENSG00000224531**		*0.16666667*		*0.166666667*			*0.3953488*
**ENSG00000174226**	*0.46407186*	*0.46060606*	*0.46407186*	*0.460606061*	*0.494047619*	*0.4767442*	*0.1511628*
**ENSG00000159352**		*0.24846626*		*0.248466258*			*0.4821429*
**ENSG00000125971**							*0.1162791*
**ENSG00000255121**	*0.10795455*		*0.10795455*			*0.1022727*	
**ENSG00000056291**	*0.30239521*		*0.30239521*		*0.463855422*	*0.5*	
**ENSG00000004399**		*0.15030675*		*0.150306748*			*0.2588235*
**ENSG00000105507**		*0.13939394*		*0.139393939*			*0.1818182*
**ENSG00000149930**		*0.5*		*0.5*			*0.297619*
**ENSG00000173258**		*0.27439024*		*0.274390244*			*0.377907*
**ENSG00000148498**		*0.11111111*		*0.111111111*			*0.1744186*
**ENSG00000125089**	*0.46363636*	*0.36666667*	*0.46363636*	*0.366666667*	*0.327380952*	*0.2764706*	*0.4545455*
**ENSG00000109265**		*0.44785276*		*0.447852761*			*0.3081395*
**ENSG00000249915**		*0.23780488*		*0.237804878*			*0.3197674*
**ENSG00000138640**	*0.36227545*	*0.35365854*	*0.36227545*	*0.353658537*	*0.333333333*	*0.3953488*	*0.4476744*
**ENSG00000102078**		*0.4537037*		*0.453703704*			*0.372093*
**ENSG00000134440**	*0.13333333*	*0.45454545*	*0.13333333*	*0.454545455*			*0.4764706*
**ENSG00000144713**	*0.39393939*	*0.12424242*	*0.39393939*	*0.124242424*	*0.083333333*	*0.1477273*	*0.0872093*
**ENSG00000136286**		*0.13793103*		*0.137931034*			*0.4011628*
**ENSG00000125827**		*0.4969697*		*0.496969697*			*0.5*
**ENSG00000255833**		*0.49390244*		*0.493902439*			*0.5*
**ENSG00000100298**		*0.44848485*		*0.448484848*			*0.2790698*
**ENSG00000196139**	*0.49101796*	*0.36931818*	*0.49101796*	*0.369318182*	*0.208333333*	*0.0813953*	*0.1555556*
**ENSG00000136877**		*0.36363636*		*0.363636364*			*0.3470588*
**ENSG00000130812**		*0.11818182*		*0.118181818*			
**ENSG00000139926**		*0.1969697*		*0.196969697*			*0.4302326*
**ENSG00000099904**		*0.1918239*		*0.191823899*			
**ENSG00000074842**		*0.08787879*		*0.087878788*			*0.2267442*
**ENSG00000129282**		*0.35454545*		*0.354545455*			*0.2797619*
**ENSG00000197208**		*0.38181818*		*0.381818182*			*0.2674419*
**ENSG00000115977**		*0.18181818*		*0.181818182*			*0.0639535*
**ENSG00000179954**		*0.33850932*		*0.338509317*			*0.3630952*
**ENSG00000137275**							*0.2616279*
**ENSG00000185056**	*0.08333333*		*0.08333333*			*0.0666667*	
**ENSG00000197084**		*0.3445122*		*0.344512195*			*0.25*
**ENSG00000168781**		*0.3*		*0.3*			*0.3895349*
**ENSG00000173714**	*0.35628743*	*0.32727273*	*0.35628743*	*0.327272727*			*0.4360465*
**ENSG00000168621**	*0.35542169*	*0.43939394*	*0.35542169*	*0.439393939*	*0.261904762*	*0.3023256*	*0.4294118*
**ENSG00000181016**	*0.48802395*	*0.38181818*	*0.48802395*	*0.381818182*	*0.133333333*	*0.0639535*	*0.0523256*

**ENSG00000033030**	0.22865854		0.22865854		0.297619048	0.4146341	
**ENSG00000117228**	0.26204819	0.38787879	0.26204819	0.387878788	0.071428571	0.0755814	0.4767442
**ENSG00000136003**		0.12222222		0.122222222	0.353658537	0.3815789	0.1222222
**ENSG00000165059**		0.22222222		0.222222222			0.2386364
**ENSG00000147874**	0.20555556	0.33030303	0.20555556	0.33030303	0.154761905	0.1117647	0.1860465
**ENSG00000161888**		0.07303371		0.073033708			
**ENSG00000124140**		0.26111111		0.261111111			0.4333333
**ENSG00000085998**		0.34545455		0.345454545			0.202381
**ENSG00000187912**		0.34242424		0.342424242			0.4302326
**ENSG00000130881**							0.4235294
**ENSG00000212743**		0.23333333		0.233333333			0.3023256
**ENSG00000173085**		0.27710843		0.277108434			
**ENSG00000136319**	0.26047904	0.49090909	0.26047904	0.490909091	0.148809524	0.2151163	0.4709302
**ENSG00000126804**		0.41515152		0.415151515			0.3546512
**ENSG00000111729**	0.41916168	0.22121212	0.41916168	0.221212121			0.0930233
**ENSG00000101290**		0.47272727		0.472727273			0.4555556
**ENSG00000175040**		0.34848485		0.348484848			0.25
**ENSG00000060140**	0.49698795	0.46060606	0.49698795	0.460606061	0.464285714	0.4186047	0.2529412
**ENSG00000256040**	0.21856287	0.43333333	0.21856287	0.433333333	0.285714286	0.4069767	0.0930233
**ENSG00000171649**	0.16477273	0.13030303	0.16477273	0.13030303	0.193181818	0.1590909	0.1470588
**ENSG00000106789**	0.15060241	0.31515152	0.15060241	0.315151515	0.277777778	0.2045455	0.3255814
**ENSG00000198848**					0.068181818		
**ENSG00000080511**		0.47752809		0.47752809			0.3888889
**ENSG00000113407**	0.4491018	0.16969697	0.4491018	0.16969697	0.320512821	0.2763158	0.2151163
**ENSG00000175868**	0.42613636	0.31515152	0.42613636	0.315151515	0.266666667	0.3	0.2965116
**ENSG00000112874**		0.34756098		0.347560976			0.4709302
**ENSG00000174606**		0.30182927		0.301829268			0.4593023
**ENSG00000147854**		0.10909091		0.109090909			0.255814
**ENSG00000178175**	0.10778443	0.13888889	0.10778443	0.138888889	0.404761905	0.4302326	
**ENSG00000146409**		0.05151515		0.051515152			0.0697674
**ENSG00000134046**		0.44848485		0.448484848			0.3430233
**ENSG00000163803**	0.29041916	0.44242424	0.29041916	0.442424242	0.125	0.1918605	0.3430233
**ENSG00000197381**		0.44242424		0.442424242			0.4360465
**ENSG00000213949**	0.28977273	0.42528736	0.28977273	0.425287356	0.244444444	0.3068182	0.5
**ENSG00000108829**		0.42424242		0.424242424			0.127907
**ENSG00000145087**		0.38109756		0.381097561			0.125
**ENSG00000159377**	0.1497006		0.1497006		0.43452381	0.4882353	
**ENSG00000108001**		0.2969697		0.296969697			0.3372093
**ENSG00000147471**		0.16969697		0.16969697			0.2848837
**ENSG00000030304**	0.27844311		0.27844311		0.053571429		
**ENSG00000248099**		0.3803681		0.380368098			0.3647059
**ENSG00000065361**		0.3969697		0.396969697			0.3662791
**ENSG00000204446**		0.42121212		0.421212121			0.3430233
**ENSG00000131781**	0.25	0.1030303	0.25	0.103030303	0.055555556	0.0681818	
**ENSG00000107731**		0.19662921		0.196629213			0.3977273
**ENSG00000022267**		0.4030303		0.403030303			0.3255814
**ENSG00000164074**	0.20658683	0.44848485	0.20658683	0.448484848	0.285714286	0.3255814	0.3255814
**ENSG00000244509**		0.33522727		0.335227273			0.4777778
**ENSG00000165757**		0.44512195		0.445121951			0.4411765
**ENSG00000166501**		0.48484848		0.484848485			0.4883721
**ENSG00000166170**		0.42727273		0.427272727			0.1569767
**ENSG00000104635**	0.41477273	0.38181818	0.41477273	0.381818182	0.188888889	0.1477273	0.372093
**ENSG00000108961**							0.3977273
**ENSG00000011677**	0.48795181		0.48795181		0.409638554	0.4069767	

**ENSG00000159387**	*0.08682635*	*0.42331288*	*0.08682635*	*0.423312883*				*0.4534884*
**ENSG00000162368**		*0.47865854*		*0.478658537*				*0.4244186*
**ENSG00000165186**		*0.4969697*		*0.496969697*				*0.3409091*
**ENSG00000179387**		*0.08787879*		*0.087878788*				
**ENSG00000167671**		*0.44512195*		*0.445121951*				*0.3058824*
**ENSG00000079156**	*0.20658683*	*0.40184049*	*0.20658683*	*0.401840491*	*0.226190476*		*0.3197674*	*0.4244186*
**ENSG00000156795**	*0.3373494*	*0.35*	*0.3373494*	*0.35*	*0.445783133*		*0.3662791*	*0.3863636*
**ENSG00000226746**	*0.28143713*	*0.06363636*	*0.28143713*	*0.063636364*				
**ENSG00000162959**		*0.07878788*		*0.078787879*				*0.1569767*
**ENSG00000182050**	*0.49700599*		*0.49700599*		*0.05952381*		*0.1117647*	
**ENSG00000119547**		*0.31666667*		*0.316666667*				*0.4886364*
**ENSG00000072849**		*0.13030303*		*0.13030303*				*0.0697674*
**ENSG00000148180**	*0.16467066*	*0.1595092*	*0.16467066*	*0.159509202*	*0.369047619*		*0.3139535*	
**ENSG00000184434**		*0.05*		*0.05*				*0.3522727*
**ENSG00000155542**	*0.36826347*	*0.35454545*	*0.36826347*	*0.354545455*	*0.43452381*		*0.3662791*	*0.0639535*
**ENSG00000100889**		*0.20909091*		*0.209090909*				*0.2857143*
**ENSG00000080824**		*0.07777778*		*0.077777778*				*0.0568182*
**ENSG00000179715**					*0.273809524*		*0.2209302*	
**ENSG00000054598**		*0.42777778*		*0.427777778*				*0.1802326*
**ENSG00000135164**	*0.24550898*		*0.24550898*		*0.345238095*		*0.4651163*	*0.0777778*
**ENSG00000231421**	*0.4011976*		*0.4011976*		*0.166666667*		*0.2666667*	
**ENSG00000118369**		*0.15151515*		*0.151515152*	*0.31547619*		*0.4709302*	*0.4883721*
**ENSG00000150687**		*0.19135802*		*0.191358025*				*0.4302326*
**ENSG00000188126**	*0.46407186*		*0.46407186*		*0.379518072*		*0.4186047*	
**ENSG00000171700**		*0.30246914*		*0.302469136*				*0.1882353*
**ENSG00000132465**								*0.3863636*
**ENSG00000171772**	*0.23333333*	*0.10606061*	*0.23333333*	*0.106060606*	*0.144444444*		*0.1590909*	*0.2616279*
**ENSG00000129007**	*0.26347305*	*0.46363636*	*0.26347305*	*0.463636364*	*0.196428571*		*0.1647059*	*0.4709302*
**ENSG00000172551**	*0.28915663*		*0.28915663*		*0.136904762*		*0.1529412*	
**ENSG00000126214**	*0.21257485*	*0.43209877*	*0.21257485*	*0.432098765*	*0.261904762*		*0.2965116*	*0.4880952*
**ENSG00000197746**	*0.19480519*	*0.30606061*	*0.19480519*	*0.306060606*	*0.202380952*		*0.2804878*	*0.4011628*
**ENSG00000166265**		*0.42424242*		*0.424242424*				*0.3488372*
**ENSG00000204314**					*0.148809524*		*0.2732558*	
**ENSG00000140391**	*0.26347305*	*0.32012195*	*0.26347305*	*0.320121951*	*0.493975904*		*0.4941176*	*0.494186*
**ENSG00000029153**		*0.13030303*		*0.13030303*				*0.1453488*
**ENSG00000143816**		*0.40909091*		*0.409090909*				*0.3837209*
**ENSG00000101407**		*0.46969697*		*0.46969697*				*0.3690476*
**ENSG00000149289**	*0.36826347*	*0.29393939*	*0.36826347*	*0.293939394*	*0.333333333*		*0.3235294*	*0.3546512*
**ENSG00000019582**		*0.12424242*		*0.124242424*				*0.3255814*
**ENSG00000060566**					*0.101190476*		*0.0872093*	*0.2840909*
**ENSG00000108821**		*0.31818182*		*0.318181818*				*0.4186047*
**ENSG00000126870**		*0.28963415*		*0.289634146*				*0.3081395*
**ENSG00000138032**	*0.35329341*		*0.35329341*		*0.416666667*		*0.4069767*	
**ENSG00000128791**		*0.45151515*		*0.451515152*				*0.4709302*
**ENSG00000167703**		*0.44242424*		*0.442424242*				*0.4651163*
**ENSG00000237289**		*0.07317073*		*0.073170732*				*0.3095238*
**ENSG00000165813**	*0.05421687*	*0.08484848*	*0.05421687*	*0.084848485*				
**ENSG00000163492**		*0.39090909*		*0.390909091*				*0.4186047*
**ENSG00000184254**		*0.43333333*		*0.433333333*				*0.4302326*
**ENSG00000253819**	*0.32777778*		*0.32777778*		*0.333333333*		*0.4883721*	
**ENSG00000111206**		*0.19090909*		*0.190909091*				*0.2616279*
**ENSG00000007372**	*0.4760479*	*0.46629213*	*0.4760479*	*0.466292135*	*0.464285714*		*0.4767442*	*0.4772727*
**ENSG00000160973**	*0.45508982*		*0.45508982*		*0.321428571*		*0.25*	
**ENSG00000185917**	*0.44011976*	*0.32407407*	*0.44011976*	*0.324074074*	*0.31547619*		*0.244186*	*0.4418605*

**ENSG00000072110**	0.05389222		0.05389222				
**ENSG00000082515**		0.44375		0.44375			0.0872093
**ENSG00000090512**		0.41158537		0.411585366			0.3546512
**ENSG00000148606**		0.2030303		0.203030303			0.4878049
**ENSG00000126952**		0.14242424		0.142424242			0.0523256
**ENSG00000133275**		0.15517241		0.155172414			0.1136364
**ENSG00000100139**					0.125	0.2560976	
**ENSG00000070785**		0.30487805		0.304878049			0.4294118
**ENSG00000197520**	0.31626506	0.13636364	0.31626506	0.136363636			0.1162791
**ENSG00000204936**		0.33636364		0.336363636			0.4534884
**ENSG00000163009**					0.05952381	0.0755814	
**ENSG00000124532**	0.10479042	0.41515152	0.10479042	0.415151515	0.226190476	0.1704545	0.1647059
**ENSG00000219607**		0.14444444		0.144444444			
**ENSG00000177992**					0.136904762	0.0872093	0.3430233
**ENSG00000172803**		0.3803681		0.380368098			0.3895349
**ENSG00000176928**		0.25151515		0.251515152			0.3255814
**ENSG00000212916**		0.27575758		0.275757576			0.4127907
**ENSG00000253958**	0.20224719	0.22222222	0.20224719	0.222222222	0.438271605	0.422619	0.4659091
**ENSG00000233927**	0.32634731		0.32634731		0.321428571	0.1860465	
**ENSG00000160948**		0.48484848		0.484848485			0.3255814
**ENSG00000113211**		0.071875		0.071875	0.488095238	0.4534884	
**ENSG00000151651**		0.1		0.1			0.1352941
**ENSG00000064300**	0.36666667	0.4054878	0.36666667	0.405487805	0.2	0.3977273	0.494186
**ENSG00000105576**		0.44478528		0.444785276			0.1395349
**ENSG00000177311**	0.13333333		0.13333333		0.4	0.3977273	
**ENSG00000170279**		0.44848485		0.448484848			0.3313953
**ENSG00000180879**	0.21111111		0.21111111		0.380952381	0.3313953	
**ENSG00000136237**		0.43125		0.43125	0.077380952	0.0639535	0.494186
**ENSG00000142583**	0.2	0.37575758	0.2	0.375757576			0.3895349
**ENSG00000178789**		0.40123457		0.401234568			0.2616279
**ENSG00000146350**		0.40606061		0.406060606			0.4011628
**ENSG00000152683**		0.38787879		0.387878788			0.4411765
**ENSG00000162496**		0.27878788		0.278787879			0.4534884
**ENSG00000180787**		0.24137931		0.24137931			0.3636364
**ENSG00000001084**		0.43939394		0.439393939			0.4534884
**ENSG00000143891**	0.09580838	0.47865854	0.09580838	0.478658537			0.1130952
**ENSG00000174282**	0.10843373	0.35454545	0.10843373	0.354545455	0.220238095	0.2267442	0.2732558
**ENSG00000177025**	0.33962264		0.33962264		0.078313253		
**ENSG00000115993**	0.10479042	0.4969697	0.10479042	0.496969697			0.4069767
**ENSG00000106976**	0.05421687		0.05421687		0.44047619	0.4127907	
**ENSG00000131446**	0.12349398		0.12349398		0.214285714	0.3235294	
**ENSG00000159871**		0.46363636		0.463636364			0.4588235
**ENSG00000197595**	0.43113772	0.17272727	0.43113772	0.172727273	0.31547619	0.2906977	0.0647059
**ENSG00000186654**		0.11212121		0.112121212			0.4825581
**ENSG00000164951**		0.42222222		0.422222222			0.3372093
**ENSG00000112337**		0.396875		0.396875			0.3235294
**ENSG00000166200**		0.46060606		0.460606061			0.3081395
**ENSG00000158716**					0.1	0.1777778	
**ENSG00000157703**	0.32934132	0.26060606	0.32934132	0.260606061	0.463414634	0.4709302	0.3953488
**ENSG00000171823**							0.3023256
**ENSG00000214402**	0.34638554	0.27272727	0.34638554	0.272727273	0.130952381	0.2117647	0.2093023
**ENSG00000204363**	0.0508982		0.0508982				
**ENSG00000115970**	0.23353293	0.46646341	0.23353293	0.466463415			0.3554217
**ENSG00000166523**		0.26060606		0.260606061			0.4593023

**ENSG00000135052**	*0.4*		*0.4*		*0.44047619*	*0.4767442*	*0.4418605*
**ENSG00000127922**		*0.33333333*		*0.333333333*			*0.127907*
**ENSG00000109775**		*0.22865854*		*0.228658537*			*0.1802326*
**ENSG00000122733**	*0.3373494*	*0.48181818*	*0.3373494*	*0.481818182*	*0.375*	*0.4069767*	*0.4534884*
**ENSG00000109654**		*0.17222222*		*0.172222222*			*0.2386364*
**ENSG00000127152**		*0.29393939*		*0.293939394*			*0.1627907*
**ENSG00000135905**	*0.46107784*	*0.4695122*	*0.46107784*	*0.469512195*	*0.475903614*	*0.2941176*	*0.3546512*
**ENSG00000171049**		*0.08333333*		*0.083333333*			*0.0888889*
**ENSG00000100416**		*0.17272727*		*0.172727273*			
**ENSG00000101425**	*0.10795455*	*0.43333333*	*0.10795455*	*0.433333333*	*0.477777778*	*0.3372093*	*0.5*
**ENSG00000140323**		*0.17088608*		*0.170886076*			*0.4883721*
**ENSG00000163328**		*0.32777778*		*0.327777778*			*0.3181818*
**ENSG00000080503**	*0.24096386*		*0.24096386*		*0.178571429*	*0.2560976*	
**ENSG00000035687**		*0.16666667*		*0.166666667*			*0.2674419*
**ENSG00000184937**	*0.17977528*	*0.28181818*	*0.17977528*	*0.281818182*	*0.295180723*	*0.4235294*	*0.3588235*
**ENSG00000120057**		*0.20606061*		*0.206060606*			*0.4418605*
**ENSG00000177494**	*0.32934132*	*0.05454545*	*0.32934132*	*0.054545455*	*0.101190476*	*0.0764706*	
**ENSG00000185130**	*0.25*		*0.25*		*0.088888889*	*0.0777778*	
**ENSG00000147160**		*0.25914634*		*0.259146341*			*0.2732558*
**ENSG00000157110**		*0.21515152*		*0.215151515*			*0.1162791*
**ENSG00000132718**	*0.31736527*	*0.38787879*	*0.31736527*	*0.387878788*	*0.279761905*	*0.2117647*	*0.2034884*
**ENSG00000124767**	*0.26946108*	*0.13333333*	*0.26946108*	*0.133333333*	*0.446428571*	*0.4651163*	*0.1918605*
**ENSG00000065413**		*0.29090909*		*0.290909091*			*0.1686047*
**ENSG00000138777**		*0.48181818*		*0.481818182*			*0.2965116*
**ENSG00000197651**					*0.279761905*	*0.244186*	
**ENSG00000156486**		*0.23636364*		*0.236363636*			*0.2209302*
**ENSG00000072062**		*0.11818182*		*0.118181818*			
**ENSG00000173818**		*0.32424242*		*0.324242424*			*0.422619*
**ENSG00000157557**		*0.43529412*		*0.435294118*			*0.2267442*
**ENSG00000164342**	*0.13855422*		*0.13855422*		*0.232142857*	*0.2965116*	
**ENSG00000123388**		*0.27300613*		*0.273006135*			*0.172619*
**ENSG00000221996**	*0.22413793*		*0.22413793*		*0.284090909*	*0.2727273*	
**ENSG00000169635**		*0.31515152*		*0.315151515*			*0.4651163*
**ENSG00000166535**		*0.26060606*		*0.260606061*			*0.4244186*
**ENSG00000188559**		*0.43597561*		*0.43597561*			*0.2209302*
**ENSG00000185219**		*0.2962963*		*0.296296296*			
**ENSG00000135205**	*0.24550898*	*0.35454545*	*0.24550898*	*0.354545455*	*0.458333333*	*0.3430233*	*0.1860465*
**ENSG00000151612**		*0.09393939*		*0.093939394*			
**ENSG00000161542**							*0.0930233*
**ENSG00000135443**		*0.49390244*		*0.493902439*			*0.4127907*
**ENSG00000164056**	*0.30838323*	*0.27878788*	*0.30838323*	*0.278787879*	*0.380952381*	*0.3154762*	*0.3197674*
**ENSG00000136859**		*0.37575758*		*0.375757576*			*0.2151163*
**ENSG00000138411**	*0.2005988*	*0.30792683*	*0.2005988*	*0.307926829*			*0.3546512*
**ENSG00000140987**	*0.41666667*	*0.37222222*	*0.41666667*	*0.372222222*	*0.155555556*	*0.1477273*	*0.3*
**ENSG00000133398**		*0.46646341*		*0.466463415*			*0.4470588*
**ENSG00000175003**		*0.43597561*		*0.43597561*	*0.126506024*	*0.1294118*	*0.2034884*
**ENSG00000108733**	*0.09580838*	*0.30232558*	*0.09580838*	*0.302325581*	*0.136904762*	*0.1511628*	*0.3181818*
**ENSG00000153820**		*0.08045977*		*0.08045977*			*0.2906977*
**ENSG00000128805**	*0.4939759*	*0.42424242*	*0.4939759*	*0.424242424*	*0.303571429*	*0.4709302*	*0.3546512*
**ENSG00000023287**	*0.17222222*		*0.17222222*				
**ENSG00000099817**		*0.35757576*		*0.357575758*	*0.365853659*	*0.3857143*	*0.4302326*
**ENSG00000105889**	*0.16111111*	*0.44242424*	*0.16111111*	*0.442424242*			*0.4588235*
**ENSG00000094963**		*0.31111111*		*0.311111111*			*0.2797619*
**ENSG00000254967**					*0.113095238*	*0.0523256*	

**ENSG00000162714**		*0.36969697*		*0.36969697*			*0.4883721*
**ENSG00000116675**		*0.44848485*		*0.448484848*			*0.1686047*
**ENSG00000173905**		*0.05182927*		*0.051829268*			*0.1046512*
**ENSG00000147649**		*0.33333333*		*0.333333333*			*0.375*
**ENSG00000148704**		*0.2969697*		*0.296969697*			*0.3953488*
**ENSG00000183077**		*0.24085366*		*0.240853659*			*0.1686047*
**ENSG00000165280**		*0.21515152*		*0.215151515*			*0.0588235*
**ENSG00000174574**		*0.37575758*		*0.375757576*	*0.098765432*	*0.1309524*	*0.3488372*
**ENSG00000183161**		*0.13636364*		*0.136363636*	*0.255952381*	*0.1686047*	*0.0988372*
**ENSG00000140968**		*0.48888889*		*0.488888889*			*0.3953488*
**ENSG00000074706**		*0.45757576*		*0.457575758*			*0.4888889*
**ENSG00000180998**		*0.16060606*		*0.160606061*			*0.2222222*
**ENSG00000181817**	*0.0873494*		*0.0873494*		*0.079268293*	*0.1144578*	
**ENSG00000073861**		*0.22727273*		*0.227272727*			
**ENSG00000180574**		*0.26993865*		*0.26993865*			*0.152439*
**ENSG00000087053**	*0.05722892*	*0.38484848*	*0.05722892*	*0.384848485*	*0.313253012*	*0.3488372*	*0.3452381*
**ENSG00000172673**		*0.5*		*0.5*			
**ENSG00000238244**		*0.43333333*		*0.433333333*			*0.4659091*
**ENSG00000177234**		*0.47272727*		*0.472727273*			*0.3895349*
**ENSG00000188467**							*0.4593023*
**ENSG00000118492**	*0.38554217*	*0.47575758*	*0.38554217*	*0.475757576*	*0.089285714*	*0.1802326*	*0.4593023*
**ENSG00000198482**		*0.35454545*		*0.354545455*			*0.4659091*
**ENSG00000198000**	*0.23652695*	*0.47530864*	*0.23652695*	*0.475308642*	*0.083333333*	*0.2383721*	*0.3571429*
**ENSG00000183741**		*0.31212121*		*0.312121212*			*0.4411765*
**ENSG00000135338**	*0.05722892*	*0.05*	*0.05722892*	*0.05*			*0.0697674*
**ENSG00000145216**		*0.15730337*		*0.157303371*			*0.1976744*
**ENSG00000115806**	*0.30838323*	*0.05792683*	*0.30838323*	*0.057926829*	*0.06547619*	*0.0872093*	*0.3352941*
**ENSG00000165948**	*0.22155689*		*0.22155689*			*0.0988372*	
**ENSG00000101577**		*0.39156627*		*0.391566265*			*0.3837209*
**ENSG00000153253**	*0.1257485*		*0.1257485*				
**ENSG00000011454**		*0.17777778*		*0.177777778*			*0.2529412*
**ENSG00000138669**							*0.3430233*
**ENSG00000184635**		*0.18888889*		*0.188888889*			*0.2840909*
**ENSG00000137204**		*0.13030303*		*0.13030303*			
**ENSG00000240224**		*0.22777778*		*0.227777778*			*0.1590909*
**ENSG00000108592**		*0.38888889*		*0.388888889*	*0.410714286*	*0.4011628*	*0.4545455*
**ENSG00000133393**		*0.42378049*		*0.423780488*			*0.3068182*
**ENSG00000139354**		*0.3*		*0.3*	*0.469879518*	*0.4302326*	*0.2386364*
**ENSG00000112972**	*0.35454545*	*0.48333333*	*0.35454545*	*0.483333333*	*0.2*	*0.2777778*	*0.2159091*
**ENSG00000118939**		*0.19090909*		*0.190909091*			*0.0523256*
**ENSG00000011021**		*0.16969697*		*0.16969697*			*0.0639535*
**ENSG00000169169**	*0.35555556*		*0.35555556*		*0.277777778*	*0.2272727*	
**ENSG00000144655**	*0.11676647*	*0.2195122*	*0.11676647*	*0.219512195*	*0.160714286*		*0.2176471*
**ENSG00000132837**	*0.39759036*	*0.4847561*	*0.39759036*	*0.484756098*	*0.172619048*	*0.2616279*	*0.2840909*
**ENSG00000198947**	*0.16292135*	*0.31111111*	*0.16292135*	*0.311111111*	*0.1*	*0.1704545*	*0.3522727*
**ENSG00000254004**		*0.25*		*0.25*			*0.125*
**ENSG00000244607**		*0.26060606*		*0.260606061*			*0.3488372*
**ENSG00000143919**		*0.15517241*		*0.155172414*			
**ENSG00000160741**		*0.26666667*		*0.266666667*			*0.2941176*
**ENSG00000075643**		*0.28797468*		*0.287974684*			*0.2678571*
**ENSG00000089351**	*0.26047904*		*0.26047904*		*0.107142857*	*0.1860465*	
**ENSG00000177663**		*0.46111111*		*0.461111111*	*0.377777778*	*0.4069767*	*0.4069767*
**ENSG00000182095**		*0.284375*		*0.284375*	*0.125*	*0.2804878*	*0.1395349*
**ENSG00000112303**		*0.26993865*		*0.26993865*	*0.333333333*	*0.3444444*	*0.3255814*

**ENSG00000106302**	*0.18072289*		*0.18072289*					
**ENSG00000186260**		*0.1*		*0.1*				*0.3863636*
**ENSG00000165325**	*0.46932515*	*0.3908046*	*0.46932515*	*0.390804598*	*0.088888889*		*0.2732558*	*0.494186*
**ENSG00000094880**		*0.35889571*		*0.358895706*				*0.4418605*
**ENSG00000147041**	*0.19760479*	*0.43902439*	*0.19760479*	*0.43902439*	*0.174698795*		*0.1555556*	*0.4186047*
**ENSG00000138190**		*0.42727273*		*0.427272727*				*0.4244186*
**ENSG00000167011**		*0.36666667*		*0.366666667*				*0.4883721*
**ENSG00000134489**		*0.38181818*		*0.381818182*				*0.3604651*
**ENSG00000109814**	*0.49698795*	*0.47865854*	*0.49698795*	*0.478658537*	*0.414634146*		*0.3588235*	*0.4333333*
**ENSG00000196296**		*0.33939394*		*0.339393939*				*0.1395349*
**ENSG00000163297**	*0.44848485*	*0.21818182*	*0.44848485*	*0.218181818*	*0.234567901*		*0.25*	
**ENSG00000105750**								*0.2333333*
**ENSG00000225607**		*0.16969697*		*0.16969697*				*0.1*
**ENSG00000168309**	*0.2754491*	*0.06969697*	*0.2754491*	*0.06969697*	*0.476190476*		*0.3953488*	*0.1845238*
**ENSG00000180219**	*0.2439759*	*0.31609195*	*0.2439759*	*0.316091954*	*0.067073171*		*0.1470588*	*0.3662791*
**ENSG00000113389**	*0.32831325*	*0.46646341*	*0.32831325*	*0.466463415*	*0.095238095*		*0.0813953*	*0.4244186*
**ENSG00000104312**	*0.08888889*	*0.05454545*	*0.08888889*	*0.054545455*	*0.222222222*		*0.125*	*0.1588235*
**ENSG00000124207**		*0.4054878*		*0.405487805*				*0.297619*
**ENSG00000068366**								*0.0681818*
**ENSG00000161996**		*0.35365854*		*0.353658537*				*0.2674419*
**ENSG00000175520**		*0.29393939*		*0.293939394*				*0.2034884*
**ENSG00000119042**	*0.35*	*0.32012195*	*0.35*	*0.320121951*				*0.2267442*
**ENSG00000070190**	*0.3253012*	*0.23939394*	*0.3253012*	*0.239393939*	*0.404761905*		*0.4702381*	*0.2108434*
**ENSG00000125703**		*0.4054878*		*0.405487805*				*0.3255814*
**ENSG00000137714**		*0.15555556*		*0.155555556*				*0.2588235*
**ENSG00000184207**		*0.46666667*		*0.466666667*				*0.3313953*
**ENSG00000100503**		*0.42424242*		*0.424242424*				*0.2674419*
**ENSG00000108602**	*0.1369863*	*0.38484848*	*0.1369863*	*0.384848485*	*0.2*		*0.0972222*	*0.4709302*
**ENSG00000105784**		*0.07878788*		*0.078787879*				*0.1807229*
**ENSG00000189332**	*0.16049383*	*0.4875*	*0.16049383*	*0.4875*	*0.142857143*		*0.1625*	*0.4216867*
**ENSG00000085978**	*0.2826087*	*0.44817073*	*0.2826087*	*0.448170732*	*0.386904762*		*0.2093023*	*0.4117647*
**ENSG00000156381**	*0.09580838*		*0.09580838*		*0.285714286*		*0.244186*	
**ENSG00000123684**		*0.30792683*		*0.307926829*				*0.297619*
**ENSG00000104973**	*0.05389222*		*0.05389222*		*0.148809524*		*0.1162791*	*0.0930233*
**ENSG00000144677**		*0.16363636*		*0.163636364*				
**ENSG00000074696**	*0.36309524*	*0.2*	*0.36309524*	*0.2*				
**ENSG00000018236**		*0.4*		*0.4*				*0.4360465*
**ENSG00000006025**	*0.0505618*	*0.34848485*	*0.0505618*	*0.348484848*				
**ENSG00000153246**	*0.3988764*	*0.42121212*	*0.3988764*	*0.421212121*	*0.272727273*		*0.4069767*	*0.3313953*
**ENSG00000175198**	*0.12222222*		*0.12222222*					
**ENSG00000165695**					*0.339285714*		*0.3081395*	
**ENSG00000173674**		*0.15740741*		*0.157407407*				*0.0813953*
**ENSG00000125872**	*0.23952096*		*0.23952096*		*0.494047619*		*0.4360465*	
**ENSG00000112200**		*0.12121212*		*0.121212121*				*0.0795455*
**ENSG00000151834**	*0.28143713*	*0.49090909*	*0.28143713*	*0.490909091*	*0.196428571*		*0.1395349*	*0.4825581*
**ENSG00000184459**	*0.05988024*		*0.05988024*		*0.154761905*		*0.2176471*	
**ENSG00000221947**	*0.11676647*	*0.43333333*	*0.11676647*	*0.433333333*	*0.071428571*		*0.1860465*	*0.3977273*
**ENSG00000163527**		*0.0969697*		*0.096969697*				
**ENSG00000060749**		*0.16666667*		*0.166666667*				
**ENSG00000229387**		*0.16666667*		*0.166666667*				*0.1686047*
**ENSG00000198919**	*0.05*	*0.18181818*	*0.05*	*0.181818182*				*0.4360465*
**ENSG00000197121**		*0.21646341*		*0.216463415*				*0.0872093*
**ENSG00000146090**	*0.28143713*	*0.47777778*	*0.28143713*	*0.477777778*	*0.279761905*		*0.3139535*	*0.3888889*
**ENSG00000028310**	*0.13068182*		*0.13068182*		*0.166666667*		*0.0909091*	*0.2616279*

**ENSG00000169718**	*0.07185629*		*0.07185629*		*0.089285714*	*0.0523256*	
**ENSG00000213494**	*0.46407186*		*0.46407186*		*0.313253012*	*0.3197674*	
**ENSG00000159450**		*0.35*		*0.35*			*0.4666667*
**ENSG00000178358**		*0.17575758*		*0.175757576*			*0.4825581*
**ENSG00000259280**	*0.43413174*	*0.33030303*	*0.43413174*	*0.33030303*	*0.363095238*	*0.4593023*	*0.372093*
**ENSG00000100991**		*0.36969697*		*0.36969697*			*0.1802326*
**ENSG00000196109**	*0.23652695*	*0.32022472*	*0.23652695*	*0.320224719*	*0.31547619*	*0.3197674*	
**ENSG00000176092**		*0.15151515*		*0.151515152*			
**ENSG00000111674**							*0.2674419*
**ENSG00000125450**	*0.31111111*	*0.22865854*	*0.31111111*	*0.228658537*	*0.1*	*0.0681818*	*0.0639535*
**ENSG00000120675**		*0.48181818*		*0.481818182*	*0.125*	*0.0523256*	*0.3511905*
**ENSG00000111670**		*0.48787879*		*0.487878788*			*0.3023256*
**ENSG00000185480**	*0.32335329*		*0.32335329*		*0.458333333*	*0.4941176*	
**ENSG00000115041**							*0.1046512*
**ENSG00000074582**	*0.14071856*		*0.14071856*		*0.136904762*	*0.1569767*	
**ENSG00000215568**		*0.42121212*		*0.421212121*			*0.4476744*
**ENSG00000079215**		*0.33636364*		*0.336363636*			*0.244186*
**ENSG00000102189**		*0.36969697*		*0.36969697*			*0.0697674*
**ENSG00000133808**	*0.27710843*		*0.27710843*			*0.0930233*	
**ENSG00000104765**	*0.34638554*	*0.44545455*	*0.34638554*	*0.445454545*	*0.119047619*	*0.1686047*	*0.4186047*
**ENSG00000185880**	*0.47005988*	*0.09883721*	*0.47005988*	*0.098837209*	*0.422619048*	*0.2848837*	*0.1463415*
**ENSG00000174236**					*0.148809524*	*0.1395349*	
**ENSG00000007174**	*0.38888889*	*0.4847561*	*0.38888889*	*0.484756098*	*0.333333333*	*0.4418605*	*0.3662791*
**ENSG00000102710**		*0.3902439*		*0.390243902*			*0.2674419*
**ENSG00000230344**		*0.47777778*		*0.477777778*			*0.4659091*
**ENSG00000186280**		*0.13414634*		*0.134146341*			
**ENSG00000188295**	*0.43113772*	*0.17378049*	*0.43113772*	*0.173780488*	*0.107142857*	*0.1511628*	*0.1395349*
**ENSG00000157119**		*0.42331288*		*0.423312883*			*0.3372093*
**ENSG00000152795**		*0.4*		*0.4*			*0.3197674*
**ENSG00000180626**	*0.13333333*	*0.12424242*	*0.13333333*	*0.124242424*	*0.494047619*	*0.4883721*	*0.0813953*
**ENSG00000157181**		*0.37804878*		*0.37804878*			*0.3433735*
**ENSG00000100528**		*0.15243902*		*0.152439024*			
**ENSG00000089159**		*0.46060606*		*0.460606061*			*0.4705882*
**ENSG00000186638**	*0.3373494*	*0.23636364*	*0.3373494*	*0.236363636*	*0.380952381*	*0.3023256*	*0.0523256*
**ENSG00000131116**	*0.23353293*		*0.23353293*		*0.265060241*	*0.2559524*	
**ENSG00000188763**		*0.18484848*		*0.184848485*			*0.122093*
**ENSG00000166148**	*0.20481928*		*0.20481928*				
**ENSG00000055208**	*0.07185629*	*0.21111111*	*0.07185629*	*0.211111111*			*0.5*
**ENSG00000177238**	*0.0748503*		*0.0748503*				
**ENSG00000166986**		*0.17575758*		*0.175757576*			*0.1058824*
**ENSG00000120071**		*0.21036585*		*0.210365854*	*0.488888889*	*0.4883721*	
**ENSG00000178605**		*0.34567901*		*0.345679012*			*0.4940476*
**ENSG00000147439**		*0.32424242*		*0.324242424*			*0.2325581*
**ENSG00000157578**	*0.39820359*		*0.39820359*		*0.255952381*	*0.372093*	
**ENSG00000172164**		*0.38888889*		*0.388888889*			*0.4534884*
**ENSG00000198910**	*0.17045455*		*0.17045455*		*0.066666667*	*0.0681818*	
**ENSG00000189269**	*0.13333333*	*0.38181818*	*0.13333333*	*0.381818182*	*0.311111111*	*0.3522727*	*0.0813953*
**ENSG00000172936**		*0.14545455*		*0.145454545*			*0.2848837*
**ENSG00000006576**		*0.14848485*		*0.148484848*	*0.130952381*	*0.2151163*	*0.1058824*
**ENSG00000182070**		*0.42424242*		*0.424242424*			*0.4352941*
**ENSG00000132763**		*0.46551724*		*0.465517241*			*0.3295455*
**ENSG00000170162**		*0.14242424*		*0.142424242*			*0.0823529*
**ENSG00000131142**		*0.06969697*		*0.06969697*			*0.0988372*
**ENSG00000077942**	*0.28143713*	*0.43939394*	*0.28143713*	*0.439393939*	*0.476190476*	*0.3470588*	*0.4418605*

**ENSG00000255152**		*0.13939394*		*0.139393939*				*0.2674419*
**ENSG00000196569**	*0.08950617*	*0.25903614*	*0.08950617*	*0.259036145*				*0.1823529*
**ENSG00000136383**		*0.29393939*		*0.293939394*				*0.4090909*
**ENSG00000162735**		*0.44444444*		*0.444444444*	*0.055555556*			*0.3522727*
**ENSG00000141068**		*0.47256098*		*0.472560976*				*0.3705882*
**ENSG00000078687**		*0.06666667*		*0.066666667*				*0.1547619*
**ENSG00000142606**		*0.35757576*		*0.357575758*				*0.3488372*
**ENSG00000170271**		*0.11818182*		*0.118181818*				*0.1046512*
**ENSG00000170860**	*0.39655172*	*0.48787879*	*0.39655172*	*0.487878788*	*0.244444444*		*0.1818182*	*0.4418605*
**ENSG00000096060**		*0.23636364*		*0.236363636*				*0.1627907*
**ENSG00000138376**	*0.17222222*	*0.41666667*	*0.17222222*	*0.416666667*	*0.366666667*		*0.3111111*	*0.4302326*
**ENSG00000149313**								*0.375*
**ENSG00000183250**		*0.38787879*		*0.387878788*				*0.3255814*
**ENSG00000120832**	*0.43678161*	*0.46969697*	*0.43678161*	*0.46969697*	*0.340909091*		*0.2325581*	*0.3255814*
**ENSG00000144445**		*0.45121951*		*0.451219512*				*0.5*
**ENSG00000175582**		*0.45505618*		*0.45505618*				*0.4222222*
**ENSG00000131061**	*0.07784431*	*0.05487805*	*0.07784431*	*0.054878049*				*0.0639535*
**ENSG00000133020**	*0.18975904*		*0.18975904*		*0.196428571*		*0.1882353*	
**ENSG00000125952**		*0.37878788*		*0.378787879*				*0.4418605*
**ENSG00000152439**	*0.165625*	*0.31097561*	*0.165625*	*0.31097561*	*0.339506173*		*0.4709302*	*0.375*
**ENSG00000070526**		*0.48484848*		*0.484848485*				*0.1764706*
**ENSG00000113272**	*0.33233533*	*0.39130435*	*0.33233533*	*0.391304348*	*0.380952381*		*0.4709302*	*0.4882353*
**ENSG00000120280**		*0.22560976*		*0.225609756*				
**ENSG00000189339**								*0.4823529*
**ENSG00000138606**	*0.46407186*	*0.14242424*	*0.46407186*	*0.142424242*				*0.2790698*
**ENSG00000154102**		*0.22222222*		*0.222222222*				*0.2613636*
**ENSG00000121903**		*0.25609756*		*0.256097561*				*0.2289157*
**ENSG00000182185**		*0.34545455*		*0.345454545*				*0.494186*
**ENSG00000085433**		*0.23636364*		*0.236363636*				*0.3372093*
**ENSG00000184814**	*0.08383234*		*0.08383234*				*0.1058824*	
**ENSG00000144063**	*0.17073171*	*0.15517241*	*0.17073171*	*0.155172414*				*0.1395349*
**ENSG00000089280**	*0.25*		*0.25*		*0.148809524*		*0.2325581*	
**ENSG00000135549**	*0.41317365*	*0.45151515*	*0.41317365*	*0.451515152*	*0.363095238*		*0.4823529*	*0.2916667*
**ENSG00000197296**		*0.23636364*		*0.236363636*				*0.4244186*
**ENSG00000102921**		*0.33333333*		*0.333333333*				*0.2906977*
**ENSG00000184162**								*0.1477273*
**ENSG00000170265**		*0.1*		*0.1*				
**ENSG00000214510**	*0.15555556*		*0.15555556*					
**ENSG00000176108**		*0.40229885*		*0.402298851*				*0.4534884*
**ENSG00000078399**		*0.38181818*		*0.381818182*				*0.2848837*
**ENSG00000112309**	*0.22155689*	*0.48181818*	*0.22155689*	*0.481818182*	*0.267857143*		*0.3023256*	*0.4360465*
**ENSG00000163909**		*0.41515152*		*0.415151515*				*0.3372093*
**ENSG00000205853**	*0.0748503*	*0.07575758*	*0.0748503*	*0.075757576*	*0.18452381*		*0.1569767*	
**ENSG00000047634**	*0.4126506*		*0.4126506*					
**ENSG00000106080**		*0.16666667*		*0.166666667*				*0.0853659*
**ENSG00000092108**	*0.16766467*		*0.16766467*		*0.317073171*		*0.3192771*	
**ENSG00000255302**		*0.12727273*		*0.127272727*				*0.2848837*
**ENSG00000243955**	*0.32335329*		*0.32335329*		*0.095238095*		*0.127907*	
**ENSG00000105989**		*0.44545455*		*0.445454545*				*0.4090909*
**ENSG00000215440**		*0.40909091*		*0.409090909*				*0.4709302*
**ENSG00000072864**		*0.43939394*		*0.439393939*				*0.4470588*
**ENSG00000165060**		*0.48888889*		*0.488888889*				*0.4882353*
**ENSG00000137310**	*0.37724551*	*0.47272727*	*0.37724551*	*0.472727273*	*0.470238095*		*0.5*	*0.5*
**ENSG00000088205**		*0.22981366*		*0.229813665*				*0.3255814*

**ENSG00000077147**		*0.45757576*		*0.457575758*				*0.4767442*
**ENSG00000140525**	*0.16467066*	*0.36060606*	*0.16467066*	*0.360606061*	*0.285714286*		*0.2848837*	*0.377907*
**ENSG00000142544**		*0.4969697*		*0.496969697*				*0.1976744*
**ENSG00000154328**		*0.38819876*		*0.388198758*	*0.159090909*		*0.1842105*	*0.2529412*
**ENSG00000170579**	*0.1741573*	*0.35757576*	*0.1741573*	*0.357575758*	*0.303571429*		*0.2267442*	*0.4825581*
**ENSG00000153283**	*0.17065868*	*0.23030303*	*0.17065868*	*0.23030303*	*0.464285714*		*0.4593023*	*0.1845238*
**ENSG00000189068**		*0.42682927*		*0.426829268*				*0.2848837*
**ENSG00000168229**		*0.28353659*		*0.283536585*				*0.2383721*
**ENSG00000189369**	*0.3*		*0.3*		*0.077777778*		*0.1022727*	
**ENSG00000197846**		*0.26136364*		*0.261363636*				*0.1818182*
**ENSG00000141682**		*0.16257669*		*0.162576687*				*0.2267442*
**ENSG00000140332**		*0.42352941*		*0.423529412*				*0.3139535*
**ENSG00000152592**		*0.28888889*		*0.288888889*				*0.1818182*
**ENSG00000065328**		*0.17878788*		*0.178787879*				*0.2674419*
**ENSG00000181378**	*0.48203593*	*0.12121212*	*0.48203593*	*0.121212121*	*0.446428571*		*0.4418605*	*0.2093023*
**ENSG00000097007**		*0.48170732*		*0.481707317*				*0.4705882*
**ENSG00000032444**	*0.20658683*		*0.20658683*		*0.488095238*		*0.4593023*	
**ENSG00000174500**		*0.28220859*		*0.282208589*				
**ENSG00000048140**		*0.26969697*		*0.26969697*				*0.5*
**ENSG00000138798**	*0.08083832*	*0.07878788*	*0.08083832*	*0.078787879*				*0.2151163*
**ENSG00000163645**	*0.26047904*		*0.26047904*					
**ENSG00000136051**		*0.2*		*0.2*	*0.058139535*		*0.1*	*0.2325581*
**ENSG00000182670**	*0.31437126*	*0.46363636*	*0.31437126*	*0.463636364*	*0.327380952*		*0.4204545*	*0.3895349*
**ENSG00000197056**	*0.30538922*		*0.30538922*		*0.238095238*		*0.2529412*	
**ENSG00000150628**	*0.41017964*		*0.41017964*		*0.386904762*		*0.3488372*	
**ENSG00000065833**								*0.0609756*
**ENSG00000120333**		*0.23809524*		*0.238095238*				*0.3081395*
**ENSG00000166311**		*0.22121212*		*0.221212121*				*0.1744186*
**ENSG00000143162**		*0.18484848*		*0.184848485*				*0.2267442*
**ENSG00000176635**	*0.13772455*		*0.13772455*					*0.1162791*
**ENSG00000079785**	*0.13173653*		*0.13173653*		*0.154761905*		*0.0639535*	
**ENSG00000075043**		*0.45151515*		*0.451515152*				
**ENSG00000173726**		*0.29268293*		*0.292682927*				*0.4529412*
**ENSG00000140955**		*0.09302326*		*0.093023256*				*0.0769231*
**ENSG00000137731**	*0.15269461*	*0.33030303*	*0.15269461*	*0.33030303*	*0.297619048*		*0.2235294*	*0.25*
**ENSG00000133488**	*0.23312883*	*0.24848485*	*0.23312883*	*0.248484848*				*0.0872093*
**ENSG00000181061**		*0.26666667*		*0.266666667*				*0.1569767*
**ENSG00000174796**		*0.43103448*		*0.431034483*				*0.4886364*
**ENSG00000157833**		*0.20606061*		*0.206060606*	*0.208333333*		*0.1860465*	*0.1104651*
**ENSG00000160716**		*0.2195122*		*0.219512195*				*0.2588235*
**ENSG00000168216**	*0.19461078*	*0.46666667*	*0.19461078*	*0.466666667*	*0.419753086*		*0.4166667*	*0.4*
**ENSG00000119973**		*0.13939394*		*0.139393939*				
**ENSG00000243772**	*0.07954545*	*0.13888889*	*0.07954545*	*0.138888889*				
**ENSG00000136937**	*0.39520958*	*0.36666667*	*0.39520958*	*0.366666667*	*0.113095238*		*0.0872093*	*0.0555556*
**ENSG00000254827**	*0.39820359*		*0.39820359*		*0.297619048*		*0.3081395*	
**ENSG00000059573**		*0.48181818*		*0.481818182*				*0.0523256*
**ENSG00000168538**		*0.1969697*		*0.196969697*				*0.1860465*
**ENSG00000048162**		*0.10606061*		*0.106060606*				
**ENSG00000004948**	*0.46604938*	*0.28527607*	*0.46604938*	*0.285276074*	*0.295180723*		*0.3231707*	*0.4647059*
**ENSG00000137154**	*0.18562874*	*0.08787879*	*0.18562874*	*0.087878788*				
**ENSG00000235478**		*0.05*		*0.05*				*0.4772727*
**ENSG00000168995**		*0.05172414*		*0.051724138*				*0.4204545*
**ENSG00000184924**		*0.44785276*		*0.447852761*				*0.4529412*
**ENSG00000145860**	*0.29341317*	*0.16666667*	*0.29341317*	*0.166666667*	*0.333333333*		*0.4705882*	*0.0588235*

**ENSG00000136143**		*0.16049383*		*0.160493827*			
**ENSG00000041802**		*0.45555556*		*0.455555556*			*0.4777778*
**ENSG00000164430**		*0.30357143*		*0.303571429*			*0.1818182*
**ENSG00000111339**		*0.47239264*		*0.472392638*			*0.3333333*
**ENSG00000136842**							*0.0568182*
**ENSG00000147408**	*0.47005988*	*0.31212121*	*0.47005988*	*0.312121212*	*0.422619048*	*0.4534884*	
**ENSG00000123444**					*0.25*	*0.2325581*	
**ENSG00000105492**	*0.43181818*		*0.43181818*		*0.433333333*	*0.4886364*	
**ENSG00000050405**	*0.33030303*	*0.45757576*	*0.33030303*	*0.457575758*	*0.285714286*	*0.244186*	*0.2613636*
**ENSG00000188706**		*0.12424242*		*0.124242424*			*0.4534884*
**ENSG00000111596**	*0.47305389*	*0.34567901*	*0.47305389*	*0.345679012*	*0.488095238*	*0.4302326*	*0.4302326*
**ENSG00000101333**		*0.05151515*		*0.051515152*			*0.2674419*
**ENSG00000166347**							*0.0588235*
**ENSG00000182704**		*0.12121212*		*0.121212121*			
**ENSG00000167785**	*0.15*	*0.33939394*	*0.15*	*0.339393939*	*0.421686747*	*0.3255814*	*0.4767442*
**ENSG00000243509**					*0.444444444*	*0.4204545*	
**ENSG00000179520**	*0.15568862*	*0.41573034*	*0.15568862*	*0.415730337*			*0.25*
**ENSG00000011275**	*0.125*	*0.05151515*	*0.125*	*0.051515152*	*0.220238095*	*0.2151163*	*0.1162791*
**ENSG00000113638**		*0.29938272*		*0.299382716*			
**ENSG00000216588**		*0.22560976*		*0.225609756*			*0.0872093*
**ENSG00000143418**		*0.35454545*		*0.354545455*			*0.4825581*
**ENSG00000130827**		*0.14242424*		*0.142424242*			*0.0882353*
**ENSG00000164821**	*0.46385542*	*0.26969697*	*0.46385542*	*0.26969697*	*0.214285714*	*0.1918605*	*0.3837209*
**ENSG00000152254**		*0.38181818*		*0.381818182*			*0.4090909*
**ENSG00000214026**		*0.15853659*		*0.158536585*			*0.1309524*
**ENSG00000155657**	*0.06024096*	*0.06666667*	*0.06024096*	*0.066666667*			*0.1477273*
**ENSG00000067191**		*0.20555556*		*0.205555556*			*0.1395349*
**ENSG00000197959**	*0.23192771*	*0.42121212*	*0.23192771*	*0.421212121*	*0.291666667*	*0.3023256*	*0.5*
**ENSG00000010671**		*0.42727273*		*0.427272727*			*0.4302326*
**ENSG00000010219**		*0.42727273*		*0.427272727*			*0.2117647*
**ENSG00000125851**	*0.11676647*	*0.15757576*	*0.11676647*	*0.157575758*	*0.148809524*	*0.2764706*	*0.127907*
**ENSG00000257127**		*0.49079755*		*0.490797546*	*0.156626506*	*0.1569767*	*0.4360465*
**ENSG00000006062**		*0.46969697*		*0.46969697*			*0.3953488*
**ENSG00000118246**	*0.2994012*	*0.22392638*	*0.2994012*	*0.22392638*		*0.0581395*	*0.2891566*
**ENSG00000108639**		*0.26060606*		*0.260606061*			*0.1511628*
**ENSG00000153179**		*0.465625*		*0.465625*			*0.3023256*
**ENSG00000139352**	*0.44879518*	*0.23101266*	*0.44879518*	*0.231012658*	*0.154761905*	*0.0581395*	*0.1219512*
**ENSG00000083799**		*0.07303371*		*0.073033708*			
**ENSG00000164694**		*0.14848485*		*0.148484848*			
**ENSG00000103160**		*0.46666667*		*0.466666667*			*0.3058824*
**ENSG00000131386**		*0.13636364*		*0.136363636*			*0.0872093*
**ENSG00000125965**					*0.238095238*	*0.25*	
**ENSG00000139269**	*0.1497006*	*0.26060606*	*0.1497006*	*0.260606061*	*0.311111111*	*0.4418605*	*0.3372093*
**ENSG00000188428**	*0.35329341*	*0.2791411*	*0.35329341*	*0.279141104*	*0.06547619*	*0.0523256*	*0.2209302*
**ENSG00000142207**		*0.26060606*		*0.260606061*			*0.4593023*
**ENSG00000184831**	*0.21910112*	*0.27878788*	*0.21910112*	*0.278787879*	*0.455555556*	*0.3068182*	*0.3372093*
**ENSG00000161574**	*0.35955056*	*0.20909091*	*0.35955056*	*0.209090909*		*0.0795455*	*0.4709302*
**ENSG00000198208**	*0.41566265*	*0.43333333*	*0.41566265*	*0.433333333*	*0.428571429*	*0.4593023*	*0.4886364*
**ENSG00000196235**	*0.14371257*		*0.14371257*		*0.493975904*	*0.4647059*	
**ENSG00000146112**	*0.12777778*		*0.12777778*		*0.355555556*	*0.3068182*	*0.1744186*
**ENSG00000092758**		*0.29393939*		*0.293939394*			*0.1046512*
**ENSG00000180332**	*0.21856287*		*0.21856287*				
**ENSG00000088682**		*0.08484848*		*0.084848485*			
**ENSG00000130477**		*0.28181818*		*0.281818182*			*0.0930233*

**ENSG00000090020**	*0.26506024*	*0.35454545*	*0.26506024*	*0.354545455*	*0.077380952*	*0.1104651*	*0.122093*
**ENSG00000101158**		*0.47272727*		*0.472727273*			*0.4127907*
**ENSG00000105855**	*0.134375*		*0.134375*		*0.44375*	*0.373494*	
**ENSG00000187997**		*0.43636364*		*0.436363636*			*0.3023256*
**ENSG00000138495**		*0.4*		*0.4*			*0.3235294*
**ENSG00000169385**							*0.0639535*
**ENSG00000174123**	*0.43712575*		*0.43712575*		*0.446428571*	*0.4767442*	*0.1176471*
**ENSG00000119820**		*0.14242424*		*0.142424242*			
**ENSG00000174564**		*0.36060606*		*0.360606061*			*0.1511628*
**ENSG00000176903**		*0.35151515*		*0.351515152*			*0.1395349*
**ENSG00000105866**		*0.33030303*		*0.33030303*			*0.2209302*
**ENSG00000056586**	*0.46111111*	*0.49393939*	*0.46111111*	*0.493939394*	*0.266666667*	*0.2159091*	*0.25*
**ENSG00000172243**		*0.16666667*		*0.166666667*	*0.291666667*	*0.4069767*	*0.2965116*
**ENSG00000187189**	*0.05555556*	*0.46067416*	*0.05555556*	*0.460674157*			*0.377907*
**ENSG00000112167**		*0.34545455*		*0.345454545*			*0.2732558*
**ENSG00000138231**		*0.31764706*		*0.317647059*	*0.053571429*		*0.4651163*
**ENSG00000039068**		*0.22121212*		*0.221212121*			*0.127907*
**ENSG00000140479**		*0.49695122*		*0.49695122*			*0.3488372*
**ENSG00000114346**		*0.40606061*		*0.406060606*			*0.3023256*
**ENSG00000153487**	*0.41477273*		*0.41477273*		*0.188888889*	*0.1818182*	
**ENSG00000174327**	*0.21666667*		*0.21666667*		*0.244444444*	*0.1818182*	
**ENSG00000116752**		*0.48181818*		*0.481818182*			*0.377907*
**ENSG00000166257**		*0.47546012*		*0.475460123*	*0.166666667*	*0.0813953*	*0.4651163*
**ENSG00000082175**	*0.3502994*	*0.3*	*0.3502994*	*0.3*			*0.4888889*
**ENSG00000074771**		*0.12921348*		*0.129213483*			*0.2444444*
**ENSG00000148482**		*0.275*		*0.275*			*0.2764706*
**ENSG00000175161**		*0.37777778*		*0.377777778*			*0.1704545*
**ENSG00000167280**		*0.37575758*		*0.375757576*			*0.3235294*
**ENSG00000006071**		*0.36585366*		*0.365853659*			*0.3953488*
**ENSG00000198369**		*0.45151515*		*0.451515152*			*0.2151163*
**ENSG00000171303**		*0.46111111*		*0.461111111*			*0.2848837*
**ENSG00000166473**		*0.45151515*		*0.451515152*			*0.4244186*
**ENSG00000135241**	*0.09659091*	*0.33030303*	*0.09659091*	*0.33030303*		*0.125*	*0.1337209*
**ENSG00000169592**		*0.48787879*		*0.487878788*			*0.3023256*
**ENSG00000113649**		*0.10909091*		*0.109090909*			*0.3255814*
**ENSG00000099810**		*0.41818182*		*0.418181818*			*0.494186*
**ENSG00000161609**	*0.41916168*	*0.25454545*	*0.41916168*	*0.254545455*	*0.445783133*	*0.4588235*	*0.1744186*
**ENSG00000053770**	*0.0988024*		*0.0988024*		*0.05952381*		
**ENSG00000112234**	*0.08385093*		*0.08385093*		*0.222891566*	*0.2891566*	
**ENSG00000178741**		*0.41212121*		*0.412121212*			*0.2151163*
**ENSG00000107560**	*0.08682635*	*0.33231707*	*0.08682635*	*0.332317073*	*0.488095238*	*0.4418605*	*0.2727273*
**ENSG00000178852**	*0.22754491*		*0.22754491*		*0.208333333*	*0.2151163*	
**ENSG00000168461**		*0.49079755*		*0.490797546*			*0.4647059*
**ENSG00000243696**		*0.36969697*		*0.36969697*			*0.3888889*
**ENSG00000120694**	*0.27245509*		*0.27245509*		*0.363095238*	*0.2965116*	
**ENSG00000197748**		*0.05952381*		*0.05952381*			*0.2333333*
**ENSG00000166006**	*0.24698795*	*0.41011236*	*0.24698795*	*0.41011236*			*0.4555556*
**ENSG00000162733**	*0.29640719*	*0.06060606*	*0.29640719*	*0.060606061*		*0.1395349*	
**ENSG00000137841**		*0.05757576*		*0.057575758*			*0.0930233*
**ENSG00000061455**							*0.1444444*
**ENSG00000107745**		*0.35757576*		*0.357575758*			*0.1746988*
**ENSG00000153208**	*0.18975904*	*0.35151515*	*0.18975904*	*0.351515152*	*0.125*	*0.1104651*	*0.2151163*
**ENSG00000162004**	*0.36196319*	*0.4969697*	*0.36196319*	*0.496969697*	*0.201219512*	*0.2073171*	*0.2209302*
**ENSG00000197619**		*0.27743902*		*0.277439024*	*0.333333333*	*0.3430233*	*0.3863636*

**ENSG00000058866**	0.43975904	0.5	0.43975904	0.5		0.0523256	0.3409091
**ENSG00000152766**							0.1162791
**ENSG00000162298**		0.06329114		0.063291139			
**ENSG00000175564**	0.0988024	0.49444444	0.0988024	0.494444444	0.327380952	0.2848837	0.4555556
**ENSG00000144821**		0.48787879		0.487878788			
**ENSG00000100591**	0.41916168	0.49393939	0.41916168	0.493939394	0.090361446	0.0755814	0.0755814
**ENSG00000134668**	0.31736527	0.24848485	0.31736527	0.248484848	0.477777778	0.3313953	0.4534884
**ENSG00000119715**		0.24848485		0.248484848			0.2209302
**ENSG00000152670**	0.31927711		0.31927711		0.321428571	0.3546512	
**ENSG00000258947**	0.37078652	0.07878788	0.37078652	0.078787879	0.375	0.244186	0.127907
**ENSG00000138068**	0.27409639		0.27409639				
**ENSG00000185736**	0.14670659	0.34756098	0.14670659	0.347560976	0.392857143	0.3953488	0.2965116
**ENSG00000243789**		0.48295455		0.482954545			0.2555556
**ENSG00000182264**	0.28443114		0.28443114		0.19047619	0.1511628	
**ENSG00000114388**	0.18862275		0.18862275		0.375	0.4825581	
**ENSG00000141441**		0.27272727		0.272727273			0.1860465
**ENSG00000163596**		0.31595092		0.31595092			0.1395349
**ENSG00000132677**		0.4969697		0.496969697			0.4222222
**ENSG00000105641**		0.17878788		0.178787879			0.0523256
**ENSG00000112343**	0.47305389		0.47305389		0.077380952	0.0639535	
**ENSG00000158055**	0.43113772	0.3030303	0.43113772	0.303030303	0.220238095	0.122093	0.4244186
**ENSG00000189051**		0.22121212		0.221212121			0.1764706
**ENSG00000221845**		0.23939394		0.239393939			0.3604651
**ENSG00000140274**		0.07272727		0.072727273			0.1477273
**ENSG00000010030**	0.26347305		0.26347305		0.303571429	0.3604651	
**ENSG00000116205**		0.41818182		0.418181818			0.3837209
**ENSG00000158321**	0.25609756		0.25609756		0.148809524		0.0568182
**ENSG00000188910**		0.14545455		0.145454545			0.3313953
**ENSG00000198765**		0.42222222		0.422222222			0.4705882
**ENSG00000172007**		0.29447853		0.294478528			0.1
**ENSG00000151665**		0.26851852		0.268518519			0.1036585
**ENSG00000187021**		0.32386364		0.323863636			0.3636364
**ENSG00000149115**		0.34848485		0.348484848			0.3953488
**ENSG00000159251**	0.05988024	0.34545455	0.05988024	0.345454545			0.4939024
**ENSG00000205364**							0.0581395
**ENSG00000141258**		0.28787879		0.287878788			0.1744186
**ENSG00000115109**		0.26363636		0.263636364	0.372093023	0.3488372	0.3546512
**ENSG00000188846**	0.23053892	0.22424242	0.23053892	0.224242424	0.5	0.4651163	0.4186047
**ENSG00000140718**	0.07185629	0.46022727	0.07185629	0.460227273			0.4941176
**ENSG00000144320**		0.48780488		0.487804878			0.3764706
**ENSG00000100372**		0.3030303		0.303030303	0.35	0.327381	0.3235294
**ENSG00000177143**		0.2030303		0.203030303			0.0639535
**ENSG00000174156**	0.16766467		0.16766467				
**ENSG00000140264**	0.31736527	0.07222222	0.31736527	0.072222222			0.244186
**ENSG00000132182**		0.48159509		0.481595092			0.4107143
**ENSG00000116117**		0.4494382		0.449438202			0.4583333
**ENSG00000146555**	0.38855422	0.475	0.38855422	0.475	0.220238095	0.2383721	0.3953488
**ENSG00000181638**	0.24444444	0.459375	0.24444444	0.459375	0.307228916	0.2906977	0.2954545
**ENSG00000105875**		0.26666667		0.266666667			0.1511628
**ENSG00000170113**	0.45808383	0.4969697	0.45808383	0.496969697	0.410714286	0.4767442	0.4767442
**ENSG00000089123**		0.17777778		0.177777778			0.2840909
**ENSG00000163577**		0.34146341		0.341463415			0.3546512
**ENSG00000172348**	0.36445783	0.13939394	0.36445783	0.139393939	0.297619048	0.2764706	0.1569767
**ENSG00000147614**		0.2969697		0.296969697			0.1627907

**ENSG00000230301**	*0.3502994*		*0.3502994*		*0.494047619*	*0.3313953*	
**ENSG00000171262**		*0.32424242*		*0.324242424*			*0.2588235*
**ENSG00000204070**		*0.4202454*		*0.420245399*			*0.4011628*
**ENSG00000124256**		*0.36890244*		*0.368902439*			*0.4825581*
**ENSG00000070501**					*0.113095238*	*0.1823529*	
**ENSG00000177679**							*0.1941176*
**ENSG00000154743**	*0.10909091*	*0.48170732*	*0.10909091*	*0.481707317*			*0.1823529*
**ENSG00000122882**		*0.06097561*		*0.06097561*			*0.2888889*
**ENSG00000130589**	*0.36144578*	*0.3*	*0.36144578*	*0.3*	*0.4*	*0.4090909*	*0.3313953*
**ENSG00000164292**		*0.42682927*		*0.426829268*			*0.2738095*
**ENSG00000183778**		*0.41212121*		*0.412121212*			*0.4767442*
**ENSG00000095303**	*0.31736527*	*0.17878788*	*0.31736527*	*0.178787879*	*0.078313253*	*0.0755814*	*0.0639535*
**ENSG00000123243**	*0.36826347*	*0.23295455*	*0.36826347*	*0.232954545*	*0.463855422*	*0.4825581*	*0.1647059*
**ENSG00000188112**		*0.15151515*		*0.151515152*			*0.3023256*
**ENSG00000120093**	*0.47005988*		*0.47005988*		*0.178571429*	*0.1588235*	
**ENSG00000147852**		*0.24444444*		*0.244444444*			
**ENSG00000213337**							*0.0681818*
**ENSG00000166402**		*0.37777778*		*0.377777778*			*0.494186*
**ENSG00000225940**	*0.26363636*		*0.26363636*		*0.148809524*	*0.1860465*	
**ENSG00000175938**		*0.10509554*		*0.105095541*			
**ENSG00000121440**		*0.47878788*		*0.478787879*			*0.2267442*
**ENSG00000118816**		*0.05617978*		*0.056179775*			*0.1704545*
**ENSG00000196642**	*0.4126506*		*0.4126506*		*0.172619048*	*0.125*	
**ENSG00000074047**	*0.39197531*	*0.29393939*	*0.39197531*	*0.293939394*			*0.4476744*
**ENSG00000250571**		*0.46969697*		*0.46969697*			*0.2954545*
**ENSG00000107902**		*0.05151515*		*0.051515152*			*0.127907*
**ENSG00000256045**		*0.40909091*		*0.409090909*			*0.2906977*
**ENSG00000170703**		*0.35454545*		*0.354545455*			
**ENSG00000081842**		*0.05151515*		*0.051515152*			*0.1162791*
**ENSG00000198315**		*0.27710843*		*0.277108434*			*0.4186047*
**ENSG00000140199**	*0.15340909*	*0.33544304*	*0.15340909*	*0.335443038*	*0.238636364*	*0.3863636*	*0.3658537*
**ENSG00000162374**		*0.08181818*		*0.081818182*			*0.2790698*
**ENSG00000250733**					*0.386904762*	*0.3546512*	
**ENSG00000164933**	*0.4760479*	*0.44848485*	*0.4760479*	*0.448484848*	*0.452380952*	*0.3488372*	*0.3571429*
**ENSG00000068724**	*0.2*	*0.2347561*	*0.2*	*0.234756098*			*0.4583333*
**ENSG00000166704**	*0.15909091*	*0.40804598*	*0.15909091*	*0.408045977*	*0.488888889*	*0.375*	*0.3430233*
**ENSG00000102547**	*0.16467066*	*0.39506173*	*0.16467066*	*0.395061728*	*0.446428571*	*0.4176471*	*0.4777778*
**ENSG00000132854**	*0.5*	*0.35889571*	*0.5*	*0.358895706*	*0.321428571*	*0.2267442*	*0.1744186*
**ENSG00000161544**		*0.36111111*		*0.361111111*			*0.1931818*
**ENSG00000091157**		*0.32727273*		*0.327272727*			*0.4886364*
**ENSG00000143324**		*0.48787879*		*0.487878788*			*0.2831325*
**ENSG00000077420**	*0.46111111*		*0.46111111*				*0.0639535*
**ENSG00000103257**		*0.27777778*		*0.277777778*			*0.2727273*
**ENSG00000121207**	*0.40718563*	*0.1030303*	*0.40718563*	*0.103030303*	*0.255952381*	*0.1395349*	
**ENSG00000126456**	*0.25748503*		*0.25748503*		*0.273809524*	*0.2151163*	
**ENSG00000187800**	*0.27777778*	*0.27134146*	*0.27777778*	*0.271341463*	*0.377777778*	*0.4204545*	*0.4534884*
**ENSG00000065534**	*0.39634146*	*0.38787879*	*0.39634146*	*0.387878788*	*0.466666667*	*0.4555556*	*0.4772727*
**ENSG00000186130**	*0.45783133*		*0.45783133*		*0.321428571*	*0.25*	*0.0764706*
**ENSG00000010072**		*0.37341772*		*0.373417722*			*0.4476744*
**ENSG00000197646**	*0.19161677*		*0.19161677*		*0.178571429*	*0.2093023*	
**ENSG00000126733**		*0.43333333*		*0.433333333*			*0.4883721*
**ENSG00000036257**	*0.1626506*	*0.48484848*	*0.1626506*	*0.484848485*	*0.285714286*	*0.3488372*	*0.4127907*
**ENSG00000182521**	*0.24444444*	*0.21348315*	*0.24444444*	*0.213483146*	*0.355555556*	*0.3863636*	
**ENSG00000183166**		*0.21666667*		*0.216666667*			*0.5*

**ENSG00000103275**		*0.31212121*		*0.312121212*	*0.053571429*		*0.0813953*	*0.1333333*
**ENSG00000116157**		*0.4695122*		*0.469512195*				*0.1744186*
**ENSG00000182492**	*0.26807229*	*0.3*	*0.26807229*	*0.3*	*0.446428571*		*0.4176471*	*0.2209302*
**ENSG00000108511**		*0.18181818*		*0.181818182*				
**ENSG00000152377**	*0.36666667*	*0.47272727*	*0.36666667*	*0.472727273*				*0.3255814*
**ENSG00000170465**		*0.40606061*		*0.406060606*				*0.1744186*
**ENSG00000135094**		*0.12121212*		*0.121212121*				*0.25*
**ENSG00000091622**		*0.46666667*		*0.466666667*				*0.3372093*
**ENSG00000138433**		*0.26363636*		*0.263636364*				*0.2906977*
**ENSG00000142556**	*0.37724551*	*0.22121212*	*0.37724551*	*0.221212121*	*0.178571429*		*0.1162791*	*0.4593023*
**ENSG00000183495**								*0.1976744*
**ENSG00000148158**		*0.37878788*		*0.378787879*				*0.3197674*
**ENSG00000214022**		*0.26969697*		*0.26969697*				*0.3522727*
**ENSG00000156709**		*0.4*		*0.4*				*0.1744186*
**ENSG00000128951**	*0.07185629*		*0.07185629*		*0.06547619*			
**ENSG00000167580**		*0.25*		*0.25*				*0.3662791*
**ENSG00000171595**	*0.39520958*		*0.39520958*		*0.253012048*		*0.1927711*	
**ENSG00000174038**		*0.22727273*		*0.227272727*				*0.4069767*
**ENSG00000140682**		*0.39655172*		*0.396551724*				*0.4318182*
**ENSG00000196460**	*0.11363636*	*0.41212121*	*0.11363636*	*0.412121212*				*0.4418605*
**ENSG00000147894**	*0.18263473*	*0.47878788*	*0.18263473*	*0.478787879*	*0.089285714*		*0.1104651*	*0.4418605*
**ENSG00000074071**		*0.13636364*		*0.136363636*				*0.3662791*
**ENSG00000145075**	*0.34444444*		*0.34444444*		*0.422619048*		*0.4011628*	
**ENSG00000177675**		*0.47222222*		*0.472222222*				*0.3522727*
**ENSG00000134326**		*0.20606061*		*0.206060606*				*0.122093*
**ENSG00000182376**		*0.14367816*		*0.143678161*				*0.3837209*
**ENSG00000184014**	*0.31137725*		*0.31137725*		*0.119047619*		*0.122093*	
**ENSG00000186532**		*0.31515152*		*0.315151515*				*0.4069767*
**ENSG00000183570**	*0.22424242*	*0.31818182*	*0.22424242*	*0.318181818*	*0.285714286*		*0.372093*	*0.2034884*
**ENSG00000138722**	*0.29192547*	*0.40804598*	*0.29192547*	*0.408045977*	*0.228915663*		*0.2083333*	
**ENSG00000011114**		*0.49393939*		*0.493939394*				*0.4360465*
**ENSG00000112079**		*0.16666667*		*0.166666667*				*0.4418605*
**ENSG00000133710**		*0.5*		*0.5*				*0.4825581*
**ENSG00000149451**		*0.44827586*		*0.448275862*				*0.4476744*
**ENSG00000131828**	*0.24251497*	*0.05681818*	*0.24251497*	*0.056818182*				*0.1785714*
**ENSG00000167395**		*0.36666667*		*0.366666667*	*0.053571429*		*0.0988372*	*0.0988372*
**ENSG00000178913**	*0.15361446*	*0.10909091*	*0.15361446*	*0.109090909*				*0.172619*
**ENSG00000173391**	*0.30555556*	*0.5*	*0.30555556*	*0.5*	*0.380952381*		*0.372093*	*0.1976744*
**ENSG00000112902**		*0.35*		*0.35*				*0.1111111*
**ENSG00000148965**	*0.27844311*		*0.27844311*		*0.089285714*		*0.0755814*	
**ENSG00000216560**		*0.21515152*		*0.215151515*				*0.0523256*
**ENSG00000158816**	*0.30555556*	*0.44886364*	*0.30555556*	*0.448863636*	*0.422619048*		*0.3662791*	*0.375*
**ENSG00000174804**		*0.44242424*		*0.442424242*				*0.4476744*
**ENSG00000168679**		*0.16969697*		*0.16969697*				*0.3139535*
**ENSG00000197415**	*0.41317365*	*0.3006135*	*0.41317365*	*0.300613497*	*0.422619048*		*0.4127907*	*0.4772727*
**ENSG00000137691**		*0.43939394*		*0.439393939*				*0.2705882*
**ENSG00000101162**		*0.1969697*		*0.196969697*				*0.2209302*
**ENSG00000150337**								*0.1976744*
**ENSG00000143340**		*0.4030303*		*0.403030303*				*0.2732558*
**ENSG00000130561**	*0.11377246*	*0.41212121*	*0.11377246*	*0.412121212*			*0.0813953*	*0.3023256*
**ENSG00000198836**		*0.46363636*		*0.463636364*				*0.297619*
**ENSG00000138442**		*0.18181818*		*0.181818182*				
**ENSG00000173976**		*0.22256098*		*0.222560976*				
**ENSG00000005156**		*0.49085366*		*0.490853659*				*0.377907*

**ENSG00000257184**		*0.38181818*		*0.381818182*			*0.2764706*
**ENSG00000164944**		*0.24242424*		*0.242424242*			*0.4883721*
**ENSG00000107036**		*0.40606061*		*0.406060606*		*0.127907*	*0.4204545*
**ENSG00000185436**		*0.45977011*		*0.459770115*			*0.4705882*
**ENSG00000166839**		*0.33333333*		*0.333333333*			*0.2151163*
**ENSG00000014257**		*0.48484848*		*0.484848485*			*0.4444444*
**ENSG00000133703**		*0.49695122*		*0.49695122*			*0.1882353*
**ENSG00000151233**		*0.41818182*		*0.418181818*			*0.4011628*
**ENSG00000178904**		*0.18888889*		*0.188888889*			*0.1022727*
**ENSG00000075568**		*0.34545455*		*0.345454545*			*0.2674419*
**ENSG00000137962**		*0.35889571*		*0.358895706*			*0.4186047*
**ENSG00000197261**		*0.23636364*		*0.236363636*			*0.3546512*
**ENSG00000110881**		*0.34146341*		*0.341463415*			*0.1337209*
**ENSG00000125651**	*0.0748503*	*0.05151515*	*0.0748503*	*0.051515152*	*0.416666667*	*0.3430233*	*0.3662791*
**ENSG00000178252**	*0.44610778*	*0.24242424*	*0.44610778*	*0.242424242*	*0.06626506*	*0.0872093*	
**ENSG00000151131**		*0.24157303*		*0.241573034*			*0.3522727*
**ENSG00000204618**		*0.28484848*		*0.284848485*			*0.3895349*
**ENSG00000156886**		*0.2816092*		*0.281609195*			*0.2386364*
**ENSG00000165091**	*0.32934132*		*0.32934132*		*0.476190476*	*0.4883721*	
**ENSG00000162383**		*0.25304878*		*0.25304878*			*0.3176471*
**ENSG00000198517**		*0.44242424*		*0.442424242*			*0.2848837*
**ENSG00000163482**		*0.37575758*		*0.375757576*			*0.0843373*
**ENSG00000027697**	*0.47402597*		*0.47402597*		*0.5*	*0.4418605*	
**ENSG00000122873**	*0.30538922*	*0.19886364*	*0.30538922*	*0.198863636*	*0.458333333*	*0.4352941*	*0.4431818*
**ENSG00000103966**		*0.47878788*		*0.478787879*			*0.1022727*
**ENSG00000173212**		*0.25766871*		*0.257668712*			*0.2647059*
**ENSG00000108389**		*0.17777778*		*0.177777778*			*0.25*
**ENSG00000011451**		*0.28484848*		*0.284848485*			
**ENSG00000112367**	*0.19461078*	*0.41818182*	*0.19461078*	*0.418181818*	*0.386904762*	*0.3837209*	*0.3662791*
**ENSG00000167799**		*0.36585366*		*0.365853659*			*0.0872093*
**ENSG00000189195**	*0.08333333*		*0.08333333*				
**ENSG00000053747**		*0.31111111*		*0.311111111*			*0.2159091*
**ENSG00000151135**		*0.45757576*		*0.457575758*			*0.3255814*
**ENSG00000166164**		*0.46575342*		*0.465753425*			*0.2236842*
**ENSG00000005801**	*0.39090909*	*0.27058824*	*0.39090909*	*0.270588235*	*0.392857143*	*0.4534884*	*0.4204545*
**ENSG00000091136**	*0.18452381*		*0.18452381*				
**ENSG00000152700**		*0.1*		*0.1*	*0.343373494*		*0.3662791*
**ENSG00000090861**		*0.05487805*		*0.054878049*			
**ENSG00000110455**					*0.422222222*	*0.2272727*	*0.0639535*
**ENSG00000110318**		*0.46036585*		*0.460365854*			*0.2325581*
**ENSG00000106346**		*0.0969697*		*0.096969697*			*0.4117647*
**ENSG00000196821**		*0.13939394*		*0.139393939*			*0.0988372*
**ENSG00000101193**		*0.46060606*		*0.460606061*			*0.3139535*
**ENSG00000112659**		*0.14848485*		*0.148484848*			
**ENSG00000066926**		*0.18787879*		*0.187878788*			*0.4825581*
**ENSG00000179813**		*0.29310345*		*0.293103448*			*0.3764706*
**ENSG00000123178**		*0.49444444*		*0.494444444*			*0.4583333*
**ENSG00000162227**	*0.36227545*		*0.36227545*		*0.125*	*0.1569767*	*0.1453488*
**ENSG00000120265**		*0.34756098*		*0.347560976*			*0.2888889*
**ENSG00000189280**	*0.21385542*		*0.21385542*		*0.464285714*	*0.4127907*	
**ENSG00000184838**	*0.42771084*	*0.05*	*0.42771084*	*0.05*			*0.0555556*
**ENSG00000166562**		*0.15*		*0.15*			*0.3636364*
**ENSG00000090975**	*0.05688623*	*0.33333333*	*0.05688623*	*0.333333333*			
**ENSG00000135999**		*0.33030303*		*0.33030303*			*0.1802326*

**ENSG00000155066**		*0.48787879*		*0.487878788*				*0.4651163*
**ENSG00000119487**	*0.45808383*	*0.40606061*	*0.45808383*	*0.406060606*	*0.43452381*		*0.4534884*	*0.3023256*
**ENSG00000170454**	*0.10555556*	*0.36666667*	*0.10555556*	*0.366666667*				*0.4069767*
**ENSG00000081692**		*0.41212121*		*0.412121212*				*0.2093023*
**ENSG00000143373**	*0.46407186*	*0.32424242*	*0.46407186*	*0.324242424*	*0.351190476*		*0.4651163*	*0.4651163*
**ENSG00000065485**		*0.39393939*		*0.393939394*				*0.4883721*
**ENSG00000104872**	*0.33832335*	*0.45402299*	*0.33832335*	*0.454022989*	*0.392857143*		*0.3139535*	*0.2727273*
**ENSG00000203780**		*0.41666667*		*0.416666667*				*0.3863636*
**ENSG00000148384**		*0.47878788*		*0.478787879*				*0.2151163*
**ENSG00000178568**		*0.44303797*		*0.443037975*				*0.4888889*
**ENSG00000079819**	*0.3502994*	*0.34545455*	*0.3502994*	*0.345454545*	*0.5*		*0.4186047*	*0.2916667*
**ENSG00000203740**	*0.07185629*		*0.07185629*		*0.291666667*		*0.2267442*	
**ENSG00000157103**	*0.2994012*	*0.30606061*	*0.2994012*	*0.306060606*	*0.375*		*0.3139535*	*0.4244186*
**ENSG00000176915**	*0.38690476*		*0.38690476*		*0.454545455*		*0.4318182*	
**ENSG00000112541**		*0.46666667*		*0.466666667*				*0.4418605*
**ENSG00000167618**					*0.243243243*		*0.2073171*	
**ENSG00000181381**		*0.42777778*		*0.427777778*				*0.3095238*
**ENSG00000164520**	*0.17065868*	*0.4*	*0.17065868*	*0.4*	*0.411111111*		*0.4888889*	*0.1590909*
**ENSG00000141485**		*0.44545455*		*0.445454545*				*0.2325581*
**ENSG00000165731**		*0.26969697*		*0.26969697*				*0.4772727*
**ENSG00000041515**	*0.22222222*	*0.44512195*	*0.22222222*	*0.445121951*	*0.318181818*		*0.3444444*	*0.2083333*
**ENSG00000184731**		*0.11212121*		*0.112121212*				*0.1976744*
**ENSG00000182952**		*0.47865854*		*0.478658537*			*0.1*	*0.3488372*
**ENSG00000030419**		*0.41515152*		*0.415151515*				*0.3197674*
**ENSG00000034063**	*0.48203593*		*0.48203593*		*0.095238095*		*0.2058824*	
**ENSG00000075213**	*0.1257485*	*0.25304878*	*0.1257485*	*0.25304878*	*0.180722892*		*0.1744186*	
**ENSG00000141034**		*0.3*		*0.3*				*0.1395349*
**ENSG00000258512**								*0.0813953*
**ENSG00000113810**		*0.4969697*		*0.496969697*				*0.2202381*
**ENSG00000110777**		*0.43636364*		*0.436363636*				*0.1162791*
**ENSG00000179604**		*0.43939394*		*0.439393939*				*0.4882353*
**ENSG00000110025**		*0.37272727*		*0.372727273*				*0.1686047*
**ENSG00000151304**		*0.25*		*0.25*				
**ENSG00000144909**	*0.33832335*	*0.08484848*	*0.33832335*	*0.084848485*	*0.160714286*		*0.1627907*	*0.0639535*
**ENSG00000143954**	*0.33532934*	*0.32727273*	*0.33532934*	*0.327272727*	*0.386904762*		*0.3546512*	*0.4588235*
**ENSG00000165490**	*0.47126437*	*0.4969697*	*0.47126437*	*0.496969697*	*0.188888889*		*0.3068182*	*0.3197674*
**ENSG00000005889**		*0.29141104*		*0.291411043*				*0.3953488*
**ENSG00000119335**								*0.0523256*
**ENSG00000117009**		*0.24539877*		*0.245398773*				*0.3493976*
**ENSG00000143995**		*0.4*		*0.4*				*0.2383721*
**ENSG00000112293**	*0.43373494*	*0.30182927*	*0.43373494*	*0.301829268*	*0.148809524*		*0.2034884*	*0.4883721*
**ENSG00000169813**	*0.25*		*0.25*		*0.288888889*		*0.3409091*	*0.3068182*
**ENSG00000113312**					*0.119047619*		*0.25*	
**ENSG00000196715**		*0.42424242*		*0.424242424*				*0.4709302*
**ENSG00000119718**		*0.42528736*		*0.425287356*				*0.1777778*
**ENSG00000132603**	*0.27777778*	*0.40340909*	*0.27777778*	*0.403409091*	*0.081395349*		*0.0697674*	*0.0988372*
**ENSG00000077238**		*0.47878788*		*0.478787879*				*0.377907*
**ENSG00000164756**		*0.43333333*		*0.433333333*				*0.4534884*
**ENSG00000100897**	*0.10479042*	*0.21818182*	*0.10479042*	*0.218181818*				*0.4294118*
**ENSG00000064652**		*0.39393939*		*0.393939394*				
**ENSG00000135775**	*0.10479042*		*0.10479042*					
**ENSG00000155621**		*0.37575758*		*0.375757576*				*0.4651163*
**ENSG00000160321**		*0.42283951*		*0.422839506*				*0.4593023*
**ENSG00000171723**	*0.26347305*		*0.26347305*		*0.44047619*		*0.494186*	*0.4825581*

**ENSG00000100399**	*0.27222222*		*0.27222222*				
**ENSG00000026103**	*0.28484848*	*0.34969325*	*0.28484848*	*0.349693252*	*0.090361446*	*0.127907*	*0.4529412*
**ENSG00000087008**		*0.28484848*		*0.284848485*			*0.2352941*
**ENSG00000163501**		*0.39325843*		*0.393258427*			*0.2727273*
**ENSG00000013810**		*0.36809816*		*0.36809816*			*0.3313953*
**ENSG00000130433**	*0.27245509*	*0.35582822*	*0.27245509*	*0.355828221*	*0.267857143*	*0.3139535*	*0.4418605*
**ENSG00000153291**		*0.23636364*		*0.236363636*	*0.142857143*	*0.1046512*	*0.2093023*
**ENSG00000160224**	*0.4*	*0.45426829*	*0.4*	*0.454268293*	*0.477777778*	*0.3863636*	*0.3662791*
**ENSG00000228006**	*0.47727273*		*0.47727273*		*0.488888889*	*0.4883721*	
**ENSG00000155508**		*0.12962963*		*0.12962963*			*0.0795455*
**ENSG00000223526**	*0.28651685*	*0.44545455*	*0.28651685*	*0.445454545*	*0.455555556*	*0.3636364*	*0.0988372*
**ENSG00000169575**	*0.28143713*	*0.2*	*0.28143713*	*0.2*	*0.095238095*	*0.244186*	*0.1627907*
**ENSG00000104228**	*0.14444444*	*0.35757576*	*0.14444444*	*0.357575758*	*0.056818182*	*0.1444444*	*0.1363636*
**ENSG00000102119**		*0.18965517*		*0.189655172*			*0.3181818*
**ENSG00000128923**	*0.25*	*0.30606061*	*0.25*	*0.306060606*			*0.5*
**ENSG00000105137**		*0.2969697*		*0.296969697*			*0.494186*
**ENSG00000204130**		*0.09756098*		*0.097560976*			*0.122093*
**ENSG00000064886**	*0.32831325*	*0.29444444*	*0.32831325*	*0.294444444*			*0.3863636*
**ENSG00000046653**		*0.13939394*		*0.139393939*	*0.178571429*	*0.1104651*	*0.4476744*
**ENSG00000182912**	*0.21666667*		*0.21666667*		*0.053571429*	*0.122093*	
**ENSG00000082074**	*0.44011976*	*0.37195122*	*0.44011976*	*0.37195122*	*0.470238095*	*0.4825581*	*0.375*
**ENSG00000179941**		*0.3117284*		*0.311728395*			*0.0941176*
**ENSG00000113719**		*0.37777778*		*0.377777778*			*0.1104651*
**ENSG00000086598**	*0.19461078*		*0.19461078*		*0.108433735*	*0.0639535*	
**ENSG00000166848**	*0.36931818*		*0.36931818*		*0.193181818*	*0.2209302*	
**ENSG00000107201**	*0.43888889*	*0.27575758*	*0.43888889*	*0.275757576*	*0.444444444*	*0.3409091*	*0.25*
**ENSG00000235942**	*0.08333333*		*0.08333333*		*0.5*	*0.4555556*	
**ENSG00000145242**		*0.375*		*0.375*			*0.4418605*
**ENSG00000114745**	*0.35329341*	*0.1*	*0.35329341*	*0.1*	*0.303571429*	*0.1976744*	*0.2325581*
**ENSG00000106462**		*0.07012195*		*0.070121951*			
**ENSG00000168209**		*0.36363636*		*0.363636364*			*0.2840909*
**ENSG00000130303**							*0.3470588*
**ENSG00000240849**		*0.33146067*		*0.331460674*			*0.2613636*
**ENSG00000132781**	*0.26946108*	*0.29310345*	*0.26946108*	*0.293103448*	*0.379518072*	*0.3837209*	*0.452381*
**ENSG00000254349**	*0.29640719*	*0.22368421*	*0.29640719*	*0.223684211*	*0.089285714*	*0.127907*	
**ENSG00000165138**		*0.27222222*		*0.272222222*			*0.4127907*
**ENSG00000115661**		*0.32727273*		*0.327272727*			*0.2790698*
**ENSG00000126583**	*0.05757576*		*0.05757576*				
**ENSG00000131732**	*0.45209581*	*0.05151515*	*0.45209581*	*0.051515152*	*0.25*	*0.2267442*	
**ENSG00000131037**					*0.107142857*	*0.1104651*	
**ENSG00000186777**		*0.1*		*0.1*			
**ENSG00000153956**	*0.45180723*	*0.25555556*	*0.45180723*	*0.255555556*	*0.391566265*	*0.3372093*	*0.2888889*
**ENSG00000206527**		*0.47878788*		*0.478787879*			*0.3430233*
**ENSG00000205328**	*0.42814371*		*0.42814371*		*0.113095238*		
**ENSG00000177707**	*0.0988024*		*0.0988024*		*0.095238095*	*0.0755814*	
**ENSG00000124440**		*0.18181818*		*0.181818182*			*0.4767442*
**ENSG00000116273**		*0.35757576*		*0.357575758*			*0.4418605*
**ENSG00000139180**		*0.2804878*		*0.280487805*			*0.3604651*
**ENSG00000166012**	*0.39204545*	*0.38787879*	*0.39204545*	*0.387878788*	*0.102272727*	*0.0988372*	*0.4941176*
**ENSG00000121310**	*0.19444444*		*0.19444444*		*0.303571429*	*0.2732558*	*0.2732558*
**ENSG00000138398**	*0.16766467*	*0.15*	*0.16766467*	*0.15*	*0.214285714*	*0.2732558*	*0.0795455*
**ENSG00000160058**		*0.15757576*		*0.157575758*			
**ENSG00000168827**	*0.38922156*	*0.43333333*	*0.38922156*	*0.433333333*	*0.133333333*	*0.25*	*0.4545455*
**ENSG00000124067**		*0.15757576*		*0.157575758*			*0.1395349*

**ENSG00000165630**	*0.19444444*		*0.19444444*					
**ENSG00000169429**	*0.31437126*	*0.40606061*	*0.31437126*	*0.406060606*				*0.3197674*
**ENSG00000164466**		*0.33333333*		*0.333333333*				*0.3294118*
**ENSG00000170486**	*0.14371257*	*0.33231707*	*0.14371257*	*0.332317073*	*0.470238095*		*0.4764706*	*0.494186*
**ENSG00000161217**	*0.46385542*	*0.43030303*	*0.46385542*	*0.43030303*	*0.19047619*		*0.1918605*	*0.2674419*
**ENSG00000197728**	*0.14204545*		*0.14204545*		*0.277777778*		*0.2674419*	
**ENSG00000100029**	*0.48837209*	*0.06976744*	*0.48837209*	*0.069767442*	*0.226190476*		*0.2386364*	
**ENSG00000105357**		*0.16363636*		*0.163636364*				*0.25*
**ENSG00000176515**	*0.46706587*	*0.28181818*	*0.46706587*	*0.281818182*	*0.261904762*		*0.3662791*	*0.3604651*
**ENSG00000165392**	*0.31736527*		*0.31736527*					
**ENSG00000113763**		*0.19090909*		*0.190909091*				*0.372093*
**ENSG00000165899**		*0.36503067*		*0.365030675*				*0.297619*
**ENSG00000169515**		*0.17272727*		*0.172727273*				*0.1363636*
**ENSG00000134285**	*0.07185629*		*0.07185629*		*0.465909091*		*0.4651163*	
**ENSG00000123975**		*0.12777778*		*0.127777778*				*0.0909091*
**ENSG00000171960**	*0.28742515*		*0.28742515*		*0.375*		*0.494186*	
**ENSG00000115275**		*0.10909091*		*0.109090909*				*0.1860465*
**ENSG00000105810**		*0.37730061*		*0.377300613*				*0.2613636*
**ENSG00000130038**	*0.17664671*	*0.42222222*	*0.17664671*	*0.422222222*				*0.4476744*
**ENSG00000111913**		*0.47222222*		*0.472222222*				*0.4764706*
**ENSG00000170613**		*0.46296296*		*0.462962963*				*0.2267442*
**ENSG00000165269**		*0.08333333*		*0.083333333*				*0.0823529*
**ENSG00000139793**	*0.17222222*		*0.17222222*					*0.1022727*
**ENSG00000160404**		*0.39393939*		*0.393939394*				*0.3430233*
**ENSG00000082269**		*0.44848485*		*0.448484848*				*0.4127907*
**ENSG00000146856**	*0.30239521*		*0.30239521*					
**ENSG00000127954**		*0.4*		*0.4*				*0.1860465*
**ENSG00000169891**		*0.45121951*		*0.451219512*				*0.1627907*
**ENSG00000186952**		*0.10909091*		*0.109090909*				*0.3197674*
**ENSG00000080293**		*0.28787879*		*0.287878788*				*0.2882353*
**ENSG00000125735**	*0.26347305*	*0.34242424*	*0.26347305*	*0.342424242*	*0.494047619*		*0.4186047*	*0.4545455*
**ENSG00000160678**		*0.33888889*		*0.338888889*				*0.1477273*
**ENSG00000119457**		*0.20555556*		*0.205555556*				*0.2045455*
**ENSG00000148985**	*0.47005988*	*0.46363636*	*0.47005988*	*0.463636364*				*0.372093*
**ENSG00000166963**		*0.3*		*0.3*				*0.3823529*
**ENSG00000080572**	*0.38922156*		*0.38922156*		*0.482142857*		*0.4764706*	
**ENSG00000118491**	*0.06287425*		*0.06287425*					
**ENSG00000176533**	*0.4011976*	*0.4969697*	*0.4011976*	*0.496969697*	*0.458333333*		*0.4825581*	*0.4777778*
**ENSG00000058335**	*0.22754491*		*0.22754491*					*0.3882353*
**ENSG00000172426**		*0.14242424*		*0.142424242*				*0.3117647*
**ENSG00000204687**								*0.2034884*
**ENSG00000121577**		*0.41925466*		*0.419254658*	*0.327380952*		*0.3*	*0.3255814*
**ENSG00000166226**	*0.38333333*	*0.2030303*	*0.38333333*	*0.203030303*	*0.377777778*		*0.4888889*	*0.1569767*
**ENSG00000182759**		*0.23030303*		*0.23030303*				*0.3662791*
**ENSG00000138385**	*0.26111111*		*0.26111111*		*0.202380952*		*0.1627907*	
**ENSG00000129933**		*0.37962963*		*0.37962963*				*0.3295455*
**ENSG00000112378**		*0.46111111*		*0.461111111*				*0.4666667*
**ENSG00000136448**		*0.44512195*		*0.445121951*				*0.4883721*
**ENSG00000134504**	*0.11377246*	*0.12121212*	*0.11377246*	*0.121212121*	*0.208333333*		*0.1511628*	*0.0697674*
**ENSG00000005189**		*0.38787879*		*0.387878788*				*0.1453488*
**ENSG00000107815**		*0.16363636*		*0.163636364*				*0.4476744*
**ENSG00000197045**		*0.15730337*		*0.157303371*				
**ENSG00000188227**		*0.22727273*		*0.227272727*				*0.3313953*
**ENSG00000135899**	*0.12777778*		*0.12777778*		*0.25*		*0.2857143*	

**ENSG00000147394**	*0.35555556*	*0.2195122*	*0.35555556*	*0.219512195*	*0.136363636*		*0.0568182*	*0.3546512*
**ENSG00000108786**	*0.34730539*		*0.34730539*		*0.402439024*		*0.4476744*	
**ENSG00000115966**	*0.18975904*	*0.11627907*	*0.18975904*	*0.11627907*	*0.125*		*0.1352941*	
**ENSG00000186715**		*0.11627907*		*0.11627907*				*0.1744186*
**ENSG00000182885**		*0.27325581*		*0.273255814*				*0.3604651*
**ENSG00000063177**	*0.23053892*		*0.23053892*		*0.313253012*		*0.2093023*	
**ENSG00000163904**	*0.49101796*	*0.34242424*	*0.49101796*	*0.342424242*	*0.321428571*		*0.4069767*	*0.4767442*
**ENSG00000151687**	*0.09281437*		*0.09281437*					
**ENSG00000243978**	*0.17065868*	*0.34848485*	*0.17065868*	*0.348484848*				
**ENSG00000181090**		*0.34242424*		*0.342424242*				*0.2965116*
**ENSG00000067798**	*0.10479042*	*0.44753086*	*0.10479042*	*0.447530864*	*0.494047619*		*0.4761905*	*0.1627907*
**ENSG00000184860**	*0.32222222*	*0.4030303*	*0.32222222*	*0.403030303*				*0.0872093*
**ENSG00000086289**		*0.36363636*		*0.363636364*	*0.133333333*		*0.0888889*	*0.4244186*
**ENSG00000121848**		*0.34444444*		*0.344444444*				*0.0930233*
**ENSG00000128340**	*0.19879518*	*0.41111111*	*0.19879518*	*0.411111111*	*0.208333333*		*0.2034884*	*0.4659091*
**ENSG00000113319**		*0.22777778*		*0.227777778*				*0.1136364*
**ENSG00000198794**	*0.47904192*	*0.15454545*	*0.47904192*	*0.154545455*	*0.428571429*		*0.3895349*	*0.4642857*
**ENSG00000255559**		*0.13888889*		*0.138888889*				*0.3522727*
**ENSG00000155974**	*0.46060606*	*0.35757576*	*0.46060606*	*0.357575758*	*0.426829268*		*0.4268293*	*0.372093*
**ENSG00000214189**	*0.0625*	*0.23333333*	*0.0625*	*0.233333333*				*0.3837209*
**ENSG00000125812**		*0.27272727*		*0.272727273*				*0.3837209*
**ENSG00000164399**	*0.11931818*		*0.11931818*		*0.444444444*		*0.4318182*	
**ENSG00000155660**		*0.2*		*0.2*				
**ENSG00000242732**		*0.17073171*		*0.170731707*				*0.0755814*
**ENSG00000126215**	*0.16666667*		*0.16666667*		*0.477777778*		*0.4090909*	
**ENSG00000112818**		*0.36363636*		*0.363636364*				*0.4318182*
**ENSG00000163714**	*0.47305389*	*0.47727273*	*0.47305389*	*0.477272727*	*0.355421687*		*0.3662791*	*0.3444444*
**ENSG00000173706**		*0.37575758*		*0.375757576*				*0.4186047*
**ENSG00000160307**	*0.3742515*	*0.09393939*	*0.3742515*	*0.093939394*	*0.125*		*0.0872093*	*0.3662791*
**ENSG00000054803**	*0.0748503*		*0.0748503*		*0.464285714*		*0.494186*	
**ENSG00000107672**	*0.10555556*	*0.25862069*	*0.10555556*	*0.25862069*	*0.136363636*		*0.1707317*	*0.4318182*
**ENSG00000115233**	*0.45454545*	*0.06441718*	*0.45454545*	*0.064417178*	*0.422222222*		*0.5*	
**ENSG00000167383**	*0.37125749*	*0.36196319*	*0.37125749*	*0.36196319*	*0.166666667*		*0.1395349*	*0.3895349*
**ENSG00000198492**	*0.25555556*		*0.25555556*		*0.222222222*		*0.2272727*	
**ENSG00000124225**	*0.29819277*	*0.1*	*0.29819277*	*0.1*	*0.494047619*		*0.4883721*	*0.2325581*
**ENSG00000133313**	*0.48314607*	*0.36585366*	*0.48314607*	*0.365853659*	*0.327380952*		*0.2790698*	*0.0813953*
**ENSG00000135083**		*0.14242424*		*0.142424242*				*0.1627907*
**ENSG00000128581**	*0.09580838*	*0.16060606*	*0.09580838*	*0.160606061*	*0.238095238*		*0.2790698*	
**ENSG00000223519**	*0.48802395*		*0.48802395*		*0.392857143*		*0.3882353*	
**ENSG00000188707**		*0.21515152*		*0.215151515*				*0.4186047*
**ENSG00000100036**	*0.06287425*	*0.48275862*	*0.06287425*	*0.482758621*	*0.398809524*		*0.4883721*	*0.4883721*
**ENSG00000166266**		*0.29885057*		*0.298850575*				
**ENSG00000177363**		*0.29090909*		*0.290909091*				*0.2732558*
**ENSG00000137413**	*0.21556886*	*0.45348837*	*0.21556886*	*0.453488372*	*0.166666667*		*0.2034884*	*0.4883721*
**ENSG00000121741**	*0.16666667*	*0.08787879*	*0.16666667*	*0.087878788*				*0.3222222*
**ENSG00000143110**		*0.34848485*		*0.348484848*				*0.2848837*
**ENSG00000258365**	*0.2005988*	*0.35955056*	*0.2005988*	*0.359550562*	*0.303571429*		*0.3837209*	*0.4090909*
**ENSG00000198742**		*0.09815951*		*0.098159509*				*0.2411765*
**ENSG00000101945**		*0.26666667*		*0.266666667*				*0.2034884*
**ENSG00000136011**	*0.40718563*	*0.27300613*	*0.40718563*	*0.273006135*	*0.494047619*		*0.4825581*	*0.4244186*
**ENSG00000152952**		*0.45731707*		*0.457317073*				*0.452381*
**ENSG00000100342**	*0.11904762*	*0.1969697*	*0.11904762*	*0.196969697*				*0.1918605*
**ENSG00000104885**		*0.10227273*		*0.102272727*				*0.244186*
**ENSG00000157510**		*0.43597561*		*0.43597561*				*0.4069767*

**ENSG00000106258**							*0.2848837*
**ENSG00000115850**		*0.10909091*		*0.109090909*			*0.1860465*
**ENSG00000108947**		*0.42236025*		*0.422360248*			*0.4294118*
**ENSG00000126814**					*0.084337349*	*0.0697674*	*0.3255814*
**ENSG00000169744**		*0.23030303*		*0.23030303*			*0.3197674*
**ENSG00000205336**	*0.21428571*	*0.46666667*	*0.21428571*	*0.466666667*	*0.397058824*	*0.3157895*	*0.244186*
**ENSG00000138119**	*0.12874251*	*0.13939394*	*0.12874251*	*0.139393939*	*0.476190476*	*0.4767442*	*0.2647059*
**ENSG00000105664**		*0.08787879*		*0.087878788*			
**ENSG00000132507**	*0.35757576*	*0.46060606*	*0.35757576*	*0.460606061*	*0.053571429*		*0.494186*
**ENSG00000144136**	*0.48203593*	*0.21515152*	*0.48203593*	*0.215151515*			
**ENSG00000134318**	*0.23652695*	*0.43636364*	*0.23652695*	*0.436363636*			*0.3662791*
**ENSG00000102021**	*0.05988024*	*0.31402439*	*0.05988024*	*0.31402439*			*0.0647059*
**ENSG00000164287**	*0.31736527*	*0.34848485*	*0.31736527*	*0.348484848*	*0.44047619*	*0.3470588*	*0.3546512*
**ENSG00000076864**		*0.15*		*0.15*			
**ENSG00000106018**		*0.39393939*		*0.393939394*			*0.25*
**ENSG00000090061**	*0.11077844*		*0.11077844*				
**ENSG00000234284**		*0.34242424*		*0.342424242*			*0.4767442*
**ENSG00000175449**	*0.45508982*	*0.35632184*	*0.45508982*	*0.356321839*	*0.327380952*	*0.3139535*	*0.4204545*
**ENSG00000187942**		*0.2183908*		*0.218390805*			*0.1823529*
**ENSG00000069966**	*0.39820359*	*0.31818182*	*0.39820359*	*0.318181818*	*0.380952381*	*0.4880952*	*0.2674419*
**ENSG00000129009**	*0.42215569*	*0.17878788*	*0.42215569*	*0.178787879*			
**ENSG00000179029**		*0.37804878*		*0.37804878*			*0.4534884*
**ENSG00000249852**		*0.10060976*		*0.100609756*			*0.2267442*
**ENSG00000136805**		*0.20909091*		*0.209090909*			*0.3430233*
**ENSG00000131791**		*0.48275862*		*0.482758621*			*0.4418605*
**ENSG00000129354**							*0.4318182*
**ENSG00000169507**		*0.36363636*		*0.363636364*			*0.4302326*
**ENSG00000119328**							*0.4318182*
**ENSG00000102908**		*0.49444444*		*0.494444444*			*0.3470588*
**ENSG00000137807**	*0.27844311*	*0.05454545*	*0.27844311*	*0.054545455*	*0.15060241*	*0.2560976*	*0.3536585*
**ENSG00000090447**		*0.45454545*		*0.454545455*			*0.2732558*
**ENSG00000144278**	*0.4491018*	*0.45757576*	*0.4491018*	*0.457575758*	*0.202380952*	*0.244186*	*0.494186*
**ENSG00000164125**	*0.4760479*	*0.27878788*	*0.4760479*	*0.278787879*	*0.053571429*		*0.1860465*
**ENSG00000212950**		*0.22727273*		*0.227272727*			*0.3604651*
**ENSG00000084234**							*0.0697674*
**ENSG00000135953**		*0.47560976*		*0.475609756*			*0.3139535*
**ENSG00000115221**		*0.43030303*		*0.43030303*			*0.4244186*
**ENSG00000102755**		*0.08484848*		*0.084848485*			*0.3023256*
**ENSG00000172548**		*0.47222222*		*0.472222222*			*0.3470588*
**ENSG00000006606**	*0.12921348*	*0.25151515*	*0.12921348*	*0.251515152*			*0.1162791*
**ENSG00000167747**	*0.22121212*	*0.35955056*	*0.22121212*	*0.359550562*	*0.31547619*	*0.3488372*	*0.3333333*
**ENSG00000157778**		*0.11212121*		*0.112121212*			
**ENSG00000159648**							*0.4941176*
**ENSG00000133835**	*0.25*	*0.45121951*	*0.25*	*0.451219512*	*0.481707317*	*0.4772727*	*0.3571429*
**ENSG00000152284**		*0.48773006*		*0.487730061*			*0.2034884*
**ENSG00000163029**	*0.49101796*	*0.31212121*	*0.49101796*	*0.312121212*		*0.0813953*	*0.1569767*
**ENSG00000197111**		*0.16463415*		*0.164634146*			
**ENSG00000121964**		*0.46111111*		*0.461111111*			*0.1590909*
**ENSG00000134049**		*0.07272727*		*0.072727273*			*0.2616279*
**ENSG00000174469**	*0.46022727*	*0.46969697*	*0.46022727*	*0.46969697*	*0.277108434*	*0.3529412*	*0.3294118*
**ENSG00000137261**	*0.17771084*	*0.43030303*	*0.17771084*	*0.43030303*	*0.119047619*	*0.1071429*	*0.3941176*
**ENSG00000197321**	*0.46107784*		*0.46107784*		*0.303571429*	*0.2965116*	
**ENSG00000107263**	*0.09550562*	*0.29393939*	*0.09550562*	*0.293939394*	*0.214285714*	*0.255814*	*0.4823529*
**ENSG00000083937**	*0.11077844*	*0.25*	*0.11077844*	*0.25*	*0.192771084*	*0.2083333*	*0.2727273*

**ENSG00000009954**		*0.18181818*		*0.181818182*			*0.127907*
**ENSG00000185666**	*0.21666667*	*0.42121212*	*0.21666667*	*0.421212121*	*0.177777778*	*0.1931818*	*0.127907*
**ENSG00000018610**		*0.41463415*		*0.414634146*			*0.2965116*
**ENSG00000168491**		*0.47272727*		*0.472727273*			*0.3430233*
**ENSG00000131051**		*0.18390805*		*0.183908046*			*0.1704545*
**ENSG00000107951**		*0.47222222*		*0.472222222*			*0.3222222*
**ENSG00000188869**	*0.30555556*		*0.30555556*		*0.311111111*	*0.4886364*	
**ENSG00000128989**		*0.11280488*		*0.112804878*			*0.4772727*
**ENSG00000119421**		*0.26666667*		*0.266666667*			*0.3895349*
**ENSG00000103423**	*0.3*	*0.35365854*	*0.3*	*0.353658537*	*0.255555556*	*0.1818182*	*0.3546512*
**ENSG00000140093**		*0.17575758*		*0.175757576*			*0.3488372*
**ENSG00000254377**		*0.16111111*		*0.161111111*			*0.4431818*
**ENSG00000171587**		*0.47272727*		*0.472727273*			*0.4651163*
**ENSG00000258986**	*0.32335329*	*0.38050314*	*0.32335329*	*0.380503145*	*0.366666667*	*0.4204545*	*0.4107143*
**ENSG00000184465**	*0.16766467*	*0.24085366*	*0.16766467*	*0.240853659*	*0.357142857*	*0.4476744*	*0.202381*
**ENSG00000169306**					*0.148809524*	*0.1785714*	
**ENSG00000159733**	*0.22222222*	*0.36111111*	*0.22222222*	*0.361111111*	*0.353658537*	*0.3780488*	*0.4302326*
**ENSG00000181754**		*0.3045977*		*0.304597701*			*0.3977273*
**ENSG00000218823**		*0.09146341*		*0.091463415*			*0.0581395*
**ENSG00000115020**		*0.13030303*		*0.13030303*			*0.0581395*
**ENSG00000151465**		*0.48850575*		*0.488505747*			*0.2386364*
**ENSG00000179846**		*0.32926829*		*0.329268293*			*0.2763158*
**ENSG00000168398**	*0.3125*	*0.28527607*	*0.3125*	*0.285276074*	*0.470238095*	*0.4883721*	*0.3953488*
**ENSG00000135093**	*0.21257485*	*0.16666667*	*0.21257485*	*0.166666667*			*0.1453488*
**ENSG00000162928**	*0.47305389*	*0.45454545*	*0.47305389*	*0.454545455*			
**ENSG00000174498**		*0.45555556*		*0.455555556*	*0.136904762*	*0.2209302*	*0.2151163*
**ENSG00000169299**	*0.33233533*	*0.12345679*	*0.33233533*	*0.12345679*	*0.265060241*	*0.2093023*	*0.1395349*
**ENSG00000006453**		*0.46969697*		*0.46969697*			*0.1802326*
**ENSG00000219016**	*0.12275449*	*0.22222222*	*0.12275449*	*0.222222222*	*0.357142857*	*0.4186047*	*0.1477273*
**ENSG00000077498**	*0.3*		*0.3*		*0.101190476*	*0.1453488*	
**ENSG00000204519**		*0.43678161*		*0.436781609*			*0.4883721*
**ENSG00000058272**		*0.08181818*		*0.081818182*			*0.3392857*
**ENSG00000135862**		*0.48850575*		*0.488505747*			*0.4431818*
**ENSG00000006432**		*0.13636364*		*0.136363636*			*0.2209302*
**ENSG00000117318**		*0.4*		*0.4*			*0.2034884*
**ENSG00000163637**	*0.30538922*	*0.37730061*	*0.30538922*	*0.377300613*	*0.363095238*	*0.3023256*	*0.5*
**ENSG00000068793**		*0.46111111*		*0.461111111*			*0.4302326*
**ENSG00000158158**		*0.06969697*		*0.06969697*			*0.0755814*
**ENSG00000184809**	*0.45*	*0.45121951*	*0.45*	*0.451219512*	*0.411111111*	*0.3522727*	*0.4244186*
**ENSG00000183401**	*0.24096386*		*0.24096386*				
**ENSG00000167113**		*0.2*		*0.2*			
**ENSG00000188404**		*0.38484848*		*0.384848485*			*0.4360465*
**ENSG00000119938**		*0.06666667*		*0.066666667*			
**ENSG00000095713**		*0.36969697*		*0.36969697*			*0.3372093*
**ENSG00000173559**	*0.08682635*		*0.08682635*		*0.375*	*0.4709302*	
**ENSG00000204632**		*0.47575758*		*0.475757576*			*0.2674419*
**ENSG00000175536**		*0.43636364*		*0.436363636*			*0.372093*
**ENSG00000108469**		*0.34545455*		*0.345454545*			*0.4302326*
**ENSG00000163395**	*0.40853659*	*0.39329268*	*0.40853659*	*0.393292683*	*0.409090909*	*0.372093*	*0.4360465*
**ENSG00000165689**	*0.0813253*	*0.44444444*	*0.0813253*	*0.444444444*		*0.0941176*	*0.25*
**ENSG00000160588**		*0.44827586*		*0.448275862*			*0.3295455*
**ENSG00000069275**		*0.41158537*		*0.411585366*			*0.4651163*
**ENSG00000121388**		*0.07471264*		*0.074712644*			
**ENSG00000172572**		*0.37575758*		*0.375757576*			*0.1511628*

**ENSG00000159423**	*0.11676647*	*0.43333333*	*0.11676647*	*0.433333333*				*0.4534884*
**ENSG00000058085**	*0.2005988*	*0.11212121*	*0.2005988*	*0.112121212*	*0.352272727*		*0.4186047*	*0.4360465*
**ENSG00000132746**	*0.5*	*0.0969697*	*0.5*	*0.096969697*	*0.125*		*0.0535714*	*0.0523256*
**ENSG00000115592**	*0.4378882*	*0.19512195*	*0.4378882*	*0.195121951*	*0.431818182*		*0.3139535*	*0.4244186*
**ENSG00000187151**	*0.32335329*		*0.32335329*		*0.458333333*		*0.4418605*	
**ENSG00000116199**		*0.46666667*		*0.466666667*				*0.3662791*
**ENSG00000170689**		*0.39655172*		*0.396551724*				*0.4090909*
**ENSG00000183773**								*0.1136364*
**ENSG00000165816**	*0.12275449*		*0.12275449*		*0.053571429*		*0.0639535*	
**ENSG00000129158**		*0.13030303*		*0.13030303*				
**ENSG00000127129**		*0.12195122*		*0.12195122*				*0.1046512*
**ENSG00000123094**	*0.32022472*	*0.26234568*	*0.32022472*	*0.262345679*	*0.311111111*		*0.2555556*	*0.4709302*
**ENSG00000114013**		*0.33231707*		*0.332317073*				*0.3372093*
**ENSG00000123297**	*0.09659091*	*0.35714286*	*0.09659091*	*0.357142857*	*0.288888889*		*0.2840909*	*0.3026316*
**ENSG00000078401**		*0.07878788*		*0.078787879*				
**ENSG00000103241**		*0.5*		*0.5*				*0.1529412*
**ENSG00000105404**	*0.17964072*		*0.17964072*		*0.18452381*		*0.2470588*	
**ENSG00000162402**		*0.20606061*		*0.206060606*				*0.0755814*
**ENSG00000077585**	*0.06626506*		*0.06626506*		*0.208333333*		*0.1569767*	
**ENSG00000042317**	*0.15060241*	*0.32727273*	*0.15060241*	*0.327272727*				*0.3197674*
**ENSG00000066382**		*0.42222222*		*0.422222222*				*0.4058824*
**ENSG00000183780**		*0.09090909*		*0.090909091*				
**ENSG00000177380**		*0.33908046*		*0.33908046*				*0.2045455*
**ENSG00000120158**		*0.44512195*		*0.445121951*				*0.4702381*
**ENSG00000134905**		*0.46666667*		*0.466666667*	*0.174698795*		*0.1860465*	*0.3023256*
**ENSG00000105499**		*0.27380952*		*0.273809524*				*0.1352941*
**ENSG00000070759**	*0.1*	*0.24848485*	*0.1*	*0.248484848*	*0.088888889*			*0.4302326*
**ENSG00000134152**		*0.12121212*		*0.121212121*				
**ENSG00000198798**		*0.05757576*		*0.057575758*				*0.4235294*
**ENSG00000007516**		*0.49090909*		*0.490909091*				*0.1511628*
**ENSG00000166825**		*0.43597561*		*0.43597561*				*0.1686047*
**ENSG00000183323**		*0.40184049*		*0.401840491*	*0.291666667*		*0.2034884*	*0.3433735*
**ENSG00000178015**		*0.16666667*		*0.166666667*				*0.4431818*
**ENSG00000108387**		*0.42424242*		*0.424242424*				*0.4411765*
**ENSG00000197587**		*0.23636364*		*0.236363636*				*0.3023256*
**ENSG00000115415**	*0.48863636*	*0.42682927*	*0.48863636*	*0.426829268*	*0.5*		*0.4545455*	*0.1104651*
**ENSG00000105483**	*0.22155689*	*0.40606061*	*0.22155689*	*0.406060606*	*0.482142857*		*0.4302326*	*0.4302326*
**ENSG00000110911**	*0.33233533*	*0.43167702*	*0.33233533*	*0.431677019*	*0.375*		*0.4418605*	*0.4534884*
**ENSG00000203721**	*0.34444444*	*0.28333333*	*0.34444444*	*0.283333333*	*0.244444444*		*0.1818182*	*0.1818182*
**ENSG00000108556**		*0.33333333*		*0.333333333*				*0.3068182*
**ENSG00000163349**		*0.49386503*		*0.493865031*				*0.3255814*
**ENSG00000181481**	*0.18333333*		*0.18333333*					
**ENSG00000095932**								*0.1627907*
**ENSG00000120533**		*0.10555556*		*0.105555556*				*0.0909091*
**ENSG00000124942**		*0.12121212*		*0.121212121*				*0.1162791*
**ENSG00000039560**	*0.35*	*0.41463415*	*0.35*	*0.414634146*	*0.420454545*		*0.3636364*	*0.4127907*
**ENSG00000197562**		*0.46969697*		*0.46969697*				*0.3546512*
**ENSG00000102572**		*0.29393939*		*0.293939394*				*0.2272727*
**ENSG00000011258**		*0.47560976*		*0.475609756*				*0.4411765*
**ENSG00000241595**		*0.24719101*		*0.247191011*				
**ENSG00000184343**	*0.09939759*		*0.09939759*		*0.44047619*		*0.3488372*	*0.3068182*
**ENSG00000125354**	*0.06927711*	*0.24382716*	*0.06927711*	*0.24382716*	*0.363095238*		*0.2209302*	*0.2848837*
**ENSG00000188694**		*0.05454545*		*0.054545455*				*0.1511628*
**ENSG00000175189**		*0.25914634*		*0.259146341*				*0.475*

**ENSG00000117016**		*0.46060606*		*0.460606061*				*0.4651163*
**ENSG00000136944**		*0.47272727*		*0.472727273*				*0.3488372*
**ENSG00000116031**								*0.0639535*
**ENSG00000142621**		*0.42727273*		*0.427272727*				*0.4302326*
**ENSG00000121570**		*0.30606061*		*0.306060606*				*0.2732558*
**ENSG00000156384**	*0.4251497*	*0.1*	*0.4251497*	*0.1*	*0.166666667*		*0.2848837*	*0.3409091*
**ENSG00000170262**		*0.13190184*		*0.13190184*				*0.1*
**ENSG00000110484**		*0.14848485*		*0.148484848*				*0.1104651*
**ENSG00000152137**	*0.12874251*	*0.24242424*	*0.12874251*	*0.242424242*				*0.2732558*
**ENSG00000116652**	*0.34444444*		*0.34444444*		*0.466666667*		*0.375*	
**ENSG00000163848**	*0.41916168*	*0.28787879*	*0.41916168*	*0.287878788*	*0.458333333*		*0.4*	*0.4593023*
**ENSG00000187855**	*0.08682635*	*0.06707317*	*0.08682635*	*0.067073171*	*0.077380952*		*0.0988372*	
**ENSG00000181784**		*0.25914634*		*0.259146341*				*0.4186047*
**ENSG00000245317**	*0.33888889*		*0.33888889*					*0.1222222*
**ENSG00000213471**	*0.2245509*		*0.2245509*		*0.380952381*		*0.377907*	
**ENSG00000198373**	*0.2005988*	*0.31212121*	*0.2005988*	*0.312121212*				*0.2439024*
**ENSG00000186086**		*0.17241379*		*0.172413793*				
**ENSG00000197299**		*0.25757576*		*0.257575758*	*0.114457831*		*0.1204819*	*0.1802326*
**ENSG00000204217**		*0.47239264*		*0.472392638*				*0.0764706*
**ENSG00000137145**	*0.36111111*	*0.43333333*	*0.36111111*	*0.433333333*				*0.3197674*
**ENSG00000178645**		*0.5*		*0.5*				*0.4222222*
**ENSG00000102978**					*0.416666667*		*0.494186*	
**ENSG00000180251**	*0.20359281*	*0.3*	*0.20359281*	*0.3*	*0.357142857*		*0.4186047*	*0.4360465*
**ENSG00000137203**	*0.14204545*	*0.16363636*	*0.14204545*	*0.163636364*	*0.233333333*		*0.3068182*	*0.0988372*
**ENSG00000092439**		*0.43030303*		*0.43030303*				*0.4651163*
**ENSG00000125995**		*0.05757576*		*0.057575758*				
**ENSG00000127220**	*0.1257485*	*0.30606061*	*0.1257485*	*0.306060606*	*0.130952381*		*0.125*	
**ENSG00000177189**		*0.15337423*		*0.153374233*				*0.0647059*
**ENSG00000072609**		*0.32941176*		*0.329411765*	*0.077777778*		*0.0681818*	*0.4069767*
**ENSG00000010322**								*0.0697674*
**ENSG00000150471**	*0.45808383*		*0.45808383*		*0.277108434*		*0.3*	
**ENSG00000087460**	*0.4251497*	*0.42121212*	*0.4251497*	*0.421212121*	*0.160714286*		*0.2235294*	*0.4360465*
**ENSG00000167315**		*0.05555556*		*0.055555556*				*0.1444444*
**ENSG00000258691**		*0.11349693*		*0.113496933*				*0.2352941*
**ENSG00000154478**		*0.3*		*0.3*				*0.3604651*
**ENSG00000122741**		*0.15243902*		*0.152439024*				*0.0813953*
**ENSG00000106038**	*0.321875*	*0.11212121*	*0.321875*	*0.112121212*	*0.43452381*		*0.494186*	*0.4277108*
**ENSG00000139405**	*0.09281437*	*0.38181818*	*0.09281437*	*0.381818182*	*0.05952381*		*0.1046512*	*0.4127907*
**ENSG00000113360**		*0.22865854*		*0.228658537*			*0.0595238*	*0.4651163*
**ENSG00000176105**		*0.46296296*		*0.462962963*				*0.4698795*
**ENSG00000158195**		*0.22727273*		*0.227272727*				
**ENSG00000116514**		*0.22121212*		*0.221212121*				*0.2093023*
**ENSG00000223953**	*0.4760479*	*0.18888889*	*0.4760479*	*0.188888889*	*0.244047619*		*0.2352941*	*0.0681818*
**ENSG00000138074**	*0.16766467*	*0.06363636*	*0.16766467*	*0.063636364*	*0.113095238*		*0.0523256*	
**ENSG00000248405**		*0.15454545*		*0.154545455*				*0.3614458*
**ENSG00000196459**		*0.38333333*		*0.383333333*				*0.2613636*
**ENSG00000180279**	*0.15432099*		*0.15432099*		*0.357142857*		*0.4823529*	
**ENSG00000169641**	*0.22754491*	*0.16666667*	*0.22754491*	*0.166666667*	*0.273809524*		*0.244186*	*0.2383721*
**ENSG00000164024**		*0.42424242*		*0.424242424*				*0.1511628*
**ENSG00000135336**	*0.08231707*	*0.43710692*	*0.08231707*	*0.437106918*				*0.3841463*
**ENSG00000198685**	*0.29819277*	*0.37272727*	*0.29819277*	*0.372727273*	*0.321428571*		*0.3488372*	*0.4011628*
**ENSG00000168404**		*0.45402299*		*0.454022989*				*0.2045455*
**ENSG00000156509**	*0.22222222*	*0.49685535*	*0.22222222*	*0.496855346*	*0.066666667*		*0.1477273*	*0.4825581*
**ENSG00000150510**								*0.372093*

**ENSG00000215066**	*0.43072289*	*0.16860465*	*0.43072289*	*0.168604651*	*0.271084337*	*0.2906977*	*0.3414634*
**ENSG00000180530**		*0.35276074*		*0.352760736*			*0.3433735*
**ENSG00000138079**	*0.17365269*	*0.34242424*	*0.17365269*	*0.342424242*	*0.345238095*	*0.4176471*	*0.4069767*
**ENSG00000172456**	*0.36526946*		*0.36526946*		*0.416666667*	*0.494186*	
**ENSG00000143183**	*0.07777778*		*0.07777778*				
**ENSG00000153815**	*0.37222222*	*0.2030303*	*0.37222222*	*0.203030303*	*0.38372093*	*0.4418605*	
**ENSG00000110786**					*0.321428571*	*0.255814*	
**ENSG00000134369**		*0.44848485*		*0.448484848*	*0.288888889*	*0.2267442*	*0.4476744*
**ENSG00000123700**	*0.30555556*		*0.30555556*				
**ENSG00000185608**	*0.47222222*		*0.47222222*		*0.464285714*	*0.439759*	
**ENSG00000127914**		*0.39490446*		*0.394904459*			*0.1704545*
**ENSG00000138801**	*0.17613636*	*0.25925926*	*0.17613636*	*0.259259259*			*0.4476744*
**ENSG00000088726**	*0.34831461*		*0.34831461*		*0.177777778*	*0.1136364*	
**ENSG00000178425**		*0.47777778*		*0.477777778*			*0.377907*
**ENSG00000197694**	*0.46407186*		*0.46407186*		*0.464285714*	*0.4825581*	
**ENSG00000213741**		*0.22777778*		*0.227777778*			*0.1136364*
**ENSG00000171984**		*0.43939394*		*0.439393939*			*0.3023256*
**ENSG00000213578**							*0.1918605*
**ENSG00000101890**	*0.15060241*		*0.15060241*		*0.125*	*0.1428571*	
**ENSG00000145824**		*0.07272727*		*0.072727273*			*0.0882353*
**ENSG00000173335**		*0.35757576*		*0.357575758*			*0.25*
**ENSG00000184305**		*0.46551724*		*0.465517241*			*0.3333333*
**ENSG00000128891**	*0.22155689*	*0.5*	*0.22155689*	*0.5*	*0.101190476*	*0.1162791*	*0.3430233*
**ENSG00000082641**	*0.43820225*		*0.43820225*		*0.174418605*	*0.1888889*	
**ENSG00000111981**							*0.3295455*
**ENSG00000257599**		*0.17073171*		*0.170731707*			*0.0647059*
**ENSG00000129521**		*0.22619048*		*0.226190476*			*0.4069767*
**ENSG00000186732**	*0.48802395*		*0.48802395*		*0.083333333*	*0.1395349*	
**ENSG00000203865**	*0.05688623*		*0.05688623*		*0.285714286*	*0.3255814*	
**ENSG00000258572**		*0.35454545*		*0.354545455*			*0.2848837*
**ENSG00000006007**		*0.25757576*		*0.257575758*			*0.3953488*
**ENSG00000197256**		*0.39090909*		*0.390909091*			*0.3604651*
**ENSG00000069869**		*0.409375*		*0.409375*			*0.3975904*
**ENSG00000151715**		*0.34146341*		*0.341463415*			*0.4418605*
**ENSG00000166068**		*0.25151515*		*0.251515152*			*0.2034884*
**ENSG00000156787**		*0.44242424*		*0.442424242*			*0.4651163*
**ENSG00000177614**							*0.0542169*
**ENSG00000008517**	*0.18484848*		*0.18484848*		*0.071428571*		
**ENSG00000197479**	*0.2994012*		*0.2994012*				
**ENSG00000184277**		*0.46363636*		*0.463636364*			
**ENSG00000072042**		*0.12121212*		*0.121212121*			*0.1918605*
**ENSG00000090266**	*0.26946108*		*0.26946108*		*0.148809524*	*0.0581395*	
**ENSG00000113492**		*0.45757576*		*0.457575758*			*0.4705882*
**ENSG00000168267**		*0.06969697*		*0.06969697*			*0.3411765*
**ENSG00000069206**		*0.34545455*		*0.345454545*			*0.3023256*
**ENSG00000131773**		*0.15151515*		*0.151515152*			
**ENSG00000111412**					*0.273809524*	*0.3837209*	*0.2790698*
**ENSG00000151690**		*0.30745342*		*0.307453416*			*0.3546512*
**ENSG00000008394**		*0.3*		*0.3*			*0.3372093*
**ENSG00000167371**		*0.17272727*		*0.172727273*			*0.0647059*
**ENSG00000139722**	*0.26347305*	*0.11212121*	*0.26347305*	*0.112121212*			*0.2209302*
**ENSG00000128607**		*0.43030303*		*0.43030303*			*0.4593023*
**ENSG00000163539**		*0.43636364*		*0.436363636*			*0.4318182*
**ENSG00000104093**	*0.48484848*	*0.1969697*	*0.48484848*	*0.196969697*	*0.446428571*	*0.4651163*	

**ENSG00000115649**	*0.22754491*	*0.4030303*	*0.22754491*	*0.403030303*	*0.255952381*		*0.25*	*0.25*
**ENSG00000163655**		*0.35555556*		*0.355555556*				*0.3977273*
**ENSG00000188620**		*0.46060606*		*0.460606061*				*0.2093023*
**ENSG00000197343**	*0.30239521*	*0.11585366*	*0.30239521*	*0.115853659*				
**ENSG00000077984**	*0.11377246*	*0.42682927*	*0.11377246*	*0.426829268*	*0.071428571*		*0.127907*	*0.125*
**ENSG00000188163**								*0.0813953*
**ENSG00000154258**		*0.31212121*		*0.312121212*				*0.4117647*
**ENSG00000087245**		*0.42424242*		*0.424242424*				*0.25*
**ENSG00000163915**								*0.1511628*
**ENSG00000133318**		*0.11666667*		*0.116666667*				
**ENSG00000131871**		*0.31515152*		*0.315151515*				*0.255814*
**ENSG00000197119**	*0.21257485*	*0.4695122*	*0.21257485*	*0.469512195*	*0.148809524*		*0.2732558*	*0.2674419*
**ENSG00000251569**	*0.10674157*		*0.10674157*					
**ENSG00000135249**	*0.46067416*		*0.46067416*				*0.1136364*	
**ENSG00000198815**	*0.43888889*	*0.13803681*	*0.43888889*	*0.13803681*				*0.3430233*
**ENSG00000198780**		*0.38125*		*0.38125*				*0.3988095*
**ENSG00000114857**		*0.33030303*		*0.33030303*				*0.4825581*
**ENSG00000132424**		*0.5*		*0.5*				*0.2906977*
**ENSG00000136541**		*0.05454545*		*0.054545455*	*0.06547619*			
**ENSG00000101216**		*0.44848485*		*0.448484848*				*0.4777778*
**ENSG00000253320**		*0.37878788*		*0.378787879*				*0.3571429*
**ENSG00000108352**		*0.33333333*		*0.333333333*				*0.3139535*
**ENSG00000101782**	*0.42777778*		*0.42777778*		*0.255555556*		*0.3181818*	
**ENSG00000103942**		*0.47839506*		*0.478395062*				*0.4767442*
**ENSG00000185024**	*0.5*	*0.33707865*	*0.5*	*0.337078652*	*0.172619048*		*0.1511628*	
**ENSG00000182899**		*0.13190184*		*0.13190184*				*0.0697674*
**ENSG00000139644**	*0.29444444*	*0.07575758*	*0.29444444*	*0.075757576*				*0.0795455*
**ENSG00000152684**	*0.28977273*	*0.40243902*	*0.28977273*	*0.402439024*	*0.244444444*		*0.3068182*	*0.4651163*
**ENSG00000232769**		*0.44207317*		*0.442073171*				*0.0639535*
**ENSG00000146678**		*0.06134969*		*0.061349693*				
**ENSG00000171657**		*0.22727273*		*0.227272727*				*0.1136364*
**ENSG00000157423**		*0.40555556*		*0.405555556*				*0.4127907*
**ENSG00000111961**	*0.23295455*	*0.4030303*	*0.23295455*	*0.403030303*	*0.416666667*		*0.4127907*	*0.372093*
**ENSG00000005981**		*0.48787879*		*0.487878788*				*0.1860465*
**ENSG00000145284**	*0.18263473*	*0.34146341*	*0.18263473*	*0.341463415*				*0.3035714*
**ENSG00000177125**		*0.12727273*		*0.127272727*				*0.3895349*
**ENSG00000163081**	*0.48802395*		*0.48802395*		*0.355421687*		*0.4011628*	
**ENSG00000077616**	*0.23652695*	*0.45555556*	*0.23652695*	*0.455555556*	*0.404761905*		*0.3662791*	*0.3522727*
**ENSG00000089091**	*0.44311377*		*0.44311377*		*0.410714286*		*0.494186*	
**ENSG00000126561**		*0.2*		*0.2*				*0.3837209*
**ENSG00000178795**		*0.32424242*		*0.324242424*				*0.2325581*
**ENSG00000178188**	*0.20658683*		*0.20658683*		*0.166666667*		*0.1337209*	
**ENSG00000124575**		*0.06363636*		*0.063636364*				
**ENSG00000165076**		*0.46060606*		*0.460606061*				*0.4767442*
**ENSG00000203877**	*0.47904192*		*0.47904192*		*0.340909091*		*0.244186*	
**ENSG00000204965**	*0.26111111*	*0.05151515*	*0.26111111*	*0.051515152*	*0.464285714*		*0.4883721*	*0.1162791*
**ENSG00000163810**		*0.47575758*		*0.475757576*				*0.1104651*
**ENSG00000108244**	*0.21556886*		*0.21556886*		*0.458333333*		*0.494186*	
**ENSG00000123545**		*0.30555556*		*0.305555556*				*0.3181818*
**ENSG00000235711**		*0.39329268*		*0.393292683*				*0.375*
**ENSG00000000003**		*0.42121212*		*0.421212121*				*0.2823529*
**ENSG00000247077**		*0.38333333*		*0.383333333*				*0.4*
**ENSG00000213265**	*0.27777778*		*0.27777778*		*0.277777778*		*0.2111111*	
**ENSG00000077044**		*0.32424242*		*0.324242424*				*0.4186047*

**ENSG00000133619**		*0.17878788*		*0.178787879*			*0.1511628*
**ENSG00000065600**	*0.13414634*	*0.35454545*	*0.13414634*	*0.354545455*	*0.081395349*	*0.0909091*	*0.372093*
**ENSG00000196305**	*0.20658683*	*0.15454545*	*0.20658683*	*0.154545455*			*0.1104651*
**ENSG00000092009**		*0.12727273*		*0.127272727*			
**ENSG00000132016**	*0.29041916*	*0.27777778*	*0.29041916*	*0.277777778*	*0.303571429*	*0.3255814*	*0.3255814*
**ENSG00000096384**	*0.12883436*		*0.12883436*		*0.345238095*	*0.2647059*	
**ENSG00000160145**	*0.41111111*	*0.3908046*	*0.41111111*	*0.390804598*	*0.454545455*	*0.4186047*	*0.4418605*
**ENSG00000082805**		*0.42727273*		*0.427272727*			*0.1363636*
**ENSG00000107077**	*0.11363636*	*0.49079755*	*0.11363636*	*0.490797546*	*0.186046512*	*0.2222222*	*0.4534884*
**ENSG00000163958**		*0.18787879*		*0.187878788*			*0.4823529*
**ENSG00000213190**	*0.32386364*	*0.22121212*	*0.32386364*	*0.221212121*	*0.088888889*	*0.0639535*	*0.1395349*
**ENSG00000178409**		*0.37777778*		*0.377777778*			*0.244186*
**ENSG00000165322**		*0.47256098*		*0.472560976*			*0.4588235*
**ENSG00000123064**							*0.2790698*
**ENSG00000158856**	*0.45808383*	*0.21515152*	*0.45808383*	*0.215151515*			
**ENSG00000116138**		*0.22727273*		*0.227272727*			*0.4476744*
**ENSG00000141576**		*0.2804878*		*0.280487805*			*0.1941176*
**ENSG00000140416**	*0.45*	*0.36666667*	*0.45*	*0.366666667*	*0.5*	*0.5*	
**ENSG00000075303**	*0.17977528*		*0.17977528*		*0.133333333*	*0.1744186*	
**ENSG00000168348**		*0.16060606*		*0.160606061*			*0.3953488*
**ENSG00000221890**		*0.42727273*		*0.427272727*			*0.2965116*
**ENSG00000155111**		*0.27575758*		*0.275757576*			*0.3430233*
**ENSG00000138085**	*0.16766467*		*0.16766467*		*0.113095238*	*0.0523256*	
**ENSG00000105699**	*0.32634731*		*0.32634731*		*0.208333333*	*0.127907*	
**ENSG00000119953**		*0.40243902*		*0.402439024*			*0.1569767*
**ENSG00000184515**	*0.25903614*		*0.25903614*				
**ENSG00000139835**							*0.1104651*
**ENSG00000198010**		*0.47701149*		*0.477011494*			*0.4642857*
**ENSG00000100600**		*0.4030303*		*0.403030303*			*0.0872093*
**ENSG00000116667**		*0.29545455*		*0.295454545*			*0.2840909*
**ENSG00000109323**		*0.47575758*		*0.475757576*			*0.4761905*
**ENSG00000082212**		*0.36363636*		*0.363636364*			*0.4709302*
**ENSG00000148600**		*0.45757576*		*0.457575758*			*0.3430233*
**ENSG00000149179**		*0.14545455*		*0.145454545*	*0.409638554*	*0.3139535*	*0.3139535*
**ENSG00000172671**	*0.13554217*	*0.13109756*	*0.13554217*	*0.131097561*	*0.178571429*	*0.2294118*	*0.1104651*
**ENSG00000137270**		*0.15454545*		*0.154545455*			*0.0930233*
**ENSG00000133250**	*0.31626506*	*0.45454545*	*0.31626506*	*0.454545455*	*0.142857143*	*0.1627907*	*0.3837209*
**ENSG00000182628**		*0.44545455*		*0.445454545*			*0.377907*
**ENSG00000160219**		*0.37195122*		*0.37195122*			*0.244186*
**ENSG00000116906**	*0.46706587*		*0.46706587*		*0.385542169*	*0.372093*	
**ENSG00000170802**		*0.31515152*		*0.315151515*			*0.4883721*
**ENSG00000164325**		*0.46060606*		*0.460606061*			*0.2848837*
**ENSG00000170323**		*0.13636364*		*0.136363636*			*0.3023256*
**ENSG00000137103**	*0.17065868*	*0.33333333*	*0.17065868*	*0.333333333*	*0.363095238*	*0.3411765*	*0.1627907*
**ENSG00000168703**		*0.36206897*		*0.362068966*			*0.1136364*
**ENSG00000013364**						*0.0853659*	
**ENSG00000187105**	*0.45180723*		*0.45180723*		*0.108433735*		
**ENSG00000157131**							*0.060241*
**ENSG00000185250**	*0.25301205*	*0.38787879*	*0.25301205*	*0.387878788*	*0.375*	*0.4411765*	*0.4011628*
**ENSG00000155100**							*0.0777778*
**ENSG00000198089**		*0.29393939*		*0.293939394*			*0.3837209*
**ENSG00000183255**		*0.45757576*		*0.457575758*			*0.4476744*
**ENSG00000143390**	*0.05389222*	*0.33333333*	*0.05389222*	*0.333333333*			*0.494186*
**ENSG00000182968**		*0.35060976*		*0.350609756*			*0.4470588*

**ENSG00000124172**		0.31515152		0.315151515				0.2209302
**ENSG00000133026**		0.37878788		0.378787879				0.3294118
**ENSG00000010810**	0.41573034	0.16666667	0.41573034	0.166666667	0.273809524		0.255814	0.3139535
**ENSG00000178695**		0.43292683		0.432926829				0.3529412
**ENSG00000124134**		0.4030303		0.403030303				0.1976744
**ENSG00000163660**	0.05722892		0.05722892					
**ENSG00000109066**	0.48275862	0.29012346	0.48275862	0.290123457	0.3		0.2954545	0.4285714
**ENSG00000110436**		0.37575758		0.375757576	0.05952381		0.0581395	0.4431818
**ENSG00000198271**								0.1470588
**ENSG00000100906**		0.3902439		0.390243902				0.2732558
**ENSG00000186188**		0.32222222		0.322222222				0.1590909
**ENSG00000165556**	0.32035928	0.49090909	0.32035928	0.490909091	0.398809524		0.4360465	0.4418605
**ENSG00000153885**		0.33888889		0.338888889				0.2272727
**ENSG00000166024**	0.08682635	0.34545455	0.08682635	0.345454545	0.321428571		0.2616279	0.4825581
**ENSG00000143344**	0.05389222	0.44848485	0.05389222	0.448484848				0.4882353
**ENSG00000203870**		0.2962963		0.296296296				0.2151163
**ENSG00000168795**		0.07878788		0.078787879				0.494186
**ENSG00000162769**		0.47727273		0.477272727				0.4886364
**ENSG00000092200**		0.32777778		0.327777778				0.3977273
**ENSG00000164458**	0.18562874	0.35757576	0.18562874	0.357575758	0.246987952		0.2732558	0.2732558
**ENSG00000148773**		0.46666667		0.466666667				0.4886364
**ENSG00000124126**		0.47272727		0.472727273				0.4634146
**ENSG00000170423**	0.35493827		0.35493827		0.488095238		0.4883721	
**ENSG00000140386**	0.22777778	0.49390244	0.22777778	0.493902439	0.454545455		0.4204545	0.3953488
**ENSG00000188522**		0.48181818		0.481818182				0.4767442
**ENSG00000134882**		0.31481481		0.314814815				0.4
**ENSG00000147912**	0.14444444	0.16969697	0.14444444	0.16969697				0.1569767
**ENSG00000166747**		0.34545455		0.345454545				0.3117647
**ENSG00000108219**	0.37272727	0.4691358	0.37272727	0.469135802	0.144578313		0.1235294	0.1337209
**ENSG00000132485**		0.48333333		0.483333333				0.2380952
**ENSG00000258171**		0.16666667		0.166666667				0.0930233
**ENSG00000240303**	0.41515152	0.11212121	0.41515152	0.112121212	0.488095238		0.3197674	0.0813953
**ENSG00000152359**	0.16467066	0.07272727	0.16467066	0.072727273	0.482142857		0.4588235	0.3588235
**ENSG00000170464**		0.28353659		0.283536585				0.4186047
**ENSG00000143437**		0.36060606		0.360606061				0.3953488
**ENSG00000020426**		0.09444444		0.094444444				0.3888889
**ENSG00000162994**		0.48181818		0.481818182				0.4534884
**ENSG00000138771**		0.22424242		0.224242424				0.2383721
**ENSG00000178028**		0.3		0.3				0.2674419
**ENSG00000135547**		0.40606061		0.406060606				0.3546512
**ENSG00000205339**		0.46363636		0.463636364				0.3630952
**ENSG00000111726**		0.16363636		0.163636364				0.494186
**ENSG00000127554**		0.25304878		0.25304878				
**ENSG00000131864**		0.48170732		0.481707317				0.2383721
**ENSG00000163517**								0.0609756
**ENSG00000197535**	0.32035928	0.37804878	0.32035928	0.37804878				0.2616279
**ENSG00000178878**		0.39090909		0.390909091				0.3372093
**ENSG00000115607**	0.18484848	0.19090909	0.18484848	0.190909091				0.4117647
**ENSG00000198783**		0.4		0.4				0.2289157
**ENSG00000123384**		0.24242424		0.242424242				0.1627907
**ENSG00000144668**		0.39329268		0.393292683				0.4651163
**ENSG00000164631**		0.14848485		0.148484848				
**ENSG00000138375**					0.198795181		0.3197674	
**ENSG00000173548**		0.3445122		0.344512195				0.4825581

**ENSG00000244482**		*0.25*		*0.25*			*0.4047619*
**ENSG00000130201**		*0.38787879*		*0.387878788*			*0.1569767*
**ENSG00000138430**		*0.45151515*		*0.451515152*			*0.3352941*
**ENSG00000163393**		*0.23033708*		*0.230337079*			*0.2386364*
**ENSG00000143337**		*0.46666667*		*0.466666667*			*0.2705882*
**ENSG00000130720**		*0.44207317*		*0.442073171*			*0.4709302*
**ENSG00000144369**		*0.25*		*0.25*			*0.2380952*
**ENSG00000107611**		*0.32121212*		*0.321212121*			*0.4651163*
**ENSG00000257335**	*0.25*		*0.25*		*0.277777778*	*0.3409091*	
**ENSG00000106341**	*0.17771084*	*0.27777778*	*0.17771084*	*0.277777778*	*0.476190476*	*0.4651163*	*0.2906977*
**ENSG00000186591**	*0.48235294*	*0.15*	*0.48235294*	*0.15*			*0.1590909*
**ENSG00000173757**							*0.0581395*
**ENSG00000213366**	*0.21987952*		*0.21987952*				
**ENSG00000181019**	*0.21666667*	*0.09509202*	*0.21666667*	*0.095092025*			*0.3529412*
**ENSG00000143498**	*0.41616766*		*0.41616766*		*0.113095238*	*0.122093*	
**ENSG00000198561**	*0.40718563*	*0.3*	*0.40718563*	*0.3*	*0.113095238*	*0.1046512*	*0.0930233*
**ENSG00000119242**	*0.42215569*	*0.37878788*	*0.42215569*	*0.378787879*	*0.101190476*	*0.1337209*	*0.2034884*
**ENSG00000145321**	*0.26347305*	*0.42378049*	*0.26347305*	*0.423780488*	*0.379518072*	*0.3313953*	*0.244186*
**ENSG00000176007**	*0.28378378*		*0.28378378*		*0.407894737*	*0.3780488*	*0.3176471*
**ENSG00000117322**	*0.07692308*	*0.19393939*	*0.07692308*	*0.193939394*	*0.071428571*	*0.1097561*	
**ENSG00000101665**							*0.1744186*
**ENSG00000198743**		*0.34146341*		*0.341463415*			*0.4651163*
**ENSG00000122545**		*0.37195122*		*0.37195122*			*0.4411765*
**ENSG00000183770**							*0.0813953*
**ENSG00000110696**	*0.13636364*	*0.44785276*	*0.13636364*	*0.447852761*	*0.188888889*	*0.0909091*	*0.0523256*
**ENSG00000179348**	*0.44186047*	*0.21472393*	*0.44186047*	*0.214723926*	*0.188888889*	*0.1704545*	*0.0783133*
**ENSG00000006074**		*0.36666667*		*0.366666667*			*0.3255814*
**ENSG00000126012**							*0.3197674*
**ENSG00000109689**		*0.0872093*		*0.087209302*			*0.3*
**ENSG00000214376**		*0.13888889*		*0.138888889*			*0.4767442*
**ENSG00000189337**		*0.43939394*		*0.439393939*			*0.2616279*
**ENSG00000091651**					*0.06547619*		
**ENSG00000168092**		*0.15454545*		*0.154545455*			*0.494186*
**ENSG00000196136**	*0.45508982*		*0.45508982*		*0.333333333*	*0.4244186*	
**ENSG00000163995**		*0.26060606*		*0.260606061*			*0.4772727*
**ENSG00000054118**		*0.07012195*		*0.070121951*			
**ENSG00000128272**	*0.0748503*		*0.0748503*				
**ENSG00000187955**		*0.43636364*		*0.436363636*			*0.372093*
**ENSG00000163098**	*0.16060606*		*0.16060606*		*0.307228916*	*0.3392857*	
**ENSG00000134954**		*0.49390244*		*0.493902439*			*0.4709302*
**ENSG00000017427**		*0.41818182*		*0.418181818*			*0.494186*
**ENSG00000173040**		*0.27575758*		*0.275757576*			*0.4593023*
**ENSG00000160208**		*0.41104294*		*0.411042945*			*0.1686047*
**ENSG00000075089**					*0.055555556*		
**ENSG00000074695**		*0.27439024*		*0.274390244*			*0.4709302*
**ENSG00000141574**		*0.49350649*		*0.493506494*			
**ENSG00000205835**		*0.3597561*		*0.359756098*			*0.4117647*
**ENSG00000180871**	*0.12349398*	*0.46969697*	*0.12349398*	*0.46969697*	*0.357142857*	*0.3139535*	*0.3139535*
**ENSG00000104237**	*0.09444444*		*0.09444444*				
**ENSG00000153822**		*0.09090909*		*0.090909091*			
**ENSG00000049192**		*0.08787879*		*0.087878788*			*0.0639535*
**ENSG00000213799**		*0.08988764*		*0.08988764*			*0.0681818*
**ENSG00000184897**	*0.29411765*	*0.10465116*	*0.29411765*	*0.104651163*			
**ENSG00000214114**	*0.11676647*	*0.12121212*	*0.11676647*	*0.121212121*	*0.303571429*	*0.244186*	*0.3488372*

**ENSG00000257315**	*0.41916168*		*0.41916168*		*0.321428571*	*0.3662791*	
**ENSG00000115216**	*0.25555556*		*0.25555556*		*0.144444444*	*0.0930233*	
**ENSG00000225885**							*0.4545455*
**ENSG00000134824**	*0.31437126*	*0.34242424*	*0.31437126*	*0.342424242*			*0.3081395*
**ENSG00000166192**		*0.22121212*		*0.221212121*			*0.3546512*
**ENSG00000170624**	*0.18965517*	*0.41818182*	*0.18965517*	*0.418181818*	*0.193181818*	*0.1555556*	*0.1904762*
**ENSG00000157211**	*0.24850299*		*0.24850299*		*0.303571429*	*0.3488372*	
**ENSG00000172752**	*0.40555556*		*0.40555556*		*0.333333333*	*0.1704545*	
**ENSG00000113522**	*0.24550898*		*0.24550898*				
**ENSG00000114544**		*0.14444444*		*0.144444444*			*0.1666667*
**ENSG00000160781**	*0.12874251*	*0.31515152*	*0.12874251*	*0.315151515*	*0.18452381*	*0.372093*	*0.255814*
**ENSG00000124557**		*0.48181818*		*0.481818182*			*0.2891566*
**ENSG00000163285**		*0.49085366*		*0.490853659*			*0.2590361*
**ENSG00000114166**		*0.2030303*		*0.203030303*			*0.4294118*
**ENSG00000115520**		*0.17682927*		*0.176829268*			*0.172619*
**ENSG00000198604**		*0.15151515*		*0.151515152*			*0.1860465*
**ENSG00000198915**		*0.16770186*		*0.167701863*	*0.444444444*	*0.4777778*	*0.4166667*
**ENSG00000137814**		*0.24233129*		*0.242331288*			*0.2857143*
**ENSG00000085382**		*0.11515152*		*0.115151515*			
**ENSG00000162729**		*0.15454545*		*0.154545455*			*0.0523256*
**ENSG00000112992**		*0.19393939*		*0.193939394*	*0.06547619*	*0.1395349*	*0.1588235*
**ENSG00000110583**	*0.08682635*	*0.44848485*	*0.08682635*	*0.448484848*	*0.05625*	*0.0882353*	*0.1411765*
**ENSG00000119138**	*0.08982036*	*0.43333333*	*0.08982036*	*0.433333333*	*0.154761905*	*0.1744186*	*0.2151163*
**ENSG00000158481**	*0.1497006*	*0.11818182*	*0.1497006*	*0.118181818*			*0.3081395*
**ENSG00000137745**		*0.06060606*		*0.060606061*			
**ENSG00000167332**		*0.13218391*		*0.132183908*			*0.2045455*
**ENSG00000139597**		*0.1097561*		*0.109756098*			*0.4534884*
**ENSG00000064763**		*0.45757576*		*0.457575758*			*0.3837209*
**ENSG00000196357**	*0.19760479*	*0.07777778*	*0.19760479*	*0.077777778*			
**ENSG00000251349**		*0.05*		*0.05*			*0.1704545*
**ENSG00000063046**	*0.45508982*	*0.12804878*	*0.45508982*	*0.12804878*	*0.19047619*	*0.2325581*	*0.3647059*
**ENSG00000151364**		*0.15757576*		*0.157575758*			*0.4418605*
**ENSG00000131127**	*0.45209581*	*0.41111111*	*0.45209581*	*0.411111111*	*0.411111111*	*0.3522727*	*0.3292683*
**ENSG00000106003**		*0.06060606*		*0.060606061*			
**ENSG00000139567**		*0.2962963*		*0.296296296*			*0.3076923*
**ENSG00000168779**		*0.36666667*		*0.366666667*			*0.494186*
**ENSG00000143870**	*0.39520958*	*0.06111111*	*0.39520958*	*0.061111111*	*0.178571429*	*0.1860465*	
**ENSG00000129250**		*0.17777778*		*0.177777778*			*0.4333333*
**ENSG00000141027**		*0.4969697*		*0.496969697*			*0.1976744*
**ENSG00000138496**		*0.43333333*		*0.433333333*			*0.3488372*
**ENSG00000126822**	*0.09146341*	*0.10606061*	*0.09146341*	*0.106060606*			
**ENSG00000198125**		*0.10060976*		*0.100609756*	*0.071428571*	*0.1*	*0.0639535*
**ENSG00000148019**		*0.5*		*0.5*			*0.3139535*
**ENSG00000221837**		*0.25568182*		*0.255681818*			*0.4888889*
**ENSG00000171533**		*0.40853659*		*0.408536585*			*0.4117647*
**ENSG00000112530**		*0.47560976*		*0.475609756*	*0.44047619*	*0.4588235*	*0.2764706*
**ENSG00000177694**		*0.30487805*		*0.304878049*			*0.304878*
**ENSG00000177946**	*0.18263473*		*0.18263473*				
**ENSG00000175600**		*0.43939394*		*0.439393939*			*0.2209302*
**ENSG00000100360**	*0.40718563*		*0.40718563*				
**ENSG00000174928**		*0.38181818*		*0.381818182*			*0.3214286*
**ENSG00000122862**		*0.08231707*		*0.082317073*			*0.4825581*
**ENSG00000101294**		*0.14848485*		*0.148484848*			
**ENSG00000153391**		*0.31212121*		*0.312121212*			

**ENSG00000139679**		*0.09444444*		*0.094444444*	*0.083333333*		*0.0755814*	
**ENSG00000187416**		*0.37878788*		*0.378787879*				*0.4593023*
**ENSG00000184486**		*0.32424242*		*0.324242424*				*0.2093023*
**ENSG00000164187**	*0.5*	*0.41212121*	*0.5*	*0.412121212*	*0.210843373*		*0.2383721*	*0.2383721*
**ENSG00000163832**	*0.22413793*	*0.08888889*	*0.22413793*	*0.088888889*	*0.238095238*		*0.2380952*	
**ENSG00000159217**		*0.47878788*		*0.478787879*				*0.2093023*
**ENSG00000154553**		*0.1*		*0.1*				*0.172619*
**ENSG00000005249**		*0.17777778*		*0.177777778*				
**ENSG00000170608**	*0.10479042*		*0.10479042*		*0.095238095*		*0.1162791*	
**ENSG00000101307**		*0.38343558*		*0.383435583*				
**ENSG00000214530**	*0.48888889*		*0.48888889*		*0.344444444*		*0.3662791*	
**ENSG00000136492**		*0.41463415*		*0.414634146*				*0.1569767*
**ENSG00000069702**	*0.12874251*	*0.44848485*	*0.12874251*	*0.448484848*	*0.422619048*		*0.4127907*	*0.4476744*
**ENSG00000197889**		*0.29444444*		*0.294444444*				*0.4*
**ENSG00000068028**		*0.15243902*		*0.152439024*	*0.071428571*		*0.1011905*	*0.1011905*
**ENSG00000146701**		*0.30606061*		*0.306060606*				*0.3197674*
**ENSG00000169139**								*0.1395349*
**ENSG00000104313**		*0.26666667*		*0.266666667*	*0.488095238*		*0.4642857*	*0.4642857*
**ENSG00000116299**		*0.21818182*		*0.218181818*				*0.0639535*
**ENSG00000168878**		*0.10365854*		*0.103658537*				*0.244186*
**ENSG00000205250**		*0.06363636*		*0.063636364*				
**ENSG00000160688**	*0.44311377*		*0.44311377*					
**ENSG00000154518**	*0.43712575*	*0.15555556*	*0.43712575*	*0.155555556*	*0.470238095*		*0.4011628*	*0.1931818*
**ENSG00000153975**		*0.30606061*		*0.306060606*				*0.4011628*
**ENSG00000110321**	*0.07865169*		*0.07865169*					
**ENSG00000181555**		*0.48787879*		*0.487878788*				*0.4294118*
**ENSG00000144815**	*0.28443114*	*0.47368421*	*0.28443114*	*0.473684211*	*0.422222222*		*0.4886364*	*0.494186*
**ENSG00000178562**								*0.494186*
**ENSG00000135540**	*0.44277108*		*0.44277108*					
**ENSG00000153094**		*0.46111111*		*0.461111111*				*0.4176471*
**ENSG00000078070**	*0.25*	*0.34146341*	*0.25*	*0.341463415*	*0.282051282*		*0.3636364*	*0.3392857*
**ENSG00000074603**	*0.19161677*	*0.38181818*	*0.19161677*	*0.381818182*	*0.267857143*		*0.3604651*	*0.3255814*
**ENSG00000162396**		*0.16969697*		*0.16969697*				*0.4767442*
**ENSG00000024422**	*0.25*	*0.12121212*	*0.25*	*0.121212121*				
**ENSG00000132518**		*0.49393939*		*0.493939394*				*0.0813953*
**ENSG00000080345**	*0.41317365*	*0.33707865*	*0.41317365*	*0.337078652*	*0.43452381*		*0.4764706*	*0.3777778*
**ENSG00000090530**	*0.23053892*		*0.23053892*		*0.476190476*		*0.3895349*	
**ENSG00000006118**		*0.42424242*		*0.424242424*				*0.4069767*
**ENSG00000124243**		*0.27222222*		*0.272222222*				*0.4333333*
**ENSG00000134852**	*0.18562874*	*0.37272727*	*0.18562874*	*0.372727273*	*0.357142857*		*0.4360465*	*0.4294118*
**ENSG00000189091**		*0.3969697*		*0.396969697*				*0.4772727*
**ENSG00000183092**	*0.32634731*		*0.32634731*		*0.345238095*		*0.3895349*	
**ENSG00000137502**	*0.06707317*	*0.5*	*0.06707317*	*0.5*	*0.113095238*		*0.1337209*	*0.3235294*
**ENSG00000106268**		*0.17272727*		*0.172727273*	*0.107142857*			
**ENSG00000143297**		*0.35454545*		*0.354545455*				*0.375*
**ENSG00000137960**		*0.21341463*		*0.213414634*				*0.25*
**ENSG00000087266**	*0.24418605*	*0.5*	*0.24418605*	*0.5*			*0.0795455*	*0.4772727*
**ENSG00000152104**		*0.18787879*		*0.187878788*				*0.4069767*
**ENSG00000136267**		*0.36363636*		*0.363636364*				*0.4651163*
**ENSG00000176890**		*0.27743902*		*0.277439024*				*0.3430233*
**ENSG00000005102**	*0.34302326*	*0.18333333*	*0.34302326*	*0.183333333*				*0.2272727*
**ENSG00000196867**		*0.2804878*		*0.280487805*				*0.2732558*
**ENSG00000167302**		*0.43373494*		*0.43373494*				*0.4*
**ENSG00000170325**		*0.41818182*		*0.418181818*				*0.4883721*

**ENSG00000179213**	*0.17065868*		*0.17065868*		*0.494047619*	*0.3895349*	
**ENSG00000197892**		*0.35365854*		*0.353658537*			*0.4333333*
**ENSG00000173464**	*0.4011976*	*0.34242424*	*0.4011976*	*0.342424242*	*0.363095238*	*0.3895349*	*0.4476744*
**ENSG00000044459**		*0.22777778*		*0.227777778*			*0.1823529*
**ENSG00000221852**		*0.3030303*		*0.303030303*			*0.2965116*
**ENSG00000159314**		*0.40606061*		*0.406060606*			*0.4302326*
**ENSG00000147853**	*0.34659091*	*0.47777778*	*0.34659091*	*0.477777778*	*0.311111111*	*0.3139535*	*0.2954545*
**ENSG00000139112**	*0.35329341*	*0.20606061*	*0.35329341*	*0.206060606*	*0.444444444*	*0.4777778*	*0.4777778*
**ENSG00000119318**		*0.10670732*		*0.106707317*		*0.0666667*	
**ENSG00000120696**		*0.1030303*		*0.103030303*	*0.4*	*0.5*	
**ENSG00000079102**		*0.48314607*		*0.483146067*			*0.1918605*
**ENSG00000237541**	*0.31578947*	*0.47878788*	*0.31578947*	*0.478787879*			*0.3255814*
**ENSG00000127920**	*0.37735849*		*0.37735849*		*0.226190476*	*0.2209302*	
**ENSG00000079691**	*0.46407186*		*0.46407186*		*0.357142857*	*0.2965116*	
**ENSG00000117983**	*0.40555556*		*0.40555556*		*0.411111111*	*0.4819277*	
**ENSG00000128829**		*0.15151515*		*0.151515152*			*0.3953488*
**ENSG00000161040**		*0.3*		*0.3*			*0.445122*
**ENSG00000136270**		*0.25786164*		*0.257861635*			*0.1686747*
**ENSG00000170899**		*0.48888889*		*0.488888889*			*0.2352941*
**ENSG00000034677**	*0.08888889*		*0.08888889*				
**ENSG00000006744**		*0.40243902*		*0.402439024*			*0.4186047*
**ENSG00000169136**	*0.13772455*	*0.37878788*	*0.13772455*	*0.378787879*			*0.4390244*
**ENSG00000166793**	*0.23952096*		*0.23952096*				
**ENSG00000120549**	*0.31927711*		*0.31927711*		*0.233333333*	*0.1333333*	
**ENSG00000092098**	*0.42215569*		*0.42215569*		*0.439759036*	*0.4244186*	*0.2*
**ENSG00000118997**	*0.15243902*		*0.15243902*				
**ENSG00000213988**							*0.4204545*
**ENSG00000150961**	*0.25449102*	*0.4030303*	*0.25449102*	*0.403030303*	*0.476190476*	*0.4593023*	*0.4593023*
**ENSG00000153767**	*0.20658683*	*0.41111111*	*0.20658683*	*0.411111111*	*0.446428571*	*0.4825581*	*0.1931818*
**ENSG00000138031**	*0.2754491*	*0.10493827*	*0.2754491*	*0.104938272*	*0.261904762*	*0.2906977*	
**ENSG00000175387**		*0.49390244*		*0.493902439*			*0.3522727*
**ENSG00000116678**	*0.16566265*	*0.3404908*	*0.16566265*	*0.340490798*	*0.19375*	*0.2256098*	*0.2674419*
**ENSG00000166275**		*0.34545455*		*0.345454545*			*0.4593023*
**ENSG00000172482**		*0.5*		*0.5*			*0.255814*
**ENSG00000242372**	*0.05988024*	*0.2625*	*0.05988024*	*0.2625*		*0.0872093*	*0.1410256*
**ENSG00000231738**	*0.13473054*		*0.13473054*		*0.375*	*0.3372093*	
**ENSG00000165972**	*0.14204545*	*0.36363636*	*0.14204545*	*0.363636364*	*0.111111111*	*0.0681818*	*0.2383721*
**ENSG00000176087**	*0.47305389*	*0.25304878*	*0.47305389*	*0.25304878*	*0.142857143*	*0.2209302*	*0.2209302*
**ENSG00000197046**		*0.16666667*		*0.166666667*			*0.3977273*
**ENSG00000149927**		*0.12777778*		*0.127777778*			
**ENSG00000253857**	*0.06321839*	*0.25454545*	*0.06321839*	*0.254545455*	*0.1*		*0.2732558*
**ENSG00000111245**	*0.15568862*		*0.15568862*				
**ENSG00000129083**	*0.21590909*		*0.21590909*		*0.284090909*	*0.3522727*	
**ENSG00000108963**		*0.16363636*		*0.163636364*			
**ENSG00000101230**		*0.13414634*		*0.134146341*			*0.255814*
**ENSG00000172939**		*0.34242424*		*0.342424242*			*0.244186*
**ENSG00000124784**	*0.46407186*		*0.46407186*		*0.488095238*	*0.4772727*	
**ENSG00000167914**	*0.44011976*	*0.16969697*	*0.44011976*	*0.16969697*	*0.482142857*	*0.4593023*	*0.127907*
**ENSG00000182674**	*0.42215569*		*0.42215569*		*0.410714286*	*0.3081395*	
**ENSG00000137558**		*0.44207317*		*0.442073171*			
**ENSG00000184182**	*0.22289157*	*0.37878788*	*0.22289157*	*0.378787879*	*0.410714286*	*0.4418605*	*0.2034884*
**ENSG00000203786**		*0.11515152*		*0.115151515*			*0.2848837*
**ENSG00000198721**	*0.15568862*	*0.37575758*	*0.15568862*	*0.375757576*			*0.2771084*
**ENSG00000003147**	*0.25748503*	*0.15853659*	*0.25748503*	*0.158536585*	*0.428571429*	*0.4647059*	*0.2034884*

**ENSG00000107957**		0.43636364		0.436363636			0.494186
**ENSG00000160345**	0.37724551		0.37724551		0.172619048		0.1569767	
**ENSG00000137411**	0.11377246		0.11377246		0.291666667		0.2034884	
**ENSG00000159958**		0.24545455		0.245454545				0.1162791
**ENSG00000234224**		0.25555556		0.255555556				
**ENSG00000138399**	0.07777778	0.08181818	0.07777778	0.081818182	0.196428571		0.2764706	0.255814
**ENSG00000104976**		0.27272727		0.272727273				0.4360465
**ENSG00000112276**		0.13939394		0.139393939				0.1511628
**ENSG00000165388**		0.22121212		0.221212121				0.2209302
**ENSG00000136643**	0.13125		0.13125		0.409090909		0.3444444	
**ENSG00000106069**	0.42814371	0.3	0.42814371	0.3	0.404761905		0.372093	0.3470588
**ENSG00000117298**		0.13939394		0.139393939				0.2613636
**ENSG00000178031**		0.42424242		0.424242424				0.3372093
**ENSG00000188897**	0.13473054		0.13473054		0.452380952		0.4709302	
**ENSG00000169188**	0.06287425	0.24848485	0.06287425	0.248484848				0.4705882
**ENSG00000153936**	0.49090909	0.34242424	0.49090909	0.342424242	0.054216867			0.195122
**ENSG00000114670**		0.26363636		0.263636364				0.4360465
**ENSG00000171148**		0.16666667		0.166666667				0.4886364
**ENSG00000163191**	0.11445783		0.11445783					
**ENSG00000176532**	0.35454545	0.18181818	0.35454545	0.181818182	0.494047619		0.4709302	
**ENSG00000196584**	0.09444444		0.09444444		0.154761905			0.2
**ENSG00000166866**		0.07272727		0.072727273	0.122222222		0.0697674	0.0988372
**ENSG00000134249**	0.41666667		0.41666667					
**ENSG00000023902**					0.056818182		0.0909091	
**ENSG00000205581**	0.47191011		0.47191011		0.233333333		0.1590909	
**ENSG00000101546**		0.37272727		0.372727273				0.4318182
**ENSG00000110844**		0.1091954		0.109195402				0.0697674
**ENSG00000140750**		0.09393939		0.093939394				
**ENSG00000162999**		0.38181818		0.381818182				0.1104651
**ENSG00000070718**	0.45481928	0.43636364	0.45481928	0.436363636	0.470238095		0.4534884	0.4534884
**ENSG00000249868**	0.23053892		0.23053892		0.267857143		0.2790698	
**ENSG00000149054**	0.33641975	0.40993789	0.33641975	0.409937888	0.482142857		0.4883721	0.3662791
**ENSG00000188175**		0.27777778		0.277777778				0.3409091
**ENSG00000112576**		0.31212121		0.312121212	0.446428571		0.4	0.2383721
**ENSG00000125869**	0.35329341		0.35329341		0.166666667		0.1686047	
**ENSG00000173567**		0.35057471		0.350574713				0.2045455
**ENSG00000108878**		0.47575758		0.475757576				0.1802326
**ENSG00000150556**	0.35628743		0.35628743		0.126506024		0.2267442	
**ENSG00000075702**		0.1969697		0.196969697				0.1918605
**ENSG00000188997**		0.47878788		0.478787879				0.4825581
**ENSG00000127366**	0.20958084		0.20958084		0.31547619		0.2848837	
**ENSG00000122678**		0.21515152		0.215151515				0.1104651
**ENSG00000185049**		0.14606742		0.146067416				0.4069767
**ENSG00000005893**		0.44785276		0.447852761				0.2882353
**ENSG00000160886**	0.31666667	0.43333333	0.31666667	0.433333333	0.444444444		0.3636364	0.3139535
**ENSG00000143514**		0.15454545		0.154545455				0.2916667
**ENSG00000143001**	0.34444444	0.16363636	0.34444444	0.163636364	0.5		0.4318182	0.1976744
**ENSG00000124459**		0.44848485		0.448484848				0.1931818
**ENSG00000159479**		0.43258427		0.43258427				0.1363636
**ENSG00000102977**	0.33532934		0.33532934					
**ENSG00000118640**		0.44545455		0.445454545	0.055555556			0.4069767
**ENSG00000178235**								0.0755814
**ENSG00000092969**		0.2125		0.2125				0.3170732
**ENSG00000188474**	0.0625	0.05487805	0.0625	0.054878049				

**ENSG00000108946**	*0.30538922*	*0.26666667*	*0.30538922*	*0.266666667*				*0.4886364*
**ENSG00000159708**	*0.29444444*		*0.29444444*					
**ENSG00000043039**	*0.44610778*	*0.34259259*	*0.44610778*	*0.342592593*	*0.482142857*		*0.4534884*	
**ENSG00000125657**		*0.45151515*		*0.451515152*				*0.3488372*
**ENSG00000061794**		*0.15151515*		*0.151515152*				*0.1918605*
**ENSG00000227124**	*0.15730337*	*0.36969697*	*0.15730337*	*0.36969697*	*0.154761905*		*0.0888889*	*0.5*
**ENSG00000011485**		*0.43333333*		*0.433333333*	*0.44047619*		*0.2790698*	*0.3705882*
**ENSG00000128534**	*0.27245509*	*0.32727273*	*0.27245509*	*0.327272727*	*0.297619048*		*0.1785714*	*0.1569767*
**ENSG00000111837**		*0.16363636*		*0.163636364*				*0.0639535*
**ENSG00000244623**		*0.43865031*		*0.438650307*				*0.2616279*
**ENSG00000162888**								*0.375*
**ENSG00000171097**	*0.07272727*	*0.18787879*	*0.07272727*	*0.187878788*			*0.0705882*	*0.2093023*
**ENSG00000188223**		*0.1125*		*0.1125*				*0.244186*
**ENSG00000139973**	*0.25555556*	*0.06969697*	*0.25555556*	*0.06969697*	*0.166666667*		*0.1136364*	*0.0639535*
**ENSG00000176678**		*0.06969697*		*0.06969697*				
**ENSG00000104804**	*0.47005988*		*0.47005988*		*0.170731707*		*0.3809524*	
**ENSG00000184304**	*0.35555556*	*0.46060606*	*0.35555556*	*0.460606061*	*0.077777778*		*0.0555556*	*0.2440476*
**ENSG00000169684**		*0.36060606*		*0.360606061*				*0.1511628*
**ENSG00000130176**	*0.28742515*		*0.28742515*		*0.130952381*		*0.0813953*	
**ENSG00000167986**	*0.23214286*		*0.23214286*					
**ENSG00000139155**		*0.47272727*		*0.472727273*				*0.3522727*
**ENSG00000163682**	*0.43181818*		*0.43181818*		*0.1*		*0.0568182*	
**ENSG00000100325**		*0.48484848*		*0.484848485*				*0.4767442*
**ENSG00000165949**	*0.48802395*		*0.48802395*		*0.458333333*		*0.4302326*	*0.4069767*
**ENSG00000089818**		*0.278125*		*0.278125*				*0.4464286*
**ENSG00000137727**	*0.44610778*	*0.45757576*	*0.44610778*	*0.457575758*	*0.279761905*		*0.1453488*	*0.4360465*
**ENSG00000070748**	*0.38719512*		*0.38719512*		*0.321428571*		*0.4825581*	
**ENSG00000148082**	*0.1746988*		*0.1746988*					
**ENSG00000160131**		*0.47239264*		*0.472392638*				*0.0666667*
**ENSG00000119004**		*0.48148148*		*0.481481481*				*0.2906977*
**ENSG00000206561**		*0.40606061*		*0.406060606*				*0.4883721*
**ENSG00000172115**		*0.26404494*		*0.264044944*				*0.4709302*
**ENSG00000213694**	*0.32934132*	*0.23333333*	*0.32934132*	*0.233333333*	*0.18452381*		*0.2093023*	*0.0930233*
**ENSG00000198682**		*0.29090909*		*0.290909091*				*0.1445783*
**ENSG00000167384**		*0.07575758*		*0.075757576*				*0.3895349*
**ENSG00000124496**	*0.24850299*	*0.33333333*	*0.24850299*	*0.333333333*				*0.1802326*
**ENSG00000176396**		*0.32317073*		*0.323170732*				*0.1569767*
**ENSG00000122223**	*0.28742515*	*0.47575758*	*0.28742515*	*0.475757576*	*0.357142857*		*0.3372093*	*0.3888889*
**ENSG00000184900**		*0.11212121*		*0.112121212*				
**ENSG00000099256**		*0.37272727*		*0.372727273*				*0.1764706*
**ENSG00000205542**	*0.0813253*		*0.0813253*					
**ENSG00000133858**	*0.11976048*		*0.11976048*					
**ENSG00000081189**		*0.41463415*		*0.414634146*				*0.494186*
**ENSG00000131183**		*0.37878788*		*0.378787879*				*0.255814*
**ENSG00000154144**	*0.27743902*	*0.09090909*	*0.27743902*	*0.090909091*				*0.1627907*
**ENSG00000185551**		*0.19090909*		*0.190909091*				*0.255814*
**ENSG00000138018**		*0.47878788*		*0.478787879*				*0.372093*
**ENSG00000111897**		*0.45341615*		*0.453416149*				*0.439759*
**ENSG00000101292**	*0.22590361*		*0.22590361*		*0.19047619*		*0.0872093*	*0.4418605*
**ENSG00000156273**		*0.48787879*		*0.487878788*				*0.4534884*
**ENSG00000167207**	*0.14156627*	*0.3908046*	*0.14156627*	*0.390804598*				*0.2261905*
**ENSG00000151553**		*0.44242424*		*0.442424242*				*0.25*
**ENSG00000101040**		*0.39444444*		*0.394444444*				*0.2159091*
**ENSG00000198556**		*0.125*		*0.125*				*0.3294118*

**ENSG00000144182**	*0.46706587*		*0.46706587*		*0.10625*		*0.1235294*	
**ENSG00000182512**	*0.34638554*	*0.38787879*	*0.34638554*	*0.387878788*	*0.457831325*		*0.4883721*	*0.3372093*
**ENSG00000197442**	*0.2962963*		*0.2962963*		*0.409638554*		*0.4761905*	
**ENSG00000197020**		*0.45555556*		*0.455555556*				*0.4090909*
**ENSG00000188822**		*0.4*		*0.4*				*0.3895349*
**ENSG00000054690**	*0.43072289*	*0.37575758*	*0.43072289*	*0.375757576*	*0.44047619*		*0.4345238*	*0.4709302*
**ENSG00000170006**		*0.05757576*		*0.057575758*				
**ENSG00000130520**		*0.07878788*		*0.078787879*				*0.0581395*
**ENSG00000138180**		*0.24848485*		*0.248484848*				*0.3414634*
**ENSG00000163430**		*0.44848485*		*0.448484848*				*0.3941176*
**ENSG00000147130**		*0.06321839*		*0.063218391*				*0.0909091*
**ENSG00000154655**		*0.5*		*0.5*				*0.4186047*
**ENSG00000163145**		*0.40797546*		*0.40797546*				*0.4111111*
**ENSG00000197863**	*0.21666667*		*0.21666667*					
**ENSG00000213551**	*0.40963855*	*0.05*	*0.40963855*	*0.05*	*0.404761905*		*0.4069767*	*0.3068182*
**ENSG00000136111**	*0.28888889*	*0.15625*	*0.28888889*	*0.15625*	*0.211111111*		*0.3181818*	*0.2560976*
**ENSG00000147402**		*0.48780488*		*0.487804878*				*0.3139535*
**ENSG00000180185**		*0.16969697*		*0.16969697*				*0.1976744*
**ENSG00000250120**		*0.05151515*		*0.051515152*				*0.1162791*
**ENSG00000188735**		*0.36363636*		*0.363636364*				*0.4883721*
**ENSG00000134444**								*0.2*
**ENSG00000068885**		*0.05*		*0.05*	*0.05952381*			
**ENSG00000155970**		*0.45348837*		*0.453488372*				*0.4444444*
**ENSG00000118096**					*0.488095238*		*0.4360465*	
**ENSG00000155307**	*0.45398773*	*0.0969697*	*0.45398773*	*0.096969697*	*0.446428571*		*0.4823529*	*0.1686047*
**ENSG00000182287**		*0.43888889*		*0.438888889*				*0.375*
**ENSG00000226593**	*0.26404494*		*0.26404494*				*0.0681818*	
**ENSG00000165934**		*0.2*		*0.2*	*0.369047619*		*0.3546512*	*0.3470588*
**ENSG00000131507**		*0.37777778*		*0.377777778*				*0.3409091*
**ENSG00000162746**	*0.36666667*	*0.05757576*	*0.36666667*	*0.057575758*	*0.494047619*		*0.3604651*	*0.2325581*
**ENSG00000128045**		*0.25454545*		*0.254545455*				*0.25*
**ENSG00000166128**		*0.109375*		*0.109375*				*0.4759036*
**ENSG00000104343**		*0.22424242*		*0.224242424*				*0.1046512*
**ENSG00000125912**		*0.43030303*		*0.43030303*				*0.4473684*
**ENSG00000169877**		*0.0969697*		*0.096969697*				*0.0930233*
**ENSG00000050748**		*0.48787879*		*0.487878788*				*0.2732558*
**ENSG00000198586**		*0.26060606*		*0.260606061*				*0.4090909*
**ENSG00000196187**	*0.20658683*	*0.35057471*	*0.20658683*	*0.350574713*	*0.06626506*		*0.1294118*	*0.2954545*
**ENSG00000137834**	*0.17964072*		*0.17964072*		*0.232142857*		*0.1860465*	*0.1931818*
**ENSG00000168658**	*0.24251497*	*0.5*	*0.24251497*	*0.5*	*0.130952381*		*0.1627907*	*0.244186*
**ENSG00000164929**		*0.20731707*		*0.207317073*				*0.0639535*
**ENSG00000153048**		*0.4030303*		*0.403030303*				*0.5*
**ENSG00000116726**		*0.18484848*		*0.184848485*				*0.3081395*
**ENSG00000170290**	*0.29518072*		*0.29518072*		*0.321428571*		*0.3895349*	
**ENSG00000148842**		*0.35227273*		*0.352272727*				*0.1022727*
**ENSG00000179071**		*0.4068323*		*0.406832298*	*0.066666667*		*0.1627907*	*0.4518072*
**ENSG00000164411**		*0.45731707*		*0.457317073*				*0.4883721*
**ENSG00000163795**		*0.40606061*		*0.406060606*				*0.0988372*
**ENSG00000105072**	*0.22727273*	*0.34242424*	*0.22727273*	*0.342424242*	*0.288888889*		*0.2674419*	*0.1104651*
**ENSG00000175267**	*0.43333333*	*0.24846626*	*0.43333333*	*0.248466258*	*0.384146341*		*0.4883721*	*0.1529412*
**ENSG00000181609**								*0.127907*
**ENSG00000197150**		*0.43636364*		*0.436363636*				*0.3197674*
**ENSG00000162576**	*0.32831325*	*0.09119497*	*0.32831325*	*0.091194969*	*0.142857143*		*0.2261905*	*0.255814*
**ENSG00000081277**	*0.05555556*	*0.18484848*	*0.05555556*	*0.184848485*	*0.077777778*			*0.1588235*

**ENSG00000154016**		*0.17241379*		*0.172413793*			*0.375*
**ENSG00000228998**		*0.26969697*		*0.26969697*			*0.3662791*
**ENSG00000139946**	*0.09281437*	*0.49056604*	*0.09281437*	*0.490566038*	*0.162650602*	*0.1162791*	*0.4666667*
**ENSG00000122254**		*0.40606061*		*0.406060606*			*0.4534884*
**ENSG00000150637**		*0.49393939*		*0.493939394*			*0.3488372*
**ENSG00000205212**		*0.21348315*		*0.213483146*			*0.2777778*
**ENSG00000151812**	*0.47904192*	*0.39240506*	*0.47904192*	*0.392405063*	*0.363095238*	*0.2906977*	*0.4647059*
**ENSG00000135931**	*0.26646707*	*0.32121212*	*0.26646707*	*0.321212121*	*0.136904762*	*0.1802326*	*0.1569767*
**ENSG00000100721**							*0.1395349*
**ENSG00000126522**	*0.08383234*	*0.08227848*	*0.08383234*	*0.082278481*	*0.172619048*	*0.1686047*	*0.1647059*
**ENSG00000181800**		*0.46666667*		*0.466666667*			*0.2906977*
**ENSG00000158882**	*0.06287425*		*0.06287425*		*0.494047619*	*0.4593023*	
**ENSG00000181333**		*0.32727273*		*0.327272727*			*0.3235294*
**ENSG00000177042**		*0.47484277*		*0.474842767*			*0.4235294*
**ENSG00000170759**		*0.27160494*		*0.271604938*			
**ENSG00000241043**		*0.38202247*		*0.382022472*			*0.3170732*
**ENSG00000104783**		*0.20606061*		*0.206060606*			*0.0930233*
**ENSG00000115286**	*0.29341317*	*0.36363636*	*0.29341317*	*0.363636364*	*0.31547619*	*0.4235294*	*0.4176471*
**ENSG00000185245**	*0.25903614*		*0.25903614*		*0.210843373*	*0.2647059*	*0.0639535*
**ENSG00000188786**		*0.23595506*		*0.235955056*			*0.3488372*
**ENSG00000179820**		*0.08333333*		*0.083333333*			
**ENSG00000163866**		*0.07272727*		*0.072727273*			*0.122093*
**ENSG00000221818**		*0.45757576*		*0.457575758*			*0.2529412*
**ENSG00000189292**		*0.38484848*		*0.384848485*			*0.4011628*
**ENSG00000255835**							*0.2727273*
**ENSG00000137767**	*0.0508982*		*0.0508982*				
**ENSG00000146839**	*0.22754491*	*0.4*	*0.22754491*	*0.4*	*0.464285714*	*0.4352941*	
**ENSG00000171786**		*0.39634146*		*0.396341463*			*0.4767442*
**ENSG00000139194**		*0.09659091*		*0.096590909*			
**ENSG00000158714**	*0.05688623*	*0.14545455*	*0.05688623*	*0.145454545*			
**ENSG00000196946**		*0.46060606*		*0.460606061*			*0.4302326*
**ENSG00000133106**	*0.31437126*	*0.49390244*	*0.31437126*	*0.493902439*	*0.475903614*	*0.4529412*	*0.4302326*
**ENSG00000171596**		*0.30909091*		*0.309090909*			*0.4709302*
**ENSG00000131467**	*0.1257485*		*0.1257485*				
**ENSG00000256713**							*0.0555556*
**ENSG00000124275**		*0.45505618*		*0.45505618*			*0.3295455*
**ENSG00000196092**		*0.28481013*		*0.284810127*			*0.304878*
**ENSG00000158966**		*0.16969697*		*0.16969697*			
**ENSG00000170421**	*0.4375*	*0.48181818*	*0.4375*	*0.481818182*	*0.098765432*	*0.2073171*	*0.3837209*
**ENSG00000105953**					*0.115853659*	*0.1176471*	*0.3488372*
**ENSG00000165055**		*0.18539326*		*0.185393258*			
**ENSG00000084676**		*0.21111111*		*0.211111111*			*0.0777778*
**ENSG00000187792**		*0.18484848*		*0.184848485*			*0.3546512*
**ENSG00000120159**	*0.30113636*		*0.30113636*		*0.322222222*	*0.3409091*	*0.3409091*
**ENSG00000139734**	*0.15868263*	*0.37222222*	*0.15868263*	*0.372222222*	*0.166666667*	*0.1162791*	*0.2045455*
**ENSG00000130559**		*0.28787879*		*0.287878788*			*0.3647059*
**ENSG00000169783**		*0.40062112*		*0.400621118*			*0.4588235*
**ENSG00000114867**		*0.28181818*		*0.281818182*			*0.3081395*
**ENSG00000049883**		*0.37058824*		*0.370588235*			*0.3255814*
**ENSG00000171320**	*0.11077844*	*0.46666667*	*0.11077844*	*0.466666667*	*0.234939759*	*0.2209302*	*0.4244186*
**ENSG00000197982**					*0.285714286*	*0.3488372*	
**ENSG00000111087**	*0.37724551*		*0.37724551*		*0.244047619*	*0.4593023*	
**ENSG00000156097**		*0.16969697*		*0.16969697*			*0.3662791*
**ENSG00000145198**	*0.1686747*		*0.1686747*		*0.146341463*	*0.1627907*	

**ENSG00000172071**					*0.481707317*	*0.4431818*	
**ENSG00000181513**	*0.39520958*	*0.0505618*	*0.39520958*	*0.050561798*	*0.208333333*	*0.25*	
**ENSG00000107331**	*0.41916168*	*0.29393939*	*0.41916168*	*0.293939394*	*0.130952381*	*0.2235294*	*0.2325581*
**ENSG00000101349**	*0.36227545*	*0.365625*	*0.36227545*	*0.365625*	*0.207317073*	*0.25*	*0.4705882*
**ENSG00000075336**	*0.24550898*	*0.06363636*	*0.24550898*	*0.063636364*	*0.119047619*	*0.2159091*	*0.1511628*
**ENSG00000111879**		*0.40853659*		*0.408536585*			*0.25*
**ENSG00000102178**		*0.14417178*		*0.144171779*			*0.0872093*
**ENSG00000103769**		*0.16111111*		*0.161111111*			*0.2840909*
**ENSG00000138028**		*0.403125*		*0.403125*			*0.2142857*
**ENSG00000128563**	*0.08988764*	*0.27272727*	*0.08988764*	*0.272727273*			*0.2616279*
**ENSG00000120685**		*0.14848485*		*0.148484848*			*0.1647059*
**ENSG00000164023**		*0.26060606*		*0.260606061*	*0.141025641*	*0.1585366*	*0.0595238*
**ENSG00000166685**	*0.45481928*		*0.45481928*		*0.345238095*	*0.3546512*	
**ENSG00000120659**		*0.48888889*		*0.488888889*			*0.3604651*
**ENSG00000118972**		*0.24233129*		*0.242331288*			*0.0988372*
**ENSG00000165300**	*0.26136364*		*0.26136364*				
**ENSG00000063854**		*0.26966292*		*0.269662921*			*0.2613636*
**ENSG00000075413**		*0.36060606*		*0.360606061*			*0.3023256*
**ENSG00000166317**		*0.11818182*		*0.118181818*			*0.1860465*
**ENSG00000141404**		*0.46060606*		*0.460606061*			*0.4883721*
**ENSG00000118260**	*0.37777778*	*0.20555556*	*0.37777778*	*0.205555556*			
**ENSG00000009790**	*0.35928144*	*0.36111111*	*0.35928144*	*0.361111111*	*0.261904762*	*0.4069767*	*0.2386364*
**ENSG00000171492**	*0.45508982*		*0.45508982*		*0.392857143*	*0.4825581*	*0.1453488*
**ENSG00000154305**		*0.09393939*		*0.093939394*			*0.1162791*
**ENSG00000137218**		*0.32424242*		*0.324242424*			*0.1046512*
**ENSG00000157637**		*0.2607362*		*0.260736196*			
**ENSG00000241123**	*0.12068966*		*0.12068966*				*0.1555556*
**ENSG00000037965**		*0.44252874*		*0.442528736*			*0.4127907*
**ENSG00000221826**	*0.11111111*		*0.11111111*				
**ENSG00000148153**		*0.08181818*		*0.081818182*			
**ENSG00000153574**		*0.23636364*		*0.236363636*			*0.1744186*
**ENSG00000131910**		*0.26060606*		*0.260606061*			
**ENSG00000011198**							*0.1470588*
**ENSG00000184470**	*0.39221557*	*0.19620253*	*0.39221557*	*0.196202532*	*0.136904762*	*0.1511628*	*0.0581395*
**ENSG00000113356**	*0.41017964*	*0.14545455*	*0.41017964*	*0.145454545*	*0.470238095*	*0.3372093*	*0.3895349*
**ENSG00000188883**		*0.17878788*		*0.178787879*			*0.1511628*
**ENSG00000112357**		*0.42901235*		*0.429012346*			*0.5*
**ENSG00000172380**		*0.47159091*		*0.471590909*			*0.4886364*
**ENSG00000143036**		*0.23030303*		*0.23030303*			
**ENSG00000105131**		*0.24712644*		*0.247126437*			*0.2045455*
**ENSG00000203951**		*0.46111111*		*0.461111111*			*0.372093*
**ENSG00000140015**		*0.49393939*		*0.493939394*			*0.3604651*
**ENSG00000099957**		*0.43888889*		*0.438888889*			*0.4767442*
**ENSG00000106144**		*0.05*		*0.05*			
**ENSG00000117222**	*0.08083832*	*0.18181818*	*0.08083832*	*0.181818182*	*0.488888889*	*0.4666667*	*0.4593023*
**ENSG00000165029**	*0.19461078*	*0.31515152*	*0.19461078*	*0.315151515*	*0.327380952*	*0.2823529*	*0.2209302*
**ENSG00000104044**	*0.12874251*		*0.12874251*		*0.357142857*	*0.3255814*	
**ENSG00000120798**	*0.06886228*		*0.06886228*				
**ENSG00000203908**		*0.13636364*		*0.136363636*			
**ENSG00000158552**		*0.32727273*		*0.327272727*			*0.244186*
**ENSG00000144895**		*0.40555556*		*0.405555556*			*0.3522727*
**ENSG00000103365**		*0.12777778*		*0.127777778*			*0.2848837*
**ENSG00000204435**	*0.29640719*	*0.31212121*	*0.29640719*	*0.312121212*	*0.404761905*	*0.4302326*	*0.4534884*
**ENSG00000140157**		*0.46111111*		*0.461111111*			*0.3837209*

**ENSG00000121270**		*0.17272727*		*0.172727273*			
**ENSG00000135480**	*0.45783133*		*0.45783133*		*0.5*	*0.494186*	
**ENSG00000091583**	*0.07784431*	*0.46363636*	*0.07784431*	*0.463636364*		*0.0535714*	*0.0639535*
**ENSG00000179409**		*0.32012195*		*0.320121951*			*0.1818182*
**ENSG00000084092**	*0.26111111*		*0.26111111*		*0.488888889*	*0.5*	
**ENSG00000213923**							*0.1395349*
**ENSG00000134398**	*0.05988024*		*0.05988024*		*0.261904762*	*0.4069767*	
**ENSG00000135956**		*0.49085366*		*0.490853659*			*0.2906977*
**ENSG00000183833**	*0.17058824*	*0.29573171*	*0.17058824*	*0.295731707*	*0.214285714*	*0.2804878*	*0.2790698*
**ENSG00000141458**					*0.368421053*	*0.2205882*	
**ENSG00000140471**		*0.48701299*		*0.487012987*			*0.4418605*
**ENSG00000154277**	*0.13888889*	*0.23333333*	*0.13888889*	*0.233333333*			*0.1511628*
**ENSG00000186716**		*0.34090909*		*0.340909091*			*0.3555556*
**ENSG00000158887**		*0.09090909*		*0.090909091*			
**ENSG00000168038**	*0.29041916*		*0.29041916*				
**ENSG00000103657**		*0.15909091*		*0.159090909*			*0.0755814*
**ENSG00000104884**		*0.31212121*		*0.312121212*			*0.1022727*
**ENSG00000188263**		*0.41477273*		*0.414772727*			*0.4534884*
**ENSG00000165915**		*0.32121212*		*0.321212121*			*0.3571429*
**ENSG00000167613**	*0.43373494*	*0.32727273*	*0.43373494*	*0.327272727*	*0.178571429*	*0.1918605*	*0.4125*
**ENSG00000213020**	*0.28333333*	*0.36060606*	*0.28333333*	*0.360606061*	*0.466666667*	*0.4444444*	*0.4705882*
**ENSG00000171714**		*0.32208589*		*0.32208589*			*0.4772727*
**ENSG00000172987**		*0.49444444*		*0.494444444*			*0.4651163*
**ENSG00000114204**		*0.07621951*		*0.076219512*			*0.3430233*
**ENSG00000258811**		*0.28395062*		*0.283950617*			*0.3717949*
**ENSG00000064995**	*0.34090909*	*0.14848485*	*0.34090909*	*0.148484848*	*0.155555556*	*0.1136364*	*0.4941176*
**ENSG00000108883**		*0.38181818*		*0.381818182*			*0.4588235*
**ENSG00000116962**		*0.15454545*		*0.154545455*			*0.3294118*
**ENSG00000086758**		*0.41818182*		*0.418181818*			*0.4235294*
**ENSG00000175329**	*0.40340909*	*0.39393939*	*0.40340909*	*0.393939394*	*0.130952381*	*0.2727273*	*0.377907*
**ENSG00000140538**		*0.39570552*		*0.395705521*			*0.4186047*
**ENSG00000138658**	*0.37078652*	*0.42424242*	*0.37078652*	*0.424242424*	*0.418604651*	*0.4360465*	*0.4360465*
**ENSG00000185222**	*0.05722892*		*0.05722892*				
**ENSG00000140262**		*0.23939394*		*0.239393939*			*0.4207317*
**ENSG00000132341**		*0.29393939*		*0.293939394*			*0.3081395*
**ENSG00000204599**		*0.12727273*		*0.127272727*			*0.372093*
**ENSG00000182257**		*0.33939394*		*0.339393939*			*0.1046512*
**ENSG00000179038**		*0.08598726*		*0.085987261*			*0.2034884*
**ENSG00000162946**	*0.34431138*	*0.38787879*	*0.34431138*	*0.387878788*	*0.333333333*	*0.3081395*	*0.4534884*
**ENSG00000106105**	*0.14371257*		*0.14371257*				
**ENSG00000162595**	*0.44610778*		*0.44610778*				
**ENSG00000135447**		*0.31212121*		*0.312121212*			*0.0639535*
**ENSG00000139193**					*0.255952381*	*0.1744186*	
**ENSG00000205138**		*0.16111111*		*0.161111111*			
**ENSG00000110080**	*0.39820359*		*0.39820359*		*0.488888889*	*0.4204545*	
**ENSG00000215475**		*0.5*		*0.5*	*0.05952381*	*0.0872093*	*0.4886364*
**ENSG00000123395**		*0.17272727*		*0.172727273*			*0.4767442*
**ENSG00000188687**	*0.16111111*	*0.38484848*	*0.16111111*	*0.384848485*	*0.388888889*	*0.3181818*	*0.1860465*
**ENSG00000019991**		*0.22424242*		*0.224242424*	*0.073170732*	*0.0581395*	*0.1511628*
**ENSG00000171877**					*0.464285714*	*0.4186047*	*0.2732558*
**ENSG00000189114**		*0.13939394*		*0.139393939*			
**ENSG00000188312**	*0.23652695*	*0.21818182*	*0.23652695*	*0.218181818*	*0.255952381*	*0.4883721*	*0.4886364*
**ENSG00000170049**		*0.49390244*		*0.493902439*			*0.1395349*
**ENSG00000100418**		*0.20909091*		*0.209090909*			*0.0639535*

**ENSG00000106392**	*0.11077844*	*0.4202454*	*0.11077844*	*0.420245399*				*0.4*
**ENSG00000204110**		*0.43055556*		*0.430555556*				*0.4594595*
**ENSG00000149021**	*0.3502994*		*0.3502994*		*0.392857143*		*0.4011628*	
**ENSG00000118579**		*0.38181818*		*0.381818182*				*0.1162791*
**ENSG00000154930**	*0.38922156*	*0.34848485*	*0.38922156*	*0.348484848*	*0.279761905*		*0.1882353*	*0.2151163*
**ENSG00000162819**	*0.21257485*	*0.24848485*	*0.21257485*	*0.248484848*	*0.261904762*		*0.3430233*	*0.4593023*
**ENSG00000206538**		*0.44444444*		*0.444444444*				*0.4883721*
**ENSG00000047849**		*0.4030303*		*0.403030303*				*0.244186*
**ENSG00000126001**		*0.2030303*		*0.203030303*				*0.244186*
**ENSG00000169758**		*0.49695122*		*0.49695122*				*0.4476744*
**ENSG00000099875**		*0.1744186*		*0.174418605*	*0.244047619*		*0.1686047*	*0.5*
**ENSG00000132004**	*0.43333333*		*0.43333333*					
**ENSG00000129255**	*0.32335329*	*0.38484848*	*0.32335329*	*0.384848485*	*0.369047619*		*0.4127907*	*0.2790698*
**ENSG00000166333**	*0.42168675*	*0.32424242*	*0.42168675*	*0.324242424*	*0.339285714*		*0.3662791*	*0.3662791*
**ENSG00000188779**		*0.22727273*		*0.227272727*				*0.2034884*
**ENSG00000174946**	*0.2245509*		*0.2245509*		*0.107142857*		*0.0755814*	
**ENSG00000134222**	*0.05688623*	*0.11890244*	*0.05688623*	*0.118902439*				
**ENSG00000163931**		*0.23837209*		*0.238372093*				*0.1511628*
**ENSG00000143013**		*0.10559006*		*0.105590062*				*0.2151163*
**ENSG00000101204**		*0.41768293*		*0.417682927*				*0.3647059*
**ENSG00000129103**		*0.26363636*		*0.263636364*				*0.3430233*
**ENSG00000198225**		*0.35795455*		*0.357954545*				*0.4767442*
**ENSG00000185933**		*0.35757576*		*0.357575758*				*0.4705882*
**ENSG00000187980**		*0.39759036*		*0.397590361*				*0.4666667*
**ENSG00000164118**		*0.37878788*		*0.378787879*				
**ENSG00000022976**	*0.28089888*	*0.28658537*	*0.28089888*	*0.286585366*	*0.428571429*		*0.3546512*	*0.4825581*
**ENSG00000204256**	*0.15757576*	*0.41818182*	*0.15757576*	*0.418181818*	*0.488095238*		*0.4302326*	*0.2906977*
**ENSG00000206503**	*0.26470588*	*0.12121212*	*0.26470588*	*0.121212121*	*0.345238095*		*0.078125*	*0.1104651*
**ENSG00000105767**		*0.4969697*		*0.496969697*				*0.1918605*
**ENSG00000079931**	*0.27840909*	*0.07272727*	*0.27840909*	*0.072727273*	*0.271084337*		*0.1845238*	*0.5*
**ENSG00000198467**	*0.23952096*	*0.16666667*	*0.23952096*	*0.166666667*	*0.053571429*		*0.0523256*	
**ENSG00000057593**		*0.12121212*		*0.121212121*				*0.0523256*
**ENSG00000110395**		*0.30909091*		*0.309090909*				*0.4069767*
**ENSG00000110195**	*0.13772455*		*0.13772455*		*0.138554217*			
**ENSG00000103037**		*0.41212121*		*0.412121212*				*0.372093*
**ENSG00000184908**	*0.38023952*		*0.38023952*					
**ENSG00000167524**		*0.07716049*		*0.077160494*				*0.1506024*
**ENSG00000198168**		*0.43333333*		*0.433333333*				*0.2619048*
**ENSG00000187741**		*0.43888889*		*0.438888889*				
**ENSG00000204536**	*0.4760479*	*0.46969697*	*0.4760479*	*0.46969697*	*0.410714286*		*0.4709302*	*0.4302326*
**ENSG00000235162**		*0.47777778*		*0.477777778*				*0.4090909*
**ENSG00000148832**		*0.15*		*0.15*				*0.0529412*
**ENSG00000188157**		*0.28834356*		*0.288343558*				*0.1818182*
**ENSG00000055483**	*0.11976048*	*0.48181818*	*0.11976048*	*0.481818182*	*0.475903614*		*0.494186*	*0.4941176*
**ENSG00000158748**	*0.4011976*		*0.4011976*		*0.15060241*		*0.0705882*	
**ENSG00000108091**		*0.49425287*		*0.494252874*				*0.5*
**ENSG00000124568**		*0.44545455*		*0.445454545*				*0.3*
**ENSG00000167645**		*0.23939394*		*0.239393939*				*0.4593023*
**ENSG00000162144**	*0.36826347*		*0.36826347*					
**ENSG00000196814**		*0.48484848*		*0.484848485*				*0.4767442*
**ENSG00000148225**	*0.10555556*	*0.41818182*	*0.10555556*	*0.418181818*	*0.162790698*		*0.2*	*0.2*
**ENSG00000171503**		*0.28484848*		*0.284848485*				*0.1569767*
**ENSG00000176601**	*0.35628743*	*0.38109756*	*0.35628743*	*0.381097561*	*0.488095238*		*0.4534884*	*0.3493976*
**ENSG00000219073**		*0.34259259*		*0.342592593*				*0.3783784*

**ENSG00000090554**		*0.18181818*		*0.181818182*			*0.122093*
**ENSG00000134160**		*0.49393939*		*0.493939394*			*0.1453488*
**ENSG00000205913**		*0.30909091*		*0.309090909*			*0.4222222*
**ENSG00000176697**	*0.12222222*	*0.33636364*	*0.12222222*	*0.336363636*	*0.204545455*	*0.4111111*	*0.372093*
**ENSG00000107018**		*0.19817073*		*0.198170732*			*0.4709302*
**ENSG00000144468**	*0.48333333*	*0.26666667*	*0.48333333*	*0.266666667*	*0.2*	*0.125*	
**ENSG00000187672**		*0.45705521*		*0.457055215*			*0.2034884*
**ENSG00000185873**		*0.26111111*		*0.261111111*			
**ENSG00000172379**		*0.42987805*		*0.429878049*			*0.077381*
**ENSG00000166225**	*0.14071856*	*0.36666667*	*0.14071856*	*0.366666667*	*0.327380952*	*0.2235294*	*0.2674419*
**ENSG00000180113**		*0.20552147*		*0.205521472*			*0.1176471*
**ENSG00000133302**		*0.19090909*		*0.190909091*			*0.2848837*
**ENSG00000183826**		*0.45454545*		*0.454545455*			*0.4*
**ENSG00000163093**	*0.10674157*		*0.10674157*				
**ENSG00000136738**		*0.09937888*		*0.099378882*			*0.054878*
**ENSG00000203666**		*0.375*		*0.375*			*0.3488372*
**ENSG00000182199**	*0.0508982*	*0.06060606*	*0.0508982*	*0.060606061*	*0.154761905*	*0.0813953*	
**ENSG00000149295**	*0.33832335*	*0.32758621*	*0.33832335*	*0.327586207*	*0.208333333*	*0.2732558*	*0.4659091*
**ENSG00000138741**		*0.31097561*		*0.31097561*			*0.4470588*
**ENSG00000163606**		*0.46226415*		*0.462264151*			*0.4545455*
**ENSG00000170185**		*0.37222222*		*0.372222222*			*0.2386364*
**ENSG00000137434**	*0.41111111*		*0.41111111*				
**ENSG00000132680**	*0.34659091*		*0.34659091*		*0.088888889*	*0.0909091*	
**ENSG00000168994**		*0.15757576*		*0.157575758*			*0.2267442*
**ENSG00000182077**	*0.39820359*		*0.39820359*		*0.321428571*	*0.3546512*	
**ENSG00000109339**	*0.46107784*	*0.48192771*	*0.46107784*	*0.481927711*	*0.313253012*	*0.3*	*0.377907*
**ENSG00000047249**	*0.14371257*	*0.05454545*	*0.14371257*	*0.054545455*			*0.4285714*
**ENSG00000183760**	*0.08383234*		*0.08383234*		*0.273809524*	*0.25*	
**ENSG00000197658**	*0.49101796*	*0.49411765*	*0.49101796*	*0.494117647*	*0.083333333*	*0.1046512*	*0.3444444*
**ENSG00000139800**		*0.20909091*		*0.209090909*			*0.0872093*
**ENSG00000116830**					*0.101190476*	*0.1569767*	
**ENSG00000166833**	*0.47701149*	*0.47878788*	*0.47701149*	*0.478787879*	*0.288888889*	*0.2613636*	*0.4244186*
**ENSG00000175356**		*0.23636364*		*0.236363636*			*0.127907*
**ENSG00000146352**	*0.30113636*	*0.21036585*	*0.30113636*	*0.210365854*	*0.455555556*	*0.2954545*	*0.125*
**ENSG00000145147**	*0.14371257*	*0.29878049*	*0.14371257*	*0.298780488*		*0.0697674*	*0.1555556*
**ENSG00000107862**	*0.17365269*	*0.11212121*	*0.17365269*	*0.112121212*	*0.214285714*	*0.2267442*	
**ENSG00000118655**	*0.15269461*	*0.21212121*	*0.15269461*	*0.212121212*	*0.119047619*	*0.2638889*	*0.1744186*
**ENSG00000174842**		*0.46385542*		*0.463855422*			
**ENSG00000183628**		*0.08333333*		*0.083333333*			
**ENSG00000181215**	*0.23053892*	*0.33939394*	*0.23053892*	*0.339393939*	*0.279761905*	*0.4302326*	*0.1744186*
**ENSG00000185055**							*0.0639535*
**ENSG00000140009**		*0.41515152*		*0.415151515*			*0.4659091*
**ENSG00000123200**		*0.32727273*		*0.327272727*			*0.3081395*
**ENSG00000196118**	*0.23652695*		*0.23652695*		*0.077380952*	*0.0988372*	
**ENSG00000152422**					*0.111111111*	*0.1590909*	
**ENSG00000166888**	*0.10778443*	*0.44848485*	*0.10778443*	*0.448484848*	*0.301204819*	*0.1976744*	*0.3764706*
**ENSG00000146054**	*0.30113636*		*0.30113636*		*0.111111111*	*0.125*	
**ENSG00000224689**		*0.15340909*		*0.153409091*			
**ENSG00000066735**		*0.47878788*		*0.478787879*			*0.3705882*
**ENSG00000158161**		*0.29090909*		*0.290909091*			*0.4244186*
**ENSG00000004809**	*0.39090909*	*0.49390244*	*0.39090909*	*0.493902439*	*0.475903614*	*0.3588235*	*0.4651163*
**ENSG00000164344**		*0.09393939*		*0.093939394*			*0.0523256*
**ENSG00000154845**		*0.19817073*		*0.198170732*			*0.4360465*
**ENSG00000104835**	*0.07185629*	*0.375*	*0.07185629*	*0.375*			*0.2954545*

**ENSG00000154133**	*0.38372093*	*0.37878788*	*0.38372093*	*0.378787879*			*0.1162791*
**ENSG00000136697**	*0.28143713*		*0.28143713*		*0.267857143*	*0.2732558*	
**ENSG00000108590**		*0.29775281*		*0.297752809*			*0.4090909*
**ENSG00000156265**	*0.46107784*	*0.48181818*	*0.46107784*	*0.481818182*	*0.297619048*	*0.327381*	*0.4593023*
**ENSG00000122970**	*0.45808383*		*0.45808383*				
**ENSG00000213999**		*0.33030303*		*0.33030303*			*0.3081395*
**ENSG00000135828**	*0.06287425*	*0.4*	*0.06287425*	*0.4*	*0.273809524*	*0.1918605*	*0.3662791*
**ENSG00000133983**		*0.29090909*		*0.290909091*			*0.0930233*
**ENSG00000197780**		*0.20121951*		*0.201219512*			*0.4069767*
**ENSG00000136870**		*0.47878788*		*0.478787879*			*0.4529412*
**ENSG00000106536**	*0.23333333*	*0.38181818*	*0.23333333*	*0.381818182*	*0.102272727*	*0.1777778*	*0.5*
**ENSG00000107105**	*0.23952096*	*0.08227848*	*0.23952096*	*0.082278481*			
**ENSG00000137752**		*0.25914634*		*0.259146341*	*0.057142857*		*0.25*
**ENSG00000178752**		*0.4*		*0.4*			*0.2333333*
**ENSG00000102003**		*0.31212121*		*0.312121212*			*0.3313953*
**ENSG00000014164**		*0.26060606*		*0.260606061*			*0.4244186*
**ENSG00000205268**		*0.31111111*		*0.311111111*			*0.5*
**ENSG00000166153**		*0.13414634*		*0.134146341*			*0.3705882*
**ENSG00000110880**		*0.49691358*		*0.49691358*			
**ENSG00000243444**		*0.2752809*		*0.275280899*			*0.3111111*
**ENSG00000167470**	*0.09375*	*0.26969697*	*0.09375*	*0.26969697*	*0.426829268*	*0.4457831*	*0.255814*
**ENSG00000185905**		*0.20909091*		*0.209090909*			*0.0639535*
**ENSG00000182132**	*0.21176471*	*0.29090909*	*0.21176471*	*0.290909091*	*0.238095238*	*0.2383721*	*0.2034884*
**ENSG00000176170**	*0.46540881*		*0.46540881*		*0.0625*	*0.0853659*	*0.0813953*
**ENSG00000164828**	*0.21987952*	*0.29393939*	*0.21987952*	*0.293939394*	*0.477777778*	*0.4318182*	*0.3255814*
**ENSG00000113593**		*0.3030303*		*0.303030303*			*0.3488372*
**ENSG00000109181**		*0.14848485*		*0.148484848*			*0.1352941*
**ENSG00000108384**		*0.2*		*0.2*			*0.2383721*
**ENSG00000186660**		*0.29090909*		*0.290909091*			*0.2093023*
**ENSG00000166851**		*0.14772727*		*0.147727273*			*0.3863636*
**ENSG00000141141**		*0.21511628*		*0.215116279*			*0.4772727*
**ENSG00000135473**		*0.06111111*		*0.061111111*			*0.25*
**ENSG00000095627**		*0.08787879*		*0.087878788*			*0.0639535*
**ENSG00000141965**		*0.32727273*		*0.327272727*			*0.4127907*
**ENSG00000100731**		*0.46111111*		*0.461111111*			*0.2732558*
**ENSG00000103313**		*0.46036585*		*0.460365854*			*0.3953488*
**ENSG00000205045**	*0.31927711*		*0.31927711*		*0.18452381*	*0.1744186*	
**ENSG00000100714**	*0.06886228*		*0.06886228*		*0.31547619*	*0.2151163*	
**ENSG00000181355**	*0.48502994*	*0.25609756*	*0.48502994*	*0.256097561*	*0.05952381*		*0.4*
**ENSG00000162086**	*0.10179641*		*0.10179641*		*0.113095238*	*0.2093023*	
**ENSG00000128654**	*0.25748503*		*0.25748503*		*0.113095238*	*0.2034884*	
**ENSG00000140545**		*0.37575758*		*0.375757576*			*0.4883721*
**ENSG00000198677**	*0.10479042*	*0.15853659*	*0.10479042*	*0.158536585*	*0.428571429*	*0.4593023*	*0.1860465*
**ENSG00000148572**		*0.32424242*		*0.324242424*			*0.2151163*
**ENSG00000099246**							*0.3197674*
**ENSG00000129474**		*0.33888889*		*0.338888889*			*0.2159091*
**ENSG00000146938**	*0.11666667*	*0.40116279*	*0.11666667*	*0.401162791*	*0.428571429*	*0.3823529*	*0.3895349*
**ENSG00000111615**		*0.41818182*		*0.418181818*			*0.494186*
**ENSG00000138166**		*0.31402439*		*0.31402439*			*0.4302326*
**ENSG00000079263**		*0.26704545*		*0.267045455*			*0.1511628*
**ENSG00000155875**	*0.33532934*	*0.22727273*	*0.33532934*	*0.227272727*	*0.494047619*	*0.4709302*	*0.2965116*
**ENSG00000204118**		*0.07777778*		*0.077777778*			*0.1081081*
**ENSG00000148408**		*0.48484848*		*0.484848485*	*0.208333333*	*0.1802326*	*0.3289474*
**ENSG00000114019**		*0.33636364*		*0.336363636*			

**ENSG00000139651**		*0.0505618*		*0.050561798*	*0.154761905*	*0.2267442*	*0.2073171*
**ENSG00000131778**		*0.33030303*		*0.33030303*			*0.1744186*
**ENSG00000003056**	*0.06287425*	*0.48447205*	*0.06287425*	*0.48447205*			*0.4883721*
**ENSG00000153214**	*0.22590361*	*0.46060606*	*0.22590361*	*0.460606061*	*0.119047619*	*0.1235294*	*0.1294118*
**ENSG00000173327**		*0.28181818*		*0.281818182*			*0.1764706*
**ENSG00000183715**		*0.34876543*		*0.348765432*			*0.4360465*
**ENSG00000111252**	*0.46084337*	*0.2*	*0.46084337*	*0.2*			*0.4534884*
**ENSG00000185201**	*0.35928144*		*0.35928144*		*0.351190476*	*0.2764706*	
**ENSG00000178971**		*0.46363636*		*0.463636364*			*0.494186*
**ENSG00000069399**	*0.26646707*		*0.26646707*		*0.398809524*	*0.377907*	
**ENSG00000242550**	*0.4*		*0.4*		*0.477777778*	*0.375*	*0.1666667*
**ENSG00000186867**		*0.15454545*		*0.154545455*			*0.3313953*
**ENSG00000169490**	*0.09580838*	*0.36060606*	*0.09580838*	*0.360606061*	*0.285714286*	*0.3197674*	*0.2383721*
**ENSG00000166508**					*0.267857143*	*0.2906977*	
**ENSG00000170745**	*0.2754491*	*0.4*	*0.2754491*	*0.4*	*0.476190476*	*0.4090909*	*0.3255814*
**ENSG00000108179**		*0.45454545*		*0.454545455*			*0.3035714*
**ENSG00000160808**		*0.11656442*		*0.116564417*			*0.2383721*
**ENSG00000174306**	*0.5*	*0.45757576*	*0.5*	*0.457575758*		*0.0930233*	*0.4777778*
**ENSG00000157106**		*0.36111111*		*0.361111111*			*0.0755814*
**ENSG00000120555**		*0.09550562*		*0.095505618*			
**ENSG00000130787**		*0.06969697*		*0.06969697*			
**ENSG00000187961**		*0.07575758*		*0.075757576*			*0.3488372*
**ENSG00000116731**	*0.15868263*	*0.32621951*	*0.15868263*	*0.326219512*	*0.231707317*	*0.2613636*	*0.4767442*
**ENSG00000162512**	*0.35329341*	*0.47878788*	*0.35329341*	*0.478787879*	*0.488095238*	*0.4418605*	*0.4418605*
**ENSG00000151748**		*0.35454545*		*0.354545455*			*0.2267442*
**ENSG00000173376**		*0.22865854*		*0.228658537*			*0.2*
**ENSG00000138303**	*0.29518072*	*0.44207317*	*0.29518072*	*0.442073171*	*0.125*	*0.1162791*	*0.2965116*
**ENSG00000186665**		*0.29393939*		*0.293939394*			*0.2325581*
**ENSG00000153786**		*0.29393939*		*0.293939394*			*0.494186*
**ENSG00000141179**	*0.43333333*	*0.21515152*	*0.43333333*	*0.215151515*	*0.056818182*	*0.1111111*	*0.1511628*
**ENSG00000215853**		*0.46341463*		*0.463414634*			*0.4360465*
**ENSG00000104613**		*0.43333333*		*0.433333333*			*0.244186*
**ENSG00000204703**		*0.08787879*		*0.087878788*			*0.0697674*
**ENSG00000124116**		*0.33030303*		*0.33030303*			*0.2906977*
**ENSG00000132535**	*0.35240964*		*0.35240964*		*0.470238095*	*0.4302326*	
**ENSG00000136193**		*0.35365854*		*0.353658537*			*0.3068182*
**ENSG00000165449**	*0.25449102*	*0.41194969*	*0.25449102*	*0.411949686*		*0.1337209*	*0.3546512*
**ENSG00000130429**		*0.06363636*		*0.063636364*			
**ENSG00000182117**		*0.15340909*		*0.153409091*			*0.375*
**ENSG00000174839**		*0.45731707*		*0.457317073*			*0.4823529*
**ENSG00000144283**		*0.31515152*		*0.315151515*			*0.2848837*
**ENSG00000232013**		*0.40804598*		*0.408045977*			*0.4659091*
**ENSG00000125611**	*0.07784431*	*0.45121951*	*0.07784431*	*0.451219512*	*0.494047619*	*0.3604651*	*0.452381*
**ENSG00000135424**	*0.39204545*	*0.06666667*	*0.39204545*	*0.066666667*	*0.31547619*	*0.3255814*	
**ENSG00000157404**		*0.13030303*		*0.13030303*			*0.0755814*
**ENSG00000139144**	*0.33536585*	*0.2347561*	*0.33536585*	*0.234756098*	*0.269230769*	*0.2926829*	*0.3313953*
**ENSG00000023191**	*0.34131737*	*0.11728395*	*0.34131737*	*0.117283951*	*0.226190476*	*0.2058824*	*0.0952381*
**ENSG00000151322**	*0.46111111*	*0.41818182*	*0.46111111*	*0.418181818*	*0.404761905*	*0.25*	*0.4651163*
**ENSG00000204614**	*0.31325301*	*0.32424242*	*0.31325301*	*0.324242424*	*0.291666667*	*0.2333333*	*0.1569767*
**ENSG00000158516**		*0.41818182*		*0.418181818*			*0.2352941*
**ENSG00000147164**		*0.3969697*		*0.396969697*			*0.3139535*
**ENSG00000111802**	*0.11377246*	*0.31818182*	*0.11377246*	*0.318181818*	*0.333333333*	*0.4767442*	*0.1162791*
**ENSG00000203797**		*0.4*		*0.4*			*0.3953488*
**ENSG00000175155**		*0.49444444*		*0.494444444*			*0.2906977*

**ENSG00000104970**		*0.25609756*		*0.256097561*				*0.1976744*
**ENSG00000170777**		*0.27272727*		*0.272727273*				*0.1569767*
**ENSG00000071246**	*0.13173653*	*0.43030303*	*0.13173653*	*0.43030303*	*0.238095238*		*0.255814*	*0.4883721*
**ENSG00000178662**		*0.45*		*0.45*				*0.2954545*
**ENSG00000135486**	*0.17365269*	*0.12777778*	*0.17365269*	*0.127777778*	*0.44047619*		*0.4069767*	
**ENSG00000057608**		*0.36111111*		*0.361111111*	*0.111111111*		*0.0681818*	*0.4204545*
**ENSG00000120217**		*0.45454545*		*0.454545455*				*0.4709302*
**ENSG00000171722**		*0.44478528*		*0.444785276*				*0.4767442*
**ENSG00000100697**	*0.16111111*	*0.38414634*	*0.16111111*	*0.384146341*				*0.4186047*
**ENSG00000163520**	*0.32777778*	*0.2247191*	*0.32777778*	*0.224719101*				*0.2222222*
**ENSG00000205133**	*0.22777778*	*0.08231707*	*0.22777778*	*0.082317073*	*0.377777778*		*0.2613636*	
**ENSG00000158869**		*0.12121212*		*0.121212121*				*0.4117647*
**ENSG00000149418**	*0.07185629*	*0.36206897*	*0.07185629*	*0.362068966*	*0.196428571*		*0.1337209*	*0.4431818*
**ENSG00000112320**		*0.25454545*		*0.254545455*				*0.4302326*
**ENSG00000077684**		*0.16969697*		*0.16969697*				
**ENSG00000198730**	*0.10778443*	*0.07471264*	*0.10778443*	*0.074712644*	*0.44047619*		*0.4941176*	*0.2159091*
**ENSG00000158258**		*0.34848485*		*0.348484848*				*0.0930233*
**ENSG00000138459**		*0.08181818*		*0.081818182*				*0.25*
**ENSG00000213995**		*0.26060606*		*0.260606061*				*0.3081395*
**ENSG00000120438**		*0.49393939*		*0.493939394*				*0.4476744*
**ENSG00000124205**								*0.1162791*
**ENSG00000156171**	*0.22754491*	*0.14242424*	*0.22754491*	*0.142424242*	*0.154761905*		*0.1976744*	*0.3294118*
**ENSG00000174358**		*0.45757576*		*0.457575758*				*0.3255814*
**ENSG00000158955**		*0.31111111*		*0.311111111*				*0.375*
**ENSG00000184508**		*0.26969697*		*0.26969697*				*0.4418605*
**ENSG00000056972**		*0.46060606*		*0.460606061*				*0.4127907*
**ENSG00000186038**	*0.23888889*		*0.23888889*		*0.238095238*		*0.255814*	
**ENSG00000138435**		*0.06969697*		*0.06969697*				*0.0697674*
**ENSG00000196367**		*0.16363636*		*0.163636364*				*0.2705882*
**ENSG00000115677**		*0.18484848*		*0.184848485*				*0.4883721*
**ENSG00000153904**		*0.42073171*		*0.420731707*				*0.3255814*
**ENSG00000144036**		*0.37272727*		*0.372727273*				*0.0595238*
**ENSG00000221880**								*0.1547619*
**ENSG00000143643**		*0.28571429*		*0.285714286*				*0.2857143*
**ENSG00000198826**		*0.31818182*		*0.318181818*				*0.2325581*
**ENSG00000120519**		*0.5*		*0.5*				*0.2857143*
**ENSG00000172497**		*0.46932515*		*0.469325153*				*0.2705882*
**ENSG00000186088**		*0.08181818*		*0.081818182*	*0.148148148*		*0.0581395*	
**ENSG00000141622**		*0.27272727*		*0.272727273*				*0.2267442*
**ENSG00000160818**	*0.13888889*	*0.25151515*	*0.13888889*	*0.251515152*	*0.11627907*		*0.0555556*	*0.4941176*
**ENSG00000100084**	*0.15868263*		*0.15868263*		*0.375*		*0.3139535*	
**ENSG00000150676**		*0.13888889*		*0.138888889*				*0.0888889*
**ENSG00000125877**		*0.08787879*		*0.087878788*				*0.1337209*
**ENSG00000105879**	*0.25581395*		*0.25581395*		*0.443181818*		*0.3068182*	
**ENSG00000104870**		*0.21818182*		*0.218181818*				*0.0639535*
**ENSG00000171853**		*0.42777778*		*0.427777778*				*0.4882353*
**ENSG00000162408**		*0.09090909*		*0.090909091*				*0.2888889*
**ENSG00000073734**		*0.49090909*		*0.490909091*				*0.494186*
**ENSG00000198898**		*0.46341463*		*0.463414634*				*0.494186*
**ENSG00000142676**	*0.05232558*	*0.35454545*	*0.05232558*	*0.354545455*				*0.4011628*
**ENSG00000197301**		*0.15151515*		*0.151515152*				*0.3411765*
**ENSG00000160161**		*0.16666667*		*0.166666667*				
**ENSG00000082397**	*0.22155689*	*0.06666667*	*0.22155689*	*0.066666667*	*0.095238095*		*0.1*	*0.1*
**ENSG00000151474**	*0.08982036*	*0.41515152*	*0.08982036*	*0.415151515*	*0.142857143*		*0.127907*	*0.494186*

**ENSG00000076053**	*0.38622754*	*0.48888889*	*0.38622754*	*0.488888889*	*0.196428571*	*0.122093*	*0.4883721*
**ENSG00000161533**	*0.07784431*	*0.34242424*	*0.07784431*	*0.342424242*	*0.168674699*	*0.1337209*	*0.3139535*
**ENSG00000224940**	*0.37125749*		*0.37125749*		*0.44047619*	*0.4825581*	
**ENSG00000186174**		*0.39329268*		*0.393292683*			*0.2325581*
**ENSG00000078747**	*0.30538922*	*0.45454545*	*0.30538922*	*0.454545455*	*0.458333333*	*0.3546512*	*0.3546512*
**ENSG00000164346**	*0.36666667*		*0.36666667*		*0.170454545*	*0.1666667*	
**ENSG00000100422**	*0.1257485*	*0.40449438*	*0.1257485*	*0.404494382*	*0.05952381*		*0.4825581*
**ENSG00000204822**	*0.14759036*		*0.14759036*				
**ENSG00000204209**	*0.40555556*		*0.40555556*		*0.466666667*	*0.3488372*	
**ENSG00000100938**	*0.1627907*	*0.05151515*	*0.1627907*	*0.051515152*	*0.172619048*	*0.1976744*	
**ENSG00000128641**							*0.0697674*
**ENSG00000105983**	*0.10479042*	*0.48787879*	*0.10479042*	*0.487878788*	*0.142857143*	*0.1686047*	*0.4709302*
**ENSG00000151640**							*0.4204545*
**ENSG00000204616**		*0.38484848*		*0.384848485*			*0.2159091*
**ENSG00000072958**		*0.23033708*		*0.230337079*			*0.125*
**ENSG00000214413**		*0.05182927*		*0.051829268*			
**ENSG00000112511**		*0.12222222*		*0.122222222*			
**ENSG00000061492**	*0.12951807*		*0.12951807*				
**ENSG00000167634**	*0.12874251*		*0.12874251*		*0.361445783*	*0.3895349*	
**ENSG00000109208**	*0.3502994*		*0.3502994*				
**ENSG00000167595**	*0.23353293*	*0.49393939*	*0.23353293*	*0.493939394*	*0.339285714*	*0.3546512*	*0.4767442*
**ENSG00000166439**		*0.44512195*		*0.445121951*			*0.2272727*
**ENSG00000125409**	*0.11077844*	*0.47878788*	*0.11077844*	*0.478787879*	*0.273809524*	*0.2142857*	*0.4186047*
**ENSG00000141198**		*0.34659091*		*0.346590909*			*0.1022727*
**ENSG00000166900**	*0.39820359*	*0.06363636*	*0.39820359*	*0.063636364*		*0.1084337*	*0.0755814*
**ENSG00000197808**	*0.22155689*		*0.22155689*		*0.21686747*	*0.1511628*	
**ENSG00000105771**	*0.27108434*	*0.20121951*	*0.27108434*	*0.201219512*	*0.303571429*	*0.0823529*	*0.0903614*
**ENSG00000163673**		*0.22727273*		*0.227272727*			*0.3255814*
**ENSG00000058799**	*0.27840909*		*0.27840909*		*0.25*	*0.2954545*	*0.1104651*
**ENSG00000172818**	*0.30838323*	*0.21818182*	*0.30838323*	*0.218181818*	*0.416666667*	*0.4593023*	*0.0523256*
**ENSG00000101981**		*0.1402439*		*0.140243902*			
**ENSG00000215252**							*0.4186047*
**ENSG00000118922**		*0.18787879*		*0.187878788*			*0.3588235*
**ENSG00000198821**		*0.13793103*		*0.137931034*			*0.3295455*
**ENSG00000132670**	*0.29041916*		*0.29041916*		*0.119047619*	*0.1686047*	
**ENSG00000177627**		*0.5*		*0.5*			*0.2954545*
**ENSG00000088832**		*0.4*		*0.4*			*0.122093*
**ENSG00000104812**	*0.29341317*	*0.1030303*	*0.29341317*	*0.103030303*	*0.238095238*	*0.2383721*	*0.0755814*
**ENSG00000170917**	*0.16167665*	*0.28963415*	*0.16167665*	*0.289634146*	*0.077380952*	*0.0697674*	*0.4767442*
**ENSG00000148943**		*0.27011494*		*0.270114943*			*0.4431818*
**ENSG00000064205**		*0.34848485*		*0.348484848*			*0.2267442*
**ENSG00000144504**	*0.06896552*	*0.26363636*	*0.06896552*	*0.263636364*			*0.2732558*
**ENSG00000203995**	*0.08383234*		*0.08383234*		*0.279761905*	*0.2848837*	
**ENSG00000166069**		*0.23170732*		*0.231707317*			*0.1686047*
**ENSG00000205352**							*0.1363636*
**ENSG00000256043**		*0.42727273*		*0.427272727*			*0.2790698*
**ENSG00000184719**	*0.23652695*	*0.37575758*	*0.23652695*	*0.375757576*	*0.5*	*0.3823529*	*0.4360465*
**ENSG00000131373**	*0.08536585*	*0.08231707*	*0.08536585*	*0.082317073*	*0.339285714*	*0.3352941*	*0.2*
**ENSG00000119392**	*0.44207317*	*0.20606061*	*0.44207317*	*0.206060606*	*0.385542169*	*0.4529412*	*0.3255814*
**ENSG00000185386**		*0.2969697*		*0.296969697*			*0.3139535*
**ENSG00000144339**	*0.21556886*	*0.13030303*	*0.21556886*	*0.13030303*			
**ENSG00000165509**	*0.37048193*		*0.37048193*		*0.5*	*0.5*	
**ENSG00000162493**		*0.34848485*		*0.348484848*			*0.2151163*
**ENSG00000145681**		*0.33536585*		*0.335365854*			*0.3666667*

**ENSG00000139899**	*0.31437126*		*0.31437126*		*0.327380952*	*0.2383721*	
**ENSG00000198339**							*0.2882353*
**ENSG00000142233**		*0.24242424*		*0.242424242*			*0.3647059*
**ENSG00000153495**	*0.33832335*		*0.33832335*		*0.220238095*	*0.3255814*	
**ENSG00000196209**		*0.1954023*		*0.195402299*			*0.3895349*
**ENSG00000000460**	*0.28484848*	*0.11666667*	*0.28484848*	*0.116666667*	*0.319277108*	*0.3662791*	
**ENSG00000138134**	*0.31137725*	*0.49390244*	*0.31137725*	*0.493902439*	*0.416666667*	*0.3604651*	*0.122093*
**ENSG00000128408**	*0.19461078*		*0.19461078*		*0.481927711*	*0.4176471*	
**ENSG00000171885**		*0.36666667*		*0.366666667*			*0.4318182*
**ENSG00000133111**		*0.16049383*		*0.160493827*			*0.2325581*
**ENSG00000204574**						*0.0523256*	*0.0523256*
**ENSG00000130511**		*0.33636364*		*0.336363636*			*0.25*
**ENSG00000171132**		*0.16363636*		*0.163636364*			*0.2732558*
**ENSG00000147036**		*0.16292135*		*0.162921348*			
**ENSG00000184916**		*0.13636364*		*0.136363636*			*0.1860465*
**ENSG00000163113**	*0.3742515*		*0.3742515*		*0.244047619*	*0.4186047*	*0.1337209*
**ENSG00000167107**		*0.32183908*		*0.32183908*			*0.125*
**ENSG00000015532**		*0.46511628*		*0.465116279*			*0.1363636*
**ENSG00000120733**		*0.4375*		*0.4375*			*0.4659091*
**ENSG00000141946**							*0.4244186*
**ENSG00000116132**	*0.08682635*	*0.27878788*	*0.08682635*	*0.278787879*	*0.083333333*	*0.122093*	*0.2093023*
**ENSG00000165678**	*0.26704545*	*0.165625*	*0.26704545*	*0.165625*			
**ENSG00000186908**	*0.23353293*	*0.22121212*	*0.23353293*	*0.221212121*	*0.226190476*	*0.1744186*	
**ENSG00000189120**	*0.26047904*	*0.34848485*	*0.26047904*	*0.348484848*			
**ENSG00000109458**		*0.35365854*		*0.353658537*			
**ENSG00000108465**		*0.3255814*		*0.325581395*			*0.3863636*
**ENSG00000152256**	*0.38333333*		*0.38333333*		*0.222222222*	*0.1818182*	*0.1802326*
**ENSG00000171988**		*0.48888889*		*0.488888889*			*0.4659091*
**ENSG00000127412**		*0.08181818*		*0.081818182*			
**ENSG00000168350**		*0.13109756*		*0.131097561*			*0.0988372*
**ENSG00000139437**		*0.11280488*		*0.112804878*			*0.0882353*
**ENSG00000125733**		*0.17272727*		*0.172727273*			*0.2034884*
**ENSG00000243135**		*0.22777778*		*0.227777778*			*0.1590909*
**ENSG00000154928**	*0.23493976*	*0.15243902*	*0.23493976*	*0.152439024*	*0.125*	*0.0813953*	
**ENSG00000164418**		*0.11111111*		*0.111111111*			
**ENSG00000163376**		*0.16975309*		*0.169753086*			
**ENSG00000111642**							*0.2906977*
**ENSG00000197948**	*0.35628743*	*0.43865031*	*0.35628743*	*0.438650307*	*0.232142857*	*0.2209302*	*0.2209302*
**ENSG00000102024**	*0.2*		*0.2*		*0.477777778*	*0.4545455*	
**ENSG00000237440**		*0.43939394*		*0.439393939*			*0.4111111*
**ENSG00000049247**	*0.3742515*		*0.3742515*		*0.30952381*	*0.3372093*	
**ENSG00000152518**		*0.16363636*		*0.163636364*			
**ENSG00000081041**		*0.36280488*		*0.362804878*			*0.5*
**ENSG00000197437**		*0.14242424*		*0.142424242*			*0.0639535*
**ENSG00000197451**		*0.27575758*		*0.275757576*			*0.3588235*
**ENSG00000197183**		*0.36363636*		*0.363636364*			*0.3197674*
**ENSG00000127955**	*0.10606061*	*0.41717791*	*0.10606061*	*0.417177914*	*0.192771084*	*0.3372093*	*0.1046512*
**ENSG00000159063**		*0.3*		*0.3*			*0.372093*
**ENSG00000130779**	*0.38181818*	*0.33333333*	*0.38181818*	*0.333333333*	*0.345238095*	*0.3837209*	*0.4090909*
**ENSG00000173209**	*0.26646707*	*0.14545455*	*0.26646707*	*0.145454545*	*0.375*	*0.372093*	*0.372093*
**ENSG00000135655**		*0.47222222*		*0.472222222*			*0.1931818*
**ENSG00000173894**		*0.22121212*		*0.221212121*			*0.3953488*
**ENSG00000167658**		*0.08484848*		*0.084848485*			
**ENSG00000214265**		*0.48181818*		*0.481818182*			*0.4651163*

**ENSG00000111142**	*0.22155689*	*0.11111111*	*0.22155689*	*0.111111111*	*0.196428571*	*0.255814*	*0.1918605*
**ENSG00000011465**	*0.22666667*		*0.22666667*				*0.0609756*
**ENSG00000073169**							*0.1976744*
**ENSG00000154640**							*0.1744186*
**ENSG00000132749**		*0.31515152*		*0.315151515*			*0.2325581*
**ENSG00000086159**	*0.07185629*		*0.07185629*		*0.494047619*	*0.4825581*	
**ENSG00000187323**		*0.45426829*		*0.454268293*			*0.4411765*
**ENSG00000169427**		*0.11212121*		*0.112121212*			
**ENSG00000167916**		*0.24242424*		*0.242424242*			*0.2616279*
**ENSG00000173124**	*0.44610778*	*0.44047619*	*0.44610778*	*0.44047619*	*0.066666667*		
**ENSG00000161904**		*0.4847561*		*0.484756098*			*0.3*
**ENSG00000164299**		*0.15151515*		*0.151515152*			*0.4512195*
**ENSG00000088538**		*0.19393939*		*0.193939394*			*0.2151163*
**ENSG00000112246**		*0.35093168*		*0.350931677*			*0.4888889*
**ENSG00000006377**							*0.1395349*
**ENSG00000119522**		*0.14329268*		*0.143292683*			*0.2965116*
**ENSG00000170088**	*0.27743902*	*0.08231707*	*0.27743902*	*0.082317073*	*0.439759036*	*0.3430233*	
**ENSG00000088053**		*0.20909091*		*0.209090909*			*0.2616279*
**ENSG00000215021**							*0.0581395*
**ENSG00000106524**		*0.34242424*		*0.342424242*			*0.4823529*
**ENSG00000169083**		*0.1402439*		*0.140243902*			
**ENSG00000182831**		*0.42613636*		*0.426136364*			*0.5*
**ENSG00000249471**		*0.47575758*		*0.475757576*			*0.2383721*
**ENSG00000134283**		*0.42222222*		*0.422222222*			*0.4090909*
**ENSG00000189320**		*0.45757576*		*0.457575758*			*0.4476744*
**ENSG00000163689**		*0.06363636*		*0.063636364*			*0.3430233*
**ENSG00000149507**	*0.08083832*	*0.42424242*	*0.08083832*	*0.424242424*	*0.18452381*	*0.2848837*	*0.4244186*
**ENSG00000198752**		*0.43636364*		*0.436363636*			*0.1337209*
**ENSG00000115590**		*0.12121212*		*0.121212121*			*0.1744186*
**ENSG00000148660**		*0.10555556*		*0.105555556*			*0.1022727*
**ENSG00000113721**	*0.26966292*	*0.49404762*	*0.26966292*	*0.494047619*	*0.101190476*	*0.0930233*	*0.1104651*
**ENSG00000185721**		*0.37575758*		*0.375757576*			
**ENSG00000136819**	*0.19879518*	*0.31212121*	*0.19879518*	*0.312121212*	*0.21686747*	*0.2882353*	*0.2954545*
**ENSG00000136250**	*0.45508982*	*0.36060606*	*0.45508982*	*0.360606061*	*0.452380952*	*0.4767442*	*0.1860465*
**ENSG00000128626**	*0.26966292*		*0.26966292*				
**ENSG00000079805**		*0.23636364*		*0.236363636*			*0.4777778*
**ENSG00000197275**	*0.32634731*	*0.35454545*	*0.32634731*	*0.354545455*	*0.488095238*	*0.4588235*	*0.4825581*
**ENSG00000085377**	*0.33532934*		*0.33532934*		*0.06547619*	*0.1162791*	*0.1860465*
**ENSG00000185634**		*0.45454545*		*0.454545455*	*0.095238095*		*0.2674419*
**ENSG00000102309**		*0.30909091*		*0.309090909*			*0.4647059*
**ENSG00000111348**		*0.11585366*		*0.115853659*			
**ENSG00000105355**	*0.13888889*		*0.13888889*		*0.095238095*	*0.1395349*	
**ENSG00000105509**	*0.25157233*	*0.43939394*	*0.25157233*	*0.439393939*			*0.4470588*
**ENSG00000140931**		*0.45555556*		*0.455555556*	*0.380952381*	*0.4418605*	*0.4772727*
**ENSG00000163947**	*0.26506024*	*0.40606061*	*0.26506024*	*0.406060606*	*0.201219512*	*0.127907*	*0.4767442*
**ENSG00000164040**		*0.27777778*		*0.277777778*			*0.4545455*
**ENSG00000169981**		*0.24848485*		*0.248484848*			
**ENSG00000115884**		*0.34117647*		*0.341176471*			
**ENSG00000187908**	*0.32183908*	*0.18484848*	*0.32183908*	*0.184848485*	*0.261363636*	*0.255814*	*0.3181818*
**ENSG00000132170**		*0.12893082*		*0.128930818*			*0.1707317*
**ENSG00000106771**		*0.49074074*		*0.490740741*	*0.303571429*	*0.2790698*	*0.3823529*
**ENSG00000187535**	*0.28888889*	*0.23939394*	*0.28888889*	*0.239393939*	*0.488888889*	*0.4404762*	*0.4360465*
**ENSG00000183621**	*0.29640719*		*0.29640719*		*0.44047619*	*0.4069767*	
**ENSG00000007968**		*0.4969697*		*0.496969697*			*0.3546512*

**ENSG00000091039**					*0.166666667*	*0.1046512*	
**ENSG00000164506**	*0.37421384*		*0.37421384*		*0.295180723*	*0.2743902*	*0.2209302*
**ENSG00000204655**	*0.17365269*	*0.31395349*	*0.17365269*	*0.313953488*			*0.2*
**ENSG00000102606**	*0.40060241*	*0.33333333*	*0.40060241*	*0.333333333*	*0.321428571*	*0.4069767*	*0.4127907*
**ENSG00000205301**		*0.22727273*		*0.227272727*			*0.1860465*
**ENSG00000100320**		*0.0969697*		*0.096969697*			*0.0639535*
**ENSG00000150776**	*0.06287425*	*0.28658537*	*0.06287425*	*0.286585366*	*0.375*	*0.3837209*	*0.4127907*
**ENSG00000157551**	*0.28947368*	*0.42073171*	*0.28947368*	*0.420731707*	*0.333333333*	*0.4534884*	*0.3409091*
**ENSG00000220702**		*0.1196319*		*0.119631902*			*0.0697674*
**ENSG00000148400**		*0.47852761*		*0.478527607*			*0.2151163*
**ENSG00000213865**							*0.0568182*
**ENSG00000211450**							*0.2151163*
**ENSG00000101850**		*0.21818182*		*0.218181818*			*0.4069767*
**ENSG00000186487**	*0.07784431*	*0.48850575*	*0.07784431*	*0.488505747*	*0.053571429*	*0.0523256*	*0.3444444*
**ENSG00000088899**	*0.11656442*	*0.35454545*	*0.11656442*	*0.354545455*			*0.0755814*
**ENSG00000135643**		*0.37195122*		*0.37195122*			*0.4534884*
**ENSG00000120708**		*0.47222222*		*0.472222222*			*0.4222222*
**ENSG00000143924**	*0.27575758*	*0.12424242*	*0.27575758*	*0.124242424*	*0.487951807*	*0.4470588*	*0.1607143*
**ENSG00000135637**	*0.19161677*	*0.10365854*	*0.19161677*	*0.103658537*			*0.1764706*
**ENSG00000068796**	*0.2797619*		*0.2797619*		*0.363636364*	*0.3809524*	
**ENSG00000100380**					*0.073170732*		*0.1136364*
**ENSG00000167754**							*0.0555556*
**ENSG00000072135**		*0.18390805*		*0.183908046*			*0.1511628*
**ENSG00000089693**	*0.41666667*		*0.41666667*		*0.317073171*	*0.3125*	
**ENSG00000103335**		*0.32121212*		*0.321212121*			*0.1036585*
**ENSG00000167858**	*0.0753012*	*0.49393939*	*0.0753012*	*0.493939394*	*0.488095238*	*0.3662791*	*0.2941176*
**ENSG00000028277**		*0.06666667*		*0.066666667*			*0.0697674*
**ENSG00000149474**		*0.48181818*		*0.481818182*	*0.313253012*	*0.2151163*	*0.0523256*
**ENSG00000156253**		*0.46060606*		*0.460606061*			*0.3863636*
**ENSG00000215244**		*0.41818182*		*0.418181818*			*0.3313953*
**ENSG00000111481**		*0.13030303*		*0.13030303*			*0.25*
**ENSG00000139289**	*0.17065868*	*0.16768293*	*0.17065868*	*0.167682927*	*0.44047619*	*0.4882353*	*0.4302326*
**ENSG00000165495**	*0.22155689*	*0.5*	*0.22155689*	*0.5*	*0.476190476*	*0.4593023*	*0.3176471*
**ENSG00000175575**	*0.30239521*		*0.30239521*		*0.196428571*	*0.1569767*	
**ENSG00000138709**	*0.45783133*	*0.35542169*	*0.45783133*	*0.355421687*	*0.422619048*	*0.3705882*	*0.3666667*
**ENSG00000101654**		*0.48484848*		*0.484848485*			*0.1686047*
**ENSG00000154027**	*0.49101796*	*0.16768293*	*0.49101796*	*0.167682927*	*0.267857143*	*0.3430233*	*0.3117647*
**ENSG00000141644**		*0.1*		*0.1*			*0.0988372*
**ENSG00000106031**		*0.0969697*		*0.096969697*			*0.4418605*
**ENSG00000150768**	*0.19461078*	*0.30606061*	*0.19461078*	*0.306060606*	*0.375*	*0.3895349*	*0.4*
**ENSG00000151729**					*0.079545455*	*0.1704545*	
**ENSG00000107882**		*0.40909091*		*0.409090909*			*0.4767442*
**ENSG00000204709**		*0.16666667*		*0.166666667*			*0.4244186*
**ENSG00000259431**					*0.25*	*0.255814*	
**ENSG00000135821**		*0.49393939*		*0.493939394*	*0.422619048*	*0.4127907*	*0.255814*
**ENSG00000084463**	*0.34730539*		*0.34730539*		*0.291666667*	*0.2732558*	
**ENSG00000135631**		*0.28181818*		*0.281818182*			*0.3988095*
**ENSG00000165806**		*0.3030303*		*0.303030303*	*0.146341463*	*0.0714286*	*0.4638554*
**ENSG00000115241**		*0.40909091*		*0.409090909*			*0.0988372*
**ENSG00000241186**	*0.44311377*	*0.37222222*	*0.44311377*	*0.372222222*	*0.369047619*	*0.372093*	*0.1444444*
**ENSG00000108443**		*0.35757576*		*0.357575758*			*0.494186*
**ENSG00000180884**		*0.47575758*		*0.475757576*			*0.4709302*
**ENSG00000134539**	*0.35*	*0.41158537*	*0.35*	*0.411585366*	*0.5*	*0.375*	*0.4186047*
**ENSG00000110975**	*0.19101124*	*0.47239264*	*0.19101124*	*0.472392638*	*0.088888889*	*0.0568182*	*0.4476744*

**ENSG00000139287**		*0.12777778*		*0.127777778*			*0.2444444*
**ENSG00000230453**		*0.17987805*		*0.179878049*			*0.1744186*
**ENSG00000104290**		*0.41158537*		*0.411585366*			*0.4886364*
**ENSG00000162341**		*0.43333333*		*0.433333333*			*0.4476744*
**ENSG00000054282**	*0.06666667*		*0.06666667*		*0.233333333*	*0.3068182*	
**ENSG00000111713**		*0.39444444*		*0.394444444*			*0.4886364*
**ENSG00000253159**		*0.20224719*		*0.202247191*			*0.1477273*
**ENSG00000099219**		*0.32424242*		*0.324242424*			*0.4764706*
**ENSG00000108582**		*0.49090909*		*0.490909091*			*0.2764706*
**ENSG00000100234**		*0.28181818*		*0.281818182*			*0.1104651*
**ENSG00000148484**	*0.23888889*	*0.09756098*	*0.23888889*	*0.097560976*	*0.066666667*		*0.0813953*
**ENSG00000197361**		*0.4*		*0.4*			*0.0697674*
**ENSG00000124253**		*0.2969697*		*0.296969697*			*0.2848837*
**ENSG00000046651**	*0.23952096*		*0.23952096*		*0.476190476*	*0.4593023*	
**ENSG00000158528**		*0.48787879*		*0.487878788*			*0.4418605*
**ENSG00000179148**	*0.16467066*	*0.42121212*	*0.16467066*	*0.421212121*	*0.244047619*	*0.1976744*	*0.3255814*
**ENSG00000182472**		*0.16666667*		*0.166666667*			*0.2383721*
**ENSG00000165997**		*0.36060606*		*0.360606061*			*0.3373494*
**ENSG00000250091**		*0.37272727*		*0.372727273*			*0.1860465*
**ENSG00000161036**							*0.4761905*
**ENSG00000169224**	*0.08333333*	*0.16969697*	*0.08333333*	*0.16969697*	*0.355555556*	*0.3409091*	*0.1511628*
**ENSG00000117971**		*0.32424242*		*0.324242424*			*0.4705882*
**ENSG00000241058**	*0.28143713*		*0.28143713*		*0.285714286*	*0.3255814*	
**ENSG00000110172**	*0.4011976*	*0.26666667*	*0.4011976*	*0.266666667*	*0.377777778*	*0.4058824*	*0.2840909*
**ENSG00000111907**	*0.31818182*	*0.21875*	*0.31818182*	*0.21875*	*0.411111111*	*0.3372093*	*0.2235294*
**ENSG00000106336**		*0.2030303*		*0.203030303*			
**ENSG00000187239**		*0.49425287*		*0.494252874*			*0.2954545*
**ENSG00000128918**	*0.08333333*	*0.46540881*	*0.08333333*	*0.465408805*	*0.133333333*	*0.1*	*0.372093*
**ENSG00000160679**		*0.46060606*		*0.460606061*			*0.3081395*
**ENSG00000163515**	*0.31111111*	*0.2030303*	*0.31111111*	*0.203030303*			
**ENSG00000143105**	*0.18263473*		*0.18263473*		*0.267857143*	*0.3313953*	
**ENSG00000133030**		*0.08888889*		*0.088888889*			*0.1453488*
**ENSG00000175785**		*0.36969697*		*0.36969697*			*0.3546512*
**ENSG00000180697**	*0.27840909*	*0.41515152*	*0.27840909*	*0.415151515*	*0.3*	*0.2727273*	*0.4244186*
**ENSG00000156689**	*0.26136364*		*0.26136364*		*0.409090909*	*0.4204545*	
**ENSG00000205085**	*0.21556886*		*0.21556886*		*0.428571429*	*0.4244186*	
**ENSG00000072201**	*0.3*	*0.15730337*	*0.3*	*0.157303371*	*0.375*	*0.3452381*	*0.1976744*
**ENSG00000198053**		*0.3*		*0.3*			*0.3895349*
**ENSG00000204713**		*0.12121212*		*0.121212121*	*0.407407407*	*0.4880952*	*0.1511628*
**ENSG00000170484**		*0.31818182*		*0.318181818*			*0.3888889*
**ENSG00000049449**	*0.19444444*	*0.48333333*	*0.19444444*	*0.483333333*	*0.244186047*	*0.2954545*	*0.2386364*
**ENSG00000136573**	*0.30120482*	*0.48780488*	*0.30120482*	*0.487804878*	*0.238095238*	*0.2965116*	
**ENSG00000143850**		*0.48484848*		*0.484848485*			*0.4318182*
**ENSG00000100170**		*0.49090909*		*0.490909091*			*0.2848837*
**ENSG00000211695**	*0.23053892*		*0.23053892*		*0.458333333*	*0.5*	
**ENSG00000140948**		*0.44099379*		*0.440993789*			*0.4883721*
**ENSG00000229676**	*0.28089888*	*0.10606061*	*0.28089888*	*0.106060606*			*0.2209302*
**ENSG00000157827**	*0.075*	*0.45757576*	*0.075*	*0.457575758*			*0.2710843*
**ENSG00000240764**		*0.20224719*		*0.202247191*			*0.1477273*
**ENSG00000178999**		*0.1030303*		*0.103030303*			*0.3546512*
**ENSG00000156738**		*0.46666667*		*0.466666667*			*0.4418605*
**ENSG00000166526**		*0.33333333*		*0.333333333*			*0.494186*
**ENSG00000139746**		*0.48484848*		*0.484848485*			*0.494186*
**ENSG00000104497**	*0.43888889*	*0.37878788*	*0.43888889*	*0.378787879*	*0.380952381*	*0.4186047*	*0.4431818*

**ENSG00000230786**	*0.24444444*	*0.22424242*	*0.24444444*	*0.224242424*	*0.155555556*		*0.1590909*	*0.4360465*
**ENSG00000160799**		*0.29651163*		*0.296511628*				*0.4204545*
**ENSG00000122121**								*0.4*
**ENSG00000077009**	*0.37037037*		*0.37037037*					
**ENSG00000211734**	*0.41573034*		*0.41573034*		*0.133333333*		*0.125*	
**ENSG00000170166**	*0.49401198*	*0.21036585*	*0.49401198*	*0.210365854*	*0.071428571*		*0.1453488*	
**ENSG00000135108**		*0.46794872*		*0.467948718*				*0.3846154*
**ENSG00000100429**		*0.33536585*		*0.335365854*				*0.1744186*
**ENSG00000089597**								*0.0529412*
**ENSG00000162614**								*0.0681818*
**ENSG00000164976**		*0.2*		*0.2*	*0.122222222*		*0.125*	*0.35*
**ENSG00000181135**	*0.20359281*	*0.13939394*	*0.20359281*	*0.139393939*	*0.255952381*		*0.2383721*	*0.2613636*
**ENSG00000248458**	*0.05120482*		*0.05120482*		*0.077380952*		*0.0823529*	
**ENSG00000214078**	*0.10555556*	*0.18292683*	*0.10555556*	*0.182926829*				*0.0988372*
**ENSG00000113658**	*0.42215569*	*0.30182927*	*0.42215569*	*0.301829268*	*0.43452381*		*0.2848837*	*0.2797619*
**ENSG00000169499**		*0.44444444*		*0.444444444*				*0.3571429*
**ENSG00000149499**		*0.32121212*		*0.321212121*				
**ENSG00000095203**		*0.39090909*		*0.390909091*				*0.1569767*
**ENSG00000154473**	*0.32335329*		*0.32335329*		*0.422619048*		*0.3313953*	
**ENSG00000185008**	*0.25*	*0.46666667*	*0.25*	*0.466666667*	*0.339285714*		*0.372093*	*0.494186*
**ENSG00000123415**	*0.32035928*	*0.47058824*	*0.32035928*	*0.470588235*	*0.398809524*		*0.4186047*	*0.4545455*
**ENSG00000112763**	*0.45508982*	*0.16111111*	*0.45508982*	*0.161111111*	*0.077380952*		*0.0697674*	*0.1802326*
**ENSG00000196262**		*0.38484848*		*0.384848485*				*0.1918605*
**ENSG00000148344**		*0.06097561*		*0.06097561*				*0.3470588*
**ENSG00000176912**		*0.47560976*		*0.475609756*				*0.1627907*
**ENSG00000141744**	*0.26047904*		*0.26047904*		*0.416666667*		*0.3941176*	
**ENSG00000075975**		*0.3969697*		*0.396969697*				*0.1428571*
**ENSG00000183066**		*0.49393939*		*0.493939394*				*0.1818182*
**ENSG00000116983**		*0.44545455*		*0.445454545*				*0.4825581*
**ENSG00000224982**		*0.19393939*		*0.193939394*				*0.3255814*
**ENSG00000064012**	*0.1741573*	*0.12222222*	*0.1741573*	*0.122222222*				*0.2965116*
**ENSG00000169432**	*0.47222222*	*0.12643678*	*0.47222222*	*0.126436782*	*0.055555556*		*0.0888889*	*0.1*
**ENSG00000147896**		*0.24242424*		*0.242424242*				*0.1802326*
**ENSG00000164144**	*0.12874251*	*0.40909091*	*0.12874251*	*0.409090909*	*0.451807229*		*0.5*	*0.2906977*
**ENSG00000028137**		*0.49689441*		*0.49689441*				*0.4647059*
**ENSG00000205189**		*0.22727273*		*0.227272727*				*0.1882353*
**ENSG00000180205**	*0.11111111*		*0.11111111*					
**ENSG00000087157**		*0.23636364*		*0.236363636*				
**ENSG00000069424**	*0.34444444*	*0.4137931*	*0.34444444*	*0.413793103*	*0.077777778*		*0.0909091*	*0.3181818*
**ENSG00000204967**		*0.05151515*		*0.051515152*				*0.1162791*
**ENSG00000136449**		*0.25757576*		*0.257575758*				*0.1453488*
**ENSG00000127328**		*0.32727273*		*0.327272727*				*0.4360465*
**ENSG00000198610**	*0.29341317*	*0.09444444*	*0.29341317*	*0.094444444*	*0.166666667*		*0.0697674*	
**ENSG00000139350**		*0.48181818*		*0.481818182*				*0.4709302*
**ENSG00000100068**		*0.13030303*		*0.13030303*	*0.267857143*		*0.2674419*	*0.4318182*
**ENSG00000145730**	*0.28742515*	*0.38202247*	*0.28742515*	*0.382022472*	*0.351190476*		*0.4011628*	*0.4825581*
**ENSG00000183023**		*0.45454545*		*0.454545455*				*0.4883721*
**ENSG00000069020**		*0.05246914*		*0.052469136*				*0.2571429*
**ENSG00000196150**		*0.38484848*		*0.384848485*				*0.5*
**ENSG00000196535**		*0.4969697*		*0.496969697*				*0.4767442*
**ENSG00000163633**		*0.19090909*		*0.190909091*				
**ENSG00000134013**	*0.37777778*	*0.4*	*0.37777778*	*0.4*	*0.444444444*		*0.4545455*	*0.4593023*
**ENSG00000183379**		*0.34848485*		*0.348484848*				*0.3777778*
**ENSG00000012171**	*0.43939394*		*0.43939394*					

**ENSG00000137486**	*0.42045455*	*0.13939394*	*0.42045455*	*0.139393939*	*0.488095238*	*0.372093*	*0.3895349*
**ENSG00000177459**		*0.38484848*		*0.384848485*			*0.4767442*
**ENSG00000161298**	*0.0813253*	*0.23888889*	*0.0813253*	*0.238888889*	*0.493975904*	*0.3953488*	*0.125*
**ENSG00000150712**		*0.22049689*		*0.220496894*			*0.2941176*
**ENSG00000214353**		*0.17272727*		*0.172727273*			*0.1395349*
**ENSG00000138686**	*0.18181818*	*0.35454545*	*0.18181818*	*0.354545455*	*0.455555556*	*0.4772727*	*0.4888889*
**ENSG00000115464**		*0.17575758*		*0.175757576*			*0.372093*
**ENSG00000146729**	*0.44382022*	*0.23939394*	*0.44382022*	*0.239393939*	*0.302325581*	*0.3295455*	*0.4069767*
**ENSG00000101004**		*0.46969697*		*0.46969697*			*0.1071429*
**ENSG00000013293**							*0.2560976*
**ENSG00000196735**	*0.40184049*	*0.15517241*	*0.40184049*	*0.155172414*	*0.445121951*	*0.4883721*	*0.402439*
**ENSG00000159921**	*0.09580838*	*0.24242424*	*0.09580838*	*0.242424242*			*0.3430233*
**ENSG00000131845**		*0.49393939*		*0.493939394*			*0.3139535*
**ENSG00000145864**		*0.32121212*		*0.321212121*			*0.494186*
**ENSG00000180071**		*0.29141104*		*0.291411043*			*0.4390244*
**ENSG00000105671**	*0.07784431*	*0.45555556*	*0.07784431*	*0.455555556*			*0.2590361*
**ENSG00000158270**		*0.20552147*		*0.205521472*			*0.3154762*
**ENSG00000205981**		*0.16111111*		*0.161111111*			*0.0568182*
**ENSG00000077235**		*0.17777778*		*0.177777778*			
**ENSG00000132846**		*0.27575758*		*0.275757576*			*0.3154762*
**ENSG00000164180**		*0.33641975*		*0.336419753*			*0.2926829*
**ENSG00000105058**							*0.1395349*
**ENSG00000136155**		*0.37272727*		*0.372727273*			*0.4534884*
**ENSG00000162897**	*0.17365269*	*0.25151515*	*0.17365269*	*0.251515152*	*0.214285714*	*0.1569767*	*0.1511628*
**ENSG00000172661**	*0.18965517*		*0.18965517*		*0.088888889*	*0.2045455*	
**ENSG00000243335**		*0.40229885*		*0.402298851*			*0.4*
**ENSG00000222009**		*0.37777778*		*0.377777778*			*0.4090909*
**ENSG00000174611**		*0.38764045*		*0.387640449*			*0.2045455*
**ENSG00000161671**		*0.23780488*		*0.237804878*			*0.4011628*
**ENSG00000158786**		*0.26060606*		*0.260606061*			*0.2965116*
**ENSG00000132950**	*0.35534591*	*0.10909091*	*0.35534591*	*0.109090909*			
**ENSG00000152495**		*0.40606061*		*0.406060606*			*0.4404762*
**ENSG00000126860**	*0.24550898*	*0.38787879*	*0.24550898*	*0.387878788*	*0.422619048*	*0.4883721*	*0.4117647*
**ENSG00000115604**	*0.16666667*	*0.28787879*	*0.16666667*	*0.287878788*			*0.4186047*
**ENSG00000135324**		*0.16060606*		*0.160606061*			*0.1453488*
**ENSG00000214694**	*0.31111111*	*0.06666667*	*0.31111111*	*0.066666667*	*0.255555556*	*0.1931818*	
**ENSG00000162813**	*0.13473054*		*0.13473054*				
**ENSG00000181222**		*0.37272727*		*0.372727273*			*0.4360465*
**ENSG00000090686**	*0.0508982*	*0.3*	*0.0508982*	*0.3*	*0.420731707*	*0.4470588*	*0.3953488*
**ENSG00000100350**		*0.23333333*		*0.233333333*			*0.3181818*
**ENSG00000198393**	*0.35955056*		*0.35955056*		*0.188888889*	*0.1477273*	
**ENSG00000103740**	*0.17065868*	*0.48170732*	*0.17065868*	*0.481707317*	*0.160714286*	*0.1744186*	*0.2790698*
**ENSG00000137764**	*0.35329341*	*0.21515152*	*0.35329341*	*0.215151515*	*0.202380952*	*0.2732558*	*0.4825581*
**ENSG00000231852**		*0.0875*		*0.0875*			
**ENSG00000151093**		*0.16060606*		*0.160606061*			*0.122093*
**ENSG00000048342**		*0.36363636*		*0.363636364*			*0.4186047*
**ENSG00000153707**	*0.26875*	*0.05*	*0.26875*	*0.05*	*0.107142857*	*0.0888889*	
**ENSG00000118600**		*0.06024096*		*0.060240964*			*0.3068182*
**ENSG00000109133**		*0.27878788*		*0.278787879*			
**ENSG00000135457**	*0.23053892*	*0.25151515*	*0.23053892*	*0.251515152*	*0.285714286*	*0.2209302*	*0.4011628*
**ENSG00000183665**	*0.17365269*		*0.17365269*		*0.101190476*	*0.1627907*	*0.2386364*
**ENSG00000023318**		*0.45757576*		*0.457575758*			*0.0639535*
**ENSG00000152910**		*0.25454545*		*0.254545455*			*0.122093*
**ENSG00000100453**	*0.35329341*	*0.22424242*	*0.35329341*	*0.224242424*	*0.267857143*	*0.2383721*	*0.2674419*

**ENSG00000141934**		*0.26687117*		*0.266871166*			*0.1428571*
**ENSG00000188613**							*0.1337209*
**ENSG00000160221**		*0.18484848*		*0.184848485*			*0.2093023*
**ENSG00000169314**					*0.107142857*	*0.0697674*	
**ENSG00000183439**		*0.3445122*		*0.344512195*			*0.3988095*
**ENSG00000134575**	*0.38787879*	*0.37878788*	*0.38787879*	*0.378787879*	*0.297619048*	*0.2616279*	*0.3863636*
**ENSG00000156958**		*0.24242424*		*0.242424242*	*0.107142857*	*0.0755814*	*0.0523256*
**ENSG00000133739**		*0.35714286*		*0.357142857*			*0.3869048*
**ENSG00000132002**		*0.18404908*		*0.18404908*			
**ENSG00000046889**		*0.38068182*		*0.380681818*			*0.5*
**ENSG00000214050**		*0.28089888*		*0.280898876*			*0.3295455*
**ENSG00000166394**	*0.21257485*	*0.33841463*	*0.21257485*	*0.338414634*			*0.1337209*
**ENSG00000101190**		*0.29754601*		*0.297546012*			*0.0952381*
**ENSG00000102384**	*0.20481928*	*0.3*	*0.20481928*	*0.3*	*0.267857143*	*0.25*	*0.2383721*
**ENSG00000130513**		*0.14848485*		*0.148484848*			*0.1976744*
**ENSG00000186073**	*0.49438202*	*0.16768293*	*0.49438202*	*0.167682927*	*0.454545455*	*0.4545455*	*0.1036585*
**ENSG00000173531**		*0.25757576*		*0.257575758*			*0.0833333*
**ENSG00000179562**		*0.19393939*		*0.193939394*			*0.3*
**ENSG00000203867**		*0.09090909*		*0.090909091*			*0.2823529*
**ENSG00000186017**		*0.29411765*		*0.294117647*			*0.1627907*
**ENSG00000125898**	*0.38764045*	*0.35151515*	*0.38764045*	*0.351515152*	*0.056818182*		*0.3023256*
**ENSG00000135903**		*0.18484848*		*0.184848485*			*0.2034884*
**ENSG00000164935**		*0.11212121*		*0.112121212*			*0.0755814*
**ENSG00000132780**		*0.4375*		*0.4375*		*0.0909091*	*0.3372093*
**ENSG00000205821**	*0.33532934*		*0.33532934*				
**ENSG00000115468**	*0.25449102*	*0.33030303*	*0.25449102*	*0.33030303*	*0.172619048*	*0.25*	*0.4709302*
**ENSG00000203685**		*0.46666667*		*0.466666667*			*0.4186047*
**ENSG00000127419**	*0.36666667*	*0.11818182*	*0.36666667*	*0.118181818*	*0.351190476*	*0.2965116*	*0.122093*
**ENSG00000040341**	*0.17964072*	*0.22727273*	*0.17964072*	*0.227272727*			*0.494186*
**ENSG00000101435**	*0.19760479*		*0.19760479*		*0.464285714*	*0.4360465*	
**ENSG00000116685**		*0.43636364*		*0.436363636*			*0.3430233*
**ENSG00000214688**		*0.23619632*		*0.236196319*			*0.4360465*
**ENSG00000116584**	*0.14071856*		*0.14071856*		*0.160714286*	*0.1569767*	
**ENSG00000152669**	*0.31097561*		*0.31097561*		*0.317073171*	*0.2375*	
**ENSG00000198108**							*0.2267442*
**ENSG00000074181**		*0.26060606*		*0.260606061*			*0.375*
**ENSG00000196242**	*0.20481928*		*0.20481928*		*0.113095238*	*0.0988372*	
**ENSG00000143140**	*0.1011236*	*0.27575758*	*0.1011236*	*0.275757576*			*0.4709302*
**ENSG00000173349**	*0.42987805*		*0.42987805*		*0.357142857*	*0.25*	
**ENSG00000241697**		*0.05*		*0.05*			*0.1704545*
**ENSG00000159388**		*0.07575758*		*0.075757576*			
**ENSG00000140406**		*0.05*		*0.05*			
**ENSG00000065029**		*0.18965517*		*0.189655172*			*0.4127907*
**ENSG00000179115**	*0.12275449*		*0.12275449*		*0.160714286*	*0.1627907*	
**ENSG00000070214**	*0.37640449*	*0.13333333*	*0.37640449*	*0.133333333*			
**ENSG00000106829**	*0.18263473*		*0.18263473*		*0.244047619*	*0.3176471*	
**ENSG00000140464**		*0.48484848*		*0.484848485*			*0.3372093*
**ENSG00000172478**	*0.15868263*	*0.46969697*	*0.15868263*	*0.46969697*	*0.477777778*	*0.3313953*	*0.4011628*
**ENSG00000103994**		*0.1*		*0.1*			*0.1477273*
**ENSG00000142698**	*0.05120482*	*0.48181818*	*0.05120482*	*0.481818182*	*0.25*	*0.3023256*	*0.4476744*
**ENSG00000169964**		*0.3*		*0.3*			
**ENSG00000105497**	*0.34659091*	*0.35*	*0.34659091*	*0.35*	*0.220238095*	*0.2674419*	*0.304878*
**ENSG00000140254**	*0.32934132*		*0.32934132*		*0.488095238*	*0.4825581*	*0.0697674*
**ENSG00000136514**		*0.35757576*		*0.357575758*			*0.2383721*

**ENSG00000118200**		*0.19393939*		*0.193939394*				*0.4825581*
**ENSG00000253537**		*0.20224719*		*0.202247191*				*0.1477273*
**ENSG00000084444**		*0.15243902*		*0.152439024*				
**ENSG00000174840**		*0.31707317*		*0.317073171*				*0.0930233*
**ENSG00000141664**		*0.22121212*		*0.221212121*				*0.3139535*
**ENSG00000164305**		*0.48295455*		*0.482954545*				*0.1890244*
**ENSG00000173889**		*0.42987805*		*0.429878049*				*0.4186047*
**ENSG00000065989**		*0.37272727*		*0.372727273*				*0.5*
**ENSG00000197375**		*0.39090909*		*0.390909091*				*0.2616279*
**ENSG00000104112**	*0.0872093*	*0.4702381*	*0.0872093*	*0.470238095*	*0.113095238*		*0.1511628*	*0.3658537*
**ENSG00000026508**		*0.3404908*		*0.340490798*				*0.4476744*
**ENSG00000174943**		*0.42236025*		*0.422360248*				*0.2916667*
**ENSG00000163453**		*0.16768293*		*0.167682927*				
**ENSG00000161203**		*0.45731707*		*0.457317073*				*0.3882353*
**ENSG00000183876**	*0.17045455*	*0.08841463*	*0.17045455*	*0.088414634*	*0.1*		*0.0888889*	
**ENSG00000255529**	*0.4491018*	*0.2*	*0.4491018*	*0.2*	*0.416666667*		*0.4883721*	*0.4244186*
**ENSG00000112365**		*0.38484848*		*0.384848485*				*0.3953488*
**ENSG00000157800**	*0.26946108*	*0.39444444*	*0.26946108*	*0.394444444*	*0.19047619*		*0.1162791*	*0.1477273*
**ENSG00000204843**	*0.43712575*		*0.43712575*		*0.5*		*0.4761905*	
**ENSG00000099960**								*0.4886364*
**ENSG00000080608**							*0.1*	
**ENSG00000119661**		*0.08841463*		*0.088414634*				*0.3647059*
**ENSG00000082781**		*0.36931818*		*0.369318182*				*0.4555556*
**ENSG00000003400**	*0.38922156*	*0.44444444*	*0.38922156*	*0.444444444*				*0.1477273*
**ENSG00000107438**		*0.23033708*		*0.230337079*				*0.1363636*
**ENSG00000075407**	*0.48795181*	*0.23333333*	*0.48795181*	*0.233333333*	*0.427710843*		*0.4825581*	*0.1590909*
**ENSG00000108064**	*0.26946108*	*0.39393939*	*0.26946108*	*0.393939394*	*0.19047619*		*0.1569767*	*0.4352941*
**ENSG00000128699**	*0.35151515*	*0.24444444*	*0.35151515*	*0.244444444*	*0.071428571*		*0.0523256*	*0.2616279*
**ENSG00000159461**		*0.4054878*		*0.405487805*				*0.4186047*
**ENSG00000149182**		*0.46363636*		*0.463636364*				*0.2705882*
**ENSG00000061676**		*0.32121212*		*0.321212121*				*0.1686047*
**ENSG00000103671**		*0.14242424*		*0.142424242*				
**ENSG00000186918**	*0.31666667*	*0.41515152*	*0.31666667*	*0.415151515*	*0.488888889*		*0.4333333*	*0.3295455*
**ENSG00000197696**		*0.39393939*		*0.393939394*				*0.1104651*
**ENSG00000165629**		*0.33030303*		*0.33030303*				*0.2616279*
**ENSG00000128513**		*0.28881988*		*0.288819876*				*0.3430233*
**ENSG00000100347**	*0.20555556*		*0.20555556*		*0.077777778*			
**ENSG00000142875**		*0.40449438*		*0.404494382*				*0.4431818*
**ENSG00000131668**		*0.3969697*		*0.396969697*				*0.2267442*
**ENSG00000161547**					*0.493975904*		*0.4883721*	
**ENSG00000162881**		*0.47777778*		*0.477777778*				*0.3666667*
**ENSG00000080189**					*0.077777778*		*0.0795455*	
**ENSG00000129167**					*0.113636364*		*0.1022727*	
**ENSG00000204542**		*0.33333333*		*0.333333333*				
**ENSG00000151503**	*0.16167665*	*0.35454545*	*0.16167665*	*0.354545455*	*0.416666667*		*0.494186*	*0.494186*
**ENSG00000176390**		*0.11111111*		*0.111111111*				*0.2674419*
**ENSG00000013441**		*0.06666667*		*0.066666667*				
**ENSG00000157219**	*0.39325843*	*0.30606061*	*0.39325843*	*0.306060606*	*0.488888889*		*0.5*	*0.1931818*
**ENSG00000137033**		*0.32317073*		*0.323170732*				*0.4011628*
**ENSG00000065618**		*0.22670807*		*0.226708075*				*0.244186*
**ENSG00000175697**		*0.32926829*		*0.329268293*				*0.3941176*
**ENSG00000179292**		*0.10606061*		*0.106060606*				*0.1860465*
**ENSG00000197472**		*0.44252874*		*0.442528736*				*0.4659091*
**ENSG00000160796**	*0.25*		*0.25*		*0.470238095*		*0.4302326*	

**ENSG00000104946**	*0.40361446*		*0.40361446*		*0.243902439*	*0.2352941*	
**ENSG00000043093**		*0.33333333*		*0.333333333*	*0.220238095*	*0.2209302*	*0.5*
**ENSG00000132681**	*0.11144578*		*0.11144578*		*0.339285714*	*0.2732558*	
**ENSG00000214944**		*0.35057471*		*0.350574713*			*0.4204545*
**ENSG00000115652**	*0.3988764*	*0.21348315*	*0.3988764*	*0.213483146*	*0.455555556*	*0.4666667*	*0.4222222*
**ENSG00000143549**	*0.1257485*	*0.09444444*	*0.1257485*	*0.094444444*	*0.053571429*		*0.4333333*
**ENSG00000166147**	*0.31137725*	*0.27439024*	*0.31137725*	*0.274390244*	*0.214285714*	*0.3928571*	*0.4882353*
**ENSG00000119681**		*0.43939394*		*0.439393939*			*0.2093023*
**ENSG00000008988**	*0.41017964*	*0.30555556*	*0.41017964*	*0.305555556*	*0.053571429*		*0.0568182*
**ENSG00000184524**		*0.45757576*		*0.457575758*			*0.2616279*
**ENSG00000104979**		*0.26923077*		*0.269230769*			*0.4069767*
**ENSG00000187556**	*0.24850299*		*0.24850299*		*0.31547619*	*0.3255814*	
**ENSG00000167186**		*0.19318182*		*0.193181818*			*0.0795455*
**ENSG00000160181**		*0.35454545*		*0.354545455*			*0.25*
**ENSG00000197008**	*0.07784431*	*0.40909091*	*0.07784431*	*0.409090909*	*0.148809524*	*0.2111111*	*0.4868421*
**ENSG00000166140**	*0.42352941*	*0.35757576*	*0.42352941*	*0.357575758*	*0.178571429*	*0.2325581*	*0.3546512*
**ENSG00000160844**		*0.26111111*		*0.261111111*			*0.4090909*
**ENSG00000086696**	*0.36144578*		*0.36144578*		*0.5*	*0.3705882*	
**ENSG00000249624**		*0.25757576*		*0.257575758*			*0.4882353*
**ENSG00000116266**	*0.28409091*	*0.1969697*	*0.28409091*	*0.196969697*	*0.244444444*	*0.2386364*	*0.246988*
**ENSG00000167419**	*0.07784431*		*0.07784431*				
**ENSG00000141524**		*0.05792683*		*0.057926829*			*0.1337209*
**ENSG00000123562**	*0.47222222*	*0.40062112*	*0.47222222*	*0.400621118*	*0.329545455*	*0.3295455*	*0.373494*
**ENSG00000204381**	*0.12275449*	*0.33333333*	*0.12275449*	*0.333333333*			
**ENSG00000113302**		*0.2030303*		*0.203030303*			*0.4418605*
**ENSG00000090534**	*0.37575758*	*0.4969697*	*0.37575758*	*0.496969697*	*0.487951807*	*0.4294118*	*0.5*
**ENSG00000146776**		*0.5*		*0.5*			*0.3313953*
**ENSG00000100079**	*0.10429448*		*0.10429448*			*0.255814*	
**ENSG00000112078**	*0.47222222*	*0.21348315*	*0.47222222*	*0.213483146*	*0.388888889*	*0.4204545*	*0.4518072*
**ENSG00000184144**		*0.43333333*		*0.433333333*			*0.3662791*
**ENSG00000188386**		*0.11212121*		*0.112121212*			*0.3604651*
**ENSG00000101331**		*0.20786517*		*0.207865169*			*0.1333333*
**ENSG00000254842**		*0.42378049*		*0.423780488*			*0.4593023*
**ENSG00000075426**		*0.48484848*		*0.484848485*			*0.4431818*
**ENSG00000101966**		*0.42331288*		*0.423312883*			*0.3023256*
**ENSG00000171246**	*0.2005988*	*0.29393939*	*0.2005988*	*0.293939394*			*0.4529412*
**ENSG00000112742**	*0.31736527*	*0.14204545*	*0.31736527*	*0.142045455*	*0.433333333*	*0.3313953*	
**ENSG00000163161**		*0.06666667*		*0.066666667*			
**ENSG00000242715**	*0.14371257*	*0.47256098*	*0.14371257*	*0.472560976*	*0.5*	*0.4186047*	*0.2906977*
**ENSG00000018699**	*0.05389222*		*0.05389222*				
**ENSG00000103852**		*0.14110429*		*0.141104294*			*0.4127907*
**ENSG00000168724**		*0.25454545*		*0.254545455*			*0.4709302*
**ENSG00000162923**		*0.27300613*		*0.273006135*			*0.1453488*
**ENSG00000125247**	*0.29885057*	*0.31460674*	*0.29885057*	*0.314606742*	*0.369047619*	*0.4111111*	*0.1818182*
**ENSG00000116857**	*0.16853933*	*0.47256098*	*0.16853933*	*0.472560976*			*0.3081395*
**ENSG00000156711**		*0.16363636*		*0.163636364*			*0.4651163*
**ENSG00000196639**	*0.31437126*	*0.22424242*	*0.31437126*	*0.224242424*	*0.077380952*	*0.0697674*	*0.244186*
**ENSG00000197249**	*0.29861111*	*0.42378049*	*0.29861111*	*0.423780488*	*0.353658537*	*0.4871795*	*0.3604651*
**ENSG00000066084**		*0.36280488*		*0.362804878*			*0.4545455*
**ENSG00000130940**							*0.0639535*
**ENSG00000187626**		*0.36969697*		*0.36969697*	*0.410714286*	*0.3837209*	*0.3837209*
**ENSG00000058600**		*0.28787879*		*0.287878788*			*0.4761905*
**ENSG00000139178**		*0.46296296*		*0.462962963*			*0.4476744*
**ENSG00000117569**		*0.46666667*		*0.466666667*			*0.3882353*

**ENSG00000213231**		*0.49090909*		*0.490909091*			*0.3058824*
**ENSG00000178538**	*0.48863636*	*0.28220859*	*0.48863636*	*0.282208589*	*0.344444444*	*0.2045455*	*0.3863636*
**ENSG00000127191**	*0.14848485*		*0.14848485*		*0.142857143*	*0.0941176*	
**ENSG00000172689**		*0.46060606*		*0.460606061*			*0.2209302*
**ENSG00000122085**		*0.21515152*		*0.215151515*			*0.4886364*
**ENSG00000198855**		*0.40909091*		*0.409090909*			*0.127907*
**ENSG00000215018**		*0.24545455*		*0.245454545*			*0.2034884*
**ENSG00000144567**	*0.23913043*	*0.4030303*	*0.23913043*	*0.403030303*	*0.446428571*	*0.4294118*	*0.4294118*
**ENSG00000175768**		*0.2*		*0.2*	*0.053571429*		*0.122093*
**ENSG00000135766**	*0.28409091*		*0.28409091*		*0.477777778*	*0.4883721*	
**ENSG00000102796**		*0.28181818*		*0.281818182*			*0.255814*
**ENSG00000065308**		*0.49390244*		*0.493902439*			*0.4886364*
**ENSG00000012061**	*0.37724551*	*0.23939394*	*0.37724551*	*0.239393939*			*0.4825581*
**ENSG00000114349**		*0.34848485*		*0.348484848*			
**ENSG00000131873**		*0.47878788*		*0.478787879*			*0.3081395*
**ENSG00000179299**		*0.31818182*		*0.318181818*			
**ENSG00000175054**		*0.15757576*		*0.157575758*			*0.122093*
**ENSG00000166965**	*0.4691358*	*0.08536585*	*0.4691358*	*0.085365854*	*0.320512821*	*0.4418605*	*0.4647059*
**ENSG00000197054**		*0.084375*		*0.084375*		*0.0833333*	
**ENSG00000131711**		*0.24431818*		*0.244318182*			*0.3953488*
**ENSG00000114023**		*0.43030303*		*0.43030303*			*0.3444444*
**ENSG00000127884**		*0.17575758*		*0.175757576*			
**ENSG00000151458**		*0.17878788*		*0.178787879*			*0.244186*
**ENSG00000189410**		*0.43292683*		*0.432926829*	*0.277777778*	*0.3522727*	*0.1453488*
**ENSG00000041357**	*0.28313253*	*0.39393939*	*0.28313253*	*0.393939394*	*0.439759036*	*0.4767442*	*0.0988372*
**ENSG00000118557**	*0.17283951*	*0.48888889*	*0.17283951*	*0.488888889*	*0.054878049*		*0.3863636*
**ENSG00000184428**	*0.47305389*	*0.28484848*	*0.47305389*	*0.284848485*	*0.071428571*	*0.0988372*	*0.3546512*
**ENSG00000125772**		*0.41111111*		*0.411111111*			*0.4666667*
**ENSG00000196247**	*0.46407186*	*0.1*	*0.46407186*	*0.1*	*0.220238095*	*0.2790698*	
**ENSG00000136141**		*0.22424242*		*0.224242424*			*0.3837209*
**ENSG00000140365**							*0.1104651*
**ENSG00000188004**		*0.14545455*		*0.145454545*			
**ENSG00000152127**		*0.43333333*		*0.433333333*			*0.4888889*
**ENSG00000154803**	*0.4047619*	*0.3*	*0.4047619*	*0.3*	*0.4*	*0.3522727*	*0.2764706*
**ENSG00000136243**	*0.11931818*	*0.1196319*	*0.11931818*	*0.119631902*			
**ENSG00000156467**		*0.46551724*		*0.465517241*			*0.3522727*
**ENSG00000103089**	*0.47865854*	*0.17816092*	*0.47865854*	*0.17816092*		*0.1162791*	
**ENSG00000166979**	*0.48888889*	*0.30792683*	*0.48888889*	*0.307926829*	*0.422222222*	*0.3666667*	*0.3081395*
**ENSG00000129911**		*0.2247191*		*0.224719101*			*0.125*
**ENSG00000124194**		*0.16969697*		*0.16969697*			*0.2616279*
**ENSG00000120616**		*0.13888889*		*0.138888889*			*0.0813953*
**ENSG00000196924**		*0.08536585*		*0.085365854*			*0.1162791*
**ENSG00000226232**					*0.166666667*	*0.1890244*	
**ENSG00000162009**		*0.42424242*		*0.424242424*			*0.1686047*
**ENSG00000196498**	*0.19886364*	*0.14606742*	*0.19886364*	*0.146067416*	*0.466666667*	*0.4318182*	*0.0909091*
**ENSG00000159023**	*0.46407186*	*0.33939394*	*0.46407186*	*0.339393939*	*0.089285714*	*0.0813953*	*0.4476744*
**ENSG00000180758**		*0.4375*		*0.4375*			*0.5*
**ENSG00000164051**	*0.25149701*		*0.25149701*		*0.267857143*	*0.3895349*	
**ENSG00000006638**		*0.17272727*		*0.172727273*			
**ENSG00000175832**	*0.0508982*		*0.0508982*		*0.398809524*	*0.3604651*	
**ENSG00000158106**		*0.19018405*		*0.190184049*			*0.2209302*
**ENSG00000166165**		*0.38414634*		*0.384146341*			*0.2325581*
**ENSG00000124795**		*0.26966292*		*0.269662921*			*0.2267442*
**ENSG00000138835**	*0.18562874*	*0.18787879*	*0.18562874*	*0.187878788*			

**ENSG00000177103**	*0.07222222*	*0.37423313*	*0.07222222*	*0.374233129*	*0.306818182*	*0.2333333*	*0.2790698*
**ENSG00000161570**	*0.41916168*	*0.05151515*	*0.41916168*	*0.051515152*	*0.34939759*	*0.3411765*	
**ENSG00000100225**		*0.29090909*		*0.290909091*			*0.2848837*
**ENSG00000172183**	*0.39820359*		*0.39820359*		*0.208333333*	*0.3255814*	
**ENSG00000241404**							*0.0813953*
**ENSG00000101746**		*0.19875776*		*0.198757764*			
**ENSG00000156110**	*0.1497006*	*0.24242424*	*0.1497006*	*0.242424242*	*0.385542169*	*0.4418605*	*0.1882353*
**ENSG00000204282**		*0.06666667*		*0.066666667*			*0.1547619*
**ENSG00000221874**		*0.20245399*		*0.202453988*			*0.0697674*
**ENSG00000091879**	*0.20359281*	*0.38181818*	*0.20359281*	*0.381818182*	*0.154761905*	*0.1627907*	*0.2647059*
**ENSG00000111358**		*0.42727273*		*0.427272727*			
**ENSG00000163053**		*0.25757576*		*0.257575758*			*0.2790698*
**ENSG00000171729**	*0.32934132*	*0.465625*	*0.32934132*	*0.465625*	*0.30952381*	*0.3529412*	*0.4337349*
**ENSG00000255423**		*0.46666667*		*0.466666667*			*0.4709302*
**ENSG00000100441**	*0.16766467*	*0.47575758*	*0.16766467*	*0.475757576*			*0.2386364*
**ENSG00000203808**		*0.1030303*		*0.103030303*			*0.1453488*
**ENSG00000103199**		*0.38787879*		*0.387878788*			*0.3023256*
**ENSG00000186288**		*0.36046512*		*0.360465116*			
**ENSG00000129204**	*0.22222222*	*0.35555556*	*0.22222222*	*0.355555556*	*0.488888889*	*0.4204545*	*0.0764706*
**ENSG00000196233**							*0.3604651*
**ENSG00000125731**		*0.17272727*		*0.172727273*			*0.1976744*
**ENSG00000111262**	*0.0873494*	*0.47865854*	*0.0873494*	*0.478658537*	*0.136904762*	*0.1162791*	*0.4011628*
**ENSG00000196776**		*0.48181818*		*0.481818182*			*0.3255814*
**ENSG00000136048**		*0.45882353*		*0.458823529*			*0.3333333*
**ENSG00000138613**		*0.44545455*		*0.445454545*			*0.4593023*
**ENSG00000103197**		*0.11666667*		*0.116666667*			
**ENSG00000111700**		*0.10555556*		*0.105555556*			*0.3068182*
**ENSG00000176155**	*0.47891566*	*0.48214286*	*0.47891566*	*0.482142857*	*0.136904762*	*0.1235294*	*0.127907*
**ENSG00000205765**		*0.06363636*		*0.063636364*			*0.0588235*
**ENSG00000134690**	*0.27245509*	*0.44242424*	*0.27245509*	*0.442424242*	*0.246987952*	*0.4186047*	*0.4186047*
**ENSG00000176148**	*0.4760479*	*0.14848485*	*0.4760479*	*0.148484848*	*0.476744186*	*0.4651163*	*0.0755814*
**ENSG00000185305**		*0.11728395*		*0.117283951*			*0.372093*
**ENSG00000076716**		*0.32727273*		*0.327272727*			*0.3882353*
**ENSG00000156298**		*0.42424242*		*0.424242424*			*0.1882353*
**ENSG00000066136**	*0.4491018*		*0.4491018*		*0.5*	*0.5*	
**ENSG00000171217**	*0.44311377*		*0.44311377*		*0.279761905*	*0.3430233*	
**ENSG00000167100**		*0.47256098*		*0.472560976*			*0.4069767*
**ENSG00000176371**		*0.23636364*		*0.236363636*			*0.0872093*
**ENSG00000004766**		*0.32424242*		*0.324242424*			*0.3662791*
**ENSG00000171608**		*0.42121212*		*0.421212121*			*0.5*
**ENSG00000205426**		*0.38333333*		*0.383333333*			*0.1363636*
**ENSG00000159516**		*0.40606061*		*0.406060606*			*0.4235294*
**ENSG00000187372**		*0.2804878*		*0.280487805*			*0.1823529*
**ENSG00000172890**	*0.22754491*	*0.28181818*	*0.22754491*	*0.281818182*	*0.095238095*	*0.122093*	*0.3837209*
**ENSG00000144747**		*0.47239264*		*0.472392638*			*0.4651163*
**ENSG00000008323**							*0.0988372*
**ENSG00000078699**	*0.16766467*	*0.16969697*	*0.16766467*	*0.16969697*			*0.3647059*
**ENSG00000197063**	*0.06287425*		*0.06287425*				
**ENSG00000172927**	*0.22155689*		*0.22155689*		*0.422619048*	*0.4593023*	
**ENSG00000151422**	*0.31437126*	*0.48181818*	*0.31437126*	*0.481818182*			*0.4886364*
**ENSG00000122133**							*0.4767442*
**ENSG00000160216**	*0.28333333*	*0.30487805*	*0.28333333*	*0.304878049*	*0.255555556*	*0.3863636*	*0.3837209*
**ENSG00000115844**	*0.13636364*		*0.13636364*		*0.122222222*	*0.1363636*	
**ENSG00000198146**		*0.15*		*0.15*			

**ENSG00000170965**		*0.35365854*		*0.353658537*			*0.2882353*
**ENSG00000125618**		*0.46363636*		*0.463636364*			*0.3546512*
**ENSG00000144535**		*0.2*		*0.2*			*0.4235294*
**ENSG00000139531**	*0.38323353*		*0.38323353*		*0.428571429*	*0.4360465*	
**ENSG00000071575**	*0.20958084*	*0.46363636*	*0.20958084*	*0.463636364*			*0.1860465*
**ENSG00000179912**	*0.06111111*		*0.06111111*		*0.111111111*	*0.0666667*	
**ENSG00000064601**	*0.14371257*		*0.14371257*		*0.482142857*	*0.4534884*	
**ENSG00000109805**	*0.35329341*	*0.49444444*	*0.35329341*	*0.494444444*	*0.458333333*	*0.494186*	*0.2674419*
**ENSG00000029534**		*0.17272727*		*0.172727273*			*0.1627907*
**ENSG00000126217**	*0.47093023*	*0.48181818*	*0.47093023*	*0.481818182*	*0.410714286*	*0.372093*	*0.4244186*
**ENSG00000114770**		*0.4969697*		*0.496969697*			*0.5*
**ENSG00000168685**		*0.42424242*		*0.424242424*			*0.4593023*
**ENSG00000104321**		*0.49444444*		*0.494444444*	*0.066666667*		*0.3662791*
**ENSG00000120594**					*0.493975904*	*0.4647059*	
**ENSG00000196878**		*0.27878788*		*0.278787879*	*0.220238095*	*0.2383721*	*0.3546512*
**ENSG00000179930**		*0.10555556*		*0.105555556*			*0.2111111*
**ENSG00000086062**		*0.48333333*		*0.483333333*			*0.4767442*
**ENSG00000143257**		*0.41212121*		*0.412121212*			*0.4651163*
**ENSG00000067369**		*0.30487805*		*0.304878049*			*0.3837209*
**ENSG00000196557**							*0.0581395*
**ENSG00000198216**		*0.35757576*		*0.357575758*			*0.5*
**ENSG00000182732**		*0.19393939*		*0.193939394*			*0.2383721*
**ENSG00000249087**		*0.21818182*		*0.218181818*			*0.2848837*
**ENSG00000100099**	*0.32934132*	*0.45555556*	*0.32934132*	*0.455555556*	*0.279761905*	*0.2151163*	*0.3295455*
**ENSG00000185862**		*0.31818182*		*0.318181818*			*0.4360465*
**ENSG00000197323**	*0.40662651*	*0.07303371*	*0.40662651*	*0.073033708*			
**ENSG00000250361**		*0.43150685*		*0.431506849*			
**ENSG00000171790**					*0.261904762*	*0.2142857*	
**ENSG00000049167**		*0.44242424*		*0.442424242*			*0.125*
**ENSG00000169509**		*0.39393939*		*0.393939394*			*0.2674419*
**ENSG00000124839**	*0.47590361*	*0.24848485*	*0.47590361*	*0.248484848*	*0.410714286*	*0.4244186*	*0.3662791*
**ENSG00000232423**		*0.065625*		*0.065625*			*0.1130952*
**ENSG00000124222**		*0.46036585*		*0.460365854*			*0.5*
**ENSG00000143801**	*0.31437126*		*0.31437126*		*0.291666667*	*0.1309524*	
**ENSG00000175854**							*0.1046512*
**ENSG00000090565**		*0.46363636*		*0.463636364*			*0.1627907*
**ENSG00000115866**	*0.20348837*		*0.20348837*		*0.422222222*	*0.2272727*	
**ENSG00000100593**		*0.375*		*0.375*			*0.0697674*
**ENSG00000138382**		*0.09509202*		*0.095092025*			
**ENSG00000186469**	*0.35329341*	*0.36111111*	*0.35329341*	*0.361111111*	*0.410714286*	*0.4011628*	*0.4204545*
**ENSG00000104626**	*0.34730539*	*0.4847561*	*0.34730539*	*0.484756098*	*0.273809524*	*0.2093023*	*0.4333333*
**ENSG00000177169**	*0.26646707*	*0.25757576*	*0.26646707*	*0.257575758*	*0.321428571*	*0.3546512*	
**ENSG00000106052**	*0.20958084*		*0.20958084*		*0.125*	*0.1153846*	
**ENSG00000182612**	*0.44011976*		*0.44011976*		*0.208333333*	*0.2906977*	
**ENSG00000204538**	*0.4625*	*0.37272727*	*0.4625*	*0.372727273*	*0.404761905*	*0.3430233*	*0.4651163*
**ENSG00000134532**	*0.47904192*		*0.47904192*		*0.288888889*	*0.2674419*	
**ENSG00000257076**		*0.3902439*		*0.390243902*			*0.2732558*
**ENSG00000156052**		*0.35*		*0.35*			*0.4244186*
**ENSG00000172346**		*0.14457831*		*0.144578313*			
**ENSG00000139083**		*0.35*		*0.35*			*0.4444444*
**ENSG00000204514**	*0.34431138*	*0.36969697*	*0.34431138*	*0.36969697*			*0.3895349*
**ENSG00000164007**		*0.4382716*		*0.438271605*			*0.4285714*
**ENSG00000121281**		*0.48787879*		*0.487878788*			*0.4277108*
**ENSG00000104738**	*0.33532934*		*0.33532934*		*0.156626506*	*0.1369048*	

**ENSG00000006451**		*0.34883721*		*0.348837209*				
**ENSG00000155545**	*0.49101796*	*0.26969697*	*0.49101796*	*0.26969697*	*0.43452381*		*0.4651163*	*0.3470588*
**ENSG00000111328**					*0.05952381*			
**ENSG00000136682**		*0.2247191*		*0.224719101*				*0.4111111*
**ENSG00000068650**	*0.15568862*	*0.41818182*	*0.15568862*	*0.418181818*	*0.160714286*		*0.0813953*	*0.4651163*
**ENSG00000111775**		*0.35*		*0.35*				*0.3068182*
**ENSG00000206262**								*0.0813953*
**ENSG00000165487**		*0.16363636*		*0.163636364*				*0.3588235*
**ENSG00000172602**		*0.46625767*		*0.466257669*				*0.4069767*
**ENSG00000168876**	*0.11077844*	*0.14545455*	*0.11077844*	*0.145454545*				*0.1046512*
**ENSG00000187456**					*0.214285714*		*0.1918605*	
**ENSG00000072310**	*0.12349398*		*0.12349398*					
**ENSG00000152642**		*0.42528736*		*0.425287356*				*0.4069767*
**ENSG00000179826**	*0.25903614*		*0.25903614*		*0.101190476*		*0.0882353*	
**ENSG00000169446**		*0.16459627*		*0.164596273*			*0.0930233*	*0.0952381*
**ENSG00000251258**		*0.21212121*		*0.212121212*				*0.0529412*
**ENSG00000182218**		*0.05487805*		*0.054878049*				
**ENSG00000162398**		*0.17878788*		*0.178787879*				*0.3953488*
**ENSG00000102901**	*0.13772455*		*0.13772455*		*0.119047619*		*0.127907*	
**ENSG00000148700**								*0.0988372*
**ENSG00000172197**		*0.06060606*		*0.060606061*				
**ENSG00000156973**		*0.22413793*		*0.224137931*				*0.1022727*
**ENSG00000141200**					*0.428571429*		*0.3546512*	
**ENSG00000162076**	*0.41317365*	*0.45757576*	*0.41317365*	*0.457575758*	*0.465116279*		*0.4545455*	*0.244186*
**ENSG00000213281**		*0.24085366*		*0.240853659*	*0.184210526*		*0.3636364*	
**ENSG00000169717**								*0.1686047*
**ENSG00000100941**		*0.23291925*		*0.232919255*				*0.1802326*
**ENSG00000170458**	*0.31609195*	*0.23636364*	*0.31609195*	*0.236363636*	*0.5*		*0.4875*	*0.1686047*
**ENSG00000184005**		*0.48255814*		*0.48255814*				*0.244186*
**ENSG00000131115**	*0.43712575*		*0.43712575*		*0.083333333*		*0.1511628*	*0.1352941*
**ENSG00000198106**	*0.25*		*0.25*		*0.431818182*		*0.3953488*	
**ENSG00000148110**		*0.28333333*		*0.283333333*				
**ENSG00000241258**	*0.28181818*	*0.37878788*	*0.28181818*	*0.378787879*	*0.333333333*		*0.3895349*	*0.494186*
**ENSG00000100461**	*0.36526946*	*0.2030303*	*0.36526946*	*0.203030303*	*0.261904762*		*0.1647059*	*0.1918605*
**ENSG00000004799**	*0.38323353*	*0.46666667*	*0.38323353*	*0.466666667*	*0.238095238*		*0.2093023*	*0.2272727*
**ENSG00000206260**		*0.11111111*		*0.111111111*				*0.1363636*
**ENSG00000164241**								*0.1104651*
**ENSG00000110848**	*0.15*	*0.34242424*	*0.15*	*0.342424242*				*0.3977273*
**ENSG00000183576**		*0.4030303*		*0.403030303*				*0.4476744*
**ENSG00000163618**		*0.37575758*		*0.375757576*				*0.3430233*
**ENSG00000169885**					*0.482142857*		*0.4767442*	
**ENSG00000254290**		*0.49090909*		*0.490909091*	*0.113095238*		*0.0930233*	*0.4545455*
**ENSG00000135912**	*0.09281437*	*0.44817073*	*0.09281437*	*0.448170732*	*0.160714286*		*0.1627907*	*0.1627907*
**ENSG00000157540**	*0.32831325*	*0.38181818*	*0.32831325*	*0.381818182*	*0.369047619*		*0.3882353*	*0.3953488*
**ENSG00000185739**		*0.48787879*		*0.487878788*				*0.4651163*
**ENSG00000125257**		*0.46969697*		*0.46969697*				*0.4705882*
**ENSG00000114209**	*0.45508982*		*0.45508982*		*0.303571429*		*0.3197674*	
**ENSG00000112214**		*0.17272727*		*0.172727273*				*0.2790698*
**ENSG00000155329**		*0.22121212*		*0.221212121*				*0.25*
**ENSG00000141720**		*0.4969697*		*0.496969697*				*0.4534884*
**ENSG00000156990**		*0.12048193*		*0.120481928*				*0.4360465*
**ENSG00000180066**		*0.06969697*		*0.06969697*				*0.0697674*
**ENSG00000170153**		*0.42121212*		*0.421212121*				*0.4127907*
**ENSG00000137504**		*0.17272727*		*0.172727273*				*0.4360465*

**ENSG00000156482**	*0.46407186*		*0.46407186*		*0.071428571*			
**ENSG00000116661**		*0.20909091*		*0.209090909*				*0.2732558*
**ENSG00000010256**								*0.0581395*
**ENSG00000167034**	*0.34883721*	*0.4137931*	*0.34883721*	*0.413793103*	*0.344444444*		*0.3214286*	*0.372093*
**ENSG00000206026**		*0.25842697*		*0.258426966*				*0.3372093*
**ENSG00000065320**								*0.4825581*
**ENSG00000171501**	*0.38484848*		*0.38484848*		*0.475903614*		*0.4638554*	
**ENSG00000165863**		*0.05454545*		*0.054545455*				*0.3837209*
**ENSG00000101844**								*0.0523256*
**ENSG00000166482**		*0.17878788*		*0.178787879*				
**ENSG00000133816**	*0.34242424*		*0.34242424*		*0.402439024*		*0.3647059*	
**ENSG00000059758**		*0.18787879*		*0.187878788*				*0.2*
**ENSG00000091317**		*0.35060976*		*0.350609756*				*0.4186047*
**ENSG00000077232**		*0.34876543*		*0.348765432*				*0.4883721*
**ENSG00000148339**	*0.33532934*	*0.16363636*	*0.33532934*	*0.163636364*				*0.244186*
**ENSG00000025156**	*0.48427673*		*0.48427673*		*0.409638554*		*0.4390244*	
**ENSG00000112081**		*0.18902439*		*0.18902439*				*0.4767442*
**ENSG00000107521**		*0.4969697*		*0.496969697*				*0.4883721*
**ENSG00000163012**	*0.3742515*		*0.3742515*					
**ENSG00000109270**	*0.40419162*	*0.26363636*	*0.40419162*	*0.263636364*	*0.392857143*		*0.494186*	*0.2034884*
**ENSG00000136379**		*0.12727273*		*0.127272727*				*0.3470588*
**ENSG00000166268**	*0.32335329*	*0.36363636*	*0.32335329*	*0.363636364*	*0.142857143*		*0.2151163*	*0.4647059*
**ENSG00000164185**		*0.25757576*		*0.257575758*				*0.3953488*
**ENSG00000095752**		*0.27586207*		*0.275862069*				*0.0581395*
**ENSG00000115827**	*0.41017964*	*0.22256098*	*0.41017964*	*0.222560976*	*0.261904762*		*0.3604651*	*0.3546512*
**ENSG00000188379**		*0.25304878*		*0.25304878*				
**ENSG00000103061**		*0.41818182*		*0.418181818*				*0.1802326*
**ENSG00000113088**		*0.22727273*		*0.227272727*				
**ENSG00000172057**	*0.43333333*		*0.43333333*		*0.409090909*		*0.2840909*	
**ENSG00000101104**	*0.44444444*	*0.39090909*	*0.44444444*	*0.390909091*	*0.388888889*		*0.4111111*	*0.3953488*
**ENSG00000102468**	*0.25149701*	*0.13496933*	*0.25149701*	*0.134969325*				*0.2151163*
**ENSG00000180875**		*0.44512195*		*0.445121951*				*0.3953488*
**ENSG00000084072**		*0.43865031*		*0.438650307*				*0.3941176*
**ENSG00000182749**		*0.49378882*		*0.49378882*				*0.3841463*
**ENSG00000157343**		*0.24848485*		*0.248484848*				*0.3546512*
**ENSG00000136153**	*0.41317365*		*0.41317365*		*0.255555556*		*0.1777778*	
**ENSG00000112852**		*0.06363636*		*0.063636364*				*0.1162791*
**ENSG00000085231**	*0.45783133*		*0.45783133*		*0.103658537*		*0.1506024*	
**ENSG00000188372**	*0.15568862*		*0.15568862*		*0.410714286*		*0.494186*	
**ENSG00000186212**		*0.20245399*		*0.202453988*				*0.2209302*
**ENSG00000203985**		*0.39090909*		*0.390909091*				*0.4011628*
**ENSG00000160214**		*0.41818182*		*0.418181818*				*0.4825581*
**ENSG00000165271**	*0.43712575*	*0.12424242*	*0.43712575*	*0.124242424*	*0.5*		*0.4418605*	*0.2151163*
**ENSG00000113889**	*0.46107784*	*0.41463415*	*0.46107784*	*0.414634146*	*0.321428571*		*0.2764706*	*0.3488372*
**ENSG00000151576**		*0.44848485*		*0.448484848*				*0.4534884*
**ENSG00000205047**	*0.23888889*	*0.09815951*	*0.23888889*	*0.098159509*	*0.101190476*			*0.1686047*
**ENSG00000160339**		*0.28787879*		*0.287878788*				*0.3444444*
**ENSG00000177303**	*0.47647059*		*0.47647059*		*0.4*		*0.4883721*	
**ENSG00000134744**		*0.13636364*		*0.136363636*				*0.1395349*
**ENSG00000110237**		*0.07228916*		*0.072289157*				
**ENSG00000214194**	*0.48888889*	*0.24390244*	*0.48888889*	*0.243902439*	*0.102272727*		*0.0581395*	*0.0529412*
**ENSG00000233276**	*0.20555556*		*0.20555556*		*0.077777778*		*0.1022727*	
**ENSG00000175711**		*0.18390805*		*0.183908046*				*0.2045455*
**ENSG00000099869**	*0.38787879*	*0.47256098*	*0.38787879*	*0.472560976*	*0.108433735*			*0.4883721*

**ENSG00000088986**	*0.17964072*	*0.30606061*	*0.17964072*	*0.306060606*	*0.053571429*			*0.4418605*
**ENSG00000138172**		*0.37272727*		*0.372727273*				*0.3953488*
**ENSG00000253276**		*0.4*		*0.4*				*0.3941176*
**ENSG00000127870**	*0.43113772*		*0.43113772*		*0.416666667*		*0.4534884*	
**ENSG00000116497**	*0.26347305*	*0.30606061*	*0.26347305*	*0.306060606*				*0.2045455*
**ENSG00000205323**		*0.32222222*		*0.322222222*				*0.3181818*
**ENSG00000220205**		*0.2969697*		*0.296969697*				*0.4534884*
**ENSG00000131473**		*0.13333333*		*0.133333333*				*0.0813953*
**ENSG00000187860**	*0.48888889*		*0.48888889*		*0.188888889*		*0.2159091*	
**ENSG00000114812**		*0.47878788*		*0.478787879*				*0.3662791*
**ENSG00000177294**		*0.27743902*		*0.277439024*				*0.2732558*
**ENSG00000197894**		*0.36931818*		*0.369318182*				
**ENSG00000079974**		*0.11515152*		*0.115151515*				*0.3604651*
**ENSG00000152291**	*0.4760479*	*0.49438202*	*0.4760479*	*0.494382022*	*0.090361446*		*0.1918605*	*0.5*
**ENSG00000115145**		*0.28888889*		*0.288888889*				*0.3488372*
**ENSG00000146540**	*0.10465116*	*0.1882716*	*0.10465116*	*0.188271605*	*0.341463415*		*0.4883721*	*0.2267442*
**ENSG00000120053**								*0.0697674*
**ENSG00000055917**	*0.17664671*	*0.3117284*	*0.17664671*	*0.311728395*				*0.2738095*
**ENSG00000166912**		*0.46969697*		*0.46969697*				*0.1395349*
**ENSG00000141994**	*0.21556886*		*0.21556886*		*0.464285714*		*0.3941176*	
**ENSG00000188130**	*0.35555556*		*0.35555556*		*0.5*		*0.3409091*	
**ENSG00000197782**		*0.11494253*		*0.114942529*				*0.1931818*
**ENSG00000031081**	*0.19760479*	*0.25294118*	*0.19760479*	*0.252941176*	*0.279761905*		*0.2848837*	*0.1744186*
**ENSG00000136560**		*0.46363636*		*0.463636364*				*0.4823529*
**ENSG00000164134**		*0.13939394*		*0.139393939*	*0.083333333*			*0.255814*
**ENSG00000188177**		*0.41158537*		*0.411585366*				*0.2267442*
**ENSG00000174231**	*0.3742515*	*0.05454545*	*0.3742515*	*0.054545455*	*0.1*		*0.0568182*	
**ENSG00000177646**	*0.29341317*	*0.29393939*	*0.29341317*	*0.293939394*	*0.44047619*		*0.4767442*	*0.4767442*
**ENSG00000253719**		*0.45121951*		*0.451219512*				*0.4411765*
**ENSG00000183479**	*0.46111111*		*0.46111111*					
**ENSG00000175548**	*0.18711656*	*0.35616438*	*0.18711656*	*0.356164384*	*0.136904762*		*0.1162791*	*0.1*
**ENSG00000150045**		*0.09090909*		*0.090909091*				
**ENSG00000204335**		*0.31111111*		*0.311111111*				*0.3977273*
**ENSG00000147862**	*0.49401198*	*0.30674847*	*0.49401198*	*0.306748466*	*0.44047619*		*0.4767442*	*0.4879518*
**ENSG00000177606**	*0.18862275*		*0.18862275*					
**ENSG00000033170**		*0.41212121*		*0.412121212*	*0.265060241*		*0.1337209*	*0.4476744*
**ENSG00000014641**	*0.11377246*		*0.11377246*		*0.166666667*		*0.2045455*	
**ENSG00000198590**	*0.16666667*		*0.16666667*				*0.2222222*	
**ENSG00000132434**		*0.39440994*		*0.394409938*	*0.409638554*		*0.4767442*	*0.4941176*
**ENSG00000108960**		*0.45757576*		*0.457575758*				*0.3352941*
**ENSG00000136731**	*0.26047904*	*0.39444444*	*0.26047904*	*0.394444444*	*0.202380952*		*0.1627907*	*0.5*
**ENSG00000184828**	*0.12222222*		*0.12222222*		*0.188888889*		*0.2*	
**ENSG00000147180**		*0.11212121*		*0.112121212*				*0.1904762*
**ENSG00000105549**	*0.38023952*		*0.38023952*		*0.13253012*		*0.2790698*	
**ENSG00000171109**		*0.2347561*		*0.234756098*				*0.4418605*
**ENSG00000120436**	*0.17777778*		*0.17777778*		*0.388888889*		*0.3555556*	
**ENSG00000117501**		*0.12777778*		*0.127777778*				
**ENSG00000165480**		*0.31212121*		*0.312121212*				*0.2228916*
**ENSG00000165943**		*0.35060976*		*0.350609756*				*0.2267442*
**ENSG00000175606**	*0.146875*	*0.5*	*0.146875*	*0.5*	*0.204819277*		*0.0941176*	*0.25*
**ENSG00000188277**		*0.47151899*		*0.471518987*				*0.4*
**ENSG00000162236**								*0.1511628*
**ENSG00000157954**		*0.35151515*		*0.351515152*				*0.4651163*
**ENSG00000114796**		*0.24534161*		*0.245341615*				*0.4*

**ENSG00000198496**	*0.20555556*		*0.20555556*		*0.311111111*	*0.2840909*	
**ENSG00000118004**	*0.41111111*		*0.41111111*		*0.1*	*0.1477273*	
**ENSG00000066654**		*0.38787879*		*0.387878788*			*0.2848837*
**ENSG00000113597**	*0.41916168*		*0.41916168*		*0.396341463*	*0.3571429*	
**ENSG00000127472**					*0.273809524*	*0.2790698*	
**ENSG00000254858**		*0.33888889*		*0.338888889*			*0.0909091*
**ENSG00000117461**	*0.3969697*	*0.36666667*	*0.3969697*	*0.366666667*	*0.355421687*	*0.2848837*	*0.2333333*
**ENSG00000137757**	*0.18562874*	*0.47777778*	*0.18562874*	*0.477777778*			*0.2045455*
**ENSG00000136689**	*0.20658683*	*0.32098765*	*0.20658683*	*0.320987654*	*0.273809524*	*0.2209302*	*0.3444444*
**ENSG00000149806**						*0.0581395*	
**ENSG00000073756**		*0.37116564*		*0.371165644*			*0.2383721*
**ENSG00000154511**		*0.1*		*0.1*			
**ENSG00000152229**		*0.1*		*0.1*			*0.4204545*
**ENSG00000108509**		*0.06111111*		*0.061111111*			*0.0909091*
**ENSG00000179837**		*0.17878788*		*0.178787879*			*0.2117647*
**ENSG00000081800**		*0.29268293*		*0.292682927*			*0.3764706*
**ENSG00000165970**		*0.47575758*		*0.475757576*			*0.4593023*
**ENSG00000176463**	*0.12275449*	*0.39772727*	*0.12275449*	*0.397727273*	*0.228395062*	*0.125*	*0.3295455*
**ENSG00000165801**		*0.14942529*		*0.149425287*			*0.4880952*
**ENSG00000185479**	*0.17065868*		*0.17065868*		*0.18452381*	*0.122093*	
**ENSG00000204316**		*0.2652439*		*0.265243902*			*0.2325581*
**ENSG00000143224**	*0.19760479*		*0.19760479*		*0.428571429*	*0.4767442*	
**ENSG00000205502**	*0.0748503*		*0.0748503*		*0.244047619*	*0.2209302*	
**ENSG00000175874**		*0.2969697*		*0.296969697*			*0.1588235*
**ENSG00000135945**		*0.47878788*		*0.478787879*			*0.3176471*
**ENSG00000029364**		*0.19090909*		*0.190909091*			
**ENSG00000018280**	*0.18562874*	*0.36666667*	*0.18562874*	*0.366666667*	*0.136904762*	*0.1117647*	*0.2674419*
**ENSG00000198862**		*0.10060976*		*0.100609756*			*0.1046512*
**ENSG00000005075**		*0.16969697*		*0.16969697*			*0.4593023*
**ENSG00000124299**		*0.27272727*		*0.272727273*			*0.1162791*
**ENSG00000158483**		*0.35393258*		*0.353932584*			*0.3409091*
**ENSG00000104219**		*0.2030303*		*0.203030303*			*0.2823529*
**ENSG00000143889**		*0.22424242*		*0.224242424*			*0.3023256*
**ENSG00000132600**	*0.38922156*		*0.38922156*		*0.226190476*	*0.1918605*	
**ENSG00000164930**	*0.24444444*	*0.48787879*	*0.24444444*	*0.487878788*	*0.422222222*	*0.3977273*	*0.3895349*
**ENSG00000258839**	*0.37078652*	*0.13939394*	*0.37078652*	*0.139393939*	*0.366666667*	*0.2325581*	*0.127907*
**ENSG00000070193**		*0.36363636*		*0.363636364*			*0.4883721*
**ENSG00000009413**	*0.37222222*	*0.44242424*	*0.37222222*	*0.442424242*	*0.488888889*	*0.4318182*	*0.4318182*
**ENSG00000162817**	*0.2994012*	*0.22727273*	*0.2994012*	*0.227272727*	*0.476190476*	*0.4476744*	*0.2093023*
**ENSG00000106305**	*0.3253012*	*0.14848485*	*0.3253012*	*0.148484848*	*0.136904762*	*0.1453488*	*0.0988372*
**ENSG00000115421**		*0.24242424*		*0.242424242*			*0.1931818*
**ENSG00000163888**		*0.22121212*		*0.221212121*			*0.4825581*
**ENSG00000137770**	*0.23053892*	*0.40229885*	*0.23053892*	*0.402298851*	*0.196428571*	*0.127907*	*0.0988372*
**ENSG00000183723**		*0.06707317*		*0.067073171*			*0.2383721*
**ENSG00000149187**		*0.1402439*		*0.140243902*			*0.3546512*
**ENSG00000172466**	*0.45505618*	*0.43258427*	*0.45505618*	*0.43258427*	*0.422619048*	*0.4360465*	*0.25*
**ENSG00000109472**	*0.22155689*		*0.22155689*		*0.303571429*	*0.255814*	
**ENSG00000185379**							*0.1104651*
**ENSG00000145569**		*0.10909091*		*0.109090909*			*0.2159091*
**ENSG00000253598**	*0.47222222*		*0.47222222*		*0.066666667*	*0.1704545*	
**ENSG00000130813**		*0.11818182*		*0.118181818*			
**ENSG00000184350**		*0.34242424*		*0.342424242*			
**ENSG00000135702**	*0.10778443*		*0.10778443*		*0.136904762*	*0.2294118*	
**ENSG00000057252**	*0.09580838*	*0.40909091*	*0.09580838*	*0.409090909*	*0.488095238*	*0.494186*	*0.4825581*

**ENSG00000124615**	*0.23295455*	*0.11042945*	*0.23295455*	*0.110429448*	*0.177777778*		*0.1860465*	*0.2034884*
**ENSG00000222033**		*0.35757576*		*0.357575758*				
**ENSG00000047648**		*0.20245399*		*0.202453988*				
**ENSG00000249967**		*0.1969697*		*0.196969697*				*0.0988372*
**ENSG00000087494**		*0.06363636*		*0.063636364*				*0.1337209*
**ENSG00000130517**		*0.4375*		*0.4375*				*0.4767442*
**ENSG00000168421**	*0.31024096*	*0.38888889*	*0.31024096*	*0.388888889*	*0.409090909*		*0.4285714*	*0.25*
**ENSG00000139726**		*0.08895706*		*0.088957055*				
**ENSG00000005100**		*0.13793103*		*0.137931034*				*0.2727273*
**ENSG00000089820**		*0.46060606*		*0.460606061*				*0.255814*
**ENSG00000167653**	*0.23636364*	*0.45757576*	*0.23636364*	*0.457575758*	*0.261904762*		*0.377907*	*0.3823529*
**ENSG00000054148**	*0.17365269*		*0.17365269*		*0.148809524*		*0.0755814*	
**ENSG00000147246**	*0.37222222*	*0.12777778*	*0.37222222*	*0.127777778*	*0.177777778*		*0.1477273*	*0.0795455*
**ENSG00000206474**	*0.17365269*		*0.17365269*				*0.1046512*	
**ENSG00000112685**		*0.27272727*		*0.272727273*				*0.2906977*
**ENSG00000166130**		*0.49090909*		*0.490909091*	*0.297619048*		*0.1666667*	*0.4593023*
**ENSG00000168269**		*0.25151515*		*0.251515152*				*0.4127907*
**ENSG00000059378**		*0.3597561*		*0.359756098*				*0.4883721*
**ENSG00000121316**	*0.4246988*	*0.32317073*	*0.4246988*	*0.323170732*	*0.476190476*		*0.4360465*	*0.1705882*
**ENSG00000124493**	*0.36826347*	*0.17222222*	*0.36826347*	*0.172222222*	*0.427710843*		*0.372093*	*0.2727273*
**ENSG00000152214**	*0.1011236*		*0.1011236*		*0.089285714*		*0.0930233*	
**ENSG00000213625**		*0.41573034*		*0.415730337*				*0.1888889*
**ENSG00000072818**	*0.31111111*		*0.31111111*		*0.307228916*		*0.1704545*	
**ENSG00000128487**		*0.42696629*		*0.426966292*				*0.1309524*
**ENSG00000161179**		*0.18181818*		*0.181818182*				*0.372093*
**ENSG00000165078**	*0.38253012*	*0.20731707*	*0.38253012*	*0.207317073*	*0.083333333*		*0.1744186*	
**ENSG00000117114**	*0.30239521*	*0.06179775*	*0.30239521*	*0.061797753*	*0.060240964*		*0.0755814*	*0.0813953*
**ENSG00000212993**	*0.20359281*	*0.29393939*	*0.20359281*	*0.293939394*	*0.363095238*		*0.2732558*	*0.2151163*
**ENSG00000232098**	*0.14371257*		*0.14371257*		*0.321428571*		*0.244186*	
**ENSG00000155093**		*0.09659091*		*0.096590909*				*0.2111111*
**ENSG00000141219**		*0.23030303*		*0.23030303*				*0.1860465*
**ENSG00000177938**	*0.43072289*		*0.43072289*		*0.301204819*		*0.3255814*	
**ENSG00000083642**		*0.46363636*		*0.463636364*				*0.1941176*
**ENSG00000186474**	*0.37356322*		*0.37356322*		*0.44047619*		*0.5*	
**ENSG00000167674**		*0.42727273*		*0.427272727*				*0.2823529*
**ENSG00000170379**		*0.45757576*		*0.457575758*				*0.4642857*
**ENSG00000160584**		*0.15*		*0.15*				*0.1477273*
**ENSG00000122034**		*0.24545455*		*0.245454545*				
**ENSG00000137200**	*0.29640719*	*0.05757576*	*0.29640719*	*0.057575758*				
**ENSG00000100427**		*0.29310345*		*0.293103448*			*0.0639535*	*0.3636364*
**ENSG00000051620**								*0.1337209*
**ENSG00000071889**		*0.20689655*		*0.206896552*				*0.2325581*
**ENSG00000137710**		*0.47575758*		*0.475757576*				*0.4659091*
**ENSG00000161692**	*0.05*	*0.2030303*	*0.05*	*0.203030303*				*0.1918605*
**ENSG00000109771**	*0.46060606*		*0.46060606*		*0.345238095*		*0.3662791*	
**ENSG00000162409**		*0.47878788*		*0.478787879*				*0.4593023*
**ENSG00000205413**								*0.1162791*
**ENSG00000164252**		*0.19886364*		*0.198863636*				*0.0581395*
**ENSG00000174738**		*0.09393939*		*0.093939394*				
**ENSG00000132704**	*0.07185629*	*0.20606061*	*0.07185629*	*0.206060606*	*0.386904762*		*0.4883721*	*0.3255814*
**ENSG00000143977**	*0.22754491*		*0.22754491*		*0.136904762*		*0.2222222*	
**ENSG00000162972**	*0.13173653*		*0.13173653*		*0.238095238*		*0.1918605*	
**ENSG00000104611**		*0.45151515*		*0.451515152*				*0.1555556*
**ENSG00000179242**	*0.24444444*	*0.18390805*	*0.24444444*	*0.183908046*	*0.133333333*		*0.1860465*	*0.3977273*

**ENSG00000188517**	*0.08383234*	*0.42987805*	*0.08383234*	*0.429878049*	*0.422619048*	*0.4360465*	*0.4302326*
**ENSG00000136297**		*0.44848485*		*0.448484848*			*0.2790698*
**ENSG00000154153**	*0.43413174*	*0.31707317*	*0.43413174*	*0.317073171*	*0.488095238*	*0.377907*	*0.4411765*
**ENSG00000178038**		*0.48863636*		*0.488636364*			*0.4534884*
**ENSG00000137869**	*0.15568862*	*0.46666667*	*0.15568862*	*0.466666667*	*0.482142857*	*0.3662791*	*0.4647059*
**ENSG00000117597**		*0.14329268*		*0.143292683*			*0.1046512*
**ENSG00000139971**		*0.42727273*		*0.427272727*			*0.4864865*
**ENSG00000181788**		*0.12804878*		*0.12804878*			
**ENSG00000157224**	*0.43413174*	*0.31515152*	*0.43413174*	*0.315151515*	*0.255952381*	*0.1860465*	*0.4011628*
**ENSG00000121351**		*0.23333333*		*0.233333333*			*0.0697674*
**ENSG00000111863**	*0.4246988*	*0.23333333*	*0.4246988*	*0.233333333*	*0.488888889*	*0.4883721*	*0.3555556*
**ENSG00000163913**	*0.14371257*		*0.14371257*				
**ENSG00000134086**	*0.11212121*	*0.36969697*	*0.11212121*	*0.36969697*	*0.232142857*	*0.2674419*	*0.2616279*
**ENSG00000205678**		*0.19318182*		*0.193181818*			*0.2954545*
**ENSG00000141314**							*0.1022727*
**ENSG00000136699**	*0.46107784*	*0.34756098*	*0.46107784*	*0.347560976*	*0.488095238*	*0.3863636*	*0.3705882*
**ENSG00000147642**	*0.43413174*	*0.08841463*	*0.43413174*	*0.088414634*	*0.476190476*	*0.4360465*	
**ENSG00000253710**		*0.45454545*		*0.454545455*			*0.4821429*
**ENSG00000099800**	*0.45808383*		*0.45808383*		*0.398809524*	*0.4883721*	
**ENSG00000152348**		*0.48170732*		*0.481707317*			*0.0581395*
**ENSG00000137440**	*0.19444444*	*0.31111111*	*0.19444444*	*0.311111111*	*0.333333333*	*0.3222222*	*0.0777778*
**ENSG00000105849**	*0.11077844*	*0.14110429*	*0.11077844*	*0.141104294*	*0.380952381*	*0.4651163*	*0.4709302*
**ENSG00000111885**		*0.4969697*		*0.496969697*			*0.4476744*
**ENSG00000187699**	*0.35329341*	*0.3*	*0.35329341*	*0.3*	*0.130952381*	*0.1395349*	*0.4772727*
**ENSG00000141580**		*0.40251572*		*0.402515723*			*0.3493976*
**ENSG00000206013**							*0.3081395*
**ENSG00000005108**		*0.36196319*		*0.36196319*			*0.1918605*
**ENSG00000132142**					*0.44047619*	*0.2616279*	
**ENSG00000162490**		*0.46511628*		*0.465116279*			*0.452381*
**ENSG00000112406**		*0.3969697*		*0.396969697*			*0.4090909*
**ENSG00000164323**		*0.5*		*0.5*			*0.3197674*
**ENSG00000186868**		*0.42121212*		*0.421212121*			*0.1046512*
**ENSG00000102743**		*0.4245283*		*0.424528302*			*0.4222222*
**ENSG00000087237**		*0.20606061*		*0.206060606*			*0.1104651*
**ENSG00000138642**		*0.06363636*		*0.063636364*			
**ENSG00000211772**		*0.43636364*		*0.436363636*			*0.122093*
**ENSG00000139343**	*0.12650602*	*0.47752809*	*0.12650602*	*0.47752809*	*0.136904762*	*0.0813953*	*0.4333333*
**ENSG00000176410**		*0.48787879*		*0.487878788*			*0.2764706*
**ENSG00000171045**		*0.10060976*		*0.100609756*			*0.122093*
**ENSG00000152192**		*0.40449438*		*0.404494382*			*0.2727273*
**ENSG00000187498**		*0.08181818*		*0.081818182*			
**ENSG00000168803**	*0.21212121*	*0.31515152*	*0.21212121*	*0.315151515*	*0.5*	*0.4883721*	*0.4883721*
**ENSG00000135597**		*0.48275862*		*0.482758621*			*0.4318182*
**ENSG00000102780**		*0.2*		*0.2*			
**ENSG00000103375**		*0.40229885*		*0.402298851*			*0.4318182*
**ENSG00000067533**		*0.05974843*		*0.059748428*			*0.0888889*
**ENSG00000254505**	*0.2005988*	*0.49090909*	*0.2005988*	*0.490909091*	*0.172619048*	*0.1976744*	*0.1976744*
**ENSG00000183580**		*0.31818182*		*0.318181818*			*0.1395349*
**ENSG00000117481**	*0.08083832*		*0.08083832*				
**ENSG00000083828**		*0.39090909*		*0.390909091*			*0.4390244*
**ENSG00000212928**	*0.10778443*	*0.14634146*	*0.10778443*	*0.146341463*	*0.119047619*	*0.0755814*	*0.0755814*
**ENSG00000180357**		*0.1091954*		*0.109195402*			
**ENSG00000153132**	*0.25*	*0.0969697*	*0.25*	*0.096969697*	*0.202380952*	*0.1162791*	*0.1744186*
**ENSG00000165105**		*0.48787879*		*0.487878788*			*0.25*

**ENSG00000120526**	*0.05988024*	*0.41104294*	*0.05988024*	*0.411042945*				*0.494186*
**ENSG00000122778**		*0.30606061*		*0.306060606*				*0.4593023*
**ENSG00000163807**	*0.14371257*		*0.14371257*					
**ENSG00000010932**	*0.20658683*	*0.35757576*	*0.20658683*	*0.357575758*	*0.30952381*		*0.244186*	*0.2965116*
**ENSG00000092607**					*0.095238095*			
**ENSG00000187783**		*0.26969697*		*0.26969697*				*0.4534884*
**ENSG00000065060**		*0.09393939*		*0.093939394*				*0.2674419*
**ENSG00000187735**		*0.1030303*		*0.103030303*				
**ENSG00000166261**		*0.37804878*		*0.37804878*				*0.2325581*
**ENSG00000205060**		*0.46363636*		*0.463636364*				*0.3081395*
**ENSG00000111678**	*0.5*		*0.5*		*0.232142857*		*0.2906977*	
**ENSG00000166479**		*0.07222222*		*0.072222222*				*0.1823529*
**ENSG00000119431**	*0.26807229*		*0.26807229*		*0.321428571*		*0.3488372*	
**ENSG00000170275**		*0.42941176*		*0.429411765*				*0.0681818*
**ENSG00000115446**	*0.13473054*		*0.13473054*		*0.267857143*		*0.2965116*	
**ENSG00000149742**	*0.22754491*		*0.22754491*		*0.446428571*		*0.3953488*	
**ENSG00000170525**		*0.48181818*		*0.481818182*				*0.4886364*
**ENSG00000151327**		*0.36111111*		*0.361111111*				*0.3977273*
**ENSG00000091542**		*0.38787879*		*0.387878788*				*0.1162791*
**ENSG00000239388**	*0.06363636*		*0.06363636*					
**ENSG00000127989**	*0.28089888*	*0.46666667*	*0.28089888*	*0.466666667*	*0.446428571*		*0.4941176*	*0.4772727*
**ENSG00000197506**	*0.07317073*	*0.18902439*	*0.07317073*	*0.18902439*				*0.3139535*
**ENSG00000198825**		*0.47575758*		*0.475757576*				*0.4058824*
**ENSG00000183421**	*0.33888889*	*0.48484848*	*0.33888889*	*0.484848485*	*0.05952381*			*0.3197674*
**ENSG00000213965**		*0.1625*		*0.1625*				*0.2083333*
**ENSG00000130684**		*0.1969697*		*0.196969697*				*0.3139535*
**ENSG00000205011**		*0.19444444*		*0.194444444*				*0.2386364*
**ENSG00000188529**		*0.44545455*		*0.445454545*				*0.3235294*
**ENSG00000117020**		*0.2804878*		*0.280487805*				*0.2674419*
**ENSG00000169031**		*0.15757576*		*0.157575758*				*0.1802326*
**ENSG00000187134**		*0.28409091*		*0.284090909*				
**ENSG00000189007**		*0.49393939*		*0.493939394*				*0.25*
**ENSG00000136875**	*0.08333333*	*0.31818182*	*0.08333333*	*0.318181818*	*0.240963855*		*0.2411765*	*0.4186047*
**ENSG00000114698**	*0.3*	*0.38636364*	*0.3*	*0.386363636*	*0.26744186*		*0.1785714*	*0.4176471*
**ENSG00000134242**		*0.2347561*		*0.234756098*				*0.4360465*
**ENSG00000188647**		*0.28484848*		*0.284848485*				*0.255814*
**ENSG00000142192**	*0.06470588*		*0.06470588*					
**ENSG00000106868**		*0.23584906*		*0.235849057*				*0.2386364*
**ENSG00000213463**		*0.14242424*		*0.142424242*				*0.4940476*
**ENSG00000147606**		*0.3*		*0.3*				
**ENSG00000187838**	*0.49101796*	*0.13939394*	*0.49101796*	*0.139393939*	*0.327380952*		*0.2383721*	*0.2151163*
**ENSG00000197928**	*0.18562874*	*0.45454545*	*0.18562874*	*0.454545455*	*0.355421687*		*0.3546512*	*0.2674419*
**ENSG00000136147**		*0.45151515*		*0.451515152*				*0.4302326*
**ENSG00000152926**	*0.3253012*		*0.3253012*		*0.136904762*		*0.1309524*	*0.0972222*
**ENSG00000196502**								*0.2159091*
**ENSG00000196090**		*0.45151515*		*0.451515152*				*0.4825581*
**ENSG00000114200**		*0.19444444*		*0.194444444*				*0.2045455*
**ENSG00000172273**	*0.32335329*	*0.43030303*	*0.32335329*	*0.43030303*	*0.244047619*		*0.3081395*	*0.3081395*
**ENSG00000167210**	*0.08682635*		*0.08682635*		*0.142857143*		*0.2732558*	
**ENSG00000186326**		*0.25914634*		*0.259146341*				*0.3604651*
**ENSG00000185896**								*0.1104651*
**ENSG00000143776**	*0.37724551*	*0.5*	*0.37724551*	*0.5*	*0.357142857*		*0.4*	*0.3081395*
**ENSG00000146374**								*0.0523256*
**ENSG00000115750**	*0.35151515*	*0.32727273*	*0.35151515*	*0.327272727*	*0.357142857*		*0.2235294*	*0.494186*

**ENSG00000143603**	*0.43113772*		*0.43113772*		*0.113095238*	*0.0639535*	
**ENSG00000154645**	*0.06586826*	*0.20606061*	*0.06586826*	*0.206060606*			
**ENSG00000177479**	*0.05389222*		*0.05389222*				
**ENSG00000198182**	*0.41616766*	*0.15151515*	*0.41616766*	*0.151515152*	*0.398809524*	*0.3313953*	*0.3313953*
**ENSG00000132204**							*0.1477273*
**ENSG00000121390**	*0.29041916*		*0.29041916*		*0.077380952*	*0.0639535*	
**ENSG00000119977**	*0.17664671*	*0.33333333*	*0.17664671*	*0.333333333*		*0.0930233*	*0.3409091*
**ENSG00000022556**		*0.49425287*		*0.494252874*			*0.2386364*
**ENSG00000115353**		*0.14242424*		*0.142424242*			*0.1453488*
**ENSG00000084453**	*0.13068182*	*0.18181818*	*0.13068182*	*0.181818182*	*0.455555556*	*0.4069767*	*0.4090909*
**ENSG00000184117**		*0.39090909*		*0.390909091*			*0.4772727*
**ENSG00000111679**	*0.27844311*		*0.27844311*		*0.202380952*	*0.2151163*	
**ENSG00000115363**	*0.35555556*		*0.35555556*		*0.375*	*0.3255814*	
**ENSG00000100105**							*0.4709302*
**ENSG00000175984**		*0.14545455*		*0.145454545*			*0.372093*
**ENSG00000258832**		*0.43820225*		*0.438202247*			*0.4318182*
**ENSG00000134042**	*0.11212121*	*0.49695122*	*0.11212121*	*0.49695122*	*0.114457831*	*0.0882353*	*0.452381*
**ENSG00000186377**						*0.0523256*	
**ENSG00000155827**	*0.34131737*		*0.34131737*		*0.337349398*	*0.2325581*	
**ENSG00000111728**	*0.06586826*	*0.47777778*	*0.06586826*	*0.477777778*	*0.119047619*		*0.4659091*
**ENSG00000184672**	*0.12048193*	*0.34848485*	*0.12048193*	*0.348484848*	*0.411111111*	*0.3181818*	*0.3658537*
**ENSG00000124529**		*0.38957055*		*0.389570552*			*0.3255814*
**ENSG00000189143**	*0.44311377*	*0.21212121*	*0.44311377*	*0.212121212*	*0.464285714*	*0.4418605*	*0.4418605*
**ENSG00000152207**		*0.42121212*		*0.421212121*			*0.3837209*
**ENSG00000244025**		*0.49444444*		*0.494444444*			*0.4431818*
**ENSG00000104325**	*0.23888889*	*0.16853933*	*0.23888889*	*0.168539326*	*0.322222222*	*0.375*	*0.3*
**ENSG00000158089**	*0.47647059*	*0.13109756*	*0.47647059*	*0.131097561*			*0.1764706*
**ENSG00000168434**		*0.11890244*		*0.118902439*			*0.25*
**ENSG00000076067**		*0.23780488*		*0.237804878*			*0.3625*
**ENSG00000023697**							*0.3295455*
**ENSG00000114646**	*0.5*		*0.5*		*0.246987952*	*0.2380952*	
**ENSG00000197586**	*0.42814371*	*0.49693252*	*0.42814371*	*0.496932515*	*0.398809524*	*0.3837209*	*0.0988372*
**ENSG00000180730**		*0.38181818*		*0.381818182*			*0.244186*
**ENSG00000134240**					*0.06547619*		
**ENSG00000237515**		*0.17977528*		*0.179775281*			*0.2386364*
**ENSG00000083838**		*0.47865854*		*0.478658537*			*0.25*
**ENSG00000155729**	*0.22754491*	*0.16049383*	*0.22754491*	*0.160493827*	*0.156626506*	*0.1686047*	*0.3630952*
**ENSG00000144893**		*0.42727273*		*0.427272727*			*0.3636364*
**ENSG00000175029**	*0.33832335*	*0.33707865*	*0.33832335*	*0.337078652*	*0.386904762*	*0.4360465*	*0.3139535*
**ENSG00000103599**		*0.25460123*		*0.254601227*			*0.377907*
**ENSG00000182247**		*0.16666667*		*0.166666667*			*0.3333333*
**ENSG00000105792**		*0.46111111*		*0.461111111*			*0.4166667*
**ENSG00000175538**		*0.4969697*		*0.496969697*			*0.4882353*
**ENSG00000040275**	*0.40963855*	*0.27272727*	*0.40963855*	*0.272727273*	*0.261904762*	*0.3*	*0.3081395*
**ENSG00000120913**		*0.35889571*		*0.358895706*			*0.4705882*
**ENSG00000006740**	*0.06586826*	*0.29268293*	*0.06586826*	*0.292682927*	*0.1875*	*0.1410256*	*0.3372093*
**ENSG00000198353**					*0.2*	*0.1785714*	
**ENSG00000116903**		*0.39393939*		*0.393939394*			*0.4534884*
**ENSG00000173402**	*0.0952381*	*0.43333333*	*0.0952381*	*0.433333333*	*0.097560976*	*0.1162791*	*0.1162791*
**ENSG00000136709**		*0.2875*		*0.2875*			*0.2732558*
**ENSG00000146477**		*0.46363636*		*0.463636364*			*0.4767442*
**ENSG00000029725**		*0.44848485*		*0.448484848*			*0.375*
**ENSG00000114923**	*0.25151515*	*0.14545455*	*0.25151515*	*0.145454545*			*0.1453488*
**ENSG00000106538**	*0.16167665*	*0.34545455*	*0.16167665*	*0.345454545*	*0.295454545*	*0.4333333*	*0.3977273*

**ENSG00000203668**		*0.36363636*		*0.363636364*			*0.2235294*
**ENSG00000145014**		*0.1993865*		*0.199386503*			*0.2470588*
**ENSG00000170893**	*0.33532934*		*0.33532934*		*0.339285714*	*0.3197674*	
**ENSG00000137824**	*0.10606061*		*0.10606061*				
**ENSG00000183688**		*0.42528736*		*0.425287356*			*0.125*
**ENSG00000198546**							*0.0988372*
**ENSG00000234127**	*0.37125749*	*0.31288344*	*0.37125749*	*0.312883436*	*0.277777778*	*0.3068182*	*0.1411765*
**ENSG00000178096**	*0.49101796*		*0.49101796*				
**ENSG00000038274**		*0.45757576*		*0.457575758*			*0.3139535*
**ENSG00000127377**		*0.06363636*		*0.063636364*			
**ENSG00000132122**		*0.39444444*		*0.394444444*			*0.2*
**ENSG00000054392**		*0.28181818*		*0.281818182*			*0.3662791*
**ENSG00000099949**	*0.46706587*	*0.16666667*	*0.46706587*	*0.166666667*			*0.25*
**ENSG00000171346**	*0.46686747*	*0.16969697*	*0.46686747*	*0.16969697*	*0.095238095*	*0.0588235*	
**ENSG00000111880**		*0.13414634*		*0.134146341*			*0.2647059*
**ENSG00000075461**		*0.31609195*		*0.316091954*			*0.3636364*
**ENSG00000170385**		*0.18390805*		*0.183908046*			*0.4772727*
**ENSG00000251537**		*0.465625*		*0.465625*			*0.1294118*
**ENSG00000054983**	*0.24698795*	*0.48757764*	*0.24698795*	*0.48757764*	*0.077380952*	*0.0872093*	*0.4651163*
**ENSG00000165113**		*0.37222222*		*0.372222222*			*0.2840909*
**ENSG00000107518**		*0.10606061*		*0.106060606*			*0.2222222*
**ENSG00000213022**		*0.44242424*		*0.442424242*			*0.255814*
**ENSG00000259003**		*0.48780488*		*0.487804878*			*0.2906977*
**ENSG00000198689**							*0.1395349*
**ENSG00000130844**	*0.36060606*	*0.25151515*	*0.36060606*	*0.251515152*	*0.43452381*	*0.4360465*	*0.2790698*
**ENSG00000137875**	*0.32758621*	*0.13636364*	*0.32758621*	*0.136363636*	*0.21875*	*0.0681818*	*0.1802326*
**ENSG00000144645**		*0.32098765*		*0.320987654*			*0.3588235*
**ENSG00000109576**		*0.0617284*		*0.061728395*			
**ENSG00000145692**		*0.31460674*		*0.314606742*			*0.125*
**ENSG00000127481**	*0.29341317*	*0.31097561*	*0.29341317*	*0.31097561*	*0.363095238*	*0.4069767*	*0.1529412*
**ENSG00000182552**	*0.08682635*	*0.24085366*	*0.08682635*	*0.240853659*	*0.267857143*	*0.3837209*	*0.1569767*
**ENSG00000183762**		*0.30246914*		*0.302469136*			*0.3313953*
**ENSG00000158050**		*0.46363636*		*0.463636364*			*0.3255814*
**ENSG00000186496**		*0.43333333*		*0.433333333*			*0.4868421*
**ENSG00000176095**		*0.45757576*		*0.457575758*			*0.2383721*
**ENSG00000140396**		*0.10555556*		*0.105555556*			*0.1477273*
**ENSG00000169403**	*0.14371257*		*0.14371257*				
**ENSG00000144229**		*0.35403727*		*0.354037267*			*0.125*
**ENSG00000163468**	*0.10479042*		*0.10479042*		*0.178571429*	*0.2209302*	*0.2444444*
**ENSG00000183828**		*0.39444444*		*0.394444444*			*0.4888889*
**ENSG00000106261**		*0.33636364*		*0.336363636*			*0.4941176*
**ENSG00000143756**		*0.44848485*		*0.448484848*			*0.4883721*
**ENSG00000172795**	*0.18263473*	*0.496875*	*0.18263473*	*0.496875*	*0.261904762*	*0.2383721*	*0.4390244*
**ENSG00000102290**		*0.25280899*		*0.252808989*			*0.2333333*
**ENSG00000132768**		*0.21515152*		*0.215151515*			*0.4476744*
**ENSG00000198842**		*0.14242424*		*0.142424242*			*0.0988372*
**ENSG00000250506**		*0.2247191*		*0.224719101*	*0.077380952*	*0.1511628*	*0.1666667*
**ENSG00000101074**		*0.14242424*		*0.142424242*			
**ENSG00000144120**		*0.19101124*		*0.191011236*	*0.162790698*	*0.1395349*	*0.1888889*
**ENSG00000198885**	*0.29341317*		*0.29341317*				
**ENSG00000138829**		*0.14044944*		*0.140449438*			*0.0681818*
**ENSG00000137038**					*0.172619048*	*0.1395349*	*0.2840909*
**ENSG00000099942**		*0.3*		*0.3*			*0.2142857*
**ENSG00000110274**	*0.10479042*	*0.35757576*	*0.10479042*	*0.357575758*			*0.4883721*

**ENSG00000119231**		*0.20552147*		*0.205521472*			*0.1176471*
**ENSG00000158406**	*0.16167665*		*0.16167665*		*0.089285714*		
**ENSG00000140575**		*0.4054878*		*0.405487805*			*0.4186047*
**ENSG00000140553**		*0.23333333*		*0.233333333*			*0.4767442*
**ENSG00000115392**		*0.10465116*		*0.104651163*			*0.0568182*
**ENSG00000127081**		*0.39444444*		*0.394444444*			*0.3181818*
**ENSG00000177432**	*0.37125749*		*0.37125749*		*0.494047619*	*0.4011628*	
**ENSG00000204366**	*0.06666667*		*0.06666667*		*0.355555556*	*0.4886364*	
**ENSG00000169598**		*0.46969697*		*0.46969697*			*0.4825581*
**ENSG00000163214**		*0.46060606*		*0.460606061*			*0.2209302*
**ENSG00000182220**							*0.1918605*
**ENSG00000143815**		*0.14848485*		*0.148484848*			*0.0666667*
**ENSG00000224236**							*0.372093*
**ENSG00000113282**		*0.42073171*		*0.420731707*			*0.2674419*
**ENSG00000175216**		*0.11818182*		*0.118181818*			*0.3895349*
**ENSG00000088305**		*0.44545455*		*0.445454545*			*0.122093*
**ENSG00000173597**		*0.4030303*		*0.403030303*			*0.4011628*
**ENSG00000138182**					*0.288888889*	*0.1818182*	
**ENSG00000135074**	*0.07831325*	*0.46666667*	*0.07831325*	*0.466666667*			*0.3488372*
**ENSG00000215375**	*0.10843373*		*0.10843373*				
**ENSG00000103035**		*0.33030303*		*0.33030303*			*0.2383721*
**ENSG00000007001**	*0.39444444*	*0.05757576*	*0.39444444*	*0.057575758*	*0.155555556*	*0.1104651*	*0.2034884*
**ENSG00000140983**		*0.08181818*		*0.081818182*			*0.3139535*
**ENSG00000109680**		*0.46273292*		*0.462732919*			
**ENSG00000243927**		*0.34545455*		*0.345454545*			*0.1647059*
**ENSG00000185189**		*0.41666667*		*0.416666667*			
**ENSG00000157020**	*0.07432432*	*0.41463415*	*0.07432432*	*0.414634146*	*0.078947368*	*0.1111111*	*0.3895349*
**ENSG00000006194**	*0.48493976*	*0.21818182*	*0.48493976*	*0.218181818*			
**ENSG00000227164**		*0.33030303*		*0.33030303*			*0.0930233*
**ENSG00000229200**	*0.45705521*		*0.45705521*				
**ENSG00000179407**	*0.30120482*		*0.30120482*		*0.226190476*	*0.2674419*	
**ENSG00000105954**		*0.26829268*		*0.268292683*			*0.1529412*
**ENSG00000173227**		*0.06134969*		*0.061349693*	*0.055555556*	*0.0568182*	*0.2209302*
**ENSG00000158014**		*0.35060976*		*0.350609756*			*0.2176471*
**ENSG00000185585**		*0.09393939*		*0.093939394*			*0.4204545*
**ENSG00000130640**	*0.18674699*		*0.18674699*				
**ENSG00000185960**		*0.34969325*		*0.349693252*	*0.43902439*	*0.4878049*	*0.372093*
**ENSG00000186998**		*0.21202532*		*0.212025316*			*0.2954545*
**ENSG00000131697**	*0.13473054*	*0.43030303*	*0.13473054*	*0.43030303*			*0.2529412*
**ENSG00000147010**	*0.06287425*		*0.06287425*				
**ENSG00000111271**	*0.16969697*		*0.16969697*		*0.428571429*	*0.4011628*	
**ENSG00000006555**		*0.06969697*		*0.06969697*			*0.4883721*
**ENSG00000139133**		*0.17378049*		*0.173780488*	*0.071428571*		*0.1686047*
**ENSG00000158042**		*0.11515152*		*0.115151515*			*0.3430233*
**ENSG00000164219**		*0.35670732*		*0.356707317*			*0.2470588*
**ENSG00000148411**		*0.06111111*		*0.061111111*			*0.3295455*
**ENSG00000205745**		*0.40606061*		*0.406060606*			*0.377907*
**ENSG00000178773**		*0.17878788*		*0.178787879*			*0.2616279*
**ENSG00000002016**	*0.43333333*	*0.45977011*	*0.43333333*	*0.459770115*	*0.311111111*	*0.3636364*	*0.4767442*
**ENSG00000160551**		*0.18181818*		*0.181818182*			
**ENSG00000177917**		*0.2030303*		*0.203030303*			*0.2790698*
**ENSG00000185087**		*0.18787879*		*0.187878788*			*0.2034884*
**ENSG00000135363**	*0.20359281*	*0.46363636*	*0.20359281*	*0.463636364*	*0.363095238*	*0.3372093*	*0.4360465*
**ENSG00000100815**		*0.43939394*		*0.439393939*	*0.102272727*	*0.1111111*	*0.4127907*

**ENSG00000136750**	*0.13772455*	*0.19178082*	*0.13772455*	*0.191780822*	*0.333333333*		*0.4651163*	*0.4166667*
**ENSG00000174844**	*0.10479042*		*0.10479042*		*0.43452381*		*0.4767442*	
**ENSG00000258315**	*0.09883721*		*0.09883721*					
**ENSG00000170145**		*0.23033708*		*0.230337079*				*0.2333333*
**ENSG00000145642**		*0.3*		*0.3*				*0.4431818*
**ENSG00000197408**		*0.39090909*		*0.390909091*				*0.3604651*
**ENSG00000126947**	*0.28915663*		*0.28915663*		*0.188888889*		*0.125*	
**ENSG00000183484**	*0.36526946*	*0.07272727*	*0.36526946*	*0.072727273*	*0.44047619*		*0.4360465*	*0.3313953*
**ENSG00000134909**		*0.43251534*		*0.432515337*				*0.4011628*
**ENSG00000124571**	*0.07865169*	*0.41515152*	*0.07865169*	*0.415151515*				*0.4186047*
**ENSG00000011132**	*0.34482759*	*0.0969697*	*0.34482759*	*0.096969697*	*0.322222222*		*0.25*	*0.1428571*
**ENSG00000123977**	*0.4491018*	*0.2*	*0.4491018*	*0.2*	*0.470238095*		*0.4593023*	*0.1111111*
**ENSG00000171456**		*0.35454545*		*0.354545455*				*0.3197674*
**ENSG00000109943**		*0.20606061*		*0.206060606*				*0.3837209*
**ENSG00000120254**		*0.3*		*0.3*				*0.4431818*
**ENSG00000111490**		*0.5*		*0.5*				*0.2674419*
**ENSG00000132334**	*0.21165644*	*0.44207317*	*0.21165644*	*0.442073171*	*0.416666667*		*0.4529412*	*0.4883721*
**ENSG00000162591**		*0.11890244*		*0.118902439*				*0.1395349*
**ENSG00000174405**	*0.14444444*	*0.0969697*	*0.14444444*	*0.096969697*				*0.1444444*
**ENSG00000141338**	*0.27245509*		*0.27245509*		*0.18452381*		*0.2034884*	
**ENSG00000134986**		*0.5*		*0.5*			*0.0755814*	*0.4294118*
**ENSG00000109832**	*0.40718563*		*0.40718563*		*0.095238095*		*0.1627907*	
**ENSG00000166819**		*0.34444444*		*0.344444444*				*0.3863636*
**ENSG00000099940**	*0.37951807*	*0.46060606*	*0.37951807*	*0.460606061*	*0.333333333*		*0.3837209*	*0.4941176*
**ENSG00000108278**	*0.49700599*		*0.49700599*		*0.43452381*		*0.4069767*	
**ENSG00000008300**		*0.12643678*		*0.126436782*				*0.0568182*
**ENSG00000111536**		*0.06060606*		*0.060606061*				*0.4476744*
**ENSG00000023330**		*0.44545455*		*0.445454545*				*0.494186*
**ENSG00000162981**		*0.34848485*		*0.348484848*				*0.4186047*
**ENSG00000156096**	*0.32777778*	*0.32727273*	*0.32777778*	*0.327272727*	*0.177777778*		*0.1818182*	*0.3895349*
**ENSG00000130208**		*0.06666667*		*0.066666667*				
**ENSG00000111783**	*0.19078947*	*0.43636364*	*0.19078947*	*0.436363636*	*0.127906977*		*0.0731707*	*0.2647059*
**ENSG00000102100**		*0.42727273*		*0.427272727*				*0.2383721*
**ENSG00000118194**								*0.0755814*
**ENSG00000176659**	*0.22530864*	*0.46511628*	*0.22530864*	*0.465116279*	*0.379518072*		*0.3488372*	*0.2380952*
**ENSG00000120820**					*0.321428571*		*0.3546512*	
**ENSG00000142669**	*0.14071856*	*0.13333333*	*0.14071856*	*0.133333333*				
**ENSG00000113249**	*0.13772455*		*0.13772455*					
**ENSG00000184845**	*0.16167665*	*0.44545455*	*0.16167665*	*0.445454545*	*0.261904762*		*0.2674419*	*0.4534884*
**ENSG00000137080**		*0.15757576*		*0.157575758*				*0.4883721*
**ENSG00000116044**		*0.37804878*		*0.37804878*				
**ENSG00000182134**	*0.46666667*	*0.48787879*	*0.46666667*	*0.487878788*	*0.477777778*		*0.3409091*	*0.4302326*
**ENSG00000048471**		*0.46932515*		*0.469325153*				*0.4529412*
**ENSG00000168955**	*0.17964072*	*0.17272727*	*0.17964072*	*0.172727273*	*0.119047619*		*0.0581395*	*0.2034884*
**ENSG00000166881**		*0.3902439*		*0.390243902*				*0.2848837*
**ENSG00000196586**		*0.4556962*		*0.455696203*				*0.3647059*
**ENSG00000100271**	*0.27586207*	*0.42727273*	*0.27586207*	*0.427272727*	*0.142857143*		*0.0609756*	*0.3255814*
**ENSG00000063169**		*0.2*		*0.2*				*0.3139535*
**ENSG00000060237**	*0.49700599*	*0.27439024*	*0.49700599*	*0.274390244*	*0.458333333*		*0.4825581*	*0.1860465*
**ENSG00000170743**		*0.47575758*		*0.475757576*				*0.3662791*
**ENSG00000196704**	*0.05688623*		*0.05688623*		*0.210843373*		*0.2906977*	
**ENSG00000164171**		*0.34545455*		*0.345454545*				*0.4888889*
**ENSG00000003436**								*0.0681818*
**ENSG00000150995**	*0.4491018*		*0.4491018*		*0.267857143*		*0.3953488*	

**ENSG00000159173**	*0.40419162*	*0.45*	*0.40419162*	*0.45*	*0.31547619*		*0.4302326*	*0.4244186*
**ENSG00000213903**	*0.06741573*	*0.45402299*	*0.06741573*	*0.454022989*	*0.144444444*		*0.2272727*	*0.1704545*
**ENSG00000175110**	*0.07777778*	*0.46666667*	*0.07777778*	*0.466666667*				
**ENSG00000215067**		*0.08181818*		*0.081818182*				*0.2093023*
**ENSG00000137975**		*0.14367816*		*0.143678161*				
**ENSG00000215397**		*0.37195122*		*0.37195122*				*0.3372093*
**ENSG00000064687**		*0.31034483*		*0.310344828*	*0.273809524*		*0.3953488*	*0.3597561*
**ENSG00000063978**	*0.08426966*	*0.17272727*	*0.08426966*	*0.172727273*	*0.097560976*		*0.1410256*	*0.1104651*
**ENSG00000182901**	*0.125*	*0.10606061*	*0.125*	*0.106060606*				*0.1453488*
**ENSG00000133985**		*0.48787879*		*0.487878788*				*0.4302326*
**ENSG00000141446**	*0.05688623*		*0.05688623*					
**ENSG00000125434**	*0.32335329*		*0.32335329*		*0.422619048*		*0.4534884*	*0.3977273*
**ENSG00000164061**		*0.48181818*		*0.481818182*				*0.2848837*
**ENSG00000068354**		*0.43251534*		*0.432515337*				*0.4244186*
**ENSG00000131435**		*0.13953488*		*0.139534884*				*0.4886364*
**ENSG00000150594**		*0.15454545*		*0.154545455*				*0.4476744*
**ENSG00000136810**		*0.48484848*		*0.484848485*			*0.0535714*	
**ENSG00000197712**		*0.46363636*		*0.463636364*				*0.4825581*
**ENSG00000082014**	*0.41916168*		*0.41916168*				*0.0697674*	
**ENSG00000172113**		*0.33231707*		*0.332317073*				*0.122093*
**ENSG00000139372**	*0.16111111*		*0.16111111*					
**ENSG00000196361**								*0.0595238*
**ENSG00000087502**		*0.20481928*		*0.204819277*				*0.3375*
**ENSG00000198873**	*0.3045977*		*0.3045977*					
**ENSG00000134438**		*0.28484848*		*0.284848485*				*0.2034884*
**ENSG00000153029**		*0.47191011*		*0.471910112*				*0.4888889*
**ENSG00000146039**		*0.2969697*		*0.296969697*				*0.3953488*
**ENSG00000157881**		*0.3*		*0.3*				*0.4545455*
**ENSG00000185052**		*0.42424242*		*0.424242424*				*0.4418605*
**ENSG00000149485**	*0.14071856*	*0.37790698*	*0.14071856*	*0.377906977*	*0.340909091*		*0.3111111*	*0.3111111*
**ENSG00000070444**		*0.05151515*		*0.051515152*				*0.1686047*
**ENSG00000103034**	*0.33532934*	*0.32424242*	*0.33532934*	*0.324242424*	*0.25*		*0.1744186*	*0.4127907*
**ENSG00000018189**	*0.34730539*	*0.4*	*0.34730539*	*0.4*				*0.4107143*
**ENSG00000149534**	*0.4760479*		*0.4760479*		*0.34939759*		*0.3372093*	*0.1453488*
**ENSG00000174450**	*0.41011236*		*0.41011236*		*0.166666667*		*0.1477273*	
**ENSG00000213639**		*0.5*		*0.5*				*0.25*
**ENSG00000198093**	*0.41317365*	*0.13333333*	*0.41317365*	*0.133333333*	*0.238095238*		*0.25*	*0.2325581*
**ENSG00000153046**	*0.45808383*	*0.29141104*	*0.45808383*	*0.291411043*	*0.232142857*		*0.1744186*	*0.1428571*
**ENSG00000140299**		*0.35757576*		*0.357575758*	*0.380952381*		*0.3684211*	*0.4476744*
**ENSG00000178074**		*0.21515152*		*0.215151515*				*0.1802326*
**ENSG00000076201**		*0.43939394*		*0.439393939*				*0.4709302*
**ENSG00000169682**		*0.28089888*		*0.280898876*				*0.0681818*
**ENSG00000183891**	*0.48333333*		*0.48333333*		*0.4*		*0.2386364*	
**ENSG00000160991**	*0.36526946*	*0.2804878*	*0.36526946*	*0.280487805*	*0.446428571*		*0.4476744*	*0.4941176*
**ENSG00000123992**		*0.10909091*		*0.109090909*				*0.2209302*
**ENSG00000107130**		*0.46060606*		*0.460606061*				*0.2261905*
**ENSG00000105366**		*0.23333333*		*0.233333333*				*0.3863636*
**ENSG00000146021**		*0.46363636*		*0.463636364*				*0.494186*
**ENSG00000122952**		*0.17575758*		*0.175757576*	*0.089285714*		*0.0755814*	
**ENSG00000009694**		*0.2030303*		*0.203030303*				*0.3352941*
**ENSG00000066468**	*0.47305389*	*0.43939394*	*0.47305389*	*0.439393939*	*0.279761905*		*0.2093023*	*0.3604651*
**ENSG00000150977**		*0.39197531*		*0.391975309*				*0.2613636*
**ENSG00000197771**		*0.46590909*		*0.465909091*				*0.4431818*
**ENSG00000168907**		*0.5*		*0.5*				*0.3636364*

**ENSG00000203392**		*0.47575758*		*0.475757576*			*0.2848837*
**ENSG00000052795**		*0.43030303*		*0.43030303*			*0.1294118*
**ENSG00000165819**	*0.17816092*	*0.47878788*	*0.17816092*	*0.478787879*			*0.2848837*
**ENSG00000197165**		*0.38271605*		*0.382716049*			*0.1084337*
**ENSG00000132693**		*0.33333333*		*0.333333333*			*0.2965116*
**ENSG00000101413**	*0.3742515*		*0.3742515*		*0.154761905*	*0.1395349*	
**ENSG00000185811**		*0.30898876*		*0.308988764*			*0.1130952*
**ENSG00000162433**	*0.06287425*	*0.4*	*0.06287425*	*0.4*	*0.086419753*	*0.1309524*	*0.3068182*
**ENSG00000180957**		*0.23170732*		*0.231707317*			*0.1918605*
**ENSG00000185818**		*0.13375796*		*0.133757962*			*0.2386364*
**ENSG00000179709**		*0.22121212*		*0.221212121*			*0.244186*
**ENSG00000114124**		*0.11875*		*0.11875*			
**ENSG00000076242**	*0.46067416*	*0.33636364*	*0.46067416*	*0.336363636*	*0.446428571*	*0.494186*	*0.0639535*
**ENSG00000215114**		*0.2752809*		*0.275280899*			*0.25*
**ENSG00000178726**		*0.36503067*		*0.365030675*			*0.255814*
**ENSG00000124882**	*0.28181818*	*0.20987654*	*0.28181818*	*0.209876543*	*0.130952381*	*0.0872093*	*0.2588235*
**ENSG00000100167**		*0.31515152*		*0.315151515*			*0.1104651*
**ENSG00000197444**	*0.10778443*	*0.46067416*	*0.10778443*	*0.460674157*	*0.077380952*	*0.1046512*	*0.1162791*
**ENSG00000168405**	*0.35*	*0.15151515*	*0.35*	*0.151515152*	*0.416666667*	*0.377907*	*0.1176471*
**ENSG00000187790**		*0.10909091*		*0.109090909*			*0.1511628*
**ENSG00000141012**		*0.31097561*		*0.31097561*			*0.5*
**ENSG00000084652**		*0.06666667*		*0.066666667*			*0.1860465*
**ENSG00000163568**	*0.06325301*		*0.06325301*		*0.297619048*	*0.4186047*	
**ENSG00000133962**		*0.43258427*		*0.43258427*			*0.0666667*
**ENSG00000173486**							*0.3*
**ENSG00000163864**	*0.38202247*	*0.28333333*	*0.38202247*	*0.283333333*	*0.377777778*	*0.3977273*	*0.3977273*
**ENSG00000153157**	*0.35628743*	*0.29090909*	*0.35628743*	*0.290909091*	*0.444444444*	*0.3837209*	*0.3546512*
**ENSG00000157625**	*0.05688623*	*0.42073171*	*0.05688623*	*0.420731707*	*0.244047619*	*0.2848837*	*0.5*
**ENSG00000174456**	*0.45808383*		*0.45808383*		*0.05952381*	*0.1337209*	*0.0777778*
**ENSG00000165591**		*0.18787879*		*0.187878788*			*0.2616279*
**ENSG00000116990**		*0.26060606*		*0.260606061*			
**ENSG00000159618**	*0.44011976*	*0.19662921*	*0.44011976*	*0.196629213*	*0.5*	*0.4176471*	*0.4555556*
**ENSG00000166295**							*0.1222222*
**ENSG00000090432**		*0.36363636*		*0.363636364*	*0.122222222*	*0.0909091*	*0.0941176*
**ENSG00000213390**		*0.36363636*		*0.363636364*			*0.3139535*
**ENSG00000121680**	*0.21022727*	*0.25151515*	*0.21022727*	*0.251515152*	*0.477777778*	*0.3636364*	*0.1411765*
**ENSG00000120949**	*0.21022727*	*0.22560976*	*0.21022727*	*0.225609756*	*0.333333333*	*0.2906977*	*0.2906977*
**ENSG00000085982**		*0.375*		*0.375*			*0.1627907*
**ENSG00000167037**		*0.30357143*		*0.303571429*			*0.4886364*
**ENSG00000147573**	*0.1257485*		*0.1257485*				
**ENSG00000127483**		*0.06666667*		*0.066666667*	*0.113095238*	*0.1686047*	
**ENSG00000105996**		*0.09090909*		*0.090909091*			
**ENSG00000026559**	*0.27245509*	*0.38484848*	*0.27245509*	*0.384848485*	*0.446428571*	*0.3255814*	*0.3255814*
**ENSG00000105398**	*0.06586826*	*0.23863636*	*0.06586826*	*0.238636364*			*0.2840909*
**ENSG00000156876**		*0.10555556*		*0.105555556*			*0.1190476*
**ENSG00000137642**	*0.18263473*	*0.44444444*	*0.18263473*	*0.444444444*			*0.4431818*
**ENSG00000140022**		*0.11046512*		*0.110465116*			
**ENSG00000106992**		*0.17272727*		*0.172727273*			*0.122093*
**ENSG00000182463**		*0.39393939*		*0.393939394*			*0.2034884*
**ENSG00000108239**		*0.43030303*		*0.43030303*			*0.2325581*
**ENSG00000232382**	*0.12359551*		*0.12359551*		*0.155555556*	*0.1111111*	
**ENSG00000151962**		*0.12727273*		*0.127272727*			*0.4883721*
**ENSG00000114978**	*0.26666667*	*0.11515152*	*0.26666667*	*0.115151515*	*0.4*	*0.25*	*0.3023256*
**ENSG00000205790**	*0.24850299*		*0.24850299*		*0.301204819*	*0.25*	

**ENSG00000198477**		*0.13068182*		*0.130681818*			*0.2650602*
**ENSG00000078668**		*0.05757576*		*0.057575758*			*0.2151163*
**ENSG00000149547**		*0.11666667*		*0.116666667*			
**ENSG00000165682**		*0.38787879*		*0.387878788*			*0.2906977*
**ENSG00000171119**	*0.30538922*		*0.30538922*		*0.439759036*	*0.4302326*	
**ENSG00000205208**		*0.38333333*		*0.383333333*			*0.2093023*
**ENSG00000103044**		*0.08333333*		*0.083333333*			
**ENSG00000175426**	*0.26047904*	*0.26666667*	*0.26047904*	*0.266666667*	*0.30952381*	*0.3023256*	*0.2034884*
**ENSG00000116883**					*0.06547619*	*0.1104651*	
**ENSG00000170412**	*0.34659091*	*0.23030303*	*0.34659091*	*0.23030303*	*0.155555556*	*0.1477273*	*0.2151163*
**ENSG00000111664**		*0.34545455*		*0.345454545*			*0.2906977*
**ENSG00000188493**	*0.12222222*	*0.10606061*	*0.12222222*	*0.106060606*	*0.355555556*	*0.2848837*	*0.1511628*
**ENSG00000177932**		*0.43820225*		*0.438202247*	*0.077380952*	*0.1046512*	*0.494186*
**ENSG00000166840**	*0.18562874*	*0.34848485*	*0.18562874*	*0.348484848*	*0.446428571*	*0.3372093*	*0.3604651*
**ENSG00000204388**	*0.22289157*		*0.22289157*		*0.060240964*	*0.0833333*	
**ENSG00000092094**	*0.47904192*		*0.47904192*		*0.428571429*	*0.4823529*	
**ENSG00000159307**		*0.38181818*		*0.381818182*			*0.1744186*
**ENSG00000144230**		*0.29938272*		*0.299382716*			*0.1927711*
**ENSG00000152315**		*0.42727273*		*0.427272727*			*0.4302326*
**ENSG00000155961**		*0.18181818*		*0.181818182*			*0.1569767*
**ENSG00000175895**		*0.08888889*		*0.088888889*			
**ENSG00000005513**		*0.49393939*		*0.493939394*			*0.372093*
**ENSG00000151914**		*0.27878788*		*0.278787879*			*0.3081395*
**ENSG00000247626**		*0.16969697*		*0.16969697*			*0.2034884*
**ENSG00000131149**		*0.36363636*		*0.363636364*			*0.494186*
**ENSG00000148218**	*0.30838323*	*0.38181818*	*0.30838323*	*0.381818182*			*0.4069767*
**ENSG00000188368**	*0.05688623*		*0.05688623*				
**ENSG00000116641**		*0.32222222*		*0.322222222*			*0.1176471*
**ENSG00000118961**		*0.40853659*		*0.408536585*			*0.3837209*
**ENSG00000204065**							*0.125*
**ENSG00000171659**	*0.12048193*	*0.18333333*	*0.12048193*	*0.183333333*		*0.0581395*	*0.0568182*
**ENSG00000171169**	*0.30239521*		*0.30239521*		*0.19047619*	*0.2616279*	
**ENSG00000065911**		*0.44444444*		*0.444444444*			*0.2727273*
**ENSG00000116288**	*0.21987952*		*0.21987952*		*0.297619048*	*0.4011628*	
**ENSG00000157538**		*0.17977528*		*0.179775281*			*0.0681818*
**ENSG00000115107**	*0.19101124*	*0.26969697*	*0.19101124*	*0.26969697*	*0.407407407*	*0.255814*	*0.3488372*
**ENSG00000119699**		*0.22727273*		*0.227272727*			*0.4651163*
**ENSG00000128039**		*0.34848485*		*0.348484848*			*0.255814*
**ENSG00000111432**		*0.34269663*		*0.342696629*			*0.4651163*
**ENSG00000092978**	*0.27777778*	*0.4494382*	*0.27777778*	*0.449438202*	*0.2*	*0.1931818*	*0.1931818*
**ENSG00000109072**	*0.34730539*		*0.34730539*		*0.273809524*	*0.2267442*	
**ENSG00000146047**		*0.08522727*		*0.085227273*			*0.0697674*
**ENSG00000165410**		*0.16158537*		*0.161585366*			*0.1764706*
**ENSG00000012223**	*0.15269461*	*0.07878788*	*0.15269461*	*0.078787879*	*0.398809524*	*0.3546512*	*0.2151163*
**ENSG00000145936**	*0.13664596*	*0.33030303*	*0.13664596*	*0.33030303*	*0.380952381*	*0.2882353*	*0.4534884*
**ENSG00000083223**	*0.10479042*	*0.47222222*	*0.10479042*	*0.472222222*	*0.5*	*0.3488372*	*0.3546512*
**ENSG00000169519**	*0.31437126*		*0.31437126*		*0.476190476*	*0.4709302*	
**ENSG00000110851**		*0.42121212*		*0.421212121*			*0.4186047*
**ENSG00000116489**	*0.1497006*	*0.27743902*	*0.1497006*	*0.277439024*			*0.1976744*
**ENSG00000205653**		*0.2030303*		*0.203030303*			*0.4418605*
**ENSG00000085063**		*0.36363636*		*0.363636364*			*0.4647059*
**ENSG00000243543**							*0.1569767*
**ENSG00000204922**	*0.46407186*	*0.27777778*	*0.46407186*	*0.277777778*	*0.071428571*	*0.0581395*	*0.0681818*
**ENSG00000146285**	*0.25287356*	*0.46666667*	*0.25287356*	*0.466666667*	*0.30952381*	*0.255814*	*0.494186*

**ENSG00000164591**							*0.122093*
**ENSG00000167881**		*0.2969697*		*0.296969697*			*0.4647059*
**ENSG00000102265**		*0.48484848*		*0.484848485*			*0.4659091*
**ENSG00000113108**	*0.49444444*	*0.25757576*	*0.49444444*	*0.257575758*	*0.133333333*	*0.1704545*	*0.2209302*
**ENSG00000170946**		*0.33030303*		*0.33030303*			*0.3488372*
**ENSG00000060709**	*0.47005988*	*0.36666667*	*0.47005988*	*0.366666667*	*0.391566265*	*0.4302326*	*0.4476744*
**ENSG00000175106**		*0.21165644*		*0.211656442*			*0.1647059*
**ENSG00000127074**					*0.446428571*	*0.3869048*	
**ENSG00000105393**		*0.20625*		*0.20625*			
**ENSG00000104490**	*0.48802395*	*0.27878788*	*0.48802395*	*0.278787879*	*0.458333333*	*0.4534884*	*0.244186*
**ENSG00000120075**		*0.36060606*		*0.360606061*			*0.3953488*
**ENSG00000198691**							*0.0755814*
**ENSG00000170099**		*0.46666667*		*0.466666667*			*0.1294118*
**ENSG00000075239**		*0.45151515*		*0.451515152*			*0.1162791*
**ENSG00000020922**	*0.33832335*		*0.33832335*		*0.482142857*	*0.3837209*	
**ENSG00000131355**		*0.06969697*		*0.06969697*			
**ENSG00000090920**		*0.21036585*		*0.210365854*			*0.2267442*
**ENSG00000163817**		*0.42378049*		*0.423780488*			*0.2117647*
**ENSG00000258223**	*0.45625*	*0.06097561*	*0.45625*	*0.06097561*	*0.202380952*	*0.1184211*	*0.1294118*
**ENSG00000129667**		*0.3*		*0.3*			*0.372093*
**ENSG00000238045**	*0.18263473*		*0.18263473*				
**ENSG00000159556**	*0.25149701*	*0.30606061*	*0.25149701*	*0.306060606*			*0.3837209*
**ENSG00000175265**		*0.22727273*		*0.227272727*			*0.1931818*
**ENSG00000203963**		*0.39444444*		*0.394444444*			*0.1704545*
**ENSG00000164674**					*0.053571429*	*0.0581395*	
**ENSG00000140044**	*0.41017964*	*0.42424242*	*0.41017964*	*0.424242424*			*0.4244186*
**ENSG00000119283**		*0.33333333*		*0.333333333*			*0.4886364*
**ENSG00000123358**		*0.0969697*		*0.096969697*			*0.1235294*
**ENSG00000130612**	*0.33030303*	*0.1030303*	*0.33030303*	*0.103030303*	*0.452380952*	*0.4418605*	*0.25*
**ENSG00000008226**		*0.1*		*0.1*			
**ENSG00000119689**							*0.4117647*
**ENSG00000146834**	*0.13473054*	*0.16969697*	*0.13473054*	*0.16969697*			
**ENSG00000100281**	*0.08383234*	*0.3597561*	*0.08383234*	*0.359756098*	*0.5*	*0.4476744*	*0.4583333*
**ENSG00000137094**	*0.125*	*0.49393939*	*0.125*	*0.493939394*	*0.291666667*	*0.4127907*	*0.4058824*
**ENSG00000196263**		*0.44848485*		*0.448484848*			*0.4127907*
**ENSG00000120149**		*0.17177914*		*0.171779141*			
**ENSG00000165895**		*0.28658537*		*0.286585366*			
**ENSG00000144455**		*0.48181818*		*0.481818182*			*0.4555556*
**ENSG00000162722**		*0.40555556*		*0.405555556*			*0.375*
**ENSG00000259458**	*0.39520958*	*0.44848485*	*0.39520958*	*0.448484848*	*0.172619048*	*0.25*	*0.2965116*
**ENSG00000010292**	*0.40804598*	*0.5*	*0.40804598*	*0.5*	*0.133333333*	*0.2613636*	*0.4767442*
**ENSG00000138131**		*0.34545455*		*0.345454545*			*0.4825581*
**ENSG00000158793**		*0.1*		*0.1*			*0.0755814*
**ENSG00000144741**	*0.37125749*	*0.47575758*	*0.37125749*	*0.475757576*	*0.476190476*	*0.4825581*	*0.3333333*
**ENSG00000096401**	*0.48203593*	*0.05*	*0.48203593*	*0.05*	*0.422619048*	*0.4593023*	
**ENSG00000108021**					*0.053571429*	*0.0697674*	
**ENSG00000106331**	*0.26966292*	*0.20909091*	*0.26966292*	*0.209090909*	*0.375*	*0.3409091*	*0.3430233*
**ENSG00000111237**		*0.33742331*		*0.337423313*			*0.1104651*
**ENSG00000106484**		*0.2*		*0.2*			*0.2383721*
**ENSG00000174015**	*0.2*		*0.2*				
**ENSG00000137675**					*0.101190476*	*0.1084337*	
**ENSG00000168785**	*0.09195402*		*0.09195402*				
**ENSG00000122691**		*0.11931818*		*0.119318182*			*0.4880952*
**ENSG00000163531**		*0.43333333*		*0.433333333*			*0.244186*

**ENSG00000127980**					*0.077777778*			
**ENSG00000101928**		*0.40606061*		*0.406060606*				*0.4352941*
**ENSG00000163694**	*0.4186747*	*0.36363636*	*0.4186747*	*0.363636364*	*0.470238095*		*0.5*	*0.494186*
**ENSG00000135119**		*0.12804878*		*0.12804878*				*0.4176471*
**ENSG00000131747**		*0.27272727*		*0.272727273*				
**ENSG00000130529**					*0.081395349*			
**ENSG00000135316**								*0.0909091*
**ENSG00000168575**								*0.0813953*
**ENSG00000179133**		*0.27564103*		*0.275641026*				*0.4011628*
**ENSG00000134815**	*0.35555556*	*0.07575758*	*0.35555556*	*0.075757576*	*0.422222222*		*0.4886364*	
**ENSG00000151929**		*0.40606061*		*0.406060606*				*0.3255814*
**ENSG00000025293**	*0.08888889*	*0.17272727*	*0.08888889*	*0.172727273*				*0.1453488*
**ENSG00000134077**	*0.5*	*0.46363636*	*0.5*	*0.463636364*	*0.446428571*		*0.4244186*	*0.4593023*
**ENSG00000143107**	*0.44011976*		*0.44011976*		*0.06547619*		*0.2093023*	
**ENSG00000090316**	*0.23333333*		*0.23333333*		*0.111111111*		*0.1666667*	
**ENSG00000093100**		*0.40606061*		*0.406060606*				*0.4593023*
**ENSG00000163374**	*0.27777778*	*0.31818182*	*0.27777778*	*0.318181818*	*0.355555556*		*0.1777778*	*0.2616279*
**ENSG00000163827**		*0.43251534*		*0.432515337*				*0.4651163*
**ENSG00000185163**		*0.13333333*		*0.133333333*				*0.2093023*
**ENSG00000072071**		*0.10606061*		*0.106060606*				*0.0813953*
**ENSG00000242028**	*0.32335329*	*0.07222222*	*0.32335329*	*0.072222222*	*0.321428571*		*0.244186*	*0.244186*
**ENSG00000188676**		*0.46319018*		*0.463190184*				*0.3235294*
**ENSG00000007350**	*0.26829268*	*0.22121212*	*0.26829268*	*0.221212121*				*0.2732558*
**ENSG00000116679**		*0.46969697*		*0.46969697*				*0.3488372*
**ENSG00000187094**	*0.32934132*		*0.32934132*		*0.25*		*0.2965116*	*0.0872093*
**ENSG00000160505**	*0.48493976*	*0.10060976*	*0.48493976*	*0.100609756*	*0.5*		*0.4883721*	*0.2093023*
**ENSG00000185630**		*0.3*		*0.3*				*0.2111111*
**ENSG00000186143**	*0.43413174*	*0.48333333*	*0.43413174*	*0.483333333*	*0.261904762*		*0.1744186*	*0.2840909*
**ENSG00000171681**	*0.26946108*		*0.26946108*		*0.415662651*		*0.3928571*	
**ENSG00000103528**		*0.49390244*		*0.493902439*				
**ENSG00000172901**	*0.37356322*	*0.5*	*0.37356322*	*0.5*	*0.446428571*		*0.4127907*	*0.2732558*
**ENSG00000133317**	*0.28614458*	*0.13690476*	*0.28614458*	*0.136904762*	*0.373493976*		*0.3488372*	*0.0930233*
**ENSG00000134375**		*0.47239264*		*0.472392638*				*0.4294118*
**ENSG00000214946**		*0.22222222*		*0.222222222*				*0.4127907*
**ENSG00000115457**		*0.05555556*		*0.055555556*				
**ENSG00000049249**								*0.4651163*
**ENSG00000169925**	*0.19760479*	*0.29444444*	*0.19760479*	*0.294444444*	*0.274390244*		*0.4127907*	*0.4204545*
**ENSG00000146587**		*0.48181818*		*0.481818182*				*0.3823529*
**ENSG00000144233**	*0.11666667*	*0.47575758*	*0.11666667*	*0.475757576*	*0.433333333*		*0.2954545*	*0.3953488*
**ENSG00000004777**	*0.42168675*		*0.42168675*		*0.255952381*		*0.2209302*	
**ENSG00000111644**		*0.19090909*		*0.190909091*				*0.3604651*
**ENSG00000136935**		*0.44444444*		*0.444444444*				*0.4090909*
**ENSG00000253305**		*0.20224719*		*0.202247191*				*0.1477273*
**ENSG00000224470**		*0.34269663*		*0.342696629*				*0.3863636*
**ENSG00000246366**		*0.0969697*		*0.096969697*				
**ENSG00000126267**	*0.11377246*		*0.11377246*		*0.136904762*		*0.1453488*	
**ENSG00000145431**		*0.33850932*		*0.338509317*	*0.089285714*		*0.127907*	*0.3414634*
**ENSG00000110092**		*0.48170732*		*0.481707317*				*0.4709302*
**ENSG00000163072**	*0.46385542*	*0.42727273*	*0.46385542*	*0.427272727*	*0.30952381*		*0.3604651*	*0.4011628*
**ENSG00000104331**		*0.13529412*		*0.135294118*	*0.311111111*		*0.2272727*	*0.3571429*
**ENSG00000120899**	*0.36111111*	*0.48181818*	*0.36111111*	*0.481818182*	*0.3*		*0.3662791*	*0.4883721*
**ENSG00000124313**		*0.08641975*		*0.086419753*				
**ENSG00000106304**		*0.26969697*		*0.26969697*				*0.0581395*
**ENSG00000255482**	*0.22155689*		*0.22155689*		*0.36746988*		*0.3023256*	

**ENSG00000123569**					*0.446428571*	*0.3662791*	
**ENSG00000198844**		*0.35151515*		*0.351515152*			*0.4593023*
**ENSG00000166455**	*0.43081761*	*0.12121212*	*0.43081761*	*0.121212121*	*0.273809524*	*0.2965116*	*0.3023256*
**ENSG00000204173**	*0.23888889*	*0.31111111*	*0.23888889*	*0.311111111*	*0.466666667*	*0.3977273*	*0.3977273*
**ENSG00000163945**	*0.21590909*	*0.34444444*	*0.21590909*	*0.344444444*	*0.438271605*	*0.4825581*	*0.4593023*
**ENSG00000137404**							*0.1744186*
**ENSG00000103351**					*0.155555556*	*0.0909091*	
**ENSG00000081177**	*0.27777778*	*0.38181818*	*0.27777778*	*0.381818182*			*0.0581395*
**ENSG00000080802**		*0.36666667*		*0.366666667*		*0.0666667*	*0.4534884*
**ENSG00000118276**		*0.47272727*		*0.472727273*			*0.3705882*
**ENSG00000179979**	*0.10179641*		*0.10179641*		*0.3*	*0.3662791*	
**ENSG00000115665**		*0.42121212*		*0.421212121*	*0.174698795*	*0.1411765*	*0.244186*
**ENSG00000163346**		*0.41818182*		*0.418181818*			*0.0588235*
**ENSG00000175073**							*0.0581395*
**ENSG00000109927**		*0.3*		*0.3*			*0.3313953*
**ENSG00000118420**		*0.26993865*		*0.26993865*			*0.1918605*
**ENSG00000102802**		*0.15151515*		*0.151515152*			*0.3313953*
**ENSG00000224130**		*0.1746988*		*0.174698795*			*0.2073171*
**ENSG00000144354**		*0.28484848*		*0.284848485*			*0.3313953*
**ENSG00000147655**	*0.28888889*	*0.23030303*	*0.28888889*	*0.23030303*	*0.055555556*		*0.1607143*
**ENSG00000138759**		*0.41111111*		*0.411111111*			*0.4875*
**ENSG00000186350**	*0.32222222*		*0.32222222*		*0.2*	*0.2444444*	
**ENSG00000181472**							*0.1777778*
**ENSG00000250685**	*0.39444444*		*0.39444444*		*0.422222222*	*0.4767442*	
**ENSG00000143748**	*0.49101796*		*0.49101796*		*0.111111111*	*0.0697674*	
**ENSG00000161551**	*0.35928144*	*0.41158537*	*0.35928144*	*0.411585366*	*0.31547619*	*0.3333333*	*0.2588235*
**ENSG00000169327**	*0.35928144*		*0.35928144*		*0.379518072*	*0.4825581*	
**ENSG00000157429**		*0.26111111*		*0.261111111*			*0.1111111*
**ENSG00000172889**		*0.22222222*		*0.222222222*	*0.220238095*	*0.244186*	*0.125*
**ENSG00000164683**		*0.30606061*		*0.306060606*			*0.25*
**ENSG00000180104**		*0.39570552*		*0.395705521*			*0.4117647*
**ENSG00000144834**	*0.4845679*	*0.23030303*	*0.4845679*	*0.23030303*	*0.457831325*	*0.4186047*	*0.3837209*
**ENSG00000134709**		*0.16111111*		*0.161111111*			*0.2045455*
**ENSG00000156232**	*0.39520958*		*0.39520958*		*0.228915663*	*0.2790698*	
**ENSG00000117407**	*0.17987805*		*0.17987805*				
**ENSG00000101109**		*0.46666667*		*0.466666667*			*0.2857143*
**ENSG00000041988**		*0.34756098*		*0.347560976*			*0.4360465*
**ENSG00000166845**		*0.32424242*		*0.324242424*			*0.2954545*
**ENSG00000063660**		*0.36111111*		*0.361111111*			*0.3604651*
**ENSG00000176896**	*0.05747126*	*0.45454545*	*0.05747126*	*0.454545455*			*0.3837209*
**ENSG00000136444**	*0.07647059*	*0.22121212*	*0.07647059*	*0.221212121*	*0.204545455*	*0.1222222*	*0.0639535*
**ENSG00000096968**	*0.10240964*		*0.10240964*		*0.457317073*	*0.3837209*	
**ENSG00000085644**		*0.43604651*		*0.436046512*			*0.1744186*
**ENSG00000153933**		*0.31132075*		*0.311320755*			*0.1585366*
**ENSG00000196391**	*0.4760479*	*0.43333333*	*0.4760479*	*0.433333333*	*0.345238095*	*0.3488372*	*0.4825581*
**ENSG00000116183**	*0.11377246*	*0.47852761*	*0.11377246*	*0.478527607*		*0.0581395*	*0.3863636*
**ENSG00000100478**	*0.14071856*		*0.14071856*		*0.470238095*	*0.372093*	
**ENSG00000042304**		*0.38235294*		*0.382352941*			*0.4886364*
**ENSG00000188211**		*0.42424242*		*0.424242424*			*0.5*
**ENSG00000131620**	*0.27844311*		*0.27844311*				
**ENSG00000162521**					*0.127906977*	*0.1136364*	
**ENSG00000134569**							*0.0930233*
**ENSG00000164284**		*0.47878788*		*0.478787879*			*0.377907*
**ENSG00000183049**		*0.47878788*		*0.478787879*			*0.4588235*

**ENSG00000164128**		*0.25757576*		*0.257575758*			*0.4883721*
**ENSG00000161973**	*0.45808383*	*0.3*	*0.45808383*	*0.3*	*0.482142857*	*0.4360465*	*0.4360465*
**ENSG00000140526**		*0.46551724*		*0.465517241*			*0.2674419*
**ENSG00000105662**		*0.38636364*		*0.386363636*			*0.1477273*
**ENSG00000109193**	*0.28963415*	*0.16666667*	*0.28963415*	*0.166666667*	*0.243902439*	*0.3058824*	
**ENSG00000112115**		*0.21111111*		*0.211111111*			*0.1569767*
**ENSG00000185532**		*0.30909091*		*0.309090909*			*0.2965116*
**ENSG00000075223**	*0.05555556*	*0.11585366*	*0.05555556*	*0.115853659*		*0.0888889*	*0.0705882*
**ENSG00000129351**		*0.36666667*		*0.366666667*			*0.3488372*
**ENSG00000174111**							*0.0755814*
**ENSG00000186204**	*0.21646341*	*0.4969697*	*0.21646341*	*0.496969697*			*0.2823529*
**ENSG00000184108**	*0.30606061*		*0.30606061*		*0.386904762*	*0.3139535*	
**ENSG00000198673**	*0.32222222*	*0.4969697*	*0.32222222*	*0.496969697*	*0.1*	*0.1444444*	*0.3647059*
**ENSG00000176101**		*0.28787879*		*0.287878788*			
**ENSG00000078177**		*0.42592593*		*0.425925926*			*0.5*
**ENSG00000172154**	*0.10479042*		*0.10479042*				
**ENSG00000183853**		*0.43636364*		*0.436363636*			*0.4476744*
**ENSG00000145592**		*0.43636364*		*0.436363636*			*0.4593023*
**ENSG00000125675**		*0.06666667*		*0.066666667*			
**ENSG00000175573**							*0.0581395*
**ENSG00000236338**		*0.08181818*		*0.081818182*			*0.0988372*
**ENSG00000165061**		*0.19631902*		*0.196319018*			
**ENSG00000188282**	*0.49101796*	*0.12121212*	*0.49101796*	*0.121212121*	*0.404761905*	*0.372093*	*0.1395349*
**ENSG00000066379**	*0.32222222*	*0.10606061*	*0.32222222*	*0.106060606*	*0.155555556*	*0.2272727*	*0.2093023*
**ENSG00000218416**		*0.35454545*		*0.354545455*			*0.1136364*
**ENSG00000189043**							*0.1555556*
**ENSG00000142188**		*0.49079755*		*0.490797546*			*0.4878049*
**ENSG00000185215**	*0.34444444*	*0.48888889*	*0.34444444*	*0.488888889*	*0.477777778*	*0.4545455*	*0.4545455*
**ENSG00000184349**		*0.39393939*		*0.393939394*			*0.4418605*
**ENSG00000243137**		*0.228125*		*0.228125*			*0.4695122*
**ENSG00000066855**	*0.07784431*		*0.07784431*				
**ENSG00000167889**		*0.33231707*		*0.332317073*			*0.4244186*
**ENSG00000183291**	*0.44610778*	*0.23939394*	*0.44610778*	*0.239393939*	*0.054216867*		
**ENSG00000009844**		*0.5*		*0.5*			*0.1555556*
**ENSG00000104432**							*0.2111111*
**ENSG00000140795**	*0.0969697*		*0.0969697*		*0.361445783*	*0.4345238*	
**ENSG00000034533**	*0.16566265*		*0.16566265*		*0.095238095*	*0.1*	
**ENSG00000013563**	*0.17964072*	*0.05882353*	*0.17964072*	*0.058823529*	*0.05952381*	*0.1162791*	*0.1477273*
**ENSG00000136158**		*0.33333333*		*0.333333333*	*0.066666667*		*0.3604651*
**ENSG00000171497**	*0.31111111*	*0.3125*	*0.31111111*	*0.3125*	*0.066666667*	*0.1976744*	*0.2235294*
**ENSG00000166262**	*0.32634731*	*0.42121212*	*0.32634731*	*0.421212121*			*0.2674419*
**ENSG00000159433**		*0.21515152*		*0.215151515*			*0.2674419*
**ENSG00000034693**		*0.17987805*		*0.179878049*			*0.1941176*
**ENSG00000032389**		*0.40606061*		*0.406060606*			*0.4651163*
**ENSG00000206129**	*0.24157303*	*0.45705521*	*0.24157303*	*0.457055215*	*0.144444444*	*0.1569767*	*0.4880952*
**ENSG00000118308**	*0.21111111*	*0.22865854*	*0.21111111*	*0.228658537*	*0.272727273*	*0.3111111*	*0.0581395*
**ENSG00000130957**		*0.45679012*		*0.456790123*			*0.2616279*
**ENSG00000147604**	*0.12777778*	*0.12424242*	*0.12777778*	*0.124242424*	*0.277777778*	*0.2159091*	*0.0639535*
**ENSG00000128383**	*0.08888889*		*0.08888889*				
**ENSG00000130300**	*0.47305389*		*0.47305389*		*0.125*	*0.2151163*	*0.1428571*
**ENSG00000204414**		*0.41477273*		*0.414772727*			
**ENSG00000100767**		*0.28181818*		*0.281818182*			*0.1117647*
**ENSG00000111254**					*0.333333333*	*0.3409091*	
**ENSG00000115008**		*0.26363636*		*0.263636364*			*0.127907*

**ENSG00000103245**	*0.46107784*	*0.20909091*	*0.46107784*	*0.209090909*	*0.196428571*	*0.2470588*	*0.2529412*
**ENSG00000123427**	*0.10493827*	*0.36666667*	*0.10493827*	*0.366666667*	*0.380952381*	*0.375*	*0.2840909*
**ENSG00000125352**	*0.16768293*		*0.16768293*		*0.178571429*	*0.125*	
**ENSG00000147536**		*0.21515152*		*0.215151515*			*0.2906977*
**ENSG00000165169**		*0.12222222*		*0.122222222*			
**ENSG00000198771**		*0.25925926*		*0.259259259*	*0.154761905*	*0.2790698*	*0.3472222*
**ENSG00000173578**	*0.18263473*	*0.37272727*	*0.18263473*	*0.372727273*	*0.398809524*	*0.4069767*	*0.4069767*
**ENSG00000070087**	*0.13953488*		*0.13953488*				
**ENSG00000138336**	*0.33030303*		*0.33030303*		*0.457831325*	*0.4069767*	
**ENSG00000118495**	*0.28742515*	*0.39506173*	*0.28742515*	*0.395061728*	*0.43452381*	*0.474359*	*0.2380952*
**ENSG00000034053**		*0.2202381*		*0.220238095*	*0.297619048*	*0.2738095*	*0.375*
**ENSG00000249773**	*0.49438202*		*0.49438202*		*0.477777778*	*0.4318182*	
**ENSG00000163293**		*0.37222222*		*0.372222222*			*0.4127907*
**ENSG00000122642**		*0.40625*		*0.40625*			*0.1818182*
**ENSG00000072121**		*0.4030303*		*0.403030303*			*0.3953488*
**ENSG00000197077**	*0.11676647*	*0.49390244*	*0.11676647*	*0.493902439*	*0.107142857*	*0.122093*	*0.4709302*
**ENSG00000058404**		*0.45*		*0.45*			*0.4090909*
**ENSG00000159111**		*0.35454545*		*0.354545455*			*0.3662791*
**ENSG00000176020**	*0.28012048*	*0.23006135*	*0.28012048*	*0.23006135*	*0.154761905*	*0.2383721*	
**ENSG00000158526**							*0.1058824*
**ENSG00000167074**		*0.26829268*		*0.268292683*			*0.3255814*
**ENSG00000008952**		*0.21666667*		*0.216666667*			*0.2906977*
**ENSG00000143839**	*0.3253012*		*0.3253012*		*0.06547619*		
**ENSG00000164167**	*0.20658683*	*0.36206897*	*0.20658683*	*0.362068966*	*0.066666667*	*0.0988372*	*0.0795455*
**ENSG00000168874**		*0.36046512*		*0.360465116*			*0.1395349*
**ENSG00000171860**		*0.3*		*0.3*			*0.1337209*
**ENSG00000167105**		*0.5*		*0.5*			*0.4036145*
**ENSG00000142512**	*0.1746988*		*0.1746988*		*0.092592593*	*0.0609756*	
**ENSG00000165171**		*0.17948718*		*0.179487179*			*0.4342105*
**ENSG00000167535**		*0.42987805*		*0.429878049*			*0.3571429*
**ENSG00000116985**	*0.16111111*	*0.29444444*	*0.16111111*	*0.294444444*			*0.1931818*
**ENSG00000185482**		*0.28181818*		*0.281818182*			*0.2267442*
**ENSG00000150867**		*0.3969697*		*0.396969697*			*0.3546512*
**ENSG00000182600**	*0.19461078*		*0.19461078*		*0.162650602*	*0.1686047*	
**ENSG00000067842**		*0.05625*		*0.05625*			*0.2235294*
**ENSG00000127022**	*0.15568862*	*0.31515152*	*0.15568862*	*0.315151515*	*0.416666667*	*0.4476744*	*0.2882353*
**ENSG00000125741**		*0.4*		*0.4*			*0.1976744*
**ENSG00000186815**	*0.1875*	*0.1954023*	*0.1875*	*0.195402299*	*0.233333333*	*0.2840909*	*0.255814*
**ENSG00000198231**		*0.3908046*		*0.390804598*			*0.4545455*
**ENSG00000103363**		*0.4875*		*0.4875*			*0.3072289*
**ENSG00000197093**		*0.43636364*		*0.436363636*			*0.372093*
**ENSG00000130935**					*0.222222222*	*0.3977273*	
**ENSG00000075340**		*0.44848485*		*0.448484848*			*0.3555556*
**ENSG00000152953**		*0.12424242*		*0.124242424*			*0.4529412*
**ENSG00000137720**	*0.07784431*	*0.32121212*	*0.07784431*	*0.321212121*			*0.1976744*
**ENSG00000112038**	*0.14371257*	*0.36890244*	*0.14371257*	*0.368902439*	*0.136904762*	*0.0523256*	*0.1022727*
**ENSG00000124507**		*0.11818182*		*0.118181818*			*0.2906977*
**ENSG00000104881**		*0.15123457*		*0.151234568*			*0.4534884*
**ENSG00000130307**		*0.40909091*		*0.409090909*			*0.25*
**ENSG00000164751**	*0.2994012*		*0.2994012*		*0.285714286*	*0.3470588*	
**ENSG00000145685**		*0.49390244*		*0.493902439*			*0.3023256*
**ENSG00000198055**							*0.0892857*
**ENSG00000184347**							*0.1882353*
**ENSG00000122585**	*0.4*		*0.4*		*0.340909091*	*0.3333333*	

**ENSG00000169347**		*0.08787879*		*0.087878788*			*0.1744186*
**ENSG00000161328**		*0.37878788*		*0.378787879*			*0.2267442*
**ENSG00000167118**		*0.13333333*		*0.133333333*			*0.0813953*
**ENSG00000162779**	*0.28443114*	*0.08695652*	*0.28443114*	*0.086956522*	*0.327380952*	*0.4651163*	*0.3705882*
**ENSG00000197363**		*0.14242424*		*0.142424242*			*0.4764706*
**ENSG00000184571**	*0.46407186*		*0.46407186*		*0.43452381*	*0.4593023*	
**ENSG00000140284**		*0.1595092*		*0.159509202*			
**ENSG00000135622**		*0.12121212*		*0.121212121*			*0.1627907*
**ENSG00000173137**		*0.4202454*		*0.420245399*			*0.3647059*
**ENSG00000182508**		*0.08231707*		*0.082317073*			
**ENSG00000144021**		*0.33333333*		*0.333333333*			*0.3837209*
**ENSG00000115548**		*0.16975309*		*0.169753086*			
**ENSG00000021300**	*0.38764045*	*0.10909091*	*0.38764045*	*0.109090909*	*0.1*	*0.1444444*	*0.1511628*
**ENSG00000091409**		*0.36969697*		*0.36969697*			*0.0988372*
**ENSG00000092051**					*0.244047619*	*0.2093023*	
**ENSG00000153113**	*0.11976048*	*0.42987805*	*0.11976048*	*0.429878049*	*0.267857143*	*0.2616279*	*0.2906977*
**ENSG00000182253**	*0.4011976*	*0.28353659*	*0.4011976*	*0.283536585*	*0.238095238*	*0.3023256*	*0.4235294*
**ENSG00000185774**		*0.41515152*		*0.415151515*	*0.178571429*	*0.1941176*	*0.3941176*
**ENSG00000180347**	*0.16363636*	*0.28823529*	*0.16363636*	*0.288235294*	*0.415662651*	*0.3197674*	*0.1511628*
**ENSG00000186951**		*0.19207317*		*0.192073171*			
**ENSG00000204010**					*0.095238095*	*0.2151163*	
**ENSG00000141098**		*0.14545455*		*0.145454545*			*0.0529412*
**ENSG00000111424**	*0.18373494*	*0.44545455*	*0.18373494*	*0.445454545*	*0.404761905*	*0.3255814*	*0.3470588*
**ENSG00000138439**		*0.41463415*		*0.414634146*			*0.3214286*
**ENSG00000188937**					*0.470238095*	*0.4411765*	
**ENSG00000128886**		*0.06666667*		*0.066666667*			*0.255814*
**ENSG00000120334**	*0.36227545*		*0.36227545*				
**ENSG00000147316**		*0.29444444*		*0.294444444*			*0.25*
**ENSG00000160117**	*0.20555556*	*0.15757576*	*0.20555556*	*0.157575758*	*0.284090909*	*0.375*	*0.3197674*
**ENSG00000103742**		*0.1030303*		*0.103030303*			*0.2209302*
**ENSG00000167984**	*0.30182927*	*0.36503067*	*0.30182927*	*0.365030675*	*0.333333333*	*0.2888889*	*0.4825581*
**ENSG00000134324**		*0.15151515*		*0.151515152*			*0.2674419*
**ENSG00000157087**	*0.4251497*	*0.47878788*	*0.4251497*	*0.478787879*	*0.345238095*	*0.3546512*	*0.3197674*
**ENSG00000183018**		*0.36111111*		*0.361111111*			*0.4772727*
**ENSG00000138363**		*0.27222222*		*0.272222222*			*0.25*
**ENSG00000120215**	*0.21666667*	*0.31176471*	*0.21666667*	*0.311764706*			*0.373494*
**ENSG00000212710**		*0.33888889*		*0.338888889*			*0.4333333*
**ENSG00000139718**		*0.22159091*		*0.221590909*			
**ENSG00000177182**							*0.3636364*
**ENSG00000135218**	*0.30538922*	*0.43292683*	*0.30538922*	*0.432926829*	*0.377777778*	*0.2840909*	*0.377907*
**ENSG00000120645**		*0.37272727*		*0.372727273*			*0.3411765*
**ENSG00000159164**		*0.21212121*		*0.212121212*	*0.077380952*	*0.1046512*	
**ENSG00000164209**	*0.12352941*	*0.06741573*	*0.12352941*	*0.06741573*	*0.26744186*	*0.1842105*	*0.0795455*
**ENSG00000074410**		*0.43597561*		*0.43597561*			*0.5*
**ENSG00000060718**	*0.10240964*	*0.17272727*	*0.10240964*	*0.172727273*			*0.2267442*
**ENSG00000164091**		*0.41212121*		*0.412121212*			*0.4767442*
**ENSG00000182963**		*0.38484848*		*0.384848485*			*0.4534884*
**ENSG00000181495**	*0.32335329*		*0.32335329*				
**ENSG00000173200**		*0.24242424*		*0.242424242*			*0.4825581*
**ENSG00000168394**		*0.06969697*		*0.06969697*			*0.1337209*
**ENSG00000153339**		*0.22727273*		*0.227272727*			*0.4476744*
**ENSG00000106723**		*0.11666667*		*0.116666667*			*0.0681818*
**ENSG00000099899**	*0.06287425*	*0.2030303*	*0.06287425*	*0.203030303*	*0.119047619*	*0.1395349*	*0.0909091*
**ENSG00000115947**	*0.28143713*	*0.40243902*	*0.28143713*	*0.402439024*	*0.5*	*0.5*	*0.4*

**ENSG00000155903**		*0.35555556*		*0.355555556*			*0.3662791*
**ENSG00000073711**	*0.48203593*		*0.48203593*		*0.19047619*	*0.2848837*	
**ENSG00000115194**							*0.0697674*
**ENSG00000107282**		*0.23333333*		*0.233333333*			*0.4431818*
**ENSG00000136695**		*0.5*		*0.5*			*0.3222222*
**ENSG00000107614**	*0.4011976*	*0.48888889*	*0.4011976*	*0.488888889*	*0.267857143*	*0.3953488*	*0.4772727*
**ENSG00000111341**		*0.35*		*0.35*			*0.1022727*
**ENSG00000106328**	*0.20359281*	*0.1*	*0.20359281*	*0.1*	*0.107142857*	*0.0529412*	*0.127907*
**ENSG00000176971**	*0.18862275*	*0.14545455*	*0.18862275*	*0.145454545*	*0.077380952*	*0.1627907*	*0.2325581*
**ENSG00000083896**		*0.45061728*		*0.450617284*			*0.4418605*
**ENSG00000132530**	*0.23353293*		*0.23353293*		*0.416666667*	*0.2882353*	
**ENSG00000160062**		*0.09509202*		*0.095092025*			*0.3139535*
**ENSG00000187650**		*0.49090909*		*0.490909091*			*0.2705882*
**ENSG00000162510**		*0.46969697*		*0.46969697*			*0.4186047*
**ENSG00000075826**		*0.32121212*		*0.321212121*			*0.3430233*
**ENSG00000197584**		*0.3969697*		*0.396969697*			*0.2616279*
**ENSG00000196456**		*0.44207317*		*0.442073171*			*0.3197674*
**ENSG00000141294**	*0.0748503*		*0.0748503*				
**ENSG00000149089**	*0.27108434*		*0.27108434*		*0.255952381*	*0.3197674*	
**ENSG00000129680**		*0.36060606*		*0.360606061*			*0.3197674*
**ENSG00000145817**	*0.41317365*	*0.25581395*	*0.41317365*	*0.255813953*		*0.0595238*	*0.402439*
**ENSG00000141646**		*0.36666667*		*0.366666667*			*0.4593023*
**ENSG00000178445**		*0.34242424*		*0.342424242*			*0.0523256*
**ENSG00000145675**		*0.43902439*		*0.43902439*			*0.4186047*
**ENSG00000165572**	*0.06111111*	*0.08484848*	*0.06111111*	*0.084848485*			
**ENSG00000221994**		*0.46319018*		*0.463190184*	*0.077380952*	*0.1104651*	*0.1511628*
**ENSG00000070761**					*0.166666667*	*0.1058824*	
**ENSG00000010803**		*0.35534591*		*0.355345912*			*0.4659091*
**ENSG00000110218**		*0.37575758*		*0.375757576*			*0.2738095*
**ENSG00000140853**	*0.46407186*	*0.40372671*	*0.46407186*	*0.403726708*	*0.375*	*0.4705882*	*0.4352941*
**ENSG00000006611**	*0.30113636*	*0.47878788*	*0.30113636*	*0.478787879*	*0.477272727*	*0.3181818*	*0.4186047*
**ENSG00000259207**		*0.33333333*		*0.333333333*			*0.2613636*
**ENSG00000138078**	*0.26347305*	*0.34242424*	*0.26347305*	*0.342424242*	*0.34939759*	*0.3882353*	*0.4069767*
**ENSG00000196418**	*0.17964072*	*0.11818182*	*0.17964072*	*0.118181818*			*0.1117647*
**ENSG00000186283**	*0.10179641*	*0.32926829*	*0.10179641*	*0.329268293*	*0.355555556*	*0.3863636*	*0.4011628*
**ENSG00000152382**		*0.34146341*		*0.341463415*			*0.372093*
**ENSG00000157093**		*0.3969697*		*0.396969697*			*0.3837209*
**ENSG00000164362**		*0.18012422*		*0.180124224*			
**ENSG00000135406**	*0.3742515*		*0.3742515*		*0.071428571*		
**ENSG00000105278**		*0.10909091*		*0.109090909*			*0.1686047*
**ENSG00000182957**	*0.43975904*	*0.38414634*	*0.43975904*	*0.384146341*	*0.416666667*	*0.4647059*	*0.4767442*
**ENSG00000104888**		*0.27272727*		*0.272727273*			*0.4883721*
**ENSG00000166924**		*0.33636364*		*0.336363636*			*0.3488372*
**ENSG00000126337**	*0.14670659*	*0.2247191*	*0.14670659*	*0.224719101*			*0.3068182*
**ENSG00000104951**	*0.17365269*		*0.17365269*		*0.345238095*	*0.4418605*	
**ENSG00000160094**		*0.40853659*		*0.408536585*			*0.3035714*
**ENSG00000140798**	*0.17964072*	*0.2030303*	*0.17964072*	*0.203030303*			*0.0581395*
**ENSG00000182916**					*0.477777778*	*0.4886364*	
**ENSG00000181092**		*0.37730061*		*0.377300613*			*0.4360465*
**ENSG00000123983**	*0.30239521*	*0.40432099*	*0.30239521*	*0.404320988*	*0.244047619*	*0.1488095*	*0.1829268*
**ENSG00000218537**	*0.21257485*	*0.11805556*	*0.21257485*	*0.118055556*	*0.482142857*	*0.4825581*	*0.2261905*
**ENSG00000164902**	*0.32934132*		*0.32934132*		*0.327380952*	*0.4011628*	
**ENSG00000168792**		*0.36969697*		*0.36969697*			*0.0581395*
**ENSG00000090263**		*0.21910112*		*0.219101124*			*0.1222222*

**ENSG00000103064**		0.41818182		0.418181818			0.1802326
**ENSG00000198933**	0.16467066		0.16467066		0.410714286	0.3546512	
**ENSG00000171135**	0.4	0.46969697	0.4	0.46969697	0.25	0.3	0.3604651
**ENSG00000169902**		0.13636364		0.136363636			0.25
**ENSG00000155636**		0.08787879		0.087878788			
**ENSG00000138041**		0.27272727		0.272727273			0.4709302
**ENSG00000073578**		0.13483146		0.134831461			
**ENSG00000125879**		0.16969697		0.16969697			0.4666667
**ENSG00000164244**		0.26162791		0.261627907			0.4825581
**ENSG00000139044**		0.46666667		0.466666667			0.2732558
**ENSG00000165879**		0.37878788		0.378787879			0.3470588
**ENSG00000081479**		0.23030303		0.23030303			0.4767442
**ENSG00000163623**	0.06111111		0.06111111				
**ENSG00000168263**	0.31818182		0.31818182		0.155555556	0.1363636	
**ENSG00000242419**		0.20224719		0.202247191			0.1477273
**ENSG00000101574**	0.38023952	0.39393939	0.38023952	0.393939394	0.142857143	0.0872093	0.255814
**ENSG00000163659**	0.07185629		0.07185629				
**ENSG00000170786**	0.23053892		0.23053892		0.101190476		
**ENSG00000147121**		0.42727273		0.427272727			0.0639535
**ENSG00000167378**		0.43030303		0.43030303			0.4883721
**ENSG00000092140**	0.06287425	0.30487805	0.06287425	0.304878049	0.31547619	0.2965116	0.3023256
**ENSG00000119703**	0.48502994	0.09393939	0.48502994	0.093939394	0.178571429	0.1941176	
**ENSG00000183486**	0.43712575		0.43712575		0.386904762	0.4360465	
**ENSG00000142197**		0.2195122		0.219512195			
**ENSG00000245869**	0.19886364		0.19886364		0.466666667	0.4222222	
**ENSG00000174938**	0.5	0.32121212	0.5	0.321212121	0.420454545	0.244186	0.2034884
**ENSG00000185900**		0.1196319		0.119631902			
**ENSG00000240053**	0.14670659	0.31212121	0.14670659	0.312121212	0.125	0.0697674	0.4534884
**ENSG00000108953**		0.09444444		0.094444444			0.3409091
**ENSG00000183784**		0.32222222		0.322222222			0.2727273
**ENSG00000168314**	0.48125	0.22424242	0.48125	0.224242424	0.280487805	0.2555556	0.3058824
**ENSG00000213413**		0.4375		0.4375			0.3170732
**ENSG00000072518**	0.28443114	0.49393939	0.28443114	0.493939394			0.127907
**ENSG00000249884**	0.2005988		0.2005988			0.0639535	
**ENSG00000171466**		0.20689655		0.206896552			
**ENSG00000131096**	0.48125		0.48125		0.273809524	0.3372093	
**ENSG00000147099**	0.24850299	0.09883721	0.24850299	0.098837209	0.238095238	0.3430233	0.4186047
**ENSG00000123737**		0.34444444		0.344444444			0.2045455
**ENSG00000165650**		0.2969697		0.296969697			
**ENSG00000163207**	0.26347305	0.0797546	0.26347305	0.079754601	0.404761905	0.377907	0.3902439
**ENSG00000044574**	0.16766467	0.43030303	0.16766467	0.43030303			0.4058824
**ENSG00000122550**	0.29341317	0.32822086	0.29341317	0.328220859	0.279761905	0.375	0.3372093
**ENSG00000048462**	0.26946108	0.40588235	0.26946108	0.405882353	0.160714286	0.2209302	0.4459459
**ENSG00000131409**		0.08484848		0.084848485			0.3953488
**ENSG00000004468**		0.44242424		0.442424242			0.4069767
**ENSG00000067141**		0.33707865		0.337078652			0.4111111
**ENSG00000253148**		0.44848485		0.448484848			0.1860465
**ENSG00000180745**		0.08536585		0.085365854			0.0930233
**ENSG00000099377**		0.35454545		0.354545455			0.0988372
**ENSG00000160072**		0.05151515		0.051515152			0.3604651
**ENSG00000073849**	0.19277108	0.45757576	0.19277108	0.457575758	0.327380952	0.372093	0.4011628
**ENSG00000116704**		0.29213483		0.292134831			0.1477273
**ENSG00000189325**	0.23952096		0.23952096		0.25	0.2267442	
**ENSG00000125107**	0.13473054		0.13473054				

**ENSG00000203880**		*0.25454545*		*0.254545455*				*0.0581395*
**ENSG00000214193**	*0.15868263*	*0.38787879*	*0.15868263*	*0.387878788*	*0.279761905*		*0.2093023*	*0.1104651*
**ENSG00000136535**		*0.15757576*		*0.157575758*				*0.1686047*
**ENSG00000184601**		*0.4054878*		*0.405487805*				*0.4235294*
**ENSG00000164236**		*0.42121212*		*0.421212121*				*0.3529412*
**ENSG00000166801**	*0.38023952*	*0.22121212*	*0.38023952*	*0.221212121*	*0.279761905*		*0.3662791*	*0.3176471*
**ENSG00000132000**	*0.06111111*	*0.45454545*	*0.06111111*	*0.454545455*			*0.0568182*	*0.3255814*
**ENSG00000165379**	*0.30681818*		*0.30681818*		*0.238636364*		*0.1818182*	
**ENSG00000119899**		*0.30555556*		*0.305555556*				*0.0941176*
**ENSG00000163399**	*0.44011976*		*0.44011976*					
**ENSG00000102401**		*0.23006135*		*0.23006135*				
**ENSG00000170074**		*0.43888889*		*0.438888889*				*0.4545455*
**ENSG00000048991**								*0.0755814*
**ENSG00000174837**		*0.32317073*		*0.323170732*				*0.2797619*
**ENSG00000215915**	*0.06886228*		*0.06886228*					*0.3662791*
**ENSG00000197919**	*0.26111111*		*0.26111111*		*0.11627907*		*0.0909091*	
**ENSG00000178927**		*0.09393939*		*0.093939394*				*0.255814*
**ENSG00000197566**		*0.10909091*		*0.109090909*				*0.2965116*
**ENSG00000138380**		*0.05757576*		*0.057575758*				*0.0909091*
**ENSG00000096696**		*0.3969697*		*0.396969697*				*0.1627907*
**ENSG00000161791**		*0.12424242*		*0.124242424*				*0.0697674*
**ENSG00000165669**		*0.34848485*		*0.348484848*				*0.1569767*
**ENSG00000102317**	*0.20731707*	*0.43333333*	*0.20731707*	*0.433333333*	*0.392857143*		*0.4244186*	*0.4244186*
**ENSG00000135333**		*0.49090909*		*0.490909091*				*0.3176471*
**ENSG00000165966**		*0.31818182*		*0.318181818*				*0.2790698*
**ENSG00000180509**	*0.08682635*	*0.45061728*	*0.08682635*	*0.450617284*	*0.404761905*		*0.4941176*	*0.4709302*
**ENSG00000122859**		*0.05151515*		*0.051515152*				*0.0755814*
**ENSG00000144306**		*0.303125*		*0.303125*				*0.2848837*
**ENSG00000157005**		*0.05757576*		*0.057575758*				*0.0813953*
**ENSG00000184178**		*0.25151515*		*0.251515152*	*0.226190476*		*0.2034884*	*0.3705882*
**ENSG00000204287**	*0.34117647*	*0.3445122*	*0.34117647*	*0.344512195*	*0.488888889*		*0.3409091*	*0.4411765*
**ENSG00000130348**	*0.11077844*	*0.46363636*	*0.11077844*	*0.463636364*			*0.0755814*	*0.1976744*
**ENSG00000148950**	*0.35555556*		*0.35555556*		*0.222222222*		*0.3409091*	
**ENSG00000251322**		*0.16768293*		*0.167682927*				*0.2267442*
**ENSG00000074966**		*0.2030303*		*0.203030303*				*0.25*
**ENSG00000175728**		*0.05151515*		*0.051515152*				
**ENSG00000253633**		*0.1875*		*0.1875*				*0.4651163*
**ENSG00000184608**	*0.46107784*	*0.43902439*	*0.46107784*	*0.43902439*	*0.363095238*		*0.3139535*	*0.3235294*
**ENSG00000121594**		*0.21818182*		*0.218181818*				*0.2965116*
**ENSG00000204104**		*0.29878049*		*0.298780488*				*0.325*
**ENSG00000090776**		*0.10802469*		*0.108024691*				
**ENSG00000187079**		*0.33939394*		*0.339393939*				*0.4418605*
**ENSG00000019144**	*0.49700599*	*0.49085366*	*0.49700599*	*0.490853659*	*0.297619048*		*0.2705882*	*0.494186*
**ENSG00000075420**	*0.29775281*	*0.38787879*	*0.29775281*	*0.387878788*	*0.266666667*		*0.2613636*	*0.3313953*
**ENSG00000141568**		*0.24545455*		*0.245454545*				*0.3977273*
**ENSG00000184489**	*0.36526946*		*0.36526946*					
**ENSG00000154760**		*0.44444444*		*0.444444444*	*0.136904762*		*0.2151163*	*0.4883721*
**ENSG00000181220**		*0.40555556*		*0.405555556*				*0.1918605*
**ENSG00000133246**	*0.31609195*		*0.31609195*		*0.166666667*		*0.1511628*	
**ENSG00000112494**	*0.47289157*	*0.25*	*0.47289157*	*0.25*	*0.476190476*		*0.4709302*	*0.25*
**ENSG00000052723**		*0.26363636*		*0.263636364*				*0.1104651*
**ENSG00000180900**	*0.47878788*		*0.47878788*		*0.36746988*		*0.3823529*	
**ENSG00000169067**		*0.23888889*		*0.238888889*				
**ENSG00000147403**	*0.41916168*	*0.05882353*	*0.41916168*	*0.058823529*	*0.060240964*		*0.1162791*	*0.1477273*

**ENSG00000086475**	*0.33908046*		*0.33908046*		*0.222222222*	*0.2777778*	*0.1555556*
**ENSG00000104447**	*0.45555556*	*0.44848485*	*0.45555556*	*0.448484848*	*0.130952381*	*0.2764706*	*0.3352941*
**ENSG00000155849**	*0.10479042*	*0.22424242*	*0.10479042*	*0.224242424*	*0.232142857*	*0.1802326*	*0.1686047*
**ENSG00000144848**		*0.28181818*		*0.281818182*			*0.3837209*
**ENSG00000161638**							*0.0872093*
**ENSG00000164176**		*0.37078652*		*0.370786517*			*0.3444444*
**ENSG00000122008**		*0.20909091*		*0.209090909*	*0.482142857*	*0.494186*	*0.3837209*
**ENSG00000189266**		*0.48181818*		*0.481818182*			*0.4593023*
**ENSG00000171812**							*0.4767442*
**ENSG00000010310**	*0.16666667*	*0.25304878*	*0.16666667*	*0.25304878*			*0.494186*
**ENSG00000161920**		*0.20909091*		*0.209090909*			*0.2790698*
**ENSG00000214855**	*0.5*		*0.5*		*0.311111111*	*0.3522727*	
**ENSG00000150938**		*0.33888889*		*0.338888889*			*0.4534884*
**ENSG00000161016**	*0.11904762*		*0.11904762*		*0.06097561*	*0.0595238*	
**ENSG00000109332**		*0.4969697*		*0.496969697*	*0.465909091*	*0.4777778*	*0.4302326*
**ENSG00000175970**		*0.45454545*		*0.454545455*			*0.4360465*
**ENSG00000114933**		*0.29444444*		*0.294444444*			*0.255814*
**ENSG00000197604**		*0.22256098*		*0.222560976*			*0.0647059*
**ENSG00000150667**		*0.14848485*		*0.148484848*			
**ENSG00000168297**		*0.34756098*		*0.347560976*			
**ENSG00000111731**	*0.22155689*		*0.22155689*				
**ENSG00000184210**	*0.29216867*		*0.29216867*		*0.125*	*0.0988372*	
**ENSG00000187554**					*0.083333333*	*0.0755814*	
**ENSG00000101557**		*0.32777778*		*0.327777778*			*0.1931818*
**ENSG00000165458**		*0.05555556*		*0.055555556*			*0.0909091*
**ENSG00000100599**		*0.43333333*		*0.433333333*			*0.3411765*
**ENSG00000168939**		*0.35555556*		*0.355555556*			*0.5*
**ENSG00000152782**		*0.28658537*		*0.286585366*			*0.4216867*
**ENSG00000160188**		*0.10606061*		*0.106060606*	*0.136904762*	*0.1860465*	*0.2906977*
**ENSG00000134115**	*0.46987952*	*0.43333333*	*0.46987952*	*0.433333333*	*0.44047619*	*0.4069767*	*0.4127907*
**ENSG00000171223**	*0.2005988*		*0.2005988*				
**ENSG00000153015**	*0.11976048*		*0.11976048*		*0.482142857*	*0.4651163*	
**ENSG00000158286**	*0.36363636*	*0.37575758*	*0.36363636*	*0.375757576*	*0.107142857*	*0.0872093*	*0.3023256*
**ENSG00000121892**							*0.2906977*
**ENSG00000215218**		*0.10670732*		*0.106707317*			*0.0988372*
**ENSG00000119711**		*0.18484848*		*0.184848485*			*0.2732558*
**ENSG00000149516**		*0.34848485*		*0.348484848*			*0.1918605*
**ENSG00000165698**	*0.31736527*	*0.42424242*	*0.31736527*	*0.424242424*			*0.3488372*
**ENSG00000239697**		*0.19090909*		*0.190909091*			*0.0823529*
**ENSG00000132155**		*0.14848485*		*0.148484848*			
**ENSG00000152056**	*0.33888889*	*0.37974684*	*0.33888889*	*0.379746835*		*0.125*	*0.3977273*
**ENSG00000079313**		*0.29754601*		*0.297546012*			*0.4390244*
**ENSG00000124613**	*0.41017964*	*0.23939394*	*0.41017964*	*0.239393939*	*0.386904762*	*0.4767442*	*0.3888889*
**ENSG00000163875**		*0.23033708*		*0.230337079*			*0.3023256*
**ENSG00000101940**	*0.36826347*		*0.36826347*		*0.125*	*0.0523256*	
**ENSG00000196371**		*0.37654321*		*0.37654321*			*0.2705882*
**ENSG00000148634**		*0.30254777*		*0.302547771*			*0.1860465*
**ENSG00000136866**		*0.4695122*		*0.469512195*			*0.3837209*
**ENSG00000162849**	*0.18888889*		*0.18888889*			*0.1022727*	
**ENSG00000116237**		*0.08484848*		*0.084848485*			*0.0755814*
**ENSG00000167608**	*0.36904762*	*0.45*	*0.36904762*	*0.45*	*0.433333333*	*0.2159091*	*0.2840909*
**ENSG00000170542**		*0.48787879*		*0.487878788*			*0.4470588*
**ENSG00000149809**		*0.38787879*		*0.387878788*			*0.2325581*
**ENSG00000101695**	*0.0508982*	*0.13030303*	*0.0508982*	*0.13030303*			

**ENSG00000007392**	*0.43712575*		*0.43712575*		*0.482142857*		*0.3895349*	
**ENSG00000142327**	*0.30838323*		*0.30838323*					
**ENSG00000145888**	*0.23652695*		*0.23652695*		*0.363095238*		*0.4117647*	
**ENSG00000130783**		*0.10606061*		*0.106060606*	*0.375*		*0.3023256*	*0.2267442*
**ENSG00000163964**								*0.0568182*
**ENSG00000095209**		*0.16292135*		*0.162921348*				*0.4*
**ENSG00000141959**	*0.23636364*	*0.43167702*	*0.23636364*	*0.431677019*	*0.375*		*0.4127907*	*0.4127907*
**ENSG00000147027**		*0.17272727*		*0.172727273*				*0.4470588*
**ENSG00000074755**		*0.40909091*		*0.409090909*				*0.377907*
**ENSG00000217340**		*0.48787879*		*0.487878788*				*0.4418605*
**ENSG00000105852**		*0.47878788*		*0.478787879*				*0.1569767*
**ENSG00000131730**		*0.31212121*		*0.312121212*				*0.0523256*
**ENSG00000160360**		*0.4689441*		*0.468944099*				*0.2882353*
**ENSG00000165192**		*0.40853659*		*0.408536585*				*0.4476744*
**ENSG00000134812**	*0.17365269*	*0.07272727*	*0.17365269*	*0.072727273*				*0.0697674*
**ENSG00000133055**		*0.4137931*		*0.413793103*				*0.2613636*
**ENSG00000104524**	*0.21257485*	*0.26363636*	*0.21257485*	*0.263636364*	*0.369047619*		*0.4011628*	*0.4011628*
**ENSG00000115226**		*0.43125*		*0.43125*				*0.0952381*
**ENSG00000116353**		*0.10909091*		*0.109090909*				*0.4883721*
**ENSG00000168496**		*0.37575758*		*0.375757576*				*0.3023256*
**ENSG00000169371**		*0.23780488*		*0.237804878*				
**ENSG00000094975**		*0.1030303*		*0.103030303*				
**ENSG00000134551**		*0.26111111*		*0.261111111*				*0.1976744*
**ENSG00000214112**	*0.09580838*		*0.09580838*					
**ENSG00000159210**	*0.06586826*	*0.32621951*	*0.06586826*	*0.326219512*				*0.244186*
**ENSG00000147127**	*0.49698795*		*0.49698795*				*0.0697674*	*0.0813953*
**ENSG00000174197**		*0.1091954*		*0.109195402*				*0.0681818*
**ENSG00000163357**		*0.31212121*		*0.312121212*				*0.0639535*
**ENSG00000169752**	*0.06024096*		*0.06024096*					
**ENSG00000158711**		*0.13636364*		*0.136363636*				*0.4127907*
**ENSG00000180953**	*0.05555556*		*0.05555556*		*0.261363636*		*0.2333333*	
**ENSG00000138785**	*0.32035928*		*0.32035928*				*0.0523256*	
**ENSG00000106077**		*0.44545455*		*0.445454545*				*0.4883721*
**ENSG00000166569**		*0.4*		*0.4*				*0.4709302*
**ENSG00000144048**	*0.48203593*		*0.48203593*		*0.25*		*0.3430233*	
**ENSG00000140937**	*0.26347305*	*0.26111111*	*0.26347305*	*0.261111111*				*0.3636364*
**ENSG00000176472**	*0.21856287*		*0.21856287*		*0.101190476*		*0.1453488*	
**ENSG00000107249**	*0.45555556*	*0.48484848*	*0.45555556*	*0.484848485*	*0.322222222*		*0.3888889*	*0.4476744*
**ENSG00000175294**	*0.47005988*		*0.47005988*		*0.464285714*		*0.3488372*	
**ENSG00000006831**		*0.4969697*		*0.496969697*				*0.4302326*
**ENSG00000235531**	*0.06666667*	*0.15757576*	*0.06666667*	*0.157575758*				*0.1*
**ENSG00000078295**	*0.07784431*	*0.43030303*	*0.07784431*	*0.43030303*				*0.4883721*
**ENSG00000160183**	*0.38922156*	*0.23333333*	*0.38922156*	*0.233333333*	*0.422619048*		*0.4709302*	*0.4302326*
**ENSG00000101347**		*0.46969697*		*0.46969697*				*0.3529412*
**ENSG00000136240**		*0.18484848*		*0.184848485*				*0.2916667*
**ENSG00000134545**		*0.16981132*		*0.169811321*				*0.3823529*
**ENSG00000244687**		*0.33146067*		*0.331460674*				*0.2613636*
**ENSG00000116209**	*0.32634731*		*0.32634731*		*0.197368421*		*0.3571429*	
**ENSG00000152583**	*0.49101796*		*0.49101796*		*0.476190476*		*0.3953488*	
**ENSG00000162222**	*0.18235294*		*0.18235294*					
**ENSG00000211689**		*0.24848485*		*0.248484848*				*0.3197674*
**ENSG00000153902**		*0.35151515*		*0.351515152*				*0.1337209*
**ENSG00000122912**		*0.06111111*		*0.061111111*				*0.1444444*
**ENSG00000151247**	*0.06586826*	*0.37962963*	*0.06586826*	*0.37962963*	*0.30952381*		*0.2470588*	*0.2222222*

**ENSG00000106603**	*0.0813253*	*0.44242424*	*0.0813253*	*0.442424242*	*0.321428571*	*0.3470588*	*0.3488372*
**ENSG00000133422**	*0.47272727*		*0.47272727*		*0.369047619*	*0.4*	
**ENSG00000119048**	*0.32335329*		*0.32335329*		*0.06547619*	*0.0581395*	*0.0581395*
**ENSG00000167995**	*0.23353293*	*0.32424242*	*0.23353293*	*0.324242424*	*0.291666667*	*0.1569767*	*0.2674419*
**ENSG00000203778**		*0.47239264*		*0.472392638*			*0.4058824*
**ENSG00000021645**	*0.26666667*	*0.24545455*	*0.26666667*	*0.245454545*	*0.488888889*	*0.3222222*	*0.2444444*
**ENSG00000049656**		*0.16969697*		*0.16969697*			
**ENSG00000181915**		*0.47777778*		*0.477777778*			*0.4*
**ENSG00000109685**		*0.09090909*		*0.090909091*			
**ENSG00000165238**	*0.24719101*	*0.06666667*	*0.24719101*	*0.066666667*	*0.428571429*	*0.4476744*	
**ENSG00000105523**		*0.41515152*		*0.415151515*			
**ENSG00000181026**	*0.14156627*	*0.10909091*	*0.14156627*	*0.109090909*	*0.255952381*	*0.25*	*0.2777778*
**ENSG00000187140**		*0.2195122*		*0.219512195*			*0.1976744*
**ENSG00000139146**							*0.2674419*
**ENSG00000152595**		*0.17791411*		*0.17791411*			*0.255814*
**ENSG00000135387**	*0.25842697*	*0.09444444*	*0.25842697*	*0.094444444*			
**ENSG00000137101**		*0.43678161*		*0.436781609*			*0.4767442*
**ENSG00000137942**							*0.060241*
**ENSG00000160201**	*0.42777778*		*0.42777778*		*0.101190476*	*0.1046512*	
**ENSG00000133121**	*0.1497006*	*0.40490798*	*0.1497006*	*0.404907975*	*0.101190476*	*0.0930233*	*0.4534884*
**ENSG00000170419**	*0.42814371*	*0.42121212*	*0.42814371*	*0.421212121*	*0.333333333*	*0.3139535*	*0.4117647*
**ENSG00000164949**	*0.31137725*	*0.36666667*	*0.31137725*	*0.366666667*	*0.172619048*	*0.2674419*	*0.2906977*
**ENSG00000215041**		*0.41212121*		*0.412121212*			*0.0697674*
**ENSG00000182022**		*0.45757576*		*0.457575758*			*0.4767442*
**ENSG00000112031**		*0.24157303*		*0.241573034*			*0.2555556*
**ENSG00000168003**	*0.05389222*	*0.24545455*	*0.05389222*	*0.245454545*	*0.180722892*	*0.1976744*	*0.1976744*
**ENSG00000119227**		*0.42682927*		*0.426829268*			*0.2965116*
**ENSG00000107187**							*0.0909091*
**ENSG00000079335**	*0.24137931*	*0.12424242*	*0.24137931*	*0.124242424*	*0.477272727*	*0.4772727*	*0.126506*
**ENSG00000168036**	*0.29375*	*0.48181818*	*0.29375*	*0.481818182*	*0.24691358*	*0.2256098*	*0.2790698*
**ENSG00000141698**		*0.24444444*		*0.244444444*			*0.1333333*
**ENSG00000119771**		*0.47256098*		*0.472560976*			*0.1704545*
**ENSG00000142494**		*0.15517241*		*0.155172414*	*0.06547619*	*0.0813953*	*0.4883721*
**ENSG00000144488**		*0.37878788*		*0.378787879*			*0.3764706*
**ENSG00000066827**		*0.3969697*		*0.396969697*			*0.4651163*
**ENSG00000124203**		*0.31987578*		*0.319875776*			*0.4204545*
**ENSG00000123739**		*0.43030303*		*0.43030303*			*0.2176471*
**ENSG00000147378**	*0.12874251*	*0.06402439*	*0.12874251*	*0.06402439*			*0.3372093*
**ENSG00000141497**		*0.05792683*		*0.057926829*			*0.2151163*
**ENSG00000112238**		*0.20606061*		*0.206060606*			
**ENSG00000204049**		*0.37575758*		*0.375757576*			*0.2790698*
**ENSG00000164823**	*0.22891566*		*0.22891566*		*0.482142857*	*0.4186047*	*0.2093023*
**ENSG00000113209**		*0.06060606*		*0.060606061*			*0.1162791*
**ENSG00000239789**		*0.25153374*		*0.251533742*	*0.101190476*		
**ENSG00000183230**		*0.21818182*		*0.218181818*			
**ENSG00000162783**	*0.23952096*		*0.23952096*		*0.113095238*	*0.0988372*	
**ENSG00000198502**		*0.32704403*		*0.327044025*			*0.3253012*
**ENSG00000114771**	*0.37125749*	*0.45757576*	*0.37125749*	*0.457575758*	*0.404761905*	*0.3081395*	*0.4069767*
**ENSG00000103187**		*0.29090909*		*0.290909091*			*0.4244186*
**ENSG00000101132**		*0.44848485*		*0.448484848*			*0.1918605*
**ENSG00000198026**		*0.11656442*		*0.116564417*			*0.2674419*
**ENSG00000078061**		*0.378125*		*0.378125*			*0.4588235*
**ENSG00000158315**	*0.46629213*	*0.44848485*	*0.46629213*	*0.448484848*	*0.290697674*	*0.25*	*0.244186*
**ENSG00000160712**	*0.38068182*	*0.23863636*	*0.38068182*	*0.238636364*	*0.422222222*	*0.4767442*	*0.1888889*

**ENSG00000163820**	*0.07784431*	*0.36666667*	*0.07784431*	*0.366666667*		*0.0988372*	*0.4883721*
**ENSG00000241106**	*0.2560241*	*0.22727273*	*0.2560241*	*0.227272727*	*0.43452381*	*0.4825581*	*0.3313953*
**ENSG00000146386**	*0.39655172*	*0.4695122*	*0.39655172*	*0.469512195*	*0.244444444*	*0.2272727*	*0.0941176*
**ENSG00000169231**	*0.24850299*		*0.24850299*				
**ENSG00000075790**		*0.27777778*		*0.277777778*			*0.3197674*
**ENSG00000152818**	*0.17222222*	*0.21472393*	*0.17222222*	*0.214723926*	*0.266666667*	*0.2555556*	*0.4666667*
**ENSG00000015475**	*0.06666667*	*0.17878788*	*0.06666667*	*0.178787879*	*0.122222222*	*0.125*	*0.122093*
**ENSG00000124343**	*0.28963415*	*0.16975309*	*0.28963415*	*0.169753086*			*0.2797619*
**ENSG00000117411**	*0.21856287*	*0.06969697*	*0.21856287*	*0.06969697*	*0.178571429*	*0.1627907*	*0.2906977*
**ENSG00000168334**	*0.44311377*	*0.23333333*	*0.44311377*	*0.233333333*	*0.220238095*	*0.2151163*	*0.1802326*
**ENSG00000119638**	*0.39444444*	*0.48484848*	*0.39444444*	*0.484848485*	*0.172619048*	*0.2571429*	*0.2117647*
**ENSG00000128908**		*0.35632184*		*0.356321839*			*0.25*
**ENSG00000204851**							*0.1686047*
**ENSG00000198732**		*0.22424242*		*0.224242424*			*0.4709302*
**ENSG00000166188**		*0.4*		*0.4*			*0.4418605*
**ENSG00000182013**	*0.27844311*	*0.32121212*	*0.27844311*	*0.321212121*	*0.19047619*	*0.1104651*	*0.127907*
**ENSG00000027847**		*0.4847561*		*0.484756098*			*0.1686047*
**ENSG00000128610**	*0.38622754*		*0.38622754*		*0.416666667*	*0.4294118*	
**ENSG00000205439**		*0.33146067*		*0.331460674*			*0.4659091*
**ENSG00000147421**		*0.31666667*		*0.316666667*			*0.4767442*
**ENSG00000159212**		*0.06134969*		*0.061349693*			
**ENSG00000138813**		*0.23888889*		*0.238888889*			*0.2159091*
**ENSG00000129038**		*0.47272727*		*0.472727273*			*0.3197674*
**ENSG00000204524**	*0.24444444*	*0.42424242*	*0.24444444*	*0.424242424*	*0.211111111*	*0.2272727*	*0.4244186*
**ENSG00000089248**		*0.11585366*		*0.115853659*			*0.4090909*
**ENSG00000205279**	*0.08383234*	*0.43333333*	*0.08383234*	*0.433333333*	*0.30952381*	*0.2906977*	*0.3023256*
**ENSG00000254870**		*0.39411765*		*0.394117647*			*0.0988372*
**ENSG00000186010**	*0.16455696*		*0.16455696*				
**ENSG00000170523**		*0.37931034*		*0.379310345*			*0.1860465*
**ENSG00000153914**		*0.08045977*		*0.08045977*			*0.1453488*
**ENSG00000169504**		*0.26993865*		*0.26993865*			*0.4767442*
**ENSG00000077454**	*0.05389222*		*0.05389222*		*0.261904762*	*0.2965116*	
**ENSG00000178919**	*0.29216867*	*0.43636364*	*0.29216867*	*0.436363636*	*0.144578313*	*0.122093*	*0.1235294*
**ENSG00000139990**	*0.2245509*	*0.33030303*	*0.2245509*	*0.33030303*			*0.0639535*
**ENSG00000117139**	*0.41017964*	*0.24545455*	*0.41017964*	*0.245454545*	*0.488095238*	*0.3705882*	*0.1976744*
**ENSG00000076108**	*0.2994012*		*0.2994012*		*0.214285714*	*0.3255814*	
**ENSG00000132478**							*0.3430233*
**ENSG00000177409**		*0.23030303*		*0.23030303*			*0.377907*
**ENSG00000204304**		*0.2183908*		*0.218390805*			*0.3372093*
**ENSG00000185019**		*0.38719512*		*0.387195122*			*0.122093*
**ENSG00000109118**		*0.20114943*		*0.201149425*			*0.0909091*
**ENSG00000166949**	*0.43975904*	*0.48780488*	*0.43975904*	*0.487804878*	*0.464285714*	*0.4302326*	*0.4529412*
**ENSG00000147586**	*0.16666667*		*0.16666667*		*0.144444444*	*0.0909091*	
**ENSG00000197070**		*0.1*		*0.1*			*0.1104651*
**ENSG00000127526**		*0.07878788*		*0.078787879*			*0.1104651*
**ENSG00000128242**					*0.36746988*	*0.2882353*	
**ENSG00000138767**		*0.14417178*		*0.144171779*			*0.1931818*
**ENSG00000186960**		*0.34090909*		*0.340909091*			
**ENSG00000184292**		*0.18787879*		*0.187878788*			*0.1453488*
**ENSG00000100242**		*0.34269663*		*0.342696629*			*0.4767442*
**ENSG00000180481**	*0.21556886*		*0.21556886*		*0.494047619*	*0.4883721*	
**ENSG00000198704**		*0.08181818*		*0.081818182*			*0.4825581*
**ENSG00000100246**		*0.19090909*		*0.190909091*			*0.0813953*
**ENSG00000182568**	*0.21257485*	*0.37804878*	*0.21257485*	*0.37804878*	*0.343373494*	*0.3197674*	*0.3837209*

**ENSG00000180773**		*0.3006135*		*0.300613497*			*0.3231707*
**ENSG00000221867**		*0.1882716*		*0.188271605*			
**ENSG00000104499**		*0.44817073*		*0.448170732*			*0.1569767*
**ENSG00000204516**	*0.19753086*		*0.19753086*		*0.209302326*	*0.1704545*	
**ENSG00000204116**		*0.17575758*		*0.175757576*			
**ENSG00000132842**		*0.18787879*		*0.187878788*			*0.1627907*
**ENSG00000165233**		*0.22424242*		*0.224242424*			*0.3095238*
**ENSG00000178105**	*0.09281437*	*0.37931034*	*0.09281437*	*0.379310345*	*0.416666667*	*0.4767442*	*0.4*
**ENSG00000196922**	*0.38622754*	*0.14848485*	*0.38622754*	*0.148484848*	*0.351190476*	*0.3352941*	*0.3488372*
**ENSG00000171792**		*0.29411765*		*0.294117647*			*0.3181818*
**ENSG00000103342**	*0.28614458*	*0.45121951*	*0.28614458*	*0.451219512*		*0.1104651*	*0.2325581*
**ENSG00000037757**		*0.40909091*		*0.409090909*			*0.4069767*
**ENSG00000114395**	*0.28443114*		*0.28443114*		*0.375*	*0.4825581*	
**ENSG00000203756**		*0.15757576*		*0.157575758*			*0.2906977*
**ENSG00000170779**		*0.19512195*		*0.195121951*			*0.372093*
**ENSG00000215193**		*0.41111111*		*0.411111111*			*0.4772727*
**ENSG00000107719**		*0.21264368*		*0.212643678*			*0.3214286*
**ENSG00000165118**	*0.20658683*	*0.23939394*	*0.20658683*	*0.239393939*	*0.422619048*	*0.4186047*	*0.2616279*
**ENSG00000101605**	*0.30538922*	*0.43636364*	*0.30538922*	*0.436363636*	*0.416666667*	*0.3546512*	*0.4107143*
**ENSG00000105650**		*0.30909091*		*0.309090909*			*0.2848837*
**ENSG00000257594**		*0.27878788*		*0.278787879*			*0.1686047*
**ENSG00000204866**	*0.08888889*	*0.36419753*	*0.08888889*	*0.364197531*	*0.122222222*		*0.4302326*
**ENSG00000169570**		*0.28888889*		*0.288888889*			*0.1444444*
**ENSG00000086300**	*0.23888889*		*0.23888889*		*0.443181818*	*0.3888889*	
**ENSG00000196260**		*0.26363636*		*0.263636364*			*0.4418605*
**ENSG00000117640**		*0.22727273*		*0.227272727*	*0.369047619*	*0.3294118*	
**ENSG00000092345**	*0.21556886*	*0.26966292*	*0.21556886*	*0.269662921*	*0.220238095*	*0.2790698*	*0.4176471*
**ENSG00000198185**	*0.35928144*		*0.35928144*		*0.369047619*	*0.2848837*	
**ENSG00000162267**		*0.11486486*		*0.114864865*			*0.0921053*
**ENSG00000049283**	*0.12345679*	*0.20606061*	*0.12345679*	*0.206060606*	*0.122222222*	*0.1158537*	
**ENSG00000148308**		*0.33536585*		*0.335365854*			*0.1802326*
**ENSG00000161850**		*0.46666667*		*0.466666667*			*0.3953488*
**ENSG00000132294**	*0.5*	*0.14848485*	*0.5*	*0.148484848*	*0.289156627*	*0.2797619*	
**ENSG00000166670**		*0.31481481*		*0.314814815*			*0.1686747*
**ENSG00000168566**		*0.35454545*		*0.354545455*			*0.4545455*
**ENSG00000237240**		*0.37931034*		*0.379310345*			*0.4593023*
**ENSG00000168386**	*0.12222222*		*0.12222222*				
**ENSG00000161572**							*0.1777778*
**ENSG00000100284**	*0.21111111*	*0.22727273*	*0.21111111*	*0.227272727*	*0.464285714*	*0.3444444*	*0.2267442*
**ENSG00000154175**	*0.23652695*	*0.4537037*	*0.23652695*	*0.453703704*	*0.214285714*	*0.3372093*	*0.4651163*
**ENSG00000175309**		*0.11818182*		*0.118181818*			*0.0872093*
**ENSG00000017260**	*0.15060241*		*0.15060241*		*0.174698795*	*0.1964286*	
**ENSG00000244411**		*0.18484848*		*0.184848485*			*0.3837209*
**ENSG00000198791**	*0.18333333*	*0.45454545*	*0.18333333*	*0.454545455*			*0.25*
**ENSG00000156966**		*0.22727273*		*0.227272727*			*0.2352941*
**ENSG00000135930**		*0.46666667*		*0.466666667*	*0.077380952*	*0.1569767*	*0.3068182*
**ENSG00000256269**	*0.07185629*		*0.07185629*		*0.253012048*	*0.25*	
**ENSG00000124731**		*0.47575758*		*0.475757576*			*0.3313953*
**ENSG00000146232**		*0.1744186*		*0.174418605*			*0.4772727*
**ENSG00000214435**		*0.26666667*		*0.266666667*			*0.3604651*
**ENSG00000124374**		*0.33939394*		*0.339393939*			*0.4111111*
**ENSG00000173473**		*0.40853659*		*0.408536585*			*0.2383721*
**ENSG00000203795**	*0.43888889*		*0.43888889*		*0.477272727*	*0.3863636*	
**ENSG00000138678**		*0.38414634*		*0.384146341*			*0.5*

**ENSG00000140307**	*0.24444444*	*0.3190184*	*0.24444444*	*0.319018405*	*0.369047619*	*0.4186047*	*0.4235294*
**ENSG00000102683**	*0.11976048*	*0.44545455*	*0.11976048*	*0.445454545*	*0.160714286*	*0.1395349*	*0.4651163*
**ENSG00000173557**		*0.33888889*		*0.338888889*			*0.4545455*
**ENSG00000112769**		*0.23780488*		*0.237804878*			*0.2616279*
**ENSG00000196792**	*0.25149701*	*0.48148148*	*0.25149701*	*0.481481481*			*0.3882353*
**ENSG00000164485**	*0.26506024*	*0.25151515*	*0.26506024*	*0.251515152*	*0.369047619*	*0.4534884*	*0.1860465*
**ENSG00000198513**					*0.202380952*	*0.2267442*	*0.2*
**ENSG00000138688**	*0.05688623*		*0.05688623*		*0.107142857*	*0.1411765*	
**ENSG00000168288**	*0.2*	*0.44444444*	*0.2*	*0.444444444*	*0.188888889*	*0.1444444*	*0.125*
**ENSG00000089006**	*0.14071856*		*0.14071856*		*0.43452381*	*0.3941176*	
**ENSG00000176774**	*0.13554217*	*0.24848485*	*0.13554217*	*0.248484848*	*0.174698795*	*0.1337209*	*0.3392857*
**ENSG00000157933**		*0.13125*		*0.13125*			*0.1511628*
**ENSG00000175787**	*0.32934132*		*0.32934132*		*0.416666667*	*0.4244186*	
**ENSG00000164508**	*0.08682635*		*0.08682635*		*0.285714286*	*0.2823529*	*0.1*
**ENSG00000141161**		*0.33908046*		*0.33908046*			*0.4090909*
**ENSG00000091137**	*0.34431138*	*0.16111111*	*0.34431138*	*0.161111111*	*0.343373494*	*0.3352941*	*0.3068182*
**ENSG00000119397**	*0.42424242*		*0.42424242*		*0.433333333*	*0.4090909*	
**ENSG00000115561**		*0.46363636*		*0.463636364*			*0.3023256*
**ENSG00000154767**	*0.39655172*	*0.4*	*0.39655172*	*0.4*	*0.088888889*		*0.4127907*
**ENSG00000105290**							*0.0639535*
**ENSG00000124092**		*0.4*		*0.4*			*0.4767442*
**ENSG00000008130**	*0.12275449*		*0.12275449*				
**ENSG00000105186**		*0.25454545*		*0.254545455*			*0.494186*
**ENSG00000153993**	*0.15868263*	*0.4030303*	*0.15868263*	*0.403030303*	*0.083333333*	*0.0755814*	*0.246988*
**ENSG00000198612**	*0.45555556*	*0.06962025*	*0.45555556*	*0.069620253*			
**ENSG00000100227**		*0.29090909*		*0.290909091*			*0.4069767*
**ENSG00000110047**	*0.0505618*	*0.45151515*	*0.0505618*	*0.451515152*	*0.44047619*	*0.4512195*	*0.4545455*
**ENSG00000154237**	*0.36419753*	*0.39393939*	*0.36419753*	*0.393939394*	*0.226190476*	*0.2906977*	*0.3313953*
**ENSG00000140451**	*0.23053892*	*0.07272727*	*0.23053892*	*0.072727273*	*0.220238095*	*0.1104651*	*0.1744186*
**ENSG00000167880**		*0.34242424*		*0.342424242*			*0.4825581*
**ENSG00000173626**	*0.07222222*	*0.35757576*	*0.07222222*	*0.357575758*			*0.3604651*
**ENSG00000243811**		*0.48181818*		*0.481818182*			*0.2616279*
**ENSG00000115170**	*0.36826347*	*0.27878788*	*0.36826347*	*0.278787879*			*0.2529412*
**ENSG00000175467**		*0.5*		*0.5*			*0.3197674*
**ENSG00000166167**		*0.49444444*		*0.494444444*			*0.1590909*
**ENSG00000137331**	*0.11077844*	*0.14545455*	*0.11077844*	*0.145454545*	*0.053571429*	*0.1569767*	*0.0581395*
**ENSG00000137492**					*0.333333333*	*0.2906977*	
**ENSG00000102910**		*0.16060606*		*0.160606061*			
**ENSG00000170967**		*0.48850575*		*0.488505747*			*0.4659091*
**ENSG00000162377**		*0.36666667*		*0.366666667*			*0.1569767*
**ENSG00000006015**		*0.4054878*		*0.405487805*			*0.3837209*
**ENSG00000124357**	*0.08895706*	*0.29393939*	*0.08895706*	*0.293939394*	*0.111111111*	*0.122093*	*0.2444444*
**ENSG00000146263**		*0.48787879*		*0.487878788*			*0.2682927*
**ENSG00000198944**		*0.17878788*		*0.178787879*			*0.1337209*
**ENSG00000062524**		*0.2808642*		*0.280864198*			*0.1704545*
**ENSG00000198853**	*0.49401198*		*0.49401198*		*0.303571429*	*0.4302326*	
**ENSG00000169550**		*0.25454545*		*0.254545455*			*0.2151163*
**ENSG00000177426**	*0.34131737*	*0.41818182*	*0.34131737*	*0.418181818*	*0.177777778*	*0.1627907*	*0.1569767*
**ENSG00000006695**	*0.48502994*	*0.37575758*	*0.48502994*	*0.375757576*	*0.380952381*	*0.4883721*	*0.2616279*
**ENSG00000110931**	*0.27844311*	*0.26666667*	*0.27844311*	*0.266666667*	*0.172619048*	*0.1511628*	*0.4709302*
**ENSG00000177054**	*0.25449102*		*0.25449102*		*0.261904762*	*0.2823529*	
**ENSG00000162877**		*0.32183908*		*0.32183908*			*0.4659091*
**ENSG00000171385**		*0.4030303*		*0.403030303*			*0.3953488*
**ENSG00000167751**		*0.26666667*		*0.266666667*			*0.1918605*

**ENSG00000111816**	*0.3*	*0.38181818*	*0.3*	*0.381818182*				*0.2058824*
**ENSG00000134644**		*0.12777778*		*0.127777778*				
**ENSG00000176994**	*0.10778443*		*0.10778443*		*0.494047619*		*0.4705882*	
**ENSG00000101266**		*0.38484848*		*0.384848485*				*0.3604651*
**ENSG00000115602**	*0.41515152*	*0.42727273*	*0.41515152*	*0.427272727*	*0.155555556*		*0.1768293*	*0.5*
**ENSG00000115091**		*0.37777778*		*0.377777778*				*0.4545455*
**ENSG00000188859**		*0.34242424*		*0.342424242*				*0.2034884*
**ENSG00000187486**		*0.37272727*		*0.372727273*				*0.4476744*
**ENSG00000132589**		*0.28787879*		*0.287878788*				*0.2965116*
**ENSG00000140623**	*0.14371257*		*0.14371257*		*0.30952381*		*0.3529412*	
**ENSG00000111665**	*0.07386364*		*0.07386364*		*0.344444444*		*0.3977273*	
**ENSG00000171451**		*0.37777778*		*0.377777778*	*0.19047619*		*0.1860465*	*0.5*
**ENSG00000242220**		*0.13888889*		*0.138888889*				
**ENSG00000103148**		*0.14634146*		*0.146341463*				*0.2965116*
**ENSG00000169242**		*0.38787879*		*0.387878788*				*0.0988372*
**ENSG00000203783**		*0.19393939*		*0.193939394*				*0.0872093*
**ENSG00000151320**	*0.38622754*	*0.45962733*	*0.38622754*	*0.459627329*	*0.166666667*		*0.0813953*	*0.3197674*
**ENSG00000204899**	*0.08383234*	*0.22222222*	*0.08383234*	*0.222222222*	*0.458333333*		*0.4476744*	
**ENSG00000040487**	*0.15454545*	*0.33529412*	*0.15454545*	*0.335294118*	*0.396341463*		*0.4825581*	
**ENSG00000077713**		*0.4847561*		*0.484756098*				*0.494186*
**ENSG00000142168**	*0.16766467*		*0.16766467*					*0.0755814*
**ENSG00000114383**		*0.08484848*		*0.084848485*				
**ENSG00000024862**	*0.22754491*	*0.28484848*	*0.22754491*	*0.284848485*			*0.1235294*	*0.2674419*
**ENSG00000164690**	*0.08083832*	*0.49393939*	*0.08083832*	*0.493939394*				*0.25*
**ENSG00000115540**		*0.17222222*		*0.172222222*				*0.2272727*
**ENSG00000054796**		*0.22777778*		*0.227777778*				*0.0555556*
**ENSG00000164405**	*0.23888889*		*0.23888889*		*0.476190476*		*0.5*	
**ENSG00000204876**	*0.4251497*		*0.4251497*		*0.357142857*		*0.4360465*	
**ENSG00000151967**					*0.101190476*		*0.0697674*	
**ENSG00000185838**		*0.38484848*		*0.384848485*				*0.3882353*
**ENSG00000163873**		*0.11212121*		*0.112121212*				*0.0588235*
**ENSG00000198121**		*0.4030303*		*0.403030303*				*0.4593023*
**ENSG00000146411**		*0.41818182*		*0.418181818*				*0.2857143*
**ENSG00000107758**		*0.08536585*		*0.085365854*				*0.4941176*
**ENSG00000153347**	*0.1497006*	*0.35185185*	*0.1497006*	*0.351851852*				*0.1860465*
**ENSG00000162757**		*0.42121212*		*0.421212121*				*0.2209302*
**ENSG00000120690**	*0.11676647*	*0.28787879*	*0.11676647*	*0.287878788*	*0.196428571*		*0.2151163*	*0.255814*
**ENSG00000064199**	*0.42814371*	*0.36280488*	*0.42814371*	*0.362804878*	*0.130952381*		*0.1453488*	*0.4186047*
**ENSG00000037474**		*0.21515152*		*0.215151515*				*0.5*
**ENSG00000112584**		*0.2030303*		*0.203030303*				*0.1802326*
**ENSG00000188917**		*0.43820225*		*0.438202247*				
**ENSG00000166337**	*0.48802395*		*0.48802395*		*0.333333333*		*0.3662791*	
**ENSG00000139890**		*0.12195122*		*0.12195122*				*0.1627907*
**ENSG00000121940**		*0.36060606*		*0.360606061*				*0.494186*
**ENSG00000180815**	*0.31736527*		*0.31736527*					
**ENSG00000100926**	*0.0617284*		*0.0617284*					
**ENSG00000164626**		*0.44817073*		*0.448170732*				*0.3488372*
**ENSG00000187609**	*0.0748503*	*0.49390244*	*0.0748503*	*0.493902439*				*0.3662791*
**ENSG00000198286**								*0.126506*
**ENSG00000003402**		*0.21910112*		*0.219101124*				*0.0581395*
**ENSG00000125975**	*0.1497006*	*0.06666667*	*0.1497006*	*0.066666667*			*0.0581395*	*0.0581395*
**ENSG00000171634**	*0.48333333*	*0.20606061*	*0.48333333*	*0.206060606*				
**ENSG00000103175**	*0.26347305*	*0.32183908*	*0.26347305*	*0.32183908*	*0.386904762*		*0.4825581*	*0.2857143*
**ENSG00000182346**	*0.05792683*		*0.05792683*		*0.410714286*		*0.3430233*	

**ENSG00000163590**		*0.48787879*		*0.487878788*			*0.4651163*
**ENSG00000010270**	*0.1*	*0.125*	*0.1*	*0.125*	*0.155555556*	*0.0777778*	*0.4880952*
**ENSG00000065923**		*0.16666667*		*0.166666667*			*0.3488372*
**ENSG00000139209**	*0.49101796*	*0.20731707*	*0.49101796*	*0.207317073*	*0.363095238*	*0.4534884*	*0.4886364*
**ENSG00000253110**	*0.40588235*		*0.40588235*		*0.487804878*	*0.4666667*	
**ENSG00000104936**	*0.07386364*		*0.07386364*		*0.155555556*	*0.1976744*	
**ENSG00000158560**		*0.12121212*		*0.121212121*			
**ENSG00000138279**	*0.14444444*	*0.06969697*	*0.14444444*	*0.06969697*			
**ENSG00000163687**	*0.2005988*		*0.2005988*		*0.373493976*	*0.4127907*	
**ENSG00000151418**		*0.06097561*		*0.06097561*			
**ENSG00000153446**		*0.31818182*		*0.318181818*			*0.2732558*
**ENSG00000111602**	*0.13772455*		*0.13772455*				
**ENSG00000174013**		*0.26969697*		*0.26969697*			*0.3928571*
**ENSG00000172318**	*0.19161677*		*0.19161677*		*0.470238095*	*0.3928571*	
**ENSG00000198646**	*0.29775281*	*0.13939394*	*0.29775281*	*0.139393939*	*0.455555556*	*0.3333333*	*0.1337209*
**ENSG00000100376**	*0.09580838*	*0.46363636*	*0.09580838*	*0.463636364*	*0.411111111*	*0.4431818*	*0.494186*
**ENSG00000204576**		*0.32727273*		*0.327272727*			*0.255814*
**ENSG00000184047**	*0.23353293*	*0.43597561*	*0.23353293*	*0.43597561*	*0.273809524*	*0.3313953*	*0.2616279*
**ENSG00000182158**		*0.34545455*		*0.345454545*			*0.4709302*
**ENSG00000222047**							*0.0523256*
**ENSG00000168675**	*0.38202247*	*0.22121212*	*0.38202247*	*0.221212121*	*0.232142857*	*0.1802326*	*0.4186047*
**ENSG00000065357**	*0.07784431*	*0.22727273*	*0.07784431*	*0.227272727*	*0.11627907*	*0.1046512*	*0.1309524*
**ENSG00000171603**		*0.05454545*		*0.054545455*			*0.2848837*
**ENSG00000165526**		*0.24545455*		*0.245454545*			*0.1569767*
**ENSG00000163635**		*0.36060606*		*0.360606061*			*0.4764706*
**ENSG00000105865**		*0.47272727*		*0.472727273*			*0.4011628*
**ENSG00000153561**		*0.30246914*		*0.302469136*			*0.0697674*
**ENSG00000079246**	*0.19879518*	*0.10606061*	*0.19879518*	*0.106060606*	*0.297619048*	*0.3411765*	*0.1294118*
**ENSG00000065526**	*0.05389222*	*0.0969697*	*0.05389222*	*0.096969697*	*0.053571429*		*0.2674419*
**ENSG00000187642**							*0.4058824*
**ENSG00000051128**	*0.09580838*		*0.09580838*		*0.43902439*	*0.3294118*	
**ENSG00000173848**	*0.15189873*	*0.35757576*	*0.15189873*	*0.357575758*	*0.179487179*	*0.0921053*	*0.3139535*
**ENSG00000156521**		*0.37195122*		*0.37195122*			
**ENSG00000089022**	*0.31024096*		*0.31024096*		*0.470238095*	*0.4883721*	
**ENSG00000197410**					*0.144444444*	*0.0909091*	
**ENSG00000005483**	*0.39820359*	*0.13636364*	*0.39820359*	*0.136363636*	*0.369047619*	*0.244186*	*0.244186*
**ENSG00000112182**	*0.08383234*	*0.45757576*	*0.08383234*	*0.457575758*	*0.321428571*	*0.4302326*	*0.4069767*
**ENSG00000204961**	*0.25*	*0.21835443*	*0.25*	*0.21835443*	*0.482142857*	*0.4941176*	*0.1162791*
**ENSG00000137251**	*0.10778443*	*0.325*	*0.10778443*	*0.325*			*0.1569767*
**ENSG00000179674**					*0.327380952*	*0.2732558*	
**ENSG00000128285**	*0.45508982*	*0.47560976*	*0.45508982*	*0.475609756*			*0.1686047*
**ENSG00000162601**		*0.43333333*		*0.433333333*			*0.4069767*
**ENSG00000241635**		*0.22777778*		*0.227777778*			*0.1590909*
**ENSG00000169194**		*0.22256098*		*0.222560976*			*0.2848837*
**ENSG00000052802**	*0.29041916*		*0.29041916*		*0.172619048*	*0.1744186*	
**ENSG00000129691**					*0.265060241*	*0.2906977*	*0.2727273*
**ENSG00000243244**		*0.41818182*		*0.418181818*			*0.494186*
**ENSG00000050165**		*0.06969697*		*0.06969697*			
**ENSG00000087884**		*0.25151515*		*0.251515152*			*0.1104651*
**ENSG00000146963**		*0.20555556*		*0.205555556*			*0.2093023*
**ENSG00000175203**		*0.16969697*		*0.16969697*			*0.3662791*
**ENSG00000164078**		*0.47878788*		*0.478787879*			*0.1918605*
**ENSG00000159399**		*0.48484848*		*0.484848485*			*0.2965116*
**ENSG00000182923**	*0.41017964*	*0.29393939*	*0.41017964*	*0.293939394*	*0.18452381*	*0.1918605*	*0.2209302*

**ENSG00000170852**		*0.37575758*		*0.375757576*	*0.285714286*	*0.4127907*	*0.4127907*
**ENSG00000239961**		*0.12804878*		*0.12804878*			*0.0777778*
**ENSG00000220256**		*0.41768293*		*0.417682927*			*0.4476744*
**ENSG00000178803**		*0.38509317*		*0.385093168*			*0.4166667*
**ENSG00000131725**	*0.08383234*		*0.08383234*		*0.380952381*	*0.4244186*	
**ENSG00000119509**		*0.44242424*		*0.442424242*	*0.380952381*	*0.2848837*	*0.0529412*
