# Supplementary material for: Insights into the regulation of human CNV-miRNAs from the view of their target genes
Source: BMC Genomics. 2012 Dec 18;13:707. doi: 10.1186/1471-2164-13-707 (PMC3582595; doi:10.1186/1471-2164-13-707)
Supplement: Additional file 6 — List of 2,624 differentially expressed genes among six population pairs comparisons selected from four HapMap ethnic populations. [file 1471-2164-13-707-S6.pdf]

Ensembl Gene ID	YRI vs. CEU	YRI vs. CHB	YRI vs. JPT	CEU vs. CHB	CEU Vs. JPT	CHB vs. JPT
**ENSG00000197969**						*yes*
**ENSG00000051596**		*yes*	*yes*	*yes*	*yes*	
**ENSG00000135750**	*yes*			*yes*	*yes*	
**ENSG00000057663**						*yes*
**ENSG00000182511**	*yes*			*yes*	*yes*	
**ENSG00000164164**		*yes*				
**ENSG00000188290**	*yes*	*yes*	*yes*	*yes*	*yes*	
**ENSG00000135919**				*yes*	*yes*	
**ENSG00000166548**		*yes*	*yes*		*yes*	
**ENSG00000105701**						*yes*
**ENSG00000169087**					*yes*	
**ENSG00000152684**			*yes*			
**ENSG00000178860**	*yes*			*yes*		
**ENSG00000157216**		*yes*				*yes*
**ENSG00000134452**		*yes*				
**ENSG00000203883**	*yes*	*yes*		*yes*	*yes*	*yes*
**ENSG00000175130**			*yes*			*yes*
**ENSG00000142539**		*yes*		*yes*		*yes*
**ENSG00000008853**		*yes*				
**ENSG00000126561**				*yes*		
**ENSG00000176978**		*yes*		*yes*		*yes*
**ENSG00000177370**			*yes*		*yes*	*yes*
**ENSG00000124575**					*yes*	*yes*
**ENSG00000131724**			*yes*		*yes*	*yes*
**ENSG00000158710**		*yes*		*yes*		*yes*
**ENSG00000171867**					*yes*	
**ENSG00000103005**				*yes*	*yes*	
**ENSG00000184408**			*yes*			*yes*
**ENSG00000168564**	*yes*			*yes*		
**ENSG00000100211**						*yes*
**ENSG00000129128**			*yes*			*yes*
**ENSG00000131196**				*yes*	*yes*	
**ENSG00000175220**				*yes*		
**ENSG00000196700**						*yes*
**ENSG00000168283**				*yes*	*yes*	
**ENSG00000204165**	*yes*			*yes*	*yes*	
**ENSG00000117394**			*yes*			
**ENSG00000114650**		*yes*				*yes*
**ENSG00000213190**	*yes*	*yes*				*yes*
**ENSG00000165322**						*yes*
**ENSG00000136161**		*yes*	*yes*			
**ENSG00000034510**	*yes*				*yes*	
**ENSG00000089639**						*yes*
**ENSG00000141956**		*yes*	*yes*			
**ENSG00000187608**	*yes*				*yes*	
**ENSG00000141576**				*yes*	*yes*	
**ENSG00000038358**						*yes*
**ENSG00000163131**				*yes*		
**ENSG00000188677**		*yes*	*yes*	*yes*	*yes*	*yes*
**ENSG00000114405**					*yes*	*yes*
**ENSG00000137628**		*yes*				*yes*

**ENSG00000170581**	*yes*					
**ENSG00000011422**		*yes*		*yes*	*yes*	
**ENSG00000122515**	*yes*			*yes*	*yes*	
**ENSG00000102805**	*yes*					
**ENSG00000138623**	*yes*		*yes*	*yes*		*yes*
**ENSG00000213015**			*yes*			
**ENSG00000081052**	*yes*	*yes*	*yes*			
**ENSG00000163607**			*yes*			
**ENSG00000100600**	*yes*			*yes*	*yes*	
**ENSG00000109323**	*yes*		*yes*	*yes*		*yes*
**ENSG00000149179**						*yes*
**ENSG00000142089**	*yes*			*yes*	*yes*	
**ENSG00000170819**		*yes*	*yes*	*yes*		
**ENSG00000196664**			*yes*		*yes*	
**ENSG00000013364**						*yes*
**ENSG00000135148**	*yes*			*yes*	*yes*	
**ENSG00000184709**		*yes*		*yes*		*yes*
**ENSG00000168264**						*yes*
**ENSG00000108576**		*yes*	*yes*	*yes*	*yes*	*yes*
**ENSG00000155846**						*yes*
**ENSG00000198089**	*yes*			*yes*	*yes*	
**ENSG00000243251**	*yes*				*yes*	
**ENSG00000161011**			*yes*	*yes*	*yes*	*yes*
**ENSG00000197978**						*yes*
**ENSG00000155760**					*yes*	
**ENSG00000133026**	*yes*			*yes*	*yes*	
**ENSG00000010810**		*yes*				
**ENSG00000135697**	*yes*			*yes*	*yes*	
**ENSG00000065154**		*yes*	*yes*			
**ENSG00000167965**						*yes*
**ENSG00000183941**			*yes*			*yes*
**ENSG00000197238**						*yes*
**ENSG00000187796**					*yes*	
**ENSG00000087086**					*yes*	
**ENSG00000143344**	*yes*	*yes*	*yes*			
**ENSG00000141522**						*yes*
**ENSG00000169026**	*yes*				*yes*	
**ENSG00000124429**	*yes*			*yes*	*yes*	
**ENSG00000124126**						*yes*
**ENSG00000125755**		*yes*		*yes*		*yes*
**ENSG00000111676**						*yes*
**ENSG00000155465**		*yes*	*yes*	*yes*	*yes*	
**ENSG00000134882**	*yes*		*yes*			
**ENSG00000140564**						*yes*
**ENSG00000003249**	*yes*		*yes*	*yes*		*yes*
**ENSG00000115904**		*yes*	*yes*			
**ENSG00000173915**	*yes*	*yes*	*yes*			
**ENSG00000164237**						*yes*
**ENSG00000123636**					*yes*	*yes*
**ENSG00000213240**		*yes*				*yes*
**ENSG00000141293**		*yes*	*yes*			
**ENSG00000115963**	*yes*			*yes*	*yes*	
**ENSG00000154642**			*yes*			*yes*
**ENSG00000178567**	*yes*			*yes*	*yes*	

**ENSG00000141378**			*yes*			
**ENSG00000099337**			*yes*	*yes*	*yes*	*yes*
**ENSG00000090376**						*yes*
**ENSG00000131153**	*yes*		*yes*			
**ENSG00000138771**	*yes*		*yes*		*yes*	*yes*
**ENSG00000205339**	*yes*					
**ENSG00000174130**		*yes*	*yes*			
**ENSG00000127084**	*yes*		*yes*	*yes*	*yes*	*yes*
**ENSG00000164330**	*yes*			*yes*		*yes*
**ENSG00000178878**			*yes*			*yes*
**ENSG00000160285**						*yes*
**ENSG00000197279**			*yes*			*yes*
**ENSG00000136026**		*yes*	*yes*			
**ENSG00000168630**					*yes*	
**ENSG00000124570**		*yes*	*yes*	*yes*	*yes*	
**ENSG00000116922**	*yes*					
**ENSG00000100300**	*yes*			*yes*	*yes*	*yes*
**ENSG00000138758**	*yes*			*yes*	*yes*	
**ENSG00000084636**		*yes*				*yes*
**ENSG00000188313**		*yes*		*yes*		*yes*
**ENSG00000184887**				*yes*		
**ENSG00000172578**		*yes*	*yes*		*yes*	
**ENSG00000181938**	*yes*					
**ENSG00000213366**	*yes*			*yes*		*yes*
**ENSG00000132471**	*yes*			*yes*		*yes*
**ENSG00000181019**	*yes*			*yes*		
**ENSG00000176381**			*yes*		*yes*	*yes*
**ENSG00000119242**		*yes*	*yes*			*yes*
**ENSG00000198561**		*yes*				
**ENSG00000008710**						*yes*
**ENSG00000170632**	*yes*	*yes*	*yes*			
**ENSG00000112679**	*yes*	*yes*	*yes*			
**ENSG00000111801**	*yes*			*yes*	*yes*	
**ENSG00000197943**	*yes*				*yes*	
**ENSG00000125505**						*yes*
**ENSG00000007237**		*yes*	*yes*		*yes*	*yes*
**ENSG00000213341**	*yes*					
**ENSG00000120129**		*yes*		*yes*		*yes*
**ENSG00000142784**						*yes*
**ENSG00000159335**	*yes*			*yes*		*yes*
**ENSG00000165188**				*yes*	*yes*	
**ENSG00000154864**	*yes*			*yes*	*yes*	
**ENSG00000142235**	*yes*	*yes*	*yes*	*yes*	*yes*	
**ENSG00000013503**	*yes*			*yes*		
**ENSG00000117322**	*yes*	*yes*		*yes*	*yes*	*yes*
**ENSG00000101665**						*yes*
**ENSG00000118508**				*yes*	*yes*	
**ENSG00000128274**		*yes*	*yes*		*yes*	*yes*
**ENSG00000126012**				*yes*		*yes*
**ENSG00000164742**	*yes*			*yes*	*yes*	
**ENSG00000104960**						*yes*
**ENSG00000102962**		*yes*	*yes*			
**ENSG00000173545**						*yes*
**ENSG00000128272**		*yes*				

**ENSG00000184164**		*yes*	*yes*			
**ENSG00000063245**				*yes*		*yes*
**ENSG00000134954**						*yes*
**ENSG00000017427**				*yes*	*yes*	
**ENSG00000100196**			*yes*		*yes*	
**ENSG00000110987**					*yes*	
**ENSG00000125148**			*yes*		*yes*	*yes*
**ENSG00000114480**		*yes*				*yes*
**ENSG00000169245**		*yes*	*yes*	*yes*	*yes*	
**ENSG00000184060**		*yes*	*yes*		*yes*	
**ENSG00000106025**				*yes*	*yes*	
**ENSG00000135898**				*yes*		
**ENSG00000186193**				*yes*		
**ENSG00000142751**						*yes*
**ENSG00000132612**						*yes*
**ENSG00000070413**						*yes*
**ENSG00000189060**			*yes*			*yes*
**ENSG00000120709**						*yes*
**ENSG00000123080**		*yes*	*yes*			
**ENSG00000132185**				*yes*		*yes*
**ENSG00000189283**						*yes*
**ENSG00000076662**	*yes*			*yes*		
**ENSG00000025039**		*yes*				*yes*
**ENSG00000182359**				*yes*		
**ENSG00000178585**	*yes*			*yes*	*yes*	
**ENSG00000118513**	*yes*			*yes*	*yes*	
**ENSG00000119138**				*yes*	*yes*	
**ENSG00000105649**	*yes*	*yes*	*yes*	*yes*		*yes*
**ENSG00000007312**		*yes*	*yes*		*yes*	
**ENSG00000139597**	*yes*			*yes*		
**ENSG00000172638**	*yes*			*yes*		
**ENSG00000151364**		*yes*	*yes*	*yes*	*yes*	
**ENSG00000136436**			*yes*			
**ENSG00000204160**				*yes*		*yes*
**ENSG00000129250**						*yes*
**ENSG00000143870**						*yes*
**ENSG00000096872**					*yes*	
**ENSG00000174996**					*yes*	
**ENSG00000128590**			*yes*			
**ENSG00000149527**	*yes*	*yes*	*yes*			
**ENSG00000104894**	*yes*			*yes*		
**ENSG00000119669**		*yes*				
**ENSG00000198042**		*yes*	*yes*			
**ENSG00000131408**	*yes*			*yes*		
**ENSG00000173480**				*yes*		
**ENSG00000105851**		*yes*		*yes*		
**ENSG00000122707**						*yes*
**ENSG00000143622**	*yes*			*yes*	*yes*	
**ENSG00000139679**	*yes*		*yes*	*yes*	*yes*	*yes*
**ENSG00000186834**	*yes*			*yes*		
**ENSG00000085733**	*yes*				*yes*	*yes*
**ENSG00000128731**	*yes*	*yes*	*yes*			
**ENSG00000159200**	*yes*			*yes*	*yes*	
**ENSG00000170608**	*yes*			*yes*	*yes*	

**ENSG00000102595**				*yes*		
**ENSG00000214530**						*yes*
**ENSG00000069702**	*yes*					
**ENSG00000101336**		*yes*	*yes*	*yes*	*yes*	
**ENSG00000116299**			*yes*			*yes*
**ENSG00000174004**		*yes*		*yes*		
**ENSG00000108753**	*yes*	*yes*		*yes*	*yes*	*yes*
**ENSG00000177683**				*yes*	*yes*	
**ENSG00000115738**			*yes*		*yes*	*yes*
**ENSG00000186111**			*yes*			*yes*
**ENSG00000076641**		*yes*	*yes*			
**ENSG00000211455**		*yes*				*yes*
**ENSG00000107679**	*yes*				*yes*	
**ENSG00000048405**			*yes*			
**ENSG00000162894**	*yes*	*yes*				
**ENSG00000196152**					*yes*	
**ENSG00000089692**	*yes*	*yes*		*yes*	*yes*	*yes*
**ENSG00000090530**	*yes*	*yes*	*yes*	*yes*	*yes*	
**ENSG00000119986**					*yes*	
**ENSG00000006118**		*yes*		*yes*		
**ENSG00000188811**						*yes*
**ENSG00000125740**		*yes*	*yes*			
**ENSG00000148229**			*yes*	*yes*		*yes*
**ENSG00000122122**						*yes*
**ENSG00000137502**					*yes*	
**ENSG00000250722**					*yes*	
**ENSG00000143297**	*yes*			*yes*	*yes*	
**ENSG00000106397**				*yes*		
**ENSG00000104064**			*yes*			
**ENSG00000102096**	*yes*			*yes*	*yes*	
**ENSG00000168061**					*yes*	*yes*
**ENSG00000149328**			*yes*			*yes*
**ENSG00000163823**		*yes*	*yes*	*yes*	*yes*	
**ENSG00000132199**		*yes*				*yes*
**ENSG00000108381**	*yes*			*yes*	*yes*	
**ENSG00000176715**		*yes*	*yes*	*yes*	*yes*	*yes*
**ENSG00000103653**	*yes*					
**ENSG00000162739**	*yes*	*yes*	*yes*	*yes*		
**ENSG00000182118**		*yes*		*yes*	*yes*	
**ENSG00000134716**		*yes*				
**ENSG00000161277**	*yes*		*yes*	*yes*		*yes*
**ENSG00000164733**		*yes*		*yes*	*yes*	
**ENSG00000212864**						*yes*
**ENSG00000185420**						*yes*
**ENSG00000166233**		*yes*	*yes*			
**ENSG00000159314**	*yes*			*yes*	*yes*	
**ENSG00000109534**			*yes*			
**ENSG00000119616**				*yes*		
**ENSG00000196544**		*yes*				
**ENSG00000140497**		*yes*				
**ENSG00000213054**		*yes*				*yes*
**ENSG00000170430**	*yes*			*yes*	*yes*	
**ENSG00000167106**		*yes*	*yes*			
**ENSG00000237541**	*yes*	*yes*	*yes*	*yes*		

**ENSG00000127920**	*yes*	*yes*		*yes*	*yes*	
**ENSG00000119411**		*yes*	*yes*	*yes*	*yes*	*yes*
**ENSG00000184678**	*yes*			*yes*	*yes*	*yes*
**ENSG00000197102**					*yes*	
**ENSG00000167513**			*yes*			*yes*
**ENSG00000177105**				*yes*		
**ENSG00000126261**	*yes*			*yes*		
**ENSG00000139998**		*yes*		*yes*		*yes*
**ENSG00000116133**	*yes*			*yes*	*yes*	
**ENSG00000140650**	*yes*		*yes*			
**ENSG00000169136**	*yes*				*yes*	
**ENSG00000069493**	*yes*			*yes*	*yes*	
**ENSG00000166793**				*yes*	*yes*	
**ENSG00000169896**						*yes*
**ENSG00000066336**			*yes*			*yes*
**ENSG00000139266**				*yes*		*yes*
**ENSG00000196374**			*yes*			*yes*
**ENSG00000203811**			*yes*		*yes*	*yes*
**ENSG00000144802**						*yes*
**ENSG00000138795**	*yes*		*yes*	*yes*	*yes*	*yes*
**ENSG00000104320**	*yes*	*yes*				
**ENSG00000035403**					*yes*	
**ENSG00000176087**						*yes*
**ENSG00000121104**						*yes*
**ENSG00000198113**	*yes*					
**ENSG00000055332**						*yes*
**ENSG00000110324**				*yes*	*yes*	
**ENSG00000160124**	*yes*					
**ENSG00000111335**	*yes*			*yes*	*yes*	
**ENSG00000162066**				*yes*		*yes*
**ENSG00000135638**			*yes*	*yes*	*yes*	
**ENSG00000003147**	*yes*	*yes*	*yes*			
**ENSG00000205020**	*yes*	*yes*	*yes*	*yes*	*yes*	
**ENSG00000185650**		*yes*	*yes*			
**ENSG00000159958**		*yes*		*yes*	*yes*	
**ENSG00000185527**	*yes*					
**ENSG00000198707**						*yes*
**ENSG00000140297**		*yes*	*yes*			
**ENSG00000176171**	*yes*			*yes*	*yes*	
**ENSG00000121769**						*yes*
**ENSG00000162511**		*yes*				
**ENSG00000197405**	*yes*			*yes*	*yes*	
**ENSG00000146223**						*yes*
**ENSG00000157916**		*yes*				
**ENSG00000001561**						*yes*
**ENSG00000130635**	*yes*	*yes*	*yes*	*yes*	*yes*	
**ENSG00000115267**				*yes*		
**ENSG00000137078**	*yes*			*yes*	*yes*	
**ENSG00000169188**						*yes*
**ENSG00000162692**	*yes*	*yes*	*yes*	*yes*	*yes*	*yes*
**ENSG00000163191**			*yes*			*yes*
**ENSG00000071539**	*yes*	*yes*	*yes*			
**ENSG00000196584**						*yes*
**ENSG00000105122**		*yes*				*yes*

**ENSG00000172785**		*yes*				
**ENSG00000172893**						*yes*
**ENSG00000023902**						*yes*
**ENSG00000108771**	*yes*	*yes*	*yes*			
**ENSG00000080823**			*yes*			
**ENSG00000117091**	*yes*				*yes*	
**ENSG00000111052**	*yes*			*yes*	*yes*	
**ENSG00000094914**						*yes*
**ENSG00000100321**		*yes*	*yes*	*yes*		
**ENSG00000070718**	*yes*				*yes*	
**ENSG00000091490**	*yes*		*yes*	*yes*	*yes*	*yes*
**ENSG00000153721**		*yes*		*yes*	*yes*	
**ENSG00000164920**			*yes*		*yes*	*yes*
**ENSG00000149054**	*yes*			*yes*		
**ENSG00000073605**	*yes*			*yes*		
**ENSG00000112576**	*yes*		*yes*			*yes*
**ENSG00000198380**		*yes*	*yes*			
**ENSG00000150556**	*yes*	*yes*	*yes*	*yes*	*yes*	*yes*
**ENSG00000075702**				*yes*	*yes*	
**ENSG00000197879**						*yes*
**ENSG00000149428**						*yes*
**ENSG00000177728**	*yes*			*yes*		*yes*
**ENSG00000109436**		*yes*		*yes*		*yes*
**ENSG00000092847**						*yes*
**ENSG00000152558**						*yes*
**ENSG00000102977**						*yes*
**ENSG00000198003**	*yes*			*yes*	*yes*	
**ENSG00000145850**	*yes*	*yes*		*yes*	*yes*	
**ENSG00000188986**				*yes*		*yes*
**ENSG00000118242**	*yes*			*yes*	*yes*	
**ENSG00000173875**						*yes*
**ENSG00000125657**				*yes*		*yes*
**ENSG00000137259**						*yes*
**ENSG00000198301**			*yes*			*yes*
**ENSG00000072571**				*yes*	*yes*	
**ENSG00000083845**						*yes*
**ENSG00000235863**	*yes*					
**ENSG00000126264**	*yes*	*yes*			*yes*	*yes*
**ENSG00000125124**			*yes*			
**ENSG00000112237**				*yes*		*yes*
**ENSG00000186462**						*yes*
**ENSG00000162517**						*yes*
**ENSG00000132382**	*yes*					
**ENSG00000021355**	*yes*			*yes*	*yes*	
**ENSG00000138035**	*yes*					
**ENSG00000124831**		*yes*		*yes*		*yes*
**ENSG00000100325**						*yes*
**ENSG00000213203**		*yes*				
**ENSG00000165949**	*yes*			*yes*	*yes*	
**ENSG00000164896**						*yes*
**ENSG00000130830**						*yes*
**ENSG00000119004**	*yes*			*yes*		
**ENSG00000126461**						*yes*
**ENSG00000004864**	*yes*				*yes*	*yes*

**ENSG00000172115**	*yes*		*yes*	*yes*	*yes*	
**ENSG00000101337**						*yes*
**ENSG00000105948**	*yes*				*yes*	
**ENSG00000131165**						*yes*
**ENSG00000184260**					*yes*	
**ENSG00000133612**			*yes*			*yes*
**ENSG00000184979**		*yes*	*yes*	*yes*		
**ENSG00000122223**	*yes*			*yes*	*yes*	
**ENSG00000100997**	*yes*	*yes*	*yes*			
**ENSG00000196126**	*yes*		*yes*	*yes*	*yes*	*yes*
**ENSG00000095794**				*yes*		
**ENSG00000187266**						*yes*
**ENSG00000081189**		*yes*				
**ENSG00000112305**						*yes*
**ENSG00000104853**						*yes*
**ENSG00000169122**				*yes*	*yes*	
**ENSG00000119943**			*yes*	*yes*	*yes*	
**ENSG00000185551**			*yes*		*yes*	*yes*
**ENSG00000144674**	*yes*					
**ENSG00000169288**						*yes*
**ENSG00000078142**						*yes*
**ENSG00000159685**			*yes*		*yes*	
**ENSG00000167207**	*yes*			*yes*	*yes*	
**ENSG00000151491**	*yes*			*yes*	*yes*	
**ENSG00000197442**		*yes*	*yes*		*yes*	
**ENSG00000239264**	*yes*	*yes*				*yes*
**ENSG00000171236**						*yes*
**ENSG00000185090**		*yes*	*yes*	*yes*	*yes*	
**ENSG00000164985**					*yes*	
**ENSG00000125962**		*yes*				
**ENSG00000130592**			*yes*			*yes*
**ENSG00000048740**						*yes*
**ENSG00000198736**	*yes*			*yes*	*yes*	
**ENSG00000115694**		*yes*	*yes*			
**ENSG00000136111**	*yes*	*yes*	*yes*	*yes*	*yes*	*yes*
**ENSG00000155307**						*yes*
**ENSG00000131669**			*yes*		*yes*	*yes*
**ENSG00000137965**				*yes*		
**ENSG00000196172**						*yes*
**ENSG00000136261**	*yes*		*yes*			
**ENSG00000168528**	*yes*		*yes*	*yes*		*yes*
**ENSG00000081059**	*yes*			*yes*	*yes*	
**ENSG00000102970**	*yes*	*yes*			*yes*	*yes*
**ENSG00000125912**						*yes*
**ENSG00000123143**						*yes*
**ENSG00000169508**	*yes*		*yes*			
**ENSG00000196187**	*yes*			*yes*	*yes*	
**ENSG00000184232**		*yes*		*yes*	*yes*	
**ENSG00000138760**	*yes*	*yes*	*yes*	*yes*	*yes*	
**ENSG00000153048**	*yes*				*yes*	*yes*
**ENSG00000156113**	*yes*		*yes*	*yes*	*yes*	
**ENSG00000066032**	*yes*			*yes*	*yes*	
**ENSG00000109861**	*yes*		*yes*	*yes*	*yes*	*yes*
**ENSG00000165684**			*yes*			

**ENSG00000166689**			*yes*		*yes*	
**ENSG00000163162**	*yes*			*yes*	*yes*	
**ENSG00000196453**				*yes*		*yes*
**ENSG00000123240**	*yes*				*yes*	
**ENSG00000121486**						*yes*
**ENSG00000150637**						*yes*
**ENSG00000154217**		*yes*		*yes*	*yes*	*yes*
**ENSG00000131187**	*yes*			*yes*	*yes*	
**ENSG00000100721**	*yes*	*yes*			*yes*	*yes*
**ENSG00000165655**		*yes*		*yes*	*yes*	
**ENSG00000145506**				*yes*		*yes*
**ENSG00000048392**						*yes*
**ENSG00000174373**					*yes*	
**ENSG00000166197**	*yes*		*yes*			
**ENSG00000177042**			*yes*			
**ENSG00000177674**	*yes*			*yes*		*yes*
**ENSG00000177951**						*yes*
**ENSG00000086967**		*yes*	*yes*			
**ENSG00000242265**	*yes*		*yes*		*yes*	*yes*
**ENSG00000125844**		*yes*				
**ENSG00000179820**	*yes*			*yes*	*yes*	
**ENSG00000138587**						*yes*
**ENSG00000110697**	*yes*			*yes*		*yes*
**ENSG00000135521**	*yes*		*yes*			
**ENSG00000101439**			*yes*	*yes*	*yes*	*yes*
**ENSG00000120992**				*yes*		
**ENSG00000196151**	*yes*		*yes*			*yes*
**ENSG00000093167**			*yes*			*yes*
**ENSG00000125246**	*yes*		*yes*			
**ENSG00000133106**	*yes*			*yes*	*yes*	
**ENSG00000168298**						*yes*
**ENSG00000131467**	*yes*				*yes*	
**ENSG00000105953**						*yes*
**ENSG00000130559**	*yes*					
**ENSG00000101445**		*yes*				*yes*
**ENSG00000161939**						*yes*
**ENSG00000197982**				*yes*		*yes*
**ENSG00000176022**				*yes*		
**ENSG00000174749**	*yes*					
**ENSG00000068383**	*yes*			*yes*	*yes*	*yes*
**ENSG00000122861**	*yes*				*yes*	*yes*
**ENSG00000103769**						*yes*
**ENSG00000101236**	*yes*			*yes*	*yes*	
**ENSG00000120162**						*yes*
**ENSG00000124491**	*yes*			*yes*	*yes*	
**ENSG00000168734**					*yes*	
**ENSG00000110917**						*yes*
**ENSG00000146376**				*yes*		*yes*
**ENSG00000123136**						*yes*
**ENSG00000010244**	*yes*			*yes*		
**ENSG00000114767**		*yes*				
**ENSG00000166317**	*yes*	*yes*			*yes*	*yes*
**ENSG00000123095**			*yes*			
**ENSG00000168495**				*yes*		

**ENSG00000009790**		*yes*	*yes*			
**ENSG00000100285**		*yes*	*yes*	*yes*	*yes*	
**ENSG00000124588**	*yes*	*yes*	*yes*			
**ENSG00000099622**				*yes*		
**ENSG00000165891**	*yes*			*yes*	*yes*	
**ENSG00000126016**	*yes*		*yes*	*yes*	*yes*	*yes*
**ENSG00000171617**		*yes*	*yes*	*yes*	*yes*	
**ENSG00000101846**				*yes*	*yes*	
**ENSG00000198033**					*yes*	*yes*
**ENSG00000148468**						*yes*
**ENSG00000147872**	*yes*					
**ENSG00000011198**	*yes*				*yes*	
**ENSG00000188958**					*yes*	
**ENSG00000100304**	*yes*	*yes*	*yes*	*yes*	*yes*	
**ENSG00000153140**						*yes*
**ENSG00000115165**	*yes*			*yes*	*yes*	
**ENSG00000111215**		*yes*	*yes*			
**ENSG00000155629**	*yes*					
**ENSG00000126107**						*yes*
**ENSG00000095906**		*yes*				
**ENSG00000180543**		*yes*	*yes*			
**ENSG00000198498**		*yes*				
**ENSG00000134061**	*yes*					
**ENSG00000090238**			*yes*			
**ENSG00000105281**			*yes*			*yes*
**ENSG00000186652**		*yes*				
**ENSG00000070831**	*yes*			*yes*	*yes*	
**ENSG00000162441**	*yes*	*yes*	*yes*			
**ENSG00000085514**						*yes*
**ENSG00000135480**	*yes*				*yes*	
**ENSG00000100364**						*yes*
**ENSG00000196923**						*yes*
**ENSG00000179409**						*yes*
**ENSG00000181031**					*yes*	
**ENSG00000110446**		*yes*		*yes*		
**ENSG00000141458**						*yes*
**ENSG00000154277**	*yes*		*yes*	*yes*	*yes*	*yes*
**ENSG00000116455**				*yes*		
**ENSG00000103657**		*yes*		*yes*		*yes*
**ENSG00000198846**			*yes*			
**ENSG00000180611**	*yes*	*yes*	*yes*	*yes*		*yes*
**ENSG00000168952**	*yes*			*yes*	*yes*	*yes*
**ENSG00000085117**				*yes*		
**ENSG00000149231**					*yes*	*yes*
**ENSG00000100083**			*yes*			*yes*
**ENSG00000071967**	*yes*		*yes*		*yes*	*yes*
**ENSG00000126353**	*yes*	*yes*		*yes*	*yes*	
**ENSG00000102221**	*yes*			*yes*	*yes*	
**ENSG00000086758**						*yes*
**ENSG00000185222**	*yes*	*yes*	*yes*			
**ENSG00000167925**						*yes*
**ENSG00000166598**						*yes*
**ENSG00000167476**	*yes*	*yes*			*yes*	*yes*
**ENSG00000132341**	*yes*			*yes*	*yes*	

**ENSG00000144746**		*yes*	*yes*			
**ENSG00000111332**			*yes*			
**ENSG00000106105**						*yes*
**ENSG00000167414**		*yes*	*yes*	*yes*	*yes*	
**ENSG00000139193**		*yes*				*yes*
**ENSG00000064787**					*yes*	
**ENSG00000105963**	*yes*		*yes*	*yes*	*yes*	
**ENSG00000137269**						*yes*
**ENSG00000101255**	*yes*		*yes*			*yes*
**ENSG00000110328**		*yes*				
**ENSG00000106392**				*yes*		
**ENSG00000111962**	*yes*		*yes*	*yes*	*yes*	*yes*
**ENSG00000168772**	*yes*	*yes*	*yes*	*yes*	*yes*	*yes*
**ENSG00000154930**		*yes*				*yes*
**ENSG00000162430**	*yes*	*yes*	*yes*	*yes*		*yes*
**ENSG00000198624**		*yes*	*yes*			
**ENSG00000099875**	*yes*	*yes*		*yes*	*yes*	*yes*
**ENSG00000127507**		*yes*	*yes*	*yes*	*yes*	
**ENSG00000071073**				*yes*	*yes*	
**ENSG00000155438**	*yes*		*yes*			
**ENSG00000174946**	*yes*			*yes*	*yes*	
**ENSG00000072210**			*yes*			
**ENSG00000153395**				*yes*		
**ENSG00000143554**		*yes*				
**ENSG00000108797**						*yes*
**ENSG00000177192**	*yes*		*yes*			*yes*
**ENSG00000184602**	*yes*				*yes*	
**ENSG00000184674**		*yes*	*yes*	*yes*	*yes*	
**ENSG00000143013**	*yes*				*yes*	*yes*
**ENSG00000107562**	*yes*			*yes*	*yes*	
**ENSG00000166821**				*yes*		
**ENSG00000060982**	*yes*			*yes*	*yes*	
**ENSG00000121236**		*yes*		*yes*		
**ENSG00000143368**						*yes*
**ENSG00000072756**	*yes*	*yes*	*yes*			
**ENSG00000122643**	*yes*			*yes*	*yes*	
**ENSG00000206503**		*yes*	*yes*		*yes*	*yes*
**ENSG00000167748**	*yes*	*yes*		*yes*	*yes*	*yes*
**ENSG00000114742**			*yes*			
**ENSG00000128271**				*yes*		*yes*
**ENSG00000105085**				*yes*		
**ENSG00000103495**						*yes*
**ENSG00000079931**		*yes*	*yes*	*yes*	*yes*	*yes*
**ENSG00000086666**		*yes*		*yes*	*yes*	*yes*
**ENSG00000134001**	*yes*	*yes*	*yes*	*yes*	*yes*	
**ENSG00000213145**					*yes*	
**ENSG00000108848**			*yes*			*yes*
**ENSG00000158828**			*yes*		*yes*	
**ENSG00000109466**						*yes*
**ENSG00000116649**			*yes*			
**ENSG00000163563**	*yes*			*yes*	*yes*	
**ENSG00000073969**				*yes*	*yes*	
**ENSG00000179344**		*yes*	*yes*			
**ENSG00000101361**	*yes*		*yes*			*yes*

**ENSG00000116574**						*yes*
**ENSG00000055483**	*yes*	*yes*	*yes*			
**ENSG00000180011**	*yes*	*yes*	*yes*			
**ENSG00000154451**	*yes*			*yes*	*yes*	
**ENSG00000136235**		*yes*				
**ENSG00000162144**		*yes*		*yes*	*yes*	
**ENSG00000116793**						*yes*
**ENSG00000171503**		*yes*	*yes*			
**ENSG00000167397**	*yes*			*yes*		
**ENSG00000085511**		*yes*		*yes*		
**ENSG00000132646**	*yes*		*yes*	*yes*	*yes*	
**ENSG00000224051**				*yes*		*yes*
**ENSG00000125637**	*yes*		*yes*	*yes*		*yes*
**ENSG00000187514**						*yes*
**ENSG00000127990**	*yes*		*yes*	*yes*	*yes*	*yes*
**ENSG00000129353**	*yes*		*yes*	*yes*	*yes*	*yes*
**ENSG00000007038**		*yes*	*yes*			
**ENSG00000198860**	*yes*			*yes*		
**ENSG00000153162**		*yes*				
**ENSG00000139116**	*yes*			*yes*	*yes*	
**ENSG00000116874**	*yes*					
**ENSG00000136717**	*yes*			*yes*	*yes*	
**ENSG00000133302**						*yes*
**ENSG00000113140**		*yes*	*yes*	*yes*		*yes*
**ENSG00000151948**	*yes*	*yes*	*yes*	*yes*	*yes*	
**ENSG00000158717**	*yes*			*yes*	*yes*	
**ENSG00000205571**	*yes*	*yes*	*yes*			
**ENSG00000173812**						*yes*
**ENSG00000136738**		*yes*				
**ENSG00000117519**	*yes*	*yes*		*yes*	*yes*	*yes*
**ENSG00000138764**			*yes*			
**ENSG00000139697**	*yes*					
**ENSG00000168994**			*yes*		*yes*	*yes*
**ENSG00000075618**	*yes*		*yes*	*yes*		*yes*
**ENSG00000110107**						*yes*
**ENSG00000143333**					*yes*	
**ENSG00000118655**	*yes*				*yes*	
**ENSG00000122557**						*yes*
**ENSG00000075234**	*yes*	*yes*	*yes*			
**ENSG00000196313**				*yes*		*yes*
**ENSG00000143179**		*yes*	*yes*			
**ENSG00000198931**		*yes*		*yes*		*yes*
**ENSG00000119922**		*yes*		*yes*		*yes*
**ENSG00000115239**	*yes*			*yes*	*yes*	
**ENSG00000007541**			*yes*	*yes*		*yes*
**ENSG00000133114**						*yes*
**ENSG00000172301**	*yes*					
**ENSG00000004478**		*yes*				
**ENSG00000095015**	*yes*			*yes*		
**ENSG00000136522**	*yes*					
**ENSG00000118496**	*yes*					
**ENSG00000156265**						*yes*
**ENSG00000108523**		*yes*	*yes*			
**ENSG00000168938**				*yes*		*yes*

**ENSG00000137752**						*yes*
**ENSG00000164978**		*yes*				
**ENSG00000172354**						*yes*
**ENSG00000014164**						*yes*
**ENSG00000164047**	*yes*	*yes*				*yes*
**ENSG00000129003**						*yes*
**ENSG00000165474**			*yes*			
**ENSG00000002586**		*yes*	*yes*			
**ENSG00000197822**					*yes*	
**ENSG00000164054**	*yes*			*yes*	*yes*	
**ENSG00000162976**				*yes*		
**ENSG00000091844**			*yes*		*yes*	
**ENSG00000166896**	*yes*	*yes*	*yes*			
**ENSG00000153064**		*yes*	*yes*	*yes*	*yes*	
**ENSG00000196743**		*yes*	*yes*			
**ENSG00000145220**		*yes*	*yes*			
**ENSG00000109181**		*yes*	*yes*	*yes*	*yes*	
**ENSG00000183386**						*yes*
**ENSG00000186603**						*yes*
**ENSG00000198925**				*yes*		*yes*
**ENSG00000164904**	*yes*	*yes*	*yes*		*yes*	
**ENSG00000088992**	*yes*	*yes*		*yes*	*yes*	*yes*
**ENSG00000111845**	*yes*	*yes*	*yes*			
**ENSG00000099246**	*yes*		*yes*			
**ENSG00000138166**	*yes*	*yes*	*yes*	*yes*	*yes*	
**ENSG00000178950**						*yes*
**ENSG00000154832**	*yes*	*yes*			*yes*	*yes*
**ENSG00000170522**				*yes*	*yes*	
**ENSG00000105568**						*yes*
**ENSG00000173566**	*yes*		*yes*	*yes*		*yes*
**ENSG00000182985**		*yes*	*yes*	*yes*	*yes*	*yes*
**ENSG00000132744**					*yes*	*yes*
**ENSG00000173581**			*yes*			*yes*
**ENSG00000167578**				*yes*		*yes*
**ENSG00000229117**					*yes*	*yes*
**ENSG00000232258**			*yes*		*yes*	
**ENSG00000166016**		*yes*	*yes*	*yes*		
**ENSG00000173327**						*yes*
**ENSG00000111252**	*yes*	*yes*	*yes*			
**ENSG00000185201**	*yes*			*yes*	*yes*	
**ENSG00000101974**						*yes*
**ENSG00000166451**		*yes*	*yes*			
**ENSG00000084073**	*yes*			*yes*		
**ENSG00000168610**	*yes*			*yes*	*yes*	
**ENSG00000172009**		*yes*		*yes*		*yes*
**ENSG00000178971**	*yes*				*yes*	
**ENSG00000069399**	*yes*		*yes*	*yes*		*yes*
**ENSG00000242550**		*yes*	*yes*	*yes*	*yes*	*yes*
**ENSG00000141013**						*yes*
**ENSG00000108179**			*yes*			
**ENSG00000197417**		*yes*	*yes*			
**ENSG00000116191**	*yes*				*yes*	*yes*
**ENSG00000164924**	*yes*			*yes*	*yes*	
**ENSG00000127528**	*yes*					

**ENSG00000141179**		*yes*	*yes*			
**ENSG00000011304**						*yes*
**ENSG00000048544**	*yes*	*yes*	*yes*			
**ENSG00000104856**				*yes*		*yes*
**ENSG00000147679**						*yes*
**ENSG00000165449**	*yes*	*yes*	*yes*			
**ENSG00000013374**	*yes*			*yes*	*yes*	
**ENSG00000165724**			*yes*			
**ENSG00000130429**	*yes*	*yes*	*yes*			
**ENSG00000071205**						*yes*
**ENSG00000153201**			*yes*	*yes*	*yes*	
**ENSG00000179403**	*yes*		*yes*	*yes*		*yes*
**ENSG00000183918**			*yes*			
**ENSG00000023191**						*yes*
**ENSG00000168005**						*yes*
**ENSG00000197930**	*yes*			*yes*	*yes*	
**ENSG00000166289**						*yes*
**ENSG00000138071**	*yes*	*yes*		*yes*	*yes*	
**ENSG00000046604**				*yes*		
**ENSG00000128422**	*yes*			*yes*	*yes*	
**ENSG00000135900**					*yes*	
**ENSG00000184588**		*yes*				
**ENSG00000077514**						*yes*
**ENSG00000082146**		*yes*	*yes*	*yes*	*yes*	
**ENSG00000160932**	*yes*			*yes*	*yes*	*yes*
**ENSG00000173262**	*yes*			*yes*	*yes*	
**ENSG00000198417**			*yes*		*yes*	*yes*
**ENSG00000006282**	*yes*	*yes*	*yes*			
**ENSG00000149554**	*yes*					
**ENSG00000178053**	*yes*					
**ENSG00000180448**						*yes*
**ENSG00000102858**						*yes*
**ENSG00000114779**			*yes*			*yes*
**ENSG00000169418**				*yes*	*yes*	
**ENSG00000198692**	*yes*	*yes*	*yes*			
**ENSG00000112320**	*yes*			*yes*	*yes*	
**ENSG00000143153**		*yes*		*yes*		*yes*
**ENSG00000114423**			*yes*			
**ENSG00000169738**			*yes*			
**ENSG00000161243**		*yes*				
**ENSG00000114948**	*yes*	*yes*		*yes*	*yes*	
**ENSG00000167202**			*yes*			*yes*
**ENSG00000110066**		*yes*	*yes*			
**ENSG00000182378**	*yes*	*yes*	*yes*			
**ENSG00000100024**		*yes*				*yes*
**ENSG00000180917**	*yes*			*yes*		
**ENSG00000155962**	*yes*			*yes*	*yes*	
**ENSG00000184886**	*yes*		*yes*			*yes*
**ENSG00000156171**			*yes*			
**ENSG00000087087**				*yes*		
**ENSG00000110002**	*yes*					
**ENSG00000118507**		*yes*	*yes*	*yes*		
**ENSG00000153904**	*yes*			*yes*	*yes*	
**ENSG00000144036**			*yes*			*yes*

**ENSG00000119686**	*yes*	*yes*			*yes*	*yes*
**ENSG00000071794**						*yes*
**ENSG00000143375**						*yes*
**ENSG00000211460**	*yes*				*yes*	
**ENSG00000102575**	*yes*		*yes*	*yes*	*yes*	*yes*
**ENSG00000186088**	*yes*			*yes*		*yes*
**ENSG00000135763**	*yes*	*yes*	*yes*			
**ENSG00000159884**						*yes*
**ENSG00000148671**		*yes*		*yes*		*yes*
**ENSG00000161395**		*yes*				
**ENSG00000147454**				*yes*	*yes*	
**ENSG00000160014**						*yes*
**ENSG00000204577**	*yes*			*yes*	*yes*	
**ENSG00000132205**			*yes*		*yes*	*yes*
**ENSG00000100219**		*yes*				*yes*
**ENSG00000082397**		*yes*	*yes*			
**ENSG00000131503**			*yes*			
**ENSG00000154611**			*yes*			*yes*
**ENSG00000151474**			*yes*		*yes*	*yes*
**ENSG00000120705**	*yes*					
**ENSG00000137825**		*yes*				
**ENSG00000112893**	*yes*			*yes*	*yes*	
**ENSG00000101187**		*yes*	*yes*	*yes*	*yes*	
**ENSG00000137868**	*yes*			*yes*	*yes*	
**ENSG00000125977**						*yes*
**ENSG00000186352**	*yes*		*yes*	*yes*	*yes*	*yes*
**ENSG00000151640**	*yes*	*yes*	*yes*		*yes*	
**ENSG00000125462**				*yes*		
**ENSG00000162337**	*yes*			*yes*		*yes*
**ENSG00000138964**			*yes*			*yes*
**ENSG00000112511**	*yes*			*yes*		*yes*
**ENSG00000178381**			*yes*			*yes*
**ENSG00000167634**			*yes*		*yes*	
**ENSG00000167632**			*yes*			*yes*
**ENSG00000197081**						*yes*
**ENSG00000133424**		*yes*	*yes*			
**ENSG00000048028**	*yes*			*yes*	*yes*	
**ENSG00000101901**			*yes*			*yes*
**ENSG00000143653**		*yes*	*yes*			
**ENSG00000141198**				*yes*		
**ENSG00000159674**	*yes*			*yes*	*yes*	
**ENSG00000059377**		*yes*				*yes*
**ENSG00000133561**		*yes*	*yes*			
**ENSG00000134864**				*yes*	*yes*	
**ENSG00000106415**		*yes*	*yes*			
**ENSG00000073060**		*yes*				
**ENSG00000027075**	*yes*			*yes*	*yes*	
**ENSG00000111737**		*yes*		*yes*		*yes*
**ENSG00000118922**			*yes*			*yes*
**ENSG00000145736**		*yes*		*yes*		
**ENSG00000145495**			*yes*			
**ENSG00000177627**				*yes*	*yes*	
**ENSG00000196653**		*yes*	*yes*			
**ENSG00000088832**			*yes*			*yes*

**ENSG00000104812**				*yes*		*yes*
**ENSG00000152990**					*yes*	
**ENSG00000100628**	*yes*			*yes*	*yes*	
**ENSG00000186860**		*yes*	*yes*			
**ENSG00000100395**	*yes*			*yes*		
**ENSG00000112977**				*yes*		*yes*
**ENSG00000121989**				*yes*	*yes*	
**ENSG00000119912**	*yes*			*yes*		
**ENSG00000205352**		*yes*				*yes*
**ENSG00000163132**		*yes*	*yes*	*yes*	*yes*	
**ENSG00000188001**	*yes*			*yes*	*yes*	
**ENSG00000196154**		*yes*		*yes*		*yes*
**ENSG00000146463**						*yes*
**ENSG00000097021**					*yes*	
**ENSG00000137709**		*yes*		*yes*		
**ENSG00000154165**				*yes*		*yes*
**ENSG00000172175**	*yes*					
**ENSG00000102781**						*yes*
**ENSG00000132819**				*yes*		*yes*
**ENSG00000164659**			*yes*		*yes*	*yes*
**ENSG00000011638**	*yes*			*yes*		
**ENSG00000205358**			*yes*		*yes*	
**ENSG00000138134**				*yes*		*yes*
**ENSG00000166173**	*yes*			*yes*	*yes*	
**ENSG00000108187**					*yes*	
**ENSG00000204574**						*yes*
**ENSG00000117133**		*yes*	*yes*			
**ENSG00000151702**	*yes*					
**ENSG00000124635**						*yes*
**ENSG00000205021**	*yes*			*yes*	*yes*	
**ENSG00000136156**	*yes*	*yes*	*yes*			
**ENSG00000141526**	*yes*			*yes*	*yes*	
**ENSG00000173702**	*yes*			*yes*	*yes*	
**ENSG00000145990**	*yes*					
**ENSG00000104419**	*yes*	*yes*			*yes*	*yes*
**ENSG00000130305**		*yes*				
**ENSG00000152256**			*yes*			*yes*
**ENSG00000013573**						*yes*
**ENSG00000112640**						*yes*
**ENSG00000163703**	*yes*			*yes*	*yes*	
**ENSG00000154928**		*yes*		*yes*		*yes*
**ENSG00000114520**					*yes*	*yes*
**ENSG00000163376**	*yes*		*yes*			
**ENSG00000102024**			*yes*		*yes*	*yes*
**ENSG00000153575**	*yes*			*yes*	*yes*	
**ENSG00000049247**		*yes*	*yes*	*yes*	*yes*	
**ENSG00000153406**		*yes*				
**ENSG00000177106**	*yes*				*yes*	
**ENSG00000076928**	*yes*			*yes*		*yes*
**ENSG00000170727**		*yes*				
**ENSG00000205220**						*yes*
**ENSG00000180263**						*yes*
**ENSG00000099385**						*yes*
**ENSG00000079432**						*yes*

**ENSG00000104375**						*yes*
**ENSG00000000938**	*yes*	*yes*	*yes*			
**ENSG00000173209**			*yes*			
**ENSG00000134107**	*yes*			*yes*	*yes*	
**ENSG00000136824**	*yes*			*yes*	*yes*	
**ENSG00000100075**						*yes*
**ENSG00000165506**		*yes*				
**ENSG00000095002**	*yes*					
**ENSG00000172081**	*yes*		*yes*			
**ENSG00000160746**			*yes*			*yes*
**ENSG00000143119**	*yes*	*yes*				
**ENSG00000164484**		*yes*	*yes*	*yes*	*yes*	*yes*
**ENSG00000105854**	*yes*		*yes*	*yes*	*yes*	*yes*
**ENSG00000152969**					*yes*	
**ENSG00000132773**			*yes*			
**ENSG00000153037**	*yes*		*yes*			
**ENSG00000184983**	*yes*			*yes*	*yes*	
**ENSG00000100241**		*yes*				*yes*
**ENSG00000146425**				*yes*		
**ENSG00000253506**			*yes*			*yes*
**ENSG00000198598**	*yes*	*yes*		*yes*	*yes*	*yes*
**ENSG00000062822**				*yes*		*yes*
**ENSG00000148935**	*yes*		*yes*			*yes*
**ENSG00000156398**		*yes*		*yes*	*yes*	*yes*
**ENSG00000204351**						*yes*
**ENSG00000115590**	*yes*		*yes*	*yes*	*yes*	
**ENSG00000198746**						*yes*
**ENSG00000104921**	*yes*			*yes*	*yes*	
**ENSG00000079805**						*yes*
**ENSG00000064692**	*yes*			*yes*	*yes*	
**ENSG00000100385**		*yes*				
**ENSG00000111348**		*yes*				*yes*
**ENSG00000189077**		*yes*		*yes*		*yes*
**ENSG00000105355**		*yes*				
**ENSG00000169981**	*yes*		*yes*			*yes*
**ENSG00000151790**				*yes*		
**ENSG00000163947**	*yes*			*yes*	*yes*	
**ENSG00000102409**		*yes*	*yes*			
**ENSG00000115884**	*yes*	*yes*				*yes*
**ENSG00000132170**					*yes*	
**ENSG00000112851**				*yes*		
**ENSG00000103111**			*yes*			*yes*
**ENSG00000007968**			*yes*	*yes*		*yes*
**ENSG00000091039**						*yes*
**ENSG00000164506**	*yes*			*yes*	*yes*	
**ENSG00000073737**		*yes*	*yes*	*yes*		*yes*
**ENSG00000148400**				*yes*		
**ENSG00000106560**		*yes*	*yes*			
**ENSG00000160785**	*yes*			*yes*		
**ENSG00000120708**			*yes*			
**ENSG00000173041**						*yes*
**ENSG00000143924**					*yes*	
**ENSG00000099849**	*yes*					
**ENSG00000156853**						*yes*

**ENSG00000148297**						*yes*
**ENSG00000100380**						*yes*
**ENSG00000214113**	*yes*	*yes*				
**ENSG00000089693**						*yes*
**ENSG00000185946**				*yes*		
**ENSG00000028277**				*yes*		*yes*
**ENSG00000133101**					*yes*	
**ENSG00000119965**	*yes*					
**ENSG00000120725**						*yes*
**ENSG00000184293**	*yes*			*yes*	*yes*	
**ENSG00000138709**	*yes*		*yes*			
**ENSG00000141644**	*yes*					
**ENSG00000124279**	*yes*		*yes*			
**ENSG00000128218**		*yes*	*yes*	*yes*	*yes*	
**ENSG00000155380**					*yes*	*yes*
**ENSG00000135924**				*yes*		
**ENSG00000135821**		*yes*				
**ENSG00000177697**		*yes*	*yes*		*yes*	
**ENSG00000163362**				*yes*		*yes*
**ENSG00000099326**		*yes*				
**ENSG00000132274**						*yes*
**ENSG00000142186**						*yes*
**ENSG00000213029**			*yes*			
**ENSG00000105373**		*yes*				*yes*
**ENSG00000162341**	*yes*		*yes*	*yes*		*yes*
**ENSG00000104290**				*yes*		*yes*
**ENSG00000196141**						*yes*
**ENSG00000197888**		*yes*	*yes*	*yes*	*yes*	
**ENSG00000085465**	*yes*			*yes*	*yes*	
**ENSG00000084207**		*yes*				*yes*
**ENSG00000099219**	*yes*			*yes*	*yes*	
**ENSG00000156976**	*yes*			*yes*		
**ENSG00000145332**			*yes*			
**ENSG00000174125**	*yes*	*yes*	*yes*			*yes*
**ENSG00000147813**		*yes*	*yes*	*yes*	*yes*	*yes*
**ENSG00000140030**				*yes*	*yes*	
**ENSG00000046651**						*yes*
**ENSG00000137441**	*yes*			*yes*	*yes*	
**ENSG00000165997**		*yes*				
**ENSG00000221983**	*yes*	*yes*	*yes*			
**ENSG00000108679**	*yes*	*yes*		*yes*	*yes*	*yes*
**ENSG00000182472**	*yes*			*yes*	*yes*	
**ENSG00000137161**						*yes*
**ENSG00000005436**	*yes*					
**ENSG00000109390**				*yes*		*yes*
**ENSG00000074660**				*yes*		
**ENSG00000187239**		*yes*				
**ENSG00000083123**						*yes*
**ENSG00000072858**	*yes*					
**ENSG00000113194**						*yes*
**ENSG00000158457**		*yes*		*yes*		
**ENSG00000172349**		*yes*				
**ENSG00000096746**						*yes*
**ENSG00000132510**				*yes*		*yes*

**ENSG00000169100**						*yes*
**ENSG00000090006**				*yes*		*yes*
**ENSG00000100055**	*yes*	*yes*	*yes*			
**ENSG00000198053**	*yes*	*yes*	*yes*			
**ENSG00000145287**	*yes*	*yes*	*yes*	*yes*	*yes*	
**ENSG00000085185**	*yes*					
**ENSG00000179163**		*yes*	*yes*	*yes*		
**ENSG00000136573**		*yes*	*yes*	*yes*		*yes*
**ENSG00000172005**					*yes*	*yes*
**ENSG00000170684**	*yes*			*yes*	*yes*	
**ENSG00000185504**						*yes*
**ENSG00000165929**		*yes*		*yes*		*yes*
**ENSG00000131653**			*yes*			
**ENSG00000157827**		*yes*	*yes*	*yes*	*yes*	
**ENSG00000156738**		*yes*	*yes*	*yes*		
**ENSG00000213339**						*yes*
**ENSG00000091527**						*yes*
**ENSG00000242574**		*yes*				
**ENSG00000206337**		*yes*	*yes*			
**ENSG00000010626**		*yes*				
**ENSG00000121966**	*yes*			*yes*		
**ENSG00000176454**				*yes*		*yes*
**ENSG00000198805**		*yes*	*yes*			
**ENSG00000170627**	*yes*	*yes*	*yes*	*yes*	*yes*	
**ENSG00000152894**					*yes*	*yes*
**ENSG00000162614**		*yes*	*yes*			
**ENSG00000134343**	*yes*			*yes*	*yes*	
**ENSG00000054611**		*yes*				
**ENSG00000169957**				*yes*		*yes*
**ENSG00000127824**	*yes*			*yes*	*yes*	
**ENSG00000086730**	*yes*			*yes*	*yes*	
**ENSG00000117000**	*yes*					
**ENSG00000174788**	*yes*					
**ENSG00000244038**						*yes*
**ENSG00000149499**						*yes*
**ENSG00000152061**					*yes*	
**ENSG00000075975**		*yes*				
**ENSG00000111671**		*yes*	*yes*			*yes*
**ENSG00000050030**	*yes*			*yes*	*yes*	*yes*
**ENSG00000187954**	*yes*	*yes*				
**ENSG00000164715**						*yes*
**ENSG00000183508**					*yes*	
**ENSG00000140398**	*yes*	*yes*	*yes*	*yes*	*yes*	
**ENSG00000005379**				*yes*	*yes*	
**ENSG00000028137**	*yes*	*yes*		*yes*	*yes*	*yes*
**ENSG00000175197**			*yes*		*yes*	*yes*
**ENSG00000175602**		*yes*				
**ENSG00000087157**						*yes*
**ENSG00000179750**		*yes*	*yes*	*yes*		
**ENSG00000115641**	*yes*			*yes*	*yes*	
**ENSG00000139350**	*yes*			*yes*	*yes*	
**ENSG00000145730**	*yes*			*yes*	*yes*	
**ENSG00000106853**	*yes*		*yes*			
**ENSG00000164849**	*yes*		*yes*	*yes*	*yes*	*yes*

**ENSG00000178922**	*yes*	*yes*				
**ENSG00000147168**				*yes*		*yes*
**ENSG00000068654**			*yes*			
**ENSG00000177459**			*yes*	*yes*		*yes*
**ENSG00000244752**	*yes*	*yes*	*yes*			
**ENSG00000235750**	*yes*			*yes*	*yes*	
**ENSG00000137959**	*yes*			*yes*	*yes*	
**ENSG00000188994**						*yes*
**ENSG00000184903**		*yes*				*yes*
**ENSG00000105383**		*yes*				*yes*
**ENSG00000184357**			*yes*			*yes*
**ENSG00000179088**			*yes*			
**ENSG00000196735**		*yes*	*yes*	*yes*	*yes*	*yes*
**ENSG00000099956**		*yes*				
**ENSG00000100410**			*yes*			
**ENSG00000141380**					*yes*	
**ENSG00000137267**	*yes*	*yes*	*yes*	*yes*	*yes*	*yes*
**ENSG00000171453**			*yes*			
**ENSG00000203879**						*yes*
**ENSG00000117143**		*yes*	*yes*			
**ENSG00000077150**		*yes*				*yes*
**ENSG00000077235**						*yes*
**ENSG00000115504**						*yes*
**ENSG00000132846**	*yes*				*yes*	
**ENSG00000128311**		*yes*				
**ENSG00000109220**						*yes*
**ENSG00000163121**	*yes*	*yes*		*yes*	*yes*	*yes*
**ENSG00000188389**		*yes*		*yes*	*yes*	
**ENSG00000117174**	*yes*		*yes*			
**ENSG00000137198**						*yes*
**ENSG00000170264**						*yes*
**ENSG00000171848**	*yes*		*yes*	*yes*		*yes*
**ENSG00000166133**						*yes*
**ENSG00000124587**	*yes*	*yes*	*yes*			
**ENSG00000115604**	*yes*		*yes*	*yes*	*yes*	*yes*
**ENSG00000144560**	*yes*			*yes*	*yes*	
**ENSG00000181222**						*yes*
**ENSG00000233224**						*yes*
**ENSG00000136205**		*yes*	*yes*			
**ENSG00000118515**		*yes*	*yes*		*yes*	*yes*
**ENSG00000122692**	*yes*					
**ENSG00000168310**				*yes*		*yes*
**ENSG00000051523**					*yes*	
**ENSG00000166750**			*yes*			*yes*
**ENSG00000048540**	*yes*	*yes*		*yes*	*yes*	*yes*
**ENSG00000167081**	*yes*					
**ENSG00000135457**					*yes*	
**ENSG00000066923**		*yes*				
**ENSG00000084774**						*yes*
**ENSG00000213983**			*yes*			
**ENSG00000179950**						*yes*
**ENSG00000133256**	*yes*			*yes*	*yes*	
**ENSG00000166394**		*yes*	*yes*	*yes*	*yes*	
**ENSG00000101190**			*yes*		*yes*	*yes*

**ENSG00000138101**		*yes*		*yes*	*yes*	
**ENSG00000187990**						*yes*
**ENSG00000188343**	*yes*	*yes*			*yes*	*yes*
**ENSG00000087077**				*yes*		
**ENSG00000240563**	*yes*	*yes*	*yes*	*yes*	*yes*	
**ENSG00000130513**	*yes*			*yes*	*yes*	
**ENSG00000240344**	*yes*	*yes*	*yes*			
**ENSG00000129277**	*yes*		*yes*	*yes*	*yes*	
**ENSG00000173531**		*yes*	*yes*			
**ENSG00000164587**						*yes*
**ENSG00000168234**			*yes*	*yes*	*yes*	*yes*
**ENSG00000125898**						*yes*
**ENSG00000243056**				*yes*		
**ENSG00000143537**				*yes*		*yes*
**ENSG00000131759**			*yes*			*yes*
**ENSG00000127419**		*yes*				
**ENSG00000147065**	*yes*					
**ENSG00000160211**						*yes*
**ENSG00000017483**		*yes*	*yes*			
**ENSG00000152689**		*yes*	*yes*			
**ENSG00000159388**	*yes*					
**ENSG00000065029**		*yes*				
**ENSG00000164638**				*yes*		
**ENSG00000115009**			*yes*		*yes*	*yes*
**ENSG00000067048**	*yes*	*yes*	*yes*			
**ENSG00000081913**	*yes*		*yes*	*yes*		*yes*
**ENSG00000158402**	*yes*			*yes*	*yes*	
**ENSG00000008256**	*yes*	*yes*	*yes*			
**ENSG00000070214**	*yes*			*yes*	*yes*	*yes*
**ENSG00000154065**						*yes*
**ENSG00000079112**		*yes*		*yes*	*yes*	
**ENSG00000106829**	*yes*			*yes*	*yes*	
**ENSG00000005194**	*yes*			*yes*		
**ENSG00000143575**			*yes*			
**ENSG00000012779**	*yes*			*yes*	*yes*	
**ENSG00000154620**		*yes*	*yes*			
**ENSG00000135838**	*yes*			*yes*	*yes*	
**ENSG00000136514**					*yes*	
**ENSG00000123329**	*yes*					
**ENSG00000118200**	*yes*			*yes*	*yes*	
**ENSG00000099910**		*yes*	*yes*			
**ENSG00000168066**	*yes*	*yes*	*yes*	*yes*	*yes*	
**ENSG00000196950**						*yes*
**ENSG00000139626**		*yes*				
**ENSG00000065989**				*yes*		*yes*
**ENSG00000136108**				*yes*		
**ENSG00000135596**		*yes*				
**ENSG00000104852**						*yes*
**ENSG00000104805**						*yes*
**ENSG00000026508**			*yes*	*yes*	*yes*	
**ENSG00000163877**						*yes*
**ENSG00000174943**						*yes*
**ENSG00000143797**	*yes*	*yes*	*yes*		*yes*	*yes*
**ENSG00000139357**						*yes*

**ENSG00000078237**	*yes*			*yes*	*yes*	
**ENSG00000163389**				*yes*	*yes*	
**ENSG00000072840**	*yes*			*yes*	*yes*	
**ENSG00000130775**	*yes*					
**ENSG00000164761**	*yes*			*yes*	*yes*	*yes*
**ENSG00000172179**	*yes*			*yes*	*yes*	
**ENSG00000183878**		*yes*	*yes*			
**ENSG00000157800**		*yes*				
**ENSG00000169413**	*yes*	*yes*				
**ENSG00000146122**	*yes*			*yes*	*yes*	*yes*
**ENSG00000080608**	*yes*					
**ENSG00000130489**			*yes*			*yes*
**ENSG00000170915**			*yes*		*yes*	*yes*
**ENSG00000119917**		*yes*				*yes*
**ENSG00000082781**		*yes*	*yes*	*yes*	*yes*	*yes*
**ENSG00000120860**	*yes*					
**ENSG00000108064**				*yes*	*yes*	
**ENSG00000169045**		*yes*		*yes*		*yes*
**ENSG00000117682**	*yes*			*yes*		*yes*
**ENSG00000131584**						*yes*
**ENSG00000151552**						*yes*
**ENSG00000128699**	*yes*	*yes*	*yes*			
**ENSG00000061676**		*yes*	*yes*	*yes*	*yes*	*yes*
**ENSG00000162385**						*yes*
**ENSG00000186918**	*yes*			*yes*	*yes*	
**ENSG00000146707**			*yes*		*yes*	*yes*
**ENSG00000198131**	*yes*		*yes*	*yes*		*yes*
**ENSG00000070770**			*yes*			*yes*
**ENSG00000108861**				*yes*		
**ENSG00000137947**			*yes*			
**ENSG00000143384**			*yes*			*yes*
**ENSG00000068137**			*yes*			*yes*
**ENSG00000107742**						*yes*
**ENSG00000005448**	*yes*					
**ENSG00000152082**						*yes*
**ENSG00000133574**	*yes*	*yes*	*yes*			
**ENSG00000125398**		*yes*		*yes*	*yes*	
**ENSG00000175283**	*yes*			*yes*		*yes*
**ENSG00000197472**		*yes*	*yes*	*yes*		
**ENSG00000107020**					*yes*	*yes*
**ENSG00000104946**				*yes*		*yes*
**ENSG00000196730**	*yes*			*yes*	*yes*	
**ENSG00000115652**		*yes*	*yes*			
**ENSG00000166147**			*yes*	*yes*	*yes*	*yes*
**ENSG00000118432**	*yes*		*yes*	*yes*	*yes*	*yes*
**ENSG00000167740**					*yes*	
**ENSG00000135925**	*yes*			*yes*	*yes*	
**ENSG00000196226**						*yes*
**ENSG00000184524**	*yes*			*yes*		
**ENSG00000127666**		*yes*	*yes*			
**ENSG00000118292**	*yes*	*yes*				
**ENSG00000185885**	*yes*			*yes*	*yes*	
**ENSG00000148450**		*yes*	*yes*			
**ENSG00000172086**		*yes*	*yes*		*yes*	

**ENSG00000117791**	*yes*		*yes*	*yes*	*yes*	*yes*
**ENSG00000112149**	*yes*	*yes*	*yes*			
**ENSG00000103202**						*yes*
**ENSG00000135829**	*yes*			*yes*	*yes*	
**ENSG00000180902**		*yes*				*yes*
**ENSG00000160593**	*yes*	*yes*	*yes*			
**ENSG00000136404**					*yes*	
**ENSG00000121552**	*yes*	*yes*				*yes*
**ENSG00000176014**				*yes*	*yes*	
**ENSG00000075426**	*yes*	*yes*		*yes*	*yes*	
**ENSG00000130164**					*yes*	*yes*
**ENSG00000148459**	*yes*	*yes*	*yes*			
**ENSG00000095370**	*yes*		*yes*	*yes*		*yes*
**ENSG00000127837**						*yes*
**ENSG00000018699**		*yes*				
**ENSG00000139291**						*yes*
**ENSG00000090104**						*yes*
**ENSG00000228314**						*yes*
**ENSG00000118985**	*yes*					
**ENSG00000058600**			*yes*			
**ENSG00000182718**			*yes*			
**ENSG00000198796**	*yes*	*yes*				*yes*
**ENSG00000136732**	*yes*			*yes*	*yes*	
**ENSG00000099341**				*yes*		*yes*
**ENSG00000226979**			*yes*			
**ENSG00000198855**			*yes*			*yes*
**ENSG00000204475**	*yes*	*yes*				
**ENSG00000145882**						*yes*
**ENSG00000135766**	*yes*					
**ENSG00000155506**						*yes*
**ENSG00000196352**		*yes*	*yes*			
**ENSG00000002587**		*yes*		*yes*		*yes*
**ENSG00000012124**	*yes*	*yes*	*yes*			
**ENSG00000102471**	*yes*			*yes*	*yes*	
**ENSG00000127884**		*yes*				*yes*
**ENSG00000151458**				*yes*	*yes*	
**ENSG00000227826**	*yes*	*yes*	*yes*	*yes*	*yes*	
**ENSG00000184428**	*yes*	*yes*	*yes*			
**ENSG00000125772**			*yes*			
**ENSG00000166401**				*yes*	*yes*	
**ENSG00000100439**	*yes*					
**ENSG00000125726**	*yes*			*yes*	*yes*	
**ENSG00000111537**	*yes*	*yes*			*yes*	*yes*
**ENSG00000169442**	*yes*	*yes*				
**ENSG00000166681**	*yes*	*yes*	*yes*		*yes*	
**ENSG00000120616**						*yes*
**ENSG00000196498**				*yes*		*yes*
**ENSG00000143479**		*yes*				
**ENSG00000183426**	*yes*		*yes*			
**ENSG00000175832**						*yes*
**ENSG00000151882**	*yes*			*yes*		
**ENSG00000166165**	*yes*	*yes*	*yes*	*yes*		*yes*
**ENSG00000113328**				*yes*	*yes*	
**ENSG00000083857**	*yes*			*yes*	*yes*	

**ENSG00000134955**	*yes*		*yes*	*yes*	*yes*	
**ENSG00000188641**				*yes*		*yes*
**ENSG00000161570**	*yes*				*yes*	
**ENSG00000172183**	*yes*				*yes*	*yes*
**ENSG00000101746**	*yes*			*yes*	*yes*	
**ENSG00000091483**	*yes*					
**ENSG00000119408**					*yes*	
**ENSG00000177556**						*yes*
**ENSG00000111358**		*yes*		*yes*		
**ENSG00000161513**		*yes*	*yes*			
**ENSG00000167123**	*yes*					
**ENSG00000185112**					*yes*	
**ENSG00000171729**	*yes*	*yes*		*yes*	*yes*	
**ENSG00000198718**						*yes*
**ENSG00000116711**					*yes*	
**ENSG00000125170**		*yes*				
**ENSG00000047644**						*yes*
**ENSG00000129204**						*yes*
**ENSG00000121064**	*yes*	*yes*	*yes*			
**ENSG00000170558**	*yes*			*yes*	*yes*	
**ENSG00000115183**	*yes*			*yes*	*yes*	
**ENSG00000117724**				*yes*	*yes*	
**ENSG00000116741**	*yes*		*yes*			*yes*
**ENSG00000196588**	*yes*			*yes*		*yes*
**ENSG00000138613**		*yes*		*yes*		*yes*
**ENSG00000134690**	*yes*			*yes*		*yes*
**ENSG00000076716**		*yes*	*yes*	*yes*	*yes*	
**ENSG00000119729**		*yes*		*yes*		*yes*
**ENSG00000135913**						*yes*
**ENSG00000137285**	*yes*	*yes*			*yes*	*yes*
**ENSG00000147324**	*yes*			*yes*	*yes*	
**ENSG00000197223**	*yes*	*yes*	*yes*			
**ENSG00000172927**	*yes*	*yes*	*yes*			
**ENSG00000112425**				*yes*		
**ENSG00000110852**	*yes*			*yes*	*yes*	
**ENSG00000140105**						*yes*
**ENSG00000150907**		*yes*	*yes*	*yes*		*yes*
**ENSG00000166823**		*yes*	*yes*			
**ENSG00000162654**	*yes*			*yes*	*yes*	
**ENSG00000160216**						*yes*
**ENSG00000221955**		*yes*	*yes*	*yes*	*yes*	
**ENSG00000160789**			*yes*			
**ENSG00000144837**	*yes*			*yes*	*yes*	
**ENSG00000179912**	*yes*			*yes*		
**ENSG00000064601**						*yes*
**ENSG00000181523**			*yes*			*yes*
**ENSG00000068097**		*yes*	*yes*			
**ENSG00000167851**	*yes*			*yes*	*yes*	
**ENSG00000181744**						*yes*
**ENSG00000198625**	*yes*			*yes*		
**ENSG00000120594**				*yes*	*yes*	
**ENSG00000100413**			*yes*			
**ENSG00000148399**						*yes*
**ENSG00000151445**			*yes*			

**ENSG00000152465**		*yes*				
**ENSG00000141384**	*yes*					
**ENSG00000186395**						*yes*
**ENSG00000100294**		*yes*				
**ENSG00000129055**					*yes*	*yes*
**ENSG00000117054**		*yes*				
**ENSG00000156313**		*yes*	*yes*	*yes*		
**ENSG00000002822**			*yes*			*yes*
**ENSG00000105193**	*yes*				*yes*	
**ENSG00000185862**	*yes*	*yes*			*yes*	
**ENSG00000115594**	*yes*	*yes*	*yes*	*yes*	*yes*	*yes*
**ENSG00000216490**				*yes*		
**ENSG00000148090**	*yes*			*yes*		
**ENSG00000163040**			*yes*		*yes*	
**ENSG00000105698**	*yes*		*yes*	*yes*		*yes*
**ENSG00000185339**				*yes*	*yes*	
**ENSG00000123131**						*yes*
**ENSG00000049167**				*yes*	*yes*	
**ENSG00000162434**	*yes*				*yes*	
**ENSG00000076924**		*yes*				
**ENSG00000166927**		*yes*	*yes*	*yes*	*yes*	
**ENSG00000164850**	*yes*			*yes*	*yes*	
**ENSG00000106991**				*yes*		
**ENSG00000125810**	*yes*			*yes*	*yes*	
**ENSG00000177169**		*yes*				
**ENSG00000126709**	*yes*			*yes*	*yes*	
**ENSG00000181192**				*yes*		
**ENSG00000142864**						*yes*
**ENSG00000101017**		*yes*				
**ENSG00000182054**		*yes*				*yes*
**ENSG00000111344**		*yes*		*yes*		
**ENSG00000128965**	*yes*	*yes*	*yes*			*yes*
**ENSG00000122884**	*yes*			*yes*	*yes*	
**ENSG00000106009**						*yes*
**ENSG00000176749**	*yes*					
**ENSG00000221869**				*yes*	*yes*	
**ENSG00000152217**		*yes*	*yes*			
**ENSG00000189171**	*yes*	*yes*	*yes*			
**ENSG00000213886**	*yes*					
**ENSG00000108773**						*yes*
**ENSG00000132541**						*yes*
**ENSG00000141510**						*yes*
**ENSG00000067057**	*yes*			*yes*	*yes*	
**ENSG00000204389**		*yes*	*yes*			
**ENSG00000175857**	*yes*	*yes*	*yes*			
**ENSG00000100345**		*yes*		*yes*		
**ENSG00000171843**	*yes*					
**ENSG00000213719**					*yes*	
**ENSG00000180776**	*yes*			*yes*	*yes*	*yes*
**ENSG00000035141**	*yes*			*yes*		
**ENSG00000164897**				*yes*		*yes*
**ENSG00000165487**				*yes*	*yes*	
**ENSG00000109756**	*yes*			*yes*	*yes*	
**ENSG00000128849**					*yes*	

**ENSG00000072310**				*yes*		
**ENSG00000204592**	*yes*	*yes*			*yes*	
**ENSG00000198890**	*yes*					
**ENSG00000170962**				*yes*	*yes*	
**ENSG00000096070**				*yes*		
**ENSG00000102901**						*yes*
**ENSG00000155755**	*yes*			*yes*	*yes*	
**ENSG00000148700**	*yes*	*yes*	*yes*			
**ENSG00000187624**	*yes*	*yes*	*yes*			
**ENSG00000120875**	*yes*		*yes*			
**ENSG00000109113**	*yes*	*yes*			*yes*	*yes*
**ENSG00000134697**			*yes*			
**ENSG00000103426**	*yes*		*yes*	*yes*		*yes*
**ENSG00000064932**						*yes*
**ENSG00000154310**				*yes*		
**ENSG00000196576**						*yes*
**ENSG00000196532**			*yes*		*yes*	*yes*
**ENSG00000128228**			*yes*			*yes*
**ENSG00000178127**						*yes*
**ENSG00000100941**			*yes*		*yes*	
**ENSG00000183955**						*yes*
**ENSG00000131089**				*yes*	*yes*	
**ENSG00000075292**						*yes*
**ENSG00000119321**						*yes*
**ENSG00000150991**					*yes*	
**ENSG00000083750**					*yes*	
**ENSG00000130402**						*yes*
**ENSG00000182108**						*yes*
**ENSG00000111540**			*yes*			*yes*
**ENSG00000148110**	*yes*	*yes*	*yes*			
**ENSG00000172915**					*yes*	
**ENSG00000186807**		*yes*		*yes*	*yes*	
**ENSG00000110848**	*yes*		*yes*	*yes*		*yes*
**ENSG00000197136**						*yes*
**ENSG00000123689**		*yes*	*yes*	*yes*	*yes*	
**ENSG00000163516**	*yes*			*yes*	*yes*	
**ENSG00000134463**						*yes*
**ENSG00000139192**						*yes*
**ENSG00000158555**	*yes*				*yes*	
**ENSG00000130066**	*yes*			*yes*	*yes*	
**ENSG00000116761**			*yes*			
**ENSG00000188636**	*yes*	*yes*	*yes*			
**ENSG00000214655**		*yes*				
**ENSG00000145703**	*yes*			*yes*	*yes*	
**ENSG00000185518**	*yes*			*yes*	*yes*	
**ENSG00000120802**	*yes*			*yes*	*yes*	
**ENSG00000185262**				*yes*	*yes*	
**ENSG00000243477**						*yes*
**ENSG00000109452**	*yes*			*yes*	*yes*	
**ENSG00000102580**						*yes*
**ENSG00000171540**				*yes*	*yes*	
**ENSG00000060069**						*yes*
**ENSG00000040633**						*yes*
**ENSG00000105640**						*yes*

**ENSG00000099991**						*yes*
**ENSG00000133321**				*yes*	*yes*	
**ENSG00000111275**			*yes*			
**ENSG00000115073**				*yes*		
**ENSG00000121413**		*yes*				*yes*
**ENSG00000171861**						*yes*
**ENSG00000167617**		*yes*	*yes*	*yes*	*yes*	*yes*
**ENSG00000091317**	*yes*			*yes*		
**ENSG00000077232**					*yes*	
**ENSG00000148339**	*yes*					
**ENSG00000154040**	*yes*	*yes*		*yes*	*yes*	
**ENSG00000135842**	*yes*	*yes*	*yes*	*yes*	*yes*	
**ENSG00000139117**	*yes*	*yes*	*yes*	*yes*	*yes*	
**ENSG00000061656**	*yes*			*yes*	*yes*	
**ENSG00000189050**				*yes*		
**ENSG00000188042**	*yes*			*yes*	*yes*	
**ENSG00000175567**			*yes*			*yes*
**ENSG00000140995**				*yes*		*yes*
**ENSG00000172716**				*yes*		
**ENSG00000137571**	*yes*		*yes*			*yes*
**ENSG00000116014**	*yes*			*yes*	*yes*	
**ENSG00000178951**	*yes*			*yes*		
**ENSG00000162896**	*yes*	*yes*	*yes*			*yes*
**ENSG00000129636**						*yes*
**ENSG00000176401**			*yes*			
**ENSG00000197635**	*yes*					
**ENSG00000196072**		*yes*				
**ENSG00000126246**	*yes*			*yes*	*yes*	
**ENSG00000221886**						*yes*
**ENSG00000198034**						*yes*
**ENSG00000171612**	*yes*		*yes*			
**ENSG00000100592**			*yes*			*yes*
**ENSG00000037897**			*yes*			*yes*
**ENSG00000102445**			*yes*			*yes*
**ENSG00000162545**		*yes*				*yes*
**ENSG00000110237**	*yes*			*yes*		
**ENSG00000174903**						*yes*
**ENSG00000138744**				*yes*		
**ENSG00000181577**						*yes*
**ENSG00000197050**		*yes*				
**ENSG00000125534**		*yes*	*yes*			*yes*
**ENSG00000117408**						*yes*
**ENSG00000138172**						*yes*
**ENSG00000184825**		*yes*				*yes*
**ENSG00000168016**	*yes*			*yes*		
**ENSG00000162924**		*yes*	*yes*			
**ENSG00000173917**				*yes*	*yes*	
**ENSG00000220205**			*yes*			*yes*
**ENSG00000109321**				*yes*		*yes*
**ENSG00000102981**		*yes*				
**ENSG00000117410**						*yes*
**ENSG00000144724**					*yes*	
**ENSG00000131788**		*yes*				
**ENSG00000165502**		*yes*				

**ENSG00000123610**		*yes*				*yes*
**ENSG00000120053**	*yes*					
**ENSG00000159147**	*yes*			*yes*	*yes*	
**ENSG00000078081**						*yes*
**ENSG00000188130**						*yes*
**ENSG00000105655**				*yes*		*yes*
**ENSG00000141994**	*yes*		*yes*			*yes*
**ENSG00000172270**			*yes*			*yes*
**ENSG00000179119**			*yes*			
**ENSG00000136560**	*yes*					
**ENSG00000163071**	*yes*		*yes*	*yes*	*yes*	*yes*
**ENSG00000146416**		*yes*	*yes*	*yes*	*yes*	*yes*
**ENSG00000120539**					*yes*	*yes*
**ENSG00000196636**		*yes*				
**ENSG00000175215**		*yes*				
**ENSG00000147862**					*yes*	
**ENSG00000177606**			*yes*			
**ENSG00000183726**					*yes*	
**ENSG00000203813**		*yes*				
**ENSG00000132434**	*yes*			*yes*	*yes*	
**ENSG00000125910**	*yes*	*yes*	*yes*	*yes*		*yes*
**ENSG00000148444**		*yes*				
**ENSG00000214517**				*yes*		*yes*
**ENSG00000213516**	*yes*		*yes*			
**ENSG00000074370**	*yes*					
**ENSG00000175606**	*yes*			*yes*	*yes*	
**ENSG00000182551**		*yes*	*yes*			
**ENSG00000151779**				*yes*		*yes*
**ENSG00000161677**						*yes*
**ENSG00000167552**					*yes*	
**ENSG00000237441**			*yes*			
**ENSG00000146242**		*yes*	*yes*	*yes*	*yes*	
**ENSG00000181035**		*yes*	*yes*			
**ENSG00000186889**						*yes*
**ENSG00000175115**			*yes*			*yes*
**ENSG00000105559**				*yes*		
**ENSG00000188486**				*yes*		*yes*
**ENSG00000135686**		*yes*				
**ENSG00000100116**		*yes*	*yes*			
**ENSG00000073756**				*yes*		
**ENSG00000182389**	*yes*			*yes*	*yes*	*yes*
**ENSG00000166831**	*yes*	*yes*	*yes*			*yes*
**ENSG00000160959**				*yes*	*yes*	
**ENSG00000105229**						*yes*
**ENSG00000113742**	*yes*			*yes*	*yes*	
**ENSG00000004975**						*yes*
**ENSG00000027869**	*yes*		*yes*	*yes*	*yes*	*yes*
**ENSG00000126391**				*yes*		*yes*
**ENSG00000124299**						*yes*
**ENSG00000152128**				*yes*	*yes*	
**ENSG00000171766**		*yes*	*yes*	*yes*		*yes*
**ENSG00000133104**		*yes*				
**ENSG00000164930**			*yes*	*yes*	*yes*	
**ENSG00000004059**						*yes*

**ENSG00000152749**		*yes*				
**ENSG00000148516**	*yes*			*yes*	*yes*	
**ENSG00000162817**	*yes*	*yes*		*yes*	*yes*	*yes*
**ENSG00000133454**	*yes*	*yes*			*yes*	*yes*
**ENSG00000039523**	*yes*			*yes*		*yes*
**ENSG00000145908**	*yes*		*yes*	*yes*		*yes*
**ENSG00000248541**			*yes*		*yes*	*yes*
**ENSG00000110944**		*yes*		*yes*		
**ENSG00000091972**	*yes*		*yes*	*yes*	*yes*	*yes*
**ENSG00000172466**	*yes*		*yes*			
**ENSG00000146859**	*yes*			*yes*	*yes*	
**ENSG00000130813**		*yes*				
**ENSG00000119599**		*yes*	*yes*			
**ENSG00000187325**	*yes*			*yes*	*yes*	*yes*
**ENSG00000137936**	*yes*	*yes*		*yes*	*yes*	*yes*
**ENSG00000117479**	*yes*					
**ENSG00000168421**	*yes*					
**ENSG00000170955**	*yes*			*yes*	*yes*	
**ENSG00000089820**		*yes*				*yes*
**ENSG00000158517**	*yes*	*yes*				
**ENSG00000135046**		*yes*				*yes*
**ENSG00000121083**						*yes*
**ENSG00000025708**		*yes*		*yes*	*yes*	
**ENSG00000059378**		*yes*		*yes*	*yes*	
**ENSG00000140379**			*yes*			
**ENSG00000168899**	*yes*			*yes*	*yes*	
**ENSG00000171865**						*yes*
**ENSG00000072818**	*yes*		*yes*	*yes*		*yes*
**ENSG00000175048**	*yes*	*yes*	*yes*			
**ENSG00000061918**		*yes*	*yes*	*yes*		
**ENSG00000091129**	*yes*			*yes*	*yes*	
**ENSG00000171467**						*yes*
**ENSG00000132005**						*yes*
**ENSG00000179588**			*yes*			*yes*
**ENSG00000141424**	*yes*			*yes*		
**ENSG00000112306**		*yes*				*yes*
**ENSG00000155093**	*yes*	*yes*		*yes*	*yes*	
**ENSG00000160584**				*yes*		
**ENSG00000196793**		*yes*	*yes*			
**ENSG00000090054**	*yes*	*yes*	*yes*			
**ENSG00000140400**	*yes*			*yes*		*yes*
**ENSG00000127418**		*yes*	*yes*	*yes*	*yes*	
**ENSG00000188315**	*yes*					
**ENSG00000167895**	*yes*					
**ENSG00000143614**			*yes*			*yes*
**ENSG00000146674**	*yes*			*yes*	*yes*	
**ENSG00000051620**	*yes*	*yes*	*yes*	*yes*	*yes*	
**ENSG00000130204**		*yes*				
**ENSG00000185404**			*yes*			*yes*
**ENSG00000071889**						*yes*
**ENSG00000141753**		*yes*		*yes*		*yes*
**ENSG00000137710**						*yes*
**ENSG00000138297**	*yes*		*yes*			
**ENSG00000130956**	*yes*	*yes*	*yes*			

**ENSG00000105397**						*yes*
**ENSG00000127423**	*yes*					
**ENSG00000132361**	*yes*		*yes*			
**ENSG00000103932**				*yes*		
**ENSG00000174871**	*yes*				*yes*	
**ENSG00000026297**	*yes*			*yes*	*yes*	*yes*
**ENSG00000110876**	*yes*			*yes*	*yes*	
**ENSG00000179603**		*yes*	*yes*			
**ENSG00000154153**	*yes*			*yes*		*yes*
**ENSG00000198087**						*yes*
**ENSG00000181788**		*yes*	*yes*	*yes*	*yes*	
**ENSG00000157224**				*yes*		
**ENSG00000126705**						*yes*
**ENSG00000111863**	*yes*	*yes*				
**ENSG00000042493**		*yes*		*yes*		*yes*
**ENSG00000174083**	*yes*			*yes*		*yes*
**ENSG00000162373**	*yes*			*yes*	*yes*	
**ENSG00000125753**						*yes*
**ENSG00000176788**	*yes*			*yes*	*yes*	
**ENSG00000105849**			*yes*			
**ENSG00000187699**		*yes*	*yes*			
**ENSG00000148655**				*yes*	*yes*	
**ENSG00000100473**	*yes*		*yes*	*yes*		
**ENSG00000197061**					*yes*	*yes*
**ENSG00000112294**	*yes*			*yes*	*yes*	
**ENSG00000112406**		*yes*				
**ENSG00000154319**	*yes*	*yes*	*yes*		*yes*	*yes*
**ENSG00000242185**	*yes*	*yes*		*yes*	*yes*	*yes*
**ENSG00000196189**		*yes*	*yes*			
**ENSG00000067533**	*yes*		*yes*			
**ENSG00000136280**	*yes*	*yes*				*yes*
**ENSG00000134717**						*yes*
**ENSG00000056736**			*yes*		*yes*	*yes*
**ENSG00000090520**						*yes*
**ENSG00000033867**		*yes*		*yes*		*yes*
**ENSG00000142684**			*yes*			
**ENSG00000184368**	*yes*		*yes*	*yes*		*yes*
**ENSG00000169692**					*yes*	
**ENSG00000160179**		*yes*	*yes*	*yes*	*yes*	*yes*
**ENSG00000168517**		*yes*				
**ENSG00000107281**				*yes*	*yes*	
**ENSG00000183742**		*yes*	*yes*			
**ENSG00000083828**		*yes*				
**ENSG00000060339**				*yes*	*yes*	
**ENSG00000111843**			*yes*			
**ENSG00000180357**						*yes*
**ENSG00000196968**	*yes*			*yes*		
**ENSG00000092607**			*yes*		*yes*	*yes*
**ENSG00000187735**		*yes*	*yes*			
**ENSG00000088038**						*yes*
**ENSG00000102554**				*yes*	*yes*	
**ENSG00000128928**		*yes*				
**ENSG00000127125**						*yes*
**ENSG00000213722**				*yes*		

**ENSG00000143624**	*yes*			*yes*		*yes*
**ENSG00000143185**			*yes*	*yes*	*yes*	*yes*
**ENSG00000148926**	*yes*			*yes*	*yes*	*yes*
**ENSG00000248098**				*yes*		*yes*
**ENSG00000151327**	*yes*	*yes*				
**ENSG00000091542**	*yes*	*yes*				
**ENSG00000163378**	*yes*			*yes*	*yes*	
**ENSG00000127989**			*yes*			
**ENSG00000176225**						*yes*
**ENSG00000198825**				*yes*	*yes*	
**ENSG00000115946**	*yes*	*yes*	*yes*			
**ENSG00000115902**		*yes*		*yes*	*yes*	
**ENSG00000067082**	*yes*			*yes*	*yes*	
**ENSG00000089195**	*yes*		*yes*	*yes*		*yes*
**ENSG00000171163**				*yes*		*yes*
**ENSG00000157303**	*yes*			*yes*	*yes*	
**ENSG00000120868**	*yes*		*yes*	*yes*		*yes*
**ENSG00000187134**					*yes*	
**ENSG00000119471**					*yes*	*yes*
**ENSG00000168374**			*yes*			
**ENSG00000183963**						*yes*
**ENSG00000197019**			*yes*			
**ENSG00000089116**		*yes*		*yes*		*yes*
**ENSG00000116560**	*yes*			*yes*		
**ENSG00000142192**	*yes*			*yes*	*yes*	
**ENSG00000145050**						*yes*
**ENSG00000138160**				*yes*		
**ENSG00000181458**	*yes*			*yes*	*yes*	
**ENSG00000177030**						*yes*
**ENSG00000132965**	*yes*			*yes*	*yes*	
**ENSG00000132635**	*yes*					
**ENSG00000171793**	*yes*					
**ENSG00000142655**						*yes*
**ENSG00000158615**			*yes*			
**ENSG00000106689**	*yes*		*yes*	*yes*	*yes*	*yes*
**ENSG00000138640**						*yes*
**ENSG00000114200**	*yes*				*yes*	
**ENSG00000136286**	*yes*			*yes*	*yes*	*yes*
**ENSG00000136877**				*yes*		*yes*
**ENSG00000133874**	*yes*	*yes*				*yes*
**ENSG00000100298**		*yes*	*yes*			
**ENSG00000130812**	*yes*			*yes*	*yes*	*yes*
**ENSG00000131002**		*yes*	*yes*			
**ENSG00000157654**				*yes*	*yes*	
**ENSG00000139926**		*yes*	*yes*	*yes*	*yes*	
**ENSG00000114268**	*yes*			*yes*	*yes*	
**ENSG00000142541**		*yes*				*yes*
**ENSG00000074842**						*yes*
**ENSG00000184117**						*yes*
**ENSG00000120942**						*yes*
**ENSG00000101161**	*yes*	*yes*	*yes*			
**ENSG00000135736**				*yes*	*yes*	
**ENSG00000160271**				*yes*		*yes*
**ENSG00000144642**	*yes*				*yes*	

**ENSG00000168916**			*yes*			*yes*
**ENSG00000164002**						*yes*
**ENSG00000167562**		*yes*	*yes*			
**ENSG00000124529**			*yes*		*yes*	*yes*
**ENSG00000117228**				*yes*	*yes*	
**ENSG00000159176**						*yes*
**ENSG00000108479**				*yes*		
**ENSG00000104325**		*yes*				
**ENSG00000158089**		*yes*				
**ENSG00000138823**		*yes*		*yes*		
**ENSG00000163002**	*yes*	*yes*	*yes*			
**ENSG00000147874**	*yes*					
**ENSG00000196365**				*yes*		*yes*
**ENSG00000102524**						*yes*
**ENSG00000175826**	*yes*			*yes*	*yes*	
**ENSG00000197586**				*yes*		
**ENSG00000067248**	*yes*					
**ENSG00000244405**	*yes*			*yes*	*yes*	
**ENSG00000012817**	*yes*	*yes*	*yes*			
**ENSG00000173085**			*yes*			
**ENSG00000106701**	*yes*			*yes*	*yes*	
**ENSG00000175029**	*yes*			*yes*	*yes*	
**ENSG00000111729**	*yes*	*yes*	*yes*			
**ENSG00000175040**				*yes*		
**ENSG00000137673**		*yes*	*yes*	*yes*	*yes*	*yes*
**ENSG00000164116**		*yes*	*yes*	*yes*		
**ENSG00000106789**	*yes*	*yes*	*yes*			
**ENSG00000204852**	*yes*				*yes*	
**ENSG00000029725**	*yes*			*yes*	*yes*	
**ENSG00000106538**	*yes*	*yes*			*yes*	*yes*
**ENSG00000052749**	*yes*	*yes*	*yes*			
**ENSG00000164691**	*yes*					
**ENSG00000183558**	*yes*			*yes*	*yes*	
**ENSG00000198431**	*yes*		*yes*			*yes*
**ENSG00000183688**	*yes*				*yes*	
**ENSG00000130508**	*yes*			*yes*	*yes*	*yes*
**ENSG00000214063**		*yes*				
**ENSG00000108829**			*yes*			
**ENSG00000115657**				*yes*		
**ENSG00000187840**		*yes*				
**ENSG00000213977**			*yes*			
**ENSG00000182979**						*yes*
**ENSG00000213930**	*yes*					
**ENSG00000111880**	*yes*	*yes*	*yes*			
**ENSG00000090971**						*yes*
**ENSG00000170385**			*yes*			*yes*
**ENSG00000184867**						*yes*
**ENSG00000137040**	*yes*		*yes*			
**ENSG00000184009**	*yes*	*yes*				
**ENSG00000134184**	*yes*	*yes*	*yes*	*yes*		*yes*
**ENSG00000072694**			*yes*			
**ENSG00000198356**						*yes*
**ENSG00000050820**			*yes*			*yes*
**ENSG00000130844**				*yes*	*yes*	

**ENSG00000162368**			*yes*		*yes*	
**ENSG00000164442**					*yes*	
**ENSG00000137875**		*yes*				
**ENSG00000179041**	*yes*		*yes*			
**ENSG00000165389**						*yes*
**ENSG00000129824**	*yes*	*yes*	*yes*			
**ENSG00000127481**						*yes*
**ENSG00000213780**						*yes*
**ENSG00000182552**						*yes*
**ENSG00000179886**				*yes*		*yes*
**ENSG00000136536**	*yes*			*yes*		
**ENSG00000072849**						*yes*
**ENSG00000108433**			*yes*			
**ENSG00000172432**			*yes*			*yes*
**ENSG00000155542**	*yes*			*yes*	*yes*	
**ENSG00000116918**				*yes*		
**ENSG00000158050**	*yes*			*yes*	*yes*	
**ENSG00000080824**	*yes*		*yes*			
**ENSG00000211456**		*yes*	*yes*			
**ENSG00000179715**		*yes*	*yes*			
**ENSG00000165782**				*yes*		*yes*
**ENSG00000169403**		*yes*	*yes*			
**ENSG00000134779**			*yes*			
**ENSG00000137168**	*yes*					
**ENSG00000129028**				*yes*	*yes*	
**ENSG00000120738**	*yes*		*yes*	*yes*	*yes*	*yes*
**ENSG00000185418**				*yes*		
**ENSG00000183828**	*yes*			*yes*		*yes*
**ENSG00000105438**	*yes*			*yes*		*yes*
**ENSG00000171700**	*yes*	*yes*				
**ENSG00000105825**		*yes*				*yes*
**ENSG00000132465**	*yes*			*yes*	*yes*	*yes*
**ENSG00000225697**	*yes*			*yes*		*yes*
**ENSG00000100596**			*yes*			
**ENSG00000140391**	*yes*				*yes*	
**ENSG00000112727**			*yes*		*yes*	*yes*
**ENSG00000107537**	*yes*	*yes*	*yes*			
**ENSG00000133142**					*yes*	
**ENSG00000140553**				*yes*		
**ENSG00000107485**		*yes*				*yes*
**ENSG00000019582**			*yes*			
**ENSG00000138032**	*yes*			*yes*	*yes*	
**ENSG00000167703**						*yes*
**ENSG00000160953**			*yes*			*yes*
**ENSG00000184575**	*yes*		*yes*			
**ENSG00000100348**		*yes*				
**ENSG00000163938**			*yes*			
**ENSG00000113657**			*yes*			
**ENSG00000133275**						*yes*
**ENSG00000135074**		*yes*	*yes*			
**ENSG00000116005**						*yes*
**ENSG00000106733**		*yes*	*yes*			
**ENSG00000101544**	*yes*	*yes*	*yes*			
**ENSG00000170345**						*yes*

**ENSG00000140368**	*yes*		*yes*	*yes*		*yes*
**ENSG00000016391**						*yes*
**ENSG00000171813**				*yes*		
**ENSG00000076003**	*yes*					
**ENSG00000151651**				*yes*		*yes*
**ENSG00000152804**		*yes*		*yes*		
**ENSG00000186998**	*yes*	*yes*	*yes*			
**ENSG00000180879**						*yes*
**ENSG00000136237**			*yes*			
**ENSG00000142583**	*yes*	*yes*	*yes*	*yes*	*yes*	
**ENSG00000108395**					*yes*	
**ENSG00000175643**		*yes*	*yes*	*yes*	*yes*	
**ENSG00000138755**			*yes*		*yes*	
**ENSG00000134762**	*yes*			*yes*	*yes*	
**ENSG00000001084**	*yes*			*yes*	*yes*	
**ENSG00000143891**				*yes*	*yes*	
**ENSG00000168395**	*yes*		*yes*			
**ENSG00000164080**						*yes*
**ENSG00000133116**		*yes*	*yes*		*yes*	
**ENSG00000178773**				*yes*		
**ENSG00000164163**	*yes*		*yes*			
**ENSG00000165732**	*yes*		*yes*			
**ENSG00000115526**	*yes*	*yes*				
**ENSG00000136783**						*yes*
**ENSG00000103507**	*yes*			*yes*		*yes*
**ENSG00000131446**	*yes*					
**ENSG00000139263**	*yes*	*yes*		*yes*	*yes*	*yes*
**ENSG00000187837**				*yes*	*yes*	
**ENSG00000107959**	*yes*			*yes*	*yes*	*yes*
**ENSG00000159256**	*yes*				*yes*	
**ENSG00000119906**						*yes*
**ENSG00000165996**	*yes*			*yes*	*yes*	*yes*
**ENSG00000158716**	*yes*	*yes*			*yes*	*yes*
**ENSG00000172992**			*yes*			*yes*
**ENSG00000175183**				*yes*		*yes*
**ENSG00000154917**						*yes*
**ENSG00000173540**						*yes*
**ENSG00000135245**	*yes*		*yes*	*yes*	*yes*	*yes*
**ENSG00000143494**	*yes*			*yes*	*yes*	
**ENSG00000128309**			*yes*			
**ENSG00000109654**						*yes*
**ENSG00000100605**				*yes*		*yes*
**ENSG00000126947**	*yes*					
**ENSG00000167136**			*yes*			
**ENSG00000149218**	*yes*		*yes*	*yes*	*yes*	
**ENSG00000197903**			*yes*	*yes*	*yes*	*yes*
**ENSG00000227500**			*yes*			
**ENSG00000080503**	*yes*		*yes*	*yes*	*yes*	
**ENSG00000073282**	*yes*			*yes*		
**ENSG00000184937**		*yes*				
**ENSG00000177494**			*yes*			
**ENSG00000104055**	*yes*	*yes*	*yes*	*yes*	*yes*	
**ENSG00000135069**	*yes*		*yes*			
**ENSG00000111490**				*yes*	*yes*	

**ENSG00000157110**			*yes*	*yes*		*yes*
**ENSG00000134986**						*yes*
**ENSG00000234745**	*yes*					
**ENSG00000138777**	*yes*			*yes*	*yes*	
**ENSG00000130158**	*yes*			*yes*		
**ENSG00000182752**						*yes*
**ENSG00000079387**	*yes*					
**ENSG00000144476**	*yes*	*yes*	*yes*		*yes*	*yes*
**ENSG00000139832**	*yes*			*yes*	*yes*	
**ENSG00000169554**	*yes*				*yes*	
**ENSG00000158373**			*yes*			*yes*
**ENSG00000187764**	*yes*					
**ENSG00000198900**	*yes*					
**ENSG00000116044**		*yes*	*yes*			
**ENSG00000168763**	*yes*			*yes*		
**ENSG00000071127**				*yes*		
**ENSG00000129932**			*yes*			*yes*
**ENSG00000133243**		*yes*				
**ENSG00000167996**					*yes*	
**ENSG00000164056**	*yes*		*yes*	*yes*		*yes*
**ENSG00000092036**	*yes*					
**ENSG00000136859**	*yes*			*yes*		
**ENSG00000138411**	*yes*	*yes*	*yes*	*yes*	*yes*	*yes*
**ENSG00000196586**			*yes*			*yes*
**ENSG00000133398**			*yes*			*yes*
**ENSG00000099817**		*yes*	*yes*		*yes*	
**ENSG00000150995**				*yes*		
**ENSG00000137491**		*yes*				
**ENSG00000111752**					*yes*	*yes*
**ENSG00000110713**	*yes*		*yes*			
**ENSG00000101079**						*yes*
**ENSG00000214897**			*yes*			*yes*
**ENSG00000183856**						*yes*
**ENSG00000173905**			*yes*			
**ENSG00000141446**		*yes*				
**ENSG00000074706**	*yes*		*yes*	*yes*		*yes*
**ENSG00000100644**	*yes*			*yes*	*yes*	
**ENSG00000142102**	*yes*		*yes*	*yes*		
**ENSG00000139372**	*yes*					
**ENSG00000008516**					*yes*	
**ENSG00000169220**						*yes*
**ENSG00000183741**						*yes*
**ENSG00000031691**		*yes*				
**ENSG00000173198**				*yes*	*yes*	
**ENSG00000198873**	*yes*			*yes*	*yes*	
**ENSG00000143641**		*yes*				
**ENSG00000163069**	*yes*			*yes*	*yes*	
**ENSG00000153029**		*yes*				
**ENSG00000119878**		*yes*	*yes*			
**ENSG00000121858**	*yes*			*yes*	*yes*	
**ENSG00000198374**						*yes*
**ENSG00000239713**					*yes*	
**ENSG00000163872**	*yes*		*yes*	*yes*		*yes*
**ENSG00000089127**	*yes*	*yes*	*yes*	*yes*	*yes*	*yes*

**ENSG00000172590**				*yes*		
**ENSG00000153814**			*yes*			
**ENSG00000112773**		*yes*	*yes*			
**ENSG00000118939**				*yes*		
**ENSG00000243364**				*yes*		
**ENSG00000182087**				*yes*		
**ENSG00000076201**						*yes*
**ENSG00000144655**				*yes*	*yes*	
**ENSG00000090372**						*yes*
**ENSG00000005844**	*yes*			*yes*	*yes*	
**ENSG00000160741**						*yes*
**ENSG00000100201**	*yes*	*yes*	*yes*			
**ENSG00000075643**		*yes*	*yes*		*yes*	
**ENSG00000112303**				*yes*		
**ENSG00000143184**			*yes*	*yes*	*yes*	*yes*
**ENSG00000127947**	*yes*			*yes*		
**ENSG00000166557**						*yes*
**ENSG00000168488**	*yes*			*yes*		*yes*
**ENSG00000206418**			*yes*			*yes*
**ENSG00000138190**		*yes*		*yes*	*yes*	
**ENSG00000185811**						*yes*
**ENSG00000089775**	*yes*			*yes*	*yes*	
**ENSG00000162433**			*yes*			
**ENSG00000196177**	*yes*			*yes*	*yes*	
**ENSG00000163297**	*yes*	*yes*	*yes*			
**ENSG00000071564**			*yes*			*yes*
**ENSG00000104312**		*yes*	*yes*			
**ENSG00000168906**			*yes*			*yes*
**ENSG00000163568**	*yes*	*yes*		*yes*	*yes*	*yes*
**ENSG00000204569**		*yes*				
**ENSG00000163864**		*yes*	*yes*			
**ENSG00000083097**						*yes*
**ENSG00000128973**		*yes*		*yes*		*yes*
**ENSG00000128604**			*yes*			*yes*
**ENSG00000176974**		*yes*				
**ENSG00000159618**	*yes*	*yes*		*yes*	*yes*	
**ENSG00000074696**	*yes*			*yes*	*yes*	
**ENSG00000173674**	*yes*					
**ENSG00000120949**						*yes*
**ENSG00000112561**			*yes*			*yes*
**ENSG00000103145**				*yes*		
**ENSG00000156876**		*yes*				
**ENSG00000198551**						*yes*
**ENSG00000136840**						*yes*
**ENSG00000137642**			*yes*			*yes*
**ENSG00000126243**		*yes*	*yes*			
**ENSG00000196507**					*yes*	
**ENSG00000145414**		*yes*				*yes*
**ENSG00000186810**	*yes*	*yes*			*yes*	*yes*
**ENSG00000068489**		*yes*				*yes*
**ENSG00000114978**	*yes*			*yes*	*yes*	
**ENSG00000121957**		*yes*				
**ENSG00000198477**						*yes*
**ENSG00000134755**	*yes*			*yes*	*yes*	

**ENSG00000240505**	*yes*			*yes*	*yes*	
**ENSG00000171234**		*yes*	*yes*	*yes*	*yes*	
**ENSG00000130479**		*yes*		*yes*		*yes*
**ENSG00000064651**	*yes*					
**ENSG00000178403**	*yes*	*yes*		*yes*	*yes*	
**ENSG00000197776**			*yes*			
**ENSG00000170412**	*yes*			*yes*	*yes*	
**ENSG00000204388**		*yes*	*yes*	*yes*		
**ENSG00000111674**	*yes*			*yes*	*yes*	
**ENSG00000163041**			*yes*			*yes*
**ENSG00000186891**		*yes*	*yes*	*yes*	*yes*	*yes*
**ENSG00000111670**		*yes*				
**ENSG00000171763**		*yes*	*yes*			
**ENSG00000104765**	*yes*					
**ENSG00000179598**		*yes*	*yes*		*yes*	*yes*
**ENSG00000116641**						*yes*
**ENSG00000156170**	*yes*				*yes*	
**ENSG00000178163**		*yes*				
**ENSG00000180354**						*yes*
**ENSG00000112796**						*yes*
**ENSG00000197006**						*yes*
**ENSG00000141985**			*yes*	*yes*		*yes*
**ENSG00000152795**	*yes*					
**ENSG00000180626**					*yes*	*yes*
**ENSG00000136040**		*yes*				
**ENSG00000131116**		*yes*		*yes*		
**ENSG00000171659**				*yes*	*yes*	
**ENSG00000065911**		*yes*	*yes*			
**ENSG00000197705**					*yes*	
**ENSG00000112964**	*yes*			*yes*	*yes*	
**ENSG00000188763**		*yes*	*yes*			
**ENSG00000168067**		*yes*				
**ENSG00000099804**						*yes*
**ENSG00000166986**						*yes*
**ENSG00000092978**	*yes*				*yes*	
**ENSG00000172164**	*yes*	*yes*	*yes*			
**ENSG00000163666**		*yes*				
**ENSG00000223496**	*yes*					
**ENSG00000124693**			*yes*			*yes*
**ENSG00000145936**	*yes*	*yes*				
**ENSG00000110851**			*yes*			*yes*
**ENSG00000180616**		*yes*				*yes*
**ENSG00000160299**						*yes*
**ENSG00000110651**		*yes*				*yes*
**ENSG00000178150**					*yes*	
**ENSG00000131844**	*yes*					
**ENSG00000102265**					*yes*	
**ENSG00000113108**	*yes*			*yes*		
**ENSG00000060709**	*yes*			*yes*	*yes*	
**ENSG00000127074**		*yes*	*yes*	*yes*	*yes*	*yes*
**ENSG00000104490**		*yes*	*yes*	*yes*	*yes*	*yes*
**ENSG00000169230**		*yes*	*yes*			
**ENSG00000142409**	*yes*		*yes*	*yes*	*yes*	*yes*
**ENSG00000115368**		*yes*				

**ENSG00000165175**	*yes*			*yes*		*yes*
**ENSG00000099308**	*yes*		*yes*	*yes*		*yes*
**ENSG00000129667**	*yes*			*yes*	*yes*	
**ENSG00000169435**				*yes*		
**ENSG00000159556**	*yes*		*yes*			*yes*
**ENSG00000164414**			*yes*			
**ENSG00000164674**	*yes*			*yes*	*yes*	
**ENSG00000105928**		*yes*	*yes*	*yes*	*yes*	
**ENSG00000140044**		*yes*				
**ENSG00000127863**	*yes*		*yes*	*yes*	*yes*	*yes*
**ENSG00000148840**	*yes*		*yes*			
**ENSG00000059122**						*yes*
**ENSG00000137094**					*yes*	*yes*
**ENSG00000186431**				*yes*		*yes*
**ENSG00000114993**	*yes*			*yes*	*yes*	*yes*
**ENSG00000137216**						*yes*
**ENSG00000010292**	*yes*	*yes*	*yes*			
**ENSG00000179958**			*yes*			
**ENSG00000154146**	*yes*			*yes*		
**ENSG00000173110**		*yes*		*yes*		*yes*
**ENSG00000164934**	*yes*					
**ENSG00000130193**						*yes*
**ENSG00000133943**			*yes*			
**ENSG00000189319**	*yes*			*yes*	*yes*	
**ENSG00000135316**	*yes*			*yes*		
**ENSG00000170231**					*yes*	*yes*
**ENSG00000125459**	*yes*		*yes*	*yes*		*yes*
**ENSG00000151929**				*yes*	*yes*	
**ENSG00000176165**	*yes*			*yes*	*yes*	
**ENSG00000047634**	*yes*		*yes*			*yes*
**ENSG00000106080**						*yes*
**ENSG00000160113**		*yes*	*yes*			
**ENSG00000215440**		*yes*	*yes*	*yes*		
**ENSG00000132912**		*yes*				
**ENSG00000189409**		*yes*	*yes*			
**ENSG00000165030**	*yes*			*yes*	*yes*	
**ENSG00000164221**		*yes*	*yes*	*yes*	*yes*	
**ENSG00000185163**		*yes*				
**ENSG00000125245**		*yes*				*yes*
**ENSG00000154328**				*yes*		
**ENSG00000116679**	*yes*		*yes*	*yes*		*yes*
**ENSG00000153283**	*yes*		*yes*			
**ENSG00000155115**				*yes*		
**ENSG00000197846**			*yes*		*yes*	*yes*
**ENSG00000141682**			*yes*			
**ENSG00000140332**	*yes*			*yes*		
**ENSG00000197905**	*yes*		*yes*	*yes*		*yes*
**ENSG00000110921**						*yes*
**ENSG00000183337**				*yes*	*yes*	
**ENSG00000174500**		*yes*	*yes*	*yes*	*yes*	
**ENSG00000178078**					*yes*	
**ENSG00000132329**						*yes*
**ENSG00000115457**		*yes*	*yes*	*yes*	*yes*	
**ENSG00000169660**	*yes*			*yes*	*yes*	

**ENSG00000117289**	*yes*	*yes*	*yes*		*yes*	*yes*
**ENSG00000169925**						*yes*
**ENSG00000049249**		*yes*				
**ENSG00000065833**	*yes*	*yes*	*yes*	*yes*	*yes*	
**ENSG00000204371**						*yes*
**ENSG00000137731**		*yes*				
**ENSG00000181061**	*yes*					
**ENSG00000179262**						*yes*
**ENSG00000204463**				*yes*		*yes*
**ENSG00000102786**	*yes*		*yes*			
**ENSG00000057019**	*yes*			*yes*	*yes*	
**ENSG00000090097**			*yes*			*yes*
**ENSG00000104331**						*yes*
**ENSG00000147443**				*yes*	*yes*	
**ENSG00000120899**	*yes*		*yes*	*yes*		*yes*
**ENSG00000104774**						*yes*
**ENSG00000160284**	*yes*	*yes*	*yes*	*yes*	*yes*	
**ENSG00000176393**						*yes*
**ENSG00000048162**			*yes*			*yes*
**ENSG00000071655**				*yes*		*yes*
**ENSG00000162594**		*yes*		*yes*		
**ENSG00000145416**						*yes*
**ENSG00000173621**		*yes*		*yes*		
**ENSG00000163346**		*yes*		*yes*		*yes*
**ENSG00000162607**	*yes*					
**ENSG00000163479**						*yes*
**ENSG00000006075**	*yes*					
**ENSG00000157326**		*yes*	*yes*			
**ENSG00000161813**	*yes*		*yes*	*yes*		
**ENSG00000142694**	*yes*	*yes*	*yes*			
**ENSG00000135077**	*yes*			*yes*	*yes*	*yes*
**ENSG00000144354**			*yes*			
**ENSG00000125868**						*yes*
**ENSG00000186350**	*yes*			*yes*	*yes*	
**ENSG00000136842**	*yes*			*yes*	*yes*	
**ENSG00000151208**		*yes*	*yes*	*yes*	*yes*	
**ENSG00000050405**		*yes*				
**ENSG00000162623**	*yes*			*yes*		
**ENSG00000035720**	*yes*			*yes*	*yes*	
**ENSG00000142178**				*yes*	*yes*	
**ENSG00000164683**						*yes*
**ENSG00000177181**	*yes*	*yes*		*yes*	*yes*	
**ENSG00000134709**		*yes*		*yes*		*yes*
**ENSG00000180104**				*yes*		
**ENSG00000148341**			*yes*			*yes*
**ENSG00000167785**	*yes*			*yes*		*yes*
**ENSG00000130707**		*yes*	*yes*			
**ENSG00000164983**	*yes*			*yes*	*yes*	
**ENSG00000196781**		*yes*				*yes*
**ENSG00000123933**		*yes*		*yes*	*yes*	
**ENSG00000113638**						*yes*
**ENSG00000104267**	*yes*		*yes*	*yes*		*yes*
**ENSG00000223865**		*yes*				
**ENSG00000176273**	*yes*					

**ENSG00000006062**			*yes*			
**ENSG00000157782**	*yes*					
**ENSG00000139352**		*yes*	*yes*	*yes*	*yes*	
**ENSG00000129351**	*yes*			*yes*	*yes*	
**ENSG00000075651**		*yes*	*yes*		*yes*	
**ENSG00000123892**			*yes*		*yes*	*yes*
**ENSG00000182224**	*yes*					
**ENSG00000178209**		*yes*	*yes*			
**ENSG00000100744**	*yes*			*yes*	*yes*	
**ENSG00000243989**						*yes*
**ENSG00000240972**					*yes*	
**ENSG00000198464**	*yes*					
**ENSG00000092758**	*yes*			*yes*	*yes*	
**ENSG00000146094**				*yes*		
**ENSG00000171914**	*yes*			*yes*	*yes*	
**ENSG00000221823**		*yes*				
**ENSG00000174123**		*yes*				
**ENSG00000198856**						*yes*
**ENSG00000185215**		*yes*	*yes*			
**ENSG00000105246**				*yes*		
**ENSG00000176903**		*yes*				
**ENSG00000188021**						*yes*
**ENSG00000148290**		*yes*				
**ENSG00000068971**	*yes*			*yes*	*yes*	*yes*
**ENSG00000187189**	*yes*					
**ENSG00000039068**		*yes*		*yes*		*yes*
**ENSG00000137575**		*yes*		*yes*	*yes*	
**ENSG00000179388**	*yes*	*yes*	*yes*	*yes*	*yes*	
**ENSG00000163082**			*yes*	*yes*	*yes*	
**ENSG00000013563**	*yes*		*yes*	*yes*		
**ENSG00000148175**			*yes*			
**ENSG00000029363**	*yes*					
**ENSG00000136158**			*yes*		*yes*	*yes*
**ENSG00000198369**					*yes*	
**ENSG00000159433**	*yes*			*yes*		
**ENSG00000131981**				*yes*		*yes*
**ENSG00000169592**						*yes*
**ENSG00000118308**		*yes*	*yes*			
**ENSG00000135452**		*yes*				
**ENSG00000106404**			*yes*			
**ENSG00000168461**		*yes*	*yes*		*yes*	
**ENSG00000115008**		*yes*	*yes*			
**ENSG00000103245**				*yes*		
**ENSG00000123427**		*yes*	*yes*	*yes*		
**ENSG00000162733**	*yes*			*yes*	*yes*	
**ENSG00000083444**					*yes*	*yes*
**ENSG00000137841**	*yes*		*yes*			*yes*
**ENSG00000159216**		*yes*		*yes*	*yes*	
**ENSG00000197619**			*yes*			
**ENSG00000171298**				*yes*		
**ENSG00000165169**						*yes*
**ENSG00000091127**	*yes*					
**ENSG00000180549**	*yes*	*yes*	*yes*	*yes*	*yes*	
**ENSG00000038427**		*yes*				*yes*

**ENSG00000198771**		*yes*	*yes*			
**ENSG00000070087**		*yes*	*yes*	*yes*	*yes*	
**ENSG00000134775**	*yes*	*yes*	*yes*	*yes*	*yes*	
**ENSG00000118495**		*yes*	*yes*	*yes*		*yes*
**ENSG00000146757**						*yes*
**ENSG00000100290**		*yes*	*yes*	*yes*	*yes*	
**ENSG00000153774**		*yes*	*yes*			
**ENSG00000159111**						*yes*
**ENSG00000151092**				*yes*	*yes*	
**ENSG00000092929**				*yes*		*yes*
**ENSG00000008952**						*yes*
**ENSG00000137166**					*yes*	*yes*
**ENSG00000100316**						*yes*
**ENSG00000140563**	*yes*			*yes*	*yes*	
**ENSG00000138061**	*yes*	*yes*	*yes*	*yes*	*yes*	
**ENSG00000075131**	*yes*					
**ENSG00000133794**	*yes*					
**ENSG00000174374**		*yes*	*yes*			
**ENSG00000158321**	*yes*			*yes*	*yes*	
**ENSG00000104881**	*yes*			*yes*		
**ENSG00000187193**			*yes*		*yes*	*yes*
**ENSG00000140743**			*yes*			
**ENSG00000141258**						*yes*
**ENSG00000205364**			*yes*			
**ENSG00000188846**	*yes*	*yes*	*yes*			
**ENSG00000100372**	*yes*			*yes*	*yes*	
**ENSG00000168071**	*yes*	*yes*				*yes*
**ENSG00000151835**	*yes*				*yes*	
**ENSG00000146072**	*yes*	*yes*			*yes*	*yes*
**ENSG00000160767**				*yes*		*yes*
**ENSG00000179134**						*yes*
**ENSG00000115548**	*yes*		*yes*			
**ENSG00000185624**	*yes*				*yes*	
**ENSG00000105875**				*yes*	*yes*	
**ENSG00000092051**	*yes*					
**ENSG00000130653**			*yes*			
**ENSG00000183093**						*yes*
**ENSG00000169155**			*yes*			
**ENSG00000145247**					*yes*	
**ENSG00000182253**		*yes*		*yes*		*yes*
**ENSG00000197932**				*yes*		
**ENSG00000130675**	*yes*	*yes*		*yes*	*yes*	*yes*
**ENSG00000124256**	*yes*	*yes*			*yes*	*yes*
**ENSG00000060762**					*yes*	
**ENSG00000128886**		*yes*		*yes*		*yes*
**ENSG00000120334**				*yes*		*yes*
**ENSG00000164292**		*yes*		*yes*	*yes*	*yes*
**ENSG00000148288**	*yes*	*yes*		*yes*	*yes*	*yes*
**ENSG00000134324**		*yes*	*yes*	*yes*	*yes*	
**ENSG00000143155**	*yes*					
**ENSG00000147852**	*yes*		*yes*	*yes*		*yes*
**ENSG00000104998**						*yes*
**ENSG00000175938**	*yes*		*yes*			*yes*
**ENSG00000125538**		*yes*	*yes*			

**ENSG00000107902**	*yes*	*yes*	*yes*			
**ENSG00000196182**						*yes*
**ENSG00000165983**	*yes*	*yes*	*yes*			
**ENSG00000173200**		*yes*	*yes*	*yes*	*yes*	
**ENSG00000115163**	*yes*			*yes*		
**ENSG00000077420**	*yes*			*yes*		*yes*
**ENSG00000103257**			*yes*			*yes*
**ENSG00000126456**				*yes*		*yes*
**ENSG00000196976**		*yes*	*yes*			
**ENSG00000010072**	*yes*			*yes*		
**ENSG00000132530**			*yes*			
**ENSG00000146828**		*yes*				
**ENSG00000122729**						*yes*
**ENSG00000152332**						*yes*
**ENSG00000115514**	*yes*				*yes*	
**ENSG00000196456**		*yes*				
**ENSG00000178445**			*yes*	*yes*	*yes*	*yes*
**ENSG00000092964**	*yes*		*yes*	*yes*	*yes*	*yes*
**ENSG00000113504**	*yes*	*yes*	*yes*	*yes*	*yes*	
**ENSG00000101210**		*yes*		*yes*	*yes*	*yes*
**ENSG00000172070**		*yes*	*yes*			
**ENSG00000196418**			*yes*			*yes*
**ENSG00000136231**			*yes*		*yes*	*yes*
**ENSG00000140682**				*yes*		*yes*
**ENSG00000146083**						*yes*
**ENSG00000141401**	*yes*			*yes*	*yes*	
**ENSG00000198331**				*yes*		
**ENSG00000134748**	*yes*					
**ENSG00000124787**	*yes*					
**ENSG00000177675**		*yes*	*yes*			
**ENSG00000184014**					*yes*	*yes*
**ENSG00000109790**				*yes*	*yes*	
**ENSG00000160213**		*yes*	*yes*			
**ENSG00000183570**			*yes*		*yes*	*yes*
**ENSG00000112079**	*yes*					
**ENSG00000160094**	*yes*				*yes*	*yes*
**ENSG00000026950**	*yes*			*yes*		
**ENSG00000123983**		*yes*		*yes*		*yes*
**ENSG00000123908**		*yes*				*yes*
**ENSG00000164938**	*yes*	*yes*	*yes*			
**ENSG00000064703**	*yes*			*yes*		
**ENSG00000126821**				*yes*		
**ENSG00000183722**	*yes*			*yes*	*yes*	
**ENSG00000198836**	*yes*				*yes*	
**ENSG00000167770**			*yes*			*yes*
**ENSG00000167283**	*yes*			*yes*	*yes*	
**ENSG00000104904**	*yes*				*yes*	
**ENSG00000166839**	*yes*	*yes*	*yes*	*yes*	*yes*	
**ENSG00000203896**		*yes*		*yes*		*yes*
**ENSG00000014257**		*yes*		*yes*		
**ENSG00000132128**		*yes*				
**ENSG00000183486**		*yes*		*yes*		*yes*
**ENSG00000023445**		*yes*		*yes*		*yes*
**ENSG00000117395**	*yes*					

**ENSG00000108953**						*yes*
**ENSG00000027697**	*yes*					
**ENSG00000122873**		*yes*				
**ENSG00000177721**	*yes*	*yes*	*yes*			
**ENSG00000213413**	*yes*			*yes*	*yes*	
**ENSG00000112282**						*yes*
**ENSG00000204394**		*yes*				
**ENSG00000126460**		*yes*				*yes*
**ENSG00000044574**	*yes*			*yes*		
**ENSG00000137076**			*yes*			*yes*
**ENSG00000100647**			*yes*			
**ENSG00000167799**						*yes*
**ENSG00000182481**	*yes*			*yes*		*yes*
**ENSG00000160326**	*yes*			*yes*	*yes*	
**ENSG00000139725**						*yes*
**ENSG00000090861**						*yes*
**ENSG00000048462**		*yes*				*yes*
**ENSG00000113369**			*yes*			
**ENSG00000110455**		*yes*		*yes*		*yes*
**ENSG00000004468**	*yes*		*yes*	*yes*	*yes*	*yes*
**ENSG00000139505**	*yes*			*yes*	*yes*	
**ENSG00000196821**				*yes*		
**ENSG00000112659**						*yes*
**ENSG00000066926**		*yes*	*yes*		*yes*	
**ENSG00000134255**	*yes*		*yes*	*yes*	*yes*	
**ENSG00000100813**						*yes*
**ENSG00000162227**				*yes*		
**ENSG00000105516**				*yes*		
**ENSG00000130222**		*yes*				
**ENSG00000155363**				*yes*		*yes*
**ENSG00000124920**			*yes*		*yes*	
**ENSG00000125629**	*yes*			*yes*	*yes*	
**ENSG00000109107**	*yes*			*yes*	*yes*	
**ENSG00000185432**	*yes*			*yes*		*yes*
**ENSG00000228300**			*yes*			*yes*
**ENSG00000148384**						*yes*
**ENSG00000099917**	*yes*			*yes*		*yes*
**ENSG00000135048**	*yes*			*yes*	*yes*	
**ENSG00000178809**	*yes*			*yes*	*yes*	
**ENSG00000174837**		*yes*	*yes*	*yes*	*yes*	
**ENSG00000159363**			*yes*			
**ENSG00000010278**	*yes*			*yes*	*yes*	
**ENSG00000011009**						*yes*
**ENSG00000102317**	*yes*					
**ENSG00000104808**	*yes*	*yes*	*yes*			
**ENSG00000144118**		*yes*	*yes*			
**ENSG00000066294**	*yes*					
**ENSG00000136463**						*yes*
**ENSG00000213672**	*yes*			*yes*	*yes*	
**ENSG00000179604**						*yes*
**ENSG00000110777**	*yes*					
**ENSG00000120742**						*yes*
**ENSG00000169398**	*yes*				*yes*	*yes*
**ENSG00000136830**		*yes*		*yes*		*yes*

**ENSG00000026751**						*yes*
**ENSG00000165490**	*yes*	*yes*				
**ENSG00000059804**	*yes*			*yes*	*yes*	
**ENSG00000088882**	*yes*		*yes*	*yes*	*yes*	*yes*
**ENSG00000117009**	*yes*			*yes*	*yes*	
**ENSG00000121594**		*yes*				*yes*
**ENSG00000132603**	*yes*		*yes*			*yes*
**ENSG00000184489**	*yes*			*yes*	*yes*	
**ENSG00000205038**		*yes*	*yes*			
**ENSG00000104824**						*yes*
**ENSG00000139687**		*yes*				
**ENSG00000197409**						*yes*
**ENSG00000102760**	*yes*		*yes*	*yes*	*yes*	*yes*
**ENSG00000123815**						*yes*
**ENSG00000181751**		*yes*				
**ENSG00000108622**			*yes*	*yes*	*yes*	
**ENSG00000174600**		*yes*				
**ENSG00000135916**			*yes*	*yes*	*yes*	
**ENSG00000064886**	*yes*		*yes*	*yes*	*yes*	*yes*
**ENSG00000174799**						*yes*
**ENSG00000157036**	*yes*			*yes*	*yes*	
**ENSG00000151792**		*yes*		*yes*	*yes*	
**ENSG00000118762**					*yes*	*yes*
**ENSG00000115138**	*yes*					
**ENSG00000107201**						*yes*
**ENSG00000132388**				*yes*		*yes*
**ENSG00000124766**	*yes*	*yes*	*yes*			
**ENSG00000177465**				*yes*	*yes*	
**ENSG00000168297**		*yes*				
**ENSG00000168209**	*yes*		*yes*	*yes*		*yes*
**ENSG00000138794**	*yes*			*yes*		
**ENSG00000243156**	*yes*	*yes*			*yes*	
**ENSG00000130303**		*yes*				
**ENSG00000149262**	*yes*					
**ENSG00000140612**		*yes*				*yes*
**ENSG00000239697**			*yes*			*yes*
**ENSG00000136021**	*yes*			*yes*	*yes*	
**ENSG00000082213**				*yes*		
**ENSG00000158806**						*yes*
**ENSG00000079313**						*yes*
**ENSG00000165025**	*yes*	*yes*			*yes*	
**ENSG00000221995**	*yes*		*yes*	*yes*		*yes*
**ENSG00000153989**			*yes*			
**ENSG00000148296**	*yes*	*yes*	*yes*			
**ENSG00000124120**	*yes*				*yes*	
**ENSG00000165475**						*yes*
**ENSG00000166012**			*yes*			
**ENSG00000105270**		*yes*	*yes*	*yes*	*yes*	
**ENSG00000121310**		*yes*	*yes*	*yes*	*yes*	
**ENSG00000089486**		*yes*	*yes*			
**ENSG00000170542**				*yes*		
**ENSG00000168827**	*yes*					
**ENSG00000139370**			*yes*			*yes*
**ENSG00000188739**					*yes*	*yes*

**ENSG00000169429**		*yes*	*yes*	*yes*	*yes*	
**ENSG00000186470**	*yes*			*yes*	*yes*	
**ENSG00000197728**	*yes*	*yes*	*yes*			
**ENSG00000100029**	*yes*		*yes*			*yes*
**ENSG00000141564**			*yes*			
**ENSG00000125484**			*yes*			
**ENSG00000134285**			*yes*			*yes*
**ENSG00000111913**	*yes*	*yes*				
**ENSG00000139793**				*yes*	*yes*	
**ENSG00000125454**		*yes*				
**ENSG00000143198**		*yes*	*yes*			
**ENSG00000082269**						*yes*
**ENSG00000159720**	*yes*	*yes*				
**ENSG00000178719**	*yes*			*yes*		*yes*
**ENSG00000104524**	*yes*			*yes*		*yes*
**ENSG00000159210**						*yes*
**ENSG00000169752**	*yes*			*yes*	*yes*	
**ENSG00000158711**	*yes*		*yes*			
**ENSG00000159753**						*yes*
**ENSG00000142039**				*yes*		
**ENSG00000160183**	*yes*	*yes*		*yes*	*yes*	
**ENSG00000166226**			*yes*			
**ENSG00000136240**			*yes*			*yes*
**ENSG00000175324**						*yes*
**ENSG00000112378**	*yes*			*yes*	*yes*	
**ENSG00000156697**			*yes*			
**ENSG00000133195**						*yes*
**ENSG00000197045**	*yes*					
**ENSG00000107815**	*yes*	*yes*	*yes*			
**ENSG00000171488**				*yes*	*yes*	
**ENSG00000077348**		*yes*		*yes*		
**ENSG00000205560**			*yes*			*yes*
**ENSG00000021645**	*yes*	*yes*	*yes*	*yes*	*yes*	
**ENSG00000049656**				*yes*		*yes*
**ENSG00000006125**						*yes*
**ENSG00000124383**			*yes*			
**ENSG00000125445**			*yes*			
**ENSG00000086289**	*yes*		*yes*	*yes*	*yes*	
**ENSG00000128340**	*yes*			*yes*	*yes*	
**ENSG00000105369**	*yes*					
**ENSG00000152443**	*yes*					
**ENSG00000103316**	*yes*	*yes*	*yes*	*yes*	*yes*	
**ENSG00000183246**	*yes*	*yes*	*yes*			
**ENSG00000075711**				*yes*		
**ENSG00000204642**	*yes*					
**ENSG00000155660**		*yes*				*yes*
**ENSG00000110660**			*yes*			
**ENSG00000100109**						*yes*
**ENSG00000168003**		*yes*	*yes*			
**ENSG00000144488**		*yes*		*yes*		
**ENSG00000163629**	*yes*			*yes*	*yes*	
**ENSG00000142494**		*yes*	*yes*			
**ENSG00000157873**	*yes*			*yes*		
**ENSG00000100365**		*yes*				*yes*

**ENSG00000025772**						*yes*
**ENSG00000100097**	*yes*			*yes*	*yes*	
**ENSG00000132313**		*yes*				
**ENSG00000239789**			*yes*			
**ENSG00000188707**	*yes*			*yes*	*yes*	
**ENSG00000143110**	*yes*				*yes*	*yes*
**ENSG00000198502**	*yes*	*yes*	*yes*	*yes*	*yes*	*yes*
**ENSG00000100292**	*yes*		*yes*	*yes*		*yes*
**ENSG00000124802**	*yes*					
**ENSG00000196329**	*yes*	*yes*	*yes*			
**ENSG00000152952**	*yes*			*yes*	*yes*	
**ENSG00000078061**						*yes*
**ENSG00000104885**				*yes*		*yes*
**ENSG00000092621**		*yes*	*yes*		*yes*	*yes*
**ENSG00000117399**		*yes*	*yes*	*yes*		*yes*
**ENSG00000172794**	*yes*	*yes*			*yes*	*yes*
**ENSG00000241106**					*yes*	*yes*
**ENSG00000197457**	*yes*	*yes*	*yes*			
**ENSG00000139174**	*yes*				*yes*	
**ENSG00000130595**	*yes*	*yes*	*yes*			
**ENSG00000108854**	*yes*	*yes*	*yes*			
**ENSG00000205336**						*yes*
**ENSG00000112290**		*yes*		*yes*		*yes*
**ENSG00000051108**			*yes*		*yes*	
**ENSG00000168246**	*yes*			*yes*	*yes*	
**ENSG00000015475**	*yes*	*yes*				
**ENSG00000135723**				*yes*		
**ENSG00000182013**			*yes*		*yes*	
**ENSG00000112658**			*yes*	*yes*		*yes*
**ENSG00000076864**			*yes*		*yes*	*yes*
**ENSG00000027847**						*yes*
**ENSG00000147421**		*yes*	*yes*	*yes*	*yes*	
**ENSG00000159212**		*yes*	*yes*	*yes*	*yes*	
**ENSG00000196405**	*yes*		*yes*	*yes*	*yes*	
**ENSG00000119698**		*yes*	*yes*			
**ENSG00000197747**	*yes*	*yes*	*yes*			
**ENSG00000168242**			*yes*		*yes*	*yes*
**ENSG00000051382**	*yes*					
**ENSG00000117139**	*yes*	*yes*		*yes*	*yes*	
**ENSG00000104131**	*yes*		*yes*			
**ENSG00000084234**	*yes*				*yes*	*yes*
**ENSG00000102755**	*yes*	*yes*	*yes*			
**ENSG00000197070**				*yes*		*yes*
**ENSG00000089327**	*yes*			*yes*	*yes*	
**ENSG00000117877**	*yes*		*yes*			
**ENSG00000217555**			*yes*		*yes*	
**ENSG00000100246**						*yes*
**ENSG00000182568**				*yes*		
**ENSG00000197111**	*yes*					
**ENSG00000105127**				*yes*		
**ENSG00000165233**		*yes*	*yes*		*yes*	
**ENSG00000183010**		*yes*				
**ENSG00000018610**	*yes*					
**ENSG00000225932**		*yes*				

**ENSG00000036448**	*yes*	*yes*	*yes*			
**ENSG00000131051**					*yes*	*yes*
**ENSG00000107719**	*yes*	*yes*	*yes*			*yes*
**ENSG00000123159**			*yes*			
**ENSG00000103043**			*yes*			
**ENSG00000011590**	*yes*	*yes*		*yes*	*yes*	*yes*
**ENSG00000114861**		*yes*				*yes*
**ENSG00000161929**	*yes*	*yes*	*yes*			
**ENSG00000151465**						*yes*
**ENSG00000064666**				*yes*		*yes*
**ENSG00000130770**		*yes*	*yes*			
**ENSG00000054267**						*yes*
**ENSG00000108375**		*yes*				*yes*
**ENSG00000006453**	*yes*			*yes*	*yes*	
**ENSG00000168386**		*yes*		*yes*		*yes*
**ENSG00000139737**			*yes*			
**ENSG00000151575**				*yes*	*yes*	
**ENSG00000138772**	*yes*				*yes*	
**ENSG00000179094**	*yes*	*yes*		*yes*	*yes*	*yes*
**ENSG00000058272**	*yes*			*yes*	*yes*	
**ENSG00000102393**			*yes*		*yes*	
**ENSG00000100284**				*yes*		
**ENSG00000135862**	*yes*		*yes*	*yes*		*yes*
**ENSG00000117318**	*yes*			*yes*		
**ENSG00000197461**		*yes*				
**ENSG00000133247**	*yes*			*yes*		*yes*
**ENSG00000158158**				*yes*		*yes*
**ENSG00000171130**	*yes*	*yes*	*yes*			*yes*
**ENSG00000198911**	*yes*			*yes*		
**ENSG00000198816**		*yes*	*yes*	*yes*	*yes*	
**ENSG00000139410**		*yes*	*yes*			
**ENSG00000188404**		*yes*				*yes*
**ENSG00000138678**						*yes*
**ENSG00000204632**			*yes*			*yes*
**ENSG00000136213**						*yes*
**ENSG00000167930**						*yes*
**ENSG00000121388**						*yes*
**ENSG00000159423**						*yes*
**ENSG00000089006**	*yes*		*yes*	*yes*		*yes*
**ENSG00000144867**			*yes*			*yes*
**ENSG00000197858**						*yes*
**ENSG00000078596**	*yes*					
**ENSG00000141002**		*yes*				*yes*
**ENSG00000108702**	*yes*					
**ENSG00000244754**	*yes*					
**ENSG00000170638**						*yes*
**ENSG00000114013**		*yes*	*yes*	*yes*	*yes*	
**ENSG00000154767**		*yes*				
**ENSG00000078401**	*yes*			*yes*	*yes*	
**ENSG00000172339**	*yes*			*yes*		
**ENSG00000197170**	*yes*					
**ENSG00000179144**	*yes*	*yes*	*yes*			
**ENSG00000154237**		*yes*				
**ENSG00000117586**				*yes*	*yes*	

**ENSG00000183780**				*yes*	*yes*	
**ENSG00000054219**						*yes*
**ENSG00000184905**	*yes*	*yes*		*yes*	*yes*	*yes*
**ENSG00000152409**	*yes*	*yes*				
**ENSG00000120158**	*yes*					
**ENSG00000105499**	*yes*	*yes*	*yes*	*yes*	*yes*	
**ENSG00000187688**						*yes*
**ENSG00000149557**	*yes*	*yes*			*yes*	*yes*
**ENSG00000137331**		*yes*	*yes*			
**ENSG00000133997**	*yes*			*yes*		
**ENSG00000124357**	*yes*			*yes*		
**ENSG00000122224**			*yes*	*yes*	*yes*	
**ENSG00000198944**				*yes*	*yes*	
**ENSG00000230463**	*yes*	*yes*	*yes*	*yes*	*yes*	*yes*
**ENSG00000181481**	*yes*	*yes*	*yes*			
**ENSG00000161249**	*yes*					
**ENSG00000239779**		*yes*				
**ENSG00000039560**					*yes*	
**ENSG00000170364**	*yes*					
**ENSG00000143772**			*yes*	*yes*	*yes*	*yes*
**ENSG00000125354**						*yes*
**ENSG00000182534**	*yes*	*yes*	*yes*			
**ENSG00000168961**						*yes*
**ENSG00000132589**		*yes*		*yes*	*yes*	
**ENSG00000137513**		*yes*	*yes*			
**ENSG00000099958**	*yes*			*yes*	*yes*	
**ENSG00000160255**	*yes*			*yes*	*yes*	
**ENSG00000188060**	*yes*			*yes*		*yes*
**ENSG00000196961**						*yes*
**ENSG00000149541**						*yes*
**ENSG00000185838**						*yes*
**ENSG00000151967**					*yes*	*yes*
**ENSG00000136878**						*yes*
**ENSG00000162302**						*yes*
**ENSG00000184640**	*yes*			*yes*	*yes*	*yes*
**ENSG00000177189**	*yes*		*yes*			
**ENSG00000111331**	*yes*			*yes*	*yes*	
**ENSG00000064199**	*yes*				*yes*	
**ENSG00000168300**	*yes*		*yes*			*yes*
**ENSG00000146833**		*yes*	*yes*			
**ENSG00000213626**	*yes*		*yes*			*yes*
**ENSG00000151806**	*yes*					
**ENSG00000118454**	*yes*				*yes*	
**ENSG00000100650**	*yes*	*yes*			*yes*	*yes*
**ENSG00000116514**						*yes*
**ENSG00000198286**					*yes*	*yes*
**ENSG00000100504**	*yes*				*yes*	*yes*
**ENSG00000134910**					*yes*	
**ENSG00000172817**		*yes*	*yes*	*yes*	*yes*	*yes*
**ENSG00000105447**		*yes*	*yes*			
**ENSG00000213402**	*yes*	*yes*				
**ENSG00000135045**						*yes*
**ENSG00000151490**	*yes*			*yes*	*yes*	
**ENSG00000104936**					*yes*	*yes*

**ENSG00000163154**				*yes*		
**ENSG00000213759**		*yes*	*yes*	*yes*	*yes*	
**ENSG00000163687**	*yes*	*yes*	*yes*	*yes*	*yes*	
**ENSG00000170571**		*yes*	*yes*		*yes*	
**ENSG00000239998**			*yes*			
**ENSG00000123700**	*yes*				*yes*	
**ENSG00000100376**		*yes*	*yes*			
**ENSG00000177548**		*yes*				
**ENSG00000197694**			*yes*	*yes*		*yes*
**ENSG00000213741**						*yes*
**ENSG00000171603**		*yes*				
**ENSG00000160410**						*yes*
**ENSG00000079246**				*yes*		
**ENSG00000185728**	*yes*					
**ENSG00000064225**	*yes*		*yes*	*yes*	*yes*	
**ENSG00000179431**		*yes*	*yes*			
**ENSG00000184634**						*yes*
**ENSG00000197153**			*yes*		*yes*	*yes*
**ENSG00000184261**	*yes*			*yes*	*yes*	
**ENSG00000129521**				*yes*	*yes*	
**ENSG00000177879**	*yes*				*yes*	
**ENSG00000166068**	*yes*			*yes*	*yes*	*yes*
**ENSG00000104972**	*yes*		*yes*	*yes*	*yes*	
**ENSG00000243244**						*yes*
**ENSG00000008517**	*yes*	*yes*		*yes*	*yes*	*yes*
**ENSG00000205213**				*yes*	*yes*	
**ENSG00000112473**						*yes*
**ENSG00000131773**	*yes*			*yes*	*yes*	
**ENSG00000170043**						*yes*
**ENSG00000008394**	*yes*	*yes*	*yes*		*yes*	*yes*
**ENSG00000139722**	*yes*				*yes*	*yes*
**ENSG00000188620**			*yes*	*yes*	*yes*	
**ENSG00000253729**					*yes*	
**ENSG00000149257**	*yes*			*yes*		
**ENSG00000204540**	*yes*		*yes*			
