# Supplementary material for: Insights into the regulation of human CNV-miRNAs from the view of their target genes
Source: BMC Genomics. 2012 Dec 18;13:707. doi: 10.1186/1471-2164-13-707 (PMC3582595; doi:10.1186/1471-2164-13-707)
Supplement: Additional file 7 — The differential expression ratios (DERs) of 1,8763 human genes that were used for TargetScan5.1 prediction. [file 1471-2164-13-707-S7.pdf]

Ensembl Gene ID	Entrez Gene ID	Gene Symbol	DERs
ENSG00000213801	399669	ZNF321	0.49
ENSG00000259662	283807	FBXL22	0.365
ENSG00000255738	2543	GAGE1	0.477
ENSG00000224420	199800	LOC199800	0.386
ENSG00000258664	163590	TOR1AIP2	0.454
ENSG00000258518	6474	SHOX2	0.583
ENSG00000259741	10771	ZMYND11	0.618
ENSG00000265969	23621	BACE1	0.553
ENSG00000262023	8629	JRK	0.629
ENSG00000262150	8000	PSCA	0.443
ENSG00000262378	54742	LY6K	0.504
ENSG00000263194	51337	C8orf55	0.502
ENSG00000204685	285033	LOC285033	0.408
ENSG00000163121	93082	LINCR	0.457
ENSG00000261229	10588	MTHFS	0.609
ENSG00000120645	440073	IQSEC3	0.436
ENSG00000010379	6540	SLC6A13	0.475
ENSG00000073614	5927	JARID1A	0.618
ENSG00000257267	10778	ZNF271	0.503
ENSG00000120647	84318	CCDC77	0.483
ENSG00000139044	283358	B4GALNT3	0.482
ENSG00000171840	4815	NINJ2	0.469
ENSG00000183060	145748	LYSMD4	0.516
ENSG00000139835	79774	GRTP1	0.458
ENSG00000154227	204219	LASS3	0.443
ENSG00000263243	3859	KRT12	0.456
ENSG00000263057	54474	KRT20	0.427
ENSG00000263309	25984	KRT23	0.474
ENSG00000262845	125115	KRT40	0.488
ENSG00000263101	85293	KRTAP3-3	0.365
ENSG00000140471	55180	LINS1	0.482
ENSG00000263296	83897	KRTAP3-2	0.36
ENSG00000262068	83896	KRTAP3-1	0.356
ENSG00000129991	7137	TNNI3	0.45
ENSG00000263236	83755	KRTAP4-12	0.393
ENSG00000167646	352909	C19orf51	0.405
ENSG00000186009	496	ATP4B	0.474
ENSG00000184497	348013	FAM70B	0.425
ENSG00000080031	5794	PTPRH	0.495
ENSG00000180089	255043	TMEM86B	0.483
ENSG00000105063	22870	SAPS1	0.512
ENSG00000160471	125965	COX6B2	0.483
ENSG00000130177	8881	CDC16	0.624
ENSG00000160472	147744	TMEM190	0.467
ENSG00000131871	55829	SELS	0.536
ENSG00000197483	89887	ZNF628	0.444
ENSG00000179943	84922	FIZ1	0.488
ENSG00000261846	344752	AADACL2	0.352
ENSG00000171425	51545	ZNF581	0.528
ENSG00000173581	29903	CCDC106	0.486
ENSG00000185792	338321	NLRP9	0.356
ENSG00000179873	204801	NLRP11	0.413

ENSG00000160505	147945	NLRP4	0.45
ENSG00000179709	126205	NLRP8	0.419
ENSG00000171487	126206	NLRP5	0.413
ENSG00000142409	126208	ZNF787	0.52
ENSG00000167685	55311	ZNF444	0.565
ENSG00000197487	85569	GALP	0.443
ENSG00000131848	79149	ZSCAN5A	0.483
ENSG00000018869	147948	ZNF582	0.482
ENSG00000196263	57573	ZNF471	0.439
ENSG00000196867	140612	ZFP28	0.527
ENSG00000197016	388566	ZNF470	0.365
ENSG00000198300	5178	PEG3	0.535
ENSG00000131864	57663	USP29	0.433
ENSG00000141946	114026	ZIM3	0.375
ENSG00000105146	6795	AURKC	0.468
ENSG00000197714	10794	ZNF460	0.527
ENSG00000178229	125919	ZNF543	0.364
ENSG00000180229	283755	LOC283755	0.415
ENSG00000256060	6399	TRAPPC2	0.525
ENSG00000152433	284306	ZNF547	0.491
ENSG00000188785	147694	ZNF548	0.49
ENSG00000186272	7565	ZNF17	0.482
ENSG00000186230	388567	ZNF749	0.398
ENSG00000178201	57191	VN1R1	0.464
ENSG00000105136	79744	ZNF419	0.57
ENSG00000121406	256051	ZNF549	0.439
ENSG00000083817	55659	ZNF416	0.486
ENSG00000171649	284307	ZIK1	0.481
ENSG00000183647	348327	ZNF530	0.414
ENSG00000213762	7693	ZNF134	0.481
ENSG00000121417	10520	ZNF211	0.485
ENSG00000180532	201516	ZSCAN4	0.482
ENSG00000204519	90233	ZNF551	0.476
ENSG00000179909	7710	ZNF154	0.506
ENSG00000083814	79891	ZNF671	0.422
ENSG00000152443	284309	ZNF776	0.505
ENSG00000083828	54807	ZNF586	0.476
ENSG00000178935	79818	ZNF552	0.468
ENSG00000198466	84914	ZNF587	0.503
ENSG00000173480	147687	ZNF417	0.451
ENSG00000152454	10172	ZNF256	0.44
ENSG00000177025	147685	C19orf18	0.442
ENSG00000166704	80095	ZNF606	0.462
ENSG00000152467	284312	ZSCAN1	0.386
ENSG00000176293	7694	ZNF135	0.482
ENSG00000121413	65982	ZSCAN18	0.569
ENSG00000181894	79673	ZNF329	0.472
ENSG00000171606	10782	ZNF274	0.523
ENSG00000083842	7554	ZNF8	0.527
ENSG00000121410	1	A1BG	0.51
ENSG00000174586	162968	ZNF497	0.377
ENSG00000083845	6193	RPS5	0.533
ENSG00000131849	7691	ZNF132	0.47
ENSG00000249471	388569	ZNF324B	0.446

ENSG00000083812	25799	ZNF324	0.465
ENSG00000083838	55663	ZNF446	0.51
ENSG00000083807	10998	SLC27A5	0.458
ENSG00000119574	84878	ZBTB45	0.491
ENSG00000130724	27243	CHMP2A	0.544
ENSG00000099326	7593	MZF1	0.634
ENSG00000267493	148046	C19orf23	0.506
ENSG00000180846	255193	C19orf34	0.4
ENSG00000167798	388503	LOC388503	0.377
ENSG00000231205	664701	ZNF826	0.522
ENSG00000113643	5917	RARS	0.527
ENSG00000081320	9262	STK17B	0.532
ENSG00000240303	84129	ACAD11	0.482
ENSG00000144395	284992	CCDC150	0.469
ENSG00000206337	10866	HCP5	0.492
ENSG00000040275	54908	CCDC99	0.514
ENSG00000119041	9330	GTF3C3	0.521
ENSG00000081307	79876	UBA5	0.512
ENSG00000235043	9524	GPSN2	0.507
ENSG00000163781	11073	TOPBP1	0.545
ENSG00000091513	7018	TF	0.603
ENSG00000169251	51068	NMD3	0.54
ENSG00000234511	133874	LOC133874	0.508
ENSG00000090402	6476	SI	0.435
ENSG00000043462	3937	LCP2	0.55
ENSG00000144867	58477	SRPRB	0.564
ENSG00000115524	23451	SF3B1	0.604
ENSG00000114200	590	BCHE	0.489
ENSG00000169064	79740	ZBBX	0.45
ENSG00000129055	25847	ANAPC13	0.589
ENSG00000114204	5276	SERPINI2	0.489
ENSG00000221972	80111	C3orf36	0.398
ENSG00000174776	151790	WDR49	0.537
ENSG00000162944	130132	RFTN2	0.51
ENSG00000145936	3779	KCNMB1	0.521
ENSG00000226259	653238	GTF2H2B	0.47
ENSG00000163785	6259	RYK	0.639
ENSG00000094755	2568	GABRP	0.465
ENSG00000247626	92935	MARS2	0.535
ENSG00000163449	92691	TMEM169	0.459
ENSG00000163536	5274	SERPINI1	0.521
ENSG00000138375	50485	SMARCAL1	0.52
ENSG00000085276	2122	EVI1	0.541
ENSG00000197756	6168	RPL37A	0.597
ENSG00000115687	23178	PASK	0.599
ENSG00000174579	55167	MSL2L1	0.489
ENSG00000114054	5096	PCCB	0.57
ENSG00000115685	5510	PPP1R7	0.633
ENSG00000184378	84517	ARPM1	0.487
ENSG00000085274	55892	MYNN	0.523
ENSG00000118017	51146	A4GNT	0.453
ENSG00000171757	151827	LRRC34	0.467
ENSG00000138231	51163	DBR1	0.497
ENSG00000146205	50636	TMEM16G	0.357

ENSG00000162972	79568	C2orf47	0.518
ENSG00000114248	79782	LRRC31	0.412
ENSG00000196141	26010	LOC26010	0.571
ENSG00000013441	1195	CLK1	0.505
ENSG00000173890	26996	GPR160	0.572
ENSG00000180871	3579	IL8RB	0.511
ENSG00000113719	57222	ERGIC1	0.545
ENSG00000163464	3577	IL8RA	0.492
ENSG00000240344	53938	PPIL3	0.42
ENSG00000179921	151306	GPBAR1	0.462
ENSG00000113749	3274	HRH2	0.414
ENSG00000163584	200916	RPL22L1	0.474
ENSG00000037241	51121	RPL26L1	0.526
ENSG00000163535	151246	SGOL2	0.623
ENSG00000113732	8992	ATP6V0E1	0.657
ENSG00000136603	6498	SKIL	0.594
ENSG00000196290	60491	NIF3L1	0.497
ENSG00000051596	84321	THOC3	0.467
ENSG00000006607	9855	FARP2	0.517
ENSG00000168917	80723	TMEM22	0.544
ENSG00000182230	202134	FAM153B	0.46
ENSG00000158220	83850	FAM62C	0.386
ENSG00000168875	8403	SOX14	0.415
ENSG00000176720	666	BOK	0.422
ENSG00000145919	91272	FAM44B	0.5
ENSG00000127831	7429	VIL1	0.473
ENSG00000119013	4709	NDUFB3	0.56
ENSG00000003402	8837	CFLAR	0.666
ENSG00000168393	1841	DTYMK	0.545
ENSG00000158234	55179	FAIM	0.507
ENSG00000180902	728294	D2HGDH	0.468
ENSG00000204099	129807	NEU4	0.477
ENSG00000229738	11017	RY1	0.559
ENSG00000188389	5133	PDCD1	0.501
ENSG00000188011	285093	FLJ33590	0.256
ENSG00000115568	7701	ZNF142	0.511
ENSG00000113194	23197	UBXD8	0.54
ENSG00000074582	617	BCS1L	0.561
ENSG00000121858	8743	TNFSF10	0.598
ENSG00000175110	56945	MRPS22	0.512
ENSG00000163481	64320	RNF25	0.43
ENSG00000074276	54825	PCDH24	0.455
ENSG00000064012	841	CASP8	0.608
ENSG00000144962	83893	SPATA16	0.379
ENSG00000184432	9276	COPB2	0.54
ENSG00000135929	1593	CYP27A1	0.511
ENSG00000115596	7475	WNT6	0.601
ENSG00000163497	54738	FEV	0.486
ENSG00000164611	9232	PTTG1	0.579
ENSG00000183674	221718	C6orf218	0.415
ENSG00000138378	6775	STAT4	0.55
ENSG00000170584	134492	NUDCD2	0.485
ENSG00000137841	5330	PLCB2	0.53
ENSG00000034533	28990	ASTE1	0.475

ENSG00000233095	3135	HLA-G	0.612
ENSG00000223980	3105	HLA-A	0.588
ENSG00000114670	79858	NEK11	0.496
ENSG00000233795	30834	ZNRD1	0.542
ENSG00000128944	90417	C15orf23	0.474
ENSG00000237829	6992	PPP1R11	0.524
ENSG00000230467	80352	RNF39	0.512
ENSG00000128928	3712	IVD	0.646
ENSG00000224168	11074	TRIM31	0.6
ENSG00000233931	135644	TRIM40	0.322
ENSG00000198585	131870	NUDT16	0.513
ENSG00000229346	10107	TRIM10	0.579
ENSG00000141504	112483	SAT2	0.524
ENSG00000224145	89870	TRIM15	0.566
ENSG00000166133	27079	RPUSD2	0.482
ENSG00000234046	7726	TRIM26	0.558
ENSG00000135362	79899	FLJ14213	0.487
ENSG00000229929	56658	TRIM39	0.466
ENSG00000118997	56171	DNAH7	0.44
ENSG00000239927	79897	RPP21	0.468
ENSG00000175104	7189	TRAF6	0.537
ENSG00000230254	3133	HLA-E	0.629
ENSG00000014257	55	ACPP	0.528
ENSG00000142687	79932	KIAA0319L	0.488
ENSG00000204625	10255	HCG9	0.453
ENSG00000226882	2794	GNL1	0.552
ENSG00000166352	119710	C11orf74	0.425
ENSG00000137880	2644	GCHFR	0.546
ENSG00000104129	55192	DNAJC17	0.444
ENSG00000234616	8629	JRK	0.629
ENSG00000141499	55135	WDR79	0.57
ENSG00000229202	80742	PRR3	0.522
ENSG00000166140	84936	ZFYVE19	0.523
ENSG00000225989	23	ABCF1	0.557
ENSG00000132703	325	APCS	0.456
ENSG00000132693	1401	CRP	0.504
ENSG00000183914	146754	DNAH2	0.46
ENSG00000166143	54866	PPP1R14D	0.461
ENSG00000100867	10202	DHRS2	0.581
ENSG00000166145	6692	SPINT1	0.547
ENSG00000151779	51594	NAG	0.505
ENSG00000052841	55761	TTC17	0.547
ENSG00000238104	5514	PPP1R10	0.598
ENSG00000104140	171177	RHOV	0.449
ENSG00000158716	54935	DUSP23	0.517
ENSG00000203942	414157	C10orf62	0.403
ENSG00000132510	23135	JMJD3	0.64
ENSG00000223775	28973	MRPS18B	0.57
ENSG00000128908	54617	INOC1	0.507
ENSG00000149084	51144	HSD17B12	0.554
ENSG00000119986	60370	AVPI1	0.533
ENSG00000234549	79969	C6orf134	0.503
ENSG00000157326	10901	DHRS4	0.494
ENSG00000167874	92162	TMEM88	0.48

ENSG00000183011	84316	LSMD1	0.496
ENSG00000166199	221120	ALKBH3	0.451
ENSG00000178997	161829	EXDL1	0.423
ENSG00000182224	124637	CYB5D1	0.529
ENSG00000187446	11261	CHP	0.549
ENSG00000262826	65123	INTS3	0.517
ENSG00000104147	11339	OIP5	0.528
ENSG00000137804	51203	NUSAP1	0.614
ENSG00000126067	5690	PSMB2	0.579
ENSG00000263163	11000	SLC27A3	0.498
ENSG00000237100	221545	C6orf136	0.518
ENSG00000261992	57459	GATAD2B	0.456
ENSG00000092853	63967	CLSPN	0.468
ENSG00000226171	8449	DHX16	0.471
ENSG00000213085	25790	CCDC19	0.415
ENSG00000110455	84680	ACCS	0.465
ENSG00000137806	51103	NDUFAF1	0.501
ENSG00000102313	347365	ITIH5L	0.384
ENSG00000079785	1653	DDX1	0.522
ENSG00000236428	170954	KIAA1949	0.563
ENSG00000146039	10050	SLC17A4	0.492
ENSG00000155265	401647	C10orf132	0.424
ENSG00000231135	9656	MDC1	0.575
ENSG00000095713	55118	CRTAC1	0.482
ENSG00000170037	116840	CNTROB	0.51
ENSG00000124568	6568	SLC17A1	0.507
ENSG00000232421	203068	TUBB	0.613
ENSG00000085552	57549	IGSF9	0.461
ENSG00000085117	3732	CD82	0.495
ENSG00000154874	284047	CCDC144B	0.558
ENSG00000100884	9362	CPNE6	0.508
ENSG00000067445	7216	TRO	0.625
ENSG00000157570	90139	TSPAN18	0.516
ENSG00000124564	10786	SLC17A3	0.492
ENSG00000118965	57539	WDR35	0.556
ENSG00000166024	27291	C10orf28	0.453
ENSG00000102349	11279	KLF8	0.433
ENSG00000197872	81553	FAM49A	0.579
ENSG00000178591	245938	DEFB125	0.444
ENSG00000132716	50717	WDR42A	0.604
ENSG00000125788	81623	DEFB126	0.416
ENSG00000179477	242	ALOX12B	0.48
ENSG00000162723	89886	SLAMF9	0.464
ENSG00000088782	140850	DEFB127	0.379
ENSG00000092850	27285	TEKT2	0.438
ENSG00000125903	140881	DEFB129	0.429
ENSG00000112337	10246	SLC17A2	0.451
ENSG00000230143	10211	FLOT1	0.631
ENSG00000186458	400830	DEFB132	0.394
ENSG00000179148	59344	ALOXE3	0.546
ENSG00000162728	3765	KCNJ9	0.481
ENSG00000019485	56981	PRDM11	0.446
ENSG00000112343	10475	TRIM38	0.612
ENSG00000162729	93185	IGSF8	0.528

ENSG00000139914	161247	FIT1	0.408
ENSG00000092010	5720	PSME1	0.543
ENSG00000124508	10385	BTN2A2	0.566
ENSG00000165591	158584	FAAH2	0.477
ENSG00000100889	5106	PCK2	0.552
ENSG00000198205	7789	ZXDA	0.5
ENSG00000119943	84795	C10orf33	0.482
ENSG00000125826	10616	RBCK1	0.615
ENSG00000155287	81894	SLC25A28	0.498
ENSG00000026950	11119	BTN3A1	0.602
ENSG00000158571	5207	PFKFB1	0.425
ENSG00000100908	51016	FAM158A	0.476
ENSG00000258465	50717	WDR42A	0.604
ENSG00000234776	143678	LOC143678	0.393
ENSG00000121680	9409	PEX16	0.593
ENSG00000107521	3257	HPS1	0.616
ENSG00000119929	51076	CUTC	0.528
ENSG00000102043	55613	MTMR8	0.547
ENSG00000169188	27301	APEX2	0.489
ENSG00000178999	9212	AURKB	0.562
ENSG00000100911	5721	PSME2	0.581
ENSG00000100897	80344	WDR23	0.574
ENSG00000165905	120071	GYLTL1B	0.513
ENSG00000014919	1355	COX15	0.578
ENSG00000126970	55906	KIAA1166	0.471
ENSG00000111801	10384	BTN3A3	0.569
ENSG00000178295	348654	GEN1	0.462
ENSG00000132681	480	ATP1A4	0.551
ENSG00000122218	1314	COPA	0.604
ENSG00000179406	285908	LOC285908	0.418
ENSG00000234068	203569	PAGE2	0.382
ENSG00000023839	1244	ABCC2	0.47
ENSG00000226075	5260	PHKG1	0.516
ENSG00000001497	81887	LAS1L	0.524
ENSG00000182518	90736	FAM104B	0.449
ENSG00000158373	3017	HIST1H2BD	0.58
ENSG00000240857	57665	RDH14	0.52
ENSG00000185013	93034	NT5C1B	0.416
ENSG00000158639	90737	PAGE5	0.449
ENSG00000112763	11120	BTN2A1	0.565
ENSG00000196966	8350	HIST1H3A	0.4
ENSG00000172987	60495	HPSE2	0.447
ENSG00000187601	28986	MAGEH1	0.483
ENSG00000124578	8369	HIST1H4G	0.426
ENSG00000247746	158880	USP51	0.48
ENSG00000256316	8350	HIST1H3A	0.4
ENSG00000189299	139628	FOXR2	0.325
ENSG00000197459	8345	HIST1H2BH	0.475
ENSG00000083750	10325	RRAGB	0.485
ENSG00000256018	8350	HIST1H3A	0.4
ENSG00000118961	60526	C2orf43	0.623
ENSG00000124557	696	BTN1A1	0.475
ENSG00000183891	130502	TTC32	0.412
ENSG00000146109	29777	ABT1	0.502

ENSG00000166157	7179	TPTE	0.455
ENSG00000084674	338	APOB	0.475
ENSG00000155659	11326	VSIG4	0.459
ENSG00000197903	85236	HIST1H2BK	0.56
ENSG00000259529	10379	IRF9	0.544
ENSG00000198339	8294	HIST1H4I	0.459
ENSG00000112812	10279	PRSS16	0.514
ENSG00000120054	1369	CPN1	0.479
ENSG00000106133	155400	NSUN5B	0.564
ENSG00000089472	9843	HEPH	0.546
ENSG00000158477	909	CD1A	0.521
ENSG00000166351	317754	A26B3	0.386
ENSG00000100918	9985	REC8	0.492
ENSG00000158481	911	CD1C	0.494
ENSG00000171311	51013	EXOSC1	0.464
ENSG00000188992	149998	LIPI	0.353
ENSG00000158485	910	CD1B	0.459
ENSG00000115761	79954	NOL10	0.461
ENSG00000124613	346157	ZNF391	0.439
ENSG00000158488	913	CD1E	0.521
ENSG00000183640	337879	KRTAP8-1	0.488
ENSG00000096654	7738	ZNF184	0.491
ENSG00000185130	8340	HIST1H2BL	0.483
ENSG00000155307	64092	SAMSN1	0.505
ENSG00000204574	23	ABCF1	0.557
ENSG00000197238	8294	HIST1H4I	0.459
ENSG00000197914	8294	HIST1H4I	0.459
ENSG00000184348	8329	HIST1H2AI	0.501
ENSG00000173960	165324	UBXD4	0.482
ENSG00000147166	26548	ITGB1BP2	0.487
ENSG00000131080	60401	EDA2R	0.468
ENSG00000188931	257177	C1orf192	0.447
ENSG00000143226	2212	FCGR2A	0.558
ENSG00000196497	79711	IPO4	0.498
ENSG00000092009	1215	CMA1	0.438
ENSG00000147133	6872	TAF1	0.567
ENSG00000145996	54901	CDKAL1	0.548
ENSG00000100448	1511	CTSG	0.511
ENSG00000100450	2999	GZMH	0.43
ENSG00000100453	3002	GZMB	0.479
ENSG00000173110	3310	HSPA6	0.572
ENSG00000197279	7718	ZNF165	0.489
ENSG00000203747	2214	FCGR3A	0.548
ENSG00000168952	29091	STXBP6	0.583
ENSG00000196812	80345	ZSCAN16	0.469
ENSG00000123965	5383	PMS2L5	0.603
ENSG00000172179	5617	PRL	0.518
ENSG00000137185	7746	ZNF193	0.502
ENSG00000117091	962	CD48	0.513
ENSG00000072506	3028	HSD17B10	0.535
ENSG00000107593	9033	PKD2L1	0.456
ENSG00000072694	2213	FCGR2B	0.511
ENSG00000115128	51639	SF3B14	0.514
ENSG00000112273	154150	HDGFL1	0.419

ENSG00000026751	57823	SLAMF7	0.482
ENSG00000173557	339778	C2orf70	0.388
ENSG00000187626	387032	ZKSCAN4	0.489
ENSG00000157884	130106	CIB4	0.378
ENSG00000189134	222698	NKAPL	0.492
ENSG00000163554	6708	SPTA1	0.472
ENSG00000137338	84547	PGBD1	0.443
ENSG00000204568	28973	MRPS18B	0.57
ENSG00000154639	1525	CXADR	0.558
ENSG00000261915	2874	GPS2	0.479
ENSG00000162520	81493	SYNC1	0.46
ENSG00000235109	64288	ZNF323	0.475
ENSG00000215041	84461	KIAA1787	0.435
ENSG00000115129	9540	TP53I3	0.546
ENSG00000166091	116173	CMTM5	0.418
ENSG00000124532	57380	MRS2	0.597
ENSG00000244682	9103	FCGR2C	0.581
ENSG00000176248	29882	ANAPC2	0.482
ENSG00000115163	1058	CENPA	0.61
ENSG00000162747	2215	FCGR3B	0.531
ENSG00000166189	79803	HPS6	0.477
ENSG00000072818	9744	CENTB1	0.52
ENSG00000092140	55632	KIAA1333	0.591
ENSG00000197616	4624	MYH6	0.447
ENSG00000176101	8636	SSNA1	0.525
ENSG00000162975	3754	KCNF1	0.451
ENSG00000150873	130813	C2orf50	0.396
ENSG00000162976	130814	PQLC3	0.457
ENSG00000132185	84824	FCRLA	0.532
ENSG00000092054	4625	MYH7	0.46
ENSG00000159055	54069	C21orf45	0.515
ENSG00000187713	94107	TMEM203	0.5
ENSG00000188566	27158	NDOR1	0.478
ENSG00000170262	56246	MRAP	0.564
ENSG00000174292	8711	TNK1	0.585
ENSG00000163563	4332	MNDA	0.504
ENSG00000147174	93953	ACRC	0.434
ENSG00000186810	2833	CXCR3	0.564
ENSG00000163564	149628	PYHIN1	0.466
ENSG00000165643	402381	SOHLH1	0.475
ENSG00000198569	142680	SLC34A3	0.441
ENSG00000129460	25983	NGDN	0.62
ENSG00000204131	340527	NHSL2	0.41
ENSG00000188229	10383	TUBB2C	0.631
ENSG00000119777	54867	TMEM214	0.488
ENSG00000135372	55226	NAT10	0.539
ENSG00000163565	3428	IFI16	0.617
ENSG00000205544	254863	C17orf61	0.488
ENSG00000188163	401565	FAM166A	0.424
ENSG00000102309	5303	PIN4	0.475
ENSG00000197768	441476	LOC441476	0.467
ENSG00000181323	374768	SPEM1	0.407
ENSG00000081721	11266	DUSP12	0.536
ENSG00000166979	59271	C21orf63	0.445

ENSG00000151116	55293	UEVLD	0.472
ENSG00000188986	25920	COBRA1	0.513
ENSG00000181284	284114	TMEM102	0.325
ENSG00000198113	54863	C9orf167	0.535
ENSG00000186871	54821	ERCC6L	0.513
ENSG00000187609	54932	FLJ20433	0.512
ENSG00000259431	79178	THTPA	0.454
ENSG00000198034	6191	RPS4X	0.551
ENSG00000170175	1140	CHRNB1	0.508
ENSG00000132517	55065	GPR172B	0.354
ENSG00000204560	8449	DHX16	0.471
ENSG00000242220	140290	TCP10L	0.451
ENSG00000167840	7775	ZNF232	0.521
ENSG00000125931	4435	CITED1	0.478
ENSG00000121691	847	CAT	0.596
ENSG00000163568	9447	AIM2	0.526
ENSG00000188747	10811	NOXA1	0.48
ENSG00000119915	83401	ELOVL3	0.37
ENSG00000175061	125144	C17orf45	0.491
ENSG00000159079	56683	C21orf59	0.496
ENSG00000111802	51567	TTRAP	0.508
ENSG00000130560	10422	UBAC1	0.54
ENSG00000169006	23620	NTSR2	0.431
ENSG00000112304	55856	THEM2	0.579
ENSG00000239887	400793	FLJ13137	0.455
ENSG00000121903	7579	ZSCAN20	0.562
ENSG00000171722	284680	C1orf111	0.379
ENSG00000213088	2532	DARC	0.495
ENSG00000148411	138151	BTBD14A	0.454
ENSG00000161929	388325	C17orf87	0.591
ENSG00000241935	112817	C10orf65	0.518
ENSG00000170255	259249	MRGPRX1	0.364
ENSG00000183695	117194	MRGPRX2	0.462
ENSG00000149089	51074	APIP	0.538
ENSG00000177054	54503	ZDHHC13	0.537
ENSG00000100473	1690	COCH	0.481
ENSG00000137337	9656	MDC1	0.575
ENSG00000117143	6675	UAP1	0.554
ENSG00000108559	4927	NUP88	0.584
ENSG00000077150	4791	NFKB2	0.637
ENSG00000213221	728489	DNLZ	0.455
ENSG00000187796	64170	CARD9	0.457
ENSG00000130653	375775	PNPLA7	0.563
ENSG00000129170	8048	CSRP3	0.466
ENSG00000162733	4921	DDR2	0.528
ENSG00000129197	84268	RPAIN	0.546
ENSG00000165684	6621	SNAPC4	0.584
ENSG00000159086	94104	C21orf66	0.568
ENSG00000176256	127540	HMGB4	0.41
ENSG00000142698	84970	C1orf94	0.358
ENSG00000163866	113444	C1orf212	0.523
ENSG00000060688	9410	WDR57	0.529
ENSG00000189280	2709	GJB5	0.51
ENSG00000172070	140809	SRXN1	0.5

ENSG00000189433	127534	GJB4	0.462
ENSG00000188910	2707	GJB3	0.604
ENSG00000178921	5198	PFAS	0.541
ENSG00000182154	64975	MRPL41	0.454
ENSG00000148399	92715	WDR85	0.457
ENSG00000108561	708	C1QBP	0.602
ENSG00000187513	2701	GJA4	0.531
ENSG00000101276	113278	C20orf54	0.465
ENSG00000142694	55194	FAM176B	0.52
ENSG00000129226	968	CD68	0.562
ENSG00000213839	51075	TXNDC14	0.496
ENSG00000005100	56919	DHX33	0.475
ENSG00000165688	23203	PMPCA	0.52
ENSG00000148384	56623	INPP5E	0.523
ENSG00000091592	22861	NLRP1	0.618
ENSG00000116885	127700	C1orf102	0.492
ENSG00000203993	85026	C9orf37	0.475
ENSG00000197056	79830	ZMYM1	0.46
ENSG00000121764	3061	HCRTR1	0.456
ENSG00000116898	64960	MRPS15	0.503
ENSG00000149090	25891	DKFZP586H2123	0.479
ENSG00000184650	146852	ODF4	0.54
ENSG00000184619	124751	KRBA2	0.37
ENSG00000263809	124751	KRBA2	0.37
ENSG00000109854	10553	HTATIP2	0.594
ENSG00000161970	6154	RPL26	0.526
ENSG00000129221	23746	AIPL1	0.466
ENSG00000119535	1441	CSF3R	0.538
ENSG00000196366	158055	C9orf163	0.464
ENSG00000185238	10196	PRMT3	0.517
ENSG00000129195	54478	FAM64A	0.515
ENSG00000180720	1132	CHRM4	0.393
ENSG00000172889	51162	EGFL7	0.498
ENSG00000165970	9152	SLC6A5	0.429
ENSG00000091622	83394	PITPNM3	0.424
ENSG00000169692	10555	AGPAT2	0.546
ENSG00000165973	4745	NELL1	0.489
ENSG00000180423	283254	HARBI1	0.425
ENSG00000165716	138311	FAM69B	0.533
ENSG00000175224	9776	KIAA0652	0.609
ENSG00000187922	414332	LCN10	0.397
ENSG00000125900	128646	SIRPD	0.391
ENSG00000161973	146849	CCDC42	0.443
ENSG00000163875	64769	C1orf149	0.501
ENSG00000101307	10326	SIRPB1	0.446
ENSG00000185156	162387	FLJ35773	0.429
ENSG00000141506	23533	PIK3R5	0.457
ENSG00000089012	55423	SIRPG	0.484
ENSG00000173137	203054	ADCK5	0.419
ENSG00000170310	9482	STX8	0.5
ENSG00000182557	201305	SPNS3	0.443
ENSG00000134697	29889	GNL2	0.518
ENSG00000160323	11093	ADAMTS13	0.455
ENSG00000166596	146845	WDR16	0.461

ENSG00000071894	29894	CPSF1	0.579
ENSG00000175213	79797	ZNF408	0.469
ENSG00000180210	2147	F2	0.484
ENSG00000166948	343641	TGM6	0.42
ENSG00000130775	9473	C1orf38	0.572
ENSG00000006071	6833	ABCC8	0.548
ENSG00000184544	201140	DHRS7C	0.441
ENSG00000147804	55630	SLC39A4	0.523
ENSG00000144057	84620	ST6GAL2	0.434
ENSG00000167741	124975	GGT6	0.432
ENSG00000149488	117532	TMC2	0.42
ENSG00000101361	10528	NOL5A	0.615
ENSG00000130768	27293	SMPDL3B	0.467
ENSG00000113296	7060	THBS4	0.501
ENSG00000161905	246	ALOX15	0.465
ENSG00000118434	81833	SPACA1	0.416
ENSG00000135766	54583	EGLN1	0.509
ENSG00000160948	51160	VPS28	0.488
ENSG00000006611	10083	USH1C	0.578
ENSG00000163749	339965	FLJ25770	0.379
ENSG00000164300	256987	SERINC5	0.703
ENSG00000149179	79096	C11orf49	0.515
ENSG00000141456	27043	PELP1	0.496
ENSG00000198203	6819	SULT1C2	0.549
ENSG00000160326	11182	SLC2A6	0.521
ENSG00000101365	3420	IDH3B	0.6
ENSG00000164299	84654	SPZ1	0.331
ENSG00000160949	4796	NFKBIL2	0.458
ENSG00000006788	8735	MYH13	0.446
ENSG00000176034	286464	CXorf59	0.439
ENSG00000187954	50626	CYHR1	0.576
ENSG00000160688	80308	FLAD1	0.572
ENSG00000198075	27233	SULT1C4	0.394
ENSG00000122224	4063	LY9	0.537
ENSG00000198173	442444	FAM47C	0.371
ENSG00000176020	386724	AMIGO3	0.373
ENSG00000185189	340371	NRBP2	0.477
ENSG00000146966	27147	DENND2A	0.584
ENSG00000133020	4626	MYH8	0.53
ENSG00000108669	9267	PSCD1	0.569
ENSG00000176732	375189	PFN4	0.439
ENSG00000130052	9754	STARD8	0.488
ENSG00000125485	64794	DDX31	0.488
ENSG00000267206	158062	LCN6	0.456
ENSG00000264424	4622	MYH4	0.392
ENSG00000154548	135295	SRrp35	0.457
ENSG00000171714	203859	TMEM16E	0.469
ENSG00000186453	653140	FLJ30851	0.373
ENSG00000152380	167555	FAM151B	0.45
ENSG00000075290	7479	WNT8B	0.469
ENSG00000255515	29104	N6AMT1	0.428
ENSG00000169403	5724	PTAFR	0.552
ENSG00000204001	138307	LCN8	0.507
ENSG00000123453	1757	SARDH	0.58

ENSG00000146281	135293	PM20D2	0.563
ENSG00000146276	2569	GABRR1	0.467
ENSG00000088882	56265	CPXM1	0.503
ENSG00000133808	84953	MICALCL	0.402
ENSG00000133597	90956	ADCK2	0.652
ENSG00000134297	51054	PLEKHA9	0.578
ENSG00000228716	1719	DHFR	0.633
ENSG00000165912	29763	PACSIN3	0.477
ENSG00000177984	389812	RP11-216L13.5	0.4
ENSG00000118816	10983	CCNI	0.578
ENSG00000108381	443	ASPA	0.47
ENSG00000129158	26297	SERGEF	0.497
ENSG00000090266	4708	NDUFB2	0.55
ENSG00000258713	128653	C20orf141	0.495
ENSG00000176095	9807	IHPK1	0.5
ENSG00000136546	6332	SCN7A	0.432
ENSG00000244187	85014	TMEM141	0.52
ENSG00000108590	51003	MED31	0.512
ENSG00000108469	9400	RECQL5	0.601
ENSG00000160973	8928	FOXH1	0.444
ENSG00000109061	4619	MYH1	0.413
ENSG00000160972	84988	PPP1R16A	0.507
ENSG00000111886	2570	GABRR2	0.453
ENSG00000227184	83481	EPPK1	0.472
ENSG00000134574	1643	DDB2	0.574
ENSG00000113318	4437	MSH3	0.533
ENSG00000178209	5339	PLEC1	0.603
ENSG00000130770	93974	ATPIF1	0.542
ENSG00000212734	388327	hCG_1985469	0.411
ENSG00000215305	64601	VPS16	0.563
ENSG00000163354	127579	DCST2	0.314
ENSG00000238105	55592	GOLGA2L1	0.461
ENSG00000187516	25763	CXorf27	0.449
ENSG00000161920	400569	MED11	0.469
ENSG00000170788	143241	DYDC1	0.438
ENSG00000181191	64219	PJA1	0.546
ENSG00000161921	58191	CXCL16	0.507
ENSG00000157764	673	BRAF	0.545
ENSG00000167701	2875	GPT	0.478
ENSG00000182134	11022	TDRKH	0.543
ENSG00000133665	84332	DYDC2	0.36
ENSG00000130772	54797	MED18	0.532
ENSG00000154646	5651	PRSS7	0.533
ENSG00000109063	4621	MYH3	0.456
ENSG00000141497	84225	ZMYND15	0.425
ENSG00000232040	114821	SCAND3	0.425
ENSG00000198833	51465	UBE2J1	0.651
ENSG00000116954	64121	RRAGC	0.522
ENSG00000185614	389119	C3orf54	0.44
ENSG00000100926	10548	TM9SF1	0.553
ENSG00000182179	7318	UBA7	0.591
ENSG00000125484	9329	GTF3C4	0.485
ENSG00000163357	149095	DCST1	0.42
ENSG00000132530	54739	XAF1	0.506

ENSG00000156234	10563	CXCL13	0.462
ENSG00000122378	84293	C10orf58	0.594
ENSG00000133028	6341	SCO1	0.479
ENSG00000090263	51650	MRPS33	0.536
ENSG00000142484	9032	TM4SF5	0.495
ENSG00000134343	63982	TMEM16C	0.477
ENSG00000166105	112937	GLB1L3	0.425
ENSG00000166788	113174	SAAL1	0.482
ENSG00000060642	55650	PIGV	0.508
ENSG00000110347	4321	MMP12	0.517
ENSG00000165695	158067	C9orf98	0.385
ENSG00000113319	5924	RASGRF2	0.474
ENSG00000167700	113655	MFSD3	0.51
ENSG00000156313	6103	RPGR	0.489
ENSG00000179914	55600	ITLN1	0.455
ENSG00000177294	162517	FBXO39	0.348
ENSG00000166295	119504	C10orf104	0.617
ENSG00000182853	284013	VMO1	0.5
ENSG00000127957	5387	PMS2L3	0.644
ENSG00000134668	90853	SPOCD1	0.521
ENSG00000182327	388323	GLTPD2	0.441
ENSG00000262304	7442	TRPV1	0.472
ENSG00000254692	10548	TM9SF1	0.553
ENSG00000172318	8708	B3GALT1	0.464
ENSG00000160959	9684	LRRC14	0.555
ENSG00000139908	283629	TSSK4	0.402
ENSG00000151611	166785	MMAA	0.54
ENSG00000196689	7442	TRPV1	0.472
ENSG00000167858	83659	TEKT1	0.42
ENSG00000142507	5694	PSMB6	0.534
ENSG00000197935	282890	ZNF311	0.466
ENSG00000054148	29085	PHPT1	0.508
ENSG00000177494	79413	ZBED2	0.461
ENSG00000154719	54148	MRPL39	0.521
ENSG00000169550	143662	MUC15	0.45
ENSG00000240891	257068	PLCXD2	0.493
ENSG00000165698	11092	C9orf9	0.473
ENSG00000142751	54707	GPN2	0.491
ENSG00000135778	84284	C1orf57	0.521
ENSG00000128524	9296	ATP6V1F	0.544
ENSG00000101958	2742	GLRA2	0.478
ENSG00000177943	158056	MAMDC4	0.419
ENSG00000169288	65008	MRPL1	0.535
ENSG00000148942	159963	SLC5A12	0.475
ENSG00000179826	117195	MRGPRX3	0.436
ENSG00000213563	414919	MGC70857	0.478
ENSG00000135749	80003	PCNXL2	0.566
ENSG00000179817	117196	MRGPRX4	0.413
ENSG00000254505	29082	CHMP4A	0.564
ENSG00000219200	440400	RNASEK	0.494
ENSG00000148965	6291	SAA4	0.488
ENSG00000129151	8424	BBOX1	0.469
ENSG00000108679	3959	LGALS3BP	0.547
ENSG00000147799	80728	KIAA1688	0.413

ENSG00000163006	165055	CCDC138	0.531
ENSG00000112462	81797	OR12D3	0.409
ENSG00000186912	5030	P2RY4	0.491
ENSG00000181544	2187	FANCB	0.487
ENSG00000168787	26529	OR12D2	0.438
ENSG00000134339	6289	SAA2	0.5
ENSG00000120500	407	ARR3	0.476
ENSG00000090273	10726	NUDC	0.594
ENSG00000257093	57189	KIAA1147	0.531
ENSG00000163072	115677	NOSTRIN	0.493
ENSG00000131730	1160	CKMT2	0.495
ENSG00000173432	6288	SAA1	0.493
ENSG00000198169	90987	ZNF251	0.489
ENSG00000154721	58494	JAM2	0.471
ENSG00000164167	11157	LSM6	0.529
ENSG00000106028	6742	SSBP1	0.629
ENSG00000180098	54952	TRSPAP1	0.505
ENSG00000254996	54882	ANKHD1	0.503
ENSG00000127191	7186	TRAF2	0.491
ENSG00000036473	5009	OTC	0.452
ENSG00000178719	2907	GRINA	0.542
ENSG00000165702	8328	GFI1B	0.403
ENSG00000213886	10537	UBD	0.48
ENSG00000118508	10981	RAB32	0.518
ENSG00000131732	84240	ZCCHC9	0.445
ENSG00000186583	375686	SPATC1	0.441
ENSG00000127362	50831	TAS2R3	0.429
ENSG00000118492	79747	C6orf103	0.36
ENSG00000127366	54429	TAS2R5	0.406
ENSG00000196378	80778	ZNF34	0.475
ENSG00000253368	388610	TNRP	0.556
ENSG00000165076	136242	LOC136242	0.404
ENSG00000178896	54512	EXOSC4	0.571
ENSG00000148308	9328	GTF3C5	0.478
ENSG00000102048	140462	ASB9	0.503
ENSG00000257335	8972	MGAM	0.445
ENSG00000159069	54461	FBXW5	0.452
ENSG00000197747	6281	S100A10	0.533
ENSG00000161016	6132	RPL8	0.554
ENSG00000174326	162515	SLC16A11	0.494
ENSG00000213523	10011	SRA1	0.496
ENSG00000163191	6282	S100A11	0.559
ENSG00000152253	57405	SPC25	0.569
ENSG00000197858	8733	GPAA1	0.579
ENSG00000138315	170392	OIT3	0.435
ENSG00000166136	4714	NDUFB8	0.62
ENSG00000132514	10462	CLEC10A	0.492
ENSG00000188373	387695	C10orf99	0.48
ENSG00000148600	92211	PCDH21	0.465
ENSG00000120509	51248	PDZD11	0.51
ENSG00000112159	23195	MDN1	0.521
ENSG00000176919	733	C8G	0.463
ENSG00000129559	4738	NEDD8	0.513
ENSG00000161944	433	ASGR2	0.477

ENSG00000188060	115273	RAB42	0.439
ENSG00000162419	10691	GMEB1	0.451
ENSG00000127774	83460	TMEM93	0.466
ENSG00000164078	4486	MST1R	0.508
ENSG00000121621	81930	KIF18A	0.466
ENSG00000143631	2312	FLG	0.46
ENSG00000184925	286256	LCN12	0.441
ENSG00000154723	522	ATP5J	0.554
ENSG00000073734	8647	ABCB11	0.496
ENSG00000258227	23601	CLEC5A	0.477
ENSG00000083454	5026	P2RX5	0.525
ENSG00000167280	64772	FLJ21865	0.585
ENSG00000138772	306	ANXA3	0.545
ENSG00000137473	83894	TTC29	0.355
ENSG00000148602	26103	LRIT1	0.416
ENSG00000100938	51292	GMPR2	0.514
ENSG00000148604	5995	RGR	0.468
ENSG00000141505	432	ASGR1	0.479
ENSG00000182220	10159	ATP6AP2	0.648
ENSG00000073737	10170	DHRS9	0.522
ENSG00000143536	49860	CRNN	0.408
ENSG00000107317	5730	PTGDS	0.602
ENSG00000257138	5726	TAS2R38	0.309
ENSG00000163202	84648	LCE3D	0.448
ENSG00000143674	84451	KIAA1804	0.436
ENSG00000197363	340385	ZNF517	0.37
ENSG00000160339	2220	FCN2	0.529
ENSG00000179091	1537	CYC1	0.587
ENSG00000185245	2811	GP1BA	0.505
ENSG00000159455	26239	LCE2B	0.462
ENSG00000167281	146713	HRNBP3	0.526
ENSG00000138756	55589	BMP2K	0.597
ENSG00000179526	81858	SHARPIN	0.464
ENSG00000174500	257144	GCET2	0.396
ENSG00000087842	8544	PIR	0.544
ENSG00000107771	54462	KIAA1128	0.553
ENSG00000131808	2488	FSHB	0.451
ENSG00000015568	84220	RGPD5	0.536
ENSG00000108528	8402	SLC25A11	0.565
ENSG00000152219	120534	C11orf46	0.495
ENSG00000185753	159013	CXorf38	0.456
ENSG00000085265	2219	FCN1	0.484
ENSG00000186226	353135	LCE1E	0.455
ENSG00000264198	22806	IKZF3	0.454
ENSG00000258223	136541	TRYX3	0.425
ENSG00000108479	2584	GALK1	0.466
ENSG00000214402	401562	FLJ45224	0.5
ENSG00000170835	1056	CEL	0.54
ENSG00000168275	388753	C1orf31	0.488
ENSG00000148362	286257	C9orf142	0.436
ENSG00000197084	353133	LCE1C	0.412
ENSG00000059588	6894	TARBP1	0.498
ENSG00000129083	1315	COPB1	0.573
ENSG00000196734	353132	LCE1B	0.489

ENSG00000182156	339221	ENPP7	0.378
ENSG00000184924	391356	LOC391356	0.478
ENSG00000102010	660	BMX	0.446
ENSG00000147789	7553	ZNF7	0.538
ENSG00000169583	9022	CLIC3	0.463
ENSG00000083457	3682	ITGAE	0.547
ENSG00000166321	25961	NUDT13	0.464
ENSG00000108523	26001	RNF167	0.488
ENSG00000163206	4184	SMCP	0.485
ENSG00000164169	90826	LOC90826	0.454
ENSG00000163207	3713	IVL	0.466
ENSG00000177791	58529	MYOZ1	0.448
ENSG00000004534	10180	RBM6	0.555
ENSG00000184148	163778	SPRR4	0.455
ENSG00000170458	929	CD14	0.55
ENSG00000169474	6698	SPRR1A	0.541
ENSG00000163209	6707	SPRR3	0.476
ENSG00000130234	59272	ACE2	0.555
ENSG00000167291	125058	TBC1D16	0.53
ENSG00000170619	28991	COMMD5	0.564
ENSG00000186919	353174	ZACN	0.367
ENSG00000137700	2542	SLC37A4	0.581
ENSG00000131495	4695	NDUFA2	0.587
ENSG00000256977	96626	LIMS3	0.438
ENSG00000172650	119385	KIAA1975	0.55
ENSG00000108515	2027	ENO3	0.501
ENSG00000169469	6699	SPRR1B	0.483
ENSG00000196475	2712	GK2	0.465
ENSG00000163216	6703	SPRR2D	0.496
ENSG00000222046	149069	DCDC2B	0.386
ENSG00000129084	5682	PSMA1	0.594
ENSG00000170946	120526	DNAJC24	0.466
ENSG00000160345	138162	C9orf116	0.537
ENSG00000241794	6700	SPRR2A	0.514
ENSG00000160055	56063	C1orf91	0.526
ENSG00000204815	83538	TTC25	0.489
ENSG00000196805	6701	SPRR2B	0.423
ENSG00000182687	8811	GALR2	0.432
ENSG00000122882	11319	ECD	0.519
ENSG00000162755	126823	KLHDC9	0.423
ENSG00000144848	64422	ATG3	0.553
ENSG00000204983	5644	PRSS1	0.404
ENSG00000235173	51236	C8orf30A	0.591
ENSG00000148950	196294	IMMP1L	0.538
ENSG00000186468	6228	RPS23	0.525
ENSG00000130055	54857	GDPD2	0.48
ENSG00000177602	83903	GSG2	0.406
ENSG00000122140	51116	MRPS2	0.481
ENSG00000159516	6706	SPRR2G	0.375
ENSG00000141519	55036	CCDC40	0.533
ENSG00000163093	129880	BBS5	0.419
ENSG00000261530	685	BTC	0.469
ENSG00000092330	26277	TINF2	0.503
ENSG00000084623	8668	EIF3I	0.542

ENSG00000092929	201294	UNC13D	0.496
ENSG00000188739	23029	RBM34	0.608
ENSG00000179832	727957	KIAA1833	0.5
ENSG00000109911	26610	ELP4	0.48
ENSG00000116350	6429	SFRS4	0.55
ENSG00000160349	3933	LCN1	0.457
ENSG00000115137	51277	RBJ	0.553
ENSG00000122136	29991	OBP2A	0.42
ENSG00000159527	114771	PGLYRP3	0.481
ENSG00000143256	5202	PFDN2	0.5
ENSG00000055208	23118	MAP3K7IP2	0.585
ENSG00000166796	3948	LDHC	0.44
ENSG00000205464	92270	LOC92270	0.512
ENSG00000163218	57115	PGLYRP4	0.47
ENSG00000142765	84958	SYTL1	0.492
ENSG00000158793	4817	NIT1	0.52
ENSG00000113141	3550	IK	0.516
ENSG00000170631	7564	ZNF16	0.447
ENSG00000147003	57393	TMEM27	0.46
ENSG00000169241	55974	RAG1AP1	0.459
ENSG00000055950	84545	MRPL43	0.514
ENSG00000239474	10324	KBTBD10	0.444
ENSG00000196968	170384	FUT11	0.44
ENSG00000116353	51102	MECR	0.459
ENSG00000174695	153339	TMEM167A	0.576
ENSG00000172586	118487	CHCHD1	0.466
ENSG00000091732	51530	ZC3HC1	0.447
ENSG00000171298	2548	GAA	0.539
ENSG00000142733	9064	MAP3K6	0.485
ENSG00000138399	79675	FASTKD1	0.505
ENSG00000163220	6280	S100A9	0.539
ENSG00000179085	54344	DPM3	0.498
ENSG00000163221	6283	S100A12	0.517
ENSG00000204657	7932	OR2H2	0.526
ENSG00000169249	8233	ZRSR2	0.532
ENSG00000256206	5682	PSMA1	0.594
ENSG00000182307	65265	C8orf33	0.509
ENSG00000148288	26301	GBGT1	0.512
ENSG00000106554	54927	CHCHD3	0.497
ENSG00000163463	200185	KRTCAP2	0.526
ENSG00000184330	338324	S100A7A	0.439
ENSG00000152422	7518	XRCC4	0.609
ENSG00000204655	4340	MOG	0.581
ENSG00000122133	5047	PAEP	0.492
ENSG00000178199	340152	ZC3H12D	0.455
ENSG00000143556	6278	S100A7	0.458
ENSG00000166800	160287	LDHAL6A	0.411
ENSG00000128607	23008	KIAA0265	0.601
ENSG00000141543	9775	EIF4A3	0.548
ENSG00000197956	6277	S100A6	0.537
ENSG00000092295	7051	TGM1	0.486
ENSG00000180549	2529	FUT7	0.56
ENSG00000188937	60506	NYX	0.459
ENSG00000107281	56654	NPDC1	0.526

ENSG00000173269	79812	MMRN2	0.522
ENSG00000003756	10181	RBM5	0.637
ENSG00000184937	7490	WT1	0.54
ENSG00000047230	56474	CTPS2	0.506
ENSG00000186104	120227	CYP2R1	0.465
ENSG00000171102	29989	OBP2B	0.421
ENSG00000196420	6276	S100A5	0.473
ENSG00000182180	51021	MRPS16	0.52
ENSG00000054179	954	ENTPD2	0.476
ENSG00000254285	3856	KRT8	0.529
ENSG00000173267	6623	SNCG	0.555
ENSG00000110680	796	CALCA	0.589
ENSG00000127412	56302	TRPV5	0.469
ENSG00000170727	23246	BOP1	0.511
ENSG00000196154	6275	S100A4	0.579
ENSG00000256453	373863	DND1	0.415
ENSG00000148671	10974	C10orf116	0.497
ENSG00000256671	96626	LIMS3	0.438
ENSG00000267080	339201	C17orf65	0.492
ENSG00000186193	89958	C9orf140	0.446
ENSG00000141527	79092	CARD14	0.536
ENSG00000197826	255119	C4orf22	0.345
ENSG00000162526	81629	TSSK3	0.485
ENSG00000100949	5875	RABGGTA	0.534
ENSG00000148296	6838	SURF6	0.5
ENSG00000149100	10480	EIF3M	0.596
ENSG00000156042	118491	TTC18	0.54
ENSG00000183054	84220	RGPD5	0.536
ENSG00000142748	8547	FCN3	0.484
ENSG00000197355	91373	UAP1L1	0.471
ENSG00000115138	5443	POMC	0.458
ENSG00000169906	795	S100G	0.467
ENSG00000196754	6273	S100A2	0.596
ENSG00000197993	3792	KEL	0.482
ENSG00000129646	84074	QRICH2	0.42
ENSG00000154479	129881	LOC129881	0.436
ENSG00000055211	116254	C6orf72	0.541
ENSG00000156239	29104	N6AMT1	0.428
ENSG00000144362	493911	PHOSPHO2	0.473
ENSG00000158516	1358	CPA2	0.518
ENSG00000155530	136332	LRGUK	0.494
ENSG00000213160	151230	KLHL23	0.493
ENSG00000188643	140576	S100A16	0.495
ENSG00000164176	10085	EDIL3	0.484
ENSG00000163607	29083	GTPBP8	0.535
ENSG00000170291	23587	C17orf81	0.5
ENSG00000161542	5635	PRPSAP1	0.522
ENSG00000120498	56159	TEX11	0.471
ENSG00000169679	699	BUB1	0.616
ENSG00000148303	6130	RPL7A	0.522
ENSG00000138385	6741	SSB	0.576
ENSG00000181523	6448	SGSH	0.605
ENSG00000127184	1350	COX7C	0.617
ENSG00000132481	91107	TRIM47	0.495

ENSG00000198862	26046	ZNF294	0.504
ENSG00000189171	6284	S100A13	0.589
ENSG00000122376	54537	FAM35A	0.491
ENSG00000163608	25871	C3orf17	0.536
ENSG00000085662	231	AKR1B1	0.58
ENSG00000204644	346171	ZFP57	0.388
ENSG00000159763	5304	PIP	0.501
ENSG00000126705	27245	AHDC1	0.499
ENSG00000204642	3134	HLA-F	0.626
ENSG00000176978	29952	DPP7	0.588
ENSG00000141569	201292	TRIM65	0.458
ENSG00000131023	9113	LATS1	0.496
ENSG00000121690	91614	DEPDC7	0.44
ENSG00000107789	9562	MINPP1	0.504
ENSG00000014123	23376	KIAA0776	0.577
ENSG00000145425	6189	RPS3A	0.546
ENSG00000204316	64978	MRPL38	0.492
ENSG00000180815	389840	MAP3K15	0.522
ENSG00000204632	3135	HLA-G	0.612
ENSG00000158525	93979	CPA5	0.408
ENSG00000143224	5498	PPOX	0.502
ENSG00000176261	339487	ZBTB8OS	0.419
ENSG00000198074	57016	AKR1B10	0.51
ENSG00000138382	29081	METTL5	0.581
ENSG00000171659	2857	GPR34	0.515
ENSG00000160678	6271	S100A1	0.483
ENSG00000186862	79955	PDZD7	0.421
ENSG00000143578	148327	CREB3L4	0.53
ENSG00000171657	27197	GPR82	0.417
ENSG00000147164	29934	SNX12	0.553
ENSG00000110696	10944	C11orf58	0.57
ENSG00000172331	669	BPGM	0.55
ENSG00000109686	152503	SH3D19	0.497
ENSG00000129673	15	AANAT	0.455
ENSG00000148290	6834	SURF1	0.581
ENSG00000188878	85302	FBF1	0.574
ENSG00000091704	1357	CPA1	0.419
ENSG00000126709	2537	IFI6	0.525
ENSG00000166343	118490	ZMYND17	0.405
ENSG00000148291	6835	SURF2	0.497
ENSG00000167740	124936	CYB5D2	0.443
ENSG00000206503	3105	HLA-A	0.588
ENSG00000143543	10899	JTB	0.609
ENSG00000196943	161424	C14orf21	0.429
ENSG00000146007	153527	ZMAT2	0.508
ENSG00000136305	27141	CIDEB	0.504
ENSG00000071909	140469	MYO3B	0.482
ENSG00000147168	3561	IL2RG	0.502
ENSG00000185803	79581	GPR172A	0.55
ENSG00000107821	81621	KAZALD1	0.575
ENSG00000120256	84918	LRP11	0.562
ENSG00000110700	6207	RPS13	0.505
ENSG00000213906	56413	LTB4R2	0.439
ENSG00000255408	56145	PCDHA3	0.436

ENSG00000198870	169436	C9orf96	0.435
ENSG00000206530	55779	WDR52	0.479
ENSG00000239521	352954	tcag7.1177	0.446
ENSG00000131831	10742	RAI2	0.456
ENSG00000213903	1241	LTB4R	0.543
ENSG00000182325	26233	FBXL6	0.498
ENSG00000059691	5188	PET112L	0.5
ENSG00000148300	57109	REXO4	0.463
ENSG00000070526	55808	ST6GALNAC1	0.455
ENSG00000177324	139105	CXorf20	0.39
ENSG00000164520	135250	RAET1E	0.429
ENSG00000129467	196883	ADCY4	0.457
ENSG00000070081	4925	NUCB2	0.535
ENSG00000177954	6232	RPS27	0.528
ENSG00000105875	29062	WDR91	0.484
ENSG00000204961	9752	PCDHA9	0.479
ENSG00000131015	80328	ULBP2	0.479
ENSG00000111981	80329	ULBP1	0.448
ENSG00000143552	91181	NUP210L	0.385
ENSG00000163611	152185	CCDC52	0.443
ENSG00000008086	6792	CDKL5	0.551
ENSG00000169418	4881	NPR1	0.556
ENSG00000158864	4720	NDUFS2	0.518
ENSG00000250506	1018	CDK3	0.489
ENSG00000156110	132	ADK	0.606
ENSG00000167880	2125	EVPL	0.487
ENSG00000066379	30834	ZNRD1	0.542
ENSG00000156261	10694	CCT8	0.626
ENSG00000086717	5475	PPEF1	0.504
ENSG00000110651	975	CD81	0.544
ENSG00000181038	124512	C17orf95	0.453
ENSG00000166171	25911	RP11-529I10.4	0.481
ENSG00000254535	132430	PABPC4L	0.4
ENSG00000158748	3362	HTR6	0.474
ENSG00000162542	255104	TMCO4	0.443
ENSG00000114113	5948	RBP2	0.417
ENSG00000161547	6427	SFRS2	0.667
ENSG00000184281	10078	TSSC4	0.464
ENSG00000152292	284948	SH2D6	0.39
ENSG00000155561	23165	NUP205	0.563
ENSG00000158869	2207	FCER1G	0.552
ENSG00000204616	11074	TRIM31	0.6
ENSG00000114115	5947	RBP1	0.504
ENSG00000255398	8843	GPR109B	0.488
ENSG00000070985	29850	TRPM5	0.419
ENSG00000129465	11035	RIPK3	0.471
ENSG00000158874	336	APOA2	0.527
ENSG00000178828	54546	RNF186	0.478
ENSG00000163864	349565	NMNAT3	0.486
ENSG00000130783	84660	CCDC62	0.46
ENSG00000149970	22866	CNKSR2	0.476
ENSG00000164509	133396	IL31RA	0.486
ENSG00000155749	130540	ALS2CR12	0.457
ENSG00000188257	5320	PLA2G2A	0.524

ENSG00000151806	60558	GUF1	0.492
ENSG00000079393	51207	DUSP13	0.5
ENSG00000254440	59351	PBOV1	0.431
ENSG00000164707	26266	SLC13A4	0.476
ENSG00000137463	84709	OSAP	0.443
ENSG00000051620	23593	HEBP2	0.538
ENSG00000127472	5322	PLA2G5	0.557
ENSG00000133958	57578	KIAA1409	0.481
ENSG00000109390	4717	NDUFC1	0.543
ENSG00000155890	287015	TRIM42	0.518
ENSG00000130787	9026	HIP1R	0.491
ENSG00000204614	135644	TRIM40	0.322
ENSG00000152104	5784	PTPN14	0.504
ENSG00000156265	56911	C21orf7	0.475
ENSG00000164758	90390	MED30	0.515
ENSG00000081148	50939	IMPG2	0.448
ENSG00000204613	10107	TRIM10	0.579
ENSG00000087206	51720	UIMC1	0.54
ENSG00000134352	3572	IL6ST	0.678
ENSG00000082146	55437	ALS2CR2	0.496
ENSG00000162543	127733	UBXD3	0.5
ENSG00000169562	2705	GJB1	0.508
ENSG00000164134	80155	NARG1	0.506
ENSG00000163285	2565	GABRG1	0.456
ENSG00000143257	9970	NR1I3	0.453
ENSG00000136840	27090	ST6GALNAC4	0.56
ENSG00000024862	25901	CCDC28A	0.538
ENSG00000117724	1063	CENPF	0.603
ENSG00000110628	5002	SLC22A18	0.524
ENSG00000196345	55888	ZNF167	0.475
ENSG00000204610	89870	TRIM15	0.566
ENSG00000164761	4982	TNFRSF11B	0.536
ENSG00000170516	170712	COX7B2	0.36
ENSG00000187581	341947	COX8C	0.351
ENSG00000184374	10584	COLEC10	0.442
ENSG00000140067	90050	C14orf152	0.5
ENSG00000100628	51676	ASB2	0.529
ENSG00000136960	5168	ENPP2	0.598
ENSG00000168894	51255	RNF181	0.511
ENSG00000144792	285349	ZNF660	0.429
ENSG00000155903	5922	RASA2	0.476
ENSG00000174173	54931	RG9MTD1	0.487
ENSG00000186448	10168	ZNF197	0.499
ENSG00000169019	54951	COMMD8	0.512
ENSG00000168890	129303	TMEM150	0.504
ENSG00000115263	2641	GCG	0.461
ENSG00000082126	58538	MPP4	0.5
ENSG00000158828	65018	PINK1	0.601
ENSG00000085871	4258	MGST2	0.546
ENSG00000089737	57062	DDX24	0.585
ENSG00000114391	6152	RPL24	0.542
ENSG00000169981	7584	ZNF35	0.472
ENSG00000155542	133383	C5orf35	0.441
ENSG00000241370	79897	RPP21	0.468

ENSG00000110619	833	CARS	0.559
ENSG00000069849	483	ATP1B3	0.556
ENSG00000196653	91392	ZNF502	0.512
ENSG00000165948	122509	FAM14B	0.463
ENSG00000186446	115560	ZNF501	0.483
ENSG00000111325	79676	OGFOD2	0.583
ENSG00000168883	10713	USP39	0.449
ENSG00000171189	2897	GRIK1	0.584
ENSG00000114126	7029	TFDP2	0.582
ENSG00000115267	64135	IFIH1	0.553
ENSG00000163807	57456	KIAA1143	0.556
ENSG00000107833	10360	NPM3	0.537
ENSG00000204592	3133	HLA-E	0.629
ENSG00000120725	64374	SIL1	0.509
ENSG00000136982	79075	DSCC1	0.593
ENSG00000163808	56992	KIF15	0.529
ENSG00000261742	283867	LOC283867	0.386
ENSG00000155792	64798	DEPDC6	0.532
ENSG00000187955	7373	COL14A1	0.586
ENSG00000138018	85465	SELI	0.512
ENSG00000135577	4829	NMBR	0.463
ENSG00000115271	25801	GCA	0.55
ENSG00000138395	65061	PFTK2	0.549
ENSG00000205978	57523	KIAA1305	0.474
ENSG00000197683	388818	KRTAP26-1	0.458
ENSG00000177335	286122	C8orf31	0.5
ENSG00000182816	337959	KRTAP13-2	0.333
ENSG00000157856	92749	C2orf39	0.381
ENSG00000198390	140258	KRTAP13-1	0.366
ENSG00000119632	83982	FAM14A	0.569
ENSG00000182196	51329	ARL6IP4	0.55
ENSG00000100441	23351	KIAA0323	0.629
ENSG00000176956	4062	LY6H	0.465
ENSG00000148334	80142	PTGES2	0.502
ENSG00000119698	57718	KIAA1622	0.537
ENSG00000244025	337970	KRTAP19-3	0.435
ENSG00000182851	338328	GPIHBP1	0.443
ENSG00000181638	286128	ZFP41	0.54
ENSG00000140093	51156	SERPINA10	0.404
ENSG00000115155	9381	OTOF	0.473
ENSG00000170099	866	SERPINA6	0.467
ENSG00000109445	27309	ZNF330	0.608
ENSG00000264668	286128	ZFP41	0.54
ENSG00000250571	2738	GLI4	0.459
ENSG00000074660	8578	SCARF1	0.481
ENSG00000100445	56948	C14orf124	0.504
ENSG00000169964	131616	TMEM42	0.48
ENSG00000185730	79943	ZNF696	0.45
ENSG00000116030	7341	SUMO1	0.651
ENSG00000197249	5265	SERPINA1	0.59
ENSG00000115290	2888	GRB14	0.485
ENSG00000131183	6569	SLC34A1	0.526
ENSG00000172172	28998	MRPL13	0.521
ENSG00000164136	3600	IL15	0.589

ENSG00000163810	7047	TGM4	0.59
ENSG00000148346	3934	LCN2	0.5
ENSG00000131187	2161	F12	0.545
ENSG00000163293	152519	NPAL1	0.473
ENSG00000055044	51602	NOP5/NOP58	0.523
ENSG00000172167	27085	MTBP	0.533
ENSG00000168878	6439	SFTPB	0.625
ENSG00000184428	116447	TOP1MT	0.473
ENSG00000198515	1259	CNGA1	0.455
ENSG00000167705	83547	RILP	0.457
ENSG00000151917	221336	C6orf65	0.467
ENSG00000172164	6641	SNTB1	0.617
ENSG00000171159	79095	C9orf16	0.642
ENSG00000074966	7294	TXK	0.529
ENSG00000115523	10578	GNLY	0.544
ENSG00000175054	545	ATR	0.505
ENSG00000175857	202309	C5orf29	0.415
ENSG00000168116	57691	KIAA1586	0.5
ENSG00000050555	10319	LAMC3	0.457
ENSG00000152932	115827	RAB3C	0.489
ENSG00000138483	84692	CCDC54	0.358
ENSG00000138439	150864	ALS2CR13	0.528
ENSG00000170054	327657	SERPINA9	0.458
ENSG00000127423	79000	C1orf135	0.507
ENSG00000182749	164091	PAQR7	0.487
ENSG00000051825	10198	MPHOSPH9	0.62
ENSG00000169507	151258	SLC38A11	0.424
ENSG00000126878	83543	C9orf58	0.553
ENSG00000243775	202459	LOC202459	0.391
ENSG00000005801	7748	ZNF195	0.487
ENSG00000104518	79792	GSDMDC1	0.485
ENSG00000075914	23016	EXOSC7	0.581
ENSG00000196923	9260	PDLIM7	0.643
ENSG00000146416	51390	AIG1	0.586
ENSG00000156787	93594	WDR67	0.482
ENSG00000131233	81025	GJA9	0.471
ENSG00000165953	145264	SERPINA12	0.407
ENSG00000100665	5267	SERPINA4	0.497
ENSG00000158315	54933	RHBDL2	0.529
ENSG00000170222	56985	C17orf48	0.468
ENSG00000138442	55759	WDR12	0.536
ENSG00000170476	51237	MGC29506	0.462
ENSG00000260880	283902	LOC283902	0.375
ENSG00000162814	128153	SPATA17	0.496
ENSG00000167311	116969	ART5	0.5
ENSG00000130921	91574	C12orf65	0.489
ENSG00000129744	417	ART1	0.497
ENSG00000145248	201780	SLC10A4	0.42
ENSG00000182223	326340	ZAR1	0.409
ENSG00000034693	8504	PEX3	0.566
ENSG00000068654	25885	POLR1A	0.464
ENSG00000168653	4725	NDUFS5	0.51
ENSG00000233670	644139	hCG_1776018	0.395
ENSG00000163710	26577	PCOLCE2	0.508

ENSG00000146094	79930	DOK3	0.473
ENSG00000001036	2519	FUCA2	0.474
ENSG00000196136	12	SERPINA3	0.534
ENSG00000260539	2734	GLG1	0.608
ENSG00000142794	84224	NBPF3	0.489
ENSG00000112208	9532	BAG2	0.547
ENSG00000114446	55081	IFT57	0.536
ENSG00000132386	5176	SERPINF1	0.548
ENSG00000092969	7042	TGFB2	0.612
ENSG00000183258	51428	DDX41	0.515
ENSG00000189376	84933	C8orf76	0.504
ENSG00000249751	641700	ECSM2	0.47
ENSG00000106976	1759	DNM1	0.513
ENSG00000142684	51042	ZNF593	0.536
ENSG00000142675	10256	CNKSR1	0.5
ENSG00000146166	51557	GLULD1	0.392
ENSG00000143353	127018	LYPLAL1	0.512
ENSG00000197465	2996	GYPE	0.479
ENSG00000146067	54540	FLJ10404	0.485
ENSG00000104529	1936	EEF1D	0.546
ENSG00000250361	2994	GYPB	0.609
ENSG00000156802	29028	ATAD2	0.563
ENSG00000144821	22989	MYH15	0.501
ENSG00000125787	2797	GNRH2	0.481
ENSG00000125901	64949	MRPS26	0.47
ENSG00000101405	5020	OXT	0.463
ENSG00000101200	551	AVP	0.464
ENSG00000178662	80034	FAM130A2	0.416
ENSG00000184209	11066	U1SNRNPBP	0.508
ENSG00000135521	84946	LTV1	0.511
ENSG00000215251	60493	FASTKD5	0.469
ENSG00000088899	9762	ProSAPiP1	0.521
ENSG00000213443	29121	CLEC2D	0.506
ENSG00000132300	55037	PTCD3	0.578
ENSG00000198171	65992	C20orf116	0.477
ENSG00000153086	130013	ACMSD	0.488
ENSG00000148985	27315	FRAG1	0.562
ENSG00000154957	7566	ZNF18	0.505
ENSG00000115339	2591	GALNT3	0.61
ENSG00000125877	3704	ITPA	0.523
ENSG00000196660	55532	SLC30A10	0.438
ENSG00000163507	57650	KIAA1524	0.5
ENSG00000133800	10894	LYVE1	0.512
ENSG00000163687	1776	DNASE1L3	0.496
ENSG00000086598	10959	TMED2	0.61
ENSG00000136628	2058	EPRS	0.626
ENSG00000130723	84726	KIAA0515	0.563
ENSG00000145348	93627	MGC16169	0.447
ENSG00000170074	285596	FAM153A	0.468
ENSG00000088836	83959	SLC4A11	0.569
ENSG00000164904	501	ALDH7A1	0.586
ENSG00000169976	83443	SF3B5	0.503
ENSG00000006740	9912	RICH2	0.545
ENSG00000111364	57696	DDX55	0.51

ENSG00000255103	643314	KIAA0754	0.465
ENSG00000111911	135114	HINT3	0.5
ENSG00000090621	8761	PABPC4	0.581
ENSG00000176601	80122	YSK4	0.441
ENSG00000156795	55093	C8orf32	0.47
ENSG00000175325	5626	PROP1	0.475
ENSG00000165959	79789	CLMN	0.5
ENSG00000066651	60487	TRMT11	0.498
ENSG00000145911	23138	N4BP3	0.512
ENSG00000130714	10585	POMT1	0.508
ENSG00000163746	57047	PLSCR2	0.429
ENSG00000145916	64777	RMND5B	0.596
ENSG00000156804	114907	FBXO32	0.599
ENSG00000188313	5359	PLSCR1	0.55
ENSG00000164182	91942	NDUFAF2	0.475
ENSG00000134571	4607	MYBPC3	0.452
ENSG00000164162	10393	ANAPC10	0.52
ENSG00000255154	11102	RPP14	0.514
ENSG00000158062	91544	UBXD5	0.492
ENSG00000145912	55651	NOLA2	0.537
ENSG00000203760	387103	C6orf173	0.448
ENSG00000167720	63826	SRR	0.553
ENSG00000006744	60528	ELAC2	0.589
ENSG00000182700	492311	LOC492311	0.509
ENSG00000120306	84418	C5orf32	0.462
ENSG00000198730	9646	CTR9	0.53
ENSG00000163515	84666	RETNLB	0.486
ENSG00000178722	285668	FLJ37543	0.405
ENSG00000163519	50852	TRAT1	0.449
ENSG00000113068	5201	PFDN1	0.516
ENSG00000164902	51808	RNUXA	0.441
ENSG00000104524	65263	PYCRL	0.458
ENSG00000196900	389320	C5orf48	0.39
ENSG00000138472	9626	GUCA1C	0.518
ENSG00000173585	10803	CCR9	0.455
ENSG00000175309	85007	AGXT2L2	0.549
ENSG00000084072	10450	PPIE	0.644
ENSG00000167721	55720	TSR1	0.579
ENSG00000093144	55862	ECHDC1	0.52
ENSG00000213231	9623	TCL1B	0.48
ENSG00000125861	64096	GFRA4	0.486
ENSG00000114487	27136	MORC1	0.432
ENSG00000145247	132299	OCIAD2	0.492
ENSG00000144891	185	AGTR1	0.604
ENSG00000149451	80332	ADAM33	0.533
ENSG00000153002	1360	CPB1	0.465
ENSG00000121988	84083	ZRANB3	0.534
ENSG00000100721	8115	TCL1A	0.533
ENSG00000153976	9955	HS3ST3A1	0.462
ENSG00000164022	9255	SCYE1	0.593
ENSG00000176092	55057	AIM1L	0.453
ENSG00000163530	151871	DPPA2	0.451
ENSG00000121570	55211	DPPA4	0.446
ENSG00000183309	9831	ZNF623	0.5

ENSG00000198754	64064	OXCT2	0.467
ENSG00000163751	1359	CPA3	0.474
ENSG00000181135	286075	ZNF707	0.432
ENSG00000163754	2992	GYG1	0.601
ENSG00000138801	9061	PAPSS1	0.556
ENSG00000197503	144360	C12orf67	0.462
ENSG00000176083	257101	ZNF683	0.439
ENSG00000130717	83549	UCK1	0.542
ENSG00000165916	5702	PSMC3	0.527
ENSG00000110328	374378	GALNTL4	0.497
ENSG00000162885	148789	B3GALNT2	0.5
ENSG00000125409	64518	TEKT3	0.377
ENSG00000114933	54891	FLJ20309	0.505
ENSG00000140522	6017	RLBP1	0.483
ENSG00000214338	9729	KIAA0408	0.402
ENSG00000140525	55215	FANCI	0.574
ENSG00000168301	200845	KCTD6	0.556
ENSG00000185915	257240	KLHL34	0.424
ENSG00000131914	79727	LIN28	0.416
ENSG00000164035	51705	EMCN	0.491
ENSG00000153093	55289	ACOXL	0.444
ENSG00000168306	8309	ACOX2	0.495
ENSG00000175106	201158	FAM18B2	0.5
ENSG00000255330	9729	KIAA0408	0.402
ENSG00000012174	51360	MBTPS2	0.506
ENSG00000206073	6318	SERPINB4	0.528
ENSG00000163263	388701	C1orf189	0.389
ENSG00000143612	25912	C1orf43	0.568
ENSG00000206072	89778	SERPINB11	0.459
ENSG00000144224	23190	UBXD2	0.635
ENSG00000255036	57653	KIAA1529	0.523
ENSG00000189367	9729	KIAA0408	0.402
ENSG00000123545	29078	C6orf66	0.517
ENSG00000057149	6317	SERPINB3	0.527
ENSG00000123500	1300	COL10A1	0.53
ENSG00000181085	225689	MAPK15	0.447
ENSG00000129596	1036	CDO1	0.504
ENSG00000166396	8710	SERPINB7	0.481
ENSG00000136731	56886	UGCGL1	0.512
ENSG00000088386	6564	SLC15A1	0.572
ENSG00000184530	352999	C6orf58	0.52
ENSG00000180921	286077	FAM83H	0.5
ENSG00000149187	10658	CUGBP1	0.654
ENSG00000197632	5055	SERPINB2	0.534
ENSG00000113811	58515	SELK	0.548
ENSG00000105894	5764	PTN	0.635
ENSG00000168389	84879	MFSD2	0.463
ENSG00000140521	5428	POLG	0.611
ENSG00000134480	902	CCNH	0.575
ENSG00000172673	387357	C6orf190	0.476
ENSG00000221926	10626	TRIM16	0.619
ENSG00000115850	3938	LCT	0.486
ENSG00000102174	5251	PHEX	0.445
ENSG00000145782	9140	ATG12	0.592

ENSG00000188820	441168	FAM26F	0.518
ENSG00000164180	153396	TMEM161B	0.608
ENSG00000223722	10410	IFITM3	0.537
ENSG00000213619	4722	NDUFS3	0.549
ENSG00000081138	1005	CDH7	0.487
ENSG00000164451	221301	FAM26D	0.423
ENSG00000145293	58478	ENOPH1	0.5
ENSG00000165644	118881	COMTD1	0.438
ENSG00000076003	4175	MCM6	0.564
ENSG00000187607	57335	ZNF286A	0.489
ENSG00000175809	158506	ZNF645	0.444
ENSG00000071991	28513	CDH19	0.477
ENSG00000184735	168400	DDX53	0.317
ENSG00000123131	10549	PRDX4	0.566
ENSG00000166479	54495	TXNDC10	0.545
ENSG00000122787	6718	AKR1D1	0.448
ENSG00000112234	26235	FBXL4	0.489
ENSG00000111832	51389	RWDD1	0.475
ENSG00000123130	23597	ACOT9	0.502
ENSG00000249242	441027	FLJ12993	0.508
ENSG00000132359	23108	GARNL4	0.541
ENSG00000140534	90381	C15orf42	0.558
ENSG00000163689	200844	C3orf67	0.519
ENSG00000144771	57408	LRTM1	0.51
ENSG00000133816	9645	MICAL2	0.658
ENSG00000150636	79839	CCDC102B	0.48
ENSG00000125843	55317	C20orf29	0.462
ENSG00000110536	114971	PTPMT1	0.577
ENSG00000166819	5346	PLIN	0.457
ENSG00000109475	6164	RPL34	0.51
ENSG00000255104	57335	ZNF286A	0.489
ENSG00000166821	8800	PEX11A	0.543
ENSG00000088888	57506	VISA	0.523
ENSG00000115866	1615	DARS	0.59
ENSG00000206052	220164	DOK6	0.486
ENSG00000179950	22827	PUF60	0.541
ENSG00000140527	56964	WDR93	0.402
ENSG00000132423	51805	COQ3	0.503
ENSG00000198856	58505	DC2	0.541
ENSG00000150637	10666	CD226	0.468
ENSG00000189283	2272	FHIT	0.561
ENSG00000138674	22872	SEC31A	0.641
ENSG00000111834	345895	RSHL3	0.284
ENSG00000125779	80025	PANK2	0.559
ENSG00000153975	221302	ZUFSP	0.42
ENSG00000164089	64850	AGXT2L1	0.457
ENSG00000172247	114900	C1QTNF4	0.424
ENSG00000148655	83938	C10orf11	0.486
ENSG00000180376	285331	CCDC66	0.447
ENSG00000221882	4995	OR3A2	0.462
ENSG00000055147	10827	FAM114A2	0.471
ENSG00000125482	7270	TTF1	0.561
ENSG00000188095	145873	MESP2	0.481
ENSG00000141255	84690	SPATA22	0.478

ENSG00000113396	28965	SLC27A6	0.483
ENSG00000214941	125150	ZSWIM7	0.546
ENSG00000125245	2841	GPR18	0.521
ENSG00000169508	1880	EBI2	0.497
ENSG00000125304	9375	TM9SF2	0.543
ENSG00000170396	91752	ZNF804A	0.534
ENSG00000196911	3841	KPNA5	0.478
ENSG00000188738	401024	FLJ44048	0.423
ENSG00000188517	84570	COL25A1	0.449
ENSG00000157823	10239	AP3S2	0.569
ENSG00000145781	51397	COMMD10	0.503
ENSG00000175198	5095	PCCA	0.565
ENSG00000152076	90557	CCDC74A	0.531
ENSG00000157703	136306	SVOPL	0.415
ENSG00000011295	54902	TTC19	0.545
ENSG00000186792	8372	HYAL3	0.49
ENSG00000114405	57415	C3orf14	0.49
ENSG00000262970	55331	PHCA	0.626
ENSG00000143575	10456	HAX1	0.525
ENSG00000169567	3094	HINT1	0.585
ENSG00000243477	24142	NAT6	0.483
ENSG00000165923	79841	AGBL2	0.421
ENSG00000242498	348110	C15orf38	0.512
ENSG00000153208	10461	MERTK	0.575
ENSG00000196569	3908	LAMA2	0.618
ENSG00000184831	79135	APOO	0.521
ENSG00000177830	66005	CHID1	0.518
ENSG00000114378	3373	HYAL1	0.545
ENSG00000262264	192134	B3GNT6	0.425
ENSG00000105929	50617	ATP6V0A4	0.483
ENSG00000262875	726	CAPN5	0.496
ENSG00000183807	221303	FAM162B	0.46
ENSG00000165182	254158	CXorf58	0.442
ENSG00000173612	222545	GPRC6A	0.49
ENSG00000174010	80311	KLHL15	0.489
ENSG00000153266	55079	FEZF2	0.465
ENSG00000185002	222546	RFXDC1	0.378
ENSG00000182054	3418	IDH2	0.59
ENSG00000136895	84253	GARNL3	0.451
ENSG00000143595	89872	AQP10	0.393
ENSG00000141027	9611	NCOR1	0.563
ENSG00000141665	201456	FBXO15	0.346
ENSG00000117118	6390	SDHB	0.584
ENSG00000153140	1070	CETN3	0.546
ENSG00000144227	11249	NXPH2	0.428
ENSG00000125247	84899	TMTC4	0.525
ENSG00000078687	57690	TNRC6C	0.571
ENSG00000075336	29090	C18orf55	0.529
ENSG00000065548	55854	ZC3H15	0.58
ENSG00000259634	4287	ATXN3	0.654
ENSG00000176055	153364	LOC153364	0.51
ENSG00000259131	153364	LOC153364	0.51
ENSG00000138802	10427	SEC24B	0.517
ENSG00000175792	8607	RUVBL1	0.546

ENSG00000067992	5165	PDK3	0.631
ENSG00000049089	1298	COL9A2	0.522
ENSG00000166347	1528	CYB5A	0.667
ENSG00000026297	8635	RNASET2	0.636
ENSG00000047936	6098	ROS1	0.479
ENSG00000164576	79685	SAP30L	0.489
ENSG00000109323	4126	MANBA	0.548
ENSG00000187773	125704	C18orf51	0.434
ENSG00000141524	11322	TMC6	0.56
ENSG00000133313	55748	CNDP2	0.534
ENSG00000259141	116068	LYSMD3	0.429
ENSG00000089053	51433	ANAPC5	0.621
ENSG00000164199	84059	GPR98	0.499
ENSG00000005059	55013	CCDC109B	0.556
ENSG00000142619	51702	PADI3	0.475
ENSG00000198542	9358	ITGBL1	0.592
ENSG00000185043	10519	CIB1	0.561
ENSG00000213471	440307	TTLL13	0.442
ENSG00000136856	29988	SLC2A8	0.468
ENSG00000102466	2259	FGF14	0.45
ENSG00000179407	165721	DNAJB8	0.37
ENSG00000089063	29058	C20orf30	0.529
ENSG00000150656	84735	CNDP1	0.483
ENSG00000138794	839	CASP6	0.549
ENSG00000198685	23434	C3orf27	0.467
ENSG00000120471	63970	P53AIP1	0.54
ENSG00000182768	51335	NGRN	0.536
ENSG00000134900	7174	TPP2	0.567
ENSG00000159339	23569	PADI4	0.563
ENSG00000123739	81579	PLA2G12A	0.483
ENSG00000163157	29765	TMOD4	0.455
ENSG00000144161	84524	ZC3H8	0.504
ENSG00000169084	207063	DHRSX	0.482
ENSG00000163239	126668	TDRD10	0.48
ENSG00000196391	342132	ZNF774	0.364
ENSG00000132646	5111	PCNA	0.589
ENSG00000134909	9743	RICS	0.522
ENSG00000164934	25879	WDSOF1	0.493
ENSG00000112237	892	CCNC	0.529
ENSG00000184956	4588	MUC6	0.472
ENSG00000099725	5616	PRKY	0.487
ENSG00000075073	6865	TACR2	0.464
ENSG00000262942	163223	ZNF676	0.436
ENSG00000163012	151112	ZSWIM2	0.342
ENSG00000002586	4267	CD99	0.606
ENSG00000187621	27004	TCL6	0.632
ENSG00000205403	3426	CFI	0.499
ENSG00000177646	28976	ACAD9	0.483
ENSG00000198788	4583	MUC2	0.463
ENSG00000187801	65243	ZNF643	0.467
ENSG00000163159	6944	VPS72	0.515
ENSG00000164465	285761	DCBLD1	0.462
ENSG00000108474	9487	PIGL	0.505
ENSG00000157741	254048	tcag7.1228	0.5

ENSG00000112486	1235	CCR6	0.513
ENSG00000139780	196541	C13orf39	0.375
ENSG00000188817	132203	S100A1L	0.341
ENSG00000151287	93081	C13orf27	0.523
ENSG00000109534	54433	NOLA1	0.515
ENSG00000176774	286514	MAGEB18	0.396
ENSG00000167862	3396	ICT1	0.501
ENSG00000176746	158809	MAGEB6	0.467
ENSG00000074964	55160	ARHGEF10L	0.54
ENSG00000166984	6953	TCP10	0.448
ENSG00000167895	147138	TMC8	0.563
ENSG00000133835	3295	HSD17B4	0.565
ENSG00000163634	80145	THOC7	0.492
ENSG00000180245	10692	RRH	0.49
ENSG00000164898	154791	C7orf55	0.561
ENSG00000114388	10641	TUSC4	0.62
ENSG00000134901	79070	KDELC1	0.474
ENSG00000124343	7499	XG	0.454
ENSG00000196152	7633	ZNF79	0.569
ENSG00000112494	54346	UNC93A	0.538
ENSG00000168152	79725	THAP9	0.423
ENSG00000188050	168433	RNF133	0.357
ENSG00000235631	378925	RNF148	0.38
ENSG00000167863	10476	ATP5H	0.555
ENSG00000125630	84172	POLR1B	0.535
ENSG00000099399	4113	MAGEB2	0.473
ENSG00000197958	6136	RPL12	0.587
ENSG00000198798	4114	MAGEB3	0.458
ENSG00000128519	50833	TAS2R16	0.463
ENSG00000120289	4115	MAGEB4	0.491
ENSG00000214107	4112	MAGEB1	0.395
ENSG00000120440	83887	TTLL2	0.417
ENSG00000169297	190	NR0B1	0.482
ENSG00000186451	353324	SPATA12	0.363
ENSG00000089094	84678	FBXL10	0.524
ENSG00000164342	7098	TLR3	0.482
ENSG00000122859	50674	NEUROG3	0.447
ENSG00000203690	6953	TCP10	0.448
ENSG00000119285	55127	HEATR1	0.594
ENSG00000170190	9121	SLC16A5	0.63
ENSG00000152082	80097	FAM128B	0.579
ENSG00000170322	4798	NFRKB	0.621
ENSG00000138798	1950	EGF	0.519
ENSG00000109794	25854	FAM149A	0.531
ENSG00000164935	81501	TM7SF4	0.434
ENSG00000188883	346689	KLRG2	0.457
ENSG00000117983	727897	MUC5B	0.533
ENSG00000136710	84317	CCDC115	0.45
ENSG00000164483	154075	SAMD3	0.495
ENSG00000197299	641	BLM	0.563
ENSG00000198523	5350	PLN	0.542
ENSG00000148606	11128	POLR3A	0.521
ENSG00000148356	90678	LRSAM1	0.508
ENSG00000136718	92856	IMP4	0.539

ENSG00000163636	9861	PSMD6	0.575
ENSG00000147647	1807	DPYS	0.439
ENSG00000164037	150159	NHEDC1	0.552
ENSG00000117148	81569	ACTL8	0.486
ENSG00000174405	3981	LIG4	0.519
ENSG00000164675	154865	IQUB	0.42
ENSG00000175745	7025	NR2F1	0.559
ENSG00000117154	84966	IGSF21	0.458
ENSG00000182511	2242	FES	0.499
ENSG00000152034	84539	MCHR2	0.367
ENSG00000167900	7083	TK1	0.547
ENSG00000125449	79637	ARMC7	0.429
ENSG00000009709	5081	PAX7	0.454
ENSG00000125458	30833	NT5C	0.482
ENSG00000059377	6916	TBXAS1	0.493
ENSG00000111879	79632	C6orf60	0.532
ENSG00000102524	10673	TNFSF13B	0.547
ENSG00000166573	2587	GALR1	0.42
ENSG00000159352	5710	PSMD4	0.641
ENSG00000256463	27164	SALL3	0.402
ENSG00000164484	114801	TMEM200A	0.443
ENSG00000078902	54472	TOLLIP	0.509
ENSG00000082516	25929	GEMIN5	0.515
ENSG00000128609	4698	NDUFA5	0.548
ENSG00000164334	340069	FAM170A	0.488
ENSG00000112249	10973	ASCC3	0.53
ENSG00000006756	414	ARSD	0.539
ENSG00000169704	2815	GP9	0.46
ENSG00000166377	374868	ATP9B	0.563
ENSG00000164830	55074	OXR1	0.526
ENSG00000240682	57461	ISY1	0.563
ENSG00000138663	51138	COPS4	0.484
ENSG00000183077	125061	AFMID	0.429
ENSG00000125611	84269	CHCHD5	0.557
ENSG00000174325	116093	DIRC1	0.421
ENSG00000248483	134187	POU5F2	0.436
ENSG00000164038	133308	NHEDC2	0.504
ENSG00000185261	285600	C5orf36	0.522
ENSG00000139714	283385	MORN3	0.404
ENSG00000157625	257397	MAP3K7IP3	0.514
ENSG00000144136	6574	SLC20A1	0.571
ENSG00000181867	94033	FTMT	0.462
ENSG00000151304	153443	SRFBP1	0.5
ENSG00000132446	53940	FTHL17	0.482
ENSG00000157399	415	ARSE	0.484
ENSG00000133302	84250	ANKRD32	0.481
ENSG00000145287	51316	PLAC8	0.503
ENSG00000164344	3818	KLKB1	0.459
ENSG00000145850	91937	TIMD4	0.429
ENSG00000062096	416	ARSF	0.489
ENSG00000125450	79902	NUP85	0.508
ENSG00000163344	10654	PMVK	0.529
ENSG00000169607	150468	CKAP2L	0.504
ENSG00000113249	26762	HAVCR1	0.438

ENSG00000173085	27235	COQ2	0.509
ENSG00000064692	9627	SNCAIP	0.488
ENSG00000088926	2160	F11	0.5
ENSG00000135077	84868	HAVCR2	0.5
ENSG00000125337	3834	KIF25	0.543
ENSG00000168412	4543	MTNR1A	0.423
ENSG00000213995	55739	FLJ10769	0.508
ENSG00000152093	55997	CFC1	0.426
ENSG00000083857	2195	FAT	0.541
ENSG00000106304	6677	SPAM1	0.536
ENSG00000166965	91433	RCCD1	0.493
ENSG00000099284	55506	H2AFY2	0.518
ENSG00000155868	9443	MED7	0.56
ENSG00000146350	221322	C6orf170	0.5
ENSG00000122490	80148	PQLC1	0.489
ENSG00000173083	10855	HPSE	0.518
ENSG00000170775	2861	GPR37	0.531
ENSG00000153303	79981	FRMD1	0.456
ENSG00000134905	79587	CARS2	0.505
ENSG00000136698	55997	CFC1	0.426
ENSG00000253626	143244	EIF5AL1	0.483
ENSG00000125538	3553	IL1B	0.61
ENSG00000138778	1062	CENPE	0.528
ENSG00000174429	137735	ABRA	0.49
ENSG00000182208	81532	HCCA2	0.421
ENSG00000183624	56941	C3orf37	0.557
ENSG00000115368	84128	WDR75	0.496
ENSG00000128513	25913	POT1	0.526
ENSG00000125571	27178	IL1F7	0.393
ENSG00000168374	378	ARF4	0.568
ENSG00000179046	205860	TRIML2	0.425
ENSG00000141759	10907	TXNL4A	0.609
ENSG00000172771	90288	C3orf25	0.468
ENSG00000170613	153745	FAM71B	0.524
ENSG00000101546	79863	C18orf22	0.468
ENSG00000205867	440021	KRTAP5-2	0.378
ENSG00000184056	26276	VPS33B	0.587
ENSG00000025156	3298	HSF2	0.559
ENSG00000130021	8226	HDHD1A	0.529
ENSG00000117984	1509	CTSD	0.565
ENSG00000169836	6870	TACR3	0.453
ENSG00000163312	113510	HEL308	0.527
ENSG00000184108	339976	TRIML1	0.447
ENSG00000164488	168002	DACT2	0.465
ENSG00000256950	5715	PSMD9	0.598
ENSG00000136688	56300	IL1F9	0.457
ENSG00000158023	144406	WDR66	0.506
ENSG00000187775	8632	DNAH17	0.447
ENSG00000153347	153643	FAM81B	0.411
ENSG00000149043	90019	SYT8	0.495
ENSG00000136696	27177	IL1F8	0.476
ENSG00000109536	2483	FRG1	0.519
ENSG00000133661	6441	SFTPD	0.441
ENSG00000104412	9694	TTC35	0.536

ENSG00000182583	26609	VCX	0.429
ENSG00000163913	55764	IFT122	0.61
ENSG00000133678	80195	C10orf57	0.495
ENSG00000163319	51023	MRPS18C	0.484
ENSG00000180817	5464	PPA1	0.531
ENSG00000198677	9652	TTC37	0.537
ENSG00000185448	158724	FAM47A	0.29
ENSG00000189132	170062	FAM47B	0.257
ENSG00000160401	286207	C9orf117	0.478
ENSG00000136697	84639	IL1F10	0.353
ENSG00000164841	157753	TMEM74	0.478
ENSG00000189129	219348	PLAC9	0.459
ENSG00000130598	7136	TNNI2	0.47
ENSG00000174417	7201	TRHR	0.412
ENSG00000177337	649446	FLJ35776	0.437
ENSG00000165164	170063	CXorf22	0.444
ENSG00000120526	84955	NUDCD1	0.543
ENSG00000164434	2173	FABP7	0.589
ENSG00000143416	8991	SELENBP1	0.525
ENSG00000187024	138428	PTRH1	0.46
ENSG00000163322	84142	FAM175A	0.525
ENSG00000138777	27068	PPA2	0.552
ENSG00000138678	84803	AGPAT9	0.496
ENSG00000183304	171482	FAM9A	0.442
ENSG00000138381	54529	ASNSD1	0.5
ENSG00000120533	56943	ENY2	0.526
ENSG00000177138	171483	FAM9B	0.413
ENSG00000172594	10924	SMPDL3A	0.544
ENSG00000149418	6768	ST14	0.584
ENSG00000151687	150709	ANKAR	0.459
ENSG00000158113	254050	LRRC43	0.49
ENSG00000146352	134829	RLBP1L2	0.419
ENSG00000156574	4838	NODAL	0.459
ENSG00000153495	121793	C13orf16	0.407
ENSG00000205038	93035	PKHD1L1	0.462
ENSG00000153498	122258	C13orf28	0.373
ENSG00000184465	253769	WDR27	0.578
ENSG00000160404	27433	TOR2A	0.467
ENSG00000173272	653784	FAM128A	0.486
ENSG00000180644	5551	PRF1	0.512
ENSG00000176383	79369	B3GNT4	0.458
ENSG00000125445	51081	MRPS7	0.488
ENSG00000196810	92070	C4orf42	0.491
ENSG00000128694	64172	OSGEPL1	0.503
ENSG00000075886	7278	TUBA3C	0.469
ENSG00000168944	153241	CCDC100	0.533
ENSG00000101850	4935	GPR143	0.431
ENSG00000128699	94101	ORMDL1	0.534
ENSG00000125457	57409	MIF4GD	0.573
ENSG00000130024	55274	PHF10	0.523
ENSG00000163040	90557	CCDC74A	0.531
ENSG00000130595	7140	TNNT3	0.473
ENSG00000064933	5378	PMS1	0.588
ENSG00000166220	219793	C10orf27	0.534

ENSG00000147654	9166	EBAG9	0.606
ENSG00000184786	6991	TCTE3	0.496
ENSG00000172568	408263	C5orf40	0.295
ENSG00000125454	60386	SLC25A19	0.45
ENSG00000147642	55638	GOLSYN	0.488
ENSG00000130023	55780	C6orf70	0.441
ENSG00000197927	29798	C2orf27	0.516
ENSG00000236699	54848	FLJ20184	0.46
ENSG00000177842	253639	ZNF620	0.5
ENSG00000112996	10884	MRPS30	0.545
ENSG00000172888	285268	ZNF621	0.491
ENSG00000127463	23065	KIAA0090	0.596
ENSG00000111907	7164	TPD52L1	0.538
ENSG00000120451	399979	SNX19	0.561
ENSG00000172548	348938	ICHTHYIN	0.445
ENSG00000186481	440482	MGC26718	0.457
ENSG00000138785	57117	INTS12	0.51
ENSG00000187699	84281	MGC13057	0.526
ENSG00000150551	116372	LYPD1	0.491
ENSG00000185594	145946	SPATA8	0.415
ENSG00000176771	344148	NAP5	0.4
ENSG00000112584	84498	FAM120B	0.543
ENSG00000214026	6150	MRPL23	0.509
ENSG00000113272	54974	THG1L	0.472
ENSG00000185087	283777	FAM169B	0.333
ENSG00000115353	6869	TACR1	0.546
ENSG00000138379	2660	MSTN	0.391
ENSG00000198246	55315	SLC29A3	0.513
ENSG00000198130	26275	HIBCH	0.558
ENSG00000151883	79668	PARP8	0.53
ENSG00000111906	51020	HDDC2	0.608
ENSG00000152127	4249	MGAT5	0.432
ENSG00000057593	2155	F7	0.476
ENSG00000244067	2938	GSTA1	0.549
ENSG00000182667	50863	HNT	0.468
ENSG00000008018	5689	PSMB1	0.621
ENSG00000182253	23336	DMN	0.553
ENSG00000126218	2159	F10	0.508
ENSG00000170385	7779	SLC30A1	0.527
ENSG00000117650	4751	NEK2	0.584
ENSG00000164794	27012	KCNV1	0.434
ENSG00000103852	64927	TTC23	0.5
ENSG00000178804	132243	H1FOO	0.37
ENSG00000243955	2938	GSTA1	0.549
ENSG00000168038	54986	ULK4	0.462
ENSG00000126231	8858	PROZ	0.458
ENSG00000106344	55131	RBM28	0.479
ENSG00000078177	55728	N4BP2	0.455
ENSG00000033030	55596	ZCCHC8	0.524
ENSG00000126226	55795	PCID2	0.486
ENSG00000206013	387733	IFITM5	0.5
ENSG00000185201	10581	IFITM2	0.561
ENSG00000005436	6936	C2orf3	0.541
ENSG00000047648	395	ARHGAP6	0.565

ENSG00000071994	5134	PDCD2	0.612
ENSG00000185885	8519	IFITM1	0.594
ENSG00000162482	22977	AKR7A3	0.545
ENSG00000104324	10404	PGCP	0.558
ENSG00000254647	3630	INS	0.454
ENSG00000142089	10410	IFITM3	0.537
ENSG00000143493	25896	INTS7	0.542
ENSG00000053371	8574	AKR7A2	0.567
ENSG00000151689	3628	INPP1	0.551
ENSG00000180176	7054	TH	0.434
ENSG00000168421	399	RHOH	0.495
ENSG00000174156	2940	GSTA3	0.451
ENSG00000174343	55584	CHRNA9	0.456
ENSG00000258839	4157	MC1R	0.492
ENSG00000125363	265	AMELX	0.444
ENSG00000184363	11187	PKP3	0.553
ENSG00000151690	54842	FLJ20160	0.476
ENSG00000258947	10381	TUBB3	0.593
ENSG00000166118	219938	SPATA19	0.439
ENSG00000164332	134510	UBLCP1	0.535
ENSG00000005302	10943	MSL3L1	0.565
ENSG00000077549	832	CAPZB	0.657
ENSG00000171729	55092	TMEM51	0.525
ENSG00000185187	59307	SIGIRR	0.563
ENSG00000183734	430	ASCL2	0.398
ENSG00000113302	3593	IL12B	0.439
ENSG00000172023	5968	REG1B	0.483
ENSG00000170893	7200	TRH	0.448
ENSG00000213949	3672	ITGA1	0.615
ENSG00000064201	10077	TSPAN32	0.451
ENSG00000164399	3562	IL3	0.418
ENSG00000143476	51514	DTL	0.538
ENSG00000115386	5967	REG1A	0.461
ENSG00000184445	9735	KNTC1	0.522
ENSG00000185101	338440	TMEM16J	0.385
ENSG00000135245	29923	HIG2	0.509
ENSG00000187094	885	CCK	0.48
ENSG00000170231	2172	FABP6	0.485
ENSG00000147677	8667	EIF3H	0.532
ENSG00000156482	6156	RPL30	0.53
ENSG00000172016	5068	REG3A	0.498
ENSG00000165630	8559	PRPF18	0.596
ENSG00000176681	9884	LRRC37A	0.514
ENSG00000205085	346653	FAM71F2	0.41
ENSG00000174749	132720	C4orf32	0.517
ENSG00000114812	7433	VIPR1	0.501
ENSG00000099260	54873	PALMD	0.545
ENSG00000138660	55435	C4orf16	0.483
ENSG00000177459	203111	C8orf47	0.519
ENSG00000156869	391059	FRRS1	0.426
ENSG00000153923	9629	CLCA3	0.424
ENSG00000238083	474170	LRRC37A2	0.603
ENSG00000101916	51311	TLR8	0.463
ENSG00000073331	80216	ALPK1	0.489

ENSG00000023191	6050	RNH1	0.545
ENSG00000221886	63920	LOC63920	0.45
ENSG00000152684	53918	PELO	0.593
ENSG00000131435	8572	PDLIM4	0.633
ENSG00000187268	171484	FAM9C	0.338
ENSG00000104356	10940	POP1	0.475
ENSG00000262334	9501	RPH3AL	0.484
ENSG00000159409	11189	TNRC4	0.509
ENSG00000162438	11330	CTRC	0.473
ENSG00000137270	8521	GCM1	0.459
ENSG00000198759	25975	EGFL6	0.434
ENSG00000104361	79815	NPAL2	0.505
ENSG00000185829	51326	ARL17P1	0.507
ENSG00000177946	92806	MGC16385	0.569
ENSG00000171695	198437	LOC198437	0.41
ENSG00000142615	63036	ELA2A	0.499
ENSG00000197208	6583	SLC22A4	0.536
ENSG00000065600	55248	TMEM206	0.482
ENSG00000215704	51032	RP11-265F14.2	0.459
ENSG00000163541	8802	SUCLG1	0.526
ENSG00000073969	4905	NSF	0.568
ENSG00000196455	30849	PIK3R4	0.598
ENSG00000117691	29937	NENF	0.598
ENSG00000138658	55345	C4orf21	0.462
ENSG00000171202	84233	TMEM126A	0.462
ENSG00000151500	29087	THYN1	0.52
ENSG00000132906	842	CASP9	0.583
ENSG00000176896	170082	LOC170082	0.533
ENSG00000118520	383	ARG1	0.462
ENSG00000123595	9367	RAB9A	0.492
ENSG00000089157	6175	RPLP0	0.59
ENSG00000154277	7345	UCHL1	0.522
ENSG00000128617	611	OPN1SW	0.476
ENSG00000127955	2770	GNAI1	0.518
ENSG00000143436	65005	MRPL9	0.554
ENSG00000151498	27034	ACAD8	0.537
ENSG00000221819	750	C16orf3	0.44
ENSG00000161328	115399	LRRC56	0.446
ENSG00000164172	4338	MOCS2	0.491
ENSG00000126856	11105	PRDM7	0.372
ENSG00000162771	149647	FAM71A	0.466
ENSG00000123685	55509	BATF3	0.507
ENSG00000008324	51188	SS18L2	0.522
ENSG00000185522	256329	C11orf35	0.422
ENSG00000134363	10468	FST	0.579
ENSG00000158955	7484	WNT9B	0.462
ENSG00000174720	51574	LARP7	0.566
ENSG00000099849	8045	RASSF7	0.61
ENSG00000136930	5695	PSMB7	0.56
ENSG00000183648	4707	NDUFB1	0.492
ENSG00000070047	57661	KIAA1542	0.48
ENSG00000185267	441549	ARMETL1	0.444
ENSG00000113525	3567	IL5	0.405
ENSG00000179673	388394	RPRML	0.432

ENSG00000116771	79814	AGMAT	0.49
ENSG00000156876	163786	SASS6	0.462
ENSG00000187522	51182	HSPA14	0.541
ENSG00000146147	90523	C6orf142	0.456
ENSG00000185507	3665	IRF7	0.52
ENSG00000203705	128387	TATDN3	0.515
ENSG00000122477	127495	LRRC39	0.488
ENSG00000260807	64788	LMF1	0.575
ENSG00000150556	130576	LOC130576	0.439
ENSG00000198336	4635	MYL4	0.616
ENSG00000099834	53841	MUPCDH	0.561
ENSG00000259207	3690	ITGB3	0.652
ENSG00000169271	8988	HSPB3	0.49
ENSG00000136918	401551	WDR38	0.5
ENSG00000163377	151647	FAM19A4	0.404
ENSG00000186854	129293	LOC129293	0.511
ENSG00000136942	11224	RPL35	0.506
ENSG00000137996	8634	RTCD1	0.575
ENSG00000168288	27249	C2orf25	0.496
ENSG00000162458	54751	FBLIM1	0.504
ENSG00000178852	124989	C17orf57	0.493
ENSG00000157119	131377	KBTBD5	0.321
ENSG00000164919	1345	COX6C	0.54
ENSG00000145649	3001	GZMA	0.47
ENSG00000137251	27283	TINAG	0.54
ENSG00000136950	81873	ARPC5L	0.543
ENSG00000164287	166979	CDC20B	0.487
ENSG00000154269	5169	ENPP3	0.514
ENSG00000170890	5319	PLA2G1B	0.44
ENSG00000152457	64421	DCLRE1C	0.592
ENSG00000244607	152206	CCDC13	0.569
ENSG00000132554	26166	RGS22	0.438
ENSG00000185523	149643	LOC149643	0.388
ENSG00000168143	222584	FAM83B	0.45
ENSG00000150676	220047	CCDC83	0.367
ENSG00000137252	3062	HCRTR2	0.479
ENSG00000111775	1337	COX6A1	0.548
ENSG00000010282	57467	HHATL	0.438
ENSG00000070031	6343	SCT	0.386
ENSG00000184898	375287	RBM43	0.403
ENSG00000170855	51499	TRIAP1	0.532
ENSG00000069696	1815	DRD4	0.46
ENSG00000177030	10522	DEAF1	0.521
ENSG00000123609	9111	NMI	0.543
ENSG00000173611	286205	C9orf126	0.451
ENSG00000164294	493869	GPX8	0.481
ENSG00000116809	7709	ZBTB17	0.571
ENSG00000111786	8683	SFRS9	0.577
ENSG00000073905	7416	VDAC1	0.638
ENSG00000123610	7130	TNFAIP6	0.581
ENSG00000152669	10309	CCNO	0.497
ENSG00000067248	54505	DHX29	0.514
ENSG00000156509	286151	FBXO43	0.404
ENSG00000147669	5440	POLR2K	0.577

ENSG00000144744	9039	UBA3	0.549
ENSG00000100600	5641	LGMN	0.531
ENSG00000162692	7412	VCAM1	0.583
ENSG00000104450	6674	SPAG1	0.52
ENSG00000039123	23517	SKIV2L2	0.538
ENSG00000112175	653	BMP5	0.565
ENSG00000144648	1238	CCBP2	0.491
ENSG00000197889	644890	MEIG1	0.417
ENSG00000174606	90806	ANGEL2	0.619
ENSG00000152463	55301	OLAH	0.477
ENSG00000183888	149563	C1orf64	0.402
ENSG00000117543	51611	DPH5	0.58
ENSG00000173641	27129	HSPB7	0.47
ENSG00000115459	84173	RBED1	0.452
ENSG00000164403	134549	SHROOM1	0.551
ENSG00000136933	10244	RABEPK	0.527
ENSG00000164404	2661	GDF9	0.525
ENSG00000079950	8417	STX7	0.615
ENSG00000186510	1187	CLCNKA	0.534
ENSG00000149196	51501	C11orf73	0.495
ENSG00000152464	10557	RPP38	0.488
ENSG00000164405	27089	UQCRQ	0.532
ENSG00000182308	285429	WDR21B	0.384
ENSG00000174226	169166	SNX31	0.434
ENSG00000149201	60494	CCDC81	0.447
ENSG00000182983	389114	ZNF662	0.475
ENSG00000177042	283232	TMEM80	0.543
ENSG00000183091	4703	NEB	0.559
ENSG00000184908	1188	CLCNKB	0.477
ENSG00000177156	6888	TALDO1	0.562
ENSG00000151376	10873	ME3	0.538
ENSG00000167272	51367	POP5	0.577
ENSG00000188848	389206	CCDC4	0.538
ENSG00000144647	84892	C3orf39	0.437
ENSG00000177225	347862	PDDC1	0.438
ENSG00000178343	152573	SHISA3	0.492
ENSG00000112299	8876	VNN1	0.51
ENSG00000159111	124995	MRPL10	0.49
ENSG00000160746	55129	TMEM16K	0.527
ENSG00000110917	9761	KIAA0152	0.599
ENSG00000141294	90506	LRRC46	0.491
ENSG00000142632	128272	ARHGEF19	0.453
ENSG00000150687	11098	PRSS23	0.572
ENSG00000077943	8516	ITGA8	0.5
ENSG00000042493	822	CAPG	0.552
ENSG00000174607	7368	UGT8	0.48
ENSG00000138653	64579	NDST4	0.5
ENSG00000141295	90507	SCRN2	0.538
ENSG00000117707	5629	PROX1	0.436
ENSG00000174599	133022	TRAM1L1	0.51
ENSG00000122971	35	ACADS	0.496
ENSG00000152670	54514	DDX4	0.424
ENSG00000184524	51286	CEND1	0.476
ENSG00000165943	64112	MOAP1	0.556

ENSG00000112303	8875	VNN2	0.516
ENSG00000157837	121665	UNQ1887	0.544
ENSG00000132881	79363	C1orf89	0.456
ENSG00000037637	54455	FBXO42	0.596
ENSG00000148481	80013	C10orf97	0.484
ENSG00000145388	57721	KIAA1627	0.542
ENSG00000150961	9871	SEC24D	0.597
ENSG00000167183	79170	ATAD4	0.517
ENSG00000262666	10712	C1orf2	0.504
ENSG00000263290	10067	SCAMP3	0.518
ENSG00000261893	1196	CLK2	0.527
ENSG00000108465	80279	CDK5RAP3	0.537
ENSG00000263324	57657	HCN3	0.509
ENSG00000262785	5313	PKLR	0.523
ENSG00000177595	55367	LRDD	0.463
ENSG00000262245	283150	FOXR1	0.411
ENSG00000263192	338657	CCDC84	0.425
ENSG00000262088	6230	RPS25	0.53
ENSG00000187144	374955	SPATA21	0.423
ENSG00000267619	51399	TRAPPC4	0.567
ENSG00000262676	2542	SLC37A4	0.581
ENSG00000146409	116843	C6orf192	0.539
ENSG00000262812	10525	HYOU1	0.538
ENSG00000213585	7416	VDAC1	0.638
ENSG00000112306	6206	RPS12	0.561
ENSG00000235178	282890	ZNF311	0.466
ENSG00000157191	55707	NECAP2	0.523
ENSG00000242022	81797	OR12D3	0.409
ENSG00000227446	26529	OR12D2	0.438
ENSG00000234347	26531	OR11A1	0.415
ENSG00000232984	26716	OR2H1	0.517
ENSG00000237112	2550	GABBR1	0.533
ENSG00000177600	6181	RPLP2	0.621
ENSG00000240038	280	AMY2B	0.423
ENSG00000135114	8638	OASL	0.583
ENSG00000231968	10537	UBD	0.48
ENSG00000229680	7932	OR2H2	0.526
ENSG00000234623	4340	MOG	0.581
ENSG00000077498	7299	TYR	0.519
ENSG00000243480	279	AMY2A	0.556
ENSG00000223852	346171	ZFP57	0.388
ENSG00000235220	3134	HLA-F	0.626
ENSG00000235346	3135	HLA-G	0.612
ENSG00000235657	3105	HLA-A	0.588
ENSG00000236949	30834	ZNRD1	0.542
ENSG00000227720	6992	PPP1R11	0.524
ENSG00000235022	80352	RNF39	0.512
ENSG00000233573	11074	TRIM31	0.6
ENSG00000164096	401152	LOC401152	0.597
ENSG00000227458	135644	TRIM40	0.322
ENSG00000144736	55164	SHQ1	0.585
ENSG00000227472	10107	TRIM10	0.579
ENSG00000005243	51226	COPZ2	0.52
ENSG00000235960	89870	TRIM15	0.566

ENSG00000145384	2169	FABP2	0.431
ENSG00000107611	8029	CUBN	0.52
ENSG00000226060	7726	TRIM26	0.558
ENSG00000232839	56658	TRIM39	0.466
ENSG00000243009	79897	RPP21	0.468
ENSG00000229252	3133	HLA-E	0.629
ENSG00000229470	2794	GNL1	0.552
ENSG00000223887	80742	PRR3	0.522
ENSG00000135185	79161	C7orf23	0.523
ENSG00000232169	23	ABCF1	0.557
ENSG00000227804	5514	PPP1R10	0.598
ENSG00000233813	28973	MRPS18B	0.57
ENSG00000229061	79969	C6orf134	0.503
ENSG00000006837	51265	CDKL3	0.484
ENSG00000181798	151477	C2orf52	0.39
ENSG00000261974	51503	CWC15	0.447
ENSG00000261946	55693	JMJD2D	0.445
ENSG00000177700	5441	POLR2L	0.59
ENSG00000237012	221545	C6orf136	0.518
ENSG00000233418	8449	DHX16	0.471
ENSG00000005471	5244	ABCB4	0.473
ENSG00000234000	170954	KIAA1949	0.563
ENSG00000236843	11270	NRM	0.523
ENSG00000225589	9656	MDC1	0.575
ENSG00000255423	55096	FLJ10213	0.481
ENSG00000121440	23024	PDZRN3	0.521
ENSG00000141293	8631	SKAP1	0.504
ENSG00000235067	203068	TUBB	0.613
ENSG00000262683	2272	FHIT	0.561
ENSG00000168930	57093	TRIM49	0.5
ENSG00000120094	3211	HOXB1	0.453
ENSG00000164109	4085	MAD2L1	0.55
ENSG00000141934	8612	PPAP2C	0.519
ENSG00000262505	51802	ACCN5	0.474
ENSG00000262635	6999	TDO2	0.498
ENSG00000263238	1519	CTSO	0.574
ENSG00000138738	11107	PRDM5	0.489
ENSG00000206379	10211	FLOT1	0.631
ENSG00000085760	4528	MTIF2	0.584
ENSG00000227231	8870	IER3	0.564
ENSG00000099866	8174	MADCAM1	0.499
ENSG00000230456	780	DDR1	0.636
ENSG00000085563	5243	ABCB1	0.56
ENSG00000141933	91978	C19orf20	0.514
ENSG00000116785	10878	CFHR3	0.494
ENSG00000197540	3004	GZMM	0.46
ENSG00000198755	4736	RPL10A	0.543
ENSG00000172270	682	BSG	0.53
ENSG00000100564	5283	PIGH	0.471
ENSG00000077616	10003	NAALAD2	0.452
ENSG00000226384	2968	GTF2H4	0.5
ENSG00000244414	3078	CFHR1	0.64
ENSG00000127419	84286	TMEM175	0.412
ENSG00000230985	57176	VARS2	0.454

ENSG00000099821	5442	POLRMT	0.547
ENSG00000225454	389376	SFTPG	0.462
ENSG00000232251	135656	DPCR1	0.476
ENSG00000225543	29113	C6orf15	0.444
ENSG00000233439	170679	PSORS1C1	0.429
ENSG00000237123	1041	CDSN	0.502
ENSG00000232127	170680	PSORS1C2	0.442
ENSG00000050730	79931	TNIP3	0.441
ENSG00000206355	54535	CCHCR1	0.631
ENSG00000081181	384	ARG2	0.573
ENSG00000099812	126353	C19orf21	0.486
ENSG00000100568	10490	VTI1B	0.511
ENSG00000186867	84109	GPR103	0.417
ENSG00000233890	6941	TCF19	0.531
ENSG00000229094	5460	POU5F1	0.546
ENSG00000228299	3107	HLA-C	0.695
ENSG00000159182	84366	C17orf92	0.45
ENSG00000129951	79948	PRG2	0.44
ENSG00000172232	566	AZU1	0.428
ENSG00000224378	4277	MICB	0.526
ENSG00000196415	5657	PRTN3	0.427
ENSG00000197561	1991	ELA2	0.439
ENSG00000197766	1675	CFD	0.534
ENSG00000230624	7919	BAT1	0.592
ENSG00000175221	10025	MED16	0.633
ENSG00000080910	3080	CFHR2	0.446
ENSG00000234668	534	ATP6V1G2	0.492
ENSG00000134365	10877	CFHR4	0.481
ENSG00000236196	4795	NFKBIL1	0.461
ENSG00000114480	2632	GBE1	0.523
ENSG00000116014	84634	KISS1R	0.451
ENSG00000223919	4049	LTA	0.506
ENSG00000228849	7124	TNF	0.524
ENSG00000236925	4050	LTB	0.52
ENSG00000065268	57418	WDR18	0.511
ENSG00000131379	84077	C3orf20	0.329
ENSG00000223465	7940	LST1	0.606
ENSG00000134389	81494	CFHR5	0.416
ENSG00000108010	10539	GLRX3	0.574
ENSG00000237103	259197	NCR3	0.564
ENSG00000153404	153478	PLEKHG4B	0.49
ENSG00000234836	199	AIF1	0.617
ENSG00000064666	1265	CNN2	0.562
ENSG00000231370	7916	BAT2	0.58
ENSG00000143278	2165	F13B	0.434
ENSG00000145214	1609	DGKQ	0.445
ENSG00000064687	10347	ABCA7	0.511
ENSG00000227761	7917	BAT3	0.576
ENSG00000066279	259266	ASPM	0.58
ENSG00000123737	5393	EXOSC9	0.501
ENSG00000180448	23526	HMHA1	0.539
ENSG00000227567	55937	APOM	0.62
ENSG00000164366	133957	CCDC127	0.492
ENSG00000228435	57827	C6orf47	0.496

ENSG00000099817	5434	POLR2E	0.569
ENSG00000233210	7918	BAT4	0.51
ENSG00000132570	84105	PCBD2	0.532
ENSG00000139988	145226	RDH12	0.425
ENSG00000073578	6389	SDHA	0.517
ENSG00000230700	1460	CSNK2B	0.51
ENSG00000152705	347732	CATSPER3	0.403
ENSG00000064932	22904	SBNO2	0.611
ENSG00000244672	58496	LY6G5B	0.556
ENSG00000226404	80741	LY6G5C	0.527
ENSG00000064835	5449	POU1F1	0.475
ENSG00000231488	7920	BAT5	0.551
ENSG00000157343	221481	C6orf81	0.477
ENSG00000243003	259215	LY6G6F	0.375
ENSG00000224393	80739	C6orf25	0.434
ENSG00000099624	513	ATP5D	0.586
ENSG00000228859	80740	LY6G6C	0.463
ENSG00000227317	23564	DDAH2	0.601
ENSG00000145217	10861	SLC26A1	0.512
ENSG00000226248	1192	CLIC1	0.536
ENSG00000230293	4439	MSH5	0.508
ENSG00000127415	3425	IDUA	0.543
ENSG00000176171	664	BNIP3	0.582
ENSG00000181433	55511	SAGE1	0.431
ENSG00000137392	1208	CLPS	0.447
ENSG00000228300	55009	C19orf24	0.477
ENSG00000197753	222662	LHFPL5	0.405
ENSG00000096171	7407	VARS	0.588
ENSG00000160953	84939	MUM1	0.52
ENSG00000231502	57819	LSM2	0.551
ENSG00000234258	3305	HSPA1L	0.463
ENSG00000115286	374291	NDUFS7	0.514
ENSG00000234475	3303	HSPA1A	0.537
ENSG00000231555	3303	HSPA1A	0.537
ENSG00000229300	50854	C6orf48	0.542
ENSG00000130005	2593	GAMT	0.505
ENSG00000234343	4758	NEU1	0.519
ENSG00000169446	93380	TMEM32	0.5
ENSG00000228263	80736	SLC44A4	0.452
ENSG00000179021	285237	C3orf38	0.5
ENSG00000227333	10919	EHMT2	0.557
ENSG00000115268	6209	RPS15	0.529
ENSG00000138686	55212	BBS7	0.462
ENSG00000231543	717	C2	0.523
ENSG00000119559	148223	C19orf25	0.492
ENSG00000178222	285498	RNF212	0.542
ENSG00000184500	5627	PROS1	0.537
ENSG00000115257	54760	PCSK4	0.418
ENSG00000136542	11227	GALNT5	0.582
ENSG00000262782	374868	ATP9B	0.563
ENSG00000234852	221527	ZBTB12	0.46
ENSG00000241534	629	CFB	0.59
ENSG00000185761	339366	ADAMTSL5	0.441
ENSG00000169379	200894	ARL13B	0.457

ENSG00000206268	7936	RDBP	0.505
ENSG00000071655	53615	MBD3	0.597
ENSG00000223493	6499	SKIV2L	0.492
ENSG00000267059	10975	UQCR	0.476
ENSG00000127540	10975	UQCR	0.476
ENSG00000163001	112942	CCDC104	0.463
ENSG00000145832	153328	LOC153328	0.467
ENSG00000165752	282974	STK32C	0.53
ENSG00000236765	1797	DOM3Z	0.616
ENSG00000226257	8859	STK19	0.565
ENSG00000130270	148229	ATP8B3	0.466
ENSG00000213401	4111	MAGEA12	0.465
ENSG00000159674	10417	SPON2	0.493
ENSG00000205669	641372	ACOT6	0.352
ENSG00000221990	116349	LOC116349	0.422
ENSG00000145839	3578	IL9	0.421
ENSG00000112053	116369	SLC26A8	0.427
ENSG00000115165	9595	PSCDBP	0.501
ENSG00000145826	3950	LECT2	0.479
ENSG00000180104	11336	EXOC3	0.516
ENSG00000213638	113179	ADAT3	0.476
ENSG00000232414	1589	CYP21A2	0.47
ENSG00000157212	22976	PAXIP1	0.543
ENSG00000164616	26223	FBXL21	0.561
ENSG00000133243	55643	BTBD2	0.51
ENSG00000178750	415117	STX19	0.357
ENSG00000263313	4314	MMP3	0.476
ENSG00000178700	200895	DHFRL1	0.522
ENSG00000262406	4321	MMP12	0.517
ENSG00000221867	4102	MAGEA3	0.461
ENSG00000231608	7148	TNXB	0.679
ENSG00000095970	54209	TREM2	0.445
ENSG00000134376	23418	CRB1	0.46
ENSG00000145220	55646	LYAR	0.56
ENSG00000168468	1388	CREBL1	0.561
ENSG00000119977	26123	TCTN3	0.575
ENSG00000148814	80313	LRRC27	0.539
ENSG00000178694	63899	NSUN3	0.5
ENSG00000147383	50814	NSDHL	0.562
ENSG00000188056	285852	TREML4	0.463
ENSG00000224200	63943	FKBPL	0.478
ENSG00000225141	80863	PRRT1	0.558
ENSG00000099840	113177	C19orf36	0.574
ENSG00000151093	54995	OXSM	0.505
ENSG00000129680	79649	MAP7D3	0.559
ENSG00000228116	9374	PPT2	0.542
ENSG00000080224	285220	EPHA6	0.442
ENSG00000226892	10665	C6orf10	0.434
ENSG00000228987	3122	HLA-DRA	0.555
ENSG00000168826	166793	ZNF509	0.419
ENSG00000236884	3123	HLA-DRB1	0.614
ENSG00000124731	54210	TREM1	0.53
ENSG00000236418	3117	HLA-DQA1	0.533
ENSG00000233209	3119	HLA-DQB1	0.68

ENSG00000007001	151531	UPP2	0.537
ENSG00000179796	116135	LRRC3B	0.414
ENSG00000223793	3118	HLA-DQA2	0.706
ENSG00000113966	84100	ARL6	0.521
ENSG00000140043	145482	PTGR2	0.462
ENSG00000153157	221711	SYCP2L	0.327
ENSG00000066230	6550	SLC9A3	0.454
ENSG00000243496	3112	HLA-DOB	0.518
ENSG00000104897	8175	SF3A2	0.584
ENSG00000223481	6891	TAP2	0.58
ENSG00000168824	27065	D4S234E	0.595
ENSG00000133983	51241	COX16	0.494
ENSG00000104899	268	AMH	0.455
ENSG00000230034	5696	PSMB8	0.535
ENSG00000165828	118471	PRAP1	0.423
ENSG00000167476	126306	JSRP1	0.44
ENSG00000153237	130940	CCDC148	0.453
ENSG00000163491	152110	NEK10	0.423
ENSG00000240118	5698	PSMB9	0.561
ENSG00000224212	6890	TAP1	0.552
ENSG00000233280	131544	DKFZp667G2110	0.486
ENSG00000080200	131544	DKFZp667G2110	0.486
ENSG00000220008	645191	hCG_2040376	0.432
ENSG00000039139	1767	DNAH5	0.537
ENSG00000226264	3109	HLA-DMB	0.573
ENSG00000241394	3108	HLA-DMA	0.551
ENSG00000230678	6046	BRD2	0.659
ENSG00000144283	8502	PKP4	0.629
ENSG00000112877	55722	CEP72	0.466
ENSG00000176155	284001	CCDC57	0.468
ENSG00000235744	3111	HLA-DOA	0.651
ENSG00000119862	29094	HSPC159	0.481
ENSG00000229685	3113	HLA-DPA1	0.611
ENSG00000133067	59352	LGR6	0.444
ENSG00000139985	8747	ADAM21	0.485
ENSG00000237710	3115	HLA-DPB1	0.543
ENSG00000148803	282969	C10orf125	0.407
ENSG00000134007	8748	ADAM20	0.513
ENSG00000141873	29985	SLC39A3	0.521
ENSG00000168818	53407	STX18	0.515
ENSG00000230930	1302	COL11A2	0.611
ENSG00000172009	7064	THOP1	0.518
ENSG00000162929	84542	KIAA1841	0.456
ENSG00000164113	132612	ADAD1	0.543
ENSG00000172006	115196	ZNF554	0.553
ENSG00000186300	148254	ZNF555	0.493
ENSG00000171853	51112	TTC15	0.54
ENSG00000172000	80032	ZNF556	0.43
ENSG00000228333	6257	RXRB	0.61
ENSG00000171970	126295	ZNF57	0.491
ENSG00000175691	58492	ZNF77	0.485
ENSG00000127884	1892	ECHS1	0.541
ENSG00000229802	7922	SLC39A7	0.474
ENSG00000104953	79816	TLE6	0.459

ENSG00000225312	7923	HSD17B8	0.52
ENSG00000228520	6015	RING1	0.576
ENSG00000184258	1038	CDR1	0.436
ENSG00000228425	6293	VPS52	0.528
ENSG00000065717	7089	TLE2	0.553
ENSG00000148832	196743	PAOX	0.536
ENSG00000170891	54360	CYTL1	0.451
ENSG00000160818	54865	GPATCH4	0.598
ENSG00000077152	29089	UBE2T	0.519
ENSG00000102239	680	BRS3	0.461
ENSG00000180066	170393	C10orf91	0.293
ENSG00000223367	6222	RPS18	0.516
ENSG00000198573	64663	SPANXC	0.481
ENSG00000102241	27336	HTATSF1	0.572
ENSG00000235155	8705	B3GALT4	0.488
ENSG00000226916	9277	WDR46	0.512
ENSG00000224782	10471	PFDN6	0.6
ENSG00000088256	2767	GNA11	0.669
ENSG00000237825	5863	RGL2	0.529
ENSG00000109471	3558	IL2	0.481
ENSG00000188818	79844	ZDHHC11	0.49
ENSG00000214367	79441	C4orf15	0.508
ENSG00000060558	2769	GNA15	0.501
ENSG00000163331	92196	DAPL1	0.381
ENSG00000125910	8698	S1PR4	0.488
ENSG00000171421	64979	MRPL36	0.507
ENSG00000183837	29944	PNMA3	0.439
ENSG00000224201	84968	PNMA6A	0.5
ENSG00000102243	51442	VGLL1	0.538
ENSG00000236490	6892	TAPBP	0.616
ENSG00000155495	9947	MAGEC1	0.464
ENSG00000145494	4726	NDUFS6	0.534
ENSG00000161082	60680	BRUNOL5	0.573
ENSG00000081248	779	CACNA1S	0.461
ENSG00000188649	387707	CC2D2B	0.413
ENSG00000232382	26339	OR5K1	0.511
ENSG00000227780	9278	ZBTB22	0.534
ENSG00000206206	1616	DAXX	0.603
ENSG00000241465	2579	GAGE7	0.446
ENSG00000124827	9247	GCM2	0.44
ENSG00000046774	51438	MAGEC2	0.461
ENSG00000146192	221472	FGD2	0.466
ENSG00000129932	83475	DOHH	0.579
ENSG00000203724	388722	C1orf53	0.533
ENSG00000138684	59067	IL21	0.463
ENSG00000182551	55256	ADI1	0.524
ENSG00000028310	65980	BRD9	0.502
ENSG00000129562	1603	DAD1	0.516
ENSG00000181004	166379	BBS12	0.513
ENSG00000164690	6469	SHH	0.461
ENSG00000161091	126321	C19orf28	0.47
ENSG00000138685	2247	FGF2	0.602
ENSG00000170917	11162	NUDT6	0.477
ENSG00000234313	10255	HCG9	0.453

ENSG00000203902	84968	PNMA6A	0.5
ENSG00000141551	1453	CSNK1D	0.607
ENSG00000196151	151525	WDSUB1	0.411
ENSG00000206562	131965	METTL6	0.51
ENSG00000120729	9499	MYOT	0.425
ENSG00000143355	56956	LHX9	0.509
ENSG00000179855	126326	GIPC3	0.52
ENSG00000006638	6915	TBXA2R	0.614
ENSG00000244476	405754	HERV-FRD	0.425
ENSG00000105298	58509	C19orf29	0.61
ENSG00000234079	253018	HCG27	0.44
ENSG00000198681	4100	MAGEA1	0.471
ENSG00000163945	57654	KIAA1530	0.518
ENSG00000171811	54777	C10orf92	0.449
ENSG00000082929	10141	C4orf6	0.462
ENSG00000171865	246243	RNASEH1	0.536
ENSG00000096088	5225	PGC	0.528
ENSG00000139990	8816	WDR22	0.575
ENSG00000105982	140545	RNF32	0.464
ENSG00000173040	132884	EVC2	0.467
ENSG00000078795	27039	PKD2L2	0.434
ENSG00000183617	116541	MRPL54	0.409
ENSG00000173976	84839	RAX2	0.557
ENSG00000262452	57405	SPC25	0.569
ENSG00000183535	378832	C21orf123	0.375
ENSG00000262563	57818	G6PC2	0.47
ENSG00000007264	4145	MATK	0.489
ENSG00000151414	140609	NEK7	0.566
ENSG00000213397	55559	UCHL5IP	0.591
ENSG00000263298	8647	ABCB11	0.496
ENSG00000095585	29760	BLNK	0.52
ENSG00000261899	51232	CRIM1	0.601
ENSG00000260383	727957	KIAA1833	0.5
ENSG00000105278	23217	ZFR2	0.406
ENSG00000261236	23246	BOP1	0.511
ENSG00000196159	79633	FAT4	0.536
ENSG00000077009	27231	ITGB1BP3	0.459
ENSG00000261132	3297	HSF1	0.62
ENSG00000179979	285464	CRIPAK	0.526
ENSG00000167657	1613	DAPK3	0.559
ENSG00000081177	55218	EXDL2	0.504
ENSG00000031003	51306	FAM13B1	0.548
ENSG00000171863	6201	RPS7	0.562
ENSG00000261698	8694	DGAT1	0.493
ENSG00000126934	5605	MAP2K2	0.616
ENSG00000119723	51004	COQ6	0.479
ENSG00000060566	84699	CREB3L3	0.364
ENSG00000261678	83482	SCRT1	0.397
ENSG00000077463	51548	SIRT6	0.498
ENSG00000236362	2579	GAGE7	0.446
ENSG00000177932	30832	ZNF354C	0.387
ENSG00000087116	9509	ADAMTS2	0.547
ENSG00000261042	79581	GPR172A	0.55
ENSG00000089847	170961	ANKRD24	0.458

ENSG00000105246	10148	EBI3	0.489
ENSG00000105248	55702	CCDC94	0.479
ENSG00000105251	56961	SHD	0.495
ENSG00000167664	126259	TMIGD2	0.412
ENSG00000261539	26233	FBXL6	0.498
ENSG00000178078	55620	STAP2	0.48
ENSG00000148824	92170	MTG1	0.501
ENSG00000008382	84954	MPND	0.458
ENSG00000215269	2579	GAGE7	0.446
ENSG00000261694	203054	ADCK5	0.419
ENSG00000151418	127124	ATP6V1G3	0.419
ENSG00000261230	29894	CPSF1	0.579
ENSG00000072840	2121	EVC	0.403
ENSG00000167671	80700	UBXD1	0.482
ENSG00000147274	27316	RBMX	0.545
ENSG00000261297	55630	SLC39A4	0.523
ENSG00000164066	27152	INTU	0.433
ENSG00000167674	84717	HDGF2	0.49
ENSG00000260313	51160	VPS28	0.488
ENSG00000028116	7444	VRK2	0.527
ENSG00000047457	1356	CP	0.501
ENSG00000118004	78989	COLEC11	0.468
ENSG00000117139	10765	JARID1B	0.629
ENSG00000167676	729359	KIAA1881	0.388
ENSG00000260716	4796	NFKBIL2	0.458
ENSG00000146410	113115	FAM54A	0.584
ENSG00000214456	440503	LSDP5	0.525
ENSG00000262217	160728	SLC5A8	0.34
ENSG00000171236	116844	LRG1	0.471
ENSG00000061492	7478	WNT8A	0.392
ENSG00000262795	3460	IFNGR2	0.56
ENSG00000151475	83447	SLC25A31	0.429
ENSG00000263160	757	TMEM50B	0.531
ENSG00000112981	8382	NME5	0.497
ENSG00000182492	633	BGN	0.551
ENSG00000205777	2543	GAGE1	0.477
ENSG00000114942	1933	EEF1B2	0.546
ENSG00000185361	126282	TNFAIP8L1	0.553
ENSG00000197555	26037	SIPA1L1	0.614
ENSG00000074842	56005	C19orf10	0.597
ENSG00000262911	54943	DNAJC28	0.427
ENSG00000262473	2618	GART	0.639
ENSG00000173762	924	CD7	0.546
ENSG00000132688	10763	NES	0.48
ENSG00000261155	2248	FGF3	0.448
ENSG00000068985	8712	PAGE1	0.443
ENSG00000141965	55527	FEM1A	0.474
ENSG00000127666	148022	TICAM1	0.529
ENSG00000261309	3768	KCNJ12	0.563
ENSG00000105355	10226	M6PRBP1	0.544
ENSG00000259857	339263	C17orf51	0.507
ENSG00000112983	10902	BRD8	0.612
ENSG00000261074	169714	QSOX2	0.47
ENSG00000127663	23030	JMJD2B	0.617

ENSG00000163395	91156	IGFN1	0.426
ENSG00000259938	1523	CUX1	0.594
ENSG00000143320	1382	CRABP2	0.543
ENSG00000112578	705	BYSL	0.538
ENSG00000101951	9506	PAGE4	0.432
ENSG00000180316	285848	PNPLA1	0.385
ENSG00000259885	10603	SH2B2	0.548
ENSG00000141574	6398	SECTM1	0.501
ENSG00000261283	79706	PRKRIP1	0.479
ENSG00000171798	85442	KNDC1	0.519
ENSG00000183671	2825	GPR1	0.473
ENSG00000262562	7639	ZNF85	0.519
ENSG00000263141	94104	C21orf66	0.568
ENSG00000071539	9319	TRIP13	0.554
ENSG00000107447	1791	DNTT	0.447
ENSG00000262938	56245	C21orf62	0.486
ENSG00000013810	10460	TACC3	0.526
ENSG00000163762	116441	TM4SF18	0.529
ENSG00000105428	148066	ZNRF4	0.454
ENSG00000267483	146713	HRNBP3	0.526
ENSG00000143303	51093	C1orf66	0.5
ENSG00000248476	571	BACH1	0.579
ENSG00000261981	339221	ENPP7	0.378
ENSG00000262762	84733	CBX2	0.477
ENSG00000130649	1571	CYP2E1	0.589
ENSG00000262572	57332	CBX8	0.468
ENSG00000169903	7104	TM4SF4	0.46
ENSG00000131373	26061	HACL1	0.5
ENSG00000112137	221692	PHACTR1	0.469
ENSG00000133398	84246	MED10	0.507
ENSG00000018408	25937	WWTR1	0.626
ENSG00000176988	158521	FMR1NB	0.348
ENSG00000261772	440073	IQSEC3	0.436
ENSG00000037474	54888	NSUN2	0.504
ENSG00000262552	240	ALOX5	0.617
ENSG00000182459	400629	FLJ35767	0.429
ENSG00000151360	55821	ALLC	0.456
ENSG00000138400	130752	MDH1B	0.485
ENSG00000262448	220972	8-Mar	0.464
ENSG00000146909	64434	NOM1	0.444
ENSG00000142731	10733	PLK4	0.603
ENSG00000115392	55120	FANCL	0.536
ENSG00000130255	25873	RPL36	0.524
ENSG00000263031	54826	GIN1	0.488
ENSG00000181396	79701	FLJ22222	0.609
ENSG00000262234	23262	HISPPD1	0.495
ENSG00000174917	125988	P117	0.474
ENSG00000118246	22868	FASTKD2	0.561
ENSG00000241399	9936	CD302	0.564
ENSG00000100632	2079	ERH	0.523
ENSG00000119636	80127	C14orf45	0.471
ENSG00000261887	90355	C5orf30	0.525
ENSG00000167733	374875	HSD11B1L	0.41
ENSG00000262254	91689	C22orf32	0.55

ENSG00000010030	51513	ETV7	0.49
ENSG00000262894	4700	NDUFA6	0.601
ENSG00000137200	23070	KIAA0082	0.515
ENSG00000197223	10438	C1D	0.494
ENSG00000262024	6942	TCF20	0.629
ENSG00000145506	85409	NKD2	0.449
ENSG00000082996	11342	RNF13	0.572
ENSG00000183654	441061	11-Mar	0.455
ENSG00000144810	1295	COL8A1	0.547
ENSG00000119711	4329	ALDH6A1	0.635
ENSG00000196365	9361	LONP1	0.478
ENSG00000143314	79590	MRPL24	0.489
ENSG00000184220	84319	C3orf26	0.515
ENSG00000171772	93426	SYCE1	0.44
ENSG00000101981	2158	F9	0.46
ENSG00000164073	256471	MFSD8	0.568
ENSG00000174898	257062	TMEM146	0.358
ENSG00000118194	7139	TNNT2	0.479
ENSG00000212123	163154	MGC24975	0.617
ENSG00000113504	10723	SLC12A7	0.563
ENSG00000141994	56931	DUS3L	0.5
ENSG00000147081	8852	AKAP4	0.48
ENSG00000118263	8609	KLF7	0.574
ENSG00000168386	11259	FILIP1L	0.564
ENSG00000145979	51256	TBC1D7	0.544
ENSG00000163576	151651	EFHB	0.38
ENSG00000187118	152100	CMC1	0.494
ENSG00000156413	2528	FUT6	0.588
ENSG00000171794	8433	UTF1	0.446
ENSG00000168884	79155	TNIP2	0.587
ENSG00000171124	2525	FUT3	0.543
ENSG00000169660	284004	HEXDC	0.492
ENSG00000153246	22925	PLA2R1	0.543
ENSG00000130383	2527	FUT5	0.496
ENSG00000174886	126328	NDUFA11	0.512
ENSG00000164074	80167	C4orf29	0.457
ENSG00000213316	4056	LTC4S	0.446
ENSG00000205659	91750	LIN52	0.459
ENSG00000147082	85417	CCNB3	0.43
ENSG00000036054	55773	TBC1D23	0.538
ENSG00000145545	6715	SRD5A1	0.602
ENSG00000151650	27287	VENTX	0.478
ENSG00000105993	10049	DNAJB6	0.594
ENSG00000169814	686	BTD	0.553
ENSG00000112984	10112	KIF20A	0.504
ENSG00000151651	101	ADAM8	0.54
ENSG00000144401	151194	FAM119A	0.559
ENSG00000138709	55132	LARP2	0.616
ENSG00000144566	5868	RAB5A	0.591
ENSG00000100650	6430	SFRS5	0.62
ENSG00000115221	3694	ITGB6	0.532
ENSG00000130377	81616	ACSBG2	0.463
ENSG00000112941	11044	POLS	0.509
ENSG00000125652	84266	ALKBH7	0.5

ENSG00000125651	2962	GTF2F1	0.602
ENSG00000161011	8878	SQSTM1	0.66
ENSG00000181240	284427	SLC25A41	0.438
ENSG00000125648	79085	SLC25A23	0.483
ENSG00000134313	57498	KIDINS220	0.613
ENSG00000119688	5826	ABCD4	0.566
ENSG00000205744	79958	DENND1C	0.477
ENSG00000118231	1421	CRYGD	0.442
ENSG00000163254	1420	CRYGC	0.45
ENSG00000182187	1419	CRYGB	0.421
ENSG00000168582	1418	CRYGA	0.45
ENSG00000198843	51714	SELT	0.522
ENSG00000114021	56954	NIT2	0.508
ENSG00000104833	10382	TUBB4	0.447
ENSG00000125726	970	CD70	0.474
ENSG00000125735	8740	TNFSF14	0.484
ENSG00000188674	389073	LOC389073	0.348
ENSG00000178927	79415	C17orf62	0.496
ENSG00000125734	56927	GPR108	0.5
ENSG00000161010	51149	C5orf45	0.481
ENSG00000159348	51706	CYB5R1	0.521
ENSG00000197620	91966	CXorf40A	0.541
ENSG00000125731	10045	SH2D3A	0.549
ENSG00000141968	7409	VAV1	0.525
ENSG00000006659	56891	LGALS14	0.49
ENSG00000105205	1178	CLC	0.432
ENSG00000174837	2015	EMR1	0.478
ENSG00000164363	348932	SLC6A18	0.41
ENSG00000119599	26094	WDR21A	0.477
ENSG00000146013	2676	GFRA3	0.449
ENSG00000048545	2978	GUCA1A	0.569
ENSG00000230522	125997	MBD3L2	0.372
ENSG00000090920	8857	FCGBP	0.492
ENSG00000013275	5704	PSMC4	0.551
ENSG00000163645	131831	C3orf44	0.409
ENSG00000206559	152098	ZCWPW2	0.386
ENSG00000206535	348801	LNP1	0.514
ENSG00000187187	339327	ZNF546	0.514
ENSG00000013288	23324	MAN2B2	0.538
ENSG00000104883	92960	PEX11G	0.439
ENSG00000198723	374877	C19orf45	0.412
ENSG00000198816	140467	ZNF358	0.473
ENSG00000090674	57192	MCOLN1	0.48
ENSG00000174521	148014	TTC9B	0.488
ENSG00000169621	200558	C2orf13	0.469
ENSG00000115020	200576	PIP5K3	0.526
ENSG00000112357	5191	PEX7	0.569
ENSG00000164362	7015	TERT	0.457
ENSG00000181458	55076	TMEM45A	0.531
ENSG00000076924	56949	XAB2	0.445
ENSG00000163646	7401	CLRN1	0.552
ENSG00000174788	126006	PCP2	0.42
ENSG00000076944	6813	STXBP2	0.513
ENSG00000129810	151648	SGOL1	0.5

ENSG00000104918	56729	RETN	0.402
ENSG00000235030	8870	IER3	0.564
ENSG00000183019	199675	C19orf59	0.425
ENSG00000181029	126003	TRAPPC5	0.432
ENSG00000104921	2208	FCER2	0.572
ENSG00000182566	339390	CLEC4G	0.481
ENSG00000168970	8681	LOC100137047-PLA2G4B	0.621
ENSG00000232995	8490	RGS5	0.607
ENSG00000144820	84873	GPR128	0.387
ENSG00000100652	6554	SLC10A1	0.461
ENSG00000163219	9938	ARHGAP25	0.572
ENSG00000234674	6941	TCF19	0.531
ENSG00000130640	10844	TUBGCP2	0.606
ENSG00000237582	5460	POU5F1	0.546
ENSG00000159166	3898	LAD1	0.56
ENSG00000151466	132320	SCLT1	0.43
ENSG00000124496	55809	TRERF1	0.532
ENSG00000225921	51406	NOL7	0.609
ENSG00000160396	147746	HIPK4	0.377
ENSG00000144407	5746	PTH2R	0.466
ENSG00000123584	4108	MAGEA9	0.594
ENSG00000090013	645	BLVRB	0.572
ENSG00000163993	6286	S100P	0.56
ENSG00000172250	94009	SERHL	0.426
ENSG00000178988	114932	MRFAP1L1	0.524
ENSG00000186222	55330	CNO	0.456
ENSG00000118197	83479	DDX59	0.541
ENSG00000136560	10010	TANK	0.583
ENSG00000104976	6618	SNAPC2	0.496
ENSG00000178531	404217	CTXN1	0.396
ENSG00000104980	10469	TIMM44	0.554
ENSG00000133980	55237	C14orf115	0.427
ENSG00000198400	4914	NTRK1	0.437
ENSG00000131142	6370	CCL25	0.468
ENSG00000174946	29909	GPR171	0.469
ENSG00000090006	8425	LTBP4	0.566
ENSG00000174944	9934	P2RY14	0.496
ENSG00000113269	55819	RNF130	0.523
ENSG00000119655	10577	NPC2	0.554
ENSG00000137877	51332	SPTBN5	0.497
ENSG00000164485	116379	IL22RA2	0.405
ENSG00000138271	53836	GPR87	0.447
ENSG00000181631	53829	P2RY13	0.47
ENSG00000167774	4701	NDUFA7	0.55
ENSG00000169313	64805	P2RY12	0.477
ENSG00000233927	6234	RPS28	0.538
ENSG00000186994	256949	KANK3	0.462
ENSG00000123815	79934	ADCK4	0.472
ENSG00000185236	9230	RAB11B	0.529
ENSG00000188493	284325	C19orf54	0.494
ENSG00000050393	63933	CCDC90A	0.493
ENSG00000099783	4670	HNRNPM	0.618
ENSG00000077312	6626	SNRPA	0.519
ENSG00000261857	8190	MIA	0.459

ENSG00000146918	54892	NCAPG2	0.507
ENSG00000197953	344752	AADACL2	0.352
ENSG00000206435	3107	HLA-C	0.695
ENSG00000114771	13	AADAC	0.492
ENSG00000133246	84106	PRAM1	0.425
ENSG00000255974	1548	CYP2A6	0.642
ENSG00000198829	56670	SUCNR1	0.522
ENSG00000198077	1549	CYP2A7	0.507
ENSG00000133250	84330	ZNF414	0.563
ENSG00000197408	1555	CYP2B6	0.547
ENSG00000197838	1553	CYP2A13	0.503
ENSG00000197446	1572	CYP2F1	0.561
ENSG00000181786	284382	MGC33407	0.421
ENSG00000170948	85509	MBD3L1	0.435
ENSG00000167600	29785	CYP2S1	0.484
ENSG00000184319	284942	MGC70863	0.4
ENSG00000159337	283748	PLA2G4D	0.444
ENSG00000165898	122961	ISCA2	0.481
ENSG00000188000	162998	OR7D2	0.411
ENSG00000027697	3459	IFNGR1	0.613
ENSG00000173013	257236	CCDC96	0.453
ENSG00000173011	93624	MGC21874	0.517
ENSG00000180537	221687	RNF182	0.506
ENSG00000185247	4110	MAGEA11	0.47
ENSG00000163217	27302	BMP10	0.441
ENSG00000188321	84527	ZNF559	0.492
ENSG00000142046	641649	TMEM91	0.368
ENSG00000109519	80273	GRPEL1	0.566
ENSG00000162924	5966	REL	0.626
ENSG00000170264	84140	FAM161A	0.59
ENSG00000183607	200504	GKN2	0.396
ENSG00000141562	26502	NARF	0.482
ENSG00000120733	51780	JMJD1B	0.586
ENSG00000188629	7730	ZNF177	0.484
ENSG00000228964	3106	HLA-B	0.594
ENSG00000123810	80776	B9D2	0.439
ENSG00000077348	56915	EXOSC5	0.482
ENSG00000248098	593	BCKDHA	0.544
ENSG00000166008	4108	MAGEA9	0.594
ENSG00000215217	134121	C5orf49	0.433
ENSG00000145526	1016	CDH18	0.484
ENSG00000198028	147741	ZNF560	0.404
ENSG00000151470	132321	C4orf33	0.538
ENSG00000130818	79088	ZNF426	0.428
ENSG00000177191	374907	B3GNT8	0.438
ENSG00000105341	55101	ATP5SL	0.594
ENSG00000184985	57537	SORCS2	0.431
ENSG00000115233	10213	PSMD14	0.575
ENSG00000237787	152118	LOC152118	0.354
ENSG00000171469	93134	ZNF561	0.504
ENSG00000114790	26084	SGEF	0.466
ENSG00000007129	90273	CEACAM21	0.546
ENSG00000187243	81557	MAGED4B	0.496
ENSG00000169605	56287	GKN1	0.432

ENSG00000124275	4552	MTRR	0.585
ENSG00000227772	4277	MICB	0.526
ENSG00000105352	1089	CEACAM4	0.486
ENSG00000007306	1087	CEACAM7	0.577
ENSG00000105388	1048	CEACAM5	0.579
ENSG00000235439	7919	BAT1	0.592
ENSG00000086548	4680	CEACAM6	0.556
ENSG00000170956	1084	CEACAM3	0.565
ENSG00000198546	118472	ZNF511	0.461
ENSG00000156009	4107	MAGEA8	0.464
ENSG00000183103	147719	LYPD4	0.338
ENSG00000142025	63946	DMRTC2	0.316
ENSG00000105372	6223	RPS19	0.579
ENSG00000159176	1465	CSRP1	0.583
ENSG00000151693	8853	DDEF2	0.52
ENSG00000178597	768239	PSAPL1	0.438
ENSG00000105404	10567	RABAC1	0.525
ENSG00000154813	285381	DPH3	0.507
ENSG00000198258	59286	UBL5	0.475
ENSG00000197021	541578	CXorf40B	0.494
ENSG00000163362	55765	C1orf106	0.526
ENSG00000130643	50632	CALY	0.413
ENSG00000100462	10419	PRMT5	0.481
ENSG00000105088	93145	OLFM2	0.486
ENSG00000133055	4608	MYBPH	0.456
ENSG00000154814	92106	OXNAD1	0.505
ENSG00000105732	64763	ZNF574	0.479
ENSG00000234920	534	ATP6V1G2	0.492
ENSG00000236346	4795	NFKBIL1	0.461
ENSG00000161055	92304	SCGB3A1	0.436
ENSG00000080511	50700	RDH8	0.411
ENSG00000130813	55337	C19orf66	0.584
ENSG00000047579	84062	DTNBP1	0.563
ENSG00000130812	83854	ANGPTL6	0.448
ENSG00000167625	116115	ZNF526	0.449
ENSG00000109736	10227	MFSD10	0.529
ENSG00000160838	149499	C1orf92	0.406
ENSG00000130810	56342	PPAN	0.462
ENSG00000155640	26148	C10orf12	0.535
ENSG00000244165	5032	P2RY11	0.438
ENSG00000130811	8666	EIF3G	0.505
ENSG00000079462	5050	PAFAH1B3	0.493
ENSG00000230279	4049	LTA	0.506
ENSG00000188368	284338	PRR19	0.457
ENSG00000230108	7124	TNF	0.524
ENSG00000238114	4050	LTB	0.52
ENSG00000105427	84518	CNFN	0.494
ENSG00000154545	81557	MAGED4B	0.496
ENSG00000230791	7940	LST1	0.606
ENSG00000168530	4632	MYL1	0.484
ENSG00000223833	259197	NCR3	0.564
ENSG00000124469	1088	CEACAM8	0.432
ENSG00000119185	9270	ITGB1BP1	0.602
ENSG00000221826	5671	PSG3	0.558

ENSG00000164256	56979	PRDM9	0.398
ENSG00000171100	4534	MTM1	0.548
ENSG00000231924	5669	PSG1	0.599
ENSG00000087269	8602	NOL14	0.552
ENSG00000117868	57488	FAM62B	0.615
ENSG00000267534	9294	S1PR2	0.457
ENSG00000235588	199	AIF1	0.617
ENSG00000105364	51073	MRPL4	0.5
ENSG00000170848	5675	PSG6	0.518
ENSG00000112619	5961	PRPH2	0.472
ENSG00000221878	5676	PSG7	0.471
ENSG00000133063	1118	CHIT1	0.466
ENSG00000231825	7916	BAT2	0.58
ENSG00000243130	5680	PSG11	0.599
ENSG00000173163	150684	COMMD1	0.5
ENSG00000090339	3383	ICAM1	0.637
ENSG00000242221	5670	PSG2	0.452
ENSG00000105371	3386	ICAM4	0.479
ENSG00000105376	7087	ICAM5	0.473
ENSG00000124279	79072	FASTKD3	0.477
ENSG00000124659	6903	TBCC	0.526
ENSG00000204941	5673	PSG5	0.463
ENSG00000243137	5672	PSG4	0.535
ENSG00000267673	112812	FDX1L	0.444
ENSG00000183668	5678	PSG9	0.586
ENSG00000119616	51077	FCF1	0.523
ENSG00000146223	285855	RPL7L1	0.536
ENSG00000076662	3385	ICAM3	0.509
ENSG00000131126	83639	TEX101	0.433
ENSG00000113100	1007	CDH9	0.447
ENSG00000137814	55142	CEP27	0.509
ENSG00000105755	23474	ETHE1	0.556
ENSG00000176472	284346	ZNF575	0.418
ENSG00000105397	7297	TYK2	0.508
ENSG00000073050	7515	XRCC1	0.536
ENSG00000169599	27247	NFU1	0.517
ENSG00000119203	51692	CPSF3	0.531
ENSG00000113303	79908	BTNL8	0.588
ENSG00000167378	126298	IRGQ	0.407
ENSG00000124444	79177	ZNF576	0.535
ENSG00000126870	55112	WDR60	0.465
ENSG00000233348	7917	BAT3	0.576
ENSG00000011422	5329	PLAUR	0.632
ENSG00000124449	56269	IRGC	0.444
ENSG00000105771	56006	C19orf61	0.463
ENSG00000092036	54930	C14orf94	0.541
ENSG00000197885	28512	NKIRAS1	0.507
ENSG00000168903	10917	BTNL3	0.498
ENSG00000159871	284348	LYPD5	0.434
ENSG00000163655	8833	GMPS	0.544
ENSG00000167637	284349	ZNF283	0.459
ENSG00000125388	2868	GRK4	0.516
ENSG00000176222	342908	ZNF404	0.395
ENSG00000165810	153579	BTNL9	0.615

ENSG00000159905	7638	ZNF221	0.439
ENSG00000042088	55775	TDP1	0.524
ENSG00000079999	9817	KEAP1	0.518
ENSG00000221821	441150	LOC441150	0.5
ENSG00000155622	9502	XAGE2	0.429
ENSG00000204920	7711	ZNF155	0.413
ENSG00000204382	9503	XAGE1D	0.5
ENSG00000186889	200728	TMEM17	0.44
ENSG00000180739	53637	S1PR5	0.476
ENSG00000159882	7773	ZNF230	0.476
ENSG00000159885	7673	ZNF222	0.418
ENSG00000267022	7766	ZNF223	0.47
ENSG00000171611	171558	PTCRA	0.575
ENSG00000178386	7766	ZNF223	0.47
ENSG00000155959	7411	VBP1	0.548
ENSG00000186026	342909	ZNF284	0.472
ENSG00000115504	23301	EHBP1	0.586
ENSG00000146233	51302	CYP39A1	0.452
ENSG00000267680	7767	ZNF224	0.579
ENSG00000137161	10695	CNPY3	0.493
ENSG00000146054	81786	TRIM7	0.566
ENSG00000256294	7768	ZNF225	0.446
ENSG00000263002	10780	ZNF234	0.567
ENSG00000106018	7434	VIPR2	0.565
ENSG00000167380	7769	ZNF226	0.531
ENSG00000082074	2533	FYB	0.569
ENSG00000261371	5175	PECAM1	0.647
ENSG00000131115	7770	ZNF227	0.445
ENSG00000204628	10399	GNB2L1	0.634
ENSG00000170293	152189	CMTM8	0.488
ENSG00000235754	55937	APOM	0.62
ENSG00000117322	1380	CR2	0.5
ENSG00000159917	9310	ZNF235	0.5
ENSG00000263304	284021	C17orf60	0.405
ENSG00000188770	26254	OPTC	0.418
ENSG00000226103	57827	C6orf47	0.496
ENSG00000159915	353355	ZNF233	0.425
ENSG00000228605	7918	BAT4	0.51
ENSG00000124713	27232	GNMT	0.456
ENSG00000204379	9503	XAGE1D	0.5
ENSG00000062370	7771	ZFP112	0.476
ENSG00000262917	11232	POLG2	0.474
ENSG00000134330	285148	IAH1	0.488
ENSG00000267508	26974	ZNF285A	0.459
ENSG00000004468	952	CD38	0.493
ENSG00000263077	1655	DDX5	0.527
ENSG00000198444	8263	F8A1	0.541
ENSG00000185990	8263	F8A1	0.541
ENSG00000167383	7772	ZNF229	0.467
ENSG00000185973	55217	TMLHE	0.47
ENSG00000113492	64902	AGXT2	0.43
ENSG00000224774	1460	CSNK2B	0.51
ENSG00000163394	886	CCKAR	0.527
ENSG00000109680	55296	TBC1D19	0.479

ENSG00000073008	5817	PVR	0.66
ENSG00000263046	90799	CCDC45	0.538
ENSG00000141560	79672	FN3KRP	0.508
ENSG00000166947	2038	EPB42	0.474
ENSG00000168016	9881	LBA1	0.522
ENSG00000186567	56971	CEACAM19	0.459
ENSG00000157036	9941	ENDOGL1	0.582
ENSG00000137440	9982	FGFBP1	0.494
ENSG00000112186	10486	CAP2	0.614
ENSG00000239497	58496	LY6G5B	0.556
ENSG00000124780	89822	KCNK17	0.407
ENSG00000111971	80741	LY6G5C	0.527
ENSG00000197308	399717	FLJ45983	0.351
ENSG00000144635	51143	DYNC1LI1	0.492
ENSG00000137441	83888	FGFBP2	0.466
ENSG00000152315	56659	KCNK13	0.434
ENSG00000142273	23624	CBLC	0.485
ENSG00000100764	5700	PSMC1	0.545
ENSG00000230475	7920	BAT5	0.551
ENSG00000185751	9502	XAGE2	0.429
ENSG00000100591	10598	AHSA1	0.562
ENSG00000187244	4059	BCAM	0.626
ENSG00000183461	9503	XAGE1D	0.5
ENSG00000067829	3421	IDH3G	0.553
ENSG00000180113	221400	TDRD6	0.419
ENSG00000167363	64122	FN3K	0.468
ENSG00000174738	9975	NR1D2	0.54
ENSG00000124587	5190	PEX6	0.525
ENSG00000197415	79674	VEPH1	0.582
ENSG00000125089	54436	SH3TC1	0.5
ENSG00000130203	348	APOE	0.629
ENSG00000166946	23582	CCNDBP1	0.523
ENSG00000169777	50834	TAS2R1	0.447
ENSG00000130208	341	APOC1	0.565
ENSG00000150756	134145	FAM173B	0.536
ENSG00000204376	9503	XAGE1D	0.5
ENSG00000095981	83795	KCNK16	0.447
ENSG00000224916	344	APOC2	0.506
ENSG00000076242	4292	MLH1	0.567
ENSG00000267467	346	APOC4	0.504
ENSG00000234906	344	APOC2	0.506
ENSG00000141556	6904	TBCD	0.617
ENSG00000146070	7941	PLA2G7	0.49
ENSG00000144451	79582	SPAG16	0.486
ENSG00000241822	259215	LY6G6F	0.375
ENSG00000231003	80739	C6orf25	0.434
ENSG00000150753	22948	CCT5	0.563
ENSG00000235925	80740	LY6G6C	0.463
ENSG00000228128	23564	DDAH2	0.601
ENSG00000113494	5618	PRLR	0.603
ENSG00000104055	9333	TGM5	0.499
ENSG00000163518	83417	FCRL4	0.544
ENSG00000170684	162979	ZNF296	0.43
ENSG00000142252	79760	GEMIN7	0.505

ENSG00000223639	1192	CLIC1	0.536
ENSG00000124334	3581	IL9R	0.571
ENSG00000196975	307	ANXA4	0.61
ENSG00000100802	60686	C14orf93	0.48
ENSG00000168803	161823	ADAL	0.538
ENSG00000237333	4439	MSH5	0.508
ENSG00000179846	284353	NKPD1	0.385
ENSG00000198208	83694	RPS6KL1	0.48
ENSG00000007255	79090	TRAPPC6A	0.535
ENSG00000171453	9533	POLR1C	0.587
ENSG00000250490	401172	FLJ33360	0.357
ENSG00000174891	51319	RSRC1	0.453
ENSG00000204375	9503	XAGE1D	0.5
ENSG00000122188	54900	LAX1	0.46
ENSG00000180879	6748	SSR4	0.538
ENSG00000104879	1158	CKM	0.492
ENSG00000137822	27229	TUBGCP4	0.621
ENSG00000104892	147700	KLC3	0.49
ENSG00000100593	145501	THSD3	0.451
ENSG00000138376	580	BARD1	0.561
ENSG00000100767	89932	PAPLN	0.554
ENSG00000160856	115352	FCRL3	0.485
ENSG00000104884	2068	ERCC2	0.526
ENSG00000067369	7158	TP53BP1	0.512
ENSG00000137207	25844	YIPF3	0.525
ENSG00000112818	4224	MEP1A	0.483
ENSG00000087008	8310	ACOX3	0.527
ENSG00000117877	10849	CD3EAP	0.526
ENSG00000052749	23223	RRP12	0.596
ENSG00000012061	2067	ERCC1	0.608
ENSG00000231945	7407	VARS	0.588
ENSG00000147403	6134	RPL10	0.646
ENSG00000234846	4758	NEU1	0.519
ENSG00000152582	79925	SPEF2	0.513
ENSG00000232180	80736	SLC44A4	0.452
ENSG00000102195	9248	GPR50	0.474
ENSG00000197721	1379	CR1L	0.486
ENSG00000115750	9014	TAF1B	0.511
ENSG00000160131	203547	LOC203547	0.542
ENSG00000182004	6635	SNRPE	0.564
ENSG00000143951	51057	LOC51057	0.507
ENSG00000241476	6757	SSX2	0.575
ENSG00000168781	9677	HISPPD2A	0.516
ENSG00000213889	147699	FLJ40125	0.5
ENSG00000168827	85476	GFM1	0.509
ENSG00000119705	81892	C14orf156	0.548
ENSG00000124380	11017	RY1	0.559
ENSG00000047365	116984	CENTD1	0.615
ENSG00000125741	80207	OPA3	0.42
ENSG00000100603	22938	SNW1	0.567
ENSG00000125746	24139	EML2	0.607
ENSG00000188981	345222	C4orf44	0.353
ENSG00000100804	5693	PSMB5	0.553
ENSG00000013563	1774	DNASE1L1	0.549

ENSG00000117335	4179	CD46	0.567
ENSG00000224143	10919	EHMT2	0.557
ENSG00000138363	471	ATIC	0.58
ENSG00000157950	6757	SSX2	0.575
ENSG00000235696	717	C2	0.523
ENSG00000010310	2696	GIPR	0.478
ENSG00000079257	56925	LXN	0.457
ENSG00000125743	6633	SNRPD2	0.547
ENSG00000124733	4201	MEA1	0.525
ENSG00000118849	5918	RARRES1	0.578
ENSG00000242335	629	CFB	0.59
ENSG00000164237	134147	CMBL	0.549
ENSG00000118855	64747	MFSD1	0.533
ENSG00000183813	1233	CCR4	0.427
ENSG00000171402	170626	XAGE3	0.395
ENSG00000237289	1159	CKMT1B	0.63
ENSG00000179304	29057	FAM156A	0.488
ENSG00000153292	266977	GPR110	0.485
ENSG00000147378	89885	FATE1	0.447
ENSG00000132704	79368	FCRL2	0.488
ENSG00000104941	81492	RSHL1	0.402
ENSG00000125755	8189	SYMPK	0.572
ENSG00000242866	161497	STRC	0.404
ENSG00000170608	3171	FOXA3	0.465
ENSG00000182646	29057	FAM156A	0.488
ENSG00000176182	339344	LOC339344	0.447
ENSG00000188425	339345	NANOS2	0.359
ENSG00000100784	9252	RPS6KA5	0.596
ENSG00000008438	8993	PGLYRP1	0.451
ENSG00000188624	388555	IGFL3	0.333
ENSG00000204866	147920	IGFL2	0.376
ENSG00000233801	7936	RDBP	0.505
ENSG00000188293	374918	IGFL1	0.375
ENSG00000155275	152992	C4orf23	0.476
ENSG00000005448	84058	WDR54	0.476
ENSG00000115998	54980	C2orf42	0.465
ENSG00000228896	6499	SKIV2L	0.492
ENSG00000168685	3575	IL7R	0.557
ENSG00000145592	6167	RPL37	0.549
ENSG00000197734	283579	C14orf178	0.366
ENSG00000063761	57143	ADCK1	0.488
ENSG00000132357	84674	CARD6	0.478
ENSG00000185313	6336	SCN10A	0.51
ENSG00000166762	117155	CATSPER2	0.547
ENSG00000169515	83987	CCDC8	0.5
ENSG00000112936	730	C7	0.499
ENSG00000124541	88745	C6orf153	0.477
ENSG00000223572	1159	CKMT1B	0.63
ENSG00000105287	25865	PRKD2	0.553
ENSG00000119640	97	ACYP1	0.517
ENSG00000147381	4103	MAGEA4	0.461
ENSG00000175711	146712	B3GNTL1	0.524
ENSG00000044090	9820	CUL7	0.577
ENSG00000133943	80017	C14orf159	0.509

ENSG00000181027	79147	FKRP	0.444
ENSG00000234798	1797	DOM3Z	0.616
ENSG00000042753	1175	AP2S1	0.593
ENSG00000130751	4861	NPAS1	0.5
ENSG00000130748	54958	TMEM160	0.489
ENSG00000155269	27201	GPR78	0.518
ENSG00000236250	8859	STK19	0.565
ENSG00000142230	10055	SAE1	0.531
ENSG00000205795	192668	CYS1	0.506
ENSG00000105321	26093	CCDC9	0.512
ENSG00000152611	133690	CAPSL	0.487
ENSG00000197405	728	C5AR1	0.484
ENSG00000134830	27202	GPR77	0.436
ENSG00000134815	9704	DHX34	0.562
ENSG00000114993	6242	RTKN	0.574
ENSG00000014641	4190	MDH1	0.556
ENSG00000137364	7172	TPMT	0.569
ENSG00000145626	133688	UGT3A1	0.593
ENSG00000073754	922	CD5L	0.464
ENSG00000109625	8532	CPZ	0.503
ENSG00000118162	11133	KPTN	0.476
ENSG00000143839	5972	REN	0.458
ENSG00000126012	8242	JARID1C	0.53
ENSG00000105373	29997	GLTSCR2	0.508
ENSG00000171495	133558	FLJ40243	0.522
ENSG00000250588	29970	SCHIP1	0.544
ENSG00000002549	51056	LAP3	0.511
ENSG00000092470	79968	WDR76	0.484
ENSG00000176393	6051	RNPEP	0.536
ENSG00000198457	1589	CYP21A2	0.47
ENSG00000178980	6415	SEPW1	0.552
ENSG00000105392	1406	CRX	0.488
ENSG00000164187	92255	LMBRD2	0.455
ENSG00000105398	6822	SULT2A1	0.528
ENSG00000169393	64100	ELSPBP1	0.452
ENSG00000105507	56344	CABP5	0.528
ENSG00000165097	221656	AOF1	0.489
ENSG00000137171	89953	KLC4	0.503
ENSG00000105499	8605	PLA2G4C	0.517
ENSG00000115274	83444	ZNHIT4	0.602
ENSG00000105486	3978	LIG1	0.507
ENSG00000092067	1053	CEBPE	0.466
ENSG00000164393	222611	GPR111	0.458
ENSG00000105483	22900	CARD8	0.537
ENSG00000153294	221393	GPR115	0.398
ENSG00000126895	554	AVPR2	0.511
ENSG00000168811	3592	IL12A	0.447
ENSG00000215612	3166	HMX1	0.54
ENSG00000178150	163071	ZNF114	0.533
ENSG00000105479	93233	CCDC114	0.398
ENSG00000124602	222643	UNC5CL	0.448
ENSG00000142227	2014	EMP3	0.582
ENSG00000109758	3083	HGFAC	0.446
ENSG00000105467	23546	SYNGR4	0.468

ENSG00000089820	393	ARHGAP4	0.493
ENSG00000124260	4109	MAGEA10	0.453
ENSG00000071553	537	ATP6AP1	0.51
ENSG00000105443	9266	PSCD2	0.494
ENSG00000124596	221443	C6orf130	0.533
ENSG00000158473	912	CD1D	0.516
ENSG00000145604	6502	SKP2	0.572
ENSG00000088002	6820	SULT2B1	0.504
ENSG00000105523	54854	FAM83E	0.392
ENSG00000162877	148811	PM20D1	0.434
ENSG00000177202	171169	SPACA4	0.378
ENSG00000063177	6141	RPL18	0.567
ENSG00000175920	285489	DOK7	0.438
ENSG00000118579	80306	MED28	0.599
ENSG00000145491	83853	ROPN1L	0.438
ENSG00000115275	7841	GCS1	0.488
ENSG00000113810	10051	SMC4	0.642
ENSG00000163257	54876	C4orf30	0.52
ENSG00000063180	770	CA11	0.503
ENSG00000011677	2556	GABRA3	0.421
ENSG00000142233	126147	LOC126147	0.5
ENSG00000176920	2524	FUT2	0.563
ENSG00000146085	4594	MUT	0.575
ENSG00000176909	284358	FLJ36070	0.435
ENSG00000236236	7148	TNXB	0.679
ENSG00000182264	284359	IZUMO1	0.44
ENSG00000039537	729	C6	0.497
ENSG00000105550	26291	FGF21	0.435
ENSG00000235956	80863	PRRT1	0.558
ENSG00000147402	55879	GABRQ	0.472
ENSG00000087076	51171	HSD17B14	0.486
ENSG00000227600	9374	PPT2	0.542
ENSG00000099999	200312	RNF215	0.347
ENSG00000187815	339559	ZNF642	0.5
ENSG00000104804	7288	TULP2	0.463
ENSG00000163956	4043	LRPAP1	0.553
ENSG00000164002	64789	C1orf176	0.47
ENSG00000104805	4924	NUCB1	0.554
ENSG00000204822	116540	MRPL53	0.475
ENSG00000135637	84865	CCDC142	0.396
ENSG00000156127	10538	BATF	0.485
ENSG00000086205	2346	FOLH1	0.602
ENSG00000187105	399671	HEATR4	0.31
ENSG00000166710	567	B2M	0.581
ENSG00000109667	56606	SLC2A9	0.524
ENSG00000087088	581	BAX	0.587
ENSG00000197172	4105	MAGEA6	0.44
ENSG00000239974	80864	EGFL8	0.538
ENSG00000206504	10255	HCG9	0.453
ENSG00000151092	55768	NGLY1	0.596
ENSG00000228892	10554	AGPAT1	0.573
ENSG00000166582	201161	PRR6	0.586
ENSG00000119686	55640	FLVCR2	0.491
ENSG00000242114	51537	MTP18	0.507

ENSG00000117010	127396	ZNF684	0.52
ENSG00000185880	140691	TRIM69	0.538
ENSG00000144036	23233	EXOC6B	0.52
ENSG00000112212	222642	BZRPL1	0.465
ENSG00000104827	1082	CGB	0.63
ENSG00000125772	56261	RP5-1022P6.2	0.592
ENSG00000167014	145645	C15orf43	0.351
ENSG00000189052	1082	CGB	0.63
ENSG00000213030	1082	CGB	0.63
ENSG00000140263	6652	SORD	0.565
ENSG00000167744	4909	NTF4	0.445
ENSG00000104848	3743	KCNA7	0.425
ENSG00000170315	7314	UBB	0.568
ENSG00000104852	6625	SNRP70	0.579
ENSG00000227277	6048	RNF5	0.596
ENSG00000237405	177	AGER	0.548
ENSG00000221916	55150	FLJ10490	0.425
ENSG00000187688	51393	TRPV2	0.482
ENSG00000169676	1816	DRD5	0.45
ENSG00000083720	5019	OXCT1	0.534
ENSG00000220890	253018	HCG27	0.44
ENSG00000198590	339883	C3orf35	0.347
ENSG00000063127	28968	SLC6A16	0.455
ENSG00000104894	951	CD37	0.5
ENSG00000174125	7096	TLR1	0.517
ENSG00000104901	27120	DKKL1	0.438
ENSG00000161609	147872	FLJ32658	0.45
ENSG00000140274	405753	DUOXA2	0.376
ENSG00000142538	113091	PTH2	0.453
ENSG00000112077	6005	RHAG	0.533
ENSG00000198049	553	AVPR1B	0.499
ENSG00000119673	10965	ACOT2	0.618
ENSG00000104872	55011	PIH1D1	0.517
ENSG00000164188	202151	RANBP3L	0.448
ENSG00000140254	90527	DUOXA1	0.448
ENSG00000161618	126133	ALDH16A1	0.508
ENSG00000124688	9587	MAD2L1BP	0.535
ENSG00000090554	2323	FLT3LG	0.582
ENSG00000071859	9130	FAM50A	0.503
ENSG00000174130	10333	TLR6	0.529
ENSG00000259919	50614	GALNT9	0.444
ENSG00000112651	51069	MRPL2	0.502
ENSG00000100029	23481	PES1	0.527
ENSG00000171984	149840	C20orf196	0.465
ENSG00000181350	388341	C17orf76	0.452
ENSG00000142534	6205	RPS11	0.58
ENSG00000089199	1114	CHGB	0.506
ENSG00000142552	57333	RCN3	0.55
ENSG00000142546	51070	NOSIP	0.491
ENSG00000126460	5639	PRRG2	0.464
ENSG00000141040	57336	ZNF287	0.576
ENSG00000126456	3661	IRF3	0.544
ENSG00000239756	5252	PHF1	0.589
ENSG00000172426	221421	C6orf206	0.446

ENSG00000185339	6948	TCN2	0.514
ENSG00000224952	5089	PBX2	0.584
ENSG00000115289	84759	PCGF1	0.621
ENSG00000167065	150290	DUSP18	0.472
ENSG00000234508	63940	GPSM3	0.56
ENSG00000197566	57547	ZNF624	0.458
ENSG00000124490	7180	CRISP2	0.42
ENSG00000150244	79097	TRIM48	0.466
ENSG00000198625	4194	MDM4	0.466
ENSG00000169169	126129	CPT1C	0.457
ENSG00000234876	4855	NOTCH4	0.497
ENSG00000170160	9720	CCDC144A	0.446
ENSG00000205765	285636	LOC285636	0.559
ENSG00000126467	60385	TSKS	0.521
ENSG00000133422	22880	MORC2	0.57
ENSG00000168026	199223	TTC21A	0.483
ENSG00000102030	8260	ARD1A	0.541
ENSG00000137860	9153	SLC28A2	0.545
ENSG00000232106	10665	C6orf10	0.434
ENSG00000183963	6525	SMTN	0.586
ENSG00000125885	84515	MCM8	0.515
ENSG00000171766	2628	GATM	0.581
ENSG00000096080	55168	MRPS18A	0.574
ENSG00000124900	84767	SPRYD5	0.352
ENSG00000104973	81857	MED25	0.539
ENSG00000234794	3122	HLA-DRA	0.555
ENSG00000198832	140606	SELM	0.515
ENSG00000171763	79029	SPATA5L1	0.591
ENSG00000218497	7637	ZNF84	0.543
ENSG00000039650	11284	PNKP	0.462
ENSG00000185133	27124	PIB5PA	0.503
ENSG00000096006	10321	CRISP3	0.459
ENSG00000228080	3123	HLA-DRB1	0.614
ENSG00000113407	6897	TARS	0.545
ENSG00000115297	3196	TLX2	0.542
ENSG00000236575	7574	ZNF26	0.489
ENSG00000144045	165545	DQX1	0.44
ENSG00000104951	259307	IL4I1	0.447
ENSG00000213024	23636	NUP62	0.576
ENSG00000161640	114132	SIGLEC11	0.513
ENSG00000133962	79820	CATSPERB	0.462
ENSG00000169136	22809	ATF5	0.591
ENSG00000232062	3117	HLA-DQA1	0.533
ENSG00000231939	3119	HLA-DQB1	0.68
ENSG00000240505	23495	TNFRSF13B	0.468
ENSG00000231526	3118	HLA-DQA2	0.706
ENSG00000117594	3290	HSD11B1	0.495
ENSG00000088766	54675	CRLS1	0.563
ENSG00000161652	126123	C19orf41	0.358
ENSG00000137767	58472	SQRDL	0.55
ENSG00000185721	4733	DRG1	0.555
ENSG00000109684	116449	MIST	0.456
ENSG00000239457	3112	HLA-DOB	0.518
ENSG00000184708	56478	EIF4ENIF1	0.467

ENSG00000228090	80739	C6orf25	0.434
ENSG00000232326	6891	TAP2	0.58
ENSG00000235452	80740	LY6G6C	0.463
ENSG00000100387	9978	RBX1	0.53
ENSG00000170950	5232	PGK2	0.436
ENSG00000102032	5973	RENBP	0.493
ENSG00000124812	167	CRISP1	0.45
ENSG00000163138	133015	C4orf28	0.517
ENSG00000233076	23564	DDAH2	0.601
ENSG00000131400	9476	NAPSA	0.473
ENSG00000062822	5424	POLD1	0.516
ENSG00000125872	164312	LRRN4	0.457
ENSG00000119650	112752	C14orf179	0.55
ENSG00000149136	6749	SSRP1	0.595
ENSG00000086967	4606	MYBPC2	0.49
ENSG00000226651	1192	CLIC1	0.536
ENSG00000142530	112703	FAM71E1	0.419
ENSG00000204653	554235	LOC554235	0.341
ENSG00000131409	94030	LRRC4B	0.38
ENSG00000100395	83746	L3MBTL2	0.585
ENSG00000009790	80342	TRAF3IP3	0.62
ENSG00000171793	1503	CTPS	0.565
ENSG00000161681	50944	SHANK1	0.442
ENSG00000231631	5696	PSMB8	0.535
ENSG00000230961	4439	MSH5	0.508
ENSG00000243958	5698	PSMB9	0.561
ENSG00000105472	6320	CLEC11A	0.591
ENSG00000142513	93650	ACPT	0.438
ENSG00000109991	5024	P2RX3	0.442
ENSG00000224748	6890	TAP1	0.552
ENSG00000167748	3816	KLK1	0.486
ENSG00000174562	55554	KLK15	0.437
ENSG00000175164	28	ABO	0.59
ENSG00000223957	4758	NEU1	0.519
ENSG00000142515	354	KLK3	0.556
ENSG00000250722	6414	SEPP1	0.57
ENSG00000183530	253143	C22orf30	0.465
ENSG00000167751	3817	KLK2	0.563
ENSG00000188467	283652	SLC24A5	0.355
ENSG00000167749	9622	KLK4	0.5
ENSG00000136059	50853	VILL	0.47
ENSG00000167754	25818	KLK5	0.481
ENSG00000165929	123036	TC2N	0.528
ENSG00000134802	29015	SLC43A3	0.563
ENSG00000100150	9681	DEPDC5	0.496
ENSG00000167755	5653	KLK6	0.505
ENSG00000129455	11202	KLK8	0.505
ENSG00000204364	717	C2	0.523
ENSG00000241296	3109	HLA-DMB	0.573
ENSG00000144655	64651	AXUD1	0.481
ENSG00000167757	11012	KLK11	0.484
ENSG00000243215	3108	HLA-DMA	0.551
ENSG00000186474	43849	KLK12	0.498
ENSG00000196976	8270	LAGE3	0.586

ENSG00000171790	200172	SLFNL1	0.506
ENSG00000167759	26085	KLK13	0.584
ENSG00000129437	43847	KLK14	0.429
ENSG00000125827	56255	TXNDC13	0.634
ENSG00000129450	27180	SIGLEC9	0.472
ENSG00000168995	27036	SIGLEC7	0.6
ENSG00000105383	945	CD33	0.501
ENSG00000179213	284369	FLJ40235	0.333
ENSG00000100170	6523	SLC5A1	0.475
ENSG00000142549	402665	LOC402665	0.407
ENSG00000105379	2109	ETFB	0.551
ENSG00000235307	6046	BRD2	0.659
ENSG00000168329	1524	CX3CR1	0.507
ENSG00000160318	125875	CLDND2	0.406
ENSG00000105374	4818	NKG7	0.495
ENSG00000179598	201164	LOC201164	0.533
ENSG00000105370	3982	LIM2	0.411
ENSG00000205856	150297	RP1-127L4.6	0.549
ENSG00000140030	8477	GPR65	0.491
ENSG00000177721	389289	C5orf39	0.4
ENSG00000260513	90827	ZNF479	0.46
ENSG00000128253	10739	RFPL2	0.452
ENSG00000142512	89790	SIGLEC10	0.561
ENSG00000204359	629	CFB	0.59
ENSG00000151388	81792	ADAMTS12	0.455
ENSG00000232957	3111	HLA-DOA	0.651
ENSG00000261900	9849	ZNF518A	0.547
ENSG00000071889	60343	FAM3A	0.543
ENSG00000236177	3113	HLA-DPA1	0.611
ENSG00000100191	6527	SLC5A4	0.44
ENSG00000236221	7148	TNXB	0.679
ENSG00000262509	29760	BLNK	0.52
ENSG00000179934	1237	CCR8	0.458
ENSG00000128276	10738	RFPL3	0.459
ENSG00000128951	1854	DUT	0.619
ENSG00000105366	27181	SIGLEC8	0.548
ENSG00000144659	54977	SLC25A38	0.534
ENSG00000100220	51493	C22orf28	0.556
ENSG00000162757	148304	C1orf74	0.451
ENSG00000183733	344018	FIGLA	0.341
ENSG00000117595	3664	IRF6	0.536
ENSG00000105492	946	SIGLEC6	0.557
ENSG00000229488	80863	PRRT1	0.558
ENSG00000226826	3115	HLA-DPB1	0.543
ENSG00000168452	9374	PPT2	0.542
ENSG00000152672	165530	CLEC4F	0.444
ENSG00000105497	7728	ZNF175	0.489
ENSG00000100225	25793	FBXO7	0.529
ENSG00000105501	8778	SIGLEC5	0.454
ENSG00000105509	3036	HAS1	0.478
ENSG00000171051	2357	FPR1	0.525
ENSG00000171049	2358	FPR2	0.554
ENSG00000187474	2359	FPR3	0.499
ENSG00000116039	525	ATP6V1B1	0.471

ENSG00000161551	84765	ZNF577	0.534
ENSG00000141030	8533	COPS3	0.542
ENSG00000038219	259282	FAM44A	0.549
ENSG00000198093	65251	ZNF649	0.4
ENSG00000100418	27351	FAM152B	0.518
ENSG00000176024	79898	ZNF613	0.428
ENSG00000256683	59348	ZNF350	0.447
ENSG00000197619	284370	ZNF615	0.459
ENSG00000232541	1302	COL11A2	0.611
ENSG00000196419	2547	XRCC6	0.636
ENSG00000168028	3921	RPSA	0.527
ENSG00000256087	9668	ZNF432	0.465
ENSG00000204611	90317	ZNF616	0.518
ENSG00000182631	51289	RXFP3	0.434
ENSG00000164175	51151	SLC45A2	0.528
ENSG00000119715	2103	ESRRB	0.464
ENSG00000243897	80864	EGFL8	0.538
ENSG00000103995	22995	CEP152	0.535
ENSG00000100281	10042	HMG2L1	0.572
ENSG00000177854	8269	TMEM187	0.49
ENSG00000227642	10554	AGPAT1	0.573
ENSG00000196214	90321	ZNF766	0.438
ENSG00000198464	147657	ZNF480	0.444
ENSG00000242110	23600	AMACR	0.611
ENSG00000134809	26519	TIMM10	0.512
ENSG00000100815	9321	TRIP11	0.566
ENSG00000167554	162963	ZNF610	0.469
ENSG00000237462	5987	TRIM27	0.637
ENSG00000197603	65250	C5orf42	0.49
ENSG00000235589	282890	ZNF311	0.466
ENSG00000167555	84436	ZNF528	0.455
ENSG00000236014	6293	VPS52	0.528
ENSG00000100138	4809	NHP2L1	0.581
ENSG00000228907	6048	RNF5	0.596
ENSG00000258405	147660	ZNF578	0.414
ENSG00000167077	150365	MEI1	0.51
ENSG00000198482	388558	ZNF808	0.436
ENSG00000167562	55762	ZNF701	0.469
ENSG00000177453	167359	MGC42105	0.429
ENSG00000042317	55812	SPATA7	0.455
ENSG00000213020	81856	ZNF611	0.417
ENSG00000189190	162966	ZNF600	0.484
ENSG00000227794	6222	RPS18	0.516
ENSG00000198538	7576	ZNF28	0.382
ENSG00000233450	3833	KIFC1	0.535
ENSG00000229058	177	AGER	0.548
ENSG00000204604	90333	ZNF468	0.445
ENSG00000044012	2981	GUCA2B	0.461
ENSG00000182986	162967	ZNF320	0.433
ENSG00000255302	23741	EID1	0.604
ENSG00000180257	125893	ZNF816A	0.42
ENSG00000224487	81797	OR12D3	0.409
ENSG00000233481	26529	OR12D2	0.438
ENSG00000149131	710	SERPING1	0.538

ENSG00000237258	26531	OR11A1	0.415
ENSG00000138593	9728	KIAA0256	0.56
ENSG00000224395	26716	OR2H1	0.517
ENSG00000143486	1939	LGTN	0.537
ENSG00000170949	90338	ZNF160	0.526
ENSG00000170954	55786	ZNF415	0.483
ENSG00000237051	2550	GABBR1	0.533
ENSG00000112659	23113	PARC	0.514
ENSG00000197937	84671	ZNF347	0.507
ENSG00000205309	56953	NT5M	0.484
ENSG00000197497	79788	ZNF665	0.458
ENSG00000197928	342926	ZNF677	0.504
ENSG00000225553	5252	PHF1	0.589
ENSG00000196131	317701	VN1R2	0.455
ENSG00000180992	64928	MRPL14	0.479
ENSG00000196417	91661	ZNF765	0.462
ENSG00000232005	5089	PBX2	0.584
ENSG00000130844	55422	ZNF331	0.494
ENSG00000144031	79998	ANKRD53	0.433
ENSG00000159958	115650	TNFRSF13C	0.466
ENSG00000100162	79019	CENPM	0.488
ENSG00000177181	284716	FAM80A	0.412
ENSG00000226492	51596	CUTA	0.507
ENSG00000066185	84217	ZMYND12	0.489
ENSG00000100297	4174	MCM5	0.606
ENSG00000100565	145497	C14orf166B	0.427
ENSG00000142405	91662	NLRP12	0.437
ENSG00000127125	79717	PPCS	0.484
ENSG00000227460	8831	SYNGAP1	0.494
ENSG00000236697	63940	GPSM3	0.56
ENSG00000172409	10978	CLP1	0.518
ENSG00000232339	4855	NOTCH4	0.497
ENSG00000224654	10537	UBD	0.48
ENSG00000227044	7932	OR2H2	0.526
ENSG00000230885	4340	MOG	0.581
ENSG00000143479	8444	DYRK3	0.541
ENSG00000124357	55577	NAGK	0.531
ENSG00000183066	164684	WBP2NL	0.393
ENSG00000008226	9940	DLEC1	0.552
ENSG00000186409	728621	RP4-692D3.1	0.454
ENSG00000206245	10665	C6orf10	0.434
ENSG00000105605	59284	CACNG7	0.442
ENSG00000236515	221504	ZBTB9	0.523
ENSG00000198125	4151	MB	0.527
ENSG00000260334	2520	GAST	0.484
ENSG00000142408	59283	CACNG8	0.476
ENSG00000160211	2539	G6PD	0.504
ENSG00000261125	9001	HAP1	0.554
ENSG00000130433	59285	CACNG6	0.5
ENSG00000221963	80830	APOL6	0.519
ENSG00000189068	284415	VSTM1	0.351
ENSG00000128313	80831	APOL5	0.446
ENSG00000261577	3728	JUP	0.548
ENSG00000137225	11131	CAPN11	0.432

ENSG00000170909	126014	OSCAR	0.479
ENSG00000100197	1565	CYP2D6	0.607
ENSG00000232099	346171	ZFP57	0.388
ENSG00000261923	5140	PDE3B	0.591
ENSG00000170906	4696	NDUFA3	0.49
ENSG00000237508	3134	HLA-F	0.626
ENSG00000226260	3122	HLA-DRA	0.555
ENSG00000166262	196951	C15orf33	0.424
ENSG00000213593	51075	TXNDC14	0.496
ENSG00000229074	3123	HLA-DRB1	0.614
ENSG00000105619	29844	TFPT	0.495
ENSG00000225890	3117	HLA-DQA1	0.533
ENSG00000262968	120227	CYP2R1	0.465
ENSG00000171960	10465	PPIH	0.519
ENSG00000105618	26121	PRPF31	0.643
ENSG00000128284	80833	APOL3	0.479
ENSG00000261163	10609	SC65	0.495
ENSG00000261258	60681	FKBP10	0.488
ENSG00000048342	57545	CC2D2A	0.545
ENSG00000235680	3135	HLA-G	0.612
ENSG00000189306	27341	CTA-126B4.3	0.56
ENSG00000262933	796	CALCA	0.589
ENSG00000249948	57733	GBA3	0.449
ENSG00000151882	56477	CCL28	0.55
ENSG00000225824	3119	HLA-DQB1	0.68
ENSG00000113569	9631	NUP155	0.507
ENSG00000260753	115024	NT5C3L	0.504
ENSG00000100336	80832	APOL4	0.57
ENSG00000183569	253190	SERHL2	0.504
ENSG00000105617	79165	LENG1	0.431
ENSG00000167608	147798	TMC4	0.5
ENSG00000134962	152831	KLB	0.46
ENSG00000231834	3105	HLA-A	0.588
ENSG00000172244	375444	C5orf34	0.494
ENSG00000231823	3118	HLA-DQA2	0.706
ENSG00000163682	6133	RPL9	0.531
ENSG00000128335	23780	APOL2	0.555
ENSG00000104047	56986	DTWD1	0.536
ENSG00000206235	6891	TAP2	0.58
ENSG00000235176	30834	ZNRD1	0.542
ENSG00000261631	317719	KLHL10	0.5
ENSG00000263341	83902	KRTAP17-1	0.405
ENSG00000263012	3884	KRT33B	0.466
ENSG00000054392	55733	HHAT	0.447
ENSG00000236443	5696	PSMB8	0.535
ENSG00000259896	55175	KLHL11	0.416
ENSG00000237812	10255	HCG9	0.453
ENSG00000100342	8542	APOL1	0.53
ENSG00000170892	79042	TSEN34	0.482
ENSG00000260245	47	ACLY	0.625
ENSG00000170889	6203	RPS9	0.601
ENSG00000115325	1796	DOK1	0.609
ENSG00000236560	6992	PPP1R11	0.524
ENSG00000112667	10591	C6orf108	0.638

ENSG00000262993	3881	KRT31	0.495
ENSG00000263022	8688	KRT37	0.446
ENSG00000237733	80352	RNF39	0.512
ENSG00000073009	8517	IKBKG	0.55
ENSG00000204577	11025	LILRB3	0.61
ENSG00000239836	5698	PSMB9	0.561
ENSG00000124370	84693	MCEE	0.508
ENSG00000225130	11074	TRIM31	0.6
ENSG00000102080	1527	TEX28	0.455
ENSG00000226173	6890	TAP1	0.552
ENSG00000142224	29949	IL19	0.484
ENSG00000260703	83538	TTC25	0.489
ENSG00000244482	79168	LILRA6	0.46
ENSG00000124383	10199	MPHOSPH10	0.5
ENSG00000112115	3605	IL17A	0.495
ENSG00000260283	1267	CNP	0.574
ENSG00000165553	58157	NGB	0.447
ENSG00000060971	30	ACAA1	0.62
ENSG00000262684	7266	DNAJC7	0.519
ENSG00000260770	28511	NKIRAS2	0.563
ENSG00000105609	10990	LILRB5	0.48
ENSG00000237046	135644	TRIM40	0.322
ENSG00000235025	10107	TRIM10	0.579
ENSG00000239329	3109	HLA-DMB	0.573
ENSG00000260320	201181	ZNF385C	0.422
ENSG00000131042	10288	LILRB2	0.535
ENSG00000140284	11001	SLC27A2	0.555
ENSG00000233599	89870	TRIM15	0.566
ENSG00000243189	3108	HLA-DMA	0.551
ENSG00000182242	1527	TEX28	0.455
ENSG00000231002	7726	TRIM26	0.558
ENSG00000260178	79132	DHX58	0.461
ENSG00000162891	50604	IL20	0.441
ENSG00000234704	6046	BRD2	0.659
ENSG00000259958	2648	KAT2A	0.514
ENSG00000226437	56658	TRIM39	0.466
ENSG00000170866	11026	LILRA3	0.506
ENSG00000187116	353514	LILRA5	0.575
ENSG00000128274	53947	A4GALT	0.483
ENSG00000082068	55100	WDR70	0.466
ENSG00000125863	8195	MKKS	0.525
ENSG00000239961	23547	LILRA4	0.49
ENSG00000242247	26286	ARFGAP3	0.517
ENSG00000143473	3756	KCNH1	0.466
ENSG00000261378	5878	RAB5C	0.596
ENSG00000144035	9027	NAT8	0.453
ENSG00000231281	253018	HCG27	0.44
ENSG00000167613	3903	LAIR1	0.605
ENSG00000183678	1485	CTAG1B	0.431
ENSG00000144034	51002	TPRKB	0.56
ENSG00000188846	9045	RPL14	0.607
ENSG00000239865	79897	RPP21	0.468
ENSG00000185254	1527	TEX28	0.455
ENSG00000230389	10866	HCP5	0.492

ENSG00000231558	3111	HLA-DOA	0.651
ENSG00000104064	2553	GABPB2	0.598
ENSG00000075292	27332	ZNF638	0.56
ENSG00000109610	6649	SOD3	0.539
ENSG00000184033	1485	CTAG1B	0.431
ENSG00000263014	127124	ATP6V1G3	0.419
ENSG00000100353	8664	EIF3D	0.541
ENSG00000228163	3113	HLA-DPA1	0.611
ENSG00000262418	5788	PTPRC	0.621
ENSG00000100266	11252	PACSIN2	0.545
ENSG00000162894	9214	FAIM3	0.574
ENSG00000171962	83450	LRRC48	0.511
ENSG00000236632	3133	HLA-E	0.629
ENSG00000229295	3115	HLA-DPB1	0.543
ENSG00000232143	2794	GNL1	0.552
ENSG00000164008	79078	C1orf50	0.526
ENSG00000100271	25809	TTLL1	0.494
ENSG00000126890	30848	CTAG2	0.515
ENSG00000166862	10369	CACNG2	0.444
ENSG00000177873	285267	ZNF619	0.488
ENSG00000167617	148170	CDC42EP5	0.472
ENSG00000100360	11020	RABL4	0.599
ENSG00000223699	1302	COL11A2	0.611
ENSG00000167618	3904	LAIR2	0.483
ENSG00000144048	8446	DUSP11	0.504
ENSG00000172995	10777	ARPP-21	0.507
ENSG00000171953	91647	ATPAF2	0.594
ENSG00000223766	80742	PRR3	0.522
ENSG00000239998	11027	LILRA2	0.604
ENSG00000177868	374969	CCDC23	0.491
ENSG00000165521	161436	EML5	0.457
ENSG00000231129	23	ABCF1	0.557
ENSG00000104972	10859	LILRB1	0.53
ENSG00000100290	638	BIK	0.516
ENSG00000100294	27349	MCAT	0.515
ENSG00000100300	706	TSPO	0.543
ENSG00000130826	1736	DKC1	0.619
ENSG00000100365	4689	NCF4	0.594
ENSG00000108591	1819	DRG2	0.541
ENSG00000100368	1439	CSF2RB	0.559
ENSG00000104974	11024	LILRA1	0.565
ENSG00000185264	339669	C22orf33	0.338
ENSG00000231321	6257	RXRB	0.61
ENSG00000186818	11006	LILRB4	0.499
ENSG00000128311	7263	TST	0.562
ENSG00000231737	5514	PPP1R10	0.598
ENSG00000146215	401262	CRIP3	0.396
ENSG00000109618	51091	SEPSECS	0.512
ENSG00000128309	4357	MPST	0.506
ENSG00000186976	64800	EFCAB6	0.456
ENSG00000167633	3811	KIR3DL1	0.542
ENSG00000226111	28973	MRPS18B	0.57
ENSG00000242019	115653	KIR3DL3	0.481
ENSG00000227402	7922	SLC39A7	0.474

ENSG00000186973	440585	hCG_23177	0.5
ENSG00000187045	164656	TMPRSS6	0.504
ENSG00000189013	3805	KIR2DL4	0.611
ENSG00000235658	79969	C6orf134	0.503
ENSG00000228357	7923	HSD17B8	0.52
ENSG00000110031	9404	LPXN	0.574
ENSG00000243772	3802	KIR2DL1	0.443
ENSG00000089048	51575	ESF1	0.505
ENSG00000125498	3802	KIR2DL1	0.443
ENSG00000221957	3809	KIR2DS4	0.408
ENSG00000243710	149465	WDR65	0.442
ENSG00000240403	3812	KIR3DL2	0.578
ENSG00000235107	6015	RING1	0.576
ENSG00000184601	400258	C14orf180	0.477
ENSG00000183473	6753	SSTR3	0.5
ENSG00000091536	51168	MYO15A	0.434
ENSG00000223618	6293	VPS52	0.528
ENSG00000186431	2204	FCAR	0.594
ENSG00000145321	2638	GC	0.433
ENSG00000100341	150379	PNPLA5	0.425
ENSG00000100055	27128	PSCD4	0.552
ENSG00000189430	9437	NCR1	0.558
ENSG00000258986	388021	TMEM179	0.36
ENSG00000163017	72	ACTG2	0.517
ENSG00000233164	221545	C6orf136	0.518
ENSG00000167634	199713	NLRP7	0.392
ENSG00000214944	64283	RGNEF	0.505
ENSG00000101247	79133	C20orf7	0.519
ENSG00000100577	2954	GSTZ1	0.528
ENSG00000146221	202500	TCTE1	0.429
ENSG00000233049	8449	DHX16	0.471
ENSG00000165533	123016	TTC8	0.55
ENSG00000022556	55655	NLRP2	0.463
ENSG00000100344	80339	PNPLA3	0.512
ENSG00000171467	24149	ZNF318	0.553
ENSG00000149124	10249	GLYAT	0.508
ENSG00000203485	64423	INF2	0.5
ENSG00000100347	25813	SAMM50	0.582
ENSG00000156689	219970	GLYATL2	0.367
ENSG00000166840	92292	GLYATL1	0.576
ENSG00000088053	51206	GP6	0.492
ENSG00000053900	29945	ANAPC4	0.545
ENSG00000229998	170954	KIAA1949	0.563
ENSG00000179178	128218	TMEM125	0.454
ENSG00000160439	112724	RDH13	0.637
ENSG00000228867	11270	NRM	0.523
ENSG00000101251	80343	SEL1L2	0.405
ENSG00000068745	51447	IHPK2	0.529
ENSG00000100079	3957	LGALS2	0.485
ENSG00000188677	29780	PARVB	0.646
ENSG00000131037	54869	EPS8L1	0.606
ENSG00000100083	26088	GGA1	0.612
ENSG00000227647	10255	HCG9	0.453
ENSG00000114956	1716	DGUOK	0.622

ENSG00000189057	374393	FAM111B	0.588
ENSG00000112742	7272	TTK	0.519
ENSG00000166801	63901	FAM111A	0.49
ENSG00000249481	221409	SPATS1	0.427
ENSG00000228575	9656	MDC1	0.575
ENSG00000115446	25972	UNC50	0.528
ENSG00000138964	64098	PARVG	0.507
ENSG00000185100	122622	ADSSL1	0.541
ENSG00000105048	7138	TNNT1	0.516
ENSG00000100092	23616	SH3BP1	0.508
ENSG00000096401	988	CDC5L	0.621
ENSG00000232575	203068	TUBB	0.613
ENSG00000123843	725	C4BPB	0.495
ENSG00000117400	4352	MPL	0.578
ENSG00000133059	25778	RIPK5	0.565
ENSG00000056291	10886	NPFFR2	0.441
ENSG00000181450	339500	ZNF678	0.528
ENSG00000117399	991	CDC20	0.549
ENSG00000143740	116841	C1orf142	0.486
ENSG00000113460	55299	BXDC2	0.508
ENSG00000177731	2314	FLII	0.583
ENSG00000238148	253018	HCG27	0.44
ENSG00000138944	85352	LL22NC03-75B3.6	0.523
ENSG00000100097	3956	LGALS1	0.598
ENSG00000049860	3074	HEXB	0.549
ENSG00000256872	79159	NOL12	0.448
ENSG00000163170	388962	BOLA3	0.477
ENSG00000254585	54551	MAGEL2	0.451
ENSG00000186654	55615	PRR5	0.573
ENSG00000091490	23231	KIAA0746	0.613
ENSG00000171462	65989	DLK2	0.481
ENSG00000100116	23464	GCAT	0.552
ENSG00000185823	23742	C15orf2	0.456
ENSG00000128739	6638	SNRPN	0.488
ENSG00000128310	8484	GALR3	0.487
ENSG00000203463	80736	SLC44A4	0.452
ENSG00000114978	55233	MOBKL1B	0.626
ENSG00000236271	10211	FLOT1	0.631
ENSG00000123838	722	C4BPA	0.46
ENSG00000248405	23779	ARHGAP8	0.554
ENSG00000100129	51386	EIF3EIP	0.536
ENSG00000214265	6638	SNRPN	0.488
ENSG00000081692	65094	JMJD4	0.466
ENSG00000197932	8263	F8A1	0.541
ENSG00000230128	8870	IER3	0.564
ENSG00000241484	23779	ARHGAP8	0.554
ENSG00000234078	780	DDR1	0.636
ENSG00000093217	9942	XYLB	0.561
ENSG00000166902	54948	MRPL16	0.535
ENSG00000143816	7483	WNT9A	0.435
ENSG00000134812	2694	GIF	0.393
ENSG00000134827	6947	TCN1	0.484
ENSG00000262605	92259	MRPS36	0.515
ENSG00000196352	1604	CD55	0.609

ENSG00000262841	1022	CDK7	0.581
ENSG00000125870	6629	SNRPB2	0.509
ENSG00000262407	202243	CCDC125	0.448
ENSG00000151445	63894	C14orf133	0.509
ENSG00000149507	219990	PLAC1L	0.321
ENSG00000100142	5435	POLR2F	0.517
ENSG00000149516	932	MS4A3	0.481
ENSG00000263302	6880	TAF9	0.613
ENSG00000186297	2558	GABRA5	0.428
ENSG00000125879	56914	OTOR	0.445
ENSG00000140025	90141	C14orf143	0.552
ENSG00000177302	7156	TOP3A	0.621
ENSG00000262511	5884	RAD17	0.597
ENSG00000109743	683	BST1	0.502
ENSG00000182256	2567	GABRG3	0.479
ENSG00000262607	440073	IQSEC3	0.436
ENSG00000104044	4948	OCA2	0.482
ENSG00000100373	7380	UPK3A	0.443
ENSG00000110077	64231	MS4A6A	0.531
ENSG00000233149	2968	GTF2H4	0.5
ENSG00000125864	631	BFSP1	0.453
ENSG00000100156	23539	SLC16A8	0.459
ENSG00000178922	81888	HYI	0.471
ENSG00000262169	2966	GTF2H2	0.486
ENSG00000077935	27127	SMC1B	0.453
ENSG00000260384	347735	SERINC2	0.533
ENSG00000128408	26150	RIBC2	0.469
ENSG00000241962	51263	MRPL30	0.518
ENSG00000077942	2192	FBLN1	0.671
ENSG00000143793	79169	C1orf35	0.457
ENSG00000164347	84340	GFM2	0.574
ENSG00000223494	57176	VARS2	0.454
ENSG00000144182	51601	LIPT1	0.512
ENSG00000183629	283768	GOLGA8G	0.452
ENSG00000263062	4671	NAIP	0.491
ENSG00000158411	129531	MITD1	0.444
ENSG00000262170	6606	SMN1	0.657
ENSG00000162910	128308	MRPL55	0.548
ENSG00000262305	8293	SERF1A	0.468
ENSG00000182257	55267	C22orf26	0.478
ENSG00000166926	245802	MS4A6E	0.447
ENSG00000166927	58475	MS4A7	0.563
ENSG00000185674	254773	LYG2	0.429
ENSG00000140104	122616	C14orf79	0.504
ENSG00000178467	54681	PH-4	0.538
ENSG00000205643	150383	C22orf40	0.441
ENSG00000130943	10343	PKDREJ	0.421
ENSG00000075234	55020	TTC38	0.579
ENSG00000125888	140836	BANF2	0.4
ENSG00000144214	129530	LYG1	0.421
ENSG00000261932	55814	BDP1	0.55
ENSG00000164346	10412	TINP1	0.521
ENSG00000104059	23359	KIAA0574	0.463
ENSG00000185115	56160	NDNL2	0.418

ENSG00000075218	51512	GTSE1	0.655
ENSG00000230072	389376	SFTPG	0.462
ENSG00000262057	64087	MCCC2	0.59
ENSG00000231624	29113	C6orf15	0.444
ENSG00000198780	26049	KIAA0888	0.5
ENSG00000231094	170679	PSORS1C1	0.429
ENSG00000237114	1041	CDSN	0.502
ENSG00000100196	11015	KDELR3	0.602
ENSG00000163631	213	ALB	0.602
ENSG00000166930	64232	MS4A5	0.403
ENSG00000234605	170680	PSORS1C2	0.442
ENSG00000100416	55687	TRMU	0.461
ENSG00000156738	931	MS4A1	0.573
ENSG00000189375	254272	TBC1D28	0.39
ENSG00000066135	9682	JMJD2A	0.601
ENSG00000223533	54535	CCHCR1	0.631
ENSG00000213339	81890	QTRT1	0.496
ENSG00000071203	54860	MS4A12	0.433
ENSG00000166664	89832	CHRFAM7A	0.451
ENSG00000108448	147166	TRIM16L	0.518
ENSG00000224941	6941	TCF19	0.531
ENSG00000230336	5460	POU5F1	0.546
ENSG00000198690	22909	MTMR15	0.54
ENSG00000142444	90580	C19orf52	0.446
ENSG00000260130	9172	MYOM2	0.496
ENSG00000100211	25776	CBY1	0.549
ENSG00000166959	83661	MS4A8B	0.431
ENSG00000262273	5407	PNLIPRP1	0.495
ENSG00000143774	2987	GUK1	0.61
ENSG00000263162	8924	HERC2	0.494
ENSG00000266200	5408	PNLIPRP2	0.517
ENSG00000262564	143379	C10orf82	0.431
ENSG00000263156	2771	GNAI2	0.516
ENSG00000188191	5575	PRKAR1B	0.551
ENSG00000263052	259217	HSPA12A	0.564
ENSG00000134160	4308	TRPM1	0.53
ENSG00000262015	7869	SEMA3B	0.595
ENSG00000171931	10517	FBXW10	0.39
ENSG00000161888	147841	SPC24	0.493
ENSG00000100242	25777	UNC84B	0.507
ENSG00000182858	79087	ALG12	0.466
ENSG00000184164	79174	CRELD2	0.496
ENSG00000130158	57572	DOCK6	0.645
ENSG00000237022	3107	HLA-C	0.695
ENSG00000183134	11251	GPR44	0.536
ENSG00000149506	22917	ZP1	0.371
ENSG00000083097	23033	DOPEY1	0.578
ENSG00000164818	54919	HEATR2	0.503
ENSG00000261869	132228	C3orf45	0.406
ENSG00000130173	55908	LOC55908	0.435
ENSG00000263093	7866	IFRD2	0.549
ENSG00000130167	26526	TSPAN16	0.52
ENSG00000125846	7692	ZNF133	0.586
ENSG00000261855	8372	HYAL3	0.49

ENSG00000105518	374882	TMEM205	0.5
ENSG00000261871	24142	NAT6	0.483
ENSG00000100427	23209	MLC1	0.487
ENSG00000224608	3106	HLA-B	0.594
ENSG00000183401	126075	LOC126075	0.414
ENSG00000183828	256281	NUDT14	0.496
ENSG00000262208	3373	HYAL1	0.545
ENSG00000105520	64748	LPPR2	0.531
ENSG00000185024	2972	BRF1	0.567
ENSG00000073146	54456	MOV10L1	0.446
ENSG00000173928	126074	C19orf39	0.459
ENSG00000261921	8692	HYAL2	0.519
ENSG00000187266	2057	EPOR	0.701
ENSG00000128383	200315	APOBEC3A	0.417
ENSG00000262485	11334	TUSC2	0.566
ENSG00000205517	57139	RGL3	0.528
ENSG00000179750	9582	APOBEC3B	0.533
ENSG00000164828	23353	UNC84A	0.571
ENSG00000263005	11186	RASSF1	0.515
ENSG00000113163	10087	COL4A3BP	0.489
ENSG00000166922	6447	SCG5	0.517
ENSG00000244509	27350	APOBEC3C	0.546
ENSG00000198003	115948	CCDC151	0.462
ENSG00000128394	200316	APOBEC3F	0.423
ENSG00000248905	342184	FMN1	0.495
ENSG00000231372	4277	MICB	0.526
ENSG00000239713	60489	APOBEC3G	0.57
ENSG00000130175	5589	PRKCSH	0.592
ENSG00000178057	25915	C3orf60	0.538
ENSG00000225859	7919	BAT1	0.592
ENSG00000013375	5238	PGM3	0.541
ENSG00000161914	115950	ZNF653	0.444
ENSG00000130159	51295	ECSIT	0.486
ENSG00000110446	51296	SLC15A3	0.471
ENSG00000178035	3615	IMPDH2	0.564
ENSG00000128159	85378	TUBGCP6	0.528
ENSG00000154025	125206	SLC5A10	0.464
ENSG00000198835	57165	GJC2	0.553
ENSG00000181873	200205	C1orf69	0.475
ENSG00000169857	57099	AVEN	0.5
ENSG00000184887	90135	BTBD6	0.475
ENSG00000132664	10621	POLR3F	0.45
ENSG00000184984	1133	CHRM5	0.451
ENSG00000100316	6122	RPL3	0.617
ENSG00000100429	83933	HDAC10	0.458
ENSG00000013725	923	CD6	0.61
ENSG00000198551	199692	ZNF627	0.5
ENSG00000102575	54	ACP5	0.517
ENSG00000197044	126068	ZNF441	0.44
ENSG00000188522	644815	FAM83G	0.45
ENSG00000177599	126069	ZNF491	0.391
ENSG00000182405	161779	PGBD4	0.468
ENSG00000226850	534	ATP6V1G2	0.492
ENSG00000171295	126070	ZNF440	0.51

ENSG00000227565	4795	NFKBIL1	0.461
ENSG00000171291	90594	ZNF439	0.432
ENSG00000231408	4049	LTA	0.506
ENSG00000013392	112611	RWDD2A	0.508
ENSG00000197054	284390	ZNF763	0.558
ENSG00000228321	7124	TNF	0.524
ENSG00000196757	90592	ZNF700	0.447
ENSG00000117407	9048	ARTN	0.562
ENSG00000223448	4050	LTB	0.52
ENSG00000154016	10750	GRAP	0.506
ENSG00000231048	7940	LST1	0.606
ENSG00000239857	51608	C7orf20	0.555
ENSG00000132010	7568	ZNF20	0.469
ENSG00000257591	90589	ZNF625	0.446
ENSG00000100324	10454	MAP3K7IP1	0.531
ENSG00000196646	7695	ZNF136	0.456
ENSG00000197857	51710	ZNF44	0.584
ENSG00000188868	147837	ZNF563	0.443
ENSG00000198342	79973	ZNF442	0.463
ENSG00000196466	90576	ZNF799	0.568
ENSG00000180855	10224	ZNF443	0.521
ENSG00000262022	163227	ZNF100	0.455
ENSG00000242852	163051	ZNF709	0.469
ENSG00000236979	259197	NCR3	0.564
ENSG00000105963	11033	CENTA1	0.609
ENSG00000196826	163051	ZNF709	0.469
ENSG00000249709	163050	ZNF564	0.552
ENSG00000188033	57474	ZNF490	0.504
ENSG00000128272	468	ATF4	0.569
ENSG00000173875	163049	ZNF791	0.493
ENSG00000187051	91582	RPS19BP1	0.49
ENSG00000182117	55505	NOLA3	0.5
ENSG00000104774	4125	MAN2B1	0.545
ENSG00000184507	256646	C15orf55	0.46
ENSG00000226618	7916	BAT2	0.58
ENSG00000229859	643834	PGA3	0.419
ENSG00000123154	84292	MORG1	0.418
ENSG00000095059	1725	DHPS	0.596
ENSG00000236802	8705	B3GALT4	0.488
ENSG00000236222	9277	WDR46	0.512
ENSG00000132768	1802	DPH2	0.521
ENSG00000262149	4542	MYO1F	0.525
ENSG00000261964	81794	ADAMTS10	0.518
ENSG00000205593	414918	FAM116B	0.464
ENSG00000176177	150350	ENTHD1	0.5
ENSG00000100351	9402	GRAP2	0.507
ENSG00000132631	140856	C20orf79	0.382
ENSG00000154099	123872	LRRC50	0.557
ENSG00000228760	7917	BAT3	0.576
ENSG00000240230	90639	COX19	0.484
ENSG00000115539	79031	PDCL3	0.511
ENSG00000132669	54453	RIN2	0.523
ENSG00000119431	81932	HDHD3	0.521
ENSG00000235692	10471	PFDN6	0.6

ENSG00000167815	7001	PRDX2	0.62
ENSG00000103168	9013	TAF1C	0.549
ENSG00000104889	10535	RNASEH2A	0.566
ENSG00000021776	9716	AQR	0.542
ENSG00000132026	83546	RTBDN	0.411
ENSG00000239900	158	ADSL	0.574
ENSG00000128165	79924	ADM2	0.482
ENSG00000100253	55586	MIOX	0.431
ENSG00000105612	1777	DNASE2	0.596
ENSG00000122986	84329	HVCN1	0.53
ENSG00000105610	10661	KLF1	0.498
ENSG00000105607	2639	GCDH	0.595
ENSG00000228736	5863	RGL2	0.529
ENSG00000231974	55937	APOM	0.62
ENSG00000228177	57827	C6orf47	0.496
ENSG00000071082	6160	RPL31	0.603
ENSG00000161860	256126	SYCE2	0.422
ENSG00000223932	7918	BAT4	0.51
ENSG00000179115	2193	FARSA	0.613
ENSG00000025770	29781	NCAPH2	0.598
ENSG00000147586	28957	MRPS28	0.513
ENSG00000232960	1460	CSNK2B	0.51
ENSG00000169435	166824	RASSF6	0.485
ENSG00000182809	1397	CRIP2	0.553
ENSG00000240433	58496	LY6G5B	0.556
ENSG00000228883	80741	LY6G5C	0.527
ENSG00000179271	90480	GADD45GIP1	0.433
ENSG00000224552	7920	BAT5	0.551
ENSG00000104907	55621	TRMT1	0.603
ENSG00000172046	10869	USP19	0.498
ENSG00000130489	9997	SCO2	0.534
ENSG00000166069	145942	TMCO5A	0.478
ENSG00000157653	257169	C9orf43	0.473
ENSG00000160877	112939	BTBD14B	0.5
ENSG00000025708	1890	TYMP	0.596
ENSG00000162931	51127	TRIM17	0.413
ENSG00000239741	259215	LY6G6F	0.375
ENSG00000171262	283742	FAM98B	0.446
ENSG00000177989	440836	LOC440836	0.592
ENSG00000173093	160762	CCDC63	0.37
ENSG00000130487	113730	KLHDC7B	0.481
ENSG00000206208	6892	TAPBP	0.616
ENSG00000104537	312	ANXA13	0.483
ENSG00000109272	5197	PF4V1	0.447
ENSG00000111358	2967	GTF2H3	0.502
ENSG00000140955	161931	ADAD2	0.435
ENSG00000163737	5196	PF4	0.482
ENSG00000203877	134701	C6orf159	0.324
ENSG00000111245	4633	MYL2	0.49
ENSG00000037757	84245	MGC3207	0.525
ENSG00000237056	9278	ZBTB22	0.534
ENSG00000104979	28974	C19orf53	0.518
ENSG00000163736	5473	PPBP	0.481
ENSG00000213145	1396	CRIP1	0.564

ENSG00000187556	342977	NANOS3	0.342
ENSG00000065615	51167	CYB5R4	0.513
ENSG00000132016	79173	C19orf57	0.484
ENSG00000231617	1616	DAXX	0.603
ENSG00000100288	1120	CHKB	0.531
ENSG00000150667	161835	FSIP1	0.429
ENSG00000163738	441024	MTHFD2L	0.483
ENSG00000166073	11245	GPR176	0.467
ENSG00000103175	58189	WFDC1	0.486
ENSG00000132000	79883	PODNL1	0.468
ENSG00000257341	1396	CRIP1	0.564
ENSG00000162441	84328	LZIC	0.528
ENSG00000132017	90379	LOC90379	0.626
ENSG00000064270	9914	ATP2C2	0.56
ENSG00000229006	5987	TRIM27	0.637
ENSG00000135324	112609	MRAP2	0.4
ENSG00000171136	117579	RLN3	0.447
ENSG00000185347	283643	C14orf80	0.397
ENSG00000104998	9466	IL27RA	0.618
ENSG00000233831	282890	ZNF311	0.466
ENSG00000100299	410	ARSA	0.505
ENSG00000135315	22832	KIAA1009	0.528
ENSG00000140319	6727	SRP14	0.515
ENSG00000250364	81797	OR12D3	0.409
ENSG00000225247	26529	OR12D2	0.438
ENSG00000223898	26531	OR11A1	0.415
ENSG00000229408	26716	OR2H1	0.517
ENSG00000164687	2171	FABP5	0.586
ENSG00000236561	4340	MOG	0.581
ENSG00000213029	10638	SPHAR	0.557
ENSG00000147588	5375	PMP2	0.431
ENSG00000123136	10212	DDX39	0.561
ENSG00000162444	116362	RBP7	0.514
ENSG00000183665	55039	TRMT12	0.487
ENSG00000172037	3913	LAMB2	0.521
ENSG00000156970	701	BUB1B	0.552
ENSG00000123143	5585	PKN1	0.518
ENSG00000147687	83940	TATDN1	0.495
ENSG00000100312	49	ACR	0.468
ENSG00000171643	170591	S100Z	0.455
ENSG00000079974	11158	RABL2B	0.552
ENSG00000160951	5731	PTGER1	0.528
ENSG00000109321	374	AREG	0.408
ENSG00000135318	4907	NT5E	0.572
ENSG00000197653	196385	DNAH10	0.476
ENSG00000099797	9524	GPSN2	0.507
ENSG00000205595	374	AREG	0.408
ENSG00000099795	4713	NDUFB7	0.559
ENSG00000140950	57707	KIAA1609	0.6
ENSG00000174808	685	BTC	0.469
ENSG00000131355	84658	EMR3	0.428
ENSG00000069248	55746	NUP133	0.545
ENSG00000234669	346171	ZFP57	0.388
ENSG00000160961	84449	ZNF333	0.568

ENSG00000127507	30817	EMR2	0.529
ENSG00000229698	3134	HLA-F	0.626
ENSG00000234599	10255	HCG9	0.453
ENSG00000169116	25849	DKFZP564O0823	0.492
ENSG00000188269	26659	OR7A5	0.462
ENSG00000104231	79752	ZFAND1	0.509
ENSG00000105143	6511	SLC1A6	0.474
ENSG00000167333	55128	TRIM68	0.513
ENSG00000089234	8315	BRAP	0.588
ENSG00000105131	79852	ABHD9	0.436
ENSG00000147684	4715	NDUFB9	0.514
ENSG00000105127	10270	AKAP8	0.574
ENSG00000011243	26993	AKAP8L	0.591
ENSG00000180785	143503	OR51E1	0.464
ENSG00000106927	259	AMBP	0.549
ENSG00000105122	64926	FLJ21438	0.491
ENSG00000161031	114770	PGLYRP2	0.386
ENSG00000132846	84327	ZBED3	0.425
ENSG00000186529	4051	CYP4F3	0.516
ENSG00000167346	56547	MMP26	0.456
ENSG00000186204	66002	CYP4F12	0.522
ENSG00000136811	4957	ODF2	0.473
ENSG00000223577	253018	HCG27	0.44
ENSG00000171942	26538	OR10H2	0.407
ENSG00000186723	26539	OR10H1	0.443
ENSG00000186115	8529	CYP4F2	0.475
ENSG00000173421	339834	CCDC36	0.471
ENSG00000171903	57834	CYP4F11	0.465
ENSG00000115590	7850	IL1R2	0.561
ENSG00000136883	113220	KIF12	0.441
ENSG00000244734	3043	HBB	0.572
ENSG00000227429	10866	HCP5	0.492
ENSG00000219073	23436	ELA3B	0.429
ENSG00000196684	84941	HSH2D	0.5
ENSG00000223609	3045	HBD	0.489
ENSG00000180938	137209	ZNF572	0.378
ENSG00000142789	10136	ELA3A	0.544
ENSG00000267068	84941	HSH2D	0.5
ENSG00000104549	6713	SQLE	0.601
ENSG00000141977	117286	CIB3	0.556
ENSG00000105058	26017	FAM32A	0.492
ENSG00000178882	144347	FAM101A	0.431
ENSG00000213934	3047	HBG1	0.461
ENSG00000127528	10365	KLF2	0.532
ENSG00000127527	58513	EPS15L1	0.602
ENSG00000174792	152816	C4orf26	0.367
ENSG00000141979	125972	CALR3	0.474
ENSG00000105072	84167	C19orf44	0.486
ENSG00000164961	9897	KIAA0196	0.519
ENSG00000263255	90649	ZNF486	0.396
ENSG00000196565	3048	HBG2	0.507
ENSG00000135801	27097	TAF5L	0.454
ENSG00000072954	79041	TMEM38A	0.455
ENSG00000111275	217	ALDH2	0.58

ENSG00000213931	3046	HBE1	0.567
ENSG00000164253	55255	WDR41	0.534
ENSG00000184881	79345	OR51B2	0.395
ENSG00000262249	199777	ZNF626	0.48
ENSG00000127533	9002	F2RL3	0.433
ENSG00000175520	50613	UBQLN3	0.459
ENSG00000056678	3833	KIFC1	0.535
ENSG00000160111	27151	CPAMD8	0.486
ENSG00000229314	5004	ORM1	0.452
ENSG00000115598	8808	IL1RL2	0.447
ENSG00000131351	93323	NY-SAR-48	0.492
ENSG00000135763	9816	KIAA0133	0.52
ENSG00000156831	286053	NSMCE2	0.504
ENSG00000233276	2876	GPX1	0.542
ENSG00000053501	55850	USE1	0.563
ENSG00000173372	712	C1QA	0.449
ENSG00000099330	79629	OCEL1	0.515
ENSG00000159189	714	C1QC	0.518
ENSG00000173369	713	C1QB	0.479
ENSG00000115602	9173	IL1RL1	0.572
ENSG00000105393	29086	C19orf62	0.518
ENSG00000160117	126549	ANKLE1	0.438
ENSG00000089022	8550	MAPKAPK5	0.632
ENSG00000130312	64981	MRPL34	0.506
ENSG00000074855	57719	TMEM16H	0.493
ENSG00000228278	5005	ORM2	0.562
ENSG00000130300	83483	PLVAP	0.466
ENSG00000130303	684	BST2	0.561
ENSG00000138768	8615	USO1	0.58
ENSG00000141971	93343	FAM125A	0.496
ENSG00000136997	4609	MYC	0.622
ENSG00000147697	56169	MLZE	0.427
ENSG00000171773	115861	NXNL1	0.566
ENSG00000167483	199786	FAM129C	0.407
ENSG00000175279	378708	APITD1	0.509
ENSG00000198270	89894	TMEM116	0.508
ENSG00000115604	8809	IL18R1	0.476
ENSG00000130479	55201	MAP1S	0.425
ENSG00000241563	1325	CORT	0.465
ENSG00000130475	23149	FCHO1	0.501
ENSG00000160049	1676	DFFA	0.526
ENSG00000004487	23028	AOF2	0.527
ENSG00000179913	10331	B3GNT3	0.484
ENSG00000248099	3640	INSL3	0.513
ENSG00000154102	404550	C16orf74	0.466
ENSG00000105639	3718	JAK3	0.611
ENSG00000115607	8807	IL18RAP	0.479
ENSG00000133742	759	CA1	0.54
ENSG00000105640	6142	RPL18A	0.529
ENSG00000105641	6528	SLC5A5	0.471
ENSG00000007080	115098	CCDC124	0.492
ENSG00000105642	3780	KCNN1	0.469
ENSG00000171530	6902	TBCA	0.542
ENSG00000096996	3594	IL12RB1	0.471

ENSG00000131143	1327	COX4I1	0.607
ENSG00000089248	10961	ERP29	0.552
ENSG00000254858	84769	FKSG24	0.469
ENSG00000105650	5143	PDE4C	0.599
ENSG00000150991	7316	UBC	0.634
ENSG00000140968	3394	IRF8	0.526
ENSG00000130518	80726	KIAA1683	0.485
ENSG00000153317	50807	DDEF1	0.556
ENSG00000130520	25804	LSM4	0.615
ENSG00000130513	9518	GDF15	0.593
ENSG00000175489	126364	LRRC25	0.467
ENSG00000130511	170463	SSBP4	0.521
ENSG00000135773	10753	CAPN9	0.531
ENSG00000105655	51477	ISYNA1	0.526
ENSG00000132274	10346	TRIM22	0.52
ENSG00000111300	80018	C12orf30	0.52
ENSG00000135346	1081	CGA	0.467
ENSG00000135953	84804	MFSD9	0.431
ENSG00000104267	760	CA2	0.522
ENSG00000221983	7311	UBA52	0.514
ENSG00000184911	728656	LOC728656	0.324
ENSG00000150990	57647	DHX37	0.492
ENSG00000006016	9244	CRLF1	0.524
ENSG00000105696	25789	TMEM59L	0.487
ENSG00000167487	55295	KLHL26	0.47
ENSG00000143994	84696	ABHD1	0.48
ENSG00000105664	1311	COMP	0.489
ENSG00000155897	114	ADCY8	0.472
ENSG00000132196	51478	HSD17B7	0.518
ENSG00000081760	65985	AACS	0.498
ENSG00000205176	254958	REXO1L1	0.444
ENSG00000147614	245972	ATP6V0D2	0.594
ENSG00000223802	2657	GDF1	0.448
ENSG00000130283	2657	GDF1	0.448
ENSG00000105669	11316	COPE	0.568
ENSG00000164893	157724	SLC7A13	0.429
ENSG00000185168	284185	C17orf55	0.403
ENSG00000159123	728656	LOC728656	0.324
ENSG00000105671	54555	DDX49	0.563
ENSG00000051128	9454	HOMER3	0.61
ENSG00000064607	10147	SFRS14	0.62
ENSG00000181634	9966	TNFSF15	0.445
ENSG00000105676	93436	ARMC6	0.501
ENSG00000249087	148898	C1orf213	0.455
ENSG00000204219	6920	TCEA3	0.517
ENSG00000106952	944	TNFSF8	0.475
ENSG00000132259	1262	CNGA4	0.447
ENSG00000135972	64965	MRPS9	0.489
ENSG00000064545	54929	TMEM161A	0.61
ENSG00000088280	55616	DDEFL1	0.592
ENSG00000213999	4207	MEF2B	0.548
ENSG00000009724	10747	MASP2	0.554
ENSG00000135973	11250	GPR45	0.445
ENSG00000170955	112464	PRKCDBP	0.492

ENSG00000135966	9392	TGFBRAP1	0.481
ENSG00000186462	4674	NAP1L2	0.487
ENSG00000164062	327	APEH	0.489
ENSG00000166311	6609	SMPD1	0.613
ENSG00000132837	29958	DMGDH	0.492
ENSG00000139370	121260	SLC15A4	0.588
ENSG00000120055	79946	C10orf95	0.465
ENSG00000138755	4283	CXCL9	0.482
ENSG00000135974	79074	C2orf49	0.49
ENSG00000267629	404037	HAPLN4	0.423
ENSG00000156219	419	ART3	0.471
ENSG00000213996	53345	TM6SF2	0.426
ENSG00000105705	57794	SF4	0.544
ENSG00000129295	23639	LRRC6	0.486
ENSG00000171824	5394	EXOSC10	0.51
ENSG00000115641	2274	FHL2	0.578
ENSG00000151948	144423	GLT1D1	0.405
ENSG00000119333	89891	WDR34	0.518
ENSG00000244560	155060	ZNF783	0.569
ENSG00000085719	8895	CPNE3	0.576
ENSG00000131263	51132	RNF12	0.595
ENSG00000132840	23743	BHMT2	0.459
ENSG00000173531	4485	MST1	0.568
ENSG00000104731	54758	KLHDC4	0.516
ENSG00000178093	83983	TSSK6	0.474
ENSG00000186010	51079	NDUFA13	0.537
ENSG00000142676	6135	RPL11	0.517
ENSG00000250067	374887	FLJ44968	0.387
ENSG00000173409	64801	ARV1	0.539
ENSG00000105717	80714	PBX4	0.39
ENSG00000146282	57038	RARS2	0.514
ENSG00000133624	79970	ZNF767	0.494
ENSG00000089639	51291	GMIP	0.468
ENSG00000125207	9271	PIWIL1	0.468
ENSG00000184221	116448	OLIG1	0.515
ENSG00000145692	635	BHMT	0.44
ENSG00000131269	22	ABCB7	0.523
ENSG00000089009	6128	RPL6	0.501
ENSG00000169248	6373	CXCL11	0.507
ENSG00000182118	375061	FAM89A	0.451
ENSG00000119147	84417	C2orf40	0.426
ENSG00000198793	2475	FRAP1	0.59
ENSG00000181896	94039	ZNF101	0.605
ENSG00000138750	53371	NUP54	0.554
ENSG00000105708	7561	ZNF14	0.51
ENSG00000081665	440515	ZNF506	0.471
ENSG00000259980	84311	MRPL45	0.488
ENSG00000261413	30837	SOCS7	0.462
ENSG00000256771	56242	ZNF253	0.506
ENSG00000160447	29941	PKN3	0.493
ENSG00000184635	81931	ZNF93	0.559
ENSG00000197124	91120	ZNF682	0.405
ENSG00000176571	168975	CNBD1	0.537
ENSG00000256229	90649	ZNF486	0.396

ENSG00000188171	199777	ZNF626	0.48
ENSG00000176566	138009	WDR21C	0.479
ENSG00000105750	7639	ZNF85	0.519
ENSG00000118620	80264	ZNF430	0.504
ENSG00000160352	148206	ZNF714	0.52
ENSG00000196705	170959	ZNF431	0.54
ENSG00000182141	7562	ZNF708	0.409
ENSG00000174990	763	CA5A	0.48
ENSG00000042832	7038	TG	0.51
ENSG00000143633	128061	C1orf131	0.465
ENSG00000197020	163227	ZNF100	0.455
ENSG00000198521	7594	ZNF43	0.549
ENSG00000160321	7757	ZNF208	0.476
ENSG00000197134	113835	ZNF257	0.401
ENSG00000196109	163223	ZNF676	0.436
ENSG00000143228	83540	NUF2	0.552
ENSG00000135336	23595	ORC3L	0.532
ENSG00000167232	7644	ZNF91	0.547
ENSG00000197372	171392	ZNF675	0.41
ENSG00000196172	148213	ZNF681	0.458
ENSG00000104312	8767	RIPK2	0.582
ENSG00000213096	9534	ZNF254	0.482
ENSG00000169021	7386	UQCRFS1	0.524
ENSG00000105171	10775	POP4	0.527
ENSG00000164329	167153	PAPD4	0.57
ENSG00000119401	22954	TRIM32	0.528
ENSG00000089169	22895	RPH3A	0.528
ENSG00000160446	84885	ZDHHC12	0.464
ENSG00000131943	83636	C19orf12	0.524
ENSG00000116906	8443	GNPAT	0.54
ENSG00000263148	492	ATP2B3	0.509
ENSG00000262919	92002	FAM58A	0.443
ENSG00000164068	63891	RNF123	0.503
ENSG00000263138	84465	MEGF11	0.459
ENSG00000078725	1620	DBC1	0.503
ENSG00000260150	10771	ZMYND11	0.618
ENSG00000262788	84916	CIRH1A	0.532
ENSG00000261677	9200	PTPLA	0.518
ENSG00000260873	6645	SNTB2	0.624
ENSG00000260045	8027	STAM	0.533
ENSG00000104320	4683	NBN	0.612
ENSG00000260314	4360	MRC1	0.547
ENSG00000259777	221074	SLC39A12	0.421
ENSG00000215641	5987	TRIM27	0.637
ENSG00000265264	26515	FXC1	0.48
ENSG00000105185	9141	PDCD5	0.489
ENSG00000111452	283383	GPR133	0.491
ENSG00000136861	55755	CDK5RAP2	0.5
ENSG00000262279	4684	NCAM1	0.659
ENSG00000138085	51374	C2orf28	0.516
ENSG00000164309	202333	CMYA5	0.551
ENSG00000169260	282890	ZNF311	0.466
ENSG00000179532	144132	DNHD1	0.449
ENSG00000262377	54970	TTC12	0.533

ENSG00000213965	390916	NUDT19	0.323
ENSG00000173809	91646	TDRD12	0.49
ENSG00000094841	139596	UPRT	0.5
ENSG00000206517	26531	OR11A1	0.415
ENSG00000021488	11136	SLC7A9	0.503
ENSG00000206516	26716	OR2H1	0.517
ENSG00000206511	2550	GABBR1	0.533
ENSG00000121289	84902	CCDC123	0.476
ENSG00000171819	10218	ANGPTL7	0.47
ENSG00000188822	1269	CNR2	0.503
ENSG00000131944	91442	C19orf40	0.504
ENSG00000167136	2021	ENDOG	0.517
ENSG00000198917	51490	C9orf114	0.518
ENSG00000120942	29914	UBIAD1	0.474
ENSG00000188529	10772	FUSIP1	0.541
ENSG00000076650	55094	GPATCH1	0.468
ENSG00000102383	158866	ZDHHC15	0.416
ENSG00000206513	10537	UBD	0.48
ENSG00000206512	7932	OR2H2	0.526
ENSG00000137345	4340	MOG	0.581
ENSG00000159128	3460	IFNGR2	0.56
ENSG00000166359	126248	WDR88	0.468
ENSG00000104325	1666	DECR1	0.563
ENSG00000130876	56301	SLC7A10	0.455
ENSG00000132275	23378	KIAA0409	0.568
ENSG00000206510	346171	ZFP57	0.388
ENSG00000089127	4938	OAS1	0.592
ENSG00000206509	3134	HLA-F	0.626
ENSG00000124299	5184	PEPD	0.531
ENSG00000116661	26232	FBXO2	0.512
ENSG00000206506	3135	HLA-G	0.612
ENSG00000124391	27189	IL17C	0.375
ENSG00000051523	1535	CYBA	0.544
ENSG00000206505	3105	HLA-A	0.588
ENSG00000171097	883	CCBL1	0.589
ENSG00000257103	26065	LSM14A	0.638
ENSG00000206502	30834	ZNRD1	0.542
ENSG00000261059	29989	OBP2B	0.421
ENSG00000102390	51260	CXorf26	0.552
ENSG00000206501	6992	PPP1R11	0.524
ENSG00000256062	28	ABO	0.59
ENSG00000261306	6838	SURF6	0.5
ENSG00000206500	80352	RNF39	0.512
ENSG00000167508	4597	MVD	0.518
ENSG00000198934	57692	MAGEE1	0.533
ENSG00000261579	6837	MED22	0.525
ENSG00000142661	127294	MYOM3	0.53
ENSG00000137397	11074	TRIM31	0.6
ENSG00000148795	1586	CYP17A1	0.504
ENSG00000111335	4939	OAS2	0.595
ENSG00000260501	6130	RPL7A	0.522
ENSG00000198598	4326	MMP17	0.476
ENSG00000126249	84306	PDCD2L	0.495
ENSG00000172524	135644	TRIM40	0.322

ENSG00000137394	10107	TRIM10	0.579
ENSG00000260692	6834	SURF1	0.581
ENSG00000175287	254295	PHYHD1	0.533
ENSG00000137384	89870	TRIM15	0.566
ENSG00000143171	6258	RXRG	0.501
ENSG00000205209	284402	LOC284402	0.382
ENSG00000089335	55900	ZNF302	0.515
ENSG00000261361	6835	SURF2	0.497
ENSG00000177692	54943	DNAJC28	0.427
ENSG00000260181	6836	SURF4	0.603
ENSG00000185669	333929	SNAI3	0.533
ENSG00000166337	6881	TAF10	0.53
ENSG00000137313	7726	TRIM26	0.558
ENSG00000153896	148103	ZNF599	0.531
ENSG00000159131	2618	GART	0.639
ENSG00000260489	169436	C9orf96	0.435
ENSG00000180884	126375	ZNF792	0.444
ENSG00000261493	57109	REXO4	0.463
ENSG00000089351	57655	GRAMD1A	0.517
ENSG00000177192	80324	PUS1	0.487
ENSG00000206495	56658	TRIM39	0.466
ENSG00000260099	11093	ADAMTS13	0.455
ENSG00000174177	348180	C16orf84	0.505
ENSG00000242726	79897	RPP21	0.468
ENSG00000266964	5348	FXYD1	0.49
ENSG00000183495	57634	EP400	0.602
ENSG00000260502	11094	C9orf7	0.59
ENSG00000206493	3133	HLA-E	0.629
ENSG00000261768	11182	SLC2A6	0.521
ENSG00000089327	53827	FXYD5	0.592
ENSG00000162763	440699	LRRC52	0.457
ENSG00000143198	4259	MGST3	0.532
ENSG00000198445	150160	CCT8L2	0.427
ENSG00000177558	148109	TMEM162	0.373
ENSG00000105699	51599	LSR	0.515
ENSG00000206492	2794	GNL1	0.552
ENSG00000105697	57817	HAMP	0.461
ENSG00000012124	933	CD22	0.59
ENSG00000177663	23765	IL17RA	0.598
ENSG00000175283	22845	DOLK	0.497
ENSG00000095319	23511	NUP188	0.618
ENSG00000188508	388533	KRTDAP	0.437
ENSG00000131174	1349	COX7B	0.552
ENSG00000143149	223	ALDH9A1	0.517
ENSG00000161249	93099	DMKN	0.486
ENSG00000206491	80742	PRR3	0.522
ENSG00000206490	23	ABCF1	0.557
ENSG00000184933	8590	OR6A2	0.436
ENSG00000149054	7762	ZNF215	0.48
ENSG00000163794	7349	UCN	0.463
ENSG00000001461	57185	NPAL3	0.538
ENSG00000115204	4358	MPV17	0.521
ENSG00000131100	529	ATP6V1E1	0.51
ENSG00000149050	7761	ZNF214	0.424

ENSG00000206489	5514	PPP1R10	0.598
ENSG00000158077	338323	NLRP14	0.37
ENSG00000189001	374897	SBSN	0.447
ENSG00000139405	84934	C12orf52	0.564
ENSG00000143183	54499	TMCO1	0.62
ENSG00000185163	317781	DDX51	0.519
ENSG00000105679	26330	GAPDHS	0.454
ENSG00000117602	11123	RCAN3	0.474
ENSG00000105677	10430	TMEM147	0.542
ENSG00000167513	81620	CDT1	0.518
ENSG00000203624	28973	MRPS18B	0.57
ENSG00000105675	495	ATP4A	0.432
ENSG00000166578	115811	IQCD	0.448
ENSG00000249115	23354	KIAA0841	0.471
ENSG00000206488	79969	C6orf134	0.503
ENSG00000129518	55837	EAPP	0.551
ENSG00000126254	79171	RBM42	0.547
ENSG00000105672	2116	ETV2	0.435
ENSG00000126267	1340	COX6B1	0.534
ENSG00000184967	79050	NOC4L	0.444
ENSG00000105668	11045	UPK1A	0.443
ENSG00000183801	283298	OLFML1	0.465
ENSG00000011590	27033	ZBTB32	0.462
ENSG00000105663	9757	MLL4	0.555
ENSG00000126246	79713	TMEM149	0.469
ENSG00000198931	353	APRT	0.572
ENSG00000213780	2968	GTF2H4	0.5
ENSG00000206487	221545	C6orf136	0.518
ENSG00000161265	199746	U2AF1L4	0.411
ENSG00000141012	2588	GALNS	0.523
ENSG00000262359	8447	DOC2B	0.434
ENSG00000206486	8449	DHX16	0.471
ENSG00000267796	55957	LIN37	0.505
ENSG00000206485	170954	KIAA1949	0.563
ENSG00000004777	115703	SNX26	0.571
ENSG00000183628	8214	DGCR6	0.438
ENSG00000188859	149297	FAM78B	0.378
ENSG00000206484	11270	NRM	0.523
ENSG00000100033	5625	PRODH	0.486
ENSG00000250799	58510	PRODH2	0.464
ENSG00000161270	4868	NPHS1	0.449
ENSG00000106804	727	C5	0.491
ENSG00000206481	9656	MDC1	0.575
ENSG00000187624	400566	LOC400566	0.437
ENSG00000137411	57176	VARS2	0.454
ENSG00000126259	84063	KIRREL2	0.467
ENSG00000167515	51693	TRAPPC2L	0.505
ENSG00000105290	333	APLP1	0.483
ENSG00000188343	137392	FAM92A1	0.568
ENSG00000183311	203068	TUBB	0.613
ENSG00000187848	22953	P2RX2	0.542
ENSG00000089060	80024	SLC24A6	0.498
ENSG00000152382	117143	TADA1L	0.474
ENSG00000115211	8890	EIF2B4	0.557

ENSG00000126264	10870	HCST	0.475
ENSG00000141252	55275	VPS53	0.521
ENSG00000011600	7305	TYROBP	0.511
ENSG00000206203	23617	TSSK2	0.516
ENSG00000126243	79414	LRFN3	0.493
ENSG00000205138	644096	LOC644096	0.579
ENSG00000143195	387597	C1orf32	0.48
ENSG00000173198	10800	CYSLTR1	0.466
ENSG00000239382	84964	ALKBH6	0.465
ENSG00000205758	9946	CRYZL1	0.545
ENSG00000169427	51305	KCNK9	0.523
ENSG00000206480	10211	FLOT1	0.631
ENSG00000166394	51700	CYB5R2	0.467
ENSG00000148843	22984	PDCD11	0.577
ENSG00000161277	199745	THAP8	0.44
ENSG00000167632	83696	NIBP	0.606
ENSG00000175206	4878	NPPA	0.485
ENSG00000120937	4879	NPPB	0.467
ENSG00000206478	8870	IER3	0.564
ENSG00000075702	284403	WDR62	0.525
ENSG00000078589	27334	P2RY10	0.473
ENSG00000143194	84944	MAEL	0.504
ENSG00000147138	84636	GPR174	0.391
ENSG00000078596	9452	ITM2A	0.536
ENSG00000105258	5438	POLR2I	0.512
ENSG00000215522	780	DDR1	0.636
ENSG00000100883	6729	SRP54	0.541
ENSG00000105254	1155	TBCB	0.544
ENSG00000177084	5426	POLE	0.546
ENSG00000122145	50945	TBX22	0.388
ENSG00000161281	1346	COX7A1	0.502
ENSG00000242259	128977	C22orf39	0.491
ENSG00000151176	196463	P76	0.449
ENSG00000196357	147929	ZNF565	0.473
ENSG00000185608	64976	MRPL40	0.518
ENSG00000070010	7353	UFD1L	0.56
ENSG00000176715	197322	ACSF3	0.556
ENSG00000182261	338322	NLRP10	0.438
ENSG00000175390	8665	EIF3F	0.546
ENSG00000181007	284406	ZFP82	0.525
ENSG00000221974	2968	GTF2H4	0.5
ENSG00000254004	339324	ZNF260	0.477
ENSG00000139410	113675	SDSL	0.496
ENSG00000129910	1013	CDH15	0.566
ENSG00000093009	8318	CDC45L	0.517
ENSG00000161298	84911	ZNF382	0.5
ENSG00000122965	9904	RBM19	0.545
ENSG00000170100	197320	ZNF778	0.468
ENSG00000189042	163081	ZNF567	0.532
ENSG00000206476	57176	VARS2	0.454
ENSG00000164951	54704	PPM2C	0.515
ENSG00000197863	388536	ZNF790	0.4
ENSG00000251247	25850	ZNF345	0.444
ENSG00000196260	389376	SFTPG	0.462

ENSG00000185869	374899	ZNF829	0.47
ENSG00000198842	92235	DUSP27	0.443
ENSG00000168631	135656	DPCR1	0.476
ENSG00000198453	374900	ZNF568	0.564
ENSG00000179409	50628	GEMIN4	0.578
ENSG00000197050	147923	ZNF420	0.475
ENSG00000167699	51031	GLOD4	0.511
ENSG00000196967	199704	ZNF585A	0.523
ENSG00000203618	2812	GP1BB	0.436
ENSG00000245680	92285	ZNF585B	0.389
ENSG00000185838	54584	GNB1L	0.479
ENSG00000143190	5451	POU2F1	0.521
ENSG00000206465	389376	SFTPG	0.462
ENSG00000188283	163087	ZNF383	0.453
ENSG00000229284	135656	DPCR1	0.476
ENSG00000184470	10587	TXNRD2	0.569
ENSG00000204542	29113	C6orf15	0.444
ENSG00000204540	170679	PSORS1C1	0.429
ENSG00000189164	84503	ZNF527	0.442
ENSG00000197912	6687	SPG7	0.596
ENSG00000138172	51063	CALHM2	0.585
ENSG00000196437	148266	ZNF569	0.457
ENSG00000206461	29113	C6orf15	0.444
ENSG00000206458	170679	PSORS1C1	0.429
ENSG00000206460	1041	CDSN	0.502
ENSG00000171827	148268	ZNF570	0.491
ENSG00000206459	170680	PSORS1C2	0.442
ENSG00000206457	54535	CCHCR1	0.631
ENSG00000197275	25788	RAD54B	0.595
ENSG00000117614	25949	SYF2	0.52
ENSG00000171817	163255	ZNF540	0.504
ENSG00000204538	170680	PSORS1C2	0.442
ENSG00000180479	51276	ZNF571	0.482
ENSG00000116691	60672	RP5-1077B9.4	0.56
ENSG00000176894	5827	PXMP2	0.51
ENSG00000204536	54535	CCHCR1	0.631
ENSG00000093010	1312	COMT	0.611
ENSG00000247077	192111	PGAM5	0.494
ENSG00000196381	163115	ZNF781	0.466
ENSG00000189144	126231	ZNF573	0.498
ENSG00000206455	6941	TCF19	0.531
ENSG00000130413	65975	STK33	0.534
ENSG00000206454	5460	POU5F1	0.546
ENSG00000164944	25962	KIAA1429	0.561
ENSG00000171861	55178	RNMTL1	0.49
ENSG00000115241	5496	PPM1G	0.539
ENSG00000167526	6137	RPL13	0.583
ENSG00000183597	128989	C22orf25	0.452
ENSG00000167642	10653	SPINT2	0.553
ENSG00000148335	28989	METTL11A	0.597
ENSG00000148175	2040	STOM	0.652
ENSG00000107960	79991	OBFC1	0.515
ENSG00000167641	94274	PPP1R14A	0.453
ENSG00000111412	79794	C12orf49	0.494

ENSG00000206452	3107	HLA-C	0.695
ENSG00000100890	9692	KIAA0391	0.535
ENSG00000178773	27132	CPNE7	0.491
ENSG00000099899	27037	HTF9C	0.44
ENSG00000179058	375759	C9orf50	0.447
ENSG00000198157	79366	NSBP1	0.589
ENSG00000167644	64073	C19orf33	0.437
ENSG00000099337	9424	KCNK6	0.466
ENSG00000206450	3106	HLA-B	0.594
ENSG00000065613	9748	SLK	0.52
ENSG00000099338	57828	C19orf15	0.459
ENSG00000104413	54845	RBM35A	0.471
ENSG00000258790	9692	KIAA0391	0.535
ENSG00000100902	5687	PSMA6	0.536
ENSG00000099341	5714	PSMD8	0.554
ENSG00000179168	199720	GGN	0.472
ENSG00000206449	4277	MICB	0.526
ENSG00000015413	1800	DPEP1	0.483
ENSG00000138002	26160	IFT172	0.485
ENSG00000128185	85359	DGCR6L	0.482
ENSG00000215425	7919	BAT1	0.592
ENSG00000166441	6157	RPL27A	0.541
ENSG00000167157	51450	PRRX2	0.508
ENSG00000148344	9536	PTGES	0.566
ENSG00000163623	4825	NKX6-1	0.441
ENSG00000174989	26259	FBXW8	0.445
ENSG00000183035	1538	CYLC1	0.522
ENSG00000196622	85376	RIMBP3	0.5
ENSG00000100906	4792	NFKBIA	0.593
ENSG00000196218	6261	RYR1	0.48
ENSG00000131165	5119	CHMP1A	0.545
ENSG00000145050	7873	ARMET	0.532
ENSG00000164291	153642	ARSK	0.47
ENSG00000185252	7625	ZNF74	0.488
ENSG00000072133	27330	RPS6KA6	0.578
ENSG00000175764	158135	TTLL11	0.439
ENSG00000099910	84861	KLHL22	0.601
ENSG00000206445	534	ATP6V1G2	0.492
ENSG00000187010	6007	RHD	0.547
ENSG00000145041	9730	VPRBP	0.559
ENSG00000178982	27335	EIF3K	0.58
ENSG00000167523	124045	C16orf55	0.439
ENSG00000132429	64208	POPDC3	0.514
ENSG00000185681	254956	MORN5	0.455
ENSG00000185324	8558	CDK10	0.649
ENSG00000174373	253959	GARNL1	0.565
ENSG00000206440	4795	NFKBIL1	0.461
ENSG00000205076	3963	LGALS7	0.469
ENSG00000178934	3963	LGALS7	0.469
ENSG00000171747	3960	LGALS4	0.498
ENSG00000164080	23132	RAD54L2	0.459
ENSG00000173503	4049	LTA	0.506
ENSG00000105339	22898	DENND3	0.615
ENSG00000104823	1891	ECH1	0.561

ENSG00000104824	3191	HNRNPL	0.619
ENSG00000206439	7124	TNF	0.524
ENSG00000206437	4050	LTB	0.52
ENSG00000175449	317671	RFESD	0.481
ENSG00000164941	55656	INTS8	0.518
ENSG00000206433	7940	LST1	0.606
ENSG00000068903	22933	SIRT2	0.515
ENSG00000155008	139322	APOOL	0.473
ENSG00000145757	83890	SPATA9	0.482
ENSG00000206344	253018	HCG27	0.44
ENSG00000241837	539	ATP5O	0.602
ENSG00000206430	259197	NCR3	0.564
ENSG00000204525	3107	HLA-C	0.695
ENSG00000198040	7637	ZNF84	0.543
ENSG00000104825	4793	NFKBIB	0.547
ENSG00000143199	55811	ADCY10	0.501
ENSG00000104835	54938	SARS2	0.514
ENSG00000164081	51368	TEX264	0.51
ENSG00000128626	6183	MRPS12	0.655
ENSG00000136682	55871	CBWD1	0.477
ENSG00000206428	199	AIF1	0.617
ENSG00000179751	342898	SYCN	0.384
ENSG00000183709	282616	IL28A	0.311
ENSG00000182393	282618	IL29	0.422
ENSG00000130755	9535	GMFG	0.51
ENSG00000206427	7916	BAT2	0.58
ENSG00000196387	7699	ZNF140	0.454
ENSG00000075399	9605	C16orf7	0.487
ENSG00000204882	2843	GPR20	0.497
ENSG00000057663	9474	ATG5	0.589
ENSG00000243927	64968	MRPS6	0.539
ENSG00000111445	5985	RFC5	0.608
ENSG00000099937	3053	SERPIND1	0.472
ENSG00000116996	57829	ZP4	0.477
ENSG00000159197	9992	KCNE2	0.434
ENSG00000105193	6217	RPS16	0.563
ENSG00000205670	54065	FAM165B	0.515
ENSG00000234745	3106	HLA-B	0.594
ENSG00000105197	92609	TIMM50	0.515
ENSG00000090932	10683	DLL3	0.484
ENSG00000157330	93190	C1orf158	0.394
ENSG00000197748	80217	C10orf79	0.41
ENSG00000176401	126272	EID2B	0.5
ENSG00000176396	163126	EID2	0.438
ENSG00000229972	401067	IQCF3	0.425
ENSG00000105198	29124	LGALS13	0.455
ENSG00000256223	7556	ZNF10	0.507
ENSG00000096155	7917	BAT3	0.576
ENSG00000133019	1131	CHRM3	0.492
ENSG00000171045	203062	TSNARE1	0.475
ENSG00000084734	2646	GCKR	0.48
ENSG00000116726	390999	PRAMEF12	0.425
ENSG00000184345	389123	IQCF2	0.343
ENSG00000248712	283152	LOC283152	0.422

ENSG00000156170	137682	C8orf38	0.56
ENSG00000173389	132141	IQCF1	0.479
ENSG00000126733	117154	DACH2	0.381
ENSG00000188076	147199	SCGB1C1	0.4
ENSG00000177947	113746	ODF3	0.427
ENSG00000120952	65122	PRAMEF2	0.481
ENSG00000184436	80764	THAP7	0.453
ENSG00000188672	6006	RHCE	0.53
ENSG00000206409	55937	APOM	0.62
ENSG00000221843	84226	C2orf16	0.408
ENSG00000114767	9136	RRP9	0.533
ENSG00000260117	283489	ZNF828	0.511
ENSG00000203623	57827	C6orf47	0.496
ENSG00000206408	7918	BAT4	0.51
ENSG00000130347	84816	RTN4IP1	0.559
ENSG00000223953	83552	MFRP	0.432
ENSG00000041880	10039	PARP3	0.496
ENSG00000187741	2175	FANCA	0.588
ENSG00000099960	6545	SLC7A4	0.555
ENSG00000151332	51562	MBIP	0.469
ENSG00000176029	56673	C11orf16	0.471
ENSG00000206406	1460	CSNK2B	0.51
ENSG00000176009	56676	ASCL3	0.44
ENSG00000188611	56624	ASAH2	0.398
ENSG00000143164	55827	IQWD1	0.501
ENSG00000107130	23413	FREQ	0.537
ENSG00000259159	83552	MFRP	0.432
ENSG00000160886	54742	LY6K	0.504
ENSG00000131781	2330	FMO5	0.562
ENSG00000175352	56675	NRIP3	0.519
ENSG00000102271	56062	KLHL4	0.482
ENSG00000239285	58496	LY6G5B	0.556
ENSG00000206404	80741	LY6G5C	0.527
ENSG00000142082	23410	SIRT3	0.618
ENSG00000132376	51763	SKIP	0.575
ENSG00000204414	1444	CSHL1	0.554
ENSG00000176834	54621	FLJ20674	0.479
ENSG00000204516	4277	MICB	0.526
ENSG00000259384	2688	GH1	0.568
ENSG00000206403	7920	BAT5	0.551
ENSG00000148834	9446	GSTO1	0.57
ENSG00000130193	51337	C8orf55	0.502
ENSG00000131778	9557	CHD1L	0.568
ENSG00000126233	57152	SLURP1	0.452
ENSG00000176714	79635	CCDC121	0.45
ENSG00000147183	53336	CPXCR1	0.5
ENSG00000229415	253970	SFTPH	0.421
ENSG00000130348	55278	QRSL1	0.58
ENSG00000065621	119391	GSTO2	0.424
ENSG00000243804	259215	LY6G6F	0.375
ENSG00000056586	54542	RC3H2	0.619
ENSG00000206396	80739	C6orf25	0.434
ENSG00000144134	11159	RABL2A	0.536
ENSG00000204178	55219	TMEM57	0.511

ENSG00000100038	8940	TOP3B	0.603
ENSG00000167656	8581	LY6D	0.474
ENSG00000104499	2765	GML	0.462
ENSG00000185627	5719	PSMD13	0.553
ENSG00000160882	1584	CYP11B1	0.499
ENSG00000206398	80740	LY6G6C	0.463
ENSG00000206395	23564	DDAH2	0.601
ENSG00000206394	1192	CLIC1	0.536
ENSG00000185917	54093	SETD4	0.567
ENSG00000130349	51250	C6orf203	0.472
ENSG00000156469	51001	MTERFD1	0.506
ENSG00000148584	29974	A1CF	0.493
ENSG00000178409	57673	KIAA1553	0.5
ENSG00000184014	23258	RAB6IP1	0.557
ENSG00000169575	7441	VPREB1	0.442
ENSG00000235569	4439	MSH5	0.508
ENSG00000137699	23650	TRIM29	0.584
ENSG00000163629	5783	PTPN13	0.515
ENSG00000143147	23432	GPR161	0.625
ENSG00000148841	85450	KIAA1754	0.453
ENSG00000179142	1585	CYP11B2	0.443
ENSG00000108622	3384	ICAM2	0.606
ENSG00000120051	159686	CCDC147	0.457
ENSG00000169548	129025	ZNF280A	0.463
ENSG00000185686	23532	PRAME	0.489
ENSG00000183032	89874	SLC25A21	0.451
ENSG00000100121	91227	GGTLC2	0.511
ENSG00000227686	7407	VARS	0.588
ENSG00000142102	80162	ATHL1	0.481
ENSG00000091483	2271	FH	0.603
ENSG00000172850	57819	LSM2	0.551
ENSG00000139767	84530	KIAA1853	0.463
ENSG00000163798	22950	SLC4A1AP	0.529
ENSG00000206383	3305	HSPA1L	0.463
ENSG00000215328	3303	HSPA1A	0.537
ENSG00000162836	51205	ACP6	0.524
ENSG00000171448	57684	ZBTB26	0.454
ENSG00000100218	27156	RTDR1	0.45
ENSG00000159228	873	CBR1	0.516
ENSG00000212866	3303	HSPA1A	0.537
ENSG00000206380	50854	C6orf48	0.542
ENSG00000115091	10096	ACTR3	0.638
ENSG00000181264	219902	TMEM136	0.578
ENSG00000159231	874	CBR3	0.533
ENSG00000141002	22980	TCF25	0.665
ENSG00000243147	9553	MRPL33	0.536
ENSG00000248487	25864	ABHD14A	0.52
ENSG00000183273	160777	CCDC60	0.391
ENSG00000184494	4758	NEU1	0.519
ENSG00000165471	4153	MBL2	0.481
ENSG00000206338	1589	CYP21A2	0.47
ENSG00000256525	11232	POLG2	0.474
ENSG00000171174	64080	RBKS	0.588
ENSG00000128322	3543	IGLL1	0.477

ENSG00000243989	95	ACY1	0.5
ENSG00000189269	51233	LOC51233	0.435
ENSG00000150275	65217	PCDH15	0.492
ENSG00000206378	80736	SLC44A4	0.452
ENSG00000129514	3169	FOXA1	0.508
ENSG00000143185	6846	XCL2	0.472
ENSG00000143184	6375	XCL1	0.436
ENSG00000159496	266747	RGL4	0.378
ENSG00000203668	1122	CHML	0.484
ENSG00000162843	128025	WDR64	0.442
ENSG00000128218	29802	VPREB3	0.476
ENSG00000143196	1805	DPT	0.6
ENSG00000206376	10919	EHMT2	0.557
ENSG00000163633	132989	C4orf36	0.552
ENSG00000204498	4795	NFKBIL1	0.461
ENSG00000121634	2703	GJA8	0.487
ENSG00000188092	51463	GPR89B	0.577
ENSG00000162244	6159	RPL29	0.557
ENSG00000250479	400916	CHCHD10	0.495
ENSG00000164086	1849	DUSP7	0.466
ENSG00000135249	60561	RINT1	0.477
ENSG00000099958	91319	DERL3	0.523
ENSG00000176435	161198	CLEC14A	0.5
ENSG00000133460	66035	SLC2A11	0.482
ENSG00000164087	25886	WDR51A	0.617
ENSG00000088205	8886	DDX18	0.639
ENSG00000206372	717	C2	0.523
ENSG00000258890	90799	CCDC45	0.538
ENSG00000227507	4050	LTB	0.52
ENSG00000023330	211	ALAS1	0.542
ENSG00000240972	4282	MIF	0.545
ENSG00000128536	222256	FLJ23834	0.477
ENSG00000133433	2953	GSTT2	0.471
ENSG00000099977	1652	DDT	0.555
ENSG00000206366	221527	ZBTB12	0.46
ENSG00000184674	2952	GSTT1	0.524
ENSG00000241253	629	CFB	0.59
ENSG00000154114	219899	TBCEL	0.496
ENSG00000197769	440738	MAP1LC3C	0.434
ENSG00000159259	8208	CHAF1B	0.522
ENSG00000204482	7940	LST1	0.606
ENSG00000118690	84071	ARMC2	0.417
ENSG00000099991	23523	CABIN1	0.57
ENSG00000239732	54106	TLR9	0.491
ENSG00000206357	7936	RDBP	0.505
ENSG00000176809	374819	LRRC37A3	0.544
ENSG00000102362	94121	SYTL4	0.6
ENSG00000198189	51170	HSD17B11	0.583
ENSG00000105835	10135	NAMPT	0.615
ENSG00000117475	8548	BLZF1	0.537
ENSG00000163803	151056	PLB1	0.377
ENSG00000100014	23384	SPECC1L	0.541
ENSG00000206353	6499	SKIV2L	0.492
ENSG00000159261	23562	CLDN14	0.45

ENSG00000170502	53343	NUDT9	0.493
ENSG00000117477	57821	C1orf114	0.452
ENSG00000100024	51733	UPB1	0.49
ENSG00000101811	1478	CSTF2	0.539
ENSG00000164308	64167	ERAP2	0.505
ENSG00000206346	1797	DOM3Z	0.616
ENSG00000122952	11130	ZWINT	0.568
ENSG00000152583	8404	SPARCL1	0.524
ENSG00000253276	168455	FLJ36031	0.556
ENSG00000204475	259197	NCR3	0.564
ENSG00000100031	2678	GGT1	0.595
ENSG00000198734	2153	F5	0.523
ENSG00000019169	8685	MARCO	0.505
ENSG00000151151	253430	IPMK	0.38
ENSG00000122873	55847	CISD1	0.498
ENSG00000183145	53820	DSCR6	0.415
ENSG00000185808	51227	PIGP	0.544
ENSG00000206342	8859	STK19	0.565
ENSG00000243452	284565	NBPF15	0.5
ENSG00000204472	199	AIF1	0.617
ENSG00000154240	201134	CCDC46	0.471
ENSG00000168237	132158	GLYCTK	0.521
ENSG00000152591	1834	DSPP	0.467
ENSG00000182670	7267	TTC3	0.642
ENSG00000186132	130355	C2orf76	0.435
ENSG00000178026	388886	C22orf36	0.43
ENSG00000029559	3381	IBSP	0.455
ENSG00000054282	10806	SDCCAG8	0.583
ENSG00000259571	414899	BLID	0.4
ENSG00000164597	10466	COG5	0.582
ENSG00000114841	25981	DNAH1	0.533
ENSG00000229353	7148	TNXB	0.679
ENSG00000188917	79979	CXorf34	0.519
ENSG00000109944	79864	C11orf63	0.447
ENSG00000100053	1417	CRYBB3	0.446
ENSG00000150526	117153	MIA2	0.471
ENSG00000244752	1415	CRYBB2	0.555
ENSG00000150337	2209	FCGR1A	0.548
ENSG00000152595	56955	MEPE	0.395
ENSG00000163806	245711	SPDYA	0.395
ENSG00000260952	9326	ZNHIT3	0.532
ENSG00000105865	11062	DUS4L	0.555
ENSG00000260164	80179	MYO19	0.527
ENSG00000232962	3111	HLA-DOA	0.651
ENSG00000058729	55781	RIOK2	0.505
ENSG00000118785	6696	SPP1	0.53
ENSG00000174175	6403	SELP	0.496
ENSG00000206331	80863	PRRT1	0.558
ENSG00000135736	92922	CCDC102A	0.457
ENSG00000206329	9374	PPT2	0.542
ENSG00000159618	221188	GPR114	0.429
ENSG00000258941	4253	CTAGE5	0.553
ENSG00000091583	350	APOH	0.465
ENSG00000224103	3113	HLA-DPA1	0.611

ENSG00000244444	80864	EGFL8	0.538
ENSG00000183941	8294	HIST1H4I	0.459
ENSG00000080293	6344	SCTR	0.47
ENSG00000171103	55006	FLJ20628	0.504
ENSG00000206324	10554	AGPAT1	0.573
ENSG00000261300	284098	PIGW	0.529
ENSG00000102384	2491	CENPI	0.571
ENSG00000260508	79893	GGNBP2	0.549
ENSG00000148541	220965	FAM13C1	0.453
ENSG00000183574	6048	RNF5	0.596
ENSG00000163075	200373	hCG_17324	0.493
ENSG00000100099	89781	HPS4	0.559
ENSG00000230708	3115	HLA-DPB1	0.543
ENSG00000182217	8294	HIST1H4I	0.459
ENSG00000111537	3458	IFNG	0.5
ENSG00000178096	51027	BOLA1	0.481
ENSG00000261508	79154	MGC4172	0.502
ENSG00000144120	80775	TMEM177	0.5
ENSG00000206320	177	AGER	0.548
ENSG00000262595	79922	MRM1	0.437
ENSG00000108878	786	CACNG1	0.478
ENSG00000173728	200159	C1orf100	0.341
ENSG00000000460	55732	C1orf112	0.513
ENSG00000100104	402055	SRRD	0.516
ENSG00000035687	159	ADSS	0.539
ENSG00000100109	24144	TFIP11	0.546
ENSG00000135587	6610	SMPD2	0.591
ENSG00000189350	165186	LOC165186	0.44
ENSG00000166250	79827	ASAM	0.508
ENSG00000023171	57476	GRAMD1B	0.535
ENSG00000206315	5089	PBX2	0.584
ENSG00000118777	9429	ABCG2	0.534
ENSG00000100122	1414	CRYBB1	0.464
ENSG00000196431	1413	CRYBA4	0.459
ENSG00000141447	114876	OSBPL1A	0.565
ENSG00000111536	55801	IL26	0.425
ENSG00000127318	50616	IL22	0.439
ENSG00000135596	64780	MICAL1	0.485
ENSG00000206314	63940	GPSM3	0.56
ENSG00000091138	1811	SLC26A3	0.509
ENSG00000206312	4855	NOTCH4	0.497
ENSG00000014914	10903	MTMR11	0.523
ENSG00000154529	79937	CNTNAP3	0.441
ENSG00000183765	11200	CHEK2	0.512
ENSG00000126953	1678	TIMM8A	0.598
ENSG00000227801	1302	COL11A2	0.611
ENSG00000205359	133482	SLCO6A1	0.383
ENSG00000157538	10311	DSCR3	0.577
ENSG00000206310	10665	C6orf10	0.434
ENSG00000189139	84075	FSCB	0.395
ENSG00000206308	3122	HLA-DRA	0.555
ENSG00000206306	3123	HLA-DRB1	0.614
ENSG00000206305	3117	HLA-DQA1	0.533
ENSG00000206302	3119	HLA-DQB1	0.68

ENSG00000130935	25926	NOL11	0.51
ENSG00000225103	3118	HLA-DQA2	0.706
ENSG00000198718	23116	KIAA0423	0.505
ENSG00000206299	6891	TAP2	0.58
ENSG00000206298	5696	PSMB8	0.535
ENSG00000091136	3912	LAMB1	0.584
ENSG00000100209	150274	HSCB	0.482
ENSG00000243594	5698	PSMB9	0.561
ENSG00000188404	6402	SELL	0.492
ENSG00000204300	338661	PMP22CD	0.356
ENSG00000234734	26165	FAM75A7	0.416
ENSG00000121644	51029	FAM152A	0.596
ENSG00000206297	6890	TAP1	0.552
ENSG00000197181	55124	PIWIL2	0.54
ENSG00000114854	7134	TNNC1	0.508
ENSG00000203667	116228	FAM36A	0.545
ENSG00000241674	3109	HLA-DMB	0.573
ENSG00000110002	4013	LOH11CR2A	0.594
ENSG00000138642	55008	HERC6	0.495
ENSG00000100249	25770	C22orf31	0.407
ENSG00000242685	3108	HLA-DMA	0.551
ENSG00000112365	9841	ZBTB24	0.504
ENSG00000227322	6257	RXRB	0.61
ENSG00000140465	1543	CYP1A1	0.531
ENSG00000215077	6046	BRD2	0.659
ENSG00000155085	221264	C6orf199	0.613
ENSG00000133115	161003	STOML3	0.451
ENSG00000100263	25807	RHBDD3	0.521
ENSG00000186665	284018	C17orf58	0.445
ENSG00000140505	1544	CYP1A2	0.529
ENSG00000170312	983	CDC2	0.602
ENSG00000111581	57122	NUP107	0.546
ENSG00000154143	116337	PANX3	0.345
ENSG00000010327	23166	STAB1	0.549
ENSG00000206292	3111	HLA-DOA	0.651
ENSG00000182885	222487	GPR97	0.478
ENSG00000154611	143471	PSMA8	0.337
ENSG00000206291	3113	HLA-DPA1	0.611
ENSG00000226614	7922	SLC39A7	0.474
ENSG00000215048	3115	HLA-DPB1	0.543
ENSG00000133103	57511	COG6	0.473
ENSG00000138646	51191	HERC5	0.511
ENSG00000120910	5533	PPP3CC	0.649
ENSG00000206290	1302	COL11A2	0.611
ENSG00000140506	79748	LMAN1L	0.442
ENSG00000203666	84288	EFCAB2	0.488
ENSG00000228712	7923	HSD17B8	0.52
ENSG00000145337	84992	PIGY	0.552
ENSG00000226788	6015	RING1	0.576
ENSG00000255072	84992	PIGY	0.552
ENSG00000141337	22901	ARSG	0.495
ENSG00000102738	10240	MRPS31	0.549
ENSG00000100276	10633	RASL10A	0.455
ENSG00000112367	9896	FIG4	0.53

ENSG00000110013	54414	SIAE	0.554
ENSG00000128250	5988	RFPL1	0.431
ENSG00000100285	4744	NEFH	0.535
ENSG00000154080	83539	CHST9	0.47
ENSG00000206289	6257	RXRB	0.61
ENSG00000135679	4193	MDM2	0.632
ENSG00000064199	53340	SPA17	0.487
ENSG00000019102	23584	VSIG2	0.5
ENSG00000168268	64943	NT5DC2	0.5
ENSG00000120662	9617	MTRF1	0.501
ENSG00000135241	50640	PNPLA8	0.528
ENSG00000225590	6293	VPS52	0.528
ENSG00000149564	90952	ESAM	0.455
ENSG00000138640	10144	FAM13A1	0.64
ENSG00000118298	23632	CA14	0.446
ENSG00000206288	7922	SLC39A7	0.474
ENSG00000070540	55062	WIPI1	0.593
ENSG00000112474	7923	HSD17B8	0.52
ENSG00000206287	6015	RING1	0.576
ENSG00000172766	79612	NARG1L	0.528
ENSG00000206286	6293	VPS52	0.528
ENSG00000177683	168451	THAP5	0.509
ENSG00000184076	29796	UCRC	0.533
ENSG00000185420	64754	SMYD3	0.514
ENSG00000163499	1412	CRYBA2	0.459
ENSG00000147996	220869	CBWD5	0.48
ENSG00000134760	1828	DSG1	0.468
ENSG00000185862	2124	EVI2B	0.486
ENSG00000096150	6222	RPS18	0.516
ENSG00000175065	147409	DSG4	0.333
ENSG00000126860	2123	EVI2A	0.509
ENSG00000206285	8705	B3GALT4	0.488
ENSG00000206284	9277	WDR46	0.512
ENSG00000184903	83943	IMMP2L	0.55
ENSG00000181378	255101	CCDC108	0.449
ENSG00000046604	1829	DSG2	0.506
ENSG00000206283	10471	PFDN6	0.6
ENSG00000141338	10351	ABCA8	0.472
ENSG00000172301	55352	C17orf79	0.51
ENSG00000206282	5863	RGL2	0.529
ENSG00000235650	6222	RPS18	0.516
ENSG00000118271	7276	TTR	0.469
ENSG00000108651	55813	UTP6	0.493
ENSG00000176635	150280	HORMAD2	0.494
ENSG00000226936	8705	B3GALT4	0.488
ENSG00000204221	9277	WDR46	0.512
ENSG00000206281	6892	TAPBP	0.616
ENSG00000162851	64216	TFB2M	0.519
ENSG00000239282	652968	LOC652968	0.505
ENSG00000180346	166815	TIGD2	0.357
ENSG00000203797	8528	DDO	0.522
ENSG00000206280	9278	ZBTB22	0.534
ENSG00000154133	54538	ROBO4	0.495
ENSG00000206279	1616	DAXX	0.603

ENSG00000070729	1258	CNGB1	0.591
ENSG00000068976	5837	PYGM	0.451
ENSG00000162852	163882	C1orf71	0.543
ENSG00000099992	83874	TBC1D10A	0.514
ENSG00000118292	79630	C1orf54	0.522
ENSG00000004809	85413	SLC22A16	0.406
ENSG00000237335	10471	PFDN6	0.6
ENSG00000159208	148523	C1orf51	0.543
ENSG00000138621	60490	PPCDC	0.5
ENSG00000204197	3833	KIFC1	0.535
ENSG00000154258	10350	ABCA9	0.554
ENSG00000155438	84365	MKI67IP	0.566
ENSG00000185158	114659	LRRC37B	0.377
ENSG00000224841	5863	RGL2	0.529
ENSG00000183230	29119	CTNNA3	0.465
ENSG00000184895	6736	SRY	0.452
ENSG00000141434	4225	MEP1B	0.432
ENSG00000129824	6192	RPS4Y1	0.51
ENSG00000117360	9129	PRPF3	0.5
ENSG00000136518	86	ACTL6A	0.533
ENSG00000090382	4069	LYZ	0.479
ENSG00000149548	80071	CCDC15	0.502
ENSG00000140365	54939	COMMD4	0.603
ENSG00000176679	90655	TGIF2LY	0.483
ENSG00000133106	94240	EPSTI1	0.548
ENSG00000136244	3569	IL6	0.518
ENSG00000151065	196513	DCP1B	0.48
ENSG00000154262	23460	ABCA6	0.464
ENSG00000196683	54543	TOMM7	0.562
ENSG00000112493	6892	TAPBP	0.616
ENSG00000151422	2241	FER	0.468
ENSG00000166226	10576	CCT2	0.597
ENSG00000120658	55068	ENOX1	0.522
ENSG00000140398	79661	NEIL1	0.503
ENSG00000198839	11179	ZNF277	0.623
ENSG00000099721	266	AMELY	0.473
ENSG00000158552	130617	ZFAND2B	0.488
ENSG00000092377	90665	TBL1Y	0.425
ENSG00000179630	144811	C13orf31	0.514
ENSG00000148634	26091	HERC4	0.565
ENSG00000070761	29105	C16orf80	0.562
ENSG00000122550	55975	KLHL7	0.561
ENSG00000229253	9278	ZBTB22	0.534
ENSG00000155115	112495	GTF3C6	0.504
ENSG00000136522	57129	MRPL47	0.521
ENSG00000140400	4123	MAN2C1	0.52
ENSG00000115657	10058	ABCB6	0.577
ENSG00000150433	219854	TMEM218	0.479
ENSG00000125995	140823	C20orf52	0.445
ENSG00000227046	1616	DAXX	0.603
ENSG00000163125	23248	KIAA0460	0.469
ENSG00000134020	157310	PEBP4	0.451
ENSG00000058056	8975	USP13	0.53
ENSG00000154263	10349	ABCA10	0.378

ENSG00000198841	112970	KTI12	0.485
ENSG00000133114	55425	KIAA1704	0.517
ENSG00000261657	115286	SLC25A26	0.487
ENSG00000197498	84154	BXDC1	0.496
ENSG00000188342	2963	GTF2F2	0.495
ENSG00000135747	93474	ZNF670	0.5
ENSG00000197472	57116	ZNF695	0.478
ENSG00000103023	221191	Klkbl4	0.398
ENSG00000181016	286006	C7orf53	0.486
ENSG00000228927	7258	TSPY1	0.538
ENSG00000161980	51728	POLR3K	0.492
ENSG00000143374	80222	TARS2	0.482
ENSG00000163106	27306	PGDS	0.473
ENSG00000136243	11097	NUPL2	0.519
ENSG00000161981	79622	C16orf33	0.534
ENSG00000174032	253512	SLC25A30	0.532
ENSG00000188295	79862	ZNF669	0.454
ENSG00000258992	7258	TSPY1	0.538
ENSG00000262914	50837	TAS2R7	0.436
ENSG00000163728	151613	TTC14	0.549
ENSG00000103152	4350	MPG	0.539
ENSG00000263210	50835	TAS2R9	0.469
ENSG00000263247	11272	PRR4	0.447
ENSG00000103148	8131	C16orf35	0.584
ENSG00000108666	64149	C17orf75	0.48
ENSG00000236424	7258	TSPY1	0.538
ENSG00000186812	84307	ZNF397	0.534
ENSG00000136235	10457	GPNMB	0.558
ENSG00000196418	7678	ZNF124	0.492
ENSG00000262955	50838	TAS2R13	0.411
ENSG00000263284	5554	PRH1	0.509
ENSG00000154415	5506	PPP1R3A	0.5
ENSG00000262859	259296	TAS2R50	0.409
ENSG00000145075	339829	CCDC39	0.44
ENSG00000130656	3050	HBZ	0.447
ENSG00000169371	10073	SNUPN	0.512
ENSG00000206177	3042	HBM	0.359
ENSG00000188536	3039	HBA1	0.573
ENSG00000163521	79411	GLB1L	0.45
ENSG00000206172	3039	HBA1	0.573
ENSG00000174015	220082	SPERT	0.543
ENSG00000215475	283514	LOC283514	0.386
ENSG00000123200	23091	ZC3H13	0.568
ENSG00000164209	91137	SLC25A46	0.517
ENSG00000154265	23461	ABCA5	0.514
ENSG00000177971	55272	IMP3	0.512
ENSG00000162714	84838	ZNF496	0.535
ENSG00000085840	4998	ORC1L	0.506
ENSG00000186496	252884	ZNF396	0.589
ENSG00000114902	28972	SPCS1	0.51
ENSG00000145777	85480	TSLP	0.522
ENSG00000153391	125476	C18orf37	0.48
ENSG00000115661	8576	STK16	0.513
ENSG00000182950	161753	ODF3L1	0.368

ENSG00000173535	8794	TNFRSF10C	0.555
ENSG00000080618	1361	CPB2	0.475
ENSG00000162711	114548	NLRP3	0.589
ENSG00000076344	8786	RGS11	0.532
ENSG00000140367	92912	UBE2Q2	0.478
ENSG00000156928	115416	C7orf30	0.52
ENSG00000127824	7277	TUBA4A	0.543
ENSG00000134987	134430	WDR36	0.529
ENSG00000236579	10255	HCG9	0.453
ENSG00000173530	8793	TNFRSF10D	0.479
ENSG00000143369	1893	ECM1	0.539
ENSG00000181778	169693	C9orf71	0.386
ENSG00000107242	8395	PIP5K1B	0.603
ENSG00000111203	55846	ITFG2	0.493
ENSG00000055957	3697	ITIH1	0.47
ENSG00000141428	83608	C18orf21	0.449
ENSG00000104689	8797	TNFRSF10A	0.537
ENSG00000170545	57228	LOC57228	0.557
ENSG00000242173	398	ARHGDIG	0.455
ENSG00000139610	1990	ELA1	0.465
ENSG00000185615	64714	PDIA2	0.603
ENSG00000103375	343	AQP8	0.45
ENSG00000141425	55197	P15RS	0.545
ENSG00000169224	148823	C1orf150	0.373
ENSG00000143382	54507	ADAMTSL4	0.519
ENSG00000108671	5717	PSMD11	0.589
ENSG00000165059	5568	PRKACG	0.446
ENSG00000171792	83695	C12orf32	0.508
ENSG00000086504	10573	MRPL28	0.515
ENSG00000186684	339761	CYP27C1	0.409
ENSG00000169752	145957	NRG4	0.33
ENSG00000163161	2071	ERCC3	0.502
ENSG00000169189	197370	NSMCE1	0.556
ENSG00000078070	56922	MCCC1	0.52
ENSG00000196242	81472	OR2C3	0.304
ENSG00000129925	58986	TMEM8	0.481
ENSG00000153822	3773	KCNJ16	0.47
ENSG00000134759	55250	ELP2	0.545
ENSG00000164211	134429	STARD4	0.583
ENSG00000171222	51282	SCAND1	0.527
ENSG00000149646	140894	C20orf152	0.476
ENSG00000197905	7004	TEAD4	0.575
ENSG00000163114	5161	PDHA2	0.443
ENSG00000163116	285555	C4orf37	0.455
ENSG00000077238	3566	IL4R	0.558
ENSG00000162722	25893	TRIM58	0.443
ENSG00000134910	3703	STT3A	0.515
ENSG00000167612	341405	ANKRD33	0.421
ENSG00000077235	2975	GTF3C1	0.54
ENSG00000056972	10758	TRAF3IP2	0.595
ENSG00000140374	2108	ETFA	0.537
ENSG00000242612	26063	DECR2	0.472
ENSG00000188732	340277	C7orf46	0.478
ENSG00000053524	23101	MCF2L2	0.44

ENSG00000130038	84766	EFCAB4B	0.398
ENSG00000196071	284521	OR2L13	0.444
ENSG00000162267	3699	ITIH3	0.46
ENSG00000139684	2098	ESD	0.613
ENSG00000196116	23424	TDRD7	0.494
ENSG00000259858	8972	MGAM	0.445
ENSG00000136143	8803	SUCLA2	0.531
ENSG00000261180	23601	CLEC5A	0.477
ENSG00000260696	5726	TAS2R38	0.309
ENSG00000224398	1460	CSNK2B	0.51
ENSG00000196335	56164	STK31	0.432
ENSG00000232758	253018	HCG27	0.44
ENSG00000075643	55034	MOCOS	0.526
ENSG00000129864	9084	VCY	0.621
ENSG00000237105	10866	HCP5	0.492
ENSG00000182183	348378	FAM159A	0.432
ENSG00000203663	26246	OR2L2	0.519
ENSG00000149735	170589	GPHA2	0.382
ENSG00000260736	136541	TRYX3	0.425
ENSG00000103037	79918	SETD6	0.475
ENSG00000168070	283129	LOC283129	0.385
ENSG00000129862	9084	VCY	0.621
ENSG00000166685	9382	COG1	0.561
ENSG00000168062	116071	BATF2	0.446
ENSG00000135063	9413	C9orf61	0.504
ENSG00000141316	124912	SPACA3	0.456
ENSG00000241132	58496	LY6G5B	0.556
ENSG00000169885	163688	CALML6	0.407
ENSG00000237495	80741	LY6G5C	0.527
ENSG00000178821	339456	TMEM52	0.415
ENSG00000136925	158427	C9orf97	0.516
ENSG00000135643	27345	KCNMB4	0.521
ENSG00000136159	55270	NUDT15	0.491
ENSG00000205609	728689	EIF3CL	0.474
ENSG00000182415	9085	CDY1	0.556
ENSG00000172468	86614	HSFY1	0.567
ENSG00000142609	85452	KIAA1751	0.542
ENSG00000135480	3855	KRT7	0.608
ENSG00000115718	5624	PROC	0.472
ENSG00000134779	25941	C18orf10	0.646
ENSG00000161992	146325	LOC146325	0.421
ENSG00000169953	86614	HSFY1	0.567
ENSG00000007541	9091	PIGQ	0.476
ENSG00000171161	79894	ZNF672	0.513
ENSG00000188603	1201	CLN3	0.573
ENSG00000121310	55268	ECHDC2	0.551
ENSG00000236063	7920	BAT5	0.551
ENSG00000138346	1763	DNA2	0.5
ENSG00000117906	5955	RCN2	0.614
ENSG00000108691	6347	CCL2	0.572
ENSG00000111247	10635	RAD51AP1	0.536
ENSG00000168061	29901	SAC3D1	0.52
ENSG00000122585	4852	NPY	0.469
ENSG00000144591	29926	GMPPA	0.485

ENSG00000140368	9051	PSTPIP1	0.459
ENSG00000105926	51678	MPP6	0.486
ENSG00000012817	8284	JARID1D	0.493
ENSG00000261473	5644	PRSS1	0.404
ENSG00000262739	5645	PRSS2	0.511
ENSG00000168060	10004	NAALADL1	0.476
ENSG00000171163	55657	ZNF692	0.559
ENSG00000170442	3892	KRT86	0.482
ENSG00000257108	283948	FLJ36208	0.444
ENSG00000258832	3892	KRT86	0.482
ENSG00000260195	2051	EPHB6	0.485
ENSG00000187730	2563	GABRD	0.467
ENSG00000120820	83468	GLT8D2	0.495
ENSG00000172156	6356	CCL11	0.502
ENSG00000108700	6355	CCL8	0.499
ENSG00000161849	3890	KRT84	0.43
ENSG00000161850	3888	KRT82	0.441
ENSG00000181374	6357	CCL13	0.556
ENSG00000185479	3854	KRT6B	0.576
ENSG00000143452	84072	HORMAD1	0.359
ENSG00000101489	56853	BRUNOL4	0.552
ENSG00000108702	6346	CCL1	0.491
ENSG00000127578	117166	WFIKKN1	0.446
ENSG00000130731	84326	C16orf13	0.55
ENSG00000042980	10863	ADAM28	0.558
ENSG00000010219	8798	DYRK4	0.504
ENSG00000101084	55969	C20orf24	0.584
ENSG00000139648	112802	KRT71	0.421
ENSG00000111727	29915	HCFC2	0.466
ENSG00000170484	121391	KRT74	0.492
ENSG00000168216	55788	LMBRD1	0.527
ENSG00000124006	23363	OBSL1	0.713
ENSG00000078142	5289	PIK3C3	0.55
ENSG00000116171	6342	SCP2	0.633
ENSG00000186049	319101	KRT73	0.482
ENSG00000260119	55503	TRPV6	0.468
ENSG00000171053	160065	PATE	0.414
ENSG00000109182	80157	FLJ21511	0.5
ENSG00000196844	399967	C11orf38	0.338
ENSG00000204711	138255	C9orf135	0.465
ENSG00000261264	56302	TRPV5	0.469
ENSG00000184730	55911	APOB48R	0.433
ENSG00000180917	55783	FLJ11171	0.441
ENSG00000173517	79834	SGK269	0.515
ENSG00000172366	84331	C16orf14	0.466
ENSG00000123999	3623	INHA	0.475
ENSG00000136936	7507	XPA	0.559
ENSG00000144589	114790	STK11IP	0.482
ENSG00000139679	10161	P2RY5	0.534
ENSG00000260557	135927	C7orf34	0.466
ENSG00000197272	246778	IL27	0.449
ENSG00000260040	3792	KEL	0.482
ENSG00000132141	10693	CCT6B	0.493
ENSG00000170122	2298	FOXD4	0.418

ENSG00000172785	55871	CBWD1	0.477
ENSG00000102854	10232	MSLN	0.478
ENSG00000198331	219844	HYLS1	0.463
ENSG00000136932	51531	C9orf156	0.591
ENSG00000134028	27299	ADAMDEC1	0.496
ENSG00000178084	170572	HTR3C	0.34
ENSG00000243696	389125	MUSTN1	0.49
ENSG00000240957	259215	LY6G6F	0.375
ENSG00000069206	8756	ADAM7	0.521
ENSG00000139180	4704	NDUFA9	0.502
ENSG00000172215	10663	CXCR6	0.51
ENSG00000203965	84455	EFCAB7	0.513
ENSG00000100739	623	BDKRB1	0.473
ENSG00000176046	26471	NUPR1	0.561
ENSG00000185069	51350	KRT76	0.436
ENSG00000185220	267002	PGBD2	0.476
ENSG00000173914	83759	RBM4B	0.533
ENSG00000198692	9086	EIF1AY	0.509
ENSG00000176476	112869	CCDC101	0.544
ENSG00000136929	55363	HEMGN	0.497
ENSG00000169994	4648	MYO7B	0.518
ENSG00000108294	5691	PSMB3	0.538
ENSG00000237459	80739	C6orf25	0.434
ENSG00000170477	3851	KRT4	0.534
ENSG00000172809	6169	RPL38	0.618
ENSG00000168303	136647	C7orf11	0.44
ENSG00000130035	26290	GALNT8	0.441
ENSG00000173578	2829	XCR1	0.444
ENSG00000197165	6799	SULT1A2	0.591
ENSG00000083067	80036	TRPM3	0.631
ENSG00000005156	3980	LIG3	0.591
ENSG00000161996	197335	WDR90	0.543
ENSG00000185640	338785	KRT79	0.415
ENSG00000170423	196374	KRT78	0.534
ENSG00000175600	79783	C7orf10	0.446
ENSG00000196502	6817	SULT1A1	0.572
ENSG00000112761	8838	WISP3	0.47
ENSG00000141720	8396	PIP4K2B	0.603
ENSG00000167377	7571	ZNF23	0.55
ENSG00000198099	127	ADH4	0.566
ENSG00000175063	11065	UBE2C	0.542
ENSG00000170421	3856	KRT8	0.529
ENSG00000095380	54187	NANS	0.488
ENSG00000183625	1232	CCR3	0.446
ENSG00000153037	6728	SRP19	0.539
ENSG00000228250	80740	LY6G6C	0.463
ENSG00000225635	23564	DDAH2	0.601
ENSG00000108296	54883	CCDC49	0.505
ENSG00000047617	57101	TMEM16B	0.439
ENSG00000162585	199990	C1orf86	0.593
ENSG00000101470	7125	TNNC2	0.482
ENSG00000110799	7450	VWF	0.486
ENSG00000074935	51175	TUBE1	0.49
ENSG00000262156	200315	APOBEC3A	0.417

ENSG00000214556	388381	LOC388381	0.358
ENSG00000162383	6512	SLC1A7	0.507
ENSG00000184110	8663	EIF3C	0.491
ENSG00000157429	7567	ZNF19	0.554
ENSG00000172955	130	ADH6	0.587
ENSG00000149823	738	C11orf2	0.511
ENSG00000173715	79703	C11orf80	0.502
ENSG00000234414	5940	RBMY1A1	0.605
ENSG00000226417	1192	CLIC1	0.536
ENSG00000197046	284266	SIGLEC15	0.483
ENSG00000137547	29088	MRPL15	0.537
ENSG00000262896	85280	KRTAP9-4	0.325
ENSG00000115548	55818	JMJD1A	0.57
ENSG00000183784	157983	C9orf66	0.464
ENSG00000107099	81704	DOCK8	0.59
ENSG00000127324	7103	TSPAN8	0.522
ENSG00000255150	493861	EID3	0.425
ENSG00000187758	124	ADH1A	0.524
ENSG00000149809	7108	TM7SF2	0.545
ENSG00000139292	8549	LGR5	0.537
ENSG00000095383	55357	TBC1D2	0.496
ENSG00000102547	81617	CAB39L	0.485
ENSG00000140835	10164	CHST4	0.527
ENSG00000104237	6101	RP1	0.402
ENSG00000121797	9034	CCRL2	0.6
ENSG00000171595	64446	DNAI2	0.553
ENSG00000116151	79906	MORN1	0.446
ENSG00000177414	148581	UBE2U	0.384
ENSG00000162385	4116	MAGOH	0.577
ENSG00000111057	3875	KRT18	0.549
ENSG00000101473	10005	ACOT8	0.495
ENSG00000185379	5892	RAD51L3	0.572
ENSG00000067182	7132	TNFRSF1A	0.55
ENSG00000136169	83852	SETDB2	0.605
ENSG00000165730	219736	STOX1	0.446
ENSG00000256646	5683	PSMA2	0.63
ENSG00000227314	4439	MSH5	0.508
ENSG00000136147	51131	PHF11	0.52
ENSG00000212643	6728	SRP19	0.539
ENSG00000012223	4057	LTF	0.554
ENSG00000133858	196441	CCDC131	0.52
ENSG00000196616	125	ADH1B	0.546
ENSG00000140057	122481	AK7	0.495
ENSG00000152234	498	ATP5A1	0.558
ENSG00000188993	339977	LRRC66	0.5
ENSG00000161999	339123	LOC339123	0.549
ENSG00000169807	9081	PRY	0.454
ENSG00000105954	64111	NPVF	0.446
ENSG00000116120	10056	FARSB	0.589
ENSG00000050344	9603	NFE2L3	0.549
ENSG00000140832	91862	MARVELD3	0.555
ENSG00000140395	80349	WDR61	0.585
ENSG00000248144	126	ADH1C	0.547
ENSG00000151131	121053	C12orf45	0.465

ENSG00000124003	116255	MOGAT1	0.404
ENSG00000101342	140711	C20orf118	0.443
ENSG00000040199	23035	PHLPPL	0.577
ENSG00000149634	128497	C20orf165	0.418
ENSG00000124257	140825	NEURL2	0.467
ENSG00000196344	131	ADH7	0.467
ENSG00000101347	25939	SAMHD1	0.547
ENSG00000106591	64983	MRPL32	0.485
ENSG00000152213	115761	ARL11	0.526
ENSG00000184661	157313	CDCA2	0.554
ENSG00000123179	84650	EBPL	0.526
ENSG00000163931	7086	TKT	0.557
ENSG00000139193	939	CD27	0.496
ENSG00000127580	84219	WDR24	0.415
ENSG00000174276	741	ZNHIT2	0.509
ENSG00000152240	115106	CCDC5	0.519
ENSG00000112280	1297	COL9A1	0.478
ENSG00000149806	2197	FAU	0.513
ENSG00000139192	55080	TAPBPL	0.567
ENSG00000204347	388419	LOC388419	0.345
ENSG00000105989	7472	WNT2	0.545
ENSG00000166426	1381	CRABP1	0.527
ENSG00000260950	400566	LOC400566	0.437
ENSG00000135900	65080	MRPL44	0.463
ENSG00000261321	359845	FAM101B	0.583
ENSG00000137574	96764	TGS1	0.492
ENSG00000103260	79006	METRN	0.523
ENSG00000173253	10655	DMRT2	0.485
ENSG00000154438	136991	ASZ1	0.408
ENSG00000157911	5192	PEX10	0.553
ENSG00000178952	7284	TUFM	0.523
ENSG00000124019	79843	FAM124B	0.452
ENSG00000254087	4067	LYN	0.649
ENSG00000080839	5933	RBL1	0.519
ENSG00000234114	54535	CCHCR1	0.631
ENSG00000110074	55572	FOXRED1	0.423
ENSG00000138813	84103	C4orf17	0.458
ENSG00000058804	55706	TMEM48	0.54
ENSG00000135905	55619	DOCK10	0.567
ENSG00000105784	154661	RUNDC3B	0.504
ENSG00000145331	93587	RG9MTD2	0.542
ENSG00000173227	91683	SYT12	0.526
ENSG00000111639	51258	MRPL51	0.544
ENSG00000122728	138474	TAF1L	0.432
ENSG00000163825	83597	RTP3	0.476
ENSG00000212122	83942	TSSK1B	0.414
ENSG00000137074	54840	APTX	0.498
ENSG00000010292	9918	NCAPD2	0.558
ENSG00000047188	64848	YTHDC2	0.566
ENSG00000165732	9188	DDX21	0.553
ENSG00000188266	123688	LOC123688	0.418
ENSG00000153015	10283	SDCCAG10	0.553
ENSG00000161204	55324	ABCF3	0.519
ENSG00000173156	29984	RHOD	0.57

ENSG00000167779	3489	IGFBP6	0.518
ENSG00000249884	7844	RNF103	0.537
ENSG00000239305	7844	RNF103	0.537
ENSG00000167780	8435	SOAT2	0.438
ENSG00000103253	84264	HAGHL	0.451
ENSG00000184178	152579	SCFD2	0.548
ENSG00000205457	24150	TP53TG3	0.448
ENSG00000262764	5519	PPP2R1B	0.671
ENSG00000136436	10241	CALCOCO2	0.564
ENSG00000241186	6997	TDGF1	0.528
ENSG00000144230	2840	GPR17	0.534
ENSG00000155621	138241	C9orf85	0.545
ENSG00000145216	81608	FIP1L1	0.464
ENSG00000101353	140699	C20orf132	0.49
ENSG00000213937	9080	CLDN9	0.522
ENSG00000111640	2597	GAPDH	0.653
ENSG00000198553	283518	KCNRG	0.476
ENSG00000103145	54985	HCFC1R1	0.554
ENSG00000262577	79796	ALG9	0.497
ENSG00000169789	9081	PRY	0.454
ENSG00000183753	9083	BPY2	0.567
ENSG00000261509	24150	TP53TG3	0.448
ENSG00000178789	124599	CD300LB	0.463
ENSG00000167850	10871	CD300C	0.468
ENSG00000171241	79801	SHCBP1	0.498
ENSG00000075303	55972	SLC25A40	0.496
ENSG00000139631	51380	CSAD	0.519
ENSG00000166548	7084	TK2	0.606
ENSG00000171681	55729	ATF7IP	0.613
ENSG00000008988	6224	RPS20	0.552
ENSG00000224379	6941	TCF19	0.531
ENSG00000134640	4544	MTNR1B	0.44
ENSG00000169684	1138	CHRNA5	0.508
ENSG00000180773	120103	SLC36A4	0.48
ENSG00000157881	55229	PANK4	0.47
ENSG00000173120	22992	FBXL11	0.604
ENSG00000078043	9063	PIAS2	0.626
ENSG00000182352	146723	C17orf77	0.365
ENSG00000131652	79228	THOC6	0.486
ENSG00000103245	64428	NARFL	0.459
ENSG00000178038	259173	ALS2CL	0.532
ENSG00000233911	5460	POU5F1	0.546
ENSG00000117971	1143	CHRNB4	0.468
ENSG00000121316	79887	FLJ22662	0.498
ENSG00000106603	55744	C7orf44	0.58
ENSG00000267229	79796	ALG9	0.497
ENSG00000008517	9235	IL32	0.537
ENSG00000182378	55344	PLCXD1	0.492
ENSG00000262028	91893	hCG_2033039	0.425
ENSG00000100749	7443	VRK1	0.542
ENSG00000262176	64776	C11orf1	0.475
ENSG00000058799	54432	YIPF1	0.53
ENSG00000108298	6143	RPL19	0.528
ENSG00000167216	83473	KATNAL2	0.58

ENSG00000263007	1410	CRYAB	0.514
ENSG00000168263	169522	KCNV2	0.424
ENSG00000139626	3695	ITGB7	0.496
ENSG00000127586	63922	CHTF18	0.5
ENSG00000143412	8416	ANXA9	0.572
ENSG00000106605	644	BLVRA	0.606
ENSG00000109270	8649	MAP2K1IP1	0.438
ENSG00000198954	26128	KIAA1279	0.55
ENSG00000150510	220108	FAM124A	0.545
ENSG00000164729	146861	AMAC1	0.429
ENSG00000105996	3199	HOXA2	0.469
ENSG00000086061	3301	DNAJA1	0.647
ENSG00000166750	162394	SLFN5	0.554
ENSG00000178605	8225	GTPBP6	0.515
ENSG00000153563	925	CD8A	0.463
ENSG00000080608	9933	KIAA0020	0.516
ENSG00000157873	8764	TNFRSF14	0.497
ENSG00000159199	516	ATP5G1	0.517
ENSG00000172716	91607	SLFN11	0.52
ENSG00000262852	3316	HSPB2	0.534
ENSG00000010295	25900	IFFO	0.588
ENSG00000262978	3316	HSPB2	0.534
ENSG00000262600	91894	C11orf52	0.478
ENSG00000062582	64951	MRPS24	0.46
ENSG00000172748	169270	ZNF596	0.472
ENSG00000110063	28960	DCPS	0.495
ENSG00000070019	2984	GUCY2C	0.418
ENSG00000167393	28227	PPP2R3B	0.447
ENSG00000014138	23649	POLA2	0.542
ENSG00000177455	930	CD19	0.484
ENSG00000175336	319	APOF	0.411
ENSG00000262277	85458	DIXDC1	0.499
ENSG00000197837	8294	HIST1H4I	0.459
ENSG00000144231	5433	POLR2D	0.591
ENSG00000111602	8914	TIMELESS	0.578
ENSG00000262204	50837	TAS2R7	0.436
ENSG00000262770	50835	TAS2R9	0.469
ENSG00000170786	195814	RDHE2	0.417
ENSG00000246705	55766	H2AFJ	0.462
ENSG00000263040	11272	PRR4	0.447
ENSG00000166004	85459	KIAA1731	0.45
ENSG00000127588	51764	GNG13	0.462
ENSG00000139287	121278	TPH2	0.391
ENSG00000106608	55665	URG4	0.483
ENSG00000206181	51224	TCEB3B	0.43
ENSG00000103227	64788	LMF1	0.575
ENSG00000167220	84064	HDHD2	0.488
ENSG00000263032	1737	DLAT	0.645
ENSG00000262101	50838	TAS2R13	0.411
ENSG00000262969	5554	PRH1	0.509
ENSG00000181195	5179	PENK	0.476
ENSG00000261993	259296	TAS2R50	0.409
ENSG00000108306	84961	FBXL20	0.635
ENSG00000172123	55106	SLFN12	0.455

ENSG00000182993	144608	C12orf60	0.506
ENSG00000217555	51192	CKLF	0.589
ENSG00000172116	926	CD8B	0.542
ENSG00000136715	79595	SAP130	0.491
ENSG00000179256	440087	LOC440087	0.494
ENSG00000111339	420	ART4	0.455
ENSG00000080298	5991	RFX3	0.484
ENSG00000224264	7407	VARS	0.588
ENSG00000111341	4256	MGP	0.513
ENSG00000168955	79853	TM4SF20	0.411
ENSG00000123219	64105	CENPK	0.544
ENSG00000111641	4839	NOL1	0.54
ENSG00000110080	6484	ST3GAL4	0.573
ENSG00000173744	3267	HRB	0.616
ENSG00000111348	397	ARHGDIB	0.521
ENSG00000176953	84901	NFATC2IP	0.617
ENSG00000233841	3107	HLA-C	0.695
ENSG00000120907	148	ADRA1A	0.586
ENSG00000139053	5149	PDE6H	0.435
ENSG00000157870	127281	C1orf93	0.446
ENSG00000117791	54996	MOSC2	0.595
ENSG00000016391	55349	CHDH	0.579
ENSG00000165091	117531	TMC1	0.418
ENSG00000042304	56918	DKFZp547H025	0.406
ENSG00000154760	146857	SLFN13	0.573
ENSG00000267228	51124	IER3IP1	0.511
ENSG00000135917	80704	SLC19A3	0.482
ENSG00000164221	153733	CCDC112	0.415
ENSG00000134049	51124	IER3IP1	0.511
ENSG00000108641	27077	B9D1	0.568
ENSG00000119523	85365	ALG2	0.538
ENSG00000262740	120379	PIH1D2	0.453
ENSG00000123977	164781	WDR69	0.422
ENSG00000215114	137886	LOC137886	0.504
ENSG00000173418	51126	NAT5	0.483
ENSG00000056736	55540	IL17RB	0.521
ENSG00000130182	84891	ZSCAN10	0.516
ENSG00000136738	8027	STAM	0.533
ENSG00000106031	3209	HOXA13	0.434
ENSG00000106038	2128	EVX1	0.461
ENSG00000122386	7755	ZNF205	0.466
ENSG00000128534	51691	LSM8	0.502
ENSG00000106049	11112	HIBADH	0.53
ENSG00000106803	10952	SEC61B	0.556
ENSG00000182544	84975	MFSD5	0.521
ENSG00000135476	9700	ESPL1	0.598
ENSG00000164850	2852	GPER	0.534
ENSG00000081870	51668	HSPB11	0.613
ENSG00000186205	64757	MOSC1	0.545
ENSG00000089505	113540	CMTM1	0.451
ENSG00000136206	285955	WBSCR19	0.474
ENSG00000103811	1512	CTSH	0.522
ENSG00000241343	6173	RPL36A	0.452
ENSG00000122711	27290	SPINK4	0.46

ENSG00000007908	6401	SELE	0.459
ENSG00000167910	1581	CYP7A1	0.397
ENSG00000167992	220001	VWCE	0.468
ENSG00000113593	23398	PPWD1	0.474
ENSG00000198223	1438	CSF2RA	0.56
ENSG00000102393	2717	GLA	0.546
ENSG00000260380	2483	FRG1	0.519
ENSG00000116212	115353	LRRC42	0.46
ENSG00000165092	216	ALDH1A1	0.557
ENSG00000185246	55015	PRPF39	0.454
ENSG00000023734	11171	STRAP	0.55
ENSG00000206549	29122	TSP50	0.447
ENSG00000123349	5204	PFDN5	0.571
ENSG00000116176	25823	TPSG1	0.466
ENSG00000106052	8887	TAX1BP1	0.646
ENSG00000023697	51071	DERA	0.575
ENSG00000166012	79101	JOSD3	0.577
ENSG00000123171	83446	CCDC70	0.427
ENSG00000157542	3763	KCNJ6	0.454
ENSG00000178381	90637	ZFAND2A	0.402
ENSG00000204444	55937	APOM	0.62
ENSG00000214160	10195	ALG3	0.506
ENSG00000103313	4210	MEFV	0.455
ENSG00000172236	7177	TPSAB1	0.631
ENSG00000260587	1795	DOCK3	0.464
ENSG00000155330	388272	C16orf87	0.571
ENSG00000100982	63935	PCIF1	0.627
ENSG00000259880	7873	ARMET	0.532
ENSG00000104714	157697	ERICH1	0.477
ENSG00000180881	84698	CAPS2	0.459
ENSG00000184029	10281	DSCR4	0.433
ENSG00000008394	4257	MGST1	0.591
ENSG00000111644	84519	ACRBP	0.509
ENSG00000164877	79778	MICALL2	0.493
ENSG00000223532	3106	HLA-B	0.594
ENSG00000126945	3188	HNRNPH2	0.527
ENSG00000225998	57819	LSM2	0.551
ENSG00000180613	170825	GSX2	0.467
ENSG00000204438	7918	BAT4	0.51
ENSG00000139637	60314	C12orf10	0.513
ENSG00000262858	2186	BPTF	0.599
ENSG00000169763	9081	PRY	0.454
ENSG00000154079	135154	C6orf57	0.46
ENSG00000156502	6832	SUPV3L1	0.507
ENSG00000166046	255394	TCP11L2	0.472
ENSG00000160808	4634	MYL3	0.515
ENSG00000095917	23430	TPSD1	0.439
ENSG00000259956	29890	RBM15B	0.511
ENSG00000058335	5923	RASGRF1	0.562
ENSG00000260623	9730	VPRBP	0.559
ENSG00000227129	4758	NEU1	0.519
ENSG00000166482	4239	MFAP4	0.504
ENSG00000159224	2695	GIP	0.465
ENSG00000263085	284018	C17orf58	0.445

ENSG00000183336	552900	BOLA2	0.562
ENSG00000262472	3838	KPNA2	0.59
ENSG00000094914	8086	AAAS	0.478
ENSG00000172352	9085	CDY1	0.556
ENSG00000071243	54556	ING3	0.531
ENSG00000104228	23087	TRIM35	0.476
ENSG00000167986	1642	DDB1	0.528
ENSG00000157551	3772	KCNJ15	0.564
ENSG00000153832	130888	FBXO36	0.482
ENSG00000183795	9083	BPY2	0.567
ENSG00000261443	23132	RAD54L2	0.459
ENSG00000141627	54808	DYM	0.476
ENSG00000163141	149428	BNIPL	0.462
ENSG00000171806	92342	C1orf156	0.503
ENSG00000262747	51321	AMZ2	0.48
ENSG00000135899	3431	SP110	0.617
ENSG00000183281	5342	PLGLB2	0.488
ENSG00000140993	91151	TIGD7	0.556
ENSG00000263074	22901	ARSG	0.495
ENSG00000160801	5745	PTHR1	0.504
ENSG00000086065	51510	CHMP5	0.55
ENSG00000262275	9120	SLC16A6	0.492
ENSG00000166557	23423	TMED3	0.512
ENSG00000162086	7627	ZNF75A	0.56
ENSG00000181625	79008	GIYD2	0.586
ENSG00000126391	83786	FRMD8	0.462
ENSG00000182919	28970	C11orf54	0.516
ENSG00000177576	497661	C18orf32	0.481
ENSG00000215472	6139	RPL17	0.621
ENSG00000089101	26074	C20orf26	0.536
ENSG00000143512	79802	HHIPL2	0.462
ENSG00000168158	4993	OR2C1	0.479
ENSG00000140987	54925	ZNF434	0.55
ENSG00000116209	9528	TMEM59	0.532
ENSG00000126947	51309	ARMCX1	0.502
ENSG00000135046	301	ANXA1	0.578
ENSG00000136371	10588	MTHFS	0.609
ENSG00000213648	6818	SULT1A3	0.567
ENSG00000182372	2055	CLN8	0.596
ENSG00000265681	6139	RPL17	0.621
ENSG00000149571	84623	KIRREL3	0.475
ENSG00000023318	23071	TXNDC4	0.614
ENSG00000229396	1616	DAXX	0.603
ENSG00000111404	79785	RERGL	0.446
ENSG00000100985	4318	MMP9	0.505
ENSG00000147853	50808	AK3	0.535
ENSG00000139144	5288	PIK3C2G	0.468
ENSG00000187790	57697	FANCM	0.517
ENSG00000259332	10588	MTHFS	0.609
ENSG00000180953	400410	ST20	0.442
ENSG00000167080	124872	B4GALNT2	0.417
ENSG00000125551	5342	PLGLB2	0.488
ENSG00000140932	146225	CMTM2	0.463
ENSG00000103343	7727	ZNF174	0.541

ENSG00000142186	57410	SCYL1	0.47
ENSG00000108055	9126	SMC3	0.629
ENSG00000139151	89869	PLCZ1	0.407
ENSG00000140379	597	BCL2A1	0.519
ENSG00000079263	11262	SP140	0.476
ENSG00000138600	84888	SPPL2A	0.556
ENSG00000229077	80736	SLC44A4	0.452
ENSG00000182218	84439	HHIPL1	0.392
ENSG00000128482	7732	RNF112	0.426
ENSG00000173401	256710	GLIPR1L1	0.463
ENSG00000103485	23475	QPRT	0.528
ENSG00000156510	80201	HKDC1	0.476
ENSG00000143498	9015	TAF1A	0.478
ENSG00000167981	146434	ZNF597	0.39
ENSG00000142494	55244	SLC47A1	0.509
ENSG00000122390	79903	FLJ14154	0.528
ENSG00000079616	3835	KIF22	0.511
ENSG00000101413	58490	C20orf77	0.535
ENSG00000136098	4752	NEK3	0.531
ENSG00000167083	2793	GNGT2	0.424
ENSG00000161509	2905	GRIN2C	0.57
ENSG00000137869	1588	CYP19A1	0.553
ENSG00000185291	3563	IL3RA	0.485
ENSG00000164880	26173	INTS1	0.563
ENSG00000139278	11010	GLIPR1	0.595
ENSG00000177938	93661	CAPZA3	0.407
ENSG00000169717	140625	ACTRT2	0.37
ENSG00000112309	135152	B3GAT2	0.5
ENSG00000108798	51225	ABI3	0.5
ENSG00000160799	151903	CCDC12	0.443
ENSG00000161513	2232	FDXR	0.557
ENSG00000167315	10449	ACAA2	0.601
ENSG00000262145	91419	XRCC6BP1	0.511
ENSG00000162641	254268	C1orf62	0.491
ENSG00000164708	5224	PGAM2	0.457
ENSG00000171872	128209	KLF17	0.356
ENSG00000103876	2184	FAH	0.526
ENSG00000007520	115939	C16orf42	0.575
ENSG00000172954	253558	LYCAT	0.465
ENSG00000122678	27434	POLM	0.467
ENSG00000178028	55929	DMAP1	0.448
ENSG00000238134	10919	EHMT2	0.557
ENSG00000119900	79627	OGFRL1	0.485
ENSG00000261345	140628	GATA5	0.458
ENSG00000135409	269	AMHR2	0.421
ENSG00000164645	219557	MGC26647	0.367
ENSG00000261417	128826	C20orf166	0.417
ENSG00000164647	26872	STEAP1	0.533
ENSG00000168056	4054	LTBP3	0.506
ENSG00000136114	55901	THSD1	0.561
ENSG00000059145	64718	UNKL	0.451
ENSG00000174403	253868	C20orf200	0.519
ENSG00000172410	10022	INSL5	0.457
ENSG00000120210	11172	INSL6	0.414

ENSG00000120211	3641	INSL4	0.484
ENSG00000002745	51384	WNT16	0.45
ENSG00000107014	6019	RLN2	0.464
ENSG00000169100	293	SLC25A6	0.614
ENSG00000240053	58496	LY6G5B	0.556
ENSG00000185404	93349	LOC93349	0.467
ENSG00000204428	80741	LY6G5C	0.527
ENSG00000172508	57571	KIAA1394	0.471
ENSG00000107018	6013	RLN1	0.443
ENSG00000107020	55848	C9orf46	0.479
ENSG00000259988	253868	C20orf200	0.519
ENSG00000188559	57186	C20orf74	0.569
ENSG00000148482	221074	SLC39A12	0.421
ENSG00000132139	246176	GAS2L2	0.375
ENSG00000067066	6672	SP100	0.648
ENSG00000234218	4277	MICB	0.526
ENSG00000106624	165	AEBP1	0.536
ENSG00000149633	85449	KIAA1755	0.455
ENSG00000172361	220136	CCDC11	0.508
ENSG00000103249	1186	CLCN7	0.569
ENSG00000121957	29899	GPSM2	0.574
ENSG00000130762	27237	ARHGEF16	0.512
ENSG00000036448	9172	MYOM2	0.496
ENSG00000120915	2053	EPHX2	0.469
ENSG00000215883	606495	LOC606495	0.485
ENSG00000204427	7920	BAT5	0.551
ENSG00000166477	123169	LEO1	0.463
ENSG00000136891	54881	TEX10	0.582
ENSG00000175634	6199	RPS6KB2	0.487
ENSG00000139155	53919	SLCO1C1	0.456
ENSG00000149476	26007	DAK	0.505
ENSG00000106066	54504	CPVL	0.595
ENSG00000110218	24145	PANX1	0.532
ENSG00000137875	10017	BCL2L10	0.432
ENSG00000213918	1773	DNASE1	0.506
ENSG00000225073	7919	BAT1	0.592
ENSG00000101425	671	BPI	0.442
ENSG00000157778	84262	PSMG3	0.452
ENSG00000185894	9083	BPY2	0.567
ENSG00000105792	79846	FLJ21062	0.498
ENSG00000169093	8623	ASMTL	0.619
ENSG00000002822	8379	MAD1L1	0.496
ENSG00000135898	9290	GPR55	0.425
ENSG00000111700	28234	SLCO1B3	0.449
ENSG00000107036	57589	KIAA1432	0.574
ENSG00000141744	5409	PNMT	0.488
ENSG00000154768	146853	C17orf50	0.367
ENSG00000161395	93210	PERLD1	0.596
ENSG00000172288	9085	CDY1	0.556
ENSG00000126602	10131	TRAP1	0.499
ENSG00000128833	55930	MYO5C	0.513
ENSG00000123901	10888	GPR83	0.439
ENSG00000139675	144983	RP11-78J21.1	0.421
ENSG00000180638	146802	SLC47A2	0.413

ENSG00000172283	9081	PRY	0.454
ENSG00000165416	10910	SUGT1	0.559
ENSG00000013364	9961	MVP	0.533
ENSG00000008311	10157	AASS	0.561
ENSG00000116221	51253	MRPL37	0.5
ENSG00000110955	506	ATP5B	0.51
ENSG00000120370	92344	SCYL1BP1	0.482
ENSG00000172086	51315	KRCC1	0.54
ENSG00000115593	150572	SMYD1	0.456
ENSG00000066697	91283	C9orf30	0.568
ENSG00000110811	10536	LEPREL2	0.486
ENSG00000262102	92126	DSEL	0.562
ENSG00000182162	286530	P2RY8	0.477
ENSG00000165496	140801	RPL10L	0.443
ENSG00000134538	10599	SLCO1B1	0.438
ENSG00000136110	11061	LECT1	0.482
ENSG00000117419	79033	PRNPIP	0.5
ENSG00000197976	8227	SFRS17A	0.577
ENSG00000149654	64405	CDH22	0.429
ENSG00000196421	284739	PRR17	0.395
ENSG00000257017	3240	HP	0.486
ENSG00000187535	9742	IFT140	0.465
ENSG00000213402	5790	PTPRCAP	0.496
ENSG00000084453	6579	SLCO1A2	0.537
ENSG00000102837	10562	OLFM4	0.462
ENSG00000159593	8883	NAE1	0.521
ENSG00000129988	3929	LBP	0.579
ENSG00000172725	57175	CORO1B	0.553
ENSG00000114859	1181	CLCN2	0.477
ENSG00000120457	3762	KCNJ5	0.58
ENSG00000088930	22803	XRN2	0.512
ENSG00000158089	79623	GALNT14	0.529
ENSG00000128989	10776	ARPP-19	0.602
ENSG00000163156	79005	SCNM1	0.471
ENSG00000262634	220134	C18orf24	0.455
ENSG00000111664	2784	GNB3	0.543
ENSG00000196433	438	ASMT	0.485
ENSG00000135914	3357	HTR2B	0.453
ENSG00000170471	57148	KIAA1219	0.551
ENSG00000135931	80210	ARMC9	0.558
ENSG00000131634	79652	TMEM204	0.508
ENSG00000108602	218	ALDH3A1	0.502
ENSG00000114026	4968	OGG1	0.607
ENSG00000121351	3375	IAPP	0.413
ENSG00000165269	364	AQP7	0.514
ENSG00000121350	79912	PYROXD1	0.502
ENSG00000106628	5425	POLD2	0.515
ENSG00000172653	256957	C17orf66	0.405
ENSG00000163586	2168	FABP1	0.448
ENSG00000226560	717	C2	0.523
ENSG00000156206	161502	C15orf26	0.451
ENSG00000144115	55258	THNSL2	0.498
ENSG00000149483	51524	TMEM138	0.545
ENSG00000004700	5965	RECQL	0.6

ENSG00000173465	10534	SSSCA1	0.491
ENSG00000137876	51187	C15orf15	0.523
ENSG00000122687	29960	FTSJ2	0.571
ENSG00000128039	79644	SRD5A3	0.492
ENSG00000106733	54981	C9orf95	0.536
ENSG00000105793	85865	GTPBP10	0.445
ENSG00000090534	7066	THPO	0.578
ENSG00000069943	9488	PIGB	0.508
ENSG00000183621	220929	ZNF438	0.508
ENSG00000123213	57486	NLN	0.658
ENSG00000134548	80763	C12orf39	0.49
ENSG00000161570	6352	CCL5	0.597
ENSG00000156966	93010	B3GNT7	0.462
ENSG00000177182	157807	RLBP1L1	0.421
ENSG00000117501	80133	C1orf129	0.414
ENSG00000111713	2998	GYS2	0.429
ENSG00000162390	26027	ACOT11	0.568
ENSG00000170145	23235	SNF1LK2	0.492
ENSG00000134627	143689	PIWIL4	0.46
ENSG00000139574	8620	NPFF	0.533
ENSG00000165501	122769	PPIL5	0.506
ENSG00000106268	4521	NUDT1	0.525
ENSG00000260916	9236	CCPG1	0.602
ENSG00000217930	51025	Magmas	0.505
ENSG00000111669	7167	TPI1	0.616
ENSG00000172073	200523	C2orf51	0.4
ENSG00000165272	360	AQP3	0.645
ENSG00000241468	9551	ATP5J2	0.538
ENSG00000122574	644150	WIPF3	0.429
ENSG00000139971	145407	C14orf37	0.444
ENSG00000106631	58498	MYL7	0.465
ENSG00000179941	79738	BBS10	0.422
ENSG00000154839	220134	C18orf24	0.455
ENSG00000184905	140597	TCEAL2	0.447
ENSG00000106266	29886	SNX8	0.465
ENSG00000007933	2328	FMO3	0.535
ENSG00000187456	201299	RDM1	0.451
ENSG00000011052	4831	NME2	0.6
ENSG00000197780	6884	TAF13	0.455
ENSG00000172345	80765	STARD5	0.497
ENSG00000256061	161582	DYX1C1	0.538
ENSG00000121073	10237	SLC35B1	0.52
ENSG00000149532	79869	FLJ12529	0.527
ENSG00000136897	54534	MRPL50	0.454
ENSG00000184515	340542	BEX5	0.436
ENSG00000120832	80298	MTERFD3	0.524
ENSG00000083544	81550	TDRD3	0.578
ENSG00000227587	534	ATP6V1G2	0.492
ENSG00000262246	79585	CORO7	0.5
ENSG00000177673	165100	C2orf57	0.425
ENSG00000111671	84727	SPSB2	0.457
ENSG00000187514	5757	PTMA	0.581
ENSG00000185554	56001	NXF2	0.423
ENSG00000010626	10233	LRRC23	0.636

ENSG00000141741	84299	C17orf37	0.511
ENSG00000186280	55693	JMJD2D	0.445
ENSG00000135390	517	ATP5G2	0.593
ENSG00000165502	6166	RPL36AL	0.514
ENSG00000165506	55172	C14orf104	0.473
ENSG00000149212	143686	SESN3	0.548
ENSG00000156973	5147	PDE6D	0.558
ENSG00000204424	259215	LY6G6F	0.375
ENSG00000178445	2731	GLDC	0.499
ENSG00000100479	5427	POLE2	0.561
ENSG00000082212	4200	ME2	0.616
ENSG00000177383	64110	MAGEF1	0.516
ENSG00000156931	23355	VPS8	0.506
ENSG00000103024	4832	NME3	0.544
ENSG00000243678	4831	NME2	0.6
ENSG00000181827	64864	RFXDC2	0.459
ENSG00000136872	229	ALDOB	0.596
ENSG00000204296	10665	C6orf10	0.434
ENSG00000150361	57626	KLHL1	0.442
ENSG00000162391	338094	FAM151A	0.425
ENSG00000151575	374618	TEX9	0.523
ENSG00000166589	1014	CDH16	0.492
ENSG00000198556	285989	ZNF789	0.495
ENSG00000111726	55907	CMAS	0.457
ENSG00000106086	84725	PLEKHA8	0.57
ENSG00000008405	1407	CRY1	0.519
ENSG00000140598	79631	EFTUD1	0.514
ENSG00000234530	4795	NFKBIL1	0.461
ENSG00000074071	65993	MRPS34	0.494
ENSG00000160213	1476	CSTB	0.586
ENSG00000261701	3250	HPR	0.464
ENSG00000160214	8568	RRP1	0.509
ENSG00000132671	6754	SSTR4	0.474
ENSG00000078900	7161	TP73	0.463
ENSG00000238130	4049	LTA	0.506
ENSG00000184313	374977	C1orf175	0.485
ENSG00000111678	113246	C12orf57	0.531
ENSG00000132661	29107	NXT1	0.534
ENSG00000110697	9600	PITPNM1	0.496
ENSG00000099139	5125	PCSK5	0.578
ENSG00000160908	84124	ZNF394	0.494
ENSG00000111679	5777	PTPN6	0.533
ENSG00000204420	80739	C6orf25	0.434
ENSG00000204490	7124	TNF	0.524
ENSG00000058091	5218	PFTK1	0.62
ENSG00000196531	4666	NACA	0.557
ENSG00000174460	170261	ZCCHC12	0.36
ENSG00000086848	79796	ALG9	0.497
ENSG00000108599	11216	AKAP10	0.506
ENSG00000140829	9785	DHX38	0.5
ENSG00000010932	2326	FMO1	0.515
ENSG00000101447	81610	FAM83D	0.463
ENSG00000161572	57151	LYZL6	0.514
ENSG00000185945	56001	NXF2	0.423

ENSG00000188659	283726	FAM154B	0.429
ENSG00000204487	4050	LTB	0.52
ENSG00000107077	23081	JMJD2C	0.559
ENSG00000135002	55312	RFK	0.566
ENSG00000162594	149233	IL23R	0.444
ENSG00000221909	221786	C7orf38	0.529
ENSG00000144535	129563	DIS3L2	0.488
ENSG00000162032	90864	SPSB3	0.579
ENSG00000010438	5646	PRSS3	0.535
ENSG00000120594	84898	PLXDC2	0.492
ENSG00000197978	440295	LOC440295	0.517
ENSG00000163440	132954	PDCL2	0.364
ENSG00000088727	64147	KIF9	0.621
ENSG00000128487	92521	SPECC1	0.537
ENSG00000095906	10101	NUBP2	0.457
ENSG00000226182	7940	LST1	0.606
ENSG00000163283	250	ALPP	0.447
ENSG00000149929	8479	HIRIP3	0.562
ENSG00000168078	55872	PBK	0.549
ENSG00000184779	6218	RPS17	0.626
ENSG00000100567	5684	PSMA3	0.565
ENSG00000113790	1962	EHHADH	0.478
ENSG00000213494	6358	CCL14	0.6
ENSG00000169592	283899	CCDC95	0.379
ENSG00000163286	251	ALPPL2	0.565
ENSG00000167985	54949	C11orf79	0.507
ENSG00000234196	221527	ZBTB12	0.46
ENSG00000153406	57407	NMRAL1	0.527
ENSG00000161574	6358	CCL14	0.6
ENSG00000243570	629	CFB	0.59
ENSG00000125823	128817	CSTL1	0.324
ENSG00000204287	3122	HLA-DRA	0.555
ENSG00000109255	10874	NMU	0.501
ENSG00000125831	140880	CST11	0.505
ENSG00000162819	148362	C1orf58	0.585
ENSG00000196648	440295	LOC440295	0.517
ENSG00000076258	2329	FMO4	0.501
ENSG00000163295	248	ALPI	0.526
ENSG00000267596	6359	CCL15	0.544
ENSG00000099769	3483	IGFALS	0.454
ENSG00000204421	80740	LY6G6C	0.463
ENSG00000125815	10047	CST8	0.428
ENSG00000063854	3029	HAGH	0.537
ENSG00000258529	79796	ALG9	0.497
ENSG00000101435	128821	CST9L	0.413
ENSG00000136045	11137	PWP1	0.58
ENSG00000182774	6218	RPS17	0.626
ENSG00000250423	57481	RP13-347D8.3	0.386
ENSG00000255561	91893	hCG_2033039	0.425
ENSG00000142945	11004	KIF2C	0.583
ENSG00000171148	10474	TADA3L	0.534
ENSG00000137720	64776	C11orf1	0.475
ENSG00000167236	6368	CCL23	0.552
ENSG00000168350	123099	DEGS2	0.47

ENSG00000137038	90871	C9orf123	0.591
ENSG00000255529	81488	GRINL1A	0.552
ENSG00000006074	6362	CCL18	0.572
ENSG00000180806	3225	HOXC9	0.504
ENSG00000163900	90407	TMEM41A	0.564
ENSG00000127989	7978	MTERF	0.466
ENSG00000226397	196415	LOC196415	0.311
ENSG00000109846	1410	CRYAB	0.514
ENSG00000151929	9531	BAG3	0.546
ENSG00000232694	646627	LOC646627	0.372
ENSG00000090989	55763	EXOC1	0.498
ENSG00000198502	3127	HLA-DRB5	0.629
ENSG00000135902	1144	CHRND	0.484
ENSG00000083535	10464	PIBF1	0.5
ENSG00000111684	10162	LPCAT3	0.585
ENSG00000155833	1539	CYLC2	0.457
ENSG00000006625	79017	C7orf24	0.542
ENSG00000136824	10592	SMC2	0.556
ENSG00000198056	5557	PRIM1	0.54
ENSG00000118308	4033	LRMP	0.533
ENSG00000173335	128822	CST9	0.442
ENSG00000169989	201798	TIGD4	0.491
ENSG00000106009	221927	C7orf27	0.432
ENSG00000167791	51475	CABP2	0.43
ENSG00000173039	5970	RELA	0.598
ENSG00000213722	23564	DDAH2	0.601
ENSG00000197119	123096	SLC25A29	0.583
ENSG00000101439	1471	CST3	0.51
ENSG00000237808	259197	NCR3	0.564
ENSG00000180185	81889	FAHD1	0.54
ENSG00000141200	84643	KIF2B	0.438
ENSG00000182326	716	C1S	0.514
ENSG00000197969	23230	VPS13A	0.63
ENSG00000084207	2950	GSTP1	0.574
ENSG00000165516	23588	KLHDC2	0.526
ENSG00000166035	3990	LIPC	0.452
ENSG00000101441	1472	CST4	0.488
ENSG00000162592	148870	CCDC27	0.458
ENSG00000196126	3123	HLA-DRB1	0.614
ENSG00000170373	1469	CST1	0.465
ENSG00000167792	4723	NDUFV1	0.504
ENSG00000170369	1470	CST2	0.439
ENSG00000102554	688	KLF5	0.584
ENSG00000121270	85320	ABCC11	0.455
ENSG00000005022	292	SLC25A5	0.548
ENSG00000123415	23583	SMUG1	0.517
ENSG00000118557	83449	PMFBP1	0.475
ENSG00000106012	23288	IQCE	0.584
ENSG00000154309	84976	DISP1	0.548
ENSG00000112852	56133	PCDHB2	0.518
ENSG00000113205	56132	PCDHB3	0.474
ENSG00000081818	56131	PCDHB4	0.476
ENSG00000196811	1146	CHRNG	0.422
ENSG00000113209	26167	PCDHB5	0.429

ENSG00000106105	2617	GARS	0.577
ENSG00000196735	3117	HLA-DQA1	0.533
ENSG00000158164	11013	TMSL8	0.526
ENSG00000113212	56129	PCDHB7	0.449
ENSG00000125962	64860	ARMCX5	0.496
ENSG00000162039	254528	C16orf73	0.402
ENSG00000221944	200765	TIGD1	0.537
ENSG00000196963	57717	PCDHB16	0.424
ENSG00000120324	56126	PCDHB10	0.468
ENSG00000135930	9470	EIF4E2	0.601
ENSG00000167194	146378	FLJ25404	0.385
ENSG00000162961	84661	DPY30	0.471
ENSG00000041353	5874	RAB27B	0.5
ENSG00000197479	56125	PCDHB11	0.438
ENSG00000185942	286183	NKAIN3	0.462
ENSG00000120328	56124	PCDHB12	0.441
ENSG00000121579	80218	NAT13	0.53
ENSG00000162040	64711	HS3ST6	0.406
ENSG00000164825	1672	DEFB1	0.492
ENSG00000137563	8836	GGH	0.524
ENSG00000094916	23468	CBX5	0.509
ENSG00000198185	55713	ZNF334	0.422
ENSG00000170367	1473	CST5	0.421
ENSG00000149435	92086	GGTLC1	0.575
ENSG00000118307	55259	CASC1	0.466
ENSG00000187372	56123	PCDHB13	0.423
ENSG00000140107	283600	C14orf68	0.383
ENSG00000120327	56122	PCDHB14	0.44
ENSG00000164822	1671	DEFA6	0.432
ENSG00000113248	56121	PCDHB15	0.407
ENSG00000237727	199	AIF1	0.617
ENSG00000213719	1192	CLIC1	0.536
ENSG00000204683	387638	C10orf113	0.422
ENSG00000130764	57470	LRRC47	0.465
ENSG00000187554	7100	TLR5	0.531
ENSG00000163060	150483	TEKT4	0.48
ENSG00000229363	7936	RDBP	0.505
ENSG00000166510	80323	CCDC68	0.452
ENSG00000164821	1669	DEFA4	0.471
ENSG00000106258	1577	CYP3A5	0.584
ENSG00000101463	79953	C20orf39	0.467
ENSG00000206047	1667	DEFA1	0.621
ENSG00000240247	1667	DEFA1	0.621
ENSG00000140986	6123	RPL3L	0.477
ENSG00000077984	8530	CST7	0.503
ENSG00000168101	84309	NUDT16L1	0.5
ENSG00000239839	1667	DEFA1	0.621
ENSG00000103723	8120	AP3B2	0.467
ENSG00000172824	283848	FLJ37464	0.457
ENSG00000140990	4716	NDUFB10	0.516
ENSG00000164816	1670	DEFA5	0.44
ENSG00000166856	11318	GPR182	0.487
ENSG00000177257	1673	DEFB4	0.564
ENSG00000140988	6187	RPS2	0.594

ENSG00000116198	9731	KIAA0562	0.515
ENSG00000106113	1395	CRHR2	0.58
ENSG00000170276	3316	HSPB2	0.534
ENSG00000254445	3316	HSPB2	0.534
ENSG00000157456	9133	CCNB2	0.555
ENSG00000149300	91894	C11orf52	0.478
ENSG00000134014	55140	ELP3	0.482
ENSG00000140105	7453	WARS	0.592
ENSG00000018610	63932	CXorf56	0.462
ENSG00000184384	84441	MAML2	0.635
ENSG00000120329	83884	SLC25A2	0.444
ENSG00000198932	9737	GPRASP1	0.506
ENSG00000178913	6879	TAF7	0.516
ENSG00000225164	7916	BAT2	0.58
ENSG00000162396	25973	PARS2	0.455
ENSG00000006555	55001	TTC22	0.538
ENSG00000073605	55876	GSDML	0.534
ENSG00000205707	144363	LYRM5	0.415
ENSG00000157578	150082	LCA5L	0.504
ENSG00000139178	51279	C1RL	0.495
ENSG00000137561	7274	TTPA	0.465
ENSG00000203730	127670	TEDDM1	0.444
ENSG00000077721	7319	UBE2A	0.593
ENSG00000171989	92483	LDHAL6B	0.385
ENSG00000180828	27319	BHLHB5	0.46
ENSG00000172817	9420	CYP7B1	0.501
ENSG00000241644	11185	INMT	0.473
ENSG00000142937	6202	RPS8	0.539
ENSG00000139194	83758	RBP5	0.433
ENSG00000204410	4439	MSH5	0.508
ENSG00000140297	9245	GCNT3	0.441
ENSG00000123094	11228	RASSF8	0.518
ENSG00000172005	4118	MAL	0.492
ENSG00000167800	347853	TBX10	0.556
ENSG00000140307	2958	GTF2A2	0.559
ENSG00000140836	463	ZFHX3	0.508
ENSG00000132744	91703	ACY3	0.453
ENSG00000123095	79365	BHLHB3	0.557
ENSG00000177243	55894	DEFB103A	0.531
ENSG00000174600	1240	CMKLR1	0.568
ENSG00000166246	146562	C16orf71	0.4
ENSG00000164871	10407	SPAG11B	0.512
ENSG00000136527	6434	SFRS10	0.622
ENSG00000143502	55061	SUSD4	0.476
ENSG00000162595	9077	DIRAS3	0.499
ENSG00000172315	112858	TP53RK	0.519
ENSG00000183340	8690	JRKL	0.496
ENSG00000262576	56111	PCDHGA4	0.442
ENSG00000179344	3119	HLA-DQB1	0.68
ENSG00000102910	83752	LONP2	0.551
ENSG00000132746	222	ALDH3B2	0.531
ENSG00000103199	26048	ZNF500	0.576
ENSG00000162398	163747	C1orf177	0.383
ENSG00000179580	146310	RNF151	0.385

ENSG00000171956	27023	FOXB1	0.389
ENSG00000232616	6499	SKIV2L	0.492
ENSG00000183751	10607	TBL3	0.513
ENSG00000188243	170622	COMMD6	0.528
ENSG00000106125	84182	FLJ22374	0.443
ENSG00000248496	114821	SCAND3	0.425
ENSG00000261934	56107	PCDHGA9	0.429
ENSG00000144029	64969	MRPS5	0.556
ENSG00000237071	5987	TRIM27	0.637
ENSG00000121211	84057	MND1	0.541
ENSG00000160218	7109	TMEM1	0.613
ENSG00000118939	7347	UCHL3	0.542
ENSG00000160870	1551	CYP3A7	0.568
ENSG00000168496	2237	FEN1	0.583
ENSG00000198876	25853	WDR40A	0.55
ENSG00000186628	123722	FSD2	0.423
ENSG00000223687	282890	ZNF311	0.466
ENSG00000178307	8834	TMEM11	0.504
ENSG00000142959	266675	BEST4	0.435
ENSG00000107165	7306	TYRP1	0.513
ENSG00000140623	124404	12-Sep	0.402
ENSG00000134824	9415	FADS2	0.53
ENSG00000197496	81031	SLC2A10	0.491
ENSG00000166866	4640	MYO1A	0.503
ENSG00000110057	81622	UNC93B1	0.439
ENSG00000160868	1576	CYP3A4	0.64
ENSG00000251394	81797	OR12D3	0.409
ENSG00000235966	26529	OR12D2	0.438
ENSG00000230780	26531	OR11A1	0.415
ENSG00000235132	26716	OR2H1	0.517
ENSG00000213139	1427	CRYGS	0.509
ENSG00000182600	389084	UNQ830	0.466
ENSG00000143001	199964	TMEM61	0.36
ENSG00000162399	7809	BSND	0.48
ENSG00000154035	256302	MGC33894	0.512
ENSG00000067836	79641	ROGDI	0.527
ENSG00000133169	55859	BEX1	0.503
ENSG00000237172	84752	MGC4655	0.54
ENSG00000106128	2692	GHRHR	0.619
ENSG00000102871	8717	TRADD	0.626
ENSG00000147206	56000	NXF3	0.442
ENSG00000156232	123720	WHDC1	0.47
ENSG00000135314	80759	KHDC1	0.459
ENSG00000214050	157574	FBXO16	0.442
ENSG00000150773	120379	PIH1D2	0.453
ENSG00000178287	10407	SPAG11B	0.512
ENSG00000069020	375449	MAST4	0.516
ENSG00000196408	124056	NOXO1	0.384
ENSG00000135722	55336	FBXL8	0.416
ENSG00000169598	1677	DFFB	0.49
ENSG00000169612	83640	FAM103A1	0.507
ENSG00000104671	10671	DCTN6	0.549
ENSG00000034971	4653	MYOC	0.458
ENSG00000169609	123207	C15orf40	0.517

ENSG00000197586	955	ENTPD6	0.522
ENSG00000125122	26231	LRRC29	0.422
ENSG00000196436	440348	LOC440348	0.494
ENSG00000100575	26520	TIMM9	0.514
ENSG00000136783	25934	NIPSNAP3A	0.491
ENSG00000090512	26998	FETUB	0.59
ENSG00000140632	84656	N-PAC	0.567
ENSG00000138075	64240	ABCG5	0.423
ENSG00000177675	283316	CD163L1	0.435
ENSG00000015520	29881	NPC1L1	0.463
ENSG00000021461	64816	CYP3A43	0.605
ENSG00000165028	55335	NIPSNAP3B	0.507
ENSG00000176797	55894	DEFB103A	0.531
ENSG00000185437	6450	SH3BGR	0.519
ENSG00000171711	1673	DEFB4	0.564
ENSG00000110717	4728	NDUFS8	0.548
ENSG00000178395	164127	C1orf65	0.429
ENSG00000168701	29100	TMEM208	0.488
ENSG00000172922	84153	RNASEH2C	0.569
ENSG00000157426	132949	AASDH	0.5
ENSG00000174945	155185	AMZ1	0.436
ENSG00000229524	7917	BAT3	0.576
ENSG00000167962	90850	ZNF598	0.5
ENSG00000116745	6121	RPE65	0.444
ENSG00000148444	23412	COMMD3	0.515
ENSG00000113905	3273	HRG	0.525
ENSG00000237541	3118	HLA-DQA2	0.706
ENSG00000113889	3827	KNG1	0.481
ENSG00000100578	9786	KIAA0586	0.564
ENSG00000150779	26521	TIMM8B	0.517
ENSG00000232632	2550	GABBR1	0.533
ENSG00000203909	340168	DPPA5	0.417
ENSG00000110719	10312	TCIRG1	0.535
ENSG00000204370	6392	SDHD	0.552
ENSG00000029153	56938	ARNTL2	0.54
ENSG00000010165	51603	KIAA0859	0.628
ENSG00000205268	5150	PDE7A	0.579
ENSG00000203908	154288	C6orf221	0.408
ENSG00000183971	283869	NPW	0.383
ENSG00000155367	333926	PPM1J	0.472
ENSG00000080007	55510	DDX43	0.42
ENSG00000183067	150084	IGSF5	0.38
ENSG00000177425	5074	PAWR	0.602
ENSG00000102409	56271	BEX4	0.537
ENSG00000241106	3112	HLA-DOB	0.518
ENSG00000182319	157285	PRAGMIN	0.477
ENSG00000118898	5493	PPL	0.532
ENSG00000183036	5121	PCP4	0.489
ENSG00000198535	145741	FAM148A	0.441
ENSG00000111701	339	APOBEC1	0.469
ENSG00000205502	388125	FAM148B	0.549
ENSG00000164978	318	NUDT2	0.45
ENSG00000078549	117	ADCYAP1R1	0.467
ENSG00000155906	55005	RMND1	0.487

ENSG00000065057	4913	NTHL1	0.518
ENSG00000102924	869	CBLN1	0.452
ENSG00000090863	2734	GLG1	0.608
ENSG00000114650	22937	SCAP	0.525
ENSG00000241945	5822	PWP2	0.494
ENSG00000254470	91056	DKFZp761E198	0.431
ENSG00000137462	7097	TLR2	0.504
ENSG00000184344	9573	GDF3	0.415
ENSG00000145428	285533	RNF175	0.487
ENSG00000187569	359787	DPPA3	0.417
ENSG00000123405	4778	NFE2	0.479
ENSG00000198178	170482	CLEC4C	0.492
ENSG00000166152	123970	C16orf78	0.38
ENSG00000104626	90459	THEX1	0.479
ENSG00000212719	339263	C17orf51	0.507
ENSG00000091106	58484	NLRC4	0.547
ENSG00000140416	7168	TPM1	0.654
ENSG00000103197	7249	TSC2	0.567
ENSG00000203805	196051	PPAPDC1A	0.46
ENSG00000131697	261734	NPHP4	0.536
ENSG00000156049	9630	GNA14	0.434
ENSG00000164972	84688	C9orf24	0.467
ENSG00000150783	56158	TEX12	0.39
ENSG00000140939	8996	NOL3	0.589
ENSG00000197580	83875	BCO2	0.483
ENSG00000167100	201191	SAMD14	0.513
ENSG00000197265	2961	GTF2E2	0.525
ENSG00000180347	223075	CCDC129	0.452
ENSG00000136271	54606	DDX56	0.484
ENSG00000214900	283551	LOC283551	0.506
ENSG00000166503	50810	HDGFRP3	0.586
ENSG00000163617	57577	KIAA1407	0.469
ENSG00000104687	2936	GSR	0.528
ENSG00000182916	56849	TCEAL7	0.456
ENSG00000262655	10418	SPON1	0.585
ENSG00000102886	79153	GDPD3	0.462
ENSG00000164430	115004	C6orf150	0.471
ENSG00000148019	84131	CEP78	0.509
ENSG00000136003	23479	ISCU	0.532
ENSG00000163918	5984	RFC4	0.606
ENSG00000033011	56052	ALG1	0.466
ENSG00000103642	114294	LACTB	0.565
ENSG00000153930	162282	ANKFN1	0.422
ENSG00000262489	22800	RRAS2	0.635
ENSG00000176473	79446	WDR25	0.449
ENSG00000146476	79624	C6orf211	0.51
ENSG00000139572	53831	GPR84	0.481
ENSG00000140600	6457	SH3GL3	0.561
ENSG00000224313	1797	DOM3Z	0.616
ENSG00000146833	89122	TRIM4	0.575
ENSG00000181092	9370	ADIPOQ	0.476
ENSG00000214929	389763	FLJ46321	0.365
ENSG00000165105	158158	RASEF	0.583
ENSG00000174236	387849	REP15	0.483

ENSG00000061794	60488	MRPS35	0.535
ENSG00000135824	85397	RGS8	0.444
ENSG00000110318	57562	KIAA1377	0.507
ENSG00000185222	51186	WBP5	0.513
ENSG00000120262	80129	C6orf97	0.471
ENSG00000153446	146556	MGC45438	0.42
ENSG00000008838	9862	MED24	0.592
ENSG00000120008	55717	BRWD2	0.458
ENSG00000196123	653319	LOC653319	0.463
ENSG00000127980	5189	PEX1	0.549
ENSG00000115827	80067	C2orf37	0.479
ENSG00000130561	6295	SAG	0.547
ENSG00000087494	5744	PTHLH	0.609
ENSG00000226033	8859	STK19	0.565
ENSG00000179044	283849	EXOC3L	0.434
ENSG00000118894	196483	FAM86A	0.364
ENSG00000214226	339210	C17orf67	0.429
ENSG00000160862	563	AZGP1	0.491
ENSG00000163923	116832	RPL39L	0.518
ENSG00000153933	8526	DGKE	0.46
ENSG00000135069	29968	PSAT1	0.549
ENSG00000175077	132112	RTP1	0.532
ENSG00000110876	6404	SELPLG	0.59
ENSG00000123106	55297	CCDC91	0.529
ENSG00000166681	27018	NGFRAP1	0.54
ENSG00000172732	80198	MUS81	0.489
ENSG00000135297	25821	MTO1	0.561
ENSG00000228913	10537	UBD	0.48
ENSG00000229185	7932	OR2H2	0.526
ENSG00000108823	6442	SGCA	0.478
ENSG00000198918	6170	RPL39	0.52
ENSG00000237834	4340	MOG	0.581
ENSG00000204264	5696	PSMB8	0.535
ENSG00000111704	79923	NANOG	0.431
ENSG00000197361	283807	FBXL22	0.365
ENSG00000078328	54715	A2BP1	0.525
ENSG00000133863	56154	TEX15	0.488
ENSG00000137691	85016	C11orf70	0.452
ENSG00000133773	29080	CCDC59	0.506
ENSG00000166529	7589	ZSCAN21	0.491
ENSG00000154678	5137	PDE1C	0.558
ENSG00000165392	7486	WRN	0.499
ENSG00000147571	1392	CRH	0.523
ENSG00000102879	11151	CORO1A	0.502
ENSG00000172476	142684	RAB40A	0.43
ENSG00000160221	8209	C21orf33	0.636
ENSG00000150787	5805	PTS	0.54
ENSG00000128050	10606	PAICS	0.655
ENSG00000064763	55711	FAR2	0.503
ENSG00000127720	84190	C12orf26	0.472
ENSG00000155393	55027	HEATR3	0.479
ENSG00000166797	84191	FAM96A	0.54
ENSG00000136514	64108	RTP4	0.452
ENSG00000157005	6750	SST	0.473

ENSG00000087502	51290	ERGIC2	0.522
ENSG00000148057	414328	C9orf103	0.463
ENSG00000134061	4064	CD180	0.495
ENSG00000185559	8788	DLK1	0.473
ENSG00000108278	9326	ZNHIT3	0.532
ENSG00000054796	23626	SPO11	0.46
ENSG00000158604	222068	TMED4	0.582
ENSG00000122735	27019	DNAI1	0.445
ENSG00000171860	719	C3AR1	0.505
ENSG00000164776	5260	PHKG1	0.516
ENSG00000171566	5356	PLRG1	0.527
ENSG00000157131	731	C8A	0.465
ENSG00000111729	50856	CLEC4A	0.544
ENSG00000067365	79091	C16orf68	0.475
ENSG00000143106	5686	PSMA5	0.546
ENSG00000175806	4482	MSRA	0.495
ENSG00000171847	55138	FAM90A1	0.414
ENSG00000169627	552900	BOLA2	0.562
ENSG00000242802	9907	KIAA0415	0.495
ENSG00000069424	8514	KCNAB2	0.608
ENSG00000226858	346171	ZFP57	0.388
ENSG00000166527	338339	CLEC4D	0.5
ENSG00000085982	55230	USP40	0.5
ENSG00000163832	54859	TMEM103	0.512
ENSG00000132207	79008	GIYD2	0.586
ENSG00000066557	55631	LRRC40	0.592
ENSG00000168961	3965	LGALS9	0.538
ENSG00000150867	5305	PIP4K2A	0.494
ENSG00000224290	55937	APOM	0.62
ENSG00000151577	1814	DRD3	0.545
ENSG00000235360	57827	C6orf47	0.496
ENSG00000125352	7737	RNF113A	0.484
ENSG00000236011	7918	BAT4	0.51
ENSG00000125356	4694	NDUFA1	0.52
ENSG00000127993	84060	DKFZP564O0523	0.55
ENSG00000077380	1781	DYNC1I2	0.52
ENSG00000184647	203074	UNQ9391	0.451
ENSG00000168913	375704	C9orf165	0.422
ENSG00000072041	55117	SLC6A15	0.499
ENSG00000137403	3134	HLA-F	0.626
ENSG00000106153	51142	CHCHD2	0.524
ENSG00000102890	79767	ELMO3	0.439
ENSG00000135845	5279	PIGC	0.659
ENSG00000101003	9837	GINS1	0.561
ENSG00000136155	8796	SCEL	0.47
ENSG00000186635	116985	CENTD2	0.571
ENSG00000023445	330	BIRC3	0.575
ENSG00000261052	6818	SULT1A3	0.567
ENSG00000185513	26013	L3MBTL	0.654
ENSG00000184857	25880	TMEM186	0.494
ENSG00000111052	8825	LIN7A	0.496
ENSG00000110075	55291	SAPS3	0.536
ENSG00000110330	329	BIRC2	0.56
ENSG00000165113	80318	GKAP1	0.527

ENSG00000183844	54097	FAM3B	0.412
ENSG00000166839	348094	ANKDD1A	0.564
ENSG00000104660	23484	LEPROTL1	0.568
ENSG00000111732	57379	AICDA	0.442
ENSG00000157927	55698	RADIL	0.514
ENSG00000121064	59342	SCPEP1	0.538
ENSG00000171060	203076	C8orf74	0.451
ENSG00000111058	79611	ACSS3	0.535
ENSG00000123384	4035	LRP1	0.584
ENSG00000090487	51324	SPG21	0.561
ENSG00000018699	55622	TTC27	0.476
ENSG00000181847	201633	VSTM3	0.329
ENSG00000159708	55282	LRRC36	0.424
ENSG00000165309	219681	ARMC3	0.453
ENSG00000101004	22981	RP4-691N24.1	0.49
ENSG00000230413	3135	HLA-G	0.612
ENSG00000120318	64411	CENTD3	0.518
ENSG00000173406	1600	DAB1	0.459
ENSG00000172465	9338	TCEAL1	0.502
ENSG00000180999	92346	C1orf105	0.476
ENSG00000186471	158798	AKAP14	0.483
ENSG00000166532	57494	FAM80B	0.597
ENSG00000186198	123264	OSTbeta	0.409
ENSG00000133640	84125	LRRIQ1	0.479
ENSG00000197696	4828	NMB	0.546
ENSG00000172543	1521	CTSW	0.499
ENSG00000140612	23478	SEC11A	0.587
ENSG00000169217	10421	CD2BP2	0.561
ENSG00000166535	144568	A2ML1	0.481
ENSG00000105778	23080	KIAA0241	0.607
ENSG00000166855	10845	CLPX	0.544
ENSG00000135413	90070	LACRT	0.357
ENSG00000228875	1460	CSNK2B	0.51
ENSG00000165115	55582	KIF27	0.514
ENSG00000171135	84522	JAGN1	0.483
ENSG00000108784	4669	NAGLU	0.533
ENSG00000172500	9158	FIBP	0.533
ENSG00000108826	51264	MRPL27	0.549
ENSG00000185177	90827	ZNF479	0.46
ENSG00000205413	54809	SAMD9	0.488
ENSG00000161634	117159	DCD	0.357
ENSG00000167964	25837	RAB26	0.593
ENSG00000254093	54984	PINX1	0.482
ENSG00000171564	2244	FGB	0.516
ENSG00000147869	9350	CER1	0.508
ENSG00000174498	9543	PUNC	0.519
ENSG00000183486	4600	MX2	0.521
ENSG00000177409	219285	SAMD9L	0.541
ENSG00000164946	158326	FREM1	0.551
ENSG00000164967	138716	C9orf23	0.44
ENSG00000110900	441631	TSPAN11	0.521
ENSG00000165118	84267	C9orf64	0.543
ENSG00000103742	57722	NOPE	0.512
ENSG00000113946	10686	CLDN16	0.454

ENSG00000137100	11258	DCTN3	0.526
ENSG00000130684	26152	ZNF337	0.579
ENSG00000156222	9154	SLC28A1	0.476
ENSG00000198398	131920	TMEM207	0.367
ENSG00000198774	9182	RASSF9	0.465
ENSG00000108786	3292	HSD17B1	0.495
ENSG00000126368	9572	NR1D1	0.554
ENSG00000101049	10110	SGK2	0.47
ENSG00000137673	4316	MMP7	0.525
ENSG00000135829	1660	DHX9	0.605
ENSG00000224320	3105	HLA-A	0.588
ENSG00000173041	340252	ZNF680	0.448
ENSG00000007171	4843	NOS2A	0.501
ENSG00000110887	1610	DAO	0.455
ENSG00000241713	58496	LY6G5B	0.556
ENSG00000068120	80347	COASY	0.545
ENSG00000188175	253012	HEPACAM2	0.425
ENSG00000235134	1589	CYP21A2	0.47
ENSG00000123485	55355	HJURP	0.517
ENSG00000137674	9313	MMP20	0.411
ENSG00000188895	339287	MSL-1	0.537
ENSG00000231325	80741	LY6G5C	0.527
ENSG00000137675	64066	MMP27	0.467
ENSG00000196247	51427	ZNF107	0.494
ENSG00000166164	29117	BRD7	0.498
ENSG00000125375	27109	ATP5S	0.631
ENSG00000160223	23308	ICOSLG	0.575
ENSG00000144481	79054	TRPM8	0.455
ENSG00000197008	7697	ZNF138	0.571
ENSG00000148450	22921	MSRB2	0.58
ENSG00000172551	118430	MUCL1	0.437
ENSG00000134183	2780	GNAT2	0.447
ENSG00000124253	5105	PCK1	0.528
ENSG00000135426	9840	KIAA0748	0.441
ENSG00000182050	25834	MGAT4C	0.467
ENSG00000004766	55610	CCDC132	0.484
ENSG00000139187	10219	KLRG1	0.52
ENSG00000235676	7920	BAT5	0.551
ENSG00000197568	11147	HHLA3	0.51
ENSG00000107929	23185	LARP5	0.624
ENSG00000138079	6519	SLC3A1	0.542
ENSG00000261149	222865	TMEM130	0.423
ENSG00000100490	8814	CDKL1	0.483
ENSG00000162600	115209	OMA1	0.574
ENSG00000167965	64223	GBL	0.449
ENSG00000180209	29895	MYLPF	0.451
ENSG00000165805	160419	C12orf50	0.388
ENSG00000204390	3305	HSPA1L	0.463
ENSG00000188958	257313	UTS2D	0.376
ENSG00000180035	197407	ZNF553	0.418
ENSG00000149292	54970	TTC12	0.533
ENSG00000179363	203562	TMEM31	0.365
ENSG00000126790	112849	C14orf149	0.465
ENSG00000198039	10793	ZNF273	0.47

ENSG00000180096	1731	1-Sep	0.437
ENSG00000152492	152137	CCDC50	0.601
ENSG00000261023	8295	TRRAP	0.56
ENSG00000203989	727940	RHOXF2B	0.556
ENSG00000142182	29947	DNMT3L	0.461
ENSG00000118113	4317	MMP8	0.473
ENSG00000198042	84549	MAK16	0.517
ENSG00000198796	115701	ALPK2	0.477
ENSG00000262860	26065	LSM14A	0.638
ENSG00000050130	51528	C14orf100	0.489
ENSG00000153060	146279	TEKT5	0.542
ENSG00000133641	91298	C12orf29	0.497
ENSG00000204389	3303	HSPA1A	0.537
ENSG00000103274	4682	NUBP1	0.528
ENSG00000203943	148418	SAMD13	0.53
ENSG00000120337	8995	TNFSF18	0.438
ENSG00000168515	10648	SCGB1D1	0.428
ENSG00000147955	10280	OPRS1	0.598
ENSG00000079134	9984	THOC1	0.501
ENSG00000157601	4599	MX1	0.531
ENSG00000072080	6694	SPP2	0.488
ENSG00000131068	117285	DEFB118	0.378
ENSG00000152926	51351	ZNF117	0.591
ENSG00000242574	3109	HLA-DMB	0.573
ENSG00000124256	81030	ZBP1	0.426
ENSG00000170456	160518	MGC24039	0.519
ENSG00000159720	9114	ATP6V0D1	0.564
ENSG00000127252	57110	HRASLS	0.582
ENSG00000162601	114803	MYSM1	0.535
ENSG00000179399	2262	GPC5	0.44
ENSG00000175899	2	A2M	0.518
ENSG00000101883	158800	RHOXF1	0.481
ENSG00000124935	10647	SCGB1D2	0.477
ENSG00000131721	84528	RHOXF2	0.533
ENSG00000206258	7148	TNXB	0.679
ENSG00000183098	10082	GPC6	0.49
ENSG00000146757	168374	ZNF92	0.52
ENSG00000204548	245934	DEFB121	0.357
ENSG00000204387	50854	C6orf48	0.542
ENSG00000166676	780776	FAM18A	0.417
ENSG00000232859	201229	LOC201229	0.506
ENSG00000174485	10260	DENND4A	0.488
ENSG00000133878	78986	DUSP26	0.488
ENSG00000138078	9581	PREPL	0.626
ENSG00000221838	9179	AP4M1	0.463
ENSG00000108272	79154	MGC4172	0.502
ENSG00000180424	245936	DEFB123	0.381
ENSG00000139160	254013	MGC50559	0.531
ENSG00000105968	94239	H2AFV	0.654
ENSG00000170439	196410	METTL7B	0.474
ENSG00000151743	196394	AMN1	0.451
ENSG00000126838	5858	PZP	0.486
ENSG00000101052	51098	IFT52	0.503
ENSG00000088320	28954	REM1	0.476

ENSG00000198707	80184	CEP290	0.522
ENSG00000166816	197257	LDHD	0.459
ENSG00000179133	256815	C10orf67	0.465
ENSG00000169919	2990	GUSB	0.548
ENSG00000166670	4319	MMP10	0.471
ENSG00000177143	1068	CETN1	0.458
ENSG00000117592	9588	PRDX6	0.608
ENSG00000079101	27098	CLUL1	0.443
ENSG00000162753	284525	SLC9A11	0.447
ENSG00000132781	4595	MUTYH	0.567
ENSG00000157985	116987	CENTG2	0.489
ENSG00000134242	26191	PTPN22	0.566
ENSG00000137976	58511	DNASE2B	0.467
ENSG00000129282	79922	MRM1	0.437
ENSG00000136286	64005	MYO1G	0.567
ENSG00000213930	2592	GALT	0.519
ENSG00000160224	326	AIRE	0.458
ENSG00000182685	283870	C16orf79	0.51
ENSG00000080166	1638	DCT	0.603
ENSG00000204386	4758	NEU1	0.519
ENSG00000132130	3975	LHX1	0.456
ENSG00000176912	494514	C18orf56	0.412
ENSG00000176890	7298	TYMS	0.597
ENSG00000184207	283871	LOC283871	0.461
ENSG00000184293	160365	CLECL1	0.444
ENSG00000179958	79077	XTP3TPA	0.503
ENSG00000123570	51209	RAB9B	0.507
ENSG00000204257	3108	HLA-DMA	0.551
ENSG00000167967	1877	E4F1	0.469
ENSG00000152689	25780	RASGRP3	0.505
ENSG00000196611	4312	MMP1	0.538
ENSG00000069482	51083	GAL	0.559
ENSG00000175467	9092	SART1	0.526
ENSG00000117133	80135	BXDC5	0.519
ENSG00000181610	51649	MRPS23	0.46
ENSG00000149968	4314	MMP3	0.476
ENSG00000163702	84818	IL17RC	0.519
ENSG00000136573	640	BLK	0.519
ENSG00000204385	80736	SLC44A4	0.452
ENSG00000189046	121642	ALKBH2	0.476
ENSG00000172456	55277	FGGY	0.525
ENSG00000150045	51348	KLRF1	0.476
ENSG00000124237	128602	C20orf85	0.423
ENSG00000168928	440387	CTRB2	0.439
ENSG00000132199	55556	ENOSF1	0.634
ENSG00000158427	11013	TMSL8	0.526
ENSG00000126522	435	ASL	0.512
ENSG00000167968	1775	DNASE1L2	0.462
ENSG00000224859	30834	ZNRD1	0.542
ENSG00000175643	116028	C16orf75	0.517
ENSG00000168925	1504	CTRB1	0.557
ENSG00000164610	6100	RP9	0.478
ENSG00000110852	9976	CLEC2B	0.517
ENSG00000198513	51062	SPG3A	0.496

ENSG00000178279	7142	TNP2	0.546
ENSG00000164975	6619	SNAPC3	0.595
ENSG00000108270	26574	AATF	0.52
ENSG00000167969	1632	DCI	0.541
ENSG00000214530	10809	STARD10	0.54
ENSG00000234058	6992	PPP1R11	0.524
ENSG00000228628	1388	CREBL1	0.561
ENSG00000172322	160364	CLEC12A	0.463
ENSG00000227171	80352	RNF39	0.512
ENSG00000166938	115752	DIS3L	0.561
ENSG00000240008	259215	LY6G6F	0.375
ENSG00000164638	222962	SLC29A4	0.471
ENSG00000197506	64078	SLC28A3	0.456
ENSG00000154328	252969	NEIL2	0.429
ENSG00000123575	139231	CXorf39	0.511
ENSG00000148702	3026	HABP2	0.479
ENSG00000149021	7356	SCGB1A1	0.464
ENSG00000115042	51011	FAHD2A	0.558
ENSG00000230060	80739	C6orf25	0.434
ENSG00000226402	11074	TRIM31	0.6
ENSG00000124196	149699	GTSF1L	0.495
ENSG00000117151	1486	CTBS	0.581
ENSG00000124191	84969	TOX2	0.544
ENSG00000145740	64924	SLC30A5	0.579
ENSG00000178460	157777	C8orf45	0.5
ENSG00000075131	54962	TIPIN	0.514
ENSG00000169957	79724	ZNF768	0.478
ENSG00000166748	123624	AGBL1	0.453
ENSG00000136449	84073	MYCBPAP	0.483
ENSG00000165682	51266	CLEC1B	0.447
ENSG00000080986	10403	NDC80	0.533
ENSG00000148377	91734	IDI2	0.429
ENSG00000135441	2647	BLOC1S1	0.506
ENSG00000256660	387837	CLEC12B	0.375
ENSG00000185565	4045	LSAMP	0.47
ENSG00000136450	6426	SFRS1	0.649
ENSG00000148156	10880	ACTL7B	0.42
ENSG00000187003	10881	ACTL7A	0.425
ENSG00000169951	92595	ZNF764	0.591
ENSG00000153066	51061	TXNDC11	0.431
ENSG00000166603	4160	MC4R	0.445
ENSG00000135437	5959	RDH5	0.482
ENSG00000166682	80975	TMPRSS5	0.448
ENSG00000150048	51267	CLEC1A	0.471
ENSG00000037042	27175	TUBG2	0.476
ENSG00000254772	1937	EEF1G	0.607
ENSG00000183153	125111	GJD3	0.473
ENSG00000168765	2948	GSTM4	0.59
ENSG00000189108	26280	IL1RAPL2	0.461
ENSG00000101596	23347	SMCHD1	0.576
ENSG00000229809	146542	ZNF688	0.524
ENSG00000172243	64581	CLEC7A	0.491
ENSG00000127928	2792	GNGT1	0.399
ENSG00000141959	5211	PFKL	0.558

ENSG00000187240	79659	DYNC2H1	0.513
ENSG00000197345	219927	MRPL21	0.458
ENSG00000168010	89849	ATG16L2	0.522
ENSG00000174446	10302	SNAPC5	0.581
ENSG00000197893	4892	NRAP	0.444
ENSG00000197162	146540	ZNF785	0.496
ENSG00000171490	26156	RSL1D1	0.622
ENSG00000174444	6124	RPL4	0.576
ENSG00000196503	132946	ARL9	0.343
ENSG00000132740	3508	IGHMBP2	0.55
ENSG00000228001	135644	TRIM40	0.322
ENSG00000261516	286075	ZNF707	0.432
ENSG00000079459	2222	FDFT1	0.608
ENSG00000171476	84525	HOPX	0.518
ENSG00000237192	10107	TRIM10	0.579
ENSG00000157060	81626	C1orf14	0.451
ENSG00000167972	21	ABCA3	0.507
ENSG00000254685	8790	FPGT	0.49
ENSG00000164989	203238	C9orf93	0.517
ENSG00000197563	23556	PIGN	0.472
ENSG00000011143	54903	MKS1	0.438
ENSG00000139323	282809	WDR51B	0.483
ENSG00000236183	80740	LY6G6C	0.463
ENSG00000213366	2946	GSTM2	0.532
ENSG00000226634	23564	DDAH2	0.601
ENSG00000145014	93109	TMEM44	0.551
ENSG00000260463	225689	MAPK15	0.447
ENSG00000151748	60485	SAV1	0.523
ENSG00000171557	2266	FGG	0.421
ENSG00000134262	10717	AP4B1	0.558
ENSG00000261380	286077	FAM83H	0.5
ENSG00000133149	56157	TEX13A	0.421
ENSG00000159753	146206	RLTPR	0.525
ENSG00000260968	23513	SCRIB	0.496
ENSG00000185482	246329	STAC3	0.467
ENSG00000198033	7278	TUBA3C	0.469
ENSG00000102595	55757	UGCGL2	0.526
ENSG00000136270	9238	TBRG4	0.541
ENSG00000041802	55341	LSG1	0.568
ENSG00000139133	84920	ALG10	0.457
ENSG00000143919	79823	C2orf34	0.5
ENSG00000143753	8560	DEGS1	0.611
ENSG00000230685	1192	CLIC1	0.536
ENSG00000260485	22827	PUF60	0.541
ENSG00000227147	89870	TRIM15	0.566
ENSG00000086827	9183	ZW10	0.495
ENSG00000048462	608	TNFRSF17	0.432
ENSG00000175334	8815	BANF1	0.505
ENSG00000223666	63943	FKBPL	0.478
ENSG00000241258	27297	RCP9	0.539
ENSG00000244045	147007	TMEM199	0.515
ENSG00000227122	80863	PRRT1	0.558
ENSG00000205323	84324	CIP29	0.452
ENSG00000102977	65057	ACD	0.5

ENSG00000178125	286187	LRRC67	0.481
ENSG00000128040	6691	SPINK2	0.465
ENSG00000114331	23527	CENTB2	0.579
ENSG00000230230	7726	TRIM26	0.558
ENSG00000175315	1474	CST6	0.52
ENSG00000259494	26589	MRPL46	0.502
ENSG00000175294	117144	CATSPER1	0.5
ENSG00000163424	152405	C3orf30	0.385
ENSG00000143748	4931	NVL	0.499
ENSG00000233948	5987	TRIM27	0.637
ENSG00000162063	899	CCNF	0.603
ENSG00000121022	10987	COPS5	0.55
ENSG00000197296	128486	C20orf142	0.578
ENSG00000185736	105	ADARB2	0.463
ENSG00000101074	140902	R3HDML	0.39
ENSG00000101605	8736	MYOM1	0.48
ENSG00000135049	23287	AGTPBP1	0.556
ENSG00000213927	10850	CCL27	0.476
ENSG00000103381	55313	FLJ11151	0.505
ENSG00000184451	2826	CCR10	0.473
ENSG00000115648	79083	MLPH	0.54
ENSG00000131055	84701	COX4I2	0.49
ENSG00000262046	27346	TMEM97	0.654
ENSG00000107669	11101	ATE1	0.554
ENSG00000260533	340371	NRBP2	0.477
ENSG00000262866	90410	IFT20	0.553
ENSG00000099256	56952	PRTFDC1	0.558
ENSG00000169902	8460	TPST1	0.516
ENSG00000117593	55157	DARS2	0.526
ENSG00000128708	8520	HAT1	0.561
ENSG00000181991	64963	MRPS11	0.555
ENSG00000231618	9374	PPT2	0.542
ENSG00000224994	56658	TRIM39	0.466
ENSG00000134184	2944	GSTM1	0.586
ENSG00000262447	7126	TNFAIP1	0.575
ENSG00000084093	5978	REST	0.419
ENSG00000121390	55269	PSPC1	0.545
ENSG00000134716	1573	CYP2J2	0.499
ENSG00000127920	2791	GNG11	0.528
ENSG00000132153	22907	DHX30	0.584
ENSG00000257594	8693	GALNT4	0.487
ENSG00000162062	80178	C16orf59	0.477
ENSG00000151023	219670	C10orf63	0.354
ENSG00000132950	9205	ZMYM5	0.532
ENSG00000231389	3113	HLA-DPA1	0.611
ENSG00000165621	27199	OXGR1	0.38
ENSG00000088325	22974	TPX2	0.559
ENSG00000006282	64847	SPATA20	0.496
ENSG00000141655	8792	TNFRSF11A	0.502
ENSG00000123561	6906	SERPINA7	0.457
ENSG00000174442	55055	ZWILCH	0.514
ENSG00000189058	347	APOD	0.518
ENSG00000113555	51294	PCDH12	0.47
ENSG00000172183	3669	ISG20	0.594

ENSG00000068784	55133	SRBD1	0.477
ENSG00000166822	124491	TMEM170A	0.553
ENSG00000172878	254042	MAP1D	0.463
ENSG00000176945	200958	MUC20	0.527
ENSG00000153044	64946	CENPH	0.51
ENSG00000214309	255374	LOC255374	0.463
ENSG00000188186	389541	C7orf59	0.446
ENSG00000261150	83481	EPPK1	0.472
ENSG00000172927	26579	MYEOV	0.46
ENSG00000267721	147007	TMEM199	0.515
ENSG00000084092	84273	C4orf14	0.492
ENSG00000013561	9604	RNF14	0.581
ENSG00000005381	4353	MPO	0.531
ENSG00000204692	81797	OR12D3	0.409
ENSG00000122679	10268	RAMP3	0.495
ENSG00000261109	5339	PLEC1	0.603
ENSG00000102981	50855	PARD6A	0.487
ENSG00000186281	150763	LOC150763	0.489
ENSG00000267556	23098	SARM1	0.549
ENSG00000162598	127795	C1orf87	0.349
ENSG00000204690	26529	OR12D2	0.438
ENSG00000145113	4585	MUC4	0.576
ENSG00000206472	26531	OR11A1	0.415
ENSG00000223865	3115	HLA-DPB1	0.543
ENSG00000206471	26716	OR2H1	0.517
ENSG00000183196	4166	CHST6	0.387
ENSG00000241863	79897	RPP21	0.468
ENSG00000119328	54942	C9orf6	0.49
ENSG00000031081	57514	CDGAP	0.548
ENSG00000081913	23239	PHLPP	0.533
ENSG00000262062	7448	VTN	0.497
ENSG00000135702	23563	CHST5	0.466
ENSG00000047315	5431	POLR2B	0.524
ENSG00000124207	1434	CSE1L	0.628
ENSG00000101608	10627	MRCL3	0.611
ENSG00000159761	388284	C16orf86	0.4
ENSG00000262186	113235	SLC46A1	0.49
ENSG00000172724	6363	CCL19	0.464
ENSG00000206466	2550	GABBR1	0.533
ENSG00000117601	462	SERPINC1	0.459
ENSG00000175189	3626	INHBC	0.557
ENSG00000232119	28985	MCTS1	0.528
ENSG00000139269	83729	INHBE	0.43
ENSG00000118680	103910	MRLC2	0.575
ENSG00000134539	3824	KLRD1	0.558
ENSG00000183421	54101	RIPK4	0.484
ENSG00000262654	9058	SLC13A2	0.465
ENSG00000255604	7448	VTN	0.497
ENSG00000160226	755	C21orf2	0.623
ENSG00000111087	2735	GLI1	0.479
ENSG00000134201	2949	GSTM5	0.568
ENSG00000149716	220064	ORAOV1	0.49
ENSG00000255819	22914	KLRK1	0.5
ENSG00000171517	23566	LPAR3	0.496

ENSG00000197102	1778	DYNC1H1	0.516
ENSG00000137077	6366	CCL21	0.505
ENSG00000205084	79583	FLJ22167	0.562
ENSG00000133135	79589	RNF128	0.487
ENSG00000179772	2307	FOXS1	0.51
ENSG00000188501	197021	LCTL	0.444
ENSG00000116791	1429	CRYZ	0.533
ENSG00000095777	53904	MYO3A	0.468
ENSG00000264253	8456	FOXN1	0.447
ENSG00000162068	4917	NTN2L	0.572
ENSG00000149305	9177	HTR3B	0.421
ENSG00000166278	717	C2	0.523
ENSG00000234495	5987	TRIM27	0.637
ENSG00000119326	8727	CTNNAL1	0.542
ENSG00000124839	64284	RAB17	0.484
ENSG00000180116	283461	C12orf40	0.436
ENSG00000197093	79690	GAL3ST4	0.474
ENSG00000146678	3484	IGFBP1	0.472
ENSG00000171155	29071	C1GALT1C1	0.518
ENSG00000166736	3359	HTR3A	0.568
ENSG00000141098	81577	GFOD2	0.465
ENSG00000100612	51635	DHRS7	0.633
ENSG00000225351	282890	ZNF311	0.466
ENSG00000261660	84875	PARP10	0.625
ENSG00000233904	3133	HLA-E	0.629
ENSG00000083782	1833	EPYC	0.471
ENSG00000169154	137362	GOT1L1	0.5
ENSG00000264058	125113	KRT222P	0.45
ENSG00000213424	125113	KRT222P	0.45
ENSG00000240592	80864	EGFL8	0.538
ENSG00000139330	11081	KERA	0.448
ENSG00000162604	83941	TM2D1	0.632
ENSG00000139329	4060	LUM	0.523
ENSG00000213809	22914	KLRK1	0.5
ENSG00000162643	126820	WDR63	0.427
ENSG00000165474	2706	GJB2	0.458
ENSG00000169231	7059	THBS3	0.531
ENSG00000102904	55815	TSNAXIP1	0.461
ENSG00000174527	283446	MYO1H	0.414
ENSG00000151962	166863	RBM46	0.463
ENSG00000167916	192666	KRT24	0.424
ENSG00000186895	2248	FGF3	0.448
ENSG00000162066	51005	AMDHD2	0.455
ENSG00000156873	5261	PHKG2	0.494
ENSG00000131620	55107	TMEM16A	0.476
ENSG00000123329	64333	ARHGAP9	0.512
ENSG00000183323	202243	CCDC125	0.448
ENSG00000069764	8399	PLA2G10	0.478
ENSG00000065457	23536	ADAT1	0.524
ENSG00000136750	2572	GAD2	0.568
ENSG00000163453	3490	IGFBP7	0.626
ENSG00000235986	2794	GNL1	0.552
ENSG00000205221	5212	VIT	0.539
ENSG00000235758	10554	AGPAT1	0.573

ENSG00000183542	8302	KLRC4	0.443
ENSG00000066923	10734	STAG3	0.477
ENSG00000196839	100	ADA	0.601
ENSG00000131477	10266	RAMP2	0.488
ENSG00000205810	3823	KLRC3	0.482
ENSG00000204897	147183	KRT25	0.367
ENSG00000149489	6094	ROM1	0.498
ENSG00000206075	5268	SERPINB5	0.537
ENSG00000232289	26531	OR11A1	0.415
ENSG00000205809	3822	KLRC2	0.5
ENSG00000229125	26716	OR2H1	0.517
ENSG00000198874	55253	TYW1	0.503
ENSG00000142185	7226	TRPM2	0.512
ENSG00000165475	51084	CRYL1	0.555
ENSG00000197641	5275	SERPINB13	0.577
ENSG00000261721	2907	GRINA	0.542
ENSG00000131044	164395	TTLL9	0.511
ENSG00000196954	837	CASP4	0.63
ENSG00000134115	27255	CNTN6	0.417
ENSG00000032742	8100	IFT88	0.492
ENSG00000260319	375686	SPATC1	0.441
ENSG00000134545	3821	KLRC1	0.542
ENSG00000007216	9058	SLC13A2	0.465
ENSG00000127990	8910	SGCE	0.522
ENSG00000069535	4129	MAOB	0.528
ENSG00000171631	5031	P2RY6	0.519
ENSG00000260189	54512	EXOSC4	0.571
ENSG00000235222	4439	MSH5	0.508
ENSG00000260226	8733	GPAA1	0.579
ENSG00000113845	51300	C3orf1	0.533
ENSG00000213246	6827	SUPT4H1	0.555
ENSG00000196118	90835	LOC90835	0.46
ENSG00000152061	9910	RABGAP1L	0.604
ENSG00000135040	60560	MAK10	0.579
ENSG00000183426	9284	NPIP	0.403
ENSG00000077420	54518	APBB1IP	0.491
ENSG00000171446	342574	KRT27	0.378
ENSG00000103591	79719	FLJ11506	0.506
ENSG00000085231	6880	TAF9	0.613
ENSG00000205923	752014	CEMP1	0.418
ENSG00000228186	80742	PRR3	0.522
ENSG00000173908	162605	KRT28	0.383
ENSG00000137757	838	CASP5	0.48
ENSG00000132330	51540	SCLY	0.639
ENSG00000065427	3735	KARS	0.576
ENSG00000186395	3858	KRT10	0.562
ENSG00000087460	2778	GNAS	0.691
ENSG00000167920	147184	TMEM99	0.411
ENSG00000232569	2550	GABBR1	0.533
ENSG00000187242	3859	KRT12	0.456
ENSG00000171431	54474	KRT20	0.427
ENSG00000108264	6871	TADA2L	0.528
ENSG00000174038	138724	C9orf131	0.5
ENSG00000175324	27257	LSM1	0.506

ENSG00000143771	29097	CNIH4	0.553
ENSG00000069812	54626	HES2	0.54
ENSG00000102901	80152	CENPT	0.483
ENSG00000100614	5494	PPM1A	0.626
ENSG00000147223	92129	RIPPLY1	0.412
ENSG00000108244	25984	KRT23	0.474
ENSG00000111196	55110	MAGOHB	0.465
ENSG00000117450	5052	PRDX1	0.553
ENSG00000064205	8839	WISP2	0.483
ENSG00000236149	23	ABCF1	0.557
ENSG00000100504	5836	PYGL	0.569
ENSG00000152942	5884	RAD17	0.597
ENSG00000137968	204962	SLC44A5	0.505
ENSG00000165966	29951	PDZRN4	0.413
ENSG00000109906	7704	ZBTB16	0.536
ENSG00000124249	60598	KCNK15	0.471
ENSG00000060140	55359	STYK1	0.524
ENSG00000151233	283464	GLT8D3	0.485
ENSG00000117448	10327	AKR1A1	0.538
ENSG00000144837	51365	PLA1A	0.518
ENSG00000166986	4141	MARS	0.623
ENSG00000103599	64799	IQCH	0.442
ENSG00000174851	10897	YIF1A	0.507
ENSG00000144488	339768	ESPNL	0.476
ENSG00000116752	10286	BCAS2	0.47
ENSG00000168040	8772	FADD	0.52
ENSG00000185664	6490	SILV	0.5
ENSG00000156735	9530	BAG4	0.484
ENSG00000184719	55328	C10orf59	0.47
ENSG00000163958	131540	ZDHHC19	0.434
ENSG00000115808	6801	STRN	0.513
ENSG00000061918	2983	GUCY1B3	0.54
ENSG00000122674	51622	C7orf28A	0.537
ENSG00000166741	4837	NNMT	0.561
ENSG00000176160	124535	HSF5	0.46
ENSG00000121377	50837	TAS2R7	0.436
ENSG00000188037	1180	CLCN1	0.49
ENSG00000085788	23259	DDHD2	0.53
ENSG00000121381	50835	TAS2R9	0.469
ENSG00000111215	11272	PRR4	0.447
ENSG00000101336	3055	HCK	0.554
ENSG00000225452	6048	RNF5	0.596
ENSG00000163959	200931	OSTalpha	0.471
ENSG00000155875	158297	FAM154A	0.361
ENSG00000107959	10531	PITRM1	0.514
ENSG00000180425	54494	C11orf71	0.479
ENSG00000161326	11072	DUSP14	0.54
ENSG00000147874	54801	FAM29A	0.462
ENSG00000257127	574028	CLLU1	0.463
ENSG00000006114	11276	AP1GBP1	0.493
ENSG00000132323	80895	ILKAP	0.493
ENSG00000214215	338809	LOC338809	0.521
ENSG00000204889	125115	KRT40	0.488
ENSG00000230514	177	AGER	0.548

ENSG00000204022	142910	LIPJ	0.5
ENSG00000206468	10537	UBD	0.48
ENSG00000121577	64091	POPDC2	0.488
ENSG00000183690	80258	EFHC2	0.551
ENSG00000206467	7932	OR2H2	0.526
ENSG00000150456	221143	N6AMT2	0.381
ENSG00000099385	9274	BCL7C	0.494
ENSG00000161217	5130	PCYT1A	0.551
ENSG00000188599	9284	NPIP	0.403
ENSG00000182333	8513	LIPF	0.433
ENSG00000232641	4340	MOG	0.581
ENSG00000230995	5514	PPP1R10	0.598
ENSG00000231887	5554	PRH1	0.509
ENSG00000212128	50838	TAS2R13	0.411
ENSG00000156968	255027	MPV17L	0.444
ENSG00000212899	85293	KRTAP3-3	0.365
ENSG00000212900	83897	KRTAP3-2	0.36
ENSG00000212901	83896	KRTAP3-1	0.356
ENSG00000212126	259296	TAS2R50	0.409
ENSG00000008869	54497	HEATR5B	0.524
ENSG00000136805	23732	C9orf4	0.467
ENSG00000256394	51802	ACCN5	0.474
ENSG00000221829	2189	FANCG	0.512
ENSG00000213123	255758	TCTEX1D2	0.45
ENSG00000197870	5544	PRB3	0.632
ENSG00000230657	5545	PRB4	0.474
ENSG00000251655	5542	PRB1	0.516
ENSG00000205678	253017	SRD5A2L2	0.355
ENSG00000183978	28958	CCDC56	0.562
ENSG00000151790	6999	TDO2	0.498
ENSG00000083223	79670	ZCCHC6	0.534
ENSG00000121335	653247	PRB2	0.55
ENSG00000204366	221527	ZBTB12	0.46
ENSG00000243649	629	CFB	0.59
ENSG00000174791	9610	RIN1	0.56
ENSG00000091428	11069	RAPGEF4	0.468
ENSG00000069509	139341	FUNDC1	0.494
ENSG00000163960	26043	UBXD7	0.564
ENSG00000147872	123	ADFP	0.596
ENSG00000213416	83755	KRTAP4-12	0.393
ENSG00000116748	270	AMPD1	0.482
ENSG00000189037	63904	DUSP21	0.432
ENSG00000139168	85437	ZCRB1	0.536
ENSG00000198271	85289	KRTAP4-5	0.358
ENSG00000103363	6923	TCEB2	0.562
ENSG00000196156	85290	KRTAP4-3	0.373
ENSG00000244537	85291	KRTAP4-2	0.432
ENSG00000198443	85285	KRTAP4-1	0.353
ENSG00000226898	10537	UBD	0.48
ENSG00000239886	83899	KRTAP9-2	0.378
ENSG00000139174	144165	PRICKLE1	0.562
ENSG00000204873	83900	KRTAP9-3	0.338
ENSG00000224319	7932	OR2H2	0.526
ENSG00000165480	221150	C13orf3	0.496

ENSG00000117054	34	ACADM	0.534
ENSG00000103355	260429	PRSS33	0.449
ENSG00000198924	9937	DCLRE1A	0.476
ENSG00000165078	57094	CPA6	0.477
ENSG00000144028	23020	ASCC3L1	0.545
ENSG00000160844	352954	tcag7.1177	0.446
ENSG00000213413	79037	PVRIG	0.476
ENSG00000227420	28973	MRPS18B	0.57
ENSG00000226589	7407	VARS	0.588
ENSG00000147050	7403	UTX	0.553
ENSG00000125037	55831	TMEM111	0.556
ENSG00000234096	4340	MOG	0.581
ENSG00000095110	120400	FAM55A	0.431
ENSG00000152910	85445	CNTNAP4	0.489
ENSG00000163961	165918	RNF168	0.396
ENSG00000250565	90423	ATP6V1E2	0.492
ENSG00000121716	29990	PILRB	0.517
ENSG00000072864	54820	NDE1	0.512
ENSG00000214097	255798	C3orf43	0.395
ENSG00000184302	4990	SIX6	0.504
ENSG00000007038	10942	PRSS21	0.466
ENSG00000241595	85280	KRTAP9-4	0.325
ENSG00000126778	6495	SIX1	0.501
ENSG00000185798	348793	WDR53	0.441
ENSG00000136250	313	AOAH	0.473
ENSG00000186860	83902	KRTAP17-1	0.405
ENSG00000223858	346171	ZFP57	0.388
ENSG00000223752	79969	C6orf134	0.503
ENSG00000204104	26146	TRAF3IP1	0.464
ENSG00000234487	3134	HLA-F	0.626
ENSG00000129317	83448	PUS7L	0.455
ENSG00000046889	80243	DEPDC2	0.442
ENSG00000108417	8688	KRT37	0.446
ENSG00000162078	124220	LOC124220	0.476
ENSG00000177398	89766	UMODL1	0.467
ENSG00000206432	645369	TTMA	0.526
ENSG00000148459	23590	PDSS1	0.517
ENSG00000144554	2177	FANCD2	0.5
ENSG00000198001	51135	IRAK4	0.476
ENSG00000162194	79081	C11orf48	0.484
ENSG00000174004	375387	LRRC33	0.41
ENSG00000080823	5891	RAGE	0.53
ENSG00000173141	78988	MRP63	0.591
ENSG00000174684	11041	B3GNT1	0.51
ENSG00000165714	118426	LOH12CR1	0.435
ENSG00000196865	374354	NHLRC2	0.538
ENSG00000240563	54596	L1TD1	0.435
ENSG00000122512	5395	PMS2	0.534
ENSG00000171360	8687	KRT38	0.469
ENSG00000174669	3177	SLC29A2	0.517
ENSG00000215570	253832	ZDHHC20	0.566
ENSG00000256043	1519	CTSO	0.574
ENSG00000236826	57819	LSM2	0.551
ENSG00000203737	9293	GPR52	0.444

ENSG00000159840	7791	ZYX	0.562
ENSG00000162076	114984	FLYWCH2	0.483
ENSG00000145431	56034	PDGFC	0.554
ENSG00000137634	54827	FAM55D	0.43
ENSG00000151665	5281	PIGF	0.584
ENSG00000137752	834	CASP1	0.646
ENSG00000236251	3305	HSPA1L	0.463
ENSG00000059122	84256	FLYWCH1	0.568
ENSG00000080572	139212	CXorf41	0.429
ENSG00000020426	4331	MNAT1	0.547
ENSG00000021300	58473	PLEKHB1	0.542
ENSG00000197079	3886	KRT35	0.436
ENSG00000152133	253635	CCDC75	0.491
ENSG00000136273	3364	HUS1	0.551
ENSG00000198015	28977	MRPL42	0.549
ENSG00000158497	57824	HMHB1	0.494
ENSG00000164047	820	CAMP	0.477
ENSG00000214756	751071	LOC751071	0.528
ENSG00000237724	3303	HSPA1A	0.537
ENSG00000138134	57559	STAMBPL1	0.484
ENSG00000126337	8689	KRT36	0.419
ENSG00000204922	790955	LOC790955	0.465
ENSG00000183150	2842	GPR19	0.495
ENSG00000162191	51035	LOC51035	0.589
ENSG00000171401	3860	KRT13	0.492
ENSG00000102898	10204	NUTF2	0.542
ENSG00000236353	5089	PBX2	0.584
ENSG00000085514	29992	PILRA	0.553
ENSG00000177628	2629	GBA	0.667
ENSG00000139531	6821	SUOX	0.539
ENSG00000165487	221154	EFHA1	0.526
ENSG00000213782	51202	DDX47	0.483
ENSG00000158683	168507	PKD1L1	0.452
ENSG00000237216	3135	HLA-G	0.612
ENSG00000181013	284083	C17orf47	0.385
ENSG00000145241	1060	CENPC1	0.528
ENSG00000038358	23644	EDC4	0.495
ENSG00000076382	10615	SPAG5	0.545
ENSG00000227858	346171	ZFP57	0.388
ENSG00000091181	3568	IL5RA	0.578
ENSG00000152939	153562	MARVELD2	0.52
ENSG00000131650	79412	KREMEN2	0.436
ENSG00000106305	7965	JTV1	0.596
ENSG00000175894	54084	C21orf29	0.5
ENSG00000166971	64400	AKTIP	0.514
ENSG00000171346	3866	KRT15	0.47
ENSG00000155428	378108	TRIM74	0.529
ENSG00000055332	5610	EIF2AK2	0.47
ENSG00000139974	145389	SLC38A6	0.51
ENSG00000035720	26228	STAP1	0.452
ENSG00000131480	314	AOC2	0.467
ENSG00000005421	5444	PON1	0.499
ENSG00000137955	5876	RABGGTB	0.592
ENSG00000086232	27102	EIF2AK1	0.62

ENSG00000175121	149708	WFDC5	0.42
ENSG00000174547	65003	MRPL11	0.509
ENSG00000229215	3105	HLA-A	0.588
ENSG00000105852	5446	PON3	0.495
ENSG00000197728	6231	RPS26	0.598
ENSG00000078487	55063	ZCWPW1	0.464
ENSG00000130414	4705	NDUFA10	0.51
ENSG00000173818	284131	FLJ35220	0.484
ENSG00000168703	128488	WFDC12	0.425
ENSG00000177119	196527	TMEM16F	0.504
ENSG00000124102	5266	PI3	0.567
ENSG00000204397	114769	COP1	0.566
ENSG00000120333	63931	MRPS14	0.594
ENSG00000124233	6406	SEMG1	0.434
ENSG00000255221	440068	INCA	0.488
ENSG00000167524	124923	FLJ25006	0.526
ENSG00000139921	81542	TXNDC1	0.589
ENSG00000227715	3105	HLA-A	0.588
ENSG00000224501	3303	HSPA1A	0.537
ENSG00000124157	6407	SEMG2	0.447
ENSG00000013583	50865	HEBP1	0.515
ENSG00000165568	83592	AKR1CL2	0.46
ENSG00000163975	4241	MFI2	0.483
ENSG00000109917	8882	ZNF259	0.506
ENSG00000255501	59082	ICEBERG	0.463
ENSG00000177363	221091	LRRN4CL	0.465
ENSG00000173261	153770	PLAC8L1	0.472
ENSG00000124107	6590	SLPI	0.513
ENSG00000137154	6194	RPS6	0.567
ENSG00000128973	54982	CLN6	0.49
ENSG00000171345	3880	KRT19	0.525
ENSG00000164049	285231	FBXW12	0.489
ENSG00000111305	83445	GSG1	0.481
ENSG00000165046	137994	LETM2	0.492
ENSG00000167394	79759	ZNF668	0.476
ENSG00000226710	50854	C6orf48	0.542
ENSG00000102683	6445	SGCG	0.44
ENSG00000136275	80099	FLJ21075	0.415
ENSG00000186847	3861	KRT14	0.496
ENSG00000052723	80143	RP5-1000E10.4	0.632
ENSG00000233641	221545	C6orf136	0.518
ENSG00000026103	355	FAS	0.655
ENSG00000169018	10116	FEM1B	0.613
ENSG00000164744	256979	SUNC1	0.325
ENSG00000177291	219770	GJD4	0.333
ENSG00000177076	340485	ASAH3L	0.362
ENSG00000169372	8738	CRADD	0.567
ENSG00000184697	9074	CLDN6	0.453
ENSG00000148513	91074	ANKRD30A	0.356
ENSG00000186832	3868	KRT16	0.48
ENSG00000236808	30834	ZNRD1	0.542
ENSG00000163705	115795	C3orf24	0.446
ENSG00000233561	8449	DHX16	0.471
ENSG00000136040	10154	PLXNC1	0.601

ENSG00000107890	22852	ANKRD26	0.555
ENSG00000091436	51776	ZAK	0.529
ENSG00000108825	80755	AARSD1	0.484
ENSG00000198765	6847	SYCP1	0.535
ENSG00000250305	57604	C8orf79	0.449
ENSG00000091262	368	ABCC6	0.572
ENSG00000254999	55845	C3orf10	0.45
ENSG00000124159	8785	MATN4	0.442
ENSG00000151006	339105	POL3S	0.441
ENSG00000227315	4758	NEU1	0.519
ENSG00000170379	285966	FAM115C	0.536
ENSG00000204356	7936	RDBP	0.505
ENSG00000254986	10072	DPP3	0.504
ENSG00000130305	55695	NSUN5	0.586
ENSG00000243489	386678	KRTAP10-11	0.329
ENSG00000164746	136288	C7orf57	0.342
ENSG00000005981	51666	ASB4	0.592
ENSG00000057468	4438	MSH4	0.446
ENSG00000108384	5889	RAD51C	0.635
ENSG00000027001	4285	MIPEP	0.57
ENSG00000187134	1645	AKR1C1	0.616
ENSG00000164125	51313	C4orf18	0.506
ENSG00000172893	1717	DHCR7	0.61
ENSG00000234243	63940	GPSM3	0.56
ENSG00000160767	10712	C1orf2	0.504
ENSG00000188959	401546	C9orf152	0.455
ENSG00000178602	150677	OTOS	0.39
ENSG00000110921	4598	MVK	0.601
ENSG00000215764	3803	KIR2DL2	0.475
ENSG00000231247	170954	KIAA1949	0.563
ENSG00000164050	5364	PLXNB1	0.583
ENSG00000185128	84218	TBC1D3	0.452
ENSG00000146755	135892	TRIM50	0.515
ENSG00000183696	7378	UPP1	0.566
ENSG00000103507	10295	BCKDK	0.503
ENSG00000175581	51642	MRPL48	0.508
ENSG00000124232	11317	RBPJL	0.45
ENSG00000158560	1780	DYNC1I1	0.528
ENSG00000144504	51281	ANKMY1	0.515
ENSG00000147121	55634	ZNF673	0.472
ENSG00000136810	7295	TXN	0.615
ENSG00000077800	8468	FKBP6	0.484
ENSG00000223355	4855	NOTCH4	0.497
ENSG00000072756	51095	TRNT1	0.531
ENSG00000086288	51314	TXNDC3	0.425
ENSG00000141564	57521	KIAA1303	0.5
ENSG00000231479	80736	SLC44A4	0.452
ENSG00000160813	221908	C7orf47	0.512
ENSG00000237403	6992	PPP1R11	0.524
ENSG00000235443	30834	ZNRD1	0.542
ENSG00000230332	80352	RNF39	0.512
ENSG00000197681	84218	TBC1D3	0.452
ENSG00000204193	255220	TXNDC8	0.425
ENSG00000080561	11043	MID2	0.507

ENSG00000185955	402573	LOC402573	0.381
ENSG00000154007	127247	ASB17	0.405
ENSG00000258484	246777	SPESP1	0.422
ENSG00000135047	1514	CTSL1	0.567
ENSG00000233314	6992	PPP1R11	0.524
ENSG00000100865	51550	CINP	0.491
ENSG00000101158	51497	TH1L	0.517
ENSG00000142327	57140	RNPEPL1	0.489
ENSG00000188352	54914	KIAA1797	0.519
ENSG00000255346	79400	NOX5	0.477
ENSG00000143207	64326	RFWD2	0.514
ENSG00000198885	150771	KIAA1754L	0.424
ENSG00000065518	4710	NDUFB4	0.493
ENSG00000196663	9895	KIAA0329	0.523
ENSG00000113924	3081	HGD	0.555
ENSG00000110245	345	APOC3	0.458
ENSG00000189169	386685	KRTAP10-12	0.369
ENSG00000132581	6388	SDF2	0.507
ENSG00000205572	8293	SERF1A	0.468
ENSG00000118137	335	APOA1	0.498
ENSG00000140718	79068	FTO	0.548
ENSG00000257961	201176	ARHGAP27	0.526
ENSG00000008256	9265	PSCD3	0.485
ENSG00000235773	11270	NRM	0.523
ENSG00000140876	283927	NUDT7	0.543
ENSG00000121152	23397	NCAPH	0.53
ENSG00000106351	3268	HRBL	0.636
ENSG00000151632	1646	AKR1C2	0.594
ENSG00000115816	10153	CEBPZ	0.533
ENSG00000223531	11074	TRIM31	0.6
ENSG00000251192	641339	ZNF674	0.398
ENSG00000168679	9122	SLC16A4	0.52
ENSG00000237881	10665	C6orf10	0.434
ENSG00000254806	90196	SYS1	0.488
ENSG00000103510	84148	MYST1	0.635
ENSG00000171724	57687	KIAA1576	0.5
ENSG00000171509	59350	RXFP1	0.442
ENSG00000142330	11132	CAPN10	0.567
ENSG00000204227	6015	RING1	0.576
ENSG00000189180	7581	ZNF33A	0.554
ENSG00000213215	26211	OR2F1	0.44
ENSG00000236967	80352	RNF39	0.512
ENSG00000147119	56548	CHST7	0.487
ENSG00000240654	338872	C1QTNF9	0.478
ENSG00000205571	6606	SMN1	0.657
ENSG00000205850	542767	PCOTH	0.415
ENSG00000102699	143	PARP4	0.54
ENSG00000224587	9656	MDC1	0.575
ENSG00000236759	10919	EHMT2	0.557
ENSG00000172890	55191	NADSYN1	0.5
ENSG00000152404	143884	CWF19L2	0.526
ENSG00000160180	7033	TFF3	0.54
ENSG00000159596	51249	TMEM69	0.452
ENSG00000141086	1506	CTRL	0.495

ENSG00000179715	91523	FAM113B	0.505
ENSG00000124251	27296	C20orf10	0.512
ENSG00000156381	122416	ANKRD9	0.531
ENSG00000005175	79657	RPAP3	0.479
ENSG00000122417	57489	ODF2L	0.567
ENSG00000160181	7032	TFF2	0.492
ENSG00000065923	84679	SLC9A7	0.495
ENSG00000224542	11074	TRIM31	0.6
ENSG00000153802	9407	TMPRSS11D	0.484
ENSG00000162222	283237	TTC9C	0.518
ENSG00000205220	5699	PSMB10	0.504
ENSG00000134200	7252	TSHB	0.458
ENSG00000224496	135644	TRIM40	0.322
ENSG00000178397	84792	MGC12966	0.482
ENSG00000160584	23387	KIAA0999	0.676
ENSG00000237703	10107	TRIM10	0.579
ENSG00000236399	135644	TRIM40	0.322
ENSG00000086015	23139	MAST2	0.618
ENSG00000229381	10107	TRIM10	0.579
ENSG00000003509	55471	C2orf56	0.488
ENSG00000230726	3122	HLA-DRA	0.555
ENSG00000139641	23344	FAM62A	0.531
ENSG00000138135	9023	CH25H	0.475
ENSG00000178127	4729	NDUFV2	0.583
ENSG00000244274	55861	DBNDD2	0.447
ENSG00000111405	8909	P11	0.473
ENSG00000257339	9842	PLEKHM1	0.56
ENSG00000184752	55967	NDUFA12	0.46
ENSG00000175575	80227	PAAF1	0.526
ENSG00000160182	7031	TFF1	0.481
ENSG00000107798	3988	LIPA	0.576
ENSG00000235259	89870	TRIM15	0.566
ENSG00000196843	10865	ARID5A	0.532
ENSG00000213398	3931	LCAT	0.559
ENSG00000176842	10265	IRX5	0.529
ENSG00000211689	445347	TARP	0.686
ENSG00000186001	84859	LRCH3	0.536
ENSG00000134248	10542	HBXIP	0.591
ENSG00000159387	79190	IRX6	0.472
ENSG00000075673	479	ATP12A	0.492
ENSG00000157017	51738	GHRL	0.548
ENSG00000235905	89870	TRIM15	0.566
ENSG00000204351	6499	SKIV2L	0.492
ENSG00000162408	79707	NOL9	0.524
ENSG00000137760	91801	ALKBH8	0.454
ENSG00000174100	84311	MRPL45	0.488
ENSG00000172058	8293	SERF1A	0.468
ENSG00000120798	7181	NR2C1	0.631
ENSG00000168002	5436	POLR2G	0.545
ENSG00000131469	6155	RPL27	0.504
ENSG00000143125	84432	PROK1	0.414
ENSG00000143105	3744	KCNA10	0.424
ENSG00000228284	3117	HLA-DQA1	0.533
ENSG00000068079	3430	IFI35	0.544

ENSG00000178226	146547	PRSS36	0.366
ENSG00000127922	7979	SHFM1	0.528
ENSG00000167525	147011	PROCA1	0.553
ENSG00000178623	2859	GPR35	0.511
ENSG00000173662	80835	TAS1R1	0.491
ENSG00000143119	963	CD53	0.543
ENSG00000160183	64699	TMPRSS3	0.471
ENSG00000162227	10629	TAF6L	0.624
ENSG00000188921	401494	PTPLAD2	0.422
ENSG00000124155	51604	PIGT	0.492
ENSG00000184502	2520	GAST	0.484
ENSG00000030304	4593	MUSK	0.516
ENSG00000227739	203068	TUBB	0.613
ENSG00000228881	7726	TRIM26	0.558
ENSG00000164535	221955	DAGLB	0.512
ENSG00000258372	51326	ARL17P1	0.507
ENSG00000042813	11055	ZPBP	0.432
ENSG00000132855	27329	ANGPTL3	0.45
ENSG00000196693	7582	ZNF33B	0.503
ENSG00000101160	1522	CTSZ	0.594
ENSG00000231286	3119	HLA-DQB1	0.68
ENSG00000196139	8644	AKR1C3	0.542
ENSG00000182628	348235	FAM33A	0.535
ENSG00000121931	55791	C1orf103	0.475
ENSG00000166126	81693	AMN	0.472
ENSG00000196636	57001	ACN9	0.5
ENSG00000231641	7726	TRIM26	0.558
ENSG00000141696	10609	SC65	0.495
ENSG00000120279	80177	MYCT1	0.449
ENSG00000114473	84223	IQCG	0.435
ENSG00000134419	6210	RPS15A	0.548
ENSG00000125703	84938	ATG4C	0.596
ENSG00000187726	374407	DNAJB13	0.446
ENSG00000185873	132724	TMPRSS11B	0.391
ENSG00000168589	83657	DYNLRB2	0.445
ENSG00000171855	3456	IFNB1	0.441
ENSG00000177047	3467	IFNW1	0.432
ENSG00000137080	3452	IFNA21	0.459
ENSG00000177992	286234	C9orf79	0.478
ENSG00000236637	3441	IFNA4	0.488
ENSG00000214042	3444	IFNA7	0.455
ENSG00000112029	26271	FBXO5	0.45
ENSG00000172062	6606	SMN1	0.657
ENSG00000101162	81027	TUBB1	0.444
ENSG00000186803	3446	IFNA10	0.448
ENSG00000156345	23552	CCRK	0.477
ENSG00000204859	3104	ZBTB48	0.49
ENSG00000257413	91746	YTHDC1	0.581
ENSG00000241233	57830	KRTAP5-8	0.405
ENSG00000170540	23204	ARL6IP1	0.529
ENSG00000165733	9790	BMS1	0.5
ENSG00000178498	196403	DTX3	0.574
ENSG00000172482	189	AGXT	0.538
ENSG00000124172	514	ATP5E	0.506

ENSG00000147885	3449	IFNA16	0.478
ENSG00000132972	56163	RNF17	0.556
ENSG00000234829	3451	IFNA17	0.456
ENSG00000110660	54733	SLC35F2	0.514
ENSG00000228083	3448	IFNA14	0.491
ENSG00000068489	55771	PRR11	0.482
ENSG00000171503	2110	ETFDH	0.563
ENSG00000196511	27010	TPK1	0.482
ENSG00000211584	55652	FLJ20489	0.61
ENSG00000225201	3133	HLA-E	0.629
ENSG00000101844	115201	ATG4A	0.531
ENSG00000254997	3846	KRTAP5-9	0.474
ENSG00000204571	440051	KRTAP5-11	0.333
ENSG00000182899	6165	RPL35A	0.625
ENSG00000156171	128338	TMEM77	0.516
ENSG00000248746	89	ACTN3	0.438
ENSG00000240771	115557	GEFT	0.427
ENSG00000167098	140732	SPAG4L	0.564
ENSG00000206301	3118	HLA-DQA2	0.706
ENSG00000042062	140876	FAM65C	0.514
ENSG00000137975	9635	CLCA2	0.573
ENSG00000116183	60676	PAPPA2	0.572
ENSG00000168569	79064	MGC3196	0.502
ENSG00000173627	403314	APOBEC4	0.44
ENSG00000112031	54516	MTRF1L	0.503
ENSG00000230308	56658	TRIM39	0.466
ENSG00000087253	54947	LPCAT2	0.586
ENSG00000185475	374395	TMEM179B	0.446
ENSG00000147596	63978	PRDM14	0.453
ENSG00000006128	6863	TAC1	0.444
ENSG00000233816	3447	IFNA13	0.428
ENSG00000228581	2794	GNL1	0.552
ENSG00000188379	3440	IFNA2	0.427
ENSG00000120242	3445	IFNA8	0.429
ENSG00000197919	3439	IFNA1	0.427
ENSG00000172478	79919	C2orf54	0.444
ENSG00000184995	338376	IFNE1	0.378
ENSG00000235017	717	C2	0.523
ENSG00000099810	4507	MTAP	0.612
ENSG00000070669	440	ASNS	0.539
ENSG00000198752	9578	CDC42BPB	0.451
ENSG00000162413	9903	KLHL21	0.535
ENSG00000167447	55181	C17orf71	0.461
ENSG00000156475	5521	PPP2R2B	0.533
ENSG00000174080	8722	CTSF	0.527
ENSG00000173212	126868	C1orf161	0.435
ENSG00000241910	3112	HLA-DOB	0.518
ENSG00000125384	5732	PTGER2	0.506
ENSG00000258085	7366	UGT2B15	0.512
ENSG00000106333	5118	PCOLCE	0.555
ENSG00000087301	57544	TXNDC16	0.516
ENSG00000257745	7365	UGT2B10	0.513
ENSG00000103121	56942	C16orf61	0.524
ENSG00000179331	54734	RAB39	0.411

ENSG00000078898	80341	BPIL1	0.388
ENSG00000101443	10406	WFDC2	0.499
ENSG00000262515	23284	LPHN3	0.558
ENSG00000171497	5481	PPID	0.57
ENSG00000134255	10390	CEPT1	0.514
ENSG00000232112	51372	CCDC72	0.505
ENSG00000088035	29929	ALG6	0.516
ENSG00000163399	476	ATP1A1	0.531
ENSG00000257437	4137	MAPT	0.645
ENSG00000164053	84126	ATRIP	0.478
ENSG00000257679	79799	UGT2A3	0.423
ENSG00000137818	6176	RPLP1	0.53
ENSG00000147889	1029	CDKN2A	0.588
ENSG00000119917	3437	IFIT3	0.535
ENSG00000017373	80725	SNIP	0.472
ENSG00000075239	38	ACAT1	0.536
ENSG00000198860	116461	TSEN15	0.579
ENSG00000241779	79897	RPP21	0.468
ENSG00000186191	149954	C20orf186	0.43
ENSG00000135502	65012	SLC26A10	0.482
ENSG00000196465	140465	MYL6B	0.529
ENSG00000087128	28983	TMPRSS11E	0.513
ENSG00000160255	3689	ITGB2	0.514
ENSG00000185745	3434	IFIT1	0.565
ENSG00000206412	2794	GNL1	0.552
ENSG00000249437	4671	NAIP	0.491
ENSG00000103490	29108	PYCARD	0.538
ENSG00000256812	84290	CAPNS2	0.46
ENSG00000243543	140870	WFDC6	0.433
ENSG00000103546	6530	SLC6A2	0.597
ENSG00000092841	4637	MYL6	0.638
ENSG00000138433	9541	CIR	0.48
ENSG00000016490	1179	CLCA1	0.455
ENSG00000135175	4951	LOC4951	0.433
ENSG00000173992	9973	CCS	0.501
ENSG00000131050	140683	C20orf70	0.42
ENSG00000143742	6726	SRP9	0.535
ENSG00000134291	79022	TMEM106C	0.531
ENSG00000205436	91828	C14orf73	0.435
ENSG00000136247	55146	ZDHHC4	0.529
ENSG00000038945	4481	MSR1	0.626
ENSG00000158901	90199	WFDC8	0.477
ENSG00000115828	25797	QPCT	0.523
ENSG00000143819	2052	EPHX1	0.562
ENSG00000106536	11281	POU6F2	0.5
ENSG00000177238	493829	TRIM72	0.317
ENSG00000169302	202374	STK32A	0.55
ENSG00000132436	63979	FIGNL1	0.508
ENSG00000170364	6419	SETMAR	0.477
ENSG00000169900	260434	PYDC1	0.454
ENSG00000197888	7367	UGT2B17	0.497
ENSG00000196620	7366	UGT2B15	0.512
ENSG00000232280	10211	FLOT1	0.631
ENSG00000016602	22802	CLCA4	0.462

ENSG00000137078	27240	SIT1	0.45
ENSG00000164123	152940	C4orf45	0.44
ENSG00000185215	7127	TNFAIP2	0.609
ENSG00000130045	158046	NXNL2	0.419
ENSG00000205730	162073	LOC162073	0.609
ENSG00000205356	25851	DKFZP434B0335	0.593
ENSG00000103528	51760	SYT17	0.501
ENSG00000141096	64180	DPEP3	0.423
ENSG00000109181	7365	UGT2B10	0.513
ENSG00000135220	79799	UGT2A3	0.423
ENSG00000141698	115024	NT5C3L	0.504
ENSG00000148082	53358	SHC3	0.468
ENSG00000176399	63951	DMRTA1	0.382
ENSG00000237599	6891	TAP2	0.58
ENSG00000023608	6617	SNAPC1	0.518
ENSG00000131059	128861	C20orf71	0.398
ENSG00000106327	7036	TFR2	0.574
ENSG00000168014	26005	C2CD3	0.565
ENSG00000233564	80742	PRR3	0.522
ENSG00000167261	64174	DPEP2	0.487
ENSG00000180205	259240	WFDC9	0.533
ENSG00000227222	8449	DHX16	0.471
ENSG00000180083	259239	WFDC11	0.349
ENSG00000257793	1394	CRHR1	0.585
ENSG00000145736	2966	GTF2H2	0.486
ENSG00000231500	6222	RPS18	0.516
ENSG00000204348	1797	DOM3Z	0.616
ENSG00000198848	1066	CES1	0.519
ENSG00000248643	5936	RBM4	0.563
ENSG00000236342	23	ABCF1	0.557
ENSG00000116815	965	CD58	0.626
ENSG00000241127	57002	C7orf36	0.457
ENSG00000139973	83851	SYT16	0.357
ENSG00000230341	170954	KIAA1949	0.563
ENSG00000143627	5313	PKLR	0.523
ENSG00000150201	53828	FXYD4	0.376
ENSG00000168634	164237	WFDC13	0.356
ENSG00000239605	285051	C2orf61	0.415
ENSG00000124116	140686	WFDC3	0.456
ENSG00000132437	1644	DDC	0.54
ENSG00000168454	84203	TXNDC2	0.349
ENSG00000166451	55839	CENPN	0.603
ENSG00000198610	1109	AKR1C4	0.447
ENSG00000078579	26281	FGF20	0.475
ENSG00000187908	1755	DMBT1	0.512
ENSG00000138182	9585	MPHOSPH1	0.542
ENSG00000169126	55130	ARMC4	0.444
ENSG00000123975	1164	CKS2	0.575
ENSG00000000419	8813	DPM1	0.534
ENSG00000226201	5696	PSMB8	0.535
ENSG00000171234	7364	UGT2B7	0.566
ENSG00000144306	79634	SCRN3	0.51
ENSG00000228854	11270	NRM	0.523
ENSG00000213689	11277	TREX1	0.57

ENSG00000064886	1117	CHI3L2	0.525
ENSG00000137496	10068	IL18BP	0.509
ENSG00000103540	9738	CP110	0.481
ENSG00000154856	147495	APCDD1	0.517
ENSG00000140015	27133	KCNH5	0.482
ENSG00000161594	317719	KLHL10	0.5
ENSG00000108846	8714	ABCC3	0.612
ENSG00000183291	9403	15-Sep	0.562
ENSG00000147592	51110	LACTB2	0.522
ENSG00000152556	5213	PFKM	0.563
ENSG00000178502	55175	KLHL11	0.416
ENSG00000237155	8870	IER3	0.564
ENSG00000170279	202865	C7orf33	0.478
ENSG00000119888	4072	TACSTD1	0.504
ENSG00000140678	3687	ITGAX	0.542
ENSG00000213759	10720	UGT2B11	0.554
ENSG00000196074	10388	SYCP2	0.467
ENSG00000116824	914	CD2	0.513
ENSG00000240508	5698	PSMB9	0.561
ENSG00000125999	92747	C20orf114	0.487
ENSG00000237900	221527	ZBTB12	0.46
ENSG00000107159	768	CA9	0.508
ENSG00000235863	8705	B3GALT4	0.488
ENSG00000227057	9277	WDR46	0.512
ENSG00000234012	9656	MDC1	0.575
ENSG00000239754	629	CFB	0.59
ENSG00000160752	2224	FDPS	0.535
ENSG00000137332	780	DDR1	0.636
ENSG00000115841	151393	FAM82A1	0.484
ENSG00000196793	8187	ZNF239	0.467
ENSG00000134265	8774	NAPG	0.536
ENSG00000135226	54490	UGT2B28	0.457
ENSG00000169495	203100	HTRA4	0.553
ENSG00000006453	55971	BAIAP2L1	0.539
ENSG00000114988	81562	LMAN2L	0.471
ENSG00000101391	51654	CDK5RAP1	0.502
ENSG00000230705	6890	TAP1	0.552
ENSG00000100519	5706	PSMC6	0.544
ENSG00000178462	79861	TUBAL3	0.48
ENSG00000103544	57020	C16orf62	0.49
ENSG00000198467	7169	TPM2	0.622
ENSG00000235291	5514	PPP1R10	0.598
ENSG00000166455	123775	C16orf46	0.583
ENSG00000096872	80173	IFT74	0.564
ENSG00000134216	27159	CHIA	0.454
ENSG00000140905	2653	GCSH	0.515
ENSG00000126785	57381	RHOJ	0.633
ENSG00000204344	8859	STK19	0.565
ENSG00000198298	220992	ZNF485	0.482
ENSG00000026559	3755	KCNG1	0.576
ENSG00000145414	92345	NAF1	0.416
ENSG00000095002	4436	MSH2	0.585
ENSG00000156886	3681	ITGAD	0.478
ENSG00000168003	6520	SLC3A2	0.575

ENSG00000160188	89765	RSPH1	0.422
ENSG00000074706	26034	PIP3-E	0.468
ENSG00000166323	160140	C11orf65	0.448
ENSG00000166473	114780	PKD1L2	0.482
ENSG00000169740	7580	ZNF32	0.485
ENSG00000156885	1339	COX6A2	0.489
ENSG00000159398	221223	CES7	0.474
ENSG00000134256	9398	IGSF2	0.45
ENSG00000156096	7363	UGT2B4	0.415
ENSG00000182810	55794	DDX28	0.646
ENSG00000164128	4886	NPY1R	0.468
ENSG00000242386	3109	HLA-DMB	0.573
ENSG00000119787	64225	ARL6IP2	0.556
ENSG00000184434	64922	LRRC19	0.476
ENSG00000173258	158399	ZNF483	0.467
ENSG00000120156	7010	TEK	0.573
ENSG00000224156	203068	TUBB	0.613
ENSG00000231044	7936	RDBP	0.505
ENSG00000106853	22949	PTGR1	0.542
ENSG00000101096	4773	NFATC2	0.538
ENSG00000177875	387856	LOC387856	0.446
ENSG00000187166	341567	H1FNT	0.538
ENSG00000204220	10471	PFDN6	0.6
ENSG00000141378	51651	PTRH2	0.493
ENSG00000164129	4889	NPY5R	0.455
ENSG00000140682	7041	TGFB1I1	0.502
ENSG00000177627	121273	C12orf54	0.489
ENSG00000145088	55840	EAF2	0.479
ENSG00000085999	8438	RAD54L	0.542
ENSG00000173610	10941	UGT2A1	0.449
ENSG00000151005	84076	TKTL2	0.366
ENSG00000178372	51806	CALML5	0.459
ENSG00000178363	810	CALML3	0.496
ENSG00000196372	79754	ASB13	0.474
ENSG00000143811	29920	PYCR2	0.507
ENSG00000167531	3906	LALBA	0.453
ENSG00000164266	6690	SPINK1	0.49
ENSG00000139579	79035	OBFC2B	0.466
ENSG00000196227	63939	C20orf177	0.535
ENSG00000166173	55323	LARP6	0.5
ENSG00000239463	3108	HLA-DMA	0.551
ENSG00000229861	28973	MRPS18B	0.57
ENSG00000180730	387914	SHISA2	0.356
ENSG00000236895	2968	GTF2H4	0.5
ENSG00000085465	5016	OVGP1	0.461
ENSG00000173597	27284	SULT1B1	0.48
ENSG00000169347	2813	GP2	0.546
ENSG00000236227	6046	BRD2	0.659
ENSG00000139343	6636	SNRPF	0.499
ENSG00000110723	23086	EXPH5	0.549
ENSG00000124215	60437	CDH26	0.508
ENSG00000135439	116986	CENTG1	0.504
ENSG00000139540	283375	SLC39A5	0.516
ENSG00000178105	1662	DDX10	0.55

ENSG00000129315	904	CCNT1	0.475
ENSG00000164265	117156	SCGB3A2	0.408
ENSG00000172336	10248	POP7	0.522
ENSG00000160256	85395	C21orf70	0.486
ENSG00000137821	54839	LRRC49	0.488
ENSG00000120160	54586	C9orf11	0.418
ENSG00000174456	400073	FLJ40142	0.53
ENSG00000116455	79084	WDR77	0.584
ENSG00000146839	7455	ZAN	0.567
ENSG00000165972	120935	CCDC38	0.409
ENSG00000109193	6783	SULT1E1	0.497
ENSG00000231257	79969	C6orf134	0.503
ENSG00000169344	7369	UMOD	0.454
ENSG00000140006	112840	WDR89	0.523
ENSG00000108848	51747	CROP	0.62
ENSG00000147896	56832	IFNK	0.374
ENSG00000116062	2956	MSH6	0.62
ENSG00000135697	53630	BCMO1	0.485
ENSG00000141577	22994	AZI1	0.488
ENSG00000122970	28981	IFT81	0.445
ENSG00000236178	57176	VARS2	0.454
ENSG00000255112	57132	CHMP1B	0.621
ENSG00000073712	10979	FERMT2	0.65
ENSG00000133710	11005	SPINK5	0.497
ENSG00000154889	65258	MPPE1	0.618
ENSG00000167536	147015	DHRS13	0.521
ENSG00000139344	144193	AMDHD1	0.477
ENSG00000197901	9356	SLC22A6	0.535
ENSG00000169340	204474	PDILT	0.395
ENSG00000225737	6499	SKIV2L	0.492
ENSG00000164764	157869	RPESP	0.467
ENSG00000084110	3034	HAL	0.492
ENSG00000185742	399947	LOC399947	0.439
ENSG00000183549	54988	ACSM5	0.564
ENSG00000059769	548645	DNAJC25	0.375
ENSG00000165215	1365	CLDN3	0.569
ENSG00000230141	3111	HLA-DOA	0.651
ENSG00000176659	284756	C20orf197	0.4
ENSG00000183747	123876	ACSM2A	0.447
ENSG00000148688	10556	RPP30	0.527
ENSG00000173660	7388	UQCRH	0.568
ENSG00000165025	6850	SYK	0.611
ENSG00000147604	6129	RPL7	0.588
ENSG00000140675	6524	SLC5A2	0.472
ENSG00000138435	1134	CHRNA1	0.555
ENSG00000244115	552891	LOC552891	0.625
ENSG00000165181	158401	C9orf84	0.477
ENSG00000129028	56906	THAP10	0.495
ENSG00000184154	220074	LRRC51	0.435
ENSG00000103067	80004	RBM35B	0.523
ENSG00000183439	391712	TRIM61	0.429
ENSG00000165171	155368	WBSCR27	0.449
ENSG00000172602	27289	RND1	0.489
ENSG00000122026	6144	RPL21	0.493

ENSG00000126545	1446	CSN1S1	0.424
ENSG00000067596	1659	DHX8	0.505
ENSG00000168384	3113	HLA-DPA1	0.611
ENSG00000168619	8749	ADAM18	0.445
ENSG00000221900	285877	DKFZp564N2472	0.452
ENSG00000223654	10211	FLOT1	0.631
ENSG00000215018	340267	COL28A1	0.511
ENSG00000153815	80790	CMIP	0.519
ENSG00000132432	23480	SEC61G	0.526
ENSG00000260842	150483	TEKT4	0.48
ENSG00000175877	135886	WBSCR28	0.365
ENSG00000229767	780	DDR1	0.636
ENSG00000250486	152756	C4orf39	0.468
ENSG00000066813	348158	ACSM2B	0.429
ENSG00000176979	166655	TRIM60	0.323
ENSG00000104213	5157	PDGFRL	0.515
ENSG00000225362	196993	LOC196993	0.423
ENSG00000140688	64755	C16orf58	0.547
ENSG00000165507	11067	C10orf10	0.566
ENSG00000143751	163859	C1orf55	0.478
ENSG00000102225	5127	PCTK1	0.644
ENSG00000066933	4649	MYO9A	0.521
ENSG00000111144	4048	LTA4H	0.579
ENSG00000165511	220979	C10orf25	0.339
ENSG00000165512	7570	ZNF22	0.559
ENSG00000173714	124857	WFIKKN2	0.436
ENSG00000012779	240	ALOX5	0.617
ENSG00000134285	51303	FKBP11	0.537
ENSG00000149452	9376	SLC22A8	0.477
ENSG00000143889	92906	HNRPLL	0.535
ENSG00000126549	6779	STATH	0.462
ENSG00000143110	128346	C1orf162	0.517
ENSG00000188976	26155	NOC2L	0.493
ENSG00000182463	128553	TSHZ2	0.511
ENSG00000005187	6296	ACSM3	0.546
ENSG00000131844	64087	MCCC2	0.59
ENSG00000122035	387496	RASL11A	0.383
ENSG00000117480	2166	FAAH	0.539
ENSG00000008294	9043	SPAG9	0.619
ENSG00000122034	2971	GTF3A	0.539
ENSG00000230763	3115	HLA-DPB1	0.543
ENSG00000132164	6538	SLC6A11	0.475
ENSG00000103066	23659	LYPLA3	0.517
ENSG00000133124	8471	IRS4	0.463
ENSG00000101890	2986	GUCY2F	0.448
ENSG00000169877	51327	ERAF	0.476
ENSG00000164654	54468	FLJ20323	0.595
ENSG00000231179	4277	MICB	0.526
ENSG00000224120	221545	C6orf136	0.518
ENSG00000049249	3604	TNFRSF9	0.554
ENSG00000205649	3347	HTN3	0.432
ENSG00000197362	136051	ZNF786	0.45
ENSG00000137960	54810	GIPC2	0.471
ENSG00000213337	51239	ANKRD39	0.42

ENSG00000204947	155054	ZNF425	0.462
ENSG00000172671	93550	ANUBL1	0.455
ENSG00000104755	2515	ADAM2	0.449
ENSG00000184999	387775	SLC22A10	0.345
ENSG00000237889	7919	BAT1	0.592
ENSG00000224246	389376	SFTPG	0.462
ENSG00000185947	10308	ZNF267	0.512
ENSG00000166743	116285	ACSM1	0.437
ENSG00000116288	11315	PARK7	0.552
ENSG00000121933	140	ADORA3	0.468
ENSG00000138161	50624	CUZD1	0.446
ENSG00000232308	135656	DPCR1	0.476
ENSG00000229432	29113	C6orf15	0.444
ENSG00000122033	219402	MTIF3	0.5
ENSG00000205456	24150	TP53TG3	0.448
ENSG00000037897	4234	METTL1	0.531
ENSG00000235487	170679	PSORS1C1	0.429
ENSG00000198889	139170	WDR40B	0.377
ENSG00000225682	1797	DOM3Z	0.616
ENSG00000197579	10210	TOPORS	0.613
ENSG00000197943	5336	PLCG2	0.544
ENSG00000176194	1149	CIDEA	0.431
ENSG00000165264	4712	NDUFB6	0.541
ENSG00000257727	10330	CNPY2	0.611
ENSG00000106399	6119	RPA3	0.588
ENSG00000100526	1033	CDKN3	0.558
ENSG00000196678	112479	EXOD1	0.5
ENSG00000176014	84617	TUBB6	0.55
ENSG00000108773	2648	KAT2A	0.514
ENSG00000137197	1041	CDSN	0.502
ENSG00000183632	24150	TP53TG3	0.448
ENSG00000215700	55629	PNRC2	0.511
ENSG00000065183	10885	WDR3	0.485
ENSG00000231377	8449	DHX16	0.471
ENSG00000224544	170680	PSORS1C2	0.442
ENSG00000005189	81691	LOC81691	0.524
ENSG00000110203	2352	FOLR3	0.494
ENSG00000228978	7124	TNF	0.524
ENSG00000169840	219409	GSX1	0.427
ENSG00000162426	50651	SLC45A1	0.434
ENSG00000231314	4050	LTB	0.52
ENSG00000182645	374355	C10orf96	0.372
ENSG00000123427	25895	FAM119B	0.515
ENSG00000100814	57820	CCNB1IP1	0.563
ENSG00000106868	64420	SUSD1	0.514
ENSG00000139515	3651	PDX1	0.535
ENSG00000197852	55924	C1orf183	0.511
ENSG00000203837	119548	PNLIPRP3	0.435
ENSG00000059758	5128	PCTK2	0.582
ENSG00000215699	10772	FUSIP1	0.541
ENSG00000234947	8859	STK19	0.565
ENSG00000166192	123228	SENP8	0.463
ENSG00000115966	1386	ATF2	0.636
ENSG00000064703	11218	DDX20	0.515

ENSG00000134107	8553	BHLHB2	0.602
ENSG00000110195	2348	FOLR1	0.6
ENSG00000117226	2635	GBP3	0.485
ENSG00000139549	50846	DHH	0.476
ENSG00000131203	3620	INDO	0.529
ENSG00000126550	3346	HTN1	0.441
ENSG00000068097	63897	HEATR6	0.528
ENSG00000235708	1302	COL11A2	0.611
ENSG00000106245	8896	BUD31	0.589
ENSG00000166166	115708	C14orf172	0.566
ENSG00000123297	10102	TSFM	0.598
ENSG00000148677	27063	ANKRD1	0.477
ENSG00000235915	7940	LST1	0.606
ENSG00000149609	128864	C20orf144	0.375
ENSG00000187583	84069	PLEKHN1	0.42
ENSG00000150750	341032	C11orf53	0.438
ENSG00000137965	10561	IFI44	0.603
ENSG00000157600	84187	TMEM164	0.566
ENSG00000182584	170487	C20orf134	0.413
ENSG00000167302	146705	C17orf56	0.579
ENSG00000107951	55149	PAPD1	0.488
ENSG00000188676	169355	INDOL1	0.4
ENSG00000116191	55103	RALGPS2	0.501
ENSG00000149742	114571	SLC22A9	0.425
ENSG00000213185	196792	FAM24B	0.424
ENSG00000155761	200162	SPAG17	0.406
ENSG00000154978	81552	ECOP	0.532
ENSG00000109205	54959	ODAM	0.476
ENSG00000178172	404203	SPINK6	0.389
ENSG00000110777	5450	POU2AF1	0.518
ENSG00000109472	1363	CPE	0.54
ENSG00000175535	5406	PNLIP	0.471
ENSG00000141385	10939	AFG3L2	0.516
ENSG00000106246	26024	PTCD1	0.486
ENSG00000214510	153218	SPINK5L3	0.433
ENSG00000203795	118670	FAM24A	0.378
ENSG00000126214	3831	KLC1	0.608
ENSG00000159899	4882	NPR2	0.541
ENSG00000121552	1475	CSTA	0.519
ENSG00000119965	80007	C10orf88	0.438
ENSG00000165457	2350	FOLR2	0.473
ENSG00000145879	84651	SPINK7	0.346
ENSG00000171385	3752	KCND3	0.56
ENSG00000160124	131076	CCDC58	0.449
ENSG00000197548	10533	ATG7	0.511
ENSG00000137707	54766	BTG4	0.433
ENSG00000104760	2267	FGL1	0.484
ENSG00000134461	54522	ANKRD16	0.524
ENSG00000102891	84560	MT4	0.491
ENSG00000168004	117245	HRASLS5	0.492
ENSG00000188290	57801	HES4	0.528
ENSG00000123416	10376	TUBA1B	0.632
ENSG00000183644	399949	FLJ46266	0.333
ENSG00000101182	5688	PSMA7	0.575

ENSG00000132600	54496	PRMT7	0.47
ENSG00000114023	26355	FAM162A	0.611
ENSG00000248919	26024	PTCD1	0.486
ENSG00000179988	118672	PSTK	0.491
ENSG00000161610	3060	HCRT	0.524
ENSG00000188234	119016	CTGLF1	0.474
ENSG00000141391	10650	SLMO1	0.495
ENSG00000167434	762	CA4	0.548
ENSG00000187021	5407	PNLIPRP1	0.495
ENSG00000236315	259197	NCR3	0.564
ENSG00000236104	9278	ZBTB22	0.534
ENSG00000102226	8237	USP11	0.542
ENSG00000117228	2633	GBP1	0.559
ENSG00000204179	26095	PTPN20B	0.536
ENSG00000225060	170954	KIAA1949	0.563
ENSG00000103310	7783	ZP2	0.458
ENSG00000167925	84514	GHDC	0.462
ENSG00000239672	4830	NME1	0.641
ENSG00000087250	4504	MT3	0.524
ENSG00000125148	4502	MT2A	0.586
ENSG00000167553	84790	TUBA1C	0.542
ENSG00000168754	51252	LOC51252	0.438
ENSG00000169715	4493	MT1E	0.524
ENSG00000162618	64123	ELTD1	0.451
ENSG00000204209	1616	DAXX	0.603
ENSG00000103316	1428	CRYM	0.537
ENSG00000187608	9636	ISG15	0.565
ENSG00000165061	79698	ZMAT4	0.453
ENSG00000249773	51373	MRPS17	0.485
ENSG00000181617	260436	C4orf7	0.434
ENSG00000178665	349075	ZNF713	0.4
ENSG00000154582	6921	TCEB1	0.577
ENSG00000135406	5630	PRPH	0.469
ENSG00000133317	85329	LGALS12	0.429
ENSG00000095574	64376	IKZF5	0.5
ENSG00000111229	10094	ARPC3	0.516
ENSG00000171209	1448	CSN3	0.459
ENSG00000137731	486	FXYD2	0.532
ENSG00000101421	128866	CHMP4B	0.496
ENSG00000113048	23107	MRPS27	0.533
ENSG00000162645	2634	GBP2	0.549
ENSG00000152147	79833	GEMIN6	0.512
ENSG00000234809	11270	NRM	0.523
ENSG00000184860	93517	HSPC105	0.458
ENSG00000233345	4439	MSH5	0.508
ENSG00000135451	10024	TROAP	0.513
ENSG00000205364	4499	MT1M	0.583
ENSG00000109208	26952	SMR3A	0.407
ENSG00000205362	4489	MT1A	0.596
ENSG00000171201	10879	SMR3B	0.458
ENSG00000086696	3294	HSD17B2	0.5
ENSG00000169688	4490	MT1B	0.614
ENSG00000100532	10668	CGRRF1	0.548
ENSG00000166896	91419	XRCC6BP1	0.511

ENSG00000133321	5920	RARRES3	0.534
ENSG00000137098	26206	SPAG8	0.506
ENSG00000186866	23275	POFUT2	0.602
ENSG00000133328	54979	HRASLS2	0.612
ENSG00000171199	58503	PROL1	0.443
ENSG00000162654	115361	GBP4	0.495
ENSG00000111231	51184	GPN3	0.496
ENSG00000176485	11145	HRASLS3	0.537
ENSG00000143799	142	PARP1	0.567
ENSG00000178401	79962	DNAJC22	0.481
ENSG00000224877	284184	C17orf89	0.455
ENSG00000198417	4494	MT1F	0.634
ENSG00000235712	6257	RXRB	0.61
ENSG00000237649	3833	KIFC1	0.535
ENSG00000237095	9656	MDC1	0.575
ENSG00000129484	10038	PARP2	0.606
ENSG00000239789	51373	MRPS17	0.485
ENSG00000126561	6776	STAT5A	0.544
ENSG00000020577	23034	SAMD4A	0.634
ENSG00000154589	23643	LY96	0.523
ENSG00000132963	51371	POMP	0.558
ENSG00000140749	10261	IGSF6	0.5
ENSG00000171195	4589	MUC7	0.522
ENSG00000147536	84296	GINS4	0.494
ENSG00000125144	4495	MT1G	0.582
ENSG00000101181	26164	GTPBP5	0.565
ENSG00000162129	81570	CLPB	0.473
ENSG00000137133	84681	HINT2	0.525
ENSG00000077264	5063	PAK3	0.499
ENSG00000157637	124565	SLC38A10	0.562
ENSG00000205358	4496	MT1H	0.519
ENSG00000154451	115362	GBP5	0.492
ENSG00000187193	4501	MT1X	0.6
ENSG00000102900	9688	NUP93	0.545
ENSG00000141371	124773	C17orf64	0.412
ENSG00000165862	5408	PNLIPRP2	0.517
ENSG00000146733	5723	PSPH	0.516
ENSG00000116882	51179	HAO2	0.46
ENSG00000165863	143379	C10orf82	0.431
ENSG00000183347	163351	GBP6	0.449
ENSG00000162869	129285	CCDC128	0.495
ENSG00000171217	49861	CLDN20	0.405
ENSG00000074771	50508	NOX3	0.417
ENSG00000087077	7205	TRIP6	0.571
ENSG00000132938	23281	KIAA0774	0.457
ENSG00000158669	137964	AGPAT6	0.533
ENSG00000137103	51754	C9orf127	0.521
ENSG00000133619	84626	KRBA1	0.452
ENSG00000231116	7407	VARS	0.588
ENSG00000224399	7922	SLC39A7	0.474
ENSG00000104763	427	ASAH1	0.622
ENSG00000101440	434	ASIP	0.448
ENSG00000185864	23117	LOC23117	0.536
ENSG00000087589	57091	CASS4	0.473

ENSG00000049883	79810	PTCD2	0.507
ENSG00000111237	51699	VPS29	0.492
ENSG00000071282	29995	LMCD1	0.575
ENSG00000173200	165631	PARP15	0.536
ENSG00000203859	3284	HSD3B2	0.447
ENSG00000128789	56984	PSMG2	0.536
ENSG00000140740	7385	UQCRC2	0.569
ENSG00000229684	203068	TUBB	0.613
ENSG00000233151	1589	CYP21A2	0.47
ENSG00000142973	1580	CYP4B1	0.504
ENSG00000087087	51593	ARS2	0.654
ENSG00000108849	5539	PPY	0.484
ENSG00000178522	258	AMBN	0.441
ENSG00000022277	51507	C20orf43	0.508
ENSG00000171488	84230	LRRC8C	0.537
ENSG00000147117	7712	ZNF157	0.437
ENSG00000131096	5697	PYY	0.536
ENSG00000118596	9194	SLC16A7	0.512
ENSG00000256500	3831	KLC1	0.608
ENSG00000147124	7592	ZNF41	0.446
ENSG00000136866	7539	ZFP37	0.457
ENSG00000109511	11199	ANXA10	0.439
ENSG00000151164	144715	RAD9B	0.477
ENSG00000155714	255762	C16orf65	0.333
ENSG00000204930	392307	C9orf128	0.338
ENSG00000091947	84336	TMEM101	0.469
ENSG00000224979	57819	LSM2	0.551
ENSG00000173193	54625	PARP14	0.617
ENSG00000135436	84070	C12orf25	0.439
ENSG00000162664	284695	ZNF326	0.547
ENSG00000165066	157848	NKX6-3	0.49
ENSG00000154473	9184	BUB3	0.652
ENSG00000256053	84334	C14orf153	0.548
ENSG00000136867	1318	SLC31A2	0.531
ENSG00000196196	646962	UNQ338	0.402
ENSG00000122718	56656	OR2S2	0.456
ENSG00000232357	7923	HSD17B8	0.52
ENSG00000233323	7148	TNXB	0.679
ENSG00000168005	144097	LOC144097	0.394
ENSG00000134470	3601	IL15RA	0.589
ENSG00000140488	60677	BRUNOL6	0.458
ENSG00000010256	7384	UQCRC1	0.563
ENSG00000221995	9220	TIAF1	0.51
ENSG00000140961	29948	OSGIN1	0.482
ENSG00000137628	55601	DDX60	0.504
ENSG00000072518	2011	MARK2	0.504
ENSG00000156697	10813	UTP14A	0.58
ENSG00000226704	3305	HSPA1L	0.463
ENSG00000231115	6015	RING1	0.576
ENSG00000235941	3303	HSPA1A	0.537
ENSG00000125046	51066	C3orf32	0.481
ENSG00000135940	1329	COX5B	0.614
ENSG00000128713	3237	HOXD11	0.46
ENSG00000144559	132001	C3orf31	0.526

ENSG00000085415	81929	SEH1L	0.526
ENSG00000130706	11047	ADRM1	0.525
ENSG00000132467	57050	UTP3	0.55
ENSG00000129103	25870	SUMF2	0.496
ENSG00000203857	3283	HSD3B1	0.449
ENSG00000160193	10785	WDR4	0.516
ENSG00000070915	6559	SLC12A3	0.529
ENSG00000132952	10208	USPL1	0.528
ENSG00000110324	3587	IL10RA	0.549
ENSG00000132965	241	ALOX5AP	0.519
ENSG00000108255	1411	CRYBA1	0.426
ENSG00000232804	3303	HSPA1A	0.537
ENSG00000142583	6518	SLC2A5	0.573
ENSG00000058600	55718	POLR3E	0.508
ENSG00000102802	84935	C13orf33	0.527
ENSG00000188996	135458	HUS1B	0.511
ENSG00000178175	167465	ZNF366	0.387
ENSG00000092621	26227	PHGDH	0.554
ENSG00000224455	6293	VPS52	0.528
ENSG00000175879	3234	HOXD8	0.488
ENSG00000112514	51596	CUTA	0.507
ENSG00000126215	7517	XRCC3	0.477
ENSG00000169087	79663	HSPBAP1	0.486
ENSG00000101901	79868	ALG13	0.539
ENSG00000139644	7009	TEGT	0.612
ENSG00000187048	1579	CYP4A11	0.551
ENSG00000171428	9	NAT1	0.529
ENSG00000137648	56649	TMPRSS4	0.461
ENSG00000103154	54550	NECAB2	0.472
ENSG00000122694	152007	C9orf19	0.576
ENSG00000234728	50854	C6orf48	0.542
ENSG00000181381	91351	DDX60L	0.468
ENSG00000125971	83658	DYNLRB1	0.558
ENSG00000185972	881	CCIN	0.455
ENSG00000006747	85477	SCIN	0.451
ENSG00000175664	122046	C13orf26	0.429
ENSG00000213714	388799	C20orf107	0.442
ENSG00000121898	119587	CPXM2	0.545
ENSG00000101639	55125	CEP192	0.51
ENSG00000130702	3911	LAMA5	0.528
ENSG00000186666	144233	BCDIN3D	0.444
ENSG00000160194	4731	NDUFV3	0.596
ENSG00000156006	10	NAT2	0.441
ENSG00000170166	3233	HOXD4	0.558
ENSG00000204174	5540	PPYR1	0.442
ENSG00000234539	1388	CREBL1	0.561
ENSG00000134240	3158	HMGCS2	0.485
ENSG00000204852	79600	TCTN1	0.476
ENSG00000168792	116236	LOC116236	0.434
ENSG00000203685	375057	C1orf95	0.465
ENSG00000128654	10651	MTX2	0.522
ENSG00000150165	728113	ANXA8L1	0.471
ENSG00000167543	90313	TP53I13	0.5
ENSG00000138463	84925	DIRC2	0.53

ENSG00000162571	254173	TTLL10	0.462
ENSG00000176125	402682	UFSP1	0.418
ENSG00000086159	363	AQP6	0.536
ENSG00000186377	260293	CYP4X1	0.447
ENSG00000087085	43	ACHE	0.57
ENSG00000186891	8784	TNFRSF18	0.53
ENSG00000051108	9709	HERPUD1	0.576
ENSG00000134193	83998	REG4	0.521
ENSG00000228691	4758	NEU1	0.519
ENSG00000115085	7535	ZAP70	0.503
ENSG00000162669	164045	HFM1	0.37
ENSG00000181444	168544	ZNF467	0.592
ENSG00000235336	80736	SLC44A4	0.452
ENSG00000176386	246184	CDC26	0.469
ENSG00000134249	11085	ADAM30	0.433
ENSG00000164751	5828	PXMP3	0.545
ENSG00000186827	7293	TNFRSF4	0.561
ENSG00000156709	9131	AIFM1	0.549
ENSG00000078808	51150	SDF4	0.581
ENSG00000167771	283248	RCOR2	0.352
ENSG00000163374	55249	YY1AP1	0.508
ENSG00000166130	121457	IKIP	0.586
ENSG00000186160	199974	CYP4Z1	0.417
ENSG00000180758	80045	GPR157	0.479
ENSG00000232045	10919	EHMT2	0.557
ENSG00000110583	79829	NAT11	0.512
ENSG00000131981	3958	LGALS3	0.579
ENSG00000187013	388407	C17orf82	0.487
ENSG00000149273	6188	RPS3	0.562
ENSG00000224740	10211	FLOT1	0.631
ENSG00000182022	51363	GALNAC4S-6ST	0.525
ENSG00000168434	91949	COG7	0.5
ENSG00000104427	51101	FAM164A	0.507
ENSG00000171612	84275	SLC25A33	0.478
ENSG00000116690	10216	PRG4	0.456
ENSG00000230907	63943	FKBPL	0.478
ENSG00000162779	126859	C1orf125	0.429
ENSG00000229071	80863	PRRT1	0.558
ENSG00000160593	120425	AMICA1	0.458
ENSG00000134460	3559	IL2RA	0.548
ENSG00000226225	6222	RPS18	0.516
ENSG00000065154	4942	OAT	0.543
ENSG00000104611	63898	SH2D4A	0.5
ENSG00000106479	643641	LOC643641	0.505
ENSG00000198646	23054	NCOA6	0.522
ENSG00000206256	9374	PPT2	0.542
ENSG00000148225	114987	WDR31	0.481
ENSG00000253506	342538	NACA2	0.395
ENSG00000125319	78995	C17orf53	0.421
ENSG00000136492	83990	BRIP1	0.481
ENSG00000132170	5468	PPARG	0.531
ENSG00000262749	83900	KRTAP9-3	0.338
ENSG00000171130	155066	ATP6V0E2	0.529
ENSG00000169876	140453	MUC17	0.567

ENSG00000126787	9787	DLGAP5	0.577
ENSG00000184163	388581	FAM132A	0.386
ENSG00000087237	1071	CETP	0.484
ENSG00000215186	55889	GOLGA6B	0.655
ENSG00000165650	118987	PDZD8	0.532
ENSG00000186807	244	ANXA8L2	0.556
ENSG00000171858	6227	RPS21	0.591
ENSG00000173638	6573	SLC19A1	0.589
ENSG00000116044	4780	NFE2L2	0.569
ENSG00000161664	92591	ASB16	0.473
ENSG00000102265	7076	TIMP1	0.574
ENSG00000223680	780	DDR1	0.636
ENSG00000163050	56997	CABC1	0.514
ENSG00000198019	2210	FCGR1B	0.472
ENSG00000160588	196264	MPZL3	0.513
ENSG00000123473	6491	STIL	0.529
ENSG00000168802	54921	CTF8	0.512
ENSG00000149243	283212	FLJ33790	0.581
ENSG00000165304	9833	MELK	0.549
ENSG00000130701	140893	C20orf151	0.457
ENSG00000106809	4969	OGN	0.437
ENSG00000083093	79728	PALB2	0.496
ENSG00000049541	5982	RFC2	0.572
ENSG00000127399	65999	LRRC61	0.467
ENSG00000139618	675	BRCA2	0.564
ENSG00000141076	84916	CIRH1A	0.532
ENSG00000149573	10205	MPZL2	0.544
ENSG00000126759	5199	CFP	0.469
ENSG00000126775	22863	KIAA0831	0.52
ENSG00000112706	3617	IMPG1	0.391
ENSG00000242038	80864	EGFL8	0.538
ENSG00000160200	875	CBS	0.534
ENSG00000188707	113763	C7orf29	0.491
ENSG00000229341	7148	TNXB	0.679
ENSG00000107902	64077	LHPP	0.449
ENSG00000238056	80863	PRRT1	0.558
ENSG00000156411	9556	C14orf2	0.606
ENSG00000106538	5919	RARRES2	0.545
ENSG00000130700	140628	GATA5	0.458
ENSG00000185231	4158	MC2R	0.551
ENSG00000140009	2100	ESR2	0.623
ENSG00000175322	162655	ZNF519	0.5
ENSG00000168658	200403	VWA3B	0.512
ENSG00000166851	5347	PLK1	0.532
ENSG00000133315	28992	MACROD1	0.547
ENSG00000234370	2968	GTF2H4	0.5
ENSG00000021355	1992	SERPINB1	0.592
ENSG00000047410	7175	TPR	0.62
ENSG00000198851	916	CD3E	0.5
ENSG00000127083	4958	OMD	0.533
ENSG00000162572	6339	SCNN1D	0.462
ENSG00000167011	375607	C7orf52	0.437
ENSG00000121542	26984	SEC22A	0.476
ENSG00000106384	346606	MOGAT3	0.398

ENSG00000174407	128826	C20orf166	0.417
ENSG00000236649	9374	PPT2	0.542
ENSG00000101187	28231	SLCO4A1	0.506
ENSG00000106819	54829	ASPN	0.475
ENSG00000138039	3973	LHCGR	0.467
ENSG00000156414	122402	TDRD9	0.472
ENSG00000134398	10595	ERN2	0.502
ENSG00000173175	111	ADCY5	0.492
ENSG00000167286	915	CD3D	0.482
ENSG00000166869	63928	CHP2	0.503
ENSG00000106823	1842	ECM2	0.52
ENSG00000092094	55644	OSGEP	0.495
ENSG00000166501	5579	PRKCB1	0.616
ENSG00000156136	1633	DCK	0.536
ENSG00000196659	150737	TTC30B	0.478
ENSG00000146243	134728	IRAK1BP1	0.5
ENSG00000100714	4522	MTHFD1	0.571
ENSG00000157111	134285	TMEM171	0.513
ENSG00000234032	57176	VARS2	0.454
ENSG00000186790	2301	FOXE3	0.429
ENSG00000119912	3416	IDE	0.611
ENSG00000149781	83706	FERMT3	0.438
ENSG00000006116	10368	CACNG3	0.499
ENSG00000166148	552	AVPR1A	0.534
ENSG00000177990	283417	DPY19L2	0.545
ENSG00000204315	63943	FKBPL	0.478
ENSG00000172421	146779	EFCAB3	0.4
ENSG00000204314	80863	PRRT1	0.558
ENSG00000170820	2492	FSHR	0.489
ENSG00000117834	200010	SLC5A9	0.43
ENSG00000164325	134288	TMEM174	0.505
ENSG00000087995	339175	METTL2A	0.323
ENSG00000226467	10554	AGPAT1	0.573
ENSG00000170542	5272	SERPINB9	0.547
ENSG00000145882	78991	PCYOX1L	0.52
ENSG00000060491	11054	OGFR	0.614
ENSG00000126756	8409	UXT	0.534
ENSG00000240389	80864	EGFL8	0.538
ENSG00000104368	5327	PLAT	0.545
ENSG00000186452	283471	TMPRSS12	0.344
ENSG00000176927	374786	EFCAB5	0.462
ENSG00000137601	4750	NEK1	0.622
ENSG00000251493	2297	FOXD1	0.589
ENSG00000124570	5269	SERPINB6	0.559
ENSG00000118600	10329	TMEM5	0.562
ENSG00000154743	80746	TSEN2	0.474
ENSG00000196456	285971	ZNF775	0.571
ENSG00000106511	4223	MEOX2	0.532
ENSG00000138050	80745	THUMPD2	0.478
ENSG00000236873	10554	AGPAT1	0.573
ENSG00000149743	83707	TRPT1	0.481
ENSG00000169905	163590	TOR1AIP2	0.454
ENSG00000006704	9569	GTF2IRD1	0.537
ENSG00000147118	7569	ZNF182	0.457

ENSG00000175445	4023	LPL	0.55
ENSG00000119973	2834	PRLHR	0.469
ENSG00000171243	25928	SOSTDC1	0.456
ENSG00000127743	27190	IL17B	0.434
ENSG00000171115	155038	GIMAP8	0.531
ENSG00000100983	2937	GSS	0.584
ENSG00000213380	84342	COG8	0.561
ENSG00000228405	6048	RNF5	0.596
ENSG00000100823	328	APEX1	0.561
ENSG00000132122	54558	SPATA6	0.519
ENSG00000183324	283677	LOC283677	0.447
ENSG00000234729	177	AGER	0.548
ENSG00000123496	3598	IL13RA2	0.501
ENSG00000106524	57037	ANKMY2	0.534
ENSG00000078814	57644	MYH7B	0.444
ENSG00000167283	10632	ATP5L	0.656
ENSG00000179144	168537	GIMAP7	0.402
ENSG00000132676	7818	DAP3	0.568
ENSG00000106400	10467	ZNHIT1	0.525
ENSG00000166183	374569	LOC374569	0.485
ENSG00000223767	6048	RNF5	0.596
ENSG00000133119	5983	RFC3	0.608
ENSG00000164331	57763	ANKRA2	0.515
ENSG00000127081	83744	ZNF484	0.427
ENSG00000133574	55303	GIMAP4	0.462
ENSG00000127084	89846	FGD3	0.472
ENSG00000142156	1291	COL6A1	0.684
ENSG00000106404	24146	CLDN15	0.51
ENSG00000231268	177	AGER	0.548
ENSG00000144191	1261	CNGA3	0.444
ENSG00000214253	51024	FIS1	0.484
ENSG00000102021	51213	LUZP4	0.437
ENSG00000124588	4835	NQO2	0.553
ENSG00000089775	7597	ZBTB25	0.527
ENSG00000185306	115749	C12orf56	0.424
ENSG00000165388	118738	ZNF488	0.391
ENSG00000128581	64792	RABL5	0.45
ENSG00000183111	389337	FLJ41603	0.55
ENSG00000186094	84871	AGBL4	0.528
ENSG00000147905	84186	ZCCHC7	0.539
ENSG00000137948	676	BRDT	0.439
ENSG00000186765	25794	FSCN2	0.495
ENSG00000110011	3338	DNAJC4	0.594
ENSG00000155636	129831	RBM45	0.489
ENSG00000133561	474344	GIMAP6	0.438
ENSG00000157181	54953	C1orf27	0.516
ENSG00000258388	80864	EGFL8	0.538
ENSG00000110925	81566	FAM130A1	0.56
ENSG00000106560	26157	GIMAP2	0.48
ENSG00000126803	3306	HSPA2	0.551
ENSG00000157315	146456	TMED6	0.464
ENSG00000040933	3631	INPP4A	0.628
ENSG00000107618	5949	RBP3	0.494
ENSG00000128802	2658	GDF2	0.449

ENSG00000108576	6532	SLC6A4	0.507
ENSG00000169972	126789	PUSL1	0.462
ENSG00000183675	26095	PTPN20B	0.536
ENSG00000213203	170575	GIMAP1	0.604
ENSG00000162373	79656	C1orf165	0.463
ENSG00000259823	646627	LOC646627	0.372
ENSG00000127054	54973	CPSF3L	0.518
ENSG00000137106	9380	GRHPR	0.593
ENSG00000196329	55340	GIMAP5	0.59
ENSG00000185068	404672	GTF2H5	0.517
ENSG00000162878	91461	LOC91461	0.407
ENSG00000137288	84300	C6orf125	0.557
ENSG00000088298	55741	EDEM2	0.528
ENSG00000241404	80864	EGFL8	0.538
ENSG00000205363	388135	C15orf59	0.548
ENSG00000167139	161514	TBC1D21	0.363
ENSG00000157303	203328	SUSD3	0.507
ENSG00000165583	6758	SSX5	0.435
ENSG00000132915	5145	PDE6A	0.525
ENSG00000237344	5089	PBX2	0.584
ENSG00000212710	64693	CTAGE1	0.436
ENSG00000101773	5932	RBBP8	0.528
ENSG00000106565	28959	TMEM176B	0.468
ENSG00000115239	51130	ASB3	0.533
ENSG00000237814	389376	SFTPG	0.462
ENSG00000106537	27075	TSPAN13	0.565
ENSG00000161896	117283	IHPK3	0.513
ENSG00000262180	10896	OCLM	0.463
ENSG00000116703	5132	PDC	0.409
ENSG00000257232	135656	DPCR1	0.476
ENSG00000134597	51634	RBMX2	0.594
ENSG00000164674	94120	SYTL3	0.567
ENSG00000225987	5089	PBX2	0.584
ENSG00000224105	29113	C6orf15	0.444
ENSG00000142173	1292	COL6A2	0.595
ENSG00000111647	23074	UHRF1BP1L	0.536
ENSG00000126752	6756	SSX1	0.548
ENSG00000056050	54969	C4orf27	0.507
ENSG00000173467	155465	AGR3	0.381
ENSG00000137274	670	BPHL	0.512
ENSG00000233734	170679	PSORS1C1	0.429
ENSG00000233490	63940	GPSM3	0.56
ENSG00000185104	11124	FAF1	0.56
ENSG00000002933	55365	TMEM176A	0.504
ENSG00000101194	63910	C20orf59	0.507
ENSG00000172425	143941	TTC36	0.373
ENSG00000175455	64770	CCDC14	0.528
ENSG00000237165	1041	CDSN	0.502
ENSG00000227246	170680	PSORS1C2	0.442
ENSG00000172031	253152	ABHD7	0.421
ENSG00000224180	54535	CCHCR1	0.631
ENSG00000138190	54536	EXOC6	0.556
ENSG00000134490	85019	C18orf45	0.515
ENSG00000204019	203413	CXorf61	0.406

ENSG00000235396	4855	NOTCH4	0.497
ENSG00000189195	284697	BTBD8	0.357
ENSG00000165584	10214	SSX3	0.548
ENSG00000070501	5423	POLB	0.533
ENSG00000237052	63940	GPSM3	0.56
ENSG00000116711	5321	PLA2G4A	0.54
ENSG00000238196	4855	NOTCH4	0.497
ENSG00000121454	89884	LHX4	0.5
ENSG00000109576	51166	AADAT	0.533
ENSG00000236672	10665	C6orf10	0.434
ENSG00000257923	1523	CUX1	0.594
ENSG00000057935	57504	MTA3	0.603
ENSG00000172775	80011	NIP30	0.479
ENSG00000096395	4295	MLN	0.481
ENSG00000120669	54937	SOHLH2	0.472
ENSG00000075089	64431	ACTR6	0.497
ENSG00000092820	7430	EZR	0.632
ENSG00000234280	10665	C6orf10	0.434
ENSG00000142178	150094	SNF1LK	0.54
ENSG00000101197	79444	BIRC7	0.473
ENSG00000204311	494513	DFNB59	0.472
ENSG00000174842	11146	GLMN	0.552
ENSG00000181019	1728	NQO1	0.612
ENSG00000135847	84320	ACBD6	0.482
ENSG00000147256	158763	RP13-102H20.1	0.482
ENSG00000233192	3118	HLA-DQA2	0.706
ENSG00000174473	442117	GALNT17	0.381
ENSG00000159289	55889	GOLGA6B	0.655
ENSG00000141101	28987	NOB1	0.504
ENSG00000183605	119559	SFXN4	0.569
ENSG00000123080	1031	CDKN2C	0.583
ENSG00000125998	128876	FAM83C	0.468
ENSG00000158806	10361	NPM2	0.438
ENSG00000151655	3698	ITIH2	0.487
ENSG00000166153	120863	DEPDC4	0.547
ENSG00000143942	494143	CHAC2	0.514
ENSG00000068912	27248	C2orf30	0.562
ENSG00000104371	27121	DKK4	0.445
ENSG00000188938	158293	FAM120AOS	0.57
ENSG00000198805	4860	NP	0.559
ENSG00000141452	29919	C18orf8	0.513
ENSG00000175768	401505	C9orf105	0.514
ENSG00000078668	7419	VDAC3	0.627
ENSG00000227993	3122	HLA-DRA	0.555
ENSG00000130363	83861	RSPH3	0.464
ENSG00000160282	10841	FTCD	0.458
ENSG00000118096	56912	C11orf60	0.503
ENSG00000156076	11197	WIF1	0.467
ENSG00000101199	55738	ARFGAP1	0.471
ENSG00000243612	3112	HLA-DOB	0.518
ENSG00000070601	22844	FRMPD1	0.451
ENSG00000266733	26083	TBC1D29	0.443
ENSG00000225967	6891	TAP2	0.58
ENSG00000159640	1636	ACE	0.495

ENSG00000258289	91612	CHURC1	0.56
ENSG00000002726	26	ABP1	0.498
ENSG00000065371	54763	ROPN1	0.5
ENSG00000158815	8822	FGF17	0.439
ENSG00000204305	177	AGER	0.548
ENSG00000154485	118856	MMP21	0.388
ENSG00000188690	7390	UROS	0.536
ENSG00000159579	89970	RSPRY1	0.541
ENSG00000129009	3671	ISLR	0.517
ENSG00000078124	55331	PHCA	0.626
ENSG00000160207	11077	HSF2BP	0.452
ENSG00000224051	80772	GLTPD1	0.449
ENSG00000206240	3123	HLA-DRB1	0.614
ENSG00000224472	6941	TCF19	0.531
ENSG00000017483	92745	SLC38A5	0.47
ENSG00000137868	64220	STRA6	0.509
ENSG00000158517	653361	NCF1	0.528
ENSG00000229937	221823	PRPS1L1	0.473
ENSG00000235068	5460	POU5F1	0.546
ENSG00000144550	151835	CPNE9	0.432
ENSG00000182612	83882	TSPAN10	0.389
ENSG00000162881	165140	OXER1	0.491
ENSG00000162882	23498	HAAO	0.54
ENSG00000185527	5148	PDE6G	0.493
ENSG00000095596	1592	CYP26A1	0.506
ENSG00000119737	10936	GPR75	0.477
ENSG00000196275	84163	GTF2IRD2	0.437
ENSG00000206237	3119	HLA-DQB1	0.68
ENSG00000230669	5696	PSMB8	0.535
ENSG00000204276	3118	HLA-DQA2	0.706
ENSG00000053770	55745	C14orf108	0.531
ENSG00000068878	23198	PSME4	0.591
ENSG00000055118	3757	KCNH2	0.583
ENSG00000242711	5698	PSMB9	0.561
ENSG00000173113	51504	HSPC152	0.498
ENSG00000154065	147463	ANKRD29	0.474
ENSG00000012504	9971	NR1H4	0.498
ENSG00000172171	79736	C17orf42	0.494
ENSG00000120693	4093	SMAD9	0.43
ENSG00000135835	57710	KIAA1614	0.421
ENSG00000198488	192134	B3GNT6	0.425
ENSG00000126432	25824	PRDX5	0.493
ENSG00000120697	29880	ALG5	0.519
ENSG00000204237	339229	C17orf90	0.602
ENSG00000107371	51010	EXOSC3	0.63
ENSG00000176209	114926	C8orf40	0.496
ENSG00000184060	55803	CENTA2	0.5
ENSG00000241386	3112	HLA-DOB	0.518
ENSG00000053747	3909	LAMA3	0.546
ENSG00000138119	26509	FER1L3	0.638
ENSG00000160993	54784	ALKBH4	0.472
ENSG00000161036	222229	LRWD1	0.514
ENSG00000150681	64407	RGS18	0.424
ENSG00000115970	63892	THADA	0.57

ENSG00000122741	79269	WDR32	0.535
ENSG00000147432	1142	CHRNB3	0.53
ENSG00000139354	283431	GAS2L3	0.536
ENSG00000228582	6891	TAP2	0.58
ENSG00000147434	8973	CHRNA6	0.494
ENSG00000005075	5439	POLR2J	0.546
ENSG00000160284	84221	C21orf56	0.477
ENSG00000134602	51765	RP6-213H19.1	0.497
ENSG00000102970	6361	CCL17	0.478
ENSG00000175756	54998	AURKAIP1	0.531
ENSG00000101203	57642	COL20A1	0.484
ENSG00000227816	6890	TAP1	0.552
ENSG00000131931	55145	THAP1	0.45
ENSG00000257365	2342	FNTB	0.57
ENSG00000225691	3107	HLA-C	0.695
ENSG00000136485	10238	WDR68	0.643
ENSG00000139977	122830	NAT12	0.551
ENSG00000185298	339230	CCDC137	0.482
ENSG00000120699	11340	EXOSC8	0.548
ENSG00000107643	5599	MAPK8	0.557
ENSG00000164694	84624	FNDC1	0.44
ENSG00000175787	169841	ZNF169	0.465
ENSG00000107949	56647	BCCIP	0.515
ENSG00000173464	122651	RNASE11	0.409
ENSG00000122696	92014	MCART1	0.542
ENSG00000151572	121601	TMEM16D	0.552
ENSG00000242092	3109	HLA-DMB	0.573
ENSG00000112664	11165	NUDT3	0.528
ENSG00000139233	84298	C12orf31	0.457
ENSG00000235715	5696	PSMB8	0.535
ENSG00000144713	6161	RPL32	0.497
ENSG00000102710	55578	FAM48A	0.607
ENSG00000136463	51204	CCDC44	0.48
ENSG00000160285	4047	LSS	0.646
ENSG00000137124	219	ALDH1B1	0.567
ENSG00000180340	2535	FZD2	0.559
ENSG00000180336	284071	FLJ35848	0.638
ENSG00000155957	51643	TMBIM4	0.54
ENSG00000153029	3140	MR1	0.658
ENSG00000174374	81554	WBSCR16	0.472
ENSG00000214087	339231	ARL16	0.407
ENSG00000137142	347252	IGFBPL1	0.4
ENSG00000122406	6125	RPL5	0.665
ENSG00000068438	24140	FTSJ1	0.586
ENSG00000127074	6003	RGS13	0.478
ENSG00000242361	3108	HLA-DMA	0.551
ENSG00000124614	6204	RPS10	0.541
ENSG00000011083	6534	SLC6A7	0.497
ENSG00000140481	80125	CCDC33	0.474
ENSG00000118094	11181	TREH	0.448
ENSG00000164105	8819	SAP30	0.644
ENSG00000130950	54754	FAM22F	0.396
ENSG00000164106	11341	SCRG1	0.485
ENSG00000243067	5698	PSMB9	0.561

ENSG00000131127	7700	ZNF141	0.452
ENSG00000161692	80174	DBF4B	0.491
ENSG00000258818	6038	RNASE4	0.602
ENSG00000235844	3113	HLA-DPA1	0.611
ENSG00000214274	283	ANG	0.509
ENSG00000090376	11213	IRAK3	0.468
ENSG00000236693	3115	HLA-DPB1	0.543
ENSG00000165694	90167	FRMD7	0.457
ENSG00000164867	4846	NOS3	0.505
ENSG00000173566	79873	NUDT18	0.488
ENSG00000101204	1137	CHRNA4	0.546
ENSG00000232367	6890	TAP1	0.552
ENSG00000232126	3106	HLA-B	0.594
ENSG00000140459	1583	CYP11A1	0.507
ENSG00000128805	58504	ARHGAP22	0.503
ENSG00000089876	55760	DHX32	0.478
ENSG00000075043	3785	KCNQ2	0.571
ENSG00000228049	246721	POLR2J2	0.521
ENSG00000182903	170960	ZNF721	0.446
ENSG00000130957	8789	FBP2	0.445
ENSG00000185359	9146	HGS	0.54
ENSG00000266173	92335	LYK5	0.595
ENSG00000165140	2203	FBP1	0.517
ENSG00000256870	160728	SLC5A8	0.34
ENSG00000154040	26256	CABYR	0.482
ENSG00000181784	6038	RNASE4	0.602
ENSG00000181562	10876	FAM12A	0.571
ENSG00000127311	92797	HELB	0.541
ENSG00000181552	64184	FAM12B	0.408
ENSG00000169413	6039	RNASE6	0.493
ENSG00000129538	6035	RNASE1	0.591
ENSG00000105808	10156	RASA4	0.551
ENSG00000234154	3109	HLA-DMB	0.573
ENSG00000120800	27340	UTP20	0.487
ENSG00000146618	222894	FERD3L	0.333
ENSG00000174227	54872	PIGG	0.511
ENSG00000183742	346389	7A5	0.618
ENSG00000229181	81797	OR12D3	0.409
ENSG00000169397	6037	RNASE3	0.461
ENSG00000168065	55867	SLC22A11	0.455
ENSG00000138629	84993	UBL7	0.388
ENSG00000102312	64840	PORCN	0.472
ENSG00000178809	375593	TRIM73	0.511
ENSG00000168509	148738	HFE2	0.389
ENSG00000164120	3248	HPGD	0.612
ENSG00000242485	55052	MRPL20	0.573
ENSG00000238289	4277	MICB	0.526
ENSG00000061656	6676	SPAG4	0.496
ENSG00000124664	25803	SPDEF	0.601
ENSG00000131097	51751	HIGD1B	0.453
ENSG00000185900	84197	FLJ23356	0.463
ENSG00000004846	340273	ABCB5	0.504
ENSG00000048740	10659	CUGBP2	0.588
ENSG00000169385	6036	RNASE2	0.484

ENSG00000165792	64745	METT11D1	0.512
ENSG00000197891	116085	SLC22A12	0.486
ENSG00000114491	7372	UMPS	0.616
ENSG00000243719	3108	HLA-DMA	0.551
ENSG00000108883	9343	EFTUD2	0.584
ENSG00000179403	64856	VWA1	0.517
ENSG00000203780	92565	FANK1	0.466
ENSG00000120437	39	ACAT2	0.52
ENSG00000176302	283150	FOXR1	0.411
ENSG00000196409	26149	ZNF658	0.5
ENSG00000223496	118460	EXOSC6	0.552
ENSG00000090861	16	AARS	0.543
ENSG00000186166	338657	CCDC84	0.425
ENSG00000234507	6046	BRD2	0.659
ENSG00000121851	84265	POLR3GL	0.528
ENSG00000215915	219293	ATAD3C	0.38
ENSG00000168594	11086	ADAM29	0.464
ENSG00000229496	7919	BAT1	0.592
ENSG00000152022	128077	LIX1L	0.545
ENSG00000124562	6631	SNRPC	0.517
ENSG00000118181	6230	RPS25	0.53
ENSG00000120438	6950	TCP1	0.659
ENSG00000122483	343099	CCDC18	0.569
ENSG00000160072	83858	ATAD3B	0.562
ENSG00000125534	79144	C20orf149	0.497
ENSG00000262814	6182	MRPL12	0.522
ENSG00000166211	121599	SPIC	0.378
ENSG00000158169	2176	FANCC	0.559
ENSG00000170634	98	ACYP2	0.491
ENSG00000267645	246721	POLR2J2	0.521
ENSG00000196655	51399	TRAPPC4	0.567
ENSG00000163517	79885	HDAC11	0.462
ENSG00000126861	4974	OMG	0.48
ENSG00000101213	5753	PTK6	0.504
ENSG00000183048	1468	SLC25A10	0.482
ENSG00000196091	4604	MYBPC1	0.54
ENSG00000006606	10344	CCL26	0.422
ENSG00000173702	56667	MUC13	0.435
ENSG00000005486	57414	RHBDD2	0.54
ENSG00000178021	388951	TSPYL6	0.392
ENSG00000019582	972	CD74	0.529
ENSG00000125508	6725	SRMS	0.413
ENSG00000150627	116966	WDR17	0.478
ENSG00000197150	11194	ABCB8	0.487
ENSG00000161040	222235	FBXL13	0.517
ENSG00000221955	84561	SLC12A8	0.493
ENSG00000134463	79746	ECHDC3	0.496
ENSG00000108592	117246	FTSJ3	0.502
ENSG00000112110	29074	MRPL18	0.513
ENSG00000164587	6208	RPS14	0.634
ENSG00000147155	10682	EBP	0.565
ENSG00000165633	196740	C10orf72	0.51
ENSG00000146453	154197	PNLDC1	0.415
ENSG00000198483	148741	ANKRD35	0.422

ENSG00000167131	388389	CCDC103	0.532
ENSG00000204161	170371	C10orf128	0.472
ENSG00000150628	132851	SPATA4	0.419
ENSG00000087884	28971	C11orf67	0.609
ENSG00000164122	140458	ASB5	0.405
ENSG00000197785	55210	ATAD3A	0.475
ENSG00000214447	388389	CCDC103	0.532
ENSG00000130368	4142	MAS1	0.464
ENSG00000087191	5705	PSMC5	0.52
ENSG00000215644	2642	GCGR	0.453
ENSG00000256269	3145	HMBS	0.561
ENSG00000145945	26240	FAM50B	0.512
ENSG00000125531	79025	C20orf195	0.463
ENSG00000128606	10234	LRRC17	0.49
ENSG00000124678	6954	TCP11	0.483
ENSG00000160075	29101	SSU72	0.587
ENSG00000170632	83787	ARMC10	0.589
ENSG00000130589	85441	PRIC285	0.531
ENSG00000197530	142678	MIB2	0.592
ENSG00000129128	60559	SPCS3	0.527
ENSG00000137936	8412	BCAR3	0.538
ENSG00000163492	285025	CCDC141	0.442
ENSG00000151364	65987	KCTD14	0.549
ENSG00000147257	2719	GPC3	0.514
ENSG00000067334	30836	DNTTIP2	0.527
ENSG00000109674	55247	NEIL3	0.534
ENSG00000243251	267004	PGBD3	0.467
ENSG00000151365	7069	THRSP	0.53
ENSG00000230900	534	ATP6V1G2	0.492
ENSG00000186141	10623	POLR3C	0.598
ENSG00000151366	4718	NDUFC2	0.529
ENSG00000225663	348262	LOC348262	0.515
ENSG00000258838	267004	PGBD3	0.467
ENSG00000070748	1103	CHAT	0.479
ENSG00000235125	4795	NFKBIL1	0.461
ENSG00000127952	51657	STYXL1	0.47
ENSG00000095464	5146	PDE6C	0.409
ENSG00000226275	4049	LTA	0.506
ENSG00000038002	175	AGA	0.61
ENSG00000223952	7124	TNF	0.524
ENSG00000213218	1443	CSH2	0.601
ENSG00000253729	5591	PRKDC	0.684
ENSG00000159063	79053	ALG8	0.561
ENSG00000148690	118924	C10orf4	0.518
ENSG00000101940	64743	WDR13	0.51
ENSG00000169020	521	ATP5I	0.536
ENSG00000236237	4050	LTB	0.52
ENSG00000182362	54059	C21orf57	0.512
ENSG00000234514	7940	LST1	0.606
ENSG00000258366	51750	RTEL1	0.581
ENSG00000172717	161142	FAM71D	0.453
ENSG00000111666	56994	CHPT1	0.544
ENSG00000172269	1798	DPAGT1	0.561
ENSG00000065665	55176	SEC61A2	0.493

ENSG00000117281	11126	CD160	0.48
ENSG00000169026	84179	MFSD7	0.542
ENSG00000174827	5174	PDZK1	0.497
ENSG00000160298	54058	C21orf58	0.494
ENSG00000139351	50511	SYCP3	0.5
ENSG00000130948	3293	HSD17B3	0.46
ENSG00000225211	259197	NCR3	0.564
ENSG00000163510	57703	KIAA1604	0.438
ENSG00000170965	10761	PLAC1	0.472
ENSG00000189409	8510	MMP23B	0.526
ENSG00000117262	51463	GPR89B	0.577
ENSG00000177679	222183	FLJ37078	0.439
ENSG00000235985	199	AIF1	0.617
ENSG00000114547	152015	ROPN1B	0.416
ENSG00000104738	4173	MCM4	0.633
ENSG00000256235	85027	MST150	0.531
ENSG00000172273	25988	MIZF	0.483
ENSG00000145908	91975	ZNF300	0.5
ENSG00000165609	11164	NUDT5	0.521
ENSG00000161057	5701	PSMC2	0.526
ENSG00000114544	54946	SLC41A3	0.539
ENSG00000136048	55332	DRAM	0.524
ENSG00000103051	25839	COG4	0.499
ENSG00000106211	3315	HSPB1	0.512
ENSG00000137513	79731	NARS2	0.532
ENSG00000162630	8707	B3GALT2	0.531
ENSG00000225748	7916	BAT2	0.58
ENSG00000243509	8771	TNFRSF6B	0.667
ENSG00000151465	8872	CDC123	0.538
ENSG00000168517	124790	HEXIM2	0.515
ENSG00000248333	984	CDC2L1	0.633
ENSG00000188372	7784	ZP3	0.447
ENSG00000160703	79671	NLRX1	0.492
ENSG00000188916	642938	C10orf141	0.343
ENSG00000163528	131474	CHCHD4	0.494
ENSG00000120860	51019	CCDC53	0.493
ENSG00000117528	5825	ABCD3	0.565
ENSG00000213199	9311	ACCN3	0.466
ENSG00000184922	752	FMNL1	0.541
ENSG00000160299	5116	PCNT	0.52
ENSG00000138294	4477	MSMB	0.521
ENSG00000136487	2689	GH2	0.556
ENSG00000075188	79023	NUP37	0.514
ENSG00000145975	222826	C6orf146	0.419
ENSG00000185480	55010	C12orf48	0.51
ENSG00000100554	51382	ATP6V1D	0.641
ENSG00000175003	6580	SLC22A1	0.442
ENSG00000185689	404220	C6orf201	0.481
ENSG00000180745	119467	CLRN3	0.412
ENSG00000170876	79188	TMEM43	0.527
ENSG00000136488	1442	CSH1	0.605
ENSG00000112499	6582	SLC22A2	0.447
ENSG00000158122	195827	C9orf21	0.518
ENSG00000198721	10455	PECI	0.521

ENSG00000182103	220382	MGC33846	0.465
ENSG00000008128	984	CDC2L1	0.633
ENSG00000156500	159091	FAM122C	0.542
ENSG00000137509	5547	PRCP	0.534
ENSG00000172367	79849	PDZD3	0.465
ENSG00000203896	54923	LIME1	0.449
ENSG00000015285	7454	WAS	0.576
ENSG00000184361	124783	C17orf46	0.453
ENSG00000081386	22869	ZNF510	0.472
ENSG00000143036	126969	SLC44A3	0.5
ENSG00000034239	79645	EFCAB1	0.429
ENSG00000196597	158431	ZNF782	0.477
ENSG00000183395	5367	PMCH	0.461
ENSG00000144908	10840	ALDH1L1	0.541
ENSG00000234651	7917	BAT3	0.576
ENSG00000159314	201176	ARHGAP27	0.526
ENSG00000138193	51196	PLCE1	0.588
ENSG00000178950	2580	GAK	0.56
ENSG00000197043	309	ANXA6	0.553
ENSG00000168333	492307	C8orf22	0.417
ENSG00000171759	5053	PAH	0.48
ENSG00000243566	80761	UPK3B	0.419
ENSG00000147481	54212	SNTG1	0.474
ENSG00000146707	22932	POMZP3	0.485
ENSG00000198670	4018	LPA	0.464
ENSG00000183260	140701	C20orf135	0.382
ENSG00000136943	1515	CTSL2	0.503
ENSG00000122194	5340	PLG	0.496
ENSG00000135205	57639	CCDC146	0.528
ENSG00000162994	130162	C2orf63	0.426
ENSG00000102145	2623	GATA1	0.504
ENSG00000147485	137902	PXDNL	0.462
ENSG00000065328	55388	MCM10	0.568
ENSG00000132613	92154	ABBA-1	0.488
ENSG00000094631	10013	HDAC6	0.599
ENSG00000163885	348807	CCDC37	0.532
ENSG00000172339	199857	ALG14	0.5
ENSG00000215790	9906	SLC35E2	0.493
ENSG00000100558	26499	PLEK2	0.507
ENSG00000152078	148534	TMEM56	0.495
ENSG00000186088	54103	tcag7.1314	0.557
ENSG00000000971	3075	CFH	0.506
ENSG00000198133	161145	C14orf83	0.554
ENSG00000198276	54963	UCKL1	0.504
ENSG00000226215	55937	APOM	0.62
ENSG00000116586	28956	RP11-336K24.9	0.512
ENSG00000226531	57827	C6orf47	0.496
ENSG00000232312	7918	BAT4	0.51
ENSG00000183684	10189	THOC4	0.517
ENSG00000122481	25950	RWDD3	0.476
ENSG00000164306	201973	CCDC111	0.488
ENSG00000169551	54967	CXorf48	0.422
ENSG00000141552	51529	ANAPC11	0.471
ENSG00000143947	6233	RPS27A	0.543

ENSG00000186334	285641	SLC36A3	0.36
ENSG00000170860	27258	LSM3	0.569
ENSG00000132698	57111	RAB25	0.482
ENSG00000186376	7626	ZNF75D	0.56
ENSG00000254726	92312	MEX3A	0.548
ENSG00000165623	221044	C10orf49	0.477
ENSG00000107537	5264	PHYH	0.532
ENSG00000151725	79682	MLF1IP	0.552
ENSG00000150722	151242	PPP1R1C	0.511
ENSG00000180767	166012	CHST13	0.477
ENSG00000180697	152065	C3orf22	0.427
ENSG00000101161	24148	PRPF6	0.581
ENSG00000124787	10799	RPP40	0.516
ENSG00000173145	64318	NOC3L	0.514
ENSG00000188641	1806	DPYD	0.555
ENSG00000185813	5833	PCYT2	0.534
ENSG00000159685	84303	CHCHD6	0.458
ENSG00000145982	10667	FARS2	0.577
ENSG00000164897	83590	TMUB1	0.43
ENSG00000133612	116988	CENTG3	0.54
ENSG00000256762	246744	STH	0.447
ENSG00000077232	54431	DNAJC10	0.603
ENSG00000165626	222389	C10orf30	0.455
ENSG00000135211	85025	TMEM60	0.509
ENSG00000117598	163404	PAP2D	0.448
ENSG00000117600	9890	LPPR4	0.478
ENSG00000228696	51326	ARL17P1	0.507
ENSG00000151729	291	SLC25A4	0.527
ENSG00000163870	131601	GPR175	0.497
ENSG00000102109	27344	PCSK1N	0.489
ENSG00000112799	9450	LY86	0.521
ENSG00000162998	2487	FRZB	0.542
ENSG00000073111	4171	MCM2	0.601
ENSG00000145888	2741	GLRA1	0.461
ENSG00000102103	10084	PQBP1	0.597
ENSG00000112539	168090	C6orf118	0.433
ENSG00000109775	55325	UFSP2	0.488
ENSG00000146926	136371	ASB10	0.525
ENSG00000179636	122664	TPPP2	0.456
ENSG00000114631	50512	PODXL2	0.44
ENSG00000205129	441054	Chr4_1746	0.526
ENSG00000033100	54480	CSGlcA-T	0.505
ENSG00000165799	84659	RNASE7	0.521
ENSG00000165801	55701	FLJ10357	0.598
ENSG00000082014	6604	SMARCD3	0.528
ENSG00000163002	129401	NUP35	0.517
ENSG00000154553	27295	PDLIM3	0.584
ENSG00000198818	113402	SFT2D1	0.556
ENSG00000183010	5831	PYCR1	0.537
ENSG00000160783	11243	PMF1	0.504
ENSG00000164304	285782	CAGE1	0.327
ENSG00000013374	51667	NUB1	0.5
ENSG00000108242	1562	CYP2C18	0.53
ENSG00000242252	632	BGLAP	0.454

ENSG00000160781	79957	PAQR6	0.496
ENSG00000169696	79058	ASPSCR1	0.49
ENSG00000187260	349136	WDR86	0.394
ENSG00000138109	1559	CYP2C9	0.602
ENSG00000068400	56850	GRIPAP1	0.47
ENSG00000127377	155051	CRYGN	0.372
ENSG00000035115	26751	SH3YL1	0.537
ENSG00000138115	1558	CYP2C8	0.53
ENSG00000168566	154007	C6orf151	0.526
ENSG00000198715	112770	C1orf85	0.516
ENSG00000092200	57096	RPGRIP1	0.478
ENSG00000169683	201255	LRRC45	0.419
ENSG00000106648	168391	GALNTL5	0.367
ENSG00000143727	52	ACP1	0.623
ENSG00000107438	9124	PDLIM1	0.611
ENSG00000178234	63917	GALNT11	0.5
ENSG00000124802	9521	EEF1E1	0.608
ENSG00000169738	51181	DCXR	0.518
ENSG00000151353	129787	TMEM18	0.525
ENSG00000100888	57680	CHD8	0.544
ENSG00000169733	5986	RFNG	0.507
ENSG00000172554	54221	SNTG2	0.452
ENSG00000068394	27238	GPKOW	0.497
ENSG00000169727	2873	GPS1	0.46
ENSG00000163467	128229	C1orf182	0.377
ENSG00000115705	7173	TPO	0.468
ENSG00000132677	57127	RHBG	0.446
ENSG00000196584	7516	XRCC2	0.47
ENSG00000012211	4007	PRICKLE3	0.487
ENSG00000165819	56339	METTL3	0.627
ENSG00000102001	778	CACNA1F	0.494
ENSG00000111845	55003	PAK1IP1	0.508
ENSG00000111843	51522	TMEM14C	0.5
ENSG00000137210	81853	TMEM14B	0.522
ENSG00000201405	148898	C1orf213	0.455
ENSG00000262199	80725	SNIP	0.472
ENSG00000263185	55852	TEX2	0.526
ENSG00000262909	64750	SMURF2	0.56
ENSG00000262122	9883	POM121	0.488
ENSG00000262696	155400	NSUN5B	0.564
ENSG00000262421	378108	TRIM74	0.529
ENSG00000262589	55695	NSUN5	0.586
ENSG00000262964	135892	TRIM50	0.515
ENSG00000262532	8468	FKBP6	0.484
ENSG00000263254	8326	FZD9	0.456
ENSG00000262694	9031	BAZ1B	0.55
ENSG00000263151	9275	BCL7B	0.543
ENSG00000263049	26608	TBL2	0.571
ENSG00000262077	51085	MLXIPL	0.503
ENSG00000262018	155382	VPS37D	0.468
ENSG00000261940	84277	DNAJC30	0.512
ENSG00000262030	114049	WBSCR22	0.543
ENSG00000263024	6804	STX1A	0.534
ENSG00000262947	83451	ABHD11	0.474

ENSG00000262073	1365	CLDN3	0.569
ENSG00000262988	1364	CLDN4	0.539
ENSG00000262499	155368	WBSCR27	0.449
ENSG00000262459	135886	WBSCR28	0.365
ENSG00000262184	2006	ELN	0.584
ENSG00000262063	3984	LIMK1	0.607
ENSG00000263344	7458	EIF4H	0.521
ENSG00000262128	7462	LAT2	0.622
ENSG00000261911	5982	RFC2	0.572
ENSG00000263226	7461	CLIP2	0.529
ENSG00000261920	9569	GTF2IRD1	0.537
ENSG00000263001	2969	GTF2I	0.472
ENSG00000261919	653361	NCF1	0.528
ENSG00000263008	81554	WBSCR16	0.472
ENSG00000262895	375593	TRIM73	0.511
ENSG00000261876	5387	PMS2L3	0.644
ENSG00000263334	3092	HIP1	0.542
ENSG00000262029	10344	CCL26	0.422
ENSG00000262744	57414	RHBDD2	0.54
ENSG00000262569	5447	POR	0.515
ENSG00000262263	51657	STYXL1	0.47
ENSG00000262847	4191	MDH2	0.598
ENSG00000262321	222183	FLJ37078	0.439
ENSG00000261910	4647	MYO7A	0.614
ENSG00000262325	4322	MMP13	0.484
ENSG00000261883	3145	HMBS	0.561
ENSG00000262124	148156	ZNF558	0.488
ENSG00000262523	23052	ENDOD1	0.6
ENSG00000262506	55216	C11orf57	0.536
ENSG00000266766	4853	NOTCH2	0.604
ENSG00000265808	9554	SEC22B	0.625
ENSG00000263850	2210	FCGR1B	0.472
ENSG00000266338	284565	NBPF15	0.5
ENSG00000265755	9984	THOC1	0.501
ENSG00000264337	51463	GPR89B	0.577
ENSG00000265111	5174	PDZK1	0.497
ENSG00000266249	11126	CD160	0.48
ENSG00000265491	27246	RNF115	0.507
ENSG00000264806	10623	POLR3C	0.598
ENSG00000266054	200035	NUDT17	0.5
ENSG00000263461	10401	PIAS3	0.521
ENSG00000263773	148741	ANKRD35	0.422
ENSG00000264162	8515	ITGA10	0.515
ENSG00000265832	8799	PEX11B	0.54
ENSG00000265241	9939	RBM8A	0.625
ENSG00000264936	284615	ANKRD34A	0.352
ENSG00000266741	84265	POLR3GL	0.528
ENSG00000265972	10628	TXNIP	0.621
ENSG00000265970	148738	HFE2	0.389
ENSG00000264343	388677	NOTCH2NL	0.539
ENSG00000266267	11163	NUDT4	0.627
ENSG00000266198	5565	PRKAB2	0.492
ENSG00000266748	2330	FMO5	0.562
ENSG00000264980	9557	CHD1L	0.568

ENSG00000266095	607	BCL9	0.501
ENSG00000265277	51205	ACP6	0.524
ENSG00000265107	2702	GJA5	0.493
ENSG00000264499	2703	GJA8	0.487
ENSG00000264776	51463	GPR89B	0.577
ENSG00000263956	200030	NBPF11	0.535
ENSG00000264648	11163	NUDT4	0.627
ENSG00000264708	9659	PDE4DIP	0.617
ENSG00000264983	388677	NOTCH2NL	0.539
ENSG00000266332	2209	FCGR1A	0.548
ENSG00000263792	8294	HIST1H4I	0.459
ENSG00000263420	8337	HIST2H2AA3	0.476
ENSG00000266225	8337	HIST2H2AA3	0.476
ENSG00000264782	8294	HIST1H4I	0.459
ENSG00000264719	8349	HIST2H2BE	0.564
ENSG00000265570	51027	BOLA1	0.481
ENSG00000265307	9900	SV2A	0.529
ENSG00000263977	10262	SF3B4	0.527
ENSG00000264524	10903	MTMR11	0.523
ENSG00000264522	56957	OTUD7B	0.484
ENSG00000264082	11311	VPS45	0.613
ENSG00000265346	81611	ANP32E	0.606
ENSG00000264654	23632	CA14	0.446
ENSG00000266334	51107	APH1A	0.494
ENSG00000266177	79630	C1orf54	0.522
ENSG00000266764	148523	C1orf51	0.543
ENSG00000266472	54460	MRPS21	0.503
ENSG00000265228	9129	PRPF3	0.5
ENSG00000266562	23248	KIAA0460	0.469
ENSG00000264606	1510	CTSE	0.486
ENSG00000263961	440712	C1orf186	0.451
ENSG00000265027	553	AVPR1B	0.499
ENSG00000263528	9641	IKBKE	0.574
ENSG00000266094	83593	RASSF5	0.536
ENSG00000265823	1939	LGTN	0.537
ENSG00000265184	8444	DYRK3	0.541
ENSG00000266641	9261	MAPKAPK2	0.607
ENSG00000266134	8838	WISP3	0.47
ENSG00000264528	51175	TUBE1	0.49
ENSG00000264440	619208	C6orf225	0.458
ENSG00000263699	3910	LAMA4	0.625
ENSG00000266292	93979	CPA5	0.408
ENSG00000264509	1357	CPA1	0.419
ENSG00000265594	95681	TSGA14	0.477
ENSG00000264872	4232	MEST	0.537
ENSG00000266159	114960	TSGA13	0.486
ENSG00000266607	5239	PGM5	0.494
ENSG00000266592	1645	AKR1C1	0.616
ENSG00000265231	1646	AKR1C2	0.594
ENSG00000265685	8644	AKR1C3	0.542
ENSG00000266359	1109	AKR1C4	0.447
ENSG00000263440	79861	TUBAL3	0.48
ENSG00000265551	253725	FAM21C	0.553
ENSG00000264842	119016	CTGLF1	0.474

ENSG00000266412	8031	NCOA4	0.525
ENSG00000263639	4477	MSMB	0.521
ENSG00000264230	244	ANXA8L2	0.556
ENSG00000262722	9721	GPRIN2	0.503
ENSG00000263480	83849	SYT15	0.5
ENSG00000266585	5831	PYCR1	0.537
ENSG00000264832	26095	PTPN20B	0.536
ENSG00000266524	2662	GDF10	0.491
ENSG00000263761	2658	GDF2	0.449
ENSG00000265203	5949	RBP3	0.494
ENSG00000265763	118738	ZNF488	0.391
ENSG00000264717	5540	PPYR1	0.442
ENSG00000266074	57597	BAHCC1	0.49
ENSG00000266662	25794	FSCN2	0.495
ENSG00000261843	10801	9-Sep	0.602
ENSG00000262673	5596	MAPK4	0.518
ENSG00000262924	9710	KIAA0355	0.537
ENSG00000262976	320	APBA1	0.466
ENSG00000262907	3800	KIF5C	0.589
ENSG00000262290	284415	VSTM1	0.351
ENSG00000262632	126014	OSCAR	0.479
ENSG00000261942	4696	NDUFA3	0.49
ENSG00000262229	29844	TFPT	0.495
ENSG00000262553	26121	PRPF31	0.643
ENSG00000262174	79165	LENG1	0.431
ENSG00000263166	147798	TMC4	0.5
ENSG00000261917	79143	MBOAT7	0.618
ENSG00000262716	79042	TSEN34	0.482
ENSG00000263076	6203	RPS9	0.601
ENSG00000262044	11025	LILRB3	0.61
ENSG00000262642	79168	LILRA6	0.46
ENSG00000262991	10990	LILRB5	0.48
ENSG00000261935	11026	LILRA3	0.506
ENSG00000262800	353514	LILRA5	0.575
ENSG00000263109	57348	TTYH1	0.444
ENSG00000263054	114823	LENG8	0.459
ENSG00000263030	148170	CDC42EP5	0.472
ENSG00000262317	3904	LAIR2	0.483
ENSG00000262004	11027	LILRA2	0.604
ENSG00000262727	10859	LILRB1	0.53
ENSG00000262900	11024	LILRA1	0.565
ENSG00000263108	11006	LILRB4	0.499
ENSG00000262335	115653	KIR3DL3	0.481
ENSG00000261912	3802	KIR2DL1	0.443
ENSG00000263208	3802	KIR2DL1	0.443
ENSG00000263100	9437	NCR1	0.558
ENSG00000262736	199713	NLRP7	0.392
ENSG00000262886	55655	NLRP2	0.463
ENSG00000262239	51206	GP6	0.492
ENSG00000261975	112724	RDH13	0.637
ENSG00000263295	54869	EPS8L1	0.606
ENSG00000262787	284415	VSTM1	0.351
ENSG00000262475	126014	OSCAR	0.479
ENSG00000263265	4696	NDUFA3	0.49

ENSG00000262367	29844	TFPT	0.495
ENSG00000263269	26121	PRPF31	0.643
ENSG00000262775	4849	CNOT3	0.549
ENSG00000262629	79165	LENG1	0.431
ENSG00000262200	147798	TMC4	0.5
ENSG00000262479	79143	MBOAT7	0.618
ENSG00000261969	79042	TSEN34	0.482
ENSG00000262723	6203	RPS9	0.601
ENSG00000261937	11026	LILRA3	0.506
ENSG00000262610	353514	LILRA5	0.575
ENSG00000262284	23547	LILRA4	0.49
ENSG00000262936	3903	LAIR1	0.605
ENSG00000263075	57292	KIR2DL5A	0.447
ENSG00000261870	3808	KIR2DS3	0.458
ENSG00000262897	3812	KIR3DL2	0.578
ENSG00000262806	2204	FCAR	0.594
ENSG00000262182	9437	NCR1	0.558
ENSG00000263026	199713	NLRP7	0.392
ENSG00000262615	55655	NLRP2	0.463
ENSG00000262069	51206	GP6	0.492
ENSG00000262982	112724	RDH13	0.637
ENSG00000262092	54869	EPS8L1	0.606
ENSG00000262720	284415	VSTM1	0.351
ENSG00000262707	126014	OSCAR	0.479
ENSG00000263286	4696	NDUFA3	0.49
ENSG00000263121	29844	TFPT	0.495
ENSG00000262203	26121	PRPF31	0.643
ENSG00000262272	4849	CNOT3	0.549
ENSG00000263099	79165	LENG1	0.431
ENSG00000262527	147798	TMC4	0.5
ENSG00000262695	79143	MBOAT7	0.618
ENSG00000262857	79042	TSEN34	0.482
ENSG00000263119	6203	RPS9	0.601
ENSG00000263233	3806	KIR2DS1	0.453
ENSG00000262161	3809	KIR2DS4	0.408
ENSG00000262680	9437	NCR1	0.558
ENSG00000262690	199713	NLRP7	0.392
ENSG00000262329	55655	NLRP2	0.463
ENSG00000261961	51206	GP6	0.492
ENSG00000262348	112724	RDH13	0.637
ENSG00000262287	54869	EPS8L1	0.606
ENSG00000263131	284415	VSTM1	0.351
ENSG00000261901	126014	OSCAR	0.479
ENSG00000262242	4696	NDUFA3	0.49
ENSG00000263170	29844	TFPT	0.495
ENSG00000262440	26121	PRPF31	0.643
ENSG00000261980	4849	CNOT3	0.549
ENSG00000263248	79165	LENG1	0.431
ENSG00000262441	147798	TMC4	0.5
ENSG00000262337	79143	MBOAT7	0.618
ENSG00000262954	79042	TSEN34	0.482
ENSG00000262567	6203	RPS9	0.601
ENSG00000262439	115653	KIR3DL3	0.481
ENSG00000262923	9437	NCR1	0.558

ENSG00000263168	199713	NLRP7	0.392
ENSG00000262175	55655	NLRP2	0.463
ENSG00000262867	51206	GP6	0.492
ENSG00000262110	112724	RDH13	0.637
ENSG00000262835	54869	EPS8L1	0.606
ENSG00000262427	284415	VSTM1	0.351
ENSG00000262945	126014	OSCAR	0.479
ENSG00000261881	4696	NDUFA3	0.49
ENSG00000262570	29844	TFPT	0.495
ENSG00000263133	26121	PRPF31	0.643
ENSG00000263078	4849	CNOT3	0.549
ENSG00000262551	79165	LENG1	0.431
ENSG00000262910	147798	TMC4	0.5
ENSG00000261944	79143	MBOAT7	0.618
ENSG00000263268	79042	TSEN34	0.482
ENSG00000262804	6203	RPS9	0.601
ENSG00000262007	3812	KIR3DL2	0.578
ENSG00000262661	9437	NCR1	0.558
ENSG00000262457	199713	NLRP7	0.392
ENSG00000262811	55655	NLRP2	0.463
ENSG00000262131	51206	GP6	0.492
ENSG00000262163	112724	RDH13	0.637
ENSG00000262467	54869	EPS8L1	0.606
ENSG00000262346	284415	VSTM1	0.351
ENSG00000263016	126014	OSCAR	0.479
ENSG00000262433	4696	NDUFA3	0.49
ENSG00000262825	29844	TFPT	0.495
ENSG00000262142	26121	PRPF31	0.643
ENSG00000262428	4849	CNOT3	0.549
ENSG00000263215	79165	LENG1	0.431
ENSG00000262109	147798	TMC4	0.5
ENSG00000261902	79143	MBOAT7	0.618
ENSG00000262137	79042	TSEN34	0.482
ENSG00000261909	6203	RPS9	0.601
ENSG00000262289	115653	KIR3DL3	0.481
ENSG00000261930	3812	KIR3DL2	0.578
ENSG00000262832	9437	NCR1	0.558
ENSG00000263068	199713	NLRP7	0.392
ENSG00000262929	55655	NLRP2	0.463
ENSG00000263239	51206	GP6	0.492
ENSG00000262207	112724	RDH13	0.637
ENSG00000262225	54869	EPS8L1	0.606
ENSG00000262701	284415	VSTM1	0.351
ENSG00000262524	126014	OSCAR	0.479
ENSG00000263333	4696	NDUFA3	0.49
ENSG00000262443	29844	TFPT	0.495
ENSG00000262534	26121	PRPF31	0.643
ENSG00000262369	4849	CNOT3	0.549
ENSG00000262168	79165	LENG1	0.431
ENSG00000262053	79143	MBOAT7	0.618
ENSG00000262868	79042	TSEN34	0.482
ENSG00000263332	6203	RPS9	0.601
ENSG00000262087	11025	LILRB3	0.61
ENSG00000262320	79168	LILRA6	0.46

ENSG00000262236	57348	TTYH1	0.444
ENSG00000262602	114823	LENG8	0.459
ENSG00000261906	94059	LENG9	0.42
ENSG00000261931	148170	CDC42EP5	0.472
ENSG00000262216	3904	LAIR2	0.483
ENSG00000262754	11027	LILRA2	0.604
ENSG00000262985	10859	LILRB1	0.53
ENSG00000262462	11024	LILRA1	0.565
ENSG00000263061	11006	LILRB4	0.499
ENSG00000261860	3811	KIR3DL1	0.542
ENSG00000262763	115653	KIR3DL3	0.481
ENSG00000263027	3802	KIR2DL1	0.443
ENSG00000262556	3802	KIR2DL1	0.443
ENSG00000263184	3812	KIR3DL2	0.578
ENSG00000262827	2204	FCAR	0.594
ENSG00000262717	9437	NCR1	0.558
ENSG00000262797	51206	GP6	0.492
ENSG00000263258	112724	RDH13	0.637
ENSG00000262878	54869	EPS8L1	0.606
ENSG00000262725	284415	VSTM1	0.351
ENSG00000262956	126014	OSCAR	0.479
ENSG00000262793	4696	NDUFA3	0.49
ENSG00000261861	29844	TFPT	0.495
ENSG00000262669	26121	PRPF31	0.643
ENSG00000262365	4849	CNOT3	0.549
ENSG00000261854	79165	LENG1	0.431
ENSG00000262807	147798	TMC4	0.5
ENSG00000262126	79143	MBOAT7	0.618
ENSG00000262357	79042	TSEN34	0.482
ENSG00000263088	6203	RPS9	0.601
ENSG00000263021	10990	LILRB5	0.48
ENSG00000262383	11026	LILRA3	0.506
ENSG00000262843	353514	LILRA5	0.575
ENSG00000261929	23547	LILRA4	0.49
ENSG00000263303	3903	LAIR1	0.605
ENSG00000262397	11006	LILRB4	0.499
ENSG00000262596	3806	KIR2DS1	0.453
ENSG00000263175	3803	KIR2DL2	0.475
ENSG00000262494	3811	KIR3DL1	0.542
ENSG00000261880	9437	NCR1	0.558
ENSG00000262009	199713	NLRP7	0.392
ENSG00000262260	55655	NLRP2	0.463
ENSG00000262729	51206	GP6	0.492
ENSG00000262244	112724	RDH13	0.637
ENSG00000262430	54869	EPS8L1	0.606
ENSG00000262928	7594	ZNF43	0.549
ENSG00000262327	55750	AGK	0.577
ENSG00000262599	57189	KIAA1147	0.531
ENSG00000262771	6742	SSBP1	0.629
ENSG00000264369	132001	C3orf31	0.526
LRG_1	1277	COL1A1	0.637
LRG_10	5479	PPIB	0.597
LRG_100	10235	RASGRP2	0.634
LRG_101	5993	RFX5	0.567

LRG_102	8625	RFXANK	0.472
LRG_103	5994	RFXAP	0.467
LRG_105	710	SERPING1	0.538
LRG_106	4068	SH2D1A	0.59
LRG_107	55343	SLC35C1	0.476
LRG_108	50485	SMARCAL1	0.52
LRG_109	3431	SP110	0.617
LRG_11	1029	CDKN2A	0.588
LRG_110	11005	SPINK5	0.497
LRG_111	6772	STAT1	0.674
LRG_113	8676	STX11	0.509
LRG_114	6892	TAPBP	0.616
LRG_115	10312	TCIRG1	0.535
LRG_116	6948	TCN2	0.514
LRG_117	7098	TLR3	0.482
LRG_118	11322	TMC6	0.56
LRG_119	147138	TMC8	0.563
LRG_12	60681	FKBP10	0.488
LRG_120	23495	TNFRSF13B	0.468
LRG_122	201294	UNC13D	0.496
LRG_123	81622	UNC93B1	0.439
LRG_124	7374	UNG	0.54
LRG_125	7454	WAS	0.576
LRG_126	7535	ZAP70	0.503
LRG_127	1604	CD55	0.609
LRG_128	695	BTK	0.506
LRG_129	5199	CFP	0.469
LRG_13	796	CALCA	0.589
LRG_130	324	APC	0.647
LRG_132	60	ACTB	0.653
LRG_133	204	AK2	0.685
LRG_134	355	FAS	0.655
LRG_135	472	ATM	0.63
LRG_136	629	CFB	0.59
LRG_139	731	C8A	0.465
LRG_140	931	MS4A1	0.573
LRG_141	959	CD40LG	0.488
LRG_142	975	CD81	0.544
LRG_144	1441	CSF3R	0.538
LRG_145	2161	F12	0.545
LRG_146	2357	FPR1	0.525
LRG_147	2538	G6PC	0.432
LRG_148	2539	G6PD	0.504
LRG_149	3078	CFHR1	0.64
LRG_15	7337	UBE3A	0.664
LRG_150	3561	IL2RG	0.502
LRG_151	3587	IL10RA	0.549
LRG_152	3588	IL10RB	0.559
LRG_154	4153	MBL2	0.481
LRG_155	4179	CD46	0.567
LRG_156	4598	MVK	0.601
LRG_157	4615	MYD88	0.559
LRG_158	4683	NBN	0.612
LRG_159	4689	NCF4	0.594

LRG_16	100	ADA	0.601
LRG_160	5277	PIGA	0.49
LRG_162	5591	PRKDC	0.684
LRG_164	6786	STIM1	0.52
LRG_165	6813	STXBP2	0.513
LRG_168	7056	THBD	0.6
LRG_169	8542	APOL1	0.53
LRG_17	57379	AICDA	0.442
LRG_171	8547	FCN3	0.484
LRG_172	9051	PSTPIP1	0.459
LRG_173	9652	TTC37	0.537
LRG_174	9663	LPIN2	0.605
LRG_175	10878	CFHR3	0.494
LRG_176	55636	CHD7	0.557
LRG_177	64127	NOD2	0.45
LRG_178	64170	CARD9	0.457
LRG_179	64581	CLEC7A	0.491
LRG_18	326	AIRE	0.458
LRG_180	83706	FERMT3	0.438
LRG_182	92579	G6PC3	0.573
LRG_183	113235	SLC46A1	0.49
LRG_184	115650	TNFRSF13C	0.466
LRG_187	2542	SLC37A4	0.581
LRG_188	3557	IL1RN	0.636
LRG_189	3702	ITK	0.527
LRG_190	4210	MEFV	0.455
LRG_193	7132	TNFRSF1A	0.55
LRG_194	8792	TNFRSF11A	0.502
LRG_195	11151	CORO1A	0.502
LRG_196	81704	DOCK8	0.59
LRG_197	114548	NLRP3	0.589
LRG_198	375790	AGRN	0.622
LRG_2	1278	COL1A2	0.58
LRG_200	2660	MSTN	0.391
LRG_201	9499	MYOT	0.425
LRG_203	6442	SGCA	0.478
LRG_204	6443	SGCB	0.569
LRG_207	6445	SGCG	0.44
LRG_208	137868	SGCZ	0.481
LRG_21	29760	BLNK	0.52
LRG_212	10269	ZMPSTE24	0.522
LRG_213	1073	CFL2	0.609
LRG_214	4763	NF1	0.713
LRG_22	712	C1QA	0.449
LRG_226	6899	TBX1	0.584
LRG_227	81494	CFHR5	0.416
LRG_228	8772	FADD	0.52
LRG_229	7187	TRAF3	0.568
LRG_23	713	C1QB	0.479
LRG_230	1285	COL4A3	0.66
LRG_231	1286	COL4A4	0.488
LRG_234	9639	ARHGEF10	0.582
LRG_236	9150	CTDP1	0.47
LRG_24	714	C1QC	0.518

LRG_241	9896	FIG4	0.53
LRG_242	8139	GAN	0.414
LRG_243	2617	GARS	0.577
LRG_248	3315	HSPB1	0.512
LRG_25	716	C1S	0.514
LRG_250	3508	IGHMBP2	0.55
LRG_251	8518	IKBKAP	0.581
LRG_252	23095	KIF1B	0.527
LRG_253	9516	LITAF	0.617
LRG_254	4000	LMNA	0.662
LRG_255	9927	MFN2	0.573
LRG_256	4359	MPZ	0.459
LRG_257	8898	MTMR2	0.595
LRG_258	10397	NDRG1	0.574
LRG_26	717	C2	0.523
LRG_260	4803	NGF	0.484
LRG_262	57449	PLEKHG5	0.458
LRG_263	5376	PMP22	0.575
LRG_264	5631	PRPS1	0.62
LRG_265	57716	PRX	0.533
LRG_266	7879	RAB7A	0.617
LRG_267	81846	SBF2	0.497
LRG_268	23064	SETX	0.583
LRG_27	718	C3	0.636
LRG_270	9990	SLC12A6	0.515
LRG_271	6663	SOX10	0.54
LRG_272	10558	SPTLC1	0.589
LRG_274	3949	LDLR	0.659
LRG_275	255738	PCSK9	0.465
LRG_276	26119	LDLRAP1	0.554
LRG_28	727	C5	0.491
LRG_283	3953	LEPR	0.627
LRG_287	3784	KCNQ1	0.596
LRG_288	3757	KCNH2	0.583
LRG_289	6331	SCN5A	0.482
LRG_29	729	C6	0.497
LRG_290	3753	KCNE1	0.466
LRG_291	9992	KCNE2	0.434
LRG_294	3394	IRF8	0.526
LRG_295	2624	GATA2	0.646
LRG_3	1281	COL3A1	0.567
LRG_30	730	C7	0.499
LRG_31	732	C8B	0.468
LRG_32	735	C9	0.446
LRG_326	9841	ZBTB24	0.504
LRG_327	287	ANK2	0.619
LRG_328	3759	KCNJ2	0.509
LRG_329	859	CAV3	0.449
LRG_33	843	CASP10	0.638
LRG_330	6330	SCN4B	0.405
LRG_331	10142	AKAP9	0.602
LRG_332	6640	SNTA1	0.518
LRG_333	3762	KCNJ5	0.58
LRG_334	775	CACNA1C	0.578

LRG_335	2000	ELF4	0.602
LRG_336	1373	CPS1	0.555
LRG_34	841	CASP8	0.608
LRG_35	930	CD19	0.484
LRG_36	919	CD247	0.494
LRG_37	915	CD3D	0.482
LRG_38	916	CD3E	0.5
LRG_39	917	CD3G	0.484
LRG_4	10491	CRTAP	0.546
LRG_40	958	CD40	0.632
LRG_41	966	CD59	0.687
LRG_42	973	CD79A	0.506
LRG_43	974	CD79B	0.505
LRG_44	925	CD8A	0.463
LRG_45	1053	CEBPE	0.466
LRG_46	1675	CFD	0.534
LRG_47	3075	CFH	0.506
LRG_48	3426	CFI	0.499
LRG_49	4261	CIITA	0.602
LRG_5	64175	LEPRE1	0.502
LRG_50	1075	CTSC	0.595
LRG_51	7852	CXCR4	0.585
LRG_52	1535	CYBA	0.544
LRG_53	1536	CYBB	0.54
LRG_54	64421	DCLRE1C	0.592
LRG_55	1736	DKC1	0.619
LRG_56	1789	DNMT3B	0.513
LRG_57	1991	ELA2	0.439
LRG_58	356	FASLG	0.578
LRG_59	2209	FCGR1A	0.548
LRG_6	477	ATP1A2	0.537
LRG_60	2214	FCGR3A	0.548
LRG_61	8456	FOXN1	0.447
LRG_62	50943	FOXP3	0.529
LRG_63	2672	GFI1	0.486
LRG_64	10456	HAX1	0.525
LRG_65	29851	ICOS	0.467
LRG_66	3459	IFNGR1	0.613
LRG_69	3543	IGLL1	0.477
LRG_7	773	CACNA1A	0.575
LRG_70	8517	IKBKG	0.55
LRG_71	3593	IL12B	0.439
LRG_73	3559	IL2RA	0.548
LRG_74	3575	IL7R	0.557
LRG_75	51135	IRAK4	0.476
LRG_76	3689	ITGB2	0.514
LRG_77	3718	JAK3	0.611
LRG_79	3981	LIG4	0.519
LRG_8	6323	SCN1A	0.433
LRG_80	56262	LRRC8A	0.536
LRG_81	28956	RP11-336K24.9	0.512
LRG_83	79083	MLPH	0.54
LRG_84	4353	MPO	0.531
LRG_85	4361	MRE11A	0.586

LRG_86	4644	MYO5A	0.634
LRG_88	4688	NCF2	0.552
LRG_89	4792	NFKBIA	0.593
LRG_90	79840	NHEJ1	0.464
LRG_91	4860	NP	0.559
LRG_92	4893	NRAS	0.526
LRG_93	84876	ORAI1	0.476
LRG_94	5551	PRF1	0.512
LRG_96	5873	RAB27A	0.62
LRG_97	5880	RAC2	0.612
LRG_98	5896	RAG1	0.45
LRG_99	5897	RAG2	0.442
ENSG00000181143	94025	MUC16	0.417
ENSG00000111181	6539	SLC6A12	0.494
ENSG00000168904	123355	LRRC28	0.602
ENSG00000002016	5893	RAD52	0.577
ENSG00000153531	113622	ADPRHL1	0.455
ENSG00000198176	7027	TFDP1	0.637
ENSG00000183087	2621	GAS6	0.609
ENSG00000154237	79705	LRRK1	0.445
ENSG00000185989	22821	RASA3	0.549
ENSG00000108107	6158	RPL28	0.579
ENSG00000108106	27338	UBE2S	0.683
ENSG00000063241	79763	ISOC2	0.482
ENSG00000090971	57106	NAT14	0.432
ENSG00000131876	6627	SNRPA1	0.591
ENSG00000171443	147807	ZNF524	0.356
ENSG00000140479	5046	PCSK6	0.613
ENSG00000063244	11338	U2AF2	0.596
ENSG00000198046	63934	ZNF667	0.451
ENSG00000197951	58491	ZNF71	0.521
ENSG00000083844	9422	ZNF264	0.507
ENSG00000131845	57343	ZNF304	0.475
ENSG00000198131	27300	ZNF544	0.489
ENSG00000171574	201514	ZNF584	0.447
ENSG00000130726	10155	TRIM28	0.519
ENSG00000130725	9040	UBE2M	0.573
ENSG00000134516	1794	DOCK2	0.518
ENSG00000170819	8419	BFSP2	0.489
ENSG00000179674	80117	ARL14	0.435
ENSG00000065413	91526	ANKRD44	0.557
ENSG00000168269	2299	FOXI1	0.467
ENSG00000182923	80254	CEP63	0.534
ENSG00000164438	30012	TLX3	0.435
ENSG00000181163	4869	NPM1	0.595
ENSG00000115425	55825	PECR	0.49
ENSG00000079246	7520	XRCC5	0.629
ENSG00000120149	4488	MSX2	0.61
ENSG00000164466	94081	SFXN1	0.494
ENSG00000155729	130535	KCTD18	0.431
ENSG00000127837	14	AAMP	0.514
ENSG00000135926	64114	TMBIM1	0.539
ENSG00000181322	347736	TXNDC6	0.361
ENSG00000163581	6514	SLC2A2	0.456

ENSG00000138356	316	AOX1	0.563
ENSG00000183072	1482	NKX2-5	0.473
ENSG00000066405	51208	CLDN18	0.551
ENSG00000114107	80321	CEP70	0.489
ENSG00000146066	192286	HIGD2A	0.597
ENSG00000175416	1212	CLTB	0.659
ENSG00000114346	1894	ECT2	0.52
ENSG00000115592	53632	PRKAG3	0.444
ENSG00000128641	4430	MYO1B	0.649
ENSG00000176753	644809	C15orf56	0.367
ENSG00000072571	3161	HMMR	0.596
ENSG00000140323	85455	DISP2	0.494
ENSG00000144339	23671	TMEFF2	0.556
ENSG00000114686	11222	MRPL3	0.562
ENSG00000110442	29099	COMMD9	0.485
ENSG00000137812	57082	CASC5	0.445
ENSG00000051180	5888	RAD51	0.544
ENSG00000175097	5897	RAG2	0.442
ENSG00000137824	55177	FAM82A2	0.512
ENSG00000104142	57617	VPS18	0.44
ENSG00000155256	118813	ZFYVE27	0.524
ENSG00000142686	127703	C1orf216	0.522
ENSG00000130119	54552	GNL3L	0.634
ENSG00000079689	10590	SCGN	0.503
ENSG00000146047	255626	HIST1H2BA	0.319
ENSG00000151348	2132	EXT2	0.586
ENSG00000103932	26015	RPAP1	0.496
ENSG00000179593	247	ALOX15B	0.5
ENSG00000175274	9537	TP53I11	0.627
ENSG00000196476	140680	C20orf96	0.372
ENSG00000143315	93183	PIGM	0.536
ENSG00000188021	29978	UBQLN2	0.543
ENSG00000186787	474343	SPIN2B	0.441
ENSG00000124610	3024	HIST1H1A	0.43
ENSG00000125841	80023	NRSN2	0.448
ENSG00000171812	1296	COL8A2	0.555
ENSG00000198366	8350	HIST1H3A	0.4
ENSG00000196176	8294	HIST1H4I	0.459
ENSG00000124529	8294	HIST1H4I	0.459
ENSG00000124693	8350	HIST1H3A	0.4
ENSG00000137259	3012	HIST1H2AE	0.441
ENSG00000163029	79677	SMC6	0.518
ENSG00000147059	54466	SPIN2A	0.465
ENSG00000196226	3018	HIST1H2BB	0.41
ENSG00000196532	8350	HIST1H3A	0.4
ENSG00000186767	139886	SPIN4	0.477
ENSG00000187837	3006	HIST1H1C	0.57
ENSG00000010704	3077	HFE	0.638
ENSG00000198881	142689	ASB12	0.434
ENSG00000179029	84314	TMEM107	0.589
ENSG00000158578	212	ALAS2	0.467
ENSG00000197061	8294	HIST1H4I	0.459
ENSG00000187475	3010	HIST1H1T	0.438
ENSG00000180596	8339	HIST1H2BG	0.564

ENSG00000180573	8334	HIST1H2AC	0.569
ENSG00000168298	3008	HIST1H1E	0.459
ENSG00000197697	8339	HIST1H2BG	0.564
ENSG00000188987	8294	HIST1H4I	0.459
ENSG00000197409	8350	HIST1H3A	0.4
ENSG00000197846	8339	HIST1H2BG	0.564
ENSG00000198518	8294	HIST1H4I	0.459
ENSG00000187990	8339	HIST1H2BG	0.564
ENSG00000168274	3012	HIST1H2AE	0.441
ENSG00000143318	844	CASQ1	0.477
ENSG00000124575	3007	HIST1H1D	0.455
ENSG00000198327	8294	HIST1H4I	0.459
ENSG00000168242	8339	HIST1H2BG	0.564
ENSG00000158406	8294	HIST1H4I	0.459
ENSG00000107554	23268	DNMBP	0.546
ENSG00000124635	8970	HIST1H2BJ	0.472
ENSG00000213928	10379	IRF9	0.544
ENSG00000186594	84981	C17orf91	0.547
ENSG00000115758	4953	ODC1	0.599
ENSG00000213341	1147	CHUK	0.504
ENSG00000185272	54033	RBM11	0.408
ENSG00000196747	8329	HIST1H2AI	0.501
ENSG00000203813	8350	HIST1H3A	0.4
ENSG00000182611	8331	HIST1H2AJ	0.481
ENSG00000215271	57594	HOMEZ	0.471
ENSG00000196374	8342	HIST1H2BM	0.446
ENSG00000120049	30819	KCNIP2	0.444
ENSG00000233822	8341	HIST1H2BN	0.473
ENSG00000198374	8329	HIST1H2AI	0.501
ENSG00000184357	3009	HIST1H1B	0.439
ENSG00000182572	8350	HIST1H3A	0.4
ENSG00000171307	84287	ZDHHC16	0.523
ENSG00000198558	8294	HIST1H4I	0.459
ENSG00000197153	8350	HIST1H3A	0.4
ENSG00000100836	8106	PABPN1	0.641
ENSG00000233224	8329	HIST1H2AI	0.501
ENSG00000196331	8348	HIST1H2BO	0.426
ENSG00000092096	51310	SLC22A17	0.578
ENSG00000117090	6504	SLAMF1	0.512
ENSG00000158423	158787	RIBC1	0.447
ENSG00000156304	57466	SFRS15	0.57
ENSG00000086758	10075	HUWE1	0.654
ENSG00000155229	64210	MMS19	0.481
ENSG00000198728	8861	LDB1	0.561
ENSG00000121905	3208	HPCA	0.427
ENSG00000213859	147040	KCTD11	0.444
ENSG00000112293	2822	GPLD1	0.628
ENSG00000187838	57048	PLSCR3	0.59
ENSG00000092108	23256	SCFD1	0.5
ENSG00000184560	201243	C17orf74	0.383
ENSG00000213983	8906	AP1G2	0.582
ENSG00000181222	5430	POLR2A	0.598
ENSG00000162510	4146	MATN1	0.559
ENSG00000179057	283284	IGSF22	0.5

ENSG00000107859	5309	PITX3	0.467
ENSG00000162511	7805	LAPTM5	0.586
ENSG00000161955	8741	TNFSF13	0.479
ENSG00000146112	170954	KIAA1949	0.563
ENSG00000165887	26287	ANKRD2	0.442
ENSG00000112312	51053	GMNN	0.559
ENSG00000179639	2205	FCER1A	0.488
ENSG00000161960	1973	EIF4A1	0.656
ENSG00000138030	3795	KHK	0.573
ENSG00000110435	8050	PDHX	0.532
ENSG00000129173	79733	E2F8	0.582
ENSG00000178971	80169	C17orf68	0.451
ENSG00000125878	6939	TCF15	0.504
ENSG00000163867	9204	ZMYM6	0.639
ENSG00000121769	2170	FABP3	0.562
ENSG00000125434	399512	SLC25A35	0.532
ENSG00000181817	84967	LSM10	0.476
ENSG00000142910	64129	TINAGL1	0.487
ENSG00000125775	27111	SDCBP2	0.436
ENSG00000162517	553115	PEF1	0.455
ENSG00000117751	5511	PPP1R8	0.517
ENSG00000101327	5173	PDYN	0.439
ENSG00000187486	3767	KCNJ11	0.394
ENSG00000154914	124739	USP43	0.446
ENSG00000132382	10514	MYBBP1A	0.479
ENSG00000125835	6628	SNRPB	0.609
ENSG00000117748	6118	RPA2	0.563
ENSG00000010072	83932	C1orf124	0.468
ENSG00000088876	79175	ZNF343	0.459
ENSG00000188176	342527	SMTNL2	0.427
ENSG00000109047	5957	RCVRN	0.473
ENSG00000118804	8987	STBD1	0.56
ENSG00000116918	7257	TSNAX	0.498
ENSG00000162946	27185	DISC1	0.619
ENSG00000158161	2140	EYA3	0.473
ENSG00000173207	1163	CKS1B	0.555
ENSG00000167702	90990	KIFC2	0.5
ENSG00000173540	29925	GMPPB	0.502
ENSG00000129235	84817	TXNDC17	0.576
ENSG00000163352	55891	LENEP	0.406
ENSG00000165169	6990	DYNLT3	0.538
ENSG00000091664	57084	SLC17A6	0.452
ENSG00000132635	64773	FAM113A	0.53
ENSG00000183161	2188	FANCF	0.454
ENSG00000131503	54882	ANKHD1	0.503
ENSG00000138303	51008	ASCC1	0.472
ENSG00000141485	284111	SLC13A5	0.405
ENSG00000183520	51118	UTP11L	0.528
ENSG00000196642	55684	C9orf86	0.511
ENSG00000187398	338645	LUZP2	0.444
ENSG00000163092	129446	XIRP2	0.456
ENSG00000146414	257218	SHPRH	0.48
ENSG00000204713	5987	TRIM27	0.637
ENSG00000153283	10225	CD96	0.489

ENSG00000183763	10293	TRAIP	0.477
ENSG00000212916	54627	KIAA1383	0.396
ENSG00000143537	8751	ADAM15	0.525
ENSG00000129219	5338	PLD2	0.496
ENSG00000144824	90102	PHLDB2	0.586
ENSG00000107745	10367	CBARA1	0.507
ENSG00000178685	84875	PARP10	0.625
ENSG00000107223	8721	EDF1	0.575
ENSG00000204694	26531	OR11A1	0.415
ENSG00000109881	91057	CCDC34	0.504
ENSG00000164076	79012	CAMKV	0.479
ENSG00000110756	11234	HPS5	0.516
ENSG00000148925	84280	BTBD10	0.463
ENSG00000196407	284486	THEM5	0.441
ENSG00000243056	8637	EIF4EBP3	0.486
ENSG00000159445	117145	THEM4	0.562
ENSG00000083099	57226	LYRM2	0.501
ENSG00000121775	55116	TMEM39B	0.46
ENSG00000158246	115572	FAM46B	0.441
ENSG00000152254	57818	G6PC2	0.47
ENSG00000120656	6883	TAF12	0.535
ENSG00000159450	7062	TCHH	0.444
ENSG00000128602	6608	SMO	0.489
ENSG00000173918	114897	C1QTNF1	0.515
ENSG00000213977	30851	TAX1BP3	0.613
ENSG00000152266	5741	PTH	0.444
ENSG00000138308	84647	PLA2G12B	0.485
ENSG00000143520	388698	FLG2	0.364
ENSG00000122884	5033	P4HA1	0.548
ENSG00000151615	5458	POU4F2	0.433
ENSG00000169519	196074	METT5D1	0.556
ENSG00000145687	23635	SSBP2	0.61
ENSG00000116329	4985	OPRD1	0.446
ENSG00000090889	24137	KIF4A	0.544
ENSG00000186517	257106	ARHGAP30	0.512
ENSG00000128578	57464	FAM40B	0.55
ENSG00000164077	84315	MON1A	0.441
ENSG00000167881	6730	SRP68	0.513
ENSG00000160051	55721	IQCC	0.412
ENSG00000152348	83734	ATG10	0.522
ENSG00000091972	4345	CD200	0.585
ENSG00000156269	84779	ARD1B	0.492
ENSG00000186265	151888	BTLA	0.438
ENSG00000113119	55374	TMCO6	0.505
ENSG00000240428	96626	LIMS3	0.438
ENSG00000182473	23265	EXOC7	0.638
ENSG00000169242	1942	EFNA1	0.54
ENSG00000122375	94233	OPN4	0.382
ENSG00000144063	7851	MALL	0.525
ENSG00000106123	2051	EPHB6	0.485
ENSG00000144061	4867	NPHP1	0.485
ENSG00000091640	9552	SPAG7	0.498
ENSG00000183615	84734	FAM167B	0.409
ENSG00000214655	23053	KIAA0913	0.539

ENSG00000165125	55503	TRPV6	0.468
ENSG00000177688	387082	SUMO4	0.48
ENSG00000074370	489	ATP2A3	0.568
ENSG00000152784	56978	PRDM8	0.43
ENSG00000198589	987	LRBA	0.573
ENSG00000213551	23234	DNAJC9	0.589
ENSG00000126010	2925	GRPR	0.488
ENSG00000166265	116159	CYYR1	0.488
ENSG00000131013	85313	PPIL4	0.5
ENSG00000146842	84928	TMEM209	0.459
ENSG00000188015	6274	S100A3	0.496
ENSG00000165131	135927	C7orf34	0.466
ENSG00000138669	5593	PRKG2	0.456
ENSG00000148297	6837	MED22	0.525
ENSG00000165120	136263	C7orf45	0.521
ENSG00000152904	9453	GGPS1	0.596
ENSG00000185499	4582	MUC1	0.655
ENSG00000205060	84912	SLC35B4	0.582
ENSG00000172469	79694	MANEA	0.451
ENSG00000189334	57402	S100A14	0.46
ENSG00000177239	11253	MAN1B1	0.569
ENSG00000143222	51506	UFC1	0.532
ENSG00000135378	79056	PRRG4	0.511
ENSG00000116957	6905	TBCE	0.559
ENSG00000102054	5931	RBBP7	0.528
ENSG00000181045	284129	SLC26A11	0.469
ENSG00000112855	23438	HARS2	0.492
ENSG00000176148	55346	TCP11L1	0.545
ENSG00000165349	84889	SLC7A3	0.465
ENSG00000000938	2268	FGR	0.481
ENSG00000132507	1984	EIF5A	0.607
ENSG00000120253	348995	NUP43	0.508
ENSG00000189221	4128	MAOA	0.62
ENSG00000157379	115817	DHRS1	0.568
ENSG00000160679	26097	C1orf77	0.604
ENSG00000107819	81855	SFXN3	0.591
ENSG00000144357	130507	UBR3	0.529
ENSG00000166689	144100	PLEKHA7	0.448
ENSG00000156253	10069	RWDD2B	0.47
ENSG00000144857	91653	BOC	0.548
ENSG00000143621	3608	ILF2	0.525
ENSG00000122861	5328	PLAU	0.601
ENSG00000161544	114757	CYGB	0.479
ENSG00000156256	10600	USP16	0.515
ENSG00000143545	5872	RAB13	0.543
ENSG00000222047	414236	C10orf55	0.35
ENSG00000070731	10610	ST6GALNAC2	0.548
ENSG00000184634	9968	MED12	0.623
ENSG00000117758	23673	STX12	0.547
ENSG00000146859	55281	TMEM140	0.5
ENSG00000166348	159195	USP54	0.527
ENSG00000182534	439921	MXRA7	0.52
ENSG00000147679	84294	UTP23	0.434
ENSG00000189184	54510	PCDH18	0.577

ENSG00000204618	80352	RNF39	0.512
ENSG00000143549	7170	TPM3	0.488
ENSG00000112378	64065	PERP	0.555
ENSG00000144290	57282	SLC4A10	0.442
ENSG00000182782	338442	GPR109A	0.576
ENSG00000048140	26262	TSPAN17	0.519
ENSG00000196338	54413	NLGN3	0.447
ENSG00000100968	4776	NFATC4	0.574
ENSG00000167889	146664	MGAT5B	0.463
ENSG00000160883	3101	HK3	0.532
ENSG00000053918	3784	KCNQ1	0.596
ENSG00000151834	2555	GABRA2	0.517
ENSG00000197635	1803	DPP4	0.607
ENSG00000155754	151254	ALS2CR11	0.569
ENSG00000136999	4856	NOV	0.561
ENSG00000118640	8673	VAMP8	0.539
ENSG00000160867	2264	FGFR4	0.583
ENSG00000163288	2560	GABRB1	0.464
ENSG00000168899	10791	VAMP5	0.622
ENSG00000181649	7262	PHLDA2	0.605
ENSG00000081154	57092	PCNP	0.493
ENSG00000078098	2191	FAP	0.492
ENSG00000107831	2253	FGF8	0.446
ENSG00000117245	57576	KIF17	0.475
ENSG00000136636	51133	KCTD3	0.488
ENSG00000173567	165082	GPR113	0.471
ENSG00000165949	3429	IFI27	0.507
ENSG00000204590	2794	GNL1	0.552
ENSG00000166471	440026	TMEM41B	0.583
ENSG00000130713	23404	EXOSC2	0.621
ENSG00000117640	56181	FAM54B	0.487
ENSG00000082438	22837	COBLL1	0.592
ENSG00000130720	84929	FIBCD1	0.457
ENSG00000174231	10594	PRPF8	0.543
ENSG00000148337	25792	CIZ1	0.591
ENSG00000114439	56987	BBX	0.561
ENSG00000136986	79139	DERL1	0.572
ENSG00000131188	80758	PRR7	0.504
ENSG00000158106	114822	RHPN1	0.487
ENSG00000112200	26036	ZNF451	0.589
ENSG00000117632	3925	STMN1	0.601
ENSG00000133789	23075	SWAP70	0.634
ENSG00000092978	55105	GPATCH2	0.501
ENSG00000158006	5051	PAFAH2	0.558
ENSG00000132670	5786	PTPRA	0.567
ENSG00000120756	5357	PLS1	0.491
ENSG00000188488	5104	SERPINA5	0.496
ENSG00000163815	7123	CLEC3B	0.464
ENSG00000126883	8021	NUP214	0.522
ENSG00000138380	79800	ALS2CR8	0.491
ENSG00000158014	7780	SLC30A2	0.495
ENSG00000158022	84676	TRIM63	0.456
ENSG00000147689	84985	FAM83A	0.552
ENSG00000170464	202052	DNAJC18	0.534

ENSG00000153147	8467	SMARCA5	0.593
ENSG00000147813	93100	NAPRT1	0.479
ENSG00000249992	25907	TMEM158	0.561
ENSG00000011376	23395	LARS2	0.573
ENSG00000007174	1770	DNAH9	0.526
ENSG00000197245	79927	GRRP1	0.417
ENSG00000133937	145258	GSC	0.48
ENSG00000114455	11148	HHLA2	0.404
ENSG00000135547	23493	HEY2	0.481
ENSG00000198221	653483	C6orf124	0.446
ENSG00000155324	65983	GRAMD3	0.526
ENSG00000119004	57404	CYP20A1	0.454
ENSG00000108963	1801	DPH1	0.466
ENSG00000027847	11285	B4GALT7	0.564
ENSG00000082258	905	CCNT2	0.652
ENSG00000167110	2801	GOLGA2	0.579
ENSG00000158458	9542	NRG2	0.576
ENSG00000049167	1161	ERCC8	0.514
ENSG00000135604	8676	STX11	0.509
ENSG00000183682	353500	BMP8A	0.542
ENSG00000163686	57406	ABHD6	0.641
ENSG00000198919	9666	DZIP3	0.63
ENSG00000163818	54585	LZTFL1	0.513
ENSG00000168297	54899	PXK	0.526
ENSG00000163599	1493	CTLA4	0.503
ENSG00000176438	161176	C14orf49	0.469
ENSG00000179886	84948	TIGD5	0.4
ENSG00000066336	6688	SPI1	0.487
ENSG00000169442	1043	CD52	0.573
ENSG00000168291	5162	PDHB	0.594
ENSG00000145794	84466	MEGF10	0.478
ENSG00000113240	57396	CLK4	0.529
ENSG00000088827	6614	SIGLEC1	0.543
ENSG00000048991	23518	R3HDM1	0.613
ENSG00000156671	142891	SAMD8	0.447
ENSG00000091482	23676	SMPX	0.448
ENSG00000168243	2786	GNG4	0.546
ENSG00000112218	81491	GPR63	0.472
ENSG00000143669	1130	LYST	0.615
ENSG00000101220	54976	C20orf27	0.599
ENSG00000243414	353376	TICAM2	0.573
ENSG00000113812	93973	ACTR8	0.469
ENSG00000114349	2779	GNAT1	0.554
ENSG00000134970	51014	TMED7	0.577
ENSG00000241322	374286	CDRT1	0.541
ENSG00000186231	114792	KLHL32	0.432
ENSG00000166401	5271	SERPINB8	0.557
ENSG00000157445	55799	CACNA2D3	0.452
ENSG00000160563	9442	MED27	0.624
ENSG00000196358	84628	NTNG2	0.462
ENSG00000198643	131177	FAM3D	0.46
ENSG00000177879	1176	AP3S1	0.562
ENSG00000153107	64682	ANAPC1	0.482
ENSG00000153064	55024	BANK1	0.483

ENSG00000166813	374654	KIF7	0.433
ENSG00000165655	84858	ZNF503	0.5
ENSG00000172901	206338	LVRN	0.452
ENSG00000116962	4811	NID1	0.603
ENSG00000123444	55709	KBTBD4	0.537
ENSG00000134882	337867	UBAC2	0.515
ENSG00000176225	25914	RTTN	0.541
ENSG00000144229	80731	THSD7B	0.48
ENSG00000138821	64116	SLC39A8	0.634
ENSG00000066583	51015	ISOC1	0.532
ENSG00000077585	7107	GPR137B	0.557
ENSG00000150540	3176	HNMT	0.621
ENSG00000188800	127391	TMCO2	0.434
ENSG00000166342	81832	NETO1	0.569
ENSG00000171873	146	ADRA1D	0.55
ENSG00000214128	155006	TMEM213	0.416
ENSG00000037749	4238	MFAP3	0.598
ENSG00000158985	56990	CDC42SE2	0.54
ENSG00000153214	84910	TMEM87B	0.52
ENSG00000068001	8692	HYAL2	0.519
ENSG00000142623	29943	PADI1	0.472
ENSG00000144152	129804	FBLN7	0.453
ENSG00000183020	161	AP2A2	0.626
ENSG00000102452	259232	NALCN	0.437
ENSG00000068028	11186	RASSF1	0.515
ENSG00000138448	3685	ITGAV	0.598
ENSG00000169570	285605	DTWD2	0.463
ENSG00000030066	23279	NUP160	0.587
ENSG00000163630	132204	SYNPR	0.404
ENSG00000215421	55628	ZNF407	0.492
ENSG00000198945	84456	L3MBTL3	0.495
ENSG00000144369	165215	FAM171B	0.5
ENSG00000214717	9189	ZBED1	0.527
ENSG00000075884	55843	ARHGAP15	0.505
ENSG00000064989	10203	CALCRL	0.55
ENSG00000004838	51364	ZMYND10	0.551
ENSG00000081800	6561	SLC13A1	0.507
ENSG00000151715	120224	TMEM45B	0.534
ENSG00000171224	219738	C10orf35	0.504
ENSG00000114656	57501	KIAA1257	0.438
ENSG00000134899	2073	ERCC5	0.563
ENSG00000111877	254394	MCM9	0.433
ENSG00000236279	154790	CLEC2L	0.4
ENSG00000182346	267012	DAOA	0.511
ENSG00000179023	127707	KLHDC7A	0.528
ENSG00000114395	11068	CYB561D2	0.497
ENSG00000114654	79825	CCDC48	0.463
ENSG00000138326	6229	RPS24	0.522
ENSG00000184838	51334	PRR16	0.462
ENSG00000072135	26469	PTPN18	0.597
ENSG00000146809	142685	ASB15	0.341
ENSG00000113083	4015	LOX	0.601
ENSG00000164039	56898	BDH2	0.5
ENSG00000077522	88	ACTN2	0.577

ENSG00000139725	54509	RHOF	0.637
ENSG00000113263	3702	ITK	0.527
ENSG00000088543	51161	C3orf18	0.471
ENSG00000154188	284	ANGPT1	0.605
ENSG00000184897	8971	H1FX	0.552
ENSG00000101544	22850	ADNP2	0.571
ENSG00000152661	2697	GJA1	0.532
ENSG00000085382	57531	HACE1	0.49
ENSG00000129071	8930	MBD4	0.638
ENSG00000104408	3646	EIF3E	0.533
ENSG00000179603	2918	GRM8	0.549
ENSG00000168772	80319	CXXC4	0.451
ENSG00000136695	26525	IL1F5	0.456
ENSG00000006757	8228	PNPLA4	0.567
ENSG00000176463	28232	SLCO3A1	0.601
ENSG00000115762	55041	PLEKHB2	0.473
ENSG00000011201	3730	KAL1	0.512
ENSG00000136689	3557	IL1RN	0.636
ENSG00000183643	145858	C15orf32	0.444
ENSG00000130592	4046	LSP1	0.526
ENSG00000185442	400451	FAM174B	0.595
ENSG00000167094	158248	TTC16	0.409
ENSG00000186439	10345	TRDN	0.45
ENSG00000126216	10426	TUBGCP3	0.581
ENSG00000168938	5480	PPIC	0.581
ENSG00000184047	56616	DIABLO	0.496
ENSG00000095370	10044	SH2D3C	0.511
ENSG00000106328	29999	FSCN3	0.423
ENSG00000159377	5692	PSMB4	0.555
ENSG00000146373	154214	RNF217	0.405
ENSG00000166228	5092	PCBD1	0.548
ENSG00000183840	2863	GPR39	0.464
ENSG00000082512	7188	TRAF5	0.54
ENSG00000073464	1183	CLCN4	0.538
ENSG00000136877	2356	FPGS	0.484
ENSG00000113282	9685	CLINT1	0.573
ENSG00000115363	84141	FAM176A	0.465
ENSG00000053372	51154	MRTO4	0.484
ENSG00000115364	9801	MRPL19	0.536
ENSG00000189337	23254	RP1-21O18.1	0.6
ENSG00000145860	153830	RNF145	0.516
ENSG00000111011	65117	RSRC2	0.588
ENSG00000170899	2941	GSTA4	0.552
ENSG00000182606	22906	TRAK1	0.646
ENSG00000143954	130120	REG3G	0.409
ENSG00000104341	55353	LAPTM4B	0.639
ENSG00000179299	79730	NSUN7	0.517
ENSG00000113312	7265	TTC1	0.52
ENSG00000172752	256076	COL29A1	0.489
ENSG00000164400	1437	CSF2	0.581
ENSG00000140995	54849	DEF8	0.472
ENSG00000151503	23310	NCAPD3	0.554
ENSG00000115947	5000	ORC4L	0.546
ENSG00000157093	131375	LYZL4	0.404

ENSG00000132541	10247	HRSP12	0.516
ENSG00000206384	131873	COL6A6	0.392
ENSG00000145365	92610	TIFA	0.561
ENSG00000145861	114898	C1QTNF2	0.5
ENSG00000135248	84691	FAM71F1	0.518
ENSG00000125510	4987	OPRL1	0.556
ENSG00000187939	8447	DOC2B	0.434
ENSG00000164609	10569	SLU7	0.527
ENSG00000089154	10985	GCN1L1	0.556
ENSG00000012660	60481	ELOVL5	0.664
ENSG00000171204	55863	TMEM126B	0.529
ENSG00000203880	55251	PCMTD2	0.51
ENSG00000174775	3265	HRAS	0.536
ENSG00000196459	6399	TRAPPC2	0.525
ENSG00000137501	54843	SYTL2	0.519
ENSG00000156486	3788	KCNS2	0.459
ENSG00000132549	157680	VPS13B	0.565
ENSG00000135218	948	CD36	0.549
ENSG00000112282	9439	MED23	0.494
ENSG00000117697	25936	NSL1	0.53
ENSG00000108379	7473	WNT3	0.485
ENSG00000113522	10111	RAD50	0.614
ENSG00000075223	10512	SEMA3C	0.599
ENSG00000116786	23207	PLEKHM2	0.516
ENSG00000162461	284723	SLC25A34	0.454
ENSG00000152455	79723	SUV39H2	0.503
ENSG00000164283	11082	ESM1	0.467
ENSG00000113088	3003	GZMK	0.475
ENSG00000089163	23409	SIRT4	0.541
ENSG00000019991	3082	HGF	0.622
ENSG00000113520	3565	IL4	0.553
ENSG00000162769	28982	FLVCR1	0.473
ENSG00000088986	8655	DYNLL1	0.529
ENSG00000080345	55183	RIF1	0.582
ENSG00000042445	54884	RETSAT	0.48
ENSG00000110871	84274	COQ5	0.448
ENSG00000153956	781	CACNA2D1	0.443
ENSG00000074266	8726	EED	0.562
ENSG00000079931	26002	MOXD1	0.526
ENSG00000144746	10550	ARL6IP5	0.614
ENSG00000163380	56203	LMOD3	0.5
ENSG00000014824	10463	SLC30A9	0.528
ENSG00000164406	116842	LEAP2	0.509
ENSG00000067113	8611	PPAP2A	0.573
ENSG00000177058	153129	SLC38A9	0.492
ENSG00000060718	1301	COL11A1	0.545
ENSG00000142627	1969	EPHA2	0.577
ENSG00000153993	223117	SEMA3D	0.447
ENSG00000164100	9348	NDST3	0.459
ENSG00000198822	2913	GRM3	0.452
ENSG00000164099	8492	PRSS12	0.491
ENSG00000135100	6927	HNF1A	0.556
ENSG00000153485	26175	C14orf109	0.556
ENSG00000185946	55599	RNPC3	0.551

ENSG00000081059	6932	TCF7	0.599
ENSG00000172399	51778	MYOZ2	0.526
ENSG00000058453	9696	CROCC	0.542
ENSG00000163421	60675	PROK2	0.43
ENSG00000177685	283229	EFCAB4A	0.424
ENSG00000169155	23099	ZBTB43	0.593
ENSG00000117122	4237	MFAP2	0.509
ENSG00000172986	727936	GLT8D4	0.424
ENSG00000135124	5025	P2RX4	0.529
ENSG00000115145	10254	STAM2	0.576
ENSG00000155090	7071	KLF10	0.559
ENSG00000173275	203523	ZNF449	0.5
ENSG00000105549	51298	THEG	0.404
ENSG00000112039	2178	FANCE	0.506
ENSG00000148773	4288	MKI67	0.628
ENSG00000152700	51128	SAR1B	0.525
ENSG00000110172	26973	CHORDC1	0.5
ENSG00000070423	55658	RNF126	0.541
ENSG00000173376	79625	C4orf31	0.522
ENSG00000099864	5064	PALM	0.496
ENSG00000118514	64577	ALDH8A1	0.476
ENSG00000170430	4255	MGMT	0.549
ENSG00000120087	3217	HOXB7	0.578
ENSG00000159184	10481	HOXB13	0.492
ENSG00000154781	51244	C3orf19	0.489
ENSG00000112041	7287	TULP1	0.474
ENSG00000198858	91300	C19orf22	0.571
ENSG00000116032	116444	GRIN3B	0.37
ENSG00000164112	132332	TMEM155	0.456
ENSG00000167468	2879	GPX4	0.521
ENSG00000175470	55844	PPP2R2D	0.466
ENSG00000096063	6732	SRPK1	0.563
ENSG00000127418	53834	FGFRL1	0.488
ENSG00000145386	890	CCNA2	0.62
ENSG00000151640	10570	DPYSL4	0.593
ENSG00000115255	92840	REEP6	0.434
ENSG00000251380	140947	C5orf20	0.384
ENSG00000136541	57471	ERMN	0.448
ENSG00000182185	5890	RAD51L1	0.522
ENSG00000079313	57455	REXO1	0.557
ENSG00000177465	122970	ACOT4	0.473
ENSG00000129968	81926	FAM108A1	0.504
ENSG00000227500	113178	SCAMP4	0.487
ENSG00000147400	1069	CETN2	0.531
ENSG00000112195	79865	TREML2	0.436
ENSG00000183856	128239	IQGAP3	0.537
ENSG00000147394	7739	ZNF185	0.512
ENSG00000096264	9436	NCR2	0.55
ENSG00000162928	5194	PEX13	0.513
ENSG00000142319	6531	SLC6A3	0.485
ENSG00000130332	51690	LSM7	0.571
ENSG00000157219	3361	HTR5A	0.432
ENSG00000099860	4616	GADD45B	0.668
ENSG00000138035	87178	PNPT1	0.516

ENSG00000170854	84864	MINA	0.569
ENSG00000134595	6658	SOX3	0.494
ENSG00000163132	4487	MSX1	0.572
ENSG00000146910	285888	CNPY1	0.39
ENSG00000115380	2202	EFEMP1	0.559
ENSG00000197977	54898	ELOVL2	0.554
ENSG00000105325	51343	FZR1	0.648
ENSG00000173209	130872	AHSA2	0.59
ENSG00000064961	10362	HMG20B	0.609
ENSG00000177733	10949	HNRNPA0	0.631
ENSG00000179542	139065	SLITRK4	0.461
ENSG00000159733	57732	ZFYVE28	0.495
ENSG00000148826	84504	NKX6-2	0.405
ENSG00000170561	153572	IRX2	0.506
ENSG00000113262	2916	GRM6	0.451
ENSG00000156050	145483	FAM161B	0.483
ENSG00000167658	1938	EEF2	0.556
ENSG00000105229	51588	PIAS4	0.521
ENSG00000163755	84343	HPS3	0.625
ENSG00000111863	84830	C6orf105	0.495
ENSG00000055813	114800	CCDC85A	0.508
ENSG00000145536	170690	ADAMTS16	0.438
ENSG00000164663	25862	USP49	0.486
ENSG00000081237	5788	PTPRC	0.621
ENSG00000124641	9477	MED20	0.584
ENSG00000136536	64844	7-Mar	0.538
ENSG00000155465	9056	SLC7A7	0.524
ENSG00000072832	1400	CRMP1	0.477
ENSG00000197177	84435	GPR123	0.341
ENSG00000145569	54491	FAM105A	0.502
ENSG00000204186	57683	ZDBF2	0.412
ENSG00000187097	957	ENTPD5	0.493
ENSG00000169908	4071	TM4SF1	0.646
ENSG00000197430	93377	OPALIN	0.416
ENSG00000134326	129607	CMPK2	0.441
ENSG00000173545	90441	ZNF622	0.573
ENSG00000147382	92002	FAM58A	0.443
ENSG00000198937	154467	C6orf129	0.5
ENSG00000134321	91543	RSAD2	0.494
ENSG00000105519	828	CAPS	0.565
ENSG00000087266	6452	SH3BP2	0.601
ENSG00000109501	7466	WFS1	0.524
ENSG00000205683	8110	DPF3	0.501
ENSG00000130545	92359	CRB3	0.391
ENSG00000114166	8850	KAT2B	0.532
ENSG00000125730	718	C3	0.636
ENSG00000119865	25927	CNRIP1	0.487
ENSG00000105202	2091	FBL	0.578
ENSG00000164530	221476	PI16	0.457
ENSG00000124523	23408	SIRT5	0.587
ENSG00000130544	79230	ZNF557	0.429
ENSG00000115421	64895	PAPOLG	0.563
ENSG00000158402	995	CDC25C	0.572
ENSG00000162702	23528	ZNF281	0.516

ENSG00000112599	2979	GUCA1B	0.504
ENSG00000163444	92703	TMEM183A	0.463
ENSG00000118193	9928	KIF14	0.522
ENSG00000182747	340146	SLC35D3	0.435
ENSG00000104938	10332	CLEC4M	0.524
ENSG00000016402	53832	IL20RA	0.479
ENSG00000048544	55173	MRPS10	0.527
ENSG00000179010	93621	MRFAP1	0.442
ENSG00000124767	2739	GLO1	0.532
ENSG00000143847	8497	PPFIA4	0.485
ENSG00000142449	84467	FBN3	0.361
ENSG00000090661	79603	LASS4	0.504
ENSG00000155984	84548	TMEM185A	0.454
ENSG00000167775	51293	CD320	0.513
ENSG00000154175	25890	ABI3BP	0.485
ENSG00000124721	1769	DNAH8	0.415
ENSG00000167785	148156	ZNF558	0.488
ENSG00000130803	57693	ZNF317	0.492
ENSG00000100678	6547	SLC8A3	0.464
ENSG00000168907	255189	PLA2G4F	0.443
ENSG00000119681	4053	LTBP2	0.586
ENSG00000050748	5601	MAPK9	0.583
ENSG00000185049	7469	WHSC2	0.547
ENSG00000174652	10781	ZNF266	0.54
ENSG00000169860	5028	P2RY1	0.462
ENSG00000185825	10134	BCAP31	0.522
ENSG00000197713	6120	RPE	0.503
ENSG00000171466	54811	ZNF562	0.481
ENSG00000122180	4656	MYOG	0.454
ENSG00000187800	375033	PEAR1	0.518
ENSG00000162927	150962	PUS10	0.528
ENSG00000105369	973	CD79A	0.506
ENSG00000174953	170506	DHX36	0.609
ENSG00000105409	478	ATP1A3	0.43
ENSG00000214013	2595	GANC	0.504
ENSG00000115484	10575	CCT4	0.533
ENSG00000144445	151050	C2orf67	0.5
ENSG00000105737	2901	GRIK5	0.62
ENSG00000105723	2931	GSK3A	0.573
ENSG00000163995	84448	ABLIM2	0.573
ENSG00000170142	7324	UBE2E1	0.527
ENSG00000040731	1008	CDH10	0.485
ENSG00000137198	2766	GMPR	0.538
ENSG00000185515	79184	BRCC3	0.582
ENSG00000105401	11140	CDC37	0.505
ENSG00000131116	126299	ZNF428	0.544
ENSG00000124459	7596	ZNF45	0.554
ENSG00000112164	2740	GLP1R	0.598
ENSG00000117036	2117	ETV3	0.504
ENSG00000080815	5663	PSEN1	0.626
ENSG00000167384	7733	ZNF180	0.455
ENSG00000091317	54918	CMTM6	0.581
ENSG00000112167	55776	C6orf64	0.471
ENSG00000163660	57018	CCNL1	0.506

ENSG00000186007	93273	LEMD1	0.42
ENSG00000007062	8842	PROM1	0.474
ENSG00000183718	84851	TRIM52	0.503
ENSG00000204052	221424	C6orf154	0.39
ENSG00000104853	1209	CLPTM1	0.568
ENSG00000182973	25904	CNOT10	0.508
ENSG00000203710	1378	CR1	0.611
ENSG00000104859	11129	SFRS16	0.548
ENSG00000119720	55051	C14orf102	0.473
ENSG00000163661	5806	PTX3	0.513
ENSG00000113600	735	C9	0.446
ENSG00000164627	221458	KIF6	0.486
ENSG00000159495	116179	TGM7	0.396
ENSG00000087338	64395	GMCL1	0.486
ENSG00000144452	26154	ABCA12	0.461
ENSG00000119718	8892	EIF2B2	0.517
ENSG00000137177	63971	KIF13A	0.511
ENSG00000166049	139135	PASD1	0.338
ENSG00000159788	6002	RGS12	0.635
ENSG00000174514	148808	MFSD4	0.614
ENSG00000132356	5562	PRKAA1	0.547
ENSG00000170266	2720	GLB1	0.54
ENSG00000244617	151516	ASPRV1	0.392
ENSG00000169564	5093	PCBP1	0.507
ENSG00000183862	1260	CNGA2	0.333
ENSG00000163534	115350	FCRL1	0.44
ENSG00000182013	55228	PNMAL1	0.488
ENSG00000141579	79755	ZNF750	0.408
ENSG00000090372	29888	STRN4	0.449
ENSG00000184194	54328	GPR173	0.415
ENSG00000128886	80237	ELL3	0.565
ENSG00000021645	9369	NRXN3	0.546
ENSG00000134375	10440	TIMM17A	0.647
ENSG00000119638	91754	NEK9	0.603
ENSG00000183853	55243	KIRREL	0.473
ENSG00000117280	8934	RAB7L1	0.583
ENSG00000242028	25764	HYPK	0.511
ENSG00000140259	4236	MFAP1	0.51
ENSG00000170498	3814	KISS1	0.487
ENSG00000169764	7360	UGP2	0.596
ENSG00000174567	127845	GOLT1A	0.407
ENSG00000115756	3241	HPCAL1	0.603
ENSG00000105447	83743	GRWD1	0.451
ENSG00000239779	23559	WBP1	0.472
ENSG00000174059	947	CD34	0.538
ENSG00000104133	80208	SPG11	0.527
ENSG00000105516	1628	DBP	0.562
ENSG00000109805	64151	NCAPG	0.607
ENSG00000105538	54922	RASIP1	0.519
ENSG00000087074	23645	PPP1R15A	0.605
ENSG00000165409	7253	TSHR	0.582
ENSG00000143977	6637	SNRPG	0.54
ENSG00000119714	8111	GPR68	0.455
ENSG00000115282	64427	TTC31	0.483

ENSG00000104826	3972	LHB	0.483
ENSG00000100012	266629	SEC14L3	0.433
ENSG00000035141	84908	FAM136A	0.539
ENSG00000133488	284904	SEC14L4	0.442
ENSG00000174123	81793	TLR10	0.52
ENSG00000130528	3270	HRC	0.463
ENSG00000145147	9353	SLIT2	0.532
ENSG00000124701	10930	APOBEC2	0.454
ENSG00000119685	23093	TTLL5	0.651
ENSG00000142541	23521	RPL13A	0.606
ENSG00000197712	92689	FAM114A1	0.523
ENSG00000196533	440712	C1orf186	0.451
ENSG00000075340	119	ADD2	0.604
ENSG00000126461	58506	SCAF1	0.452
ENSG00000089195	51605	TRMT6	0.53
ENSG00000126457	3276	PRMT1	0.52
ENSG00000121895	80008	TMEM156	0.456
ENSG00000010361	80199	FUZ	0.487
ENSG00000112655	5754	PTK7	0.517
ENSG00000151876	26272	FBXO4	0.463
ENSG00000166920	84419	C15orf48	0.451
ENSG00000100078	50487	PLA2G3	0.485
ENSG00000123689	50486	G0S2	0.532
ENSG00000105053	51231	VRK3	0.613
ENSG00000105357	79784	MYH14	0.627
ENSG00000157796	57728	WDR19	0.505
ENSG00000170382	10446	LRRN2	0.57
ENSG00000131398	3748	KCNC3	0.444
ENSG00000172404	150353	DNAJB7	0.414
ENSG00000161677	126119	JOSD2	0.367
ENSG00000130827	55558	PLXNA3	0.54
ENSG00000167747	84798	C19orf48	0.491
ENSG00000156575	10394	PRG3	0.457
ENSG00000186652	5553	PRG2	0.45
ENSG00000100401	5905	RANGAP1	0.573
ENSG00000119699	7043	TGFB3	0.561
ENSG00000169035	5650	KLK7	0.486
ENSG00000157869	9364	RAB28	0.545
ENSG00000102178	8266	UBL4A	0.499
ENSG00000010803	22955	SCMH1	0.5
ENSG00000128245	7533	YWHAH	0.57
ENSG00000172262	7690	ZNF131	0.559
ENSG00000100413	171568	POLR3H	0.542
ENSG00000154803	201163	FLCN	0.47
ENSG00000149150	8501	SLC43A1	0.526
ENSG00000116035	25806	VAX2	0.456
ENSG00000135624	10574	CCT7	0.551
ENSG00000185666	8224	SYN3	0.46
ENSG00000100417	5372	PMM1	0.527
ENSG00000105568	5518	PPP2R1A	0.52
ENSG00000127129	1907	EDN2	0.463
ENSG00000187091	5333	PLCD1	0.534
ENSG00000175329	91464	ISX	0.376
ENSG00000214872	219537	SMTNL1	0.354

ENSG00000156587	9246	UBE2L6	0.542
ENSG00000167766	55769	ZNF83	0.516
ENSG00000198911	6721	SREBF2	0.493
ENSG00000137216	55362	TMEM63B	0.534
ENSG00000100292	3162	HMOX1	0.552
ENSG00000156958	2585	GALK2	0.565
ENSG00000177096	150368	FAM109B	0.463
ENSG00000183172	91689	C22orf32	0.55
ENSG00000184983	4700	NDUFA6	0.601
ENSG00000100722	79882	ZC3H14	0.637
ENSG00000065978	4904	YBX1	0.597
ENSG00000151881	64417	C5orf28	0.494
ENSG00000132623	63926	ANKRD5	0.471
ENSG00000135625	1961	EGR4	0.528
ENSG00000114784	10289	EIF1B	0.514
ENSG00000116127	7840	ALMS1	0.634
ENSG00000112116	112744	IL17F	0.405
ENSG00000168032	956	ENTPD3	0.527
ENSG00000121897	11019	LIAS	0.514
ENSG00000159374	130951	C2orf65	0.475
ENSG00000112118	4172	MCM3	0.577
ENSG00000117385	64175	LEPRE1	0.502
ENSG00000162892	11009	IL24	0.512
ENSG00000100348	25828	TXN2	0.576
ENSG00000167614	57348	TTYH1	0.444
ENSG00000096384	3326	HSP90AB1	0.637
ENSG00000007350	8277	TKTL1	0.569
ENSG00000138592	9101	USP8	0.527
ENSG00000160219	139716	GAB3	0.567
ENSG00000100362	5816	PVALB	0.473
ENSG00000164010	114625	ERMAP	0.447
ENSG00000159307	80274	SCUBE1	0.434
ENSG00000186732	758	MPPED1	0.474
ENSG00000089123	55617	TASP1	0.49
ENSG00000153575	114791	TUBGCP5	0.467
ENSG00000100385	3560	IL2RB	0.518
ENSG00000182795	79098	C1orf116	0.557
ENSG00000133466	114904	C1QTNF6	0.474
ENSG00000128340	5880	RAC2	0.612
ENSG00000068793	23191	CYFIP1	0.576
ENSG00000242689	1270	CNTF	0.455
ENSG00000113456	5810	RAD1	0.611
ENSG00000185010	2157	F8	0.491
ENSG00000131899	3996	LLGL1	0.525
ENSG00000124574	89845	ABCC10	0.615
ENSG00000197629	219972	MPEG1	0.535
ENSG00000172940	9390	SLC22A13	0.444
ENSG00000179455	7681	MKRN3	0.426
ENSG00000165555	122945	C14orf148	0.454
ENSG00000189060	3005	H1F0	0.573
ENSG00000066322	64834	ELOVL1	0.556
ENSG00000144671	9389	SLC22A14	0.455
ENSG00000135636	8291	DYSF	0.52
ENSG00000163626	285521	COX18	0.476

ENSG00000159479	112950	MED8	0.588
ENSG00000142208	207	AKT1	0.527
ENSG00000198198	23334	KIAA0467	0.561
ENSG00000128346	84645	C22orf23	0.377
ENSG00000177427	125170	SMCR7	0.391
ENSG00000214827	4515	MTCP1	0.607
ENSG00000149534	2206	MS4A2	0.524
ENSG00000005700	25998	IBTK	0.567
ENSG00000184381	8398	PLA2G6	0.582
ENSG00000099814	283638	KIAA0284	0.578
ENSG00000185414	51263	MRPL30	0.518
ENSG00000110079	51338	MS4A4A	0.457
ENSG00000166428	122618	PLD4	0.495
ENSG00000185567	113146	AHNAK2	0.534
ENSG00000166928	84689	MS4A14	0.441
ENSG00000115514	10190	TXNDC9	0.578
ENSG00000168135	3761	KCNJ4	0.549
ENSG00000099203	11018	TMED1	0.515
ENSG00000146242	7162	TPBG	0.538
ENSG00000118420	90025	UBE2CBP	0.504
ENSG00000100425	23774	BRD1	0.573
ENSG00000149474	57325	CSRP2BP	0.589
ENSG00000171928	51030	FAM18B	0.472
ENSG00000169918	161725	OTUD7A	0.518
ENSG00000175344	1139	CHRNA7	0.486
ENSG00000141127	5636	PRPSAP2	0.54
ENSG00000089091	55184	C20orf12	0.473
ENSG00000006118	54972	TMEM132A	0.528
ENSG00000134153	56851	C15orf24	0.534
ENSG00000122008	51426	POLK	0.531
ENSG00000197933	55552	ZNF823	0.474
ENSG00000089050	10741	RBBP9	0.56
ENSG00000198218	54870	QRICH1	0.494
ENSG00000128463	51234	TMEM85	0.536
ENSG00000154358	84033	OBSCN	0.431
ENSG00000110448	921	CD5	0.476
ENSG00000117408	9670	IPO13	0.52
ENSG00000188130	6300	MAPK12	0.478
ENSG00000167987	55048	VPS37C	0.533
ENSG00000176454	254531	LPCAT4	0.599
ENSG00000104435	11075	STMN2	0.548
ENSG00000132004	84261	FBXW9	0.435
ENSG00000175265	23015	GOLGA8A	0.55
ENSG00000125821	92675	DTD1	0.515
ENSG00000123144	79002	C19orf43	0.527
ENSG00000198356	439	ASNA1	0.53
ENSG00000215252	23015	GOLGA8A	0.55
ENSG00000117410	533	ATP6V0B	0.551
ENSG00000164683	23462	HEY1	0.618
ENSG00000073067	54905	CYP2W1	0.434
ENSG00000182979	9112	MTA1	0.59
ENSG00000105613	22983	MAST1	0.494
ENSG00000100359	27352	SGSM3	0.593
ENSG00000134146	89978	ATPBD4	0.524

ENSG00000152359	134359	C5orf37	0.447
ENSG00000079557	173	AFM	0.46
ENSG00000128285	2847	MCHR1	0.467
ENSG00000179284	199699	DAND5	0.467
ENSG00000154370	81559	TRIM11	0.527
ENSG00000100372	10478	SLC25A17	0.545
ENSG00000171603	22883	CLSTN1	0.519
ENSG00000148229	54107	POLE3	0.559
ENSG00000196236	63929	XPNPEP3	0.511
ENSG00000169429	3576	IL8	0.698
ENSG00000205560	1375	CPT1B	0.579
ENSG00000168148	8290	HIST3H3	0.394
ENSG00000181218	92815	HIST3H2A	0.485
ENSG00000163162	284996	RNF149	0.489
ENSG00000175746	400360	C15orf54	0.325
ENSG00000177105	391	RHOG	0.54
ENSG00000168418	93107	KCNG4	0.45
ENSG00000008735	23542	MAPK8IP2	0.527
ENSG00000105011	55723	ASF1B	0.498
ENSG00000173614	64802	NMNAT1	0.484
ENSG00000167113	51117	COQ4	0.465
ENSG00000181104	2149	F2R	0.532
ENSG00000130939	10277	UBE4B	0.634
ENSG00000124882	2069	EREG	0.533
ENSG00000112837	9096	TBX18	0.485
ENSG00000132109	6737	TRIM21	0.496
ENSG00000184986	80757	TMEM121	0.47
ENSG00000160994	126402	CCDC105	0.405
ENSG00000135317	57231	SNX14	0.525
ENSG00000105135	10994	ILVBL	0.596
ENSG00000114316	7375	USP4	0.627
ENSG00000164695	92421	CHMP4C	0.45
ENSG00000072958	8907	AP1M1	0.512
ENSG00000214046	79086	C19orf42	0.514
ENSG00000175518	143630	UBQLNL	0.392
ENSG00000121236	117854	TRIM6	0.44
ENSG00000130311	79016	DDA1	0.482
ENSG00000130299	84705	GTPBP3	0.526
ENSG00000119392	2733	GLE1	0.527
ENSG00000176731	401466	C8orf59	0.535
ENSG00000130313	25796	PGLS	0.541
ENSG00000130309	79709	GLT25D1	0.461
ENSG00000135316	10492	SYNCRIP	0.673
ENSG00000132256	85363	TRIM5	0.464
ENSG00000185015	377677	CA13	0.504
ENSG00000131153	51659	GINS2	0.54
ENSG00000197694	6709	SPTAN1	0.627
ENSG00000105643	27106	ARRDC2	0.488
ENSG00000105647	5296	PIK3R2	0.526
ENSG00000135744	183	AGT	0.496
ENSG00000216490	10437	IFI30	0.586
ENSG00000105649	5864	RAB3A	0.461
ENSG00000130517	54858	PGPEP1	0.512
ENSG00000095397	25861	DFNB31	0.554

ENSG00000179546	3352	HTR1D	0.416
ENSG00000164879	761	CA3	0.459
ENSG00000198301	55153	SDAD1	0.492
ENSG00000105700	79036	C19orf50	0.525
ENSG00000006015	55049	C19orf60	0.534
ENSG00000184992	140707	BRI3BP	0.543
ENSG00000175262	148345	C1orf127	0.365
ENSG00000138073	10113	PREB	0.507
ENSG00000143643	79573	TTC13	0.505
ENSG00000180878	160298	C11orf42	0.39
ENSG00000107874	79004	CUEDC2	0.505
ENSG00000213204	154313	C6orf165	0.377
ENSG00000176678	2300	FOXL1	0.428
ENSG00000185860	339512	C1orf110	0.439
ENSG00000184156	3786	KCNQ3	0.461
ENSG00000117152	5999	RGS4	0.613
ENSG00000176623	51115	FAM82B	0.503
ENSG00000187969	389874	ZCCHC13	0.391
ENSG00000205929	56245	C21orf62	0.486
ENSG00000151952	121256	TMEM132D	0.437
ENSG00000160161	148113	CILP2	0.438
ENSG00000148219	23245	ASTN2	0.528
ENSG00000169245	3627	CXCL10	0.5
ENSG00000105726	57130	ATP13A1	0.486
ENSG00000057757	57095	C1orf128	0.51
ENSG00000143248	8490	RGS5	0.607
ENSG00000129493	25938	HEATR5A	0.557
ENSG00000196268	284443	ZNF493	0.448
ENSG00000105173	898	CCNE1	0.582
ENSG00000136869	7099	TLR4	0.458
ENSG00000198597	9745	ZNF536	0.47
ENSG00000243646	3588	IL10RB	0.559
ENSG00000186326	388531	RGS9BP	0.4
ENSG00000151413	80224	NUBPL	0.486
ENSG00000189266	55629	PNRC2	0.511
ENSG00000179588	161882	ZFPM1	0.427
ENSG00000104415	8840	WISP1	0.539
ENSG00000084774	790	CAD	0.57
ENSG00000166333	3611	ILK	0.543
ENSG00000186675	139599	MAGEE2	0.479
ENSG00000105220	2821	GPI	0.545
ENSG00000116670	10459	MAD2L2	0.454
ENSG00000142279	126374	WTIP	0.418
ENSG00000142677	58985	IL22RA1	0.451
ENSG00000168661	90075	ZNF30	0.511
ENSG00000185436	163702	IL28RA	0.408
ENSG00000089356	5349	FXYD3	0.585
ENSG00000008513	6482	ST3GAL1	0.554
ENSG00000214435	57412	AS3MT	0.495
ENSG00000155100	51633	OTUD6B	0.488
ENSG00000215568	128954	GAB4	0.39
ENSG00000066827	57623	ZFAT	0.519
ENSG00000105695	4099	MAG	0.523
ENSG00000069998	27440	CECR5	0.503

ENSG00000138100	57159	TRIM54	0.516
ENSG00000093072	51816	CECR1	0.49
ENSG00000076685	22978	NT5C2	0.543
ENSG00000158042	63875	MRPL17	0.51
ENSG00000165240	538	ATP7A	0.466
ENSG00000170748	27288	RBMXL2	0.559
ENSG00000183785	51807	TUBA8	0.441
ENSG00000167595	148137	C19orf55	0.43
ENSG00000184979	11274	USP18	0.526
ENSG00000159147	29980	DONSON	0.514
ENSG00000148835	6877	TAF5	0.544
ENSG00000198604	11177	BAZ1A	0.573
ENSG00000173915	84833	USMG5	0.489
ENSG00000179300	203430	ZCCHC5	0.387
ENSG00000147145	2846	LPAR4	0.43
ENSG00000164953	91147	TMEM67	0.521
ENSG00000186017	84924	ZNF566	0.485
ENSG00000104472	54108	CHRAC1	0.53
ENSG00000135094	10993	SDS	0.521
ENSG00000089116	64211	LHX5	0.445
ENSG00000143167	10223	GPA33	0.507
ENSG00000116688	9927	MFN2	0.573
ENSG00000165288	254065	BRWD3	0.49
ENSG00000184113	7122	CLDN5	0.463
ENSG00000079112	1015	CDH17	0.459
ENSG00000181666	284459	HKR1	0.482
ENSG00000204539	1041	CDSN	0.502
ENSG00000092020	55012	PPP2R3C	0.478
ENSG00000164949	2669	GEM	0.568
ENSG00000099889	421	ARVCF	0.562
ENSG00000176915	23141	ANKLE2	0.653
ENSG00000163795	130557	ZNF513	0.489
ENSG00000171471	643246	MAP1LC3B2	0.359
ENSG00000167645	90522	YIF1B	0.464
ENSG00000198821	919	CD247	0.494
ENSG00000177370	29928	TIMM22	0.51
ENSG00000099901	5902	RANBP1	0.591
ENSG00000131171	6451	SH3BGRL	0.608
ENSG00000204531	5460	POU5F1	0.546
ENSG00000156162	286148	DPY19L4	0.511
ENSG00000104814	11184	MAP4K1	0.554
ENSG00000119421	4702	NDUFA8	0.534
ENSG00000136816	27348	TOR1B	0.519
ENSG00000165259	139324	HDX	0.444
ENSG00000136819	51759	C9orf78	0.474
ENSG00000119446	92400	RBM18	0.506
ENSG00000148187	92399	MRRF	0.507
ENSG00000162496	9249	DHRS3	0.547
ENSG00000147180	7552	ZNF711	0.456
ENSG00000158792	124044	SPATA2L	0.492
ENSG00000124429	79983	POF1B	0.464
ENSG00000175305	9134	CCNE2	0.574
ENSG00000090924	64857	PLEKHG2	0.514
ENSG00000164082	2912	GRM2	0.468

ENSG00000196235	6829	SUPT5H	0.535
ENSG00000177951	51272	BET1L	0.475
ENSG00000112297	202	AIM1	0.57
ENSG00000180509	3753	KCNE1	0.466
ENSG00000099949	8216	LZTR1	0.502
ENSG00000076706	4162	MCAM	0.641
ENSG00000143158	25874	BRP44	0.545
ENSG00000176871	55884	WSB2	0.57
ENSG00000243943	84450	ZNF512	0.537
ENSG00000235718	83552	MFRP	0.432
ENSG00000198576	23237	ARC	0.488
ENSG00000167653	8000	PSCA	0.443
ENSG00000159212	54102	CLIC6	0.529
ENSG00000175895	79666	PLEKHF2	0.497
ENSG00000136940	5082	PDCL	0.527
ENSG00000089220	5037	PEBP1	0.618
ENSG00000175356	57758	SCUBE2	0.5
ENSG00000180155	66004	LYNX1	0.54
ENSG00000174740	140886	PABPC5	0.412
ENSG00000130707	445	ASS1	0.558
ENSG00000180929	118442	GPR62	0.404
ENSG00000007312	974	CD79B	0.505
ENSG00000136327	26257	NKX2-8	0.459
ENSG00000160932	4061	LY6E	0.515
ENSG00000204991	84501	SPIRE2	0.518
ENSG00000213760	534	ATP6V1G2	0.492
ENSG00000054277	23596	OPN3	0.539
ENSG00000204086	29935	RPA4	0.423
ENSG00000159256	23515	MORC3	0.483
ENSG00000000005	64102	TNMD	0.45
ENSG00000174371	9156	EXO1	0.535
ENSG00000112335	8724	SNX3	0.613
ENSG00000164307	51752	ERAP1	0.608
ENSG00000180287	200150	PLD5	0.452
ENSG00000247596	11344	TWF2	0.526
ENSG00000099994	56241	SUSD2	0.492
ENSG00000109929	6309	SC5DL	0.61
ENSG00000092208	8487	SIP1	0.584
ENSG00000137642	6653	SORL1	0.574
ENSG00000163064	2019	EN1	0.441
ENSG00000183137	285753	C6orf182	0.511
ENSG00000168481	203190	LGI3	0.455
ENSG00000122870	80114	BICC1	0.452
ENSG00000111530	55832	CAND1	0.613
ENSG00000168484	6440	SFTPC	0.602
ENSG00000155368	1622	DBI	0.606
ENSG00000204469	7916	BAT2	0.58
ENSG00000109971	3312	HSPA8	0.666
ENSG00000135535	8763	CD164	0.587
ENSG00000171227	140738	TMEM37	0.521
ENSG00000100095	23544	SEZ6L	0.621
ENSG00000183558	8337	HIST2H2AA3	0.476
ENSG00000203812	8337	HIST2H2AA3	0.476
ENSG00000128203	57168	ASPHD2	0.507

ENSG00000184678	8349	HIST2H2BE	0.564
ENSG00000118762	5311	PKD2	0.538
ENSG00000128294	8459	TPST2	0.526
ENSG00000197170	5718	PSMD12	0.585
ENSG00000102387	54457	TAF7L	0.418
ENSG00000179397	257044	C1orf101	0.484
ENSG00000133107	7223	TRPC4	0.541
ENSG00000010671	695	BTK	0.506
ENSG00000154217	26207	PITPNC1	0.546
ENSG00000120686	51569	UFM1	0.504
ENSG00000010322	11188	NISCH	0.536
ENSG00000100219	7494	XBP1	0.572
ENSG00000091128	22798	LAMB4	0.5
ENSG00000196704	51321	AMZ2	0.48
ENSG00000175782	55508	SLC35E3	0.502
ENSG00000140854	10300	KATNB1	0.575
ENSG00000120690	1997	ELF1	0.598
ENSG00000138311	22891	ZNF365	0.513
ENSG00000165572	89890	KBTBD6	0.472
ENSG00000120696	84078	KBTBD7	0.46
ENSG00000134755	1824	DSC2	0.565
ENSG00000134765	1823	DSC1	0.436
ENSG00000120458	79684	C11orf61	0.466
ENSG00000140859	3801	KIFC3	0.537
ENSG00000145723	54826	GIN1	0.488
ENSG00000100319	55954	ZMAT5	0.472
ENSG00000100325	84164	ASCC2	0.512
ENSG00000171121	27094	KCNMB3	0.461
ENSG00000108950	54757	FAM20A	0.55
ENSG00000134757	1830	DSG3	0.476
ENSG00000102760	28984	C13orf15	0.522
ENSG00000102763	23078	KIAA0564	0.574
ENSG00000121864	51193	ZNF639	0.488
ENSG00000178718	54913	RPP25	0.508
ENSG00000102780	160851	DGKH	0.516
ENSG00000111186	81029	WNT5B	0.512
ENSG00000151062	93589	CACNA2D4	0.5
ENSG00000244005	9054	NFS1	0.542
ENSG00000159648	374739	TEPP	0.407
ENSG00000067646	7544	ZFY	0.466
ENSG00000127337	8089	YEATS4	0.461
ENSG00000108176	56521	DNAJC12	0.514
ENSG00000211460	7247	TSN	0.6
ENSG00000145743	64839	FBXL17	0.565
ENSG00000115649	27013	C2orf24	0.556
ENSG00000120675	29103	DNAJC15	0.539
ENSG00000136732	2995	GYPC	0.512
ENSG00000102804	8848	TSC22D1	0.564
ENSG00000117862	51060	TXNDC12	0.473
ENSG00000006652	3475	IFRD1	0.599
ENSG00000136717	274	BIN1	0.619
ENSG00000103021	29070	CCDC113	0.486
ENSG00000083635	26747	NUFIP1	0.62
ENSG00000168067	5871	MAP4K2	0.477

ENSG00000127325	144453	BEST3	0.562
ENSG00000120889	8795	TNFRSF10B	0.653
ENSG00000016864	55830	GLT8D1	0.555
ENSG00000163516	55139	ANKZF1	0.5
ENSG00000173214	91749	KIAA1919	0.577
ENSG00000133895	4221	MEN1	0.548
ENSG00000086506	3049	HBQ1	0.47
ENSG00000173548	257364	SNX33	0.509
ENSG00000173546	1464	CSPG4	0.586
ENSG00000114904	6787	NEK4	0.538
ENSG00000154330	5239	PGM5	0.494
ENSG00000138347	84665	MYPN	0.489
ENSG00000205981	131118	DNAJC19	0.557
ENSG00000167196	26263	FBXO22	0.507
ENSG00000134744	23318	ZCCHC11	0.579
ENSG00000108984	5608	MAP2K6	0.597
ENSG00000054356	5798	PTPRN	0.514
ENSG00000111206	2305	FOXM1	0.551
ENSG00000152495	814	CAMK4	0.512
ENSG00000182168	8633	UNC5C	0.454
ENSG00000155666	79831	JMJD5	0.428
ENSG00000141424	25800	SLC39A6	0.597
ENSG00000196876	6334	SCN8A	0.45
ENSG00000169193	90693	CCDC126	0.556
ENSG00000136141	23143	LRCH1	0.457
ENSG00000111218	56341	PRMT8	0.456
ENSG00000135269	26136	TES	0.587
ENSG00000134013	4017	LOXL2	0.598
ENSG00000116157	2882	GPX7	0.542
ENSG00000176597	84002	B3GNT5	0.531
ENSG00000166598	7184	HSP90B1	0.64
ENSG00000175084	1674	DES	0.565
ENSG00000111224	57097	PARP11	0.44
ENSG00000162377	65260	C1orf163	0.522
ENSG00000047249	51606	ATP6V1H	0.534
ENSG00000169181	146395	GSG1L	0.386
ENSG00000140386	49855	SCAPER	0.632
ENSG00000131043	25980	C20orf4	0.486
ENSG00000055955	3700	ITIH4	0.562
ENSG00000118972	8074	FGF23	0.415
ENSG00000111241	2251	FGF6	0.461
ENSG00000107282	320	APBA1	0.466
ENSG00000136146	29079	MED4	0.46
ENSG00000141219	55028	C17orf80	0.477
ENSG00000143457	55204	GOLPH3L	0.534
ENSG00000175193	55486	PARL	0.484
ENSG00000150477	57536	KIAA1328	0.403
ENSG00000134940	56	ACRV1	0.602
ENSG00000147509	8601	RGS20	0.497
ENSG00000135443	3891	KRT85	0.476
ENSG00000170454	9119	KRT75	0.509
ENSG00000105928	1687	DFNA5	0.503
ENSG00000072182	55515	ACCN4	0.464
ENSG00000123989	79586	CHPF	0.505

ENSG00000186081	3852	KRT5	0.512
ENSG00000163166	55677	IWS1	0.504
ENSG00000170486	140807	KRT72	0.392
ENSG00000172867	3849	KRT2	0.467
ENSG00000167768	3848	KRT1	0.49
ENSG00000168398	624	BDKRB2	0.512
ENSG00000086189	27292	DIMT1L	0.685
ENSG00000172137	794	CALB2	0.491
ENSG00000198887	23137	SMC5	0.565
ENSG00000067704	55699	IARS2	0.548
ENSG00000060339	55749	CCAR1	0.554
ENSG00000152214	6014	RIT2	0.45
ENSG00000101457	116092	DNTTIP1	0.528
ENSG00000163131	1520	CTSS	0.583
ENSG00000152217	26040	SETBP1	0.493
ENSG00000111254	10566	AKAP3	0.481
ENSG00000173898	6712	SPTBN2	0.457
ENSG00000120992	10434	LYPLA1	0.607
ENSG00000198783	91603	ZNF830	0.417
ENSG00000114923	6508	SLC4A3	0.505
ENSG00000132874	8170	SLC14A2	0.455
ENSG00000141469	6563	SLC14A1	0.477
ENSG00000143387	1513	CTSK	0.561
ENSG00000149636	79980	DSN1	0.48
ENSG00000106785	9830	TRIM14	0.627
ENSG00000125691	9349	RPL23	0.611
ENSG00000102543	81602	CDADC1	0.476
ENSG00000103042	55238	SLC38A7	0.58
ENSG00000171310	50515	CHST11	0.546
ENSG00000185483	4919	ROR1	0.586
ENSG00000112769	3910	LAMA4	0.625
ENSG00000136197	79020	C7orf25	0.551
ENSG00000103740	23205	ACSBG1	0.503
ENSG00000118873	25782	RAB3GAP2	0.575
ENSG00000152229	9050	PSTPIP2	0.491
ENSG00000147437	2796	GNRH1	0.467
ENSG00000111319	6337	SCNN1A	0.593
ENSG00000168488	11273	ATXN2L	0.571
ENSG00000165526	84881	RPUSD4	0.507
ENSG00000153790	136895	C7orf31	0.43
ENSG00000103266	10273	STUB1	0.539
ENSG00000107625	79009	DDX50	0.524
ENSG00000072163	55679	LIMS2	0.508
ENSG00000163071	132671	SPATA18	0.541
ENSG00000132801	140831	ZSWIM3	0.365
ENSG00000143379	9869	SETDB1	0.601
ENSG00000136144	55213	RCBTB1	0.514
ENSG00000171951	7857	SCG2	0.495
ENSG00000085449	57590	WDFY1	0.5
ENSG00000139291	55266	TMEM19	0.514
ENSG00000064218	58524	DMRT3	0.528
ENSG00000174332	148979	GLIS1	0.376
ENSG00000103254	65990	FAM173A	0.463
ENSG00000162004	124093	CCDC78	0.377

ENSG00000149527	9651	PLCH2	0.49
ENSG00000107362	51104	FAM108B1	0.525
ENSG00000167851	11314	CD300A	0.57
ENSG00000123178	57213	C13orf1	0.502
ENSG00000001626	1080	CFTR	0.595
ENSG00000138823	4547	MTTP	0.437
ENSG00000100979	5360	PLTP	0.527
ENSG00000086300	29887	SNX10	0.527
ENSG00000196296	487	ATP2A1	0.483
ENSG00000204977	10206	TRIM13	0.542
ENSG00000006327	51330	TNFRSF12A	0.505
ENSG00000136709	55339	WDR33	0.503
ENSG00000149792	740	MRPL49	0.538
ENSG00000101638	29906	ST8SIA5	0.46
ENSG00000119125	9615	GDA	0.52
ENSG00000070190	27071	DAPP1	0.52
ENSG00000141161	146862	UNC45B	0.369
ENSG00000170791	79145	CHCHD7	0.513
ENSG00000006634	10926	DBF4	0.544
ENSG00000136104	79621	RNASEH2B	0.613
ENSG00000008516	64386	MMP25	0.57
ENSG00000136378	11173	ADAMTS7	0.563
ENSG00000144468	84236	RHBDD1	0.571
ENSG00000165325	159989	CCDC67	0.587
ENSG00000240694	10687	PNMA2	0.549
ENSG00000143409	55793	FAM63A	0.479
ENSG00000165996	9200	PTPLA	0.518
ENSG00000162636	284611	FAM102B	0.587
ENSG00000118702	2691	GHRH	0.456
ENSG00000168958	56947	MFF	0.521
ENSG00000106006	3203	HOXA6	0.453
ENSG00000122862	5552	SRGN	0.575
ENSG00000122592	3204	HOXA7	0.592
ENSG00000008277	53616	ADAM22	0.633
ENSG00000211452	1733	DIO1	0.447
ENSG00000091651	23594	ORC6L	0.495
ENSG00000172932	338692	ANKRD13D	0.493
ENSG00000139055	121506	ERP27	0.5
ENSG00000146540	84310	C7orf50	0.533
ENSG00000134533	85004	RERG	0.545
ENSG00000213625	54741	LEPROT	0.525
ENSG00000115009	6364	CCL20	0.543
ENSG00000145358	115265	DDIT4L	0.492
ENSG00000153820	80309	SPHKAP	0.333
ENSG00000162639	113802	C1orf59	0.5
ENSG00000187957	92737	DNER	0.482
ENSG00000101343	51340	CRNKL1	0.509
ENSG00000135517	4284	MIP	0.446
ENSG00000085644	7760	ZNF213	0.464
ENSG00000173825	220359	TIGD3	0.397
ENSG00000153827	9320	TRIP12	0.526
ENSG00000162241	283130	SLC25A45	0.476
ENSG00000166006	3747	KCNC2	0.432
ENSG00000108733	5193	PEX12	0.491

ENSG00000119953	10285	SMNDC1	0.488
ENSG00000106686	55064	C9orf68	0.451
ENSG00000184574	57121	LPAR5	0.531
ENSG00000167258	51755	CRKRS	0.585
ENSG00000006194	10127	ZNF263	0.534
ENSG00000143107	163479	FNDC7	0.324
ENSG00000111653	51147	ING4	0.556
ENSG00000116266	6814	STXBP3	0.516
ENSG00000175482	57804	POLD4	0.512
ENSG00000100442	2287	FKBP3	0.508
ENSG00000013503	55703	POLR3B	0.488
ENSG00000131771	84152	PPP1R1B	0.44
ENSG00000007516	8938	BAIAP3	0.514
ENSG00000175166	5708	PSMD2	0.511
ENSG00000143443	54964	C1orf56	0.522
ENSG00000075142	6717	SRI	0.619
ENSG00000076067	5939	RBMS2	0.586
ENSG00000131748	10948	STARD3	0.523
ENSG00000126746	171017	ZNF384	0.513
ENSG00000140931	123920	CMTM3	0.512
ENSG00000086666	54469	ZFAND6	0.552
ENSG00000120586	4360	MRC1	0.547
ENSG00000101407	9675	KIAA0406	0.508
ENSG00000119509	27130	INVS	0.573
ENSG00000042429	9440	MED17	0.517
ENSG00000096968	3717	JAK2	0.557
ENSG00000102401	51566	ARMCX3	0.531
ENSG00000090581	84572	GNPTG	0.462
ENSG00000111615	11103	KRR1	0.567
ENSG00000113597	80006	C5orf44	0.506
ENSG00000075945	22920	KIFAP3	0.527
ENSG00000205352	54458	PRR13	0.493
ENSG00000103351	23059	CLUAP1	0.571
ENSG00000152763	79819	WDR78	0.493
ENSG00000129636	81533	ITFG1	0.51
ENSG00000104112	29106	SCG3	0.416
ENSG00000111783	5992	RFX4	0.412
ENSG00000167984	197358	NLRC3	0.469
ENSG00000162949	92291	CAPN13	0.553
ENSG00000120903	1135	CHRNA2	0.452
ENSG00000138594	29766	TMOD3	0.483
ENSG00000129270	79148	MMP28	0.489
ENSG00000176595	9920	KBTBD11	0.513
ENSG00000120217	29126	CD274	0.436
ENSG00000069966	10681	GNB5	0.616
ENSG00000089692	3902	LAG3	0.479
ENSG00000101017	958	CD40	0.632
ENSG00000102967	1723	DHODH	0.49
ENSG00000151702	2313	FLI1	0.554
ENSG00000126952	55998	NXF5	0.368
ENSG00000162591	1953	MEGF6	0.535
ENSG00000175182	131408	FAM131A	0.524
ENSG00000100726	9894	TELO2	0.575
ENSG00000140830	54957	TXNL4B	0.502

ENSG00000151704	3758	KCNJ1	0.501
ENSG00000121940	23155	CLCC1	0.552
ENSG00000163155	388695	LYSMD1	0.463
ENSG00000198873	2869	GRK5	0.632
ENSG00000117899	23184	MESDC2	0.526
ENSG00000141736	2064	ERBB2	0.602
ENSG00000139734	81624	DIAPH3	0.469
ENSG00000204301	4855	NOTCH4	0.497
ENSG00000154305	375056	MIA3	0.575
ENSG00000121067	8405	SPOP	0.573
ENSG00000241697	8577	TMEFF1	0.53
ENSG00000174370	219833	C11orf45	0.427
ENSG00000170681	347273	MURC	0.436
ENSG00000111665	83461	CDCA3	0.472
ENSG00000158109	127262	TPRG1L	0.467
ENSG00000154832	30827	CXXC1	0.589
ENSG00000163882	5437	POLR2H	0.569
ENSG00000080189	51006	SLC35C2	0.497
ENSG00000176973	23625	FAM89B	0.598
ENSG00000183354	158358	KIAA2026	0.534
ENSG00000173442	254102	EHBP1L1	0.481
ENSG00000134851	55858	TMEM165	0.542
ENSG00000166432	84460	ZMAT1	0.496
ENSG00000120215	2315	MLANA	0.577
ENSG00000172349	3603	IL16	0.619
ENSG00000176532	222171	PRR15	0.491
ENSG00000137033	90865	IL33	0.448
ENSG00000090539	8646	CHRD	0.545
ENSG00000172663	80194	TMEM134	0.492
ENSG00000116213	49856	WDR8	0.482
ENSG00000172071	9451	EIF2AK3	0.534
ENSG00000196371	2526	FUT4	0.539
ENSG00000187049	51259	TMEM216	0.482
ENSG00000182035	149685	ADIG	0.543
ENSG00000111716	3945	LDHB	0.587
ENSG00000170653	11016	ATF7	0.505
ENSG00000121361	3764	KCNJ8	0.548
ENSG00000069431	10060	ABCC9	0.583
ENSG00000115053	4691	NCL	0.563
ENSG00000134042	83876	MRO	0.559
ENSG00000171596	10316	NMUR1	0.45
ENSG00000173338	10089	KCNK7	0.441
ENSG00000241058	221078	NSUN6	0.468
ENSG00000148908	6001	RGS10	0.627
ENSG00000151923	7073	TIAL1	0.637
ENSG00000106633	2645	GCK	0.44
ENSG00000111674	2026	ENO2	0.548
ENSG00000197991	64881	PCDH20	0.483
ENSG00000125813	5075	PAX1	0.452
ENSG00000141738	2886	GRB7	0.504
ENSG00000094963	2327	FMO2	0.492
ENSG00000137871	54816	ZNF280D	0.479
ENSG00000141642	55520	ELAC1	0.43
ENSG00000171320	157570	ESCO2	0.569

ENSG00000166592	6236	RRAD	0.591
ENSG00000134072	8536	CAMK1	0.578
ENSG00000168140	114990	VASN	0.47
ENSG00000166595	51647	FAM96B	0.521
ENSG00000151136	121551	BTBD11	0.554
ENSG00000147316	79648	MCPH1	0.464
ENSG00000072858	54847	SIDT1	0.473
ENSG00000197136	399909	PCNXL3	0.354
ENSG00000197776	122773	KLHDC1	0.5
ENSG00000009765	389434	IYD	0.345
ENSG00000215021	11331	PHB2	0.539
ENSG00000091879	285	ANGPT2	0.541
ENSG00000205581	3150	HMGN1	0.593
ENSG00000204335	389058	SP5	0.408
ENSG00000180818	3226	HOXC10	0.498
ENSG00000154975	56934	CA10	0.432
ENSG00000162148	220004	C11orf66	0.455
ENSG00000171551	9427	ECEL1	0.452
ENSG00000106100	10392	NOD1	0.477
ENSG00000172789	3222	HOXC5	0.461
ENSG00000161405	22806	IKZF3	0.454
ENSG00000127914	10142	AKAP9	0.602
ENSG00000188386	5535	PPP3R2	0.455
ENSG00000172828	23491	CES3	0.479
ENSG00000198825	22876	INPP5F	0.544
ENSG00000103569	366	AQP9	0.492
ENSG00000197520	400823	FAM177B	0.455
ENSG00000198353	3221	HOXC4	0.467
ENSG00000129277	6351	CCL4	0.515
ENSG00000166845	162681	C18orf54	0.552
ENSG00000185760	56479	KCNQ5	0.463
ENSG00000155363	4343	MOV10	0.542
ENSG00000149926	83723	FAM57B	0.408
ENSG00000198736	51734	SEPX1	0.545
ENSG00000025423	8630	HSD17B6	0.579
ENSG00000149925	226	ALDOA	0.551
ENSG00000120254	25902	MTHFD1L	0.605
ENSG00000176358	255061	TAC4	0.404
ENSG00000158296	64849	SLC13A3	0.552
ENSG00000139547	8608	RDH16	0.472
ENSG00000243725	7268	TTC4	0.505
ENSG00000168096	124401	ANKS3	0.417
ENSG00000166863	6866	TAC3	0.502
ENSG00000186075	124626	ZPBP2	0.423
ENSG00000149231	79780	CCDC82	0.583
ENSG00000154930	84532	ACSS1	0.54
ENSG00000152936	160492	IFLTD1	0.426
ENSG00000116717	1647	GADD45A	0.578
ENSG00000109016	25979	DHRS7B	0.514
ENSG00000123096	8082	SSPN	0.596
ENSG00000198855	11153	FICD	0.521
ENSG00000150768	1737	DLAT	0.645
ENSG00000134825	746	C11orf10	0.519
ENSG00000100987	30813	VSX1	0.464

ENSG00000165525	9147	SDCCAG1	0.544
ENSG00000076201	25930	PTPN23	0.464
ENSG00000064102	55726	C12orf11	0.517
ENSG00000168081	5368	PNOC	0.494
ENSG00000075856	9733	SART3	0.585
ENSG00000113838	55171	TBCCD1	0.484
ENSG00000034152	5606	MAP2K3	0.627
ENSG00000169174	255738	PCSK9	0.465
ENSG00000108344	5709	PSMD3	0.546
ENSG00000064115	51768	TM7SF3	0.539
ENSG00000090520	51726	DNAJB11	0.592
ENSG00000152683	55676	SLC30A6	0.611
ENSG00000186638	347240	KIF24	0.5
ENSG00000145192	197	AHSG	0.529
ENSG00000127554	2671	GFER	0.527
ENSG00000069667	6095	RORA	0.545
ENSG00000064726	53339	BTBD1	0.514
ENSG00000102878	3299	HSF4	0.488
ENSG00000129003	54832	VPS13C	0.614
ENSG00000168918	3635	INPP5D	0.57
ENSG00000127561	9143	SYNGR3	0.491
ENSG00000152944	9412	MED21	0.563
ENSG00000198286	84433	CARD11	0.395
ENSG00000166292	55273	TMEM100	0.51
ENSG00000005812	26224	FBXL3	0.545
ENSG00000065054	9351	SLC9A3R2	0.464
ENSG00000144026	84874	ZNF514	0.471
ENSG00000198912	339448	C1orf174	0.481
ENSG00000141179	58488	PCTP	0.473
ENSG00000180964	90843	TCEAL8	0.484
ENSG00000163067	7549	ZNF2	0.466
ENSG00000135828	6041	RNASEL	0.432
ENSG00000216937	221016	CCDC7	0.475
ENSG00000221986	343263	MYBPHL	0.469
ENSG00000150782	3606	IL18	0.502
ENSG00000164976	57462	KIAA1161	0.5
ENSG00000204065	340543	TCEAL5	0.548
ENSG00000149922	6911	TBX6	0.534
ENSG00000169594	646	BNC1	0.485
ENSG00000172803	254122	SNX32	0.494
ENSG00000146555	221935	SDK1	0.579
ENSG00000024526	55635	DEPDC1	0.558
ENSG00000147421	79618	HMBOX1	0.491
ENSG00000066926	2235	FECH	0.557
ENSG00000155380	6566	SLC16A1	0.621
ENSG00000104695	5516	PPP2CB	0.57
ENSG00000150076	79741	C10orf68	0.411
ENSG00000070785	8891	EIF2B3	0.491
ENSG00000183691	9241	NOG	0.437
ENSG00000134440	4677	NARS	0.529
ENSG00000127241	5648	MASP1	0.573
ENSG00000221968	3995	FADS3	0.601
ENSG00000172159	257019	FRMD3	0.561
ENSG00000103657	8925	HERC1	0.541

ENSG00000077044	8527	DGKD	0.526
ENSG00000135838	80896	NPL	0.498
ENSG00000151576	79691	QTRTD1	0.487
ENSG00000168411	55159	RFWD3	0.485
ENSG00000197892	23303	KIF13B	0.517
ENSG00000171720	8841	HDAC3	0.539
ENSG00000179041	23212	RRS1	0.523
ENSG00000147576	137872	ADHFE1	0.515
ENSG00000170627	121355	GTSF1	0.474
ENSG00000133142	79921	TCEAL4	0.507
ENSG00000157168	3084	NRG1	0.644
ENSG00000167994	5866	RAB3IL1	0.482
ENSG00000101146	8480	RAE1	0.622
ENSG00000166526	7551	ZNF3	0.575
ENSG00000133687	83857	TMTC1	0.564
ENSG00000166803	9768	KIAA0101	0.621
ENSG00000187288	63924	CIDEC	0.488
ENSG00000103671	9325	TRIP4	0.533
ENSG00000135018	29979	UBQLN1	0.586
ENSG00000133872	51669	TMEM66	0.541
ENSG00000162337	4041	LRP5	0.499
ENSG00000140451	80119	PIF1	0.507
ENSG00000204394	7407	VARS	0.588
ENSG00000168676	146212	KCTD19	0.434
ENSG00000167995	7439	BEST1	0.483
ENSG00000111046	4618	MYF6	0.448
ENSG00000166523	26253	CLEC4E	0.448
ENSG00000177082	84942	WDR73	0.484
ENSG00000168404	197259	MLKL	0.567
ENSG00000242515	54575	UGT1A10	0.452
ENSG00000106355	23658	LSM5	0.576
ENSG00000197614	8076	MFAP5	0.572
ENSG00000174255	7634	ZNF80	0.412
ENSG00000167165	54578	UGT1A6	0.49
ENSG00000103707	123263	MTFMT	0.443
ENSG00000103089	79152	FA2H	0.494
ENSG00000244474	54657	UGT1A4	0.544
ENSG00000168394	6890	TAP1	0.552
ENSG00000090530	55214	LEPREL1	0.5
ENSG00000116793	10745	PHTF1	0.591
ENSG00000181722	26137	ZBTB20	0.603
ENSG00000114646	10675	CSPG5	0.544
ENSG00000103091	79726	WDR59	0.607
ENSG00000163701	132014	IL17RE	0.471
ENSG00000138615	8483	CILP	0.484
ENSG00000172728	84750	FUT10	0.575
ENSG00000124092	140690	CTCFL	0.383
ENSG00000181754	57463	AMIGO1	0.466
ENSG00000169085	254778	C8orf46	0.505
ENSG00000156097	83873	GPR61	0.435
ENSG00000170191	140838	NANP	0.566
ENSG00000180318	8092	ALX1	0.49
ENSG00000154920	146956	EME1	0.537
ENSG00000013573	1663	DDX11	0.598

ENSG00000136297	221938	MMD2	0.412
ENSG00000166508	4176	MCM7	0.6
ENSG00000122642	11328	FKBP9	0.514
ENSG00000204392	57819	LSM2	0.551
ENSG00000146587	57786	RBAK	0.547
ENSG00000131018	23345	SYNE1	0.569
ENSG00000133636	4922	NTS	0.441
ENSG00000149503	3619	INCENP	0.475
ENSG00000159713	51673	TPPP3	0.452
ENSG00000129696	80185	C8orf41	0.446
ENSG00000116761	1491	CTH	0.585
ENSG00000123560	5354	PLP1	0.45
ENSG00000213853	2013	EMP2	0.547
ENSG00000175602	11007	CCDC85B	0.615
ENSG00000175592	8061	FOSL1	0.502
ENSG00000176387	3291	HSD11B2	0.47
ENSG00000196262	5478	PPIA	0.62
ENSG00000122643	51251	NT5C3	0.493
ENSG00000167107	80221	ACSF2	0.526
ENSG00000114279	2257	FGF12	0.6
ENSG00000172175	10892	MALT1	0.646
ENSG00000196562	55959	SULF2	0.513
ENSG00000124939	4246	SCGB2A1	0.398
ENSG00000180483	245932	DEFB119	0.456
ENSG00000184292	4070	TACSTD2	0.582
ENSG00000182379	11247	NXPH4	0.508
ENSG00000110484	4250	SCGB2A2	0.471
ENSG00000204388	3303	HSPA1A	0.537
ENSG00000107937	23560	GTPBP4	0.549
ENSG00000162174	80150	ASRGL1	0.515
ENSG00000135424	3679	ITGA7	0.577
ENSG00000186603	84842	HPDL	0.452
ENSG00000158270	81035	COLEC12	0.49
ENSG00000175573	83638	C11orf68	0.5
ENSG00000131470	29893	PSMC3IP	0.584
ENSG00000101057	4605	MYBL2	0.546
ENSG00000179918	22928	SEPHS2	0.548
ENSG00000132749	9633	MTL5	0.433
ENSG00000110848	969	CD69	0.506
ENSG00000136444	55316	RSAD1	0.497
ENSG00000101294	81502	HM13	0.486
ENSG00000167208	124460	SNX20	0.46
ENSG00000183831	339416	ANKRD45	0.427
ENSG00000168505	2637	GBX2	0.443
ENSG00000183779	80139	ZNF703	0.485
ENSG00000122304	5620	PRM2	0.434
ENSG00000076248	7374	UNG	0.54
ENSG00000132321	79781	IQCA1	0.476
ENSG00000122507	27241	BBS9	0.635
ENSG00000137692	84259	DCUN1D5	0.457
ENSG00000166562	90701	SEC11C	0.48
ENSG00000198612	10920	COPS8	0.611
ENSG00000134443	2922	GRP	0.48
ENSG00000134438	30062	RAX	0.442

ENSG00000074695	3998	LMAN1	0.553
ENSG00000178772	1370	CPN2	0.455
ENSG00000183655	64410	KLHL25	0.49
ENSG00000109083	90410	IFT20	0.553
ENSG00000164620	285613	RELL2	0.514
ENSG00000131462	7283	TUBG1	0.542
ENSG00000204256	6046	BRD2	0.659
ENSG00000101542	28316	CDH20	0.465
ENSG00000131747	7153	TOP2A	0.618
ENSG00000068137	79990	PLEKHH3	0.47
ENSG00000137070	3590	IL11RA	0.538
ENSG00000122299	29066	ZC3H7A	0.428
ENSG00000135404	967	CD63	0.57
ENSG00000106290	6878	TAF6	0.501
ENSG00000162620	127255	LRRIQ3	0.462
ENSG00000155034	80028	FBXL18	0.63
ENSG00000075624	60	ACTB	0.653
ENSG00000139131	51067	YARS2	0.466
ENSG00000171560	2243	FGA	0.538
ENSG00000005893	3920	LAMP2	0.62
ENSG00000131746	84951	TNS4	0.449
ENSG00000149596	57158	JPH2	0.492
ENSG00000156858	78994	PRR14	0.546
ENSG00000134057	891	CCNB1	0.588
ENSG00000173950	152002	C3orf21	0.518
ENSG00000175548	84920	ALG10	0.457
ENSG00000049283	55040	EPN3	0.468
ENSG00000139117	144402	CPNE8	0.6
ENSG00000048028	57646	USP28	0.496
ENSG00000116783	51086	TNNI3K	0.449
ENSG00000087365	10992	SF3B2	0.533
ENSG00000149016	64852	TUT1	0.449
ENSG00000140543	55070	DET1	0.534
ENSG00000184203	5504	PPP1R2	0.627
ENSG00000166997	245812	CNPY4	0.462
ENSG00000004139	23098	SARM1	0.549
ENSG00000058085	3918	LAMC2	0.607
ENSG00000141664	54877	ZCCHC2	0.528
ENSG00000124074	84080	C16orf48	0.431
ENSG00000175115	55690	PACS1	0.438
ENSG00000097021	11332	ACOT7	0.607
ENSG00000044459	54875	CNTLN	0.502
ENSG00000162599	4774	NFIA	0.594
ENSG00000140694	5073	PARN	0.495
ENSG00000107672	54780	NSMCE4A	0.587
ENSG00000132824	10955	SERINC3	0.624
ENSG00000185883	527	ATP6V0C	0.584
ENSG00000116774	56944	OLFML3	0.472
ENSG00000134202	2947	GSTM3	0.577
ENSG00000140511	145864	HAPLN3	0.462
ENSG00000168734	11142	PKIG	0.538
ENSG00000124831	9208	LRRFIP1	0.636
ENSG00000170962	80310	PDGFD	0.525
ENSG00000188906	120892	LRRK2	0.526

ENSG00000198758	79574	EPS8L3	0.453
ENSG00000162344	9965	FGF19	0.436
ENSG00000170579	9229	DLGAP1	0.571
ENSG00000132329	10267	RAMP1	0.506
ENSG00000170967	414301	DDI1	0.365
ENSG00000034713	11345	GABARAPL2	0.538
ENSG00000184182	140739	UBE2F	0.555
ENSG00000150471	23284	LPHN3	0.558
ENSG00000175591	5029	P2RY2	0.487
ENSG00000133138	54885	TBC1D8B	0.501
ENSG00000167230	284099	C17orf78	0.359
ENSG00000162623	127253	TYW3	0.472
ENSG00000164116	2982	GUCY1A3	0.522
ENSG00000147465	6770	STAR	0.455
ENSG00000163703	78987	CRELD1	0.528
ENSG00000006283	8913	CACNA1G	0.601
ENSG00000144843	141	ADPRH	0.505
ENSG00000157045	123803	NTAN1	0.57
ENSG00000109101	8456	FOXN1	0.447
ENSG00000164542	23366	KIAA0895	0.463
ENSG00000187017	83715	ESPN	0.452
ENSG00000172382	83886	PRSS27	0.341
ENSG00000155876	10670	RRAGA	0.525
ENSG00000124479	4693	NDP	0.478
ENSG00000175984	163259	DENND2C	0.515
ENSG00000089682	55285	RBM41	0.486
ENSG00000134551	5554	PRH1	0.509
ENSG00000137764	5607	MAP2K5	0.691
ENSG00000135070	81689	ISCA1	0.61
ENSG00000123374	1017	CDK2	0.633
ENSG00000132780	4678	NASP	0.59
ENSG00000144485	55502	HES6	0.522
ENSG00000179008	317761	C14orf39	0.386
ENSG00000215788	8718	TNFRSF25	0.684
ENSG00000076043	25996	REXO2	0.518
ENSG00000144021	9391	CIAO1	0.562
ENSG00000182890	2747	GLUD2	0.584
ENSG00000150459	10284	SAP18	0.648
ENSG00000147535	84513	PPAPDC1B	0.575
ENSG00000174744	25855	BRMS1	0.508
ENSG00000166848	54386	TERF2IP	0.534
ENSG00000097096	84144	SYDE2	0.396
ENSG00000109107	230	ALDOC	0.557
ENSG00000131738	3884	KRT33B	0.466
ENSG00000142867	8915	BCL10	0.513
ENSG00000131737	3885	KRT34	0.474
ENSG00000183833	89876	C3orf15	0.512
ENSG00000094796	3881	KRT31	0.495
ENSG00000111261	54682	MANSC1	0.53
ENSG00000131467	10197	PSME3	0.627
ENSG00000147548	54904	WHSC1L1	0.54
ENSG00000185085	80789	INTS5	0.538
ENSG00000005001	64063	PRSS22	0.506
ENSG00000152766	118932	ANKRD22	0.408

ENSG00000108759	3882	KRT32	0.465
ENSG00000137145	55667	DENND4C	0.532
ENSG00000168286	57215	THAP11	0.515
ENSG00000164048	51385	ZNF589	0.62
ENSG00000100505	114088	TRIM9	0.449
ENSG00000033800	8554	PIAS1	0.619
ENSG00000175203	10540	DCTN2	0.524
ENSG00000158445	3745	KCNB1	0.495
ENSG00000184613	4753	NELL2	0.517
ENSG00000119878	9419	CRIPT	0.509
ENSG00000121101	56155	TEX14	0.448
ENSG00000124134	3787	KCNS1	0.501
ENSG00000103496	6810	STX4	0.502
ENSG00000162607	7398	USP1	0.575
ENSG00000127564	9088	PKMYT1	0.557
ENSG00000065361	2065	ERBB3	0.632
ENSG00000156463	153769	SH3RF2	0.492
ENSG00000165084	116328	C8orf34	0.454
ENSG00000197183	140688	C20orf112	0.586
ENSG00000154359	91694	LONRF1	0.52
ENSG00000116701	4688	NCF2	0.552
ENSG00000105854	5445	PON2	0.573
ENSG00000146904	2041	EPHA1	0.556
ENSG00000133706	51520	LARS	0.522
ENSG00000171403	3857	KRT9	0.423
ENSG00000162073	124222	PAQR4	0.476
ENSG00000112473	7922	SLC39A7	0.474
ENSG00000128422	3872	KRT17	0.547
ENSG00000022976	55778	ZNF839	0.573
ENSG00000167397	79001	VKORC1	0.522
ENSG00000095627	56165	TDRD1	0.435
ENSG00000140350	8125	ANP32A	0.643
ENSG00000134070	3656	IRAK2	0.558
ENSG00000204228	7923	HSD17B8	0.52
ENSG00000182957	221178	SPATA13	0.541
ENSG00000110243	116519	APOA5	0.46
ENSG00000188542	285193	DUSP28	0.435
ENSG00000161267	622	BDH1	0.555
ENSG00000175175	22843	PPM1E	0.498
ENSG00000198420	9747	FAM115A	0.621
ENSG00000179869	154664	ABCA13	0.479
ENSG00000113851	51185	CRBN	0.513
ENSG00000174483	582	BBS1	0.514
ENSG00000157014	9797	TATDN2	0.517
ENSG00000116521	10067	SCAMP3	0.518
ENSG00000170925	56156	TEX13B	0.459
ENSG00000107679	59338	PLEKHA1	0.522
ENSG00000101842	340547	VSIG1	0.444
ENSG00000197429	3652	IPP	0.513
ENSG00000229117	6171	RPL41	0.518
ENSG00000087302	51637	C14orf166	0.54
ENSG00000223501	6293	VPS52	0.528
ENSG00000166509	10143	CLEC3A	0.49
ENSG00000004864	10165	SLC25A13	0.559

ENSG00000186153	51741	WWOX	0.599
ENSG00000177508	79191	IRX3	0.5
ENSG00000173812	10209	EIF1	0.64
ENSG00000154027	26289	AK5	0.474
ENSG00000075407	7587	ZNF37A	0.47
ENSG00000198863	146923	RUNDC1	0.504
ENSG00000078053	273	AMPH	0.496
ENSG00000177301	3737	KCNA2	0.446
ENSG00000106638	26608	TBL2	0.571
ENSG00000124067	6560	SLC12A4	0.646
ENSG00000173805	9001	HAP1	0.554
ENSG00000104723	7991	TUSC3	0.64
ENSG00000137807	9493	KIF23	0.531
ENSG00000176444	1196	CLK2	0.527
ENSG00000170290	6588	SLN	0.501
ENSG00000106336	26261	FBXO24	0.434
ENSG00000176108	79643	CHMP6	0.468
ENSG00000128791	57045	TWSG1	0.522
ENSG00000130988	9104	RGN	0.491
ENSG00000182107	161291	TMEM30B	0.527
ENSG00000184900	6612	SUMO3	0.581
ENSG00000009950	51085	MLXIPL	0.503
ENSG00000185621	89782	LMLN	0.526
ENSG00000166446	124359	CDYL2	0.448
ENSG00000183255	754	PTTG1IP	0.589
ENSG00000164051	79714	CCDC51	0.516
ENSG00000166033	5654	HTRA1	0.524
ENSG00000163833	51725	FBXO40	0.442
ENSG00000101448	57119	SPINLW1	0.51
ENSG00000182791	55231	CCDC87	0.447
ENSG00000175564	7352	UCP3	0.574
ENSG00000147883	1030	CDKN2B	0.489
ENSG00000142856	23421	ITGB3BP	0.573
ENSG00000087495	116154	PHACTR3	0.474
ENSG00000186354	286223	C9orf47	0.532
ENSG00000213694	1903	S1PR3	0.543
ENSG00000198242	6147	RPL23A	0.572
ENSG00000151849	55835	CENPJ	0.495
ENSG00000149311	472	ATM	0.63
ENSG00000180305	140832	WFDC10A	0.378
ENSG00000182931	280664	WFDC10B	0.388
ENSG00000041988	90326	THAP3	0.547
ENSG00000198680	286319	TUSC1	0.517
ENSG00000173230	2804	GOLGB1	0.597
ENSG00000160606	116238	TLCD1	0.522
ENSG00000151846	5042	PABPC3	0.488
ENSG00000071462	114049	WBSCR22	0.543
ENSG00000135506	10956	OS9	0.578
ENSG00000160602	284086	NEK8	0.479
ENSG00000133316	54663	WDR74	0.569
ENSG00000055130	8454	CUL1	0.565
ENSG00000164713	25798	BRI3	0.54
ENSG00000169490	83877	TM2D2	0.551
ENSG00000124217	27304	MOCS3	0.478

ENSG00000135842	116496	FAM129A	0.585
ENSG00000072736	4775	NFATC3	0.635
ENSG00000140691	79798	ARMC5	0.47
ENSG00000155975	137492	VPS37A	0.515
ENSG00000149380	283208	P4HA3	0.482
ENSG00000188554	4077	NBR1	0.537
ENSG00000167257	257160	RNF214	0.489
ENSG00000198498	55319	C4orf43	0.443
ENSG00000243709	10637	LEFTY1	0.482
ENSG00000074527	59277	NTN4	0.497
ENSG00000104321	8989	TRPA1	0.52
ENSG00000132591	26284	ERAL1	0.515
ENSG00000173947	128344	C1orf88	0.441
ENSG00000163406	6565	SLC15A2	0.525
ENSG00000237441	5863	RGL2	0.529
ENSG00000160190	54020	SLC37A1	0.498
ENSG00000182674	9312	KCNB2	0.522
ENSG00000145734	55814	BDP1	0.55
ENSG00000197140	203102	ADAM32	0.421
ENSG00000146574	51622	C7orf28A	0.537
ENSG00000147601	7013	TERF1	0.629
ENSG00000130427	2056	EPO	0.513
ENSG00000184261	56660	KCNK12	0.444
ENSG00000108021	54906	C10orf18	0.522
ENSG00000159461	267	AMFR	0.55
ENSG00000116459	515	ATP5F1	0.571
ENSG00000189143	1364	CLDN4	0.539
ENSG00000020256	55734	ZFP64	0.589
ENSG00000134253	80263	TRIM45	0.5
ENSG00000160191	5152	PDE9A	0.496
ENSG00000152484	219333	USP12	0.563
ENSG00000135469	93058	COQ10A	0.475
ENSG00000135446	1019	CDK4	0.545
ENSG00000174482	158038	LINGO2	0.438
ENSG00000122729	48	ACO1	0.536
ENSG00000170419	222008	VSTM2A	0.444
ENSG00000155660	9601	PDIA4	0.614
ENSG00000143768	7044	LEFTY2	0.481
ENSG00000187634	148398	SAMD11	0.455
ENSG00000145103	286676	ILDR1	0.491
ENSG00000049247	10911	UTS2	0.562
ENSG00000135222	1447	CSN2	0.464
ENSG00000241685	10552	ARPC1A	0.508
ENSG00000128656	1123	CHN1	0.531
ENSG00000148090	549	AUH	0.538
ENSG00000149357	55004	C11orf59	0.504
ENSG00000087263	55239	OGFOD1	0.509
ENSG00000162616	11080	DNAJB4	0.561
ENSG00000169884	7480	WNT10B	0.515
ENSG00000107201	23586	DDX58	0.498
ENSG00000166165	1152	CKB	0.53
ENSG00000100523	80821	DDHD1	0.564
ENSG00000064787	8537	BCAS1	0.501
ENSG00000179761	51268	PIPOX	0.485

ENSG00000196505	54834	GDAP2	0.425
ENSG00000174437	488	ATP2A2	0.631
ENSG00000111012	1594	CYP27B1	0.485
ENSG00000110200	25906	C11orf51	0.495
ENSG00000146426	26230	TIAM2	0.526
ENSG00000162456	148930	KNCN	0.378
ENSG00000240021	84066	C1orf49	0.443
ENSG00000019186	1591	CYP24A1	0.431
ENSG00000054938	25884	CHRDL2	0.47
ENSG00000137959	10964	IFI44L	0.51
ENSG00000102038	6594	SMARCA1	0.616
ENSG00000189050	51136	RNFT1	0.534
ENSG00000125124	583	BBS2	0.524
ENSG00000137714	2230	FDX1	0.545
ENSG00000111145	2004	ELK3	0.6
ENSG00000167550	121268	RHEBL1	0.54
ENSG00000122025	2322	FLT3	0.505
ENSG00000101134	55816	DOK5	0.503
ENSG00000231925	6892	TAPBP	0.616
ENSG00000166170	9529	BAG5	0.569
ENSG00000164326	9607	CARTPT	0.471
ENSG00000131061	84905	ZNF341	0.521
ENSG00000087586	6790	AURKA	0.654
ENSG00000167552	7846	TUBA1A	0.525
ENSG00000122126	4952	OCRL	0.609
ENSG00000183814	286826	LIN9	0.523
ENSG00000204381	143903	LAYN	0.517
ENSG00000011638	57146	TMEM159	0.528
ENSG00000112695	1347	COX7A2	0.542
ENSG00000134452	84893	FBXO18	0.479
ENSG00000151033	119180	LYZL2	0.5
ENSG00000175311	257629	ANKS4B	0.383
ENSG00000112697	55754	TMEM30A	0.542
ENSG00000145309	85438	C4orf35	0.426
ENSG00000161649	146894	CD300LG	0.58
ENSG00000146729	2631	GBAS	0.533
ENSG00000173757	6777	STAT5B	0.632
ENSG00000165462	401	PHOX2A	0.441
ENSG00000137726	53826	FXYD6	0.496
ENSG00000186283	64222	TOR3A	0.502
ENSG00000139508	283537	SLC46A3	0.52
ENSG00000162139	10825	NEU3	0.551
ENSG00000187778	10445	MCRS1	0.505
ENSG00000196453	27153	ZNF777	0.575
ENSG00000137491	11309	SLCO2B1	0.592
ENSG00000146731	908	CCT6A	0.568
ENSG00000131979	2643	GCH1	0.576
ENSG00000120802	7112	TMPO	0.636
ENSG00000005108	221981	THSD7A	0.501
ENSG00000119938	5507	PPP1R3C	0.561
ENSG00000119457	57864	SLC46A2	0.435
ENSG00000110844	25766	PRPF40B	0.4
ENSG00000171492	55144	LRRC8D	0.537
ENSG00000185332	284186	TMEM105	0.415

ENSG00000198673	338811	FAM19A2	0.341
ENSG00000188816	3167	HMX2	0.443
ENSG00000103150	23417	MLYCD	0.498
ENSG00000182871	80781	COL18A1	0.538
ENSG00000062038	1001	CDH3	0.542
ENSG00000137941	79739	TTLL7	0.453
ENSG00000204176	83849	SYT15	0.5
ENSG00000122705	1211	CLTA	0.644
ENSG00000057252	6646	SOAT1	0.538
ENSG00000101464	128869	PIGU	0.482
ENSG00000182533	859	CAV3	0.449
ENSG00000175048	79683	ZDHHC14	0.523
ENSG00000161800	29127	RACGAP1	0.526
ENSG00000112699	2762	GMDS	0.577
ENSG00000180914	5021	OXTR	0.477
ENSG00000070950	56852	RAD18	0.549
ENSG00000225697	65010	SLC26A6	0.485
ENSG00000103047	79613	TMCO7	0.45
ENSG00000136875	9128	PRPF4	0.601
ENSG00000168447	6338	SCNN1B	0.471
ENSG00000049239	9563	H6PD	0.561
ENSG00000165646	6571	SLC18A2	0.495
ENSG00000157150	7079	TIMP4	0.473
ENSG00000188807	199953	TMEM201	0.594
ENSG00000086730	7462	LAT2	0.622
ENSG00000242441	11036	GTF2A1L	0.439
ENSG00000073910	10129	FRY	0.664
ENSG00000213588	221504	ZBTB9	0.523
ENSG00000134453	84991	RBM17	0.508
ENSG00000075568	23505	TMEM131	0.51
ENSG00000176022	126792	B3GALT6	0.559
ENSG00000176340	1351	COX8A	0.537
ENSG00000103356	124454	EARS2	0.556
ENSG00000178449	84987	C12orf62	0.458
ENSG00000164385	154386	C6orf195	0.385
ENSG00000139624	91012	LASS5	0.483
ENSG00000147408	55790	CSGALNACT1	0.522
ENSG00000213676	1388	CREBL1	0.561
ENSG00000124535	56897	WRNIP1	0.48
ENSG00000004779	4706	NDUFAB1	0.551
ENSG00000178974	55030	FBXO34	0.521
ENSG00000165669	63877	C10orf84	0.484
ENSG00000131069	55902	ACSS2	0.506
ENSG00000221949	283416	C12orf61	0.39
ENSG00000116218	7827	NPHS2	0.447
ENSG00000214022	29803	REPIN1	0.494
ENSG00000136267	1607	DGKB	0.475
ENSG00000162782	163589	TDRD5	0.37
ENSG00000182508	340596	LHFPL1	0.362
ENSG00000070718	10947	AP3M2	0.476
ENSG00000168439	10963	STIP1	0.563
ENSG00000104613	55174	INTS10	0.591
ENSG00000132612	27183	VPS4A	0.498
ENSG00000134594	9363	RAB33A	0.528

ENSG00000146247	55023	PHIP	0.566
ENSG00000123268	466	ATF1	0.53
ENSG00000097046	8317	CDC7	0.524
ENSG00000203791	399818	METTL10	0.474
ENSG00000145741	689	BTF3	0.626
ENSG00000197779	347344	ZNF81	0.469
ENSG00000102078	9016	SLC25A14	0.559
ENSG00000206527	201562	PTPLB	0.544
ENSG00000221988	9374	PPT2	0.542
ENSG00000149257	871	SERPINH1	0.542
ENSG00000065534	4638	MYLK	0.564
ENSG00000141446	114799	ESCO1	0.548
ENSG00000158865	115584	SLC5A11	0.433
ENSG00000132603	51388	NIP7	0.502
ENSG00000107581	8661	EIF3A	0.599
ENSG00000036565	6570	SLC18A1	0.458
ENSG00000221994	57232	ZNF630	0.436
ENSG00000140750	55114	ARHGAP17	0.488
ENSG00000174206	144577	C12orf66	0.513
ENSG00000118418	9324	HMGN3	0.565
ENSG00000160201	7307	U2AF1	0.544
ENSG00000075975	23609	MKRN2	0.552
ENSG00000135457	7024	TFCP2	0.59
ENSG00000101190	10732	TCFL5	0.529
ENSG00000107623	2662	GDF10	0.491
ENSG00000132155	5894	RAF1	0.561
ENSG00000138160	3832	KIF11	0.538
ENSG00000108578	642	BLMH	0.563
ENSG00000178104	9659	PDE4DIP	0.617
ENSG00000101191	11083	DIDO1	0.596
ENSG00000103018	80777	CYB5B	0.603
ENSG00000165233	84270	C9orf89	0.5
ENSG00000173457	26472	PPP1R14B	0.481
ENSG00000147443	9046	DOK2	0.454
ENSG00000180228	8575	PRKRA	0.511
ENSG00000067221	9399	STOML1	0.564
ENSG00000106541	10551	AGR2	0.474
ENSG00000180219	196472	FAM71C	0.312
ENSG00000152804	3087	HHEX	0.576
ENSG00000085741	7481	WNT11	0.523
ENSG00000116260	5768	QSOX1	0.55
ENSG00000155850	1836	SLC26A2	0.545
ENSG00000164296	81789	TIGD6	0.432
ENSG00000101000	10544	PROCR	0.564
ENSG00000165238	65268	WNK2	0.56
ENSG00000160963	136227	EMID2	0.561
ENSG00000140848	221184	CPNE2	0.413
ENSG00000158636	56946	C11orf30	0.468
ENSG00000180772	186	AGTR2	0.589
ENSG00000087916	11254	SLC6A14	0.459
ENSG00000242372	3692	EIF6	0.522
ENSG00000173838	162333	10-Mar	0.416
ENSG00000160202	1409	CRYAA	0.504
ENSG00000171126	170850	KCNG3	0.469

ENSG00000088726	55287	TMEM40	0.457
ENSG00000113721	5159	PDGFRB	0.528
ENSG00000101198	128414	NKAIN4	0.474
ENSG00000079150	51661	FKBP7	0.545
ENSG00000137507	2615	LRRC32	0.516
ENSG00000182450	50801	KCNK4	0.43
ENSG00000133104	23111	SPG20	0.563
ENSG00000137267	7280	TUBB2A	0.573
ENSG00000136021	55681	SCYL2	0.533
ENSG00000160999	10603	SH2B2	0.548
ENSG00000048052	9734	HDAC9	0.507
ENSG00000005961	3674	ITGA2B	0.608
ENSG00000107404	1855	DVL1	0.539
ENSG00000122484	79871	RPAP2	0.441
ENSG00000155657	7273	TTN	0.537
ENSG00000133101	8900	CCNA1	0.516
ENSG00000176208	79915	ATAD5	0.5
ENSG00000180440	400120	C13orf36	0.424
ENSG00000151657	22944	KIN	0.475
ENSG00000102931	23568	ARL2BP	0.597
ENSG00000160991	80228	ORAI2	0.569
ENSG00000162576	54587	MXRA8	0.385
ENSG00000131668	56033	BARX1	0.417
ENSG00000186577	221491	C6orf1	0.449
ENSG00000137474	4647	MYO7A	0.614
ENSG00000126001	11190	CEP250	0.587
ENSG00000005194	57019	CIAPIN1	0.583
ENSG00000113722	1044	CDX1	0.468
ENSG00000164104	3148	HMGB2	0.574
ENSG00000165629	509	ATP5C1	0.637
ENSG00000137285	347733	TUBB2B	0.518
ENSG00000100557	55195	C14orf105	0.417
ENSG00000120925	81790	RNF170	0.444
ENSG00000213654	63940	GPSM3	0.56
ENSG00000125991	51614	ERGIC3	0.61
ENSG00000112096	6648	SOD2	0.643
ENSG00000106714	79937	CNTNAP3	0.441
ENSG00000133110	10631	POSTN	0.53
ENSG00000168522	2339	FNTA	0.615
ENSG00000148120	84909	C9orf3	0.532
ENSG00000105849	221830	TWISTNB	0.495
ENSG00000110375	7379	UPK2	0.477
ENSG00000214078	8904	CPNE1	0.511
ENSG00000033170	2530	FUT8	0.545
ENSG00000108588	57003	CCDC47	0.509
ENSG00000074201	1207	CLNS1A	0.516
ENSG00000131779	8799	PEX11B	0.54
ENSG00000177994	129852	C2orf73	0.4
ENSG00000132680	22889	KIAA0907	0.545
ENSG00000178301	282679	AQP11	0.39
ENSG00000048649	51773	RSF1	0.523
ENSG00000064995	6882	TAF11	0.556
ENSG00000117505	1810	DR1	0.627
ENSG00000165383	474354	LRRC18	0.393

ENSG00000131095	2670	GFAP	0.477
ENSG00000133256	5158	PDE6B	0.464
ENSG00000183434	51270	TFDP3	0.425
ENSG00000177354	118461	C10orf71	0.372
ENSG00000186364	200035	NUDT17	0.5
ENSG00000165794	29986	SLC39A2	0.451
ENSG00000086589	55696	RBM22	0.509
ENSG00000023572	51022	GLRX2	0.515
ENSG00000198691	24	ABCA4	0.504
ENSG00000105819	9512	PMPCB	0.53
ENSG00000165704	3251	HPRT1	0.553
ENSG00000129187	1635	DCTD	0.641
ENSG00000154764	7476	WNT7A	0.495
ENSG00000146701	4191	MDH2	0.598
ENSG00000115310	57142	RTN4	0.608
ENSG00000178645	282966	C10orf53	0.393
ENSG00000161714	113026	PLCD3	0.536
ENSG00000105821	27000	DNAJC2	0.522
ENSG00000162687	343450	KCNT2	0.562
ENSG00000145901	10318	TNIP1	0.56
ENSG00000197114	84619	ZGPAT	0.557
ENSG00000169139	7336	UBE2V2	0.504
ENSG00000189091	23450	SF3B3	0.575
ENSG00000168538	60684	C4orf41	0.505
ENSG00000176273	159371	TMEM20	0.523
ENSG00000189056	5649	RELN	0.489
ENSG00000151468	83643	CCDC3	0.5
ENSG00000125520	56731	SLC2A4RG	0.547
ENSG00000164889	6522	SLC4A2	0.523
ENSG00000157368	146433	IL34	0.423
ENSG00000160803	56893	UBQLN4	0.433
ENSG00000165494	51585	PCF11	0.556
ENSG00000203950	26071	FAM127B	0.455
ENSG00000196743	2760	GM2A	0.679
ENSG00000157423	54768	HYDIN	0.476
ENSG00000070476	79364	ZXDC	0.532
ENSG00000137500	60492	CCDC90B	0.503
ENSG00000159650	131669	UROC1	0.446
ENSG00000214113	57128	LYRM4	0.481
ENSG00000171703	6919	TCEA2	0.528
ENSG00000113140	6678	SPARC	0.594
ENSG00000164323	57587	KIAA1430	0.559
ENSG00000061676	10787	NCKAP1	0.567
ENSG00000109771	55805	LRP2BP	0.467
ENSG00000186352	353322	ANKRD37	0.505
ENSG00000168491	256309	CCDC110	0.432
ENSG00000176381	285800	PRR18	0.321
ENSG00000165804	51222	ZNF219	0.471
ENSG00000124784	83732	RIOK1	0.45
ENSG00000096696	1832	DSP	0.535
ENSG00000153162	654	BMP6	0.555
ENSG00000169750	5881	RAC3	0.443
ENSG00000243279	11230	PRAF2	0.504
ENSG00000163468	7203	CCT3	0.563

ENSG00000196998	11152	WDR45	0.561
ENSG00000124786	51000	SLC35B3	0.522
ENSG00000125462	10485	C1orf61	0.599
ENSG00000169718	64118	DUS1L	0.485
ENSG00000059573	5832	ALDH18A1	0.515
ENSG00000179362	283651	C15orf21	0.359
ENSG00000060237	65125	WNK1	0.636
ENSG00000068305	4205	MEF2A	0.605
ENSG00000139842	8451	CUL4A	0.57
ENSG00000185896	3916	LAMP1	0.656
ENSG00000140470	170691	ADAMTS17	0.493
ENSG00000150401	55208	DCUN1D2	0.502
ENSG00000082805	23085	ERC1	0.584
ENSG00000183475	140460	ASB7	0.469
ENSG00000184254	220	ALDH1A3	0.525
ENSG00000129990	6861	SYT5	0.515
ENSG00000160469	84446	BRSK1	0.516
ENSG00000133247	84787	SUV420H2	0.457
ENSG00000095752	3589	IL11	0.559
ENSG00000131873	22856	CHSY1	0.58
ENSG00000169062	65110	UPF3A	0.62
ENSG00000179922	147808	ZNF784	0.44
ENSG00000213015	51157	ZNF580	0.47
ENSG00000184277	80213	TM2D3	0.525
ENSG00000182318	342945	ZSCAN22	0.414
ENSG00000138411	57520	HECW2	0.573
ENSG00000120137	79646	PANK3	0.49
ENSG00000184347	6586	SLIT3	0.569
ENSG00000113971	27031	NPHP3	0.552
ENSG00000197121	80055	PGAP1	0.58
ENSG00000091527	55573	CDV3	0.636
ENSG00000213186	286827	TRIM59	0.522
ENSG00000186432	3840	KPNA4	0.528
ENSG00000163590	151742	PPM1L	0.426
ENSG00000121871	22865	SLITRK3	0.48
ENSG00000115540	25843	MOBKL3	0.574
ENSG00000154917	51560	RAB6B	0.551
ENSG00000174640	6578	SLCO2A1	0.507
ENSG00000118242	55686	MREG	0.526
ENSG00000114209	11235	PDCD10	0.546
ENSG00000115520	80219	COQ10B	0.493
ENSG00000152430	66037	BOLL	0.447
ENSG00000154928	2047	EPHB1	0.54
ENSG00000114019	51421	AMOTL2	0.539
ENSG00000174611	339855	KY	0.5
ENSG00000204764	64901	RANBP17	0.495
ENSG00000173905	27333	GOLIM4	0.527
ENSG00000144583	57574	4-Mar	0.423
ENSG00000115896	5334	PLCL1	0.511
ENSG00000119042	23314	SATB2	0.577
ENSG00000073711	5523	PPP2R3A	0.59
ENSG00000122085	130916	MTERFD2	0.488
ENSG00000156427	8817	FGF18	0.595
ENSG00000072803	23291	FBXW11	0.566

ENSG00000115461	3488	IGFBP5	0.659
ENSG00000118245	7141	TNP1	0.481
ENSG00000178074	205327	C2orf69	0.504
ENSG00000162971	129450	C2orf60	0.539
ENSG00000079308	7145	TNS1	0.623
ENSG00000113742	80315	CPEB4	0.558
ENSG00000072786	6793	STK10	0.573
ENSG00000114098	25852	ARMC8	0.578
ENSG00000115677	3069	HDLBP	0.617
ENSG00000008952	7095	SEC62	0.575
ENSG00000170091	51617	HMP19	0.416
ENSG00000168246	92181	UBTD2	0.469
ENSG00000174705	285590	SH3PXD2B	0.504
ENSG00000173889	80012	PHC3	0.545
ENSG00000184845	1812	DRD1	0.456
ENSG00000120129	1843	DUSP1	0.614
ENSG00000118007	10274	STAG1	0.529
ENSG00000168385	4735	2-Sep	0.574
ENSG00000145920	10814	CPLX2	0.469
ENSG00000163577	56648	EIF5A2	0.492
ENSG00000164463	153222	C5orf41	0.565
ENSG00000127838	25953	PNKD	0.592
ENSG00000013297	5010	CLDN11	0.449
ENSG00000158186	22808	MRAS	0.458
ENSG00000115942	4999	ORC2L	0.498
ENSG00000113734	662	BNIP1	0.591
ENSG00000115694	10494	STK25	0.568
ENSG00000018280	6556	SLC11A1	0.62
ENSG00000012171	7869	SEMA3B	0.595
ENSG00000155744	285172	FAM126B	0.52
ENSG00000113739	8614	STC2	0.583
ENSG00000176946	51078	THAP4	0.508
ENSG00000158163	199221	DZIP1L	0.4
ENSG00000082153	9689	BZW1	0.488
ENSG00000144579	58190	CTDSP1	0.51
ENSG00000170085	375484	C5orf25	0.44
ENSG00000135913	57695	USP37	0.53
ENSG00000122203	57179	KIAA1191	0.456
ENSG00000168395	84289	ING5	0.444
ENSG00000175414	285598	ARL10	0.431
ENSG00000075420	64778	FNDC3B	0.58
ENSG00000115556	84812	PLCD4	0.483
ENSG00000003400	843	CASP10	0.638
ENSG00000183770	668	FOXL2	0.498
ENSG00000144959	57552	AADACL1	0.511
ENSG00000146083	22838	RNF44	0.549
ENSG00000163482	27148	STK36	0.536
ENSG00000169760	22871	NLGN1	0.474
ENSG00000169258	114787	GPRIN1	0.49
ENSG00000074317	6620	SNCB	0.465
ENSG00000177694	254827	NAALADL2	0.439
ENSG00000177565	79718	TBL1XR1	0.546
ENSG00000197584	10242	KCNMB2	0.492
ENSG00000172667	64393	ZMAT3	0.555

ENSG00000121879	5290	PIK3CA	0.527
ENSG00000135925	80326	WNT10A	0.476
ENSG00000017260	27032	ATP2C1	0.617
ENSG00000145864	2561	GABRB2	0.477
ENSG00000022355	2554	GABRA1	0.457
ENSG00000113327	2566	GABRG2	0.478
ENSG00000113328	900	CCNG1	0.515
ENSG00000173559	64859	OBFC2A	0.462
ENSG00000188549	388115	C15orf52	0.476
ENSG00000038274	27430	MAT2B	0.555
ENSG00000179431	24147	FJX1	0.512
ENSG00000166326	54765	TRIM44	0.547
ENSG00000179241	143458	LDLRAD3	0.542
ENSG00000140320	22893	BAHD1	0.478
ENSG00000169105	113189	CHST14	0.459
ENSG00000128891	90416	C15orf57	0.5
ENSG00000196950	57181	SLC39A10	0.542
ENSG00000145934	57451	ODZ2	0.462
ENSG00000146463	9202	ZMYM4	0.602
ENSG00000196353	131034	CPNE4	0.507
ENSG00000129244	482	ATP1B2	0.501
ENSG00000141510	7157	TP53	0.609
ENSG00000166349	5896	RAG1	0.45
ENSG00000138246	23317	DNAJC13	0.53
ENSG00000111913	9750	FAM65B	0.569
ENSG00000148948	57689	LRRC4C	0.5
ENSG00000071575	28951	TRIB2	0.596
ENSG00000166181	8539	API5	0.643
ENSG00000155252	55361	PI4K2A	0.593
ENSG00000184083	54954	FAM120C	0.463
ENSG00000108947	1949	EFNB3	0.541
ENSG00000162981	151354	FAM84A	0.537
ENSG00000196632	65267	WNK3	0.523
ENSG00000171160	118812	MORN4	0.452
ENSG00000128917	54567	DLL4	0.448
ENSG00000128965	79094	CHAC1	0.525
ENSG00000020129	23154	NCDN	0.562
ENSG00000079691	55604	LRRC16A	0.533
ENSG00000170004	1107	CHD3	0.604
ENSG00000102302	2245	FGD1	0.485
ENSG00000116819	339488	TFAP2E	0.395
ENSG00000137815	23168	RTF1	0.54
ENSG00000170049	9196	KCNAB3	0.455
ENSG00000170043	58485	TRAPPC1	0.461
ENSG00000134698	192670	EIF2C4	0.497
ENSG00000158710	8407	TAGLN2	0.614
ENSG00000052850	60529	ALX4	0.421
ENSG00000092847	26523	EIF2C1	0.473
ENSG00000092445	7301	TYRO3	0.613
ENSG00000126070	192669	EIF2C3	0.504
ENSG00000174197	23269	MGA	0.489
ENSG00000137802	23005	MAPKBP1	0.501
ENSG00000134323	4613	MYCN	0.555
ENSG00000119946	26507	CNNM1	0.465

ENSG00000138131	84171	LOXL4	0.523
ENSG00000177807	3766	KCNJ10	0.472
ENSG00000204271	169981	SPIN3	0.548
ENSG00000116863	54936	ADPRHL2	0.516
ENSG00000019505	57586	SYT13	0.468
ENSG00000177764	85364	ZCCHC3	0.547
ENSG00000177732	6666	SOX12	0.475
ENSG00000175264	8534	CHST1	0.49
ENSG00000129535	4901	NRL	0.564
ENSG00000179111	84667	HES7	0.415
ENSG00000181830	55343	SLC35C1	0.476
ENSG00000179094	5187	PER1	0.569
ENSG00000120053	2805	GOT1	0.555
ENSG00000101255	57761	TRIB3	0.529
ENSG00000121671	1408	CRY2	0.494
ENSG00000198455	158586	ZXDB	0.454
ENSG00000119919	159296	NKX2-3	0.42
ENSG00000132031	4148	MATN3	0.497
ENSG00000131089	23229	ARHGEF9	0.546
ENSG00000116871	55700	MAP7D1	0.511
ENSG00000068697	9741	LAPTM4A	0.57
ENSG00000018625	477	ATP1A2	0.537
ENSG00000121653	9479	MAPK8IP1	0.54
ENSG00000198018	57089	ENTPD7	0.471
ENSG00000220205	6844	VAMP2	0.588
ENSG00000184675	139285	FAM123B	0.356
ENSG00000162735	5824	PEX19	0.604
ENSG00000125875	128637	TBC1D20	0.511
ENSG00000115884	6382	SDC1	0.607
ENSG00000196544	54785	C17orf59	0.417
ENSG00000101266	1457	CSNK2A1	0.618
ENSG00000055917	23369	PUM2	0.628
ENSG00000054118	9967	THRAP3	0.488
ENSG00000102053	340554	ZC3H12B	0.509
ENSG00000170745	3790	KCNS3	0.521
ENSG00000092098	55072	RNF31	0.462
ENSG00000143878	388	RHOB	0.509
ENSG00000147065	4478	MSN	0.551
ENSG00000106610	64940	STAG3L4	0.477
ENSG00000182952	10473	HMGN4	0.639
ENSG00000143867	130497	OSR1	0.482
ENSG00000162736	23385	NCSTN	0.502
ENSG00000162734	8682	PEA15	0.635
ENSG00000181315	79692	ZNF322A	0.547
ENSG00000196787	8329	HIST1H2AI	0.501
ENSG00000107566	10613	ERLIN1	0.563
ENSG00000171786	4807	NHLH1	0.46
ENSG00000162738	57216	VANGL2	0.459
ENSG00000137393	255488	RNF144B	0.554
ENSG00000119778	54454	ATAD2B	0.512
ENSG00000124313	23096	IQSEC2	0.461
ENSG00000172201	3400	ID4	0.6
ENSG00000162739	114836	SLAMF6	0.531
ENSG00000155304	6782	HSPA13	0.612

ENSG00000182591	337880	KRTAP11-1	0.4
ENSG00000156299	7074	TIAM1	0.635
ENSG00000172197	154141	MBOAT1	0.43
ENSG00000095485	55280	CWF19L1	0.49
ENSG00000072501	8243	SMC1A	0.624
ENSG00000112242	1871	E2F3	0.576
ENSG00000129473	599	BCL2L2	0.511
ENSG00000180530	8204	NRIP1	0.591
ENSG00000155313	29761	USP25	0.581
ENSG00000143882	245973	ATP6V1C2	0.518
ENSG00000169083	367	AR	0.586
ENSG00000124766	6659	SOX4	0.656
ENSG00000198315	7745	ZNF192	0.462
ENSG00000143870	10130	PDIA6	0.659
ENSG00000196072	282991	BLOC1S2	0.52
ENSG00000204569	5514	PPP1R10	0.598
ENSG00000119782	2281	FKBP1B	0.563
ENSG00000079482	4983	OPHN1	0.499
ENSG00000139910	4857	NOVA1	0.56
ENSG00000152954	140767	NRSN1	0.424
ENSG00000100842	10278	EFS	0.558
ENSG00000110427	25758	C11orf41	0.482
ENSG00000171303	3777	KCNK3	0.55
ENSG00000146038	51473	DCDC2	0.575
ENSG00000213699	54978	C2orf18	0.481
ENSG00000166090	64806	IL25	0.395
ENSG00000137343	79969	C6orf134	0.503
ENSG00000162522	57648	KIAA1522	0.508
ENSG00000176165	2290	FOXG1	0.457
ENSG00000120029	79591	C10orf76	0.575
ENSG00000085063	966	CD59	0.687
ENSG00000147162	8473	OGT	0.66
ENSG00000134684	8565	YARS	0.57
ENSG00000184304	5587	PRKD1	0.516
ENSG00000110429	26273	FBXO3	0.546
ENSG00000157851	56896	DPYSL5	0.533
ENSG00000160097	252995	FNDC5	0.504
ENSG00000142149	30811	HUNK	0.429
ENSG00000084764	22924	MAPRE3	0.575
ENSG00000135363	4005	LMO2	0.517
ENSG00000135387	4076	CAPRIN1	0.617
ENSG00000116514	127544	RNF19B	0.609
ENSG00000074319	7251	TSG101	0.525
ENSG00000004455	204	AK2	0.685
ENSG00000242732	340526	RGAG4	0.451
ENSG00000169016	1876	E2F6	0.506
ENSG00000060656	10076	PTPRU	0.501
ENSG00000204564	221545	C6orf136	0.518
ENSG00000169992	57555	NLGN2	0.533
ENSG00000166197	9221	NOLC1	0.627
ENSG00000161958	2256	FGF11	0.527
ENSG00000198435	441478	NRARP	0.472
ENSG00000129250	10749	KIF1C	0.576
ENSG00000196208	9687	GREB1	0.553

ENSG00000166016	25841	ABTB2	0.513
ENSG00000130559	157922	CAMSAP1	0.656
ENSG00000174282	57659	ZBTB4	0.541
ENSG00000116525	55223	TRIM62	0.541
ENSG00000162745	25903	OLFML2B	0.508
ENSG00000179119	144108	SPTY2D1	0.492
ENSG00000160094	149076	ZNF362	0.479
ENSG00000129204	9098	USP6	0.527
ENSG00000172943	23133	PHF8	0.444
ENSG00000198929	9722	NOS1AP	0.466
ENSG00000134686	1912	PHC2	0.529
ENSG00000151117	144110	TMEM86A	0.564
ENSG00000084693	60509	AGBL5	0.5
ENSG00000135374	2001	ELF5	0.558
ENSG00000107862	8729	GBF1	0.511
ENSG00000165802	26012	NELF	0.516
ENSG00000162706	57863	CADM3	0.597
ENSG00000110786	84867	PTPN5	0.466
ENSG00000147099	55869	HDAC8	0.566
ENSG00000134324	23175	LPIN1	0.624
ENSG00000134644	9698	PUM1	0.587
ENSG00000112308	81688	C6orf62	0.649
ENSG00000135373	26298	EHF	0.512
ENSG00000165886	80019	UBTD1	0.467
ENSG00000161956	26168	SENP3	0.612
ENSG00000159082	8867	SYNJ1	0.569
ENSG00000121904	114784	CSMD2	0.458
ENSG00000238227	90120	C9orf69	0.442
ENSG00000152332	127933	UHMK1	0.512
ENSG00000092051	84502	JPH4	0.409
ENSG00000107187	8022	LHX3	0.465
ENSG00000029725	9135	RABEP1	0.585
ENSG00000165661	169714	QSOX2	0.47
ENSG00000026508	960	CD44	0.681
ENSG00000196792	29966	STRN3	0.546
ENSG00000084628	79570	NKAIN1	0.465
ENSG00000196230	203068	TUBB	0.613
ENSG00000166833	89797	NAV2	0.582
ENSG00000135365	51317	PHF21A	0.545
ENSG00000214193	79729	C1orf113	0.471
ENSG00000059915	5662	PSD	0.486
ENSG00000116544	58512	DLGAP3	0.448
ENSG00000121766	51538	ZCCHC17	0.466
ENSG00000196182	83931	STK40	0.547
ENSG00000125898	83541	FAM110A	0.463
ENSG00000157613	90993	CREB3L1	0.584
ENSG00000165724	116225	ZMYND19	0.478
ENSG00000149091	8525	DGKZ	0.507
ENSG00000072849	51009	DERL2	0.423
ENSG00000129255	9526	MPDU1	0.525
ENSG00000168528	347735	SERINC2	0.533
ENSG00000101282	343637	RSPO4	0.402
ENSG00000197070	92714	ARRDC1	0.485
ENSG00000125818	9491	PSMF1	0.618

ENSG00000148396	9919	SEC16A	0.506
ENSG00000110436	6506	SLC1A2	0.504
ENSG00000125895	55321	C20orf46	0.455
ENSG00000108961	29098	RANGRF	0.538
ENSG00000181090	79813	EHMT1	0.486
ENSG00000198844	22899	ARHGEF15	0.545
ENSG00000101298	9751	SNPH	0.538
ENSG00000129245	9513	FXR2	0.585
ENSG00000179314	23302	WSCD1	0.524
ENSG00000088832	2280	FKBP1A	0.633
ENSG00000084636	1307	COL16A1	0.577
ENSG00000110492	4192	MDK	0.538
ENSG00000166579	81565	NDEL1	0.509
ENSG00000148400	4851	NOTCH1	0.5
ENSG00000088833	55968	NSFL1C	0.64
ENSG00000110497	55626	AMBRA1	0.569
ENSG00000133026	4628	MYH10	0.64
ENSG00000163873	2899	GRIK3	0.461
ENSG00000163874	80149	ZC3H12A	0.478
ENSG00000163877	79753	SNIP1	0.458
ENSG00000132388	7326	UBE2G1	0.597
ENSG00000163879	7802	DNALI1	0.47
ENSG00000198053	140885	SIRPA	0.669
ENSG00000065320	9423	NTN1	0.442
ENSG00000175220	392	ARHGAP1	0.631
ENSG00000125834	140901	STK35	0.487
ENSG00000125780	7053	TGM3	0.48
ENSG00000169218	284654	RSPO1	0.453
ENSG00000116922	54955	C1orf109	0.475
ENSG00000175216	9793	CKAP5	0.546
ENSG00000138760	950	SCARB2	0.597
ENSG00000116903	149371	EXOC8	0.5
ENSG00000183317	284656	EPHA10	0.4
ENSG00000177034	345778	MTX3	0.516
ENSG00000135334	55122	AKIRIN2	0.565
ENSG00000134569	4038	LRP4	0.548
ENSG00000118432	1268	CNR1	0.566
ENSG00000007237	8522	GAS7	0.64
ENSG00000115665	60482	SLC5A7	0.402
ENSG00000160325	11094	C9orf7	0.59
ENSG00000111880	8732	RNGTT	0.632
ENSG00000196449	79693	YRDC	0.509
ENSG00000158156	55113	XKR8	0.48
ENSG00000188786	4520	MTF1	0.556
ENSG00000039319	9765	ZFYVE16	0.528
ENSG00000204084	3633	INPP5B	0.56
ENSG00000138771	57619	SHROOM3	0.545
ENSG00000149182	84364	ARFGAP2	0.531
ENSG00000141480	409	ARRB2	0.529
ENSG00000130962	5638	PRRG1	0.492
ENSG00000138758	55752	11-Sep	0.66
ENSG00000154640	10950	BTG3	0.653
ENSG00000099194	6319	SCD	0.617
ENSG00000146278	10957	PNRC1	0.533

ENSG00000088881	57593	EBF4	0.439
ENSG00000135968	9648	GCC2	0.524
ENSG00000125492	56751	BARHL1	0.449
ENSG00000123454	1621	DBH	0.458
ENSG00000183431	10946	SF3A3	0.552
ENSG00000047597	7504	XK	0.485
ENSG00000154642	54149	C21orf91	0.451
ENSG00000117713	8289	ARID1A	0.626
ENSG00000165168	1536	CYBB	0.54
ENSG00000121753	576	BAI2	0.472
ENSG00000198399	50618	ITSN2	0.628
ENSG00000150316	51503	CWC15	0.447
ENSG00000129152	4654	MYOD1	0.484
ENSG00000126698	22826	DNAJC8	0.605
ENSG00000160685	51043	ZBTB7B	0.481
ENSG00000197702	55742	PARVA	0.591
ENSG00000129159	3746	KCNC1	0.452
ENSG00000151224	4143	MAT1A	0.487
ENSG00000183386	2275	FHL3	0.491
ENSG00000154645	140578	CHODL	0.432
ENSG00000090776	1947	EFNB1	0.549
ENSG00000122223	51744	CD244	0.443
ENSG00000130766	83667	SESN2	0.541
ENSG00000116991	57568	SIPA1L2	0.572
ENSG00000187079	7003	TEAD1	0.519
ENSG00000101955	8406	SRPX	0.533
ENSG00000130054	27112	FAM155B	0.5
ENSG00000118496	84085	FBXO30	0.5
ENSG00000185668	5453	POU3F1	0.547
ENSG00000128591	2318	FLNC	0.496
ENSG00000204138	65979	PHACTR4	0.505
ENSG00000214114	26292	MYCBP	0.647
ENSG00000133794	406	ARNTL	0.533
ENSG00000158813	1896	EDA	0.647
ENSG00000160293	7410	VAV2	0.544
ENSG00000138767	246175	CNOT6L	0.582
ENSG00000046653	2824	GPM6B	0.615
ENSG00000025039	58528	RRAGD	0.598
ENSG00000153201	5903	RANBP2	0.614
ENSG00000108219	81619	TSPAN14	0.485
ENSG00000170365	4086	SMAD1	0.585
ENSG00000204160	84243	ZDHHC18	0.501
ENSG00000006530	55750	AGK	0.577
ENSG00000161526	29115	SAP30BP	0.482
ENSG00000198648	27347	STK39	0.597
ENSG00000135299	22881	ANKRD6	0.564
ENSG00000180198	1104	RCC1	0.577
ENSG00000168209	54541	DDIT4	0.61
ENSG00000175793	2810	SFN	0.639
ENSG00000046647	54960	GEMIN8	0.505
ENSG00000089289	3476	IGBP1	0.527
ENSG00000148719	54788	DNAJB12	0.667
ENSG00000151612	152485	ZNF827	0.566
ENSG00000158769	50848	F11R	0.553

ENSG00000165699	7248	TSC1	0.529
ENSG00000128604	3663	IRF5	0.582
ENSG00000035862	7077	TIMP2	0.59
ENSG00000109956	27087	B3GAT1	0.46
ENSG00000169925	8019	BRD3	0.536
ENSG00000172292	253782	LASS6	0.593
ENSG00000178217	387694	SH2D4B	0.372
ENSG00000176971	387758	FIBIN	0.571
ENSG00000143365	6097	RORC	0.497
ENSG00000152822	2911	GRM1	0.59
ENSG00000130150	158747	MOSPD2	0.564
ENSG00000184007	8073	PTP4A2	0.668
ENSG00000204688	26716	OR2H1	0.517
ENSG00000197417	23729	SHPK	0.461
ENSG00000064419	23534	TNPO3	0.649
ENSG00000138759	80144	FRAS1	0.52
ENSG00000040531	1497	CTNS	0.582
ENSG00000196363	11091	WDR5	0.541
ENSG00000132470	3691	ITGB4	0.638
ENSG00000084676	8648	NCOA1	0.62
ENSG00000120519	84068	SLC10A7	0.516
ENSG00000205213	55366	LGR4	0.513
ENSG00000156298	7102	TSPAN7	0.521
ENSG00000121774	10657	KHDRBS1	0.602
ENSG00000141503	50488	MINK1	0.642
ENSG00000171302	124583	CANT1	0.581
ENSG00000161940	255877	BCL6B	0.548
ENSG00000144827	55347	ABHD10	0.522
ENSG00000135960	10913	EDAR	0.505
ENSG00000131910	8431	NR0B2	0.521
ENSG00000148943	55327	LIN7C	0.562
ENSG00000175707	126695	C1orf172	0.42
ENSG00000204681	2550	GABBR1	0.533
ENSG00000176697	627	BDNF	0.506
ENSG00000156026	90550	CCDC109A	0.466
ENSG00000186350	6256	RXRA	0.645
ENSG00000165175	58526	MID1IP1	0.546
ENSG00000172985	344558	SH3RF3	0.473
ENSG00000183337	54880	BCOR	0.511
ENSG00000186522	151011	10-Sep	0.603
ENSG00000158457	340348	TSPAN33	0.56
ENSG00000113108	10307	APBB3	0.504
ENSG00000090020	6548	SLC9A1	0.52
ENSG00000130635	1289	COL5A1	0.615
ENSG00000158773	7391	USF1	0.434
ENSG00000197601	84188	FAR1	0.58
ENSG00000165195	5277	PIGA	0.49
ENSG00000110768	2965	GTF2H1	0.602
ENSG00000025800	23633	KPNA6	0.597
ENSG00000158467	23382	AHCYL2	0.604
ENSG00000198492	51441	YTHDF2	0.483
ENSG00000108556	1145	CHRNE	0.535
ENSG00000169509	54544	CRCT1	0.421
ENSG00000133818	22800	RRAS2	0.635

ENSG00000135750	3775	KCNK1	0.586
ENSG00000165197	2277	FIGF	0.475
ENSG00000159023	2035	EPB41	0.553
ENSG00000132535	1742	DLG4	0.516
ENSG00000166135	55662	HIF1AN	0.539
ENSG00000198142	65124	ANKRD57	0.497
ENSG00000151617	1909	EDNRA	0.57
ENSG00000154727	2551	GABPA	0.494
ENSG00000066382	744	MPPED2	0.534
ENSG00000142784	23038	WDTC1	0.603
ENSG00000081479	4036	LRP2	0.431
ENSG00000134333	3939	LDHA	0.558
ENSG00000084652	200081	TXLNA	0.516
ENSG00000143624	65123	INTS3	0.517
ENSG00000180182	9282	MED14	0.603
ENSG00000075891	5076	PAX2	0.571
ENSG00000182771	2894	GRID1	0.492
ENSG00000160050	79140	CCDC28B	0.575
ENSG00000173894	84733	CBX2	0.477
ENSG00000142192	351	APP	0.604
ENSG00000179632	84232	MAF1	0.383
ENSG00000163291	152559	PAQR3	0.472
ENSG00000164168	55751	TMEM184C	0.486
ENSG00000141570	57332	CBX8	0.468
ENSG00000072778	37	ACADVL	0.541
ENSG00000128585	4289	MKLN1	0.514
ENSG00000132475	3020	H3F3A	0.521
ENSG00000062650	23063	WAPAL	0.597
ENSG00000176087	113829	SLC35A4	0.463
ENSG00000130558	10439	OLFM1	0.614
ENSG00000138092	79172	CENPO	0.49
ENSG00000186501	84065	TMEM222	0.573
ENSG00000141582	8535	CBX4	0.513
ENSG00000044446	5256	PHKA2	0.563
ENSG00000160271	5900	RALGDS	0.57
ENSG00000108518	5216	PFN1	0.543
ENSG00000166317	79933	SYNPO2L	0.439
ENSG00000138031	109	ADCY3	0.596
ENSG00000203727	389432	SAMD5	0.545
ENSG00000168264	359948	IRF2BP2	0.607
ENSG00000106459	4899	NRF1	0.588
ENSG00000196422	9858	KIAA0649	0.532
ENSG00000173726	9804	TOMM20	0.597
ENSG00000111961	23328	SASH1	0.582
ENSG00000143217	81607	PVRL4	0.431
ENSG00000128567	5420	PODXL	0.552
ENSG00000243364	1945	EFNA4	0.536
ENSG00000163297	118429	ANTXR2	0.569
ENSG00000132478	85451	UNK	0.571
ENSG00000124486	8239	USP9X	0.589
ENSG00000119906	55719	FAM178A	0.58
ENSG00000176986	9632	SEC24C	0.535
ENSG00000143590	1944	EFNA3	0.576
ENSG00000203784	149018	LELP1	0.425

ENSG00000074356	55421	C17orf85	0.503
ENSG00000253304	399474	TMEM200B	0.452
ENSG00000111962	10090	UST	0.566
ENSG00000173698	10149	GPR64	0.487
ENSG00000151623	4306	NR3C2	0.507
ENSG00000112182	60468	BACH2	0.503
ENSG00000186591	7328	UBE2H	0.594
ENSG00000007372	5080	PAX6	0.501
ENSG00000122367	11155	LDB3	0.584
ENSG00000221866	91584	PLXNA4	0.431
ENSG00000004660	84254	CAMKK1	0.545
ENSG00000169239	11238	CA5B	0.591
ENSG00000183242	51352	WIT1	0.525
ENSG00000215301	1654	DDX3X	0.661
ENSG00000004975	1856	DVL2	0.594
ENSG00000082458	1741	DLG3	0.598
ENSG00000170390	166614	DCLK2	0.548
ENSG00000054267	51742	ARID4B	0.553
ENSG00000108509	23125	CAMTA2	0.544
ENSG00000143554	11000	SLC27A3	0.498
ENSG00000135341	6885	MAP3K7	0.654
ENSG00000091986	151887	CCDC80	0.627
ENSG00000182287	8905	AP1S2	0.562
ENSG00000107779	657	BMPR1A	0.555
ENSG00000040633	79142	PHF23	0.475
ENSG00000131558	60412	EXOC4	0.531
ENSG00000163462	80128	TRIM46	0.418
ENSG00000116478	3065	HDAC1	0.577
ENSG00000152270	5140	PDE3B	0.591
ENSG00000132471	23558	WBP2	0.516
ENSG00000143614	57459	GATAD2B	0.456
ENSG00000129654	2302	FOXJ1	0.41
ENSG00000147044	8573	CASK	0.636
ENSG00000154734	9510	ADAMTS1	0.538
ENSG00000166507	8509	NDST2	0.501
ENSG00000158796	9191	DEDD	0.571
ENSG00000198837	9909	DENND4B	0.496
ENSG00000120314	54853	WDR55	0.531
ENSG00000138675	2250	FGF5	0.54
ENSG00000095539	57715	SEMA4G	0.545
ENSG00000175826	23399	DULLARD	0.509
ENSG00000135333	2045	EPHA7	0.473
ENSG00000148660	818	CAMK2G	0.634
ENSG00000175130	65108	MARCKSL1	0.561
ENSG00000148672	2746	GLUD1	0.612
ENSG00000188487	387755	INSC	0.36
ENSG00000185262	283991	FAM100B	0.513
ENSG00000145681	1404	HAPLN1	0.499
ENSG00000154736	11096	ADAMTS5	0.503
ENSG00000152785	651	BMP3	0.457
ENSG00000160741	200186	CRTC2	0.424
ENSG00000160058	55108	BSDC1	0.607
ENSG00000169895	94056	SYAP1	0.507
ENSG00000119772	1788	DNMT3A	0.497

ENSG00000181773	2827	GPR3	0.514
ENSG00000158195	10163	WASF2	0.519
ENSG00000086712	55787	CXorf15	0.484
ENSG00000128510	51200	CPA4	0.502
ENSG00000110693	55553	SOX6	0.494
ENSG00000060749	79832	QSER1	0.489
ENSG00000138670	153020	RASGEF1B	0.508
ENSG00000172461	10690	FUT9	0.468
ENSG00000181856	6517	SLC2A4	0.449
ENSG00000145715	5921	RASA1	0.611
ENSG00000138279	310	ANXA7	0.573
ENSG00000143570	27173	SLC39A1	0.508
ENSG00000143258	27005	USP21	0.505
ENSG00000181541	10586	MAB21L2	0.546
ENSG00000006047	51087	YBX2	0.497
ENSG00000074755	23140	ZZEF1	0.588
ENSG00000107816	84445	LZTS2	0.621
ENSG00000175931	63893	UBE2O	0.496
ENSG00000198682	9060	PAPSS2	0.603
ENSG00000176884	2902	GRIN1	0.576
ENSG00000178826	135932	TMEM139	0.52
ENSG00000169891	9185	REPS2	0.495
ENSG00000138138	84896	ATAD1	0.515
ENSG00000185000	8694	DGAT1	0.493
ENSG00000122786	800	CALD1	0.688
ENSG00000176102	1479	CSTF3	0.541
ENSG00000138101	1838	DTNB	0.515
ENSG00000162521	5928	RBBP4	0.601
ENSG00000143553	23557	SNAPIN	0.447
ENSG00000120265	5110	PCMT1	0.623
ENSG00000184481	4303	FOXO4	0.491
ENSG00000106477	95681	TSGA14	0.477
ENSG00000147010	30011	SH3KBP1	0.585
ENSG00000148248	6836	SURF4	0.603
ENSG00000110422	10114	HIPK3	0.625
ENSG00000170616	83482	SCRT1	0.397
ENSG00000185722	51479	ANKFY1	0.488
ENSG00000188158	4810	NHS	0.525
ENSG00000107758	5532	PPP3CB	0.627
ENSG00000009780	199870	FAM76A	0.477
ENSG00000214140	768206	PRCD	0.474
ENSG00000152268	10418	SPON1	0.585
ENSG00000161533	51	ACOX1	0.596
ENSG00000204969	56146	PCDHA2	0.546
ENSG00000035403	7414	VCL	0.609
ENSG00000158850	8703	B4GALT3	0.518
ENSG00000173681	256643	CXorf23	0.389
ENSG00000011405	5286	PIK3C2A	0.562
ENSG00000204965	56143	PCDHA5	0.356
ENSG00000106484	4232	MEST	0.537
ENSG00000107807	3195	TLX1	0.456
ENSG00000081842	56142	PCDHA6	0.426
ENSG00000102098	10389	SCML2	0.499
ENSG00000250120	56139	PCDHA10	0.424

ENSG00000138136	10660	LBX1	0.438
ENSG00000185009	26985	AP3M1	0.531
ENSG00000166167	8945	BTRC	0.613
ENSG00000158859	9507	ADAMTS4	0.494
ENSG00000251664	56137	PCDHA12	0.538
ENSG00000070495	23210	JMJD6	0.616
ENSG00000080802	4850	CNOT4	0.642
ENSG00000158747	4681	NBL1	0.553
ENSG00000173674	1964	EIF1AX	0.606
ENSG00000156650	23522	MYST4	0.619
ENSG00000138650	57575	PCDH10	0.444
ENSG00000143970	55252	ASXL2	0.525
ENSG00000177468	167826	OLIG3	0.36
ENSG00000118503	7128	TNFAIP3	0.632
ENSG00000204619	6992	PPP1R11	0.524
ENSG00000164754	5885	RAD21	0.574
ENSG00000177189	6197	RPS6KA3	0.596
ENSG00000136535	10716	TBR1	0.485
ENSG00000151012	23657	SLC7A11	0.609
ENSG00000106992	203	AK1	0.6
ENSG00000132563	51308	REEP2	0.489
ENSG00000012963	55148	UBR7	0.526
ENSG00000196917	27198	GPR81	0.422
ENSG00000139726	8562	DENR	0.515
ENSG00000084731	3797	KIF3C	0.546
ENSG00000092931	79157	MFSD11	0.471
ENSG00000011114	55727	BTBD7	0.543
ENSG00000151014	25819	CCRN4L	0.5
ENSG00000112379	57221	KIAA1244	0.548
ENSG00000169914	23252	OTUD3	0.517
ENSG00000109381	1998	ELF2	0.535
ENSG00000160408	30815	ST6GALNAC6	0.496
ENSG00000120738	1958	EGR1	0.555
ENSG00000168906	4144	MAT2A	0.602
ENSG00000120705	2107	ETF1	0.592
ENSG00000158258	64084	CLSTN2	0.478
ENSG00000113763	90249	UNC5A	0.534
ENSG00000164756	169026	SLC30A8	0.418
ENSG00000115993	66008	TRAK2	0.615
ENSG00000084733	10890	RAB10	0.553
ENSG00000113013	3313	HSPA9	0.641
ENSG00000114120	55186	SLC25A36	0.624
ENSG00000115486	2677	GGCX	0.572
ENSG00000182197	2131	EXT1	0.575
ENSG00000138468	57337	SENP7	0.488
ENSG00000129757	1028	CDKN1C	0.651
ENSG00000175093	92369	SPSB4	0.443
ENSG00000155893	92370	ACPL2	0.54
ENSG00000177570	401474	SAMD12	0.422
ENSG00000185219	353274	ZNF445	0.509
ENSG00000113761	23567	ZNF346	0.547
ENSG00000147130	9203	ZMYM3	0.528
ENSG00000175785	145270	PRIMA1	0.51
ENSG00000139722	79720	VPS37B	0.505

ENSG00000135597	85021	REPS1	0.524
ENSG00000177311	253461	ZBTB38	0.533
ENSG00000109158	2557	GABRA4	0.456
ENSG00000162545	55450	CAMK2N1	0.563
ENSG00000150967	23457	ABCB9	0.549
ENSG00000234127	7726	TRIM26	0.558
ENSG00000155755	65062	ALS2CR4	0.525
ENSG00000082482	3776	KCNK2	0.473
ENSG00000107829	6468	FBXW4	0.523
ENSG00000205531	4676	NAP1L4	0.544
ENSG00000095015	4214	MAP3K1	0.505
ENSG00000204599	56658	TRIM39	0.466
ENSG00000172007	83452	RAB33B	0.463
ENSG00000145391	80854	SETD7	0.486
ENSG00000183114	163933	FAM43B	0.537
ENSG00000158825	978	CDA	0.499
ENSG00000156273	571	BACH1	0.579
ENSG00000066422	27107	ZBTB11	0.546
ENSG00000089723	78990	OTUB2	0.44
ENSG00000145246	57205	ATP10D	0.566
ENSG00000114125	9616	RNF7	0.508
ENSG00000167106	399665	FAM102A	0.539
ENSG00000196782	55534	MAML3	0.538
ENSG00000182504	79598	CEP97	0.398
ENSG00000165671	64324	NSD1	0.501
ENSG00000153130	60592	SCOC	0.516
ENSG00000153132	1047	CLGN	0.508
ENSG00000147140	4841	NONO	0.57
ENSG00000144815	91775	FAM55C	0.564
ENSG00000003393	57679	ALS2	0.553
ENSG00000155545	166968	MIER3	0.568
ENSG00000144802	64332	NFKBIZ	0.55
ENSG00000171169	203245	NAIF1	0.417
ENSG00000146006	26045	LRRTM2	0.454
ENSG00000148339	114789	SLC25A25	0.41
ENSG00000145244	10699	CORIN	0.458
ENSG00000146386	58527	C6orf115	0.504
ENSG00000169228	53917	RAB24	0.493
ENSG00000112406	51696	HECA	0.524
ENSG00000189410	400745	SH2D5	0.451
ENSG00000198408	10724	MGEA5	0.614
ENSG00000164440	167838	TXLNB	0.427
ENSG00000109436	23158	TBC1D9	0.589
ENSG00000164442	10370	CITED2	0.599
ENSG00000107164	8939	FUBP3	0.496
ENSG00000170044	131368	ZPLD1	0.488
ENSG00000167703	124935	SLC43A2	0.439
ENSG00000009844	51534	VTA1	0.592
ENSG00000127483	50809	HP1BP3	0.499
ENSG00000156284	9073	CLDN8	0.454
ENSG00000170017	214	ALCAM	0.606
ENSG00000130711	59335	PRDM12	0.46
ENSG00000175066	256356	GK5	0.448
ENSG00000170153	57484	RNF150	0.448

ENSG00000158887	4359	MPZ	0.459
ENSG00000139899	643866	CBLN3	0.394
ENSG00000169223	10960	LMAN2	0.605
ENSG00000143252	6391	SDHC	0.576
ENSG00000114127	54464	XRN1	0.614
ENSG00000205339	10527	IPO7	0.643
ENSG00000021762	114879	OSBPL5	0.55
ENSG00000162430	57190	SEPN1	0.482
ENSG00000145632	10769	PLK2	0.528
ENSG00000155760	8324	FZD7	0.597
ENSG00000184611	90134	KCNH7	0.495
ENSG00000169220	10636	RGS14	0.586
ENSG00000168887	388969	C2orf68	0.49
ENSG00000112414	57211	GPR126	0.509
ENSG00000196482	2104	ESRRG	0.557
ENSG00000075151	8672	EIF4G3	0.594
ENSG00000204576	80742	PRR3	0.522
ENSG00000182263	55137	FIGN	0.522
ENSG00000114423	868	CBLB	0.616
ENSG00000090975	57605	PITPNM2	0.535
ENSG00000198055	2870	GRK6	0.623
ENSG00000163812	51304	ZDHHC3	0.571
ENSG00000170961	3037	HAS2	0.503
ENSG00000178764	22882	ZHX2	0.564
ENSG00000204217	659	BMPR2	0.586
ENSG00000166483	7465	WEE1	0.622
ENSG00000135605	7006	TEC	0.48
ENSG00000113758	1627	DBN1	0.594
ENSG00000163596	130026	ICA1L	0.488
ENSG00000010818	3097	HIVEP2	0.572
ENSG00000120727	51247	PAIP2	0.542
ENSG00000113448	5144	PDE4D	0.623
ENSG00000014164	23144	ZC3H3	0.513
ENSG00000170482	9963	SLC23A1	0.464
ENSG00000168874	84913	ATOH8	0.54
ENSG00000170185	84640	USP38	0.518
ENSG00000115525	8869	ST3GAL5	0.553
ENSG00000167716	124997	WDR81	0.471
ENSG00000153253	6328	SCN3A	0.427
ENSG00000117298	1889	ECE1	0.577
ENSG00000213071	80350	LPAL2	0.537
ENSG00000133812	81846	SBF2	0.497
ENSG00000109171	57606	SLAIN2	0.618
ENSG00000189007	134637	ADAT2	0.513
ENSG00000144935	7220	TRPC1	0.601
ENSG00000163814	64866	CDCP1	0.525
ENSG00000174574	79647	AKIRIN1	0.492
ENSG00000067533	51018	RRP15	0.539
ENSG00000110713	4928	NUP98	0.64
ENSG00000204839	642475	C8orf73	0.393
ENSG00000196776	961	CD47	0.664
ENSG00000075539	285527	FRYL	0.58
ENSG00000127603	23499	MACF1	0.647
ENSG00000167711	5345	SERPINF2	0.504

ENSG00000188582	344838	PAQR9	0.473
ENSG00000163714	23350	SR140	0.602
ENSG00000148926	133	ADM	0.557
ENSG00000112419	9749	PHACTR2	0.588
ENSG00000175087	149420	PDIK1L	0.453
ENSG00000133805	272	AMPD3	0.576
ENSG00000112232	202559	KHDRBS2	0.433
ENSG00000136531	6326	SCN2A	0.429
ENSG00000100697	23405	DICER1	0.665
ENSG00000144426	65065	NBEAL1	0.357
ENSG00000186532	114826	SMYD4	0.46
ENSG00000183955	387893	SETD8	0.579
ENSG00000144791	8994	LIMD1	0.569
ENSG00000162551	249	ALPL	0.465
ENSG00000168743	255743	NPNT	0.54
ENSG00000112245	7803	PTP4A1	0.634
ENSG00000035499	55789	DEPDC1B	0.483
ENSG00000211456	22908	SACM1L	0.549
ENSG00000131508	7322	UBE2D2	0.644
ENSG00000175040	9435	CHST2	0.533
ENSG00000152128	81615	TMEM163	0.539
ENSG00000185019	22888	UBOX5	0.537
ENSG00000132383	6117	RPA1	0.597
ENSG00000111912	135112	NCOA7	0.525
ENSG00000118482	23469	PHF3	0.624
ENSG00000188026	353116	RILPL1	0.559
ENSG00000185924	146760	RTN4RL1	0.458
ENSG00000184840	54732	TMED9	0.554
ENSG00000181744	205428	C3orf58	0.513
ENSG00000110315	50862	RNF141	0.51
ENSG00000126882	286336	FAM78A	0.488
ENSG00000138443	10152	ABI2	0.637
ENSG00000164181	79993	ELOVL7	0.496
ENSG00000065559	6416	MAP2K4	0.605
ENSG00000160539	84814	PPAPDC3	0.48
ENSG00000076864	5909	RAP1GAP	0.618
ENSG00000118495	5325	PLAGL1	0.627
ENSG00000072952	10335	MRVI1	0.55
ENSG00000109180	54940	OCIAD1	0.597
ENSG00000146005	84249	PSD2	0.388
ENSG00000114698	57088	PLSCR4	0.502
ENSG00000177374	3090	HIC1	0.455
ENSG00000130695	64793	CCDC21	0.47
ENSG00000152818	7402	UTRN	0.571
ENSG00000173166	65059	RAPH1	0.537
ENSG00000070366	23293	SMG6	0.551
ENSG00000163817	54716	SLC6A20	0.449
ENSG00000142669	83442	SH3BGRL3	0.513
ENSG00000088854	25943	C20orf194	0.549
ENSG00000163684	11102	RPP14	0.514
ENSG00000188725	643155	C5orf43	0.482
ENSG00000178562	940	CD28	0.555
ENSG00000144285	6323	SCN1A	0.433
ENSG00000088812	8455	ATRN	0.633

ENSG00000185129	5813	PURA	0.666
ENSG00000197451	3182	HNRNPAB	0.55
ENSG00000163600	29851	ICOS	0.467
ENSG00000146374	84870	RSPO3	0.458
ENSG00000174963	84107	ZIC4	0.432
ENSG00000068796	3796	KIF2A	0.584
ENSG00000118518	81847	RNF146	0.481
ENSG00000164163	6059	ABCE1	0.566
ENSG00000110321	1982	EIF4G2	0.576
ENSG00000135298	577	BAI3	0.556
ENSG00000163909	26508	HEYL	0.485
ENSG00000113368	4001	LMNB1	0.567
ENSG00000116983	51440	HPCAL4	0.544
ENSG00000113070	1839	HBEGF	0.627
ENSG00000182512	51218	GLRX5	0.534
ENSG00000169432	6335	SCN9A	0.439
ENSG00000152977	7545	ZIC1	0.496
ENSG00000104522	7264	TSTA3	0.573
ENSG00000163820	79443	FYCO1	0.483
ENSG00000141258	9905	SGSM2	0.603
ENSG00000118257	8828	NRP2	0.656
ENSG00000164164	54726	OTUD4	0.607
ENSG00000116985	656	BMP8B	0.524
ENSG00000155011	27123	DKK2	0.471
ENSG00000165915	91252	SLC39A13	0.497
ENSG00000173926	115123	3-Mar	0.499
ENSG00000050767	91522	COL23A1	0.454
ENSG00000125430	9953	HS3ST3B1	0.485
ENSG00000043514	54802	TRIT1	0.526
ENSG00000109099	5376	PMP22	0.575
ENSG00000070444	4335	MNT	0.493
ENSG00000071794	6596	HLTF	0.541
ENSG00000001617	6405	SEMA3F	0.613
ENSG00000198939	80108	ZFP2	0.412
ENSG00000127804	79066	METT10D	0.488
ENSG00000138668	3184	HNRNPD	0.668
ENSG00000170242	55031	USP47	0.566
ENSG00000152767	10160	FARP1	0.614
ENSG00000112214	9457	FHL5	0.483
ENSG00000155016	113612	CYP2U1	0.484
ENSG00000165637	7417	VDAC2	0.572
ENSG00000117682	79947	DHDDS	0.451
ENSG00000111816	2444	FRK	0.439
ENSG00000145780	56929	FEM1C	0.451
ENSG00000105887	136319	MTPN	0.522
ENSG00000007168	5048	PAFAH1B1	0.659
ENSG00000102572	8428	STK24	0.654
ENSG00000107263	2889	RAPGEF1	0.473
ENSG00000165917	5913	RAPSN	0.431
ENSG00000181072	1129	CHRM2	0.405
ENSG00000227372	57212	KIAA0495	0.57
ENSG00000205279	613212	CTXN3	0.488
ENSG00000064651	6558	SLC12A2	0.546
ENSG00000116990	4610	MYCL1	0.608

ENSG00000129657	6397	SEC14L1	0.599
ENSG00000114353	2771	GNAI2	0.516
ENSG00000101222	25876	SPEF1	0.482
ENSG00000168309	11170	FAM107A	0.574
ENSG00000132361	23277	KIAA0664	0.515
ENSG00000152795	9987	HNRPDL	0.692
ENSG00000138795	51176	LEF1	0.595
ENSG00000138814	5530	PPP3CA	0.611
ENSG00000157680	9162	DGKI	0.472
ENSG00000180900	23513	SCRIB	0.496
ENSG00000187189	23270	TSPYL4	0.525
ENSG00000153094	10018	BCL2L11	0.551
ENSG00000088387	23348	DOCK9	0.611
ENSG00000152894	5796	PTPRK	0.572
ENSG00000111817	29940	DSE	0.529
ENSG00000050165	27122	DKK3	0.554
ENSG00000146263	253714	C6orf167	0.636
ENSG00000125817	1059	CENPB	0.515
ENSG00000143569	9898	UBAP2L	0.662
ENSG00000101224	994	CDC25B	0.578
ENSG00000198830	3151	HMGN2	0.537
ENSG00000178033	254228	FAM26E	0.447
ENSG00000117676	6195	RPS6KA1	0.552
ENSG00000182158	64764	CREB3L2	0.551
ENSG00000140519	51458	RHCG	0.449
ENSG00000165186	139411	PTCHD1	0.482
ENSG00000171451	92126	DSEL	0.562
ENSG00000138829	2201	FBN2	0.58
ENSG00000184486	5454	POU3F2	0.451
ENSG00000131236	10487	CAP1	0.563
ENSG00000114251	7474	WNT5A	0.581
ENSG00000184640	10801	9-Sep	0.602
ENSG00000146267	84553	C6orf168	0.496
ENSG00000145284	79966	SCD5	0.52
ENSG00000187672	26059	ERC2	0.462
ENSG00000136720	9394	HS6ST1	0.491
ENSG00000109919	23788	MTCH2	0.541
ENSG00000144724	5793	PTPRG	0.558
ENSG00000166825	290	ANPEP	0.524
ENSG00000156113	3778	KCNMA1	0.614
ENSG00000074416	11343	MGLL	0.539
ENSG00000081189	4208	MEF2C	0.638
ENSG00000060762	51660	BRP44L	0.549
ENSG00000101236	11237	RNF24	0.583
ENSG00000125246	171425	CLYBL	0.5
ENSG00000131238	5538	PPT1	0.562
ENSG00000132424	25957	SFRS18	0.637
ENSG00000145808	171019	ADAMTS19	0.475
ENSG00000071242	6196	RPS6KA2	0.638
ENSG00000139800	85416	ZIC5	0.439
ENSG00000109320	4790	NFKB1	0.594
ENSG00000170677	9306	SOCS6	0.551
ENSG00000092421	57556	SEMA6A	0.609
ENSG00000141668	147381	CBLN2	0.44

ENSG00000140548	374655	ZNF710	0.574
ENSG00000117000	6018	RLF	0.514
ENSG00000144228	339745	SPOPL	0.515
ENSG00000084073	10269	ZMPSTE24	0.522
ENSG00000186687	90624	LYRM7	0.56
ENSG00000163618	8618	CADPS	0.492
ENSG00000123552	85015	USP45	0.5
ENSG00000143515	57198	ATP8B2	0.488
ENSG00000122778	57670	KIAA1549	0.422
ENSG00000058262	29927	SEC61A1	0.504
ENSG00000005889	7543	ZFX	0.567
ENSG00000164574	55568	GALNT10	0.593
ENSG00000155097	528	ATP6V1C1	0.604
ENSG00000168702	53353	LRP1B	0.425
ENSG00000171867	5621	PRNP	0.62
ENSG00000117115	11240	PADI2	0.487
ENSG00000109920	23360	FNBP4	0.543
ENSG00000163946	23272	C3orf63	0.599
ENSG00000185033	10509	SEMA4B	0.518
ENSG00000171864	23627	PRND	0.441
ENSG00000113356	10622	POLR3G	0.537
ENSG00000164929	79870	BAALC	0.508
ENSG00000101265	9770	RASSF2	0.544
ENSG00000186197	128178	EDARADD	0.404
ENSG00000146858	92092	ZC3HAV1L	0.446
ENSG00000158987	51735	RAPGEF6	0.519
ENSG00000114383	11334	TUSC2	0.566
ENSG00000175699	256369	C14orf48	0.483
ENSG00000160712	3570	IL6R	0.601
ENSG00000176018	116068	LYSMD3	0.429
ENSG00000164930	8323	FZD6	0.587
ENSG00000089057	9962	SLC23A2	0.631
ENSG00000172869	1657	DMXL1	0.535
ENSG00000132394	60678	EEFSEC	0.465
ENSG00000113196	9421	HAND1	0.452
ENSG00000102230	9468	PCYT1B	0.563
ENSG00000155506	23367	LARP1	0.583
ENSG00000164932	115908	CTHRC1	0.554
ENSG00000116977	3964	LGALS8	0.649
ENSG00000115919	8942	KYNU	0.654
ENSG00000179348	2624	GATA2	0.646
ENSG00000109332	7323	UBE2D3	0.629
ENSG00000169291	126669	SHE	0.452
ENSG00000101868	5422	POLA1	0.513
ENSG00000084070	64744	SMAP2	0.504
ENSG00000213066	11116	FGFR1OP	0.627
ENSG00000164933	81034	SLC25A32	0.531
ENSG00000075785	7879	RAB7A	0.617
ENSG00000151208	9231	DLG5	0.601
ENSG00000047932	57120	GOPC	0.569
ENSG00000149177	5795	PTPRJ	0.6
ENSG00000140575	8826	IQGAP1	0.624
ENSG00000163947	50650	ARHGEF3	0.564
ENSG00000217128	96459	FNIP1	0.492

ENSG00000179051	55920	RCC2	0.542
ENSG00000188177	376940	ZC3H6	0.565
ENSG00000101290	8760	CDS2	0.551
ENSG00000160714	55585	UBE2Q1	0.507
ENSG00000180011	284273	ZADH2	0.608
ENSG00000145779	25816	TNFAIP8	0.599
ENSG00000179981	10194	TSHZ1	0.482
ENSG00000176406	9699	RIMS2	0.561
ENSG00000206026	284274	C18orf62	0.442
ENSG00000099282	23555	TSPAN15	0.469
ENSG00000101493	9658	ZNF516	0.544
ENSG00000004848	170302	ARX	0.512
ENSG00000109089	30850	CDR2L	0.523
ENSG00000143398	8394	PIP5K1A	0.572
ENSG00000130856	7776	ZNF236	0.605
ENSG00000155508	9337	CNOT8	0.619
ENSG00000114999	150465	TTL	0.51
ENSG00000163635	6314	ATXN7	0.557
ENSG00000170633	80196	RNF34	0.543
ENSG00000180901	23510	KCTD2	0.542
ENSG00000140577	64784	CRTC3	0.545
ENSG00000146963	51631	LUC7L2	0.599
ENSG00000153989	116150	NUS1	0.559
ENSG00000043039	8538	BARX2	0.46
ENSG00000196376	222553	SLC35F1	0.409
ENSG00000164398	23305	ACSL6	0.558
ENSG00000169306	11141	IL1RAPL1	0.493
ENSG00000134897	54841	BIVM	0.49
ENSG00000111860	387119	C6orf204	0.513
ENSG00000197971	4155	MBP	0.601
ENSG00000144730	54756	IL17RD	0.546
ENSG00000160716	1141	CHRNB2	0.446
ENSG00000121964	79712	GTDC1	0.608
ENSG00000198814	2710	GK	0.647
ENSG00000189308	132660	LIN54	0.436
ENSG00000108639	9144	SYNGR2	0.528
ENSG00000145354	493856	CISD2	0.513
ENSG00000113369	57561	ARRDC3	0.504
ENSG00000144366	51454	GULP1	0.592
ENSG00000125255	6555	SLC10A2	0.487
ENSG00000197467	1305	COL13A1	0.57
ENSG00000160710	103	ADAR	0.533
ENSG00000125266	1948	EFNB2	0.629
ENSG00000140564	5045	FURIN	0.551
ENSG00000134884	55082	ARGLU1	0.543
ENSG00000204442	728215	FAM155A	0.593
ENSG00000064393	28996	HIPK2	0.588
ENSG00000112238	59336	PRDM13	0.444
ENSG00000130396	4301	MLLT4	0.608
ENSG00000147650	29967	LRP12	0.584
ENSG00000113391	83989	FAM172A	0.52
ENSG00000169554	9839	ZEB2	0.522
ENSG00000111875	25842	ASF1A	0.627
ENSG00000139826	84945	ABHD13	0.507

ENSG00000112246	6492	SIM1	0.444
ENSG00000126062	11070	TMEM115	0.481
ENSG00000169946	23414	ZFPM2	0.479
ENSG00000163637	166336	PRICKLE2	0.42
ENSG00000007402	9254	CACNA2D2	0.53
ENSG00000041515	23026	MYO16	0.5
ENSG00000163638	56999	ADAMTS9	0.536
ENSG00000157500	26060	APPL1	0.526
ENSG00000182500	84876	ORAI1	0.476
ENSG00000172780	339122	RAB43	0.509
ENSG00000138792	2028	ENPEP	0.532
ENSG00000189159	51155	HN1	0.513
ENSG00000079819	2037	EPB41L2	0.597
ENSG00000170325	56980	PRDM10	0.571
ENSG00000143603	3782	KCNN3	0.537
ENSG00000196547	4122	MAN2A2	0.608
ENSG00000159423	8659	ALDH4A1	0.631
ENSG00000169714	7555	CNBP	0.534
ENSG00000174672	9024	BRSK2	0.429
ENSG00000185950	8660	IRS2	0.614
ENSG00000187498	1282	COL4A1	0.603
ENSG00000198947	1756	DMD	0.604
ENSG00000145476	285440	CYP4V2	0.548
ENSG00000143373	57592	ZNF687	0.473
ENSG00000170624	6444	SGCD	0.612
ENSG00000131196	4772	NFATC1	0.635
ENSG00000106299	8976	WASL	0.566
ENSG00000188612	6613	SUMO2	0.58
ENSG00000111885	4121	MAN1A1	0.579
ENSG00000140553	55898	UNC45A	0.552
ENSG00000108175	57178	ZMIZ1	0.575
ENSG00000204262	1290	COL5A2	0.581
ENSG00000134871	1284	COL4A2	0.624
ENSG00000127481	23352	UBR4	0.587
ENSG00000164418	2898	GRIK2	0.56
ENSG00000143393	5298	PI4KB	0.631
ENSG00000060069	9150	CTDP1	0.47
ENSG00000163346	57326	PBXIP1	0.624
ENSG00000101825	25878	MXRA5	0.499
ENSG00000184508	374659	HDDC3	0.413
ENSG00000108179	10105	PPIF	0.611
ENSG00000183943	5613	PRKX	0.547
ENSG00000139832	55647	RAB20	0.49
ENSG00000059378	64761	PARP12	0.53
ENSG00000115008	3552	IL1A	0.583
ENSG00000165424	219654	ZCCHC24	0.626
ENSG00000084234	334	APLP2	0.657
ENSG00000006459	80853	JHDM1D	0.517
ENSG00000184557	9021	SOCS3	0.601
ENSG00000163348	90780	PYGO2	0.461
ENSG00000136854	6812	STXBP1	0.573
ENSG00000087157	9489	PGS1	0.476
ENSG00000198901	9055	PRC1	0.521
ENSG00000146938	57502	NLGN4X	0.468

ENSG00000160691	6464	SHC1	0.61
ENSG00000136002	50649	ARHGEF4	0.57
ENSG00000116984	4548	MTR	0.526
ENSG00000042286	84883	AIFM2	0.534
ENSG00000184545	1850	DUSP8	0.534
ENSG00000055163	26999	CYFIP2	0.616
ENSG00000156521	219743	TYSND1	0.512
ENSG00000174839	201627	FAM116A	0.463
ENSG00000110801	5715	PSMD9	0.598
ENSG00000143390	5993	RFX5	0.567
ENSG00000114735	51409	HEMK1	0.602
ENSG00000147655	340419	RSPO2	0.371
ENSG00000178184	84552	PARD6G	0.546
ENSG00000205302	6643	SNX2	0.574
ENSG00000153487	3621	ING1	0.575
ENSG00000079332	56681	SAR1A	0.633
ENSG00000088448	55608	ANKRD10	0.535
ENSG00000125447	23163	GGA3	0.563
ENSG00000163681	7871	SLMAP	0.571
ENSG00000138449	30061	SLC40A1	0.581
ENSG00000102606	8874	ARHGEF7	0.617
ENSG00000114737	1154	CISH	0.517
ENSG00000112562	64094	SMOC2	0.517
ENSG00000101846	412	STS	0.594
ENSG00000152102	130074	FAM168B	0.515
ENSG00000111897	57515	SERINC1	0.529
ENSG00000185518	9899	SV2B	0.509
ENSG00000110987	605	BCL7A	0.554
ENSG00000168769	54790	TET2	0.514
ENSG00000175727	22877	MLXIP	0.593
ENSG00000147027	83604	TMEM47	0.579
ENSG00000135549	5570	PKIB	0.545
ENSG00000048405	168850	ZNF800	0.486
ENSG00000140557	8128	ST8SIA2	0.465
ENSG00000133606	23608	MKRN1	0.599
ENSG00000088538	1795	DOCK3	0.464
ENSG00000122359	311	ANXA11	0.585
ENSG00000172731	55222	LRRC20	0.509
ENSG00000173575	1106	CHD2	0.532
ENSG00000125637	23550	PSD4	0.525
ENSG00000186340	7058	THBS2	0.55
ENSG00000064652	28966	SNX24	0.512
ENSG00000148730	1979	EIF4EBP2	0.595
ENSG00000101849	6907	TBL1X	0.658
ENSG00000182968	6656	SOX1	0.456
ENSG00000004059	381	ARF5	0.543
ENSG00000107719	27143	KIAA1274	0.471
ENSG00000138316	140766	ADAMTS14	0.427
ENSG00000196323	29068	ZBTB44	0.475
ENSG00000068650	23250	ATP11A	0.626
ENSG00000185127	387263	C6orf120	0.566
ENSG00000182175	56963	RGMA	0.492
ENSG00000136068	2317	FLNB	0.607
ENSG00000146950	357	SHROOM2	0.53

ENSG00000134917	11095	ADAMTS8	0.53
ENSG00000170915	85315	PAQR8	0.537
ENSG00000106331	5078	PAX4	0.568
ENSG00000188580	154215	NKAIN2	0.433
ENSG00000096093	114327	EFHC1	0.507
ENSG00000163683	201895	C4orf34	0.416
ENSG00000047644	55841	WWC3	0.516
ENSG00000159399	3099	HK2	0.616
ENSG00000126217	23263	MCF2L	0.579
ENSG00000166224	8879	SGPL1	0.64
ENSG00000135074	8728	ADAM19	0.61
ENSG00000131002	246126	CYorf15A	0.566
ENSG00000078140	3093	UBE2K	0.569
ENSG00000151292	1456	CSNK1G3	0.501
ENSG00000139719	65082	VPS33A	0.432
ENSG00000197157	27044	SND1	0.521
ENSG00000164588	348980	HCN1	0.447
ENSG00000065308	9697	TRAM2	0.604
ENSG00000168036	1499	CTNNB1	0.555
ENSG00000198719	28514	DLL1	0.536
ENSG00000143442	23126	POGZ	0.574
ENSG00000185551	7026	NR2F2	0.624
ENSG00000039600	11063	SOX30	0.451
ENSG00000121892	23244	PDS5A	0.631
ENSG00000140450	91947	ARRDC4	0.513
ENSG00000138780	79807	GSTCD	0.481
ENSG00000177885	2885	GRB2	0.576
ENSG00000140443	3480	IGF1R	0.643
ENSG00000168916	57507	ZNF608	0.518
ENSG00000177728	9772	KIAA0195	0.553
ENSG00000167244	3481	IGF2	0.63
ENSG00000155858	134353	LSM11	0.472
ENSG00000101871	4281	MID1	0.599
ENSG00000198570	343035	RD3	0.384
ENSG00000107736	64072	CDH23	0.569
ENSG00000112592	6908	TBP	0.515
ENSG00000164796	114788	CSMD3	0.444
ENSG00000163914	6010	RHO	0.528
ENSG00000164330	1879	EBF1	0.601
ENSG00000128594	64101	LRRC4	0.47
ENSG00000004399	23129	PLXND1	0.597
ENSG00000174697	3952	LEP	0.454
ENSG00000123684	9926	LPGAT1	0.551
ENSG00000111725	5564	PRKAB1	0.61
ENSG00000183715	4978	OPCML	0.57
ENSG00000122966	11113	CIT	0.544
ENSG00000106348	3614	IMPDH1	0.524
ENSG00000177303	57513	CASKIN2	0.533
ENSG00000180543	85453	TSPYL5	0.529
ENSG00000147649	92140	MTDH	0.638
ENSG00000163694	54502	RBM47	0.521
ENSG00000143375	57530	CGN	0.544
ENSG00000176204	80059	LRRTM4	0.486
ENSG00000165209	55342	STRBP	0.595

ENSG00000172765	23023	TMCC1	0.636
ENSG00000080854	22997	IGSF9B	0.548
ENSG00000016082	3670	ISL1	0.5
ENSG00000112144	22858	ICK	0.486
ENSG00000106991	2022	ENG	0.567
ENSG00000170214	147	ADRA1B	0.475
ENSG00000166086	83700	JAM3	0.558
ENSG00000143367	7286	TUFT1	0.524
ENSG00000138386	4664	NAB1	0.616
ENSG00000073350	3993	LLGL2	0.518
ENSG00000121989	92	ACVR2A	0.517
ENSG00000132561	4147	MATN2	0.501
ENSG00000104447	7227	TRPS1	0.541
ENSG00000164093	5308	PITX2	0.461
ENSG00000112146	26268	FBXO9	0.621
ENSG00000170234	114825	PWWP2A	0.518
ENSG00000169933	9758	FRMPD4	0.449
ENSG00000072682	8974	P4HA2	0.554
ENSG00000173436	440574	C1orf151	0.509
ENSG00000163697	323	APBB2	0.613
ENSG00000148204	286204	CRB2	0.41
ENSG00000115419	2744	GLS	0.686
ENSG00000135127	92558	CCDC64	0.499
ENSG00000101911	5634	PRPS2	0.552
ENSG00000107738	64115	C10orf54	0.557
ENSG00000119522	57706	DENND1A	0.45
ENSG00000151276	9223	MAGI1	0.538
ENSG00000143376	81609	SNX27	0.598
ENSG00000066032	1496	CTNNA2	0.478
ENSG00000135083	79616	CCNJL	0.505
ENSG00000196664	51284	TLR7	0.429
ENSG00000197746	5660	PSAP	0.606
ENSG00000111737	11021	RAB35	0.548
ENSG00000142634	79180	EFHD2	0.545
ENSG00000151474	55691	FRMD4A	0.576
ENSG00000205542	7114	TMSB4X	0.583
ENSG00000066027	5525	PPP2R5A	0.562
ENSG00000164171	3673	ITGA2	0.54
ENSG00000187391	9863	MAGI2	0.531
ENSG00000118507	9465	AKAP7	0.6
ENSG00000204406	55777	MBD5	0.528
ENSG00000162951	347730	LRRTM1	0.392
ENSG00000003249	79007	DBNDD1	0.488
ENSG00000196132	4661	MYT1	0.565
ENSG00000128595	813	CALU	0.643
ENSG00000197375	6584	SLC22A5	0.526
ENSG00000141013	2622	GAS8	0.475
ENSG00000178403	63973	NEUROG2	0.374
ENSG00000144741	115286	SLC25A26	0.487
ENSG00000151502	112936	VPS26B	0.547
ENSG00000104375	6788	STK3	0.583
ENSG00000001084	2729	GCLC	0.6
ENSG00000065809	83641	FAM107B	0.57
ENSG00000106689	9355	LHX2	0.559

ENSG00000135999	26122	EPC2	0.463
ENSG00000137504	58487	CREBZF	0.587
ENSG00000122863	9469	CHST3	0.615
ENSG00000107742	9806	SPOCK2	0.554
ENSG00000162772	467	ATF3	0.58
ENSG00000179071	220388	CCDC89	0.348
ENSG00000125347	3659	IRF1	0.552
ENSG00000119408	10783	NEK6	0.562
ENSG00000144749	26018	LRIG1	0.569
ENSG00000168280	3800	KIF5C	0.589
ENSG00000124743	401265	KLHL31	0.512
ENSG00000137269	55227	LRRC1	0.53
ENSG00000117620	23443	SLC35A3	0.552
ENSG00000116138	23341	DNAJC16	0.604
ENSG00000114857	4820	NKTR	0.633
ENSG00000108433	9570	GOSR2	0.644
ENSG00000156875	64645	HIAT1	0.448
ENSG00000064042	22998	LIMCH1	0.57
ENSG00000004897	996	CDC27	0.663
ENSG00000197312	84301	DDI2	0.36
ENSG00000089159	5829	PXN	0.579
ENSG00000145362	287	ANK2	0.619
ENSG00000136931	2516	NR5A1	0.458
ENSG00000148200	2649	NR6A1	0.608
ENSG00000164258	4724	NDUFS4	0.523
ENSG00000163376	84541	KBTBD8	0.458
ENSG00000165934	53981	CPSF2	0.54
ENSG00000185585	169611	OLFML2A	0.539
ENSG00000185305	54622	ARL15	0.459
ENSG00000137992	1629	DBT	0.625
ENSG00000140090	123041	SLC24A4	0.439
ENSG00000187123	130574	LYPD6	0.551
ENSG00000178996	112574	SNX18	0.496
ENSG00000183662	407738	FAM19A1	0.414
ENSG00000114853	92999	ZBTB47	0.579
ENSG00000163378	285203	C3orf64	0.459
ENSG00000169194	3596	IL13	0.476
ENSG00000136935	2800	GOLGA1	0.621
ENSG00000176407	56888	KCMF1	0.547
ENSG00000152284	83439	TCF7L1	0.502
ENSG00000135097	4440	MSI1	0.491
ENSG00000131437	11127	KIF3A	0.485
ENSG00000065526	23013	SPEN	0.589
ENSG00000079335	8556	CDC14A	0.601
ENSG00000144747	7110	TMF1	0.634
ENSG00000073921	8301	PICALM	0.655
ENSG00000146151	54511	HMGCLL1	0.517
ENSG00000197594	5167	ENPP1	0.552
ENSG00000152291	10618	TGOLN2	0.702
ENSG00000141279	9520	NPEPPS	0.579
ENSG00000143494	79805	VASH2	0.481
ENSG00000177106	64787	EPS8L2	0.495
ENSG00000181061	25994	HIGD1A	0.57
ENSG00000181656	54112	GPR88	0.43

ENSG00000119414	5537	PPP6C	0.558
ENSG00000145349	817	CAMK2D	0.625
ENSG00000022840	9921	RNF10	0.552
ENSG00000162694	2135	EXTL2	0.523
ENSG00000109132	8929	PHOX2B	0.496
ENSG00000034677	25897	RNF19A	0.516
ENSG00000118523	1490	CTGF	0.578
ENSG00000162695	148867	SLC30A7	0.553
ENSG00000198944	134548	ANKRD43	0.446
ENSG00000108424	3837	KPNB1	0.693
ENSG00000186472	27445	PCLO	0.573
ENSG00000124749	81578	COL21A1	0.495
ENSG00000114541	23150	FRMD4B	0.622
ENSG00000044574	3309	HSPA5	0.587
ENSG00000165219	26130	GAPVD1	0.6
ENSG00000152465	9397	NMT2	0.64
ENSG00000198933	9755	TBKBP1	0.517
ENSG00000072364	27125	AFF4	0.52
ENSG00000066455	9950	GOLGA5	0.512
ENSG00000073861	30009	TBX21	0.461
ENSG00000136643	26750	RPS6KC1	0.52
ENSG00000170989	1901	S1PR1	0.524
ENSG00000070756	26986	PABPC1	0.576
ENSG00000170381	9723	SEMA3E	0.422
ENSG00000118733	118427	OLFM3	0.426
ENSG00000006025	114881	OSBPL7	0.519
ENSG00000100604	1113	CHGA	0.455
ENSG00000075213	10371	SEMA3A	0.503
ENSG00000163788	54861	SNRK	0.589
ENSG00000157782	9478	CABP1	0.528
ENSG00000124406	10396	ATP8A1	0.493
ENSG00000180801	79642	ARSJ	0.452
ENSG00000164924	7534	YWHAZ	0.637
ENSG00000100605	3705	ITPK1	0.626
ENSG00000148468	221061	FAM171A1	0.539
ENSG00000187098	4286	MITF	0.572
ENSG00000175970	84747	UNC119B	0.467
ENSG00000119487	79109	MAPKAP1	0.524
ENSG00000174804	8322	FZD4	0.511
ENSG00000166575	65084	TMEM135	0.5
ENSG00000177542	79751	SLC25A22	0.472
ENSG00000164659	222223	KIAA1324L	0.513
ENSG00000189120	80320	SP6	0.413
ENSG00000167182	6668	SP2	0.535
ENSG00000151914	667	DST	0.657
ENSG00000120963	51123	ZNF706	0.513
ENSG00000055070	26099	C1orf144	0.568
ENSG00000108439	55163	PNPO	0.542
ENSG00000109861	1075	CTSC	0.595
ENSG00000083307	79977	GRHL2	0.456
ENSG00000053108	23105	FSTL4	0.486
ENSG00000114861	27086	FOXP1	0.577
ENSG00000167081	5090	PBX3	0.56
ENSG00000135164	9988	DMTF1	0.505

ENSG00000113583	56951	C5orf15	0.534
ENSG00000104490	83988	NCALD	0.553
ENSG00000112319	2070	EYA4	0.495
ENSG00000157895	64897	C12orf43	0.468
ENSG00000165983	9317	PTER	0.514
ENSG00000168959	2915	GRM5	0.545
ENSG00000148484	6251	RSU1	0.623
ENSG00000048392	50484	RRM2B	0.474
ENSG00000172403	171024	SYNPO2	0.646
ENSG00000163412	317649	EIF4E3	0.574
ENSG00000089041	5027	P2RX7	0.502
ENSG00000145390	54532	USP53	0.452
ENSG00000086991	50507	NOX4	0.482
ENSG00000196814	89853	FAM125B	0.603
ENSG00000162980	26225	ARL5A	0.509
ENSG00000118526	6943	TCF21	0.467
ENSG00000163602	23429	RYBP	0.607
ENSG00000113558	6500	SKP1	0.662
ENSG00000136944	4010	LMX1B	0.456
ENSG00000028839	9519	TBPL1	0.501
ENSG00000104517	51366	UBR5	0.611
ENSG00000082641	4779	NFE2L1	0.599
ENSG00000138735	8654	PDE5A	0.549
ENSG00000177697	977	CD151	0.532
ENSG00000182389	785	CACNB4	0.437
ENSG00000108468	10951	CBX1	0.566
ENSG00000005469	54677	CROT	0.492
ENSG00000177125	403341	ZBTB34	0.458
ENSG00000136828	9649	RALGPS1	0.549
ENSG00000113575	5515	PPP2CA	0.552
ENSG00000146411	154091	SLC2A12	0.511
ENSG00000198890	55170	PRMT6	0.5
ENSG00000162631	22854	NTNG1	0.544
ENSG00000118515	6446	SGK1	0.562
ENSG00000159363	23400	ATP13A2	0.459
ENSG00000107614	1787	TRDMT1	0.479
ENSG00000214063	7106	TSPAN4	0.565
ENSG00000131389	6533	SLC6A6	0.597
ENSG00000173917	3212	HOXB2	0.55
ENSG00000134215	10451	VAV3	0.558
ENSG00000105556	54531	MIER2	0.594
ENSG00000165359	203522	DDX26B	0.427
ENSG00000110931	10645	CAMKK2	0.617
ENSG00000129946	25759	SHC2	0.5
ENSG00000120093	3213	HOXB3	0.48
ENSG00000113805	5067	CNTN3	0.535
ENSG00000119048	7320	UBE2B	0.603
ENSG00000155096	51582	AZIN1	0.604
ENSG00000099804	997	CDC34	0.525
ENSG00000237190	91368	CDKN2AIPNL	0.406
ENSG00000157827	114793	FMNL2	0.581
ENSG00000196504	55660	PRPF40A	0.645
ENSG00000136859	23452	ANGPTL2	0.618
ENSG00000160307	6285	S100B	0.501

ENSG00000043143	23338	PHF15	0.6
ENSG00000185008	6092	ROBO2	0.517
ENSG00000007866	7005	TEAD3	0.525
ENSG00000169855	6091	ROBO1	0.552
ENSG00000120075	3215	HOXB5	0.565
ENSG00000108511	3216	HOXB6	0.579
ENSG00000026025	7431	VIM	0.556
ENSG00000011304	5725	PTBP1	0.618
ENSG00000115355	55704	CCDC88A	0.608
ENSG00000164111	308	ANXA5	0.547
ENSG00000120068	3218	HOXB8	0.451
ENSG00000170689	3219	HOXB9	0.493
ENSG00000164615	819	CAMLG	0.501
ENSG00000112339	10767	HBS1L	0.557
ENSG00000108001	253738	EBF3	0.545
ENSG00000145833	9879	DDX46	0.507
ENSG00000177917	151188	ARL6IP6	0.545
ENSG00000072042	51109	RDH11	0.61
ENSG00000116017	1820	ARID3A	0.493
ENSG00000177519	56475	RPRM	0.5
ENSG00000144278	114805	GALNT13	0.528
ENSG00000181904	134553	C5orf24	0.547
ENSG00000182087	91304	C19orf6	0.581
ENSG00000175161	253559	CADM2	0.46
ENSG00000096060	2289	FKBP5	0.532
ENSG00000113621	79770	TXNDC15	0.5
ENSG00000176769	256536	TCERG1L	0.426
ENSG00000162989	3760	KCNJ3	0.517
ENSG00000206538	389136	VGLL3	0.487
ENSG00000154783	152273	FGD5	0.45
ENSG00000153234	4929	NR4A2	0.599
ENSG00000083937	25978	CHMP2B	0.604
ENSG00000069011	5307	PITX1	0.565
ENSG00000113648	9555	H2AFY	0.628
ENSG00000118046	6794	STK11	0.556
ENSG00000072121	23503	ZFYVE26	0.583
ENSG00000163320	8545	CGGBP1	0.529
ENSG00000167470	90007	MIDN	0.538
ENSG00000099622	1153	CIRBP	0.646
ENSG00000115159	2820	GPD2	0.559
ENSG00000118513	4602	MYB	0.649
ENSG00000177463	7182	NR2C2	0.477
ENSG00000071626	26528	DAZAP1	0.512
ENSG00000175105	55279	ZNF654	0.5
ENSG00000198689	10479	SLC9A6	0.514
ENSG00000044524	2042	EPHA3	0.564
ENSG00000249915	10016	PDCD6	0.657
ENSG00000115266	10297	APC2	0.54
ENSG00000138741	7222	TRPC3	0.505
ENSG00000181588	399664	MEX3D	0.58
ENSG00000131368	64432	MRPS25	0.55
ENSG00000181965	4762	NEUROG1	0.436
ENSG00000145824	9547	CXCL14	0.529
ENSG00000071564	6929	TCF3	0.67

ENSG00000177888	360023	ZBTB41	0.419
ENSG00000131381	64145	ZFYVE20	0.524
ENSG00000143952	51542	VPS54	0.502
ENSG00000119661	83544	DNAL1	0.51
ENSG00000133275	1455	CSNK1G2	0.58
ENSG00000138041	57223	SMEK2	0.479
ENSG00000132406	85013	TMEM128	0.459
ENSG00000120708	7045	TGFBI	0.538
ENSG00000186487	23040	MYT1L	0.486
ENSG00000099875	2872	MKNK2	0.536
ENSG00000156030	91748	C14orf43	0.523
ENSG00000123612	130399	ACVR1C	0.458
ENSG00000065000	8943	AP3D1	0.601
ENSG00000101997	28952	CCDC22	0.514
ENSG00000215386	388815	C21orf34	0.505
ENSG00000159692	1487	CTBP1	0.631
ENSG00000115170	90	ACVR1	0.573
ENSG00000131375	23473	CAPN7	0.569
ENSG00000104885	84444	DOT1L	0.446
ENSG00000197329	57162	PELI1	0.547
ENSG00000049768	50943	FOXP3	0.529
ENSG00000185650	677	ZFP36L1	0.621
ENSG00000113658	4090	SMAD5	0.573
ENSG00000072110	87	ACTN1	0.652
ENSG00000138185	953	ENTPD1	0.57
ENSG00000116852	23046	KIF21B	0.526
ENSG00000112062	1432	MAPK14	0.612
ENSG00000213463	55333	SYNJ2BP	0.511
ENSG00000137166	116113	FOXP4	0.527
ENSG00000185818	339983	NAT8L	0.444
ENSG00000176619	84823	LMNB2	0.544
ENSG00000131378	23180	RFTN1	0.538
ENSG00000176490	148252	DIRAS1	0.469
ENSG00000186480	3638	INSIG1	0.633
ENSG00000104969	6449	SGTA	0.503
ENSG00000119844	54812	AFTPH	0.505
ENSG00000152377	6695	SPOCK1	0.524
ENSG00000049769	89801	PPP1R3F	0.475
ENSG00000119725	57862	ZNF410	0.538
ENSG00000133997	10001	MED6	0.645
ENSG00000090316	10296	MAEA	0.493
ENSG00000135541	54806	AHI1	0.607
ENSG00000111837	4117	MAK	0.475
ENSG00000171813	170394	PWWP2B	0.495
ENSG00000131370	9467	SH3BP5	0.63
ENSG00000164778	2020	EN2	0.489
ENSG00000171368	11076	TPPP	0.463
ENSG00000146021	26249	KLHL3	0.522
ENSG00000179833	9792	SERTAD2	0.594
ENSG00000182195	23641	LDOC1	0.505
ENSG00000152953	55351	STK32B	0.51
ENSG00000133985	23508	TTC9	0.542
ENSG00000068383	3632	INPP5A	0.535
ENSG00000115902	6509	SLC1A4	0.63

ENSG00000006432	4293	MAP3K9	0.589
ENSG00000184863	155435	RBM33	0.563
ENSG00000112561	7942	TFEB	0.568
ENSG00000125912	56926	NCLN	0.573
ENSG00000141526	9123	SLC16A3	0.644
ENSG00000092345	1618	DAZL	0.48
ENSG00000115183	85461	TANC1	0.566
ENSG00000033867	9497	SLC4A7	0.632
ENSG00000080822	56650	CLDND1	0.511
ENSG00000141905	4782	NFIC	0.564
ENSG00000123933	10608	MXD4	0.609
ENSG00000077157	4660	PPP1R12B	0.571
ENSG00000113430	50805	IRX4	0.473
ENSG00000107443	54619	CCNJ	0.462
ENSG00000100731	22990	PCNX	0.618
ENSG00000038382	7204	TRIO	0.658
ENSG00000102245	959	CD40LG	0.488
ENSG00000154822	23228	PLCL2	0.601
ENSG00000145375	166378	SPATA5	0.396
ENSG00000129675	9459	ARHGEF6	0.531
ENSG00000186111	23396	PIP5K1C	0.527
ENSG00000063587	10838	ZNF275	0.57
ENSG00000102069	389898	UBE2NL	0.402
ENSG00000100439	63874	ABHD4	0.495
ENSG00000123636	29994	BAZ2B	0.507
ENSG00000185985	84631	SLITRK2	0.41
ENSG00000011523	23177	CEP68	0.594
ENSG00000132702	60484	HAPLN2	0.445
ENSG00000131374	9779	TBC1D5	0.614
ENSG00000144597	85403	EAF1	0.58
ENSG00000221870	9142	CXorf1	0.434
ENSG00000115464	9736	USP34	0.658
ENSG00000164056	10252	SPRY1	0.573
ENSG00000206561	8292	COLQ	0.464
ENSG00000151458	57182	ANKRD50	0.551
ENSG00000138069	5861	RAB1A	0.63
ENSG00000137218	10817	FRS3	0.488
ENSG00000132692	63827	BCAN	0.621
ENSG00000167654	85300	ATCAY	0.427
ENSG00000124593	29964	PRICKLE4	0.507
ENSG00000178951	51341	ZBTB7A	0.616
ENSG00000102081	2332	FMR1	0.593
ENSG00000174137	152877	FAM53A	0.434
ENSG00000171408	27115	PDE7B	0.472
ENSG00000096070	27154	BRPF3	0.557
ENSG00000105255	79187	FSD1	0.5
ENSG00000138071	10097	ACTR2	0.657
ENSG00000141985	6455	SH3GL1	0.505
ENSG00000167670	10036	CHAF1A	0.617
ENSG00000176783	80230	RUFY1	0.507
ENSG00000163950	7884	SLBP	0.559
ENSG00000105983	64327	LMBR1	0.535
ENSG00000137193	5292	PIM1	0.6
ENSG00000198369	200734	SPRED2	0.62

ENSG00000063978	6047	RNF4	0.528
ENSG00000167680	10501	SEMA6B	0.484
ENSG00000142002	91039	DPP9	0.533
ENSG00000095951	3096	HIVEP1	0.548
ENSG00000164070	22824	HSPA4L	0.525
ENSG00000143995	4211	MEIS1	0.511
ENSG00000169045	3187	HNRNPH1	0.667
ENSG00000065491	55633	TBC1D22B	0.47
ENSG00000100626	57452	GALNTL1	0.537
ENSG00000164151	23379	KIAA0947	0.497
ENSG00000168936	92305	TMEM129	0.534
ENSG00000182568	6304	SATB1	0.513
ENSG00000105426	5802	PTPRS	0.528
ENSG00000067842	492	ATP2B3	0.509
ENSG00000171365	1184	CLCN5	0.553
ENSG00000112130	9025	RNF8	0.55
ENSG00000143319	81875	ISG20L2	0.62
ENSG00000029363	9774	BCLAF1	0.621
ENSG00000078401	1906	EDN1	0.506
ENSG00000125386	8603	C4orf8	0.556
ENSG00000112576	896	CCND3	0.632
ENSG00000156925	7547	ZIC3	0.491
ENSG00000160633	6294	SAFB	0.629
ENSG00000057019	131566	DCBLD2	0.552
ENSG00000154124	90268	FAM105B	0.612
ENSG00000154122	56172	ANKH	0.492
ENSG00000129682	2258	FGF13	0.547
ENSG00000152969	152789	JAKMIP1	0.51
ENSG00000081277	5317	PKP1	0.581
ENSG00000114744	51122	COMMD2	0.451
ENSG00000155966	2334	AFF2	0.615
ENSG00000054219	4065	LY75	0.549
ENSG00000143971	54465	ETAA1	0.478
ENSG00000130675	3110	MNX1	0.528
ENSG00000183580	23194	FBXL7	0.504
ENSG00000029364	55334	SLC39A9	0.57
ENSG00000183155	5877	RABIF	0.558
ENSG00000182732	9628	RGS6	0.553
ENSG00000068078	2261	FGFR3	0.56
ENSG00000176887	6664	SOX11	0.554
ENSG00000095587	7093	TLL2	0.543
ENSG00000163508	8320	EOMES	0.46
ENSG00000183960	131096	KCNH8	0.513
ENSG00000117153	59349	KLHL12	0.471
ENSG00000127022	821	CANX	0.635
ENSG00000009335	9690	UBE3C	0.531
ENSG00000179165	222659	PXT1	0.333
ENSG00000118260	1385	CREB1	0.644
ENSG00000112078	222658	KCTD20	0.54
ENSG00000135525	9053	MAP7	0.57
ENSG00000119866	53335	BCL11A	0.632
ENSG00000137413	129685	TAF8	0.535
ENSG00000070087	5217	PFN2	0.569
ENSG00000112139	266727	MDGA1	0.551

ENSG00000159346	51094	ADIPOR1	0.518
ENSG00000161021	9794	MAML1	0.524
ENSG00000130829	1852	DUSP9	0.526
ENSG00000010404	3423	IDS	0.739
ENSG00000143321	3068	HDGF	0.639
ENSG00000196428	9819	TSC22D2	0.675
ENSG00000221823	5534	PPP3R1	0.535
ENSG00000031823	8498	RANBP3	0.612
ENSG00000163512	64343	AZI2	0.493
ENSG00000161013	11282	MGAT4B	0.5
ENSG00000151692	9781	RNF144A	0.52
ENSG00000145555	4651	MYO10	0.611
ENSG00000120742	27230	SERP1	0.582
ENSG00000077147	56889	TM9SF3	0.537
ENSG00000087903	5990	RFX2	0.499
ENSG00000100647	9766	KIAA0247	0.551
ENSG00000168924	3954	LETM1	0.513
ENSG00000157227	4323	MMP14	0.652
ENSG00000115738	3398	ID2	0.564
ENSG00000158352	57477	SHROOM4	0.496
ENSG00000156639	60685	ZFAND3	0.485
ENSG00000130382	4298	MLLT1	0.442
ENSG00000197442	4217	MAP3K5	0.526
ENSG00000143294	5546	PRCC	0.513
ENSG00000206560	23243	ANKRD28	0.547
ENSG00000088247	8570	KHSRP	0.574
ENSG00000115946	56902	PNO1	0.601
ENSG00000082898	7514	XPO1	0.542
ENSG00000197324	26020	LRP10	0.561
ENSG00000094880	8697	CDC23	0.557
ENSG00000163251	7855	FZD5	0.573
ENSG00000116833	2494	NR5A2	0.573
ENSG00000112079	11329	STK38	0.663
ENSG00000074211	5522	PPP2R2C	0.596
ENSG00000183826	114781	BTBD9	0.556
ENSG00000109685	7468	WHSC1	0.6
ENSG00000153250	5937	RBMS1	0.667
ENSG00000125733	9322	TRIP10	0.533
ENSG00000154174	9868	TOMM70A	0.604
ENSG00000105204	9149	DYRK1B	0.505
ENSG00000138413	3417	IDH1	0.58
ENSG00000164040	10424	PGRMC2	0.579
ENSG00000122824	170685	NUDT10	0.435
ENSG00000171105	3643	INSR	0.6
ENSG00000139890	161253	REM2	0.481
ENSG00000196368	55190	NUDT11	0.516
ENSG00000077684	79960	PHF17	0.555
ENSG00000181788	6478	SIAH2	0.545
ENSG00000104880	23370	ARHGEF18	0.506
ENSG00000027869	9047	SH2D2A	0.512
ENSG00000130758	4294	MAP3K10	0.485
ENSG00000078295	108	ADCY2	0.578
ENSG00000105219	79935	CNTD2	0.483
ENSG00000112081	6428	SFRS3	0.67

ENSG00000100461	55147	RBM23	0.491
ENSG00000032444	10908	PNPLA6	0.512
ENSG00000144642	27303	RBMS3	0.572
ENSG00000105221	208	AKT2	0.602
ENSG00000076826	57662	KIAA1543	0.518
ENSG00000144893	116931	MED12L	0.434
ENSG00000124762	1026	CDKN1A	0.608
ENSG00000090659	30835	CD209	0.534
ENSG00000189369	23708	GSPT2	0.526
ENSG00000179222	9500	MAGED1	0.583
ENSG00000160392	126526	C19orf47	0.464
ENSG00000124772	57699	CPNE5	0.5
ENSG00000197226	23061	TBC1D9B	0.624
ENSG00000105223	23646	PLD3	0.538
ENSG00000142459	115704	EVI5L	0.527
ENSG00000171017	80131	LRRC8E	0.451
ENSG00000076984	5609	MAP2K7	0.567
ENSG00000198732	64093	SMOC1	0.477
ENSG00000105227	57716	PRX	0.533
ENSG00000197019	29950	SERTAD1	0.493
ENSG00000160460	57731	SPTBN4	0.474
ENSG00000078018	4133	MAP2	0.55
ENSG00000170871	9778	KIAA0232	0.533
ENSG00000087274	118	ADD1	0.621
ENSG00000066044	1994	ELAVL1	0.591
ENSG00000101974	286410	ATP11C	0.496
ENSG00000010017	10048	RANBP9	0.62
ENSG00000155629	118788	PIK3AP1	0.551
ENSG00000132405	57533	TBC1D14	0.448
ENSG00000105245	9253	NUMBL	0.538
ENSG00000120709	51307	FAM53C	0.543
ENSG00000130821	6535	SLC6A8	0.629
ENSG00000167772	51129	ANGPTL4	0.471
ENSG00000198663	221477	C6orf89	0.605
ENSG00000086544	80271	ITPKC	0.56
ENSG00000146090	255426	RASGEF1C	0.392
ENSG00000159173	7135	TNNI1	0.499
ENSG00000099785	51257	2-Mar	0.494
ENSG00000167578	53916	RAB4B	0.515
ENSG00000049656	81037	CLPTM1L	0.546
ENSG00000171570	112398	EGLN2	0.493
ENSG00000142347	4542	MYO1F	0.525
ENSG00000152601	4154	MBNL1	0.636
ENSG00000142303	81794	ADAMTS10	0.518
ENSG00000118200	23271	CAMSAP1L1	0.622
ENSG00000167601	558	AXL	0.592
ENSG00000143797	129642	MBOAT2	0.515
ENSG00000163513	7048	TGFBR2	0.637
ENSG00000105323	11100	HNRNPUL1	0.575
ENSG00000142039	90324	CCDC97	0.538
ENSG00000105329	7040	TGFB1	0.629
ENSG00000176788	10409	BASP1	0.585
ENSG00000174307	23612	PHLDA3	0.495
ENSG00000166887	23339	VPS39	0.491

ENSG00000165861	53349	ZFYVE1	0.489
ENSG00000181467	5912	RAP2B	0.552
ENSG00000137409	23787	MTCH1	0.498
ENSG00000144644	339896	GADL1	0.326
ENSG00000112149	9308	CD83	0.55
ENSG00000008083	3720	JARID2	0.621
ENSG00000131459	9945	GFPT2	0.52
ENSG00000169604	84168	ANTXR1	0.543
ENSG00000196526	60312	AFAP1	0.511
ENSG00000103978	25963	TMEM87A	0.609
ENSG00000154162	1010	CDH12	0.517
ENSG00000196233	84458	LCOR	0.461
ENSG00000127445	5300	PIN1	0.491
ENSG00000113300	57472	CNOT6	0.469
ENSG00000080573	50509	COL5A3	0.531
ENSG00000101986	215	ABCD1	0.482
ENSG00000028277	5452	POU2F2	0.612
ENSG00000119682	9870	KIAA0317	0.521
ENSG00000151789	79750	ZNF385D	0.425
ENSG00000092529	825	CAPN3	0.582
ENSG00000160570	162989	DEDD2	0.492
ENSG00000115361	33	ACADL	0.539
ENSG00000105722	2077	ERF	0.519
ENSG00000079432	23152	CIC	0.53
ENSG00000213390	84986	ARHGAP19	0.549
ENSG00000196549	4311	MME	0.577
ENSG00000167619	284339	TMEM145	0.415
ENSG00000119707	58517	RBM25	0.589
ENSG00000105429	1954	MEGF8	0.473
ENSG00000079435	3991	LIPE	0.568
ENSG00000079385	634	CEACAM1	0.622
ENSG00000131446	4245	MGAT1	0.547
ENSG00000182247	7325	UBE2E2	0.535
ENSG00000115365	10314	LANCL1	0.589
ENSG00000103994	64397	ZFP106	0.488
ENSG00000144645	114884	OSBPL10	0.634
ENSG00000114805	23007	PLCH1	0.565
ENSG00000132694	9826	ARHGEF11	0.547
ENSG00000021826	1373	CPS1	0.555
ENSG00000198380	2673	GFPT1	0.593
ENSG00000141568	3607	FOXK2	0.537
ENSG00000112624	23506	KIAA0240	0.577
ENSG00000092531	8773	SNAP23	0.636
ENSG00000007944	29116	MYLIP	0.57
ENSG00000134369	89796	NAV1	0.661
ENSG00000161847	125950	RAVER1	0.482
ENSG00000141580	56270	WDR45L	0.53
ENSG00000180979	255252	LRRC57	0.415
ENSG00000188687	57835	SLC4A5	0.463
ENSG00000176531	653583	PHLDB3	0.441
ENSG00000124788	6310	ATXN1	0.587
ENSG00000063601	8776	MTMR1	0.628
ENSG00000119596	56252	YLPM1	0.649
ENSG00000105767	199731	CADM4	0.619

ENSG00000174928	285315	C3orf33	0.5
ENSG00000169359	9197	SLC33A1	0.593
ENSG00000113361	1004	CDH6	0.577
ENSG00000159388	7832	BTG2	0.614
ENSG00000065989	5141	PDE4A	0.619
ENSG00000001561	22875	ENPP4	0.542
ENSG00000112796	59084	ENPP5	0.478
ENSG00000170340	10678	B3GNT2	0.518
ENSG00000152642	23171	GPD1L	0.551
ENSG00000169282	7881	KCNAB1	0.596
ENSG00000130734	84971	ATG4D	0.452
ENSG00000172348	10231	RCAN2	0.502
ENSG00000141542	10966	RAB40B	0.553
ENSG00000128881	146057	TTBK2	0.509
ENSG00000122176	2331	FMOD	0.512
ENSG00000129347	65095	KRI1	0.433
ENSG00000112183	221662	RBM24	0.49
ENSG00000155961	116442	RAB39B	0.471
ENSG00000114739	93	ACVR2B	0.5
ENSG00000159459	197131	UBR1	0.572
ENSG00000113360	29102	RNASEN	0.547
ENSG00000129355	1032	CDKN2D	0.578
ENSG00000114850	6747	SSR3	0.539
ENSG00000187122	6585	SLIT1	0.572
ENSG00000112902	9037	SEMA5A	0.593
ENSG00000188783	5549	PRELP	0.57
ENSG00000129353	57153	SLC44A2	0.591
ENSG00000137221	93643	TJAP1	0.566
ENSG00000174748	6138	RPL15	0.596
ENSG00000153291	9481	SLC25A27	0.548
ENSG00000058668	493	ATP2B4	0.655
ENSG00000163545	81788	NUAK2	0.575
ENSG00000163659	25976	TIPARP	0.565
ENSG00000178568	2066	ERBB4	0.594
ENSG00000143297	83416	FCRL5	0.542
ENSG00000115977	22848	AAK1	0.66
ENSG00000162873	55220	KLHDC8A	0.444
ENSG00000119689	1743	DLST	0.59
ENSG00000164626	8645	KCNK5	0.523
ENSG00000069399	602	BCL3	0.597
ENSG00000197386	3064	HTT	0.576
ENSG00000168939	10251	SPRY3	0.407
ENSG00000030419	22807	IKZF2	0.444
ENSG00000124333	6845	VAMP7	0.525
ENSG00000130202	5819	PVRL2	0.544
ENSG00000130204	10452	TOMM40	0.534
ENSG00000137842	80021	TMEM62	0.475
ENSG00000204843	1639	DCTN1	0.613
ENSG00000129474	84962	JUB	0.572
ENSG00000151694	6868	ADAM17	0.631
ENSG00000178567	9852	EPM2AIP1	0.497
ENSG00000112640	5528	PPP2R5D	0.58
ENSG00000137414	51439	FAM8A1	0.491
ENSG00000007047	57787	MARK4	0.587

ENSG00000117266	5129	PCTK3	0.519
ENSG00000168779	6474	SHOX2	0.583
ENSG00000124789	9972	NUP153	0.522
ENSG00000153071	1601	DAB2	0.632
ENSG00000151090	7068	THRB	0.493
ENSG00000058673	9877	ZC3H11A	0.581
ENSG00000140265	146050	ZSCAN29	0.463
ENSG00000130201	90332	EXOC3L2	0.465
ENSG00000029993	3149	HMGB3	0.531
ENSG00000183873	6331	SCN5A	0.482
ENSG00000134308	10971	YWHAQ	0.624
ENSG00000170801	94031	HTRA3	0.515
ENSG00000119630	5228	PGF	0.56
ENSG00000177853	9849	ZNF518A	0.547
ENSG00000100596	9517	SPTLC2	0.585
ENSG00000178053	4291	MLF1	0.557
ENSG00000165879	10023	FRAT1	0.491
ENSG00000198700	55705	IPO9	0.573
ENSG00000198668	801	CALM1	0.703
ENSG00000181274	23401	FRAT2	0.557
ENSG00000115507	5013	OTX1	0.462
ENSG00000067840	57595	PDZD4	0.446
ENSG00000077092	5915	RARB	0.667
ENSG00000133401	23037	PDZD2	0.483
ENSG00000069122	221395	GPR116	0.576
ENSG00000125740	2354	FOSB	0.531
ENSG00000125744	6253	RTN2	0.561
ENSG00000169851	5099	PCDH7	0.587
ENSG00000166963	4130	MAP1A	0.647
ENSG00000100601	8846	ALKBH1	0.527
ENSG00000143842	9580	SOX13	0.58
ENSG00000125753	7408	VASP	0.507
ENSG00000171522	5734	PTGER4	0.592
ENSG00000198910	3897	L1CAM	0.56
ENSG00000146122	23500	DAAM2	0.505
ENSG00000169762	202018	TAPT1	0.57
ENSG00000119684	27030	MLH3	0.57
ENSG00000124702	116138	KLHDC3	0.564
ENSG00000177051	23403	FBXO46	0.466
ENSG00000134317	29841	GRHL1	0.539
ENSG00000177045	147912	SIX5	0.419
ENSG00000059728	4084	MXD1	0.527
ENSG00000104936	1760	DMPK	0.574
ENSG00000130032	79057	PRRG3	0.459
ENSG00000185800	1762	DMWD	0.602
ENSG00000145495	10299	6-Mar	0.589
ENSG00000124571	57510	XPO5	0.513
ENSG00000170604	26145	IRF2BP1	0.481
ENSG00000104967	4858	NOVA2	0.517
ENSG00000113384	64083	GOLPH3	0.529
ENSG00000124440	64344	HIF3A	0.569
ENSG00000100813	22985	ACIN1	0.514
ENSG00000115414	2335	FN1	0.667
ENSG00000077097	7155	TOP2B	0.567

ENSG00000011485	5536	PPP5C	0.593
ENSG00000143845	55224	ETNK2	0.519
ENSG00000150712	54545	MTMR12	0.486
ENSG00000160014	801	CALM1	0.703
ENSG00000160013	5739	PTGIR	0.471
ENSG00000197380	147906	DACT3	0.52
ENSG00000172059	8462	KLF11	0.574
ENSG00000167004	2923	PDIA3	0.561
ENSG00000124615	4337	MOCS1	0.557
ENSG00000119703	79696	FAM164C	0.491
ENSG00000160007	2909	GRLF1	0.621
ENSG00000184205	64061	TSPYL2	0.552
ENSG00000130749	23211	ZC3H4	0.509
ENSG00000158715	85414	SLC45A3	0.556
ENSG00000105327	27113	BBC3	0.508
ENSG00000163431	25802	LMOD1	0.558
ENSG00000116001	7072	TIA1	0.678
ENSG00000171848	6241	RRM2	0.577
ENSG00000146072	27242	TNFRSF21	0.603
ENSG00000168356	11280	SCN11A	0.536
ENSG00000118160	6543	SLC8A2	0.468
ENSG00000140264	10169	SERF2	0.548
ENSG00000198087	23607	CD2AP	0.552
ENSG00000056097	51663	ZFR	0.616
ENSG00000063169	29998	GLTSCR1	0.467
ENSG00000024422	30846	EHD2	0.605
ENSG00000154274	55286	C4orf19	0.496
ENSG00000181826	768211	RELL1	0.48
ENSG00000102287	2564	GABRE	0.511
ENSG00000171314	5223	PGAM1	0.672
ENSG00000170275	10491	CRTAP	0.546
ENSG00000211448	1734	DIO2	0.579
ENSG00000170348	10972	TMED10	0.623
ENSG00000171877	84978	FRMD5	0.566
ENSG00000133065	254428	SLC41A1	0.464
ENSG00000179933	55017	C14orf119	0.454
ENSG00000151967	29970	SCHIP1	0.544
ENSG00000156564	57497	LRFN2	0.438
ENSG00000092068	23428	SLC7A8	0.637
ENSG00000065882	23216	TBC1D1	0.589
ENSG00000170734	5429	POLH	0.473
ENSG00000143850	22874	PLEKHA6	0.48
ENSG00000144674	2803	GOLGA4	0.594
ENSG00000173705	26032	SUSD5	0.455
ENSG00000166734	113201	CASC4	0.511
ENSG00000114742	57599	WDR48	0.614
ENSG00000170345	2353	FOS	0.596
ENSG00000161558	55260	TMEM143	0.471
ENSG00000137770	51496	CTDSPL2	0.569
ENSG00000105438	10945	KDELR1	0.528
ENSG00000105464	2906	GRIN2D	0.457
ENSG00000124795	7913	DEK	0.554
ENSG00000182324	3770	KCNJ14	0.462
ENSG00000104131	8669	EIF3J	0.581

ENSG00000142235	114783	LMTK3	0.431
ENSG00000153558	25827	FBXL2	0.508
ENSG00000124818	221391	OPN5	0.362
ENSG00000063176	56848	SPHK2	0.582
ENSG00000163435	1999	ELF3	0.557
ENSG00000174502	115019	SLC26A9	0.456
ENSG00000116005	51449	PCYOX1	0.562
ENSG00000105552	587	BCAT2	0.563
ENSG00000076356	5362	PLXNA2	0.569
ENSG00000172432	54676	GTPBP2	0.514
ENSG00000105559	57664	PLEKHA4	0.49
ENSG00000140044	122953	JDP2	0.507
ENSG00000100003	23541	SEC14L2	0.529
ENSG00000158615	84919	PPP1R15B	0.484
ENSG00000152620	133686	C5orf33	0.54
ENSG00000109787	51274	KLF3	0.508
ENSG00000031691	55166	CENPQ	0.486
ENSG00000203879	2664	GDI1	0.536
ENSG00000133056	5287	PIK3C2B	0.527
ENSG00000114745	64689	GORASP1	0.496
ENSG00000104812	2997	GYS1	0.516
ENSG00000003137	56603	CYP26B1	0.502
ENSG00000113387	10923	SUB1	0.614
ENSG00000015133	440193	CCDC88C	0.477
ENSG00000178177	254251	LCORL	0.5
ENSG00000169862	1501	CTNND2	0.539
ENSG00000117016	9783	RIMS3	0.575
ENSG00000163235	7039	TGFA	0.611
ENSG00000182836	345557	PLCXD3	0.466
ENSG00000177380	8541	PPFIA3	0.546
ENSG00000071127	9948	WDR1	0.649
ENSG00000066136	4802	NFYC	0.597
ENSG00000140279	50506	DUOX2	0.435
ENSG00000074219	8463	TEAD2	0.568
ENSG00000001167	4800	NFYA	0.599
ENSG00000104888	57030	SLC17A7	0.584
ENSG00000143862	127829	ARL8A	0.496
ENSG00000008118	57172	CAMK1G	0.524
ENSG00000071537	6400	SEL1L	0.6
ENSG00000100796	55671	SMEK1	0.531
ENSG00000126464	57479	PRR12	0.447
ENSG00000100036	339665	SLC35E4	0.457
ENSG00000135638	2016	EMX1	0.551
ENSG00000113389	4883	NPR3	0.551
ENSG00000144668	3680	ITGA9	0.499
ENSG00000184792	23762	OSBP2	0.463
ENSG00000144040	94097	SFXN5	0.524
ENSG00000196961	160	AP2A1	0.52
ENSG00000079215	6507	SLC1A3	0.51
ENSG00000163486	23380	SRGAP2	0.544
ENSG00000104960	53635	PTOV1	0.533
ENSG00000185070	23768	FLRT2	0.551
ENSG00000204673	84335	AKT1S1	0.5
ENSG00000144677	10217	CTDSPL	0.655

ENSG00000104154	7782	SLC30A4	0.499
ENSG00000138942	91445	RNF185	0.429
ENSG00000104164	26258	PLDN	0.582
ENSG00000112715	7422	VEGFA	0.682
ENSG00000182541	3985	LIMK2	0.653
ENSG00000153560	7342	UBP1	0.513
ENSG00000100100	113791	PIK3IP1	0.595
ENSG00000112964	2690	GHR	0.519
ENSG00000183908	219527	LRRC55	0.395
ENSG00000100105	23598	PATZ1	0.668
ENSG00000142528	25888	ZNF473	0.575
ENSG00000178163	85460	ZNF518B	0.536
ENSG00000134817	187	APLNR	0.472
ENSG00000149115	85456	TNKS1BP1	0.473
ENSG00000133935	11161	C14orf1	0.63
ENSG00000133030	23164	MPRIP	0.614
ENSG00000131408	7376	NR1H2	0.54
ENSG00000198089	9814	SFI1	0.661
ENSG00000137872	80031	SEMA6D	0.531
ENSG00000115307	550	AUP1	0.511
ENSG00000100393	2033	EP300	0.56
ENSG00000213023	84258	SYT3	0.49
ENSG00000163531	23114	NFASC	0.572
ENSG00000179862	163732	CITED4	0.491
ENSG00000164190	25836	NIPBL	0.587
ENSG00000241878	23761	PISD	0.519
ENSG00000135631	26056	RAB11FIP5	0.536
ENSG00000104177	50804	MYEF2	0.511
ENSG00000002587	9957	HS3ST1	0.597
ENSG00000143466	9641	IKBKE	0.574
ENSG00000129451	5655	KLK10	0.558
ENSG00000125845	650	BMP2	0.597
ENSG00000008197	83741	TFAP2D	0.355
ENSG00000101323	54363	HAO1	0.468
ENSG00000135632	10322	SMYD5	0.497
ENSG00000163539	23122	CLASP2	0.61
ENSG00000100403	23264	ZC3H7B	0.635
ENSG00000167074	7008	TEF	0.538
ENSG00000183864	10766	TOB2	0.552
ENSG00000100410	84844	PHF5A	0.448
ENSG00000008196	7021	TFAP2B	0.53
ENSG00000100412	50	ACO2	0.539
ENSG00000126903	8273	SLC10A3	0.522
ENSG00000182621	23236	PLCB1	0.607
ENSG00000172534	3054	HCFC1	0.585
ENSG00000185774	80333	KCNIP4	0.532
ENSG00000170927	5314	PKHD1	0.536
ENSG00000166147	2200	FBN1	0.562
ENSG00000116031	50489	CD207	0.47
ENSG00000109705	579	NKX3-2	0.492
ENSG00000172346	27254	CSDC2	0.48
ENSG00000115317	27429	HTRA2	0.576
ENSG00000100234	7078	TIMP3	0.65
ENSG00000133424	9215	LARGE	0.558

ENSG00000136653	83593	RASSF5	0.536
ENSG00000035928	5981	RFC1	0.623
ENSG00000117597	27042	C1orf107	0.592
ENSG00000184216	3654	IRAK1	0.594
ENSG00000101333	5332	PLCB4	0.565
ENSG00000100284	10043	TOM1	0.538
ENSG00000168314	4336	MOBP	0.542
ENSG00000112658	6722	SRF	0.592
ENSG00000185634	399694	SHC4	0.421
ENSG00000071246	22846	VASH1	0.499
ENSG00000112972	3157	HMGCS1	0.582
ENSG00000197273	2980	GUCA2A	0.481
ENSG00000137449	132864	CPEB2	0.558
ENSG00000115318	84695	LOXL3	0.471
ENSG00000100147	79879	CCDC134	0.405
ENSG00000198815	22887	FOXJ3	0.592
ENSG00000066427	4287	ATXN3	0.654
ENSG00000108551	51655	RASD1	0.481
ENSG00000166200	9318	COPS2	0.612
ENSG00000013523	23357	ANGEL1	0.573
ENSG00000144043	113419	TEX261	0.583
ENSG00000100167	55964	3-Sep	0.471
ENSG00000166793	219539	YPEL4	0.414
ENSG00000070778	11099	PTPN21	0.633
ENSG00000082196	114899	C1QTNF3	0.474
ENSG00000179820	91663	MYADM	0.559
ENSG00000156599	25921	ZDHHC5	0.535
ENSG00000108557	10743	RAI1	0.424
ENSG00000126583	5582	PRKCG	0.457
ENSG00000100302	23551	RASD2	0.409
ENSG00000169057	4204	MECP2	0.608
ENSG00000198951	4668	NAGA	0.576
ENSG00000072310	6720	SREBF1	0.524
ENSG00000100320	23543	RBM9	0.663
ENSG00000163145	114905	C1QTNF7	0.467
ENSG00000039560	26064	RAI14	0.533
ENSG00000162889	9261	MAPKAPK2	0.607
ENSG00000156603	219541	MED19	0.463
ENSG00000119669	64207	C14orf4	0.463
ENSG00000198894	85457	KIAA1737	0.518
ENSG00000170011	25924	MYRIP	0.491
ENSG00000100207	6942	TCF20	0.629
ENSG00000101349	57144	PAK7	0.494
ENSG00000211450	280636	C11orf31	0.598
ENSG00000088038	4849	CNOT3	0.549
ENSG00000082497	56256	SERTAD4	0.535
ENSG00000140285	2252	FGF7	0.538
ENSG00000109819	10891	PPARGC1A	0.506
ENSG00000165548	57156	TMEM63C	0.4
ENSG00000198561	1500	CTNND1	0.641
ENSG00000170248	10015	PDCD6IP	0.54
ENSG00000163013	150726	FBXO41	0.54
ENSG00000100227	84271	POLDIP3	0.59
ENSG00000136634	3586	IL10	0.458

ENSG00000104043	79895	ATP8B4	0.481
ENSG00000172239	10605	PAIP1	0.631
ENSG00000100345	4627	MYH9	0.531
ENSG00000177108	283576	ZDHHC22	0.465
ENSG00000146216	84630	TTBK1	0.487
ENSG00000100243	1727	CYB5R3	0.513
ENSG00000132639	6616	SNAP25	0.56
ENSG00000009830	29954	POMT2	0.483
ENSG00000175662	146691	TOM1L2	0.509
ENSG00000164007	149461	CLDN19	0.537
ENSG00000184144	6900	CNTN2	0.453
ENSG00000109606	1665	DHX15	0.581
ENSG00000112992	23530	NNT	0.605
ENSG00000137204	10864	SLC22A7	0.574
ENSG00000100350	80020	FOXRED2	0.496
ENSG00000101384	182	JAG1	0.664
ENSG00000124374	400961	PAIP2B	0.523
ENSG00000109814	7358	UGDH	0.538
ENSG00000181982	91050	CCDC149	0.456
ENSG00000135622	10505	SEMA4F	0.549
ENSG00000117625	55758	RCOR3	0.512
ENSG00000167615	114823	LENG8	0.459
ENSG00000132640	22903	BTBD3	0.558
ENSG00000182909	94059	LENG9	0.42
ENSG00000153012	55203	LGI2	0.448
ENSG00000172296	55304	SPTLC3	0.442
ENSG00000162896	5284	PIGR	0.511
ENSG00000141034	79018	C17orf39	0.51
ENSG00000100304	23170	TTLL12	0.531
ENSG00000118564	26234	FBXL5	0.564
ENSG00000164318	133584	EGFLAM	0.479
ENSG00000117394	6513	SLC2A1	0.58
ENSG00000117222	5929	RBBP5	0.46
ENSG00000157593	347734	SLC35B2	0.465
ENSG00000101230	140862	C20orf82	0.485
ENSG00000100379	79734	KCTD17	0.49
ENSG00000124356	10617	STAMBP	0.535
ENSG00000205838	153657	TTC23L	0.479
ENSG00000196924	2316	FLNA	0.619
ENSG00000172936	4615	MYD88	0.559
ENSG00000186660	80829	ZFP91	0.576
ENSG00000038210	55300	PI4K2B	0.505
ENSG00000130540	25830	SULT4A1	0.447
ENSG00000180667	55432	YOD1	0.485
ENSG00000168228	29063	ZCCHC4	0.58
ENSG00000123836	5208	PFKFB2	0.59
ENSG00000166897	114794	ELFN2	0.492
ENSG00000100060	4242	MFNG	0.608
ENSG00000100065	29775	CARD10	0.632
ENSG00000135338	167691	LCA5	0.545
ENSG00000198478	83699	SH3BGRL2	0.543
ENSG00000113594	3977	LIFR	0.532
ENSG00000172939	9943	OXSR1	0.536
ENSG00000140157	81614	NIPA2	0.495

ENSG00000125503	54776	PPP1R12C	0.471
ENSG00000110042	23220	DTX4	0.522
ENSG00000091542	54890	ALKBH5	0.506
ENSG00000172264	140733	MACROD2	0.512
ENSG00000170113	123606	NIPA1	0.508
ENSG00000071073	11320	MGAT4A	0.5
ENSG00000187605	200424	TET3	0.48
ENSG00000110048	5007	OSBP	0.542
ENSG00000145623	9180	OSMR	0.554
ENSG00000171617	8507	ENC1	0.65
ENSG00000156140	9508	ADAMTS3	0.483
ENSG00000196284	8464	SUPT3H	0.585
ENSG00000188636	84247	LDOC1L	0.481
ENSG00000100106	11078	TRIOBP	0.619
ENSG00000166889	219988	PATL1	0.592
ENSG00000182636	4692	NDN	0.473
ENSG00000124813	860	RUNX2	0.619
ENSG00000184990	10572	SIVA1	0.623
ENSG00000133069	9911	TMCC2	0.478
ENSG00000168724	134218	DNAJC21	0.576
ENSG00000100124	129138	ANKRD54	0.4
ENSG00000114302	5576	PRKAR2A	0.592
ENSG00000125848	23767	FLRT3	0.517
ENSG00000083123	594	BCKDHB	0.625
ENSG00000089177	55614	KIF16B	0.48
ENSG00000053254	1112	FOXN3	0.64
ENSG00000196872	343990	C2orf55	0.457
ENSG00000166900	6809	STX3	0.596
ENSG00000164327	253260	RICTOR	0.589
ENSG00000065911	10797	MTHFD2	0.58
ENSG00000114062	7337	UBE3A	0.664
ENSG00000056487	112885	PHF21B	0.443
ENSG00000100139	85377	MICALL1	0.573
ENSG00000132466	26057	ANKRD17	0.551
ENSG00000206190	57194	ATP10A	0.549
ENSG00000166206	2562	GABRB3	0.512
ENSG00000093000	10762	NUP50	0.637
ENSG00000112782	53405	CLIC5	0.563
ENSG00000100146	6663	SOX10	0.54
ENSG00000144681	6769	STAC	0.53
ENSG00000100364	23313	C22orf9	0.6
ENSG00000143761	375	ARF1	0.596
ENSG00000112773	55603	FAM46A	0.57
ENSG00000125851	5126	PCSK2	0.52
ENSG00000100151	9463	PICK1	0.508
ENSG00000177479	10425	ARIH2	0.607
ENSG00000100376	55007	FAM118A	0.476
ENSG00000128298	80115	BAIAP2L2	0.478
ENSG00000125868	11034	DSTN	0.613
ENSG00000176994	140775	SMCR8	0.526
ENSG00000176974	6470	SHMT1	0.562
ENSG00000142949	5792	PTPRF	0.635
ENSG00000130638	25814	ATXN10	0.632
ENSG00000185022	23764	MAFF	0.62

ENSG00000198792	25829	TMEM184B	0.534
ENSG00000186951	5465	PPARA	0.565
ENSG00000034053	321	APBA2	0.562
ENSG00000213923	1454	CSNK1E	0.576
ENSG00000170779	55038	CDCA4	0.564
ENSG00000104067	7082	TJP1	0.585
ENSG00000129351	3609	ILF3	0.69
ENSG00000100201	10521	DDX17	0.635
ENSG00000075275	9620	CELSR1	0.566
ENSG00000079805	1785	DNM2	0.581
ENSG00000075240	23151	GRAMD4	0.523
ENSG00000100422	64781	CERK	0.523
ENSG00000100206	11144	DMC1	0.504
ENSG00000126091	6487	ST3GAL3	0.5
ENSG00000184916	3714	JAG2	0.579
ENSG00000142453	10498	CARM1	0.523
ENSG00000158417	9669	EIF5B	0.67
ENSG00000130733	78992	YIPF2	0.594
ENSG00000178252	11180	WDR6	0.482
ENSG00000127616	6597	SMARCA4	0.652
ENSG00000197461	5154	PDGFA	0.609
ENSG00000100216	56993	TOMM22	0.508
ENSG00000125850	58495	OVOL2	0.546
ENSG00000100221	9929	JOSD1	0.532
ENSG00000219438	25817	FAM19A5	0.507
ENSG00000100226	9567	GTPBP1	0.626
ENSG00000130164	3949	LDLR	0.659
ENSG00000125871	92667	C20orf72	0.5
ENSG00000166961	219995	MS4A15	0.458
ENSG00000135945	51455	REV1	0.478
ENSG00000100426	9889	ZBED4	0.491
ENSG00000197256	25959	KANK2	0.585
ENSG00000113161	3156	HMGCR	0.608
ENSG00000169926	51621	KLF13	0.454
ENSG00000198355	415116	PIM3	0.466
ENSG00000178149	55152	DALRD3	0.564
ENSG00000110107	27339	PRPF19	0.53
ENSG00000100246	10126	DNAL4	0.518
ENSG00000198826	9824	ARHGAP11A	0.483
ENSG00000221890	23467	NPTXR	0.545
ENSG00000183741	23466	CBX6	0.58
ENSG00000166923	26585	GREM1	0.585
ENSG00000144218	3899	AFF3	0.534
ENSG00000198838	6263	RYR3	0.499
ENSG00000196361	1995	ELAVL3	0.498
ENSG00000110108	79073	TMEM109	0.537
ENSG00000073150	56666	PANX2	0.469
ENSG00000073169	83642	RP3-402G11.5	0.538
ENSG00000100307	23492	CBX7	0.568
ENSG00000130176	1264	CNN1	0.501
ENSG00000100311	5155	PDGFB	0.628
ENSG00000170500	164832	LONRF2	0.5
ENSG00000130165	84337	ELOF1	0.442
ENSG00000134152	79768	C15orf29	0.513

ENSG00000179364	23241	PACS2	0.579
ENSG00000100321	9145	SYNGR1	0.621
ENSG00000140199	9990	SLC12A6	0.515
ENSG00000128268	4248	MGAT3	0.534
ENSG00000100335	54471	SMCR7L	0.602
ENSG00000065833	4199	ME1	0.623
ENSG00000115526	9486	CHST10	0.55
ENSG00000196576	23654	PLXNB2	0.618
ENSG00000072134	22905	EPN2	0.551
ENSG00000100346	8911	CACNA1I	0.575
ENSG00000105583	51398	C19orf56	0.49
ENSG00000146250	167681	PRSS35	0.472
ENSG00000105576	30000	TNPO2	0.602
ENSG00000065609	9892	SNAP91	0.476
ENSG00000171608	5293	PIK3CD	0.632
ENSG00000100239	9701	SAPS2	0.557
ENSG00000185052	57419	SLC24A3	0.549
ENSG00000170485	4862	NPAS2	0.636
ENSG00000100241	6305	SBF1	0.583
ENSG00000171223	3726	JUNB	0.589
ENSG00000159251	70	ACTC1	0.495
ENSG00000133477	113828	FAM83F	0.442
ENSG00000100354	23112	TNRC6B	0.549
ENSG00000198146	54989	ZNF770	0.486
ENSG00000148218	210	ALAD	0.572
ENSG00000186073	84529	C15orf41	0.528
ENSG00000179218	811	CALR	0.663
ENSG00000076554	7163	TPD52	0.6
ENSG00000196588	57591	MKL1	0.627
ENSG00000179262	5886	RAD23A	0.616
ENSG00000134138	4212	MEIS2	0.542
ENSG00000008441	4784	NFIX	0.521
ENSG00000204634	11138	TBC1D8	0.596
ENSG00000186298	5501	PPP1CC	0.529
ENSG00000100380	6767	ST13	0.621
ENSG00000138835	5998	RGS3	0.607
ENSG00000160888	9592	IER2	0.552
ENSG00000166068	161742	SPRED1	0.612
ENSG00000141837	773	CACNA1A	0.575
ENSG00000145703	10788	IQGAP2	0.546
ENSG00000172575	10125	RASGRP1	0.524
ENSG00000158435	55571	C2orf29	0.504
ENSG00000137801	7057	THBS1	0.659
ENSG00000178585	56998	CTNNBIP1	0.511
ENSG00000132003	65249	ZSWIM4	0.416
ENSG00000175854	375757	C9orf119	0.525
ENSG00000163735	6374	CXCL5	0.57
ENSG00000111249	23316	CUX2	0.472
ENSG00000167323	6786	STIM1	0.52
ENSG00000132024	54862	CC2D1A	0.625
ENSG00000116574	58480	RHOU	0.566
ENSG00000176853	157769	FAM91A1	0.536
ENSG00000205189	65986	ZBTB10	0.525
ENSG00000128829	440275	EIF2AK4	0.512

ENSG00000090686	84196	USP48	0.568
ENSG00000175874	200407	CREG2	0.39
ENSG00000198324	144717	FAM109A	0.425
ENSG00000132005	5989	RFX1	0.459
ENSG00000164684	619279	ZNF704	0.5
ENSG00000111252	10019	SH2B3	0.578
ENSG00000072062	5566	PRKACA	0.495
ENSG00000167325	6240	RRM1	0.607
ENSG00000076641	55824	PAG1	0.584
ENSG00000071054	9448	MAP4K4	0.6
ENSG00000072071	22859	LPHN1	0.612
ENSG00000204842	6311	ATXN2	0.547
ENSG00000104081	90427	BMF	0.478
ENSG00000251322	85358	SHANK3	0.465
ENSG00000164983	157378	TMEM65	0.508
ENSG00000185344	23545	ATP6V0A2	0.571
ENSG00000154429	126731	C1orf96	0.56
ENSG00000167114	10999	SLC27A4	0.518
ENSG00000170881	11236	RNF139	0.484
ENSG00000143632	58	ACTA1	0.469
ENSG00000145708	1393	CRHBP	0.488
ENSG00000123159	10755	GIPC1	0.496
ENSG00000132002	3337	DNAJB1	0.579
ENSG00000164252	55109	AGGF1	0.603
ENSG00000137843	56924	PAK6	0.472
ENSG00000167118	81605	URM1	0.513
ENSG00000142798	3339	HSPG2	0.588
ENSG00000157657	114991	ZNF618	0.541
ENSG00000163743	25898	RCHY1	0.592
ENSG00000105137	85360	SYDE1	0.624
ENSG00000074181	4854	NOTCH3	0.56
ENSG00000141867	23476	BRD4	0.599
ENSG00000103194	9100	USP10	0.572
ENSG00000170873	9788	MTSS1	0.62
ENSG00000011451	58525	WIZ	0.597
ENSG00000177352	64925	CCDC71	0.46
ENSG00000185909	200942	KLHDC8B	0.468
ENSG00000171954	126410	CYP4F22	0.321
ENSG00000054523	23095	KIF1B	0.527
ENSG00000188315	375341	C3orf62	0.434
ENSG00000167460	7171	TPM4	0.598
ENSG00000179195	144348	ZNF664	0.514
ENSG00000167461	4218	RAB8A	0.541
ENSG00000104497	64089	SNX16	0.475
ENSG00000103196	83716	CRISPLD2	0.498
ENSG00000174796	152815	THAP6	0.53
ENSG00000196498	9612	NCOR2	0.656
ENSG00000115594	3554	IL1R1	0.588
ENSG00000135776	23456	ABCB10	0.491
ENSG00000184672	138046	RALYL	0.469
ENSG00000138769	8999	CDKL2	0.474
ENSG00000070831	998	CDC42	0.625
ENSG00000153786	55625	ZDHHC7	0.503
ENSG00000138757	9908	G3BP2	0.627

ENSG00000127526	79939	SLC35E1	0.599
ENSG00000105085	9441	MED26	0.495
ENSG00000162552	54361	WNT4	0.485
ENSG00000184677	9923	ZBTB40	0.552
ENSG00000135709	9764	KIAA0513	0.535
ENSG00000070886	2046	EPHA8	0.495
ENSG00000099331	4650	MYO9B	0.63
ENSG00000067560	387	RHOA	0.573
ENSG00000173334	10221	TRIB1	0.582
ENSG00000131149	23199	KIAA0182	0.596
ENSG00000168672	157638	FAM84B	0.556
ENSG00000133216	2048	EPHB2	0.676
ENSG00000127220	79575	ABHD8	0.417
ENSG00000143641	2590	GALNT2	0.607
ENSG00000145022	6988	TCTA	0.543
ENSG00000106948	80709	AKNA	0.491
ENSG00000177614	79605	PGBD5	0.514
ENSG00000073060	949	SCARB1	0.58
ENSG00000153310	51571	FAM49B	0.603
ENSG00000130477	23025	UNC13A	0.484
ENSG00000135775	22796	COG2	0.526
ENSG00000131148	10328	COX4NB	0.51
ENSG00000156194	5470	PPEF2	0.474
ENSG00000171540	23440	OTP	0.463
ENSG00000142655	5195	PEX14	0.56
ENSG00000099308	23031	MAST3	0.507
ENSG00000132842	8546	AP3B1	0.583
ENSG00000130940	54897	CASZ1	0.515
ENSG00000115616	6549	SLC9A2	0.449
ENSG00000130522	3727	JUND	0.627
ENSG00000145029	84276	NICN1	0.496
ENSG00000169641	7798	LUZP1	0.544
ENSG00000105656	8178	ELL	0.529
ENSG00000125944	10236	HNRNPR	0.615
ENSG00000105701	23770	FKBP8	0.589
ENSG00000103241	2294	FOXF1	0.492
ENSG00000105662	23373	CRTC1	0.558
ENSG00000188994	23036	ZNF292	0.595
ENSG00000100478	11154	AP4S1	0.554
ENSG00000136888	9550	ATP6V1G1	0.513
ENSG00000173402	1605	DAG1	0.603
ENSG00000005007	5976	UPF1	0.589
ENSG00000157693	203197	C9orf91	0.52
ENSG00000119280	84886	C1orf198	0.552
ENSG00000120948	23435	TARDBP	0.578
ENSG00000170417	130827	TMEM182	0.531
ENSG00000132294	23167	EFR3A	0.587
ENSG00000107872	79176	FBXL15	0.444
ENSG00000123124	11059	WWP1	0.59
ENSG00000051009	84067	FAM160A2	0.477
ENSG00000135148	10906	TRAFD1	0.552
ENSG00000125945	80818	ZNF436	0.516
ENSG00000145685	10184	LHFPL2	0.529
ENSG00000092148	25831	HECTD1	0.609

ENSG00000041982	3371	TNC	0.603
ENSG00000164061	8927	BSN	0.419
ENSG00000113273	411	ARSB	0.512
ENSG00000184388	645974	PABPC1L2B	0.342
ENSG00000186288	645974	PABPC1L2B	0.342
ENSG00000103264	79791	FBXO31	0.599
ENSG00000139364	114795	TMEM132B	0.447
ENSG00000173064	283450	C12orf51	0.525
ENSG00000204116	53344	CHIC1	0.532
ENSG00000184162	126382	NR2C2AP	0.487
ENSG00000140941	81631	MAP1LC3B	0.613
ENSG00000130287	1463	NCAN	0.495
ENSG00000187664	404037	HAPLN4	0.423
ENSG00000140948	23174	ZCCHC14	0.613
ENSG00000147100	6567	SLC16A2	0.508
ENSG00000129933	23383	KIAA0892	0.618
ENSG00000154118	57338	JPH3	0.47
ENSG00000007968	1870	E2F2	0.457
ENSG00000173077	50514	1-Dec	0.478
ENSG00000165071	137835	TMEM71	0.417
ENSG00000186143	339779	C2orf53	0.411
ENSG00000117318	3399	ID3	0.546
ENSG00000167491	54815	GATAD2A	0.515
ENSG00000119335	6418	SET	0.671
ENSG00000205927	10215	OLIG2	0.552
ENSG00000138074	8884	SLC5A6	0.529
ENSG00000129292	51105	PHF20L1	0.587
ENSG00000138107	10121	ACTR1A	0.571
ENSG00000182752	5069	PAPPA	0.568
ENSG00000064547	9170	LPAR2	0.547
ENSG00000050030	340533	KIAA2022	0.515
ENSG00000071051	8440	NCK2	0.597
ENSG00000111432	11211	FZD10	0.489
ENSG00000170289	54714	CNGB3	0.418
ENSG00000119283	440730	TRIM67	0.451
ENSG00000011009	11313	LYPLA2	0.509
ENSG00000115652	80146	UXS1	0.538
ENSG00000103257	8140	SLC7A5	0.591
ENSG00000110171	10612	TRIM3	0.591
ENSG00000156103	4325	MMP16	0.631
ENSG00000060709	23504	RIMBP2	0.448
ENSG00000179295	5781	PTPN11	0.675
ENSG00000152413	9456	HOMER1	0.514
ENSG00000137331	8870	IER3	0.564
ENSG00000172530	54971	BANP	0.448
ENSG00000111450	2054	STX2	0.587
ENSG00000204580	780	DDR1	0.636
ENSG00000132341	5901	RAN	0.608
ENSG00000105176	8725	C19orf2	0.622
ENSG00000132254	23647	ARFIP2	0.512
ENSG00000160445	10444	ZER1	0.541
ENSG00000121297	57616	TSHZ3	0.533
ENSG00000164823	734	OSGIN2	0.62
ENSG00000168813	22847	ZNF507	0.469

ENSG00000107021	54662	TBC1D13	0.54
ENSG00000157306	79178	THTPA	0.454
ENSG00000107882	51684	SUFU	0.61
ENSG00000105186	84079	ANKRD27	0.518
ENSG00000129480	112487	C14orf126	0.543
ENSG00000155926	6503	SLA	0.584
ENSG00000131941	85415	RHPN2	0.442
ENSG00000158545	124245	ZC3H18	0.515
ENSG00000171206	81603	TRIM8	0.531
ENSG00000204624	57540	PTCHD2	0.455
ENSG00000061936	6433	SFRS8	0.548
ENSG00000130881	4037	LRP3	0.511
ENSG00000245848	1050	CEBPA	0.545
ENSG00000153879	1054	CEBPG	0.525
ENSG00000104419	10397	NDRG1	0.574
ENSG00000138175	403	ARL3	0.621
ENSG00000124302	64377	CHST8	0.389
ENSG00000100852	394	ARHGAP5	0.529
ENSG00000153885	79047	KCTD15	0.562
ENSG00000104327	793	CALB1	0.523
ENSG00000156398	118980	SFXN2	0.472
ENSG00000132879	93611	FBXO44	0.447
ENSG00000166398	9710	KIAA0355	0.537
ENSG00000166272	54838	C10orf26	0.592
ENSG00000142188	757	TMEM50B	0.531
ENSG00000162761	4009	LMX1A	0.32
ENSG00000085224	546	ATRX	0.466
ENSG00000180694	169200	TMEM64	0.505
ENSG00000136802	56262	LRRC8A	0.536
ENSG00000126261	10054	UBA2	0.507
ENSG00000123119	64168	NECAB1	0.429
ENSG00000177169	8408	ULK1	0.511
ENSG00000151320	9472	AKAP6	0.545
ENSG00000166275	119032	C10orf32	0.485
ENSG00000158717	115992	RNF166	0.553
ENSG00000166340	1200	TPP1	0.621
ENSG00000135144	1840	DTX1	0.51
ENSG00000155099	55529	TMEM55A	0.453
ENSG00000111344	8437	RASAL1	0.462
ENSG00000105711	6324	SCN1B	0.479
ENSG00000151322	64067	NPAS3	0.507
ENSG00000115194	7781	SLC30A3	0.499
ENSG00000147606	115111	SLC26A7	0.544
ENSG00000158055	57822	GRHL3	0.438
ENSG00000162490	374946	C1orf187	0.522
ENSG00000106780	1955	MEGF9	0.591
ENSG00000148842	54805	CNNM2	0.574
ENSG00000105698	7392	USF2	0.562
ENSG00000102158	84061	MAGT1	0.478
ENSG00000119402	26190	FBXW2	0.608
ENSG00000183307	27439	CECR6	0.422
ENSG00000123064	79039	DDX54	0.482
ENSG00000129521	112399	EGLN3	0.48
ENSG00000079102	862	RUNX1T1	0.636

ENSG00000099954	27443	CECR2	0.523
ENSG00000182902	83733	SLC25A18	0.453
ENSG00000131773	10656	KHDRBS3	0.512
ENSG00000099968	23786	BCL2L13	0.554
ENSG00000165389	171546	C14orf147	0.619
ENSG00000102144	5230	PGK1	0.65
ENSG00000119403	26147	PHF19	0.511
ENSG00000170743	143425	SYT9	0.475
ENSG00000184454	400746	C1orf130	0.366
ENSG00000147724	51059	FAM135B	0.435
ENSG00000133226	10250	SRRM1	0.575
ENSG00000159140	6651	SON	0.62
ENSG00000243156	57553	MICAL3	0.54
ENSG00000166387	8495	PPFIBP2	0.607
ENSG00000129515	58533	SNX6	0.498
ENSG00000177000	4524	MTHFR	0.578
ENSG00000181031	9501	RPH3AL	0.484
ENSG00000011021	1185	CLCN6	0.48
ENSG00000215193	55670	PEX26	0.489
ENSG00000148798	9118	INA	0.497
ENSG00000115207	2976	GTF3C2	0.582
ENSG00000148343	84895	FAM73B	0.495
ENSG00000156374	84108	PCGF6	0.444
ENSG00000056558	7185	TRAF1	0.544
ENSG00000169436	169044	COL22A1	0.505
ENSG00000143157	57645	POGK	0.481
ENSG00000165410	1073	CFL2	0.609
ENSG00000169504	25932	CLIC4	0.638
ENSG00000070413	9993	DGCR2	0.56
ENSG00000183688	359845	FAM101B	0.583
ENSG00000100056	8220	DGCR14	0.593
ENSG00000100075	6576	SLC25A1	0.57
ENSG00000119397	11064	CEP110	0.494
ENSG00000187325	51616	TAF9B	0.557
ENSG00000181392	163183	C19orf46	0.421
ENSG00000070371	8218	CLTCL1	0.521
ENSG00000167130	57171	DOLPP1	0.525
ENSG00000129993	863	CBFA2T3	0.475
ENSG00000105270	25999	CLIP3	0.494
ENSG00000100084	7290	HIRA	0.53
ENSG00000083444	5351	PLOD1	0.512
ENSG00000167635	7705	ZNF146	0.5
ENSG00000205726	6453	ITSN1	0.608
ENSG00000142065	57677	ZFP14	0.435
ENSG00000123908	27161	EIF2C2	0.546
ENSG00000119383	5524	PPP2R4	0.625
ENSG00000119396	51552	RAB14	0.659
ENSG00000167695	79850	FAM57A	0.506
ENSG00000020633	864	RUNX3	0.577
ENSG00000167522	29123	ANKRD11	0.54
ENSG00000166402	7275	TUB	0.592
ENSG00000166405	79608	RIC3	0.486
ENSG00000215012	79680	C22orf29	0.462
ENSG00000169398	5747	PTK2	0.568

ENSG00000151327	283635	FAM177A1	0.512
ENSG00000089225	6910	TBX5	0.542
ENSG00000188227	390927	ZNF793	0.415
ENSG00000135111	6926	TBX3	0.553
ENSG00000123066	23389	MED13L	0.622
ENSG00000166407	4004	LMO1	0.523
ENSG00000198182	84775	ZNF607	0.441
ENSG00000105738	23094	SIPA1L3	0.58
ENSG00000107954	9148	NEURL	0.554
ENSG00000167693	64359	NXN	0.524
ENSG00000011332	8193	DPF1	0.461
ENSG00000107957	9644	SH3PXD2A	0.527
ENSG00000090615	2802	GOLGA3	0.501
ENSG00000120949	943	TNFRSF8	0.518
ENSG00000188483	389792	IER5L	0.562
ENSG00000128191	54487	DGCR8	0.625
ENSG00000117616	57035	C1orf63	0.599
ENSG00000136848	153090	DAB2IP	0.573
ENSG00000159842	29	ABR	0.607
ENSG00000028137	7133	TNFRSF1B	0.577
ENSG00000135119	84900	RNFT2	0.561
ENSG00000099904	29801	ZDHHC8	0.444
ENSG00000072609	55743	CHFR	0.536
ENSG00000135116	8739	HRK	0.5
ENSG00000198626	6262	RYR2	0.545
ENSG00000048707	55187	VPS13D	0.655
ENSG00000040608	65078	RTN4R	0.443
ENSG00000171777	115727	RASGRP4	0.486
ENSG00000187772	389421	LIN28B	0.48
ENSG00000198771	92241	RCSD1	0.452
ENSG00000112276	11149	BVES	0.507
ENSG00000166444	6764	ST5	0.562
ENSG00000137310	6941	TCF19	0.531
ENSG00000163624	1040	CDS1	0.588
ENSG00000088992	54997	TESC	0.529
ENSG00000244486	91179	SCARF2	0.507
ENSG00000163625	23001	WDFY3	0.618
ENSG00000135108	23014	FBXO21	0.609
ENSG00000099917	51586	MED15	0.485
ENSG00000179837	29890	RBM15B	0.511
ENSG00000197965	9019	MPZL1	0.637
ENSG00000130402	81	ACTN4	0.53
ENSG00000168348	84684	INSM2	0.408
ENSG00000125618	7849	PAX8	0.669
ENSG00000136827	1861	TOR1A	0.545
ENSG00000182472	147968	CAPN12	0.483
ENSG00000106852	26468	LHX6	0.508
ENSG00000057657	639	PRDM1	0.499
ENSG00000089250	4842	NOS1	0.54
ENSG00000241973	5297	PI4KA	0.489
ENSG00000164292	22836	RHOBTB3	0.672
ENSG00000022567	57210	SLC45A4	0.521
ENSG00000184811	286753	TUSC5	0.375
ENSG00000108953	7531	YWHAE	0.617

ENSG00000161243	126433	FBXO27	0.495
ENSG00000171435	283455	KSR2	0.446
ENSG00000130669	10298	PAK4	0.601
ENSG00000187239	23048	FNBP1	0.618
ENSG00000128011	57622	LRFN1	0.415
ENSG00000179134	55095	SAMD4B	0.468
ENSG00000063322	55588	MED29	0.468
ENSG00000128016	7538	ZFP36	0.549
ENSG00000184489	11156	PTP4A3	0.561
ENSG00000198743	6526	SLC5A3	0.602
ENSG00000167193	1398	CRK	0.58
ENSG00000115226	64838	FNDC4	0.511
ENSG00000156384	119392	C10orf78	0.486
ENSG00000099940	9342	SNAP29	0.468
ENSG00000197879	4641	MYO1C	0.569
ENSG00000099942	1399	CRKL	0.603
ENSG00000158805	92822	ZNF276	0.493
ENSG00000138639	83478	ARHGAP24	0.515
ENSG00000118985	22936	ELL2	0.592
ENSG00000095303	5742	PTGS1	0.591
ENSG00000188419	1121	CHM	0.462
ENSG00000166452	56672	C11orf17	0.495
ENSG00000175426	5122	PCSK1	0.441
ENSG00000110395	867	CBL	0.516
ENSG00000109339	5602	MAPK10	0.555
ENSG00000177963	60626	RIC8A	0.492
ENSG00000148358	57720	GPR107	0.622
ENSG00000100916	84312	BRMS1L	0.512
ENSG00000173456	79102	RNF26	0.505
ENSG00000131791	5565	PRKAB2	0.492
ENSG00000181790	575	BAI1	0.475
ENSG00000164815	5001	ORC5L	0.585
ENSG00000180875	64388	GREM2	0.469
ENSG00000175348	56674	TMEM9B	0.493
ENSG00000169635	23119	HIC2	0.597
ENSG00000185651	7332	UBE2L3	0.652
ENSG00000164938	94241	TP53INP1	0.566
ENSG00000153113	831	CAST	0.661
ENSG00000036672	9099	USP2	0.553
ENSG00000128228	23753	SDF2L1	0.505
ENSG00000159216	861	RUNX1	0.717
ENSG00000187416	375612	LHFPL3	0.396
ENSG00000198964	259230	SGMS1	0.516
ENSG00000156467	7381	UQCRB	0.589
ENSG00000198522	11321	GPN1	0.511
ENSG00000100027	29799	YPEL1	0.518
ENSG00000135090	51347	TAOK3	0.608
ENSG00000100030	5594	MAPK1	0.564
ENSG00000005483	55904	MLL5	0.525
ENSG00000100034	9647	PPM1F	0.575
ENSG00000198563	7919	BAT1	0.592
ENSG00000154096	7070	THY1	0.595
ENSG00000090097	57060	PCBP4	0.517
ENSG00000110400	5818	PVRL1	0.559

ENSG00000102290	27328	PCDH11X	0.444
ENSG00000157978	26119	LDLRAP1	0.554
ENSG00000007314	6329	SCN4A	0.505
ENSG00000164494	57107	PDSS2	0.541
ENSG00000115084	80255	SLC35F5	0.467
ENSG00000136352	7080	NKX2-1	0.565
ENSG00000162493	10630	PDPN	0.634
ENSG00000198807	5083	PAX9	0.483
ENSG00000174238	5306	PITPNA	0.617
ENSG00000156471	9791	PTDSS1	0.57
ENSG00000198477	140883	ZNF280B	0.478
ENSG00000119760	9913	SUPT7L	0.601
ENSG00000185532	5592	PRKG1	0.523
ENSG00000111707	64426	SUDS3	0.492
ENSG00000186310	4675	NAP1L3	0.51
ENSG00000116128	607	BCL9	0.501
ENSG00000147202	1730	DIAPH2	0.66
ENSG00000135250	6733	SRPK2	0.672
ENSG00000112320	55084	SOBP	0.534
ENSG00000186130	10773	ZBTB6	0.457
ENSG00000146285	256380	SCML4	0.549
ENSG00000116731	7799	PRDM2	0.688
ENSG00000184232	220323	OAF	0.541
ENSG00000137709	25833	POU2F3	0.51
ENSG00000151338	145282	MIPOL1	0.513
ENSG00000169439	6383	SDC2	0.602
ENSG00000136478	55852	TEX2	0.526
ENSG00000011454	23637	RABGAP1	0.628
ENSG00000156395	22986	SORCS3	0.429
ENSG00000128266	2781	GNAZ	0.48
ENSG00000177613	23283	CSTF2T	0.573
ENSG00000107984	22943	DKK1	0.499
ENSG00000186716	613	BCR	0.639
ENSG00000196914	23365	ARHGEF12	0.579
ENSG00000143178	9095	TBX19	0.463
ENSG00000175497	57628	DPP10	0.473
ENSG00000108018	114815	SORCS1	0.515
ENSG00000143140	2702	GJA5	0.493
ENSG00000165194	57526	PCDH19	0.434
ENSG00000187792	7621	ZNF70	0.521
ENSG00000169314	150248	C22orf15	0.564
ENSG00000081087	28962	OSTM1	0.504
ENSG00000158019	9577	BRE	0.604
ENSG00000143153	481	ATP1B1	0.593
ENSG00000112333	7101	NR2E1	0.465
ENSG00000172493	4299	AFF1	0.628
ENSG00000099953	4320	MMP11	0.557
ENSG00000099956	6598	SMARCB1	0.528
ENSG00000108654	1655	DDX5	0.527
ENSG00000139874	6751	SSTR1	0.452
ENSG00000226979	4049	LTA	0.506
ENSG00000000003	7105	TSPAN6	0.597
ENSG00000100934	10484	SEC23A	0.564
ENSG00000146776	222255	ATXN7L1	0.57

ENSG00000143156	29922	NME7	0.52
ENSG00000135537	246269	LACE1	0.536
ENSG00000232810	7124	TNF	0.524
ENSG00000108039	7511	XPNPEP1	0.56
ENSG00000125633	54520	CCDC93	0.627
ENSG00000118689	2309	FOXO3	0.632
ENSG00000108854	64750	SMURF2	0.56
ENSG00000008282	6856	SYPL1	0.601
ENSG00000075426	2355	FOSL2	0.638
ENSG00000143702	9859	CEP170	0.586
ENSG00000099998	2687	GGT5	0.513
ENSG00000080546	27244	SESN1	0.545
ENSG00000120063	10672	GNA13	0.535
ENSG00000159263	6493	SIM2	0.587
ENSG00000128271	135	ADORA2A	0.506
ENSG00000164088	132160	PPM1M	0.523
ENSG00000117479	10560	SLC19A2	0.519
ENSG00000138867	83606	C22orf13	0.492
ENSG00000159267	3141	HLCS	0.536
ENSG00000125629	51141	INSIG2	0.552
ENSG00000100028	6634	SNRPD3	0.533
ENSG00000105851	5294	PIK3CG	0.517
ENSG00000005249	5577	PRKAR2B	0.517
ENSG00000105856	26959	HBP1	0.59
ENSG00000182400	122553	TRAPPC6B	0.444
ENSG00000168646	8313	AXIN2	0.609
ENSG00000164091	80335	WDR82	0.513
ENSG00000115107	55240	STEAP3	0.52
ENSG00000072401	7321	UBE2D1	0.578
ENSG00000100941	5411	PNN	0.64
ENSG00000113441	4012	LNPEP	0.453
ENSG00000213639	5500	PPP1CB	0.589
ENSG00000152592	1758	DMP1	0.471
ENSG00000108064	7019	TFAM	0.615
ENSG00000167037	129049	SGSM1	0.5
ENSG00000125170	55715	DOK4	0.576
ENSG00000154127	84959	UBASH3B	0.596
ENSG00000100068	91355	LRP5L	0.461
ENSG00000100077	157	ADRBK2	0.552
ENSG00000145721	167410	LIX1	0.548
ENSG00000133454	84700	MYO18B	0.453
ENSG00000150527	4253	CTAGE5	0.553
ENSG00000165443	84457	PHYHIPL	0.485
ENSG00000168487	649	BMP1	0.606
ENSG00000174136	285704	RGMB	0.561
ENSG00000154229	5578	PRKCA	0.686
ENSG00000075790	55973	BCAP29	0.628
ENSG00000153922	1105	CHD1	0.535
ENSG00000126950	59353	TMEM35	0.494
ENSG00000163811	23160	WDR43	0.534
ENSG00000127334	8445	DYRK2	0.617
ENSG00000148700	120	ADD3	0.676
ENSG00000185250	285755	PPIL6	0.46
ENSG00000075461	27092	CACNG4	0.53

ENSG00000159164	9900	SV2A	0.529
ENSG00000102385	1821	DRP2	0.503
ENSG00000179456	10472	ZNF238	0.584
ENSG00000205336	9289	GPR56	0.579
ENSG00000088179	5775	PTPN4	0.555
ENSG00000198265	9931	HELZ	0.533
ENSG00000091137	5172	SLC26A4	0.457
ENSG00000105879	79872	CBLL1	0.465
ENSG00000163930	8314	BAP1	0.475
ENSG00000204463	7917	BAT3	0.576
ENSG00000113532	7903	ST8SIA4	0.517
ENSG00000169184	4330	MN1	0.542
ENSG00000180957	23760	PITPNB	0.541
ENSG00000165449	220963	SLC16A9	0.479
ENSG00000111554	56890	MDM1	0.567
ENSG00000108091	8030	CCDC6	0.526
ENSG00000179270	388939	C2orf71	0.35
ENSG00000173930	353189	SLCO4C1	0.442
ENSG00000115295	79745	CLIP4	0.516
ENSG00000179361	10620	ARID3B	0.498
ENSG00000151150	288	ANK3	0.596
ENSG00000165355	254170	FBXO33	0.43
ENSG00000115109	57669	EPB41L5	0.487
ENSG00000179335	1198	CLK3	0.526
ENSG00000165379	145581	LRFN5	0.423
ENSG00000179476	122525	C14orf28	0.5
ENSG00000010319	56920	SEMA3G	0.469
ENSG00000127314	5908	RAP1B	0.56
ENSG00000091140	1738	DLD	0.556
ENSG00000179454	54813	KLHL28	0.503
ENSG00000145730	5066	PAM	0.606
ENSG00000166257	55800	SCN3B	0.529
ENSG00000154059	55364	IMPACT	0.514
ENSG00000166261	7753	ZNF202	0.564
ENSG00000168490	9796	PHYHIP	0.465
ENSG00000168495	661	POLR3D	0.527
ENSG00000198795	25925	ZNF521	0.556
ENSG00000163113	56957	OTUD7B	0.484
ENSG00000159873	150275	CCDC117	0.579
ENSG00000171634	2186	BPTF	0.599
ENSG00000179151	80153	EDC3	0.468
ENSG00000183579	84133	ZNRF3	0.471
ENSG00000141380	6760	SS18	0.626
ENSG00000183762	83999	KREMEN1	0.541
ENSG00000153187	3192	HNRNPU	0.647
ENSG00000186998	129080	EMID1	0.49
ENSG00000150893	341640	FREM2	0.449
ENSG00000120685	80209	C13orf23	0.516
ENSG00000104635	23516	SLC39A14	0.568
ENSG00000182481	3838	KPNA2	0.59
ENSG00000182944	2130	EWSR1	0.597
ENSG00000103653	1445	CSK	0.532
ENSG00000144118	5899	RALB	0.61
ENSG00000154144	84897	TBRG1	0.612

ENSG00000188811	387921	NHLRC3	0.504
ENSG00000183722	10186	LHFP	0.507
ENSG00000091129	4897	NRCAM	0.595
ENSG00000141384	6875	TAF4B	0.507
ENSG00000163083	3625	INHBB	0.507
ENSG00000074047	2736	GLI2	0.535
ENSG00000072422	9886	RHOBTB1	0.526
ENSG00000150907	2308	FOXO1	0.613
ENSG00000171885	361	AQP4	0.558
ENSG00000138641	8916	HERC3	0.459
ENSG00000196932	219623	TMEM26	0.388
ENSG00000213578	594855	CPLX3	0.421
ENSG00000120896	10174	SORBS3	0.584
ENSG00000150347	84159	ARID5B	0.541
ENSG00000115112	29842	TFCP2L1	0.498
ENSG00000108932	9120	SLC16A6	0.492
ENSG00000108946	5573	PRKAR1A	0.648
ENSG00000074054	23332	CLASP1	0.526
ENSG00000100296	8563	THOC5	0.533
ENSG00000182010	219790	RTKN2	0.464
ENSG00000170558	1000	CDH2	0.576
ENSG00000143401	81611	ANP32E	0.606
ENSG00000154146	4900	NRGN	0.533
ENSG00000120688	11193	WBP4	0.604
ENSG00000186575	4771	NF2	0.607
ENSG00000177432	266812	NAP1L5	0.536
ENSG00000134762	1825	DSC3	0.517
ENSG00000162849	55083	KIF26B	0.447
ENSG00000140497	10066	SCAMP2	0.516
ENSG00000181915	84890	ADO	0.583
ENSG00000146360	2830	GPR6	0.465
ENSG00000122877	1959	EGR2	0.513
ENSG00000112290	8936	WASF1	0.466
ENSG00000117362	51107	APH1A	0.494
ENSG00000100314	164633	CABP7	0.5
ENSG00000178802	4351	MPI	0.544
ENSG00000105877	8701	DNAH11	0.541
ENSG00000128590	4189	DNAJB9	0.588
ENSG00000148572	29982	NRBF2	0.47
ENSG00000068831	10235	RASGRP2	0.634
ENSG00000145725	23262	HISPPD1	0.495
ENSG00000171988	221037	JMJD1C	0.551
ENSG00000178761	57184	C15orf17	0.541
ENSG00000100330	8897	MTMR3	0.599
ENSG00000131242	84440	RAB11FIP4	0.57
ENSG00000173114	54674	LRRN3	0.568
ENSG00000163939	55193	PBRM1	0.582
ENSG00000164649	55536	CDCA7L	0.471
ENSG00000120913	64236	PDLIM2	0.493
ENSG00000118276	9331	B4GALT6	0.561
ENSG00000128512	9732	DOCK4	0.494
ENSG00000178741	9377	COX5A	0.575
ENSG00000171109	55669	MFN1	0.627
ENSG00000128342	3976	LIF	0.525

ENSG00000099985	5008	OSM	0.477
ENSG00000153339	22878	KIAA1012	0.516
ENSG00000135678	1368	CPM	0.512
ENSG00000185477	285513	GPRIN3	0.488
ENSG00000198794	192683	SCAMP5	0.492
ENSG00000163501	3549	IHH	0.492
ENSG00000145335	6622	SNCA	0.598
ENSG00000187736	79840	NHEJ1	0.464
ENSG00000136237	9771	RAPGEF5	0.555
ENSG00000213901	151295	SLC23A3	0.5
ENSG00000241852	541565	C8orf58	0.521
ENSG00000165476	221035	REEP3	0.479
ENSG00000099995	10291	SF3A1	0.612
ENSG00000101695	54941	RNF125	0.505
ENSG00000138722	22915	MMRN1	0.469
ENSG00000111605	11052	CPSF6	0.561
ENSG00000178691	23512	SUZ12	0.526
ENSG00000134758	51444	RNF138	0.526
ENSG00000155111	23097	CDC2L6	0.563
ENSG00000141441	64762	FAM59A	0.521
ENSG00000114450	59345	GNB4	0.543
ENSG00000167173	56905	C15orf39	0.592
ENSG00000006831	79602	ADIPOR2	0.519
ENSG00000023516	11215	AKAP11	0.625
ENSG00000158941	57805	KIAA1967	0.491
ENSG00000181751	90355	C5orf30	0.525
ENSG00000120659	8600	TNFSF11	0.51
ENSG00000112874	83594	NUDT12	0.5
ENSG00000197705	57565	KLHL14	0.508
ENSG00000153207	25909	AHCTF1	0.516
ENSG00000166960	374864	C18orf34	0.395
ENSG00000168066	7536	SF1	0.626
ENSG00000165478	220296	HEPACAM	0.379
ENSG00000198739	347731	LRRTM3	0.485
ENSG00000166225	10818	FRS2	0.497
ENSG00000099715	27328	PCDH11X	0.444
ENSG00000166159	654429	LRTM2	0.438
ENSG00000184349	1946	EFNA5	0.453
ENSG00000134955	219855	SLC37A2	0.4
ENSG00000166188	57567	ZNF319	0.5
ENSG00000179813	144809	C13orf30	0.5
ENSG00000103005	79650	C16orf57	0.544
ENSG00000122591	84668	FAM126A	0.601
ENSG00000155052	129684	CNTNAP5	0.582
ENSG00000144567	79137	FAM134A	0.644
ENSG00000151067	775	CACNA1C	0.578
ENSG00000126858	55288	RHOT1	0.574
ENSG00000096717	23411	SIRT1	0.484
ENSG00000123505	262	AMD1	0.625
ENSG00000151773	160857	CCDC122	0.455
ENSG00000147439	55909	BIN3	0.479
ENSG00000101746	8715	NOL4	0.465
ENSG00000163104	56916	SMARCAD1	0.473
ENSG00000169213	5865	RAB3B	0.529

ENSG00000102996	4324	MMP15	0.508
ENSG00000244462	10137	RBM12	0.591
ENSG00000198961	9867	PJA2	0.545
ENSG00000112893	4124	MAN2A1	0.537
ENSG00000131051	9584	RBM39	0.593
ENSG00000134769	1837	DTNA	0.659
ENSG00000136521	4711	NDUFB5	0.532
ENSG00000179388	1960	EGR3	0.519
ENSG00000134717	91408	BTF3L4	0.602
ENSG00000198925	79065	ATG9A	0.515
ENSG00000180332	386618	KCTD4	0.445
ENSG00000165495	63876	PKNOX2	0.597
ENSG00000157077	9372	ZFYVE9	0.555
ENSG00000169375	25942	SIN3A	0.5
ENSG00000114757	51555	PEX5L	0.446
ENSG00000007384	64285	RHBDF1	0.482
ENSG00000141314	162494	RHBDL3	0.553
ENSG00000146802	64418	TMEM168	0.535
ENSG00000154222	200014	CC2D1B	0.468
ENSG00000164603	154743	C7orf60	0.523
ENSG00000166974	10982	MAPRE2	0.624
ENSG00000127328	117177	RAB3IP	0.566
ENSG00000163110	10611	PDLIM5	0.683
ENSG00000136152	83548	COG3	0.524
ENSG00000164604	54329	GPR85	0.428
ENSG00000169410	5780	PTPN9	0.53
ENSG00000103034	65009	NDRG4	0.511
ENSG00000009413	5980	REV3L	0.521
ENSG00000114374	8287	USP9Y	0.491
ENSG00000128573	93986	FOXP2	0.54
ENSG00000010244	7756	ZNF207	0.608
ENSG00000007392	55692	LUC7L	0.532
ENSG00000114416	8087	FXR1	0.627
ENSG00000025293	51230	PHF20	0.633
ENSG00000149547	9538	EI24	0.596
ENSG00000171219	55561	CDC42BPG	0.473
ENSG00000136231	10643	IGF2BP3	0.584
ENSG00000141429	2589	GALNT1	0.606
ENSG00000135924	3300	DNAJB2	0.527
ENSG00000004478	2288	FKBP4	0.599
ENSG00000136167	3936	LCP1	0.545
ENSG00000138696	658	BMPR1B	0.468
ENSG00000134748	84950	PRPF38A	0.514
ENSG00000110047	10938	EHD1	0.646
ENSG00000181449	6657	SOX2	0.59
ENSG00000067048	8653	DDX3Y	0.563
ENSG00000058063	23200	ATP11B	0.525
ENSG00000147457	91782	CHMP7	0.54
ENSG00000139629	11226	GALNT6	0.5
ENSG00000050438	9498	SLC4A8	0.488
ENSG00000102445	80183	C13orf18	0.588
ENSG00000187866	116224	FAM122A	0.534
ENSG00000103126	8312	AXIN1	0.483
ENSG00000179774	220202	ATOH7	0.48

ENSG00000155592	342357	ZKSCAN2	0.408
ENSG00000182601	9951	HS3ST4	0.439
ENSG00000183878	7404	UTY	0.541
ENSG00000110046	23130	ATG2A	0.508
ENSG00000111596	4848	CNOT2	0.645
ENSG00000043093	54165	DCUN1D1	0.55
ENSG00000135272	29969	MDFIC	0.563
ENSG00000164548	29896	TRA2A	0.62
ENSG00000096746	3189	HNRNPH3	0.64
ENSG00000176749	8851	CDK5R1	0.593
ENSG00000176658	4642	MYO1D	0.526
ENSG00000105967	22797	TFEC	0.5
ENSG00000123992	23549	DNPEP	0.621
ENSG00000138698	5910	RAP1GDS1	0.559
ENSG00000103202	4833	NME4	0.553
ENSG00000123700	3759	KCNJ2	0.509
ENSG00000169758	123591	C15orf27	0.516
ENSG00000103522	50615	IL21R	0.511
ENSG00000011105	10867	TSPAN9	0.536
ENSG00000088367	2036	EPB41L1	0.57
ENSG00000125398	6662	SOX9	0.598
ENSG00000133195	201266	SLC39A11	0.513
ENSG00000023287	9821	RB1CC1	0.599
ENSG00000143384	4170	MCL1	0.673
ENSG00000078081	27074	LAMP3	0.526
ENSG00000078369	2782	GNB1	0.619
ENSG00000068971	5526	PPP2R5B	0.532
ENSG00000139567	94	ACVRL1	0.533
ENSG00000143420	2029	ENSA	0.662
ENSG00000135503	91	ACVR1B	0.663
ENSG00000102468	3356	HTR2A	0.571
ENSG00000111696	51559	NT5DC3	0.478
ENSG00000204130	55680	RUFY2	0.497
ENSG00000161835	160622	GRASP	0.416
ENSG00000090565	9727	RAB11FIP3	0.536
ENSG00000134986	9315	C5orf13	0.59
ENSG00000197217	9583	ENTPD4	0.593
ENSG00000047578	23247	KIAA0556	0.524
ENSG00000172578	89857	KLHL6	0.534
ENSG00000180616	6752	SSTR2	0.509
ENSG00000159556	64843	ISL2	0.466
ENSG00000123358	3164	NR4A1	0.654
ENSG00000149554	1111	CHEK1	0.575
ENSG00000082556	4986	OPRK1	0.459
ENSG00000136842	7111	TMOD1	0.583
ENSG00000114796	54800	KLHL24	0.627
ENSG00000169967	10746	MAP3K2	0.516
ENSG00000162378	79699	ZYG11B	0.571
ENSG00000118971	894	CCND2	0.621
ENSG00000169180	23214	XPO6	0.569
ENSG00000072195	10290	SPEG	0.513
ENSG00000165246	22829	NLGN4Y	0.462
ENSG00000078237	57103	C12orf5	0.56
ENSG00000175137	80851	SH3BP5L	0.419

ENSG00000103326	6650	SOLH	0.494
ENSG00000213465	402	ARL2	0.529
ENSG00000105971	858	CAV2	0.616
ENSG00000123395	60673	C12orf44	0.473
ENSG00000080845	22839	DLGAP4	0.616
ENSG00000167767	144501	KRT80	0.495
ENSG00000133193	84923	FAM104A	0.449
ENSG00000188322	388228	SBK1	0.464
ENSG00000108684	40	ACCN1	0.458
ENSG00000127329	5787	PTPRB	0.534
ENSG00000180834	79929	MAP6D1	0.451
ENSG00000110025	29907	SNX15	0.492
ENSG00000105974	857	CAV1	0.614
ENSG00000168785	10098	TSPAN5	0.53
ENSG00000010810	2534	FYN	0.669
ENSG00000139372	6996	TDG	0.536
ENSG00000125107	23019	CNOT1	0.599
ENSG00000136937	4686	NCBP1	0.564
ENSG00000139687	5925	RB1	0.621
ENSG00000114770	10057	ABCC5	0.577
ENSG00000105976	4233	MET	0.649
ENSG00000205426	3887	KRT81	0.492
ENSG00000118707	60436	TGIF2	0.604
ENSG00000179604	23580	CDC42EP4	0.601
ENSG00000197562	57799	RAB40C	0.489
ENSG00000129595	64097	EPB41L4A	0.519
ENSG00000159167	6781	STC1	0.571
ENSG00000188647	375743	PTAR1	0.525
ENSG00000151247	1977	EIF4E	0.594
ENSG00000188760	130612	TMEM198	0.495
ENSG00000181291	124842	TMEM132E	0.43
ENSG00000187735	6917	TCEA1	0.537
ENSG00000134982	324	APC	0.647
ENSG00000006451	5898	RALA	0.55
ENSG00000138336	80312	TET1	0.412
ENSG00000165072	256691	MAMDC2	0.5
ENSG00000173933	5936	RBM4	0.563
ENSG00000198898	830	CAPZA2	0.551
ENSG00000065883	8621	CDC2L5	0.638
ENSG00000164024	23173	METAP1	0.552
ENSG00000178919	2304	FOXE1	0.571
ENSG00000101082	84174	SLA2	0.452
ENSG00000140382	10363	HMG20A	0.522
ENSG00000120837	4801	NFYB	0.592
ENSG00000056661	7703	PCGF2	0.61
ENSG00000070882	26031	OSBPL3	0.585
ENSG00000119138	687	KLF9	0.618
ENSG00000213533	375346	TMEM110	0.491
ENSG00000198431	7296	TXNRD1	0.589
ENSG00000169783	84894	LINGO1	0.438
ENSG00000145191	8893	EIF2B5	0.528
ENSG00000132872	6860	SYT4	0.464
ENSG00000163935	51460	SFMBT1	0.496
ENSG00000066739	55102	ATG2B	0.494

ENSG00000102531	22862	FNDC3A	0.569
ENSG00000186442	3850	KRT3	0.459
ENSG00000101079	57446	NDRG3	0.552
ENSG00000109832	29118	DDX25	0.437
ENSG00000151079	3742	KCNA6	0.421
ENSG00000068615	65055	REEP1	0.598
ENSG00000167202	23102	TBC1D2B	0.627
ENSG00000109184	23142	DCUN1D4	0.62
ENSG00000141540	94015	TTYH2	0.462
ENSG00000174348	127435	PODN	0.472
ENSG00000116106	2043	EPHA4	0.532
ENSG00000111262	3736	KCNA1	0.426
ENSG00000185652	4908	NTF3	0.489
ENSG00000086200	51194	IPO11	0.558
ENSG00000136425	10518	CIB2	0.599
ENSG00000004866	7982	ST7	0.623
ENSG00000104722	4741	NEFM	0.501
ENSG00000166411	3419	IDH3A	0.59
ENSG00000147459	80005	DOCK5	0.512
ENSG00000161202	1857	DVL3	0.61
ENSG00000163933	91869	RFT1	0.527
ENSG00000082293	1310	COL19A1	0.508
ENSG00000010278	928	CD9	0.561
ENSG00000092871	117584	RFFL	0.615
ENSG00000135903	5077	PAX3	0.577
ENSG00000064309	50937	CDON	0.448
ENSG00000008323	55200	PLEKHG6	0.494
ENSG00000106571	2737	GLI3	0.513
ENSG00000152223	57724	KIAA1632	0.567
ENSG00000157184	1376	CPT2	0.58
ENSG00000124104	90203	SNX21	0.534
ENSG00000157933	6497	SKI	0.513
ENSG00000143437	405	ARNT	0.608
ENSG00000164736	64321	SOX17	0.487
ENSG00000203778	619208	C6orf225	0.458
ENSG00000140983	89941	RHOT2	0.57
ENSG00000161203	1173	AP2M1	0.52
ENSG00000002834	3927	LASP1	0.533
ENSG00000125166	2806	GOT2	0.557
ENSG00000100744	51527	C14orf129	0.402
ENSG00000198650	6898	TAT	0.533
ENSG00000150394	1006	CDH8	0.578
ENSG00000063046	1975	EIF4B	0.594
ENSG00000206579	114786	XKR4	0.413
ENSG00000158966	57685	CACHD1	0.503
ENSG00000196169	124602	KIF19	0.429
ENSG00000104756	54793	KCTD9	0.533
ENSG00000106588	5683	PSMA2	0.63
ENSG00000172795	167227	DCP2	0.542
ENSG00000161381	57125	PLXDC1	0.555
ENSG00000140403	55466	DNAJA4	0.502
ENSG00000157193	7804	LRP8	0.613
ENSG00000197798	79607	FAM118B	0.586
ENSG00000135048	23670	TMEM2	0.523

ENSG00000163069	6443	SGCB	0.569
ENSG00000167904	137695	TMEM68	0.528
ENSG00000149639	140710	C20orf117	0.534
ENSG00000173653	9986	RCE1	0.584
ENSG00000111077	23371	TENC1	0.501
ENSG00000163081	151278	CCDC140	0.396
ENSG00000173599	5091	PC	0.489
ENSG00000122566	3181	HNRNPA2B1	0.559
ENSG00000157916	11079	RER1	0.655
ENSG00000090060	10914	PAPOLA	0.621
ENSG00000140937	1009	CDH11	0.587
ENSG00000111321	4055	LTBR	0.522
ENSG00000123983	2181	ACSL3	0.666
ENSG00000168612	90204	ZSWIM1	0.46
ENSG00000064601	5476	CTSA	0.562
ENSG00000152049	23704	KCNE4	0.508
ENSG00000182934	6734	SRPR	0.589
ENSG00000136928	9568	GABBR2	0.611
ENSG00000152056	130340	AP1S3	0.527
ENSG00000173451	83591	THAP2	0.512
ENSG00000107104	23189	KANK1	0.607
ENSG00000221818	64641	EBF2	0.436
ENSG00000143006	63948	DMRTB1	0.34
ENSG00000162298	84447	SYVN1	0.551
ENSG00000127585	146330	FBXL16	0.573
ENSG00000162437	55225	RAVER2	0.466
ENSG00000102753	3839	KPNA3	0.581
ENSG00000137090	1761	DMRT1	0.434
ENSG00000135919	5270	SERPINE2	0.49
ENSG00000002746	23072	HECW1	0.553
ENSG00000171444	4163	MCC	0.524
ENSG00000139190	6843	VAMP1	0.581
ENSG00000179776	1003	CDH5	0.499
ENSG00000136381	3658	IREB2	0.481
ENSG00000122565	11335	CBX3	0.534
ENSG00000164197	285671	RNF180	0.512
ENSG00000036257	8452	CUL3	0.561
ENSG00000080503	6595	SMARCA2	0.639
ENSG00000080371	23011	RAB21	0.525
ENSG00000162434	3716	JAK1	0.592
ENSG00000152242	147339	C18orf25	0.525
ENSG00000109189	64854	USP46	0.553
ENSG00000170703	284076	TTLL6	0.383
ENSG00000141622	494470	RNF165	0.518
ENSG00000221914	5520	PPP2R2A	0.573
ENSG00000186479	401190	RGS7BP	0.425
ENSG00000115561	51652	VPS24	0.537
ENSG00000136051	23325	KIAA1033	0.611
ENSG00000178188	25970	SH2B1	0.57
ENSG00000163827	79442	LRRC2	0.461
ENSG00000121749	64786	TBC1D15	0.49
ENSG00000067191	782	CACNB1	0.618
ENSG00000153006	285672	SFRS12IP1	0.469
ENSG00000165323	120114	FAT3	0.558

ENSG00000073598	54752	FNDC8	0.413
ENSG00000167778	84926	SPRYD3	0.459
ENSG00000165138	203286	ANKS6	0.564
ENSG00000128045	65997	RASL11B	0.471
ENSG00000164543	9263	STK17A	0.665
ENSG00000166747	164	AP1G1	0.54
ENSG00000134531	2012	EMP1	0.656
ENSG00000135473	9924	PAN2	0.505
ENSG00000143418	29956	LASS2	0.504
ENSG00000150086	2904	GRIN2B	0.503
ENSG00000188120	1617	DAZ1	0.468
ENSG00000005020	8935	SKAP2	0.605
ENSG00000153561	64795	RMND5A	0.606
ENSG00000157388	776	CACNA1D	0.493
ENSG00000069329	55737	VPS35	0.531
ENSG00000049192	11174	ADAMTS6	0.486
ENSG00000014216	823	CAPN1	0.54
ENSG00000172794	326624	RAB37	0.52
ENSG00000147852	7436	VLDLR	0.545
ENSG00000181690	5324	PLAG1	0.509
ENSG00000080644	1136	CHRNA3	0.557
ENSG00000080709	3781	KCNN2	0.475
ENSG00000104765	665	BNIP3L	0.6
ENSG00000155130	4082	MARCKS	0.607
ENSG00000139651	283337	ZNF740	0.404
ENSG00000204291	1306	COL15A1	0.517
ENSG00000116675	9829	DNAJC6	0.536
ENSG00000196591	3066	HDAC2	0.56
ENSG00000110944	51561	IL23A	0.575
ENSG00000170581	6773	STAT2	0.595
ENSG00000105991	3198	HOXA1	0.494
ENSG00000197921	388585	HES5	0.382
ENSG00000144460	57624	KIAA1486	0.368
ENSG00000150455	114609	TIRAP	0.522
ENSG00000169047	3667	IRS1	0.566
ENSG00000136044	55198	APPL2	0.498
ENSG00000116141	4139	MARK1	0.541
ENSG00000092964	1808	DPYSL2	0.542
ENSG00000081052	1286	COL4A4	0.488
ENSG00000173020	156	ADRBK1	0.574
ENSG00000152503	55521	TRIM36	0.466
ENSG00000105997	3200	HOXA3	0.446
ENSG00000185787	10933	MORF4L1	0.514
ENSG00000169031	1285	COL4A3	0.66
ENSG00000164031	79982	DNAJB14	0.478
ENSG00000107372	7763	ZFAND5	0.607
ENSG00000141750	342667	STAC2	0.495
ENSG00000106799	7046	TGFBR1	0.547
ENSG00000197576	3201	HOXA4	0.501
ENSG00000166002	56935	C11orf75	0.516
ENSG00000116678	3953	LEPR	0.627
ENSG00000106004	3202	HOXA5	0.502
ENSG00000172819	5916	RARG	0.632
ENSG00000144233	83607	AMMECR1L	0.483

ENSG00000084463	51729	WBP11	0.567
ENSG00000101363	63905	MANBAL	0.532
ENSG00000164219	5229	PGGT1B	0.461
ENSG00000078399	3205	HOXA9	0.56
ENSG00000122692	55234	SMU1	0.465
ENSG00000104331	54928	IMPAD1	0.505
ENSG00000102786	26512	INTS6	0.601
ENSG00000072657	29953	TRHDE	0.47
ENSG00000197122	6714	SRC	0.588
ENSG00000169122	90362	FAM110B	0.488
ENSG00000253293	3206	HOXA10	0.549
ENSG00000169682	83985	SPNS1	0.454
ENSG00000149798	10435	CDC42EP2	0.571
ENSG00000151490	5800	PTPRO	0.557
ENSG00000127152	64919	BCL11B	0.494
ENSG00000072201	84708	LNX1	0.504
ENSG00000125686	5469	MED1	0.559
ENSG00000159202	65264	UBE2Z	0.519
ENSG00000175387	4087	SMAD2	0.648
ENSG00000133884	5977	DPF2	0.518
ENSG00000107249	169792	GLIS3	0.5
ENSG00000005513	30812	SOX8	0.414
ENSG00000005073	3207	HOXA11	0.573
ENSG00000164849	115330	GPR146	0.433
ENSG00000196557	8912	CACNA1H	0.489
ENSG00000183576	84193	SETD3	0.533
ENSG00000015592	81551	STMN4	0.486
ENSG00000117411	8704	B4GALT2	0.527
ENSG00000184828	201501	ZBTB7C	0.394
ENSG00000184588	5142	PDE4B	0.639
ENSG00000086062	2683	B4GALT1	0.626
ENSG00000153823	55022	PID1	0.517
ENSG00000157540	1859	DYRK1A	0.635
ENSG00000151491	2059	EPS8	0.563
ENSG00000185960	6473	SHOX	0.45
ENSG00000140795	91807	MYLK3	0.533
ENSG00000139668	115825	WDFY2	0.526
ENSG00000143363	58497	PRUNE	0.643
ENSG00000166619	10904	BLCAP	0.591
ENSG00000136279	28988	DBNL	0.527
ENSG00000119508	8013	NR4A3	0.616
ENSG00000111642	1108	CHD4	0.603
ENSG00000213658	27040	LAT	0.556
ENSG00000176422	283377	SPRYD4	0.434
ENSG00000135423	27165	GLS2	0.48
ENSG00000074590	9891	NUAK1	0.545
ENSG00000184408	3751	KCND2	0.43
ENSG00000181585	259236	TMIE	0.549
ENSG00000137575	6386	SDCBP	0.571
ENSG00000010539	7752	ZNF200	0.574
ENSG00000134030	9811	KIAA0427	0.578
ENSG00000106688	6505	SLC1A1	0.584
ENSG00000134186	55119	PRPF38B	0.509
ENSG00000102796	79758	DHRS12	0.576

ENSG00000107262	573	BAG1	0.576
ENSG00000142606	79258	MMEL1	0.439
ENSG00000136630	3142	HLX	0.511
ENSG00000172830	54961	SSH3	0.641
ENSG00000109220	26511	CHIC2	0.514
ENSG00000101665	4092	SMAD7	0.554
ENSG00000106025	23554	TSPAN12	0.476
ENSG00000143507	11221	DUSP10	0.58
ENSG00000159214	149473	CCDC24	0.466
ENSG00000136874	55014	STX17	0.482
ENSG00000109065	26151	NAT9	0.511
ENSG00000090061	8812	CCNK	0.464
ENSG00000145194	9718	ECE2	0.485
ENSG00000204439	57827	C6orf47	0.496
ENSG00000006125	163	AP2B1	0.577
ENSG00000103275	7329	UBE2I	0.638
ENSG00000134853	5156	PDGFRA	0.651
ENSG00000048540	55885	LMO3	0.503
ENSG00000118473	84251	SGIP1	0.515
ENSG00000182149	9798	KIAA0174	0.603
ENSG00000159217	10642	IGF2BP1	0.587
ENSG00000112305	60682	SMAP1	0.547
ENSG00000166123	84706	GPT2	0.548
ENSG00000163053	151473	SLC16A14	0.495
ENSG00000120899	2185	PTK2B	0.584
ENSG00000138166	1847	DUSP5	0.578
ENSG00000053438	4826	NNAT	0.495
ENSG00000198010	9228	DLGAP2	0.548
ENSG00000000457	57147	SCYL3	0.563
ENSG00000171532	4761	NEUROD2	0.496
ENSG00000204435	1460	CSNK2B	0.51
ENSG00000205808	403313	PPAPDC2	0.462
ENSG00000069345	10294	DNAJA2	0.525
ENSG00000106993	55664	CDC37L1	0.5
ENSG00000169330	23251	KIAA1024	0.463
ENSG00000109066	54868	TMEM104	0.458
ENSG00000175505	23529	CLCF1	0.465
ENSG00000163888	94032	CAMK2N2	0.409
ENSG00000172613	5883	RAD9A	0.544
ENSG00000213626	81606	LBH	0.514
ENSG00000113595	373	TRIM23	0.577
ENSG00000035681	8439	NSMAF	0.563
ENSG00000197622	56882	CDC42SE1	0.568
ENSG00000253797	9724	UTP14C	0.552
ENSG00000132821	128434	VSTM2L	0.404
ENSG00000196517	6536	SLC6A9	0.495
ENSG00000104728	9639	ARHGEF10	0.582
ENSG00000197471	6693	SPN	0.65
ENSG00000139200	196500	C12orf53	0.462
ENSG00000081014	23431	AP4E1	0.522
ENSG00000172531	5499	PPP1CA	0.55
ENSG00000183723	146223	CMTM4	0.571
ENSG00000111652	50813	COPS7A	0.485
ENSG00000153814	221895	JAZF1	0.5

ENSG00000127954	79689	STEAP4	0.427
ENSG00000157404	3815	KIT	0.516
ENSG00000086102	4799	NFX1	0.588
ENSG00000114867	1981	EIF4G1	0.606
ENSG00000213190	10962	MLLT11	0.53
ENSG00000198963	6096	RORB	0.459
ENSG00000157554	2078	ERG	0.594
ENSG00000198959	7052	TGM2	0.657
ENSG00000101670	9388	LIPG	0.527
ENSG00000124140	57468	SLC12A5	0.478
ENSG00000142611	63976	PRDM16	0.479
ENSG00000146592	9586	CREB5	0.522
ENSG00000135720	1783	DYNC1LI2	0.596
ENSG00000152760	200132	TCTEX1D1	0.353
ENSG00000186417	342035	GLDN	0.446
ENSG00000170374	121340	SP7	0.468
ENSG00000185591	6667	SP1	0.49
ENSG00000116205	127428	C1orf83	0.5
ENSG00000076108	11176	BAZ2A	0.616
ENSG00000104093	23312	DMXL2	0.559
ENSG00000173868	162466	PHOSPHO1	0.36
ENSG00000134954	2113	ETS1	0.536
ENSG00000119121	140803	TRPM6	0.5
ENSG00000136100	51028	VPS36	0.553
ENSG00000157214	261729	STEAP2	0.523
ENSG00000072210	224	ALDH3A2	0.63
ENSG00000184867	9823	ARMCX2	0.472
ENSG00000196937	10447	FAM3C	0.569
ENSG00000103495	4150	MAZ	0.556
ENSG00000167306	4645	MYO5B	0.531
ENSG00000089693	8079	MLF2	0.519
ENSG00000079841	22999	RIMS1	0.496
ENSG00000198740	22834	ZNF652	0.544
ENSG00000198517	7975	MAFK	0.47
ENSG00000141150	91608	RASL10B	0.491
ENSG00000129534	55320	C14orf106	0.492
ENSG00000124160	57727	NCOA5	0.597
ENSG00000128872	29767	TMOD2	0.463
ENSG00000197111	5094	PCBP2	0.656
ENSG00000139154	121536	AEBP2	0.444
ENSG00000106278	5803	PTPRZ1	0.498
ENSG00000188827	84464	BTBD12	0.484
ENSG00000172379	9915	ARNT2	0.479
ENSG00000172572	5139	PDE3A	0.556
ENSG00000164855	202915	TMEM184A	0.5
ENSG00000167085	5245	PHB	0.585
ENSG00000156515	3098	HK1	0.533
ENSG00000069956	5597	MAPK6	0.565
ENSG00000135932	51719	CAB39	0.509
ENSG00000141644	4152	MBD1	0.561
ENSG00000159335	5763	PTMS	0.54
ENSG00000135916	81618	ITM2C	0.524
ENSG00000198846	9760	TOX	0.554
ENSG00000167371	112476	PRRT2	0.454

ENSG00000178538	767	CA8	0.487
ENSG00000139289	22822	PHLDA1	0.655
ENSG00000197646	80380	PDCD1LG2	0.45
ENSG00000173991	8557	TCAP	0.464
ENSG00000160796	23218	NBEAL2	0.49
ENSG00000157557	2114	ETS2	0.632
ENSG00000172660	8148	TAF15	0.543
ENSG00000250510	27239	GPR162	0.46
ENSG00000187109	4673	NAP1L1	0.661
ENSG00000198160	57708	MIER1	0.555
ENSG00000020922	4361	MRE11A	0.586
ENSG00000136379	58489	FAM108C1	0.496
ENSG00000157216	23648	SSBP3	0.479
ENSG00000197535	4644	MYO5A	0.634
ENSG00000103888	57214	KIAA1199	0.525
ENSG00000099219	79956	ERMP1	0.515
ENSG00000162144	220002	CYBASC3	0.5
ENSG00000128052	3791	KDR	0.541
ENSG00000171316	55636	CHD7	0.557
ENSG00000139915	161357	MDGA2	0.412
ENSG00000066629	2009	EML1	0.548
ENSG00000103502	10423	CDIPT	0.511
ENSG00000136099	5100	PCDH8	0.436
ENSG00000165995	783	CACNB2	0.585
ENSG00000116132	5396	PRRX1	0.532
ENSG00000182040	124590	USH1G	0.415
ENSG00000102893	5257	PHKB	0.608
ENSG00000139625	7786	MAP3K12	0.582
ENSG00000106069	1124	CHN2	0.606
ENSG00000118946	27253	PCDH17	0.514
ENSG00000139546	6895	TARBP2	0.559
ENSG00000111785	55188	RIC8B	0.508
ENSG00000005339	1387	CREBBP	0.573
ENSG00000047346	56204	KIAA1370	0.528
ENSG00000064300	4804	NGFR	0.47
ENSG00000182575	11248	NXPH3	0.475
ENSG00000120885	1191	CLU	0.631
ENSG00000185658	54014	BRWD1	0.558
ENSG00000148123	54886	RP11-35N6.1	0.496
ENSG00000169856	3175	ONECUT1	0.453
ENSG00000081803	93664	CADPS2	0.45
ENSG00000166415	256764	WDR72	0.495
ENSG00000007545	57585	CRAMP1L	0.526
ENSG00000162104	115	ADCY9	0.631
ENSG00000140406	59274	MESDC1	0.523
ENSG00000183117	64478	CSMD1	0.527
ENSG00000137766	440279	UNC13C	0.512
ENSG00000085433	22911	WDR47	0.516
ENSG00000111667	8078	USP5	0.506
ENSG00000135045	55071	C9orf40	0.536
ENSG00000162783	51278	IER5	0.533
ENSG00000156017	138199	C9orf41	0.512
ENSG00000198216	777	CACNA1E	0.515
ENSG00000185739	6345	SRL	0.437

ENSG00000168876	54851	ANKRD49	0.49
ENSG00000197860	54557	SGTB	0.558
ENSG00000187147	55182	RNF220	0.467
ENSG00000111711	51026	GOLT1B	0.491
ENSG00000090447	7023	TFAP4	0.496
ENSG00000206053	90861	HN1L	0.642
ENSG00000213741	6235	RPS29	0.525
ENSG00000168748	766	CA7	0.485
ENSG00000126603	84662	GLIS2	0.469
ENSG00000170777	89882	TPD52L3	0.5
ENSG00000172840	57546	PDP2	0.515
ENSG00000166025	154810	AMOTL1	0.592
ENSG00000147419	55246	CCDC25	0.495
ENSG00000165271	65083	NOL6	0.46
ENSG00000131724	3597	IL13RA1	0.645
ENSG00000134852	9575	CLOCK	0.583
ENSG00000151135	90488	C12orf23	0.524
ENSG00000062598	63916	ELMO2	0.605
ENSG00000138834	23162	MAPK8IP3	0.614
ENSG00000136881	570	BAAT	0.458
ENSG00000137713	5519	PPP2R1B	0.671
ENSG00000141639	5596	MAPK4	0.518
ENSG00000150593	27250	PDCD4	0.625
ENSG00000147854	115426	UHRF2	0.469
ENSG00000166450	283659	PRTG	0.448
ENSG00000157224	9069	CLDN12	0.491
ENSG00000198363	444	ASPH	0.638
ENSG00000136193	9805	SCRN1	0.537
ENSG00000181555	29072	SETD2	0.58
ENSG00000069869	4734	NEDD4	0.477
ENSG00000091039	114882	OSBPL8	0.577
ENSG00000215717	56900	TMEM167B	0.457
ENSG00000174943	253980	KCTD13	0.543
ENSG00000083290	9706	ULK2	0.613
ENSG00000148516	6935	ZEB1	0.599
ENSG00000101438	140679	SLC32A1	0.448
ENSG00000101442	79913	ACTR5	0.55
ENSG00000173327	4296	MAP3K11	0.54
ENSG00000165997	221079	ARL5B	0.485
ENSG00000182580	2049	EPHB3	0.554
ENSG00000149218	23052	ENDOD1	0.6
ENSG00000125820	4821	NKX2-2	0.493
ENSG00000184226	5101	PCDH9	0.603
ENSG00000125798	3170	FOXA2	0.57
ENSG00000101445	26051	PPP1R16B	0.53
ENSG00000106263	8662	EIF3B	0.585
ENSG00000110958	10728	PTGES3	0.557
ENSG00000106080	55033	FKBP14	0.482
ENSG00000183496	84206	MEX3B	0.45
ENSG00000149930	9344	TAOK2	0.587
ENSG00000112851	55914	ERBB2IP	0.508
ENSG00000111676	1822	ATN1	0.591
ENSG00000138587	55329	MNS1	0.482
ENSG00000011258	54799	MBTD1	0.4

ENSG00000111728	6489	ST8SIA1	0.47
ENSG00000178726	7056	THBD	0.6
ENSG00000125810	22918	CD93	0.552
ENSG00000111731	9847	KIAA0528	0.538
ENSG00000125812	64412	GZF1	0.51
ENSG00000012822	57658	CALCOCO1	0.536
ENSG00000165659	1602	DACH1	0.521
ENSG00000126106	79639	TMEM53	0.453
ENSG00000196652	23660	ZKSCAN5	0.547
ENSG00000175556	79836	LONRF3	0.508
ENSG00000144524	64708	COPS7B	0.482
ENSG00000141646	4089	SMAD4	0.608
ENSG00000125814	63908	NAPB	0.517
ENSG00000121104	81558	FAM117A	0.52
ENSG00000106636	10652	YKT6	0.591
ENSG00000140262	6938	TCF12	0.595
ENSG00000109670	55294	FBXW7	0.497
ENSG00000011260	51096	UTP18	0.513
ENSG00000186063	64853	AIDA	0.492
ENSG00000204899	440145	C13orf37	0.543
ENSG00000077458	143684	FAM76B	0.54
ENSG00000136122	79866	C13orf34	0.524
ENSG00000103423	9093	DNAJA3	0.549
ENSG00000198729	81706	PPP1R14C	0.468
ENSG00000197343	79027	ZNF655	0.605
ENSG00000152518	678	ZFP36L2	0.655
ENSG00000152527	130271	PLEKHH2	0.504
ENSG00000013016	30845	EHD3	0.507
ENSG00000176624	51320	MEX3C	0.527
ENSG00000239389	56136	PCDHA13	0.5
ENSG00000187323	1630	DCC	0.466
ENSG00000139163	55500	ETNK1	0.587
ENSG00000136504	11143	MYST2	0.527
ENSG00000158125	7498	XDH	0.487
ENSG00000058404	816	CAMK2B	0.63
ENSG00000187210	2650	GCNT1	0.493
ENSG00000187068	285382	C3orf70	0.522
ENSG00000165322	94134	ARHGAP12	0.542
ENSG00000165152	84302	C9orf125	0.443
ENSG00000134532	6660	SOX5	0.469
ENSG00000161573	6360	CCL16	0.47
ENSG00000128849	84952	CGNL1	0.57
ENSG00000083520	22894	DIS3	0.544
ENSG00000180354	222166	C7orf41	0.566
ENSG00000106772	158471	PRUNE2	0.514
ENSG00000137878	145781	Gcom1	0.535
ENSG00000166037	9702	CEP57	0.62
ENSG00000073803	9175	MAP3K13	0.524
ENSG00000180233	223082	ZNRF2	0.479
ENSG00000123364	3229	HOXC13	0.473
ENSG00000136213	55501	CHST12	0.506
ENSG00000106003	3955	LFNG	0.506
ENSG00000123388	3227	HOXC11	0.501
ENSG00000213445	6494	SIPA1	0.502

ENSG00000160216	56894	AGPAT3	0.549
ENSG00000248383	56135	PCDHAC1	0.466
ENSG00000107341	54926	UBE2R2	0.524
ENSG00000060982	586	BCAT1	0.581
ENSG00000149927	8448	DOC2A	0.499
ENSG00000078114	10529	NEBL	0.637
ENSG00000137073	55833	UBAP2	0.582
ENSG00000186908	23390	ZDHHC17	0.567
ENSG00000128683	2571	GAD1	0.568
ENSG00000134046	8932	MBD2	0.632
ENSG00000108061	8036	SHOC2	0.508
ENSG00000103415	3163	HMOX2	0.59
ENSG00000197757	3223	HOXC6	0.565
ENSG00000101751	11201	POLI	0.505
ENSG00000157240	8321	FZD1	0.568
ENSG00000117523	23215	BAT2D1	0.649
ENSG00000100811	7528	YY1	0.673
ENSG00000204103	9935	MAFB	0.523
ENSG00000198900	7150	TOP1	0.602
ENSG00000153707	5789	PTPRD	0.599
ENSG00000168079	286133	SCARA5	0.458
ENSG00000198785	116443	GRIN3A	0.492
ENSG00000089486	29965	C16orf5	0.491
ENSG00000170006	201799	TMEM154	0.46
ENSG00000101856	10857	PGRMC1	0.572
ENSG00000243232	56134	PCDHAC2	0.413
ENSG00000167797	10263	CDK2AP2	0.532
ENSG00000077713	203427	SLC25A43	0.542
ENSG00000128918	8854	ALDH1A2	0.549
ENSG00000164144	27236	ARFIP1	0.53
ENSG00000135821	2752	GLUL	0.632
ENSG00000163898	200879	LIPH	0.41
ENSG00000197037	221785	ZNF498	0.472
ENSG00000214575	64506	CPEB1	0.456
ENSG00000163904	59343	SENP2	0.506
ENSG00000011347	9066	SYT7	0.57
ENSG00000141198	10040	TOM1L1	0.529
ENSG00000155189	55326	AGPAT5	0.507
ENSG00000037965	3224	HOXC8	0.435
ENSG00000153443	124402	FAM100A	0.459
ENSG00000102858	23295	MGRN1	0.491
ENSG00000137845	102	ADAM10	0.622
ENSG00000118922	11278	KLF12	0.652
ENSG00000176542	205717	KIAA2018	0.582
ENSG00000128923	54629	FAM63B	0.538
ENSG00000032219	5926	ARID4A	0.538
ENSG00000087053	8898	MTMR2	0.595
ENSG00000136111	9882	TBC1D4	0.58
ENSG00000157450	54778	RNF111	0.46
ENSG00000073792	10644	IGF2BP2	0.542
ENSG00000142864	26135	SERBP1	0.673
ENSG00000134780	747	DAGLA	0.437
ENSG00000124181	5335	PLCG1	0.554
ENSG00000110851	11108	PRDM4	0.571

ENSG00000137460	85462	FHDC1	0.467
ENSG00000204682	399726	C10orf114	0.488
ENSG00000138036	51626	DYNC2LI1	0.597
ENSG00000137776	79811	SLTM	0.488
ENSG00000153914	140890	SFRS12	0.514
ENSG00000162959	51072	MEMO1	0.511
ENSG00000115468	80303	EFHD1	0.518
ENSG00000078403	8028	MLLT10	0.608
ENSG00000150594	150	ADRA2A	0.463
ENSG00000119927	57678	GPAM	0.497
ENSG00000241553	10093	ARPC4	0.619
ENSG00000166860	9880	ZBTB39	0.482
ENSG00000204120	26058	GIGYF2	0.579
ENSG00000124920	745	C11orf9	0.578
ENSG00000170759	3799	KIF5B	0.567
ENSG00000108813	1748	DLX4	0.572
ENSG00000158301	114928	GPRASP2	0.5
ENSG00000204956	56114	PCDHGA1	0.435
ENSG00000157483	4643	MYO1E	0.618
ENSG00000197771	79892	C10orf119	0.492
ENSG00000135486	3178	HNRNPA1	0.605
ENSG00000075035	9671	WSCD2	0.414
ENSG00000115806	26003	GORASP2	0.638
ENSG00000196628	6925	TCF4	0.678
ENSG00000064195	1747	DLX3	0.452
ENSG00000133703	3845	KRAS	0.662
ENSG00000005884	3675	ITGA3	0.587
ENSG00000157470	145773	FAM81A	0.568
ENSG00000109654	23321	TRIM2	0.633
ENSG00000104442	55156	ARMC1	0.52
ENSG00000172977	10524	KAT5	0.567
ENSG00000167799	254552	NUDT8	0.538
ENSG00000139182	9746	CLSTN3	0.444
ENSG00000114573	523	ATP6V1A	0.628
ENSG00000165891	144455	E2F7	0.577
ENSG00000114648	23276	KLHL18	0.581
ENSG00000150764	85458	DIXDC1	0.499
ENSG00000172380	55970	GNG12	0.53
ENSG00000172057	94103	ORMDL3	0.568
ENSG00000140299	663	BNIP2	0.487
ENSG00000136295	80727	TTYH3	0.476
ENSG00000103035	5713	PSMD7	0.558
ENSG00000254245	56112	PCDHGA3	0.583
ENSG00000067955	865	CBFB	0.622
ENSG00000149972	53942	CNTN5	0.446
ENSG00000253953	8641	PCDHGB4	0.504
ENSG00000253767	5098	PCDHGC3	0.568
ENSG00000067798	89795	NAV3	0.606
ENSG00000123104	3709	ITPR2	0.599
ENSG00000182718	302	ANXA2	0.621
ENSG00000116729	79971	GPR177	0.505
ENSG00000166260	1353	COX11	0.581
ENSG00000244405	2119	ETV5	0.583
ENSG00000143126	1952	CELSR2	0.546

ENSG00000174306	23051	ZHX3	0.553
ENSG00000253305	56100	PCDHGB6	0.392
ENSG00000253846	56106	PCDHGA10	0.445
ENSG00000254122	56099	PCDHGB7	0.513
ENSG00000058866	1608	DGKG	0.493
ENSG00000166263	252983	STXBP4	0.518
ENSG00000116133	1718	DHCR24	0.53
ENSG00000136153	4008	LMO7	0.5
ENSG00000139197	5830	PEX5	0.567
ENSG00000066855	9650	MTFR1	0.544
ENSG00000125149	80262	C16orf70	0.454
ENSG00000173846	1263	PLK3	0.567
ENSG00000153714	286343	C9orf150	0.483
ENSG00000015676	23386	NUDCD3	0.564
ENSG00000155366	389	RHOC	0.569
ENSG00000021574	6683	SPAST	0.576
ENSG00000240583	358	AQP1	0.576
ENSG00000253873	5098	PCDHGC3	0.568
ENSG00000186918	55893	ZNF395	0.575
ENSG00000107186	8777	MPDZ	0.557
ENSG00000165006	51271	UBAP1	0.597
ENSG00000109265	57482	KIAA1211	0.536
ENSG00000198586	9874	TLK1	0.608
ENSG00000064655	2139	EYA2	0.494
ENSG00000103942	9455	HOMER2	0.522
ENSG00000128915	79664	NARG2	0.494
ENSG00000001631	889	KRIT1	0.611
ENSG00000150776	55216	C11orf57	0.536
ENSG00000111790	26127	FGFR1OP2	0.641
ENSG00000196470	6477	SIAH1	0.568
ENSG00000104290	7976	FZD3	0.487
ENSG00000186416	55922	NKRF	0.511
ENSG00000120616	80314	EPC1	0.609
ENSG00000253159	26025	PCDHGA12	0.529
ENSG00000067715	6857	SYT1	0.559
ENSG00000157110	11030	RBPMS	0.662
ENSG00000102921	9683	N4BP1	0.639
ENSG00000091164	9352	TXNL1	0.578
ENSG00000166881	23306	TMEM194A	0.57
ENSG00000117533	8674	VAMP4	0.609
ENSG00000125354	23157	6-Sep	0.667
ENSG00000178695	115207	KCTD12	0.621
ENSG00000165029	19	ABCA1	0.646
ENSG00000240184	5098	PCDHGC3	0.568
ENSG00000170647	83935	TMEM133	0.471
ENSG00000197142	51703	ACSL5	0.517
ENSG00000242419	56098	PCDHGC4	0.456
ENSG00000082175	5241	PGR	0.474
ENSG00000183798	90187	EMILIN3	0.465
ENSG00000240764	5098	PCDHGC3	0.568
ENSG00000211455	23012	STK38L	0.61
ENSG00000146535	2768	GNA12	0.542
ENSG00000162402	23358	USP24	0.569
ENSG00000131504	1729	DIAPH1	0.605

ENSG00000108924	3131	HLF	0.634
ENSG00000012232	2137	EXTL3	0.567
ENSG00000108342	1440	CSF3	0.475
ENSG00000118900	29855	UBN1	0.619
ENSG00000091157	23335	WDR7	0.544
ENSG00000177575	9332	CD163	0.551
ENSG00000124177	84181	CHD6	0.559
ENSG00000108960	23531	MMD	0.517
ENSG00000156976	1974	EIF4A2	0.564
ENSG00000005882	5164	PDK2	0.6
ENSG00000149485	3992	FADS1	0.653
ENSG00000101040	23613	ZMYND8	0.636
ENSG00000005810	23077	MYCBP2	0.592
ENSG00000137672	7225	TRPC6	0.529
ENSG00000131016	9590	AKAP12	0.536
ENSG00000196781	7088	TLE1	0.605
ENSG00000058272	4659	PPP1R12A	0.604
ENSG00000171914	83660	TLN2	0.503
ENSG00000181472	57621	ZBTB2	0.496
ENSG00000196581	55966	AJAP1	0.558
ENSG00000171587	1826	DSCAM	0.484
ENSG00000184185	3768	KCNJ12	0.563
ENSG00000103184	9717	SEC14L5	0.516
ENSG00000253958	137075	CLDN23	0.521
ENSG00000001629	54467	ANKIB1	0.589
ENSG00000147324	9258	MFHAS1	0.506
ENSG00000085978	55054	ATG16L1	0.465
ENSG00000172818	5017	OVOL1	0.485
ENSG00000111481	22818	COPZ1	0.517
ENSG00000172757	1072	CFL1	0.532
ENSG00000168283	648	BMI1	0.56
ENSG00000135723	29109	FHOD1	0.5
ENSG00000184307	254887	ZDHHC23	0.478
ENSG00000162909	824	CAPN2	0.624
ENSG00000143333	6004	RGS16	0.609
ENSG00000109046	26118	WSB1	0.696
ENSG00000123600	79828	METTL8	0.483
ENSG00000110841	8496	PPFIBP1	0.588
ENSG00000204267	6891	TAP2	0.58
ENSG00000164600	63974	NEUROD6	0.45
ENSG00000165527	382	ARF6	0.615
ENSG00000119820	84272	YIPF4	0.569
ENSG00000102935	23090	ZNF423	0.497
ENSG00000090238	83719	YPEL3	0.579
ENSG00000156052	2776	GNAQ	0.564
ENSG00000197959	26052	DNM3	0.509
ENSG00000166888	6778	STAT6	0.635
ENSG00000134243	6272	SORT1	0.534
ENSG00000177511	51046	ST8SIA3	0.489
ENSG00000102882	5595	MAPK3	0.521
ENSG00000145423	6423	SFRP2	0.503
ENSG00000185088	51065	RPS27L	0.566
ENSG00000155066	150696	PROM2	0.446
ENSG00000119547	9480	ONECUT2	0.491

ENSG00000166128	51762	RAB8B	0.535
ENSG00000073849	6480	ST6GAL1	0.617
ENSG00000157259	57798	GATAD1	0.609
ENSG00000147570	85479	DNAJC5B	0.486
ENSG00000147573	84675	TRIM55	0.466
ENSG00000115760	57448	BIRC6	0.56
ENSG00000196090	11122	PTPRT	0.483
ENSG00000196155	25894	PLEKHG4	0.484
ENSG00000156218	57188	ADAMTSL3	0.499
ENSG00000161642	25946	ZNF385A	0.5
ENSG00000087510	7022	TFAP2C	0.555
ENSG00000087448	57542	KLHDC5	0.6
ENSG00000074410	771	CA12	0.661
ENSG00000177469	284119	PTRF	0.586
ENSG00000183160	338773	TMEM119	0.496
ENSG00000033627	535	ATP6V0A1	0.546
ENSG00000173281	79660	PPP1R3B	0.472
ENSG00000128059	5471	PPAT	0.584
ENSG00000161638	3678	ITGA5	0.559
ENSG00000205423	255919	TMEM188	0.45
ENSG00000091831	2099	ESR1	0.676
ENSG00000162407	8613	PPAP2B	0.616
ENSG00000089818	25977	NECAP1	0.53
ENSG00000110880	23603	CORO1C	0.547
ENSG00000198799	9860	LRIG2	0.507
ENSG00000173273	8658	TNKS	0.659
ENSG00000081923	5205	ATP8B1	0.509
ENSG00000110721	1119	CHKA	0.507
ENSG00000141068	8844	KSR1	0.571
ENSG00000106261	7586	ZKSCAN1	0.639
ENSG00000106341	10842	C7orf16	0.463
ENSG00000101144	655	BMP7	0.605
ENSG00000162409	5563	PRKAA2	0.505
ENSG00000033122	57554	LRRC7	0.5
ENSG00000121060	7706	TRIM25	0.493
ENSG00000151532	143187	VTI1A	0.545
ENSG00000164970	203259	C9orf25	0.504
ENSG00000183092	57596	BEGAIN	0.496
ENSG00000015171	10771	ZMYND11	0.618
ENSG00000077327	9576	SPAG6	0.54
ENSG00000100997	26090	ABHD12	0.504
ENSG00000070214	23446	SLC44A1	0.589
ENSG00000035664	23604	DAPK2	0.562
ENSG00000110066	51111	SUV420H1	0.569
ENSG00000049759	23327	NEDD4L	0.615
ENSG00000106829	7091	TLE4	0.638
ENSG00000059804	6515	SLC2A3	0.679
ENSG00000150093	3688	ITGB1	0.689
ENSG00000100485	6655	SOS2	0.62
ENSG00000164916	221937	FOXK1	0.5
ENSG00000108821	1277	COL1A1	0.637
ENSG00000028528	6642	SNX1	0.67
ENSG00000125351	65109	UPF3B	0.521
ENSG00000065970	55810	FOXJ2	0.523

ENSG00000126107	79654	HECTD3	0.502
ENSG00000113916	604	BCL6	0.632
ENSG00000123338	3071	NCKAP1L	0.507
ENSG00000179104	160335	TMTC2	0.496
ENSG00000105953	4967	OGDH	0.542
ENSG00000165617	51339	DACT1	0.498
ENSG00000145012	4026	LPP	0.595
ENSG00000100592	23002	DAAM1	0.522
ENSG00000157734	79856	SNX22	0.5
ENSG00000148737	6934	TCF7L2	0.644
ENSG00000205250	1874	E2F4	0.636
ENSG00000137693	10413	YAP1	0.51
ENSG00000169118	53944	CSNK1G1	0.567
ENSG00000121274	64282	PAPD5	0.516
ENSG00000240065	5698	PSMB9	0.561
ENSG00000141140	80179	MYO19	0.527
ENSG00000008710	5310	PKD1	0.617
ENSG00000071967	79901	CYBRD1	0.512
ENSG00000142875	5567	PRKACB	0.604
ENSG00000147862	4781	NFIB	0.667
ENSG00000081026	260425	MAGI3	0.543
ENSG00000171067	53838	C11orf24	0.587
ENSG00000145675	5295	PIK3R1	0.629
ENSG00000120875	1846	DUSP4	0.601
ENSG00000116754	9295	SFRS11	0.6
ENSG00000183044	18	ABAT	0.652
ENSG00000188001	285386	TPRG1	0.485
ENSG00000143028	284612	SYPL2	0.385
ENSG00000180357	23060	ZNF609	0.56
ENSG00000180304	4947	OAZ2	0.559
ENSG00000183638	94137	RP1L1	0.315
ENSG00000162650	127002	ATXN7L2	0.333
ENSG00000122756	1271	CNTFR	0.482
ENSG00000124193	6431	SFRS6	0.637
ENSG00000149294	4684	NCAM1	0.659
ENSG00000132819	55544	RBM38	0.532
ENSG00000166831	348093	RBPMS2	0.416
ENSG00000066468	2263	FGFR2	0.641
ENSG00000234545	257415	FAM133B	0.407
ENSG00000133704	10526	IPO8	0.496
ENSG00000143514	7159	TP53BP2	0.538
ENSG00000073282	8626	TP63	0.635
ENSG00000126351	7067	THRA	0.682
ENSG00000121281	113	ADCY7	0.574
ENSG00000167105	162461	TMEM92	0.466
ENSG00000140650	5373	PMM2	0.557
ENSG00000241839	80301	PLEKHO2	0.52
ENSG00000123360	5153	PDE1B	0.463
ENSG00000121058	8161	COIL	0.569
ENSG00000015532	64132	XYLT2	0.481
ENSG00000110888	65981	CAPRIN2	0.531
ENSG00000196507	85012	TCEAL3	0.509
ENSG00000174780	6731	SRP72	0.645
ENSG00000139737	122060	SLAIN1	0.478

ENSG00000168090	10980	COPS6	0.596
ENSG00000123562	9643	MORF4L2	0.564
ENSG00000084112	54434	SSH1	0.58
ENSG00000115041	30818	KCNIP3	0.563
ENSG00000103710	51285	RASL12	0.45
ENSG00000135447	5502	PPP1R1A	0.507
ENSG00000151240	22982	DIP2C	0.594
ENSG00000174151	284613	CYB561D1	0.596
ENSG00000171056	83595	SOX7	0.578
ENSG00000090470	10081	PDCD7	0.554
ENSG00000175893	340481	ZDHHC21	0.608
ENSG00000087299	79944	L2HGDH	0.475
ENSG00000124151	8202	NCOA3	0.638
ENSG00000187555	7874	USP7	0.59
ENSG00000105810	1021	CDK6	0.571
ENSG00000136160	1910	EDNRB	0.568
ENSG00000169221	26000	TBC1D10B	0.51
ENSG00000152558	114908	TMEM123	0.528
ENSG00000094975	51430	C1orf9	0.514
ENSG00000156508	1915	EEF1A1	0.592
ENSG00000166716	9640	ZNF592	0.541
ENSG00000138617	54956	PARP16	0.485
ENSG00000163347	9076	CLDN1	0.512
ENSG00000121057	8165	AKAP1	0.64
ENSG00000136383	57538	ALPK3	0.492
ENSG00000170852	25948	KBTBD2	0.592
ENSG00000131653	84231	TRAF7	0.541
ENSG00000218823	56903	PAPOLB	0.407
ENSG00000165119	3190	HNRNPK	0.569
ENSG00000049323	4052	LTBP1	0.591
ENSG00000196083	3556	IL1RAP	0.583
ENSG00000106692	2218	FKTN	0.477
ENSG00000122515	83637	ZMIZ2	0.606
ENSG00000065135	2773	GNAI3	0.578
ENSG00000171044	286046	XKR6	0.549
ENSG00000152192	5457	POU4F1	0.529
ENSG00000152193	79596	RNF219	0.511
ENSG00000104643	66036	MTMR9	0.614
ENSG00000111752	1911	PHC1	0.482
ENSG00000139746	64062	RBM26	0.608
ENSG00000099250	8829	NRP1	0.636
ENSG00000108829	55379	LRRC59	0.512
ENSG00000167971	57524	CASKIN1	0.514
ENSG00000118454	81573	ANKRD13C	0.529
ENSG00000074696	51495	PTPLAD1	0.576
ENSG00000005955	79893	GGNBP2	0.549
ENSG00000116254	26038	CHD5	0.551
ENSG00000182831	29035	C16orf72	0.578
ENSG00000073417	5151	PDE8A	0.564
ENSG00000108349	22794	CASC3	0.514
ENSG00000003056	4074	M6PR	0.577
ENSG00000183454	2903	GRIN2A	0.499
ENSG00000138614	81556	C15orf44	0.449
ENSG00000181619	64582	GPR135	0.555

ENSG00000157954	26100	WIPI2	0.603
ENSG00000101557	9097	USP14	0.577
ENSG00000108352	51195	RAPGEFL1	0.437
ENSG00000159714	29800	ZDHHC1	0.524
ENSG00000185697	4603	MYBL1	0.556
ENSG00000108788	6945	MLX	0.627
ENSG00000139146	58516	FAM60A	0.561
ENSG00000074621	9187	SLC24A1	0.632
ENSG00000148143	58499	ZNF462	0.569
ENSG00000136158	10253	SPRY2	0.542
ENSG00000153944	124540	MSI2	0.588
ENSG00000186187	84937	ZNRF1	0.585
ENSG00000133874	79845	RNF122	0.461
ENSG00000180611	151963	C3orf59	0.486
ENSG00000162415	57643	ZSWIM5	0.456
ENSG00000188042	10123	ARL4C	0.647
ENSG00000178235	114798	SLITRK1	0.422
ENSG00000184564	84189	SLITRK6	0.521
ENSG00000166111	55530	SVOP	0.471
ENSG00000165300	26050	SLITRK5	0.474
ENSG00000154319	83648	FAM167A	0.605
ENSG00000130147	23677	SH3BP4	0.5
ENSG00000117586	7292	TNFSF4	0.492
ENSG00000116337	271	AMPD2	0.534
ENSG00000177485	10009	ZBTB33	0.52
ENSG00000081019	54665	RSBN1	0.541
ENSG00000196715	154807	VKORC1L1	0.558
ENSG00000170776	11214	AKAP13	0.636
ENSG00000182199	6472	SHMT2	0.599
ENSG00000156687	137970	UNC5D	0.365
ENSG00000050628	5733	PTGER3	0.695
ENSG00000175073	80124	VCPIP1	0.44
ENSG00000087095	51701	NLK	0.513
ENSG00000171475	147179	WIPF2	0.605
ENSG00000125355	55026	FAM70A	0.518
ENSG00000175550	10589	DRAP1	0.527
ENSG00000135093	84749	USP30	0.599
ENSG00000120549	56243	KIAA1217	0.577
ENSG00000174718	55196	C12orf35	0.476
ENSG00000103769	8766	RAB11A	0.589
ENSG00000146676	5814	PURB	0.617
ENSG00000104205	23678	SGK3	0.511
ENSG00000182108	28955	DEXI	0.504
ENSG00000151746	636	BICD1	0.558
ENSG00000038532	23274	CLEC16A	0.5
ENSG00000074657	55205	ZNF532	0.498
ENSG00000124225	56937	PMEPA1	0.546
ENSG00000198836	4976	OPA1	0.603
ENSG00000119318	5887	RAD23B	0.58
ENSG00000005844	3683	ITGAL	0.515
ENSG00000177606	3725	JUN	0.673
ENSG00000157890	84465	MEGF11	0.459
ENSG00000039523	79567	FAM65A	0.575
ENSG00000141699	162427	FAM134C	0.522

ENSG00000180891	404093	CUEDC1	0.483
ENSG00000088451	23483	TGDS	0.526
ENSG00000132485	9406	ZRANB2	0.577
ENSG00000136457	1101	CHAD	0.459
ENSG00000152749	160897	GPR180	0.512
ENSG00000140807	85407	NKD1	0.508
ENSG00000125285	11166	SOX21	0.44
ENSG00000125257	10257	ABCC4	0.491
ENSG00000149295	1813	DRD2	0.623
ENSG00000137745	4322	MMP13	0.484
ENSG00000131759	5914	RARA	0.617
ENSG00000136826	9314	KLF4	0.593
ENSG00000004948	799	CALCR	0.542
ENSG00000136574	2626	GATA4	0.493
ENSG00000185338	8651	SOCS1	0.638
ENSG00000147475	11160	ERLIN2	0.535
ENSG00000173473	6599	SMARCC1	0.684
ENSG00000110090	1374	CPT1A	0.617
ENSG00000189067	9516	LITAF	0.617
ENSG00000076321	27252	KLHL20	0.592
ENSG00000136280	83605	CCM2	0.521
ENSG00000087470	10059	DNM1L	0.569
ENSG00000164985	11168	PSIP1	0.606
ENSG00000176274	401612	MCART6	0.46
ENSG00000139970	6252	RTN1	0.595
ENSG00000107863	57584	ARHGAP21	0.548
ENSG00000205937	10921	RNPS1	0.584
ENSG00000109084	27346	TMEM97	0.654
ENSG00000139324	160418	TMTC3	0.54
ENSG00000176105	7525	YES1	0.622
ENSG00000124942	79026	AHNAK	0.612
ENSG00000124126	57580	PREX1	0.503
ENSG00000204371	10919	EHMT2	0.557
ENSG00000141433	116	ADCYAP1	0.5
ENSG00000114315	3280	HES1	0.62
ENSG00000172061	131578	LRRC15	0.5
ENSG00000083799	1540	CYLD	0.666
ENSG00000148053	4915	NTRK2	0.63
ENSG00000132142	31	ACACA	0.577
ENSG00000163359	1293	COL6A3	0.534
ENSG00000169955	65988	ZNF747	0.502
ENSG00000136451	7716	VEZF1	0.662
ENSG00000172020	2596	GAP43	0.574
ENSG00000182095	84629	TNRC18	0.554
ENSG00000134873	9071	CLDN10	0.513
ENSG00000197406	1735	DIO3	0.465
ENSG00000184602	8303	SNN	0.57
ENSG00000172260	257194	NEGR1	0.574
ENSG00000169032	5604	MAP2K1	0.544
ENSG00000078304	5527	PPP2R5C	0.636
ENSG00000197948	89848	FCHSD1	0.486
ENSG00000101892	23439	ATP1B4	0.462
ENSG00000049130	4254	KITLG	0.517
ENSG00000140538	4916	NTRK3	0.609

ENSG00000119812	25940	FAM98A	0.522
ENSG00000144847	152404	IGSF11	0.434
ENSG00000147471	11212	PROSC	0.635
ENSG00000109079	7126	TNFAIP1	0.575
ENSG00000185633	56901	NDUFA4L2	0.486
ENSG00000115840	8604	SLC25A12	0.559
ENSG00000116251	6146	RPL22	0.66
ENSG00000134874	22873	DZIP1	0.567
ENSG00000156853	115509	ZNF689	0.537
ENSG00000139318	1848	DUSP6	0.616
ENSG00000188761	440603	BCL2L15	0.536
ENSG00000102974	10664	CTCF	0.545
ENSG00000176641	220441	RNF152	0.51
ENSG00000133657	79572	ATP13A3	0.593
ENSG00000121083	140735	DYNLL2	0.463
ENSG00000057294	5318	PKP2	0.552
ENSG00000124209	57403	RAB22A	0.619
ENSG00000141753	3487	IGFBP4	0.532
ENSG00000156453	5097	PCDH1	0.601
ENSG00000175376	84285	EIF1AD	0.528
ENSG00000047056	22884	WDR37	0.549
ENSG00000103342	2935	GSPT1	0.561
ENSG00000135862	3915	LAMC1	0.605
ENSG00000117155	117178	SSX2IP	0.634
ENSG00000132823	51526	C20orf111	0.6
ENSG00000123572	203447	NRK	0.463
ENSG00000134709	51361	HOOK1	0.49
ENSG00000143756	23219	FBXO28	0.592
ENSG00000020181	25960	GPR124	0.574
ENSG00000100503	51199	NIN	0.491
ENSG00000048471	92017	SNX29	0.521
ENSG00000185112	131583	FAM43A	0.463
ENSG00000135414	10220	GDF11	0.534
ENSG00000158290	8450	CUL4B	0.603
ENSG00000124194	78997	GDAP1L1	0.383
ENSG00000132205	84034	EMILIN2	0.468
ENSG00000173391	4973	OLR1	0.491
ENSG00000179912	22864	R3HDM2	0.536
ENSG00000101577	9663	LPIN2	0.605
ENSG00000126353	1236	CCR7	0.479
ENSG00000125968	3397	ID1	0.581
ENSG00000124164	9217	VAPB	0.622
ENSG00000196199	54737	MPHOSPH8	0.529
ENSG00000134444	57614	KIAA1468	0.476
ENSG00000175229	89792	GAL3ST3	0.458
ENSG00000156860	64319	FBRS	0.483
ENSG00000081791	9812	KIAA0141	0.585
ENSG00000204252	3111	HLA-DOA	0.651
ENSG00000164619	168667	BMPER	0.446
ENSG00000108797	8506	CNTNAP1	0.5
ENSG00000114638	7348	UPK1B	0.546
ENSG00000123353	29095	ORMDL2	0.511
ENSG00000073584	6605	SMARCE1	0.595
ENSG00000171552	598	BCL2L1	0.606

ENSG00000118655	64858	DCLRE1B	0.495
ENSG00000108799	2145	EZH1	0.633
ENSG00000150938	51232	CRIM1	0.601
ENSG00000139112	23710	GABARAPL1	0.602
ENSG00000138083	6496	SIX3	0.447
ENSG00000163349	204851	HIPK1	0.595
ENSG00000149480	9219	MTA2	0.487
ENSG00000121578	8702	B4GALT4	0.561
ENSG00000139116	55605	KIF21A	0.461
ENSG00000173068	54796	BNC2	0.535
ENSG00000070961	490	ATP2B1	0.638
ENSG00000185352	266722	HS6ST3	0.583
ENSG00000116237	23463	ICMT	0.601
ENSG00000101076	3172	HNF4A	0.63
ENSG00000164733	1508	CTSB	0.617
ENSG00000124198	10564	ARFGEF2	0.616
ENSG00000170577	10736	SIX2	0.555
ENSG00000135392	85406	DNAJC14	0.474
ENSG00000134121	10752	CHL1	0.459
ENSG00000067057	5214	PFKP	0.521
ENSG00000123342	4327	MMP19	0.488
ENSG00000139793	10150	MBNL2	0.609
ENSG00000104221	55290	BRF2	0.53
ENSG00000080603	10847	SRCAP	0.608
ENSG00000121741	7750	ZMYM2	0.617
ENSG00000157502	139221	MUM1L1	0.449
ENSG00000110092	595	CCND1	0.633
ENSG00000149499	256364	EML3	0.576
ENSG00000146826	55262	C7orf43	0.476
ENSG00000186260	57496	MKL2	0.492
ENSG00000157766	176	ACAN	0.611
ENSG00000124120	79183	TTPAL	0.485
ENSG00000178965	127254	C1orf173	0.414
ENSG00000144355	1745	DLX1	0.458
ENSG00000243335	27342	RABGEF1	0.505
ENSG00000164692	1278	COL1A2	0.58
ENSG00000156675	80223	RAB11FIP1	0.554
ENSG00000158292	387509	GPR153	0.571
ENSG00000164742	107	ADCY1	0.606
ENSG00000005379	9256	BZRAP1	0.524
ENSG00000171055	9637	FEZ2	0.659
ENSG00000103449	6299	SALL1	0.489
ENSG00000121207	9227	LRAT	0.427
ENSG00000124222	8675	STX16	0.669
ENSG00000125249	5911	RAP2A	0.605
ENSG00000176142	55254	TMEM39A	0.512
ENSG00000065150	3843	IPO5	0.615
ENSG00000103460	27324	TOX3	0.535
ENSG00000108094	8453	CUL2	0.542
ENSG00000177426	7050	TGIF1	0.547
ENSG00000170473	84305	WIBG	0.496
ENSG00000185875	79896	THNSL1	0.483
ENSG00000135870	149041	RC3H1	0.436
ENSG00000115844	1746	DLX2	0.596

ENSG00000173852	23333	DPY19L1	0.622
ENSG00000065357	1606	DGKA	0.588
ENSG00000113552	10007	GNPDA1	0.557
ENSG00000154710	27342	RABGEF1	0.505
ENSG00000101306	85366	MYLK2	0.454
ENSG00000151025	57512	GPR158	0.422
ENSG00000070759	10420	TESK2	0.516
ENSG00000139445	121643	FOXN4	0.517
ENSG00000177200	80205	CHD9	0.624
ENSG00000171132	5581	PRKCE	0.549
ENSG00000162065	57465	TBC1D24	0.472
ENSG00000173208	225	ABCD2	0.438
ENSG00000091409	3655	ITGA6	0.625
ENSG00000149599	128853	DUSP15	0.477
ENSG00000122557	64224	HERPUD2	0.517
ENSG00000137478	9873	FCHSD2	0.531
ENSG00000103429	51283	BFAR	0.503
ENSG00000134058	1022	CDK7	0.581
ENSG00000137834	4091	SMAD6	0.632
ENSG00000075618	6624	FSCN1	0.572
ENSG00000171791	596	BCL2	0.667
ENSG00000106144	835	CASP2	0.638
ENSG00000141084	57610	RANBP10	0.558
ENSG00000151229	114134	SLC2A13	0.568
ENSG00000174996	64837	KLC2	0.502
ENSG00000166949	4088	SMAD3	0.705
ENSG00000076351	113235	SLC46A1	0.49
ENSG00000197651	196477	C12orf12	0.34
ENSG00000047849	4134	MAP4	0.648
ENSG00000106609	55069	C7orf42	0.489
ENSG00000011275	54476	RNF216	0.562
ENSG00000141956	63977	PRDM15	0.527
ENSG00000134207	148281	SYT6	0.445
ENSG00000188778	155	ADRB3	0.534
ENSG00000107295	6456	SH3GL2	0.481
ENSG00000121743	2700	GJA3	0.41
ENSG00000119537	2531	KDSR	0.527
ENSG00000213420	221914	GPC2	0.527
ENSG00000121742	10804	GJB6	0.482
ENSG00000075388	2249	FGF4	0.52
ENSG00000122545	989	7-Sep	0.544
ENSG00000197323	51592	TRIM33	0.647
ENSG00000119541	9525	VPS4B	0.513
ENSG00000126524	51119	SBDS	0.446
ENSG00000215440	79716	NPEPL1	0.618
ENSG00000140526	11057	ABHD2	0.614
ENSG00000187840	1978	EIF4EBP1	0.55
ENSG00000222040	151	ADRA2B	0.469
ENSG00000163389	56983	KTELC1	0.474
ENSG00000127995	64921	CASD1	0.517
ENSG00000124214	6780	STAU1	0.61
ENSG00000178031	92949	ADAMTSL1	0.516
ENSG00000131507	80762	NDFIP1	0.558
ENSG00000174903	81876	RAB1B	0.493

ENSG00000171862	5728	PTEN	0.649
ENSG00000122733	23349	KIAA1045	0.51
ENSG00000011465	1634	DCN	0.6
ENSG00000110906	83892	KCTD10	0.555
ENSG00000187678	81848	SPRY4	0.59
ENSG00000061938	10188	TNK2	0.634
ENSG00000132849	10207	INADL	0.543
ENSG00000080824	3320	HSP90AA1	0.664
ENSG00000137094	25822	DNAJB5	0.585
ENSG00000113578	2246	FGF1	0.583
ENSG00000131475	84313	VPS25	0.471
ENSG00000066777	10565	ARFGEF1	0.603
ENSG00000157064	23057	NMNAT2	0.484
ENSG00000126562	65266	WNK4	0.5
ENSG00000089597	23193	GANAB	0.575
ENSG00000095794	1390	CREM	0.629
ENSG00000179889	23042	PDXDC1	0.544
ENSG00000140992	5170	PDPK1	0.595
ENSG00000173821	57674	RNF213	0.597
ENSG00000135052	51280	GOLM1	0.505
ENSG00000158050	1844	DUSP2	0.524
ENSG00000084090	56910	STARD7	0.538
ENSG00000162624	431707	LHX8	0.364
ENSG00000165806	840	CASP7	0.52
ENSG00000146674	3486	IGFBP3	0.585
ENSG00000145819	23092	ARHGAP26	0.549
ENSG00000165280	7415	VCP	0.602
ENSG00000106771	23731	C9orf5	0.615
ENSG00000072274	7037	TFRC	0.632
ENSG00000174871	254263	CNIH2	0.42
ENSG00000088356	81572	PDRG1	0.477
ENSG00000151148	89910	UBE3B	0.628
ENSG00000101331	140706	C20orf160	0.487
ENSG00000101098	140730	RIMS4	0.49
ENSG00000138162	10579	TACC2	0.609
ENSG00000122547	80820	EEPD1	0.549
ENSG00000108375	54894	RNF43	0.461
ENSG00000165376	9075	CLDN2	0.462
ENSG00000060138	8531	CSDA	0.595
ENSG00000015153	10138	YAF2	0.51
ENSG00000131626	8500	PPFIA1	0.659
ENSG00000166913	7529	YWHAB	0.637
ENSG00000167977	54442	KCTD5	0.489
ENSG00000133639	694	BTG1	0.638
ENSG00000198081	7541	ZFP161	0.579
ENSG00000108389	9110	MTMR4	0.586
ENSG00000144619	152330	CNTN4	0.549
ENSG00000140153	91833	WDR20	0.519
ENSG00000082397	23136	EPB41L3	0.582
ENSG00000152256	5163	PDK1	0.522
ENSG00000134283	51535	PPHLN1	0.591
ENSG00000076053	10179	RBM7	0.5
ENSG00000164045	993	CDC25A	0.599
ENSG00000116698	9887	SMG7	0.606

ENSG00000167978	23524	SRRM2	0.629
ENSG00000109103	9094	UNC119	0.512
ENSG00000136205	64759	TNS3	0.535
ENSG00000102189	8411	EEA1	0.53
ENSG00000162923	80232	WDR26	0.573
ENSG00000108387	5414	4-Sep	0.537
ENSG00000113580	2908	NR3C1	0.679
ENSG00000172458	53342	IL17D	0.442
ENSG00000157617	25966	C2CD2	0.535
ENSG00000102870	23361	ZNF629	0.487
ENSG00000132953	64328	XPO4	0.492
ENSG00000055732	55283	MCOLN3	0.482
ENSG00000116016	2034	EPAS1	0.622
ENSG00000166780	89927	C16orf45	0.539
ENSG00000110237	9828	ARHGEF17	0.469
ENSG00000095203	54566	EPB41L4B	0.572
ENSG00000150281	1489	CTF1	0.48
ENSG00000135956	55654	TMEM127	0.492
ENSG00000124228	55661	DDX27	0.587
ENSG00000159784	9715	FAM131B	0.452
ENSG00000138495	10063	COX17	0.546
ENSG00000166783	9665	KIAA0430	0.554
ENSG00000139083	2120	ETV6	0.544
ENSG00000179292	256472	TMEM151A	0.396
ENSG00000158321	26053	AUTS2	0.545
ENSG00000174807	57124	CD248	0.489
ENSG00000099364	54620	FBXL19	0.469
ENSG00000121380	79370	BCL2L14	0.49
ENSG00000103479	5934	RBL2	0.603
ENSG00000125675	2892	GRIA3	0.569
ENSG00000145242	2044	EPHA5	0.557
ENSG00000150457	26524	LATS2	0.549
ENSG00000011426	54443	ANLN	0.529
ENSG00000126581	8678	BECN1	0.584
ENSG00000171396	84616	KRTAP4-4	0.35
ENSG00000132326	8864	PER2	0.609
ENSG00000163704	285368	PRRT3	0.452
ENSG00000070018	4040	LRP6	0.549
ENSG00000054967	84957	RELT	0.444
ENSG00000173276	49854	ZNF295	0.533
ENSG00000213281	4893	NRAS	0.526
ENSG00000101337	9777	TM9SF4	0.59
ENSG00000175938	93129	ORAI3	0.531
ENSG00000173598	11163	NUDT4	0.627
ENSG00000111540	5869	RAB5B	0.514
ENSG00000054965	23201	FAM168A	0.565
ENSG00000184371	1435	CSF1	0.615
ENSG00000099381	9739	SETD1A	0.5
ENSG00000100625	51804	SIX4	0.521
ENSG00000185274	64409	WBSCR17	0.434
ENSG00000166987	114785	MBD6	0.466
ENSG00000242265	23089	PEG10	0.598
ENSG00000009307	7812	CSDE1	0.584
ENSG00000085733	2017	CTTN	0.62

ENSG00000154655	91133	L3MBTL4	0.528
ENSG00000101109	6789	STK4	0.551
ENSG00000174013	200933	FBXO45	0.576
ENSG00000163964	54965	PIGX	0.493
ENSG00000158528	55607	PPP1R9A	0.538
ENSG00000133392	4629	MYH11	0.615
ENSG00000111266	80824	DUSP16	0.517
ENSG00000070159	5774	PTPN3	0.57
ENSG00000124201	57169	ZNFX1	0.472
ENSG00000153904	23576	DDAH1	0.529
ENSG00000132854	163782	KANK4	0.398
ENSG00000126003	5326	PLAGL2	0.563
ENSG00000068024	9759	HDAC4	0.543
ENSG00000101346	23509	POFUT1	0.514
ENSG00000177889	7334	UBE2N	0.671
ENSG00000088756	79822	ARHGAP28	0.535
ENSG00000182985	23705	CADM1	0.627
ENSG00000141141	11056	DDX52	0.533
ENSG00000243444	114299	PALM2	0.468
ENSG00000145817	81555	YIPF5	0.51
ENSG00000168710	10768	AHCYL1	0.66
ENSG00000043591	153	ADRB1	0.482
ENSG00000111269	1389	CREBL2	0.598
ENSG00000103111	22879	MON1B	0.468
ENSG00000125676	57187	THOC2	0.587
ENSG00000111276	1027	CDKN1B	0.617
ENSG00000174576	266743	NPAS4	0.425
ENSG00000165813	55088	C10orf118	0.491
ENSG00000151239	5756	TWF1	0.605
ENSG00000178878	81575	APOLD1	0.481
ENSG00000155849	9844	ELMO1	0.521
ENSG00000183166	83698	CALN1	0.515
ENSG00000174007	84984	C3orf34	0.516
ENSG00000144852	8856	NR1I2	0.532
ENSG00000108100	219771	CCNY	0.537
ENSG00000136754	10006	ABI1	0.602
ENSG00000101350	9371	KIF3B	0.534
ENSG00000157654	445815	PALM2-AKAP2	0.579
ENSG00000142871	3491	CYR61	0.612
ENSG00000180370	5062	PAK2	0.648
ENSG00000099365	112755	STX1B	0.451
ENSG00000129007	91860	CALML4	0.585
ENSG00000171680	57449	PLEKHG5	0.458
ENSG00000196313	9883	POM121	0.488
ENSG00000119231	205564	SENP5	0.625
ENSG00000123411	64375	IKZF4	0.469
ENSG00000124212	5740	PTGIS	0.6
ENSG00000183775	57528	KCTD16	0.418
ENSG00000067082	1316	KLF6	0.634
ENSG00000120251	2891	GRIA2	0.493
ENSG00000143786	149111	CNIH3	0.443
ENSG00000117174	54680	ZNHIT6	0.534
ENSG00000171456	171023	ASXL1	0.605
ENSG00000126814	57570	TRMT5	0.546

ENSG00000180447	2619	GAS1	0.561
ENSG00000171502	255631	COL24A1	0.451
ENSG00000133393	123811	C16orf63	0.55
ENSG00000196730	1612	DAPK1	0.623
ENSG00000139428	326625	MMAB	0.529
ENSG00000186314	153768	PRELID2	0.4
ENSG00000033178	55236	UBA6	0.604
ENSG00000120332	63923	TNN	0.503
ENSG00000171150	9655	SOCS5	0.581
ENSG00000108753	6928	HNF1B	0.553
ENSG00000204231	6257	RXRB	0.61
ENSG00000162105	22941	SHANK2	0.587
ENSG00000143093	85369	FAM40A	0.431
ENSG00000013588	9052	GPRC5A	0.524
ENSG00000174516	246330	PELI3	0.523
ENSG00000103222	4363	ABCC1	0.646
ENSG00000241978	445815	PALM2-AKAP2	0.579
ENSG00000102678	2254	FGF9	0.542
ENSG00000157514	1831	TSC22D3	0.631
ENSG00000120833	8835	SOCS2	0.581
ENSG00000116641	85440	DOCK7	0.533
ENSG00000140873	170692	ADAMTS18	0.517
ENSG00000158470	9334	B4GALT5	0.598
ENSG00000075711	1739	DLG1	0.656
ENSG00000151835	26278	SACS	0.527
ENSG00000180398	90411	MCFD2	0.593
ENSG00000103494	23322	RPGRIP1L	0.48
ENSG00000175582	5870	RAB6A	0.641
ENSG00000152578	2893	GRIA4	0.409
ENSG00000177283	8325	FZD8	0.489
ENSG00000137809	22801	ITGA11	0.49
ENSG00000189079	196528	ARID2	0.567
ENSG00000137573	23213	SULF1	0.588
ENSG00000167395	9726	ZNF646	0.486
ENSG00000197818	23315	SLC9A8	0.522
ENSG00000139926	122786	FRMD6	0.504
ENSG00000146834	56257	MEPCE	0.481
ENSG00000007202	9703	KIAA0100	0.578
ENSG00000127863	55504	TNFRSF19	0.557
ENSG00000131482	2538	G6PC	0.432
ENSG00000173482	5797	PTPRM	0.574
ENSG00000162704	10092	ARPC5	0.526
ENSG00000171843	4300	MLLT3	0.547
ENSG00000077782	2260	FGFR1	0.684
ENSG00000005238	80256	KIAA1539	0.646
ENSG00000139218	9169	SFRS2IP	0.622
ENSG00000197106	388662	SLC6A17	0.545
ENSG00000004799	5166	PDK4	0.492
ENSG00000063660	2817	GPC1	0.578
ENSG00000164741	10395	DLC1	0.642
ENSG00000106346	84132	USP42	0.538
ENSG00000182359	143879	KBTBD3	0.455
ENSG00000163428	116064	LRRC58	0.557
ENSG00000103647	10391	CORO2B	0.523

ENSG00000163430	11167	FSTL1	0.48
ENSG00000116396	3749	KCNC4	0.525
ENSG00000149313	60496	AASDHPPT	0.584
ENSG00000171246	4884	NPTX1	0.47
ENSG00000173801	3728	JUP	0.548
ENSG00000116147	7143	TNR	0.474
ENSG00000170515	5036	PA2G4	0.601
ENSG00000086289	54749	EPDR1	0.539
ENSG00000088305	1789	DNMT3B	0.513
ENSG00000198105	57209	ZNF248	0.519
ENSG00000158480	9825	SPATA2	0.589
ENSG00000188763	8326	FZD9	0.456
ENSG00000124226	55905	RNF114	0.613
ENSG00000111371	81539	SLC38A1	0.57
ENSG00000009954	9031	BAZ1B	0.55
ENSG00000152402	2977	GUCY1A2	0.49
ENSG00000198722	10497	UNC13B	0.514
ENSG00000184005	256435	ST6GALNAC3	0.451
ENSG00000162775	64783	RBM15	0.521
ENSG00000165124	79987	SVEP1	0.557
ENSG00000143344	23179	RGL1	0.527
ENSG00000124216	6615	SNAI1	0.515
ENSG00000117069	81849	ST6GALNAC5	0.497
ENSG00000136758	10730	YME1L1	0.601
ENSG00000124145	6385	SDC4	0.57
ENSG00000166924	222950	C7orf51	0.4
ENSG00000159592	60313	GPBP1L1	0.494
ENSG00000204070	90196	SYS1	0.488
ENSG00000134294	54407	SLC38A2	0.587
ENSG00000184787	7327	UBE2G2	0.548
ENSG00000106483	6424	SFRP4	0.533
ENSG00000010270	83930	STARD3NL	0.472
ENSG00000186469	54331	GNG2	0.651
ENSG00000091009	54439	RBM27	0.569
ENSG00000206418	201475	RAB12	0.557
ENSG00000159792	5681	PSKH1	0.5
ENSG00000101966	331	XIAP	0.533
ENSG00000155980	3798	KIF5A	0.504
ENSG00000109111	6830	SUPT6H	0.58
ENSG00000139438	84915	C12orf34	0.458
ENSG00000139209	55089	SLC38A4	0.433
ENSG00000108395	4591	TRIM37	0.545
ENSG00000181924	51287	CHCHD8	0.521
ENSG00000111199	59341	TRPV4	0.437
ENSG00000106635	9275	BCL7B	0.543
ENSG00000162188	2785	GNG3	0.481
ENSG00000103489	64131	XYLT1	0.499
ENSG00000214753	221092	HNRNPUL2	0.525
ENSG00000172845	6670	SP3	0.536
ENSG00000145016	9711	KIAA0226	0.617
ENSG00000052344	5652	PRSS8	0.505
ENSG00000142892	10026	PIGK	0.517
ENSG00000089902	23186	RCOR1	0.527
ENSG00000135482	84872	ZC3H10	0.43

ENSG00000144840	285282	RABL3	0.571
ENSG00000122068	84248	FYTTD1	0.594
ENSG00000113649	10915	TCERG1	0.551
ENSG00000134198	10100	TSPAN2	0.496
ENSG00000136238	5879	RAC1	0.643
ENSG00000166908	79837	PIP4K2C	0.473
ENSG00000185670	79842	ZBTB3	0.455
ENSG00000077454	4034	LRCH4	0.62
ENSG00000153767	2960	GTF2E1	0.516
ENSG00000143815	3930	LBR	0.553
ENSG00000138604	26035	GLCE	0.518
ENSG00000101367	22919	MAPRE1	0.615
ENSG00000138430	29789	OLA1	0.513
ENSG00000101745	23253	ANKRD12	0.629
ENSG00000101972	10735	STAG2	0.602
ENSG00000087245	4313	MMP2	0.55
ENSG00000240849	387521	TMEM189	0.5
ENSG00000134259	4803	NGF	0.484
ENSG00000079337	10411	RAPGEF3	0.499
ENSG00000137819	54852	PAQR5	0.452
ENSG00000173218	81839	VANGL1	0.542
ENSG00000102221	9767	PHF16	0.48
ENSG00000130294	547	KIF1A	0.565
ENSG00000110675	55531	ELMOD1	0.472
ENSG00000137571	81796	SLCO5A1	0.466
ENSG00000118729	845	CASQ2	0.468
ENSG00000108828	10493	VAT1	0.554
ENSG00000169129	84632	AFAP1L2	0.438
ENSG00000006377	1750	DLX6	0.47
ENSG00000145087	9515	STXBP5L	0.515
ENSG00000105880	1749	DLX5	0.454
ENSG00000174111	30837	SOCS7	0.462
ENSG00000087303	22795	NID2	0.459
ENSG00000109113	83871	RAB34	0.58
ENSG00000178573	4094	MAF	0.627
ENSG00000107897	91452	ACBD5	0.464
ENSG00000139433	51228	GLTP	0.492
ENSG00000146469	7432	VIP	0.46
ENSG00000017797	10928	RALBP1	0.58
ENSG00000196396	5770	PTPN1	0.647
ENSG00000144455	285362	SUMF1	0.504
ENSG00000108830	8153	RND2	0.574
ENSG00000036549	26009	ZZZ3	0.53
ENSG00000205208	201725	C4orf46	0.5
ENSG00000198642	55958	KLHL9	0.562
ENSG00000139437	84260	TCHP	0.531
ENSG00000107140	7016	TESK1	0.537
ENSG00000089280	2521	FUS	0.62
ENSG00000100644	3091	HIF1A	0.568
ENSG00000154380	55740	ENAH	0.552
ENSG00000177551	4808	NHLH2	0.517
ENSG00000157106	23049	SMG1	0.537
ENSG00000198121	1902	LPAR1	0.598
ENSG00000051341	10721	POLQ	0.611

ENSG00000163393	55356	SLC22A15	0.507
ENSG00000180263	55785	FGD6	0.51
ENSG00000165731	5979	RET	0.596
ENSG00000006715	27072	VPS41	0.554
ENSG00000083896	91746	YTHDC1	0.581
ENSG00000198756	23127	GLT25D2	0.526
ENSG00000175567	7351	UCP2	0.588
ENSG00000162231	10482	NXF1	0.548
ENSG00000143933	801	CALM1	0.703
ENSG00000175866	10458	BAIAP2	0.63
ENSG00000115825	23683	PRKD3	0.567
ENSG00000140396	10499	NCOA2	0.558
ENSG00000185811	10320	IKZF1	0.616
ENSG00000154845	9989	PPP4R1	0.525
ENSG00000012048	672	BRCA1	0.603
ENSG00000168229	5729	PTGDR	0.489
ENSG00000106723	10927	SPIN1	0.51
ENSG00000061273	51564	HDAC7	0.513
ENSG00000162804	25992	SNED1	0.58
ENSG00000166266	8065	CUL5	0.579
ENSG00000153982	284161	GDPD1	0.506
ENSG00000152092	460	ASTN1	0.455
ENSG00000117461	8503	PIK3R3	0.643
ENSG00000187140	27022	FOXD3	0.412
ENSG00000147526	6867	TACC1	0.623
ENSG00000136813	23392	KIAA0368	0.601
ENSG00000137101	971	CD72	0.516
ENSG00000136240	11014	KDELR2	0.617
ENSG00000149577	51092	SIDT2	0.576
ENSG00000124171	84612	PARD6B	0.52
ENSG00000124243	55653	BCAS4	0.5
ENSG00000099246	22931	RAB18	0.564
ENSG00000052795	57600	FNIP2	0.537
ENSG00000170537	79905	TMC7	0.444
ENSG00000182872	8241	RBM10	0.564
ENSG00000077254	23032	USP33	0.626
ENSG00000028203	55591	VEZT	0.485
ENSG00000101166	51012	SLMO2	0.519
ENSG00000117472	10103	TSPAN1	0.506
ENSG00000111424	7421	VDR	0.633
ENSG00000140332	7090	TLE3	0.594
ENSG00000149308	4863	NPAT	0.574
ENSG00000124203	128611	ZNF831	0.492
ENSG00000139436	9815	GIT2	0.532
ENSG00000106330	64598	MOSPD3	0.511
ENSG00000124205	1908	EDN3	0.56
ENSG00000091844	26575	RGS17	0.466
ENSG00000169826	55454	CSGALNACT2	0.563
ENSG00000175155	388403	YPEL2	0.479
ENSG00000162777	79961	DENND2D	0.518
ENSG00000162236	6811	STX5	0.492
ENSG00000198915	221002	RASGEF1A	0.451
ENSG00000137522	55298	RNF121	0.482
ENSG00000152779	387700	SLC16A12	0.389

ENSG00000108406	79665	DHX40	0.495
ENSG00000135454	2583	B4GALNT1	0.495
ENSG00000143630	57657	HCN3	0.509
ENSG00000180998	283554	GPR137C	0.435
ENSG00000116667	81563	C1orf21	0.541
ENSG00000139219	1280	COL2A1	0.537
ENSG00000197565	1288	COL4A6	0.629
ENSG00000152782	53354	PANK1	0.536
ENSG00000239306	10432	RBM14	0.551
ENSG00000108292	4302	MLLT6	0.563
ENSG00000116406	80267	EDEM3	0.581
ENSG00000085998	55624	POMGNT1	0.481
ENSG00000137135	84904	C9orf100	0.545
ENSG00000174469	26047	CNTNAP2	0.563
ENSG00000116273	148479	PHF13	0.45
ENSG00000101126	23394	ADNP	0.549
ENSG00000109756	9693	RAPGEF2	0.652
ENSG00000067167	23471	TRAM1	0.65
ENSG00000107105	1993	ELAVL2	0.5
ENSG00000097033	51100	SH3GLB1	0.642
ENSG00000197930	30001	ERO1L	0.472
ENSG00000120159	79886	C9orf82	0.501
ENSG00000143061	3321	IGSF3	0.55
ENSG00000101558	9218	VAPA	0.553
ENSG00000150051	283078	MKX	0.504
ENSG00000137055	9373	PLAA	0.538
ENSG00000079387	29843	SENP1	0.442
ENSG00000169813	3185	HNRNPF	0.519
ENSG00000176428	155382	VPS37D	0.468
ENSG00000176410	84277	DNAJC30	0.512
ENSG00000141367	1213	CLTC	0.579
ENSG00000139613	6601	SMARCC2	0.613
ENSG00000006007	51573	GDE1	0.558
ENSG00000007923	55735	DNAJC11	0.508
ENSG00000100664	1983	EIF5	0.676
ENSG00000165566	219287	FAM123A	0.496
ENSG00000146576	79034	C7orf26	0.607
ENSG00000139505	9107	MTMR6	0.52
ENSG00000113657	1809	DPYSL3	0.544
ENSG00000167264	54920	DUS2L	0.562
ENSG00000119899	26503	SLC17A5	0.527
ENSG00000139496	9818	NUPL1	0.53
ENSG00000106070	2887	GRB10	0.603
ENSG00000164054	51246	SHISA5	0.483
ENSG00000163328	151556	GPR155	0.527
ENSG00000169499	59339	PLEKHA2	0.493
ENSG00000104313	2138	EYA1	0.466
ENSG00000111142	10988	METAP2	0.565
ENSG00000163171	10602	CDC42EP3	0.631
ENSG00000076604	9618	TRAF4	0.574
ENSG00000176049	9832	JAKMIP2	0.501
ENSG00000157087	491	ATP2B2	0.566
ENSG00000153936	9653	HS2ST1	0.589
ENSG00000166454	23300	ATMIN	0.588

ENSG00000134247	5738	PTGFRN	0.541
ENSG00000150054	143098	MPP7	0.545
ENSG00000173848	10276	NET1	0.57
ENSG00000104219	51201	ZDHHC2	0.541
ENSG00000154864	63895	FAM38B	0.484
ENSG00000198797	57795	FAM5B	0.457
ENSG00000143013	8543	LMO4	0.608
ENSG00000156535	135228	CD109	0.62
ENSG00000214517	51400	PPME1	0.572
ENSG00000160613	9159	PCSK7	0.483
ENSG00000171735	23261	CAMTA1	0.506
ENSG00000187764	10507	SEMA4D	0.522
ENSG00000164631	7559	ZNF12	0.514
ENSG00000132932	51761	ATP8A2	0.567
ENSG00000106236	4885	NPTX2	0.523
ENSG00000198791	29883	CNOT7	0.557
ENSG00000065243	5586	PKN2	0.592
ENSG00000137497	4926	NUMA1	0.614
ENSG00000106462	2146	EZH2	0.56
ENSG00000181852	10193	RNF41	0.577
ENSG00000095787	51322	WAC	0.615
ENSG00000177981	140461	ASB8	0.486
ENSG00000106089	6804	STX1A	0.534
ENSG00000107562	6387	CXCL12	0.607
ENSG00000154001	5529	PPP2R5E	0.556
ENSG00000075413	4140	MARK3	0.577
ENSG00000103550	400506	C16orf88	0.528
ENSG00000173065	55731	C17orf63	0.501
ENSG00000174628	124152	IQCK	0.654
ENSG00000115935	7456	WIPF1	0.565
ENSG00000121481	6045	RNF2	0.505
ENSG00000178860	9242	MSC	0.492
ENSG00000146830	64599	GIGYF1	0.479
ENSG00000196367	8295	TRRAP	0.56
ENSG00000167528	121274	ZNF641	0.591
ENSG00000181409	9625	AATK	0.508
ENSG00000168615	8754	ADAM9	0.564
ENSG00000183918	4068	SH2D1A	0.59
ENSG00000180488	374986	FAM73A	0.5
ENSG00000198252	6815	STYX	0.565
ENSG00000171357	541468	C1orf190	0.422
ENSG00000151553	57700	FAM160B1	0.504
ENSG00000121486	81627	C1orf25	0.498
ENSG00000076513	88455	ANKRD13A	0.561
ENSG00000167191	51704	GPRC5B	0.608
ENSG00000137076	7094	TLN1	0.511
ENSG00000054793	10079	ATP9A	0.626
ENSG00000188153	1287	COL4A5	0.518
ENSG00000178202	143888	KDELC2	0.487
ENSG00000139620	54934	C12orf41	0.484
ENSG00000062716	81671	TMEM49	0.5
ENSG00000106077	83451	ABHD11	0.474
ENSG00000127870	6049	RNF6	0.604
ENSG00000162614	91624	NEXN	0.496

ENSG00000132589	2319	FLOT2	0.568
ENSG00000141404	2774	GNAL	0.56
ENSG00000107551	83937	RASSF4	0.562
ENSG00000132825	5509	PPP1R3D	0.55
ENSG00000100522	64841	GNPNAT1	0.516
ENSG00000173786	1267	CNP	0.574
ENSG00000154945	91369	ANKRD40	0.472
ENSG00000165434	283209	PGM2L1	0.586
ENSG00000106078	23242	COBL	0.528
ENSG00000049245	9341	VAMP3	0.62
ENSG00000153721	154043	CNKSR3	0.525
ENSG00000174233	112	ADCY6	0.487
ENSG00000137947	2959	GTF2B	0.567
ENSG00000087258	2775	GNAO1	0.576
ENSG00000009694	10178	ODZ1	0.438
ENSG00000101400	6640	SNTA1	0.518
ENSG00000120162	79817	MOBKL2B	0.523
ENSG00000132128	10489	LRRC41	0.573
ENSG00000049246	8863	PER3	0.498
ENSG00000139645	283373	ANKRD52	0.429
ENSG00000003987	9108	MTMR7	0.496
ENSG00000114268	5210	PFKFB4	0.477
ENSG00000078699	9139	CBFA2T2	0.569
ENSG00000147894	203228	C9orf72	0.507
ENSG00000132964	1024	CDK8	0.582
ENSG00000175538	10008	KCNE3	0.521
ENSG00000165832	142940	TRUB1	0.605
ENSG00000178776	389336	C5orf46	0.482
ENSG00000167535	784	CACNB3	0.584
ENSG00000101115	57167	SALL4	0.463
ENSG00000168539	1128	CHRM1	0.4
ENSG00000198742	57154	SMURF1	0.549
ENSG00000137944	56267	CCBL2	0.546
ENSG00000162613	8880	FUBP1	0.652
ENSG00000054654	23224	SYNE2	0.515
ENSG00000095739	25805	BAMBI	0.544
ENSG00000111799	1303	COL12A1	0.607
ENSG00000107518	26033	ATRNL1	0.52
ENSG00000149289	85463	ZC3H12C	0.484
ENSG00000077514	10714	POLD3	0.526
ENSG00000127688	8139	GAN	0.414
ENSG00000165023	54769	DIRAS2	0.451
ENSG00000132970	10810	WASF3	0.517
ENSG00000137710	5962	RDX	0.624
ENSG00000186318	23621	BACE1	0.553
ENSG00000109118	57649	PHF12	0.496
ENSG00000179242	1002	CDH4	0.555
ENSG00000174243	9416	DDX23	0.575
ENSG00000003989	6542	SLC7A2	0.53
ENSG00000187720	79875	THSD4	0.481
ENSG00000158158	26504	CNNM4	0.488
ENSG00000213079	22828	RBM16	0.519
ENSG00000197321	6840	SVIL	0.595
ENSG00000117481	387338	NSUN4	0.582

ENSG00000134287	377	ARF3	0.59
ENSG00000108423	51174	TUBD1	0.542
ENSG00000167005	11051	NUDT21	0.576
ENSG00000168763	26505	CNNM3	0.493
ENSG00000057608	2665	GDI2	0.614
ENSG00000168256	28511	NKIRAS2	0.563
ENSG00000170088	201931	TMEM192	0.459
ENSG00000125967	63941	NECAB3	0.539
ENSG00000107175	10488	CREB3	0.511
ENSG00000121039	157506	RDH10	0.583
ENSG00000163126	200539	ANKRD23	0.472
ENSG00000129422	57509	MTUS1	0.588
ENSG00000075391	9462	RASAL2	0.576
ENSG00000139537	85478	CCDC65	0.508
ENSG00000062485	1431	CS	0.54
ENSG00000049540	2006	ELN	0.584
ENSG00000163041	3020	H3F3A	0.521
ENSG00000198162	10905	MAN1A2	0.609
ENSG00000108443	6198	RPS6KB1	0.511
ENSG00000141232	10140	TOB1	0.546
ENSG00000171940	7764	ZNF217	0.567
ENSG00000165406	220972	8-Mar	0.464
ENSG00000125084	7471	WNT1	0.436
ENSG00000197381	104	ADARB1	0.621
ENSG00000181418	23109	DDN	0.474
ENSG00000183508	54855	FAM46C	0.478
ENSG00000141401	3613	IMPA2	0.537
ENSG00000181929	5571	PRKAG1	0.52
ENSG00000139266	92979	9-Mar	0.606
ENSG00000114013	942	CD86	0.576
ENSG00000187595	201181	ZNF385C	0.422
ENSG00000169071	4920	ROR2	0.478
ENSG00000101888	55916	NXT2	0.563
ENSG00000197587	127343	DMBX1	0.421
ENSG00000070610	57704	GBA2	0.507
ENSG00000114270	1294	COL7A1	0.603
ENSG00000176076	23630	KCNE1L	0.434
ENSG00000068366	2182	ACSL4	0.533
ENSG00000103064	9057	SLC7A6	0.626
ENSG00000063015	124925	SEZ6	0.477
ENSG00000110274	22897	CEP164	0.57
ENSG00000197024	57541	ZNF398	0.548
ENSG00000066654	55623	THUMPD1	0.585
ENSG00000116473	5906	RAP1A	0.541
ENSG00000122420	5737	PTGFR	0.458
ENSG00000167548	8085	MLL2	0.499
ENSG00000116285	54206	ERRFI1	0.49
ENSG00000157103	6529	SLC6A1	0.468
ENSG00000130429	10095	ARPC1B	0.615
ENSG00000187961	339451	KLHL17	0.468
ENSG00000116539	55870	ASH1L	0.497
ENSG00000125378	652	BMP4	0.566
ENSG00000090054	10558	SPTLC1	0.589
ENSG00000139517	222484	LNX2	0.485

ENSG00000186184	51082	POLR1D	0.544
ENSG00000130699	6874	TAF4	0.559
ENSG00000079277	8569	MKNK1	0.527
ENSG00000137727	57569	ARHGAP20	0.536
ENSG00000137265	3662	IRF4	0.585
ENSG00000052802	6307	SC4MOL	0.586
ENSG00000138081	80204	FBXO11	0.568
ENSG00000175832	2118	ETV4	0.486
ENSG00000036828	846	CASR	0.544
ENSG00000182827	64746	ACBD3	0.576
ENSG00000101132	5203	PFDN4	0.57
ENSG00000165556	1045	CDX2	0.486
ENSG00000172661	253725	FAM21C	0.553
ENSG00000132434	55915	LANCL2	0.546
ENSG00000168758	54910	SEMA4C	0.592
ENSG00000115875	6432	SFRS7	0.67
ENSG00000106244	11333	PDAP1	0.589
ENSG00000139636	55716	LMBR1L	0.477
ENSG00000106415	113263	GLCCI1	0.601
ENSG00000165757	57608	KIAA1462	0.511
ENSG00000149657	149986	LSM14B	0.489
ENSG00000101412	1869	E2F1	0.585
ENSG00000005102	4222	MEOX1	0.473
ENSG00000175318	196996	GRAMD2	0.538
ENSG00000166439	254225	RNF169	0.467
ENSG00000101417	11264	PXMP4	0.49
ENSG00000167941	50964	SOST	0.441
ENSG00000166435	143570	XRRA1	0.462
ENSG00000003147	3382	ICA1	0.639
ENSG00000188215	123879	DCUN1D3	0.5
ENSG00000108774	5878	RAB5C	0.596
ENSG00000196639	3269	HRH1	0.548
ENSG00000142599	473	RERE	0.624
ENSG00000180628	84333	PCGF5	0.591
ENSG00000177103	57453	DSCAML1	0.475
ENSG00000170260	7988	ZNF212	0.504
ENSG00000089558	23415	KCNH4	0.444
ENSG00000108861	1845	DUSP3	0.632
ENSG00000143079	55917	CTTNBP2NL	0.522
ENSG00000038295	7092	TLL1	0.473
ENSG00000124098	116151	C20orf108	0.518
ENSG00000112685	55770	EXOC2	0.467
ENSG00000107968	1326	MAP3K8	0.569
ENSG00000196411	2050	EPHB4	0.58
ENSG00000152520	255967	PAN3	0.544
ENSG00000131711	4131	MAP1B	0.523
ENSG00000119314	9991	ROD1	0.58
ENSG00000160917	10898	CPSF4	0.521
ENSG00000101935	9949	AMMECR1	0.538
ENSG00000161647	4356	MPP3	0.47
ENSG00000165458	3636	INPPL1	0.515
ENSG00000104343	55284	UBE2W	0.508
ENSG00000134245	7482	WNT2B	0.537
ENSG00000116679	10625	IVNS1ABP	0.617

ENSG00000102755	2321	FLT1	0.613
ENSG00000243978	57529	RGAG1	0.38
ENSG00000197045	2764	GMFB	0.602
ENSG00000134109	9695	EDEM1	0.542
ENSG00000007341	54879	ST7L	0.489
ENSG00000114030	3836	KPNA1	0.617
ENSG00000176907	56892	C8orf4	0.482
ENSG00000134278	56907	SPIRE1	0.571
ENSG00000101938	91851	CHRDL1	0.492
ENSG00000188157	375790	AGRN	0.622
ENSG00000117114	23266	LPHN2	0.561
ENSG00000171388	8862	APLN	0.533
ENSG00000116194	9068	ANGPTL1	0.517
ENSG00000122121	7512	XPNPEP2	0.518
ENSG00000196104	50859	SPOCK3	0.496
ENSG00000125970	22913	RALY	0.524
ENSG00000175215	10106	CTDSP2	0.606
ENSG00000125459	55154	MSTO1	0.521
ENSG00000170832	84669	USP32	0.523
ENSG00000196177	36	ACADSB	0.496
ENSG00000184402	26039	SS18L1	0.521
ENSG00000119471	84263	HSDL2	0.588
ENSG00000104332	6422	SFRP1	0.603
ENSG00000147533	51125	GOLGA7	0.513
ENSG00000137817	56965	PARP6	0.535
ENSG00000175606	54968	TMEM70	0.565
ENSG00000197006	51108	METTL9	0.518
ENSG00000122584	30010	NXPH1	0.387
ENSG00000196277	2917	GRM7	0.517
ENSG00000108852	4355	MPP2	0.612
ENSG00000189043	4697	NDUFA4	0.518
ENSG00000142961	148932	MOBKL2C	0.492
ENSG00000123352	65244	SPATS2	0.537
ENSG00000184743	25923	ATL3	0.486
ENSG00000116199	9917	FAM20B	0.608
ENSG00000170802	3344	FOXN2	0.472
ENSG00000123472	64756	ATPAF1	0.596
ENSG00000118363	9789	SPCS2	0.537
ENSG00000188706	51114	ZDHHC9	0.538
ENSG00000154518	518	ATP5G3	0.587
ENSG00000106443	9678	PHF14	0.457
ENSG00000104369	56704	JPH1	0.487
ENSG00000092607	6913	TBX15	0.469
ENSG00000144560	9686	VGLL4	0.606
ENSG00000144320	80856	KIAA1715	0.566
ENSG00000101624	79959	CEP76	0.51
ENSG00000140945	1012	CDH13	0.518
ENSG00000135519	23416	KCNH3	0.443
ENSG00000133318	10313	RTN3	0.54
ENSG00000104381	54332	GDAP1	0.477
ENSG00000103056	55512	SMPD3	0.476
ENSG00000165868	259217	HSPA12A	0.564
ENSG00000163840	151636	DTX3L	0.508
ENSG00000143322	27	ABL2	0.486

ENSG00000112701	26054	SENP6	0.575
ENSG00000181220	155061	ZNF746	0.471
ENSG00000125977	8894	EIF2S2	0.581
ENSG00000164270	3360	HTR4	0.626
ENSG00000062725	10513	APPBP2	0.629
ENSG00000159658	9813	KIAA0494	0.638
ENSG00000137747	84000	TMPRSS13	0.55
ENSG00000101180	11255	HRH3	0.569
ENSG00000112511	5252	PHF1	0.589
ENSG00000148158	401548	SNX30	0.455
ENSG00000130703	9885	OSBPL2	0.603
ENSG00000139514	6541	SLC7A1	0.664
ENSG00000168610	6774	STAT3	0.609
ENSG00000101444	191	AHCY	0.57
ENSG00000184939	146198	ZFP90	0.568
ENSG00000107854	80351	TNKS2	0.522
ENSG00000137558	51050	PI15	0.461
ENSG00000187164	57698	KIAA1598	0.49
ENSG00000121005	83690	CRISPLD1	0.473
ENSG00000049618	57492	ARID1B	0.489
ENSG00000122042	5412	UBL3	0.595
ENSG00000175354	5771	PTPN2	0.639
ENSG00000230989	3281	HSBP1	0.615
ENSG00000078747	83737	ITCH	0.634
ENSG00000102781	84056	KATNAL1	0.554
ENSG00000171282	57597	BAHCC1	0.49
ENSG00000077274	827	CAPN6	0.552
ENSG00000189403	3146	HMGB1	0.628
ENSG00000106460	54664	TMEM106B	0.552
ENSG00000175267	146177	VWA3A	0.374
ENSG00000029534	286	ANK1	0.611
ENSG00000128714	3239	HOXD13	0.519
ENSG00000161791	91010	FMNL3	0.552
ENSG00000122707	8434	RECK	0.649
ENSG00000161654	124801	LSM12	0.562
ENSG00000119321	23307	FKBP15	0.613
ENSG00000169252	154	ADRB2	0.51
ENSG00000018189	22902	RUFY3	0.665
ENSG00000169247	79628	SH3TC2	0.483
ENSG00000164749	3174	HNF4G	0.445
ENSG00000137486	408	ARRB1	0.549
ENSG00000103319	29904	EEF2K	0.56
ENSG00000077279	1641	DCX	0.524
ENSG00000213614	3073	HEXA	0.618
ENSG00000135655	9958	USP15	0.563
ENSG00000143067	90874	ZNF697	0.475
ENSG00000078061	369	ARAF	0.516
ENSG00000115073	10120	ACTR1B	0.492
ENSG00000115904	6654	SOS1	0.593
ENSG00000128710	3236	HOXD10	0.467
ENSG00000108256	57532	NUFIP2	0.569
ENSG00000141349	92579	G6PC3	0.573
ENSG00000160551	57551	TAOK1	0.484
ENSG00000075415	5250	SLC25A3	0.535

ENSG00000164379	94234	FOXQ1	0.495
ENSG00000106683	3984	LIMK1	0.607
ENSG00000137273	2295	FOXF2	0.527
ENSG00000054598	2296	FOXC1	0.535
ENSG00000128652	3232	HOXD3	0.536
ENSG00000184009	71	ACTG1	0.624
ENSG00000083312	3842	TNPO1	0.669
ENSG00000085185	63035	BCORL1	0.486
ENSG00000122482	84146	ZNF644	0.573
ENSG00000167566	57701	KIAA1602	0.389
ENSG00000154478	2849	GPR26	0.329
ENSG00000091656	79776	ZFHX4	0.512
ENSG00000140743	1039	CDR2	0.527
ENSG00000135472	23017	FAIM2	0.547
ENSG00000196850	160760	PPTC7	0.598
ENSG00000101460	84557	MAP1LC3A	0.523
ENSG00000157152	6854	SYN2	0.542
ENSG00000215712	729515	C6orf35	0.5
ENSG00000128645	3231	HOXD1	0.476
ENSG00000143772	3707	ITPKB	0.521
ENSG00000156011	23362	PSD3	0.602
ENSG00000108840	10014	HDAC5	0.535
ENSG00000196586	4646	MYO6	0.594
ENSG00000168477	7148	TNXB	0.679
ENSG00000243244	11037	STON1	0.501
ENSG00000167580	359	AQP2	0.49
ENSG00000197283	8831	SYNGAP1	0.494
ENSG00000108262	28964	GIT1	0.492
ENSG00000106682	7458	EIF4H	0.521
ENSG00000039068	999	CDH1	0.592
ENSG00000160199	5316	PKNOX1	0.611
ENSG00000136868	1317	SLC31A1	0.529
ENSG00000082684	54437	SEMA5B	0.446
ENSG00000177098	6330	SCN4B	0.405
ENSG00000134250	4853	NOTCH2	0.604
ENSG00000180008	122809	SOCS4	0.457
ENSG00000143801	5664	PSEN2	0.603
ENSG00000061987	23041	MON2	0.523
ENSG00000140943	8720	MBTPS1	0.574
ENSG00000187676	145173	B3GALTL	0.494
ENSG00000122254	9956	HS3ST2	0.489
ENSG00000168175	93487	MAPK1IP1L	0.604
ENSG00000122644	10124	ARL4A	0.53
ENSG00000103404	57478	USP31	0.603
ENSG00000078804	58476	TP53INP2	0.5
ENSG00000173210	22885	ABLIM3	0.538
ENSG00000166828	6340	SCNN1G	0.46
ENSG00000121068	6909	TBX2	0.584
ENSG00000149575	6327	SCN2B	0.511
ENSG00000171033	5569	PKIA	0.519
ENSG00000159921	10020	GNE	0.551
ENSG00000006468	2115	ETV1	0.629
ENSG00000171621	80176	SPSB1	0.5
ENSG00000121075	9496	TBX4	0.424

ENSG00000170144	220988	HNRNPA3	0.633
ENSG00000120868	317	APAF1	0.612
ENSG00000110881	41	ACCN2	0.536
ENSG00000166233	25820	ARIH1	0.622
ENSG00000100711	79038	ZFYVE21	0.491
ENSG00000066117	6602	SMARCD1	0.601
ENSG00000165188	138065	RNF183	0.444
ENSG00000129116	23022	PALLD	0.639
ENSG00000132463	2926	GRSF1	0.632
ENSG00000174721	143282	FGFBP3	0.41
ENSG00000011566	8491	MAP4K3	0.544
ENSG00000104432	3574	IL7	0.501
ENSG00000095564	9044	BTAF1	0.527
ENSG00000103365	23062	GGA2	0.684
ENSG00000030110	578	BAK1	0.547
ENSG00000131067	2686	GGT7	0.566
ENSG00000137075	152006	RNF38	0.542
ENSG00000168675	753	C18orf1	0.63
ENSG00000167588	2819	GPD1	0.56
ENSG00000103160	83693	HSDL1	0.543
ENSG00000130340	51429	SNX9	0.542
ENSG00000103044	3038	HAS3	0.557
ENSG00000167770	55611	OTUB1	0.592
ENSG00000008056	6853	SYN1	0.452
ENSG00000160087	118424	UBE2J2	0.51
ENSG00000088808	23368	PPP1R13B	0.475
ENSG00000196220	9901	SRGAP3	0.56
ENSG00000145949	340156	MYLK4	0.545
ENSG00000162367	6886	TAL1	0.576
ENSG00000140463	585	BBS4	0.52
ENSG00000198720	124930	ANKRD13B	0.462
ENSG00000168591	79089	TMUB2	0.468
ENSG00000096433	3710	ITPR3	0.619
ENSG00000170370	2018	EMX2	0.462
ENSG00000065485	10954	PDIA5	0.536
ENSG00000103353	56061	UBFD1	0.496
ENSG00000149679	81928	CABLES2	0.516
ENSG00000106366	5054	SERPINE1	0.61
ENSG00000107560	22841	RAB11FIP2	0.576
ENSG00000101654	8731	RNMT	0.568
ENSG00000108506	57508	INTS2	0.425
ENSG00000157510	134265	AFAP1L1	0.548
ENSG00000157107	115548	FCHO2	0.504
ENSG00000050405	51474	LIMA1	0.532
ENSG00000018510	8540	AGPS	0.536
ENSG00000139597	90634	N4BP2L1	0.638
ENSG00000087152	56970	ATXN7L3	0.413
ENSG00000107864	22849	CPEB3	0.458
ENSG00000111110	57460	PPM1H	0.518
ENSG00000083168	7994	MYST3	0.576
ENSG00000078269	8871	SYNJ2	0.591
ENSG00000166847	84516	DCTN5	0.554
ENSG00000140853	84166	NLRC5	0.525
ENSG00000158555	81544	GDPD5	0.563

ENSG00000167549	84940	CORO6	0.442
ENSG00000072315	7224	TRPC5	0.395
ENSG00000106367	1174	AP1S1	0.56
ENSG00000159322	83440	ADPGK	0.513
ENSG00000161813	113251	LARP4	0.568
ENSG00000128564	7425	VGF	0.486
ENSG00000108510	9969	MED13	0.61
ENSG00000173542	92597	MOBKL1A	0.469
ENSG00000145439	84869	CBR4	0.517
ENSG00000198060	54708	5-Mar	0.503
ENSG00000126500	23769	FLRT1	0.505
ENSG00000126767	2002	ELK1	0.614
ENSG00000126777	3895	KTN1	0.598
ENSG00000170525	5209	PFKFB3	0.566
ENSG00000183570	54039	PCBP3	0.487
ENSG00000244754	10443	N4BP2L2	0.624
ENSG00000162368	51727	CMPK1	0.494
ENSG00000164284	134266	GRPEL2	0.5
ENSG00000168807	6645	SNTB2	0.624
ENSG00000141298	85464	SSH2	0.551
ENSG00000189319	9679	FAM53B	0.548
ENSG00000197557	92104	TTC30A	0.439
ENSG00000083642	23047	PDS5B	0.58
ENSG00000154447	57630	SH3RF1	0.552
ENSG00000066084	57609	DIP2B	0.549
ENSG00000108312	7343	UBTF	0.59
ENSG00000186564	2306	FOXD2	0.504
ENSG00000128655	50940	PDE11A	0.467
ENSG00000126016	154796	AMOT	0.519
ENSG00000067141	4756	NEO1	0.518
ENSG00000122257	5930	RBBP6	0.644
ENSG00000101188	4923	NTSR1	0.465
ENSG00000106665	7461	CLIP2	0.529
ENSG00000008300	1951	CELSR3	0.544
ENSG00000067900	6093	ROCK1	0.577
ENSG00000147246	3358	HTR2C	0.554
ENSG00000056277	55609	ZNF280C	0.422
ENSG00000101189	55257	C20orf20	0.491
ENSG00000110344	9354	UBE4A	0.524
ENSG00000168137	55209	SETD5	0.552
ENSG00000185432	25840	METTL7A	0.637
ENSG00000110911	4891	SLC11A2	0.617
ENSG00000146872	11011	TLK2	0.581
ENSG00000080493	8671	SLC4A4	0.654
ENSG00000127080	64768	IPPK	0.427
ENSG00000196092	5079	PAX5	0.43
ENSG00000165660	23172	FAM175B	0.556
ENSG00000019995	54764	ZRANB1	0.597
ENSG00000090905	27327	TNRC6A	0.578
ENSG00000151893	143384	C10orf46	0.562
ENSG00000133121	90627	STARD13	0.517
ENSG00000143776	8476	CDC42BPA	0.637
ENSG00000196935	57522	SRGAP1	0.515
ENSG00000113712	1452	CSNK1A1	0.686

ENSG00000185046	56899	ANKS1B	0.476
ENSG00000138622	10021	HCN4	0.429
ENSG00000050426	25875	LETMD1	0.531
ENSG00000156642	27020	NPTN	0.549
ENSG00000179915	9378	NRXN1	0.559
ENSG00000143337	26092	TOR1AIP1	0.622
ENSG00000069702	7049	TGFBR3	0.558
ENSG00000130224	57631	LRCH2	0.436
ENSG00000118058	4297	MLL	0.627
ENSG00000185963	23299	BICD2	0.633
ENSG00000004939	6521	SLC4A1	0.514
ENSG00000175029	1488	CTBP2	0.639
ENSG00000183023	6546	SLC8A1	0.592
ENSG00000167088	6632	SNRPD1	0.602
ENSG00000171533	4135	MAP6	0.592
ENSG00000164338	84135	UTP15	0.555
ENSG00000188613	340719	NANOS1	0.523
ENSG00000103855	80381	CD276	0.547
ENSG00000065675	5588	PRKCQ	0.564
ENSG00000066735	26153	KIF26A	0.504
ENSG00000104365	3551	IKBKB	0.627
ENSG00000179841	9495	AKAP5	0.457
ENSG00000126804	22890	ZBTB1	0.586
ENSG00000213672	51517	NCKIPSD	0.583
ENSG00000011028	9902	MRC2	0.594
ENSG00000100991	26133	TRPC4AP	0.492
ENSG00000155846	133522	PPARGC1B	0.511
ENSG00000158201	171586	ABHD3	0.491
ENSG00000185504	80233	C17orf70	0.484
ENSG00000147416	526	ATP6V1B2	0.547
ENSG00000135837	9857	CEP350	0.652
ENSG00000062282	84649	DGAT2	0.52
ENSG00000132604	7014	TERF2	0.53
ENSG00000184271	5463	POU6F1	0.569
ENSG00000172915	26960	NBEA	0.538
ENSG00000198382	7405	UVRAG	0.53
ENSG00000184575	11260	XPOT	0.561
ENSG00000183283	9802	DAZAP2	0.61
ENSG00000079156	114880	OSBPL6	0.549
ENSG00000102024	5358	PLS3	0.534
ENSG00000061337	11178	LZTS1	0.657
ENSG00000077809	2969	GTF2I	0.472
ENSG00000173511	7423	VEGFB	0.551
ENSG00000173486	2286	FKBP2	0.539
ENSG00000126822	26030	PLEKHG3	0.588
ENSG00000130338	56995	TULP4	0.513
ENSG00000143924	27436	EML4	0.536
ENSG00000168546	2675	GFRA2	0.56
ENSG00000109572	1182	CLCN3	0.593
ENSG00000101752	57534	MIB1	0.622
ENSG00000107938	26098	C10orf137	0.571
ENSG00000165782	90809	TMEM55B	0.458
ENSG00000146433	57583	TMEM181	0.518
ENSG00000156959	375323	LHFPL4	0.394

ENSG00000162374	1996	ELAVL4	0.486
ENSG00000139946	57161	PELI2	0.505
ENSG00000141448	2627	GATA6	0.503
ENSG00000163719	64419	MTMR14	0.469
ENSG00000146425	6993	DYNLT1	0.562
ENSG00000102908	10725	NFAT5	0.588
ENSG00000129038	4016	LOXL1	0.484
ENSG00000182446	55666	NPLOC4	0.495
ENSG00000130227	23039	XPO7	0.588
ENSG00000070269	54916	C14orf101	0.522
ENSG00000134508	91768	CABLES1	0.519
ENSG00000198879	57713	SFMBT2	0.364
ENSG00000131669	4814	NINJ1	0.536
ENSG00000108582	1362	CPD	0.591
ENSG00000123243	80760	ITIH5	0.491
ENSG00000168795	9925	ZBTB5	0.532
ENSG00000198948	9848	MFAP3L	0.584
ENSG00000204310	10554	AGPAT1	0.573
ENSG00000161904	221496	LEMD2	0.529
ENSG00000142700	63950	DMRTA2	0.462
ENSG00000125966	10893	MMP24	0.627
ENSG00000137492	5612	PRKRIR	0.521
ENSG00000101193	54994	C20orf11	0.52
ENSG00000119979	404636	FAM45A	0.479
ENSG00000070182	6710	SPTB	0.607
ENSG00000180660	4081	MAB21L1	0.453
ENSG00000133083	9201	DCLK1	0.515
ENSG00000149582	84866	TMEM25	0.528
ENSG00000135677	2799	GNS	0.649
ENSG00000106546	196	AHR	0.536
ENSG00000073756	5743	PTGS2	0.565
ENSG00000003096	90293	KLHL13	0.481
ENSG00000182578	1436	CSF1R	0.532
ENSG00000165675	10495	ENOX2	0.573
ENSG00000071189	23161	SNX13	0.606
ENSG00000115944	9167	COX7A2L	0.518
ENSG00000030582	2896	GRN	0.63
ENSG00000147912	26267	FBXO10	0.532
ENSG00000116580	54856	GON4L	0.56
ENSG00000101782	8780	RIOK3	0.656
ENSG00000149658	54915	YTHDF1	0.477
ENSG00000108587	9527	GOSR1	0.624
ENSG00000204308	6048	RNF5	0.596
ENSG00000170921	26115	TANC2	0.5
ENSG00000124493	2914	GRM4	0.513
ENSG00000101019	55245	UQCC	0.537
ENSG00000165588	5015	OTX2	0.458
ENSG00000131725	54521	WDR44	0.462
ENSG00000167178	57611	ISLR2	0.421
ENSG00000198373	11060	WWP2	0.607
ENSG00000123091	26994	RNF11	0.505
ENSG00000143324	9213	XPR1	0.568
ENSG00000164691	117289	TAGAP	0.525
ENSG00000085831	22996	TTC39A	0.608

ENSG00000109586	51809	GALNT7	0.556
ENSG00000182704	25987	TSKU	0.49
ENSG00000174106	23592	LEMD3	0.508
ENSG00000116095	65977	PLEKHA3	0.548
ENSG00000147255	3547	IGSF1	0.489
ENSG00000176390	51379	CRLF3	0.539
ENSG00000070367	10640	EXOC5	0.527
ENSG00000048828	23196	FAM120A	0.62
ENSG00000095139	372	ARCN1	0.509
ENSG00000158856	2039	EPB49	0.521
ENSG00000162670	339479	FAM5C	0.465
ENSG00000160208	23076	RRP1B	0.602
ENSG00000137309	3159	HMGA1	0.608
ENSG00000144712	23066	CAND2	0.497
ENSG00000019144	23187	PHLDB1	0.59
ENSG00000179520	246213	SLC17A8	0.429
ENSG00000168575	6575	SLC20A2	0.516
ENSG00000174099	253827	MSRB3	0.593
ENSG00000173153	2101	ESRRA	0.55
ENSG00000197724	5253	PHF2	0.578
ENSG00000133111	5994	RFXAP	0.467
ENSG00000149260	726	CAPN5	0.496
ENSG00000067208	7813	EVI5	0.586
ENSG00000102934	51090	PLLP	0.569
ENSG00000102962	6367	CCL22	0.491
ENSG00000186566	23131	GPATCH8	0.566
ENSG00000149948	8091	HMGA2	0.557
ENSG00000168071	283234	CCDC88B	0.393
ENSG00000158079	138639	PTPDC1	0.476
ENSG00000139998	376267	RAB15	0.587
ENSG00000006210	6376	CX3CL1	0.555
ENSG00000085832	2060	EPS15	0.596
ENSG00000156983	7862	BRPF1	0.489
ENSG00000107338	6461	SHB	0.575
ENSG00000135823	10228	STX6	0.64
ENSG00000160145	8997	KALRN	0.562
ENSG00000204304	5089	PBX2	0.584
ENSG00000198909	4215	MAP3K3	0.523
ENSG00000196712	4763	NF1	0.713
ENSG00000221978	81669	CCNL2	0.458
ENSG00000151812	341880	SLC35F4	0.38
ENSG00000158863	64760	FAM160B2	0.517
ENSG00000144711	9922	IQSEC1	0.633
ENSG00000090857	55066	PDPR	0.503
ENSG00000070808	815	CAMK2A	0.508
ENSG00000213240	388677	NOTCH2NL	0.539
ENSG00000116741	5997	RGS2	0.561
ENSG00000154511	388650	FAM69A	0.509
ENSG00000148110	84641	HIATL1	0.5
ENSG00000125952	4149	MAX	0.655
ENSG00000164107	9464	HAND2	0.426
ENSG00000164117	26269	FBXO8	0.503
ENSG00000143033	22823	MTF2	0.627
ENSG00000162302	8986	RPS6KA4	0.512

ENSG00000136490	80774	LIMD2	0.476
ENSG00000117859	114883	OSBPL9	0.484
ENSG00000168172	84376	HOOK3	0.504
ENSG00000124507	29993	PACSIN1	0.474
ENSG00000116750	51377	UCHL5	0.578
ENSG00000164118	80817	KIAA1712	0.553
ENSG00000168453	55806	HR	0.531
ENSG00000073670	4185	ADAM11	0.493
ENSG00000110367	1656	DDX6	0.455
ENSG00000070814	6949	TCOF1	0.596
ENSG00000132182	23225	NUP210	0.64
ENSG00000149269	5058	PAK1	0.553
ENSG00000160683	643	CXCR5	0.555
ENSG00000122691	7291	TWIST1	0.526
ENSG00000123728	57826	RAP2C	0.566
ENSG00000173452	256130	TMEM196	0.384
ENSG00000107485	2625	GATA3	0.582
ENSG00000186174	283149	BCL9L	0.5
ENSG00000182963	10052	GJC1	0.547
ENSG00000105855	3696	ITGB8	0.574
ENSG00000168476	80346	REEP4	0.498
ENSG00000076770	55796	MBNL3	0.461
ENSG00000146457	9589	WTAP	0.645
ENSG00000102978	5432	POLR2C	0.601
ENSG00000196821	64771	C6orf106	0.591
ENSG00000120805	400	ARL1	0.62
ENSG00000117289	10628	TXNIP	0.621
ENSG00000145451	8001	GLRA3	0.536
ENSG00000117500	50999	TMED5	0.58
ENSG00000181039	284615	ANKRD34A	0.352
ENSG00000127946	3092	HIP1	0.542
ENSG00000137266	63027	SLC22A23	0.614
ENSG00000131795	9939	RBM8A	0.625
ENSG00000198231	11325	DDX42	0.538
ENSG00000110076	9379	NRXN2	0.511
ENSG00000132718	23208	SYT11	0.539
ENSG00000065060	54887	UHRF1BP1	0.547
ENSG00000143622	6016	RIT1	0.569
ENSG00000164651	221833	SP8	0.477
ENSG00000150625	2823	GPM6A	0.539
ENSG00000105866	6671	SP4	0.479
ENSG00000143127	8515	ITGA10	0.515
ENSG00000148429	9712	USP6NL	0.48
ENSG00000148848	8038	ADAM12	0.593
ENSG00000173706	57493	HEG1	0.609
ENSG00000149428	10525	HYOU1	0.538
ENSG00000064999	23294	ANKS1A	0.543
ENSG00000116747	6738	TROVE2	0.674
ENSG00000127948	5447	POR	0.515
ENSG00000115306	6711	SPTBN1	0.701
ENSG00000138180	55165	CEP55	0.525
ENSG00000137942	54874	FNBP1L	0.525
ENSG00000068354	4943	TBC1D25	0.485
ENSG00000171723	10243	GPHN	0.537

ENSG00000070614	3340	NDST1	0.588
ENSG00000131788	10401	PIAS3	0.521
ENSG00000168994	221749	C6orf145	0.556
ENSG00000197081	3482	IGF2R	0.606
ENSG00000185920	5727	PTCH1	0.627
ENSG00000148426	254427	C10orf47	0.485
ENSG00000112739	8899	PRPF4B	0.648
ENSG00000076716	2239	GPC4	0.576
ENSG00000151461	26019	UPF2	0.509
ENSG00000150630	7424	VEGFC	0.519
ENSG00000116584	9181	ARHGEF2	0.629
ENSG00000171992	11346	SYNPO	0.538
ENSG00000108604	6603	SMARCD2	0.569
ENSG00000161048	222236	NAPEPLD	0.528
ENSG00000101216	26205	GMEB2	0.564
ENSG00000102317	5935	RBM3	0.604
ENSG00000131094	10882	C1QL1	0.572
ENSG00000181192	55526	DHTKD1	0.48
ENSG00000163848	7707	ZNF148	0.61
ENSG00000172992	79877	DCAKD	0.545
ENSG00000221869	1052	CEBPD	0.646
ENSG00000154493	118611	C10orf90	0.564
ENSG00000187231	91404	SESTD1	0.519
ENSG00000156531	84295	PHF6	0.553
ENSG00000023909	2730	GCLM	0.543
ENSG00000197457	50861	STMN3	0.486
ENSG00000136448	4836	NMT1	0.659
ENSG00000218336	55714	ODZ3	0.497
ENSG00000157350	6483	ST3GAL2	0.468
ENSG00000132912	51164	DCTN4	0.537
ENSG00000182150	375748	C9orf102	0.358
ENSG00000114520	8723	SNX4	0.614
ENSG00000188486	3014	H2AFX	0.627
ENSG00000157353	197258	FUK	0.433
ENSG00000144331	151126	ZNF385B	0.515
ENSG00000187714	6572	SLC18A3	0.434
ENSG00000172375	9854	C2CD2L	0.525
ENSG00000144909	114885	OSBPL11	0.513
ENSG00000151718	80014	WWC2	0.519
ENSG00000111670	79158	GNPTAB	0.575
ENSG00000188997	283219	KCTD21	0.412
ENSG00000118369	57558	USP35	0.443
ENSG00000197444	55753	OGDHL	0.483
ENSG00000181513	79777	ACBD4	0.474
ENSG00000072415	64398	MPP5	0.517
ENSG00000137962	9411	ARHGAP29	0.528
ENSG00000211445	2878	GPX3	0.567
ENSG00000227345	8505	PARG	0.557
ENSG00000165244	195828	ZNF367	0.477
ENSG00000033327	9846	GAB2	0.559
ENSG00000172350	64137	ABCG4	0.477
ENSG00000156504	159090	FAM122B	0.523
ENSG00000170027	7532	YWHAG	0.484
ENSG00000146700	136853	SRCRB4D	0.423

ENSG00000185624	5034	P4HB	0.6
ENSG00000186834	10614	HEXIM1	0.634
ENSG00000146197	222663	SCUBE3	0.491
ENSG00000101246	10139	ARFRP1	0.557
ENSG00000185619	10336	PCGF3	0.594
ENSG00000170035	10477	UBE2E3	0.538
ENSG00000115232	3676	ITGA4	0.591
ENSG00000081377	8555	CDC14B	0.664
ENSG00000149256	26011	ODZ4	0.522
ENSG00000138293	8031	NCOA4	0.525
ENSG00000168556	3622	ING2	0.572
ENSG00000182552	201965	RWDD4A	0.431
ENSG00000108231	9211	LGI1	0.468
ENSG00000091073	113878	DTX2	0.492
ENSG00000163479	6746	SSR2	0.534
ENSG00000132334	5791	PTPRE	0.563
ENSG00000183049	57118	CAMK1D	0.48
ENSG00000065029	7629	ZNF76	0.524
ENSG00000117525	2152	F3	0.535
ENSG00000168993	10815	CPLX1	0.456
ENSG00000134001	1965	EIF2S1	0.635
ENSG00000101945	6839	SUV39H1	0.516
ENSG00000101928	56180	MOSPD1	0.512
ENSG00000146477	6581	SLC22A3	0.472
ENSG00000123240	10133	OPTN	0.638
ENSG00000017427	3479	IGF1	0.638
ENSG00000165490	220042	C11orf82	0.504
ENSG00000130584	140685	ZBTB46	0.496
ENSG00000101150	7165	TPD52L2	0.515
ENSG00000173320	56977	STOX2	0.55
ENSG00000164303	133121	ENPP6	0.398
ENSG00000137502	27314	RAB30	0.56
ENSG00000141522	396	ARHGDIA	0.586
ENSG00000225190	9842	PLEKHM1	0.56
ENSG00000162992	4760	NEUROD1	0.423
ENSG00000168310	3660	IRF2	0.537
ENSG00000139352	429	ASCL1	0.623
ENSG00000179088	374470	C12orf42	0.465
ENSG00000138434	6744	SSFA2	0.536
ENSG00000117519	1266	CNN3	0.537
ENSG00000168300	115294	PCMTD1	0.557
ENSG00000101152	80331	DNAJC5	0.52
ENSG00000160305	23181	DIP2A	0.5
ENSG00000163884	28999	KLF15	0.428
ENSG00000120088	1394	CRHR1	0.585
ENSG00000198624	26112	CCDC69	0.546
ENSG00000164305	836	CASP3	0.529
ENSG00000147488	9705	ST18	0.48
ENSG00000085511	4216	MAP3K4	0.597
ENSG00000153046	9425	CDYL	0.659
ENSG00000008130	65220	NADK	0.65
ENSG00000137494	338699	ANKRD42	0.475
ENSG00000054690	57475	PLEKHH1	0.562
ENSG00000117569	58155	PTBP2	0.506

ENSG00000112033	5467	PPARD	0.622
ENSG00000160789	4000	LMNA	0.662
ENSG00000196700	57473	ZNF512B	0.593
ENSG00000127947	5782	PTPN12	0.624
ENSG00000123643	206358	SLC36A1	0.512
ENSG00000164896	10922	FASTK	0.602
ENSG00000186868	4137	MAPT	0.645
ENSG00000115252	5136	PDE1A	0.503
ENSG00000130590	140700	SAMD10	0.5
ENSG00000151726	2180	ACSL1	0.6
ENSG00000086475	22929	SEPHS1	0.622
ENSG00000150672	1740	DLG2	0.461
ENSG00000026652	56895	AGPAT4	0.466
ENSG00000086570	2196	FAT2	0.46
ENSG00000162627	51375	SNX7	0.533
ENSG00000124785	51299	NRN1	0.524
ENSG00000187257	222194	RSBN1L	0.542
ENSG00000114554	5361	PLXNA1	0.627
ENSG00000120071	284058	KIAA1267	0.567
ENSG00000006576	57157	PHTF2	0.65
ENSG00000124491	2162	F13A1	0.525
ENSG00000112530	135138	PACRG	0.54
ENSG00000108239	23232	TBC1D12	0.487
ENSG00000119969	3070	HELLS	0.523
ENSG00000112531	9444	QKI	0.698
ENSG00000126768	10245	TIMM17B	0.525
ENSG00000124782	6239	RREB1	0.585
ENSG00000187531	51547	SIRT7	0.548
ENSG00000033050	10061	ABCF2	0.619
ENSG00000155511	2890	GRIA1	0.557
ENSG00000112541	10846	PDE10A	0.532
ENSG00000124783	6745	SSR1	0.628
ENSG00000160785	9673	SLC25A44	0.558
ENSG00000162999	142679	DUSP19	0.507
ENSG00000164458	6862	T	0.454
ENSG00000197063	4097	MAFG	0.567
ENSG00000102100	7355	SLC35A2	0.608
ENSG00000102096	11040	PIM2	0.54
ENSG00000068308	55593	OTUD5	0.539
ENSG00000185269	147111	NOTUM	0.443
ENSG00000102057	3750	KCND1	0.472
ENSG00000092199	3183	HNRNPC	0.648
ENSG00000106615	6009	RHEB	0.625
ENSG00000198952	23381	SMG5	0.558
ENSG00000106617	51422	PRKAG2	0.545
ENSG00000169689	201254	STRA13	0.581
ENSG00000163472	84283	TMEM79	0.451
ENSG00000068323	7030	TFE3	0.581
ENSG00000239264	81567	TXNDC5	0.516
ENSG00000147144	90060	CCDC120	0.504
ENSG00000188428	63915	MUTED	0.527
ENSG00000095637	10580	SORBS1	0.594
ENSG00000092201	11198	SUPT16H	0.525
ENSG00000189292	285016	FAM150B	0.409

ENSG00000055609	58508	MLL3	0.605
ENSG00000137203	7020	TFAP2A	0.624
ENSG00000017621	79917	MAGIX	0.421
ENSG00000129472	84932	RAB2B	0.481
ENSG00000102007	5355	PLP2	0.559
ENSG00000133627	57180	ACTR3B	0.527
ENSG00000092203	9878	TOX4	0.583
ENSG00000111846	2651	GCNT2	0.478
ENSG00000102003	6855	SYP	0.461
ENSG00000130226	1804	DPP6	0.483
ENSG00000130508	7837	PXDN	0.59
ENSG00000116604	4209	MEF2D	0.55
ENSG00000165821	6297	SALL2	0.52
ENSG00000169710	2194	FASN	0.535
ENSG00000150403	55002	TMCO3	0.549
ENSG00000133265	23640	HSPBP1	0.489
ENSG00000218891	163033	ZNF579	0.421
ENSG00000198824	283489	ZNF828	0.511
ENSG00000063245	29924	EPN1	0.463
ENSG00000185418	123283	TARSL2	0.573
ENSG00000198440	147949	ZNF583	0.478
ENSG00000196724	147686	ZNF418	0.414
ENSG00000129048	51554	CCRL1	0.466
ENSG00000144868	66000	TMEM108	0.546
ENSG00000169255	8706	B3GALNT1	0.548
ENSG00000196542	165679	C3orf57	0.338
ENSG00000182132	30820	KCNIP1	0.492
ENSG00000144381	3329	HSPD1	0.592
ENSG00000115541	3336	HSPE1	0.534
ENSG00000115457	3485	IGFBP2	0.547
ENSG00000163466	10109	ARPC2	0.612
ENSG00000163558	5584	PRKCI	0.644
ENSG00000013293	57709	SLC7A14	0.457
ENSG00000158092	4690	NCK1	0.617
ENSG00000174564	53833	IL20RB	0.471
ENSG00000168397	23192	ATG4B	0.496
ENSG00000075651	5337	PLD1	0.602
ENSG00000135912	9654	TTLL4	0.57
ENSG00000171450	8941	CDK5R2	0.456
ENSG00000118322	23120	ATP10B	0.54
ENSG00000145863	2559	GABRA6	0.417
ENSG00000168497	8436	SDPR	0.495
ENSG00000148408	774	CACNA1B	0.477
ENSG00000113645	23286	WWC1	0.58
ENSG00000158526	90121	TSR2	0.469
ENSG00000158714	56833	SLAMF8	0.528
ENSG00000186648	90668	LRRC16B	0.403
ENSG00000120057	6425	SFRP5	0.459
ENSG00000137825	3706	ITPKA	0.46
ENSG00000062524	4058	LTK	0.585
ENSG00000102316	10916	MAGED2	0.611
ENSG00000132518	3000	GUCY2D	0.459
ENSG00000163032	7447	VSNL1	0.564
ENSG00000054116	27095	TRAPPC3	0.582

ENSG00000118960	64342	HS1BP3	0.534
ENSG00000186470	11118	BTN3A2	0.614
ENSG00000066294	8832	CD84	0.61
ENSG00000163026	80304	C2orf44	0.508
ENSG00000142168	6647	SOD1	0.57
ENSG00000181704	286451	YIPF6	0.633
ENSG00000146049	353219	KAAG1	0.373
ENSG00000116497	64766	S100PBP	0.498
ENSG00000176058	286262	C9orf75	0.409
ENSG00000182896	339168	TMEM95	0.415
ENSG00000121900	113452	TMEM54	0.507
ENSG00000134318	9475	ROCK2	0.568
ENSG00000148840	23082	PPRC1	0.527
ENSG00000162746	127943	FCRLB	0.455
ENSG00000212864	727800	RNF208	0.45
ENSG00000107147	57582	KCNT1	0.508
ENSG00000112294	7915	ALDH5A1	0.576
ENSG00000142920	113451	ADC	0.504
ENSG00000137261	9856	KIAA0319	0.459
ENSG00000118217	22926	ATF6	0.597
ENSG00000180787	124961	ZFP3	0.455
ENSG00000239697	8742	TNFSF12	0.49
ENSG00000162512	9672	SDC3	0.51
ENSG00000137404	11270	NRM	0.523
ENSG00000180626	84622	ZNF594	0.412
ENSG00000198574	117157	SH2D1B	0.5
ENSG00000138080	11117	EMILIN1	0.472
ENSG00000067177	5255	PHKA1	0.505
ENSG00000138028	10669	CGREF1	0.45
ENSG00000165689	10807	SDCCAG3	0.497
ENSG00000101280	51378	ANGPT4	0.441
ENSG00000167842	79003	MIS12	0.466
ENSG00000129194	6665	SOX15	0.575
ENSG00000116560	6421	SFPQ	0.675
ENSG00000198920	9851	KIAA0753	0.505
ENSG00000134690	55143	CDCA8	0.508
ENSG00000185090	149175	MANEAL	0.466
ENSG00000197982	127687	C1orf122	0.449
ENSG00000197859	9719	ADAMTSL2	0.47
ENSG00000189298	80317	ZKSCAN3	0.505
ENSG00000125414	4620	MYH2	0.478
ENSG00000075826	25956	SEC31B	0.499
ENSG00000046651	8481	OFD1	0.498
ENSG00000167723	162514	TRPV3	0.485
ENSG00000169756	3987	LIMS1	0.593
ENSG00000055483	57602	USP36	0.542
ENSG00000148935	2620	GAS2	0.467
ENSG00000213213	84960	KIAA1984	0.4
ENSG00000112425	7957	EPM2A	0.586
ENSG00000138764	901	CCNG2	0.652
ENSG00000143450	51686	OAZ3	0.526
ENSG00000134575	53	ACP2	0.52
ENSG00000198168	258010	SVIP	0.539
ENSG00000154654	4685	NCAM2	0.472

ENSG00000149328	89944	GLB1L2	0.551
ENSG00000108839	239	ALOX12	0.472
ENSG00000198746	63906	GPATCH3	0.466
ENSG00000165678	27069	GHITM	0.594
ENSG00000144834	29114	TAGLN3	0.515
ENSG00000172497	134526	ACOT12	0.41
ENSG00000114529	79669	C3orf52	0.52
ENSG00000182255	3739	KCNA4	0.447
ENSG00000164506	134957	STXBP5	0.484
ENSG00000183780	148641	SLC35F3	0.505
ENSG00000213265	114960	TSGA13	0.486
ENSG00000174595	136259	KLF14	0.449
ENSG00000071205	79658	ARHGAP10	0.455
ENSG00000107331	20	ABCA2	0.605
ENSG00000196150	58500	ZNF250	0.453
ENSG00000203782	4014	LOR	0.436
ENSG00000138459	55032	SLC35A5	0.489
ENSG00000108405	5023	P2RX1	0.459
ENSG00000182866	3932	LCK	0.611
ENSG00000143546	6279	S100A8	0.539
ENSG00000049449	5954	RCN1	0.548
ENSG00000038427	1462	VCAN	0.674
ENSG00000141576	114804	RNF157	0.517
ENSG00000138398	9360	PPIG	0.595
ENSG00000170296	11337	GABARAP	0.545
ENSG00000175868	797	CALCB	0.485
ENSG00000163606	131450	CD200R1	0.532
ENSG00000170445	3035	HARS	0.532
ENSG00000131828	5160	PDHA1	0.528
ENSG00000107815	56652	C10orf2	0.537
ENSG00000181885	1366	CLDN7	0.493
ENSG00000176170	8877	SPHK1	0.498
ENSG00000197448	373156	GSTK1	0.515
ENSG00000185122	3297	HSF1	0.62
ENSG00000129667	79651	RHBDF2	0.475
ENSG00000132522	2874	GPS2	0.479
ENSG00000047634	6322	SCML1	0.505
ENSG00000122783	78996	C7orf49	0.567
ENSG00000184368	256714	MAP7D2	0.4
ENSG00000102104	6247	RS1	0.519
ENSG00000166169	27343	POLL	0.455
ENSG00000170270	84520	C14orf142	0.509
ENSG00000011198	51099	ABHD5	0.607
ENSG00000158882	84134	TOMM40L	0.507
ENSG00000179152	285343	C3orf23	0.517
ENSG00000163281	132789	GNPDA2	0.496
ENSG00000117215	26279	PLA2G2D	0.498
ENSG00000158786	64600	PLA2G2F	0.46
ENSG00000084754	3030	HADHA	0.619
ENSG00000164512	79722	ANKRD55	0.48
ENSG00000044115	1495	CTNNA1	0.713
ENSG00000138029	3032	HADHB	0.561
ENSG00000090432	79594	MUL1	0.473
ENSG00000136908	8818	DPM2	0.546

ENSG00000244038	1650	DDOST	0.563
ENSG00000064313	6873	TAF2	0.5
ENSG00000179387	255520	ELMOD2	0.509
ENSG00000213347	83463	MXD3	0.473
ENSG00000042781	7399	USH2A	0.442
ENSG00000169230	27166	PRELID1	0.51
ENSG00000062194	65056	GPBP1	0.57
ENSG00000248485	654790	PCP4L1	0.402
ENSG00000117643	57134	MAN1C1	0.577
ENSG00000170448	152518	NFXL1	0.474
ENSG00000015479	9782	MATR3	0.588
ENSG00000097007	25	ABL1	0.601
ENSG00000166478	7702	ZNF143	0.579
ENSG00000109452	8821	INPP4B	0.512
ENSG00000025434	10062	NR1H3	0.549
ENSG00000158008	2134	EXTL1	0.454
ENSG00000109458	2549	GAB1	0.499
ENSG00000129749	57053	CHRNA10	0.479
ENSG00000187824	388335	TMEM220	0.477
ENSG00000111328	8099	CDK2AP1	0.534
ENSG00000112210	51715	RAB23	0.518
ENSG00000184584	340061	TMEM173	0.518
ENSG00000165156	11244	ZHX1	0.513
ENSG00000110514	8567	MADD	0.588
ENSG00000150977	196383	RILPL2	0.48
ENSG00000181804	285195	SLC9A9	0.504
ENSG00000171604	51523	CXXC5	0.569
ENSG00000170180	2993	GYPA	0.588
ENSG00000152952	5352	PLOD2	0.595
ENSG00000123607	79809	TTC21B	0.456
ENSG00000164161	64399	HHIP	0.5
ENSG00000111361	1967	EIF2B1	0.512
ENSG00000132305	10989	IMMT	0.524
ENSG00000115839	22930	RAB3GAP1	0.612
ENSG00000162813	10380	BPNT1	0.446
ENSG00000113073	83697	SLC4A9	0.469
ENSG00000006695	1352	COX10	0.488
ENSG00000169131	6940	ZNF354A	0.516
ENSG00000164023	166929	SGMS2	0.557
ENSG00000164244	133619	PRRC1	0.564
ENSG00000132622	116835	HSPA12B	0.462
ENSG00000239704	284040	CDRT4	0.5
ENSG00000138796	3033	HADH	0.614
ENSG00000178425	221294	NT5DC1	0.493
ENSG00000102172	6611	SMS	0.539
ENSG00000189241	7259	TSPYL1	0.515
ENSG00000107290	23064	SETX	0.583
ENSG00000179564	132228	C3orf45	0.406
ENSG00000214706	7866	IFRD2	0.549
ENSG00000122779	8805	TRIM24	0.56
ENSG00000166823	55897	MESP1	0.584
ENSG00000170425	136	ADORA2B	0.515
ENSG00000121966	7852	CXCR4	0.585
ENSG00000130066	6303	SAT1	0.615

ENSG00000043355	7546	ZIC2	0.54
ENSG00000088826	54498	SMOX	0.607
ENSG00000198108	337876	CHSY3	0.454
ENSG00000154556	8470	SORBS2	0.593
ENSG00000086619	56605	ERO1LB	0.506
ENSG00000130741	1968	EIF2S3	0.56
ENSG00000136699	55627	SMPD4	0.575
ENSG00000187715	166348	KLHDC6	0.429
ENSG00000170162	245806	VGLL2	0.449
ENSG00000105939	56829	ZC3HAV1	0.586
ENSG00000146376	93663	ARHGAP18	0.565
ENSG00000105948	79989	TTC26	0.446
ENSG00000167861	283987	C17orf28	0.423
ENSG00000163902	6184	RPN1	0.533
ENSG00000170271	10826	C5orf4	0.603
ENSG00000003436	7035	TFPI	0.617
ENSG00000120280	80231	CXorf21	0.439
ENSG00000056998	8908	GYG2	0.587
ENSG00000170522	79071	ELOVL6	0.608
ENSG00000082515	29093	MRPL22	0.552
ENSG00000168542	1281	COL3A1	0.567
ENSG00000136830	64855	FAM129B	0.531
ENSG00000239388	142686	ASB14	0.439
ENSG00000181789	22820	COPG	0.478
ENSG00000164185	133923	ZNF474	0.458
ENSG00000089685	332	BIRC5	0.619
ENSG00000106302	23553	HYAL4	0.444
ENSG00000158104	3242	HPD	0.482
ENSG00000174840	201626	PDE12	0.469
ENSG00000178171	205147	FAM123C	0.429
ENSG00000179059	132625	ZFP42	0.464
ENSG00000157800	84255	SLC37A3	0.45
ENSG00000114738	7867	MAPKAPK3	0.625
ENSG00000179562	79571	GCC1	0.523
ENSG00000170571	133418	EMB	0.6
ENSG00000115350	56655	POLE4	0.527
ENSG00000130779	6249	CLIP1	0.566
ENSG00000136807	1025	CDK9	0.541
ENSG00000107731	219699	UNC5B	0.509
ENSG00000096092	28978	TMEM14A	0.559
ENSG00000004961	3052	HCCS	0.573
ENSG00000198211	10381	TUBB3	0.593
ENSG00000040487	54896	PQLC2	0.485
ENSG00000182173	283989	TSEN54	0.558
ENSG00000165055	55798	METTL2B	0.487
ENSG00000174915	81490	PTDSS2	0.479
ENSG00000171700	10287	RGS19	0.51
ENSG00000162688	178	AGL	0.552
ENSG00000115415	6772	STAT1	0.674
ENSG00000093183	9117	SEC22C	0.537
ENSG00000128596	64753	CCDC136	0.562
ENSG00000164920	116039	OSR2	0.526
ENSG00000122435	54482	CCDC76	0.522
ENSG00000172340	8801	SUCLG2	0.592

ENSG00000034510	9168	TMSB10	0.564
ENSG00000115963	390	RND3	0.586
ENSG00000100599	79890	RIN3	0.637
ENSG00000164402	23176	8-Sep	0.567
ENSG00000109133	55161	TMEM33	0.516
ENSG00000186106	157567	ANKRD46	0.513
ENSG00000180432	1582	CYP8B1	0.463
ENSG00000185519	348487	FAM131C	0.415
ENSG00000155329	54819	ZCCHC10	0.485
ENSG00000143499	56950	SMYD2	0.566
ENSG00000170606	3308	HSPA4	0.67
ENSG00000123892	23682	RAB38	0.483
ENSG00000177666	57104	PNPLA2	0.598
ENSG00000163605	151987	PPP4R2	0.414
ENSG00000002919	29916	SNX11	0.562
ENSG00000155087	4956	ODF1	0.46
ENSG00000181781	284451	ODF3L2	0.418
ENSG00000099822	610	HCN2	0.48
ENSG00000182742	3214	HOXB4	0.427
ENSG00000160310	3275	PRMT2	0.632
ENSG00000070404	10272	FSTL3	0.537
ENSG00000113615	10802	SEC24A	0.582
ENSG00000099625	255057	C19orf26	0.456
ENSG00000179097	3355	HTR1F	0.474
ENSG00000063438	57491	AHRR	0.483
ENSG00000022267	2273	FHL1	0.633
ENSG00000138688	84162	KIAA1109	0.63
ENSG00000129911	83855	KLF16	0.455
ENSG00000143851	5778	PTPN7	0.531
ENSG00000184160	152	ADRA2C	0.47
ENSG00000176903	9240	PNMA1	0.545
ENSG00000172081	126308	MOBKL2A	0.515
ENSG00000104886	55111	PLEKHJ1	0.492
ENSG00000032389	7260	TSSC1	0.479
ENSG00000104904	4946	OAZ1	0.565
ENSG00000099800	26517	TIMM13	0.485
ENSG00000176533	2788	GNG7	0.581
ENSG00000130997	353497	POLN	0.436
ENSG00000156920	139378	GPR112	0.4
ENSG00000163382	128240	APOA1BP	0.445
ENSG00000213047	163486	DENND1B	0.561
ENSG00000153395	79888	LPCAT1	0.561
ENSG00000112559	4188	MDFI	0.478
ENSG00000104964	166	AES	0.544
ENSG00000156711	5603	MAPK13	0.578
ENSG00000111859	4739	NEDD9	0.617
ENSG00000011132	9546	APBA3	0.562
ENSG00000080819	1371	CPOX	0.526
ENSG00000023228	4719	NDUFS1	0.554
ENSG00000064225	10402	ST3GAL6	0.6
ENSG00000170549	79192	IRX1	0.427
ENSG00000116857	252839	TMEM9	0.464
ENSG00000172738	221468	TMEM217	0.41
ENSG00000203772	503542	SPRN	0.561

ENSG00000114948	8745	ADAM23	0.525
ENSG00000130254	9667	SAFB2	0.583
ENSG00000172590	122704	MRPL52	0.467
ENSG00000243667	116143	WDR92	0.46
ENSG00000154153	54463	FAM134B	0.592
ENSG00000171119	4902	NRTN	0.425
ENSG00000101977	4168	MCF2	0.566
ENSG00000144895	83939	EIF2A	0.531
ENSG00000130822	139728	PNCK	0.333
ENSG00000163249	151195	CCNYL1	0.567
ENSG00000125656	8192	CLPP	0.521
ENSG00000125657	8744	TNFSF9	0.517
ENSG00000145990	54438	GFOD1	0.536
ENSG00000115956	5341	PLEK	0.565
ENSG00000167565	29946	SERTAD3	0.485
ENSG00000155093	5799	PTPRN2	0.567
ENSG00000160410	92799	SHKBP1	0.486
ENSG00000137168	51645	PPIL1	0.482
ENSG00000114354	10342	TFG	0.565
ENSG00000103966	30844	EHD4	0.526
ENSG00000131386	117248	GALNTL2	0.549
ENSG00000163485	134	ADORA1	0.522
ENSG00000076928	9138	ARHGEF1	0.557
ENSG00000127452	54850	FBXL12	0.516
ENSG00000037280	2324	FLT4	0.493
ENSG00000133048	1116	CHI3L1	0.609
ENSG00000198753	5365	PLXNB3	0.505
ENSG00000013619	10046	MAMLD1	0.471
ENSG00000189377	284340	CXCL17	0.405
ENSG00000130816	1786	DNMT1	0.562
ENSG00000124466	27076	LYPD3	0.528
ENSG00000168214	3516	RBPJ	0.623
ENSG00000104783	3783	KCNN4	0.542
ENSG00000184343	26576	SRPK3	0.475
ENSG00000140326	146059	CDAN1	0.525
ENSG00000146063	90933	TRIM41	0.467
ENSG00000155962	1193	CLIC2	0.522
ENSG00000129354	10053	AP1M2	0.574
ENSG00000153551	112616	CMTM7	0.556
ENSG00000163673	85443	DCLK3	0.391
ENSG00000102181	83692	CD99L2	0.628
ENSG00000104856	5971	RELB	0.546
ENSG00000174899	152078	C3orf55	0.474
ENSG00000109689	57620	STIM2	0.52
ENSG00000168806	9836	LCMT2	0.519
ENSG00000082213	55322	C5orf22	0.517
ENSG00000102119	2010	EMD	0.51
ENSG00000104881	10848	PPP1R13L	0.534
ENSG00000165914	145567	TTC7B	0.474
ENSG00000177464	2828	GPR4	0.543
ENSG00000139880	64403	CDH24	0.54
ENSG00000011478	54814	QPCTL	0.445
ENSG00000113638	23548	TTC33	0.485
ENSG00000102125	6901	TAZ	0.53

ENSG00000158711	2005	ELK4	0.523
ENSG00000093167	9209	LRRFIP2	0.579
ENSG00000198892	149345	SHISA4	0.421
ENSG00000169744	9079	LDB2	0.503
ENSG00000105281	6510	SLC1A5	0.537
ENSG00000105419	56917	MEIS3	0.451
ENSG00000069275	64710	NUCKS1	0.572
ENSG00000133961	8650	NUMB	0.558
ENSG00000105402	8775	NAPA	0.561
ENSG00000151552	5860	QDPR	0.549
ENSG00000169299	55276	PGM2	0.51
ENSG00000100629	145508	C14orf145	0.536
ENSG00000068885	57560	IFT80	0.531
ENSG00000174951	2523	FUT1	0.528
ENSG00000112977	1611	DAP	0.532
ENSG00000104808	27294	DHDH	0.496
ENSG00000087086	2512	FTL	0.586
ENSG00000183207	10856	RUVBL2	0.549
ENSG00000165417	2957	GTF2A1	0.476
ENSG00000104863	64130	LIN7B	0.456
ENSG00000130529	54795	TRPM4	0.462
ENSG00000128242	9514	GAL3ST1	0.434
ENSG00000170075	9283	GPR37L1	0.458
ENSG00000137857	53905	DUOX1	0.571
ENSG00000104870	2217	FCGRT	0.586
ENSG00000116096	6697	SPR	0.514
ENSG00000126458	6237	RRAS	0.516
ENSG00000126453	83596	BCL2L12	0.393
ENSG00000196188	1510	CTSE	0.486
ENSG00000138606	90525	SHF	0.469
ENSG00000196878	3914	LAMB3	0.571
ENSG00000109790	51088	KLHL5	0.576
ENSG00000104946	79735	TBC1D17	0.475
ENSG00000117013	9132	KCNQ4	0.428
ENSG00000054983	2581	GALC	0.606
ENSG00000142539	6689	SPIB	0.523
ENSG00000101311	55612	FERMT1	0.558
ENSG00000161671	284361	C19orf63	0.478
ENSG00000168334	165904	XIRP1	0.388
ENSG00000074803	6557	SLC12A1	0.466
ENSG00000140092	10516	FBLN5	0.512
ENSG00000128254	25775	C22orf24	0.455
ENSG00000135617	84279	C2orf7	0.467
ENSG00000100433	54207	KCNK10	0.466
ENSG00000089916	55668	C14orf118	0.489
ENSG00000142556	80110	ZNF614	0.593
ENSG00000127124	59269	HIVEP3	0.436
ENSG00000181577	221416	C6orf223	0.444
ENSG00000141026	55090	MED9	0.46
ENSG00000133027	10400	PEMT	0.483
ENSG00000152990	166647	GPR125	0.51
ENSG00000125869	24141	C20orf103	0.464
ENSG00000235568	150372	NFAM1	0.482
ENSG00000125505	79143	MBOAT7	0.618

ENSG00000112759	2030	SLC29A1	0.605
ENSG00000140287	3067	HDC	0.462
ENSG00000164011	51058	ZNF691	0.429
ENSG00000146232	4794	NFKBIE	0.536
ENSG00000130830	4354	MPP1	0.551
ENSG00000117395	10969	EBNA1BP2	0.528
ENSG00000124608	57505	AARS2	0.453
ENSG00000118402	6785	ELOVL4	0.463
ENSG00000253313	149466	C1orf210	0.391
ENSG00000128283	11135	CDC42EP1	0.496
ENSG00000066056	7075	TIE1	0.495
ENSG00000183513	493753	C2orf64	0.495
ENSG00000157765	10568	SLC34A2	0.452
ENSG00000241360	57026	PDXP	0.451
ENSG00000165775	65991	FUNDC2	0.454
ENSG00000135951	80705	TSGA10	0.47
ENSG00000178537	788	SLC25A20	0.501
ENSG00000128731	8924	HERC2	0.494
ENSG00000125844	6238	RRBP1	0.651
ENSG00000089006	27131	SNX5	0.576
ENSG00000183484	29933	GPR132	0.436
ENSG00000054611	25771	TBC1D22A	0.593
ENSG00000166912	54893	MTMR10	0.43
ENSG00000176928	51301	GCNT4	0.456
ENSG00000110104	79080	CCDC86	0.545
ENSG00000105514	9545	RAB3D	0.455
ENSG00000170638	80305	TRABD	0.473
ENSG00000101310	10483	SEC23B	0.576
ENSG00000185386	5600	MAPK11	0.602
ENSG00000172053	5859	QARS	0.547
ENSG00000039987	54831	BEST2	0.444
ENSG00000095066	29911	HOOK2	0.506
ENSG00000119411	54836	BSPRY	0.489
ENSG00000081051	174	AFP	0.474
ENSG00000100258	91289	LMF2	0.566
ENSG00000122012	22987	SV2C	0.434
ENSG00000104903	4066	LYL1	0.48
ENSG00000124875	6372	CXCL6	0.49
ENSG00000163739	2919	CXCL1	0.554
ENSG00000104957	81576	CCDC130	0.482
ENSG00000163734	2921	CXCL3	0.529
ENSG00000081041	2920	CXCL2	0.548
ENSG00000168118	5867	RAB4A	0.621
ENSG00000167112	26995	TRUB2	0.464
ENSG00000168778	79867	TCTN2	0.473
ENSG00000123146	976	CD97	0.556
ENSG00000164220	2151	F2RL2	0.457
ENSG00000170323	2167	FABP4	0.493
ENSG00000164251	2150	F2RL1	0.624
ENSG00000133731	3612	IMPA1	0.539
ENSG00000103187	23406	COTL1	0.542
ENSG00000135686	79786	KLHL36	0.465
ENSG00000167123	51148	CERCAM	0.529
ENSG00000167332	81285	OR51E2	0.474

ENSG00000111271	80724	ACAD10	0.488
ENSG00000113231	8622	PDE8B	0.523
ENSG00000119242	80212	CCDC92	0.526
ENSG00000085872	10523	CHERP	0.574
ENSG00000133739	85444	LRRCC1	0.5
ENSG00000127511	23309	SIN3B	0.563
ENSG00000142657	5226	PGD	0.563
ENSG00000133740	1875	E2F5	0.559
ENSG00000160113	2063	NR2F6	0.629
ENSG00000130307	83878	USHBP1	0.4
ENSG00000130304	376497	SLC27A1	0.468
ENSG00000145020	275	AMT	0.524
ENSG00000138744	27163	NAAA	0.559
ENSG00000168830	3354	HTR1E	0.449
ENSG00000103248	64779	MTHFSD	0.472
ENSG00000111850	57150	C6orf162	0.525
ENSG00000181035	284439	SLC25A42	0.5
ENSG00000110148	887	CCKBR	0.496
ENSG00000064490	8625	RFXANK	0.472
ENSG00000116649	6723	SRM	0.563
ENSG00000166313	322	APBB1	0.476
ENSG00000137312	10211	FLOT1	0.631
ENSG00000138111	79847	TMEM180	0.515
ENSG00000164414	10559	SLC35A1	0.565
ENSG00000011007	6924	TCEB3	0.587
ENSG00000152409	133746	JMY	0.529
ENSG00000159110	3455	IFNAR2	0.62
ENSG00000117308	2582	GALE	0.553
ENSG00000166289	79156	PLEKHF1	0.468
ENSG00000132286	26515	FXC1	0.48
ENSG00000117305	3155	HMGCL	0.556
ENSG00000178904	147991	DPY19L3	0.5
ENSG00000142166	3454	IFNAR1	0.517
ENSG00000179163	2517	FUCA1	0.535
ENSG00000185630	5087	PBX1	0.649
ENSG00000111331	4940	OAS3	0.489
ENSG00000116663	26270	FBXO6	0.448
ENSG00000105707	3249	HPN	0.496
ENSG00000153902	163175	LGI4	0.455
ENSG00000221946	53822	FXYD7	0.466
ENSG00000177674	57085	AGTRAP	0.507
ENSG00000163793	285126	DNAJC5G	0.404
ENSG00000166341	8642	DCHS1	0.56
ENSG00000001460	90529	C1orf201	0.5
ENSG00000095261	5711	PSMD5	0.565
ENSG00000148341	56904	SH3GLB2	0.491
ENSG00000015475	637	BID	0.597
ENSG00000186815	53373	TPCN1	0.544
ENSG00000182870	50614	GALNT9	0.444
ENSG00000205155	55851	PSENEN	0.465
ENSG00000143179	7371	UCK2	0.552
ENSG00000004776	126393	HSPB6	0.48
ENSG00000167604	84807	NFKBID	0.504
ENSG00000183808	389677	RBM12B	0.475

ENSG00000116685	90231	KIAA2013	0.516
ENSG00000095321	1384	CRAT	0.543
ENSG00000115234	9784	SNX17	0.517
ENSG00000126247	826	CAPNS1	0.538
ENSG00000186020	57711	ZNF529	0.485
ENSG00000197808	92283	ZNF461	0.337
ENSG00000174016	169966	FAM46D	0.362
ENSG00000184702	2812	GP1BB	0.436
ENSG00000184058	6899	TBX1	0.584
ENSG00000148180	2934	GSN	0.629
ENSG00000120784	22835	ZFP30	0.484
ENSG00000185933	255022	CALHM1	0.415
ENSG00000115216	29959	NRBP1	0.47
ENSG00000157992	200634	KRTCAP3	0.451
ENSG00000143162	8804	CREG1	0.546
ENSG00000130244	147965	FAM98C	0.453
ENSG00000148331	140459	ASB6	0.482
ENSG00000196767	5456	POU3F4	0.451
ENSG00000065618	1308	COL17A1	0.478
ENSG00000085377	5550	PREP	0.576
ENSG00000136878	10868	USP20	0.504
ENSG00000198393	7574	ZNF26	0.489
ENSG00000006712	54623	PAF1	0.496
ENSG00000173221	2745	GLRX	0.596
ENSG00000183773	150209	AIFM3	0.455
ENSG00000183726	23585	TMEM50A	0.5
ENSG00000155816	56776	FMN2	0.571
ENSG00000159200	1827	RCAN1	0.62
ENSG00000099957	9127	P2RX6	0.507
ENSG00000182901	6000	RGS7	0.521
ENSG00000161179	150223	YDJC	0.47
ENSG00000161180	164592	CCDC116	0.442
ENSG00000100023	23759	PPIL2	0.637
ENSG00000156172	157657	C8orf37	0.451
ENSG00000179083	286499	FAM133A	0.464
ENSG00000143155	261726	TIPRL	0.474
ENSG00000178607	2081	ERN1	0.496
ENSG00000117009	8564	KMO	0.576
ENSG00000114779	84836	ABHD14B	0.526
ENSG00000213064	375035	SFT2D2	0.517
ENSG00000091127	54517	PUS7	0.522
ENSG00000100228	9609	RAB36	0.472
ENSG00000025796	11231	SEC63	0.611
ENSG00000142197	9980	DOPEY2	0.583
ENSG00000152137	26353	HSPB8	0.5
ENSG00000145332	57563	KLHL8	0.566
ENSG00000102359	27286	SRPX2	0.568
ENSG00000108370	8787	RGS9	0.492
ENSG00000007952	27035	NOX1	0.575
ENSG00000109943	56253	CRTAM	0.449
ENSG00000172209	2845	GPR22	0.434
ENSG00000117020	10000	AKT3	0.621
ENSG00000174132	345757	FAM174A	0.489
ENSG00000143368	10262	SF3B4	0.527

ENSG00000010318	51533	PHF7	0.576
ENSG00000171094	238	ALK	0.561
ENSG00000119950	4601	MXI1	0.601
ENSG00000134489	59340	HRH4	0.533
ENSG00000163644	152926	PPM1K	0.534
ENSG00000136631	11311	VPS45	0.613
ENSG00000159625	84229	CCDC135	0.443
ENSG00000134504	284252	KCTD1	0.544
ENSG00000023902	51177	PLEKHO1	0.495
ENSG00000185340	10634	GAS2L1	0.586
ENSG00000140474	25989	ULK3	0.508
ENSG00000100280	162	AP1B1	0.504
ENSG00000102743	10166	SLC25A15	0.537
ENSG00000184117	8508	NIPSNAP1	0.592
ENSG00000154134	64221	ROBO3	0.488
ENSG00000168438	51362	CDC40	0.575
ENSG00000078618	4898	NRD1	0.555
ENSG00000143653	51097	SCCPDH	0.584
ENSG00000171823	144699	FBXL14	0.5
ENSG00000187145	54460	MRPS21	0.503
ENSG00000141431	80816	ASXL3	0.525
ENSG00000151778	387923	SERP2	0.562
ENSG00000163938	26354	GNL3	0.517
ENSG00000070770	1459	CSNK2A2	0.586
ENSG00000008853	23221	RHOBTB2	0.519
ENSG00000133112	7178	TPT1	0.621
ENSG00000112394	117247	SLC16A10	0.471
ENSG00000181938	64785	GINS3	0.578
ENSG00000149557	9638	FEZ1	0.626
ENSG00000172466	7572	ZNF24	0.574
ENSG00000167930	83986	ITFG3	0.5
ENSG00000110934	51411	BIN2	0.464
ENSG00000053702	83714	NRIP2	0.403
ENSG00000165060	2395	FXN	0.535
ENSG00000108187	64081	PBLD	0.5
ENSG00000078246	7289	TULP3	0.628
ENSG00000119139	9414	TJP2	0.547
ENSG00000006042	26022	TMEM98	0.532
ENSG00000154620	9087	TMSB4Y	0.469
ENSG00000134775	80206	FHOD3	0.503
ENSG00000147454	51312	SLC25A37	0.675
ENSG00000163872	55689	YEATS2	0.516
ENSG00000047621	57102	C12orf4	0.527
ENSG00000167034	4824	NKX3-1	0.561
ENSG00000136156	9445	ITM2B	0.606
ENSG00000108688	6354	CCL7	0.487
ENSG00000101335	10398	MYL9	0.508
ENSG00000170523	3889	KRT83	0.447
ENSG00000067606	5590	PRKCZ	0.58
ENSG00000205420	3853	KRT6A	0.664
ENSG00000140391	10099	TSPAN3	0.607
ENSG00000122912	8034	SLC25A16	0.599
ENSG00000189182	374454	KRT77	0.451
ENSG00000136161	1102	RCBTB2	0.537

ENSG00000197894	128	ADH5	0.604
ENSG00000132313	51318	MRPL35	0.518
ENSG00000153233	5801	PTPRR	0.566
ENSG00000152207	57105	CYSLTR2	0.414
ENSG00000110060	83480	PUS3	0.524
ENSG00000136938	10541	ANP32B	0.598
ENSG00000146670	113130	CDCA5	0.5
ENSG00000162300	7542	ZFPL1	0.636
ENSG00000079739	5236	PGM1	0.562
ENSG00000130037	3741	KCNA5	0.466
ENSG00000122641	3624	INHBA	0.579
ENSG00000163823	1230	CCR1	0.6
ENSG00000129625	7905	REEP5	0.615
ENSG00000163932	5580	PRKCD	0.535
ENSG00000106789	7464	CORO2A	0.509
ENSG00000162384	54987	C1orf123	0.52
ENSG00000160791	1234	CCR5	0.508
ENSG00000136052	84102	SLC41A2	0.517
ENSG00000172115	54205	CYCS	0.579
ENSG00000103269	9028	RHBDL1	0.463
ENSG00000170412	55890	GPRC5C	0.506
ENSG00000173621	78999	LRFN4	0.5
ENSG00000073536	54475	NLE1	0.578
ENSG00000085491	29957	SLC25A24	0.5
ENSG00000041357	5685	PSMA4	0.569
ENSG00000119514	79695	GALNT12	0.524
ENSG00000162433	205	AK3L1	0.547
ENSG00000162069	146439	CCDC64B	0.431
ENSG00000186047	220107	DLEU7	0.41
ENSG00000077063	83992	CTTNBP2	0.418
ENSG00000118705	6185	RPN2	0.611
ENSG00000177548	79874	RABEP2	0.614
ENSG00000007376	113000	RPUSD1	0.385
ENSG00000082269	57579	FAM135A	0.423
ENSG00000173349	84826	SFT2D3	0.477
ENSG00000186074	146722	CD300LF	0.481
ENSG00000164032	3015	H2AFZ	0.588
ENSG00000078967	51619	UBE2D4	0.56
ENSG00000162817	79762	C1orf115	0.497
ENSG00000122958	9559	VPS26A	0.534
ENSG00000147364	26260	FBXO25	0.477
ENSG00000106013	56311	ANKRD7	0.438
ENSG00000102984	55565	ZNF821	0.55
ENSG00000109062	9368	SLC9A3R1	0.525
ENSG00000159210	11267	SNF8	0.482
ENSG00000166484	5598	MAPK7	0.554
ENSG00000123191	540	ATP7B	0.525
ENSG00000180190	157695	C8orf42	0.527
ENSG00000136026	10970	CKAP4	0.582
ENSG00000198026	63925	ZNF335	0.593
ENSG00000119801	51646	YPEL5	0.512
ENSG00000132792	56259	CTNNBL1	0.518
ENSG00000106034	79974	C7orf58	0.519
ENSG00000198960	54470	ARMCX6	0.556

ENSG00000092439	54822	TRPM7	0.59
ENSG00000120158	10171	RCL1	0.523
ENSG00000171208	81831	NETO2	0.538
ENSG00000052126	54477	PLEKHA5	0.508
ENSG00000036530	10858	CYP46A1	0.421
ENSG00000175463	374403	TBC1D10C	0.423
ENSG00000143434	10500	SEMA6C	0.453
ENSG00000140280	256586	LYSMD2	0.426
ENSG00000173404	3642	INSM1	0.447
ENSG00000136108	26586	CKAP2	0.601
ENSG00000010610	920	CD4	0.591
ENSG00000104388	5862	RAB2A	0.704
ENSG00000185928	79447	C16orf53	0.523
ENSG00000173692	5707	PSMD1	0.58
ENSG00000183527	8624	PSMG1	0.554
ENSG00000163154	79626	TNFAIP8L2	0.486
ENSG00000174938	26470	SEZ6L2	0.558
ENSG00000069974	5873	RAB27A	0.62
ENSG00000175544	57010	CABP4	0.441
ENSG00000137040	26953	RANBP6	0.523
ENSG00000168077	51435	SCARA3	0.518
ENSG00000174939	253982	ASPHD1	0.495
ENSG00000136870	7743	ZNF189	0.538
ENSG00000131966	55860	ACTR10	0.519
ENSG00000134996	26578	OSTF1	0.523
ENSG00000140798	94160	ABCC12	0.554
ENSG00000149932	124446	TMEM219	0.529
ENSG00000196405	51466	EVL	0.531
ENSG00000168282	4247	MGAT2	0.584
ENSG00000110711	9049	AIP	0.543
ENSG00000116299	57535	KIAA1324	0.469
ENSG00000116704	23169	SLC35D1	0.587
ENSG00000153574	22934	RPIA	0.54
ENSG00000101452	60625	DHX35	0.47
ENSG00000172831	8824	CES2	0.576
ENSG00000155827	56254	RNF20	0.472
ENSG00000116489	829	CAPZA1	0.557
ENSG00000006075	6348	CCL3	0.61
ENSG00000081985	3595	IL12RB2	0.467
ENSG00000120278	57480	PLEKHG1	0.535
ENSG00000031698	6301	SARS	0.517
ENSG00000113211	56130	PCDHB6	0.463
ENSG00000182093	7485	WRB	0.515
ENSG00000101474	57136	C20orf3	0.541
ENSG00000174799	9662	CEP135	0.558
ENSG00000124422	23326	USP22	0.591
ENSG00000198908	80823	BHLHB9	0.505
ENSG00000175183	1466	CSRP2	0.574
ENSG00000178075	54762	GRAMD1C	0.486
ENSG00000001630	1595	CYP51A1	0.604
ENSG00000214021	26140	TTLL3	0.56
ENSG00000115474	3769	KCNJ13	0.476
ENSG00000136770	64215	DNAJC1	0.526
ENSG00000066248	25791	NGEF	0.6

ENSG00000107651	11196	SEC23IP	0.602
ENSG00000132793	64900	LPIN3	0.353
ENSG00000121210	23240	KIAA0922	0.504
ENSG00000134222	84722	PSRC1	0.483
ENSG00000136404	53346	TM6SF1	0.5
ENSG00000149923	5531	PPP4C	0.492
ENSG00000139220	8499	PPFIA2	0.479
ENSG00000102805	1203	CLN5	0.607
ENSG00000104299	55756	INTS9	0.513
ENSG00000183778	10317	B3GALT5	0.483
ENSG00000143921	64241	ABCG8	0.461
ENSG00000166886	4665	NAB2	0.569
ENSG00000103174	51172	NAGPA	0.526
ENSG00000133134	84707	BEX2	0.5
ENSG00000138095	10128	LRPPRC	0.567
ENSG00000135740	6553	SLC9A5	0.498
ENSG00000023041	64429	ZDHHC6	0.505
ENSG00000100994	5834	PYGB	0.523
ENSG00000138613	83464	APH1B	0.503
ENSG00000140455	9960	USP3	0.522
ENSG00000156990	285367	RPUSD3	0.484
ENSG00000100483	79609	C14orf138	0.484
ENSG00000182240	25825	BACE2	0.501
ENSG00000176371	54993	ZSCAN2	0.439
ENSG00000172733	29942	PURG	0.448
ENSG00000187889	199920	C1orf168	0.491
ENSG00000172638	30008	EFEMP2	0.546
ENSG00000166794	5479	PPIB	0.597
ENSG00000138032	5495	PPM1B	0.592
ENSG00000021852	732	C8B	0.468
ENSG00000111049	4617	MYF5	0.439
ENSG00000106701	83856	FSD1L	0.529
ENSG00000126088	7389	UROD	0.598
ENSG00000153048	23589	CARHSP1	0.507
ENSG00000167996	2495	FTH1	0.614
ENSG00000074603	54878	DPP8	0.494
ENSG00000095209	55151	TMEM38B	0.486
ENSG00000184886	284098	PIGW	0.529
ENSG00000101882	79576	NKAP	0.5
ENSG00000117560	356	FASLG	0.578
ENSG00000166669	80063	ATF7IP2	0.511
ENSG00000102471	54602	NDFIP2	0.581
ENSG00000123307	58158	NEUROD4	0.479
ENSG00000155158	158219	TTC39B	0.537
ENSG00000012983	11183	MAP4K5	0.591
ENSG00000127249	84239	ATP13A4	0.488
ENSG00000179583	4261	CIITA	0.602
ENSG00000069493	29121	CLEC2D	0.506
ENSG00000215262	157855	KCNU1	0.36
ENSG00000179965	51333	ZNF771	0.62
ENSG00000159723	181	AGRP	0.47
ENSG00000094804	990	CDC6	0.601
ENSG00000184517	162239	ZFP1	0.508
ENSG00000139132	121512	FGD4	0.504

ENSG00000174021	2787	GNG5	0.487
ENSG00000178966	80010	RMI1	0.52
ENSG00000175646	5619	PRM1	0.424
ENSG00000167207	64127	NOD2	0.45
ENSG00000213865	56260	C8orf44	0.427
ENSG00000144476	57007	CXCR7	0.544
ENSG00000050820	9564	BCAR1	0.497
ENSG00000123576	80712	ESX1	0.348
ENSG00000101574	64863	METTL4	0.481
ENSG00000183287	147372	CCBE1	0.537
ENSG00000178732	2814	GP5	0.529
ENSG00000141682	5366	PMAIP1	0.603
ENSG00000067064	3422	IDI1	0.573
ENSG00000070061	8518	IKBKAP	0.581
ENSG00000120334	91687	CENPL	0.5
ENSG00000076555	32	ACACB	0.633
ENSG00000105825	7980	TFPI2	0.553
ENSG00000158286	388591	RNF207	0.5
ENSG00000167419	4025	LPO	0.433
ENSG00000162341	219931	TPCN2	0.571
ENSG00000153774	10428	CFDP1	0.624
ENSG00000198768	164284	APCDD1L	0.388
ENSG00000175595	2072	ERCC4	0.543
ENSG00000165685	120939	C12orf59	0.449
ENSG00000105829	10282	BET1	0.531
ENSG00000184012	7113	TMPRSS2	0.524
ENSG00000181026	64782	AEN	0.493
ENSG00000104218	79848	CSPP1	0.562
ENSG00000185278	84614	ZBTB37	0.489
ENSG00000147231	55086	CXorf57	0.541
ENSG00000126773	64430	C14orf135	0.497
ENSG00000132773	114034	TOE1	0.51
ENSG00000153898	255231	MCOLN2	0.489
ENSG00000134056	92259	MRPS36	0.515
ENSG00000140545	4240	MFGE8	0.537
ENSG00000204248	1302	COL11A2	0.611
ENSG00000185149	4887	NPY2R	0.586
ENSG00000164114	79884	MAP9	0.505
ENSG00000103512	23420	NOMO1	0.597
ENSG00000129691	9070	ASH2L	0.509
ENSG00000149541	26229	B3GAT3	0.535
ENSG00000132763	25974	MMACHC	0.467
ENSG00000103549	9810	RNF40	0.566
ENSG00000121594	941	CD80	0.513
ENSG00000018236	1272	CNTN1	0.575
ENSG00000133131	79710	MORC4	0.483
ENSG00000085721	54700	RRN3	0.497
ENSG00000148735	79949	C10orf81	0.465
ENSG00000186523	85002	FAM86B1	0.5
ENSG00000087111	94005	PIGS	0.454
ENSG00000176563	124817	CNTD1	0.395
ENSG00000173171	4580	MTX1	0.537
ENSG00000131969	145447	ABHD12B	0.403
ENSG00000162642	148423	C1orf52	0.467

ENSG00000025772	10953	TOMM34	0.59
ENSG00000198088	54830	NUP62CL	0.486
ENSG00000119729	23433	RHOQ	0.667
ENSG00000175197	1649	DDIT3	0.545
ENSG00000165282	84720	PIGO	0.538
ENSG00000180776	253832	ZDHHC20	0.566
ENSG00000065802	51665	ASB1	0.556
ENSG00000099377	80270	HSD3B7	0.451
ENSG00000116161	27101	CACYBP	0.611
ENSG00000172113	10201	NME6	0.5
ENSG00000109738	2743	GLRB	0.534
ENSG00000139173	84216	TMEM117	0.452
ENSG00000147224	5631	PRPS1	0.62
ENSG00000101680	284217	LAMA1	0.442
ENSG00000160233	81543	LRRC3	0.427
ENSG00000114503	22916	NCBP2	0.615
ENSG00000107796	59	ACTA2	0.588
ENSG00000165283	30968	STOML2	0.532
ENSG00000119227	80235	PIGZ	0.476
ENSG00000137656	84811	BUD13	0.522
ENSG00000082701	2932	GSK3B	0.558
ENSG00000084444	57613	KIAA1467	0.549
ENSG00000168000	26580	BSCL2	0.583
ENSG00000160179	9619	ABCG1	0.587
ENSG00000131471	8639	AOC3	0.481
ENSG00000155886	25769	SLC24A2	0.469
ENSG00000134086	7428	VHL	0.517
ENSG00000156150	257	ALX3	0.459
ENSG00000170903	84437	KIAA1826	0.565
ENSG00000138152	118663	BTBD16	0.433
ENSG00000159588	149483	CCDC17	0.417
ENSG00000186150	164153	UBL4B	0.457
ENSG00000147113	79742	CXorf36	0.497
ENSG00000027075	5583	PRKCH	0.566
ENSG00000149600	149951	COMMD7	0.566
ENSG00000164124	55314	TMEM144	0.548
ENSG00000068724	57217	TTC7A	0.581
ENSG00000144354	83879	CDCA7	0.561
ENSG00000166925	81628	TSC22D4	0.513
ENSG00000110244	337	APOA4	0.438
ENSG00000175395	219749	ZNF25	0.554
ENSG00000168502	23255	KIAA0802	0.509
ENSG00000173588	51134	CCDC41	0.498
ENSG00000109163	2798	GNRHR	0.543
ENSG00000139211	347902	AMIGO2	0.529
ENSG00000131323	7187	TRAF3	0.568
ENSG00000057704	57458	TMCC3	0.493
ENSG00000101843	5716	PSMD10	0.533
ENSG00000185164	283820	NOMO2	0.5
ENSG00000185053	137868	SGCZ	0.481
ENSG00000050327	7984	ARHGEF5	0.505
ENSG00000114982	55683	KIAA1310	0.489
ENSG00000177272	3738	KCNA3	0.464
ENSG00000102218	6102	RP2	0.5

ENSG00000120539	84930	MASTL	0.478
ENSG00000172216	1051	CEBPB	0.59
ENSG00000174165	254359	ZDHHC24	0.454
ENSG00000198853	9853	RUSC2	0.49
ENSG00000158483	55199	FAM86C	0.458
ENSG00000119922	3433	IFIT2	0.548
ENSG00000168092	5049	PAFAH1B2	0.474
ENSG00000141756	60681	FKBP10	0.488
ENSG00000180353	3059	HCLS1	0.517
ENSG00000099204	3983	ABLIM1	0.627
ENSG00000147123	54539	NDUFB11	0.534
ENSG00000175928	57633	LRRN1	0.459
ENSG00000164715	22853	LMTK2	0.487
ENSG00000157020	6396	SEC13	0.537
ENSG00000167186	10229	COQ7	0.558
ENSG00000160185	53347	UBASH3A	0.468
ENSG00000152778	24138	IFIT5	0.557
ENSG00000112038	4988	OPRM1	0.548
ENSG00000196187	9725	TMEM63A	0.649
ENSG00000169896	3684	ITGAM	0.585
ENSG00000168461	11031	RAB31	0.661
ENSG00000103534	79838	TMC5	0.49
ENSG00000198183	51297	PLUNC	0.45
ENSG00000155970	286097	EFHA2	0.479
ENSG00000150995	3708	ITPR1	0.618
ENSG00000187742	79048	SECISBP2	0.474
ENSG00000168843	56884	FSTL5	0.436
ENSG00000077080	51412	ACTL6B	0.517
ENSG00000149591	6876	TAGLN	0.544
ENSG00000131473	47	ACLY	0.625
ENSG00000172354	2783	GNB2	0.543
ENSG00000136014	84101	USP44	0.458
ENSG00000130985	7317	UBA1	0.5
ENSG00000173226	9657	IQCB1	0.551
ENSG00000166448	222865	TMEM130	0.423
ENSG00000120341	89866	SEC16B	0.567
ENSG00000137831	55075	UACA	0.579
ENSG00000138061	1545	CYP1B1	0.625
ENSG00000116830	8458	TTF2	0.5
ENSG00000160753	23623	RUSC1	0.505
ENSG00000148680	3363	HTR7	0.507
ENSG00000168259	7266	DNAJC7	0.519
ENSG00000145416	55016	1-Mar	0.489
ENSG00000126821	81537	SGPP1	0.534
ENSG00000184988	113277	TMEM106A	0.462
ENSG00000135452	6302	TSPAN31	0.599
ENSG00000130222	10912	GADD45G	0.501
ENSG00000106392	56913	C1GALT1	0.498
ENSG00000175906	379	ARL4D	0.571
ENSG00000134258	79679	VTCN1	0.452
ENSG00000146648	1956	EGFR	0.656
ENSG00000040341	27067	STAU2	0.543
ENSG00000109466	11275	KLHL2	0.524
ENSG00000136319	91875	TTC5	0.415

ENSG00000165030	4783	NFIL3	0.566
ENSG00000151892	2674	GFRA1	0.463
ENSG00000145040	90226	UCN2	0.455
ENSG00000112679	56940	DUSP22	0.472
ENSG00000108771	79132	DHX58	0.461
ENSG00000148154	7357	UGCG	0.601
ENSG00000143891	130589	GALM	0.513
ENSG00000213057	400798	C1orf220	0.395
ENSG00000103061	84138	SLC7A6OS	0.473
ENSG00000135407	10677	AVIL	0.509
ENSG00000116668	54823	C1orf26	0.486
ENSG00000196305	3376	IARS	0.545
ENSG00000170265	8427	ZNF282	0.528
ENSG00000102897	57149	LYRM1	0.541
ENSG00000067225	5315	PKM2	0.552
ENSG00000054803	140689	CBLN4	0.471
ENSG00000134108	55207	ARL8B	0.51
ENSG00000145868	81545	FBXO38	0.561
ENSG00000100528	10175	CNIH	0.533
ENSG00000074800	2023	ENO1	0.576
ENSG00000196981	54554	WDR5B	0.509
ENSG00000078674	5108	PCM1	0.615
ENSG00000165338	143279	HECTD2	0.524
ENSG00000163214	90957	DHX57	0.456
ENSG00000122122	54440	SASH3	0.519
ENSG00000139350	121441	NEDD1	0.523
ENSG00000146828	56996	SLC12A9	0.492
ENSG00000029639	51106	TFB1M	0.45
ENSG00000139263	121227	LRIG3	0.422
ENSG00000138496	83666	PARP9	0.507
ENSG00000135698	10200	MPHOSPH6	0.505
ENSG00000118407	27145	FILIP1	0.496
ENSG00000165185	158405	KIAA1958	0.465
ENSG00000116874	10352	WARS2	0.518
ENSG00000148153	58493	C9orf80	0.538
ENSG00000101138	1477	CSTF1	0.581
ENSG00000204856	29902	C12orf24	0.524
ENSG00000170836	8493	PPM1D	0.524
ENSG00000143341	83872	HMCN1	0.484
ENSG00000197147	23507	LRRC8B	0.504
ENSG00000131686	765	CA6	0.439
ENSG00000161653	162417	NAGS	0.425
ENSG00000231852	1589	CYP21A2	0.47
ENSG00000132464	10117	ENAM	0.5
ENSG00000186642	5138	PDE2A	0.534
ENSG00000132465	3512	IGJ	0.476
ENSG00000141376	54828	BCAS3	0.514
ENSG00000198554	11169	WDHD1	0.618
ENSG00000129566	7011	TEP1	0.48
ENSG00000128709	3235	HOXD9	0.549
ENSG00000198000	55035	NOL8	0.524
ENSG00000204175	9721	GPRIN2	0.503
ENSG00000102034	2000	ELF4	0.602
ENSG00000120694	10808	HSPH1	0.581

ENSG00000133105	122042	RXFP2	0.407
ENSG00000177150	125228	C18orf19	0.507
ENSG00000162366	10158	PDZK1IP1	0.496
ENSG00000169871	81844	TRIM56	0.457
ENSG00000134077	25917	THUMPD3	0.577
ENSG00000106397	8985	PLOD3	0.57
ENSG00000160654	917	CD3G	0.484
ENSG00000152154	130733	TMEM178	0.478
ENSG00000143340	148753	FAM163A	0.413
ENSG00000092758	1299	COL9A3	0.513
ENSG00000133116	9365	KL	0.466
ENSG00000136261	28969	BZW2	0.496
ENSG00000126653	84081	CCDC55	0.452
ENSG00000122335	84947	SERAC1	0.493
ENSG00000108309	10900	RUNDC3A	0.502
ENSG00000149761	84304	NUDT22	0.479
ENSG00000165807	145376	C14orf50	0.489
ENSG00000205629	51451	LCMT1	0.475
ENSG00000137275	8737	RIPK1	0.536
ENSG00000183735	29110	TBK1	0.525
ENSG00000013306	51629	SLC25A39	0.549
ENSG00000153179	283349	RASSF3	0.503
ENSG00000137054	64425	POLR1E	0.492
ENSG00000140464	5371	PML	0.676
ENSG00000002330	572	BAD	0.562
ENSG00000173264	56834	GPR137	0.575
ENSG00000008283	1534	CYB561	0.654
ENSG00000204645	6759	SSX4	0.42
ENSG00000198946	6759	SSX4	0.42
ENSG00000141458	4864	NPC1	0.619
ENSG00000165275	158234	RG9MTD3	0.513
ENSG00000128563	79706	PRKRIP1	0.479
ENSG00000160209	8566	PDXK	0.629
ENSG00000165672	10935	PRDX3	0.6
ENSG00000162676	2672	GFI1	0.486
ENSG00000147251	139818	DOCK11	0.594
ENSG00000125965	8200	GDF5	0.447
ENSG00000176153	2877	GPX2	0.513
ENSG00000173826	81033	KCNH6	0.577
ENSG00000090104	5996	RGS1	0.565
ENSG00000181481	84282	RNF135	0.515
ENSG00000180329	124808	CCDC43	0.431
ENSG00000168234	125488	TTC39C	0.504
ENSG00000088682	57017	COQ9	0.535
ENSG00000138623	8482	SEMA7A	0.531
ENSG00000165102	138050	HGSNAT	0.517
ENSG00000101210	1917	EEF1A2	0.549
ENSG00000082781	3693	ITGB5	0.626
ENSG00000160294	8888	MCM3AP	0.629
ENSG00000164808	23514	KIAA0146	0.5
ENSG00000171004	90161	HS6ST2	0.508
ENSG00000157349	11269	DDX19B	0.527
ENSG00000149262	92105	INTS4	0.538
ENSG00000138207	5950	RBP4	0.544

ENSG00000225830	2074	ERCC6	0.526
ENSG00000163520	2199	FBLN2	0.506
ENSG00000164591	91977	MYOZ3	0.488
ENSG00000168872	55308	DDX19A	0.559
ENSG00000134371	79577	CDC73	0.489
ENSG00000150760	1793	DOCK1	0.538
ENSG00000165795	57447	NDRG2	0.628
ENSG00000121848	27246	RNF115	0.507
ENSG00000130958	11046	SLC35D2	0.546
ENSG00000130956	22927	HABP4	0.537
ENSG00000168564	55602	CDKN2AIP	0.503
ENSG00000164885	1020	CDK5	0.543
ENSG00000019549	6591	SNAI2	0.51
ENSG00000154767	7508	XPC	0.574
ENSG00000136011	55576	STAB2	0.462
ENSG00000103043	55697	VAC14	0.577
ENSG00000127951	10875	FGL2	0.565
ENSG00000023892	50619	DEF6	0.495
ENSG00000134590	8933	FAM127A	0.5
ENSG00000203883	54345	SOX18	0.479
ENSG00000185345	5071	PARK2	0.477
ENSG00000177556	475	ATOX1	0.504
ENSG00000145907	10146	G3BP1	0.629
ENSG00000109762	83891	SNX25	0.538
ENSG00000196189	64218	SEMA4A	0.495
ENSG00000132911	56923	NMUR2	0.409
ENSG00000114626	80325	ABTB1	0.533
