# Supplementary material for: Insights into the regulation of human CNV-miRNAs from the view of their target genes
Source: BMC Genomics. 2012 Dec 18;13:707. doi: 10.1186/1471-2164-13-707 (PMC3582595; doi:10.1186/1471-2164-13-707)
Supplement: Additional file 8 — The 21 and eight CNV-miRNA-families of Pan troglodytes and Mus musculus,respectively. As there are no miRNA targets from the TargetScan prediction for Pan troglodyte, the miRNA-family IDs were represented by Rfam identifiers (http://rfam.sanger.ac.uk/). [file 1471-2164-13-707-S8.pdf]

Species	miRNA Family ID	miRNA
Pan troglodytes	MIPF0000007	ptr-mir-181a-2; ptr-mir-181b-2
	MIPF0000029	ptr-mir-133a-2
	MIPF0000038	ptr-mir-1-1
	MIPF0000042	ptr-mir-204
	MIPF0000061	ptr-mir-365-1
	MIPF0000082	ptr-mir-193b
	MIPF0000163	ptr-mir-320b-1
	MIPF0000219	ptr-mir-484
	MIPF0000317	ptr-mir-548i-4
	MIPF0000329	ptr-mir-423
	MIPF0000445	ptr-mir-1225
	MIPF0000456	ptr-mir-1302-7
	MIPF0000465	ptr-mir-661
	MIPF0000511	ptr-mir-942
	MIPF0000553	ptr-mir-618
	MIPF0000569	ptr-mir-1244-4
	MIPF0000587	ptr-mir-1282-1
	MIPF0000622	ptr-mir-1248
	MIPF0000672	ptr-mir-617
	MIPF0000689	ptr-mir-1236
	MIPF0000691	ptr-mir-1293
Mus musculus	GGAGACG	mmu-miR-139-3p
	CUACAGU	mmu-miR-139-5p
	UGAAAUG	mmu-miR-203
	AUACAUA	mmu-miR-297b-3p
	UGUAUGU	mmu-miR-297b-5p
	AGCACCA	mmu-miR-29b;mmu-miR-29c
	GGCAUCU	mmu-miR-680
	ACGCGGG	mmu-miR-700
