# Supplementary material for: Insights into the regulation of human CNV-miRNAs from the view of their target genes
Source: BMC Genomics. 2012 Dec 18;13:707. doi: 10.1186/1471-2164-13-707 (PMC3582595; doi:10.1186/1471-2164-13-707)
Supplement: Additional file 1 — The 63,428 regulatory relationships among 541 miRNA families and 9,174 target genes. [file 1471-2164-13-707-S1.pdf]

Ensembl Gene ID	miRNA Family ID
ENSG00000000003	CCAGUGC
ENSG00000000003	CUCUUCC
ENSG00000000003	UCCAGUU
ENSG00000000003	UUCAAAU
ENSG00000000005	AUUCAUU
ENSG00000000457	CUUUGGU
ENSG00000000457	GAGGUAG
ENSG00000000457	UUGGCAC
ENSG00000000938	UAUAAAG
ENSG00000001084	ACAGUAU
ENSG00000001084	AGGGUCU
ENSG00000001084	CUGGAAA
ENSG00000001084	GGAAUGU
ENSG00000001084	GUUAAUG
ENSG00000001084	UACAAAG
ENSG00000001084	UCCAGAG
ENSG00000001084	UCUGGAA
ENSG00000001084	UUGGUCC
ENSG00000001167	AAGUGCA
ENSG00000001167	AAUCUCU
ENSG00000001167	AGUGGUU
ENSG00000001167	CUUCACC
ENSG00000001167	GACCCUG
ENSG00000001167	UGAAAUG
ENSG00000001167	UGAGAAG
ENSG00000001167	UGCCAGC
ENSG00000001460	GAGAACC
ENSG00000001561	AAGUGCA
ENSG00000001561	AGUGCAA
ENSG00000001561	GUGCAAA
ENSG00000001561	UACAAAG
ENSG00000001617	AAUACUG
ENSG00000001617	AGCCAGU
ENSG00000001617	AUAUUAU
ENSG00000001617	CCAGUGU
ENSG00000001617	UGUCUUU
ENSG00000001626	AGUGCCG
ENSG00000001626	AGUGCCU
ENSG00000001629	AAGGCAC
ENSG00000001629	AAGUGCA
ENSG00000001629	ACACACU
ENSG00000001629	ACAUUAC
ENSG00000001629	CAUGGGU
ENSG00000001629	GAGUAUU
ENSG00000001629	GGGUGGU
ENSG00000001629	GGUGGUU
ENSG00000001629	UCAAGUA
ENSG00000001629	UCACAGU
ENSG00000001629	UGGCCAC
ENSG00000001630	UCAUUCG
ENSG00000001631	AAAGCUG
ENSG00000001631	AAUUAUU
ENSG00000001631	ACACACU
ENSG00000001631	AUAAAGU
ENSG00000001631	AUAUAAC
ENSG00000001631	CUCAAAC
ENSG00000001631	UGAAAUG
ENSG00000001631	UGAGAAG
ENSG00000001631	UGAGAAU
ENSG00000001631	UGUGCUU
ENSG00000001631	UUCAAAU
ENSG00000002016	GUCCAGA

ENSG00000002330	UUCCGGC
ENSG00000002587	ACUGUAG
ENSG00000002587	AUGGCAC
ENSG00000002587	GGAUUUC
ENSG00000002587	GUGAAUG
ENSG00000002587	UACAAAG
ENSG00000002746	AUUGCAC
ENSG00000002746	GAGGUAU
ENSG00000002746	GUAAACA
ENSG00000002746	UGCAUAG
ENSG00000002746	UGCAUAU
ENSG00000002834	AAAGUGC
ENSG00000002834	AAGUGCA
ENSG00000002834	ACAGGUG
ENSG00000002834	AGCACCA
ENSG00000002834	AGGCAGG
ENSG00000002834	AGGGAGG
ENSG00000002834	CACAGUG
ENSG00000002834	CACCAGC
ENSG00000002834	CCAGGAG
ENSG00000002834	CUUUGGU
ENSG00000002834	GAGAUGA
ENSG00000002834	GUGCUUG
ENSG00000002834	UCACAGU
ENSG00000002834	UCCAGUU
ENSG00000002834	UCCCUGU
ENSG00000002834	UGAAAUG
ENSG00000002834	UGAGUCU
ENSG00000002834	UGAUCCA
ENSG00000002834	UGUGCUU
ENSG00000002919	GAGCCCU
ENSG00000003056	AAAGUGC
ENSG00000003056	AACAUUC
ENSG00000003056	AAGUGCA
ENSG00000003056	AGUGCAA
ENSG00000003056	CCAGUGC
ENSG00000003056	CCCUGAG
ENSG00000003056	GAGAUGA
ENSG00000003056	UCCCUUG
ENSG00000003096	AGCAAAA
ENSG00000003096	AGCAGCU
ENSG00000003096	GCCCUUA
ENSG00000003096	UGUGCUU
ENSG00000003137	AAAGUGC
ENSG00000003137	AAGAACC
ENSG00000003137	ACAAUAU
ENSG00000003137	ACAUUCA
ENSG00000003137	ACCCGUA
ENSG00000003137	AGCAGCG
ENSG00000003137	AGCAGCU
ENSG00000003137	AGUACUG
ENSG00000003137	AUAAAGU
ENSG00000003137	AUAAUAC
ENSG00000003137	CAAAACU
ENSG00000003137	CAGACAG
ENSG00000003137	CAUAUUG
ENSG00000003137	CUCACAC
ENSG00000003137	GGCAGGG
ENSG00000003137	GUGGGGA
ENSG00000003137	UAAUGCU
ENSG00000003137	UUGUGUC
ENSG00000003137	UUUCAAC
ENSG00000003147	CUUUGGU
ENSG00000003147	GACCCUG

ENSG00000003147	UGGCUGC
ENSG00000003249	GUGCUUG
ENSG00000003249	UGUGCUU
ENSG00000003393	AACAUUC
ENSG00000003393	AGCAGCU
ENSG00000003393	AUCCUUU
ENSG00000003393	CCAGUGC
ENSG00000003393	UCAAGUA
ENSG00000003400	ACAUUCA
ENSG00000003400	UCCAGUU
ENSG00000003436	CACAGUG
ENSG00000003436	GGCUCAG
ENSG00000003436	UCACAGG
ENSG00000003987	ACAUUCA
ENSG00000003987	UUCUAAU
ENSG00000003989	AGCAAAA
ENSG00000003989	AGCUGCC
ENSG00000003989	CAGCAAA
ENSG00000003989	CUCCAUU
ENSG00000003989	CUUACAG
ENSG00000003989	GAAACAU
ENSG00000003989	GGAAUGU
ENSG00000003989	GGCAGUG
ENSG00000003989	GGCAUUG
ENSG00000003989	UUGGUCC
ENSG00000004059	ACCACAG
ENSG00000004059	AGCACCA
ENSG00000004059	AGGCAGG
ENSG00000004059	GGAGUCC
ENSG00000004139	CUGGACU
ENSG00000004139	GGCAGGG
ENSG00000004142	AGGCAGG
ENSG00000004142	CAGCUGG
ENSG00000004142	CCCCUGG
ENSG00000004142	CUGCAGG
ENSG00000004142	CUUGGAG
ENSG00000004142	GCAGCAU
ENSG00000004142	GGGUCUU
ENSG00000004142	GUGACAG
ENSG00000004142	UGCCCUG
ENSG00000004142	UUCUGCA
ENSG00000004142	UUGGAGG
ENSG00000004399	AAAGAAU
ENSG00000004399	AUAAAGU
ENSG00000004399	AUAUAAC
ENSG00000004399	CACAGUG
ENSG00000004399	CCAGUGC
ENSG00000004399	GAGGUAG
ENSG00000004399	GAGGUAU
ENSG00000004399	GGAUGAG
ENSG00000004399	UAAUAUC
ENSG00000004399	UCACAGG
ENSG00000004399	UCCAGUU
ENSG00000004455	AAAGCUG
ENSG00000004455	AAGGCAC
ENSG00000004455	AGAUAAA
ENSG00000004455	AGUGCUG
ENSG00000004455	CAGUUAC
ENSG00000004455	CCCUGUU
ENSG00000004455	CCUUCUG
ENSG00000004455	CUGCCCC
ENSG00000004455	CUUUGGU
ENSG00000004455	GCAGCAU
ENSG00000004455	GGCAAGA

ENSG00000004455	UCACAGU
ENSG00000004455	UGGAGAU
ENSG00000004455	UUGGUCC
ENSG00000004478	AAAGGUA
ENSG00000004478	AGCAAAA
ENSG00000004478	CAAAACU
ENSG00000004478	GCCUGGA
ENSG00000004478	UACAAAG
ENSG00000004478	UGCUCAC
ENSG00000004660	AAUUUUA
ENSG00000004660	CUGCUCA
ENSG00000004660	GCAGCAU
ENSG00000004660	GGAGGCA
ENSG00000004660	GGAUGAG
ENSG00000004660	GGUGGGC
ENSG00000004776	CUGGCUC
ENSG00000004779	AUACAAG
ENSG00000004799	CAGUGCA
ENSG00000004799	UAUCAGA
ENSG00000004799	UGAUCCA
ENSG00000004799	UUGACAC
ENSG00000004799	UUGAUCA
ENSG00000004838	GGGGCAG
ENSG00000004838	UCUGGAG
ENSG00000004848	AAAAGUA
ENSG00000004848	AAAGUAC
ENSG00000004848	AACAGUC
ENSG00000004848	AAGUGCA
ENSG00000004848	ACAUUAC
ENSG00000004848	AGUGCAA
ENSG00000004848	AUACAAG
ENSG00000004848	CUACAAA
ENSG00000004848	CUUUGGU
ENSG00000004848	UACAAAG
ENSG00000004848	UACAGUU
ENSG00000004848	UCACAGU
ENSG00000004848	UCCCUUG
ENSG00000004864	UCCAGAG
ENSG00000004866	AAAGCUG
ENSG00000004866	GGGGGAA
ENSG00000004866	UUCUUGU
ENSG00000004897	AAAUCCC
ENSG00000004897	ACAUUAC
ENSG00000004897	ACAUUCA
ENSG00000004897	AGCAAAA
ENSG00000004897	CUCUUCC
ENSG00000004897	GAAUUGU
ENSG00000004897	GCAGCAU
ENSG00000004897	GGAUUUC
ENSG00000004897	GGAUUUU
ENSG00000004897	UAAGACU
ENSG00000004897	UCACACA
ENSG00000004897	UCACAGG
ENSG00000004897	UCACAGU
ENSG00000004897	UCCUUGC
ENSG00000004897	UGCAAAG
ENSG00000004939	UUGGCAA
ENSG00000004939	UUUCAAC
ENSG00000004948	AAAUCCC
ENSG00000004948	ACCCUGU
ENSG00000004948	AGCACCA
ENSG00000004948	AGCUGCC
ENSG00000004948	CUGGAAA
ENSG00000004948	GAGUCUU

ENSG00000004948	GGAGUCC
ENSG00000004948	GGCAAGA
ENSG00000004948	GUUAAUG
ENSG00000004948	UAUAAAG
ENSG00000004948	UGAAAGG
ENSG00000004948	UUCCUAG
ENSG00000004948	UUCUAAU
ENSG00000004948	UUGGCAA
ENSG00000004948	UUGGCAC
ENSG00000004948	UUGUGAC
ENSG00000004961	AAUACUG
ENSG00000004975	CACAGUG
ENSG00000004975	UAUUGCU
ENSG00000004975	UCACAGU
ENSG00000005001	UACAAAG
ENSG00000005007	AAAGAAU
ENSG00000005007	CACAGUG
ENSG00000005007	CCUGCUG
ENSG00000005007	CUACAAA
ENSG00000005007	CUCACAC
ENSG00000005007	CUCCCAA
ENSG00000005007	CUCCCAC
ENSG00000005007	CUCUUCC
ENSG00000005007	GAAAACA
ENSG00000005007	GAAGGAA
ENSG00000005007	GAGAUGA
ENSG00000005007	GCUACAU
ENSG00000005007	UAAUAUC
ENSG00000005007	UACAAAG
ENSG00000005007	UCUGGAA
ENSG00000005007	UCUGGAG
ENSG00000005020	AAAGCUG
ENSG00000005020	ACUGGCU
ENSG00000005020	AGCAAAA
ENSG00000005020	AUAUAAC
ENSG00000005020	GGGGGAA
ENSG00000005020	UCAUUUG
ENSG00000005020	UCCCUCU
ENSG00000005020	UGGGGGA
ENSG00000005020	UUCUAAU
ENSG00000005020	UUGGCAA
ENSG00000005073	AACACUG
ENSG00000005073	AAGUCAC
ENSG00000005073	AUAAUAC
ENSG00000005073	CACACCU
ENSG00000005073	CUUUGGU
ENSG00000005073	GGCAGGG
ENSG00000005073	GGUGGGC
ENSG00000005073	GUAAACA
ENSG00000005073	UCACAUU
ENSG00000005073	UCCCUGU
ENSG00000005073	UCCUUGC
ENSG00000005102	AUGUAAU
ENSG00000005102	GGCUCAG
ENSG00000005102	GUCCAGA
ENSG00000005108	GUGCAAA
ENSG00000005194	AGCAGCA
ENSG00000005238	AAAAGUA
ENSG00000005238	AGGGAGG
ENSG00000005238	CAGGUGA
ENSG00000005238	CCAGGAG
ENSG00000005238	CUGACUC
ENSG00000005238	CUGCCCC
ENSG00000005238	GAGGUAG

ENSG00000005238	GAGGUAU
ENSG00000005238	GAGUCUU
ENSG00000005238	GCCUGGA
ENSG00000005238	GGAGGCA
ENSG00000005238	GGCCCUG
ENSG00000005238	GUGGCCC
ENSG00000005238	UAUAAAG
ENSG00000005238	UCAAGUA
ENSG00000005238	UCCUGAG
ENSG00000005238	UGGCCCU
ENSG00000005238	UUUCAAC
ENSG00000005249	AUAUAAC
ENSG00000005249	AUCACUA
ENSG00000005249	AUUGCAC
ENSG00000005249	GGCAUUG
ENSG00000005249	UGCAUAU
ENSG00000005339	AAAAAUC
ENSG00000005339	AAAGAAU
ENSG00000005339	AACAUUC
ENSG00000005339	AAGUCUU
ENSG00000005339	AAGUGUG
ENSG00000005339	AAUACUG
ENSG00000005339	AAUGUUG
ENSG00000005339	AAUUGCU
ENSG00000005339	AAUUUUA
ENSG00000005339	ACAAUAU
ENSG00000005339	ACAUAGA
ENSG00000005339	AGAUGUG
ENSG00000005339	AGUGAUC
ENSG00000005339	AUACAAG
ENSG00000005339	AUAUAAC
ENSG00000005339	AUAUUAU
ENSG00000005339	CUAUACA
ENSG00000005339	CUGUUGA
ENSG00000005339	GAAAACA
ENSG00000005339	GAGUUGA
ENSG00000005339	GUUAAUG
ENSG00000005339	UAAUACA
ENSG00000005339	UACAAAG
ENSG00000005339	UAUAAAG
ENSG00000005339	UCCUAUA
ENSG00000005339	UGCAUAG
ENSG00000005339	UGUCUGC
ENSG00000005339	UGUCUGG
ENSG00000005339	UGUUGAA
ENSG00000005339	UUCAAGC
ENSG00000005379	AAGGCAC
ENSG00000005379	ACCACAG
ENSG00000005379	AGCAAAA
ENSG00000005379	AGUGCAA
ENSG00000005379	AUAAUAC
ENSG00000005379	CAGUGCA
ENSG00000005379	CCAGUGU
ENSG00000005379	CCCACCG
ENSG00000005379	CCCACCU
ENSG00000005379	CCUUCUU
ENSG00000005379	CUCCCAA
ENSG00000005379	CUUGGAG
ENSG00000005379	GAGGCAG
ENSG00000005379	GCUUCCU
ENSG00000005379	GUGCAAA
ENSG00000005379	UCCCACC
ENSG00000005379	UGGAGAU
ENSG00000005469	AGUGGUU

ENSG00000005469	GAGGCUG
ENSG00000005469	GUGCUUG
ENSG00000005469	UGCAUAG
ENSG00000005469	UGUUGAA
ENSG00000005469	UUGAGGC
ENSG00000005483	AAAACUG
ENSG00000005483	AAAAGUA
ENSG00000005483	AAAGUAC
ENSG00000005483	AAUGCCC
ENSG00000005483	ACAGUAU
ENSG00000005483	ACAUUAC
ENSG00000005483	CCAGUGC
ENSG00000005513	AAAGUUU
ENSG00000005513	UCACAGG
ENSG00000005513	UCACAGU
ENSG00000005700	UAUUGCU
ENSG00000005810	AAAAAUC
ENSG00000005810	AAAUGGU
ENSG00000005810	AAAUUUC
ENSG00000005810	ACAUUCA
ENSG00000005810	CACUGUU
ENSG00000005810	CUCAAAC
ENSG00000005810	GAGGCAG
ENSG00000005810	UGACAUC
ENSG00000005810	UGUAUAA
ENSG00000005812	ACAGGUG
ENSG00000005812	UCCAGUU
ENSG00000005844	ACUCCAG
ENSG00000005844	UCUCCAA
ENSG00000005882	CACUGUU
ENSG00000005882	GUCUCUG
ENSG00000005882	UGAGGAC
ENSG00000005884	AAGGCAC
ENSG00000005884	ACAGUAC
ENSG00000005884	ACAGUAU
ENSG00000005884	ACAUUCA
ENSG00000005884	AGUAGUU
ENSG00000005884	AUACAAG
ENSG00000005884	CAGUAGA
ENSG00000005884	CAGUAGU
ENSG00000005884	CCAGUGC
ENSG00000005884	CCCUGUC
ENSG00000005884	CUACAGU
ENSG00000005884	CUGCCCC
ENSG00000005884	GAGAAGA
ENSG00000005884	GCUACAU
ENSG00000005884	GGCUCAG
ENSG00000005884	GGGGCAG
ENSG00000005884	GGUGGGC
ENSG00000005884	UACAAAG
ENSG00000005884	UGAGAAG
ENSG00000005889	AACUACU
ENSG00000005889	ACACACC
ENSG00000005889	AGGGAGG
ENSG00000005889	AUGUAAU
ENSG00000005889	CAAAGUG
ENSG00000005889	CUACAGU
ENSG00000005889	CUUCACC
ENSG00000005889	GCAUCCC
ENSG00000005889	GUCAGUU
ENSG00000005889	GUGCUUG
ENSG00000005889	UAAUAGU
ENSG00000005889	UAUAUGA
ENSG00000005889	UCAAGUA

ENSG00000005889	UCACACA
ENSG00000005889	UUUGCAA
ENSG00000005889	UUUUUGC
ENSG00000005893	AAAAAUC
ENSG00000005893	AGCCAGU
ENSG00000005893	GUUAGGA
ENSG00000005893	UAAUGCU
ENSG00000005955	AAGGCGC
ENSG00000005955	AUGGCUU
ENSG00000005955	CAGUAGU
ENSG00000005955	UCACAUU
ENSG00000005961	CAGCAGG
ENSG00000006007	AAAAAUC
ENSG00000006007	AUGCAUU
ENSG00000006007	CUGCCCC
ENSG00000006007	GCUAGCA
ENSG00000006007	GGUGGUU
ENSG00000006007	GUGCUUG
ENSG00000006007	UGUGCUU
ENSG00000006015	CAGCUGG
ENSG00000006025	AAGGCAC
ENSG00000006025	CUGCAGG
ENSG00000006025	CUGCCCC
ENSG00000006025	GGAGGCA
ENSG00000006025	GGGGAGC
ENSG00000006025	UGGGGGA
ENSG00000006042	AGUGGUU
ENSG00000006042	GAUUGUA
ENSG00000006047	AAAACUG
ENSG00000006047	ACUGGAU
ENSG00000006047	AUGGCUU
ENSG00000006047	CCUUCUG
ENSG00000006047	CCUUCUU
ENSG00000006047	CUCUGGG
ENSG00000006047	GAGGUAG
ENSG00000006047	GAGUCUU
ENSG00000006047	GAGUUGG
ENSG00000006047	GGAGGAG
ENSG00000006047	GGCUCAG
ENSG00000006047	UCCUGAG
ENSG00000006047	UCUCAAG
ENSG00000006047	UGAGUCU
ENSG00000006062	AAAGUGC
ENSG00000006062	AAGUGCU
ENSG00000006062	AGUGCAA
ENSG00000006062	AGUGCUU
ENSG00000006062	CAAAGUG
ENSG00000006062	GAGAACC
ENSG00000006062	GGCAGGG
ENSG00000006062	GUGCAAA
ENSG00000006062	UCACAGG
ENSG00000006062	UCACAGU
ENSG00000006062	UGCAAAG
ENSG00000006062	UUGAGGC
ENSG00000006075	GAGGUAG
ENSG00000006118	AGCACCA
ENSG00000006125	AAGUGCA
ENSG00000006125	AAUCUCU
ENSG00000006125	ACAAGGU
ENSG00000006125	ACCAGCA
ENSG00000006125	ACUCAGG
ENSG00000006125	AGUGGUU
ENSG00000006125	AUGAGCU
ENSG00000006125	GGUUACC

ENSG00000006125	UUCUGCA
ENSG00000006125	UUGGCAC
ENSG00000006125	UUGUGUC
ENSG00000006194	CUUUGGU
ENSG00000006210	ACUGCAG
ENSG00000006210	AGCACCA
ENSG00000006210	AGGGUUG
ENSG00000006210	AUGGCAC
ENSG00000006210	CAGCAGA
ENSG00000006283	UAUUGCU
ENSG00000006283	UUCUGCA
ENSG00000006327	CAAAUGC
ENSG00000006327	GUGCAAA
ENSG00000006377	AAAGUAU
ENSG00000006377	ACAAUAU
ENSG00000006377	ACUCCAG
ENSG00000006377	GCCUGGA
ENSG00000006377	UAUAUGA
ENSG00000006377	UCAACAG
ENSG00000006377	UCCAGUU
ENSG00000006432	AAGUGCA
ENSG00000006432	AUCCCUU
ENSG00000006432	GGAAGAC
ENSG00000006432	GUCUCUG
ENSG00000006432	GUGGCCC
ENSG00000006432	UAUCAGA
ENSG00000006432	UGGCCCU
ENSG00000006432	UGGCUGC
ENSG00000006451	ACAUUCA
ENSG00000006451	CAGUAGA
ENSG00000006451	GGCUCAG
ENSG00000006459	AAAACUG
ENSG00000006459	AAAUCCC
ENSG00000006459	AACAGUC
ENSG00000006459	AACCUGU
ENSG00000006459	AAGACAU
ENSG00000006459	AAUCUCA
ENSG00000006459	ACCCAUU
ENSG00000006459	ACCUGGA
ENSG00000006459	ACUCCAG
ENSG00000006459	ACUGGCC
ENSG00000006459	ACUGUAG
ENSG00000006459	ACUGUUU
ENSG00000006459	AGCAGCU
ENSG00000006459	AGCCAGU
ENSG00000006459	AGGUUAU
ENSG00000006459	AGUGCAA
ENSG00000006459	AGUGGUU
ENSG00000006459	CACAGGG
ENSG00000006459	CAGGCUC
ENSG00000006459	CAGUCUG
ENSG00000006459	CAGUGCA
ENSG00000006459	CCCUGUC
ENSG00000006459	CUACAAA
ENSG00000006459	CUAGUAA
ENSG00000006459	CUCCAGC
ENSG00000006459	CUGCAGG
ENSG00000006459	CUUUGGU
ENSG00000006459	GCCCUUA
ENSG00000006459	GCUAGCA
ENSG00000006459	GGAAGAC
ENSG00000006459	GGCAGUG
ENSG00000006459	GGGUGUU
ENSG00000006459	GGUUUAC

ENSG00000006459	GUAACAG
ENSG00000006459	GUCCAGA
ENSG00000006459	GUGUGGC
ENSG00000006459	UAUAAAG
ENSG00000006459	UAUUGCU
ENSG00000006459	UGCAUAG
ENSG00000006459	UGCAUAU
ENSG00000006459	UGCUGAC
ENSG00000006459	UGGAUAA
ENSG00000006459	UGGGACA
ENSG00000006459	UUGGUCC
ENSG00000006459	UUGGUUC
ENSG00000006468	AACAGUC
ENSG00000006468	AAGGCAC
ENSG00000006468	AAGUGCA
ENSG00000006468	AAGUGCU
ENSG00000006468	AAUGCCC
ENSG00000006468	AAUGUUG
ENSG00000006468	ACAGUAC
ENSG00000006468	ACAGUAU
ENSG00000006468	ACUGGCU
ENSG00000006468	AGCAGCA
ENSG00000006468	AGCAUUC
ENSG00000006468	AGCUGCC
ENSG00000006468	AGUGCUG
ENSG00000006468	AUAUUAU
ENSG00000006468	CAGCUGG
ENSG00000006468	CAGUAGA
ENSG00000006468	CAGUCCA
ENSG00000006468	CAGUGCA
ENSG00000006468	GGAUUUU
ENSG00000006468	GUCACUC
ENSG00000006468	GUGAAUG
ENSG00000006468	GUGCAAA
ENSG00000006468	UAAUGCU
ENSG00000006468	UACAAAG
ENSG00000006468	UCACAUU
ENSG00000006468	UCAUACA
ENSG00000006468	UCAUUUG
ENSG00000006468	UCCCACC
ENSG00000006468	UGAAGCU
ENSG00000006468	UGCAUAU
ENSG00000006468	UGCCAGC
ENSG00000006468	UGGAUAA
ENSG00000006468	UGGGUAC
ENSG00000006530	AGCAGCA
ENSG00000006530	UUGGUCC
ENSG00000006576	AAAGAGC
ENSG00000006576	AAAGCUG
ENSG00000006576	AAAGUGC
ENSG00000006576	AAGUGCA
ENSG00000006576	CUUUGGU
ENSG00000006576	UUGGUCC
ENSG00000006634	UAAUAUC
ENSG00000006634	UAUAAAG
ENSG00000006652	AAAAACC
ENSG00000006695	UGAAAUG
ENSG00000006712	CUGGGCA
ENSG00000006715	UCACAAG
ENSG00000006715	UCCUGAG
ENSG00000006757	AAAGAAU
ENSG00000006831	AAAGAGC
ENSG00000006831	AAAGUGC
ENSG00000006831	AACCUGG

ENSG00000006831	AAGGCAC
ENSG00000006831	AAGUGCA
ENSG00000006831	ACAUUAC
ENSG00000006831	AGCCAGU
ENSG00000006831	AUACAAG
ENSG00000006831	AUAUAAC
ENSG00000006831	CAAAGUG
ENSG00000006831	GAGGUAG
ENSG00000006831	GUGCUUG
ENSG00000006831	GUGUGGC
ENSG00000006831	UCUGGAA
ENSG00000006831	UGUGUGG
ENSG00000007047	AAAAACC
ENSG00000007047	AAAGUGC
ENSG00000007047	AACGGCU
ENSG00000007047	ACAAGGU
ENSG00000007047	ACUGGAU
ENSG00000007047	AGGCAGG
ENSG00000007047	CAAAGUG
ENSG00000007047	CAGCAGG
ENSG00000007047	CAGCUGG
ENSG00000007047	CAGGAAC
ENSG00000007047	CAGGUGA
ENSG00000007047	CCAGUGU
ENSG00000007047	CGUUGGC
ENSG00000007047	CUCCAGC
ENSG00000007047	CUGCAGC
ENSG00000007047	CUGCAGG
ENSG00000007047	GAAGGAA
ENSG00000007047	GCAGGUU
ENSG00000007047	GCCUGGG
ENSG00000007047	GCGGGUG
ENSG00000007047	GGCAGCG
ENSG00000007047	GGGGCAG
ENSG00000007047	GGUGCGG
ENSG00000007047	UGCCAGC
ENSG00000007047	UGGAGAU
ENSG00000007047	UGGGACA
ENSG00000007047	UUCUGCA
ENSG00000007062	AAAAGUA
ENSG00000007062	ACACACU
ENSG00000007062	GUAAACA
ENSG00000007168	AAAGUGC
ENSG00000007168	AAAUUUC
ENSG00000007168	AACAUUC
ENSG00000007168	AAGGCAC
ENSG00000007168	AAGUGCA
ENSG00000007168	ACAUUCA
ENSG00000007168	ACUGUAG
ENSG00000007168	AGUGCAA
ENSG00000007168	AUCACUA
ENSG00000007168	CACACCG
ENSG00000007168	CACACCU
ENSG00000007168	CAGUCUG
ENSG00000007168	CCCUGAG
ENSG00000007168	CUGGGCA
ENSG00000007168	GCACCAU
ENSG00000007168	GCAGAAG
ENSG00000007168	GCAUCCC
ENSG00000007168	GGAAUGU
ENSG00000007168	GUAACAG
ENSG00000007168	UAAUAUC
ENSG00000007168	UCACCAC
ENSG00000007168	UCUAGAG

ENSG00000007168	UGCCAGC
ENSG00000007168	UGGUACC
ENSG00000007168	UGUGUGG
ENSG00000007174	CUGCAGG
ENSG00000007202	AAAAAUC
ENSG00000007202	ACUGCAG
ENSG00000007202	CUGCAGC
ENSG00000007202	GGGUCUU
ENSG00000007202	UUGAUCA
ENSG00000007237	AAAACUG
ENSG00000007237	AAAGUAA
ENSG00000007237	AAAGUAC
ENSG00000007237	ACACACC
ENSG00000007237	ACACACU
ENSG00000007237	ACAUUCA
ENSG00000007237	ACCCAUU
ENSG00000007237	AGCACCA
ENSG00000007237	AGGUAGU
ENSG00000007237	AUGGCUU
ENSG00000007237	CAGCUGG
ENSG00000007237	CUAGUAA
ENSG00000007237	GAGGUAU
ENSG00000007237	GAUUGGU
ENSG00000007237	GCCUGAU
ENSG00000007237	GCUGGUG
ENSG00000007237	UCCUGAG
ENSG00000007237	UGCAAAG
ENSG00000007312	GUCUCUG
ENSG00000007314	AAGACAU
ENSG00000007314	CCUUCUG
ENSG00000007314	GGGGAGC
ENSG00000007314	GGGGCAG
ENSG00000007314	UAAGACG
ENSG00000007314	UAAGACU
ENSG00000007314	UCCCUUU
ENSG00000007341	AGCACCA
ENSG00000007341	AGGGAGG
ENSG00000007341	AUGCAUU
ENSG00000007341	CACACCG
ENSG00000007341	CACACCU
ENSG00000007341	CCCCUGG
ENSG00000007341	CUCACAC
ENSG00000007341	CUGCCCC
ENSG00000007341	CUGGGCA
ENSG00000007341	GACCCUG
ENSG00000007341	GAGACCU
ENSG00000007341	GCAGAAG
ENSG00000007341	GCUUCCU
ENSG00000007341	GGCGCCU
ENSG00000007341	UCACAUU
ENSG00000007341	UGCCAGC
ENSG00000007350	CUGCAGG
ENSG00000007372	AAAAGUA
ENSG00000007372	AAAGUUU
ENSG00000007372	AAGAACC
ENSG00000007372	CAAAUGC
ENSG00000007372	GAUUGGU
ENSG00000007372	UAUAAUA
ENSG00000007372	UAUAUGA
ENSG00000007372	UCAUGAU
ENSG00000007372	UGGAUAA
ENSG00000007372	UUCUUGU
ENSG00000007372	UUGAUCA
ENSG00000007372	UUGGCAA

ENSG00000007372	UUGGCAC
ENSG00000007372	UUGUUCG
ENSG00000007376	AAGGCAC
ENSG00000007384	AAGGCAC
ENSG00000007384	GGCAGCG
ENSG00000007384	GGUGGUC
ENSG00000007392	CACUCCU
ENSG00000007392	GACCCUG
ENSG00000007392	GGCUCAG
ENSG00000007402	AAAGUUU
ENSG00000007402	ACAAGGU
ENSG00000007402	ACACACU
ENSG00000007402	ACAUUCA
ENSG00000007402	CAGCUGG
ENSG00000007402	CAGUCCA
ENSG00000007402	CAGUUAC
ENSG00000007402	CUACAGU
ENSG00000007402	CUGGGGG
ENSG00000007402	CUUCACC
ENSG00000007402	GAGAAAU
ENSG00000007402	GAGCCCC
ENSG00000007402	GAGGGGC
ENSG00000007402	GGCAGUG
ENSG00000007402	GGGGGAA
ENSG00000007402	UACAGUU
ENSG00000007402	UCACACA
ENSG00000007402	UCUCCAA
ENSG00000007402	UGAAGUG
ENSG00000007402	UGGGGGA
ENSG00000007402	UUGGCAA
ENSG00000007402	UUUUUGC
ENSG00000007516	UGAAGCU
ENSG00000007545	AAAAACC
ENSG00000007545	AAAGUGC
ENSG00000007545	AUAUUAU
ENSG00000007545	AUGCACC
ENSG00000007545	AUGGCUU
ENSG00000007545	CAAAGUG
ENSG00000007545	CCCUGAG
ENSG00000007545	UAUAAUA
ENSG00000007866	AAUUUUA
ENSG00000007866	ACAGUAC
ENSG00000007866	AGGGAGG
ENSG00000007866	AUAAUAC
ENSG00000007866	CCCCCAG
ENSG00000007866	GAGCCCU
ENSG00000007866	GAGGUAG
ENSG00000007866	GAGUGUG
ENSG00000007866	GGAGUGU
ENSG00000007866	UCCCACA
ENSG00000007923	AAAAACC
ENSG00000007923	AGAUAAA
ENSG00000007923	AUCCUUG
ENSG00000007923	CCUGCUG
ENSG00000007923	UAUAAAG
ENSG00000007944	AAAGUAU
ENSG00000007944	AAAGUGC
ENSG00000007944	AAGGCAC
ENSG00000007944	AAGUCUU
ENSG00000007944	AAUCUCA
ENSG00000007944	AAUGCCC
ENSG00000007944	AUAUAAC
ENSG00000007944	AUGUCUG
ENSG00000007944	AUUGCAC

ENSG00000007944	GUGACAG
ENSG00000007944	GUGCCAC
ENSG00000007944	UAAUAUC
ENSG00000007944	UAUAUGA
ENSG00000007944	UGCAAAG
ENSG00000007944	UUGACAC
ENSG00000007952	AAAUUUC
ENSG00000007968	AAAACUG
ENSG00000007968	AAAGUGC
ENSG00000007968	AACAAAC
ENSG00000007968	AAGUGCU
ENSG00000007968	ACUCAGG
ENSG00000007968	AGUGCAA
ENSG00000007968	AGUGCUG
ENSG00000007968	CAGCUGG
ENSG00000007968	CCCUGAG
ENSG00000007968	CUACAAA
ENSG00000007968	CUCAGGG
ENSG00000007968	CUCUGGG
ENSG00000007968	GAGGUAG
ENSG00000007968	GCUACAU
ENSG00000007968	GCUGGUG
ENSG00000007968	GGCAAGA
ENSG00000007968	GUGAGGU
ENSG00000007968	GUGUCAC
ENSG00000007968	UACAAAG
ENSG00000007968	UCCCUGU
ENSG00000008056	ACCACAG
ENSG00000008056	GAGAAAU
ENSG00000008056	GUGCAAA
ENSG00000008056	GUGGGGA
ENSG00000008083	AAAAACC
ENSG00000008083	AAAAGUA
ENSG00000008083	AAGUAGC
ENSG00000008083	ACAUUCA
ENSG00000008083	ACCCUGU
ENSG00000008083	ACUCCAG
ENSG00000008083	AGCACCA
ENSG00000008083	AGCAGCA
ENSG00000008083	AGCAGCG
ENSG00000008083	AGCUGCC
ENSG00000008083	AGGCAGG
ENSG00000008083	AUAAAGU
ENSG00000008083	AUCCCUU
ENSG00000008083	AUGGAUU
ENSG00000008083	AUUGCAC
ENSG00000008083	CCAUUAC
ENSG00000008083	CUCCAUU
ENSG00000008083	GCUGGUG
ENSG00000008083	GGAUUUC
ENSG00000008083	GGCUCUG
ENSG00000008083	GUGCAAA
ENSG00000008083	UCAAGUA
ENSG00000008083	UCACAUU
ENSG00000008083	UCUCACC
ENSG00000008083	UGCAAAG
ENSG00000008083	UGCAUAG
ENSG00000008083	UUCUGCA
ENSG00000008118	AUGGCUU
ENSG00000008118	GACCUGG
ENSG00000008118	GAGACCU
ENSG00000008130	AAGUGUG
ENSG00000008130	GGAAUGU
ENSG00000008130	UGUGUGG

ENSG00000008196	AAAUGGU
ENSG00000008196	AACUACU
ENSG00000008196	AAGAACC
ENSG00000008196	AAGGCGC
ENSG00000008196	AAGUGCA
ENSG00000008196	AAUUAUU
ENSG00000008196	ACUGUUU
ENSG00000008196	AGCCCUU
ENSG00000008196	AUAAAGU
ENSG00000008196	AUACAAG
ENSG00000008196	AUGUAAU
ENSG00000008196	CCAUUAC
ENSG00000008196	GAGGUAU
ENSG00000008196	GCAGAAG
ENSG00000008196	GCCCUGU
ENSG00000008196	GGAUUUU
ENSG00000008196	GGUUGUC
ENSG00000008196	GUGGCCC
ENSG00000008196	UCUUGAG
ENSG00000008196	UGCCCUG
ENSG00000008196	UUGGUUC
ENSG00000008197	AGCAGCA
ENSG00000008197	CAGCAGA
ENSG00000008197	CAGCAGG
ENSG00000008277	GUAAACA
ENSG00000008282	CAGCAAA
ENSG00000008282	CAGUAGU
ENSG00000008282	CCAUUAC
ENSG00000008282	CUUACAG
ENSG00000008282	GUAGUGU
ENSG00000008282	UGUGCUU
ENSG00000008282	UUGGCAA
ENSG00000008283	ACUCAGC
ENSG00000008283	GAAAACA
ENSG00000008300	AAAAGUA
ENSG00000008300	AAAGGUA
ENSG00000008300	AAGGCAC
ENSG00000008300	ACAUUCA
ENSG00000008300	AGUAACA
ENSG00000008300	AUGGCAC
ENSG00000008300	CAGACAG
ENSG00000008300	CUGCAGC
ENSG00000008300	GCUGGUG
ENSG00000008300	GGAAGCC
ENSG00000008300	GGUUACC
ENSG00000008323	ACCAGCA
ENSG00000008323	GAGGUAU
ENSG00000008323	GCCUGGG
ENSG00000008441	AAAAACC
ENSG00000008441	AAAUUUC
ENSG00000008441	AAGGCAC
ENSG00000008441	AAUCUCU
ENSG00000008441	AAUGUUG
ENSG00000008441	ACAUUAC
ENSG00000008441	ACCCGGC
ENSG00000008441	ACCCUGU
ENSG00000008441	AGCACCA
ENSG00000008441	AGGGAGG
ENSG00000008441	AGUACUG
ENSG00000008441	AUAAUAC
ENSG00000008441	AUGCAUU
ENSG00000008441	AUUGCAC
ENSG00000008441	CAGCUGG
ENSG00000008441	CAGUGCA

ENSG00000008441	CCAUUAC
ENSG00000008441	CCCACCG
ENSG00000008441	CCCACCU
ENSG00000008441	CCCUGUU
ENSG00000008441	CGAGGAC
ENSG00000008441	CGUUGGC
ENSG00000008441	CUCAAAC
ENSG00000008441	CUCCCAC
ENSG00000008441	CUGCAGC
ENSG00000008441	CUGGCUC
ENSG00000008441	CUUGUGU
ENSG00000008441	GACCCUG
ENSG00000008441	GCGGGUG
ENSG00000008441	GGACCUG
ENSG00000008441	GGAGAGA
ENSG00000008441	GGAUUUU
ENSG00000008441	GGCAUUG
ENSG00000008441	GGCUGCG
ENSG00000008441	UACAAAG
ENSG00000008441	UAUAAUA
ENSG00000008441	UAUUGCU
ENSG00000008441	UCACAUU
ENSG00000008441	UCCCACC
ENSG00000008441	UCCGCUC
ENSG00000008441	UGCAGCU
ENSG00000008441	UGCAUUG
ENSG00000008441	UGUAUAA
ENSG00000008441	UUGGUUC
ENSG00000008441	UUGUUCG
ENSG00000008441	UUUUUGC
ENSG00000008513	UGCUGAC
ENSG00000008516	UCCAGAG
ENSG00000008710	AAGUGCU
ENSG00000008710	AGUGCUG
ENSG00000008710	UGAGGAC
ENSG00000008735	CACACCG
ENSG00000008735	CACACCU
ENSG00000008853	CCCUGAG
ENSG00000008952	AACAGUC
ENSG00000008952	AAGGCGC
ENSG00000008952	AAGUCUU
ENSG00000008952	ACAGUAC
ENSG00000008952	ACAGUAU
ENSG00000008952	ACAUAGA
ENSG00000008952	AUAAUAC
ENSG00000008952	CAGUCCA
ENSG00000008952	CUGCUCA
ENSG00000008952	CUUACAG
ENSG00000008952	GCCUGGA
ENSG00000008952	GCUGGAU
ENSG00000008952	GGAAUGU
ENSG00000008952	GGAUUUC
ENSG00000008952	GGAUUUU
ENSG00000008952	UAAGACU
ENSG00000008952	UACAAAG
ENSG00000008952	UCCAGUU
ENSG00000008952	UGAAGCU
ENSG00000008952	UGAGAAG
ENSG00000008952	UGAGAAU
ENSG00000008952	UUCCUAG
ENSG00000008952	UUGGCAC
ENSG00000008952	UUUCAAC
ENSG00000009307	AGCAGCA
ENSG00000009307	CUUCACC

ENSG00000009307	GCAGAAG
ENSG00000009307	UGUGCUU
ENSG00000009307	UUGAUCA
ENSG00000009335	AAAGUGC
ENSG00000009335	ACAUUCA
ENSG00000009335	AGUGCCU
ENSG00000009335	AUAAAGU
ENSG00000009335	CUACAAA
ENSG00000009335	UAUUGCU
ENSG00000009335	UUGGCAA
ENSG00000009413	AAAAGUA
ENSG00000009413	AAAGUAC
ENSG00000009413	AACAAAC
ENSG00000009413	AAUGCCC
ENSG00000009413	AGCAAAA
ENSG00000009413	CACAAUG
ENSG00000009413	GCACCAU
ENSG00000009413	GUAACAG
ENSG00000009413	UACAAAG
ENSG00000009413	UGUCUUU
ENSG00000009413	UUCCAUA
ENSG00000009694	AAGGCAC
ENSG00000009694	AUUGCAC
ENSG00000009694	CUUGUGU
ENSG00000009694	GAGGUAU
ENSG00000009694	GAUAUGU
ENSG00000009694	GCCUGAU
ENSG00000009694	GGCAUUG
ENSG00000009694	GGCUCAG
ENSG00000009694	GGUAGAC
ENSG00000009694	UAUAAUA
ENSG00000009694	UCAACAG
ENSG00000009694	UCAUUUG
ENSG00000009694	UCCUGAG
ENSG00000009694	UGCAAAG
ENSG00000009694	UGGAUAA
ENSG00000009694	UUCUGCA
ENSG00000009765	AAGUUGU
ENSG00000009765	CACUCCU
ENSG00000009765	GGCUCUG
ENSG00000009780	AACCUGG
ENSG00000009780	ACCUGGA
ENSG00000009780	CUAUACA
ENSG00000009780	UACAAAG
ENSG00000009830	CCUGCUG
ENSG00000009830	CUGGGGG
ENSG00000009830	GACCCUG
ENSG00000009830	GCUUCCU
ENSG00000009830	GGAGUGU
ENSG00000009830	GGGGGAA
ENSG00000009830	UCCCUUU
ENSG00000009830	UGGGGGA
ENSG00000009830	UUCCUCG
ENSG00000009844	AAAAGUA
ENSG00000009844	AACUGGU
ENSG00000009844	AGCACCA
ENSG00000009844	GAAAACA
ENSG00000009844	GCAGGUU
ENSG00000009844	GGACUGC
ENSG00000009844	UGGACUG
ENSG00000009950	CUGCUCA
ENSG00000009954	AAACUGU
ENSG00000009954	ACCCUGU
ENSG00000009954	ACUGUAG

ENSG00000009954	AUACAAG
ENSG00000009954	CCCUGUU
ENSG00000009954	CUACAAA
ENSG00000009954	GCAACUU
ENSG00000009954	GCAGGAC
ENSG00000009954	GGUGGGC
ENSG00000009954	UACAAAG
ENSG00000009954	UAUUACC
ENSG00000009954	UGAAAUG
ENSG00000010017	AAACUGU
ENSG00000010017	AACAAAC
ENSG00000010017	AACUACU
ENSG00000010017	AAGACAU
ENSG00000010017	ACAGUAU
ENSG00000010017	ACAUCAC
ENSG00000010017	ACUCAGC
ENSG00000010017	ACUGUUU
ENSG00000010017	AUAUAAC
ENSG00000010017	AUGUAAU
ENSG00000010017	AUUGCAC
ENSG00000010017	GAGUCUU
ENSG00000010017	GAUUGUA
ENSG00000010017	GUCUACU
ENSG00000010017	UAAGACG
ENSG00000010017	UAAGACU
ENSG00000010017	UACAAAG
ENSG00000010017	UACAGUU
ENSG00000010017	UAUUGCU
ENSG00000010017	UCAAGUA
ENSG00000010017	UCAUUUG
ENSG00000010017	UCCUGAG
ENSG00000010017	UGAGUCU
ENSG00000010072	GCAACUU
ENSG00000010244	AAUCUCA
ENSG00000010244	AAUCUCU
ENSG00000010244	AUGUAAU
ENSG00000010244	CAGCUGG
ENSG00000010244	CAGGAAC
ENSG00000010244	CAGUUAC
ENSG00000010244	GAGAAAU
ENSG00000010244	GUAUGUU
ENSG00000010244	UAUUACC
ENSG00000010244	UCACAAG
ENSG00000010244	UCUCCAA
ENSG00000010270	AAAGAGC
ENSG00000010270	AGGCAGG
ENSG00000010270	AUAUUAU
ENSG00000010270	GAGGUAG
ENSG00000010270	UACAAAG
ENSG00000010270	UCACACA
ENSG00000010278	CCUGCUG
ENSG00000010278	CUCAGGG
ENSG00000010278	CUGCUCA
ENSG00000010278	GGAUUUC
ENSG00000010278	UGUCUUU
ENSG00000010318	GGAGAGA
ENSG00000010319	ACAGUAC
ENSG00000010319	CUCUGGG
ENSG00000010319	CUGGGCA
ENSG00000010319	GAGAACC
ENSG00000010319	GAGAACU
ENSG00000010319	GCCUGGA
ENSG00000010319	GUCACUC
ENSG00000010319	UCAUUUG

ENSG00000010322	AGCAGCG
ENSG00000010322	AGCAGCU
ENSG00000010361	GAGACGC
ENSG00000010404	AGGGAGG
ENSG00000010404	CUGACUC
ENSG00000010404	CUGCUCA
ENSG00000010404	GGAAGAC
ENSG00000010539	ACAUUCA
ENSG00000010539	AGGUAGU
ENSG00000010539	GAGCUAA
ENSG00000010539	GUAAACA
ENSG00000010539	GUGAGGU
ENSG00000010610	ACAUUCA
ENSG00000010671	AGCUUAU
ENSG00000010704	CAGGAAC
ENSG00000010803	AAAGGUA
ENSG00000010803	CCUUCAU
ENSG00000010803	CUCCAGC
ENSG00000010803	GGGGUGC
ENSG00000010803	UAUAAAG
ENSG00000010803	UGAGAAG
ENSG00000010803	UGCUGAC
ENSG00000010810	AAAGAGG
ENSG00000010810	AAUACUG
ENSG00000010810	AUGCAUU
ENSG00000010810	UUGGCAC
ENSG00000010818	AAAAAUC
ENSG00000010818	AAAGUAC
ENSG00000010818	AAAGUAU
ENSG00000010818	AACUACU
ENSG00000010818	AAGAACC
ENSG00000010818	AAGUGCU
ENSG00000010818	ACAUAGA
ENSG00000010818	AUGAUGA
ENSG00000010818	CAAAGCA
ENSG00000010818	CAGCAAA
ENSG00000010818	GAAACAU
ENSG00000010818	UAUUACC
ENSG00000010818	UCACAGG
ENSG00000010818	UCACAGU
ENSG00000010818	UCACAUU
ENSG00000010818	UCAUGAU
ENSG00000010818	UUGGUCC
ENSG00000010818	UUGUUCG
ENSG00000011007	UCACAUU
ENSG00000011009	AAUACUG
ENSG00000011009	ACAUUCA
ENSG00000011009	AGCAGCA
ENSG00000011009	AGCAGCU
ENSG00000011009	CAGGCUC
ENSG00000011009	CCCUGAG
ENSG00000011009	CUGGGCA
ENSG00000011009	GGCUCAG
ENSG00000011009	GUGAGGU
ENSG00000011009	UCCUGAG
ENSG00000011021	AAAAAUC
ENSG00000011021	AAGGCAC
ENSG00000011021	CUGGAAA
ENSG00000011021	CUUUGGU
ENSG00000011021	GGCUCAG
ENSG00000011021	GUGCAAA
ENSG00000011021	UACAAAG
ENSG00000011021	UUCAAAU
ENSG00000011028	CACAGGG

ENSG00000011028	CUCCAGC
ENSG00000011028	GGCUCAG
ENSG00000011028	UCACAUU
ENSG00000011028	UUGGUCC
ENSG00000011105	AAAGUGC
ENSG00000011105	AAGUGCA
ENSG00000011105	AGCACCA
ENSG00000011105	AGCAGCA
ENSG00000011105	AGGGAGG
ENSG00000011105	AUGUCUG
ENSG00000011105	CUUUGGU
ENSG00000011105	GAAGGAA
ENSG00000011105	GAGGUAU
ENSG00000011105	GCAGCAU
ENSG00000011105	GCCUGGG
ENSG00000011105	GGAAUGU
ENSG00000011105	GGUGGGC
ENSG00000011105	GUGCUUG
ENSG00000011105	UCCUGAG
ENSG00000011105	UGUGCUU
ENSG00000011105	UUGGCAC
ENSG00000011114	AAAAGUA
ENSG00000011114	AAAGUAC
ENSG00000011114	AAAGUAU
ENSG00000011114	AACAGUC
ENSG00000011114	AAGGCAC
ENSG00000011114	AAGUGCU
ENSG00000011114	ACUUGGA
ENSG00000011114	AGCACCA
ENSG00000011114	AGCCCUU
ENSG00000011114	AGGACAC
ENSG00000011114	AGUACUG
ENSG00000011114	AUGCAUU
ENSG00000011114	CACAGGG
ENSG00000011114	CUAUACA
ENSG00000011114	CUCACAC
ENSG00000011114	CUUUGGU
ENSG00000011114	GAGUGUG
ENSG00000011114	GAUUGUC
ENSG00000011114	GCCUGAU
ENSG00000011114	GUAAACA
ENSG00000011114	GUAGUGU
ENSG00000011114	UCAAGUA
ENSG00000011114	UCACAAG
ENSG00000011114	UGCAUAU
ENSG00000011114	UGCCAGC
ENSG00000011114	UUAUGCA
ENSG00000011114	UUCUGCA
ENSG00000011114	UUGGUUC
ENSG00000011132	AAGGCAC
ENSG00000011132	CACAGUG
ENSG00000011198	AAGGCAC
ENSG00000011201	AACUCUA
ENSG00000011201	CUACAGU
ENSG00000011201	GUUAAUG
ENSG00000011258	AAUUGCU
ENSG00000011258	ACAUCAC
ENSG00000011258	AUAAUAC
ENSG00000011258	AUGCAUU
ENSG00000011258	GUGACUG
ENSG00000011258	UCCUGAG
ENSG00000011258	UUGACAC
ENSG00000011260	UAAGACG
ENSG00000011260	UAAGACU

ENSG00000011275	AAAGAAU
ENSG00000011275	AAGGCAC
ENSG00000011275	AAGUGCU
ENSG00000011275	ACACACC
ENSG00000011275	ACACACU
ENSG00000011275	AGUAGUU
ENSG00000011275	AUACAAG
ENSG00000011275	CAAAGUG
ENSG00000011275	CAGUAGU
ENSG00000011275	CUCCAGC
ENSG00000011275	GUGCAAA
ENSG00000011275	UCCAGUU
ENSG00000011304	AAAACUG
ENSG00000011304	AAACUGU
ENSG00000011304	AAAGUAU
ENSG00000011304	AAAGUGC
ENSG00000011304	AAUACUG
ENSG00000011304	AAUCUCU
ENSG00000011304	AAUUUUA
ENSG00000011304	ACUGGCC
ENSG00000011304	CAGCUGG
ENSG00000011304	CUUUGGU
ENSG00000011304	GGAAUGU
ENSG00000011304	UAAUACA
ENSG00000011304	UAUAAAG
ENSG00000011304	UAUAAUA
ENSG00000011304	UAUUGCU
ENSG00000011304	UCUGGAA
ENSG00000011304	UGCAUAG
ENSG00000011304	UUUGCAA
ENSG00000011304	UUUGCAC
ENSG00000011332	AUGGCUU
ENSG00000011332	UUGAGGC
ENSG00000011347	AAAGAGG
ENSG00000011347	AAAGUGC
ENSG00000011347	AGCACCA
ENSG00000011347	AUAAAGU
ENSG00000011347	AUCCUUU
ENSG00000011347	CAAAGCA
ENSG00000011347	CACCAGC
ENSG00000011347	CCCACCU
ENSG00000011347	CUACAAA
ENSG00000011347	CUGGCUC
ENSG00000011347	GAGGCAG
ENSG00000011347	GAGGUAG
ENSG00000011347	GAGGUAU
ENSG00000011347	GGAGGCA
ENSG00000011347	GGGUUUA
ENSG00000011347	UAGAGGA
ENSG00000011347	UAUGGUU
ENSG00000011347	UCACAAG
ENSG00000011347	UCAUUUG
ENSG00000011347	UCCCACC
ENSG00000011347	UCUGGAG
ENSG00000011347	UUUGCAC
ENSG00000011347	UUUUUGC
ENSG00000011376	CUGGGGG
ENSG00000011405	AAAGAGG
ENSG00000011405	AGCAGCU
ENSG00000011405	CUCAAAC
ENSG00000011405	CUUUGGU
ENSG00000011405	GCACCAU
ENSG00000011405	GGAAUGU
ENSG00000011405	GUCAGUU

ENSG00000011405	GUGCAAA
ENSG00000011405	GUUAGGA
ENSG00000011405	UCAAGUA
ENSG00000011405	UUCAAGC
ENSG00000011405	UUGGCAC
ENSG00000011405	UUGGUCC
ENSG00000011426	ACUGCAG
ENSG00000011426	AGCAGCA
ENSG00000011426	AGCAGCG
ENSG00000011451	AACGGAA
ENSG00000011451	ACAUAGA
ENSG00000011451	AGUGUGC
ENSG00000011451	CCCUGAG
ENSG00000011451	CUAUACA
ENSG00000011451	CUCUUCC
ENSG00000011451	CUGGAAA
ENSG00000011451	GAGAAAU
ENSG00000011451	GGAAUGU
ENSG00000011451	GGCAGGG
ENSG00000011451	GGCAUUG
ENSG00000011451	GGCUCAG
ENSG00000011451	GGGAGCU
ENSG00000011451	GGUCUAG
ENSG00000011451	GUAGUGU
ENSG00000011451	UCCAGAG
ENSG00000011451	UCCCACC
ENSG00000011451	UCUGGAA
ENSG00000011451	UGCAAAG
ENSG00000011454	AAGGUGC
ENSG00000011454	AAUUUUA
ENSG00000011454	ACAUUCA
ENSG00000011454	AGUGCCU
ENSG00000011454	AGUGCUU
ENSG00000011454	AUAAAGU
ENSG00000011454	AUACAAG
ENSG00000011454	CACAAUG
ENSG00000011454	CACACCU
ENSG00000011454	CAGUAGA
ENSG00000011454	CCAGUGC
ENSG00000011454	CUUACAG
ENSG00000011454	GGCAGCG
ENSG00000011454	GGUUUAC
ENSG00000011454	UCACACA
ENSG00000011454	UGCAUAU
ENSG00000011454	UUGGCAA
ENSG00000011454	UUGGCAC
ENSG00000011465	CCAGUAC
ENSG00000011465	UAAUACA
ENSG00000011478	CUCUUCC
ENSG00000011485	AAAGUAA
ENSG00000011485	AAUGCCC
ENSG00000011485	GGGGUCC
ENSG00000011485	UAUUGCU
ENSG00000011485	UGCCCUG
ENSG00000011523	AAAGCUG
ENSG00000011523	AAGGCAC
ENSG00000011523	ACAUUAC
ENSG00000011523	AGCAGCA
ENSG00000011523	AUUCAUU
ENSG00000011523	CACAAUG
ENSG00000011523	GGCUCUG
ENSG00000011523	GGGUUUA
ENSG00000011523	GUGCUUG
ENSG00000011523	UCAAGUA

ENSG00000011523	UCACAAG
ENSG00000011523	UCCCUUG
ENSG00000011523	UCCUGAG
ENSG00000011566	AGGUAGU
ENSG00000011566	CCAGUGU
ENSG00000011566	CUUGUGU
ENSG00000011566	GAGGUAU
ENSG00000011638	AAGUGCA
ENSG00000011638	UCAACAG
ENSG00000012048	AACAGUC
ENSG00000012048	AAUUAUU
ENSG00000012048	AUUCAUU
ENSG00000012048	CCUUCAU
ENSG00000012048	CUGGGGG
ENSG00000012048	GGAAGAC
ENSG00000012048	GGGAUCG
ENSG00000012048	UACAAAG
ENSG00000012048	UGGAUAA
ENSG00000012048	UGGGAUC
ENSG00000012048	UGUGCUU
ENSG00000012171	ACAUUCA
ENSG00000012171	GCUACAU
ENSG00000012171	GUUAAUG
ENSG00000012171	UAUUGCU
ENSG00000012171	UCACAGG
ENSG00000012171	UCACAGU
ENSG00000012232	AAAAAUC
ENSG00000012232	AAGGCAC
ENSG00000012232	AGCAGCA
ENSG00000012232	AGCAGCG
ENSG00000012232	AGCAGCU
ENSG00000012232	AUAUAAC
ENSG00000012232	CAGUGCA
ENSG00000012232	CCAGUGU
ENSG00000012232	CUGCUCA
ENSG00000012232	GCCCUGU
ENSG00000012232	GGAAGAC
ENSG00000012232	GUGUGGC
ENSG00000012232	UAAUAGU
ENSG00000012232	UACAAAG
ENSG00000012232	UGCCCUG
ENSG00000012232	UGUGCUU
ENSG00000012660	GGCCCUG
ENSG00000012660	GGGCCCC
ENSG00000012822	AAGGCAC
ENSG00000012822	AUCAUAC
ENSG00000012822	GGGGCAG
ENSG00000012822	UAUUGCU
ENSG00000012963	AAGGCAC
ENSG00000012963	AGCCCUU
ENSG00000012963	AGGCAGG
ENSG00000012963	AUCCCUU
ENSG00000012963	AUGCAUU
ENSG00000012963	CACAGGG
ENSG00000012963	CAGCAGA
ENSG00000012963	CCCUGAG
ENSG00000012963	GCUUCCU
ENSG00000012963	GGAAGCC
ENSG00000012963	UCACAGG
ENSG00000012983	CACAGUG
ENSG00000013016	AAGGCAC
ENSG00000013016	ACUUGGA
ENSG00000013016	CACAGUG
ENSG00000013016	CCUGCUG

ENSG00000013016	CUUGGAG
ENSG00000013016	UCACAGU
ENSG00000013016	UGCCAGC
ENSG00000013293	UGAAAUG
ENSG00000013297	AAGGCAC
ENSG00000013297	ACCCGUA
ENSG00000013297	CAGGAAC
ENSG00000013297	CAGUCUG
ENSG00000013297	GGAGAGA
ENSG00000013297	UAUAAAG
ENSG00000013297	UCAUUUG
ENSG00000013306	AAGGCAC
ENSG00000013306	UUGGUCC
ENSG00000013503	UUCUAAU
ENSG00000013523	CCCCUGG
ENSG00000013523	CUUUGGU
ENSG00000013523	GAGCUAA
ENSG00000013523	GCUGGAU
ENSG00000013523	GGCUCAG
ENSG00000013523	GUCUCUG
ENSG00000013523	UCUGGAA
ENSG00000013523	UGAUCCA
ENSG00000013573	AAUUGCU
ENSG00000013588	CCAGUGU
ENSG00000013588	CUCCCAC
ENSG00000013588	CUUACAG
ENSG00000013588	UAUUGCU
ENSG00000013619	UCACAGG
ENSG00000013619	UGCAUAU
ENSG00000014164	GAGGUAG
ENSG00000014164	GAGGUAU
ENSG00000014164	UGCUGAC
ENSG00000014216	AAGGCAC
ENSG00000014216	AGGCAGG
ENSG00000014216	CUGCAGG
ENSG00000014216	GAGGCAG
ENSG00000014216	UGCAGCU
ENSG00000014824	UAUAAAG
ENSG00000015133	CACAGUG
ENSG00000015133	CCAGUGC
ENSG00000015133	CCAGUGU
ENSG00000015133	UAUUACC
ENSG00000015133	UCCAGAG
ENSG00000015133	UUGGCAC
ENSG00000015153	AAAGAAU
ENSG00000015153	AAAGUAC
ENSG00000015153	ACAAUAU
ENSG00000015153	ACAGUAC
ENSG00000015153	ACCAGCA
ENSG00000015153	ACUGUGU
ENSG00000015153	AGUAGAC
ENSG00000015153	AUAAAGU
ENSG00000015153	CACAAUG
ENSG00000015153	CAGCAGG
ENSG00000015153	CCAGUGU
ENSG00000015153	CUACAGU
ENSG00000015153	CUAGUAA
ENSG00000015153	CUCAAAC
ENSG00000015153	CUUACAG
ENSG00000015153	CUUGUAU
ENSG00000015153	GAGGAUA
ENSG00000015153	GAGGUAG
ENSG00000015153	GCAGCAU
ENSG00000015153	GUGCAAA

ENSG00000015153	UACAAAG
ENSG00000015153	UCACAAG
ENSG00000015153	UCCUAUA
ENSG00000015153	UCUCAAG
ENSG00000015153	UCUGGAA
ENSG00000015153	UGUGUGG
ENSG00000015153	UUCCUAG
ENSG00000015153	UUGGCAA
ENSG00000015171	AAAAACC
ENSG00000015171	AACGGCU
ENSG00000015171	AAUGCCC
ENSG00000015171	AAUUAUU
ENSG00000015171	ACCCUGU
ENSG00000015171	ACUUGGA
ENSG00000015171	AGCAGCU
ENSG00000015171	AGUAACA
ENSG00000015171	AUACAAG
ENSG00000015171	AUGCAUU
ENSG00000015171	CACAAUG
ENSG00000015171	CCCUGUU
ENSG00000015171	CCUGCUG
ENSG00000015171	GCUGGUG
ENSG00000015171	GGAAUGU
ENSG00000015171	GUGUCAC
ENSG00000015171	GUUAGGA
ENSG00000015171	UAUAAAG
ENSG00000015171	UAUUGCU
ENSG00000015171	UCUGGAA
ENSG00000015171	UGCAGCU
ENSG00000015171	UGCAUAG
ENSG00000015475	AAGGCAC
ENSG00000015479	GGCUCAG
ENSG00000015532	CCCCUGG
ENSG00000015532	CUGUUGA
ENSG00000015532	UGCAUAG
ENSG00000015592	AGGAUGU
ENSG00000015592	AGUGCUG
ENSG00000015592	AUGGCUU
ENSG00000015592	CCAGUGC
ENSG00000015592	CUCCCAC
ENSG00000015592	CUGGAAA
ENSG00000015592	UCCCACC
ENSG00000015592	UGGCCAC
ENSG00000015676	AUUGCAC
ENSG00000015676	CAGCUGG
ENSG00000015676	GAAAACA
ENSG00000015676	UAUUGCU
ENSG00000015676	UGCAAAG
ENSG00000016082	AAGUCUU
ENSG00000016082	AGCACCA
ENSG00000016082	CUAUACA
ENSG00000016082	CUUCACC
ENSG00000016082	CUUUGGU
ENSG00000016082	GAGACGC
ENSG00000016082	GAUUGUA
ENSG00000016082	GCACCAU
ENSG00000016082	GGCAAGA
ENSG00000016082	UCACAGG
ENSG00000016082	UGUCUUU
ENSG00000016082	UUGGCAA
ENSG00000016082	UUGGCAC
ENSG00000016402	UGUCUGC
ENSG00000016864	GAGAAAU
ENSG00000017260	AAGUGCU

ENSG00000017260	ACAGUAC
ENSG00000017260	ACAGUAU
ENSG00000017260	AGUGAUC
ENSG00000017260	AUGCCUU
ENSG00000017260	CAAAGUG
ENSG00000017260	CACCACC
ENSG00000017260	CAGCAGG
ENSG00000017260	GCCCUUA
ENSG00000017260	GGUGGUU
ENSG00000017260	UGCAUAG
ENSG00000017260	UGCAUAU
ENSG00000017260	UGCCAGC
ENSG00000017260	UGCCCUG
ENSG00000017260	UUCAAAU
ENSG00000017260	UUCCUAG
ENSG00000017260	UUUCAAC
ENSG00000017260	UUUGCAA
ENSG00000017427	AAAGCUG
ENSG00000017427	AAAUUUC
ENSG00000017427	AAGGUGC
ENSG00000017427	ACUCCAG
ENSG00000017427	ACUGGAU
ENSG00000017427	AGCAGCA
ENSG00000017427	AGCAGCG
ENSG00000017427	AGCAGCU
ENSG00000017427	AGGCAGG
ENSG00000017427	AGGUAGU
ENSG00000017427	AUAUAAC
ENSG00000017427	AUGCAUU
ENSG00000017427	CACAGUG
ENSG00000017427	CAGACAG
ENSG00000017427	CAGUAGA
ENSG00000017427	CAGUAGU
ENSG00000017427	CCAGCAU
ENSG00000017427	CUACAAA
ENSG00000017427	CUCCAGC
ENSG00000017427	CUUCACC
ENSG00000017427	CUUUGGU
ENSG00000017427	GAGGUAG
ENSG00000017427	GAGGUAU
ENSG00000017427	GCACCAU
ENSG00000017427	GCUACAU
ENSG00000017427	GGGUUUA
ENSG00000017427	GUAAACA
ENSG00000017427	UCAAGUA
ENSG00000017427	UCACACA
ENSG00000017427	UCACAGG
ENSG00000017427	UCACAGU
ENSG00000017427	UCUCAAG
ENSG00000017427	UGAAGUG
ENSG00000017427	UGACCUA
ENSG00000017427	UGGCCAC
ENSG00000017427	UUCUAAU
ENSG00000017621	ACCACAG
ENSG00000017621	AGGAUGU
ENSG00000017621	CACACCG
ENSG00000017621	CACACCU
ENSG00000017621	CUCACAC
ENSG00000017621	CUGACUC
ENSG00000017621	GUGACUG
ENSG00000017797	AAGGCAC
ENSG00000017797	ACAGGUG
ENSG00000017797	ACAGUAC
ENSG00000017797	ACAGUAU

ENSG00000017797	ACAUUCA
ENSG00000017797	AGGUAGU
ENSG00000017797	AGUGCAA
ENSG00000017797	CAGUGCA
ENSG00000017797	CUCCAGC
ENSG00000017797	CUGUUGA
ENSG00000017797	CUUCUCU
ENSG00000017797	GAGGCAG
ENSG00000017797	GGUUUAC
ENSG00000017797	UAGAGGA
ENSG00000017797	UAUUGCU
ENSG00000017797	UCUGGAA
ENSG00000017797	UGUUGAA
ENSG00000017797	UUGAUCA
ENSG00000017797	UUGGUUC
ENSG00000018189	AAGUCAC
ENSG00000018189	AUCCUUG
ENSG00000018189	CUACAGU
ENSG00000018189	GAAGGAA
ENSG00000018189	GAGGUAG
ENSG00000018189	UACAGUU
ENSG00000018236	AAGGCAC
ENSG00000018280	AAAGAGG
ENSG00000018280	CUGCCCC
ENSG00000018280	CUGGAAA
ENSG00000018280	GACCUGG
ENSG00000018280	GCCCUUA
ENSG00000018280	GGAGGAG
ENSG00000018280	GGGGAGC
ENSG00000018280	UGCCCUG
ENSG00000018510	AACCUGU
ENSG00000018510	CAGCUGG
ENSG00000018625	CUCAAAC
ENSG00000018625	CUUACAG
ENSG00000018625	GGCAGUG
ENSG00000019144	AAGGCAC
ENSG00000019144	AAGGCGC
ENSG00000019144	AAUACUG
ENSG00000019144	CCAGGAG
ENSG00000019144	CCUUCUG
ENSG00000019144	CUGCUCA
ENSG00000019144	CUUUGGU
ENSG00000019144	GGAGAGA
ENSG00000019144	GGCUCUG
ENSG00000019144	UACAAAG
ENSG00000019144	UGGUACC
ENSG00000019144	UUGGCAA
ENSG00000019144	UUGGCAC
ENSG00000019186	UCCCUGU
ENSG00000019505	AGGGAGG
ENSG00000019505	CUCAGGG
ENSG00000019505	GGAGGCA
ENSG00000019505	GUGCUUG
ENSG00000019505	UCACAAG
ENSG00000019505	UCUGGAA
ENSG00000019505	UGAUCCA
ENSG00000019549	GGAAUGU
ENSG00000019991	ACUGGCU
ENSG00000019991	UACAAAG
ENSG00000019995	AAAGUGC
ENSG00000019995	ACUGUAG
ENSG00000019995	AGGACAC
ENSG00000019995	AGUGCUU
ENSG00000019995	AUGCAUU

ENSG00000019995	CUUACAG
ENSG00000019995	GCUGGUG
ENSG00000019995	GUGCUUG
ENSG00000019995	UACAAAG
ENSG00000019995	UAUCAGA
ENSG00000019995	UCACAGG
ENSG00000019995	UCACAGU
ENSG00000019995	UCCCACA
ENSG00000019995	UCCUAUA
ENSG00000019995	UCCUGAG
ENSG00000019995	UGAAGUG
ENSG00000019995	UGGAUAA
ENSG00000019995	UGUGCUU
ENSG00000019995	UUCCUAG
ENSG00000019995	UUGUUCG
ENSG00000020129	AAAGCUG
ENSG00000020129	ACGCACU
ENSG00000020129	CCUGCUG
ENSG00000020129	CGGCCUG
ENSG00000020129	CUGGCUC
ENSG00000020129	GAAACAU
ENSG00000020129	GCUGGAU
ENSG00000020129	GGACCUG
ENSG00000020129	GGCAGGG
ENSG00000020129	GGCAGUG
ENSG00000020129	GGCGGGG
ENSG00000020129	GGGUGUU
ENSG00000020129	UGCCAGC
ENSG00000020129	UGGGGGA
ENSG00000020181	AAGGCAC
ENSG00000020181	AGCAGCA
ENSG00000020181	AUAAUAC
ENSG00000020181	AUAUUAU
ENSG00000020181	AUGUGGG
ENSG00000020181	AUUGCAC
ENSG00000020181	CAUAUUG
ENSG00000020181	CCAGUGU
ENSG00000020181	CUCCAGC
ENSG00000020181	CUUUGGU
ENSG00000020181	GCAGCAU
ENSG00000020181	GGAAGAC
ENSG00000020181	GUAAACA
ENSG00000020181	UAAUACA
ENSG00000020181	UAAUAUC
ENSG00000020181	UACAAAG
ENSG00000020181	UCAUUCG
ENSG00000020181	UCCCUCU
ENSG00000020181	UGCAAAG
ENSG00000020181	UGCAUAG
ENSG00000020181	UGCAUAU
ENSG00000020181	UUGGCAA
ENSG00000020181	UUUGCAA
ENSG00000020256	AACAAAC
ENSG00000020633	AAGUGCA
ENSG00000020633	AAGUGCU
ENSG00000020633	AAGUUGU
ENSG00000020633	ACAAUAU
ENSG00000020633	ACUGUAG
ENSG00000020633	AGUGCUG
ENSG00000020633	CAAAGCA
ENSG00000020633	CAAAGUG
ENSG00000020633	CAGGAAC
ENSG00000020633	CCAGUGC
ENSG00000020633	CCAGUGU

ENSG00000020633	CUACAAA
ENSG00000020633	CUGGGGG
ENSG00000020633	GUGCAAA
ENSG00000020633	UACAAAG
ENSG00000020633	UGCAAAG
ENSG00000020922	GAGAAAU
ENSG00000020922	UGCAUAG
ENSG00000021574	AAAAAUC
ENSG00000021574	AAUACUG
ENSG00000021574	AGCACCA
ENSG00000021574	AUACAAG
ENSG00000021574	UAUUACC
ENSG00000021574	UGUGCUU
ENSG00000021574	UUGGCAA
ENSG00000021645	AAGUGUG
ENSG00000021645	AUGCAUU
ENSG00000021645	GAUAUGU
ENSG00000021645	UGUGUCA
ENSG00000021762	AAGUGCU
ENSG00000021762	AGCAUUC
ENSG00000021762	GGAAGAC
ENSG00000021762	GGCUCUG
ENSG00000021762	GUGCCUG
ENSG00000021826	CACAAUG
ENSG00000021826	GCACCAU
ENSG00000021826	UCACAAG
ENSG00000021852	AUGGCAC
ENSG00000022267	ACUGCAG
ENSG00000022267	CAGGCUC
ENSG00000022267	GAGCCCC
ENSG00000022267	GGCGCCU
ENSG00000022355	AAUCUCA
ENSG00000022355	AGCCCUU
ENSG00000022355	GCCCUUA
ENSG00000022355	UAAGACG
ENSG00000022355	UAAGACU
ENSG00000022567	AAAGUGC
ENSG00000022567	AAGUGCA
ENSG00000022567	GAGGUAG
ENSG00000022840	AAAAAUC
ENSG00000022840	ACUCAAA
ENSG00000022840	AUUGCAC
ENSG00000022840	CAGUAGU
ENSG00000022840	CUGGAAA
ENSG00000022840	GAGCUAA
ENSG00000022840	GCAACUU
ENSG00000022840	UAUUGCU
ENSG00000022840	UUCUUGU
ENSG00000022840	UUGGUUC
ENSG00000022976	UAUAAAG
ENSG00000023041	AUGGCAC
ENSG00000023041	AUGGCUU
ENSG00000023228	AAAACUG
ENSG00000023287	AAAGUGC
ENSG00000023287	AACAUUC
ENSG00000023287	AAGGCAC
ENSG00000023287	AAGUGCU
ENSG00000023287	ACAGGUG
ENSG00000023287	AGUGCUU
ENSG00000023287	AUAAAGU
ENSG00000023287	CAAAUGC
ENSG00000023287	CACUCCU
ENSG00000023287	GCUGGUG
ENSG00000023287	GUUAAUG

ENSG00000023287	UCACAAG
ENSG00000023287	UUCAAAU
ENSG00000023516	AAAGGUA
ENSG00000023516	ACAUUAC
ENSG00000023516	AGCAGCG
ENSG00000023516	AGCAGCU
ENSG00000023516	CCAGUGC
ENSG00000023516	GCUGGUG
ENSG00000023516	GGAAUGU
ENSG00000023572	GUUCAUG
ENSG00000023892	UCUCAAG
ENSG00000023902	GAGGUAG
ENSG00000023902	GAGGUAU
ENSG00000023902	UUUUUGC
ENSG00000023909	ACCCUGU
ENSG00000023909	AUUGCAC
ENSG00000023909	CUUUGGU
ENSG00000023909	GGCAAGA
ENSG00000023909	UCCAGAG
ENSG00000023909	UUCUUGU
ENSG00000024422	AGCACCA
ENSG00000024422	GAGCCCC
ENSG00000024422	GGCAGGG
ENSG00000024422	GGGGAGC
ENSG00000024526	GAGAAAU
ENSG00000025039	AAAGUGC
ENSG00000025039	AAGUGCU
ENSG00000025293	AAAGGUA
ENSG00000025293	AAUGUUG
ENSG00000025293	ACAUUAC
ENSG00000025293	ACCUGGA
ENSG00000025293	AGCAGCA
ENSG00000025293	CACAGUG
ENSG00000025293	CAGUGCA
ENSG00000025293	CCCUGAG
ENSG00000025293	CUCACAC
ENSG00000025293	GGCUCUG
ENSG00000025293	GUGCUUG
ENSG00000025293	GUUAAUG
ENSG00000025293	UAUUGCU
ENSG00000025293	UCAAGUA
ENSG00000025293	UGCAAAG
ENSG00000025293	UGCAGCU
ENSG00000025293	UGUGCUU
ENSG00000025293	UUCAAGC
ENSG00000025293	UUUGCAC
ENSG00000025423	AAAGAAU
ENSG00000025434	GGAAUGU
ENSG00000025772	AAGGCAC
ENSG00000025772	CCCACCU
ENSG00000025772	GAGAACC
ENSG00000025796	GGAAUGU
ENSG00000025800	AAAGAAU
ENSG00000025800	AAAGAGC
ENSG00000025800	AAAGAGG
ENSG00000025800	AAGUCAC
ENSG00000025800	ACAGUAU
ENSG00000025800	ACUCAGC
ENSG00000025800	AGGCAGG
ENSG00000025800	AGGGAGG
ENSG00000025800	AUACAAG
ENSG00000025800	AUAUAAC
ENSG00000025800	AUGACAC
ENSG00000025800	CACAGUG

ENSG00000025800	CACCACC
ENSG00000025800	CAGCAGA
ENSG00000025800	CAGCUGG
ENSG00000025800	CAGGAAC
ENSG00000025800	CAGUAGA
ENSG00000025800	CAGUAGU
ENSG00000025800	CAUGGAU
ENSG00000025800	CCCCUGG
ENSG00000025800	CCCUGAG
ENSG00000025800	CUAGUCC
ENSG00000025800	CUCUUCC
ENSG00000025800	CUGGAAA
ENSG00000025800	GAAGGAA
ENSG00000025800	GAGAACC
ENSG00000025800	GAGAACU
ENSG00000025800	GAGGCAG
ENSG00000025800	GCAACUU
ENSG00000025800	GCACCAU
ENSG00000025800	GGAAGAC
ENSG00000025800	GGAGGCA
ENSG00000025800	GGCAGGG
ENSG00000025800	GGCCCUG
ENSG00000025800	GGGAUCG
ENSG00000025800	GGGUCGG
ENSG00000025800	GGUGGUC
ENSG00000025800	GGUGGUU
ENSG00000025800	GUAAACA
ENSG00000025800	GUCUACU
ENSG00000025800	UAAUAUC
ENSG00000025800	UCACAGU
ENSG00000025800	UCACCAC
ENSG00000025800	UCUGGAA
ENSG00000025800	UGACAUC
ENSG00000025800	UGACCUA
ENSG00000025800	UGAUCCA
ENSG00000025800	UGGCCAC
ENSG00000025800	UGGGAUC
ENSG00000025800	UGUGUGG
ENSG00000025800	UUCAAAU
ENSG00000025800	UUCAAGC
ENSG00000025800	UUCUUGU
ENSG00000025800	UUGACAC
ENSG00000025800	UUGGUCC
ENSG00000025800	UUUCAAC
ENSG00000026025	AAAACUG
ENSG00000026025	AAAGCUG
ENSG00000026025	AAGGCAC
ENSG00000026025	CUGGAAA
ENSG00000026025	GCUGGUG
ENSG00000026025	UGAAGCU
ENSG00000026508	CAGUAGA
ENSG00000026508	CAGUAGU
ENSG00000026508	CUUCACC
ENSG00000026652	AGCACCA
ENSG00000026652	GAGACCU
ENSG00000026652	UCCCUUG
ENSG00000026652	UCCCUUU
ENSG00000026652	UUGGAGG
ENSG00000027075	UCACAGG
ENSG00000027075	UCACAGU
ENSG00000027847	GCUUCCU
ENSG00000027869	AACAAAC
ENSG00000027869	AGCAGCA
ENSG00000027869	AGCCAGU

ENSG00000027869	CAGACAG
ENSG00000027869	GCAGCAU
ENSG00000027869	GGCUCAG
ENSG00000028137	AGCUGCC
ENSG00000028137	CAGCUGG
ENSG00000028137	CAGUGCA
ENSG00000028137	GAGGUAG
ENSG00000028137	GAGGUAU
ENSG00000028137	GUGCAAA
ENSG00000028203	ACAGUAC
ENSG00000028203	ACAGUAU
ENSG00000028203	AGCCCUU
ENSG00000028203	GCCCUUA
ENSG00000028277	ACAUUCA
ENSG00000028277	AGCACCA
ENSG00000028277	AUCCUUU
ENSG00000028277	CAAAACU
ENSG00000028277	CACUCCU
ENSG00000028277	CAGCUGG
ENSG00000028277	CCCCCAG
ENSG00000028277	CUGGAAA
ENSG00000028277	CUGGCUC
ENSG00000028277	CUGUUGA
ENSG00000028277	CUUUGGU
ENSG00000028277	GAGGUAG
ENSG00000028277	GAGGUAU
ENSG00000028277	GCCCUGU
ENSG00000028277	GGAGGCA
ENSG00000028277	GUCACUC
ENSG00000028277	UCUGGAA
ENSG00000028277	UCUGGAG
ENSG00000028277	UGAAGCU
ENSG00000028277	UGGAGAU
ENSG00000028277	UUCCUCG
ENSG00000028277	UUGUGAC
ENSG00000028528	ACUCCAG
ENSG00000028528	AGCAAAA
ENSG00000028528	AGCCAGU
ENSG00000028528	AGGGAGG
ENSG00000028528	AGUAACA
ENSG00000028528	AUAUUAU
ENSG00000028528	AUGGCAC
ENSG00000028528	CAGGUGA
ENSG00000028528	GACCUGG
ENSG00000028528	GAGGUAG
ENSG00000028528	GCACCAU
ENSG00000028528	GGAAGCC
ENSG00000028528	GGCAAGA
ENSG00000028528	GGCAGCG
ENSG00000028528	GUAACAG
ENSG00000028528	UAUUACC
ENSG00000028528	UGAGAAU
ENSG00000028528	UGUAUAA
ENSG00000028528	UUCAAGC
ENSG00000028528	UUGGUCC
ENSG00000028839	ACAUUCA
ENSG00000028839	AGUGCAA
ENSG00000028839	GUAAACA
ENSG00000028839	UUGGUCC
ENSG00000029363	AAAUGGU
ENSG00000029363	AAUCCUU
ENSG00000029363	ACAAGGU
ENSG00000029363	ACUCAAA
ENSG00000029363	AUCCUUU

ENSG00000029363	AUGACAC
ENSG00000029363	CAAAGCA
ENSG00000029363	CACACCU
ENSG00000029363	CCCUGUC
ENSG00000029363	CCCUGUU
ENSG00000029363	CUUUGGU
ENSG00000029363	GAGCUAA
ENSG00000029363	GUAAACA
ENSG00000029363	UACAAAG
ENSG00000029363	UCACAGG
ENSG00000029363	UCACAGU
ENSG00000029363	UCCCUGU
ENSG00000029363	UGCAAAG
ENSG00000029363	UUGGUUC
ENSG00000029364	AAAUGGU
ENSG00000029364	AGCACCA
ENSG00000029364	AGCAGCA
ENSG00000029364	AGUGGUU
ENSG00000029364	CAGCAGA
ENSG00000029364	CUGGACU
ENSG00000029364	GGUUGUC
ENSG00000029364	UCCCUGU
ENSG00000029364	UGAGAAU
ENSG00000029364	UUGGCAC
ENSG00000029534	ACAUCAC
ENSG00000029534	ACAUUCA
ENSG00000029534	ACUGCAG
ENSG00000029534	ACUGUAG
ENSG00000029534	AGCACCA
ENSG00000029534	AGCAUUC
ENSG00000029534	AGGAGCU
ENSG00000029534	AGGCAGG
ENSG00000029534	CACAGUG
ENSG00000029534	CUACAGU
ENSG00000029534	CUCUAGC
ENSG00000029534	CUCUGGG
ENSG00000029534	CUGGCUC
ENSG00000029534	CUUACAG
ENSG00000029534	CUUCUCU
ENSG00000029534	GGAAGCC
ENSG00000029534	GGAUUUU
ENSG00000029534	GUGUGGC
ENSG00000029534	UACAGUU
ENSG00000029534	UCACAGU
ENSG00000029534	UGCAUAU
ENSG00000029534	UGUGCUU
ENSG00000029534	UGUGUGG
ENSG00000029639	ACUGUAG
ENSG00000029725	AAAGGUA
ENSG00000029725	AAGUGCU
ENSG00000029725	AAUACUG
ENSG00000029725	AGCAGCU
ENSG00000029725	AGCAUUC
ENSG00000029725	AGGACAC
ENSG00000029725	AGUGCUU
ENSG00000029725	AUUCAUU
ENSG00000029725	CAGUUAC
ENSG00000029725	CAUAUUG
ENSG00000029725	CUUGUGU
ENSG00000029725	GUGCUUG
ENSG00000029725	UAAUAUC
ENSG00000029725	UACAGUU
ENSG00000029725	UUCUUGU
ENSG00000029725	UUGUGAC

ENSG00000029993	AAAGUGC
ENSG00000029993	AAGUGCA
ENSG00000029993	AGCAUUC
ENSG00000029993	AUGCCUU
ENSG00000029993	AUGUAAU
ENSG00000029993	CAAAGUG
ENSG00000029993	CACCACC
ENSG00000029993	CAGCAAA
ENSG00000029993	CAGCAGA
ENSG00000029993	CAGUGCA
ENSG00000029993	CCCUGAG
ENSG00000029993	GAAACAU
ENSG00000029993	GCAUCCC
ENSG00000029993	GGCAGCG
ENSG00000029993	GUAAACA
ENSG00000029993	UAAUGCU
ENSG00000029993	UAUUACC
ENSG00000029993	UCAAGUA
ENSG00000029993	UCACAGU
ENSG00000029993	UCAUUUG
ENSG00000029993	UGCCAGC
ENSG00000030066	AGCACCA
ENSG00000030066	CACCAGC
ENSG00000030066	GUCAGUU
ENSG00000030110	AAAGCUG
ENSG00000030110	AAUGUUG
ENSG00000030110	AGCACCA
ENSG00000030110	CUGCAGC
ENSG00000030110	GCACCAU
ENSG00000030419	AAAGCUG
ENSG00000030419	AAGGCAC
ENSG00000030419	AAUACUG
ENSG00000030419	AUGCAUU
ENSG00000030419	AUUGCAC
ENSG00000030419	CACAGUG
ENSG00000030419	CAGUUAC
ENSG00000030419	CUCACAC
ENSG00000030419	CUUGGAG
ENSG00000030419	CUUUGGU
ENSG00000030419	GAGAAAU
ENSG00000030419	GAGGUAG
ENSG00000030419	UCCAGUU
ENSG00000030419	UCCCUUG
ENSG00000030419	UGAGAAG
ENSG00000030419	UGCAGCU
ENSG00000030419	UGCAUAG
ENSG00000030419	UGCUGAC
ENSG00000030419	UUCUAAU
ENSG00000030582	CACAGGG
ENSG00000030582	UGGCCAC
ENSG00000031691	CAGACAG
ENSG00000031691	UAAUAUC
ENSG00000031698	UAUUACC
ENSG00000031823	AAAAGUA
ENSG00000031823	AAAGUUU
ENSG00000031823	AAGUCUU
ENSG00000031823	ACAUUCA
ENSG00000031823	AGCAGCA
ENSG00000031823	AUGACAC
ENSG00000031823	AUGAUGA
ENSG00000031823	AUGAUGC
ENSG00000031823	AUGUGCC
ENSG00000031823	CAAAGCA
ENSG00000031823	CCAGUGU

ENSG00000031823	CUUACAG
ENSG00000031823	GAAGGAA
ENSG00000031823	GAGAAGA
ENSG00000031823	GUGCCAC
ENSG00000031823	UGAGAAG
ENSG00000032219	AAAGUUU
ENSG00000032219	AUAAUAC
ENSG00000032219	AUGUCUG
ENSG00000032219	CUGGGCA
ENSG00000032219	GAAAACA
ENSG00000032219	GCCCUUA
ENSG00000032219	GCUUCCU
ENSG00000032219	GGCAGCG
ENSG00000032219	GGCAGUG
ENSG00000032219	UAAUGCU
ENSG00000032219	UCCUAUA
ENSG00000032219	UCUAGAG
ENSG00000032219	UGCAUAG
ENSG00000032219	UGCAUAU
ENSG00000032219	UUGGCAA
ENSG00000032219	UUGGCAC
ENSG00000032389	UCUCAAG
ENSG00000032444	AGCAGCA
ENSG00000032444	AGCAGCG
ENSG00000032444	AGCAGCU
ENSG00000032444	CAGCAGG
ENSG00000032444	CAGUCCA
ENSG00000032444	CCAGUGC
ENSG00000032444	CCAGUGU
ENSG00000032444	UGUGUGG
ENSG00000033050	AAAAACC
ENSG00000033050	AAGGCAC
ENSG00000033050	ACAUCAC
ENSG00000033050	AGGCAGG
ENSG00000033050	AUUGCAC
ENSG00000033050	CUGGGGG
ENSG00000033050	GAGGCUG
ENSG00000033050	GCAGCAU
ENSG00000033050	GCAGGAC
ENSG00000033050	GGGCCCC
ENSG00000033050	GUCCAGA
ENSG00000033050	UCCUGAG
ENSG00000033122	AAGGCAC
ENSG00000033122	CCAGCAU
ENSG00000033122	GAGGCUG
ENSG00000033122	GGCAGUG
ENSG00000033122	GUGCUUG
ENSG00000033122	UGUGCUU
ENSG00000033122	UUGGUCC
ENSG00000033170	GCACCAU
ENSG00000033178	GAGAAAU
ENSG00000033178	UCACAUU
ENSG00000033178	UCCCUUG
ENSG00000033178	UUUGCAC
ENSG00000033178	UUUUUGC
ENSG00000033327	AAAGAAU
ENSG00000033327	AAGUGCU
ENSG00000033327	AGCAAAA
ENSG00000033327	AGCACCA
ENSG00000033327	CAGCAAA
ENSG00000033327	CCCUGAG
ENSG00000033327	CCUGCUG
ENSG00000033327	CCUGUAC
ENSG00000033327	CCUUCAU

ENSG00000033327	CUGGCUC
ENSG00000033327	CUUUGGU
ENSG00000033327	GAGGCUG
ENSG00000033327	GAGGUAG
ENSG00000033327	GGACCUG
ENSG00000033327	GUGCUUG
ENSG00000033327	GUGGCCC
ENSG00000033327	UCCCACC
ENSG00000033327	UUCCUAG
ENSG00000033627	AGGCAGG
ENSG00000033627	CACCACC
ENSG00000033627	UCAUGAU
ENSG00000033627	UCCCUGU
ENSG00000033800	AUACAAG
ENSG00000033867	AAAGUGC
ENSG00000033867	AAGGCAC
ENSG00000033867	AAGUGCA
ENSG00000033867	AGCAGCA
ENSG00000033867	AGUGCUU
ENSG00000033867	GGAUUUC
ENSG00000033867	GGCUCUG
ENSG00000033867	UCCUGAG
ENSG00000033867	UUGGCAA
ENSG00000034053	AAAGCUG
ENSG00000034053	ACACACC
ENSG00000034053	ACACACU
ENSG00000034053	AGUGAUC
ENSG00000034053	CACUCCU
ENSG00000034053	GCCUGGG
ENSG00000034053	UACAAAG
ENSG00000034053	UAGUGAG
ENSG00000034053	UCACAGU
ENSG00000034053	UUGGCAC
ENSG00000034063	AACUACU
ENSG00000034063	AAGGCAC
ENSG00000034063	AAGUGCU
ENSG00000034063	AGUGCUU
ENSG00000034063	AUCCCUU
ENSG00000034063	AUGGCUU
ENSG00000034063	CCCUGUC
ENSG00000034063	CUGCAGC
ENSG00000034063	GAGAACC
ENSG00000034063	GAGGUAG
ENSG00000034063	GUCUGCC
ENSG00000034063	UCAUUUG
ENSG00000034063	UCCAGUU
ENSG00000034063	UCCCUGU
ENSG00000034152	AGCUUAU
ENSG00000034152	AUACAAG
ENSG00000034152	GUGCAAA
ENSG00000034510	CACAGUG
ENSG00000034510	UCACAGU
ENSG00000034677	AAGGCAC
ENSG00000034677	AUACAAG
ENSG00000034677	CUGCAGC
ENSG00000034677	GCACCAU
ENSG00000034677	GCAGCAU
ENSG00000034713	CAAAUGC
ENSG00000034713	UCCAGUU
ENSG00000035141	GUCCAGA
ENSG00000035403	AGCACCA
ENSG00000035403	AUAAAGU
ENSG00000035403	AUUGCAC
ENSG00000035403	CAAAUGC

ENSG00000035403	CUAUACA
ENSG00000035403	CUGCAGG
ENSG00000035403	CUUUGGU
ENSG00000035403	GGAAGAC
ENSG00000035403	GGAGGCA
ENSG00000035403	GGCAGUG
ENSG00000035403	UAAUAUC
ENSG00000035403	UGCAUUG
ENSG00000035403	UUGGCAA
ENSG00000035403	UUGGCAC
ENSG00000035499	ACUCAAA
ENSG00000035499	AGUAACA
ENSG00000035499	CCAGUGU
ENSG00000035499	GUAACAG
ENSG00000035499	UCACAGU
ENSG00000035664	AAAAAUC
ENSG00000035664	CAAAGUG
ENSG00000035664	CAGACAG
ENSG00000035664	GCUGGUG
ENSG00000035664	UCACAGG
ENSG00000035664	UGCAAAG
ENSG00000035681	AAGUCAC
ENSG00000035681	GGUUUAC
ENSG00000035681	UUGGUUC
ENSG00000035862	AAAGUGC
ENSG00000035862	AACAAAC
ENSG00000035862	AAGUGCA
ENSG00000035862	AGUGCAA
ENSG00000035862	AUAAUAC
ENSG00000035862	AUGACAC
ENSG00000035862	CAAAGCA
ENSG00000035862	CAAAUGC
ENSG00000035862	CAGUCUG
ENSG00000035862	CCAGGAG
ENSG00000035862	CUGCUCA
ENSG00000035862	GCUACAU
ENSG00000035862	GGUGGGC
ENSG00000035862	GUAAACA
ENSG00000035862	UAAUACA
ENSG00000035862	UACAAAG
ENSG00000035862	UCUCAAG
ENSG00000035862	UGACAUC
ENSG00000035862	UUUCAAC
ENSG00000035928	AAAUUUC
ENSG00000035928	CACCACC
ENSG00000035928	CUACAAA
ENSG00000036257	AAAAACC
ENSG00000036257	AAAGUGC
ENSG00000036257	AAGUGCU
ENSG00000036257	AAUCACU
ENSG00000036257	AAUCCUU
ENSG00000036257	AAUCUCA
ENSG00000036257	ACAAUAU
ENSG00000036257	ACAUCAC
ENSG00000036257	ACAUUCA
ENSG00000036257	AGCAAAA
ENSG00000036257	AGCUGCC
ENSG00000036257	AGUGCAA
ENSG00000036257	AGUGGUU
ENSG00000036257	AUACAAG
ENSG00000036257	AUAUAAC
ENSG00000036257	AUCCUUG
ENSG00000036257	AUGAGCU
ENSG00000036257	AUUGCAC

ENSG00000036257	CAAAGCA
ENSG00000036257	CAAAGUG
ENSG00000036257	CUAUACA
ENSG00000036257	GACCUGG
ENSG00000036257	GAGGCUG
ENSG00000036257	GGAAGCC
ENSG00000036257	GUCUACU
ENSG00000036257	GUGCUUG
ENSG00000036257	UAGACUG
ENSG00000036257	UCAACAG
ENSG00000036257	UGAAGCU
ENSG00000036257	UGUGCUU
ENSG00000036257	UUGGAGG
ENSG00000036530	GAGGUAG
ENSG00000036549	AAAAAUC
ENSG00000036549	AAAGAAU
ENSG00000036549	AAAGAGG
ENSG00000036549	ACACACC
ENSG00000036549	ACAUCAC
ENSG00000036549	ACAUUAC
ENSG00000036549	ACAUUCA
ENSG00000036549	AGCAGCU
ENSG00000036549	AGGCAGG
ENSG00000036549	AGUGCUG
ENSG00000036549	AGUGCUU
ENSG00000036549	AUGAGCU
ENSG00000036549	AUGAUGC
ENSG00000036549	CAAAACU
ENSG00000036549	CACAGUG
ENSG00000036549	CAGUUAC
ENSG00000036549	CUACAAA
ENSG00000036549	CUCACAC
ENSG00000036549	GAAACAU
ENSG00000036549	GAGGCAG
ENSG00000036549	GCAGAAG
ENSG00000036549	GGAAUGU
ENSG00000036549	GUCAGUU
ENSG00000036549	UCACACA
ENSG00000036549	UCACAGU
ENSG00000036565	CAGGUGA
ENSG00000036565	GCAGAAG
ENSG00000036672	AGCAGCA
ENSG00000036672	AGCAGCU
ENSG00000036672	GGCUCAG
ENSG00000036828	AAUACUG
ENSG00000036828	AGCAGCG
ENSG00000036828	AUAAUAC
ENSG00000037280	CUCUUCC
ENSG00000037749	GCACCAU
ENSG00000037965	AAAGCGC
ENSG00000037965	AACAAAC
ENSG00000037965	ACAAUAU
ENSG00000037965	AGCAGCA
ENSG00000037965	AGGCAGG
ENSG00000037965	AGUGAUC
ENSG00000037965	AGUGCAA
ENSG00000037965	AUACAAG
ENSG00000037965	AUUGCAC
ENSG00000037965	CUAUACA
ENSG00000037965	GUAUGUU
ENSG00000037965	UAGGUAU
ENSG00000037965	UAUUGCU
ENSG00000037965	UCAUAGA
ENSG00000037965	UCCCUUG

ENSG00000038210	CCCUGAG
ENSG00000038210	GUAAACA
ENSG00000038210	UCCCUGU
ENSG00000038274	AAAGUGC
ENSG00000038274	AAAGUUU
ENSG00000038274	AUAAAGU
ENSG00000038274	CAAGAGC
ENSG00000038274	CACAAUG
ENSG00000038274	UGCAAAG
ENSG00000038274	UUGAUCA
ENSG00000038295	AGCAGCA
ENSG00000038295	AGUAGUU
ENSG00000038295	GCACCAU
ENSG00000038295	GGCUCAG
ENSG00000038382	AAUACUG
ENSG00000038382	CAGGUGA
ENSG00000038382	UAUUACC
ENSG00000038427	AAGGCAC
ENSG00000038532	ACAAGGU
ENSG00000038532	ACAUUAC
ENSG00000038532	AUUGCAC
ENSG00000038532	CCCUGAG
ENSG00000038532	GUCUUGC
ENSG00000038532	UCAUUUG
ENSG00000039068	AACAAAC
ENSG00000039068	ACUCAAA
ENSG00000039068	CUUUGGU
ENSG00000039068	UGGCUGC
ENSG00000039319	GAGGUAG
ENSG00000039319	UCAUUUG
ENSG00000039319	UCCUGAG
ENSG00000039319	UCUUGAG
ENSG00000039523	AUAUUAU
ENSG00000039523	GGAGGCA
ENSG00000039560	AAAAAUC
ENSG00000039560	AGUAACA
ENSG00000039560	CACAGUG
ENSG00000039560	CUUUGGU
ENSG00000039560	GAGGCAG
ENSG00000039560	GGAUUUC
ENSG00000039560	GUAAACA
ENSG00000039560	GUUAAUG
ENSG00000039560	UCCCUUG
ENSG00000039560	UCCUGAG
ENSG00000039560	UGCAUAU
ENSG00000039560	UGUUGAA
ENSG00000039560	UUGGAGG
ENSG00000039600	UGCAUAU
ENSG00000039600	UUCAAAU
ENSG00000039987	AGGCAGG
ENSG00000040341	GGCAAGA
ENSG00000040487	GAGGUAG
ENSG00000040487	GAGGUAU
ENSG00000040531	AAGGCAC
ENSG00000040531	ACCAGCA
ENSG00000040531	CACACCU
ENSG00000040531	CACCAGC
ENSG00000040531	CCCACCU
ENSG00000040531	GAGGUAG
ENSG00000040531	GGCAGGG
ENSG00000040531	GGCCCUG
ENSG00000040531	GUGAGGU
ENSG00000040531	UUGGAGG
ENSG00000040608	GUAAACA

ENSG00000040608	UCUGGAA
ENSG00000040633	AAAAAUC
ENSG00000040633	CCCUGAG
ENSG00000040633	GACAACU
ENSG00000040633	GGCUCAG
ENSG00000040731	AAAAAUC
ENSG00000041357	UGAAAUG
ENSG00000041515	AGCACCA
ENSG00000041515	AGUGUGC
ENSG00000041515	CUACAAA
ENSG00000041515	GUGCUUG
ENSG00000041515	UACAAAG
ENSG00000041515	UAUAAAG
ENSG00000041515	UGUGCUU
ENSG00000041982	AAAAGUA
ENSG00000041982	AAAGUAC
ENSG00000041982	AUAUUAU
ENSG00000041982	AUGACAC
ENSG00000041982	CUCUGGG
ENSG00000041982	GUGCUUG
ENSG00000041982	UGUCUUU
ENSG00000041988	CAGCAGG
ENSG00000041988	GGGUUUA
ENSG00000042286	AUUGCAC
ENSG00000042286	GAGGCUG
ENSG00000042286	UAUUGCU
ENSG00000042429	GUGCUUG
ENSG00000042429	UUCAAAU
ENSG00000042445	CUGACUC
ENSG00000042781	AAUACUG
ENSG00000042781	UUGACAC
ENSG00000042781	UUGGCAA
ENSG00000043039	AACAAAC
ENSG00000043039	AAGGCAC
ENSG00000043039	AAGUCUU
ENSG00000043039	AAGUGCA
ENSG00000043039	AGUGCUG
ENSG00000043039	CAGCAAA
ENSG00000043039	UACAAAG
ENSG00000043093	AAGUGCU
ENSG00000043093	CAGCAGA
ENSG00000043093	GAGGAUA
ENSG00000043093	GUGCAAA
ENSG00000043093	GUGCUUG
ENSG00000043093	UUGGCAA
ENSG00000043143	AAAGAAU
ENSG00000043143	AACAAAC
ENSG00000043143	AAUUGCU
ENSG00000043143	ACAUCAC
ENSG00000043143	ACAUUCA
ENSG00000043143	ACUGGCC
ENSG00000043143	AGCAGCU
ENSG00000043143	AGUGCUU
ENSG00000043143	AUAUUAU
ENSG00000043143	AUCCUUU
ENSG00000043143	AUUGCAC
ENSG00000043143	CACAGUG
ENSG00000043143	CUGGGCA
ENSG00000043143	CUGGGGG
ENSG00000043143	CUUUGGU
ENSG00000043143	GAAAACA
ENSG00000043143	GAAACAU
ENSG00000043143	GAAGGAA
ENSG00000043143	GAGAAAU

ENSG00000043143	GAGCCCC
ENSG00000043143	GAGCUAA
ENSG00000043143	GGGCCCC
ENSG00000043143	GUGUCAC
ENSG00000043143	UACAAAG
ENSG00000043143	UAUUGCU
ENSG00000043143	UCCCUGU
ENSG00000043143	UCUCCAA
ENSG00000043143	UGCAAAG
ENSG00000043143	UGGCCCU
ENSG00000043143	UGUGCUU
ENSG00000043143	UUGGCAA
ENSG00000043143	UUGGCAC
ENSG00000043143	UUGUGAC
ENSG00000043143	UUUGCAA
ENSG00000043355	UUGGCAA
ENSG00000043355	UUGGCAC
ENSG00000043514	CCUGCUG
ENSG00000043514	UCUGGAA
ENSG00000043591	AAGUGCU
ENSG00000043591	ACAGUAU
ENSG00000043591	AGUAACA
ENSG00000043591	AGUAGUU
ENSG00000043591	AUGGAUU
ENSG00000043591	CAGUAGU
ENSG00000043591	CAGUUAC
ENSG00000043591	GAGGUAG
ENSG00000043591	GAGGUAU
ENSG00000043591	GCACCAU
ENSG00000043591	GUAAACA
ENSG00000043591	GUGAGGU
ENSG00000043591	UAAUAUC
ENSG00000043591	UAUUGCU
ENSG00000043591	UGUGCUU
ENSG00000044115	AACACUG
ENSG00000044115	CUGCAGC
ENSG00000044115	UUGGUUC
ENSG00000044446	CACAGGG
ENSG00000044446	CAGACAG
ENSG00000044446	GUCACUC
ENSG00000044446	UGUGUCA
ENSG00000044459	CAAAGCA
ENSG00000044459	UCAUACA
ENSG00000044524	AAGGCAC
ENSG00000044524	ACAUUAC
ENSG00000044524	UAUAAAG
ENSG00000044574	AAGACAU
ENSG00000044574	ACAUUCA
ENSG00000044574	CAAAUGC
ENSG00000044574	CCAGUAC
ENSG00000044574	CCAGUGU
ENSG00000044574	GGUAGAC
ENSG00000044574	GUAAACA
ENSG00000044574	GUCACUC
ENSG00000044574	GUGUCAC
ENSG00000044574	UAUGGUU
ENSG00000044574	UUUCAAC
ENSG00000046647	AAUUUUA
ENSG00000046647	AUAAAGU
ENSG00000046647	UGUGUCA
ENSG00000046651	CUGGGCA
ENSG00000046653	AGCCCUU
ENSG00000046653	AUCACUA
ENSG00000046653	GGAAGCC

ENSG00000046653	GUUAGGA
ENSG00000046653	UACAAAG
ENSG00000046653	UUGGCAC
ENSG00000047056	ACAUUCA
ENSG00000047056	ACUGUUU
ENSG00000047056	AGGUAGU
ENSG00000047056	AUGUAAU
ENSG00000047056	AUUGCAC
ENSG00000047056	CUCAAAC
ENSG00000047056	CUGGACU
ENSG00000047056	GAGGUAG
ENSG00000047056	GAGGUAU
ENSG00000047056	GCUACAU
ENSG00000047056	GGCAGUG
ENSG00000047056	UAAUAUC
ENSG00000047056	UCACAGG
ENSG00000047056	UCACAGU
ENSG00000047056	UCCUGAG
ENSG00000047056	UCUGGAA
ENSG00000047056	UCUGGAG
ENSG00000047056	UGUCUUU
ENSG00000047249	AUCCUUU
ENSG00000047249	UGAGAAU
ENSG00000047346	UCCAGUU
ENSG00000047346	UGUGCUU
ENSG00000047578	ACAGUAU
ENSG00000047578	AGCUGCC
ENSG00000047578	CCCUGAG
ENSG00000047578	UAAUAUC
ENSG00000047578	UGCAUAU
ENSG00000047597	AAAGUUU
ENSG00000047597	GAGGCAG
ENSG00000047597	GAGGUAG
ENSG00000047597	GAGGUAU
ENSG00000047621	GGAAUGU
ENSG00000047634	UGCAUAU
ENSG00000047644	AAAUUUC
ENSG00000047644	AAGGCAC
ENSG00000047644	AAGUCAC
ENSG00000047644	ACUGCAG
ENSG00000047644	AUAUUAU
ENSG00000047644	CCCUGUU
ENSG00000047644	GCCCUGU
ENSG00000047644	GCCUGAU
ENSG00000047644	UAAUAGU
ENSG00000047644	UCACAAG
ENSG00000047644	UCCCACC
ENSG00000047644	UCCCUUG
ENSG00000047644	UUUGCAC
ENSG00000047849	AAACUGU
ENSG00000047849	AAGUCAC
ENSG00000047849	CCAGGAG
ENSG00000047849	CCAGUGU
ENSG00000047849	GAAGGUC
ENSG00000047849	GCAGCAU
ENSG00000047849	UGCAGCU
ENSG00000047849	UGCUCAC
ENSG00000047932	ACUAGAU
ENSG00000047932	ACUCAAA
ENSG00000047932	CCCUGAG
ENSG00000047932	CUUUGGU
ENSG00000047932	GAGGUAG
ENSG00000047932	GAGGUAU
ENSG00000047932	UAUAAUA

ENSG00000048028	GCUGUCU
ENSG00000048028	UAUAAAG
ENSG00000048028	UAUUGCU
ENSG00000048052	UAAUACA
ENSG00000048140	CUGGACU
ENSG00000048392	AAGGCAC
ENSG00000048392	CAAAGCA
ENSG00000048392	UAUUGCU
ENSG00000048392	UCACACA
ENSG00000048405	ACAUCAC
ENSG00000048405	AGUGCUU
ENSG00000048405	GAUUGUC
ENSG00000048471	AAAGAGC
ENSG00000048471	AACAAAC
ENSG00000048471	AGAUAAA
ENSG00000048471	AGUGGUU
ENSG00000048471	AUAAUAC
ENSG00000048471	GCUACAU
ENSG00000048471	GUAAACA
ENSG00000048471	GUAACAG
ENSG00000048471	UAUAAAG
ENSG00000048471	UUGGCAC
ENSG00000048540	AAAUGGU
ENSG00000048540	AAGGCAC
ENSG00000048540	AAGUGCU
ENSG00000048540	ACAGUAC
ENSG00000048540	ACAGUAU
ENSG00000048540	ACCAGCA
ENSG00000048540	ACUAGAU
ENSG00000048540	AGUGCUG
ENSG00000048540	AUGGCAC
ENSG00000048540	CACCAGC
ENSG00000048540	CAGGUGA
ENSG00000048540	CAGUAGU
ENSG00000048540	CUCUUCC
ENSG00000048540	GAUUGUC
ENSG00000048540	GGGGUGC
ENSG00000048540	GGGGUGG
ENSG00000048540	GUAGUGU
ENSG00000048540	GUGCUUG
ENSG00000048540	GUGUCAC
ENSG00000048540	UGUGUCA
ENSG00000048540	UUCAAAU
ENSG00000048544	UGCUCAC
ENSG00000048649	AAGUGCU
ENSG00000048649	ACUGGCC
ENSG00000048649	AGUGCUU
ENSG00000048649	GACCAUG
ENSG00000048649	UAUAAAG
ENSG00000048707	AAAGAGG
ENSG00000048707	AGCAGCU
ENSG00000048707	CCUGCUG
ENSG00000048707	CUUGGAG
ENSG00000048707	GUCUUGC
ENSG00000048707	GUGCAAA
ENSG00000048707	GUGGCCC
ENSG00000048707	UCAUUUG
ENSG00000048707	UGUGUCA
ENSG00000048707	UUGUGUC
ENSG00000048828	AAAGAGG
ENSG00000048828	AACGGCU
ENSG00000048828	AACUACU
ENSG00000048828	AAGUUGU
ENSG00000048828	AAUCACU

ENSG00000048828	ACACACC
ENSG00000048828	AUCACUA
ENSG00000048828	CAUAUUG
ENSG00000048828	CCUUCAU
ENSG00000048828	CUUCACC
ENSG00000048828	GGCAGGG
ENSG00000048828	UAUAUGA
ENSG00000048828	UCAUUCG
ENSG00000048828	UGCAAAG
ENSG00000048828	UGCAUUG
ENSG00000048828	UUUGCAC
ENSG00000048991	AUACAAG
ENSG00000049130	AAAAGUA
ENSG00000049130	ACAGUAC
ENSG00000049130	ACUCCAG
ENSG00000049130	AGUAACA
ENSG00000049130	CACAGUG
ENSG00000049130	CACUCCU
ENSG00000049130	CCUGCUG
ENSG00000049130	CUGACUC
ENSG00000049130	GUAACAG
ENSG00000049130	UCACAGG
ENSG00000049130	UCAUUUG
ENSG00000049130	UGGAGAU
ENSG00000049167	AAAGUUU
ENSG00000049192	AAAAAUC
ENSG00000049192	AAAGGUA
ENSG00000049192	AACAGUC
ENSG00000049192	AAUGCCC
ENSG00000049192	ACAUUCA
ENSG00000049192	ACCACAG
ENSG00000049192	AGAUAAA
ENSG00000049192	AGCAGCA
ENSG00000049192	AGUGGUU
ENSG00000049192	AUGGAUU
ENSG00000049192	CACAGGG
ENSG00000049192	CACUCCU
ENSG00000049192	CCAGCAU
ENSG00000049192	CUCUGGG
ENSG00000049192	CUGGAAA
ENSG00000049192	GAAACAU
ENSG00000049192	GAGGUAG
ENSG00000049192	GAGGUAU
ENSG00000049192	GAGUAUU
ENSG00000049192	GCCUGGA
ENSG00000049192	GCUACAU
ENSG00000049192	GGCUCAG
ENSG00000049192	GGCUCUG
ENSG00000049192	GGUUACC
ENSG00000049192	GUCCAGA
ENSG00000049192	GUGAGGU
ENSG00000049192	UAUAAUA
ENSG00000049192	UCAAGUA
ENSG00000049192	UCACAGG
ENSG00000049192	UCACAGU
ENSG00000049192	UCACAUU
ENSG00000049192	UCUCCAA
ENSG00000049192	UCUGGAA
ENSG00000049192	UGGCCAC
ENSG00000049239	CCCCCAG
ENSG00000049245	AGGCAGG
ENSG00000049245	CUUUGGU
ENSG00000049245	GCAGGUU
ENSG00000049245	GUAGUGU

ENSG00000049245	UGCAUAU
ENSG00000049245	UUGGUCC
ENSG00000049246	AAGGCAC
ENSG00000049246	CUUCUCU
ENSG00000049246	GAAGGAA
ENSG00000049246	GAGCCCC
ENSG00000049246	GCAGCAU
ENSG00000049246	GUAAACA
ENSG00000049247	ACAUAGA
ENSG00000049283	UCCAGUU
ENSG00000049283	UUGUGAC
ENSG00000049319	AGCAGCU
ENSG00000049319	CAGCAGA
ENSG00000049319	CAGCAGG
ENSG00000049323	CAGUGCA
ENSG00000049323	UUGGUCC
ENSG00000049449	GAGGUAG
ENSG00000049540	ACAGUAC
ENSG00000049540	ACAGUAU
ENSG00000049540	ACAUUCA
ENSG00000049540	AGUACUG
ENSG00000049540	GGGGUCC
ENSG00000049540	GGUGGGC
ENSG00000049540	GGUUUAC
ENSG00000049540	UCUCCAA
ENSG00000049540	UGGGUAC
ENSG00000049618	AAGUUGU
ENSG00000049618	AGCCAGU
ENSG00000049618	AGUGCUU
ENSG00000049618	AUAAAGU
ENSG00000049618	CACAGUG
ENSG00000049618	CUCACAC
ENSG00000049618	GAAGGUC
ENSG00000049618	UCACACA
ENSG00000049618	UGAAAUG
ENSG00000049656	GAAACAU
ENSG00000049656	GAGAAAU
ENSG00000049656	UGAUCCA
ENSG00000049656	UUGAUCA
ENSG00000049656	UUGGCAC
ENSG00000049759	AAAAGUA
ENSG00000049759	AAUACUG
ENSG00000049759	AGUGCCU
ENSG00000049759	AUAAAGU
ENSG00000049759	AUACCUC
ENSG00000049759	AUUCAUU
ENSG00000049759	AUUGCAC
ENSG00000049759	CAAAUGC
ENSG00000049759	GGCUCAG
ENSG00000049759	GUAAACA
ENSG00000049759	UCACAUU
ENSG00000049759	UCCAGUU
ENSG00000049759	UGAAAUG
ENSG00000049768	AGGCAGG
ENSG00000049768	CAUGGAU
ENSG00000049769	AAAAAUC
ENSG00000049769	AAAGCUG
ENSG00000049769	AUAAUAC
ENSG00000049769	AUAUAAC
ENSG00000049769	CCAGCAU
ENSG00000049769	CUCCAGC
ENSG00000049769	GCAGGUU
ENSG00000049769	GGGUAAG
ENSG00000049769	GUUAAUG

ENSG00000049769	UAUAAUA
ENSG00000049769	UCAUAGA
ENSG00000049769	UCCAGAG
ENSG00000050030	AAACUGU
ENSG00000050030	AAAGCUG
ENSG00000050030	AAAGUAU
ENSG00000050030	AACUACU
ENSG00000050030	AAUCACU
ENSG00000050030	ACCACAG
ENSG00000050030	AGCAGCU
ENSG00000050030	AGGCAGG
ENSG00000050030	AGUGAUC
ENSG00000050030	AGUGCAA
ENSG00000050030	AUCACUA
ENSG00000050030	AUGACAC
ENSG00000050030	CUACAAA
ENSG00000050030	CUACAGU
ENSG00000050030	CUAGUAG
ENSG00000050030	GAAACAU
ENSG00000050030	GAGAUGA
ENSG00000050030	GAGGUAG
ENSG00000050030	GAGGUAU
ENSG00000050030	GCACCAU
ENSG00000050030	GGGGUGG
ENSG00000050030	GGGUGGU
ENSG00000050030	GGUGGUU
ENSG00000050030	UCACAGG
ENSG00000050030	UCACAGU
ENSG00000050030	UCCAGUU
ENSG00000050030	UCCCACC
ENSG00000050030	UGUGCUU
ENSG00000050030	UUCAAAU
ENSG00000050030	UUGACAC
ENSG00000050165	AGGAGCU
ENSG00000050165	AUACAAG
ENSG00000050165	GAGGUAG
ENSG00000050165	GUGCAAA
ENSG00000050165	UUAUGCA
ENSG00000050327	UCACAUU
ENSG00000050405	AAACUGU
ENSG00000050405	AAGUGCA
ENSG00000050405	GCCCUUA
ENSG00000050426	ACUGCAG
ENSG00000050426	ACUUGGA
ENSG00000050426	AGUGCUU
ENSG00000050426	AUGCAUU
ENSG00000050426	CCCACCU
ENSG00000050426	CUGCAGG
ENSG00000050426	UUGGUCC
ENSG00000050438	AAGGCAC
ENSG00000050438	ACUUGGA
ENSG00000050438	AUGAUGA
ENSG00000050438	CCAGUGU
ENSG00000050438	CCCUGAG
ENSG00000050438	CUUACAG
ENSG00000050438	GCUGGUG
ENSG00000050438	GUCUUGC
ENSG00000050438	UACAGUU
ENSG00000050438	UCCCUGU
ENSG00000050628	AAAGUGC
ENSG00000050628	AAUUAUU
ENSG00000050628	CAAAGCA
ENSG00000050628	CACAGUG
ENSG00000050628	UCUAGAG

ENSG00000050628	UUCCAUA
ENSG00000050628	UUGGCAA
ENSG00000050628	UUGUGUC
ENSG00000050748	UGCUCAC
ENSG00000050767	AUAUUAU
ENSG00000050767	CAGGUGA
ENSG00000050767	UACAAAG
ENSG00000050767	UAUUACC
ENSG00000050820	GAUUGGU
ENSG00000051009	AUUGCAC
ENSG00000051009	GAUUGUC
ENSG00000051009	UCUCCAA
ENSG00000051180	GACCUGG
ENSG00000051180	GUGACUG
ENSG00000051341	AAAGUGC
ENSG00000051341	AAGUGCU
ENSG00000051341	AGCAAAA
ENSG00000051341	GAGGCAG
ENSG00000051341	UUUCAAC
ENSG00000052126	CCAGUAC
ENSG00000052344	CAGUCCA
ENSG00000052344	CCGGAGC
ENSG00000052344	GGAGUCC
ENSG00000052344	GGCAAGA
ENSG00000052344	GGCUCAG
ENSG00000052795	AAAGAGC
ENSG00000052795	AACAUUC
ENSG00000052795	AAGGCAC
ENSG00000052795	CUUUGGU
ENSG00000052795	UAAGACG
ENSG00000052795	UAAGACU
ENSG00000052795	UCCUGAG
ENSG00000052802	AAAAAUC
ENSG00000052802	AAGUGCA
ENSG00000052802	AGUGCAA
ENSG00000052802	GUCAGUU
ENSG00000052802	UCACAUU
ENSG00000052802	UUCUGCA
ENSG00000052850	AAAGUGC
ENSG00000052850	AAGUGCU
ENSG00000052850	AGUGCUG
ENSG00000053108	AAAAAUC
ENSG00000053108	AAAGUGC
ENSG00000053108	AAAUUUC
ENSG00000053108	AAGGCAC
ENSG00000053108	AGCAGCU
ENSG00000053108	AGGCAGG
ENSG00000053108	AUAAAGU
ENSG00000053108	AUCAUAC
ENSG00000053108	CCAGUGU
ENSG00000053108	GCAGCAU
ENSG00000053108	GGCAGGG
ENSG00000053108	GUCUGCC
ENSG00000053108	UAUAAAG
ENSG00000053108	UGCCCUG
ENSG00000053108	UGUUGAA
ENSG00000053254	AAAAACC
ENSG00000053254	AAAGUAU
ENSG00000053254	AAAGUUU
ENSG00000053254	AAAUCCC
ENSG00000053254	AAAUUUC
ENSG00000053254	AACAGUC
ENSG00000053254	AACUGGU
ENSG00000053254	AAGUCUU

ENSG00000053254	AAUUUUA
ENSG00000053254	ACAAUAU
ENSG00000053254	ACACACC
ENSG00000053254	ACAUUAC
ENSG00000053254	ACUCCAG
ENSG00000053254	AGCACCA
ENSG00000053254	AGGACAC
ENSG00000053254	AGGUUAU
ENSG00000053254	AGUAGUU
ENSG00000053254	AGUGCUG
ENSG00000053254	AUCAUAC
ENSG00000053254	AUGCAUU
ENSG00000053254	CAAGAGC
ENSG00000053254	CACAAUG
ENSG00000053254	CACAGUG
ENSG00000053254	CAGGCUC
ENSG00000053254	CCCUGAG
ENSG00000053254	CUAUACA
ENSG00000053254	CUGGGCA
ENSG00000053254	CUUGGAG
ENSG00000053254	CUUUGGU
ENSG00000053254	GAGGUAU
ENSG00000053254	GCACCAU
ENSG00000053254	GCAGGUU
ENSG00000053254	GGAGGAG
ENSG00000053254	GUCAACA
ENSG00000053254	GUGCUUG
ENSG00000053254	UAGUGAG
ENSG00000053254	UCACAAG
ENSG00000053254	UCACAGU
ENSG00000053254	UCUGGAG
ENSG00000053254	UGAAGCU
ENSG00000053254	UGAGAAU
ENSG00000053254	UGAUCCA
ENSG00000053254	UGCAUAG
ENSG00000053254	UGCAUAU
ENSG00000053254	UGCCCUG
ENSG00000053254	UGCUGAC
ENSG00000053254	UGGCCAC
ENSG00000053254	UUAUGCA
ENSG00000053254	UUGAGGC
ENSG00000053254	UUGGCAC
ENSG00000053254	UUGGUCC
ENSG00000053372	CAGCAAA
ENSG00000053438	ACAAUAU
ENSG00000053438	CCCUGUU
ENSG00000053438	CUGGCUC
ENSG00000053438	GAUCAGA
ENSG00000053438	GCUGGUG
ENSG00000053438	GGUGGUC
ENSG00000053438	UCCCUGU
ENSG00000053702	UCACAGU
ENSG00000053918	UGCCCUG
ENSG00000054116	AAAGAGG
ENSG00000054116	UGUCUUU
ENSG00000054118	AGCAAAA
ENSG00000054118	CACCAGC
ENSG00000054118	CAGCAGG
ENSG00000054118	GAAAACA
ENSG00000054118	GAAACAU
ENSG00000054118	GCUGGUG
ENSG00000054118	UAUAAAG
ENSG00000054118	UUGGUCC
ENSG00000054219	AAUACUG

ENSG00000054219	GAGGUAG
ENSG00000054219	GAGGUAU
ENSG00000054219	UGCAUUG
ENSG00000054267	AAUUAUU
ENSG00000054267	CAAAGUG
ENSG00000054267	CAGUAGU
ENSG00000054267	UCACAAG
ENSG00000054267	UGCCAGC
ENSG00000054267	UGUGCUU
ENSG00000054277	AAAAAUC
ENSG00000054356	AAAAGUA
ENSG00000054356	ACACACU
ENSG00000054356	UCUAGAG
ENSG00000054356	UGCUCAC
ENSG00000054523	AAAUGGU
ENSG00000054523	AAGUCUU
ENSG00000054523	ACUCAAA
ENSG00000054523	AGCAAAA
ENSG00000054523	AUCCCUU
ENSG00000054523	AUGUGCC
ENSG00000054523	AUUGCAC
ENSG00000054523	CAAAGCA
ENSG00000054523	CAGCAGG
ENSG00000054523	CCCUGUC
ENSG00000054523	CCCUGUU
ENSG00000054523	CUAUACA
ENSG00000054523	CUGACUC
ENSG00000054523	GCAGGUU
ENSG00000054523	GGAGAGA
ENSG00000054523	GGCAAGA
ENSG00000054523	GGUUACC
ENSG00000054523	GUCUGCC
ENSG00000054523	GUGCAAA
ENSG00000054523	GUGCCAC
ENSG00000054523	GUUAGGA
ENSG00000054523	UACAAAG
ENSG00000054523	UGCAUUG
ENSG00000054523	UUGGCAA
ENSG00000054523	UUUGCAA
ENSG00000054598	AAAGUAU
ENSG00000054598	UACAGUU
ENSG00000054598	UCCCUUG
ENSG00000054598	UUGGUCC
ENSG00000054611	UUGGCAA
ENSG00000054654	AAGUCUU
ENSG00000054654	AUGCACC
ENSG00000054654	UUGGCAA
ENSG00000054654	UUGGCAC
ENSG00000054690	AGCAGCA
ENSG00000054690	CACAGUG
ENSG00000054690	CAGCAAA
ENSG00000054690	CAGUAGU
ENSG00000054690	CCAGUGU
ENSG00000054690	GAGGUAG
ENSG00000054690	GUCAGUU
ENSG00000054793	AAAAGUA
ENSG00000054793	AAAGAAU
ENSG00000054793	CACAGUG
ENSG00000054793	CAGCAGA
ENSG00000054793	CCUGCUG
ENSG00000054793	GUGUCAC
ENSG00000054793	UCAAGUA
ENSG00000054793	UGAGUCU
ENSG00000054793	UGUGUCA

ENSG00000054793	UUGGCAA
ENSG00000054793	UUGGCAC
ENSG00000054803	AAGGCAC
ENSG00000054938	UAAUAUC
ENSG00000054965	AAAGUGC
ENSG00000054965	ACUCCAG
ENSG00000054965	AGCAGCU
ENSG00000054965	AGUAGUU
ENSG00000054965	AUAAAGU
ENSG00000054965	CCAGCAU
ENSG00000054965	CUCCAGC
ENSG00000054965	GACCCUG
ENSG00000054965	GAGGCAG
ENSG00000054965	UAAGACU
ENSG00000054965	UACAAAG
ENSG00000054965	UGCAAAG
ENSG00000054965	UGCAUAG
ENSG00000054965	UGCAUAU
ENSG00000054967	AUGCAUU
ENSG00000054967	CCAGGAG
ENSG00000054967	CCCACCG
ENSG00000054967	CUGCAGC
ENSG00000054967	GGCAGGG
ENSG00000054967	GGCUCUG
ENSG00000054967	GGGCCCC
ENSG00000054967	UCCCUUU
ENSG00000054967	UGCAGCU
ENSG00000054983	AGGUAGU
ENSG00000055070	AAAUCCC
ENSG00000055070	AACAAAC
ENSG00000055070	AAGGCGC
ENSG00000055070	AAUGUUG
ENSG00000055070	ACUGGCU
ENSG00000055070	AGCCCUU
ENSG00000055070	AGCUGCC
ENSG00000055070	AGGAGCU
ENSG00000055070	AGUGCAA
ENSG00000055070	AUUCAUU
ENSG00000055070	CAAAUGC
ENSG00000055070	CAGGCUC
ENSG00000055070	CCCUGAG
ENSG00000055070	CUGCAGG
ENSG00000055070	CUGCCCC
ENSG00000055070	CUGGAAA
ENSG00000055070	CUUUGGU
ENSG00000055070	GAAACAU
ENSG00000055070	GACAACU
ENSG00000055070	GCAGGUU
ENSG00000055070	GCUGGUG
ENSG00000055070	GGCAGUG
ENSG00000055070	GGCUCAG
ENSG00000055070	GGCUCUG
ENSG00000055070	GGGGAGC
ENSG00000055070	GGGUGUU
ENSG00000055070	GUCUGCC
ENSG00000055070	GUGGGGA
ENSG00000055070	GUUAAUG
ENSG00000055070	UAUAAAG
ENSG00000055070	UAUUGCU
ENSG00000055070	UCCCUUU
ENSG00000055070	UCUGGAA
ENSG00000055070	UGCAAAG
ENSG00000055070	UGGGACA
ENSG00000055130	AUACAAG

ENSG00000055130	CUAUACA
ENSG00000055130	GAAACAU
ENSG00000055130	UCCUGAG
ENSG00000055163	AGCAAAA
ENSG00000055163	AUUCAUU
ENSG00000055163	CAGCUGG
ENSG00000055163	CUGCAGG
ENSG00000055163	GAAACAU
ENSG00000055163	GGGGAGC
ENSG00000055163	UCACAAG
ENSG00000055163	UGCCCUG
ENSG00000055163	UGGAGAU
ENSG00000055483	UGCAGCU
ENSG00000055609	AAACUGU
ENSG00000055609	AAAGUAA
ENSG00000055609	AAAGUGC
ENSG00000055609	AACACUG
ENSG00000055609	AAGUGCU
ENSG00000055609	AAUACUG
ENSG00000055609	ACAUCAC
ENSG00000055609	AGAUAAA
ENSG00000055609	AGCAGCA
ENSG00000055609	AGCAGCU
ENSG00000055609	AGUAACA
ENSG00000055609	AGUGCUG
ENSG00000055609	CAAGAGC
ENSG00000055609	CACAGUG
ENSG00000055609	CAGUCUG
ENSG00000055609	CAUGGAU
ENSG00000055609	CUGACUC
ENSG00000055609	CUGCAGC
ENSG00000055609	CUGCCCC
ENSG00000055609	CUUACAG
ENSG00000055609	GCAGGUU
ENSG00000055609	GCUGGUG
ENSG00000055609	GUCAGUU
ENSG00000055609	UACAAAG
ENSG00000055609	UCAAGUA
ENSG00000055609	UCACAGG
ENSG00000055609	UCAUUCG
ENSG00000055609	UCCUUGC
ENSG00000055609	UUCUGCA
ENSG00000055609	UUGGUCC
ENSG00000055609	UUGGUUC
ENSG00000055732	AAAAAUC
ENSG00000055732	ACUCAGC
ENSG00000055813	ACACACC
ENSG00000055813	ACACACU
ENSG00000055813	ACAGUAC
ENSG00000055813	CAGUGCA
ENSG00000055917	AACUGGU
ENSG00000055917	AAUCACU
ENSG00000055917	AAUUAUU
ENSG00000055917	ACAGUAC
ENSG00000055917	ACAGUAU
ENSG00000055917	AGCCAGU
ENSG00000055917	AUCAUAC
ENSG00000055917	CAAAGCA
ENSG00000055917	CAGUCCA
ENSG00000055917	GGUUUAC
ENSG00000055917	UCCAGAG
ENSG00000055955	GGCCCUG
ENSG00000056097	AAACUGU
ENSG00000056097	AAAGUAA

ENSG00000056097	CAAAUGC
ENSG00000056277	AAACUGU
ENSG00000056277	AAGUUGU
ENSG00000056277	AAUACUG
ENSG00000056277	ACUGCAU
ENSG00000056277	GCACCAU
ENSG00000056277	GGUGGGC
ENSG00000056277	GUCCAGA
ENSG00000056277	UCACAGU
ENSG00000056277	UGUGCUU
ENSG00000056277	UGUUGAA
ENSG00000056345	CUGGCUC
ENSG00000056345	GAGGUAG
ENSG00000056345	GAGGUAU
ENSG00000056345	GCUACAU
ENSG00000056345	UCACAAG
ENSG00000056487	AAGACAU
ENSG00000056487	GAGAAAU
ENSG00000056487	GGCAAGA
ENSG00000056487	GUUCAUG
ENSG00000056487	UAAGACG
ENSG00000056487	UAAGACU
ENSG00000056487	UUGGCAA
ENSG00000056487	UUGUUCG
ENSG00000056487	UUUUUGC
ENSG00000056558	AGGCAGG
ENSG00000056558	CUGGAAA
ENSG00000056558	UCUGGAA
ENSG00000056558	UGCCAGC
ENSG00000056661	AAGGCAC
ENSG00000056661	ACAUUCA
ENSG00000056661	CUGGGGG
ENSG00000056661	GGCAUUG
ENSG00000056661	UAUAAAG
ENSG00000056661	UAUUACC
ENSG00000056661	UCACAGG
ENSG00000056661	UUCCUAG
ENSG00000056661	UUGGUUC
ENSG00000056998	GAGGUAG
ENSG00000057019	AGCAGCA
ENSG00000057019	AGUGCAA
ENSG00000057019	AUACAAG
ENSG00000057019	CACAGUG
ENSG00000057019	CUGCAGC
ENSG00000057019	CUUUGGU
ENSG00000057019	GAUUGUA
ENSG00000057019	GAUUGUC
ENSG00000057019	GGCUCAG
ENSG00000057019	GUGCAAA
ENSG00000057019	GUGCUUG
ENSG00000057019	UAAUACA
ENSG00000057019	UCAAGUA
ENSG00000057019	UGAAGUG
ENSG00000057019	UGCAAAG
ENSG00000057019	UUUGCAA
ENSG00000057019	UUUGCAC
ENSG00000057252	CUUUGGU
ENSG00000057294	ACAUUCA
ENSG00000057294	UAAUAUC
ENSG00000057608	UCCAGAG
ENSG00000057608	UUGGUCC
ENSG00000057657	AAAGAAU
ENSG00000057657	AAAGUAA
ENSG00000057657	AAUGCCC

ENSG00000057657	ACAUUCA
ENSG00000057657	AGCAAAA
ENSG00000057657	CACUGUU
ENSG00000057657	GAGGUAG
ENSG00000057657	GAGGUAU
ENSG00000057657	GCCCUGU
ENSG00000057657	UACAAAG
ENSG00000057657	UGUUGAA
ENSG00000057657	UUGGCAA
ENSG00000057657	UUUGCAA
ENSG00000057704	AAGGCAC
ENSG00000057704	AUUGCAC
ENSG00000057704	GGCAGUG
ENSG00000057704	UCACAUU
ENSG00000057757	CAAAGCA
ENSG00000057757	UUCUGCA
ENSG00000058063	AGCAGCU
ENSG00000058063	CACAGUG
ENSG00000058063	GCUACAU
ENSG00000058063	GGAGUCC
ENSG00000058063	UUUGCAA
ENSG00000058085	AGCACCA
ENSG00000058085	CUCACAC
ENSG00000058085	GAGAAAU
ENSG00000058262	AAAGAAU
ENSG00000058262	GGAAUGU
ENSG00000058262	GUGACAG
ENSG00000058262	GUGCUUG
ENSG00000058262	UCUGGAG
ENSG00000058262	UGAGAAG
ENSG00000058262	UGUGCUU
ENSG00000058262	UUUUUGC
ENSG00000058272	AGCAGCU
ENSG00000058272	AGUGGUU
ENSG00000058272	AUAAUAC
ENSG00000058272	CAUAUUG
ENSG00000058272	CCAGUGC
ENSG00000058272	CCAGUGU
ENSG00000058272	GCUAGCA
ENSG00000058272	GUGACUG
ENSG00000058272	GUGCAAA
ENSG00000058272	UAAUAUC
ENSG00000058272	UGCAUAU
ENSG00000058272	UUGGCAA
ENSG00000058272	UUGGCAC
ENSG00000058404	CUGCAGC
ENSG00000058404	CUGCAGG
ENSG00000058404	GAGGCAG
ENSG00000058453	AAAGGUA
ENSG00000058453	UACAAAG
ENSG00000058668	AAAGAAU
ENSG00000058668	AACCUGU
ENSG00000058668	AAGGCAC
ENSG00000058668	AAGUCAC
ENSG00000058668	AGCACCA
ENSG00000058668	AGCAGCU
ENSG00000058668	AGUGCUU
ENSG00000058668	AUAAAGU
ENSG00000058668	AUGCACC
ENSG00000058668	AUGGCUU
ENSG00000058668	GAGGUAG
ENSG00000058668	GAGGUAU
ENSG00000058668	GCACCAU
ENSG00000058668	GUGCUUG

ENSG00000058668	UAUAAAG
ENSG00000058668	UCCUGAG
ENSG00000058668	UGCAUAG
ENSG00000058668	UGUCUUU
ENSG00000058668	UUCAAAU
ENSG00000058668	UUGGCAC
ENSG00000058668	UUUGCAA
ENSG00000058668	UUUGCAC
ENSG00000058673	ACAGGUG
ENSG00000058673	AGGAGCU
ENSG00000058673	GAGGUAU
ENSG00000058673	GCUGUCU
ENSG00000058673	UCCAGUU
ENSG00000058866	ACAAUAU
ENSG00000058866	AUAUAAC
ENSG00000058866	AUAUUAU
ENSG00000058866	CAGGUGA
ENSG00000058866	GAGAACU
ENSG00000058866	GAGCCCU
ENSG00000058866	GGAAUGU
ENSG00000058866	GGGGCAG
ENSG00000058866	UCUCAAG
ENSG00000058866	UUAGAGA
ENSG00000059378	CCAGUGU
ENSG00000059378	GUGACAG
ENSG00000059573	AAAGUAU
ENSG00000059728	AAAGUGC
ENSG00000059728	AAGGCAC
ENSG00000059728	AAGUCUU
ENSG00000059728	AAGUUGU
ENSG00000059728	ACAGUAC
ENSG00000059728	ACAUUAC
ENSG00000059728	ACUCAAA
ENSG00000059728	ACUGUUU
ENSG00000059728	AGCCCUU
ENSG00000059728	AGCUGCC
ENSG00000059728	AGUGCAA
ENSG00000059728	AUAUAAC
ENSG00000059728	CAAAGUG
ENSG00000059728	GAGCCCU
ENSG00000059728	GAGGUAG
ENSG00000059728	GAGGUAU
ENSG00000059728	GCCCUUA
ENSG00000059728	GCUGGUG
ENSG00000059728	GGGUUUA
ENSG00000059728	GUGCAAA
ENSG00000059728	UCCCUCU
ENSG00000059728	UCUGGAA
ENSG00000059728	UGCAAAG
ENSG00000059728	UGCUGAC
ENSG00000059728	UGGAGAU
ENSG00000059728	UUCUAAU
ENSG00000059728	UUGACAC
ENSG00000059728	UUGUGAC
ENSG00000059728	UUUCAAC
ENSG00000059804	AAAAAUC
ENSG00000059804	AAGUGUG
ENSG00000059804	AAUACUG
ENSG00000059804	AGCACCA
ENSG00000059804	AGCAGCG
ENSG00000059804	AGCAGCU
ENSG00000059804	AGGGAGG
ENSG00000059804	CAAAGUG
ENSG00000059804	CAGUGCA

ENSG00000059804	CAUGGAU
ENSG00000059804	GUGACAG
ENSG00000059915	AAAGUGC
ENSG00000059915	AAGUGCA
ENSG00000059915	AGUGCAA
ENSG00000059915	CCUGUAC
ENSG00000059915	CUGCCCC
ENSG00000059915	GCCCUGU
ENSG00000059915	GUGCAAA
ENSG00000059915	UGCCCUG
ENSG00000060069	AAAAACC
ENSG00000060069	AAAAGUA
ENSG00000060069	ACAUCAC
ENSG00000060069	CUUUGGU
ENSG00000060069	GGUGGGC
ENSG00000060069	UACAAAG
ENSG00000060069	UUGGCAA
ENSG00000060069	UUGGCAC
ENSG00000060138	ACCAGCA
ENSG00000060138	CACCAGC
ENSG00000060138	CCAGCAU
ENSG00000060237	AAAGCGC
ENSG00000060237	AAAGUGC
ENSG00000060237	AACAUUC
ENSG00000060237	AAGGCAC
ENSG00000060237	AAUUUUA
ENSG00000060237	ACUGUUU
ENSG00000060237	CAAAACU
ENSG00000060237	CAAAGCA
ENSG00000060237	CAGUAGA
ENSG00000060237	CAGUAGU
ENSG00000060237	CCAGCAU
ENSG00000060237	GCAGAAG
ENSG00000060237	GCAGCAU
ENSG00000060237	GGGGUCC
ENSG00000060237	GGUUUAC
ENSG00000060237	UAAUACA
ENSG00000060237	UAAUAUC
ENSG00000060237	UAAUGCU
ENSG00000060237	UCAAGUA
ENSG00000060237	UCACAGU
ENSG00000060237	UCAUUCG
ENSG00000060237	UCCCUUU
ENSG00000060237	UGACCUA
ENSG00000060339	UUUGCAA
ENSG00000060656	AAAGCGC
ENSG00000060656	AAGCGCU
ENSG00000060656	CAGUAGA
ENSG00000060656	GAGGCAG
ENSG00000060656	GAGGUAG
ENSG00000060656	GAUUGUC
ENSG00000060656	GGCACGG
ENSG00000060656	UACAGUU
ENSG00000060656	UUCUGCA
ENSG00000060656	UUGAGGC
ENSG00000060709	AAAAACC
ENSG00000060709	AAAAGUA
ENSG00000060709	AAGGCAC
ENSG00000060709	ACACACC
ENSG00000060709	ACAGUAC
ENSG00000060709	AUGGCAC
ENSG00000060709	AUUGCAC
ENSG00000060709	CAGCAGA
ENSG00000060709	CAGUCUG

ENSG00000060709	CGAGGAC
ENSG00000060709	CGAGGAG
ENSG00000060709	CUAGUAG
ENSG00000060709	CUCCCAA
ENSG00000060709	CUCCCAC
ENSG00000060709	GAGCUGC
ENSG00000060709	GCAUCCC
ENSG00000060709	GCUACAU
ENSG00000060709	GGGGGAA
ENSG00000060709	GUGCUUG
ENSG00000060709	GUGGGGA
ENSG00000060709	UCCCUGU
ENSG00000060709	UGAAAGG
ENSG00000060709	UGGACUG
ENSG00000060709	UGGAGAU
ENSG00000060709	UGGGGGA
ENSG00000060709	UGUAUAA
ENSG00000060709	UUCAAGC
ENSG00000060709	UUCUUGU
ENSG00000060718	AUAUUAU
ENSG00000060749	AAGUGCU
ENSG00000060749	AAGUGUG
ENSG00000060749	ACAUUCA
ENSG00000060749	AGUGCAA
ENSG00000060749	AGUGCUG
ENSG00000060749	AGUGCUU
ENSG00000060749	AUAAAGU
ENSG00000060749	AUGCAUU
ENSG00000060749	AUGGCUU
ENSG00000060749	AUUGCAC
ENSG00000060749	CUAGUAG
ENSG00000060749	CUGGACU
ENSG00000060749	GCACCAU
ENSG00000060749	GCUUCCU
ENSG00000060749	GGUGGUC
ENSG00000060749	GGUGGUU
ENSG00000060749	GUCCAGA
ENSG00000060749	UAAUACA
ENSG00000060749	UACAAAG
ENSG00000060749	UAUUGCU
ENSG00000060749	UCCUGAG
ENSG00000060749	UGGCUGC
ENSG00000060749	UGUGCUU
ENSG00000060749	UGUUGAA
ENSG00000060749	UUCUUGU
ENSG00000060749	UUGGCAA
ENSG00000060762	AAGGCAC
ENSG00000060762	UAUUGCU
ENSG00000060982	AAGUGCA
ENSG00000060982	AAGUGCU
ENSG00000060982	AAUCUCA
ENSG00000060982	AAUUAUU
ENSG00000060982	AGAUAAA
ENSG00000060982	AGUGCAA
ENSG00000060982	CACACCG
ENSG00000060982	CACACCU
ENSG00000060982	CAGACAG
ENSG00000060982	GAAUUGU
ENSG00000060982	GAGGUAU
ENSG00000060982	GGAGGCA
ENSG00000060982	GUGAAUG
ENSG00000060982	GUGCCAC
ENSG00000060982	GUGCUUG
ENSG00000060982	UAUAAUA

ENSG00000060982	UCACACA
ENSG00000060982	UCCCUUU
ENSG00000060982	UGCAGCU
ENSG00000060982	UGCUGAC
ENSG00000060982	UGUUGAA
ENSG00000060982	UUGAUCA
ENSG00000061273	AGUGGUU
ENSG00000061273	AUGUGGG
ENSG00000061273	CCUUCUU
ENSG00000061273	CUCCCAC
ENSG00000061273	GAGAUGA
ENSG00000061273	GCACCAU
ENSG00000061273	GUGCAAA
ENSG00000061273	UACAAAG
ENSG00000061273	UCACAUU
ENSG00000061273	UCCAGAG
ENSG00000061273	UCCUGAG
ENSG00000061273	UGCAAAG
ENSG00000061273	UUGGCAC
ENSG00000061337	AAGGCAC
ENSG00000061337	AGCAGCU
ENSG00000061337	AGGGAGG
ENSG00000061337	AUUCAUU
ENSG00000061337	GAGAAAU
ENSG00000061337	GGCAUUG
ENSG00000061676	AAUGCCC
ENSG00000061676	UAUAUGA
ENSG00000061936	GAAACAU
ENSG00000061936	GCUGGUG
ENSG00000061936	UCACAGG
ENSG00000061936	UCACAGU
ENSG00000061938	CUCUGGG
ENSG00000061938	CUGGAAA
ENSG00000061938	CUGGCUC
ENSG00000061938	GGAAGAC
ENSG00000061938	GGCCCUG
ENSG00000061938	UCUGGAA
ENSG00000061987	AAAAGUA
ENSG00000061987	AACAUUC
ENSG00000061987	AAGUCUU
ENSG00000061987	ACAGUAU
ENSG00000061987	ACAUCAC
ENSG00000061987	AGCUGCC
ENSG00000061987	CAAGAGC
ENSG00000061987	CUGACUC
ENSG00000061987	GAGGUAG
ENSG00000061987	GAGGUAU
ENSG00000061987	GCUACAU
ENSG00000061987	GGCAGUG
ENSG00000061987	GGUUACC
ENSG00000061987	UACAGUU
ENSG00000061987	UCACAGG
ENSG00000061987	UCACAUU
ENSG00000061987	UCCCUUG
ENSG00000061987	UCUCAAG
ENSG00000061987	UGCAUAU
ENSG00000062038	GAUUGUC
ENSG00000062194	AAAGCUG
ENSG00000062194	UCACAUU
ENSG00000062282	AACAUUC
ENSG00000062282	AAGGCAC
ENSG00000062282	AGCAUUC
ENSG00000062282	CACCACC
ENSG00000062282	GGUUUAC

ENSG00000062282	UAUAAUA
ENSG00000062485	AAAAACC
ENSG00000062485	AGCAUUC
ENSG00000062485	AGUGCCU
ENSG00000062485	CAGUGCA
ENSG00000062485	CAGUUAC
ENSG00000062485	GGAGUGU
ENSG00000062485	GGGUUUA
ENSG00000062485	GGUUUAC
ENSG00000062485	GUUAAUG
ENSG00000062485	UCAACAG
ENSG00000062485	UCUAGAG
ENSG00000062485	UGCAUUG
ENSG00000062485	UUUGCAA
ENSG00000062485	UUUGCAC
ENSG00000062524	ACUGCAG
ENSG00000062598	AAGGCAC
ENSG00000062598	AGCACCA
ENSG00000062598	AGCAGCA
ENSG00000062598	AUAUAAC
ENSG00000062598	CUGGCUC
ENSG00000062598	GAAGGAA
ENSG00000062598	GAGCCCU
ENSG00000062598	GAGUAUU
ENSG00000062598	GGCAUUG
ENSG00000062598	GGGCCCC
ENSG00000062598	UGGCCAC
ENSG00000062598	UGUAUAA
ENSG00000062650	AAAGUAC
ENSG00000062650	AAGACAU
ENSG00000062650	ACUGUAG
ENSG00000062650	AGCAGCA
ENSG00000062650	AUGGCUU
ENSG00000062650	CAGCUGG
ENSG00000062650	CCAGUGU
ENSG00000062650	CGUUGGC
ENSG00000062650	CUCCAGC
ENSG00000062650	CUUUGGU
ENSG00000062650	GGAAGAC
ENSG00000062650	UACAAAG
ENSG00000062650	UCAAGUA
ENSG00000062650	UUCUAAU
ENSG00000062716	AAAUGGU
ENSG00000062716	ACAUCAC
ENSG00000062716	GGAAUGU
ENSG00000062716	UCAUUCG
ENSG00000062725	AACACUG
ENSG00000062725	CACAGUG
ENSG00000062725	CAGGAAC
ENSG00000062725	CCAGUGU
ENSG00000062725	GCACCAU
ENSG00000062725	GCUACAU
ENSG00000062725	UCACAGG
ENSG00000062725	UCCAGUU
ENSG00000062725	UCUAGAG
ENSG00000062725	UUGGAGG
ENSG00000063015	CAAAGUG
ENSG00000063015	CUCUGGG
ENSG00000063015	CUGGCUC
ENSG00000063015	CUUUGGU
ENSG00000063015	UACAAAG
ENSG00000063015	UCUUGAG
ENSG00000063046	AAGUGCU
ENSG00000063046	AAUUUUA

ENSG00000063046	AGCAGCA
ENSG00000063046	AGUGCUU
ENSG00000063046	CUACAGU
ENSG00000063046	CUAGGCU
ENSG00000063046	CUCCAGC
ENSG00000063046	CUCCCAC
ENSG00000063046	CUCUGGG
ENSG00000063046	CUGGCUC
ENSG00000063046	GCAGCAU
ENSG00000063046	GGAUUUU
ENSG00000063046	GUCUUGC
ENSG00000063046	UACAAAG
ENSG00000063046	UCAUUUG
ENSG00000063046	UCCAGUU
ENSG00000063046	UCUCCAA
ENSG00000063046	UGGAGAU
ENSG00000063046	UGUCUGC
ENSG00000063046	UUGGAGG
ENSG00000063169	GGAAGAC
ENSG00000063169	GGAGGCA
ENSG00000063169	GGCAAGA
ENSG00000063169	UUCAAAU
ENSG00000063176	AAUUGCU
ENSG00000063176	AUUGCAC
ENSG00000063176	GGAAUGU
ENSG00000063176	GGAGGCA
ENSG00000063176	GUGCAAA
ENSG00000063176	UAUUGCU
ENSG00000063176	UGCAUAU
ENSG00000063176	UUUCAAC
ENSG00000063241	UGGGAUC
ENSG00000063244	AAUUGCU
ENSG00000063244	AGCAAAA
ENSG00000063244	CACUCCU
ENSG00000063244	CCCCCAG
ENSG00000063244	GUCACUC
ENSG00000063244	UCCCUCU
ENSG00000063244	UGGCCAC
ENSG00000063245	GUAGUGU
ENSG00000063322	AAAAAUC
ENSG00000063322	AUUGCAC
ENSG00000063322	CCGGAGC
ENSG00000063322	CCUGCUG
ENSG00000063322	CUGCCCC
ENSG00000063322	CUUGGGC
ENSG00000063322	GCCUGGG
ENSG00000063322	GCUUCCU
ENSG00000063322	GGGGAGC
ENSG00000063322	GGGGCAG
ENSG00000063322	GGGGUCC
ENSG00000063322	GGGUGGU
ENSG00000063322	GUGGGGA
ENSG00000063322	UCACACA
ENSG00000063322	UCAUUCG
ENSG00000063322	UCCUUGC
ENSG00000063438	CCCUGAG
ENSG00000063587	AAUUGCU
ENSG00000063587	AUAAAGU
ENSG00000063587	AUGGCUU
ENSG00000063587	CAAAGCA
ENSG00000063587	CUCCAGC
ENSG00000063587	GAGAUGA
ENSG00000063587	GAGGUAU
ENSG00000063587	GGCAGUG

ENSG00000063587	GUUCAUG
ENSG00000063587	UAAUAUC
ENSG00000063601	AAUUGCU
ENSG00000063601	UCAAGUA
ENSG00000063660	ACAAGGU
ENSG00000063660	AGCCCUU
ENSG00000063660	AUACAAG
ENSG00000063660	UUGGCAC
ENSG00000063978	AAAGUGC
ENSG00000063978	AAGGUGC
ENSG00000063978	CACCACC
ENSG00000063978	CACUCCU
ENSG00000063978	CCUUCAU
ENSG00000063978	GAGGCAG
ENSG00000063978	GGAGUCC
ENSG00000063978	UCCUGAG
ENSG00000063978	UUCAAAU
ENSG00000064042	AAAGGUA
ENSG00000064042	ACAUUCA
ENSG00000064042	ACUGCAU
ENSG00000064042	AUCAUAC
ENSG00000064042	CAAAGUG
ENSG00000064042	UAUAAAG
ENSG00000064042	UGUAUAA
ENSG00000064042	UUCUAAU
ENSG00000064102	AAAAAUC
ENSG00000064115	AAAGGUA
ENSG00000064195	AAGUUGU
ENSG00000064195	AAUGCCC
ENSG00000064195	AUAAAGU
ENSG00000064195	AUAUAAC
ENSG00000064195	CCAGGAG
ENSG00000064195	CCAGUAC
ENSG00000064195	CUUCUCU
ENSG00000064195	GCCCUUA
ENSG00000064195	GUCUUGC
ENSG00000064195	GUGCAAA
ENSG00000064195	UCUGGAG
ENSG00000064195	UGAGAAG
ENSG00000064195	UGCAAAG
ENSG00000064195	UUGGUUC
ENSG00000064218	UCCUGAG
ENSG00000064225	AAAACUG
ENSG00000064225	UCACAGG
ENSG00000064225	UCACAGU
ENSG00000064300	AUGUGGG
ENSG00000064300	GGAAUGU
ENSG00000064300	GGCUCUG
ENSG00000064300	UCACAGG
ENSG00000064300	UGAGUCU
ENSG00000064309	AAAGGUA
ENSG00000064309	AACACUG
ENSG00000064309	AAUACUG
ENSG00000064309	ACAUCAC
ENSG00000064309	AUAAAGU
ENSG00000064309	AUAUAAC
ENSG00000064309	AUGACAC
ENSG00000064309	CACAAUG
ENSG00000064309	CAGUCCA
ENSG00000064309	CAGUGCA
ENSG00000064309	CAGUUAC
ENSG00000064309	CUCUUCC
ENSG00000064309	GAGAACU
ENSG00000064309	GCUACAU

ENSG00000064309	GGAAUGU
ENSG00000064309	GUGACAG
ENSG00000064309	UAAUAUC
ENSG00000064309	UCUGGAA
ENSG00000064309	UGACCUA
ENSG00000064309	UGCAUAG
ENSG00000064313	ACCACAG
ENSG00000064393	AAAAGUA
ENSG00000064393	AAAGCUG
ENSG00000064393	AAAGUAC
ENSG00000064393	AAAUUUC
ENSG00000064393	AACACUG
ENSG00000064393	AAGGCAC
ENSG00000064393	AAGUGUG
ENSG00000064393	AAUACUG
ENSG00000064393	AAUGCCC
ENSG00000064393	AAUUAUU
ENSG00000064393	ACAGUAC
ENSG00000064393	ACAUUCA
ENSG00000064393	ACGUCAU
ENSG00000064393	ACUGUUU
ENSG00000064393	AGAUAAA
ENSG00000064393	AGCAGCG
ENSG00000064393	AGCAGCU
ENSG00000064393	AGCAUUC
ENSG00000064393	AGCCAGU
ENSG00000064393	AGCCCUU
ENSG00000064393	AGGUUAU
ENSG00000064393	AUAAAGU
ENSG00000064393	AUAAUAC
ENSG00000064393	AUACAAG
ENSG00000064393	AUACCUC
ENSG00000064393	AUGAGCU
ENSG00000064393	AUGAUGA
ENSG00000064393	AUGGAUU
ENSG00000064393	AUGUAAU
ENSG00000064393	AUUCAUU
ENSG00000064393	CACAGUG
ENSG00000064393	CACCACC
ENSG00000064393	CACUCCU
ENSG00000064393	CAGUAGA
ENSG00000064393	CAGUUAC
ENSG00000064393	CAUGGAU
ENSG00000064393	CCAGUGC
ENSG00000064393	CCAGUGU
ENSG00000064393	CCCUGUU
ENSG00000064393	CUACAGU
ENSG00000064393	CUAUACA
ENSG00000064393	CUCCAUU
ENSG00000064393	CUUGGAG
ENSG00000064393	CUUGUAU
ENSG00000064393	CUUGUGU
ENSG00000064393	CUUUGGU
ENSG00000064393	GAAGGAA
ENSG00000064393	GAGCUAA
ENSG00000064393	GAGCUGC
ENSG00000064393	GAGGUAG
ENSG00000064393	GAGGUAU
ENSG00000064393	GCAGCAU
ENSG00000064393	GCCCUGU
ENSG00000064393	GCUACAU
ENSG00000064393	GCUUCCU
ENSG00000064393	GGAAGAC
ENSG00000064393	GGAAUGU

ENSG00000064393	GGUUGUC
ENSG00000064393	GGUUUAC
ENSG00000064393	GUCAGUU
ENSG00000064393	GUGCAAA
ENSG00000064393	UACAAAG
ENSG00000064393	UAUAAAG
ENSG00000064393	UAUAAUA
ENSG00000064393	UAUUACC
ENSG00000064393	UAUUGCU
ENSG00000064393	UCAAGUA
ENSG00000064393	UCACAGG
ENSG00000064393	UCACAUU
ENSG00000064393	UCAUGAU
ENSG00000064393	UCCCUGU
ENSG00000064393	UCCUGAG
ENSG00000064393	UCUAGAG
ENSG00000064393	UGAGGAC
ENSG00000064393	UGCAUAU
ENSG00000064393	UGCCCUG
ENSG00000064393	UUGAGGC
ENSG00000064393	UUGGUUC
ENSG00000064393	UUGUGUC
ENSG00000064419	AAGACAU
ENSG00000064419	CACAGUG
ENSG00000064419	CAGGAAC
ENSG00000064419	CUCCCAC
ENSG00000064419	GAUUGUA
ENSG00000064419	GAUUGUC
ENSG00000064419	UCCCACC
ENSG00000064419	UGGGACA
ENSG00000064419	UUCUAAU
ENSG00000064490	UUGGUUC
ENSG00000064547	CACAGGG
ENSG00000064547	GAGAUGA
ENSG00000064601	AAGUGCU
ENSG00000064601	AUAAAGU
ENSG00000064601	UCCUGAG
ENSG00000064601	UUCCAUA
ENSG00000064651	AAGGCAC
ENSG00000064651	AAGUAGC
ENSG00000064651	AAUCCUU
ENSG00000064651	AGCAGCG
ENSG00000064651	AGCAGCU
ENSG00000064651	AUCCCUU
ENSG00000064651	AUUGCAC
ENSG00000064651	CAAAGCA
ENSG00000064651	GGAAGAC
ENSG00000064651	GGCAGUG
ENSG00000064651	GUGCUUG
ENSG00000064651	UACAAAG
ENSG00000064651	UCACAGG
ENSG00000064651	UCACAGU
ENSG00000064651	UCCCUUG
ENSG00000064651	UGUGCUU
ENSG00000064652	AGCACCA
ENSG00000064652	UGAAAUG
ENSG00000064652	UGCCCUG
ENSG00000064655	AAGGCAC
ENSG00000064655	AUGCCUU
ENSG00000064655	GAUUGUA
ENSG00000064655	GUAAACA
ENSG00000064726	CAAAUGC
ENSG00000064787	AUAAUAC
ENSG00000064787	UAAUACA

ENSG00000064961	AAGGUGC
ENSG00000064989	AAUCCUU
ENSG00000064989	UAUCAGA
ENSG00000064995	GCAACUU
ENSG00000064999	AACAAAC
ENSG00000064999	AACAUUC
ENSG00000064999	AAUGCCC
ENSG00000064999	ACAGUAC
ENSG00000064999	ACAGUAU
ENSG00000064999	AGCAGCG
ENSG00000064999	CACAGUG
ENSG00000064999	CCUUCAU
ENSG00000064999	GAUUGGU
ENSG00000064999	GGGUCUU
ENSG00000064999	GUAGUGU
ENSG00000064999	UCACAGG
ENSG00000064999	UCACAGU
ENSG00000065000	AAAGUGC
ENSG00000065000	AAUCACU
ENSG00000065000	AGCAAAA
ENSG00000065000	GGAAUGU
ENSG00000065029	CCCUGAG
ENSG00000065029	UAUUGCU
ENSG00000065054	CAGCAGG
ENSG00000065054	GAAACAU
ENSG00000065060	AAUACUG
ENSG00000065060	ACAUUCA
ENSG00000065060	CCAGUAC
ENSG00000065060	CUCUGGG
ENSG00000065060	CUUUGGU
ENSG00000065060	GAGGUAU
ENSG00000065060	GGUGGUU
ENSG00000065060	GUAAACA
ENSG00000065060	UCACAGU
ENSG00000065060	UGAAAUG
ENSG00000065060	UUCAAGC
ENSG00000065060	UUCCUCG
ENSG00000065060	UUGGCAC
ENSG00000065135	AAGGCAC
ENSG00000065135	AAUACUG
ENSG00000065135	ACAGUAC
ENSG00000065135	CAGACAG
ENSG00000065135	GAAAACA
ENSG00000065135	GACCAUG
ENSG00000065135	GCAGCAU
ENSG00000065135	GGUGGUC
ENSG00000065135	GUUCAUG
ENSG00000065135	UGCAUAG
ENSG00000065135	UGUGCUU
ENSG00000065135	UUGGCAA
ENSG00000065135	UUGGCAC
ENSG00000065150	AACAUUC
ENSG00000065150	AAGAACC
ENSG00000065150	ACACACC
ENSG00000065150	ACAGUAC
ENSG00000065150	ACAUUCA
ENSG00000065150	AGUACUG
ENSG00000065150	AUGCAUU
ENSG00000065150	AUGUCUG
ENSG00000065150	AUUCAUU
ENSG00000065150	CAGACAG
ENSG00000065150	CAGUGCA
ENSG00000065150	GGAAGCC
ENSG00000065150	UCACAUU

ENSG00000065150	UCAUUUG
ENSG00000065150	UCCCUUG
ENSG00000065150	UCCCUUU
ENSG00000065150	UGACAUC
ENSG00000065150	UGAGGAC
ENSG00000065150	UGCUCAC
ENSG00000065150	UGUGUGG
ENSG00000065150	UUGGAGG
ENSG00000065243	AAACUGU
ENSG00000065243	AAAGUGC
ENSG00000065243	AAGUGCU
ENSG00000065243	AAUUAUU
ENSG00000065243	ACAUUCA
ENSG00000065243	AGUGAUC
ENSG00000065243	AGUGCUG
ENSG00000065243	CAAAGUG
ENSG00000065243	CAAAUGC
ENSG00000065243	CAGCAGA
ENSG00000065243	CCAGUGU
ENSG00000065243	GAGGUAG
ENSG00000065243	GGCAAGA
ENSG00000065243	UAGACUG
ENSG00000065243	UAGUGAG
ENSG00000065243	UAUUGCU
ENSG00000065243	UCACAGG
ENSG00000065243	UCACAGU
ENSG00000065243	UCCAGUU
ENSG00000065243	UCCUGAG
ENSG00000065243	UGCAUAG
ENSG00000065243	UGGCCAC
ENSG00000065243	UGUCUGC
ENSG00000065243	UUCUUGU
ENSG00000065243	UUGAGGC
ENSG00000065243	UUGGCAA
ENSG00000065243	UUGGCAC
ENSG00000065308	AAAAGUA
ENSG00000065308	ACCAGCA
ENSG00000065308	AGCACCA
ENSG00000065308	AUUGCAC
ENSG00000065308	CAAAACU
ENSG00000065308	CACCAGC
ENSG00000065308	CCUUCAU
ENSG00000065308	CCUUCUG
ENSG00000065308	GAGAUGA
ENSG00000065308	GGAGGAG
ENSG00000065308	GUAAACA
ENSG00000065308	UAAUAGU
ENSG00000065308	UAUUGCU
ENSG00000065308	UCCCUUU
ENSG00000065308	UGAAAUG
ENSG00000065308	UGAGAAG
ENSG00000065308	UGCAUUG
ENSG00000065308	UGCUCAC
ENSG00000065308	UUGGCAC
ENSG00000065308	UUGGUUC
ENSG00000065320	AAAGUGC
ENSG00000065320	ACCCGGC
ENSG00000065320	AGUGCCU
ENSG00000065320	AUAAUAC
ENSG00000065320	AUAUAAC
ENSG00000065320	AUAUUAU
ENSG00000065320	AUUCAUU
ENSG00000065320	CCAGCAU
ENSG00000065320	CCCCUGG

ENSG00000065320	CUCAGGG
ENSG00000065320	CUGGAAA
ENSG00000065320	CUGUUGA
ENSG00000065320	GAGGUAG
ENSG00000065320	GGGAGCU
ENSG00000065320	GGGCCCC
ENSG00000065320	GGUGGUU
ENSG00000065320	GUCACUC
ENSG00000065320	GUCAUUG
ENSG00000065320	GUGCCUG
ENSG00000065320	GUGUCAC
ENSG00000065320	UAAUACA
ENSG00000065320	UCACAGU
ENSG00000065320	UGCAUUG
ENSG00000065357	AAUACUG
ENSG00000065357	GAAGGAA
ENSG00000065357	GAGGCAG
ENSG00000065357	GGCAGCG
ENSG00000065361	GAGAUGA
ENSG00000065361	GUGCAAA
ENSG00000065361	UCCAGUU
ENSG00000065413	UUCAAAU
ENSG00000065413	UUCUAAU
ENSG00000065485	GAGGCAG
ENSG00000065485	UCACAGG
ENSG00000065491	AAAAACC
ENSG00000065491	AAAGAGG
ENSG00000065491	AAUACUG
ENSG00000065491	ACCCAUU
ENSG00000065491	AGCCCUU
ENSG00000065491	AGUGAUC
ENSG00000065491	AGUGGUU
ENSG00000065491	CACAGUG
ENSG00000065491	CUACAAA
ENSG00000065491	GAGAAAU
ENSG00000065491	GAGAAGA
ENSG00000065491	GAUCAGA
ENSG00000065491	GCCUGAU
ENSG00000065491	GCUACAU
ENSG00000065491	GGAGGAG
ENSG00000065491	GGCUCAG
ENSG00000065491	GGGAACG
ENSG00000065491	UAAGACG
ENSG00000065491	UAAGACU
ENSG00000065491	UACAAAG
ENSG00000065491	UAUAAAG
ENSG00000065491	UGUCUUU
ENSG00000065491	UUGGCAC
ENSG00000065526	AAAACUG
ENSG00000065526	ACACACU
ENSG00000065526	AGCAGCA
ENSG00000065526	CAAAACU
ENSG00000065526	GUGCAAA
ENSG00000065526	UACAAAG
ENSG00000065526	UGCAAAG
ENSG00000065526	UUGGCAA
ENSG00000065534	AAGUGCA
ENSG00000065534	AAGUGCU
ENSG00000065559	AAAAACC
ENSG00000065559	AAAGAGC
ENSG00000065559	AAUGUUG
ENSG00000065559	AAUUGCU
ENSG00000065559	ACCAGCA
ENSG00000065559	AGCACCA

ENSG00000065559	AGCAGCA
ENSG00000065559	AGCAGCG
ENSG00000065559	AGCAGCU
ENSG00000065559	AGCCCUU
ENSG00000065559	AGCUGCC
ENSG00000065559	AUAAUAC
ENSG00000065559	AUCCCUU
ENSG00000065559	AUGGCUU
ENSG00000065559	CCAGUAC
ENSG00000065559	CCAGUGU
ENSG00000065559	CCCUGUU
ENSG00000065559	CUAUACA
ENSG00000065559	CUGCAGG
ENSG00000065559	CUGGGCA
ENSG00000065559	CUUACAG
ENSG00000065559	CUUCUCU
ENSG00000065559	GACCUGG
ENSG00000065559	GGAUGAG
ENSG00000065559	GGCAGCG
ENSG00000065559	GUCAACA
ENSG00000065559	UACAGUU
ENSG00000065559	UAUUACC
ENSG00000065559	UAUUGCU
ENSG00000065559	UCACAGG
ENSG00000065559	UCCUUGC
ENSG00000065559	UGCCCUG
ENSG00000065559	UGUCUGG
ENSG00000065559	UUCUAAU
ENSG00000065609	AACACUG
ENSG00000065609	AAUGUUG
ENSG00000065609	AGCUGCC
ENSG00000065609	AUUGCAC
ENSG00000065609	UAUUGCU
ENSG00000065609	UCAACAG
ENSG00000065609	UGAAAUG
ENSG00000065609	UGUAUGU
ENSG00000065618	UUCCUAG
ENSG00000065675	UAGUGAG
ENSG00000065675	UCUCAAG
ENSG00000065802	CAGUCCA
ENSG00000065802	GUGAAUG
ENSG00000065802	UUGGUCC
ENSG00000065809	ACUCAAA
ENSG00000065809	AGCUUAU
ENSG00000065809	AUAAAGU
ENSG00000065809	CCAGUGC
ENSG00000065809	CUGCUCA
ENSG00000065809	GAAAACA
ENSG00000065809	GGAAGAC
ENSG00000065809	GGCUCAG
ENSG00000065809	GUCUCUG
ENSG00000065809	UCACAAG
ENSG00000065809	UCCAGUU
ENSG00000065809	UCCCUUU
ENSG00000065809	UCCUGAG
ENSG00000065833	UGCAUAU
ENSG00000065833	UUUGCAA
ENSG00000065833	UUUGCAC
ENSG00000065882	AAGGCAC
ENSG00000065882	AGCAAAA
ENSG00000065882	CUUCUCU
ENSG00000065882	GAGAAGA
ENSG00000065882	GCCUGGA
ENSG00000065882	UGAGAAG

ENSG00000065883	AAAAAUC
ENSG00000065883	AAAGUAC
ENSG00000065883	AGAUAAA
ENSG00000065883	AUGUGCC
ENSG00000065883	UACAAAG
ENSG00000065883	UCAAGUA
ENSG00000065883	UGAAAUG
ENSG00000065883	UGCAUAG
ENSG00000065883	UUGGUCC
ENSG00000065911	AGCUGCC
ENSG00000065911	GUGAGGU
ENSG00000065911	UGCAUUG
ENSG00000065911	UUGGUUC
ENSG00000065970	AAAAGUA
ENSG00000065970	AACAUUC
ENSG00000065970	AAGUGCU
ENSG00000065970	ACAUCAC
ENSG00000065970	AUAAAGU
ENSG00000065970	CAAAGCA
ENSG00000065970	CACCACC
ENSG00000065970	CUUCUCU
ENSG00000065970	GAAACAU
ENSG00000065970	GAGACCU
ENSG00000065970	GCAGCAU
ENSG00000065970	UCACCAC
ENSG00000065970	UGAGAAU
ENSG00000065970	UGCAAAG
ENSG00000065978	GAGAAAU
ENSG00000065978	UGAGAAG
ENSG00000065978	UGAGAAU
ENSG00000065989	AAGGCAC
ENSG00000065989	ACAGUAC
ENSG00000065989	ACCCUGU
ENSG00000065989	AUCCCUU
ENSG00000065989	AUGGCUU
ENSG00000065989	CCCACCU
ENSG00000065989	GCUUCCU
ENSG00000065989	UAAUACA
ENSG00000065989	UAUAAUA
ENSG00000065989	UAUUGCU
ENSG00000065989	UCCCUUG
ENSG00000066027	UACAAAG
ENSG00000066027	UCAAGUA
ENSG00000066032	AUACAAG
ENSG00000066032	UGUGCUU
ENSG00000066032	UUUUUGC
ENSG00000066044	AAAAGUA
ENSG00000066044	AAAGGUA
ENSG00000066044	AAAGUAA
ENSG00000066044	AACAUUC
ENSG00000066044	AACUGGU
ENSG00000066044	ACUCAGC
ENSG00000066044	ACUGUAG
ENSG00000066044	AUACAAG
ENSG00000066044	AUUCAUU
ENSG00000066044	CAAAUGC
ENSG00000066044	CACAAUG
ENSG00000066044	CCCUGAG
ENSG00000066044	CUACAAA
ENSG00000066044	GAGUGUG
ENSG00000066044	GAGUUGA
ENSG00000066044	GAGUUGG
ENSG00000066044	GGCAAGA
ENSG00000066044	GGUCUAG

ENSG00000066044	GUCAACA
ENSG00000066044	GUGCAAA
ENSG00000066044	GUUCAUG
ENSG00000066044	UAAUAGU
ENSG00000066044	UCAUUCG
ENSG00000066044	UCCAGAG
ENSG00000066044	UCUGGAA
ENSG00000066044	UGAGAAG
ENSG00000066044	UGAGAAU
ENSG00000066044	UUCAAAU
ENSG00000066044	UUCCAUA
ENSG00000066044	UUCUGCA
ENSG00000066044	UUGGCAA
ENSG00000066044	UUGGCAC
ENSG00000066044	UUUCAAC
ENSG00000066056	CAGCAGA
ENSG00000066084	AAAUUUC
ENSG00000066084	AAGGCAC
ENSG00000066084	AAGUCUU
ENSG00000066084	AAUUAUU
ENSG00000066084	ACAUUCA
ENSG00000066084	AGCACCA
ENSG00000066084	GAGACCU
ENSG00000066084	GCACCAU
ENSG00000066084	GGGGCAG
ENSG00000066084	GUCCAGA
ENSG00000066084	UAAUAGU
ENSG00000066084	UCUGGAA
ENSG00000066084	UUAGGAU
ENSG00000066117	AACACUG
ENSG00000066117	AACGGAA
ENSG00000066117	AAUACUG
ENSG00000066117	ACAGUAC
ENSG00000066117	ACCCGUA
ENSG00000066117	AGGCAGG
ENSG00000066117	CACAGGG
ENSG00000066117	CACUCCU
ENSG00000066117	CAGUGCA
ENSG00000066117	CAGUUAC
ENSG00000066117	CCCACCG
ENSG00000066117	CCCACCU
ENSG00000066117	CCUGCUG
ENSG00000066117	CUCCAGC
ENSG00000066117	CUCCCAA
ENSG00000066117	CUCCCAC
ENSG00000066117	CUGGGCA
ENSG00000066117	GACCUGG
ENSG00000066117	GAGGGGC
ENSG00000066117	GCAUCCC
ENSG00000066117	GCUGUCU
ENSG00000066117	GGAAGAC
ENSG00000066117	GGGGAGC
ENSG00000066117	GGGGUGG
ENSG00000066117	GGGUUUA
ENSG00000066117	GUCAGUU
ENSG00000066117	GUCUCUG
ENSG00000066117	GUGGGGA
ENSG00000066117	UCAACAG
ENSG00000066117	UCCCACC
ENSG00000066117	UGAAGCU
ENSG00000066117	UGAGUCU
ENSG00000066136	AGCCUGC
ENSG00000066136	GCACCAU
ENSG00000066136	UAUUGCU

ENSG00000066136	UGAAGUG
ENSG00000066136	UGCAUUG
ENSG00000066136	UGCCAGC
ENSG00000066248	AAUACUG
ENSG00000066294	UUAGGAU
ENSG00000066322	UAUUGCU
ENSG00000066336	UAAUGCU
ENSG00000066382	AAAAAUC
ENSG00000066382	AACACUG
ENSG00000066382	AAUUUUA
ENSG00000066382	AGUGCAA
ENSG00000066382	AUAAAGU
ENSG00000066382	AUAUAAC
ENSG00000066382	CACAGUG
ENSG00000066382	CAGUGCA
ENSG00000066382	UAUAAAG
ENSG00000066382	UGCAUAG
ENSG00000066405	GCUGUCU
ENSG00000066405	UAAUAUC
ENSG00000066422	AAGGCAC
ENSG00000066422	AAGUGCA
ENSG00000066422	ACAUCAC
ENSG00000066422	CAAAUGC
ENSG00000066422	CUUGUGU
ENSG00000066422	GUGCUUG
ENSG00000066422	UGCAAAG
ENSG00000066427	CCCUGAG
ENSG00000066427	CUUUGGU
ENSG00000066427	UUUGCAA
ENSG00000066455	CCCUGAG
ENSG00000066455	UCCCUGU
ENSG00000066468	AACAUUC
ENSG00000066468	AUAAUAC
ENSG00000066468	AUAUAAC
ENSG00000066468	CCAGCAU
ENSG00000066468	UGCAUAG
ENSG00000066583	AGCAGCA
ENSG00000066629	ACAAUAU
ENSG00000066629	ACAUUCA
ENSG00000066629	ACUUGGA
ENSG00000066629	CCAGUGC
ENSG00000066629	CCUGCUG
ENSG00000066629	CCUUCUU
ENSG00000066629	CUAUACA
ENSG00000066629	GACCAUG
ENSG00000066629	GCAGGUU
ENSG00000066629	GGUCUAG
ENSG00000066629	GUCUACU
ENSG00000066629	UCACAGU
ENSG00000066654	AAGGCAC
ENSG00000066654	AAGUAGC
ENSG00000066654	CAGCUGG
ENSG00000066735	AAGGCAC
ENSG00000066735	UACAAAG
ENSG00000066735	UUGGCAA
ENSG00000066735	UUGGCAC
ENSG00000066739	AACAUUC
ENSG00000066739	ACAUUCA
ENSG00000066739	ACUGCAU
ENSG00000066739	ACUUGGA
ENSG00000066739	AUAAAGU
ENSG00000066739	AUGUCUG
ENSG00000066739	CACCAGC
ENSG00000066739	CAGCAAA

ENSG00000066739	CAUGGAU
ENSG00000066739	GAGAAAU
ENSG00000066739	GGCAGCG
ENSG00000066739	GUCUGCC
ENSG00000066739	UCAUUCG
ENSG00000066739	UGAGAAG
ENSG00000066739	UGAGAAU
ENSG00000066739	UGUCUGC
ENSG00000066739	UUGACAC
ENSG00000066739	UUGGUUC
ENSG00000066739	UUGUUCG
ENSG00000066777	AAGUUGU
ENSG00000066777	ACAGUAU
ENSG00000066777	AGUGCAA
ENSG00000066777	AGUGUGC
ENSG00000066777	CACAGUG
ENSG00000066777	UCCUGAG
ENSG00000066827	AUUCAUU
ENSG00000066855	AGAUAAA
ENSG00000066855	CAGCAGA
ENSG00000066855	CAGCAGG
ENSG00000066855	GGCAAGA
ENSG00000066926	AAAGUUU
ENSG00000066926	UACAAAG
ENSG00000067048	AAAAGUA
ENSG00000067048	AAAGAAU
ENSG00000067048	AAAGUAU
ENSG00000067048	AAGGCAC
ENSG00000067048	AAGUGCA
ENSG00000067048	ACAGUAC
ENSG00000067048	AGCAAAA
ENSG00000067048	AGCCUGC
ENSG00000067048	AUGGCUU
ENSG00000067048	CAGCUGG
ENSG00000067048	CCAGUGU
ENSG00000067048	CCUGCUG
ENSG00000067057	AAGUGCU
ENSG00000067057	AUAAAGU
ENSG00000067057	UACAAAG
ENSG00000067064	GAAAACA
ENSG00000067082	ACAUUCA
ENSG00000067082	GCAUCCC
ENSG00000067082	GGAGGCA
ENSG00000067082	UGGCUGC
ENSG00000067113	AGCAGCA
ENSG00000067113	AGCAGCG
ENSG00000067113	AGCAGCU
ENSG00000067141	AAAAAUC
ENSG00000067141	AACAAAC
ENSG00000067141	AACAUUC
ENSG00000067141	AAGUCUU
ENSG00000067141	AAGUGUG
ENSG00000067141	AAUACUG
ENSG00000067141	AAUCUCA
ENSG00000067141	AAUCUCU
ENSG00000067141	AAUUAUU
ENSG00000067141	ACAUUCA
ENSG00000067141	AUAAUAC
ENSG00000067141	AUGGAUU
ENSG00000067141	CACAAUG
ENSG00000067141	CACAGGG
ENSG00000067141	CAGCAGA
ENSG00000067141	CAGCUGG
ENSG00000067141	CCCUGAG

ENSG00000067141	CUCACAC
ENSG00000067141	CUGCAGG
ENSG00000067141	CUUUGGU
ENSG00000067141	GAGCUGC
ENSG00000067141	GAUUGGU
ENSG00000067141	GCCUGGA
ENSG00000067141	GCUGUCU
ENSG00000067141	GGAUUUU
ENSG00000067141	GGCCCUG
ENSG00000067141	UCACAGU
ENSG00000067141	UCUGGAA
ENSG00000067167	AGAUAAA
ENSG00000067167	CUUUGGU
ENSG00000067167	UAAGACU
ENSG00000067167	UACAAAG
ENSG00000067167	UCAAGUA
ENSG00000067177	AAGGCAC
ENSG00000067177	ACAUUCA
ENSG00000067177	UGAAAUG
ENSG00000067191	AAGUGCA
ENSG00000067191	AGCAGCA
ENSG00000067191	CAGACAG
ENSG00000067191	CAGCAGG
ENSG00000067191	CCCUGAG
ENSG00000067191	GGCAGUG
ENSG00000067191	GGGGCAG
ENSG00000067191	UUGGCAC
ENSG00000067191	UUGGUUC
ENSG00000067191	UUUUUGC
ENSG00000067208	AAAGCUG
ENSG00000067208	ACUGUUU
ENSG00000067208	AGCAGCA
ENSG00000067208	AUCCCUU
ENSG00000067208	AUGCACC
ENSG00000067208	AUGGCAC
ENSG00000067208	AUGGCUU
ENSG00000067208	CAAAUGC
ENSG00000067208	CAAGAGC
ENSG00000067208	CACAGUG
ENSG00000067208	CAGUUAC
ENSG00000067208	CUGGAAA
ENSG00000067208	GUAAACA
ENSG00000067208	GUCUACU
ENSG00000067208	GUGCAAA
ENSG00000067208	UCACAGU
ENSG00000067208	UCAUACA
ENSG00000067208	UCUCCAA
ENSG00000067208	UUCUAAU
ENSG00000067208	UUGGCAC
ENSG00000067208	UUGUGUC
ENSG00000067208	UUUCAAC
ENSG00000067208	UUUGCAA
ENSG00000067221	GGGCCCC
ENSG00000067221	GUAAACA
ENSG00000067221	GUCCAGA
ENSG00000067221	UGCCCUG
ENSG00000067225	GCCUGGA
ENSG00000067533	AACACUG
ENSG00000067533	CAGCUGG
ENSG00000067533	CUCCAUU
ENSG00000067533	CUGGCUC
ENSG00000067533	CUUGGGC
ENSG00000067533	GAGCCCC
ENSG00000067533	GCUGUCU

ENSG00000067533	GGCUGCG
ENSG00000067533	GUCUCUG
ENSG00000067533	UCAACAG
ENSG00000067533	UGGCUGC
ENSG00000067533	UUCUUGU
ENSG00000067533	UUGGUCC
ENSG00000067560	AAAACUG
ENSG00000067560	AAUACUG
ENSG00000067560	ACCACAG
ENSG00000067560	AUCAUAC
ENSG00000067560	UCAUACA
ENSG00000067606	UGCAUAU
ENSG00000067646	AAAGUUU
ENSG00000067646	GCAACUU
ENSG00000067704	AUGCCUU
ENSG00000067715	AAAAACC
ENSG00000067715	AAAACUG
ENSG00000067715	AAAGAGG
ENSG00000067715	AACAUUC
ENSG00000067715	AAGUGCA
ENSG00000067715	AAGUUGU
ENSG00000067715	AAUACUG
ENSG00000067715	ACUGGCC
ENSG00000067715	AGUGCCG
ENSG00000067715	AUGGCUU
ENSG00000067715	CAGUGCA
ENSG00000067715	GAAAACA
ENSG00000067715	GAGGUAG
ENSG00000067715	GCAGAAG
ENSG00000067715	GGAAUGU
ENSG00000067715	UAAGACU
ENSG00000067715	UAAUAGU
ENSG00000067715	UAUAAAG
ENSG00000067715	UCACAGU
ENSG00000067715	UCCUAUA
ENSG00000067715	UGAAGUG
ENSG00000067715	UGUAUAA
ENSG00000067715	UUGGUCC
ENSG00000067798	AAGGCAC
ENSG00000067798	ACACACC
ENSG00000067798	ACAGUAU
ENSG00000067798	ACUGUUU
ENSG00000067798	AGCCCUU
ENSG00000067798	AGCCUGC
ENSG00000067798	AGUAGAC
ENSG00000067798	AUUGCAC
ENSG00000067798	CACAGUG
ENSG00000067798	CAGUAGA
ENSG00000067798	CUACAAA
ENSG00000067798	CUCUAGC
ENSG00000067798	GCACCAU
ENSG00000067798	GGAAUGU
ENSG00000067798	GUGCUUG
ENSG00000067798	UAAUGCU
ENSG00000067798	UAGACUG
ENSG00000067798	UGCAUAG
ENSG00000067798	UGCUCAC
ENSG00000067798	UUGGCAC
ENSG00000067840	AGCUGCC
ENSG00000067840	AGGAGCU
ENSG00000067840	CCCCCAG
ENSG00000067840	CCCUGUC
ENSG00000067840	GAGCUGC
ENSG00000067840	GGCAAGA

ENSG00000067840	UGUGCUU
ENSG00000067840	UUGGCAA
ENSG00000067842	AAAGAGC
ENSG00000067842	AGAUAAA
ENSG00000067842	AGCAGCA
ENSG00000067842	AUGGCUU
ENSG00000067842	CCCUGAG
ENSG00000067842	CUGCUCA
ENSG00000067842	GCCCUUA
ENSG00000067842	UACAAAG
ENSG00000067842	UCCCUGU
ENSG00000067842	UCUCAAG
ENSG00000067842	UUGUUCG
ENSG00000067900	AGUGCUU
ENSG00000067900	AUGGCUU
ENSG00000067900	CAAAGCA
ENSG00000067900	CCAGUGU
ENSG00000067900	GAGGUAU
ENSG00000067900	UCCAGUU
ENSG00000067900	UUGGCAA
ENSG00000067955	AUCCCUU
ENSG00000067955	GCUACAU
ENSG00000067955	UCACAGU
ENSG00000068001	UACAAAG
ENSG00000068024	AAAGCUG
ENSG00000068024	AAAGUAA
ENSG00000068024	AAAGUGC
ENSG00000068024	AACUCUA
ENSG00000068024	AAGUAGC
ENSG00000068024	AAGUGCU
ENSG00000068024	AAUACUG
ENSG00000068024	AAUGCCC
ENSG00000068024	ACCCUGU
ENSG00000068024	ACUGUUU
ENSG00000068024	AGCAUUC
ENSG00000068024	AGCUGCC
ENSG00000068024	AGUGGUU
ENSG00000068024	AGUGUGC
ENSG00000068024	AUAAAGU
ENSG00000068024	AUACAAG
ENSG00000068024	AUACCUC
ENSG00000068024	AUGGCUU
ENSG00000068024	CAAAGUG
ENSG00000068024	CACAGUG
ENSG00000068024	CAGCAAA
ENSG00000068024	CGCCUCC
ENSG00000068024	CUACAAA
ENSG00000068024	CUGGAAA
ENSG00000068024	CUGGACU
ENSG00000068024	CUUACAG
ENSG00000068024	CUUUGGU
ENSG00000068024	GAGAAAU
ENSG00000068024	GCUGGUG
ENSG00000068024	GGAAUGU
ENSG00000068024	GGCAUUG
ENSG00000068024	GGGCCCC
ENSG00000068024	GGGUAAG
ENSG00000068024	GUCAGUU
ENSG00000068024	GUGCAAA
ENSG00000068024	GUGCCUG
ENSG00000068024	UAUAAAG
ENSG00000068024	UCAACAG
ENSG00000068024	UCACAAG
ENSG00000068024	UGGGUGA

ENSG00000068028	CUCUGGG
ENSG00000068078	AAAAACC
ENSG00000068078	AAAACUG
ENSG00000068078	AAAGGUA
ENSG00000068078	ACAUUCA
ENSG00000068078	CAAAACU
ENSG00000068078	GGCUCAG
ENSG00000068078	UACAAAG
ENSG00000068137	CCCUGUC
ENSG00000068137	CUGCAGG
ENSG00000068137	GCCCUGU
ENSG00000068305	ACAGUAC
ENSG00000068305	AGAUAAA
ENSG00000068305	AGCCCUU
ENSG00000068305	AUAUAAC
ENSG00000068305	AUAUUAU
ENSG00000068305	CUACAAA
ENSG00000068305	GUGCAAA
ENSG00000068305	UACAAAG
ENSG00000068305	UAUUGCU
ENSG00000068305	UCAUAGA
ENSG00000068308	ACAUUAC
ENSG00000068308	CCCACCU
ENSG00000068308	CCCCUGG
ENSG00000068308	CUCCCAC
ENSG00000068308	GGAAUGU
ENSG00000068308	GGAGGAG
ENSG00000068308	GGCUCAG
ENSG00000068308	GGGUCUU
ENSG00000068308	GGUGGGC
ENSG00000068308	GUCAACA
ENSG00000068308	GUGCCAC
ENSG00000068308	UGGCUGC
ENSG00000068323	AAAGCGC
ENSG00000068323	GGAAUGU
ENSG00000068323	GGGAGCU
ENSG00000068323	GGGGAGC
ENSG00000068323	GGUGGUU
ENSG00000068323	UUGGUCC
ENSG00000068354	AAAUGGU
ENSG00000068354	ACCACAG
ENSG00000068354	AGGCAGG
ENSG00000068354	CAGGAAC
ENSG00000068354	GGCAGGG
ENSG00000068354	GGCAGUG
ENSG00000068354	GGGGCAG
ENSG00000068354	UGCAAAG
ENSG00000068354	UGCCAGC
ENSG00000068366	AAAGUGC
ENSG00000068366	AAGUGCA
ENSG00000068366	AGCAGCA
ENSG00000068366	AGCAGCG
ENSG00000068366	GGAUUUC
ENSG00000068366	UCCAGUU
ENSG00000068383	AACAAAC
ENSG00000068383	AAUCACU
ENSG00000068383	AAUUUUA
ENSG00000068383	ACAUUCA
ENSG00000068383	ACUGGAU
ENSG00000068383	ACUUGGA
ENSG00000068383	AGCAGCU
ENSG00000068383	AGUAACA
ENSG00000068383	AUAUAAC
ENSG00000068383	CUCCCAA

ENSG00000068383	CUCCCAC
ENSG00000068383	CUGGAAA
ENSG00000068383	GAGGUAG
ENSG00000068383	GAGGUAU
ENSG00000068383	GCACCAU
ENSG00000068383	GUGAGGU
ENSG00000068383	UACAAAG
ENSG00000068383	UAUAAAG
ENSG00000068383	UCACAUU
ENSG00000068383	UCACCAC
ENSG00000068383	UUGGCAC
ENSG00000068615	AACAAAC
ENSG00000068615	AAGACAU
ENSG00000068615	AAGGCAC
ENSG00000068615	AGUGCAA
ENSG00000068615	AUGUCUG
ENSG00000068615	CAAAGCA
ENSG00000068615	CGGUGCU
ENSG00000068615	GAGGUAG
ENSG00000068615	GAGGUAU
ENSG00000068615	GGAUGAG
ENSG00000068615	GGCUCAG
ENSG00000068615	GUGCAAA
ENSG00000068615	UAAUACA
ENSG00000068615	UACAAAG
ENSG00000068615	UCCUGAG
ENSG00000068615	UGCAUUG
ENSG00000068615	UGCCAGC
ENSG00000068615	UUGGCAA
ENSG00000068615	UUGGCAC
ENSG00000068650	AAAGUGC
ENSG00000068650	ACAGGUG
ENSG00000068650	ACAUUCA
ENSG00000068650	AUAUAAC
ENSG00000068650	AUGCACC
ENSG00000068650	AUUGCAC
ENSG00000068650	CUGCCCC
ENSG00000068650	CUUUGGU
ENSG00000068650	GUGCAAA
ENSG00000068650	UACAAAG
ENSG00000068650	UAUUGCU
ENSG00000068650	UGCAAAG
ENSG00000068650	UUCUAAU
ENSG00000068697	AAAGUGC
ENSG00000068697	AAGUGCA
ENSG00000068697	GAUUGUC
ENSG00000068697	UGUCUUU
ENSG00000068724	CUGCAGC
ENSG00000068793	AUACAAG
ENSG00000068796	AAGGCAC
ENSG00000068796	AGCAGCA
ENSG00000068796	GAGGUAU
ENSG00000068796	UGUGCUU
ENSG00000068831	AGGCAGG
ENSG00000068831	GGCCCUG
ENSG00000068831	UCUGGAA
ENSG00000068831	UGGCCCU
ENSG00000068885	AAAACUG
ENSG00000068971	AAAGCUG
ENSG00000068971	CCCCUGG
ENSG00000069011	CAAAGUG
ENSG00000069011	CACAGGG
ENSG00000069011	GCAACUU
ENSG00000069011	GCGGGUG

ENSG00000069011	GUAAACA
ENSG00000069011	GUGCAAA
ENSG00000069011	UAUGGUU
ENSG00000069011	UGCAAAG
ENSG00000069122	AGUGCAA
ENSG00000069122	CUAUACA
ENSG00000069275	AUGGCUU
ENSG00000069329	AAAUUUC
ENSG00000069329	AAGGCAC
ENSG00000069329	GUUAAUG
ENSG00000069329	UAAUAGU
ENSG00000069345	AAAACUG
ENSG00000069345	AAAAGUA
ENSG00000069345	AAAGCUG
ENSG00000069345	AACAGUC
ENSG00000069345	ACCCAUU
ENSG00000069345	AGCAGCA
ENSG00000069345	AGCAGCG
ENSG00000069345	AGCUUAU
ENSG00000069345	AGUGCUU
ENSG00000069345	GCAGGUU
ENSG00000069345	GUGACAG
ENSG00000069345	GUGCAAA
ENSG00000069345	UCAAGUA
ENSG00000069345	UGAAAGG
ENSG00000069345	UGCAAAG
ENSG00000069345	UUAUGCA
ENSG00000069345	UUCAAGC
ENSG00000069399	CACAGUG
ENSG00000069399	CUGCCCC
ENSG00000069399	GUGCAAA
ENSG00000069399	UACAAAG
ENSG00000069399	UAUUACC
ENSG00000069399	UCACAGG
ENSG00000069431	CCAUUAC
ENSG00000069493	UUGGCAA
ENSG00000069667	AAGUGCU
ENSG00000069667	AGAUGUG
ENSG00000069702	AAAACUG
ENSG00000069702	AAUGUUG
ENSG00000069702	ACCUGGA
ENSG00000069702	AGCAGCA
ENSG00000069702	CAAAGUG
ENSG00000069702	GCAGCAU
ENSG00000069702	GGAAUGU
ENSG00000069869	ACCCUGU
ENSG00000069869	CACAGUG
ENSG00000069869	CUCAAAC
ENSG00000069956	AAAGAAU
ENSG00000069956	ACUGUGU
ENSG00000069956	CACAGUG
ENSG00000069956	CACUGUU
ENSG00000069956	CUAUACA
ENSG00000069956	GAGGUAU
ENSG00000069956	GCUGGUG
ENSG00000069956	UGCAUUG
ENSG00000069966	AAGUGCU
ENSG00000069966	UCCCACA
ENSG00000069974	GGCAAGA
ENSG00000069998	GAGGCAG
ENSG00000070018	AAAGCUG
ENSG00000070018	AAGGCAC
ENSG00000070018	ACAUUCA
ENSG00000070018	AGCCCUU

ENSG00000070018	AUCGUAC
ENSG00000070018	AUGCCUU
ENSG00000070018	CAAAGCA
ENSG00000070018	CACAGUG
ENSG00000070018	CCAGUAC
ENSG00000070018	CGUACCG
ENSG00000070018	CUCCAGC
ENSG00000070018	CUUGGGC
ENSG00000070018	GAGAAAU
ENSG00000070018	GAGGUAU
ENSG00000070018	GAUUGUC
ENSG00000070018	GCACCAU
ENSG00000070018	GGAAGCC
ENSG00000070018	GGACUGC
ENSG00000070018	GGAGGAG
ENSG00000070018	GGGCCCC
ENSG00000070018	GUCUGCC
ENSG00000070018	UAAGACG
ENSG00000070018	UAAGACU
ENSG00000070018	UAUGGUU
ENSG00000070018	UAUUGCU
ENSG00000070018	UCACAGG
ENSG00000070018	UCACAGU
ENSG00000070018	UGGACUG
ENSG00000070018	UGGCCAC
ENSG00000070018	UUGGAGG
ENSG00000070018	UUGGCAC
ENSG00000070018	UUGUGUC
ENSG00000070061	GAGGUAG
ENSG00000070087	AAAUUUC
ENSG00000070087	ACAAGGU
ENSG00000070087	ACACACU
ENSG00000070087	ACUCCAG
ENSG00000070087	AGUGCCG
ENSG00000070087	AGUGCCU
ENSG00000070087	AGUGCUG
ENSG00000070087	CAGUUAC
ENSG00000070087	CUCACAC
ENSG00000070087	CUCCCAA
ENSG00000070087	GAGGCUG
ENSG00000070087	GAUUGUA
ENSG00000070087	GGAAGAC
ENSG00000070087	GGAAUGU
ENSG00000070087	GGCAUUG
ENSG00000070087	GUAACAG
ENSG00000070087	UCACAAG
ENSG00000070087	UUGGUUC
ENSG00000070159	AAGUAGC
ENSG00000070159	AGCAGCG
ENSG00000070159	AGCAGCU
ENSG00000070159	CACAGUG
ENSG00000070159	CAGUAGA
ENSG00000070159	CAGUAGU
ENSG00000070159	UCACAGG
ENSG00000070159	UCACAGU
ENSG00000070159	UCCAGUU
ENSG00000070159	UCCCACA
ENSG00000070159	UCCUGAG
ENSG00000070182	AAAUGGU
ENSG00000070182	CCCUGAG
ENSG00000070182	CUAGUAA
ENSG00000070182	GCUUCCU
ENSG00000070182	GGAGGAG
ENSG00000070182	GGGGAGC

ENSG00000070182	GGGGUGG
ENSG00000070182	GGGUUUA
ENSG00000070182	UCCCUGU
ENSG00000070182	UGCAGCU
ENSG00000070182	UGGACUG
ENSG00000070182	UGGCUGC
ENSG00000070190	GACCAUG
ENSG00000070214	ACCCGUA
ENSG00000070214	UCCCUGU
ENSG00000070214	UGGCUGC
ENSG00000070269	AAGGCAC
ENSG00000070269	AAGUCUU
ENSG00000070269	AAUUAUU
ENSG00000070269	ACAUCAC
ENSG00000070269	ACUCCAG
ENSG00000070269	ACUGGCC
ENSG00000070269	ACUGGCU
ENSG00000070269	AGCUUAU
ENSG00000070269	AGGACAC
ENSG00000070269	AUGAGCU
ENSG00000070269	CAAGAGC
ENSG00000070269	CACCAGC
ENSG00000070269	CAGACAG
ENSG00000070269	CAGGCUC
ENSG00000070269	CCUUCAU
ENSG00000070269	CUAUACA
ENSG00000070269	CUGCAGC
ENSG00000070269	CUGCAGG
ENSG00000070269	GAAUUGU
ENSG00000070269	GAGACCU
ENSG00000070269	GAUCAGA
ENSG00000070269	GGCAGCG
ENSG00000070269	GGGGGAA
ENSG00000070269	GUGACUG
ENSG00000070269	UAUGGUU
ENSG00000070269	UCCCUCU
ENSG00000070269	UGAAAGG
ENSG00000070269	UGACAUC
ENSG00000070269	UGCAUUG
ENSG00000070269	UUGACAC
ENSG00000070269	UUGAUCA
ENSG00000070366	AAAAAUC
ENSG00000070366	CCCCCAG
ENSG00000070366	GGAAGAC
ENSG00000070366	GGCUCAG
ENSG00000070366	GGCUCUG
ENSG00000070366	GGGUGGU
ENSG00000070366	GGUUUAC
ENSG00000070366	UGUCUGC
ENSG00000070367	AACUACU
ENSG00000070367	AGCAGCA
ENSG00000070367	AGUAACA
ENSG00000070367	AUGCCUU
ENSG00000070367	AUGGCUU
ENSG00000070367	AUUGCAC
ENSG00000070367	CAGCAGA
ENSG00000070367	CAGCAGG
ENSG00000070367	CUGCUCA
ENSG00000070367	CUGGCUC
ENSG00000070367	GAGGCAG
ENSG00000070367	GCAGCAU
ENSG00000070367	GGCUCAG
ENSG00000070367	GGCUCUG
ENSG00000070367	GGCUGCG

ENSG00000070367	GUAACAG
ENSG00000070367	GUGCUUG
ENSG00000070367	UAAUAUC
ENSG00000070367	UAUCAGA
ENSG00000070367	UCAACAG
ENSG00000070367	UCACAUU
ENSG00000070367	UCAUUUG
ENSG00000070367	UGUGCUU
ENSG00000070367	UUGACAC
ENSG00000070371	AGCAAAA
ENSG00000070371	CAGGCUC
ENSG00000070371	GGCUCAG
ENSG00000070371	UAAUAUC
ENSG00000070404	AAGGCAC
ENSG00000070413	AAAAAUC
ENSG00000070413	ACAGUAC
ENSG00000070413	AUGGAUU
ENSG00000070413	AUGGCAC
ENSG00000070413	CUCACAC
ENSG00000070413	GGAUGAG
ENSG00000070413	GGCAUUG
ENSG00000070413	UCUCACC
ENSG00000070413	UCUCCAA
ENSG00000070413	UGAAAUG
ENSG00000070413	UUGGCAA
ENSG00000070413	UUGGCAC
ENSG00000070423	UAUAAAG
ENSG00000070444	AAGUCUU
ENSG00000070444	AAUCUCU
ENSG00000070444	ACUCCAG
ENSG00000070444	AGAUGUG
ENSG00000070444	AGCAGCA
ENSG00000070444	AGCAGCG
ENSG00000070444	AGCAGCU
ENSG00000070444	AGCCAGU
ENSG00000070444	AGGGUUG
ENSG00000070444	AGUGCUU
ENSG00000070444	AUUCAUU
ENSG00000070444	CACAGGG
ENSG00000070444	CACUGUU
ENSG00000070444	CAGGAAC
ENSG00000070444	CAGUCUG
ENSG00000070444	CAUGGAU
ENSG00000070444	CCUUCUU
ENSG00000070444	CUGGACU
ENSG00000070444	GACCCUG
ENSG00000070444	GAGGUAG
ENSG00000070444	GAGGUAU
ENSG00000070444	GGAAUGU
ENSG00000070444	GGGGAGC
ENSG00000070444	GGGGUCC
ENSG00000070444	GUAAACA
ENSG00000070444	GUGUGGC
ENSG00000070444	UAUUACC
ENSG00000070444	UCACAGU
ENSG00000070444	UCAUUCG
ENSG00000070444	UCCCACA
ENSG00000070444	UGUGUGG
ENSG00000070476	ACCCAUU
ENSG00000070476	GAGACCU
ENSG00000070476	GUGACUG
ENSG00000070476	UCACACA
ENSG00000070476	UCACCAC
ENSG00000070495	AAGGCAC

ENSG00000070495	CCCCUGG
ENSG00000070495	UGGCUGC
ENSG00000070610	ACUGGCC
ENSG00000070610	ACUGGCU
ENSG00000070610	CUCAGGG
ENSG00000070610	GAGGCUG
ENSG00000070610	GAUUGUA
ENSG00000070610	GAUUGUC
ENSG00000070610	GGCUCAG
ENSG00000070614	AAAAACC
ENSG00000070614	AACGGAA
ENSG00000070614	AACGGCU
ENSG00000070614	AAGGCAC
ENSG00000070614	AAUACUG
ENSG00000070614	ACAGGUG
ENSG00000070614	ACAUUAC
ENSG00000070614	AGCACCA
ENSG00000070614	AGUGGUU
ENSG00000070614	AUAUAAC
ENSG00000070614	CAGGAAC
ENSG00000070614	CCAGUGC
ENSG00000070614	CUCUAGC
ENSG00000070614	GAAGGAA
ENSG00000070614	GAGGCAG
ENSG00000070614	GAGGGGC
ENSG00000070614	GAGUAUU
ENSG00000070614	GAUUGUA
ENSG00000070614	GCACCAU
ENSG00000070614	GGAGGAG
ENSG00000070614	GGCAGUG
ENSG00000070614	GGCAUUG
ENSG00000070614	GUCUCUG
ENSG00000070614	UACAAAG
ENSG00000070614	UAUUGCU
ENSG00000070614	UCACAUU
ENSG00000070614	UCAUAGA
ENSG00000070614	UUGGCAC
ENSG00000070718	CAGCAAA
ENSG00000070718	GAGAACU
ENSG00000070718	UCCAGUU
ENSG00000070718	UGACCUA
ENSG00000070731	GCAGAAG
ENSG00000070756	AAAGUUU
ENSG00000070756	AAUUUUA
ENSG00000070756	GGCAUUG
ENSG00000070756	UACAAAG
ENSG00000070756	UAUAAUA
ENSG00000070756	UUUGCAC
ENSG00000070759	AAGUGCU
ENSG00000070759	AGUGCAA
ENSG00000070759	GGAUUUC
ENSG00000070759	GUAGUGU
ENSG00000070759	GUGCAAA
ENSG00000070759	UGCAUAU
ENSG00000070759	UUGGUUC
ENSG00000070770	UCACAUU
ENSG00000070778	AAAGUGC
ENSG00000070778	AAGUGCU
ENSG00000070778	GUAAACA
ENSG00000070778	GUGCAAA
ENSG00000070778	UUCUUGU
ENSG00000070785	ACAGGUG
ENSG00000070785	GGUGGGC
ENSG00000070808	ACCAGCA

ENSG00000070808	AUAAAGU
ENSG00000070808	CAGACAG
ENSG00000070808	CAGUGCA
ENSG00000070808	CCAGCAU
ENSG00000070808	CUCUUCC
ENSG00000070808	GAGGCAG
ENSG00000070808	UAUAAAG
ENSG00000070808	UAUUACC
ENSG00000070808	UAUUGCU
ENSG00000070808	UCAACAG
ENSG00000070808	UCCAGAG
ENSG00000070808	UGAGAAU
ENSG00000070808	UGCAAAG
ENSG00000070808	UUUCAAC
ENSG00000070808	UUUGCAA
ENSG00000070808	UUUGCAC
ENSG00000070814	GUUCAUG
ENSG00000070814	UCACACA
ENSG00000070814	UCAUUUG
ENSG00000070831	AACACUG
ENSG00000070831	AAGGUGC
ENSG00000070831	AUACAAG
ENSG00000070831	AUGCCUU
ENSG00000070831	AUUGCAC
ENSG00000070831	CACCAGC
ENSG00000070831	CUCACAC
ENSG00000070831	GCAACUU
ENSG00000070831	GCAGAAG
ENSG00000070831	GGAAUGU
ENSG00000070831	GGCUCUG
ENSG00000070831	GUCAUUG
ENSG00000070831	UAUUGCU
ENSG00000070831	UCAACAG
ENSG00000070831	UCACAGU
ENSG00000070831	UCUGGAG
ENSG00000070831	UGCAAAG
ENSG00000070831	UGCAUAU
ENSG00000070831	UUGGUUC
ENSG00000070882	AAAGAAU
ENSG00000070882	AAAGCUG
ENSG00000070882	AAGACAU
ENSG00000070882	ACCAGCA
ENSG00000070882	ACUCCAG
ENSG00000070882	AGCACCA
ENSG00000070882	AUGCACC
ENSG00000070882	CAGCAGA
ENSG00000070882	CCAGCAU
ENSG00000070882	CUCCAGC
ENSG00000070882	CUUUGGU
ENSG00000070882	GAAUUGU
ENSG00000070882	GAGGUAG
ENSG00000070882	GCUGUCU
ENSG00000070882	GUUAAUG
ENSG00000070882	UCAUUUG
ENSG00000070882	UGGGUGA
ENSG00000070882	UUGGUUC
ENSG00000070886	AAUUAUU
ENSG00000070886	ACCCUGU
ENSG00000070886	AGUGCUG
ENSG00000070886	AUAAUAC
ENSG00000070886	AUUGCAC
ENSG00000070886	CCCUGUU
ENSG00000070886	CUGCAGG
ENSG00000070886	CUGUUGA

ENSG00000070886	GAAGGAA
ENSG00000070886	GAGGAUA
ENSG00000070886	GGCUCAG
ENSG00000070886	GUGCUUG
ENSG00000070886	UAUAAUA
ENSG00000070886	UAUUGCU
ENSG00000070886	UCUCACC
ENSG00000070886	UGUGCUU
ENSG00000070886	UUCCAUA
ENSG00000070886	UUUGCAC
ENSG00000070886	UUUUUGC
ENSG00000070950	UGCAAAG
ENSG00000070961	AAAAGUA
ENSG00000070961	AAAGUAU
ENSG00000070961	AACAUUC
ENSG00000070961	AAGUAGC
ENSG00000070961	AAGUGUG
ENSG00000070961	CACAGUG
ENSG00000070961	CAGCAGA
ENSG00000070961	CAGUCUG
ENSG00000070961	CUGCUCA
ENSG00000070961	UGAAGUG
ENSG00000070961	UGCAGCU
ENSG00000071051	AAAACUG
ENSG00000071051	AACAUUC
ENSG00000071051	AUAAUAC
ENSG00000071051	AUGGCAC
ENSG00000071051	AUGUGCC
ENSG00000071051	AUGUGGG
ENSG00000071051	CCCUGUC
ENSG00000071051	CCCUGUU
ENSG00000071051	CCUUCUU
ENSG00000071051	GGAAUGU
ENSG00000071051	GGCAAGA
ENSG00000071051	GUAAACA
ENSG00000071051	GUGCCAC
ENSG00000071051	UAUUACC
ENSG00000071051	UGAGAAG
ENSG00000071054	AAAACUG
ENSG00000071054	AACAAAC
ENSG00000071054	AACACUG
ENSG00000071054	AACUACU
ENSG00000071054	AAGACAU
ENSG00000071054	ACAUUAC
ENSG00000071054	ACAUUCA
ENSG00000071054	AGCACCA
ENSG00000071054	AGUGCAA
ENSG00000071054	AUAAUAC
ENSG00000071054	AUAUUAU
ENSG00000071054	AUGCAUU
ENSG00000071054	CAGUCUG
ENSG00000071054	GAGGUAG
ENSG00000071054	GAGGUAU
ENSG00000071054	GGCAGUG
ENSG00000071054	GUAACAG
ENSG00000071054	UACAAAG
ENSG00000071054	UAUAAAG
ENSG00000071054	UAUUACC
ENSG00000071054	UGGCCAC
ENSG00000071054	UGUCUGC
ENSG00000071054	UGUCUUU
ENSG00000071054	UUGGCAC
ENSG00000071073	AAAUUUC
ENSG00000071073	AAGACAU

ENSG00000071073	AAGGCAC
ENSG00000071073	AGGUAGU
ENSG00000071073	AGUACUG
ENSG00000071073	AUAUAAC
ENSG00000071073	AUGCAUU
ENSG00000071073	CAGCAAA
ENSG00000071073	CAGUAGA
ENSG00000071073	CCCUGAG
ENSG00000071073	GAGGUAG
ENSG00000071073	GAGGUAU
ENSG00000071073	GUAGUGU
ENSG00000071073	UAAUAGU
ENSG00000071073	UACAGUU
ENSG00000071073	UCAUACA
ENSG00000071073	UCUCAAG
ENSG00000071073	UCUCACC
ENSG00000071073	UGUCUUU
ENSG00000071073	UUGACAC
ENSG00000071073	UUGUUCG
ENSG00000071127	AAACUGU
ENSG00000071127	AAAGCUG
ENSG00000071127	AAGUGCA
ENSG00000071127	CCCUGAG
ENSG00000071127	CUGGAAA
ENSG00000071127	GUAAACA
ENSG00000071127	UCUGGAA
ENSG00000071127	UGUGCUU
ENSG00000071189	AAUGUUG
ENSG00000071189	ACAUUAC
ENSG00000071189	AUUGCAC
ENSG00000071189	CACCAGC
ENSG00000071189	GCACCAU
ENSG00000071189	GUGCUUG
ENSG00000071189	UAAGACG
ENSG00000071189	UAAGACU
ENSG00000071205	UCAUUUG
ENSG00000071242	AAAGUGC
ENSG00000071242	AAAUCCC
ENSG00000071242	AAGUGCU
ENSG00000071242	AAUACUG
ENSG00000071242	AGUGCAA
ENSG00000071242	AGUGCUG
ENSG00000071242	CAAAGUG
ENSG00000071242	CUACAAA
ENSG00000071242	UCAAGUA
ENSG00000071242	UUGGUUC
ENSG00000071246	AAUACUG
ENSG00000071246	AGCUGCC
ENSG00000071246	AGGCAGG
ENSG00000071246	CUGGCUC
ENSG00000071246	GAGCUGC
ENSG00000071246	GCUACAU
ENSG00000071246	GGAGGAG
ENSG00000071246	GGCUCUG
ENSG00000071246	GUAAACA
ENSG00000071246	UGCCAGC
ENSG00000071462	UUCUAAU
ENSG00000071537	ACAGUAU
ENSG00000071537	ACAUUCA
ENSG00000071537	ACUGCAG
ENSG00000071537	ACUGCAU
ENSG00000071537	AGCAGCU
ENSG00000071537	AGUGGUU
ENSG00000071537	AUCCCUU

ENSG00000071537	CCCUGAG
ENSG00000071537	CUUCACC
ENSG00000071537	GGAGGCA
ENSG00000071537	GGCAGGG
ENSG00000071537	GUCACUC
ENSG00000071537	GUCAUUG
ENSG00000071537	UACAAAG
ENSG00000071537	UCACCAC
ENSG00000071537	UGAUCCA
ENSG00000071537	UUCCAUA
ENSG00000071564	AAAAACC
ENSG00000071564	AAGGCAC
ENSG00000071564	AAUGUUG
ENSG00000071564	AAUUAUU
ENSG00000071564	AGCAGCA
ENSG00000071564	AUACAAG
ENSG00000071564	CAAAGUG
ENSG00000071564	CAGCAGA
ENSG00000071564	GAGGCAG
ENSG00000071564	GCUACAU
ENSG00000071564	UACAAAG
ENSG00000071564	UAUAAAG
ENSG00000071564	UUGAGGC
ENSG00000071575	AACAGUC
ENSG00000071575	AACAUUC
ENSG00000071575	AAGGUGC
ENSG00000071575	ACCCGUA
ENSG00000071575	ACUGGCC
ENSG00000071575	AGCUGCC
ENSG00000071575	AGGACAC
ENSG00000071575	AGUAACA
ENSG00000071575	CCCUGAG
ENSG00000071575	CCUUCUG
ENSG00000071575	GAGGUAG
ENSG00000071575	GAGGUAU
ENSG00000071575	GCACCAU
ENSG00000071575	GCUUCCU
ENSG00000071575	GUGACAG
ENSG00000071575	UAAUACA
ENSG00000071575	UCACAAG
ENSG00000071575	UCAUACA
ENSG00000071575	UCCCUGU
ENSG00000071626	AAAAGUA
ENSG00000071626	AAAGUUU
ENSG00000071626	GAUUGUA
ENSG00000071626	UGUGCUU
ENSG00000071794	AAUUAUU
ENSG00000071794	UUGGUCC
ENSG00000071967	ACUGUUU
ENSG00000071967	AGCAGCU
ENSG00000071967	AUAAUAC
ENSG00000071967	AUAUAAC
ENSG00000071967	AUGUAAU
ENSG00000071967	CAGCAGA
ENSG00000071967	UAUAAUA
ENSG00000071967	UCACAUU
ENSG00000071967	UCUAGAG
ENSG00000071967	UGAAAGG
ENSG00000071967	UUGACAC
ENSG00000072042	GGCAGUG
ENSG00000072042	GGGUCUU
ENSG00000072062	CAAAACU
ENSG00000072062	CUCCCAC
ENSG00000072062	GGCAGGG

ENSG00000072062	UAAUAGU
ENSG00000072062	UCCCACC
ENSG00000072062	UUUCAAC
ENSG00000072071	AAAGCUG
ENSG00000072071	AAGGCAC
ENSG00000072071	ACCCUGU
ENSG00000072071	AGCAAAA
ENSG00000072071	AGCAGCA
ENSG00000072071	AGCAGCG
ENSG00000072071	AGCAGCU
ENSG00000072071	AGGGAGG
ENSG00000072071	AUACAAG
ENSG00000072071	AUGAGCU
ENSG00000072071	AUGGCAC
ENSG00000072071	CAGCAAA
ENSG00000072071	CAGCUGG
ENSG00000072071	CCCUGUC
ENSG00000072071	CUGGGGG
ENSG00000072071	CUUCUCU
ENSG00000072071	CUUUGGU
ENSG00000072071	GAGACCU
ENSG00000072071	GAUUGUC
ENSG00000072071	GCUGGUG
ENSG00000072071	GGCAGCG
ENSG00000072071	GGCAGUG
ENSG00000072071	GGCUGCG
ENSG00000072071	GGGAACG
ENSG00000072071	GGGGCAG
ENSG00000072071	GGGGUGG
ENSG00000072071	GUCUCUG
ENSG00000072071	GUGCUUG
ENSG00000072071	UCAAGUA
ENSG00000072071	UGAAAUG
ENSG00000072071	UGGCUGC
ENSG00000072071	UGUGCUU
ENSG00000072071	UUCCAUA
ENSG00000072071	UUCUUGU
ENSG00000072071	UUGGCAA
ENSG00000072071	UUGGCAC
ENSG00000072071	UUGUUCG
ENSG00000072071	UUUUUGC
ENSG00000072110	AUAAUAC
ENSG00000072110	AUCAUAC
ENSG00000072110	AUGCACC
ENSG00000072110	GUAAACA
ENSG00000072110	GUGCCUG
ENSG00000072110	UCCUAUA
ENSG00000072110	UUCCUAG
ENSG00000072121	AAGAACC
ENSG00000072121	AGCACCA
ENSG00000072121	AGUGCAA
ENSG00000072121	AGUGCUU
ENSG00000072121	AUGGCAC
ENSG00000072121	GAGGUAU
ENSG00000072121	GUAAACA
ENSG00000072121	GUGCAAA
ENSG00000072121	UCCAGUU
ENSG00000072121	UGAAAGG
ENSG00000072121	UGUGCUU
ENSG00000072121	UUGUUCG
ENSG00000072134	AAACUGU
ENSG00000072134	AAAGCUG
ENSG00000072134	AAGGCAC
ENSG00000072134	ACAGUAC

ENSG00000072134	ACAGUAU
ENSG00000072134	ACAUUAC
ENSG00000072134	ACAUUCA
ENSG00000072134	ACUCAAA
ENSG00000072134	AGAUAAA
ENSG00000072134	AUAAAGU
ENSG00000072134	AUGACAC
ENSG00000072134	AUGCCUU
ENSG00000072134	AUGUGCC
ENSG00000072134	CAGUGCA
ENSG00000072134	CUCACAC
ENSG00000072134	GACCUGG
ENSG00000072134	GAUCAGA
ENSG00000072134	GCCUGGG
ENSG00000072134	GUAAACA
ENSG00000072134	GUCAACA
ENSG00000072134	GUGCAAA
ENSG00000072134	UCACAUU
ENSG00000072134	UCAUGAU
ENSG00000072134	UUCUGCA
ENSG00000072135	UGAUCCA
ENSG00000072163	CCUUCAU
ENSG00000072163	GGCCCUG
ENSG00000072182	CUCCAGC
ENSG00000072182	GGGAGCU
ENSG00000072182	GUGCAAA
ENSG00000072182	UAUAAAG
ENSG00000072195	CAGCUGG
ENSG00000072195	GAGGUAG
ENSG00000072195	GAGGUAU
ENSG00000072195	GCUGGAU
ENSG00000072195	GCUGGUG
ENSG00000072195	GGAAUGU
ENSG00000072201	UCUGGAA
ENSG00000072201	UCUGGAG
ENSG00000072210	CAGACAG
ENSG00000072210	GGAAGCC
ENSG00000072274	AACACUG
ENSG00000072274	AAGGCAC
ENSG00000072274	AAUUAUU
ENSG00000072274	ACAGUAU
ENSG00000072274	ACAUUAC
ENSG00000072274	ACUGGAU
ENSG00000072274	CAAAACU
ENSG00000072274	CACUGUU
ENSG00000072274	CAGUGCA
ENSG00000072274	CCAGUAC
ENSG00000072274	CUGGAAA
ENSG00000072274	CUUUGGU
ENSG00000072274	GAGGCAG
ENSG00000072274	GCAACUU
ENSG00000072274	GCUUCCU
ENSG00000072274	GUAACAG
ENSG00000072274	GUCACUC
ENSG00000072274	GUCAUUG
ENSG00000072274	UCCAGUU
ENSG00000072274	UGGAGAU
ENSG00000072274	UGGCCAC
ENSG00000072274	UUCAAGC
ENSG00000072274	UUGGCAA
ENSG00000072274	UUGGCAC
ENSG00000072274	UUGUGAC
ENSG00000072274	UUUGCAC
ENSG00000072310	AUAAAGU

ENSG00000072310	CAAAACU
ENSG00000072310	UAUAAAG
ENSG00000072310	UCAUUUG
ENSG00000072310	UCUCAAG
ENSG00000072310	UUUGCAA
ENSG00000072315	UCCCUUU
ENSG00000072315	UGUAUAA
ENSG00000072364	AAAGUAC
ENSG00000072364	AAAGUGC
ENSG00000072364	AAGGCAC
ENSG00000072364	AAUUGCU
ENSG00000072364	ACAGUAC
ENSG00000072364	AGCACCA
ENSG00000072364	AGCAGCA
ENSG00000072364	AUGACAC
ENSG00000072364	AUGCAUU
ENSG00000072364	AUGGCAC
ENSG00000072364	AUUGCAC
ENSG00000072364	CAAAGUG
ENSG00000072364	CAGUAGA
ENSG00000072364	CCUUCAU
ENSG00000072364	GAGCCCU
ENSG00000072364	GAUUGUC
ENSG00000072364	GCAACGA
ENSG00000072364	GCACCAU
ENSG00000072364	GCUGUCU
ENSG00000072364	GGCAGUG
ENSG00000072364	GGUUGAC
ENSG00000072364	GUGCUUG
ENSG00000072364	UAUAAAG
ENSG00000072364	UCAUACA
ENSG00000072364	UCCCACC
ENSG00000072364	UGAAAGG
ENSG00000072364	UGAUCCA
ENSG00000072364	UGUAUGU
ENSG00000072364	UGUGCUU
ENSG00000072401	AAUACUG
ENSG00000072401	AGUGCAA
ENSG00000072401	CAGUGCA
ENSG00000072401	UCAAGUA
ENSG00000072415	AGCAGCU
ENSG00000072415	CCAGUGC
ENSG00000072415	CCAGUGU
ENSG00000072415	UCACAUU
ENSG00000072415	UUCAAGC
ENSG00000072415	UUCCUAG
ENSG00000072422	AAAGCUG
ENSG00000072422	AAAGUAC
ENSG00000072422	AAAGUAU
ENSG00000072422	AGAUAAA
ENSG00000072422	AGCCCUU
ENSG00000072422	AUAUUAU
ENSG00000072422	AUGGCAC
ENSG00000072422	CAGGCUC
ENSG00000072422	CAGUGCA
ENSG00000072422	CCAUUAC
ENSG00000072422	CUUUGGU
ENSG00000072422	GAAACAU
ENSG00000072422	GGUUGUC
ENSG00000072422	GUGCUUG
ENSG00000072422	GUGGCCC
ENSG00000072422	UAAUACA
ENSG00000072422	UAAUGCU
ENSG00000072422	UACAAAG

ENSG00000072422	UGAUCCA
ENSG00000072422	UGCCCUG
ENSG00000072422	UUCAAAU
ENSG00000072422	UUCUAAU
ENSG00000072422	UUGAUCA
ENSG00000072422	UUGGCAC
ENSG00000072422	UUGGUUC
ENSG00000072501	AAUCCUU
ENSG00000072501	AGCAAAA
ENSG00000072501	AGCCCUU
ENSG00000072501	AGUGCUU
ENSG00000072501	AUCACUA
ENSG00000072501	CAAAUGC
ENSG00000072501	CAGACAG
ENSG00000072501	CAGUCCA
ENSG00000072501	CUCAAAC
ENSG00000072501	CUCACAC
ENSG00000072501	CUUUGGU
ENSG00000072501	GAGGUAU
ENSG00000072501	GUGCCUG
ENSG00000072501	GUGGCCC
ENSG00000072501	UCUAGAG
ENSG00000072501	UCUCCAA
ENSG00000072501	UCUUGAG
ENSG00000072501	UGAAGCU
ENSG00000072501	UGACAUC
ENSG00000072501	UGCCCUG
ENSG00000072501	UUGGAGG
ENSG00000072571	UCAACAG
ENSG00000072609	AAAGCUG
ENSG00000072609	GUAAACA
ENSG00000072609	UCCAGAG
ENSG00000072609	UGAGAAG
ENSG00000072609	UGUGCUU
ENSG00000072609	UUCAAGC
ENSG00000072657	AGGCAGG
ENSG00000072657	AUGGCAC
ENSG00000072657	GAGGUAG
ENSG00000072657	GCAGGAC
ENSG00000072657	GCUGGAU
ENSG00000072657	GUAACAG
ENSG00000072657	GUGCUUG
ENSG00000072657	UGCAUAG
ENSG00000072657	UGCAUAU
ENSG00000072657	UUUGCAC
ENSG00000072682	AAGGCAC
ENSG00000072682	CUUUGGU
ENSG00000072682	GAGGUAG
ENSG00000072682	GAGGUAU
ENSG00000072682	UUUUUGC
ENSG00000072736	AAAGUAU
ENSG00000072778	AAGGCAC
ENSG00000072778	GAGGCAG
ENSG00000072786	AAAAAUC
ENSG00000072786	UACAAAG
ENSG00000072786	UCACAGG
ENSG00000072786	UCACAGU
ENSG00000072786	UCCUGAG
ENSG00000072786	UGCCAGC
ENSG00000072803	AAAGUGC
ENSG00000072803	AAAUUUC
ENSG00000072803	AACAGUC
ENSG00000072803	AAGUCUU
ENSG00000072803	AAGUGCA

ENSG00000072803	AAUACUG
ENSG00000072803	ACUCAGC
ENSG00000072803	AGUAGUU
ENSG00000072803	AGUGCAA
ENSG00000072803	AGUGCUU
ENSG00000072803	AUAAUAC
ENSG00000072803	CCAGUGC
ENSG00000072803	CUCCCAA
ENSG00000072803	CUGACUC
ENSG00000072803	GGAGGCA
ENSG00000072803	GUCCAGA
ENSG00000072803	GUGCAAA
ENSG00000072803	UACAAAG
ENSG00000072803	UAGACUG
ENSG00000072803	UAUUACC
ENSG00000072803	UGCAAAG
ENSG00000072803	UGCUCAC
ENSG00000072803	UGGGUGA
ENSG00000072803	UUCAAAU
ENSG00000072803	UUGGCAC
ENSG00000072803	UUGGUCC
ENSG00000072832	ACACACC
ENSG00000072832	GUGCAAA
ENSG00000072849	AAGUGCU
ENSG00000072849	AGUGCUU
ENSG00000072849	AUAAAGU
ENSG00000072849	UUGGCAC
ENSG00000072858	AGCACCA
ENSG00000072858	AGCAGCA
ENSG00000072858	GAGAUGA
ENSG00000072952	AUCCUUG
ENSG00000072952	AUGGCAC
ENSG00000072952	CACACCU
ENSG00000072952	GCCUGGA
ENSG00000072952	GCCUGGG
ENSG00000072952	GGGUAAG
ENSG00000072952	GUGACAG
ENSG00000072952	GUGACUG
ENSG00000072952	GUGCCUG
ENSG00000072952	UGAAAUG
ENSG00000072952	UGACAUC
ENSG00000072952	UGAUCCA
ENSG00000072952	UGUCUUU
ENSG00000072952	UUCAAAU
ENSG00000072952	UUUCAAC
ENSG00000072958	GGGAGCU
ENSG00000073060	CAGUAGA
ENSG00000073060	CCAGUAC
ENSG00000073060	GAGUGUG
ENSG00000073060	UCCAGUU
ENSG00000073067	GGCCCUG
ENSG00000073150	AAAGUGC
ENSG00000073150	AAGUGCA
ENSG00000073150	GAGGUAG
ENSG00000073150	GAUAUGU
ENSG00000073150	GUUAAUG
ENSG00000073150	UAUUGCU
ENSG00000073169	CAGACAG
ENSG00000073169	CAUGGAU
ENSG00000073282	AAAAGUA
ENSG00000073282	AAGUGCU
ENSG00000073282	AGUGCAA
ENSG00000073282	AUUGCAC
ENSG00000073282	GCAACUU

ENSG00000073282	GUGCAAA
ENSG00000073282	UAGACUG
ENSG00000073282	UAUUGCU
ENSG00000073282	UCUGGAG
ENSG00000073282	UGAAAUG
ENSG00000073282	UUUGCAC
ENSG00000073350	AAAACUG
ENSG00000073350	AAACUGU
ENSG00000073350	AGUGUGC
ENSG00000073350	CAGUAGA
ENSG00000073350	CAGUAGU
ENSG00000073350	CUACAAA
ENSG00000073350	GUAGUGU
ENSG00000073350	GUGCAAA
ENSG00000073417	AUUGCAC
ENSG00000073417	CAGUAGA
ENSG00000073417	CAGUAGU
ENSG00000073417	GUAGUGU
ENSG00000073417	UGCAUUG
ENSG00000073464	AAGGUGC
ENSG00000073464	CUCUGGG
ENSG00000073464	CUUUGGU
ENSG00000073464	GAGAAAU
ENSG00000073464	GCAUCCC
ENSG00000073464	GUGACUG
ENSG00000073464	UUGGAGG
ENSG00000073464	UUUGCAA
ENSG00000073536	UUGGUCC
ENSG00000073584	AAAGCUG
ENSG00000073584	AAAUGGU
ENSG00000073584	GAAACAU
ENSG00000073584	GGCUCUG
ENSG00000073584	UGUCUUU
ENSG00000073598	AGCAGCU
ENSG00000073598	GCUGGUG
ENSG00000073598	GGCAGUG
ENSG00000073670	AAGACAU
ENSG00000073670	AGCUGCC
ENSG00000073670	CCCUGAG
ENSG00000073670	CUUUGGU
ENSG00000073670	GAGUUGA
ENSG00000073670	GCUACAU
ENSG00000073670	GGAAGCC
ENSG00000073670	GUAAACA
ENSG00000073670	UCACAGG
ENSG00000073670	UGAAAGG
ENSG00000073670	UUGGUUC
ENSG00000073711	AAAGUGC
ENSG00000073711	ACUCCAG
ENSG00000073711	AGGAUGU
ENSG00000073711	AGUGCUG
ENSG00000073711	AGUGCUU
ENSG00000073711	AGUGGUU
ENSG00000073711	CCAGUAC
ENSG00000073711	CCUUCUG
ENSG00000073711	CUUCACC
ENSG00000073711	GAGAUGA
ENSG00000073711	GAGGAUA
ENSG00000073711	GGCAGGG
ENSG00000073711	GUCUCUG
ENSG00000073711	UCAAGUA
ENSG00000073711	UCACAUU
ENSG00000073711	UCCAGAG
ENSG00000073711	UCUGGAA

ENSG00000073711	UCUGGAG
ENSG00000073756	ACAGUAU
ENSG00000073756	AGAUGUG
ENSG00000073756	GAGAUGA
ENSG00000073756	GAGCUGC
ENSG00000073756	GAGGCAG
ENSG00000073792	AAAAACC
ENSG00000073792	AACACUG
ENSG00000073792	AACCUGU
ENSG00000073792	AAGGUGC
ENSG00000073792	AAUCUCU
ENSG00000073792	AUCCCUU
ENSG00000073792	CAAGAGC
ENSG00000073792	CACUGUU
ENSG00000073792	CAGGUGA
ENSG00000073792	CAGUAGA
ENSG00000073792	CAGUCUG
ENSG00000073792	CCUUCUG
ENSG00000073792	CUGGGGG
ENSG00000073792	CUGUUGA
ENSG00000073792	GAGGCUG
ENSG00000073792	GAGGUAU
ENSG00000073792	GCCUGGA
ENSG00000073792	GCCUGGG
ENSG00000073792	GCUACAU
ENSG00000073792	GCUGGAU
ENSG00000073792	GCUGGUG
ENSG00000073792	UAGAGGA
ENSG00000073792	UUCAAGC
ENSG00000073792	UUGGUUC
ENSG00000073803	ACAGUAC
ENSG00000073803	ACAGUAU
ENSG00000073803	AGUGCAA
ENSG00000073803	AUUGCAC
ENSG00000073803	UACAAAG
ENSG00000073803	UACAGUU
ENSG00000073803	UAUUGCU
ENSG00000073849	CCAGUGU
ENSG00000073849	CCUGCUG
ENSG00000073849	UUCAAGC
ENSG00000073861	AGCACCA
ENSG00000073861	CCUUCUU
ENSG00000073861	UCUCCAA
ENSG00000073910	AGCAGCA
ENSG00000073910	AGCAGCG
ENSG00000073910	CUUUGGU
ENSG00000073910	UAUUGCU
ENSG00000073921	AAUUGCU
ENSG00000073921	ACAUUCA
ENSG00000073921	ACCCAUU
ENSG00000073921	ACUGCAG
ENSG00000073921	ACUGCAU
ENSG00000073921	AUUCAUU
ENSG00000073921	CAAAGCA
ENSG00000073921	GGAAGCC
ENSG00000073921	GGAAUGU
ENSG00000073921	GUAGUGU
ENSG00000073921	UCAACAG
ENSG00000073921	UCACAUU
ENSG00000073921	UCAUUUG
ENSG00000073921	UGGAUAA
ENSG00000074047	ACAUUCA
ENSG00000074047	GAGGUAU
ENSG00000074047	GCUACAU

ENSG00000074047	GCUGGUG
ENSG00000074047	GUAAACA
ENSG00000074054	AAAGCUG
ENSG00000074054	AAUUUUA
ENSG00000074054	ACUCAAA
ENSG00000074054	AGCAGCA
ENSG00000074054	AGCCCUU
ENSG00000074054	AUGGAUU
ENSG00000074054	AUGGCUU
ENSG00000074054	CAGCAGA
ENSG00000074054	CCUUCAU
ENSG00000074054	GAAACAU
ENSG00000074054	GAGACCU
ENSG00000074054	GAUUGUC
ENSG00000074054	GUAACAG
ENSG00000074054	GUCUCUG
ENSG00000074181	AUACAAG
ENSG00000074181	CUCCAUU
ENSG00000074181	CUGGAAA
ENSG00000074181	GGAAUGU
ENSG00000074181	UACAAAG
ENSG00000074181	UCUGGAA
ENSG00000074181	UGAAGUG
ENSG00000074201	CUACAAA
ENSG00000074211	AAAGAAU
ENSG00000074211	AACAAAC
ENSG00000074211	ACAAGGU
ENSG00000074211	ACACACC
ENSG00000074211	ACACACU
ENSG00000074211	AUGCACC
ENSG00000074211	GAUUGUA
ENSG00000074211	GAUUGUC
ENSG00000074211	GUAACAG
ENSG00000074211	GUCCAGA
ENSG00000074211	GUGCUUG
ENSG00000074211	UAAGACG
ENSG00000074211	UAUAAUA
ENSG00000074211	UCAACAG
ENSG00000074211	UCAUUCG
ENSG00000074211	UGUGCUU
ENSG00000074211	UUGUGAC
ENSG00000074219	CACUCCU
ENSG00000074219	CCCACCU
ENSG00000074219	GGCUCUG
ENSG00000074219	GGGGUCC
ENSG00000074219	GUGAGGU
ENSG00000074266	ACAGUAC
ENSG00000074317	AUUCAUU
ENSG00000074317	CAUGGAU
ENSG00000074317	CUGGCUC
ENSG00000074317	CUGGGGG
ENSG00000074317	GCUACAU
ENSG00000074317	GCUGGUG
ENSG00000074317	GGCUCUG
ENSG00000074317	UGUGCUU
ENSG00000074319	AAAGUGC
ENSG00000074319	AAGUGCA
ENSG00000074356	AUAAUAC
ENSG00000074356	AUACAAG
ENSG00000074356	CAAGAGC
ENSG00000074356	CACAAUG
ENSG00000074356	CACAGUG
ENSG00000074356	CAGGUGA
ENSG00000074356	CCUUCUU

ENSG00000074356	CUAUACA
ENSG00000074356	CUGCAGG
ENSG00000074356	CUGCUCA
ENSG00000074356	CUUUGGU
ENSG00000074356	GAGAAGA
ENSG00000074356	GAGCUGC
ENSG00000074356	GCUGGUG
ENSG00000074356	GGAUGAG
ENSG00000074356	GUAACAG
ENSG00000074356	UAAUACA
ENSG00000074356	UAGUGAG
ENSG00000074356	UCUGGAG
ENSG00000074356	UGAGAAG
ENSG00000074370	CAGCUGG
ENSG00000074370	GAGGCAG
ENSG00000074370	UGACAUC
ENSG00000074410	CAAAGCA
ENSG00000074410	CACAGUG
ENSG00000074410	GCUGUCU
ENSG00000074410	GUCCAGA
ENSG00000074416	AAAGUGC
ENSG00000074416	AUAAAGU
ENSG00000074416	AUAUUAU
ENSG00000074416	AUUGCAC
ENSG00000074416	GAAAACA
ENSG00000074416	GAGGGGC
ENSG00000074416	GAGGUAG
ENSG00000074416	GCCCUUA
ENSG00000074416	GGCAGGG
ENSG00000074416	UAUUGCU
ENSG00000074527	AAGUGCU
ENSG00000074527	AGUGCUU
ENSG00000074590	AAAACUG
ENSG00000074590	ACUCAAA
ENSG00000074590	AGAUGUG
ENSG00000074590	AGCAGCA
ENSG00000074590	AUGCCUU
ENSG00000074590	CAGCAGG
ENSG00000074590	CUCAAAC
ENSG00000074590	CUGGGCA
ENSG00000074590	GAAAACA
ENSG00000074590	GAGGCAG
ENSG00000074590	GCAGCAU
ENSG00000074590	GCCUGGG
ENSG00000074590	GGGUCUU
ENSG00000074590	GGUGGUU
ENSG00000074590	GUAAACA
ENSG00000074590	GUGCAAA
ENSG00000074590	UGGCUGC
ENSG00000074590	UGGGUGA
ENSG00000074590	UUGGCAC
ENSG00000074603	AAAACUG
ENSG00000074603	AUGGCAC
ENSG00000074621	AGCAGCA
ENSG00000074621	UGAAAUG
ENSG00000074657	AAAGUGC
ENSG00000074657	AAGAACC
ENSG00000074657	AAGACAU
ENSG00000074657	AGCACCA
ENSG00000074657	AGCAGCA
ENSG00000074657	AGCCCUU
ENSG00000074657	AGUACUG
ENSG00000074657	AUUGCAC
ENSG00000074657	CAAGAGC

ENSG00000074657	CUACAGU
ENSG00000074657	CUGCCCC
ENSG00000074657	GAGAAAU
ENSG00000074657	GCAUCCC
ENSG00000074657	GGCUCAG
ENSG00000074657	UAAUACA
ENSG00000074657	UACAGUU
ENSG00000074657	UUGGCAA
ENSG00000074657	UUGGCAC
ENSG00000074695	AAAUGGU
ENSG00000074695	AGCAGCU
ENSG00000074695	CUUUGGU
ENSG00000074695	GGUUACC
ENSG00000074695	UCCCUCU
ENSG00000074696	AAAGCUG
ENSG00000074696	CUCAAAC
ENSG00000074696	UAAUAGU
ENSG00000074696	UGCAAAG
ENSG00000074696	UGUUGAA
ENSG00000074755	AAAAACC
ENSG00000074755	AUGUCUG
ENSG00000074755	GUCACUC
ENSG00000074755	UAUUGCU
ENSG00000074755	UCCUGAG
ENSG00000074755	UGCAUAG
ENSG00000074755	UGUCUGC
ENSG00000074755	UUGGCAA
ENSG00000074755	UUUCAAC
ENSG00000074800	AGCUGCC
ENSG00000074800	GAGCUGC
ENSG00000074803	GAUCAGA
ENSG00000075035	AAUCCUU
ENSG00000075035	ACAUUCA
ENSG00000075035	AGCACCA
ENSG00000075035	AUGGCUU
ENSG00000075035	CCCCUGG
ENSG00000075035	CUCUUCC
ENSG00000075035	GACCUGG
ENSG00000075035	GAGCCCC
ENSG00000075035	GCAGGAC
ENSG00000075035	GGAAUGU
ENSG00000075035	GGAGAGA
ENSG00000075035	GGCAGUG
ENSG00000075035	UGACCUA
ENSG00000075142	AAUGUUG
ENSG00000075142	GUGACAG
ENSG00000075151	AGGCAGG
ENSG00000075151	GAGGCAG
ENSG00000075213	AACCUGU
ENSG00000075213	AAUGCCC
ENSG00000075213	ACUGCAG
ENSG00000075213	AGGUAGU
ENSG00000075213	AGUAGAC
ENSG00000075213	AUUGCAC
ENSG00000075213	CAAAGCA
ENSG00000075213	CAGUAGA
ENSG00000075213	CCUGUAC
ENSG00000075213	CUCAGGG
ENSG00000075213	CUCCAGC
ENSG00000075213	GGUAGAC
ENSG00000075213	UCUAGAG
ENSG00000075213	UGCUGAC
ENSG00000075223	AACAUUC
ENSG00000075240	AAAAACC

ENSG00000075240	AAUUAUU
ENSG00000075240	ACAGUAU
ENSG00000075240	AGGAUGU
ENSG00000075240	AUACAAG
ENSG00000075240	AUGGCUU
ENSG00000075240	AUUGCAC
ENSG00000075240	CAGACAG
ENSG00000075240	GUCAGUU
ENSG00000075240	UAUAAUA
ENSG00000075240	UAUUGCU
ENSG00000075240	UCCCUUU
ENSG00000075240	UGCAAAG
ENSG00000075275	AAAGUAU
ENSG00000075275	AAGACAU
ENSG00000075275	AAGUCUU
ENSG00000075275	AGCAGCU
ENSG00000075275	AUAAAGU
ENSG00000075275	AUAUAAC
ENSG00000075275	GUGCAAA
ENSG00000075275	UAAGACG
ENSG00000075275	UAAGACU
ENSG00000075275	UCAAGUA
ENSG00000075275	UCCAGUU
ENSG00000075275	UUGGCAA
ENSG00000075340	GCAGCAU
ENSG00000075388	AAAGUGC
ENSG00000075388	AAGUGCA
ENSG00000075391	AAAAAUC
ENSG00000075391	AACCUGG
ENSG00000075391	AACUCUA
ENSG00000075391	ACAUUAC
ENSG00000075391	ACUGCAG
ENSG00000075391	AGUGCUU
ENSG00000075391	AUAUUAU
ENSG00000075391	UUUCAAC
ENSG00000075407	UCCCACC
ENSG00000075413	AGCACCA
ENSG00000075413	AGUGCAA
ENSG00000075413	GAAAACA
ENSG00000075413	GUAGUGU
ENSG00000075413	UAUUGCU
ENSG00000075415	AAAAGUA
ENSG00000075415	AACACUG
ENSG00000075415	AUAUAAC
ENSG00000075415	CUACAGU
ENSG00000075420	AAGGUGC
ENSG00000075420	AAUGCCC
ENSG00000075420	AGCAGCA
ENSG00000075420	AGCAUUC
ENSG00000075420	AGUACUG
ENSG00000075420	AUAUUAU
ENSG00000075420	AUGGCAC
ENSG00000075420	CACAAUG
ENSG00000075420	CCCUGAG
ENSG00000075420	CUUUGGU
ENSG00000075420	GAGAUGA
ENSG00000075420	GAGGUAG
ENSG00000075420	GAGGUAU
ENSG00000075420	GAUAUGU
ENSG00000075420	GCUACAU
ENSG00000075420	GGCAGUG
ENSG00000075420	GUAAACA
ENSG00000075420	GUGCAAA
ENSG00000075420	UACAGUU

ENSG00000075420	UUCAAAU
ENSG00000075420	UUUUUGC
ENSG00000075426	AAGGCAC
ENSG00000075426	AAGGUGC
ENSG00000075426	ACGCUCA
ENSG00000075426	AUUGCAC
ENSG00000075426	CAGCAAA
ENSG00000075426	GAGACGC
ENSG00000075426	GCCUGGG
ENSG00000075426	GCUGGUG
ENSG00000075426	GGAGAGA
ENSG00000075426	UGAAAUG
ENSG00000075426	UUGGCAA
ENSG00000075461	AGCACCA
ENSG00000075461	AGCAGCU
ENSG00000075461	CUCAAAC
ENSG00000075461	GAGGUAG
ENSG00000075539	AAGGUGC
ENSG00000075539	AGCAGCA
ENSG00000075539	AGCAUUC
ENSG00000075539	AUUGCAC
ENSG00000075539	CAGUGCA
ENSG00000075539	CUGCAGC
ENSG00000075539	GAGAACU
ENSG00000075539	GAUCAGA
ENSG00000075539	GUCUCUG
ENSG00000075539	GUGCUUG
ENSG00000075539	UCACACA
ENSG00000075539	UCACAGU
ENSG00000075539	UUCAAAU
ENSG00000075539	UUGGUUC
ENSG00000075568	AAAGUAC
ENSG00000075618	AAAGAGG
ENSG00000075618	AUACAAG
ENSG00000075618	CUGGGGG
ENSG00000075618	GGCUCAG
ENSG00000075618	UACAAAG
ENSG00000075618	UUCUGCA
ENSG00000075618	UUGGUCC
ENSG00000075624	UCCUGAG
ENSG00000075651	GAAAACA
ENSG00000075651	GAGCUGC
ENSG00000075651	UUGAUCA
ENSG00000075711	AAAGAGG
ENSG00000075711	AACAAAC
ENSG00000075711	AAUCUCU
ENSG00000075711	AGCACCA
ENSG00000075711	AGGCAGG
ENSG00000075711	AUAAAGU
ENSG00000075711	AUGUAAU
ENSG00000075711	GGAAUGU
ENSG00000075711	GGGUAAG
ENSG00000075711	UACAAAG
ENSG00000075711	UAUAAAG
ENSG00000075711	UCACAUU
ENSG00000075711	UCCCUUG
ENSG00000075711	UCCCUUU
ENSG00000075711	UGGAUAA
ENSG00000075711	UUCCUAG
ENSG00000075785	AAAGUAA
ENSG00000075785	AAAUGGU
ENSG00000075785	AAUACUG
ENSG00000075785	AUAUUAU
ENSG00000075785	CAAAACU

ENSG00000075785	GAAGGAA
ENSG00000075785	GAGAUGA
ENSG00000075785	UGCAUAG
ENSG00000075790	GAGGUAG
ENSG00000075790	GAGGUAU
ENSG00000075790	UUGAGGC
ENSG00000075826	GAGGUAG
ENSG00000075856	UACAAAG
ENSG00000075884	AAAAAUC
ENSG00000075884	AGAUGUG
ENSG00000075891	ACACACC
ENSG00000075891	AGCAGCA
ENSG00000075891	CAAAGCA
ENSG00000075891	CACACCG
ENSG00000075891	CAGACAG
ENSG00000075891	CCCCCAG
ENSG00000075891	CUACAGU
ENSG00000075891	GAUUGUA
ENSG00000075891	GAUUGUC
ENSG00000075891	GUGCUUG
ENSG00000075891	GUGUCAC
ENSG00000075891	UGCAAAG
ENSG00000075891	UGCAUAU
ENSG00000075891	UGUGCUU
ENSG00000075891	UGUGUCA
ENSG00000075891	UGUGUGG
ENSG00000075891	UUUGCAC
ENSG00000075891	UUUUUGC
ENSG00000075945	AUGCAUU
ENSG00000075975	AAUUUUA
ENSG00000075975	GGGUUUA
ENSG00000075975	UACAGUU
ENSG00000075975	UAUUGCU
ENSG00000076043	UACAAAG
ENSG00000076043	UGCAUUG
ENSG00000076053	CCCUGAG
ENSG00000076053	UGAAAGG
ENSG00000076053	UUCCUCG
ENSG00000076067	UUUGCAA
ENSG00000076108	AAACUGU
ENSG00000076108	AAAGUUU
ENSG00000076108	AAAUUUC
ENSG00000076108	AAGUCAC
ENSG00000076108	ACACACU
ENSG00000076108	ACAGUAC
ENSG00000076108	ACAUCAC
ENSG00000076108	ACAUUAC
ENSG00000076108	ACAUUCA
ENSG00000076108	ACCCGUA
ENSG00000076108	ACUGGCC
ENSG00000076108	ACUGGCU
ENSG00000076108	AGCAGCA
ENSG00000076108	AGCAGCU
ENSG00000076108	AGUAGUU
ENSG00000076108	AGUGCAA
ENSG00000076108	AGUGCCU
ENSG00000076108	AUAAAGU
ENSG00000076108	AUAUAAC
ENSG00000076108	AUGCAUU
ENSG00000076108	AUUGCAC
ENSG00000076108	CAAAUGC
ENSG00000076108	CAGCAGA
ENSG00000076108	CAGUAGU
ENSG00000076108	CAGUGCA

ENSG00000076108	CCAGUGC
ENSG00000076108	CCCUGAG
ENSG00000076108	CCUGCUG
ENSG00000076108	CUACAGU
ENSG00000076108	CUGCCCC
ENSG00000076108	GAGGCUG
ENSG00000076108	GAUUGGU
ENSG00000076108	GCGGGUG
ENSG00000076108	GGAAGCC
ENSG00000076108	GGCAGUG
ENSG00000076108	GUCCAGA
ENSG00000076108	GUGCCUG
ENSG00000076108	UAUAAAG
ENSG00000076108	UAUUGCU
ENSG00000076108	UCCAGUU
ENSG00000076108	UCCCUUU
ENSG00000076108	UUCAAAU
ENSG00000076108	UUGAGGC
ENSG00000076108	UUGAUCA
ENSG00000076108	UUGGUUC
ENSG00000076201	AAAGAGC
ENSG00000076201	AGCAAAA
ENSG00000076248	AGCAGCA
ENSG00000076321	AAAGUGC
ENSG00000076321	AAGUGCA
ENSG00000076321	AGUGCAA
ENSG00000076321	GAGGCAG
ENSG00000076321	GUGCAAA
ENSG00000076321	UUCUGCA
ENSG00000076321	UUGAGGC
ENSG00000076351	GAGGCUG
ENSG00000076351	GUGCCUG
ENSG00000076351	UUGGCAC
ENSG00000076356	AAAGUUU
ENSG00000076356	AACAAAC
ENSG00000076356	AAUACUG
ENSG00000076356	ACAAUAU
ENSG00000076356	ACAUCAC
ENSG00000076356	ACUCAGG
ENSG00000076356	ACUGCAG
ENSG00000076356	ACUGCAU
ENSG00000076356	AGCAGCA
ENSG00000076356	AGGCAGG
ENSG00000076356	AUAUUAU
ENSG00000076356	CAGGCUC
ENSG00000076356	CAUAUUG
ENSG00000076356	CCAGUGC
ENSG00000076356	CUCAGGG
ENSG00000076356	CUCUUCC
ENSG00000076356	CUUUGGU
ENSG00000076356	GAAAACA
ENSG00000076356	GAAGGUC
ENSG00000076356	GAGGCUG
ENSG00000076356	GGAAGCC
ENSG00000076356	GGCAAGA
ENSG00000076356	GUAAACA
ENSG00000076356	GUCUUGC
ENSG00000076356	GUGAAUG
ENSG00000076356	GUGUCAC
ENSG00000076356	UAAUACA
ENSG00000076356	UAGGGCC
ENSG00000076356	UAUAAUA
ENSG00000076356	UAUUGCU
ENSG00000076356	UCAAGUA

ENSG00000076356	UGCAGCU
ENSG00000076356	UGGAUAA
ENSG00000076356	UGUGCUU
ENSG00000076356	UUGGUCC
ENSG00000076513	AACUCUA
ENSG00000076513	AAGGCAC
ENSG00000076513	CAGUGCA
ENSG00000076513	CUUGUAU
ENSG00000076513	GACCUGG
ENSG00000076513	GAGAAAU
ENSG00000076513	GUGAGGU
ENSG00000076513	UAUUGCU
ENSG00000076513	UCCCUUG
ENSG00000076513	UUCAAAU
ENSG00000076554	AAAGCUG
ENSG00000076554	AAAGGUA
ENSG00000076554	AAGAACC
ENSG00000076554	CACUCCU
ENSG00000076554	CUCUAGC
ENSG00000076554	CUCUUCC
ENSG00000076554	GAGACCU
ENSG00000076554	GUCACUC
ENSG00000076554	UGCAGCU
ENSG00000076554	UUGGUUC
ENSG00000076555	CCCUGAG
ENSG00000076604	AGCACCA
ENSG00000076604	AGCCCUU
ENSG00000076604	AUGGCUU
ENSG00000076604	AUGUCUG
ENSG00000076604	CAGUCCA
ENSG00000076604	CCCCUGG
ENSG00000076604	CCUGCUG
ENSG00000076604	CUGCCCC
ENSG00000076604	CUUGGGC
ENSG00000076604	GGCUCUG
ENSG00000076604	GGGGUCC
ENSG00000076604	GGGUGGU
ENSG00000076604	GUCUGCC
ENSG00000076604	UCUCAAG
ENSG00000076604	UGGGACA
ENSG00000076604	UGUCUGC
ENSG00000076641	AGCCCUU
ENSG00000076641	AUGCAUU
ENSG00000076641	AUGUGCC
ENSG00000076641	CCAGGAG
ENSG00000076641	CCAUUAC
ENSG00000076641	GAGGCAG
ENSG00000076641	GAGGUAU
ENSG00000076641	GCAGCAU
ENSG00000076641	UACAAAG
ENSG00000076641	UCAUACA
ENSG00000076641	UCAUUUG
ENSG00000076641	UCCUAUA
ENSG00000076641	UGCAUUG
ENSG00000076641	UGGCCAC
ENSG00000076641	UUAGAGA
ENSG00000076641	UUUCAAC
ENSG00000076685	UGAAGUG
ENSG00000076706	UGCAAAG
ENSG00000076716	AAACUGU
ENSG00000076716	AAGGCAC
ENSG00000076716	AUGCCUU
ENSG00000076716	GAGCUAA
ENSG00000076716	UCAAGUA

ENSG00000076716	UCACAUU
ENSG00000076770	AAAUGGU
ENSG00000076770	AACACUG
ENSG00000076770	ACUCCAG
ENSG00000076770	UCUCCAA
ENSG00000076826	AAUUAUU
ENSG00000076826	CCCUGAG
ENSG00000076826	GAGCCCC
ENSG00000076826	GAGCCCU
ENSG00000076826	GCAGGAC
ENSG00000076864	GGCAGGG
ENSG00000076864	UGUGCUU
ENSG00000076928	AAGGCAC
ENSG00000076928	CCCUGAG
ENSG00000076984	AAAGUUU
ENSG00000076984	AACACUG
ENSG00000076984	AGCAGCU
ENSG00000076984	CACAGUG
ENSG00000076984	CAGCUGG
ENSG00000076984	CCCCUGG
ENSG00000076984	CCCUGAG
ENSG00000076984	CCUGCUG
ENSG00000076984	CUUUGGU
ENSG00000076984	GAAGGUC
ENSG00000076984	GCUGGUG
ENSG00000076984	GCUGUCU
ENSG00000076984	GGCCCUG
ENSG00000076984	GGGGUGG
ENSG00000076984	UACAAAG
ENSG00000076984	UCACAGG
ENSG00000076984	UCACAGU
ENSG00000077044	GCACCAU
ENSG00000077063	AGGCAGG
ENSG00000077080	ACAUCAC
ENSG00000077092	AAAGGUA
ENSG00000077092	AAAGUGC
ENSG00000077092	AGCACCA
ENSG00000077092	AGCCAGU
ENSG00000077092	AGUGCUG
ENSG00000077092	AUGGCUU
ENSG00000077092	CAGUUAC
ENSG00000077092	CCAGGAG
ENSG00000077092	UAUAUGA
ENSG00000077092	UGCCAGC
ENSG00000077092	UUGGUCC
ENSG00000077097	AAAAACC
ENSG00000077097	AAACUGU
ENSG00000077097	UCAUUUG
ENSG00000077147	AACAAAC
ENSG00000077147	AAUCUCU
ENSG00000077147	ACAGUAU
ENSG00000077147	ACAUUAC
ENSG00000077147	ACAUUCA
ENSG00000077147	AUGAGCU
ENSG00000077147	CAGUUAC
ENSG00000077147	CCAGUGU
ENSG00000077147	CCUUCAU
ENSG00000077147	CUUCACC
ENSG00000077147	CUUUGGU
ENSG00000077147	GGCAAGA
ENSG00000077147	UACAAAG
ENSG00000077147	UAUUACC
ENSG00000077157	AAAAAUC
ENSG00000077157	AAAGUGC

ENSG00000077157	ACCCAUU
ENSG00000077157	ACUGUUU
ENSG00000077157	AGCAGCA
ENSG00000077157	AGGUAGU
ENSG00000077157	CAGCUGG
ENSG00000077157	CUCCAGC
ENSG00000077157	CUGCAGC
ENSG00000077157	GAGGCAG
ENSG00000077157	GAGGUAG
ENSG00000077157	GCUUCCU
ENSG00000077157	GGAGAGA
ENSG00000077157	GGCAAGA
ENSG00000077157	GGGGUCC
ENSG00000077157	GUGACAG
ENSG00000077157	UAAUAUC
ENSG00000077157	UCACAAG
ENSG00000077157	UGCAUAU
ENSG00000077157	UUCAAAU
ENSG00000077157	UUCUGCA
ENSG00000077254	AGUGCAA
ENSG00000077254	CAAAACU
ENSG00000077254	CUACAAA
ENSG00000077254	GUGCAAA
ENSG00000077254	UACAAAG
ENSG00000077274	AACAAAC
ENSG00000077274	ACUGGCU
ENSG00000077274	AGCAGCA
ENSG00000077274	AGCAGCU
ENSG00000077274	AGGAGCU
ENSG00000077274	CAGGCUC
ENSG00000077274	CAUGGAU
ENSG00000077274	CUCCCAC
ENSG00000077274	CUGACUC
ENSG00000077274	GAAGGUC
ENSG00000077274	GAGUGUG
ENSG00000077274	GGAGGAG
ENSG00000077274	GGAGGCA
ENSG00000077274	GGUGGUU
ENSG00000077274	UCACAUU
ENSG00000077274	UCUCAAG
ENSG00000077274	UGCAGCU
ENSG00000077274	UGGGGGA
ENSG00000077274	UGUGUGG
ENSG00000077274	UUGAUCA
ENSG00000077279	AAAUUUC
ENSG00000077279	AACACUG
ENSG00000077279	AACAUUC
ENSG00000077279	AACCUGU
ENSG00000077279	AACUACU
ENSG00000077279	AAUCCUU
ENSG00000077279	AAUUUUA
ENSG00000077279	ACCUGGA
ENSG00000077279	AGAUAAA
ENSG00000077279	AUAUUAU
ENSG00000077279	AUCCCUU
ENSG00000077279	AUCCUUG
ENSG00000077279	AUCCUUU
ENSG00000077279	AUGGCAC
ENSG00000077279	AUUGCAC
ENSG00000077279	CAAAGCA
ENSG00000077279	CAAGAGC
ENSG00000077279	CACAGGG
ENSG00000077279	CACCAGC
ENSG00000077279	CCCUGUU

ENSG00000077279	CUAGGCU
ENSG00000077279	CUCACAC
ENSG00000077279	CUGGACU
ENSG00000077279	GAAUUGU
ENSG00000077279	GACCCUG
ENSG00000077279	GCAGAAG
ENSG00000077279	GCCCUGU
ENSG00000077279	GCUACAU
ENSG00000077279	GGAUUUC
ENSG00000077279	GGCAGUG
ENSG00000077279	GGGAGCU
ENSG00000077279	GGGGAGC
ENSG00000077279	GGUGGGC
ENSG00000077279	GUAAACA
ENSG00000077279	GUGACAG
ENSG00000077279	GUGCUUG
ENSG00000077279	UCCCUUU
ENSG00000077279	UCUAGAG
ENSG00000077279	UCUCACC
ENSG00000077279	UCUCCAA
ENSG00000077279	UGAGAAU
ENSG00000077279	UGCAAAG
ENSG00000077279	UGCCCUG
ENSG00000077279	UGGAGAU
ENSG00000077279	UGUCUGG
ENSG00000077279	UGUCUUU
ENSG00000077279	UGUGCUU
ENSG00000077279	UUCAAGC
ENSG00000077279	UUCUAAU
ENSG00000077279	UUCUUGU
ENSG00000077279	UUGAUCA
ENSG00000077279	UUUGCAA
ENSG00000077279	UUUGCAC
ENSG00000077279	UUUUUGC
ENSG00000077327	AUAUUAU
ENSG00000077327	GUGAAUG
ENSG00000077454	AAGGCAC
ENSG00000077454	AGCAGCG
ENSG00000077454	AGGAUGU
ENSG00000077454	CCAGCAU
ENSG00000077454	CCAGGAG
ENSG00000077454	CUCUUCC
ENSG00000077454	CUUUGGU
ENSG00000077454	GAGGCUG
ENSG00000077454	GGGCCCC
ENSG00000077454	GGUGGGC
ENSG00000077454	UGUCUUU
ENSG00000077458	ACAAUAU
ENSG00000077458	ACUGUAG
ENSG00000077458	AGAUGUG
ENSG00000077458	AGCAUUC
ENSG00000077458	AGUAGUU
ENSG00000077458	AUUGCAC
ENSG00000077458	CACAGGG
ENSG00000077458	UAUUGCU
ENSG00000077458	UGAAGUG
ENSG00000077458	UGCAUAU
ENSG00000077514	GUGCUUG
ENSG00000077514	UGUGCUU
ENSG00000077522	CUUUGGU
ENSG00000077585	AGCAUUC
ENSG00000077585	AGUACUG
ENSG00000077585	AUGCAUU
ENSG00000077585	UAUAAAG

ENSG00000077585	UUAUGCA
ENSG00000077684	AGCAUUC
ENSG00000077684	AUAAUAC
ENSG00000077684	AUGGCUU
ENSG00000077684	AUUGCAC
ENSG00000077684	CAAAACU
ENSG00000077684	CCUUCAU
ENSG00000077684	GGAAGAC
ENSG00000077684	GGCUCUG
ENSG00000077684	GUGCUUG
ENSG00000077684	UAAGACU
ENSG00000077684	UAUAAAG
ENSG00000077684	UCACAUU
ENSG00000077684	UCUCACC
ENSG00000077684	UGAAAGG
ENSG00000077684	UGACAUC
ENSG00000077684	UGCAAAG
ENSG00000077684	UGCAUAG
ENSG00000077684	UGCAUAU
ENSG00000077684	UGCUGAC
ENSG00000077684	UUCUUGU
ENSG00000077684	UUGACAC
ENSG00000077684	UUGAUCA
ENSG00000077713	AUCCUUU
ENSG00000077713	UCACAGG
ENSG00000077713	UGAUCCA
ENSG00000077782	AAAAGUA
ENSG00000077782	AAACUGU
ENSG00000077782	AAGACAU
ENSG00000077782	AAGUAGC
ENSG00000077782	AAUCUCU
ENSG00000077782	ACCAGCA
ENSG00000077782	ACUCAGC
ENSG00000077782	ACUCAGG
ENSG00000077782	CCAGGAG
ENSG00000077782	CCCCCAG
ENSG00000077782	CCCUGAG
ENSG00000077782	CUCUGGG
ENSG00000077782	GGCAGGG
ENSG00000077782	GGGCCCC
ENSG00000077782	UAAUAUC
ENSG00000077782	UAUAUGA
ENSG00000077782	UCAUACA
ENSG00000077782	UCAUAGA
ENSG00000077782	UCCUGAG
ENSG00000077782	UGAGGAC
ENSG00000077782	UGGUACC
ENSG00000077782	UUGGUCC
ENSG00000077782	UUGUGUC
ENSG00000077809	AUAUUAU
ENSG00000077809	CAUGGAU
ENSG00000077809	CUACAAA
ENSG00000077809	CUUCUCU
ENSG00000077809	GAGGUAG
ENSG00000077809	GAGGUAU
ENSG00000077809	GUCACUC
ENSG00000077809	UCACAGU
ENSG00000077809	UGAAAGG
ENSG00000077809	UGUAUAA
ENSG00000077809	UUGAGGC
ENSG00000078018	AAAGUUU
ENSG00000078018	ACUGUGU
ENSG00000078018	CAAGAGC
ENSG00000078018	CUAUACA

ENSG00000078018	UGGCCAC
ENSG00000078018	UUUGCAC
ENSG00000078053	AUGACAC
ENSG00000078053	CAAAGCA
ENSG00000078061	GAGGCAG
ENSG00000078061	GUGCUUG
ENSG00000078061	UGGAGAU
ENSG00000078061	UGUGCUU
ENSG00000078081	AAGUUGU
ENSG00000078081	CUGGCUC
ENSG00000078081	GGCUCUG
ENSG00000078081	UCUGGAG
ENSG00000078098	ACAGGUG
ENSG00000078114	AAAUUUC
ENSG00000078114	AACAAAC
ENSG00000078114	AAGGCAC
ENSG00000078114	AGCACCA
ENSG00000078114	CUUUGGU
ENSG00000078114	GUGACAG
ENSG00000078114	UAAUAGU
ENSG00000078114	UCUCAAG
ENSG00000078114	UGCAUAG
ENSG00000078114	UGCAUAU
ENSG00000078114	UGUGCUU
ENSG00000078140	AAGAACC
ENSG00000078140	AAGUAGC
ENSG00000078140	AAUUUUA
ENSG00000078140	AGCACCA
ENSG00000078140	AGCAGCU
ENSG00000078140	CAAAACU
ENSG00000078140	CAAAGCA
ENSG00000078140	CACAGGG
ENSG00000078140	CACAGUG
ENSG00000078140	CAGCAGA
ENSG00000078140	GAAACAU
ENSG00000078140	GCACCAU
ENSG00000078140	GCAGAAG
ENSG00000078140	GGCAAGA
ENSG00000078140	GUAAACA
ENSG00000078140	UAUUGCU
ENSG00000078140	UCACACA
ENSG00000078140	UCUCCAA
ENSG00000078140	UGAGAAG
ENSG00000078140	UGUGUCA
ENSG00000078140	UUGGCAA
ENSG00000078140	UUGGCAC
ENSG00000078237	AAAUCCC
ENSG00000078237	AUCCCUU
ENSG00000078237	CCAGUGC
ENSG00000078237	UCCCUUU
ENSG00000078246	UCUCAAG
ENSG00000078269	UCUCCAA
ENSG00000078269	UGUCUUU
ENSG00000078295	AACUGGU
ENSG00000078295	AAUACUG
ENSG00000078295	AGUGCAA
ENSG00000078295	AUGCACC
ENSG00000078295	CAGUGCA
ENSG00000078295	GAGGCAG
ENSG00000078295	GCACCAU
ENSG00000078295	UACAAAG
ENSG00000078295	UAUCAGA
ENSG00000078295	UAUUGCU
ENSG00000078295	UCCUGAG

ENSG00000078295	UGAGGAC
ENSG00000078295	UGUGCUU
ENSG00000078295	UUGGCAA
ENSG00000078304	AAUACUG
ENSG00000078304	ACUGGCC
ENSG00000078304	AGCAGCA
ENSG00000078304	CCCUGAG
ENSG00000078304	GCAGCAU
ENSG00000078304	GUAUGUU
ENSG00000078304	UAUUGCU
ENSG00000078304	UGGCCAC
ENSG00000078369	AGGUUAU
ENSG00000078369	CUACAGU
ENSG00000078369	GCAUCCC
ENSG00000078369	GUGCUUG
ENSG00000078369	UAUUACC
ENSG00000078369	UGUGCUU
ENSG00000078369	UUUCAAC
ENSG00000078399	AAAGGUA
ENSG00000078399	CUAUACA
ENSG00000078399	GAGGUAG
ENSG00000078399	GAGGUAU
ENSG00000078399	GUGCAAA
ENSG00000078399	UUGGUCC
ENSG00000078401	AGUGCAA
ENSG00000078401	GGUAGAC
ENSG00000078401	GUGAGGU
ENSG00000078403	AAAAAUC
ENSG00000078403	AGUGCAA
ENSG00000078403	CAGCAGG
ENSG00000078403	CUACAGU
ENSG00000078403	GAGGUAG
ENSG00000078403	GAGGUAU
ENSG00000078403	UACAAAG
ENSG00000078403	UCACAAG
ENSG00000078618	ACUGCAG
ENSG00000078674	AACACUG
ENSG00000078674	AAGACAU
ENSG00000078674	ACAUCAC
ENSG00000078674	CACAGUG
ENSG00000078674	CAGUCUG
ENSG00000078699	AAAUGGU
ENSG00000078699	ACAUUCA
ENSG00000078699	AGCAAAA
ENSG00000078699	AUGCACC
ENSG00000078699	CAAAGCA
ENSG00000078699	CAGCAGA
ENSG00000078699	CCUGCUG
ENSG00000078699	CUACAGU
ENSG00000078699	CUGGAAA
ENSG00000078699	CUUUGGU
ENSG00000078699	GACUUCC
ENSG00000078699	GAGCUGC
ENSG00000078699	GCCUGGA
ENSG00000078699	GCUGGUG
ENSG00000078699	GGAGGAG
ENSG00000078699	GGGAGCU
ENSG00000078699	GGGGAGC
ENSG00000078699	GUGCAAA
ENSG00000078699	UCCUAUA
ENSG00000078699	UGGCCAC
ENSG00000078699	UUAUGCA
ENSG00000078747	AAGACAU
ENSG00000078747	AAGUGCA

ENSG00000078747	AGAUGUG
ENSG00000078747	AGUGCUG
ENSG00000078747	CAAAGUG
ENSG00000078747	CAGCAGG
ENSG00000078747	CCAGUGU
ENSG00000078747	CUUGGGC
ENSG00000078747	GUGCAAA
ENSG00000078747	UAGGGCC
ENSG00000078747	UGGAGAU
ENSG00000078747	UGGGAUC
ENSG00000078804	AAAAAUC
ENSG00000078804	AAGGCAC
ENSG00000078804	AAGUGCU
ENSG00000078804	AAUUUUA
ENSG00000078804	ACACACC
ENSG00000078804	ACACACU
ENSG00000078804	ACAGUAC
ENSG00000078804	ACAGUAU
ENSG00000078804	AGUGCUG
ENSG00000078804	AUAUUAU
ENSG00000078804	CAAGAGC
ENSG00000078804	CUAUACA
ENSG00000078804	GAGCCCC
ENSG00000078804	GAGCCCU
ENSG00000078804	GCUACAU
ENSG00000078804	GCUGGUG
ENSG00000078804	GGGGAGC
ENSG00000078804	GGUGGUU
ENSG00000078804	GUAGUGU
ENSG00000078804	GUCUGCC
ENSG00000078804	UCAAGUA
ENSG00000078804	UCUCCAA
ENSG00000078804	UGAAAGG
ENSG00000078804	UGAGUCU
ENSG00000078804	UGCAUAG
ENSG00000078804	UGCAUAU
ENSG00000078804	UGUCUGC
ENSG00000078804	UUGGCAA
ENSG00000078967	GUGAAUG
ENSG00000078967	UUGGCAA
ENSG00000078967	UUGGCAC
ENSG00000079102	AAAUCCC
ENSG00000079102	AAUCUCU
ENSG00000079102	ACCUGGA
ENSG00000079102	ACUGCAG
ENSG00000079102	ACUGGCU
ENSG00000079102	AGGGAGG
ENSG00000079102	AUAAAGU
ENSG00000079102	AUAUUAU
ENSG00000079102	CAAAGUG
ENSG00000079102	CACAGGG
ENSG00000079102	CAGCUGG
ENSG00000079102	CUGCAGG
ENSG00000079102	CUGUUGA
ENSG00000079102	CUUCACC
ENSG00000079102	GAGAACU
ENSG00000079102	GGAGGAG
ENSG00000079102	GGAUGAG
ENSG00000079102	GGGUCUU
ENSG00000079102	GUCUACU
ENSG00000079102	GUUAAUG
ENSG00000079102	UCACAGG
ENSG00000079102	UGACCUA
ENSG00000079102	UUGUGAC

ENSG00000079102	UUUUUGC
ENSG00000079112	AACAUUC
ENSG00000079112	UUCAAAU
ENSG00000079150	UGCAAAG
ENSG00000079150	UUUGCAA
ENSG00000079156	GAAACAU
ENSG00000079156	UGCAUAG
ENSG00000079215	AAAGUAA
ENSG00000079215	CAGUCCA
ENSG00000079215	GGGAGCU
ENSG00000079215	UACAAAG
ENSG00000079246	UAUAAAG
ENSG00000079277	AGCAGCG
ENSG00000079277	AGCAGCU
ENSG00000079277	CAAAGUG
ENSG00000079277	GGAAGAC
ENSG00000079277	UGCAAAG
ENSG00000079277	UUAUGCA
ENSG00000079308	AAAGUAA
ENSG00000079308	AAAGUAC
ENSG00000079308	AAAGUGC
ENSG00000079308	AAGUGCU
ENSG00000079308	AGCUUAU
ENSG00000079308	AGUGCUU
ENSG00000079308	AUAUAAC
ENSG00000079308	AUAUUAU
ENSG00000079308	AUGUCUG
ENSG00000079308	CCUUCUU
ENSG00000079308	CUUUGGU
ENSG00000079308	GAGACCU
ENSG00000079308	GAGGCAG
ENSG00000079308	GGCAUUG
ENSG00000079308	GGCUCAG
ENSG00000079308	GGGAGCU
ENSG00000079308	UACAAAG
ENSG00000079308	UUCUUGU
ENSG00000079308	UUGGCAA
ENSG00000079308	UUGGCAC
ENSG00000079313	ACUGUUU
ENSG00000079313	CACUGUU
ENSG00000079313	UAUUGCU
ENSG00000079332	AAAACUG
ENSG00000079332	AAUCACU
ENSG00000079332	CACCACC
ENSG00000079332	CCAGUGC
ENSG00000079332	CCAGUGU
ENSG00000079332	CUCCAGC
ENSG00000079332	CUGGACU
ENSG00000079332	GAGAAGA
ENSG00000079332	GAGACGC
ENSG00000079332	GGAGAGA
ENSG00000079332	GUCCAGA
ENSG00000079332	GUGCAAA
ENSG00000079332	UCACAAG
ENSG00000079332	UCACCAC
ENSG00000079332	UCUCCAA
ENSG00000079335	ACAGUAC
ENSG00000079335	ACAGUAU
ENSG00000079335	CAGUAGU
ENSG00000079335	CUAUACA
ENSG00000079335	GCAGCAU
ENSG00000079335	UCCCUCU
ENSG00000079335	UCCCUUU
ENSG00000079337	CACAGGG

ENSG00000079337	CUCCAGC
ENSG00000079385	CAGUUAC
ENSG00000079385	GUAAACA
ENSG00000079387	AAAAGUA
ENSG00000079387	AAAGUAU
ENSG00000079387	ACAUAGA
ENSG00000079387	ACAUUCA
ENSG00000079387	AGAUGUG
ENSG00000079387	AGCACCA
ENSG00000079387	AGCAGCU
ENSG00000079387	AGCCCUU
ENSG00000079387	AUGACAC
ENSG00000079387	CAGUUAC
ENSG00000079387	CUCUUCC
ENSG00000079387	GAAACAU
ENSG00000079387	GAGCCCU
ENSG00000079387	GAGGCUG
ENSG00000079387	GCUGGAU
ENSG00000079387	GGAGUGU
ENSG00000079387	GGGUCUU
ENSG00000079387	GGUGGUC
ENSG00000079387	GUGAAUG
ENSG00000079387	UCACAGG
ENSG00000079387	UCACAGU
ENSG00000079387	UCCAGUU
ENSG00000079387	UCCCUGU
ENSG00000079387	UGCAAAG
ENSG00000079387	UGGGAUC
ENSG00000079387	UGUGCUU
ENSG00000079387	UUCCUAG
ENSG00000079387	UUUGCAA
ENSG00000079432	AAAGUAC
ENSG00000079432	AAAGUGC
ENSG00000079432	AACCUGG
ENSG00000079432	AACCUGU
ENSG00000079432	AAGUGCA
ENSG00000079432	AAUCUCA
ENSG00000079432	AAUCUCU
ENSG00000079432	AGGUUAU
ENSG00000079432	AUAAAGU
ENSG00000079432	AUGGCUU
ENSG00000079432	CAAAGUG
ENSG00000079432	CCUUCUU
ENSG00000079432	CUCCAGC
ENSG00000079432	CUCGGCG
ENSG00000079432	CUCUGGG
ENSG00000079432	GAGCCCC
ENSG00000079432	GCAGGAC
ENSG00000079432	GUGGCCC
ENSG00000079432	UCCCUGU
ENSG00000079435	AAGGCAC
ENSG00000079435	AGCAGCA
ENSG00000079482	AAGUCAC
ENSG00000079482	AAGUGUG
ENSG00000079482	GAGCUGC
ENSG00000079482	UCCUAUA
ENSG00000079482	UUGGCAA
ENSG00000079557	UCACCAC
ENSG00000079689	GAGUCUU
ENSG00000079691	GUGCAAA
ENSG00000079691	UCAAGUA
ENSG00000079739	AAGGCAC
ENSG00000079805	AAAGUGC
ENSG00000079805	AAGGCAC

ENSG00000079805	AAGUGCA
ENSG00000079805	AGUGCAA
ENSG00000079805	CAAAGUG
ENSG00000079805	CUUACAG
ENSG00000079805	UAAUAGU
ENSG00000079805	UCCCUUG
ENSG00000079819	AGCAAAA
ENSG00000079819	CUAUACA
ENSG00000079819	UCUGGAA
ENSG00000079841	AAAAACC
ENSG00000079841	AAAAAUC
ENSG00000079841	AAACUGU
ENSG00000079841	AAGACAU
ENSG00000079841	AGCCCUU
ENSG00000079841	AUAAAGU
ENSG00000079841	AUGGCUU
ENSG00000079841	AUGUCUG
ENSG00000079841	CACAGGG
ENSG00000079841	CCAGUGC
ENSG00000079841	CCAGUGU
ENSG00000079841	GAAGGAA
ENSG00000079841	GAUUGUA
ENSG00000079841	GCAGAAG
ENSG00000079841	GCCUGGG
ENSG00000079841	GUGCUUG
ENSG00000079841	UAAGACG
ENSG00000079841	UCACAAG
ENSG00000079841	UCAUUUG
ENSG00000079841	UGCAGCU
ENSG00000079841	UGUGCUU
ENSG00000079931	CUACAAA
ENSG00000080189	CAGGUGA
ENSG00000080189	UCACACA
ENSG00000080345	AUACAAG
ENSG00000080345	UAUAAAG
ENSG00000080371	AGAUAAA
ENSG00000080371	GUCUCUG
ENSG00000080371	GUGCAAA
ENSG00000080371	UACAAAG
ENSG00000080371	UGCAUAU
ENSG00000080371	UUUGCAC
ENSG00000080493	AAAGUGC
ENSG00000080493	AAUACUG
ENSG00000080493	ACAGUAC
ENSG00000080493	ACAGUAU
ENSG00000080493	ACUGCAU
ENSG00000080493	AGCAGCG
ENSG00000080493	AGCAGCU
ENSG00000080493	AGCCCUU
ENSG00000080493	AUAAAGU
ENSG00000080493	CAAAACU
ENSG00000080493	CAGCAGA
ENSG00000080493	CAGCAGG
ENSG00000080493	CCUGCUG
ENSG00000080493	CUGCAGG
ENSG00000080493	GAAGGAA
ENSG00000080493	GAGGCAG
ENSG00000080493	GAGGUAG
ENSG00000080493	GAGUUGA
ENSG00000080493	GAGUUGG
ENSG00000080493	GCAGGAC
ENSG00000080493	GCAGGUU
ENSG00000080493	GCUACAU
ENSG00000080493	GGAAGAC

ENSG00000080493	GGCAGCG
ENSG00000080493	GGCUCUG
ENSG00000080493	GUGACUG
ENSG00000080493	UAGGCCG
ENSG00000080493	UCCCUUU
ENSG00000080493	UGCAUAU
ENSG00000080493	UGGGACA
ENSG00000080493	UUCAAGC
ENSG00000080493	UUCUAAU
ENSG00000080493	UUGUUCG
ENSG00000080503	AAGACAU
ENSG00000080503	AAUUUUA
ENSG00000080503	ACAAUAU
ENSG00000080503	CACAGUG
ENSG00000080503	CAGGCUC
ENSG00000080503	CUACAAA
ENSG00000080503	UGCAAAG
ENSG00000080503	UUCCAUA
ENSG00000080546	AGCAGCA
ENSG00000080546	AUGGCAC
ENSG00000080573	AGGCAGG
ENSG00000080573	AGUGCUU
ENSG00000080573	CCAGUGU
ENSG00000080573	GCACCAU
ENSG00000080573	UAAUACA
ENSG00000080573	UUAGGAU
ENSG00000080573	UUGGUCC
ENSG00000080603	AACACUG
ENSG00000080603	CUUUGGU
ENSG00000080603	GUGGGGA
ENSG00000080644	GUCUCUG
ENSG00000080644	UCAUUUG
ENSG00000080709	AAGUGCA
ENSG00000080709	ACAAUAU
ENSG00000080709	AUGAUGA
ENSG00000080709	AUGAUGC
ENSG00000080709	GACCAUG
ENSG00000080709	UGACAUC
ENSG00000080802	AAAGCUG
ENSG00000080802	AAUACUG
ENSG00000080802	AGGUUAU
ENSG00000080802	GUCCAGA
ENSG00000080815	AGUGCUU
ENSG00000080815	CAGUGCA
ENSG00000080815	CUUUGGU
ENSG00000080815	GUGCUUG
ENSG00000080815	UCCUGAG
ENSG00000080815	UGAGAAU
ENSG00000080819	AAGGCAC
ENSG00000080819	AAUCUCU
ENSG00000080822	AAGGCAC
ENSG00000080822	UAUAAAG
ENSG00000080824	AACAAAC
ENSG00000080824	GAAAACA
ENSG00000080845	AAAAAUC
ENSG00000080845	AAAGCUG
ENSG00000080845	AGCCCUU
ENSG00000080845	AGGGAGG
ENSG00000080845	AUGUGCC
ENSG00000080845	CACACCU
ENSG00000080845	CUCUAGC
ENSG00000080845	CUUGUAU
ENSG00000080845	GAAGGAA
ENSG00000080845	GAGGGGC

ENSG00000080845	GAGGUAG
ENSG00000080845	UCACACA
ENSG00000080845	UGCAGCU
ENSG00000080845	UGCAUAU
ENSG00000080854	AACACUG
ENSG00000080854	ACCAGCA
ENSG00000080854	AGCCUGC
ENSG00000080854	CUGCAGC
ENSG00000080854	GAGCUGC
ENSG00000080854	GCAGCAU
ENSG00000080854	GCCUGGA
ENSG00000080854	UCUCCAA
ENSG00000080854	UGCAAAG
ENSG00000081014	ACUGUGU
ENSG00000081014	AGCACCA
ENSG00000081014	AUGUAAU
ENSG00000081014	CUCCCAC
ENSG00000081014	UUGGCAC
ENSG00000081019	AAAGUAA
ENSG00000081019	AAAUGGU
ENSG00000081019	AAUCACU
ENSG00000081019	ACAAUAU
ENSG00000081019	ACAUUAC
ENSG00000081019	CACAGUG
ENSG00000081019	GAGAAGA
ENSG00000081019	GCUGGAU
ENSG00000081019	UAAUACA
ENSG00000081019	UAUAAAG
ENSG00000081019	UCAUGAU
ENSG00000081019	UCCUGAG
ENSG00000081019	UGAGAAG
ENSG00000081019	UGCAUAG
ENSG00000081019	UGCAUAU
ENSG00000081026	AAGACAU
ENSG00000081026	AAGUCUU
ENSG00000081026	AGUGCUU
ENSG00000081026	CAGUAGA
ENSG00000081026	CCUUCAU
ENSG00000081026	GUAAACA
ENSG00000081026	UUGGCAC
ENSG00000081041	AAGACAU
ENSG00000081041	CACAGUG
ENSG00000081051	CUCUUCC
ENSG00000081052	AAUUAUU
ENSG00000081052	AUGCAUU
ENSG00000081052	CUCCCAA
ENSG00000081052	CUCCCAC
ENSG00000081052	CUGCAGG
ENSG00000081052	GACUUCC
ENSG00000081052	GAGAAAU
ENSG00000081052	GCCCUUA
ENSG00000081052	GGGGAGC
ENSG00000081052	GUGAAUG
ENSG00000081052	UAAUAUC
ENSG00000081052	UCCCUGU
ENSG00000081052	UUCAAGC
ENSG00000081052	UUGUGAC
ENSG00000081052	UUUCAAC
ENSG00000081059	AUGCAUU
ENSG00000081059	CUGCAGG
ENSG00000081059	GACCUGG
ENSG00000081059	GAGGCUG
ENSG00000081059	GUGGGGA
ENSG00000081059	UGAAGCU

ENSG00000081087	AAAAGUA
ENSG00000081087	AAGUGCU
ENSG00000081087	AGUGCUU
ENSG00000081087	AGUGGUU
ENSG00000081087	CAGUUAC
ENSG00000081087	GUAAACA
ENSG00000081087	UAUUGCU
ENSG00000081154	AAAGGUA
ENSG00000081154	GAGCUAA
ENSG00000081189	AAAGUAC
ENSG00000081189	AACAAAC
ENSG00000081189	AAGUGCU
ENSG00000081189	ACACACU
ENSG00000081189	ACUGUUU
ENSG00000081189	AGUGCUU
ENSG00000081189	AUAUAAC
ENSG00000081189	AUGGCUU
ENSG00000081189	AUGUAAU
ENSG00000081189	CGACCCA
ENSG00000081189	GAGGCAG
ENSG00000081189	GCAGGUU
ENSG00000081189	GUAACAG
ENSG00000081189	GUUAAUG
ENSG00000081189	UAAUAUC
ENSG00000081189	UCAACAG
ENSG00000081189	UCACAGG
ENSG00000081189	UCACAGU
ENSG00000081189	UGCAUAG
ENSG00000081189	UUCAAAU
ENSG00000081237	UCUUGAG
ENSG00000081277	AAUACUG
ENSG00000081277	CUGCAGG
ENSG00000081377	AAAAGUA
ENSG00000081377	AAAGUAC
ENSG00000081377	AAGGCAC
ENSG00000081377	ACUGCAG
ENSG00000081377	AUACAAG
ENSG00000081377	CAAAUGC
ENSG00000081377	CACAGUG
ENSG00000081377	CCCUGAG
ENSG00000081377	CCUUCAU
ENSG00000081377	CUUUGGU
ENSG00000081377	GAGGUAG
ENSG00000081377	UCACAGU
ENSG00000081377	UGCCCUG
ENSG00000081377	UGUGCUU
ENSG00000081479	ACAGUAU
ENSG00000081479	AUAAAGU
ENSG00000081479	AUGUGCC
ENSG00000081479	CAGUAGA
ENSG00000081479	UACAAAG
ENSG00000081479	UCCCUUG
ENSG00000081479	UGCAAAG
ENSG00000081791	GUCUCUG
ENSG00000081791	UCACACA
ENSG00000081791	UCUCACC
ENSG00000081800	GUUAAUG
ENSG00000081803	ACAUCAC
ENSG00000081803	ACUGCAG
ENSG00000081803	AUACAAG
ENSG00000081803	CUAGUCC
ENSG00000081803	GGCAUUG
ENSG00000081803	GGGUUUA
ENSG00000081803	UAUUGCU

ENSG00000081842	AAAGAAU
ENSG00000081842	AAGUGCA
ENSG00000081842	AGCAGCG
ENSG00000081842	AGCAGCU
ENSG00000081842	AUAAUAC
ENSG00000081842	CAAAGUG
ENSG00000081842	GAGGCAG
ENSG00000081842	UAAUACA
ENSG00000081842	UAAUAGU
ENSG00000081842	UACAAAG
ENSG00000081842	UAUAAUA
ENSG00000081842	UCAACAG
ENSG00000081842	UCACAUU
ENSG00000081842	UCAUACA
ENSG00000081842	UGCAUAG
ENSG00000081842	UGCAUAU
ENSG00000081842	UGUGCUU
ENSG00000081842	UUUGCAA
ENSG00000081842	UUUGCAC
ENSG00000081853	AAAACUG
ENSG00000081853	CACCAGC
ENSG00000081853	GGAGGCA
ENSG00000081853	UGCAUAG
ENSG00000081853	UGGCCAC
ENSG00000081923	GGAGAGA
ENSG00000081923	GUAAACA
ENSG00000081923	UAAUAUC
ENSG00000081923	UCCCUUG
ENSG00000081923	UCCCUUU
ENSG00000081923	UCCUGAG
ENSG00000081985	UCCCUUU
ENSG00000082153	ACAGUAC
ENSG00000082153	ACAGUAU
ENSG00000082153	ACAUUAC
ENSG00000082153	AGCCCUU
ENSG00000082153	AGGACAC
ENSG00000082153	AGGGAGG
ENSG00000082153	AUAAAGU
ENSG00000082153	AUCACUA
ENSG00000082153	AUGGCAC
ENSG00000082153	CCUGUAC
ENSG00000082153	GCCCUUA
ENSG00000082153	UACAGUU
ENSG00000082153	UAUUACC
ENSG00000082153	UGCAAAG
ENSG00000082153	UUUGCAA
ENSG00000082175	AAAGCUG
ENSG00000082175	AACCUGU
ENSG00000082175	CAAGAGC
ENSG00000082175	GCAACUU
ENSG00000082175	UCAAGUA
ENSG00000082175	UCACAUU
ENSG00000082175	UGUUGAA
ENSG00000082196	AAGUCUU
ENSG00000082196	UGCAAAG
ENSG00000082196	UUCUUGU
ENSG00000082213	CUGGAAA
ENSG00000082213	UCUGGAA
ENSG00000082258	AGCAGCU
ENSG00000082258	AUGUAAU
ENSG00000082258	GUAUGUU
ENSG00000082258	UCAUACA
ENSG00000082258	UCUCCAA
ENSG00000082258	UGACCUA

ENSG00000082258	UGUAUGU
ENSG00000082269	GAGGUAG
ENSG00000082269	GAGGUAU
ENSG00000082293	AAACUGU
ENSG00000082293	AAAGUGC
ENSG00000082293	AAGUGCA
ENSG00000082293	ACCAGCA
ENSG00000082293	AGUGCAA
ENSG00000082293	CUUGUGU
ENSG00000082293	GAGGUAU
ENSG00000082293	GCACCAU
ENSG00000082293	GUGCAAA
ENSG00000082293	GUGCCUG
ENSG00000082293	UAGGUAU
ENSG00000082293	UCACAGG
ENSG00000082293	UGCAAAG
ENSG00000082397	AAGUUGU
ENSG00000082397	UCACCAC
ENSG00000082397	UUGGCAC
ENSG00000082438	AAGUGCA
ENSG00000082458	AAAGUAA
ENSG00000082458	AACACUG
ENSG00000082458	AGCAGCU
ENSG00000082458	AGUGCUU
ENSG00000082458	AUGAUGA
ENSG00000082458	AUGGAUU
ENSG00000082458	AUGUCUG
ENSG00000082458	CAGGUGA
ENSG00000082458	CAGUCCA
ENSG00000082458	CUCCAUU
ENSG00000082458	CUCUGGG
ENSG00000082458	CUCUUCC
ENSG00000082458	GAAGGUC
ENSG00000082458	GAGCUGC
ENSG00000082458	GCAACUU
ENSG00000082458	GCAGGAC
ENSG00000082458	GGGGUGG
ENSG00000082458	GGUUUAC
ENSG00000082458	GUCCAGA
ENSG00000082458	GUCUCUG
ENSG00000082458	UCCAGAG
ENSG00000082458	UGUCUUU
ENSG00000082458	UUGGUUC
ENSG00000082458	UUGUUCG
ENSG00000082458	UUUGCAC
ENSG00000082482	CAGUCUG
ENSG00000082482	UAGGUAU
ENSG00000082482	UCACAGG
ENSG00000082497	AGAUAAA
ENSG00000082497	CCUGCUG
ENSG00000082512	AAACUGU
ENSG00000082512	GGUCUAG
ENSG00000082515	CAAAACU
ENSG00000082556	AAAAGUA
ENSG00000082556	AAGACAU
ENSG00000082556	ACUGCAG
ENSG00000082556	AUCCCUU
ENSG00000082556	CCAUUAC
ENSG00000082641	AAAGCGC
ENSG00000082641	AAAUCCC
ENSG00000082641	ACACGGG
ENSG00000082641	ACUCAAA
ENSG00000082641	ACUCCAG
ENSG00000082641	AGAUAAA

ENSG00000082641	AGCAGCA
ENSG00000082641	AGCAGCG
ENSG00000082641	AGCAUUC
ENSG00000082641	AGCCCUU
ENSG00000082641	AUGGCAC
ENSG00000082641	CAAAGCA
ENSG00000082641	CAGUUAC
ENSG00000082641	CCCCCAG
ENSG00000082641	CUCCAGC
ENSG00000082641	GAGUCUU
ENSG00000082641	GGCAGGG
ENSG00000082641	GGCAGUG
ENSG00000082641	GGGGCAG
ENSG00000082641	UCCAGUU
ENSG00000082641	UCUCCAA
ENSG00000082641	UGAGAAG
ENSG00000082641	UGUGCUU
ENSG00000082684	ACUGGAU
ENSG00000082684	AGCACCA
ENSG00000082684	AGCAGCA
ENSG00000082684	CAGCAGA
ENSG00000082684	GGGGCAG
ENSG00000082684	UCCUGAG
ENSG00000082701	CCAGUGU
ENSG00000082781	UUGGUUC
ENSG00000082805	CACCAGC
ENSG00000082805	CAGCAGG
ENSG00000082805	GACCAUG
ENSG00000082805	GAUUGGU
ENSG00000082805	GGAAUGU
ENSG00000082805	GGUGGGC
ENSG00000082805	GUUAAUG
ENSG00000082898	AAAGCUG
ENSG00000082898	AAAUUUC
ENSG00000082898	AAGUAGC
ENSG00000082898	AUACAAG
ENSG00000082898	AUGCACC
ENSG00000082898	CACUCCU
ENSG00000082898	CUCAAAC
ENSG00000082898	GAAAACA
ENSG00000082898	GAGUUGA
ENSG00000082898	GAGUUGG
ENSG00000082898	GAUAUGU
ENSG00000082898	GUUAAUG
ENSG00000082898	UACAAAG
ENSG00000082898	UCACAGG
ENSG00000082898	UCACAGU
ENSG00000082898	UGAGGAC
ENSG00000082898	UUGGUCC
ENSG00000083099	AAGUGCA
ENSG00000083099	AGUGCUU
ENSG00000083099	CAAAUGC
ENSG00000083099	CAGGUGA
ENSG00000083099	GAGGCUG
ENSG00000083099	UCCCUCU
ENSG00000083099	UUGUGAC
ENSG00000083099	UUUGCAA
ENSG00000083123	CUGGAAA
ENSG00000083123	UCUGGAA
ENSG00000083123	UGGCCAC
ENSG00000083168	AGCUGCC
ENSG00000083168	CAGACAG
ENSG00000083168	CCUGCUG
ENSG00000083168	CCUUCUG

ENSG00000083168	GCUGGUG
ENSG00000083168	UCAACAG
ENSG00000083168	UCACACA
ENSG00000083168	UCUGGAG
ENSG00000083168	UGAAAUG
ENSG00000083290	AAGGCAC
ENSG00000083290	AAUACUG
ENSG00000083290	GUGCCUG
ENSG00000083307	AAAGUGC
ENSG00000083307	AAGUGCU
ENSG00000083307	AGUGCUG
ENSG00000083307	AGUGCUU
ENSG00000083307	CAAAGUG
ENSG00000083307	CCUGUAC
ENSG00000083307	CUUGUAU
ENSG00000083307	GAGAUGA
ENSG00000083307	GAGGGGC
ENSG00000083307	GAGGUAU
ENSG00000083307	GGCAGUG
ENSG00000083307	GUAAACA
ENSG00000083307	GUAACAG
ENSG00000083307	UAUAAAG
ENSG00000083307	UUGGCAA
ENSG00000083312	AACACUG
ENSG00000083312	AAUCACU
ENSG00000083312	ACCAGCA
ENSG00000083312	ACUGCAG
ENSG00000083312	AGCAGCG
ENSG00000083312	AGCUUAU
ENSG00000083312	AGGGUUG
ENSG00000083312	AGUGCAA
ENSG00000083312	AUAUAAC
ENSG00000083312	AUCAUAC
ENSG00000083312	AUGUGCC
ENSG00000083312	CAGUGCA
ENSG00000083312	CAUGGGU
ENSG00000083312	CCUUCAU
ENSG00000083312	CUGCAGC
ENSG00000083312	CUUCACC
ENSG00000083312	GAGGCAG
ENSG00000083312	GAUUGUA
ENSG00000083312	GCAGAAG
ENSG00000083312	GCAGCAU
ENSG00000083312	GCAGGUU
ENSG00000083312	GGAAUGU
ENSG00000083312	GGUUUAC
ENSG00000083312	GUAAACA
ENSG00000083312	GUAACAG
ENSG00000083312	GUGGCCC
ENSG00000083312	UCAAGUA
ENSG00000083312	UCCCUGU
ENSG00000083312	UCCUGAG
ENSG00000083312	UGAUCCA
ENSG00000083312	UGCAGCU
ENSG00000083312	UGUCUUU
ENSG00000083444	GGCAGUG
ENSG00000083444	UAAUAGU
ENSG00000083520	AAAAAUC
ENSG00000083520	AAAGUAA
ENSG00000083520	CCCUGAG
ENSG00000083520	GUCACUC
ENSG00000083635	CAGCAAA
ENSG00000083642	AAAACUG
ENSG00000083642	ACUGCAU

ENSG00000083642	CACAGUG
ENSG00000083642	CAGUAGA
ENSG00000083642	UUGGUUC
ENSG00000083799	AAAACUG
ENSG00000083799	AAAGAGG
ENSG00000083799	AACAUUC
ENSG00000083799	AAUUAUU
ENSG00000083799	ACAUUCA
ENSG00000083799	AGCAGCA
ENSG00000083799	CAAAACU
ENSG00000083799	CACCACC
ENSG00000083799	CUACAAA
ENSG00000083799	CUCAAAC
ENSG00000083799	GAAAACA
ENSG00000083799	GCAGAAG
ENSG00000083799	GCUUCCU
ENSG00000083799	GGAAGCC
ENSG00000083799	UAGACUG
ENSG00000083799	UCUCAAG
ENSG00000083799	UGCCAGC
ENSG00000083799	UUGGAGG
ENSG00000083799	UUGGCAA
ENSG00000083799	UUGGUCC
ENSG00000083799	UUGGUUC
ENSG00000083844	GAGUCUU
ENSG00000083896	AAAGAGG
ENSG00000083896	AAAUCCC
ENSG00000083896	AACACUG
ENSG00000083896	AAGUUGU
ENSG00000083896	ACACACU
ENSG00000083896	ACUGCAG
ENSG00000083896	AGCAGCG
ENSG00000083896	AGCAGCU
ENSG00000083896	AGUGCUU
ENSG00000083896	CAGUGCA
ENSG00000083896	CCUUCUG
ENSG00000083896	GAGGCUG
ENSG00000083896	GCAACUU
ENSG00000083896	GCAGCAU
ENSG00000083896	GCUACAU
ENSG00000083896	GCUUCCU
ENSG00000083896	GGACUGC
ENSG00000083896	GGCAGUG
ENSG00000083896	GUGCAAA
ENSG00000083896	UUGGAGG
ENSG00000083937	AAGGCAC
ENSG00000083937	AGGCAGG
ENSG00000083937	AUGGCUU
ENSG00000083937	CUUUGGU
ENSG00000084070	AAAGCUG
ENSG00000084070	ACAGGUG
ENSG00000084070	CUUUGGU
ENSG00000084070	GAGGUAG
ENSG00000084070	GCUGGUG
ENSG00000084073	AACACUG
ENSG00000084073	AAGGCAC
ENSG00000084073	GAAGGAA
ENSG00000084073	UUGGCAA
ENSG00000084090	AAAAAUC
ENSG00000084090	AAGGCAC
ENSG00000084090	CAAGAGC
ENSG00000084090	GGAAUGU
ENSG00000084090	UUGGCAC
ENSG00000084112	AAGUCUU

ENSG00000084112	GAGGUAG
ENSG00000084112	UGUGCUU
ENSG00000084112	UUCUUGU
ENSG00000084234	AAAGAAU
ENSG00000084234	AAUACUG
ENSG00000084234	ACAGUAC
ENSG00000084234	ACAGUAU
ENSG00000084234	ACAUUCA
ENSG00000084234	AGUACUG
ENSG00000084234	AUAUUAU
ENSG00000084234	AUCAUAC
ENSG00000084234	CAGUAGU
ENSG00000084234	GGUUACC
ENSG00000084234	UCACAGG
ENSG00000084234	UGCAUAG
ENSG00000084444	ACAAUAU
ENSG00000084444	CCCUGAG
ENSG00000084444	GAGGUAG
ENSG00000084463	CCAGUGC
ENSG00000084463	UACAAAG
ENSG00000084628	AGCACCA
ENSG00000084628	CAAAGCA
ENSG00000084628	CUCUGGG
ENSG00000084628	GAAAACA
ENSG00000084628	GGAGGCA
ENSG00000084628	GUGUGGC
ENSG00000084628	UAUAAAG
ENSG00000084628	UAUUGCU
ENSG00000084636	AACAUUC
ENSG00000084636	AGCACCA
ENSG00000084636	UCACAAG
ENSG00000084652	AAGUGCU
ENSG00000084652	ACAUCAC
ENSG00000084652	AGCAGCU
ENSG00000084652	AGGCAGG
ENSG00000084652	AGUGCCU
ENSG00000084652	AUCCUUU
ENSG00000084652	CAGCAGG
ENSG00000084652	CCCCCAG
ENSG00000084652	CCCCUGG
ENSG00000084652	GAGGUAG
ENSG00000084652	GCCUGAU
ENSG00000084652	GCCUGGA
ENSG00000084652	GCCUGGG
ENSG00000084652	GGAGGAG
ENSG00000084652	GGUUUAC
ENSG00000084652	GUGCCUG
ENSG00000084652	UCAUUUG
ENSG00000084652	UGAGAAG
ENSG00000084676	AAAAGUA
ENSG00000084676	AAACUGU
ENSG00000084676	AAAGUAA
ENSG00000084676	AAUCUCA
ENSG00000084676	AAUUAUU
ENSG00000084676	ACAAUAU
ENSG00000084676	ACACACC
ENSG00000084676	ACACACU
ENSG00000084676	AGCAUUC
ENSG00000084676	AGCCCUU
ENSG00000084676	AGCUGCC
ENSG00000084676	AUAUAAC
ENSG00000084676	AUGGCUU
ENSG00000084676	AUUGCAC
ENSG00000084676	CACAGUG

ENSG00000084676	CCUUCAU
ENSG00000084676	GAUUGUA
ENSG00000084676	GAUUGUC
ENSG00000084676	GCUGGUG
ENSG00000084676	GGCAGUG
ENSG00000084676	GGCUCAG
ENSG00000084676	GGCUGCG
ENSG00000084676	GGUAGAC
ENSG00000084676	UAUUGCU
ENSG00000084676	UCACAGU
ENSG00000084676	UCUCAAG
ENSG00000084676	UCUGGAA
ENSG00000084676	UGCAUUG
ENSG00000084676	UGGCUGC
ENSG00000084676	UGUAUAA
ENSG00000084676	UGUAUGU
ENSG00000084676	UUCUUGU
ENSG00000084693	AGCUGCC
ENSG00000084693	AGGGAGG
ENSG00000084693	UAGGGCC
ENSG00000084693	UUCCUCG
ENSG00000084731	AGGCAGG
ENSG00000084731	CUGGGGG
ENSG00000084731	GCCCUGU
ENSG00000084731	GCCUGGG
ENSG00000084731	GUAAACA
ENSG00000084731	UGUGCUU
ENSG00000084733	AGUGCUU
ENSG00000084733	AGUGGUU
ENSG00000084733	CACUGUU
ENSG00000084733	CUCUUCC
ENSG00000084733	GCUAGCA
ENSG00000084754	AAGGCAC
ENSG00000084764	AAAAAUC
ENSG00000084764	AAAAGUA
ENSG00000084764	AACGGAA
ENSG00000084764	AAGUGCA
ENSG00000084764	ACCACAG
ENSG00000084764	AGUGCAA
ENSG00000084764	CAAAGCA
ENSG00000084764	CCAGUAC
ENSG00000084774	AGCAGCU
ENSG00000084774	CAGCUGG
ENSG00000084774	CUGCCCC
ENSG00000084774	UCCUUGC
ENSG00000085063	CUGGCUC
ENSG00000085063	GCCUGGA
ENSG00000085063	GGCUCUG
ENSG00000085185	AAACUGU
ENSG00000085185	AAGGCGC
ENSG00000085185	ACAAUAU
ENSG00000085185	AGCACCA
ENSG00000085185	CAGUCUG
ENSG00000085185	GAGAUGA
ENSG00000085185	GCUCGGU
ENSG00000085185	UAAUGCU
ENSG00000085185	UAUUGCU
ENSG00000085185	UCACAGU
ENSG00000085185	UUCUGCA
ENSG00000085185	UUCUUGU
ENSG00000085185	UUGGUCC
ENSG00000085185	UUGUUCG
ENSG00000085224	AAAAACC
ENSG00000085224	AAAAAUC

ENSG00000085224	AAACUGU
ENSG00000085224	AACUACU
ENSG00000085224	AACUCUA
ENSG00000085224	AAGUCUU
ENSG00000085224	AAUACUG
ENSG00000085224	ACAUCAC
ENSG00000085224	ACAUUAC
ENSG00000085224	AGCAAAA
ENSG00000085224	AGUGCAA
ENSG00000085224	AUACAAG
ENSG00000085224	AUAUUAU
ENSG00000085224	CAAAGUG
ENSG00000085224	CAGCAGA
ENSG00000085224	CAGCAGG
ENSG00000085224	CAGCUGG
ENSG00000085224	CAGUAGU
ENSG00000085224	CUAGGCU
ENSG00000085224	CUUCACC
ENSG00000085224	GAGAAGA
ENSG00000085224	GCAACUU
ENSG00000085224	GCUGGAU
ENSG00000085224	GGACUGC
ENSG00000085224	GGCAUUG
ENSG00000085224	GUCAUUG
ENSG00000085224	UACAAAG
ENSG00000085224	UACAGUU
ENSG00000085224	UCACAGG
ENSG00000085224	UCACAGU
ENSG00000085224	UCCAGUU
ENSG00000085224	UGACAUC
ENSG00000085224	UGAGAAG
ENSG00000085224	UGAGGAC
ENSG00000085224	UGCCAGC
ENSG00000085224	UGGACUG
ENSG00000085224	UGUGCUU
ENSG00000085224	UUGGUCC
ENSG00000085365	ACAAUAU
ENSG00000085365	ACAGUAC
ENSG00000085365	ACAGUAU
ENSG00000085365	AGCUGCC
ENSG00000085365	CAAAACU
ENSG00000085365	CACCACC
ENSG00000085365	CCAGUAC
ENSG00000085365	GGCAGCG
ENSG00000085365	GUAAACA
ENSG00000085365	GUUAAUG
ENSG00000085365	UCAAGUA
ENSG00000085365	UGUCUUU
ENSG00000085377	AGCCCUU
ENSG00000085382	AAAUGGU
ENSG00000085382	AUAUAAC
ENSG00000085382	AUAUUAU
ENSG00000085382	CAGUAGA
ENSG00000085433	AGCAGCA
ENSG00000085433	UGUGCUU
ENSG00000085433	UUGGCAA
ENSG00000085449	AAUUUUA
ENSG00000085449	AGCACCA
ENSG00000085449	GCAGAAG
ENSG00000085449	UGCUCAC
ENSG00000085491	GAGGUAG
ENSG00000085511	AGCAGCA
ENSG00000085511	AUGGCAC
ENSG00000085644	CAAAGUG

ENSG00000085721	AUUGCAC
ENSG00000085721	CUCUUCC
ENSG00000085733	AAAGUAU
ENSG00000085733	AUAUUAU
ENSG00000085733	GCUACAU
ENSG00000085733	GUAGUGU
ENSG00000085741	GGUGGGC
ENSG00000085831	ACAUCAC
ENSG00000085831	ACAUUCA
ENSG00000085831	AGGAGCU
ENSG00000085831	AGGCAGG
ENSG00000085831	GGCAGGG
ENSG00000085831	GUAAACA
ENSG00000085831	GUCCAGA
ENSG00000085831	UCACAGU
ENSG00000085831	UUCUAAU
ENSG00000085831	UUCUGCA
ENSG00000085832	AAAACUG
ENSG00000085832	AACAAAC
ENSG00000085832	AAUACUG
ENSG00000085832	AAUCUCA
ENSG00000085832	AAUUUUA
ENSG00000085832	ACAUUCA
ENSG00000085832	AGCACCA
ENSG00000085832	AGCCCUU
ENSG00000085832	AGGUAGU
ENSG00000085832	CAGGAAC
ENSG00000085832	CCAGUGC
ENSG00000085832	CCUUCAU
ENSG00000085832	GUAUUCU
ENSG00000085832	GUCAACA
ENSG00000085832	UUCAAAU
ENSG00000085832	UUGGUCC
ENSG00000085872	UCACAGG
ENSG00000085872	UCACAGU
ENSG00000085978	AAAGUAU
ENSG00000085978	AUAAAGU
ENSG00000085978	GAGGUAG
ENSG00000085978	GAGGUAU
ENSG00000085978	GUAGUGU
ENSG00000085978	UGCCCUG
ENSG00000085978	UUGGCAA
ENSG00000085998	ACAGUAC
ENSG00000085998	CCAGUGU
ENSG00000086062	AAAAAUC
ENSG00000086062	CCCUGAG
ENSG00000086062	CUACAAA
ENSG00000086062	GACCCUG
ENSG00000086062	UCAAGUA
ENSG00000086102	ACACACU
ENSG00000086102	CUGGACU
ENSG00000086102	UCACAGU
ENSG00000086189	UGUGCGG
ENSG00000086200	AAAGUAA
ENSG00000086200	ACUGUAG
ENSG00000086200	CUUUGGU
ENSG00000086200	GAGGUAU
ENSG00000086200	GGAAGAC
ENSG00000086200	GGGCCCC
ENSG00000086200	UACAAAG
ENSG00000086289	AAAGUAA
ENSG00000086289	AGGAGCU
ENSG00000086289	GUGCCAC
ENSG00000086289	UAGGGCC

ENSG00000086300	AGUAACA
ENSG00000086300	UUCCAUA
ENSG00000086475	AAUCACU
ENSG00000086475	AAUGUUG
ENSG00000086475	ACAAUAU
ENSG00000086475	AGCUGCC
ENSG00000086475	GGCAAGA
ENSG00000086475	UUGGCAA
ENSG00000086506	ACUCAAA
ENSG00000086544	CACAGUG
ENSG00000086544	CUGCAGC
ENSG00000086544	CUGCAGG
ENSG00000086544	UUGGUUC
ENSG00000086570	AAAGUGC
ENSG00000086570	AGGGUUG
ENSG00000086570	CACUCCU
ENSG00000086570	CAGACAG
ENSG00000086570	CUCUUCC
ENSG00000086570	CUUGUGU
ENSG00000086570	GCUACAU
ENSG00000086570	GGCAGUG
ENSG00000086570	GUGCCUG
ENSG00000086570	UUGUGUC
ENSG00000086589	AAAAAUC
ENSG00000086589	AGAUGUG
ENSG00000086589	CUACAAA
ENSG00000086589	GUUAAUG
ENSG00000086619	UCAAGUA
ENSG00000086619	UGCAUAU
ENSG00000086666	AUAAUAC
ENSG00000086712	AGUAGUU
ENSG00000086712	CACCACC
ENSG00000086712	CAGUAGU
ENSG00000086712	GGUAGAC
ENSG00000086712	GUGCAAA
ENSG00000086730	UAAUGCU
ENSG00000086758	AUAUAAC
ENSG00000086991	AUUGCAC
ENSG00000086991	UCAACAG
ENSG00000087053	AAAGUGC
ENSG00000087053	ACAGUAU
ENSG00000087053	AGCUGCC
ENSG00000087053	AUAAAGU
ENSG00000087053	AUGGCUU
ENSG00000087053	CUACAGU
ENSG00000087053	GAUUGUA
ENSG00000087053	GGCUCUG
ENSG00000087053	GUCAGUU
ENSG00000087053	UACAGUU
ENSG00000087053	UUUGCAA
ENSG00000087074	CUACAAA
ENSG00000087086	AGCUGCC
ENSG00000087095	AAAGAGC
ENSG00000087095	AAAGUAA
ENSG00000087095	AAAUCCC
ENSG00000087095	AAGUGUG
ENSG00000087095	AAGUUGU
ENSG00000087095	AAUGUUG
ENSG00000087095	AAUUAUU
ENSG00000087095	ACAGUAU
ENSG00000087095	ACAUUCA
ENSG00000087095	AGUGUGC
ENSG00000087095	AUACAAG
ENSG00000087095	AUGCACC

ENSG00000087095	AUGUAAU
ENSG00000087095	CAAAGUG
ENSG00000087095	CACCACC
ENSG00000087095	CAGGUGA
ENSG00000087095	CAGUAGA
ENSG00000087095	CAGUGCA
ENSG00000087095	CCAGGAG
ENSG00000087095	CUAUACA
ENSG00000087095	CUCUGGG
ENSG00000087095	GAGGUAG
ENSG00000087095	GAUUGGU
ENSG00000087095	GCUACAU
ENSG00000087095	GGCUCAG
ENSG00000087095	GUAAACA
ENSG00000087095	GUCCAGA
ENSG00000087095	GUGCAAA
ENSG00000087095	UACAAAG
ENSG00000087095	UACAGUU
ENSG00000087095	UCACAUU
ENSG00000087095	UCAUACA
ENSG00000087095	UGAAGUG
ENSG00000087095	UGUAUAA
ENSG00000087095	UUCAAGC
ENSG00000087111	GGCUCAG
ENSG00000087152	AAUCUCA
ENSG00000087152	AAUGCCC
ENSG00000087152	ACCACAG
ENSG00000087152	ACCAGCA
ENSG00000087152	ACCCAUU
ENSG00000087152	AGCAGCA
ENSG00000087152	AGCCUGC
ENSG00000087152	AGGGAGG
ENSG00000087152	AUCCCUU
ENSG00000087152	AUGGAUU
ENSG00000087152	CACCAGC
ENSG00000087152	CAGACAG
ENSG00000087152	CAGCAGA
ENSG00000087152	CCAGCAU
ENSG00000087152	CCAGUAC
ENSG00000087152	CCCCCAG
ENSG00000087152	CCCCUGG
ENSG00000087152	CCUGUAC
ENSG00000087152	CUCUUCC
ENSG00000087152	CUGCCCC
ENSG00000087152	CUGCUCA
ENSG00000087152	CUGGGCA
ENSG00000087152	CUUGGGC
ENSG00000087152	GAGCCCU
ENSG00000087152	GAGCUGC
ENSG00000087152	GAGGCUG
ENSG00000087152	GCCUGGA
ENSG00000087152	GCCUGGG
ENSG00000087152	GCGGGUG
ENSG00000087152	GGAGAGA
ENSG00000087152	GGCAGUG
ENSG00000087152	GGCUCAG
ENSG00000087152	GGGAGCU
ENSG00000087152	GGGGAGC
ENSG00000087152	GUCCAGA
ENSG00000087152	GUGCCUG
ENSG00000087152	GUGGCCC
ENSG00000087152	UCACAUU
ENSG00000087152	UCCAGUU
ENSG00000087152	UCCCACC

ENSG00000087152	UCUCAAG
ENSG00000087152	UCUCACC
ENSG00000087152	UGAAGCU
ENSG00000087152	UGCUGAC
ENSG00000087152	UUCAAAU
ENSG00000087157	CUGCAGC
ENSG00000087157	GGCUCUG
ENSG00000087157	UGCAGCU
ENSG00000087245	AAGUGCA
ENSG00000087245	AGCACCA
ENSG00000087245	CAAAGCA
ENSG00000087245	CCAGUGC
ENSG00000087245	UGACAUC
ENSG00000087245	UGUCUUU
ENSG00000087258	AAGUCAC
ENSG00000087258	AAUUAUU
ENSG00000087258	ACAUUAC
ENSG00000087258	CUGCUCA
ENSG00000087258	CUUACAG
ENSG00000087258	UACAAAG
ENSG00000087258	UAUUACC
ENSG00000087258	UUGGCAA
ENSG00000087258	UUGGCAC
ENSG00000087263	CAAGAGC
ENSG00000087266	CCUGCUG
ENSG00000087266	GGCUCUG
ENSG00000087274	AAAGAAU
ENSG00000087274	AAUUUUA
ENSG00000087274	CUCUUCC
ENSG00000087274	GUCAUUG
ENSG00000087274	UACAAAG
ENSG00000087274	UCACAGU
ENSG00000087274	UGCAUAG
ENSG00000087274	UGCAUUG
ENSG00000087274	UUGUGAC
ENSG00000087299	AAGGCAC
ENSG00000087299	ACCUGGA
ENSG00000087299	CUUGUAU
ENSG00000087299	GAGGUAG
ENSG00000087299	GAGGUAU
ENSG00000087299	GGAUGAG
ENSG00000087299	GGUUUAC
ENSG00000087299	GUGAAUG
ENSG00000087299	UACAAAG
ENSG00000087299	UGAAGUG
ENSG00000087299	UGGAGAU
ENSG00000087299	UUCCAUA
ENSG00000087299	UUGGAGG
ENSG00000087302	AAAGAAU
ENSG00000087303	ACAGUAC
ENSG00000087303	ACAGUAU
ENSG00000087303	AGCACCA
ENSG00000087303	GAGGUAG
ENSG00000087303	UGUGCUU
ENSG00000087338	UUCAAAU
ENSG00000087338	UUCUUGU
ENSG00000087338	UUUCAAC
ENSG00000087365	AAGAACC
ENSG00000087365	UACAAAG
ENSG00000087448	AGCUGCC
ENSG00000087448	CUUUGGU
ENSG00000087448	GGCCCUG
ENSG00000087448	UUCAAGC
ENSG00000087448	UUGGUCC

ENSG00000087448	UUUCAAC
ENSG00000087470	AGCAGCU
ENSG00000087470	AUGCAUU
ENSG00000087470	UGAAAUG
ENSG00000087470	UUCUGCA
ENSG00000087495	CUUCUCU
ENSG00000087510	AAAGCUG
ENSG00000087510	UCAAGUA
ENSG00000087510	UUCAAGC
ENSG00000087586	UAUUGCU
ENSG00000087903	CAAAACU
ENSG00000087903	GUAAACA
ENSG00000087916	UACAAAG
ENSG00000088038	AAAACUG
ENSG00000088038	AUUCAUU
ENSG00000088038	GCUUCCU
ENSG00000088038	GGCAGGG
ENSG00000088038	GGGGAGC
ENSG00000088038	GGUUUAC
ENSG00000088179	AAGUGCA
ENSG00000088179	AGUGCAA
ENSG00000088179	AGUGCUG
ENSG00000088179	AUAAAGU
ENSG00000088247	AAAACUG
ENSG00000088247	AAAGUAA
ENSG00000088247	ACAUUCA
ENSG00000088247	AUUCAUU
ENSG00000088247	GGAAUGU
ENSG00000088247	UCACAGG
ENSG00000088247	UCACAGU
ENSG00000088247	UCACAUU
ENSG00000088247	UCAUUCG
ENSG00000088247	UUGUGAC
ENSG00000088305	AAAACUG
ENSG00000088305	AAACUGU
ENSG00000088305	AACUCUA
ENSG00000088305	AAGGCAC
ENSG00000088305	AAUACUG
ENSG00000088305	CCUGCUG
ENSG00000088305	UACAAAG
ENSG00000088305	UCAAGUA
ENSG00000088305	UCCUGAG
ENSG00000088305	UGAAAUG
ENSG00000088305	UGCAGCU
ENSG00000088305	UGUAUAA
ENSG00000088356	AGUGCCU
ENSG00000088356	AUUCAUU
ENSG00000088356	CAGCAGG
ENSG00000088356	CUUGUGU
ENSG00000088356	GGAAUGU
ENSG00000088356	UUGUGUC
ENSG00000088367	AAAAAUC
ENSG00000088367	AAAAGUA
ENSG00000088367	ACAGUAC
ENSG00000088367	ACAUCAC
ENSG00000088367	ACCAGCA
ENSG00000088367	ACUGUUU
ENSG00000088367	AGCAGCA
ENSG00000088367	AGUGCAA
ENSG00000088367	CACUGUU
ENSG00000088367	CAGCUGG
ENSG00000088367	CAGUGCA
ENSG00000088367	CCUUCUU
ENSG00000088367	CUCUGGG

ENSG00000088367	CUGGCUC
ENSG00000088367	CUUCUCU
ENSG00000088367	CUUGGAG
ENSG00000088367	GAGUAUU
ENSG00000088367	GCAGAAG
ENSG00000088367	GCAGGAC
ENSG00000088367	GUCUACU
ENSG00000088367	GUCUGCC
ENSG00000088367	GUCUUGC
ENSG00000088367	GUGACAG
ENSG00000088367	GUGGGGA
ENSG00000088367	UAAUAUC
ENSG00000088367	UAUAAUA
ENSG00000088367	UCACAGU
ENSG00000088367	UCCCUCU
ENSG00000088367	UGCAAAG
ENSG00000088367	UGCUCAC
ENSG00000088367	UGGGGGA
ENSG00000088367	UGUGCUU
ENSG00000088387	CAGCAGG
ENSG00000088387	UGUGCUU
ENSG00000088448	GCUACAU
ENSG00000088448	UGCAUAG
ENSG00000088451	GAGGUAG
ENSG00000088451	UAUAAAG
ENSG00000088538	AAAAACC
ENSG00000088538	AAAUCCC
ENSG00000088538	ACAAUAU
ENSG00000088538	ACAGUAC
ENSG00000088538	ACAGUAU
ENSG00000088538	ACCUGGA
ENSG00000088538	ACUGCAG
ENSG00000088538	AGGUAGU
ENSG00000088538	CAGCAAA
ENSG00000088538	CAGUAGA
ENSG00000088538	CCCUGAG
ENSG00000088538	CUGCAGG
ENSG00000088538	GAGGUAG
ENSG00000088538	GGCAGUG
ENSG00000088538	UACAAAG
ENSG00000088538	UGCUCAC
ENSG00000088543	AAGUGCU
ENSG00000088543	CUGGGGG
ENSG00000088543	UCCCUCU
ENSG00000088682	AAGACAU
ENSG00000088726	GAAGGAA
ENSG00000088726	GGUGGUC
ENSG00000088756	AGGUAGU
ENSG00000088756	CUGGAAA
ENSG00000088756	GAGGUAU
ENSG00000088756	GCACCAU
ENSG00000088756	GCAGAAG
ENSG00000088756	GUGAGGU
ENSG00000088756	UCCAGUU
ENSG00000088808	AGCACCA
ENSG00000088808	AUAUAAC
ENSG00000088808	CACCACC
ENSG00000088808	CUUUGGU
ENSG00000088808	GCACCAU
ENSG00000088808	GCUGUCU
ENSG00000088808	UCACAUU
ENSG00000088808	UGUCUGC
ENSG00000088808	UUGGCAA
ENSG00000088812	AAAAACC

ENSG00000088812	AACACUG
ENSG00000088812	AAGUGCA
ENSG00000088812	AAUUUUA
ENSG00000088812	ACUCCAG
ENSG00000088812	ACUGCAU
ENSG00000088812	ACUGUAG
ENSG00000088812	AGCACCA
ENSG00000088812	AGUAACA
ENSG00000088812	AGUGCAA
ENSG00000088812	AUGGCAC
ENSG00000088812	AUGUAAU
ENSG00000088812	CAGGCUC
ENSG00000088812	CCUGCUG
ENSG00000088812	CUAGGCU
ENSG00000088812	CUGCUCA
ENSG00000088812	GAAGGAA
ENSG00000088812	GACCAUG
ENSG00000088812	GAGGCUG
ENSG00000088812	GGAAGAC
ENSG00000088812	UAAGACG
ENSG00000088812	UAUGGUU
ENSG00000088812	UGCUGAC
ENSG00000088812	UGUAUAA
ENSG00000088812	UGUGCUU
ENSG00000088812	UUGUGUC
ENSG00000088812	UUUGCAA
ENSG00000088826	AGAUAAA
ENSG00000088827	CACCACC
ENSG00000088832	AAAACUG
ENSG00000088832	AAUCACU
ENSG00000088832	ACCAGCA
ENSG00000088832	AGCAGCA
ENSG00000088832	AUCACUA
ENSG00000088832	AUGGCUU
ENSG00000088832	CUACAAA
ENSG00000088832	GUAGUGU
ENSG00000088832	UGCAGCU
ENSG00000088833	AAAAACC
ENSG00000088833	CUGGACU
ENSG00000088833	GCAACUU
ENSG00000088833	GGACUGC
ENSG00000088833	UGGACUG
ENSG00000088833	UUUUUGC
ENSG00000088854	AAAAACC
ENSG00000088854	AAAAGUA
ENSG00000088854	AAGGCAC
ENSG00000088854	AAGUAGC
ENSG00000088854	ACAGUAC
ENSG00000088854	ACAGUAU
ENSG00000088854	ACUGCAG
ENSG00000088854	ACUGUGU
ENSG00000088854	AUGUGCC
ENSG00000088854	AUUGCAC
ENSG00000088854	CAGCAAA
ENSG00000088854	CAGUAGU
ENSG00000088854	CAUAUUG
ENSG00000088854	CUACAGU
ENSG00000088854	GAGGUAG
ENSG00000088854	GAGGUAU
ENSG00000088854	GGCACGG
ENSG00000088854	GUGCUUG
ENSG00000088854	UACAAAG
ENSG00000088854	UACAGUU
ENSG00000088854	UCCCUUG

ENSG00000088854	UGAAGCU
ENSG00000088854	UGAGAAG
ENSG00000088854	UGUGCUU
ENSG00000088854	UUGAGGC
ENSG00000088854	UUGGCAA
ENSG00000088854	UUGGUCC
ENSG00000088876	UCCCACA
ENSG00000088881	AGUGCUG
ENSG00000088881	CAAGAGC
ENSG00000088881	CACAGUG
ENSG00000088881	CCCUGAG
ENSG00000088881	CUGGGCA
ENSG00000088881	UGUGUGG
ENSG00000088986	GUCUCUG
ENSG00000088992	AAAAACC
ENSG00000088992	AGAUAAA
ENSG00000089006	GAGGUAU
ENSG00000089041	AAAGAAU
ENSG00000089041	UCCCUUU
ENSG00000089050	AAAAAUC
ENSG00000089057	AAAAACC
ENSG00000089057	AAAACUG
ENSG00000089057	AAACUGU
ENSG00000089057	AAAGAAU
ENSG00000089057	AAAGUAA
ENSG00000089057	AAAGUAC
ENSG00000089057	AACACUG
ENSG00000089057	AACAGUC
ENSG00000089057	AAGAACC
ENSG00000089057	AAGUUGU
ENSG00000089057	AAUACUG
ENSG00000089057	ACAAUAU
ENSG00000089057	ACACACC
ENSG00000089057	ACACACU
ENSG00000089057	ACAGUAC
ENSG00000089057	ACUAGAU
ENSG00000089057	ACUGCAG
ENSG00000089057	ACUGCAU
ENSG00000089057	ACUGGCC
ENSG00000089057	ACUGGCU
ENSG00000089057	ACUGUUU
ENSG00000089057	AUAAAGU
ENSG00000089057	AUACAAG
ENSG00000089057	AUUCAUU
ENSG00000089057	CAGCAAA
ENSG00000089057	CAGUAGU
ENSG00000089057	CCAGUAC
ENSG00000089057	CCCUGAG
ENSG00000089057	CCUUCUU
ENSG00000089057	CUACAGU
ENSG00000089057	CUCAAAC
ENSG00000089057	CUGCUCA
ENSG00000089057	CUGGAAA
ENSG00000089057	CUGGGCA
ENSG00000089057	CUUCACC
ENSG00000089057	GACCAUG
ENSG00000089057	GAGAAAU
ENSG00000089057	GAGGCUG
ENSG00000089057	GCAUCCC
ENSG00000089057	GGAAUGU
ENSG00000089057	GGGUAAG
ENSG00000089057	GUAUUCU
ENSG00000089057	UCACAAG
ENSG00000089057	UCCCUCU

ENSG00000089057	UCCCUGU
ENSG00000089091	AAUUAUU
ENSG00000089116	CUUUGGU
ENSG00000089123	AGCAGCA
ENSG00000089154	CAAAGCA
ENSG00000089154	UUUCAAC
ENSG00000089159	AACAGUC
ENSG00000089159	AAUCUCU
ENSG00000089159	CACAGUG
ENSG00000089159	CAGUAGU
ENSG00000089159	CCAGUGU
ENSG00000089159	GCUGGAU
ENSG00000089159	GGCAUUG
ENSG00000089159	GGGGUGC
ENSG00000089159	UAUUGCU
ENSG00000089159	UCACAGU
ENSG00000089159	UCCAGUU
ENSG00000089159	UCCUGAG
ENSG00000089159	UGUGCUU
ENSG00000089163	UCCCUGU
ENSG00000089177	AAGGCAC
ENSG00000089177	CAAAGCA
ENSG00000089177	GCAGAAG
ENSG00000089177	UAAUAUC
ENSG00000089177	UCAUUUG
ENSG00000089177	UCCUGAG
ENSG00000089195	CAUAUUG
ENSG00000089195	UAUUGCU
ENSG00000089220	UUCAAAU
ENSG00000089225	AAGAACC
ENSG00000089225	CCCUGUU
ENSG00000089225	GAAACAU
ENSG00000089225	GAGGUAG
ENSG00000089250	AUGCACC
ENSG00000089250	GAGGCAG
ENSG00000089250	GCCCUUA
ENSG00000089280	UACAAAG
ENSG00000089280	UCCAGUU
ENSG00000089280	UGGGUAC
ENSG00000089289	AUGAUGA
ENSG00000089289	UUGACAC
ENSG00000089356	GAGAUGA
ENSG00000089486	CAAAGCA
ENSG00000089486	CACAGGG
ENSG00000089486	CACAGUG
ENSG00000089486	CUACAAA
ENSG00000089486	GAAGGAA
ENSG00000089486	GAGAAAU
ENSG00000089486	GGAAGCC
ENSG00000089486	GGAGUCC
ENSG00000089486	GGCAAGA
ENSG00000089486	GGCAGUG
ENSG00000089486	GUGACAG
ENSG00000089486	UCACAGG
ENSG00000089486	UCACAGU
ENSG00000089486	UUGGUCC
ENSG00000089558	CAGACAG
ENSG00000089558	GACCAUG
ENSG00000089558	GCAGAAG
ENSG00000089558	GCCUGGG
ENSG00000089558	GUCCAGA
ENSG00000089597	AGCAGCU
ENSG00000089597	CAAAACU
ENSG00000089597	CAGGUGA

ENSG00000089597	CCAGUGU
ENSG00000089597	GGGGUGG
ENSG00000089682	UCCUGAG
ENSG00000089685	UGAAAUG
ENSG00000089692	UGCUGAC
ENSG00000089693	CCCUGAG
ENSG00000089693	UAAUAUC
ENSG00000089693	UUCCAUA
ENSG00000089723	AGCACCA
ENSG00000089723	CAGCAGA
ENSG00000089723	CUGGCUC
ENSG00000089818	AGCUGCC
ENSG00000089818	AGUGAUC
ENSG00000089818	CAAAUGC
ENSG00000089818	GAGAAAU
ENSG00000089818	GAUUGUC
ENSG00000089818	GCACCAU
ENSG00000089818	UAUUGCU
ENSG00000089818	UCACAGG
ENSG00000089902	AAAAGUA
ENSG00000089902	AAAGUGC
ENSG00000089902	AAGGCAC
ENSG00000089902	AAGGUGC
ENSG00000089902	AAUUAUU
ENSG00000089902	ACAAGGU
ENSG00000089902	ACAUCAC
ENSG00000089902	ACUGUUU
ENSG00000089902	AGCACCA
ENSG00000089902	AGUAACA
ENSG00000089902	AUAUAAC
ENSG00000089902	AUGGCUU
ENSG00000089902	CAGUAGU
ENSG00000089902	CUUGGAG
ENSG00000089902	CUUUGGU
ENSG00000089902	GAAAACA
ENSG00000089902	GCAGAAG
ENSG00000089902	GGAGGAG
ENSG00000089902	GUAAACA
ENSG00000089902	GUGCUUG
ENSG00000089902	UAUAAUA
ENSG00000089902	UCAAGUA
ENSG00000089902	UCACAUU
ENSG00000089902	UGCAUAG
ENSG00000089902	UGGAUAA
ENSG00000089902	UUCUGCA
ENSG00000089902	UUUUUGC
ENSG00000089916	UUGGCAC
ENSG00000090020	AGCAGCU
ENSG00000090020	AGCUGCC
ENSG00000090020	GGUUGUC
ENSG00000090020	GUGCAAA
ENSG00000090020	UUGGUUC
ENSG00000090054	AAGGCAC
ENSG00000090054	AAUUAUU
ENSG00000090054	AAUUUUA
ENSG00000090054	AGCAGCA
ENSG00000090054	UCACAAG
ENSG00000090060	AAAAGUA
ENSG00000090060	AACCUGU
ENSG00000090060	AAGUCAC
ENSG00000090060	AAGUGCA
ENSG00000090060	ACAAGGU
ENSG00000090060	ACCUGGA
ENSG00000090060	ACUCCAG

ENSG00000090060	CAAAGCA
ENSG00000090060	CCCUGAG
ENSG00000090060	CUGGACU
ENSG00000090060	GACUUCC
ENSG00000090060	GGAGUGU
ENSG00000090060	UACAAAG
ENSG00000090061	AAAGUAA
ENSG00000090061	ACAGUAU
ENSG00000090061	CACAGUG
ENSG00000090061	CCAGUGC
ENSG00000090061	CCUGCUG
ENSG00000090061	UCACACA
ENSG00000090061	UCUAGAG
ENSG00000090061	UUUGCAA
ENSG00000090097	AAAACUG
ENSG00000090097	AAACUGU
ENSG00000090097	AGCAGCA
ENSG00000090097	GAGAACU
ENSG00000090097	GGAGUCC
ENSG00000090097	UCCAGUU
ENSG00000090097	UGAGAAU
ENSG00000090104	UCACAGU
ENSG00000090238	CAGUGCA
ENSG00000090238	UCACAGU
ENSG00000090269	UAUGGUU
ENSG00000090316	AAAGUAC
ENSG00000090316	ACAUUCA
ENSG00000090316	CUUUGGU
ENSG00000090316	GAGUAUU
ENSG00000090316	UACAAAG
ENSG00000090316	UUCAAGC
ENSG00000090372	AGCACCA
ENSG00000090372	CACCACC
ENSG00000090372	CAGGAAC
ENSG00000090372	CCUUCUG
ENSG00000090372	GAGGCUG
ENSG00000090372	GGGUUUA
ENSG00000090372	UAUAAAG
ENSG00000090432	CCCUGAG
ENSG00000090447	AAAAAUC
ENSG00000090447	AAGGCAC
ENSG00000090447	AAGUGCA
ENSG00000090447	ACAGUAC
ENSG00000090447	AGUGCUU
ENSG00000090447	AUAAUAC
ENSG00000090447	UACAGUU
ENSG00000090447	UAUAAUA
ENSG00000090470	CAAAACU
ENSG00000090470	GUCACUC
ENSG00000090520	AGCACCA
ENSG00000090530	AACAUUC
ENSG00000090530	AACCUGG
ENSG00000090530	GUUAAUG
ENSG00000090539	AGCCAGU
ENSG00000090539	CUCAGGG
ENSG00000090539	CUGCUCA
ENSG00000090565	AAAAAUC
ENSG00000090565	AAAGUAU
ENSG00000090565	AAUCUCU
ENSG00000090565	UACAGUU
ENSG00000090565	UUGAUCA
ENSG00000090581	AGCAUUC
ENSG00000090615	AAAGCUG
ENSG00000090615	AAAGUAA

ENSG00000090615	AAAGUUU
ENSG00000090615	ACUCAAA
ENSG00000090615	ACUGUUU
ENSG00000090615	CAGACAG
ENSG00000090615	CCUGUAC
ENSG00000090615	UAAUGCU
ENSG00000090615	UAUAAUA
ENSG00000090615	UAUUGCU
ENSG00000090615	UCUCCAA
ENSG00000090615	UGGAGAU
ENSG00000090615	UUCAAAU
ENSG00000090615	UUCAAGC
ENSG00000090615	UUCUAAU
ENSG00000090615	UUUCAAC
ENSG00000090659	AGUGGUU
ENSG00000090659	GCAACUU
ENSG00000090659	GCUGGAU
ENSG00000090659	GUGAAUG
ENSG00000090661	CCCUGUC
ENSG00000090686	AAGGCAC
ENSG00000090686	AGUGCAA
ENSG00000090686	CUCUUCC
ENSG00000090686	GAAACAU
ENSG00000090686	UGAAAGG
ENSG00000090686	UGACAUC
ENSG00000090686	UUGACAC
ENSG00000090776	AAAAACC
ENSG00000090776	AAAGUGC
ENSG00000090776	AAGGCAC
ENSG00000090776	AAGUGCA
ENSG00000090776	ACAUUAC
ENSG00000090776	ACCUGGA
ENSG00000090776	AGCAGCU
ENSG00000090776	AGGCAGG
ENSG00000090776	AGGGAGG
ENSG00000090776	AUACAAG
ENSG00000090776	CAAAGUG
ENSG00000090776	CAAGAGC
ENSG00000090776	CAGCUGG
ENSG00000090776	CCAGGAG
ENSG00000090776	CCCACCU
ENSG00000090776	CUACAAA
ENSG00000090776	CUCCCAC
ENSG00000090776	CUCUGGG
ENSG00000090776	CUCUUCC
ENSG00000090776	CUGCAGC
ENSG00000090776	CUGCAGG
ENSG00000090776	CUGGGCA
ENSG00000090776	GAGGCAG
ENSG00000090776	GCUUCCU
ENSG00000090776	GGAGGCA
ENSG00000090776	GGCAAGA
ENSG00000090776	GGCAGGG
ENSG00000090776	GGGGGAA
ENSG00000090776	UAAGACG
ENSG00000090776	UAAGACU
ENSG00000090776	UACAAAG
ENSG00000090776	UAUAAAG
ENSG00000090776	UCCCACC
ENSG00000090776	UUCUAAU
ENSG00000090857	AGCAAAA
ENSG00000090857	AGCAGCA
ENSG00000090857	CCCUGAG
ENSG00000090857	GAGGUAU

ENSG00000090857	GCUUCCU
ENSG00000090857	GGCAAGA
ENSG00000090857	GUGAGGU
ENSG00000090889	AACAUUC
ENSG00000090889	CUGCUCA
ENSG00000090905	AAAGUGC
ENSG00000090905	AAGUGCA
ENSG00000090905	AAGUGCU
ENSG00000090905	ACAGUAU
ENSG00000090905	AGUGCAA
ENSG00000090905	AUACAAG
ENSG00000090905	CUCCAGC
ENSG00000090905	GGAAGAC
ENSG00000090905	GUGCAAA
ENSG00000090905	UCCUGAG
ENSG00000090905	UGCAAAG
ENSG00000090905	UGCAUAG
ENSG00000090905	UGGCCAC
ENSG00000090905	UUGGCAA
ENSG00000090905	UUGGCAC
ENSG00000090924	AACAAAC
ENSG00000090971	CAGUGCA
ENSG00000090975	AGCACCA
ENSG00000090975	AGGCAGG
ENSG00000090975	AGUGCAA
ENSG00000090975	AGUGCUG
ENSG00000090975	AUUGCAC
ENSG00000090975	CACAGUG
ENSG00000090975	CAGUUAC
ENSG00000090975	CUUUGGU
ENSG00000090975	GAAACAU
ENSG00000090975	GAGGGGC
ENSG00000090975	GAUUGUA
ENSG00000090975	GAUUGUC
ENSG00000090975	GCACCAU
ENSG00000090975	GCUACAU
ENSG00000090975	GGGUGUU
ENSG00000090975	GUGCAAA
ENSG00000090975	UAUUGCU
ENSG00000090975	UCACACA
ENSG00000090975	UCACAGU
ENSG00000090975	UUGGUCC
ENSG00000090975	UUGGUUC
ENSG00000091009	AACAUUC
ENSG00000091009	AAUUAUU
ENSG00000091009	ACUCAAA
ENSG00000091009	GUAGUGU
ENSG00000091009	UAAUACA
ENSG00000091009	UCCCUCU
ENSG00000091039	ACAAUAU
ENSG00000091039	AUGGCAC
ENSG00000091039	CAAAACU
ENSG00000091039	CACUGUU
ENSG00000091039	CAGUCCA
ENSG00000091039	GGAAUGU
ENSG00000091039	UAUUGCU
ENSG00000091039	UCAUUUG
ENSG00000091039	UCCUAUA
ENSG00000091039	UCCUGAG
ENSG00000091073	AUUGCAC
ENSG00000091073	GAGGUAU
ENSG00000091073	UAUUGCU
ENSG00000091073	UGCAAAG
ENSG00000091127	AAGGCAC

ENSG00000091128	CUCCAGC
ENSG00000091129	AACACUG
ENSG00000091129	AAGACAU
ENSG00000091129	AAGGCAC
ENSG00000091129	CAGCAAA
ENSG00000091129	GCCCUGU
ENSG00000091137	AACAAAC
ENSG00000091137	UCAAGUA
ENSG00000091137	UUCAAGC
ENSG00000091140	AAAGUUU
ENSG00000091140	CAGUCCA
ENSG00000091140	GAAACAU
ENSG00000091140	UAAGACU
ENSG00000091140	UUCUAAU
ENSG00000091140	UUGAUCA
ENSG00000091157	AGUGCUG
ENSG00000091157	GAGGCAG
ENSG00000091157	UCACAGU
ENSG00000091164	CCAGUGU
ENSG00000091164	GUCUCUG
ENSG00000091317	CUUUGGU
ENSG00000091317	GAAACAU
ENSG00000091317	GAGAAAU
ENSG00000091317	GCAGAAG
ENSG00000091409	ACAUUCA
ENSG00000091409	ACUGUAG
ENSG00000091409	AGCACCA
ENSG00000091409	CAGUAGA
ENSG00000091409	CAGUAGU
ENSG00000091482	AUAUAAC
ENSG00000091527	AACACUG
ENSG00000091527	AAGUUGU
ENSG00000091527	ACUGGCU
ENSG00000091527	AGCAGCA
ENSG00000091527	AGGUAGU
ENSG00000091527	CAGCAAA
ENSG00000091527	CAGUAGA
ENSG00000091527	CCAGCAU
ENSG00000091527	CCAGUAC
ENSG00000091527	GAGGUAU
ENSG00000091527	GCAGCAU
ENSG00000091527	GCCUGAU
ENSG00000091527	GGAUUUC
ENSG00000091527	UCACAAG
ENSG00000091527	UGAGAAU
ENSG00000091527	UGAUCCA
ENSG00000091527	UUGGCAA
ENSG00000091527	UUGGCAC
ENSG00000091542	AAGUGCU
ENSG00000091542	ACUGGCC
ENSG00000091542	ACUUGGA
ENSG00000091542	AGCAAAA
ENSG00000091542	AGUGCUG
ENSG00000091542	AUAUUAU
ENSG00000091542	CUACAAA
ENSG00000091542	CUCUAGC
ENSG00000091542	GCAACUU
ENSG00000091542	UUCCAUA
ENSG00000091542	UUGGUUC
ENSG00000091640	AGCAGCA
ENSG00000091640	AGCAGCG
ENSG00000091640	AGCAGCU
ENSG00000091640	AUAAUAC
ENSG00000091640	UAAUACA

ENSG00000091640	UCACACA
ENSG00000091651	GGAAGCC
ENSG00000091656	AAAGCUG
ENSG00000091656	AACAGUC
ENSG00000091656	AAGUCAC
ENSG00000091656	AAGUGCA
ENSG00000091656	AAGUGCU
ENSG00000091656	AAUACUG
ENSG00000091656	AAUGUUG
ENSG00000091656	AAUUAUU
ENSG00000091656	AAUUUUA
ENSG00000091656	ACAAUAU
ENSG00000091656	ACAUUAC
ENSG00000091656	ACAUUCA
ENSG00000091656	ACUGCAG
ENSG00000091656	ACUGUAG
ENSG00000091656	AGCAGCA
ENSG00000091656	AGCAGCG
ENSG00000091656	AGCAUUC
ENSG00000091656	AGUAACA
ENSG00000091656	AUAAAGU
ENSG00000091656	AUAAUAC
ENSG00000091656	AUACAAG
ENSG00000091656	AUAUAAC
ENSG00000091656	AUCCCUU
ENSG00000091656	AUGACAC
ENSG00000091656	AUGUAAU
ENSG00000091656	AUUGCAC
ENSG00000091656	CAAAGCA
ENSG00000091656	CAAAUGC
ENSG00000091656	CACACCU
ENSG00000091656	CACAGGG
ENSG00000091656	CACAGUG
ENSG00000091656	CACUGUU
ENSG00000091656	CAGCAAA
ENSG00000091656	CAUAUUG
ENSG00000091656	CCAGCAU
ENSG00000091656	CCAGGAG
ENSG00000091656	CCUUCUG
ENSG00000091656	CUCACAC
ENSG00000091656	CUCAGGG
ENSG00000091656	CUUACAG
ENSG00000091656	CUUCACC
ENSG00000091656	CUUUGGU
ENSG00000091656	GACAACU
ENSG00000091656	GACCUGG
ENSG00000091656	GAGGCUG
ENSG00000091656	GAGUUGG
ENSG00000091656	GCACCAU
ENSG00000091656	GGAAUGU
ENSG00000091656	GGAGGCA
ENSG00000091656	GGCAAGA
ENSG00000091656	GGCAGUG
ENSG00000091656	GGCUGCG
ENSG00000091656	GGUCUAG
ENSG00000091656	GGUUUAC
ENSG00000091656	GUAACAG
ENSG00000091656	GUGACAG
ENSG00000091656	GUUAGGA
ENSG00000091656	GUUCAUG
ENSG00000091656	UACAAAG
ENSG00000091656	UAUAUGA
ENSG00000091656	UCACAGU
ENSG00000091656	UCAUGAU

ENSG00000091656	UCCAGAG
ENSG00000091656	UCCAGUU
ENSG00000091656	UCCCUCU
ENSG00000091656	UCCCUUU
ENSG00000091656	UCCUGAG
ENSG00000091656	UCUCAAG
ENSG00000091656	UGAAAUG
ENSG00000091656	UGAGAAU
ENSG00000091656	UGCAUAU
ENSG00000091656	UGCAUUG
ENSG00000091656	UGGUACC
ENSG00000091656	UUAGAGA
ENSG00000091656	UUAUGCA
ENSG00000091656	UUCAAGC
ENSG00000091656	UUCCUAG
ENSG00000091656	UUGGAGG
ENSG00000091656	UUGGCAC
ENSG00000091656	UUGGUUC
ENSG00000091656	UUGUGAC
ENSG00000091656	UUGUUCG
ENSG00000091656	UUUGCAA
ENSG00000091656	UUUGCAC
ENSG00000091656	UUUUUGC
ENSG00000091664	CAAAGCA
ENSG00000091831	AAAGAGG
ENSG00000091831	AAGAACC
ENSG00000091831	ACAUUCA
ENSG00000091831	ACCCAUU
ENSG00000091831	ACUGUGU
ENSG00000091831	AGCAAAA
ENSG00000091831	AGUGCUG
ENSG00000091831	AUACAAG
ENSG00000091831	AUAUAAC
ENSG00000091831	AUAUUAU
ENSG00000091831	CAAAGCA
ENSG00000091831	CAGUGCA
ENSG00000091831	CUUGUAU
ENSG00000091831	GAGAAAU
ENSG00000091831	GAUAUGU
ENSG00000091831	GAUUGUC
ENSG00000091831	GCCCUGU
ENSG00000091831	GGAGUGU
ENSG00000091831	GGCAGCG
ENSG00000091831	GGUUUAC
ENSG00000091831	UCCAGUU
ENSG00000091831	UCCCUUU
ENSG00000091831	UCCUAUA
ENSG00000091831	UGAAAUG
ENSG00000091831	UGCCCUG
ENSG00000091831	UGUCUGC
ENSG00000091831	UGUCUGG
ENSG00000091844	CACAGUG
ENSG00000091844	CAGGAAC
ENSG00000091844	UCACAGG
ENSG00000091879	AAGUCAC
ENSG00000091879	GUGACAG
ENSG00000091879	UUGUGAC
ENSG00000091972	AGUAGAC
ENSG00000091972	AUAUAAC
ENSG00000091986	AAAGUUU
ENSG00000091986	AGGCAGG
ENSG00000092020	AGCAUUC
ENSG00000092051	AGCCCUU
ENSG00000092051	AGGCAGG

ENSG00000092051	AGGGAGG
ENSG00000092051	AUGUAAU
ENSG00000092051	CACUGUU
ENSG00000092051	CCUUCAU
ENSG00000092051	CUGACUC
ENSG00000092051	GGAGGCA
ENSG00000092051	GUCCAGA
ENSG00000092051	UAAUAUC
ENSG00000092051	UCUAGAG
ENSG00000092068	AAAAGUA
ENSG00000092068	AAAGUAA
ENSG00000092068	AAGGCAC
ENSG00000092068	AAUUUUA
ENSG00000092068	ACAUCAC
ENSG00000092068	ACCAGCA
ENSG00000092068	CUUUGGU
ENSG00000092068	GGCGGGG
ENSG00000092068	UACAAAG
ENSG00000092068	UAUUACC
ENSG00000092068	UGGGUAC
ENSG00000092068	UUCCAUA
ENSG00000092068	UUCUGCA
ENSG00000092068	UUGGCAA
ENSG00000092068	UUGGCAC
ENSG00000092068	UUGGUCC
ENSG00000092096	AGGGAGG
ENSG00000092096	GAAACAU
ENSG00000092096	GGAGGAG
ENSG00000092098	GCUGGAU
ENSG00000092098	UCCUGAG
ENSG00000092108	AAAAAUC
ENSG00000092148	AACACUG
ENSG00000092148	AAGUGUG
ENSG00000092148	ACUGUAG
ENSG00000092148	AGCAAAA
ENSG00000092148	AGCAGCA
ENSG00000092148	AGCAGCG
ENSG00000092148	AGUGCAA
ENSG00000092148	AGUGUGC
ENSG00000092148	AUAAUAC
ENSG00000092148	AUACAAG
ENSG00000092148	CAGCAAA
ENSG00000092199	AAAGAAU
ENSG00000092199	AAAGAGC
ENSG00000092199	AAAGCUG
ENSG00000092199	AAGUCAC
ENSG00000092199	ACAGUAU
ENSG00000092199	AUCCUUU
ENSG00000092199	CAGGCUC
ENSG00000092199	GGUUUAC
ENSG00000092199	GUAACAG
ENSG00000092199	UAAGACG
ENSG00000092199	UAGAGGA
ENSG00000092199	UCACAAG
ENSG00000092199	UGGAGAU
ENSG00000092199	UGUUGAA
ENSG00000092199	UUCUAAU
ENSG00000092199	UUGAUCA
ENSG00000092199	UUGGAGG
ENSG00000092201	AAAGCUG
ENSG00000092201	AAGUCAC
ENSG00000092201	ACAGGUG
ENSG00000092201	AGCAGCA
ENSG00000092201	GAUUGGU

ENSG00000092201	GCCUGGA
ENSG00000092201	GGUGGGC
ENSG00000092201	UACAGUU
ENSG00000092201	UUGGUCC
ENSG00000092203	ACUGGCU
ENSG00000092203	CAGCAAA
ENSG00000092203	CAGCAGA
ENSG00000092203	CUAGGCU
ENSG00000092203	CUUCACC
ENSG00000092203	CUUGGAG
ENSG00000092203	CUUUGGU
ENSG00000092203	GGCCCUG
ENSG00000092203	UGGCCCU
ENSG00000092208	AGCACCA
ENSG00000092345	GAUUGGU
ENSG00000092345	UUCAAAU
ENSG00000092421	AACAGUC
ENSG00000092421	AAGUCUU
ENSG00000092421	AAUUAUU
ENSG00000092421	ACAUCAC
ENSG00000092421	ACAUUAC
ENSG00000092421	AGCCUGC
ENSG00000092421	AUGACAC
ENSG00000092421	AUGGAUU
ENSG00000092421	CACAAUG
ENSG00000092421	CACAGUG
ENSG00000092421	CAGACAG
ENSG00000092421	CAGCAGG
ENSG00000092421	CAGUCCA
ENSG00000092421	CCAGCAU
ENSG00000092421	CCAGUGU
ENSG00000092421	CUCACAC
ENSG00000092421	CUUCACC
ENSG00000092421	GCAGCAU
ENSG00000092421	GCUGGUG
ENSG00000092421	GCUGUCU
ENSG00000092421	GGCAUUG
ENSG00000092421	GGCUCAG
ENSG00000092421	GUAACAG
ENSG00000092421	GUUAAUG
ENSG00000092421	GUUAGGA
ENSG00000092421	UAUUACC
ENSG00000092421	UCACACA
ENSG00000092421	UGAAAUG
ENSG00000092421	UGACAUC
ENSG00000092421	UGGAGAU
ENSG00000092421	UUAUGCA
ENSG00000092421	UUGGCAC
ENSG00000092439	AUGGCUU
ENSG00000092445	AAUUUUA
ENSG00000092445	ACCACAG
ENSG00000092445	CAGACAG
ENSG00000092445	UGCCAGC
ENSG00000092445	UGGGACA
ENSG00000092529	AUGCCUU
ENSG00000092529	CAGACAG
ENSG00000092529	GAGGUAG
ENSG00000092529	GAGGUAU
ENSG00000092529	UCCUGAG
ENSG00000092531	CUUUGGU
ENSG00000092531	GAGGUAG
ENSG00000092531	GAGGUAU
ENSG00000092531	UUGGCAC
ENSG00000092607	AUUCAUU

ENSG00000092607	CGUGCAU
ENSG00000092607	CUACAAA
ENSG00000092607	UAAUAUC
ENSG00000092607	UAUUGCU
ENSG00000092607	UGCAUUG
ENSG00000092607	UGGAGAU
ENSG00000092607	UGGCUGC
ENSG00000092607	UGUGCUU
ENSG00000092607	UUGGCAC
ENSG00000092758	GAGGUAG
ENSG00000092758	GAGGUAU
ENSG00000092847	AAAGGUA
ENSG00000092847	AAGGCAC
ENSG00000092847	AAGUGCU
ENSG00000092847	ACCCAUU
ENSG00000092847	AGAUGUG
ENSG00000092847	AGCAAAA
ENSG00000092847	AGCAGCA
ENSG00000092847	AGCAGCU
ENSG00000092847	AGGACAC
ENSG00000092847	AGUAGAC
ENSG00000092847	AGUGCCG
ENSG00000092847	AUGGAUU
ENSG00000092847	CACCAGC
ENSG00000092847	CAGCAAA
ENSG00000092847	CAGCAGA
ENSG00000092847	CAGCAGG
ENSG00000092847	CAGCUGG
ENSG00000092847	CCAGUGC
ENSG00000092847	CCAGUGU
ENSG00000092847	CUACAAA
ENSG00000092847	CUCCCAC
ENSG00000092847	CUGGCUC
ENSG00000092847	CUGGGGG
ENSG00000092847	CUUGUGU
ENSG00000092847	GAAACAU
ENSG00000092847	GAGCUGC
ENSG00000092847	GAGGUAG
ENSG00000092847	GAGGUAU
ENSG00000092847	GCAGCAU
ENSG00000092847	GCUUCCU
ENSG00000092847	GGAAGAC
ENSG00000092847	GGAAUGU
ENSG00000092847	GGAGAGA
ENSG00000092847	GGAGUGU
ENSG00000092847	GGAUUUC
ENSG00000092847	GGUGGUU
ENSG00000092847	GGUUACC
ENSG00000092847	GUAAACA
ENSG00000092847	GUAACAG
ENSG00000092847	GUGAAUG
ENSG00000092847	GUGAGGU
ENSG00000092847	GUGCAAA
ENSG00000092847	UAUAAAG
ENSG00000092847	UCCCUCU
ENSG00000092847	UGCAUAG
ENSG00000092847	UGCAUAU
ENSG00000092847	UGUGCUU
ENSG00000092847	UUCUGCA
ENSG00000092847	UUGGCAA
ENSG00000092847	UUGGCAC
ENSG00000092847	UUGGUCC
ENSG00000092847	UUUGCAA
ENSG00000092847	UUUGCAC

ENSG00000092871	AACAAAC
ENSG00000092871	AAUCUCA
ENSG00000092871	AGCUUAU
ENSG00000092871	AGUGGUU
ENSG00000092871	AUAAAGU
ENSG00000092871	CAGCAGA
ENSG00000092871	GUAACAG
ENSG00000092871	GUGCUUG
ENSG00000092871	UACAAAG
ENSG00000092871	UAUUACC
ENSG00000092871	UGCAUAG
ENSG00000092871	UGCAUAU
ENSG00000092871	UGUGCUU
ENSG00000092931	ACUCCAG
ENSG00000092931	GUAAACA
ENSG00000092931	UCCCUUU
ENSG00000092964	AAAUCCC
ENSG00000092964	ACACACC
ENSG00000092964	ACAUUCA
ENSG00000092964	ACUGCAG
ENSG00000092964	ACUGGCU
ENSG00000092964	AGCACCA
ENSG00000092964	AGCAUUC
ENSG00000092964	AGGAUGU
ENSG00000092964	AGUGGUU
ENSG00000092964	AUAUAAC
ENSG00000092964	AUAUUAU
ENSG00000092964	CACAGGG
ENSG00000092964	CAGCAAA
ENSG00000092964	CAGGCUC
ENSG00000092964	CAGGUGA
ENSG00000092964	GAAACAU
ENSG00000092964	GAGCUGC
ENSG00000092964	GCAGGUU
ENSG00000092964	GGAAGAC
ENSG00000092964	GGCUGCG
ENSG00000092964	GUCCAGA
ENSG00000092964	UCACAGG
ENSG00000092964	UCCAGUU
ENSG00000092964	UGGCUGC
ENSG00000092964	UUAGAGA
ENSG00000092964	UUAGGAU
ENSG00000092964	UUCAAAU
ENSG00000092978	GCACCAU
ENSG00000093000	AAUCACU
ENSG00000093000	ACCCUGU
ENSG00000093000	AGCAGCG
ENSG00000093000	AGCAGCU
ENSG00000093000	AUGGAUU
ENSG00000093000	CCCUGAG
ENSG00000093000	GACCCUG
ENSG00000093000	GGAAUGU
ENSG00000093000	UCUUGAG
ENSG00000093000	UUGGCAC
ENSG00000093072	GCCCUGU
ENSG00000093134	AUGUGCC
ENSG00000093134	CUUGUAU
ENSG00000093134	UUCCAUA
ENSG00000093167	AAGGCAC
ENSG00000093183	ACAUCAC
ENSG00000094796	UCCUUGC
ENSG00000094804	UCACAAG
ENSG00000094880	AAGUGCU
ENSG00000094880	AAUCUCA

ENSG00000094880	AAUCUCU
ENSG00000094880	AGCAGCA
ENSG00000094880	GCAGCAU
ENSG00000094963	CACAAUG
ENSG00000094963	GAGUCUU
ENSG00000094963	GGAUUUU
ENSG00000094963	GGCAUUG
ENSG00000094975	AAGGCAC
ENSG00000094975	AAGGUGC
ENSG00000094975	AAGUGCU
ENSG00000094975	CACAGUG
ENSG00000094975	UCACAGG
ENSG00000094975	UCACAUU
ENSG00000094975	UUGACAC
ENSG00000095015	AAGGCAC
ENSG00000095015	ACUGGCU
ENSG00000095015	CAGUCCA
ENSG00000095015	CUACAAA
ENSG00000095015	CUUUGGU
ENSG00000095015	GAGUCUU
ENSG00000095015	GGAAUGU
ENSG00000095015	GGCAAGA
ENSG00000095015	UAUAAAG
ENSG00000095015	UGAGAAU
ENSG00000095015	UGAGUCU
ENSG00000095066	UCACAUU
ENSG00000095139	AAAACUG
ENSG00000095139	AAAAGUA
ENSG00000095139	AAAGUAA
ENSG00000095139	CAAAACU
ENSG00000095139	CCCUGAG
ENSG00000095139	CCUGCUG
ENSG00000095139	CUUUGGU
ENSG00000095139	GUGAAUG
ENSG00000095139	UAUAAUA
ENSG00000095139	UAUCAGA
ENSG00000095139	UAUUACC
ENSG00000095139	UCCCUUG
ENSG00000095139	UCCUUGC
ENSG00000095139	UUCUUGU
ENSG00000095139	UUGGCAC
ENSG00000095203	AAGGCAC
ENSG00000095203	ACAUUCA
ENSG00000095203	AGCAGCA
ENSG00000095203	AGCAGCG
ENSG00000095203	AGCAGCU
ENSG00000095203	AGCUGCC
ENSG00000095203	AGGCAGG
ENSG00000095203	CAAGAGC
ENSG00000095203	GCUGGUG
ENSG00000095203	GGCAGGG
ENSG00000095203	UACAGUU
ENSG00000095203	UGCAAAG
ENSG00000095203	UGGAGAU
ENSG00000095203	UUGGCAC
ENSG00000095209	UCACAUU
ENSG00000095261	AAGGCAC
ENSG00000095303	AGCUGCC
ENSG00000095303	GACCCUG
ENSG00000095321	AAGGCAC
ENSG00000095370	AAAAGUA
ENSG00000095370	AAAGUUU
ENSG00000095397	AUAUAAC
ENSG00000095397	CUCUGGG

ENSG00000095485	AAAGAGG
ENSG00000095485	AACAGUC
ENSG00000095485	AAUGUUG
ENSG00000095485	AGGCAGG
ENSG00000095485	GAGGCAG
ENSG00000095485	GGCAGGG
ENSG00000095485	UCCCUUG
ENSG00000095485	UCCCUUU
ENSG00000095539	AAAGUGC
ENSG00000095539	AGGACAC
ENSG00000095539	AUGUGCC
ENSG00000095539	AUUCAUU
ENSG00000095539	CAGGCUC
ENSG00000095539	CCCACCU
ENSG00000095539	CUACAAA
ENSG00000095539	CUGCAGC
ENSG00000095539	CUGCCCC
ENSG00000095539	GAGCCCU
ENSG00000095539	GAGGCUG
ENSG00000095539	GAGGUAG
ENSG00000095539	GAGGUAU
ENSG00000095539	GAUUGUA
ENSG00000095539	GAUUGUC
ENSG00000095539	GCCCUUA
ENSG00000095539	GCCUGGA
ENSG00000095539	GGACCUG
ENSG00000095539	GGAGGAG
ENSG00000095539	GGCUCAG
ENSG00000095539	GUGAGGU
ENSG00000095539	GUGCCAC
ENSG00000095539	UGCAUAU
ENSG00000095564	CAGCAGG
ENSG00000095564	GGAAUGU
ENSG00000095587	AAAGCUG
ENSG00000095587	AAUGCCC
ENSG00000095587	ACCACAG
ENSG00000095587	AUGCAUU
ENSG00000095587	AUGUAAU
ENSG00000095587	CAAAACU
ENSG00000095587	GGGAGCU
ENSG00000095587	UUUGCAC
ENSG00000095627	GAGGCAG
ENSG00000095637	AAAUGGU
ENSG00000095637	AAUUAUU
ENSG00000095637	ACUGCAG
ENSG00000095637	AGAUAAA
ENSG00000095637	AUAAAGU
ENSG00000095637	AUAUUAU
ENSG00000095637	CUGACUC
ENSG00000095637	CUGGAAA
ENSG00000095637	CUUCUCU
ENSG00000095637	GAGCUAA
ENSG00000095637	GAGGCUG
ENSG00000095637	UACAGUU
ENSG00000095637	UCAACAG
ENSG00000095637	UCAUUUG
ENSG00000095637	UCCCUUG
ENSG00000095637	UCCUGAG
ENSG00000095637	UGGAGAU
ENSG00000095637	UGGGACA
ENSG00000095637	UGUAUAA
ENSG00000095637	UGUGCUU
ENSG00000095637	UUCAAAU
ENSG00000095739	AAGUGCU

ENSG00000095739	AGAUAAA
ENSG00000095739	AUAAAGU
ENSG00000095739	CAGCAGA
ENSG00000095739	CCAGCAU
ENSG00000095752	AAAAACC
ENSG00000095752	AAAGUAA
ENSG00000095752	UCACAUU
ENSG00000095787	AAAGUGC
ENSG00000095787	AAUCACU
ENSG00000095787	AAUUAUU
ENSG00000095787	AGUGCCU
ENSG00000095787	AUACAAG
ENSG00000095787	AUGGCUU
ENSG00000095787	CAGACAG
ENSG00000095787	CCCCUGG
ENSG00000095787	CUCCCAC
ENSG00000095787	GGUUUAC
ENSG00000095787	GUCUACU
ENSG00000095787	GUGCAAA
ENSG00000095787	UACAAAG
ENSG00000095787	UAGGUAU
ENSG00000095787	UGCAGCU
ENSG00000095794	AUCCUUU
ENSG00000095794	CAAAGCA
ENSG00000095794	CUACAAA
ENSG00000095794	GAGGUAG
ENSG00000095951	AAGUGUG
ENSG00000095951	AAUUAUU
ENSG00000095951	AUACAAG
ENSG00000095951	AUUGCAC
ENSG00000095951	GUAAACA
ENSG00000095951	GUGCUUG
ENSG00000096060	AAAGCUG
ENSG00000096060	AACACUG
ENSG00000096060	ACCCGUA
ENSG00000096060	ACUCCAG
ENSG00000096060	AGCAGCA
ENSG00000096060	CACUGUU
ENSG00000096060	CAGCAGG
ENSG00000096060	CUGGACU
ENSG00000096060	GAGUUGA
ENSG00000096060	GAGUUGG
ENSG00000096060	GCUUCCU
ENSG00000096060	GGAGUGU
ENSG00000096060	UCCAGAG
ENSG00000096060	UCCUGAG
ENSG00000096060	UGAAAUG
ENSG00000096063	AGCAGCA
ENSG00000096070	AAGUCAC
ENSG00000096070	ACAAUAU
ENSG00000096070	ACAGUAU
ENSG00000096070	ACCUGGA
ENSG00000096070	ACUGUGU
ENSG00000096070	AGCAGCU
ENSG00000096070	AGUGAUC
ENSG00000096070	AUAAAGU
ENSG00000096070	AUAUUAU
ENSG00000096070	CACAAUG
ENSG00000096070	CAGCAGA
ENSG00000096070	CAGCAGG
ENSG00000096070	CUACAAA
ENSG00000096070	CUUUGGU
ENSG00000096070	GGUGGUU
ENSG00000096070	GUGCUUG

ENSG00000096070	UCUAGAG
ENSG00000096070	UGUAUAA
ENSG00000096070	UGUGCUU
ENSG00000096070	UUGGCAA
ENSG00000096092	AAUACUG
ENSG00000096093	AAGAACC
ENSG00000096093	GAGCUGC
ENSG00000096264	AAUUUUA
ENSG00000096384	CUAUACA
ENSG00000096433	ACAUUAC
ENSG00000096433	AGUGCUU
ENSG00000096433	GUAGUGU
ENSG00000096696	AAAGUUU
ENSG00000096696	UAUUGCU
ENSG00000096696	UGCCAGC
ENSG00000096717	AAAGAGC
ENSG00000096717	AUGGCUU
ENSG00000096717	CACAGUG
ENSG00000096717	CCAGUGU
ENSG00000096717	UAAUGCU
ENSG00000096717	UCCCUUG
ENSG00000096717	UGCAUAU
ENSG00000096717	UUGGUCC
ENSG00000096746	AAAAAUC
ENSG00000096746	AAAAGUA
ENSG00000096746	AAAGUUU
ENSG00000096746	AACAUUC
ENSG00000096746	AAGUCAC
ENSG00000096746	CAAAGUG
ENSG00000096746	CCUUCAU
ENSG00000096746	CUAGUAA
ENSG00000096746	CUCCCAA
ENSG00000096746	CUGUUGA
ENSG00000096746	UCACAUU
ENSG00000096746	UCCCACA
ENSG00000096968	UUCAAAU
ENSG00000097007	AAAGAGG
ENSG00000097007	AGGUAGU
ENSG00000097007	UGAAAUG
ENSG00000097021	CUUUGGU
ENSG00000097021	UGGCCAC
ENSG00000097033	AAUUAUU
ENSG00000097033	ACUGCAG
ENSG00000097033	AGCACCA
ENSG00000097033	AUAUAAC
ENSG00000097033	CACAAUG
ENSG00000097033	CUUUGGU
ENSG00000097033	GUAGUGU
ENSG00000097033	UAGUGAG
ENSG00000097033	UGUCUUU
ENSG00000097046	GCACCAU
ENSG00000097096	AAGUGCU
ENSG00000097096	AGCAGCG
ENSG00000097096	GUUAAUG
ENSG00000097096	UGCAUUG
ENSG00000099194	AAAAAUC
ENSG00000099194	AAAACUG
ENSG00000099194	AACAAAC
ENSG00000099194	AAGGCAC
ENSG00000099194	ACUGGAU
ENSG00000099194	ACUGUAG
ENSG00000099194	AGCAUUC
ENSG00000099194	AGUAGUU
ENSG00000099194	CAAAACU

ENSG00000099194	CAGUAGA
ENSG00000099194	CCCACCG
ENSG00000099194	CCCACCU
ENSG00000099194	CCCCUGG
ENSG00000099194	CCCUGAG
ENSG00000099194	CUUACAG
ENSG00000099194	GACCCUG
ENSG00000099194	GAGCUAA
ENSG00000099194	GAGGCAG
ENSG00000099194	GAGGUAU
ENSG00000099194	GGAAUGU
ENSG00000099194	UCUCAAG
ENSG00000099194	UGCCAGC
ENSG00000099194	UUCAAAU
ENSG00000099203	GACCAUG
ENSG00000099204	AGCCCUU
ENSG00000099204	GGCAAGA
ENSG00000099204	GGCAGUG
ENSG00000099204	UUGGCAA
ENSG00000099219	AGUAACA
ENSG00000099219	CAGUGCA
ENSG00000099219	GCUUCCU
ENSG00000099219	UCAAGUA
ENSG00000099219	UUCAAGC
ENSG00000099246	ACUGUGU
ENSG00000099246	AUGUGCC
ENSG00000099250	AAGGCAC
ENSG00000099250	ACAUUCA
ENSG00000099250	AGCCAGU
ENSG00000099250	AGCCCUU
ENSG00000099250	AGUAGUU
ENSG00000099250	AGUGCAA
ENSG00000099250	CCAGUGC
ENSG00000099250	GAGAAAU
ENSG00000099250	GUUCAUG
ENSG00000099250	UAUUGCU
ENSG00000099250	UCACAAG
ENSG00000099250	UCCUGAG
ENSG00000099250	UGAAGCU
ENSG00000099250	UGAGAAU
ENSG00000099250	UUGGAGG
ENSG00000099282	AAAAAUC
ENSG00000099282	AAGGCAC
ENSG00000099290	GCAGAAG
ENSG00000099308	AAAGUGC
ENSG00000099308	CAAAGUG
ENSG00000099308	GUAAACA
ENSG00000099308	UACAAAG
ENSG00000099308	UAUAAAG
ENSG00000099308	UCACACA
ENSG00000099308	UGGGACA
ENSG00000099331	UACAAAG
ENSG00000099331	UUGGUCC
ENSG00000099364	AACACUG
ENSG00000099364	AGCAAAA
ENSG00000099364	AGCUGCC
ENSG00000099364	AGUAACA
ENSG00000099364	AUACAAG
ENSG00000099364	AUGUGGG
ENSG00000099364	CAGUCCA
ENSG00000099364	CAGUGCA
ENSG00000099364	CCAGGAG
ENSG00000099364	CCAGUAC
ENSG00000099364	CCCUGUU

ENSG00000099364	CUCCCAC
ENSG00000099364	CUGGGCA
ENSG00000099364	CUUUGGU
ENSG00000099364	GAGGUAG
ENSG00000099364	GAGGUAU
ENSG00000099364	GGAAGAC
ENSG00000099364	GGAGGAG
ENSG00000099364	GGAGUCC
ENSG00000099364	GGCUCAG
ENSG00000099364	GGCUGCG
ENSG00000099364	UCCCACC
ENSG00000099364	UCCCUGU
ENSG00000099364	UUCCAUA
ENSG00000099364	UUGGUCC
ENSG00000099365	AUGUGCC
ENSG00000099365	CCUGCUG
ENSG00000099365	GCCUGGA
ENSG00000099365	GGGGGAA
ENSG00000099365	UAUUGCU
ENSG00000099377	GGACCUG
ENSG00000099381	AAAAAUC
ENSG00000099381	AAUCACU
ENSG00000099381	ACAAUAU
ENSG00000099381	AUAAAGU
ENSG00000099381	UAUAAAG
ENSG00000099622	AAGAACC
ENSG00000099622	ACAAUAU
ENSG00000099622	CAGGCUC
ENSG00000099622	UCAGCGG
ENSG00000099622	UCUUGAG
ENSG00000099625	AGCCUGC
ENSG00000099625	CAGGCUC
ENSG00000099625	CUCUAGC
ENSG00000099715	CAAAGCA
ENSG00000099715	CCUGCUG
ENSG00000099715	CUGGGCA
ENSG00000099715	UUGGCAC
ENSG00000099785	AUAAAGU
ENSG00000099785	AUUCAUU
ENSG00000099785	UAUAAAG
ENSG00000099800	GGCUCAG
ENSG00000099804	AACAAAC
ENSG00000099804	AGGUAGU
ENSG00000099804	GAGGUAU
ENSG00000099814	UCCUGAG
ENSG00000099822	ACAUUCA
ENSG00000099822	AUGGCUU
ENSG00000099822	GGGGUGG
ENSG00000099860	UCUCCAA
ENSG00000099864	AGCACCA
ENSG00000099875	AGCAAAA
ENSG00000099875	CCCUGAG
ENSG00000099875	GAGCCCU
ENSG00000099875	GAUUGUC
ENSG00000099875	UCCUGAG
ENSG00000099875	UGCAUAG
ENSG00000099889	AGCACCA
ENSG00000099889	CAGCAGG
ENSG00000099889	CUUACAG
ENSG00000099889	UACAAAG
ENSG00000099901	UCCCUUG
ENSG00000099904	AAAAACC
ENSG00000099904	CAAGAGC
ENSG00000099904	CAGCUGG

ENSG00000099904	CUCCCAC
ENSG00000099904	GGCAGGG
ENSG00000099904	GGGGCAG
ENSG00000099904	GUGCUUG
ENSG00000099904	GUUAGGA
ENSG00000099904	UCCAGUU
ENSG00000099904	UCCCACA
ENSG00000099904	UGAAAGG
ENSG00000099904	UGUGCUU
ENSG00000099917	AAAUUUC
ENSG00000099917	AGAUAAA
ENSG00000099917	AGUGCAA
ENSG00000099917	CAGGCUC
ENSG00000099917	CCCUGAG
ENSG00000099917	GCAGAAG
ENSG00000099940	AAGGCAC
ENSG00000099940	UGCAAAG
ENSG00000099940	UUUGCAA
ENSG00000099942	AAAGCUG
ENSG00000099942	AACUCUA
ENSG00000099942	AAGUCAC
ENSG00000099942	ACAGGUG
ENSG00000099942	ACUGUUU
ENSG00000099942	AGCAGCA
ENSG00000099942	AUAUUAU
ENSG00000099942	AUCCCUU
ENSG00000099942	CAAGAGC
ENSG00000099942	CACAGUG
ENSG00000099942	CACUGUU
ENSG00000099942	CAGCAGG
ENSG00000099942	CAGUUAC
ENSG00000099942	CUUCUCU
ENSG00000099942	GAGAAGA
ENSG00000099942	GCUACAU
ENSG00000099942	GCUUCCU
ENSG00000099942	GUAAACA
ENSG00000099942	GUGCUUG
ENSG00000099942	UAAUACA
ENSG00000099942	UAUAAAG
ENSG00000099942	UAUUGCU
ENSG00000099942	UCCCACA
ENSG00000099942	UCCCACC
ENSG00000099942	UGAGAAG
ENSG00000099942	UGUGCUU
ENSG00000099942	UUGGCAA
ENSG00000099942	UUGGCAC
ENSG00000099949	UCCCUGU
ENSG00000099953	AUGGCUU
ENSG00000099953	CAGGUGA
ENSG00000099953	GAGGUAG
ENSG00000099953	GAGGUAU
ENSG00000099953	GUAUGUU
ENSG00000099954	AAAGUUU
ENSG00000099954	ACAAUAU
ENSG00000099954	ACAUUCA
ENSG00000099954	AUACCUC
ENSG00000099954	CUGCUCA
ENSG00000099954	GAUUGUC
ENSG00000099956	AUACAAG
ENSG00000099956	CUAUACA
ENSG00000099956	GGAAUGU
ENSG00000099957	GGGGUGG
ENSG00000099968	AAAGUAA
ENSG00000099968	AAAGUAC

ENSG00000099968	ACAAGGU
ENSG00000099968	GCAGCAU
ENSG00000099968	UAUUGCU
ENSG00000099985	AAGUGCA
ENSG00000099985	ACAAUAU
ENSG00000099985	CGUGGCC
ENSG00000099985	GGAGGAG
ENSG00000099985	UAUAAAG
ENSG00000099985	UGUGUCA
ENSG00000099994	GGGUGUU
ENSG00000099995	AGGAGCU
ENSG00000099995	CAGGCUC
ENSG00000099995	GAGCCCU
ENSG00000099995	UCAUUCG
ENSG00000099998	CUCUGGG
ENSG00000099998	CUGGGCA
ENSG00000100003	AAUUUUA
ENSG00000100003	GUAAACA
ENSG00000100003	UCCCUGU
ENSG00000100003	UUCCUAG
ENSG00000100003	UUGGCAC
ENSG00000100012	GUCUCUG
ENSG00000100023	AAAGCUG
ENSG00000100027	AAAGUGC
ENSG00000100027	CAAAGUG
ENSG00000100027	CCAGUGC
ENSG00000100027	CCAGUGU
ENSG00000100027	CUCACAC
ENSG00000100027	CUCUAGC
ENSG00000100027	GCAGAAG
ENSG00000100027	UCAAGUA
ENSG00000100027	UCACACA
ENSG00000100027	UUGGUUC
ENSG00000100028	CAAAGCA
ENSG00000100028	CUCAAAC
ENSG00000100028	UCUCAAG
ENSG00000100030	AAAGCUG
ENSG00000100030	AAAGUAC
ENSG00000100030	AAAUUUC
ENSG00000100030	AACGGCU
ENSG00000100030	AAGGCAC
ENSG00000100030	AAGUGCA
ENSG00000100030	AAUUAUU
ENSG00000100030	AAUUUUA
ENSG00000100030	ACAAUAU
ENSG00000100030	ACAGUAC
ENSG00000100030	ACUUGGA
ENSG00000100030	AGCAAAA
ENSG00000100030	AGCAUUC
ENSG00000100030	AGUGCAA
ENSG00000100030	AGUGCUG
ENSG00000100030	AGUGCUU
ENSG00000100030	AGUGGUU
ENSG00000100030	AUAUAAC
ENSG00000100030	CAAAACU
ENSG00000100030	CACAGGG
ENSG00000100030	CAGGUGA
ENSG00000100030	CUAUACA
ENSG00000100030	GAGAAAU
ENSG00000100030	GAGAUGA
ENSG00000100030	GAGCCCU
ENSG00000100030	GCUAGCA
ENSG00000100030	GGAAUGU
ENSG00000100030	GGAGGCA

ENSG00000100030	GUCACUC
ENSG00000100030	GUGCAAA
ENSG00000100030	GUUCAUG
ENSG00000100030	UAAUACA
ENSG00000100030	UCACCAC
ENSG00000100030	UCCCUUG
ENSG00000100030	UCCCUUU
ENSG00000100030	UGAAAGG
ENSG00000100030	UGCUGAC
ENSG00000100030	UGGACUG
ENSG00000100030	UGGAUAA
ENSG00000100030	UUCCUAG
ENSG00000100034	AAGGCAC
ENSG00000100034	ACUGCAG
ENSG00000100034	ACUGCAU
ENSG00000100034	CACCACC
ENSG00000100034	CUGGAAA
ENSG00000100034	CUGGCUC
ENSG00000100034	GGCAGGG
ENSG00000100034	GGUUUAC
ENSG00000100034	UAUAUGA
ENSG00000100034	UUGACAC
ENSG00000100034	UUGGCAC
ENSG00000100036	AGCAGCU
ENSG00000100036	AGGCAGG
ENSG00000100036	CCAGUGC
ENSG00000100036	CCAGUGU
ENSG00000100036	CCCCCAG
ENSG00000100036	GCCUGGA
ENSG00000100036	UGAGGAC
ENSG00000100036	UUGAGGC
ENSG00000100036	UUGGCAC
ENSG00000100056	AAGGCAC
ENSG00000100056	AAGUAGC
ENSG00000100056	ACAGGUG
ENSG00000100056	ACUCAGC
ENSG00000100056	CAGGCUC
ENSG00000100060	CCUUCAU
ENSG00000100060	UGGCUGC
ENSG00000100065	AGCAGCA
ENSG00000100065	GGCUCAG
ENSG00000100065	GUGCAAA
ENSG00000100065	UGCAGCU
ENSG00000100068	AGAUGUG
ENSG00000100068	CAGUAGA
ENSG00000100068	CCAGUAC
ENSG00000100068	GCAGGUU
ENSG00000100068	UGGCCAC
ENSG00000100075	AAGGCAC
ENSG00000100075	ACCCUGU
ENSG00000100075	GAGGCAG
ENSG00000100075	GGUGGUC
ENSG00000100075	UCCUGAG
ENSG00000100077	AAGUGCA
ENSG00000100077	CAGCAGA
ENSG00000100077	CAGUAGA
ENSG00000100077	CCAGUGU
ENSG00000100077	CUCCAUU
ENSG00000100077	GAGGUAG
ENSG00000100077	UAUAAAG
ENSG00000100077	UGUGCUU
ENSG00000100078	ACCCAUU
ENSG00000100078	CAGCAGG
ENSG00000100084	AAAAAUC

ENSG00000100084	AAUUAUU
ENSG00000100084	AGCAAAA
ENSG00000100084	CUAGUAA
ENSG00000100084	CUAGUAG
ENSG00000100084	GUUCAUG
ENSG00000100084	UCAUUUG
ENSG00000100084	UUCCUAG
ENSG00000100095	CUCUGGG
ENSG00000100095	GUCUCUG
ENSG00000100100	AAAAGUA
ENSG00000100100	AAGGCAC
ENSG00000100100	AUAUAAC
ENSG00000100100	GAGGUAG
ENSG00000100100	GGAAGCC
ENSG00000100101	GGAAGCC
ENSG00000100105	AAAACUG
ENSG00000100105	AAAGAGC
ENSG00000100105	AAUCUCU
ENSG00000100105	AAUUAUU
ENSG00000100105	CAGUAGA
ENSG00000100105	CAGUAGU
ENSG00000100105	GUGACUG
ENSG00000100105	UAUGGUU
ENSG00000100105	UCACAGG
ENSG00000100105	UCACAGU
ENSG00000100105	UUCCAUA
ENSG00000100106	AAGGUGC
ENSG00000100106	AUAUAAC
ENSG00000100106	CCUGCUG
ENSG00000100106	CUUCACC
ENSG00000100106	GAGGUAG
ENSG00000100106	GUGUGGC
ENSG00000100106	UAUAAAG
ENSG00000100106	UCACAUU
ENSG00000100106	UCCCUCU
ENSG00000100106	UGUGUGG
ENSG00000100124	AAGUGCU
ENSG00000100124	AGUGCUG
ENSG00000100124	CUGCCCC
ENSG00000100124	GCUGGUG
ENSG00000100139	AGUGCUU
ENSG00000100139	CCCUGAG
ENSG00000100146	AGAUAAA
ENSG00000100146	GAAUUGU
ENSG00000100146	GCUACAU
ENSG00000100146	UAAUGCU
ENSG00000100147	CUGCAGC
ENSG00000100147	CUGCAGG
ENSG00000100147	GAGCCCU
ENSG00000100147	GUCUCUG
ENSG00000100151	AGGCAGG
ENSG00000100151	AGGGAGG
ENSG00000100151	CAGCAGG
ENSG00000100151	CUCCCAC
ENSG00000100151	GAGGCAG
ENSG00000100151	GGAGGCA
ENSG00000100151	UUAUGCA
ENSG00000100167	AAGUCAC
ENSG00000100167	ACAUUCA
ENSG00000100167	AGCCCUU
ENSG00000100167	CUCACAC
ENSG00000100167	GGCAGUG
ENSG00000100167	UAUUGCU
ENSG00000100167	UCCCUCU

ENSG00000100167	UGAAAUG
ENSG00000100201	ACCAGCA
ENSG00000100201	ACUGGAU
ENSG00000100201	AUACCUC
ENSG00000100201	GCCUGAU
ENSG00000100201	GGAAUGU
ENSG00000100201	UCAAGUA
ENSG00000100201	UCCAGUU
ENSG00000100206	AAAGUAA
ENSG00000100206	UCACCAC
ENSG00000100207	AAGUCUU
ENSG00000100207	AAGUGCU
ENSG00000100207	ACUGUUU
ENSG00000100207	AGUGCUG
ENSG00000100207	AUCCCUU
ENSG00000100207	CACCACC
ENSG00000100207	CCUUCUU
ENSG00000100207	GAAAACA
ENSG00000100207	GAAACAU
ENSG00000100207	GAGAUGA
ENSG00000100207	GUCACUC
ENSG00000100207	GUGCUUG
ENSG00000100207	UACAAAG
ENSG00000100207	UCAUUUG
ENSG00000100207	UCCCUUU
ENSG00000100207	UGUGCUU
ENSG00000100207	UGUGUCA
ENSG00000100207	UUGUGAC
ENSG00000100216	AUGUGCC
ENSG00000100216	CAGCAGA
ENSG00000100216	CAGCAGG
ENSG00000100216	GUGCCAC
ENSG00000100216	UAAUAUC
ENSG00000100219	AAUUGCU
ENSG00000100221	AAAUGGU
ENSG00000100221	AACAAAC
ENSG00000100221	AAGUGCU
ENSG00000100221	ACUCCAG
ENSG00000100221	AGCACCA
ENSG00000100221	AGUGCUG
ENSG00000100221	AUGGCUU
ENSG00000100221	CCCUGUC
ENSG00000100221	CUUGGGC
ENSG00000100221	GAGGCAG
ENSG00000100221	GCAGCAU
ENSG00000100221	GCCCUUA
ENSG00000100221	GGAAUGU
ENSG00000100221	GGAGGCA
ENSG00000100221	GUCAUUG
ENSG00000100221	UAAUAUC
ENSG00000100221	UCCCUGU
ENSG00000100221	UGAAAUG
ENSG00000100221	UGCAUAG
ENSG00000100221	UGCAUAU
ENSG00000100221	UUGGUUC
ENSG00000100226	AAAAACC
ENSG00000100226	AACAAAC
ENSG00000100226	ACUUGGA
ENSG00000100226	AGCAGCA
ENSG00000100226	AGCUUAU
ENSG00000100226	AUAAUAC
ENSG00000100226	CAGUCCA
ENSG00000100226	CUACAAA
ENSG00000100226	CUGGAAA

ENSG00000100226	CUGGGCA
ENSG00000100226	CUUUGGU
ENSG00000100226	GAUUGUC
ENSG00000100226	GGCAGGG
ENSG00000100226	GGCUCAG
ENSG00000100226	GGGUGGU
ENSG00000100226	UAUAAAG
ENSG00000100226	UAUAAUA
ENSG00000100226	UUGGCAA
ENSG00000100226	UUGGCAC
ENSG00000100227	AACAAAC
ENSG00000100227	CUGCCCC
ENSG00000100227	GGAAUGU
ENSG00000100228	UUGGCAA
ENSG00000100234	AAACUGU
ENSG00000100234	AAAUUUC
ENSG00000100234	ACAAUAU
ENSG00000100234	ACACACU
ENSG00000100234	ACAGUAC
ENSG00000100234	ACAGUAU
ENSG00000100234	ACAUUAC
ENSG00000100234	CAGCAGA
ENSG00000100234	GAAAACA
ENSG00000100234	GAGCUAA
ENSG00000100234	GCCUGGG
ENSG00000100234	GCUACAU
ENSG00000100234	GGCAGCG
ENSG00000100234	UUAUGCA
ENSG00000100234	UUUGCAA
ENSG00000100239	AAAGUGC
ENSG00000100239	AAGGCAC
ENSG00000100239	AAGUGCA
ENSG00000100239	AGGUAGU
ENSG00000100239	AGUGCAA
ENSG00000100239	CAAAGUG
ENSG00000100239	CACCAGC
ENSG00000100239	GUGCAAA
ENSG00000100239	UAUUGCU
ENSG00000100241	AAUACUG
ENSG00000100241	GUAAACA
ENSG00000100243	AAAGUUU
ENSG00000100243	AGGCAGG
ENSG00000100246	AUAUAAC
ENSG00000100246	CCCUGAG
ENSG00000100246	GCCUGGG
ENSG00000100258	AAGGCAC
ENSG00000100280	GGGGUGG
ENSG00000100284	AAGGCAC
ENSG00000100284	CUCCCAA
ENSG00000100284	CUCCCAC
ENSG00000100284	GGCAGUG
ENSG00000100292	CUGCAGG
ENSG00000100296	CAGGUGA
ENSG00000100296	CCCACCU
ENSG00000100296	UGUCUUU
ENSG00000100302	AACAAAC
ENSG00000100302	AAGGCAC
ENSG00000100302	ACAGUAU
ENSG00000100302	CUGGCUC
ENSG00000100302	GCAGAAG
ENSG00000100302	UACAGUU
ENSG00000100304	AGCAGCU
ENSG00000100304	GCAGAAG
ENSG00000100304	GCUGGUG

ENSG00000100307	AAACGGU
ENSG00000100307	AAGUCAC
ENSG00000100307	AAUUUUA
ENSG00000100307	AGAUAAA
ENSG00000100307	AGCAAAA
ENSG00000100307	AGUACUG
ENSG00000100307	AUUCAUU
ENSG00000100307	CCCUGAG
ENSG00000100307	CUAUACA
ENSG00000100307	CUUGUGU
ENSG00000100307	CUUUGGU
ENSG00000100307	GACAACU
ENSG00000100307	GACCUGG
ENSG00000100307	GACUUCC
ENSG00000100307	GAGACCU
ENSG00000100307	GCUGUCU
ENSG00000100307	GGAAGCC
ENSG00000100307	GGGAACG
ENSG00000100307	GUGCAAA
ENSG00000100307	UCAUUUG
ENSG00000100307	UCUGGAA
ENSG00000100307	UGCAAAG
ENSG00000100307	UGUCUUU
ENSG00000100307	UGUGCUU
ENSG00000100307	UUGGCAA
ENSG00000100307	UUGGUUC
ENSG00000100311	AAUCACU
ENSG00000100311	ACAAUAU
ENSG00000100311	AGCACCA
ENSG00000100311	AUCACUA
ENSG00000100311	CACUCCU
ENSG00000100311	CAGUCUG
ENSG00000100311	GAGGUAG
ENSG00000100311	GCACCAU
ENSG00000100311	UACAAAG
ENSG00000100314	AUAAAGU
ENSG00000100314	CUGCAGG
ENSG00000100314	GUGGCCC
ENSG00000100314	GUGUGGC
ENSG00000100314	UGAAAUG
ENSG00000100319	UAUAAAG
ENSG00000100320	AAACUGU
ENSG00000100320	AACAAAC
ENSG00000100320	ACUGUAG
ENSG00000100320	AGCAGCU
ENSG00000100320	AGCCAGU
ENSG00000100320	AGCUGCC
ENSG00000100320	AGGACAC
ENSG00000100320	AGUGCAA
ENSG00000100320	AUGGCUU
ENSG00000100320	AUUGCAC
ENSG00000100320	CAAGAGC
ENSG00000100320	CACAAUG
ENSG00000100320	CAGUGCA
ENSG00000100320	CUACAAA
ENSG00000100320	CUAUACA
ENSG00000100320	CUCUGGG
ENSG00000100320	CUGGGCA
ENSG00000100320	CUUCUCU
ENSG00000100320	GACCCUG
ENSG00000100320	GAGGUAG
ENSG00000100320	GCAUCCC
ENSG00000100320	GGCCCUG
ENSG00000100320	GGUGGUC

ENSG00000100320	GUCUCUG
ENSG00000100320	GUCUGCC
ENSG00000100320	UACAGUU
ENSG00000100320	UAGUGAG
ENSG00000100320	UAUAAAG
ENSG00000100320	UCCUAUA
ENSG00000100320	UGGGUGA
ENSG00000100320	UGUCUGC
ENSG00000100320	UUAGAGA
ENSG00000100320	UUAUGCA
ENSG00000100320	UUCCUCG
ENSG00000100320	UUGUGUC
ENSG00000100321	AAGUCAC
ENSG00000100321	ACUGGAU
ENSG00000100321	CACCAGC
ENSG00000100321	CCUGCUG
ENSG00000100321	CCUUCUG
ENSG00000100321	CUAUACA
ENSG00000100321	CUUCACC
ENSG00000100321	GGUGGGC
ENSG00000100321	UAUUACC
ENSG00000100321	UCUCCAA
ENSG00000100321	UGAGUCU
ENSG00000100321	UGUGCUU
ENSG00000100325	AAGGCGC
ENSG00000100325	ACUGUGU
ENSG00000100325	CUCAGGG
ENSG00000100330	AAAGAAU
ENSG00000100330	AAAGUAA
ENSG00000100330	AAAGUAC
ENSG00000100330	AAAGUAU
ENSG00000100330	AAGACAU
ENSG00000100330	AAGUGCA
ENSG00000100330	AAGUGCU
ENSG00000100330	ACAGGUG
ENSG00000100330	ACAUCAC
ENSG00000100330	ACCCGUA
ENSG00000100330	ACCCUGU
ENSG00000100330	AGGAGCU
ENSG00000100330	AGGUAGU
ENSG00000100330	AGUGCUU
ENSG00000100330	CAAAGCA
ENSG00000100330	CAAAGUG
ENSG00000100330	CAUGGAU
ENSG00000100330	CCAGUGC
ENSG00000100330	CCAGUGU
ENSG00000100330	CCCUGAG
ENSG00000100330	CCCUGUC
ENSG00000100330	CCCUGUU
ENSG00000100330	CUACAAA
ENSG00000100330	CUGACUC
ENSG00000100330	CUGCCCC
ENSG00000100330	GAGCUGC
ENSG00000100330	GAGGUAG
ENSG00000100330	GAGUCUU
ENSG00000100330	GAUUGGU
ENSG00000100330	GCCUGGA
ENSG00000100330	GGAUUUC
ENSG00000100330	GGAUUUU
ENSG00000100330	GGCUCAG
ENSG00000100330	GGUGGUU
ENSG00000100330	GUCUGCC
ENSG00000100330	GUGGCCC
ENSG00000100330	UAAGACG

ENSG00000100330	UAAGACU
ENSG00000100330	UACAAAG
ENSG00000100330	UAUAUGA
ENSG00000100330	UCACACA
ENSG00000100330	UCCCUGU
ENSG00000100330	UCCUGAG
ENSG00000100330	UCCUUGC
ENSG00000100330	UGAGUCU
ENSG00000100330	UUAGGAU
ENSG00000100330	UUGACAC
ENSG00000100330	UUGGCAC
ENSG00000100330	UUGGUUC
ENSG00000100330	UUUUUGC
ENSG00000100335	AACAAAC
ENSG00000100335	AAGUUGU
ENSG00000100335	ACCCUGU
ENSG00000100335	AGGUAGU
ENSG00000100335	AUUCAUU
ENSG00000100335	CACAGUG
ENSG00000100335	CACCAGC
ENSG00000100335	CUCUUCC
ENSG00000100335	CUUCACC
ENSG00000100335	GAAACAU
ENSG00000100335	GAGGCAG
ENSG00000100335	GAGGUAG
ENSG00000100335	GGAGAGA
ENSG00000100335	GUGAGGU
ENSG00000100335	UCAUUCG
ENSG00000100335	UUCUAAU
ENSG00000100345	AUAUAAC
ENSG00000100345	AUUGCAC
ENSG00000100345	CCAGUGU
ENSG00000100345	GGACCUG
ENSG00000100345	GGCAGUG
ENSG00000100345	GUAGUGU
ENSG00000100345	GUGAGGU
ENSG00000100345	UUGGUCC
ENSG00000100346	AAACUGU
ENSG00000100346	AGCCCUU
ENSG00000100346	CAGCAAA
ENSG00000100346	CAGCAGG
ENSG00000100346	CUGGAAA
ENSG00000100346	CUGGCUC
ENSG00000100346	GAGGUAG
ENSG00000100346	GAGGUAU
ENSG00000100346	GCCCUUA
ENSG00000100346	GCUGUCU
ENSG00000100346	GGCCCUG
ENSG00000100346	GGCUCUG
ENSG00000100346	GUGCUUG
ENSG00000100346	UAUUGCU
ENSG00000100346	UCCUGAG
ENSG00000100348	AGCAGCA
ENSG00000100348	CUACAAA
ENSG00000100348	CUGCUCA
ENSG00000100350	CUUUGGU
ENSG00000100350	UUGGCAC
ENSG00000100354	AAAGCUG
ENSG00000100354	AAAUUUC
ENSG00000100354	AAGAACC
ENSG00000100354	AAGUCUU
ENSG00000100354	AAGUGCA
ENSG00000100354	AAGUGCU
ENSG00000100354	AAUCCUU

ENSG00000100354	AAUGCCC
ENSG00000100354	ACAGUAU
ENSG00000100354	ACAUAGA
ENSG00000100354	ACAUCAC
ENSG00000100354	ACAUUCA
ENSG00000100354	ACCCGGC
ENSG00000100354	ACCCUGU
ENSG00000100354	ACUGGCU
ENSG00000100354	ACUGUGU
ENSG00000100354	AGCAAAA
ENSG00000100354	AGCACCA
ENSG00000100354	AGCAGCG
ENSG00000100354	AGCAGCU
ENSG00000100354	AGCCCUU
ENSG00000100354	AGCCUGC
ENSG00000100354	AGCUGCC
ENSG00000100354	AGGACAC
ENSG00000100354	AGUACUG
ENSG00000100354	AGUGCCU
ENSG00000100354	AUAUAAC
ENSG00000100354	AUGAUGA
ENSG00000100354	AUGAUGC
ENSG00000100354	AUGGCAC
ENSG00000100354	AUGGCUU
ENSG00000100354	AUGUCUG
ENSG00000100354	AUUGCAC
ENSG00000100354	CAAAGCA
ENSG00000100354	CAAGAGC
ENSG00000100354	CACAAUG
ENSG00000100354	CACCACC
ENSG00000100354	CAGCAAA
ENSG00000100354	CAGCAGA
ENSG00000100354	CAGUCUG
ENSG00000100354	CAGUUAC
ENSG00000100354	CAUAUUG
ENSG00000100354	CCAGGAG
ENSG00000100354	CCCUGUU
ENSG00000100354	CCUUCAU
ENSG00000100354	CUCCCAC
ENSG00000100354	CUCUAGC
ENSG00000100354	CUGGGCA
ENSG00000100354	CUUGUAU
ENSG00000100354	CUUUGGU
ENSG00000100354	GAAGGAA
ENSG00000100354	GAGAAGA
ENSG00000100354	GAGCUAA
ENSG00000100354	GAGGCAG
ENSG00000100354	GAGGCUG
ENSG00000100354	GAGGGGC
ENSG00000100354	GAGGUAU
ENSG00000100354	GAGUUGA
ENSG00000100354	GAGUUGG
ENSG00000100354	GCACCAU
ENSG00000100354	GCAGCAU
ENSG00000100354	GCAGGUU
ENSG00000100354	GCCUGGA
ENSG00000100354	GCUACAU
ENSG00000100354	GCUAGCA
ENSG00000100354	GCUGGUG
ENSG00000100354	GCUUCCU
ENSG00000100354	GGAAGAC
ENSG00000100354	GGACGGA
ENSG00000100354	GGAUUUU
ENSG00000100354	GGCAGUG

ENSG00000100354	GGCAUUG
ENSG00000100354	GGUAGAC
ENSG00000100354	GGUUUAC
ENSG00000100354	GUCAGUU
ENSG00000100354	GUCCAGA
ENSG00000100354	GUCUCUG
ENSG00000100354	GUCUUGC
ENSG00000100354	GUGACUG
ENSG00000100354	GUGCCAC
ENSG00000100354	UAUUGCU
ENSG00000100354	UCACAAG
ENSG00000100354	UCCCUUG
ENSG00000100354	UGAAAGG
ENSG00000100354	UGAAGUG
ENSG00000100354	UGAGAAU
ENSG00000100354	UGCAUAG
ENSG00000100354	UGCAUAU
ENSG00000100354	UGGAGAU
ENSG00000100354	UGUAUGU
ENSG00000100354	UUCAAGC
ENSG00000100354	UUGGAGG
ENSG00000100354	UUGGUCC
ENSG00000100354	UUGGUUC
ENSG00000100354	UUGUUCG
ENSG00000100359	UCCUGAG
ENSG00000100362	UCCAGAG
ENSG00000100364	ACAGGUG
ENSG00000100364	CUUUGGU
ENSG00000100364	GAGGCAG
ENSG00000100364	GAGGUAG
ENSG00000100364	GAGGUAU
ENSG00000100364	GCUGGUG
ENSG00000100364	GGAAGCC
ENSG00000100364	GGCAGCG
ENSG00000100364	GGUGGGC
ENSG00000100364	GUGAGGU
ENSG00000100364	UCUGGAA
ENSG00000100364	UCUGGAG
ENSG00000100364	UGAAAUG
ENSG00000100364	UGGCUGC
ENSG00000100372	UGCUGAC
ENSG00000100376	ACUGUUU
ENSG00000100376	CCCUGAG
ENSG00000100376	CUUUGGU
ENSG00000100376	GAGGUAG
ENSG00000100376	GAGGUAU
ENSG00000100376	GGUUACC
ENSG00000100376	UCAAGUA
ENSG00000100376	UUCAAGC
ENSG00000100376	UUGGCAA
ENSG00000100376	UUGGCAC
ENSG00000100376	UUUCAAC
ENSG00000100379	ACCCUGU
ENSG00000100379	AGGCAGG
ENSG00000100379	GAGGCAG
ENSG00000100379	GAGGUAG
ENSG00000100379	GAGGUAU
ENSG00000100379	GUGAGGU
ENSG00000100379	UAUUACC
ENSG00000100380	ACUGCAG
ENSG00000100380	GAAAACA
ENSG00000100380	GAAGGUC
ENSG00000100380	GGUGGUC
ENSG00000100380	UAUUGCU

ENSG00000100380	UGAGAAG
ENSG00000100385	GCCCUGU
ENSG00000100393	AAAGAAU
ENSG00000100393	AAUACUG
ENSG00000100393	ACAAGGU
ENSG00000100393	ACAAUAU
ENSG00000100393	AGCUGCC
ENSG00000100393	AGUGAUC
ENSG00000100393	CUGCCCC
ENSG00000100393	UCACACA
ENSG00000100401	AGCAGCU
ENSG00000100401	CUCCAGC
ENSG00000100403	AAGUGCA
ENSG00000100403	AAGUGUG
ENSG00000100403	ACACACC
ENSG00000100403	ACACACU
ENSG00000100403	ACAUUAC
ENSG00000100403	ACCACAG
ENSG00000100403	ACUAGAU
ENSG00000100403	AUAAUAC
ENSG00000100403	AUAUAAC
ENSG00000100403	AUAUUAU
ENSG00000100403	AUGCAUU
ENSG00000100403	CAAAGUG
ENSG00000100403	CAGACAG
ENSG00000100403	CAGCUGG
ENSG00000100403	CAGGCUC
ENSG00000100403	CCCACCU
ENSG00000100403	CUCUUCC
ENSG00000100403	CUGCAGC
ENSG00000100403	CUGGAAA
ENSG00000100403	GAGAAAU
ENSG00000100403	GCAGCAU
ENSG00000100403	GCAUCCC
ENSG00000100403	GCCUGAU
ENSG00000100403	GCUGGUG
ENSG00000100403	GGAAUGU
ENSG00000100403	GGCCCUG
ENSG00000100403	GGCGACA
ENSG00000100403	GGGCCCC
ENSG00000100403	GGGGUCC
ENSG00000100403	GUAUUCU
ENSG00000100403	GUGGGGA
ENSG00000100403	UAAUAUC
ENSG00000100403	UAUAAUA
ENSG00000100403	UCAAGUA
ENSG00000100403	UCUAGAG
ENSG00000100403	UCUGGAA
ENSG00000100403	UCUGGAG
ENSG00000100403	UGAAGCU
ENSG00000100410	CUGCAGC
ENSG00000100410	GCAGCAU
ENSG00000100410	GGCAGCG
ENSG00000100412	AAUCCUU
ENSG00000100412	UGAAAUG
ENSG00000100413	GAAGGAA
ENSG00000100417	AGCAGCA
ENSG00000100422	CUGCCCC
ENSG00000100422	CUUCUCU
ENSG00000100422	UCUCAAG
ENSG00000100422	UUUGCAC
ENSG00000100425	AAAGUUU
ENSG00000100425	UAAUGCU
ENSG00000100425	UAUUGCU

ENSG00000100425	UGUAUAA
ENSG00000100426	AAGGCAC
ENSG00000100426	AGGAGCU
ENSG00000100426	AUGGCUU
ENSG00000100426	CAGUGCA
ENSG00000100426	CCCUGUC
ENSG00000100426	GACAACU
ENSG00000100426	GUAAACA
ENSG00000100426	UAUAAAG
ENSG00000100426	UCACCAC
ENSG00000100426	UCCAGAG
ENSG00000100426	UGCAAAG
ENSG00000100426	UUCUAAU
ENSG00000100433	CACAGUG
ENSG00000100439	AAGGCAC
ENSG00000100439	AGCACCA
ENSG00000100439	AGGCAGG
ENSG00000100439	AGGGAGG
ENSG00000100439	CAAAGCA
ENSG00000100439	CAGACAG
ENSG00000100439	CAGCUGG
ENSG00000100439	CAGUAGU
ENSG00000100439	CUGGCUC
ENSG00000100439	GAAGGAA
ENSG00000100439	UAAUAUC
ENSG00000100439	UACAAAG
ENSG00000100442	GUAAACA
ENSG00000100461	AAAGUUU
ENSG00000100461	AUCCCUU
ENSG00000100461	AUGGCUU
ENSG00000100461	CAGACAG
ENSG00000100461	CAGCAGA
ENSG00000100461	CCAGUGC
ENSG00000100461	CCAGUGU
ENSG00000100461	GAGGCAG
ENSG00000100461	GGAGGCA
ENSG00000100461	UUGGUCC
ENSG00000100478	AAGGCAC
ENSG00000100478	CUUCUCU
ENSG00000100483	AGGCAGG
ENSG00000100483	GGGUAAG
ENSG00000100485	AAACUGU
ENSG00000100485	AAGAACC
ENSG00000100485	CAAAGCA
ENSG00000100485	CCAGUGC
ENSG00000100485	UAAGACG
ENSG00000100503	GAGGUAU
ENSG00000100503	UCCCUGU
ENSG00000100503	UGCCCUG
ENSG00000100505	AAAAACC
ENSG00000100505	AAAGAAU
ENSG00000100505	ACCCAUU
ENSG00000100505	AGCACCA
ENSG00000100505	CAGCUGG
ENSG00000100505	GUAAACA
ENSG00000100505	UCCUGAG
ENSG00000100505	UGCUGAC
ENSG00000100522	AACUCUA
ENSG00000100522	CACCAGC
ENSG00000100522	CUUUGGU
ENSG00000100522	GCAGCAU
ENSG00000100522	GGAAUGU
ENSG00000100522	GUCAUUG
ENSG00000100522	UUGGUUC

ENSG00000100523	UACAAAG
ENSG00000100528	AGUGCAA
ENSG00000100557	AAGAACC
ENSG00000100592	ACUGUUU
ENSG00000100592	CACUGUU
ENSG00000100592	GGAAUGU
ENSG00000100592	GGCAGUG
ENSG00000100592	GUGCAAA
ENSG00000100592	GUGCUUG
ENSG00000100596	AAAGAAU
ENSG00000100596	AAAGCUG
ENSG00000100596	AAGGCAC
ENSG00000100596	AAGUGUG
ENSG00000100596	AGCACCA
ENSG00000100596	AGCCAGU
ENSG00000100596	CACACCU
ENSG00000100596	CUGGCUC
ENSG00000100596	GGCUCAG
ENSG00000100596	GUGACUG
ENSG00000100596	UACAAAG
ENSG00000100596	UAUAAAG
ENSG00000100596	UUGGUCC
ENSG00000100596	UUGUGAC
ENSG00000100599	UCCCUUU
ENSG00000100601	CAUGGAU
ENSG00000100601	GAAAACA
ENSG00000100601	GAGGUAG
ENSG00000100601	GGCAAGA
ENSG00000100604	AAAGCUG
ENSG00000100604	AUUGCAC
ENSG00000100604	UAUUGCU
ENSG00000100604	UCUAGAG
ENSG00000100605	AGUGCAA
ENSG00000100605	CAAAGCA
ENSG00000100605	CAGUAGA
ENSG00000100605	CAGUGCA
ENSG00000100605	CUCUGGG
ENSG00000100605	GAGCUGC
ENSG00000100605	GUAAACA
ENSG00000100605	UGACAUC
ENSG00000100605	UGGAGAU
ENSG00000100605	UUGGAGG
ENSG00000100625	AACAGUC
ENSG00000100625	AAUGCCC
ENSG00000100625	ACAGUAU
ENSG00000100625	ACAUUCA
ENSG00000100625	AGCAGCA
ENSG00000100625	AGUGCAA
ENSG00000100625	AGUGUGC
ENSG00000100625	AUGGCUU
ENSG00000100625	CAUAUUG
ENSG00000100625	CUACAAA
ENSG00000100625	GGGUAAG
ENSG00000100625	GUCCAGA
ENSG00000100625	UACAGUU
ENSG00000100625	UGCAUAG
ENSG00000100625	UGGGUAC
ENSG00000100625	UUCAAGC
ENSG00000100625	UUCCUAG
ENSG00000100625	UUGGUUC
ENSG00000100625	UUUGCAA
ENSG00000100626	ACAUUCA
ENSG00000100626	AGCAAAA
ENSG00000100626	AGGGAGG

ENSG00000100626	AGUGGUU
ENSG00000100626	CUUUGGU
ENSG00000100626	UCCAGAG
ENSG00000100626	UUGGUCC
ENSG00000100629	AUAAAGU
ENSG00000100644	AAAGUGC
ENSG00000100644	AAGUGCA
ENSG00000100644	ACAAUAU
ENSG00000100644	CCAGUGU
ENSG00000100644	GCUGGUG
ENSG00000100644	GUUAAUG
ENSG00000100644	UAAUAGU
ENSG00000100644	UAGACUG
ENSG00000100647	AAAGCUG
ENSG00000100647	AAAGUGC
ENSG00000100647	AAGGCAC
ENSG00000100647	ACAGGUG
ENSG00000100647	ACAUUCA
ENSG00000100647	ACCCUGU
ENSG00000100647	AGUGGUU
ENSG00000100647	AUAUAAC
ENSG00000100647	CACACCG
ENSG00000100647	CAGUUAC
ENSG00000100647	CCCUGAG
ENSG00000100647	CUCCAGC
ENSG00000100647	CUGCAGC
ENSG00000100647	CUUCACC
ENSG00000100647	CUUUGGU
ENSG00000100647	GAGCUGC
ENSG00000100647	GCCCUUA
ENSG00000100647	GUAAACA
ENSG00000100647	UCACACA
ENSG00000100647	UCACAGU
ENSG00000100647	UCACCAC
ENSG00000100647	UGAAAUG
ENSG00000100647	UGACAUC
ENSG00000100647	UGCAUAG
ENSG00000100647	UGCAUAU
ENSG00000100647	UUCUGCA
ENSG00000100664	AAAGAAU
ENSG00000100664	AAGUCUU
ENSG00000100664	AAGUGCU
ENSG00000100664	AAUUUUA
ENSG00000100664	AGAUGUG
ENSG00000100664	CAAAGUG
ENSG00000100664	CACAGUG
ENSG00000100664	CCUGCUG
ENSG00000100664	CUUUGGU
ENSG00000100664	GCAACUU
ENSG00000100664	GUGAAUG
ENSG00000100664	UAAGACG
ENSG00000100664	UAAGACU
ENSG00000100664	UAAUAUC
ENSG00000100664	UACAAAG
ENSG00000100664	UGCUCAC
ENSG00000100664	UGUAUAA
ENSG00000100664	UUGGCAC
ENSG00000100678	AUGACAC
ENSG00000100678	UGCCCUG
ENSG00000100697	AAAGCUG
ENSG00000100697	AAGAACC
ENSG00000100697	AAGGCAC
ENSG00000100697	AAGUGCA
ENSG00000100697	AAGUUGU

ENSG00000100697	AGCACCA
ENSG00000100697	AGCAGCA
ENSG00000100697	AGUGCCU
ENSG00000100697	AUAAUAC
ENSG00000100697	AUGAGCU
ENSG00000100697	AUGUCUG
ENSG00000100697	CAGCAAA
ENSG00000100697	CAGUGCA
ENSG00000100697	CCCUGAG
ENSG00000100697	CUGCUCA
ENSG00000100697	CUUUGGU
ENSG00000100697	GAGGUAG
ENSG00000100697	GAUAUGU
ENSG00000100697	GCUACAU
ENSG00000100697	GGAGUGU
ENSG00000100697	GGCAAGA
ENSG00000100697	GUGCAAA
ENSG00000100697	GUUAGGA
ENSG00000100697	UAAUACA
ENSG00000100697	UAUUACC
ENSG00000100697	UGAAAUG
ENSG00000100697	UGAGAAU
ENSG00000100697	UGAUCCA
ENSG00000100697	UGCAGCU
ENSG00000100697	UGGUACC
ENSG00000100697	UUCCUCG
ENSG00000100697	UUGAGGC
ENSG00000100697	UUUCAAC
ENSG00000100711	AAGGCAC
ENSG00000100711	AUACAAG
ENSG00000100711	CAUAUUG
ENSG00000100722	AUAUUAU
ENSG00000100722	CAUAUUG
ENSG00000100722	UACAAAG
ENSG00000100726	CUACAAA
ENSG00000100726	UACAAAG
ENSG00000100731	AAAGAGG
ENSG00000100731	AAUCUCA
ENSG00000100731	AUCCUUG
ENSG00000100731	GACCAUG
ENSG00000100731	GAGGCUG
ENSG00000100731	GCACCAU
ENSG00000100731	GCUGGUG
ENSG00000100731	GGCAGUG
ENSG00000100731	GGCUGCG
ENSG00000100731	GUUCAUG
ENSG00000100731	UAGGUAU
ENSG00000100731	UCACAGG
ENSG00000100731	UCCCUGU
ENSG00000100731	UCUCAAG
ENSG00000100731	UGCAUAG
ENSG00000100731	UGCAUUG
ENSG00000100731	UGGCUGC
ENSG00000100731	UGUUGAA
ENSG00000100731	UUCAAAU
ENSG00000100731	UUCCUCG
ENSG00000100731	UUGGCAC
ENSG00000100744	AGCAAAA
ENSG00000100744	AUUCAUU
ENSG00000100744	CUUUGGU
ENSG00000100744	GUGCUUG
ENSG00000100744	UGUGCUU
ENSG00000100796	AAAAACC
ENSG00000100796	ACUCAGG

ENSG00000100796	AGGCAGG
ENSG00000100796	AUAUUAU
ENSG00000100796	AUGCACC
ENSG00000100796	CCCUGUU
ENSG00000100796	CUCAGGG
ENSG00000100796	CUGCAGC
ENSG00000100796	CUGCAGG
ENSG00000100796	GAGAAAU
ENSG00000100796	GAUUGUA
ENSG00000100796	GGCAUUG
ENSG00000100796	UAUUGCU
ENSG00000100796	UCCAGAG
ENSG00000100796	UCCCUGU
ENSG00000100796	UGCCCUG
ENSG00000100796	UUCCAUA
ENSG00000100796	UUGGCAC
ENSG00000100811	AAAAAUC
ENSG00000100811	ACAGGUG
ENSG00000100811	ACAUUCA
ENSG00000100811	ACUGCAG
ENSG00000100811	ACUGCAU
ENSG00000100811	ACUUGGA
ENSG00000100811	AGCACCA
ENSG00000100811	AUGCACC
ENSG00000100811	AUGCAUU
ENSG00000100811	CACUGUU
ENSG00000100811	GGCAGUG
ENSG00000100811	UAUAAAG
ENSG00000100811	UGAAGUG
ENSG00000100813	ACUGCAG
ENSG00000100813	AUAAAGU
ENSG00000100813	CUGCAGG
ENSG00000100813	UAUAAAG
ENSG00000100813	UGCAGCU
ENSG00000100836	ACACACU
ENSG00000100836	UCCAGAG
ENSG00000100842	AAAGCUG
ENSG00000100842	AGGCAGG
ENSG00000100842	GAGAUGA
ENSG00000100842	GUGCCUG
ENSG00000100842	UCCAGAG
ENSG00000100852	AAAGUAU
ENSG00000100852	CCAGUAC
ENSG00000100852	GUGCAAA
ENSG00000100852	UCAACAG
ENSG00000100852	UGUGCUU
ENSG00000100916	AAGUGCA
ENSG00000100916	AUGGCAC
ENSG00000100931	UUGGCAC
ENSG00000100934	GUGCCAC
ENSG00000100934	UUGUUCG
ENSG00000100941	AAAAGUA
ENSG00000100941	AAAGUAC
ENSG00000100941	AAGGCAC
ENSG00000100941	AUCCUUU
ENSG00000100941	GUAAACA
ENSG00000100941	UACAAAG
ENSG00000100941	UUAUGCA
ENSG00000100968	AGCACCA
ENSG00000100968	CACACCU
ENSG00000100968	UCUCCAA
ENSG00000100968	UCUGGAG
ENSG00000100979	CUUCUCU
ENSG00000100987	AAGUGCU

ENSG00000100987	AGUGCUU
ENSG00000100987	CUACAAA
ENSG00000100991	AAAGUUU
ENSG00000100991	AGGCAGG
ENSG00000100991	AUAAAGU
ENSG00000100991	GUUAAUG
ENSG00000100991	UUGGCAA
ENSG00000100994	CCAGCAU
ENSG00000100997	CACCACC
ENSG00000100997	CAGUCUG
ENSG00000100997	UCCUGAG
ENSG00000101000	UGCCAGC
ENSG00000101017	CUCUGGG
ENSG00000101017	UCCAGUU
ENSG00000101019	AAAAAUC
ENSG00000101019	AAACUGU
ENSG00000101019	AAUGUUG
ENSG00000101019	AUGUAAU
ENSG00000101019	CACUCCU
ENSG00000101019	CAGCAGA
ENSG00000101019	GUCAGUU
ENSG00000101019	UCACACA
ENSG00000101019	UCACAGG
ENSG00000101019	UCACAGU
ENSG00000101019	UGGGGGA
ENSG00000101040	AAAAACC
ENSG00000101040	AACAUUC
ENSG00000101040	ACAUUAC
ENSG00000101040	AUAAUAC
ENSG00000101040	GUAGUGU
ENSG00000101040	UGACAUC
ENSG00000101057	AGCACCA
ENSG00000101057	GAGAUGA
ENSG00000101057	GAGGGGC
ENSG00000101057	GCAGAAG
ENSG00000101076	CUCUGGG
ENSG00000101076	GAGCCCC
ENSG00000101076	GGAAUGU
ENSG00000101079	AAAGAAU
ENSG00000101079	ACCUGGA
ENSG00000101079	CCCUGAG
ENSG00000101079	GGAGGAG
ENSG00000101079	GGAGUGU
ENSG00000101079	GGAUGAG
ENSG00000101079	GGCAAGA
ENSG00000101079	UGAAAUG
ENSG00000101082	CAGCAGG
ENSG00000101082	CUGUUGA
ENSG00000101082	GGAGAGA
ENSG00000101082	GGCCCUG
ENSG00000101082	UGGCCCU
ENSG00000101098	AAAGCUG
ENSG00000101098	AACAAAC
ENSG00000101098	AACCUGG
ENSG00000101098	AGGCAGG
ENSG00000101098	AGGGAGG
ENSG00000101098	AUCCUUG
ENSG00000101098	AUGAGCU
ENSG00000101098	AUGUGCC
ENSG00000101098	CAGCAGA
ENSG00000101098	CAGCAGG
ENSG00000101098	CAGGUGA
ENSG00000101098	CCAGGAG
ENSG00000101098	CGGAUCC

ENSG00000101098	CUGCUCA
ENSG00000101098	CUGGAAA
ENSG00000101098	CUGGCUC
ENSG00000101098	GAGCCCC
ENSG00000101098	GAGCUAA
ENSG00000101098	GAGGCAG
ENSG00000101098	GCACCAU
ENSG00000101098	GCAGGUU
ENSG00000101098	GCCCUUA
ENSG00000101098	GCCUGGG
ENSG00000101098	GCUGGUG
ENSG00000101098	GGAAUGU
ENSG00000101098	GGAGAGA
ENSG00000101098	GGCAGCG
ENSG00000101098	GGCUCAG
ENSG00000101098	GGCUGCG
ENSG00000101098	GGGAGCU
ENSG00000101098	GGGGAGC
ENSG00000101098	GGGGUCC
ENSG00000101098	GUGCUUG
ENSG00000101098	UAUUGCU
ENSG00000101098	UCACAGG
ENSG00000101098	UCACAGU
ENSG00000101098	UCCUUGC
ENSG00000101098	UCUGGAA
ENSG00000101098	UCUGGAG
ENSG00000101098	UGCAAAG
ENSG00000101098	UGGAGAU
ENSG00000101098	UGUGCUU
ENSG00000101098	UUGGCAC
ENSG00000101109	AAAGAGG
ENSG00000101109	AAAGCGC
ENSG00000101109	AAAGCUG
ENSG00000101109	AAGGCAC
ENSG00000101109	AAGGUGC
ENSG00000101109	AAGUCUU
ENSG00000101109	ACAGUAC
ENSG00000101109	ACAGUAU
ENSG00000101109	AGCAUUC
ENSG00000101109	AGCCCUU
ENSG00000101109	AGGAGCU
ENSG00000101109	AGUGCCU
ENSG00000101109	CCUGUAC
ENSG00000101109	GAGCUGC
ENSG00000101109	GGAGGAG
ENSG00000101109	GGAUGAG
ENSG00000101109	GUGCCUG
ENSG00000101109	GUGGGGA
ENSG00000101109	GUUCAUG
ENSG00000101109	UCAAGUA
ENSG00000101109	UCACAAG
ENSG00000101109	UCAUACA
ENSG00000101109	UGCUGAC
ENSG00000101109	UGGACUG
ENSG00000101109	UUAGAGA
ENSG00000101109	UUCAAGC
ENSG00000101115	GAUUGUA
ENSG00000101115	GAUUGUC
ENSG00000101126	ACUAGAU
ENSG00000101126	AGGUUAU
ENSG00000101126	CUAUACA
ENSG00000101126	GAUUGUA
ENSG00000101126	GUCCAGA
ENSG00000101126	GUCUACU

ENSG00000101126	UAAUACA
ENSG00000101126	UACAAAG
ENSG00000101126	UAUAAUA
ENSG00000101126	UAUUACC
ENSG00000101126	UUUCAAC
ENSG00000101132	UAUAAAG
ENSG00000101132	UCAAGUA
ENSG00000101132	UCAUUUG
ENSG00000101132	UUAUGCA
ENSG00000101134	GAAGGAA
ENSG00000101138	GUGAAUG
ENSG00000101144	GUGACAG
ENSG00000101144	UAUUACC
ENSG00000101146	UACAAAG
ENSG00000101150	ACUGCAU
ENSG00000101150	CACAGUG
ENSG00000101150	GAGAAAU
ENSG00000101150	UCCUGAG
ENSG00000101152	AAUACUG
ENSG00000101152	ACUGCAG
ENSG00000101152	GAAGGAA
ENSG00000101152	GGAAUGU
ENSG00000101152	GUUCAUG
ENSG00000101166	CAAAUGC
ENSG00000101166	GGCUCUG
ENSG00000101166	UGAAAUG
ENSG00000101166	UUGGUCC
ENSG00000101180	CACACCU
ENSG00000101180	CAGCUGG
ENSG00000101180	GAAAACA
ENSG00000101180	GGGAGCU
ENSG00000101180	GGGCGUG
ENSG00000101188	AAAGAGG
ENSG00000101188	CCAUUAC
ENSG00000101188	UUCAAAU
ENSG00000101189	AAAAACC
ENSG00000101189	AAAAAUC
ENSG00000101189	AAAACUG
ENSG00000101189	ACAGUAU
ENSG00000101189	CUACAAA
ENSG00000101189	CUGCAGC
ENSG00000101189	CUGCAGG
ENSG00000101189	GAGGCAG
ENSG00000101189	GUGCUUG
ENSG00000101189	UACAGUU
ENSG00000101189	UCCAGUU
ENSG00000101189	UUCUGCA
ENSG00000101189	UUGGUCC
ENSG00000101190	GGAGUCC
ENSG00000101191	CCAGGAG
ENSG00000101191	CUUCACC
ENSG00000101193	AAGGCAC
ENSG00000101193	ACUGCAU
ENSG00000101193	AGCACCA
ENSG00000101193	AUAUUAU
ENSG00000101193	UCCUAUA
ENSG00000101193	UGAAAUG
ENSG00000101193	UGAUCCA
ENSG00000101193	UUCAAGC
ENSG00000101193	UUCCUAG
ENSG00000101198	GAGGCUG
ENSG00000101210	GGACCUG
ENSG00000101210	GGCGGGG
ENSG00000101216	AAAACUG

ENSG00000101216	AAACUGU
ENSG00000101216	AAAGUAC
ENSG00000101216	ACAGUAU
ENSG00000101216	ACUCAAA
ENSG00000101216	CAAAGUG
ENSG00000101216	CUUCUCU
ENSG00000101216	UACAAAG
ENSG00000101216	UGCAAAG
ENSG00000101220	GGAAGCC
ENSG00000101220	GGCAGGG
ENSG00000101222	CUCCAGC
ENSG00000101222	CUGGAAA
ENSG00000101222	GUCUCUG
ENSG00000101222	UCUGGAA
ENSG00000101224	AGCAGCA
ENSG00000101224	AGUGCUU
ENSG00000101224	CAGUGCA
ENSG00000101224	UCCCUUU
ENSG00000101230	AAAGUGC
ENSG00000101230	CAGUGCA
ENSG00000101230	UACAAAG
ENSG00000101230	UCACAUU
ENSG00000101230	UCCAGAG
ENSG00000101230	UUUGCAC
ENSG00000101236	AACAGUC
ENSG00000101236	CACUCCU
ENSG00000101236	CAGCUGG
ENSG00000101236	CAGUCUG
ENSG00000101236	CUGGCUC
ENSG00000101236	CUUCUCU
ENSG00000101236	CUUUGGU
ENSG00000101236	GCUGGUG
ENSG00000101236	UCACAAG
ENSG00000101236	UCAUGAU
ENSG00000101236	UUGGUUC
ENSG00000101246	AAAGUUU
ENSG00000101246	CCCCCAG
ENSG00000101246	UGUCUUU
ENSG00000101255	AAGGCAC
ENSG00000101255	UCUUGAG
ENSG00000101255	UUGGCAA
ENSG00000101265	ACACACU
ENSG00000101265	AGCAGCA
ENSG00000101265	AGUGCAA
ENSG00000101265	CCAGUGC
ENSG00000101265	GAGAAAU
ENSG00000101265	GCAGAAG
ENSG00000101265	GUGCUUG
ENSG00000101265	UCCAGUU
ENSG00000101265	UUGAUCA
ENSG00000101266	AAAGAAU
ENSG00000101266	AAAUCCC
ENSG00000101266	AAUCUCU
ENSG00000101266	UCUGGAG
ENSG00000101280	GGCUCAG
ENSG00000101282	GAAACAU
ENSG00000101282	GGCAGUG
ENSG00000101282	GGGCCCC
ENSG00000101282	UGCAAAG
ENSG00000101290	ACUCAAA
ENSG00000101290	AGCAGCA
ENSG00000101290	CAAAGCA
ENSG00000101290	CAGCAGA
ENSG00000101290	CAGCAGG

ENSG00000101290	CUGCCCC
ENSG00000101290	GACUUCC
ENSG00000101294	AAACUGU
ENSG00000101294	AACCUGG
ENSG00000101294	CCUUCUG
ENSG00000101298	AGUGCAA
ENSG00000101298	CAAAGCA
ENSG00000101298	CAGACAG
ENSG00000101298	CAGUGCA
ENSG00000101298	CCAGUGC
ENSG00000101298	GACUUCC
ENSG00000101298	GAGGCUG
ENSG00000101298	GCAGAAG
ENSG00000101298	GGAGGCA
ENSG00000101298	GUGCAAA
ENSG00000101298	UCAUACA
ENSG00000101298	UGCAAAG
ENSG00000101298	UGCAUAG
ENSG00000101298	UGCAUAU
ENSG00000101298	UUGGAGG
ENSG00000101306	AACCUGU
ENSG00000101306	CCUGUAC
ENSG00000101306	CCUUCUG
ENSG00000101306	GAGCCCC
ENSG00000101306	GGGGAGC
ENSG00000101306	GGGUGGU
ENSG00000101306	GGUGGUC
ENSG00000101306	UGUGUGG
ENSG00000101310	AGCUGCC
ENSG00000101311	AAAUUUC
ENSG00000101311	CAAAACU
ENSG00000101321	AACACUG
ENSG00000101321	AGCACCA
ENSG00000101323	AACAGUC
ENSG00000101323	ACCCAUU
ENSG00000101323	CACAGUG
ENSG00000101323	CCAGGAG
ENSG00000101323	GUCUCUG
ENSG00000101323	UAAUAUC
ENSG00000101323	UCAAGUA
ENSG00000101323	UUCCUAG
ENSG00000101327	AACUGGU
ENSG00000101327	AAUGCCC
ENSG00000101327	AGCAUUC
ENSG00000101327	CUGCUCA
ENSG00000101331	AAGUAGC
ENSG00000101331	AGCUGCC
ENSG00000101331	AGGUAGU
ENSG00000101331	CCAGGAG
ENSG00000101331	GAGGAUA
ENSG00000101331	GCAGGAC
ENSG00000101331	GCAUCCC
ENSG00000101331	GGAAGCC
ENSG00000101331	GGAGGAG
ENSG00000101333	AAUGCCC
ENSG00000101333	AUGGCAC
ENSG00000101333	UUAGAGA
ENSG00000101335	GGCGGGG
ENSG00000101337	AACACUG
ENSG00000101337	ACAUUCA
ENSG00000101337	CAGGAAC
ENSG00000101337	CCAGGAG
ENSG00000101337	CCUGCUG
ENSG00000101337	CUACAGU

ENSG00000101337	CUCUUCC
ENSG00000101337	GUGCCAC
ENSG00000101337	GUGCCUG
ENSG00000101337	UCCAGUU
ENSG00000101337	UUGGCAC
ENSG00000101343	AAAAAUC
ENSG00000101346	AAUUUUA
ENSG00000101346	AGCCCUU
ENSG00000101346	GGCAGUG
ENSG00000101346	GGCUCUG
ENSG00000101349	AAAGUAC
ENSG00000101349	AAUACUG
ENSG00000101349	ACAUUCA
ENSG00000101349	AGCAGCA
ENSG00000101349	AUGGCUU
ENSG00000101349	GCUAGCA
ENSG00000101350	AAAACUG
ENSG00000101350	AAAGAGG
ENSG00000101350	AAAGGUA
ENSG00000101350	AAAGUGC
ENSG00000101350	AAGACAU
ENSG00000101350	AAGUGCU
ENSG00000101350	AGCACCA
ENSG00000101350	AGCAGCA
ENSG00000101350	AGCAGCU
ENSG00000101350	AGUGCUU
ENSG00000101350	AUGAGCU
ENSG00000101350	AUGCAUU
ENSG00000101350	AUGUAAU
ENSG00000101350	AUUGCAC
ENSG00000101350	CUCAAAC
ENSG00000101350	GAAGGUC
ENSG00000101350	GACCUGG
ENSG00000101350	GAGAAGA
ENSG00000101350	GCAGCAU
ENSG00000101350	GCCUGGA
ENSG00000101350	GGGAACG
ENSG00000101350	UAUUACC
ENSG00000101350	UAUUGCU
ENSG00000101350	UCCUGAG
ENSG00000101350	UGAUCCA
ENSG00000101350	UGCCAGC
ENSG00000101350	UUCAAGC
ENSG00000101350	UUCCUAG
ENSG00000101350	UUCUAAU
ENSG00000101350	UUGACAC
ENSG00000101350	UUGGCAA
ENSG00000101350	UUUCAAC
ENSG00000101363	AAGGCAC
ENSG00000101363	CCAGGAG
ENSG00000101363	CUGGCUC
ENSG00000101367	AAAAACC
ENSG00000101367	AAAGUAA
ENSG00000101367	AAAGUAC
ENSG00000101367	AAAGUGC
ENSG00000101367	AAGGCAC
ENSG00000101367	AAGUGCA
ENSG00000101367	AAUCUCA
ENSG00000101367	ACACACC
ENSG00000101367	ACCCUGU
ENSG00000101367	ACUCAAA
ENSG00000101367	ACUGUAG
ENSG00000101367	AGCAGCA
ENSG00000101367	CAAAGUG

ENSG00000101367	CAGUAGA
ENSG00000101367	CAGUAGU
ENSG00000101367	CUCAAAC
ENSG00000101367	GAGUAUU
ENSG00000101367	GCUGGUG
ENSG00000101367	GGAAGCC
ENSG00000101367	GUUAAUG
ENSG00000101367	UAAUACA
ENSG00000101367	UGCAAAG
ENSG00000101367	UGCAUUG
ENSG00000101367	UUGGCAA
ENSG00000101367	UUGGCAC
ENSG00000101384	ACCACAG
ENSG00000101384	AGUGAUC
ENSG00000101384	AGUGGUU
ENSG00000101384	CACAGUG
ENSG00000101384	CAGUAGA
ENSG00000101384	GGCAGUG
ENSG00000101384	UGCAAAG
ENSG00000101384	UGCAUAU
ENSG00000101384	UGCUGAC
ENSG00000101384	UUGUGAC
ENSG00000101384	UUUGCAA
ENSG00000101384	UUUGCAC
ENSG00000101400	AAGGCAC
ENSG00000101400	AUGGCUU
ENSG00000101400	UCACAGG
ENSG00000101400	UGCUCAC
ENSG00000101407	GAUCGAC
ENSG00000101407	UGUGUGG
ENSG00000101407	UUCUAAU
ENSG00000101412	AAAGCUG
ENSG00000101412	AAGUGCA
ENSG00000101412	AAGUGCU
ENSG00000101412	CAAAGCA
ENSG00000101412	CAAAGUG
ENSG00000101412	CAGUCUG
ENSG00000101412	CCUUCAU
ENSG00000101412	CUCCAUU
ENSG00000101412	GAGCCCC
ENSG00000101417	AAGGCAC
ENSG00000101417	GAGAAGA
ENSG00000101417	GCUGGAU
ENSG00000101417	GGAGUGU
ENSG00000101417	GUGACUG
ENSG00000101417	GUGAGGU
ENSG00000101417	UCCAGAG
ENSG00000101417	UCCUAUA
ENSG00000101417	UGAGAAG
ENSG00000101417	UGCAUAG
ENSG00000101417	UGCAUAU
ENSG00000101417	UUAGAGA
ENSG00000101438	AAGCGCU
ENSG00000101438	AUUGCAC
ENSG00000101438	CACCACC
ENSG00000101438	CUGGAAA
ENSG00000101438	GAAAACA
ENSG00000101438	UCCUGAG
ENSG00000101442	CCAGGAG
ENSG00000101442	CUGGGCA
ENSG00000101442	GAGAAAU
ENSG00000101444	AAAGUAC
ENSG00000101444	ACAGUAU
ENSG00000101444	CAGCAGA

ENSG00000101444	CAGCUGG
ENSG00000101445	AAAGCUG
ENSG00000101445	AAGGCGC
ENSG00000101445	AAGUGUG
ENSG00000101445	AAUUAUU
ENSG00000101445	ACAGUAC
ENSG00000101445	ACUCCAG
ENSG00000101445	AGCUGCC
ENSG00000101445	AGGUAGU
ENSG00000101445	AUAUUAU
ENSG00000101445	CACAGGG
ENSG00000101445	CAGCAAA
ENSG00000101445	CAGGUGA
ENSG00000101445	CCCACCU
ENSG00000101445	CUGCAGC
ENSG00000101445	CUGCAGG
ENSG00000101445	GAGGUAG
ENSG00000101445	GCGGAGG
ENSG00000101445	GGAAGCC
ENSG00000101445	GGCAGCG
ENSG00000101445	GGGGUGG
ENSG00000101445	GUCUGCC
ENSG00000101445	UACAAAG
ENSG00000101445	UAUUGCU
ENSG00000101445	UCACAGG
ENSG00000101445	UCACAUU
ENSG00000101448	CACCAGC
ENSG00000101452	AUAAAGU
ENSG00000101457	GGGGUGC
ENSG00000101460	ACCACAG
ENSG00000101460	AGGCAGG
ENSG00000101460	CUGCAGC
ENSG00000101460	CUGCAGG
ENSG00000101460	GGCAGGG
ENSG00000101460	UUCUGCA
ENSG00000101464	CUGCUCA
ENSG00000101474	CCAGUGU
ENSG00000101493	AAAAACC
ENSG00000101493	AAAACUG
ENSG00000101493	AAACUGU
ENSG00000101493	AAAGCUG
ENSG00000101493	AAAGGUA
ENSG00000101493	AACACUG
ENSG00000101493	AAUUAUU
ENSG00000101493	AGCCCUU
ENSG00000101493	AGGUAGU
ENSG00000101493	AGUAACA
ENSG00000101493	AUAAUAC
ENSG00000101493	AUGUGCC
ENSG00000101493	CACAAUG
ENSG00000101493	CCAGUGC
ENSG00000101493	CCAUUAC
ENSG00000101493	CCCUGUU
ENSG00000101493	CUACAAA
ENSG00000101493	CUAGUAA
ENSG00000101493	GAGGUAG
ENSG00000101493	GAGGUAU
ENSG00000101493	GCCCUGU
ENSG00000101493	GGAAUGU
ENSG00000101493	GUUAGGA
ENSG00000101493	UACAAAG
ENSG00000101493	UAUAAUA
ENSG00000101493	UAUUACC
ENSG00000101493	UCAAGUA

ENSG00000101493	UCACACA
ENSG00000101493	UCACAGG
ENSG00000101493	UCACAGU
ENSG00000101493	UCCAGUU
ENSG00000101493	UCCUGAG
ENSG00000101493	UGCAUUG
ENSG00000101493	UUAGGAU
ENSG00000101493	UUUGCAA
ENSG00000101493	UUUGCAC
ENSG00000101493	UUUUUGC
ENSG00000101542	UAUUGCU
ENSG00000101542	UCCAGAG
ENSG00000101542	UGAUCCA
ENSG00000101544	CGUGCAU
ENSG00000101557	AAAGCUG
ENSG00000101557	AGAUAAA
ENSG00000101557	AGCAGCA
ENSG00000101557	CUAGUAG
ENSG00000101557	CUUUGGU
ENSG00000101557	GAUUGUA
ENSG00000101557	GAUUGUC
ENSG00000101557	UGUAUAA
ENSG00000101557	UUCCAUA
ENSG00000101557	UUGAUCA
ENSG00000101558	AAAGGUA
ENSG00000101558	AACAGUC
ENSG00000101558	AACAUUC
ENSG00000101558	AACCGUU
ENSG00000101558	AAGUGCU
ENSG00000101558	ACAUUAC
ENSG00000101558	AUACCUC
ENSG00000101558	CAUAUUG
ENSG00000101558	CCAGUAC
ENSG00000101558	CUUGUAU
ENSG00000101558	GCAACUU
ENSG00000101558	GCACCAU
ENSG00000101558	GCAGGAC
ENSG00000101558	GGCAUUG
ENSG00000101558	GUAAACA
ENSG00000101558	GUAACAG
ENSG00000101558	GUCAACA
ENSG00000101558	GUCCAGA
ENSG00000101558	UAUAUGA
ENSG00000101558	UCCAGUU
ENSG00000101558	UCCUGAG
ENSG00000101558	UCUUGAG
ENSG00000101558	UGACAUC
ENSG00000101558	UGCUCAC
ENSG00000101558	UGGGACA
ENSG00000101558	UUCUAAU
ENSG00000101558	UUCUUGU
ENSG00000101558	UUUGCAC
ENSG00000101574	UCACAAG
ENSG00000101577	AAUACUG
ENSG00000101577	ACACACU
ENSG00000101577	ACUGGAU
ENSG00000101577	AGUACUG
ENSG00000101577	AUGGAUU
ENSG00000101577	GAAAACA
ENSG00000101577	GACAACU
ENSG00000101577	GAGAAGA
ENSG00000101577	GCACCAU
ENSG00000101577	GCAGAAG
ENSG00000101577	GGAUUUU

ENSG00000101577	GUCAUUG
ENSG00000101577	UCACAGG
ENSG00000101577	UCACAGU
ENSG00000101577	UCAUUUG
ENSG00000101577	UCUGGAA
ENSG00000101624	AACUACU
ENSG00000101624	AAUUUUA
ENSG00000101638	AAUUGCU
ENSG00000101638	GUGCUUG
ENSG00000101638	UAUAAAG
ENSG00000101654	AAUACUG
ENSG00000101654	ACAUUAC
ENSG00000101654	ACUGUAG
ENSG00000101654	CUUCUCU
ENSG00000101665	AACAUUC
ENSG00000101665	AAGAACC
ENSG00000101665	AAUCUCA
ENSG00000101665	ACAUUCA
ENSG00000101665	AGCAGCG
ENSG00000101665	AGCAGCU
ENSG00000101665	AGUAGUU
ENSG00000101665	AUAUUAU
ENSG00000101665	CAGCAAA
ENSG00000101665	CCAGGAG
ENSG00000101665	CUCAAAC
ENSG00000101665	CUUCUCU
ENSG00000101665	GGCAUUG
ENSG00000101665	GUUCAUG
ENSG00000101665	UAUAAAG
ENSG00000101665	UCAUUUG
ENSG00000101665	UCUCAAG
ENSG00000101665	UGAGAAU
ENSG00000101665	UGCAUUG
ENSG00000101665	UGGUACC
ENSG00000101665	UUGGCAA
ENSG00000101665	UUGGCAC
ENSG00000101670	CAGCAGA
ENSG00000101670	CAGCAGG
ENSG00000101670	UUGGCAA
ENSG00000101680	GAGGUAU
ENSG00000101695	ACAGUAC
ENSG00000101695	AGCAAAA
ENSG00000101695	AGCCCUU
ENSG00000101695	AUAAAGU
ENSG00000101695	CAGCAAA
ENSG00000101695	CCUGUAC
ENSG00000101695	GCAGCAU
ENSG00000101695	GCCCUUA
ENSG00000101695	UAAUAGU
ENSG00000101695	UACAGUU
ENSG00000101695	UCCCUCU
ENSG00000101695	UUUGCAA
ENSG00000101745	AAAGAAU
ENSG00000101745	AAAUUUC
ENSG00000101745	AACUACU
ENSG00000101745	AACUCUA
ENSG00000101745	AAGUGCA
ENSG00000101745	AAUCACU
ENSG00000101745	AAUCCUU
ENSG00000101745	ACCCUGU
ENSG00000101745	AGCCCUU
ENSG00000101745	AGUGCAA
ENSG00000101745	AGUGCUU
ENSG00000101745	AUAAAGU

ENSG00000101745	AUACAAG
ENSG00000101745	AUCCCUU
ENSG00000101745	CCAGUGC
ENSG00000101745	CUUUGGU
ENSG00000101745	GACCAUG
ENSG00000101745	GCCCUUA
ENSG00000101745	GUGCAAA
ENSG00000101745	GUGCCAC
ENSG00000101745	GUUCAUG
ENSG00000101745	UAAUAUC
ENSG00000101745	UAUAAUA
ENSG00000101745	UCAUGAU
ENSG00000101745	UCAUUCG
ENSG00000101745	UCCCUUU
ENSG00000101745	UCCUGAG
ENSG00000101745	UCCUUGC
ENSG00000101745	UUCUGCA
ENSG00000101746	AAAGUGC
ENSG00000101746	AACAUUC
ENSG00000101746	AAGGCAC
ENSG00000101746	AAGUGCA
ENSG00000101746	ACAUUCA
ENSG00000101746	AGUGCAA
ENSG00000101746	CACAGUG
ENSG00000101746	CAGUUAC
ENSG00000101746	GAUUGUC
ENSG00000101746	GUCACUC
ENSG00000101746	GUGCAAA
ENSG00000101746	UCACAGU
ENSG00000101751	AAGUAGC
ENSG00000101751	ACAUUAC
ENSG00000101751	AUUCAUU
ENSG00000101751	CCUUCUG
ENSG00000101751	CCUUCUU
ENSG00000101751	UCAUUCG
ENSG00000101751	UCUGGAA
ENSG00000101752	AGAUGUG
ENSG00000101752	AGCACCA
ENSG00000101752	AGCAGCG
ENSG00000101752	AGCAGCU
ENSG00000101752	AGUAGAC
ENSG00000101752	AGUGCAA
ENSG00000101752	CCCUGAG
ENSG00000101752	CUCCCAA
ENSG00000101752	CUUACAG
ENSG00000101752	GCACCAU
ENSG00000101752	GUAACAG
ENSG00000101752	GUGCAAA
ENSG00000101752	UAGGUAU
ENSG00000101752	UCCUAUA
ENSG00000101752	UCCUGAG
ENSG00000101752	UUCAAGC
ENSG00000101782	AGCACCA
ENSG00000101782	AGGUAGU
ENSG00000101782	CACUCCU
ENSG00000101782	CUGGAAA
ENSG00000101782	GAAAACA
ENSG00000101782	GAGGUAG
ENSG00000101782	GCACCAU
ENSG00000101782	UGAAAUG
ENSG00000101825	AAGUCUU
ENSG00000101825	CUUGUAU
ENSG00000101842	CAGGAAC
ENSG00000101843	CUGGAAA

ENSG00000101846	CCUUCAU
ENSG00000101846	UCAUUUG
ENSG00000101846	UCUGGAA
ENSG00000101849	AAAACUG
ENSG00000101849	AAAGUGC
ENSG00000101849	AACUGGU
ENSG00000101849	AAGUGCA
ENSG00000101849	ACAAGGU
ENSG00000101849	ACCCUGU
ENSG00000101849	AUGAGCU
ENSG00000101849	CAAAACU
ENSG00000101849	CAAAGCA
ENSG00000101849	CAAAGUG
ENSG00000101849	CCUGUAC
ENSG00000101849	CUGGCUC
ENSG00000101849	CUUACAG
ENSG00000101849	GAAGGAA
ENSG00000101849	GAGCUAA
ENSG00000101849	GCCCUUA
ENSG00000101849	GCUGGUG
ENSG00000101849	GGAGGAG
ENSG00000101849	GGUUUAC
ENSG00000101849	GUAAACA
ENSG00000101849	UACAGUU
ENSG00000101849	UCACACA
ENSG00000101849	UCUAGAG
ENSG00000101849	UUGGCAA
ENSG00000101849	UUGGUCC
ENSG00000101849	UUGGUUC
ENSG00000101856	AACACUG
ENSG00000101856	CAAAGCA
ENSG00000101856	UAGAGGA
ENSG00000101856	UUAGAGA
ENSG00000101856	UUCUAAU
ENSG00000101868	AAGUAGC
ENSG00000101868	GGAAUGU
ENSG00000101871	AAAAAUC
ENSG00000101871	AAACUGU
ENSG00000101871	AAAGUGC
ENSG00000101871	AACCUGU
ENSG00000101871	AACUCUA
ENSG00000101871	AAGGCAC
ENSG00000101871	AAGUGCA
ENSG00000101871	AAUUAUU
ENSG00000101871	ACCUGGA
ENSG00000101871	ACUGCAG
ENSG00000101871	ACUGUAG
ENSG00000101871	AUUCAUU
ENSG00000101871	CAAAGUG
ENSG00000101871	CCUGUAC
ENSG00000101871	UACAAAG
ENSG00000101871	UAUAAUA
ENSG00000101871	UGGUACC
ENSG00000101882	AAGGCAC
ENSG00000101888	AAGGCAC
ENSG00000101888	AAGGUGC
ENSG00000101888	AGCUGCC
ENSG00000101888	AUGGCUU
ENSG00000101888	GAGGUAG
ENSG00000101888	GCUGGAU
ENSG00000101892	AAAAAUC
ENSG00000101892	AAAGAAU
ENSG00000101892	AAUGUUG
ENSG00000101892	AGCAGCA

ENSG00000101892	CCCCUGG
ENSG00000101892	CCCUGUU
ENSG00000101892	CUGGCUC
ENSG00000101892	UCCCACA
ENSG00000101892	UCCCUGU
ENSG00000101892	UGAAGCU
ENSG00000101892	UGAUCCA
ENSG00000101911	CUUUGGU
ENSG00000101911	UUGGCAA
ENSG00000101928	AAUCCUU
ENSG00000101928	ACAUUCA
ENSG00000101928	AUCCUUG
ENSG00000101928	CAGUCCA
ENSG00000101928	CAGUGCA
ENSG00000101928	GAUCAGA
ENSG00000101928	GGAGUGU
ENSG00000101928	UAUAAAG
ENSG00000101935	AAACUGU
ENSG00000101935	AACGGAA
ENSG00000101935	AAGUCAC
ENSG00000101935	ACAGUAC
ENSG00000101935	ACAGUAU
ENSG00000101935	ACAUCAC
ENSG00000101935	ACUAGAU
ENSG00000101935	ACUCCAG
ENSG00000101935	AGCACCA
ENSG00000101935	AGCAGCA
ENSG00000101935	AGGCAGG
ENSG00000101935	AUCACUA
ENSG00000101935	AUCCUUU
ENSG00000101935	CAGUGCA
ENSG00000101935	CUUUGGU
ENSG00000101935	GACCUGG
ENSG00000101935	GAGAUGA
ENSG00000101935	GCAGGAC
ENSG00000101935	GGCUCAG
ENSG00000101935	GUGACUG
ENSG00000101935	UAUUACC
ENSG00000101935	UAUUGCU
ENSG00000101935	UCAAGUA
ENSG00000101935	UGCAUAU
ENSG00000101935	UGCUGAC
ENSG00000101935	UGGGUAC
ENSG00000101935	UGUGCGG
ENSG00000101935	UUGGUUC
ENSG00000101938	AACCUGG
ENSG00000101938	AGCCUGC
ENSG00000101938	CACUGUU
ENSG00000101938	CAGUCCA
ENSG00000101938	CUGCAGC
ENSG00000101938	CUGUUGA
ENSG00000101938	UAGGGCC
ENSG00000101938	UGCAGCU
ENSG00000101938	UUCUUGU
ENSG00000101945	AGUGCAA
ENSG00000101945	CCCUGAG
ENSG00000101945	CCUUCUG
ENSG00000101945	GCAGAAG
ENSG00000101945	GUGCCUG
ENSG00000101945	UCCCUCU
ENSG00000101945	UCCCUGU
ENSG00000101945	UUCAAGC
ENSG00000101945	UUCCUAG
ENSG00000101945	UUUCAAC

ENSG00000101955	ACAUCAC
ENSG00000101955	UACAAAG
ENSG00000101966	AAAGUGC
ENSG00000101966	AACAAAC
ENSG00000101966	AAGACAU
ENSG00000101966	AAGUAGC
ENSG00000101966	AAUACUG
ENSG00000101966	AAUCCUU
ENSG00000101966	AAUCUCA
ENSG00000101966	ACAUUCA
ENSG00000101966	AGCAAAA
ENSG00000101966	AGGCAGG
ENSG00000101966	AGUAGUU
ENSG00000101966	AUAAAGU
ENSG00000101966	GAAACAU
ENSG00000101966	GAUUGUC
ENSG00000101966	GGAAGAC
ENSG00000101966	GGUCUAG
ENSG00000101966	GUUAGGA
ENSG00000101966	UAUAAUA
ENSG00000101966	UCAACAG
ENSG00000101966	UCACAGG
ENSG00000101966	UCAUUUG
ENSG00000101966	UUCUAAU
ENSG00000101966	UUGAUCA
ENSG00000101966	UUGGCAC
ENSG00000101972	AAAGUAA
ENSG00000101972	AACAAAC
ENSG00000101972	AACCUGG
ENSG00000101972	AAGACAU
ENSG00000101972	AAGUUGU
ENSG00000101972	ACAUUAC
ENSG00000101972	ACUGGAU
ENSG00000101972	AGCACCA
ENSG00000101972	CAAAACU
ENSG00000101972	CACUGUU
ENSG00000101972	CAGCAGA
ENSG00000101972	CUCUUCC
ENSG00000101972	CUGCCCC
ENSG00000101972	CUUCACC
ENSG00000101972	GAAGGAA
ENSG00000101972	GAGUUGA
ENSG00000101972	UAUAAAG
ENSG00000101972	UCCCACA
ENSG00000101972	UCCCACC
ENSG00000101972	UCUCAAG
ENSG00000101972	UGUAUAA
ENSG00000101972	UUAGAGA
ENSG00000101974	AAAUUUC
ENSG00000101974	ACAUCAC
ENSG00000101974	ACCCAUU
ENSG00000101974	AUUGCAC
ENSG00000101974	CAAAUGC
ENSG00000101974	CUUUGGU
ENSG00000101974	GGCAUUG
ENSG00000101974	UCCUGAG
ENSG00000101977	AAGGCAC
ENSG00000101986	AAGGCAC
ENSG00000101986	CCUUCAU
ENSG00000101986	CUUUGGU
ENSG00000101986	GGCAGUG
ENSG00000101986	UUGGCAA
ENSG00000101997	AAACUGU
ENSG00000101997	GAGCCCU

ENSG00000101997	GGCAGGG
ENSG00000102003	AGAUAAA
ENSG00000102003	AUAAUAC
ENSG00000102003	CACAGGG
ENSG00000102003	CAGCAAA
ENSG00000102003	GAGACCU
ENSG00000102003	GCAGGAC
ENSG00000102003	GGCUCAG
ENSG00000102003	GGGGCAG
ENSG00000102003	GGUGGUU
ENSG00000102003	UCACAGG
ENSG00000102003	UCUGGAA
ENSG00000102003	UCUGGAG
ENSG00000102003	UUGUUCG
ENSG00000102007	AAGGCAC
ENSG00000102007	GCAGAAG
ENSG00000102007	UGGCCAC
ENSG00000102024	AAUACUG
ENSG00000102024	AUGCACC
ENSG00000102034	AGGUAGU
ENSG00000102034	CUGGGCA
ENSG00000102034	GAGGUAG
ENSG00000102038	CAAAGCA
ENSG00000102038	UAAUACA
ENSG00000102038	UUCAAAU
ENSG00000102053	ACAUUAC
ENSG00000102053	AGCAGCA
ENSG00000102053	AGGCAGG
ENSG00000102053	AUACAAG
ENSG00000102053	CACACCG
ENSG00000102053	CACAGUG
ENSG00000102053	CCCUGAG
ENSG00000102053	GAGGCAG
ENSG00000102053	GCAGCAU
ENSG00000102053	GGCAGUG
ENSG00000102053	GUCAUUG
ENSG00000102053	GUGACAG
ENSG00000102053	GUGCCAC
ENSG00000102053	GUGUCAC
ENSG00000102053	UCACAGG
ENSG00000102053	UCACAGU
ENSG00000102053	UCACAUU
ENSG00000102053	UCAUGAU
ENSG00000102053	UCUCAAG
ENSG00000102053	UGCAGCU
ENSG00000102054	UGCAAAG
ENSG00000102057	ACUCCAG
ENSG00000102057	CUGCAGC
ENSG00000102057	CUGGACU
ENSG00000102057	GUCCAGA
ENSG00000102057	UAUUGCU
ENSG00000102057	UGCAUAU
ENSG00000102057	UGUGCUU
ENSG00000102069	AAAUGGU
ENSG00000102069	AACAAAC
ENSG00000102069	AACUGGU
ENSG00000102069	AAUCACU
ENSG00000102069	AGCUGCC
ENSG00000102069	AGUGCUU
ENSG00000102069	AUGUCUG
ENSG00000102069	CCAUUAC
ENSG00000102069	CCUUCAU
ENSG00000102069	UAAGACU
ENSG00000102069	UAGACUG

ENSG00000102069	UCACAAG
ENSG00000102069	UCACAGG
ENSG00000102069	UCAUUCG
ENSG00000102069	UCCAGAG
ENSG00000102069	UGUCUGG
ENSG00000102078	AUGCCUU
ENSG00000102081	AAGGCAC
ENSG00000102081	ACAGUAC
ENSG00000102081	ACAUUCA
ENSG00000102081	AUCAUAC
ENSG00000102081	AUGCAUU
ENSG00000102081	CAAGAGC
ENSG00000102081	CUGUUGA
ENSG00000102081	CUUACAG
ENSG00000102081	CUUUGGU
ENSG00000102081	GAAGGUC
ENSG00000102081	GAGGCAG
ENSG00000102081	UCAUACA
ENSG00000102081	UGCAUAG
ENSG00000102081	UUGGCAC
ENSG00000102096	AUGGCUU
ENSG00000102096	CCCUGUC
ENSG00000102096	CCCUGUU
ENSG00000102096	CUGGGCA
ENSG00000102096	GUCAACA
ENSG00000102096	UCCCUGU
ENSG00000102096	UGGGAUC
ENSG00000102098	AGUGCAA
ENSG00000102098	CAGUGCA
ENSG00000102098	GGCAGUG
ENSG00000102098	GUGCAAA
ENSG00000102098	UCCCUUG
ENSG00000102098	UGCAAAG
ENSG00000102098	UGCAUAG
ENSG00000102098	UGCAUAU
ENSG00000102100	AACAAAC
ENSG00000102100	GCAGGUU
ENSG00000102100	UGAUCCA
ENSG00000102100	UUGGCAA
ENSG00000102100	UUGGCAC
ENSG00000102104	UGAAAUG
ENSG00000102119	AAGGCAC
ENSG00000102125	CCCUGAG
ENSG00000102144	AAAGUAU
ENSG00000102144	AACAAAC
ENSG00000102144	AAUGCCC
ENSG00000102144	AUGACAC
ENSG00000102144	CAGGAAC
ENSG00000102144	GAGAUGA
ENSG00000102144	GGGAUCG
ENSG00000102144	UGGGAUC
ENSG00000102144	UUCCAUA
ENSG00000102158	GAUUGUA
ENSG00000102158	UGGAUAA
ENSG00000102172	UCACAUU
ENSG00000102178	CUGCCCC
ENSG00000102181	AAAGCUG
ENSG00000102189	AAAGUGC
ENSG00000102189	AAGACAU
ENSG00000102189	AGGUAGU
ENSG00000102189	AUGCACC
ENSG00000102189	CAAAGUG
ENSG00000102189	GAGGUAG
ENSG00000102189	GGCAGUG

ENSG00000102189	GUGCAAA
ENSG00000102189	UAAGACG
ENSG00000102189	UCACAUU
ENSG00000102189	UCCAGUU
ENSG00000102189	UGAUCCA
ENSG00000102189	UGUGUGG
ENSG00000102189	UUGGCAA
ENSG00000102189	UUGGCAC
ENSG00000102189	UUGUGAC
ENSG00000102189	UUGUGUC
ENSG00000102218	AAUCUCA
ENSG00000102218	UAUUACC
ENSG00000102218	UCAAGUA
ENSG00000102221	AAAAGUA
ENSG00000102221	AAAGUAU
ENSG00000102221	AACAAAC
ENSG00000102221	AAUACUG
ENSG00000102221	AAUUAUU
ENSG00000102221	AAUUUUA
ENSG00000102230	AAAGUGC
ENSG00000102230	AGCACCA
ENSG00000102230	AGGGAGG
ENSG00000102230	AGGUAGU
ENSG00000102230	AGUGCAA
ENSG00000102230	CAAAGCA
ENSG00000102230	CAAAGUG
ENSG00000102230	CAUAUUG
ENSG00000102230	CUUGGGC
ENSG00000102230	GAGGUAG
ENSG00000102230	GCAGGUU
ENSG00000102230	UAUAAAG
ENSG00000102230	UCACAAG
ENSG00000102230	UCCAGUU
ENSG00000102230	UCCCUUU
ENSG00000102230	UGCCCUG
ENSG00000102245	AGGCAGG
ENSG00000102245	AUGUCUG
ENSG00000102245	UAUAAAG
ENSG00000102245	UGUCUGC
ENSG00000102287	AAGUCAC
ENSG00000102287	ACCCAUU
ENSG00000102287	AGCAGCA
ENSG00000102287	CACAGGG
ENSG00000102287	CAGCAGA
ENSG00000102287	GAGGCUG
ENSG00000102287	GGCAUUG
ENSG00000102287	UCACACA
ENSG00000102287	UCACAGG
ENSG00000102287	UGCUGAC
ENSG00000102287	UUGAGGC
ENSG00000102290	ACACACU
ENSG00000102290	ACCACAG
ENSG00000102290	CCUGCUG
ENSG00000102290	CUGGAAA
ENSG00000102290	CUGGGCA
ENSG00000102290	UCCUAUA
ENSG00000102290	UUGGCAC
ENSG00000102302	AAAGUGC
ENSG00000102302	AAUACUG
ENSG00000102302	AGCAUUC
ENSG00000102302	CAUGGGU
ENSG00000102302	CCAGCAU
ENSG00000102302	GGAGGCA
ENSG00000102302	GGCCCUG

ENSG00000102302	UGACUGU
ENSG00000102302	UGGCCCU
ENSG00000102302	UUGUGAC
ENSG00000102316	UGGCUGC
ENSG00000102317	CCAGUAC
ENSG00000102317	CUUGUGU
ENSG00000102317	GAUCAGA
ENSG00000102317	UAUAAAG
ENSG00000102317	UCCAGUU
ENSG00000102317	UCCUUGC
ENSG00000102317	UGCAGCU
ENSG00000102359	CUGGCUC
ENSG00000102385	AAAGAGC
ENSG00000102385	AACAAAC
ENSG00000102385	ACCACAG
ENSG00000102385	AGCACCA
ENSG00000102385	AGGUUAU
ENSG00000102385	CACAGUG
ENSG00000102385	CAGCAAA
ENSG00000102385	CAGCAGG
ENSG00000102385	CAGUCCA
ENSG00000102385	CUGGAAA
ENSG00000102385	GAGGUAU
ENSG00000102385	GGAGAGA
ENSG00000102385	GUGACUG
ENSG00000102385	UAGUGAG
ENSG00000102385	UAUAAAG
ENSG00000102387	AGCAGCA
ENSG00000102401	AAUGCCC
ENSG00000102401	CAGCAAA
ENSG00000102445	AGCAGCA
ENSG00000102445	AGCAGCG
ENSG00000102445	AGCAGCU
ENSG00000102445	UCUCAAG
ENSG00000102452	UGCUGAC
ENSG00000102468	ACAAUAU
ENSG00000102468	CAAAUGC
ENSG00000102468	CUGCUCA
ENSG00000102471	GGAAUGU
ENSG00000102531	AAGACAU
ENSG00000102531	AAUCUCU
ENSG00000102531	ACAUUAC
ENSG00000102531	ACAUUCA
ENSG00000102531	AGCAGCU
ENSG00000102531	CAAAGCA
ENSG00000102531	CAAAGUG
ENSG00000102531	GAGGUAU
ENSG00000102531	GUGCAAA
ENSG00000102531	UAAGACG
ENSG00000102531	UAAGACU
ENSG00000102531	UCACAGG
ENSG00000102531	UCACAGU
ENSG00000102531	UGCAAAG
ENSG00000102543	AAGUGCA
ENSG00000102572	AAAGCUG
ENSG00000102572	AAUCACU
ENSG00000102572	ACAAGGU
ENSG00000102572	ACUCCAG
ENSG00000102572	AGCAUUC
ENSG00000102572	AGGAGCU
ENSG00000102572	AUACAAG
ENSG00000102572	AUGCACC
ENSG00000102572	AUGUAAU
ENSG00000102572	AUGUGCC

ENSG00000102572	CUACAGU
ENSG00000102572	GAGGUAG
ENSG00000102572	GAGGUAU
ENSG00000102572	UAUAAAG
ENSG00000102572	UGGCCAC
ENSG00000102572	UGUGCUU
ENSG00000102572	UUAUGCA
ENSG00000102572	UUCUAAU
ENSG00000102606	AACAUUC
ENSG00000102606	AAGUGCA
ENSG00000102606	AAGUUGU
ENSG00000102606	ACAAUAU
ENSG00000102606	ACAUUAC
ENSG00000102606	AGCAAAA
ENSG00000102606	AUGCAUU
ENSG00000102606	CUUUGGU
ENSG00000102606	GAAAACA
ENSG00000102606	GCUAGCA
ENSG00000102606	UUGGCAC
ENSG00000102678	AAAGUUU
ENSG00000102678	AAAUUUC
ENSG00000102678	AAGUGCU
ENSG00000102678	AUGCAUU
ENSG00000102678	AUGGCAC
ENSG00000102678	CACCAGC
ENSG00000102678	CCAGCAU
ENSG00000102678	CCAGGAG
ENSG00000102678	CCCACCG
ENSG00000102678	GCAGAAG
ENSG00000102678	GGCAGCG
ENSG00000102678	UGUCUUU
ENSG00000102678	UUAUGCA
ENSG00000102678	UUGGCAC
ENSG00000102743	GGAAGAC
ENSG00000102753	AAAAGUA
ENSG00000102753	AAAGAGC
ENSG00000102753	AAGUGCA
ENSG00000102753	ACAGUAC
ENSG00000102753	ACAGUAU
ENSG00000102753	AGCAGCA
ENSG00000102753	AGCAGCG
ENSG00000102753	AUCAUAC
ENSG00000102753	AUCCCUU
ENSG00000102753	AUGUGCC
ENSG00000102753	CAGCAGG
ENSG00000102753	CAGGAAC
ENSG00000102753	CAGUUAC
ENSG00000102753	GAGAAAU
ENSG00000102753	GAGUUGA
ENSG00000102753	GCAGCAU
ENSG00000102753	GGCUCAG
ENSG00000102753	GGUGGUC
ENSG00000102753	GGUGGUU
ENSG00000102753	UAAGACG
ENSG00000102753	UACAGUU
ENSG00000102753	UAUAAAG
ENSG00000102753	UCACAUU
ENSG00000102753	UGAUCCA
ENSG00000102753	UGCAAAG
ENSG00000102753	UGCAGCU
ENSG00000102753	UGCAUAU
ENSG00000102753	UUGGCAC
ENSG00000102755	ACAUUCA
ENSG00000102755	AGCAGCU

ENSG00000102755	AUAAUAC
ENSG00000102760	AUAUAAC
ENSG00000102760	UACAAAG
ENSG00000102763	AAUUAUU
ENSG00000102780	AGCACCA
ENSG00000102781	GAGGUAU
ENSG00000102781	GAUCAGA
ENSG00000102781	GGUAGAC
ENSG00000102781	UGCAAAG
ENSG00000102786	AACAGUC
ENSG00000102786	AAGGCAC
ENSG00000102786	AAUACUG
ENSG00000102786	AGUGAUC
ENSG00000102786	AUACAAG
ENSG00000102786	AUGUGCC
ENSG00000102786	CACUGUU
ENSG00000102786	GGAUUUU
ENSG00000102786	GGUUUAC
ENSG00000102786	GUAUGUU
ENSG00000102786	GUGCUUG
ENSG00000102786	UGAGAAU
ENSG00000102786	UGGCCAC
ENSG00000102786	UGUAUGU
ENSG00000102786	UUGACAC
ENSG00000102786	UUGGCAC
ENSG00000102786	UUUUUGC
ENSG00000102796	GAGCUGC
ENSG00000102796	GGAUUUC
ENSG00000102804	AAGUGCA
ENSG00000102804	CUAUACA
ENSG00000102804	UGAAAGG
ENSG00000102805	CUGCAGC
ENSG00000102858	AAAGCUG
ENSG00000102858	ACAGUAC
ENSG00000102858	ACAGUAU
ENSG00000102858	GCUGUCU
ENSG00000102858	GGGGUCC
ENSG00000102858	UAUUACC
ENSG00000102870	AACACUG
ENSG00000102870	ACUCAAA
ENSG00000102870	AGCAGCG
ENSG00000102870	CACUGUU
ENSG00000102870	CCAGUGC
ENSG00000102870	CUCUGGG
ENSG00000102870	CUUUGGU
ENSG00000102870	GAGACCU
ENSG00000102870	GCUACAU
ENSG00000102870	UAUGGUU
ENSG00000102870	UCCCUUG
ENSG00000102870	UGAGAAU
ENSG00000102870	UUGGAGG
ENSG00000102878	GGAAGCC
ENSG00000102882	AACAGUC
ENSG00000102882	AGCCCUU
ENSG00000102882	AGGCAGG
ENSG00000102882	AUAUUAU
ENSG00000102882	CUCAGGG
ENSG00000102882	GGAAUGU
ENSG00000102882	UUCUAAU
ENSG00000102893	AUGUAAU
ENSG00000102893	GUAACAG
ENSG00000102893	UGCAUAG
ENSG00000102897	CUGGAAA
ENSG00000102908	AAAUCCC

ENSG00000102908	AAGACAU
ENSG00000102908	AAGGUGC
ENSG00000102908	AAGUGCA
ENSG00000102908	ACCACAG
ENSG00000102908	ACCCUGU
ENSG00000102908	ACUGGAU
ENSG00000102908	ACUGUUU
ENSG00000102908	AGCACCA
ENSG00000102908	AGCCAGU
ENSG00000102908	AGCUGCC
ENSG00000102908	AGUGGUU
ENSG00000102908	AUUCAUU
ENSG00000102908	AUUGCAC
ENSG00000102908	CAAAACU
ENSG00000102908	CACAGUG
ENSG00000102908	CAGACAG
ENSG00000102908	CAGCAAA
ENSG00000102908	CCUGUAC
ENSG00000102908	CCUUCUG
ENSG00000102908	CCUUCUU
ENSG00000102908	CUACAGU
ENSG00000102908	CUCAGGG
ENSG00000102908	CUGCAGC
ENSG00000102908	CUGCAGG
ENSG00000102908	CUGGAAA
ENSG00000102908	CUGGCUC
ENSG00000102908	GAGAAGA
ENSG00000102908	GAGUGUG
ENSG00000102908	GAUCAGA
ENSG00000102908	GCAUCCC
ENSG00000102908	GCCUGAU
ENSG00000102908	GCUACAU
ENSG00000102908	GCUGGUG
ENSG00000102908	GGAGUGU
ENSG00000102908	GGCAAGA
ENSG00000102908	GGCAUUG
ENSG00000102908	GGCUCAG
ENSG00000102908	GGGAACG
ENSG00000102908	GGGUUUA
ENSG00000102908	GGUAGAC
ENSG00000102908	GGUCUAG
ENSG00000102908	GGUUGAC
ENSG00000102908	GUAUUCU
ENSG00000102908	GUCCAGA
ENSG00000102908	GUUCAUG
ENSG00000102908	UAAUGCU
ENSG00000102908	UACAGUU
ENSG00000102908	UAUCAGA
ENSG00000102908	UCACAGU
ENSG00000102908	UCCCUUG
ENSG00000102908	UCCCUUU
ENSG00000102908	UGAACGG
ENSG00000102908	UGACCUA
ENSG00000102908	UGACUGU
ENSG00000102908	UGAGAAG
ENSG00000102908	UGCAGCU
ENSG00000102908	UGGCCAC
ENSG00000102908	UUAUGCA
ENSG00000102908	UUCAAGC
ENSG00000102908	UUCCAUA
ENSG00000102908	UUCUUGU
ENSG00000102908	UUGAUCA
ENSG00000102908	UUGGUCC
ENSG00000102908	UUGGUUC

ENSG00000102908	UUUGCAA
ENSG00000102908	UUUGCAC
ENSG00000102921	AAGUGCA
ENSG00000102921	ACCAGCA
ENSG00000102921	AGCAGCG
ENSG00000102921	AGCAGCU
ENSG00000102921	AGCCCUU
ENSG00000102921	AGCCUGC
ENSG00000102921	AGGAGCU
ENSG00000102921	AGUGCAA
ENSG00000102921	CAAGAGC
ENSG00000102921	CACAGUG
ENSG00000102921	CUUCACC
ENSG00000102921	GAAAACA
ENSG00000102921	GUCUUGC
ENSG00000102921	GUGACUG
ENSG00000102921	UAAUGCU
ENSG00000102921	UCACAGU
ENSG00000102921	UGAGAAU
ENSG00000102921	UGUGCUU
ENSG00000102921	UUGGCAA
ENSG00000102921	UUGGCAC
ENSG00000102931	AAAAGUA
ENSG00000102931	GAGUAUU
ENSG00000102931	UAUAAUA
ENSG00000102934	AGUGCUG
ENSG00000102934	AGUGCUU
ENSG00000102935	AAUACUG
ENSG00000102935	AAUCUCA
ENSG00000102935	AAUGUUG
ENSG00000102935	ACAUCAC
ENSG00000102935	AGCAGCA
ENSG00000102935	AGCAGCU
ENSG00000102935	AUGACAC
ENSG00000102935	AUGUAAU
ENSG00000102935	CAAAUGC
ENSG00000102935	GAUUGGU
ENSG00000102935	GCAGGAC
ENSG00000102935	UAAUAUC
ENSG00000102935	UCACAUU
ENSG00000102935	UCAUUUG
ENSG00000102935	UCCAGUU
ENSG00000102935	UCCCUUG
ENSG00000102935	UGCAAAG
ENSG00000102935	UUCAAAU
ENSG00000102962	AGCUUAU
ENSG00000102962	AUAAUAC
ENSG00000102962	CUGCAGC
ENSG00000102962	GGAGGAG
ENSG00000102962	GGGGAGC
ENSG00000102962	GUCUCUG
ENSG00000102962	GUGACAG
ENSG00000102962	GUGGGGA
ENSG00000102962	UAUAAUA
ENSG00000102962	UUGGAGG
ENSG00000102967	AAAGAAU
ENSG00000102967	UCCUUGC
ENSG00000102974	ACAGUAC
ENSG00000102974	AGUGGUU
ENSG00000102974	GGUUUAC
ENSG00000102974	GUAUGUU
ENSG00000102974	UCACACA
ENSG00000102974	UCUGGAG
ENSG00000102974	UGACCUA

ENSG00000102978	UAUUGCU
ENSG00000102978	UGUGCUU
ENSG00000102984	AGAUAAA
ENSG00000102996	ACAGUAC
ENSG00000102996	ACAGUAU
ENSG00000102996	CUGGCUC
ENSG00000102996	CUUUGGU
ENSG00000102996	UCCCACA
ENSG00000102996	UCCCACC
ENSG00000102996	UUGGUCC
ENSG00000103005	AAAACUG
ENSG00000103005	GGUGGGC
ENSG00000103005	UUGGAGG
ENSG00000103018	ACUGUGU
ENSG00000103018	CACUGUU
ENSG00000103018	GGAUUUU
ENSG00000103018	GGCUCUG
ENSG00000103021	GAAGGUC
ENSG00000103034	AAAAGUA
ENSG00000103034	ACAGUAC
ENSG00000103034	ACAGUAU
ENSG00000103034	ACCAGCA
ENSG00000103034	AUGGCUU
ENSG00000103034	GGCUCAG
ENSG00000103034	GUGCUUG
ENSG00000103034	UCACAUU
ENSG00000103034	UGUGCUU
ENSG00000103035	AAGAACC
ENSG00000103035	CCCUGAG
ENSG00000103035	GAAGGUC
ENSG00000103035	GUAAACA
ENSG00000103035	UACAAAG
ENSG00000103042	CUUCACC
ENSG00000103042	GUCCAGA
ENSG00000103042	UCCAGAG
ENSG00000103043	AAAGAGG
ENSG00000103044	AAAAAUC
ENSG00000103044	AAAGAGG
ENSG00000103044	ACCCAUU
ENSG00000103044	ACCCUGU
ENSG00000103044	AGCCCUU
ENSG00000103044	AGUGCUU
ENSG00000103044	AUAAAGU
ENSG00000103044	CCCACCG
ENSG00000103044	CCCACCU
ENSG00000103044	CCCUGUU
ENSG00000103044	CUCCCAC
ENSG00000103044	GACCCUG
ENSG00000103044	GAUUGUC
ENSG00000103044	GCACCAU
ENSG00000103044	GCCCUUA
ENSG00000103044	GGAUUUC
ENSG00000103044	GGCUCAG
ENSG00000103044	UACAAAG
ENSG00000103044	UAUAAAG
ENSG00000103044	UAUUGCU
ENSG00000103044	UCCCACC
ENSG00000103044	UCCCUCU
ENSG00000103044	UCCCUUU
ENSG00000103044	UGAGAAU
ENSG00000103044	UUGACAC
ENSG00000103044	UUGGUUC
ENSG00000103047	CCUGCUG
ENSG00000103047	UGCCAGC

ENSG00000103056	AAAACUG
ENSG00000103056	AAAGAAU
ENSG00000103056	AAGGCAC
ENSG00000103056	ACAAUAU
ENSG00000103056	AGCACCA
ENSG00000103056	AUGGCAC
ENSG00000103056	CACAGUG
ENSG00000103056	CAGCAAA
ENSG00000103056	CAGCAGA
ENSG00000103056	CUUUGGU
ENSG00000103056	GAUUGUA
ENSG00000103056	GAUUGUC
ENSG00000103056	GGCAGCG
ENSG00000103056	GUGCUUG
ENSG00000103056	UAAUAUC
ENSG00000103056	UACAAAG
ENSG00000103056	UAUCAGA
ENSG00000103056	UCACAGU
ENSG00000103056	UCCUGAG
ENSG00000103056	UGGCUGC
ENSG00000103056	UGGGUGA
ENSG00000103056	UUCUAAU
ENSG00000103061	UCACAUU
ENSG00000103064	AAAGGUA
ENSG00000103064	ACUGCAG
ENSG00000103064	AGCACCA
ENSG00000103064	AGCAGCU
ENSG00000103064	CACAGGG
ENSG00000103064	CAGACAG
ENSG00000103064	CCCACCU
ENSG00000103064	CCCUGAG
ENSG00000103064	CCUUCAU
ENSG00000103064	CUGACUC
ENSG00000103064	GAGCCCU
ENSG00000103064	GGAAUGU
ENSG00000103064	GGCAGUG
ENSG00000103064	GGCUCAG
ENSG00000103064	GGCUCUG
ENSG00000103064	GUAAACA
ENSG00000103064	GUGAGGU
ENSG00000103064	UCAAGUA
ENSG00000103064	UCCUGAG
ENSG00000103064	UGACAUC
ENSG00000103064	UGCCAGC
ENSG00000103089	AUACAAG
ENSG00000103089	UAUAAAG
ENSG00000103089	UUCAAAU
ENSG00000103089	UUUCAAC
ENSG00000103091	AAUUUUA
ENSG00000103111	AGCAGCA
ENSG00000103111	GCAGGUU
ENSG00000103126	AAGGCAC
ENSG00000103126	CACAGUG
ENSG00000103126	GAGAAAU
ENSG00000103126	GGCAGGG
ENSG00000103126	UAUUGCU
ENSG00000103126	UCACACA
ENSG00000103150	AACAAAC
ENSG00000103160	AAAGUUU
ENSG00000103160	AAUCACU
ENSG00000103160	AUAAAGU
ENSG00000103160	AUCACUA
ENSG00000103160	GAGAAAU
ENSG00000103160	UCACACA

ENSG00000103160	UUGGCAA
ENSG00000103174	AGGCAGG
ENSG00000103174	CAGCAGA
ENSG00000103174	UCAAGUA
ENSG00000103174	UUGGCAA
ENSG00000103174	UUGGCAC
ENSG00000103184	AAGUCAC
ENSG00000103184	CCUUCUG
ENSG00000103184	GAGGUAG
ENSG00000103184	GAGGUAU
ENSG00000103187	AAGGCAC
ENSG00000103187	GCUGGUG
ENSG00000103194	ACUGUGU
ENSG00000103194	AUAAAGU
ENSG00000103194	GACAACU
ENSG00000103194	GGCGACA
ENSG00000103196	ACAUCAC
ENSG00000103196	CAAAGCA
ENSG00000103196	CAGCAAA
ENSG00000103196	CCCUGUU
ENSG00000103196	CUCCCAC
ENSG00000103196	UGUCUGC
ENSG00000103196	UGUCUGG
ENSG00000103196	UUCAAAU
ENSG00000103202	AAGGCAC
ENSG00000103202	AAUUUUA
ENSG00000103202	AGGUAGU
ENSG00000103202	GAGGUAG
ENSG00000103222	AAGUCAC
ENSG00000103222	ACUGCAU
ENSG00000103222	CAGGAAC
ENSG00000103222	CCAGUGC
ENSG00000103222	GAGGCAG
ENSG00000103222	GGAAGAC
ENSG00000103222	GGCUGCG
ENSG00000103222	UCCAGUU
ENSG00000103222	UUGGUCC
ENSG00000103226	UCUCACC
ENSG00000103241	AAAGUGC
ENSG00000103241	AAAGUUU
ENSG00000103241	ACAGUAC
ENSG00000103241	ACAGUAU
ENSG00000103241	AGUGCCU
ENSG00000103241	AUAUAAC
ENSG00000103241	CAGCAGA
ENSG00000103241	CAGUAGA
ENSG00000103241	CAGUGCA
ENSG00000103241	GUGUCAC
ENSG00000103241	UAAUACA
ENSG00000103241	UACAGUU
ENSG00000103248	CUGGCUC
ENSG00000103254	GAGUCUU
ENSG00000103257	CAGUGCA
ENSG00000103257	CGUACCG
ENSG00000103264	AAGACAU
ENSG00000103264	AAGUGCA
ENSG00000103266	UGCUCAC
ENSG00000103269	GGCUCAG
ENSG00000103275	AAAGUUU
ENSG00000103275	AAUACUG
ENSG00000103275	ACCCUGU
ENSG00000103275	AUCCCUU
ENSG00000103275	GAAGGAA
ENSG00000103275	GACCCUG

ENSG00000103275	UGAAAUG
ENSG00000103319	AAAACUG
ENSG00000103319	AACCUGU
ENSG00000103319	AGAUAAA
ENSG00000103319	AGCAAAA
ENSG00000103319	AGGUAGU
ENSG00000103319	AUAAAGU
ENSG00000103319	CAAAGCA
ENSG00000103319	CAGUCCA
ENSG00000103319	CUCAAAC
ENSG00000103319	CUCUGGG
ENSG00000103319	GAGGUAG
ENSG00000103319	GGAAUGU
ENSG00000103319	GGCAGUG
ENSG00000103319	UAGAGGA
ENSG00000103319	UGCCCUG
ENSG00000103319	UUGAUCA
ENSG00000103319	UUGGUUC
ENSG00000103319	UUUCAAC
ENSG00000103326	AAAAAUC
ENSG00000103326	AAAGUGC
ENSG00000103326	AUAAAGU
ENSG00000103326	AUGGCUU
ENSG00000103326	GAGGUAU
ENSG00000103326	UCACAGG
ENSG00000103326	UCACAGU
ENSG00000103326	UCCUGAG
ENSG00000103326	UGCAUAG
ENSG00000103326	UGCAUAU
ENSG00000103342	AAAGCUG
ENSG00000103342	ACAGUAU
ENSG00000103342	UCACAGU
ENSG00000103351	AAUCACU
ENSG00000103351	UCCCUUG
ENSG00000103353	AAAGUAC
ENSG00000103353	AAAGUGC
ENSG00000103353	AAAUGGU
ENSG00000103353	AAGUGCA
ENSG00000103353	AAGUGCU
ENSG00000103353	AAUCUCU
ENSG00000103353	ACUGGCU
ENSG00000103353	AGCACCA
ENSG00000103353	AGCAGCA
ENSG00000103353	AGCCUGC
ENSG00000103353	AGGACAC
ENSG00000103353	AGUGCUU
ENSG00000103353	AUGGCAC
ENSG00000103353	AUGUGGG
ENSG00000103353	CAGUCCA
ENSG00000103353	CAGUCUG
ENSG00000103353	CAGUGCA
ENSG00000103353	CCAGCAU
ENSG00000103353	CCUUCAU
ENSG00000103353	CUGCAGG
ENSG00000103353	CUUUGGU
ENSG00000103353	GAAACAU
ENSG00000103353	GAGGUAG
ENSG00000103353	GAGGUAU
ENSG00000103353	GCAACUU
ENSG00000103353	GCACCAU
ENSG00000103353	GCAGAAG
ENSG00000103353	GCUGGAU
ENSG00000103353	GGCCCUG
ENSG00000103353	GUGCAAA

ENSG00000103353	UCACAGG
ENSG00000103353	UCACAGU
ENSG00000103353	UGCAUAG
ENSG00000103353	UGCAUAU
ENSG00000103353	UGGACUG
ENSG00000103353	UGGCCCU
ENSG00000103353	UUGGCAC
ENSG00000103353	UUGGUCC
ENSG00000103353	UUUGCAA
ENSG00000103353	UUUGCAC
ENSG00000103356	GAAGGUC
ENSG00000103365	AGCAUUC
ENSG00000103365	AGGCAGG
ENSG00000103365	CACAGUG
ENSG00000103365	CUUGGAG
ENSG00000103365	GAAGGAA
ENSG00000103365	GCAGAAG
ENSG00000103365	GGAUGAG
ENSG00000103365	GGCAGGG
ENSG00000103365	GGGAGCU
ENSG00000103365	UCACCAC
ENSG00000103365	UUGGCAC
ENSG00000103404	AAGGCGC
ENSG00000103404	AAGUCUU
ENSG00000103404	AAUUAUU
ENSG00000103404	AGCACCA
ENSG00000103404	AGCAGCA
ENSG00000103404	AUACCUC
ENSG00000103404	AUGGCUU
ENSG00000103404	CAAAGCA
ENSG00000103404	CAAAUGC
ENSG00000103404	CACAGGG
ENSG00000103404	CACCACC
ENSG00000103404	CCAGUGC
ENSG00000103404	CUGCUCA
ENSG00000103404	CUUUGGU
ENSG00000103404	GACUUCC
ENSG00000103404	GAGAAAU
ENSG00000103404	GAGUAUU
ENSG00000103404	GCAGAAG
ENSG00000103404	GCAGGUU
ENSG00000103404	GCCCUUA
ENSG00000103404	GGCAGGG
ENSG00000103404	GUGCUUG
ENSG00000103404	UAAUACA
ENSG00000103404	UAAUGCU
ENSG00000103404	UCACAAG
ENSG00000103404	UCACAUU
ENSG00000103404	UCACCAC
ENSG00000103404	UGCAGCU
ENSG00000103404	UGCAUAG
ENSG00000103404	UGCAUAU
ENSG00000103404	UGCCCUG
ENSG00000103404	UGUGCUU
ENSG00000103415	AAGUCAC
ENSG00000103415	CCAGCAU
ENSG00000103415	CUGGCUC
ENSG00000103423	UCAUUUG
ENSG00000103423	UCCAGUU
ENSG00000103423	UGCAGCU
ENSG00000103423	UUUGCAC
ENSG00000103426	AAAGUUU
ENSG00000103426	GGCAAGA
ENSG00000103429	AGCAGCA

ENSG00000103429	CAAAGCA
ENSG00000103429	UUCCUAG
ENSG00000103449	AAAGUGC
ENSG00000103449	AACUCUA
ENSG00000103449	AAGUGCA
ENSG00000103449	AAUGCCC
ENSG00000103449	ACAGUAC
ENSG00000103449	AGCAGCG
ENSG00000103449	AGCAGCU
ENSG00000103449	CAGCAGG
ENSG00000103449	CUAGUCC
ENSG00000103449	GCAGCAU
ENSG00000103449	GGCAUUG
ENSG00000103449	GUUAAUG
ENSG00000103449	UAAUGCU
ENSG00000103449	UACAGUU
ENSG00000103449	UCAAGUA
ENSG00000103449	UUAGAGA
ENSG00000103449	UUUGCAA
ENSG00000103460	AAAGUAU
ENSG00000103460	AAGUGCU
ENSG00000103460	ACAUUAC
ENSG00000103460	AGAUAAA
ENSG00000103460	CCAGUGU
ENSG00000103460	GUGCUUG
ENSG00000103460	UCAUACA
ENSG00000103460	UGAAAUG
ENSG00000103460	UGUGCUU
ENSG00000103460	UUGGCAC
ENSG00000103479	AGCAAAA
ENSG00000103479	AUUGCAC
ENSG00000103479	CUAGUAA
ENSG00000103489	AAAACUG
ENSG00000103489	AAAGUGC
ENSG00000103489	AAGUCAC
ENSG00000103489	ACAGGUG
ENSG00000103489	ACAGUAC
ENSG00000103489	ACAUUAC
ENSG00000103489	ACAUUCA
ENSG00000103489	AGCCCUU
ENSG00000103489	AUAAAGU
ENSG00000103489	AUAUUAU
ENSG00000103489	AUGACAC
ENSG00000103489	AUGCAUU
ENSG00000103489	CAAAUGC
ENSG00000103489	CACCACC
ENSG00000103489	CACUGUU
ENSG00000103489	CAGCAGA
ENSG00000103489	CAGCAGG
ENSG00000103489	CAGUUAC
ENSG00000103489	CUCUGGG
ENSG00000103489	CUUCUCU
ENSG00000103489	CUUUGGU
ENSG00000103489	GAGCCCU
ENSG00000103489	GAGGCAG
ENSG00000103489	GAGGUAG
ENSG00000103489	GAGGUAU
ENSG00000103489	GCAGAAG
ENSG00000103489	GGAGAGA
ENSG00000103489	GGCUCAG
ENSG00000103489	GGUAGAC
ENSG00000103489	GGUUGUC
ENSG00000103489	GUCACUC
ENSG00000103489	GUGAAUG

ENSG00000103489	GUGCAAA
ENSG00000103489	GUGCCAC
ENSG00000103489	UACAGUU
ENSG00000103489	UCACAUU
ENSG00000103489	UCACCAC
ENSG00000103489	UGAAAUG
ENSG00000103489	UGCAUAG
ENSG00000103489	UGGCUGC
ENSG00000103489	UUCAAAU
ENSG00000103489	UUGGUCC
ENSG00000103489	UUGUGAC
ENSG00000103489	UUUGCAA
ENSG00000103494	AAAGAGC
ENSG00000103494	AAAUGGU
ENSG00000103494	AACAAAC
ENSG00000103494	AAUCACU
ENSG00000103494	AAUUAUU
ENSG00000103494	AAUUUUA
ENSG00000103494	ACAGUAU
ENSG00000103494	AGCAUUC
ENSG00000103494	AGCCUGC
ENSG00000103494	AGGAGCU
ENSG00000103494	AGGUAGU
ENSG00000103494	AUCCUUU
ENSG00000103494	CACAGUG
ENSG00000103494	CUCCAUU
ENSG00000103494	GAAUUGU
ENSG00000103494	GCAGCAU
ENSG00000103494	UCACAAG
ENSG00000103494	UCACACA
ENSG00000103494	UCACAGG
ENSG00000103494	UGCAAAG
ENSG00000103494	UUUGCAA
ENSG00000103494	UUUUUGC
ENSG00000103495	AGCACCA
ENSG00000103495	CAGACAG
ENSG00000103495	CAGGAAC
ENSG00000103495	CUGCCCC
ENSG00000103495	UCUGGAG
ENSG00000103496	GGCUCUG
ENSG00000103496	UACAAAG
ENSG00000103502	CUGACUC
ENSG00000103502	GGGGAGC
ENSG00000103512	UCUCACC
ENSG00000103522	CUGCAGG
ENSG00000103522	UCUCACC
ENSG00000103534	CACAGGG
ENSG00000103549	CAGGCUC
ENSG00000103549	CCCUGAG
ENSG00000103549	GGAAUGU
ENSG00000103550	AGCACCA
ENSG00000103550	AUGGCUU
ENSG00000103550	CCCUGAG
ENSG00000103550	GAGCUGC
ENSG00000103569	CCAUUAC
ENSG00000103569	GUCCAGA
ENSG00000103647	AAUCACU
ENSG00000103647	CUCUGGG
ENSG00000103647	GCAGCAU
ENSG00000103647	GGAGGCA
ENSG00000103647	GUCUCUG
ENSG00000103647	UGGGUGA
ENSG00000103653	AAAAAUC
ENSG00000103653	AAUGCCC

ENSG00000103653	CUUGUAU
ENSG00000103653	UAUUACC
ENSG00000103653	UUCUUGU
ENSG00000103657	AAACUGU
ENSG00000103671	UAUAAAG
ENSG00000103707	UCCUUGC
ENSG00000103710	AGCAGCA
ENSG00000103710	CUGCAGC
ENSG00000103710	CUGCAGG
ENSG00000103710	GCAGCAU
ENSG00000103710	GUAAACA
ENSG00000103710	UUCUGCA
ENSG00000103740	UGAAGCU
ENSG00000103769	CUAUACA
ENSG00000103769	GACCUGG
ENSG00000103769	GCUGGUG
ENSG00000103855	ACCAGCA
ENSG00000103855	AGCACCA
ENSG00000103855	AGCAGCU
ENSG00000103855	GAGAAAU
ENSG00000103855	GAGAACC
ENSG00000103855	GAGGUAU
ENSG00000103855	GCACCAU
ENSG00000103855	GUGAGGU
ENSG00000103888	AAUCUCA
ENSG00000103888	AGCACCA
ENSG00000103888	AGUGAUC
ENSG00000103888	AGUGGUU
ENSG00000103888	AUAAUAC
ENSG00000103888	GGUAGAC
ENSG00000103888	GUCUCUG
ENSG00000103888	UAUAAUA
ENSG00000103888	UCACAGG
ENSG00000103888	UCACAGU
ENSG00000103888	UGGCCAC
ENSG00000103932	CUCAAAC
ENSG00000103942	ACUGCAU
ENSG00000103942	AGCCCUU
ENSG00000103942	GCCCUUA
ENSG00000103942	GGUAGAC
ENSG00000103942	UAUGGUU
ENSG00000103942	UCCCACA
ENSG00000103942	UGCCCUG
ENSG00000103966	CCAGUGU
ENSG00000103966	GGCAGUG
ENSG00000103978	AGCAGCA
ENSG00000103978	AUUGCAC
ENSG00000103978	UGUGUCA
ENSG00000103994	AGCUGCC
ENSG00000103994	GAGCUGC
ENSG00000103994	GGAGGCA
ENSG00000103994	UUCCAUA
ENSG00000104043	CUGCCCC
ENSG00000104043	GAGGUAG
ENSG00000104043	GUGCUUG
ENSG00000104043	UACAAAG
ENSG00000104067	AAGGCAC
ENSG00000104067	AAUACUG
ENSG00000104067	AUGUGCC
ENSG00000104067	GCUGGUG
ENSG00000104067	GGGUAAG
ENSG00000104067	GUGCCAC
ENSG00000104067	UAUUGCU
ENSG00000104067	UGCAUAG

ENSG00000104081	AAAAAUC
ENSG00000104081	AAGGCAC
ENSG00000104081	ACAUUCA
ENSG00000104081	CAGCAGG
ENSG00000104081	CCUGCUG
ENSG00000104081	CUCAGGG
ENSG00000104081	GAGCCCC
ENSG00000104081	GAGCCCU
ENSG00000104081	GAGGCAG
ENSG00000104081	GAGGGGC
ENSG00000104081	GGAGAGA
ENSG00000104081	GGCUCAG
ENSG00000104081	GGGUCUU
ENSG00000104081	UCCAGAG
ENSG00000104081	UCCUGAG
ENSG00000104081	UGAGAAU
ENSG00000104081	UGCCCUG
ENSG00000104081	UUGACAC
ENSG00000104093	UGCAUAG
ENSG00000104093	UGGCCAC
ENSG00000104112	AUGUCUG
ENSG00000104131	AGCAUUC
ENSG00000104131	UUGGCAA
ENSG00000104131	UUGGCAC
ENSG00000104133	UUCUAAU
ENSG00000104142	UAAUGCU
ENSG00000104154	AAGGCAC
ENSG00000104154	ACUGCAG
ENSG00000104154	CUGCAGG
ENSG00000104154	GAGGUAG
ENSG00000104154	GAUUGUC
ENSG00000104154	GGAAGAC
ENSG00000104154	GGUUUAC
ENSG00000104154	UACAAAG
ENSG00000104154	UGUCUUU
ENSG00000104164	AGGUAGU
ENSG00000104164	GAGGUAG
ENSG00000104164	GAUAUGU
ENSG00000104164	GAUCAGA
ENSG00000104164	GCCUGAU
ENSG00000104177	AUUCAUU
ENSG00000104177	GGAAUGU
ENSG00000104205	AAGUCUU
ENSG00000104205	AAUGCCC
ENSG00000104205	CAGUAGU
ENSG00000104205	CAGUCCA
ENSG00000104205	CUUUGGU
ENSG00000104205	GAAACAU
ENSG00000104205	GAGGCAG
ENSG00000104205	GCAGAAG
ENSG00000104205	GUAAACA
ENSG00000104205	UAUUACC
ENSG00000104205	UGCAAAG
ENSG00000104205	UGCAUAU
ENSG00000104205	UUGGCAA
ENSG00000104205	UUGGCAC
ENSG00000104205	UUUGCAA
ENSG00000104218	AAGGCAC
ENSG00000104219	AAGGCAC
ENSG00000104219	ACAAGGU
ENSG00000104219	AGUGCAA
ENSG00000104219	AUACAAG
ENSG00000104219	GAGAUGA
ENSG00000104219	GAUUGUC

ENSG00000104219	GCUGGAU
ENSG00000104219	UGUGCUU
ENSG00000104221	ACAAUAU
ENSG00000104221	AUACAAG
ENSG00000104221	AUAUUAU
ENSG00000104221	UAAUAUC
ENSG00000104290	AAAAAUC
ENSG00000104290	AAAAGUA
ENSG00000104290	AAAGCUG
ENSG00000104290	UUCUAAU
ENSG00000104290	UUGGCAA
ENSG00000104299	ACUGUAG
ENSG00000104313	AAAGUGC
ENSG00000104313	AACAUUC
ENSG00000104313	AAGGCAC
ENSG00000104313	ACAAUAU
ENSG00000104313	AGCAGCG
ENSG00000104313	AGCAGCU
ENSG00000104313	AUGUAAU
ENSG00000104313	CAAAGUG
ENSG00000104313	UACAAAG
ENSG00000104313	UAUUACC
ENSG00000104313	UGACAUC
ENSG00000104313	UUGGUCC
ENSG00000104321	CAGGAAC
ENSG00000104321	UGACAUC
ENSG00000104327	ACAGUAU
ENSG00000104327	ACUGGCC
ENSG00000104327	AGUGCAA
ENSG00000104327	AUCACUA
ENSG00000104327	UGGAUAA
ENSG00000104331	AAAUGGU
ENSG00000104331	AAGUCUU
ENSG00000104331	AAUGCCC
ENSG00000104331	AGGUAGU
ENSG00000104331	AUGACAC
ENSG00000104331	CUCAAAC
ENSG00000104331	CUUUGGU
ENSG00000104331	GUGCUUG
ENSG00000104331	UCACAAG
ENSG00000104331	UCUUGAG
ENSG00000104331	UUAUGCA
ENSG00000104331	UUCCAUA
ENSG00000104331	UUCUUGU
ENSG00000104332	ACAGUAU
ENSG00000104332	CAAAACU
ENSG00000104332	CAAAGCA
ENSG00000104332	CACAGUG
ENSG00000104332	UCACAUU
ENSG00000104332	UGAAAUG
ENSG00000104341	AAUUGCU
ENSG00000104343	AAAGAAU
ENSG00000104343	AAAGUGC
ENSG00000104343	AAGGCAC
ENSG00000104343	ACAUUCA
ENSG00000104343	ACCUGGA
ENSG00000104343	ACUGUAG
ENSG00000104343	AGUGCAA
ENSG00000104343	AUACAAG
ENSG00000104343	CAAAACU
ENSG00000104343	CAGUAGU
ENSG00000104343	CAGUGCA
ENSG00000104343	CCCUGAG
ENSG00000104343	CUAUACA

ENSG00000104343	CUGCAGC
ENSG00000104343	CUGGACU
ENSG00000104343	GACCUGG
ENSG00000104343	GCAGCAU
ENSG00000104343	GCUAGCA
ENSG00000104343	GGCAGGG
ENSG00000104343	GGGGUGC
ENSG00000104343	GGGGUGG
ENSG00000104343	GUCAGUU
ENSG00000104343	UAAUACA
ENSG00000104343	UAUAAAG
ENSG00000104343	UAUUGCU
ENSG00000104343	UCACAGU
ENSG00000104343	UCACCAC
ENSG00000104343	UCAUACA
ENSG00000104343	UCAUAGA
ENSG00000104343	UCCAGUU
ENSG00000104343	UUUGCAA
ENSG00000104343	UUUGCAC
ENSG00000104365	AGCAGCG
ENSG00000104365	AGCAGCU
ENSG00000104365	AUAAUAC
ENSG00000104365	CCAGUGC
ENSG00000104365	CCAGUGU
ENSG00000104369	AAACUGU
ENSG00000104369	AAGGCAC
ENSG00000104369	UAAGACU
ENSG00000104369	UACAGUU
ENSG00000104375	CCUUCAU
ENSG00000104375	UUGGUUC
ENSG00000104381	AAAGCUG
ENSG00000104381	GGAGGCA
ENSG00000104381	UAGGUAU
ENSG00000104381	UCUGGAG
ENSG00000104381	UGAAAUG
ENSG00000104381	UUCCUAG
ENSG00000104388	UGUCUUU
ENSG00000104408	AAUUUUA
ENSG00000104415	AGCAGCA
ENSG00000104419	CUUUGGU
ENSG00000104419	GGCAGGG
ENSG00000104419	UAAUACA
ENSG00000104419	UACAAAG
ENSG00000104419	UGCAAAG
ENSG00000104419	UUGGCAA
ENSG00000104419	UUGGCAC
ENSG00000104419	UUGGUCC
ENSG00000104432	AAAGUAA
ENSG00000104432	ACUGUGU
ENSG00000104432	UUGGAGG
ENSG00000104435	UCAUACA
ENSG00000104442	AAAACUG
ENSG00000104442	AAGGCAC
ENSG00000104442	ACUGGCC
ENSG00000104442	CACCACC
ENSG00000104442	GAGGCAG
ENSG00000104442	UAUUGCU
ENSG00000104442	UCACACA
ENSG00000104442	UCCUGAG
ENSG00000104442	UGCAAAG
ENSG00000104442	UGUGCGU
ENSG00000104442	UUGGCAA
ENSG00000104442	UUGGUUC
ENSG00000104447	AAAACUG

ENSG00000104447	AAAGAGG
ENSG00000104447	AAAGUAC
ENSG00000104447	AAAGUGC
ENSG00000104447	AACAUUC
ENSG00000104447	AACUACU
ENSG00000104447	AAGGCAC
ENSG00000104447	AAGUAGC
ENSG00000104447	AAGUGCA
ENSG00000104447	AAUGUUG
ENSG00000104447	ACAAUAU
ENSG00000104447	ACACACU
ENSG00000104447	ACCACAG
ENSG00000104447	ACUAGAU
ENSG00000104447	ACUGCAG
ENSG00000104447	ACUGCAU
ENSG00000104447	ACUGUAG
ENSG00000104447	AGAUGUG
ENSG00000104447	AGCCCUU
ENSG00000104447	AGGAGCU
ENSG00000104447	AGGCAGG
ENSG00000104447	AGUGCAA
ENSG00000104447	AGUGCUU
ENSG00000104447	AUCACUA
ENSG00000104447	AUCCUUG
ENSG00000104447	AUGUAAU
ENSG00000104447	AUGUGCC
ENSG00000104447	CAAAGUG
ENSG00000104447	CAGCAGG
ENSG00000104447	CAGGCUC
ENSG00000104447	CAGUCUG
ENSG00000104447	CAGUGCA
ENSG00000104447	CAGUUAC
ENSG00000104447	CAUGGAU
ENSG00000104447	CCCUGAG
ENSG00000104447	CCUUCAU
ENSG00000104447	CCUUCUG
ENSG00000104447	CCUUCUU
ENSG00000104447	CUACAAA
ENSG00000104447	CUCCCAA
ENSG00000104447	CUCCCAC
ENSG00000104447	CUGACUC
ENSG00000104447	GAGAUGA
ENSG00000104447	GAGGAUA
ENSG00000104447	GAUCAGA
ENSG00000104447	GCACCAU
ENSG00000104447	GCAGGUU
ENSG00000104447	GCAUCCC
ENSG00000104447	GCCUGAU
ENSG00000104447	GCUACAU
ENSG00000104447	GGAAUGU
ENSG00000104447	GGCAUUG
ENSG00000104447	GGUGGUC
ENSG00000104447	GGUGGUU
ENSG00000104447	GGUUACC
ENSG00000104447	GUAAACA
ENSG00000104447	GUCAACA
ENSG00000104447	GUGCAAA
ENSG00000104447	UAUUACC
ENSG00000104447	UAUUGCU
ENSG00000104447	UCAAGUA
ENSG00000104447	UCAUUCG
ENSG00000104447	UCCCUGU
ENSG00000104447	UCCCUUG
ENSG00000104447	UCCCUUU

ENSG00000104447	UCCUAUA
ENSG00000104447	UGCAAAG
ENSG00000104447	UGCAUAG
ENSG00000104447	UGCAUAU
ENSG00000104447	UGCAUUG
ENSG00000104447	UGUGCUU
ENSG00000104447	UUCCUAG
ENSG00000104447	UUCUGCA
ENSG00000104447	UUCUUGU
ENSG00000104447	UUGAGGC
ENSG00000104447	UUGGAGG
ENSG00000104447	UUGUGAC
ENSG00000104447	UUGUUCG
ENSG00000104447	UUUGCAA
ENSG00000104447	UUUUUGC
ENSG00000104472	GCUGUCU
ENSG00000104490	AACAGUC
ENSG00000104490	AUUCAUU
ENSG00000104490	CACAAUG
ENSG00000104490	GAUUGUA
ENSG00000104490	GGCUCAG
ENSG00000104490	GUAAACA
ENSG00000104490	GUGCAAA
ENSG00000104490	UCACAGU
ENSG00000104490	UCCAGUU
ENSG00000104490	UGCAAAG
ENSG00000104490	UGCUCAC
ENSG00000104490	UUCCUCG
ENSG00000104490	UUGGCAC
ENSG00000104497	AAGUGCA
ENSG00000104497	GAGGUAG
ENSG00000104517	AAGAACC
ENSG00000104517	ACACACU
ENSG00000104517	AGAUAAA
ENSG00000104517	AGUAGUU
ENSG00000104517	CUUUGGU
ENSG00000104517	GCUGUCU
ENSG00000104517	GGAAUGU
ENSG00000104517	UCACAGU
ENSG00000104517	UCAUGAU
ENSG00000104517	UGCUCAC
ENSG00000104517	UGGCUGC
ENSG00000104522	CCCUGAG
ENSG00000104522	UCCCUGU
ENSG00000104522	UGAGGAC
ENSG00000104613	AAAAAUC
ENSG00000104635	AAAACUG
ENSG00000104635	AACCUGG
ENSG00000104635	AACUCUA
ENSG00000104635	AAUACUG
ENSG00000104635	ACCUGGA
ENSG00000104635	ACUGUGU
ENSG00000104635	CAGCAAA
ENSG00000104635	CUUACAG
ENSG00000104635	UUGGUUC
ENSG00000104643	AAGAACC
ENSG00000104643	ACAUUCA
ENSG00000104643	CCAGUGC
ENSG00000104643	CCUGUAC
ENSG00000104643	CUCUGGG
ENSG00000104643	CUGGCUC
ENSG00000104643	GAGGUAU
ENSG00000104643	GUCUACU
ENSG00000104643	UAAUAGU

ENSG00000104643	UCACAUU
ENSG00000104643	UCUCCAA
ENSG00000104643	UGAGAAG
ENSG00000104695	AACAGUC
ENSG00000104695	CAGCAGG
ENSG00000104722	AAAGUAA
ENSG00000104722	GAGAACC
ENSG00000104722	GAGGUAG
ENSG00000104722	GAGGUAU
ENSG00000104722	GCACCAU
ENSG00000104722	GUAAACA
ENSG00000104722	GUGAGGU
ENSG00000104722	UCUGGAG
ENSG00000104722	UGUCUGG
ENSG00000104723	AAAUCCC
ENSG00000104723	GUAAACA
ENSG00000104725	AUGAGCU
ENSG00000104725	GGCAGCG
ENSG00000104725	UAUUGCU
ENSG00000104725	UCUGGAA
ENSG00000104728	AACAGUC
ENSG00000104728	AGCACCA
ENSG00000104728	AGCAGCU
ENSG00000104728	AGUGCUG
ENSG00000104756	AAAGCUG
ENSG00000104756	ACAAUAU
ENSG00000104756	CUUACAG
ENSG00000104756	CUUCUCU
ENSG00000104756	GUGACAG
ENSG00000104756	GUGCUUG
ENSG00000104756	UGAAAUG
ENSG00000104756	UGCAUAG
ENSG00000104756	UUCUGCA
ENSG00000104756	UUGGUUC
ENSG00000104765	AGCCCUU
ENSG00000104765	AUGGCAC
ENSG00000104765	CAAAGUG
ENSG00000104783	UAUUACC
ENSG00000104808	UGUCUUU
ENSG00000104812	AAGGCAC
ENSG00000104812	ACACACU
ENSG00000104814	GGGGUGC
ENSG00000104826	UGAGAAG
ENSG00000104853	AAAUCCC
ENSG00000104853	CUUCACC
ENSG00000104853	GGCAGGG
ENSG00000104853	GUCCAGA
ENSG00000104856	GGAAGAC
ENSG00000104859	AGCAGCA
ENSG00000104859	GCAGCAU
ENSG00000104863	AAUACUG
ENSG00000104863	ACUGUAG
ENSG00000104870	GGCUCAG
ENSG00000104880	AACACUG
ENSG00000104880	AAGUGCU
ENSG00000104880	AUGGCAC
ENSG00000104881	GGAGGCA
ENSG00000104885	AAAAAUC
ENSG00000104885	AAAACUG
ENSG00000104885	AAAGUAA
ENSG00000104885	AAUACUG
ENSG00000104885	ACAGUAC
ENSG00000104885	AGCACCA
ENSG00000104885	CUUUGGU

ENSG00000104885	GAGGUAG
ENSG00000104885	GAGGUAU
ENSG00000104885	UAUAAAG
ENSG00000104885	UCACAGG
ENSG00000104885	UCACAGU
ENSG00000104885	UCCUUGC
ENSG00000104885	UGCAUAG
ENSG00000104885	UGCAUAU
ENSG00000104885	UUGGUCC
ENSG00000104886	UCACAGG
ENSG00000104886	UCACAGU
ENSG00000104888	AUAAAGU
ENSG00000104888	CCAGGAG
ENSG00000104888	CUGGAAA
ENSG00000104888	GGAGGCA
ENSG00000104888	GGCAGGG
ENSG00000104888	GGGGCAG
ENSG00000104888	UCCCUUU
ENSG00000104888	UGAGAAU
ENSG00000104888	UGGCCAC
ENSG00000104903	CCUUCUU
ENSG00000104904	ACAAUAU
ENSG00000104904	UUUGCAC
ENSG00000104936	GGAAUGU
ENSG00000104936	GUGGGGA
ENSG00000104938	ACUGUGU
ENSG00000104946	AAAGUGC
ENSG00000104957	AAGGCAC
ENSG00000104960	CACCAGC
ENSG00000104960	CUGGGCA
ENSG00000104964	AAGGCAC
ENSG00000104967	AGGGAGG
ENSG00000104967	CACAGGG
ENSG00000104967	UCACACA
ENSG00000104969	CUGGCUC
ENSG00000104969	GCAGGUU
ENSG00000104969	GCUGGUG
ENSG00000104969	GGCAGUG
ENSG00000104969	GGCUCUG
ENSG00000104983	UACAAAG
ENSG00000105011	AGGGAGG
ENSG00000105011	UAAUAUC
ENSG00000105053	CAGCAGG
ENSG00000105085	AAACUGU
ENSG00000105085	AACUGGU
ENSG00000105085	AAGGCAC
ENSG00000105085	ACUGUAG
ENSG00000105085	AGCAAAA
ENSG00000105085	AGCACCA
ENSG00000105085	AGCAGCG
ENSG00000105085	AGCAGCU
ENSG00000105085	CCUUCUG
ENSG00000105085	CUCCCAC
ENSG00000105085	GGUGGUU
ENSG00000105085	GUGCCUG
ENSG00000105085	UGCAAAG
ENSG00000105135	CUUGGGC
ENSG00000105137	AACUGGU
ENSG00000105137	AAGUGCA
ENSG00000105137	AAGUGCU
ENSG00000105137	AGCAGCA
ENSG00000105137	GGCAGCG
ENSG00000105137	UCACAGU
ENSG00000105137	UGCUCAC

ENSG00000105173	AGCAGCG
ENSG00000105173	AGCAGCU
ENSG00000105173	GCAGCAU
ENSG00000105176	AAAACUG
ENSG00000105176	CAGCUGG
ENSG00000105186	AUGUGCC
ENSG00000105186	GUGCCAC
ENSG00000105186	UAUAAAG
ENSG00000105186	UGUGCUU
ENSG00000105186	UUCCAUA
ENSG00000105186	UUGGCAA
ENSG00000105202	UAAUAGU
ENSG00000105204	AUGGCUU
ENSG00000105204	CAGUGCA
ENSG00000105204	GGGCCCC
ENSG00000105216	AAUUUUA
ENSG00000105216	AGUGCUG
ENSG00000105216	CCAGUGC
ENSG00000105216	UCAUGAU
ENSG00000105216	UUGGCAC
ENSG00000105219	CAGGCUC
ENSG00000105219	CCAGCAU
ENSG00000105219	CUCCAGC
ENSG00000105219	GAGAAGA
ENSG00000105219	GGAAGAC
ENSG00000105219	GGAGGCA
ENSG00000105219	GGGUGGU
ENSG00000105219	UGAGGAC
ENSG00000105220	CUCUGGG
ENSG00000105221	AAGGCAC
ENSG00000105221	AAUACUG
ENSG00000105221	AGGCAGG
ENSG00000105221	CUGGGCA
ENSG00000105221	GAAGGAA
ENSG00000105221	GAGGUAG
ENSG00000105221	GCAGAAG
ENSG00000105221	GGAAGCC
ENSG00000105221	GGAGGCA
ENSG00000105221	GGCACGG
ENSG00000105221	GUAACAG
ENSG00000105221	UGAAAUG
ENSG00000105221	UUCUGCA
ENSG00000105223	CCAGCAU
ENSG00000105223	CCCCCAG
ENSG00000105223	GAGGUAG
ENSG00000105223	GGGGUGG
ENSG00000105227	GGGGAGC
ENSG00000105227	UAGUGAG
ENSG00000105227	UGGGACA
ENSG00000105229	AGCACCA
ENSG00000105229	GUCCAGA
ENSG00000105229	UCCAGAG
ENSG00000105245	AAAAGUA
ENSG00000105245	AAAGUAC
ENSG00000105245	AACACUG
ENSG00000105245	ACACACC
ENSG00000105245	AGCCCUU
ENSG00000105245	AGUGGUU
ENSG00000105245	CCCUGAG
ENSG00000105245	CUUGUAU
ENSG00000105245	GAGACCU
ENSG00000105245	GAGGUAG
ENSG00000105245	GAGGUAU
ENSG00000105245	GGGUGGU

ENSG00000105245	GGUGGGC
ENSG00000105245	UAAUACA
ENSG00000105245	UAUAAUA
ENSG00000105245	UCUGGAA
ENSG00000105245	UGGCCCU
ENSG00000105245	UGGCUGC
ENSG00000105245	UGUGCUU
ENSG00000105255	AGCAGCA
ENSG00000105255	CCAGUGU
ENSG00000105270	AAGGCAC
ENSG00000105270	AAUCUCU
ENSG00000105270	GACCAUG
ENSG00000105270	GAGCUGC
ENSG00000105270	GGUGGUC
ENSG00000105270	UCAUACA
ENSG00000105270	UCCCUGU
ENSG00000105281	CCCUGAG
ENSG00000105323	AAAAGUA
ENSG00000105323	ACUGCAU
ENSG00000105323	CAGUUAC
ENSG00000105323	UGCAAAG
ENSG00000105325	CACAAUG
ENSG00000105327	AGCACCA
ENSG00000105327	AUUCAUU
ENSG00000105327	CCCUGAG
ENSG00000105327	CUACAGU
ENSG00000105327	CUGACUC
ENSG00000105327	CUGGCUC
ENSG00000105327	GAAACAU
ENSG00000105327	GAGAUGA
ENSG00000105327	GAGGCAG
ENSG00000105327	GCUACAU
ENSG00000105327	GGCUCAG
ENSG00000105327	GGGGGAA
ENSG00000105327	GGUGGGC
ENSG00000105327	UCACAGU
ENSG00000105327	UCAUUCG
ENSG00000105327	UCCCACC
ENSG00000105327	UCCUGAG
ENSG00000105329	GCGGGGC
ENSG00000105329	GGCGGGG
ENSG00000105357	GGGGGAA
ENSG00000105357	UGGGGGA
ENSG00000105369	GAGGCAG
ENSG00000105401	CAAAGCA
ENSG00000105402	CUGCAGC
ENSG00000105402	UGCAGCU
ENSG00000105409	AAAGAGC
ENSG00000105409	AGGGAGG
ENSG00000105419	GAGGUAG
ENSG00000105426	GGAAUGU
ENSG00000105426	UAGACUG
ENSG00000105426	UGACUGU
ENSG00000105426	UGUCUUU
ENSG00000105429	AGUGCCU
ENSG00000105429	CCCUGAG
ENSG00000105429	GACCCUG
ENSG00000105429	GAUUGUC
ENSG00000105429	GCCCUGU
ENSG00000105429	GUGCCUG
ENSG00000105429	UGAGAAU
ENSG00000105438	CAAGAGC
ENSG00000105438	CCCCUGG
ENSG00000105438	CUACAAA

ENSG00000105438	UAUAAAG
ENSG00000105438	UCCUGAG
ENSG00000105438	UUCAAGC
ENSG00000105438	UUGGCAA
ENSG00000105438	UUGGCAC
ENSG00000105447	CUUUGGU
ENSG00000105464	AAAAACC
ENSG00000105464	AACACUG
ENSG00000105464	ACCCGGC
ENSG00000105464	CUCACAC
ENSG00000105464	GAGCUGC
ENSG00000105464	GCGGGGC
ENSG00000105464	GGAGGCA
ENSG00000105464	GGGAGCU
ENSG00000105464	GGUGGGC
ENSG00000105464	GGUUUAC
ENSG00000105464	GUGUGGC
ENSG00000105464	UGGAGAU
ENSG00000105464	UGUGUGG
ENSG00000105514	CCCUGAG
ENSG00000105516	AAAAAUC
ENSG00000105519	GACCUGG
ENSG00000105538	UAUUGCU
ENSG00000105538	UUCAAGC
ENSG00000105549	GGGUGUU
ENSG00000105552	CUUUGGU
ENSG00000105552	GCCCUUA
ENSG00000105552	GGCAUUG
ENSG00000105552	UUCAAAU
ENSG00000105552	UUGGCAA
ENSG00000105552	UUGGCAC
ENSG00000105556	AAAGUGC
ENSG00000105556	AGCAGCU
ENSG00000105556	AUGGCUU
ENSG00000105556	GAGGUAU
ENSG00000105556	GCUGGUG
ENSG00000105556	GGCUCUG
ENSG00000105556	GGUGCGG
ENSG00000105556	GUAAACA
ENSG00000105556	UCACAGG
ENSG00000105556	UCACAGU
ENSG00000105556	UGAAGUG
ENSG00000105556	UUCAAAU
ENSG00000105556	UUGGCAC
ENSG00000105559	AAAGUUU
ENSG00000105559	UGGCUGC
ENSG00000105568	AGCAGCA
ENSG00000105568	GACCAUG
ENSG00000105576	AAAUGGU
ENSG00000105576	AACUCUA
ENSG00000105576	AAUCUCA
ENSG00000105576	ACUCAAA
ENSG00000105576	AUGGCUU
ENSG00000105576	CACUCCU
ENSG00000105576	CAGCAGA
ENSG00000105576	CAGGCUC
ENSG00000105576	CUCCCAC
ENSG00000105576	GAGACCU
ENSG00000105576	GCAGAAG
ENSG00000105576	GGAAUGU
ENSG00000105576	UCCAGAG
ENSG00000105576	UGAGAAG
ENSG00000105583	AGGCAGG
ENSG00000105583	GGCAGGG

ENSG00000105613	UAUAUGA
ENSG00000105643	CUGGACU
ENSG00000105647	GUAAACA
ENSG00000105647	UUGGAGG
ENSG00000105649	GACAACU
ENSG00000105656	AAAACUG
ENSG00000105656	AACAUUC
ENSG00000105656	AAUACUG
ENSG00000105656	ACAGUAC
ENSG00000105656	AGCAUUC
ENSG00000105656	AGGGAGG
ENSG00000105656	CCCUGAG
ENSG00000105656	GGAGGCA
ENSG00000105656	GGGGAGC
ENSG00000105656	GUAACAG
ENSG00000105656	UCUCCAA
ENSG00000105656	UUGGCAC
ENSG00000105662	AAAGGUA
ENSG00000105662	AACAGUC
ENSG00000105662	AAGUCUU
ENSG00000105662	ACAGUAU
ENSG00000105662	GGCAGUG
ENSG00000105662	UAUUACC
ENSG00000105662	UCACAGU
ENSG00000105662	UCCAGUU
ENSG00000105662	UGCAAAG
ENSG00000105662	UGCAUAG
ENSG00000105662	UGCAUAU
ENSG00000105662	UUGGCAA
ENSG00000105695	UCCUGAG
ENSG00000105698	AAAGAAU
ENSG00000105698	ACACACC
ENSG00000105698	AUUGCAC
ENSG00000105698	CAGCUGG
ENSG00000105698	CCCUGUC
ENSG00000105698	CUGGGGG
ENSG00000105698	GAGGCAG
ENSG00000105698	GAGGGGC
ENSG00000105698	GGAGGCA
ENSG00000105698	UAUUGCU
ENSG00000105698	UCCCUCU
ENSG00000105698	UGGGGGA
ENSG00000105700	CUCCAGC
ENSG00000105700	GGCUCUG
ENSG00000105701	CUGCCCC
ENSG00000105701	GCCUGGG
ENSG00000105701	GGGCCCC
ENSG00000105707	CAAAACU
ENSG00000105711	AACUACU
ENSG00000105711	CAAAACU
ENSG00000105711	CAGCAGA
ENSG00000105711	CUGCCCC
ENSG00000105711	GCAGAAG
ENSG00000105711	GCUGGUG
ENSG00000105711	GUGCAAA
ENSG00000105711	UAUUGCU
ENSG00000105711	UCAUGAU
ENSG00000105722	ACAUUCA
ENSG00000105722	AGCAAAA
ENSG00000105722	AGCAGCU
ENSG00000105722	AGGGAGG
ENSG00000105722	CAGCAAA
ENSG00000105722	CAGCUGG
ENSG00000105722	CCCCUGG

ENSG00000105722	CCUGCUG
ENSG00000105722	CUCCAGC
ENSG00000105722	CUGGGCA
ENSG00000105722	GAGGGGC
ENSG00000105722	GAUUGUA
ENSG00000105722	GGAGGCA
ENSG00000105722	GGGGUGG
ENSG00000105722	UCCAGUU
ENSG00000105722	UCUCCAA
ENSG00000105723	AGGGAGG
ENSG00000105723	GAGGCUG
ENSG00000105726	AAGGUGC
ENSG00000105737	UCAUAGA
ENSG00000105738	AAAGUGC
ENSG00000105738	AACUCUA
ENSG00000105738	AAGUGCU
ENSG00000105738	AAUCUCA
ENSG00000105738	CACAGUG
ENSG00000105738	UAUUGCU
ENSG00000105738	UCACAGU
ENSG00000105738	UCUCAAG
ENSG00000105738	UUAGAGA
ENSG00000105738	UUUGCAA
ENSG00000105738	UUUGCAC
ENSG00000105767	AUAUAAC
ENSG00000105767	AUGGCUU
ENSG00000105767	GCCUGGA
ENSG00000105767	GGAGAGA
ENSG00000105767	GGAGGAG
ENSG00000105767	GGCAGUG
ENSG00000105767	GGUGGUC
ENSG00000105767	UAAUAUC
ENSG00000105767	UGCAGCU
ENSG00000105767	UUUGCAA
ENSG00000105810	AACACUG
ENSG00000105810	ACCCUGU
ENSG00000105810	ACUCAGC
ENSG00000105810	AGCAGCA
ENSG00000105810	AGCUUAU
ENSG00000105810	AGUGUGC
ENSG00000105810	AUUGCAC
ENSG00000105810	CACAGGG
ENSG00000105810	CAGCAGG
ENSG00000105810	CAGUGCA
ENSG00000105810	CUCACAC
ENSG00000105810	CUCUUCC
ENSG00000105810	CUGGCUC
ENSG00000105810	CUUCUCU
ENSG00000105810	CUUGGAG
ENSG00000105810	GCAGCAU
ENSG00000105810	GCUGGUG
ENSG00000105810	GGAAGCC
ENSG00000105810	GGAAUGU
ENSG00000105810	GGCAGCG
ENSG00000105810	GUGCCUG
ENSG00000105810	UAAGACU
ENSG00000105810	UCAACAG
ENSG00000105810	UCACAGG
ENSG00000105810	UCACAGU
ENSG00000105810	UCCUGAG
ENSG00000105810	UCCUUGC
ENSG00000105810	UGCAGCU
ENSG00000105810	UUCAAGC
ENSG00000105810	UUGGCAA

ENSG00000105819	AUAAUAC
ENSG00000105821	UAUAAAG
ENSG00000105825	UCACAUU
ENSG00000105829	GGAAUGU
ENSG00000105849	AUGUCUG
ENSG00000105849	GAAGGAA
ENSG00000105851	ACCAGCA
ENSG00000105851	CAGCAGA
ENSG00000105854	CAGUAGA
ENSG00000105855	ACAGUAU
ENSG00000105855	ACCCUGU
ENSG00000105855	AGCAGCU
ENSG00000105855	AGCUGCC
ENSG00000105855	AGUAACA
ENSG00000105855	AGUAGUU
ENSG00000105855	AGUGCAA
ENSG00000105855	AUAAAGU
ENSG00000105855	AUGCAUU
ENSG00000105855	AUGCCUU
ENSG00000105855	CAGCAGA
ENSG00000105855	CAGCAGG
ENSG00000105855	CUCACAC
ENSG00000105855	GAGGUAG
ENSG00000105855	GCUACAU
ENSG00000105855	GGCAGUG
ENSG00000105855	GUCAUUG
ENSG00000105855	GUGACAG
ENSG00000105855	GUGACUG
ENSG00000105855	UACAGUU
ENSG00000105855	UCAUAGA
ENSG00000105855	UGUAUGU
ENSG00000105856	AAGGCAC
ENSG00000105856	ACCACAG
ENSG00000105856	AGUAACA
ENSG00000105856	GAUUGUA
ENSG00000105856	GGUUUAC
ENSG00000105856	UGAAAUG
ENSG00000105856	UUGGUUC
ENSG00000105856	UUUCAAC
ENSG00000105866	AAGUGCA
ENSG00000105866	ACCACAG
ENSG00000105866	AUGUAAU
ENSG00000105866	CACAGUG
ENSG00000105866	UGCAAAG
ENSG00000105877	AAAGAAU
ENSG00000105877	CCUGCUG
ENSG00000105877	GGAGGCA
ENSG00000105879	CUCUGGG
ENSG00000105879	UCACAAG
ENSG00000105880	AAGGCAC
ENSG00000105880	GCACCAU
ENSG00000105880	UCAUAGA
ENSG00000105887	AACACUG
ENSG00000105887	AAGGCAC
ENSG00000105887	AAUUAUU
ENSG00000105887	ACAGUAU
ENSG00000105887	ACAUUCA
ENSG00000105887	AGCAGCU
ENSG00000105887	CAAAGCA
ENSG00000105887	CAGUCCA
ENSG00000105887	CCAUUAC
ENSG00000105887	CUUUGGU
ENSG00000105887	GAGGUAG
ENSG00000105887	GAGGUAU

ENSG00000105887	GCAGAAG
ENSG00000105887	GGAAUGU
ENSG00000105887	GGGAGCU
ENSG00000105887	GUGACAG
ENSG00000105887	GUGAGGU
ENSG00000105887	UCAAGUA
ENSG00000105887	UCUCCAA
ENSG00000105887	UUCUGCA
ENSG00000105887	UUGUUCG
ENSG00000105887	UUUGCAA
ENSG00000105887	UUUGCAC
ENSG00000105928	AGCAUUC
ENSG00000105939	AGUGGUU
ENSG00000105939	GGAGAGA
ENSG00000105948	AAGGCAC
ENSG00000105953	UCCCUGU
ENSG00000105953	UGCAAAG
ENSG00000105953	UUGGCAA
ENSG00000105967	AAAUCCC
ENSG00000105967	AGCAGCA
ENSG00000105967	AUCCCUU
ENSG00000105967	CACCAGC
ENSG00000105967	CCAGCAU
ENSG00000105967	UCCCUUG
ENSG00000105967	UUCUUGU
ENSG00000105971	AGUGCAA
ENSG00000105971	CAGUAGA
ENSG00000105971	CAGUAGU
ENSG00000105971	CAGUGCA
ENSG00000105971	GACAACU
ENSG00000105974	AAAGUAA
ENSG00000105974	AGCAAAA
ENSG00000105974	AUGGAUU
ENSG00000105974	CCAGUGU
ENSG00000105974	UGAAAUG
ENSG00000105976	ACAGUAC
ENSG00000105976	ACAGUAU
ENSG00000105976	CACAGUG
ENSG00000105976	CAGUGCA
ENSG00000105976	GCAGGUU
ENSG00000105976	GUGGCCC
ENSG00000105976	UCACAGG
ENSG00000105976	UCACAGU
ENSG00000105983	AGCUUAU
ENSG00000105983	CUGCUCA
ENSG00000105983	GAAGGAA
ENSG00000105983	GAGAAAU
ENSG00000105983	GGAAUGU
ENSG00000105983	GUGCAAA
ENSG00000105983	UGUCUGC
ENSG00000105983	UGUCUGG
ENSG00000105991	ACCCGUA
ENSG00000105991	AUUCAUU
ENSG00000105991	GAUUGUA
ENSG00000105997	AAAGCGC
ENSG00000105997	ACACACC
ENSG00000105997	ACACACU
ENSG00000105997	ACCAGCA
ENSG00000105997	ACGUCAU
ENSG00000105997	AGAUAAA
ENSG00000105997	AGCAGCA
ENSG00000105997	CAAAGCA
ENSG00000105997	CCUUCUG
ENSG00000105997	CUGACUC

ENSG00000105997	GUUCAUG
ENSG00000105997	UAAUAUC
ENSG00000105997	UCACCAC
ENSG00000105997	UGACUGU
ENSG00000105997	UGCAAAG
ENSG00000105997	UUGUUCG
ENSG00000106003	AAUACUG
ENSG00000106003	ACAAUAU
ENSG00000106003	CUGACUC
ENSG00000106003	UCCAGAG
ENSG00000106003	UCCCUGU
ENSG00000106003	UCCCUUG
ENSG00000106003	UCCCUUU
ENSG00000106003	UGAUCCA
ENSG00000106003	UGGAUAA
ENSG00000106004	AAUACUG
ENSG00000106004	AGUAACA
ENSG00000106004	AGUGCAA
ENSG00000106004	CAGGCUC
ENSG00000106004	CUUCACC
ENSG00000106004	CUUGGAG
ENSG00000106004	UCACAGG
ENSG00000106004	UCACAGU
ENSG00000106004	UCAUGAU
ENSG00000106006	UGCAAAG
ENSG00000106013	UGAAAUG
ENSG00000106025	AAGACAU
ENSG00000106025	ACAGUAC
ENSG00000106025	CCCUGAG
ENSG00000106025	UAAGACG
ENSG00000106025	UAAGACU
ENSG00000106025	UCACAUU
ENSG00000106034	GAGGUAG
ENSG00000106069	AAUACUG
ENSG00000106069	AUAAUAC
ENSG00000106070	AAAGGUA
ENSG00000106070	AGCAGCA
ENSG00000106070	AGUGAUC
ENSG00000106070	AGUGCAA
ENSG00000106070	CAAAUGC
ENSG00000106070	CACAGGG
ENSG00000106070	CAGCAGG
ENSG00000106070	CAGGCUC
ENSG00000106070	CAGUCCA
ENSG00000106070	CCAGUGC
ENSG00000106070	CCUUCUU
ENSG00000106070	GAGGCAG
ENSG00000106070	GCAGAAG
ENSG00000106070	GGCAAGA
ENSG00000106070	GUGAGGU
ENSG00000106070	GUGCAAA
ENSG00000106070	UAAUACA
ENSG00000106070	UAUAAAG
ENSG00000106070	UCAACAG
ENSG00000106070	UCACAGG
ENSG00000106070	UCCCUGU
ENSG00000106077	UGCCAGC
ENSG00000106077	UGUCUUU
ENSG00000106078	AACCUGG
ENSG00000106078	AACCUGU
ENSG00000106078	UUGGCAC
ENSG00000106080	AAGUCUU
ENSG00000106080	UUCAAAU
ENSG00000106080	UUUCAAC

ENSG00000106089	AAAAAUC
ENSG00000106089	AAAUUUC
ENSG00000106089	AAUACUG
ENSG00000106089	ACUGCAG
ENSG00000106089	ACUGCAU
ENSG00000106089	AGCACCA
ENSG00000106089	AGCAGCA
ENSG00000106089	CACAGUG
ENSG00000106089	CUUUGGU
ENSG00000106089	GCACCAU
ENSG00000106089	GGCAGUG
ENSG00000106089	UCACAGU
ENSG00000106089	UGGCUGC
ENSG00000106100	AACAAAC
ENSG00000106123	UCCCUUG
ENSG00000106144	CCCUGAG
ENSG00000106144	UGGAUAA
ENSG00000106144	UUGGCAA
ENSG00000106236	AUAAUAC
ENSG00000106236	AUAUAAC
ENSG00000106236	CACAGUG
ENSG00000106236	CUACAAA
ENSG00000106236	UAUAAAG
ENSG00000106236	UUGGCAA
ENSG00000106236	UUGGCAC
ENSG00000106244	AAAAACC
ENSG00000106244	AACAUUC
ENSG00000106244	ACAUUCA
ENSG00000106244	AGCAAAA
ENSG00000106244	AGCAGCA
ENSG00000106244	AGCAGCG
ENSG00000106244	CACAGGG
ENSG00000106244	CAGGCUC
ENSG00000106244	CUCAAAC
ENSG00000106244	CUCCCAC
ENSG00000106244	GAGGGGC
ENSG00000106244	GCCUGGA
ENSG00000106244	GGCAGGG
ENSG00000106244	GGUUACC
ENSG00000106244	GUGCCAC
ENSG00000106261	AGCAGCA
ENSG00000106261	AGUGAUC
ENSG00000106261	CUUCUCU
ENSG00000106261	GCAGCAU
ENSG00000106261	GGGGGAA
ENSG00000106261	UAGACUG
ENSG00000106261	UUGGUUC
ENSG00000106263	AAGGCAC
ENSG00000106263	GUCCAGA
ENSG00000106263	UCCAGAG
ENSG00000106278	AAAUUUC
ENSG00000106278	UAUAAAG
ENSG00000106290	UACAAAG
ENSG00000106299	AAAAGUA
ENSG00000106299	AGCAGCA
ENSG00000106299	AGUGCUU
ENSG00000106299	AUGAUGA
ENSG00000106299	CCAGUGC
ENSG00000106299	GAAUUGU
ENSG00000106299	GAGGUAG
ENSG00000106299	GGUAGAC
ENSG00000106302	UCCCUUU
ENSG00000106328	AAGUUGU
ENSG00000106330	AAAUCCC

ENSG00000106330	CACAGUG
ENSG00000106330	UCACAGU
ENSG00000106331	AGGCAGG
ENSG00000106331	CAGGUGA
ENSG00000106331	GGCAGGG
ENSG00000106331	UAUUACC
ENSG00000106336	UGGGGGA
ENSG00000106341	AAAGAGG
ENSG00000106341	CUCAGGG
ENSG00000106346	AGCACCA
ENSG00000106346	GAGUCUU
ENSG00000106346	GCAGCAU
ENSG00000106346	UGAGUCU
ENSG00000106348	AAGUGUG
ENSG00000106348	AGCACCA
ENSG00000106348	AGGCAGG
ENSG00000106348	AGUGUGC
ENSG00000106348	GAGGCAG
ENSG00000106348	GCACCAU
ENSG00000106348	GGCAGGG
ENSG00000106348	UGCAAAG
ENSG00000106348	UGCAGCU
ENSG00000106355	UACAAAG
ENSG00000106355	UCAUGAU
ENSG00000106355	UCCCUUG
ENSG00000106366	AAGUCAC
ENSG00000106366	ACAAGGU
ENSG00000106366	ACCCUGU
ENSG00000106366	AUGACAC
ENSG00000106366	CAGUGCA
ENSG00000106366	CCAGUGU
ENSG00000106366	GAGAUGA
ENSG00000106366	GGCAGUG
ENSG00000106366	GUAAACA
ENSG00000106366	UCCAGUU
ENSG00000106366	UGAAGUG
ENSG00000106367	AACCUGG
ENSG00000106367	AACCUGU
ENSG00000106367	GAGGUAG
ENSG00000106367	UCCCUCU
ENSG00000106392	AAGGCAC
ENSG00000106392	UCACAGG
ENSG00000106392	UCACAGU
ENSG00000106397	AAGGCAC
ENSG00000106415	AAUACUG
ENSG00000106415	ACAUUCA
ENSG00000106415	ACUGUUU
ENSG00000106415	CUAUACA
ENSG00000106415	UGCAUAG
ENSG00000106443	GGAUGAG
ENSG00000106443	UAUAAAG
ENSG00000106459	AAAUUUC
ENSG00000106459	ACUGUGU
ENSG00000106459	AGGUUAU
ENSG00000106459	AUACAAG
ENSG00000106459	AUUGCAC
ENSG00000106459	CAAAACU
ENSG00000106459	CUCACAC
ENSG00000106459	GACCCUG
ENSG00000106459	GGAGGCA
ENSG00000106459	UUGGCAA
ENSG00000106460	ACAUUAC
ENSG00000106460	CAAAACU
ENSG00000106460	GUAAACA

ENSG00000106460	UCACAAG
ENSG00000106460	UCAUUUG
ENSG00000106460	UGACAUC
ENSG00000106460	UGUAUAA
ENSG00000106460	UUCUAAU
ENSG00000106460	UUGACAC
ENSG00000106462	ACAGUAU
ENSG00000106462	GAGGUAG
ENSG00000106462	UACAGUU
ENSG00000106477	ACUGUAG
ENSG00000106477	UAAUGCU
ENSG00000106477	UCUUGAG
ENSG00000106477	UGACAUC
ENSG00000106483	AGAUGUG
ENSG00000106483	UCACAUU
ENSG00000106483	UGUCUGC
ENSG00000106484	ACAUUCA
ENSG00000106484	AGCACCA
ENSG00000106484	AUUCAUU
ENSG00000106484	GAGGUAU
ENSG00000106484	GCACCAU
ENSG00000106484	UAAUAGU
ENSG00000106484	UCAUUUG
ENSG00000106541	AACAUUC
ENSG00000106541	GUAACAG
ENSG00000106546	CACUCCU
ENSG00000106546	GAGAAAU
ENSG00000106546	GAGGCAG
ENSG00000106546	GUGAGGU
ENSG00000106546	UACAAAG
ENSG00000106546	UUGGCAA
ENSG00000106546	UUGUUCG
ENSG00000106571	AGGGUUG
ENSG00000106571	AGUGGUU
ENSG00000106571	GCAGGAC
ENSG00000106571	GGUUUAC
ENSG00000106571	UACAAAG
ENSG00000106571	UCCCUCU
ENSG00000106571	UGAAAUG
ENSG00000106571	UGACAUC
ENSG00000106571	UUCUUGU
ENSG00000106588	AAAAACC
ENSG00000106588	AAUUUUA
ENSG00000106588	GUCAUUG
ENSG00000106609	AAAACUG
ENSG00000106609	AAGGCAC
ENSG00000106609	AAGGUGC
ENSG00000106609	AGUGCCU
ENSG00000106609	AUCAUAC
ENSG00000106609	AUGCACC
ENSG00000106609	CACAGUG
ENSG00000106609	CAGCAGA
ENSG00000106609	CUGCAGG
ENSG00000106609	CUUUGGU
ENSG00000106609	GAUUGGU
ENSG00000106609	GUAUUCU
ENSG00000106609	GUGCCUG
ENSG00000106609	UAUAAAG
ENSG00000106609	UCAAGUA
ENSG00000106609	UCACAGG
ENSG00000106609	UCACAGU
ENSG00000106609	UGCUGAC
ENSG00000106609	UGGCCCU
ENSG00000106609	UGUCUUU

ENSG00000106610	AAAUUUC
ENSG00000106610	AAGUGUG
ENSG00000106610	AGAUAAA
ENSG00000106610	AUAAUAC
ENSG00000106610	AUGGAUU
ENSG00000106610	CACACCU
ENSG00000106610	CAGACAG
ENSG00000106610	CAGCUGG
ENSG00000106610	CCUUCUG
ENSG00000106610	GGGUGUU
ENSG00000106610	GUAUUCU
ENSG00000106610	GUUAAUG
ENSG00000106610	UAAUAGU
ENSG00000106610	UAAUAUC
ENSG00000106610	UCAAGUA
ENSG00000106610	UGAAAUG
ENSG00000106610	UGAAGUG
ENSG00000106610	UGUCUUU
ENSG00000106615	AACAAAC
ENSG00000106615	AAUGCCC
ENSG00000106615	ACAUUAC
ENSG00000106615	AGUAGUU
ENSG00000106615	AUAUAAC
ENSG00000106615	CCAGUGU
ENSG00000106615	UAAUGCU
ENSG00000106615	UCAUUUG
ENSG00000106617	AAAAAUC
ENSG00000106617	AGGACAC
ENSG00000106617	CAGUGCA
ENSG00000106617	CAUAUUG
ENSG00000106617	GAGGCAG
ENSG00000106617	UCAAGUA
ENSG00000106633	AAAAACC
ENSG00000106635	AAAAAUC
ENSG00000106635	AGCAGCU
ENSG00000106635	AUGUCUG
ENSG00000106635	CAGGCUC
ENSG00000106635	CAGUGCA
ENSG00000106635	CUUCACC
ENSG00000106635	GAGCUGC
ENSG00000106635	UUCCAUA
ENSG00000106636	AGCCCUU
ENSG00000106636	GACAACU
ENSG00000106636	GGCAGGG
ENSG00000106636	UCAUUUG
ENSG00000106638	CUCCCAC
ENSG00000106665	AACACUG
ENSG00000106665	AAUACUG
ENSG00000106665	CAAAGUG
ENSG00000106665	GAUUGUC
ENSG00000106665	GCCUGGG
ENSG00000106665	GUUAAUG
ENSG00000106665	UACAAAG
ENSG00000106665	UCACAAG
ENSG00000106665	UCUCCAA
ENSG00000106682	AAAGUAC
ENSG00000106682	AAAGUAU
ENSG00000106682	AAAGUGC
ENSG00000106682	AAAUGGU
ENSG00000106682	ACUAGAU
ENSG00000106682	CAGCAAA
ENSG00000106682	CUGCAGG
ENSG00000106682	GCAGGUU
ENSG00000106682	UGUCUUU

ENSG00000106683	AAGUGCA
ENSG00000106683	AUAAUAC
ENSG00000106683	CACAGUG
ENSG00000106683	CCCACCU
ENSG00000106683	GAGAUGA
ENSG00000106683	GCUGGUG
ENSG00000106683	UCACAGG
ENSG00000106686	AAUUAUU
ENSG00000106688	AACACUG
ENSG00000106688	ACAGUAU
ENSG00000106688	CUUUGGU
ENSG00000106688	GAGAAAU
ENSG00000106688	UCAAGUA
ENSG00000106689	AAAACUG
ENSG00000106689	AAACUGU
ENSG00000106689	AAAGAAU
ENSG00000106689	AAUGUUG
ENSG00000106689	ACAGUAU
ENSG00000106689	CAAAACU
ENSG00000106692	GGGUAAG
ENSG00000106692	UACAAAG
ENSG00000106701	AACACUG
ENSG00000106701	AAGGCAC
ENSG00000106714	UUUGCAA
ENSG00000106723	AAACUGU
ENSG00000106723	AACAUUC
ENSG00000106723	AAGUCUU
ENSG00000106723	ACAUCAC
ENSG00000106723	CACCAGC
ENSG00000106723	CACUCCU
ENSG00000106723	CAGGCUC
ENSG00000106723	CAGUGCA
ENSG00000106723	CCAGUGC
ENSG00000106723	CCUUCUG
ENSG00000106723	CUAUACA
ENSG00000106723	CUUCACC
ENSG00000106723	GGCAGCG
ENSG00000106723	GGCUCAG
ENSG00000106723	GGGUUUA
ENSG00000106723	UAUAAAG
ENSG00000106723	UCACACA
ENSG00000106723	UCACAUU
ENSG00000106723	UCACCAC
ENSG00000106723	UGACAUC
ENSG00000106723	UUCAAAU
ENSG00000106723	UUGGCAA
ENSG00000106771	AAGAACC
ENSG00000106771	AAGGCAC
ENSG00000106771	AAUACUG
ENSG00000106771	AAUCUCU
ENSG00000106771	AGCAGCA
ENSG00000106771	AGCAGCG
ENSG00000106771	AGGUAGU
ENSG00000106771	CAAAGUG
ENSG00000106771	CACAGGG
ENSG00000106771	CCAGUGU
ENSG00000106771	CUAUACA
ENSG00000106771	CUUUGGU
ENSG00000106771	GCUGGUG
ENSG00000106771	UAAUGCU
ENSG00000106771	UCACAGG
ENSG00000106771	UUGGCAC
ENSG00000106771	UUUCAAC
ENSG00000106772	ACACACU

ENSG00000106772	AGGUAGU
ENSG00000106772	AUGGCAC
ENSG00000106772	CAAAGUG
ENSG00000106772	GAUAUGU
ENSG00000106772	UUCUUGU
ENSG00000106780	AAAAGUA
ENSG00000106780	AAUUAUU
ENSG00000106780	ACAAUAU
ENSG00000106780	ACAGUAC
ENSG00000106780	ACAUUAC
ENSG00000106780	AGGCAGG
ENSG00000106780	AUGGCUU
ENSG00000106780	CACCAGC
ENSG00000106780	CAGGAAC
ENSG00000106780	GAGAUGA
ENSG00000106780	GAGGCAG
ENSG00000106780	GAGUAUU
ENSG00000106780	UACAGUU
ENSG00000106780	UAGUGAG
ENSG00000106780	UCAAGUA
ENSG00000106780	UCAUACA
ENSG00000106780	UGUCUGG
ENSG00000106780	UUCUGCA
ENSG00000106785	GAGAAAU
ENSG00000106785	GUCUCUG
ENSG00000106789	CCCUGAG
ENSG00000106799	AAAGCUG
ENSG00000106799	AACCUGG
ENSG00000106799	ACCUGGA
ENSG00000106799	AGCUGCC
ENSG00000106799	AGUGGUU
ENSG00000106799	AUAAUAC
ENSG00000106799	CACAGUG
ENSG00000106799	CCCCUGG
ENSG00000106799	CUUACAG
ENSG00000106799	GAGACCU
ENSG00000106799	GAGGUAU
ENSG00000106799	GGAUUUC
ENSG00000106799	GGAUUUU
ENSG00000106799	GUGAGGU
ENSG00000106799	GUGCCAC
ENSG00000106799	GUUAAUG
ENSG00000106799	UAAGACG
ENSG00000106799	UAAGACU
ENSG00000106799	UAUCAGA
ENSG00000106799	UCACAGU
ENSG00000106799	UCAUAGA
ENSG00000106799	UCUUGAG
ENSG00000106799	UUGGUCC
ENSG00000106829	AAAAGUA
ENSG00000106829	AAACUGU
ENSG00000106829	AAAGUGC
ENSG00000106829	AAGGCAC
ENSG00000106829	AAGUGCU
ENSG00000106829	ACAUCAC
ENSG00000106829	ACCACAG
ENSG00000106829	ACUGUAG
ENSG00000106829	AGCAGCG
ENSG00000106829	AGCAGCU
ENSG00000106829	CAAAGUG
ENSG00000106829	CAGCAAA
ENSG00000106829	CAGCAGG
ENSG00000106829	CAGUCCA
ENSG00000106829	CUAGUAA

ENSG00000106829	CUAGUAG
ENSG00000106829	UGACAUC
ENSG00000106829	UUGAGGC
ENSG00000106829	UUGAUCA
ENSG00000106852	AAAGGUA
ENSG00000106852	AACAAAC
ENSG00000106852	AACACUG
ENSG00000106852	ACUGUAG
ENSG00000106852	AGGGUUG
ENSG00000106852	AUGUAAU
ENSG00000106852	CAAAGCA
ENSG00000106852	CAGACAG
ENSG00000106852	CAGCAGA
ENSG00000106852	CUGGGCA
ENSG00000106852	GAAAACA
ENSG00000106852	GGUUGUC
ENSG00000106852	GUAACAG
ENSG00000106852	UAUUACC
ENSG00000106852	UCCAGAG
ENSG00000106852	UCCCACA
ENSG00000106852	UGAAAGG
ENSG00000106852	UGCAUUG
ENSG00000106852	UGCCAGC
ENSG00000106948	AUACAAG
ENSG00000106948	AUAUAAC
ENSG00000106948	AUUCAUU
ENSG00000106948	CUUCUCU
ENSG00000106948	GCAGGAC
ENSG00000106948	GGCAGGG
ENSG00000106948	GGCUCAG
ENSG00000106948	UCAUUUG
ENSG00000106948	UGGCCAC
ENSG00000106991	CCUGCUG
ENSG00000106991	GGCUCUG
ENSG00000106991	GGGGUGC
ENSG00000106991	GGGGUGG
ENSG00000106992	AGUGCUG
ENSG00000106992	AGUGCUU
ENSG00000106992	GAAAACA
ENSG00000106992	GCUUCCU
ENSG00000106992	GGAAGCC
ENSG00000106992	GGCAGGG
ENSG00000106992	GGCGGGG
ENSG00000106993	AAGUGCA
ENSG00000106993	AUAAAGU
ENSG00000107021	AAGGCAC
ENSG00000107021	CACAGUG
ENSG00000107021	CUUCUCU
ENSG00000107021	GAGGCAG
ENSG00000107021	UCAAGUA
ENSG00000107104	AAUACUG
ENSG00000107104	AAUUUUA
ENSG00000107104	AGCAGCA
ENSG00000107104	AGCAGCG
ENSG00000107104	AUGGCAC
ENSG00000107105	AAAGAGG
ENSG00000107105	AACAUUC
ENSG00000107105	AAGUGCA
ENSG00000107105	AAUCUCA
ENSG00000107105	ACAAUAU
ENSG00000107105	ACAUUAC
ENSG00000107105	ACAUUCA
ENSG00000107105	ACCCUGU
ENSG00000107105	ACUGCAG

ENSG00000107105	ACUGCAU
ENSG00000107105	AGUAACA
ENSG00000107105	AUCACUA
ENSG00000107105	AUCAUAC
ENSG00000107105	AUGAGCU
ENSG00000107105	CAGGUGA
ENSG00000107105	CCAGGAG
ENSG00000107105	CCUGUAC
ENSG00000107105	CCUUCUU
ENSG00000107105	CUCACAC
ENSG00000107105	CUUACAG
ENSG00000107105	CUUUGGU
ENSG00000107105	GAUCAGA
ENSG00000107105	GCAGGAC
ENSG00000107105	GGCAAGA
ENSG00000107105	GUAUUCU
ENSG00000107105	GUGCAAA
ENSG00000107105	GUUAAUG
ENSG00000107105	UCACAGG
ENSG00000107105	UCACAGU
ENSG00000107105	UGAAGCU
ENSG00000107105	UGCAUAG
ENSG00000107105	UUAGGAU
ENSG00000107105	UUAUGCA
ENSG00000107105	UUCUUGU
ENSG00000107105	UUGGCAA
ENSG00000107105	UUGGCAC
ENSG00000107105	UUGUUCG
ENSG00000107140	CCGGAGC
ENSG00000107140	UGAAAUG
ENSG00000107147	UGUGCUU
ENSG00000107164	AACGGAA
ENSG00000107164	CACAGUG
ENSG00000107164	CUUGGAG
ENSG00000107164	GACUUCC
ENSG00000107164	GGAAUGU
ENSG00000107164	UAAGACU
ENSG00000107164	UCCCUUU
ENSG00000107164	UUGGAGG
ENSG00000107175	AAUCACU
ENSG00000107175	UGAAAUG
ENSG00000107186	AAAGUGC
ENSG00000107186	UUCUAAU
ENSG00000107187	AAAGAGC
ENSG00000107187	AAGUCUU
ENSG00000107187	UGAAGUG
ENSG00000107187	UUGGCAA
ENSG00000107187	UUGGCAC
ENSG00000107201	AUUCAUU
ENSG00000107223	CAGCAGG
ENSG00000107249	AAAACUG
ENSG00000107249	AAAUCCC
ENSG00000107249	AAGUGCA
ENSG00000107249	AAUUAUU
ENSG00000107249	ACAUUAC
ENSG00000107249	ACAUUCA
ENSG00000107249	AGAUGUG
ENSG00000107249	AGCACCA
ENSG00000107249	AUGACAC
ENSG00000107249	AUGUGCC
ENSG00000107249	AUUCAUU
ENSG00000107249	CAAAGUG
ENSG00000107249	CCUUCAU
ENSG00000107249	CUAUACA

ENSG00000107249	CUCCCAA
ENSG00000107249	GAAGGAA
ENSG00000107249	GAUCAGA
ENSG00000107249	GUGACAG
ENSG00000107249	UAAUAGU
ENSG00000107249	UAUAAUA
ENSG00000107249	UCACAAG
ENSG00000107249	UCAUGAU
ENSG00000107249	UCCAGAG
ENSG00000107249	UGACAUC
ENSG00000107249	UGUAUAA
ENSG00000107249	UUGACAC
ENSG00000107262	CACUCCU
ENSG00000107262	GAGAACU
ENSG00000107262	GCUGGUG
ENSG00000107262	UCCAGAG
ENSG00000107262	UGAGAAG
ENSG00000107262	UGAGAAU
ENSG00000107263	AAAGUGC
ENSG00000107263	AAGGCAC
ENSG00000107263	AAUACUG
ENSG00000107263	ACUGUUU
ENSG00000107263	AGCUGCC
ENSG00000107263	AUAAAGU
ENSG00000107263	AUAUUAU
ENSG00000107263	AUGUCUG
ENSG00000107263	GAUUGUC
ENSG00000107263	GUCUGCC
ENSG00000107263	UCUUGAG
ENSG00000107263	UGUCUGC
ENSG00000107282	GCAGCAU
ENSG00000107290	UCACAUU
ENSG00000107295	GCAGCAU
ENSG00000107295	UGCAUAU
ENSG00000107331	AAGGCAC
ENSG00000107331	UUGGCAA
ENSG00000107331	UUGGUCC
ENSG00000107338	AAAAACC
ENSG00000107338	AAAGCUG
ENSG00000107338	AACCUGG
ENSG00000107338	AACCUGU
ENSG00000107338	AGCAGCU
ENSG00000107338	AGCAUUC
ENSG00000107338	AUAUUAU
ENSG00000107338	CACAAUG
ENSG00000107338	CCUGUAC
ENSG00000107338	CUCCAGC
ENSG00000107338	CUGGCUC
ENSG00000107338	CUUUGGU
ENSG00000107338	GAAAACA
ENSG00000107338	GGAUUUC
ENSG00000107338	GGAUUUU
ENSG00000107338	GGCAGCG
ENSG00000107338	GGGAGCU
ENSG00000107338	GGGGUGC
ENSG00000107338	UAGAGGA
ENSG00000107338	UCACAGG
ENSG00000107338	UCACAGU
ENSG00000107338	UCCUUGC
ENSG00000107338	UGAAAGG
ENSG00000107338	UGCAAAG
ENSG00000107338	UUGUUCG
ENSG00000107338	UUUGCAA
ENSG00000107341	AAAGUAU

ENSG00000107341	AAGUAGC
ENSG00000107341	AAGUGCU
ENSG00000107341	ACACACU
ENSG00000107341	ACCAGCA
ENSG00000107341	AUGCACC
ENSG00000107341	CAGACAG
ENSG00000107341	GAAAACA
ENSG00000107341	GAGACCU
ENSG00000107341	GAUCAGA
ENSG00000107341	GAUUGUA
ENSG00000107341	GCAGCAU
ENSG00000107341	GCUGGUG
ENSG00000107341	GGCAGGG
ENSG00000107341	GUAAACA
ENSG00000107341	UCACCAC
ENSG00000107341	UCCCUUU
ENSG00000107341	UUGGCAA
ENSG00000107341	UUGGUUC
ENSG00000107341	UUUUUGC
ENSG00000107362	CCUUCAU
ENSG00000107362	GCAACUU
ENSG00000107362	UUUGCAA
ENSG00000107372	AACACUG
ENSG00000107372	AAGAACC
ENSG00000107372	AAGACAU
ENSG00000107372	AAUACUG
ENSG00000107372	AAUCCUU
ENSG00000107372	AAUGUUG
ENSG00000107372	ACAUUAC
ENSG00000107372	ACAUUCA
ENSG00000107372	ACCACAG
ENSG00000107372	ACCCUGU
ENSG00000107372	ACUGCAG
ENSG00000107372	ACUGCAU
ENSG00000107372	AGAUAAA
ENSG00000107372	AGUAGAC
ENSG00000107372	AGUGCAA
ENSG00000107372	AGUGCCG
ENSG00000107372	AGUGCCU
ENSG00000107372	AUAAUAC
ENSG00000107372	AUACCUC
ENSG00000107372	AUAUAAC
ENSG00000107372	AUGUGCC
ENSG00000107372	CAAAACU
ENSG00000107372	CAGUCUG
ENSG00000107372	CAGUUAC
ENSG00000107372	CCAUUAC
ENSG00000107372	CCCUGUU
ENSG00000107372	CCUGCUG
ENSG00000107372	CUCUUCC
ENSG00000107372	CUGCAGC
ENSG00000107372	CUGCAGG
ENSG00000107372	CUGCUCA
ENSG00000107372	CUUCACC
ENSG00000107372	CUUGUAU
ENSG00000107372	GACCCUG
ENSG00000107372	GAGCUGC
ENSG00000107372	GCAGAAG
ENSG00000107372	GCUGGAU
ENSG00000107372	GCUUCCU
ENSG00000107372	GGUAGAC
ENSG00000107372	GUAUGUU
ENSG00000107372	GUCUACU
ENSG00000107372	GUGCAAA

ENSG00000107372	UACAGUU
ENSG00000107372	UCACAAG
ENSG00000107372	UCAUACA
ENSG00000107372	UCCCUCU
ENSG00000107372	UCUUGAG
ENSG00000107372	UGAAAGG
ENSG00000107372	UGUAUGU
ENSG00000107372	UUCCAUA
ENSG00000107372	UUGGCAA
ENSG00000107372	UUUGCAA
ENSG00000107404	AGCAGCU
ENSG00000107404	CUACAGU
ENSG00000107443	AAAGUGC
ENSG00000107443	AACAUUC
ENSG00000107443	AAGUGUG
ENSG00000107443	ACAGUAC
ENSG00000107443	ACUGUAG
ENSG00000107443	AGCCCUU
ENSG00000107443	CACAGUG
ENSG00000107443	CCAGUGC
ENSG00000107443	CCUGCUG
ENSG00000107443	CUUUGGU
ENSG00000107443	GAGGUAU
ENSG00000107443	GCAGCAU
ENSG00000107443	GGCAAGA
ENSG00000107443	GUAGUGU
ENSG00000107443	UAAUACA
ENSG00000107443	UCAUAGA
ENSG00000107443	UCCCUGU
ENSG00000107443	UGCAUAG
ENSG00000107443	UUCAAGC
ENSG00000107443	UUGGCAC
ENSG00000107443	UUGGUUC
ENSG00000107485	AUGGCUU
ENSG00000107485	CCUUCAU
ENSG00000107485	UCACAGG
ENSG00000107485	UCACAGU
ENSG00000107485	UGUAUAA
ENSG00000107518	ACAAUAU
ENSG00000107518	ACAUUCA
ENSG00000107518	AGCAAAA
ENSG00000107518	AUGCAUU
ENSG00000107518	CACAAUG
ENSG00000107518	CUCCAUU
ENSG00000107518	GCAACUU
ENSG00000107518	GGCAUUG
ENSG00000107518	GUAAACA
ENSG00000107518	UAAUAUC
ENSG00000107518	UUCCAUA
ENSG00000107551	GUAAACA
ENSG00000107551	UCAUUCG
ENSG00000107554	UAUAAAG
ENSG00000107560	ACAUUCA
ENSG00000107560	CAGCUGG
ENSG00000107560	CUGGAAA
ENSG00000107560	UCACAGG
ENSG00000107560	UCACAGU
ENSG00000107560	UCAUUCG
ENSG00000107560	UCUGGAA
ENSG00000107560	UGCAUAG
ENSG00000107560	UGCUGAC
ENSG00000107562	ACAAUAU
ENSG00000107562	ACAGUAC
ENSG00000107562	AUGAUGA

ENSG00000107562	CUCAAAC
ENSG00000107562	GAAUUGU
ENSG00000107562	UCUCAAG
ENSG00000107562	UGCAAAG
ENSG00000107566	AAAUUUC
ENSG00000107566	AAUACUG
ENSG00000107566	CAAAUGC
ENSG00000107566	CAGCUGG
ENSG00000107566	CAGUGCA
ENSG00000107566	CCAGUGU
ENSG00000107566	CCCUGUU
ENSG00000107566	CUUGGGC
ENSG00000107566	GAAAACA
ENSG00000107566	GCAGCAU
ENSG00000107566	UACAGUU
ENSG00000107566	UCAAGUA
ENSG00000107566	UCCCACA
ENSG00000107566	UCCCACC
ENSG00000107566	UGAUCCA
ENSG00000107566	UUGGCAA
ENSG00000107581	CUACAAA
ENSG00000107614	CAAAGUG
ENSG00000107614	UAAGACU
ENSG00000107614	UAUUACC
ENSG00000107623	AAAGUAU
ENSG00000107623	ACAGUAC
ENSG00000107623	AUAUUAU
ENSG00000107623	UACAAAG
ENSG00000107625	UUCUUGU
ENSG00000107625	UUUGCAA
ENSG00000107651	GAGAACC
ENSG00000107672	AUCAUAC
ENSG00000107672	UCAUACA
ENSG00000107679	AGCACCA
ENSG00000107679	CUUUGGU
ENSG00000107679	GACCCUG
ENSG00000107719	AGCUGCC
ENSG00000107719	AGUAGUU
ENSG00000107719	CAGUAGU
ENSG00000107719	GAGGUAU
ENSG00000107719	UGCCAGC
ENSG00000107731	UUCCUAG
ENSG00000107736	ACAUUCA
ENSG00000107736	GGAAGCC
ENSG00000107738	AAAUUUC
ENSG00000107738	AGCAGCA
ENSG00000107738	CAAAUGC
ENSG00000107738	CAGCAGA
ENSG00000107738	CAGCUGG
ENSG00000107738	CCAGCAU
ENSG00000107738	GAAAACA
ENSG00000107742	AAAAAUC
ENSG00000107742	AACACUG
ENSG00000107742	AACAUUC
ENSG00000107742	AAGACAU
ENSG00000107742	AAGGCAC
ENSG00000107742	AAGUAGC
ENSG00000107742	ACAAGGU
ENSG00000107742	CAGGCUC
ENSG00000107742	CAGUUAC
ENSG00000107742	CCAGGAG
ENSG00000107742	UAUUGCU
ENSG00000107742	UCAAGUA
ENSG00000107742	UCUCAAG

ENSG00000107745	UGCAAAG
ENSG00000107758	AUAUUAU
ENSG00000107758	GUAAACA
ENSG00000107758	UAAGACG
ENSG00000107758	UAAGACU
ENSG00000107779	AGCAGCA
ENSG00000107779	AGCAGCG
ENSG00000107779	AGCAGCU
ENSG00000107779	AGUACUG
ENSG00000107779	AUGGCUU
ENSG00000107779	CAGCAGA
ENSG00000107779	CUCCAUU
ENSG00000107779	GGCAGCG
ENSG00000107779	UCACAGG
ENSG00000107779	UCACAGU
ENSG00000107779	UCCCUUG
ENSG00000107779	UCUCCAA
ENSG00000107796	CACAGUG
ENSG00000107807	AAGUGCU
ENSG00000107807	CACAGUG
ENSG00000107807	GGUUACC
ENSG00000107807	UAUAAAG
ENSG00000107815	CUGGAAA
ENSG00000107816	GGGUUUA
ENSG00000107816	UUGGUCC
ENSG00000107819	GGAAGCC
ENSG00000107829	CUGGCUC
ENSG00000107829	UUCAAAU
ENSG00000107831	AAAAACC
ENSG00000107854	AAAGAAU
ENSG00000107854	AAAGCUG
ENSG00000107854	AAGUGCU
ENSG00000107854	AGUGCUG
ENSG00000107854	CAAAGCA
ENSG00000107854	GAAAACA
ENSG00000107854	UCAAGUA
ENSG00000107854	UCUGGAA
ENSG00000107859	CCCUGUU
ENSG00000107859	UCCGCGC
ENSG00000107862	AAAGUGC
ENSG00000107862	AAGUGCA
ENSG00000107862	CAAAGUG
ENSG00000107862	GGAGGAG
ENSG00000107863	AAAAAUC
ENSG00000107863	AAGUCAC
ENSG00000107863	ACAAUAU
ENSG00000107863	CCAGUGC
ENSG00000107863	CUGGCUC
ENSG00000107863	GAGGCAG
ENSG00000107863	GCAGAAG
ENSG00000107863	GGAAUGU
ENSG00000107863	UCAAGUA
ENSG00000107863	UCCAGUU
ENSG00000107864	AAAAGUA
ENSG00000107864	AAACUGU
ENSG00000107864	AAAGAGG
ENSG00000107864	AAAGGUA
ENSG00000107864	AAAGUGC
ENSG00000107864	AAGUCUU
ENSG00000107864	AAGUGCU
ENSG00000107864	ACAGUAU
ENSG00000107864	ACAUCAC
ENSG00000107864	ACUGGAU
ENSG00000107864	AGCACCA

ENSG00000107864	AGCAGCG
ENSG00000107864	AGCAGCU
ENSG00000107864	AUACAAG
ENSG00000107864	AUGAGCU
ENSG00000107864	CACAGUG
ENSG00000107864	CACUCCU
ENSG00000107864	CAGUAGA
ENSG00000107864	CCAGCAU
ENSG00000107864	CUGCAGC
ENSG00000107864	GAGGUAG
ENSG00000107864	GAUUGUC
ENSG00000107864	GUGCAAA
ENSG00000107864	UAUAAAG
ENSG00000107864	UAUUGCU
ENSG00000107864	UCAAGUA
ENSG00000107864	UCACAGU
ENSG00000107864	UGACUGU
ENSG00000107864	UGAGAAU
ENSG00000107864	UGCAAAG
ENSG00000107864	UGCAGCU
ENSG00000107864	UUAUGCA
ENSG00000107864	UUCCUAG
ENSG00000107864	UUCUAAU
ENSG00000107864	UUGAUCA
ENSG00000107864	UUUGCAA
ENSG00000107872	CAGUCCA
ENSG00000107872	UGAAGCU
ENSG00000107874	UCCCUCU
ENSG00000107882	AAGUCAC
ENSG00000107882	AGGCAGG
ENSG00000107882	AUGGCUU
ENSG00000107882	CAGGAAC
ENSG00000107882	CUCUAGC
ENSG00000107882	CUGCAGC
ENSG00000107882	CUGCAGG
ENSG00000107882	CUUACAG
ENSG00000107882	GAAGGAA
ENSG00000107882	GAAUUGU
ENSG00000107882	GAGACCU
ENSG00000107882	GAGGCUG
ENSG00000107882	GCAGCAU
ENSG00000107882	GGCAGGG
ENSG00000107882	UAAUGCU
ENSG00000107882	UACAAAG
ENSG00000107897	AAAGUGC
ENSG00000107897	AACUACU
ENSG00000107897	AAGUGCU
ENSG00000107897	AGUGCUU
ENSG00000107897	GUAGUGU
ENSG00000107897	UGCAAAG
ENSG00000107897	UUUCAAC
ENSG00000107937	CAAAGCA
ENSG00000107938	AAGGCAC
ENSG00000107938	CACAGUG
ENSG00000107938	UCACAGG
ENSG00000107938	UUCAAGC
ENSG00000107954	CACAAUG
ENSG00000107954	CUGCAGC
ENSG00000107954	UCACAAG
ENSG00000107957	AAAAAUC
ENSG00000107957	AAAAGUA
ENSG00000107957	AAAGUAU
ENSG00000107957	AAAGUUU
ENSG00000107957	AAAUUUC

ENSG00000107957	AAUACUG
ENSG00000107957	ACAGUAC
ENSG00000107957	ACAGUAU
ENSG00000107957	ACUGCAG
ENSG00000107957	AGCAGCU
ENSG00000107957	AGCUGCC
ENSG00000107957	AGUAACA
ENSG00000107957	AGUACUG
ENSG00000107957	AUAUAAC
ENSG00000107957	AUGCACC
ENSG00000107957	AUGCCUU
ENSG00000107957	AUGUGCC
ENSG00000107957	CACAAUG
ENSG00000107957	CAGCAGA
ENSG00000107957	CAGCUGG
ENSG00000107957	CAGUAGA
ENSG00000107957	CAGUAGU
ENSG00000107957	CAGUGCA
ENSG00000107957	CCAGCAU
ENSG00000107957	CCAGUGU
ENSG00000107957	CCUUCUU
ENSG00000107957	CUAGUAA
ENSG00000107957	CUGGCUC
ENSG00000107957	CUUUGGU
ENSG00000107957	GAGAAAU
ENSG00000107957	GAGAUGA
ENSG00000107957	GCAGAAG
ENSG00000107957	GCUGGAU
ENSG00000107957	GGAAGCC
ENSG00000107957	GGACCUG
ENSG00000107957	GGCUCAG
ENSG00000107957	GGGUGGU
ENSG00000107957	GUCCAGA
ENSG00000107957	GUGAAUG
ENSG00000107957	GUGGCCC
ENSG00000107957	GUUAAUG
ENSG00000107957	UAAUACA
ENSG00000107957	UAAUGCU
ENSG00000107957	UACAGUU
ENSG00000107957	UCAAGUA
ENSG00000107957	UCACAUU
ENSG00000107957	UGAGAAG
ENSG00000107957	UUCAAAU
ENSG00000107957	UUCCAUA
ENSG00000107957	UUCCUCG
ENSG00000107968	AAAGUGC
ENSG00000107968	AAGUGCA
ENSG00000107968	CAAAGUG
ENSG00000107968	GAGAAAU
ENSG00000107984	AAAGAAU
ENSG00000107984	AAGUGCA
ENSG00000107984	AAGUGCU
ENSG00000107984	ACUCCAG
ENSG00000107984	ACUGCAG
ENSG00000107984	ACUGCAU
ENSG00000107984	GCAGCAU
ENSG00000107984	GUGCCAC
ENSG00000108001	AAAUGGU
ENSG00000108001	AACAAAC
ENSG00000108001	AACUACU
ENSG00000108001	AAGGCAC
ENSG00000108001	AAUGUUG
ENSG00000108001	AGCAAAA
ENSG00000108001	AGUGUGC

ENSG00000108001	AUACAAG
ENSG00000108001	AUGUGGG
ENSG00000108001	CACCACC
ENSG00000108001	CAGACAG
ENSG00000108001	CAGCAGA
ENSG00000108001	CUAUACA
ENSG00000108001	CUCUAGC
ENSG00000108001	GAAACAU
ENSG00000108001	GAAUUGU
ENSG00000108001	GGCAAGA
ENSG00000108001	GGCUCAG
ENSG00000108001	GGGUCUU
ENSG00000108001	GUAAACA
ENSG00000108001	GUCAGUU
ENSG00000108001	GUUAAUG
ENSG00000108001	UAGGCCG
ENSG00000108001	UCACAGG
ENSG00000108001	UCACCAC
ENSG00000108001	UGUAUAA
ENSG00000108001	UUGUUCG
ENSG00000108001	UUUGCAA
ENSG00000108018	AAAAAUC
ENSG00000108018	AAAGCUG
ENSG00000108018	AACAAAC
ENSG00000108018	AAGGCAC
ENSG00000108018	ACAUCAC
ENSG00000108018	ACUGGCC
ENSG00000108018	ACUGGCU
ENSG00000108018	AGCUGCC
ENSG00000108018	AGUAGUU
ENSG00000108018	CAAAGCA
ENSG00000108018	CAAAGUG
ENSG00000108018	CACUCCU
ENSG00000108018	CCAUUAC
ENSG00000108018	CCUGUAC
ENSG00000108018	CUCAGGG
ENSG00000108018	CUGGAAA
ENSG00000108018	GAAAACA
ENSG00000108018	GAGAAGA
ENSG00000108018	GAGUGUG
ENSG00000108018	GAUCAGA
ENSG00000108018	GCUUCCU
ENSG00000108018	GGAAGCC
ENSG00000108018	UCCCUGU
ENSG00000108018	UCCUUGC
ENSG00000108018	UGUCUGC
ENSG00000108018	UGUCUGG
ENSG00000108018	UGUGUCA
ENSG00000108021	AGCAAAA
ENSG00000108021	AUCAUAC
ENSG00000108039	ACCUGGA
ENSG00000108039	AGCACCA
ENSG00000108039	CCUUCUG
ENSG00000108061	AAAGUGC
ENSG00000108061	AAGUGCA
ENSG00000108061	ACAUUCA
ENSG00000108061	GCAACUU
ENSG00000108064	AGUAGUU
ENSG00000108064	AUGCAUU
ENSG00000108091	AAGGCAC
ENSG00000108091	AAGUCAC
ENSG00000108091	AAGUCUU
ENSG00000108091	AAUCACU
ENSG00000108091	ACAGGUG

ENSG00000108091	ACAUUCA
ENSG00000108091	ACUGGCU
ENSG00000108091	AGCAAAA
ENSG00000108091	AGCAGCG
ENSG00000108091	AGCAGCU
ENSG00000108091	AGGGUCU
ENSG00000108091	AGUGCAA
ENSG00000108091	AUAUAAC
ENSG00000108091	AUGCAUU
ENSG00000108091	CAAAGUG
ENSG00000108091	CACAGUG
ENSG00000108091	CAGCAGA
ENSG00000108091	CCUUCUG
ENSG00000108091	CUACAAA
ENSG00000108091	CUAGUAA
ENSG00000108091	CUAGUAG
ENSG00000108091	CUGGCUC
ENSG00000108091	GAAGGAA
ENSG00000108091	GAGUGUG
ENSG00000108091	GCAGCAU
ENSG00000108091	GGAAGCC
ENSG00000108091	GGAGUGU
ENSG00000108091	GGCUGCG
ENSG00000108091	GGUUGUC
ENSG00000108091	GUAAACA
ENSG00000108091	GUCUCUG
ENSG00000108091	GUGCAAA
ENSG00000108091	UAUAAAG
ENSG00000108091	UCCCUUU
ENSG00000108091	UGAAGCU
ENSG00000108091	UGCAAAG
ENSG00000108091	UGGCUGC
ENSG00000108091	UUAGAGA
ENSG00000108091	UUUCAAC
ENSG00000108094	AAUUAUU
ENSG00000108094	ACACACC
ENSG00000108094	ACAUUAC
ENSG00000108094	ACGCUCA
ENSG00000108094	CAGCAGA
ENSG00000108094	GUAAACA
ENSG00000108100	AAGGCAC
ENSG00000108100	UAUUGCU
ENSG00000108106	UUAGAGA
ENSG00000108107	UAUUGCU
ENSG00000108175	AAAAAUC
ENSG00000108175	AAAGCUG
ENSG00000108175	AAAGUAA
ENSG00000108175	AAAGUAC
ENSG00000108175	AAAUCCC
ENSG00000108175	AACUACU
ENSG00000108175	AAUGUUG
ENSG00000108175	ACCAGCA
ENSG00000108175	AGCACCA
ENSG00000108175	AUACAAG
ENSG00000108175	AUACCUC
ENSG00000108175	AUAUUAU
ENSG00000108175	CACAAUG
ENSG00000108175	CACCAGC
ENSG00000108175	CAGCAAA
ENSG00000108175	CUCUGGG
ENSG00000108175	CUUGGAG
ENSG00000108175	GCUGGUG
ENSG00000108175	GGAAGAC
ENSG00000108175	GGUUACC

ENSG00000108175	GGUUGUC
ENSG00000108175	GUGCUUG
ENSG00000108175	UCACAAG
ENSG00000108175	UCAUGAU
ENSG00000108175	UCCCUUG
ENSG00000108175	UGAAAUG
ENSG00000108175	UGAAGCU
ENSG00000108175	UGAGUCU
ENSG00000108175	UGCAUAG
ENSG00000108175	UGCAUAU
ENSG00000108175	UGUGCUU
ENSG00000108175	UGUUGAA
ENSG00000108175	UUCCAUA
ENSG00000108175	UUGAUCA
ENSG00000108175	UUGGUUC
ENSG00000108175	UUUGCAA
ENSG00000108176	AAUUAUU
ENSG00000108179	AAGGCAC
ENSG00000108179	ACUCAGG
ENSG00000108179	AGCAGCA
ENSG00000108179	CACAGUG
ENSG00000108179	UCACAGG
ENSG00000108179	UCACAGU
ENSG00000108187	CAAAACU
ENSG00000108219	ACCAGCA
ENSG00000108219	AGCAUUC
ENSG00000108219	AUAAAGU
ENSG00000108219	CAGUAGA
ENSG00000108219	CAGUAGU
ENSG00000108219	CCCUGAG
ENSG00000108219	GUCUCUG
ENSG00000108219	UAAUGCU
ENSG00000108219	UAUAAAG
ENSG00000108219	UUGGCAA
ENSG00000108219	UUGGCAC
ENSG00000108231	AAAAGUA
ENSG00000108231	AUGCAUU
ENSG00000108231	GGCAGUG
ENSG00000108231	UUGGCAA
ENSG00000108231	UUGGCAC
ENSG00000108239	AAAGAGG
ENSG00000108239	AAAGGUA
ENSG00000108239	AAAGUAA
ENSG00000108239	AAGUCUU
ENSG00000108239	AAGUGCA
ENSG00000108239	AGUGGUU
ENSG00000108239	AUUCAUU
ENSG00000108239	AUUGCAC
ENSG00000108239	GAGGCAG
ENSG00000108239	GUGCAAA
ENSG00000108239	GUGCUUG
ENSG00000108239	UAAUACA
ENSG00000108239	UAUUGCU
ENSG00000108239	UCAUUUG
ENSG00000108239	UCCAGUU
ENSG00000108239	UGCAAAG
ENSG00000108239	UGCAUUG
ENSG00000108239	UGUGCUU
ENSG00000108239	UUAUGCA
ENSG00000108239	UUCAAGC
ENSG00000108256	AAAGUGC
ENSG00000108256	AAGGCAC
ENSG00000108256	AAGGCGC
ENSG00000108256	AAGGUGC

ENSG00000108256	AAGUCAC
ENSG00000108256	AAGUGCA
ENSG00000108256	AAGUGCU
ENSG00000108256	AAUACUG
ENSG00000108256	ACAGUAC
ENSG00000108256	ACAGUAU
ENSG00000108256	ACAUUCA
ENSG00000108256	ACUGCAU
ENSG00000108256	ACUGGCC
ENSG00000108256	ACUGGCU
ENSG00000108256	AGCACCA
ENSG00000108256	AGCAGCA
ENSG00000108256	AGCAGCG
ENSG00000108256	AGCAGCU
ENSG00000108256	AGGCAGG
ENSG00000108256	AGUGGUU
ENSG00000108256	AGUGUGC
ENSG00000108256	AUACCUC
ENSG00000108256	AUGCAUU
ENSG00000108256	AUGGCAC
ENSG00000108256	AUUGCAC
ENSG00000108256	CAAAACU
ENSG00000108256	CAGCAGA
ENSG00000108256	CAGGAAC
ENSG00000108256	CAGUAGU
ENSG00000108256	CAGUCUG
ENSG00000108256	CCAGUGU
ENSG00000108256	CCUGCUG
ENSG00000108256	CUGGACU
ENSG00000108256	CUUCACC
ENSG00000108256	CUUCUCU
ENSG00000108256	CUUGUGU
ENSG00000108256	GAAUUGU
ENSG00000108256	GAGUGUG
ENSG00000108256	GAGUUGA
ENSG00000108256	GAGUUGG
ENSG00000108256	GCAGAAG
ENSG00000108256	GCAGCAU
ENSG00000108256	GCUACAU
ENSG00000108256	GGACUGC
ENSG00000108256	GGCGACA
ENSG00000108256	GGUUUAC
ENSG00000108256	GUAAACA
ENSG00000108256	GUAUUCU
ENSG00000108256	GUGAGGU
ENSG00000108256	GUGCAAA
ENSG00000108256	GUGCUUG
ENSG00000108256	UAAUGCU
ENSG00000108256	UACAGUU
ENSG00000108256	UAGACUG
ENSG00000108256	UAGUGAG
ENSG00000108256	UCAACAG
ENSG00000108256	UCUGGAA
ENSG00000108256	UCUGGAG
ENSG00000108256	UGAGGAC
ENSG00000108256	UGCAUAG
ENSG00000108256	UGGACUG
ENSG00000108256	UGGCCCU
ENSG00000108256	UGUGUGG
ENSG00000108256	UGUUGAA
ENSG00000108256	UUAUGCA
ENSG00000108256	UUGGCAA
ENSG00000108256	UUGGCAC
ENSG00000108256	UUGGUUC

ENSG00000108256	UUGUGUC
ENSG00000108256	UUGUUCG
ENSG00000108256	UUUGCAA
ENSG00000108262	ACUGGCU
ENSG00000108262	AGCAGCA
ENSG00000108262	AGGCAGG
ENSG00000108262	AGUGGUU
ENSG00000108262	AUGACAC
ENSG00000108262	AUGUGGG
ENSG00000108262	CACCACC
ENSG00000108262	CAGGCUC
ENSG00000108262	CAGUUAC
ENSG00000108262	CAUGGGU
ENSG00000108262	CCAGUGC
ENSG00000108262	CCCACCU
ENSG00000108262	CUCCAGC
ENSG00000108262	CUGGCUC
ENSG00000108262	GCUGGUG
ENSG00000108262	GGAAUGU
ENSG00000108262	GGAGUGU
ENSG00000108262	GGGGAGC
ENSG00000108262	GUGAGGU
ENSG00000108262	GUGGCCC
ENSG00000108262	UCCAGUU
ENSG00000108262	UCUGGAG
ENSG00000108262	UGGCUGC
ENSG00000108262	UGGGGGA
ENSG00000108262	UGUCUGG
ENSG00000108292	AAAAACC
ENSG00000108292	AAAGUGC
ENSG00000108292	AACAUUC
ENSG00000108292	AAGUCUU
ENSG00000108292	AGCAGCA
ENSG00000108292	AGCCCUU
ENSG00000108292	AGGGUCU
ENSG00000108292	AGUACUG
ENSG00000108292	AGUGCAA
ENSG00000108292	AGUGCCU
ENSG00000108292	AUAUUAU
ENSG00000108292	AUGCACC
ENSG00000108292	CACUCCU
ENSG00000108292	CAGUAGA
ENSG00000108292	CAGUAGU
ENSG00000108292	CAGUGCA
ENSG00000108292	CCAGUGC
ENSG00000108292	CUCACAC
ENSG00000108292	CUCCAGC
ENSG00000108292	CUUGGAG
ENSG00000108292	GAGCUAA
ENSG00000108292	GAGUAUU
ENSG00000108292	GCAACUU
ENSG00000108292	GCCCUUA
ENSG00000108292	GCUACAU
ENSG00000108292	GCUGGUG
ENSG00000108292	GGGCCCC
ENSG00000108292	GGGGCAG
ENSG00000108292	GGUGGGC
ENSG00000108292	GUCAUUG
ENSG00000108292	GUGCAAA
ENSG00000108292	UAAUAUC
ENSG00000108292	UAUCAGA
ENSG00000108292	UCAACAG
ENSG00000108292	UCUCCAA
ENSG00000108292	UGGGUGA

ENSG00000108292	UUAUGCA
ENSG00000108292	UUGGAGG
ENSG00000108309	CAGUCUG
ENSG00000108309	UCACAGU
ENSG00000108312	AAGUGCA
ENSG00000108312	ACUGUAG
ENSG00000108312	AUAUAAC
ENSG00000108312	CCAGUGC
ENSG00000108312	CCUGCUG
ENSG00000108312	CUACAAA
ENSG00000108312	CUCCCAC
ENSG00000108312	CUGCAGG
ENSG00000108312	CUGCCCC
ENSG00000108312	CUUACAG
ENSG00000108312	GAAAACA
ENSG00000108312	GACAACU
ENSG00000108312	GAGAAAU
ENSG00000108312	GCAGAAG
ENSG00000108312	GCCUGGG
ENSG00000108312	GGCAGGG
ENSG00000108312	GGGGGAA
ENSG00000108312	GGUGGGC
ENSG00000108312	GGUUGUC
ENSG00000108312	UCAAGUA
ENSG00000108312	UCCAGUU
ENSG00000108342	AACACUG
ENSG00000108342	ACUUGGA
ENSG00000108342	AGAUAAA
ENSG00000108342	CCCACGU
ENSG00000108344	GACCCUG
ENSG00000108344	GAGAACU
ENSG00000108349	AAGGCAC
ENSG00000108349	AAGUCAC
ENSG00000108349	AAUUUUA
ENSG00000108349	ACAUUAC
ENSG00000108349	AUGCAUU
ENSG00000108349	CACACCU
ENSG00000108349	CCCCUGG
ENSG00000108349	CUACAAA
ENSG00000108349	GACAACU
ENSG00000108349	GAGGCAG
ENSG00000108349	UCACAGU
ENSG00000108349	UGCAUUG
ENSG00000108349	UUGGUCC
ENSG00000108352	AAAGCUG
ENSG00000108352	AAAGUGC
ENSG00000108352	AAGUGCU
ENSG00000108352	ACAAGGU
ENSG00000108352	AGCACCA
ENSG00000108352	AGCUGCC
ENSG00000108352	AGUGCUU
ENSG00000108352	AUACAAG
ENSG00000108352	CCCACCU
ENSG00000108352	CCCUGAG
ENSG00000108352	CUCCCAC
ENSG00000108352	GCCUGGG
ENSG00000108352	GGGGUGG
ENSG00000108352	GUGCAAA
ENSG00000108352	GUGCUUG
ENSG00000108352	UCCCACC
ENSG00000108352	UCCCUGU
ENSG00000108352	UGCAAAG
ENSG00000108352	UGUAUAA
ENSG00000108370	AAGGCAC

ENSG00000108375	ACAUUCA
ENSG00000108375	AGCAAAA
ENSG00000108375	AGCAGCA
ENSG00000108375	AGUGCCG
ENSG00000108375	AUAUUAU
ENSG00000108375	AUGGCUU
ENSG00000108375	AUGUGGG
ENSG00000108375	UCACAUU
ENSG00000108375	UGGAUAA
ENSG00000108375	UGUCUGG
ENSG00000108375	UUCAAGC
ENSG00000108379	ACACACC
ENSG00000108379	CCCCUGG
ENSG00000108379	CUUCACC
ENSG00000108379	UCCAGAG
ENSG00000108379	UGCAAAG
ENSG00000108387	AAGGCGC
ENSG00000108387	GUCUCUG
ENSG00000108387	UGAGUCU
ENSG00000108387	UUCCUCG
ENSG00000108389	AGCACCA
ENSG00000108389	AGCAGCA
ENSG00000108389	GCAGCAU
ENSG00000108389	UAUUGCU
ENSG00000108389	UCACAGU
ENSG00000108395	AACAAAC
ENSG00000108395	AGCAGCA
ENSG00000108395	AGCAGCG
ENSG00000108395	UUGGCAC
ENSG00000108405	GAGGUAG
ENSG00000108406	AAAAAUC
ENSG00000108406	ACAAUAU
ENSG00000108406	AUACAAG
ENSG00000108406	CAAAGCA
ENSG00000108406	CUUUGGU
ENSG00000108406	GUAAACA
ENSG00000108406	GUUAAUG
ENSG00000108406	UAUAAAG
ENSG00000108406	UAUUGCU
ENSG00000108406	UCCUGAG
ENSG00000108423	GACAACU
ENSG00000108423	UUGACAC
ENSG00000108424	AACAUUC
ENSG00000108424	AAGGCGC
ENSG00000108424	ACACACU
ENSG00000108424	CACUCCU
ENSG00000108424	CCAGUGC
ENSG00000108424	CCAGUGU
ENSG00000108424	CUUUGGU
ENSG00000108424	GGUGGGC
ENSG00000108424	UCACACA
ENSG00000108424	UCCAGUU
ENSG00000108424	UGAAAUG
ENSG00000108424	UUGGCAA
ENSG00000108424	UUUGCAA
ENSG00000108433	AAUGUUG
ENSG00000108433	AGAUAAA
ENSG00000108433	CACAGGG
ENSG00000108433	CAGCAGG
ENSG00000108433	CUCACAC
ENSG00000108433	GAAACAU
ENSG00000108433	UCACAGG
ENSG00000108433	UCUCACC
ENSG00000108433	UGACAUC

ENSG00000108439	CAGCAAA
ENSG00000108439	CUGGCUC
ENSG00000108439	UAUUACC
ENSG00000108443	AAGGCAC
ENSG00000108443	AUGAGCU
ENSG00000108443	AUGGCUU
ENSG00000108443	CACAGUG
ENSG00000108443	CACCAGC
ENSG00000108443	GGAAGAC
ENSG00000108443	UCACAGU
ENSG00000108443	UGCAGCU
ENSG00000108443	UGCAUAG
ENSG00000108468	AAAAAUC
ENSG00000108468	AACACUG
ENSG00000108468	AAGACAU
ENSG00000108468	ACACACC
ENSG00000108468	ACACACU
ENSG00000108468	AGCACCA
ENSG00000108468	CUGCAGG
ENSG00000108468	CUGGACU
ENSG00000108468	CUGGCUC
ENSG00000108468	CUUCUCU
ENSG00000108468	GUGACAG
ENSG00000108468	UAAGACG
ENSG00000108468	UAAGACU
ENSG00000108468	UCUCAAG
ENSG00000108468	UCUGGAA
ENSG00000108468	UCUGGAG
ENSG00000108468	UGGAGAU
ENSG00000108468	UGUGCUU
ENSG00000108506	AAUUAUU
ENSG00000108506	GCAGGUU
ENSG00000108506	UAUUGCU
ENSG00000108509	AAAGUGC
ENSG00000108509	AAGGCAC
ENSG00000108509	ACAUUCA
ENSG00000108509	AUGUGGG
ENSG00000108509	UCUGGAG
ENSG00000108509	UGUAUGU
ENSG00000108510	AAACUGU
ENSG00000108510	AAGACAU
ENSG00000108510	AAUUGCU
ENSG00000108510	AGGGAGG
ENSG00000108510	AUACAAG
ENSG00000108510	GAGAACC
ENSG00000108510	GAGGAUA
ENSG00000108510	GUGCAAA
ENSG00000108510	UAAGACU
ENSG00000108510	UAAUACA
ENSG00000108510	UACAAAG
ENSG00000108510	UCACAGU
ENSG00000108510	UCAUACA
ENSG00000108510	UUGUUCG
ENSG00000108511	AAGACAU
ENSG00000108511	CCAGUGC
ENSG00000108511	CCAGUGU
ENSG00000108511	CUACAAA
ENSG00000108511	GAGCUAA
ENSG00000108511	GAGUCUU
ENSG00000108511	GCUUCCU
ENSG00000108511	GUCCAGA
ENSG00000108511	UCCAGUU
ENSG00000108518	AAAAACC
ENSG00000108518	CCCCCAG

ENSG00000108518	GGCAGCG
ENSG00000108518	UUGGCAA
ENSG00000108518	UUGGCAC
ENSG00000108551	AAGUGCA
ENSG00000108551	AAUGCCC
ENSG00000108551	AGUGCAA
ENSG00000108556	AGCAGCA
ENSG00000108556	AGUGUGC
ENSG00000108556	GUAGUGU
ENSG00000108556	GUUAAUG
ENSG00000108557	AACAUUC
ENSG00000108557	AAGAACC
ENSG00000108557	ACAUUCA
ENSG00000108557	AGGACAC
ENSG00000108557	UAAUACA
ENSG00000108557	UAUAAUA
ENSG00000108557	UGAGGAC
ENSG00000108578	AAAGAAU
ENSG00000108578	AAAGUUU
ENSG00000108578	GCACCAU
ENSG00000108578	GCAGCAU
ENSG00000108582	ACAUUCA
ENSG00000108582	ACUCCAG
ENSG00000108582	AGUGCUU
ENSG00000108582	AUGGCUU
ENSG00000108582	CACAGUG
ENSG00000108582	CAGUAGU
ENSG00000108582	CAGUGCA
ENSG00000108582	CCAUUAC
ENSG00000108582	CUAGUAA
ENSG00000108582	CUCCAUU
ENSG00000108582	GAGGUAG
ENSG00000108582	GGGUUUA
ENSG00000108582	UAAUACA
ENSG00000108582	UCACAGG
ENSG00000108582	UCACAGU
ENSG00000108582	UCUGGAG
ENSG00000108587	AAGUGCA
ENSG00000108587	ACAAUAU
ENSG00000108587	CAAAUGC
ENSG00000108587	CCAGUGU
ENSG00000108587	CUCACAC
ENSG00000108587	CUCCCAC
ENSG00000108587	GAGAACC
ENSG00000108587	GGCAUUG
ENSG00000108587	GUCACUC
ENSG00000108587	UAUAAAG
ENSG00000108587	UCACACA
ENSG00000108587	UUCUGCA
ENSG00000108588	UAUUGCU
ENSG00000108604	ACCAGCA
ENSG00000108604	CCCUGAG
ENSG00000108604	CCUGUAC
ENSG00000108604	GAGAUGA
ENSG00000108604	GUAAACA
ENSG00000108604	GUGCAAA
ENSG00000108604	UGCAUAU
ENSG00000108639	AAAGCUG
ENSG00000108639	GGGUGUU
ENSG00000108639	UCUCCAA
ENSG00000108654	AAAGUGC
ENSG00000108654	AACACUG
ENSG00000108654	AAGUGCA
ENSG00000108654	AAUCACU

ENSG00000108654	ACAAUAU
ENSG00000108654	ACUGCAG
ENSG00000108654	CAGGAAC
ENSG00000108654	UAUAAAG
ENSG00000108684	AGAUGUG
ENSG00000108684	CCCACCU
ENSG00000108684	CUUGGGC
ENSG00000108684	GGGUCUU
ENSG00000108684	UAAUAUC
ENSG00000108684	UGCAUAG
ENSG00000108688	GAGGUAU
ENSG00000108733	UGAGAAG
ENSG00000108753	AAUACUG
ENSG00000108753	ACUGCAG
ENSG00000108753	AGUAACA
ENSG00000108753	AUUGCAC
ENSG00000108753	GUAACAG
ENSG00000108753	UCAAGUA
ENSG00000108759	GGCUCUG
ENSG00000108759	UCCUUGC
ENSG00000108771	ACUGCAG
ENSG00000108774	AAGGUGC
ENSG00000108774	AAGUGCA
ENSG00000108774	AAGUGCU
ENSG00000108774	AUAUUAU
ENSG00000108774	GAGAAAU
ENSG00000108774	GCAGGUU
ENSG00000108774	GGCUCAG
ENSG00000108774	GGUUACC
ENSG00000108774	UAAGACG
ENSG00000108774	UCCAGUU
ENSG00000108774	UGUUGAA
ENSG00000108774	UUGGUCC
ENSG00000108774	UUGGUUC
ENSG00000108788	AAAAAUC
ENSG00000108788	AGCAAAA
ENSG00000108788	UUGGCAC
ENSG00000108797	AGCAGCA
ENSG00000108797	GCAGCAU
ENSG00000108797	GGCAGUG
ENSG00000108797	UGCAUAU
ENSG00000108797	UUUGCAA
ENSG00000108799	AGUGCUU
ENSG00000108799	CAGUCUG
ENSG00000108799	CAGUGCA
ENSG00000108799	GAGGUAG
ENSG00000108799	GCAGGAC
ENSG00000108799	UCACAAG
ENSG00000108799	UCCUGAG
ENSG00000108813	GCAGGUU
ENSG00000108813	GGGGUGC
ENSG00000108813	UCUGGAG
ENSG00000108819	AAUACUG
ENSG00000108819	CAAGAGC
ENSG00000108819	CAGUGCA
ENSG00000108819	GGAGGCA
ENSG00000108819	GGCAGGG
ENSG00000108819	GUCCAGA
ENSG00000108819	GUCUGCC
ENSG00000108819	UCCAGAG
ENSG00000108819	UCCCUUG
ENSG00000108819	UGAAGCU
ENSG00000108819	UGAGGAC
ENSG00000108819	UUGGCAA

ENSG00000108819	UUGGCAC
ENSG00000108819	UUGGUCC
ENSG00000108821	AAAACUG
ENSG00000108821	AGAUAAA
ENSG00000108821	AGGUAGU
ENSG00000108821	CUCAAAC
ENSG00000108821	CUGGGCA
ENSG00000108821	GAGAUGA
ENSG00000108821	GAGGUAG
ENSG00000108821	GGGUCUU
ENSG00000108821	GGGUGGU
ENSG00000108821	UUCAAGC
ENSG00000108821	UUGGUCC
ENSG00000108821	UUGGUUC
ENSG00000108828	AGCAGCA
ENSG00000108828	CCCUGUC
ENSG00000108828	CUUUGGU
ENSG00000108828	GAGCUGC
ENSG00000108828	GGAAUGU
ENSG00000108828	GGGGAGC
ENSG00000108828	GUAAACA
ENSG00000108828	UCCUGAG
ENSG00000108828	UUGACAC
ENSG00000108828	UUGGCAA
ENSG00000108828	UUGGCAC
ENSG00000108829	AAAAGUA
ENSG00000108829	AGUAGAC
ENSG00000108829	CAGCAGA
ENSG00000108829	CAGCAGG
ENSG00000108829	CCAGCAU
ENSG00000108829	CUACAAA
ENSG00000108829	GACCAUG
ENSG00000108829	GAGGCAG
ENSG00000108829	GCAGGUU
ENSG00000108829	GGAAGCC
ENSG00000108829	GUAGUGU
ENSG00000108829	GUCCAGA
ENSG00000108829	GUGACAG
ENSG00000108829	GUUAAUG
ENSG00000108829	UAAUGCU
ENSG00000108829	UACAAAG
ENSG00000108829	UAUAAAG
ENSG00000108829	UGUGUCA
ENSG00000108829	UUCCAUA
ENSG00000108829	UUGUGUC
ENSG00000108830	AAAGAGG
ENSG00000108830	AAUUAUU
ENSG00000108830	ACUGGCU
ENSG00000108830	AGUGCAA
ENSG00000108830	CAAAACU
ENSG00000108830	CACACCU
ENSG00000108830	CAGCAGA
ENSG00000108830	CCCCCAG
ENSG00000108830	CUCCCAC
ENSG00000108830	CUGGGGG
ENSG00000108830	GAAAACA
ENSG00000108830	GAAGGUC
ENSG00000108830	GCUACAU
ENSG00000108830	GGGGGAA
ENSG00000108830	UCCCUGU
ENSG00000108830	UGGGGGA
ENSG00000108830	UUCCAUA
ENSG00000108839	CAGCAGA
ENSG00000108840	AAGGCAC

ENSG00000108840	AGGACAC
ENSG00000108840	CACAGUG
ENSG00000108840	CACCAGC
ENSG00000108840	CUUUGGU
ENSG00000108840	GAGGCAG
ENSG00000108840	GGUUUAC
ENSG00000108840	GUGACUG
ENSG00000108840	UAAUAGU
ENSG00000108840	UACAAAG
ENSG00000108840	UUGGUUC
ENSG00000108852	AGUGGUU
ENSG00000108852	CACAGUG
ENSG00000108852	UACAAAG
ENSG00000108854	AAUACUG
ENSG00000108854	AGCAGCA
ENSG00000108854	AGCAGCG
ENSG00000108854	AGCAUUC
ENSG00000108854	AGUAGUU
ENSG00000108861	AAAUGGU
ENSG00000108861	AAGGCAC
ENSG00000108861	AGUGCCU
ENSG00000108861	CCUGCUG
ENSG00000108861	UGCAUAU
ENSG00000108861	UGGGACA
ENSG00000108861	UUCCUAG
ENSG00000108924	AAGGCAC
ENSG00000108924	AAGUGCU
ENSG00000108924	AAUACUG
ENSG00000108924	AAUCUCA
ENSG00000108924	ACAUUCA
ENSG00000108924	ACCACAG
ENSG00000108924	ACUCAGG
ENSG00000108924	ACUGUAG
ENSG00000108924	AGCACCA
ENSG00000108924	AGGUAGU
ENSG00000108924	AGUGCAA
ENSG00000108924	AUAAUAC
ENSG00000108924	AUAUAAC
ENSG00000108924	AUAUUAU
ENSG00000108924	AUGGCAC
ENSG00000108924	AUGUCUG
ENSG00000108924	AUUCAUU
ENSG00000108924	CAGUGCA
ENSG00000108924	CCAGUGU
ENSG00000108924	CUCUAGC
ENSG00000108924	CUUCUCU
ENSG00000108924	GAGGUAG
ENSG00000108924	GAUUGGU
ENSG00000108924	GCAGCAU
ENSG00000108924	GUAAACA
ENSG00000108924	GUGCAAA
ENSG00000108924	GUGCUUG
ENSG00000108924	GUGUCAC
ENSG00000108924	UAGGUAU
ENSG00000108924	UAUAAUA
ENSG00000108924	UCCCUCU
ENSG00000108924	UCCUAUA
ENSG00000108924	UGGGUGA
ENSG00000108924	UGUAUAA
ENSG00000108924	UGUCUUU
ENSG00000108924	UGUGCUU
ENSG00000108924	UGUGUCA
ENSG00000108924	UUCCUAG
ENSG00000108924	UUGGUCC

ENSG00000108932	UCAAGUA
ENSG00000108932	UCCCUUG
ENSG00000108946	AAAGCUG
ENSG00000108946	AACAAAC
ENSG00000108946	AAUACUG
ENSG00000108946	AGUAACA
ENSG00000108946	AUAUUAU
ENSG00000108946	CACUCCU
ENSG00000108946	CACUGUU
ENSG00000108946	CUCCCAA
ENSG00000108946	GCAACUU
ENSG00000108946	GUAAACA
ENSG00000108946	GUCACUC
ENSG00000108947	AAAUUUC
ENSG00000108947	AAGAACC
ENSG00000108947	AAGGCAC
ENSG00000108947	ACAGUAC
ENSG00000108947	ACAGUAU
ENSG00000108947	AGGAGCU
ENSG00000108947	CCUGCUG
ENSG00000108947	GAGGCAG
ENSG00000108947	GAGGUAU
ENSG00000108947	GCUGGUG
ENSG00000108947	GCUUCCU
ENSG00000108947	GGGAACG
ENSG00000108947	GGGGUGG
ENSG00000108947	UACAGUU
ENSG00000108947	UCCAGUU
ENSG00000108947	UCCCUUG
ENSG00000108947	UGUCUGG
ENSG00000108947	UUCAAAU
ENSG00000108950	AGUGCUU
ENSG00000108953	AAAAGUA
ENSG00000108953	AAAGAAU
ENSG00000108953	AAAGAGG
ENSG00000108953	AAAGCUG
ENSG00000108953	AACCUGG
ENSG00000108953	AACCUGU
ENSG00000108953	AAUGUUG
ENSG00000108953	ACCAGCA
ENSG00000108953	AGCAAAA
ENSG00000108953	AGCACCA
ENSG00000108953	CAGGAAC
ENSG00000108953	CAGUAGU
ENSG00000108953	CCAGUAC
ENSG00000108953	GGCAAGA
ENSG00000108953	GUUAAUG
ENSG00000108953	UAAUGCU
ENSG00000108953	UCAAGUA
ENSG00000108953	UGUUGAA
ENSG00000108960	AAGACAU
ENSG00000108960	AAUGUUG
ENSG00000108960	AGCAGCA
ENSG00000108960	CACAGUG
ENSG00000108960	GAGGUAU
ENSG00000108960	UACAAAG
ENSG00000108960	UCACAGG
ENSG00000108960	UUGGCAA
ENSG00000108961	AUAAAGU
ENSG00000108961	UAUAAAG
ENSG00000108963	GAGGCUG
ENSG00000108984	AGCACCA
ENSG00000109016	UCCCACA
ENSG00000109016	UCCCACC

ENSG00000109046	AACGGAA
ENSG00000109046	ACAGUAC
ENSG00000109046	ACAGUAU
ENSG00000109046	AGAUAAA
ENSG00000109046	AGCAGCG
ENSG00000109046	CACAGUG
ENSG00000109046	CAGUAGA
ENSG00000109046	CUGCAGC
ENSG00000109046	CUUUGGU
ENSG00000109046	GCAGGUU
ENSG00000109046	UAAUACA
ENSG00000109046	UAUUGCU
ENSG00000109046	UCACAGG
ENSG00000109046	UGUAUGU
ENSG00000109047	AGGAGCU
ENSG00000109062	GGCUCAG
ENSG00000109065	CUGCAGG
ENSG00000109065	CUGCCCC
ENSG00000109065	GCAGGAC
ENSG00000109065	UGCAGCU
ENSG00000109066	AACCUGG
ENSG00000109066	AAGGCAC
ENSG00000109066	CAGGUGA
ENSG00000109066	CCCACCG
ENSG00000109066	GAGGCUG
ENSG00000109066	GGCAGUG
ENSG00000109066	GUGUGGC
ENSG00000109066	UGCCAGC
ENSG00000109066	UGUGUGG
ENSG00000109066	UUGGAGG
ENSG00000109079	AAAGUGC
ENSG00000109079	AAGUGCU
ENSG00000109079	AGCACCA
ENSG00000109079	AGCAGCA
ENSG00000109079	CAAAGUG
ENSG00000109079	CUGCAGG
ENSG00000109079	GCAGAAG
ENSG00000109079	GGGGUGG
ENSG00000109079	UACAAAG
ENSG00000109083	UUGUUCG
ENSG00000109084	AGCAAAA
ENSG00000109084	AGCAGCU
ENSG00000109084	CAGCAGA
ENSG00000109084	CAGCAGG
ENSG00000109084	GGCAGUG
ENSG00000109084	UGGGGGA
ENSG00000109084	UUUCAAC
ENSG00000109084	UUUUUGC
ENSG00000109089	AAGGCAC
ENSG00000109089	AAUACUG
ENSG00000109089	AAUCUCU
ENSG00000109089	AUGGCUU
ENSG00000109089	CACAGUG
ENSG00000109089	CAGCAGG
ENSG00000109089	CCCUGAG
ENSG00000109089	GACCUGG
ENSG00000109089	GGGUGGU
ENSG00000109089	UCAAGUA
ENSG00000109089	UCACAGU
ENSG00000109089	UCCAGUU
ENSG00000109089	UGCAUUG
ENSG00000109099	AUGUAAU
ENSG00000109099	CCAGUGC
ENSG00000109099	CCAGUGU

ENSG00000109099	GAGUCUU
ENSG00000109099	GUCAACA
ENSG00000109099	UCCAGUU
ENSG00000109101	UCCUUGC
ENSG00000109101	UGACCUA
ENSG00000109103	CUCAGGG
ENSG00000109103	GAAAACA
ENSG00000109107	CCAGGAG
ENSG00000109111	AACACUG
ENSG00000109111	ACUGUUU
ENSG00000109111	CACUGUU
ENSG00000109113	AGUGCAA
ENSG00000109113	CAGCAGG
ENSG00000109113	GCCUGGG
ENSG00000109113	GUGCAAA
ENSG00000109113	UAAUGCU
ENSG00000109113	UUGGCAA
ENSG00000109113	UUGGCAC
ENSG00000109113	UUGGUCC
ENSG00000109118	AAUGCCC
ENSG00000109118	AGUGCAA
ENSG00000109118	AUGUGCC
ENSG00000109118	CAAAUGC
ENSG00000109118	UGCAAAG
ENSG00000109132	AAACUGU
ENSG00000109132	AAAGCUG
ENSG00000109132	AACAAAC
ENSG00000109132	AACAUUC
ENSG00000109132	ACAUUCA
ENSG00000109132	AGCAGCG
ENSG00000109132	AGGAUGU
ENSG00000109132	AUUCAUU
ENSG00000109132	CAAAGCA
ENSG00000109132	CCCUGAG
ENSG00000109132	CUUGGAG
ENSG00000109132	GAGAACU
ENSG00000109132	GCUGGUG
ENSG00000109132	GUGCCUG
ENSG00000109132	GUUAAUG
ENSG00000109132	UAAUAUC
ENSG00000109132	UGAGUCU
ENSG00000109132	UGGACUG
ENSG00000109133	AAUACUG
ENSG00000109158	AACUACU
ENSG00000109158	AGCAUUC
ENSG00000109158	CUGACUC
ENSG00000109158	GGUGGGC
ENSG00000109158	GUGCCAC
ENSG00000109158	UAAUAGU
ENSG00000109158	UCCCACA
ENSG00000109158	UCCCACC
ENSG00000109163	GCCCUUA
ENSG00000109163	UUAGGAU
ENSG00000109171	AACCUGG
ENSG00000109171	AACCUGU
ENSG00000109171	AAGGCAC
ENSG00000109171	ACCACAG
ENSG00000109171	AGCCCUU
ENSG00000109171	CAGCAAA
ENSG00000109171	CAGGCUC
ENSG00000109171	CCUGUAC
ENSG00000109171	CUAGUAA
ENSG00000109171	CUUUGGU
ENSG00000109171	GACCAUG

ENSG00000109171	GAUUGUC
ENSG00000109171	GCUACAU
ENSG00000109171	GGAAGCC
ENSG00000109171	GUCAUUG
ENSG00000109171	UCCUGAG
ENSG00000109171	UCUAGAG
ENSG00000109171	UGCAUAG
ENSG00000109171	UGCAUAU
ENSG00000109171	UGUGCUU
ENSG00000109171	UUCUGCA
ENSG00000109171	UUCUUGU
ENSG00000109180	AAAAACC
ENSG00000109180	AAACUGU
ENSG00000109180	ACAAUAU
ENSG00000109180	GCUGGAU
ENSG00000109180	UAUAAUA
ENSG00000109180	UCAACAG
ENSG00000109180	UGAAGCU
ENSG00000109184	AAUACUG
ENSG00000109184	AAUCUCA
ENSG00000109184	ACAAUAU
ENSG00000109184	ACAGUAU
ENSG00000109184	ACAUCAC
ENSG00000109184	AGCACCA
ENSG00000109184	AGCAGCA
ENSG00000109184	AGCAUUC
ENSG00000109184	AGUGCAA
ENSG00000109184	AUGGCUU
ENSG00000109184	AUGUAAU
ENSG00000109184	CAAAGCA
ENSG00000109184	CAGUGCA
ENSG00000109184	CUCUAGC
ENSG00000109184	CUUACAG
ENSG00000109184	CUUUGGU
ENSG00000109184	GAGGUAU
ENSG00000109184	GCAGCAU
ENSG00000109184	GCUGGUG
ENSG00000109184	UCACCAC
ENSG00000109184	UGAAAUG
ENSG00000109184	UGAGGAC
ENSG00000109184	UUGGUCC
ENSG00000109189	AAGUGCU
ENSG00000109189	AGAUAAA
ENSG00000109189	AGCUGCC
ENSG00000109189	AUAAAGU
ENSG00000109189	CACAGUG
ENSG00000109189	CCAGUGC
ENSG00000109189	CCAGUGU
ENSG00000109189	CUUCACC
ENSG00000109189	GAGAUGA
ENSG00000109189	UCACAGG
ENSG00000109189	UCACAUU
ENSG00000109189	UCCAGUU
ENSG00000109189	UGCCCUG
ENSG00000109220	AAGGCAC
ENSG00000109220	AGCACCA
ENSG00000109220	CCAGGAG
ENSG00000109220	GCACCAU
ENSG00000109220	UCACCAC
ENSG00000109265	AAAAAUC
ENSG00000109265	AAAGGUA
ENSG00000109265	AACACUG
ENSG00000109265	ACAUUAC
ENSG00000109265	AUACAAG

ENSG00000109265	AUUCAUU
ENSG00000109265	CAGUCUG
ENSG00000109265	GAAGGUC
ENSG00000109265	GGCAAGA
ENSG00000109265	UCACAAG
ENSG00000109265	UGAAAGG
ENSG00000109265	UGUCUUU
ENSG00000109265	UUCUAAU
ENSG00000109265	UUGGCAA
ENSG00000109320	AAAUCCC
ENSG00000109320	ACAAUAU
ENSG00000109320	AGCAUUC
ENSG00000109320	GCAGAAG
ENSG00000109320	UUGAUCA
ENSG00000109332	AAAGCUG
ENSG00000109332	AAGACAU
ENSG00000109332	ACAGUAC
ENSG00000109332	AGGUUAU
ENSG00000109332	AUAAUAC
ENSG00000109332	AUAUUAU
ENSG00000109332	AUGAGCU
ENSG00000109332	CAGUGCA
ENSG00000109332	CAGUUAC
ENSG00000109332	CUCCCAC
ENSG00000109332	GGAAGAC
ENSG00000109332	GGGGAGC
ENSG00000109332	GGUUUAC
ENSG00000109332	GUAAACA
ENSG00000109332	UAAUACA
ENSG00000109332	UCACAUU
ENSG00000109332	UGACAUC
ENSG00000109332	UGUGUCA
ENSG00000109332	UUCUGCA
ENSG00000109332	UUGAUCA
ENSG00000109339	AGGCAGG
ENSG00000109339	CAAAGCA
ENSG00000109381	AACACUG
ENSG00000109381	AAUACUG
ENSG00000109381	ACUGUGU
ENSG00000109381	AUACAAG
ENSG00000109381	UCCAGUU
ENSG00000109381	UUCAAAU
ENSG00000109381	UUGGUCC
ENSG00000109436	AACAUUC
ENSG00000109436	AAGUGCA
ENSG00000109436	ACAUUCA
ENSG00000109436	GCAGGUU
ENSG00000109436	UAUAAAG
ENSG00000109452	UUCCUAG
ENSG00000109452	UUGUGUC
ENSG00000109458	AAUACUG
ENSG00000109466	UUGGCAA
ENSG00000109466	UUGGCAC
ENSG00000109501	AAGUGCU
ENSG00000109501	AGUGCUU
ENSG00000109572	GGCUCAG
ENSG00000109572	UCACAUU
ENSG00000109572	UUCUUGU
ENSG00000109586	AAAGAGG
ENSG00000109586	AAAGUUU
ENSG00000109586	AACCUGG
ENSG00000109586	AACCUGU
ENSG00000109586	AAGUGUG
ENSG00000109586	AAUCACU

ENSG00000109586	ACAGUAC
ENSG00000109586	ACAGUAU
ENSG00000109586	AGUGAUC
ENSG00000109586	AGUGCUU
ENSG00000109586	AUACAAG
ENSG00000109586	AUGCAUU
ENSG00000109586	CAGCAGG
ENSG00000109586	CAGUCCA
ENSG00000109586	CCCUGAG
ENSG00000109586	CCUUCAU
ENSG00000109586	GAAGGAA
ENSG00000109586	GAGUGUG
ENSG00000109586	UCAAGUA
ENSG00000109586	UGAAAUG
ENSG00000109586	UGCAGCU
ENSG00000109586	UGUUGAA
ENSG00000109586	UUUCAAC
ENSG00000109606	AAGUUGU
ENSG00000109606	CUACAGU
ENSG00000109606	GGAAUGU
ENSG00000109606	GGAGGCA
ENSG00000109606	GUAACAG
ENSG00000109606	GUCUACU
ENSG00000109606	UUCUAAU
ENSG00000109606	UUGUGUC
ENSG00000109654	AACACUG
ENSG00000109654	AAGGUGC
ENSG00000109654	AAGUGCU
ENSG00000109654	ACCCUGU
ENSG00000109654	AGCAGCA
ENSG00000109654	AGUGCUG
ENSG00000109654	AUGCCUU
ENSG00000109654	AUGGCAC
ENSG00000109654	CACAGGG
ENSG00000109654	CUUUGGU
ENSG00000109654	GCCCUUA
ENSG00000109654	GGUUGAC
ENSG00000109654	UAAUAUC
ENSG00000109654	UAAUGCU
ENSG00000109654	UCAUGAU
ENSG00000109654	UCCCUUU
ENSG00000109654	UGCUGAC
ENSG00000109670	AAACUGU
ENSG00000109670	AAAGCUG
ENSG00000109670	AAGUCUU
ENSG00000109670	ACACACU
ENSG00000109670	ACAGUAC
ENSG00000109670	ACAGUAU
ENSG00000109670	ACCACAG
ENSG00000109670	ACUGUGU
ENSG00000109670	AGCACCA
ENSG00000109670	AGCAGCG
ENSG00000109670	AGCAGCU
ENSG00000109670	AGCUGCC
ENSG00000109670	AUCCCUU
ENSG00000109670	AUGCAUU
ENSG00000109670	CACAGUG
ENSG00000109670	CACCACC
ENSG00000109670	CACCAGC
ENSG00000109670	CAGUCUG
ENSG00000109670	CCAGCAU
ENSG00000109670	CUGUUGA
ENSG00000109670	GACAACU
ENSG00000109670	GAGGCAG

ENSG00000109670	GAGUUGA
ENSG00000109670	GAGUUGG
ENSG00000109670	GAUUGUA
ENSG00000109670	GAUUGUC
ENSG00000109670	GCAGCAU
ENSG00000109670	GGAAGCC
ENSG00000109670	GGAAUGU
ENSG00000109670	UAGAGGA
ENSG00000109670	UCACAGG
ENSG00000109670	UCCCUUU
ENSG00000109670	UGACAUC
ENSG00000109670	UGGCCAC
ENSG00000109670	UGGGACA
ENSG00000109670	UUCCUCG
ENSG00000109670	UUCUAAU
ENSG00000109685	AAAAACC
ENSG00000109685	AACAAAC
ENSG00000109685	ACAAUAU
ENSG00000109685	ACAUCAC
ENSG00000109685	ACAUUCA
ENSG00000109685	ACUCCAG
ENSG00000109685	ACUUGGA
ENSG00000109685	AGCAAAA
ENSG00000109685	AGCAGCU
ENSG00000109685	AUCCCUU
ENSG00000109685	AUGCAUU
ENSG00000109685	CAAAGCA
ENSG00000109685	CAGGCUC
ENSG00000109685	CUCUUCC
ENSG00000109685	CUGCAGC
ENSG00000109685	CUUGGAG
ENSG00000109685	GAUUGUA
ENSG00000109685	GAUUGUC
ENSG00000109685	GCCUGGA
ENSG00000109685	GUAUGUU
ENSG00000109685	UACAAAG
ENSG00000109685	UAGAGGA
ENSG00000109685	UCACAAG
ENSG00000109685	UGGAGAU
ENSG00000109685	UUCCUAG
ENSG00000109685	UUGACAC
ENSG00000109689	ACAUUCA
ENSG00000109689	CAGUAGU
ENSG00000109689	UCACAGU
ENSG00000109705	AAAGUGC
ENSG00000109705	ACAUAGA
ENSG00000109705	ACAUUCA
ENSG00000109705	CACAGUG
ENSG00000109705	CAUGGAU
ENSG00000109705	CCAGGAG
ENSG00000109705	UAUAAAG
ENSG00000109738	AUGGCUU
ENSG00000109756	AAAAGUA
ENSG00000109756	AAAGUAU
ENSG00000109756	AAAGUGC
ENSG00000109756	AACACUG
ENSG00000109756	AAGACAU
ENSG00000109756	AAGGCAC
ENSG00000109756	AAGUGCA
ENSG00000109756	AAGUGCU
ENSG00000109756	ACAGGUG
ENSG00000109756	ACAGUAU
ENSG00000109756	ACAUCAC
ENSG00000109756	ACAUUCA

ENSG00000109756	ACCAGCA
ENSG00000109756	AGCAGCA
ENSG00000109756	AGCAGCG
ENSG00000109756	AGCAGCU
ENSG00000109756	AGGCAGG
ENSG00000109756	AGUAGAC
ENSG00000109756	AUGCACC
ENSG00000109756	CACAGUG
ENSG00000109756	CACCAGC
ENSG00000109756	CCGGAGC
ENSG00000109756	CUUCACC
ENSG00000109756	GAGAAGA
ENSG00000109756	GAUCAGA
ENSG00000109756	GGAAUGU
ENSG00000109756	GGAGGCA
ENSG00000109756	GGCAGCG
ENSG00000109756	UCAACAG
ENSG00000109756	UCACAGG
ENSG00000109756	UCACAUU
ENSG00000109756	UCCAGUU
ENSG00000109756	UGUGCUU
ENSG00000109756	UUCUAAU
ENSG00000109756	UUCUGCA
ENSG00000109756	UUCUUGU
ENSG00000109762	AAAGAGG
ENSG00000109771	AUGAUGA
ENSG00000109771	UAUAAAG
ENSG00000109771	UCUUGAG
ENSG00000109771	UGUUGAA
ENSG00000109787	AAAAAUC
ENSG00000109787	AAAGUGC
ENSG00000109787	AACAUUC
ENSG00000109787	AAUACUG
ENSG00000109787	ACAUUAC
ENSG00000109787	ACAUUCA
ENSG00000109787	AGCCCUU
ENSG00000109787	AGCCUGC
ENSG00000109787	AGUACUG
ENSG00000109787	AUAAUAC
ENSG00000109787	AUGACAC
ENSG00000109787	AUGUAAU
ENSG00000109787	AUUGCAC
ENSG00000109787	CACAGUG
ENSG00000109787	CCCUGAG
ENSG00000109787	CCUGCUG
ENSG00000109787	CCUUCAU
ENSG00000109787	GACCAUG
ENSG00000109787	GACCCUG
ENSG00000109787	GCAGGUU
ENSG00000109787	GCAUCCC
ENSG00000109787	GGCAAGA
ENSG00000109787	GGUUUAC
ENSG00000109787	GUCACUC
ENSG00000109787	GUCAUUG
ENSG00000109787	GUCUCUG
ENSG00000109787	GUGCUUG
ENSG00000109787	GUGUCAC
ENSG00000109787	GUUAAUG
ENSG00000109787	UAAUAUC
ENSG00000109787	UAUAAAG
ENSG00000109787	UAUAUGA
ENSG00000109787	UCCCUUU
ENSG00000109787	UCCUGAG
ENSG00000109787	UCUCCAA

ENSG00000109787	UGACAUC
ENSG00000109787	UGGACUG
ENSG00000109787	UGUCUUU
ENSG00000109787	UGUGCUU
ENSG00000109787	UUGACAC
ENSG00000109787	UUGGCAC
ENSG00000109790	UCACAUU
ENSG00000109805	CUUUGGU
ENSG00000109814	AAAAACC
ENSG00000109814	AAAGUGC
ENSG00000109814	UACAAAG
ENSG00000109814	UCAUUUG
ENSG00000109814	UCUAGAG
ENSG00000109819	AAAAACC
ENSG00000109819	AACAAAC
ENSG00000109819	AACAUUC
ENSG00000109819	ACCCAUU
ENSG00000109819	ACUGGCC
ENSG00000109819	AGCAGCU
ENSG00000109819	AGGACAC
ENSG00000109819	AUAAUAC
ENSG00000109819	AUAUAAC
ENSG00000109819	CAAAACU
ENSG00000109819	CAGUGCA
ENSG00000109819	CAUGGGU
ENSG00000109819	CUCCAUU
ENSG00000109819	GAGGUAG
ENSG00000109819	GAUUGUC
ENSG00000109819	GCUGGUG
ENSG00000109819	GGCAGGG
ENSG00000109819	GUAACAG
ENSG00000109819	UAAUAGU
ENSG00000109819	UACAGUU
ENSG00000109819	UAUAAUA
ENSG00000109819	UAUUACC
ENSG00000109819	UCACACA
ENSG00000109819	UCCCUUG
ENSG00000109819	UGCAUAG
ENSG00000109819	UGUGCUU
ENSG00000109832	CUCACAC
ENSG00000109832	UCUCACC
ENSG00000109861	AGCUGCC
ENSG00000109861	CCAUUAC
ENSG00000109861	CUUGGAG
ENSG00000109861	GAGGUAG
ENSG00000109861	GCUAGCA
ENSG00000109861	UCAUGAU
ENSG00000109861	UCUAGAG
ENSG00000109861	UCUCAAG
ENSG00000109861	UGAAAUG
ENSG00000109861	UUGGAGG
ENSG00000109881	AGCUUAU
ENSG00000109881	UUAGAGA
ENSG00000109919	AAAGCGC
ENSG00000109919	AACAUUC
ENSG00000109919	AAGACAU
ENSG00000109919	AGCAAAA
ENSG00000109919	CACUGUU
ENSG00000109919	CUGCCCC
ENSG00000109919	GUCACUC
ENSG00000109919	UUGGCAC
ENSG00000109919	UUUUUGC
ENSG00000109920	AAUACUG
ENSG00000109920	AUUGCAC

ENSG00000109920	GAGAAAU
ENSG00000109920	GAGCUGC
ENSG00000109929	AUCCUUG
ENSG00000109929	AUGCCUU
ENSG00000109929	UAAUACA
ENSG00000109929	UAUCAGA
ENSG00000109929	UCCUGAG
ENSG00000109943	AUUGCAC
ENSG00000109943	GUAGUGU
ENSG00000109956	AAAAACC
ENSG00000109956	AAUUUUA
ENSG00000109956	AGGGUCU
ENSG00000109956	CAGUCCA
ENSG00000109956	GAGGUAG
ENSG00000109956	GAGGUAU
ENSG00000109956	GCUUCCU
ENSG00000109956	GUAAACA
ENSG00000109956	GUGCUUG
ENSG00000109956	UAUAAAG
ENSG00000109956	UGGGAUC
ENSG00000109971	AAUGCCC
ENSG00000109971	UGCAUUG
ENSG00000110025	CAGUUAC
ENSG00000110025	GGCAGUG
ENSG00000110025	UCCAGUU
ENSG00000110025	UUGGUCC
ENSG00000110042	AAAAACC
ENSG00000110042	AAGAACC
ENSG00000110042	ACGUCAU
ENSG00000110042	AGCACCA
ENSG00000110042	AUAUAAC
ENSG00000110042	CAAAGCA
ENSG00000110042	CCCUGAG
ENSG00000110042	CCUUCUU
ENSG00000110042	GAGGUAG
ENSG00000110042	GAGGUAU
ENSG00000110042	GAGUUGG
ENSG00000110042	GAUCAGA
ENSG00000110042	GCUGGUG
ENSG00000110042	GCUUCCU
ENSG00000110042	UCACAGU
ENSG00000110042	UCAUUUG
ENSG00000110042	UCCAGAG
ENSG00000110042	UCCCUCU
ENSG00000110042	UGCUGAC
ENSG00000110042	UUCAAAU
ENSG00000110046	AAAGUGC
ENSG00000110046	AAGUGCA
ENSG00000110047	AUAUUAU
ENSG00000110047	CUUGUGU
ENSG00000110047	UAAUGCU
ENSG00000110047	UCAAGUA
ENSG00000110047	UUCUUGU
ENSG00000110048	AGCACCA
ENSG00000110048	AUAAUAC
ENSG00000110048	AUACAAG
ENSG00000110048	AUAUAAC
ENSG00000110048	GAGGCUG
ENSG00000110048	GCUUCCU
ENSG00000110048	GGAAUGU
ENSG00000110048	GGCUCAG
ENSG00000110048	UAUAAUA
ENSG00000110048	UCACCAC
ENSG00000110048	UCCAGAG

ENSG00000110060	AAGGCAC
ENSG00000110066	AAAAGUA
ENSG00000110066	AAGUGCA
ENSG00000110066	AGCACCA
ENSG00000110066	AGUGCAA
ENSG00000110066	AUUGCAC
ENSG00000110066	UCACAGG
ENSG00000110066	UCACAGU
ENSG00000110076	AAAGUUU
ENSG00000110076	AACAAAC
ENSG00000110076	CAGACAG
ENSG00000110076	CUGGAAA
ENSG00000110076	GAGGCAG
ENSG00000110076	GGCAGUG
ENSG00000110076	GGGUGUU
ENSG00000110076	UUCCGGC
ENSG00000110076	UUCCUCG
ENSG00000110079	CUGUUGA
ENSG00000110090	AUCCCUU
ENSG00000110090	GAAGGAA
ENSG00000110090	UUGGUUC
ENSG00000110092	AAAGUGC
ENSG00000110092	AAGUGCU
ENSG00000110092	AAUCACU
ENSG00000110092	AAUGCCC
ENSG00000110092	ACUGGCC
ENSG00000110092	ACUGGCU
ENSG00000110092	AGAUGUG
ENSG00000110092	AGCAGCG
ENSG00000110092	AGCAGCU
ENSG00000110092	AGUGCUU
ENSG00000110092	AUAAAGU
ENSG00000110092	AUAUUAU
ENSG00000110092	AUGACAC
ENSG00000110092	CAGCAAA
ENSG00000110092	CCUUCUG
ENSG00000110092	CUGGAAA
ENSG00000110092	GAGGUAG
ENSG00000110092	GAGGUAU
ENSG00000110092	GCUGGAU
ENSG00000110092	GGAAUGU
ENSG00000110092	GGCAGUG
ENSG00000110092	GUUAAUG
ENSG00000110092	UAAUGCU
ENSG00000110092	UAUAUGA
ENSG00000110092	UCACAUU
ENSG00000110092	UCUAGAG
ENSG00000110092	UGCAUUG
ENSG00000110092	UUGAUCA
ENSG00000110092	UUGGCAC
ENSG00000110092	UUGGUUC
ENSG00000110104	AAGGCAC
ENSG00000110104	CUCUUCC
ENSG00000110107	ACUAGAU
ENSG00000110107	CACAGUG
ENSG00000110107	GAGAUGA
ENSG00000110107	UACAAAG
ENSG00000110107	UCACAGG
ENSG00000110107	UCACAGU
ENSG00000110107	UCCCACC
ENSG00000110107	UCCCUUG
ENSG00000110107	UGAAAUG
ENSG00000110108	ACUGCAG
ENSG00000110108	AGCAGCU

ENSG00000110108	UCACAUU
ENSG00000110148	AGUGCUG
ENSG00000110171	AAGAACC
ENSG00000110171	AAGUGCA
ENSG00000110171	ACAUUCA
ENSG00000110171	AGUGCAA
ENSG00000110171	AGUGCUG
ENSG00000110171	AUAAAGU
ENSG00000110171	CAAAGCA
ENSG00000110171	UAUUGCU
ENSG00000110171	UGAAGCU
ENSG00000110172	AGCAGCA
ENSG00000110200	AGCAGCU
ENSG00000110237	AAGGCAC
ENSG00000110237	AAGUGCA
ENSG00000110237	AAUACUG
ENSG00000110237	ACUGUAG
ENSG00000110237	AGUGCUU
ENSG00000110237	CAGUGCA
ENSG00000110237	CCAGUGC
ENSG00000110237	CUUUGGU
ENSG00000110237	GGCCCUG
ENSG00000110237	GGUAGAC
ENSG00000110237	UGGCCCU
ENSG00000110237	UGUCUUU
ENSG00000110237	UUGGAGG
ENSG00000110243	UGCAAAG
ENSG00000110243	UUUGCAA
ENSG00000110244	CAGACAG
ENSG00000110274	GAGACCU
ENSG00000110274	GAGGUAG
ENSG00000110274	GAGGUAU
ENSG00000110315	AAAGAAU
ENSG00000110315	AAGACAU
ENSG00000110315	AAGGCAC
ENSG00000110315	CACAGUG
ENSG00000110315	CAGCAGA
ENSG00000110315	CUUGGAG
ENSG00000110315	GCACCAU
ENSG00000110315	GGAAUGU
ENSG00000110315	GUGCCAC
ENSG00000110315	UAUUGCU
ENSG00000110315	UCACAGG
ENSG00000110315	UCUGGAA
ENSG00000110321	AAAGUGC
ENSG00000110321	AAGUGCA
ENSG00000110321	ACAGUAC
ENSG00000110321	AGCAGCA
ENSG00000110321	AGUGGUU
ENSG00000110321	AUAAUAC
ENSG00000110321	AUACAAG
ENSG00000110321	AUAUAAC
ENSG00000110321	AUGUAAU
ENSG00000110321	CAAAGUG
ENSG00000110321	CACACCG
ENSG00000110321	CACACCU
ENSG00000110321	CAGGCUC
ENSG00000110321	CAGUUAC
ENSG00000110321	CCAUUAC
ENSG00000110321	CUACAAA
ENSG00000110321	GAGGUAG
ENSG00000110321	GUAUGUU
ENSG00000110321	GUGCAAA
ENSG00000110321	UAAGACG

ENSG00000110321	UAAGACU
ENSG00000110321	UAUAAUA
ENSG00000110321	UCACACA
ENSG00000110321	UCCUUGC
ENSG00000110321	UGGAUAA
ENSG00000110344	CACAGUG
ENSG00000110344	CUAGUCC
ENSG00000110344	GAGGCAG
ENSG00000110344	UCACAGU
ENSG00000110344	UGGCUGC
ENSG00000110344	UUCCUCG
ENSG00000110367	AAAACUG
ENSG00000110367	AAAGAGG
ENSG00000110367	AAAUCCC
ENSG00000110367	AACAGUC
ENSG00000110367	AAGUGCA
ENSG00000110367	AAGUGCU
ENSG00000110367	AAUACUG
ENSG00000110367	AAUCACU
ENSG00000110367	AAUCCUU
ENSG00000110367	AAUGUUG
ENSG00000110367	ACCAGCA
ENSG00000110367	ACUCAGG
ENSG00000110367	ACUGCAG
ENSG00000110367	ACUGGCU
ENSG00000110367	AGAUAAA
ENSG00000110367	AGCACCA
ENSG00000110367	AGCAGCU
ENSG00000110367	AGCUGCC
ENSG00000110367	CAAAGCA
ENSG00000110367	CAAAGUG
ENSG00000110367	CACAGUG
ENSG00000110367	CACUCCU
ENSG00000110367	CCAGUGC
ENSG00000110367	CCAGUGU
ENSG00000110367	CCUUCAU
ENSG00000110367	CUGCUCA
ENSG00000110367	CUUGUGU
ENSG00000110367	GAGAUGA
ENSG00000110367	GAGCCCU
ENSG00000110367	GAGCUGC
ENSG00000110367	GAGGGGC
ENSG00000110367	GAGUGUG
ENSG00000110367	GCCUGGA
ENSG00000110367	GCCUGGG
ENSG00000110367	GGAUGAG
ENSG00000110367	GGGGGAA
ENSG00000110367	GUAACAG
ENSG00000110367	GUGCCAC
ENSG00000110367	GUGCUUG
ENSG00000110367	GUGUGGC
ENSG00000110367	UACAGUU
ENSG00000110367	UAUAUGA
ENSG00000110367	UCAUAGA
ENSG00000110367	UCCAGUU
ENSG00000110367	UGACCUA
ENSG00000110367	UGCAUAU
ENSG00000110367	UGCCAGC
ENSG00000110367	UGGAGAU
ENSG00000110367	UGGGACA
ENSG00000110367	UGUGUCA
ENSG00000110375	AGGGAGG
ENSG00000110395	AAAUCCC
ENSG00000110395	AACAAAC

ENSG00000110395	AAGUCAC
ENSG00000110395	AAUCUCA
ENSG00000110395	AGCAGCU
ENSG00000110395	AGUGCUG
ENSG00000110395	AGUGCUU
ENSG00000110395	AGUGUGC
ENSG00000110395	AUACAAG
ENSG00000110395	AUAUAAC
ENSG00000110395	AUGCACC
ENSG00000110395	CAGUAGA
ENSG00000110395	CAUGGGU
ENSG00000110395	CCAGUGU
ENSG00000110395	CUCCAUU
ENSG00000110395	CUUUGGU
ENSG00000110395	GAGAUGA
ENSG00000110395	GAGGUAG
ENSG00000110395	GAGGUAU
ENSG00000110395	GCAGGUU
ENSG00000110395	GCUACAU
ENSG00000110395	GGACUGC
ENSG00000110395	GGAGGCA
ENSG00000110395	GGAGUGU
ENSG00000110395	GGCAAGA
ENSG00000110395	GGCAGGG
ENSG00000110395	GGGGAGC
ENSG00000110395	GUCAACA
ENSG00000110395	GUGACAG
ENSG00000110395	GUGAGGU
ENSG00000110395	GUGCUUG
ENSG00000110395	UCAACAG
ENSG00000110395	UCUCAAG
ENSG00000110395	UGAAAGG
ENSG00000110395	UGAAAUG
ENSG00000110395	UGGACUG
ENSG00000110395	UGGGACA
ENSG00000110395	UGUGCUU
ENSG00000110395	UGUUGAA
ENSG00000110395	UUGGAGG
ENSG00000110400	AAAAGUA
ENSG00000110400	AAUACUG
ENSG00000110400	AGCACCA
ENSG00000110400	AGCAGCA
ENSG00000110400	AUGGCUU
ENSG00000110400	AUUGCAC
ENSG00000110400	CCAGGAG
ENSG00000110400	CCAGUGC
ENSG00000110400	CCAGUGU
ENSG00000110400	CUGGCUC
ENSG00000110400	GAGGCAG
ENSG00000110400	GCAGCAU
ENSG00000110400	GCUACAU
ENSG00000110400	GGAAGCC
ENSG00000110400	GGCAGGG
ENSG00000110400	GGGCGUG
ENSG00000110400	GGGGUGG
ENSG00000110400	GUGCUUG
ENSG00000110400	UAUAAAG
ENSG00000110400	UAUUGCU
ENSG00000110400	UCCAGUU
ENSG00000110400	UGUGCUU
ENSG00000110422	AAAGCUG
ENSG00000110422	AACAUUC
ENSG00000110422	AAGUGCA
ENSG00000110422	AGCAAAA

ENSG00000110422	AGUGCUG
ENSG00000110422	AUGGCUU
ENSG00000110422	AUUGCAC
ENSG00000110422	CAGCAGA
ENSG00000110422	CGUGUCU
ENSG00000110422	CUAGGCU
ENSG00000110422	CUUGUGU
ENSG00000110422	GUGCCAC
ENSG00000110422	UGCAGCU
ENSG00000110427	AAAGUAC
ENSG00000110427	AACAAAC
ENSG00000110427	AACACUG
ENSG00000110427	AAGUGCA
ENSG00000110427	ACUGGCC
ENSG00000110427	AGCACCA
ENSG00000110427	AGUAACA
ENSG00000110427	AGUACUG
ENSG00000110427	AGUAGAC
ENSG00000110427	AUGACAC
ENSG00000110427	AUUCAUU
ENSG00000110427	CACAGUG
ENSG00000110427	CAGCAAA
ENSG00000110427	CAUGGGU
ENSG00000110427	CCAGCAU
ENSG00000110427	CUAUACA
ENSG00000110427	CUCAGGG
ENSG00000110427	CUUUGGU
ENSG00000110427	GGCAAGA
ENSG00000110427	GGCUCAG
ENSG00000110427	GUUCAUG
ENSG00000110427	UAUCAGA
ENSG00000110427	UCAAGUA
ENSG00000110427	UCAUUCG
ENSG00000110427	UGAAGCU
ENSG00000110427	UGGACUG
ENSG00000110427	UGGGUGA
ENSG00000110427	UUCUAAU
ENSG00000110427	UUGUUCG
ENSG00000110429	AAAAAUC
ENSG00000110429	CAGCAGA
ENSG00000110429	CAGGAAC
ENSG00000110429	GAUUGUC
ENSG00000110429	UAUAAAG
ENSG00000110435	AGCACCA
ENSG00000110436	AAAAGUA
ENSG00000110436	AAAGUGC
ENSG00000110436	AACAGUC
ENSG00000110436	AAGUGCA
ENSG00000110436	ACAUUAC
ENSG00000110436	ACUGCAU
ENSG00000110436	ACUGUGU
ENSG00000110436	AGCACCA
ENSG00000110436	AUGAUGC
ENSG00000110436	AUGGCAC
ENSG00000110436	CAAAGUG
ENSG00000110436	CACUGUU
ENSG00000110436	CUCCAGC
ENSG00000110436	GAUCAGA
ENSG00000110436	GCUACAU
ENSG00000110436	UAGUGAG
ENSG00000110436	UGCAUAG
ENSG00000110436	UGCCAGC
ENSG00000110442	ACCAGCA
ENSG00000110448	GACCUGG

ENSG00000110448	UACAAAG
ENSG00000110484	UCAACAG
ENSG00000110484	UUUCAAC
ENSG00000110492	AAAGCUG
ENSG00000110492	AAGGCAC
ENSG00000110492	AUCCCUU
ENSG00000110492	GGGGUGG
ENSG00000110497	AAAAACC
ENSG00000110497	AAUACUG
ENSG00000110497	ACCUGGA
ENSG00000110497	CAGCUGG
ENSG00000110497	CCCACCG
ENSG00000110497	CCCACCU
ENSG00000110497	CUCCCAC
ENSG00000110497	GAGGGGC
ENSG00000110497	GUGAGGU
ENSG00000110497	UACAAAG
ENSG00000110497	UACAGUU
ENSG00000110497	UCCCACC
ENSG00000110497	UCCCUCU
ENSG00000110497	UCUCCAA
ENSG00000110514	ACAUUCA
ENSG00000110675	AAUACUG
ENSG00000110675	ACUGCAU
ENSG00000110675	CAUGGGU
ENSG00000110675	GAGAAAU
ENSG00000110675	GCUAGCA
ENSG00000110675	GUAACAG
ENSG00000110675	UCACAGG
ENSG00000110675	UCACAGU
ENSG00000110675	UGAAAUG
ENSG00000110693	AACCUGU
ENSG00000110693	AUAUUAU
ENSG00000110693	GAUUGUC
ENSG00000110693	UCACAAG
ENSG00000110693	UGAGAAU
ENSG00000110693	UUCAAGC
ENSG00000110693	UUGUUCG
ENSG00000110693	UUUGCAA
ENSG00000110711	AGAUAAA
ENSG00000110713	AGCCCUU
ENSG00000110713	GACCAUG
ENSG00000110713	GCCCUUA
ENSG00000110713	UCCAGAG
ENSG00000110721	AGCAGCU
ENSG00000110721	AGUAACA
ENSG00000110721	GUAAACA
ENSG00000110721	UCACAGG
ENSG00000110721	UCACAGU
ENSG00000110721	UCAUACA
ENSG00000110756	AAGUGCA
ENSG00000110756	CCUGCUG
ENSG00000110768	ACCCUGU
ENSG00000110768	ACUGCAG
ENSG00000110768	AGUGCAA
ENSG00000110768	GUAAACA
ENSG00000110786	CAGACAG
ENSG00000110786	CAGCAAA
ENSG00000110786	UAUAAAG
ENSG00000110786	UCCCACA
ENSG00000110786	UGUCUGC
ENSG00000110801	CAGCUGG
ENSG00000110801	CUCUUCC
ENSG00000110801	CUUUGGU

ENSG00000110801	GUAAACA
ENSG00000110841	AACAGUC
ENSG00000110841	AAGACAU
ENSG00000110841	ACCAGCA
ENSG00000110841	AUAUAAC
ENSG00000110841	CAGCUGG
ENSG00000110841	GAGAAAU
ENSG00000110841	GCAACUU
ENSG00000110841	UCACCAC
ENSG00000110844	GGACUGC
ENSG00000110848	AAGUGCA
ENSG00000110851	AAGUGCA
ENSG00000110851	ACAUUAC
ENSG00000110851	AGCAAAA
ENSG00000110851	AGCAGCG
ENSG00000110851	AGCAGCU
ENSG00000110851	AGUGCUU
ENSG00000110851	AUAUAAC
ENSG00000110851	CCCACCG
ENSG00000110851	CCCACCU
ENSG00000110851	UAGAGGA
ENSG00000110851	UUCCUAG
ENSG00000110871	UUCAAAU
ENSG00000110880	AACACUG
ENSG00000110880	AAUACUG
ENSG00000110880	ACUGCAU
ENSG00000110880	ACUGGAU
ENSG00000110880	AGUGCCU
ENSG00000110880	AGUGCUG
ENSG00000110880	CACAGUG
ENSG00000110880	CCCUGAG
ENSG00000110880	CUUGGGC
ENSG00000110880	GAUUGUA
ENSG00000110880	GAUUGUC
ENSG00000110880	GCUGUCU
ENSG00000110880	GGAAGCC
ENSG00000110880	GGCAGUG
ENSG00000110880	UAUAAAG
ENSG00000110880	UCAUUUG
ENSG00000110880	UUGACAC
ENSG00000110880	UUGAUCA
ENSG00000110880	UUGGCAC
ENSG00000110880	UUGGUCC
ENSG00000110880	UUGGUUC
ENSG00000110881	ACAGUAC
ENSG00000110881	CCCUGAG
ENSG00000110881	CCUGCUG
ENSG00000110881	CUUUGGU
ENSG00000110881	GCUUCCU
ENSG00000110881	GGGAGCU
ENSG00000110881	GGGGCAG
ENSG00000110881	GUGCUUG
ENSG00000110881	UCACAGG
ENSG00000110881	UCACAGU
ENSG00000110881	UGUGCUU
ENSG00000110888	AAGUGCU
ENSG00000110888	AGUGCUG
ENSG00000110888	AGUGCUU
ENSG00000110888	GCUACAU
ENSG00000110888	GGCAUUG
ENSG00000110888	GUGCAAA
ENSG00000110888	UACAGUU
ENSG00000110888	UCACAGG
ENSG00000110888	UCACAGU

ENSG00000110906	AACUACU
ENSG00000110906	ACAGUAU
ENSG00000110906	AGCCUGC
ENSG00000110906	AGGGUUG
ENSG00000110906	CUCCAGC
ENSG00000110906	CUUUGGU
ENSG00000110906	GAGAACC
ENSG00000110906	GAGGUAG
ENSG00000110906	UACAAAG
ENSG00000110906	UCUCACC
ENSG00000110906	UUCCAUA
ENSG00000110911	ACUGGCU
ENSG00000110911	CAGGCUC
ENSG00000110911	CUGACUC
ENSG00000110911	CUGGCUC
ENSG00000110911	GUGCAAA
ENSG00000110911	UCCCUCU
ENSG00000110911	UCCUGAG
ENSG00000110931	AAGGUGC
ENSG00000110931	AGCACCA
ENSG00000110931	AGCCCUU
ENSG00000110931	AGUAGAC
ENSG00000110931	AUGUAAU
ENSG00000110931	AUUGCAC
ENSG00000110931	CAAGAGC
ENSG00000110931	CAGCUGG
ENSG00000110931	CUUCUCU
ENSG00000110931	CUUUGGU
ENSG00000110931	GCACCAU
ENSG00000110931	GCUUCCU
ENSG00000110931	GUAAACA
ENSG00000110931	UAUUGCU
ENSG00000110931	UGUGCUU
ENSG00000110931	UUGGCAA
ENSG00000110931	UUGGCAC
ENSG00000110934	AGCCUGC
ENSG00000110944	UCCCUUG
ENSG00000110944	UCCCUUU
ENSG00000110958	AAAUCCC
ENSG00000110958	AGUGCAA
ENSG00000110958	CAGUGCA
ENSG00000110958	GUGCAAA
ENSG00000110975	AGUGCAA
ENSG00000110975	CUUUGGU
ENSG00000110975	UGCAUAU
ENSG00000110975	UUGGAGG
ENSG00000110987	AACAUUC
ENSG00000110987	AAGGCAC
ENSG00000110987	AAGUGCA
ENSG00000110987	AAGUGCU
ENSG00000110987	AGCAGCA
ENSG00000110987	AGCCCUU
ENSG00000110987	AUGCACC
ENSG00000110987	CACCACC
ENSG00000110987	CCAGUGC
ENSG00000110987	CUUGGAG
ENSG00000110987	GAGCCCU
ENSG00000110987	GAGGUAG
ENSG00000110987	GAGGUAU
ENSG00000110987	GGAAUGU
ENSG00000110987	GGAUUUC
ENSG00000110987	GUGCAAA
ENSG00000110987	UCACAGG
ENSG00000110987	UCACAGU

ENSG00000110987	UCACCAC
ENSG00000110987	UCCCUUG
ENSG00000110987	UCCCUUU
ENSG00000110987	UCCUGAG
ENSG00000110987	UGAGAAG
ENSG00000110987	UGCCAGC
ENSG00000110987	UUUGCAA
ENSG00000111011	AAAGUAC
ENSG00000111011	UACAAAG
ENSG00000111012	AGCCAGU
ENSG00000111046	UACAAAG
ENSG00000111049	AAAGUGC
ENSG00000111049	AGGCAGG
ENSG00000111077	CUGCAGC
ENSG00000111077	UGCAGCU
ENSG00000111077	UGCUCAC
ENSG00000111110	AAAGCUG
ENSG00000111110	AGCAGCA
ENSG00000111110	AGCAGCG
ENSG00000111110	AGCAGCU
ENSG00000111110	AUAUAAC
ENSG00000111110	AUUGCAC
ENSG00000111110	CCCCUGG
ENSG00000111110	CCUUCAU
ENSG00000111110	UACAAAG
ENSG00000111110	UCAAGUA
ENSG00000111110	UCACAGU
ENSG00000111110	UCCUGAG
ENSG00000111110	UUGGUCC
ENSG00000111142	ACAUUAC
ENSG00000111142	AUGGAUU
ENSG00000111142	CAAAACU
ENSG00000111142	CCAGGAG
ENSG00000111142	UGGAUAA
ENSG00000111142	UGUCUUU
ENSG00000111145	GUGCAAA
ENSG00000111181	GGCCCUG
ENSG00000111186	AGCAGCA
ENSG00000111186	AUAAUAC
ENSG00000111186	AUACAAG
ENSG00000111186	AUAUAAC
ENSG00000111199	UCCUGAG
ENSG00000111199	UGAAAUG
ENSG00000111206	CCUGCUG
ENSG00000111206	GGAUUUC
ENSG00000111218	GCAGCAU
ENSG00000111224	AAAAGUA
ENSG00000111224	CAAAGCA
ENSG00000111241	GUGCAAA
ENSG00000111249	AAGUGCA
ENSG00000111249	AGCCUGC
ENSG00000111249	AGUGCUU
ENSG00000111249	AUACAAG
ENSG00000111249	AUCCUUU
ENSG00000111249	AUGGCUU
ENSG00000111249	CAGACAG
ENSG00000111249	CAGUCUG
ENSG00000111249	CUACAAA
ENSG00000111249	CUCACAC
ENSG00000111249	CUGGAAA
ENSG00000111249	GAGUCUU
ENSG00000111249	UAAUAUC
ENSG00000111249	UGCAUAG
ENSG00000111249	UGGAUAA

ENSG00000111249	UUAGAGA
ENSG00000111249	UUCUAAU
ENSG00000111249	UUGGUCC
ENSG00000111252	AAACUGU
ENSG00000111252	AAGGCAC
ENSG00000111252	AAUCCUU
ENSG00000111252	ACAGUAU
ENSG00000111252	ACAUUAC
ENSG00000111252	AGAUAAA
ENSG00000111252	AGGCAGG
ENSG00000111252	AUACAAG
ENSG00000111252	AUCCUUU
ENSG00000111252	AUGGAUU
ENSG00000111252	CAGUGCA
ENSG00000111252	CCCUGAG
ENSG00000111252	CCUUCUG
ENSG00000111252	CUCUGGG
ENSG00000111252	CUUUGGU
ENSG00000111252	GAAUUGU
ENSG00000111252	GAGGUAG
ENSG00000111252	GAGGUAU
ENSG00000111252	GCAGAAG
ENSG00000111252	GCAGGUU
ENSG00000111252	GCUUCCU
ENSG00000111252	GGUCUAG
ENSG00000111252	GUGACUG
ENSG00000111252	UCAACAG
ENSG00000111252	UCACAUU
ENSG00000111252	UCCCUGU
ENSG00000111252	UCCUGAG
ENSG00000111252	UGAAAGG
ENSG00000111252	UGACUGU
ENSG00000111252	UGCAUAG
ENSG00000111252	UGCAUAU
ENSG00000111252	UGCAUUG
ENSG00000111254	GGCUCUG
ENSG00000111254	UCCUUGC
ENSG00000111261	GAAGGAA
ENSG00000111262	AAGACAU
ENSG00000111262	AAGGCAC
ENSG00000111262	AAGUGCA
ENSG00000111262	AAGUGCU
ENSG00000111262	AAUGCCC
ENSG00000111262	ACAGUAU
ENSG00000111262	AGCAUUC
ENSG00000111262	AGCUUAU
ENSG00000111262	AGGCAGG
ENSG00000111262	AUGCAUU
ENSG00000111262	AUUGCAC
ENSG00000111262	CAGUCCA
ENSG00000111262	CCCUGAG
ENSG00000111262	CCUUCUG
ENSG00000111262	CUGUUGA
ENSG00000111262	CUUUGGU
ENSG00000111262	GAAAACA
ENSG00000111262	GCACCAU
ENSG00000111262	GCCCUGU
ENSG00000111262	GCUACAU
ENSG00000111262	GGAGGCA
ENSG00000111262	GGUUACC
ENSG00000111262	UAAUGCU
ENSG00000111262	UAUAAAG
ENSG00000111262	UAUUGCU
ENSG00000111262	UCCAGUU

ENSG00000111262	UCCCUGU
ENSG00000111262	UCCCUUG
ENSG00000111262	UCCCUUU
ENSG00000111262	UCUCACC
ENSG00000111262	UGGGUAC
ENSG00000111262	UGUCUGC
ENSG00000111262	UGUGCUU
ENSG00000111262	UUGGUCC
ENSG00000111266	AAGGUGC
ENSG00000111266	CUGGCUC
ENSG00000111266	GAGGUAU
ENSG00000111266	GGCUCAG
ENSG00000111269	AAGACAU
ENSG00000111269	AGCAGCU
ENSG00000111269	AGCUUAU
ENSG00000111269	CACUCCU
ENSG00000111269	CAGCAGA
ENSG00000111269	CUGGCUC
ENSG00000111269	CUUGUGU
ENSG00000111269	GAAAACA
ENSG00000111269	GCUGGAU
ENSG00000111269	GUGCAAA
ENSG00000111269	GUUCAUG
ENSG00000111269	UCAAGUA
ENSG00000111269	UUGGUUC
ENSG00000111271	CUGCAGC
ENSG00000111276	AGGUAGU
ENSG00000111276	AGUGCUU
ENSG00000111319	UCCUUGC
ENSG00000111321	AGCACCA
ENSG00000111321	CAGUCCA
ENSG00000111328	ACAUCAC
ENSG00000111328	UAUUACC
ENSG00000111331	GGGGUCC
ENSG00000111344	AGCAGCU
ENSG00000111344	AGUGCAA
ENSG00000111344	CAGUGCA
ENSG00000111361	CAGUCUG
ENSG00000111371	AAUGUUG
ENSG00000111371	AGUGCUG
ENSG00000111371	AUACAAG
ENSG00000111371	AUCACUA
ENSG00000111371	AUGAUGA
ENSG00000111371	GCUGGUG
ENSG00000111371	UCAAGUA
ENSG00000111371	UGACUGU
ENSG00000111371	UGCAUAG
ENSG00000111371	UGUGCUU
ENSG00000111424	AAGGCAC
ENSG00000111424	ACAUUAC
ENSG00000111424	CCCUGAG
ENSG00000111424	CUGCCCC
ENSG00000111424	GAAAACA
ENSG00000111424	GACUUCC
ENSG00000111424	UCACAUU
ENSG00000111424	UGCAGCU
ENSG00000111432	AAAGAAU
ENSG00000111432	AACAUUC
ENSG00000111432	AGCCCUU
ENSG00000111432	AGUGCUU
ENSG00000111432	AUAUUAU
ENSG00000111432	CAGCAAA
ENSG00000111432	CCAUUAC
ENSG00000111432	UACAAAG

ENSG00000111432	UUCCAUA
ENSG00000111450	AAGGCAC
ENSG00000111450	CUUGGGC
ENSG00000111450	UACAAAG
ENSG00000111481	ACUCAGC
ENSG00000111481	CAGCAAA
ENSG00000111481	CCCUGAG
ENSG00000111481	GAGGCAG
ENSG00000111530	AAUCCUU
ENSG00000111530	CUACAAA
ENSG00000111540	AAAACUG
ENSG00000111540	AAGUGCA
ENSG00000111540	ACAUUAC
ENSG00000111540	ACUGCAG
ENSG00000111540	AGCCCUU
ENSG00000111540	AGUGCAA
ENSG00000111540	AGUGUGC
ENSG00000111540	CAAAUGC
ENSG00000111540	CAGCAGA
ENSG00000111540	CAGCAGG
ENSG00000111540	CCCCCAG
ENSG00000111540	CCCCUGG
ENSG00000111540	CUGCAGG
ENSG00000111540	CUGCCCC
ENSG00000111540	CUUUGGU
ENSG00000111540	GAGGCAG
ENSG00000111540	GCCUGGA
ENSG00000111540	GCCUGGG
ENSG00000111540	GCUAGCA
ENSG00000111540	GGAGGCA
ENSG00000111540	GGCUCAG
ENSG00000111540	GGGAGCU
ENSG00000111540	GGGGUCC
ENSG00000111540	GUCUUGC
ENSG00000111540	GUGCAAA
ENSG00000111540	UAAUACA
ENSG00000111540	UACAAAG
ENSG00000111540	UAUAAUA
ENSG00000111540	UGCUGAC
ENSG00000111554	AUGUGCC
ENSG00000111554	GUGACAG
ENSG00000111596	AAAAAUC
ENSG00000111596	AAAUGGU
ENSG00000111596	AACAAAC
ENSG00000111596	AACAGUC
ENSG00000111596	AAGGCAC
ENSG00000111596	AAGUCUU
ENSG00000111596	ACAUUCA
ENSG00000111596	ACCAGCA
ENSG00000111596	AGGCAGG
ENSG00000111596	AGUGCAA
ENSG00000111596	AGUGCUG
ENSG00000111596	AGUGCUU
ENSG00000111596	AUAAUAC
ENSG00000111596	AUAUAAC
ENSG00000111596	AUGGAUU
ENSG00000111596	AUGUGGG
ENSG00000111596	AUUCAUU
ENSG00000111596	AUUGCAC
ENSG00000111596	CACACCU
ENSG00000111596	CACAGGG
ENSG00000111596	CAGCAGG
ENSG00000111596	CCCCUGG
ENSG00000111596	CUCAAAC

ENSG00000111596	CUGCAGC
ENSG00000111596	CUGCAGG
ENSG00000111596	CUGCUCA
ENSG00000111596	CUUCACC
ENSG00000111596	GAAGGAA
ENSG00000111596	GACCCUG
ENSG00000111596	GAGAAGA
ENSG00000111596	GAGGUAG
ENSG00000111596	GAGGUAU
ENSG00000111596	GCAGGAC
ENSG00000111596	GCAUCCC
ENSG00000111596	GCCUGAU
ENSG00000111596	GCUACAU
ENSG00000111596	GGCCCUG
ENSG00000111596	GGCGACA
ENSG00000111596	GGCUGCG
ENSG00000111596	GUCAGUU
ENSG00000111596	GUCUGCC
ENSG00000111596	UAUAAUA
ENSG00000111596	UCCUAUA
ENSG00000111596	UCUGGAG
ENSG00000111596	UGAAGCU
ENSG00000111596	UGCCAGC
ENSG00000111596	UGCUCAC
ENSG00000111596	UGGCCCU
ENSG00000111596	UGGGUAC
ENSG00000111596	UGUCUGC
ENSG00000111596	UGUGCUU
ENSG00000111596	UUCAAAU
ENSG00000111596	UUCUGCA
ENSG00000111605	AACAAAC
ENSG00000111605	AACAGUC
ENSG00000111605	AAUUGCU
ENSG00000111605	AGAUAAA
ENSG00000111605	AUCAUAC
ENSG00000111605	CAUAUUG
ENSG00000111605	CUAUACA
ENSG00000111605	CUCUUCC
ENSG00000111605	CUUUGGU
ENSG00000111605	GCUGGAU
ENSG00000111605	GUAUUCU
ENSG00000111605	GUCUUGC
ENSG00000111605	GUUCAUG
ENSG00000111605	UAUUACC
ENSG00000111605	UCACACA
ENSG00000111605	UCAUUUG
ENSG00000111605	UCCAGUU
ENSG00000111605	UCCCUGU
ENSG00000111605	UGCAAAG
ENSG00000111605	UGCUGAC
ENSG00000111605	UGUCUUU
ENSG00000111605	UUGGCAC
ENSG00000111615	AAUCCUU
ENSG00000111615	AUCCUUU
ENSG00000111615	CUCAAAC
ENSG00000111642	AUACAAG
ENSG00000111642	GAGGUAG
ENSG00000111642	GUAACAG
ENSG00000111652	AGCAGCA
ENSG00000111652	CUGGGGG
ENSG00000111652	GGCUCUG
ENSG00000111652	GUAGUGU
ENSG00000111652	UCAUAGA
ENSG00000111653	AAACUGU

ENSG00000111653	CAAAGCA
ENSG00000111653	CAGCAGG
ENSG00000111665	UAAUAUC
ENSG00000111667	GGCAUUG
ENSG00000111667	UCACAUU
ENSG00000111667	UUGGCAA
ENSG00000111667	UUGGCAC
ENSG00000111670	AACUCUA
ENSG00000111670	CCUUCUU
ENSG00000111670	CUCUAGC
ENSG00000111670	GAGGUAU
ENSG00000111670	GGAAUGU
ENSG00000111670	UAUAAAG
ENSG00000111670	UCAUGAU
ENSG00000111674	CAGCUGG
ENSG00000111676	AAUUAUU
ENSG00000111676	AGGAUGU
ENSG00000111676	AUAUAAC
ENSG00000111676	GAGGAUA
ENSG00000111676	UUGGUUC
ENSG00000111696	GAGGCUG
ENSG00000111696	GGCAGCG
ENSG00000111696	UCUGGAG
ENSG00000111696	UGAGGAC
ENSG00000111696	UUGGCAC
ENSG00000111707	AAAGUUU
ENSG00000111707	ACUCCAG
ENSG00000111707	AGGUAGU
ENSG00000111707	CACAGGG
ENSG00000111707	UUCUAAU
ENSG00000111711	AAAGUUU
ENSG00000111711	AACAUUC
ENSG00000111711	AGCAGCA
ENSG00000111711	CUACAAA
ENSG00000111711	GAGGUAG
ENSG00000111711	GAGGUAU
ENSG00000111711	UGUGCUU
ENSG00000111716	UUGUUCG
ENSG00000111725	AAUACUG
ENSG00000111725	AGUGCAA
ENSG00000111725	GUGUGGC
ENSG00000111725	UAUUGCU
ENSG00000111725	UGCUGAC
ENSG00000111728	AAGGCAC
ENSG00000111728	ACCCUGU
ENSG00000111728	ACUUGGA
ENSG00000111728	CUGCUCA
ENSG00000111728	UCUAGAG
ENSG00000111731	UCAACAG
ENSG00000111731	UCAAGUA
ENSG00000111731	UGCAAAG
ENSG00000111737	AACGGCU
ENSG00000111737	AGCAAAA
ENSG00000111737	AGGGUCU
ENSG00000111737	CAGUCCA
ENSG00000111737	CAGUGCA
ENSG00000111737	CCUUCUU
ENSG00000111737	CUGCAGC
ENSG00000111737	CUGCAGG
ENSG00000111737	CUGGAAA
ENSG00000111737	GAUUGUA
ENSG00000111737	GAUUGUC
ENSG00000111737	GGCAGCG
ENSG00000111737	GUCUCUG

ENSG00000111737	UCACAUU
ENSG00000111737	UCCGCUC
ENSG00000111737	UUGGCAC
ENSG00000111752	AAAGAAU
ENSG00000111752	ACCAGCA
ENSG00000111752	ACUCCAG
ENSG00000111752	ACUGGAU
ENSG00000111752	AGCACCA
ENSG00000111752	AGCUGCC
ENSG00000111752	AGGCAGG
ENSG00000111752	AUCCUUU
ENSG00000111752	CACCAGC
ENSG00000111752	CUCAGGG
ENSG00000111752	CUGCAGC
ENSG00000111752	CUGGCUC
ENSG00000111752	GCAUCCC
ENSG00000111752	GGCAGGG
ENSG00000111752	GGCUCAG
ENSG00000111752	UAGACUG
ENSG00000111752	UGGACUG
ENSG00000111783	ACCCAUU
ENSG00000111783	GUGCAAA
ENSG00000111785	AACAAAC
ENSG00000111785	ACUGCAG
ENSG00000111785	AGCAGCU
ENSG00000111785	AGUGGUU
ENSG00000111785	CAAAGCA
ENSG00000111785	CAGGUGA
ENSG00000111785	CCAGCAU
ENSG00000111785	GAGAAAU
ENSG00000111785	GGCAGUG
ENSG00000111785	GUUAAUG
ENSG00000111785	UCUCAAG
ENSG00000111785	UGCAGCU
ENSG00000111785	UGCCAGC
ENSG00000111785	UUUCAAC
ENSG00000111790	AGUAGUU
ENSG00000111790	AUCCUUG
ENSG00000111790	AUCCUUU
ENSG00000111790	CAAAACU
ENSG00000111790	UCCUGAG
ENSG00000111799	CACAAUG
ENSG00000111799	CAGUAGA
ENSG00000111799	GGCUCUG
ENSG00000111799	GUGCGCA
ENSG00000111799	UCACAAG
ENSG00000111816	AUGGCUU
ENSG00000111816	UCAACAG
ENSG00000111817	UUUGCAA
ENSG00000111817	UUUGCAC
ENSG00000111837	AAAGAAU
ENSG00000111837	ACAGUAU
ENSG00000111837	AGCAGCA
ENSG00000111837	UCAACAG
ENSG00000111837	UCCAGAG
ENSG00000111837	UUGGCAC
ENSG00000111846	ACAAUAU
ENSG00000111846	ACAUUCA
ENSG00000111846	AGUAGUU
ENSG00000111846	AUAUUAU
ENSG00000111846	CUUCUCU
ENSG00000111846	GAGAAGA
ENSG00000111846	GUGAGGU
ENSG00000111846	UCAACAG

ENSG00000111846	UCCCUUG
ENSG00000111850	CUGCAGC
ENSG00000111850	UGCAGCU
ENSG00000111859	CCCUGAG
ENSG00000111860	AGCAGCA
ENSG00000111860	AGUAGUU
ENSG00000111863	CUUGGAG
ENSG00000111875	AAAAAUC
ENSG00000111875	AAGGCAC
ENSG00000111875	ACUGCAU
ENSG00000111875	AGCUUAU
ENSG00000111875	CUACAGU
ENSG00000111875	GAGAAGA
ENSG00000111877	AUGCCUU
ENSG00000111880	AAAACUG
ENSG00000111880	GGAAUGU
ENSG00000111880	UAUAAAG
ENSG00000111885	ACAUUAC
ENSG00000111885	AUGUGCC
ENSG00000111885	GAGGCUG
ENSG00000111885	GUCUCUG
ENSG00000111885	UAGAGGA
ENSG00000111885	UUUGCAC
ENSG00000111885	UUUUUGC
ENSG00000111897	AAUACUG
ENSG00000111897	ACCAGCA
ENSG00000111912	AAGGCAC
ENSG00000111912	AAGUGCA
ENSG00000111912	AAUACUG
ENSG00000111912	ACAGUAU
ENSG00000111912	ACUGCAU
ENSG00000111912	AGUGCUG
ENSG00000111912	CACAGUG
ENSG00000111912	CAGUUAC
ENSG00000111912	GAGGCAG
ENSG00000111912	UCACAGG
ENSG00000111912	UCCCUUG
ENSG00000111913	AAUUUUA
ENSG00000111913	AGCACCA
ENSG00000111913	CAAAUGC
ENSG00000111913	CACAGUG
ENSG00000111913	CCCUGAG
ENSG00000111913	GAGAACC
ENSG00000111913	GAGAACU
ENSG00000111913	GCACCAU
ENSG00000111913	UACAAAG
ENSG00000111913	UAUAAAG
ENSG00000111961	AAAGCUG
ENSG00000111961	AAGAACC
ENSG00000111961	AAGGCAC
ENSG00000111961	AAUGUUG
ENSG00000111961	ACAGUAU
ENSG00000111961	ACAUUCA
ENSG00000111961	CAUAUUG
ENSG00000111961	UUCUUGU
ENSG00000111962	AGGUUAU
ENSG00000111962	AUAAUAC
ENSG00000111962	AUGUAAU
ENSG00000111962	AUGUGGG
ENSG00000111962	CACAGUG
ENSG00000111962	CUCCCAC
ENSG00000111962	GUAAACA
ENSG00000111962	UAAUGCU
ENSG00000111962	UCAUGAU

ENSG00000111962	UUGUUCG
ENSG00000112033	AAAAACC
ENSG00000112033	AAAGUGC
ENSG00000112033	AGCACCA
ENSG00000112033	AGUGCUU
ENSG00000112033	AUAUUAU
ENSG00000112033	CAGUGCA
ENSG00000112033	CUUUGGU
ENSG00000112033	GAGAAGA
ENSG00000112033	UUGGAGG
ENSG00000112038	GAGGUAG
ENSG00000112039	AAUUAUU
ENSG00000112041	AGAUGUG
ENSG00000112041	GUGACUG
ENSG00000112062	AGCUGCC
ENSG00000112062	CCCUGAG
ENSG00000112062	GGAGAGA
ENSG00000112062	UCACAGU
ENSG00000112062	UCCUAUA
ENSG00000112062	UGAAGUG
ENSG00000112062	UGGAGAU
ENSG00000112078	GCACCAU
ENSG00000112078	GCAUCCC
ENSG00000112078	GCCUGGA
ENSG00000112078	GCCUGGG
ENSG00000112078	UCCCUUU
ENSG00000112078	UGAAAGG
ENSG00000112079	AAGGCAC
ENSG00000112079	AAGUGCA
ENSG00000112079	AUAAAGU
ENSG00000112079	GCACCAU
ENSG00000112079	GUGCAAA
ENSG00000112079	UGUGCUU
ENSG00000112081	AAAGUAC
ENSG00000112081	AAAGUAU
ENSG00000112081	ACCUGGA
ENSG00000112081	AGCUUAU
ENSG00000112081	AUGGCUU
ENSG00000112081	CUACAAA
ENSG00000112081	CUCACAC
ENSG00000112081	CUGGACU
ENSG00000112081	GCACCAU
ENSG00000112081	GGAAUGU
ENSG00000112081	UGCAUUG
ENSG00000112096	CAAAGCA
ENSG00000112096	CUACAAA
ENSG00000112116	AAUUUUA
ENSG00000112118	UGCAAAG
ENSG00000112130	AACAAAC
ENSG00000112130	CAGGCUC
ENSG00000112130	GCCUGGA
ENSG00000112130	GGCUCAG
ENSG00000112130	GGCUGCG
ENSG00000112130	GUCUCUG
ENSG00000112130	UAAUAUC
ENSG00000112130	UGCAAAG
ENSG00000112130	UGGCUGC
ENSG00000112139	AAAGAAU
ENSG00000112139	AACACUG
ENSG00000112139	AAGGUGC
ENSG00000112139	ACUGCAG
ENSG00000112139	AGGAGCU
ENSG00000112139	CACAGUG
ENSG00000112139	CAGUCUG

ENSG00000112139	CCCCUGG
ENSG00000112139	CCCUGAG
ENSG00000112139	CCUUCUG
ENSG00000112139	CUGGGCA
ENSG00000112139	CUUCUCU
ENSG00000112139	GAGGCUG
ENSG00000112139	GGAGGAG
ENSG00000112139	GGCCCUG
ENSG00000112139	GUGCUUG
ENSG00000112139	UAAUAUC
ENSG00000112139	UCACAGU
ENSG00000112139	UGGGACA
ENSG00000112139	UGGUACC
ENSG00000112144	AACAUUC
ENSG00000112144	AAGUUGU
ENSG00000112144	AAUACUG
ENSG00000112144	AUGGCAC
ENSG00000112144	AUUGCAC
ENSG00000112144	CAUAUUG
ENSG00000112144	CUACAGU
ENSG00000112144	GAAAACA
ENSG00000112144	GAGGUAU
ENSG00000112144	UAUUGCU
ENSG00000112144	UCUGGAA
ENSG00000112144	UGCAUAU
ENSG00000112144	UUGGUCC
ENSG00000112146	CAAAACU
ENSG00000112146	CCAGUGC
ENSG00000112146	CCAGUGU
ENSG00000112146	UCUUGAG
ENSG00000112146	UGAAAGG
ENSG00000112146	UUCUUGU
ENSG00000112149	AAUUAUU
ENSG00000112149	AGUGCUG
ENSG00000112149	CACACCG
ENSG00000112149	CACACCU
ENSG00000112149	CUCACAC
ENSG00000112149	CUGGGCA
ENSG00000112149	GGUUGAC
ENSG00000112149	GGUUUAC
ENSG00000112149	UCACACA
ENSG00000112149	UCCCACA
ENSG00000112149	UCCCACC
ENSG00000112164	CCAGGAG
ENSG00000112164	UCUUGAG
ENSG00000112164	UGACCUA
ENSG00000112164	UUCCUCG
ENSG00000112167	ACACACU
ENSG00000112182	AAAGAAU
ENSG00000112182	AAAGGUA
ENSG00000112182	AAAGUAU
ENSG00000112182	AAAUGGU
ENSG00000112182	AACCUGG
ENSG00000112182	AAGAACC
ENSG00000112182	AAGGCAC
ENSG00000112182	AAUACUG
ENSG00000112182	AAUGUUG
ENSG00000112182	AAUUAUU
ENSG00000112182	ACAGGUG
ENSG00000112182	ACAGUAC
ENSG00000112182	ACAUUAC
ENSG00000112182	ACAUUCA
ENSG00000112182	ACCCUGU
ENSG00000112182	ACUGCAG

ENSG00000112182	AGCAGCA
ENSG00000112182	AGGCAGG
ENSG00000112182	AGGGUUG
ENSG00000112182	AGUGCAA
ENSG00000112182	AUAUAAC
ENSG00000112182	AUGCAUU
ENSG00000112182	AUGGAUU
ENSG00000112182	AUGGCAC
ENSG00000112182	AUGUCUG
ENSG00000112182	CAAAACU
ENSG00000112182	CACACCU
ENSG00000112182	CAGACAG
ENSG00000112182	CAGUGCA
ENSG00000112182	CCAUUAC
ENSG00000112182	CCCCCAG
ENSG00000112182	CCCUGUC
ENSG00000112182	CCCUGUU
ENSG00000112182	CCUGCUG
ENSG00000112182	CUGCAGC
ENSG00000112182	CUGGAAA
ENSG00000112182	GAGGCAG
ENSG00000112182	GAGGGGC
ENSG00000112182	GCAGCAU
ENSG00000112182	GGAGUGU
ENSG00000112182	GGAUGAG
ENSG00000112182	GGCAUUG
ENSG00000112182	GGGAGCU
ENSG00000112182	GGGGAGC
ENSG00000112182	GUAAACA
ENSG00000112182	GUAGUGU
ENSG00000112182	GUCAUUG
ENSG00000112182	GUGACAG
ENSG00000112182	GUUCAUG
ENSG00000112182	UACAGUU
ENSG00000112182	UAGAGGA
ENSG00000112182	UAUUGCU
ENSG00000112182	UCACAUU
ENSG00000112182	UCAUACA
ENSG00000112182	UCAUAGA
ENSG00000112182	UCCAGAG
ENSG00000112182	UCCCUGU
ENSG00000112182	UCUGGAA
ENSG00000112182	UCUGGAG
ENSG00000112182	UGCAGCU
ENSG00000112182	UGCAUAG
ENSG00000112182	UUCAAGC
ENSG00000112182	UUGAGGC
ENSG00000112182	UUGGCAA
ENSG00000112182	UUGGCAC
ENSG00000112182	UUGUUCG
ENSG00000112183	AAAACUG
ENSG00000112183	AAUUUUA
ENSG00000112183	ACCAGCA
ENSG00000112183	AUAAAGU
ENSG00000112183	CACCAGC
ENSG00000112183	CAUAUUG
ENSG00000112183	CCAGUGC
ENSG00000112183	CCAGUGU
ENSG00000112183	CUUGUAU
ENSG00000112183	CUUUGGU
ENSG00000112183	GAUUGUA
ENSG00000112183	GUCAACA
ENSG00000112183	GUGCCUG
ENSG00000112183	UAAUAUC

ENSG00000112183	UAUUGCU
ENSG00000112183	UCCCUUU
ENSG00000112183	UGUAUGU
ENSG00000112183	UGUCUGC
ENSG00000112183	UGUCUGG
ENSG00000112183	UUGUUCG
ENSG00000112195	AGGACAC
ENSG00000112200	AACAAAC
ENSG00000112200	UAAUAUC
ENSG00000112200	UGUGUGG
ENSG00000112210	AAUACUG
ENSG00000112210	UUGGCAA
ENSG00000112214	CAGCAGA
ENSG00000112214	UAGUGAG
ENSG00000112218	AGCAGCG
ENSG00000112218	AGCAGCU
ENSG00000112232	AAAUUUC
ENSG00000112232	AACACUG
ENSG00000112232	CAGUUAC
ENSG00000112232	GCUACAU
ENSG00000112232	UCAUUUG
ENSG00000112232	UCCUAUA
ENSG00000112232	UUCCUAG
ENSG00000112232	UUUCAAC
ENSG00000112238	AAGGCAC
ENSG00000112238	AAUUGCU
ENSG00000112238	UUCAAAU
ENSG00000112242	AAAGCUG
ENSG00000112242	AAAGUGC
ENSG00000112242	AACAAAC
ENSG00000112242	AAGACAU
ENSG00000112242	AAGGCAC
ENSG00000112242	ACCCUGU
ENSG00000112242	ACUGUUU
ENSG00000112242	AGAUAAA
ENSG00000112242	AGCAGCG
ENSG00000112242	AGCCCUU
ENSG00000112242	AGUACUG
ENSG00000112242	AUGCACC
ENSG00000112242	AUGCAUU
ENSG00000112242	AUUGCAC
ENSG00000112242	CACAGUG
ENSG00000112242	CACCACC
ENSG00000112242	CACUGUU
ENSG00000112242	CAGGUGA
ENSG00000112242	CAGUAGA
ENSG00000112242	CAGUGCA
ENSG00000112242	CCAGUAC
ENSG00000112242	CCAGUGU
ENSG00000112242	CCCUGAG
ENSG00000112242	GAGCCCU
ENSG00000112242	GAGGCAG
ENSG00000112242	GCCCUUA
ENSG00000112242	GCUGGUG
ENSG00000112242	GGAAGCC
ENSG00000112242	GGCACGG
ENSG00000112242	GUAAACA
ENSG00000112242	GUAACAG
ENSG00000112242	UCCAGUU
ENSG00000112242	UGCAUAG
ENSG00000112242	UGCAUAU
ENSG00000112242	UGCCCUG
ENSG00000112242	UGGUACC
ENSG00000112242	UUCAAAU

ENSG00000112242	UUGACAC
ENSG00000112242	UUGGAGG
ENSG00000112245	AACAUUC
ENSG00000112245	AAGUUGU
ENSG00000112245	AAUACUG
ENSG00000112245	AAUCUCA
ENSG00000112245	ACAAUAU
ENSG00000112245	ACAGUAU
ENSG00000112245	AGAUGUG
ENSG00000112245	CAAAGCA
ENSG00000112245	CAGACAG
ENSG00000112245	CCCUGUC
ENSG00000112245	CCCUGUU
ENSG00000112245	CUCCCAA
ENSG00000112245	CUCCCAC
ENSG00000112245	GCACCAU
ENSG00000112245	GCUGGUG
ENSG00000112245	GGAAGCC
ENSG00000112245	GGUCUAG
ENSG00000112245	GUGCAAA
ENSG00000112245	GUGCUUG
ENSG00000112245	UAAGACU
ENSG00000112245	UAAUAGU
ENSG00000112245	UCAAGUA
ENSG00000112245	UCCCACA
ENSG00000112245	UCCCUGU
ENSG00000112245	UGUCUGC
ENSG00000112245	UGUCUGG
ENSG00000112245	UUGGCAA
ENSG00000112246	GCUGGUG
ENSG00000112246	UCCUGAG
ENSG00000112276	CAGUCUG
ENSG00000112276	GCUGGUG
ENSG00000112276	UCAUACA
ENSG00000112282	UUAGAGA
ENSG00000112290	AAUACUG
ENSG00000112290	ACAAUAU
ENSG00000112290	AGCACCA
ENSG00000112290	AGCUGCC
ENSG00000112290	UUCAAAU
ENSG00000112293	CCAGGAG
ENSG00000112294	AGCUGCC
ENSG00000112294	UUGGUCC
ENSG00000112297	AGCACCA
ENSG00000112305	AGCAGCU
ENSG00000112305	CACAGUG
ENSG00000112305	GCACCAU
ENSG00000112305	GGAAUGU
ENSG00000112305	UCAUACA
ENSG00000112308	AAUACUG
ENSG00000112308	ACAGUAC
ENSG00000112308	ACAGUAU
ENSG00000112308	AUUGCAC
ENSG00000112308	CAGUAGU
ENSG00000112308	CUAUACA
ENSG00000112308	UGCAGCU
ENSG00000112312	AAAAAUC
ENSG00000112319	AAAAAUC
ENSG00000112319	AAAGCUG
ENSG00000112319	AAGACAU
ENSG00000112319	AAGUCAC
ENSG00000112319	AGGUAGU
ENSG00000112319	AUAAAGU
ENSG00000112319	CAGCAAA

ENSG00000112319	CAGCUGG
ENSG00000112319	CAGUCUG
ENSG00000112319	CUCAGGG
ENSG00000112319	GCAUCCC
ENSG00000112319	GGAAUGU
ENSG00000112319	GUAACAG
ENSG00000112319	GUCUUGC
ENSG00000112319	UAAGACG
ENSG00000112319	UAAGACU
ENSG00000112319	UGAAAUG
ENSG00000112319	UGAGGAC
ENSG00000112319	UGUUGAA
ENSG00000112319	UUGGUCC
ENSG00000112320	AAAAACC
ENSG00000112320	AAAGCUG
ENSG00000112320	AAAGGUA
ENSG00000112320	AAAGUAU
ENSG00000112320	AAAGUGC
ENSG00000112320	AAAUUUC
ENSG00000112320	AAUUGCU
ENSG00000112320	ACAAGGU
ENSG00000112320	ACAGUAU
ENSG00000112320	AGCAGCG
ENSG00000112320	AGCAGCU
ENSG00000112320	AUGGCAC
ENSG00000112320	AUUGCAC
ENSG00000112320	CAGCUGG
ENSG00000112320	CCCUGUU
ENSG00000112320	GAAACAU
ENSG00000112320	GAGGAUA
ENSG00000112320	GAUUGUC
ENSG00000112320	GCUGGUG
ENSG00000112320	GUAAACA
ENSG00000112320	UACAAAG
ENSG00000112320	UCACAAG
ENSG00000112320	UGAAAGG
ENSG00000112320	UUCUAAU
ENSG00000112333	AAAGUAC
ENSG00000112333	AAGUGCU
ENSG00000112333	AGUGCUG
ENSG00000112333	CUUUGGU
ENSG00000112333	UCACAAG
ENSG00000112335	UAUAAAG
ENSG00000112339	AAAUCCC
ENSG00000112339	AAUCUCA
ENSG00000112339	UAAUACA
ENSG00000112339	UUGGCAA
ENSG00000112378	AAAUGGU
ENSG00000112378	GGUUUAC
ENSG00000112379	AAAUUUC
ENSG00000112379	AAGGCAC
ENSG00000112379	ACAGUAC
ENSG00000112379	ACCCAUU
ENSG00000112379	CCCUGAG
ENSG00000112379	GAGAAAU
ENSG00000112379	UCCCUGU
ENSG00000112394	GAGGUAG
ENSG00000112406	AAAGUGC
ENSG00000112406	AAGACAU
ENSG00000112406	AAGUGCA
ENSG00000112414	CACAGUG
ENSG00000112414	UUCCAUA
ENSG00000112419	AAACUGU
ENSG00000112419	ACAGUAU

ENSG00000112419	ACUUGGA
ENSG00000112419	AGCACCA
ENSG00000112419	AGCAGCG
ENSG00000112419	AGCAGCU
ENSG00000112419	CAGGAAC
ENSG00000112419	CUGGAAA
ENSG00000112419	GAAUUGU
ENSG00000112419	GACCUGG
ENSG00000112419	GAGGUAG
ENSG00000112419	GAGGUAU
ENSG00000112419	GCACCAU
ENSG00000112419	GGAUUUC
ENSG00000112419	GUAAACA
ENSG00000112419	UCACAUU
ENSG00000112419	UUCAAAU
ENSG00000112425	GGAGGCA
ENSG00000112473	AAUCACU
ENSG00000112511	AAAGUGC
ENSG00000112511	AACAAAC
ENSG00000112511	CAGGAAC
ENSG00000112511	GCCUGGG
ENSG00000112511	GUAACAG
ENSG00000112511	GUGAAUG
ENSG00000112511	GUGAGGU
ENSG00000112511	UCAUACA
ENSG00000112530	ACAGUAC
ENSG00000112530	ACAUCAC
ENSG00000112530	UGACAUC
ENSG00000112531	AACACUG
ENSG00000112531	AACAGUC
ENSG00000112531	ACAAUAU
ENSG00000112531	ACAGUAC
ENSG00000112531	ACAGUAU
ENSG00000112531	ACAUUCA
ENSG00000112531	AGUGCAA
ENSG00000112531	CAGCAAA
ENSG00000112531	CCUGCUG
ENSG00000112531	GGAAUGU
ENSG00000112531	GUGCAAA
ENSG00000112531	UAAGACG
ENSG00000112531	UCACAUU
ENSG00000112531	UCAUGAU
ENSG00000112531	UGCUGAC
ENSG00000112531	UUCAAAU
ENSG00000112531	UUGGUCC
ENSG00000112541	AACAUUC
ENSG00000112541	ACAUUCA
ENSG00000112541	CAGCAAA
ENSG00000112559	CACAGUG
ENSG00000112559	GAGGUAG
ENSG00000112559	UCACAGG
ENSG00000112559	UCACAGU
ENSG00000112561	AAGGCAC
ENSG00000112561	CAAAGCA
ENSG00000112561	UCACACA
ENSG00000112562	AAAGUAA
ENSG00000112562	AAAGUAU
ENSG00000112562	AAUUUUA
ENSG00000112562	AGCAAAA
ENSG00000112562	AGUGGUU
ENSG00000112562	GUAUGUU
ENSG00000112562	UCACAGG
ENSG00000112562	UCACAGU
ENSG00000112576	AGCAGCG

ENSG00000112576	AGCAGCU
ENSG00000112576	AGUGCAA
ENSG00000112576	CACCAGC
ENSG00000112576	GUCAACA
ENSG00000112576	UCCCUCU
ENSG00000112592	AAAAACC
ENSG00000112592	AAUACUG
ENSG00000112592	AGCAGCA
ENSG00000112592	CACAGUG
ENSG00000112592	GGAAUGU
ENSG00000112592	UCACAGU
ENSG00000112599	AACCUGG
ENSG00000112599	CUGCUCA
ENSG00000112599	UGCUCAC
ENSG00000112624	AAAGUAA
ENSG00000112624	AAAGUGC
ENSG00000112624	AACAGUC
ENSG00000112624	AAUCUCA
ENSG00000112624	AAUCUCU
ENSG00000112624	AGUGCUU
ENSG00000112624	AUGGAUU
ENSG00000112624	AUUGCAC
ENSG00000112624	CUAUACA
ENSG00000112624	CUUUGGU
ENSG00000112624	GAAGGUC
ENSG00000112624	GAUUGUA
ENSG00000112624	GGUUGUC
ENSG00000112624	UAUUGCU
ENSG00000112624	UCAAGUA
ENSG00000112624	UCCCUGU
ENSG00000112624	UCUCCAA
ENSG00000112624	UGGCUGC
ENSG00000112624	UUCAAGC
ENSG00000112624	UUCCUAG
ENSG00000112624	UUGGCAC
ENSG00000112624	UUGGUUC
ENSG00000112624	UUUCAAC
ENSG00000112640	CACAGGG
ENSG00000112640	CUGCAGG
ENSG00000112640	CUUUGGU
ENSG00000112640	GGCCCUG
ENSG00000112640	GGGUGGU
ENSG00000112640	GGUGGUC
ENSG00000112640	GUGGCCC
ENSG00000112640	UUGGUCC
ENSG00000112655	AGCAGCG
ENSG00000112655	CCUUCAU
ENSG00000112655	GGUUACC
ENSG00000112658	AAAGAAU
ENSG00000112658	AAUACUG
ENSG00000112658	ACAGUAC
ENSG00000112658	ACAGUAU
ENSG00000112658	ACAUAGA
ENSG00000112658	ACAUUCA
ENSG00000112658	AGCUGCC
ENSG00000112658	AUAAAGU
ENSG00000112658	CCCUGAG
ENSG00000112658	CCUGUAC
ENSG00000112658	CUCAAAC
ENSG00000112658	CUCUUCC
ENSG00000112658	CUGCUCA
ENSG00000112658	CUGGCUC
ENSG00000112658	CUGGGCA
ENSG00000112658	CUUUGGU

ENSG00000112658	GACUUCC
ENSG00000112658	GAGGGGC
ENSG00000112658	GCAACUU
ENSG00000112658	GCAUCCC
ENSG00000112658	GGCACGG
ENSG00000112658	GGGGUGG
ENSG00000112658	UACAGUU
ENSG00000112658	UCCCUCU
ENSG00000112658	UGAAAGG
ENSG00000112658	UGCUCAC
ENSG00000112658	UGUCUUU
ENSG00000112679	UUGGCAA
ENSG00000112679	UUGGCAC
ENSG00000112685	CUGCAGC
ENSG00000112685	GAGAAAU
ENSG00000112695	UGAUCCA
ENSG00000112697	ACAUUAC
ENSG00000112697	ACUGGCC
ENSG00000112697	GUGUGGC
ENSG00000112699	CUACAAA
ENSG00000112701	CUAGUAA
ENSG00000112701	CUAGUAG
ENSG00000112701	CUGGAAA
ENSG00000112715	AAAAAUC
ENSG00000112715	AAAGUGC
ENSG00000112715	AACAUUC
ENSG00000112715	AAGUGCU
ENSG00000112715	AAUUAUU
ENSG00000112715	AGCAGCG
ENSG00000112715	AGCAGCU
ENSG00000112715	AGUGGUU
ENSG00000112715	CAAAGUG
ENSG00000112715	CCAGUGC
ENSG00000112715	CCAGUGU
ENSG00000112715	GAUCAGA
ENSG00000112715	GGAAUGU
ENSG00000112715	UAUUACC
ENSG00000112715	UCACACA
ENSG00000112715	UCAUUUG
ENSG00000112715	UGAAAUG
ENSG00000112727	AAAGAGC
ENSG00000112739	AAAGGUA
ENSG00000112739	AAGGCGC
ENSG00000112739	ACAAUAU
ENSG00000112739	ACCAGCA
ENSG00000112739	ACUGUUU
ENSG00000112739	AGCAGCU
ENSG00000112739	AGCUUAU
ENSG00000112739	AGGCAGG
ENSG00000112739	AUGCCUU
ENSG00000112739	CAAGAGC
ENSG00000112739	CACACCU
ENSG00000112739	CAGGCUC
ENSG00000112739	CUCAAAC
ENSG00000112739	CUUCACC
ENSG00000112739	GAAGGAA
ENSG00000112739	GAGCUGC
ENSG00000112739	GCAACUU
ENSG00000112739	GCACCAU
ENSG00000112739	GCUGUCU
ENSG00000112739	UACAAAG
ENSG00000112739	UCACAAG
ENSG00000112739	UCACAUU
ENSG00000112739	UGAAAGG

ENSG00000112759	AAGGCAC
ENSG00000112769	AUCCCUU
ENSG00000112769	CACUCCU
ENSG00000112769	CAGUGCA
ENSG00000112773	AGUGGUU
ENSG00000112773	CCCUGAG
ENSG00000112773	CUUUGGU
ENSG00000112773	GGCAGUG
ENSG00000112773	GUGCAAA
ENSG00000112773	UCACAUU
ENSG00000112782	AAACUGU
ENSG00000112782	AAAGGUA
ENSG00000112782	AACACUG
ENSG00000112782	AGGCAGG
ENSG00000112782	AUCCCUU
ENSG00000112782	CUCUUCC
ENSG00000112782	GAAAACA
ENSG00000112782	GAGAAAU
ENSG00000112782	GAGGCAG
ENSG00000112782	GCUUCCU
ENSG00000112782	GGAGGCA
ENSG00000112782	UGUUGAA
ENSG00000112782	UUGGCAA
ENSG00000112782	UUGGCAC
ENSG00000112796	AUAAAGU
ENSG00000112796	UGCAAAG
ENSG00000112837	ACAGUAC
ENSG00000112837	UACAGUU
ENSG00000112851	AGCCCUU
ENSG00000112851	AGUGGUU
ENSG00000112851	CUUUGGU
ENSG00000112851	UCCUAUA
ENSG00000112855	GUCUCUG
ENSG00000112874	AAAUUUC
ENSG00000112874	AGAUAAA
ENSG00000112874	AUAUUAU
ENSG00000112893	CACAGUG
ENSG00000112893	UAUUGCU
ENSG00000112893	UCAAGUA
ENSG00000112893	UCACAGU
ENSG00000112893	UGUGCUU
ENSG00000112902	AAUUAUU
ENSG00000112902	ACUGGCU
ENSG00000112902	ACUGUAG
ENSG00000112902	AGUGCCU
ENSG00000112902	GCAGAAG
ENSG00000112902	GGAGGCA
ENSG00000112902	GGCAGCG
ENSG00000112902	GGCUCAG
ENSG00000112902	GUCAACA
ENSG00000112902	UCCUGAG
ENSG00000112902	UGAGGAC
ENSG00000112902	UGGGACA
ENSG00000112902	UGUGCUU
ENSG00000112902	UUCCAUA
ENSG00000112902	UUGGCAA
ENSG00000112902	UUGGCAC
ENSG00000112964	AAAGUAC
ENSG00000112964	ACAUUAC
ENSG00000112964	AGCAGCG
ENSG00000112964	AGCAGCU
ENSG00000112964	AUGGCUU
ENSG00000112964	GAGGUAG
ENSG00000112964	UUCAAAU

ENSG00000112972	AGAUAAA
ENSG00000112972	AGCACCA
ENSG00000112972	AGCCCUU
ENSG00000112972	AUAAAGU
ENSG00000112972	AUGCACC
ENSG00000112972	GAGCCCU
ENSG00000112972	GAGGUAU
ENSG00000112972	GCCCUUA
ENSG00000112972	GUGCCAC
ENSG00000112972	UACAAAG
ENSG00000112972	UCACAGG
ENSG00000112972	UGCAAAG
ENSG00000112977	AAGGCAC
ENSG00000112977	GGAAUGU
ENSG00000112992	AAAGUUU
ENSG00000112992	GAGGUAG
ENSG00000113013	AAUACUG
ENSG00000113013	ACUGCAG
ENSG00000113013	ACUGCAU
ENSG00000113013	CAGGAAC
ENSG00000113070	AAAAACC
ENSG00000113070	AACAAAC
ENSG00000113070	AGCACCA
ENSG00000113070	AGUAACA
ENSG00000113070	AUAUAAC
ENSG00000113070	AUAUUAU
ENSG00000113070	CACAGUG
ENSG00000113070	UCACAGG
ENSG00000113070	UCCCUUG
ENSG00000113070	UGAAAUG
ENSG00000113070	UUGACAC
ENSG00000113073	ACUCCAG
ENSG00000113083	UCCAGUU
ENSG00000113088	AAUUUUA
ENSG00000113108	GAGGUAU
ENSG00000113108	GGAUUUU
ENSG00000113108	UAUAAAG
ENSG00000113119	AAAAAUC
ENSG00000113140	AACAAAC
ENSG00000113140	AGCAGCU
ENSG00000113140	CUCCCAC
ENSG00000113140	CUUGGAG
ENSG00000113140	GUUAAUG
ENSG00000113140	UCAUGAU
ENSG00000113140	UUCUAAU
ENSG00000113140	UUGGAGG
ENSG00000113161	ACCAGCA
ENSG00000113161	UCACAGG
ENSG00000113161	UCACAGU
ENSG00000113161	UGCAUUG
ENSG00000113196	AAAAACC
ENSG00000113196	AAGUGCA
ENSG00000113196	GAGGUAU
ENSG00000113211	ACAUUCA
ENSG00000113231	UCACAGG
ENSG00000113240	AAUGUUG
ENSG00000113240	UACAAAG
ENSG00000113240	UGACUGU
ENSG00000113262	GGAAGCC
ENSG00000113263	AAGUGCA
ENSG00000113263	UAAUGCU
ENSG00000113263	UGAAGCU
ENSG00000113273	CUGACUC
ENSG00000113273	GCACCAU

ENSG00000113273	GGCUCAG
ENSG00000113273	GUCUGCC
ENSG00000113273	UGAAAUG
ENSG00000113273	UGGAUAA
ENSG00000113273	UGUCUGC
ENSG00000113282	AAAAGUA
ENSG00000113282	UCAUGAU
ENSG00000113300	AAGUGCA
ENSG00000113300	AAGUGCU
ENSG00000113300	AGCACCA
ENSG00000113300	AUGAUGC
ENSG00000113300	CACAGUG
ENSG00000113300	CAGUGCA
ENSG00000113300	CCAGCAU
ENSG00000113300	GGCAGUG
ENSG00000113300	GGCUCAG
ENSG00000113300	UCAUGAU
ENSG00000113312	AGGGAGG
ENSG00000113327	AAAAGUA
ENSG00000113327	ACAAUAU
ENSG00000113327	UUCAAGC
ENSG00000113328	AAAGGUA
ENSG00000113328	CACAGUG
ENSG00000113328	UUGGUUC
ENSG00000113356	AAGUGCU
ENSG00000113356	ACUCAAA
ENSG00000113356	CUUUGGU
ENSG00000113356	GACCUGG
ENSG00000113356	GUAAACA
ENSG00000113356	UUCAAGC
ENSG00000113356	UUGGCAA
ENSG00000113356	UUGGCAC
ENSG00000113356	UUUCAAC
ENSG00000113360	AUUCAUU
ENSG00000113360	CACAGUG
ENSG00000113360	UCACAGU
ENSG00000113360	UCAUUCG
ENSG00000113361	ACUAGAU
ENSG00000113361	ACUGCAG
ENSG00000113361	GGAAGCC
ENSG00000113361	UUCUAAU
ENSG00000113368	GUGCUUG
ENSG00000113368	UGUGCUU
ENSG00000113369	AGCAUUC
ENSG00000113369	AGUGCAA
ENSG00000113369	CUACAGU
ENSG00000113369	CUUCACC
ENSG00000113369	GGCAAGA
ENSG00000113369	UUGGCAA
ENSG00000113369	UUGGCAC
ENSG00000113369	UUUUUGC
ENSG00000113384	CAGCAGA
ENSG00000113384	CUUUGGU
ENSG00000113384	GGAAUGU
ENSG00000113384	UCACAAG
ENSG00000113384	UUCAAGC
ENSG00000113387	AUAAAGU
ENSG00000113387	AUGGCUU
ENSG00000113387	CUUACAG
ENSG00000113387	GAAAACA
ENSG00000113387	UACAAAG
ENSG00000113389	UGAAAGG
ENSG00000113389	UGUCUUU
ENSG00000113391	CACAGUG

ENSG00000113391	CAGCAAA
ENSG00000113391	CAUAUUG
ENSG00000113391	GACCCUG
ENSG00000113391	GAUCAGA
ENSG00000113391	UAUUGCU
ENSG00000113391	UCAACAG
ENSG00000113391	UCACAGG
ENSG00000113391	UCACAGU
ENSG00000113391	UGGGUAC
ENSG00000113391	UUGACAC
ENSG00000113391	UUUGCAA
ENSG00000113430	AAGUGCU
ENSG00000113430	AGUGCUG
ENSG00000113430	AGUGUGC
ENSG00000113430	GAGUGUG
ENSG00000113430	GUAAACA
ENSG00000113430	GUGCAAA
ENSG00000113441	AGCACCA
ENSG00000113441	AGUGCAA
ENSG00000113441	CCCUGAG
ENSG00000113441	GGCCCUG
ENSG00000113441	GUGCAAA
ENSG00000113441	UGCAAAG
ENSG00000113441	UUUGCAC
ENSG00000113448	AAAGCUG
ENSG00000113448	AAAGUUU
ENSG00000113448	AACAUUC
ENSG00000113448	AAGGCAC
ENSG00000113448	AAUGCCC
ENSG00000113448	ACACACC
ENSG00000113448	ACACACU
ENSG00000113448	ACAGGUG
ENSG00000113448	ACAUUAC
ENSG00000113448	ACUGUUU
ENSG00000113448	AGGCAGG
ENSG00000113448	AUAUUAU
ENSG00000113448	AUGCAUU
ENSG00000113448	CAGGAAC
ENSG00000113448	CCAGUGC
ENSG00000113448	CUACAGU
ENSG00000113448	CUGCAGC
ENSG00000113448	CUGGGCA
ENSG00000113448	GAAGGUC
ENSG00000113448	GAGAAAU
ENSG00000113448	GAGCGCC
ENSG00000113448	GAUUGUA
ENSG00000113448	GAUUGUC
ENSG00000113448	GCAGCAU
ENSG00000113448	GGCAAGA
ENSG00000113448	GGUUACC
ENSG00000113448	GUAACAG
ENSG00000113448	GUCAGUU
ENSG00000113448	GUGACAG
ENSG00000113448	GUGAGGU
ENSG00000113448	GUGUCAC
ENSG00000113448	UACAGUU
ENSG00000113448	UAUUGCU
ENSG00000113448	UCAUACA
ENSG00000113448	UCAUUCG
ENSG00000113448	UCUCACC
ENSG00000113448	UGCAGCU
ENSG00000113448	UGCAUAG
ENSG00000113448	UGCAUUG
ENSG00000113448	UUGGCAA

ENSG00000113448	UUGUUCG
ENSG00000113448	UUUGCAC
ENSG00000113456	CACAAUG
ENSG00000113520	AUAUAAC
ENSG00000113520	UAUAAAG
ENSG00000113522	CAGCUGG
ENSG00000113532	AAAUUUC
ENSG00000113532	AAGGCAC
ENSG00000113532	AAGUGCA
ENSG00000113532	AAUGCCC
ENSG00000113532	ACUCAAA
ENSG00000113532	AGCCCUU
ENSG00000113532	CCCUGAG
ENSG00000113532	CUUGUAU
ENSG00000113532	CUUUGGU
ENSG00000113532	GAGAACU
ENSG00000113532	GAGAUGA
ENSG00000113532	GAGUUGA
ENSG00000113532	GAGUUGG
ENSG00000113532	GCAGCAU
ENSG00000113532	GCAGGUU
ENSG00000113532	GUGCAAA
ENSG00000113532	GUGCUUG
ENSG00000113532	GUGGCCC
ENSG00000113532	UCAAGUA
ENSG00000113532	UCACACA
ENSG00000113532	UGAAAUG
ENSG00000113532	UGCUGAC
ENSG00000113552	AAUACUG
ENSG00000113552	CACAGGG
ENSG00000113552	GGAGUGU
ENSG00000113552	UAAUAGU
ENSG00000113558	AAGUGUG
ENSG00000113558	AAGUUGU
ENSG00000113558	AAUCCUU
ENSG00000113558	ACAAUAU
ENSG00000113558	AGUGCAA
ENSG00000113558	CAGUGCA
ENSG00000113558	CUCAAAC
ENSG00000113558	CUUACAG
ENSG00000113558	GCAACUU
ENSG00000113558	GGAAGAC
ENSG00000113558	GUUAAUG
ENSG00000113558	UAAUACA
ENSG00000113558	UACAGUU
ENSG00000113558	UCAUGAU
ENSG00000113575	AAAAGUA
ENSG00000113575	AAAGUAA
ENSG00000113575	AAAGUGC
ENSG00000113575	AACUACU
ENSG00000113575	AAUACUG
ENSG00000113575	AAUGUUG
ENSG00000113575	ACUGUAG
ENSG00000113575	AGCAAAA
ENSG00000113575	CCCUGAG
ENSG00000113575	CUCUUCC
ENSG00000113575	CUGUUGA
ENSG00000113575	GUAUUCU
ENSG00000113575	UCAUUCG
ENSG00000113578	AAACUGU
ENSG00000113578	GAGAUGA
ENSG00000113578	GCUAGCA
ENSG00000113578	GGAGUGU
ENSG00000113578	UCACAGU

ENSG00000113578	UCUGGAG
ENSG00000113578	UGAGGAC
ENSG00000113580	AAAGCUG
ENSG00000113580	AAGGUGC
ENSG00000113580	ACCCAUU
ENSG00000113580	AGCCCUU
ENSG00000113580	AGCUGCC
ENSG00000113580	AUGGCAC
ENSG00000113580	GGCCCUG
ENSG00000113580	GUAAACA
ENSG00000113580	GUAGUGU
ENSG00000113580	UCCCUUG
ENSG00000113583	AGCACCA
ENSG00000113583	CACAGUG
ENSG00000113583	UCACAGU
ENSG00000113583	UGUGCUU
ENSG00000113594	ACAGUAC
ENSG00000113594	ACCCAUU
ENSG00000113594	AGUAACA
ENSG00000113594	AGUAGUU
ENSG00000113594	AGUGGUU
ENSG00000113594	AUAUUAU
ENSG00000113594	CACAGUG
ENSG00000113594	CCCUGAG
ENSG00000113594	CUCCAUU
ENSG00000113594	CUUUGGU
ENSG00000113594	GAGAUGA
ENSG00000113594	GGAAUGU
ENSG00000113594	GGCUCUG
ENSG00000113594	UAGGGCC
ENSG00000113594	UAUUGCU
ENSG00000113594	UCAAGUA
ENSG00000113594	UGAAGUG
ENSG00000113594	UGUGCUU
ENSG00000113595	AUGCACC
ENSG00000113595	CACAGUG
ENSG00000113595	GUAACAG
ENSG00000113595	UCACAGG
ENSG00000113597	AUGCAUU
ENSG00000113600	GGCAUUG
ENSG00000113615	AACACUG
ENSG00000113615	UCACAGU
ENSG00000113621	AAAAACC
ENSG00000113621	CUGGAAA
ENSG00000113638	UCACAAG
ENSG00000113645	GCUGGUG
ENSG00000113648	AAGACAU
ENSG00000113648	AGCACCA
ENSG00000113648	CAGUGCA
ENSG00000113649	GGCUCAG
ENSG00000113649	UUCAAAU
ENSG00000113657	AAAGCUG
ENSG00000113657	ACUCAAA
ENSG00000113657	CACCACC
ENSG00000113657	CCCACCG
ENSG00000113657	CCCACCU
ENSG00000113657	CCUUCUG
ENSG00000113657	CUCCCAC
ENSG00000113657	CUUCUCU
ENSG00000113657	GAGCUGC
ENSG00000113657	GAGGCUG
ENSG00000113657	GAGGUAG
ENSG00000113657	GAGGUAU
ENSG00000113657	UCCCACC

ENSG00000113657	UGCUCAC
ENSG00000113657	UGGCUGC
ENSG00000113657	UUGAGGC
ENSG00000113658	AAAGUGC
ENSG00000113658	AAAGUUU
ENSG00000113658	AAGUGCA
ENSG00000113658	ACAGUAU
ENSG00000113658	CACAAUG
ENSG00000113658	CACAGUG
ENSG00000113658	CAGCAAA
ENSG00000113658	CAGGUGA
ENSG00000113658	GAGUGUG
ENSG00000113658	GUGCAAA
ENSG00000113658	UACAAAG
ENSG00000113658	UUCUGCA
ENSG00000113712	AAAAACC
ENSG00000113712	AAAUCCC
ENSG00000113712	AUGCACC
ENSG00000113712	CACAAUG
ENSG00000113712	CAGCUGG
ENSG00000113712	CAGUCCA
ENSG00000113712	CUUACAG
ENSG00000113712	GUUAAUG
ENSG00000113712	GUUCAUG
ENSG00000113712	UACAGUU
ENSG00000113712	UCACAAG
ENSG00000113712	UGUGUCA
ENSG00000113712	UUGGUUC
ENSG00000113721	AAAAAUC
ENSG00000113721	AGCACCA
ENSG00000113721	CAGCUGG
ENSG00000113721	GAGGCAG
ENSG00000113721	GGCAGGG
ENSG00000113721	GUAAACA
ENSG00000113721	UAAUAUC
ENSG00000113721	UCCCUUG
ENSG00000113721	UGUAUAA
ENSG00000113722	CUGGGGG
ENSG00000113722	GAGGCUG
ENSG00000113722	GGAGGAG
ENSG00000113734	CUCUUCC
ENSG00000113734	CUUCUCU
ENSG00000113739	AGAUAAA
ENSG00000113739	AGUGGUU
ENSG00000113739	CAGACAG
ENSG00000113739	CCCCCAG
ENSG00000113739	GGACGGA
ENSG00000113739	GGAGGAG
ENSG00000113739	UACAAAG
ENSG00000113739	UGACAUC
ENSG00000113739	UGGAUAA
ENSG00000113739	UUGGCAC
ENSG00000113742	AAAGAAU
ENSG00000113742	AAAGAGC
ENSG00000113742	AAAGUAC
ENSG00000113742	AACACUG
ENSG00000113742	AAGUCUU
ENSG00000113742	AAGUGCA
ENSG00000113742	AAGUGUG
ENSG00000113742	ACCAGCA
ENSG00000113742	AGCAGCU
ENSG00000113742	AGCAUUC
ENSG00000113742	AGGAUGU
ENSG00000113742	AGUAGAC

ENSG00000113742	AGUGCAA
ENSG00000113742	AGUGUGC
ENSG00000113742	AUAAUAC
ENSG00000113742	AUACAAG
ENSG00000113742	AUCCUUG
ENSG00000113742	AUGUGCC
ENSG00000113742	CAAAGCA
ENSG00000113742	CACACCU
ENSG00000113742	CACAGUG
ENSG00000113742	CACCAGC
ENSG00000113742	CACUGUU
ENSG00000113742	CAGCAGA
ENSG00000113742	CAGUGCA
ENSG00000113742	CCAGUGC
ENSG00000113742	CCCGUCC
ENSG00000113742	CCUUCUU
ENSG00000113742	CUCACAC
ENSG00000113742	CUGCAGC
ENSG00000113742	CUGCAGG
ENSG00000113742	CUUACAG
ENSG00000113742	CUUCUCU
ENSG00000113742	GAGGUAG
ENSG00000113742	GUGCAAA
ENSG00000113742	GUGCCUG
ENSG00000113742	UACAAAG
ENSG00000113742	UAUAAAG
ENSG00000113742	UCAAGUA
ENSG00000113742	UCACACA
ENSG00000113742	UCACAGU
ENSG00000113742	UCAUUCG
ENSG00000113742	UGAAAUG
ENSG00000113742	UGCAUAG
ENSG00000113742	UGCAUAU
ENSG00000113742	UGCCCUG
ENSG00000113742	UGUAUAA
ENSG00000113742	UUCUGCA
ENSG00000113742	UUCUUGU
ENSG00000113742	UUGGCAC
ENSG00000113758	AAAAGUA
ENSG00000113758	AAAGCUG
ENSG00000113758	CAAAGUG
ENSG00000113758	CGUGCAU
ENSG00000113758	GCCCUGU
ENSG00000113758	GGACCUG
ENSG00000113758	GGAUUUU
ENSG00000113758	GGCCCUG
ENSG00000113758	GGGGUCC
ENSG00000113758	GUGCAAA
ENSG00000113758	GUGCUUG
ENSG00000113758	UACAAAG
ENSG00000113758	UAUUGCU
ENSG00000113758	UCCCACC
ENSG00000113761	ACCACAG
ENSG00000113761	CCAGGAG
ENSG00000113761	GAGGCAG
ENSG00000113761	GCACCAU
ENSG00000113763	AAAGCUG
ENSG00000113763	AUAUAAC
ENSG00000113763	GAGAUGA
ENSG00000113763	GAGGUAG
ENSG00000113763	GCUGGUG
ENSG00000113763	GUGCCUG
ENSG00000113763	UGCAUAG
ENSG00000113763	UGCAUAU

ENSG00000113805	AAGGCAC
ENSG00000113805	ACACACC
ENSG00000113805	ACACACU
ENSG00000113805	AUUCAUU
ENSG00000113805	CAUGGAU
ENSG00000113805	UAUAAAG
ENSG00000113805	UUGGUUC
ENSG00000113812	AUUCAUU
ENSG00000113838	UAAUAUC
ENSG00000113851	UACAAAG
ENSG00000113916	AAGGCAC
ENSG00000113916	ACCCUGU
ENSG00000113916	CUUCACC
ENSG00000113916	CUUUGGU
ENSG00000113916	GUAAACA
ENSG00000113916	UUGGUUC
ENSG00000113971	AUUGCAC
ENSG00000113971	UAUAAAG
ENSG00000114013	AGAUAAA
ENSG00000114013	GAGGUAG
ENSG00000114013	GAGUAUU
ENSG00000114019	AAAAACC
ENSG00000114019	AACAAAC
ENSG00000114019	AAUACUG
ENSG00000114019	ACUGUUU
ENSG00000114019	AGGAGCU
ENSG00000114019	AUAUUAU
ENSG00000114019	CAAAUGC
ENSG00000114019	CACAAUG
ENSG00000114019	CAGCAAA
ENSG00000114019	CCAGCAU
ENSG00000114019	CUGGCUC
ENSG00000114019	CUUUGGU
ENSG00000114019	GGAAUGU
ENSG00000114019	GUCCAGA
ENSG00000114019	UCACAAG
ENSG00000114019	UCACAGG
ENSG00000114019	UCACAGU
ENSG00000114019	UCCAGUU
ENSG00000114019	UUGGCAA
ENSG00000114019	UUGGCAC
ENSG00000114030	AAAGUAC
ENSG00000114030	AAAGUUU
ENSG00000114030	AAGUCUU
ENSG00000114030	AAGUGCA
ENSG00000114030	AAGUGCU
ENSG00000114030	ACAGGUG
ENSG00000114030	ACAGUAC
ENSG00000114030	ACUAGAU
ENSG00000114030	AGAUGUG
ENSG00000114030	AGCAAAA
ENSG00000114030	AGCAGCA
ENSG00000114030	AGCAUUC
ENSG00000114030	AGCCUGC
ENSG00000114030	AGUAACA
ENSG00000114030	AGUACUG
ENSG00000114030	AGUAGUU
ENSG00000114030	AGUGCUU
ENSG00000114030	AUGAUGA
ENSG00000114030	CAAAGCA
ENSG00000114030	CAGCAGG
ENSG00000114030	CAGUGCA
ENSG00000114030	CCUUCUG
ENSG00000114030	CUUCUCU

ENSG00000114030	GAAGGUC
ENSG00000114030	GAGCGCC
ENSG00000114030	GAGGUAG
ENSG00000114030	GAGGUAU
ENSG00000114030	GCAGAAG
ENSG00000114030	GCAGCAU
ENSG00000114030	GCUACAU
ENSG00000114030	GGAGUCC
ENSG00000114030	GGCUGCG
ENSG00000114030	GGUUGAC
ENSG00000114030	GGUUUAC
ENSG00000114030	GUAACAG
ENSG00000114030	GUGCGCA
ENSG00000114030	GUGCUUG
ENSG00000114030	UCACAUU
ENSG00000114030	UCCAGAG
ENSG00000114030	UCCCUUG
ENSG00000114030	UCUCCAA
ENSG00000114030	UGAGAAU
ENSG00000114030	UGUCUUU
ENSG00000114030	UGUGCUU
ENSG00000114030	UUGGCAA
ENSG00000114062	AAAAAUC
ENSG00000114062	AACAUUC
ENSG00000114062	AAGUGCA
ENSG00000114062	AAGUGCU
ENSG00000114062	ACUGUUU
ENSG00000114062	AGGGAGG
ENSG00000114062	CACUGUU
ENSG00000114062	CUACAAA
ENSG00000114062	CUUUGGU
ENSG00000114062	GAUUGUC
ENSG00000114062	GGAGGCA
ENSG00000114062	GUUCAUG
ENSG00000114062	UCAAGUA
ENSG00000114062	UGUGCUU
ENSG00000114062	UUGUUCG
ENSG00000114098	AAAAACC
ENSG00000114098	AAAAGUA
ENSG00000114098	ACAAUAU
ENSG00000114098	AGUGCUG
ENSG00000114098	AGUGCUU
ENSG00000114098	AUUCAUU
ENSG00000114098	CACCACC
ENSG00000114098	CCAGCAU
ENSG00000114098	CUACAGU
ENSG00000114098	CUUCACC
ENSG00000114098	GUGCAAA
ENSG00000114098	UCACAGG
ENSG00000114098	UCACCAC
ENSG00000114098	UCAUUCG
ENSG00000114107	AAUCCUU
ENSG00000114107	UGAAAGG
ENSG00000114120	AAAGUGC
ENSG00000114120	AUGGCAC
ENSG00000114120	AUUGCAC
ENSG00000114120	CAAAGUG
ENSG00000114120	UCCAGUU
ENSG00000114120	UGUGCUU
ENSG00000114120	UUGGUCC
ENSG00000114125	AAUUUUA
ENSG00000114125	GAGGUAG
ENSG00000114127	AAAGUAU
ENSG00000114127	AAAGUUU

ENSG00000114127	AAGGCAC
ENSG00000114127	AGCAAAA
ENSG00000114127	AGCACCA
ENSG00000114127	AUUGCAC
ENSG00000114127	CAAAACU
ENSG00000114127	CCUUCAU
ENSG00000114127	CUUUGGU
ENSG00000114127	GCUGGUG
ENSG00000114127	UCCCUUG
ENSG00000114127	UGCAAAG
ENSG00000114127	UGGUACC
ENSG00000114127	UGUAUAA
ENSG00000114127	UUUCAAC
ENSG00000114127	UUUGCAC
ENSG00000114166	UAUUGCU
ENSG00000114166	UUUGCAA
ENSG00000114209	AAAGAAU
ENSG00000114209	AAGGCGC
ENSG00000114209	GUAAACA
ENSG00000114209	UACAAAG
ENSG00000114209	UAUAAUA
ENSG00000114209	UCAAGUA
ENSG00000114209	UGCAGCU
ENSG00000114209	UUCAAGC
ENSG00000114251	AAAGAAU
ENSG00000114251	AACACUG
ENSG00000114251	AAUGCCC
ENSG00000114251	AAUUUUA
ENSG00000114251	AGAUAAA
ENSG00000114251	AGAUGUG
ENSG00000114251	AGGUUAU
ENSG00000114251	AGUGCUG
ENSG00000114251	AGUGCUU
ENSG00000114251	AUAAUAC
ENSG00000114251	AUGCAUU
ENSG00000114251	CAGCAAA
ENSG00000114251	UAAUAUC
ENSG00000114251	UCAAGUA
ENSG00000114251	UGAAGCU
ENSG00000114251	UGUAUAA
ENSG00000114251	UUUGCAC
ENSG00000114268	AAAGAAU
ENSG00000114268	AUUGCAC
ENSG00000114268	CAGCAGG
ENSG00000114268	GGUGGGC
ENSG00000114268	UAUUGCU
ENSG00000114268	UCACAUU
ENSG00000114270	AAUGCCC
ENSG00000114270	AGCAGCU
ENSG00000114270	CACAAUG
ENSG00000114270	CUCUAGC
ENSG00000114270	GCACCAU
ENSG00000114279	CAGUAGA
ENSG00000114279	CUUUGGU
ENSG00000114279	GGAUUUC
ENSG00000114279	GGAUUUU
ENSG00000114302	AAUGCCC
ENSG00000114302	UGGACUG
ENSG00000114302	UGGAGAU
ENSG00000114315	AAGGCGC
ENSG00000114315	AGUGCAA
ENSG00000114315	CUUUGGU
ENSG00000114315	UACAAAG
ENSG00000114315	UUGGCAA

ENSG00000114315	UUGGCAC
ENSG00000114316	UCACCAC
ENSG00000114346	AAGUCAC
ENSG00000114346	AAGUGCU
ENSG00000114349	AGGGAGG
ENSG00000114349	CAGGUGA
ENSG00000114349	GUCAACA
ENSG00000114349	UAUUGCU
ENSG00000114349	UGGCCAC
ENSG00000114353	AAAGUUU
ENSG00000114353	AAGGCAC
ENSG00000114353	ACACACU
ENSG00000114353	AGCCCUU
ENSG00000114353	AGGUUAU
ENSG00000114353	CAGCAGG
ENSG00000114353	CAGGUGA
ENSG00000114353	CCCUGUC
ENSG00000114353	CCCUGUU
ENSG00000114353	CUGCCCC
ENSG00000114353	CUGGAAA
ENSG00000114353	GAGGGGC
ENSG00000114353	GCCUGGG
ENSG00000114353	GCUACAU
ENSG00000114353	GCUGGUG
ENSG00000114353	GGCACGG
ENSG00000114353	GGCAGCG
ENSG00000114353	GGCAGGG
ENSG00000114353	GGCAGUG
ENSG00000114353	GUGGCCC
ENSG00000114353	UCAACGG
ENSG00000114353	UCAUUUG
ENSG00000114353	UCCCUGU
ENSG00000114353	UCUGGAA
ENSG00000114353	UGUCUGC
ENSG00000114353	UGUGCUU
ENSG00000114353	UUCAAAU
ENSG00000114353	UUCCUAG
ENSG00000114353	UUCUUGU
ENSG00000114354	GUAGUGU
ENSG00000114374	ACAUUCA
ENSG00000114374	AGCCAGU
ENSG00000114374	GUGAAUG
ENSG00000114374	UACAAAG
ENSG00000114374	UGGACUG
ENSG00000114374	UUGGUUC
ENSG00000114374	UUGUGAC
ENSG00000114383	AACGGCU
ENSG00000114383	AAGUGCU
ENSG00000114383	AGCAAAA
ENSG00000114383	CACCAGC
ENSG00000114383	GAGGUAG
ENSG00000114383	GUGGCCC
ENSG00000114383	UACAAAG
ENSG00000114383	UGAAAUG
ENSG00000114395	GAGGCAG
ENSG00000114416	AAAAAUC
ENSG00000114416	AAAGCUG
ENSG00000114416	AAAGUGC
ENSG00000114416	AAAGUUU
ENSG00000114416	AACAAAC
ENSG00000114416	AACAGUC
ENSG00000114416	ACUGCAU
ENSG00000114416	ACUGUAG
ENSG00000114416	AGCAGCU

ENSG00000114416	AGCCAGU
ENSG00000114416	AGUAGUU
ENSG00000114416	AGUGGUU
ENSG00000114416	AUGUAAU
ENSG00000114416	CAGUAGA
ENSG00000114416	CAUAUUG
ENSG00000114416	CCAGUGC
ENSG00000114416	CCAGUGU
ENSG00000114416	CUGCAGC
ENSG00000114416	CUGCAGG
ENSG00000114416	CUUUGGU
ENSG00000114416	GGAAGAC
ENSG00000114416	GGAGGAG
ENSG00000114416	GGCAAGA
ENSG00000114416	GUAAACA
ENSG00000114416	GUAACAG
ENSG00000114416	GUGCAAA
ENSG00000114416	UAAUGCU
ENSG00000114416	UCCAGUU
ENSG00000114416	UCUGGAG
ENSG00000114416	UGCAUAG
ENSG00000114416	UUCCUAG
ENSG00000114416	UUCCUCG
ENSG00000114416	UUGGCAC
ENSG00000114423	AAUUAUU
ENSG00000114423	GCUAGCA
ENSG00000114423	UCACAGU
ENSG00000114439	CACAAUG
ENSG00000114450	AACACUG
ENSG00000114450	AGCACCA
ENSG00000114450	AUGCCUU
ENSG00000114450	CAAAACU
ENSG00000114450	CUCCAGC
ENSG00000114450	GAAAACA
ENSG00000114450	GCACCAU
ENSG00000114450	UCACAGG
ENSG00000114450	UCACAGU
ENSG00000114450	UUGGUCC
ENSG00000114455	AAUCCUU
ENSG00000114503	UUUGCAC
ENSG00000114520	AAGGCAC
ENSG00000114520	ACCCUGU
ENSG00000114520	AGCACCA
ENSG00000114520	GCUAGCA
ENSG00000114520	GUGCUUG
ENSG00000114520	UCACAGG
ENSG00000114529	GAGGUAG
ENSG00000114541	AAAGAAU
ENSG00000114541	ACAAUAU
ENSG00000114541	CUAUACA
ENSG00000114541	GAAUUGU
ENSG00000114554	AAAAAUC
ENSG00000114554	AAAGUGC
ENSG00000114554	AAGUGCU
ENSG00000114554	AAUCUCA
ENSG00000114554	ACAGUAC
ENSG00000114554	AGCAAAA
ENSG00000114554	AGUGCUU
ENSG00000114554	CAGUUAC
ENSG00000114554	CCCUGAG
ENSG00000114554	CUAGUAA
ENSG00000114554	CUAGUAG
ENSG00000114554	CUCUGGG
ENSG00000114554	GAGGUAU

ENSG00000114554	GCUAGCA
ENSG00000114554	GGAAGAC
ENSG00000114554	GGAAGCC
ENSG00000114554	GUAAACA
ENSG00000114554	GUGCUUG
ENSG00000114554	UCAACAG
ENSG00000114554	UCCCACA
ENSG00000114554	UCCCACC
ENSG00000114554	UGAGGAC
ENSG00000114573	AAAGCUG
ENSG00000114573	AAAUCCC
ENSG00000114573	AACACUG
ENSG00000114573	AAUCUCA
ENSG00000114573	ACAGUAC
ENSG00000114573	ACAGUAU
ENSG00000114573	ACUGCAU
ENSG00000114573	AGCACCA
ENSG00000114573	AUUCAUU
ENSG00000114573	CACAGUG
ENSG00000114573	CACCACC
ENSG00000114573	CUCAAAC
ENSG00000114573	CUGGAAA
ENSG00000114573	GGAAGCC
ENSG00000114573	GGAGGCA
ENSG00000114573	UACAGUU
ENSG00000114573	UCAUACA
ENSG00000114626	CCCUGAG
ENSG00000114638	AGCACCA
ENSG00000114638	CCAGCAU
ENSG00000114646	AAAGUAC
ENSG00000114648	AAGUGCU
ENSG00000114648	ACCUGGA
ENSG00000114648	ACUGCAG
ENSG00000114648	ACUGGAU
ENSG00000114648	AGGCAGG
ENSG00000114648	AGUGCUU
ENSG00000114648	AUUGCAC
ENSG00000114648	CACAGUG
ENSG00000114648	CAGUGCA
ENSG00000114648	CCAGUGC
ENSG00000114648	CUGCAGC
ENSG00000114648	CUGCUCA
ENSG00000114648	CUGGAAA
ENSG00000114648	GCAGCAU
ENSG00000114648	GCUGGUG
ENSG00000114648	GGAGGAG
ENSG00000114648	GGCAGCG
ENSG00000114648	GUCUGCC
ENSG00000114648	UAGAGGA
ENSG00000114648	UUGGCAA
ENSG00000114648	UUGGCAC
ENSG00000114648	UUGGUUC
ENSG00000114654	GAGGCUG
ENSG00000114656	UCCUGAG
ENSG00000114686	AAUUUUA
ENSG00000114698	GGCCCUG
ENSG00000114698	GUGGCCC
ENSG00000114735	CAGGUGA
ENSG00000114735	GGGGAGC
ENSG00000114735	GUCACUC
ENSG00000114737	AAAAAUC
ENSG00000114737	CAGCUGG
ENSG00000114737	GGAAGAC
ENSG00000114737	GGCUCUG

ENSG00000114738	ACUCAGG
ENSG00000114738	UCACAGU
ENSG00000114739	AAAGCUG
ENSG00000114739	AAAGUAC
ENSG00000114739	AACAAAC
ENSG00000114739	AACGCCU
ENSG00000114739	AACUCUA
ENSG00000114739	AAGACAU
ENSG00000114739	AAGGCAC
ENSG00000114739	AAUACUG
ENSG00000114739	AAUGCCC
ENSG00000114739	AAUGUUG
ENSG00000114739	ACAAGGU
ENSG00000114739	ACAUUAC
ENSG00000114739	AGCAAAA
ENSG00000114739	AGCAGCG
ENSG00000114739	AGGUUAU
ENSG00000114739	AGUACUG
ENSG00000114739	AUGGCGC
ENSG00000114739	AUGUAAU
ENSG00000114739	AUGUCUG
ENSG00000114739	CACAGGG
ENSG00000114739	CACUGUU
ENSG00000114739	CAGACAG
ENSG00000114739	CAGUCCA
ENSG00000114739	CAGUGCA
ENSG00000114739	CCCCCAG
ENSG00000114739	CCUGCUG
ENSG00000114739	CUCACAC
ENSG00000114739	CUUACAG
ENSG00000114739	CUUCACC
ENSG00000114739	GAGAAAU
ENSG00000114739	GAGGUAG
ENSG00000114739	GAGGUAU
ENSG00000114739	GAGUCUU
ENSG00000114739	GCAACUU
ENSG00000114739	GCAGAAG
ENSG00000114739	GCAUCCC
ENSG00000114739	GGAAGCC
ENSG00000114739	GGAAUGU
ENSG00000114739	GGGGUGC
ENSG00000114739	GGGGUGG
ENSG00000114739	GUUAAUG
ENSG00000114739	UAAGACU
ENSG00000114739	UAAUGCU
ENSG00000114739	UAGGCCG
ENSG00000114739	UCAAGUA
ENSG00000114739	UCACACA
ENSG00000114739	UCACAGG
ENSG00000114739	UCACAGU
ENSG00000114739	UCAUUUG
ENSG00000114739	UCUAGAG
ENSG00000114739	UGAAAGG
ENSG00000114739	UGAAAUG
ENSG00000114739	UGAGAAG
ENSG00000114739	UGAGUCU
ENSG00000114739	UGAUCCA
ENSG00000114739	UGCAGCU
ENSG00000114739	UGCUGAC
ENSG00000114739	UGUCUGC
ENSG00000114739	UGUUGAA
ENSG00000114739	UUAGAGA
ENSG00000114739	UUCCUAG
ENSG00000114739	UUCCUCG

ENSG00000114739	UUCUAAU
ENSG00000114739	UUCUGCA
ENSG00000114739	UUGAGGC
ENSG00000114739	UUGGCAC
ENSG00000114739	UUUGCAC
ENSG00000114742	AAGGCAC
ENSG00000114742	AUAUUAU
ENSG00000114742	GCUUCCU
ENSG00000114742	GUGCUUG
ENSG00000114742	UGCAUAU
ENSG00000114742	UGUGCUU
ENSG00000114744	AUUCAUU
ENSG00000114744	UCAUUUG
ENSG00000114745	CACAGUG
ENSG00000114745	CCAGUAC
ENSG00000114745	UCACAGG
ENSG00000114745	UGUAUAA
ENSG00000114757	AAGUGCA
ENSG00000114757	AGCAAAA
ENSG00000114757	AUGCACC
ENSG00000114757	GUGCAAA
ENSG00000114757	GUGCUUG
ENSG00000114757	UGCAUAU
ENSG00000114757	UUGGUCC
ENSG00000114770	AAACUGU
ENSG00000114770	AAAGCUG
ENSG00000114770	ACAAUAU
ENSG00000114770	ACAGUAU
ENSG00000114770	AUAAAGU
ENSG00000114770	CACAGUG
ENSG00000114770	CCAGUGU
ENSG00000114770	CCCACCU
ENSG00000114770	CCCUGAG
ENSG00000114770	GAGCUGC
ENSG00000114770	GAGGCAG
ENSG00000114770	GAGGUAG
ENSG00000114770	GAGGUAU
ENSG00000114770	GGCAGGG
ENSG00000114770	UAAUAUC
ENSG00000114770	UACAGUU
ENSG00000114770	UAUAAAG
ENSG00000114779	AGGCAGG
ENSG00000114784	UUCCAUA
ENSG00000114796	CACUCCU
ENSG00000114796	CCCUGAG
ENSG00000114796	CUGGCUC
ENSG00000114796	CUUUGGU
ENSG00000114796	GAGGUAG
ENSG00000114796	GAGGUAU
ENSG00000114796	GAGUAUU
ENSG00000114796	GCUACAU
ENSG00000114796	GGCUCAG
ENSG00000114796	GUGAGGU
ENSG00000114796	UCCUGAG
ENSG00000114796	UCUGGAA
ENSG00000114805	GUAACAG
ENSG00000114805	UUCUUGU
ENSG00000114805	UUGGCAA
ENSG00000114850	AAGUUGU
ENSG00000114850	ACCACAG
ENSG00000114850	ACUGUGU
ENSG00000114850	GAGAACC
ENSG00000114850	GGCAGGG
ENSG00000114850	GGCUGCG

ENSG00000114850	UCAACAG
ENSG00000114850	UGGCUGC
ENSG00000114853	AAAGUGC
ENSG00000114853	AACAAAC
ENSG00000114853	AAGGUGC
ENSG00000114853	AAGUGCU
ENSG00000114853	ACCUGGA
ENSG00000114853	AGCACCA
ENSG00000114853	AGCUUAU
ENSG00000114853	AGGCAGG
ENSG00000114853	AGUGCUU
ENSG00000114853	AUAAAGU
ENSG00000114853	CCCUGAG
ENSG00000114853	CUCUUCC
ENSG00000114853	CUGCCCC
ENSG00000114853	GACAACU
ENSG00000114853	GCCUGGA
ENSG00000114853	GCUGGUG
ENSG00000114853	GGAAGCC
ENSG00000114853	GGGGUCC
ENSG00000114853	GUGCAAA
ENSG00000114853	UAUUGCU
ENSG00000114853	UGAGGAC
ENSG00000114853	UGCAAAG
ENSG00000114853	UGUCUGG
ENSG00000114857	AAGGUGC
ENSG00000114857	ACUGUAG
ENSG00000114857	AGCACCA
ENSG00000114857	AUACAAG
ENSG00000114857	CAAAGCA
ENSG00000114857	CACAGUG
ENSG00000114857	CUACAAA
ENSG00000114857	GCAGCAU
ENSG00000114857	GUAAACA
ENSG00000114857	UCUUGAG
ENSG00000114857	UUCAAGC
ENSG00000114857	UUCUAAU
ENSG00000114857	UUCUUGU
ENSG00000114861	AAAUCCC
ENSG00000114861	AAAUGGU
ENSG00000114861	AAGACAU
ENSG00000114861	AAGUGCA
ENSG00000114861	AAGUGCU
ENSG00000114861	AAUACUG
ENSG00000114861	ACAGUAC
ENSG00000114861	ACAGUAU
ENSG00000114861	ACAUUAC
ENSG00000114861	ACCAGCA
ENSG00000114861	ACUGUGU
ENSG00000114861	AGAUAAA
ENSG00000114861	AGCUGCC
ENSG00000114861	AGUACUG
ENSG00000114861	AGUGCAA
ENSG00000114861	AUAAAGU
ENSG00000114861	AUAAUAC
ENSG00000114861	AUCACUA
ENSG00000114861	AUGGCAC
ENSG00000114861	AUUCAUU
ENSG00000114861	CUACAAA
ENSG00000114861	CUACAGU
ENSG00000114861	CUCCCAA
ENSG00000114861	CUCCCAC
ENSG00000114861	GAGGAUA
ENSG00000114861	GAGGUAG

ENSG00000114861	GAGGUAU
ENSG00000114861	GCAACUU
ENSG00000114861	UACAAAG
ENSG00000114861	UAUAAAG
ENSG00000114861	UCAACAG
ENSG00000114861	UCACACA
ENSG00000114861	UCAUUCG
ENSG00000114861	UGAAAUG
ENSG00000114861	UGGGAUC
ENSG00000114861	UUGAUCA
ENSG00000114861	UUGGUCC
ENSG00000114867	AAAUUUC
ENSG00000114867	CCCCCAG
ENSG00000114904	CUGGACU
ENSG00000114904	GGCAGCG
ENSG00000114904	GUAAACA
ENSG00000114923	CAAAGCA
ENSG00000114923	GAGGCAG
ENSG00000114948	AUUGCAC
ENSG00000114982	CUCCCAA
ENSG00000114982	GCAGGAC
ENSG00000114999	AGCAGCA
ENSG00000114999	AGGUAGU
ENSG00000114999	CCCCCAG
ENSG00000114999	GAGAACC
ENSG00000114999	GAGCUAA
ENSG00000114999	GAGGUAG
ENSG00000114999	GAGGUAU
ENSG00000114999	GGGGCAG
ENSG00000114999	UCUCAAG
ENSG00000115008	AUUCAUU
ENSG00000115008	CUUACAG
ENSG00000115008	GAGUCUU
ENSG00000115008	UCAUUUG
ENSG00000115008	UCUUGAG
ENSG00000115009	AUGAUGA
ENSG00000115041	CCCUGAG
ENSG00000115041	CCUUCUG
ENSG00000115041	GAGAACC
ENSG00000115041	GAGAACU
ENSG00000115041	GAGACGC
ENSG00000115041	GAGCUGC
ENSG00000115041	GUGCUUG
ENSG00000115053	AACAUUC
ENSG00000115073	UACAAAG
ENSG00000115073	UAUUACC
ENSG00000115084	AGGCAGG
ENSG00000115084	CUCUUCC
ENSG00000115084	GAUUGUC
ENSG00000115084	GCAGGUU
ENSG00000115084	GUCUCUG
ENSG00000115084	UCGCUGC
ENSG00000115084	UUAGAGA
ENSG00000115107	AAGGCAC
ENSG00000115107	GAGAAGA
ENSG00000115107	GAGGUAG
ENSG00000115107	GAGGUAU
ENSG00000115107	GGCAUUG
ENSG00000115107	GGGGCAG
ENSG00000115107	GUGAGGU
ENSG00000115107	UGAGAAG
ENSG00000115109	AAAGUGC
ENSG00000115109	AAUUUUA
ENSG00000115109	UAAUACA

ENSG00000115109	UCCUGAG
ENSG00000115112	AAAGCUG
ENSG00000115112	AGCAGCU
ENSG00000115112	CUUCUCU
ENSG00000115112	GAGAAGA
ENSG00000115112	GAGGCAG
ENSG00000115112	GUCCAGA
ENSG00000115112	UCUCCAA
ENSG00000115112	UGAGAAG
ENSG00000115145	AGCAGCU
ENSG00000115145	GUGCUUG
ENSG00000115159	AGUAACA
ENSG00000115159	CACAGUG
ENSG00000115159	CAGUCCA
ENSG00000115159	CAGUUAC
ENSG00000115159	CUCAAAC
ENSG00000115159	UAUAAAG
ENSG00000115159	UCCCUCU
ENSG00000115159	UCUGGAA
ENSG00000115159	UGCAGCU
ENSG00000115170	AAAAAUC
ENSG00000115170	AAAAGUA
ENSG00000115170	AACAGUC
ENSG00000115170	AAUGUUG
ENSG00000115170	AGUAACA
ENSG00000115170	AUACAAG
ENSG00000115170	CAGUGCA
ENSG00000115170	GUAACAG
ENSG00000115170	UAUUGCU
ENSG00000115170	UGCUGAC
ENSG00000115170	UUCCUAG
ENSG00000115170	UUGGCAA
ENSG00000115170	UUGGCAC
ENSG00000115183	ACAGUAC
ENSG00000115183	ACAGUAU
ENSG00000115183	ACCAGCA
ENSG00000115183	AGAUAAA
ENSG00000115183	AGCACCA
ENSG00000115183	AGUGCAA
ENSG00000115183	CAGUAGU
ENSG00000115183	CCAGCAU
ENSG00000115183	UAAGACU
ENSG00000115183	UUCCAUA
ENSG00000115194	CUUUGGU
ENSG00000115194	GAGGCUG
ENSG00000115194	GGCAGUG
ENSG00000115194	GGGCCCC
ENSG00000115194	UGCAUAG
ENSG00000115207	AUGAGCU
ENSG00000115207	CCAGUAC
ENSG00000115207	CCUUCAU
ENSG00000115207	CUCCCAA
ENSG00000115207	UAUAAAG
ENSG00000115207	UCAAGUA
ENSG00000115216	AAUACUG
ENSG00000115226	AAGAACC
ENSG00000115226	AAGACAU
ENSG00000115226	AGUGCAA
ENSG00000115226	CACAGUG
ENSG00000115226	CAGGUGA
ENSG00000115226	UCACAGU
ENSG00000115232	AAGUGCA
ENSG00000115232	AGUGCAA
ENSG00000115232	CCAGUGU

ENSG00000115232	CUUGUAU
ENSG00000115232	UAGUGAG
ENSG00000115232	UGAGUCU
ENSG00000115234	CACAGGG
ENSG00000115234	UUGGCAA
ENSG00000115234	UUGGCAC
ENSG00000115252	CUGGCUC
ENSG00000115252	UCCCUCU
ENSG00000115255	CAGGUGA
ENSG00000115266	GAGGUAG
ENSG00000115266	GAUUGUC
ENSG00000115282	CAAAUGC
ENSG00000115282	UCUUGAG
ENSG00000115295	AAGUGCA
ENSG00000115295	AGUGCUU
ENSG00000115295	AUAAUAC
ENSG00000115295	AUUGCAC
ENSG00000115295	CAGGAAC
ENSG00000115306	AAAGGUA
ENSG00000115306	AAAGUGC
ENSG00000115306	AACAUUC
ENSG00000115306	ACAGGUG
ENSG00000115306	ACAGUAU
ENSG00000115306	ACAUUAC
ENSG00000115306	ACUAGAU
ENSG00000115306	ACUUGGA
ENSG00000115306	AGAUAAA
ENSG00000115306	AGCAAAA
ENSG00000115306	AGCACCA
ENSG00000115306	AGUAACA
ENSG00000115306	AUAAAGU
ENSG00000115306	AUAUAAC
ENSG00000115306	CACAGUG
ENSG00000115306	CCAGUAC
ENSG00000115306	CCUUCUG
ENSG00000115306	CUGCAGC
ENSG00000115306	CUUGGAG
ENSG00000115306	GAGAAAU
ENSG00000115306	GAGCUGC
ENSG00000115306	GAUUGUA
ENSG00000115306	GAUUGUC
ENSG00000115306	GCAGAAG
ENSG00000115306	GCAGCAU
ENSG00000115306	GCUGGAU
ENSG00000115306	GGCUGCG
ENSG00000115306	GGGUUUA
ENSG00000115306	GUCCAGA
ENSG00000115306	GUGACAG
ENSG00000115306	GUGUGGC
ENSG00000115306	UACAGUU
ENSG00000115306	UCAUUCG
ENSG00000115306	UCCCUCU
ENSG00000115306	UCCUAUA
ENSG00000115306	UCUGGAG
ENSG00000115306	UGCAUAG
ENSG00000115306	UGCAUAU
ENSG00000115306	UGGCUGC
ENSG00000115306	UUGGUCC
ENSG00000115306	UUGGUUC
ENSG00000115307	CUCUGGG
ENSG00000115307	CUGCCCC
ENSG00000115307	GAAAACA
ENSG00000115307	GGCUCUG
ENSG00000115307	GGGGAGC

ENSG00000115310	AUGCAUU
ENSG00000115317	AGGAGCU
ENSG00000115317	AUAUAAC
ENSG00000115317	CCAUUAC
ENSG00000115317	CUCUAGC
ENSG00000115317	GAGGCUG
ENSG00000115317	GCUGGUG
ENSG00000115317	GGACCUG
ENSG00000115317	GGUGGUU
ENSG00000115317	UAGGUAU
ENSG00000115318	AUACCUC
ENSG00000115318	AUAUAAC
ENSG00000115318	GAGGUAG
ENSG00000115318	GAGGUAU
ENSG00000115318	GGCAGCG
ENSG00000115318	GGUGGUC
ENSG00000115318	GGUGGUU
ENSG00000115318	UCCUGAG
ENSG00000115350	AAAGCUG
ENSG00000115350	UUGAUCA
ENSG00000115355	AAAGUAC
ENSG00000115355	AACCUGU
ENSG00000115355	AAGGCAC
ENSG00000115355	AAGGUGC
ENSG00000115355	AGCACCA
ENSG00000115355	AGGACAC
ENSG00000115355	CAAAUGC
ENSG00000115355	CACAGUG
ENSG00000115355	CCAGUAC
ENSG00000115355	GAGAAAU
ENSG00000115355	GUAGUGU
ENSG00000115355	GUCUUGC
ENSG00000115355	GUGAGGU
ENSG00000115355	GUGCAAA
ENSG00000115355	GUGCUUG
ENSG00000115355	UGAAAGG
ENSG00000115355	UGGCCCU
ENSG00000115355	UGUGCUU
ENSG00000115355	UUAGGAU
ENSG00000115355	UUCAAAU
ENSG00000115355	UUCUUGU
ENSG00000115361	AGGAUGU
ENSG00000115361	AUGUGGG
ENSG00000115361	CCAGGAG
ENSG00000115361	UCUUGAG
ENSG00000115363	AUAAUAC
ENSG00000115363	CCUUCAU
ENSG00000115363	UAAUACA
ENSG00000115363	UUUCAAC
ENSG00000115364	AGCAAAA
ENSG00000115364	GGAUUUC
ENSG00000115365	ACCCUGU
ENSG00000115365	CCCUGUU
ENSG00000115365	UGAGAAG
ENSG00000115365	UGAGAAU
ENSG00000115365	UUGUGAC
ENSG00000115380	ACUAGAU
ENSG00000115380	CAAAGCA
ENSG00000115380	CUUACAG
ENSG00000115380	CUUUGGU
ENSG00000115380	GAGUGUG
ENSG00000115380	UUCUAAU
ENSG00000115414	ACAGUAC
ENSG00000115414	CAGUAGU

ENSG00000115414	CUUGGAG
ENSG00000115414	GAGUUGA
ENSG00000115414	UGUCUUU
ENSG00000115414	UUGGCAA
ENSG00000115415	AGGAUGU
ENSG00000115419	AAAACUG
ENSG00000115419	AAGUGCU
ENSG00000115419	ACUCCAG
ENSG00000115419	ACUGCAU
ENSG00000115419	AGUAACA
ENSG00000115419	AUCACUA
ENSG00000115419	CCCUGAG
ENSG00000115419	CUGGACU
ENSG00000115419	CUUGGGC
ENSG00000115419	GAGGCUG
ENSG00000115419	GCUAGCA
ENSG00000115419	GUCUCUG
ENSG00000115419	UAUUGCU
ENSG00000115419	UCACAUU
ENSG00000115419	UCCCUGU
ENSG00000115419	UGCAUAG
ENSG00000115419	UUGGUCC
ENSG00000115421	UUCUGCA
ENSG00000115425	UUUCAAC
ENSG00000115457	CGGUGCU
ENSG00000115461	AACUACU
ENSG00000115461	AAGUUGU
ENSG00000115461	ACAGUAC
ENSG00000115461	ACUGGCC
ENSG00000115461	ACUGGCU
ENSG00000115461	AGGGAGG
ENSG00000115461	AGUGCAA
ENSG00000115461	AUGUAAU
ENSG00000115461	CACCAGC
ENSG00000115461	CAGUCUG
ENSG00000115461	CUCCAUU
ENSG00000115461	CUGCUCA
ENSG00000115461	CUGGCUC
ENSG00000115461	CUUCACC
ENSG00000115461	GACAACU
ENSG00000115461	GGAAUGU
ENSG00000115461	GGAGGAG
ENSG00000115461	GGCUCAG
ENSG00000115461	GGGGUGC
ENSG00000115461	UCACCAC
ENSG00000115461	UCCCUCU
ENSG00000115461	UGGGGGA
ENSG00000115461	UGUAUGU
ENSG00000115461	UGUCUGC
ENSG00000115461	UGUCUGG
ENSG00000115464	AAAACUG
ENSG00000115464	AAAUUUC
ENSG00000115464	AAGUGUG
ENSG00000115464	AAUUUUA
ENSG00000115464	ACAUCAC
ENSG00000115464	ACCACAG
ENSG00000115464	AGGACAC
ENSG00000115464	CACAAUG
ENSG00000115464	CAGACAG
ENSG00000115464	CUUGUGU
ENSG00000115464	GCCCUUA
ENSG00000115464	GUAUUCU
ENSG00000115464	GUGCUUG
ENSG00000115464	UGCCCUG

ENSG00000115464	UGUGCUU
ENSG00000115464	UUCAAGC
ENSG00000115464	UUCCUCG
ENSG00000115468	CAGCAGG
ENSG00000115468	CAGUUAC
ENSG00000115468	UACAAAG
ENSG00000115474	CAGCAGA
ENSG00000115484	CAAAUGC
ENSG00000115484	UAAUAUC
ENSG00000115486	ACUGGCU
ENSG00000115486	GUGACUG
ENSG00000115486	UGGCUGC
ENSG00000115507	AGCAGCA
ENSG00000115507	CCAGUGU
ENSG00000115507	GGCGGGG
ENSG00000115514	UACAAAG
ENSG00000115520	AAAAGUA
ENSG00000115520	AACUACU
ENSG00000115520	AAGUAGC
ENSG00000115520	CUGGACU
ENSG00000115520	UAAUAUC
ENSG00000115525	AACAAAC
ENSG00000115525	AAGUCUU
ENSG00000115525	AGUGCUU
ENSG00000115526	UAUUGCU
ENSG00000115526	UUGGCAA
ENSG00000115540	AGCAGCG
ENSG00000115540	AGCAGCU
ENSG00000115540	AGUGCUU
ENSG00000115540	GAGGUAG
ENSG00000115540	GAUUGUA
ENSG00000115540	GAUUGUC
ENSG00000115540	GUGCCAC
ENSG00000115540	GUUAAUG
ENSG00000115540	UAUAAAG
ENSG00000115540	UGAUCCA
ENSG00000115541	GGAAUGU
ENSG00000115556	CAGUCCA
ENSG00000115556	UCCCUUG
ENSG00000115561	ACAUUCA
ENSG00000115561	GAGUGUG
ENSG00000115561	GCAGAAG
ENSG00000115561	GGAGUGU
ENSG00000115561	GUAGUGU
ENSG00000115592	AGUGCCU
ENSG00000115592	GGCCCUG
ENSG00000115594	CAGGUGA
ENSG00000115594	GGCUCAG
ENSG00000115594	GUGCAAA
ENSG00000115594	UCUCCAA
ENSG00000115616	AAAGUGC
ENSG00000115616	AAGGCAC
ENSG00000115616	AAGUGCU
ENSG00000115616	AGUGCAA
ENSG00000115616	AGUGCUU
ENSG00000115616	GCUUCCU
ENSG00000115616	GUGCAAA
ENSG00000115616	UAUAAUA
ENSG00000115616	UCACCAC
ENSG00000115616	UCUCCAA
ENSG00000115616	UUCAAGC
ENSG00000115616	UUGGCAA
ENSG00000115649	AAAAACC
ENSG00000115649	AAACUGU

ENSG00000115649	ACUGUUU
ENSG00000115649	CCAGGAG
ENSG00000115649	UACAAAG
ENSG00000115652	AAACUGU
ENSG00000115652	AAAGUGC
ENSG00000115652	AAGUCUU
ENSG00000115652	AAGUGCA
ENSG00000115652	AGUGAUC
ENSG00000115652	AGUGCAA
ENSG00000115652	AUGACAC
ENSG00000115652	AUGCAUU
ENSG00000115652	CAGUAGU
ENSG00000115652	UCAUGAU
ENSG00000115665	AUGGCUU
ENSG00000115665	GUGCAAA
ENSG00000115677	AAUCUCA
ENSG00000115677	AAUCUCU
ENSG00000115677	AUUCAUU
ENSG00000115677	AUUGCAC
ENSG00000115677	CAGCAGA
ENSG00000115677	CAGCAGG
ENSG00000115677	CCAGUGC
ENSG00000115677	GAGGUAG
ENSG00000115677	GAGGUAU
ENSG00000115677	UCAUGAU
ENSG00000115677	UCCAGUU
ENSG00000115677	UGCAUAG
ENSG00000115677	UGCUGAC
ENSG00000115694	ACAGUAC
ENSG00000115694	GAAAACA
ENSG00000115694	UGAAAGG
ENSG00000115738	AAAGAGC
ENSG00000115738	AAAGAGG
ENSG00000115738	AACUCUA
ENSG00000115738	AGCAUUC
ENSG00000115738	GCAGAAG
ENSG00000115756	AUGCAUU
ENSG00000115756	CUUCACC
ENSG00000115758	CUACAAA
ENSG00000115760	AAGUGCA
ENSG00000115760	ACAGGUG
ENSG00000115760	AGGUAGU
ENSG00000115760	AGUGCAA
ENSG00000115760	AGUGGUU
ENSG00000115760	AUACAAG
ENSG00000115760	CACAAUG
ENSG00000115760	CUCACAC
ENSG00000115760	CUUACAG
ENSG00000115760	GUAAACA
ENSG00000115760	GUGCUUG
ENSG00000115760	UCCCUUG
ENSG00000115760	UCCCUUU
ENSG00000115760	UCUCACC
ENSG00000115760	UUCCUAG
ENSG00000115762	AUGCACC
ENSG00000115762	AUGCAUU
ENSG00000115762	UAUUGCU
ENSG00000115762	UCCUGAG
ENSG00000115762	UGCCCUG
ENSG00000115806	AGCAGCA
ENSG00000115806	CAGUUAC
ENSG00000115806	CCUUCUG
ENSG00000115806	GGCAGUG
ENSG00000115806	GUGCCAC

ENSG00000115806	UAGUGAG
ENSG00000115806	UUGGUUC
ENSG00000115825	ACAUUAC
ENSG00000115825	AGGUUAU
ENSG00000115825	AGUGCCU
ENSG00000115825	AUGGCUU
ENSG00000115825	CAAAUGC
ENSG00000115825	GAAGGAA
ENSG00000115825	GGCUCAG
ENSG00000115825	GUGUCAC
ENSG00000115825	UUAUGCA
ENSG00000115839	GCUGGUG
ENSG00000115840	AGUGCAA
ENSG00000115840	GUGCAAA
ENSG00000115840	UGCAGCU
ENSG00000115844	AAGGCAC
ENSG00000115844	AAUCUCA
ENSG00000115844	AUACAAG
ENSG00000115844	CAGUAGA
ENSG00000115844	CAGUAGU
ENSG00000115844	CUCUUCC
ENSG00000115844	GUCUCUG
ENSG00000115844	UACAAAG
ENSG00000115844	UUUGCAA
ENSG00000115844	UUUGCAC
ENSG00000115844	UUUUUGC
ENSG00000115875	AAAUCCC
ENSG00000115875	AAUCUCU
ENSG00000115875	ACAUCAC
ENSG00000115875	AGCCUGC
ENSG00000115875	CACCAGC
ENSG00000115875	GGUUGAC
ENSG00000115875	GUGAAUG
ENSG00000115875	GUGCCAC
ENSG00000115875	UCAACAG
ENSG00000115875	UGCAAAG
ENSG00000115884	AAAAACC
ENSG00000115884	AAGUGCA
ENSG00000115884	AAGUGCU
ENSG00000115884	ACCCAUU
ENSG00000115884	ACCCUGU
ENSG00000115884	ACUGUAG
ENSG00000115884	AGCAAAA
ENSG00000115884	AGGAGCU
ENSG00000115884	AUAUUAU
ENSG00000115884	CCCUGUU
ENSG00000115884	CUCACAC
ENSG00000115884	CUUUGGU
ENSG00000115884	GCAGAAG
ENSG00000115884	GCUGUCU
ENSG00000115884	UAUUACC
ENSG00000115884	UCCAGAG
ENSG00000115884	UUGGCAA
ENSG00000115896	AAUACUG
ENSG00000115896	GUGCAAA
ENSG00000115896	GUUAAUG
ENSG00000115896	UAAUAGU
ENSG00000115896	UUCAAAU
ENSG00000115902	AAGGCAC
ENSG00000115902	AAUUUUA
ENSG00000115902	AGGCAGG
ENSG00000115902	CAAAGCA
ENSG00000115902	GAGCUAA
ENSG00000115902	GAGGUAG

ENSG00000115902	GAGGUAU
ENSG00000115902	UCAUUUG
ENSG00000115904	AACAGUC
ENSG00000115904	ACAUUCA
ENSG00000115904	ACCACAG
ENSG00000115904	AGCAGCA
ENSG00000115904	AUAAUAC
ENSG00000115904	AUCCCUU
ENSG00000115904	CCCUGUC
ENSG00000115904	CCUGCUG
ENSG00000115904	CUUUGGU
ENSG00000115904	GCAGCAU
ENSG00000115904	GGCAGCG
ENSG00000115904	GUAAACA
ENSG00000115904	UAAUAUC
ENSG00000115904	UAAUGCU
ENSG00000115904	UCACAUU
ENSG00000115904	UUGGCAA
ENSG00000115904	UUGGCAC
ENSG00000115919	ACUAGAU
ENSG00000115919	AGAUGUG
ENSG00000115919	UGAAAUG
ENSG00000115935	AAAGAAU
ENSG00000115935	AACACUG
ENSG00000115935	AUGGCUU
ENSG00000115935	CUGGCUC
ENSG00000115935	CUUUGGU
ENSG00000115935	GCACCAU
ENSG00000115935	GUAAACA
ENSG00000115935	UUCAAAU
ENSG00000115935	UUGGCAA
ENSG00000115935	UUGGCAC
ENSG00000115942	AAGGCAC
ENSG00000115942	CACUGUU
ENSG00000115942	CAGCUGG
ENSG00000115942	CUCCAGC
ENSG00000115942	UCCAGAG
ENSG00000115942	UCCAGUU
ENSG00000115942	UCUGGAA
ENSG00000115942	UGAAAGG
ENSG00000115944	AAGUGCA
ENSG00000115944	AGUGCAA
ENSG00000115946	CUGGCUC
ENSG00000115946	UUCUUGU
ENSG00000115947	UAUAAAG
ENSG00000115956	ACAUUCA
ENSG00000115963	AAAGUGC
ENSG00000115977	AAGGCAC
ENSG00000115977	AAGUGCA
ENSG00000115977	ACAUUAC
ENSG00000115977	ACCCUGU
ENSG00000115977	ACCUGGA
ENSG00000115977	ACUGGCC
ENSG00000115977	ACUGGCU
ENSG00000115977	AGGAGCU
ENSG00000115977	AGGCAGG
ENSG00000115977	AGGUAGU
ENSG00000115977	AUGGCAC
ENSG00000115977	AUGUCUG
ENSG00000115977	AUUGCAC
ENSG00000115977	CAGCAAA
ENSG00000115977	CAGCUGG
ENSG00000115977	CCAGCAU
ENSG00000115977	CCAUUAC

ENSG00000115977	CCUGCUG
ENSG00000115977	CCUGUAC
ENSG00000115977	CUCCAGC
ENSG00000115977	CUGGGGG
ENSG00000115977	CUUGGAG
ENSG00000115977	CUUGGGC
ENSG00000115977	CUUUGGU
ENSG00000115977	GAAGGAA
ENSG00000115977	GACCAUG
ENSG00000115977	GACUUCC
ENSG00000115977	GAGGUAG
ENSG00000115977	GAUAUGU
ENSG00000115977	GCAGGUU
ENSG00000115977	GCUACAU
ENSG00000115977	GGACCUG
ENSG00000115977	GGAGGAG
ENSG00000115977	GGUCUAG
ENSG00000115977	GUAUGUU
ENSG00000115977	GUCAUUG
ENSG00000115977	GUGCAAA
ENSG00000115977	UAAUGCU
ENSG00000115977	UAGAGGA
ENSG00000115977	UCACAGG
ENSG00000115977	UCACAGU
ENSG00000115977	UCAUAGA
ENSG00000115977	UCAUUUG
ENSG00000115977	UCCCUUG
ENSG00000115977	UCGCUGC
ENSG00000115977	UGAAGCU
ENSG00000115977	UGCAUAG
ENSG00000115977	UGCCAGC
ENSG00000115977	UGUCUGG
ENSG00000115977	UUGGUCC
ENSG00000115993	AGCAAAA
ENSG00000115993	AUCCUUG
ENSG00000115993	AUCCUUU
ENSG00000115993	AUGCAUU
ENSG00000115993	AUUGCAC
ENSG00000115993	CAAAGCA
ENSG00000115993	GCCUGAU
ENSG00000115993	UGAGAAU
ENSG00000115993	UGCAUAG
ENSG00000116001	AAGGCAC
ENSG00000116001	AGAUAAA
ENSG00000116001	UUCUGCA
ENSG00000116001	UUUGCAC
ENSG00000116005	AAAAAUC
ENSG00000116005	AAUCACU
ENSG00000116005	UCACAAG
ENSG00000116016	AAAGUAC
ENSG00000116016	AAAGUUU
ENSG00000116016	AGUGCUU
ENSG00000116016	AUGUGCC
ENSG00000116016	CAGUAGA
ENSG00000116016	CAGUAGU
ENSG00000116016	CUUGUAU
ENSG00000116016	CUUUGGU
ENSG00000116016	GACCUGG
ENSG00000116016	GAUCAGA
ENSG00000116016	GCAACUU
ENSG00000116016	GCUUCCU
ENSG00000116016	GUCAGUU
ENSG00000116016	UAAUAUC
ENSG00000116016	UAAUGCU

ENSG00000116016	UCAUGAU
ENSG00000116017	CAGUGCA
ENSG00000116017	CCCUGAG
ENSG00000116017	GAGGUAG
ENSG00000116017	GAGGUAU
ENSG00000116017	GUAAACA
ENSG00000116017	UCAAGUA
ENSG00000116017	UCCCUGU
ENSG00000116031	GCAGCAU
ENSG00000116031	UGGGACA
ENSG00000116032	UGUAUAA
ENSG00000116035	GUCUCUG
ENSG00000116035	UUCUUGU
ENSG00000116095	AAGUGCA
ENSG00000116095	UGCAUAU
ENSG00000116096	AGGCAGG
ENSG00000116106	AACUACU
ENSG00000116106	CAAGAGC
ENSG00000116106	CAUGGAU
ENSG00000116106	GAGGUAG
ENSG00000116106	GAGGUAU
ENSG00000116106	GUCACUC
ENSG00000116106	UAAUACA
ENSG00000116106	UACAAAG
ENSG00000116106	UCCUUGC
ENSG00000116106	UGCAUUG
ENSG00000116127	AAAAACC
ENSG00000116128	AAAGUAA
ENSG00000116128	ACAUUCA
ENSG00000116128	CUAUACA
ENSG00000116128	CUCAAAC
ENSG00000116128	UCCCUUU
ENSG00000116128	UCUCAAG
ENSG00000116128	UGCAGCU
ENSG00000116132	AAAGUGC
ENSG00000116132	AAGUGUG
ENSG00000116132	AAUUUUA
ENSG00000116132	ACAGUAC
ENSG00000116132	AGGGAGG
ENSG00000116132	AGUGCUG
ENSG00000116132	CAAAGCA
ENSG00000116132	CAAAGUG
ENSG00000116132	CAGCAGA
ENSG00000116132	CCUGCUG
ENSG00000116132	CUACAAA
ENSG00000116132	GAGGUAG
ENSG00000116132	GAGGUAU
ENSG00000116132	GGAGGAG
ENSG00000116132	GUCACUC
ENSG00000116132	GUGAGGU
ENSG00000116132	UAAUAGU
ENSG00000116132	UACAAAG
ENSG00000116132	UUGGUCC
ENSG00000116133	AAGGCAC
ENSG00000116133	CCUUCAU
ENSG00000116133	GACCUGG
ENSG00000116133	GGCUCUG
ENSG00000116133	GGGGCAG
ENSG00000116133	UUAGGAU
ENSG00000116133	UUGGCAA
ENSG00000116133	UUGGCAC
ENSG00000116138	AAAGUGC
ENSG00000116138	ACUGGCC
ENSG00000116138	AGCAGCA

ENSG00000116138	AGCAGCG
ENSG00000116138	AGCUGCC
ENSG00000116138	AGCUUAU
ENSG00000116138	AGGUUAU
ENSG00000116138	AGUGCCU
ENSG00000116138	AUGGCUU
ENSG00000116138	CACAAUG
ENSG00000116138	CAGUGCA
ENSG00000116138	CCUUCUU
ENSG00000116138	GAGGCAG
ENSG00000116138	GUGCCUG
ENSG00000116138	UACAAAG
ENSG00000116138	UCACAAG
ENSG00000116138	UCCUUGC
ENSG00000116138	UUGUUCG
ENSG00000116141	AAGGCAC
ENSG00000116141	CCUGUAC
ENSG00000116141	CUAUACA
ENSG00000116141	GACAACU
ENSG00000116141	UAUUGCU
ENSG00000116141	UCCUGAG
ENSG00000116147	CAGCAGA
ENSG00000116147	CAUAUUG
ENSG00000116147	CCUUCAU
ENSG00000116147	UAUAAAG
ENSG00000116157	AGCACCA
ENSG00000116157	GCACCAU
ENSG00000116161	CCAGUAC
ENSG00000116161	CUGGAAA
ENSG00000116161	UGCAGCU
ENSG00000116194	AACAUUC
ENSG00000116194	CAAAACU
ENSG00000116194	UGAAAUG
ENSG00000116199	AAUCCUU
ENSG00000116199	AGUGCAA
ENSG00000116199	AUCCUUG
ENSG00000116199	CACCACC
ENSG00000116199	GAUUGUC
ENSG00000116199	GGCUCAG
ENSG00000116199	UGUGCUU
ENSG00000116205	ACUCAGG
ENSG00000116205	CAGUUAC
ENSG00000116205	CCAUUAC
ENSG00000116205	CCCCUGG
ENSG00000116205	CUCAGGG
ENSG00000116205	CUCCAUU
ENSG00000116205	CUGCAGG
ENSG00000116205	CUUCUCU
ENSG00000116205	GAAGGUC
ENSG00000116205	GAGAAAU
ENSG00000116205	GCACCAU
ENSG00000116205	GUGACAG
ENSG00000116205	UAAGACU
ENSG00000116205	UAGAGGA
ENSG00000116205	UGGGGGA
ENSG00000116205	UUAGAGA
ENSG00000116205	UUCAAAU
ENSG00000116205	UUCCUAG
ENSG00000116205	UUCUGCA
ENSG00000116213	UACAAAG
ENSG00000116218	UUGUGAC
ENSG00000116237	AAAGAAU
ENSG00000116237	AAAGUGC
ENSG00000116237	AAGGCAC

ENSG00000116237	ACCCGUA
ENSG00000116237	CCUUCUG
ENSG00000116237	CUUUGGU
ENSG00000116237	GAGGUAG
ENSG00000116237	GAGGUAU
ENSG00000116237	GCCUGGG
ENSG00000116237	GUGAGGU
ENSG00000116237	UAUUGCU
ENSG00000116237	UCACAUU
ENSG00000116237	UGCUGAC
ENSG00000116251	ACUGCAG
ENSG00000116251	AUACAAG
ENSG00000116251	GCUGGAU
ENSG00000116254	AAAGUAA
ENSG00000116254	AAAGUGC
ENSG00000116254	AACCUGU
ENSG00000116254	AAGUCUU
ENSG00000116254	AAGUGCA
ENSG00000116254	ACACACC
ENSG00000116254	ACACACU
ENSG00000116254	AGCAGCA
ENSG00000116254	AGUGCAA
ENSG00000116254	CAGCAAA
ENSG00000116254	CGUGGCC
ENSG00000116254	GGCAGGG
ENSG00000116254	GUAAACA
ENSG00000116254	GUGCAAA
ENSG00000116254	GUGGCCC
ENSG00000116254	UACAAAG
ENSG00000116254	UCAAGUA
ENSG00000116254	UCUGGAA
ENSG00000116260	AACAAAC
ENSG00000116266	CAAAGCA
ENSG00000116266	UCAUACA
ENSG00000116273	ACUGCAG
ENSG00000116273	AGCAGCU
ENSG00000116273	AGCUGCC
ENSG00000116273	AUGGCUU
ENSG00000116273	CGUGCAU
ENSG00000116273	CUUUGGU
ENSG00000116273	GUAAACA
ENSG00000116273	UACAAAG
ENSG00000116273	UCACAGG
ENSG00000116273	UCACAGU
ENSG00000116273	UGCAGCU
ENSG00000116273	UGCAUAG
ENSG00000116273	UGUCUGG
ENSG00000116273	UUGGCAC
ENSG00000116285	AAUCCUU
ENSG00000116285	ACCAGCA
ENSG00000116285	AUCCUUU
ENSG00000116285	CAGCAGA
ENSG00000116285	CAGCAGG
ENSG00000116285	CUUGUGU
ENSG00000116285	GCAGAAG
ENSG00000116285	GUGCUUG
ENSG00000116285	UAGUGAG
ENSG00000116285	UCAUUUG
ENSG00000116285	UGUGCUU
ENSG00000116299	UUGGCAA
ENSG00000116299	UUGGCAC
ENSG00000116329	GAGGGGC
ENSG00000116337	AACACUG
ENSG00000116337	UCCCACA

ENSG00000116396	ACAGUAU
ENSG00000116396	AGCAGCA
ENSG00000116396	AUGUGCC
ENSG00000116396	AUUGCAC
ENSG00000116396	CAGACAG
ENSG00000116396	GAGGUAG
ENSG00000116396	GCAGCAU
ENSG00000116396	GGCUCUG
ENSG00000116396	GGGGCAG
ENSG00000116396	UGUCUUU
ENSG00000116406	ACAGUAC
ENSG00000116406	AUGGCUU
ENSG00000116406	CACAGUG
ENSG00000116406	CUGACUC
ENSG00000116406	CUUCACC
ENSG00000116406	CUUUGGU
ENSG00000116406	GAGGUAG
ENSG00000116406	GAGGUAU
ENSG00000116406	GCAACUU
ENSG00000116406	GGCAGUG
ENSG00000116406	GGUAGAC
ENSG00000116406	GGUUUAC
ENSG00000116406	GUAGUGU
ENSG00000116406	GUCAACA
ENSG00000116406	UAUAAAG
ENSG00000116406	UCACAGG
ENSG00000116406	UCAUUCG
ENSG00000116406	UGUAUAA
ENSG00000116406	UUCUAAU
ENSG00000116406	UUGGCAA
ENSG00000116406	UUGGCAC
ENSG00000116406	UUGGUUC
ENSG00000116459	ACUGGCC
ENSG00000116473	AAAGCUG
ENSG00000116473	ACCCAUU
ENSG00000116473	ACUCAAA
ENSG00000116473	AGUGCUG
ENSG00000116473	AUGCACC
ENSG00000116473	CAAAGCA
ENSG00000116473	CUGGCUC
ENSG00000116473	CUUCACC
ENSG00000116473	GAGCUGC
ENSG00000116473	GAGUUGG
ENSG00000116473	GGAAUGU
ENSG00000116473	GUGCUUG
ENSG00000116473	UCAUUCG
ENSG00000116473	UUAUGCA
ENSG00000116478	AUAUAAC
ENSG00000116478	UAUUACC
ENSG00000116489	AAGUCUU
ENSG00000116497	AACCUGU
ENSG00000116497	UCACAUU
ENSG00000116514	AGUGUGC
ENSG00000116514	UGUGCUU
ENSG00000116521	UCCAGUU
ENSG00000116525	AAAAAUC
ENSG00000116525	AAUACUG
ENSG00000116525	CUGGAAA
ENSG00000116525	GGAUGAG
ENSG00000116539	AACAUUC
ENSG00000116539	AAGUGCU
ENSG00000116539	AAUACUG
ENSG00000116539	ACAGUAC
ENSG00000116539	ACAGUAU

ENSG00000116539	ACUAGAU
ENSG00000116539	AGAUAAA
ENSG00000116539	AGCAGCG
ENSG00000116539	AGGACAC
ENSG00000116539	AGUGCUG
ENSG00000116539	AUAUUAU
ENSG00000116539	AUGCAUU
ENSG00000116539	CAAAACU
ENSG00000116539	CCCACCU
ENSG00000116539	CUGCUCA
ENSG00000116539	CUUCUCU
ENSG00000116539	GAGAUGA
ENSG00000116539	GAUUGUA
ENSG00000116539	GGAAUGU
ENSG00000116539	GGCGGGG
ENSG00000116539	GUCUCUG
ENSG00000116539	GUUAGGA
ENSG00000116539	UAUUGCU
ENSG00000116539	UCAACAG
ENSG00000116539	UCACACA
ENSG00000116539	UGUCUUU
ENSG00000116539	UUCCAUA
ENSG00000116539	UUGGCAC
ENSG00000116539	UUGGUCC
ENSG00000116539	UUUUUGC
ENSG00000116544	ACAGUAC
ENSG00000116544	CACAGUG
ENSG00000116544	UCACAGU
ENSG00000116544	UUGGUCC
ENSG00000116560	AACACUG
ENSG00000116560	ACUGCAU
ENSG00000116574	AAGUAGC
ENSG00000116574	CAAAGCA
ENSG00000116574	UGCAUAG
ENSG00000116574	UGCAUAU
ENSG00000116580	CCAGUGC
ENSG00000116580	GCAUCCC
ENSG00000116584	AAAAAUC
ENSG00000116584	AUGGCUU
ENSG00000116584	CAGUAGU
ENSG00000116584	CUUUGGU
ENSG00000116584	GAAAACA
ENSG00000116584	GGGCCCC
ENSG00000116584	GUAGUGU
ENSG00000116584	UGGUACC
ENSG00000116584	UUGGUUC
ENSG00000116604	AAAAAUC
ENSG00000116604	AAAACUG
ENSG00000116604	AACCUGG
ENSG00000116604	AACUCUA
ENSG00000116604	AAGGUGC
ENSG00000116604	AAUACUG
ENSG00000116604	AAUCUCA
ENSG00000116604	AAUUAUU
ENSG00000116604	ACAAUAU
ENSG00000116604	ACAGUAC
ENSG00000116604	ACAGUAU
ENSG00000116604	ACUCCAG
ENSG00000116604	ACUUGGA
ENSG00000116604	AGGGAGG
ENSG00000116604	CAAAACU
ENSG00000116604	CAAAGUG
ENSG00000116604	CAAGAGC
ENSG00000116604	CAGCAGA

ENSG00000116604	CCAGGAG
ENSG00000116604	CCAGUGC
ENSG00000116604	CCAGUGU
ENSG00000116604	CCCUGAG
ENSG00000116604	CUGCAGC
ENSG00000116604	CUGCCCC
ENSG00000116604	CUGGACU
ENSG00000116604	GAGGUAG
ENSG00000116604	GAUUGUA
ENSG00000116604	GCCUGGA
ENSG00000116604	GGACUGC
ENSG00000116604	GGAGGAG
ENSG00000116604	GGAGUGU
ENSG00000116604	GGGAGCU
ENSG00000116604	GGGCCCC
ENSG00000116604	GUAAACA
ENSG00000116604	GUCAACA
ENSG00000116604	GUCUCUG
ENSG00000116604	GUGCAAA
ENSG00000116604	UACAGUU
ENSG00000116604	UAUUACC
ENSG00000116604	UGCAAAG
ENSG00000116604	UGGACUG
ENSG00000116604	UGGGACA
ENSG00000116604	UGGGUGA
ENSG00000116604	UUCAAAU
ENSG00000116604	UUGACAC
ENSG00000116604	UUGGCAA
ENSG00000116604	UUGGCAC
ENSG00000116604	UUGGUUC
ENSG00000116604	UUUCAAC
ENSG00000116641	AAAUUUC
ENSG00000116641	AACAUUC
ENSG00000116641	ACAUUCA
ENSG00000116641	UCACAUU
ENSG00000116649	CAGACAG
ENSG00000116663	GCCUGGA
ENSG00000116667	AAAAACC
ENSG00000116667	AAGGUGC
ENSG00000116667	AGCAGCG
ENSG00000116667	AGCAGCU
ENSG00000116667	AGUAACA
ENSG00000116667	AGUGCUU
ENSG00000116667	AUGUAAU
ENSG00000116667	CAAAGCA
ENSG00000116667	CACAGUG
ENSG00000116667	CACCACC
ENSG00000116667	CAGACAG
ENSG00000116667	CAGUAGU
ENSG00000116667	CCUGUAC
ENSG00000116667	CUCUUCC
ENSG00000116667	CUGGACU
ENSG00000116667	CUUCUCU
ENSG00000116667	GAAAACA
ENSG00000116667	GAUUGUA
ENSG00000116667	GAUUGUC
ENSG00000116667	GCAGAAG
ENSG00000116667	GGAAGAC
ENSG00000116667	GGAUGAG
ENSG00000116667	GGGGUGC
ENSG00000116667	GGGGUGG
ENSG00000116667	GUGACAG
ENSG00000116667	UAAUAGU
ENSG00000116667	UACAGUU

ENSG00000116667	UCACAGU
ENSG00000116667	UCAUAGA
ENSG00000116667	UGAAAGG
ENSG00000116667	UGAGAAU
ENSG00000116667	UGGCUGC
ENSG00000116667	UGUCUUU
ENSG00000116667	UUCAAGC
ENSG00000116668	GAGGUAG
ENSG00000116670	ACUUGGA
ENSG00000116675	AAAGGUA
ENSG00000116675	ACACACU
ENSG00000116675	AUAUUAU
ENSG00000116675	AUGCCUU
ENSG00000116675	CUGCAGG
ENSG00000116675	GAUUGUA
ENSG00000116675	GGGGUCC
ENSG00000116675	GUCAGUU
ENSG00000116675	GUCCAGA
ENSG00000116675	UAAUAUC
ENSG00000116675	UCACAAG
ENSG00000116675	UGACAUC
ENSG00000116675	UGUCUUU
ENSG00000116675	UUCAAAU
ENSG00000116675	UUGACAC
ENSG00000116678	AGUGAUC
ENSG00000116678	CCUUCUU
ENSG00000116678	GACCUGG
ENSG00000116678	UAGACUG
ENSG00000116678	UCAUGAU
ENSG00000116678	UUCUAAU
ENSG00000116679	AUCACUA
ENSG00000116679	CCUUCAU
ENSG00000116679	GGAUUUC
ENSG00000116679	UAUAAAG
ENSG00000116679	UUGUGUC
ENSG00000116685	AAGGCAC
ENSG00000116688	GCAGCAU
ENSG00000116698	AAAGCUG
ENSG00000116698	AACAUUC
ENSG00000116698	ACACACU
ENSG00000116698	AGCAGCU
ENSG00000116698	CAAAGCA
ENSG00000116698	UGCAGCU
ENSG00000116698	UUUCAAC
ENSG00000116701	GAAACAU
ENSG00000116704	GAUUGUA
ENSG00000116717	AAUCCUU
ENSG00000116729	AAAGCUG
ENSG00000116729	AGCAAAA
ENSG00000116729	GAGAAGA
ENSG00000116729	UGGACUG
ENSG00000116731	AAAACUG
ENSG00000116731	AAGGCAC
ENSG00000116731	ACUGCAG
ENSG00000116731	AGCCCUU
ENSG00000116731	AGGAGCU
ENSG00000116731	CUGGAAA
ENSG00000116731	CUUCACC
ENSG00000116731	GGGUGGU
ENSG00000116731	GUCUACU
ENSG00000116731	GUGCCUG
ENSG00000116731	UACAGUU
ENSG00000116731	UAUAAAG
ENSG00000116731	UCACAUU

ENSG00000116731	UGCAUAU
ENSG00000116741	AAAGUAU
ENSG00000116741	AAAUUUC
ENSG00000116741	GUAAACA
ENSG00000116747	CAAGAGC
ENSG00000116747	CACAGUG
ENSG00000116747	GAGUAUU
ENSG00000116747	UCACAGU
ENSG00000116750	AUGAUGC
ENSG00000116750	AUGCAUU
ENSG00000116750	GCAGGUU
ENSG00000116754	AAACUGU
ENSG00000116754	ACAUCAC
ENSG00000116754	ACUGUAG
ENSG00000116754	AUGACAC
ENSG00000116754	AUGGCAC
ENSG00000116754	CUUGUAU
ENSG00000116761	AAAAAUC
ENSG00000116774	GGAUUUU
ENSG00000116774	UAAUGCU
ENSG00000116783	GAGACCU
ENSG00000116783	GCUGUCU
ENSG00000116783	GUCAGUU
ENSG00000116786	AUAUAAC
ENSG00000116786	CUGCCCC
ENSG00000116793	AAAAGUA
ENSG00000116819	AUUGCAC
ENSG00000116819	GCCUGGA
ENSG00000116819	GUGCUUG
ENSG00000116819	UCAAGUA
ENSG00000116830	GAGCCCU
ENSG00000116833	AAAGAAU
ENSG00000116833	AAUCUCA
ENSG00000116833	ACCCUGU
ENSG00000116833	CACAGUG
ENSG00000116833	CUUGGAG
ENSG00000116833	GUAAACA
ENSG00000116833	GUCUACU
ENSG00000116833	UCACAGG
ENSG00000116833	UGAUCCA
ENSG00000116852	AACAGUC
ENSG00000116852	AAGGCAC
ENSG00000116852	AAUGCCC
ENSG00000116852	ACUCCAG
ENSG00000116852	AGCAAAA
ENSG00000116852	AGCAGCU
ENSG00000116852	AGGCAGG
ENSG00000116852	CAGCAAA
ENSG00000116852	CAGGCUC
ENSG00000116852	CUGCCCC
ENSG00000116852	CUUACAG
ENSG00000116852	CUUCACC
ENSG00000116852	CUUCUCU
ENSG00000116852	GAGGCAG
ENSG00000116852	GAGGUAG
ENSG00000116852	GAGGUAU
ENSG00000116852	GCCCUUA
ENSG00000116852	GCUUCCU
ENSG00000116852	GGAGGCA
ENSG00000116852	GGCUCAG
ENSG00000116852	GUAAACA
ENSG00000116852	GUGAGGU
ENSG00000116852	GUGCUUG
ENSG00000116852	UCAAGUA

ENSG00000116852	UCACAGU
ENSG00000116857	AUGGCUU
ENSG00000116863	ACUGGCU
ENSG00000116863	UAUUGCU
ENSG00000116863	UUGGUCC
ENSG00000116871	AAAAACC
ENSG00000116871	CUGGCUC
ENSG00000116871	UCACAUU
ENSG00000116871	UCUGGAA
ENSG00000116874	AAAGCUG
ENSG00000116874	AGCUGCC
ENSG00000116903	AGUGAUC
ENSG00000116903	AUGGCAC
ENSG00000116903	UUCAAAU
ENSG00000116918	CUUUGGU
ENSG00000116918	UGCUGAC
ENSG00000116922	CCCCUGG
ENSG00000116922	GACCUGG
ENSG00000116922	GGACCUG
ENSG00000116922	UCUGGAA
ENSG00000116957	AAAUGGU
ENSG00000116962	AGCACCA
ENSG00000116962	GUAAACA
ENSG00000116962	UAUAAAG
ENSG00000116977	AAUCACU
ENSG00000116977	ACAGGUG
ENSG00000116977	UUGAUCA
ENSG00000116983	ACCACAG
ENSG00000116983	AGCAGCA
ENSG00000116983	AGGCAGG
ENSG00000116983	AUGAGCU
ENSG00000116983	CAAAGCA
ENSG00000116983	CACCAGC
ENSG00000116983	CUCUUCC
ENSG00000116983	CUGCCCC
ENSG00000116983	CUUGGGC
ENSG00000116983	GGAAGAC
ENSG00000116983	GGACCUG
ENSG00000116983	GGCAGGG
ENSG00000116983	GGUGGUC
ENSG00000116983	GUCCAGA
ENSG00000116983	GUCUCUG
ENSG00000116983	GUGAGGU
ENSG00000116983	UCCAGAG
ENSG00000116983	UGGCCCU
ENSG00000116983	UUGGUUC
ENSG00000116984	CUCAAAC
ENSG00000116984	CUUUGGU
ENSG00000116984	GAGGUAU
ENSG00000116984	GUGAGGU
ENSG00000116984	UCAUUUG
ENSG00000116984	UCCAGUU
ENSG00000116984	UCUCACC
ENSG00000116984	UCUCCAA
ENSG00000116984	UUCUGCA
ENSG00000116985	CACUCCU
ENSG00000116985	CAGCUGG
ENSG00000116985	CCUGCUG
ENSG00000116985	CUGGACU
ENSG00000116985	GCUGUCU
ENSG00000116985	GUCACUC
ENSG00000116985	UCACAGG
ENSG00000116990	AAAGUGC
ENSG00000116990	AAAUUUC

ENSG00000116990	AAUCACU
ENSG00000116990	ACUGUAG
ENSG00000116990	AGCUGCC
ENSG00000116990	AUGCAUU
ENSG00000116990	CACCACC
ENSG00000116990	CUGGAAA
ENSG00000116990	CUUCACC
ENSG00000116990	GAGGUAG
ENSG00000116990	GAGGUAU
ENSG00000116990	GAGUGUG
ENSG00000116990	GGAUGAG
ENSG00000116990	GGGAUCG
ENSG00000116990	UCCCUUG
ENSG00000116990	UGAGAAG
ENSG00000116990	UGAGAAU
ENSG00000116990	UGCAAAG
ENSG00000116990	UGGGAUC
ENSG00000116990	UGUGUGG
ENSG00000116990	UUCAAAU
ENSG00000116990	UUUGCAA
ENSG00000116991	AAAAAUC
ENSG00000116991	AAAGUAA
ENSG00000116991	AAAGUAC
ENSG00000116991	AACACUG
ENSG00000116991	AACUACU
ENSG00000116991	AAUUGCU
ENSG00000116991	ACUGUAG
ENSG00000116991	AGAUAAA
ENSG00000116991	AGCAGCG
ENSG00000116991	AGCAGCU
ENSG00000116991	AGUGCAA
ENSG00000116991	CAAAGCA
ENSG00000116991	CUAUACA
ENSG00000116991	CUCAAAC
ENSG00000116991	GCACCAU
ENSG00000116991	GCAGCAU
ENSG00000116991	GUUCAUG
ENSG00000116991	UACAAAG
ENSG00000116991	UAUAAAG
ENSG00000117000	ACUCAAA
ENSG00000117000	CAAAGCA
ENSG00000117000	GGAAGCC
ENSG00000117000	UCAAGUA
ENSG00000117000	UGGGUGA
ENSG00000117009	GAGCCCU
ENSG00000117013	AAUACUG
ENSG00000117013	UCAAGUA
ENSG00000117016	AAUACUG
ENSG00000117016	ACAUAGA
ENSG00000117016	ACUGUAG
ENSG00000117016	AGCAGCA
ENSG00000117016	AGCCCUU
ENSG00000117016	AGGCAGG
ENSG00000117016	AGUGUGC
ENSG00000117016	AUGCAUU
ENSG00000117016	CAAAGCA
ENSG00000117016	CAGCAGG
ENSG00000117016	CAGCUGG
ENSG00000117016	CUGGAAA
ENSG00000117016	CUUUGGU
ENSG00000117016	GAGCCCC
ENSG00000117016	GAGGCAG
ENSG00000117016	GAGGUAG
ENSG00000117016	GAGGUAU

ENSG00000117016	GCAGGAC
ENSG00000117016	GCUGGUG
ENSG00000117016	GGCAGUG
ENSG00000117016	GGCAUUG
ENSG00000117016	UAUUGCU
ENSG00000117016	UCACAGG
ENSG00000117016	UCCCUCU
ENSG00000117016	UGCCAGC
ENSG00000117016	UGGGACA
ENSG00000117016	UGUCUUU
ENSG00000117016	UGUGCUU
ENSG00000117016	UUCAAAU
ENSG00000117016	UUGGCAA
ENSG00000117016	UUGGCAC
ENSG00000117020	AAAGUGC
ENSG00000117036	UUCUAAU
ENSG00000117069	AAUACUG
ENSG00000117069	UCACACA
ENSG00000117090	UGCAAAG
ENSG00000117114	AAAAAUC
ENSG00000117114	AACAAAC
ENSG00000117114	AAGUCUU
ENSG00000117114	AAUUAUU
ENSG00000117114	ACAAUAU
ENSG00000117114	ACAGUAC
ENSG00000117114	ACAGUAU
ENSG00000117114	AGCAGCA
ENSG00000117114	AGGCAGG
ENSG00000117114	AUACAAG
ENSG00000117114	AUAUAAC
ENSG00000117114	CAGCAGA
ENSG00000117114	CCUGCUG
ENSG00000117114	GAAGGAA
ENSG00000117114	GGAAGAC
ENSG00000117114	GGAUUUU
ENSG00000117114	GUAACAG
ENSG00000117114	GUGCAAA
ENSG00000117114	UACAGUU
ENSG00000117114	UAUAAAG
ENSG00000117114	UAUAAUA
ENSG00000117114	UCAUGAU
ENSG00000117114	UCCCACA
ENSG00000117114	UGAGAAG
ENSG00000117114	UGUAUAA
ENSG00000117114	UUGGCAC
ENSG00000117115	AAAAAUC
ENSG00000117115	AGCUGCC
ENSG00000117115	CUCCAGC
ENSG00000117115	CUCUGGG
ENSG00000117115	CUCUUCC
ENSG00000117115	GCCUGGA
ENSG00000117115	GGGGUGG
ENSG00000117115	UUUGCAA
ENSG00000117115	UUUGCAC
ENSG00000117122	AGCACCA
ENSG00000117152	AAGGUGC
ENSG00000117152	AGGGUCU
ENSG00000117152	CUCCAUU
ENSG00000117152	GAGUCUU
ENSG00000117152	GCAGCAU
ENSG00000117153	AAAGUGC
ENSG00000117153	CCAGUGC
ENSG00000117153	GUGCCUG
ENSG00000117155	AAUCUCA

ENSG00000117155	ACAAUAU
ENSG00000117155	ACACACC
ENSG00000117155	ACAGUAC
ENSG00000117155	ACCCUGU
ENSG00000117155	AGUGCUG
ENSG00000117155	CAAGAGC
ENSG00000117155	CCCUGUU
ENSG00000117155	CUCAAAC
ENSG00000117155	CUUUGGU
ENSG00000117155	UACAAAG
ENSG00000117155	UCACAGG
ENSG00000117155	UCACAGU
ENSG00000117155	UUCCUAG
ENSG00000117174	CAAAACU
ENSG00000117174	UAGUGAG
ENSG00000117215	AACCUGU
ENSG00000117222	AAGUCAC
ENSG00000117222	CCAGCAU
ENSG00000117222	GUCCAGA
ENSG00000117222	UCACAGG
ENSG00000117222	UCCCUUU
ENSG00000117222	UCCUUGC
ENSG00000117222	UGCAUAG
ENSG00000117222	UGCAUAU
ENSG00000117222	UUCCAUA
ENSG00000117245	CUCUGGG
ENSG00000117266	AAGGCAC
ENSG00000117266	ACAGUAC
ENSG00000117266	CACAGUG
ENSG00000117266	GGAGGCA
ENSG00000117266	UCACAGG
ENSG00000117266	UCACAGU
ENSG00000117266	UUGGCAC
ENSG00000117280	UGAGAAU
ENSG00000117289	AGCAGCA
ENSG00000117289	AGUGCAA
ENSG00000117289	CAAAGUG
ENSG00000117289	CAGUGCA
ENSG00000117298	AAGGCAC
ENSG00000117298	ACAGUAC
ENSG00000117298	ACAGUAU
ENSG00000117298	AGGACAC
ENSG00000117298	AGGUUAU
ENSG00000117298	AGUGCCG
ENSG00000117298	AGUGCCU
ENSG00000117298	AUAAUAC
ENSG00000117298	CCAGUGC
ENSG00000117298	CCCUGAG
ENSG00000117298	CUACAGU
ENSG00000117298	GUAUUCU
ENSG00000117298	GUGCCAC
ENSG00000117298	UAAUACA
ENSG00000117298	UCACAGG
ENSG00000117298	UCACAGU
ENSG00000117298	UCCAGUU
ENSG00000117298	UCCCUGU
ENSG00000117298	UCCCUUG
ENSG00000117298	UCCCUUU
ENSG00000117305	GAGUUGG
ENSG00000117308	GAGGUAG
ENSG00000117318	AAAGGUA
ENSG00000117318	AAGUCAC
ENSG00000117318	UAUAAAG
ENSG00000117318	UCACAGU

ENSG00000117318	UCUCCAA
ENSG00000117362	AAAAACC
ENSG00000117362	CUUCACC
ENSG00000117362	UGGAGAU
ENSG00000117385	AUCCUUG
ENSG00000117385	CACCAGC
ENSG00000117385	UCACCAC
ENSG00000117394	AACAGUC
ENSG00000117394	AACUGGU
ENSG00000117394	ACUCCAG
ENSG00000117394	AGCUGCC
ENSG00000117394	AGUGCAA
ENSG00000117394	AUAUAAC
ENSG00000117394	CAGUGCA
ENSG00000117394	CCAGUGC
ENSG00000117394	CUAUACA
ENSG00000117394	CUCAGGG
ENSG00000117394	GUCUUGC
ENSG00000117394	UAUAAAG
ENSG00000117394	UUCUAAU
ENSG00000117395	UUGGUUC
ENSG00000117408	CUUUGGU
ENSG00000117410	UCCAGUU
ENSG00000117411	CAGUGCA
ENSG00000117411	GAAAACA
ENSG00000117411	GGCAGUG
ENSG00000117411	GUGCAAA
ENSG00000117461	AAAAACC
ENSG00000117461	AAAGAAU
ENSG00000117461	AAGUCAC
ENSG00000117461	AAGUGCU
ENSG00000117461	AAUCACU
ENSG00000117461	AGCACCA
ENSG00000117461	AGGGAGG
ENSG00000117461	AUGGCUU
ENSG00000117461	AUGUCUG
ENSG00000117461	CAGCUGG
ENSG00000117461	CAGUGCA
ENSG00000117461	CUCUUCC
ENSG00000117461	CUGCAGG
ENSG00000117461	CUGUUGA
ENSG00000117461	CUUUGGU
ENSG00000117461	GAAACAU
ENSG00000117461	GCUGGAU
ENSG00000117461	GCUUCCU
ENSG00000117461	GGAAGAC
ENSG00000117461	GGAAUGU
ENSG00000117461	GGCUCAG
ENSG00000117461	GUCUGCC
ENSG00000117461	UACAAAG
ENSG00000117461	UAUUGCU
ENSG00000117461	UCAAGUA
ENSG00000117461	UCACAUU
ENSG00000117461	UGCAUUG
ENSG00000117461	UUCUGCA
ENSG00000117461	UUGGUUC
ENSG00000117472	GGGAUCG
ENSG00000117472	UGGGAUC
ENSG00000117479	AAAACUG
ENSG00000117479	AAAGAGG
ENSG00000117479	ACAGUAU
ENSG00000117479	CUUCUCU
ENSG00000117479	CUUUGGU
ENSG00000117479	UAUUGCU

ENSG00000117479	UGGAUAA
ENSG00000117481	AGCAUUC
ENSG00000117481	AUCCCUU
ENSG00000117481	CCUGCUG
ENSG00000117481	CUCCCAA
ENSG00000117481	UACAAAG
ENSG00000117481	UGAGUCU
ENSG00000117481	UUCCUAG
ENSG00000117500	ACAGUAU
ENSG00000117500	AUUGCAC
ENSG00000117500	CACAGUG
ENSG00000117500	CUGGAAA
ENSG00000117500	GAGGUAG
ENSG00000117500	UCACAGU
ENSG00000117500	UCAUGAU
ENSG00000117500	UGCAUUG
ENSG00000117500	UUUCAAC
ENSG00000117505	AAUUUUA
ENSG00000117505	CUUUGGU
ENSG00000117519	AAGGCAC
ENSG00000117519	ACAGUAU
ENSG00000117519	CACAGUG
ENSG00000117519	UAUAAAG
ENSG00000117519	UCACAGG
ENSG00000117519	UUAUGCA
ENSG00000117523	AAUACUG
ENSG00000117523	ACAGUAU
ENSG00000117523	ACCUGGA
ENSG00000117523	ACUAGAU
ENSG00000117523	AGCAGCG
ENSG00000117523	AGCAGCU
ENSG00000117523	AGCUGCC
ENSG00000117523	AGUACUG
ENSG00000117523	AUCAUAC
ENSG00000117523	AUGGCUU
ENSG00000117523	CAGCAGA
ENSG00000117523	CCUGUAC
ENSG00000117523	CUGCAGC
ENSG00000117523	CUGCAGG
ENSG00000117523	GAGGAUA
ENSG00000117523	GUUAAUG
ENSG00000117523	UAGAGGA
ENSG00000117523	UAUAAUA
ENSG00000117523	UCACAGU
ENSG00000117523	UCAUACA
ENSG00000117523	UCUGGAA
ENSG00000117523	UGGUACC
ENSG00000117523	UUCUGCA
ENSG00000117525	AAAGUUU
ENSG00000117525	AAAUCCC
ENSG00000117525	AGUAGUU
ENSG00000117525	CAGUUAC
ENSG00000117525	GUCAGUU
ENSG00000117525	UAUAAAG
ENSG00000117525	UCACAGU
ENSG00000117533	ACUGGAU
ENSG00000117533	AUCCUUG
ENSG00000117533	GCAGCAU
ENSG00000117533	GGCAAGA
ENSG00000117533	UCUCAAG
ENSG00000117533	UCUUGAG
ENSG00000117560	GAGGUAG
ENSG00000117560	GAGGUAU
ENSG00000117569	AAAAACC

ENSG00000117569	AAAAGUA
ENSG00000117569	AAAGUAU
ENSG00000117569	AAGACAU
ENSG00000117569	ACACACU
ENSG00000117569	ACAUUAC
ENSG00000117569	AUGCACC
ENSG00000117569	CAGUCUG
ENSG00000117569	CCCACCG
ENSG00000117569	CCCACCU
ENSG00000117569	CUUUGGU
ENSG00000117569	GAGUGUG
ENSG00000117569	UAAGACG
ENSG00000117569	UCCCACC
ENSG00000117569	UUCUAAU
ENSG00000117569	UUGGUCC
ENSG00000117586	UAUUACC
ENSG00000117586	UCUGGAG
ENSG00000117597	AAAGUGC
ENSG00000117597	AAGUGCU
ENSG00000117597	AGCCCUU
ENSG00000117597	CAGCAGA
ENSG00000117597	CAGCAGG
ENSG00000117597	CAGCUGG
ENSG00000117597	GAGCUGC
ENSG00000117597	GAGGAUA
ENSG00000117597	GGCUCAG
ENSG00000117597	GGGUAAG
ENSG00000117597	UUCUUGU
ENSG00000117616	AAGUGCA
ENSG00000117616	CCCACCG
ENSG00000117616	UCCCACC
ENSG00000117620	AGGACAC
ENSG00000117620	GCAGCAU
ENSG00000117620	UCACAGG
ENSG00000117620	UCACAGU
ENSG00000117620	UGCAUAG
ENSG00000117625	AACACUG
ENSG00000117625	AUCCUUU
ENSG00000117625	CAAAGCA
ENSG00000117625	GCUGGUG
ENSG00000117625	UGCAAAG
ENSG00000117625	UUUGCAA
ENSG00000117632	ACUGGCC
ENSG00000117640	AGCAGCA
ENSG00000117643	GAAAACA
ENSG00000117676	AAAGUGC
ENSG00000117676	AAGGCAC
ENSG00000117676	AAGUGCU
ENSG00000117676	CACACCG
ENSG00000117676	CACACCU
ENSG00000117676	CCCUGAG
ENSG00000117676	CUCACAC
ENSG00000117676	GCUGGUG
ENSG00000117676	GUGCCUG
ENSG00000117682	AAAUCCC
ENSG00000117682	AAGGCAC
ENSG00000117682	AAUCACU
ENSG00000117682	AAUCCUU
ENSG00000117682	ACCCUGU
ENSG00000117682	AGCAAAA
ENSG00000117682	AGCAGCA
ENSG00000117682	CAAAGUG
ENSG00000117682	CAGCAGA
ENSG00000117682	CUCCAGC

ENSG00000117682	CUUGUGU
ENSG00000117682	GAUUGGU
ENSG00000117682	GAUUGUA
ENSG00000117682	GGAGGAG
ENSG00000117682	GGAGGCA
ENSG00000117682	GGCAGGG
ENSG00000117682	GGCUCUG
ENSG00000117682	GUCAACA
ENSG00000117682	GUGCCAC
ENSG00000117682	GUGUGGC
ENSG00000117682	UCAACAG
ENSG00000117682	UGCCCUG
ENSG00000117682	UGGGGGA
ENSG00000117682	UGUGUGG
ENSG00000117697	AACAAAC
ENSG00000117697	AGUAACA
ENSG00000117697	UGAGAAU
ENSG00000117713	AAAGAGG
ENSG00000117713	AAAGGUA
ENSG00000117713	AAUCUCA
ENSG00000117713	AUAAAGU
ENSG00000117713	CACACCG
ENSG00000117713	CCUGUAC
ENSG00000117713	GAGACCU
ENSG00000117713	GGGUGUU
ENSG00000117713	GGUGGUU
ENSG00000117713	GUAUGUU
ENSG00000117748	AAGUGCA
ENSG00000117751	AAAGGUA
ENSG00000117751	AAAGUUU
ENSG00000117751	ACAGGUG
ENSG00000117751	AUGUAAU
ENSG00000117751	GUUAGGA
ENSG00000117751	UACAAAG
ENSG00000117758	AGCAAAA
ENSG00000117758	AUACAAG
ENSG00000117758	CUCCAUU
ENSG00000117758	GUGCAAA
ENSG00000117758	UACAAAG
ENSG00000117758	UGCAAAG
ENSG00000117859	AAAUUUC
ENSG00000117859	AUCCCUU
ENSG00000117859	CCAGUAC
ENSG00000117859	UUCUAAU
ENSG00000117862	CCUGCUG
ENSG00000117899	GGGCCCC
ENSG00000118007	ACAGUAU
ENSG00000118007	CUCUAGC
ENSG00000118007	GCUGGUG
ENSG00000118007	UCACAGU
ENSG00000118046	AAAGUGC
ENSG00000118046	AAGUGCA
ENSG00000118046	AUAAAGU
ENSG00000118046	CAGUAGU
ENSG00000118046	CUCUGGG
ENSG00000118046	GGAAGAC
ENSG00000118046	UAUUGCU
ENSG00000118058	AACAAAC
ENSG00000118058	AAGGCAC
ENSG00000118058	AAGUGCA
ENSG00000118058	AAUUAUU
ENSG00000118058	ACUGGCC
ENSG00000118058	AGUAACA
ENSG00000118058	AGUGCAA

ENSG00000118058	AGUGCUG
ENSG00000118058	AUAAUAC
ENSG00000118058	CAAAUGC
ENSG00000118058	CACAGUG
ENSG00000118058	CAGGCUC
ENSG00000118058	CAGUAGU
ENSG00000118058	CUAGUCC
ENSG00000118058	CUCACAC
ENSG00000118058	GACUUCC
ENSG00000118058	GGGGGAA
ENSG00000118058	GGUCUAG
ENSG00000118058	GUAGUGU
ENSG00000118058	GUCAACA
ENSG00000118058	GUUCAUG
ENSG00000118058	UAUAAAG
ENSG00000118058	UAUUGCU
ENSG00000118058	UCACACA
ENSG00000118058	UCAUAGA
ENSG00000118058	UCCUAUA
ENSG00000118058	UCUCAAG
ENSG00000118058	UGAAGUG
ENSG00000118058	UGGCCAC
ENSG00000118058	UGUGCUU
ENSG00000118058	UUAGGAU
ENSG00000118058	UUGACAC
ENSG00000118160	AAAGUUU
ENSG00000118160	ACACACU
ENSG00000118160	AGAUGUG
ENSG00000118160	AGGUAGU
ENSG00000118160	AUGCACC
ENSG00000118160	AUGGCAC
ENSG00000118160	CCCUGAG
ENSG00000118160	CUCUGGG
ENSG00000118160	GAGGUAG
ENSG00000118160	GCAACUU
ENSG00000118160	GCAGCAU
ENSG00000118160	GGAAUGU
ENSG00000118160	GGCAGCG
ENSG00000118160	UCAACAG
ENSG00000118160	UUGGUCC
ENSG00000118193	AGUGCCU
ENSG00000118193	UUCAAAU
ENSG00000118200	AGCAGCU
ENSG00000118200	AGUGCUU
ENSG00000118200	GAGUUGG
ENSG00000118200	UCCUGAG
ENSG00000118200	UGAAGUG
ENSG00000118217	AACACUG
ENSG00000118242	AAGUCUU
ENSG00000118242	UCAAGUA
ENSG00000118242	UCCUGAG
ENSG00000118242	UUCAAGC
ENSG00000118242	UUUCAAC
ENSG00000118245	ACAUUCA
ENSG00000118245	GAGAUGA
ENSG00000118257	AAAUGGU
ENSG00000118257	CACAGUG
ENSG00000118260	AAGUGCA
ENSG00000118260	ACAUUCA
ENSG00000118260	GGAGGCA
ENSG00000118267	AAAUGGU
ENSG00000118267	UUCUGCA
ENSG00000118267	UUGUUCG
ENSG00000118276	AAAGCUG

ENSG00000118276	AAAGUGC
ENSG00000118276	AAAUCCC
ENSG00000118276	AAGUGCU
ENSG00000118276	AUGCAUU
ENSG00000118276	CCCUGAG
ENSG00000118276	GGCUCUG
ENSG00000118322	UAAGACU
ENSG00000118322	UCACAGU
ENSG00000118363	AAAGCUG
ENSG00000118363	AAAUUUC
ENSG00000118363	AACAUUC
ENSG00000118363	AAGUUGU
ENSG00000118363	ACACACU
ENSG00000118363	ACUUGGA
ENSG00000118363	AGUACUG
ENSG00000118363	CAGCAGG
ENSG00000118363	GAAAACA
ENSG00000118363	GGCAGUG
ENSG00000118363	UAUAAAG
ENSG00000118363	UCAACAG
ENSG00000118363	UCACAUU
ENSG00000118363	UCAUACA
ENSG00000118369	AGGACAC
ENSG00000118369	AGGCAGG
ENSG00000118369	GAGGCAG
ENSG00000118402	GAGGUAG
ENSG00000118407	UCAUUUG
ENSG00000118412	AUACAAG
ENSG00000118412	CAGCAAA
ENSG00000118418	AGCACCA
ENSG00000118418	UACAAAG
ENSG00000118420	UGCUGAC
ENSG00000118432	AAAGAGC
ENSG00000118432	AAAGAGG
ENSG00000118432	AAAGUAU
ENSG00000118432	AACCUGG
ENSG00000118432	AAUUUUA
ENSG00000118432	ACAUUCA
ENSG00000118432	ACCAGCA
ENSG00000118432	AGCACCA
ENSG00000118432	AGUGCAA
ENSG00000118432	AGUGCUG
ENSG00000118432	AGUGCUU
ENSG00000118432	AUUGCAC
ENSG00000118432	CACAGUG
ENSG00000118432	CAGUGCA
ENSG00000118432	CCAGUGC
ENSG00000118432	GAGAAAU
ENSG00000118432	GUCCAGA
ENSG00000118432	UCACACA
ENSG00000118432	UCACAGU
ENSG00000118432	UGAAGCU
ENSG00000118432	UGGCCAC
ENSG00000118454	AAGGCAC
ENSG00000118454	AAUGUUG
ENSG00000118454	ACAUAGA
ENSG00000118454	AGUGCUG
ENSG00000118454	CAGUAGU
ENSG00000118454	CCAGUGU
ENSG00000118454	CUACAGU
ENSG00000118454	CUAUACA
ENSG00000118454	GAAUUGU
ENSG00000118454	GAUCAGA
ENSG00000118454	GCAUCCC

ENSG00000118454	GGAAUGU
ENSG00000118454	GGACCUG
ENSG00000118454	GGAGUGU
ENSG00000118454	GUCAACA
ENSG00000118454	GUUAAUG
ENSG00000118454	UAAGACG
ENSG00000118454	UAAGACU
ENSG00000118454	UAUAAAG
ENSG00000118454	UAUAUGA
ENSG00000118454	UCAACAG
ENSG00000118454	UCAUUCG
ENSG00000118454	UCCCUGU
ENSG00000118454	UCCCUUG
ENSG00000118454	UGAAAUG
ENSG00000118454	UGGAUAA
ENSG00000118473	AAGUGCA
ENSG00000118473	AAUACUG
ENSG00000118473	AAUCCUU
ENSG00000118473	AUCCUUG
ENSG00000118473	CAGGAAC
ENSG00000118473	CCCUGUU
ENSG00000118473	CCUUCUG
ENSG00000118473	GGAAUGU
ENSG00000118473	GUGCCAC
ENSG00000118482	ACAGUAC
ENSG00000118482	ACAGUAU
ENSG00000118482	AUUGCAC
ENSG00000118482	UAUUGCU
ENSG00000118495	AAAAAUC
ENSG00000118495	AAAUCCC
ENSG00000118495	AGAUAAA
ENSG00000118495	AGCAGCA
ENSG00000118495	AGCAGCG
ENSG00000118495	AUGGCUU
ENSG00000118495	CCCUGAG
ENSG00000118495	UCACAUU
ENSG00000118496	AAGGCAC
ENSG00000118496	ACCCUGU
ENSG00000118496	AGCCCUU
ENSG00000118496	CCAGCAU
ENSG00000118496	CCCUGUC
ENSG00000118496	GCAACUU
ENSG00000118496	GUCACUC
ENSG00000118496	GUCCAGA
ENSG00000118496	GUGCUUG
ENSG00000118503	AAGGUGC
ENSG00000118503	AGCAGCU
ENSG00000118503	AGGUUAU
ENSG00000118503	CAGCAGA
ENSG00000118503	CAGCAGG
ENSG00000118503	CCCUGAG
ENSG00000118503	GAGGUAG
ENSG00000118503	GCACCAU
ENSG00000118503	UCAUACA
ENSG00000118507	ACUGCAU
ENSG00000118507	AGUGCAA
ENSG00000118507	UGCAAAG
ENSG00000118507	UGCAUAG
ENSG00000118513	AGCAGCG
ENSG00000118513	AGCAGCU
ENSG00000118513	CAGCAAA
ENSG00000118513	GGCAUUG
ENSG00000118513	UGCAUAG
ENSG00000118513	UUGGCAA

ENSG00000118513	UUGGCAC
ENSG00000118514	AAAAGUA
ENSG00000118514	AACAUUC
ENSG00000118515	AAAAAUC
ENSG00000118515	AAGGCAC
ENSG00000118515	AGCAGCG
ENSG00000118515	AGCAGCU
ENSG00000118515	AUUGCAC
ENSG00000118515	CAGCUGG
ENSG00000118515	GGAAGAC
ENSG00000118515	GGGGCAG
ENSG00000118515	GUGUCAC
ENSG00000118515	UAUUGCU
ENSG00000118515	UCACAUU
ENSG00000118515	UGCAAAG
ENSG00000118515	UUCUAAU
ENSG00000118515	UUGGUCC
ENSG00000118518	AAAACUG
ENSG00000118518	UCCCACC
ENSG00000118523	AAGGUGC
ENSG00000118523	AGAUAAA
ENSG00000118523	GUCCAGA
ENSG00000118523	GUGCAAA
ENSG00000118523	UCAAGUA
ENSG00000118523	UUGGUCC
ENSG00000118526	AAAACUG
ENSG00000118526	GAGAACU
ENSG00000118564	AAGUCAC
ENSG00000118564	AGCCUGC
ENSG00000118564	AGUAGAC
ENSG00000118564	CUGCUCA
ENSG00000118640	AGCAGCA
ENSG00000118640	GCAGCAU
ENSG00000118655	CAGCUGG
ENSG00000118655	CUGGCUC
ENSG00000118655	GCAGAAG
ENSG00000118655	GCUUCCU
ENSG00000118655	GGCUCUG
ENSG00000118655	UUGGCAA
ENSG00000118655	UUGGCAC
ENSG00000118689	AAAGCUG
ENSG00000118689	ACUAGAU
ENSG00000118689	ACUGUAG
ENSG00000118689	AGCACCA
ENSG00000118689	AGGAGCU
ENSG00000118689	AUCACUA
ENSG00000118689	CACUGUU
ENSG00000118689	CCUGCUG
ENSG00000118689	CUAUACA
ENSG00000118689	CUUCACC
ENSG00000118689	CUUUGGU
ENSG00000118689	GAGAAAU
ENSG00000118689	GAGUGUG
ENSG00000118689	GCACCAU
ENSG00000118689	GCUACAU
ENSG00000118689	GGAGUGU
ENSG00000118689	GGCAGGG
ENSG00000118689	GUAAACA
ENSG00000118689	GUCUCUG
ENSG00000118689	GUGCCAC
ENSG00000118689	GUGUGGC
ENSG00000118689	UACAAAG
ENSG00000118689	UAUAUGA
ENSG00000118689	UCACAAG

ENSG00000118689	UCUGGAA
ENSG00000118689	UCUGGAG
ENSG00000118689	UGCAUAG
ENSG00000118689	UGCUGAC
ENSG00000118689	UGGAGAU
ENSG00000118689	UUAUGCA
ENSG00000118689	UUCCAUA
ENSG00000118689	UUGAGGC
ENSG00000118689	UUGGAGG
ENSG00000118689	UUGGCAC
ENSG00000118689	UUGUGUC
ENSG00000118689	UUUGCAA
ENSG00000118689	UUUGCAC
ENSG00000118702	AAUUUUA
ENSG00000118705	UCACAGG
ENSG00000118705	UCACAGU
ENSG00000118705	UCUGGAA
ENSG00000118707	AACAUUC
ENSG00000118707	ACAGUAC
ENSG00000118707	ACAGUAU
ENSG00000118707	ACCACAG
ENSG00000118707	ACUUGGA
ENSG00000118707	AGCACCA
ENSG00000118707	AGUGAUC
ENSG00000118707	CACAGUG
ENSG00000118707	CAGCAGG
ENSG00000118707	CAGCUGG
ENSG00000118707	CUGCAGG
ENSG00000118707	GCACCAU
ENSG00000118707	GCAGCAU
ENSG00000118707	GCCUGGA
ENSG00000118707	GGAAUGU
ENSG00000118707	GGAGUCC
ENSG00000118707	UACAGUU
ENSG00000118707	UCCUGAG
ENSG00000118707	UGAUCCA
ENSG00000118707	UGCUCAC
ENSG00000118707	UGUGCUU
ENSG00000118707	UUGACAC
ENSG00000118729	AAGGCAC
ENSG00000118729	GGCUCUG
ENSG00000118733	AAGACAU
ENSG00000118733	AAGGCAC
ENSG00000118733	AGUGCUG
ENSG00000118733	CACAAUG
ENSG00000118733	GGUAGAC
ENSG00000118733	GGUGGUC
ENSG00000118733	GGUUACC
ENSG00000118733	GUCUCUG
ENSG00000118733	UAUAAAG
ENSG00000118733	UCACAGG
ENSG00000118733	UCACAGU
ENSG00000118762	CAUGGAU
ENSG00000118762	GACCAUG
ENSG00000118762	UGAAGUG
ENSG00000118804	AGCAAAA
ENSG00000118873	UACAAAG
ENSG00000118900	AAAACUG
ENSG00000118900	AAGGCAC
ENSG00000118900	AAUCUCU
ENSG00000118900	AUCACUA
ENSG00000118900	AUGCAUU
ENSG00000118900	AUGUCUG
ENSG00000118900	CACCACC

ENSG00000118900	CUACAAA
ENSG00000118900	CUCCAGC
ENSG00000118900	CUGCUCA
ENSG00000118900	CUUUGGU
ENSG00000118900	GAAACAU
ENSG00000118900	GAGGCAG
ENSG00000118900	GGAAUGU
ENSG00000118900	GGCAAGA
ENSG00000118900	GUGCAAA
ENSG00000118900	UAAGACU
ENSG00000118900	UCCCUGU
ENSG00000118922	AAAAGUA
ENSG00000118922	AAACUGU
ENSG00000118922	AAAGAGC
ENSG00000118922	AAAGUAU
ENSG00000118922	AACUCUA
ENSG00000118922	AAGUGCA
ENSG00000118922	AAUACUG
ENSG00000118922	AAUUGCU
ENSG00000118922	AAUUUUA
ENSG00000118922	ACAAUAU
ENSG00000118922	ACACACC
ENSG00000118922	ACAUUAC
ENSG00000118922	ACGCUCA
ENSG00000118922	ACUGCAU
ENSG00000118922	AGCACCA
ENSG00000118922	AGCCCUU
ENSG00000118922	AGGGUCU
ENSG00000118922	AGUAGUU
ENSG00000118922	AUAUAAC
ENSG00000118922	AUCAUAC
ENSG00000118922	AUGCACC
ENSG00000118922	AUGCCUU
ENSG00000118922	CAAGAGC
ENSG00000118922	CAGACAG
ENSG00000118922	CAGCUGG
ENSG00000118922	CAGGCUC
ENSG00000118922	CAGUAGU
ENSG00000118922	CAGUCUG
ENSG00000118922	CCAGUGC
ENSG00000118922	CCAGUGU
ENSG00000118922	CCAUUAC
ENSG00000118922	CUACAAA
ENSG00000118922	CUAUACA
ENSG00000118922	CUGCCCC
ENSG00000118922	CUUUGGU
ENSG00000118922	GAGUAUU
ENSG00000118922	GCACCAU
ENSG00000118922	GCCUGGG
ENSG00000118922	GGCAGGG
ENSG00000118922	GGGUGUU
ENSG00000118922	GGUGGUC
ENSG00000118922	GGUUACC
ENSG00000118922	GUCUUGC
ENSG00000118922	GUGACAG
ENSG00000118922	GUGAGGU
ENSG00000118922	GUGCAAA
ENSG00000118922	GUGCUUG
ENSG00000118922	GUUCAUG
ENSG00000118922	UCACAGG
ENSG00000118922	UCACAGU
ENSG00000118922	UCAUAGA
ENSG00000118922	UCAUUCG
ENSG00000118922	UCCCACA

ENSG00000118922	UCCUUGC
ENSG00000118922	UGAAGUG
ENSG00000118922	UGAGAAU
ENSG00000118922	UGGCCAC
ENSG00000118922	UUGACAC
ENSG00000118922	UUGUUCG
ENSG00000118946	AAGACAU
ENSG00000118946	ACUCAAA
ENSG00000118946	ACUGUAG
ENSG00000118946	AGCAGCA
ENSG00000118946	AUAUUAU
ENSG00000118946	AUGCACC
ENSG00000118946	CUCAAAC
ENSG00000118946	GAUUGUC
ENSG00000118946	GCAGCAU
ENSG00000118946	GCUUCCU
ENSG00000118946	GGCUCAG
ENSG00000118946	GUCAUUG
ENSG00000118946	UCAUAGA
ENSG00000118946	UCCCACA
ENSG00000118960	AAGGCAC
ENSG00000118971	AAACUGU
ENSG00000118971	AAAGAAU
ENSG00000118971	AAAGCUG
ENSG00000118971	AAAGUGC
ENSG00000118971	AAAUGGU
ENSG00000118971	AAGUGCU
ENSG00000118971	AAUUGCU
ENSG00000118971	ACAUUAC
ENSG00000118971	ACCAGCA
ENSG00000118971	AGUGCUU
ENSG00000118971	CAAAGUG
ENSG00000118971	CACACCU
ENSG00000118971	CACCAGC
ENSG00000118971	CCAGUAC
ENSG00000118971	CCCUGUU
ENSG00000118971	GAAACAU
ENSG00000118971	GAGGAUA
ENSG00000118971	GAGGUAG
ENSG00000118971	GAUCAGA
ENSG00000118971	GCACCAU
ENSG00000118971	GCCCUGU
ENSG00000118971	GGUUUAC
ENSG00000118971	GUCCAGA
ENSG00000118971	GUGAGGU
ENSG00000118971	UAUAAAG
ENSG00000118971	UCAAGUA
ENSG00000118971	UCCAGUU
ENSG00000118971	UGCAUAG
ENSG00000118971	UGCAUAU
ENSG00000118971	UGGACUG
ENSG00000118971	UUGGCAA
ENSG00000118971	UUGGCAC
ENSG00000118972	CAAAUGC
ENSG00000118972	UAUAAAG
ENSG00000118985	AACACUG
ENSG00000118985	AACCUGG
ENSG00000118985	AACCUGU
ENSG00000118985	AAGACAU
ENSG00000118985	AAGGCAC
ENSG00000118985	AAGUAGC
ENSG00000118985	AAGUCUU
ENSG00000118985	ACAGUAU
ENSG00000118985	AGCACCA

ENSG00000118985	AGCAGCA
ENSG00000118985	AGCAGCG
ENSG00000118985	AUAUAAC
ENSG00000118985	CAAAACU
ENSG00000118985	CACAGUG
ENSG00000118985	CUACAGU
ENSG00000118985	CUCUUCC
ENSG00000118985	GAUUGUA
ENSG00000118985	GCUUCCU
ENSG00000118985	GGCAGCG
ENSG00000118985	GGUUUAC
ENSG00000118985	GUCAGUU
ENSG00000118985	UAAUGCU
ENSG00000118985	UCACAGU
ENSG00000118985	UCACAUU
ENSG00000118985	UCCCACA
ENSG00000118985	UCCCACC
ENSG00000118985	UCCCUUG
ENSG00000118985	UGCAUAG
ENSG00000118985	UGCAUAU
ENSG00000118985	UGCAUUG
ENSG00000118985	UGUCUUU
ENSG00000118985	UGUGCUU
ENSG00000118985	UUGGCAA
ENSG00000118985	UUGGCAC
ENSG00000118985	UUUGCAA
ENSG00000119004	UAUAAAG
ENSG00000119042	AAAGCUG
ENSG00000119042	AAUGCCC
ENSG00000119042	ACCACAG
ENSG00000119042	AGCAGCU
ENSG00000119042	CACAGUG
ENSG00000119042	CUGCAGC
ENSG00000119042	UCACAGG
ENSG00000119042	UCCCUGU
ENSG00000119042	UCCCUUU
ENSG00000119042	UGCAGCU
ENSG00000119042	UGCCCUG
ENSG00000119042	UUUGCAA
ENSG00000119048	AACCUGU
ENSG00000119048	AAGGCAC
ENSG00000119048	AAGUGCA
ENSG00000119048	ACAUUAC
ENSG00000119048	AUAUAAC
ENSG00000119048	AUAUUAU
ENSG00000119048	AUGAUGA
ENSG00000119048	AUGUAAU
ENSG00000119048	CACACCG
ENSG00000119048	CACACCU
ENSG00000119048	CAGCAGG
ENSG00000119048	CUGCUCA
ENSG00000119048	GAAAACA
ENSG00000119048	UACAGUU
ENSG00000119048	UCACACA
ENSG00000119048	UUCCAUA
ENSG00000119121	GAGAAAU
ENSG00000119121	GAGGUAG
ENSG00000119121	GAGGUAU
ENSG00000119121	GUGCAAA
ENSG00000119121	UCAAGUA
ENSG00000119121	UUCAAGC
ENSG00000119125	GUAAACA
ENSG00000119125	UUAUGCA
ENSG00000119138	AGCAAAA

ENSG00000119138	CAGCAAA
ENSG00000119138	GAGGUAG
ENSG00000119138	UCCUGAG
ENSG00000119139	AAGGCAC
ENSG00000119139	AUGGCUU
ENSG00000119227	CACAGUG
ENSG00000119231	AAAGCUG
ENSG00000119231	AAAGUGC
ENSG00000119231	AAGACAU
ENSG00000119231	AAGUGUG
ENSG00000119231	AAUACUG
ENSG00000119231	ACUGCAU
ENSG00000119231	AGCAGCA
ENSG00000119231	AGCAGCG
ENSG00000119231	AGCAGCU
ENSG00000119231	AGUAACA
ENSG00000119231	AGUGCCU
ENSG00000119231	AGUGCUG
ENSG00000119231	AUAAAGU
ENSG00000119231	CCAGGAG
ENSG00000119231	CCUGCUG
ENSG00000119231	CUGCUCA
ENSG00000119231	CUGGGCA
ENSG00000119231	GAGAAAU
ENSG00000119231	GAGGUAG
ENSG00000119231	GAGGUAU
ENSG00000119231	GCACCAU
ENSG00000119231	GGCAGCG
ENSG00000119231	GUAAACA
ENSG00000119231	GUAACAG
ENSG00000119231	GUAUUCU
ENSG00000119231	GUGGCCC
ENSG00000119231	UCAUUUG
ENSG00000119231	UGUUGAA
ENSG00000119231	UUGAGGC
ENSG00000119231	UUGGAGG
ENSG00000119242	AUAAAGU
ENSG00000119242	CACAGUG
ENSG00000119242	UCACAGU
ENSG00000119242	UUGGCAA
ENSG00000119242	UUGGCAC
ENSG00000119280	CAGGUGA
ENSG00000119280	CUGGGCA
ENSG00000119280	GCAACUU
ENSG00000119280	UCUGGAA
ENSG00000119280	UUGGCAA
ENSG00000119280	UUGGCAC
ENSG00000119283	AAAAAUC
ENSG00000119283	AAAACUG
ENSG00000119283	AAGAACC
ENSG00000119283	ACAUCAC
ENSG00000119283	ACCCAUU
ENSG00000119283	AGCUGCC
ENSG00000119283	AGGAGCU
ENSG00000119283	CAGUUAC
ENSG00000119283	GAGCUGC
ENSG00000119283	GAGGCAG
ENSG00000119283	GAGGUAG
ENSG00000119283	GAGGUAU
ENSG00000119283	GGAAGAC
ENSG00000119283	GUGGGGA
ENSG00000119283	UAAGACG
ENSG00000119283	UAUUGCU
ENSG00000119283	UCAUGAU

ENSG00000119283	UCCUGAG
ENSG00000119283	UGCUCAC
ENSG00000119283	UGCUGAC
ENSG00000119283	UUUGCAA
ENSG00000119314	AAAGCUG
ENSG00000119314	ACAGUAU
ENSG00000119314	AGCACCA
ENSG00000119314	AGUGGUU
ENSG00000119314	CUUUGGU
ENSG00000119314	GAGAUGA
ENSG00000119314	GUAAACA
ENSG00000119314	UUGGUCC
ENSG00000119318	AAAGCUG
ENSG00000119318	AAAGUGC
ENSG00000119318	AAGUGCU
ENSG00000119318	ACAUCAC
ENSG00000119318	ACAUUCA
ENSG00000119318	ACUGCAG
ENSG00000119318	ACUGCAU
ENSG00000119318	ACUGUGU
ENSG00000119318	AGCAGCA
ENSG00000119318	AGGUAGU
ENSG00000119318	AUCCCUU
ENSG00000119318	AUGAGCU
ENSG00000119318	CCAGUGC
ENSG00000119318	CUAGUAA
ENSG00000119318	CUAGUAG
ENSG00000119318	CUCACAC
ENSG00000119318	CUCCAUU
ENSG00000119318	GAGCCCC
ENSG00000119318	GAGCCCU
ENSG00000119318	GAUUGUC
ENSG00000119318	GGCAUUG
ENSG00000119318	UAAUAUC
ENSG00000119318	UCACAAG
ENSG00000119318	UCUGGAA
ENSG00000119318	UCUGGAG
ENSG00000119318	UGAAGUG
ENSG00000119318	UGGCCCU
ENSG00000119318	UGUGUGG
ENSG00000119318	UUGGCAA
ENSG00000119318	UUGGCAC
ENSG00000119318	UUGGUUC
ENSG00000119321	AAGGCAC
ENSG00000119321	CUUCACC
ENSG00000119335	AACAAAC
ENSG00000119335	AAUGCCC
ENSG00000119335	ACAUAGA
ENSG00000119335	AGGAUGU
ENSG00000119335	AGUAGAC
ENSG00000119335	AUGCACC
ENSG00000119335	CAAAGCA
ENSG00000119335	CACAGGG
ENSG00000119335	CUCAAAC
ENSG00000119335	GGCAGGG
ENSG00000119335	GUAACAG
ENSG00000119335	UGCUGAC
ENSG00000119335	UUGUGUC
ENSG00000119383	CUGGAAA
ENSG00000119383	CUUUGGU
ENSG00000119383	GUGCAAA
ENSG00000119383	GUGCUUG
ENSG00000119383	UGUGCUU
ENSG00000119383	UUGGUCC

ENSG00000119392	GCUGUCU
ENSG00000119396	AACCUGG
ENSG00000119396	AACCUGU
ENSG00000119396	AAGGCAC
ENSG00000119396	ACAGGUG
ENSG00000119396	AGCAUUC
ENSG00000119396	AUUGCAC
ENSG00000119396	CAGACAG
ENSG00000119396	CAGCAGA
ENSG00000119396	GCAGGAC
ENSG00000119396	UACAAAG
ENSG00000119396	UCACAGG
ENSG00000119396	UCACAGU
ENSG00000119396	UCCAGUU
ENSG00000119396	UCCCUGU
ENSG00000119396	UGAAAGG
ENSG00000119397	AGGUAGU
ENSG00000119397	GAGGUAU
ENSG00000119397	GUGAGGU
ENSG00000119402	AGCACCA
ENSG00000119402	CACCAGC
ENSG00000119402	CAGCAGA
ENSG00000119402	CCCCUGG
ENSG00000119402	CUUUGGU
ENSG00000119402	GAGAACC
ENSG00000119402	GAUUGUA
ENSG00000119402	GAUUGUC
ENSG00000119402	GCAUCCC
ENSG00000119402	GGUGGUC
ENSG00000119402	GUAUGUU
ENSG00000119402	GUUCAUG
ENSG00000119402	UCUCAAG
ENSG00000119402	UGAAGCU
ENSG00000119402	UUCCUAG
ENSG00000119402	UUUGCAC
ENSG00000119403	AACAAAC
ENSG00000119403	AAUUAUU
ENSG00000119403	AGCAGCU
ENSG00000119403	AGGCAGG
ENSG00000119403	CCUUCUG
ENSG00000119403	CUUUGGU
ENSG00000119403	GAUUGUC
ENSG00000119403	GCCUGGG
ENSG00000119403	GGAGUGU
ENSG00000119403	GGCAGUG
ENSG00000119403	UCACAUU
ENSG00000119403	UCCCACA
ENSG00000119403	UGAAAUG
ENSG00000119403	UUCUAAU
ENSG00000119403	UUGGUUC
ENSG00000119408	AAUCCUU
ENSG00000119408	CACAGUG
ENSG00000119408	CCUUCAU
ENSG00000119408	CUUCACC
ENSG00000119408	UCAAGUA
ENSG00000119408	UCACAGG
ENSG00000119408	UCCCUUU
ENSG00000119411	ACCUGGA
ENSG00000119414	AAAGUGC
ENSG00000119414	AAGUGCU
ENSG00000119414	AGCAGCA
ENSG00000119414	CAAAGUG
ENSG00000119414	CAGCAAA
ENSG00000119414	CAGCAGA

ENSG00000119414	CAGCAGG
ENSG00000119414	GGCAAGA
ENSG00000119421	AAGUCAC
ENSG00000119446	AAAGUAC
ENSG00000119457	AAGUGCU
ENSG00000119457	AGUGCUU
ENSG00000119471	AUCCUUG
ENSG00000119471	AUCCUUU
ENSG00000119471	UCAAGUA
ENSG00000119471	UGCAUAU
ENSG00000119471	UUCAAGC
ENSG00000119487	AAAAAUC
ENSG00000119487	AAAGAAU
ENSG00000119487	AAAGAGC
ENSG00000119487	AAAGAGG
ENSG00000119487	CUACAAA
ENSG00000119508	AAGUCAC
ENSG00000119508	AUUCAUU
ENSG00000119508	AUUGCAC
ENSG00000119508	GGAAUGU
ENSG00000119508	UAAUAUC
ENSG00000119508	UAGAGGA
ENSG00000119509	CAGUGCA
ENSG00000119514	AAGGCAC
ENSG00000119522	AAAUGGU
ENSG00000119522	CUUUGGU
ENSG00000119522	GCUGGUG
ENSG00000119522	GGUUUAC
ENSG00000119522	GUGCAAA
ENSG00000119522	UAUUGCU
ENSG00000119522	UUCAAGC
ENSG00000119537	AGCAGCG
ENSG00000119537	AGCAGCU
ENSG00000119537	AUCCUUU
ENSG00000119537	UGCAUAU
ENSG00000119537	UGGGAUC
ENSG00000119541	AAGGCAC
ENSG00000119541	ACAAGGU
ENSG00000119541	AUUGCAC
ENSG00000119541	CACAGUG
ENSG00000119541	CUUUGGU
ENSG00000119541	UGUCUUU
ENSG00000119547	AACCUGG
ENSG00000119547	AAGAACC
ENSG00000119547	AAGCGCU
ENSG00000119547	AAGGCAC
ENSG00000119547	AAUACUG
ENSG00000119547	AAUCACU
ENSG00000119547	ACACACC
ENSG00000119547	ACACACU
ENSG00000119547	ACAGUAC
ENSG00000119547	ACUCAGC
ENSG00000119547	ACUGCAU
ENSG00000119547	ACUGGCU
ENSG00000119547	ACUGUGU
ENSG00000119547	AGCACCA
ENSG00000119547	AGCAGCA
ENSG00000119547	AGCAGCU
ENSG00000119547	AGGGUUG
ENSG00000119547	AUACCUC
ENSG00000119547	AUCCCUU
ENSG00000119547	AUGCACC
ENSG00000119547	CAAAUGC
ENSG00000119547	CAAGAGC

ENSG00000119547	CACAGGG
ENSG00000119547	CACCAGC
ENSG00000119547	CAGUUAC
ENSG00000119547	CCAGUGC
ENSG00000119547	CCCUGAG
ENSG00000119547	CCCUGUC
ENSG00000119547	CUCCAGC
ENSG00000119547	CUUACAG
ENSG00000119547	GAAUUGU
ENSG00000119547	GAGCUGC
ENSG00000119547	GAGUGUG
ENSG00000119547	GAGUUGA
ENSG00000119547	GAGUUGG
ENSG00000119547	GAUUGGU
ENSG00000119547	GCACCAU
ENSG00000119547	GCCCUGU
ENSG00000119547	GCUUCCU
ENSG00000119547	GGAGGCA
ENSG00000119547	GGCAGUG
ENSG00000119547	GGGCCCC
ENSG00000119547	GUAAACA
ENSG00000119547	GUAUUCU
ENSG00000119547	GUCCAGA
ENSG00000119547	GUGACAG
ENSG00000119547	UACAAAG
ENSG00000119547	UCAUUCG
ENSG00000119547	UCCAGUU
ENSG00000119547	UCCCUGU
ENSG00000119547	UCUCAAG
ENSG00000119547	UCUCACC
ENSG00000119547	UCUGGAG
ENSG00000119547	UCUUGAG
ENSG00000119547	UGAAAGG
ENSG00000119547	UGAAGCU
ENSG00000119547	UGCAUAG
ENSG00000119547	UGCAUUG
ENSG00000119547	UGCUCAC
ENSG00000119547	UGUCUGC
ENSG00000119547	UUCAAGC
ENSG00000119596	AAAGUUU
ENSG00000119596	ACAGUAC
ENSG00000119596	ACAGUAU
ENSG00000119596	AUACCUC
ENSG00000119596	AUGAUGA
ENSG00000119596	GUGACAG
ENSG00000119614	AAUUUUA
ENSG00000119614	ACACACU
ENSG00000119614	ACAUUAC
ENSG00000119614	CACAGGG
ENSG00000119614	UAUUACC
ENSG00000119614	UCACAGG
ENSG00000119614	UCUCACC
ENSG00000119614	UUCAAAU
ENSG00000119630	AAGGCAC
ENSG00000119630	CAAGAGC
ENSG00000119630	CAGCAGA
ENSG00000119630	CUGGCUC
ENSG00000119630	UCCUGAG
ENSG00000119638	AAGUGUG
ENSG00000119661	AGAUGUG
ENSG00000119661	AGCACCA
ENSG00000119661	AGGCAGG
ENSG00000119661	AUAAAGU
ENSG00000119661	AUGGCUU

ENSG00000119661	CAAAGCA
ENSG00000119661	CUGUUGA
ENSG00000119661	GAGGCAG
ENSG00000119661	GAGGUAG
ENSG00000119661	GAGUUGG
ENSG00000119661	GAUUGUA
ENSG00000119661	GAUUGUC
ENSG00000119661	GCUACAU
ENSG00000119661	GUGCUUG
ENSG00000119661	UGUAUAA
ENSG00000119661	UGUUGAA
ENSG00000119661	UUAGAGA
ENSG00000119669	AACACUG
ENSG00000119669	AAGGCAC
ENSG00000119669	ACAUUAC
ENSG00000119669	ACCAGCA
ENSG00000119669	ACUGGCC
ENSG00000119669	AGCAGCA
ENSG00000119669	CAAAUGC
ENSG00000119669	CACCAGC
ENSG00000119669	CGUGCAU
ENSG00000119669	UAAGACU
ENSG00000119669	UAUAAAG
ENSG00000119669	UAUUACC
ENSG00000119669	UGUAUAA
ENSG00000119669	UUGGCAA
ENSG00000119681	AAAAAUC
ENSG00000119681	GAAGGAA
ENSG00000119681	GGUGGUC
ENSG00000119681	GGUGGUU
ENSG00000119682	AGCAGCU
ENSG00000119682	AUGGCUU
ENSG00000119682	CAGUAGU
ENSG00000119682	CCAGGAG
ENSG00000119682	CCCUGAG
ENSG00000119682	GACCAUG
ENSG00000119682	GAGAAGA
ENSG00000119682	GCUGGAU
ENSG00000119682	GCUGGUG
ENSG00000119682	UCCAGUU
ENSG00000119682	UUGGCAA
ENSG00000119682	UUGGCAC
ENSG00000119684	AGCAGCA
ENSG00000119684	CCUUCUU
ENSG00000119684	UGAAAGG
ENSG00000119684	UUCUAAU
ENSG00000119685	AAAGAGC
ENSG00000119689	AGCAAAA
ENSG00000119689	AGCAGCA
ENSG00000119689	CAAAGCA
ENSG00000119689	GAGAAGA
ENSG00000119689	GAGGUAG
ENSG00000119689	GGUUACC
ENSG00000119689	GUGCUUG
ENSG00000119689	UGUGCUU
ENSG00000119699	AUACAAG
ENSG00000119699	CCAGGAG
ENSG00000119699	GCUUCCU
ENSG00000119703	CAAAGUG
ENSG00000119703	CUUACAG
ENSG00000119703	GGAAUGU
ENSG00000119703	GGAGGCA
ENSG00000119703	GGGUUUA
ENSG00000119703	UCUUGAG

ENSG00000119703	UGAGGAC
ENSG00000119707	UAAUAUC
ENSG00000119707	UAUAAAG
ENSG00000119707	UGAAAUG
ENSG00000119714	GUGACAG
ENSG00000119718	AGCAGCA
ENSG00000119720	AUAAUAC
ENSG00000119720	AUAUAAC
ENSG00000119720	AUGUAAU
ENSG00000119725	CCAGGAG
ENSG00000119725	UGCAGCU
ENSG00000119729	CCCUGAG
ENSG00000119760	AAAGCUG
ENSG00000119760	UCAUGAU
ENSG00000119760	UGCCCUG
ENSG00000119772	ACAGUAU
ENSG00000119772	AGCACCA
ENSG00000119772	CAGUAGU
ENSG00000119772	CUUCUCU
ENSG00000119772	GCACCAU
ENSG00000119772	GUAACAG
ENSG00000119772	UAUUGCU
ENSG00000119778	AAGAACC
ENSG00000119778	AAGGCAC
ENSG00000119778	ACAGUAC
ENSG00000119778	ACAGUAU
ENSG00000119778	ACUGCAG
ENSG00000119778	CAAAGUG
ENSG00000119778	CAGUAGU
ENSG00000119778	CCAUUAC
ENSG00000119778	GAGGUAG
ENSG00000119778	GGCUCUG
ENSG00000119778	UACAGUU
ENSG00000119778	UGCAUAU
ENSG00000119782	AAGGCAC
ENSG00000119782	AUGUCUG
ENSG00000119782	CAAGAGC
ENSG00000119782	CUCCAGC
ENSG00000119801	AUGGCAC
ENSG00000119812	AAAAACC
ENSG00000119812	CACACCU
ENSG00000119812	UCACAGG
ENSG00000119812	UCACAGU
ENSG00000119820	CACAAUG
ENSG00000119820	CUUUGGU
ENSG00000119820	UUGGCAA
ENSG00000119844	ACAUUCA
ENSG00000119844	AGGACAC
ENSG00000119844	AGUGGUU
ENSG00000119844	CCAGUGU
ENSG00000119865	GAUUGUC
ENSG00000119865	GUCCAGA
ENSG00000119866	AAAAGUA
ENSG00000119866	AAACGGU
ENSG00000119866	AAAGGUA
ENSG00000119866	AAAUUUC
ENSG00000119866	AACGGAA
ENSG00000119866	AAGAACC
ENSG00000119866	AAGGCAC
ENSG00000119866	AAUGUUG
ENSG00000119866	AAUUAUU
ENSG00000119866	ACAGUAU
ENSG00000119866	ACUCAAA
ENSG00000119866	ACUGUAG

ENSG00000119866	ACUGUGU
ENSG00000119866	AGAUAAA
ENSG00000119866	AGGUAGU
ENSG00000119866	AGUACUG
ENSG00000119866	AGUGCAA
ENSG00000119866	AGUGGUU
ENSG00000119866	AGUGUGC
ENSG00000119866	AUAAAGU
ENSG00000119866	AUAUAAC
ENSG00000119866	AUCCUUG
ENSG00000119866	AUGAUGA
ENSG00000119866	AUGCAUU
ENSG00000119866	AUGGCUU
ENSG00000119866	CAAAGUG
ENSG00000119866	CACCACC
ENSG00000119866	CAGACAG
ENSG00000119866	CAGUGCA
ENSG00000119866	CUGGAAA
ENSG00000119866	CUUGUAU
ENSG00000119866	CUUUGGU
ENSG00000119866	GACAACU
ENSG00000119866	GAGAACC
ENSG00000119866	GAGAACU
ENSG00000119866	GAGUGUG
ENSG00000119866	GAUUGUC
ENSG00000119866	GCAACUU
ENSG00000119866	GCUGGAU
ENSG00000119866	GGAAUGU
ENSG00000119866	GGCAGCG
ENSG00000119866	GGGGCAG
ENSG00000119866	GUAAACA
ENSG00000119866	UACAAAG
ENSG00000119866	UAGGUAU
ENSG00000119866	UAUAUGA
ENSG00000119866	UAUUGCU
ENSG00000119866	UCACAGG
ENSG00000119866	UCACAGU
ENSG00000119866	UCCCUUG
ENSG00000119866	UCCCUUU
ENSG00000119866	UCCUUGC
ENSG00000119866	UGAAGUG
ENSG00000119866	UGCAUAG
ENSG00000119866	UGCAUUG
ENSG00000119866	UGUGCUU
ENSG00000119866	UUCAAAU
ENSG00000119866	UUCAAGC
ENSG00000119866	UUCCUAG
ENSG00000119866	UUGGCAC
ENSG00000119866	UUGGUCC
ENSG00000119866	UUUCAAC
ENSG00000119878	AAGUGCA
ENSG00000119899	AGGGAGG
ENSG00000119899	GCCUGGG
ENSG00000119899	GUAGUGU
ENSG00000119899	UACAAAG
ENSG00000119899	UAUAAAG
ENSG00000119899	UGUUGAA
ENSG00000119899	UUGGUUC
ENSG00000119906	AAAACUG
ENSG00000119906	AAAUUUC
ENSG00000119906	AAGGCAC
ENSG00000119906	AAGUGCA
ENSG00000119906	AAUACUG
ENSG00000119906	AAUCUCU

ENSG00000119906	ACAUUCA
ENSG00000119906	ACCUGGA
ENSG00000119906	AGCAGCU
ENSG00000119906	AGGUAGU
ENSG00000119906	AGUGCAA
ENSG00000119906	AGUGGUU
ENSG00000119906	CAAAGUG
ENSG00000119906	CACAAUG
ENSG00000119906	CACCACC
ENSG00000119906	CAGUGCA
ENSG00000119906	CAGUUAC
ENSG00000119906	CCAGUGU
ENSG00000119906	CUACAAA
ENSG00000119906	CUGACUC
ENSG00000119906	CUUGGAG
ENSG00000119906	GACCUGG
ENSG00000119906	GAGAACC
ENSG00000119906	GUGCAAA
ENSG00000119906	UUCAAGC
ENSG00000119919	ACACGGG
ENSG00000119919	CCUGUAC
ENSG00000119919	CCUUCAU
ENSG00000119919	GGCAGCG
ENSG00000119919	GUAGUGU
ENSG00000119922	UUGGUCC
ENSG00000119927	AAAAGUA
ENSG00000119927	AAGGCAC
ENSG00000119927	AGCACCA
ENSG00000119927	AUGCAUU
ENSG00000119927	AUGGCAC
ENSG00000119927	CUUUGGU
ENSG00000119927	GAAAACA
ENSG00000119927	GAGGCAG
ENSG00000119927	GGAAGCC
ENSG00000119927	GGUUGAC
ENSG00000119927	GUGCUUG
ENSG00000119927	UAUUGCU
ENSG00000119927	UGUCUUU
ENSG00000119938	GAAGGAA
ENSG00000119946	AAGAACC
ENSG00000119946	ACUCCAG
ENSG00000119946	CAGGCUC
ENSG00000119946	CCCUGAG
ENSG00000119946	CUGGCUC
ENSG00000119946	CUGGGCA
ENSG00000119946	CUUUGGU
ENSG00000119946	GACCUGG
ENSG00000119946	GAGAUGA
ENSG00000119946	GCAGAAG
ENSG00000119946	GGGUAAG
ENSG00000119946	GUGACAG
ENSG00000119946	GUGCCUG
ENSG00000119946	GUGGCCC
ENSG00000119946	UCCAGAG
ENSG00000119946	UGCAUUG
ENSG00000119946	UGGAGAU
ENSG00000119946	UGGCUGC
ENSG00000119946	UUGGUUC
ENSG00000119950	AAGACAU
ENSG00000119950	GGCUCAG
ENSG00000119953	CUAUACA
ENSG00000119953	GAGAUGA
ENSG00000119969	GGAAGAC
ENSG00000119969	UGUAUAA

ENSG00000119979	AAAGUGC
ENSG00000119979	AAGUGCA
ENSG00000119979	AGUGCAA
ENSG00000119979	CAAAGCA
ENSG00000119979	UACAAAG
ENSG00000119979	UCAAGUA
ENSG00000120029	ACCAGCA
ENSG00000120029	AGCAGCU
ENSG00000120029	CAGCAAA
ENSG00000120029	CUGCUCA
ENSG00000120029	CUGGAAA
ENSG00000120029	GGGAGCU
ENSG00000120029	GUAAACA
ENSG00000120029	UCUCCAA
ENSG00000120029	UUCAAGC
ENSG00000120049	AGCACCA
ENSG00000120049	CAAAGCA
ENSG00000120049	CACCACC
ENSG00000120049	CCCCCAG
ENSG00000120049	CUGCCCC
ENSG00000120049	GACCUGG
ENSG00000120049	GCACCAU
ENSG00000120049	UACAAAG
ENSG00000120049	UGCAAAG
ENSG00000120053	AGGCAGG
ENSG00000120053	GGUGGUC
ENSG00000120053	GGUGGUU
ENSG00000120057	AAGGCAC
ENSG00000120063	AAACUGU
ENSG00000120063	AGCCCUU
ENSG00000120063	AUGUAAU
ENSG00000120063	CACAGUG
ENSG00000120063	CAGGAAC
ENSG00000120063	GGCAAGA
ENSG00000120063	UCACAGU
ENSG00000120068	ACAUUCA
ENSG00000120068	AGGCAGG
ENSG00000120068	AGGUAGU
ENSG00000120068	CACAGUG
ENSG00000120068	GUAAACA
ENSG00000120068	UCACAGG
ENSG00000120071	AAAGAAU
ENSG00000120071	AAAUUUC
ENSG00000120071	AACAAAC
ENSG00000120071	AAGACAU
ENSG00000120071	AAUGCCC
ENSG00000120071	AUACAAG
ENSG00000120071	AUGAUGA
ENSG00000120071	AUGAUGC
ENSG00000120071	AUGCAUU
ENSG00000120071	CAAAGCA
ENSG00000120071	CUUCUCU
ENSG00000120071	GCUACAU
ENSG00000120071	GUGCUUG
ENSG00000120071	UAAUGCU
ENSG00000120071	UAUAAAG
ENSG00000120071	UGAAGCU
ENSG00000120071	UGCAUAG
ENSG00000120071	UGUGCUU
ENSG00000120071	UGUUGAA
ENSG00000120075	AACACUG
ENSG00000120075	ACCACAG
ENSG00000120075	CAGUCCA
ENSG00000120075	UAUAAAG

ENSG00000120075	UCCCACC
ENSG00000120087	ACAUAGA
ENSG00000120088	AGAUAAA
ENSG00000120088	AGGCAGG
ENSG00000120088	GGCAGUG
ENSG00000120088	UGCUCAC
ENSG00000120088	UUGGUUC
ENSG00000120093	AAAGUAA
ENSG00000120093	AAAGUUU
ENSG00000120093	AAGUCAC
ENSG00000120093	AAGUGCU
ENSG00000120093	ACAAUAU
ENSG00000120093	AGUGCAA
ENSG00000120093	AUGCCUU
ENSG00000120093	CAAGAGC
ENSG00000120093	CACAGUG
ENSG00000120093	CCAUUAC
ENSG00000120093	CCCUGAG
ENSG00000120093	CCCUGUU
ENSG00000120093	CUAUACA
ENSG00000120093	CUCACAC
ENSG00000120093	GAGCCCU
ENSG00000120093	GUGCUUG
ENSG00000120093	GUUCAUG
ENSG00000120093	UCACAGU
ENSG00000120093	UCCCUCU
ENSG00000120093	UGUGCUU
ENSG00000120093	UUCAAAU
ENSG00000120129	ACAGUAU
ENSG00000120129	GAGGUAG
ENSG00000120137	AAAGAGC
ENSG00000120137	AAGUGCA
ENSG00000120137	ACUGGCU
ENSG00000120137	AGCAGCU
ENSG00000120137	AGCUUAU
ENSG00000120137	AGUACUG
ENSG00000120137	AGUGCAA
ENSG00000120137	AUCCUUG
ENSG00000120137	AUUGCAC
ENSG00000120137	CUUUGGU
ENSG00000120137	GAGGCUG
ENSG00000120137	UGCAUUG
ENSG00000120149	AACUCUA
ENSG00000120149	UUCAAAU
ENSG00000120158	UUGGUCC
ENSG00000120159	AAGUCUU
ENSG00000120159	UCCUGAG
ENSG00000120162	AAAGCUG
ENSG00000120162	AAAUUUC
ENSG00000120162	AACAAAC
ENSG00000120162	AACACUG
ENSG00000120162	ACAUUCA
ENSG00000120162	AGAUAAA
ENSG00000120162	AGCAGCG
ENSG00000120162	AGCAGCU
ENSG00000120162	AGCCCUU
ENSG00000120162	AGUGCAA
ENSG00000120162	CAAAACU
ENSG00000120162	CACAGGG
ENSG00000120162	CACUGUU
ENSG00000120162	CAGUCUG
ENSG00000120162	CCAGUAC
ENSG00000120162	GAAGGAA
ENSG00000120162	GAGGUAG

ENSG00000120162	GCUAGCA
ENSG00000120162	GUCCAGA
ENSG00000120162	UCCAGUU
ENSG00000120162	UCCCUCU
ENSG00000120162	UGAAAUG
ENSG00000120162	UUCAAAU
ENSG00000120162	UUGGCAA
ENSG00000120215	GUUCAUG
ENSG00000120217	UGAGAAU
ENSG00000120251	AAACUGU
ENSG00000120251	AAGUCUU
ENSG00000120251	AAGUGUG
ENSG00000120251	ACAUUCA
ENSG00000120251	ACUCAAA
ENSG00000120251	ACUGGAU
ENSG00000120251	ACUGUAG
ENSG00000120251	AGAUGUG
ENSG00000120251	AGCCAGU
ENSG00000120251	AGUGUGC
ENSG00000120251	CAUGGAU
ENSG00000120251	CCUGUAC
ENSG00000120251	GGAAGCC
ENSG00000120251	GGAGUCC
ENSG00000120251	GUGCUUG
ENSG00000120251	UCAUUCG
ENSG00000120251	UCUCACC
ENSG00000120251	UCUGGAA
ENSG00000120251	UGUGCUU
ENSG00000120251	UUCAAAU
ENSG00000120253	UAGUGAG
ENSG00000120254	CUUUGGU
ENSG00000120265	ACUGCAU
ENSG00000120265	AGCAGCU
ENSG00000120265	GGAAGAC
ENSG00000120265	UUGGCAC
ENSG00000120278	CCCACCU
ENSG00000120280	UGAAAUG
ENSG00000120314	AUAUUAU
ENSG00000120314	GCUGGUG
ENSG00000120314	GUCUUGC
ENSG00000120332	AGUGGUU
ENSG00000120332	GGUUUAC
ENSG00000120332	UUAUGCA
ENSG00000120334	GAAAACA
ENSG00000120341	CAAAACU
ENSG00000120341	GCCUGGG
ENSG00000120458	AAUCCUU
ENSG00000120458	UAUUGCU
ENSG00000120519	AAGUCUU
ENSG00000120519	AGCUUAU
ENSG00000120519	CACAGUG
ENSG00000120519	UAAGACG
ENSG00000120519	UCACACA
ENSG00000120519	UCACAGU
ENSG00000120539	AUAAAGU
ENSG00000120549	AACUACU
ENSG00000120549	ACAGUAC
ENSG00000120549	AGCCCUU
ENSG00000120549	AGUGCAA
ENSG00000120549	AUGUGCC
ENSG00000120549	CAAAUGC
ENSG00000120549	CACAAUG
ENSG00000120549	CAGUGCA
ENSG00000120549	CUUUGGU

ENSG00000120549	UUGGCAC
ENSG00000120586	UGCCAGC
ENSG00000120616	AGCACCA
ENSG00000120616	AGCUGCC
ENSG00000120616	AUCCCUU
ENSG00000120616	UCCCUUG
ENSG00000120616	UGCAUAG
ENSG00000120656	AAAGUAC
ENSG00000120656	ACAUUAC
ENSG00000120659	AAAGUGC
ENSG00000120659	AAGUGCA
ENSG00000120659	ACAUUAC
ENSG00000120659	CAAAGUG
ENSG00000120675	AGGGUCU
ENSG00000120685	AAAAACC
ENSG00000120685	AAAGUUU
ENSG00000120685	AACAUUC
ENSG00000120685	ACAGUAC
ENSG00000120685	ACAGUAU
ENSG00000120685	AGCACCA
ENSG00000120685	CAGCAGA
ENSG00000120685	CAGCAGG
ENSG00000120685	CUACAGU
ENSG00000120685	GAAAACA
ENSG00000120685	UUGUUCG
ENSG00000120686	UGAAAGG
ENSG00000120688	ACAUAGA
ENSG00000120688	GAGCUGC
ENSG00000120688	UAAUACA
ENSG00000120690	CCUUCAU
ENSG00000120694	UCACAAG
ENSG00000120696	UUCUAAU
ENSG00000120705	AACAAAC
ENSG00000120705	AAGGCAC
ENSG00000120705	AAUCUCA
ENSG00000120705	ACCCAUU
ENSG00000120705	AUACAAG
ENSG00000120705	CUACAAA
ENSG00000120705	GCUGGAU
ENSG00000120705	GGCAGGG
ENSG00000120705	UAUGGUU
ENSG00000120708	UACAAAG
ENSG00000120708	UAUUACC
ENSG00000120709	AAAACUG
ENSG00000120709	ACACACU
ENSG00000120709	AGCACCA
ENSG00000120709	AGGGUCU
ENSG00000120709	CAGCAGG
ENSG00000120709	CCCUGAG
ENSG00000120709	CUCAGGG
ENSG00000120709	CUGGGCA
ENSG00000120709	GAGCCCC
ENSG00000120709	GAGCCCU
ENSG00000120709	GGAAGAC
ENSG00000120709	GGAGGCA
ENSG00000120709	GGCAGCG
ENSG00000120709	GGGUAAG
ENSG00000120709	GUGGCCC
ENSG00000120709	GUGUGGC
ENSG00000120709	UCACACA
ENSG00000120709	UUCUAAU
ENSG00000120709	UUGGCAA
ENSG00000120709	UUGGCAC
ENSG00000120709	UUUCAAC

ENSG00000120727	AAAACUG
ENSG00000120727	AAACUGU
ENSG00000120727	AAGUUGU
ENSG00000120727	CAAAACU
ENSG00000120727	UCACACA
ENSG00000120738	AACAGUC
ENSG00000120738	AACAUUC
ENSG00000120738	AAGGCAC
ENSG00000120738	ACAUUCA
ENSG00000120738	ACUCAGC
ENSG00000120738	ACUCAGG
ENSG00000120738	CAGUAGU
ENSG00000120742	AAGUAGC
ENSG00000120742	ACUUGGA
ENSG00000120742	AGGCAGG
ENSG00000120742	AGUGCCU
ENSG00000120742	AGUGUGC
ENSG00000120742	AUGACAC
ENSG00000120742	CACAGGG
ENSG00000120742	CAGGAAC
ENSG00000120742	GAAACAU
ENSG00000120742	GAGUGUG
ENSG00000120742	GGAAGAC
ENSG00000120742	UACAAAG
ENSG00000120742	UCACAGU
ENSG00000120756	AAAGAAU
ENSG00000120756	GAGGCAG
ENSG00000120756	GUAAACA
ENSG00000120784	ACAUUCA
ENSG00000120802	CUACAAA
ENSG00000120805	AAGUGCA
ENSG00000120805	ACAUUCA
ENSG00000120805	AGUAGUU
ENSG00000120805	AGUGUGC
ENSG00000120805	CUUUGGU
ENSG00000120805	GUAGUGU
ENSG00000120805	UCAUUUG
ENSG00000120805	UUCCAUA
ENSG00000120833	AGCUGCC
ENSG00000120833	CUACAGU
ENSG00000120833	GUAACAG
ENSG00000120833	UGCAUAG
ENSG00000120833	UGCAUAU
ENSG00000120833	UUCUUGU
ENSG00000120833	UUUGCAC
ENSG00000120837	AAAAGUA
ENSG00000120837	AAACUGU
ENSG00000120837	AAAGGUA
ENSG00000120837	AACAAAC
ENSG00000120837	AACACUG
ENSG00000120837	AACUCUA
ENSG00000120837	AAUCUCU
ENSG00000120837	AUAAUAC
ENSG00000120837	AUUGCAC
ENSG00000120837	CACCAGC
ENSG00000120837	CACUGUU
ENSG00000120837	CAGGAAC
ENSG00000120837	GCAACUU
ENSG00000120837	GUAAACA
ENSG00000120837	GUCUACU
ENSG00000120837	GUUAAUG
ENSG00000120837	UAAUACA
ENSG00000120837	UAAUAUC
ENSG00000120837	UAUUGCU

ENSG00000120837	UCACCAC
ENSG00000120837	UCAUGAU
ENSG00000120837	UGCAUUG
ENSG00000120837	UUCUGCA
ENSG00000120868	AAACUGU
ENSG00000120868	AAUUAUU
ENSG00000120868	AUGAUGA
ENSG00000120868	CACAGUG
ENSG00000120868	GUGCAAA
ENSG00000120868	UCACAGU
ENSG00000120875	GAGGUAG
ENSG00000120875	GAGGUAU
ENSG00000120875	UUCAAGC
ENSG00000120885	ACUGCAG
ENSG00000120885	CCUGCUG
ENSG00000120885	CUGCAGC
ENSG00000120889	AUAUAAC
ENSG00000120889	CAUAUUG
ENSG00000120896	CAAAGCA
ENSG00000120896	CAGCUGG
ENSG00000120896	CCCUGUU
ENSG00000120896	UUGGUCC
ENSG00000120899	AGGCAGG
ENSG00000120899	CGUGCAU
ENSG00000120899	GUGCAAA
ENSG00000120899	UCGUGCA
ENSG00000120903	UCACCAC
ENSG00000120913	AUGUGGG
ENSG00000120913	CAGCAGG
ENSG00000120913	CAGGAAC
ENSG00000120913	GGAAGCC
ENSG00000120913	GUGCCUG
ENSG00000120913	GUGGGGA
ENSG00000120913	UGUGUGG
ENSG00000120913	UUGAUCA
ENSG00000120913	UUGUGUC
ENSG00000120925	AUCCCUU
ENSG00000120948	AAAAAUC
ENSG00000120948	AAGUCUU
ENSG00000120948	AAUACUG
ENSG00000120948	ACAAGGU
ENSG00000120948	ACACACC
ENSG00000120948	ACAUUCA
ENSG00000120948	AGCAUUC
ENSG00000120948	AGUGCUU
ENSG00000120948	AUGCACC
ENSG00000120948	CAAAGCA
ENSG00000120948	CAGCAAA
ENSG00000120948	CCUUCUG
ENSG00000120948	CUGCAGC
ENSG00000120948	GCAGCAU
ENSG00000120948	GUCUCUG
ENSG00000120948	UCACAGG
ENSG00000120948	UCACAGU
ENSG00000120948	UCUCACC
ENSG00000120948	UGAAGUG
ENSG00000120949	UCCCUUG
ENSG00000120949	UCCCUUU
ENSG00000120963	ACAAUAU
ENSG00000120963	ACCCUGU
ENSG00000120963	AGCAGCU
ENSG00000120963	CAAAGUG
ENSG00000120963	CAGUAGA
ENSG00000120963	CCAGUGU

ENSG00000120963	CUGCAGG
ENSG00000120963	GAAUUGU
ENSG00000120963	GCAGGUU
ENSG00000120963	GGACUGC
ENSG00000120963	GUAAACA
ENSG00000120963	UCAACAG
ENSG00000120963	UGCUGAC
ENSG00000120963	UGGACUG
ENSG00000120963	UGUGUCA
ENSG00000120963	UUUUUGC
ENSG00000120992	AGCACCA
ENSG00000120992	AUGAUGC
ENSG00000120992	AUGCAUU
ENSG00000120992	UGCAAAG
ENSG00000120992	UUUGCAA
ENSG00000121005	CUCUUCC
ENSG00000121005	CUGCAGG
ENSG00000121005	GAAACAU
ENSG00000121005	GAGUAUU
ENSG00000121005	GCAGGAC
ENSG00000121005	UACAAAG
ENSG00000121005	UGCCAGC
ENSG00000121039	AAGACAU
ENSG00000121039	ACUGUAG
ENSG00000121039	AGCAAAA
ENSG00000121039	CUACAAA
ENSG00000121039	GAGCUGC
ENSG00000121039	UAAUACA
ENSG00000121039	UACAAAG
ENSG00000121039	UUCCAUA
ENSG00000121039	UUGGCAC
ENSG00000121057	AAAUGGU
ENSG00000121057	CCAGUGC
ENSG00000121057	CUCCAGC
ENSG00000121057	CUUGUAU
ENSG00000121057	GUGCAAA
ENSG00000121057	UCCCUUG
ENSG00000121057	UCUCCAA
ENSG00000121057	UUUGCAC
ENSG00000121058	GAAGGAA
ENSG00000121058	GAGGUAU
ENSG00000121058	GGAAUGU
ENSG00000121058	UGAAAGG
ENSG00000121060	CUACAAA
ENSG00000121060	CUGACUC
ENSG00000121060	CUUUGGU
ENSG00000121060	GAGCUGC
ENSG00000121060	GCAGAAG
ENSG00000121067	ACAGUAC
ENSG00000121068	AAAGCUG
ENSG00000121068	UAAUACA
ENSG00000121068	UGCAUAG
ENSG00000121075	CCCUGAG
ENSG00000121075	CUGCAGC
ENSG00000121075	UCUCCAA
ENSG00000121083	CAGUCCA
ENSG00000121083	CAGUGCA
ENSG00000121101	UGAUCCA
ENSG00000121104	AAAGUGC
ENSG00000121104	AAGGCAC
ENSG00000121104	AAGUGCU
ENSG00000121104	AGCUGCC
ENSG00000121104	CUUUGGU
ENSG00000121104	GAGAUGA

ENSG00000121104	GAGCUGC
ENSG00000121104	UGUCUUU
ENSG00000121104	UUCUAAU
ENSG00000121207	AAUCACU
ENSG00000121207	ACACACC
ENSG00000121207	GCCUGAU
ENSG00000121207	GGAAUGU
ENSG00000121207	UGAAAUG
ENSG00000121210	AAAGUGC
ENSG00000121236	UGCAUUG
ENSG00000121274	AAAUUUC
ENSG00000121274	AAGGCAC
ENSG00000121274	AAGUCUU
ENSG00000121274	AAGUGUG
ENSG00000121274	AAUCCUU
ENSG00000121274	ACAUCAC
ENSG00000121274	ACCCUGU
ENSG00000121274	ACUGUGU
ENSG00000121274	CACAAUG
ENSG00000121274	CACACCU
ENSG00000121274	CACCACC
ENSG00000121274	CAGUCUG
ENSG00000121274	CAUAUUG
ENSG00000121274	CCAGCAU
ENSG00000121274	CCAUUAC
ENSG00000121274	CCCUGUU
ENSG00000121274	CCUUCAU
ENSG00000121274	CUCCAGC
ENSG00000121274	CUCCCAA
ENSG00000121274	GAUCAGA
ENSG00000121274	GAUUGGU
ENSG00000121274	GCAGCAU
ENSG00000121274	GCUGGUG
ENSG00000121274	GUAAACA
ENSG00000121274	GUCAGUU
ENSG00000121274	UCACAAG
ENSG00000121274	UCACAGG
ENSG00000121274	UCACAUU
ENSG00000121274	UCAUGAU
ENSG00000121274	UCCCACA
ENSG00000121274	UGAGGAC
ENSG00000121274	UGAUCCA
ENSG00000121274	UGCAAAG
ENSG00000121274	UUAGAGA
ENSG00000121274	UUAUGCA
ENSG00000121274	UUGAGGC
ENSG00000121274	UUGGUCC
ENSG00000121274	UUUGCAA
ENSG00000121281	AGCCCUU
ENSG00000121281	AGUGCUU
ENSG00000121281	CACAGUG
ENSG00000121281	GUGCAAA
ENSG00000121281	UGAAGCU
ENSG00000121297	AAAGAGG
ENSG00000121297	AAAGUAC
ENSG00000121297	AAAGUAU
ENSG00000121297	AAAGUGC
ENSG00000121297	AAGUGCU
ENSG00000121297	AAUCACU
ENSG00000121297	AAUUUUA
ENSG00000121297	ACUCAGC
ENSG00000121297	ACUCAGG
ENSG00000121297	AGAUAAA
ENSG00000121297	AGAUGUG

ENSG00000121297	AGUAACA
ENSG00000121297	AGUGCUU
ENSG00000121297	AUGUGCC
ENSG00000121297	CAGCUGG
ENSG00000121297	CUUGGGC
ENSG00000121297	GAGAAAU
ENSG00000121297	GGUUACC
ENSG00000121297	GUGCCAC
ENSG00000121297	UAUAAAG
ENSG00000121297	UCACAUU
ENSG00000121297	UGCAGCU
ENSG00000121297	UUCUGCA
ENSG00000121361	AAGUGCU
ENSG00000121380	AUGAGCU
ENSG00000121380	GAGCUGC
ENSG00000121380	GAGUAUU
ENSG00000121388	AAAAGUA
ENSG00000121388	AAAGUAA
ENSG00000121388	AAGUGCA
ENSG00000121388	ACAUUAC
ENSG00000121388	CAGUCUG
ENSG00000121388	CCUUCAU
ENSG00000121388	CUCACAC
ENSG00000121388	GAGUGUG
ENSG00000121388	GGAGUGU
ENSG00000121388	UCACAGG
ENSG00000121388	UCACAGU
ENSG00000121388	UCUCACC
ENSG00000121388	UUGGCAC
ENSG00000121481	AAGUGCA
ENSG00000121481	ACAUUAC
ENSG00000121481	ACAUUCA
ENSG00000121481	AGUGCAA
ENSG00000121481	AUGGCAC
ENSG00000121481	GUGCAAA
ENSG00000121481	UAAUAUC
ENSG00000121481	UGAGAAU
ENSG00000121486	CUCCCAA
ENSG00000121486	UACAGUU
ENSG00000121486	UGAAAUG
ENSG00000121578	GCUGUCU
ENSG00000121578	UCUCACC
ENSG00000121594	GAGAACC
ENSG00000121653	AAACUGU
ENSG00000121653	ACAAUAU
ENSG00000121653	AUAUUAU
ENSG00000121653	AUGGCAC
ENSG00000121671	CACAGGG
ENSG00000121671	CAGCAGG
ENSG00000121671	CCCACCU
ENSG00000121671	CUCUGGG
ENSG00000121671	CUCUUCC
ENSG00000121671	CUGCAGC
ENSG00000121671	CUUCUCU
ENSG00000121671	GAAGGAA
ENSG00000121671	GAGGCUG
ENSG00000121671	GAGGUAG
ENSG00000121671	GGAAGAC
ENSG00000121671	GGAAGCC
ENSG00000121671	GGCUCAG
ENSG00000121671	UCACAAG
ENSG00000121671	UCACAGG
ENSG00000121671	UCUGGAG
ENSG00000121671	UGCUCAC

ENSG00000121671	UGGCUGC
ENSG00000121741	AAAAACC
ENSG00000121741	ACAGUAU
ENSG00000121741	ACAUUAC
ENSG00000121741	AGCAGCG
ENSG00000121741	AGCAGCU
ENSG00000121741	AGCAUUC
ENSG00000121741	CAAAACU
ENSG00000121741	CAGUAGA
ENSG00000121741	CUACAAA
ENSG00000121741	GCUACAU
ENSG00000121741	UAAUGCU
ENSG00000121741	UACAAAG
ENSG00000121741	UGGGUAC
ENSG00000121741	UGGUACC
ENSG00000121742	UAUAAAG
ENSG00000121742	UGAAAUG
ENSG00000121743	AACUCUA
ENSG00000121743	AUGACAC
ENSG00000121743	CAAAGCA
ENSG00000121743	UUGGCAC
ENSG00000121749	GGAAUGU
ENSG00000121749	GUAAACA
ENSG00000121753	CAAAGUG
ENSG00000121753	CUGGCUC
ENSG00000121753	GAGUGUG
ENSG00000121766	AGCACCA
ENSG00000121766	CAGCUGG
ENSG00000121766	CUCCCAA
ENSG00000121766	GGCAGCG
ENSG00000121766	GGCAGUG
ENSG00000121766	UACAAAG
ENSG00000121766	UGCAAAG
ENSG00000121769	GAGGCAG
ENSG00000121769	UGACCUA
ENSG00000121774	AAAGGUA
ENSG00000121774	AGUAACA
ENSG00000121774	AUACAAG
ENSG00000121774	AUGUCUG
ENSG00000121774	UCCCUUU
ENSG00000121775	AACAAAC
ENSG00000121848	AAAUUUC
ENSG00000121864	GAAGGAA
ENSG00000121871	AAAAGUA
ENSG00000121871	AAAGCUG
ENSG00000121871	AACAUUC
ENSG00000121871	AAGGCAC
ENSG00000121871	AAGUGCU
ENSG00000121871	ACAUUAC
ENSG00000121871	ACUGCAU
ENSG00000121871	AGUGCUU
ENSG00000121871	AUGCACC
ENSG00000121871	AUGGCAC
ENSG00000121871	CAGCAAA
ENSG00000121871	CUACAAA
ENSG00000121871	CUAUACA
ENSG00000121871	GAGAACC
ENSG00000121871	GAGAACU
ENSG00000121871	GCAGCAU
ENSG00000121871	GUGCCAC
ENSG00000121871	UACAAAG
ENSG00000121879	AAGGCAC
ENSG00000121879	ACCCUGU
ENSG00000121879	CCCUGUU

ENSG00000121892	AAAAGUA
ENSG00000121892	AAAGAAU
ENSG00000121892	AAAGCUG
ENSG00000121892	ACUCCAG
ENSG00000121892	AGCUGCC
ENSG00000121892	CAAAUGC
ENSG00000121892	UGACUGU
ENSG00000121895	UUCCAUA
ENSG00000121897	GAAGGUC
ENSG00000121900	AUAAAGU
ENSG00000121904	AAAAAUC
ENSG00000121904	AAAGUAC
ENSG00000121904	AAUCCUU
ENSG00000121904	ACAAUAU
ENSG00000121904	ACUGCAU
ENSG00000121904	AGAUAAA
ENSG00000121904	AUAAUAC
ENSG00000121904	CCCCUGG
ENSG00000121904	CUCCAGC
ENSG00000121904	CUGCAGC
ENSG00000121904	CUGCCCC
ENSG00000121904	CUGGGCA
ENSG00000121904	GAAAACA
ENSG00000121904	GCGGGGC
ENSG00000121904	GGAAGCC
ENSG00000121904	GGCAGGG
ENSG00000121904	GGCGGGG
ENSG00000121904	GGGGAGC
ENSG00000121904	UACAAAG
ENSG00000121904	UAUAAUA
ENSG00000121904	UAUUACC
ENSG00000121904	UGGUACC
ENSG00000121905	AGGGAGG
ENSG00000121905	CCCCCAG
ENSG00000121905	UGGGGGA
ENSG00000121940	AACAUUC
ENSG00000121940	AAUUGCU
ENSG00000121940	GCUAGCA
ENSG00000121964	AAAAACC
ENSG00000121964	AAAGAAU
ENSG00000121964	AAAGUGC
ENSG00000121964	AACAGUC
ENSG00000121964	AACCUGG
ENSG00000121964	AACCUGU
ENSG00000121964	AAGUGCU
ENSG00000121964	AAGUGUG
ENSG00000121964	AGCCCUU
ENSG00000121964	AGGACAC
ENSG00000121964	AUACAAG
ENSG00000121964	AUGACAC
ENSG00000121964	CAGGCUC
ENSG00000121964	CGAGGAC
ENSG00000121964	CUGCAGC
ENSG00000121964	CUGCAGG
ENSG00000121964	GAUCAGA
ENSG00000121964	GCACCAU
ENSG00000121964	GCUUCCU
ENSG00000121964	GGCCCUG
ENSG00000121964	GGCUGCG
ENSG00000121964	GGGUGGU
ENSG00000121964	GUGACUG
ENSG00000121964	GUGGCCC
ENSG00000121964	GUGUCAC
ENSG00000121964	UAAUACA

ENSG00000121964	UACAAAG
ENSG00000121964	UCUGGAA
ENSG00000121964	UGAAGCU
ENSG00000121964	UGAGGAC
ENSG00000121964	UGGCCCU
ENSG00000121964	UGUGUCA
ENSG00000121964	UUGUGAC
ENSG00000121966	CAGUCUG
ENSG00000121989	AAUACUG
ENSG00000121989	ACUGCAU
ENSG00000121989	ACUGGCU
ENSG00000121989	ACUGUGU
ENSG00000121989	AGCAGCG
ENSG00000121989	AGCUUAU
ENSG00000121989	AGGGAGG
ENSG00000121989	AGUAGUU
ENSG00000121989	AUACCUC
ENSG00000121989	CAAAUGC
ENSG00000121989	CAGCAAA
ENSG00000121989	CAUAUUG
ENSG00000121989	CCAGUGC
ENSG00000121989	CCAGUGU
ENSG00000121989	GAGGUAG
ENSG00000121989	GCUGGUG
ENSG00000121989	GUAGUGU
ENSG00000121989	GUCAUUG
ENSG00000121989	GUGUCAC
ENSG00000121989	UCACAGU
ENSG00000121989	UGAAAUG
ENSG00000121989	UGCAUAG
ENSG00000121989	UGUGUCA
ENSG00000121989	UGUUGAA
ENSG00000122008	AGUGCUU
ENSG00000122012	AUGGCUU
ENSG00000122025	ACAUAGA
ENSG00000122025	AGCAGCA
ENSG00000122025	AGUGAUC
ENSG00000122025	CAUAUUG
ENSG00000122025	UACAAAG
ENSG00000122025	UAGUGAG
ENSG00000122042	AAAGAAU
ENSG00000122042	ACAAUAU
ENSG00000122042	ACAGGUG
ENSG00000122042	ACAUCAC
ENSG00000122042	ACUGCAG
ENSG00000122042	AGCAGCA
ENSG00000122042	CUAUACA
ENSG00000122042	CUCCCAC
ENSG00000122042	CUGCUCA
ENSG00000122042	GAUCAGA
ENSG00000122042	GCAGAAG
ENSG00000122042	GCAGCAU
ENSG00000122042	GUGCAAA
ENSG00000122042	GUGCCAC
ENSG00000122042	UCCAGUU
ENSG00000122042	UCCCACA
ENSG00000122042	UGCAUAU
ENSG00000122042	UGUGCUU
ENSG00000122042	UUCAAAU
ENSG00000122068	AAAGUAC
ENSG00000122068	AACCUGU
ENSG00000122068	GCAUCCC
ENSG00000122068	GUAACAG
ENSG00000122085	AACCUGG

ENSG00000122085	AUAAAGU
ENSG00000122121	AACGGCU
ENSG00000122121	AGGAUGU
ENSG00000122122	GAGGUAU
ENSG00000122126	AGCAGCA
ENSG00000122126	AGCAGCG
ENSG00000122126	AUAAUAC
ENSG00000122126	CACUGUU
ENSG00000122126	GAGGCAG
ENSG00000122126	GUGCAAA
ENSG00000122126	UGCAAAG
ENSG00000122176	AGCAAAA
ENSG00000122176	AUGAGCU
ENSG00000122176	CAGCAAA
ENSG00000122176	GGCAGGG
ENSG00000122176	UCUGGAG
ENSG00000122180	CACAAUG
ENSG00000122180	CCCCCAG
ENSG00000122180	GAGACCU
ENSG00000122180	UCCUGAG
ENSG00000122180	UGGCCCU
ENSG00000122203	AAGUGCA
ENSG00000122203	ACUCCAG
ENSG00000122203	UUCCUAG
ENSG00000122223	GAGCCCU
ENSG00000122223	GCACCAU
ENSG00000122254	AAAGUAC
ENSG00000122254	GUAACAG
ENSG00000122254	GUGCUUG
ENSG00000122254	UGUGCUU
ENSG00000122257	AAAGCUG
ENSG00000122257	AAAGUUU
ENSG00000122257	AGCAAAA
ENSG00000122257	AUAAAGU
ENSG00000122299	AAUCCUU
ENSG00000122299	CAGCAAA
ENSG00000122304	CUCAGGG
ENSG00000122335	GGAAGAC
ENSG00000122359	GCCCUGU
ENSG00000122359	UCCUGAG
ENSG00000122359	UUGGCAA
ENSG00000122359	UUGGCAC
ENSG00000122367	AAAACUG
ENSG00000122367	ACUGCAG
ENSG00000122367	AGCAAAA
ENSG00000122367	AUCCUUU
ENSG00000122367	CUUGGAG
ENSG00000122367	GAAACAU
ENSG00000122367	GAGGUAG
ENSG00000122367	UAUAAAG
ENSG00000122367	UCAUUUG
ENSG00000122367	UGCAAAG
ENSG00000122367	UUCCUCG
ENSG00000122375	GUGACAG
ENSG00000122420	AUACAAG
ENSG00000122420	UCACAUU
ENSG00000122420	UGAAGUG
ENSG00000122435	AAAUUUC
ENSG00000122482	AACAGUC
ENSG00000122482	AACAUUC
ENSG00000122482	AUGCACC
ENSG00000122482	CAUGGAU
ENSG00000122482	GAGAAAU
ENSG00000122482	GAGGUAG

ENSG00000122482	GUGAGGU
ENSG00000122482	UAAUGCU
ENSG00000122482	UGUUGAA
ENSG00000122482	UUCCAUA
ENSG00000122484	CUUGGAG
ENSG00000122507	GCUUCCU
ENSG00000122515	ACUUGGA
ENSG00000122515	CAGACAG
ENSG00000122515	CUUCACC
ENSG00000122515	GGAGUCC
ENSG00000122515	GUGCUUG
ENSG00000122515	UAAUAGU
ENSG00000122515	UGUGCUU
ENSG00000122545	AACACUG
ENSG00000122545	ACAAUAU
ENSG00000122545	ACUGUGU
ENSG00000122545	AUGGCAC
ENSG00000122545	GGUGGUC
ENSG00000122545	UUGGCAA
ENSG00000122547	CAAAACU
ENSG00000122547	UCACAGU
ENSG00000122547	UUCAAAU
ENSG00000122547	UUUCAAC
ENSG00000122557	AAAGUAC
ENSG00000122557	AGCUGCC
ENSG00000122557	AGUAGUU
ENSG00000122557	AUGCAUU
ENSG00000122557	AUUCAUU
ENSG00000122557	CACUGUU
ENSG00000122557	GAGCUGC
ENSG00000122565	AAAACUG
ENSG00000122565	AAAGUAC
ENSG00000122565	AAAGUAU
ENSG00000122565	AACAUUC
ENSG00000122565	CAAAACU
ENSG00000122565	CAAAGUG
ENSG00000122565	CAAAUGC
ENSG00000122565	CACCAGC
ENSG00000122565	CACUGUU
ENSG00000122565	CCAGUGC
ENSG00000122565	CUACAAA
ENSG00000122565	UACAGUU
ENSG00000122565	UCACCAC
ENSG00000122566	AAACUGU
ENSG00000122566	AAAGUAA
ENSG00000122566	AACAUUC
ENSG00000122566	AACCUGG
ENSG00000122566	AACCUGU
ENSG00000122566	ACGCACU
ENSG00000122566	ACUGCAU
ENSG00000122566	ACUGGCU
ENSG00000122566	AGCAGCA
ENSG00000122566	AUCACUA
ENSG00000122566	CUCGCUG
ENSG00000122566	CUCUGGG
ENSG00000122566	CUGCAGC
ENSG00000122566	CUGCUCA
ENSG00000122566	CUGGGCA
ENSG00000122566	GCAGCAU
ENSG00000122566	GGCUCUG
ENSG00000122566	GGUUGAC
ENSG00000122566	UAAGACU
ENSG00000122566	UCACAAG
ENSG00000122566	UGAAAUG

ENSG00000122566	UGAGAAG
ENSG00000122566	UGAGAAU
ENSG00000122566	UGCAUAU
ENSG00000122566	UUCAAAU
ENSG00000122584	AAUCCUU
ENSG00000122584	AGCAGCA
ENSG00000122584	AGCAGCU
ENSG00000122584	AUGUGCC
ENSG00000122591	AAGUCUU
ENSG00000122591	CCUUCAU
ENSG00000122591	GGAUUUC
ENSG00000122592	CUACAAA
ENSG00000122592	GCUACAU
ENSG00000122592	GGGAGCU
ENSG00000122592	UACAAAG
ENSG00000122641	AUGGCUU
ENSG00000122642	CUCAGGG
ENSG00000122643	GAGUGUG
ENSG00000122643	GGAGUGU
ENSG00000122644	AAAGCUG
ENSG00000122644	AAGUGCA
ENSG00000122644	AGCACCA
ENSG00000122644	AGUGCAA
ENSG00000122644	AUAAAGU
ENSG00000122644	CAGGAAC
ENSG00000122644	UAUAAAG
ENSG00000122644	UGCAAAG
ENSG00000122644	UGCAUAG
ENSG00000122644	UUAGAGA
ENSG00000122644	UUCUAAU
ENSG00000122691	AUGCAUU
ENSG00000122691	AUUGCAC
ENSG00000122691	GCAGAAG
ENSG00000122691	UAUUGCU
ENSG00000122691	UGAGAAU
ENSG00000122691	UGCAUUG
ENSG00000122692	AAUUAUU
ENSG00000122692	AGUGCUG
ENSG00000122692	UGAAAUG
ENSG00000122705	AACUCUA
ENSG00000122707	AAACUGU
ENSG00000122707	AAAGUAU
ENSG00000122707	ACAUUCA
ENSG00000122707	AGCAGCG
ENSG00000122707	AGCAGCU
ENSG00000122707	GAUUGUA
ENSG00000122707	UGCAUUG
ENSG00000122707	UGUCUUU
ENSG00000122707	UGUGUGG
ENSG00000122729	AAAGGUA
ENSG00000122729	UUCAAAU
ENSG00000122733	AAGAACC
ENSG00000122733	CACAGUG
ENSG00000122733	CUUGGGC
ENSG00000122733	CUUUGGU
ENSG00000122733	GAAACAU
ENSG00000122733	GAGUUGA
ENSG00000122733	GCUUCCU
ENSG00000122733	GGAAGCC
ENSG00000122733	GGCAGGG
ENSG00000122733	GGCUCUG
ENSG00000122733	GGGGGAA
ENSG00000122733	GUGCUUG
ENSG00000122733	UAGUGAG

ENSG00000122733	UCACAGU
ENSG00000122733	UGCAGCU
ENSG00000122733	UGGCUGC
ENSG00000122733	UGUCUGG
ENSG00000122733	UGUGCUU
ENSG00000122756	AAUACUG
ENSG00000122756	ACUGUGU
ENSG00000122756	AGCUUAU
ENSG00000122756	CUUUGGU
ENSG00000122756	GAGAACC
ENSG00000122756	GAGAACU
ENSG00000122756	GGGCCCC
ENSG00000122756	GGGGCAG
ENSG00000122756	GGUGGGC
ENSG00000122756	UGAAAUG
ENSG00000122756	UUCAAAU
ENSG00000122778	AACAGUC
ENSG00000122778	AACUCUA
ENSG00000122778	AAGUGCU
ENSG00000122778	ACAUUCA
ENSG00000122778	ACUUGGA
ENSG00000122778	AGCACCA
ENSG00000122778	AGCAGCA
ENSG00000122778	AGCAUUC
ENSG00000122778	AGUGCUG
ENSG00000122778	AUGGCUU
ENSG00000122778	CAAAGCA
ENSG00000122778	CACAGUG
ENSG00000122778	CUACAGU
ENSG00000122778	CUAGUAG
ENSG00000122778	CUGCUCA
ENSG00000122778	CUUUGGU
ENSG00000122778	GAGGCUG
ENSG00000122778	GAGGUAG
ENSG00000122778	GAUUGUA
ENSG00000122778	GCUUCCU
ENSG00000122778	UCACAGU
ENSG00000122778	UCAUAGA
ENSG00000122778	UCUCACC
ENSG00000122778	UCUCCAA
ENSG00000122778	UGAAGUG
ENSG00000122778	UGCAGCU
ENSG00000122778	UGCAUAG
ENSG00000122778	UGCAUAU
ENSG00000122778	UGUGCUU
ENSG00000122778	UUCCAUA
ENSG00000122778	UUGGCAA
ENSG00000122778	UUGGUCC
ENSG00000122778	UUGGUUC
ENSG00000122779	UGUCUUU
ENSG00000122783	UUGGUUC
ENSG00000122786	AAGUGCA
ENSG00000122786	ACUGCAU
ENSG00000122786	AUGCACC
ENSG00000122786	CAGUAGU
ENSG00000122786	CUCUAGC
ENSG00000122786	GAGGUAU
ENSG00000122786	GCAGAAG
ENSG00000122786	GUAAACA
ENSG00000122786	GUGCAAA
ENSG00000122786	UAAUAUC
ENSG00000122786	UAGACUG
ENSG00000122786	UCUUGAG
ENSG00000122786	UGCAAAG

ENSG00000122824	AAAGAGC
ENSG00000122824	AUGAGCU
ENSG00000122824	GGAGGCA
ENSG00000122824	GUGCUUG
ENSG00000122824	UACAAAG
ENSG00000122824	UGUGCUU
ENSG00000122824	UUUUUGC
ENSG00000122861	GAGAUGA
ENSG00000122862	CUUUGGU
ENSG00000122863	AAAGAGC
ENSG00000122863	AAGUCAC
ENSG00000122863	ACCCUGU
ENSG00000122863	CAAAGCA
ENSG00000122863	CCUUCUG
ENSG00000122863	CUCAGGG
ENSG00000122863	CUCUUCC
ENSG00000122863	CUGGAAA
ENSG00000122863	GAGAAGA
ENSG00000122863	GAGGUAG
ENSG00000122863	GAGGUAU
ENSG00000122863	UCCUGAG
ENSG00000122863	UGAGAAG
ENSG00000122863	UGCCAGC
ENSG00000122863	UGGGUAC
ENSG00000122863	UUCAAAU
ENSG00000122863	UUGAGGC
ENSG00000122870	CUUUGGU
ENSG00000122877	AAAAGUA
ENSG00000122877	AAAGUAU
ENSG00000122877	AAGGCAC
ENSG00000122877	CUACAAA
ENSG00000122877	UACAAAG
ENSG00000122877	UCACACA
ENSG00000122884	GAAACAU
ENSG00000122884	UCCAGUU
ENSG00000122912	UUGGCAA
ENSG00000122912	UUGGCAC
ENSG00000122958	CCAGUGC
ENSG00000122966	AAAGGUA
ENSG00000122966	AAAGUGC
ENSG00000122966	AAGUGCA
ENSG00000122966	AAUCCUU
ENSG00000122966	ACUGCAG
ENSG00000122966	AGUAACA
ENSG00000122966	AGUGCAA
ENSG00000122966	CAAAGUG
ENSG00000122966	CAGCAAA
ENSG00000122966	CAGCUGG
ENSG00000122966	CCUGUAC
ENSG00000122966	CUGCAGC
ENSG00000122966	CUGCAGG
ENSG00000122966	CUGGAAA
ENSG00000122966	CUUCUCU
ENSG00000122966	CUUGUAU
ENSG00000122966	GAGCUGC
ENSG00000122966	GUGCAAA
ENSG00000122966	UAUAAAG
ENSG00000122966	UGUUGAA
ENSG00000123064	CAGGUGA
ENSG00000123064	GCAGGAC
ENSG00000123064	UAUUACC
ENSG00000123066	AAAGUAU
ENSG00000123066	AAGUCUU
ENSG00000123066	AAGUGCU

ENSG00000123066	ACAAGGU
ENSG00000123066	AGUGCUU
ENSG00000123066	AUAUAAC
ENSG00000123066	AUUCAUU
ENSG00000123066	CCUUCAU
ENSG00000123066	UAUAUGA
ENSG00000123066	UCAAGUA
ENSG00000123066	UCACAAG
ENSG00000123066	UGAGAAU
ENSG00000123066	UUUGCAA
ENSG00000123091	AUCCUUU
ENSG00000123091	CCAGUGU
ENSG00000123091	GGCUCAG
ENSG00000123096	CAAAUGC
ENSG00000123096	GUUAAUG
ENSG00000123096	UGCAAAG
ENSG00000123096	UUUGCAA
ENSG00000123104	AAAAAUC
ENSG00000123104	CACUCCU
ENSG00000123104	CACUGUU
ENSG00000123104	GAGCUAA
ENSG00000123104	GGUUGUC
ENSG00000123104	GUGACAG
ENSG00000123104	UGAAAGG
ENSG00000123104	UGGCUGC
ENSG00000123104	UUGGCAC
ENSG00000123119	AACACUG
ENSG00000123119	CUCAAAC
ENSG00000123119	CUGGAAA
ENSG00000123119	GGAAGAC
ENSG00000123119	GGAAUGU
ENSG00000123119	GGACUGC
ENSG00000123119	UAUAAAG
ENSG00000123119	UAUAAUA
ENSG00000123119	UGGACUG
ENSG00000123124	AAAACUG
ENSG00000123124	AGCAGCA
ENSG00000123124	AGCUUAU
ENSG00000123124	GGAAGAC
ENSG00000123124	GUGCAAA
ENSG00000123124	UGCAUAG
ENSG00000123124	UUUGCAA
ENSG00000123144	AAGUGCU
ENSG00000123146	AAAGCUG
ENSG00000123159	GAGGUAG
ENSG00000123159	GAGGUAU
ENSG00000123159	GUGGGGA
ENSG00000123159	UCACAGG
ENSG00000123159	UCACAGU
ENSG00000123178	AACAGUC
ENSG00000123178	AGGACAC
ENSG00000123178	CUUACAG
ENSG00000123191	CAGGCUC
ENSG00000123191	GAGGUAG
ENSG00000123191	GAGGUAU
ENSG00000123240	ACUGUAG
ENSG00000123240	CUUUGGU
ENSG00000123243	GUCAUUG
ENSG00000123243	UACAAAG
ENSG00000123268	AACAUUC
ENSG00000123268	UCCAGUU
ENSG00000123268	UGACCUA
ENSG00000123307	UCACAGU
ENSG00000123307	UCUGGAA

ENSG00000123307	UGCAUAU
ENSG00000123307	UGUGCUU
ENSG00000123307	UUGGCAA
ENSG00000123338	AGUGAUC
ENSG00000123338	AUGAUGC
ENSG00000123338	UAAUAUC
ENSG00000123338	UUGAUCA
ENSG00000123342	AGGGAGG
ENSG00000123342	AGUGCCU
ENSG00000123342	CUGGGCA
ENSG00000123342	CUUCUCU
ENSG00000123342	GGCAUUG
ENSG00000123352	AAUUAUU
ENSG00000123352	GAGGCAG
ENSG00000123352	GAUCAGA
ENSG00000123352	UCCAGUU
ENSG00000123353	AACUGGU
ENSG00000123353	AGCUGCC
ENSG00000123353	AUGGCUU
ENSG00000123353	GUAUGUU
ENSG00000123353	GUUCAUG
ENSG00000123353	UGAGAAG
ENSG00000123353	UGGGUGA
ENSG00000123353	UUGUUCG
ENSG00000123358	AAAUCCC
ENSG00000123358	AAGGCAC
ENSG00000123360	ACCUGGA
ENSG00000123360	ACUUGGA
ENSG00000123360	CAAAGCA
ENSG00000123360	CAAAUGC
ENSG00000123360	CACAGUG
ENSG00000123360	CCUUCUU
ENSG00000123360	CUCUGGG
ENSG00000123360	CUGGGGG
ENSG00000123360	CUUGGAG
ENSG00000123360	GACCCUG
ENSG00000123360	GAGCCCC
ENSG00000123360	GAGGGGC
ENSG00000123360	GUGGGGA
ENSG00000123360	UAUUGCU
ENSG00000123360	UCCAGAG
ENSG00000123360	UGACAUC
ENSG00000123360	UGGGGGA
ENSG00000123360	UUGGAGG
ENSG00000123364	AAAAACC
ENSG00000123364	ACAUCAC
ENSG00000123364	ACUGUUU
ENSG00000123364	AGCACCA
ENSG00000123364	AGCAGCA
ENSG00000123364	AGCAGCG
ENSG00000123364	AGCAGCU
ENSG00000123364	CACAAUG
ENSG00000123364	CACUGUU
ENSG00000123364	GAGGCAG
ENSG00000123364	GGAUUUC
ENSG00000123364	GGAUUUU
ENSG00000123364	GUCAUUG
ENSG00000123364	UACAAAG
ENSG00000123364	UGCAUUG
ENSG00000123364	UUUGCAA
ENSG00000123364	UUUGCAC
ENSG00000123364	UUUUUGC
ENSG00000123374	AAGGUGC
ENSG00000123374	AAGUGCU

ENSG00000123374	CCUUCUG
ENSG00000123374	UACAAAG
ENSG00000123388	ACUGCAU
ENSG00000123388	AGCAGCA
ENSG00000123388	AGGACAC
ENSG00000123388	AGGCAGG
ENSG00000123388	AUGCACC
ENSG00000123388	CACACCU
ENSG00000123388	CCCUGUC
ENSG00000123388	CUAGGCU
ENSG00000123388	GAGGUAG
ENSG00000123388	GGGGAGC
ENSG00000123388	UCACAUU
ENSG00000123388	UGCAUAG
ENSG00000123388	UUCAAAU
ENSG00000123388	UUUGCAC
ENSG00000123395	GGAGAGA
ENSG00000123395	UCCAGAG
ENSG00000123411	AAAAAUC
ENSG00000123411	AAGUCUU
ENSG00000123411	AAGUGCA
ENSG00000123411	AAUCUCA
ENSG00000123411	ACAGGUG
ENSG00000123411	AGCCAGU
ENSG00000123411	AGUGCAA
ENSG00000123411	AUUCAUU
ENSG00000123411	CCAGUAC
ENSG00000123411	CCCUGAG
ENSG00000123411	CCUGCUG
ENSG00000123411	CCUUCAU
ENSG00000123411	CUUACAG
ENSG00000123411	CUUUGGU
ENSG00000123411	GACCCUG
ENSG00000123411	GAGGUAU
ENSG00000123411	GCUACAU
ENSG00000123411	GGGUUUA
ENSG00000123411	GUCUCUG
ENSG00000123411	GUGGCCC
ENSG00000123411	UACAGUU
ENSG00000123411	UAUUGCU
ENSG00000123411	UCCAGAG
ENSG00000123411	UUGGUUC
ENSG00000123411	UUGUUCG
ENSG00000123444	UUCUGCA
ENSG00000123454	GGUGGGC
ENSG00000123454	UCCCUUU
ENSG00000123472	AAGUCUU
ENSG00000123472	AGCUUAU
ENSG00000123472	CACAGUG
ENSG00000123472	GAGGUAU
ENSG00000123472	GGCAGGG
ENSG00000123472	UCACAGU
ENSG00000123472	UUCAAGC
ENSG00000123505	AGCAUUC
ENSG00000123505	AUAAUAC
ENSG00000123505	CACCACC
ENSG00000123505	GAAGGAA
ENSG00000123505	UCACAGG
ENSG00000123505	UCACAGU
ENSG00000123505	UGAGGAC
ENSG00000123505	UGUCUGC
ENSG00000123505	UGUCUGG
ENSG00000123505	UUUGCGA
ENSG00000123505	UUUUUGC

ENSG00000123552	AAGGCAC
ENSG00000123552	CCCUGAG
ENSG00000123552	GCAGAAG
ENSG00000123552	UCACAGG
ENSG00000123552	UCACAGU
ENSG00000123552	UCCUGAG
ENSG00000123552	UCUGGAA
ENSG00000123560	AGCACCA
ENSG00000123560	AUCCUUG
ENSG00000123560	CAGUGCA
ENSG00000123560	GCACCAU
ENSG00000123560	UAUAAAG
ENSG00000123560	UCCUUGC
ENSG00000123562	AAAAACC
ENSG00000123562	CCUUCAU
ENSG00000123562	GCACCAU
ENSG00000123562	UUGGCAA
ENSG00000123572	ACAGUAU
ENSG00000123572	ACAUUAC
ENSG00000123572	AGGUAGU
ENSG00000123572	AGUAACA
ENSG00000123572	CACAGUG
ENSG00000123572	CUCCAUU
ENSG00000123572	GAGGUAG
ENSG00000123572	GAGGUAU
ENSG00000123572	GUAAACA
ENSG00000123572	GUGUCAC
ENSG00000123572	GUUAGGA
ENSG00000123572	UAGAGGA
ENSG00000123572	UCACAGG
ENSG00000123572	UCACAUU
ENSG00000123572	UGAGGAC
ENSG00000123572	UGUGUCA
ENSG00000123572	UUAGGAU
ENSG00000123576	AAAACUG
ENSG00000123600	AAACUGU
ENSG00000123600	AAAGAGG
ENSG00000123600	AAAGGUA
ENSG00000123600	AAAUCCC
ENSG00000123600	AAUUAUU
ENSG00000123600	ACAGUAC
ENSG00000123600	ACUGCAG
ENSG00000123600	ACUGCAU
ENSG00000123600	AGAUAAA
ENSG00000123600	AGUAACA
ENSG00000123600	AGUGGUU
ENSG00000123600	CAAAACU
ENSG00000123600	CAGUCCA
ENSG00000123600	CUGUUGA
ENSG00000123600	GCUUCCU
ENSG00000123600	GGCAUUG
ENSG00000123600	GGGAGCU
ENSG00000123600	GGGUCUU
ENSG00000123600	GGGUGUU
ENSG00000123600	GGUUGAC
ENSG00000123600	GGUUGUC
ENSG00000123600	GUCAUUG
ENSG00000123600	GUGAGGU
ENSG00000123600	GUGCCAC
ENSG00000123600	GUGUCAC
ENSG00000123600	UCAACAG
ENSG00000123600	UCACAAG
ENSG00000123600	UCAUACA
ENSG00000123600	UCAUUCG

ENSG00000123600	UCUGGAG
ENSG00000123600	UGAAAUG
ENSG00000123600	UGCUGAC
ENSG00000123600	UGUGUCA
ENSG00000123600	UUGGCAA
ENSG00000123600	UUGGCAC
ENSG00000123600	UUGGUUC
ENSG00000123600	UUGUGUC
ENSG00000123607	GCUGGAU
ENSG00000123612	AAGUGCA
ENSG00000123612	ACAGUAC
ENSG00000123612	ACUGUAG
ENSG00000123612	AGGACAC
ENSG00000123612	AGGCAGG
ENSG00000123612	AGUGCUU
ENSG00000123612	CAAGAGC
ENSG00000123612	CACAGUG
ENSG00000123612	CCCUGAG
ENSG00000123612	CUUUGGU
ENSG00000123612	GAGGUAU
ENSG00000123612	GUGACAG
ENSG00000123612	UAUUGCU
ENSG00000123612	UCAUACA
ENSG00000123612	UCAUUCG
ENSG00000123612	UCUAGAG
ENSG00000123612	UGACAUC
ENSG00000123612	UGCAAAG
ENSG00000123612	UUCCAUA
ENSG00000123612	UUGGUUC
ENSG00000123636	AACAAAC
ENSG00000123636	AACAUUC
ENSG00000123636	AACUACU
ENSG00000123636	AAGGCAC
ENSG00000123636	AAUACUG
ENSG00000123636	ACAAUAU
ENSG00000123636	ACAGUAC
ENSG00000123636	ACCCUGU
ENSG00000123636	ACUGUAG
ENSG00000123636	AGGCAGG
ENSG00000123636	AGUGGUU
ENSG00000123636	CAGUAGA
ENSG00000123636	CAGUGCA
ENSG00000123636	CAUGGAU
ENSG00000123636	CCCUGUC
ENSG00000123636	CUACAAA
ENSG00000123636	CUUGUAU
ENSG00000123636	GAGAACC
ENSG00000123636	GAGGAUA
ENSG00000123636	GCAGCAU
ENSG00000123636	GCUGGUG
ENSG00000123636	GUGCCAC
ENSG00000123636	UAGAGGA
ENSG00000123636	UCACAGG
ENSG00000123636	UCACAGU
ENSG00000123636	UGGGUGA
ENSG00000123636	UGUGCUU
ENSG00000123636	UUCAAGC
ENSG00000123636	UUUCAAC
ENSG00000123643	AGCACCA
ENSG00000123643	AUAAAGU
ENSG00000123643	CUUUGGU
ENSG00000123643	GAGGCAG
ENSG00000123643	GAUUGUA
ENSG00000123643	GGAGGAG

ENSG00000123643	GGCUCUG
ENSG00000123643	GGGGAGC
ENSG00000123684	AAAGUGC
ENSG00000123684	AAGUGCA
ENSG00000123684	AUUCAUU
ENSG00000123684	GAGGUAG
ENSG00000123684	GAUUGUC
ENSG00000123684	GCAACUU
ENSG00000123684	GGUUUAC
ENSG00000123684	GUGCAAA
ENSG00000123684	UACAAAG
ENSG00000123684	UCCCACA
ENSG00000123684	UGCAUAG
ENSG00000123684	UUGGUCC
ENSG00000123684	UUGGUUC
ENSG00000123689	AGCAAAA
ENSG00000123689	AGCAGCA
ENSG00000123689	CAGCAAA
ENSG00000123689	UUCAAAU
ENSG00000123700	AAGGCAC
ENSG00000123700	AGCAGCG
ENSG00000123700	GAUUGUC
ENSG00000123700	GGAAUGU
ENSG00000123700	GGCUCAG
ENSG00000123700	UUGGUUC
ENSG00000123728	AAAGCUG
ENSG00000123728	AGCAGCA
ENSG00000123728	CAAAGCA
ENSG00000123728	CCUGCUG
ENSG00000123728	CUUUGGU
ENSG00000123728	GAGCUGC
ENSG00000123728	GCAGCAU
ENSG00000123728	GGCUCAG
ENSG00000123728	GGUUUAC
ENSG00000123728	UAAUACA
ENSG00000123836	CACCACC
ENSG00000123836	GGAAUGU
ENSG00000123836	GGCAGGG
ENSG00000123836	GGGUCUU
ENSG00000123836	UACAGUU
ENSG00000123836	UAGUGAG
ENSG00000123836	UGCUGAC
ENSG00000123836	UUGGCAA
ENSG00000123892	AACACUG
ENSG00000123908	ACAUUAC
ENSG00000123908	AUGGCAC
ENSG00000123908	CCCUGAG
ENSG00000123908	GAUUGUA
ENSG00000123908	GGACGGA
ENSG00000123933	AAGGCAC
ENSG00000123933	AAUACUG
ENSG00000123933	AGGCAGG
ENSG00000123933	AUAUUAU
ENSG00000123933	AUGGCAC
ENSG00000123933	CACUCCU
ENSG00000123933	CAGCAAA
ENSG00000123933	CCCACCG
ENSG00000123933	CCCACCU
ENSG00000123933	CCCUGAG
ENSG00000123933	CUCCCAC
ENSG00000123933	GAGACGC
ENSG00000123933	GAGCUGC
ENSG00000123933	GAGGGGC
ENSG00000123933	GCUGGUG

ENSG00000123933	GGAAUGU
ENSG00000123933	UAAUACA
ENSG00000123933	UCCCACC
ENSG00000123933	UGCUCAC
ENSG00000123983	AAAAACC
ENSG00000123983	AAAUGGU
ENSG00000123983	AAUCACU
ENSG00000123983	AAUCCUU
ENSG00000123983	ACUGUAG
ENSG00000123983	AGCAAAA
ENSG00000123983	AUCCCUU
ENSG00000123983	CACUGUU
ENSG00000123983	CAGUUAC
ENSG00000123983	CUCAAAC
ENSG00000123983	GUGCAAA
ENSG00000123983	UGAAAUG
ENSG00000123983	UGAGAAG
ENSG00000123983	UUCAAGC
ENSG00000123983	UUGAUCA
ENSG00000123983	UUGUUCG
ENSG00000123989	AGCAGCG
ENSG00000123992	AAGUCUU
ENSG00000123992	CAGCUGG
ENSG00000123992	UCUCCAA
ENSG00000124067	CACAAUG
ENSG00000124074	CCUUCUG
ENSG00000124074	UGGCCCU
ENSG00000124092	AUUCAUU
ENSG00000124098	CCCUGAG
ENSG00000124098	CUGGCUC
ENSG00000124098	GGAAUGU
ENSG00000124098	GGCUCAG
ENSG00000124098	UCACAUU
ENSG00000124098	UCCCUGU
ENSG00000124098	UUCUGCA
ENSG00000124104	AAAGUGC
ENSG00000124104	AAGUGCU
ENSG00000124104	AGUGCUU
ENSG00000124104	CUGGAAA
ENSG00000124104	GAGAACU
ENSG00000124104	GGGGCAG
ENSG00000124104	UCUGGAA
ENSG00000124104	UUGAGGC
ENSG00000124120	AACAAAC
ENSG00000124120	AACCUGG
ENSG00000124120	AAGUGCU
ENSG00000124120	AUUGCAC
ENSG00000124120	GAGAACC
ENSG00000124120	GAGAACU
ENSG00000124120	GGAAUGU
ENSG00000124120	UCCAGAG
ENSG00000124120	UGAAGCU
ENSG00000124120	UUGGCAC
ENSG00000124126	AAAGUGC
ENSG00000124126	AAGUGCA
ENSG00000124126	AGUAGAC
ENSG00000124126	AUAAAGU
ENSG00000124126	AUAUAAC
ENSG00000124126	CCAGUGC
ENSG00000124126	GGAAUGU
ENSG00000124126	UUGGUCC
ENSG00000124126	UUUCAAC
ENSG00000124134	CUCAGGG
ENSG00000124140	AAACGGU

ENSG00000124140	AAAUGGU
ENSG00000124140	AACAAAC
ENSG00000124140	AACGGAA
ENSG00000124140	AAGAACC
ENSG00000124140	AAGGCAC
ENSG00000124140	AAGUCAC
ENSG00000124140	AAUACUG
ENSG00000124140	AAUUAUU
ENSG00000124140	ACAUUCA
ENSG00000124140	AGCAUUC
ENSG00000124140	AGUGCAA
ENSG00000124140	AGUGCCU
ENSG00000124140	AUAAAGU
ENSG00000124140	AUAAUAC
ENSG00000124140	CCCCCAG
ENSG00000124140	CCUGUAC
ENSG00000124140	CUACAGU
ENSG00000124140	CUGCAGC
ENSG00000124140	CUUUGGU
ENSG00000124140	GCAACUU
ENSG00000124140	GCCCUGU
ENSG00000124140	GGAGAGA
ENSG00000124140	GGGAGCU
ENSG00000124140	GUGAGGU
ENSG00000124140	GUGCCUG
ENSG00000124140	UAUAAUA
ENSG00000124140	UAUUGCU
ENSG00000124140	UCAACAG
ENSG00000124140	UCAACGG
ENSG00000124140	UGAAAUG
ENSG00000124140	UUUGCAA
ENSG00000124145	AAGGCAC
ENSG00000124145	AAGUCAC
ENSG00000124145	AGUAACA
ENSG00000124145	CUCCAGC
ENSG00000124145	CUUCUCU
ENSG00000124145	GUAACAG
ENSG00000124145	GUAGUGU
ENSG00000124151	AAAAGUA
ENSG00000124151	AACAAAC
ENSG00000124151	AAGUGCA
ENSG00000124151	AAGUGCU
ENSG00000124151	AGCAAAA
ENSG00000124151	AGCACCA
ENSG00000124151	AGUGCAA
ENSG00000124151	AGUGCUU
ENSG00000124151	AUAAAGU
ENSG00000124151	AUUGCAC
ENSG00000124151	CAAAGUG
ENSG00000124151	CUACAAA
ENSG00000124151	CUUUGGU
ENSG00000124151	GUAAACA
ENSG00000124151	UAAUACA
ENSG00000124151	UCACAAG
ENSG00000124151	UGACCUA
ENSG00000124151	UGCAUUG
ENSG00000124151	UUCUUGU
ENSG00000124160	CACAGUG
ENSG00000124160	CCUGCUG
ENSG00000124160	CUCCAUU
ENSG00000124160	CUGCUCA
ENSG00000124160	GGAGGAG
ENSG00000124160	UCACAGU
ENSG00000124164	AAAUUUC

ENSG00000124164	ACAUAGA
ENSG00000124164	AGCAGCA
ENSG00000124164	AUCAUAC
ENSG00000124164	GAUAUGU
ENSG00000124164	GCUACAU
ENSG00000124164	GGCAAGA
ENSG00000124164	GUCAUUG
ENSG00000124164	GUGACAG
ENSG00000124164	UCACAGU
ENSG00000124164	UGACUGU
ENSG00000124164	UGUGUGG
ENSG00000124164	UUGGUCC
ENSG00000124164	UUGUGUC
ENSG00000124171	AAAACUG
ENSG00000124171	AAAGCUG
ENSG00000124171	AAAGUAU
ENSG00000124171	AAAUUUC
ENSG00000124171	AAGUGCA
ENSG00000124171	AGCAGCA
ENSG00000124171	AGCAGCG
ENSG00000124171	AGGCAGG
ENSG00000124171	AGGUAGU
ENSG00000124171	AGUGGUU
ENSG00000124171	AGUGUGC
ENSG00000124171	AUAAUAC
ENSG00000124171	CACAGUG
ENSG00000124171	CCUUCAU
ENSG00000124171	GAGGUAU
ENSG00000124171	GCACCAU
ENSG00000124171	GCAGGUU
ENSG00000124171	GGCAGUG
ENSG00000124171	GGCUCUG
ENSG00000124171	GUAGUGU
ENSG00000124171	UAGUGAG
ENSG00000124177	AGCAGCA
ENSG00000124177	GACCCUG
ENSG00000124177	GCAGCAU
ENSG00000124177	UAGGGCC
ENSG00000124177	UGUAUAA
ENSG00000124177	UGUGCUU
ENSG00000124181	AAAGCUG
ENSG00000124181	AAGAACC
ENSG00000124181	AUAAUAC
ENSG00000124181	AUGGCUU
ENSG00000124181	CUUCUCU
ENSG00000124181	GAGGCAG
ENSG00000124181	GGCAGUG
ENSG00000124181	GUAAACA
ENSG00000124181	UGUGCUU
ENSG00000124181	UUGGCAA
ENSG00000124181	UUGGCAC
ENSG00000124181	UUUUUGC
ENSG00000124193	CAAAGUG
ENSG00000124193	GCUGGUG
ENSG00000124193	GGGUCUU
ENSG00000124193	UAUAAAG
ENSG00000124193	UAUUGCU
ENSG00000124193	UGACCUA
ENSG00000124193	UUCCAUA
ENSG00000124193	UUGGCAC
ENSG00000124194	AAGGCAC
ENSG00000124194	CCAGGAG
ENSG00000124194	GGAAUGU
ENSG00000124194	UCACACA

ENSG00000124198	AAGGCAC
ENSG00000124198	ACUGCAG
ENSG00000124198	AGCACCA
ENSG00000124198	AUAUAAC
ENSG00000124198	AUUGCAC
ENSG00000124198	CAUAUUG
ENSG00000124198	CUAUACA
ENSG00000124198	UCUCAAG
ENSG00000124198	UGACAUC
ENSG00000124201	AGUGCUU
ENSG00000124201	CUUGUGU
ENSG00000124201	GCAGAAG
ENSG00000124201	GGAAGCC
ENSG00000124201	GGACCUG
ENSG00000124201	UCUGGAA
ENSG00000124201	UGUGCUU
ENSG00000124201	UUCUUGU
ENSG00000124203	AAUUGCU
ENSG00000124203	ACAAUAU
ENSG00000124203	AGCAGCA
ENSG00000124203	AGUAACA
ENSG00000124203	AUGACAC
ENSG00000124203	CAGCAAA
ENSG00000124203	GGUUACC
ENSG00000124203	UGUCUGG
ENSG00000124203	UUGGCAC
ENSG00000124205	CACAAUG
ENSG00000124205	CUUCUCU
ENSG00000124205	UCACAAG
ENSG00000124205	UGGGAUC
ENSG00000124208	ACUGUGU
ENSG00000124208	AGAUGUG
ENSG00000124208	AGUGGUU
ENSG00000124208	AUGUGCC
ENSG00000124208	CACAGUG
ENSG00000124208	UCACAGG
ENSG00000124209	AAAGAGC
ENSG00000124209	AAGGUGC
ENSG00000124209	AAGUCUU
ENSG00000124209	ACUCAGG
ENSG00000124209	AGCAUUC
ENSG00000124209	CAGGAAC
ENSG00000124209	CCAUUAC
ENSG00000124209	CCCUGAG
ENSG00000124209	CUACAAA
ENSG00000124209	CUGGGCA
ENSG00000124209	CUUGUAU
ENSG00000124209	GAGGUAG
ENSG00000124209	GCACCAU
ENSG00000124209	GCCUGGA
ENSG00000124209	GCCUGGG
ENSG00000124209	GCUACAU
ENSG00000124209	GCUGGUG
ENSG00000124209	GUCUACU
ENSG00000124209	UAAGACG
ENSG00000124209	UAAGACU
ENSG00000124209	UCCAGAG
ENSG00000124209	UCCUGAG
ENSG00000124209	UCUGGAG
ENSG00000124209	UGCAAAG
ENSG00000124209	UUCCUAG
ENSG00000124209	UUGAUCA
ENSG00000124209	UUGGCAA
ENSG00000124212	CAGCUGG

ENSG00000124212	CAGGUGA
ENSG00000124212	CUCCCAC
ENSG00000124212	CUGGCUC
ENSG00000124212	GCCUGGA
ENSG00000124212	GCCUGGG
ENSG00000124212	GGAAGCC
ENSG00000124212	GGGUUUA
ENSG00000124212	GUGCCUG
ENSG00000124212	GUGUGGC
ENSG00000124212	UCCCUGU
ENSG00000124212	UCCCUUG
ENSG00000124212	UCCCUUU
ENSG00000124212	UGCCAGC
ENSG00000124212	UUCUGCA
ENSG00000124214	AUGUAAU
ENSG00000124214	CACUGUU
ENSG00000124214	GGGGAGC
ENSG00000124214	GUCCAGA
ENSG00000124216	ACAGGUG
ENSG00000124216	AUUGCAC
ENSG00000124216	CACAGUG
ENSG00000124216	UCACAGU
ENSG00000124216	UGCAUAU
ENSG00000124217	CAAAGCA
ENSG00000124217	UUGAGGC
ENSG00000124222	AGCACCA
ENSG00000124222	AGUGCUU
ENSG00000124222	CAAAACU
ENSG00000124222	CUUGUGU
ENSG00000124222	GCACCAU
ENSG00000124222	GUGACUG
ENSG00000124222	GUGUCAC
ENSG00000124222	UCACAGG
ENSG00000124222	UGAAAUG
ENSG00000124222	UGAAGCU
ENSG00000124222	UUGGUUC
ENSG00000124225	AAAACUG
ENSG00000124225	AAACUGU
ENSG00000124225	AAAGAAU
ENSG00000124225	AAAGUUU
ENSG00000124225	AAGGCAC
ENSG00000124225	ACACACC
ENSG00000124225	ACUCAAA
ENSG00000124225	AGCAUUC
ENSG00000124225	AGCCCUU
ENSG00000124225	AGGCAGG
ENSG00000124225	AUAUUAU
ENSG00000124225	AUGUCUG
ENSG00000124225	CAAAACU
ENSG00000124225	CAAGAGC
ENSG00000124225	CCUUCUG
ENSG00000124225	GAAUUGU
ENSG00000124225	GUAACAG
ENSG00000124225	UAAUAGU
ENSG00000124225	UACAGUU
ENSG00000124225	UAUUGCU
ENSG00000124225	UGUCUUU
ENSG00000124225	UUCUAAU
ENSG00000124225	UUGGCAC
ENSG00000124225	UUUGCAC
ENSG00000124226	AAGGCAC
ENSG00000124226	AAGUGCA
ENSG00000124226	CCCUGUC
ENSG00000124226	GGAAGCC

ENSG00000124226	UAAGACU
ENSG00000124226	UCUGGAG
ENSG00000124228	CACAGGG
ENSG00000124228	GACUUCC
ENSG00000124228	UCCCACA
ENSG00000124228	UCCCACC
ENSG00000124243	UCUCCAA
ENSG00000124243	UUGACAC
ENSG00000124302	CUGGAAA
ENSG00000124302	GUGCUUG
ENSG00000124302	UCUGGAA
ENSG00000124302	UGUGCUU
ENSG00000124313	AAGUGCA
ENSG00000124313	ACAAGGU
ENSG00000124313	ACAUUCA
ENSG00000124313	ACUGCAG
ENSG00000124313	AGGAGCU
ENSG00000124313	AGGCAGG
ENSG00000124313	AUAAUAC
ENSG00000124313	AUGGCUU
ENSG00000124313	CUCCAUU
ENSG00000124313	CUCCCAC
ENSG00000124313	CUGGACU
ENSG00000124313	CUGGCUC
ENSG00000124313	GAGCCCC
ENSG00000124313	GCCUGAU
ENSG00000124313	GGAGAGA
ENSG00000124313	GGCAAGA
ENSG00000124313	GUGUGGC
ENSG00000124313	UGCAUUG
ENSG00000124313	UUGAUCA
ENSG00000124333	AAGGCAC
ENSG00000124333	AAUUAUU
ENSG00000124333	AGCAGCA
ENSG00000124356	AUGGCUU
ENSG00000124356	CUACAGU
ENSG00000124356	UUCAAAU
ENSG00000124374	AAAGUAA
ENSG00000124374	AGAUGUG
ENSG00000124374	CACAGUG
ENSG00000124374	CUCAAAC
ENSG00000124374	GCUUCCU
ENSG00000124374	UCACACA
ENSG00000124374	UCACAGU
ENSG00000124374	UUGGUUC
ENSG00000124406	AAGGUGC
ENSG00000124406	ACAGUAU
ENSG00000124406	AGCAUUC
ENSG00000124406	AGUGCUU
ENSG00000124406	AUGAGCU
ENSG00000124406	AUGCAUU
ENSG00000124406	AUGUCUG
ENSG00000124406	CACAAUG
ENSG00000124406	CAGCAGA
ENSG00000124406	CUGUUGA
ENSG00000124406	CUUUGGU
ENSG00000124406	GAAUUGU
ENSG00000124406	UCACAAG
ENSG00000124406	UCCUGAG
ENSG00000124406	UGCAGCU
ENSG00000124406	UGCAUUG
ENSG00000124406	UGUUGAA
ENSG00000124406	UUGGCAC
ENSG00000124422	CAGCAGA

ENSG00000124429	UGAAAGG
ENSG00000124440	AGUGCCU
ENSG00000124440	GAGCCCC
ENSG00000124440	GAGGUAU
ENSG00000124440	GAGUGUG
ENSG00000124440	GCACCAU
ENSG00000124440	GGAGAGA
ENSG00000124440	GGAGUGU
ENSG00000124440	UAUGGUU
ENSG00000124440	UCCUGAG
ENSG00000124440	UCUAGAG
ENSG00000124440	UGGCUGC
ENSG00000124459	CUUCUCU
ENSG00000124466	CACAGUG
ENSG00000124466	CAGCAGA
ENSG00000124466	CAGUCCA
ENSG00000124466	GUGCCAC
ENSG00000124466	UCACAGU
ENSG00000124479	AACUCUA
ENSG00000124479	CAGUGCA
ENSG00000124486	AAAAGUA
ENSG00000124486	AAAGUAC
ENSG00000124486	AACACUG
ENSG00000124486	AAGACAU
ENSG00000124486	ACAUUCA
ENSG00000124486	ACCACAG
ENSG00000124486	ACUGCAG
ENSG00000124486	AGCCAGU
ENSG00000124486	AGUAGUU
ENSG00000124486	CAGUAGU
ENSG00000124486	CUAUACA
ENSG00000124486	CUGCAGC
ENSG00000124486	GAAUUGU
ENSG00000124486	GACUUCC
ENSG00000124486	GAGGCAG
ENSG00000124486	GCCUGGG
ENSG00000124486	GGACUGC
ENSG00000124486	GUGAAUG
ENSG00000124486	UAUUACC
ENSG00000124486	UUGGUUC
ENSG00000124486	UUGUGAC
ENSG00000124491	AAAGCUG
ENSG00000124491	CAAGAGC
ENSG00000124491	CUGCAGG
ENSG00000124491	GGCAGGG
ENSG00000124491	UAAUGCU
ENSG00000124491	UUGGCAC
ENSG00000124491	UUUGCAA
ENSG00000124493	AAAAACC
ENSG00000124493	ACACACU
ENSG00000124493	AGCACCA
ENSG00000124493	GGCCCUG
ENSG00000124493	UUUGCAC
ENSG00000124493	UUUUUGC
ENSG00000124507	AACUCUA
ENSG00000124507	ACAGGUG
ENSG00000124507	ACCACAG
ENSG00000124507	ACCAGCA
ENSG00000124507	AUACCUC
ENSG00000124507	CAAAUGC
ENSG00000124507	CACCAGC
ENSG00000124507	CUGGAAA
ENSG00000124507	CUUUGGU
ENSG00000124507	GAGACCU

ENSG00000124507	GCAGCAU
ENSG00000124507	GCCUGGG
ENSG00000124507	GGGGAGC
ENSG00000124507	UCUGGAA
ENSG00000124507	UGCAUUG
ENSG00000124507	UGCUGAC
ENSG00000124507	UUGUUCG
ENSG00000124523	AAAGAAU
ENSG00000124529	GGCCCUG
ENSG00000124535	GUGCAAA
ENSG00000124571	AAAAAUC
ENSG00000124571	AAAUUUC
ENSG00000124571	AAGGCAC
ENSG00000124571	AAGUCAC
ENSG00000124571	ACCUGGA
ENSG00000124571	AGGAUGU
ENSG00000124571	CUGGACU
ENSG00000124571	GAGAUGA
ENSG00000124571	GGCAGUG
ENSG00000124571	GUGAGGU
ENSG00000124571	GUGCCUG
ENSG00000124571	UGUGCUU
ENSG00000124574	AUAUUAU
ENSG00000124575	AAAGAGC
ENSG00000124593	AGCUGCC
ENSG00000124593	CAGCAAA
ENSG00000124593	CCCACGU
ENSG00000124593	CCCCUGG
ENSG00000124593	GGAAGCC
ENSG00000124593	GGUAGAC
ENSG00000124593	UCCCUCU
ENSG00000124608	CUGGCUC
ENSG00000124608	GGCUCAG
ENSG00000124610	AAAGAGC
ENSG00000124615	GAGAAGA
ENSG00000124615	UGAGAAG
ENSG00000124635	AAAGAGC
ENSG00000124635	GGCUCUG
ENSG00000124641	GGCAGGG
ENSG00000124693	AAAGAGC
ENSG00000124693	GGGUGGU
ENSG00000124701	CAGCAGG
ENSG00000124701	GUCUCUG
ENSG00000124702	AGCACCA
ENSG00000124702	CAGCAGG
ENSG00000124702	GAGGUAU
ENSG00000124702	UAUAAAG
ENSG00000124721	AAAGUUU
ENSG00000124743	AUUGCAC
ENSG00000124743	CACAGUG
ENSG00000124743	GAGUCUU
ENSG00000124743	GCUUCCU
ENSG00000124743	UCACAGG
ENSG00000124743	UCACAGU
ENSG00000124743	UGAGUCU
ENSG00000124743	UGGCCAC
ENSG00000124749	ACUGCAU
ENSG00000124749	GCACCAU
ENSG00000124749	UCACAGG
ENSG00000124749	UUCAAGC
ENSG00000124762	AACAGUC
ENSG00000124762	AAGUGCA
ENSG00000124762	AGCUGCC
ENSG00000124762	AGUGCAA

ENSG00000124762	CCUGCUG
ENSG00000124762	GAGCUGC
ENSG00000124762	GAGGUAG
ENSG00000124766	AAAAGUA
ENSG00000124766	AAAGCUG
ENSG00000124766	AAAGUGC
ENSG00000124766	AACAGUC
ENSG00000124766	AACUCUA
ENSG00000124766	AAGUGCA
ENSG00000124766	AAUCCUU
ENSG00000124766	ACAUAGA
ENSG00000124766	ACUCAAA
ENSG00000124766	AGAUGUG
ENSG00000124766	AGCCCUU
ENSG00000124766	AGCCUGC
ENSG00000124766	AGUGGUU
ENSG00000124766	AUACAAG
ENSG00000124766	AUCCCUU
ENSG00000124766	CAUAUUG
ENSG00000124766	CCAGCAU
ENSG00000124766	CCAGUGC
ENSG00000124766	CCAGUGU
ENSG00000124766	CUCUAGC
ENSG00000124766	GAAUUGU
ENSG00000124766	GAGUUGA
ENSG00000124766	GAGUUGG
ENSG00000124766	GCUGGUG
ENSG00000124766	GGCAGUG
ENSG00000124766	GUAAACA
ENSG00000124766	GUGCCUG
ENSG00000124766	UAAUACA
ENSG00000124766	UCAACAG
ENSG00000124766	UGGCCAC
ENSG00000124766	UUGGUCC
ENSG00000124767	UUCUAAU
ENSG00000124772	AAGAACC
ENSG00000124772	AAGGCAC
ENSG00000124772	AGGCAGG
ENSG00000124772	GGAGGAG
ENSG00000124772	GGCAGGG
ENSG00000124782	ACAGUAC
ENSG00000124782	ACAGUAU
ENSG00000124782	AUGGCAC
ENSG00000124782	AUUCAUU
ENSG00000124782	CAGUAGA
ENSG00000124782	CAGUAGU
ENSG00000124782	CUCACAC
ENSG00000124782	GGAAGAC
ENSG00000124782	UAAUGCU
ENSG00000124782	UCACACA
ENSG00000124782	UCACAUU
ENSG00000124782	UUCCAUA
ENSG00000124782	UUGGUUC
ENSG00000124783	AAGACAU
ENSG00000124783	AAGUGUG
ENSG00000124783	AAGUUGU
ENSG00000124783	ACACACU
ENSG00000124783	ACAUUAC
ENSG00000124783	ACUCAAA
ENSG00000124783	AGGGUCU
ENSG00000124783	AGGUAGU
ENSG00000124783	AGUAGUU
ENSG00000124783	AGUGUGC
ENSG00000124783	CACAAUG

ENSG00000124783	CUGCUCA
ENSG00000124783	GAAUUGU
ENSG00000124783	GCAGGUU
ENSG00000124783	GGUUUAC
ENSG00000124783	GUAUUCU
ENSG00000124783	GUGCUUG
ENSG00000124783	GUGUGGC
ENSG00000124783	UAGGGCC
ENSG00000124783	UAUUGCU
ENSG00000124783	UGCAAAG
ENSG00000124783	UGCUCAC
ENSG00000124783	UGUGCUU
ENSG00000124783	UUCUGCA
ENSG00000124783	UUCUUGU
ENSG00000124783	UUGGUUC
ENSG00000124784	AAAAAUC
ENSG00000124785	AACAUUC
ENSG00000124785	AGCAAAA
ENSG00000124785	AUAAUAC
ENSG00000124785	GGCAGUG
ENSG00000124785	GUGCUUG
ENSG00000124785	UGGCUGC
ENSG00000124785	UUGGCAC
ENSG00000124786	CUUUGGU
ENSG00000124788	AAAUCCC
ENSG00000124788	AACAGUC
ENSG00000124788	AAGUGCU
ENSG00000124788	AAGUGUG
ENSG00000124788	ACAUUCA
ENSG00000124788	ACUCCAG
ENSG00000124788	AGCACCA
ENSG00000124788	AGUGCCU
ENSG00000124788	AGUGCUG
ENSG00000124788	AGUGCUU
ENSG00000124788	AUCCCUU
ENSG00000124788	AUGCCUU
ENSG00000124788	AUUCAUU
ENSG00000124788	CACAAUG
ENSG00000124788	CACUGUU
ENSG00000124788	CAGUGCA
ENSG00000124788	CCCUGAG
ENSG00000124788	CUACAAA
ENSG00000124788	CUACAGU
ENSG00000124788	GAAACAU
ENSG00000124788	GGAUUUC
ENSG00000124788	GUAAACA
ENSG00000124788	GUGGCCC
ENSG00000124788	UAAUAUC
ENSG00000124788	UCAACAG
ENSG00000124788	UCACAUU
ENSG00000124788	UCAUUUG
ENSG00000124788	UGCAAAG
ENSG00000124788	UGCCCUG
ENSG00000124788	UUGGCAA
ENSG00000124788	UUGUUCG
ENSG00000124789	AAAUUUC
ENSG00000124789	AAGUCAC
ENSG00000124789	ACAAUAU
ENSG00000124789	ACAGGUG
ENSG00000124789	ACCACAG
ENSG00000124789	ACUGUAG
ENSG00000124789	AUGGAUU
ENSG00000124789	CACCACC
ENSG00000124789	GAUCAGA

ENSG00000124789	GCAACUU
ENSG00000124789	GGCAUUG
ENSG00000124789	GGUUUAC
ENSG00000124789	GUUAAUG
ENSG00000124789	UAAUACA
ENSG00000124789	UAAUAUC
ENSG00000124789	UCAAGUA
ENSG00000124789	UCACAGG
ENSG00000124789	UCACAGU
ENSG00000124789	UUCAAGC
ENSG00000124795	AAAGAAU
ENSG00000124795	AUAAAGU
ENSG00000124795	AUACAAG
ENSG00000124795	CAGCAGA
ENSG00000124795	CAGCAGG
ENSG00000124795	GCUGGUG
ENSG00000124795	GUAUUCU
ENSG00000124813	ACUGGCU
ENSG00000124813	AGCCCUU
ENSG00000124813	AGUGCUG
ENSG00000124813	CUGCAGC
ENSG00000124813	GUGCCUG
ENSG00000124813	UAUAUGA
ENSG00000124813	UCACAUU
ENSG00000124813	UUAGAGA
ENSG00000124813	UUCAAGC
ENSG00000124818	AAAGGUA
ENSG00000124818	AGCUGCC
ENSG00000124818	AUGCAUU
ENSG00000124818	UCACAGG
ENSG00000124818	UCACAGU
ENSG00000124818	UUGUGUC
ENSG00000124831	GUAACAG
ENSG00000124875	AAGACAU
ENSG00000124882	CAAAGCA
ENSG00000124882	CCUUCAU
ENSG00000124882	GAGAAAU
ENSG00000124920	AAAGCUG
ENSG00000124920	AAGUGCU
ENSG00000124920	ACUCCAG
ENSG00000124920	AGCAGCU
ENSG00000124920	AGUGCUU
ENSG00000124920	CAAAGCA
ENSG00000124920	CCAGUGC
ENSG00000124920	CUGCUCA
ENSG00000124920	CUUUGGU
ENSG00000124920	GUAACAG
ENSG00000124920	GUCCAGA
ENSG00000124920	UACAAAG
ENSG00000124920	UGCCCUG
ENSG00000124939	UUUCAAC
ENSG00000124942	AAUACUG
ENSG00000124942	ACAUUCA
ENSG00000124942	AGUGCUG
ENSG00000124942	GCUGGAU
ENSG00000124942	GUAAACA
ENSG00000125084	AAAAAUC
ENSG00000125084	AAUUAUU
ENSG00000125084	ACACACC
ENSG00000125084	AGGGAGG
ENSG00000125084	GAGGUAG
ENSG00000125084	GGCAGUG
ENSG00000125084	GGGGCAG
ENSG00000125084	GUGCAAA

ENSG00000125084	UUCUGCA
ENSG00000125107	AAAUUUC
ENSG00000125107	AACAUUC
ENSG00000125107	AAUCCUU
ENSG00000125107	ACAUUCA
ENSG00000125107	ACUGGCU
ENSG00000125107	AUACAAG
ENSG00000125107	CUACAAA
ENSG00000125107	CUACAGU
ENSG00000125107	UCACAGG
ENSG00000125107	UCACAGU
ENSG00000125107	UGGCCAC
ENSG00000125124	UUCAAGC
ENSG00000125149	AAAGUGC
ENSG00000125149	ACACACC
ENSG00000125149	ACACACU
ENSG00000125149	CUAUACA
ENSG00000125149	CUUUGGU
ENSG00000125149	GGAAGAC
ENSG00000125149	GUAGUGU
ENSG00000125149	GUGCAAA
ENSG00000125149	GUGCUUG
ENSG00000125149	UCAAGUA
ENSG00000125149	UCACAAG
ENSG00000125149	UGUCUUU
ENSG00000125149	UGUGCUU
ENSG00000125149	UUGGCAA
ENSG00000125149	UUGGCAC
ENSG00000125166	AAAGCUG
ENSG00000125166	GGUUGUC
ENSG00000125166	GGUUUAC
ENSG00000125166	GUAAACA
ENSG00000125166	UCACCAC
ENSG00000125166	UUGGUUC
ENSG00000125170	ACAGUAU
ENSG00000125170	AUAUUAU
ENSG00000125170	CAGCAGG
ENSG00000125170	CCUUCAU
ENSG00000125170	CCUUCUG
ENSG00000125170	CUCCAGC
ENSG00000125170	GUGCCAC
ENSG00000125170	UGCAUUG
ENSG00000125170	UUCUGCA
ENSG00000125170	UUGGCAC
ENSG00000125246	AAUCUCU
ENSG00000125246	UCAUGAU
ENSG00000125249	AAGGCAC
ENSG00000125249	AGCCCUU
ENSG00000125249	AUGGCUU
ENSG00000125249	CAGGCUC
ENSG00000125249	CUUUGGU
ENSG00000125249	GAUUGUA
ENSG00000125255	UCACAAG
ENSG00000125255	UGAAAUG
ENSG00000125255	UUUCAAC
ENSG00000125257	CCCUGAG
ENSG00000125257	GAGAUGA
ENSG00000125257	UCCUGAG
ENSG00000125257	UGUGCUU
ENSG00000125266	AAAAACC
ENSG00000125266	AAAGUGC
ENSG00000125266	AAGUGCU
ENSG00000125266	AGCAGCU
ENSG00000125266	AGUAACA

ENSG00000125266	AGUGCAA
ENSG00000125266	AUCCCUU
ENSG00000125266	AUGACAC
ENSG00000125266	AUGGCUU
ENSG00000125266	AUUCAUU
ENSG00000125266	CAGUGCA
ENSG00000125266	CUUCACC
ENSG00000125266	GAGAACC
ENSG00000125266	GAUUGUC
ENSG00000125266	GCAGCAU
ENSG00000125266	GUCAUUG
ENSG00000125266	UACAAAG
ENSG00000125266	UAGACUG
ENSG00000125266	UAUUGCU
ENSG00000125266	UCACAGG
ENSG00000125266	UCCCUGU
ENSG00000125266	UGAGUCU
ENSG00000125266	UGCAAAG
ENSG00000125266	UGCAUAG
ENSG00000125266	UGCAUAU
ENSG00000125266	UGUGCUU
ENSG00000125266	UUCUGCA
ENSG00000125266	UUGGCAC
ENSG00000125266	UUUGCAA
ENSG00000125285	AUAUAAC
ENSG00000125285	AUAUUAU
ENSG00000125285	CAGGCUC
ENSG00000125285	GAAUUGU
ENSG00000125285	UCACAAG
ENSG00000125285	UCAUUCG
ENSG00000125285	UUGUUCG
ENSG00000125347	AAAGUGC
ENSG00000125347	AAGGCAC
ENSG00000125347	CCUUCAU
ENSG00000125347	UCACAUU
ENSG00000125351	AAGGCAC
ENSG00000125351	CUUUGGU
ENSG00000125351	GGCUGCG
ENSG00000125354	AAAGCUG
ENSG00000125354	ACUCAAA
ENSG00000125354	CAGUCCA
ENSG00000125354	GAGGCAG
ENSG00000125354	GGAGGCA
ENSG00000125354	GGGGUGG
ENSG00000125354	GUAAACA
ENSG00000125355	ACAAGGU
ENSG00000125355	ACCAGCA
ENSG00000125355	GGAUUUU
ENSG00000125355	GGGGUGC
ENSG00000125355	UCCCUUG
ENSG00000125355	UUUGCAC
ENSG00000125378	CAAAACU
ENSG00000125378	UAUAAAG
ENSG00000125378	UUUGCAC
ENSG00000125386	AAAAGUA
ENSG00000125386	AAGACAU
ENSG00000125386	AAGUAGC
ENSG00000125386	ACAAUAU
ENSG00000125386	AGCCAGU
ENSG00000125386	AGUACUG
ENSG00000125386	CAGUAGA
ENSG00000125386	CUUACAG
ENSG00000125386	GUAUUCU
ENSG00000125386	GUUAAUG

ENSG00000125386	UAAUACA
ENSG00000125386	UAAUAUC
ENSG00000125386	UUCAAAU
ENSG00000125386	UUCUGCA
ENSG00000125398	AAAGAGC
ENSG00000125398	AAAGAGG
ENSG00000125398	AAAGUUU
ENSG00000125398	AGAUAAA
ENSG00000125398	AUAUUAU
ENSG00000125398	GAAAACA
ENSG00000125398	GGAAUGU
ENSG00000125398	GGUUACC
ENSG00000125398	UAAUAUC
ENSG00000125398	UCAUACA
ENSG00000125398	UGUAUAA
ENSG00000125398	UUUGCAA
ENSG00000125414	UCACAUU
ENSG00000125430	AGGCAGG
ENSG00000125430	GAGUGUG
ENSG00000125430	GUGCUUG
ENSG00000125430	GUGUGGC
ENSG00000125430	UAGUGAG
ENSG00000125430	UAUAAAG
ENSG00000125434	UGGCCAC
ENSG00000125447	AAGUCAC
ENSG00000125447	AGCAAAA
ENSG00000125447	AGCAGCA
ENSG00000125447	AGGGUCU
ENSG00000125447	AGGUAGU
ENSG00000125447	CAGCAAA
ENSG00000125447	CUGGAAA
ENSG00000125447	GAGGUAG
ENSG00000125447	GCAGCAU
ENSG00000125447	GGGCCCC
ENSG00000125447	GUCUCUG
ENSG00000125447	UCAAGUA
ENSG00000125447	UCACACA
ENSG00000125459	AUGAUGC
ENSG00000125459	CCCACCG
ENSG00000125459	CCCACCU
ENSG00000125459	CUCUUCC
ENSG00000125459	UCCCACA
ENSG00000125459	UGCAGCU
ENSG00000125462	GAGUCUU
ENSG00000125492	CAUGGGU
ENSG00000125492	GGCCCUG
ENSG00000125492	UCAUUUG
ENSG00000125492	UUUGCAC
ENSG00000125503	AGCAAAA
ENSG00000125503	AGCAGCU
ENSG00000125503	AUGGCUU
ENSG00000125503	CAGCAAA
ENSG00000125503	GCAACUU
ENSG00000125503	GUCACUC
ENSG00000125503	GUGCCUG
ENSG00000125503	GUGCUUG
ENSG00000125503	UGUGCUU
ENSG00000125503	UUGGCAC
ENSG00000125505	GGCUCAG
ENSG00000125510	GACCUGG
ENSG00000125510	GCACCAU
ENSG00000125520	AAUUGCU
ENSG00000125520	AGAUGUG
ENSG00000125520	CAAAGUG

ENSG00000125520	GUGCAAA
ENSG00000125520	UAUAAAG
ENSG00000125520	UCAACAG
ENSG00000125520	UGCAAAG
ENSG00000125618	ACAGUAC
ENSG00000125618	ACAGUAU
ENSG00000125618	CUCCCAC
ENSG00000125618	CUGGACU
ENSG00000125618	GAGUCUU
ENSG00000125618	GCUGGAU
ENSG00000125618	GGAGGAG
ENSG00000125618	GGUUUAC
ENSG00000125618	GUGACUG
ENSG00000125618	GUGCCUG
ENSG00000125618	UACAGUU
ENSG00000125618	UCAUUUG
ENSG00000125618	UGAGUCU
ENSG00000125618	UGUGUGG
ENSG00000125618	UUGGAGG
ENSG00000125618	UUGUGUC
ENSG00000125629	AAGGCAC
ENSG00000125629	AAUUUUA
ENSG00000125629	ACUGCAU
ENSG00000125629	AGUGCUG
ENSG00000125629	AUACCUC
ENSG00000125629	GGAUUUU
ENSG00000125629	GUGCAAA
ENSG00000125629	UAAUACA
ENSG00000125629	UCAUAGA
ENSG00000125629	UCUGGAA
ENSG00000125629	UUGUUCG
ENSG00000125629	UUUUUGC
ENSG00000125633	ACAGGUG
ENSG00000125633	CCUUCAU
ENSG00000125633	CUGCUCA
ENSG00000125633	GAGAAGA
ENSG00000125633	GAGGUAG
ENSG00000125633	UGAGAAG
ENSG00000125633	UGCUCAC
ENSG00000125637	ACCCAUU
ENSG00000125637	CAGCUGG
ENSG00000125637	GGAGGAG
ENSG00000125637	UGGCUGC
ENSG00000125656	UCACAGU
ENSG00000125657	GAGGUAU
ENSG00000125675	AUGGCUU
ENSG00000125675	AUUGCAC
ENSG00000125675	GAGGCUG
ENSG00000125675	UACAAAG
ENSG00000125675	UAGUGAG
ENSG00000125675	UCACAGU
ENSG00000125675	UUCCAUA
ENSG00000125675	UUGGCAA
ENSG00000125676	AGAUAAA
ENSG00000125676	GAGAAAU
ENSG00000125676	GAGGUAG
ENSG00000125676	GAGGUAU
ENSG00000125676	UACAAAG
ENSG00000125676	UGAGAAG
ENSG00000125676	UGAGAAU
ENSG00000125676	UGUGCUU
ENSG00000125686	AAAGUAA
ENSG00000125686	AAAGUAU
ENSG00000125686	AAUACUG

ENSG00000125686	ACCCUGU
ENSG00000125686	AGCAGCA
ENSG00000125686	AGCAUUC
ENSG00000125686	AGCCCUU
ENSG00000125686	AGGCAGG
ENSG00000125686	AGGGAGG
ENSG00000125686	AUGGCAC
ENSG00000125686	AUGGCUU
ENSG00000125686	CAAAUGC
ENSG00000125686	CACAGUG
ENSG00000125686	CAGUGCA
ENSG00000125686	CCCUGUC
ENSG00000125686	GAGAAAU
ENSG00000125686	GGAAUGU
ENSG00000125686	GGAUUUC
ENSG00000125686	GGCAGGG
ENSG00000125686	GGCUGCG
ENSG00000125686	GUCACUC
ENSG00000125686	GUGUCAC
ENSG00000125686	UCCCUGU
ENSG00000125686	UCCUUGC
ENSG00000125686	UCUCCAA
ENSG00000125686	UGGCUGC
ENSG00000125686	UGGGACA
ENSG00000125686	UGUGCUU
ENSG00000125686	UUCAAAU
ENSG00000125686	UUGAUCA
ENSG00000125691	UACAAAG
ENSG00000125730	UGAAGCU
ENSG00000125733	AAGUGCA
ENSG00000125733	AGCAGCA
ENSG00000125733	AUAAAGU
ENSG00000125733	GUGGCCC
ENSG00000125740	AAGUCAC
ENSG00000125740	ACAGUAU
ENSG00000125740	AUAAUAC
ENSG00000125740	AUAUAAC
ENSG00000125740	CACCAGC
ENSG00000125740	CAGUGCA
ENSG00000125740	CCUGCUG
ENSG00000125740	CUAGGCU
ENSG00000125740	GCAGAAG
ENSG00000125740	GGAAUGU
ENSG00000125740	GGCAGUG
ENSG00000125740	UAUAAUA
ENSG00000125740	UCACAUU
ENSG00000125744	AAAUCCC
ENSG00000125744	AAGUGCA
ENSG00000125744	CAGCAGG
ENSG00000125744	CCCCUGG
ENSG00000125744	CGCCUCC
ENSG00000125744	CUCCAGC
ENSG00000125744	GGCGCCU
ENSG00000125744	UAUUACC
ENSG00000125744	UGCAAAG
ENSG00000125753	AAAGUGC
ENSG00000125753	AAAUUUC
ENSG00000125753	AGCCAGU
ENSG00000125753	AGCUGCC
ENSG00000125753	AGGCAGG
ENSG00000125753	CAGCAGA
ENSG00000125753	CAGCAGG
ENSG00000125753	GAGGCAG
ENSG00000125753	GGAAGCC

ENSG00000125753	GGAAUGU
ENSG00000125753	GGCAGGG
ENSG00000125775	CUGCAGG
ENSG00000125780	CUGCAGC
ENSG00000125780	GCAGCAU
ENSG00000125798	AAAACUG
ENSG00000125798	AACACUG
ENSG00000125798	UAAUAUC
ENSG00000125810	AAUCUCA
ENSG00000125810	AUCCUUU
ENSG00000125810	CAGUCCA
ENSG00000125810	UCACACA
ENSG00000125810	UCCUGAG
ENSG00000125810	UUCCUAG
ENSG00000125810	UUUGCAC
ENSG00000125812	AACAAAC
ENSG00000125812	CAGCUGG
ENSG00000125812	CCUUCUU
ENSG00000125812	CUACAAA
ENSG00000125812	UACAAAG
ENSG00000125812	UCACAGG
ENSG00000125812	UUGGUCC
ENSG00000125813	UGCUGAC
ENSG00000125814	AAUACUG
ENSG00000125814	CUUUGGU
ENSG00000125814	GCACCAU
ENSG00000125814	GGUUACC
ENSG00000125814	UCUCAAG
ENSG00000125814	UGCAUAU
ENSG00000125814	UUGGUUC
ENSG00000125816	AUUGCAC
ENSG00000125817	AGCACCA
ENSG00000125817	AGCCCUU
ENSG00000125817	AUGGCUU
ENSG00000125817	CUAGGCU
ENSG00000125817	CUGCAGC
ENSG00000125817	GAGCCCU
ENSG00000125817	GAGGCUG
ENSG00000125817	GGGGUCC
ENSG00000125817	GUGCUUG
ENSG00000125817	UGCAGCU
ENSG00000125817	UGGCUGC
ENSG00000125817	UGUGCUU
ENSG00000125818	AACACUG
ENSG00000125818	ACAUUCA
ENSG00000125818	ACUGCAU
ENSG00000125818	AGGAGCU
ENSG00000125818	CAGCAGG
ENSG00000125818	CCCCCAG
ENSG00000125818	GCAGCAU
ENSG00000125818	GCUGGUG
ENSG00000125818	GGCAGCG
ENSG00000125818	GGGUCUU
ENSG00000125818	UCUUGAG
ENSG00000125818	UGACAUC
ENSG00000125818	UUGAGGC
ENSG00000125820	AAAAAUC
ENSG00000125820	AAAGUGC
ENSG00000125820	CUCACAC
ENSG00000125820	GGAUUUU
ENSG00000125820	GUAAACA
ENSG00000125820	UACAAAG
ENSG00000125820	UCACACA
ENSG00000125820	UCUCCAA

ENSG00000125820	UUGGUCC
ENSG00000125820	UUUGCAC
ENSG00000125821	CUUUGGU
ENSG00000125834	AAAGAAU
ENSG00000125834	AAAGUUU
ENSG00000125834	AAGGCAC
ENSG00000125834	AAGUUGU
ENSG00000125834	ACCAGCA
ENSG00000125834	AGCACCA
ENSG00000125834	AGCCAGU
ENSG00000125834	AUUCAUU
ENSG00000125834	CACAGUG
ENSG00000125834	CACCACC
ENSG00000125834	CCUGUAC
ENSG00000125834	CUGGGGG
ENSG00000125834	CUUCUCU
ENSG00000125834	CUUGGAG
ENSG00000125834	GAUAUGU
ENSG00000125834	GGUGGGC
ENSG00000125834	GUCCAGA
ENSG00000125834	GUGAGGU
ENSG00000125834	UAUUGCU
ENSG00000125834	UCACAUU
ENSG00000125834	UUGGCAC
ENSG00000125835	AACAAAC
ENSG00000125841	GAGAUGA
ENSG00000125844	AAGGCAC
ENSG00000125844	AUGGCUU
ENSG00000125844	AUUGCAC
ENSG00000125844	GGAAUGU
ENSG00000125845	AAAGUGC
ENSG00000125845	AAGUCUU
ENSG00000125845	AUAAAGU
ENSG00000125845	UGAGAAU
ENSG00000125845	UUCAAAU
ENSG00000125845	UUUCAAC
ENSG00000125848	AAAGCUG
ENSG00000125848	AAGGCAC
ENSG00000125848	ACAAGGU
ENSG00000125848	ACUCAGC
ENSG00000125848	ACUCAGG
ENSG00000125848	ACUGGCC
ENSG00000125848	AUGGCAC
ENSG00000125848	CAAAGUG
ENSG00000125848	CACAGUG
ENSG00000125848	CCAGUGC
ENSG00000125848	CCAUUAC
ENSG00000125848	CUUUGGU
ENSG00000125848	GAUUGUA
ENSG00000125848	GAUUGUC
ENSG00000125848	GGGCGUA
ENSG00000125848	GUCAACA
ENSG00000125848	GUGCUUG
ENSG00000125848	UAUAUGA
ENSG00000125848	UCACAGG
ENSG00000125848	UGUGCUU
ENSG00000125848	UUGGUUC
ENSG00000125848	UUUGCAC
ENSG00000125850	AAGGCAC
ENSG00000125850	GUAAACA
ENSG00000125850	UCAUUUG
ENSG00000125851	AAAAACC
ENSG00000125851	AACUGGU
ENSG00000125851	AAUUGCU

ENSG00000125851	AUCAUAC
ENSG00000125851	GACAACU
ENSG00000125851	GAGAAGA
ENSG00000125851	GCUGGUG
ENSG00000125851	UCACAGG
ENSG00000125851	UUGGUUC
ENSG00000125868	AAAGUAA
ENSG00000125868	GGAAGCC
ENSG00000125869	AAAGUGC
ENSG00000125869	UGCAUAG
ENSG00000125871	AAACUGU
ENSG00000125871	CUGGGCA
ENSG00000125871	UCAACAG
ENSG00000125875	AAGGCAC
ENSG00000125875	AAGUGCU
ENSG00000125875	ACCAGCA
ENSG00000125875	AGCAAAA
ENSG00000125875	AGCAGCA
ENSG00000125875	AGUGCUU
ENSG00000125875	CUCCCAA
ENSG00000125875	CUUCUCU
ENSG00000125875	GAAGGAA
ENSG00000125875	GAAGGUC
ENSG00000125875	GACCCUG
ENSG00000125875	GGAGAGA
ENSG00000125875	GGCAGGG
ENSG00000125875	UGGCUGC
ENSG00000125878	AAAAAUC
ENSG00000125878	GGGUCUU
ENSG00000125878	UCACACA
ENSG00000125895	AGCAGCG
ENSG00000125895	AGCAGCU
ENSG00000125895	CAGCAGA
ENSG00000125895	GCAGAAG
ENSG00000125898	CAGCAAA
ENSG00000125898	GAAGGAA
ENSG00000125898	GGGUAAG
ENSG00000125912	CCCUGAG
ENSG00000125912	UAUUGCU
ENSG00000125912	UCCCACA
ENSG00000125944	UCAUGAU
ENSG00000125944	UGAAAUG
ENSG00000125944	UUCAAGC
ENSG00000125945	AAAAACC
ENSG00000125945	AGCAGCA
ENSG00000125945	UUGGUCC
ENSG00000125952	AAAGCUG
ENSG00000125952	AGCUGCC
ENSG00000125952	AGGGAGG
ENSG00000125952	CAAGAGC
ENSG00000125952	CAGGAAC
ENSG00000125952	GGAAGCC
ENSG00000125952	GGCUCUG
ENSG00000125952	UAUUGCU
ENSG00000125952	UUCAAAU
ENSG00000125952	UUGGUUC
ENSG00000125954	AGUAGUU
ENSG00000125954	CAAAGCA
ENSG00000125954	GUUAAUG
ENSG00000125954	UCCUGAG
ENSG00000125965	CCAGUGC
ENSG00000125965	GGAAGAC
ENSG00000125966	AAAGUAU
ENSG00000125966	AAGUGCU

ENSG00000125966	AAUCACU
ENSG00000125966	ACCAGCA
ENSG00000125966	AGCACCA
ENSG00000125966	AGUGCUG
ENSG00000125966	AGUGCUU
ENSG00000125966	AUAAAGU
ENSG00000125966	AUCACUA
ENSG00000125966	CAGCAGG
ENSG00000125966	CAGUAGU
ENSG00000125966	CCAGGAG
ENSG00000125966	GAGCCCU
ENSG00000125966	GAUCAGA
ENSG00000125966	GCAGCAU
ENSG00000125966	GCCCUGU
ENSG00000125966	UACAAAG
ENSG00000125966	UCCUGAG
ENSG00000125966	UGAAAUG
ENSG00000125966	UUUGCAA
ENSG00000125967	AAUUAUU
ENSG00000125967	AGGCAGG
ENSG00000125967	CUGGGGG
ENSG00000125967	GGCAGGG
ENSG00000125968	AACAAAC
ENSG00000125968	AUACAAG
ENSG00000125968	GAGAAGA
ENSG00000125968	UGAGAAG
ENSG00000125968	UUCCUCG
ENSG00000125970	AAAAACC
ENSG00000125970	AAUCUCU
ENSG00000125970	ACUGGCU
ENSG00000125970	CCUGCUG
ENSG00000125970	UGCCAGC
ENSG00000125977	AAAACUG
ENSG00000125977	AACCUGU
ENSG00000125977	AAUCACU
ENSG00000125977	AUAAUAC
ENSG00000125977	GAGGUAG
ENSG00000125977	GAGGUAU
ENSG00000125977	GGCAGCG
ENSG00000125977	GUCCAGA
ENSG00000125977	GUGAGGU
ENSG00000125977	UCAACAG
ENSG00000125977	UCACAGU
ENSG00000125977	UGCAAAG
ENSG00000125977	UGCAUUG
ENSG00000125977	UUUGCAA
ENSG00000125991	AACCUGG
ENSG00000126001	GGGUUUA
ENSG00000126003	AAACGGU
ENSG00000126003	AAAGAGG
ENSG00000126003	AAAGUGC
ENSG00000126003	AAAGUUU
ENSG00000126003	AACAAAC
ENSG00000126003	AACACUG
ENSG00000126003	AACAGUC
ENSG00000126003	AAGGCAC
ENSG00000126003	AAGUGUG
ENSG00000126003	ACUUGGA
ENSG00000126003	AGCUGCC
ENSG00000126003	AUAAAGU
ENSG00000126003	AUAUAAC
ENSG00000126003	AUGGAUU
ENSG00000126003	CAAAGUG
ENSG00000126003	CACACCU

ENSG00000126003	CACAGUG
ENSG00000126003	CACUGUU
ENSG00000126003	CAGUCCA
ENSG00000126003	CCAGGAG
ENSG00000126003	CCCUGAG
ENSG00000126003	CUCCAGC
ENSG00000126003	CUGCAGG
ENSG00000126003	CUGGGCA
ENSG00000126003	GAAAACA
ENSG00000126003	GAAGGUC
ENSG00000126003	GAGAUGA
ENSG00000126003	GAGGCAG
ENSG00000126003	GAGGUAG
ENSG00000126003	GCAGGUU
ENSG00000126003	GCUGGUG
ENSG00000126003	GCUUCCU
ENSG00000126003	GGACCUG
ENSG00000126003	GGGGGAA
ENSG00000126003	GUCAGUU
ENSG00000126003	GUCUUGC
ENSG00000126003	GUGUGGC
ENSG00000126003	UAUUACC
ENSG00000126003	UCACAGG
ENSG00000126003	UCACAGU
ENSG00000126003	UGAAAUG
ENSG00000126003	UGAUCCA
ENSG00000126010	UUGGAGG
ENSG00000126016	AAGGCAC
ENSG00000126016	AAUCCUU
ENSG00000126016	AAUUAUU
ENSG00000126016	AGCAAAA
ENSG00000126016	AGCAGCA
ENSG00000126016	AGCUGCC
ENSG00000126016	AUAUUAU
ENSG00000126016	CCAGGAG
ENSG00000126016	GAGAAGA
ENSG00000126016	GGAAUGU
ENSG00000126016	UAUAUGA
ENSG00000126016	UCCUGAG
ENSG00000126016	UGAAAUG
ENSG00000126016	UGAGAAG
ENSG00000126062	AGUGGUU
ENSG00000126062	GGCUCAG
ENSG00000126062	GUAGUGU
ENSG00000126062	UCCCUCU
ENSG00000126062	UUGGCAC
ENSG00000126070	AUUGCAC
ENSG00000126070	CUGGAAA
ENSG00000126070	GAAACAU
ENSG00000126070	GAGGUAG
ENSG00000126070	GAGGUAU
ENSG00000126070	GCAACUU
ENSG00000126070	GUAUGUU
ENSG00000126070	UCCUUGC
ENSG00000126070	UGCAUAG
ENSG00000126070	UGGCUGC
ENSG00000126070	UGUGCUU
ENSG00000126088	CUGGAAA
ENSG00000126091	AUAAUAC
ENSG00000126091	AUCCCUU
ENSG00000126091	CUUCUCU
ENSG00000126091	GCUUCCU
ENSG00000126091	GGCCCUG
ENSG00000126091	GGGUAAG

ENSG00000126091	GUCUCUG
ENSG00000126091	UAAUACA
ENSG00000126091	UAUUGCU
ENSG00000126091	UCACAAG
ENSG00000126091	UGGCCCU
ENSG00000126091	UUGGCAC
ENSG00000126106	CAGCAGA
ENSG00000126106	CAGCAGG
ENSG00000126107	AAGUAGC
ENSG00000126107	GGGGCAG
ENSG00000126107	UCAAGUA
ENSG00000126216	AACAAAC
ENSG00000126216	AUAUAAC
ENSG00000126216	GUAAACA
ENSG00000126217	ACAAUAU
ENSG00000126217	ACACACC
ENSG00000126217	ACACACU
ENSG00000126217	ACCACAG
ENSG00000126217	CACAGUG
ENSG00000126247	AAGGCAC
ENSG00000126247	UUGGCAC
ENSG00000126261	AAAUGGU
ENSG00000126261	AACCUGG
ENSG00000126261	AACCUGU
ENSG00000126261	UACAAAG
ENSG00000126351	AAAGUGC
ENSG00000126351	AAGUGCA
ENSG00000126351	CAAAGUG
ENSG00000126351	CCCUGUC
ENSG00000126351	GCCCUGU
ENSG00000126351	GGAGAGA
ENSG00000126351	GGAGGAG
ENSG00000126353	AGCUUAU
ENSG00000126353	GAGGUAU
ENSG00000126453	CCCUGAG
ENSG00000126457	AAAAACC
ENSG00000126458	AAGGCAC
ENSG00000126461	GUCCAGA
ENSG00000126461	GUGGGGA
ENSG00000126461	UCAUGAU
ENSG00000126464	AAAAAUC
ENSG00000126464	AAUGCCC
ENSG00000126464	ACACACC
ENSG00000126464	ACAUAGA
ENSG00000126464	ACAUUAC
ENSG00000126464	AUAUAAC
ENSG00000126464	CACAGUG
ENSG00000126464	CCCCCAG
ENSG00000126464	CUAUACA
ENSG00000126464	GGGGCAG
ENSG00000126464	UGCCCUG
ENSG00000126500	ACAGGUG
ENSG00000126500	AGGUAGU
ENSG00000126500	AUGUCUG
ENSG00000126500	UCCAGUU
ENSG00000126524	GGCAGCG
ENSG00000126524	UUGGCAA
ENSG00000126524	UUGGCAC
ENSG00000126562	AGUGGUU
ENSG00000126562	CAGCUGG
ENSG00000126581	AAAGUGC
ENSG00000126581	AAUCUCU
ENSG00000126581	AUAAAGU
ENSG00000126581	CAAAACU

ENSG00000126581	UACAAAG
ENSG00000126583	AGCAGCU
ENSG00000126583	GCAUCCC
ENSG00000126583	UCUGGAA
ENSG00000126603	AACCUGU
ENSG00000126603	AACUCUA
ENSG00000126603	AAGGCAC
ENSG00000126603	AAUACUG
ENSG00000126603	AGCACCA
ENSG00000126603	CCAGUGC
ENSG00000126603	GCAACUU
ENSG00000126603	GCACCAU
ENSG00000126603	GCCCUGU
ENSG00000126603	GCCUGGG
ENSG00000126603	GGAAUGU
ENSG00000126603	GUCUUGC
ENSG00000126603	GUGCAAA
ENSG00000126603	UAUAAAG
ENSG00000126603	UCCCUCU
ENSG00000126603	UUUGCAA
ENSG00000126653	AUAAAGU
ENSG00000126653	UGUGCUU
ENSG00000126698	AAAAAUC
ENSG00000126698	AAGUCAC
ENSG00000126698	UGGGUAC
ENSG00000126746	AAAAACC
ENSG00000126746	AAAUCCC
ENSG00000126746	AAAUGGU
ENSG00000126746	AUACAAG
ENSG00000126746	AUAUAAC
ENSG00000126746	AUGCAUU
ENSG00000126746	UAUAAAG
ENSG00000126767	AAAAAUC
ENSG00000126767	ACCUGGA
ENSG00000126767	ACUCCAG
ENSG00000126767	ACUGGCU
ENSG00000126767	AGACGGG
ENSG00000126767	AGUACUG
ENSG00000126767	AUGGCUU
ENSG00000126767	CAUGGAU
ENSG00000126767	CCCACCG
ENSG00000126767	CCCACCU
ENSG00000126767	CCCCCAG
ENSG00000126767	CUCAGGG
ENSG00000126767	CUCCAUU
ENSG00000126767	CUCCCAA
ENSG00000126767	GACCUGG
ENSG00000126767	GAGACCU
ENSG00000126767	GGAGAGA
ENSG00000126767	UCCCACA
ENSG00000126767	UCCCACC
ENSG00000126768	CCCUGAG
ENSG00000126768	GAGGUAG
ENSG00000126768	UGCCCUG
ENSG00000126773	AACACUG
ENSG00000126777	AAAACUG
ENSG00000126777	AGCAAAA
ENSG00000126777	CAAAACU
ENSG00000126777	CAGUAGA
ENSG00000126777	CCAUUAC
ENSG00000126777	UAAUACA
ENSG00000126777	UACAAAG
ENSG00000126777	UAUAAUA
ENSG00000126777	UUGGCAC

ENSG00000126804	GGAAGAC
ENSG00000126804	GUGCCAC
ENSG00000126804	UCUCCAA
ENSG00000126814	AAAAGUA
ENSG00000126814	AAAGUAA
ENSG00000126814	AAUCACU
ENSG00000126814	AGGCAGG
ENSG00000126814	CACCAGC
ENSG00000126814	GCAGGAC
ENSG00000126814	GGAGGAG
ENSG00000126814	GGCUCUG
ENSG00000126814	GUCCAGA
ENSG00000126814	UCCUUGC
ENSG00000126814	UUAGAGA
ENSG00000126814	UUCCUAG
ENSG00000126814	UUGGAGG
ENSG00000126821	AAGGCAC
ENSG00000126821	UCACAGU
ENSG00000126822	AAAGUAC
ENSG00000126822	AAUUAUU
ENSG00000126822	AGUGGUU
ENSG00000126822	CAGCAGA
ENSG00000126822	CAGCAGG
ENSG00000126822	GCCUGGA
ENSG00000126822	UAUUGCU
ENSG00000126822	UGAAAUG
ENSG00000126822	UUGGCAA
ENSG00000126858	AAUACUG
ENSG00000126858	ACAGUAC
ENSG00000126882	AAAGUUU
ENSG00000126882	AAGGCAC
ENSG00000126882	AAGUGCU
ENSG00000126882	ACAGUAC
ENSG00000126882	AGUGCAA
ENSG00000126882	CAAAGCA
ENSG00000126882	CAAGAGC
ENSG00000126882	CACAGUG
ENSG00000126882	GCUGGAU
ENSG00000126882	GGUUGUC
ENSG00000126882	UAUUGCU
ENSG00000126882	UCACAGG
ENSG00000126882	UGCUGAC
ENSG00000126882	UGUGCUU
ENSG00000126882	UUGGCAC
ENSG00000126883	UAAUAGU
ENSG00000126903	CUUUGGU
ENSG00000126903	UGCAUAG
ENSG00000126950	AAAGAAU
ENSG00000126950	AAAUUUC
ENSG00000126950	ACACACU
ENSG00000126950	AGAUAAA
ENSG00000126950	CUGCUCA
ENSG00000126950	GAAGGUC
ENSG00000126950	GCAGCAU
ENSG00000126950	GCCUGGG
ENSG00000126950	UAUAAAG
ENSG00000126950	UGACAUC
ENSG00000126952	CCUUCUG
ENSG00000126952	GAGACCU
ENSG00000127022	AACACUG
ENSG00000127022	AAGGUGC
ENSG00000127022	ACUGUUU
ENSG00000127022	AGUGCAA
ENSG00000127022	CACUCCU

ENSG00000127022	CAGUGCA
ENSG00000127022	CCUUCAU
ENSG00000127022	CUACAGU
ENSG00000127022	GGAGUGU
ENSG00000127022	GUCACUC
ENSG00000127022	UAUAAAG
ENSG00000127022	UGCAUAG
ENSG00000127080	AAAUUUC
ENSG00000127080	AACAUUC
ENSG00000127080	AAUUUUA
ENSG00000127080	ACAAUAU
ENSG00000127080	AGCAGCA
ENSG00000127080	AGCAGCG
ENSG00000127080	AGCAGCU
ENSG00000127080	AGUGCUG
ENSG00000127080	AGUGCUU
ENSG00000127080	CAGUAGU
ENSG00000127080	CCAGUAC
ENSG00000127080	CCUGCUG
ENSG00000127080	GAGCCCU
ENSG00000127080	GGUGGUC
ENSG00000127080	GGUGGUU
ENSG00000127080	UCAUAGA
ENSG00000127080	UCCAGUU
ENSG00000127080	UUAGAGA
ENSG00000127124	AAGGCAC
ENSG00000127124	ACUGCAU
ENSG00000127129	ACAUUAC
ENSG00000127152	AAACUGU
ENSG00000127152	AAAGAAU
ENSG00000127152	AAAUCCC
ENSG00000127152	AAGACAU
ENSG00000127152	AAGGCAC
ENSG00000127152	AAGUCUU
ENSG00000127152	AAGUGCU
ENSG00000127152	AAUACUG
ENSG00000127152	AAUCUCA
ENSG00000127152	AAUGCCC
ENSG00000127152	ACACACC
ENSG00000127152	ACACACU
ENSG00000127152	ACAGUAC
ENSG00000127152	ACAGUAU
ENSG00000127152	ACUGCAG
ENSG00000127152	ACUGUAG
ENSG00000127152	AGCAGCG
ENSG00000127152	AGCUUAU
ENSG00000127152	AGUGCCG
ENSG00000127152	AUAAUAC
ENSG00000127152	AUACAAG
ENSG00000127152	AUAUAAC
ENSG00000127152	AUAUUAU
ENSG00000127152	AUCCCUU
ENSG00000127152	AUGGCAC
ENSG00000127152	CAGCAGA
ENSG00000127152	CAGCAGG
ENSG00000127152	CAGUAGA
ENSG00000127152	CAGUUAC
ENSG00000127152	CCUGUAC
ENSG00000127152	CUAUACA
ENSG00000127152	CUUGUAU
ENSG00000127152	GAAAACA
ENSG00000127152	GAAUUGU
ENSG00000127152	GCUACAU
ENSG00000127152	GGAAUGU

ENSG00000127152	GGCAGCG
ENSG00000127152	GGCAGUG
ENSG00000127152	GGGUCUU
ENSG00000127152	GUCAACA
ENSG00000127152	GUGAAUG
ENSG00000127152	GUGACAG
ENSG00000127152	UAAGACG
ENSG00000127152	UAAGACU
ENSG00000127152	UAAUAUC
ENSG00000127152	UAUUACC
ENSG00000127152	UAUUGCU
ENSG00000127152	UCAACAG
ENSG00000127152	UCAUACA
ENSG00000127152	UCCAGAG
ENSG00000127152	UCCCUUU
ENSG00000127152	UGCAUUG
ENSG00000127152	UGUAUGU
ENSG00000127152	UGUGCUU
ENSG00000127152	UGUGUCA
ENSG00000127152	UUCAAGC
ENSG00000127152	UUGUGUC
ENSG00000127152	UUUCAAC
ENSG00000127152	UUUGCAC
ENSG00000127220	ACAAGGU
ENSG00000127220	AGAUGUG
ENSG00000127220	GAUUGUC
ENSG00000127220	GCAGGUU
ENSG00000127220	GGAAGAC
ENSG00000127241	UCCUGAG
ENSG00000127249	UCCCUUU
ENSG00000127314	AACCUGU
ENSG00000127314	AACUACU
ENSG00000127314	AAGCGCU
ENSG00000127314	ACCACAG
ENSG00000127314	AGCCCUU
ENSG00000127314	AGUGGUU
ENSG00000127314	AUGUAAU
ENSG00000127314	CCCUGUU
ENSG00000127314	CUACAGU
ENSG00000127314	CUGGCUC
ENSG00000127314	CUUUGGU
ENSG00000127314	GACCCUG
ENSG00000127314	GCACCAU
ENSG00000127314	GCAGAAG
ENSG00000127314	GGCAUUG
ENSG00000127314	GUCUCUG
ENSG00000127314	UAAUGCU
ENSG00000127314	UCACAGU
ENSG00000127314	UCACAUU
ENSG00000127314	UCCCUGU
ENSG00000127314	UCUCCAA
ENSG00000127314	UCUGGAG
ENSG00000127314	UGAGAAU
ENSG00000127314	UGCAAAG
ENSG00000127314	UGGACUG
ENSG00000127314	UUCUUGU
ENSG00000127325	AACUGGU
ENSG00000127328	AAGUCUU
ENSG00000127328	AGGAUGU
ENSG00000127328	AGUGUGC
ENSG00000127328	GUCACUC
ENSG00000127328	UGCAAAG
ENSG00000127329	AAAGCUG
ENSG00000127329	ACAAUAU

ENSG00000127329	ACUGGCU
ENSG00000127329	CACAGUG
ENSG00000127329	CAGACAG
ENSG00000127329	CUCCCAA
ENSG00000127329	GGAGUGU
ENSG00000127329	UCAACAG
ENSG00000127329	UCACAAG
ENSG00000127329	UCACACA
ENSG00000127329	UGAAAUG
ENSG00000127334	AAAAGUA
ENSG00000127334	AAAGUUU
ENSG00000127334	AAAUCCC
ENSG00000127334	AAAUUUC
ENSG00000127334	AACAAAC
ENSG00000127334	AACCUGU
ENSG00000127334	AAGACAU
ENSG00000127334	AAGGCAC
ENSG00000127334	AAUGCCC
ENSG00000127334	ACACACC
ENSG00000127334	ACACACU
ENSG00000127334	ACAUUCA
ENSG00000127334	ACUGUUU
ENSG00000127334	AGCAGCA
ENSG00000127334	CAAAUGC
ENSG00000127334	CUUUGGU
ENSG00000127334	GAAAACA
ENSG00000127334	GAGGUAG
ENSG00000127334	GAGGUAU
ENSG00000127334	GCCCUGU
ENSG00000127334	GGCUCAG
ENSG00000127334	UAAGACG
ENSG00000127334	UAAGACU
ENSG00000127334	UAUGGUU
ENSG00000127334	UCACAUU
ENSG00000127334	UCCUGAG
ENSG00000127334	UGCAGCU
ENSG00000127334	UGCCCUG
ENSG00000127334	UUGAGGC
ENSG00000127334	UUGGCAA
ENSG00000127337	GUGCUUG
ENSG00000127359	AAAGUAU
ENSG00000127359	AAGGCAC
ENSG00000127359	ACAAGGU
ENSG00000127359	CAAAACU
ENSG00000127359	GAGGUAG
ENSG00000127359	GAGGUAU
ENSG00000127359	UAUUGCU
ENSG00000127359	UGAAAUG
ENSG00000127359	UUGGUCC
ENSG00000127418	AACAAAC
ENSG00000127418	AACUCUA
ENSG00000127418	AUAUUAU
ENSG00000127418	CUCAAAC
ENSG00000127418	UAAUAUC
ENSG00000127445	AAUACUG
ENSG00000127445	ACUGUGU
ENSG00000127445	AGUGGUU
ENSG00000127452	GAGGUAU
ENSG00000127481	AGAUAAA
ENSG00000127481	GGAAGCC
ENSG00000127483	AAAUUUC
ENSG00000127483	AACAAAC
ENSG00000127483	AAGACAU
ENSG00000127483	AUUCAUU

ENSG00000127483	CACAGGG
ENSG00000127483	GGAAGCC
ENSG00000127483	GGAAUGU
ENSG00000127483	GGGAUCG
ENSG00000127483	GUGCCAC
ENSG00000127483	GUGCUUG
ENSG00000127483	UAAGACG
ENSG00000127483	UAAGACU
ENSG00000127483	UCCUAUA
ENSG00000127483	UCUCCAA
ENSG00000127483	UGGGAUC
ENSG00000127483	UGUGCUU
ENSG00000127483	UUCAAAU
ENSG00000127483	UUUCAAC
ENSG00000127511	CAAAACU
ENSG00000127526	AAGGUGC
ENSG00000127526	AUACAAG
ENSG00000127526	AUGCACC
ENSG00000127526	CCAGUGC
ENSG00000127526	CCAGUGU
ENSG00000127526	UCCAGUU
ENSG00000127526	UGUCUUU
ENSG00000127554	GGCUCUG
ENSG00000127561	AAAGAAU
ENSG00000127561	AUACAAG
ENSG00000127561	GGAAGCC
ENSG00000127561	GUCUUGC
ENSG00000127564	UUCAAAU
ENSG00000127585	AAUACUG
ENSG00000127585	ACAAUAU
ENSG00000127585	ACUGGCU
ENSG00000127585	AGGUUAU
ENSG00000127585	CUACAGU
ENSG00000127585	CUCUGGG
ENSG00000127585	CUGGAAA
ENSG00000127585	CUGUUGA
ENSG00000127585	CUUUGGU
ENSG00000127585	GACUUCC
ENSG00000127585	GGAAGAC
ENSG00000127585	GGAAGCC
ENSG00000127585	GGGGUCC
ENSG00000127585	GGGGUGG
ENSG00000127585	UAAUACA
ENSG00000127585	UAAUAUC
ENSG00000127585	UAUAAAG
ENSG00000127585	UCUGGAA
ENSG00000127585	UGCAUUG
ENSG00000127585	UGCCAGC
ENSG00000127585	UUCCUCG
ENSG00000127603	AAGGCAC
ENSG00000127603	ACUGCAU
ENSG00000127603	AGUAACA
ENSG00000127603	UCACACA
ENSG00000127603	UCACAUU
ENSG00000127616	CCAGUGC
ENSG00000127616	CCAGUGU
ENSG00000127616	CUACAGU
ENSG00000127616	GGAAUGU
ENSG00000127688	AUUGCAC
ENSG00000127688	GAGGUAU
ENSG00000127688	GUAAACA
ENSG00000127688	GUGCUUG
ENSG00000127688	UAUUGCU
ENSG00000127688	UGUGCUU

ENSG00000127688	UUGGCAA
ENSG00000127804	CAAAGCA
ENSG00000127804	GAAGGAA
ENSG00000127804	GUGCCUG
ENSG00000127804	UUGAUCA
ENSG00000127837	CACACCG
ENSG00000127837	CACACCU
ENSG00000127837	CACCAGC
ENSG00000127837	CUCACAC
ENSG00000127838	AAGGCAC
ENSG00000127838	ACUGUGU
ENSG00000127838	CAGUCCA
ENSG00000127838	CCCCUGG
ENSG00000127838	GAGGUAG
ENSG00000127838	UAUUGCU
ENSG00000127838	UUGGCAA
ENSG00000127863	AAAGUAA
ENSG00000127863	AAAGUAC
ENSG00000127863	CAGCUGG
ENSG00000127863	CCAGUAC
ENSG00000127863	CUCCAGC
ENSG00000127863	GUCCAGA
ENSG00000127863	UCACAAG
ENSG00000127870	AAAGUGC
ENSG00000127870	AAGUGCU
ENSG00000127870	AGUGCUU
ENSG00000127870	GAUUGUC
ENSG00000127914	UUGUGAC
ENSG00000127946	AAAAACC
ENSG00000127946	AAGGCAC
ENSG00000127946	AGAUAAA
ENSG00000127946	AGCACCA
ENSG00000127946	AGUGAUC
ENSG00000127946	AUGAUGA
ENSG00000127946	CACAGUG
ENSG00000127946	CUCACAC
ENSG00000127946	GGAAGAC
ENSG00000127946	GGGGUCC
ENSG00000127946	UCACAGG
ENSG00000127946	UCACAGU
ENSG00000127946	UGAGAAG
ENSG00000127947	AAAAAUC
ENSG00000127947	AACCUGG
ENSG00000127947	AACCUGU
ENSG00000127947	AGGCAGG
ENSG00000127947	AUACCUC
ENSG00000127947	CCUGUAC
ENSG00000127947	CUACAAA
ENSG00000127947	CUUACAG
ENSG00000127947	GACCAUG
ENSG00000127947	GUAACAG
ENSG00000127948	AAUUAUU
ENSG00000127948	GAAAACA
ENSG00000127951	CAGUAGU
ENSG00000127954	AAAGUUU
ENSG00000127954	GAAGGAA
ENSG00000127954	GAAGGUC
ENSG00000127954	UUUGCAC
ENSG00000127995	UAUAAAG
ENSG00000127995	UCACAUU
ENSG00000127995	UUCAAAU
ENSG00000128011	AAAGUUU
ENSG00000128011	AAGGCAC
ENSG00000128011	AGUGCUG

ENSG00000128011	AUACAAG
ENSG00000128011	CAGUGCA
ENSG00000128011	CGUGUCU
ENSG00000128011	CUAGUCC
ENSG00000128011	GCGGAGG
ENSG00000128011	GGACCUG
ENSG00000128011	GGGCCCC
ENSG00000128011	UUGGAGG
ENSG00000128011	UUGGUUC
ENSG00000128016	AAAAAUC
ENSG00000128016	AGCACCA
ENSG00000128016	AUAAAGU
ENSG00000128016	AUAUAAC
ENSG00000128016	CGACCCA
ENSG00000128016	CUGCCCC
ENSG00000128016	GCUACAU
ENSG00000128016	GUUAAUG
ENSG00000128016	UACAAAG
ENSG00000128016	UCACAGG
ENSG00000128016	UCACAGU
ENSG00000128016	UUGGCAA
ENSG00000128045	AAGUGCA
ENSG00000128045	CAAAGCA
ENSG00000128045	UCUGGAA
ENSG00000128052	AGCAGCA
ENSG00000128052	AUAUUAU
ENSG00000128052	CCAGCAU
ENSG00000128052	GAAGGAA
ENSG00000128052	GUGAAUG
ENSG00000128052	GUGCCAC
ENSG00000128052	UAAUAUC
ENSG00000128052	UACAAAG
ENSG00000128059	CUUUGGU
ENSG00000128059	GGUUUAC
ENSG00000128059	UCCCUGU
ENSG00000128059	UUGGUUC
ENSG00000128191	AAAACUG
ENSG00000128191	CUGGCUC
ENSG00000128191	CUUUGGU
ENSG00000128191	GGAGGCA
ENSG00000128191	GGCAAGA
ENSG00000128191	UAAGACU
ENSG00000128191	UUGGUUC
ENSG00000128203	AUACAAG
ENSG00000128228	AACAUUC
ENSG00000128228	AAGGCAC
ENSG00000128228	CUACAAA
ENSG00000128242	AAUACUG
ENSG00000128245	AGCAGCA
ENSG00000128245	GCAGCAU
ENSG00000128254	GAGCCCC
ENSG00000128266	AAAGAAU
ENSG00000128266	AUGUGGG
ENSG00000128266	CACAGGG
ENSG00000128266	CAGCAAA
ENSG00000128266	CAGCUGG
ENSG00000128266	GUUCAUG
ENSG00000128266	UAUAAAG
ENSG00000128266	UCACAGG
ENSG00000128266	UGCAUAU
ENSG00000128266	UGGCCAC
ENSG00000128266	UUUUUGC
ENSG00000128268	AAUACUG
ENSG00000128268	ACACACC

ENSG00000128268	AGCAGCU
ENSG00000128268	AUAUUAU
ENSG00000128268	AUGCACC
ENSG00000128268	CCAGUGU
ENSG00000128268	CUUCUCU
ENSG00000128268	GGGGAGC
ENSG00000128268	UAAUAUC
ENSG00000128268	UACAAAG
ENSG00000128268	UGAAAUG
ENSG00000128271	AGCAGCA
ENSG00000128271	CUCCAGC
ENSG00000128271	GAAGGAA
ENSG00000128271	GCAGGAC
ENSG00000128283	GGGGUGG
ENSG00000128285	AUGAUGC
ENSG00000128285	GGCAGGG
ENSG00000128294	AUAUUAU
ENSG00000128298	CUGGGCA
ENSG00000128298	UACAAAG
ENSG00000128340	GGGGUGC
ENSG00000128342	AAAAAUC
ENSG00000128342	AAAGUGC
ENSG00000128342	AAGUGCU
ENSG00000128342	AGCACCA
ENSG00000128342	AGCAGCU
ENSG00000128342	AGGAGCU
ENSG00000128342	AUGACAC
ENSG00000128342	CUGCCCC
ENSG00000128342	GACCCUG
ENSG00000128342	GAGACCU
ENSG00000128342	GAGCUGC
ENSG00000128342	GCCUGGG
ENSG00000128342	GCUUCCU
ENSG00000128342	GUAACAG
ENSG00000128342	GUCAGUU
ENSG00000128342	GUCUGCC
ENSG00000128342	UAAUAUC
ENSG00000128342	UCAAGUA
ENSG00000128342	UCUGGAA
ENSG00000128342	UGCCAGC
ENSG00000128346	UCCCUCU
ENSG00000128422	GAGGCUG
ENSG00000128422	GUCAACA
ENSG00000128463	ACAUUAC
ENSG00000128510	ACACACC
ENSG00000128510	GAGGUAU
ENSG00000128510	GCAGGAC
ENSG00000128510	GGAAUGU
ENSG00000128510	UUAGGAU
ENSG00000128512	ACAAGGU
ENSG00000128512	ACAUUCA
ENSG00000128512	ACUGCAG
ENSG00000128512	AGUGCCU
ENSG00000128512	GUGCAAA
ENSG00000128512	UUGGCAA
ENSG00000128512	UUGGCAC
ENSG00000128563	GGCUCAG
ENSG00000128564	CAAAACU
ENSG00000128564	GAGGGGC
ENSG00000128564	UUCCUCG
ENSG00000128567	AACAUUC
ENSG00000128567	ACAUUCA
ENSG00000128567	CAGCAAA
ENSG00000128567	CAGUGCA

ENSG00000128567	CCAGUGC
ENSG00000128567	CCCUGUC
ENSG00000128567	CUCCAGC
ENSG00000128567	CUGGGCA
ENSG00000128567	GAUUGUC
ENSG00000128567	GCAACUU
ENSG00000128567	GCUGUCU
ENSG00000128567	GGCAGUG
ENSG00000128567	UACAAAG
ENSG00000128567	UUGGCAA
ENSG00000128567	UUGGCAC
ENSG00000128573	AAAAACC
ENSG00000128573	AAAAAUC
ENSG00000128573	AAAUGGU
ENSG00000128573	AACACUG
ENSG00000128573	AACAGUC
ENSG00000128573	AACUACU
ENSG00000128573	ACAUUAC
ENSG00000128573	ACCAGCA
ENSG00000128573	AGGUAGU
ENSG00000128573	AGUGGUU
ENSG00000128573	AUACAAG
ENSG00000128573	AUUCAUU
ENSG00000128573	AUUGCAC
ENSG00000128573	CAAAGCA
ENSG00000128573	CACAGUG
ENSG00000128573	CAGCAGG
ENSG00000128573	CAUGGAU
ENSG00000128573	CUUUGGU
ENSG00000128573	GAGUUGG
ENSG00000128573	GAUAUGU
ENSG00000128573	GGAGUGU
ENSG00000128573	GGCAGUG
ENSG00000128573	GGCUGCG
ENSG00000128573	GGUAGAC
ENSG00000128573	GGUUGUC
ENSG00000128573	GUGCUUG
ENSG00000128573	GUUCAUG
ENSG00000128573	UAUAUGA
ENSG00000128573	UCACAAG
ENSG00000128573	UCAUGAU
ENSG00000128573	UGACUGU
ENSG00000128573	UGCAGCU
ENSG00000128573	UGGCCAC
ENSG00000128573	UGGCUGC
ENSG00000128573	UUAGGAU
ENSG00000128573	UUGGCAA
ENSG00000128573	UUGGCAC
ENSG00000128573	UUGGUUC
ENSG00000128578	UUCUGCA
ENSG00000128585	AUCCCUU
ENSG00000128585	CAGACAG
ENSG00000128585	CUACAAA
ENSG00000128585	CUUUGGU
ENSG00000128585	GAGAAAU
ENSG00000128585	GGGAUCG
ENSG00000128585	GUCUCUG
ENSG00000128585	GUGUGGC
ENSG00000128585	UCCUAUA
ENSG00000128585	UGAAGCU
ENSG00000128585	UGGCCAC
ENSG00000128585	UGGUACC
ENSG00000128585	UUCUGCA
ENSG00000128585	UUCUUGU

ENSG00000128590	AAGUGCA
ENSG00000128590	ACUGGCC
ENSG00000128590	ACUGGCU
ENSG00000128590	AGAUAAA
ENSG00000128590	GAGGUAG
ENSG00000128590	GAGGUAU
ENSG00000128590	GCAGAAG
ENSG00000128590	UAAUACA
ENSG00000128590	UAAUAUC
ENSG00000128590	UCACAGG
ENSG00000128590	UCACAGU
ENSG00000128590	UUGGCAA
ENSG00000128591	AAAGCUG
ENSG00000128591	CUCACAC
ENSG00000128591	GGAGGCA
ENSG00000128591	UCACACA
ENSG00000128594	AAAGCUG
ENSG00000128594	AAAUUUC
ENSG00000128594	ACAAUAU
ENSG00000128594	ACAGGUG
ENSG00000128594	ACAUCAC
ENSG00000128594	ACAUUAC
ENSG00000128594	ACUGCAG
ENSG00000128594	AUAUAAC
ENSG00000128594	AUUGCAC
ENSG00000128594	CUGCAGG
ENSG00000128594	CUGCCCC
ENSG00000128594	GAGAAAU
ENSG00000128594	GCAGGAC
ENSG00000128594	UAUUGCU
ENSG00000128594	UCACAUU
ENSG00000128594	UCUCACC
ENSG00000128594	UGACAUC
ENSG00000128594	UGCAAAG
ENSG00000128594	UGCAUUG
ENSG00000128594	UUCAAAU
ENSG00000128595	AACACUG
ENSG00000128595	AAGGCAC
ENSG00000128595	AAUACUG
ENSG00000128595	AGCAGCA
ENSG00000128595	AGUGGUU
ENSG00000128595	CAGUAGA
ENSG00000128595	CAGUAGU
ENSG00000128595	CUCAAAC
ENSG00000128595	CUCCCAA
ENSG00000128595	GAGGAUA
ENSG00000128595	GAGGUAG
ENSG00000128595	GAGGUAU
ENSG00000128595	GGAAGAC
ENSG00000128595	GGAGGAG
ENSG00000128595	GUGCUUG
ENSG00000128596	GGGUAAG
ENSG00000128596	UGGGUAC
ENSG00000128602	GAAGGAA
ENSG00000128602	UCCCACA
ENSG00000128602	UGAAGCU
ENSG00000128604	AGCUGCC
ENSG00000128604	GCUUCCU
ENSG00000128604	UGCCAGC
ENSG00000128641	GGUUUAC
ENSG00000128641	UAUUGCU
ENSG00000128645	AGCAGCA
ENSG00000128645	AGCAGCG
ENSG00000128645	AGCAGCU

ENSG00000128645	AGUGCAA
ENSG00000128645	CCCUGAG
ENSG00000128645	CUUUGGU
ENSG00000128645	GAGGUAG
ENSG00000128645	GAUUGUA
ENSG00000128645	GUGCAAA
ENSG00000128645	UAUUACC
ENSG00000128645	UCCCUGU
ENSG00000128645	UUGGUCC
ENSG00000128652	AACUACU
ENSG00000128652	CACCAGC
ENSG00000128655	AAAAACC
ENSG00000128655	AAAACUG
ENSG00000128655	ACAUUCA
ENSG00000128655	ACUGGCU
ENSG00000128655	AGAUGUG
ENSG00000128655	AGGGUUG
ENSG00000128655	AUGGCAC
ENSG00000128655	AUGUGGG
ENSG00000128655	CAGCAGA
ENSG00000128655	CAGUAGA
ENSG00000128655	CAGUAGU
ENSG00000128655	CUGCAGC
ENSG00000128655	CUGCAGG
ENSG00000128655	GAGAAAU
ENSG00000128655	GCAGAAG
ENSG00000128655	GGAGGCA
ENSG00000128655	GGCUCUG
ENSG00000128655	GGUUGAC
ENSG00000128655	UACAAAG
ENSG00000128655	UCCUAUA
ENSG00000128655	UGAAAUG
ENSG00000128655	UGACAUC
ENSG00000128655	UGGGACA
ENSG00000128655	UGUCUGG
ENSG00000128655	UGUUGAA
ENSG00000128655	UUCUUGU
ENSG00000128655	UUGGCAA
ENSG00000128655	UUGUUCG
ENSG00000128656	GAAACAU
ENSG00000128683	AUACAAG
ENSG00000128683	AUAUAAC
ENSG00000128683	CUUUGGU
ENSG00000128683	GGCUCAG
ENSG00000128709	CCCUGAG
ENSG00000128710	ACCCUGU
ENSG00000128710	AUUGCAC
ENSG00000128710	GUGCUUG
ENSG00000128710	UAUUGCU
ENSG00000128710	UGCAGCU
ENSG00000128714	AAGUCUU
ENSG00000128714	ACUGGCU
ENSG00000128714	GAUUGUC
ENSG00000128714	GCCUGGA
ENSG00000128714	GGGGGAA
ENSG00000128714	UCACAAG
ENSG00000128731	AUUGCAC
ENSG00000128791	AUUCAUU
ENSG00000128829	AAAUUUC
ENSG00000128829	AACAUUC
ENSG00000128829	AUAUUAU
ENSG00000128829	UAUAUGA
ENSG00000128849	AGCAGCA
ENSG00000128849	CCCCCAG

ENSG00000128849	CUGCUCA
ENSG00000128849	GAGGUAG
ENSG00000128849	GAUUGUA
ENSG00000128849	GUGCCUG
ENSG00000128872	AAAUGGU
ENSG00000128872	ACUCAAA
ENSG00000128872	UCACAGG
ENSG00000128872	UUGGCAA
ENSG00000128881	AACACUG
ENSG00000128881	UUGGCAA
ENSG00000128881	UUGGCAC
ENSG00000128881	UUUCAAC
ENSG00000128886	AGCAGCU
ENSG00000128891	UCACACA
ENSG00000128891	UGAUCCA
ENSG00000128891	UGGGACA
ENSG00000128915	AAAGAGC
ENSG00000128915	AAGGCAC
ENSG00000128915	GGAUGAG
ENSG00000128915	UCCCUGU
ENSG00000128917	AAGGCAC
ENSG00000128917	AAUGUUG
ENSG00000128917	AAUUAUU
ENSG00000128917	AGCAGCA
ENSG00000128917	AGUGCUU
ENSG00000128917	AUACAAG
ENSG00000128917	AUAUUAU
ENSG00000128917	CACAGUG
ENSG00000128917	CAGUCUG
ENSG00000128917	CAGUGCA
ENSG00000128917	GAAGGAA
ENSG00000128917	UAUAAAG
ENSG00000128917	UCACAGG
ENSG00000128917	UCACAGU
ENSG00000128917	UCCUGAG
ENSG00000128917	UGCAUAG
ENSG00000128918	ACAAUAU
ENSG00000128918	AUGAGCU
ENSG00000128918	UAUAAAG
ENSG00000128918	UGGAUAA
ENSG00000128923	AAAGUUU
ENSG00000128923	AAGUGCA
ENSG00000128923	AGCAGCG
ENSG00000128923	AGCAGCU
ENSG00000128923	AGCUUAU
ENSG00000128923	CUUUGGU
ENSG00000128923	GAAGGAA
ENSG00000128923	GACCCUG
ENSG00000128923	GAUUGUC
ENSG00000128923	GCUGGUG
ENSG00000128923	GUCACUC
ENSG00000128923	GUGCUUG
ENSG00000128923	UCCAGAG
ENSG00000128923	UUGGUUC
ENSG00000128965	AGCAGCA
ENSG00000128965	AGCAGCG
ENSG00000128965	AGCAGCU
ENSG00000128965	CAGCAGA
ENSG00000128965	CAGCAGG
ENSG00000128965	CUUGGAG
ENSG00000128965	UUCAAGC
ENSG00000129003	AAGUGCA
ENSG00000129007	AAAAGUA
ENSG00000129007	AAAGUAC

ENSG00000129007	AACAAAC
ENSG00000129007	AGUGUGC
ENSG00000129007	AUGCAUU
ENSG00000129007	AUGUAAU
ENSG00000129007	AUUCAUU
ENSG00000129007	CACAAUG
ENSG00000129007	CAGUUAC
ENSG00000129007	GCUGGUG
ENSG00000129007	GGUGGUC
ENSG00000129007	GGUGGUU
ENSG00000129007	GUUAAUG
ENSG00000129007	UAUUACC
ENSG00000129007	UGCAUUG
ENSG00000129007	UGUCUUU
ENSG00000129007	UGUGCUU
ENSG00000129007	UUCAAAU
ENSG00000129007	UUCCAUA
ENSG00000129038	CCCUGAG
ENSG00000129038	UCCCUGU
ENSG00000129048	UUAGGAU
ENSG00000129071	UGCAAAG
ENSG00000129116	AAAGUAC
ENSG00000129116	AAAGUGC
ENSG00000129116	AAGUGCU
ENSG00000129116	AGUGCUG
ENSG00000129116	CAGGUGA
ENSG00000129116	CUAGUCC
ENSG00000129116	GAGCUAA
ENSG00000129152	AAAAGUA
ENSG00000129152	AAAGUAU
ENSG00000129152	AGAUAAA
ENSG00000129152	CAAAGCA
ENSG00000129152	UACAAAG
ENSG00000129159	AAAAAUC
ENSG00000129159	AAAACUG
ENSG00000129159	AACUCUA
ENSG00000129159	AAUUUUA
ENSG00000129159	ACUCAGG
ENSG00000129159	ACUGUUU
ENSG00000129159	AGAUAAA
ENSG00000129159	AGCUGCC
ENSG00000129159	AUGUGCC
ENSG00000129159	CAAAGCA
ENSG00000129159	CACACCU
ENSG00000129159	CACCAGC
ENSG00000129159	CAGUCUG
ENSG00000129159	CAUGGGU
ENSG00000129159	CCAGCAU
ENSG00000129159	CCCCCAG
ENSG00000129159	CUGACUC
ENSG00000129159	CUGGAAA
ENSG00000129159	CUUCUCU
ENSG00000129159	GAGAAAU
ENSG00000129159	GAGAACC
ENSG00000129159	GAGAUGA
ENSG00000129159	GAGCCCC
ENSG00000129159	GAGGCUG
ENSG00000129159	GAGGUAG
ENSG00000129159	GAGUUGA
ENSG00000129159	GCAGGAC
ENSG00000129159	GCAGGUU
ENSG00000129159	GGAGAGA
ENSG00000129159	GGCAGCG
ENSG00000129159	GGGGUGG

ENSG00000129159	GGGUUUA
ENSG00000129159	GGUAGAC
ENSG00000129159	GUGGCCC
ENSG00000129159	UAUUGCU
ENSG00000129159	UCACAGG
ENSG00000129159	UCUAGAG
ENSG00000129159	UCUGGAA
ENSG00000129159	UGAAAGG
ENSG00000129159	UGAAAUG
ENSG00000129159	UUGGAGG
ENSG00000129159	UUGGUUC
ENSG00000129173	AAAGUUU
ENSG00000129187	AAUUAUU
ENSG00000129187	UGAAAGG
ENSG00000129194	GCCUGGG
ENSG00000129204	AAAUUUC
ENSG00000129204	AACUCUA
ENSG00000129204	AAGUGCA
ENSG00000129204	ACAUCAC
ENSG00000129204	AGAUAAA
ENSG00000129204	AGCCCUU
ENSG00000129204	AGUAGAC
ENSG00000129204	AGUGCAA
ENSG00000129204	AUACAAG
ENSG00000129204	CAGGAAC
ENSG00000129204	CAUAUUG
ENSG00000129204	GAGGUAG
ENSG00000129204	UACAGUU
ENSG00000129204	UGCAUUG
ENSG00000129204	UGUGCUU
ENSG00000129204	UUCUAAU
ENSG00000129219	UACAAAG
ENSG00000129235	GUUAAUG
ENSG00000129244	ACAGUAC
ENSG00000129244	ACAGUAU
ENSG00000129244	ACUGCAG
ENSG00000129244	AGGGAGG
ENSG00000129244	AUAUAAC
ENSG00000129244	CAAAGUG
ENSG00000129244	CAGUAGA
ENSG00000129244	CCCCCAG
ENSG00000129244	GGCAGGG
ENSG00000129244	GGGGGAA
ENSG00000129244	UACAGUU
ENSG00000129244	UGGCCAC
ENSG00000129245	AAGGCAC
ENSG00000129245	ACAAGGU
ENSG00000129245	ACCCUGU
ENSG00000129245	ACUGUAG
ENSG00000129245	AGCAGCU
ENSG00000129245	CACAGUG
ENSG00000129245	CAGUCUG
ENSG00000129245	CCCACCG
ENSG00000129245	CCCACCU
ENSG00000129245	CCCCUGG
ENSG00000129245	CCCUGUU
ENSG00000129245	CUCCAGC
ENSG00000129245	CUCCCAC
ENSG00000129245	GAGGAUA
ENSG00000129245	GGAGGAG
ENSG00000129245	GGGUGUU
ENSG00000129245	GUGCCAC
ENSG00000129245	UCAUACA
ENSG00000129245	UCCCACC

ENSG00000129250	AACACUG
ENSG00000129250	ACCACAG
ENSG00000129250	AGCAGCA
ENSG00000129250	AGCAGCU
ENSG00000129250	CAGACAG
ENSG00000129250	CUUUGGU
ENSG00000129250	UUGGUUC
ENSG00000129255	ACCAGCA
ENSG00000129255	CACCAGC
ENSG00000129255	CAGGAAC
ENSG00000129255	GGCCCUG
ENSG00000129255	GGGGUCC
ENSG00000129270	CACCAGC
ENSG00000129277	CAGGUGA
ENSG00000129292	AAAACUG
ENSG00000129292	AAAGCUG
ENSG00000129292	AAAGGUA
ENSG00000129292	ACAGUAC
ENSG00000129292	ACAGUAU
ENSG00000129292	ACUAGAU
ENSG00000129292	AGAUAAA
ENSG00000129292	AUGUGGG
ENSG00000129292	CAAAACU
ENSG00000129292	CAAGAGC
ENSG00000129292	CACUCCU
ENSG00000129292	CAGUCUG
ENSG00000129292	CCAGCAU
ENSG00000129292	CUAGGCU
ENSG00000129292	CUUUGGU
ENSG00000129292	GAGCUAA
ENSG00000129292	GAGGCAG
ENSG00000129292	GCAACUU
ENSG00000129292	GUAUUCU
ENSG00000129292	GUCACUC
ENSG00000129292	UAAUAUC
ENSG00000129292	UCAAGUA
ENSG00000129292	UCAUGAU
ENSG00000129292	UGAAGCU
ENSG00000129292	UUCAAAU
ENSG00000129292	UUGGCAA
ENSG00000129292	UUGGUUC
ENSG00000129347	ACUGUAG
ENSG00000129347	CUCAGGG
ENSG00000129347	CUGACUC
ENSG00000129347	UUUGCAC
ENSG00000129351	AACUCUA
ENSG00000129351	AAGUUGU
ENSG00000129351	ACAUUCA
ENSG00000129351	ACUCAAA
ENSG00000129351	CAGACAG
ENSG00000129351	CUUCACC
ENSG00000129351	GAAACAU
ENSG00000129351	GCCUGAU
ENSG00000129351	GGACGGA
ENSG00000129351	GGAGUCC
ENSG00000129351	GUCUACU
ENSG00000129351	UAUAAAG
ENSG00000129351	UCACAAG
ENSG00000129351	UCACAGG
ENSG00000129351	UCCCACA
ENSG00000129351	UCCCACC
ENSG00000129353	AAGGCAC
ENSG00000129353	GGAAUGU
ENSG00000129353	GGCAGUG

ENSG00000129353	UUCCAUA
ENSG00000129353	UUGGCAC
ENSG00000129354	AAGGCAC
ENSG00000129355	AACCGUU
ENSG00000129355	AAGUCUU
ENSG00000129422	AAGUGCA
ENSG00000129422	AAUACUG
ENSG00000129422	AGGUUAU
ENSG00000129422	AGUGCUU
ENSG00000129422	CCCUGAG
ENSG00000129422	CUACAAA
ENSG00000129422	CUGGAAA
ENSG00000129422	GAGGUAG
ENSG00000129422	GUGCAAA
ENSG00000129422	UCCAGUU
ENSG00000129422	UUGGUUC
ENSG00000129451	AGCAGCU
ENSG00000129451	AGUGGUU
ENSG00000129451	GGGUGGU
ENSG00000129451	UCACACA
ENSG00000129472	AAAGAGG
ENSG00000129472	GUGCAAA
ENSG00000129472	UGCAUAU
ENSG00000129473	AAAGUGC
ENSG00000129473	AAGACAU
ENSG00000129473	AAGUGCA
ENSG00000129473	ACCCUGU
ENSG00000129473	ACUGGCC
ENSG00000129473	AGCACCA
ENSG00000129473	AGUGGUU
ENSG00000129473	AUGGCUU
ENSG00000129473	CAAAUGC
ENSG00000129473	CAAGAGC
ENSG00000129473	CAGCAAA
ENSG00000129473	CAGCAGA
ENSG00000129473	CAGCAGG
ENSG00000129473	CAGUCCA
ENSG00000129473	CCCACCU
ENSG00000129473	CCCUGAG
ENSG00000129473	CUCAGGG
ENSG00000129473	CUCUGGG
ENSG00000129473	CUGGACU
ENSG00000129473	CUGGCUC
ENSG00000129473	CUGGGCA
ENSG00000129473	GAGAACC
ENSG00000129473	GAGCUAA
ENSG00000129473	GCAGCAU
ENSG00000129473	GCAGGAC
ENSG00000129473	GGCAGGG
ENSG00000129473	GGCUCAG
ENSG00000129473	GGGAGCU
ENSG00000129473	GGGGAGC
ENSG00000129473	GUCUCUG
ENSG00000129473	GUGAGGU
ENSG00000129473	GUGGGGA
ENSG00000129473	UAAUACA
ENSG00000129473	UAUAAAG
ENSG00000129473	UCCCUGU
ENSG00000129473	UCCUGAG
ENSG00000129473	UCUAGAG
ENSG00000129473	UCUCAAG
ENSG00000129473	UUGGUCC
ENSG00000129474	AAAGUGC
ENSG00000129474	AAGUGCU

ENSG00000129474	AGUGCUG
ENSG00000129474	CAAAGUG
ENSG00000129474	CUUUGGU
ENSG00000129474	GGGUCUU
ENSG00000129480	AGCCAGU
ENSG00000129480	AUGAGCU
ENSG00000129480	GUGACUG
ENSG00000129480	UUGGCAA
ENSG00000129493	AUACAAG
ENSG00000129493	CAAAUGC
ENSG00000129515	AAGGCAC
ENSG00000129515	ACAAUAU
ENSG00000129515	AGCCCUU
ENSG00000129515	AUAAAGU
ENSG00000129515	AUCAUAC
ENSG00000129515	CCAGUGC
ENSG00000129515	CCAGUGU
ENSG00000129515	CUACAAA
ENSG00000129515	GAGGUAU
ENSG00000129515	GAGUGUG
ENSG00000129515	GGAGUGU
ENSG00000129515	GGCAGCG
ENSG00000129515	GGUUUAC
ENSG00000129515	UCCAGUU
ENSG00000129521	AAGUGCA
ENSG00000129521	ACCACAG
ENSG00000129521	AGUGCAA
ENSG00000129521	AUAAAGU
ENSG00000129521	CUUUGGU
ENSG00000129534	AAAGUUU
ENSG00000129534	UCACAAG
ENSG00000129535	AAAGCUG
ENSG00000129535	CGCCUCC
ENSG00000129535	GUGACAG
ENSG00000129566	CAGGCUC
ENSG00000129595	GCAGGUU
ENSG00000129595	UCACAGG
ENSG00000129595	UUCAAAU
ENSG00000129625	ACUGCAU
ENSG00000129636	AUACAAG
ENSG00000129654	AACACUG
ENSG00000129654	ACCUGGA
ENSG00000129654	ACUGUGU
ENSG00000129654	GACCUGG
ENSG00000129654	GAGACCU
ENSG00000129657	GAGGUAG
ENSG00000129657	GAGGUAU
ENSG00000129657	GUGCUUG
ENSG00000129657	UACAAAG
ENSG00000129657	UAUUACC
ENSG00000129657	UGUGCUU
ENSG00000129667	CACAGGG
ENSG00000129675	AAAAACC
ENSG00000129675	AAGUCUU
ENSG00000129675	AGUGUGC
ENSG00000129675	AUGGCUU
ENSG00000129675	CACAGUG
ENSG00000129675	CUGCCCC
ENSG00000129675	GAGUAUU
ENSG00000129675	UCAUUUG
ENSG00000129675	UGCAUAU
ENSG00000129682	CACUGUU
ENSG00000129682	UGUCUUU
ENSG00000129691	UCACAGG

ENSG00000129696	GUAACAG
ENSG00000129749	CUGGAAA
ENSG00000129749	GGAGAGA
ENSG00000129749	UCUGGAA
ENSG00000129757	AAAGCUG
ENSG00000129757	CCAGUGU
ENSG00000129757	GCUACAU
ENSG00000129757	GGCAGUG
ENSG00000129757	GGCUCAG
ENSG00000129757	UCAAGUA
ENSG00000129757	UGUCUUU
ENSG00000129911	AGGCAGG
ENSG00000129911	CCCUGAG
ENSG00000129933	AAGGCAC
ENSG00000129933	AAUGCCC
ENSG00000129933	AGCACCA
ENSG00000129933	AGUACUG
ENSG00000129946	ACUCCAG
ENSG00000129946	CACUCCU
ENSG00000129968	UAUUGCU
ENSG00000129990	CAAAGCA
ENSG00000129990	GAUUGUA
ENSG00000129993	AGCAGCA
ENSG00000129993	AGCAGCG
ENSG00000129993	AGCAGCU
ENSG00000129993	AGGUAGU
ENSG00000129993	CACAGUG
ENSG00000129993	CAGUAGA
ENSG00000129993	CAGUAGU
ENSG00000129993	GAGGAUA
ENSG00000129993	GAGGUAG
ENSG00000129993	GAUUGUC
ENSG00000129993	GCUAGCA
ENSG00000129993	GGACCUG
ENSG00000129993	UAGAGGA
ENSG00000129993	UCACAGU
ENSG00000129993	UUGGAGG
ENSG00000130032	AAUUAUU
ENSG00000130032	GACAACU
ENSG00000130032	UUGGCAC
ENSG00000130037	GGAAUGU
ENSG00000130054	AAAGAGC
ENSG00000130054	AACAUUC
ENSG00000130054	AAGUCUU
ENSG00000130054	AAGUGCU
ENSG00000130054	AGUGCUG
ENSG00000130054	CACAGUG
ENSG00000130054	CCUUCAU
ENSG00000130054	CUGCAGG
ENSG00000130054	CUGGAAA
ENSG00000130054	CUGGGCA
ENSG00000130054	CUUUGGU
ENSG00000130054	GACAACU
ENSG00000130054	GAGCUGC
ENSG00000130054	GCCUGGG
ENSG00000130054	GGGGCAG
ENSG00000130054	GUCUGCC
ENSG00000130054	UCUGGAG
ENSG00000130054	UGGAGAU
ENSG00000130066	CCAGUGU
ENSG00000130119	CUUGGAG
ENSG00000130119	UCCAGAG
ENSG00000130147	ACUGCAG
ENSG00000130147	ACUGCAU

ENSG00000130147	CCCUGAG
ENSG00000130147	CUUUGGU
ENSG00000130147	GAUUGGU
ENSG00000130147	GCUACAU
ENSG00000130147	GGUUUAC
ENSG00000130147	UGCAUAU
ENSG00000130147	UGUGCUU
ENSG00000130150	AAAGAAU
ENSG00000130150	ACAUUCA
ENSG00000130150	CAAAGCA
ENSG00000130150	GGCAUUG
ENSG00000130150	GGCUGCG
ENSG00000130150	GGUGGUU
ENSG00000130150	UUGGCAA
ENSG00000130150	UUGGCAC
ENSG00000130164	AGUGGUU
ENSG00000130164	CAAAGUG
ENSG00000130164	GUAAACA
ENSG00000130164	UACAAAG
ENSG00000130164	UCACAGU
ENSG00000130164	UCCUGAG
ENSG00000130164	UGCAAAG
ENSG00000130164	UUUGCAA
ENSG00000130165	CUCUGGG
ENSG00000130165	GCUGGUG
ENSG00000130165	GGAAUGU
ENSG00000130165	GUCUCUG
ENSG00000130165	UCCAGUU
ENSG00000130176	AAAGUGC
ENSG00000130176	AAGUGCU
ENSG00000130176	AGCAGCA
ENSG00000130176	CCAGUGC
ENSG00000130176	CCAGUGU
ENSG00000130176	UGUGCUU
ENSG00000130201	AGCAGCA
ENSG00000130201	CAGCAGA
ENSG00000130201	CAGCAGG
ENSG00000130202	AAGGCAC
ENSG00000130202	AGCAGCA
ENSG00000130202	CAGCAGG
ENSG00000130202	CUCCCAA
ENSG00000130202	GCCUGGA
ENSG00000130202	GGCUCAG
ENSG00000130202	GGGGAGC
ENSG00000130204	CCCUGAG
ENSG00000130204	UAUAAAG
ENSG00000130204	UUCCAUA
ENSG00000130222	GAUCAGA
ENSG00000130224	AACUACU
ENSG00000130224	AAGUGCA
ENSG00000130224	ACUCAAA
ENSG00000130224	AGUGCAA
ENSG00000130224	AUGGCUU
ENSG00000130224	CUACAAA
ENSG00000130224	GACAACU
ENSG00000130224	GGCAGUG
ENSG00000130224	GUAUUCU
ENSG00000130224	GUGCAAA
ENSG00000130224	GUGCCAC
ENSG00000130224	UAAGACG
ENSG00000130224	UACAAAG
ENSG00000130224	UCACAUU
ENSG00000130226	AAUGUUG
ENSG00000130226	ACAAGGU

ENSG00000130226	ACAUUCA
ENSG00000130226	CAUGGAU
ENSG00000130226	CUACAGU
ENSG00000130226	GGAUGAG
ENSG00000130226	GUGCUUG
ENSG00000130227	AGGCAGG
ENSG00000130227	AUACAAG
ENSG00000130227	CAGCAAA
ENSG00000130244	AAAUUUC
ENSG00000130254	GGAAUGU
ENSG00000130287	CACAGUG
ENSG00000130287	CCCUGAG
ENSG00000130287	GACCUGG
ENSG00000130287	GGCUCAG
ENSG00000130287	GGGGUGG
ENSG00000130287	GUGCUUG
ENSG00000130287	UACAAAG
ENSG00000130294	AAGACAU
ENSG00000130294	AAUUAUU
ENSG00000130294	CUGCAGC
ENSG00000130294	CUGCAGG
ENSG00000130294	GACCUGG
ENSG00000130294	GCAGGUU
ENSG00000130294	UAUAAAG
ENSG00000130294	UGGCUGC
ENSG00000130299	CUUUGGU
ENSG00000130304	AAGGCAC
ENSG00000130307	ACUCCAG
ENSG00000130309	CCCUGUU
ENSG00000130309	GCCCUGU
ENSG00000130311	AAAAACC
ENSG00000130313	UAAUAUC
ENSG00000130332	AAUUUUA
ENSG00000130338	ACAUUCA
ENSG00000130338	AGCAAAA
ENSG00000130338	AUAAUAC
ENSG00000130338	CAGUAGU
ENSG00000130338	CUUACAG
ENSG00000130338	GACAACU
ENSG00000130338	GAGGUAU
ENSG00000130338	GCAGCAU
ENSG00000130338	GCUGGUG
ENSG00000130338	GGUGGUU
ENSG00000130338	UAUAAAG
ENSG00000130338	UAUUGCU
ENSG00000130338	UCUAGAG
ENSG00000130338	UCUGGAA
ENSG00000130338	UGCAGCU
ENSG00000130338	UGCAUUG
ENSG00000130338	UUGGUCC
ENSG00000130340	AAAAACC
ENSG00000130340	AACAAAC
ENSG00000130340	AAGGCAC
ENSG00000130340	ACUGUAG
ENSG00000130340	AGUGCUU
ENSG00000130340	AUACAAG
ENSG00000130340	AUAUUAU
ENSG00000130340	CUUACAG
ENSG00000130340	UAUAUGA
ENSG00000130340	UCACACA
ENSG00000130340	UGAGAAG
ENSG00000130382	AAAACUG
ENSG00000130382	AAAGUGC
ENSG00000130382	AACAAAC

ENSG00000130382	AAGGCAC
ENSG00000130382	AAGUGCA
ENSG00000130382	AGUGCUG
ENSG00000130382	CACAAUG
ENSG00000130382	CCUGUAC
ENSG00000130382	GAGUAUU
ENSG00000130382	GGCAGUG
ENSG00000130382	UACAAAG
ENSG00000130382	UCACAAG
ENSG00000130382	UUCAAAU
ENSG00000130382	UUGGCAC
ENSG00000130382	UUGUUCG
ENSG00000130382	UUUCAAC
ENSG00000130396	AGCUGCC
ENSG00000130396	GAGGUAG
ENSG00000130396	GGCCCUG
ENSG00000130402	AAAGUAC
ENSG00000130402	AAAGUAU
ENSG00000130402	AAAUCCC
ENSG00000130402	AAGGCAC
ENSG00000130402	AUAAUAC
ENSG00000130402	AUAUAAC
ENSG00000130402	CUGCCCC
ENSG00000130402	GGGUGGU
ENSG00000130402	GGUGGUU
ENSG00000130402	GGUUUAC
ENSG00000130402	UGCAUAU
ENSG00000130427	GGAGUGU
ENSG00000130429	CAAAGCA
ENSG00000130429	CCAGCAU
ENSG00000130429	UGAAGCU
ENSG00000130477	AAGUCAC
ENSG00000130477	AGCAGCA
ENSG00000130477	AGCAGCG
ENSG00000130477	AGCAGCU
ENSG00000130477	AUGCAUU
ENSG00000130477	AUGGCUU
ENSG00000130477	CUCUGGG
ENSG00000130477	CUUUGGU
ENSG00000130477	GCCUGGG
ENSG00000130477	GGCAGCG
ENSG00000130477	GGCGGGG
ENSG00000130477	GUGCAAA
ENSG00000130477	GUGCUUG
ENSG00000130477	GUUAAUG
ENSG00000130477	UGUGCUU
ENSG00000130477	UUGUUCG
ENSG00000130477	UUUGCAA
ENSG00000130508	AAAGUAA
ENSG00000130508	GAGGUAG
ENSG00000130508	UCACAUU
ENSG00000130508	UGAGAAG
ENSG00000130508	UGCAUAG
ENSG00000130508	UGCAUAU
ENSG00000130517	GGGGCAG
ENSG00000130522	AAAAACC
ENSG00000130522	GAAACAU
ENSG00000130522	GAGGCUG
ENSG00000130522	GGAAUGU
ENSG00000130522	GGCUGCG
ENSG00000130522	GGGGUCC
ENSG00000130522	UUGUUCG
ENSG00000130528	GAGAAAU
ENSG00000130529	CCAGUGU

ENSG00000130540	AAAAAUC
ENSG00000130540	AAAUUUC
ENSG00000130540	ACAGUAU
ENSG00000130540	CCAGCAU
ENSG00000130540	UGUCUUU
ENSG00000130540	UUGGUUC
ENSG00000130544	UCCCACA
ENSG00000130544	UCCCACC
ENSG00000130545	UCACACA
ENSG00000130558	AACACUG
ENSG00000130558	AACAGUC
ENSG00000130558	AGAUAAA
ENSG00000130558	GUCAUUG
ENSG00000130559	ACAUUCA
ENSG00000130559	AGCAGCA
ENSG00000130559	CAGUAGA
ENSG00000130559	GGCAGUG
ENSG00000130559	UCAAGUA
ENSG00000130584	AAAAACC
ENSG00000130584	AAGUGCA
ENSG00000130584	ACUGCAG
ENSG00000130584	AGCAAAA
ENSG00000130584	AGCACCA
ENSG00000130584	AGCAGCA
ENSG00000130584	AGCAGCG
ENSG00000130584	AGCAGCU
ENSG00000130584	CCAGUGU
ENSG00000130584	CUGCAGC
ENSG00000130584	GGUUACC
ENSG00000130584	UCCUGAG
ENSG00000130590	AGUGCUG
ENSG00000130590	CACAGUG
ENSG00000130590	CUGGCUC
ENSG00000130590	GGGGUCC
ENSG00000130590	UCACAGU
ENSG00000130590	UGCCAGC
ENSG00000130592	CCCCUGG
ENSG00000130635	AAAGAAU
ENSG00000130635	AACAAAC
ENSG00000130635	AACAUUC
ENSG00000130635	ACAAUAU
ENSG00000130635	ACAUUCA
ENSG00000130635	AUGGCUU
ENSG00000130635	CACAGUG
ENSG00000130635	CACUGUU
ENSG00000130635	CUGGGGG
ENSG00000130635	CUUUGGU
ENSG00000130635	GAGAUGA
ENSG00000130635	GGCAAGA
ENSG00000130635	GGGGUCC
ENSG00000130635	GUCCAGA
ENSG00000130635	UCAAGUA
ENSG00000130635	UCACAGG
ENSG00000130635	UCACAGU
ENSG00000130635	UGACCUA
ENSG00000130635	UUGGCAA
ENSG00000130635	UUGGCAC
ENSG00000130638	AACUGGU
ENSG00000130638	AAUCACU
ENSG00000130638	CUGGAAA
ENSG00000130638	GAGAAAU
ENSG00000130638	UGAGUCU
ENSG00000130669	AAAACUG
ENSG00000130669	AAAGAGG

ENSG00000130669	ACAAUAU
ENSG00000130669	ACAUUCA
ENSG00000130669	CAGUAGA
ENSG00000130669	CUUUGGU
ENSG00000130669	GGCUCAG
ENSG00000130669	UCAUGAU
ENSG00000130669	UCUAGAG
ENSG00000130675	AACACUG
ENSG00000130675	ACAGUAC
ENSG00000130675	ACAUUCA
ENSG00000130675	AUACAAG
ENSG00000130675	CCAGCAU
ENSG00000130675	UCAAGUA
ENSG00000130675	UCCAGAG
ENSG00000130695	AACAAAC
ENSG00000130695	ACCCGUA
ENSG00000130695	CCCUGAG
ENSG00000130695	UAUAAAG
ENSG00000130695	UGAGAAU
ENSG00000130699	AAAAACC
ENSG00000130699	AAAGUAC
ENSG00000130699	AAUACUG
ENSG00000130699	AAUUUUA
ENSG00000130699	AGUGCAA
ENSG00000130699	AUAUAAC
ENSG00000130699	AUGUGCC
ENSG00000130699	CACAGUG
ENSG00000130699	CAGUGCA
ENSG00000130699	GGAUUUC
ENSG00000130699	UCAUUUG
ENSG00000130699	UCCUAUA
ENSG00000130699	UUGGCAA
ENSG00000130699	UUGGCAC
ENSG00000130703	ACAUUAC
ENSG00000130703	AUCCUUG
ENSG00000130703	UAAUGCU
ENSG00000130703	UCCCACA
ENSG00000130703	UUGAUCA
ENSG00000130703	UUGGCAC
ENSG00000130707	UACAAAG
ENSG00000130711	AAAGUGC
ENSG00000130711	AACUCUA
ENSG00000130711	AAUUUUA
ENSG00000130711	GAAAACA
ENSG00000130711	GACCAUG
ENSG00000130711	UAAUAUC
ENSG00000130711	UACAAAG
ENSG00000130713	CAUGGGU
ENSG00000130720	CUGCCCC
ENSG00000130725	AACAUUC
ENSG00000130725	AGUAACA
ENSG00000130725	AUUCAUU
ENSG00000130725	GAGGCAG
ENSG00000130726	AAAUCCC
ENSG00000130733	AAAAGUA
ENSG00000130733	AUAUAAC
ENSG00000130733	CUGCCCC
ENSG00000130733	UGCAUAU
ENSG00000130734	ACAGUAC
ENSG00000130734	CCAGUGU
ENSG00000130734	CCCUGAG
ENSG00000130734	GGCCCUG
ENSG00000130741	AACACUG
ENSG00000130749	AAAAAUC

ENSG00000130749	AAUACUG
ENSG00000130749	ACCACAG
ENSG00000130749	ACUGCAU
ENSG00000130749	AUACAAG
ENSG00000130749	AUCCUUU
ENSG00000130749	CAGGUGA
ENSG00000130749	CGGAUCC
ENSG00000130749	CUGGAAA
ENSG00000130749	GAUUGUA
ENSG00000130749	GAUUGUC
ENSG00000130749	GCACCAU
ENSG00000130749	GCAUCCC
ENSG00000130749	GCCUGAU
ENSG00000130749	GGCAGUG
ENSG00000130749	UCUGGAG
ENSG00000130749	UUAGAGA
ENSG00000130749	UUUGCAC
ENSG00000130758	ACAUUCA
ENSG00000130758	GCCCUGU
ENSG00000130758	UAAUGCU
ENSG00000130758	UGGCUGC
ENSG00000130766	AAAGUGC
ENSG00000130766	AACACUG
ENSG00000130766	AGCAAAA
ENSG00000130766	GGAGUGU
ENSG00000130766	UCACACA
ENSG00000130766	UCACAGG
ENSG00000130766	UCACAUU
ENSG00000130766	UGGGUGA
ENSG00000130766	UUGGCAA
ENSG00000130779	CCAGUGC
ENSG00000130803	UUCUGCA
ENSG00000130816	AUAAAGU
ENSG00000130816	GGAGAGA
ENSG00000130821	AAAGCUG
ENSG00000130821	AUGGCUU
ENSG00000130821	GCUGGUG
ENSG00000130821	GGUGGGC
ENSG00000130821	UAUAAAG
ENSG00000130822	CAGCAGA
ENSG00000130822	CAGUCCA
ENSG00000130827	CAAAGCA
ENSG00000130827	GCAGAAG
ENSG00000130827	UAUUGCU
ENSG00000130829	AAAGUAA
ENSG00000130829	AACAGUC
ENSG00000130829	AAGACAU
ENSG00000130829	AGAUGUG
ENSG00000130829	AGGCAGG
ENSG00000130829	AGUAACA
ENSG00000130829	AGUGAUC
ENSG00000130829	GAGGCAG
ENSG00000130829	GAGGUAG
ENSG00000130829	GAGGUAU
ENSG00000130829	GGCAGGG
ENSG00000130829	GUAACAG
ENSG00000130829	GUGUGGC
ENSG00000130830	UUGGCAA
ENSG00000130830	UUGGCAC
ENSG00000130856	AACAAAC
ENSG00000130856	AAUACUG
ENSG00000130856	ACAUUCA
ENSG00000130856	AUGGCUU
ENSG00000130856	CUGACUC

ENSG00000130856	CUUUGGU
ENSG00000130856	UCCUGAG
ENSG00000130856	UGUGCUU
ENSG00000130881	ACUGUGU
ENSG00000130881	GCGGGGC
ENSG00000130881	GUAUUCU
ENSG00000130881	UACAAAG
ENSG00000130881	UCCCUGU
ENSG00000130939	AGCAAAA
ENSG00000130939	CAGCAAA
ENSG00000130939	CUUUGGU
ENSG00000130939	UAAUACA
ENSG00000130940	AACAAAC
ENSG00000130940	AAUACUG
ENSG00000130940	ACACACC
ENSG00000130940	ACACACU
ENSG00000130940	ACAUAGA
ENSG00000130940	ACUGUAG
ENSG00000130940	AGCAGCA
ENSG00000130940	AUGAGCU
ENSG00000130940	AUGGCUU
ENSG00000130940	AUGUGCC
ENSG00000130940	CAAAACU
ENSG00000130940	CAGUGCA
ENSG00000130940	CCCCUGG
ENSG00000130940	CGAGGAG
ENSG00000130940	CUCAAAC
ENSG00000130940	GAGCUAA
ENSG00000130940	GGCUCUG
ENSG00000130940	GUGCAAA
ENSG00000130940	UACAAAG
ENSG00000130940	UAUAAAG
ENSG00000130940	UUGUUCG
ENSG00000130940	UUUUUGC
ENSG00000130956	AGGUAGU
ENSG00000130956	GAGGUAG
ENSG00000130958	GAGGUAU
ENSG00000130962	AAAGCUG
ENSG00000130962	AAAGGUA
ENSG00000130962	AGUGGUU
ENSG00000130962	CAGUGCA
ENSG00000130962	GGGUUUA
ENSG00000130962	GUAGUGU
ENSG00000130962	UAUAAAG
ENSG00000130962	UCACAGU
ENSG00000130962	UGCAGCU
ENSG00000130985	ACAAGGU
ENSG00000130988	CAAGAGC
ENSG00000130997	ACUCCAG
ENSG00000130997	GCCUGGG
ENSG00000131002	ACCACAG
ENSG00000131002	AGUAGUU
ENSG00000131002	GGUAGAC
ENSG00000131002	GUGCAAA
ENSG00000131013	AGCAAAA
ENSG00000131016	ACAAUAU
ENSG00000131016	AUACAAG
ENSG00000131016	GAUUGUC
ENSG00000131018	AAACUGU
ENSG00000131018	AGCAAAA
ENSG00000131043	ACCAGCA
ENSG00000131043	CAGCAGG
ENSG00000131043	UCUCCAA
ENSG00000131051	AAAGAGC

ENSG00000131051	ACACACU
ENSG00000131051	AGUGGUU
ENSG00000131051	GUCUUGC
ENSG00000131051	UAUAUGA
ENSG00000131061	UCACACA
ENSG00000131067	CCCUGAG
ENSG00000131067	GAGCUGC
ENSG00000131067	GCAUCCC
ENSG00000131067	GGGGAGC
ENSG00000131067	GGGGUCC
ENSG00000131067	UGCAAAG
ENSG00000131069	GACCUGG
ENSG00000131069	UGGCCCU
ENSG00000131089	AAAGAAU
ENSG00000131089	AAUCACU
ENSG00000131089	ACUGCAG
ENSG00000131089	AGCAGCA
ENSG00000131089	AGUGCUU
ENSG00000131089	AUCACUA
ENSG00000131089	CAAAGCA
ENSG00000131089	CAAAUGC
ENSG00000131089	CACUGUU
ENSG00000131089	CCAGCAU
ENSG00000131089	GAAUUGU
ENSG00000131089	GAGUUGA
ENSG00000131089	GAUUGUC
ENSG00000131089	GUCCAGA
ENSG00000131089	GUGUCAC
ENSG00000131089	UCCAGUU
ENSG00000131089	UUUCAAC
ENSG00000131089	UUUUUGC
ENSG00000131094	CUUUGGU
ENSG00000131094	UUGGUCC
ENSG00000131095	CUCCAGC
ENSG00000131095	UCCCUCU
ENSG00000131116	UCACACA
ENSG00000131148	AAAAAUC
ENSG00000131148	AAAGUAU
ENSG00000131148	AAGGCAC
ENSG00000131148	CACAGGG
ENSG00000131148	GUAUUCU
ENSG00000131148	UAAUAGU
ENSG00000131148	UAUAAAG
ENSG00000131149	AAAAAUC
ENSG00000131149	AAACUGU
ENSG00000131149	AAAGUAC
ENSG00000131149	AAAGUAU
ENSG00000131149	AACUGGU
ENSG00000131149	AAUACUG
ENSG00000131149	AAUCCUU
ENSG00000131149	ACAAGGU
ENSG00000131149	ACAAUAU
ENSG00000131149	ACAGUAC
ENSG00000131149	ACCAGCA
ENSG00000131149	ACUGGCC
ENSG00000131149	ACUGGCU
ENSG00000131149	AGAUAAA
ENSG00000131149	AGCAGCA
ENSG00000131149	AGCCCUU
ENSG00000131149	AGUGCAA
ENSG00000131149	AUAAUAC
ENSG00000131149	AUCCUUU
ENSG00000131149	AUGGCAC
ENSG00000131149	AUGGCUU

ENSG00000131149	CAAAGCA
ENSG00000131149	CCUUCAU
ENSG00000131149	CGGCCUG
ENSG00000131149	CUAUACA
ENSG00000131149	CUCAAAC
ENSG00000131149	CUGCCCC
ENSG00000131149	GAUUGUA
ENSG00000131149	GAUUGUC
ENSG00000131149	GCCUGGA
ENSG00000131149	GGAAGAC
ENSG00000131149	GGCAGGG
ENSG00000131149	UAAUAUC
ENSG00000131149	UACAAAG
ENSG00000131149	UCACAGG
ENSG00000131149	UCCUGAG
ENSG00000131149	UCUGGAA
ENSG00000131149	UGCCAGC
ENSG00000131149	UUAGGAU
ENSG00000131153	AGAUGUG
ENSG00000131171	UAUAAAG
ENSG00000131171	UGAAAGG
ENSG00000131171	UGAAGCU
ENSG00000131188	ACACACC
ENSG00000131188	CACACCU
ENSG00000131188	GUAACAG
ENSG00000131188	UCACACA
ENSG00000131196	AGCAAAA
ENSG00000131196	CAGCAAA
ENSG00000131196	CUACAAA
ENSG00000131196	GAAAACA
ENSG00000131196	UACAAAG
ENSG00000131196	UAUUGCU
ENSG00000131196	UGUGCUU
ENSG00000131196	UUGGUUC
ENSG00000131236	AGGCAGG
ENSG00000131236	CACAGGG
ENSG00000131236	CCAGUGC
ENSG00000131236	CUUUGGU
ENSG00000131236	GAGGUAG
ENSG00000131236	GGAAUGU
ENSG00000131236	UAGAGGA
ENSG00000131236	UCACAGG
ENSG00000131236	UGCAUAG
ENSG00000131236	UUGGUCC
ENSG00000131238	AACUACU
ENSG00000131238	UCAACAG
ENSG00000131242	AAAAAUC
ENSG00000131242	AAAGUGC
ENSG00000131242	AAGGCAC
ENSG00000131242	AAGUGCA
ENSG00000131242	AAUCCUU
ENSG00000131242	ACAGUAU
ENSG00000131242	ACCAGCA
ENSG00000131242	AUGGCAC
ENSG00000131242	CAAAGUG
ENSG00000131242	CAGCAAA
ENSG00000131242	CCUGUAC
ENSG00000131242	CUCUUCC
ENSG00000131242	CUGCAGC
ENSG00000131242	CUGCAGG
ENSG00000131242	CUUCUCU
ENSG00000131242	CUUUGGU
ENSG00000131242	GAGGUAG
ENSG00000131242	GAGUGUG

ENSG00000131242	GCAGCAU
ENSG00000131242	GGAAGAC
ENSG00000131242	GGCAGUG
ENSG00000131242	UACAAAG
ENSG00000131242	UACAGUU
ENSG00000131242	UCCAGAG
ENSG00000131242	UCUCAAG
ENSG00000131242	UGAAAGG
ENSG00000131242	UGCAAAG
ENSG00000131242	UUGGUUC
ENSG00000131323	ACAAUAU
ENSG00000131368	AAAAGUA
ENSG00000131368	AAACUGU
ENSG00000131368	AAAGCUG
ENSG00000131368	AAGUGCU
ENSG00000131368	AAUACUG
ENSG00000131368	ACAAUAU
ENSG00000131368	AGUGCUG
ENSG00000131368	AUUGCAC
ENSG00000131368	CAGUCUG
ENSG00000131368	CAGUGCA
ENSG00000131368	CUUGGGC
ENSG00000131368	GAGUAUU
ENSG00000131368	GCUGGAU
ENSG00000131368	GGCAUUG
ENSG00000131368	UAAUACA
ENSG00000131368	UGGGACA
ENSG00000131368	UGUAUAA
ENSG00000131368	UUCUUGU
ENSG00000131370	AAAGUGC
ENSG00000131370	AAGACAU
ENSG00000131370	UAUUACC
ENSG00000131370	UGUAUAA
ENSG00000131370	UUGGCAA
ENSG00000131374	AUGGCUU
ENSG00000131374	CAGUGCA
ENSG00000131374	GGAAUGU
ENSG00000131375	AGCACCA
ENSG00000131375	AUGUAAU
ENSG00000131375	AUUCAUU
ENSG00000131375	CUGGGCA
ENSG00000131375	UCACAGU
ENSG00000131378	CAAAGCA
ENSG00000131378	UAUUGCU
ENSG00000131378	UGCCCUG
ENSG00000131378	UUCUGCA
ENSG00000131378	UUGGCAC
ENSG00000131381	AAAGAGG
ENSG00000131381	AAAGUGC
ENSG00000131381	ACAAGGU
ENSG00000131381	ACUGCAG
ENSG00000131381	ACUGUAG
ENSG00000131381	AGCACCA
ENSG00000131381	AGUGCAA
ENSG00000131381	AUAAAGU
ENSG00000131381	AUACCUC
ENSG00000131381	CUGACUC
ENSG00000131381	GGUUUAC
ENSG00000131381	GUAGUGU
ENSG00000131381	GUGCCUG
ENSG00000131381	UCCCUUG
ENSG00000131381	UCCCUUU
ENSG00000131386	GAGCUGC
ENSG00000131386	GAGGUAG

ENSG00000131389	AAAAACC
ENSG00000131389	AAAGCUG
ENSG00000131389	AACCUGG
ENSG00000131389	AAGGUGC
ENSG00000131389	AAUACUG
ENSG00000131389	AAUCUCU
ENSG00000131389	AAUGUUG
ENSG00000131389	AAUUGCU
ENSG00000131389	ACAGGUG
ENSG00000131389	ACAUAGA
ENSG00000131389	ACUGCAG
ENSG00000131389	ACUGCAU
ENSG00000131389	AGCAAAA
ENSG00000131389	AGUGGUU
ENSG00000131389	AUAAAGU
ENSG00000131389	AUCAUAC
ENSG00000131389	AUGGCAC
ENSG00000131389	CACAGUG
ENSG00000131389	CAGCAGG
ENSG00000131389	CUGGAAA
ENSG00000131389	CUUGUAU
ENSG00000131389	CUUUGGU
ENSG00000131389	GCCUGGG
ENSG00000131389	GGAGGCA
ENSG00000131389	GGUCUAG
ENSG00000131389	GUAAACA
ENSG00000131389	GUGACAG
ENSG00000131389	UAUUGCU
ENSG00000131389	UCAAGUA
ENSG00000131389	UCACAGU
ENSG00000131389	UCUGGAA
ENSG00000131389	UGAGAAU
ENSG00000131389	UGUAUAA
ENSG00000131389	UGUCUUU
ENSG00000131389	UGUGCUU
ENSG00000131389	UUCAAAU
ENSG00000131389	UUGGCAA
ENSG00000131389	UUGGCAC
ENSG00000131389	UUGGUCC
ENSG00000131398	AACCUGG
ENSG00000131408	AAGGUGC
ENSG00000131408	GGAAGAC
ENSG00000131437	AAAGAAU
ENSG00000131437	AAGGCAC
ENSG00000131437	CUACAGU
ENSG00000131437	GUAAACA
ENSG00000131437	UCACAGG
ENSG00000131437	UCAUUUG
ENSG00000131437	UGAGAAU
ENSG00000131446	AGUGGUU
ENSG00000131446	CACAGUG
ENSG00000131446	CUUUGGU
ENSG00000131446	UUGGUUC
ENSG00000131459	AAAACUG
ENSG00000131459	AAGGCAC
ENSG00000131459	AGCAAAA
ENSG00000131459	CACAGUG
ENSG00000131459	CAGUAGU
ENSG00000131459	CAGUUAC
ENSG00000131459	CUAUACA
ENSG00000131459	UAUAAAG
ENSG00000131459	UCACAGG
ENSG00000131459	UGCAUAU
ENSG00000131459	UGGGUAC

ENSG00000131462	AGUGCUU
ENSG00000131467	AAAAACC
ENSG00000131467	AAAGUAC
ENSG00000131467	AAUUUUA
ENSG00000131467	AGCAGCA
ENSG00000131467	AGCCAGU
ENSG00000131467	CCCCUGG
ENSG00000131467	CUCCAGC
ENSG00000131467	GAGAAAU
ENSG00000131467	GCAUCCC
ENSG00000131467	GGCCCUG
ENSG00000131467	GUAAACA
ENSG00000131467	GUAACAG
ENSG00000131467	UAGACUG
ENSG00000131467	UCCUGAG
ENSG00000131467	UCUUGAG
ENSG00000131467	UGACAUC
ENSG00000131467	UGGCCCU
ENSG00000131470	UCCAGAG
ENSG00000131471	GAGCUGC
ENSG00000131473	UCACAGG
ENSG00000131473	UCACAGU
ENSG00000131475	AGCACCA
ENSG00000131475	AGCAGCU
ENSG00000131475	CACCAGC
ENSG00000131475	CUGCAGC
ENSG00000131475	GACCCUG
ENSG00000131475	GGUUUAC
ENSG00000131475	UGAGUCU
ENSG00000131482	AAAAGUA
ENSG00000131482	AAAGUAA
ENSG00000131482	AAUACUG
ENSG00000131482	ACUGCAU
ENSG00000131482	AGCACCA
ENSG00000131482	AGUAACA
ENSG00000131482	GGAGUGU
ENSG00000131503	AAAAAUC
ENSG00000131503	AAAUCCC
ENSG00000131503	GCUACAU
ENSG00000131504	AACAGUC
ENSG00000131504	AAGGCAC
ENSG00000131504	AGCAGCA
ENSG00000131504	AUGCACC
ENSG00000131504	CAGCAAA
ENSG00000131504	CAGCAGG
ENSG00000131504	CCAGGAG
ENSG00000131504	CUGCAGG
ENSG00000131504	CUUUGGU
ENSG00000131504	GGCUCAG
ENSG00000131504	UCCUUGC
ENSG00000131504	UGAAGCU
ENSG00000131504	UGCAAAG
ENSG00000131504	UUUGCAA
ENSG00000131507	AAGGCAC
ENSG00000131507	AAUGUUG
ENSG00000131507	AAUUUUA
ENSG00000131507	AUAUAAC
ENSG00000131507	CAAGAGC
ENSG00000131507	CCAGCAU
ENSG00000131507	CUGCAGG
ENSG00000131507	CUGCCCC
ENSG00000131507	CUUACAG
ENSG00000131507	GAAAACA
ENSG00000131507	GCAGGAC

ENSG00000131507	GCAGGUU
ENSG00000131507	GCUACAU
ENSG00000131507	GUUAGGA
ENSG00000131507	UGAGAAG
ENSG00000131507	UGAGAAU
ENSG00000131507	UGCCAGC
ENSG00000131508	ACUGUAG
ENSG00000131508	AGGUUAU
ENSG00000131508	AUGCAUU
ENSG00000131508	AUGUAAU
ENSG00000131508	GUAAACA
ENSG00000131508	UCAUAGA
ENSG00000131508	UGACUGU
ENSG00000131558	AAGUUGU
ENSG00000131558	CUCAAAC
ENSG00000131558	UCUCAAG
ENSG00000131558	UGAGAAG
ENSG00000131626	AAAAACC
ENSG00000131626	AAAAAUC
ENSG00000131626	AAAGUAC
ENSG00000131626	ACAAUAU
ENSG00000131626	ACAGUAU
ENSG00000131626	ACUGUGU
ENSG00000131626	AGUGUGC
ENSG00000131626	AUACAAG
ENSG00000131626	GGCAGUG
ENSG00000131626	GGGUAAG
ENSG00000131626	GUAGUGU
ENSG00000131653	AAAAACC
ENSG00000131653	AAAGAAU
ENSG00000131653	AAAGCUG
ENSG00000131653	AAAGGUA
ENSG00000131653	AACAAAC
ENSG00000131653	ACCUGGA
ENSG00000131653	AUGAGCU
ENSG00000131653	CAGUCUG
ENSG00000131653	CGUACCG
ENSG00000131653	CUGCUCA
ENSG00000131653	GAGCUGC
ENSG00000131653	GAGUAUU
ENSG00000131653	GUGCCUG
ENSG00000131668	AAGUCAC
ENSG00000131668	GUGCCUG
ENSG00000131668	GUGUCAC
ENSG00000131669	CUGGGCA
ENSG00000131669	GGCCCUG
ENSG00000131686	AAAUUUC
ENSG00000131711	AAGGCAC
ENSG00000131711	ACAUCAC
ENSG00000131711	AGUGCAA
ENSG00000131711	AUAAUAC
ENSG00000131711	AUCAUAC
ENSG00000131711	CAGUGCA
ENSG00000131711	CUGCAGG
ENSG00000131711	CUUUGGU
ENSG00000131711	GCUGUCU
ENSG00000131711	GGGUGUU
ENSG00000131711	GUGCAAA
ENSG00000131711	UACAGUU
ENSG00000131711	UCAAGUA
ENSG00000131711	UCACAGG
ENSG00000131724	AUGAGCU
ENSG00000131724	GGCUCUG
ENSG00000131725	CCAGUGC

ENSG00000131725	UGAGAAG
ENSG00000131725	UGAGAAU
ENSG00000131725	UGGUACC
ENSG00000131725	UGUGCUU
ENSG00000131737	GAGAAAU
ENSG00000131737	UCCUUGC
ENSG00000131738	AAAGUUU
ENSG00000131738	UUUGCAA
ENSG00000131746	UCUUGAG
ENSG00000131746	UGGCCAC
ENSG00000131747	ACAGUAU
ENSG00000131748	GACCCUG
ENSG00000131748	GAGGCAG
ENSG00000131748	GGCUCUG
ENSG00000131748	GUAAACA
ENSG00000131748	UACAAAG
ENSG00000131759	AAAAACC
ENSG00000131759	AGGCAGG
ENSG00000131759	AUGGCUU
ENSG00000131759	CACAGUG
ENSG00000131759	CAGUAGA
ENSG00000131759	CCAGUAC
ENSG00000131759	CCCGUCC
ENSG00000131759	CCUUCAU
ENSG00000131759	GUAACAG
ENSG00000131759	GUGCUUG
ENSG00000131759	UCACAGG
ENSG00000131771	GCCCUGU
ENSG00000131771	GGCAGGG
ENSG00000131771	UGCCCUG
ENSG00000131773	AAAGUUU
ENSG00000131773	ACAGUAU
ENSG00000131773	CAGACAG
ENSG00000131773	GUUCAUG
ENSG00000131773	UACAAAG
ENSG00000131773	UCACAAG
ENSG00000131773	UGGAGAU
ENSG00000131779	AAAAGUA
ENSG00000131788	AACAUUC
ENSG00000131788	AAGGUGC
ENSG00000131788	ACAUUCA
ENSG00000131788	AUAUAAC
ENSG00000131788	CUUUGGU
ENSG00000131788	GGAAUGU
ENSG00000131788	UCACAAG
ENSG00000131791	AAAUCCC
ENSG00000131791	AGCAGCU
ENSG00000131791	AGUGCUG
ENSG00000131791	AUUGCAC
ENSG00000131791	CUGCAGC
ENSG00000131791	GAGAACC
ENSG00000131791	GAGGUAG
ENSG00000131791	GCAGAAG
ENSG00000131791	GCAGCAU
ENSG00000131791	GGCUCUG
ENSG00000131791	GUCCAGA
ENSG00000131791	UGCAUAG
ENSG00000131791	UGCAUAU
ENSG00000131795	ACUCAAA
ENSG00000131795	ACUGGCC
ENSG00000131795	CCUUCUG
ENSG00000131795	GCUGGAU
ENSG00000131795	GGCAAGA
ENSG00000131795	GUGUCAC

ENSG00000131795	UAUUACC
ENSG00000131795	UGCUGAC
ENSG00000131795	UGGCUGC
ENSG00000131795	UGUGUCA
ENSG00000131828	ACUGCAU
ENSG00000131845	UGGCCAC
ENSG00000131873	AAAGUAC
ENSG00000131873	ACUGCAG
ENSG00000131873	ACUGCAU
ENSG00000131873	AGCACCA
ENSG00000131873	AUGGCUU
ENSG00000131873	CCAGCAU
ENSG00000131873	CUUUGGU
ENSG00000131873	UCAAGUA
ENSG00000131873	UGGAGAU
ENSG00000131873	UUCAAGC
ENSG00000131873	UUGGAGG
ENSG00000131873	UUGUUCG
ENSG00000131876	UACAAAG
ENSG00000131876	UUCAAAU
ENSG00000131899	AACAAAC
ENSG00000131899	ACACACC
ENSG00000131899	CUACAGU
ENSG00000131899	UAAUAUC
ENSG00000131899	UGCAAAG
ENSG00000131899	UUUCAAC
ENSG00000131910	UACAAAG
ENSG00000131910	UAUUACC
ENSG00000131941	AACACUG
ENSG00000131941	ACAGUAU
ENSG00000131941	AUAUUAU
ENSG00000131941	GAGUAUU
ENSG00000131941	UUGGCAA
ENSG00000131966	AGGUAGU
ENSG00000131966	GAGGUAG
ENSG00000131966	UGAAAUG
ENSG00000131969	AAGUCUU
ENSG00000131979	CUGCCCC
ENSG00000132002	AACAUUC
ENSG00000132002	AAUCCUU
ENSG00000132002	ACAUUCA
ENSG00000132002	AUCCCUU
ENSG00000132002	AUCCUUG
ENSG00000132002	CAGUCCA
ENSG00000132002	CAGUCUG
ENSG00000132002	CUUUGGU
ENSG00000132002	UAUUGCU
ENSG00000132002	UCCCUUU
ENSG00000132002	UGAAAGG
ENSG00000132002	UUGGUCC
ENSG00000132003	AUGGCUU
ENSG00000132003	CCAGUGC
ENSG00000132003	CCAGUGU
ENSG00000132003	CCCUGAG
ENSG00000132003	GAGGUAU
ENSG00000132003	GAGUUGA
ENSG00000132003	GAGUUGG
ENSG00000132003	GGGGUGC
ENSG00000132003	UAAUAUC
ENSG00000132003	UACAAAG
ENSG00000132003	UCCUGAG
ENSG00000132003	UGAAAUG
ENSG00000132004	AGCACCA
ENSG00000132005	AAAAACC

ENSG00000132005	AAAGGUA
ENSG00000132005	AACACUG
ENSG00000132005	AAGGCAC
ENSG00000132005	AAUUGCU
ENSG00000132005	ACAAUAU
ENSG00000132005	AUACAAG
ENSG00000132005	AUUGCAC
ENSG00000132005	CCAUUAC
ENSG00000132005	CUACAAA
ENSG00000132005	CUGGGCA
ENSG00000132005	GUGCAAA
ENSG00000132005	UCACAGG
ENSG00000132005	UCACCAC
ENSG00000132005	UGCAAAG
ENSG00000132005	UGCAGCU
ENSG00000132005	UGUGUCA
ENSG00000132024	AAGUGCU
ENSG00000132024	AGUGCUU
ENSG00000132024	CAGCUGG
ENSG00000132024	CUUUGGU
ENSG00000132024	GAUUGUA
ENSG00000132024	GUGCAAA
ENSG00000132024	GUGCUUG
ENSG00000132031	AAUUAUU
ENSG00000132031	CACAGUG
ENSG00000132031	GGCUCAG
ENSG00000132031	UCACAGG
ENSG00000132109	GAGGCAG
ENSG00000132128	CAGGAAC
ENSG00000132128	CUGGGCA
ENSG00000132128	GCCUGGG
ENSG00000132128	GGCAGGG
ENSG00000132128	UGAAAGG
ENSG00000132128	UUGGUUC
ENSG00000132142	AAUACUG
ENSG00000132142	AGCACCA
ENSG00000132142	AGCAGCA
ENSG00000132142	AGUGCUG
ENSG00000132142	AGUGGUU
ENSG00000132142	GCACCAU
ENSG00000132142	GGAAGCC
ENSG00000132142	GGCAGGG
ENSG00000132142	GGGGCAG
ENSG00000132142	UCAACAG
ENSG00000132142	UGUUGAA
ENSG00000132142	UUCCUCG
ENSG00000132142	UUGUGAC
ENSG00000132155	AAAAACC
ENSG00000132155	AGCAGCA
ENSG00000132155	AGCAGCG
ENSG00000132155	AGCAGCU
ENSG00000132155	GGGGCAG
ENSG00000132155	UCCUUGC
ENSG00000132182	AAGGCAC
ENSG00000132182	AGCAGCA
ENSG00000132182	CACAGUG
ENSG00000132182	CCAGUGU
ENSG00000132182	GGCUCAG
ENSG00000132182	UCAAGUA
ENSG00000132182	UCACAGG
ENSG00000132182	UCACAGU
ENSG00000132182	UCCCUGU
ENSG00000132205	AAAGCUG
ENSG00000132205	AUAUUAU

ENSG00000132205	CCCUGUU
ENSG00000132205	UCCCUGU
ENSG00000132205	UGUCUUU
ENSG00000132205	UUUGCAC
ENSG00000132254	AAAACUG
ENSG00000132254	AACUGGU
ENSG00000132254	AGCUGCC
ENSG00000132254	AGUGCUG
ENSG00000132254	AGUGCUU
ENSG00000132254	AGUGGUU
ENSG00000132254	CUUCUCU
ENSG00000132254	CUUUGGU
ENSG00000132254	GAAGGAA
ENSG00000132254	GAGCUGC
ENSG00000132254	GGGUGGU
ENSG00000132254	UGAAAGG
ENSG00000132254	UGACCUA
ENSG00000132254	UGGCCAC
ENSG00000132254	UGGCUGC
ENSG00000132254	UUCCUAG
ENSG00000132254	UUGGUCC
ENSG00000132256	UGUUGAA
ENSG00000132286	CCUUCUU
ENSG00000132294	GGAUGAG
ENSG00000132294	GUGCAAA
ENSG00000132294	UCUCAAG
ENSG00000132294	UGACAUC
ENSG00000132305	UCACAAG
ENSG00000132313	UCCCUUU
ENSG00000132321	AUAUAAC
ENSG00000132326	AAAAAUC
ENSG00000132326	AAAGCUG
ENSG00000132326	AACAAAC
ENSG00000132326	AACAUUC
ENSG00000132329	AAUCACU
ENSG00000132334	AAAAACC
ENSG00000132334	AAAAAUC
ENSG00000132334	AAAGUAU
ENSG00000132334	AACAUUC
ENSG00000132334	AAGGCAC
ENSG00000132334	ACAUUCA
ENSG00000132334	AGGCAGG
ENSG00000132334	AUAAAGU
ENSG00000132334	AUGUAAU
ENSG00000132334	CAAAUGC
ENSG00000132334	CAGCAAA
ENSG00000132334	CCAGUGU
ENSG00000132334	GAAACAU
ENSG00000132334	GACCAUG
ENSG00000132334	GAUCAGA
ENSG00000132334	GCUGGUG
ENSG00000132334	GGCAUUG
ENSG00000132334	GUCAACA
ENSG00000132334	UGCAUAG
ENSG00000132334	UUCUAAU
ENSG00000132334	UUGGCAA
ENSG00000132334	UUGGCAC
ENSG00000132341	AAAACUG
ENSG00000132341	ACAUCAC
ENSG00000132341	ACUGCAG
ENSG00000132341	AGUAACA
ENSG00000132341	CACAGGG
ENSG00000132341	CACCACC
ENSG00000132341	CACUCCU

ENSG00000132341	CCUUCAU
ENSG00000132341	CUCCAGC
ENSG00000132341	CUGCAGC
ENSG00000132341	GCAGCAU
ENSG00000132341	GCAGGUU
ENSG00000132341	GCAUCCC
ENSG00000132341	UCACAGG
ENSG00000132341	UGAAAUG
ENSG00000132341	UGACAUC
ENSG00000132356	AAAAACC
ENSG00000132356	CAGUGCA
ENSG00000132356	GCAACUU
ENSG00000132356	GCCCUGU
ENSG00000132356	GGAAGCC
ENSG00000132361	AAAAACC
ENSG00000132361	AAAGUGC
ENSG00000132361	ACAUUCA
ENSG00000132361	AGCAGCA
ENSG00000132361	AUUCAUU
ENSG00000132361	CUGCCCC
ENSG00000132361	GAAACAU
ENSG00000132361	GACCUGG
ENSG00000132361	GAGAAAU
ENSG00000132361	GCAGGUU
ENSG00000132361	GGUUUAC
ENSG00000132361	GUGGGGA
ENSG00000132361	UGAAAUG
ENSG00000132361	UUCAAAU
ENSG00000132382	CUUGUAU
ENSG00000132383	CAGUCUG
ENSG00000132383	GAAGGAA
ENSG00000132383	UAUCAGA
ENSG00000132388	AAAUGGU
ENSG00000132388	AACUGGU
ENSG00000132388	AAGGCAC
ENSG00000132388	AAGGUGC
ENSG00000132388	AAUUAUU
ENSG00000132388	ACAGGUG
ENSG00000132388	ACAUCAC
ENSG00000132388	AGCCAGU
ENSG00000132388	AGCUGCC
ENSG00000132388	AGUGAUC
ENSG00000132388	AUUGCAC
ENSG00000132388	CAAGAGC
ENSG00000132388	CCAGCAU
ENSG00000132388	CCAGUGU
ENSG00000132388	CCUUCAU
ENSG00000132388	CUUCUCU
ENSG00000132388	GAAGGUC
ENSG00000132388	GAGAAGA
ENSG00000132388	GAGACCU
ENSG00000132388	GCAGGAC
ENSG00000132388	GGAAGAC
ENSG00000132388	GGAGUGU
ENSG00000132388	GGCAGUG
ENSG00000132388	GUAAACA
ENSG00000132388	GUGACAG
ENSG00000132388	GUGUCAC
ENSG00000132388	GUUAGGA
ENSG00000132388	UCCUGAG
ENSG00000132388	UCUCAAG
ENSG00000132388	UCUCCAA
ENSG00000132388	UGCAGCU
ENSG00000132388	UGCAUAG

ENSG00000132388	UGCAUAU
ENSG00000132388	UGGUACC
ENSG00000132388	UGUGCUU
ENSG00000132394	CUGGGCA
ENSG00000132394	GAGGCUG
ENSG00000132405	AAAACUG
ENSG00000132405	AAAGUAC
ENSG00000132405	AAUUUUA
ENSG00000132405	ACAUUCA
ENSG00000132405	ACUCCAG
ENSG00000132405	CCAGUGC
ENSG00000132405	UGGAUAA
ENSG00000132406	AACAAAC
ENSG00000132406	AAGGCAC
ENSG00000132424	ACAUCAC
ENSG00000132424	ACAUUAC
ENSG00000132424	AGCCCUU
ENSG00000132424	CAUGGAU
ENSG00000132424	GCCCUUA
ENSG00000132434	AACAAAC
ENSG00000132434	AAGAACC
ENSG00000132434	GAAACAU
ENSG00000132434	GUGUCAC
ENSG00000132434	UGUCUUU
ENSG00000132434	UGUGUCA
ENSG00000132434	UUUGCAC
ENSG00000132463	AAAGUUU
ENSG00000132463	AAGGCAC
ENSG00000132463	ACAUUAC
ENSG00000132463	CAGGAAC
ENSG00000132463	CAGUCCA
ENSG00000132463	CCCUGAG
ENSG00000132463	CUUUGGU
ENSG00000132463	GACAACU
ENSG00000132463	GAGAACC
ENSG00000132463	GAGAACU
ENSG00000132463	GGCAGUG
ENSG00000132463	GGUUUAC
ENSG00000132463	UAAUAUC
ENSG00000132463	UAGGUAU
ENSG00000132463	UCAUUUG
ENSG00000132463	UGAAAUG
ENSG00000132463	UGAAGCU
ENSG00000132463	UGGACUG
ENSG00000132463	UUCCUAG
ENSG00000132463	UUGGUUC
ENSG00000132464	UUUGCAC
ENSG00000132465	GGAGAGA
ENSG00000132466	AAAACUG
ENSG00000132466	ACACACC
ENSG00000132466	ACAGUAU
ENSG00000132466	AUGUAAU
ENSG00000132466	CUAUACA
ENSG00000132466	CUCACAC
ENSG00000132466	GAAGGUC
ENSG00000132466	GUCAGUU
ENSG00000132466	UACAGUU
ENSG00000132466	UCACAUU
ENSG00000132466	UGCUCAC
ENSG00000132470	AAGUGCU
ENSG00000132470	GGUUACC
ENSG00000132470	UUGGUUC
ENSG00000132471	AACUGGU
ENSG00000132471	AUAUUAU

ENSG00000132471	GGAAUGU
ENSG00000132471	GGCAGGG
ENSG00000132471	GGGUUUA
ENSG00000132471	GUGCAAA
ENSG00000132471	UAAUAUC
ENSG00000132475	AAAAACC
ENSG00000132475	AAAACUG
ENSG00000132475	AAACUGU
ENSG00000132475	AAAGCUG
ENSG00000132475	AAAGUAU
ENSG00000132475	AAAUCCC
ENSG00000132475	AAAUUUC
ENSG00000132475	AACAGUC
ENSG00000132475	ACACACC
ENSG00000132475	ACACACU
ENSG00000132475	ACAUCAC
ENSG00000132475	ACCCUGU
ENSG00000132475	AGCCCUU
ENSG00000132475	AUAUAAC
ENSG00000132475	AUCCCUU
ENSG00000132475	AUGAUGC
ENSG00000132475	AUUGCAC
ENSG00000132475	CACACCU
ENSG00000132475	CACUCCU
ENSG00000132475	CCUGUAC
ENSG00000132475	CCUUCUG
ENSG00000132475	CUACAAA
ENSG00000132475	CUAGUAA
ENSG00000132475	CUAGUAG
ENSG00000132475	CUGGACU
ENSG00000132475	CUUACAG
ENSG00000132475	CUUGUAU
ENSG00000132475	GAAACAU
ENSG00000132475	GACCCUG
ENSG00000132475	GAGAAGA
ENSG00000132475	GAGCCCU
ENSG00000132475	GCCCUUA
ENSG00000132475	GGAAUGU
ENSG00000132475	GGUAGAC
ENSG00000132475	GGUUUAC
ENSG00000132475	GUCAACA
ENSG00000132475	UAAGACG
ENSG00000132475	UAAUAGU
ENSG00000132475	UACAAAG
ENSG00000132475	UAUAAUA
ENSG00000132475	UAUUGCU
ENSG00000132475	UCACAGU
ENSG00000132475	UGAGAAG
ENSG00000132475	UGAGGAC
ENSG00000132475	UGCAAAG
ENSG00000132475	UGCAUAU
ENSG00000132475	UGCUCAC
ENSG00000132475	UUAUGCA
ENSG00000132478	AAGACAU
ENSG00000132478	AAGUGUG
ENSG00000132478	ACACACC
ENSG00000132478	ACACACU
ENSG00000132478	AGCACCA
ENSG00000132478	AGCCCUU
ENSG00000132478	AGCUGCC
ENSG00000132478	AGUGCUU
ENSG00000132478	GAGCCCC
ENSG00000132478	GAGCCCU
ENSG00000132478	GCUAGCA

ENSG00000132478	GGAAGCC
ENSG00000132478	GUGCUUG
ENSG00000132478	UCGUGCA
ENSG00000132478	UUGGUCC
ENSG00000132485	AAGGCAC
ENSG00000132485	AUGCACC
ENSG00000132485	CUUGUGU
ENSG00000132485	GGCCCUG
ENSG00000132485	GUCUACU
ENSG00000132485	GUGCAAA
ENSG00000132485	UAAUACA
ENSG00000132485	UGGCCCU
ENSG00000132485	UUCUUGU
ENSG00000132507	AACAAAC
ENSG00000132507	CUGCAGG
ENSG00000132507	GCCUGAU
ENSG00000132518	GCCUGGG
ENSG00000132518	GGGGUCC
ENSG00000132522	UGGGACA
ENSG00000132535	CAGUCCA
ENSG00000132535	CUCAAAC
ENSG00000132535	GCAGCAU
ENSG00000132535	GGAAUGU
ENSG00000132535	GGAGGCA
ENSG00000132535	GGGGUGC
ENSG00000132535	GUCUCUG
ENSG00000132535	UCAAGUA
ENSG00000132535	UCUGGAA
ENSG00000132535	UGAGAAU
ENSG00000132541	CCAUUAC
ENSG00000132549	AUACAAG
ENSG00000132561	AAGGCAC
ENSG00000132561	AAUUUUA
ENSG00000132563	AAGGCAC
ENSG00000132563	CAGCAAA
ENSG00000132563	CAGCAGG
ENSG00000132563	CCAGUGU
ENSG00000132563	GCAGAAG
ENSG00000132563	GGGUGGU
ENSG00000132589	CAGUGCA
ENSG00000132589	CCCUGAG
ENSG00000132589	GAGAACU
ENSG00000132589	GCAGCAU
ENSG00000132591	AGCAGCU
ENSG00000132591	CUCAGGG
ENSG00000132591	GAGCUAA
ENSG00000132603	UCAUGAU
ENSG00000132603	UUCAAAU
ENSG00000132603	UUCUUGU
ENSG00000132604	ACAGUAC
ENSG00000132604	GUGCCUG
ENSG00000132604	UUCCUAG
ENSG00000132612	CACUCCU
ENSG00000132612	CUCCCAC
ENSG00000132612	GUCUCUG
ENSG00000132612	UAUAAAG
ENSG00000132612	UCCCACC
ENSG00000132622	AGGCAGG
ENSG00000132623	AUUCAUU
ENSG00000132635	AUGCCUU
ENSG00000132635	GUGACAG
ENSG00000132639	AAUCACU
ENSG00000132639	ACAUCAC
ENSG00000132639	AGCAUUC

ENSG00000132639	CUACAAA
ENSG00000132639	GCUGGUG
ENSG00000132639	GGAAUGU
ENSG00000132639	UCAUUCG
ENSG00000132639	UGCAUAG
ENSG00000132640	AAGUUGU
ENSG00000132640	ACAAUAU
ENSG00000132640	AGUGCCU
ENSG00000132640	CACUGUU
ENSG00000132640	CAUAUUG
ENSG00000132640	CCAGUGU
ENSG00000132640	CUCUGGG
ENSG00000132640	GAGGUAG
ENSG00000132640	GAGGUAU
ENSG00000132640	GCUAGCA
ENSG00000132640	GGGUGGU
ENSG00000132640	UAAUGCU
ENSG00000132640	UCAACAG
ENSG00000132640	UCCUGAG
ENSG00000132640	UGCAUUG
ENSG00000132640	UUCAAGC
ENSG00000132670	AACAAAC
ENSG00000132670	UACAAAG
ENSG00000132680	AUGCAUU
ENSG00000132680	UACAAAG
ENSG00000132692	AACAGUC
ENSG00000132692	GAAACAU
ENSG00000132692	UCACAGG
ENSG00000132692	UCACAGU
ENSG00000132692	UCCCUGU
ENSG00000132692	UUCCAUA
ENSG00000132694	AAAGUGC
ENSG00000132694	AAGUGCA
ENSG00000132694	ACAUUAC
ENSG00000132694	CACAGUG
ENSG00000132694	UGAAAGG
ENSG00000132702	AAUGCCC
ENSG00000132702	CAGUUAC
ENSG00000132702	GUUAAUG
ENSG00000132718	AAAGAAU
ENSG00000132718	AAAGCUG
ENSG00000132718	AAGGCGC
ENSG00000132718	AGGGAGG
ENSG00000132718	AUGGAUU
ENSG00000132718	CAUGGAU
ENSG00000132718	CUCAAAC
ENSG00000132718	GACCAUG
ENSG00000132718	GAGGCAG
ENSG00000132718	GAGGUAG
ENSG00000132718	GAGGUAU
ENSG00000132718	GGGUCUU
ENSG00000132718	GUCACUC
ENSG00000132718	GUGACUG
ENSG00000132718	UCAUUUG
ENSG00000132718	UCCAGAG
ENSG00000132718	UCCCACA
ENSG00000132718	UCCUGAG
ENSG00000132718	UGAGAAG
ENSG00000132718	UGGAGAU
ENSG00000132749	AAAGUAU
ENSG00000132749	AAGUGCA
ENSG00000132763	GAGCUGC
ENSG00000132773	UUUUUGC
ENSG00000132780	AAAGAAU

ENSG00000132780	ACAUUAC
ENSG00000132780	GCACCAU
ENSG00000132792	CAAAACU
ENSG00000132793	AAAGCUG
ENSG00000132793	AGGCAGG
ENSG00000132801	AGCAGCA
ENSG00000132819	AAAAAUC
ENSG00000132819	AUAUUAU
ENSG00000132819	CCCUGAG
ENSG00000132819	GAGGUAG
ENSG00000132819	GAGGUAU
ENSG00000132819	UUCUAAU
ENSG00000132821	CAAAACU
ENSG00000132821	CCGAGCC
ENSG00000132821	CCUGCUG
ENSG00000132821	CUGCUCA
ENSG00000132821	GGGUGGU
ENSG00000132821	GUGCAAA
ENSG00000132821	UACAAAG
ENSG00000132821	UGGCUGC
ENSG00000132823	AUUGCAC
ENSG00000132823	CACAGUG
ENSG00000132823	UAUUGCU
ENSG00000132824	AUAUUAU
ENSG00000132825	AAGGCAC
ENSG00000132825	AGCACCA
ENSG00000132825	AUUGCAC
ENSG00000132825	CCAGUGC
ENSG00000132825	UCCUGAG
ENSG00000132825	UGACCUA
ENSG00000132842	GUCCAGA
ENSG00000132842	GUGCCAC
ENSG00000132842	UAUAAAG
ENSG00000132842	UGAAAGG
ENSG00000132849	AAUGUUG
ENSG00000132849	GAAAACA
ENSG00000132849	GUUAAUG
ENSG00000132849	UAAUGCU
ENSG00000132849	UUGUGAC
ENSG00000132854	AAAAGUA
ENSG00000132854	AAAGUAU
ENSG00000132854	AAGGCAC
ENSG00000132854	AUGUGCC
ENSG00000132854	CUACAAA
ENSG00000132854	GAGGCAG
ENSG00000132854	GGAAUGU
ENSG00000132854	GGAGGAG
ENSG00000132854	GGAGUCC
ENSG00000132854	GUAUGUU
ENSG00000132854	UACAAAG
ENSG00000132854	UAUUGCU
ENSG00000132854	UGACAUC
ENSG00000132854	UUCUAAU
ENSG00000132854	UUGACAC
ENSG00000132854	UUGUUCG
ENSG00000132872	AAAACUG
ENSG00000132872	AAAGUAA
ENSG00000132872	AAAGUAU
ENSG00000132872	AGCCCUU
ENSG00000132872	AUGCAUU
ENSG00000132872	CAGUGCA
ENSG00000132872	CUCAAAC
ENSG00000132872	CUUUGGU
ENSG00000132872	UGCAUAG

ENSG00000132874	GCAGAAG
ENSG00000132874	GGGAGCU
ENSG00000132879	UCUCCAA
ENSG00000132879	UGAAAUG
ENSG00000132911	CACAGUG
ENSG00000132912	AAGGCAC
ENSG00000132912	AAGUGCU
ENSG00000132912	ACAUUAC
ENSG00000132932	CAAAGCA
ENSG00000132932	CACACCG
ENSG00000132932	CUCCCAA
ENSG00000132932	CUCCCAC
ENSG00000132932	GGCUGCG
ENSG00000132932	UCACACA
ENSG00000132953	AAUGCCC
ENSG00000132953	ACUGCAU
ENSG00000132953	AGCAGCA
ENSG00000132953	AGCAGCG
ENSG00000132953	AGGACAC
ENSG00000132953	AGUGCAA
ENSG00000132953	CACAAUG
ENSG00000132953	CCUUCAU
ENSG00000132953	GACCAUG
ENSG00000132953	UAAUAUC
ENSG00000132953	UACAGUU
ENSG00000132953	UAGUGAG
ENSG00000132953	UCACACA
ENSG00000132953	UCCCUUU
ENSG00000132953	UGAAAUG
ENSG00000132953	UGACCUA
ENSG00000132953	UUCCUCG
ENSG00000132953	UUGGUCC
ENSG00000132964	AAACUGU
ENSG00000132964	AAGUAGC
ENSG00000132964	ACAUUCA
ENSG00000132964	AUGCAUU
ENSG00000132964	CAGUGCA
ENSG00000132964	CUGCAGC
ENSG00000132964	UCACAGG
ENSG00000132964	UCACAGU
ENSG00000132964	UGCAUUG
ENSG00000132970	AAGACAU
ENSG00000132970	ACAUCAC
ENSG00000132970	AGAUGUG
ENSG00000132970	AUGGAUU
ENSG00000132970	GCAGCAU
ENSG00000132970	GUGCUUG
ENSG00000132970	UCACAAG
ENSG00000132970	UCACAUU
ENSG00000132970	UGUGCUU
ENSG00000133026	AAAAAUC
ENSG00000133026	AAAGCUG
ENSG00000133026	AAAUUUC
ENSG00000133026	AACAUUC
ENSG00000133026	AAGACAU
ENSG00000133026	ACAGUAU
ENSG00000133026	ACAUUAC
ENSG00000133026	ACAUUCA
ENSG00000133026	ACUUGGA
ENSG00000133026	AGCCAGU
ENSG00000133026	AUACAAG
ENSG00000133026	AUAUAAC
ENSG00000133026	CAAAGCA
ENSG00000133026	CACAGUG

ENSG00000133026	CCAGUAC
ENSG00000133026	CUGCCCC
ENSG00000133026	CUUACAG
ENSG00000133026	GAUAUGU
ENSG00000133026	GGUGGUU
ENSG00000133026	GUAAACA
ENSG00000133026	GUCACUC
ENSG00000133026	UAAGACG
ENSG00000133026	UAAGACU
ENSG00000133026	UACAAAG
ENSG00000133026	UCAACAG
ENSG00000133026	UCACAGG
ENSG00000133026	UGUCUGG
ENSG00000133027	AAGGCAC
ENSG00000133030	AAUACUG
ENSG00000133030	AGCUUAU
ENSG00000133030	GUGCAAA
ENSG00000133030	UAAUACA
ENSG00000133048	GGCUCAG
ENSG00000133056	AAAACUG
ENSG00000133056	ACAGUAC
ENSG00000133056	AGGACAC
ENSG00000133056	GCUUCCU
ENSG00000133056	GGCUCUG
ENSG00000133056	GUUAGGA
ENSG00000133056	UCAUUUG
ENSG00000133056	UCCCUUG
ENSG00000133056	UCCUAUA
ENSG00000133056	UUGACAC
ENSG00000133065	AAAGUGC
ENSG00000133065	AAGACAU
ENSG00000133065	AAGUGCA
ENSG00000133065	CUCAAAC
ENSG00000133065	CUGCUCA
ENSG00000133065	GAGGCUG
ENSG00000133065	GAUUGUA
ENSG00000133065	GGAGGCA
ENSG00000133065	GGCUCAG
ENSG00000133065	UAAGACG
ENSG00000133065	UAAGACU
ENSG00000133065	UAUCAGA
ENSG00000133065	UGACAUC
ENSG00000133065	UGUAUAA
ENSG00000133065	UUGGUCC
ENSG00000133069	ACUCCAG
ENSG00000133069	CCCUGAG
ENSG00000133069	GAGGCUG
ENSG00000133069	UCCAGAG
ENSG00000133069	UCCCUGU
ENSG00000133083	AACAGUC
ENSG00000133083	ACUGCAU
ENSG00000133083	AGCAGCG
ENSG00000133083	AGCAGCU
ENSG00000133083	AUGCCUU
ENSG00000133083	CUCAAAC
ENSG00000133083	GAGUUGA
ENSG00000133083	GAUCAGA
ENSG00000133083	GCACCAU
ENSG00000133083	GCAGCAU
ENSG00000133083	GCUACAU
ENSG00000133083	GUGCUUG
ENSG00000133083	UACAAAG
ENSG00000133083	UAUUGCU
ENSG00000133083	UUCAAAU

ENSG00000133083	UUCAAGC
ENSG00000133083	UUCUGCA
ENSG00000133083	UUGAGGC
ENSG00000133083	UUGGUUC
ENSG00000133101	AUAUUAU
ENSG00000133104	AAGUAGC
ENSG00000133104	AGCUUAU
ENSG00000133104	GUGCAAA
ENSG00000133104	UUCAAAU
ENSG00000133104	UUCUAAU
ENSG00000133105	AGCUGCC
ENSG00000133105	AGUGCAA
ENSG00000133107	CUACAAA
ENSG00000133110	AAAGUUU
ENSG00000133111	AAAGUAA
ENSG00000133111	CAAAUGC
ENSG00000133112	CAGUCCA
ENSG00000133116	ACCCUGU
ENSG00000133121	AAAAAUC
ENSG00000133121	ACAAUAU
ENSG00000133121	ACCCUGU
ENSG00000133121	AGCAAAA
ENSG00000133121	AGUGCAA
ENSG00000133121	AUAUUAU
ENSG00000133121	AUUGCAC
ENSG00000133121	CCCUGUC
ENSG00000133121	CUCCAGC
ENSG00000133121	GCAGAAG
ENSG00000133121	UCCCUGU
ENSG00000133121	UGUCUUU
ENSG00000133121	UUGACAC
ENSG00000133121	UUGGUCC
ENSG00000133121	UUGGUUC
ENSG00000133131	AAGGCAC
ENSG00000133134	ACAAUAU
ENSG00000133138	GUGCUUG
ENSG00000133142	UUCUGCA
ENSG00000133193	AAAGAAU
ENSG00000133193	AAUUGCU
ENSG00000133193	AGCAAAA
ENSG00000133193	AGGUAGU
ENSG00000133193	CAAAGCA
ENSG00000133193	CAGCAAA
ENSG00000133193	GAGGUAG
ENSG00000133193	GCAGCAU
ENSG00000133193	GUAAACA
ENSG00000133193	GUGCAAA
ENSG00000133193	UCACAGG
ENSG00000133193	UCACAGU
ENSG00000133193	UGAAAUG
ENSG00000133195	AAUCACU
ENSG00000133195	AGUGCUU
ENSG00000133195	CACAGUG
ENSG00000133195	GUAAACA
ENSG00000133216	AAAAAUC
ENSG00000133216	AAAGGUA
ENSG00000133216	AACAAAC
ENSG00000133216	AGCCCUU
ENSG00000133216	CGCCCUU
ENSG00000133216	CUCCCAA
ENSG00000133216	CUUUGGU
ENSG00000133216	GUAAACA
ENSG00000133216	GUGAAUG
ENSG00000133216	UCACAGU

ENSG00000133216	UCACAUU
ENSG00000133216	UGCAUAU
ENSG00000133226	AAAUUUC
ENSG00000133226	AACAAAC
ENSG00000133226	CUCAAAC
ENSG00000133247	AUGGCUU
ENSG00000133247	CCCUGAG
ENSG00000133247	UCAAGUA
ENSG00000133247	UCCUGAG
ENSG00000133256	AAAAACC
ENSG00000133265	GCCUGGG
ENSG00000133275	AAGUCUU
ENSG00000133275	CUGGACU
ENSG00000133275	CUUUGGU
ENSG00000133275	GCAGCAU
ENSG00000133275	GUUAAUG
ENSG00000133275	UCAUUUG
ENSG00000133316	CAAAGUG
ENSG00000133316	UACAAAG
ENSG00000133318	AAAAGUA
ENSG00000133318	AAAGUAC
ENSG00000133318	ACCACAG
ENSG00000133318	CAGGCUC
ENSG00000133318	CAGUUAC
ENSG00000133318	GGAAGCC
ENSG00000133318	GGAAUGU
ENSG00000133318	GGGGCAG
ENSG00000133318	UAAUACA
ENSG00000133392	AAGGCAC
ENSG00000133392	UAGUGAG
ENSG00000133393	AUCCUUG
ENSG00000133393	CUCUUCC
ENSG00000133393	GAGGUAG
ENSG00000133393	GAGGUAU
ENSG00000133393	GUCUCUG
ENSG00000133401	AACACUG
ENSG00000133401	AAGGCAC
ENSG00000133401	AAGUGUG
ENSG00000133401	AUAUUAU
ENSG00000133401	AUGCACC
ENSG00000133401	AUGCCUU
ENSG00000133401	CAUAUUG
ENSG00000133401	GACUUCC
ENSG00000133401	GGAUUUC
ENSG00000133401	GGAUUUU
ENSG00000133401	UAUUGCU
ENSG00000133401	UCCUGAG
ENSG00000133401	UUGGCAA
ENSG00000133424	AACAGUC
ENSG00000133424	CAGUCUG
ENSG00000133424	CCAGUGC
ENSG00000133424	GGGCCCC
ENSG00000133454	CUGGCUC
ENSG00000133454	GGCUCUG
ENSG00000133454	UCUUGAG
ENSG00000133466	AGCACCA
ENSG00000133466	UCUAGAG
ENSG00000133477	CCCUGAG
ENSG00000133477	CUCUUCC
ENSG00000133477	GUAAACA
ENSG00000133477	GUGCAAA
ENSG00000133488	CUCCAGC
ENSG00000133606	AAGUGCU
ENSG00000133606	ACACACU

ENSG00000133606	ACCACAG
ENSG00000133606	ACCUGGA
ENSG00000133606	AGUGCUU
ENSG00000133606	CAGUAGU
ENSG00000133606	CUUACAG
ENSG00000133606	GAGACCU
ENSG00000133606	GGUUACC
ENSG00000133606	GUGCAAA
ENSG00000133606	UACAAAG
ENSG00000133627	AGGACAC
ENSG00000133627	AUGGCUU
ENSG00000133627	UCAUACA
ENSG00000133636	AAAAAUC
ENSG00000133639	AAAGGUA
ENSG00000133639	ACCACAG
ENSG00000133639	AGCUGCC
ENSG00000133639	CUUCACC
ENSG00000133639	GCUAGCA
ENSG00000133639	UACAAAG
ENSG00000133639	UCACAAG
ENSG00000133639	UCACAGU
ENSG00000133639	UCCAGUU
ENSG00000133639	UGAUCCA
ENSG00000133639	UUCCAUA
ENSG00000133639	UUCUAAU
ENSG00000133657	AAGUGCA
ENSG00000133657	AAUGCCC
ENSG00000133657	ACAUCAC
ENSG00000133657	ACUCAAA
ENSG00000133657	AGUGAUC
ENSG00000133657	AGUGCAA
ENSG00000133657	AUACAAG
ENSG00000133657	AUGGCAC
ENSG00000133657	CCCUGAG
ENSG00000133657	UCACAAG
ENSG00000133657	UCAUAGA
ENSG00000133657	UGCAGCU
ENSG00000133657	UGCAUAG
ENSG00000133657	UUCAAGC
ENSG00000133657	UUGACAC
ENSG00000133687	AAAGUAC
ENSG00000133703	AAAGUAC
ENSG00000133703	AAAUUUC
ENSG00000133703	AAUACUG
ENSG00000133703	AAUCUCU
ENSG00000133703	AAUGUUG
ENSG00000133703	ACUCAAA
ENSG00000133703	ACUGGAU
ENSG00000133703	ACUGGCU
ENSG00000133703	AGCAAAA
ENSG00000133703	AUACAAG
ENSG00000133703	AUGCACC
ENSG00000133703	CUACAAA
ENSG00000133703	CUAUACA
ENSG00000133703	GCACCAU
ENSG00000133703	GGAAUGU
ENSG00000133703	GGCAUUG
ENSG00000133703	GUAAACA
ENSG00000133703	GUCAUUG
ENSG00000133703	GUUAAUG
ENSG00000133703	GUUCAUG
ENSG00000133703	UACAGUU
ENSG00000133703	UAUUACC
ENSG00000133703	UCACAAG

ENSG00000133703	UCAUGAU
ENSG00000133703	UGCAUAU
ENSG00000133703	UUCCUAG
ENSG00000133703	UUGUGAC
ENSG00000133704	AAGGCAC
ENSG00000133704	ACAGUAC
ENSG00000133704	ACAGUAU
ENSG00000133704	ACCCUGU
ENSG00000133704	CACUGUU
ENSG00000133704	GCUGGUG
ENSG00000133704	GUGCUUG
ENSG00000133704	UACAGUU
ENSG00000133704	UCACAUU
ENSG00000133706	UCUCCAA
ENSG00000133731	UGAAAUG
ENSG00000133739	UGGAGAU
ENSG00000133740	AAGGCAC
ENSG00000133740	UUGGCAC
ENSG00000133789	ACAGUAC
ENSG00000133789	ACAGUAU
ENSG00000133789	AGCAGCA
ENSG00000133794	AAAAACC
ENSG00000133794	AUGGCUU
ENSG00000133794	CUUACAG
ENSG00000133794	UGAAAUG
ENSG00000133805	AAAGUGC
ENSG00000133805	AAGGCAC
ENSG00000133805	AGUAACA
ENSG00000133805	GAGGCUG
ENSG00000133812	AACAUUC
ENSG00000133812	AUACAAG
ENSG00000133812	CACAGGG
ENSG00000133812	GGUGGUU
ENSG00000133812	UAUAAAG
ENSG00000133818	AAAGUAU
ENSG00000133818	AUCCCUU
ENSG00000133818	GGAAGAC
ENSG00000133818	UGAGAAU
ENSG00000133872	CAGCAAA
ENSG00000133874	AAAAAUC
ENSG00000133874	AAAUUUC
ENSG00000133874	AGCACCA
ENSG00000133874	CCAGUAC
ENSG00000133874	GAGGCAG
ENSG00000133874	GGAGGCA
ENSG00000133874	GUAAACA
ENSG00000133874	UUAGAGA
ENSG00000133884	ACCAGCA
ENSG00000133884	ACCCUGU
ENSG00000133884	CCCUGAG
ENSG00000133884	GAGGUAG
ENSG00000133884	GUGAAUG
ENSG00000133884	GUGGCCC
ENSG00000133884	UAGGGCC
ENSG00000133895	UUCCUCG
ENSG00000133935	AAAAACC
ENSG00000133935	AAAGUAC
ENSG00000133935	AGCAGCA
ENSG00000133935	AGCCCUU
ENSG00000133935	CAGCAGA
ENSG00000133935	CUCUGGG
ENSG00000133935	CUGGAAA
ENSG00000133935	GAAGGUC
ENSG00000133935	UACAAAG

ENSG00000133935	UAUUACC
ENSG00000133935	UCCCACA
ENSG00000133937	GUGCAAA
ENSG00000133961	AAUACUG
ENSG00000133961	UUGGCAA
ENSG00000133961	UUGGCAC
ENSG00000133985	AAAGUGC
ENSG00000133985	AAGGCAC
ENSG00000133985	AAGUAGC
ENSG00000133985	AAGUGCU
ENSG00000133985	AAUCUCA
ENSG00000133985	AAUCUCU
ENSG00000133985	AGCCAGU
ENSG00000133985	AGCUGCC
ENSG00000133985	AGGCAGG
ENSG00000133985	AUUCAUU
ENSG00000133985	CAAAGCA
ENSG00000133985	CACAGUG
ENSG00000133985	CAGUCUG
ENSG00000133985	CCAGUGU
ENSG00000133985	CCUUCUG
ENSG00000133985	CUAGGCU
ENSG00000133985	CUGCAGC
ENSG00000133985	CUGCAGG
ENSG00000133985	GCUUCCU
ENSG00000133985	GUCAUUG
ENSG00000133985	GUGAAUG
ENSG00000133985	UAUUGCU
ENSG00000133985	UGUAUGU
ENSG00000133985	UGUGUGG
ENSG00000133985	UGUUGAA
ENSG00000133997	GAGGUAG
ENSG00000133997	GAGUCUU
ENSG00000134001	AAAGUGC
ENSG00000134001	AAGGCAC
ENSG00000134001	AAUUUUA
ENSG00000134001	ACUGGAU
ENSG00000134001	CUGGAAA
ENSG00000134001	GAAGGAA
ENSG00000134001	UCCCACA
ENSG00000134001	UCCCACC
ENSG00000134001	UGGACUG
ENSG00000134001	UUAGGAU
ENSG00000134001	UUGGCAA
ENSG00000134001	UUGGCAC
ENSG00000134013	AUGACAC
ENSG00000134013	UCCUGAG
ENSG00000134030	ACAUUCA
ENSG00000134030	AGCAGCA
ENSG00000134030	AGCUGCC
ENSG00000134030	CAAAGCA
ENSG00000134030	CAAAGUG
ENSG00000134030	CUCCCAC
ENSG00000134030	CUGGGGG
ENSG00000134030	GCAGAAG
ENSG00000134030	GGCAAGA
ENSG00000134030	GGCCCUG
ENSG00000134030	GGGCCCC
ENSG00000134030	GGGGCAG
ENSG00000134030	GGGGUCC
ENSG00000134030	GGUUUAC
ENSG00000134030	UCCCACA
ENSG00000134030	UCUGGAG
ENSG00000134030	UGGCCCU

ENSG00000134030	UUCAAGC
ENSG00000134042	AAGUGUG
ENSG00000134042	CCUGCUG
ENSG00000134042	GGAUUUU
ENSG00000134046	AAAAAUC
ENSG00000134046	AAACUGU
ENSG00000134046	ACAUCAC
ENSG00000134046	ACCCUGU
ENSG00000134046	ACUCAAA
ENSG00000134046	AGUGCUG
ENSG00000134046	AGUGCUU
ENSG00000134046	CAGUAGA
ENSG00000134046	CAUAUUG
ENSG00000134046	CUUGGGC
ENSG00000134046	GGAAGAC
ENSG00000134046	GGCAUUG
ENSG00000134046	GUGAAUG
ENSG00000134046	UACAGUU
ENSG00000134056	UGUGCUU
ENSG00000134057	AAUUUUA
ENSG00000134058	CCAGUGU
ENSG00000134058	GUCCAGA
ENSG00000134058	UACAAAG
ENSG00000134070	AGCAGCG
ENSG00000134070	AGCAGCU
ENSG00000134072	UCCCUUG
ENSG00000134075	AACAUUC
ENSG00000134075	AGCAGCA
ENSG00000134075	UUCAAGC
ENSG00000134077	AAGGCAC
ENSG00000134086	ACAUCAC
ENSG00000134108	UCCCUUU
ENSG00000134109	ACAUUAC
ENSG00000134109	CAGUAGA
ENSG00000134109	CAUAUUG
ENSG00000134109	CCCACCU
ENSG00000134109	CUGCAGG
ENSG00000134109	GAGCCCU
ENSG00000134109	UACAAAG
ENSG00000134109	UCUGGAA
ENSG00000134109	UGAAAGG
ENSG00000134109	UGCCCUG
ENSG00000134121	ACCCUGU
ENSG00000134121	AGGGUUG
ENSG00000134121	AUAUUAU
ENSG00000134121	AUGCCUU
ENSG00000134121	CACAGGG
ENSG00000134121	GACCCUG
ENSG00000134121	UCAUGAU
ENSG00000134121	UGUGCUU
ENSG00000134121	UUGGCAA
ENSG00000134138	AAAACUG
ENSG00000134138	AAAAGUA
ENSG00000134138	AAAGCUG
ENSG00000134138	AACAUUC
ENSG00000134138	AAGUCUU
ENSG00000134138	ACAAUAU
ENSG00000134138	ACAUAGA
ENSG00000134138	AGCUGCC
ENSG00000134138	AGUAGUU
ENSG00000134138	AGUGUGC
ENSG00000134138	AUACAAG
ENSG00000134138	CAAAGUG
ENSG00000134138	CACAGUG

ENSG00000134138	CAGCAGA
ENSG00000134138	CAGUAGU
ENSG00000134138	CUGGAAA
ENSG00000134138	GAAACAU
ENSG00000134138	GAGAAAU
ENSG00000134138	GAGGUAG
ENSG00000134138	GAGGUAU
ENSG00000134138	GCUGUCU
ENSG00000134138	GUAAACA
ENSG00000134138	GUAACAG
ENSG00000134138	GUAUUCU
ENSG00000134138	UAAUAGU
ENSG00000134138	UACAGUU
ENSG00000134138	UAGUGAG
ENSG00000134138	UAUAAAG
ENSG00000134138	UCAACAG
ENSG00000134138	UCACAGU
ENSG00000134138	UCAUACA
ENSG00000134138	UCUGGAA
ENSG00000134138	UGUCUGG
ENSG00000134138	UUGGUCC
ENSG00000134138	UUUCAAC
ENSG00000134146	UUCAAAU
ENSG00000134152	AAGGCAC
ENSG00000134152	ACAAUAU
ENSG00000134152	AGCUGCC
ENSG00000134152	AGUAGUU
ENSG00000134152	AGUGCAA
ENSG00000134152	CAGCAAA
ENSG00000134152	GAGGUAG
ENSG00000134152	GUAAACA
ENSG00000134152	UAGAGGA
ENSG00000134152	UCAUAGA
ENSG00000134152	UUCCUAG
ENSG00000134153	UGACCUA
ENSG00000134186	AAGGCAC
ENSG00000134186	UGAUCCA
ENSG00000134198	AGAUGUG
ENSG00000134198	GAGAAAU
ENSG00000134198	GAGGUAG
ENSG00000134198	GAUUGUA
ENSG00000134198	GAUUGUC
ENSG00000134198	GUAAACA
ENSG00000134198	UGUCUGC
ENSG00000134198	UGUCUGG
ENSG00000134198	UUCCAUA
ENSG00000134202	AAAGAGC
ENSG00000134207	AAAGUUU
ENSG00000134207	AACAAAC
ENSG00000134207	AAGACAU
ENSG00000134207	AAGUGCA
ENSG00000134207	ACAUCAC
ENSG00000134207	AUAAAGU
ENSG00000134207	CAAAACU
ENSG00000134207	CACUCCU
ENSG00000134207	GAAACAU
ENSG00000134207	GCAGCAU
ENSG00000134207	GGCAAGA
ENSG00000134207	GGGUAAG
ENSG00000134207	GGUGGUU
ENSG00000134207	GUGAAUG
ENSG00000134207	UCUGGAG
ENSG00000134207	UGCAGCU
ENSG00000134207	UUCAAAU

ENSG00000134207	UUGGAGG
ENSG00000134207	UUUCAAC
ENSG00000134215	AACAUUC
ENSG00000134215	AGCCCUU
ENSG00000134215	CACAGUG
ENSG00000134215	CCCUGAG
ENSG00000134215	GAGGUAG
ENSG00000134215	GAGGUAU
ENSG00000134215	GGCAAGA
ENSG00000134215	GGCAGGG
ENSG00000134215	GUAAACA
ENSG00000134215	GUCAGUU
ENSG00000134215	UAAGACG
ENSG00000134215	UGAAAUG
ENSG00000134215	UGACAUC
ENSG00000134215	UGCCCUG
ENSG00000134215	UGUCUUU
ENSG00000134215	UUCUUGU
ENSG00000134222	AGUGGUU
ENSG00000134243	AGCAGCA
ENSG00000134243	AUAAAGU
ENSG00000134243	AUAUAAC
ENSG00000134243	CAAAGCA
ENSG00000134243	CAAAUGC
ENSG00000134243	CAGUCUG
ENSG00000134243	CCCUGAG
ENSG00000134243	CCUGCUG
ENSG00000134243	CUAUACA
ENSG00000134243	CUGCUCA
ENSG00000134243	CUGGCUC
ENSG00000134243	CUUGGAG
ENSG00000134243	CUUUGGU
ENSG00000134243	GACCCUG
ENSG00000134243	GGAAGAC
ENSG00000134243	GGAAGCC
ENSG00000134243	GGUGGGC
ENSG00000134243	UAAUAGU
ENSG00000134243	UACAGUU
ENSG00000134243	UCCCUUG
ENSG00000134243	UCCUGAG
ENSG00000134243	UUGGCAC
ENSG00000134243	UUGGUUC
ENSG00000134245	CUUGUGU
ENSG00000134245	GAGGUAU
ENSG00000134245	GCAACUU
ENSG00000134247	AAAGUGC
ENSG00000134247	AAAUGGU
ENSG00000134247	AAGUGCA
ENSG00000134247	AAGUUGU
ENSG00000134247	AAUUGCU
ENSG00000134247	ACAGUAU
ENSG00000134247	AGUGCAA
ENSG00000134247	AGUGCCG
ENSG00000134247	CAAAGCA
ENSG00000134247	CACAGUG
ENSG00000134247	CAGUCUG
ENSG00000134247	CCCACCG
ENSG00000134247	CUGGGCA
ENSG00000134247	CUUUGGU
ENSG00000134247	GAGAACU
ENSG00000134247	GAGCGCC
ENSG00000134247	GGAAGAC
ENSG00000134247	GGCUGCG
ENSG00000134247	UCCCACC

ENSG00000134247	UGCAAAG
ENSG00000134247	UGCAUAG
ENSG00000134247	UGCAUAU
ENSG00000134247	UGCCCUG
ENSG00000134247	UGCUCAC
ENSG00000134247	UGGCUGC
ENSG00000134247	UGGUACC
ENSG00000134247	UUAGAGA
ENSG00000134250	AAAAAUC
ENSG00000134250	AAGGUGC
ENSG00000134250	AGCACCA
ENSG00000134250	CAGCAGA
ENSG00000134250	CAGCAGG
ENSG00000134250	CCUUCAU
ENSG00000134250	CUGGGCA
ENSG00000134250	CUUUGGU
ENSG00000134250	GCAGCAU
ENSG00000134250	GGCAGUG
ENSG00000134250	UGUCUGG
ENSG00000134250	UUGUUCG
ENSG00000134253	GAGAAAU
ENSG00000134258	CUGGCUC
ENSG00000134259	AGGGAGG
ENSG00000134259	GAGGUAG
ENSG00000134278	AAAGUUU
ENSG00000134278	AACUACU
ENSG00000134278	AAGGCAC
ENSG00000134278	AGUGCAA
ENSG00000134278	AUAUUAU
ENSG00000134278	GAGGUAG
ENSG00000134278	GAGGUAU
ENSG00000134278	GCAGGUU
ENSG00000134278	GUGAAUG
ENSG00000134278	GUGCAAA
ENSG00000134278	GUUCAUG
ENSG00000134278	UUCAAGC
ENSG00000134278	UUGGCAC
ENSG00000134283	AACACUG
ENSG00000134283	CUGUUGA
ENSG00000134283	GAGGCAG
ENSG00000134287	ACAUUCA
ENSG00000134287	ACUGCAG
ENSG00000134287	CACAGUG
ENSG00000134287	CAGUUAC
ENSG00000134287	CCUGCUG
ENSG00000134287	CUCAGGG
ENSG00000134287	GCAUCCC
ENSG00000134287	GGGGUCC
ENSG00000134287	UGGCCAC
ENSG00000134294	AAGAACC
ENSG00000134294	ACAUUCA
ENSG00000134294	AGUGCUU
ENSG00000134294	AGUGGUU
ENSG00000134294	AUGGAUU
ENSG00000134294	CCAGUGC
ENSG00000134294	GAGAUGA
ENSG00000134294	GGAAGAC
ENSG00000134294	GUAAACA
ENSG00000134294	UCUGGAA
ENSG00000134308	AAAAGUA
ENSG00000134308	AAAGCUG
ENSG00000134308	AAAGUAC
ENSG00000134308	AACUGGU
ENSG00000134308	AAUACUG

ENSG00000134308	AGCAGCA
ENSG00000134308	GUAUUCU
ENSG00000134308	UAUAAAG
ENSG00000134308	UGAAAUG
ENSG00000134317	ACCCGUA
ENSG00000134317	CCCUGAG
ENSG00000134317	GGUUUAC
ENSG00000134317	GUAAACA
ENSG00000134317	UCCCUGU
ENSG00000134317	UGUAUGU
ENSG00000134318	AAGGCAC
ENSG00000134318	AGUGCAA
ENSG00000134321	UGACCUA
ENSG00000134323	AAAGUAC
ENSG00000134323	AAAGUUU
ENSG00000134323	AAUACUG
ENSG00000134323	AUAAAGU
ENSG00000134323	AUACAAG
ENSG00000134323	CUCAAAC
ENSG00000134323	UAAUAUC
ENSG00000134323	UACAAAG
ENSG00000134323	UACAGUU
ENSG00000134323	UCAUACA
ENSG00000134323	UCCAGUU
ENSG00000134323	UGCAAAG
ENSG00000134323	UGUAUAA
ENSG00000134323	UUCAAAU
ENSG00000134324	AAAAGUA
ENSG00000134324	AAAGUAA
ENSG00000134324	AAGGCAC
ENSG00000134324	AGCAAAA
ENSG00000134324	AUCCUUG
ENSG00000134324	GAGGCUG
ENSG00000134324	GGAUUUU
ENSG00000134324	UCACAGG
ENSG00000134326	AAGUGCA
ENSG00000134333	GGCAGUG
ENSG00000134333	UUAUGCA
ENSG00000134369	AAGGCAC
ENSG00000134369	AAGGUGC
ENSG00000134369	AAGUAGC
ENSG00000134369	ACCCUGU
ENSG00000134369	ACUGGCU
ENSG00000134369	AGCAGCA
ENSG00000134369	AGCAGCG
ENSG00000134369	AGCAGCU
ENSG00000134369	AGCCAGU
ENSG00000134369	AGUAACA
ENSG00000134369	AUCCUUG
ENSG00000134369	CAGCAGA
ENSG00000134369	CAGGCUC
ENSG00000134369	CCAGGAG
ENSG00000134369	CCAGUGC
ENSG00000134369	CUCAAAC
ENSG00000134369	CUCUGGG
ENSG00000134369	CUGGGCA
ENSG00000134369	CUUCACC
ENSG00000134369	CUUCUCU
ENSG00000134369	GAAGGUC
ENSG00000134369	GACCUGG
ENSG00000134369	GAGACCU
ENSG00000134369	GAGGCAG
ENSG00000134369	GAGGGGC
ENSG00000134369	GAGUUGG

ENSG00000134369	GAUUGUA
ENSG00000134369	GAUUGUC
ENSG00000134369	GCAACUU
ENSG00000134369	GCACCAU
ENSG00000134369	GCAGAAG
ENSG00000134369	GCAGCAU
ENSG00000134369	GGAUGAG
ENSG00000134369	GGGUUUA
ENSG00000134369	GGUUACC
ENSG00000134369	GUAAACA
ENSG00000134369	UCAAGUA
ENSG00000134369	UCCAGAG
ENSG00000134369	UCCCACC
ENSG00000134369	UCCCUGU
ENSG00000134369	UCUCAAG
ENSG00000134369	UGCAUAG
ENSG00000134369	UGCAUAU
ENSG00000134369	UUCUGCA
ENSG00000134371	CCUUCUU
ENSG00000134375	UUUCAAC
ENSG00000134438	CAGGAAC
ENSG00000134440	AUGCAUU
ENSG00000134443	CAGCAAA
ENSG00000134444	AAGGCAC
ENSG00000134444	ACAUUAC
ENSG00000134444	CUUUGGU
ENSG00000134444	GCUGGUG
ENSG00000134444	GUGCAAA
ENSG00000134444	UAAUACA
ENSG00000134452	AAAAGUA
ENSG00000134452	ACUGGCU
ENSG00000134452	GAGAAAU
ENSG00000134453	AUAUUAU
ENSG00000134453	UCUCCAA
ENSG00000134489	GAAAACA
ENSG00000134504	AUGGCUU
ENSG00000134508	AAAGUGC
ENSG00000134508	AAGUGCA
ENSG00000134508	ACAUAGA
ENSG00000134508	CACAGUG
ENSG00000134508	CAGUAGU
ENSG00000134508	CCAGUAC
ENSG00000134508	UCCAGUU
ENSG00000134508	UGUGCUU
ENSG00000134508	UUGGCAC
ENSG00000134516	GAGAUGA
ENSG00000134516	GUCAGUU
ENSG00000134531	AAUACUG
ENSG00000134531	ACAGUAU
ENSG00000134531	ACUAGAU
ENSG00000134531	AGCACCA
ENSG00000134531	AUCCCUU
ENSG00000134531	GGCAAGA
ENSG00000134531	UAUAAAG
ENSG00000134531	UCCCUCU
ENSG00000134532	AACAAAC
ENSG00000134532	AAGUGCA
ENSG00000134532	ACCCAUU
ENSG00000134532	ACUGGCC
ENSG00000134532	ACUGGCU
ENSG00000134532	ACUGUGU
ENSG00000134532	AGCAGCA
ENSG00000134532	AGCAGCG
ENSG00000134532	AGUAACA

ENSG00000134532	AGUGCAA
ENSG00000134532	AGUGCUU
ENSG00000134532	CACAAUG
ENSG00000134532	CACCAGC
ENSG00000134532	CAGGUGA
ENSG00000134532	CAGUGCA
ENSG00000134532	CUACAGU
ENSG00000134532	GGCAUUG
ENSG00000134532	GGGUAAG
ENSG00000134532	GUAACAG
ENSG00000134532	GUGCAAA
ENSG00000134532	UAAGACG
ENSG00000134532	UACAGUU
ENSG00000134532	UCACAAG
ENSG00000134532	UCUAGAG
ENSG00000134532	UGAAGUG
ENSG00000134532	UGAGAAU
ENSG00000134532	UGUGCUU
ENSG00000134532	UUGGCAA
ENSG00000134533	AUGUAAU
ENSG00000134533	UACAAAG
ENSG00000134533	UGCAUUG
ENSG00000134551	CUCCAGC
ENSG00000134569	AAAUUUC
ENSG00000134569	AAGUGCA
ENSG00000134569	AAUCUCU
ENSG00000134569	ACAUUCA
ENSG00000134569	ACUGGCC
ENSG00000134569	AGGACAC
ENSG00000134569	AGUGCAA
ENSG00000134569	AUAUUAU
ENSG00000134569	AUCAUAC
ENSG00000134569	CAGUGCA
ENSG00000134569	CCAGUGC
ENSG00000134569	CCAGUGU
ENSG00000134569	CCCUGAG
ENSG00000134569	CCUUCAU
ENSG00000134569	CUCCAGC
ENSG00000134569	GAGGCUG
ENSG00000134569	UGCCAGC
ENSG00000134569	UGGAGAU
ENSG00000134569	UUCUUGU
ENSG00000134575	AGUGGUU
ENSG00000134575	CCCACCU
ENSG00000134575	GGGGAGC
ENSG00000134590	AAGGCAC
ENSG00000134594	AUACAAG
ENSG00000134595	CACACCU
ENSG00000134595	UAUAAAG
ENSG00000134644	AAGUGUG
ENSG00000134644	AAUUAUU
ENSG00000134644	ACAAGGU
ENSG00000134644	AGUAGAC
ENSG00000134644	AGUGUGC
ENSG00000134644	CUACAAA
ENSG00000134644	CUAUACA
ENSG00000134644	UGUGCUU
ENSG00000134684	GGUAGAC
ENSG00000134684	UCCCACA
ENSG00000134684	UCCCUUU
ENSG00000134684	UUCAAAU
ENSG00000134686	AAGGCGC
ENSG00000134686	ACCUGGA
ENSG00000134686	AGCCAGU

ENSG00000134686	CAAAGCA
ENSG00000134686	CACAGUG
ENSG00000134686	CAGCAGG
ENSG00000134686	CAGUCCA
ENSG00000134686	CCAGUGC
ENSG00000134686	CCCUGAG
ENSG00000134686	CUACAAA
ENSG00000134686	CUUCUCU
ENSG00000134686	GAGCCCU
ENSG00000134686	GCCUGGG
ENSG00000134686	GGAGUCC
ENSG00000134686	GGGGAGC
ENSG00000134686	UAAUGCU
ENSG00000134686	UCACACA
ENSG00000134686	UCACAGU
ENSG00000134686	UCAUUUG
ENSG00000134690	GAGGUAG
ENSG00000134690	UUGGUCC
ENSG00000134698	ACAUUCA
ENSG00000134698	AGCAGCA
ENSG00000134698	AGGACAC
ENSG00000134698	GAGGUAG
ENSG00000134698	GAGGUAU
ENSG00000134698	GCUAGCA
ENSG00000134698	UACAGUU
ENSG00000134709	AAGUCUU
ENSG00000134709	AGGUAGU
ENSG00000134709	AGUAACA
ENSG00000134709	CACAGGG
ENSG00000134709	GAGGUAG
ENSG00000134709	UAAUGCU
ENSG00000134717	AAAACUG
ENSG00000134717	AAGUGCA
ENSG00000134717	AAUACUG
ENSG00000134717	AUAUAAC
ENSG00000134717	CUCAAAC
ENSG00000134717	GAGGUAG
ENSG00000134717	UGCCAGC
ENSG00000134744	AAAGUUU
ENSG00000134744	UAUAAAG
ENSG00000134748	AGCAGCA
ENSG00000134748	GUGAAUG
ENSG00000134748	UGUUGAA
ENSG00000134755	AGCACCA
ENSG00000134757	UUCCAUA
ENSG00000134758	AACUACU
ENSG00000134758	AAUCUCA
ENSG00000134758	ACUGUUU
ENSG00000134758	AGCACCA
ENSG00000134758	AGCAGCG
ENSG00000134758	AGCAGCU
ENSG00000134758	AUAUUAU
ENSG00000134758	AUGGCAC
ENSG00000134758	CUGGCUC
ENSG00000134758	GCACCAU
ENSG00000134758	GGAAUGU
ENSG00000134758	GUCAACA
ENSG00000134758	UAAUAUC
ENSG00000134758	UCAAGUA
ENSG00000134762	AAGGUGC
ENSG00000134762	ACUGCAU
ENSG00000134762	UAUAAAG
ENSG00000134762	UGACUGU
ENSG00000134765	CACACCU

ENSG00000134769	AAAGCUG
ENSG00000134769	ACUAGAU
ENSG00000134769	AUGGCAC
ENSG00000134769	UGCAUAU
ENSG00000134775	UGUGCUU
ENSG00000134780	ACAUUAC
ENSG00000134780	AGCCUGC
ENSG00000134780	AUACAAG
ENSG00000134780	AUAUUAU
ENSG00000134780	AUGGCAC
ENSG00000134780	CCCUGAG
ENSG00000134780	CUGCAGC
ENSG00000134780	GAGGUAG
ENSG00000134780	GGCACGG
ENSG00000134780	GGCUCAG
ENSG00000134780	GGCUCUG
ENSG00000134780	GGCUGCG
ENSG00000134780	GUAAACA
ENSG00000134780	GUCUCUG
ENSG00000134780	UCCAGUU
ENSG00000134780	UGCAUAG
ENSG00000134780	UGCAUAU
ENSG00000134780	UGGCUGC
ENSG00000134780	UGGGGGA
ENSG00000134780	UGUGCUU
ENSG00000134817	ACCACAG
ENSG00000134817	CACAGUG
ENSG00000134817	CUGGGCA
ENSG00000134817	GAGCCCC
ENSG00000134817	GUCUCUG
ENSG00000134817	GUCUUGC
ENSG00000134825	UACAAAG
ENSG00000134851	CUACAAA
ENSG00000134852	AAAAAUC
ENSG00000134852	AAAACUG
ENSG00000134852	AAACUGU
ENSG00000134852	AAAGUGC
ENSG00000134852	AACCUGG
ENSG00000134852	AACCUGU
ENSG00000134852	AAGGCAC
ENSG00000134852	AGUGCAA
ENSG00000134852	AUACCUC
ENSG00000134852	AUCAUAC
ENSG00000134852	CAGUGCA
ENSG00000134852	CUGACUC
ENSG00000134852	CUGGACU
ENSG00000134852	GGCAGUG
ENSG00000134852	GUAACAG
ENSG00000134852	GUGACAG
ENSG00000134852	UCUUGAG
ENSG00000134852	UGACAUC
ENSG00000134852	UGAGAAU
ENSG00000134852	UGGACUG
ENSG00000134852	UGUUGAA
ENSG00000134852	UUCAAAU
ENSG00000134852	UUGGCAC
ENSG00000134853	AAGUGCA
ENSG00000134853	AGCAGCU
ENSG00000134853	AGGUAGU
ENSG00000134853	AGUGCAA
ENSG00000134853	AGUGGUU
ENSG00000134853	AUGUGCC
ENSG00000134853	CAGUAGU
ENSG00000134853	CUCUUCC

ENSG00000134853	CUUCACC
ENSG00000134853	GAAAACA
ENSG00000134853	GACUUCC
ENSG00000134853	GAGAUGA
ENSG00000134853	GAUUGUA
ENSG00000134853	GGCUCAG
ENSG00000134853	GUAUUCU
ENSG00000134853	GUGCUUG
ENSG00000134853	GUUAAUG
ENSG00000134853	UAUUGCU
ENSG00000134853	UCAACAG
ENSG00000134853	UCAAGUA
ENSG00000134853	UCACAAG
ENSG00000134853	UCACAGU
ENSG00000134853	UCAUUCG
ENSG00000134853	UCCCUCU
ENSG00000134853	UGAAAUG
ENSG00000134853	UGUAUAA
ENSG00000134853	UUCAAGC
ENSG00000134853	UUGGCAC
ENSG00000134871	CUUUGGU
ENSG00000134871	GAGGUAG
ENSG00000134871	GAGGUAU
ENSG00000134873	AAAAAUC
ENSG00000134873	AGCCCUU
ENSG00000134873	GUUAAUG
ENSG00000134874	AAGGCAC
ENSG00000134874	GAGGUAG
ENSG00000134874	UACAGUU
ENSG00000134874	UUGAGGC
ENSG00000134882	AAAAACC
ENSG00000134882	AGCAAAA
ENSG00000134884	AAUACUG
ENSG00000134884	ACCAGCA
ENSG00000134884	UAAGACG
ENSG00000134884	UCAACAG
ENSG00000134884	UGAAGCU
ENSG00000134897	CUACAAA
ENSG00000134897	GAGAACU
ENSG00000134897	GUGCUUG
ENSG00000134897	UCCAGUU
ENSG00000134897	UGUGCUU
ENSG00000134899	AUUCAUU
ENSG00000134917	CUGCAGG
ENSG00000134917	GAGGUAU
ENSG00000134917	UGCAUAG
ENSG00000134917	UGCAUAU
ENSG00000134917	UUUGCAA
ENSG00000134940	GCCUGAU
ENSG00000134954	AAGGCAC
ENSG00000134954	ACAGUAC
ENSG00000134954	ACUGGCC
ENSG00000134954	ACUGGCU
ENSG00000134954	AGCAAAA
ENSG00000134954	AGCAGCU
ENSG00000134954	AUGUGCC
ENSG00000134954	CCAGUGC
ENSG00000134954	CCCUGAG
ENSG00000134954	CUACAGU
ENSG00000134954	CUCCCAC
ENSG00000134954	CUUUGGU
ENSG00000134954	GAGACCU
ENSG00000134954	GGAGGCA
ENSG00000134954	GGGGUCC

ENSG00000134954	GUGCCAC
ENSG00000134954	UAAGACU
ENSG00000134954	UCCAGUU
ENSG00000134954	UCUGGAG
ENSG00000134954	UGCAUUG
ENSG00000134954	UGCCAGC
ENSG00000134954	UGUAUAA
ENSG00000134954	UUUGCAA
ENSG00000134954	UUUGCAC
ENSG00000134955	CUCAGGG
ENSG00000134955	UGAGGAC
ENSG00000134970	AAUGUUG
ENSG00000134970	AUAUUAU
ENSG00000134982	AAAGAGC
ENSG00000134982	AAAGUAU
ENSG00000134982	AAGACAU
ENSG00000134982	ACUGUGU
ENSG00000134982	AGUAACA
ENSG00000134982	AUACAAG
ENSG00000134982	AUGCACC
ENSG00000134982	CAAAGCA
ENSG00000134982	CAGUUAC
ENSG00000134982	CCCUGAG
ENSG00000134982	CUCCCAA
ENSG00000134982	GAAACAU
ENSG00000134982	GGGUAAG
ENSG00000134982	GUGAAUG
ENSG00000134982	GUUAAUG
ENSG00000134982	UAAGACG
ENSG00000134982	UAAGACU
ENSG00000134982	UCACAAG
ENSG00000134982	UCACAGG
ENSG00000134982	UCACAGU
ENSG00000134982	UCAUUCG
ENSG00000134982	UCCAGAG
ENSG00000134982	UCUCACC
ENSG00000134982	UGAAAUG
ENSG00000134982	UGCAUAU
ENSG00000134982	UUUGCAA
ENSG00000134982	UUUGCAC
ENSG00000134986	AAAACUG
ENSG00000134986	AAGACAU
ENSG00000134986	AAGGUGC
ENSG00000134986	AAUACUG
ENSG00000134986	AAUUAUU
ENSG00000134986	ACAUCAC
ENSG00000134986	ACCACAG
ENSG00000134986	CCCACCU
ENSG00000134986	CCUUCUU
ENSG00000134986	GCACCAU
ENSG00000134986	GCAGCAU
ENSG00000134986	GGCAGCG
ENSG00000134986	GGCUCAG
ENSG00000134986	GUAAACA
ENSG00000134986	UACAAAG
ENSG00000134986	UAUAUGA
ENSG00000134986	UCACAGG
ENSG00000134996	GGAAUGU
ENSG00000135018	AAGUCAC
ENSG00000135045	AAAGUAU
ENSG00000135045	AAUCUCU
ENSG00000135045	UAUAAAG
ENSG00000135045	UGCAUAU
ENSG00000135048	AAAGCUG

ENSG00000135048	AAAUGGU
ENSG00000135048	AACAGUC
ENSG00000135048	AGCCCUU
ENSG00000135048	AGUGCCU
ENSG00000135048	GAGGUAU
ENSG00000135048	GUCAUUG
ENSG00000135048	UUGAGGC
ENSG00000135052	ACAUUAC
ENSG00000135052	CACAGUG
ENSG00000135052	UCACAGG
ENSG00000135070	UCCUGAG
ENSG00000135074	AAGGCAC
ENSG00000135074	ACAGUAC
ENSG00000135074	AGCACCA
ENSG00000135074	AUGGCUU
ENSG00000135074	AUUCAUU
ENSG00000135074	CAAAGCA
ENSG00000135074	CACAGUG
ENSG00000135074	CUCCCAC
ENSG00000135074	CUCUGGG
ENSG00000135074	CUGCUCA
ENSG00000135074	GCACCAU
ENSG00000135074	GCCCUGU
ENSG00000135074	GCUGUCU
ENSG00000135074	GGCCCUG
ENSG00000135074	GGCUCAG
ENSG00000135074	UCACAGU
ENSG00000135074	UCAUUUG
ENSG00000135074	UCCAGUU
ENSG00000135074	UGCAUAU
ENSG00000135074	UUGGCAA
ENSG00000135074	UUGGCAC
ENSG00000135074	UUUGCAC
ENSG00000135083	AAAUCCC
ENSG00000135083	AAUACUG
ENSG00000135083	ACUGGCC
ENSG00000135083	AGCAGCA
ENSG00000135083	CAUAUUG
ENSG00000135083	CCCUGAG
ENSG00000135083	GAGGUAG
ENSG00000135083	GGCAGUG
ENSG00000135083	GUGAGGU
ENSG00000135083	UCCUGAG
ENSG00000135083	UUCAAGC
ENSG00000135083	UUUCAAC
ENSG00000135090	AAAGUAA
ENSG00000135090	AGCAGCU
ENSG00000135090	CAGCAGA
ENSG00000135090	CUCUUCC
ENSG00000135090	GGAAUGU
ENSG00000135090	GGCUGCG
ENSG00000135090	UACAAAG
ENSG00000135090	UCCUGAG
ENSG00000135090	UCUCCAA
ENSG00000135090	UGUGCUU
ENSG00000135093	AACAAAC
ENSG00000135093	AGCAAAA
ENSG00000135093	CUACAAA
ENSG00000135093	CUGGCUC
ENSG00000135093	UUCUUGU
ENSG00000135094	UGGAUAA
ENSG00000135097	AACAAAC
ENSG00000135097	AACGGAA
ENSG00000135097	AACUCUA

ENSG00000135097	ACAGUAC
ENSG00000135097	ACAGUAU
ENSG00000135097	ACAUUCA
ENSG00000135097	ACUCAGC
ENSG00000135097	AGAUGUG
ENSG00000135097	AGGGAGG
ENSG00000135097	AGUAACA
ENSG00000135097	CAGCUGG
ENSG00000135097	CCCCUGG
ENSG00000135097	CCCUGAG
ENSG00000135097	CGGCCUG
ENSG00000135097	CUCACAC
ENSG00000135097	CUGCAGG
ENSG00000135097	CUGGAAA
ENSG00000135097	CUGGCUC
ENSG00000135097	CUUACAG
ENSG00000135097	CUUCUCU
ENSG00000135097	GCUGGUG
ENSG00000135097	GGCUGCG
ENSG00000135097	GGGGUCC
ENSG00000135097	UACAGUU
ENSG00000135097	UCACAUU
ENSG00000135097	UCUCCAA
ENSG00000135097	UGCAGCU
ENSG00000135097	UGGAGAU
ENSG00000135097	UUGGAGG
ENSG00000135097	UUGUGUC
ENSG00000135097	UUUCAAC
ENSG00000135100	AAAAGUA
ENSG00000135100	CUGCAGG
ENSG00000135108	AAACUGU
ENSG00000135108	ACAGGUG
ENSG00000135108	ACCACAG
ENSG00000135108	AGCAUUC
ENSG00000135108	AGUGCCU
ENSG00000135108	AUUCAUU
ENSG00000135108	CACAGUG
ENSG00000135108	CACCACC
ENSG00000135108	CAGCAAA
ENSG00000135108	CAGGUGA
ENSG00000135108	GAGACCU
ENSG00000135108	GGAGGCA
ENSG00000135108	GGCAUUG
ENSG00000135108	GUAGUGU
ENSG00000135108	UAUAAAG
ENSG00000135108	UCACCAC
ENSG00000135108	UGCAGCU
ENSG00000135108	UGUCUUU
ENSG00000135111	AAAAAUC
ENSG00000135111	AAAGUGC
ENSG00000135111	AAAUUUC
ENSG00000135111	AAGUGCA
ENSG00000135111	ACAUCAC
ENSG00000135111	AUUGCAC
ENSG00000135111	CAAAGUG
ENSG00000135111	CAUAUUG
ENSG00000135111	CCUUCAU
ENSG00000135111	UAAUAGU
ENSG00000135111	UAUUGCU
ENSG00000135111	UGAAAUG
ENSG00000135111	UGCAUAG
ENSG00000135116	AGGACAC
ENSG00000135116	CACAGGG
ENSG00000135116	UCUCCAA

ENSG00000135116	UGUAUGU
ENSG00000135119	AGGCAGG
ENSG00000135119	AGUAACA
ENSG00000135119	GAUUGUA
ENSG00000135119	GCCCUGU
ENSG00000135119	GUCUUGC
ENSG00000135119	UGAAAGG
ENSG00000135119	UGCCCUG
ENSG00000135124	AAGUGCU
ENSG00000135124	AGUGCUU
ENSG00000135127	AAAAAUC
ENSG00000135127	ACAUUCA
ENSG00000135127	AGCAGCA
ENSG00000135127	AGCAGCU
ENSG00000135127	AGGCAGG
ENSG00000135127	AGGGAGG
ENSG00000135127	AUUCAUU
ENSG00000135127	CCUGCUG
ENSG00000135127	GUGCUUG
ENSG00000135127	UAUAAAG
ENSG00000135127	UCAUUUG
ENSG00000135127	UGUGCUU
ENSG00000135127	UUCAAAU
ENSG00000135127	UUCUAAU
ENSG00000135127	UUUCAAC
ENSG00000135144	AAAGAGC
ENSG00000135144	CACAGUG
ENSG00000135144	CCCCUGG
ENSG00000135144	CUGGGCA
ENSG00000135144	GAGUUGA
ENSG00000135144	GAGUUGG
ENSG00000135144	GCCUGGA
ENSG00000135144	GCUGGUG
ENSG00000135144	GGAGGAG
ENSG00000135144	UCCCUUU
ENSG00000135144	UCUCCAA
ENSG00000135148	AAGUGCU
ENSG00000135148	ACAAGGU
ENSG00000135148	AGCACCA
ENSG00000135148	AGUGCUG
ENSG00000135164	AACUACU
ENSG00000135164	AAGACAU
ENSG00000135164	ACAAUAU
ENSG00000135164	CUCCCAC
ENSG00000135164	GUAGUGU
ENSG00000135164	UGCCAGC
ENSG00000135213	AAAGAGG
ENSG00000135213	AAAUUUC
ENSG00000135213	AACAGUC
ENSG00000135213	AAUCUCA
ENSG00000135213	AGCUUAU
ENSG00000135213	CACAGUG
ENSG00000135213	CUCCAGC
ENSG00000135213	CUCCCAA
ENSG00000135213	CUCCCAC
ENSG00000135213	UCAAGUA
ENSG00000135213	UCACAGU
ENSG00000135218	UUCAAAU
ENSG00000135222	UCCAGUU
ENSG00000135248	GAGAUGA
ENSG00000135248	UUCAAGC
ENSG00000135250	AAAGAGG
ENSG00000135250	AACUACU
ENSG00000135250	ACAGUAC

ENSG00000135250	AUUGCAC
ENSG00000135250	CAGUGCA
ENSG00000135250	GACAACU
ENSG00000135250	UAAUACA
ENSG00000135269	ACAGUAC
ENSG00000135269	ACAGUAU
ENSG00000135269	CUUUGGU
ENSG00000135272	AGAUAAA
ENSG00000135272	AUAAUAC
ENSG00000135272	CUCCAGC
ENSG00000135272	UAAUACA
ENSG00000135272	UGAGAAU
ENSG00000135298	AAAAACC
ENSG00000135298	AAAAGUA
ENSG00000135298	AAAGUAC
ENSG00000135298	AAAGUAU
ENSG00000135298	AACAAAC
ENSG00000135298	AACAUUC
ENSG00000135298	AUUGCAC
ENSG00000135298	CAGCAGA
ENSG00000135298	CAGGCUC
ENSG00000135298	CUGCAGC
ENSG00000135298	GCAGCAU
ENSG00000135298	GUCAGUU
ENSG00000135298	GUGCUUG
ENSG00000135298	UACAAAG
ENSG00000135298	UAUUACC
ENSG00000135298	UAUUGCU
ENSG00000135298	UCAUUUG
ENSG00000135298	UGCAUUG
ENSG00000135298	UUGGCAA
ENSG00000135299	CAGCAGG
ENSG00000135299	CUCUUCC
ENSG00000135299	UACAGUU
ENSG00000135316	AAAUGGU
ENSG00000135316	AAGUGUG
ENSG00000135316	AAGUUGU
ENSG00000135316	GUGUGGC
ENSG00000135316	UGCCAGC
ENSG00000135317	AAAAGUA
ENSG00000135333	ACAGUAU
ENSG00000135333	AGCAGCG
ENSG00000135333	AGCAGCU
ENSG00000135333	AGUGCAA
ENSG00000135333	AUGUGGG
ENSG00000135333	CCAGUGU
ENSG00000135333	GAAAACA
ENSG00000135333	GAGAAAU
ENSG00000135333	GGGAUCG
ENSG00000135333	UACAGUU
ENSG00000135333	UCAAGUA
ENSG00000135333	UGGACUG
ENSG00000135333	UGGGAUC
ENSG00000135333	UUAGAGA
ENSG00000135333	UUGGCAA
ENSG00000135333	UUGUGAC
ENSG00000135334	AGUGGUU
ENSG00000135334	CAGCAAA
ENSG00000135334	UAAUACA
ENSG00000135334	UGAAAUG
ENSG00000135334	UGACAUC
ENSG00000135338	AACAUUC
ENSG00000135338	AAGUCUU
ENSG00000135341	AACACUG

ENSG00000135341	AAGUCAC
ENSG00000135341	AGCAGCA
ENSG00000135341	AGCAGCG
ENSG00000135341	CAGCAAA
ENSG00000135341	UCAAGUA
ENSG00000135341	UCAUUUG
ENSG00000135341	UGUCUUU
ENSG00000135363	ACACACC
ENSG00000135363	ACACACU
ENSG00000135363	AUUGCAC
ENSG00000135363	UGUAUAA
ENSG00000135365	AACACUG
ENSG00000135365	AACGGCU
ENSG00000135365	AACUACU
ENSG00000135365	AAUACUG
ENSG00000135365	AAUUUUA
ENSG00000135365	ACACACC
ENSG00000135365	ACUGUUU
ENSG00000135365	AGCACCA
ENSG00000135365	AGCAGCA
ENSG00000135365	AGCCCUU
ENSG00000135365	AGUAACA
ENSG00000135365	AUAAUAC
ENSG00000135365	AUACAAG
ENSG00000135365	AUGACAC
ENSG00000135365	CACACCU
ENSG00000135365	CACCAGC
ENSG00000135365	CAGCAGG
ENSG00000135365	CCCCCAG
ENSG00000135365	CCCGUCC
ENSG00000135365	CGUGGCC
ENSG00000135365	CUCCAUU
ENSG00000135365	CUCUGGG
ENSG00000135365	CUGCAGC
ENSG00000135365	CUGCAGG
ENSG00000135365	CUGGAAA
ENSG00000135365	CUGGCUC
ENSG00000135365	CUUUGGU
ENSG00000135365	GAGGCUG
ENSG00000135365	GAUCAGA
ENSG00000135365	GCCCUUA
ENSG00000135365	GCUGGUG
ENSG00000135365	GGAAGAC
ENSG00000135365	GGACUGC
ENSG00000135365	GGAGGCA
ENSG00000135365	GUAACAG
ENSG00000135365	GUGCCAC
ENSG00000135365	UAAUAGU
ENSG00000135365	UACAAAG
ENSG00000135365	UAUAAAG
ENSG00000135365	UAUAAUA
ENSG00000135365	UCCAGAG
ENSG00000135365	UCCAGUU
ENSG00000135365	UCUCCAA
ENSG00000135365	UCUGGAA
ENSG00000135365	UGAAGCU
ENSG00000135365	UGCAUAG
ENSG00000135365	UGCAUAU
ENSG00000135365	UGCCCUG
ENSG00000135365	UGUAUAA
ENSG00000135365	UGUCUUU
ENSG00000135365	UUAGGAU
ENSG00000135365	UUCAAGC
ENSG00000135365	UUGAUCA

ENSG00000135365	UUGGCAA
ENSG00000135365	UUGGUUC
ENSG00000135365	UUUCAAC
ENSG00000135365	UUUUUGC
ENSG00000135373	AAAACUG
ENSG00000135373	AAAAGUA
ENSG00000135373	AAACUGU
ENSG00000135373	AAUUAUU
ENSG00000135373	ACUGUGU
ENSG00000135373	CACAAUG
ENSG00000135373	GUAGUGU
ENSG00000135373	UAAUGCU
ENSG00000135373	UAUCAGA
ENSG00000135373	UCACAAG
ENSG00000135373	UCACAGG
ENSG00000135373	UCACAGU
ENSG00000135373	UGAAAUG
ENSG00000135374	ACAGUAC
ENSG00000135374	ACAGUAU
ENSG00000135374	AGUGCCU
ENSG00000135374	GAGCUGC
ENSG00000135374	GUCAGUU
ENSG00000135374	UACAGUU
ENSG00000135378	UCAACAG
ENSG00000135387	AAAAAUC
ENSG00000135387	AAAGUAA
ENSG00000135387	AAAGUGC
ENSG00000135387	AACUCUA
ENSG00000135387	AAGUGCA
ENSG00000135387	AAUCCUU
ENSG00000135387	ACCACAG
ENSG00000135387	ACUGGCC
ENSG00000135387	AGAUAAA
ENSG00000135387	AGGACAC
ENSG00000135387	CAAAGCA
ENSG00000135387	CAGCAGG
ENSG00000135387	GGUUGUC
ENSG00000135387	GUCAUUG
ENSG00000135387	GUGCAAA
ENSG00000135387	UACAAAG
ENSG00000135387	UAGUGAG
ENSG00000135387	UAUAAAG
ENSG00000135387	UAUGGUU
ENSG00000135387	UCAACAG
ENSG00000135387	UCCAGUU
ENSG00000135387	UCCCUUU
ENSG00000135387	UGAAAGG
ENSG00000135387	UGAAAUG
ENSG00000135392	AAAAAUC
ENSG00000135392	AACAAAC
ENSG00000135392	AGCCCUU
ENSG00000135392	CCCUGAG
ENSG00000135392	UCCCACA
ENSG00000135392	UGCCAGC
ENSG00000135404	AAAAACC
ENSG00000135404	AACCUGG
ENSG00000135407	UUGGCAC
ENSG00000135414	AGGGUCU
ENSG00000135414	AUUGCAC
ENSG00000135423	ACUGCAG
ENSG00000135423	AGCAGCA
ENSG00000135423	AGCAGCG
ENSG00000135423	AGCAGCU
ENSG00000135423	UCUUGAG

ENSG00000135424	CAGCAAA
ENSG00000135443	GAAACAU
ENSG00000135443	GGAAGCC
ENSG00000135443	UUCCAUA
ENSG00000135446	CAGCAAA
ENSG00000135446	UGUAUAA
ENSG00000135447	AAAAACC
ENSG00000135447	AUCACUA
ENSG00000135447	CAGCAGA
ENSG00000135447	CCUUCUU
ENSG00000135447	GCAGAAG
ENSG00000135452	UCUCAAG
ENSG00000135454	ACUGGCU
ENSG00000135454	ACUUGGA
ENSG00000135454	AUGGCUU
ENSG00000135454	CUGCCCC
ENSG00000135454	CUUUGGU
ENSG00000135454	GUGUCAC
ENSG00000135454	UGAAGUG
ENSG00000135454	UUGGCAA
ENSG00000135457	GAAGGAA
ENSG00000135469	AAAAACC
ENSG00000135469	AAUUUUA
ENSG00000135469	UAUAAAG
ENSG00000135472	AAAGUGC
ENSG00000135472	ACUGGCC
ENSG00000135472	ACUUGGA
ENSG00000135472	CAAAUGC
ENSG00000135472	CAGCAAA
ENSG00000135472	CAGCUGG
ENSG00000135472	CCCCCAG
ENSG00000135472	CUCUGGG
ENSG00000135472	UUCCUCG
ENSG00000135472	UUCUGCA
ENSG00000135473	CAAAACU
ENSG00000135473	GCACCAU
ENSG00000135473	UAUAAAG
ENSG00000135482	AAAAAUC
ENSG00000135482	ACACACC
ENSG00000135482	AGCCCUU
ENSG00000135482	AGUGCUG
ENSG00000135482	CAGACAG
ENSG00000135482	CAGUCUG
ENSG00000135482	CCAGUGC
ENSG00000135482	CUUCUCU
ENSG00000135482	CUUUGGU
ENSG00000135482	GAGCCCU
ENSG00000135482	GGAGUGU
ENSG00000135482	GGCCCUG
ENSG00000135482	UCCCUCU
ENSG00000135486	ACAUUAC
ENSG00000135486	AGCAGCA
ENSG00000135486	AGCAGCG
ENSG00000135486	AUACAAG
ENSG00000135486	CCCUGUU
ENSG00000135486	CCUGCUG
ENSG00000135486	CUCUGGG
ENSG00000135486	CUUCUCU
ENSG00000135486	GAUCAGA
ENSG00000135486	GGCUCUG
ENSG00000135486	GUCACUC
ENSG00000135486	GUGCUUG
ENSG00000135486	UCAACAG
ENSG00000135486	UCACAAG

ENSG00000135486	UCAGCGG
ENSG00000135486	UGGCUGC
ENSG00000135486	UGUGCUU
ENSG00000135503	AAAAACC
ENSG00000135503	AAAAGUA
ENSG00000135503	AAAGUGC
ENSG00000135503	AACAAAC
ENSG00000135503	ACCAGCA
ENSG00000135503	ACUCCAG
ENSG00000135503	AGCUGCC
ENSG00000135503	AUGGCUU
ENSG00000135503	CACCACC
ENSG00000135503	CACCAGC
ENSG00000135503	CAGCAGG
ENSG00000135503	CCAGUGC
ENSG00000135503	CCAGUGU
ENSG00000135503	CUCAAAC
ENSG00000135503	CUCCCAA
ENSG00000135503	CUGCUCA
ENSG00000135503	CUGGGCA
ENSG00000135503	CUUUGGU
ENSG00000135503	GAGGUAG
ENSG00000135503	GAUCAGA
ENSG00000135503	GGCUCUG
ENSG00000135503	GUGAAUG
ENSG00000135503	UACAAAG
ENSG00000135503	UACAGUU
ENSG00000135503	UCCAGUU
ENSG00000135503	UCCCUCU
ENSG00000135503	UGAAGUG
ENSG00000135503	UGCAUAG
ENSG00000135503	UGCAUAU
ENSG00000135503	UGCUCAC
ENSG00000135503	UUCCUAG
ENSG00000135503	UUGGUUC
ENSG00000135506	UCCAGUU
ENSG00000135517	AGCAAAA
ENSG00000135517	CAGCAAA
ENSG00000135519	AGCCUGC
ENSG00000135519	CCCUGAG
ENSG00000135519	GCCCUGU
ENSG00000135519	UGCCCUG
ENSG00000135525	AAAAAUC
ENSG00000135525	AAAGUGC
ENSG00000135525	AAGGCAC
ENSG00000135525	AAGUGCA
ENSG00000135525	AAUUUUA
ENSG00000135525	AGUGCAA
ENSG00000135525	CUUUGGU
ENSG00000135525	UUCAAGC
ENSG00000135525	UUGGUUC
ENSG00000135535	AACAAAC
ENSG00000135535	AGCAGCA
ENSG00000135535	CACUGUU
ENSG00000135535	UCCCACA
ENSG00000135537	AAAGUGC
ENSG00000135537	AUGAUGA
ENSG00000135537	CAAAGUG
ENSG00000135537	GUCCAGA
ENSG00000135537	UCCAGAG
ENSG00000135541	AAUGCCC
ENSG00000135541	UCUCACC
ENSG00000135547	AAGUGCA
ENSG00000135547	GAGAAAU

ENSG00000135547	UCUCCAA
ENSG00000135549	AUUCAUU
ENSG00000135549	UCAUUUG
ENSG00000135597	AACGGCU
ENSG00000135597	CACAGUG
ENSG00000135597	GAGCUGC
ENSG00000135597	UCACAGG
ENSG00000135597	UCACAGU
ENSG00000135604	CAGUGCA
ENSG00000135604	GUGCUUG
ENSG00000135605	CUCUUCC
ENSG00000135605	UUCAAAU
ENSG00000135617	GGAAUGU
ENSG00000135622	AGUAGUU
ENSG00000135622	AUCCUUG
ENSG00000135622	CACAGGG
ENSG00000135622	GAAGGUC
ENSG00000135622	GGAGUCC
ENSG00000135622	UCCCACC
ENSG00000135622	UGCAUAG
ENSG00000135622	UGCAUAU
ENSG00000135624	UCCUUGC
ENSG00000135625	AAUUGCU
ENSG00000135625	UGCAAAG
ENSG00000135631	AAGGCAC
ENSG00000135631	AGCAGCA
ENSG00000135631	CAAAGUG
ENSG00000135631	CAGCAAA
ENSG00000135631	GGAAGAC
ENSG00000135631	GGGGCAG
ENSG00000135631	UGCCCUG
ENSG00000135631	UGGCCAC
ENSG00000135632	AAGGCAC
ENSG00000135632	AGCAGCA
ENSG00000135632	AGGGUCU
ENSG00000135632	CACCACC
ENSG00000135632	CAGCAGA
ENSG00000135632	CUGGAAA
ENSG00000135632	UCACCAC
ENSG00000135632	UCUGGAA
ENSG00000135632	UCUGGAG
ENSG00000135636	GGAGGAG
ENSG00000135638	AUCACUA
ENSG00000135638	CUGGAAA
ENSG00000135638	GCUGGAU
ENSG00000135655	AAGUGCA
ENSG00000135655	AGCAGCU
ENSG00000135655	AGGUAGU
ENSG00000135655	AGUACUG
ENSG00000135655	AUACAAG
ENSG00000135655	AUGGCUU
ENSG00000135655	GUAAACA
ENSG00000135655	UAUAAAG
ENSG00000135655	UGGGACA
ENSG00000135655	UUGGCAA
ENSG00000135655	UUGGCAC
ENSG00000135655	UUGUGAC
ENSG00000135655	UUUUUGC
ENSG00000135677	AAAGCUG
ENSG00000135677	AAGGCAC
ENSG00000135677	AAGUGCA
ENSG00000135677	AAUCACU
ENSG00000135677	AAUUUUA
ENSG00000135677	AGCCCUU

ENSG00000135677	AUUGCAC
ENSG00000135677	CACAGUG
ENSG00000135677	CAGCUGG
ENSG00000135677	GACCUGG
ENSG00000135677	GAGGCUG
ENSG00000135677	GAGGUAG
ENSG00000135677	GAGGUAU
ENSG00000135677	GAGUUGA
ENSG00000135677	GCAGCAU
ENSG00000135677	UCACAGG
ENSG00000135677	UUCAAAU
ENSG00000135677	UUCUAAU
ENSG00000135677	UUUCAAC
ENSG00000135678	AACCUGU
ENSG00000135678	AGGUAGU
ENSG00000135678	CAAAUGC
ENSG00000135678	CCCUGAG
ENSG00000135678	GAGGAUA
ENSG00000135678	GAGGUAG
ENSG00000135678	GCUUCCU
ENSG00000135678	UCAAGUA
ENSG00000135678	UCACAUU
ENSG00000135686	AAAGCUG
ENSG00000135698	AGGAUGU
ENSG00000135709	AAGUGCA
ENSG00000135709	AAGUGCU
ENSG00000135709	CUCUGGG
ENSG00000135709	UCACAAG
ENSG00000135709	UUGGCAA
ENSG00000135720	AGGCAGG
ENSG00000135720	CUUUGGU
ENSG00000135720	GUAGUGU
ENSG00000135720	GUGCAAA
ENSG00000135720	UUGGUCC
ENSG00000135720	UUGGUUC
ENSG00000135723	AAUACUG
ENSG00000135723	UCACACA
ENSG00000135740	AGCUGCC
ENSG00000135740	UGAGGAC
ENSG00000135744	CUGCUCA
ENSG00000135750	AAAGAGC
ENSG00000135750	AAAGAGG
ENSG00000135750	AUAAAGU
ENSG00000135775	AAAAAUC
ENSG00000135775	AAAGUUU
ENSG00000135775	ACAAUAU
ENSG00000135775	UUCCAUA
ENSG00000135776	AAGUUGU
ENSG00000135776	CAAAACU
ENSG00000135776	CAGUGCA
ENSG00000135776	GUCAUUG
ENSG00000135776	UCAUUUG
ENSG00000135821	AAAAAUC
ENSG00000135821	AGUAACA
ENSG00000135821	AGUGGUU
ENSG00000135821	CUAGUCC
ENSG00000135821	GGAAGCC
ENSG00000135821	UAGUGAG
ENSG00000135821	UAUAAAG
ENSG00000135821	UUUUUGC
ENSG00000135823	AAAGUGC
ENSG00000135823	AAGUUGU
ENSG00000135823	AAUCUCA
ENSG00000135823	ACUAGAU

ENSG00000135823	CAGUUAC
ENSG00000135823	CCUUCUU
ENSG00000135823	CUUACAG
ENSG00000135823	GCCCUUA
ENSG00000135823	GGAAUGU
ENSG00000135823	GGCUCUG
ENSG00000135823	GUAAACA
ENSG00000135823	UAAUGCU
ENSG00000135823	UAUAAAG
ENSG00000135823	UCACAGG
ENSG00000135823	UGAGAAG
ENSG00000135828	GAGAACU
ENSG00000135837	AAACGGU
ENSG00000135837	AAAGUAA
ENSG00000135837	AAAGUAC
ENSG00000135837	AACAAAC
ENSG00000135837	AAGGCAC
ENSG00000135837	AAUCUCU
ENSG00000135837	ACACACC
ENSG00000135837	ACAGUAU
ENSG00000135837	ACAUUCA
ENSG00000135837	ACUGGAU
ENSG00000135837	AGCAGCA
ENSG00000135837	AGCAGCG
ENSG00000135837	AGCAGCU
ENSG00000135837	AGGCAGG
ENSG00000135837	AGGUAGU
ENSG00000135837	AUGGCUU
ENSG00000135837	CAAGAGC
ENSG00000135837	CAGUUAC
ENSG00000135837	CUUUGGU
ENSG00000135837	GCAGAAG
ENSG00000135837	GCCUGAU
ENSG00000135837	GCCUGGA
ENSG00000135837	GGAGGCA
ENSG00000135837	GGUUUAC
ENSG00000135837	UAUAAAG
ENSG00000135837	UCCUGAG
ENSG00000135837	UGCAUUG
ENSG00000135837	UGGCUGC
ENSG00000135837	UGUCUUU
ENSG00000135837	UUCAAGC
ENSG00000135837	UUCCUAG
ENSG00000135838	GAGAAAU
ENSG00000135842	GGUUACC
ENSG00000135842	UCUCCAA
ENSG00000135842	UUCUUGU
ENSG00000135862	AAAGUAC
ENSG00000135862	AAAUUUC
ENSG00000135862	AAUCUCA
ENSG00000135862	ACAUUCA
ENSG00000135862	ACCACAG
ENSG00000135862	ACUGGCU
ENSG00000135862	AGCACCA
ENSG00000135862	AGUGCAA
ENSG00000135862	CCAGUGU
ENSG00000135862	CUUCUCU
ENSG00000135862	GGAGUGU
ENSG00000135862	GUGUCAC
ENSG00000135862	GUGUGGC
ENSG00000135862	UAAUAGU
ENSG00000135862	UACAAAG
ENSG00000135862	UAUAUGA
ENSG00000135862	UCAUGAU

ENSG00000135862	UGAGAAG
ENSG00000135862	UGCAUAG
ENSG00000135862	UGCAUUG
ENSG00000135870	AAACUGU
ENSG00000135870	AACAGUC
ENSG00000135870	AAGUCUU
ENSG00000135870	ACAGUAC
ENSG00000135870	ACAGUAU
ENSG00000135870	AGUGCUU
ENSG00000135870	AGUGGUU
ENSG00000135870	AUACCUC
ENSG00000135870	AUCCUUG
ENSG00000135870	AUGAGCU
ENSG00000135870	AUGAUGC
ENSG00000135870	AUGUGCC
ENSG00000135870	CAAAGCA
ENSG00000135870	CACUGUU
ENSG00000135870	CCAGUGC
ENSG00000135870	CCUUCUG
ENSG00000135870	CCUUCUU
ENSG00000135870	CGGGGAU
ENSG00000135870	CUGACUC
ENSG00000135870	CUGCAGC
ENSG00000135870	CUGCAGG
ENSG00000135870	CUGGGCA
ENSG00000135870	CUUUGGU
ENSG00000135870	GAAUUGU
ENSG00000135870	GAGGCAG
ENSG00000135870	GAUUGGU
ENSG00000135870	GCAACUU
ENSG00000135870	GCAGCAU
ENSG00000135870	GCAGGAC
ENSG00000135870	GCAGGUU
ENSG00000135870	GGCAGGG
ENSG00000135870	GGCUCUG
ENSG00000135870	GGGUAAG
ENSG00000135870	GUCACUC
ENSG00000135870	GUCAGUU
ENSG00000135870	UAGACUG
ENSG00000135870	UAUUACC
ENSG00000135870	UCAUUCG
ENSG00000135870	UCCAGUU
ENSG00000135870	UCCCUUU
ENSG00000135870	UGACUGU
ENSG00000135870	UGGACUG
ENSG00000135870	UUGAGGC
ENSG00000135903	ACUGCAG
ENSG00000135903	ACUGCAU
ENSG00000135903	UAUAAAG
ENSG00000135912	GAGGUAG
ENSG00000135912	GAGGUAU
ENSG00000135913	AAAGUAA
ENSG00000135913	AAAGUGC
ENSG00000135913	AAGUCAC
ENSG00000135913	AGCACCA
ENSG00000135913	AGCUGCC
ENSG00000135913	AGUGCCG
ENSG00000135913	CCAGGAG
ENSG00000135913	CCAGUGC
ENSG00000135913	CCAGUGU
ENSG00000135913	CCCUGAG
ENSG00000135913	CUCCAUU
ENSG00000135913	GAGCUGC
ENSG00000135913	GCAACUU

ENSG00000135913	GUGGCCC
ENSG00000135913	UCCAGUU
ENSG00000135913	UUGAUCA
ENSG00000135913	UUGGUUC
ENSG00000135913	UUUCAAC
ENSG00000135916	AAGGCAC
ENSG00000135916	AGGGAGG
ENSG00000135916	AUACAAG
ENSG00000135916	CUUUGGU
ENSG00000135916	GUGCUUG
ENSG00000135916	UACAAAG
ENSG00000135916	UGUGCUU
ENSG00000135916	UUGGUUC
ENSG00000135919	AGUAGUU
ENSG00000135919	CAGUAGA
ENSG00000135924	GUCUCUG
ENSG00000135924	UAAGACU
ENSG00000135925	AGUGCAA
ENSG00000135925	CUGGACU
ENSG00000135925	GUGCAAA
ENSG00000135926	CUUCACC
ENSG00000135932	AACAUUC
ENSG00000135932	AACCGUU
ENSG00000135932	AAUACUG
ENSG00000135932	AAUUUUA
ENSG00000135932	AGCAGCA
ENSG00000135932	AGCAGCU
ENSG00000135932	GUGCCUG
ENSG00000135932	UAAUGCU
ENSG00000135945	AAAAACC
ENSG00000135945	AAAACUG
ENSG00000135945	AAUACUG
ENSG00000135945	ACAGUAC
ENSG00000135945	ACAGUAU
ENSG00000135945	AUACCUC
ENSG00000135945	CACUGUU
ENSG00000135945	CAGGCUC
ENSG00000135945	CCUGUAC
ENSG00000135945	CUACAGU
ENSG00000135945	GCUACAU
ENSG00000135945	UACAGUU
ENSG00000135945	UGCAAAG
ENSG00000135945	UUGGCAA
ENSG00000135945	UUUGCAA
ENSG00000135951	AGAUAAA
ENSG00000135956	ACAUUCA
ENSG00000135956	AGCACCA
ENSG00000135956	AUGACAC
ENSG00000135956	CACAGGG
ENSG00000135956	CAGUGCA
ENSG00000135956	CCUGCUG
ENSG00000135956	CUCUGGG
ENSG00000135956	GAGAUGA
ENSG00000135956	UAUAUGA
ENSG00000135956	UCCUAUA
ENSG00000135956	UCCUGAG
ENSG00000135956	UUGGCAA
ENSG00000135956	UUGGCAC
ENSG00000135960	AAAGAGG
ENSG00000135960	ACAGUAU
ENSG00000135960	ACAUUCA
ENSG00000135960	ACUGGCU
ENSG00000135960	AGCAGCA
ENSG00000135960	AUGAUGA

ENSG00000135960	CACAGUG
ENSG00000135960	CAGCAAA
ENSG00000135960	GGAUUUC
ENSG00000135960	GGCUCAG
ENSG00000135960	UAAGACG
ENSG00000135960	UCACAGU
ENSG00000135968	AAAGUGC
ENSG00000135968	AACAAAC
ENSG00000135968	AAGUGCA
ENSG00000135968	ACAGUAC
ENSG00000135968	AGCACCA
ENSG00000135968	AGCAGCA
ENSG00000135968	AGUGCAA
ENSG00000135968	CACUGUU
ENSG00000135968	CAGCAAA
ENSG00000135968	CAGCAGA
ENSG00000135968	CAGCAGG
ENSG00000135968	GUGCAAA
ENSG00000135968	UAAUAGU
ENSG00000135968	UGCAAAG
ENSG00000135999	AACAAAC
ENSG00000135999	AACAUUC
ENSG00000135999	AAGAACC
ENSG00000135999	AAGUCAC
ENSG00000135999	AAGUGCA
ENSG00000135999	AAGUUGU
ENSG00000135999	AGGUAGU
ENSG00000135999	AGUGAUC
ENSG00000135999	AUCCCUU
ENSG00000135999	AUGAUGA
ENSG00000135999	CAUAUUG
ENSG00000135999	CUUACAG
ENSG00000135999	GAAAACA
ENSG00000135999	GAUAUGU
ENSG00000135999	GUGCAAA
ENSG00000135999	UACAAAG
ENSG00000135999	UCAUGAU
ENSG00000135999	UCCCUGU
ENSG00000135999	UCCCUUU
ENSG00000135999	UGAAAUG
ENSG00000135999	UGCAAAG
ENSG00000136002	AAGGCAC
ENSG00000136002	AUGGCUU
ENSG00000136002	CUGGCUC
ENSG00000136002	UGGAGAU
ENSG00000136002	UGUGUCA
ENSG00000136011	AUGGCUU
ENSG00000136011	CACAGUG
ENSG00000136011	GAGGUAG
ENSG00000136011	GAGGUAU
ENSG00000136014	GAGGUAG
ENSG00000136014	GAGGUAU
ENSG00000136021	UACAAAG
ENSG00000136026	UGCAUAU
ENSG00000136026	UUGGUCC
ENSG00000136044	AUAUUAU
ENSG00000136044	GAUUGUC
ENSG00000136044	UUGGUCC
ENSG00000136051	CACAGUG
ENSG00000136051	CUCACAC
ENSG00000136051	GUAAACA
ENSG00000136051	UCACAGU
ENSG00000136051	UCUCACC
ENSG00000136051	UUAUGCA

ENSG00000136052	AGUGGUU
ENSG00000136068	UACAAAG
ENSG00000136068	UACAGUU
ENSG00000136068	UCACAUU
ENSG00000136099	AAUACUG
ENSG00000136099	ACAGUAC
ENSG00000136099	UUGGCAC
ENSG00000136099	UUUGCAA
ENSG00000136100	AAGACAU
ENSG00000136100	AGCACCA
ENSG00000136100	CCCUGAG
ENSG00000136100	CUAUACA
ENSG00000136100	UUCAAAU
ENSG00000136104	UUUCAAC
ENSG00000136108	UGAAAUG
ENSG00000136111	AGGCAGG
ENSG00000136111	CAGUAGU
ENSG00000136111	CUACAAA
ENSG00000136111	GAAACAU
ENSG00000136111	GGCAGGG
ENSG00000136111	GUCAGUU
ENSG00000136111	UACAAAG
ENSG00000136111	UGGAGAU
ENSG00000136111	UUCAAGC
ENSG00000136111	UUGGAGG
ENSG00000136111	UUGGCAC
ENSG00000136122	AGCCCUU
ENSG00000136122	GCAACUU
ENSG00000136141	GGAUUUU
ENSG00000136144	GUAAACA
ENSG00000136144	UUCAAGC
ENSG00000136144	UUCUAAU
ENSG00000136146	ACUGGAU
ENSG00000136152	AAAAACC
ENSG00000136152	ACAAUAU
ENSG00000136152	AUAAAGU
ENSG00000136152	GAAACAU
ENSG00000136152	GGCAUUG
ENSG00000136152	UAUAAAG
ENSG00000136152	UUGGCAA
ENSG00000136152	UUGGCAC
ENSG00000136153	ACUGCAG
ENSG00000136153	AGCAGCA
ENSG00000136153	AGUGCUU
ENSG00000136153	AUAUUAU
ENSG00000136153	CAGCAAA
ENSG00000136153	UACAAAG
ENSG00000136153	UGUGCUU
ENSG00000136156	AUUGCAC
ENSG00000136158	AAAACUG
ENSG00000136158	AAUUUUA
ENSG00000136158	CACAGUG
ENSG00000136158	GAAAACA
ENSG00000136158	GGAGUGU
ENSG00000136158	UAAUAUC
ENSG00000136158	UAUAAAG
ENSG00000136158	UAUAAUA
ENSG00000136158	UCACAAG
ENSG00000136158	UCACAGG
ENSG00000136158	UUAUGCA
ENSG00000136160	AAGUGCA
ENSG00000136160	AGUGCUU
ENSG00000136160	CCCACCU
ENSG00000136160	GAGAACC

ENSG00000136160	GAGAACU
ENSG00000136160	GCUGGAU
ENSG00000136161	AGCCCUU
ENSG00000136167	AAAGUAA
ENSG00000136167	AAGGCAC
ENSG00000136167	GUAAACA
ENSG00000136167	UUGGCAC
ENSG00000136193	AAUCCUU
ENSG00000136193	ACUGCAG
ENSG00000136193	ACUGGAU
ENSG00000136193	AGCUUAU
ENSG00000136193	AUCCUUU
ENSG00000136193	CUCAAAC
ENSG00000136193	GCAGGAC
ENSG00000136193	GCUGGUG
ENSG00000136193	UAAGACG
ENSG00000136197	CUUUGGU
ENSG00000136205	AUACAAG
ENSG00000136205	AUUCAUU
ENSG00000136205	CUAUACA
ENSG00000136205	CUGCAGC
ENSG00000136205	GACCCUG
ENSG00000136205	GCAGCAU
ENSG00000136205	UACAGUU
ENSG00000136205	UCAUUUG
ENSG00000136205	UUGGCAA
ENSG00000136213	AUAUUAU
ENSG00000136213	GGCAGUG
ENSG00000136213	UGCAAAG
ENSG00000136231	ACAUUCA
ENSG00000136231	AGGUAGU
ENSG00000136231	GAGGUAU
ENSG00000136231	GCCUGGA
ENSG00000136231	GCCUGGG
ENSG00000136231	UCCCUUG
ENSG00000136231	UCCCUUU
ENSG00000136231	UGUCUUU
ENSG00000136237	AAAGUGC
ENSG00000136237	AACACUG
ENSG00000136237	AACAGUC
ENSG00000136237	AAUUAUU
ENSG00000136237	AAUUUUA
ENSG00000136237	ACUCAGC
ENSG00000136237	AGGUAGU
ENSG00000136237	AGUGCUG
ENSG00000136237	AGUGCUU
ENSG00000136237	CAGCAAA
ENSG00000136237	GAGAAGA
ENSG00000136237	GAUUGUA
ENSG00000136237	GAUUGUC
ENSG00000136237	GGUAGAC
ENSG00000136237	UAAUGCU
ENSG00000136237	UAGGUAU
ENSG00000136237	UGAGAAG
ENSG00000136237	UGCAAAG
ENSG00000136237	UGGCCAC
ENSG00000136238	AAUACUG
ENSG00000136238	AUACAAG
ENSG00000136238	UAUUGCU
ENSG00000136238	UCAUACA
ENSG00000136238	UGCAUUG
ENSG00000136238	UUGGCAC
ENSG00000136240	AAGGCAC
ENSG00000136240	ACACACC

ENSG00000136240	CCUUCUU
ENSG00000136240	GGAAUGU
ENSG00000136240	GGCAAGA
ENSG00000136240	UUAGGAU
ENSG00000136261	AUGGCUU
ENSG00000136261	GAGGUAG
ENSG00000136261	UGUGCUU
ENSG00000136267	AAUUAUU
ENSG00000136267	AUCACUA
ENSG00000136267	CUUUGGU
ENSG00000136267	GGUGGGC
ENSG00000136267	UUAUGCA
ENSG00000136279	CACCACC
ENSG00000136279	CACUGUU
ENSG00000136279	CUUUGGU
ENSG00000136279	UUGGUCC
ENSG00000136280	AAUUAUU
ENSG00000136280	CAAAACU
ENSG00000136280	CACAGUG
ENSG00000136280	CAGUAGA
ENSG00000136280	UCACAGG
ENSG00000136280	UCACAGU
ENSG00000136295	ACAGUAC
ENSG00000136295	ACCAGCA
ENSG00000136295	CAGCAAA
ENSG00000136295	GCCUGGG
ENSG00000136295	GUGAGGU
ENSG00000136295	UAGUGAG
ENSG00000136295	UGCAUAG
ENSG00000136295	UGCAUAU
ENSG00000136295	UGUGCUU
ENSG00000136295	UUGGCAA
ENSG00000136295	UUGGCAC
ENSG00000136295	UUGGUCC
ENSG00000136297	UAAUACA
ENSG00000136319	AAUACUG
ENSG00000136327	CUCUGGG
ENSG00000136352	AAAGAAU
ENSG00000136352	UGUGUCA
ENSG00000136352	UUGACAC
ENSG00000136352	UUGUGUC
ENSG00000136378	GCACCAU
ENSG00000136378	GGUGGGC
ENSG00000136379	CCAGUGC
ENSG00000136379	UAUAAAG
ENSG00000136381	AAAUGGU
ENSG00000136381	ACACACU
ENSG00000136381	ACUGUUU
ENSG00000136381	CAGUAGU
ENSG00000136381	CAUGGAU
ENSG00000136381	CUUACAG
ENSG00000136381	UACAAAG
ENSG00000136381	UAUGGUU
ENSG00000136383	AAUUUUA
ENSG00000136383	AUUGCAC
ENSG00000136383	CCCCCAG
ENSG00000136383	CUGCAGC
ENSG00000136383	GAGGUAG
ENSG00000136383	GCCUGGA
ENSG00000136383	GGUGGGC
ENSG00000136383	GUAAACA
ENSG00000136383	UAUUGCU
ENSG00000136383	UGCAUUG
ENSG00000136404	UCACAAG

ENSG00000136425	AGCCCUU
ENSG00000136425	GAGCCCU
ENSG00000136425	UCACAUU
ENSG00000136425	UGAAAGG
ENSG00000136425	UGCAUAU
ENSG00000136425	UGCUGAC
ENSG00000136444	CCCCUGG
ENSG00000136448	AAAGAGC
ENSG00000136448	AAGGCAC
ENSG00000136448	ACAAGGU
ENSG00000136448	ACAUUCA
ENSG00000136448	AGCAGCU
ENSG00000136448	AGCCCUU
ENSG00000136448	AGGAUGU
ENSG00000136448	AGGGAGG
ENSG00000136448	AUAAUAC
ENSG00000136448	AUAUAAC
ENSG00000136448	AUCCUUG
ENSG00000136448	CAGCAGA
ENSG00000136448	CAGCAGG
ENSG00000136448	CAGCUGG
ENSG00000136448	CAGUUAC
ENSG00000136448	CCGGUUC
ENSG00000136448	CUUGGAG
ENSG00000136448	CUUUGGU
ENSG00000136448	GAAAACA
ENSG00000136448	GCUGGUG
ENSG00000136448	GGUGGGC
ENSG00000136448	GUCCAGA
ENSG00000136448	GUCUGCC
ENSG00000136448	UCCCUUU
ENSG00000136448	UCUCACC
ENSG00000136448	UGAAGCU
ENSG00000136448	UUGAUCA
ENSG00000136448	UUGGCAA
ENSG00000136448	UUGGUCC
ENSG00000136451	AAAACUG
ENSG00000136451	AAACUGU
ENSG00000136451	AAAGGUA
ENSG00000136451	AAGGCAC
ENSG00000136451	AAUACUG
ENSG00000136451	AAUUAUU
ENSG00000136451	GGAAUGU
ENSG00000136451	UAUCAGA
ENSG00000136451	UCUCCAA
ENSG00000136451	UGUAUAA
ENSG00000136457	CAAAGCA
ENSG00000136457	CAGUAGA
ENSG00000136457	CAGUAGU
ENSG00000136478	AAGGUGC
ENSG00000136478	AUGGCUU
ENSG00000136478	UUGGCAA
ENSG00000136490	AAGGCAC
ENSG00000136490	ACAAUAU
ENSG00000136490	AGUGCAA
ENSG00000136490	CAGUGCA
ENSG00000136490	CCAGUGU
ENSG00000136490	CCCUGAG
ENSG00000136490	CUGACUC
ENSG00000136490	CUGCAGC
ENSG00000136490	GAAUUGU
ENSG00000136490	GACCCUG
ENSG00000136490	GAGCCCU
ENSG00000136490	GCUGGAU

ENSG00000136490	GGGAGCU
ENSG00000136490	GUGCAAA
ENSG00000136490	UCCUUGC
ENSG00000136490	UCGUGCA
ENSG00000136490	UGCAAAG
ENSG00000136490	UGCUGAC
ENSG00000136504	AAGGCAC
ENSG00000136504	ACAGUAC
ENSG00000136504	ACAGUAU
ENSG00000136504	ACCACAG
ENSG00000136504	ACUGUAG
ENSG00000136504	AGGUUAU
ENSG00000136504	CACAGUG
ENSG00000136504	CUGCAGC
ENSG00000136504	CUGCAGG
ENSG00000136504	CUGGCUC
ENSG00000136504	GAGAAAU
ENSG00000136504	GAGAACC
ENSG00000136504	GAGAUGA
ENSG00000136504	GCAGGUU
ENSG00000136504	GGCAGGG
ENSG00000136504	GUAGUGU
ENSG00000136504	GUGACUG
ENSG00000136504	UCACAAG
ENSG00000136504	UCGCUGC
ENSG00000136504	UUGGCAC
ENSG00000136521	AAAGAGC
ENSG00000136521	AAAGAGG
ENSG00000136521	AGCAAAA
ENSG00000136531	AACAGUC
ENSG00000136531	ACUGCAG
ENSG00000136531	AUGCACC
ENSG00000136531	AUGGCUU
ENSG00000136531	CAGCAGA
ENSG00000136531	CAGCAGG
ENSG00000136531	CUCAAAC
ENSG00000136531	GUCAACA
ENSG00000136531	GUCAGUU
ENSG00000136531	UAUAAUA
ENSG00000136531	UAUUGCU
ENSG00000136531	UGCAAAG
ENSG00000136531	UGCUCAC
ENSG00000136531	UUGGCAA
ENSG00000136531	UUGGCAC
ENSG00000136535	AAAAACC
ENSG00000136535	AACACUG
ENSG00000136535	AACUGGU
ENSG00000136535	ACAAUAU
ENSG00000136535	CACAGGG
ENSG00000136535	GAGACCU
ENSG00000136535	GUGCCUG
ENSG00000136535	UCACAGU
ENSG00000136535	UUGUUCG
ENSG00000136536	ACAGUAC
ENSG00000136536	CUACAGU
ENSG00000136536	GACAACU
ENSG00000136541	GAAUUGU
ENSG00000136541	GUUAAUG
ENSG00000136574	AAAUUUC
ENSG00000136574	AAGACAU
ENSG00000136574	AAUACUG
ENSG00000136574	AGAUAAA
ENSG00000136574	AGCAGCA
ENSG00000136574	AGCCAGU

ENSG00000136574	CAGCAGA
ENSG00000136574	GGAGUCC
ENSG00000136574	UAAGACG
ENSG00000136574	UAAGACU
ENSG00000136574	UCAAGUA
ENSG00000136574	UCUGGAA
ENSG00000136630	CACAGUG
ENSG00000136630	GUAAACA
ENSG00000136630	UCACAGU
ENSG00000136631	ACCUGGA
ENSG00000136631	GGAAUGU
ENSG00000136634	GAGGUAG
ENSG00000136634	UAUAAAG
ENSG00000136634	UCACAGG
ENSG00000136634	UCACAGU
ENSG00000136634	UUCUAAU
ENSG00000136636	AGCACCA
ENSG00000136636	GUAAACA
ENSG00000136643	ACAUUCA
ENSG00000136643	UCCCUUG
ENSG00000136653	ACCAGCA
ENSG00000136653	AGCAGCA
ENSG00000136653	AGCAUUC
ENSG00000136653	CAGCAGA
ENSG00000136653	GAAAACA
ENSG00000136653	GAGAAAU
ENSG00000136653	GCAGCAU
ENSG00000136653	GGAGUGU
ENSG00000136653	UCACAGG
ENSG00000136653	UCACAGU
ENSG00000136653	UCCUGAG
ENSG00000136653	UGCAAAG
ENSG00000136653	UGUGCUU
ENSG00000136689	GGAGUGU
ENSG00000136695	AGCAGCA
ENSG00000136699	UCACAGG
ENSG00000136699	UUGGUCC
ENSG00000136709	AAAAAUC
ENSG00000136709	UAAUACA
ENSG00000136717	AGCAGCU
ENSG00000136720	AAAGCUG
ENSG00000136720	ACAUUCA
ENSG00000136720	AUGGCUU
ENSG00000136720	AUUCAUU
ENSG00000136720	GGGUUUA
ENSG00000136720	UCAAGUA
ENSG00000136720	UCAUUCG
ENSG00000136720	UCCAGUU
ENSG00000136732	CCUGCUG
ENSG00000136754	AAGUGCA
ENSG00000136754	ACAUUCA
ENSG00000136754	AGGUUAU
ENSG00000136754	AUAUUAU
ENSG00000136754	AUGCAUU
ENSG00000136754	CUUUGGU
ENSG00000136754	GGUUUAC
ENSG00000136754	UAAGACG
ENSG00000136754	UAAGACU
ENSG00000136754	UAUAAAG
ENSG00000136754	UCAUGAU
ENSG00000136754	UGGGACA
ENSG00000136758	AAAGUAA
ENSG00000136758	AACAAAC
ENSG00000136758	AAGGCAC

ENSG00000136758	AAUACUG
ENSG00000136758	ACCAGCA
ENSG00000136758	ACUGCAG
ENSG00000136758	UCACAAG
ENSG00000136758	UGAGAAU
ENSG00000136770	GAGGUAG
ENSG00000136770	GAGGUAU
ENSG00000136802	AACACUG
ENSG00000136802	AACGGAA
ENSG00000136802	AAUACUG
ENSG00000136802	ACUGGCC
ENSG00000136802	GGAAUGU
ENSG00000136802	UAAGACG
ENSG00000136802	UAAGACU
ENSG00000136802	UGUAUAA
ENSG00000136802	UGUCUUU
ENSG00000136807	CAGUCCA
ENSG00000136807	GGAAUGU
ENSG00000136807	GGACCUG
ENSG00000136813	AAAGCUG
ENSG00000136813	AUGAUGC
ENSG00000136813	AUGGCAC
ENSG00000136813	GCUACAU
ENSG00000136816	GGAUUUC
ENSG00000136816	GUGCAAA
ENSG00000136816	UAUUGCU
ENSG00000136819	AAAAGUA
ENSG00000136826	AAUACUG
ENSG00000136826	AAUUAUU
ENSG00000136826	ACCACAG
ENSG00000136826	AGAUAAA
ENSG00000136826	AGCACCA
ENSG00000136826	AGUGCUG
ENSG00000136826	AUGGCUU
ENSG00000136826	CACAGUG
ENSG00000136826	GCACCAU
ENSG00000136826	GCAGCAU
ENSG00000136826	GCUUCCU
ENSG00000136826	GUCUGCC
ENSG00000136826	UACAAAG
ENSG00000136826	UAUUGCU
ENSG00000136826	UCAAGUA
ENSG00000136826	UCCAGUU
ENSG00000136826	UCCUUGC
ENSG00000136826	UGUCUGC
ENSG00000136826	UUGUUCG
ENSG00000136827	AAAACUG
ENSG00000136827	GAAACAU
ENSG00000136827	GAGCCCU
ENSG00000136827	GGGAGCU
ENSG00000136827	UCCUUGC
ENSG00000136828	AAAGUAC
ENSG00000136828	AACAAAC
ENSG00000136828	AACACUG
ENSG00000136828	AAGGCAC
ENSG00000136828	AAGUGUG
ENSG00000136828	ACAGUAC
ENSG00000136828	ACAGUAU
ENSG00000136828	ACAUUAC
ENSG00000136828	ACUCAGC
ENSG00000136828	ACUGCAU
ENSG00000136828	ACUUGGA
ENSG00000136828	AGCACCA
ENSG00000136828	AGUGCAA

ENSG00000136828	AGUGGUU
ENSG00000136828	AUACAAG
ENSG00000136828	AUGCAUU
ENSG00000136828	CAAAGUG
ENSG00000136828	CAGUCCA
ENSG00000136828	CUUGUGU
ENSG00000136828	GAGAUGA
ENSG00000136828	GAGGCAG
ENSG00000136828	GAGGUAG
ENSG00000136828	GAGGUAU
ENSG00000136828	GCUGGAU
ENSG00000136828	GCUGGUG
ENSG00000136828	GGAGUGU
ENSG00000136828	GGCAGUG
ENSG00000136828	GGGGUGC
ENSG00000136828	GUAACAG
ENSG00000136828	GUCACUC
ENSG00000136828	GUGACUG
ENSG00000136828	GUGCCAC
ENSG00000136828	UACAGUU
ENSG00000136828	UCACAGU
ENSG00000136828	UUGGCAA
ENSG00000136830	AAGGCAC
ENSG00000136830	CCCUGAG
ENSG00000136842	AAGGCAC
ENSG00000136842	ACCCUGU
ENSG00000136842	AGUGCAA
ENSG00000136842	AUAAAGU
ENSG00000136842	UACAGUU
ENSG00000136842	UAUAAAG
ENSG00000136842	UCCAGUU
ENSG00000136842	UGGCCAC
ENSG00000136848	AAAAAUC
ENSG00000136848	AAAAGUA
ENSG00000136848	AAUUGCU
ENSG00000136848	ACAUAGA
ENSG00000136848	ACGCUCA
ENSG00000136848	AGAUAAA
ENSG00000136848	AGCACCA
ENSG00000136848	AUUGCAC
ENSG00000136848	CCAGCAU
ENSG00000136848	CGGCCUG
ENSG00000136848	CUGGCUC
ENSG00000136848	CUUGGAG
ENSG00000136848	GCUGUCU
ENSG00000136848	GGAGAGA
ENSG00000136848	GUCUUGC
ENSG00000136848	GUGCGCA
ENSG00000136848	UAAUAUC
ENSG00000136848	UACAAAG
ENSG00000136848	UAUUACC
ENSG00000136848	UAUUGCU
ENSG00000136848	UCACAUU
ENSG00000136848	UGCAAAG
ENSG00000136848	UGCAUUG
ENSG00000136848	UGGAGAU
ENSG00000136848	UUUGCAA
ENSG00000136848	UUUGCAC
ENSG00000136848	UUUUUGC
ENSG00000136854	AACACUG
ENSG00000136854	AGCAGCA
ENSG00000136854	AGUGCUU
ENSG00000136854	CUUUGGU
ENSG00000136854	GUAAACA

ENSG00000136854	GUGCUUG
ENSG00000136854	UUCAAGC
ENSG00000136854	UUGGAGG
ENSG00000136859	AAAAACC
ENSG00000136859	ACAAGGU
ENSG00000136859	AUGGCUU
ENSG00000136859	AUUGCAC
ENSG00000136859	GAGAAAU
ENSG00000136859	GAGGUAG
ENSG00000136859	GAGGUAU
ENSG00000136859	GUCAUUG
ENSG00000136859	UAUUGCU
ENSG00000136859	UCCCUUU
ENSG00000136868	AAAGUAA
ENSG00000136868	AAAGUAC
ENSG00000136868	AACAGUC
ENSG00000136868	AACAUUC
ENSG00000136868	AACUGGU
ENSG00000136868	AAUCUCA
ENSG00000136868	AGCACCA
ENSG00000136868	AGCAGCU
ENSG00000136868	AGGUAGU
ENSG00000136868	CAAAACU
ENSG00000136868	CAAAGCA
ENSG00000136868	CAUGGGU
ENSG00000136868	CCUUCUG
ENSG00000136868	CUGUUGA
ENSG00000136868	GAGGUAG
ENSG00000136868	GAUUGUA
ENSG00000136868	GCCCUGU
ENSG00000136868	GGAAUGU
ENSG00000136868	GGGUUUA
ENSG00000136868	GUCAACA
ENSG00000136868	UCCCACA
ENSG00000136868	UCCCACC
ENSG00000136868	UGCCCUG
ENSG00000136868	UUCAAAU
ENSG00000136868	UUGGUUC
ENSG00000136868	UUGUUCG
ENSG00000136869	UGACAUC
ENSG00000136870	AAGGCAC
ENSG00000136870	CUCCCAA
ENSG00000136874	ACUGGAU
ENSG00000136874	ACUGGCU
ENSG00000136874	CCUUCUU
ENSG00000136874	GAGGCAG
ENSG00000136874	GAGGUAG
ENSG00000136874	GAGGUAU
ENSG00000136874	GAGUCUU
ENSG00000136874	GAGUUGG
ENSG00000136874	GCCUGAU
ENSG00000136874	GGCUCAG
ENSG00000136874	GGUGGGC
ENSG00000136874	UAUAAUA
ENSG00000136874	UAUUGCU
ENSG00000136874	UUGACAC
ENSG00000136874	UUGAGGC
ENSG00000136875	CACAAUG
ENSG00000136877	GGAGGAG
ENSG00000136878	GAGGUAU
ENSG00000136881	AAGACAU
ENSG00000136881	UAGACUG
ENSG00000136888	UAAUGCU
ENSG00000136888	UGGCUGC

ENSG00000136908	AAAGCUG
ENSG00000136908	AGCUGCC
ENSG00000136928	AAAAACC
ENSG00000136928	AAAGUGC
ENSG00000136928	AAGUGCA
ENSG00000136928	AAUACUG
ENSG00000136928	ACAUUAC
ENSG00000136928	AGCCCUU
ENSG00000136928	AGCUGCC
ENSG00000136928	AGGCAGG
ENSG00000136928	AUUCAUU
ENSG00000136928	CACAGUG
ENSG00000136928	CACUGUU
ENSG00000136928	CAGUCCA
ENSG00000136928	CCUGCUG
ENSG00000136928	CUCUGGG
ENSG00000136928	CUUGGGC
ENSG00000136928	CUUUGGU
ENSG00000136928	GAGGUAG
ENSG00000136928	GGAAGCC
ENSG00000136928	GGAAUGU
ENSG00000136928	GGCAGUG
ENSG00000136928	GGCCCUG
ENSG00000136928	GGGCCCC
ENSG00000136928	GUGAGGU
ENSG00000136928	UAUUGCU
ENSG00000136928	UCCAGUU
ENSG00000136928	UCCCUUG
ENSG00000136928	UCUGGAA
ENSG00000136928	UGAAAUG
ENSG00000136928	UGAAGCU
ENSG00000136928	UUGGUCC
ENSG00000136928	UUGUGUC
ENSG00000136928	UUUGCAC
ENSG00000136931	AAUUGCU
ENSG00000136931	CAGGCUC
ENSG00000136931	GGCUCAG
ENSG00000136935	AAUACUG
ENSG00000136935	ACUGCAU
ENSG00000136935	GCACCAU
ENSG00000136935	GGUUUAC
ENSG00000136935	GUAGUGU
ENSG00000136935	UACAGUU
ENSG00000136935	UCACAGG
ENSG00000136935	UCACAGU
ENSG00000136937	AAAGUUU
ENSG00000136937	AGCAUUC
ENSG00000136937	GGAAUGU
ENSG00000136937	GUGCCUG
ENSG00000136938	AAAGUAA
ENSG00000136938	GGAAUGU
ENSG00000136940	ACAGUAU
ENSG00000136940	GAGACCU
ENSG00000136940	GCUUCCU
ENSG00000136940	GUAAACA
ENSG00000136944	ACAAGGU
ENSG00000136944	ACAGGUG
ENSG00000136944	AGGACAC
ENSG00000136944	AUGGCUU
ENSG00000136944	CACAGUG
ENSG00000136944	CUCUGGG
ENSG00000136944	CUGGCUC
ENSG00000136944	GAGUGUG
ENSG00000136944	GGAAGCC

ENSG00000136944	GGCAGGG
ENSG00000136944	GGCGGGG
ENSG00000136944	GGGGUGG
ENSG00000136944	GGGUGGU
ENSG00000136944	GUGUGGC
ENSG00000136944	UACAAAG
ENSG00000136944	UGGCUGC
ENSG00000136944	UUCCAUA
ENSG00000136944	UUCUGCA
ENSG00000136986	UACAAAG
ENSG00000136986	UGAGAAU
ENSG00000136999	UCCUGAG
ENSG00000137033	ACUUGGA
ENSG00000137033	GGUUGAC
ENSG00000137033	UAUGGUU
ENSG00000137040	AAAGUGC
ENSG00000137054	CUGGCUC
ENSG00000137055	AGUGCAA
ENSG00000137055	GUGCAAA
ENSG00000137055	UCACAGU
ENSG00000137070	ACCAGCA
ENSG00000137070	CACCAGC
ENSG00000137073	GACAACU
ENSG00000137073	UACAAAG
ENSG00000137073	UCACAUU
ENSG00000137075	ACAGUAU
ENSG00000137075	ACAUCAC
ENSG00000137075	AGUGUGC
ENSG00000137075	AUCAUAC
ENSG00000137075	CAAAUGC
ENSG00000137075	CCAGUGU
ENSG00000137075	CUAGUAA
ENSG00000137075	CUUUGGU
ENSG00000137075	GACCCUG
ENSG00000137075	GAGGUAG
ENSG00000137075	GAGGUAU
ENSG00000137075	GCUGGUG
ENSG00000137075	GGAAUGU
ENSG00000137075	GGUUACC
ENSG00000137075	GUAGUGU
ENSG00000137075	GUGCAAA
ENSG00000137075	GUGCUUG
ENSG00000137075	UAUUGCU
ENSG00000137075	UCACAGU
ENSG00000137075	UGUGCUU
ENSG00000137075	UUGGAGG
ENSG00000137075	UUGGUUC
ENSG00000137076	AAGGCAC
ENSG00000137076	AAUACUG
ENSG00000137076	AGCAGCG
ENSG00000137076	UAAUACA
ENSG00000137090	AAAUUUC
ENSG00000137090	AACAUUC
ENSG00000137090	UCUCAAG
ENSG00000137094	AAAAACC
ENSG00000137094	AACAUUC
ENSG00000137094	AACCUGU
ENSG00000137094	ACUGGAU
ENSG00000137094	AGCUGCC
ENSG00000137094	CCCCUGG
ENSG00000137094	GAGAAAU
ENSG00000137094	GUCAUUG
ENSG00000137094	UACAAAG
ENSG00000137094	UAUUGCU

ENSG00000137094	UCAAGUA
ENSG00000137101	AAAACUG
ENSG00000137101	AAUUAUU
ENSG00000137101	CUGGCUC
ENSG00000137101	GCCUGGA
ENSG00000137101	GCCUGGG
ENSG00000137135	AAAGCUG
ENSG00000137135	GGGAGCU
ENSG00000137135	UCUGGAA
ENSG00000137135	UGACCUA
ENSG00000137145	GUGCAAA
ENSG00000137145	UCAUGAU
ENSG00000137145	UCCCACA
ENSG00000137166	AAAGUUU
ENSG00000137166	ACAGGUG
ENSG00000137166	ACAGUAC
ENSG00000137166	AGGGAGG
ENSG00000137166	AUGUGGG
ENSG00000137166	CACAGUG
ENSG00000137166	CCCCCAG
ENSG00000137166	CCUGCUG
ENSG00000137166	CUUCUCU
ENSG00000137166	GAGCCCC
ENSG00000137166	GAGUCUU
ENSG00000137166	GCCUGGG
ENSG00000137166	GCGGGUG
ENSG00000137166	GCUGGUG
ENSG00000137166	GCUUCCU
ENSG00000137166	GGCGACA
ENSG00000137166	GGGCCCC
ENSG00000137166	GGGGUCC
ENSG00000137166	GGUGGUC
ENSG00000137166	GGUGGUU
ENSG00000137166	GGUUUAC
ENSG00000137166	GUAAACA
ENSG00000137166	UAAUAUC
ENSG00000137166	UAGAGGA
ENSG00000137166	UAUUGCU
ENSG00000137166	UCACAGG
ENSG00000137166	UCACAGU
ENSG00000137166	UCUCACC
ENSG00000137166	UGCAGCU
ENSG00000137166	UGGCCAC
ENSG00000137166	UUCCGGC
ENSG00000137166	UUGGUCC
ENSG00000137166	UUGGUUC
ENSG00000137166	UUUUUGC
ENSG00000137168	UUGACAC
ENSG00000137168	UUGGCAC
ENSG00000137177	GUGCAAA
ENSG00000137193	AGCAGCA
ENSG00000137193	GGCUCAG
ENSG00000137193	UCUGGAA
ENSG00000137198	AUAUUAU
ENSG00000137198	GGGCCCC
ENSG00000137203	AAAGAAU
ENSG00000137203	AAAGCGC
ENSG00000137203	AAAGUAA
ENSG00000137203	AAGACAU
ENSG00000137203	ACUGUAG
ENSG00000137203	AUUGCAC
ENSG00000137203	CACAAUG
ENSG00000137203	CAGCUGG
ENSG00000137203	GCCUGGG

ENSG00000137203	GCUACAU
ENSG00000137203	GGGUAAG
ENSG00000137203	UCAAGUA
ENSG00000137203	UCACAAG
ENSG00000137203	UCCCUUG
ENSG00000137203	UCCCUUU
ENSG00000137203	UGAAAGG
ENSG00000137204	AAUGUUG
ENSG00000137204	GGAUGAG
ENSG00000137216	AAUUAUU
ENSG00000137216	CUCCAGC
ENSG00000137216	GGCUCUG
ENSG00000137216	GUGCAAA
ENSG00000137216	UGGGGGA
ENSG00000137218	AAAAAUC
ENSG00000137218	AAACUGU
ENSG00000137218	AAUUAUU
ENSG00000137218	CACAGUG
ENSG00000137218	UCACAGG
ENSG00000137218	UCACAGU
ENSG00000137221	AAAUUUC
ENSG00000137221	AGUAACA
ENSG00000137221	CUGGGGG
ENSG00000137221	GUAACAG
ENSG00000137221	UCCAGAG
ENSG00000137221	UCUAGAG
ENSG00000137259	AAAGAGC
ENSG00000137261	GAGCCCU
ENSG00000137265	AGCAGCA
ENSG00000137265	GAGAAAU
ENSG00000137265	UCACAGG
ENSG00000137265	UCCCUGU
ENSG00000137266	AAGUCUU
ENSG00000137266	GUAAACA
ENSG00000137267	AGCACCA
ENSG00000137269	AAAAGUA
ENSG00000137269	AAAGUAU
ENSG00000137269	ACAGUAC
ENSG00000137269	ACAGUAU
ENSG00000137269	AGAUAAA
ENSG00000137269	AGCUGCC
ENSG00000137269	AUACAAG
ENSG00000137269	AUGGCAC
ENSG00000137269	CUAUACA
ENSG00000137269	GGAAGAC
ENSG00000137273	AAACUGU
ENSG00000137273	AAAGUAU
ENSG00000137273	AAGUGCU
ENSG00000137273	AAUACUG
ENSG00000137273	GAGUAUU
ENSG00000137273	GGUAGAC
ENSG00000137275	CAAAACU
ENSG00000137285	AGCACCA
ENSG00000137285	AGGACAC
ENSG00000137285	GACCAUG
ENSG00000137309	AGCAGCA
ENSG00000137309	AGGUAGU
ENSG00000137309	CAGCAGG
ENSG00000137309	CUGCAGC
ENSG00000137309	GAGGUAG
ENSG00000137309	GCAGCAU
ENSG00000137309	UCCAGAG
ENSG00000137310	CUCCAUU
ENSG00000137310	CUUUGGU

ENSG00000137310	GGCAGGG
ENSG00000137310	UGGCCAC
ENSG00000137310	UUGGCAA
ENSG00000137310	UUGGCAC
ENSG00000137312	AAGGCAC
ENSG00000137331	GAGUUGA
ENSG00000137331	GUAGUGU
ENSG00000137343	AACACUG
ENSG00000137343	AAUCUCA
ENSG00000137343	AGUGUGC
ENSG00000137343	CAAAGCA
ENSG00000137343	CUCACAC
ENSG00000137343	CUCCCAA
ENSG00000137343	CUUCUCU
ENSG00000137343	GACCCUG
ENSG00000137343	GAGUGUG
ENSG00000137343	GGGAGCU
ENSG00000137343	GUGCUUG
ENSG00000137343	UACAAAG
ENSG00000137343	UCACAAG
ENSG00000137343	UCCCUGU
ENSG00000137343	UGUGCUU
ENSG00000137343	UUGGCAC
ENSG00000137393	AGCACCA
ENSG00000137393	AGCAGCG
ENSG00000137393	AGUGCCU
ENSG00000137393	CCCCUGG
ENSG00000137393	CCCUGAG
ENSG00000137393	CUGGACU
ENSG00000137393	GAGGCAG
ENSG00000137393	UUCUAAU
ENSG00000137393	UUGGCAA
ENSG00000137404	CCCUGAG
ENSG00000137409	CAAAGCA
ENSG00000137409	GUAGUGU
ENSG00000137409	UACAAAG
ENSG00000137413	CAAAUGC
ENSG00000137413	CCUUCUG
ENSG00000137413	CCUUCUU
ENSG00000137414	AAAGUAU
ENSG00000137414	AACACUG
ENSG00000137414	AUCACUA
ENSG00000137414	CUUUGGU
ENSG00000137414	GGCAUUG
ENSG00000137414	UACAAAG
ENSG00000137414	UCUGGAG
ENSG00000137414	UUAGAGA
ENSG00000137414	UUCCAUA
ENSG00000137414	UUGGCAA
ENSG00000137414	UUGGCAC
ENSG00000137449	AAAACUG
ENSG00000137449	AAAGAAU
ENSG00000137449	AAAUUUC
ENSG00000137449	AAGUGCA
ENSG00000137449	AAGUGCU
ENSG00000137449	AAGUGUG
ENSG00000137449	AAUCUCU
ENSG00000137449	ACAAUAU
ENSG00000137449	ACAGUAC
ENSG00000137449	ACAGUAU
ENSG00000137449	ACCUGGA
ENSG00000137449	AGCAGCG
ENSG00000137449	AGCAGCU
ENSG00000137449	AGGGUUG

ENSG00000137449	AGUGCAA
ENSG00000137449	AGUGUGC
ENSG00000137449	AUACAAG
ENSG00000137449	AUAUAAC
ENSG00000137449	AUGACAC
ENSG00000137449	AUGCACC
ENSG00000137449	AUGGCAC
ENSG00000137449	CAAAACU
ENSG00000137449	CACAGGG
ENSG00000137449	CACUGUU
ENSG00000137449	CAGCAGA
ENSG00000137449	CAGUCUG
ENSG00000137449	CAUAUUG
ENSG00000137449	CAUGGGU
ENSG00000137449	CCAGUAC
ENSG00000137449	CCUUCUG
ENSG00000137449	CUCCAUU
ENSG00000137449	CUUCACC
ENSG00000137449	GAAAACA
ENSG00000137449	GAGAUGA
ENSG00000137449	GAUUGUC
ENSG00000137449	GCAACUU
ENSG00000137449	GCAGAAG
ENSG00000137449	GGAAGAC
ENSG00000137449	GGAGAGA
ENSG00000137449	GGAUUUC
ENSG00000137449	GGCAGCG
ENSG00000137449	GGCAGGG
ENSG00000137449	GGCAGUG
ENSG00000137449	GGCAUUG
ENSG00000137449	GGCUGCG
ENSG00000137449	GUAAACA
ENSG00000137449	GUGACUG
ENSG00000137449	UCAAGUA
ENSG00000137449	UCUCCAA
ENSG00000137449	UCUGGAA
ENSG00000137449	UCUGGAG
ENSG00000137449	UCUUGAG
ENSG00000137449	UGAAGUG
ENSG00000137449	UGACUGU
ENSG00000137449	UGAGGAC
ENSG00000137449	UGCAAAG
ENSG00000137449	UGGAUAA
ENSG00000137449	UGUGCUU
ENSG00000137449	UUAUGCA
ENSG00000137449	UUCCAUA
ENSG00000137449	UUCCUCG
ENSG00000137449	UUGAGGC
ENSG00000137449	UUGGUUC
ENSG00000137460	AAAGGUA
ENSG00000137460	AAAUGGU
ENSG00000137460	AAGUCUU
ENSG00000137460	AAUUAUU
ENSG00000137460	AGCAGCA
ENSG00000137460	AGCAGCG
ENSG00000137460	AGCAGCU
ENSG00000137460	CACAGGG
ENSG00000137460	UCACACA
ENSG00000137460	UCUCCAA
ENSG00000137460	UGCCAGC
ENSG00000137474	CUGCAGG
ENSG00000137474	UGCCCUG
ENSG00000137474	UUAUGCA
ENSG00000137478	AAGGCAC

ENSG00000137478	AAGGUGC
ENSG00000137478	ACUGUAG
ENSG00000137478	AGCAGCA
ENSG00000137478	AGCAGCG
ENSG00000137478	GAUUGUA
ENSG00000137478	GAUUGUC
ENSG00000137478	UGCAAAG
ENSG00000137478	UGUGCUU
ENSG00000137486	GCUGGUG
ENSG00000137486	GGUAGAC
ENSG00000137486	UGAGAAU
ENSG00000137491	AACCUGG
ENSG00000137491	UCCUGAG
ENSG00000137492	AUACAAG
ENSG00000137492	GGAAUGU
ENSG00000137492	GUAAACA
ENSG00000137494	AGGACAC
ENSG00000137494	CUGCUCA
ENSG00000137494	GAGUUGG
ENSG00000137497	AACAUUC
ENSG00000137497	AAGGCAC
ENSG00000137497	GAGCUGC
ENSG00000137497	GCCCUUA
ENSG00000137497	GCCUGGG
ENSG00000137497	GGGAGCU
ENSG00000137500	UUUCAAC
ENSG00000137501	AGCAGCA
ENSG00000137502	AAAGUGC
ENSG00000137502	GAAGGUC
ENSG00000137502	GUGCCUG
ENSG00000137502	UUGGUCC
ENSG00000137504	AAAGAGC
ENSG00000137504	AAAGAGG
ENSG00000137504	AACAAAC
ENSG00000137504	AACCUGG
ENSG00000137504	AAGUCAC
ENSG00000137504	AAGUGCA
ENSG00000137504	ACCAGCA
ENSG00000137504	ACCCAUU
ENSG00000137504	ACUCAGC
ENSG00000137504	ACUGCAG
ENSG00000137504	ACUGCAU
ENSG00000137504	ACUUGGA
ENSG00000137504	AGCAGCA
ENSG00000137504	AGCAGCU
ENSG00000137504	AGUAGAC
ENSG00000137504	AUCAUAC
ENSG00000137504	AUGACAC
ENSG00000137504	CAGCAGG
ENSG00000137504	CAGCUGG
ENSG00000137504	CAGUAGA
ENSG00000137504	CAGUAGU
ENSG00000137504	CAGUCCA
ENSG00000137504	CAGUUAC
ENSG00000137504	CCCGUCC
ENSG00000137504	CUCUUCC
ENSG00000137504	CUGCCCC
ENSG00000137504	CUUCACC
ENSG00000137504	CUUUGGU
ENSG00000137504	GAAACAU
ENSG00000137504	GACCAUG
ENSG00000137504	GAGAAGA
ENSG00000137504	GAGAUGA
ENSG00000137504	GAGCCCC

ENSG00000137504	GAGCCCU
ENSG00000137504	GAGGGGC
ENSG00000137504	GCAGAAG
ENSG00000137504	GCAGGUU
ENSG00000137504	GCUGGAU
ENSG00000137504	GCUGGUG
ENSG00000137504	GGACUGC
ENSG00000137504	GGCAGGG
ENSG00000137504	GGGAACG
ENSG00000137504	GGUUUAC
ENSG00000137504	GUCAGUU
ENSG00000137504	GUCAUUG
ENSG00000137504	GUGCAAA
ENSG00000137504	GUGGCCC
ENSG00000137504	UAGGUAU
ENSG00000137504	UAUUACC
ENSG00000137504	UCACAAG
ENSG00000137504	UCAUACA
ENSG00000137504	UCAUUCG
ENSG00000137504	UGCAGCU
ENSG00000137504	UGCAUUG
ENSG00000137504	UGGACUG
ENSG00000137504	UGUGCUU
ENSG00000137504	UUAGAGA
ENSG00000137504	UUCAAGC
ENSG00000137504	UUGGUUC
ENSG00000137504	UUGUGAC
ENSG00000137504	UUUGCAA
ENSG00000137504	UUUGCAC
ENSG00000137504	UUUUUGC
ENSG00000137507	CAGCUGG
ENSG00000137507	GAGGCAG
ENSG00000137507	UACAAAG
ENSG00000137522	AAGUCAC
ENSG00000137522	ACUGGCU
ENSG00000137522	AUUCAUU
ENSG00000137522	CCAGGAG
ENSG00000137522	CCCACCG
ENSG00000137522	CCCACCU
ENSG00000137522	CCCUGAG
ENSG00000137522	CUCCAUU
ENSG00000137522	CUGGCUC
ENSG00000137522	GAGCCCC
ENSG00000137522	GAGUCUU
ENSG00000137522	GGCAGGG
ENSG00000137522	GGGGUCC
ENSG00000137522	GUGGCCC
ENSG00000137522	UCCCACA
ENSG00000137522	UCCCACC
ENSG00000137522	UCCUGAG
ENSG00000137522	UGAGAAG
ENSG00000137522	UGCCCUG
ENSG00000137522	UGCUGAC
ENSG00000137558	ACCCGUA
ENSG00000137558	AGAUAAA
ENSG00000137558	AGCUUAU
ENSG00000137558	GGUUUAC
ENSG00000137558	UACAGUU
ENSG00000137571	AAGGCAC
ENSG00000137571	GAGGUAG
ENSG00000137571	GAGGUAU
ENSG00000137571	GUGCUUG
ENSG00000137571	UCACAGU
ENSG00000137571	UGCAUAU

ENSG00000137573	AAAGUAU
ENSG00000137573	AAAGUGC
ENSG00000137573	AAGUCUU
ENSG00000137573	AAGUGCA
ENSG00000137573	AGUGGUU
ENSG00000137573	CAAAGUG
ENSG00000137573	CAGCAGG
ENSG00000137573	CAGUGCA
ENSG00000137573	CCAGUGC
ENSG00000137573	CCUUCAU
ENSG00000137573	GAAUUGU
ENSG00000137573	GAGGUAG
ENSG00000137573	GAGGUAU
ENSG00000137573	UAUUGCU
ENSG00000137573	UCCAGUU
ENSG00000137573	UCCUGAG
ENSG00000137573	UCUAGAG
ENSG00000137573	UGCCAGC
ENSG00000137575	AAGGCGC
ENSG00000137575	AUGGCUU
ENSG00000137575	UCACAUU
ENSG00000137642	AAAAAUC
ENSG00000137642	ACUCAAA
ENSG00000137642	CAUAUUG
ENSG00000137642	GCAACUU
ENSG00000137656	CAAAACU
ENSG00000137672	AGCACCA
ENSG00000137672	UCAAGUA
ENSG00000137692	GUAACAG
ENSG00000137693	AGUAGUU
ENSG00000137693	CAGUAGU
ENSG00000137693	GUAACAG
ENSG00000137709	AGAUAAA
ENSG00000137709	AUGGCUU
ENSG00000137709	CACACCG
ENSG00000137709	UCACACA
ENSG00000137709	UUGGCAC
ENSG00000137710	AACUGGU
ENSG00000137710	AAGUGCA
ENSG00000137710	AGGUAGU
ENSG00000137710	AGUGCAA
ENSG00000137710	AGUGCUU
ENSG00000137710	CUGUUGA
ENSG00000137710	GAGGUAG
ENSG00000137710	GAGGUAU
ENSG00000137710	GGCAAGA
ENSG00000137710	GGUGGUC
ENSG00000137710	UAAUAGU
ENSG00000137710	UGUCUGC
ENSG00000137710	UGUUGAA
ENSG00000137710	UUGGCAA
ENSG00000137710	UUGGCAC
ENSG00000137710	UUUCAAC
ENSG00000137713	AAGAACC
ENSG00000137713	AAUCUCA
ENSG00000137713	CCCUGAG
ENSG00000137713	CUGGAAA
ENSG00000137713	GAAGGUC
ENSG00000137713	GGAAUGU
ENSG00000137713	UACAAAG
ENSG00000137713	UUCCAUA
ENSG00000137714	AAUUAUU
ENSG00000137714	UACAAAG
ENSG00000137726	AGGGAGG

ENSG00000137726	UAUUGCU
ENSG00000137726	UGCUGAC
ENSG00000137727	AAAAGUA
ENSG00000137727	ACUCAAA
ENSG00000137727	AGCAGCG
ENSG00000137727	AGCAGCU
ENSG00000137727	CAGUAGU
ENSG00000137727	CAGUGCA
ENSG00000137727	GAGGUAG
ENSG00000137745	UCACAGG
ENSG00000137745	UCACAGU
ENSG00000137745	UGAAGCU
ENSG00000137747	GAGUAUU
ENSG00000137747	GCCUGGG
ENSG00000137747	UCCCUGU
ENSG00000137764	CAAAGCA
ENSG00000137766	AACAUUC
ENSG00000137766	AACUGGU
ENSG00000137766	CAUGGAU
ENSG00000137766	CUUCACC
ENSG00000137766	UCACAGG
ENSG00000137766	UCUAGAG
ENSG00000137766	UUUGCAC
ENSG00000137770	AAAGGUA
ENSG00000137770	AAAGUGC
ENSG00000137770	AACAGUC
ENSG00000137770	ACUGGCU
ENSG00000137770	ACUGUAG
ENSG00000137770	AGCAAAA
ENSG00000137770	AGGUUAU
ENSG00000137770	AGUGCCG
ENSG00000137770	AUGCCUU
ENSG00000137770	AUGUAAU
ENSG00000137770	CAGUCUG
ENSG00000137770	CUACAAA
ENSG00000137770	CUGUUGA
ENSG00000137770	CUUCUCU
ENSG00000137770	GAGGUAU
ENSG00000137770	GAUUGUA
ENSG00000137770	GUAAACA
ENSG00000137770	GUGCAAA
ENSG00000137770	GUUAAUG
ENSG00000137770	UCAUUUG
ENSG00000137770	UGAAGCU
ENSG00000137770	UGUUGAA
ENSG00000137770	UUAGAGA
ENSG00000137770	UUCCAUA
ENSG00000137770	UUCCUAG
ENSG00000137770	UUUUUGC
ENSG00000137776	AACAGUC
ENSG00000137776	AAUACUG
ENSG00000137776	ACAGUAC
ENSG00000137776	ACCACAG
ENSG00000137776	ACUCAGC
ENSG00000137776	ACUCAGG
ENSG00000137776	AGGUUAU
ENSG00000137776	AUAAUAC
ENSG00000137776	CCUUCUG
ENSG00000137776	CUAUACA
ENSG00000137776	GAAGGUC
ENSG00000137776	GAGCUAA
ENSG00000137776	UUCUGCA
ENSG00000137801	AAUUAUU
ENSG00000137801	ACAGUAC

ENSG00000137801	GAGGUAG
ENSG00000137801	GAGGUAU
ENSG00000137801	UCACACA
ENSG00000137802	AAAAACC
ENSG00000137802	AAAAGUA
ENSG00000137802	AACAGUC
ENSG00000137802	ACCCUGU
ENSG00000137802	AGCACCA
ENSG00000137802	AGCCCUU
ENSG00000137802	AUGGCUU
ENSG00000137802	CAAAUGC
ENSG00000137802	CAGCUGG
ENSG00000137802	CUGCAGC
ENSG00000137802	GCAGAAG
ENSG00000137802	GGAAUGU
ENSG00000137802	GGAGGCA
ENSG00000137802	GGGAGCU
ENSG00000137802	UAUAAAG
ENSG00000137802	UCCCACA
ENSG00000137802	UGCAGCU
ENSG00000137802	UUCAAAU
ENSG00000137807	AAGUCAC
ENSG00000137807	AAGUGCA
ENSG00000137807	AGCAGCG
ENSG00000137807	AGCAGCU
ENSG00000137807	CUUUGGU
ENSG00000137809	AGCACCA
ENSG00000137809	AGCCCUU
ENSG00000137809	GUGCAAA
ENSG00000137812	AAUUUUA
ENSG00000137815	AAAUGGU
ENSG00000137815	AACAGUC
ENSG00000137815	ACUGCAG
ENSG00000137815	AGCAGCA
ENSG00000137815	AGUACUG
ENSG00000137815	CAGCAGG
ENSG00000137815	CUUUGGU
ENSG00000137815	GGCAGUG
ENSG00000137815	UACAAAG
ENSG00000137815	UAUUACC
ENSG00000137815	UCACAUU
ENSG00000137815	UUCUUGU
ENSG00000137817	CAGUCCA
ENSG00000137817	GGGCCCC
ENSG00000137817	UACAAAG
ENSG00000137819	AAACUGU
ENSG00000137819	AAGUGCA
ENSG00000137819	AAUUUUA
ENSG00000137819	ACCAGCA
ENSG00000137819	CAUAUUG
ENSG00000137819	CCUUCAU
ENSG00000137819	GAAAACA
ENSG00000137819	UCACAAG
ENSG00000137824	UUGUUCG
ENSG00000137825	GCAGGAC
ENSG00000137831	AAGGCAC
ENSG00000137831	UUCCUAG
ENSG00000137834	AAAGUGC
ENSG00000137834	AUAUUAU
ENSG00000137834	AUUGCAC
ENSG00000137834	CAAAGUG
ENSG00000137842	AUUCAUU
ENSG00000137842	CAGUAGA
ENSG00000137842	CAGUAGU

ENSG00000137842	CAGUCCA
ENSG00000137842	UCAUUUG
ENSG00000137843	AAGUGCA
ENSG00000137843	AAUACUG
ENSG00000137843	AGGCAGG
ENSG00000137843	AGUGCAA
ENSG00000137843	CGGCCUG
ENSG00000137843	CUUUGGU
ENSG00000137843	GAGGCAG
ENSG00000137843	GUCUCUG
ENSG00000137843	GUGGCCC
ENSG00000137843	UCACAAG
ENSG00000137843	UCACAGU
ENSG00000137843	UCCCUGU
ENSG00000137843	UGGAGAU
ENSG00000137843	UGGCCCU
ENSG00000137843	UUGGAGG
ENSG00000137845	AAGAACC
ENSG00000137845	CAUAUUG
ENSG00000137845	CUUUGGU
ENSG00000137845	UCCUGAG
ENSG00000137845	UCUCCAA
ENSG00000137845	UGCAUAU
ENSG00000137845	UUGGCAA
ENSG00000137857	CCAGCAU
ENSG00000137871	ACUGGAU
ENSG00000137872	ACCAGCA
ENSG00000137872	AGCAGCG
ENSG00000137872	AGCAGCU
ENSG00000137872	CACAGUG
ENSG00000137872	CUUUGGU
ENSG00000137872	GUGAAUG
ENSG00000137872	GUGCAAA
ENSG00000137872	UGGAUAA
ENSG00000137872	UUCAAGC
ENSG00000137878	AUGUAAU
ENSG00000137878	GCCUGGG
ENSG00000137878	UAUCAGA
ENSG00000137878	UCUGGAG
ENSG00000137878	UUCUGCA
ENSG00000137941	AGCAAAA
ENSG00000137942	AAAGUGC
ENSG00000137942	AAGGCAC
ENSG00000137942	AAGUGCA
ENSG00000137942	ACUGUAG
ENSG00000137942	AGCCCUU
ENSG00000137942	AUAUAAC
ENSG00000137942	AUGCACC
ENSG00000137942	GCAGCAU
ENSG00000137942	UUGGCAA
ENSG00000137944	AAAAGUA
ENSG00000137944	AAAGUUU
ENSG00000137944	GGUUUAC
ENSG00000137944	UCACAAG
ENSG00000137944	UCACAGG
ENSG00000137944	UGCAAAG
ENSG00000137944	UGGGUGA
ENSG00000137944	UUCCUCG
ENSG00000137947	CUACAAA
ENSG00000137947	GCUACAU
ENSG00000137947	GGAAUGU
ENSG00000137959	CCUUCAU
ENSG00000137959	UUGAGGC
ENSG00000137962	AAGUGCU

ENSG00000137962	AUACAAG
ENSG00000137962	AUGCAUU
ENSG00000137962	AUGUAAU
ENSG00000137962	AUUGCAC
ENSG00000137962	CCAGUGC
ENSG00000137962	CCAGUGU
ENSG00000137962	CUCAAAC
ENSG00000137962	GGCCCUG
ENSG00000137962	UCCUAUA
ENSG00000137962	UUGGCAC
ENSG00000137962	UUUGCAA
ENSG00000137992	AACAUUC
ENSG00000137992	AGCACCA
ENSG00000137992	AUAUAAC
ENSG00000137992	AUCAUAC
ENSG00000137992	AUUGCAC
ENSG00000137992	CUGGACU
ENSG00000137992	CUUUGGU
ENSG00000137992	GCACCAU
ENSG00000137992	GCCUGAU
ENSG00000137992	GUGCCUG
ENSG00000137992	UAUAAAG
ENSG00000137992	UCUCCAA
ENSG00000137992	UGCAAAG
ENSG00000137992	UGUCUUU
ENSG00000138028	CUGCAGC
ENSG00000138029	UUCCUAG
ENSG00000138030	CUCUGGG
ENSG00000138031	AAAAAUC
ENSG00000138031	ACUGUAG
ENSG00000138031	AGCAAAA
ENSG00000138031	CAAGAGC
ENSG00000138031	CACAGUG
ENSG00000138032	AAUACUG
ENSG00000138032	ACAAUAU
ENSG00000138032	ACAUCAC
ENSG00000138032	GAAAACA
ENSG00000138032	UCAAGUA
ENSG00000138035	AAACUGU
ENSG00000138036	AAAACUG
ENSG00000138036	AAUUGCU
ENSG00000138036	AAUUUUA
ENSG00000138036	ACAUCAC
ENSG00000138036	CCAGUGC
ENSG00000138036	CCAGUGU
ENSG00000138036	UAUAAAG
ENSG00000138036	UCAUUUG
ENSG00000138036	UCCAGUU
ENSG00000138041	AAUUUUA
ENSG00000138041	AGCACCA
ENSG00000138041	CACCACC
ENSG00000138041	CAGACAG
ENSG00000138041	CCAGGAG
ENSG00000138041	CUACAAA
ENSG00000138041	GCACCAU
ENSG00000138041	UCACAUU
ENSG00000138041	UCCUGAG
ENSG00000138041	UCUCCAA
ENSG00000138061	CACAGGG
ENSG00000138061	UCACAGU
ENSG00000138069	AAUCUCU
ENSG00000138069	ACACACC
ENSG00000138069	ACACACU
ENSG00000138069	ACAGUAU

ENSG00000138069	ACCAGCA
ENSG00000138069	AGCAAAA
ENSG00000138069	AGUGCAA
ENSG00000138069	AUAUAAC
ENSG00000138069	CUGUUGA
ENSG00000138069	CUUACAG
ENSG00000138069	GACCAUG
ENSG00000138069	GCUACAU
ENSG00000138069	GCUGUCU
ENSG00000138069	GGAGGCA
ENSG00000138069	GUAGUGU
ENSG00000138069	GUCUUGC
ENSG00000138069	GUGACAG
ENSG00000138069	UACAGUU
ENSG00000138069	UAUAAAG
ENSG00000138069	UAUAUGA
ENSG00000138069	UCUUGAG
ENSG00000138069	UGAGUCU
ENSG00000138069	UGCAGCU
ENSG00000138069	UUCUGCA
ENSG00000138071	ACUUGGA
ENSG00000138071	AGCAGCG
ENSG00000138071	AGCAGCU
ENSG00000138071	CAGACAG
ENSG00000138071	CCAGCAU
ENSG00000138073	CAGCAAA
ENSG00000138074	GAGGUAG
ENSG00000138074	UCCCUCU
ENSG00000138080	UGUCUUU
ENSG00000138081	AAGUGCU
ENSG00000138081	ACAAUAU
ENSG00000138081	ACAUUCA
ENSG00000138081	ACCCAUU
ENSG00000138081	ACUCCAG
ENSG00000138081	ACUGUUU
ENSG00000138081	AGAUGUG
ENSG00000138081	AGCUUAU
ENSG00000138081	AUAUUAU
ENSG00000138081	CAGGCUC
ENSG00000138081	CAGUGCA
ENSG00000138081	CAGUUAC
ENSG00000138081	GACCAUG
ENSG00000138081	GCAGAAG
ENSG00000138081	GCUAGCA
ENSG00000138081	GGCAGGG
ENSG00000138081	UAAUGCU
ENSG00000138081	UACAAAG
ENSG00000138081	UAUGGUU
ENSG00000138081	UCACAUU
ENSG00000138081	UCCUGAG
ENSG00000138081	UGAAAUG
ENSG00000138081	UGGGACA
ENSG00000138081	UUCCAUA
ENSG00000138083	AAAGUAU
ENSG00000138083	GCAGGUU
ENSG00000138083	GUGCUUG
ENSG00000138092	AAAGUGC
ENSG00000138092	AGUGCAA
ENSG00000138092	UCCAGAG
ENSG00000138092	UCUGGAG
ENSG00000138095	CUGGAAA
ENSG00000138100	GUCUCUG
ENSG00000138101	AGCCUGC
ENSG00000138101	CUCUGGG

ENSG00000138101	GAGAUGA
ENSG00000138101	GGGGGAA
ENSG00000138101	UGGGGGA
ENSG00000138107	AAAGUGC
ENSG00000138107	AACAAAC
ENSG00000138107	AAUACUG
ENSG00000138107	ACUGCAG
ENSG00000138107	AGCAGCA
ENSG00000138107	AGUGGUU
ENSG00000138107	CUUGGGC
ENSG00000138107	CUUUGGU
ENSG00000138107	GGCAGGG
ENSG00000138107	GGCAGUG
ENSG00000138107	GUAAACA
ENSG00000138107	UUGGUCC
ENSG00000138111	AGCCCUU
ENSG00000138111	CAGACAG
ENSG00000138111	CUGCAGC
ENSG00000138111	GGCUCAG
ENSG00000138111	GGGGUGG
ENSG00000138131	AGCACCA
ENSG00000138131	CAAAGCA
ENSG00000138131	GAGGUAG
ENSG00000138131	GAGGUAU
ENSG00000138131	GCUUCCU
ENSG00000138131	UCUCAAG
ENSG00000138131	UUUGCAA
ENSG00000138136	AUUGCAC
ENSG00000138136	UAUUGCU
ENSG00000138138	AGUAGUU
ENSG00000138138	CAGUAGA
ENSG00000138138	CUACAGU
ENSG00000138138	CUUCACC
ENSG00000138138	UCAUGAU
ENSG00000138152	CUGGAAA
ENSG00000138160	AAAUCCC
ENSG00000138160	GAGAAAU
ENSG00000138160	UCCAGAG
ENSG00000138162	AAGGCAC
ENSG00000138162	CAGGAAC
ENSG00000138162	CAGUAGU
ENSG00000138162	GGCAAGA
ENSG00000138166	AAUUAUU
ENSG00000138166	ACAGUAC
ENSG00000138166	ACAGUAU
ENSG00000138166	ACAUUCA
ENSG00000138166	AUGGCUU
ENSG00000138166	CACAGUG
ENSG00000138166	CAGUAGU
ENSG00000138166	CCUUCAU
ENSG00000138166	GGUCUAG
ENSG00000138166	GUGCUUG
ENSG00000138166	UCACAGU
ENSG00000138166	UCUGGAA
ENSG00000138166	UUGGUCC
ENSG00000138175	AAUACUG
ENSG00000138175	AAUCUCA
ENSG00000138175	AAUCUCU
ENSG00000138175	AAUUUUA
ENSG00000138175	ACAUUCA
ENSG00000138175	CAGUUAC
ENSG00000138175	CCCACCG
ENSG00000138175	CCCACCU
ENSG00000138175	CUCCAUU

ENSG00000138175	CUCCCAC
ENSG00000138175	GUCAUUG
ENSG00000138175	UAAUACA
ENSG00000138175	UCCCACA
ENSG00000138175	UCCCACC
ENSG00000138175	UGUGCUU
ENSG00000138175	UUAUGCA
ENSG00000138175	UUCCAUA
ENSG00000138175	UUGGUCC
ENSG00000138180	AAAAAUC
ENSG00000138180	AGUGCAA
ENSG00000138180	CAGUGCA
ENSG00000138180	GUGCAAA
ENSG00000138185	AAAUGGU
ENSG00000138185	AAUACUG
ENSG00000138185	AGCAUUC
ENSG00000138185	CUCCAGC
ENSG00000138185	CUCCAUU
ENSG00000138185	GGAGAGA
ENSG00000138185	UCAACAG
ENSG00000138185	UGGGACA
ENSG00000138207	AGAUAAA
ENSG00000138246	AGUGCUU
ENSG00000138246	CACAGUG
ENSG00000138246	GGAGGAG
ENSG00000138246	UCACAGG
ENSG00000138246	UCCCUUG
ENSG00000138279	AAGGCAC
ENSG00000138279	UACAAAG
ENSG00000138293	AAAGAAU
ENSG00000138293	AAGGCAC
ENSG00000138293	ACUCAGC
ENSG00000138293	AGCACCA
ENSG00000138293	AUGCACC
ENSG00000138293	GCACCAU
ENSG00000138293	GUCACUC
ENSG00000138293	UCAAGUA
ENSG00000138303	AGUGCUU
ENSG00000138308	GAAACAU
ENSG00000138311	ACUGGCC
ENSG00000138311	CUUUGGU
ENSG00000138311	UUCAAAU
ENSG00000138316	GAGGUAG
ENSG00000138316	GAGGUAU
ENSG00000138316	GUAAACA
ENSG00000138316	GUGCUUG
ENSG00000138316	UGUGCUU
ENSG00000138326	AAAAAUC
ENSG00000138326	CUCCAGC
ENSG00000138336	AACCUGU
ENSG00000138336	AACUCUA
ENSG00000138336	AGCAGCU
ENSG00000138336	AUCCCUU
ENSG00000138336	AUGCACC
ENSG00000138336	AUGGCAC
ENSG00000138336	CACAGUG
ENSG00000138336	CCUUCAU
ENSG00000138336	CUUGUAU
ENSG00000138336	GAUUGGU
ENSG00000138336	GGCAUUG
ENSG00000138336	GUAAACA
ENSG00000138347	AAUUGCU
ENSG00000138347	CAGCAGG
ENSG00000138356	UAGAGGA

ENSG00000138380	AUGUAAU
ENSG00000138380	UCCAGUU
ENSG00000138386	AACACUG
ENSG00000138386	AACUACU
ENSG00000138386	ACAUUAC
ENSG00000138386	ACAUUCA
ENSG00000138386	AGUAGAC
ENSG00000138386	CAGUGCA
ENSG00000138386	CCAGUGC
ENSG00000138386	CUACAGU
ENSG00000138386	GAGGCAG
ENSG00000138386	GGAAUGU
ENSG00000138386	UACAAAG
ENSG00000138386	UCAUGAU
ENSG00000138386	UUCAAGC
ENSG00000138398	AAAACUG
ENSG00000138411	AAAAAUC
ENSG00000138411	AAUUAUU
ENSG00000138411	ACAUUCA
ENSG00000138411	ACUGUGU
ENSG00000138411	AGUGCUU
ENSG00000138411	AUAAAGU
ENSG00000138411	CUUUGGU
ENSG00000138411	GAUAUGU
ENSG00000138411	GUGCAAA
ENSG00000138411	UGGUACC
ENSG00000138413	GAGUGUG
ENSG00000138413	UAUUGCU
ENSG00000138413	UUGGUCC
ENSG00000138430	AAAGCUG
ENSG00000138430	AAUCACU
ENSG00000138430	ACACACC
ENSG00000138430	ACAGUAU
ENSG00000138430	ACUGUAG
ENSG00000138430	ACUGUGU
ENSG00000138430	AGCUGCC
ENSG00000138430	AGUACUG
ENSG00000138430	AUGCAUU
ENSG00000138430	CAAAUGC
ENSG00000138430	CACUGUU
ENSG00000138430	CAGGCUC
ENSG00000138430	CAGUUAC
ENSG00000138430	CUACAAA
ENSG00000138430	CUGCCCC
ENSG00000138430	GAGUGUG
ENSG00000138430	GGGUCUU
ENSG00000138430	UAUGGUU
ENSG00000138430	UCAUACA
ENSG00000138430	UCCUGAG
ENSG00000138430	UCUGGAG
ENSG00000138430	UGCAGCU
ENSG00000138430	UGUGCUU
ENSG00000138430	UUGGAGG
ENSG00000138430	UUUUUGC
ENSG00000138434	AGUAGUU
ENSG00000138434	AUACCUC
ENSG00000138434	UUCAAGC
ENSG00000138443	AAACUGU
ENSG00000138443	ACAUUAC
ENSG00000138443	ACUGGCU
ENSG00000138443	AGUACUG
ENSG00000138443	AUCCUUG
ENSG00000138443	AUCCUUU
ENSG00000138443	AUGGCAC

ENSG00000138443	CUCUGGG
ENSG00000138443	GAGAAAU
ENSG00000138443	GGAAGAC
ENSG00000138443	GGAAUGU
ENSG00000138443	GGAUUUC
ENSG00000138443	UACAAAG
ENSG00000138443	UCAUUCG
ENSG00000138443	UCUGGAG
ENSG00000138443	UGCAAAG
ENSG00000138443	UUGUUCG
ENSG00000138448	ACUCAAA
ENSG00000138448	UGAGAAG
ENSG00000138448	UGGGUGA
ENSG00000138449	AAGUGCA
ENSG00000138449	ACAAUAU
ENSG00000138449	GUAACAG
ENSG00000138459	AAAUUUC
ENSG00000138468	AAAGAAU
ENSG00000138468	AAAUGGU
ENSG00000138468	ACAGUAU
ENSG00000138468	AUUCAUU
ENSG00000138468	GAGAAAU
ENSG00000138468	UAAUAUC
ENSG00000138468	UCAUACA
ENSG00000138468	UGCAAAG
ENSG00000138468	UGCAUAG
ENSG00000138468	UGCAUAU
ENSG00000138495	UAUAAAG
ENSG00000138495	UCAUUCG
ENSG00000138496	AAGGCAC
ENSG00000138587	AGUGGUU
ENSG00000138587	UGACAUC
ENSG00000138592	GUGCAAA
ENSG00000138594	UCCAGUU
ENSG00000138594	UCCCUUG
ENSG00000138604	AAAACUG
ENSG00000138604	AAGGCAC
ENSG00000138604	ACAUUAC
ENSG00000138604	AGCAGCA
ENSG00000138604	AGCAGCG
ENSG00000138604	AUAAAGU
ENSG00000138604	AUUGCAC
ENSG00000138604	CUUGUGU
ENSG00000138604	GAUUGUC
ENSG00000138604	GGAAUGU
ENSG00000138604	GUCAACA
ENSG00000138604	GUGUCAC
ENSG00000138604	UAUUGCU
ENSG00000138604	UUCCAUA
ENSG00000138604	UUGGUUC
ENSG00000138606	CACAGUG
ENSG00000138613	AAGGCAC
ENSG00000138614	CUGGAAA
ENSG00000138614	UCCUGAG
ENSG00000138615	GUGACAG
ENSG00000138615	UUCAAGC
ENSG00000138617	CAAAACU
ENSG00000138617	CCAGGAG
ENSG00000138617	GUAAACA
ENSG00000138617	UGGCCCU
ENSG00000138617	UUUGCAA
ENSG00000138622	AAUCACU
ENSG00000138622	ACCUGGA
ENSG00000138622	CACAGUG

ENSG00000138622	CAGUAGA
ENSG00000138622	CCCUGAG
ENSG00000138622	CUAUACA
ENSG00000138622	GACCUGG
ENSG00000138622	GUGCUUG
ENSG00000138622	UCACAGU
ENSG00000138623	UCACAGU
ENSG00000138639	AAAAAUC
ENSG00000138639	AAAGUAU
ENSG00000138639	AACACUG
ENSG00000138639	AACUACU
ENSG00000138639	AAGUGCU
ENSG00000138639	AGCAUUC
ENSG00000138639	AUAUAAC
ENSG00000138639	AUGGAUU
ENSG00000138639	CACAAUG
ENSG00000138639	CAGGUGA
ENSG00000138639	CUUUGGU
ENSG00000138639	UCAUUUG
ENSG00000138639	UCCAGUU
ENSG00000138639	UGAAAGG
ENSG00000138639	UGAAGCU
ENSG00000138639	UGGUACC
ENSG00000138639	UUGGCAC
ENSG00000138639	UUGGUCC
ENSG00000138641	AAUCACU
ENSG00000138641	AUUGCAC
ENSG00000138641	CAGCAGA
ENSG00000138641	UAUUGCU
ENSG00000138641	UGCAUAG
ENSG00000138641	UGCAUAU
ENSG00000138650	ACACACU
ENSG00000138650	ACAGUAC
ENSG00000138650	ACAGUAU
ENSG00000138650	AGUGCUU
ENSG00000138650	AUAAUAC
ENSG00000138650	AUUGCAC
ENSG00000138650	CCUGCUG
ENSG00000138650	CUCAGGG
ENSG00000138650	CUUUGGU
ENSG00000138650	GGCUCAG
ENSG00000138650	UACAGUU
ENSG00000138650	UCACACA
ENSG00000138650	UGAAAUG
ENSG00000138650	UGCAUUG
ENSG00000138650	UGGAGAU
ENSG00000138650	UGUAUAA
ENSG00000138650	UUGAUCA
ENSG00000138650	UUGGAGG
ENSG00000138650	UUUCAAC
ENSG00000138650	UUUGCAA
ENSG00000138668	AAAGAGC
ENSG00000138668	AAGACAU
ENSG00000138668	AAGUUGU
ENSG00000138668	ACAAUAU
ENSG00000138668	ACAUCAC
ENSG00000138668	ACCCUGU
ENSG00000138668	ACGUCAU
ENSG00000138668	AUACAAG
ENSG00000138668	AUGACAC
ENSG00000138668	CAAAUGC
ENSG00000138668	CCCUGUU
ENSG00000138668	CUACAAA
ENSG00000138668	GAGAACC

ENSG00000138668	UGCAAAG
ENSG00000138669	AAAAAUC
ENSG00000138670	ACCAGCA
ENSG00000138670	ACUCAAA
ENSG00000138670	AGCAGCG
ENSG00000138670	AGCAGCU
ENSG00000138670	CACAGUG
ENSG00000138670	CAGCAAA
ENSG00000138675	AUGCAUU
ENSG00000138675	CUAUACA
ENSG00000138675	CUUUGGU
ENSG00000138675	GAGGUAG
ENSG00000138675	GGUUACC
ENSG00000138675	UCAUAGA
ENSG00000138675	UCCUGAG
ENSG00000138675	UGAAAUG
ENSG00000138688	CACAGUG
ENSG00000138696	AACAAAC
ENSG00000138696	ACAGUAC
ENSG00000138696	AGUACUG
ENSG00000138696	AGUGCAA
ENSG00000138696	AUAUUAU
ENSG00000138696	CCCUGAG
ENSG00000138696	CUGCCCC
ENSG00000138696	GGCUCAG
ENSG00000138696	GUAACAG
ENSG00000138696	UCACAUU
ENSG00000138696	UGAAGCU
ENSG00000138696	UGUGCUU
ENSG00000138696	UUGGCAC
ENSG00000138696	UUGUUCG
ENSG00000138698	AGCACCA
ENSG00000138698	CACUGUU
ENSG00000138698	GUGCUUG
ENSG00000138698	UGUGCUU
ENSG00000138698	UUGACAC
ENSG00000138722	AGAUAAA
ENSG00000138722	AGAUGUG
ENSG00000138735	AAAGGUA
ENSG00000138735	AAGGCAC
ENSG00000138735	AAGUUGU
ENSG00000138735	AAUCUCA
ENSG00000138735	ACACACU
ENSG00000138735	ACAUAGA
ENSG00000138735	AGUGCCG
ENSG00000138735	CCAGUGC
ENSG00000138735	UUCUAAU
ENSG00000138735	UUGUUCG
ENSG00000138741	AAAAAUC
ENSG00000138741	AAGACAU
ENSG00000138741	AUAUUAU
ENSG00000138741	CCCUGUC
ENSG00000138741	CUUGGAG
ENSG00000138741	UAUUGCU
ENSG00000138741	UGUGCUU
ENSG00000138741	UUCAAGC
ENSG00000138744	UGCAGCU
ENSG00000138757	AAGGCAC
ENSG00000138757	ACUGCAU
ENSG00000138757	AGCAGCU
ENSG00000138757	AGUAACA
ENSG00000138757	AGUGCAA
ENSG00000138757	CAGCAGA
ENSG00000138757	CAGUAGA

ENSG00000138757	GAGGGGC
ENSG00000138757	GAGUGUG
ENSG00000138757	GCACCAU
ENSG00000138757	GCAGCAU
ENSG00000138757	GGAAGAC
ENSG00000138757	GGAAUGU
ENSG00000138757	GGAGGAG
ENSG00000138757	GGAGUGU
ENSG00000138757	GUGCAAA
ENSG00000138757	UCAUACA
ENSG00000138757	UGCAGCU
ENSG00000138757	UGUGUCA
ENSG00000138758	AAGGCAC
ENSG00000138758	ACAUUCA
ENSG00000138758	ACUGCAG
ENSG00000138758	ACUGCAU
ENSG00000138758	ACUGUGU
ENSG00000138758	AGCAGCA
ENSG00000138758	AUACAAG
ENSG00000138758	AUCCUUG
ENSG00000138758	AUGCAUU
ENSG00000138758	CAGGUGA
ENSG00000138758	CCUUCAU
ENSG00000138758	CUCACAC
ENSG00000138758	CUGACUC
ENSG00000138758	GCUGGUG
ENSG00000138758	GGGUAAG
ENSG00000138758	GUAACAG
ENSG00000138758	GUGAAUG
ENSG00000138758	UACAGUU
ENSG00000138758	UCACACA
ENSG00000138758	UCACAGG
ENSG00000138758	UCACAGU
ENSG00000138758	UCCCUGU
ENSG00000138758	UGCAAAG
ENSG00000138758	UGCAGCU
ENSG00000138758	UGCAUAG
ENSG00000138758	UGCCAGC
ENSG00000138758	UGGCUGC
ENSG00000138758	UGGGUAC
ENSG00000138758	UUCUAAU
ENSG00000138758	UUGGCAC
ENSG00000138759	AAAUCCC
ENSG00000138759	AAGAACC
ENSG00000138759	AAGGCAC
ENSG00000138759	ACUCAAA
ENSG00000138759	AGCACCA
ENSG00000138759	AGUGCUU
ENSG00000138759	CAAAGCA
ENSG00000138759	CCCUGAG
ENSG00000138759	CUCCAGC
ENSG00000138759	CUGGAAA
ENSG00000138759	CUGGGGG
ENSG00000138759	GAGCUAA
ENSG00000138759	GAGGCAG
ENSG00000138759	GAGGUAU
ENSG00000138759	GAGUUGG
ENSG00000138759	GCCUGAU
ENSG00000138759	GGAAUGU
ENSG00000138759	GGUUGAC
ENSG00000138759	GUGCUUG
ENSG00000138759	UCCCUUG
ENSG00000138760	AAAAGUA
ENSG00000138760	AACACUG

ENSG00000138760	ACCAGCA
ENSG00000138760	ACCCUGU
ENSG00000138760	CAGCAAA
ENSG00000138760	GAUUGUA
ENSG00000138760	GAUUGUC
ENSG00000138760	GGAUUUC
ENSG00000138760	GUGACAG
ENSG00000138760	UACAAAG
ENSG00000138760	UCACAAG
ENSG00000138760	UCACAGG
ENSG00000138760	UGAAAUG
ENSG00000138760	UUGGCAA
ENSG00000138764	AUGGCUU
ENSG00000138767	AAAGCUG
ENSG00000138767	AAAGUGC
ENSG00000138767	AAGUGCA
ENSG00000138767	ACAGGUG
ENSG00000138767	ACAGUAU
ENSG00000138767	ACCCAUU
ENSG00000138767	ACUGUGU
ENSG00000138767	AGCAAAA
ENSG00000138767	AGCAGCG
ENSG00000138767	AGCCCUU
ENSG00000138767	AGCUGCC
ENSG00000138767	AGGCAGG
ENSG00000138767	AGUGCAA
ENSG00000138767	AGUGCCU
ENSG00000138767	AGUGCUG
ENSG00000138767	AUACCUC
ENSG00000138767	AUCACUA
ENSG00000138767	AUCAUAC
ENSG00000138767	AUGCACC
ENSG00000138767	AUGGCAC
ENSG00000138767	CACACCU
ENSG00000138767	CAGGAAC
ENSG00000138767	CCAGUAC
ENSG00000138767	CCAGUGU
ENSG00000138767	CUAUACA
ENSG00000138767	GAGGAUA
ENSG00000138767	GAGGUAG
ENSG00000138767	GAGGUAU
ENSG00000138767	GCAGCAU
ENSG00000138767	GCAGGAC
ENSG00000138767	GCCUGAU
ENSG00000138767	GGCAGCG
ENSG00000138767	GGCAGUG
ENSG00000138767	GGGGCAG
ENSG00000138767	GUGCUUG
ENSG00000138767	UAUUGCU
ENSG00000138767	UCAACAG
ENSG00000138767	UCACAGG
ENSG00000138767	UCAUACA
ENSG00000138767	UCCAGAG
ENSG00000138767	UCCAGUU
ENSG00000138767	UGAAAGG
ENSG00000138767	UGCAAAG
ENSG00000138767	UGCAUAU
ENSG00000138767	UGCUCAC
ENSG00000138767	UUGACAC
ENSG00000138767	UUGGCAA
ENSG00000138767	UUGGCAC
ENSG00000138769	AAUCUCU
ENSG00000138769	ACAUUCA
ENSG00000138769	AGCACCA

ENSG00000138771	AGCAGCA
ENSG00000138771	CUCAAAC
ENSG00000138771	CUUUGGU
ENSG00000138771	GAAGGAA
ENSG00000138771	UCACAAG
ENSG00000138771	UCCUGAG
ENSG00000138780	AAAAGUA
ENSG00000138780	AAUCACU
ENSG00000138780	ACCAGCA
ENSG00000138780	GAUCAGA
ENSG00000138780	GCAGCAU
ENSG00000138792	AAAGCUG
ENSG00000138792	AGUACUG
ENSG00000138792	CAAGAGC
ENSG00000138792	GUGAAUG
ENSG00000138792	UCACAGG
ENSG00000138792	UCACAGU
ENSG00000138795	AAGUGCU
ENSG00000138795	ACUCAAA
ENSG00000138795	AGUGCUG
ENSG00000138795	CUCAAAC
ENSG00000138795	CUCCAGC
ENSG00000138795	GAUUGUA
ENSG00000138796	AAGGCAC
ENSG00000138814	AAAAAUC
ENSG00000138814	AACAGUC
ENSG00000138814	ACAAGGU
ENSG00000138814	ACAAUAU
ENSG00000138814	ACCCGUA
ENSG00000138814	AGCUUAU
ENSG00000138814	AGGCAGG
ENSG00000138814	AGUAACA
ENSG00000138814	AUAAAGU
ENSG00000138814	AUGCACC
ENSG00000138814	AUGCAUU
ENSG00000138814	CAAAGCA
ENSG00000138814	CAGCAAA
ENSG00000138814	CCAGUAC
ENSG00000138814	CUACAAA
ENSG00000138814	CUUGGGC
ENSG00000138814	GCACCAU
ENSG00000138814	GGCAAGA
ENSG00000138814	GUAAACA
ENSG00000138814	GUAGUGU
ENSG00000138814	GUCUUGC
ENSG00000138814	GUUCAUG
ENSG00000138814	UAAUAUC
ENSG00000138814	UACAGUU
ENSG00000138814	UAUAAAG
ENSG00000138814	UCACAAG
ENSG00000138814	UCACAUU
ENSG00000138814	UGCAUAG
ENSG00000138814	UUCAAAU
ENSG00000138814	UUCAAGC
ENSG00000138814	UUGGCAA
ENSG00000138814	UUGGCAC
ENSG00000138814	UUUCAAC
ENSG00000138814	UUUGCAA
ENSG00000138821	AAAGGUA
ENSG00000138823	AUGUAAU
ENSG00000138823	GUAAACA
ENSG00000138823	UACAAAG
ENSG00000138829	AGCUGCC
ENSG00000138829	AUUGCAC

ENSG00000138829	UAUUGCU
ENSG00000138829	UUGGUCC
ENSG00000138834	AAAGCUG
ENSG00000138834	ACAUCAC
ENSG00000138834	CACAGUG
ENSG00000138834	CUUCUCU
ENSG00000138834	GAGAAAU
ENSG00000138834	GGCGGGG
ENSG00000138834	GGGAGCU
ENSG00000138834	GUGCUUG
ENSG00000138834	UAUUACC
ENSG00000138834	UCACAGU
ENSG00000138834	UCCUGAG
ENSG00000138834	UGUGCUU
ENSG00000138835	CGUACCG
ENSG00000138835	UCCUAUA
ENSG00000138835	UUCCUAG
ENSG00000138867	AAAGUAC
ENSG00000138867	AAGGCAC
ENSG00000138867	AGUGGUU
ENSG00000138867	AUCCCUU
ENSG00000138867	UACAAAG
ENSG00000138867	UCCCUUU
ENSG00000138867	UCCUGAG
ENSG00000138942	AACACUG
ENSG00000138942	AUCCCUU
ENSG00000138942	GUCUCUG
ENSG00000138942	GUGGGGA
ENSG00000138942	UAGUGAG
ENSG00000138942	UCACAAG
ENSG00000138942	UCCCUUU
ENSG00000139055	ACUUGGA
ENSG00000139083	ACUGGCC
ENSG00000139083	AUGUAAU
ENSG00000139083	CACACCU
ENSG00000139083	CAGCUGG
ENSG00000139083	CAGUCCA
ENSG00000139083	CCAGUGU
ENSG00000139083	GAAAACA
ENSG00000139083	GAGAAAU
ENSG00000139083	GCAACUU
ENSG00000139083	GCAGCAU
ENSG00000139083	GCUGUCU
ENSG00000139083	GGUGGGC
ENSG00000139083	GUCUGCC
ENSG00000139083	UAUAAAG
ENSG00000139083	UAUAAUA
ENSG00000139083	UCCUGAG
ENSG00000139083	UGCAAAG
ENSG00000139083	UGGCCCU
ENSG00000139083	UGUCUGC
ENSG00000139112	AAUUAUU
ENSG00000139112	AGCAAAA
ENSG00000139112	AGCAGCG
ENSG00000139112	CAGCUGG
ENSG00000139112	GAGAUGA
ENSG00000139112	UUGGUCC
ENSG00000139116	ACAUAGA
ENSG00000139116	AGCAGCG
ENSG00000139116	AGCAGCU
ENSG00000139116	CUUUGGU
ENSG00000139116	UACAAAG
ENSG00000139116	UCCUGAG
ENSG00000139116	UUGGUCC

ENSG00000139117	GUGCUUG
ENSG00000139117	UAUUGCU
ENSG00000139131	UGAGAAG
ENSG00000139132	AUAAAGU
ENSG00000139132	GGAAUGU
ENSG00000139146	AAAGUGC
ENSG00000139146	AAAGUUU
ENSG00000139146	AAGUGCA
ENSG00000139146	AAUACUG
ENSG00000139146	AAUGCCC
ENSG00000139146	ACACACU
ENSG00000139146	ACUGCAU
ENSG00000139146	AGCAGCA
ENSG00000139146	AGCAUUC
ENSG00000139146	CAAAGUG
ENSG00000139146	CAGUAGA
ENSG00000139146	CUGCAGC
ENSG00000139146	GAAUUGU
ENSG00000139146	GAGAUGA
ENSG00000139146	GCAGCAU
ENSG00000139146	GGCAAGA
ENSG00000139146	GGUCUAG
ENSG00000139146	UAAGACU
ENSG00000139146	UCCAGAG
ENSG00000139146	UGAUCCA
ENSG00000139146	UUGGCAA
ENSG00000139146	UUGGCAC
ENSG00000139154	AAAACUG
ENSG00000139154	AAAGUGC
ENSG00000139154	AAGACAU
ENSG00000139154	AAGGUGC
ENSG00000139154	AAGUGCU
ENSG00000139154	ACACACU
ENSG00000139154	ACAGUAU
ENSG00000139154	ACAUAGA
ENSG00000139154	ACUGCAG
ENSG00000139154	AGGAUGU
ENSG00000139154	AUAAUAC
ENSG00000139154	AUAUAAC
ENSG00000139154	AUCCCUU
ENSG00000139154	AUCCUUG
ENSG00000139154	AUCCUUU
ENSG00000139154	CACUGUU
ENSG00000139154	CAGUAGU
ENSG00000139154	CCUUCAU
ENSG00000139154	CUACAAA
ENSG00000139154	CUAGUCC
ENSG00000139154	CUGCAGC
ENSG00000139154	CUGCAGG
ENSG00000139154	CUGGAAA
ENSG00000139154	CUUUGGU
ENSG00000139154	GAAACAU
ENSG00000139154	GAAUUGU
ENSG00000139154	GCUGGUG
ENSG00000139154	GGCAGCG
ENSG00000139154	GUGUGGC
ENSG00000139154	UACAAAG
ENSG00000139154	UCACAUU
ENSG00000139154	UCCAGAG
ENSG00000139154	UCUGGAG
ENSG00000139154	UGAAGUG
ENSG00000139154	UGCAAAG
ENSG00000139154	UUCUGCA
ENSG00000139154	UUGGCAC

ENSG00000139154	UUGUUCG
ENSG00000139154	UUUGCAC
ENSG00000139163	AACACUG
ENSG00000139163	AAGGCAC
ENSG00000139163	AGCCUGC
ENSG00000139163	AGUGCAA
ENSG00000139163	AGUGCUG
ENSG00000139163	AUAAUAC
ENSG00000139163	AUCCCUU
ENSG00000139163	CAAAGCA
ENSG00000139163	CACUGUU
ENSG00000139163	CAGUGCA
ENSG00000139163	CAUAUUG
ENSG00000139163	CUACAAA
ENSG00000139163	CUCUUCC
ENSG00000139163	CUUCACC
ENSG00000139163	GAGGUAG
ENSG00000139163	GAGGUAU
ENSG00000139163	GGCUCAG
ENSG00000139163	GGUCUAG
ENSG00000139163	GUGCAAA
ENSG00000139163	UAGAGGA
ENSG00000139163	UCCAGUU
ENSG00000139163	UGCAAAG
ENSG00000139163	UGCAUAU
ENSG00000139173	AAGGCAC
ENSG00000139173	UCUCAAG
ENSG00000139182	ACAUUAC
ENSG00000139182	GAAAACA
ENSG00000139182	GAGCUGC
ENSG00000139190	AGCAGCA
ENSG00000139190	GCAGCAU
ENSG00000139190	GCAGGAC
ENSG00000139190	GCCUGGG
ENSG00000139190	GUGCAAA
ENSG00000139190	UCCCUUG
ENSG00000139190	UGAAAUG
ENSG00000139197	AAAAAUC
ENSG00000139197	AGCACCA
ENSG00000139197	AGCAGCA
ENSG00000139197	CGUACCG
ENSG00000139197	GAGGCAG
ENSG00000139200	AGGAGCU
ENSG00000139200	AUAAUAC
ENSG00000139200	CACCACC
ENSG00000139200	CCCACCG
ENSG00000139200	CCCACCU
ENSG00000139200	CUCCCAC
ENSG00000139200	CUGGCUC
ENSG00000139200	GAGUGUG
ENSG00000139200	GGCUCAG
ENSG00000139200	UAAUACA
ENSG00000139200	UCACCAC
ENSG00000139200	UCCCACA
ENSG00000139200	UCCCACC
ENSG00000139200	UGCAAAG
ENSG00000139209	AAUACUG
ENSG00000139209	CUUUGGU
ENSG00000139209	GGAAGAC
ENSG00000139209	UCACAGU
ENSG00000139211	UUGGUUC
ENSG00000139218	AAAGAAU
ENSG00000139218	AGCACCA
ENSG00000139218	AGCAGCA

ENSG00000139218	CAGUAGU
ENSG00000139218	GCAGCAU
ENSG00000139218	GUGCUUG
ENSG00000139218	UAUUGCU
ENSG00000139219	CAGUCUG
ENSG00000139219	GCACCAU
ENSG00000139219	GGAAGAC
ENSG00000139219	GUCUGCC
ENSG00000139220	AGUGCAA
ENSG00000139220	AGUGCUG
ENSG00000139263	GAGGUAU
ENSG00000139266	ACCAGCA
ENSG00000139266	AGGAGCU
ENSG00000139266	CUGGGGG
ENSG00000139266	CUUCACC
ENSG00000139266	GAGGUAG
ENSG00000139266	GAGGUAU
ENSG00000139266	GCCCUGU
ENSG00000139266	GGAGUCC
ENSG00000139266	GGCAUUG
ENSG00000139266	UAUUGCU
ENSG00000139266	UGCAUAU
ENSG00000139266	UGCCAGC
ENSG00000139266	UGCCCUG
ENSG00000139266	UUAUGCA
ENSG00000139289	ACAGUAU
ENSG00000139289	ACAUCAC
ENSG00000139289	CUGCCCC
ENSG00000139289	CUUACAG
ENSG00000139289	GCAACUU
ENSG00000139289	GCAGAAG
ENSG00000139289	GGUGGUU
ENSG00000139289	GUGCAAA
ENSG00000139289	UACAGUU
ENSG00000139289	UCCAGAG
ENSG00000139289	UGGUACC
ENSG00000139289	UUGUUCG
ENSG00000139291	UUCUAAU
ENSG00000139318	CCCUGAG
ENSG00000139318	CUUUGGU
ENSG00000139318	UGCAUUG
ENSG00000139318	UGCUCAC
ENSG00000139318	UUGAGGC
ENSG00000139324	GCACCAU
ENSG00000139324	UCAAGUA
ENSG00000139350	AAUACUG
ENSG00000139352	AAAGUUU
ENSG00000139352	AAAUUUC
ENSG00000139352	AACUGGU
ENSG00000139352	AAGACAU
ENSG00000139352	ACAUUAC
ENSG00000139352	AGCUGCC
ENSG00000139352	AUAAUAC
ENSG00000139352	GUGCAAA
ENSG00000139352	GUUAGGA
ENSG00000139352	UAAUAUC
ENSG00000139352	UACAAAG
ENSG00000139352	UAUUACC
ENSG00000139352	UCACAAG
ENSG00000139352	UGCAAAG
ENSG00000139352	UUAGGAU
ENSG00000139352	UUGUUCG
ENSG00000139352	UUUGCAC
ENSG00000139364	AAAGGUA

ENSG00000139364	ACAUUCA
ENSG00000139364	CAAAGCA
ENSG00000139364	GAAGGAA
ENSG00000139364	GAGAUGA
ENSG00000139364	GCUACAU
ENSG00000139364	GGGGUCC
ENSG00000139364	GUGCUUG
ENSG00000139364	GUGGGGA
ENSG00000139364	UGCUGAC
ENSG00000139364	UGGAUAA
ENSG00000139364	UGUGCUU
ENSG00000139372	AAGGCAC
ENSG00000139372	ACAGUAU
ENSG00000139372	CUGCAGC
ENSG00000139372	GUAAACA
ENSG00000139428	AAAGGUA
ENSG00000139428	AGGACAC
ENSG00000139428	AUACAAG
ENSG00000139428	CUCACAC
ENSG00000139428	GUCAUUG
ENSG00000139428	UGGGAUC
ENSG00000139433	AAGGCAC
ENSG00000139433	GGGUUUA
ENSG00000139433	UCACAGG
ENSG00000139433	UGAAAUG
ENSG00000139436	AAAGUGC
ENSG00000139436	AAGAACC
ENSG00000139436	AAGGCAC
ENSG00000139436	AAGGUGC
ENSG00000139436	AAGUGCA
ENSG00000139436	AGUGCAA
ENSG00000139436	AUAAAGU
ENSG00000139436	CUUGGAG
ENSG00000139436	CUUUGGU
ENSG00000139436	GGGCCCC
ENSG00000139436	GUGCAAA
ENSG00000139436	UAUUGCU
ENSG00000139436	UCCUAUA
ENSG00000139436	UGCAUAG
ENSG00000139436	UGCAUAU
ENSG00000139436	UUCCUAG
ENSG00000139436	UUGGCAC
ENSG00000139437	CUCUGGG
ENSG00000139437	CUGGGCA
ENSG00000139438	AACAUUC
ENSG00000139438	ACAGUAC
ENSG00000139438	ACAGUAU
ENSG00000139438	AGUGCAA
ENSG00000139438	AGUGCUG
ENSG00000139438	AGUGGUU
ENSG00000139438	CACAGUG
ENSG00000139438	CUCACAC
ENSG00000139438	CUCUUCC
ENSG00000139438	CUUCACC
ENSG00000139438	CUUGGAG
ENSG00000139438	GUGCUUG
ENSG00000139438	GUUAGGA
ENSG00000139438	UCACAGU
ENSG00000139438	UCUCACC
ENSG00000139438	UGGAGAU
ENSG00000139438	UGUGCGG
ENSG00000139438	UGUGCGU
ENSG00000139445	AAAUUUC
ENSG00000139445	AGUGCUU

ENSG00000139496	AAGGCAC
ENSG00000139496	CCAGUGC
ENSG00000139496	CCAGUGU
ENSG00000139496	CUUCACC
ENSG00000139496	GAGGCAG
ENSG00000139496	GCAUCCC
ENSG00000139496	UGCAUAG
ENSG00000139496	UGCCCUG
ENSG00000139505	AAAGUAC
ENSG00000139505	AAGGCAC
ENSG00000139505	ACAAUAU
ENSG00000139505	CAGUCCA
ENSG00000139505	CAGUGCA
ENSG00000139505	CUAUACA
ENSG00000139505	GAUAUGU
ENSG00000139505	UAUAUGA
ENSG00000139505	UCCCUUG
ENSG00000139508	AAGUGCU
ENSG00000139514	AAGGCAC
ENSG00000139514	AUACAAG
ENSG00000139514	CAGCAGA
ENSG00000139514	CAGUAGU
ENSG00000139514	CUAGUAA
ENSG00000139514	CUAGUAG
ENSG00000139514	CUCAAAC
ENSG00000139514	CUUUGGU
ENSG00000139514	GACCCUG
ENSG00000139514	GCACCAU
ENSG00000139514	GCUUCCU
ENSG00000139514	GGGUCUU
ENSG00000139514	UCCAGUU
ENSG00000139514	UCUCAAG
ENSG00000139517	AAGGCAC
ENSG00000139517	AAUGUUG
ENSG00000139517	UUCAAGC
ENSG00000139517	UUCCAUA
ENSG00000139517	UUGGCAC
ENSG00000139537	CACAGGG
ENSG00000139537	UAAUAGU
ENSG00000139537	UCACAGG
ENSG00000139546	AGCAGCA
ENSG00000139546	AGGCAGG
ENSG00000139546	CAGUCUG
ENSG00000139546	GAGGUAG
ENSG00000139546	UGUGUCA
ENSG00000139547	UCCCUGU
ENSG00000139567	CACAAUG
ENSG00000139567	CUCUGGG
ENSG00000139567	CUGCAGG
ENSG00000139567	UCCCUGU
ENSG00000139567	UGGCUGC
ENSG00000139597	AGCACCA
ENSG00000139597	CCAGCAU
ENSG00000139597	GACCAUG
ENSG00000139597	GCACCAU
ENSG00000139613	AGGAGCU
ENSG00000139613	AGUAGUU
ENSG00000139613	CAGUAGU
ENSG00000139620	CCAUUAC
ENSG00000139620	UCCCUUU
ENSG00000139624	AAUCACU
ENSG00000139624	CAAGAGC
ENSG00000139625	AAAGUGC
ENSG00000139625	AGUGCAA

ENSG00000139625	AUGUGGG
ENSG00000139625	GUGCAAA
ENSG00000139625	UCACAGU
ENSG00000139629	CUAGGCU
ENSG00000139629	UCACAGG
ENSG00000139636	AGUGCUU
ENSG00000139636	CACAGUG
ENSG00000139645	AAAACUG
ENSG00000139645	AAAGCUG
ENSG00000139645	AACACUG
ENSG00000139645	AACCUGG
ENSG00000139645	AAGGUGC
ENSG00000139645	AAGUGCU
ENSG00000139645	AAUACUG
ENSG00000139645	ACAGGUG
ENSG00000139645	ACAGUAC
ENSG00000139645	ACAGUAU
ENSG00000139645	ACAUUCA
ENSG00000139645	ACCACAG
ENSG00000139645	ACUGGCU
ENSG00000139645	ACUGUGU
ENSG00000139645	AGCACCA
ENSG00000139645	AGCCCUU
ENSG00000139645	AGCUGCC
ENSG00000139645	AGGAGCU
ENSG00000139645	AGGGAGG
ENSG00000139645	AGGGUCU
ENSG00000139645	AGUAGUU
ENSG00000139645	AGUGCAA
ENSG00000139645	AGUGCUU
ENSG00000139645	AGUGGUU
ENSG00000139645	AUGCACC
ENSG00000139645	AUGGCUU
ENSG00000139645	CAAAUGC
ENSG00000139645	CACACCG
ENSG00000139645	CACACCU
ENSG00000139645	CACAGGG
ENSG00000139645	CACCAGC
ENSG00000139645	CAGACAG
ENSG00000139645	CAGCAGA
ENSG00000139645	CAGCAGG
ENSG00000139645	CAGGCUC
ENSG00000139645	CAGUAGU
ENSG00000139645	CAGUGCA
ENSG00000139645	CAUAUUG
ENSG00000139645	CCAGGAG
ENSG00000139645	CCCCUGG
ENSG00000139645	CCUGCUG
ENSG00000139645	CUCACAC
ENSG00000139645	CUCAGGG
ENSG00000139645	CUCCCAA
ENSG00000139645	CUCCCAC
ENSG00000139645	CUCUGGG
ENSG00000139645	CUGCAGC
ENSG00000139645	CUGCAGG
ENSG00000139645	CUGGACU
ENSG00000139645	CUUACAG
ENSG00000139645	CUUCUCU
ENSG00000139645	CUUUGGU
ENSG00000139645	GAAUUGU
ENSG00000139645	GAGCUGC
ENSG00000139645	GAGGGGC
ENSG00000139645	GAGGUAG
ENSG00000139645	GAGGUAU

ENSG00000139645	GAGUGUG
ENSG00000139645	GAUUGUA
ENSG00000139645	GAUUGUC
ENSG00000139645	GCACCAU
ENSG00000139645	GCAGCAU
ENSG00000139645	GCCCUUA
ENSG00000139645	GCCUGGA
ENSG00000139645	GCUACAU
ENSG00000139645	GGAGAGA
ENSG00000139645	GGAGGAG
ENSG00000139645	GGAGGCA
ENSG00000139645	GGAUUUU
ENSG00000139645	GGCAAGA
ENSG00000139645	GGCAGGG
ENSG00000139645	GGCAGUG
ENSG00000139645	GGCUCAG
ENSG00000139645	GGGGUGG
ENSG00000139645	GGUGGGC
ENSG00000139645	GGUUACC
ENSG00000139645	GUCUGCC
ENSG00000139645	GUCUUGC
ENSG00000139645	GUGACAG
ENSG00000139645	GUGCUUG
ENSG00000139645	UACAGUU
ENSG00000139645	UAUUGCU
ENSG00000139645	UCAAGUA
ENSG00000139645	UCACACA
ENSG00000139645	UCACAGG
ENSG00000139645	UCACAUU
ENSG00000139645	UCAUGAU
ENSG00000139645	UCCAGUU
ENSG00000139645	UCCCUGU
ENSG00000139645	UGCAUAG
ENSG00000139645	UGCAUAU
ENSG00000139645	UGCUCAC
ENSG00000139645	UGGGACA
ENSG00000139645	UGGGUAC
ENSG00000139645	UGUCUGC
ENSG00000139645	UUCUAAU
ENSG00000139645	UUGGCAC
ENSG00000139645	UUGGUCC
ENSG00000139645	UUGGUUC
ENSG00000139651	AAAAGUA
ENSG00000139651	CACCACC
ENSG00000139651	CAGCAGA
ENSG00000139651	CAGCUGG
ENSG00000139651	CAGUAGA
ENSG00000139651	CAGUAGU
ENSG00000139651	CAGUCUG
ENSG00000139651	CCAGGAG
ENSG00000139651	CCAGUGC
ENSG00000139651	CCAGUGU
ENSG00000139651	CCCACCG
ENSG00000139651	CCCACCU
ENSG00000139651	CCUGUAC
ENSG00000139651	CUCCCAC
ENSG00000139651	GAGCUGC
ENSG00000139651	GAGGUAG
ENSG00000139651	GGGAGCU
ENSG00000139651	GGGCCCC
ENSG00000139651	GGUGGGC
ENSG00000139651	UAUAAAG
ENSG00000139651	UCCCACC
ENSG00000139651	UGCCAGC

ENSG00000139651	UUCCUCG
ENSG00000139651	UUGGUCC
ENSG00000139668	AAAGUGC
ENSG00000139668	CAAAGCA
ENSG00000139668	GUGCAAA
ENSG00000139668	UGAAGCU
ENSG00000139668	UGUAUAA
ENSG00000139687	AAAGUGC
ENSG00000139687	AAGUGCA
ENSG00000139687	CAGUAGA
ENSG00000139687	GAGGUAG
ENSG00000139687	UCCUGAG
ENSG00000139719	CUGCAGC
ENSG00000139719	CUGCAGG
ENSG00000139722	AAAAAUC
ENSG00000139722	AAGGCAC
ENSG00000139722	ACAGUAU
ENSG00000139722	AGUGCAA
ENSG00000139722	CCCUGAG
ENSG00000139722	GUGCAAA
ENSG00000139722	UCCCUGU
ENSG00000139725	GGCCCUG
ENSG00000139726	AAUCCUU
ENSG00000139726	UACAGUU
ENSG00000139726	UCCCUUU
ENSG00000139726	UGGCCAC
ENSG00000139726	UUGGCAC
ENSG00000139734	UUCAAGC
ENSG00000139734	UUUCAAC
ENSG00000139737	AACAUUC
ENSG00000139737	UGUGCUU
ENSG00000139746	AAAAACC
ENSG00000139746	AAAGAAU
ENSG00000139746	AACAUUC
ENSG00000139746	AAGAACC
ENSG00000139746	ACAUAGA
ENSG00000139746	ACAUUCA
ENSG00000139746	UCAUACA
ENSG00000139746	UGCAUAG
ENSG00000139793	AAGACAU
ENSG00000139793	AAGUCAC
ENSG00000139793	AGCAGCG
ENSG00000139793	AGCAGCU
ENSG00000139793	AGGUAGU
ENSG00000139793	AUCACUA
ENSG00000139793	CAGCUGG
ENSG00000139793	CAGUUAC
ENSG00000139793	GUAAACA
ENSG00000139793	GUGCAAA
ENSG00000139793	UAUAAUA
ENSG00000139793	UAUAUGA
ENSG00000139793	UGCAAAG
ENSG00000139793	UGGUACC
ENSG00000139800	ACAUUAC
ENSG00000139800	AGCACCA
ENSG00000139800	CUCUUCC
ENSG00000139800	GCACCAU
ENSG00000139826	AAAUGGU
ENSG00000139826	AAGACAU
ENSG00000139826	AUGGCAC
ENSG00000139826	AUUGCAC
ENSG00000139826	CAAAGUG
ENSG00000139826	CAGUUAC
ENSG00000139826	GAGGUAU

ENSG00000139826	GCUGGAU
ENSG00000139826	GUCAGUU
ENSG00000139826	UAUUGCU
ENSG00000139826	UGGAUAA
ENSG00000139832	UCACAGU
ENSG00000139832	UCCUGAG
ENSG00000139842	ACAUUAC
ENSG00000139842	CUUUGGU
ENSG00000139842	UUGGCAC
ENSG00000139874	CACACCU
ENSG00000139874	GAGAACC
ENSG00000139874	GUUCAUG
ENSG00000139874	UGAAAGG
ENSG00000139874	UGAAGCU
ENSG00000139880	UCACAGU
ENSG00000139890	AAGAACC
ENSG00000139890	GACCAUG
ENSG00000139890	GGCGACA
ENSG00000139890	UUCCUAG
ENSG00000139890	UUGGUUC
ENSG00000139899	CAGGCUC
ENSG00000139899	CUGCAGG
ENSG00000139910	AAAACUG
ENSG00000139910	AAAAGUA
ENSG00000139910	AAAGGUA
ENSG00000139910	AAAGUAC
ENSG00000139910	AAAGUAU
ENSG00000139910	AACUCUA
ENSG00000139910	ACAGGUG
ENSG00000139910	ACAGUAU
ENSG00000139910	ACUCAGG
ENSG00000139910	ACUGCAU
ENSG00000139910	ACUGGCC
ENSG00000139910	AGCAGCU
ENSG00000139910	AGCCAGU
ENSG00000139910	AGGAUGU
ENSG00000139910	AGUAGUU
ENSG00000139910	AUUCAUU
ENSG00000139910	CACAGUG
ENSG00000139910	CAGCUGG
ENSG00000139910	CUCCAUU
ENSG00000139910	GAGAUGA
ENSG00000139910	GAGGCUG
ENSG00000139910	GAGGUAG
ENSG00000139910	GAGUAUU
ENSG00000139910	GCAACUU
ENSG00000139910	GCUGGUG
ENSG00000139910	GGCAGGG
ENSG00000139910	GGGUCUU
ENSG00000139910	GUAAACA
ENSG00000139910	GUGCUUG
ENSG00000139910	GUUAAUG
ENSG00000139910	GUUCAUG
ENSG00000139910	UAAUGCU
ENSG00000139910	UAUUGCU
ENSG00000139910	UCACAGG
ENSG00000139910	UCCAGAG
ENSG00000139910	UCCCUGU
ENSG00000139910	UCUCAAG
ENSG00000139910	UGAAGUG
ENSG00000139910	UGACUGU
ENSG00000139910	UGAGGAC
ENSG00000139910	UGAUCCA
ENSG00000139910	UGCAUAG

ENSG00000139910	UGCAUAU
ENSG00000139910	UGCAUUG
ENSG00000139910	UGGCUGC
ENSG00000139910	UGGGAUC
ENSG00000139910	UGUUGAA
ENSG00000139910	UUCUGCA
ENSG00000139915	AAAGAGG
ENSG00000139915	AAGGCAC
ENSG00000139915	AGUGCAA
ENSG00000139915	AGUGCUU
ENSG00000139915	CAGGCUC
ENSG00000139915	CAGUGCA
ENSG00000139915	CCCUGUU
ENSG00000139915	GAAACAU
ENSG00000139915	GGUUGAC
ENSG00000139915	GUGAGGU
ENSG00000139915	GUGCUUG
ENSG00000139915	UAUAAAG
ENSG00000139915	UGCAUAG
ENSG00000139915	UGCAUAU
ENSG00000139915	UUCUAAU
ENSG00000139915	UUGACAC
ENSG00000139915	UUGGUUC
ENSG00000139926	AACACUG
ENSG00000139926	AAGUGCA
ENSG00000139926	CUGACUC
ENSG00000139926	UACAAAG
ENSG00000139926	UCACAGG
ENSG00000139926	UCACAGU
ENSG00000139946	ACUGUAG
ENSG00000139946	AGCAGCA
ENSG00000139946	AGCCCUU
ENSG00000139946	CACAGUG
ENSG00000139946	CCCUGAG
ENSG00000139946	CUGCAGC
ENSG00000139946	CUUCACC
ENSG00000139946	CUUGUAU
ENSG00000139946	GAGGCAG
ENSG00000139946	GCAGCAU
ENSG00000139946	GCAUCCC
ENSG00000139946	GUGACAG
ENSG00000139946	UCACAGG
ENSG00000139946	UCCCUGU
ENSG00000139946	UUCAAAU
ENSG00000139946	UUCUGCA
ENSG00000139970	AGUACUG
ENSG00000139970	GUGCAAA
ENSG00000139970	UCAAGUA
ENSG00000139998	ACCAGCA
ENSG00000139998	ACUCAGC
ENSG00000139998	AGCACCA
ENSG00000139998	AGGCAGG
ENSG00000139998	CACCAGC
ENSG00000139998	CCAGUGC
ENSG00000139998	CUGCAGC
ENSG00000139998	CUUCUCU
ENSG00000139998	GAAGGAA
ENSG00000139998	GAGGCAG
ENSG00000139998	GAGGUAG
ENSG00000139998	GCAGCAU
ENSG00000139998	GCAGGUU
ENSG00000139998	GGCAGGG
ENSG00000139998	UCACAGG
ENSG00000139998	UGAAAUG

ENSG00000139998	UGCUCAC
ENSG00000140044	AAAGUAU
ENSG00000140044	AACAUUC
ENSG00000140044	AACUGGU
ENSG00000140044	ACUCCAG
ENSG00000140044	AUGUGCC
ENSG00000140044	CAGCAAA
ENSG00000140044	CUACAGU
ENSG00000140044	CUCCAGC
ENSG00000140044	CUUUGGU
ENSG00000140044	GUGCUUG
ENSG00000140044	UGUGCUU
ENSG00000140044	UUUUUGC
ENSG00000140090	AAGUGCA
ENSG00000140090	AAUACUG
ENSG00000140090	AGCACCA
ENSG00000140090	CAAAGUG
ENSG00000140090	GUGCAAA
ENSG00000140090	GUGCUUG
ENSG00000140092	UCACAGG
ENSG00000140092	UCACAGU
ENSG00000140153	AAGUGCU
ENSG00000140153	UAAUACA
ENSG00000140153	UAUAAUA
ENSG00000140153	UGCAGCU
ENSG00000140157	AAACUGU
ENSG00000140157	AGUGCAA
ENSG00000140157	GUCAUUG
ENSG00000140157	GUUAAUG
ENSG00000140157	UUGGUCC
ENSG00000140199	AACAGUC
ENSG00000140199	AGCAAAA
ENSG00000140199	AUGGCUU
ENSG00000140199	AUGUAAU
ENSG00000140199	CUGCAGC
ENSG00000140199	GAUUGGU
ENSG00000140199	GGAGUGU
ENSG00000140199	GGAUUUU
ENSG00000140199	GGCUCUG
ENSG00000140199	UAAUAUC
ENSG00000140199	UAUUGCU
ENSG00000140199	UUGAGGC
ENSG00000140199	UUGGCAC
ENSG00000140259	AGUGCUU
ENSG00000140259	CUUGUGU
ENSG00000140262	AAGACAU
ENSG00000140262	ACAAUAU
ENSG00000140262	ACACGGG
ENSG00000140262	ACUCCAG
ENSG00000140262	ACUGCAG
ENSG00000140262	ACUGCAU
ENSG00000140262	AGGUUAU
ENSG00000140262	AUGCAUU
ENSG00000140262	CUACAGU
ENSG00000140262	GAUUGUA
ENSG00000140262	GAUUGUC
ENSG00000140262	GCAGAAG
ENSG00000140262	GCUGGUG
ENSG00000140262	GGAAGAC
ENSG00000140262	GGCAGUG
ENSG00000140262	UAAGACG
ENSG00000140262	UAAGACU
ENSG00000140262	UAUGGUU
ENSG00000140264	AAAAAUC

ENSG00000140264	AACACUG
ENSG00000140264	AAUACUG
ENSG00000140264	ACUCAGG
ENSG00000140264	ACUGGCU
ENSG00000140264	AGGGUUG
ENSG00000140264	CGGUGCU
ENSG00000140264	CUCAGGG
ENSG00000140264	CUGGCUC
ENSG00000140264	GAAGGAA
ENSG00000140264	GGACCUG
ENSG00000140264	GGAGAGA
ENSG00000140264	GGCAAGA
ENSG00000140264	GGGGUGC
ENSG00000140264	GGGGUGG
ENSG00000140264	GUGGCCC
ENSG00000140264	UAAUACA
ENSG00000140264	UUCAAGC
ENSG00000140265	AAGACAU
ENSG00000140265	AUCCCUU
ENSG00000140265	GGCAGUG
ENSG00000140279	GAGGCAG
ENSG00000140279	GCCUGGG
ENSG00000140280	AGUGCAA
ENSG00000140285	AAAGUUU
ENSG00000140285	AACAGUC
ENSG00000140285	AACUGGU
ENSG00000140285	AGCAGCG
ENSG00000140285	AGCAGCU
ENSG00000140285	AGUAGUU
ENSG00000140285	AGUGCUG
ENSG00000140285	CAGUAGA
ENSG00000140285	CAGUCCA
ENSG00000140285	CCUGCUG
ENSG00000140285	GAUCAGA
ENSG00000140285	GAUUGUC
ENSG00000140285	GCAGCAU
ENSG00000140285	GCUGGAU
ENSG00000140285	GUCUGCC
ENSG00000140285	UCAUGAU
ENSG00000140285	UGUCUGC
ENSG00000140285	UGUCUGG
ENSG00000140285	UUCUUGU
ENSG00000140285	UUGAUCA
ENSG00000140285	UUUCAAC
ENSG00000140287	UCACAAG
ENSG00000140299	AUGGCUU
ENSG00000140299	CAGUCUG
ENSG00000140299	UAUUGCU
ENSG00000140299	UGCUCAC
ENSG00000140320	AAAGAAU
ENSG00000140320	AAAGCUG
ENSG00000140320	AACACUG
ENSG00000140320	AAGGCAC
ENSG00000140320	AAGUGCU
ENSG00000140320	AAUCUCA
ENSG00000140320	ACCAGCA
ENSG00000140320	AGCACCA
ENSG00000140320	AGCUUAU
ENSG00000140320	AGGGAGG
ENSG00000140320	AGUGCUG
ENSG00000140320	AUGGAUU
ENSG00000140320	CACAGUG
ENSG00000140320	CAGCAGA
ENSG00000140320	CAGCAGG

ENSG00000140320	CAUGGAU
ENSG00000140320	CUACAAA
ENSG00000140320	CUCAAAC
ENSG00000140320	GAGGUAG
ENSG00000140320	GAGGUAU
ENSG00000140320	GGCAAGA
ENSG00000140320	GGCAGGG
ENSG00000140320	GUGAGGU
ENSG00000140320	GUGCUUG
ENSG00000140320	GUGGCCC
ENSG00000140320	GUGUGGC
ENSG00000140320	UACAAAG
ENSG00000140320	UCACAGU
ENSG00000140320	UCCCUUG
ENSG00000140320	UCCUUGC
ENSG00000140320	UCUCAAG
ENSG00000140320	UGAAAUG
ENSG00000140320	UGGGUGA
ENSG00000140320	UGUGCUU
ENSG00000140320	UUCAAGC
ENSG00000140320	UUCUGCA
ENSG00000140320	UUGGUUC
ENSG00000140323	GAGGCUG
ENSG00000140326	CUCCCAA
ENSG00000140332	CACCACC
ENSG00000140332	CCCUGAG
ENSG00000140332	CUCUGGG
ENSG00000140332	GAGCUGC
ENSG00000140332	GCUACAU
ENSG00000140332	UCACAAG
ENSG00000140332	UUCAAAU
ENSG00000140332	UUCUAAU
ENSG00000140350	AGGUUAU
ENSG00000140350	UCCCACA
ENSG00000140382	AUGGCUU
ENSG00000140382	CAGCUGG
ENSG00000140382	CCUUCUG
ENSG00000140382	CUUUGGU
ENSG00000140382	GGCAUUG
ENSG00000140382	UACAAAG
ENSG00000140382	UUCCAUA
ENSG00000140382	UUGGUUC
ENSG00000140382	UUUCAAC
ENSG00000140386	UAUAAAG
ENSG00000140391	AGUGCAA
ENSG00000140396	AAGUUGU
ENSG00000140396	CCAGUGU
ENSG00000140396	GGAGAGA
ENSG00000140396	GUGAAUG
ENSG00000140396	UAAUACA
ENSG00000140403	ACCAGCA
ENSG00000140403	AGCAGCU
ENSG00000140403	AGGAGCU
ENSG00000140403	CUAGUCC
ENSG00000140403	CUUUGGU
ENSG00000140403	GUCAACA
ENSG00000140403	GUGCCAC
ENSG00000140403	UCAACAG
ENSG00000140403	UUCCAUA
ENSG00000140406	AAAAAUC
ENSG00000140406	AAAGGUA
ENSG00000140406	AACAUUC
ENSG00000140406	AAUCACU
ENSG00000140406	CACAGUG

ENSG00000140406	GAAACAU
ENSG00000140406	GAGGUAG
ENSG00000140406	GAGGUAU
ENSG00000140406	GCUACAU
ENSG00000140406	UAAUAGU
ENSG00000140406	UCACAGU
ENSG00000140443	AAAAGUA
ENSG00000140443	AAAGCUG
ENSG00000140443	AAAUCCC
ENSG00000140443	AAAUGGU
ENSG00000140443	AACAAAC
ENSG00000140443	AACACUG
ENSG00000140443	ACAUAGA
ENSG00000140443	ACCCGUA
ENSG00000140443	ACUAGAU
ENSG00000140443	ACUGGAU
ENSG00000140443	ACUGUUU
ENSG00000140443	AGCAAAA
ENSG00000140443	AGCAGCA
ENSG00000140443	AGCAGCG
ENSG00000140443	AGUACUG
ENSG00000140443	AGUGGUU
ENSG00000140443	AUACAAG
ENSG00000140443	AUAUAAC
ENSG00000140443	AUCCUUU
ENSG00000140443	AUGCCUU
ENSG00000140443	AUGUGCC
ENSG00000140443	CAAAGCA
ENSG00000140443	CUACAAA
ENSG00000140443	CUACAGU
ENSG00000140443	CUAGUAA
ENSG00000140443	CUUGGAG
ENSG00000140443	CUUGUAU
ENSG00000140443	GAAGGUC
ENSG00000140443	GACCUGG
ENSG00000140443	GAGAAAU
ENSG00000140443	GAGAUGA
ENSG00000140443	GAGCCCU
ENSG00000140443	GAGGUAU
ENSG00000140443	GCACCAU
ENSG00000140443	GGAAGAC
ENSG00000140443	GGAUGAG
ENSG00000140443	GGCAGCG
ENSG00000140443	GGCCCUG
ENSG00000140443	GGUAGAC
ENSG00000140443	GGUUACC
ENSG00000140443	GUAAACA
ENSG00000140443	GUAUUCU
ENSG00000140443	GUCAACA
ENSG00000140443	GUCUGCC
ENSG00000140443	GUCUUGC
ENSG00000140443	GUGAGGU
ENSG00000140443	GUGUCAC
ENSG00000140443	GUUCAUG
ENSG00000140443	UACAAAG
ENSG00000140443	UCAUAGA
ENSG00000140443	UCCAGUU
ENSG00000140443	UCUGGAA
ENSG00000140443	UGCAUAG
ENSG00000140443	UGGCCAC
ENSG00000140443	UGGCCCU
ENSG00000140443	UGUCUGC
ENSG00000140443	UUAGGAU
ENSG00000140443	UUCUUGU

ENSG00000140443	UUGGCAC
ENSG00000140443	UUGGUCC
ENSG00000140443	UUGUUCG
ENSG00000140450	AGCACCA
ENSG00000140450	AGCAGCA
ENSG00000140450	AGCAGCU
ENSG00000140450	AUAAUAC
ENSG00000140450	AUUGCAC
ENSG00000140450	GAGGUAG
ENSG00000140450	GCACCAU
ENSG00000140450	UCCUGAG
ENSG00000140451	CUUCUCU
ENSG00000140455	AUGUGCC
ENSG00000140463	UCUGGAA
ENSG00000140463	UCUGGAG
ENSG00000140464	AAGGCAC
ENSG00000140464	CUGGCUC
ENSG00000140464	GGCUCAG
ENSG00000140464	UGGAGAU
ENSG00000140470	AUAUUAU
ENSG00000140470	CAAAACU
ENSG00000140470	GUCUCUG
ENSG00000140470	GUGACUG
ENSG00000140470	GUGCAAA
ENSG00000140470	UUCAAAU
ENSG00000140474	CCCUGAG
ENSG00000140479	CUUUGGU
ENSG00000140479	UAUAAAG
ENSG00000140497	AAAGUGC
ENSG00000140497	AAGGCAC
ENSG00000140497	AAGUGCA
ENSG00000140511	AAAAACC
ENSG00000140519	CUGCAGC
ENSG00000140519	CUGGGGG
ENSG00000140519	GAGCCCU
ENSG00000140526	AAAGUGC
ENSG00000140526	AAAUCCC
ENSG00000140526	AAGUGCA
ENSG00000140526	ACCACAG
ENSG00000140526	ACUGGAU
ENSG00000140526	ACUGUGU
ENSG00000140526	AGCAAAA
ENSG00000140526	AGCCUGC
ENSG00000140526	AUGGAUU
ENSG00000140526	CAAAGCA
ENSG00000140526	CAGCUGG
ENSG00000140526	CUGGGCA
ENSG00000140526	CUUGUAU
ENSG00000140526	GAAGGAA
ENSG00000140526	GAGAAGA
ENSG00000140526	GAGACCU
ENSG00000140526	GAGAUGA
ENSG00000140526	GAGGCUG
ENSG00000140526	GAUUGUA
ENSG00000140526	GAUUGUC
ENSG00000140526	GCAGCAU
ENSG00000140526	GGAAGCC
ENSG00000140526	GUAAACA
ENSG00000140526	UAAUGCU
ENSG00000140526	UACAAAG
ENSG00000140526	UAUCAGA
ENSG00000140526	UAUGGUU
ENSG00000140526	UAUUGCU
ENSG00000140526	UCAAGUA

ENSG00000140526	UCACAGG
ENSG00000140526	UCACAGU
ENSG00000140526	UGAAGCU
ENSG00000140526	UGAGAAG
ENSG00000140526	UGCAAAG
ENSG00000140526	UGCAGCU
ENSG00000140526	UGUGUGG
ENSG00000140526	UUCUUGU
ENSG00000140526	UUGUGUC
ENSG00000140538	AAGUCUU
ENSG00000140538	ACAAUAU
ENSG00000140538	AUAAAGU
ENSG00000140538	CACAGUG
ENSG00000140538	CACUCCU
ENSG00000140538	CUAUACA
ENSG00000140538	CUCUUCC
ENSG00000140538	GAGGUAG
ENSG00000140538	GAGGUAU
ENSG00000140538	GGAGGAG
ENSG00000140538	GUCUCUG
ENSG00000140538	UCCAGAG
ENSG00000140538	UCCCUUG
ENSG00000140538	UCCUAUA
ENSG00000140538	UGACAUC
ENSG00000140543	UCCCACA
ENSG00000140545	AGCUGCC
ENSG00000140548	CAGUAGU
ENSG00000140548	CCAGUGU
ENSG00000140548	GAGGUAG
ENSG00000140548	GGCUCAG
ENSG00000140548	GGUCUAG
ENSG00000140548	UUGGUCC
ENSG00000140553	CAGUAGU
ENSG00000140553	CUACAGU
ENSG00000140557	AAAUCCC
ENSG00000140557	AAGGCAC
ENSG00000140557	AAUUAUU
ENSG00000140557	ACAAGGU
ENSG00000140557	ACAGGUG
ENSG00000140557	ACUCAGC
ENSG00000140557	ACUGGCU
ENSG00000140557	ACUGUUU
ENSG00000140557	AUAAAGU
ENSG00000140557	AUACAAG
ENSG00000140557	CACACCU
ENSG00000140557	CCAGGAG
ENSG00000140557	CCUUCUU
ENSG00000140557	CUCUGGG
ENSG00000140557	CUGGGGG
ENSG00000140557	CUUCACC
ENSG00000140557	GCAGAAG
ENSG00000140557	GCAGGUU
ENSG00000140557	GCCCUUA
ENSG00000140557	GUGCCAC
ENSG00000140557	UAAUACA
ENSG00000140557	UAAUGCU
ENSG00000140557	UAGACUG
ENSG00000140557	UAUAAAG
ENSG00000140557	UAUAAUA
ENSG00000140557	UCAUUUG
ENSG00000140557	UCCCACA
ENSG00000140557	UGAAAGG
ENSG00000140564	AAGGCAC
ENSG00000140564	AGCAGCA

ENSG00000140564	AGCCCUU
ENSG00000140564	AGCUGCC
ENSG00000140564	CACAGGG
ENSG00000140564	CACAGUG
ENSG00000140564	CACCACC
ENSG00000140564	CCAUUAC
ENSG00000140564	CCCUGAG
ENSG00000140564	CUCAAAC
ENSG00000140564	CUGCCCC
ENSG00000140564	CUUGGAG
ENSG00000140564	CUUUGGU
ENSG00000140564	GAAAACA
ENSG00000140564	GAUUGUA
ENSG00000140564	GAUUGUC
ENSG00000140564	GCACCAU
ENSG00000140564	GCAGCAU
ENSG00000140564	GGAGGCA
ENSG00000140564	GGCUCAG
ENSG00000140564	GUGCAAA
ENSG00000140564	UGCAUAU
ENSG00000140564	UGGAGAU
ENSG00000140564	UUGGUCC
ENSG00000140575	CAGUCCA
ENSG00000140575	UACAAAG
ENSG00000140575	UCAUGAU
ENSG00000140577	AAAGUGC
ENSG00000140577	AGCAGCA
ENSG00000140577	AGUGGUU
ENSG00000140577	AUACAAG
ENSG00000140577	CAGUGCA
ENSG00000140577	GAGGCAG
ENSG00000140577	GGGGUGC
ENSG00000140577	GGGUAAG
ENSG00000140577	GUUAGGA
ENSG00000140577	UAUAAAG
ENSG00000140650	AGGGAGG
ENSG00000140650	CCCUGAG
ENSG00000140691	CCUUCUG
ENSG00000140694	AAUUUUA
ENSG00000140743	AAUACUG
ENSG00000140743	UCAUAGA
ENSG00000140750	CACUGUU
ENSG00000140750	GCAGCAU
ENSG00000140750	GCUUCCU
ENSG00000140795	AGCAGCA
ENSG00000140795	CAGCAGA
ENSG00000140795	CCUUCUG
ENSG00000140795	GAGCUAA
ENSG00000140798	GUCAUUG
ENSG00000140807	AAUGCCC
ENSG00000140807	AGCAGCA
ENSG00000140807	AGCAGCG
ENSG00000140807	AUGCCUU
ENSG00000140807	GAGGUAG
ENSG00000140830	CAGCUGG
ENSG00000140848	AAAAAUC
ENSG00000140853	AGGCAGG
ENSG00000140853	GAGGCAG
ENSG00000140853	UUGAGGC
ENSG00000140854	AGCAGCA
ENSG00000140859	AAAAAUC
ENSG00000140873	AAGUGCU
ENSG00000140873	AGCAGCA
ENSG00000140873	AGUGCUU

ENSG00000140873	CCAGCAU
ENSG00000140873	GCACCAU
ENSG00000140873	GUGCAAA
ENSG00000140873	UCAACAG
ENSG00000140873	UCAAGUA
ENSG00000140873	UUCAAGC
ENSG00000140873	UUGGCAC
ENSG00000140931	CAAAUGC
ENSG00000140937	AAGGCGC
ENSG00000140937	AAUACUG
ENSG00000140937	ACAGUAU
ENSG00000140937	ACAUAGA
ENSG00000140937	ACAUUAC
ENSG00000140937	AGCAAAA
ENSG00000140937	AGUAACA
ENSG00000140937	CAAGAGC
ENSG00000140937	CACAGUG
ENSG00000140937	CAGCAGA
ENSG00000140937	CAGCAGG
ENSG00000140937	GGAAGCC
ENSG00000140937	UCAAGUA
ENSG00000140937	UCACAGG
ENSG00000140937	UUCAAAU
ENSG00000140937	UUCAAGC
ENSG00000140941	AAAGCUG
ENSG00000140941	AUCCCUU
ENSG00000140941	GCAACUU
ENSG00000140943	AAAGUGC
ENSG00000140943	CUUUGGU
ENSG00000140945	AACAUUC
ENSG00000140945	ACAUUCA
ENSG00000140945	AGCCCUU
ENSG00000140945	GUAAACA
ENSG00000140945	UGCAAAG
ENSG00000140945	UGCAUAG
ENSG00000140945	UGCAUAU
ENSG00000140945	UGCUCAC
ENSG00000140945	UUGAGGC
ENSG00000140945	UUUGCAA
ENSG00000140948	AAAGAAU
ENSG00000140948	AAAGUAA
ENSG00000140948	AAAGUAU
ENSG00000140948	AACAAAC
ENSG00000140948	AAGGCAC
ENSG00000140948	AAGUCUU
ENSG00000140948	AAUACUG
ENSG00000140948	AAUCCUU
ENSG00000140948	ACAGUAU
ENSG00000140948	ACAUUAC
ENSG00000140948	ACAUUCA
ENSG00000140948	ACCAGCA
ENSG00000140948	ACUGUGU
ENSG00000140948	ACUUGGA
ENSG00000140948	AGCUGCC
ENSG00000140948	AUAAUAC
ENSG00000140948	CUCCAUU
ENSG00000140948	CUGUUGA
ENSG00000140948	GAGGCUG
ENSG00000140948	GAUUGUC
ENSG00000140948	GCCCUGU
ENSG00000140948	GCUGGUG
ENSG00000140948	GGCUCAG
ENSG00000140948	GUAAACA
ENSG00000140948	GUCAGUU

ENSG00000140948	GUGUCAC
ENSG00000140948	UACAAAG
ENSG00000140948	UCAUUCG
ENSG00000140948	UCCCUUU
ENSG00000140948	UGACAUC
ENSG00000140948	UGCAUAG
ENSG00000140948	UGCAUAU
ENSG00000140948	UGCCCUG
ENSG00000140948	UGUGUCA
ENSG00000140948	UUGGCAC
ENSG00000140948	UUGUGUC
ENSG00000140983	CCCUGAG
ENSG00000140983	UGAGAAU
ENSG00000140992	AACUACU
ENSG00000140992	AAUACUG
ENSG00000140992	ACAAUAU
ENSG00000140992	AGAUAAA
ENSG00000140992	AUAAUAC
ENSG00000140992	AUAUUAU
ENSG00000140992	CACAGUG
ENSG00000140992	CAGUUAC
ENSG00000140992	CGUGGCC
ENSG00000140992	CUGGAAA
ENSG00000140992	GACCCUG
ENSG00000140992	GGCAGCG
ENSG00000140992	GGUGGUC
ENSG00000140992	GUCAGUU
ENSG00000140992	GUGGCCC
ENSG00000140992	UAUUACC
ENSG00000140992	UCACAGU
ENSG00000140992	UCCAGAG
ENSG00000140992	UCUGGAA
ENSG00000140992	UGACUGU
ENSG00000140992	UGCAUUG
ENSG00000140992	UGCUGAC
ENSG00000140992	UUGUGUC
ENSG00000140992	UUGUUCG
ENSG00000140995	AGCACCA
ENSG00000141013	ACUGGAU
ENSG00000141013	CUCAGGG
ENSG00000141013	CUGCAGC
ENSG00000141013	GUGGGGA
ENSG00000141026	ACCUGGA
ENSG00000141026	CAGUUAC
ENSG00000141034	AAAACUG
ENSG00000141034	AAACUGU
ENSG00000141034	AAAGCUG
ENSG00000141034	AAAGUGC
ENSG00000141034	AGCAAAA
ENSG00000141034	AGCACCA
ENSG00000141034	AGGGUCU
ENSG00000141034	AUGUAAU
ENSG00000141034	CAAAUGC
ENSG00000141034	CUCCCAA
ENSG00000141034	CUCCCAC
ENSG00000141034	CUGACUC
ENSG00000141034	GAAAACA
ENSG00000141034	GCUGGUG
ENSG00000141034	GGCAUUG
ENSG00000141034	GGCUCUG
ENSG00000141034	GGUUGAC
ENSG00000141034	GUAAACA
ENSG00000141034	UGCCCUG
ENSG00000141034	UUCAAAU

ENSG00000141034	UUGGCAA
ENSG00000141034	UUGGCAC
ENSG00000141034	UUUCAAC
ENSG00000141068	AAAGAGG
ENSG00000141068	ACUCCAG
ENSG00000141068	ACUGGAU
ENSG00000141068	AGUGCCG
ENSG00000141068	AGUGCCU
ENSG00000141068	CACUCCU
ENSG00000141068	CUGGACU
ENSG00000141068	CUUUGGU
ENSG00000141068	GCAGAAG
ENSG00000141068	GCUACAU
ENSG00000141068	GUUAAUG
ENSG00000141068	UAAUGCU
ENSG00000141068	UGCAAAG
ENSG00000141068	UUCAAAU
ENSG00000141084	AAGGCAC
ENSG00000141084	AAUCUCU
ENSG00000141084	ACUCAAA
ENSG00000141084	ACUCAGC
ENSG00000141084	AGAUAAA
ENSG00000141084	AGGUAGU
ENSG00000141084	AUAUAAC
ENSG00000141084	AUCACUA
ENSG00000141084	CAAAACU
ENSG00000141084	CACCAGC
ENSG00000141084	CAGCUGG
ENSG00000141084	CAUGGGU
ENSG00000141084	CCAGUGU
ENSG00000141084	CUCCAGC
ENSG00000141084	CUCCCAC
ENSG00000141084	GAAACAU
ENSG00000141084	GAAGGAA
ENSG00000141084	GAGUUGA
ENSG00000141084	GCUACAU
ENSG00000141084	GGAGGAG
ENSG00000141084	GUAAACA
ENSG00000141084	GUGCUUG
ENSG00000141084	UAAUAUC
ENSG00000141084	UCAAGUA
ENSG00000141084	UCCAGAG
ENSG00000141084	UGCCAGC
ENSG00000141084	UGGACUG
ENSG00000141084	UGGGACA
ENSG00000141084	UGGGUAC
ENSG00000141084	UGUCUUU
ENSG00000141084	UGUGCUU
ENSG00000141084	UUGUGUC
ENSG00000141127	GCAACUU
ENSG00000141127	GCAGAAG
ENSG00000141127	UGGCCCU
ENSG00000141140	AAGGCAC
ENSG00000141140	CUUUGGU
ENSG00000141140	GGCAAGA
ENSG00000141140	UGGCCCU
ENSG00000141140	UGUCUUU
ENSG00000141140	UUGGCAA
ENSG00000141140	UUGGCAC
ENSG00000141141	ACCACAG
ENSG00000141141	CACCACC
ENSG00000141141	CCUUCAU
ENSG00000141141	UAUCAGA
ENSG00000141150	AAAACUG

ENSG00000141150	ACAUUCA
ENSG00000141150	ACCCGGC
ENSG00000141150	ACCCUGU
ENSG00000141150	AGCUGCC
ENSG00000141150	AGGGAGG
ENSG00000141150	AGUGAUC
ENSG00000141150	CAAAACU
ENSG00000141150	CCCUGAG
ENSG00000141150	CUCUGGG
ENSG00000141150	CUGCCCC
ENSG00000141150	CUGGGGG
ENSG00000141150	GACCCUG
ENSG00000141150	GAGCCCU
ENSG00000141150	GAGGGGC
ENSG00000141150	GAGGUAG
ENSG00000141150	GAUCAGA
ENSG00000141150	GCCCUGU
ENSG00000141150	GUCUCUG
ENSG00000141150	GUGGGGA
ENSG00000141150	UCCAGAG
ENSG00000141150	UGGGACA
ENSG00000141150	UUCUAAU
ENSG00000141150	UUGUGUC
ENSG00000141161	ACACACC
ENSG00000141179	CACAAUG
ENSG00000141179	GACUUCC
ENSG00000141179	GAGAAAU
ENSG00000141198	AAGGCAC
ENSG00000141198	GUGCAAA
ENSG00000141198	UCCUGAG
ENSG00000141219	GUGCAAA
ENSG00000141232	AAGACAU
ENSG00000141232	AAUACUG
ENSG00000141232	ACUCAAA
ENSG00000141232	CAGUCCA
ENSG00000141232	CUGACUC
ENSG00000141232	UGAAGCU
ENSG00000141232	UGUCUUU
ENSG00000141232	UUGGCAA
ENSG00000141258	AGGCAGG
ENSG00000141258	AGUGCUG
ENSG00000141258	CAGGCUC
ENSG00000141258	CCCUGAG
ENSG00000141258	GCAGAAG
ENSG00000141279	AACGCCU
ENSG00000141279	AACUACU
ENSG00000141279	AAUUGCU
ENSG00000141279	AUCAUAC
ENSG00000141279	AUUCAUU
ENSG00000141279	CACAGUG
ENSG00000141279	CAGCUGG
ENSG00000141279	CAGGAAC
ENSG00000141279	CAUAUUG
ENSG00000141279	GAAUUGU
ENSG00000141279	GAGUGUG
ENSG00000141279	UAUUACC
ENSG00000141279	UCACACA
ENSG00000141279	UCACAGG
ENSG00000141279	UCUGGAA
ENSG00000141298	AAAAACC
ENSG00000141298	AAAGGUA
ENSG00000141298	AAAGUAU
ENSG00000141298	AAGGCAC
ENSG00000141298	AAGUCAC

ENSG00000141298	AAUACUG
ENSG00000141298	AAUGCCC
ENSG00000141298	AGCAAAA
ENSG00000141298	AGGAGCU
ENSG00000141298	AGUGUGC
ENSG00000141298	AUAAAGU
ENSG00000141298	AUACAAG
ENSG00000141298	AUCACUA
ENSG00000141298	CCAGGAG
ENSG00000141298	CCUGCUG
ENSG00000141298	CCUUCUU
ENSG00000141298	CUCCAUU
ENSG00000141298	CUCUGGG
ENSG00000141298	CUGCCCC
ENSG00000141298	CUGGGGG
ENSG00000141298	GAGACCU
ENSG00000141298	GAGGCUG
ENSG00000141298	GCAGCAU
ENSG00000141298	GCAGGAC
ENSG00000141298	GGCUCAG
ENSG00000141298	GGGGCAG
ENSG00000141298	UCACCAC
ENSG00000141298	UGCAGCU
ENSG00000141298	UGCAUUG
ENSG00000141298	UUGGCAA
ENSG00000141298	UUGGCAC
ENSG00000141314	AAAUUUC
ENSG00000141314	AAGGCAC
ENSG00000141314	AGGGAGG
ENSG00000141314	CUCUGGG
ENSG00000141314	GGCAAGA
ENSG00000141314	UUUGCAA
ENSG00000141349	GGAGUGU
ENSG00000141349	UCACAGU
ENSG00000141367	AAAAACC
ENSG00000141367	AAACUGU
ENSG00000141367	AAAGGUA
ENSG00000141367	AAGUUGU
ENSG00000141367	ACAAUAU
ENSG00000141367	AGCAAAA
ENSG00000141367	AGCAUUC
ENSG00000141367	AGGUUAU
ENSG00000141367	AGUGAUC
ENSG00000141367	AGUGCAA
ENSG00000141367	AUGAUGC
ENSG00000141367	CAAAACU
ENSG00000141367	CAGUCCA
ENSG00000141367	CCAGUAC
ENSG00000141367	CUCAGGG
ENSG00000141367	CUCUUCC
ENSG00000141367	CUGGCUC
ENSG00000141367	CUUCACC
ENSG00000141367	GAGAAGA
ENSG00000141367	GUCUUGC
ENSG00000141367	GUGCAAA
ENSG00000141367	UAGAGGA
ENSG00000141367	UAUAAAG
ENSG00000141367	UCACAAG
ENSG00000141367	UCCCACA
ENSG00000141367	UGAAGUG
ENSG00000141367	UGAGAAG
ENSG00000141367	UGAUCCA
ENSG00000141367	UGCAUAG
ENSG00000141367	UGUCUGC

ENSG00000141367	UGUCUGG
ENSG00000141367	UGUCUUU
ENSG00000141367	UUUCAAC
ENSG00000141376	CUCUUCC
ENSG00000141380	AAAGCUG
ENSG00000141380	ACAAUAU
ENSG00000141380	AGUGCUG
ENSG00000141380	AUACAAG
ENSG00000141380	CCUUCAU
ENSG00000141380	GAGUUGA
ENSG00000141380	GGAAUGU
ENSG00000141380	GGCUGCG
ENSG00000141380	GUAGUGU
ENSG00000141380	UCUAGAG
ENSG00000141380	UGCAUAG
ENSG00000141380	UGGCUGC
ENSG00000141384	AGUGCAA
ENSG00000141384	AUAAUAC
ENSG00000141384	AUACAAG
ENSG00000141384	CAGUGCA
ENSG00000141384	CUUUGGU
ENSG00000141384	GAAACAU
ENSG00000141384	GGCAGUG
ENSG00000141384	UACAAAG
ENSG00000141401	AUUGCAC
ENSG00000141401	UAUUGCU
ENSG00000141401	UGCAUUG
ENSG00000141404	CUACAAA
ENSG00000141404	GAGGUAG
ENSG00000141404	GGUGGUC
ENSG00000141404	UUCUGCA
ENSG00000141424	UGACCUA
ENSG00000141429	AAAGUAC
ENSG00000141429	AACAAAC
ENSG00000141429	AAUCCUU
ENSG00000141429	AAUCUCU
ENSG00000141429	ACAGUAC
ENSG00000141429	ACAGUAU
ENSG00000141429	ACAUUAC
ENSG00000141429	AGCAGCA
ENSG00000141429	AGUGUGC
ENSG00000141429	CCCUGUU
ENSG00000141429	CUCUUCC
ENSG00000141429	CUUGGAG
ENSG00000141429	CUUGUAU
ENSG00000141429	GGAGUGU
ENSG00000141429	GUGCAAA
ENSG00000141429	UAUUGCU
ENSG00000141429	UCACAGG
ENSG00000141429	UCACAGU
ENSG00000141429	UUCUAAU
ENSG00000141429	UUUCAAC
ENSG00000141431	ACUGCAG
ENSG00000141433	AGUGCUG
ENSG00000141433	CGUGGCC
ENSG00000141433	CUCUAGC
ENSG00000141433	UAGGGCC
ENSG00000141433	UGCAGCU
ENSG00000141441	AAGGCAC
ENSG00000141441	AGCAGCA
ENSG00000141441	AGCAGCG
ENSG00000141441	CACACCU
ENSG00000141441	UGCAUAG
ENSG00000141446	UCCUGAG

ENSG00000141446	UGGCCCU
ENSG00000141448	AUUCAUU
ENSG00000141448	AUUGCAC
ENSG00000141448	CCCUGUU
ENSG00000141448	GGAAGAC
ENSG00000141448	UUGUUCG
ENSG00000141458	AUGGCUU
ENSG00000141458	AUUGCAC
ENSG00000141469	GACCAUG
ENSG00000141469	UAAUACA
ENSG00000141480	CCAGUGU
ENSG00000141480	UAAUGCU
ENSG00000141485	GAGUGUG
ENSG00000141503	AAAUGGU
ENSG00000141503	AAGUGCU
ENSG00000141503	ACAUUCA
ENSG00000141503	AGCAAAA
ENSG00000141503	AGCAGCA
ENSG00000141503	AGGGUCU
ENSG00000141503	AGUGCUU
ENSG00000141503	CAGCAGG
ENSG00000141503	CAGGCUC
ENSG00000141503	CCAGUGC
ENSG00000141503	CCAGUGU
ENSG00000141503	CCCCCAG
ENSG00000141503	CCCCUGG
ENSG00000141503	CUCACAC
ENSG00000141503	CUCCAUU
ENSG00000141503	CUGCCCC
ENSG00000141503	CUGCUCA
ENSG00000141503	GCUGGUG
ENSG00000141503	GGAGUGU
ENSG00000141503	GGCCCUG
ENSG00000141503	GGGAGCU
ENSG00000141503	GGGGAGC
ENSG00000141503	GGUUGAC
ENSG00000141503	GUGCAAA
ENSG00000141503	UGCUCAC
ENSG00000141503	UGGCUGC
ENSG00000141510	AAAAAUC
ENSG00000141510	AAAGUUU
ENSG00000141510	AGAUGUG
ENSG00000141510	AGCUGCC
ENSG00000141510	CACACCU
ENSG00000141510	GAGGUAG
ENSG00000141510	GAGGUAU
ENSG00000141522	AAGGCAC
ENSG00000141522	AAUACUG
ENSG00000141522	AGCAGCA
ENSG00000141522	CUUUGGU
ENSG00000141522	GGAGGCA
ENSG00000141522	UAGUGAG
ENSG00000141522	UUGGUCC
ENSG00000141526	GGAAUGU
ENSG00000141526	UCCCACC
ENSG00000141540	AACACUG
ENSG00000141540	ACAGUAU
ENSG00000141540	AGCACCA
ENSG00000141540	AGGCAGG
ENSG00000141540	AGUGGUU
ENSG00000141540	AUGCCUU
ENSG00000141540	CAGGUGA
ENSG00000141540	GAGGCUG
ENSG00000141540	GGAAGCC

ENSG00000141540	GGUUUAC
ENSG00000141540	UCUGGAA
ENSG00000141542	AGCAGCA
ENSG00000141542	CUUUGGU
ENSG00000141542	GGCAUUG
ENSG00000141542	UUGGCAA
ENSG00000141542	UUGGCAC
ENSG00000141568	AAAAAUC
ENSG00000141568	AAAGAAU
ENSG00000141568	AAAGUGC
ENSG00000141568	AACAAAC
ENSG00000141568	AAGUGCA
ENSG00000141568	AAGUGCU
ENSG00000141568	AAUGUUG
ENSG00000141568	ACCACAG
ENSG00000141568	AGUGCUG
ENSG00000141568	AGUGUGC
ENSG00000141568	AUGAUGA
ENSG00000141568	AUGAUGC
ENSG00000141568	CACAGGG
ENSG00000141568	CAGUGCA
ENSG00000141568	CAUAUUG
ENSG00000141568	CCCUGAG
ENSG00000141568	GAUUGUC
ENSG00000141568	GUCAACA
ENSG00000141568	UCACACA
ENSG00000141568	UGAAAUG
ENSG00000141568	UGCAGCU
ENSG00000141568	UUGGCAA
ENSG00000141568	UUGGUCC
ENSG00000141568	UUUGCAC
ENSG00000141570	GAGUUGA
ENSG00000141570	UGAAAUG
ENSG00000141576	GGAAUGU
ENSG00000141579	AAUUAUU
ENSG00000141580	AUGGCUU
ENSG00000141580	CACCAGC
ENSG00000141580	GUGCCUG
ENSG00000141580	UACAAAG
ENSG00000141580	UCAUGAU
ENSG00000141580	UUUGCAA
ENSG00000141582	AAUACUG
ENSG00000141582	ACAGUAC
ENSG00000141582	ACAUAGA
ENSG00000141582	AGCAGCG
ENSG00000141582	AGCAGCU
ENSG00000141582	AGCUUAU
ENSG00000141582	CUAGUAA
ENSG00000141582	CUCAAAC
ENSG00000141582	GGAAGCC
ENSG00000141582	UAAUAUC
ENSG00000141582	UAUAAUA
ENSG00000141582	UCAACAG
ENSG00000141582	UUCAAGC
ENSG00000141582	UUUGCAA
ENSG00000141622	AAAACUG
ENSG00000141622	AAACUGU
ENSG00000141622	AAAGAGC
ENSG00000141622	AAAGAGG
ENSG00000141622	AAGGCAC
ENSG00000141622	ACACACC
ENSG00000141622	ACCCUGU
ENSG00000141622	ACUAGAU
ENSG00000141622	ACUGGCU

ENSG00000141622	AGCACCA
ENSG00000141622	AGCAUUC
ENSG00000141622	AGGAGCU
ENSG00000141622	AGGGAGG
ENSG00000141622	AGGGUCU
ENSG00000141622	AUAAAGU
ENSG00000141622	AUACAAG
ENSG00000141622	AUCCUUG
ENSG00000141622	AUCGUAC
ENSG00000141622	CAAAGCA
ENSG00000141622	CAAAUGC
ENSG00000141622	CAGCUGG
ENSG00000141622	CGUACCG
ENSG00000141622	CUACAAA
ENSG00000141622	CUCCCAA
ENSG00000141622	CUCUAGC
ENSG00000141622	GAAACAU
ENSG00000141622	GACCUGG
ENSG00000141622	GAGAUGA
ENSG00000141622	GAGGCAG
ENSG00000141622	GAGGCUG
ENSG00000141622	GAGGUAG
ENSG00000141622	GAGUUGA
ENSG00000141622	GAUUGUA
ENSG00000141622	GAUUGUC
ENSG00000141622	GCUACAU
ENSG00000141622	GGACCUG
ENSG00000141622	GGAGGCA
ENSG00000141622	GGAGUGU
ENSG00000141622	GGAUUUC
ENSG00000141622	GGCUGCG
ENSG00000141622	GGGCCCC
ENSG00000141622	GGGGUGC
ENSG00000141622	GGGGUGG
ENSG00000141622	GUUCAUG
ENSG00000141622	UAUAAUA
ENSG00000141622	UCACACA
ENSG00000141622	UCACCAC
ENSG00000141622	UCCCUUG
ENSG00000141622	UGCAAAG
ENSG00000141622	UGCAUAG
ENSG00000141622	UGCAUUG
ENSG00000141622	UUCUAAU
ENSG00000141622	UUUGCAA
ENSG00000141622	UUUGCAC
ENSG00000141639	AAAACUG
ENSG00000141639	AAAGUGC
ENSG00000141639	AAGGCAC
ENSG00000141639	AAGUGCA
ENSG00000141639	AGCAGCU
ENSG00000141639	AUGGCAC
ENSG00000141639	GCUGGUG
ENSG00000141639	UGCAUAG
ENSG00000141642	CAGGAAC
ENSG00000141644	ACCAGCA
ENSG00000141644	AGCAGCA
ENSG00000141644	CAGGUGA
ENSG00000141644	CUGCAGC
ENSG00000141644	UGCAGCU
ENSG00000141646	AAAGUAA
ENSG00000141646	AAAUCCC
ENSG00000141646	AAGGCAC
ENSG00000141646	AAGUGCA
ENSG00000141646	ACAGUAU

ENSG00000141646	AGCAGCU
ENSG00000141646	AGGAGCU
ENSG00000141646	AGUAGAC
ENSG00000141646	AGUGCAA
ENSG00000141646	AUAAAGU
ENSG00000141646	AUACAAG
ENSG00000141646	AUGGCUU
ENSG00000141646	AUGUAAU
ENSG00000141646	CAGCAGA
ENSG00000141646	CAGCUGG
ENSG00000141646	CAUAUUG
ENSG00000141646	CCCUGAG
ENSG00000141646	CCUUCUG
ENSG00000141646	CCUUCUU
ENSG00000141646	CUCAAAC
ENSG00000141646	CUGGAAA
ENSG00000141646	CUUUGGU
ENSG00000141646	GAAAACA
ENSG00000141646	GAGAAAU
ENSG00000141646	GAGAACC
ENSG00000141646	GAUAUGU
ENSG00000141646	GCAACUU
ENSG00000141646	GCAGAAG
ENSG00000141646	GCCUGAU
ENSG00000141646	GGAAUGU
ENSG00000141646	GGCAGUG
ENSG00000141646	GGGGCAG
ENSG00000141646	GUCUGCC
ENSG00000141646	GUCUUGC
ENSG00000141646	GUGCAAA
ENSG00000141646	UACAAAG
ENSG00000141646	UAUAAAG
ENSG00000141646	UCAAGUA
ENSG00000141646	UCCCUUU
ENSG00000141646	UCUAGAG
ENSG00000141646	UCUGGAA
ENSG00000141646	UGCAAAG
ENSG00000141646	UGUCUUU
ENSG00000141646	UGUGUCA
ENSG00000141646	UUCAAGC
ENSG00000141646	UUGUGUC
ENSG00000141664	AGCAGCA
ENSG00000141664	CAAAGUG
ENSG00000141664	CAGUGCA
ENSG00000141664	GGAAGCC
ENSG00000141664	GUUAAUG
ENSG00000141664	UCCAGUU
ENSG00000141664	UCUAGAG
ENSG00000141668	AAAACUG
ENSG00000141668	AAUUGCU
ENSG00000141668	ACCCAUU
ENSG00000141668	CACAGGG
ENSG00000141668	CGUGCAU
ENSG00000141668	CUAUACA
ENSG00000141668	GGUUACC
ENSG00000141668	GUGCAAA
ENSG00000141668	UCACAGG
ENSG00000141668	UCAUUUG
ENSG00000141668	UCGUGCA
ENSG00000141668	UCUUGAG
ENSG00000141668	UGCUCAC
ENSG00000141668	UGGAUAA
ENSG00000141668	UUGGUUC
ENSG00000141682	GAGGUAG

ENSG00000141682	GAGGUAU
ENSG00000141682	GUAGUGU
ENSG00000141682	UCACAUU
ENSG00000141699	AAGUGCU
ENSG00000141699	AUACAAG
ENSG00000141699	CAAGAGC
ENSG00000141699	CAGCAAA
ENSG00000141699	CUCCCAA
ENSG00000141699	GAGGAUA
ENSG00000141699	GGAGGAG
ENSG00000141699	GGCUCUG
ENSG00000141699	UCACAAG
ENSG00000141699	UCACAGG
ENSG00000141699	UCACAGU
ENSG00000141699	UCAUACA
ENSG00000141699	UCUCCAA
ENSG00000141736	AAAAACC
ENSG00000141736	UUUGCAA
ENSG00000141738	ACUGGCC
ENSG00000141738	UACAAAG
ENSG00000141750	CUGGGGG
ENSG00000141750	GAAACAU
ENSG00000141750	GGCAGCG
ENSG00000141750	GGCAGUG
ENSG00000141750	GGCAUUG
ENSG00000141750	GUGUGGC
ENSG00000141750	UAUUGCU
ENSG00000141750	UCAAGUA
ENSG00000141750	UGAGAAU
ENSG00000141750	UUCAAGC
ENSG00000141753	AAGACAU
ENSG00000141753	CCUGCUG
ENSG00000141753	CUGCAGC
ENSG00000141753	CUGGAAA
ENSG00000141753	GGAGGCA
ENSG00000141756	CAGCAGA
ENSG00000141837	AAAACUG
ENSG00000141837	ACAAGGU
ENSG00000141837	AGGGUUG
ENSG00000141837	CACAGUG
ENSG00000141837	CUGCAGC
ENSG00000141837	GCAGGAC
ENSG00000141837	UAAUAGU
ENSG00000141837	UCACAGG
ENSG00000141837	UCUCAAG
ENSG00000141837	UUCAAAU
ENSG00000141837	UUUCAAC
ENSG00000141837	UUUUUGC
ENSG00000141867	AAAUUUC
ENSG00000141867	AACACUG
ENSG00000141867	ACAUAGA
ENSG00000141867	CUGGAAA
ENSG00000141867	GAAGGAA
ENSG00000141867	GGCUCUG
ENSG00000141867	UCCCUUU
ENSG00000141867	UCUGGAA
ENSG00000141867	UUCCAUA
ENSG00000141867	UUGGCAA
ENSG00000141905	AGCAGCA
ENSG00000141905	CUCCCAA
ENSG00000141905	GAGAUGA
ENSG00000141956	AUUGCAC
ENSG00000141956	CAGCAAA
ENSG00000141956	CAGUGCA

ENSG00000141956	CCAGCAU
ENSG00000141956	CUACAGU
ENSG00000141985	AAUACUG
ENSG00000141985	GGAAUGU
ENSG00000141985	GUGCUUG
ENSG00000141985	UAAUACA
ENSG00000141985	UGUGCUU
ENSG00000142002	AAAGAAU
ENSG00000142002	CAGUGCA
ENSG00000142002	CCCCUGG
ENSG00000142002	CCCUGAG
ENSG00000142002	GGUGGUU
ENSG00000142002	UAUAAAG
ENSG00000142002	UGUGCUU
ENSG00000142039	AAAGAGG
ENSG00000142039	ACUCAGG
ENSG00000142039	CUGGGGG
ENSG00000142039	GGAGUGU
ENSG00000142039	GGCAGGG
ENSG00000142039	GGGGAGC
ENSG00000142039	UCCCACA
ENSG00000142039	UCCCACC
ENSG00000142065	GAGGCAG
ENSG00000142065	GAGUGUG
ENSG00000142065	GCUAGCA
ENSG00000142065	UCACAUU
ENSG00000142149	AAAAGUA
ENSG00000142149	AAAGAAU
ENSG00000142149	AAAGUAC
ENSG00000142149	AACAUUC
ENSG00000142149	AAUUAUU
ENSG00000142149	AGCUGCC
ENSG00000142149	AUAAUAC
ENSG00000142149	AUCCUUG
ENSG00000142149	AUGUCUG
ENSG00000142149	CACAGUG
ENSG00000142149	UAUAAUA
ENSG00000142149	UCACAGG
ENSG00000142149	UCACAGU
ENSG00000142149	UCAUAGA
ENSG00000142149	UCUGGAA
ENSG00000142149	UGCAUUG
ENSG00000142149	UUGGUUC
ENSG00000142166	GAAAACA
ENSG00000142168	GGAAUGU
ENSG00000142188	AAGGCAC
ENSG00000142188	AAGUGCA
ENSG00000142188	AAUUAUU
ENSG00000142188	AGCUGCC
ENSG00000142188	AUCCCUU
ENSG00000142188	AUUGCAC
ENSG00000142188	CUAGUCC
ENSG00000142188	CUGGCUC
ENSG00000142188	GAGCUGC
ENSG00000142188	UAUUGCU
ENSG00000142188	UUGGCAC
ENSG00000142192	AAAGUGC
ENSG00000142192	AAGUGCU
ENSG00000142192	UACAGUU
ENSG00000142197	ACAAGGU
ENSG00000142208	AAAAAUC
ENSG00000142208	AAGUGCU
ENSG00000142235	AAGUUGU
ENSG00000142235	GGCAGUG

ENSG00000142235	UCAUUUG
ENSG00000142235	UUGGCAA
ENSG00000142235	UUGGCAC
ENSG00000142279	AAAGAAU
ENSG00000142279	AAGUCAC
ENSG00000142279	AGCAGCU
ENSG00000142303	ACAGUAC
ENSG00000142303	AGCACCA
ENSG00000142303	AGUGCUG
ENSG00000142303	CACAGUG
ENSG00000142303	CUGGGGG
ENSG00000142303	CUUCACC
ENSG00000142303	UCACAGG
ENSG00000142319	AACAGUC
ENSG00000142347	GAGGUAG
ENSG00000142347	GGCAGGG
ENSG00000142449	CAGUGCA
ENSG00000142453	AAGUGUG
ENSG00000142453	ACAUUCA
ENSG00000142453	AGGGUCU
ENSG00000142453	GCAGCAU
ENSG00000142453	GCUGUCU
ENSG00000142453	GUCAGUU
ENSG00000142453	GUGAGGU
ENSG00000142453	UCAUUUG
ENSG00000142453	UUGACAC
ENSG00000142453	UUGGUUC
ENSG00000142459	AAAUGGU
ENSG00000142459	AGUGUGC
ENSG00000142459	CUGCAGG
ENSG00000142459	CUUUGGU
ENSG00000142459	UUGGCAC
ENSG00000142459	UUGGUUC
ENSG00000142528	AAGUGCA
ENSG00000142528	CUGGCUC
ENSG00000142528	GAGGUAG
ENSG00000142528	GAGGUAU
ENSG00000142539	UCUGGAA
ENSG00000142541	AACAGUC
ENSG00000142541	GGAGGAG
ENSG00000142556	CCAGUGC
ENSG00000142599	AACUACU
ENSG00000142599	AAGGCAC
ENSG00000142599	AAGUCAC
ENSG00000142599	AAUCUCA
ENSG00000142599	ACUGUGU
ENSG00000142599	AGCACCA
ENSG00000142599	AUAAAGU
ENSG00000142599	AUGUAAU
ENSG00000142599	CAAAUGC
ENSG00000142599	CACAGUG
ENSG00000142599	CAGUCCA
ENSG00000142599	CUUGGAG
ENSG00000142599	CUUUGGU
ENSG00000142599	GCAGAAG
ENSG00000142599	GGAAUGU
ENSG00000142599	GUAGUGU
ENSG00000142599	UACAAAG
ENSG00000142599	UAGUGAG
ENSG00000142599	UCACAAG
ENSG00000142599	UCCCUUU
ENSG00000142599	UGUCUGC
ENSG00000142599	UUCAAAU
ENSG00000142599	UUCCUAG

ENSG00000142599	UUUUUGC
ENSG00000142606	GGGCGUG
ENSG00000142606	UGAAGCU
ENSG00000142611	AACAAAC
ENSG00000142611	AACAUUC
ENSG00000142611	AAGUGCU
ENSG00000142611	AAUCACU
ENSG00000142611	AAUCUCA
ENSG00000142611	AAUGCCC
ENSG00000142611	AAUUAUU
ENSG00000142611	ACAGUAC
ENSG00000142611	ACUGUUU
ENSG00000142611	AGCUGCC
ENSG00000142611	AGUGCUU
ENSG00000142611	AUAUUAU
ENSG00000142611	AUCACUA
ENSG00000142611	AUGGCUU
ENSG00000142611	AUUCAUU
ENSG00000142611	AUUGCAC
ENSG00000142611	CACAAUG
ENSG00000142611	CACAGUG
ENSG00000142611	CAGCAGG
ENSG00000142611	CCAGUGC
ENSG00000142611	CCAGUGU
ENSG00000142611	CCUUCAU
ENSG00000142611	CUAUACA
ENSG00000142611	CUCUUCC
ENSG00000142611	CUUCACC
ENSG00000142611	GAAAACA
ENSG00000142611	GAUAUGU
ENSG00000142611	GAUUGUA
ENSG00000142611	GAUUGUC
ENSG00000142611	GGGUUUA
ENSG00000142611	GGUUUAC
ENSG00000142611	GUAACAG
ENSG00000142611	UACAGUU
ENSG00000142611	UAUUGCU
ENSG00000142611	UCACAGU
ENSG00000142611	UCCCACA
ENSG00000142611	UGCAUAG
ENSG00000142611	UGGCCCU
ENSG00000142611	UGUAUAA
ENSG00000142611	UGUCUGC
ENSG00000142611	UGUCUGG
ENSG00000142611	UUGGCAA
ENSG00000142611	UUGGCAC
ENSG00000142611	UUGGUCC
ENSG00000142611	UUGUUCG
ENSG00000142623	CUCCCAC
ENSG00000142623	CUCUGGG
ENSG00000142627	AAGUGCU
ENSG00000142634	AAGUAGC
ENSG00000142634	AAUUUUA
ENSG00000142634	AUCGUAC
ENSG00000142634	AUUCAUU
ENSG00000142634	GAGGUAG
ENSG00000142634	GCUUCCU
ENSG00000142634	GUGAGGU
ENSG00000142634	UGCAUAG
ENSG00000142634	UGCAUAU
ENSG00000142634	UGUAUAA
ENSG00000142655	UAUUACC
ENSG00000142655	UCACACA
ENSG00000142657	GGAAUGU

ENSG00000142669	AAUGUUG
ENSG00000142669	CCUUCAU
ENSG00000142669	GCGGGGC
ENSG00000142669	GGAAUGU
ENSG00000142669	UUGGCAA
ENSG00000142669	UUGGCAC
ENSG00000142677	GGCAGGG
ENSG00000142686	CUCCAGC
ENSG00000142686	UCUCCAA
ENSG00000142700	AAAAACC
ENSG00000142700	AAGUGCA
ENSG00000142700	ACUGGCU
ENSG00000142700	AUAUAAC
ENSG00000142700	CAAAGUG
ENSG00000142700	CUGGCUC
ENSG00000142700	CUUGUAU
ENSG00000142700	GCAACGA
ENSG00000142700	GCACCAU
ENSG00000142700	GGCUCAG
ENSG00000142700	UAAUACA
ENSG00000142700	UGCAAAG
ENSG00000142784	AAACUGU
ENSG00000142784	AAAUGGU
ENSG00000142784	AAGUUGU
ENSG00000142784	ACUGCAG
ENSG00000142784	AGCAGCA
ENSG00000142784	AGCAGCU
ENSG00000142784	AGCUGCC
ENSG00000142784	CACAGUG
ENSG00000142784	CCAGUGU
ENSG00000142784	CCUUCAU
ENSG00000142784	CUGCAGG
ENSG00000142784	CUGCCCC
ENSG00000142784	CUUCACC
ENSG00000142784	CUUUGGU
ENSG00000142784	GCCCUUA
ENSG00000142784	GGAGGAG
ENSG00000142784	GGCUCAG
ENSG00000142784	GGGCCCC
ENSG00000142784	GUCUCUG
ENSG00000142784	UCACCAC
ENSG00000142784	UGGCCCU
ENSG00000142798	ACCACAG
ENSG00000142798	AGCACCA
ENSG00000142798	AGCAGCG
ENSG00000142798	AGCAGCU
ENSG00000142798	CAGCAAA
ENSG00000142798	CAGGCUC
ENSG00000142798	CGGGGAU
ENSG00000142798	CUUACAG
ENSG00000142798	GAGCUGC
ENSG00000142798	UACAAAG
ENSG00000142856	AAAGUUU
ENSG00000142864	AAAGCUG
ENSG00000142864	AAAUCCC
ENSG00000142864	AACUGGU
ENSG00000142864	ACACACC
ENSG00000142864	ACCAGCA
ENSG00000142864	AGCACCA
ENSG00000142864	AGCAGCA
ENSG00000142864	AGGGUUG
ENSG00000142864	AUAAAGU
ENSG00000142864	AUCAUAC
ENSG00000142864	AUGGCAC

ENSG00000142864	CAAAACU
ENSG00000142864	CAAAGCA
ENSG00000142864	CAAAGUG
ENSG00000142864	CACACCG
ENSG00000142864	CACAGUG
ENSG00000142864	CACCAGC
ENSG00000142864	CAGUCUG
ENSG00000142864	CAGUGCA
ENSG00000142864	CCAGUGC
ENSG00000142864	CUCUGGG
ENSG00000142864	CUUUGGU
ENSG00000142864	GAGAAGA
ENSG00000142864	GCAGAAG
ENSG00000142864	GCCCUUA
ENSG00000142864	GCUGUCU
ENSG00000142864	GGAGGCA
ENSG00000142864	GGUUGUC
ENSG00000142864	GUGCUUG
ENSG00000142864	UAUUACC
ENSG00000142864	UAUUGCU
ENSG00000142864	UCACAGG
ENSG00000142864	UCAUGAU
ENSG00000142864	UCAUUUG
ENSG00000142864	UGUCUGC
ENSG00000142864	UGUGCUU
ENSG00000142864	UUCAAGC
ENSG00000142864	UUGGUUC
ENSG00000142864	UUGUUCG
ENSG00000142864	UUUGCAA
ENSG00000142867	UUUCAAC
ENSG00000142871	AAUUAUU
ENSG00000142871	CCAGUAC
ENSG00000142871	GCUACAU
ENSG00000142871	UCCAGUU
ENSG00000142875	AAAGUGC
ENSG00000142875	AAGUGCU
ENSG00000142875	GUCAGUU
ENSG00000142892	AAAGCUG
ENSG00000142892	UCCCACC
ENSG00000142910	CUGGGGG
ENSG00000142920	UAAGACU
ENSG00000142920	UGCAUAG
ENSG00000142920	UGCAUAU
ENSG00000142949	AAAAAUC
ENSG00000142949	AACAAAC
ENSG00000142949	ACAAGGU
ENSG00000142949	ACAUUAC
ENSG00000142949	ACUGUGU
ENSG00000142949	AUGAUGC
ENSG00000142949	AUGCACC
ENSG00000142949	AUGGCUU
ENSG00000142949	CCAGUAC
ENSG00000142949	CCGGAGC
ENSG00000142949	GAGAAGA
ENSG00000142949	GGACCUG
ENSG00000142949	GGCCCUG
ENSG00000142949	GUCUUGC
ENSG00000142949	UGGCCCU
ENSG00000142949	UUAUGCA
ENSG00000142961	AAAGCUG
ENSG00000142961	AAGUCUU
ENSG00000142961	AGGCAGG
ENSG00000142961	AUAAAGU
ENSG00000142961	GGAAUGU

ENSG00000142961	GGAGGCA
ENSG00000142961	GGCUCAG
ENSG00000142961	GGGAGCU
ENSG00000143006	AUUCAUU
ENSG00000143006	CCUGCUG
ENSG00000143006	GCAGAAG
ENSG00000143006	GCCUGGG
ENSG00000143006	UCAUUCG
ENSG00000143013	AAAGAGC
ENSG00000143013	AAGGCAC
ENSG00000143013	AAGGUGC
ENSG00000143013	ACAAUAU
ENSG00000143013	ACUAGAU
ENSG00000143013	AUACAAG
ENSG00000143013	CCAGUGC
ENSG00000143013	CUACAAA
ENSG00000143013	CUCCCAA
ENSG00000143013	CUCCCAC
ENSG00000143013	GAAACAU
ENSG00000143013	GGGAUCG
ENSG00000143013	UGCCCUG
ENSG00000143013	UGGGAUC
ENSG00000143013	UUCUGCA
ENSG00000143013	UUGUGAC
ENSG00000143028	ACUGUUU
ENSG00000143028	CACUGUU
ENSG00000143028	CAGUAGA
ENSG00000143028	CCUUCAU
ENSG00000143028	CGGCCUG
ENSG00000143028	GAAAACA
ENSG00000143028	GCUGUCU
ENSG00000143032	UGGGGGA
ENSG00000143033	ACCCUGU
ENSG00000143033	AGCUGCC
ENSG00000143033	UCACACA
ENSG00000143033	UGAGAAG
ENSG00000143061	AAGGUGC
ENSG00000143061	AAUACUG
ENSG00000143061	ACAGGUG
ENSG00000143061	ACCUGGA
ENSG00000143061	ACUGGCC
ENSG00000143061	AGAUGUG
ENSG00000143061	AGUGGUU
ENSG00000143061	AUAUAAC
ENSG00000143061	CAAAGCA
ENSG00000143061	CAAAGUG
ENSG00000143061	CACAGUG
ENSG00000143061	CUACAAA
ENSG00000143061	GAAAACA
ENSG00000143061	GCACCAU
ENSG00000143061	UUCCAUA
ENSG00000143061	UUGGCAC
ENSG00000143061	UUUUUGC
ENSG00000143067	AAGUCUU
ENSG00000143067	AAGUGCU
ENSG00000143067	CAGCAAA
ENSG00000143067	CUACAGU
ENSG00000143067	CUAUACA
ENSG00000143067	GAGAACC
ENSG00000143067	GAGGUAG
ENSG00000143067	UCAAGUA
ENSG00000143067	UGGCCCU
ENSG00000143067	UGUGCUU
ENSG00000143067	UUGGCAC

ENSG00000143079	CAGUAGA
ENSG00000143079	CUGGAAA
ENSG00000143079	GGAAUGU
ENSG00000143079	GUUCAUG
ENSG00000143079	UCACAUU
ENSG00000143079	UCUGGAA
ENSG00000143079	UUCUGCA
ENSG00000143079	UUGGUUC
ENSG00000143093	ACUGCAG
ENSG00000143093	CUGCAGC
ENSG00000143093	GCAGCAU
ENSG00000143107	UGCCCUG
ENSG00000143126	AAACUGU
ENSG00000143126	AAAGUGC
ENSG00000143126	AAGUGCA
ENSG00000143126	ACUGGCC
ENSG00000143126	AUAUUAU
ENSG00000143126	CAAAGUG
ENSG00000143126	CAGCAAA
ENSG00000143126	CAGCAGA
ENSG00000143126	CCCUGAG
ENSG00000143126	CCUUCUG
ENSG00000143126	CUCUUCC
ENSG00000143126	CUGCCCC
ENSG00000143126	GAGGCUG
ENSG00000143126	GCAGCAU
ENSG00000143126	GCAGGAC
ENSG00000143126	GUCUUGC
ENSG00000143126	GUGCUUG
ENSG00000143126	GUUCAUG
ENSG00000143126	UUCCUCG
ENSG00000143126	UUGGCAC
ENSG00000143127	AAGUCUU
ENSG00000143127	ACUCAAA
ENSG00000143127	AGCUGCC
ENSG00000143127	CAGCAGA
ENSG00000143127	CUCAGGG
ENSG00000143127	CUGUUGA
ENSG00000143127	CUUGUGU
ENSG00000143127	GAAUUGU
ENSG00000143127	GCAGAAG
ENSG00000143127	GGCAGUG
ENSG00000143127	GUCUCUG
ENSG00000143127	UCCUUGC
ENSG00000143127	UGCCAGC
ENSG00000143140	GAGGCUG
ENSG00000143140	UCACAGG
ENSG00000143140	UCACAGU
ENSG00000143140	UCCAGUU
ENSG00000143153	AAAUGGU
ENSG00000143153	AAUUAUU
ENSG00000143153	ACAUUCA
ENSG00000143153	AGCACCA
ENSG00000143153	CAAGAGC
ENSG00000143153	CUUUGGU
ENSG00000143153	UGCAUUG
ENSG00000143153	UUCUUGU
ENSG00000143153	UUGGUUC
ENSG00000143153	UUUCAAC
ENSG00000143155	AAGACAU
ENSG00000143155	UCACAUU
ENSG00000143156	AGUGCAA
ENSG00000143156	CCAGGAG
ENSG00000143157	AAAGUUU

ENSG00000143157	CCAGUGU
ENSG00000143157	CUGCAGG
ENSG00000143157	GGAAGAC
ENSG00000143157	UCCAGUU
ENSG00000143158	CAGGAAC
ENSG00000143162	AUGGCUU
ENSG00000143167	AAUUUUA
ENSG00000143167	CAAAGCA
ENSG00000143178	AAAUGGU
ENSG00000143178	ACAGGUG
ENSG00000143178	UCACAGG
ENSG00000143179	AGUAGUU
ENSG00000143179	UUGGCAC
ENSG00000143217	AAUACUG
ENSG00000143217	AGGCAGG
ENSG00000143217	CACACCU
ENSG00000143217	CAGCAGG
ENSG00000143217	CUCCAGC
ENSG00000143217	CUCUGGG
ENSG00000143217	CUGGGGG
ENSG00000143217	GAGCCCU
ENSG00000143217	GGAGGCA
ENSG00000143217	GGGGUCC
ENSG00000143217	UUGGAGG
ENSG00000143222	AGCAGCU
ENSG00000143248	AGUAACA
ENSG00000143248	GGUUACC
ENSG00000143248	GUUAAUG
ENSG00000143252	AAAGCUG
ENSG00000143252	AAUCUCU
ENSG00000143252	CUCAGGG
ENSG00000143252	CUCUAGC
ENSG00000143252	CUUCUCU
ENSG00000143252	GCCCUUA
ENSG00000143252	GGCAGUG
ENSG00000143252	UCCCUCU
ENSG00000143258	AAAUUUC
ENSG00000143258	GAGGUAG
ENSG00000143258	GAGGUAU
ENSG00000143258	GUCUCUG
ENSG00000143294	AAAAAUC
ENSG00000143294	GGGGUCC
ENSG00000143294	UUUCAAC
ENSG00000143297	AUUCAUU
ENSG00000143297	UCAUUCG
ENSG00000143315	AUCCUUU
ENSG00000143318	CUGCCCC
ENSG00000143319	AAAUUUC
ENSG00000143319	AAGGCAC
ENSG00000143319	GCACCAU
ENSG00000143321	AAAAACC
ENSG00000143321	AAUUUUA
ENSG00000143321	AGCACCA
ENSG00000143321	AGCAGCA
ENSG00000143321	CAGACAG
ENSG00000143321	CAGCAAA
ENSG00000143321	CAGCAGG
ENSG00000143321	CAGGAAC
ENSG00000143321	CUACAGU
ENSG00000143321	GAAACAU
ENSG00000143321	UGGGUGA
ENSG00000143321	UGUCUGG
ENSG00000143321	UUUUUGC
ENSG00000143322	AAGUGCU

ENSG00000143322	AAGUGUG
ENSG00000143322	AAUCCUU
ENSG00000143322	AAUUUUA
ENSG00000143322	ACAAUAU
ENSG00000143322	ACACACU
ENSG00000143322	ACAUCAC
ENSG00000143322	ACAUUCA
ENSG00000143322	AGCAAAA
ENSG00000143322	AGGUAGU
ENSG00000143322	AGUGUGC
ENSG00000143322	AUUGCAC
ENSG00000143322	CAGACAG
ENSG00000143322	CAGUAGA
ENSG00000143322	CAGUAGU
ENSG00000143322	CAGUGCA
ENSG00000143322	CCCUGAG
ENSG00000143322	CCCUGUU
ENSG00000143322	CUUUGGU
ENSG00000143322	GACAACU
ENSG00000143322	GAGCCCU
ENSG00000143322	GAGGUAU
ENSG00000143322	GAUUGUC
ENSG00000143322	GCAGCAU
ENSG00000143322	GCUAGCA
ENSG00000143322	GGAAUGU
ENSG00000143322	GUAGUGU
ENSG00000143322	GUGCUUG
ENSG00000143322	UCACAGG
ENSG00000143322	UCACAUU
ENSG00000143322	UCCCUGU
ENSG00000143322	UCUCCAA
ENSG00000143322	UCUUGAG
ENSG00000143322	UGUGCUU
ENSG00000143322	UUAUGCA
ENSG00000143322	UUGGCAA
ENSG00000143322	UUGGUCC
ENSG00000143324	AAAAGUA
ENSG00000143324	AAAGAGG
ENSG00000143324	AACUGGU
ENSG00000143324	AAGUAGC
ENSG00000143324	AGCAGCA
ENSG00000143324	AGUGCCU
ENSG00000143324	AUUGCAC
ENSG00000143324	CAAAACU
ENSG00000143324	CACAGUG
ENSG00000143324	CCAGUGC
ENSG00000143324	CCGGUUC
ENSG00000143324	CCUUCAU
ENSG00000143324	CUGGAAA
ENSG00000143324	GAAGGUC
ENSG00000143324	GAGGUAU
ENSG00000143324	GAUUGGU
ENSG00000143324	GAUUGUC
ENSG00000143324	UCCCACC
ENSG00000143324	UUGGAGG
ENSG00000143324	UUGGCAA
ENSG00000143324	UUUCAAC
ENSG00000143333	ACAGUAU
ENSG00000143333	AGGAGCU
ENSG00000143333	CUCUUCC
ENSG00000143333	UUGUUCG
ENSG00000143337	GAGGCAG
ENSG00000143337	UCCUAUA
ENSG00000143337	UGCAUAG

ENSG00000143340	CUGCAGC
ENSG00000143341	CCAGUGC
ENSG00000143344	AAUACUG
ENSG00000143344	ACAGUAC
ENSG00000143344	ACUGGCC
ENSG00000143344	ACUGGCU
ENSG00000143344	AGCAGCU
ENSG00000143344	AUAUAAC
ENSG00000143344	AUUGCAC
ENSG00000143344	GUAAACA
ENSG00000143363	AACAAAC
ENSG00000143363	AGCAGCU
ENSG00000143363	AUGCAUU
ENSG00000143363	CAAAACU
ENSG00000143363	CUUUGGU
ENSG00000143363	GCUACAU
ENSG00000143363	UACAAAG
ENSG00000143365	AAGUGCA
ENSG00000143365	AUGGCAC
ENSG00000143365	CCUUCUG
ENSG00000143365	GAGGCUG
ENSG00000143365	GAGGUAG
ENSG00000143365	UGUGUCA
ENSG00000143367	AGCAGCA
ENSG00000143367	AGGGAGG
ENSG00000143367	CAGCAGA
ENSG00000143367	GAAGGAA
ENSG00000143368	UUGGUCC
ENSG00000143373	AAGGCAC
ENSG00000143373	ACUGCAG
ENSG00000143373	CUGCAGG
ENSG00000143373	UAUUACC
ENSG00000143373	UGAAAGG
ENSG00000143375	AAGGCAC
ENSG00000143375	GAGGUAU
ENSG00000143375	GGGGCAG
ENSG00000143375	GUGCAAA
ENSG00000143375	UCCCUGU
ENSG00000143376	AAAAACC
ENSG00000143376	AAAAAUC
ENSG00000143376	AAAGGUA
ENSG00000143376	AAAGUUU
ENSG00000143376	AACACUG
ENSG00000143376	ACAAGGU
ENSG00000143376	ACAUUAC
ENSG00000143376	ACUGCAU
ENSG00000143376	ACUGGCC
ENSG00000143376	AGAUAAA
ENSG00000143376	AGUGCAA
ENSG00000143376	AGUGGUU
ENSG00000143376	CAGUGCA
ENSG00000143376	CCCUGAG
ENSG00000143376	CCCUGUU
ENSG00000143376	CCUGCUG
ENSG00000143376	CCUUCAU
ENSG00000143376	CUCAGGG
ENSG00000143376	CUGGGCA
ENSG00000143376	CUUCACC
ENSG00000143376	GAGGUAU
ENSG00000143376	GCAGGUU
ENSG00000143376	GGCUCAG
ENSG00000143376	GGUUACC
ENSG00000143376	GUAAACA
ENSG00000143376	GUGCAAA

ENSG00000143376	GUUAAUG
ENSG00000143376	UACAGUU
ENSG00000143376	UAUUGCU
ENSG00000143376	UCAUAGA
ENSG00000143376	UCUGGAA
ENSG00000143376	UGCAAAG
ENSG00000143376	UGCAGCU
ENSG00000143376	UGCCCUG
ENSG00000143376	UUAGAGA
ENSG00000143379	AGCACCA
ENSG00000143379	AGGAGCU
ENSG00000143379	GCUAGCA
ENSG00000143379	UUCAAGC
ENSG00000143384	AAAAAUC
ENSG00000143384	AAAGGUA
ENSG00000143384	AAGACAU
ENSG00000143384	AAUUAUU
ENSG00000143384	ACAUUCA
ENSG00000143384	AGCACCA
ENSG00000143384	AGGCAGG
ENSG00000143384	AGUACUG
ENSG00000143384	AUGGCUU
ENSG00000143384	AUUGCAC
ENSG00000143384	GCACCAU
ENSG00000143384	GGCAGGG
ENSG00000143384	UCCCUGU
ENSG00000143384	UUGGUCC
ENSG00000143387	GCACCAU
ENSG00000143390	AAGUGCA
ENSG00000143390	ACCUGGA
ENSG00000143390	CACAGGG
ENSG00000143390	CCCUGAG
ENSG00000143390	CCUUCUG
ENSG00000143390	GAUUGGU
ENSG00000143390	UCCCACA
ENSG00000143390	UCCCUCU
ENSG00000143393	AAAAAUC
ENSG00000143393	AAUACUG
ENSG00000143393	ACCACAG
ENSG00000143393	AGCAGCA
ENSG00000143393	CUCCCAC
ENSG00000143393	GCAGCAU
ENSG00000143393	UCCCUGU
ENSG00000143393	UCUGGAG
ENSG00000143393	UUCAAAU
ENSG00000143398	AAGUCUU
ENSG00000143398	AAUGUUG
ENSG00000143398	ACAUCAC
ENSG00000143398	ACAUUAC
ENSG00000143398	ACUGGCU
ENSG00000143398	CAGCAAA
ENSG00000143398	CCUUCUG
ENSG00000143398	CUCCCAC
ENSG00000143398	CUUGUAU
ENSG00000143398	GGCAGCG
ENSG00000143398	GGCAGUG
ENSG00000143398	UCCCACC
ENSG00000143398	UCCCUCU
ENSG00000143398	UCCUGAG
ENSG00000143398	UUCUUGU
ENSG00000143401	AAAGAGC
ENSG00000143401	AAAGUAU
ENSG00000143401	AAAGUUU
ENSG00000143401	AUGCACC

ENSG00000143401	CAAAGUG
ENSG00000143401	GGAAUGU
ENSG00000143401	GGAGGCA
ENSG00000143401	UAUAAAG
ENSG00000143401	UCUGGAG
ENSG00000143401	UGAAAUG
ENSG00000143401	UUCCUAG
ENSG00000143409	CUCAGGG
ENSG00000143409	GGGUGGU
ENSG00000143409	UACAAAG
ENSG00000143409	UGCCCUG
ENSG00000143409	UGGGUGA
ENSG00000143418	AAAUGGU
ENSG00000143418	CUUUGGU
ENSG00000143418	GGAAUGU
ENSG00000143418	GGCUCUG
ENSG00000143418	GGUUUAC
ENSG00000143418	UCUGGAG
ENSG00000143418	UGGGUGA
ENSG00000143418	UUGGCAA
ENSG00000143418	UUGGCAC
ENSG00000143418	UUGGUCC
ENSG00000143420	AUAAUAC
ENSG00000143420	CUAUACA
ENSG00000143420	CUCCAUU
ENSG00000143420	GAAAACA
ENSG00000143420	GAGAUGA
ENSG00000143434	AAGGCAC
ENSG00000143434	CUGGCUC
ENSG00000143437	ACAUAGA
ENSG00000143437	ACCCUGU
ENSG00000143437	ACUGUAG
ENSG00000143437	AGCACCA
ENSG00000143437	AUAUUAU
ENSG00000143437	AUGGCUU
ENSG00000143437	GACCCUG
ENSG00000143437	GGCUGCG
ENSG00000143437	GUCAUUG
ENSG00000143437	UACAGUU
ENSG00000143437	UCACACA
ENSG00000143437	UCACAUU
ENSG00000143437	UUCCAUA
ENSG00000143437	UUGGUUC
ENSG00000143437	UUUUUGC
ENSG00000143442	AAGACAU
ENSG00000143442	ACAGUAC
ENSG00000143442	CACAGGG
ENSG00000143442	GAGGUAG
ENSG00000143442	GAGGUAU
ENSG00000143442	GCUACAU
ENSG00000143442	GUCCAGA
ENSG00000143442	UAAGACG
ENSG00000143442	UAAGACU
ENSG00000143442	UAUAAAG
ENSG00000143442	UCACAUU
ENSG00000143442	UCCAGAG
ENSG00000143442	UGAAGCU
ENSG00000143443	AAAAAUC
ENSG00000143443	AUAUUAU
ENSG00000143443	CUUCUCU
ENSG00000143443	GGGUUUA
ENSG00000143450	AUUGCAC
ENSG00000143457	AAAAAUC
ENSG00000143457	GUCUCUG

ENSG00000143458	AAACUGU
ENSG00000143466	AGGGAGG
ENSG00000143466	CCAGGAG
ENSG00000143466	GAGGUAG
ENSG00000143466	UAAUGCU
ENSG00000143469	AAAAAUC
ENSG00000143469	AAAGUAU
ENSG00000143469	AACCUGU
ENSG00000143469	AAGUGCU
ENSG00000143469	AAUCUCA
ENSG00000143469	ACAGUAC
ENSG00000143469	ACAGUAU
ENSG00000143469	ACAUCAC
ENSG00000143469	AGCAAAA
ENSG00000143469	AGCACCA
ENSG00000143469	AUGAGCU
ENSG00000143469	CACUGUU
ENSG00000143469	CCUGCUG
ENSG00000143469	CUGGAAA
ENSG00000143469	CUUUGGU
ENSG00000143469	GCUUCCU
ENSG00000143469	UAUAAAG
ENSG00000143469	UGAAAGG
ENSG00000143469	UGACAUC
ENSG00000143469	UGAGGAC
ENSG00000143469	UUAGAGA
ENSG00000143469	UUCUAAU
ENSG00000143469	UUUCAAC
ENSG00000143494	AAAAACC
ENSG00000143494	AAGACAU
ENSG00000143494	AAGUGCA
ENSG00000143494	AGCACCA
ENSG00000143494	AUGGCUU
ENSG00000143494	CAGUGCA
ENSG00000143494	CUUGUGU
ENSG00000143494	GAGGUAG
ENSG00000143494	GAGGUAU
ENSG00000143494	GGGUAAG
ENSG00000143494	GUGCUUG
ENSG00000143494	UAAUAUC
ENSG00000143494	UGCAAAG
ENSG00000143494	UGCAUAG
ENSG00000143494	UGCUGAC
ENSG00000143499	UGCAUAG
ENSG00000143507	ACAAGGU
ENSG00000143507	ACAUUCA
ENSG00000143507	UAUUGCU
ENSG00000143514	AAUCCUU
ENSG00000143514	GUCAUUG
ENSG00000143515	AAACUGU
ENSG00000143515	ACAUUCA
ENSG00000143515	AUAAUAC
ENSG00000143515	CAGCAGG
ENSG00000143515	CUCUGGG
ENSG00000143515	GAGAACC
ENSG00000143515	GAGGGGC
ENSG00000143515	GCAGGAC
ENSG00000143515	GUAAACA
ENSG00000143515	GUGGCCC
ENSG00000143515	GUGUCAC
ENSG00000143515	UAUAAAG
ENSG00000143515	UCUCACC
ENSG00000143515	UGGUACC
ENSG00000143515	UUCCUCG

ENSG00000143515	UUUGCAC
ENSG00000143520	CUUGGAG
ENSG00000143520	UCAUAGA
ENSG00000143520	UUGUUCG
ENSG00000143537	UAUUGCU
ENSG00000143545	GUGCAAA
ENSG00000143546	GAGGUAU
ENSG00000143549	AAACGGU
ENSG00000143549	AACAUUC
ENSG00000143549	AAGUCAC
ENSG00000143549	AAGUUGU
ENSG00000143549	AGCAGCU
ENSG00000143549	GGGGCAG
ENSG00000143549	UCCAGAG
ENSG00000143553	AGCCAGU
ENSG00000143553	CAGUGCA
ENSG00000143553	CCAGUGC
ENSG00000143553	GUAAACA
ENSG00000143554	UACAAAG
ENSG00000143554	UAUUACC
ENSG00000143569	AAAUUUC
ENSG00000143569	AGGACAC
ENSG00000143569	CACCACC
ENSG00000143569	CCAUUAC
ENSG00000143569	UCCUAUA
ENSG00000143569	UGAAGCU
ENSG00000143570	GAAACAU
ENSG00000143570	GUGCUUG
ENSG00000143570	UGAAAUG
ENSG00000143570	UGUGCUU
ENSG00000143570	UUGGCAA
ENSG00000143590	AAGUCAC
ENSG00000143590	AAUGCCC
ENSG00000143590	ACAUUAC
ENSG00000143590	CAAAGCA
ENSG00000143590	CUAGUCC
ENSG00000143590	CUUCACC
ENSG00000143590	GAGGGGC
ENSG00000143590	GGGGUCC
ENSG00000143590	GUAAACA
ENSG00000143590	GUGGGGA
ENSG00000143590	UAGACUG
ENSG00000143590	UAUUGCU
ENSG00000143590	UCACAGG
ENSG00000143590	UCACAGU
ENSG00000143590	UCUCCAA
ENSG00000143590	UGCAUAG
ENSG00000143590	UGCAUAU
ENSG00000143590	UGUGCGU
ENSG00000143603	GGCCCUG
ENSG00000143603	UAAGACG
ENSG00000143603	UAAGACU
ENSG00000143614	AAAAAUC
ENSG00000143614	AAAAGUA
ENSG00000143614	AAAGGUA
ENSG00000143614	AAAGUAA
ENSG00000143614	AAAGUGC
ENSG00000143614	AACCGUU
ENSG00000143614	AAGGCAC
ENSG00000143614	AAGUGCU
ENSG00000143614	AAUACUG
ENSG00000143614	AAUCUCA
ENSG00000143614	AAUCUCU
ENSG00000143614	ACAAUAU

ENSG00000143614	ACAUUAC
ENSG00000143614	ACCACAG
ENSG00000143614	ACUCCAG
ENSG00000143614	ACUGCAU
ENSG00000143614	ACUUGGA
ENSG00000143614	AGGACAC
ENSG00000143614	AGGGUCU
ENSG00000143614	AGUGCAA
ENSG00000143614	AGUGCUG
ENSG00000143614	AGUGCUU
ENSG00000143614	AUAAUAC
ENSG00000143614	AUCACUA
ENSG00000143614	AUCAUAC
ENSG00000143614	AUCCCUU
ENSG00000143614	AUGCCUU
ENSG00000143614	AUGGCAC
ENSG00000143614	AUUCAUU
ENSG00000143614	CAAAGCA
ENSG00000143614	CACCACC
ENSG00000143614	CAGCAAA
ENSG00000143614	CAGUGCA
ENSG00000143614	CCAGUAC
ENSG00000143614	CCCACCU
ENSG00000143614	CUCGGCG
ENSG00000143614	GAAACAU
ENSG00000143614	GCUGGUG
ENSG00000143614	GGAGGCA
ENSG00000143614	GGAGUGU
ENSG00000143614	GGCAAGA
ENSG00000143614	GGCAGUG
ENSG00000143614	GGCUCUG
ENSG00000143614	GGGGAGC
ENSG00000143614	GGGUCUU
ENSG00000143614	GGGUGGU
ENSG00000143614	UAAUGCU
ENSG00000143614	UAGGGCC
ENSG00000143614	UAGGUAU
ENSG00000143614	UCACAAG
ENSG00000143614	UCACCAC
ENSG00000143614	UCAUACA
ENSG00000143614	UCCCACC
ENSG00000143614	UCCCUUG
ENSG00000143614	UCUGGAG
ENSG00000143614	UGUAUAA
ENSG00000143614	UGUCUUU
ENSG00000143614	UUCAAAU
ENSG00000143614	UUGACAC
ENSG00000143614	UUUGCAA
ENSG00000143614	UUUGCAC
ENSG00000143621	UUUCAAC
ENSG00000143622	AAAGAGC
ENSG00000143622	ACUGGAU
ENSG00000143622	CUGGACU
ENSG00000143622	UCACAGG
ENSG00000143622	UGGACUG
ENSG00000143622	UUCUAAU
ENSG00000143624	AAAAACC
ENSG00000143624	CAGUCCA
ENSG00000143624	GUCAUUG
ENSG00000143630	ACUUGGA
ENSG00000143630	CUAGGCU
ENSG00000143630	GAGAAGA
ENSG00000143630	GUGCUUG
ENSG00000143630	UGAGAAG

ENSG00000143632	AACAAAC
ENSG00000143632	CACAGGG
ENSG00000143632	UCCUGAG
ENSG00000143641	AAAGUAC
ENSG00000143641	AAAGUAU
ENSG00000143641	AAAGUUU
ENSG00000143641	AAUACUG
ENSG00000143641	ACUGUGU
ENSG00000143641	GACAACU
ENSG00000143641	GACCAUG
ENSG00000143641	GAGAAGA
ENSG00000143641	GAGGUAG
ENSG00000143641	GGCAGGG
ENSG00000143641	GUGACAG
ENSG00000143641	GUGUCAC
ENSG00000143641	UGCAAAG
ENSG00000143641	UGUGUCA
ENSG00000143641	UUCAAGC
ENSG00000143641	UUCCAUA
ENSG00000143641	UUGGCAA
ENSG00000143641	UUUCAAC
ENSG00000143641	UUUGCAA
ENSG00000143643	UGUAUAA
ENSG00000143643	UUCAAGC
ENSG00000143653	UGAAAUG
ENSG00000143669	AAAAGUA
ENSG00000143669	AAUCACU
ENSG00000143669	GUAAACA
ENSG00000143702	AAAGUGC
ENSG00000143702	AACACUG
ENSG00000143702	AUGGCUU
ENSG00000143702	GAUAUGU
ENSG00000143702	GUAACAG
ENSG00000143702	GUGCAAA
ENSG00000143702	UCAUGAU
ENSG00000143702	UCCAGAG
ENSG00000143702	UCUGGAG
ENSG00000143702	UUGGCAC
ENSG00000143756	CAGUUAC
ENSG00000143756	GUGCAAA
ENSG00000143761	AAAACUG
ENSG00000143761	AAAGCUG
ENSG00000143761	ACAUUAC
ENSG00000143761	AGUGCUU
ENSG00000143761	AUUGCAC
ENSG00000143761	CUCCAGC
ENSG00000143761	UACAGUU
ENSG00000143761	UGCAUAU
ENSG00000143761	UUCUAAU
ENSG00000143768	AAGUGCA
ENSG00000143768	AGUGCUU
ENSG00000143772	AAAGUGC
ENSG00000143772	AAGGCAC
ENSG00000143772	AGUGCAA
ENSG00000143772	CAAAGUG
ENSG00000143772	CUUGUAU
ENSG00000143772	GGGGCAG
ENSG00000143772	GUAGUGU
ENSG00000143772	GUCACUC
ENSG00000143772	GUGCAAA
ENSG00000143772	UUGUUCG
ENSG00000143776	AAAAACC
ENSG00000143776	AAAACUG
ENSG00000143776	AAACUGU

ENSG00000143776	AACUGGU
ENSG00000143776	ACUGUGU
ENSG00000143776	AGCACCA
ENSG00000143776	AUACAAG
ENSG00000143776	AUUGCAC
ENSG00000143776	CAAAACU
ENSG00000143776	GAGAAAU
ENSG00000143776	GAGAACU
ENSG00000143776	UAAUAUC
ENSG00000143776	UAUUGCU
ENSG00000143776	UCCCACC
ENSG00000143776	UGGCCAC
ENSG00000143776	UUGGCAC
ENSG00000143786	AGCAGCA
ENSG00000143786	CAGGAAC
ENSG00000143786	UCACAGG
ENSG00000143786	UGUAUAA
ENSG00000143797	AAGACAU
ENSG00000143797	AAGGCGC
ENSG00000143797	AAGGUGC
ENSG00000143797	AAUACUG
ENSG00000143797	AGUGCCU
ENSG00000143797	AUUCAUU
ENSG00000143797	AUUGCAC
ENSG00000143797	CAGUGCA
ENSG00000143797	GACCCUG
ENSG00000143797	GAGAUGA
ENSG00000143797	GGGUUUA
ENSG00000143797	UAUUGCU
ENSG00000143797	UGGGAUC
ENSG00000143801	AAAACUG
ENSG00000143801	CUAUACA
ENSG00000143815	AAGUCAC
ENSG00000143815	GAGGUAG
ENSG00000143815	GAGGUAU
ENSG00000143842	AAAAAUC
ENSG00000143842	AAAGAGG
ENSG00000143842	CCCCCAG
ENSG00000143842	CCCCUGG
ENSG00000143842	CUUGGAG
ENSG00000143842	GAGCUAA
ENSG00000143842	GAGGUAG
ENSG00000143845	AGUACUG
ENSG00000143845	CCCUGAG
ENSG00000143845	CUUCACC
ENSG00000143845	GAGCCCU
ENSG00000143845	GAGGUAG
ENSG00000143845	GGUUGAC
ENSG00000143845	UAAUGCU
ENSG00000143845	UCUCCAA
ENSG00000143845	UGAAGUG
ENSG00000143845	UUGGCAA
ENSG00000143845	UUGGCAC
ENSG00000143847	ACACACU
ENSG00000143847	ACAGUAU
ENSG00000143847	AGUACUG
ENSG00000143847	CUCACAC
ENSG00000143847	UGAGAAG
ENSG00000143850	AAAGAGC
ENSG00000143850	AAGGCAC
ENSG00000143850	AAGUCUU
ENSG00000143850	AAUACUG
ENSG00000143850	AGCAAAA
ENSG00000143850	AGCUGCC

ENSG00000143850	AUACAAG
ENSG00000143850	AUAUUAU
ENSG00000143850	AUGUGCC
ENSG00000143850	AUUGCAC
ENSG00000143850	CCUGCUG
ENSG00000143850	CUGCCCC
ENSG00000143850	CUGGCUC
ENSG00000143850	CUUUGGU
ENSG00000143850	GAGCUGC
ENSG00000143850	GGGGUGG
ENSG00000143850	UCACAGG
ENSG00000143850	UCACAGU
ENSG00000143850	UGAAAGG
ENSG00000143850	UGCAUAU
ENSG00000143850	UGGCCCU
ENSG00000143850	UGUAUAA
ENSG00000143850	UGUCUUU
ENSG00000143851	CUCUUCC
ENSG00000143858	GAGGUAG
ENSG00000143858	GCAGCAU
ENSG00000143862	AAAAACC
ENSG00000143862	AAAUGGU
ENSG00000143862	ACAAUAU
ENSG00000143862	ACACACC
ENSG00000143862	ACACACU
ENSG00000143862	ACUGUAG
ENSG00000143862	CAGCAGG
ENSG00000143862	CAGUUAC
ENSG00000143862	GAGAACC
ENSG00000143862	GAGAACU
ENSG00000143862	GCAGCAU
ENSG00000143862	GGGGUCC
ENSG00000143862	GUGCAAA
ENSG00000143862	UACAGUU
ENSG00000143867	AAAAGUA
ENSG00000143867	AAAGUAA
ENSG00000143867	AAUACUG
ENSG00000143867	AGCUUAU
ENSG00000143867	AUAAAGU
ENSG00000143867	CAGGCUC
ENSG00000143867	CCAGUGU
ENSG00000143867	GGGGAGC
ENSG00000143870	ACAAUAU
ENSG00000143870	AGCAGCA
ENSG00000143870	CUCCCAA
ENSG00000143870	CUGGAAA
ENSG00000143870	GAGAUGA
ENSG00000143870	UAAUAUC
ENSG00000143870	UCACAUU
ENSG00000143870	UUCUUGU
ENSG00000143878	AGCUUAU
ENSG00000143878	AUGGCAC
ENSG00000143878	AUGUAAU
ENSG00000143878	UAAUAUC
ENSG00000143878	UGCAAAG
ENSG00000143882	AACACUG
ENSG00000143882	CCAUUAC
ENSG00000143882	CUUGGGC
ENSG00000143882	GAGGCUG
ENSG00000143882	GUAUUCU
ENSG00000143882	UCUCAAG
ENSG00000143882	UUCCAUA
ENSG00000143882	UUCUUGU
ENSG00000143891	GCCUGGA

ENSG00000143921	ACUGCAG
ENSG00000143921	CUGCAGC
ENSG00000143921	UGUCUUU
ENSG00000143924	AAAGUUU
ENSG00000143924	AUGGCAC
ENSG00000143924	AUUCAUU
ENSG00000143924	CAGUAGU
ENSG00000143924	CUUCACC
ENSG00000143924	GGAAUGU
ENSG00000143924	GGCUCAG
ENSG00000143924	GUCAGUU
ENSG00000143924	UAAGACG
ENSG00000143924	UCACACA
ENSG00000143924	UCACAUU
ENSG00000143924	UCAUUCG
ENSG00000143924	UGAGAAG
ENSG00000143924	UUCUAAU
ENSG00000143924	UUCUGCA
ENSG00000143924	UUGAGGC
ENSG00000143933	AAAGUUU
ENSG00000143933	ACCAGCA
ENSG00000143933	ACUUGGA
ENSG00000143933	AUAAUAC
ENSG00000143933	AUACAAG
ENSG00000143933	AUGGAUU
ENSG00000143933	CAUAUUG
ENSG00000143933	CUAUACA
ENSG00000143933	GAGGCAG
ENSG00000143933	GGAAUGU
ENSG00000143933	GGAGGAG
ENSG00000143933	GGAUUUC
ENSG00000143933	GUGCCAC
ENSG00000143933	UAAUACA
ENSG00000143933	UACAAAG
ENSG00000143933	UCAACAG
ENSG00000143933	UCCCUCU
ENSG00000143933	UGCAUAU
ENSG00000143933	UGGAUAA
ENSG00000143933	UGUUGAA
ENSG00000143933	UUGACAC
ENSG00000143933	UUGAUCA
ENSG00000143933	UUUGCAC
ENSG00000143952	AGCAUUC
ENSG00000143952	AUUGCAC
ENSG00000143952	CAAAGCA
ENSG00000143952	GGAUUUC
ENSG00000143952	GGAUUUU
ENSG00000143952	UGCAAAG
ENSG00000143952	UGGGACA
ENSG00000143954	AUGAUGA
ENSG00000143970	AAGGUGC
ENSG00000143970	AAGUCUU
ENSG00000143970	AAGUGCU
ENSG00000143970	CUCAGGG
ENSG00000143970	GACAACU
ENSG00000143970	GGAAUGU
ENSG00000143970	GUAUUCU
ENSG00000143970	GUGACAG
ENSG00000143970	GUGCAAA
ENSG00000143970	UCCUGAG
ENSG00000143970	UCUGGAA
ENSG00000143971	CUCACAC
ENSG00000143971	GAGAAAU
ENSG00000143971	UCUCACC

ENSG00000143977	AACAAAC
ENSG00000143995	AAAAGUA
ENSG00000143995	AAAGUAU
ENSG00000143995	AAAUUUC
ENSG00000143995	AACAGUC
ENSG00000143995	AAGACAU
ENSG00000143995	AAUGCCC
ENSG00000143995	ACAGUAU
ENSG00000143995	ACUAGAU
ENSG00000143995	ACUCAAA
ENSG00000143995	AUGAUGC
ENSG00000143995	AUGCAUU
ENSG00000143995	GAUUGGU
ENSG00000143995	GCAGGAC
ENSG00000143995	GGAAUGU
ENSG00000143995	GGAGGAG
ENSG00000143995	GGGUGGU
ENSG00000143995	GGUGGUU
ENSG00000143995	GUCAACA
ENSG00000143995	GUGACAG
ENSG00000143995	UAAGACG
ENSG00000143995	UAAGACU
ENSG00000143995	UAAUACA
ENSG00000143995	UAUAAUA
ENSG00000143995	UCCAGUU
ENSG00000143995	UCCCUUG
ENSG00000143995	UCCCUUU
ENSG00000143995	UCUGGAA
ENSG00000143995	UCUGGAG
ENSG00000143995	UGGAUAA
ENSG00000143995	UGUUGAA
ENSG00000143995	UUCAAAU
ENSG00000143995	UUCAAGC
ENSG00000143995	UUCCAUA
ENSG00000143995	UUGGUCC
ENSG00000143995	UUGUUCG
ENSG00000143995	UUUGCAA
ENSG00000143995	UUUUUGC
ENSG00000144021	CUGGGGG
ENSG00000144021	UCCCUUG
ENSG00000144026	UAUAAAG
ENSG00000144040	AAAGUAU
ENSG00000144040	AAAGUGC
ENSG00000144040	AAGUGCA
ENSG00000144040	AGAUAAA
ENSG00000144040	GCCUGGA
ENSG00000144040	UAUUGCU
ENSG00000144040	UCACAUU
ENSG00000144040	UGUGCUU
ENSG00000144040	UUGGCAA
ENSG00000144043	AAGGCAC
ENSG00000144043	ACUUGGA
ENSG00000144043	AGCAAAA
ENSG00000144043	AGUGCUU
ENSG00000144043	CACUCCU
ENSG00000144043	CUUACAG
ENSG00000144043	GAGAACU
ENSG00000144043	GAGGUAG
ENSG00000144043	GAGGUAU
ENSG00000144043	GCCUGGA
ENSG00000144043	GGCAAGA
ENSG00000144043	GUCAACA
ENSG00000144043	GUGCCAC
ENSG00000144043	UUCAAGC

ENSG00000144061	UAUAAAG
ENSG00000144063	UUCAAGC
ENSG00000144063	UUUCAAC
ENSG00000144118	GAGGUAG
ENSG00000144118	GGCUCUG
ENSG00000144118	UAUAAAG
ENSG00000144118	UGCCAGC
ENSG00000144118	UUGGCAA
ENSG00000144119	AAAAACC
ENSG00000144119	UAAUGCU
ENSG00000144119	UGCAAAG
ENSG00000144152	GAGAAAU
ENSG00000144218	AAAAACC
ENSG00000144218	AACUCUA
ENSG00000144218	AAGUGUG
ENSG00000144218	ACAUCAC
ENSG00000144218	ACUCAAA
ENSG00000144218	ACUGUGU
ENSG00000144218	AGAUAAA
ENSG00000144218	AGUGCAA
ENSG00000144218	AUACAAG
ENSG00000144218	AUCCUUG
ENSG00000144218	AUCCUUU
ENSG00000144218	CACAAUG
ENSG00000144218	CACAGUG
ENSG00000144218	CCUUCAU
ENSG00000144218	CUACAGU
ENSG00000144218	CUUGGGC
ENSG00000144218	GCAGAAG
ENSG00000144218	GGCAGUG
ENSG00000144218	GGCUGCG
ENSG00000144218	GUAACAG
ENSG00000144218	GUCUACU
ENSG00000144218	GUGACUG
ENSG00000144218	UACAGUU
ENSG00000144218	UAUAAAG
ENSG00000144218	UAUAAUA
ENSG00000144218	UCCCACC
ENSG00000144218	UGCCCUG
ENSG00000144218	UUGGUUC
ENSG00000144228	AAAAGUA
ENSG00000144228	AAGUCAC
ENSG00000144228	AAGUGCA
ENSG00000144228	ACCUGGA
ENSG00000144228	ACUGGCC
ENSG00000144228	AGUGCUU
ENSG00000144228	AUCCUUU
ENSG00000144228	AUGGCAC
ENSG00000144228	CACAGUG
ENSG00000144228	CAGCAAA
ENSG00000144228	CAGUCUG
ENSG00000144228	CCUUCAU
ENSG00000144228	CUGGACU
ENSG00000144228	CUUACAG
ENSG00000144228	GACCUGG
ENSG00000144228	GGAAGAC
ENSG00000144228	GGCAAGA
ENSG00000144228	UGGCCCU
ENSG00000144228	UGGUACC
ENSG00000144228	UGUGCUU
ENSG00000144228	UUAUGCA
ENSG00000144228	UUGGCAA
ENSG00000144228	UUGGUCC
ENSG00000144229	GAUUGUC

ENSG00000144229	GGAUUUC
ENSG00000144233	AACGGAA
ENSG00000144233	AACUGGU
ENSG00000144233	AAGGCAC
ENSG00000144233	AGCACCA
ENSG00000144233	CUCAGGG
ENSG00000144233	CUUGGGC
ENSG00000144233	GAGGUAG
ENSG00000144233	GCUAGCA
ENSG00000144233	UAAUACA
ENSG00000144233	UUUUUGC
ENSG00000144278	AAGUUGU
ENSG00000144278	AUGGCUU
ENSG00000144278	AUGUGCC
ENSG00000144278	GUGCUUG
ENSG00000144278	UCAUUUG
ENSG00000144285	ACCACAG
ENSG00000144285	CUCAAAC
ENSG00000144285	GGAGUCC
ENSG00000144285	GGCAGUG
ENSG00000144285	GUCAGUU
ENSG00000144285	UAAUGCU
ENSG00000144285	UACAAAG
ENSG00000144285	UAUUGCU
ENSG00000144285	UGCUCAC
ENSG00000144285	UUGGCAA
ENSG00000144285	UUGGCAC
ENSG00000144290	GUCAACA
ENSG00000144290	UCAUAGA
ENSG00000144320	AACACUG
ENSG00000144320	AAGGCAC
ENSG00000144320	AUGUGCC
ENSG00000144320	CGUACCG
ENSG00000144320	GUGCCUG
ENSG00000144320	UACAGUU
ENSG00000144320	UCAUUCG
ENSG00000144320	UGGCCCU
ENSG00000144320	UUCCUAG
ENSG00000144320	UUUCAAC
ENSG00000144331	CCCUGUU
ENSG00000144331	UCAAGUA
ENSG00000144331	UCCCUGU
ENSG00000144331	UUCAAGC
ENSG00000144339	GAAACAU
ENSG00000144354	AAAGUGC
ENSG00000144354	AAGGCAC
ENSG00000144355	AAAACUG
ENSG00000144355	AAAGAGC
ENSG00000144355	AAAUUUC
ENSG00000144355	ACAUAGA
ENSG00000144355	CUCAAAC
ENSG00000144355	GUGCAAA
ENSG00000144355	UCUCAAG
ENSG00000144355	UGACAUC
ENSG00000144355	UUGACAC
ENSG00000144355	UUGAUCA
ENSG00000144357	GCAGCAU
ENSG00000144357	UGAGAAU
ENSG00000144366	ACAAUAU
ENSG00000144366	AUAAAGU
ENSG00000144366	UAUUGCU
ENSG00000144369	GCAACUU
ENSG00000144381	GGAAUGU
ENSG00000144426	AAAGUGC

ENSG00000144426	AGAUGUG
ENSG00000144445	AGCAAAA
ENSG00000144445	GAAGGAA
ENSG00000144445	GACAACU
ENSG00000144445	GUCAGUU
ENSG00000144445	UAUCAGA
ENSG00000144452	AAUGUUG
ENSG00000144452	ACACACC
ENSG00000144452	UUCUAAU
ENSG00000144455	AAAGUGC
ENSG00000144455	AAGGCAC
ENSG00000144455	AAGUGCA
ENSG00000144455	CAAAGUG
ENSG00000144455	CUGCCCC
ENSG00000144460	AAAGUAC
ENSG00000144460	AAUUAUU
ENSG00000144460	ACAGUAC
ENSG00000144460	ACAUAGA
ENSG00000144460	CAGCAAA
ENSG00000144460	UCUCCAA
ENSG00000144460	UUGGCAC
ENSG00000144468	AGCACCA
ENSG00000144476	AUAAAGU
ENSG00000144485	AGGGAGG
ENSG00000144524	AAACUGU
ENSG00000144524	AGCAGCA
ENSG00000144524	AUAUAAC
ENSG00000144524	CCCUGAG
ENSG00000144524	CCUGUAC
ENSG00000144524	CUCUGGG
ENSG00000144524	CUGCCCC
ENSG00000144524	GAGCCCC
ENSG00000144524	GAGUCUU
ENSG00000144524	GGCAGUG
ENSG00000144524	GUAAACA
ENSG00000144524	UCAAGUA
ENSG00000144524	UGAGUCU
ENSG00000144524	UGUUGAA
ENSG00000144560	AAAUUUC
ENSG00000144560	ACAAUAU
ENSG00000144560	ACCAGCA
ENSG00000144560	ACGCACU
ENSG00000144560	CAGUAGA
ENSG00000144560	CAGUAGU
ENSG00000144560	CAGUCCA
ENSG00000144560	CUACAAA
ENSG00000144560	GGAAUGU
ENSG00000144560	GUGCAAA
ENSG00000144560	UCCAGUU
ENSG00000144567	AGCAGCA
ENSG00000144567	AGGCAGG
ENSG00000144567	AGGGAGG
ENSG00000144567	AGUGCCU
ENSG00000144567	AUAAAGU
ENSG00000144567	CCCUGAG
ENSG00000144567	GAGGAUA
ENSG00000144567	GAGGCAG
ENSG00000144567	GGAGGCA
ENSG00000144567	GGCAGGG
ENSG00000144567	GUGCCUG
ENSG00000144567	UGGGACA
ENSG00000144579	AAUCCUU
ENSG00000144579	AUCCUUU
ENSG00000144579	CAGCAGA

ENSG00000144579	CCCACCU
ENSG00000144579	CCCCCAG
ENSG00000144579	CUGCCCC
ENSG00000144579	GGCAGCG
ENSG00000144579	GGGAGCU
ENSG00000144579	GGGGCAG
ENSG00000144579	UAUAUGA
ENSG00000144579	UCCCUCU
ENSG00000144579	UUGGCAA
ENSG00000144579	UUGGCAC
ENSG00000144583	AAAAGUA
ENSG00000144583	AAAGUAC
ENSG00000144583	AAAGUGC
ENSG00000144583	UACAAAG
ENSG00000144583	UGUGCUU
ENSG00000144583	UUUCAAC
ENSG00000144597	AAAAAUC
ENSG00000144597	AAGGCAC
ENSG00000144597	AGUGGUU
ENSG00000144597	CCCUGAG
ENSG00000144597	GUCCAGA
ENSG00000144597	UAUAAAG
ENSG00000144597	UCAAGUA
ENSG00000144597	UCCCUGU
ENSG00000144597	UCCUAUA
ENSG00000144619	AAAGAAU
ENSG00000144619	AAUACUG
ENSG00000144619	AAUGCCC
ENSG00000144619	AAUGUUG
ENSG00000144619	ACAUAGA
ENSG00000144619	AGCAUUC
ENSG00000144619	AUAAUAC
ENSG00000144619	AUGCAUU
ENSG00000144619	CAAAACU
ENSG00000144619	CAGACAG
ENSG00000144619	CUUUGGU
ENSG00000144619	GUAAACA
ENSG00000144619	UACAAAG
ENSG00000144619	UCCAGUU
ENSG00000144619	UGAGGAC
ENSG00000144619	UGCAUUG
ENSG00000144619	UGUAUAA
ENSG00000144619	UUGAGGC
ENSG00000144642	AAAAAUC
ENSG00000144642	AAGGCAC
ENSG00000144642	AGCUUAU
ENSG00000144642	CCAGCAU
ENSG00000144642	GGAAGAC
ENSG00000144642	GUGACAG
ENSG00000144642	UACAGUU
ENSG00000144642	UAUUGCU
ENSG00000144644	AAUGUUG
ENSG00000144644	CUCUGGG
ENSG00000144644	CUGGGGG
ENSG00000144644	GGAAUGU
ENSG00000144645	AGCAAAA
ENSG00000144645	UACAAAG
ENSG00000144645	UCACAGU
ENSG00000144645	UUGGCAC
ENSG00000144668	AGUGCUU
ENSG00000144668	CAGUGCA
ENSG00000144668	CCCCUGG
ENSG00000144668	CCCUGAG
ENSG00000144668	GACCUGG

ENSG00000144668	GAGAAGA
ENSG00000144668	GGAAGAC
ENSG00000144668	GUAACAG
ENSG00000144668	GUGACUG
ENSG00000144668	GUGCUUG
ENSG00000144668	UAGGUAU
ENSG00000144668	UAUUGCU
ENSG00000144671	GGCAUUG
ENSG00000144671	UGAGAAU
ENSG00000144674	ACAGUAC
ENSG00000144674	ACAGUAU
ENSG00000144674	AGCAAAA
ENSG00000144674	AGCAGCA
ENSG00000144674	CAGUAGU
ENSG00000144674	GAGGUAG
ENSG00000144674	GAGGUAU
ENSG00000144674	GGUUUAC
ENSG00000144677	AAAGUGC
ENSG00000144677	AAGGUGC
ENSG00000144677	ACCCUGU
ENSG00000144677	ACUGCAG
ENSG00000144677	AGCAGCA
ENSG00000144677	AGCAGCG
ENSG00000144677	AGCAGCU
ENSG00000144677	AUGUCUG
ENSG00000144677	CACAGUG
ENSG00000144677	CAGCUGG
ENSG00000144677	CCCUGUC
ENSG00000144677	CCUGUAC
ENSG00000144677	CUGGCUC
ENSG00000144677	GAAGGAA
ENSG00000144677	GAGUGUG
ENSG00000144677	GGAGUGU
ENSG00000144677	GGCAGCG
ENSG00000144677	GGCAGGG
ENSG00000144677	GUAACAG
ENSG00000144677	GUGAAUG
ENSG00000144677	GUGCUUG
ENSG00000144677	UAUUGCU
ENSG00000144677	UGUCUGG
ENSG00000144677	UGUGCUU
ENSG00000144677	UGUGUGG
ENSG00000144677	UUGGAGG
ENSG00000144677	UUGGCAA
ENSG00000144677	UUGGCAC
ENSG00000144681	ACAGUAU
ENSG00000144681	ACUGUUU
ENSG00000144681	CACAGUG
ENSG00000144681	CUUUGGU
ENSG00000144681	GGUUGAC
ENSG00000144681	UCACAGU
ENSG00000144681	UUGGUUC
ENSG00000144711	AACAAAC
ENSG00000144711	AACUCUA
ENSG00000144711	AAGUGCU
ENSG00000144711	AGGAUGU
ENSG00000144711	CAAAGUG
ENSG00000144711	CUGGAAA
ENSG00000144711	GCUGGUG
ENSG00000144711	UAGACUG
ENSG00000144711	UGAAAGG
ENSG00000144711	UGCAAAG
ENSG00000144711	UUAGGAU
ENSG00000144711	UUUGCAA

ENSG00000144712	AAGGCAC
ENSG00000144712	UCCUGAG
ENSG00000144724	AAAGUAA
ENSG00000144724	ACUGUGU
ENSG00000144724	AGGCAGG
ENSG00000144724	AGUAACA
ENSG00000144724	CAAAUGC
ENSG00000144724	CAGUAGA
ENSG00000144724	CAGUAGU
ENSG00000144724	UAAGACG
ENSG00000144724	UAAGACU
ENSG00000144730	AGCACCA
ENSG00000144730	AUGUCUG
ENSG00000144730	CACUGUU
ENSG00000144730	CUGGAAA
ENSG00000144730	GGCAUUG
ENSG00000144730	UACAAAG
ENSG00000144730	UCACAAG
ENSG00000144730	UCACAUU
ENSG00000144730	UGCCAGC
ENSG00000144741	AAAGGUA
ENSG00000144741	UAUAAAG
ENSG00000144741	UCAUUUG
ENSG00000144741	UGGUACC
ENSG00000144741	UGUCUUU
ENSG00000144746	AAUUUUA
ENSG00000144746	UUUGCAA
ENSG00000144747	AAUCUCU
ENSG00000144747	UGCCCUG
ENSG00000144749	AAAGUGC
ENSG00000144749	AAAUUUC
ENSG00000144749	AAGGCAC
ENSG00000144749	AAUACUG
ENSG00000144749	GAGGUAG
ENSG00000144749	GAGGUAU
ENSG00000144749	GUGCAAA
ENSG00000144749	GUGCUUG
ENSG00000144749	UAUAAAG
ENSG00000144749	UUGGCAC
ENSG00000144749	UUUCAAC
ENSG00000144785	AAAAACC
ENSG00000144791	GAGGUAG
ENSG00000144791	GAGGUAU
ENSG00000144791	GCACCAU
ENSG00000144791	GUGAGGU
ENSG00000144791	GUGGCCC
ENSG00000144791	UGGCCCU
ENSG00000144791	UUGUGUC
ENSG00000144791	UUUGCAA
ENSG00000144802	AAAUGGU
ENSG00000144802	AAGACAU
ENSG00000144802	ACCUGGA
ENSG00000144802	AUAAUAC
ENSG00000144802	GAGUGUG
ENSG00000144802	GGUCUAG
ENSG00000144802	UAUAAUA
ENSG00000144815	AACCUGG
ENSG00000144815	AACCUGU
ENSG00000144815	UCCUAUA
ENSG00000144815	UUCAAGC
ENSG00000144815	UUGGUUC
ENSG00000144824	UAUUGCU
ENSG00000144824	UUCUUGU
ENSG00000144827	AGCAGCU

ENSG00000144827	CACAAUG
ENSG00000144827	CUCUUCC
ENSG00000144827	UUGUGAC
ENSG00000144834	UGCAUAU
ENSG00000144840	UGCAAAG
ENSG00000144840	UGCAUAG
ENSG00000144840	UUUGCAA
ENSG00000144843	CUGCUCA
ENSG00000144843	GAGUCUU
ENSG00000144847	AAAGAAU
ENSG00000144847	CCCUGAG
ENSG00000144847	UUGGCAC
ENSG00000144852	ACCACAG
ENSG00000144852	UCCUGAG
ENSG00000144857	AUAUAAC
ENSG00000144868	AUAAAGU
ENSG00000144893	ACCACAG
ENSG00000144893	CUGGAAA
ENSG00000144893	GUGCAAA
ENSG00000144893	GUGCUUG
ENSG00000144895	UUUGCAC
ENSG00000144909	AAAGUAA
ENSG00000144909	AACACUG
ENSG00000144909	AAUUGCU
ENSG00000144909	AGCAAAA
ENSG00000144909	AUAAUAC
ENSG00000144909	AUAUUAU
ENSG00000144909	CAGCAAA
ENSG00000144909	GAUUGGU
ENSG00000144909	GUCUUGC
ENSG00000144909	UCACAGG
ENSG00000144909	UCACAGU
ENSG00000144935	AAAGAGG
ENSG00000144935	AAAUCCC
ENSG00000144935	AAUUAUU
ENSG00000144959	CAAAGCA
ENSG00000144959	CAGUGCA
ENSG00000144959	CCAGUGU
ENSG00000144959	GGCAGUG
ENSG00000144959	UUCAAGC
ENSG00000145012	AACACUG
ENSG00000145012	AACAUUC
ENSG00000145012	AAGUGUG
ENSG00000145012	ACAUUCA
ENSG00000145012	AGCAGCA
ENSG00000145012	AGCAGCG
ENSG00000145012	AGCAGCU
ENSG00000145012	AGCUGCC
ENSG00000145012	AGGUUAU
ENSG00000145012	CACUGUU
ENSG00000145012	CAGCAAA
ENSG00000145012	CAGUUAC
ENSG00000145012	CCCUGAG
ENSG00000145012	CUACAAA
ENSG00000145012	CUUUGGU
ENSG00000145012	GAGCUGC
ENSG00000145012	GUAAACA
ENSG00000145012	GUGAAUG
ENSG00000145012	UCCCUGU
ENSG00000145016	AAAGUGC
ENSG00000145016	AAGGCAC
ENSG00000145016	AAGUGCA
ENSG00000145016	AAGUGCU
ENSG00000145016	ACAUUCA

ENSG00000145016	AGCAGCA
ENSG00000145016	AGGCAGG
ENSG00000145016	AGUGCUU
ENSG00000145016	CAAAGUG
ENSG00000145016	CAGCAGA
ENSG00000145016	CAGUGCA
ENSG00000145016	GAGGCAG
ENSG00000145016	GUCAGUU
ENSG00000145016	GUCUUGC
ENSG00000145016	UGCAAAG
ENSG00000145020	GAGGUAG
ENSG00000145022	CAGCAGA
ENSG00000145022	GAGGCAG
ENSG00000145029	AUGCAUU
ENSG00000145029	CUCCCAC
ENSG00000145029	GAGAACC
ENSG00000145040	GCCUGGA
ENSG00000145040	GGCAAGA
ENSG00000145087	AAAACUG
ENSG00000145087	AAGUGCU
ENSG00000145087	AAUCCUU
ENSG00000145087	AAUUGCU
ENSG00000145087	ACAGUAC
ENSG00000145087	ACAGUAU
ENSG00000145087	AGCCCUU
ENSG00000145087	AUGAUGA
ENSG00000145087	AUUGCAC
ENSG00000145087	CACAAUG
ENSG00000145087	CAGGCUC
ENSG00000145087	CAGUGCA
ENSG00000145087	CCCUGAG
ENSG00000145087	CUCCAUU
ENSG00000145087	CUGGCUC
ENSG00000145087	GGCCCUG
ENSG00000145087	GUCAUUG
ENSG00000145087	GUUAAUG
ENSG00000145087	UCCCUGU
ENSG00000145087	UGCAUAU
ENSG00000145087	UUCAAGC
ENSG00000145087	UUGGCAA
ENSG00000145087	UUGGUUC
ENSG00000145087	UUUCAAC
ENSG00000145103	UGAGAAU
ENSG00000145147	AAAAAUC
ENSG00000145191	AAUACUG
ENSG00000145191	AGCAGCA
ENSG00000145191	CCCUGAG
ENSG00000145192	CAAGAGC
ENSG00000145194	AAAAAUC
ENSG00000145194	GGAGGCA
ENSG00000145194	UCACAGU
ENSG00000145194	UUGGCAC
ENSG00000145242	AAAGUGC
ENSG00000145242	AAGUGCA
ENSG00000145242	CAAAGUG
ENSG00000145244	AACCUGG
ENSG00000145244	AAGUGCU
ENSG00000145244	AGGUAGU
ENSG00000145244	GCUGGUG
ENSG00000145246	AAAGUUU
ENSG00000145246	CCAGCAU
ENSG00000145246	CCAUUAC
ENSG00000145246	UGAGAAU
ENSG00000145284	AAAGUAA

ENSG00000145284	AAAGUAU
ENSG00000145284	AAAGUGC
ENSG00000145284	AAGUGCU
ENSG00000145284	AAUCACU
ENSG00000145284	AUGUCUG
ENSG00000145284	CCUUCAU
ENSG00000145284	GCAACUU
ENSG00000145284	GGAUGAG
ENSG00000145284	UGUCUGG
ENSG00000145309	AAAGAAU
ENSG00000145332	UUGGCAA
ENSG00000145335	CACAAUG
ENSG00000145335	CUGGGCA
ENSG00000145335	GACCCUG
ENSG00000145335	UCACAAG
ENSG00000145335	UGAAGCU
ENSG00000145335	UGCAUAG
ENSG00000145349	AAACUGU
ENSG00000145349	ACUGCAG
ENSG00000145349	AUGGCAC
ENSG00000145349	CACUGUU
ENSG00000145349	CAGCAGA
ENSG00000145349	CUGUUGA
ENSG00000145349	GAAACAU
ENSG00000145349	GCAGGUU
ENSG00000145349	GGCAGGG
ENSG00000145349	GGCUCAG
ENSG00000145349	UCAACAG
ENSG00000145349	UCACAAG
ENSG00000145349	UCACAGU
ENSG00000145349	UUCAAAU
ENSG00000145349	UUCCUCG
ENSG00000145354	UAAGACG
ENSG00000145354	UAAGACU
ENSG00000145354	UGUUGAA
ENSG00000145358	CUACAAA
ENSG00000145362	AAAAAUC
ENSG00000145362	AAGACAU
ENSG00000145362	AAGUGCA
ENSG00000145362	ACCACAG
ENSG00000145362	CAGUGCA
ENSG00000145362	CCUUCAU
ENSG00000145362	CCUUCUU
ENSG00000145362	CUCACAC
ENSG00000145362	CUUCACC
ENSG00000145362	UAAUACA
ENSG00000145362	UCAUAGA
ENSG00000145362	UCUCACC
ENSG00000145362	UUGGUCC
ENSG00000145365	CAGGUGA
ENSG00000145375	AAAGAAU
ENSG00000145375	AAGUGCA
ENSG00000145375	AGGGUCU
ENSG00000145375	UGAAAUG
ENSG00000145386	AAAAACC
ENSG00000145390	AAAGUGC
ENSG00000145390	AACACUG
ENSG00000145390	AAGUGCU
ENSG00000145390	AGCAGCA
ENSG00000145390	AGCAGCG
ENSG00000145390	AGCAGCU
ENSG00000145390	AGUGCUU
ENSG00000145391	AACUCUA
ENSG00000145391	AAUACUG

ENSG00000145391	AGUGCAA
ENSG00000145391	AUGCACC
ENSG00000145391	AUGGCUU
ENSG00000145391	AUUGCAC
ENSG00000145391	CACUGUU
ENSG00000145391	CAGACAG
ENSG00000145391	CUACAAA
ENSG00000145391	GAAACAU
ENSG00000145391	GAUUGUA
ENSG00000145391	GGGCCCC
ENSG00000145391	GGUUGUC
ENSG00000145391	UACAAAG
ENSG00000145391	UAUUGCU
ENSG00000145391	UCCAGAG
ENSG00000145391	UGCCCUG
ENSG00000145391	UGGACUG
ENSG00000145391	UUCCUAG
ENSG00000145391	UUCUGCA
ENSG00000145391	UUGAUCA
ENSG00000145391	UUGGCAC
ENSG00000145391	UUGUGUC
ENSG00000145416	GUAGUGU
ENSG00000145423	AAGUCAC
ENSG00000145423	ACAGGUG
ENSG00000145423	ACUCAGG
ENSG00000145423	AUAUUAU
ENSG00000145423	GAAAACA
ENSG00000145423	GUUAAUG
ENSG00000145423	UACAGUU
ENSG00000145439	UAGGUAU
ENSG00000145439	UUGGCAA
ENSG00000145451	AAAAACC
ENSG00000145451	AAAGCUG
ENSG00000145451	GGCAGUG
ENSG00000145476	GGAGAGA
ENSG00000145476	GUCAGUU
ENSG00000145495	AAAAACC
ENSG00000145495	AAAGUGC
ENSG00000145495	AAUACUG
ENSG00000145495	ACUAGAU
ENSG00000145495	CAGCAGG
ENSG00000145495	UAAUACA
ENSG00000145495	UAAUAUC
ENSG00000145495	UACAAAG
ENSG00000145536	UAUAAAG
ENSG00000145555	AAAGCUG
ENSG00000145555	AAAGUAC
ENSG00000145555	AAAGUAU
ENSG00000145555	AGUGCAA
ENSG00000145555	AGUGGUU
ENSG00000145555	AUAAUAC
ENSG00000145555	CAGCAAA
ENSG00000145555	CAGCAGA
ENSG00000145555	CAGUAGA
ENSG00000145555	CAGUAGU
ENSG00000145555	CUUUGGU
ENSG00000145555	GCUACAU
ENSG00000145555	GGUAGAC
ENSG00000145555	GUUAAUG
ENSG00000145555	UAUAAUA
ENSG00000145555	UCCCUUG
ENSG00000145555	UGGGAUC
ENSG00000145569	UAAUGCU
ENSG00000145623	AAUUUUA

ENSG00000145623	AGGUAGU
ENSG00000145632	CGUACCG
ENSG00000145632	UAUUGCU
ENSG00000145632	UCACAGG
ENSG00000145675	AAAGCUG
ENSG00000145675	AAAGUGC
ENSG00000145675	AAGUGCA
ENSG00000145675	ACCAGCA
ENSG00000145675	AGCACCA
ENSG00000145675	AGCAGCG
ENSG00000145675	AUAUUAU
ENSG00000145675	CAAAACU
ENSG00000145675	CACAGUG
ENSG00000145675	CAGUAGA
ENSG00000145675	CAGUAGU
ENSG00000145675	CUGCCCC
ENSG00000145675	CUGGGCA
ENSG00000145675	CUUGGGC
ENSG00000145675	GAAGGUC
ENSG00000145675	GACCCUG
ENSG00000145675	GACUUCC
ENSG00000145675	GAGCCCU
ENSG00000145675	GCACCAU
ENSG00000145675	GCAGCAU
ENSG00000145675	GGAGGCA
ENSG00000145675	GGCAUUG
ENSG00000145675	GUCAUUG
ENSG00000145675	GUGCUUG
ENSG00000145675	UAAUGCU
ENSG00000145675	UAUCAGA
ENSG00000145675	UCAUAGA
ENSG00000145675	UCCCUCU
ENSG00000145675	UCUCACC
ENSG00000145675	UGAAAGG
ENSG00000145675	UGCAGCU
ENSG00000145675	UGCAUAG
ENSG00000145675	UGUCUGG
ENSG00000145675	UUCAAAU
ENSG00000145675	UUGGCAA
ENSG00000145675	UUGGCAC
ENSG00000145675	UUUCAAC
ENSG00000145681	ACAGUAC
ENSG00000145681	ACAGUAU
ENSG00000145681	CACAGUG
ENSG00000145685	CAGGAAC
ENSG00000145685	CUCUUCC
ENSG00000145685	CUUGUGU
ENSG00000145685	GAGAAGA
ENSG00000145685	GCUACAU
ENSG00000145685	GGCUCAG
ENSG00000145685	GUAAACA
ENSG00000145685	GUCCAGA
ENSG00000145685	UAUUGCU
ENSG00000145685	UCCAGUU
ENSG00000145685	UGAGAAG
ENSG00000145685	UGUAUGU
ENSG00000145687	CUGACUC
ENSG00000145687	GUAAACA
ENSG00000145687	UACAAAG
ENSG00000145687	UAUAAAG
ENSG00000145687	UCCUAUA
ENSG00000145703	AAGGCAC
ENSG00000145703	ACAUUCA
ENSG00000145703	AGUGCAA

ENSG00000145703	CAGUAGU
ENSG00000145703	GAAUUGU
ENSG00000145703	UGGGUAC
ENSG00000145703	UUCAAAU
ENSG00000145703	UUGGUCC
ENSG00000145708	AAAGUUU
ENSG00000145708	AAUACUG
ENSG00000145708	CUACAAA
ENSG00000145715	CAAGAGC
ENSG00000145715	GGCUCAG
ENSG00000145715	UCACACA
ENSG00000145715	UCCAGUU
ENSG00000145721	AAGGCAC
ENSG00000145721	AGAUAAA
ENSG00000145721	UGGCUGC
ENSG00000145721	UUCUAAU
ENSG00000145723	AUUCAUU
ENSG00000145725	AGCACCA
ENSG00000145725	GUAAACA
ENSG00000145725	UGCAGCU
ENSG00000145730	AAAAAUC
ENSG00000145730	AACAGUC
ENSG00000145730	AAGGCAC
ENSG00000145730	AGCAGCA
ENSG00000145730	AGCAGCG
ENSG00000145730	UACAGUU
ENSG00000145730	UAUUACC
ENSG00000145734	UUCAAGC
ENSG00000145741	AAAGAAU
ENSG00000145741	AAAGAGC
ENSG00000145741	AGCAGCA
ENSG00000145741	AGCAGCG
ENSG00000145741	UGCAAAG
ENSG00000145743	GAUUGUC
ENSG00000145743	GUAAACA
ENSG00000145743	UAUAAAG
ENSG00000145779	AAGUCUU
ENSG00000145779	CAGCAGG
ENSG00000145779	UUGGUUC
ENSG00000145780	AAAGUGC
ENSG00000145780	AAGACAU
ENSG00000145780	AAGUGCU
ENSG00000145780	AAUUAUU
ENSG00000145780	AGAUAAA
ENSG00000145780	AGCCCUU
ENSG00000145780	AGUGCUG
ENSG00000145780	CACAGGG
ENSG00000145780	GAGCCCU
ENSG00000145780	GCAGCAU
ENSG00000145780	GCAGGAC
ENSG00000145780	GGCAAGA
ENSG00000145780	UAAGACG
ENSG00000145780	UAAGACU
ENSG00000145780	UAUAUGA
ENSG00000145780	UAUGGUU
ENSG00000145780	UCACAGG
ENSG00000145780	UGAAAGG
ENSG00000145780	UUGGCAA
ENSG00000145780	UUGGCAC
ENSG00000145794	ACACACC
ENSG00000145794	ACACACU
ENSG00000145794	AUAAUAC
ENSG00000145794	AUACAAG
ENSG00000145794	AUAUUAU

ENSG00000145794	AUGCACC
ENSG00000145794	CUACAAA
ENSG00000145794	CUUUGGU
ENSG00000145794	UAUAAUA
ENSG00000145808	AAAACUG
ENSG00000145808	AGUGCAA
ENSG00000145808	CAGUGCA
ENSG00000145808	CUUGUGU
ENSG00000145808	GGCAGGG
ENSG00000145808	GUGAAUG
ENSG00000145808	UACAGUU
ENSG00000145808	UAUAAAG
ENSG00000145808	UCAAGUA
ENSG00000145808	UUGGUCC
ENSG00000145817	AAAGCUG
ENSG00000145817	AAAGUAA
ENSG00000145817	AAAUCCC
ENSG00000145817	AGUGAUC
ENSG00000145817	CAGGAAC
ENSG00000145817	GCAGGAC
ENSG00000145817	GCUGGUG
ENSG00000145817	UAGUGAG
ENSG00000145817	UGUAUAA
ENSG00000145819	AAAACUG
ENSG00000145819	AAAGAAU
ENSG00000145819	AAAGCUG
ENSG00000145819	AAAGUAA
ENSG00000145819	AAAUCCC
ENSG00000145819	AAUACUG
ENSG00000145819	AAUCACU
ENSG00000145819	AAUUAUU
ENSG00000145819	ACAGUAC
ENSG00000145819	ACUGUAG
ENSG00000145819	AGUGCUU
ENSG00000145819	AUCAUAC
ENSG00000145819	CAGCAGA
ENSG00000145819	CAGCAGG
ENSG00000145819	GAAACAU
ENSG00000145819	GAGUCUU
ENSG00000145819	GGAGUCC
ENSG00000145819	GGCAGUG
ENSG00000145819	GGCUGCG
ENSG00000145819	GGGGCAG
ENSG00000145819	GGUUACC
ENSG00000145819	GUCAUUG
ENSG00000145819	UACAGUU
ENSG00000145819	UCACACA
ENSG00000145819	UCCAGUU
ENSG00000145819	UCCUGAG
ENSG00000145819	UGGCUGC
ENSG00000145824	AAAAACC
ENSG00000145824	AAAACUG
ENSG00000145824	AAAGAAU
ENSG00000145824	AAAGCUG
ENSG00000145824	AGUGCUU
ENSG00000145824	AUAAAGU
ENSG00000145824	CUGGAAA
ENSG00000145824	UAUAAAG
ENSG00000145824	UAUUACC
ENSG00000145824	UCUGGAA
ENSG00000145833	ACAAUAU
ENSG00000145833	AGCACCA
ENSG00000145833	AGUGAUC
ENSG00000145833	GUAAACA

ENSG00000145860	ACUGUUU
ENSG00000145860	UCACACA
ENSG00000145860	UCAUACA
ENSG00000145861	CCCUGUU
ENSG00000145861	GGAUUUC
ENSG00000145861	GGAUUUU
ENSG00000145863	GAGGUAG
ENSG00000145863	GAGGUAU
ENSG00000145864	AAAUGGU
ENSG00000145864	AACAUUC
ENSG00000145864	AAUUGCU
ENSG00000145864	ACAGUAC
ENSG00000145864	ACAGUAU
ENSG00000145864	ACCCUGU
ENSG00000145864	AGUGCAA
ENSG00000145864	AUGCAUU
ENSG00000145864	AUGGCUU
ENSG00000145864	GGGUGUU
ENSG00000145864	GUGCAAA
ENSG00000145864	GUGCUUG
ENSG00000145864	UAUGGUU
ENSG00000145864	UCACAUU
ENSG00000145864	UGCAUUG
ENSG00000145864	UGCUCAC
ENSG00000145864	UGCUGAC
ENSG00000145864	UGUAUGU
ENSG00000145864	UUCCUAG
ENSG00000145864	UUUGCAA
ENSG00000145864	UUUGCAC
ENSG00000145868	AAGGCAC
ENSG00000145901	AGCAAAA
ENSG00000145901	GGAUUUC
ENSG00000145901	GGAUUUU
ENSG00000145901	GGGGCAG
ENSG00000145901	GUGCAAA
ENSG00000145907	AGAUAAA
ENSG00000145920	AAAGUAA
ENSG00000145920	AAAGUAC
ENSG00000145920	AAAGUAU
ENSG00000145920	AUGGAUU
ENSG00000145920	AUUGCAC
ENSG00000145920	CCAGCAU
ENSG00000145920	CCCCCAG
ENSG00000145920	CCCUGUC
ENSG00000145920	CUCUAGC
ENSG00000145920	CUCUGGG
ENSG00000145920	CUGGGCA
ENSG00000145920	GAAAACA
ENSG00000145920	GACCAUG
ENSG00000145920	GACCUGG
ENSG00000145920	GAGAAGA
ENSG00000145920	GAUAUGU
ENSG00000145920	GCCCUGU
ENSG00000145920	GCCUGGG
ENSG00000145920	GGAAGCC
ENSG00000145920	GGCAGGG
ENSG00000145920	GGCCCUG
ENSG00000145920	GGGGCAG
ENSG00000145920	GGGGUCC
ENSG00000145920	GUCAUUG
ENSG00000145920	GUGUCAC
ENSG00000145920	UACAAAG
ENSG00000145920	UCAUACA
ENSG00000145920	UCCCUUU

ENSG00000145920	UGAGGAC
ENSG00000145920	UGCCCUG
ENSG00000145920	UGUGUCA
ENSG00000145934	AAAAACC
ENSG00000145934	AACAAAC
ENSG00000145934	ACCCUGU
ENSG00000145934	ACUCAAA
ENSG00000145934	AGCAGCG
ENSG00000145934	AGCAGCU
ENSG00000145934	AUAUUAU
ENSG00000145934	CACAAUG
ENSG00000145934	GAUUGUA
ENSG00000145934	GUGACUG
ENSG00000145934	UACAAAG
ENSG00000145934	UGUAUGU
ENSG00000145949	ACUGGCU
ENSG00000145949	AGCAGCA
ENSG00000145949	GGAGGAG
ENSG00000145949	UCCCUUG
ENSG00000145949	UGAAAUG
ENSG00000145949	UGCAAAG
ENSG00000145949	UUCUAAU
ENSG00000145949	UUUGCAA
ENSG00000145990	CACAGUG
ENSG00000145990	UCACAGU
ENSG00000146005	AGCAAAA
ENSG00000146005	AGGCAGG
ENSG00000146005	CCUGCUG
ENSG00000146005	CUACAAA
ENSG00000146005	GAAUUGU
ENSG00000146005	GCUGGUG
ENSG00000146005	GGUGGUC
ENSG00000146005	GGUGGUU
ENSG00000146005	UGAGAAG
ENSG00000146005	UGCUCAC
ENSG00000146005	UGUGCUU
ENSG00000146006	AACACUG
ENSG00000146006	AGGUUAU
ENSG00000146006	AGUGCAA
ENSG00000146006	AUAUAAC
ENSG00000146006	AUGGAUU
ENSG00000146006	GAGAACU
ENSG00000146006	GCAGAAG
ENSG00000146006	GCUAGCA
ENSG00000146006	GUGCAAA
ENSG00000146006	GUGUGGC
ENSG00000146006	UAGACUG
ENSG00000146006	UGAAGUG
ENSG00000146021	AAAAAUC
ENSG00000146021	AAAGAAU
ENSG00000146021	AAAGUAA
ENSG00000146021	AAUACUG
ENSG00000146021	AAUCUCA
ENSG00000146021	ACAUUAC
ENSG00000146021	ACCUGGA
ENSG00000146021	AGAUAAA
ENSG00000146021	AGUAGAC
ENSG00000146021	AUUGCAC
ENSG00000146021	CAAAGUG
ENSG00000146021	CCAGUAC
ENSG00000146021	CCAGUGU
ENSG00000146021	GCAACUU
ENSG00000146021	GGAGAGA
ENSG00000146021	UAUUGCU

ENSG00000146021	UCACAAG
ENSG00000146021	UCACAGG
ENSG00000146021	UGAAAGG
ENSG00000146021	UGAUCCA
ENSG00000146021	UGCAUAG
ENSG00000146021	UGCAUAU
ENSG00000146038	AAUACUG
ENSG00000146038	GGAUUUU
ENSG00000146047	AAAGAGC
ENSG00000146049	GAUUGGU
ENSG00000146063	GGCAGUG
ENSG00000146066	UUCAAGC
ENSG00000146072	AAAGCUG
ENSG00000146072	AACAAAC
ENSG00000146072	ACUGGCC
ENSG00000146072	CACCACC
ENSG00000146072	CAGACAG
ENSG00000146072	CUCCAGC
ENSG00000146072	CUUUGGU
ENSG00000146072	UGCAAAG
ENSG00000146083	AAAAACC
ENSG00000146083	AAAAAUC
ENSG00000146083	AAAACUG
ENSG00000146083	AAAAGUA
ENSG00000146083	AACACUG
ENSG00000146083	AAUGCCC
ENSG00000146083	ACAAUAU
ENSG00000146083	ACAGUAC
ENSG00000146083	ACAGUAU
ENSG00000146083	ACAUCAC
ENSG00000146083	ACCACAG
ENSG00000146083	ACCUGGA
ENSG00000146083	ACUUGGA
ENSG00000146083	AGCAUUC
ENSG00000146083	AGCUGCC
ENSG00000146083	AGGAUGU
ENSG00000146083	AUAUUAU
ENSG00000146083	AUCAUAC
ENSG00000146083	AUCCCUU
ENSG00000146083	AUGUGCC
ENSG00000146083	CAGCAAA
ENSG00000146083	CAGCUGG
ENSG00000146083	CAGUGCA
ENSG00000146083	CCCUGAG
ENSG00000146083	CUACAAA
ENSG00000146083	CUCAAAC
ENSG00000146083	CUGCAGC
ENSG00000146083	CUGCCCC
ENSG00000146083	CUGGGGG
ENSG00000146083	CUUUGGU
ENSG00000146083	GACCCUG
ENSG00000146083	GAGAAAU
ENSG00000146083	GAGGCAG
ENSG00000146083	GAGGUAG
ENSG00000146083	GAGGUAU
ENSG00000146083	GAGUCUU
ENSG00000146083	GCAACUU
ENSG00000146083	GCAUCCC
ENSG00000146083	GCUACAU
ENSG00000146083	GGCAGUG
ENSG00000146083	GGGUGGU
ENSG00000146083	GGUGGUU
ENSG00000146083	GUAAACA
ENSG00000146083	GUGCAAA

ENSG00000146083	UAAGACU
ENSG00000146083	UAGUGAG
ENSG00000146083	UAUAAUA
ENSG00000146083	UCAACAG
ENSG00000146083	UCAUACA
ENSG00000146083	UCAUUUG
ENSG00000146083	UCUCAAG
ENSG00000146083	UUCUAAU
ENSG00000146083	UUGGAGG
ENSG00000146083	UUUCAAC
ENSG00000146090	CUCUUCC
ENSG00000146090	GAGGCAG
ENSG00000146112	GGGAGCU
ENSG00000146112	UCCCUCU
ENSG00000146122	AAGGCAC
ENSG00000146122	ACCUGGA
ENSG00000146122	AGGACAC
ENSG00000146122	CACUGUU
ENSG00000146122	CCCUGAG
ENSG00000146122	CCCUGUC
ENSG00000146122	CCUUCUU
ENSG00000146122	GCUGGUG
ENSG00000146122	GUGCCAC
ENSG00000146122	UAAUAUC
ENSG00000146122	UAUAAAG
ENSG00000146122	UCCCUGU
ENSG00000146122	UGGAGAU
ENSG00000146143	AGCAAAA
ENSG00000146151	AACUACU
ENSG00000146151	ACACACU
ENSG00000146151	AGAUAAA
ENSG00000146151	AUAAAGU
ENSG00000146151	CUUGGAG
ENSG00000146151	UAUUGCU
ENSG00000146151	UUGGCAA
ENSG00000146197	AAGGCAC
ENSG00000146197	ACUCAAA
ENSG00000146197	AUGGCUU
ENSG00000146197	AUUGCAC
ENSG00000146197	CCCCCAG
ENSG00000146197	CUUUGGU
ENSG00000146197	GAGGUAG
ENSG00000146197	GGUGGGC
ENSG00000146216	AACAUUC
ENSG00000146216	ACAUUCA
ENSG00000146216	CACACCU
ENSG00000146216	CCAGUGU
ENSG00000146216	CUGCAGC
ENSG00000146216	CUGGGCA
ENSG00000146216	GAAGGAA
ENSG00000146216	GAGCCCU
ENSG00000146216	GAGUGUG
ENSG00000146216	GAUUGUA
ENSG00000146216	GAUUGUC
ENSG00000146216	GGCAAGA
ENSG00000146216	UCCCUUG
ENSG00000146216	UCCCUUU
ENSG00000146216	UCCUGAG
ENSG00000146232	AAGUCUU
ENSG00000146232	CUGCAGC
ENSG00000146242	AAACUGU
ENSG00000146242	UUGAGGC
ENSG00000146247	AACAUUC
ENSG00000146247	AGCAGCA

ENSG00000146250	AGAUGUG
ENSG00000146250	AGUGGUU
ENSG00000146250	AUUCAUU
ENSG00000146250	CUCUGGG
ENSG00000146250	CUGGAAA
ENSG00000146250	UCUGGAA
ENSG00000146263	AAGUCAC
ENSG00000146263	AUAAAGU
ENSG00000146267	AAAACUG
ENSG00000146267	ACACACC
ENSG00000146267	ACACACU
ENSG00000146267	ACAGGUG
ENSG00000146267	ACAGUAC
ENSG00000146267	AGCACCA
ENSG00000146267	AGGUAGU
ENSG00000146267	CAAAACU
ENSG00000146267	CACAGUG
ENSG00000146267	CCAUUAC
ENSG00000146267	GAGGCAG
ENSG00000146267	GAGGUAG
ENSG00000146267	GAGGUAU
ENSG00000146267	GGAGAGA
ENSG00000146267	GGAGGAG
ENSG00000146267	GUGAGGU
ENSG00000146267	UCACACA
ENSG00000146267	UCCAGUU
ENSG00000146267	UGCAAAG
ENSG00000146267	UUGACAC
ENSG00000146267	UUUGCAC
ENSG00000146278	AAUCUCA
ENSG00000146278	AAUUAUU
ENSG00000146278	ACAAUAU
ENSG00000146278	AGUGCAA
ENSG00000146278	CAGUAGA
ENSG00000146278	GAUUGUA
ENSG00000146278	GAUUGUC
ENSG00000146285	AAGGCAC
ENSG00000146285	AGUGCAA
ENSG00000146285	CAGUCUG
ENSG00000146285	CUUCUCU
ENSG00000146285	GCAGCAU
ENSG00000146285	GUCUGCC
ENSG00000146285	UCAAGUA
ENSG00000146285	UGUUGAA
ENSG00000146285	UUGGCAC
ENSG00000146285	UUUUUGC
ENSG00000146360	AACACUG
ENSG00000146360	ACUGUUU
ENSG00000146360	AGCAGCU
ENSG00000146360	AUAAAGU
ENSG00000146360	CACUGUU
ENSG00000146360	CAGCAGA
ENSG00000146373	GGCAUUG
ENSG00000146374	AAAGUAU
ENSG00000146374	AAUGUUG
ENSG00000146374	AGCAGCG
ENSG00000146374	AGCAGCU
ENSG00000146374	CCUUCUU
ENSG00000146374	GCAGCAU
ENSG00000146374	GGAAUGU
ENSG00000146376	AUGGCAC
ENSG00000146386	GAGAAAU
ENSG00000146386	UAUUACC
ENSG00000146411	AAAACUG

ENSG00000146411	AGGUAGU
ENSG00000146411	AUAUAAC
ENSG00000146411	GAGGUAG
ENSG00000146411	GAGGUAU
ENSG00000146411	UGAAAGG
ENSG00000146414	AUGCACC
ENSG00000146425	AAGACAU
ENSG00000146425	AGCACCA
ENSG00000146425	UAAGACG
ENSG00000146425	UAAGACU
ENSG00000146426	AAUGCCC
ENSG00000146433	AAGGCAC
ENSG00000146433	AGCAUUC
ENSG00000146433	CAAAUGC
ENSG00000146433	UCCCUUG
ENSG00000146433	UGCAUAG
ENSG00000146433	UUCAAAU
ENSG00000146457	AAAGCUG
ENSG00000146457	AAGGCAC
ENSG00000146457	AAGUCUU
ENSG00000146457	AAUUUUA
ENSG00000146457	ACAAGGU
ENSG00000146457	ACAUUAC
ENSG00000146457	ACUUGGA
ENSG00000146457	GGCUCAG
ENSG00000146457	GGGGAGC
ENSG00000146457	UCCCACC
ENSG00000146457	UCCCUCU
ENSG00000146457	UGACUGU
ENSG00000146463	AAAAAUC
ENSG00000146463	AAAGCUG
ENSG00000146463	AAUACUG
ENSG00000146463	ACCACAG
ENSG00000146463	AGCUGCC
ENSG00000146463	CACAAUG
ENSG00000146463	CACAGUG
ENSG00000146463	GAGAAAU
ENSG00000146463	GAGGGGC
ENSG00000146463	GCACCAU
ENSG00000146463	UGAAAUG
ENSG00000146463	UUAUGCA
ENSG00000146469	CCUUCAU
ENSG00000146469	GUAAACA
ENSG00000146469	UAUUACC
ENSG00000146469	UCACAAG
ENSG00000146469	UUGGUUC
ENSG00000146477	AAGGCAC
ENSG00000146477	ACAAGGU
ENSG00000146477	CUUGGGC
ENSG00000146477	GAGAAAU
ENSG00000146477	GAGAUGA
ENSG00000146477	GGGGUCC
ENSG00000146477	GGGUCUU
ENSG00000146477	GUGACAG
ENSG00000146477	GUGCAAA
ENSG00000146477	UCCCUUU
ENSG00000146477	UGAGAAG
ENSG00000146477	UGGCUGC
ENSG00000146535	AACAGUC
ENSG00000146535	AGCAGCA
ENSG00000146535	AGCAGCU
ENSG00000146535	AGCAUUC
ENSG00000146535	AGUAGUU
ENSG00000146535	GAAGGAA

ENSG00000146535	UCCUGAG
ENSG00000146535	UUUUUGC
ENSG00000146540	CAGCUGG
ENSG00000146555	AGCACCA
ENSG00000146574	UAUUGCU
ENSG00000146574	UCCAGUU
ENSG00000146576	AAGGCAC
ENSG00000146576	ACUGUUU
ENSG00000146576	CCCUGAG
ENSG00000146587	UCCCUGU
ENSG00000146592	AAACUGU
ENSG00000146592	AACAAAC
ENSG00000146592	AAGUGCU
ENSG00000146592	ACAUCAC
ENSG00000146592	ACUCAAA
ENSG00000146592	AGCACCA
ENSG00000146592	AGCAGCU
ENSG00000146592	AGGACAC
ENSG00000146592	AUGGAUU
ENSG00000146592	CACUGUU
ENSG00000146592	CAUGGAU
ENSG00000146592	CUCCCAC
ENSG00000146592	CUCUUCC
ENSG00000146592	CUUUGGU
ENSG00000146592	GAGAAAU
ENSG00000146592	GAGACCU
ENSG00000146592	GAGAUGA
ENSG00000146592	GCAGCAU
ENSG00000146592	GCUGUCU
ENSG00000146592	GCUUCCU
ENSG00000146592	GGAGGCA
ENSG00000146592	GGAUUUC
ENSG00000146592	GGCAGUG
ENSG00000146592	GUGCAAA
ENSG00000146592	GUGUGGC
ENSG00000146592	UAAUAGU
ENSG00000146592	UAUUGCU
ENSG00000146592	UCCAGUU
ENSG00000146592	UCCCACA
ENSG00000146592	UGACCUA
ENSG00000146592	UGCAUAG
ENSG00000146592	UGCAUAU
ENSG00000146592	UGCAUUG
ENSG00000146592	UGCUGAC
ENSG00000146592	UGGAGAU
ENSG00000146592	UGGUACC
ENSG00000146592	UGUGUGG
ENSG00000146592	UUCUAAU
ENSG00000146592	UUGGUCC
ENSG00000146592	UUGGUUC
ENSG00000146592	UUUCAAC
ENSG00000146648	UUGGUCC
ENSG00000146670	AGCCCUU
ENSG00000146674	AGCCAGU
ENSG00000146674	CACCACC
ENSG00000146674	GGCAGUG
ENSG00000146674	UUGGUUC
ENSG00000146676	AAAAACC
ENSG00000146676	AAAGCUG
ENSG00000146676	AAAUUUC
ENSG00000146676	AACAUUC
ENSG00000146676	AAGUGCU
ENSG00000146676	AAGUGUG
ENSG00000146676	ACAGUAU

ENSG00000146676	ACAUCAC
ENSG00000146676	ACCCUGU
ENSG00000146676	ACUGCAU
ENSG00000146676	ACUGGCU
ENSG00000146676	AGCAGCA
ENSG00000146676	AGCUGCC
ENSG00000146676	AGCUUAU
ENSG00000146676	AGUGCUG
ENSG00000146676	AGUGCUU
ENSG00000146676	AGUGGUU
ENSG00000146676	AGUGUGC
ENSG00000146676	AUCCUUG
ENSG00000146676	AUGCCUU
ENSG00000146676	AUGGAUU
ENSG00000146676	CAAAGUG
ENSG00000146676	CAGCAAA
ENSG00000146676	CAGCUGG
ENSG00000146676	CCCUGUC
ENSG00000146676	CCUUCUG
ENSG00000146676	CUAUACA
ENSG00000146676	CUCAAAC
ENSG00000146676	CUCCAUU
ENSG00000146676	CUCUGGG
ENSG00000146676	CUGCAGC
ENSG00000146676	GAAACAU
ENSG00000146676	GAAGGUC
ENSG00000146676	GACCCUG
ENSG00000146676	GAGGAUA
ENSG00000146676	GAGGUAG
ENSG00000146676	GAGGUAU
ENSG00000146676	GAUUGGU
ENSG00000146676	GAUUGUA
ENSG00000146676	GCAACGA
ENSG00000146676	GCCUGAU
ENSG00000146676	GCUGGAU
ENSG00000146676	GGAAGAC
ENSG00000146676	GGACCUG
ENSG00000146676	GGAGGAG
ENSG00000146676	GGAUUUC
ENSG00000146676	GGCUCAG
ENSG00000146676	GGCUCUG
ENSG00000146676	GGUCUAG
ENSG00000146676	GUAGUGU
ENSG00000146676	GUGAAUG
ENSG00000146676	GUGCUUG
ENSG00000146676	UAAGACG
ENSG00000146676	UAAUAUC
ENSG00000146676	UACAGUU
ENSG00000146676	UAGUGAG
ENSG00000146676	UAUAAAG
ENSG00000146676	UAUUGCU
ENSG00000146676	UCUGGAA
ENSG00000146676	UCUGGAG
ENSG00000146676	UGAAGUG
ENSG00000146676	UGAGAAG
ENSG00000146676	UGAGAAU
ENSG00000146676	UGAUCCA
ENSG00000146676	UGCAUUG
ENSG00000146676	UGUAUAA
ENSG00000146676	UGUGCUU
ENSG00000146676	UGUUGAA
ENSG00000146676	UUAGGAU
ENSG00000146676	UUCAAGC
ENSG00000146676	UUGUUCG

ENSG00000146700	AGCACCA
ENSG00000146700	GCACCAU
ENSG00000146700	UCCCUCU
ENSG00000146700	UUGGCAC
ENSG00000146701	AUGAUGC
ENSG00000146729	GGAUUUU
ENSG00000146731	UAUUGCU
ENSG00000146776	AAAAACC
ENSG00000146776	AAAAGUA
ENSG00000146776	AAGGUGC
ENSG00000146776	AAGUGCA
ENSG00000146776	AAGUGCU
ENSG00000146776	AAUUAUU
ENSG00000146776	ACAAUAU
ENSG00000146776	ACCAGCA
ENSG00000146776	ACUCAAA
ENSG00000146776	ACUGUAG
ENSG00000146776	AGGGUCU
ENSG00000146776	AGUAACA
ENSG00000146776	AGUGCAA
ENSG00000146776	AUACAAG
ENSG00000146776	AUGCAUU
ENSG00000146776	AUGGCUU
ENSG00000146776	CAGUAGU
ENSG00000146776	CAGUGCA
ENSG00000146776	CAGUUAC
ENSG00000146776	CCAGCAU
ENSG00000146776	CCAGUGU
ENSG00000146776	CUUUGGU
ENSG00000146776	GAUAUGU
ENSG00000146776	GAUCAGA
ENSG00000146776	GUCAGUU
ENSG00000146776	UAUAAUA
ENSG00000146776	UAUUACC
ENSG00000146776	UCCAGAG
ENSG00000146776	UCCCUUU
ENSG00000146776	UGAGAAU
ENSG00000146776	UGUAUAA
ENSG00000146776	UUCCUAG
ENSG00000146776	UUGAUCA
ENSG00000146802	AAAGUGC
ENSG00000146802	AAGGCAC
ENSG00000146802	AAGUAGC
ENSG00000146802	AAGUGCA
ENSG00000146802	AUGGCUU
ENSG00000146802	CAAAGUG
ENSG00000146802	GGCAAGA
ENSG00000146802	UGCAUAG
ENSG00000146809	AGGGAGG
ENSG00000146826	AAUACUG
ENSG00000146826	AAUCUCA
ENSG00000146826	AGUGCUU
ENSG00000146826	CACCACC
ENSG00000146826	CAGCAGA
ENSG00000146826	CAGGCUC
ENSG00000146826	GGAAGCC
ENSG00000146826	GGGAGCU
ENSG00000146826	GGGGUGG
ENSG00000146826	GUAAACA
ENSG00000146826	UGCCCUG
ENSG00000146828	AAGGCAC
ENSG00000146828	GAGGUAG
ENSG00000146828	GAGGUAU
ENSG00000146830	AAAGUGC

ENSG00000146830	AACACUG
ENSG00000146830	AACAGUC
ENSG00000146830	AAGUGCU
ENSG00000146830	AAUACUG
ENSG00000146830	ACACACC
ENSG00000146830	AGCAGCA
ENSG00000146830	AGGCAGG
ENSG00000146830	AGUGCCU
ENSG00000146830	AGUGCUG
ENSG00000146830	CAGGCUC
ENSG00000146830	CAGUUAC
ENSG00000146830	CUCAGGG
ENSG00000146830	CUGGGGG
ENSG00000146830	CUUUGGU
ENSG00000146830	GCAACUU
ENSG00000146830	GGCAGUG
ENSG00000146830	GGCUCAG
ENSG00000146830	GGCUGCG
ENSG00000146830	GGGAGCU
ENSG00000146830	GGGCCCC
ENSG00000146830	GGGGUGG
ENSG00000146830	GGGUAAG
ENSG00000146830	GGUGGGC
ENSG00000146830	GUAACAG
ENSG00000146830	UAUUGCU
ENSG00000146830	UCACAGG
ENSG00000146830	UCCAGUU
ENSG00000146830	UGCAUUG
ENSG00000146830	UGGCUGC
ENSG00000146830	UGGGUGA
ENSG00000146830	UUAGAGA
ENSG00000146834	AACAGUC
ENSG00000146834	ACAGUAC
ENSG00000146834	CACAGUG
ENSG00000146834	CAGCAAA
ENSG00000146834	CAGUCCA
ENSG00000146834	UCACAGG
ENSG00000146834	UGUGCUU
ENSG00000146842	AGGGUUG
ENSG00000146858	AAAAGUA
ENSG00000146858	CAGCAGA
ENSG00000146858	UGCCCUG
ENSG00000146859	GUGUGGC
ENSG00000146872	AAAACUG
ENSG00000146872	AAAGCUG
ENSG00000146872	AACAUUC
ENSG00000146872	AACUCUA
ENSG00000146872	AAUUAUU
ENSG00000146872	ACAGGUG
ENSG00000146872	ACAGUAC
ENSG00000146872	ACAUUCA
ENSG00000146872	ACUGCAU
ENSG00000146872	ACUGGCU
ENSG00000146872	AGCUGCC
ENSG00000146872	AGUGCCG
ENSG00000146872	AUGGAUU
ENSG00000146872	CACAGUG
ENSG00000146872	CAGUCUG
ENSG00000146872	CCCUGAG
ENSG00000146872	CUCCCAC
ENSG00000146872	CUGGACU
ENSG00000146872	CUGGCUC
ENSG00000146872	GAAAACA
ENSG00000146872	GAGAUGA

ENSG00000146872	GAGCUGC
ENSG00000146872	GCAACUU
ENSG00000146872	GCUGUCU
ENSG00000146872	GGAAUGU
ENSG00000146872	GGACUGC
ENSG00000146872	GGAGGAG
ENSG00000146872	UACAAAG
ENSG00000146872	UCAAGUA
ENSG00000146872	UCACAGG
ENSG00000146872	UGAAAUG
ENSG00000146872	UGAGAAU
ENSG00000146872	UGAGUCU
ENSG00000146872	UGUGCUU
ENSG00000146872	UUCAAGC
ENSG00000146872	UUCCUCG
ENSG00000146904	AGCAGCA
ENSG00000146904	UACAAAG
ENSG00000146910	UGACCUA
ENSG00000146938	AAAAAUC
ENSG00000146938	AAGGCAC
ENSG00000146938	ACAUUAC
ENSG00000146938	ACUGUAG
ENSG00000146938	ACUGUGU
ENSG00000146938	CAGGAAC
ENSG00000146938	CAGGCUC
ENSG00000146938	CAUAUUG
ENSG00000146938	CUACAAA
ENSG00000146938	CUCUUCC
ENSG00000146938	CUUUGGU
ENSG00000146938	GAUAUGU
ENSG00000146938	GCUAGCA
ENSG00000146938	GGCAGGG
ENSG00000146938	UAUUGCU
ENSG00000146938	UCACAAG
ENSG00000146938	UCACAGU
ENSG00000146938	UCAUAGA
ENSG00000146938	UUCCAUA
ENSG00000146938	UUUGCAC
ENSG00000146950	AAGUGUG
ENSG00000146950	UCACAGG
ENSG00000146963	AAGUGCU
ENSG00000146963	AGUGCUU
ENSG00000146963	AUGUAAU
ENSG00000146963	CCAGCAU
ENSG00000146963	UCAUUUG
ENSG00000146963	UCUCCAA
ENSG00000146963	UGCUCAC
ENSG00000146963	UUCAAAU
ENSG00000147010	AAGGCAC
ENSG00000147010	GCCUGAU
ENSG00000147010	UUGGCAA
ENSG00000147027	ACAUUCA
ENSG00000147027	AGGACAC
ENSG00000147027	CUACAAA
ENSG00000147027	CUAGGCU
ENSG00000147027	GCUAGCA
ENSG00000147027	GUCAGUU
ENSG00000147027	GUGCAAA
ENSG00000147027	UACAAAG
ENSG00000147036	AUACAAG
ENSG00000147036	CACAGUG
ENSG00000147036	CCUUCAU
ENSG00000147036	CUCAGGG
ENSG00000147036	GAGAACC

ENSG00000147036	GAUUGGU
ENSG00000147036	GCAGCAU
ENSG00000147036	UUCAAGC
ENSG00000147036	UUCUGCA
ENSG00000147044	AAUUAUU
ENSG00000147044	CCUGCUG
ENSG00000147044	CUCUAGC
ENSG00000147044	GAGAACU
ENSG00000147044	GUCUCUG
ENSG00000147044	UAUAAAG
ENSG00000147044	UGCUGAC
ENSG00000147059	GUCUACU
ENSG00000147065	AUAAUAC
ENSG00000147065	CCAGCAU
ENSG00000147065	GGAGGAG
ENSG00000147065	UUCUAAU
ENSG00000147065	UUGGUCC
ENSG00000147099	CAGGAAC
ENSG00000147099	CUGGAAA
ENSG00000147099	GGGGUGC
ENSG00000147100	AAGUGUG
ENSG00000147100	AAUGCCC
ENSG00000147100	AGAUGUG
ENSG00000147100	AGCACCA
ENSG00000147100	AGCUGCC
ENSG00000147100	AGUGUGC
ENSG00000147100	CUUCACC
ENSG00000147100	GGCAAGA
ENSG00000147100	GGCAGUG
ENSG00000147100	UCACAUU
ENSG00000147100	UCCAGUU
ENSG00000147100	UCUGGAG
ENSG00000147100	UGAAAGG
ENSG00000147113	GGAGGCA
ENSG00000147123	GAGCCCC
ENSG00000147130	AAAAACC
ENSG00000147130	AAAUGGU
ENSG00000147130	AACAAAC
ENSG00000147130	ACAAUAU
ENSG00000147130	AGCACCA
ENSG00000147130	AGGCAGG
ENSG00000147130	AUACAAG
ENSG00000147130	CAGCAAA
ENSG00000147130	CAGCUGG
ENSG00000147130	GAAACAU
ENSG00000147130	GACAACU
ENSG00000147130	GGCAGGG
ENSG00000147130	GGCUCAG
ENSG00000147130	UAUAAAG
ENSG00000147130	UUUUUGC
ENSG00000147140	AAAGCUG
ENSG00000147140	ACCACAG
ENSG00000147140	ACCCUGU
ENSG00000147140	AUGCACC
ENSG00000147140	CCCUGUU
ENSG00000147140	CUAGUCC
ENSG00000147140	CUCCAGC
ENSG00000147140	GAUUGUA
ENSG00000147140	GCACCAU
ENSG00000147140	UCCCUUG
ENSG00000147144	AAAGAGC
ENSG00000147144	AAAUCCC
ENSG00000147144	CCAGUGC
ENSG00000147144	CCCCCAG

ENSG00000147144	CUCUAGC
ENSG00000147144	GAAGGAA
ENSG00000147144	GGCAGGG
ENSG00000147144	UCACAGU
ENSG00000147144	UCCAGUU
ENSG00000147144	UCCCUUG
ENSG00000147144	UCCCUUU
ENSG00000147145	CAGUAGA
ENSG00000147145	GACCUGG
ENSG00000147162	AACACUG
ENSG00000147162	AAUCACU
ENSG00000147162	ACAUUCA
ENSG00000147162	ACCAGCA
ENSG00000147162	AGCAGCA
ENSG00000147162	AGUGGUU
ENSG00000147162	CACCAGC
ENSG00000147162	CAGCAAA
ENSG00000147162	CCAGGAG
ENSG00000147162	CUGGAAA
ENSG00000147162	GCAGCAU
ENSG00000147162	GCAGGAC
ENSG00000147162	GGAAGAC
ENSG00000147162	GGAUUUC
ENSG00000147162	GGCUCAG
ENSG00000147162	GGUUUAC
ENSG00000147162	UCCCUUU
ENSG00000147162	UUCUGCA
ENSG00000147162	UUGAGGC
ENSG00000147162	UUGGCAA
ENSG00000147162	UUGGCAC
ENSG00000147180	GUGCAAA
ENSG00000147202	AAGAACC
ENSG00000147202	AAGGCAC
ENSG00000147202	ACUCCAG
ENSG00000147202	AGCACCA
ENSG00000147202	AGCAGCU
ENSG00000147202	AGGUAGU
ENSG00000147202	AUGGCUU
ENSG00000147202	CCUUCUG
ENSG00000147202	CUCAAAC
ENSG00000147202	CUGGAAA
ENSG00000147202	CUUUGGU
ENSG00000147202	GACCCUG
ENSG00000147202	GGUUUAC
ENSG00000147202	UAUCAGA
ENSG00000147202	UCCAGAG
ENSG00000147202	UCCCUGU
ENSG00000147202	UCUGGAA
ENSG00000147202	UGACCUA
ENSG00000147202	UUGGCAA
ENSG00000147202	UUGGCAC
ENSG00000147202	UUGGUUC
ENSG00000147202	UUGUGAC
ENSG00000147224	AAGGCAC
ENSG00000147224	CUCCCAA
ENSG00000147231	UCAUUUG
ENSG00000147246	AAGGCAC
ENSG00000147246	AAUCUCA
ENSG00000147246	ACAUCAC
ENSG00000147246	CAAAUGC
ENSG00000147246	CUGCAGC
ENSG00000147246	UCACAUU
ENSG00000147246	UGAAAUG
ENSG00000147246	UUCCUAG

ENSG00000147251	ACCCUGU
ENSG00000147255	ACCCUGU
ENSG00000147255	GCCUGGA
ENSG00000147255	GCCUGGG
ENSG00000147255	GUGCCUG
ENSG00000147316	CUUCUCU
ENSG00000147316	GUGAGGU
ENSG00000147324	AAACUGU
ENSG00000147324	AAAUCCC
ENSG00000147324	AAAUUUC
ENSG00000147324	AAGUAGC
ENSG00000147324	AGAUAAA
ENSG00000147324	AGAUGUG
ENSG00000147324	AUAUUAU
ENSG00000147324	AUUGCAC
ENSG00000147324	CACAGUG
ENSG00000147324	CUUGGAG
ENSG00000147324	GAAAACA
ENSG00000147324	GAGAAGA
ENSG00000147324	GCAGAAG
ENSG00000147324	GGAGGAG
ENSG00000147324	UCAAGUA
ENSG00000147324	UCACAGU
ENSG00000147324	UGAGAAG
ENSG00000147324	UGCAUAG
ENSG00000147324	UUGGAGG
ENSG00000147324	UUGGCAC
ENSG00000147364	ACAAGGU
ENSG00000147364	UAUUACC
ENSG00000147382	CAGCUGG
ENSG00000147382	CUUCUCU
ENSG00000147382	CUUUGGU
ENSG00000147394	AUUCAUU
ENSG00000147400	CAAAUGC
ENSG00000147408	CUUUGGU
ENSG00000147416	AACACUG
ENSG00000147416	AGCAGCA
ENSG00000147416	AUUGCAC
ENSG00000147416	GAAAACA
ENSG00000147416	GGAAUGU
ENSG00000147419	AAAGUGC
ENSG00000147419	AAGACAU
ENSG00000147419	AAGUGCU
ENSG00000147419	CAAAGUG
ENSG00000147419	UCCAGUU
ENSG00000147419	UUGGUUC
ENSG00000147421	AAAGGUA
ENSG00000147421	GCACCAU
ENSG00000147421	UAAUACA
ENSG00000147437	CCUUCUG
ENSG00000147439	AACAAAC
ENSG00000147439	GAGGUAG
ENSG00000147439	GGACGGA
ENSG00000147443	GGAGGAG
ENSG00000147454	UCAAGUA
ENSG00000147457	ACUGCAG
ENSG00000147457	AGUGAUC
ENSG00000147457	AUUGCAC
ENSG00000147457	CAGCUGG
ENSG00000147457	CUGCAGG
ENSG00000147457	GAUUGUA
ENSG00000147457	GAUUGUC
ENSG00000147457	GGCUCAG
ENSG00000147457	GGUGGGC

ENSG00000147457	UAUAAAG
ENSG00000147457	UGAGAAG
ENSG00000147457	UGGGUGA
ENSG00000147457	UGUAUAA
ENSG00000147457	UGUGUGG
ENSG00000147459	CAGCAGG
ENSG00000147459	CUCAGGG
ENSG00000147459	GUAUUCU
ENSG00000147465	AGCAUUC
ENSG00000147471	AGUGCAA
ENSG00000147471	CUGGGCA
ENSG00000147471	UCACAUU
ENSG00000147471	UCCAGAG
ENSG00000147471	UGCAUAG
ENSG00000147475	AAAAAUC
ENSG00000147475	AAAAGUA
ENSG00000147475	AAUUUUA
ENSG00000147475	AGCACCA
ENSG00000147475	AGCAGCA
ENSG00000147475	AGCAGCG
ENSG00000147475	AGCAGCU
ENSG00000147475	AGGGAGG
ENSG00000147475	AGGUAGU
ENSG00000147475	CAGCAGG
ENSG00000147475	GAAACAU
ENSG00000147475	GGAGGCA
ENSG00000147475	UGCAAAG
ENSG00000147475	UUCUGCA
ENSG00000147488	AAUUAUU
ENSG00000147488	ACAUUAC
ENSG00000147488	AUACAAG
ENSG00000147488	GUGAAUG
ENSG00000147488	UACAGUU
ENSG00000147488	UGCAUUG
ENSG00000147488	UGUGCUU
ENSG00000147488	UUCCUAG
ENSG00000147509	GUGCUUG
ENSG00000147526	AAGGCAC
ENSG00000147526	AAGUGCA
ENSG00000147526	ACUGCAU
ENSG00000147526	AGCACCA
ENSG00000147526	CACAGUG
ENSG00000147526	CAGCAAA
ENSG00000147526	CAGUAGU
ENSG00000147526	GAUUGUA
ENSG00000147526	GAUUGUC
ENSG00000147526	GCCCUGU
ENSG00000147526	GGCAAGA
ENSG00000147526	GUGCAAA
ENSG00000147526	GUGCUUG
ENSG00000147526	UCUCCAA
ENSG00000147526	UGGAUAA
ENSG00000147533	AUGGCAC
ENSG00000147533	CAGCAGA
ENSG00000147533	GAGGUAG
ENSG00000147533	GGCUCUG
ENSG00000147535	AAAGUAC
ENSG00000147535	GUAAACA
ENSG00000147548	CCUUCAU
ENSG00000147548	UAGACUG
ENSG00000147570	GAGAAAU
ENSG00000147570	UCACAGG
ENSG00000147570	UCACAGU
ENSG00000147573	CCUGCUG

ENSG00000147573	CUUUGGU
ENSG00000147573	UUGGUCC
ENSG00000147576	AUCCUUG
ENSG00000147601	AGUGAUC
ENSG00000147606	AAAGAAU
ENSG00000147606	GGUUGUC
ENSG00000147606	GUCAGUU
ENSG00000147606	UGCAUAU
ENSG00000147649	AGUGCCU
ENSG00000147649	CACAGUG
ENSG00000147649	GCUGUCU
ENSG00000147649	GUGCCUG
ENSG00000147650	AAAUCCC
ENSG00000147650	ACAUUCA
ENSG00000147650	AGUGCUU
ENSG00000147650	GUGCAAA
ENSG00000147650	UAUUGCU
ENSG00000147655	AACAUUC
ENSG00000147655	ACAGUAC
ENSG00000147655	ACAGUAU
ENSG00000147655	AGGUAGU
ENSG00000147655	UAAUGCU
ENSG00000147655	UGCAAAG
ENSG00000147655	UUUGCAA
ENSG00000147676	AAAUGGU
ENSG00000147676	AUAAUAC
ENSG00000147676	CAAAUGC
ENSG00000147676	UACAAAG
ENSG00000147676	UCACAGG
ENSG00000147676	UCACAGU
ENSG00000147676	UGUGUGG
ENSG00000147676	UUGGCAC
ENSG00000147676	UUGUUCG
ENSG00000147679	UACAAAG
ENSG00000147689	GACAACU
ENSG00000147724	AAAAAUC
ENSG00000147724	AAAGAGG
ENSG00000147724	AACCUGG
ENSG00000147724	AACCUGU
ENSG00000147724	AAGGCAC
ENSG00000147724	AGCAGCU
ENSG00000147724	AGCCCUU
ENSG00000147724	CAGACAG
ENSG00000147724	CAGCAGG
ENSG00000147724	CCCUGAG
ENSG00000147724	CCUUCUG
ENSG00000147724	CUUUGGU
ENSG00000147724	GAGGCAG
ENSG00000147724	GCACCAU
ENSG00000147724	GGCAAGA
ENSG00000147724	UAUUGCU
ENSG00000147724	UGCAGCU
ENSG00000147724	UGCAUAG
ENSG00000147813	CUCACAC
ENSG00000147813	UCACACA
ENSG00000147852	AAUACUG
ENSG00000147852	CUAUACA
ENSG00000147852	GGUUACC
ENSG00000147852	UUGGCAC
ENSG00000147854	ACAAUAU
ENSG00000147854	AGGUAGU
ENSG00000147854	AGUAGUU
ENSG00000147854	GAGGCAG
ENSG00000147854	GGCAGUG

ENSG00000147854	UCCUGAG
ENSG00000147862	AAAAGUA
ENSG00000147862	AAAGAAU
ENSG00000147862	AAAGUAC
ENSG00000147862	AAAGUGC
ENSG00000147862	AAAUCCC
ENSG00000147862	AAAUGGU
ENSG00000147862	AACAGUC
ENSG00000147862	AACUACU
ENSG00000147862	AAGGCAC
ENSG00000147862	ACACACU
ENSG00000147862	ACAUUAC
ENSG00000147862	ACUGGAU
ENSG00000147862	ACUGUGU
ENSG00000147862	AGAUAAA
ENSG00000147862	AGCAAAA
ENSG00000147862	AGCACCA
ENSG00000147862	AGCAGCU
ENSG00000147862	AGCCUGC
ENSG00000147862	AGCUGCC
ENSG00000147862	AGGACAC
ENSG00000147862	AGUAACA
ENSG00000147862	AGUGCAA
ENSG00000147862	AUGAGCU
ENSG00000147862	AUGGAUU
ENSG00000147862	CACAGUG
ENSG00000147862	CAGCAAA
ENSG00000147862	CAGCUGG
ENSG00000147862	CAGGAAC
ENSG00000147862	CAUGGAU
ENSG00000147862	CCAGUAC
ENSG00000147862	CCCUGAG
ENSG00000147862	CCCUGUC
ENSG00000147862	CCCUGUU
ENSG00000147862	CUACAAA
ENSG00000147862	CUACAGU
ENSG00000147862	CUAUACA
ENSG00000147862	CUCAGGG
ENSG00000147862	CUCCAUU
ENSG00000147862	CUGGCUC
ENSG00000147862	CUUUGGU
ENSG00000147862	GAAUUGU
ENSG00000147862	GAGCUGC
ENSG00000147862	GAUAUGU
ENSG00000147862	GCAACUU
ENSG00000147862	GCACCAU
ENSG00000147862	GCAGCAU
ENSG00000147862	GCUGGAU
ENSG00000147862	GGAAGAC
ENSG00000147862	GUCACUC
ENSG00000147862	GUGAGGU
ENSG00000147862	UACAAAG
ENSG00000147862	UAUAAAG
ENSG00000147862	UAUAUGA
ENSG00000147862	UAUCAGA
ENSG00000147862	UCACAGG
ENSG00000147862	UCACAGU
ENSG00000147862	UCCAGUU
ENSG00000147862	UCCCUGU
ENSG00000147862	UCGUGCA
ENSG00000147862	UCUGGAG
ENSG00000147862	UGAAAGG
ENSG00000147862	UGAAGCU
ENSG00000147862	UGACUGU

ENSG00000147862	UGAGGAC
ENSG00000147862	UGCAAAG
ENSG00000147862	UGCAUAU
ENSG00000147862	UGCCAGC
ENSG00000147862	UGGCUGC
ENSG00000147862	UGUCUGC
ENSG00000147862	UGUUGAA
ENSG00000147862	UUAGGAU
ENSG00000147862	UUCAAGC
ENSG00000147862	UUGGUCC
ENSG00000147862	UUUGCAC
ENSG00000147883	AAUUAUU
ENSG00000147883	UACAGUU
ENSG00000147883	UUGUUCG
ENSG00000147894	ACUGUAG
ENSG00000147894	CACUGUU
ENSG00000147912	AAAGUGC
ENSG00000147912	AAGGCAC
ENSG00000147912	AAGUGCA
ENSG00000147912	AAGUGCU
ENSG00000147912	AGCAGCA
ENSG00000147912	CAGCUGG
ENSG00000147912	GAGAAGA
ENSG00000147912	GGCAGUG
ENSG00000147912	UGAGAAG
ENSG00000148053	AAGUGCA
ENSG00000148053	AAUUAUU
ENSG00000148053	ACUCCAG
ENSG00000148053	AGGUUAU
ENSG00000148053	AUGGAUU
ENSG00000148053	AUGUCUG
ENSG00000148053	CAAAGCA
ENSG00000148053	CAGACAG
ENSG00000148053	CAUGGAU
ENSG00000148053	CGAGGAC
ENSG00000148053	CUUCUCU
ENSG00000148053	GAGAAGA
ENSG00000148053	GAUCGAC
ENSG00000148053	GCAACUU
ENSG00000148053	GCUGGUG
ENSG00000148053	GCUGUCU
ENSG00000148053	GGAAGCC
ENSG00000148053	GGAUUUC
ENSG00000148053	GGAUUUU
ENSG00000148053	GGGUCUU
ENSG00000148053	GUGCAAA
ENSG00000148053	UGCUGAC
ENSG00000148053	UGUCUGC
ENSG00000148090	CUUUGGU
ENSG00000148110	AAAACUG
ENSG00000148110	AAGGCAC
ENSG00000148110	AAUCCUU
ENSG00000148110	AGCAAAA
ENSG00000148110	AGCACCA
ENSG00000148110	AGGCAGG
ENSG00000148110	CAGCAAA
ENSG00000148110	GCACCAU
ENSG00000148110	GCAGCAU
ENSG00000148110	UACAAAG
ENSG00000148110	UCAUACA
ENSG00000148120	AAAGAAU
ENSG00000148120	GUCCAGA
ENSG00000148120	UCCCUCU
ENSG00000148123	CACAGUG

ENSG00000148123	GCUACAU
ENSG00000148123	UCACAGU
ENSG00000148123	UCAUACA
ENSG00000148143	AGUGCUU
ENSG00000148143	CCAGUGC
ENSG00000148153	UUGGCAA
ENSG00000148153	UUGGCAC
ENSG00000148154	AAAACUG
ENSG00000148154	AAUACUG
ENSG00000148158	AAGUGCU
ENSG00000148158	AGCAAAA
ENSG00000148158	AGCUGCC
ENSG00000148158	AUUGCAC
ENSG00000148158	CAGCAGG
ENSG00000148158	CAGUCCA
ENSG00000148158	CCAGCAU
ENSG00000148158	CUUUGGU
ENSG00000148158	GCAGCAU
ENSG00000148158	GCUGGUG
ENSG00000148158	GGAUUUU
ENSG00000148158	GGCAGCG
ENSG00000148158	GGCAGUG
ENSG00000148158	UAGAGGA
ENSG00000148158	UCACAUU
ENSG00000148158	UCCAGAG
ENSG00000148158	UCUCAAG
ENSG00000148158	UGCCAGC
ENSG00000148180	AAGGCAC
ENSG00000148187	GGCUCUG
ENSG00000148200	UCACAUU
ENSG00000148200	UGUUGAA
ENSG00000148204	CUGGCUC
ENSG00000148204	GAGGUAG
ENSG00000148204	GGCUCUG
ENSG00000148204	GUGAGGU
ENSG00000148204	UCCCUGU
ENSG00000148204	UGCCAGC
ENSG00000148218	CAGGAAC
ENSG00000148218	CUCCCAA
ENSG00000148218	CUCCCAC
ENSG00000148218	CUUCUCU
ENSG00000148218	CUUGGGC
ENSG00000148218	CUUUGGU
ENSG00000148218	UGAUCCA
ENSG00000148218	UUCAAGC
ENSG00000148218	UUGAUCA
ENSG00000148218	UUGGUUC
ENSG00000148219	UAAUGCU
ENSG00000148229	AGCACCA
ENSG00000148248	AAAGUUU
ENSG00000148248	AAGGCAC
ENSG00000148248	AAUACUG
ENSG00000148248	ACAGUAC
ENSG00000148248	AGGGAGG
ENSG00000148248	AUAAAGU
ENSG00000148248	CACAGUG
ENSG00000148248	CAGGUGA
ENSG00000148248	CUCAAAC
ENSG00000148248	GAGGUAG
ENSG00000148248	GAGGUAU
ENSG00000148248	GUAAACA
ENSG00000148248	GUGCCUG
ENSG00000148248	UCACAGU
ENSG00000148248	UCCCACC

ENSG00000148248	UCUCAAG
ENSG00000148248	UGCUCAC
ENSG00000148248	UUCAAAU
ENSG00000148248	UUUCAAC
ENSG00000148297	AAAGAGC
ENSG00000148331	AGCUGCC
ENSG00000148331	CUGCAGC
ENSG00000148331	GGCUGCG
ENSG00000148331	UGGCUGC
ENSG00000148337	AAAAACC
ENSG00000148339	AAAAAUC
ENSG00000148339	AACUCUA
ENSG00000148339	ACAUUCA
ENSG00000148339	AGGACAC
ENSG00000148339	CCUUCUG
ENSG00000148339	CUGGAAA
ENSG00000148339	GAGAUGA
ENSG00000148339	GCAGGAC
ENSG00000148339	GGAAGCC
ENSG00000148339	GGAAUGU
ENSG00000148339	GUUAGGA
ENSG00000148339	UCCAGUU
ENSG00000148339	UCUGGAA
ENSG00000148339	UUCAAAU
ENSG00000148339	UUUGCAA
ENSG00000148339	UUUGCAC
ENSG00000148341	UUGGCAA
ENSG00000148343	ACACACU
ENSG00000148343	ACAUUCA
ENSG00000148343	AGUGCAA
ENSG00000148343	CUGCAGG
ENSG00000148343	GGCAGUG
ENSG00000148343	UGUGCGU
ENSG00000148358	AAAGCUG
ENSG00000148358	ACUCAGG
ENSG00000148358	AGCUGCC
ENSG00000148358	AUGCACC
ENSG00000148358	CCCUGAG
ENSG00000148358	GCACCAU
ENSG00000148358	GGAGUGU
ENSG00000148358	GUGUCAC
ENSG00000148358	GUUAGGA
ENSG00000148396	AACAGUC
ENSG00000148396	GGCAGUG
ENSG00000148396	UCUUGAG
ENSG00000148396	UUAGGAU
ENSG00000148400	AACAAAC
ENSG00000148400	ACAUCAC
ENSG00000148400	AUAAUAC
ENSG00000148400	CAGUUAC
ENSG00000148400	GUAAACA
ENSG00000148400	UACAGUU
ENSG00000148400	UAUUGCU
ENSG00000148400	UCAUGAU
ENSG00000148408	AAAACUG
ENSG00000148426	AACACUG
ENSG00000148426	ACUCCAG
ENSG00000148426	CUCCAGC
ENSG00000148426	UUCAAAU
ENSG00000148426	UUUCAAC
ENSG00000148429	AAUACUG
ENSG00000148429	ACAGUAC
ENSG00000148429	ACAUUCA
ENSG00000148429	AGCACCA

ENSG00000148429	GCACCAU
ENSG00000148429	UCACAUU
ENSG00000148429	UUGGCAC
ENSG00000148468	AAGGCAC
ENSG00000148468	ACUUGGA
ENSG00000148468	AGCAGCU
ENSG00000148468	UGCAUAG
ENSG00000148468	UUGGCAC
ENSG00000148483	AAUACUG
ENSG00000148483	CCUUCAU
ENSG00000148484	AAAGCUG
ENSG00000148484	AAAGUUU
ENSG00000148484	AGCUGCC
ENSG00000148484	GGUUACC
ENSG00000148484	UGAGAAG
ENSG00000148484	UUCUGCA
ENSG00000148484	UUGGCAA
ENSG00000148484	UUGGCAC
ENSG00000148516	AGUGCAA
ENSG00000148516	CAGUAGU
ENSG00000148516	CUCACAC
ENSG00000148516	CUCCCAC
ENSG00000148516	GUAGUGU
ENSG00000148516	UCACAUU
ENSG00000148572	AAGUGUG
ENSG00000148572	AAUACUG
ENSG00000148572	AUGCACC
ENSG00000148572	GUAAACA
ENSG00000148572	GUGUGGC
ENSG00000148572	UCACAGG
ENSG00000148572	UCCCUUG
ENSG00000148660	AAAAACC
ENSG00000148660	AACAUUC
ENSG00000148660	ACAUUCA
ENSG00000148660	ACCAGCA
ENSG00000148660	ACCCUGU
ENSG00000148660	ACUCAGG
ENSG00000148660	ACUGUUU
ENSG00000148660	AGCACCA
ENSG00000148660	AGGGUCU
ENSG00000148660	AUGCCUU
ENSG00000148660	CACCAGC
ENSG00000148660	CACUCCU
ENSG00000148660	CAGCAGA
ENSG00000148660	CAGCUGG
ENSG00000148660	CCAGCAU
ENSG00000148660	CCCACCU
ENSG00000148660	CGUGCAU
ENSG00000148660	GAGGCAG
ENSG00000148660	GCAGCAU
ENSG00000148660	GGCUCUG
ENSG00000148660	GGGCGUG
ENSG00000148660	GUAACAG
ENSG00000148660	GUCACUC
ENSG00000148660	UCCAGAG
ENSG00000148660	UCCUAUA
ENSG00000148660	UGAAGCU
ENSG00000148660	UGCAUUG
ENSG00000148660	UGCUGAC
ENSG00000148672	AAAAAUC
ENSG00000148672	AAAUUUC
ENSG00000148672	AGCAGCA
ENSG00000148672	AGUAGUU
ENSG00000148672	CAGUUAC

ENSG00000148672	GCAGCAU
ENSG00000148672	GGUUACC
ENSG00000148672	GUAAACA
ENSG00000148672	UGAGAAG
ENSG00000148680	UGUGCUU
ENSG00000148700	AAAUCCC
ENSG00000148700	AUUCAUU
ENSG00000148700	GGCUCAG
ENSG00000148700	UAAUACA
ENSG00000148700	UUGUGAC
ENSG00000148704	AACAUUC
ENSG00000148704	AAGAACC
ENSG00000148704	ACAUUCA
ENSG00000148704	AGCAGCU
ENSG00000148704	AGGGAGG
ENSG00000148704	AUUCAUU
ENSG00000148704	CACCAGC
ENSG00000148704	CUGCUCA
ENSG00000148704	GACUUCC
ENSG00000148704	GGACCUG
ENSG00000148704	GGAGGCA
ENSG00000148704	GUGUCAC
ENSG00000148704	UAAUAUC
ENSG00000148704	UCCCACA
ENSG00000148704	UUGUUCG
ENSG00000148719	AAGGCAC
ENSG00000148719	ACUUGGA
ENSG00000148719	CAGUGCA
ENSG00000148719	CCCCCAG
ENSG00000148719	CUGGGGG
ENSG00000148719	GACCCUG
ENSG00000148719	GCCCUGU
ENSG00000148719	GGAUUUC
ENSG00000148719	GGCAGCG
ENSG00000148719	GGGGCAG
ENSG00000148719	UAUUGCU
ENSG00000148719	UCAUGAU
ENSG00000148719	UCUCCAA
ENSG00000148719	UGUCUUU
ENSG00000148719	UUCUAAU
ENSG00000148719	UUCUGCA
ENSG00000148730	AGCUUAU
ENSG00000148730	UUGGCAA
ENSG00000148730	UUGGCAC
ENSG00000148735	GGGAUCG
ENSG00000148737	AACAGUC
ENSG00000148737	CAGUCUG
ENSG00000148737	CUACAAA
ENSG00000148737	UACAAAG
ENSG00000148773	CACACCU
ENSG00000148798	AAUUUUA
ENSG00000148798	AGCACCA
ENSG00000148798	UGAAAUG
ENSG00000148798	UGCAUAU
ENSG00000148798	UUGGCAC
ENSG00000148826	UACAAAG
ENSG00000148835	AAACUGU
ENSG00000148840	UGCAUAG
ENSG00000148842	AAAAACC
ENSG00000148842	AAAACUG
ENSG00000148842	AGCAGCG
ENSG00000148842	AGCAGCU
ENSG00000148842	CAGCAGA
ENSG00000148842	CUUUGGU

ENSG00000148842	GCAGCAU
ENSG00000148842	UCCUGAG
ENSG00000148842	UUGGCAA
ENSG00000148842	UUGGCAC
ENSG00000148842	UUGGUCC
ENSG00000148848	AGCACCA
ENSG00000148848	AUGGCUU
ENSG00000148848	CUUCACC
ENSG00000148848	GUGCAAA
ENSG00000148848	UCAAGUA
ENSG00000148848	UCCCUUU
ENSG00000148848	UCUGGAA
ENSG00000148848	UUCAAGC
ENSG00000148848	UUUGCAA
ENSG00000148908	UCCUGAG
ENSG00000148925	AAUUUUA
ENSG00000148925	CUUUGGU
ENSG00000148926	ACAUUCA
ENSG00000148926	AUUGCAC
ENSG00000148926	GAGAAAU
ENSG00000148926	GGCUCAG
ENSG00000148926	UUCCUCG
ENSG00000148935	AAGGCAC
ENSG00000148935	UUGGUCC
ENSG00000148943	AAAGCUG
ENSG00000148943	AACUACU
ENSG00000148943	AAUGCCC
ENSG00000148943	AAUGUUG
ENSG00000148943	ACAUUCA
ENSG00000148943	ACUCAAA
ENSG00000148943	ACUGUAG
ENSG00000148943	ACUGUUU
ENSG00000148943	AUACAAG
ENSG00000148943	AUAUAAC
ENSG00000148943	AUGACAC
ENSG00000148943	AUGCACC
ENSG00000148943	CACAGUG
ENSG00000148943	CAGGAAC
ENSG00000148943	CCAGUGC
ENSG00000148943	CUACAAA
ENSG00000148943	CUUUGGU
ENSG00000148943	GAAGGAA
ENSG00000148943	GAUUGUA
ENSG00000148943	GAUUGUC
ENSG00000148943	GCACCAU
ENSG00000148943	GCAGAAG
ENSG00000148943	GGAAUGU
ENSG00000148943	UACAAAG
ENSG00000148943	UGAAAUG
ENSG00000148943	UGACAUC
ENSG00000148943	UGCAGCU
ENSG00000148943	UGCCCUG
ENSG00000148943	UGCUGAC
ENSG00000148943	UGGCUGC
ENSG00000148943	UUGGUCC
ENSG00000148943	UUUCAAC
ENSG00000148948	CACAAUG
ENSG00000148948	CAGUAGA
ENSG00000148948	GAAACAU
ENSG00000148948	GUCAUUG
ENSG00000148948	UCAUUUG
ENSG00000149016	UAUUGCU
ENSG00000149091	CACACCG
ENSG00000149091	CACACCU

ENSG00000149091	GGCAGUG
ENSG00000149091	UGAAAUG
ENSG00000149115	AAAGUGC
ENSG00000149115	AAGUGCA
ENSG00000149115	AUGUGCC
ENSG00000149115	CAAAGUG
ENSG00000149115	GUGCCUG
ENSG00000149115	UACAAAG
ENSG00000149150	UAUAAAG
ENSG00000149150	UCCCUUG
ENSG00000149177	AAAGUGC
ENSG00000149177	AAGGCAC
ENSG00000149177	AAGUGCA
ENSG00000149177	AAUCACU
ENSG00000149177	AAUCCUU
ENSG00000149177	AAUGCCC
ENSG00000149177	AGCAGCA
ENSG00000149177	AGCCAGU
ENSG00000149177	AGUGCAA
ENSG00000149177	AUGCCUU
ENSG00000149177	AUUGCAC
ENSG00000149177	CAAAGUG
ENSG00000149177	CAGUAGA
ENSG00000149177	CAUAUUG
ENSG00000149177	GGAAGCC
ENSG00000149177	GUUAAUG
ENSG00000149177	UCACAGG
ENSG00000149177	UCACAGU
ENSG00000149177	UUCAAAU
ENSG00000149182	AACAGUC
ENSG00000149182	AGCAGCA
ENSG00000149182	AGCAGCU
ENSG00000149182	AGCCCUU
ENSG00000149182	GGCAGGG
ENSG00000149182	GUCCAGA
ENSG00000149182	UGGCCAC
ENSG00000149182	UGGGGGA
ENSG00000149218	AAGGCAC
ENSG00000149218	ACACACU
ENSG00000149218	AGUGCAA
ENSG00000149231	CUUCUCU
ENSG00000149231	UAAUACA
ENSG00000149231	UCAUGAU
ENSG00000149256	ACAAGGU
ENSG00000149256	ACAUCAC
ENSG00000149256	AGCUUAU
ENSG00000149256	AUGAGCU
ENSG00000149256	AUUCAUU
ENSG00000149256	CAGUCCA
ENSG00000149256	CCAGUGC
ENSG00000149256	CUCAAAC
ENSG00000149256	CUCCAGC
ENSG00000149256	CUGCCCC
ENSG00000149256	CUUUGGU
ENSG00000149256	GAGAAAU
ENSG00000149256	GCUGUCU
ENSG00000149256	GGCAUUG
ENSG00000149256	GUCAACA
ENSG00000149256	UAUCAGA
ENSG00000149256	UCACAGG
ENSG00000149256	UCAUUUG
ENSG00000149256	UCCCUUG
ENSG00000149256	UCUAGAG
ENSG00000149256	UCUCAAG

ENSG00000149256	UGAGAAG
ENSG00000149256	UGAGAAU
ENSG00000149256	UGAGUCU
ENSG00000149256	UGCAAAG
ENSG00000149256	UGGAGAU
ENSG00000149256	UGUCUUU
ENSG00000149256	UUAUGCA
ENSG00000149256	UUGGCAC
ENSG00000149256	UUUUUGC
ENSG00000149257	AGCACCA
ENSG00000149260	AAUCUCA
ENSG00000149260	CAGACAG
ENSG00000149260	CAGCAGG
ENSG00000149260	CCAGGAG
ENSG00000149260	GCCCUUA
ENSG00000149260	UCACACA
ENSG00000149260	UUGGUCC
ENSG00000149262	UCUCAAG
ENSG00000149269	CACAAUG
ENSG00000149269	GAGGCUG
ENSG00000149269	GAGGUAG
ENSG00000149269	GCUACAU
ENSG00000149269	UCUUGAG
ENSG00000149269	UUGGCAC
ENSG00000149289	AAAGGUA
ENSG00000149289	AAGGUGC
ENSG00000149289	ACAGUAC
ENSG00000149289	ACUAGAU
ENSG00000149289	AGUGCAA
ENSG00000149289	AUCCCUU
ENSG00000149289	CACAGUG
ENSG00000149289	CAGUGCA
ENSG00000149289	CCAGUAC
ENSG00000149289	CUACAAA
ENSG00000149289	CUAUACA
ENSG00000149289	GAGCUGC
ENSG00000149289	GCACCAU
ENSG00000149289	GGCAAGA
ENSG00000149289	GUGCUUG
ENSG00000149289	GUGUCAC
ENSG00000149289	UACAAAG
ENSG00000149289	UCCCUCU
ENSG00000149289	UCCUGAG
ENSG00000149289	UCCUUGC
ENSG00000149289	UGAGAAG
ENSG00000149289	UGCAUAG
ENSG00000149289	UGCAUAU
ENSG00000149289	UGCUGAC
ENSG00000149289	UUAGGAU
ENSG00000149289	UUGGCAC
ENSG00000149294	AAAAAUC
ENSG00000149294	AAAGUAA
ENSG00000149294	AAAGUAC
ENSG00000149294	AAAUUUC
ENSG00000149294	AACACUG
ENSG00000149294	AAUACUG
ENSG00000149294	ACUCAGC
ENSG00000149294	ACUCAGG
ENSG00000149294	ACUGCAU
ENSG00000149294	AGGAGCU
ENSG00000149294	AGUACUG
ENSG00000149294	AUACAAG
ENSG00000149294	AUGACAC
ENSG00000149294	CACAGUG

ENSG00000149294	CAGUGCA
ENSG00000149294	CAGUUAC
ENSG00000149294	CCAGGAG
ENSG00000149294	CUCCAUU
ENSG00000149294	CUCUUCC
ENSG00000149294	GCAGAAG
ENSG00000149294	GGCAUUG
ENSG00000149294	GUUAAUG
ENSG00000149294	UACAAAG
ENSG00000149294	UCACACA
ENSG00000149294	UCACAGG
ENSG00000149294	UCCGCUC
ENSG00000149294	UCCUGAG
ENSG00000149294	UGAAAUG
ENSG00000149294	UGAGAAU
ENSG00000149294	UGCAAAG
ENSG00000149294	UGCAUAG
ENSG00000149294	UGCAUAU
ENSG00000149294	UGUUGAA
ENSG00000149294	UUGGCAA
ENSG00000149294	UUGGCAC
ENSG00000149295	AACACUG
ENSG00000149295	ACUGCAG
ENSG00000149295	CUGGGGG
ENSG00000149295	CUUUGGU
ENSG00000149295	GAGCUGC
ENSG00000149295	GGCAGGG
ENSG00000149308	AAGGCAC
ENSG00000149308	AAGUGCA
ENSG00000149308	AUGGAUU
ENSG00000149308	AUGGCUU
ENSG00000149308	GCAGGUU
ENSG00000149308	GGCUCAG
ENSG00000149308	UACAGUU
ENSG00000149308	UGCAUUG
ENSG00000149311	UCUGGAG
ENSG00000149313	AAGACAU
ENSG00000149313	AUAUUAU
ENSG00000149313	AUGAUGA
ENSG00000149313	AUGAUGC
ENSG00000149328	CCCUGAG
ENSG00000149357	GAGGCUG
ENSG00000149380	CUUCUCU
ENSG00000149380	UCCCUCU
ENSG00000149397	GGUUACC
ENSG00000149428	AAAGUAC
ENSG00000149428	ACAUUCA
ENSG00000149428	ACCACAG
ENSG00000149428	ACUCAAA
ENSG00000149428	AGUGAUC
ENSG00000149428	CACAGUG
ENSG00000149428	CAGUAGA
ENSG00000149428	GAGAAGA
ENSG00000149428	GGCUCUG
ENSG00000149428	UCACAGU
ENSG00000149428	UCUCAAG
ENSG00000149474	GGAGUCC
ENSG00000149480	AAAAACC
ENSG00000149480	AAACUGU
ENSG00000149480	CAGUGCA
ENSG00000149480	GAAAACA
ENSG00000149480	GGCAGUG
ENSG00000149480	UGCCAGC
ENSG00000149485	AACAUUC

ENSG00000149485	AGUGGUU
ENSG00000149485	CACAGUG
ENSG00000149499	CAGCAGG
ENSG00000149499	GGAAUGU
ENSG00000149499	GGCUCAG
ENSG00000149499	UAUUGCU
ENSG00000149503	CAGCAGG
ENSG00000149527	UCCUGAG
ENSG00000149534	AAAGUUU
ENSG00000149541	GAGGUAG
ENSG00000149547	AAAAAUC
ENSG00000149547	AACCUGG
ENSG00000149547	AGGCAGG
ENSG00000149547	AUGGCAC
ENSG00000149547	CAGUCCA
ENSG00000149547	GGCAGUG
ENSG00000149547	UACAAAG
ENSG00000149547	UUUGCAA
ENSG00000149547	UUUGCAC
ENSG00000149554	AGCAGCG
ENSG00000149554	AGCAGCU
ENSG00000149554	CUCUAGC
ENSG00000149557	UAAGACU
ENSG00000149575	AAAUGGU
ENSG00000149575	AACAUUC
ENSG00000149575	AAGGCAC
ENSG00000149575	ACUCAAA
ENSG00000149575	AGCCAGU
ENSG00000149575	AGGCAGG
ENSG00000149575	AUAAUAC
ENSG00000149575	AUAUAAC
ENSG00000149575	AUGCACC
ENSG00000149575	AUGGCUU
ENSG00000149575	CAGCAAA
ENSG00000149575	CAGCAGA
ENSG00000149575	CAGCUGG
ENSG00000149575	CAGUCCA
ENSG00000149575	CCCUGAG
ENSG00000149575	CUUUGGU
ENSG00000149575	GAGAUGA
ENSG00000149575	GCCCUUA
ENSG00000149575	GGAAGAC
ENSG00000149575	GGACCUG
ENSG00000149575	GGCCCUG
ENSG00000149575	GGGGUCC
ENSG00000149575	GGGGUGC
ENSG00000149575	GUCACUC
ENSG00000149575	GUGCUUG
ENSG00000149575	GUGGCCC
ENSG00000149575	UAAGACG
ENSG00000149575	UAAGACU
ENSG00000149575	UAUAAUA
ENSG00000149575	UGGCCCU
ENSG00000149575	UUGGUCC
ENSG00000149575	UUGUUCG
ENSG00000149577	AACACUG
ENSG00000149577	AGCACCA
ENSG00000149577	AGCAGCA
ENSG00000149577	AUGGCAC
ENSG00000149577	CCUGCUG
ENSG00000149577	GGUUGUC
ENSG00000149577	GUAAACA
ENSG00000149577	UGAGAAG
ENSG00000149582	AAGUGCU

ENSG00000149582	AGCAAAA
ENSG00000149582	AGUAACA
ENSG00000149582	CAGCAAA
ENSG00000149582	GCAGCAU
ENSG00000149582	GUGCAAA
ENSG00000149582	GUGCUUG
ENSG00000149582	UCACAGU
ENSG00000149582	UGCAUAG
ENSG00000149591	AGCUGCC
ENSG00000149591	AGGCAGG
ENSG00000149596	UAUUGCU
ENSG00000149596	UCCCACC
ENSG00000149596	UGGCCAC
ENSG00000149599	AAGGCAC
ENSG00000149599	CAGCAGG
ENSG00000149600	AUGGCAC
ENSG00000149636	AAAGAAU
ENSG00000149636	UUCAAAU
ENSG00000149639	AAAAACC
ENSG00000149639	AUGACAC
ENSG00000149639	CUACAAA
ENSG00000149639	GCAGGAC
ENSG00000149639	GCUGGUG
ENSG00000149639	UACAGUU
ENSG00000149657	AAGGUGC
ENSG00000149657	AAGUGCA
ENSG00000149657	AGCACCA
ENSG00000149657	AGUGGUU
ENSG00000149657	CUACAAA
ENSG00000149657	GUAAACA
ENSG00000149658	AAAGCUG
ENSG00000149658	ACUCCAG
ENSG00000149658	AGUAACA
ENSG00000149658	AUACAAG
ENSG00000149658	CAGCAAA
ENSG00000149658	CUACAGU
ENSG00000149658	CUUGUAU
ENSG00000149658	GUAACAG
ENSG00000149658	UAGAGGA
ENSG00000149679	ACCACAG
ENSG00000149679	CAGCAGA
ENSG00000149679	GGCAGCG
ENSG00000149679	GGUUUAC
ENSG00000149679	UAUUGCU
ENSG00000149679	UCACAGU
ENSG00000149679	UGCAAAG
ENSG00000149679	UUGGUUC
ENSG00000149761	AUAAAGU
ENSG00000149792	AAAAACC
ENSG00000149792	AGCUUAU
ENSG00000149792	UAAUAGU
ENSG00000149792	UGCAUUG
ENSG00000149792	UGUAUAA
ENSG00000149798	ACCAGCA
ENSG00000149798	AGCAGCA
ENSG00000149798	AUUGCAC
ENSG00000149798	UAUUGCU
ENSG00000149922	GUGCCUG
ENSG00000149923	CACAGUG
ENSG00000149925	GGAGUGU
ENSG00000149926	AGCACCA
ENSG00000149926	GGAAGCC
ENSG00000149927	ACACACU
ENSG00000149927	GGGGCAG

ENSG00000149927	GUAAACA
ENSG00000149927	UGCAUAG
ENSG00000149927	UGGGUAC
ENSG00000149930	CUGCCCC
ENSG00000149930	GAGAUGA
ENSG00000149930	GCACCAU
ENSG00000149930	UUGACAC
ENSG00000149932	CCCUGAG
ENSG00000149948	AAAGUAC
ENSG00000149948	AAAGUGC
ENSG00000149948	AACAGUC
ENSG00000149948	AAUGCCC
ENSG00000149948	ACUUGGA
ENSG00000149948	AGCAGCA
ENSG00000149948	AGCAGCG
ENSG00000149948	AGCAGCU
ENSG00000149948	AUUGCAC
ENSG00000149948	CAGCAAA
ENSG00000149948	CAGUGCA
ENSG00000149948	CUACAAA
ENSG00000149948	CUCCCAA
ENSG00000149948	GAUUGUC
ENSG00000149948	GUCCAGA
ENSG00000149948	UCAACAG
ENSG00000149948	UCACAUU
ENSG00000149948	UCCCACA
ENSG00000149948	UCUGGAG
ENSG00000149948	UUAUGCA
ENSG00000149972	ACACACU
ENSG00000149972	UGCAUAU
ENSG00000149972	UUGACAC
ENSG00000150051	AAAGUAA
ENSG00000150051	AAGGCAC
ENSG00000150051	CCCACGU
ENSG00000150051	UCACAUU
ENSG00000150051	UCAUACA
ENSG00000150054	GGAAUGU
ENSG00000150054	UAUAAAG
ENSG00000150076	CAGCAAA
ENSG00000150086	ACUCCAG
ENSG00000150086	CCAGGAG
ENSG00000150086	CCCACCU
ENSG00000150086	GGGAACG
ENSG00000150086	UCACCAC
ENSG00000150093	AGCACCA
ENSG00000150093	UCUCAAG
ENSG00000150281	AAAACUG
ENSG00000150281	UACAAAG
ENSG00000150316	AAACUGU
ENSG00000150316	GAAAACA
ENSG00000150316	UAUUACC
ENSG00000150347	AAGGCAC
ENSG00000150347	AGUGCAA
ENSG00000150347	AUUGCAC
ENSG00000150347	CAAAGCA
ENSG00000150347	CUAUACA
ENSG00000150347	CUCAAAC
ENSG00000150347	GCAACUU
ENSG00000150347	GUAGUGU
ENSG00000150347	UUGGCAC
ENSG00000150394	UCUAGAG
ENSG00000150394	UUUUUGC
ENSG00000150401	AAAAAUC
ENSG00000150401	AAGGCAC

ENSG00000150401	CUCAGGG
ENSG00000150401	GAGGUAG
ENSG00000150401	UGUAUAA
ENSG00000150403	AAGGCAC
ENSG00000150455	AGUGUGC
ENSG00000150455	CUGGGGG
ENSG00000150455	GGAGUCC
ENSG00000150455	GUAGUGU
ENSG00000150457	AAUACUG
ENSG00000150457	AGCAGCU
ENSG00000150457	GAGAAAU
ENSG00000150457	GCAGCAU
ENSG00000150457	GUAAACA
ENSG00000150457	GUGCCAC
ENSG00000150459	CAUAUUG
ENSG00000150459	UUCUUGU
ENSG00000150471	GCAGAAG
ENSG00000150471	UAAUACA
ENSG00000150471	UCUGGAG
ENSG00000150477	CAAGAGC
ENSG00000150477	UGAAAGG
ENSG00000150527	AAAACUG
ENSG00000150527	AAACUGU
ENSG00000150527	AGCAAAA
ENSG00000150527	AGUAACA
ENSG00000150527	CAGUUAC
ENSG00000150527	CCAUUAC
ENSG00000150527	GUAACAG
ENSG00000150540	CAGGAAC
ENSG00000150540	CAGUAGA
ENSG00000150540	GUCAGUU
ENSG00000150540	UGGAUAA
ENSG00000150593	AAAGCUG
ENSG00000150593	AAAGGUA
ENSG00000150593	AACACUG
ENSG00000150593	AAGACAU
ENSG00000150593	ACAGUAU
ENSG00000150593	ACUUGGA
ENSG00000150593	AGAUGUG
ENSG00000150593	AGCAGCG
ENSG00000150593	AGUGCUU
ENSG00000150593	AUGCCUU
ENSG00000150593	CAAAGCA
ENSG00000150593	CAGACAG
ENSG00000150593	CUACAAA
ENSG00000150593	CUAGUCC
ENSG00000150593	CUGGAAA
ENSG00000150593	CUUACAG
ENSG00000150593	UACAAAG
ENSG00000150593	UAUAAAG
ENSG00000150593	UCAACAG
ENSG00000150593	UCACAUU
ENSG00000150593	UCAUUUG
ENSG00000150593	UCCCUGU
ENSG00000150593	UGAAAGG
ENSG00000150594	ACUGUUU
ENSG00000150594	AUGGCUU
ENSG00000150594	CACAGGG
ENSG00000150594	CACCAGC
ENSG00000150594	GGCAGGG
ENSG00000150594	UCCCUGU
ENSG00000150594	UGCAAAG
ENSG00000150594	UGCAUUG
ENSG00000150594	UUCAAGC

ENSG00000150594	UUGAUCA
ENSG00000150625	AACUCUA
ENSG00000150625	ACUGCAG
ENSG00000150625	AUUGCAC
ENSG00000150625	CAAAGCA
ENSG00000150625	CUACAAA
ENSG00000150625	GCUACAU
ENSG00000150625	UCCCUUU
ENSG00000150625	UGCUCAC
ENSG00000150625	UUCUUGU
ENSG00000150625	UUGGCAC
ENSG00000150630	AUAAUAC
ENSG00000150630	UUGAUCA
ENSG00000150672	AAAAAUC
ENSG00000150672	AAAGCUG
ENSG00000150672	AAUUAUU
ENSG00000150672	ACAUUCA
ENSG00000150672	ACUCCAG
ENSG00000150672	AGCACCA
ENSG00000150672	AGCCCUU
ENSG00000150672	AGUGCUG
ENSG00000150672	AUAAAGU
ENSG00000150672	AUGGCUU
ENSG00000150672	CACAGUG
ENSG00000150672	CCAGCAU
ENSG00000150672	CUACAAA
ENSG00000150672	CUCUUCC
ENSG00000150672	CUGGGCA
ENSG00000150672	GAAACAU
ENSG00000150672	GAGCCCU
ENSG00000150672	GAUUGUA
ENSG00000150672	GAUUGUC
ENSG00000150672	GCAACUU
ENSG00000150672	GCACCAU
ENSG00000150672	GCUACAU
ENSG00000150672	GCUGUCU
ENSG00000150672	GGAAUGU
ENSG00000150672	GGAGUGU
ENSG00000150672	GGUGGUU
ENSG00000150672	GUAAACA
ENSG00000150672	GUGCUUG
ENSG00000150672	GUGUGGC
ENSG00000150672	UACAAAG
ENSG00000150672	UCUCACC
ENSG00000150672	UGACUGU
ENSG00000150672	UGUGCUU
ENSG00000150672	UGUGUCA
ENSG00000150672	UUGAUCA
ENSG00000150712	AAAAACC
ENSG00000150712	AAAUUUC
ENSG00000150712	AACCUGG
ENSG00000150712	AAGGCAC
ENSG00000150712	AAUCACU
ENSG00000150712	ACAAUAU
ENSG00000150712	ACAGUAU
ENSG00000150712	ACUUGGA
ENSG00000150712	AGUGCAA
ENSG00000150712	AGUGCUG
ENSG00000150712	AGUGCUU
ENSG00000150712	AUAUAAC
ENSG00000150712	CAGCAGA
ENSG00000150712	CUUGGAG
ENSG00000150712	GAGGUAG
ENSG00000150712	GGAAUGU

ENSG00000150712	GUGCAAA
ENSG00000150712	GUGCCUG
ENSG00000150712	GUGCUUG
ENSG00000150712	UGCAUAU
ENSG00000150760	GGCAAGA
ENSG00000150764	AAAGAAU
ENSG00000150764	AAAGCUG
ENSG00000150764	AACACUG
ENSG00000150764	AAGAACC
ENSG00000150764	ACAGUAU
ENSG00000150764	AGCAAAA
ENSG00000150764	AGCAGCG
ENSG00000150764	AGCAGCU
ENSG00000150764	GAGAAAU
ENSG00000150764	GCUGUCU
ENSG00000150764	GGCAGUG
ENSG00000150764	UCCCACA
ENSG00000150764	UCUCCAA
ENSG00000150764	UGCAAAG
ENSG00000150764	UGUAUAA
ENSG00000150764	UUCCAUA
ENSG00000150764	UUCUAAU
ENSG00000150764	UUGGCAA
ENSG00000150768	CUGGACU
ENSG00000150776	AAUCUCA
ENSG00000150776	AGCACCA
ENSG00000150776	AGGUAGU
ENSG00000150776	CAAAGCA
ENSG00000150776	CCCUGAG
ENSG00000150776	CUCUUCC
ENSG00000150776	GAGAAGA
ENSG00000150776	GAGGUAG
ENSG00000150776	GGAAUGU
ENSG00000150776	UGAGAAG
ENSG00000150776	UGUGCUU
ENSG00000150782	AAUUUUA
ENSG00000150893	AAUACUG
ENSG00000150893	CUAGGCU
ENSG00000150893	GAGGUAG
ENSG00000150893	GAGGUAU
ENSG00000150893	GGCUCAG
ENSG00000150893	GUCUUGC
ENSG00000150893	UGUCUUU
ENSG00000150907	AAAAAUC
ENSG00000150907	AACAAAC
ENSG00000150907	AACAGUC
ENSG00000150907	AACCUGG
ENSG00000150907	AACCUGU
ENSG00000150907	AAUGUUG
ENSG00000150907	ACAGUAU
ENSG00000150907	AGCAGCA
ENSG00000150907	AGUAGAC
ENSG00000150907	AUAUUAU
ENSG00000150907	AUGGCAC
ENSG00000150907	CAGACAG
ENSG00000150907	CUACAGU
ENSG00000150907	CUUUGGU
ENSG00000150907	GCAUCCC
ENSG00000150907	GCUGUCU
ENSG00000150907	GUAACAG
ENSG00000150907	GUAGUGU
ENSG00000150907	UAGUGAG
ENSG00000150907	UAUAAUA
ENSG00000150907	UCCAGUU

ENSG00000150907	UCUGGAA
ENSG00000150907	UGCAUAG
ENSG00000150907	UGUGUCA
ENSG00000150907	UUGGCAA
ENSG00000150907	UUGUGUC
ENSG00000150907	UUUGCAA
ENSG00000150938	AAAAGUA
ENSG00000150938	AAAGAGG
ENSG00000150938	AAAGUAA
ENSG00000150938	AAAGUGC
ENSG00000150938	AAGGUGC
ENSG00000150938	AAGUCAC
ENSG00000150938	AAGUGCA
ENSG00000150938	ACAUUCA
ENSG00000150938	CAGUAGU
ENSG00000150938	GGAAUGU
ENSG00000150938	GUGCUUG
ENSG00000150938	UACAAAG
ENSG00000150967	ACUGGCU
ENSG00000150967	AGGUAGU
ENSG00000150967	GAGGUAG
ENSG00000150967	GCAGGAC
ENSG00000150967	UCACAGG
ENSG00000150967	UCACAGU
ENSG00000150977	CUGCAGC
ENSG00000150995	AGUGCAA
ENSG00000150995	UCAAGUA
ENSG00000151012	CAGUGCA
ENSG00000151012	CCAGUGC
ENSG00000151012	GUCUUGC
ENSG00000151012	UCUCCAA
ENSG00000151012	UGCAUUG
ENSG00000151012	UGUCUUU
ENSG00000151012	UUCCUAG
ENSG00000151014	AGUGCAA
ENSG00000151014	CUGGAAA
ENSG00000151014	GCUGGAU
ENSG00000151014	GUGCAAA
ENSG00000151014	UAAGACG
ENSG00000151025	AAGUUGU
ENSG00000151025	ACAGUAU
ENSG00000151025	AUGCAUU
ENSG00000151025	AUUGCAC
ENSG00000151025	GGCAGUG
ENSG00000151025	UAUUGCU
ENSG00000151025	UUGUGUC
ENSG00000151033	UCUCCAA
ENSG00000151062	CUGGACU
ENSG00000151062	UGCUGAC
ENSG00000151067	AAAAAUC
ENSG00000151067	AAAACUG
ENSG00000151067	AAAGCUG
ENSG00000151067	AAAGGUA
ENSG00000151067	AAAUUUC
ENSG00000151067	AACCUGU
ENSG00000151067	AAGUGCA
ENSG00000151067	AAUACUG
ENSG00000151067	AAUGCCC
ENSG00000151067	ACAUUAC
ENSG00000151067	ACCCAUU
ENSG00000151067	ACUCCAG
ENSG00000151067	AGUGGUU
ENSG00000151067	AUAAUAC
ENSG00000151067	AUAUUAU

ENSG00000151067	AUUCAUU
ENSG00000151067	AUUGCAC
ENSG00000151067	CACCACC
ENSG00000151067	CAGCAAA
ENSG00000151067	CCCCCAG
ENSG00000151067	CCGGUUC
ENSG00000151067	CCUUCUU
ENSG00000151067	CUGGCUC
ENSG00000151067	CUUGGAG
ENSG00000151067	GACCUGG
ENSG00000151067	GACUUCC
ENSG00000151067	GAGAAGA
ENSG00000151067	GAGAUGA
ENSG00000151067	GCAGCAU
ENSG00000151067	GCCUGGA
ENSG00000151067	GGCCCUG
ENSG00000151067	GUAAACA
ENSG00000151067	UAAUACA
ENSG00000151067	UAAUGCU
ENSG00000151067	UACAGUU
ENSG00000151067	UAUUGCU
ENSG00000151067	UCAACAG
ENSG00000151067	UCAAGUA
ENSG00000151067	UCACACA
ENSG00000151067	UCCCUGU
ENSG00000151067	UGAAGCU
ENSG00000151067	UGAGAAG
ENSG00000151067	UGCAAAG
ENSG00000151067	UGCAGCU
ENSG00000151067	UGCAUAG
ENSG00000151067	UGCAUAU
ENSG00000151067	UGGGACA
ENSG00000151067	UUGGCAC
ENSG00000151067	UUUGCAC
ENSG00000151079	AAGGCAC
ENSG00000151079	ACUCAGC
ENSG00000151079	AGGGAGG
ENSG00000151079	CAAAGCA
ENSG00000151079	GAGGUAG
ENSG00000151079	UGCAGCU
ENSG00000151079	UGCAUAU
ENSG00000151090	AAAGUAC
ENSG00000151090	AAAGUAU
ENSG00000151090	AAAGUGC
ENSG00000151090	AAAUCCC
ENSG00000151090	AAAUUUC
ENSG00000151090	AAGGCAC
ENSG00000151090	AAUCCUU
ENSG00000151090	ACACACC
ENSG00000151090	ACAGUAC
ENSG00000151090	ACUGUUU
ENSG00000151090	AGCAAAA
ENSG00000151090	AGUGCUU
ENSG00000151090	AUACAAG
ENSG00000151090	AUAUUAU
ENSG00000151090	AUGGCUU
ENSG00000151090	AUUCAUU
ENSG00000151090	CAAAUGC
ENSG00000151090	CACACCG
ENSG00000151090	CACAGUG
ENSG00000151090	CAGUAGU
ENSG00000151090	CAUGGGU
ENSG00000151090	CUGCAGG
ENSG00000151090	CUGGAAA

ENSG00000151090	CUUGUGU
ENSG00000151090	GAUUGUA
ENSG00000151090	GCAGCAU
ENSG00000151090	GCCCUGU
ENSG00000151090	GCUACAU
ENSG00000151090	GGAAUGU
ENSG00000151090	GGGAGCU
ENSG00000151090	GUCUUGC
ENSG00000151090	GUGCAAA
ENSG00000151090	UACAAAG
ENSG00000151090	UAUUGCU
ENSG00000151090	UCACAGU
ENSG00000151090	UCACAUU
ENSG00000151090	UCUGGAA
ENSG00000151090	UGAAAUG
ENSG00000151090	UGAGGAC
ENSG00000151090	UGCAAAG
ENSG00000151090	UGCCCUG
ENSG00000151090	UGGGUAC
ENSG00000151090	UGUCUUU
ENSG00000151090	UGUGCUU
ENSG00000151090	UUCAAAU
ENSG00000151090	UUUCAAC
ENSG00000151117	AGCACCA
ENSG00000151117	AGUAACA
ENSG00000151117	AUGUGCC
ENSG00000151117	CAAAGCA
ENSG00000151117	CAGACAG
ENSG00000151117	CAGGCUC
ENSG00000151117	CCCUGAG
ENSG00000151117	GCACCAU
ENSG00000151117	GUCAUUG
ENSG00000151117	GUGCCUG
ENSG00000151117	UGAGGAC
ENSG00000151117	UGCAUUG
ENSG00000151117	UUGGCAA
ENSG00000151117	UUGGCAC
ENSG00000151135	AAAGGUA
ENSG00000151135	AACAUUC
ENSG00000151135	AAGGCAC
ENSG00000151135	CCUUCAU
ENSG00000151135	GUAACAG
ENSG00000151136	CAAAUGC
ENSG00000151148	CUGGAAA
ENSG00000151148	GUCAACA
ENSG00000151148	UCUGGAA
ENSG00000151148	UGCAUAG
ENSG00000151150	AAGGCGC
ENSG00000151150	AAGGUGC
ENSG00000151150	ACUGCAU
ENSG00000151150	CAGCAGA
ENSG00000151150	CCAGUAC
ENSG00000151150	CCAGUGC
ENSG00000151150	GAGGCAG
ENSG00000151150	GCAGCAU
ENSG00000151150	GCCCUUA
ENSG00000151150	UGCAAAG
ENSG00000151150	UGCAGCU
ENSG00000151150	UUGGCAA
ENSG00000151208	AAGUGCA
ENSG00000151208	ACAGUAC
ENSG00000151208	AGUGCAA
ENSG00000151208	AUUGCAC
ENSG00000151208	UCCCUUU

ENSG00000151208	UGACCUA
ENSG00000151224	AGCACCA
ENSG00000151224	GGCUCAG
ENSG00000151224	GGGAGCU
ENSG00000151224	UGGCUGC
ENSG00000151229	AAGGCAC
ENSG00000151229	AGCAGCU
ENSG00000151229	GCAGCAU
ENSG00000151229	GGCAGUG
ENSG00000151229	UUCAAGC
ENSG00000151239	AAAGCUG
ENSG00000151239	CAGCAGG
ENSG00000151239	UAUAAAG
ENSG00000151239	UGAAAUG
ENSG00000151239	UUAUGCA
ENSG00000151239	UUCAAGC
ENSG00000151240	AAAGAGC
ENSG00000151240	AAAGAGG
ENSG00000151240	AAAGCUG
ENSG00000151240	AAGGUGC
ENSG00000151240	ACAUUAC
ENSG00000151240	AUAAUAC
ENSG00000151240	AUAUUAU
ENSG00000151240	AUGGCUU
ENSG00000151240	CGUACCG
ENSG00000151240	UAAUACA
ENSG00000151240	UACAAAG
ENSG00000151247	AAAGAAU
ENSG00000151247	AGCAGCA
ENSG00000151247	AGCAGCG
ENSG00000151247	AGCAGCU
ENSG00000151247	AUCACUA
ENSG00000151247	CACUGUU
ENSG00000151247	CAGUCCA
ENSG00000151247	CUCCCAA
ENSG00000151247	CUUCACC
ENSG00000151247	CUUUGGU
ENSG00000151247	UCACACA
ENSG00000151247	UGAAAUG
ENSG00000151276	ACAUUAC
ENSG00000151276	GGCUCAG
ENSG00000151276	UCACAUU
ENSG00000151276	UUGGCAC
ENSG00000151292	ACAUUCA
ENSG00000151292	GAGAUGA
ENSG00000151320	AAGGCAC
ENSG00000151320	AAUCUCU
ENSG00000151320	AAUUAUU
ENSG00000151320	ACUGUGU
ENSG00000151320	AGCUGCC
ENSG00000151320	AUACAAG
ENSG00000151320	AUGGAUU
ENSG00000151320	CACCAGC
ENSG00000151320	CCCCCAG
ENSG00000151320	CUUGGGC
ENSG00000151320	GAAGGAA
ENSG00000151320	GAGGUAG
ENSG00000151320	GAUUGUC
ENSG00000151320	GGCAGUG
ENSG00000151320	GUCAUUG
ENSG00000151320	UCUUGAG
ENSG00000151320	UGCAUAG
ENSG00000151320	UGGGACA
ENSG00000151320	UUCCUCG

ENSG00000151320	UUCUUGU
ENSG00000151322	AAAGAAU
ENSG00000151322	AAAGUGC
ENSG00000151322	AAGUGCU
ENSG00000151322	AAUUAUU
ENSG00000151322	GUAUUCU
ENSG00000151322	UCACAGG
ENSG00000151322	UUGAUCA
ENSG00000151327	AAAGUAU
ENSG00000151327	AAUUUUA
ENSG00000151327	AGUGCUU
ENSG00000151327	CACAGUG
ENSG00000151327	UGCAUAG
ENSG00000151327	UGCAUAU
ENSG00000151338	AAUCACU
ENSG00000151338	ACUGUUU
ENSG00000151338	AGAUGUG
ENSG00000151338	AGUGGUU
ENSG00000151338	CAAAGUG
ENSG00000151338	CACCAGC
ENSG00000151338	CAGCAAA
ENSG00000151338	CAGCAGA
ENSG00000151338	CAGCAGG
ENSG00000151338	CUGGAAA
ENSG00000151338	GAAGGAA
ENSG00000151338	GUGCAAA
ENSG00000151338	UCUGGAG
ENSG00000151338	UGAAGUG
ENSG00000151348	AGCCAGU
ENSG00000151413	AAUUUUA
ENSG00000151413	UUGUGAC
ENSG00000151413	UUUCAAC
ENSG00000151458	AACACUG
ENSG00000151458	AAGGUGC
ENSG00000151458	ACUGCAU
ENSG00000151458	AGUGCUU
ENSG00000151458	AUGCACC
ENSG00000151458	GGAUUUU
ENSG00000151458	GGCAGCG
ENSG00000151458	UACAGUU
ENSG00000151458	UCAAGUA
ENSG00000151458	UUCAAAU
ENSG00000151461	AAUUGCU
ENSG00000151461	AGAUGUG
ENSG00000151461	GAGAAGA
ENSG00000151461	UGAAGCU
ENSG00000151468	UAGACUG
ENSG00000151468	UCCCUUG
ENSG00000151474	AAAACUG
ENSG00000151474	AAAAGUA
ENSG00000151474	AAAGUGC
ENSG00000151474	AACCUGU
ENSG00000151474	AAGGCAC
ENSG00000151474	AAGUGUG
ENSG00000151474	AAUCUCU
ENSG00000151474	ACAAUAU
ENSG00000151474	ACUGUUU
ENSG00000151474	AGCACCA
ENSG00000151474	AGUGCUG
ENSG00000151474	AGUGCUU
ENSG00000151474	AUACAAG
ENSG00000151474	AUGGCUU
ENSG00000151474	AUGUAAU
ENSG00000151474	CACAGUG

ENSG00000151474	CCAGGAG
ENSG00000151474	CUCACAC
ENSG00000151474	CUUUGGU
ENSG00000151474	GACAACU
ENSG00000151474	GAGGCAG
ENSG00000151474	GCUGGUG
ENSG00000151474	GGCAGCG
ENSG00000151474	GGCAGUG
ENSG00000151474	GGGAGCU
ENSG00000151474	GGGUAAG
ENSG00000151474	GUAAACA
ENSG00000151474	GUCCAGA
ENSG00000151474	GUGAGGU
ENSG00000151474	UAUAAUA
ENSG00000151474	UCAUUUG
ENSG00000151474	UCCCUUG
ENSG00000151474	UGACAUC
ENSG00000151474	UGCAAAG
ENSG00000151474	UGCAUAU
ENSG00000151474	UGUGCUU
ENSG00000151474	UUGAGGC
ENSG00000151474	UUGGCAA
ENSG00000151474	UUGGCAC
ENSG00000151474	UUUGCAC
ENSG00000151490	AAAGUGC
ENSG00000151490	AAGUCUU
ENSG00000151490	ACUGUAG
ENSG00000151490	GAGGUAG
ENSG00000151490	UGCUCAC
ENSG00000151490	UGGCCAC
ENSG00000151491	AAUGCCC
ENSG00000151491	AUUGCAC
ENSG00000151491	CUGACUC
ENSG00000151491	CUUACAG
ENSG00000151491	GGCAUUG
ENSG00000151491	UAUUGCU
ENSG00000151491	UCCCUUG
ENSG00000151491	UCCCUUU
ENSG00000151502	AAAGAAU
ENSG00000151502	AACACUG
ENSG00000151502	ACUGCAG
ENSG00000151502	CUGCAGC
ENSG00000151502	GAAACAU
ENSG00000151502	GAGGUAG
ENSG00000151502	GGAGGCA
ENSG00000151502	UGCAGCU
ENSG00000151502	UUGGCAA
ENSG00000151502	UUGGCAC
ENSG00000151503	UAUAAAG
ENSG00000151514	AACAGUC
ENSG00000151514	CUACAAA
ENSG00000151532	AAAACUG
ENSG00000151532	AAAGAAU
ENSG00000151532	AAAUUUC
ENSG00000151532	AACAGUC
ENSG00000151532	AAGACAU
ENSG00000151532	AAUCACU
ENSG00000151532	AAUCUCU
ENSG00000151532	AAUUAUU
ENSG00000151532	AAUUGCU
ENSG00000151532	ACUCAGC
ENSG00000151532	ACUGUUU
ENSG00000151532	AGCAGCA
ENSG00000151532	AGCCCUU

ENSG00000151532	AGCCUGC
ENSG00000151532	AUUCAUU
ENSG00000151532	CAGCAGG
ENSG00000151532	CCAUUAC
ENSG00000151532	CUCAGGG
ENSG00000151532	GAAAACA
ENSG00000151532	GAGUUGG
ENSG00000151532	GCCCUUA
ENSG00000151532	GGUGGGC
ENSG00000151532	GUCAGUU
ENSG00000151532	GUGGGGA
ENSG00000151532	UAAUGCU
ENSG00000151532	UCACCAC
ENSG00000151532	UGAAGCU
ENSG00000151532	UGUCUUU
ENSG00000151532	UUCAAAU
ENSG00000151532	UUGUUCG
ENSG00000151552	AAGGCAC
ENSG00000151553	AAAGUGC
ENSG00000151553	AAAUGGU
ENSG00000151553	AAUCACU
ENSG00000151553	AGCAGCA
ENSG00000151553	AGGCAGG
ENSG00000151553	AUCACUA
ENSG00000151553	GAUUGUC
ENSG00000151553	GGAGGCA
ENSG00000151553	UCCUGAG
ENSG00000151553	UGCCCUG
ENSG00000151576	CAAAUGC
ENSG00000151576	UAAUAUC
ENSG00000151576	UCAACAG
ENSG00000151612	ACUCCAG
ENSG00000151612	AGCUGCC
ENSG00000151612	CACAGUG
ENSG00000151612	CAGCUGG
ENSG00000151612	CUCCAGC
ENSG00000151612	CUUGUAU
ENSG00000151612	CUUGUGU
ENSG00000151612	GAAGGUC
ENSG00000151612	GAUUGUC
ENSG00000151612	GCAGAAG
ENSG00000151612	GCAGGUU
ENSG00000151612	GGAAUGU
ENSG00000151612	GGGUCUU
ENSG00000151612	GUAGUGU
ENSG00000151612	UCACAGU
ENSG00000151612	UGCAGCU
ENSG00000151612	UUCUUGU
ENSG00000151615	AUGUAAU
ENSG00000151615	CAGCAGG
ENSG00000151617	AAAUGGU
ENSG00000151617	CAGUCUG
ENSG00000151617	UCACAGU
ENSG00000151617	UCCUAUA
ENSG00000151617	UGAAAUG
ENSG00000151617	UUGGCAC
ENSG00000151623	AAACGGU
ENSG00000151623	AAUUAUU
ENSG00000151623	AGUACUG
ENSG00000151623	CUUACAG
ENSG00000151623	GUAAACA
ENSG00000151623	UACAAAG
ENSG00000151623	UCCCUUG
ENSG00000151623	UCCCUUU

ENSG00000151623	UGGGACA
ENSG00000151640	AGCAGCU
ENSG00000151657	AACAAAC
ENSG00000151657	AUACAAG
ENSG00000151657	CAGUAGA
ENSG00000151657	UUCAAAU
ENSG00000151657	UUUCAAC
ENSG00000151692	AAGGCAC
ENSG00000151692	ACCUGGA
ENSG00000151692	AGGAUGU
ENSG00000151692	AGUGCUU
ENSG00000151692	AUGGCUU
ENSG00000151692	CCAGCAU
ENSG00000151692	CCCUGAG
ENSG00000151692	CUUUGGU
ENSG00000151692	GCUGGUG
ENSG00000151692	GGGGCAG
ENSG00000151692	GGUUUAC
ENSG00000151692	GUAUUCU
ENSG00000151692	UAGUGAG
ENSG00000151692	UCUCAAG
ENSG00000151692	UGAAAUG
ENSG00000151692	UGCAUAU
ENSG00000151692	UUCAAAU
ENSG00000151692	UUCUAAU
ENSG00000151692	UUGACAC
ENSG00000151694	ACCACAG
ENSG00000151694	ACCAGCA
ENSG00000151694	CAGUGCA
ENSG00000151694	CCAGCAU
ENSG00000151694	GCACCAU
ENSG00000151694	UACAAAG
ENSG00000151694	UACAGUU
ENSG00000151702	UUGUGAC
ENSG00000151704	AGCAGCU
ENSG00000151715	AAAGUAC
ENSG00000151718	AAAGUGC
ENSG00000151718	AACCUGG
ENSG00000151718	AACCUGU
ENSG00000151718	AAGUGCA
ENSG00000151718	ACAUUAC
ENSG00000151718	ACCCUGU
ENSG00000151718	AGCUGCC
ENSG00000151718	AUACAAG
ENSG00000151718	CAGUCCA
ENSG00000151718	CCUUCAU
ENSG00000151718	CUAGGCU
ENSG00000151718	GAAAACA
ENSG00000151718	GAAACAU
ENSG00000151718	GUGAAUG
ENSG00000151718	GUUCAUG
ENSG00000151718	UAUAAAG
ENSG00000151718	UCACAUU
ENSG00000151718	UCCAGUU
ENSG00000151718	UCCUGAG
ENSG00000151718	UGACCUA
ENSG00000151718	UUGUGAC
ENSG00000151718	UUGUUCG
ENSG00000151726	AACAUUC
ENSG00000151726	AAGGCAC
ENSG00000151726	ACAUUCA
ENSG00000151726	CAAAACU
ENSG00000151726	GCAGAAG
ENSG00000151726	GGCAGUG

ENSG00000151726	GGUUUAC
ENSG00000151726	GUGCAAA
ENSG00000151726	UAUAAAG
ENSG00000151726	UCCUAUA
ENSG00000151746	GUGCUUG
ENSG00000151746	UGUGCUU
ENSG00000151773	CCUUCUG
ENSG00000151773	GUCAUUG
ENSG00000151778	AAGACAU
ENSG00000151789	AAUUAUU
ENSG00000151789	AUUGCAC
ENSG00000151789	UAUUGCU
ENSG00000151812	GUAAACA
ENSG00000151812	UCACAGG
ENSG00000151812	UCACAGU
ENSG00000151834	ACAUAGA
ENSG00000151835	AAUUGCU
ENSG00000151835	CUUUGGU
ENSG00000151835	UUGGUUC
ENSG00000151846	AAAGUUU
ENSG00000151846	AAUUUUA
ENSG00000151846	CAAAGUG
ENSG00000151846	UAUAAUA
ENSG00000151849	UCAUGAU
ENSG00000151876	AAUUAUU
ENSG00000151881	UGGAUAA
ENSG00000151892	UCACAAG
ENSG00000151893	AAAGUGC
ENSG00000151893	AAGGCAC
ENSG00000151893	AAGUGCA
ENSG00000151893	AAUGCCC
ENSG00000151893	AGCAGCG
ENSG00000151893	AGCAGCU
ENSG00000151893	AGUGCUU
ENSG00000151893	CAAAGUG
ENSG00000151893	CCAGUGC
ENSG00000151893	CUGCCCC
ENSG00000151893	GAGUUGG
ENSG00000151893	GAUUGUC
ENSG00000151893	UACAAAG
ENSG00000151893	UCCUGAG
ENSG00000151893	UUGGUCC
ENSG00000151914	CUGCAGC
ENSG00000151914	CUGCAGG
ENSG00000151914	GAGGUAU
ENSG00000151923	AUACAAG
ENSG00000151923	CCUUCAU
ENSG00000151923	CUAUACA
ENSG00000151923	CUUCACC
ENSG00000151923	GAAUUGU
ENSG00000151923	UCACCAC
ENSG00000151952	AUAUUAU
ENSG00000151952	CUUACAG
ENSG00000151952	UACAGUU
ENSG00000151952	UAUAAAG
ENSG00000151952	UGAUCCA
ENSG00000151967	AACAUUC
ENSG00000151967	ACAUUCA
ENSG00000151967	ACCACAG
ENSG00000151967	ACUGUAG
ENSG00000151967	AGCACCA
ENSG00000151967	AGGCAGG
ENSG00000151967	AGGUAGU
ENSG00000151967	AGUACUG

ENSG00000151967	CUACAGU
ENSG00000151967	GAGGCAG
ENSG00000151967	GUCAACA
ENSG00000151967	UGUUGAA
ENSG00000152049	AUACAAG
ENSG00000152049	CCCCUGG
ENSG00000152049	GCACCAU
ENSG00000152049	GUGACAG
ENSG00000152049	UCAAGUA
ENSG00000152056	AAAGUAA
ENSG00000152056	AUCCCUU
ENSG00000152056	CAAAACU
ENSG00000152056	CAGCAAA
ENSG00000152056	CAGCAGA
ENSG00000152056	CAGCAGG
ENSG00000152056	GAAAACA
ENSG00000152056	UACAAAG
ENSG00000152056	UCAAGUA
ENSG00000152056	UUCAAAU
ENSG00000152056	UUGGCAA
ENSG00000152056	UUGGCAC
ENSG00000152056	UUGGUUC
ENSG00000152092	ACAAUAU
ENSG00000152092	AUACCUC
ENSG00000152092	CAAGAGC
ENSG00000152092	CCAUUAC
ENSG00000152092	CCUUCUU
ENSG00000152092	CUCUGGG
ENSG00000152092	CUGGGCA
ENSG00000152092	GCUUCCU
ENSG00000152092	GGCAGCG
ENSG00000152092	UAAUAGU
ENSG00000152092	UAAUGCU
ENSG00000152092	UCCCUGU
ENSG00000152092	UUGGCAA
ENSG00000152092	UUGGCAC
ENSG00000152102	AAAACUG
ENSG00000152102	AAAAGUA
ENSG00000152102	AAAGUAA
ENSG00000152102	AAAGUAU
ENSG00000152102	AAGUGCA
ENSG00000152102	AAGUGCU
ENSG00000152102	AAGUUGU
ENSG00000152102	ACAAUAU
ENSG00000152102	ACUGGCU
ENSG00000152102	AGCACCA
ENSG00000152102	AGUGAUC
ENSG00000152102	AUACAAG
ENSG00000152102	AUGAUGC
ENSG00000152102	AUGCAUU
ENSG00000152102	AUGCCUU
ENSG00000152102	CAGUCCA
ENSG00000152102	CUGCAGC
ENSG00000152102	CUGGCUC
ENSG00000152102	GAAAACA
ENSG00000152102	GCAGGUU
ENSG00000152102	GGAAUGU
ENSG00000152102	GGAGGCA
ENSG00000152102	GGAUUUC
ENSG00000152102	UAAGACG
ENSG00000152102	UAGUGAG
ENSG00000152102	UAUAAAG
ENSG00000152102	UCCCUUG
ENSG00000152102	UGAUCCA

ENSG00000152102	UUUGCAA
ENSG00000152102	UUUGCAC
ENSG00000152128	GUGCUUG
ENSG00000152128	UGCAAAG
ENSG00000152128	UUGGCAC
ENSG00000152137	UGAAAUG
ENSG00000152137	UUGGUUC
ENSG00000152154	UGCAUAG
ENSG00000152154	UGUGCUU
ENSG00000152192	AACACUG
ENSG00000152192	AAGUGUG
ENSG00000152192	ACAAUAU
ENSG00000152192	AGUAGAC
ENSG00000152192	AGUGCUG
ENSG00000152192	AGUGCUU
ENSG00000152192	CAGUCUG
ENSG00000152192	CCCUGUU
ENSG00000152192	CUAUACA
ENSG00000152192	GUCUCUG
ENSG00000152192	GUGCAAA
ENSG00000152192	UAGACUG
ENSG00000152192	UGCAUAG
ENSG00000152192	UGGGACA
ENSG00000152192	UUCAAAU
ENSG00000152192	UUGGUCC
ENSG00000152193	ACAUAGA
ENSG00000152193	AGUGCUU
ENSG00000152193	AUGACAC
ENSG00000152193	GUGCUUG
ENSG00000152193	UCACAUU
ENSG00000152207	UGGAGAU
ENSG00000152214	AAUUUUA
ENSG00000152217	GGCAUUG
ENSG00000152223	AAGGCAC
ENSG00000152223	ACUCAAA
ENSG00000152223	CAGCAAA
ENSG00000152223	CAGCAGG
ENSG00000152223	GAGAUGA
ENSG00000152223	GCUUCCU
ENSG00000152223	GGCUCUG
ENSG00000152223	GUCACUC
ENSG00000152223	UCACAUU
ENSG00000152223	UGAGAAG
ENSG00000152223	UGAGAAU
ENSG00000152229	AAAUCCC
ENSG00000152229	CAAAGUG
ENSG00000152229	UCCUGAG
ENSG00000152229	UUUGCAA
ENSG00000152242	AACACUG
ENSG00000152242	AACAGUC
ENSG00000152242	AAGUGCA
ENSG00000152242	AAUGCCC
ENSG00000152242	AAUUAUU
ENSG00000152242	ACUCCAG
ENSG00000152242	ACUGCAU
ENSG00000152242	AGCAAAA
ENSG00000152242	AUCACUA
ENSG00000152242	AUCAUAC
ENSG00000152242	AUUGCAC
ENSG00000152242	CAAAUGC
ENSG00000152242	CACAGUG
ENSG00000152242	CAGCAGA
ENSG00000152242	CCAGCAU
ENSG00000152242	CCAGUGC

ENSG00000152242	CCAGUGU
ENSG00000152242	CUCCAGC
ENSG00000152242	CUGGAAA
ENSG00000152242	CUUUGGU
ENSG00000152242	GAAGGUC
ENSG00000152242	GCACCAU
ENSG00000152242	GCAGAAG
ENSG00000152242	GGGUCUU
ENSG00000152242	UCAAGUA
ENSG00000152242	UCAUACA
ENSG00000152242	UCAUGAU
ENSG00000152242	UGAAGCU
ENSG00000152242	UGCAUUG
ENSG00000152242	UUAUGCA
ENSG00000152242	UUGGUUC
ENSG00000152254	UGAGAAG
ENSG00000152256	AAUCCUU
ENSG00000152256	CAGUGCA
ENSG00000152256	UCACAGU
ENSG00000152266	AAAGAAU
ENSG00000152266	GCAGCAU
ENSG00000152268	ACAAGGU
ENSG00000152268	CUUUGGU
ENSG00000152268	GAAAACA
ENSG00000152268	UGUUGAA
ENSG00000152270	AAAGUGC
ENSG00000152270	AAGUGCA
ENSG00000152270	ACAGUAU
ENSG00000152270	AUGCACC
ENSG00000152270	CACAGUG
ENSG00000152270	GCAGCAU
ENSG00000152270	UCACAGU
ENSG00000152270	UCCUGAG
ENSG00000152270	UGCAUAG
ENSG00000152284	AACAGUC
ENSG00000152284	AAUUUUA
ENSG00000152284	ACACACC
ENSG00000152284	ACACACU
ENSG00000152284	AGGGUCU
ENSG00000152284	CAGUCCA
ENSG00000152284	CUGGGCA
ENSG00000152284	GCAACUU
ENSG00000152284	GUCCAGA
ENSG00000152284	UCCAGAG
ENSG00000152284	UCCCUUU
ENSG00000152284	UUGUGAC
ENSG00000152291	AAAGUGC
ENSG00000152291	AAGUGCA
ENSG00000152291	ACAAGGU
ENSG00000152291	AGGGAGG
ENSG00000152291	AGUGCAA
ENSG00000152291	CAAAGUG
ENSG00000152291	CACAGUG
ENSG00000152291	CCAGCAU
ENSG00000152291	CUUUGGU
ENSG00000152291	GCAGGUU
ENSG00000152291	GUGCAAA
ENSG00000152291	UCCCUGU
ENSG00000152291	UCCCUUU
ENSG00000152291	UUGGUCC
ENSG00000152332	AAAAAUC
ENSG00000152332	ACUGCAG
ENSG00000152332	CUACAGU
ENSG00000152332	GGAAGAC

ENSG00000152332	GUGCAAA
ENSG00000152348	AUCCUUG
ENSG00000152359	AACAUUC
ENSG00000152377	AAGUCUU
ENSG00000152377	AGCCCUU
ENSG00000152377	AGCCUGC
ENSG00000152377	AUGGCUU
ENSG00000152377	CUGCAGG
ENSG00000152377	CUUCUCU
ENSG00000152377	GACCUGG
ENSG00000152377	GGAAGCC
ENSG00000152377	GUUAAUG
ENSG00000152377	UAAUGCU
ENSG00000152377	UACAGUU
ENSG00000152377	UGAGAAG
ENSG00000152377	UGAGAAU
ENSG00000152377	UGGUACC
ENSG00000152377	UUCCUAG
ENSG00000152402	CUGGGCA
ENSG00000152402	GAGUCUU
ENSG00000152402	GCCUGAU
ENSG00000152402	GUGCUUG
ENSG00000152402	UAUUGCU
ENSG00000152402	UUGAUCA
ENSG00000152409	AUUGCAC
ENSG00000152409	GGAAGAC
ENSG00000152413	AGCUGCC
ENSG00000152413	CACUCCU
ENSG00000152413	UCAAGUA
ENSG00000152428	AGGUAGU
ENSG00000152430	AGCUGCC
ENSG00000152430	AGCUUAU
ENSG00000152430	AGUAACA
ENSG00000152430	GAGCUGC
ENSG00000152430	GCUGGAU
ENSG00000152430	GGAAUGU
ENSG00000152430	UCACAAG
ENSG00000152430	UGAAGCU
ENSG00000152455	UGGCCAC
ENSG00000152465	AAAAACC
ENSG00000152465	AAAGUAC
ENSG00000152465	AACAGUC
ENSG00000152465	ACAUUCA
ENSG00000152465	AGCAAAA
ENSG00000152465	AGUAACA
ENSG00000152465	AUGAUGA
ENSG00000152465	AUGUGCC
ENSG00000152465	CAAAGUG
ENSG00000152465	CUCCCAC
ENSG00000152465	CUUUGGU
ENSG00000152465	GAAGGAA
ENSG00000152465	GAGAAGA
ENSG00000152465	GCAGCAU
ENSG00000152465	GGAAUGU
ENSG00000152465	GGCAGUG
ENSG00000152465	GUGCAAA
ENSG00000152465	GUGCUUG
ENSG00000152465	UACAAAG
ENSG00000152465	UCUAGAG
ENSG00000152465	UUCAAAU
ENSG00000152465	UUGGCAA
ENSG00000152465	UUUGCAA
ENSG00000152484	GUGCAAA
ENSG00000152495	AUGCAUU

ENSG00000152495	UGCAUUG
ENSG00000152503	AAAGCUG
ENSG00000152503	GUCUACU
ENSG00000152518	AGCUUAU
ENSG00000152518	GCUGGUG
ENSG00000152518	GGAAUGU
ENSG00000152518	GGUGGUU
ENSG00000152518	UCCAGAG
ENSG00000152520	AAAGAAU
ENSG00000152520	AAGGCAC
ENSG00000152520	AAGUCUU
ENSG00000152520	AAGUGCA
ENSG00000152520	AAUACUG
ENSG00000152520	AGUAACA
ENSG00000152520	CCAGUGU
ENSG00000152520	CUUCACC
ENSG00000152520	GGCGACA
ENSG00000152520	GUAACAG
ENSG00000152520	GUCUCUG
ENSG00000152520	GUGACUG
ENSG00000152520	GUGCUUG
ENSG00000152520	UAUAAAG
ENSG00000152520	UCACAGG
ENSG00000152520	UCACAGU
ENSG00000152520	UCCUGAG
ENSG00000152520	UGAAGUG
ENSG00000152520	UGUGCUU
ENSG00000152520	UUGGUCC
ENSG00000152527	AACAUUC
ENSG00000152527	AUGUGGG
ENSG00000152527	CAAAGCA
ENSG00000152527	CAAAGUG
ENSG00000152527	CAGCAAA
ENSG00000152527	CAGGCUC
ENSG00000152527	UCACAGU
ENSG00000152527	UCACAUU
ENSG00000152527	UCAUGAU
ENSG00000152527	UGAAAGG
ENSG00000152558	AAAGUGC
ENSG00000152558	AAGACAU
ENSG00000152558	AAGUGCU
ENSG00000152558	AGCAGCU
ENSG00000152558	CACAGUG
ENSG00000152558	CCAGUGC
ENSG00000152558	CCAGUGU
ENSG00000152558	CCCUGAG
ENSG00000152558	GACCCUG
ENSG00000152558	UCACAGU
ENSG00000152558	UCCAGUU
ENSG00000152558	UUGGUCC
ENSG00000152578	AAAGAAU
ENSG00000152578	AAAUUUC
ENSG00000152578	AAGAACC
ENSG00000152578	AAUUAUU
ENSG00000152578	ACAAUAU
ENSG00000152578	AGCAAAA
ENSG00000152578	AGUGAUC
ENSG00000152578	AUUGCAC
ENSG00000152578	CAGUUAC
ENSG00000152578	CCGAGCC
ENSG00000152578	CUUACAG
ENSG00000152578	GAAGGUC
ENSG00000152578	GAAUUGU
ENSG00000152578	GCAGCAU

ENSG00000152578	GGGUUUA
ENSG00000152578	GGUGGGC
ENSG00000152578	GUGCUUG
ENSG00000152578	GUUAAUG
ENSG00000152578	UACAGUU
ENSG00000152578	UAGUGAG
ENSG00000152578	UAUUACC
ENSG00000152578	UAUUGCU
ENSG00000152578	UCACAGG
ENSG00000152578	UGGGUAC
ENSG00000152578	UGUGCUU
ENSG00000152578	UUAUGCA
ENSG00000152578	UUGGUUC
ENSG00000152578	UUUUUGC
ENSG00000152592	GAGGUAG
ENSG00000152592	GAGGUAU
ENSG00000152592	UACAAAG
ENSG00000152592	UAUUACC
ENSG00000152601	AAAGUAA
ENSG00000152601	AACAAAC
ENSG00000152601	AACAUUC
ENSG00000152601	AACUCUA
ENSG00000152601	AAGGUGC
ENSG00000152601	AAGUCAC
ENSG00000152601	AAUUAUU
ENSG00000152601	ACAUUCA
ENSG00000152601	ACCCGUA
ENSG00000152601	ACUCAAA
ENSG00000152601	AGAUGUG
ENSG00000152601	AGCUUAU
ENSG00000152601	AUCCUUU
ENSG00000152601	AUGUAAU
ENSG00000152601	CAAGAGC
ENSG00000152601	CAGUAGA
ENSG00000152601	CUUACAG
ENSG00000152601	CUUUGGU
ENSG00000152601	GAAGGAA
ENSG00000152601	GACCUGG
ENSG00000152601	GCCUGAU
ENSG00000152601	GGAGUGU
ENSG00000152601	GGUCUAG
ENSG00000152601	GUAAACA
ENSG00000152601	GUCAGUU
ENSG00000152601	GUCUGCC
ENSG00000152601	GUGCAAA
ENSG00000152601	GUGCUUG
ENSG00000152601	UAAUAUC
ENSG00000152601	UAUAAUA
ENSG00000152601	UCAUGAU
ENSG00000152601	UCCCUUG
ENSG00000152601	UCCCUUU
ENSG00000152601	UCUCAAG
ENSG00000152601	UGAGAAU
ENSG00000152601	UGAUCCA
ENSG00000152601	UGUCUGC
ENSG00000152601	UGUCUGG
ENSG00000152601	UUAUGCA
ENSG00000152601	UUCUAAU
ENSG00000152601	UUGGCAC
ENSG00000152601	UUGUUCG
ENSG00000152620	AAAGAAU
ENSG00000152620	AUAAAGU
ENSG00000152620	UAUAAAG
ENSG00000152642	ACAGGUG

ENSG00000152642	AGCCCUU
ENSG00000152642	AGUGGUU
ENSG00000152642	CAGCAGA
ENSG00000152642	GGUGGUU
ENSG00000152642	GUGCUUG
ENSG00000152642	UAAUGCU
ENSG00000152642	UCACAGG
ENSG00000152642	UCACAGU
ENSG00000152642	UGUGCUU
ENSG00000152661	AACUGGU
ENSG00000152661	ACAGUAU
ENSG00000152661	GUAAACA
ENSG00000152661	UACAGUU
ENSG00000152661	UUCAAGC
ENSG00000152683	CUACAAA
ENSG00000152683	UCAUGAU
ENSG00000152683	UGCAAAG
ENSG00000152700	AUGAUGA
ENSG00000152749	AACAAAC
ENSG00000152749	AAUUAUU
ENSG00000152749	AUCCUUG
ENSG00000152749	AUCCUUU
ENSG00000152749	CAUGGGU
ENSG00000152749	CCAGUGC
ENSG00000152749	CCAGUGU
ENSG00000152749	CUUGGGC
ENSG00000152749	GUUAAUG
ENSG00000152749	UAUUGCU
ENSG00000152749	UGAAAUG
ENSG00000152749	UUUCAAC
ENSG00000152760	AAAGUGC
ENSG00000152760	AAGUGCA
ENSG00000152760	AAUUGCU
ENSG00000152760	CAAGAGC
ENSG00000152760	CUGCUCA
ENSG00000152760	GCCCUGU
ENSG00000152760	UGAAAGG
ENSG00000152760	UGACCUA
ENSG00000152763	UUCUAAU
ENSG00000152766	ACUGGCU
ENSG00000152766	CUGGGGG
ENSG00000152766	GGAUUUU
ENSG00000152767	AAAAGUA
ENSG00000152767	AAGGCAC
ENSG00000152767	ACUGGCC
ENSG00000152767	ACUGGCU
ENSG00000152767	AGCAAAA
ENSG00000152767	GAGGUAG
ENSG00000152767	GUGAGGU
ENSG00000152767	UCAUUUG
ENSG00000152767	UCCCUGU
ENSG00000152767	UCCCUUG
ENSG00000152767	UUGGCAA
ENSG00000152767	UUGGUCC
ENSG00000152778	AAAACUG
ENSG00000152778	UUCCUAG
ENSG00000152779	AAAGUGC
ENSG00000152779	AAGUGCU
ENSG00000152779	CCUGCUG
ENSG00000152779	GAAAACA
ENSG00000152779	GAGACCU
ENSG00000152782	ACGUCAU
ENSG00000152782	AGCAGCA
ENSG00000152782	AUGGCUU

ENSG00000152782	CAAAACU
ENSG00000152782	CACAGUG
ENSG00000152782	CAGCAAA
ENSG00000152782	CAGCAGG
ENSG00000152782	GAUCAGA
ENSG00000152782	GGAUUUU
ENSG00000152782	UCACAGG
ENSG00000152784	CAAAUGC
ENSG00000152784	CGUGCAU
ENSG00000152784	UAUAAAG
ENSG00000152784	UAUAUGA
ENSG00000152785	AAAGCUG
ENSG00000152785	AAGGCAC
ENSG00000152785	AGCUUAU
ENSG00000152785	AUGCAUU
ENSG00000152785	CACAGUG
ENSG00000152785	UCACAGG
ENSG00000152785	UCCUGAG
ENSG00000152795	AAAAACC
ENSG00000152795	AAGUCUU
ENSG00000152795	GUCAACA
ENSG00000152795	UACAGUU
ENSG00000152795	UCUCCAA
ENSG00000152795	UGUAUAA
ENSG00000152804	CAGUGCA
ENSG00000152804	CUACAAA
ENSG00000152804	GUCAGUU
ENSG00000152818	AUACAAG
ENSG00000152818	GAGGUAU
ENSG00000152822	ACAUUAC
ENSG00000152822	ACAUUCA
ENSG00000152822	ACUGCAG
ENSG00000152822	AUACAAG
ENSG00000152822	CUACAGU
ENSG00000152822	GUGCUUG
ENSG00000152822	UUGACAC
ENSG00000152822	UUGGCAC
ENSG00000152894	AUUGCAC
ENSG00000152894	CUUUGGU
ENSG00000152894	GUGCUUG
ENSG00000152894	UGAUCCA
ENSG00000152894	UGUGCUU
ENSG00000152904	AAAAAUC
ENSG00000152904	UGGAUAA
ENSG00000152936	UUGUGAC
ENSG00000152944	CAAGAGC
ENSG00000152952	UUGGCAA
ENSG00000152953	ACUCAGC
ENSG00000152953	GAGGGGC
ENSG00000152954	AAAAAUC
ENSG00000152954	AAGUGCA
ENSG00000152954	AGCACCA
ENSG00000152954	AGUGCAA
ENSG00000152954	AUGGCUU
ENSG00000152969	AAGGCAC
ENSG00000152969	AUUGCAC
ENSG00000152969	UGCAAAG
ENSG00000152977	AAACUGU
ENSG00000152977	AAAGUGC
ENSG00000152977	AAAUUUC
ENSG00000152977	ACUAGAU
ENSG00000152977	ACUGCAG
ENSG00000152977	ACUGCAU
ENSG00000152977	ACUGUAG

ENSG00000152977	AUACAAG
ENSG00000152977	AUAUAAC
ENSG00000152977	AUCACUA
ENSG00000152977	CAGCAGG
ENSG00000152977	CUACAGU
ENSG00000152977	CUGGAAA
ENSG00000152977	GCAGGUU
ENSG00000152977	GGUUGUC
ENSG00000152977	GUAACAG
ENSG00000152977	GUCUGCC
ENSG00000152977	UAAUACA
ENSG00000152977	UAGACUG
ENSG00000152977	UCAUUUG
ENSG00000152977	UCUUGAG
ENSG00000152977	UGCAAAG
ENSG00000152977	UGGGACA
ENSG00000152977	UGGUACC
ENSG00000152977	UGUCUGC
ENSG00000152977	UUAGAGA
ENSG00000152977	UUGACAC
ENSG00000152977	UUUGCAC
ENSG00000152990	GGAAUGU
ENSG00000153006	AAAAGUA
ENSG00000153006	AAAGCUG
ENSG00000153006	AAGGCAC
ENSG00000153006	AAUUGCU
ENSG00000153006	AUCACUA
ENSG00000153006	CAAAGCA
ENSG00000153006	CCAGGAG
ENSG00000153006	CCCUGAG
ENSG00000153006	CUUCUCU
ENSG00000153006	CUUUGGU
ENSG00000153006	UCACAGU
ENSG00000153006	UUGAGGC
ENSG00000153012	AAAAGUA
ENSG00000153012	AGCACCA
ENSG00000153012	CAAAGCA
ENSG00000153012	CCCUGAG
ENSG00000153012	GUCCAGA
ENSG00000153046	AACUACU
ENSG00000153046	ACUGUAG
ENSG00000153046	AGUGGUU
ENSG00000153046	GAGGUAG
ENSG00000153046	GAGGUAU
ENSG00000153046	GAUUGUA
ENSG00000153046	UCACAGG
ENSG00000153046	UCACAGU
ENSG00000153046	UCCCACA
ENSG00000153046	UCUCACC
ENSG00000153048	AAGGCAC
ENSG00000153048	ACUCAGC
ENSG00000153064	UAUAAUA
ENSG00000153071	AAAGUGC
ENSG00000153071	AAGAACC
ENSG00000153071	AAGUGCA
ENSG00000153071	GAGUUGA
ENSG00000153094	AAAGUGC
ENSG00000153094	AAGGCAC
ENSG00000153094	AAGUGCU
ENSG00000153094	ACAGUAC
ENSG00000153094	ACAGUAU
ENSG00000153094	ACUGCAG
ENSG00000153094	AGUGAUC
ENSG00000153094	AGUGCAA

ENSG00000153094	AUACAAG
ENSG00000153094	CAGCAGA
ENSG00000153094	CUGCAGC
ENSG00000153094	GAAACAU
ENSG00000153094	UAGUGAG
ENSG00000153094	UAUUGCU
ENSG00000153094	UCAUAGA
ENSG00000153094	UUCCAUA
ENSG00000153107	UACAAAG
ENSG00000153113	AAAGAAU
ENSG00000153113	AAAGUUU
ENSG00000153113	AAGUCAC
ENSG00000153113	ACAGGUG
ENSG00000153113	ACUGUGU
ENSG00000153113	AUAAAGU
ENSG00000153113	CACCAGC
ENSG00000153113	CAGGUGA
ENSG00000153113	CUUACAG
ENSG00000153113	UACAAAG
ENSG00000153113	UGCAAAG
ENSG00000153130	AUAAAGU
ENSG00000153130	UCACAAG
ENSG00000153130	UUGGUCC
ENSG00000153130	UUUGCAA
ENSG00000153132	AUUGCAC
ENSG00000153132	UAUUGCU
ENSG00000153132	UGCAUUG
ENSG00000153147	GUAAACA
ENSG00000153162	UACAAAG
ENSG00000153162	UUCUAAU
ENSG00000153179	AGGUAGU
ENSG00000153187	AAAUGGU
ENSG00000153187	AACAAAC
ENSG00000153187	AACAGUC
ENSG00000153187	AACAUUC
ENSG00000153187	AAUACUG
ENSG00000153187	ACACACU
ENSG00000153187	ACAGUAC
ENSG00000153187	ACUGGAU
ENSG00000153187	ACUGGCU
ENSG00000153187	AGCCAGU
ENSG00000153187	AGCCCUU
ENSG00000153187	AGCUUAU
ENSG00000153187	AUGAGCU
ENSG00000153187	AUGGCUU
ENSG00000153187	AUUGCAC
ENSG00000153187	CACAAUG
ENSG00000153187	CAGCAGA
ENSG00000153187	CAGCAGG
ENSG00000153187	CCAGUAC
ENSG00000153187	CGUGCAU
ENSG00000153187	CUACAGU
ENSG00000153187	CUCCCAA
ENSG00000153187	CUCCCAC
ENSG00000153187	CUGGGGG
ENSG00000153187	CUUGGGC
ENSG00000153187	CUUGUGU
ENSG00000153187	GAAGGUC
ENSG00000153187	GAGAAAU
ENSG00000153187	GAGUCUU
ENSG00000153187	GCAGAAG
ENSG00000153187	GGAUUUC
ENSG00000153187	GGAUUUU
ENSG00000153187	GUGACAG

ENSG00000153187	GUGCAAA
ENSG00000153187	UACAGUU
ENSG00000153187	UAUAAAG
ENSG00000153187	UAUUGCU
ENSG00000153187	UCGUGCA
ENSG00000153187	UCUCCAA
ENSG00000153187	UGCAAAG
ENSG00000153187	UGGAGAU
ENSG00000153187	UGGGUAC
ENSG00000153187	UGGGUGA
ENSG00000153187	UUCUUGU
ENSG00000153187	UUGGAGG
ENSG00000153201	AAAGGUA
ENSG00000153201	ACCCAUU
ENSG00000153201	ACUGCAG
ENSG00000153201	ACUGCAU
ENSG00000153201	AGGUAGU
ENSG00000153201	AGUAGAC
ENSG00000153201	AUAAAGU
ENSG00000153201	CACAAUG
ENSG00000153201	CACACCG
ENSG00000153201	CACACCU
ENSG00000153201	CAUAUUG
ENSG00000153201	CUACAAA
ENSG00000153201	GACAACU
ENSG00000153201	GAGGUAU
ENSG00000153201	GCUACAU
ENSG00000153201	UAAUACA
ENSG00000153201	UAUAAAG
ENSG00000153201	UAUUGCU
ENSG00000153201	UCACACA
ENSG00000153201	UCCCUUU
ENSG00000153201	UGCAUAG
ENSG00000153201	UUGGUUC
ENSG00000153201	UUUCAAC
ENSG00000153207	AACCUGU
ENSG00000153207	AAGUCUU
ENSG00000153207	ACAAUAU
ENSG00000153207	ACCUGGA
ENSG00000153207	GUGCCUG
ENSG00000153207	UAAUAUC
ENSG00000153207	UCACACA
ENSG00000153214	AGCAGCA
ENSG00000153233	UGUGCUU
ENSG00000153234	AAAGUUU
ENSG00000153234	AACAGUC
ENSG00000153234	AACUACU
ENSG00000153234	AAGUGCU
ENSG00000153234	AAUGUUG
ENSG00000153234	AAUUUUA
ENSG00000153234	ACAUAGA
ENSG00000153234	AGUGCCU
ENSG00000153234	AUGGCAC
ENSG00000153234	CCCACCU
ENSG00000153234	GAGAAAU
ENSG00000153234	GGAAUGU
ENSG00000153234	GGACGGA
ENSG00000153234	GGCAGUG
ENSG00000153234	UAUAAAG
ENSG00000153234	UAUAAUA
ENSG00000153234	UCUGGAA
ENSG00000153250	AAAACUG
ENSG00000153250	AAACUGU
ENSG00000153250	AACACUG

ENSG00000153250	AAGACAU
ENSG00000153250	AAGGCAC
ENSG00000153250	ACAAGGU
ENSG00000153250	CAAAACU
ENSG00000153250	CAGCAAA
ENSG00000153250	GAUUGUA
ENSG00000153250	GAUUGUC
ENSG00000153250	UAUAAAG
ENSG00000153250	UUGAUCA
ENSG00000153253	ACCACAG
ENSG00000153253	GUCAGUU
ENSG00000153253	UACAAAG
ENSG00000153253	UGCAUAG
ENSG00000153253	UGCAUAU
ENSG00000153253	UUGGCAA
ENSG00000153253	UUGGCAC
ENSG00000153283	AUUCAUU
ENSG00000153291	AAAGAGC
ENSG00000153291	AAGUGCU
ENSG00000153291	AGUGCUG
ENSG00000153291	CUGCCCC
ENSG00000153291	GAGGUAG
ENSG00000153310	AAAGCUG
ENSG00000153310	AAGUCAC
ENSG00000153310	AAUACUG
ENSG00000153310	ACAUCAC
ENSG00000153310	ACGCACU
ENSG00000153310	AUCACUA
ENSG00000153310	CAGCAGG
ENSG00000153310	GCAGAAG
ENSG00000153310	UACAAAG
ENSG00000153310	UCAAGUA
ENSG00000153310	UUGGCAA
ENSG00000153339	AAAAAUC
ENSG00000153339	AAAGUAU
ENSG00000153339	AACACUG
ENSG00000153339	AAGUUGU
ENSG00000153339	AAUUAUU
ENSG00000153339	ACAGUAC
ENSG00000153339	AUGGCUU
ENSG00000153339	CACAGUG
ENSG00000153339	CAGCAGA
ENSG00000153339	CUAUACA
ENSG00000153339	UACAGUU
ENSG00000153339	UAUAAAG
ENSG00000153339	UCACAGG
ENSG00000153339	UGUAUAA
ENSG00000153339	UUCAAAU
ENSG00000153339	UUUGCAA
ENSG00000153339	UUUGCAC
ENSG00000153395	CACAGUG
ENSG00000153395	UCACAGG
ENSG00000153395	UGUGCUU
ENSG00000153443	AGCUGCC
ENSG00000153443	GGGGUGC
ENSG00000153485	AAUUUUA
ENSG00000153487	AAGUGCA
ENSG00000153487	AGUGCAA
ENSG00000153531	AGUGUGC
ENSG00000153531	CACACCG
ENSG00000153531	CACACCU
ENSG00000153531	UCACACA
ENSG00000153551	UUGGCAA
ENSG00000153558	CUUUGGU

ENSG00000153558	GACCCUG
ENSG00000153558	UGUGCUU
ENSG00000153558	UUGGUCC
ENSG00000153560	AAAGGUA
ENSG00000153560	AAUGCCC
ENSG00000153560	ACACACU
ENSG00000153560	ACUGGCU
ENSG00000153560	CAGUCUG
ENSG00000153560	CUUCACC
ENSG00000153560	CUUGGAG
ENSG00000153560	GAUUGUA
ENSG00000153560	UGAAAUG
ENSG00000153560	UUAGGAU
ENSG00000153561	AAAGUUU
ENSG00000153561	AAUUAUU
ENSG00000153561	ACUGCAU
ENSG00000153561	AUGCACC
ENSG00000153561	CAGUGCA
ENSG00000153561	CUCACAC
ENSG00000153561	UACAAAG
ENSG00000153561	UGAAAUG
ENSG00000153561	UGCAUUG
ENSG00000153561	UUCUGCA
ENSG00000153561	UUGAUCA
ENSG00000153574	AAGGCAC
ENSG00000153574	UUUUUGC
ENSG00000153575	AAUUUUA
ENSG00000153707	AAAGAGC
ENSG00000153707	AAAGGUA
ENSG00000153707	AACAUUC
ENSG00000153707	AAGGCAC
ENSG00000153707	AAGUGCA
ENSG00000153707	AAGUGCU
ENSG00000153707	AGCACCA
ENSG00000153707	AGUGCAA
ENSG00000153707	AGUGCUU
ENSG00000153707	AUAUUAU
ENSG00000153707	AUGCAUU
ENSG00000153707	AUGGCUU
ENSG00000153707	CACACCU
ENSG00000153707	CAGCAGA
ENSG00000153707	CAGCAGG
ENSG00000153707	CAGUCUG
ENSG00000153707	CAGUGCA
ENSG00000153707	CCCCUGG
ENSG00000153707	CCCUGUC
ENSG00000153707	CCUUCUG
ENSG00000153707	CUGUUGA
ENSG00000153707	CUUCUCU
ENSG00000153707	GAGGUAG
ENSG00000153707	GGAAGCC
ENSG00000153707	GGAUUUC
ENSG00000153707	GGCAGUG
ENSG00000153707	GGGUGUU
ENSG00000153707	GUGUCAC
ENSG00000153707	UAUUGCU
ENSG00000153707	UCCCACA
ENSG00000153707	UCCCUGU
ENSG00000153707	UGCAUAG
ENSG00000153707	UGGAUAA
ENSG00000153707	UGGGUGA
ENSG00000153707	UUGGUCC
ENSG00000153714	AGCAGCU
ENSG00000153714	AUUGCAC

ENSG00000153714	CCCUGAG
ENSG00000153714	GGUUUAC
ENSG00000153721	AACAAAC
ENSG00000153721	AAGGCAC
ENSG00000153721	AUACAAG
ENSG00000153767	AAAGUUU
ENSG00000153767	ACAAGGU
ENSG00000153767	UAAUACA
ENSG00000153767	UAUCAGA
ENSG00000153774	UCACAUU
ENSG00000153786	AAGGCAC
ENSG00000153786	ACAUUCA
ENSG00000153786	AGUGCAA
ENSG00000153786	AUGUAAU
ENSG00000153786	CCCUGAG
ENSG00000153786	CUUUGGU
ENSG00000153786	GGGAGCU
ENSG00000153786	UCCCUGU
ENSG00000153786	UUUGCAA
ENSG00000153789	CCCUGAG
ENSG00000153790	AUCCUUU
ENSG00000153790	GAGGCUG
ENSG00000153790	UUCUUGU
ENSG00000153814	AAAGUGC
ENSG00000153814	AACAUUC
ENSG00000153814	AAGGCAC
ENSG00000153814	AAGUGCU
ENSG00000153814	AGCACCA
ENSG00000153814	AGGGUUG
ENSG00000153814	AGUGCUU
ENSG00000153814	AUAUUAU
ENSG00000153814	CUAUACA
ENSG00000153814	GAGAACU
ENSG00000153814	UCACAUU
ENSG00000153814	UUGGCAC
ENSG00000153820	UUUCAAC
ENSG00000153823	AAGGCAC
ENSG00000153823	AUGCAUU
ENSG00000153823	GGCAGUG
ENSG00000153823	GUGAAUG
ENSG00000153823	UUAUGCA
ENSG00000153823	UUGGCAA
ENSG00000153823	UUGGCAC
ENSG00000153827	AUCCCUU
ENSG00000153827	CAAAGCA
ENSG00000153827	CAGCAAA
ENSG00000153827	GCCUGAU
ENSG00000153879	AAGGCAC
ENSG00000153879	AAUCUCA
ENSG00000153879	CACACCG
ENSG00000153879	CAGGAAC
ENSG00000153879	CAGUGCA
ENSG00000153879	CUCACAC
ENSG00000153879	GAGGCAG
ENSG00000153879	UCAAGUA
ENSG00000153885	AAUACUG
ENSG00000153885	ACUGUUU
ENSG00000153885	AGAUAAA
ENSG00000153885	AGCACCA
ENSG00000153885	AUGGCAC
ENSG00000153885	CCUGCUG
ENSG00000153885	CUACAGU
ENSG00000153885	CUAGUCC
ENSG00000153885	CUCGCUG

ENSG00000153885	CUCUGGG
ENSG00000153885	CUGGGCA
ENSG00000153885	GAGAACU
ENSG00000153885	GGAAGCC
ENSG00000153885	GGACCUG
ENSG00000153885	GGAGGAG
ENSG00000153885	GGAGUCC
ENSG00000153885	GGCUGCG
ENSG00000153885	GGGGUGG
ENSG00000153885	GGUGGGC
ENSG00000153885	UCCUGAG
ENSG00000153885	UCUGGAA
ENSG00000153885	UGCUGAC
ENSG00000153885	UUGGUCC
ENSG00000153898	AAAUUUC
ENSG00000153902	CAGCAGA
ENSG00000153904	AAAGUAC
ENSG00000153904	ACAGUAC
ENSG00000153904	CACAGUG
ENSG00000153904	CAGUAGA
ENSG00000153904	GAUUGUC
ENSG00000153904	GCAACUU
ENSG00000153904	GCAGAAG
ENSG00000153904	UGGAGAU
ENSG00000153904	UUGGCAA
ENSG00000153914	AGCAGCU
ENSG00000153914	AUGCAUU
ENSG00000153914	CUAUACA
ENSG00000153914	GAGGUAG
ENSG00000153914	GAGGUAU
ENSG00000153914	GAUUGUC
ENSG00000153914	GGAUUUC
ENSG00000153914	GGAUUUU
ENSG00000153914	UAUCAGA
ENSG00000153914	UGUGCUU
ENSG00000153922	AAGGCAC
ENSG00000153922	AAUACUG
ENSG00000153922	ACAUUCA
ENSG00000153922	AGUAACA
ENSG00000153922	AUGGCUU
ENSG00000153922	CAGUGCA
ENSG00000153922	CUGGAAA
ENSG00000153922	GAAACAU
ENSG00000153922	GGCAGUG
ENSG00000153922	GGCUGCG
ENSG00000153922	UAUUGCU
ENSG00000153922	UCAAGUA
ENSG00000153922	UGACAUC
ENSG00000153922	UGCAUAG
ENSG00000153922	UGGCUGC
ENSG00000153922	UUGACAC
ENSG00000153936	AAAGCUG
ENSG00000153936	AAAUGGU
ENSG00000153936	AAGGCAC
ENSG00000153936	AAUACUG
ENSG00000153936	ACAGGUG
ENSG00000153936	ACAUUCA
ENSG00000153936	ACCACAG
ENSG00000153936	AGAUGUG
ENSG00000153936	AUGCAUU
ENSG00000153936	CAAAGUG
ENSG00000153936	CACACCU
ENSG00000153936	CAGCAGG
ENSG00000153936	CAGUAGA

ENSG00000153936	CUACAGU
ENSG00000153936	GAGGUAG
ENSG00000153936	GAGGUAU
ENSG00000153936	GGAGUCC
ENSG00000153936	GGCAGUG
ENSG00000153936	GUGACAG
ENSG00000153936	GUGAGGU
ENSG00000153936	GUGCAAA
ENSG00000153936	UACAAAG
ENSG00000153936	UCAAGUA
ENSG00000153936	UCACAGG
ENSG00000153936	UCCCACA
ENSG00000153936	UCCCACC
ENSG00000153936	UCUCCAA
ENSG00000153936	UGCUCAC
ENSG00000153936	UGUUGAA
ENSG00000153936	UUCAAGC
ENSG00000153944	AUGCCUU
ENSG00000153944	CUCACAC
ENSG00000153944	CUUACAG
ENSG00000153944	GAGAAGA
ENSG00000153944	GUAAACA
ENSG00000153944	UCACAAG
ENSG00000153944	UCACCAC
ENSG00000153944	UCAUAGA
ENSG00000153944	UCUCACC
ENSG00000153944	UGAGAAG
ENSG00000153944	UUGUGAC
ENSG00000153956	AGCAUUC
ENSG00000153956	AUGAUGC
ENSG00000153982	AAAAAUC
ENSG00000153982	ACAUUCA
ENSG00000153982	ACUGCAU
ENSG00000153982	AUGGCUU
ENSG00000153982	GAGGUAG
ENSG00000153982	GAGGUAU
ENSG00000153982	UACAAAG
ENSG00000153982	UCUGGAG
ENSG00000153989	AAAAACC
ENSG00000153989	AAAGAAU
ENSG00000153989	AAAGUAA
ENSG00000153989	AGUGCAA
ENSG00000153989	AUGCACC
ENSG00000153989	CACUGUU
ENSG00000153989	CCAGUGC
ENSG00000153989	GUGCAAA
ENSG00000153989	UGCAAAG
ENSG00000153989	UGUUGAA
ENSG00000153989	UUCAAGC
ENSG00000153989	UUGGCAA
ENSG00000153989	UUGGUCC
ENSG00000153993	GAGAAAU
ENSG00000153993	GUGAGGU
ENSG00000153993	UCACACA
ENSG00000154001	AAAGUAA
ENSG00000154001	AAAGUUU
ENSG00000154001	AACAGUC
ENSG00000154001	AAUCCUU
ENSG00000154001	ACAGUAC
ENSG00000154001	ACAGUAU
ENSG00000154001	ACAUUCA
ENSG00000154001	ACUCAGC
ENSG00000154001	ACUGUAG
ENSG00000154001	AGAUGUG

ENSG00000154001	AGCAUUC
ENSG00000154001	AGUAGUU
ENSG00000154001	AGUGCUG
ENSG00000154001	AUCCUUU
ENSG00000154001	AUGCACC
ENSG00000154001	CAGUAGA
ENSG00000154001	GAAACAU
ENSG00000154001	GUUAGGA
ENSG00000154001	UAAUACA
ENSG00000154001	UGAAGUG
ENSG00000154001	UGAGAAG
ENSG00000154001	UUAGAGA
ENSG00000154001	UUGGUCC
ENSG00000154027	UCCAGAG
ENSG00000154059	AAGGCAC
ENSG00000154059	GAAACAU
ENSG00000154059	GGAUUUU
ENSG00000154059	UCUAGAG
ENSG00000154059	UGAAAGG
ENSG00000154059	UUCAAAU
ENSG00000154096	GCAGCAU
ENSG00000154096	UCCAGAG
ENSG00000154096	UCUGGAA
ENSG00000154118	AAAAAUC
ENSG00000154118	AAACUGU
ENSG00000154118	AAAGGUA
ENSG00000154118	ACAUUAC
ENSG00000154118	ACUUGGA
ENSG00000154118	AGGCAGG
ENSG00000154118	AUGCAUU
ENSG00000154118	CAAAGCA
ENSG00000154118	CAGUGCA
ENSG00000154118	CCAGUGU
ENSG00000154118	GGCAGUG
ENSG00000154118	UCCCUUG
ENSG00000154118	UGCAUAU
ENSG00000154118	UUCAAGC
ENSG00000154118	UUUUUGC
ENSG00000154122	AAAGUAC
ENSG00000154122	AAAGUAU
ENSG00000154122	AAUACUG
ENSG00000154122	ACUGCAG
ENSG00000154122	ACUGCAU
ENSG00000154122	CAAAACU
ENSG00000154122	CCCUGAG
ENSG00000154122	CCUGCUG
ENSG00000154122	CUUUGGU
ENSG00000154122	GAUUGUC
ENSG00000154122	GGAUUUC
ENSG00000154122	GGAUUUU
ENSG00000154122	GGCAUUG
ENSG00000154122	UAUAAAG
ENSG00000154124	AACAGUC
ENSG00000154124	AGCCAGU
ENSG00000154124	CAGUCUG
ENSG00000154124	CUGGGCA
ENSG00000154124	UACAGUU
ENSG00000154124	UCACAGG
ENSG00000154124	UGUGCUU
ENSG00000154127	AAAGAGG
ENSG00000154127	AAAGUAA
ENSG00000154127	AAAGUAC
ENSG00000154127	ACAAUAU
ENSG00000154127	AUUGCAC

ENSG00000154127	CAUAUUG
ENSG00000154127	CCCUGAG
ENSG00000154127	CUCACAC
ENSG00000154127	GCCUGGG
ENSG00000154127	GUAUUCU
ENSG00000154127	GUCAUUG
ENSG00000154127	UCACACA
ENSG00000154127	UCCCUGU
ENSG00000154127	UCCUGAG
ENSG00000154127	UGACUGU
ENSG00000154127	UUGGUUC
ENSG00000154134	GAUCAGA
ENSG00000154144	AUCCCUU
ENSG00000154144	CCUUCUU
ENSG00000154146	AGGCAGG
ENSG00000154146	CCAGGAG
ENSG00000154146	CUCUGGG
ENSG00000154146	GCCUGGG
ENSG00000154146	GCGGAGG
ENSG00000154146	GCGGGGC
ENSG00000154146	UCACAUU
ENSG00000154153	AAGGCAC
ENSG00000154162	CUCUUCC
ENSG00000154162	UAUUGCU
ENSG00000154174	AAAGAGG
ENSG00000154174	AAAGCUG
ENSG00000154174	AUGUAAU
ENSG00000154174	GGAAUGU
ENSG00000154174	UACAAAG
ENSG00000154174	UGUCUUU
ENSG00000154174	UUGGUUC
ENSG00000154175	AGGGAGG
ENSG00000154175	AUACAAG
ENSG00000154175	AUUCAUU
ENSG00000154188	UAUAAAG
ENSG00000154217	UCCUGAG
ENSG00000154222	AGCAGCG
ENSG00000154222	AGCAGCU
ENSG00000154222	CUCCCAA
ENSG00000154222	CUCCCAC
ENSG00000154222	GAGCUGC
ENSG00000154222	GCAGCAU
ENSG00000154222	GUGACAG
ENSG00000154222	UCCCACA
ENSG00000154222	UCCCACC
ENSG00000154222	UCCUUGC
ENSG00000154222	UGUGCUU
ENSG00000154222	UUAUGCA
ENSG00000154229	AAAGUAC
ENSG00000154229	AAAGUUU
ENSG00000154229	AAGAACC
ENSG00000154229	AAGUCUU
ENSG00000154229	AAUACUG
ENSG00000154229	AAUCUCA
ENSG00000154229	AAUCUCU
ENSG00000154229	ACUGCAG
ENSG00000154229	ACUGUAG
ENSG00000154229	AGGGUUG
ENSG00000154229	AUAAAGU
ENSG00000154229	AUAAUAC
ENSG00000154229	AUGCAUU
ENSG00000154229	AUGGCAC
ENSG00000154229	CCUUCUG
ENSG00000154229	CCUUCUU

ENSG00000154229	CUGCAGC
ENSG00000154229	CUUUGGU
ENSG00000154229	GCUUCCU
ENSG00000154229	GGCUCAG
ENSG00000154229	GGCUCUG
ENSG00000154229	GUCUACU
ENSG00000154229	UAUAAUA
ENSG00000154229	UCAUUUG
ENSG00000154229	UUCCUAG
ENSG00000154229	UUGAUCA
ENSG00000154229	UUUGCAA
ENSG00000154237	AGCAGCG
ENSG00000154237	AGCAGCU
ENSG00000154237	AUUCAUU
ENSG00000154274	CCCUGAG
ENSG00000154274	CUCUGGG
ENSG00000154274	GAUUGUC
ENSG00000154274	UCCCUGU
ENSG00000154274	UGGGUGA
ENSG00000154305	AACAAAC
ENSG00000154305	AAUCCUU
ENSG00000154305	ACAUUAC
ENSG00000154305	CAAAGUG
ENSG00000154305	CUACAAA
ENSG00000154305	GUAACAG
ENSG00000154305	UACAAAG
ENSG00000154319	UAUUGCU
ENSG00000154319	UGACCUA
ENSG00000154319	UUGGCAA
ENSG00000154330	UGGCCCU
ENSG00000154342	AGCAGCG
ENSG00000154342	AGCAGCU
ENSG00000154342	CACAGUG
ENSG00000154342	GCAGCAU
ENSG00000154342	UCACAGG
ENSG00000154342	UCACAGU
ENSG00000154358	GUGAGGU
ENSG00000154359	UGCAAAG
ENSG00000154370	CUUUGGU
ENSG00000154380	AAACUGU
ENSG00000154380	AACACUG
ENSG00000154380	AACAUUC
ENSG00000154380	AAGUGUG
ENSG00000154380	AAUCUCU
ENSG00000154380	AAUUGCU
ENSG00000154380	ACAGUAC
ENSG00000154380	ACAGUAU
ENSG00000154380	ACUCAGC
ENSG00000154380	ACUCAGG
ENSG00000154380	ACUUGGA
ENSG00000154380	AGCACCA
ENSG00000154380	AGCAUUC
ENSG00000154380	AGUGAUC
ENSG00000154380	AGUGUGC
ENSG00000154380	AUGAUGA
ENSG00000154380	AUGCAUU
ENSG00000154380	CAAGAGC
ENSG00000154380	CACCAGC
ENSG00000154380	CCAGUAC
ENSG00000154380	CUCCCAC
ENSG00000154380	CUGCUCA
ENSG00000154380	CUGGACU
ENSG00000154380	GAAAACA
ENSG00000154380	GAGAAAU

ENSG00000154380	GAGCUGC
ENSG00000154380	GAGGUAU
ENSG00000154380	GCAGGUU
ENSG00000154380	GGCAAGA
ENSG00000154380	GUAAACA
ENSG00000154380	GUGACAG
ENSG00000154380	GUGUCAC
ENSG00000154380	UAAGACG
ENSG00000154380	UAAGACU
ENSG00000154380	UCACAGG
ENSG00000154380	UCACAGU
ENSG00000154380	UCACAUU
ENSG00000154380	UCAUAGA
ENSG00000154380	UCAUGAU
ENSG00000154380	UCCCUGU
ENSG00000154380	UCCUGAG
ENSG00000154380	UCUGGAA
ENSG00000154380	UCUGGAG
ENSG00000154380	UGAAAUG
ENSG00000154380	UGAAGCU
ENSG00000154380	UGAGGAC
ENSG00000154380	UGCAUAU
ENSG00000154380	UUAGGAU
ENSG00000154380	UUGUUCG
ENSG00000154429	GAGGUAG
ENSG00000154429	GAUUGUC
ENSG00000154447	AAGGCAC
ENSG00000154447	ACAAGGU
ENSG00000154447	ACAGUAC
ENSG00000154447	UCACAGU
ENSG00000154478	GACCAUG
ENSG00000154478	GAGAAAU
ENSG00000154478	GAUUGUC
ENSG00000154478	UAUUGCU
ENSG00000154478	UGAAAUG
ENSG00000154478	UGCUCAC
ENSG00000154478	UUCAAAU
ENSG00000154493	AAAAAUC
ENSG00000154493	AUGGCUU
ENSG00000154511	AGCAGCU
ENSG00000154511	UCACAGG
ENSG00000154511	UCACAGU
ENSG00000154518	AUGACAC
ENSG00000154518	CACAGUG
ENSG00000154518	CUACAGU
ENSG00000154518	UAAUAUC
ENSG00000154518	UCACAGU
ENSG00000154556	AAAGCUG
ENSG00000154556	AACACUG
ENSG00000154556	AAGUCUU
ENSG00000154620	AUGGCAC
ENSG00000154620	CAGACAG
ENSG00000154640	AAAAACC
ENSG00000154640	AAAAAUC
ENSG00000154640	AAGGUGC
ENSG00000154640	ACAAUAU
ENSG00000154640	ACUCAAA
ENSG00000154640	AUAUAAC
ENSG00000154640	GCAACUU
ENSG00000154640	UGCAUAU
ENSG00000154640	UUUGCAA
ENSG00000154642	AAAGAGC
ENSG00000154642	AAAGAGG
ENSG00000154642	AAAGCUG

ENSG00000154642	AACACUG
ENSG00000154642	AACAGUC
ENSG00000154642	AAGGCAC
ENSG00000154642	AAUUAUU
ENSG00000154642	ACCAGCA
ENSG00000154642	AGCACCA
ENSG00000154642	AGCAUUC
ENSG00000154642	AUAAAGU
ENSG00000154642	AUUCAUU
ENSG00000154642	CUGGAAA
ENSG00000154642	GGAUUUU
ENSG00000154642	GUAAACA
ENSG00000154642	UGCUCAC
ENSG00000154642	UUAUGCA
ENSG00000154642	UUCAAGC
ENSG00000154645	AAAGGUA
ENSG00000154645	AUCCUUU
ENSG00000154645	CUUACAG
ENSG00000154645	GGGAGCU
ENSG00000154645	GGGGAGC
ENSG00000154645	UGCAAAG
ENSG00000154654	UUGGUCC
ENSG00000154655	AAUCUCA
ENSG00000154655	ACAUCAC
ENSG00000154655	AGCACCA
ENSG00000154655	CUUUGGU
ENSG00000154655	GUGCUUG
ENSG00000154655	UCACACA
ENSG00000154655	UGUGCUU
ENSG00000154710	AAACUGU
ENSG00000154710	ACAUAGA
ENSG00000154710	AUGUCUG
ENSG00000154710	CAGCAGA
ENSG00000154710	CCCCUGG
ENSG00000154710	CUUUGGU
ENSG00000154727	AAAGAGC
ENSG00000154727	AAAGAGG
ENSG00000154727	AAGUCUU
ENSG00000154727	AAGUGUG
ENSG00000154727	AAUCUCU
ENSG00000154727	ACCUGGA
ENSG00000154727	AGCAGCA
ENSG00000154727	AUGCACC
ENSG00000154727	CAAAGUG
ENSG00000154727	GAGGUAG
ENSG00000154727	UAAUACA
ENSG00000154727	UCCUGAG
ENSG00000154727	UGACUGU
ENSG00000154727	UGCAAAG
ENSG00000154727	UGUAUAA
ENSG00000154727	UGUGCUU
ENSG00000154734	AGCAGCA
ENSG00000154734	AGCAGCG
ENSG00000154734	AGCAGCU
ENSG00000154734	CCUUCUG
ENSG00000154734	CUAGUAG
ENSG00000154734	GAAGGAA
ENSG00000154734	GAGGUAG
ENSG00000154734	GAGGUAU
ENSG00000154734	GGACCUG
ENSG00000154734	UAUAUGA
ENSG00000154734	UAUUACC
ENSG00000154736	AAACUGU
ENSG00000154736	AAAGUGC

ENSG00000154736	AACAGUC
ENSG00000154736	ACCACAG
ENSG00000154736	ACUGUUU
ENSG00000154736	AGCACCA
ENSG00000154736	AGCAGCA
ENSG00000154736	AGCUGCC
ENSG00000154736	CCAGUAC
ENSG00000154736	CCAGUGC
ENSG00000154736	CUGCCCC
ENSG00000154736	CUUUGGU
ENSG00000154736	GAGGUAG
ENSG00000154736	GAGGUAU
ENSG00000154736	GCUGGUG
ENSG00000154736	GUAAACA
ENSG00000154736	UAAUAGU
ENSG00000154736	UAUAAUA
ENSG00000154736	UCACAGU
ENSG00000154736	UUGGUCC
ENSG00000154764	AGCAGCA
ENSG00000154764	UAAUAUC
ENSG00000154764	UAUUGCU
ENSG00000154767	AGAUAAA
ENSG00000154767	CUGCAGC
ENSG00000154781	UCUGGAG
ENSG00000154783	AAGUGCU
ENSG00000154783	AGUGCUU
ENSG00000154783	AUAAAGU
ENSG00000154803	AGCAAAA
ENSG00000154822	AGUGCAA
ENSG00000154822	CAAAGCA
ENSG00000154822	GUGCAAA
ENSG00000154822	GUGCUUG
ENSG00000154822	UCACAGG
ENSG00000154822	UGCAAAG
ENSG00000154832	AACAAAC
ENSG00000154832	AAUUUUA
ENSG00000154845	ACCAGCA
ENSG00000154845	CAGGAAC
ENSG00000154845	CCAGCAU
ENSG00000154845	GGAAGAC
ENSG00000154845	UCAAGUA
ENSG00000154864	AAGGCAC
ENSG00000154864	AAGUCAC
ENSG00000154864	GUGCUUG
ENSG00000154864	UGAGAAG
ENSG00000154864	UUUUUGC
ENSG00000154914	AUAUUAU
ENSG00000154917	AAAGUAA
ENSG00000154917	AAAGUAU
ENSG00000154917	AACAGUC
ENSG00000154917	AAUUAUU
ENSG00000154917	AAUUUUA
ENSG00000154917	ACACACC
ENSG00000154917	ACACACU
ENSG00000154917	AGCACCA
ENSG00000154917	AGUAACA
ENSG00000154917	AUACAAG
ENSG00000154917	AUAUAAC
ENSG00000154917	AUAUUAU
ENSG00000154917	AUGCAUU
ENSG00000154917	CAAAACU
ENSG00000154917	CACAAUG
ENSG00000154917	CCCUGAG
ENSG00000154917	CUUCACC

ENSG00000154917	GCUUCCU
ENSG00000154917	GGAAGCC
ENSG00000154917	GGCAAGA
ENSG00000154917	GUAACAG
ENSG00000154917	UACAAAG
ENSG00000154917	UCACAAG
ENSG00000154917	UGAGAAG
ENSG00000154917	UGUAUAA
ENSG00000154917	UUGGCAC
ENSG00000154920	CAAAUGC
ENSG00000154928	AAGACAU
ENSG00000154928	CAGCAAA
ENSG00000154928	UUUUUGC
ENSG00000154930	AAAGUAU
ENSG00000154945	AACAUUC
ENSG00000154945	AACCUGG
ENSG00000154945	AACCUGU
ENSG00000154945	AGUAACA
ENSG00000154945	GCACCAU
ENSG00000154945	GUCAACA
ENSG00000154945	UUGGUUC
ENSG00000154975	UACAAAG
ENSG00000155011	ACAUUAC
ENSG00000155011	CACAGUG
ENSG00000155011	CCUGUAC
ENSG00000155011	GCUACAU
ENSG00000155011	UCACAGG
ENSG00000155011	UUGGCAA
ENSG00000155016	CUCUUCC
ENSG00000155016	GAGGCAG
ENSG00000155034	AGCACCA
ENSG00000155034	AGCAGCG
ENSG00000155034	CAAAUGC
ENSG00000155034	GCAGAAG
ENSG00000155034	GCAGCAU
ENSG00000155034	UGCUGAC
ENSG00000155034	UUAGAGA
ENSG00000155052	UCUUGAG
ENSG00000155052	UUCCUAG
ENSG00000155066	CACAGGG
ENSG00000155066	CUUCUCU
ENSG00000155066	UCUGGAA
ENSG00000155066	UCUGGAG
ENSG00000155087	AGCUGCC
ENSG00000155087	GAGCUGC
ENSG00000155090	AAGUGCA
ENSG00000155090	CAAAGUG
ENSG00000155090	UAUAAAG
ENSG00000155093	AGAUAAA
ENSG00000155093	UUGGCAA
ENSG00000155093	UUGGCAC
ENSG00000155096	AAAACUG
ENSG00000155096	AAACUGU
ENSG00000155096	AAUCCUU
ENSG00000155096	ACUGUGU
ENSG00000155096	AGCCCUU
ENSG00000155096	AUACAAG
ENSG00000155096	AUCAUAC
ENSG00000155096	AUGUAAU
ENSG00000155096	AUUGCAC
ENSG00000155096	CUACAGU
ENSG00000155096	UAUUGCU
ENSG00000155096	UCAUACA
ENSG00000155097	AAAGGUA

ENSG00000155097	AACAAAC
ENSG00000155097	ACACACU
ENSG00000155097	ACAUCAC
ENSG00000155097	AUGUCUG
ENSG00000155097	CAAAACU
ENSG00000155097	CUUCUCU
ENSG00000155097	GAUUGUC
ENSG00000155097	GGGUGUU
ENSG00000155097	GUGACAG
ENSG00000155097	UGCAUAG
ENSG00000155097	UUUGCAC
ENSG00000155099	AGCAGCG
ENSG00000155099	AGCAGCU
ENSG00000155099	UAUUGCU
ENSG00000155099	UGCAUAG
ENSG00000155100	AUUCAUU
ENSG00000155100	UCCAGAG
ENSG00000155111	AAAACUG
ENSG00000155111	AAAAGUA
ENSG00000155111	AAAGUUU
ENSG00000155111	AAGUGCU
ENSG00000155111	AAUACUG
ENSG00000155111	CAUAUUG
ENSG00000155111	CCCUGAG
ENSG00000155111	CUAUACA
ENSG00000155111	GAGAAAU
ENSG00000155111	GAUCAGA
ENSG00000155111	GCACCAU
ENSG00000155111	GCAGAAG
ENSG00000155111	GCUGGAU
ENSG00000155111	GCUGGUG
ENSG00000155111	GUCUACU
ENSG00000155111	GUGCAAA
ENSG00000155111	UAUUGCU
ENSG00000155111	UGCAUUG
ENSG00000155111	UUAGGAU
ENSG00000155111	UUGUGUC
ENSG00000155130	AAAAACC
ENSG00000155130	AAAGUAU
ENSG00000155130	AAAGUUU
ENSG00000155130	AAAUGGU
ENSG00000155130	AAGUCAC
ENSG00000155130	ACAGUAU
ENSG00000155130	ACAUUCA
ENSG00000155130	ACCUGGA
ENSG00000155130	ACUGCAG
ENSG00000155130	AGUGUGC
ENSG00000155130	AUACAAG
ENSG00000155130	AUCACUA
ENSG00000155130	CAAAGCA
ENSG00000155130	CACAGUG
ENSG00000155130	CCAGUAC
ENSG00000155130	CCUGCUG
ENSG00000155130	CCUUCUG
ENSG00000155130	CUAUACA
ENSG00000155130	CUCCAGC
ENSG00000155130	CUGGAAA
ENSG00000155130	GAGAUGA
ENSG00000155130	GCACCAU
ENSG00000155130	GCUACAU
ENSG00000155130	GCUGGUG
ENSG00000155130	GGCAGGG
ENSG00000155130	GGGAUCG
ENSG00000155130	GGUGGUC

ENSG00000155130	GGUUGAC
ENSG00000155130	GGUUGUC
ENSG00000155130	GGUUUAC
ENSG00000155130	GUAAACA
ENSG00000155130	GUGCUUG
ENSG00000155130	GUGGGGA
ENSG00000155130	UAAGACU
ENSG00000155130	UAAUAGU
ENSG00000155130	UACAAAG
ENSG00000155130	UAUGGUU
ENSG00000155130	UCACAGU
ENSG00000155130	UCCUAUA
ENSG00000155130	UCUCAAG
ENSG00000155130	UCUGGAA
ENSG00000155130	UCUGGAG
ENSG00000155130	UGCUCAC
ENSG00000155130	UGGGAUC
ENSG00000155130	UGUGCUU
ENSG00000155130	UUGUGUC
ENSG00000155158	ACAAUAU
ENSG00000155189	AAAGAAU
ENSG00000155189	AAAUUUC
ENSG00000155189	AAGGCAC
ENSG00000155189	AUAUAAC
ENSG00000155189	CAGGAAC
ENSG00000155189	CCAGCAU
ENSG00000155189	UCACACA
ENSG00000155189	UCAUGAU
ENSG00000155189	UCUUGAG
ENSG00000155189	UUUGCAC
ENSG00000155229	GAUUGUC
ENSG00000155252	ACAUUCA
ENSG00000155252	AUUCAUU
ENSG00000155252	GAUUGUC
ENSG00000155252	GGGGCAG
ENSG00000155252	GUGCUUG
ENSG00000155252	UGUGCUU
ENSG00000155252	UUGGCAA
ENSG00000155256	CAGGAAC
ENSG00000155304	AAGAACC
ENSG00000155304	AGUGGUU
ENSG00000155304	CUUUGGU
ENSG00000155304	GGCAUUG
ENSG00000155304	UUGGCAA
ENSG00000155304	UUGGCAC
ENSG00000155313	AAAACUG
ENSG00000155313	ACCCUGU
ENSG00000155313	AGCAGCG
ENSG00000155313	AGCAGCU
ENSG00000155313	CAGUCUG
ENSG00000155313	UCAAGUA
ENSG00000155313	UCACAGG
ENSG00000155313	UUUGCAA
ENSG00000155313	UUUGCAC
ENSG00000155324	AGCAGCA
ENSG00000155324	CAGCAGG
ENSG00000155324	UGCUGAC
ENSG00000155329	AAAGUAA
ENSG00000155329	AAGGCAC
ENSG00000155363	GUAAACA
ENSG00000155366	AAGUGCU
ENSG00000155366	AGUGCUG
ENSG00000155366	AUAAAGU
ENSG00000155368	AACAAAC

ENSG00000155380	AGCACCA
ENSG00000155380	UUCUAAU
ENSG00000155465	UGAAGCU
ENSG00000155506	AAAACUG
ENSG00000155506	AACUACU
ENSG00000155506	AAGACAU
ENSG00000155506	AAGGCAC
ENSG00000155506	AAUCACU
ENSG00000155506	AAUGUUG
ENSG00000155506	ACAGUAC
ENSG00000155506	ACCACAG
ENSG00000155506	AGCAGCG
ENSG00000155506	AGCAGCU
ENSG00000155506	AUACAAG
ENSG00000155506	AUAUAAC
ENSG00000155506	AUAUUAU
ENSG00000155506	AUCCUUU
ENSG00000155506	CAGCAGG
ENSG00000155506	CUGGAAA
ENSG00000155506	CUUUGGU
ENSG00000155506	GAAAACA
ENSG00000155506	GAGAAAU
ENSG00000155506	GAGAUGA
ENSG00000155506	GCAACUU
ENSG00000155506	GCAGAAG
ENSG00000155506	GCCUGGA
ENSG00000155506	GCUGGAU
ENSG00000155506	GGGAGCU
ENSG00000155506	GUAAACA
ENSG00000155506	UAAUACA
ENSG00000155506	UCCAGAG
ENSG00000155506	UGCAUAG
ENSG00000155506	UUCCUCG
ENSG00000155506	UUGGUCC
ENSG00000155506	UUGGUUC
ENSG00000155508	AAUACUG
ENSG00000155508	ACCAGCA
ENSG00000155508	AGCACCA
ENSG00000155508	GACAACU
ENSG00000155508	GCUGGUG
ENSG00000155508	UCAUUUG
ENSG00000155508	UCCUGAG
ENSG00000155511	AAAGAGC
ENSG00000155511	AAUCCUU
ENSG00000155511	AUCCUUU
ENSG00000155511	CACAGUG
ENSG00000155511	GAGAAAU
ENSG00000155511	GGAAGCC
ENSG00000155511	UAUUGCU
ENSG00000155511	UGAGAAG
ENSG00000155511	UGAGAAU
ENSG00000155511	UUCUUGU
ENSG00000155511	UUGGCAA
ENSG00000155545	AAAGCUG
ENSG00000155545	AAGUGCA
ENSG00000155545	AAGUGCU
ENSG00000155545	ACAUCAC
ENSG00000155545	AGAUAAA
ENSG00000155545	AGCACCA
ENSG00000155545	AGUGGUU
ENSG00000155545	AUAAAGU
ENSG00000155545	AUUGCAC
ENSG00000155545	CAUGGAU
ENSG00000155545	CCAGUGC

ENSG00000155545	CCAGUGU
ENSG00000155545	CCUUCAU
ENSG00000155545	CUUUGGU
ENSG00000155545	GAAUUGU
ENSG00000155545	GGAUUUU
ENSG00000155545	UCCAGUU
ENSG00000155545	UGCAUAG
ENSG00000155592	AAAAAUC
ENSG00000155592	AAGACAU
ENSG00000155592	AUUCAUU
ENSG00000155592	GAGGUAU
ENSG00000155592	GAUCAGA
ENSG00000155592	GCACCAU
ENSG00000155592	UCACAGU
ENSG00000155592	UCCAGAG
ENSG00000155592	UCUAGAG
ENSG00000155629	AAGACAU
ENSG00000155629	AAUCACU
ENSG00000155629	ACCAGCA
ENSG00000155629	AUCCUUU
ENSG00000155629	CAGCAGG
ENSG00000155629	UAUAAAG
ENSG00000155629	UGGAGAU
ENSG00000155657	ACAGUAC
ENSG00000155657	AUGCCUU
ENSG00000155657	CAAAGUG
ENSG00000155657	CUUACAG
ENSG00000155657	UUGAGGC
ENSG00000155660	AAUUUUA
ENSG00000155666	CCUUCUG
ENSG00000155729	AAAGUUU
ENSG00000155729	AUGCAUU
ENSG00000155744	AAUCUCU
ENSG00000155744	ACAUCAC
ENSG00000155744	AUGGAUU
ENSG00000155744	CACUCCU
ENSG00000155744	GAGGCAG
ENSG00000155744	GGAAGAC
ENSG00000155744	UCACAGU
ENSG00000155744	UGUUGAA
ENSG00000155754	AAAUGGU
ENSG00000155755	AAAAACC
ENSG00000155755	AACACUG
ENSG00000155755	AAUACUG
ENSG00000155755	ACAUUCA
ENSG00000155755	GGAUUUC
ENSG00000155755	GUGACAG
ENSG00000155755	UCAACAG
ENSG00000155755	UGAAAUG
ENSG00000155755	UUCUUGU
ENSG00000155760	AAAGUGC
ENSG00000155760	CAAAUGC
ENSG00000155760	CACAGUG
ENSG00000155760	CUGGAAA
ENSG00000155760	CUGGGCA
ENSG00000155760	GACCCUG
ENSG00000155760	GGAAUGU
ENSG00000155760	UAUGGUU
ENSG00000155760	UCACAGU
ENSG00000155760	UCCAGUU
ENSG00000155760	UGAAGUG
ENSG00000155760	UGGCCCU
ENSG00000155760	UUCAAAU
ENSG00000155760	UUGUGAC

ENSG00000155816	UGAAAUG
ENSG00000155827	GGAAGAC
ENSG00000155846	CAUAUUG
ENSG00000155846	GAAAACA
ENSG00000155846	GAGGUAG
ENSG00000155846	GUAAACA
ENSG00000155849	AUGUCUG
ENSG00000155849	CACAGUG
ENSG00000155849	GUAAACA
ENSG00000155849	GUGCUUG
ENSG00000155849	UAGACUG
ENSG00000155849	UUGGCAC
ENSG00000155849	UUGGUCC
ENSG00000155850	ACUGGCU
ENSG00000155850	UUCAAAU
ENSG00000155858	AGCACCA
ENSG00000155858	GAGGCAG
ENSG00000155858	GAGGUAG
ENSG00000155858	GAGGUAU
ENSG00000155858	GCACCAU
ENSG00000155858	GUGAGGU
ENSG00000155876	AGCAGCA
ENSG00000155876	CAAAGCA
ENSG00000155876	UAUAAAG
ENSG00000155886	AUGGCUU
ENSG00000155893	AAGUGCA
ENSG00000155893	AAGUGCU
ENSG00000155893	AGUGCUG
ENSG00000155893	CAAAGUG
ENSG00000155893	CAGCAGA
ENSG00000155926	GAGAAGA
ENSG00000155926	GAUCAGA
ENSG00000155926	GCUUCCU
ENSG00000155926	GUGUCAC
ENSG00000155926	UAAUGCU
ENSG00000155926	UCCCACC
ENSG00000155926	UUCUUGU
ENSG00000155961	ACUGGAU
ENSG00000155961	ACUGGCU
ENSG00000155961	AUACAAG
ENSG00000155961	AUGGCUU
ENSG00000155961	CAGUGCA
ENSG00000155961	GUAAACA
ENSG00000155961	UACAGUU
ENSG00000155961	UCAUUCG
ENSG00000155961	UGAGAAU
ENSG00000155961	UGGAUAA
ENSG00000155962	AGUGGUU
ENSG00000155966	AAAGAGG
ENSG00000155966	AACAGUC
ENSG00000155966	AAUCCUU
ENSG00000155966	ACCCAUU
ENSG00000155966	ACUGCAU
ENSG00000155966	ACUGGCU
ENSG00000155966	ACUGUAG
ENSG00000155966	AGCACCA
ENSG00000155966	AGGACAC
ENSG00000155966	AGGUAGU
ENSG00000155966	AGUGAUC
ENSG00000155966	CACCAGC
ENSG00000155966	CAGCAAA
ENSG00000155966	CAGGUGA
ENSG00000155966	CCCACCU
ENSG00000155966	CUCAGGG

ENSG00000155966	CUGGCUC
ENSG00000155966	CUGUUGA
ENSG00000155966	CUUCUCU
ENSG00000155966	GACAACU
ENSG00000155966	GACUUCC
ENSG00000155966	GAGAAGA
ENSG00000155966	GAGGUAG
ENSG00000155966	GCAGGUU
ENSG00000155966	GGCAGUG
ENSG00000155966	GGCUCAG
ENSG00000155966	GGGUGUU
ENSG00000155966	GUGCCUG
ENSG00000155966	UCAACAG
ENSG00000155966	UCCAGUU
ENSG00000155966	UCCCACC
ENSG00000155966	UGAGAAG
ENSG00000155966	UGGCCCU
ENSG00000155966	UGGGUGA
ENSG00000155966	UUCCAUA
ENSG00000155966	UUGGUUC
ENSG00000155966	UUUCAAC
ENSG00000155970	CACAGUG
ENSG00000155975	UAUAAAG
ENSG00000155975	UUUGCAA
ENSG00000155980	AAAGUGC
ENSG00000155980	AAGUGCA
ENSG00000155980	ACCACAG
ENSG00000155980	AGCAGCG
ENSG00000155980	GCAGCAU
ENSG00000155980	GCAGGUU
ENSG00000155980	UCCCUCU
ENSG00000155980	UGCAGCU
ENSG00000155984	ACACACC
ENSG00000155984	AGCAAAA
ENSG00000155984	GAGAUGA
ENSG00000156011	AAGGUGC
ENSG00000156011	AAUCCUU
ENSG00000156011	ACAGUAC
ENSG00000156011	ACCAGCA
ENSG00000156011	AUGCACC
ENSG00000156011	AUGGCAC
ENSG00000156011	AUGGCUU
ENSG00000156011	CCAGUAC
ENSG00000156011	CCUUCAU
ENSG00000156011	CUCCAUU
ENSG00000156011	CUGGACU
ENSG00000156011	CUUUGGU
ENSG00000156011	GAAAACA
ENSG00000156011	GAGACCU
ENSG00000156011	GAGAUGA
ENSG00000156011	GGCUCUG
ENSG00000156011	GUAAACA
ENSG00000156011	GUCCAGA
ENSG00000156011	GUCUACU
ENSG00000156011	UAAGACU
ENSG00000156011	UAUAAAG
ENSG00000156011	UCAAGUA
ENSG00000156011	UCCAGUU
ENSG00000156011	UGCAUAU
ENSG00000156011	UUCAAAU
ENSG00000156011	UUCUAAU
ENSG00000156011	UUGGUCC
ENSG00000156017	GUUAGGA
ENSG00000156017	UGUGUCA

ENSG00000156017	UUGUGUC
ENSG00000156026	AAGGCAC
ENSG00000156026	AGCCCUU
ENSG00000156026	GGAUUUU
ENSG00000156030	AAAAAUC
ENSG00000156030	AAAAGUA
ENSG00000156030	AAAGCUG
ENSG00000156030	AACAUUC
ENSG00000156030	AAGGCAC
ENSG00000156030	AAGUAGC
ENSG00000156030	ACAAUAU
ENSG00000156030	ACAUAGA
ENSG00000156030	AGCACCA
ENSG00000156030	AGCAGCA
ENSG00000156030	AGCAGCU
ENSG00000156030	AGGAUGU
ENSG00000156030	AUCCCUU
ENSG00000156030	AUGGCUU
ENSG00000156030	AUGUAAU
ENSG00000156030	CCCCUGG
ENSG00000156030	CCCUGAG
ENSG00000156030	CCUGCUG
ENSG00000156030	CCUGUAC
ENSG00000156030	CCUUCUU
ENSG00000156030	CUCCCAA
ENSG00000156030	CUGCAGC
ENSG00000156030	CUUCUCU
ENSG00000156030	GAAACAU
ENSG00000156030	GAGACCU
ENSG00000156030	GCACCAU
ENSG00000156030	GGCAGUG
ENSG00000156030	GGCUCUG
ENSG00000156030	GUAAACA
ENSG00000156030	GUGCUUG
ENSG00000156030	UAAUAUC
ENSG00000156030	UAUAUGA
ENSG00000156030	UCCAGAG
ENSG00000156030	UCCCUUU
ENSG00000156030	UGAGAAG
ENSG00000156030	UGGGACA
ENSG00000156030	UGUGCUU
ENSG00000156030	UUCAAAU
ENSG00000156050	AAUUUUA
ENSG00000156052	AUGGCAC
ENSG00000156052	UCCCUCU
ENSG00000156097	GAUUGUC
ENSG00000156097	GUCCAGA
ENSG00000156103	AAAGCUG
ENSG00000156103	AACAGUC
ENSG00000156103	AAUACUG
ENSG00000156103	AAUCUCA
ENSG00000156103	AGCAGCU
ENSG00000156103	AUUGCAC
ENSG00000156103	CAUGGAU
ENSG00000156103	CCCACCU
ENSG00000156103	CUCCCAA
ENSG00000156103	CUGACUC
ENSG00000156103	CUGCAGG
ENSG00000156103	CUUCACC
ENSG00000156103	CUUUGGU
ENSG00000156103	GAGAACU
ENSG00000156103	GCAGGAC
ENSG00000156103	GGAAGCC
ENSG00000156103	GGCUCUG

ENSG00000156103	GGGUCUU
ENSG00000156103	GGUGGUC
ENSG00000156103	GUCAGUU
ENSG00000156103	GUUAGGA
ENSG00000156103	UCAAGUA
ENSG00000156103	UCACACA
ENSG00000156103	UCCCACC
ENSG00000156103	UCUCAAG
ENSG00000156103	UGCUGAC
ENSG00000156103	UGGAGAU
ENSG00000156103	UGGCCCU
ENSG00000156103	UGUAUAA
ENSG00000156113	AACAGUC
ENSG00000156113	AAUGUUG
ENSG00000156113	ACACACC
ENSG00000156113	ACAUUCA
ENSG00000156113	ACCACAG
ENSG00000156113	ACCAGCA
ENSG00000156113	ACUGCAG
ENSG00000156113	AGCACCA
ENSG00000156113	AGUAGUU
ENSG00000156113	AGUGAUC
ENSG00000156113	AUGUGGG
ENSG00000156113	CAGGCUC
ENSG00000156113	CAUGGGU
ENSG00000156113	CCAGCAU
ENSG00000156113	CUAUACA
ENSG00000156113	CUGCAGC
ENSG00000156113	CUGCAGG
ENSG00000156113	GAAGGAA
ENSG00000156113	GACCAUG
ENSG00000156113	GCAGGAC
ENSG00000156113	GGAAGCC
ENSG00000156113	GGAUUUU
ENSG00000156113	GGCUCUG
ENSG00000156113	GGGUGGU
ENSG00000156113	GGUGGGC
ENSG00000156113	GGUUGAC
ENSG00000156113	GGUUGUC
ENSG00000156113	GUAUGUU
ENSG00000156113	GUCAACA
ENSG00000156113	GUGCCAC
ENSG00000156113	UAUAAAG
ENSG00000156113	UCACAAG
ENSG00000156113	UCCAGAG
ENSG00000156113	UGAAAGG
ENSG00000156113	UUCAAAU
ENSG00000156113	UUCCAUA
ENSG00000156113	UUUGCAA
ENSG00000156113	UUUGCAC
ENSG00000156140	AUGCAUU
ENSG00000156140	CAGUAGA
ENSG00000156140	GUAGUGU
ENSG00000156140	UGCAUUG
ENSG00000156150	GGAGGCA
ENSG00000156162	AGCAAAA
ENSG00000156162	AGCAGCA
ENSG00000156162	GGCUCUG
ENSG00000156162	UAAUACA
ENSG00000156162	UCAUGAU
ENSG00000156162	UCCAGUU
ENSG00000156172	GGCAGUG
ENSG00000156194	ACAGGUG
ENSG00000156194	AGUGGUU

ENSG00000156194	AUAAAGU
ENSG00000156218	ACAGUAU
ENSG00000156218	ACUGGCU
ENSG00000156218	AGCAGCA
ENSG00000156218	AGUGCUG
ENSG00000156218	AUACAAG
ENSG00000156218	CCAGUGU
ENSG00000156218	CUUCACC
ENSG00000156218	GCAGCAU
ENSG00000156218	GCUGGUG
ENSG00000156218	GUGCAAA
ENSG00000156218	UAUUACC
ENSG00000156253	AAAAAUC
ENSG00000156256	GUCAGUU
ENSG00000156269	UCUCCAA
ENSG00000156273	AAAGAAU
ENSG00000156273	AAGUUGU
ENSG00000156273	ACAGUAC
ENSG00000156273	AGGUAGU
ENSG00000156273	CAAAGCA
ENSG00000156273	CCCUGAG
ENSG00000156273	GAGGUAU
ENSG00000156273	GUAAACA
ENSG00000156273	GUAGUGU
ENSG00000156273	UAUUACC
ENSG00000156273	UCUGGAG
ENSG00000156273	UGACAUC
ENSG00000156273	UGGCCAC
ENSG00000156273	UGUCUUU
ENSG00000156273	UUUGCAC
ENSG00000156284	CCUUCUU
ENSG00000156284	CUCUGGG
ENSG00000156284	GGAUUUC
ENSG00000156284	UAUCAGA
ENSG00000156284	UGCAAAG
ENSG00000156298	ACAAUAU
ENSG00000156298	CACAAUG
ENSG00000156299	AAAGCUG
ENSG00000156299	AAAGUUU
ENSG00000156299	AAGAACC
ENSG00000156299	ACAUAGA
ENSG00000156299	AGCACCA
ENSG00000156299	AGCUGCC
ENSG00000156299	AUGGCAC
ENSG00000156299	CAAAACU
ENSG00000156299	CCCUGUU
ENSG00000156299	CUCACAC
ENSG00000156299	GUGCAAA
ENSG00000156299	GUGUCAC
ENSG00000156299	UACAAAG
ENSG00000156299	UCACACA
ENSG00000156299	UCUCCAA
ENSG00000156299	UCUGGAA
ENSG00000156299	UGACAUC
ENSG00000156299	UGUGUCA
ENSG00000156299	UUCAAAU
ENSG00000156299	UUGACAC
ENSG00000156299	UUGGCAA
ENSG00000156299	UUGGCAC
ENSG00000156304	AAUUGCU
ENSG00000156374	AUGCAUU
ENSG00000156374	CCCUGAG
ENSG00000156374	GCAGAAG
ENSG00000156384	AACAUUC

ENSG00000156384	UCUCAAG
ENSG00000156384	UCUGGAG
ENSG00000156395	AAGUUGU
ENSG00000156395	ACAGUAC
ENSG00000156395	AGGCAGG
ENSG00000156395	AUUGCAC
ENSG00000156395	CAAAACU
ENSG00000156395	GGCAGGG
ENSG00000156395	GUAAACA
ENSG00000156395	GUGACAG
ENSG00000156395	UACAGUU
ENSG00000156395	UCAACAG
ENSG00000156395	UCCAGUU
ENSG00000156395	UGUCUGG
ENSG00000156398	AGGGAGG
ENSG00000156398	GGAGGCA
ENSG00000156398	UCCCUUG
ENSG00000156427	AAAAAUC
ENSG00000156427	CUCUGGG
ENSG00000156427	CUGGGGG
ENSG00000156427	CUUUGGU
ENSG00000156427	GUCAACA
ENSG00000156427	UCAAGUA
ENSG00000156427	UUCAAGC
ENSG00000156453	ACCAGCA
ENSG00000156453	AGCCCUU
ENSG00000156453	AGGCAGG
ENSG00000156453	CAGCAGG
ENSG00000156453	CCAGGAG
ENSG00000156453	CCCACCU
ENSG00000156453	CCCCCAG
ENSG00000156453	CUUUGGU
ENSG00000156453	GACCCUG
ENSG00000156453	GGGGUGC
ENSG00000156463	GAGGCAG
ENSG00000156466	AAAAAUC
ENSG00000156466	AACACUG
ENSG00000156466	AAUUAUU
ENSG00000156466	ACAUUAC
ENSG00000156466	ACUGUAG
ENSG00000156466	AUAUUAU
ENSG00000156466	CACAGUG
ENSG00000156466	CAGUGCA
ENSG00000156466	GAAUUGU
ENSG00000156466	GAGGUAU
ENSG00000156466	GCAUCCC
ENSG00000156466	GGAAUGU
ENSG00000156466	GGGGAGC
ENSG00000156466	GGUAGAC
ENSG00000156466	GUAACAG
ENSG00000156466	UCACAGG
ENSG00000156466	UUCUUGU
ENSG00000156466	UUUGCAC
ENSG00000156467	AAAGAGG
ENSG00000156467	ACAUCAC
ENSG00000156467	AGCUUAU
ENSG00000156467	AUUCAUU
ENSG00000156467	GUAUGUU
ENSG00000156467	GUCAGUU
ENSG00000156467	UGAAGUG
ENSG00000156467	UUCAAAU
ENSG00000156467	UUUCAAC
ENSG00000156471	AAGGCAC
ENSG00000156471	ACCAGCA

ENSG00000156471	AUGGCAC
ENSG00000156486	ACACACC
ENSG00000156486	AUCCUUG
ENSG00000156486	UGCAAAG
ENSG00000156486	UGCUGAC
ENSG00000156504	ACAAUAU
ENSG00000156504	AGCAGCA
ENSG00000156504	AGGCAGG
ENSG00000156504	CAAAGCA
ENSG00000156504	CUUCUCU
ENSG00000156504	UCCCUUU
ENSG00000156508	AAAAGUA
ENSG00000156508	AAAUGGU
ENSG00000156508	AAGUUGU
ENSG00000156508	AUGCAUU
ENSG00000156508	CCAUUAC
ENSG00000156508	CCUUCUU
ENSG00000156508	CUCAAAC
ENSG00000156508	GAAGGUC
ENSG00000156508	GAGACCU
ENSG00000156508	GGCAGGG
ENSG00000156508	GGGGUGC
ENSG00000156508	GGGUCUU
ENSG00000156508	UUCCAUA
ENSG00000156508	UUGGUCC
ENSG00000156515	AAAGUUU
ENSG00000156515	AAGUGCU
ENSG00000156515	AGUGCUG
ENSG00000156515	UACAAAG
ENSG00000156521	AAGGCAC
ENSG00000156521	CCCUGAG
ENSG00000156521	CUGGAAA
ENSG00000156521	GAGUCUU
ENSG00000156521	GGCCCUG
ENSG00000156521	UGAGUCU
ENSG00000156531	AAAGUGC
ENSG00000156531	AACCUGU
ENSG00000156531	AAGUGCU
ENSG00000156531	AUUCAUU
ENSG00000156531	GGGUAAG
ENSG00000156531	UAAUAGU
ENSG00000156531	UUCAAGC
ENSG00000156535	GGUCUAG
ENSG00000156535	UCUGGAA
ENSG00000156564	ACAUCAC
ENSG00000156564	CCCUGAG
ENSG00000156564	GGCUCAG
ENSG00000156564	UCCCUGU
ENSG00000156575	AGGGUCU
ENSG00000156587	CCCCUGG
ENSG00000156599	AAUUAUU
ENSG00000156599	ACACACC
ENSG00000156599	ACACACU
ENSG00000156599	AUAUAAC
ENSG00000156599	AUUGCAC
ENSG00000156599	CACACCU
ENSG00000156599	CCUGCUG
ENSG00000156599	CUCAGGG
ENSG00000156599	CUUUGGU
ENSG00000156599	GGUGGGC
ENSG00000156599	UAUUGCU
ENSG00000156599	UGAGUCU
ENSG00000156599	UUGGCAA
ENSG00000156599	UUGGCAC

ENSG00000156603	AGCUGCC
ENSG00000156603	CCUUCUU
ENSG00000156603	CUGGACU
ENSG00000156603	CUUGUGU
ENSG00000156603	GAGCUGC
ENSG00000156603	GGGUGGU
ENSG00000156603	UUCUUGU
ENSG00000156639	AAAGUUU
ENSG00000156639	AAAUCCC
ENSG00000156639	AACAUUC
ENSG00000156639	AAGGCAC
ENSG00000156639	ACAAGGU
ENSG00000156639	ACAGUAU
ENSG00000156639	ACUCCAG
ENSG00000156639	ACUGCAG
ENSG00000156639	AGUAACA
ENSG00000156639	CACCAGC
ENSG00000156639	CAGCAGA
ENSG00000156639	CCAGCAU
ENSG00000156639	CUACAGU
ENSG00000156639	CUCCAGC
ENSG00000156639	CUGGCUC
ENSG00000156639	GAGCUAA
ENSG00000156639	GAUUGGU
ENSG00000156639	GCUAGCA
ENSG00000156639	GGUUUAC
ENSG00000156639	UAAUAUC
ENSG00000156639	UAGUGAG
ENSG00000156639	UCACAAG
ENSG00000156639	UCACAGG
ENSG00000156639	UCACAGU
ENSG00000156639	UGAAAGG
ENSG00000156639	UGCAUAG
ENSG00000156639	UGUCUGG
ENSG00000156639	UUUCAAC
ENSG00000156642	ACAUUCA
ENSG00000156642	ACUAGAU
ENSG00000156642	ACUGUAG
ENSG00000156642	AGCCCUU
ENSG00000156642	AGUGCAA
ENSG00000156642	AUCACUA
ENSG00000156642	GCCCUUA
ENSG00000156642	GGCAGCG
ENSG00000156642	GUGCAAA
ENSG00000156642	UAUAAAG
ENSG00000156642	UGCAUAU
ENSG00000156650	AAAAAUC
ENSG00000156650	AAAGCUG
ENSG00000156650	AACACUG
ENSG00000156650	AAGACAU
ENSG00000156650	AAUUGCU
ENSG00000156650	AGCUGCC
ENSG00000156650	AUGGCUU
ENSG00000156650	GCAACUU
ENSG00000156650	UACAAAG
ENSG00000156650	UAUAUGA
ENSG00000156650	UGGCCCU
ENSG00000156671	GAGUCUU
ENSG00000156675	AAAGGUA
ENSG00000156675	AAAGUGC
ENSG00000156675	AACCUGU
ENSG00000156675	AAGUGCU
ENSG00000156675	AAUCUCU
ENSG00000156675	CACAGUG

ENSG00000156675	CAGCAAA
ENSG00000156675	CUACAAA
ENSG00000156675	GAGAAGA
ENSG00000156675	GAGAUGA
ENSG00000156675	GGCAUUG
ENSG00000156675	GGUUUAC
ENSG00000156675	UACAGUU
ENSG00000156675	UCACAGU
ENSG00000156675	UCCAGAG
ENSG00000156687	AAAGAGC
ENSG00000156687	AAAGAGG
ENSG00000156687	AGAUAAA
ENSG00000156687	AUGGCAC
ENSG00000156687	CUUACAG
ENSG00000156687	GAGAAAU
ENSG00000156687	GAGAACC
ENSG00000156687	GCUGGUG
ENSG00000156687	GUAAACA
ENSG00000156687	UACAAAG
ENSG00000156687	UCCUGAG
ENSG00000156687	UUGGUUC
ENSG00000156687	UUUCAAC
ENSG00000156711	CUCCCAA
ENSG00000156853	GAGGUAU
ENSG00000156853	UCCCACA
ENSG00000156858	ACUAGAU
ENSG00000156860	AAAAAUC
ENSG00000156860	ACUUGGA
ENSG00000156860	AGCACCA
ENSG00000156860	AGCCCUU
ENSG00000156860	AUAUAAC
ENSG00000156860	CAGUCUG
ENSG00000156860	CCUGCUG
ENSG00000156860	CUCUGGG
ENSG00000156860	CUGGCUC
ENSG00000156860	CUGGGGG
ENSG00000156860	CUUUGGU
ENSG00000156860	GAGCCCC
ENSG00000156860	GAGGCAG
ENSG00000156860	GAGGGGC
ENSG00000156860	GGAGGCA
ENSG00000156860	GGGUGGU
ENSG00000156860	UAGUGAG
ENSG00000156875	AAAGCUG
ENSG00000156875	AUAAAGU
ENSG00000156875	CUAUACA
ENSG00000156875	GAGAAAU
ENSG00000156875	GUAUUCU
ENSG00000156875	UAUAAAG
ENSG00000156875	UCAAGUA
ENSG00000156875	UCACAUU
ENSG00000156920	UCACAAG
ENSG00000156925	AAAGCUG
ENSG00000156925	AAGACAU
ENSG00000156925	GAGAAAU
ENSG00000156925	GAUAUGU
ENSG00000156925	UAAGACU
ENSG00000156925	UCCUGAG
ENSG00000156925	UUGGCAA
ENSG00000156925	UUGGCAC
ENSG00000156958	ACUGUGU
ENSG00000156959	AAAGUAU
ENSG00000156959	ACCCUGU
ENSG00000156959	AGCAGCA

ENSG00000156959	CAGACAG
ENSG00000156959	GACCCUG
ENSG00000156959	GACCUGG
ENSG00000156959	GGCAGGG
ENSG00000156959	GGCUCUG
ENSG00000156959	GGUGGGC
ENSG00000156959	GUCUCUG
ENSG00000156959	UCAAGUA
ENSG00000156959	UCCAGAG
ENSG00000156959	UCCCUUU
ENSG00000156959	UGAAGCU
ENSG00000156959	UUAUGCA
ENSG00000156976	AACAGUC
ENSG00000156976	AUGCAUU
ENSG00000156976	CACAAUG
ENSG00000156976	CAGCAAA
ENSG00000156976	GUGCAAA
ENSG00000156976	UAAUACA
ENSG00000156976	UGGCUGC
ENSG00000156983	ACAGUAC
ENSG00000156983	ACUGGCC
ENSG00000156983	CAGUCCA
ENSG00000156983	CAGUGCA
ENSG00000156983	CAGUUAC
ENSG00000156983	CCCCUGG
ENSG00000156983	CCCUGAG
ENSG00000156983	UACAAAG
ENSG00000156983	UACAGUU
ENSG00000156990	GAGGUAG
ENSG00000157014	AGCACCA
ENSG00000157020	AAGGCAC
ENSG00000157045	CUUUGGU
ENSG00000157064	AAAACUG
ENSG00000157064	AAGAACC
ENSG00000157064	ACAUUCA
ENSG00000157064	AGCAGCA
ENSG00000157064	AGCAGCG
ENSG00000157064	AGCAGCU
ENSG00000157064	AGGCAGG
ENSG00000157064	CACUGUU
ENSG00000157064	CCAGUAC
ENSG00000157064	CGCCUCC
ENSG00000157064	CUCUAGC
ENSG00000157064	GAGGCAG
ENSG00000157064	GAGGCUG
ENSG00000157064	UAGAGGA
ENSG00000157064	UCCUGAG
ENSG00000157064	UCUAGAG
ENSG00000157064	UCUCCAA
ENSG00000157064	UGAAAUG
ENSG00000157064	UGACUGU
ENSG00000157077	AGCUGCC
ENSG00000157077	AGUAACA
ENSG00000157077	GUAACAG
ENSG00000157077	GUGCAAA
ENSG00000157077	UGCAUAG
ENSG00000157087	AAAGUGC
ENSG00000157087	AAGUGCU
ENSG00000157087	AAUUAUU
ENSG00000157087	ACAGGUG
ENSG00000157087	AGUGCUG
ENSG00000157087	AUGGCUU
ENSG00000157087	CAAAGUG
ENSG00000157087	CUCUGGG

ENSG00000157087	GGAAGAC
ENSG00000157087	GGAAGCC
ENSG00000157087	GUGACUG
ENSG00000157087	UCAUUCG
ENSG00000157087	UCUCACC
ENSG00000157087	UGUCUUU
ENSG00000157087	UUGUGAC
ENSG00000157087	UUUGCAA
ENSG00000157093	UUCUGCA
ENSG00000157103	AAAAACC
ENSG00000157103	AAAAAUC
ENSG00000157103	AAACUGU
ENSG00000157103	ACUGCAU
ENSG00000157103	ACUGUAG
ENSG00000157103	AGCUGCC
ENSG00000157103	AUAUUAU
ENSG00000157103	CUACAAA
ENSG00000157103	GAGGUAG
ENSG00000157103	UAAUACA
ENSG00000157103	UAUAAAG
ENSG00000157103	UAUAUGA
ENSG00000157103	UCACACA
ENSG00000157103	UCACAGG
ENSG00000157103	UCAUAGA
ENSG00000157103	UCAUUUG
ENSG00000157106	AAACUGU
ENSG00000157106	AAAGGUA
ENSG00000157106	AAAGUAU
ENSG00000157106	AAUCACU
ENSG00000157106	AAUCCUU
ENSG00000157106	ACAUCAC
ENSG00000157106	AUCCCUU
ENSG00000157106	AUCCUUG
ENSG00000157106	AUUCAUU
ENSG00000157106	CAAGAGC
ENSG00000157106	CACAGGG
ENSG00000157106	CACAGUG
ENSG00000157106	CAGGUGA
ENSG00000157106	CUGGAAA
ENSG00000157106	GACCCUG
ENSG00000157106	GCCCUGU
ENSG00000157106	GCCUGAU
ENSG00000157106	GGAAGAC
ENSG00000157106	GGAAUGU
ENSG00000157106	GGGCGUA
ENSG00000157106	GUCUUGC
ENSG00000157106	GUGUGGC
ENSG00000157106	UCACCAC
ENSG00000157106	UGCAGCU
ENSG00000157106	UGCAUUG
ENSG00000157106	UGCCCUG
ENSG00000157106	UUCCUCG
ENSG00000157107	AACCUGG
ENSG00000157107	AAGGCAC
ENSG00000157107	AAGUGCA
ENSG00000157107	ACUGCAG
ENSG00000157107	AUAUUAU
ENSG00000157107	AUUGCAC
ENSG00000157107	CAAAGCA
ENSG00000157107	CUACAGU
ENSG00000157107	GCUGUCU
ENSG00000157107	UAUUGCU
ENSG00000157110	AAAAAUC
ENSG00000157110	AAAAGUA

ENSG00000157110	AAAGUAC
ENSG00000157110	AAGUCUU
ENSG00000157110	ACACACC
ENSG00000157110	AGCAUUC
ENSG00000157110	CAAAACU
ENSG00000157110	CACACCU
ENSG00000157110	CCAGUGU
ENSG00000157110	GACCAUG
ENSG00000157110	GAGGCUG
ENSG00000157110	GAUUGUC
ENSG00000157110	GUCUUGC
ENSG00000157110	UAAUACA
ENSG00000157110	UAUAAAG
ENSG00000157110	UCACACA
ENSG00000157110	UCCAGUU
ENSG00000157110	UGAUCCA
ENSG00000157110	UUCAAGC
ENSG00000157150	ACUGGCU
ENSG00000157152	ACUGCAU
ENSG00000157152	AUAUAAC
ENSG00000157152	CAGACAG
ENSG00000157152	CAGUCCA
ENSG00000157152	CUGUUGA
ENSG00000157152	GAGAACU
ENSG00000157152	GUAAACA
ENSG00000157152	UAUUGCU
ENSG00000157152	UGCAUAG
ENSG00000157152	UUCAAAU
ENSG00000157168	GGAUUUC
ENSG00000157184	AGCACCA
ENSG00000157184	UGGCUGC
ENSG00000157184	UUCUGCA
ENSG00000157193	AUCCUUU
ENSG00000157193	CAGCAGA
ENSG00000157193	CAGCAGG
ENSG00000157193	GUUAAUG
ENSG00000157214	AAGGCAC
ENSG00000157214	UGAGAAU
ENSG00000157216	AAAAAUC
ENSG00000157216	AAACUGU
ENSG00000157216	AUUGCAC
ENSG00000157216	UAUUGCU
ENSG00000157216	UCCAGUU
ENSG00000157216	UGAAAUG
ENSG00000157216	UGCAUAG
ENSG00000157216	UGCAUAU
ENSG00000157219	AUGCAUU
ENSG00000157224	AAUCCUU
ENSG00000157224	AGCAGCG
ENSG00000157224	GAGGUAU
ENSG00000157227	AAAAAUC
ENSG00000157227	AAUUAUU
ENSG00000157227	ACAUUAC
ENSG00000157227	ACAUUCA
ENSG00000157227	AUAUAAC
ENSG00000157227	GAGGCUG
ENSG00000157227	GGCUCAG
ENSG00000157227	UCAAGUA
ENSG00000157227	UCCUGAG
ENSG00000157227	UGCCAGC
ENSG00000157227	UUGGUCC
ENSG00000157240	AUAUAAC
ENSG00000157240	AUGAGCU
ENSG00000157240	CAGGUGA

ENSG00000157240	CUUACAG
ENSG00000157240	UACAAAG
ENSG00000157240	UUCCAUA
ENSG00000157259	AAAGUAA
ENSG00000157259	UCUAGAG
ENSG00000157306	AAAUCCC
ENSG00000157306	AACUACU
ENSG00000157306	ACUCAGC
ENSG00000157306	AGGAGCU
ENSG00000157306	AGGGAGG
ENSG00000157306	CAGCUGG
ENSG00000157306	GAGGCUG
ENSG00000157306	GAGGGGC
ENSG00000157306	GGAGAGA
ENSG00000157306	GGAGGCA
ENSG00000157306	GGAUUUU
ENSG00000157306	GUGCUUG
ENSG00000157306	UCCUAUA
ENSG00000157306	UGUGCUU
ENSG00000157349	GAGGUAU
ENSG00000157350	AAUACUG
ENSG00000157350	AAUUAUU
ENSG00000157350	ACUGUUU
ENSG00000157350	CUCUUCC
ENSG00000157350	CUGCAGC
ENSG00000157350	CUUUGGU
ENSG00000157350	GAUUGGU
ENSG00000157350	GCAGCAU
ENSG00000157350	GCUUCCU
ENSG00000157350	GGCAAGA
ENSG00000157350	UCACAGG
ENSG00000157350	UCACAGU
ENSG00000157350	UUGGAGG
ENSG00000157350	UUGGCAA
ENSG00000157350	UUGGCAC
ENSG00000157353	GAGAACC
ENSG00000157353	GCUUCCU
ENSG00000157368	UAAUAGU
ENSG00000157379	CUUCUCU
ENSG00000157388	AGUGCCG
ENSG00000157388	GAGGCAG
ENSG00000157388	GAGGUAG
ENSG00000157388	GAGGUAU
ENSG00000157388	GAUAUGU
ENSG00000157388	UUGAGGC
ENSG00000157404	AAAGUUU
ENSG00000157404	AAGACAU
ENSG00000157404	AAGGUGC
ENSG00000157404	AAGUGCA
ENSG00000157404	ACUGGCC
ENSG00000157404	ACUGGCU
ENSG00000157404	ACUGUAG
ENSG00000157404	ACUGUUU
ENSG00000157404	AGUGCAA
ENSG00000157404	AUAAAGU
ENSG00000157404	AUAUAAC
ENSG00000157404	AUCCUUU
ENSG00000157404	CAAAACU
ENSG00000157404	CAGUGCA
ENSG00000157404	CCAGUGU
ENSG00000157404	GUGCAAA
ENSG00000157404	GUGCUUG
ENSG00000157404	UACAAAG
ENSG00000157404	UAUAAAG

ENSG00000157404	UGCAAAG
ENSG00000157404	UGUGCUU
ENSG00000157404	UUCAAAU
ENSG00000157423	AAUCCUU
ENSG00000157445	AAAAGUA
ENSG00000157450	AGCAAAA
ENSG00000157450	AGCAGCA
ENSG00000157450	AGCAGCU
ENSG00000157450	AUACAAG
ENSG00000157450	AUGUGCC
ENSG00000157450	CAAAGCA
ENSG00000157450	CAGCAAA
ENSG00000157450	GGAAUGU
ENSG00000157450	UACAAAG
ENSG00000157450	UCACAGG
ENSG00000157450	UCACAGU
ENSG00000157450	UGCAAAG
ENSG00000157450	UUGGUUC
ENSG00000157470	AAAAGUA
ENSG00000157470	AAAUUUC
ENSG00000157470	AAGGCAC
ENSG00000157470	AUUCAUU
ENSG00000157470	AUUGCAC
ENSG00000157470	CUUUGGU
ENSG00000157470	GCAGCAU
ENSG00000157470	UAUUGCU
ENSG00000157470	UCACAGU
ENSG00000157470	UGCAAAG
ENSG00000157470	UUCAAAU
ENSG00000157470	UUGGCAA
ENSG00000157470	UUGGCAC
ENSG00000157483	AAGGCAC
ENSG00000157483	ACAGUAC
ENSG00000157483	ACCUGGA
ENSG00000157483	UAUAUGA
ENSG00000157500	AAUACUG
ENSG00000157500	AAUCCUU
ENSG00000157500	ACAAUAU
ENSG00000157500	AGCAAAA
ENSG00000157500	AUACAAG
ENSG00000157500	CAGCAAA
ENSG00000157500	CUGCAGG
ENSG00000157500	GAGAACC
ENSG00000157500	GCAGAAG
ENSG00000157500	GCAGCAU
ENSG00000157500	GUGCAAA
ENSG00000157500	UUGGCAA
ENSG00000157500	UUGGCAC
ENSG00000157502	AGUGCUU
ENSG00000157502	CAGCAGA
ENSG00000157502	GCAGAAG
ENSG00000157502	GGAAUGU
ENSG00000157502	UCAACAG
ENSG00000157502	UCAAGUA
ENSG00000157510	AGGCAGG
ENSG00000157510	GAGCUAA
ENSG00000157510	GGCAGGG
ENSG00000157514	AAAGCUG
ENSG00000157514	AACAAAC
ENSG00000157514	AAUCUCU
ENSG00000157514	ACUCAGC
ENSG00000157514	AGCACCA
ENSG00000157514	AUGCAUU
ENSG00000157514	CACCAGC

ENSG00000157514	CUGCCCC
ENSG00000157514	CUGGAAA
ENSG00000157514	GAGAAGA
ENSG00000157514	GAGACCU
ENSG00000157514	GCUACAU
ENSG00000157514	GGCUCAG
ENSG00000157514	GGCUCUG
ENSG00000157514	UCCAGAG
ENSG00000157514	UCCCACA
ENSG00000157514	UGAAGCU
ENSG00000157514	UUGGCAC
ENSG00000157540	AAAGUGC
ENSG00000157540	AACAAAC
ENSG00000157540	AAGACAU
ENSG00000157540	AAGGCAC
ENSG00000157540	AAGUGCA
ENSG00000157540	AAUUAUU
ENSG00000157540	ACAGUAC
ENSG00000157540	ACAGUAU
ENSG00000157540	ACAUUAC
ENSG00000157540	ACCAGCA
ENSG00000157540	ACCCAUU
ENSG00000157540	ACUAGAU
ENSG00000157540	ACUGCAU
ENSG00000157540	ACUGGCC
ENSG00000157540	AGCAAAA
ENSG00000157540	AGCAGCU
ENSG00000157540	AGGUUAU
ENSG00000157540	AGUACUG
ENSG00000157540	AUGACAC
ENSG00000157540	AUGUGCC
ENSG00000157540	CAAAACU
ENSG00000157540	CAAAGCA
ENSG00000157540	CAAAGUG
ENSG00000157540	CAGUGCA
ENSG00000157540	CCUGUAC
ENSG00000157540	CUCCCAA
ENSG00000157540	CUGGACU
ENSG00000157540	CUUACAG
ENSG00000157540	CUUGUGU
ENSG00000157540	CUUUGGU
ENSG00000157540	GAGGCAG
ENSG00000157540	GAGGUAG
ENSG00000157540	GCUACAU
ENSG00000157540	GGACCUG
ENSG00000157540	GGACUGC
ENSG00000157540	GGUCUAG
ENSG00000157540	GGUUACC
ENSG00000157540	GUGAGGU
ENSG00000157540	GUGCUUG
ENSG00000157540	UAAUACA
ENSG00000157540	UACAGUU
ENSG00000157540	UAUAAUA
ENSG00000157540	UCAAGUA
ENSG00000157540	UCACAGG
ENSG00000157540	UCACAGU
ENSG00000157540	UCCAGUU
ENSG00000157540	UGAAAUG
ENSG00000157540	UGAGGAC
ENSG00000157540	UGGACUG
ENSG00000157540	UGGUACC
ENSG00000157540	UGUGUCA
ENSG00000157540	UUGUGUC
ENSG00000157540	UUUGCAA

ENSG00000157554	AAAACUG
ENSG00000157554	CACAGUG
ENSG00000157554	GAUUGUC
ENSG00000157554	GUAGUGU
ENSG00000157554	UCACAGG
ENSG00000157557	AAAAAUC
ENSG00000157557	AAAGCUG
ENSG00000157557	AAAUCCC
ENSG00000157557	AAUACUG
ENSG00000157557	AGCUGCC
ENSG00000157557	CAAGAGC
ENSG00000157557	CCAGUGC
ENSG00000157557	CCAGUGU
ENSG00000157557	CUUACAG
ENSG00000157557	GAAAACA
ENSG00000157557	UCCAGUU
ENSG00000157557	UGAAAUG
ENSG00000157557	UGCAUUG
ENSG00000157557	UGCCAGC
ENSG00000157557	UUUGCAC
ENSG00000157593	AAGGCAC
ENSG00000157593	AGCAGCA
ENSG00000157593	CCAGGAG
ENSG00000157593	GUGCUUG
ENSG00000157613	AGUGGUU
ENSG00000157613	GGCAGUG
ENSG00000157613	GGGUCUU
ENSG00000157613	GUCUCUG
ENSG00000157613	UACAAAG
ENSG00000157617	AAAGUAA
ENSG00000157617	ACAGUAU
ENSG00000157617	AUAAAGU
ENSG00000157617	AUGCAUU
ENSG00000157617	AUGGCUU
ENSG00000157617	CAAAGUG
ENSG00000157617	CUUUGGU
ENSG00000157617	GUUCAUG
ENSG00000157617	UAAGACU
ENSG00000157617	UAAUAUC
ENSG00000157617	UAUUGCU
ENSG00000157617	UCACAGG
ENSG00000157617	UGCAUAG
ENSG00000157617	UUGGCAA
ENSG00000157654	AAAAAUC
ENSG00000157654	AAAGCUG
ENSG00000157654	AACAUUC
ENSG00000157654	AAUACUG
ENSG00000157654	ACACACC
ENSG00000157654	ACUGCAU
ENSG00000157654	AUCCCUU
ENSG00000157654	AUGGCUU
ENSG00000157654	CAAAACU
ENSG00000157654	CACUCCU
ENSG00000157654	CAGCAAA
ENSG00000157654	CAUAUUG
ENSG00000157654	CCAUUAC
ENSG00000157654	GAGAAAU
ENSG00000157654	GCAGCAU
ENSG00000157654	GGCAGCG
ENSG00000157654	GGUCUAG
ENSG00000157654	GUGACUG
ENSG00000157654	UACAAAG
ENSG00000157654	UAGUGAG
ENSG00000157654	UCUGGAA

ENSG00000157654	UGGCUGC
ENSG00000157654	UUAGGAU
ENSG00000157657	AAAAACC
ENSG00000157657	AAAGGUA
ENSG00000157657	AAAGUAU
ENSG00000157657	AAAGUGC
ENSG00000157657	AAAUGGU
ENSG00000157657	AAGGCAC
ENSG00000157657	AAGGUGC
ENSG00000157657	AAGUCAC
ENSG00000157657	AAGUGCA
ENSG00000157657	AAUGCCC
ENSG00000157657	ACAGUAU
ENSG00000157657	ACAUUCA
ENSG00000157657	ACUGGCC
ENSG00000157657	AGCAAAA
ENSG00000157657	AGCAGCU
ENSG00000157657	AGCUGCC
ENSG00000157657	AGUGCCU
ENSG00000157657	AGUGCUU
ENSG00000157657	AUAUUAU
ENSG00000157657	AUGAGCU
ENSG00000157657	CACACCU
ENSG00000157657	CACAGGG
ENSG00000157657	CAGACAG
ENSG00000157657	CAGCUGG
ENSG00000157657	CAGUGCA
ENSG00000157657	CCAGGAG
ENSG00000157657	CCAGUGU
ENSG00000157657	CCCUGAG
ENSG00000157657	CUACAAA
ENSG00000157657	CUUUGGU
ENSG00000157657	GAAGGUC
ENSG00000157657	GAGAUGA
ENSG00000157657	GAGCUAA
ENSG00000157657	GAGGUAU
ENSG00000157657	GAGUCUU
ENSG00000157657	GCAGAAG
ENSG00000157657	GCCCUGU
ENSG00000157657	GCCUGAU
ENSG00000157657	GCUACAU
ENSG00000157657	GGACCUG
ENSG00000157657	GGCCCUG
ENSG00000157657	GUAGUGU
ENSG00000157657	GUGACUG
ENSG00000157657	UAAUACA
ENSG00000157657	UACAGUU
ENSG00000157657	UAUAAAG
ENSG00000157657	UAUUACC
ENSG00000157657	UCACCAC
ENSG00000157657	UCCCUCU
ENSG00000157657	UCCUGAG
ENSG00000157657	UCUCAAG
ENSG00000157657	UGCCCUG
ENSG00000157657	UGGCUGC
ENSG00000157657	UUGGUUC
ENSG00000157657	UUGUGUC
ENSG00000157680	AGCUGCC
ENSG00000157680	AGGCAGG
ENSG00000157680	CAGGUGA
ENSG00000157680	CAGUAGA
ENSG00000157680	CUCCAUU
ENSG00000157680	GAGCUGC
ENSG00000157680	GCCCUUA

ENSG00000157680	GCUGGAU
ENSG00000157680	GGAAGCC
ENSG00000157680	GGAGAGA
ENSG00000157680	GUUCAUG
ENSG00000157693	CAAAGCA
ENSG00000157693	CUUCUCU
ENSG00000157693	UCACAGG
ENSG00000157693	UCACAGU
ENSG00000157734	ACUGGCC
ENSG00000157734	GUAACAG
ENSG00000157734	UCACAUU
ENSG00000157765	AAGGCAC
ENSG00000157766	AAGGCAC
ENSG00000157766	ACAUUCA
ENSG00000157766	AUGUGGG
ENSG00000157766	CAGGAAC
ENSG00000157766	UGUCUUU
ENSG00000157766	UUCAAAU
ENSG00000157766	UUUCAAC
ENSG00000157782	AGGCAGG
ENSG00000157782	CACAGUG
ENSG00000157782	GCAGGAC
ENSG00000157782	GCGGAGG
ENSG00000157782	GGCGCCU
ENSG00000157782	GGGGUGG
ENSG00000157782	GGUGGGC
ENSG00000157782	UCACAGG
ENSG00000157782	UCACAGU
ENSG00000157782	UCCAGUU
ENSG00000157796	AAUUUUA
ENSG00000157800	GGAAUGU
ENSG00000157827	AAGGCAC
ENSG00000157827	AUACAAG
ENSG00000157827	AUAUAAC
ENSG00000157827	CUAUACA
ENSG00000157827	GAUUGUA
ENSG00000157827	UAUUACC
ENSG00000157827	UUGGUUC
ENSG00000157851	AAGUGCU
ENSG00000157851	AGUGCCU
ENSG00000157851	AUGCAUU
ENSG00000157851	CAAAGUG
ENSG00000157851	GGAGGAG
ENSG00000157851	GUGCAAA
ENSG00000157851	UGCAUAG
ENSG00000157869	UGUGUGG
ENSG00000157890	AAAGCUG
ENSG00000157890	AUAUUAU
ENSG00000157890	AUCCCUU
ENSG00000157890	CACAGUG
ENSG00000157890	GAGGUAG
ENSG00000157890	GAGGUAU
ENSG00000157890	GCUUCCU
ENSG00000157890	UACAAAG
ENSG00000157890	UAUUGCU
ENSG00000157890	UCAAGUA
ENSG00000157890	UCAUAGA
ENSG00000157890	UCCAGUU
ENSG00000157890	UGAAAGG
ENSG00000157890	UGAGAAU
ENSG00000157890	UGCCCUG
ENSG00000157890	UUCUUGU
ENSG00000157895	AGGGAGG
ENSG00000157895	GCCCUGU

ENSG00000157895	UCUGGAA
ENSG00000157916	AACUACU
ENSG00000157916	AUAAUAC
ENSG00000157916	CAGUCCA
ENSG00000157916	GAGUAUU
ENSG00000157916	GGCUCAG
ENSG00000157916	GGUUUAC
ENSG00000157916	GUGUCAC
ENSG00000157916	UACAAAG
ENSG00000157916	UGAGUCU
ENSG00000157916	UGUGUCA
ENSG00000157916	UUGGUUC
ENSG00000157933	AAAGUGC
ENSG00000157933	AACAAAC
ENSG00000157933	AAGGCAC
ENSG00000157933	AAGUGCA
ENSG00000157933	AGCACCA
ENSG00000157933	AUGGCUU
ENSG00000157933	CAAAGUG
ENSG00000157933	CAGUAGA
ENSG00000157933	CUGCAGC
ENSG00000157933	UAAUGCU
ENSG00000157933	UGUGCUU
ENSG00000157933	UUGUUCG
ENSG00000157954	AAAGAAU
ENSG00000157954	AAAUCCC
ENSG00000157954	AAAUGGU
ENSG00000157954	AAGGCAC
ENSG00000157954	AAUUUUA
ENSG00000157954	AGCAGCG
ENSG00000157954	AGCAGCU
ENSG00000157954	AGCCAGU
ENSG00000157954	CAAAGUG
ENSG00000157954	CACAGGG
ENSG00000157954	CCAGUGU
ENSG00000157954	CGAGGAG
ENSG00000157954	CUUCACC
ENSG00000157954	CUUUGGU
ENSG00000157954	GAGGUAU
ENSG00000157954	GGAAUGU
ENSG00000157954	GUCUCUG
ENSG00000157954	UCACAGG
ENSG00000157954	UCACCAC
ENSG00000157954	UCAUAGA
ENSG00000157954	UGCAAAG
ENSG00000157954	UGUGCUU
ENSG00000157978	AAGGCAC
ENSG00000157978	AGCACCA
ENSG00000157978	GAAACAU
ENSG00000157978	UUCAAAU
ENSG00000157992	UAUUACC
ENSG00000158006	AGCAAAA
ENSG00000158006	GAGUAUU
ENSG00000158006	GUCUGCC
ENSG00000158008	AGUGGUU
ENSG00000158008	GAUUGGU
ENSG00000158014	UCACACA
ENSG00000158019	CCAGGAG
ENSG00000158019	GGAGAGA
ENSG00000158019	UGCUGAC
ENSG00000158019	UGGAGAU
ENSG00000158022	UUUGCAA
ENSG00000158042	CCAGGAG
ENSG00000158042	GGAUUUC

ENSG00000158050	AGCACCA
ENSG00000158050	CACAGGG
ENSG00000158050	GCAGAAG
ENSG00000158050	GGAGUGU
ENSG00000158050	GGUUGUC
ENSG00000158050	UAUAAAG
ENSG00000158055	AAUGUUG
ENSG00000158055	AGUAACA
ENSG00000158055	GUGUGGC
ENSG00000158055	UAUUACC
ENSG00000158055	UGCAUAG
ENSG00000158055	UUUUUGC
ENSG00000158079	AUGGCAC
ENSG00000158079	UAAGACG
ENSG00000158079	UAAGACU
ENSG00000158092	CAGUCUG
ENSG00000158104	GGGCGUG
ENSG00000158106	CCCCUGG
ENSG00000158106	CUGGGGG
ENSG00000158109	AAGUGCA
ENSG00000158109	GUGCAAA
ENSG00000158109	UGCAAAG
ENSG00000158125	AACUGGU
ENSG00000158125	GAGAACC
ENSG00000158125	UCAUUUG
ENSG00000158156	AGCUUAU
ENSG00000158156	GAGGUAU
ENSG00000158158	ACCCAUU
ENSG00000158158	AGCAAAA
ENSG00000158158	AUACAAG
ENSG00000158158	AUUGCAC
ENSG00000158158	CAAAACU
ENSG00000158158	CAGGAAC
ENSG00000158158	CUUUGGU
ENSG00000158158	GACCCUG
ENSG00000158158	GCUUCCU
ENSG00000158158	GGAAGAC
ENSG00000158158	GGGCCCC
ENSG00000158158	GGGGAGC
ENSG00000158158	GUGACAG
ENSG00000158158	UUGGUCC
ENSG00000158161	AGGAGCU
ENSG00000158161	UCCAGUU
ENSG00000158163	AUAAUAC
ENSG00000158163	UCUCAAG
ENSG00000158186	AAAUGGU
ENSG00000158186	ACACACC
ENSG00000158186	AUGGCUU
ENSG00000158186	CAGCAAA
ENSG00000158186	GCUGGUG
ENSG00000158186	GGAAUGU
ENSG00000158186	UCCAGAG
ENSG00000158186	UGCAAAG
ENSG00000158186	UUGGCAA
ENSG00000158186	UUUGCAA
ENSG00000158195	AAUCCUU
ENSG00000158195	AGCACCA
ENSG00000158195	CCCCCAG
ENSG00000158195	CUACAAA
ENSG00000158195	CUGCCCC
ENSG00000158195	GAGAACC
ENSG00000158195	GAGAACU
ENSG00000158195	GCUACAU
ENSG00000158195	GGAUGAG

ENSG00000158195	GGCUGCG
ENSG00000158195	UACAAAG
ENSG00000158195	UUGGCAA
ENSG00000158195	UUGGCAC
ENSG00000158195	UUGGUCC
ENSG00000158195	UUGGUUC
ENSG00000158201	CUACAAA
ENSG00000158201	UUGGUUC
ENSG00000158246	CAAAGUG
ENSG00000158246	GUGCAAA
ENSG00000158258	CACAGUG
ENSG00000158258	UACAAAG
ENSG00000158258	UGGAGAU
ENSG00000158270	ACUGUUU
ENSG00000158270	AUAUUAU
ENSG00000158270	CAGUGCA
ENSG00000158270	GGAUUUU
ENSG00000158270	UGGGAUC
ENSG00000158286	GAUUGUA
ENSG00000158286	UCACAGG
ENSG00000158286	UCACAGU
ENSG00000158290	AACACUG
ENSG00000158290	ACAGUAU
ENSG00000158290	GGUGGUU
ENSG00000158290	UGGAGAU
ENSG00000158292	CUUUGGU
ENSG00000158292	GUGCUUG
ENSG00000158292	UGUGCUU
ENSG00000158296	AGCAGCG
ENSG00000158296	AGCAGCU
ENSG00000158301	CUUUGGU
ENSG00000158301	GAGAACC
ENSG00000158301	GGUGGUU
ENSG00000158301	UUGGCAA
ENSG00000158301	UUGGCAC
ENSG00000158321	AAAGCUG
ENSG00000158321	AAUACUG
ENSG00000158321	AGCAAAA
ENSG00000158321	AUGGCAC
ENSG00000158321	CAGCAAA
ENSG00000158321	UAUAUGA
ENSG00000158352	AACACUG
ENSG00000158352	AAGGCAC
ENSG00000158352	ACUCAGC
ENSG00000158352	AGCACCA
ENSG00000158352	AGGACAC
ENSG00000158352	CUACAGU
ENSG00000158352	GAGCUGC
ENSG00000158352	GGAGGAG
ENSG00000158352	UAGAGGA
ENSG00000158352	UGCAAAG
ENSG00000158352	UGCAGCU
ENSG00000158402	CUGCUCA
ENSG00000158406	GGCCCUG
ENSG00000158417	AAUACUG
ENSG00000158417	CAUGGAU
ENSG00000158417	CCAGUGU
ENSG00000158423	UUGUUCG
ENSG00000158435	AAGGCAC
ENSG00000158435	CACUGUU
ENSG00000158445	GCAACUU
ENSG00000158445	GCAGAAG
ENSG00000158457	CUGGCUC
ENSG00000158457	GUAAACA

ENSG00000158457	GUCAACA
ENSG00000158457	UAUUGCU
ENSG00000158457	UUCCAUA
ENSG00000158458	AAAAAUC
ENSG00000158467	AAUCACU
ENSG00000158467	ACCACAG
ENSG00000158467	ACUAGAU
ENSG00000158467	AGCAGCU
ENSG00000158467	CAGGAAC
ENSG00000158467	CUCUGGG
ENSG00000158467	GGAGGCA
ENSG00000158467	GGCAGUG
ENSG00000158467	GGGGAGC
ENSG00000158467	GGGUAAG
ENSG00000158467	UCCUGAG
ENSG00000158470	AAAUUUC
ENSG00000158470	AAUCACU
ENSG00000158470	ACAAUAU
ENSG00000158470	AGCUGCC
ENSG00000158470	AGUGCAA
ENSG00000158470	AUAUAAC
ENSG00000158470	AUGAGCU
ENSG00000158470	AUGGCUU
ENSG00000158470	AUGUAAU
ENSG00000158470	CCCCUGG
ENSG00000158470	CUCCCAC
ENSG00000158470	GCCCUGU
ENSG00000158470	GUAAACA
ENSG00000158470	GUGCAAA
ENSG00000158470	GUGGGGA
ENSG00000158470	UAUUGCU
ENSG00000158470	UCCCACA
ENSG00000158470	UCUUGAG
ENSG00000158470	UGUGCGU
ENSG00000158480	AAAAAUC
ENSG00000158480	ACAGUAC
ENSG00000158480	ACAGUAU
ENSG00000158480	AGCCCUU
ENSG00000158480	CAAAGCA
ENSG00000158480	CCUUCUG
ENSG00000158480	CUCUAGC
ENSG00000158480	GAGGUAG
ENSG00000158480	GUGUGGC
ENSG00000158480	UCACAGU
ENSG00000158483	GGAUGAG
ENSG00000158526	CUGGGCA
ENSG00000158528	AAGUGCU
ENSG00000158528	AAUCACU
ENSG00000158528	AAUCCUU
ENSG00000158528	AAUUUUA
ENSG00000158528	AUAUAAC
ENSG00000158528	CACAGGG
ENSG00000158528	CACAGUG
ENSG00000158528	CCAGUGC
ENSG00000158528	GAUUGGU
ENSG00000158528	UAUUGCU
ENSG00000158528	UCACACA
ENSG00000158528	UGCAUUG
ENSG00000158545	AAAGAAU
ENSG00000158545	GAGAAAU
ENSG00000158545	UGGCUGC
ENSG00000158555	CUCCAGC
ENSG00000158555	CUGCAGG
ENSG00000158555	GCCCUGU

ENSG00000158555	GGCAGCG
ENSG00000158555	GGCUCAG
ENSG00000158555	GUGCUUG
ENSG00000158555	UGUGCUU
ENSG00000158578	CAGCUGG
ENSG00000158615	AACACUG
ENSG00000158615	AACCUGG
ENSG00000158615	AAGUGCA
ENSG00000158615	AAUUGCU
ENSG00000158615	ACCUGGA
ENSG00000158615	AGCUGCC
ENSG00000158615	AGUGCUG
ENSG00000158615	GAGGUAU
ENSG00000158615	GUGACAG
ENSG00000158615	UCAAGUA
ENSG00000158636	AAGUGCA
ENSG00000158636	AAGUGCU
ENSG00000158636	ACAGUAU
ENSG00000158636	GUCACUC
ENSG00000158636	UAUUGCU
ENSG00000158636	UUUGCAA
ENSG00000158710	AUAUUAU
ENSG00000158710	CUGCAGC
ENSG00000158710	UCCAGUU
ENSG00000158711	ACAUCAC
ENSG00000158714	CCAGUGU
ENSG00000158715	AUUGCAC
ENSG00000158715	CAGCAGA
ENSG00000158715	GGGGUGC
ENSG00000158715	UAUUGCU
ENSG00000158715	UCCUGAG
ENSG00000158715	UGCAAAG
ENSG00000158717	AAAAAUC
ENSG00000158717	AUGGCUU
ENSG00000158717	AUUGCAC
ENSG00000158717	UGUCUUU
ENSG00000158717	UUCCUAG
ENSG00000158747	AAAGUGC
ENSG00000158747	AAGUGCA
ENSG00000158747	AUAUUAU
ENSG00000158747	CACAGGG
ENSG00000158747	CAGCAGG
ENSG00000158747	CAGCUGG
ENSG00000158747	CCAGUGC
ENSG00000158747	CUGACUC
ENSG00000158769	AGCACCA
ENSG00000158769	CAGGUGA
ENSG00000158769	CUGGAAA
ENSG00000158769	UACAAAG
ENSG00000158769	UCUCCAA
ENSG00000158769	UCUGGAA
ENSG00000158773	AAUUUUA
ENSG00000158773	CAGUGCA
ENSG00000158773	GAAGGAA
ENSG00000158773	GGCAGUG
ENSG00000158786	AGCCUGC
ENSG00000158792	AGCACCA
ENSG00000158796	AACAAAC
ENSG00000158796	AGCACCA
ENSG00000158796	AGCAGCA
ENSG00000158796	AGCCCUU
ENSG00000158796	AGGGAGG
ENSG00000158796	CAUGGGU
ENSG00000158796	CCAGGAG

ENSG00000158796	CUGCAGG
ENSG00000158796	CUUUGGU
ENSG00000158796	UCCAGAG
ENSG00000158796	UUGGUUC
ENSG00000158805	AAUUAUU
ENSG00000158805	CACCAGC
ENSG00000158805	UACAAAG
ENSG00000158805	UAUUACC
ENSG00000158813	ACAUUAC
ENSG00000158813	AGCUGCC
ENSG00000158813	CAGGCUC
ENSG00000158813	CUCCCAA
ENSG00000158813	CUGGCUC
ENSG00000158813	GAGGCAG
ENSG00000158813	GAGGUAG
ENSG00000158813	GGAGAGA
ENSG00000158813	GGAGUCC
ENSG00000158813	GGCUCAG
ENSG00000158813	GGCUCUG
ENSG00000158813	UGAAAUG
ENSG00000158813	UGCUGAC
ENSG00000158813	UUUGCAA
ENSG00000158825	AGGAUGU
ENSG00000158825	UAUAAUA
ENSG00000158850	AAAUCCC
ENSG00000158850	GGGGUCC
ENSG00000158856	ACAUUCA
ENSG00000158856	GGAAGCC
ENSG00000158856	UCACACA
ENSG00000158859	AAAAGUA
ENSG00000158859	ACACACC
ENSG00000158859	AGCCUGC
ENSG00000158859	CCCUGAG
ENSG00000158859	GAGGUAU
ENSG00000158859	GGAUUUC
ENSG00000158859	GGAUUUU
ENSG00000158859	GGCAGGG
ENSG00000158859	GGGGCAG
ENSG00000158859	UCAUACA
ENSG00000158859	UCCCUGU
ENSG00000158859	UUCCUAG
ENSG00000158863	AAGUGCA
ENSG00000158863	AAUGCCC
ENSG00000158863	AGACGGG
ENSG00000158863	CAGCUGG
ENSG00000158863	CCAGGAG
ENSG00000158863	CUGCAGG
ENSG00000158863	GAGGUAG
ENSG00000158863	GAGGUAU
ENSG00000158863	GCAGGUU
ENSG00000158863	GGACCUG
ENSG00000158863	GGCUCUG
ENSG00000158863	GGCUGCG
ENSG00000158863	GGGGUGG
ENSG00000158863	GUGAGGU
ENSG00000158863	UAUUGCU
ENSG00000158863	UCCUUGC
ENSG00000158863	UGGCUGC
ENSG00000158863	UUCCUCG
ENSG00000158863	UUGGCAA
ENSG00000158863	UUUGCAA
ENSG00000158863	UUUGCAC
ENSG00000158865	GUAAACA
ENSG00000158882	AGCCCUU

ENSG00000158887	AAAAAUC
ENSG00000158887	ACGUCAU
ENSG00000158887	CAUGGGU
ENSG00000158887	CCGGAGC
ENSG00000158887	CUGCUCA
ENSG00000158887	CUGGAAA
ENSG00000158887	CUGGCUC
ENSG00000158887	CUUCUCU
ENSG00000158887	GGAAUGU
ENSG00000158887	GGCAGGG
ENSG00000158887	GUCAGUU
ENSG00000158887	GUCAUUG
ENSG00000158887	UCCCUUU
ENSG00000158887	UCUUGAG
ENSG00000158887	UGGGGGA
ENSG00000158941	AAAGAGG
ENSG00000158941	ACCACAG
ENSG00000158941	CUUUGGU
ENSG00000158941	GGUUUAC
ENSG00000158941	UUGGUUC
ENSG00000158966	AAUACUG
ENSG00000158966	AAUUUUA
ENSG00000158966	AGCAGCU
ENSG00000158966	AGUAGAC
ENSG00000158966	AUCCUUU
ENSG00000158966	GAGAUGA
ENSG00000158966	GGCAGGG
ENSG00000158966	GGCUCAG
ENSG00000158966	UCAUUUG
ENSG00000158985	AAAGUAU
ENSG00000158985	AUGCCUU
ENSG00000158985	CUACAAA
ENSG00000158985	CUCAAAC
ENSG00000158985	GUAUGUU
ENSG00000158987	AUGGCUU
ENSG00000158987	GUAAACA
ENSG00000158987	GUGCAAA
ENSG00000158987	UCAUUUG
ENSG00000159023	AAAGUGC
ENSG00000159023	AACAUUC
ENSG00000159023	AACUACU
ENSG00000159023	ACAGUAU
ENSG00000159023	ACAUUCA
ENSG00000159023	ACCUGGA
ENSG00000159023	ACUGUGU
ENSG00000159023	AGUGCCU
ENSG00000159023	AUACAAG
ENSG00000159023	CACAGUG
ENSG00000159023	CCCUGAG
ENSG00000159023	CUCUUCC
ENSG00000159023	GAGAUGA
ENSG00000159023	GAGGUAG
ENSG00000159023	GAGGUAU
ENSG00000159023	GCAACUU
ENSG00000159023	GCCUGGG
ENSG00000159023	GGGGAGC
ENSG00000159023	GGGUGGU
ENSG00000159023	UAAUACA
ENSG00000159023	UAUCAGA
ENSG00000159023	UAUUGCU
ENSG00000159023	UCACACA
ENSG00000159023	UCACAGG
ENSG00000159023	UCAUUUG
ENSG00000159023	UCCCUGU

ENSG00000159023	UCCUAUA
ENSG00000159023	UUGGUCC
ENSG00000159082	ACAAUAU
ENSG00000159082	ACUGUAG
ENSG00000159082	AGUAGUU
ENSG00000159082	AGUGGUU
ENSG00000159082	CACAGGG
ENSG00000159082	GGCAGUG
ENSG00000159082	UAUUGCU
ENSG00000159082	UCACAGU
ENSG00000159110	UGCAUAG
ENSG00000159140	AACACUG
ENSG00000159140	AAGCGCU
ENSG00000159140	CACUCCU
ENSG00000159140	CUUUGGU
ENSG00000159140	UGAAAUG
ENSG00000159147	CUUUGGU
ENSG00000159147	UUCCUCG
ENSG00000159164	AAAAACC
ENSG00000159164	AAGACAU
ENSG00000159164	AGCUGCC
ENSG00000159164	AGUGUGC
ENSG00000159164	CUACAGU
ENSG00000159164	CUCACAC
ENSG00000159164	GAGCUGC
ENSG00000159164	GAGGCUG
ENSG00000159164	GCUGGUG
ENSG00000159164	GUGCAAA
ENSG00000159164	GUGCCUG
ENSG00000159164	GUGCUUG
ENSG00000159164	UACAAAG
ENSG00000159164	UAUUGCU
ENSG00000159164	UCACACA
ENSG00000159164	UCACAGU
ENSG00000159164	UGCAAAG
ENSG00000159167	AAGUGCA
ENSG00000159167	ACUGUUU
ENSG00000159167	AGUGCAA
ENSG00000159167	GACCCUG
ENSG00000159167	GUAAACA
ENSG00000159173	AAUGUUG
ENSG00000159173	ACCUGGA
ENSG00000159173	CCCACCU
ENSG00000159173	GGGUUUA
ENSG00000159173	UCCCUCU
ENSG00000159173	UGAGUCU
ENSG00000159173	UGGCUGC
ENSG00000159184	UCCCACC
ENSG00000159200	AAGGCAC
ENSG00000159202	AAAGCUG
ENSG00000159202	AACACUG
ENSG00000159202	AAGACAU
ENSG00000159202	AAGGUGC
ENSG00000159202	AUAAAGU
ENSG00000159202	CACUGUU
ENSG00000159202	CCUUCUU
ENSG00000159202	CUUUGGU
ENSG00000159202	GAUUGUC
ENSG00000159202	GUCACUC
ENSG00000159202	UAUAAAG
ENSG00000159202	UAUUGCU
ENSG00000159202	UGCAUAU
ENSG00000159202	UUCAAAU
ENSG00000159202	UUGGUCC

ENSG00000159210	AAGGCAC
ENSG00000159212	GGAUUUU
ENSG00000159214	AAAUUUC
ENSG00000159214	UGUCUGG
ENSG00000159214	UGUGCUU
ENSG00000159216	AAAGUGC
ENSG00000159216	AAGUGCU
ENSG00000159216	AAGUUGU
ENSG00000159216	ACAGUAC
ENSG00000159216	ACAUUCA
ENSG00000159216	ACUCAAA
ENSG00000159216	ACUGCAG
ENSG00000159216	ACUGCAU
ENSG00000159216	AGUAGUU
ENSG00000159216	AGUGCCG
ENSG00000159216	AGUGCCU
ENSG00000159216	CAAAGUG
ENSG00000159216	CACAGUG
ENSG00000159216	CAGCAGA
ENSG00000159216	CAGCAGG
ENSG00000159216	CCUGCUG
ENSG00000159216	CUGCAGC
ENSG00000159216	GAGUUGA
ENSG00000159216	GCAUCCC
ENSG00000159216	UACAAAG
ENSG00000159216	UCACAUU
ENSG00000159216	UCAUUUG
ENSG00000159216	UGAAGCU
ENSG00000159216	UGACCUA
ENSG00000159216	UUCAAAU
ENSG00000159216	UUCUAAU
ENSG00000159217	AAAGUAU
ENSG00000159217	AACAAAC
ENSG00000159217	AAGGCAC
ENSG00000159217	AAGUGCA
ENSG00000159217	AAGUGCU
ENSG00000159217	AAUUGCU
ENSG00000159217	ACUGGCC
ENSG00000159217	ACUGGCU
ENSG00000159217	ACUGUGU
ENSG00000159217	AGCUGCC
ENSG00000159217	AGGAGCU
ENSG00000159217	AGGUAGU
ENSG00000159217	AGUGCAA
ENSG00000159217	AGUGCUU
ENSG00000159217	AUAUAAC
ENSG00000159217	AUCCUUU
ENSG00000159217	AUGGCUU
ENSG00000159217	CAAAGUG
ENSG00000159217	CACCACC
ENSG00000159217	CCAGUGC
ENSG00000159217	CCCCCAG
ENSG00000159217	CCCCUGG
ENSG00000159217	CCCUGUC
ENSG00000159217	CCUGCUG
ENSG00000159217	CUCAGGG
ENSG00000159217	CUCCAUU
ENSG00000159217	CUCCCAA
ENSG00000159217	CUCUGGG
ENSG00000159217	CUGGAAA
ENSG00000159217	CUGGCUC
ENSG00000159217	CUGGGCA
ENSG00000159217	CUUCUCU
ENSG00000159217	CUUUGGU

ENSG00000159217	GCUUCCU
ENSG00000159217	GGAGGAG
ENSG00000159217	GGAGGCA
ENSG00000159217	GGCUCAG
ENSG00000159217	GUCUGCC
ENSG00000159217	GUGAGGU
ENSG00000159217	UCAACAG
ENSG00000159217	UCAUACA
ENSG00000159217	UCUCCAA
ENSG00000159217	UGCAGCU
ENSG00000159217	UGCUGAC
ENSG00000159217	UGUCUUU
ENSG00000159217	UUGGCAA
ENSG00000159217	UUGGCAC
ENSG00000159251	AAACUGU
ENSG00000159251	AUAAUAC
ENSG00000159251	GGUGGUU
ENSG00000159251	GGUUUAC
ENSG00000159251	UUGUGUC
ENSG00000159256	UACAAAG
ENSG00000159263	AACACUG
ENSG00000159263	AAUGCCC
ENSG00000159263	AUUGCAC
ENSG00000159263	GAGGUAG
ENSG00000159267	CUCCAGC
ENSG00000159267	GAGAAAU
ENSG00000159267	GGCAUUG
ENSG00000159267	GUGCCUG
ENSG00000159267	UCCCUUG
ENSG00000159267	UCUGGAA
ENSG00000159267	UGCAUAG
ENSG00000159267	UUGUUCG
ENSG00000159307	UACAGUU
ENSG00000159322	CUUUGGU
ENSG00000159322	UAUAAAG
ENSG00000159322	UGGCUGC
ENSG00000159335	AGGCAGG
ENSG00000159335	AUACAAG
ENSG00000159335	CAGCUGG
ENSG00000159335	GCUGUCU
ENSG00000159335	UCUGGAG
ENSG00000159346	AAAGCUG
ENSG00000159346	AAGUGUG
ENSG00000159346	AAUCUCA
ENSG00000159346	AGUGUGC
ENSG00000159346	CUACAAA
ENSG00000159346	GACAACU
ENSG00000159346	GCCUGGA
ENSG00000159346	UACAAAG
ENSG00000159363	CCAGUGU
ENSG00000159363	GGAGUGU
ENSG00000159363	GGCAGGG
ENSG00000159374	AUCCUUG
ENSG00000159374	UCCUGAG
ENSG00000159377	UACAAAG
ENSG00000159388	AAAAACC
ENSG00000159388	AAAGUAC
ENSG00000159388	AAAGUAU
ENSG00000159388	AACAAAC
ENSG00000159388	AACAGUC
ENSG00000159388	AAGGCAC
ENSG00000159388	AGCACCA
ENSG00000159388	AGCAGCA
ENSG00000159388	AGCUUAU

ENSG00000159388	CACAGUG
ENSG00000159388	CAGUCCA
ENSG00000159388	CUGCUCA
ENSG00000159388	CUUUGGU
ENSG00000159388	GAGAACC
ENSG00000159388	GAGAACU
ENSG00000159388	GAGGUAG
ENSG00000159388	GAGGUAU
ENSG00000159388	GCAGCAU
ENSG00000159388	UACAAAG
ENSG00000159388	UCAACAG
ENSG00000159388	UCACAGG
ENSG00000159388	UCAUUUG
ENSG00000159388	UCCCUGU
ENSG00000159388	UGUGCUU
ENSG00000159388	UUCCAUA
ENSG00000159399	CCCUGAG
ENSG00000159399	CUCUUCC
ENSG00000159399	CUUCUCU
ENSG00000159399	CUUUGGU
ENSG00000159399	GAGGUAG
ENSG00000159399	GAGGUAU
ENSG00000159423	CUCAAAC
ENSG00000159423	UGGCUGC
ENSG00000159445	UCUGGAG
ENSG00000159450	AAUUAUU
ENSG00000159459	AAAUUUC
ENSG00000159459	AUGGCUU
ENSG00000159459	CUUUGGU
ENSG00000159459	UCACAGU
ENSG00000159459	UUGGCAA
ENSG00000159459	UUGGCAC
ENSG00000159459	UUUGCAC
ENSG00000159461	AAAGUAU
ENSG00000159461	ACAUAGA
ENSG00000159461	AGCAAAA
ENSG00000159461	AGCACCA
ENSG00000159461	AUAAUAC
ENSG00000159461	CAGCUGG
ENSG00000159479	CUGACUC
ENSG00000159479	UGCUGAC
ENSG00000159495	AGGGAGG
ENSG00000159556	CAUGGGU
ENSG00000159556	GAGUCUU
ENSG00000159556	GGAAUGU
ENSG00000159556	UGAGUCU
ENSG00000159556	UGGGUGA
ENSG00000159588	ACAUUCA
ENSG00000159592	AAUGUUG
ENSG00000159592	AGUAGUU
ENSG00000159592	CAGUAGU
ENSG00000159592	UCCCUUU
ENSG00000159625	GGCAGUG
ENSG00000159648	CCAGGAG
ENSG00000159650	CUCUGGG
ENSG00000159658	AAGGCAC
ENSG00000159658	AAGUGCA
ENSG00000159658	AAUUGCU
ENSG00000159658	AUAUUAU
ENSG00000159658	AUGCACC
ENSG00000159658	CAGGAAC
ENSG00000159658	GCCUGAU
ENSG00000159658	GGCUCAG
ENSG00000159658	GGUUGUC

ENSG00000159658	GUCACUC
ENSG00000159658	UAAUAUC
ENSG00000159658	UACAAAG
ENSG00000159658	UAUGGUU
ENSG00000159658	UCAAGUA
ENSG00000159658	UGCAGCU
ENSG00000159658	UUCUAAU
ENSG00000159692	AAAACUG
ENSG00000159692	AAACUGU
ENSG00000159692	AAAUGGU
ENSG00000159692	AAGACAU
ENSG00000159692	AAUGUUG
ENSG00000159692	AAUUAUU
ENSG00000159692	ACUGUGU
ENSG00000159692	AGGACAC
ENSG00000159692	AUUCAUU
ENSG00000159692	GAGAAGA
ENSG00000159692	GCUAGCA
ENSG00000159692	GGAUUUU
ENSG00000159692	GGUUACC
ENSG00000159692	UAUUGCU
ENSG00000159692	UCCUGAG
ENSG00000159692	UGAGAAG
ENSG00000159692	UGUGUGG
ENSG00000159713	AGCAGCU
ENSG00000159713	GGUGGGC
ENSG00000159714	AAAGUGC
ENSG00000159714	AAGUGCA
ENSG00000159714	UAUUGCU
ENSG00000159714	UGCAUAG
ENSG00000159723	UUGGUCC
ENSG00000159733	AAUCCUU
ENSG00000159733	CCAUUAC
ENSG00000159784	AAAAAUC
ENSG00000159784	ACCUGGA
ENSG00000159784	ACUGGCC
ENSG00000159784	AGCACCA
ENSG00000159784	AGGGAGG
ENSG00000159784	AUGCACC
ENSG00000159784	CAAGAGC
ENSG00000159784	CAGACAG
ENSG00000159784	CCCUGAG
ENSG00000159784	CUCUUCC
ENSG00000159784	CUGCCCC
ENSG00000159784	GACCUGG
ENSG00000159784	GUAAACA
ENSG00000159784	UAAGACU
ENSG00000159784	UCAUAGA
ENSG00000159784	UCAUUCG
ENSG00000159788	UCUUGAG
ENSG00000159792	ACAGUAU
ENSG00000159792	AGCAGCA
ENSG00000159792	AGCAGCG
ENSG00000159792	AGCAGCU
ENSG00000159792	AGGCAGG
ENSG00000159792	AUGGCGC
ENSG00000159792	AUGGCUU
ENSG00000159792	CUCUGGG
ENSG00000159792	CUGGCUC
ENSG00000159792	GAGGCAG
ENSG00000159792	GGAGGCA
ENSG00000159792	GGCAGGG
ENSG00000159792	GGCGCCU
ENSG00000159792	GGCUCAG

ENSG00000159792	UAAUGCU
ENSG00000159792	UACAAAG
ENSG00000159792	UCACACA
ENSG00000159842	AACAGUC
ENSG00000159842	AACAUUC
ENSG00000159842	AAGGCAC
ENSG00000159842	ACAAGGU
ENSG00000159842	ACUCAAA
ENSG00000159842	AUAAUAC
ENSG00000159842	AUCCUUG
ENSG00000159842	AUCCUUU
ENSG00000159842	CAAAGCA
ENSG00000159842	CUCCAGC
ENSG00000159842	GAAACAU
ENSG00000159842	GACCCUG
ENSG00000159842	GGCAGUG
ENSG00000159842	GGGAGCU
ENSG00000159842	UCAAGUA
ENSG00000159842	UCCAGUU
ENSG00000159842	UGCAUUG
ENSG00000159842	UUCCUCG
ENSG00000159842	UUUGCAC
ENSG00000159873	AACAGUC
ENSG00000159873	AAUACUG
ENSG00000159873	ACAUUAC
ENSG00000159873	ACAUUCA
ENSG00000159873	ACUCAAA
ENSG00000159873	CAGACAG
ENSG00000159873	GAGAAAU
ENSG00000159873	GCUAGCA
ENSG00000159873	GGCUCAG
ENSG00000159873	UCACAGG
ENSG00000159873	UUCUUGU
ENSG00000159873	UUGGCAA
ENSG00000159873	UUGGUCC
ENSG00000159873	UUGUUCG
ENSG00000159921	AAAAGUA
ENSG00000159921	AAAGUAC
ENSG00000159921	AUAUUAU
ENSG00000159921	GGAAUGU
ENSG00000159921	UUGGCAA
ENSG00000160007	AAAGUGC
ENSG00000160007	AACAUUC
ENSG00000160007	AAGUGCA
ENSG00000160007	ACAAUAU
ENSG00000160007	ACCACAG
ENSG00000160007	AGCAAAA
ENSG00000160007	AGCAGCA
ENSG00000160007	AGUGCAA
ENSG00000160007	AUAAAGU
ENSG00000160007	AUACAAG
ENSG00000160007	AUAUAAC
ENSG00000160007	CAGUCCA
ENSG00000160007	CUGCAGC
ENSG00000160007	GAGGCUG
ENSG00000160007	GCUGGUG
ENSG00000160007	GGCAGCG
ENSG00000160007	GUUAAUG
ENSG00000160007	UAAUAUC
ENSG00000160007	UACAAAG
ENSG00000160007	UAGGCCG
ENSG00000160007	UAUAAAG
ENSG00000160007	UCAACAG
ENSG00000160007	UGAGAAG

ENSG00000160007	UUCAAAU
ENSG00000160007	UUGUUCG
ENSG00000160007	UUUCAAC
ENSG00000160013	CCCCUGG
ENSG00000160013	GGGGUGG
ENSG00000160014	AAAGAGC
ENSG00000160014	AAAGCUG
ENSG00000160014	ACAAUAU
ENSG00000160014	AGCACCA
ENSG00000160014	AGCUGCC
ENSG00000160014	AGGCAGG
ENSG00000160014	AGGUAGU
ENSG00000160014	AUUGCAC
ENSG00000160014	CACAGGG
ENSG00000160014	CACUGUU
ENSG00000160014	CUGGGCA
ENSG00000160014	CUUCACC
ENSG00000160014	GCCUGGG
ENSG00000160014	GGAGUGU
ENSG00000160014	GGGGCAG
ENSG00000160014	UAUUGCU
ENSG00000160014	UCACAGG
ENSG00000160014	UCACAGU
ENSG00000160014	UGCAGCU
ENSG00000160050	AAGUCAC
ENSG00000160050	AGCACCA
ENSG00000160050	CACAGUG
ENSG00000160050	CUGGGGG
ENSG00000160050	UCACAGU
ENSG00000160051	CUUCUCU
ENSG00000160051	UGCCAGC
ENSG00000160058	AAAAACC
ENSG00000160058	AAAGAAU
ENSG00000160058	AGCACCA
ENSG00000160058	AGCAGCA
ENSG00000160058	AGGGAGG
ENSG00000160058	AUAAUAC
ENSG00000160058	AUAUAAC
ENSG00000160058	CAGCAGA
ENSG00000160058	CAGCUGG
ENSG00000160058	CCUUCUU
ENSG00000160058	CUACAAA
ENSG00000160058	CUCCCAC
ENSG00000160058	CUCUGGG
ENSG00000160058	CUGCAGC
ENSG00000160058	CUGGGGG
ENSG00000160058	GCAGCAU
ENSG00000160058	GGGGAGC
ENSG00000160058	GGGUGUU
ENSG00000160058	UGAAGUG
ENSG00000160058	UGGGGGA
ENSG00000160058	UGGGUGA
ENSG00000160058	UUGAUCA
ENSG00000160058	UUGGCAC
ENSG00000160058	UUGGUCC
ENSG00000160058	UUGUGUC
ENSG00000160087	GGUUUAC
ENSG00000160087	UUGGUUC
ENSG00000160094	AAAAAUC
ENSG00000160094	AAAGAGC
ENSG00000160094	AAAGUGC
ENSG00000160094	AACAGUC
ENSG00000160094	AACGGAA
ENSG00000160094	AACUGGU

ENSG00000160094	AAGUGCU
ENSG00000160094	AAUACUG
ENSG00000160094	ACAAUAU
ENSG00000160094	ACAGUAU
ENSG00000160094	AGCACCA
ENSG00000160094	AGCAGCA
ENSG00000160094	AGCAGCG
ENSG00000160094	AGCAGCU
ENSG00000160094	AGGGUCU
ENSG00000160094	AGUAACA
ENSG00000160094	AUGGCUU
ENSG00000160094	CAAAACU
ENSG00000160094	CUGCUCA
ENSG00000160094	CUUCACC
ENSG00000160094	CUUUGGU
ENSG00000160094	GAGAAAU
ENSG00000160094	GAGGUAG
ENSG00000160094	GCCUGGA
ENSG00000160094	GCCUGGG
ENSG00000160094	GGGUGUU
ENSG00000160094	GGUAGAC
ENSG00000160094	GUCUGCC
ENSG00000160094	UAAUAUC
ENSG00000160094	UACAAAG
ENSG00000160094	UACAGUU
ENSG00000160094	UAGACUG
ENSG00000160094	UCACAAG
ENSG00000160094	UCCUAUA
ENSG00000160094	UUGGUCC
ENSG00000160097	AAGUCAC
ENSG00000160097	AGCACCA
ENSG00000160097	AGCAGCU
ENSG00000160097	AGCCCUU
ENSG00000160097	AUGGCUU
ENSG00000160097	CAGGCUC
ENSG00000160097	CCAGGAG
ENSG00000160097	GAGAUGA
ENSG00000160097	GAGGCAG
ENSG00000160097	GCACCAU
ENSG00000160097	GGAGAGA
ENSG00000160097	GGAUGAG
ENSG00000160097	GGCAGUG
ENSG00000160097	GGCUCAG
ENSG00000160097	GGGGCAG
ENSG00000160097	UGAGGAC
ENSG00000160097	UGGGUGA
ENSG00000160097	UUUGCAA
ENSG00000160097	UUUGCAC
ENSG00000160113	UUGGUUC
ENSG00000160145	AGCUGCC
ENSG00000160145	AUGGCUU
ENSG00000160145	CAAAACU
ENSG00000160145	GAGCUGC
ENSG00000160145	GGCUGCG
ENSG00000160145	GUGCUUG
ENSG00000160145	UGGCUGC
ENSG00000160161	AGCACCA
ENSG00000160179	UUCCUAG
ENSG00000160185	AAAGUGC
ENSG00000160190	UUCAAAU
ENSG00000160191	AAAAAUC
ENSG00000160199	AAUCCUU
ENSG00000160199	ACAGUAC
ENSG00000160199	ACAUUCA

ENSG00000160199	AGCAAAA
ENSG00000160199	AUCCUUU
ENSG00000160199	CAGGCUC
ENSG00000160199	CCAGCAU
ENSG00000160199	CUCUAGC
ENSG00000160199	GAAACAU
ENSG00000160199	UAUAAAG
ENSG00000160199	UCGCUGC
ENSG00000160199	UGAAAGG
ENSG00000160199	UUGGUCC
ENSG00000160201	CUACAAA
ENSG00000160202	CUCAAAC
ENSG00000160208	AAUUUUA
ENSG00000160208	GAGGUAG
ENSG00000160208	GUGCUUG
ENSG00000160208	UACAAAG
ENSG00000160208	UCCUUGC
ENSG00000160208	UUGGCAA
ENSG00000160209	AAGGCAC
ENSG00000160216	ACCCUGU
ENSG00000160216	ACUGGCC
ENSG00000160216	AGUGCAA
ENSG00000160216	CACAGUG
ENSG00000160216	CACCAGC
ENSG00000160216	GAUUGUA
ENSG00000160216	GGCUCAG
ENSG00000160216	GUUAAUG
ENSG00000160216	UAAUAUC
ENSG00000160216	UAUUGCU
ENSG00000160216	UCACAGG
ENSG00000160216	UCCAGUU
ENSG00000160216	UGUGCUU
ENSG00000160219	CAAAGCA
ENSG00000160233	AAGGCAC
ENSG00000160233	CUGGAAA
ENSG00000160271	AAGGCAC
ENSG00000160271	AAGUGCU
ENSG00000160271	CUUUGGU
ENSG00000160271	GCAGAAG
ENSG00000160271	GGCAGUG
ENSG00000160271	UUGGCAA
ENSG00000160293	AAAGUGC
ENSG00000160293	AGCAGCA
ENSG00000160293	AGUGCAA
ENSG00000160293	CAAAGUG
ENSG00000160293	CAAGAGC
ENSG00000160293	CAGUGCA
ENSG00000160293	GGCUCUG
ENSG00000160293	GUAAACA
ENSG00000160293	GUGCAAA
ENSG00000160293	UACAAAG
ENSG00000160293	UCACAGG
ENSG00000160294	UCACAUU
ENSG00000160305	AAGUGCA
ENSG00000160305	AGUGCAA
ENSG00000160305	AUGGCAC
ENSG00000160305	CAGCAGA
ENSG00000160305	GUGCAAA
ENSG00000160307	AAAGAGG
ENSG00000160307	CAAAGCA
ENSG00000160307	GCUUCCU
ENSG00000160310	CUGGAAA
ENSG00000160310	UCACAGG
ENSG00000160325	AAGGCAC

ENSG00000160325	AGCACCA
ENSG00000160325	GAGGUAG
ENSG00000160325	GGUAGAC
ENSG00000160392	AACAGUC
ENSG00000160392	AUAUUAU
ENSG00000160392	GAAAACA
ENSG00000160392	GAGAAAU
ENSG00000160408	AAAUCCC
ENSG00000160408	ACCCUGU
ENSG00000160408	AGUGCCU
ENSG00000160408	AUAAAGU
ENSG00000160408	CCCUGAG
ENSG00000160408	GACCCUG
ENSG00000160408	GGCUCUG
ENSG00000160408	UACAAAG
ENSG00000160410	GGCAGUG
ENSG00000160445	ACACACC
ENSG00000160445	ACACACU
ENSG00000160445	AGCACCA
ENSG00000160445	AGCAGCA
ENSG00000160445	AGCAGCG
ENSG00000160445	AGCAGCU
ENSG00000160445	AGGCAGG
ENSG00000160445	AUAAUAC
ENSG00000160445	AUAUAAC
ENSG00000160445	AUCAUAC
ENSG00000160445	CAAAACU
ENSG00000160445	CAGGCUC
ENSG00000160445	CUGGGCA
ENSG00000160445	GCAGAAG
ENSG00000160445	GCCUGGG
ENSG00000160445	GCUGGUG
ENSG00000160445	GCUUCCU
ENSG00000160445	GGCAGUG
ENSG00000160445	GGCUCAG
ENSG00000160445	GGGGCAG
ENSG00000160445	GGUGGGC
ENSG00000160445	UCACACA
ENSG00000160445	UCAUACA
ENSG00000160445	UUGGCAA
ENSG00000160445	UUGGCAC
ENSG00000160460	AGUGUGC
ENSG00000160460	CACCAGC
ENSG00000160460	GAGGUAG
ENSG00000160460	GAGGUAU
ENSG00000160460	GCAGCAU
ENSG00000160460	GCGGGGC
ENSG00000160460	GGCUCUG
ENSG00000160460	GUGAGGU
ENSG00000160469	AGGCAGG
ENSG00000160469	CCCUGUC
ENSG00000160469	CUUGGGC
ENSG00000160469	GAGCCCC
ENSG00000160469	GCCCUGU
ENSG00000160469	GGCCCUG
ENSG00000160469	UUGUGUC
ENSG00000160539	AGUGCUG
ENSG00000160539	CCCCUGG
ENSG00000160539	CUGGGGG
ENSG00000160551	AAUUAUU
ENSG00000160551	AGCAGCU
ENSG00000160551	AGGCAGG
ENSG00000160551	AUGACAC
ENSG00000160551	AUGGCAC

ENSG00000160551	CACAGUG
ENSG00000160551	CAGACAG
ENSG00000160551	CAGCAGA
ENSG00000160551	CAGCAGG
ENSG00000160551	CAGUAGA
ENSG00000160551	GUCAUUG
ENSG00000160551	UCACAGU
ENSG00000160551	UGACAUC
ENSG00000160551	UUGGCAA
ENSG00000160551	UUGGCAC
ENSG00000160563	UAUAAAG
ENSG00000160570	UCCCUUU
ENSG00000160570	UCCUGAG
ENSG00000160602	GGUGGUC
ENSG00000160602	UCCAGAG
ENSG00000160606	UUUCAAC
ENSG00000160613	AAAGCUG
ENSG00000160613	CCCUGAG
ENSG00000160613	GCAGAAG
ENSG00000160613	UCCCUGU
ENSG00000160633	AUGGCAC
ENSG00000160633	GGCAGCG
ENSG00000160633	UACAAAG
ENSG00000160633	UAUUACC
ENSG00000160654	ACCUGGA
ENSG00000160679	ACAGUAU
ENSG00000160683	AAGACAU
ENSG00000160683	ACUGUAG
ENSG00000160683	CCUGUAC
ENSG00000160683	GAAACAU
ENSG00000160683	GACCUGG
ENSG00000160683	GACUUCC
ENSG00000160683	GCUUCCU
ENSG00000160683	GUCUCUG
ENSG00000160683	UGACCUA
ENSG00000160685	AAGACAU
ENSG00000160685	AAGGCAC
ENSG00000160685	ACUCAGC
ENSG00000160685	AGUGCAA
ENSG00000160685	CCAGCAU
ENSG00000160685	GAGGGGC
ENSG00000160685	GCGGAGG
ENSG00000160685	GGCGGGG
ENSG00000160685	GGGGCAG
ENSG00000160685	GGGGUCC
ENSG00000160685	GUGCAAA
ENSG00000160685	UGAAAUG
ENSG00000160685	UGCAUAG
ENSG00000160685	UGCAUAU
ENSG00000160685	UUCAAGC
ENSG00000160685	UUGGUCC
ENSG00000160685	UUUCAAC
ENSG00000160691	AAGGCAC
ENSG00000160691	AAUACUG
ENSG00000160691	CCAGGAG
ENSG00000160691	GAUUGUC
ENSG00000160691	GGAGGCA
ENSG00000160691	GUCUACU
ENSG00000160691	UCCCUUG
ENSG00000160691	UGUAUAA
ENSG00000160691	UUGGCAC
ENSG00000160710	AAAGUGC
ENSG00000160710	AAGUGCA
ENSG00000160710	AAUGCCC

ENSG00000160710	AGCAAAA
ENSG00000160710	CAGACAG
ENSG00000160712	AAAGCUG
ENSG00000160712	GGCCCUG
ENSG00000160714	AAAGGUA
ENSG00000160714	AAAGUUU
ENSG00000160714	ACCACAG
ENSG00000160714	ACCAGCA
ENSG00000160714	AGGCAGG
ENSG00000160714	AUCAUAC
ENSG00000160714	AUCCUUU
ENSG00000160714	CAGUCUG
ENSG00000160714	CCAGUGU
ENSG00000160714	CUAGUCC
ENSG00000160714	GAGAAAU
ENSG00000160714	GAGGCAG
ENSG00000160714	GCCUGGA
ENSG00000160714	GCUGGUG
ENSG00000160714	GGCAGCG
ENSG00000160714	GGUAGAC
ENSG00000160714	GGUGGUU
ENSG00000160714	GUGCCUG
ENSG00000160714	UACAAAG
ENSG00000160714	UCCAGAG
ENSG00000160714	UCUGGAG
ENSG00000160714	UUCAAAU
ENSG00000160714	UUGGUCC
ENSG00000160716	AGUGCAA
ENSG00000160716	CAGUGCA
ENSG00000160716	GGGGCAG
ENSG00000160716	UGUGUCA
ENSG00000160741	AAGUGCU
ENSG00000160741	AGUGCUU
ENSG00000160741	CAAAGUG
ENSG00000160741	CAGACAG
ENSG00000160741	CAGGCUC
ENSG00000160741	GGCUCUG
ENSG00000160741	UCCCUUG
ENSG00000160753	CAGUCCA
ENSG00000160785	AGCACCA
ENSG00000160785	AGUGCAA
ENSG00000160785	GCUAGCA
ENSG00000160785	GUAGUGU
ENSG00000160785	UCACAGG
ENSG00000160785	UCACAGU
ENSG00000160789	ACUGUAG
ENSG00000160789	CACAGGG
ENSG00000160789	CUCAGGG
ENSG00000160789	GCACCAU
ENSG00000160789	UUGGUUC
ENSG00000160791	CAAAACU
ENSG00000160796	CAAAACU
ENSG00000160796	GACUUCC
ENSG00000160796	GAGGGGC
ENSG00000160796	GGGCCCC
ENSG00000160796	GUGCAAA
ENSG00000160803	UACAAAG
ENSG00000160867	UAUAAAG
ENSG00000160867	UUCAAAU
ENSG00000160883	UAUUGCU
ENSG00000160888	CCCUGAG
ENSG00000160888	GACCCUG
ENSG00000160888	GGGGGAA
ENSG00000160917	AAAAAUC

ENSG00000160917	CAGCAGA
ENSG00000160917	GAGGUAG
ENSG00000160932	UCCUGAG
ENSG00000160963	CACUCCU
ENSG00000160963	CUCCAGC
ENSG00000160963	UUCAAAU
ENSG00000160967	AAAAAUC
ENSG00000160967	AAUCACU
ENSG00000160967	AAUCUCU
ENSG00000160967	ACCACAG
ENSG00000160967	AGUGUGC
ENSG00000160967	CUACAAA
ENSG00000160967	GCCCUGU
ENSG00000160967	GUGCAAA
ENSG00000160967	UAAUGCU
ENSG00000160967	UCCUGAG
ENSG00000160967	UGCCCUG
ENSG00000160967	UUAGAGA
ENSG00000160991	CCCCCAG
ENSG00000160994	AGGGAGG
ENSG00000160999	CUUCACC
ENSG00000161013	AAAAAUC
ENSG00000161013	AAAGUAA
ENSG00000161013	AAAGUAC
ENSG00000161013	AAAGUAU
ENSG00000161013	AAGUCAC
ENSG00000161013	AGCACCA
ENSG00000161013	AUAAAGU
ENSG00000161013	AUGCAUU
ENSG00000161013	AUGUGGG
ENSG00000161013	CAAAUGC
ENSG00000161013	CACAGUG
ENSG00000161013	CACCAGC
ENSG00000161013	CCAGUGC
ENSG00000161013	GGCGGGG
ENSG00000161013	GGGGCAG
ENSG00000161013	GUGGGGA
ENSG00000161013	UAUUACC
ENSG00000161013	UCCAGUU
ENSG00000161021	AAAACUG
ENSG00000161021	AACAAAC
ENSG00000161021	AAGGCAC
ENSG00000161021	AAGUGCA
ENSG00000161021	AAGUGCU
ENSG00000161021	AGUAGUU
ENSG00000161021	AGUGCAA
ENSG00000161021	AUAAAGU
ENSG00000161021	AUGACAC
ENSG00000161021	AUGGCAC
ENSG00000161021	CAAAGCA
ENSG00000161021	CUGCAGC
ENSG00000161021	CUGGGGG
ENSG00000161021	GCAGCAU
ENSG00000161021	GCUGGUG
ENSG00000161021	GUGCAAA
ENSG00000161021	UAAUAGU
ENSG00000161021	UAUAAUA
ENSG00000161021	UCACAUU
ENSG00000161021	UGUAUAA
ENSG00000161048	AGGCAGG
ENSG00000161048	CACAGUG
ENSG00000161048	GAGGCAG
ENSG00000161048	GAGGUAG
ENSG00000161179	CUGCAGC

ENSG00000161180	CAGCAGA
ENSG00000161202	ACCAGCA
ENSG00000161202	ACCCUGU
ENSG00000161202	AUCCUUG
ENSG00000161202	CCCUGAG
ENSG00000161202	GAGGCUG
ENSG00000161202	GAGGGGC
ENSG00000161202	GCAGGUU
ENSG00000161202	GGCCCUG
ENSG00000161202	GGCUGCG
ENSG00000161202	UAGGUAU
ENSG00000161202	UCCCUUG
ENSG00000161202	UGCAUAG
ENSG00000161202	UGGCUGC
ENSG00000161203	AACUGGU
ENSG00000161203	ACUGGCU
ENSG00000161203	CAAAGCA
ENSG00000161203	CUCACAC
ENSG00000161203	CUUUGGU
ENSG00000161203	UGGGGGA
ENSG00000161203	UUGGUCC
ENSG00000161243	AAGGCAC
ENSG00000161243	AGCAGCA
ENSG00000161243	AGGGAGG
ENSG00000161267	AGCACCA
ENSG00000161267	GGCCCUG
ENSG00000161381	CAGCAAA
ENSG00000161381	GAGUCUU
ENSG00000161381	GGGAGCU
ENSG00000161381	UGAGUCU
ENSG00000161381	UUCAAGC
ENSG00000161405	AGCAGCU
ENSG00000161405	CAAAGCA
ENSG00000161526	GAGCUGC
ENSG00000161526	GCUUCCU
ENSG00000161526	GGGGUGC
ENSG00000161526	GGGGUGG
ENSG00000161526	GUAAACA
ENSG00000161526	GUGGGGA
ENSG00000161526	UCCGCUC
ENSG00000161526	UUGACAC
ENSG00000161533	AAAAACC
ENSG00000161533	AACUACU
ENSG00000161533	AAUUGCU
ENSG00000161533	AGCAGCA
ENSG00000161533	CAGCAGG
ENSG00000161533	GAAAACA
ENSG00000161533	GUCAACA
ENSG00000161533	GUUCAUG
ENSG00000161533	UAUAAAG
ENSG00000161533	UGAAAGG
ENSG00000161544	CCUUCUG
ENSG00000161544	GCUUCCU
ENSG00000161544	GGGCCCC
ENSG00000161544	GGGUCUU
ENSG00000161544	GGUUACC
ENSG00000161544	UACAAAG
ENSG00000161558	AAGGCAC
ENSG00000161558	AGGUAGU
ENSG00000161558	GAGAAAU
ENSG00000161558	GAGGUAG
ENSG00000161573	GGAAGAC
ENSG00000161573	UUCCUCG
ENSG00000161638	AGGGAGG

ENSG00000161638	AUGUCUG
ENSG00000161638	CACAGUG
ENSG00000161638	CAGCUGG
ENSG00000161638	CCUUCAU
ENSG00000161638	GGCCCUG
ENSG00000161638	GUCUCUG
ENSG00000161638	UAUUGCU
ENSG00000161638	UCAAGUA
ENSG00000161638	UCACAGG
ENSG00000161638	UCACAGU
ENSG00000161638	UCCUGAG
ENSG00000161638	UGCAAAG
ENSG00000161638	UGCAGCU
ENSG00000161638	UGGCCCU
ENSG00000161642	AAAGAAU
ENSG00000161642	AAAGUGC
ENSG00000161642	AAGCGCU
ENSG00000161642	AAGUGCU
ENSG00000161642	AGUGCUG
ENSG00000161642	AGUGCUU
ENSG00000161642	AUGGCUU
ENSG00000161642	CAAAGUG
ENSG00000161642	CACAGUG
ENSG00000161642	CCCACCG
ENSG00000161642	CCCACCU
ENSG00000161642	CCCUGAG
ENSG00000161642	CCUUCUG
ENSG00000161642	CUGCCCC
ENSG00000161642	CUUGGGC
ENSG00000161642	GACCAUG
ENSG00000161642	GCGGGGC
ENSG00000161642	GGAGAGA
ENSG00000161642	GGAGUCC
ENSG00000161642	GGCAGGG
ENSG00000161642	GGCGGGG
ENSG00000161642	GGGGGAA
ENSG00000161642	GGGGUGG
ENSG00000161642	GGUGCGG
ENSG00000161642	GGUGGGC
ENSG00000161642	GUGAGGU
ENSG00000161642	UAUUGCU
ENSG00000161642	UCACAGU
ENSG00000161642	UCCCACC
ENSG00000161642	UGGACUG
ENSG00000161642	UUGGUCC
ENSG00000161642	UUUGCGA
ENSG00000161642	UUUUUGC
ENSG00000161647	AAAGAAU
ENSG00000161647	CUUUGGU
ENSG00000161647	UACAAAG
ENSG00000161647	UGGUACC
ENSG00000161649	UCUGGAG
ENSG00000161653	AUGGCUU
ENSG00000161653	CUGGGCA
ENSG00000161653	GAGCCCU
ENSG00000161653	GCCUGGG
ENSG00000161653	UUGGUCC
ENSG00000161654	AAAAACC
ENSG00000161654	AAAACUG
ENSG00000161654	AAACUGU
ENSG00000161654	ACAAGGU
ENSG00000161654	AGCCAGU
ENSG00000161654	AGGCAGG
ENSG00000161654	AUCCCUU

ENSG00000161654	CACAGUG
ENSG00000161654	CGCCUCC
ENSG00000161654	CGGCCUG
ENSG00000161654	CUCUAGC
ENSG00000161654	GAGACCU
ENSG00000161654	GCUGGAU
ENSG00000161654	GGGUUUA
ENSG00000161654	GUGACUG
ENSG00000161654	UAAUAUC
ENSG00000161654	UAUAAAG
ENSG00000161654	UCACAGU
ENSG00000161654	UCCCUCU
ENSG00000161654	UCCUGAG
ENSG00000161654	UGACUGU
ENSG00000161654	UGCCCUG
ENSG00000161654	UGGGUGA
ENSG00000161654	UUCAAGC
ENSG00000161654	UUGAUCA
ENSG00000161654	UUGUUCG
ENSG00000161671	CCAGUGU
ENSG00000161671	GGAGAGA
ENSG00000161677	GAGGCAG
ENSG00000161714	GAGGCUG
ENSG00000161791	AAAGUGC
ENSG00000161791	AAGUGCA
ENSG00000161791	AGUGCAA
ENSG00000161791	AGUGCCU
ENSG00000161791	CAAAGUG
ENSG00000161791	CAGCAGA
ENSG00000161791	CAGUAGA
ENSG00000161791	CUCUAGC
ENSG00000161791	GCCUGGG
ENSG00000161791	GGGGUGG
ENSG00000161791	GUGCCUG
ENSG00000161791	GUGUGGC
ENSG00000161791	UCCUUGC
ENSG00000161791	UGUGUGG
ENSG00000161791	UGUUGAA
ENSG00000161800	AAGUGCA
ENSG00000161800	GUGCAAA
ENSG00000161800	UCCCACC
ENSG00000161800	UGCAAAG
ENSG00000161813	AAGUCAC
ENSG00000161813	AAGUGCU
ENSG00000161813	AAUCCUU
ENSG00000161813	AGCACCA
ENSG00000161813	CACAAUG
ENSG00000161813	CACAGUG
ENSG00000161813	CCAGUGC
ENSG00000161813	CUCACAC
ENSG00000161813	CUCCAGC
ENSG00000161813	CUGGCUC
ENSG00000161813	GAGAUGA
ENSG00000161813	GGAAGAC
ENSG00000161813	GGAAUGU
ENSG00000161813	GUAAACA
ENSG00000161813	UACAGUU
ENSG00000161813	UCACAAG
ENSG00000161813	UCACAGG
ENSG00000161813	UCACAGU
ENSG00000161813	UCCAGUU
ENSG00000161813	UCCUAUA
ENSG00000161813	UGUUGAA
ENSG00000161813	UUGACAC

ENSG00000161835	AAAGCUG
ENSG00000161835	AACAGUC
ENSG00000161835	AAGGCAC
ENSG00000161835	ACCAGCA
ENSG00000161835	AGCCCUU
ENSG00000161835	GCCCUUA
ENSG00000161847	AAGGCAC
ENSG00000161847	AGGCAGG
ENSG00000161847	CUGCAGC
ENSG00000161847	GAAGGAA
ENSG00000161847	GCUGGUG
ENSG00000161847	GGCUCUG
ENSG00000161847	GUCUCUG
ENSG00000161847	UACAAAG
ENSG00000161904	AAGGCAC
ENSG00000161904	GGAGGCA
ENSG00000161904	GUGAAUG
ENSG00000161904	UAUUGCU
ENSG00000161940	AAUCUCU
ENSG00000161940	ACCAGCA
ENSG00000161940	AGCAAAA
ENSG00000161940	CAGACAG
ENSG00000161940	CAGCAAA
ENSG00000161940	CAGCAGA
ENSG00000161940	UCCAGAG
ENSG00000161955	UCCAGUU
ENSG00000161956	AUAUAAC
ENSG00000161956	AUCCUUU
ENSG00000161956	CACCACC
ENSG00000161956	GCAGGAC
ENSG00000161956	GGCAGGG
ENSG00000161956	GGCAUUG
ENSG00000161958	CUGGCUC
ENSG00000161958	GAGGUAG
ENSG00000161958	GAGGUAU
ENSG00000161958	GGCCCUG
ENSG00000161958	GUCCAGA
ENSG00000161958	GUCUCUG
ENSG00000161958	UAUUGCU
ENSG00000161958	UGCUGAC
ENSG00000161958	UGGCCCU
ENSG00000161960	AAAAAUC
ENSG00000161960	AUACAAG
ENSG00000161960	AUCCCUU
ENSG00000161960	CCCCUGG
ENSG00000161960	CCUUCUG
ENSG00000162004	GCUUCCU
ENSG00000162065	ACCAGCA
ENSG00000162065	CACCAGC
ENSG00000162065	CAGGCUC
ENSG00000162069	AAAGCUG
ENSG00000162073	UCCUGAG
ENSG00000162104	AAGGCAC
ENSG00000162104	AAGUGUG
ENSG00000162104	AAUACUG
ENSG00000162104	ACACACC
ENSG00000162104	ACACACU
ENSG00000162104	ACCACAG
ENSG00000162104	ACUGGCC
ENSG00000162104	ACUGGCU
ENSG00000162104	AGGUAGU
ENSG00000162104	CUUUGGU
ENSG00000162104	GAGGUAG
ENSG00000162104	GCUUCCU

ENSG00000162104	GGAAGAC
ENSG00000162104	GGAUGAG
ENSG00000162104	GGCUCAG
ENSG00000162104	GUAGUGU
ENSG00000162104	GUGCAAA
ENSG00000162104	UAUUGCU
ENSG00000162104	UGAUCCA
ENSG00000162105	AAAGUGC
ENSG00000162105	AACAAAC
ENSG00000162105	AACAGUC
ENSG00000162105	AACCUGG
ENSG00000162105	AAGACAU
ENSG00000162105	AAGUGCA
ENSG00000162105	AAUUAUU
ENSG00000162105	AAUUGCU
ENSG00000162105	ACACACU
ENSG00000162105	ACAGUAC
ENSG00000162105	ACAUAGA
ENSG00000162105	ACCUGGA
ENSG00000162105	ACUGUUU
ENSG00000162105	AGCAAAA
ENSG00000162105	AGGAGCU
ENSG00000162105	AGUAGAC
ENSG00000162105	AUACAAG
ENSG00000162105	AUAUAAC
ENSG00000162105	AUGUAAU
ENSG00000162105	CACUGUU
ENSG00000162105	CAGACAG
ENSG00000162105	CAGCAAA
ENSG00000162105	CUAGUAG
ENSG00000162105	CUGGGCA
ENSG00000162105	CUUUGGU
ENSG00000162105	GAAAACA
ENSG00000162105	GAAACAU
ENSG00000162105	GACCUGG
ENSG00000162105	GAGGUAU
ENSG00000162105	GCAGAAG
ENSG00000162105	GCAGGUU
ENSG00000162105	GCAUCCC
ENSG00000162105	GCUGGUG
ENSG00000162105	GUCAUUG
ENSG00000162105	GUGCUUG
ENSG00000162105	GUGGCCC
ENSG00000162105	UAAUGCU
ENSG00000162105	UACAGUU
ENSG00000162105	UAUAAUA
ENSG00000162105	UAUUGCU
ENSG00000162105	UCAACAG
ENSG00000162105	UGAAGUG
ENSG00000162105	UGACCUA
ENSG00000162105	UGCAUUG
ENSG00000162105	UGCCAGC
ENSG00000162105	UGUAUAA
ENSG00000162105	UUAGAGA
ENSG00000162105	UUGGCAA
ENSG00000162105	UUGGUUC
ENSG00000162105	UUGUGAC
ENSG00000162105	UUUGCAA
ENSG00000162139	CAGGUGA
ENSG00000162139	CUACAAA
ENSG00000162144	AAGUCAC
ENSG00000162144	AGCAGCU
ENSG00000162144	CACAGGG
ENSG00000162148	AAGUAGC

ENSG00000162174	GCUAGCA
ENSG00000162174	UUCAAAU
ENSG00000162188	CAGGUGA
ENSG00000162188	CUCCAUU
ENSG00000162188	GUGCUUG
ENSG00000162188	UGUGCUU
ENSG00000162188	UUCAAAU
ENSG00000162231	AAAAGUA
ENSG00000162231	ACCCGUA
ENSG00000162231	AGGAGCU
ENSG00000162231	CACAGUG
ENSG00000162231	CCCACCU
ENSG00000162231	CUCUGGG
ENSG00000162231	CUGGCUC
ENSG00000162231	GGCGGGG
ENSG00000162231	GGGCGUG
ENSG00000162231	GGGGUGG
ENSG00000162231	GUCUCUG
ENSG00000162231	GUGCCUG
ENSG00000162231	UCACAGU
ENSG00000162236	AGUGCCU
ENSG00000162236	CAGCAGA
ENSG00000162236	CAGGCUC
ENSG00000162236	CUCCCAA
ENSG00000162236	GGCUCAG
ENSG00000162236	GUCUUGC
ENSG00000162236	UCACAGU
ENSG00000162236	UUGGCAA
ENSG00000162236	UUGGCAC
ENSG00000162236	UUGGUCC
ENSG00000162241	CACCAGC
ENSG00000162290	AAAAGUA
ENSG00000162290	AAAGUAA
ENSG00000162290	AAGGCAC
ENSG00000162290	AAUCCUU
ENSG00000162290	ACAUUAC
ENSG00000162290	ACUCAAA
ENSG00000162290	AGAUAAA
ENSG00000162290	AGCAGCG
ENSG00000162290	AGCAGCU
ENSG00000162290	AGUGCUG
ENSG00000162290	AGUGCUU
ENSG00000162290	AUGGCUU
ENSG00000162290	AUUGCAC
ENSG00000162290	CAAAGCA
ENSG00000162290	CAGCAAA
ENSG00000162290	CAGGCUC
ENSG00000162290	CAGUGCA
ENSG00000162290	CCAUUAC
ENSG00000162290	CCCUGAG
ENSG00000162290	CUUGGGC
ENSG00000162290	CUUUGGU
ENSG00000162290	GAGAAAU
ENSG00000162290	GCUAGCA
ENSG00000162290	GGUUGAC
ENSG00000162290	GUAAACA
ENSG00000162290	UAAUAUC
ENSG00000162290	UAUUGCU
ENSG00000162290	UCAUAGA
ENSG00000162290	UCCCUGU
ENSG00000162290	UCCCUUU
ENSG00000162290	UGCAUAG
ENSG00000162290	UGCAUUG
ENSG00000162290	UGCUGAC

ENSG00000162290	UGGGGGA
ENSG00000162290	UUGGUCC
ENSG00000162290	UUGGUUC
ENSG00000162298	AAUACUG
ENSG00000162298	ACUGCAG
ENSG00000162298	ACUGUUU
ENSG00000162298	CACAGGG
ENSG00000162298	GAGCUAA
ENSG00000162298	GAGGGGC
ENSG00000162298	GAGUUGA
ENSG00000162298	GAGUUGG
ENSG00000162298	GGCAGUG
ENSG00000162298	GGGAGCU
ENSG00000162298	GUAACAG
ENSG00000162298	GUUAAUG
ENSG00000162298	UAAUGCU
ENSG00000162298	UACAAAG
ENSG00000162298	UGUGUGG
ENSG00000162300	AUAAAGU
ENSG00000162302	AAAAAUC
ENSG00000162302	AAAGUGC
ENSG00000162302	AAGGCAC
ENSG00000162302	AUAAAGU
ENSG00000162302	AUUGCAC
ENSG00000162302	CACAGUG
ENSG00000162302	CUUUGGU
ENSG00000162302	GGCAGUG
ENSG00000162302	GUGACAG
ENSG00000162302	UAUAAAG
ENSG00000162337	CAUAUUG
ENSG00000162337	GUUCAUG
ENSG00000162337	UUGUUCG
ENSG00000162341	AAGGCAC
ENSG00000162344	AAGUGCU
ENSG00000162366	UCACAGG
ENSG00000162366	UCACAGU
ENSG00000162367	AAAAGUA
ENSG00000162367	AAAGAGC
ENSG00000162367	AAAGAGG
ENSG00000162367	AAAGGUA
ENSG00000162367	AAAGUAC
ENSG00000162367	AAAGUGC
ENSG00000162367	AAGGCAC
ENSG00000162367	AAGUGCU
ENSG00000162367	ACAGUAU
ENSG00000162367	ACCUGGA
ENSG00000162367	AGUGCUG
ENSG00000162367	CACAGUG
ENSG00000162367	CACCACC
ENSG00000162367	CUACAAA
ENSG00000162367	GCAGAAG
ENSG00000162367	GCCCUUA
ENSG00000162367	GGCAGGG
ENSG00000162367	GGCCCUG
ENSG00000162367	GGGCCCC
ENSG00000162367	UCACAAG
ENSG00000162367	UCACCAC
ENSG00000162367	UUCUGCA
ENSG00000162368	AAAGCUG
ENSG00000162368	AAAGUGC
ENSG00000162368	AAGGCAC
ENSG00000162368	AGCACCA
ENSG00000162368	AGCAGCA
ENSG00000162368	AGCAGCG

ENSG00000162368	AGUGCAA
ENSG00000162368	CAAAGUG
ENSG00000162374	AACACUG
ENSG00000162374	AAUACUG
ENSG00000162374	AAUCCUU
ENSG00000162374	ACUGGCU
ENSG00000162374	AUCCUUU
ENSG00000162377	UUUCAAC
ENSG00000162378	AAACUGU
ENSG00000162378	AAUCUCA
ENSG00000162378	ACUGUUU
ENSG00000162378	AGCUUAU
ENSG00000162378	AGUGGUU
ENSG00000162378	AUGGCUU
ENSG00000162378	CAGUCUG
ENSG00000162378	CGUGCAU
ENSG00000162378	GGCAGUG
ENSG00000162378	UGCUCAC
ENSG00000162378	UGUGCUU
ENSG00000162378	UUAGAGA
ENSG00000162384	ACUCAGG
ENSG00000162402	AAGGCAC
ENSG00000162402	AAGGUGC
ENSG00000162402	AUGUCUG
ENSG00000162402	CACUCCU
ENSG00000162402	CAGCAGA
ENSG00000162402	CAGUCUG
ENSG00000162402	CUACAGU
ENSG00000162402	GAGGUAG
ENSG00000162402	GCAGGUU
ENSG00000162402	GGCAGUG
ENSG00000162402	GUGCAAA
ENSG00000162402	UAAGACG
ENSG00000162402	UACAAAG
ENSG00000162402	UACAGUU
ENSG00000162402	UCACAUU
ENSG00000162402	UCUCCAA
ENSG00000162402	UUCCAUA
ENSG00000162402	UUGGCAA
ENSG00000162407	AAGUCAC
ENSG00000162407	AAGUCUU
ENSG00000162407	ACAUAGA
ENSG00000162407	ACAUUAC
ENSG00000162407	ACAUUCA
ENSG00000162407	AGCAGCA
ENSG00000162407	AGGAUGU
ENSG00000162407	AGUGCUG
ENSG00000162407	AUAAUAC
ENSG00000162407	AUUGCAC
ENSG00000162407	CAGCAGA
ENSG00000162407	CAGUAGA
ENSG00000162407	CAGUGCA
ENSG00000162407	CUCUAGC
ENSG00000162407	GCUGUCU
ENSG00000162407	GGACGGA
ENSG00000162407	UCACAGU
ENSG00000162409	AAGGCAC
ENSG00000162409	CAGUCUG
ENSG00000162409	CCCUGAG
ENSG00000162409	GAGGUAG
ENSG00000162409	GAGGUAU
ENSG00000162409	GUGCAAA
ENSG00000162409	UAAUACA
ENSG00000162409	UAGACUG

ENSG00000162409	UCAAGUA
ENSG00000162409	UUCAAGC
ENSG00000162409	UUGGCAA
ENSG00000162415	AUUCAUU
ENSG00000162415	CUGCAGG
ENSG00000162415	GAGGCAG
ENSG00000162415	GAGGUAG
ENSG00000162415	GAGGUAU
ENSG00000162415	GGAGGCA
ENSG00000162415	UAAGACU
ENSG00000162415	UACAGUU
ENSG00000162415	UCAAGUA
ENSG00000162415	UUCUGCA
ENSG00000162430	AAAGCUG
ENSG00000162430	AAGACAU
ENSG00000162430	ACUGGCC
ENSG00000162430	CUACAAA
ENSG00000162430	GAAGGAA
ENSG00000162430	GAGAAAU
ENSG00000162430	GAGCCCC
ENSG00000162430	GGAUUUC
ENSG00000162430	GGCCCUG
ENSG00000162430	GGCUCAG
ENSG00000162430	UCACAGU
ENSG00000162430	UGAAAUG
ENSG00000162430	UGCAAAG
ENSG00000162430	UGGCCCU
ENSG00000162430	UGGCUGC
ENSG00000162430	UUCUGCA
ENSG00000162433	AAGGCAC
ENSG00000162434	AGGAGCU
ENSG00000162434	AUAAAGU
ENSG00000162434	GAGCUAA
ENSG00000162434	GUAAACA
ENSG00000162434	UGGCCAC
ENSG00000162437	AAGGCAC
ENSG00000162437	AUGGCUU
ENSG00000162437	CAGCAGA
ENSG00000162437	GGCAGCG
ENSG00000162437	GUGAGGU
ENSG00000162437	UAAUAUC
ENSG00000162437	UGCAUAU
ENSG00000162437	UUGGCAA
ENSG00000162437	UUGGCAC
ENSG00000162456	GUGCCUG
ENSG00000162456	UCCCACC
ENSG00000162461	AGUGUGC
ENSG00000162461	CUCUGGG
ENSG00000162461	GAGUGUG
ENSG00000162461	GGCAGGG
ENSG00000162461	GUAAACA
ENSG00000162461	GUGCAAA
ENSG00000162490	AGUGCUG
ENSG00000162490	CAGUAGA
ENSG00000162490	CUGCAGC
ENSG00000162493	ACUGCAU
ENSG00000162493	CAGGAAC
ENSG00000162493	CCAGUGC
ENSG00000162493	CUGCUCA
ENSG00000162493	CUUCUCU
ENSG00000162493	GUCUACU
ENSG00000162496	UGCAAAG
ENSG00000162510	AUAUUAU
ENSG00000162511	GAUUGUC

ENSG00000162512	GGAGGCA
ENSG00000162517	UGGCCCU
ENSG00000162521	AGUGGUU
ENSG00000162521	CAAAACU
ENSG00000162521	CUCAAAC
ENSG00000162521	GGUGGUU
ENSG00000162521	GUAACAG
ENSG00000162521	UCACAGG
ENSG00000162521	UCACAGU
ENSG00000162522	AAAGAAU
ENSG00000162522	AAAGUGC
ENSG00000162522	AAGUGCU
ENSG00000162522	ACUCAAA
ENSG00000162522	AGGCAGG
ENSG00000162522	AGUGCUU
ENSG00000162522	CAGCAGA
ENSG00000162522	CAGCAGG
ENSG00000162522	CUGCAGG
ENSG00000162522	CUUGGAG
ENSG00000162522	GAGCUGC
ENSG00000162522	GGCCCUG
ENSG00000162522	GUAAACA
ENSG00000162522	GUGCUUG
ENSG00000162522	UCUGGAA
ENSG00000162522	UCUGGAG
ENSG00000162522	UGCAUAG
ENSG00000162522	UGCAUAU
ENSG00000162522	UGUCUGC
ENSG00000162522	UGUGCUU
ENSG00000162522	UUGAGGC
ENSG00000162522	UUGGUCC
ENSG00000162545	AAGUGCA
ENSG00000162545	AGCCCUU
ENSG00000162545	AGUGGUU
ENSG00000162545	CUCACAC
ENSG00000162545	CUUUGGU
ENSG00000162545	GCCCUGU
ENSG00000162545	UCACACA
ENSG00000162545	UCUUGAG
ENSG00000162545	UGCAAAG
ENSG00000162545	UGGGGGA
ENSG00000162545	UGUCUUU
ENSG00000162551	AAAGUUU
ENSG00000162551	CAGUCUG
ENSG00000162551	CUUUGGU
ENSG00000162551	UGAGAAU
ENSG00000162551	UUGGUUC
ENSG00000162552	AAAAACC
ENSG00000162552	AAAGAAU
ENSG00000162552	ACUGUUU
ENSG00000162552	AGAUAAA
ENSG00000162552	AGCAGCG
ENSG00000162552	AGCAGCU
ENSG00000162552	CUGGCUC
ENSG00000162552	CUUUGGU
ENSG00000162552	GAGACCU
ENSG00000162552	GCAGCAU
ENSG00000162552	GGAGGCA
ENSG00000162552	GGCAGCG
ENSG00000162552	GGCCCUG
ENSG00000162552	GGCUCAG
ENSG00000162552	UCCCUUG
ENSG00000162552	UCCCUUU
ENSG00000162552	UCGUGCA

ENSG00000162552	UGAGGAC
ENSG00000162552	UUGGUCC
ENSG00000162576	CCAGGAG
ENSG00000162591	AGCACCA
ENSG00000162599	AAGUGCU
ENSG00000162599	AUACAAG
ENSG00000162599	CACACCU
ENSG00000162599	CUUACAG
ENSG00000162599	GCACCAU
ENSG00000162599	UCAACAG
ENSG00000162599	UUAGAGA
ENSG00000162607	AUCACUA
ENSG00000162613	AAAGUAU
ENSG00000162613	AAUACUG
ENSG00000162613	ACUGUAG
ENSG00000162613	AGUGAUC
ENSG00000162613	CAAAGCA
ENSG00000162613	CACAAUG
ENSG00000162613	CAGUAGA
ENSG00000162613	GCAUCCC
ENSG00000162613	GCUGGAU
ENSG00000162613	UGCAAAG
ENSG00000162614	AGUAGUU
ENSG00000162614	CUAGUAG
ENSG00000162614	UGGCCCU
ENSG00000162616	CAGUGCA
ENSG00000162620	GGCAUUG
ENSG00000162623	GCUGUCU
ENSG00000162624	AAAGUGC
ENSG00000162624	AAGUGCU
ENSG00000162627	UCCAGAG
ENSG00000162627	UUGGUUC
ENSG00000162631	AAAGAAU
ENSG00000162631	ACACACU
ENSG00000162631	ACUGGCU
ENSG00000162631	AUAAUAC
ENSG00000162631	AUGUAAU
ENSG00000162631	CACAAUG
ENSG00000162631	CAGCAAA
ENSG00000162631	CAGCUGG
ENSG00000162631	CUUUGGU
ENSG00000162631	GAUUGUC
ENSG00000162631	GUAAACA
ENSG00000162631	UAAUACA
ENSG00000162631	UAUUACC
ENSG00000162631	UGCCAGC
ENSG00000162631	UUAUGCA
ENSG00000162636	CUUGUAU
ENSG00000162639	CAGGAAC
ENSG00000162642	CACAGUG
ENSG00000162650	AAAGUUU
ENSG00000162650	GAGGUAU
ENSG00000162650	GCCUGGG
ENSG00000162650	GGAGGAG
ENSG00000162670	AAAAACC
ENSG00000162670	AAAAGUA
ENSG00000162670	AAUUUUA
ENSG00000162670	AUGUAAU
ENSG00000162670	CAGCAAA
ENSG00000162670	CUACAAA
ENSG00000162670	GUCAGUU
ENSG00000162670	GUGCUUG
ENSG00000162670	GUUCAUG
ENSG00000162670	UACAAAG

ENSG00000162670	UAGACUG
ENSG00000162670	UAGAGGA
ENSG00000162670	UAUAAUA
ENSG00000162670	UGUGCUU
ENSG00000162676	AAUACUG
ENSG00000162687	AAAUGGU
ENSG00000162688	CCUUCUU
ENSG00000162694	AACAGUC
ENSG00000162694	AAGGCAC
ENSG00000162695	AAGACAU
ENSG00000162695	ACAGUAU
ENSG00000162695	ACUGCAG
ENSG00000162695	AGCAGCU
ENSG00000162695	AGUGCCU
ENSG00000162695	CCAGCAU
ENSG00000162695	GAGGUAG
ENSG00000162695	GUAGUGU
ENSG00000162695	GUCAACA
ENSG00000162695	UGAAAUG
ENSG00000162702	AACAUUC
ENSG00000162702	GAAACAU
ENSG00000162702	UAAUACA
ENSG00000162704	AAAGCUG
ENSG00000162704	CACAGGG
ENSG00000162704	GGCAUUG
ENSG00000162704	UCAUUCG
ENSG00000162704	UCCAGUU
ENSG00000162704	UUGGUCC
ENSG00000162706	CAAAGCA
ENSG00000162706	CUCCAGC
ENSG00000162706	CUCCCAC
ENSG00000162706	GCCUGGA
ENSG00000162706	GGCCCUG
ENSG00000162706	GGGCCCC
ENSG00000162706	GGGGAGC
ENSG00000162706	UGGCUGC
ENSG00000162734	AAAACUG
ENSG00000162734	AACAGUC
ENSG00000162734	AAGGCAC
ENSG00000162734	ACACACC
ENSG00000162734	ACACACU
ENSG00000162734	ACUGCAG
ENSG00000162734	AUGAGCU
ENSG00000162734	CAAAACU
ENSG00000162734	CAGACAG
ENSG00000162734	CAGGCUC
ENSG00000162734	CAGUCUG
ENSG00000162734	CUGCAGC
ENSG00000162734	GAGCUAA
ENSG00000162734	GGAAUGU
ENSG00000162734	GGCAGUG
ENSG00000162734	GGCUCAG
ENSG00000162734	GUGCAAA
ENSG00000162734	GUGCGCA
ENSG00000162734	GUUAAUG
ENSG00000162734	GUUAGGA
ENSG00000162734	UAAUAUC
ENSG00000162734	UAAUGCU
ENSG00000162734	UGCAAAG
ENSG00000162734	UGCAUAU
ENSG00000162735	ACUCCAG
ENSG00000162735	GACCCUG
ENSG00000162735	GGCUCAG
ENSG00000162735	GUGUGGC

ENSG00000162735	UGCUGAC
ENSG00000162736	CCAGUGC
ENSG00000162736	CCAGUGU
ENSG00000162736	GGGGUCC
ENSG00000162736	UCCAGUU
ENSG00000162738	AAAAACC
ENSG00000162738	AAACUGU
ENSG00000162738	AAUCUCA
ENSG00000162738	ACAAGGU
ENSG00000162738	AGCCCUU
ENSG00000162738	AGGCAGG
ENSG00000162738	AGUGCUG
ENSG00000162738	AGUGCUU
ENSG00000162738	AUAUAAC
ENSG00000162738	AUAUUAU
ENSG00000162738	CAAAUGC
ENSG00000162738	CCCUGAG
ENSG00000162738	CUCCCAC
ENSG00000162738	GACAACU
ENSG00000162738	GACCCUG
ENSG00000162738	GAGGCAG
ENSG00000162738	GAGGUAG
ENSG00000162738	GAGGUAU
ENSG00000162738	GCCCUUA
ENSG00000162738	GUGAGGU
ENSG00000162738	UCACACA
ENSG00000162738	UCACAGU
ENSG00000162738	UCCUAUA
ENSG00000162738	UGUCUUU
ENSG00000162738	UUGGCAA
ENSG00000162738	UUGGCAC
ENSG00000162739	AGGAGCU
ENSG00000162739	CCAGGAG
ENSG00000162739	CUGGAAA
ENSG00000162739	GAGGCUG
ENSG00000162739	GAGGUAG
ENSG00000162745	AAGGUGC
ENSG00000162745	ACCAGCA
ENSG00000162745	CUACAAA
ENSG00000162745	UUUGCAC
ENSG00000162746	ACCACAG
ENSG00000162761	AAGUGCU
ENSG00000162761	AAGUUGU
ENSG00000162761	ACACACC
ENSG00000162761	ACACACU
ENSG00000162761	ACAUUCA
ENSG00000162761	ACUCAAA
ENSG00000162761	AGCACCA
ENSG00000162761	CAGUCUG
ENSG00000162761	CCCACCU
ENSG00000162761	CUCACAC
ENSG00000162761	CUUUGGU
ENSG00000162761	GAAACAU
ENSG00000162761	GAGGUAG
ENSG00000162761	GCAUCCC
ENSG00000162761	GUAUUCU
ENSG00000162761	GUGAGGU
ENSG00000162761	GUUAGGA
ENSG00000162761	UAAGACU
ENSG00000162761	UACAGUU
ENSG00000162761	UAUAAUA
ENSG00000162761	UGAGAAU
ENSG00000162761	UGCAUAG
ENSG00000162761	UGCAUAU

ENSG00000162761	UGCCCUG
ENSG00000162769	UUCAAGC
ENSG00000162769	UUUCAAC
ENSG00000162772	AUGGCUU
ENSG00000162772	GAAACAU
ENSG00000162772	UCACAGG
ENSG00000162772	UCACAGU
ENSG00000162775	AAAGAGG
ENSG00000162775	CUUCUCU
ENSG00000162775	GGCAUUG
ENSG00000162775	UUCCUCG
ENSG00000162777	ACCACAG
ENSG00000162777	UCACCAC
ENSG00000162782	UCAUGAU
ENSG00000162783	AAAAACC
ENSG00000162783	AAAUCCC
ENSG00000162783	ACUGUAG
ENSG00000162783	CCCUGUC
ENSG00000162783	GCUUCCU
ENSG00000162783	GUAAACA
ENSG00000162783	GUCUGCC
ENSG00000162783	UAAUACA
ENSG00000162783	UGACCUA
ENSG00000162804	AAGUCUU
ENSG00000162804	GAGGGGC
ENSG00000162813	GGAAUGU
ENSG00000162817	AUGUGCC
ENSG00000162849	AAAGUGC
ENSG00000162849	AAGGCAC
ENSG00000162849	AAGUGCU
ENSG00000162849	GCACCAU
ENSG00000162849	UGUCUUU
ENSG00000162849	UUUGCAA
ENSG00000162873	AGUGCAA
ENSG00000162873	CACAGUG
ENSG00000162873	GGAUUUC
ENSG00000162873	UGUGCUU
ENSG00000162889	CCCUGAG
ENSG00000162889	CUUUGGU
ENSG00000162889	GUCUCUG
ENSG00000162889	UCAUACA
ENSG00000162892	GGUCUAG
ENSG00000162896	ACCAGCA
ENSG00000162896	GAUUGUA
ENSG00000162896	GUGACAG
ENSG00000162909	AAGGCAC
ENSG00000162909	AAGUCUU
ENSG00000162909	UAAUAUC
ENSG00000162923	AAAUGGU
ENSG00000162923	AAGGCGC
ENSG00000162923	AGCAAAA
ENSG00000162923	AGCACCA
ENSG00000162923	AUAAAGU
ENSG00000162923	AUACAAG
ENSG00000162923	GCACCAU
ENSG00000162923	GUGCAAA
ENSG00000162923	UCACAAG
ENSG00000162923	UCUCAAG
ENSG00000162923	UGCAAAG
ENSG00000162923	UGCAUAG
ENSG00000162923	UUCCUAG
ENSG00000162923	UUUGCAC
ENSG00000162927	UCCUGAG
ENSG00000162927	UGAUCCA

ENSG00000162928	AGCAAAA
ENSG00000162928	AGCAGCG
ENSG00000162928	AGCAGCU
ENSG00000162928	CAGCAAA
ENSG00000162946	GCAGCAU
ENSG00000162946	GGCUCUG
ENSG00000162949	UCCCACA
ENSG00000162951	AAAGCUG
ENSG00000162951	AAGACAU
ENSG00000162951	UACAAAG
ENSG00000162951	UCCUGAG
ENSG00000162959	AGUGCAA
ENSG00000162959	CAAAACU
ENSG00000162959	CCCUGAG
ENSG00000162959	GUGCAAA
ENSG00000162959	UAAUACA
ENSG00000162971	GUGACUG
ENSG00000162971	UGAGAAG
ENSG00000162971	UGCAGCU
ENSG00000162971	UGUCUUU
ENSG00000162980	AAAGCUG
ENSG00000162980	ACAUUCA
ENSG00000162980	CAGUAGA
ENSG00000162980	CCAGCAU
ENSG00000162980	GAGGUAG
ENSG00000162980	UCCCUUU
ENSG00000162980	UCCUGAG
ENSG00000162980	UUUGCAC
ENSG00000162980	UUUUUGC
ENSG00000162981	AAAUGGU
ENSG00000162981	AAUUUUA
ENSG00000162981	ACUGGCC
ENSG00000162981	AUGGAUU
ENSG00000162981	CCCCCAG
ENSG00000162981	CUGGAAA
ENSG00000162981	GAGAAAU
ENSG00000162981	GCUGGUG
ENSG00000162981	GGAAUGU
ENSG00000162981	UCUGGAA
ENSG00000162981	UCUGGAG
ENSG00000162981	UGACAUC
ENSG00000162981	UGUUGAA
ENSG00000162989	AAAACUG
ENSG00000162989	AUUGCAC
ENSG00000162989	GUAAACA
ENSG00000162989	UAUUGCU
ENSG00000162992	AAGGCAC
ENSG00000162992	AGCAGCU
ENSG00000162992	CAGUGCA
ENSG00000162992	GGUUUAC
ENSG00000162992	UACAAAG
ENSG00000162992	UAUAAAG
ENSG00000162992	UGCAUAU
ENSG00000162992	UUUGCAC
ENSG00000162999	AUCACUA
ENSG00000162999	AUGACAC
ENSG00000162999	GAAAACA
ENSG00000162999	GCUGGAU
ENSG00000162999	UAUAAAG
ENSG00000163013	AAAGUGC
ENSG00000163013	AAGUGCU
ENSG00000163013	ACACACC
ENSG00000163013	ACACACU
ENSG00000163013	ACAUUCA

ENSG00000163013	AGCCUGC
ENSG00000163013	AGCUGCC
ENSG00000163013	AGUGCUG
ENSG00000163013	AUGACAC
ENSG00000163013	CAAAGUG
ENSG00000163013	CACAGGG
ENSG00000163013	CCCCUGG
ENSG00000163013	CUGGGGG
ENSG00000163013	GAGCCCU
ENSG00000163013	GAGCUGC
ENSG00000163013	GAGGCAG
ENSG00000163013	GAUUGUA
ENSG00000163013	GAUUGUC
ENSG00000163013	GCAGGAC
ENSG00000163013	GCCUGAU
ENSG00000163013	GCCUGGA
ENSG00000163013	GCUGGUG
ENSG00000163013	GGCAGUG
ENSG00000163013	GGCCCUG
ENSG00000163013	GUCACUC
ENSG00000163013	GUCCAGA
ENSG00000163013	GUGCUUG
ENSG00000163013	GUGGGGA
ENSG00000163013	UCACCAC
ENSG00000163013	UCCCUCU
ENSG00000163013	UCCUGAG
ENSG00000163013	UGGAUAA
ENSG00000163013	UGGCCCU
ENSG00000163013	UGGGACA
ENSG00000163013	UGUGCUU
ENSG00000163013	UUGGCAA
ENSG00000163013	UUGGCAC
ENSG00000163026	CAGACAG
ENSG00000163029	AGCAAAA
ENSG00000163029	CAAAGCA
ENSG00000163029	CUCAAAC
ENSG00000163029	CUUACAG
ENSG00000163032	AAGGCAC
ENSG00000163032	AGCUGCC
ENSG00000163032	GAGGUAG
ENSG00000163032	GAGGUAU
ENSG00000163041	AAAAACC
ENSG00000163041	AAAGUAU
ENSG00000163041	AGCAAAA
ENSG00000163041	CAAAUGC
ENSG00000163041	CAGGAAC
ENSG00000163041	CUACAAA
ENSG00000163041	CUUACAG
ENSG00000163041	GACAACU
ENSG00000163041	GCUACAU
ENSG00000163041	GUGAAUG
ENSG00000163041	UAAUAGU
ENSG00000163041	UAGGUAU
ENSG00000163053	AAGGCAC
ENSG00000163053	AGCCCUU
ENSG00000163053	GAGGUAG
ENSG00000163053	GCACCAU
ENSG00000163053	GUAAACA
ENSG00000163053	UGAGAAU
ENSG00000163064	CAGCAAA
ENSG00000163067	AAGUCAC
ENSG00000163067	AAGUGCA
ENSG00000163069	AGUGCAA
ENSG00000163069	CAAAGUG

ENSG00000163069	GAUUGGU
ENSG00000163069	GUGCAAA
ENSG00000163071	AAAUGGU
ENSG00000163071	AACUCUA
ENSG00000163071	UGUGUGG
ENSG00000163081	AAACUGU
ENSG00000163081	AUCCUUG
ENSG00000163081	UGUCUUU
ENSG00000163081	UUCCUAG
ENSG00000163083	AAAAAUC
ENSG00000163083	AAGUGCU
ENSG00000163083	AAUUAUU
ENSG00000163083	ACCCUGU
ENSG00000163083	CAAAUGC
ENSG00000163083	CAGCUGG
ENSG00000163083	GAAACAU
ENSG00000163083	GGCAGUG
ENSG00000163083	GUGCAAA
ENSG00000163083	GUGCUUG
ENSG00000163083	UAUAAUA
ENSG00000163083	UCAUUCG
ENSG00000163083	UCAUUUG
ENSG00000163083	UCCAGUU
ENSG00000163083	UGCAUAU
ENSG00000163083	UUCAAGC
ENSG00000163083	UUUCAAC
ENSG00000163092	AAUUUUA
ENSG00000163104	AAAACUG
ENSG00000163104	ACACACC
ENSG00000163104	ACACACU
ENSG00000163104	AUACAAG
ENSG00000163104	CCAGUGC
ENSG00000163104	CCAGUGU
ENSG00000163104	GUUAAUG
ENSG00000163104	UAAUGCU
ENSG00000163104	UCAACAG
ENSG00000163104	UCACAAG
ENSG00000163110	AAAGUGC
ENSG00000163110	AAGUGCU
ENSG00000163110	ACUGUUU
ENSG00000163110	AGCAAAA
ENSG00000163110	AGCAGCA
ENSG00000163110	AGGGAGG
ENSG00000163110	AUAAAGU
ENSG00000163110	AUGCCUU
ENSG00000163110	AUGUGCC
ENSG00000163110	CAAAGCA
ENSG00000163110	CACAAUG
ENSG00000163110	CACAGGG
ENSG00000163110	GAAAACA
ENSG00000163110	GAGAAAU
ENSG00000163110	GCUGGUG
ENSG00000163110	GGGAGCU
ENSG00000163110	GGGGCAG
ENSG00000163110	GGUGGUU
ENSG00000163110	UCACAGU
ENSG00000163110	UUUCAAC
ENSG00000163113	AAAGCUG
ENSG00000163113	AAAGUAU
ENSG00000163113	AAGUGCU
ENSG00000163113	AUGGCUU
ENSG00000163113	CAGGCUC
ENSG00000163113	CAGUCCA
ENSG00000163113	CCCACCU

ENSG00000163113	CCUUCUU
ENSG00000163113	CUUUGGU
ENSG00000163113	GGAGUGU
ENSG00000163113	GUGCUUG
ENSG00000163113	UAUUGCU
ENSG00000163113	UCAUUCG
ENSG00000163113	UCUCCAA
ENSG00000163113	UGCAUAG
ENSG00000163113	UGCAUAU
ENSG00000163113	UUGGUUC
ENSG00000163126	AACCUGU
ENSG00000163126	AGGGAGG
ENSG00000163126	AGGGUUG
ENSG00000163126	CACAGGG
ENSG00000163126	GAGGCUG
ENSG00000163126	GGAAUGU
ENSG00000163126	UCCAGAG
ENSG00000163131	CCUGCUG
ENSG00000163131	UACAAAG
ENSG00000163132	AACAAAC
ENSG00000163132	ACAGUAC
ENSG00000163132	ACAUAGA
ENSG00000163132	AGCUUAU
ENSG00000163132	AUAAUAC
ENSG00000163132	AUAUUAU
ENSG00000163132	AUCCCUU
ENSG00000163145	AUCACUA
ENSG00000163145	AUUCAUU
ENSG00000163145	CACAGGG
ENSG00000163145	CUGGGGG
ENSG00000163145	UCAUUUG
ENSG00000163145	UUCAAGC
ENSG00000163154	UCUGGAA
ENSG00000163155	CAGUGCA
ENSG00000163155	CUUCUCU
ENSG00000163155	GCUACAU
ENSG00000163155	UCUUGAG
ENSG00000163162	UUCCUCG
ENSG00000163166	UACAAAG
ENSG00000163171	AAAUCCC
ENSG00000163171	AAGGCAC
ENSG00000163171	ACAGUAC
ENSG00000163171	ACAGUAU
ENSG00000163171	AUAUAAC
ENSG00000163171	AUGUAAU
ENSG00000163171	CUGCAGC
ENSG00000163171	CUGCAGG
ENSG00000163171	GCAGAAG
ENSG00000163171	GUCUCUG
ENSG00000163171	UACAGUU
ENSG00000163171	UGCAAAG
ENSG00000163214	GAGGUAG
ENSG00000163235	AAUUUUA
ENSG00000163235	ACAGUAU
ENSG00000163235	ACCACAG
ENSG00000163235	AGUGCAA
ENSG00000163235	UAUUGCU
ENSG00000163235	UCAUUCG
ENSG00000163249	UCACAGG
ENSG00000163249	UCACAGU
ENSG00000163251	AAACGGU
ENSG00000163251	AAAGGUA
ENSG00000163251	AACGGAA
ENSG00000163251	AAGAACC

ENSG00000163251	AAGGCAC
ENSG00000163251	AAGUCAC
ENSG00000163251	AAUCUCU
ENSG00000163251	AAUGCCC
ENSG00000163251	ACAUUAC
ENSG00000163251	ACCCGUA
ENSG00000163251	AGCACCA
ENSG00000163251	AUAUAAC
ENSG00000163251	CAAAGCA
ENSG00000163251	CACACCU
ENSG00000163251	CCAGGAG
ENSG00000163251	CCCCCAG
ENSG00000163251	CUGGACU
ENSG00000163251	GCACCAU
ENSG00000163251	GCCCUGU
ENSG00000163251	GUAACAG
ENSG00000163251	GUGCCUG
ENSG00000163251	UCACAGG
ENSG00000163251	UCAUUUG
ENSG00000163251	UGCCCUG
ENSG00000163251	UUCUAAU
ENSG00000163251	UUGGCAA
ENSG00000163281	AAGGCAC
ENSG00000163281	AUAAAGU
ENSG00000163281	AUUGCAC
ENSG00000163281	UGCAUAU
ENSG00000163288	GCAGCAU
ENSG00000163288	UGAAGUG
ENSG00000163291	AACUACU
ENSG00000163291	AAGUAGC
ENSG00000163291	CAGCAAA
ENSG00000163291	UGCAGCU
ENSG00000163297	AAUUAUU
ENSG00000163297	AGCCCUU
ENSG00000163297	AUAUUAU
ENSG00000163297	CAGCUGG
ENSG00000163297	CCCUGAG
ENSG00000163297	CUAUACA
ENSG00000163297	CUUCACC
ENSG00000163297	CUUGGAG
ENSG00000163297	GAGCCCU
ENSG00000163297	GUCAUUG
ENSG00000163297	UCACAGU
ENSG00000163297	UGCAAAG
ENSG00000163297	UGGAGAU
ENSG00000163297	UUAUGCA
ENSG00000163297	UUGUGAC
ENSG00000163320	AAUACUG
ENSG00000163320	ACAGUAC
ENSG00000163320	ACUCCAG
ENSG00000163320	UCUGGAA
ENSG00000163320	UGAAGCU
ENSG00000163328	AAGUUGU
ENSG00000163328	AAUGUUG
ENSG00000163328	ACUGCAG
ENSG00000163328	AGAUAAA
ENSG00000163328	CUAGGCU
ENSG00000163328	GUGAAUG
ENSG00000163328	UACAAAG
ENSG00000163328	UCACAAG
ENSG00000163328	UCAUUUG
ENSG00000163328	UGAGAAG
ENSG00000163328	UGAUCCA
ENSG00000163328	UUCUGCA

ENSG00000163346	AGCCCUU
ENSG00000163346	CAGUGCA
ENSG00000163347	AGAUAAA
ENSG00000163347	AUACAAG
ENSG00000163347	AUCACUA
ENSG00000163347	CACACCU
ENSG00000163347	GUGAGGU
ENSG00000163348	AAAGCUG
ENSG00000163348	CAAGAGC
ENSG00000163348	CUUCUCU
ENSG00000163348	CUUUGGU
ENSG00000163348	GAGGUAG
ENSG00000163348	GAGGUAU
ENSG00000163348	UACAAAG
ENSG00000163348	UUCAAGC
ENSG00000163349	AACUCUA
ENSG00000163349	AAGGCAC
ENSG00000163349	AAUCACU
ENSG00000163349	AGAUAAA
ENSG00000163349	AUAUUAU
ENSG00000163349	AUGCAUU
ENSG00000163349	AUGGAUU
ENSG00000163349	AUUGCAC
ENSG00000163349	CCAUUAC
ENSG00000163349	CUACAGU
ENSG00000163349	CUUUGGU
ENSG00000163349	GGUUUAC
ENSG00000163349	GUAAACA
ENSG00000163349	GUGCAAA
ENSG00000163349	UAGUGAG
ENSG00000163349	UCAAGUA
ENSG00000163349	UUCUAAU
ENSG00000163349	UUGGUUC
ENSG00000163352	AUCCUUG
ENSG00000163352	CAAAGCA
ENSG00000163352	GAGUCUU
ENSG00000163359	AAAGUUU
ENSG00000163359	AGUGCAA
ENSG00000163359	CAGUGCA
ENSG00000163359	GAGGUAU
ENSG00000163359	GCACCAU
ENSG00000163376	AAUACUG
ENSG00000163376	AGCACCA
ENSG00000163376	CUACAGU
ENSG00000163376	GAGGUAU
ENSG00000163376	UCACAGU
ENSG00000163376	UGCAUAG
ENSG00000163378	CAGCAAA
ENSG00000163378	GAGGUAG
ENSG00000163378	GAGGUAU
ENSG00000163378	GUAAACA
ENSG00000163380	GCUGUCU
ENSG00000163382	CCCACCU
ENSG00000163389	AAGGCAC
ENSG00000163389	GAGGCAG
ENSG00000163389	GAGGUAG
ENSG00000163389	GAGGUAU
ENSG00000163389	GUAAACA
ENSG00000163389	UCACAGG
ENSG00000163389	UCCUGAG
ENSG00000163393	AACACUG
ENSG00000163393	AAGGCAC
ENSG00000163393	CUUUGGU
ENSG00000163406	UCUUGAG

ENSG00000163412	AAAGAAU
ENSG00000163412	AAGUCUU
ENSG00000163412	ACCAGCA
ENSG00000163412	AGUGCAA
ENSG00000163412	CCAGCAU
ENSG00000163412	GGAAUGU
ENSG00000163412	UGGGACA
ENSG00000163421	AUAAUAC
ENSG00000163428	ACUCAAA
ENSG00000163428	AGCAGCA
ENSG00000163428	AGCAGCG
ENSG00000163428	AGCAGCU
ENSG00000163428	AUGAUGA
ENSG00000163428	CUAGUAG
ENSG00000163428	GUAAACA
ENSG00000163428	UCAUGAU
ENSG00000163428	UUCCUAG
ENSG00000163428	UUCUAAU
ENSG00000163430	AAGGCAC
ENSG00000163430	AAUACUG
ENSG00000163430	AUUGCAC
ENSG00000163430	CUCUUCC
ENSG00000163430	GGCAGGG
ENSG00000163430	UAUUGCU
ENSG00000163430	UCUCCAA
ENSG00000163430	UGCAAAG
ENSG00000163431	AGCAGCA
ENSG00000163431	CUGCCCC
ENSG00000163431	GCUGUCU
ENSG00000163431	GUGACUG
ENSG00000163431	GUGAGGU
ENSG00000163431	UGGCCCU
ENSG00000163431	UGUAUAA
ENSG00000163431	UUGGCAA
ENSG00000163431	UUGGCAC
ENSG00000163435	CUGCAGG
ENSG00000163435	UCACAGG
ENSG00000163435	UUGGAGG
ENSG00000163435	UUGGUCC
ENSG00000163444	AAAGAAU
ENSG00000163444	AACAAAC
ENSG00000163462	AUGUGCC
ENSG00000163462	CAGCUGG
ENSG00000163462	CAUGGGU
ENSG00000163462	UCACAGU
ENSG00000163462	UUGGCAC
ENSG00000163466	UUGAUCA
ENSG00000163468	GUCAGUU
ENSG00000163472	CUCUUCC
ENSG00000163472	GGCUCUG
ENSG00000163472	UCACCAC
ENSG00000163479	CUGGAAA
ENSG00000163479	GGAGUCC
ENSG00000163479	UCUGGAA
ENSG00000163482	AAUCUCU
ENSG00000163482	CUUCUCU
ENSG00000163482	UACAAAG
ENSG00000163485	GGCUCAG
ENSG00000163486	AAGUUGU
ENSG00000163486	ACAUUCA
ENSG00000163486	AGCACCA
ENSG00000163486	AGCCCUU
ENSG00000163486	AUACAAG
ENSG00000163486	CAAAGCA

ENSG00000163486	CCAGGAG
ENSG00000163486	GGAAGAC
ENSG00000163486	GGAAUGU
ENSG00000163486	GGAUUUC
ENSG00000163486	GUGCUUG
ENSG00000163486	UAAGACU
ENSG00000163486	UACAAAG
ENSG00000163486	UCACAGG
ENSG00000163486	UCAUGAU
ENSG00000163486	UCCCUGU
ENSG00000163486	UGAAGUG
ENSG00000163501	AAUUUUA
ENSG00000163501	AGCAGCG
ENSG00000163501	AGCAGCU
ENSG00000163501	CAGCAGA
ENSG00000163501	CCAGGAG
ENSG00000163501	GAGUGUG
ENSG00000163501	GCAGCAU
ENSG00000163501	GGAGUGU
ENSG00000163501	UACAAAG
ENSG00000163501	UAUUACC
ENSG00000163501	UCACAUU
ENSG00000163508	AUGGCUU
ENSG00000163508	AUUGCAC
ENSG00000163508	CUGCAGC
ENSG00000163508	GCAGCAU
ENSG00000163512	AUGCAUU
ENSG00000163512	CAGCUGG
ENSG00000163512	CAUAUUG
ENSG00000163512	CCAUUAC
ENSG00000163512	GAUUGUA
ENSG00000163512	GGGUUUA
ENSG00000163512	GUUCAUG
ENSG00000163512	UGCAUAU
ENSG00000163513	AAAGCUG
ENSG00000163513	ACAGUAU
ENSG00000163513	AGCUUAU
ENSG00000163513	AGUAGUU
ENSG00000163513	AUAUAAC
ENSG00000163513	AUGGCUU
ENSG00000163513	CUUUGGU
ENSG00000163513	UAAUGCU
ENSG00000163513	UCCCUUG
ENSG00000163513	UUCCAUA
ENSG00000163516	ACAGUAC
ENSG00000163520	UCACAGU
ENSG00000163531	AAAGAGC
ENSG00000163531	AACACUG
ENSG00000163531	AAGGCAC
ENSG00000163531	AAGUCUU
ENSG00000163531	AAUACUG
ENSG00000163531	ACCCUGU
ENSG00000163531	ACUGGCU
ENSG00000163531	AUACAAG
ENSG00000163531	CACAGGG
ENSG00000163531	CCCUGUC
ENSG00000163531	CUCCCAA
ENSG00000163531	CUGCAGC
ENSG00000163531	CUGCAGG
ENSG00000163531	CUGGAAA
ENSG00000163531	CUUUGGU
ENSG00000163531	GAGAACU
ENSG00000163531	GAGGCAG
ENSG00000163531	GGCAGGG

ENSG00000163531	GUCAGUU
ENSG00000163531	GUCUCUG
ENSG00000163531	GUGCCUG
ENSG00000163531	GUGGCCC
ENSG00000163531	GUGGGGA
ENSG00000163531	GUUAAUG
ENSG00000163531	UAAUACA
ENSG00000163531	UAAUGCU
ENSG00000163531	UACAGUU
ENSG00000163531	UCACAGU
ENSG00000163531	UCCCUGU
ENSG00000163531	UCUGGAA
ENSG00000163531	UGCAUAG
ENSG00000163531	UGCAUAU
ENSG00000163531	UGCUCAC
ENSG00000163531	UUCAAAU
ENSG00000163531	UUGGCAA
ENSG00000163531	UUGGUUC
ENSG00000163531	UUGUGUC
ENSG00000163534	CAUGGAU
ENSG00000163539	AAAGCUG
ENSG00000163539	AAUGUUG
ENSG00000163539	AGCAGCU
ENSG00000163539	AGCAUUC
ENSG00000163539	AUGCACC
ENSG00000163539	AUGUAAU
ENSG00000163539	CACAAUG
ENSG00000163539	CAGUAGA
ENSG00000163539	CUUCUCU
ENSG00000163539	GAGGUAG
ENSG00000163539	GGAAGAC
ENSG00000163539	UAAUACA
ENSG00000163539	UAUUGCU
ENSG00000163539	UCACAAG
ENSG00000163539	UCACAUU
ENSG00000163539	UGCAGCU
ENSG00000163539	UGCCAGC
ENSG00000163539	UUCAAGC
ENSG00000163545	AAGUCUU
ENSG00000163545	AGCAGCU
ENSG00000163545	GAGAUGA
ENSG00000163545	GUGGCCC
ENSG00000163545	UCAAGUA
ENSG00000163545	UUCAAGC
ENSG00000163545	UUUCAAC
ENSG00000163558	AUGGCAC
ENSG00000163558	UCUGGAA
ENSG00000163577	AAAGCUG
ENSG00000163577	AGCAGCA
ENSG00000163577	CAAGAGC
ENSG00000163577	CCAGUAC
ENSG00000163577	CCCUGAG
ENSG00000163577	CUAUACA
ENSG00000163577	CUUUGGU
ENSG00000163577	GAGAACC
ENSG00000163577	GAGAACU
ENSG00000163577	GAGUUGG
ENSG00000163577	GGCAAGA
ENSG00000163577	GGGGUCC
ENSG00000163577	GUAAACA
ENSG00000163577	UCUAGAG
ENSG00000163577	UGAGAAG
ENSG00000163577	UGAGAAU
ENSG00000163577	UGCUCAC

ENSG00000163577	UGGCCAC
ENSG00000163577	UUCAAAU
ENSG00000163577	UUGAGGC
ENSG00000163577	UUGGCAA
ENSG00000163581	UAUAAUA
ENSG00000163590	AGCUGCC
ENSG00000163590	CUCCCAC
ENSG00000163590	UCCCACA
ENSG00000163590	UGAAAGG
ENSG00000163596	AUUCAUU
ENSG00000163596	CUAGGCU
ENSG00000163596	GGUUACC
ENSG00000163596	GUGCAAA
ENSG00000163596	UAAUGCU
ENSG00000163596	UGCAAAG
ENSG00000163596	UGCCCUG
ENSG00000163596	UGGCUGC
ENSG00000163596	UUAUGCA
ENSG00000163599	CAUGGAU
ENSG00000163599	UCCUGAG
ENSG00000163600	ACUGUAG
ENSG00000163600	AGGUAGU
ENSG00000163600	GAGGUAG
ENSG00000163600	UCACAGG
ENSG00000163600	UCACAGU
ENSG00000163600	UUCAAAU
ENSG00000163602	AAACUGU
ENSG00000163602	AAAGAGC
ENSG00000163602	AAGGCAC
ENSG00000163602	AGCACCA
ENSG00000163602	AGCAUUC
ENSG00000163602	AGGCAGG
ENSG00000163602	AGGUUAU
ENSG00000163602	AGUAGAC
ENSG00000163602	AUAUUAU
ENSG00000163602	AUGGCUU
ENSG00000163602	CACCAGC
ENSG00000163602	CAGCAGA
ENSG00000163602	CCCUGAG
ENSG00000163602	CUCCAUU
ENSG00000163602	CUUUGGU
ENSG00000163602	GAUUGUA
ENSG00000163602	GAUUGUC
ENSG00000163602	GCUGUCU
ENSG00000163602	GGAAUGU
ENSG00000163602	GGAGGCA
ENSG00000163602	GUAACAG
ENSG00000163602	GUGCCAC
ENSG00000163602	GUGCUUG
ENSG00000163602	UCACAGG
ENSG00000163602	UCACAGU
ENSG00000163602	UCAUACA
ENSG00000163602	UCGUGCA
ENSG00000163602	UGUGCUU
ENSG00000163602	UUCUAAU
ENSG00000163602	UUGACAC
ENSG00000163605	AAUACUG
ENSG00000163606	GAGGUAU
ENSG00000163618	AAGGCAC
ENSG00000163618	CAUAUUG
ENSG00000163618	GAGAAGA
ENSG00000163618	UAUAAAG
ENSG00000163618	UAUUGCU
ENSG00000163618	UGAGAAU

ENSG00000163624	CACAGUG
ENSG00000163624	GAAACAU
ENSG00000163624	UCACAGG
ENSG00000163625	AAAGUGC
ENSG00000163625	AGCUGCC
ENSG00000163625	AUCCCUU
ENSG00000163625	CAAAGCA
ENSG00000163625	GUGCAAA
ENSG00000163625	UCCCUUU
ENSG00000163625	UGCUGAC
ENSG00000163626	AAUCCUU
ENSG00000163626	UUCAAAU
ENSG00000163630	CUACAAA
ENSG00000163635	AACAUUC
ENSG00000163635	ACUCAAA
ENSG00000163635	CCAGUGC
ENSG00000163635	CUCACAC
ENSG00000163635	GUCCAGA
ENSG00000163635	UCCAGAG
ENSG00000163637	AAAAAUC
ENSG00000163637	AAAGCGC
ENSG00000163637	AACAAAC
ENSG00000163637	AACUACU
ENSG00000163637	AAGCGCU
ENSG00000163637	AAUUUUA
ENSG00000163637	ACAUCAC
ENSG00000163637	ACGUCAU
ENSG00000163637	ACUAGAU
ENSG00000163637	AGCACCA
ENSG00000163637	AUACAAG
ENSG00000163637	CAAAACU
ENSG00000163637	CCAGGAG
ENSG00000163637	CUAUACA
ENSG00000163637	GAAAACA
ENSG00000163637	GAAACAU
ENSG00000163637	GAGAAAU
ENSG00000163637	GGUUGUC
ENSG00000163637	GUCUCUG
ENSG00000163637	GUCUGCC
ENSG00000163637	UCACACA
ENSG00000163637	UCACAGU
ENSG00000163637	UCAUUUG
ENSG00000163637	UCUCAAG
ENSG00000163637	UGACAUC
ENSG00000163637	UGCUGAC
ENSG00000163637	UUUGCAA
ENSG00000163638	AAGGCAC
ENSG00000163638	AUCCUUG
ENSG00000163638	AUCCUUU
ENSG00000163638	AUUGCAC
ENSG00000163638	GAGGUAU
ENSG00000163638	GUAAACA
ENSG00000163638	UUAGGAU
ENSG00000163644	UUGGUUC
ENSG00000163659	AGUGCUU
ENSG00000163659	CUACAAA
ENSG00000163659	GCUACAU
ENSG00000163659	GUAGUGU
ENSG00000163659	UUUUUGC
ENSG00000163660	GUCUGCC
ENSG00000163660	UCCAGUU
ENSG00000163661	AGCACCA
ENSG00000163661	CUUUGGU
ENSG00000163661	UACAAAG

ENSG00000163673	AAUCUCA
ENSG00000163673	CAGGCUC
ENSG00000163673	GGCUCAG
ENSG00000163681	AAAGUUU
ENSG00000163681	AAGUCUU
ENSG00000163681	AGGGAGG
ENSG00000163681	GUGCAAA
ENSG00000163681	UACAAAG
ENSG00000163681	UAGGCCG
ENSG00000163681	UCUAGAG
ENSG00000163681	UGCCAGC
ENSG00000163683	AGCAAAA
ENSG00000163683	CGUGCAU
ENSG00000163683	UCGUGCA
ENSG00000163683	UUGGUCC
ENSG00000163684	AUAAAGU
ENSG00000163684	AUGACAC
ENSG00000163684	CAAAACU
ENSG00000163684	CCCCUGG
ENSG00000163684	CCCUGUU
ENSG00000163684	CUGCAGC
ENSG00000163684	CUGCAGG
ENSG00000163684	GAGAAGA
ENSG00000163684	GUUAAUG
ENSG00000163684	UAUAAAG
ENSG00000163684	UGACAUC
ENSG00000163684	UGAGAAG
ENSG00000163684	UGGCCCU
ENSG00000163684	UUCCAUA
ENSG00000163684	UUCUGCA
ENSG00000163684	UUGAUCA
ENSG00000163684	UUUGCAC
ENSG00000163686	AUUCAUU
ENSG00000163686	CUCAGGG
ENSG00000163694	ACAGUAC
ENSG00000163694	ACAGUAU
ENSG00000163694	CACACCU
ENSG00000163694	CAGUAGA
ENSG00000163694	CUUCACC
ENSG00000163694	GGAGUGU
ENSG00000163694	GUAGUGU
ENSG00000163694	GUGCUUG
ENSG00000163694	UCAAGUA
ENSG00000163694	UCACACA
ENSG00000163694	UCAUACA
ENSG00000163694	UCCAGUU
ENSG00000163694	UUGUUCG
ENSG00000163697	AAGGCAC
ENSG00000163697	AAGUGCU
ENSG00000163697	AGCUGCC
ENSG00000163697	AGUGCUG
ENSG00000163697	CAAAGCA
ENSG00000163697	CAAAGUG
ENSG00000163697	GGCAAGA
ENSG00000163697	UGCAUAG
ENSG00000163697	UGCAUAU
ENSG00000163701	CUCCAGC
ENSG00000163703	GCAGCAU
ENSG00000163703	GGCAGCG
ENSG00000163703	GGCAGGG
ENSG00000163704	AAAGAGG
ENSG00000163704	ACCCUGU
ENSG00000163704	CUUUGGU
ENSG00000163704	UUGGCAA

ENSG00000163714	AAGACAU
ENSG00000163714	AAGUGCA
ENSG00000163714	AGCACCA
ENSG00000163714	AGCAGCA
ENSG00000163714	CAAAGCA
ENSG00000163714	CACAAUG
ENSG00000163714	CAUAUUG
ENSG00000163714	CCCACCG
ENSG00000163714	CUACAGU
ENSG00000163714	GGGGCAG
ENSG00000163714	UCCCACA
ENSG00000163714	UCUGGAA
ENSG00000163714	UGAAAUG
ENSG00000163714	UGAGAAG
ENSG00000163714	UGAGAAU
ENSG00000163714	UUGGCAA
ENSG00000163719	AUGGCUU
ENSG00000163719	CAGCUGG
ENSG00000163719	CAGGUGA
ENSG00000163719	CAGUGCA
ENSG00000163719	GCAGGUU
ENSG00000163719	GGCUCAG
ENSG00000163719	GUGUCAC
ENSG00000163719	UGUGUCA
ENSG00000163734	AAGACAU
ENSG00000163735	GUGUCAC
ENSG00000163735	UCACAUU
ENSG00000163739	AAGACAU
ENSG00000163743	AAUCCUU
ENSG00000163743	CACUGUU
ENSG00000163743	GACCCUG
ENSG00000163743	GCCCUUA
ENSG00000163743	GUCACUC
ENSG00000163743	GUGACAG
ENSG00000163743	UUAUGCA
ENSG00000163755	GAAGGAA
ENSG00000163755	UUCAAAU
ENSG00000163788	AACACUG
ENSG00000163788	AAUGCCC
ENSG00000163788	ACAAUAU
ENSG00000163788	AGCACCA
ENSG00000163788	AGCUGCC
ENSG00000163788	AUACAAG
ENSG00000163788	AUGGCUU
ENSG00000163788	CAGUGCA
ENSG00000163788	GACCAUG
ENSG00000163788	UGGCCCU
ENSG00000163793	CUGGGCA
ENSG00000163795	CCCCCAG
ENSG00000163811	AACACUG
ENSG00000163811	CUCCAUU
ENSG00000163812	AACAUUC
ENSG00000163812	AAGGCAC
ENSG00000163812	ACAUUCA
ENSG00000163812	AGGCAGG
ENSG00000163812	AGUGCUU
ENSG00000163812	AUUGCAC
ENSG00000163812	CAGCAGA
ENSG00000163812	GCAGCAU
ENSG00000163812	GGAAGCC
ENSG00000163812	UAUUGCU
ENSG00000163812	UGCAUUG
ENSG00000163812	UUCCAUA
ENSG00000163814	AAGGCAC

ENSG00000163814	AAUUAUU
ENSG00000163814	CAGCAAA
ENSG00000163814	GAGCCCC
ENSG00000163815	UGCAAAG
ENSG00000163815	UUUGCAA
ENSG00000163817	GAGCUAA
ENSG00000163817	GAGUUGG
ENSG00000163818	GCCUGAU
ENSG00000163818	UCCAGAG
ENSG00000163820	AAGUGCA
ENSG00000163820	ACAGGUG
ENSG00000163820	GUGCUUG
ENSG00000163820	UCCUGAG
ENSG00000163820	UGUGCUU
ENSG00000163820	UUGGCAA
ENSG00000163820	UUGGCAC
ENSG00000163823	UGUCUUU
ENSG00000163827	UCUAGAG
ENSG00000163827	UUGGUCC
ENSG00000163833	GUCUUGC
ENSG00000163840	AAGUGCA
ENSG00000163840	AUGGCUU
ENSG00000163840	GGCAGGG
ENSG00000163840	UGGCCAC
ENSG00000163848	AACAAAC
ENSG00000163848	AAGGCAC
ENSG00000163848	AAGUGCU
ENSG00000163848	AAUACUG
ENSG00000163848	AAUCCUU
ENSG00000163848	AAUGUUG
ENSG00000163848	ACAAUAU
ENSG00000163848	ACUCAAA
ENSG00000163848	ACUGUAG
ENSG00000163848	AGCAUUC
ENSG00000163848	AGCCAGU
ENSG00000163848	AGCCCUU
ENSG00000163848	AGUGCAA
ENSG00000163848	AUACAAG
ENSG00000163848	AUAUAAC
ENSG00000163848	AUAUUAU
ENSG00000163848	AUGCCUU
ENSG00000163848	AUUGCAC
ENSG00000163848	CACAAUG
ENSG00000163848	CAGACAG
ENSG00000163848	CAUAUUG
ENSG00000163848	CCAGUAC
ENSG00000163848	CCCUGAG
ENSG00000163848	CCUUCAU
ENSG00000163848	CUACAGU
ENSG00000163848	CUAUACA
ENSG00000163848	CUCCCAA
ENSG00000163848	CUGGAAA
ENSG00000163848	CUUGGGC
ENSG00000163848	GAGAACU
ENSG00000163848	GAGAUGA
ENSG00000163848	GAUUGUA
ENSG00000163848	GAUUGUC
ENSG00000163848	GCCCUUA
ENSG00000163848	GCUGGUG
ENSG00000163848	GGAAGAC
ENSG00000163848	GUAAACA
ENSG00000163848	GUGACAG
ENSG00000163848	UCACAGU
ENSG00000163848	UCCCUGU

ENSG00000163848	UCCCUUU
ENSG00000163848	UGAGAAG
ENSG00000163848	UGGUACC
ENSG00000163848	UGUCUGC
ENSG00000163848	UGUCUUU
ENSG00000163848	UUCCUAG
ENSG00000163848	UUUCAAC
ENSG00000163867	CUCAAAC
ENSG00000163872	AAGGCAC
ENSG00000163873	AAACUGU
ENSG00000163873	ACAUCAC
ENSG00000163873	AGAUGUG
ENSG00000163873	AGUAACA
ENSG00000163873	AUGGCUU
ENSG00000163873	AUUCAUU
ENSG00000163873	CAGUUAC
ENSG00000163873	CCAGUGU
ENSG00000163873	CCUUCUG
ENSG00000163873	CUGCAGC
ENSG00000163873	CUUUGGU
ENSG00000163873	GAUUGGU
ENSG00000163873	GCAUCCC
ENSG00000163873	GGAGGCA
ENSG00000163873	GGCAAGA
ENSG00000163873	GGGAACG
ENSG00000163873	GGGUCUU
ENSG00000163873	GUGACUG
ENSG00000163873	GUGCUUG
ENSG00000163873	UCACAGU
ENSG00000163873	UCCAGAG
ENSG00000163873	UCUAGAG
ENSG00000163873	UGCAUAG
ENSG00000163873	UGCAUAU
ENSG00000163873	UGUGCUU
ENSG00000163873	UGUGUGG
ENSG00000163873	UUCUGCA
ENSG00000163873	UUGGUUC
ENSG00000163874	ACAGUAC
ENSG00000163874	CUACAGU
ENSG00000163874	GGCAGCG
ENSG00000163874	UCAACAG
ENSG00000163874	UUGGUUC
ENSG00000163877	AAGGCAC
ENSG00000163877	AAUUUUA
ENSG00000163877	AGUGCAA
ENSG00000163877	CUUUGGU
ENSG00000163877	GUGCAAA
ENSG00000163879	CUACAAA
ENSG00000163879	UAUUACC
ENSG00000163882	AAAAGUA
ENSG00000163884	AAUUAUU
ENSG00000163884	AUGCAUU
ENSG00000163884	GAAGGAA
ENSG00000163884	UACAGUU
ENSG00000163884	UCAUUUG
ENSG00000163884	UCUAGAG
ENSG00000163888	AAAAAUC
ENSG00000163888	AAAGAGG
ENSG00000163888	AAAGUGC
ENSG00000163888	AAGUGCA
ENSG00000163888	ACACACU
ENSG00000163888	CACUCCU
ENSG00000163888	GGGGAGC
ENSG00000163898	GAGGUAG

ENSG00000163898	UGACAUC
ENSG00000163898	UGCCCUG
ENSG00000163898	UUAGAGA
ENSG00000163902	CACAGUG
ENSG00000163902	UCACAGU
ENSG00000163904	AACAUUC
ENSG00000163904	CAGCAGG
ENSG00000163904	GAGGUAG
ENSG00000163904	UCCAGUU
ENSG00000163909	ACUCCAG
ENSG00000163909	CAGUCUG
ENSG00000163909	CUCAGGG
ENSG00000163909	CUCUGGG
ENSG00000163909	CUGGGCA
ENSG00000163909	GGCUCUG
ENSG00000163914	ACUCCAG
ENSG00000163914	ACUGUGU
ENSG00000163914	CAAAGUG
ENSG00000163914	GGUGGGC
ENSG00000163930	AACACUG
ENSG00000163930	GAGGGGC
ENSG00000163930	GGCAAGA
ENSG00000163930	UCCUGAG
ENSG00000163932	AACUACU
ENSG00000163932	UCACAGG
ENSG00000163932	UCACAGU
ENSG00000163933	ACCACAG
ENSG00000163933	CUUCUCU
ENSG00000163935	AAAGUGC
ENSG00000163935	AAGUGCA
ENSG00000163938	UCACAAG
ENSG00000163939	AAAAGUA
ENSG00000163939	ACAGUAU
ENSG00000163939	AGUGGUU
ENSG00000163939	AUAAAGU
ENSG00000163939	CCAGUGC
ENSG00000163939	CCAGUGU
ENSG00000163939	GUAAACA
ENSG00000163939	UCAUGAU
ENSG00000163939	UGCAAAG
ENSG00000163939	UGGGAUC
ENSG00000163946	AAAACUG
ENSG00000163946	AAUUUUA
ENSG00000163946	ACAGGUG
ENSG00000163946	GAGGUAG
ENSG00000163946	GAGGUAU
ENSG00000163946	GUUAAUG
ENSG00000163946	UCAUGAU
ENSG00000163947	AAAGUGC
ENSG00000163947	AAAGUUU
ENSG00000163947	AAUACUG
ENSG00000163947	ACAGGUG
ENSG00000163947	ACAGUAU
ENSG00000163947	ACUGUAG
ENSG00000163947	AGUAGUU
ENSG00000163947	AGUGCUG
ENSG00000163947	AUACAAG
ENSG00000163947	CAGUUAC
ENSG00000163947	GCAGGAC
ENSG00000163947	GGAAUGU
ENSG00000163947	GGCAGUG
ENSG00000163947	GUAAACA
ENSG00000163947	UACAGUU
ENSG00000163947	UCCAGAG

ENSG00000163947	UGAAAUG
ENSG00000163947	UUGGCAC
ENSG00000163950	AAUUUUA
ENSG00000163950	UUUGCAC
ENSG00000163950	UUUUUGC
ENSG00000163964	AAAUUUC
ENSG00000163964	UUCCUCG
ENSG00000163995	AAAAACC
ENSG00000163995	UCCAGUU
ENSG00000164007	CUUCUCU
ENSG00000164007	GCUGGAU
ENSG00000164010	GAGGCUG
ENSG00000164010	GUAAACA
ENSG00000164011	CCCUGAG
ENSG00000164023	UUGGCAA
ENSG00000164023	UUGGUUC
ENSG00000164024	AAAAAUC
ENSG00000164024	AAAGUGC
ENSG00000164024	AAGUGCU
ENSG00000164024	ACCUGGA
ENSG00000164024	CUCUGGG
ENSG00000164024	CUUCUCU
ENSG00000164024	GAGGCAG
ENSG00000164024	GGCAGUG
ENSG00000164024	UCACAAG
ENSG00000164024	UCCCUUU
ENSG00000164024	UGCAUAG
ENSG00000164031	AAGGCAC
ENSG00000164031	CUGCUCA
ENSG00000164032	CAGUCCA
ENSG00000164039	UGCUCAC
ENSG00000164040	ACAGUAC
ENSG00000164040	ACAUUAC
ENSG00000164040	AUGCAUU
ENSG00000164040	AUGGCAC
ENSG00000164040	CUGCAGC
ENSG00000164040	CUGCAGG
ENSG00000164040	CUUUGGU
ENSG00000164040	GGCAGCG
ENSG00000164040	GGCAGUG
ENSG00000164040	GUAGUGU
ENSG00000164040	UACAAAG
ENSG00000164040	UACAGUU
ENSG00000164040	UCAAGUA
ENSG00000164040	UGGCCAC
ENSG00000164040	UUGGUUC
ENSG00000164045	AAAGUUU
ENSG00000164045	AACACUG
ENSG00000164045	AAUGCCC
ENSG00000164045	ACUCAAA
ENSG00000164045	AGCUUAU
ENSG00000164045	AGGUAGU
ENSG00000164045	CUCAAAC
ENSG00000164045	GAGAAAU
ENSG00000164045	GGCAGUG
ENSG00000164048	CCCCUGG
ENSG00000164051	GGAGGAG
ENSG00000164054	GUGGCCC
ENSG00000164054	UGCAUUG
ENSG00000164054	UUGGUCC
ENSG00000164056	AAGGCAC
ENSG00000164056	ACAAUAU
ENSG00000164056	AGCACCA
ENSG00000164056	AUACAAG

ENSG00000164056	GUGUCAC
ENSG00000164056	UAAUAUC
ENSG00000164056	UGUCUUU
ENSG00000164056	UUGUGAC
ENSG00000164061	AAAGAGG
ENSG00000164061	AAAGUUU
ENSG00000164061	AAAUCCC
ENSG00000164061	AACAGUC
ENSG00000164061	ACAAGGU
ENSG00000164061	ACUCCAG
ENSG00000164061	AGGAGCU
ENSG00000164061	AGGGAGG
ENSG00000164061	AUGGCUU
ENSG00000164061	CAAAGCA
ENSG00000164061	CACAAUG
ENSG00000164061	CAGCUGG
ENSG00000164061	CCAGUGC
ENSG00000164061	CCCACCU
ENSG00000164061	CCCUGAG
ENSG00000164061	CGGUGCU
ENSG00000164061	CGUGCAU
ENSG00000164061	CUCACAC
ENSG00000164061	CUCCCAA
ENSG00000164061	CUCUUCC
ENSG00000164061	CUGACUC
ENSG00000164061	GAGAAAU
ENSG00000164061	GAGGUAG
ENSG00000164061	GAGUCUU
ENSG00000164061	GCAGAAG
ENSG00000164061	GGAAUGU
ENSG00000164061	GGAGGAG
ENSG00000164061	GGCUCAG
ENSG00000164061	GGGCCCC
ENSG00000164061	GGGGCAG
ENSG00000164061	GUAAACA
ENSG00000164061	GUCCAGA
ENSG00000164061	GUGCAAA
ENSG00000164061	GUUAGGA
ENSG00000164061	UAUUGCU
ENSG00000164061	UCACACA
ENSG00000164061	UCCAGUU
ENSG00000164061	UCCCUGU
ENSG00000164061	UCGUGCA
ENSG00000164061	UCUCAAG
ENSG00000164061	UCUCACC
ENSG00000164061	UCUCCAA
ENSG00000164061	UGCAAAG
ENSG00000164061	UGCAUAG
ENSG00000164061	UGCAUAU
ENSG00000164061	UGCCAGC
ENSG00000164061	UGGAGAU
ENSG00000164061	UGUAUAA
ENSG00000164061	UGUGCUU
ENSG00000164061	UUUGCAA
ENSG00000164070	AACUACU
ENSG00000164070	CAGUGCA
ENSG00000164076	AGCAAAA
ENSG00000164076	CUCAGGG
ENSG00000164076	UAGGGCC
ENSG00000164076	UCCCACA
ENSG00000164076	UGCCAGC
ENSG00000164076	UUCUGCA
ENSG00000164077	UAAUGCU
ENSG00000164082	CUCCAGC

ENSG00000164088	AACCUGU
ENSG00000164088	AGGACAC
ENSG00000164088	GAGAACU
ENSG00000164091	AAAAAUC
ENSG00000164091	AAAGUGC
ENSG00000164091	AAGACAU
ENSG00000164091	AAUACUG
ENSG00000164091	ACUGCAU
ENSG00000164091	ACUGGCC
ENSG00000164091	ACUGGCU
ENSG00000164091	AGUAACA
ENSG00000164091	AUAUAAC
ENSG00000164091	AUGUAAU
ENSG00000164091	CAAAACU
ENSG00000164091	CAAAGUG
ENSG00000164091	CAAAUGC
ENSG00000164091	CACAGGG
ENSG00000164091	CACUCCU
ENSG00000164091	CAGACAG
ENSG00000164091	CAGCAAA
ENSG00000164091	CAGCAGA
ENSG00000164091	CAGCAGG
ENSG00000164091	GAAACAU
ENSG00000164091	GAGCUGC
ENSG00000164091	GCUAGCA
ENSG00000164091	GGAAGAC
ENSG00000164091	GGGAGCU
ENSG00000164091	UCACACA
ENSG00000164091	UUGGCAA
ENSG00000164093	AAAAGUA
ENSG00000164093	AAAGUAA
ENSG00000164093	AGUGGUU
ENSG00000164093	AUCCUUU
ENSG00000164093	UAUAAAG
ENSG00000164093	UGACAUC
ENSG00000164093	UUGACAC
ENSG00000164099	AGCAGCA
ENSG00000164099	UUCCAUA
ENSG00000164100	UGCCAGC
ENSG00000164104	UACAGUU
ENSG00000164104	UGAAGCU
ENSG00000164107	AACAUUC
ENSG00000164107	AAGUGCA
ENSG00000164107	ACAUUCA
ENSG00000164107	AUUGCAC
ENSG00000164107	CACAGUG
ENSG00000164107	GGAAUGU
ENSG00000164107	GGUUUAC
ENSG00000164111	AAGGCAC
ENSG00000164111	CACAAUG
ENSG00000164112	AGCAGCU
ENSG00000164112	CUGCUCA
ENSG00000164112	GGAGUCC
ENSG00000164114	ACUGCAG
ENSG00000164114	AGAUAAA
ENSG00000164114	UCAAGUA
ENSG00000164116	AGUGCUU
ENSG00000164116	UAAUACA
ENSG00000164117	ACAAUAU
ENSG00000164117	ACAUUAC
ENSG00000164117	ACCAGCA
ENSG00000164117	UGUGCUU
ENSG00000164118	AGGAUGU
ENSG00000164118	AGUGCUG

ENSG00000164118	GACUUCC
ENSG00000164118	GUAAACA
ENSG00000164118	GUUCAUG
ENSG00000164124	GCUGGUG
ENSG00000164142	AAGGCAC
ENSG00000164142	AGCAGCU
ENSG00000164142	CAGGAAC
ENSG00000164142	UGCAUAU
ENSG00000164144	AAGGCAC
ENSG00000164144	GUCAGUU
ENSG00000164151	AAGACAU
ENSG00000164151	AAGGCAC
ENSG00000164151	AGCAAAA
ENSG00000164151	CAAAGUG
ENSG00000164151	CUGCAGG
ENSG00000164151	GCAGCAU
ENSG00000164151	UCAAGUA
ENSG00000164161	CCAGUGC
ENSG00000164163	AACACUG
ENSG00000164163	UGCAAAG
ENSG00000164163	UUGGCAA
ENSG00000164164	AAGGCAC
ENSG00000164164	AAUACUG
ENSG00000164164	AAUCACU
ENSG00000164164	AGCACCA
ENSG00000164164	AGCAGCA
ENSG00000164164	AGCAGCG
ENSG00000164164	AGCAGCU
ENSG00000164164	AGUAACA
ENSG00000164164	AUACAAG
ENSG00000164164	AUUGCAC
ENSG00000164164	CAAAGUG
ENSG00000164164	CAAAUGC
ENSG00000164164	CCAGUGC
ENSG00000164164	CUACAAA
ENSG00000164164	CUCACAC
ENSG00000164164	CUGCCCC
ENSG00000164164	CUUCACC
ENSG00000164164	CUUCUCU
ENSG00000164164	CUUUGGU
ENSG00000164164	GGAAGCC
ENSG00000164164	GUCAGUU
ENSG00000164164	UAAUAUC
ENSG00000164164	UCCAGAG
ENSG00000164164	UCCUGAG
ENSG00000164164	UCUCACC
ENSG00000164164	UCUCCAA
ENSG00000164164	UGAAAGG
ENSG00000164164	UGAGAAU
ENSG00000164164	UGCAUAU
ENSG00000164164	UUGACAC
ENSG00000164168	AAGACAU
ENSG00000164168	AAGUCUU
ENSG00000164168	AUACAAG
ENSG00000164168	UGAAAGG
ENSG00000164168	UUCCAUA
ENSG00000164171	AGCCAGU
ENSG00000164171	CACAGUG
ENSG00000164171	GGACUGC
ENSG00000164171	UCACAGG
ENSG00000164171	UCCUGAG
ENSG00000164181	AAGUCUU
ENSG00000164181	GAAGGAA
ENSG00000164181	GAUUGUA

ENSG00000164181	GCAGAAG
ENSG00000164185	AGAUAAA
ENSG00000164190	AAAAAUC
ENSG00000164190	AAACUGU
ENSG00000164190	AAAGAAU
ENSG00000164190	AACAAAC
ENSG00000164190	AACAUUC
ENSG00000164190	AAGAACC
ENSG00000164190	AAUUAUU
ENSG00000164190	ACAUCAC
ENSG00000164190	ACAUUCA
ENSG00000164190	ACUGCAG
ENSG00000164190	AGGUUAU
ENSG00000164190	AGUAACA
ENSG00000164190	AUACAAG
ENSG00000164190	AUCCCUU
ENSG00000164190	CACAGUG
ENSG00000164190	CUAUACA
ENSG00000164190	CUCCCAC
ENSG00000164190	CUGGAAA
ENSG00000164190	GCCUGGA
ENSG00000164190	GGCUGCG
ENSG00000164190	UACAAAG
ENSG00000164190	UAUUGCU
ENSG00000164190	UCAAGUA
ENSG00000164190	UCAUAGA
ENSG00000164190	UCCCACA
ENSG00000164190	UCCCUUU
ENSG00000164190	UGACCUA
ENSG00000164190	UGCCAGC
ENSG00000164190	UGGCUGC
ENSG00000164190	UGUAUAA
ENSG00000164190	UUCAAAU
ENSG00000164190	UUCAAGC
ENSG00000164190	UUGGCAA
ENSG00000164197	AAGUGCA
ENSG00000164197	AAUCACU
ENSG00000164197	ACCCUGU
ENSG00000164197	AGUGCAA
ENSG00000164197	AUAAAGU
ENSG00000164197	AUGCAUU
ENSG00000164197	GAUAUGU
ENSG00000164197	UAUUGCU
ENSG00000164219	AAAGAAU
ENSG00000164219	AUCACUA
ENSG00000164219	AUGGCUU
ENSG00000164219	CUGGCUC
ENSG00000164219	GAUUGUC
ENSG00000164219	GGCUCAG
ENSG00000164219	GUAAACA
ENSG00000164219	UCUUGAG
ENSG00000164219	UUGGCAC
ENSG00000164220	GGCAGUG
ENSG00000164244	AAGGCAC
ENSG00000164244	GGAAUGU
ENSG00000164251	CAAAACU
ENSG00000164252	CUUCACC
ENSG00000164252	UCACAGG
ENSG00000164252	UCACAGU
ENSG00000164258	AAAGGUA
ENSG00000164258	CACAGUG
ENSG00000164270	AGCAGCG
ENSG00000164270	AGGCAGG
ENSG00000164270	CAAGAGC

ENSG00000164270	CCUGCUG
ENSG00000164270	GAGGUAG
ENSG00000164270	GCUGGAU
ENSG00000164270	GGACCUG
ENSG00000164283	AUAUAAC
ENSG00000164284	AAAUCCC
ENSG00000164284	AAUGCCC
ENSG00000164284	AGGUAGU
ENSG00000164284	CUCUGGG
ENSG00000164284	GAGGUAG
ENSG00000164284	GCUUCCU
ENSG00000164284	UGAAAGG
ENSG00000164284	UUCUUGU
ENSG00000164292	AAAGCUG
ENSG00000164292	CAGGCUC
ENSG00000164292	UCCCUUG
ENSG00000164292	UGCAAAG
ENSG00000164296	AAUGUUG
ENSG00000164303	AGGCAGG
ENSG00000164303	AGUGAUC
ENSG00000164303	GAUUGUA
ENSG00000164303	GAUUGUC
ENSG00000164303	UGAAGCU
ENSG00000164303	UUGUGAC
ENSG00000164305	GAGGUAU
ENSG00000164305	GCUGGUG
ENSG00000164305	UUGAGGC
ENSG00000164307	CAGGAAC
ENSG00000164307	GGGUUUA
ENSG00000164318	CUACAAA
ENSG00000164318	CUGGAAA
ENSG00000164318	GAGCUAA
ENSG00000164318	UCCCUUG
ENSG00000164318	UCUGGAA
ENSG00000164318	UGCAGCU
ENSG00000164323	AAAAGUA
ENSG00000164326	AAUUUUA
ENSG00000164327	AAGUGCA
ENSG00000164327	AAUCACU
ENSG00000164327	ACCACAG
ENSG00000164327	ACUGCAU
ENSG00000164327	ACUGUAG
ENSG00000164327	AGCAGCA
ENSG00000164327	AGCAGCU
ENSG00000164327	AGGUAGU
ENSG00000164327	AGUGCUU
ENSG00000164327	AUGUCUG
ENSG00000164327	CACAGGG
ENSG00000164327	CACAGUG
ENSG00000164327	CAGCUGG
ENSG00000164327	CAGUCCA
ENSG00000164327	CUACAAA
ENSG00000164327	CUGGAAA
ENSG00000164327	GAGGUAG
ENSG00000164327	GAGGUAU
ENSG00000164327	GCUGGAU
ENSG00000164327	GGAAUGU
ENSG00000164327	GGCUCUG
ENSG00000164327	GGUCUAG
ENSG00000164327	UCACAGG
ENSG00000164327	UGAAGUG
ENSG00000164327	UGACCUA
ENSG00000164327	UGCAUAG
ENSG00000164327	UGGUACC

ENSG00000164327	UUGGAGG
ENSG00000164327	UUGGCAA
ENSG00000164327	UUGGUCC
ENSG00000164327	UUUUUGC
ENSG00000164330	AAAAGUA
ENSG00000164330	AAAGGUA
ENSG00000164330	AAAGUAU
ENSG00000164330	AAAUCCC
ENSG00000164330	AAGGCAC
ENSG00000164330	AAUCUCU
ENSG00000164330	ACAUUAC
ENSG00000164330	AGGUAGU
ENSG00000164330	AGUAGUU
ENSG00000164330	AUAAUAC
ENSG00000164330	AUGGAUU
ENSG00000164330	CAGACAG
ENSG00000164330	CAGUAGU
ENSG00000164330	GCAGCAU
ENSG00000164330	GCCUGGA
ENSG00000164330	GCUGGUG
ENSG00000164330	GUCACUC
ENSG00000164330	UACAAAG
ENSG00000164330	UAGUGAG
ENSG00000164330	UAUAUGA
ENSG00000164330	UCACAUU
ENSG00000164330	UGCAUAU
ENSG00000164330	UUGGCAC
ENSG00000164338	AAAUCCC
ENSG00000164338	GAGGUAG
ENSG00000164379	AAAGUGC
ENSG00000164379	AAGUGCA
ENSG00000164379	CACAGUG
ENSG00000164379	CAGUAGU
ENSG00000164379	CCCUGAG
ENSG00000164379	CUUUGGU
ENSG00000164379	UUGGCAA
ENSG00000164385	CAGCAGG
ENSG00000164398	GAGGUAU
ENSG00000164398	GGCUCAG
ENSG00000164398	UAUUGCU
ENSG00000164400	AUAUAAC
ENSG00000164402	CCCACCU
ENSG00000164402	GUGAAUG
ENSG00000164402	UAUUACC
ENSG00000164406	UUCCAUA
ENSG00000164411	UCUGGAA
ENSG00000164414	UUGGCAA
ENSG00000164418	AAAAAUC
ENSG00000164418	AAAUUUC
ENSG00000164418	ACACACC
ENSG00000164418	ACACACU
ENSG00000164418	ACAUUCA
ENSG00000164418	ACGCACU
ENSG00000164418	ACUGUGU
ENSG00000164418	AGUGCAA
ENSG00000164418	CACACCG
ENSG00000164418	CACACCU
ENSG00000164418	CAGCAAA
ENSG00000164418	CAGUCCA
ENSG00000164418	GAAACAU
ENSG00000164418	GAGGUAG
ENSG00000164418	GAGGUAU
ENSG00000164418	GGUGGUC
ENSG00000164418	GGUGGUU

ENSG00000164418	UACAAAG
ENSG00000164418	UGACAUC
ENSG00000164418	UGCAUAG
ENSG00000164418	UGUCUUU
ENSG00000164418	UGUGCUU
ENSG00000164418	UGUUGAA
ENSG00000164418	UUGUUCG
ENSG00000164438	AAAAGUA
ENSG00000164438	AAAGAAU
ENSG00000164438	AAAGUAC
ENSG00000164438	AAAGUAU
ENSG00000164438	AUAAUAC
ENSG00000164438	UACAAAG
ENSG00000164440	ACCCUGU
ENSG00000164440	GCUGGAU
ENSG00000164440	UGGCUGC
ENSG00000164440	UGUUGAA
ENSG00000164442	AAAUUUC
ENSG00000164442	CACAGUG
ENSG00000164442	GUUCAUG
ENSG00000164442	UGAAAUG
ENSG00000164458	GAUUGUA
ENSG00000164458	GCAGAAG
ENSG00000164463	AAAUUUC
ENSG00000164463	AAGGCAC
ENSG00000164463	AAGUCAC
ENSG00000164463	AAGUGCA
ENSG00000164463	ACAAGGU
ENSG00000164463	ACAGUAC
ENSG00000164463	ACAGUAU
ENSG00000164463	AGCAGCG
ENSG00000164463	AGCAUUC
ENSG00000164463	AUCAUAC
ENSG00000164463	CACAGUG
ENSG00000164463	CCUUCUG
ENSG00000164463	CUGCAGC
ENSG00000164463	GGCAGGG
ENSG00000164463	GGCAGUG
ENSG00000164463	UAGACUG
ENSG00000164463	UCAACAG
ENSG00000164463	UCACAGG
ENSG00000164463	UCCUAUA
ENSG00000164463	UCGCUGC
ENSG00000164463	UGGUACC
ENSG00000164466	GUAAACA
ENSG00000164494	AAAAAUC
ENSG00000164494	AAAGGUA
ENSG00000164494	AAGUGUG
ENSG00000164494	ACCACAG
ENSG00000164494	ACUCAGC
ENSG00000164494	AGUGUGC
ENSG00000164494	AUGUAAU
ENSG00000164494	CCUUCUU
ENSG00000164494	CUCCAGC
ENSG00000164494	GAAAACA
ENSG00000164494	GCUGUCU
ENSG00000164494	GGCGCCU
ENSG00000164494	GUAAACA
ENSG00000164494	GUCAACA
ENSG00000164494	GUGCUUG
ENSG00000164494	GUUAAUG
ENSG00000164494	UCACAGG
ENSG00000164494	UGACCUA
ENSG00000164494	UGCAGCU

ENSG00000164494	UGCUGAC
ENSG00000164494	UGUGCUU
ENSG00000164494	UUCUGCA
ENSG00000164494	UUCUUGU
ENSG00000164506	GAGGUAG
ENSG00000164506	GAGGUAU
ENSG00000164508	AAAGAGC
ENSG00000164512	AUGGCUU
ENSG00000164530	CCUUCAU
ENSG00000164542	CAAAGCA
ENSG00000164542	GCACCAU
ENSG00000164543	CAAAGUG
ENSG00000164543	GAUCAGA
ENSG00000164543	UUCCUAG
ENSG00000164548	AAAUUUC
ENSG00000164548	CCUUCAU
ENSG00000164548	GAAAACA
ENSG00000164574	AAAUGGU
ENSG00000164574	AGCAAAA
ENSG00000164574	CAGCUGG
ENSG00000164574	GAGAACU
ENSG00000164574	GCAGGUU
ENSG00000164574	GGAUUUC
ENSG00000164574	GGCAGGG
ENSG00000164574	UGCAAAG
ENSG00000164588	AAAUGGU
ENSG00000164588	AAGACAU
ENSG00000164588	ACAAUAU
ENSG00000164588	AGCAGCA
ENSG00000164588	AUAUUAU
ENSG00000164588	AUGCCUU
ENSG00000164588	CCCUGAG
ENSG00000164588	GACCAUG
ENSG00000164588	GGAAUGU
ENSG00000164588	GUCUUGC
ENSG00000164591	CAGGCUC
ENSG00000164600	ACACACU
ENSG00000164600	ACUGUUU
ENSG00000164600	CACUGUU
ENSG00000164600	GUGAAUG
ENSG00000164600	UCAUUUG
ENSG00000164600	UUCCUCG
ENSG00000164603	AAAAACC
ENSG00000164603	AAAAAUC
ENSG00000164603	AAAGUGC
ENSG00000164603	AAUUGCU
ENSG00000164603	ACAUUCA
ENSG00000164603	AUGUAAU
ENSG00000164603	CACAGUG
ENSG00000164603	CUGACUC
ENSG00000164603	GAGGUAG
ENSG00000164603	GAGGUAU
ENSG00000164603	GUCAACA
ENSG00000164603	GUGCUUG
ENSG00000164603	UCCCACA
ENSG00000164603	UGUCUUU
ENSG00000164603	UUGUUCG
ENSG00000164604	AAAGAAU
ENSG00000164604	AAAGUAU
ENSG00000164604	AAAUUUC
ENSG00000164604	AAGGCAC
ENSG00000164604	AAGUCUU
ENSG00000164604	AAUCCUU
ENSG00000164604	AAUUGCU

ENSG00000164604	ACAAGGU
ENSG00000164604	ACAGUAU
ENSG00000164604	AGCACCA
ENSG00000164604	AGUGGUU
ENSG00000164604	AUUGCAC
ENSG00000164604	CAAAUGC
ENSG00000164604	CAGUAGA
ENSG00000164604	CAGUAGU
ENSG00000164604	CUGCUCA
ENSG00000164604	CUUCUCU
ENSG00000164604	GCAGAAG
ENSG00000164604	GUAGUGU
ENSG00000164604	GUGCCUG
ENSG00000164604	GUGCUUG
ENSG00000164604	UACAAAG
ENSG00000164604	UAGGGCC
ENSG00000164604	UCACACA
ENSG00000164604	UCUCAAG
ENSG00000164604	UCUUGAG
ENSG00000164604	UGCAUAU
ENSG00000164604	UGUGCUU
ENSG00000164604	UUCUAAU
ENSG00000164604	UUGGCAA
ENSG00000164604	UUUGCAA
ENSG00000164609	GAGAUGA
ENSG00000164609	UGUUGAA
ENSG00000164615	GAGACCU
ENSG00000164615	UCACAAG
ENSG00000164619	AAAAACC
ENSG00000164619	AAUGCCC
ENSG00000164619	AGAUAAA
ENSG00000164619	AGGCAGG
ENSG00000164619	AUAAUAC
ENSG00000164619	AUACAAG
ENSG00000164619	AUGGCUU
ENSG00000164619	CUCCAGC
ENSG00000164619	CUCUUCC
ENSG00000164619	CUUACAG
ENSG00000164619	GGCUCUG
ENSG00000164619	GUGCAAA
ENSG00000164619	UAGGGCC
ENSG00000164619	UAUAAUA
ENSG00000164619	UCCAGAG
ENSG00000164619	UCUCCAA
ENSG00000164619	UGAAAUG
ENSG00000164619	UGGACUG
ENSG00000164619	UUAGGAU
ENSG00000164620	UAUUGCU
ENSG00000164626	CCCCUGG
ENSG00000164626	CUGGCUC
ENSG00000164626	GAGAACC
ENSG00000164626	GGGGAGC
ENSG00000164626	GUGGGGA
ENSG00000164627	AGGAGCU
ENSG00000164631	CAGUCCA
ENSG00000164631	CCCUGAG
ENSG00000164631	UCCCUGU
ENSG00000164649	AAUUGCU
ENSG00000164649	AGCAGCA
ENSG00000164649	AUUGCAC
ENSG00000164649	CAGCAGG
ENSG00000164649	CCAGUGC
ENSG00000164649	GAAGGAA
ENSG00000164649	GUGCUUG

ENSG00000164651	AAAGUGC
ENSG00000164651	AAUUAUU
ENSG00000164651	ACAUUAC
ENSG00000164651	ACUGGAU
ENSG00000164651	ACUUGGA
ENSG00000164651	AGCAAAA
ENSG00000164651	AUACAAG
ENSG00000164651	AUGCAUU
ENSG00000164651	CAAAACU
ENSG00000164651	CAAAUGC
ENSG00000164651	CACACCG
ENSG00000164651	CACACCU
ENSG00000164651	CAGCAAA
ENSG00000164651	CAGGAAC
ENSG00000164651	CAUAUUG
ENSG00000164651	CCAUUAC
ENSG00000164651	CUCACAC
ENSG00000164651	CUCCAUU
ENSG00000164651	GACAACU
ENSG00000164651	GAGAACU
ENSG00000164651	GAGGUAG
ENSG00000164651	GCAGAAG
ENSG00000164651	GCCUGAU
ENSG00000164651	GGCUCUG
ENSG00000164651	GGGAUCG
ENSG00000164651	GGGUUUA
ENSG00000164651	UCAACAG
ENSG00000164651	UCAUUUG
ENSG00000164651	UCCAGAG
ENSG00000164651	UCCAGUU
ENSG00000164651	UCUCACC
ENSG00000164651	UGCAUAG
ENSG00000164651	UGCAUAU
ENSG00000164651	UGCCAGC
ENSG00000164651	UUUGCAA
ENSG00000164651	UUUGCAC
ENSG00000164651	UUUUUGC
ENSG00000164659	AAGGCAC
ENSG00000164659	AAUCUCU
ENSG00000164659	CAGUGCA
ENSG00000164659	GAGUUGG
ENSG00000164659	UUGGCAC
ENSG00000164663	GAAGGAA
ENSG00000164663	GUAUGUU
ENSG00000164663	UGUAUGU
ENSG00000164683	GUCAACA
ENSG00000164684	AAAAAUC
ENSG00000164684	AAAGUGC
ENSG00000164684	AACAUUC
ENSG00000164684	AAGGUGC
ENSG00000164684	AAGUGCA
ENSG00000164684	AUAUUAU
ENSG00000164684	AUGCAUU
ENSG00000164684	AUGGCUU
ENSG00000164684	CCCUGAG
ENSG00000164684	CCUGCUG
ENSG00000164684	GUGCAAA
ENSG00000164684	UCCUAUA
ENSG00000164684	UGCAUAG
ENSG00000164684	UGUAUGU
ENSG00000164684	UUGGCAA
ENSG00000164684	UUUUUGC
ENSG00000164691	AGCUUAU
ENSG00000164691	AGGAGCU

ENSG00000164691	GUAGUGU
ENSG00000164692	AGGUAGU
ENSG00000164692	AUACAAG
ENSG00000164692	CUCACAC
ENSG00000164692	GAGGUAU
ENSG00000164692	GGAAGAC
ENSG00000164695	AAUUGCU
ENSG00000164695	CCAGGAG
ENSG00000164713	ACAUUAC
ENSG00000164713	UACAAAG
ENSG00000164715	ACAUUCA
ENSG00000164715	AGUGCAA
ENSG00000164715	AUGGCUU
ENSG00000164715	CACAGUG
ENSG00000164715	GGCUCAG
ENSG00000164715	UUCCUAG
ENSG00000164733	AAAGCUG
ENSG00000164733	CAGCAGG
ENSG00000164733	CCCUGUC
ENSG00000164733	GUCUUGC
ENSG00000164733	UCCUUGC
ENSG00000164733	UUGGCAC
ENSG00000164736	AACACUG
ENSG00000164736	AAGUGUG
ENSG00000164736	GGAUUUC
ENSG00000164736	UCAAGUA
ENSG00000164741	AACUCUA
ENSG00000164741	AAGAACC
ENSG00000164741	ACAAUAU
ENSG00000164741	ACAGUAC
ENSG00000164741	ACAGUAU
ENSG00000164741	AGCAGCU
ENSG00000164741	AGUGCAA
ENSG00000164741	AGUGUGC
ENSG00000164741	CACUCCU
ENSG00000164741	CCAGUGC
ENSG00000164741	CCAGUGU
ENSG00000164741	CCUUCUG
ENSG00000164741	GCUUCCU
ENSG00000164741	GUGCAAA
ENSG00000164741	GUGCCAC
ENSG00000164741	UACAAAG
ENSG00000164741	UACAGUU
ENSG00000164742	AAAGUAU
ENSG00000164742	AACAGUC
ENSG00000164742	AAGGCAC
ENSG00000164742	AAUUAUU
ENSG00000164742	ACAGUAU
ENSG00000164742	AGCAUUC
ENSG00000164742	AGGACAC
ENSG00000164742	AUAAUAC
ENSG00000164742	AUGGCUU
ENSG00000164742	CAGUAGU
ENSG00000164742	CCCUGAG
ENSG00000164742	CUGCUCA
ENSG00000164742	GCCUGGG
ENSG00000164742	GCUGGUG
ENSG00000164742	GGUGGGC
ENSG00000164742	GUGCUUG
ENSG00000164742	UAAUACA
ENSG00000164742	UACAAAG
ENSG00000164742	UACAGUU
ENSG00000164742	UAUAAUA
ENSG00000164742	UAUUGCU

ENSG00000164742	UGCAUAU
ENSG00000164742	UGGUACC
ENSG00000164742	UGUGCUU
ENSG00000164742	UUAGAGA
ENSG00000164749	AGCACCA
ENSG00000164749	CACAGUG
ENSG00000164749	GGCAGUG
ENSG00000164749	GUAAACA
ENSG00000164754	AACAUUC
ENSG00000164754	CUACAAA
ENSG00000164754	UCAUGAU
ENSG00000164754	UGUCUUU
ENSG00000164756	AAGUAGC
ENSG00000164756	ACAGGUG
ENSG00000164756	AGAUGUG
ENSG00000164756	AGUAACA
ENSG00000164756	AUGAGCU
ENSG00000164756	AUGGCAC
ENSG00000164756	CAAAGUG
ENSG00000164756	CACUGUU
ENSG00000164756	CAGCAGA
ENSG00000164756	CAGCAGG
ENSG00000164756	CUGGACU
ENSG00000164756	GAGCUGC
ENSG00000164756	GGACUGC
ENSG00000164778	AAAAAUC
ENSG00000164778	AACAUUC
ENSG00000164778	AAGGCAC
ENSG00000164778	AAGUUGU
ENSG00000164778	AAUUAUU
ENSG00000164778	ACAAUAU
ENSG00000164778	ACCACAG
ENSG00000164778	AGCAGCA
ENSG00000164778	AUUCAUU
ENSG00000164778	AUUGCAC
ENSG00000164778	CACAAUG
ENSG00000164778	CACACCU
ENSG00000164778	CACAGUG
ENSG00000164778	CAGCAAA
ENSG00000164778	CAGCAGA
ENSG00000164778	CUUUGGU
ENSG00000164778	GCAGCAU
ENSG00000164778	GGCCCUG
ENSG00000164778	UAAUACA
ENSG00000164778	UAUUGCU
ENSG00000164778	UCACAAG
ENSG00000164778	UCACAGG
ENSG00000164778	UCAUAGA
ENSG00000164778	UCAUUUG
ENSG00000164778	UGAAAUG
ENSG00000164778	UGGACUG
ENSG00000164778	UGGCCCU
ENSG00000164796	AAGUGUG
ENSG00000164796	ACAUCAC
ENSG00000164796	AGUGUGC
ENSG00000164796	AUAAAGU
ENSG00000164796	CAGGAAC
ENSG00000164796	GUGCUUG
ENSG00000164796	UAUUGCU
ENSG00000164796	UCCAGUU
ENSG00000164796	UGUGCUU
ENSG00000164808	UCACAGG
ENSG00000164815	AGCAAAA
ENSG00000164815	AUGGCUU

ENSG00000164815	CCUGCUG
ENSG00000164815	UAUAAUA
ENSG00000164815	UGAAGCU
ENSG00000164823	AAACUGU
ENSG00000164823	AAAGAAU
ENSG00000164823	AAGUAGC
ENSG00000164823	AGCCAGU
ENSG00000164823	CCAGUAC
ENSG00000164823	CCAGUGU
ENSG00000164823	GAAAACA
ENSG00000164823	GUCCAGA
ENSG00000164823	UCACACA
ENSG00000164823	UUCAAGC
ENSG00000164849	AAGUGCU
ENSG00000164849	AAUACUG
ENSG00000164855	AAAUUUC
ENSG00000164855	AAUUAUU
ENSG00000164855	ACUGUAG
ENSG00000164855	CACCACC
ENSG00000164855	CUACAGU
ENSG00000164855	GUGACUG
ENSG00000164855	UAUUGCU
ENSG00000164855	UGUGCUU
ENSG00000164855	UUUGCAA
ENSG00000164879	UGCAAAG
ENSG00000164885	AUAAAGU
ENSG00000164889	AAUUAUU
ENSG00000164896	AAAGUGC
ENSG00000164896	AAGUGCA
ENSG00000164896	AGUGCAA
ENSG00000164896	CAAAGUG
ENSG00000164896	GUGCAAA
ENSG00000164916	AAAACUG
ENSG00000164916	AAUACUG
ENSG00000164916	ACAUUCA
ENSG00000164916	ACUGUAG
ENSG00000164916	AGCAGCA
ENSG00000164916	AGCAGCG
ENSG00000164916	AUCAUAC
ENSG00000164916	AUGGCUU
ENSG00000164916	UAAUAGU
ENSG00000164916	UAAUAUC
ENSG00000164916	UAAUGCU
ENSG00000164916	UCAUACA
ENSG00000164916	UCCUGAG
ENSG00000164916	UGUCUGC
ENSG00000164916	UGUCUGG
ENSG00000164920	AUGAGCU
ENSG00000164924	AAAACUG
ENSG00000164924	AAAAGUA
ENSG00000164924	AAAGCUG
ENSG00000164924	AACCGUU
ENSG00000164924	AACCUGU
ENSG00000164924	AACUGGU
ENSG00000164924	ACCACAG
ENSG00000164924	ACUGGCC
ENSG00000164924	ACUGGCU
ENSG00000164924	AUCCCUU
ENSG00000164924	AUUCAUU
ENSG00000164924	CACAAUG
ENSG00000164924	CAUAUUG
ENSG00000164924	CAUGGAU
ENSG00000164924	CCCUGUC
ENSG00000164924	CUGGCUC

ENSG00000164924	GGCUCUG
ENSG00000164924	GGUCUAG
ENSG00000164924	GUCUACU
ENSG00000164924	GUUAGGA
ENSG00000164924	UAAUACA
ENSG00000164924	UAAUGCU
ENSG00000164924	UACAAAG
ENSG00000164924	UCAUGAU
ENSG00000164924	UCAUUUG
ENSG00000164924	UCCCUUU
ENSG00000164924	UCUCAAG
ENSG00000164924	UGUGCUU
ENSG00000164924	UUGUUCG
ENSG00000164929	ACAUUAC
ENSG00000164929	CACAGGG
ENSG00000164929	UCACAGG
ENSG00000164930	CCAGUGC
ENSG00000164930	GUGCAAA
ENSG00000164932	CUUUGGU
ENSG00000164932	GAGGUAG
ENSG00000164933	AUGGCUU
ENSG00000164933	UUCUAAU
ENSG00000164938	AAGGCAC
ENSG00000164938	AAGUGCU
ENSG00000164938	ACAGUAC
ENSG00000164938	ACAGUAU
ENSG00000164938	AGCACCA
ENSG00000164938	AGUGCUG
ENSG00000164938	AGUGUGC
ENSG00000164938	CAAAGUG
ENSG00000164938	CCCUGAG
ENSG00000164938	CCUUCAU
ENSG00000164938	GACCAUG
ENSG00000164938	GGCUCAG
ENSG00000164938	GUAAACA
ENSG00000164938	GUGAGGU
ENSG00000164938	GUGCAAA
ENSG00000164938	GUUCAUG
ENSG00000164938	UCACAAG
ENSG00000164938	UCCCUUU
ENSG00000164938	UGCAUAG
ENSG00000164938	UUGGCAC
ENSG00000164949	AAAAACC
ENSG00000164953	AAAAAUC
ENSG00000164970	AACCUGG
ENSG00000164970	AAGGCAC
ENSG00000164970	AAUACUG
ENSG00000164970	ACAAGGU
ENSG00000164970	ACACACC
ENSG00000164970	ACACGGG
ENSG00000164970	AGCAGCA
ENSG00000164970	AUAUUAU
ENSG00000164970	CACACCU
ENSG00000164970	CAGCAAA
ENSG00000164970	CUACAAA
ENSG00000164970	CUCAGGG
ENSG00000164970	GCAGAAG
ENSG00000164970	GCAGCAU
ENSG00000164970	GGAAGCC
ENSG00000164970	GGAGUGU
ENSG00000164970	GGCUCUG
ENSG00000164970	GGGGCAG
ENSG00000164970	UGACUGU
ENSG00000164970	UUGGUUC

ENSG00000164970	UUGUGUC
ENSG00000164976	GAGGCAG
ENSG00000164976	GUGCUUG
ENSG00000164976	GUUAGGA
ENSG00000164976	UCCCACC
ENSG00000164976	UGAAAGG
ENSG00000164976	UUCUGCA
ENSG00000164983	ACAUUAC
ENSG00000164983	AGCCCUU
ENSG00000164983	CAAAGCA
ENSG00000164983	CUAUACA
ENSG00000164983	GAGAAGA
ENSG00000164983	GAGGUAG
ENSG00000164983	GAGGUAU
ENSG00000164983	GCACCAU
ENSG00000164983	GUAUUCU
ENSG00000164983	UAGGUAU
ENSG00000164983	UUGUUCG
ENSG00000164985	AAAGAGG
ENSG00000164985	ACACACC
ENSG00000164985	ACUGCAG
ENSG00000164985	ACUGCAU
ENSG00000164985	AGUAACA
ENSG00000164985	AUGCACC
ENSG00000164985	CAAAUGC
ENSG00000164985	CAGCAGA
ENSG00000164985	CAGCAGG
ENSG00000164985	GUUAAUG
ENSG00000164985	UAAUACA
ENSG00000164985	UAUAAAG
ENSG00000165006	AACAAAC
ENSG00000165006	AGCAAAA
ENSG00000165006	AGCAGCA
ENSG00000165006	AGGACAC
ENSG00000165006	AGUGCUG
ENSG00000165006	AUAAUAC
ENSG00000165006	GGCAGGG
ENSG00000165023	AAAGAAU
ENSG00000165023	AAAGUAU
ENSG00000165023	ACAUCAC
ENSG00000165023	AUGGCUU
ENSG00000165023	CAAAGUG
ENSG00000165023	CACAGUG
ENSG00000165023	CCAGGAG
ENSG00000165023	CUUACAG
ENSG00000165023	GAUAUGU
ENSG00000165023	GGAGGAG
ENSG00000165023	GUCUUGC
ENSG00000165023	GUGCUUG
ENSG00000165023	UCCCACA
ENSG00000165023	UGUGCUU
ENSG00000165023	UUCAAAU
ENSG00000165029	AAAGUUU
ENSG00000165029	AAGGCAC
ENSG00000165029	AAGUGCU
ENSG00000165029	AAUACUG
ENSG00000165029	AGCAGCU
ENSG00000165029	AGUGCAA
ENSG00000165029	AGUGCUU
ENSG00000165029	AGUGGUU
ENSG00000165029	AUGGCAC
ENSG00000165029	CAGUAGU
ENSG00000165029	CCAGUGU
ENSG00000165029	CCAUUAC

ENSG00000165029	CUGCCCC
ENSG00000165029	CUUUGGU
ENSG00000165029	GGAAUGU
ENSG00000165029	GUGCUUG
ENSG00000165029	UCAAGUA
ENSG00000165029	UCACAGG
ENSG00000165029	UCACAUU
ENSG00000165029	UUGGCAC
ENSG00000165029	UUUGCAC
ENSG00000165030	CAGUAGU
ENSG00000165030	UGAAAUG
ENSG00000165055	UCACAAG
ENSG00000165060	UGUCUUU
ENSG00000165071	AAAUGGU
ENSG00000165071	ACAUUCA
ENSG00000165072	ACUGCAU
ENSG00000165072	CCCUGAG
ENSG00000165072	GUAACAG
ENSG00000165072	UGCAAAG
ENSG00000165084	AUGCAUU
ENSG00000165102	CAGACAG
ENSG00000165119	AAUGUUG
ENSG00000165119	ACAUUAC
ENSG00000165119	AGAUAAA
ENSG00000165119	CAGCAAA
ENSG00000165119	CCUUCAU
ENSG00000165119	CUUCACC
ENSG00000165119	GGAAUGU
ENSG00000165120	AAAGAGC
ENSG00000165124	AACAAAC
ENSG00000165124	AGCACCA
ENSG00000165124	CAGCAGA
ENSG00000165124	GAGAUGA
ENSG00000165124	GCACCAU
ENSG00000165124	GCAGCAU
ENSG00000165124	UAAUAUC
ENSG00000165125	AAGUGCU
ENSG00000165131	GACCUGG
ENSG00000165131	GGCCCUG
ENSG00000165138	CUCUGGG
ENSG00000165138	CUGGGCA
ENSG00000165138	UUUGCAA
ENSG00000165152	AGUGCCU
ENSG00000165152	CCAGUGC
ENSG00000165152	GAGGCAG
ENSG00000165152	GUGUGGC
ENSG00000165152	UGUGUGG
ENSG00000165156	AGUGGUU
ENSG00000165156	CACAGUG
ENSG00000165156	UUGGCAA
ENSG00000165156	UUGGUCC
ENSG00000165168	CAGCUGG
ENSG00000165168	GGCAGCG
ENSG00000165168	UCAUUCG
ENSG00000165168	UGCAAAG
ENSG00000165168	UGUUGAA
ENSG00000165168	UUAGAGA
ENSG00000165169	GACUUCC
ENSG00000165175	AAAACUG
ENSG00000165175	AAGGCAC
ENSG00000165175	CACAAUG
ENSG00000165175	CCUUCAU
ENSG00000165175	GUGCAAA
ENSG00000165175	UGUGCGG

ENSG00000165175	UGUGCGU
ENSG00000165175	UUGGUUC
ENSG00000165185	AACCUGU
ENSG00000165185	AAGGCAC
ENSG00000165186	AAGUGCU
ENSG00000165186	AGUGUGC
ENSG00000165186	AUUCAUU
ENSG00000165186	CACUGUU
ENSG00000165186	GCUACAU
ENSG00000165186	GGAGAGA
ENSG00000165186	UCUCAAG
ENSG00000165188	AGCCCUU
ENSG00000165188	CUACAAA
ENSG00000165188	CUUCUCU
ENSG00000165188	UUGGCAA
ENSG00000165194	AAAGAGC
ENSG00000165194	AAGGCAC
ENSG00000165194	AGAUAAA
ENSG00000165194	AGGUAGU
ENSG00000165194	AUAAAGU
ENSG00000165194	AUAAUAC
ENSG00000165194	AUACAAG
ENSG00000165194	AUAUAAC
ENSG00000165194	CAAGAGC
ENSG00000165194	CACUGUU
ENSG00000165194	CAGACAG
ENSG00000165194	CAGCUGG
ENSG00000165194	CUACAAA
ENSG00000165194	CUGGAAA
ENSG00000165194	CUGUUGA
ENSG00000165194	GACAACU
ENSG00000165194	GAGGUAG
ENSG00000165194	GAGGUAU
ENSG00000165194	GUAAACA
ENSG00000165194	UAUAAAG
ENSG00000165194	UAUAAUA
ENSG00000165194	UAUUACC
ENSG00000165194	UGACCUA
ENSG00000165194	UGGCCCU
ENSG00000165194	UGUGUCA
ENSG00000165194	UGUUGAA
ENSG00000165195	AAGUGCU
ENSG00000165195	AGCAGCU
ENSG00000165195	CUGCUCA
ENSG00000165195	CUGGGCA
ENSG00000165195	GAGGUAG
ENSG00000165195	GGAUUUC
ENSG00000165195	GGCUCUG
ENSG00000165195	UAUCAGA
ENSG00000165197	CAAAGCA
ENSG00000165197	GCAACUU
ENSG00000165197	GUGACAG
ENSG00000165197	UUGGCAA
ENSG00000165209	AAAAGUA
ENSG00000165209	CCUUCAU
ENSG00000165209	GAGGUAG
ENSG00000165209	GAGGUAU
ENSG00000165209	UCACAAG
ENSG00000165209	UCACAGG
ENSG00000165209	UCACAGU
ENSG00000165209	UGCAUUG
ENSG00000165219	AAAACUG
ENSG00000165219	AAAGAGG
ENSG00000165219	AAGGCAC

ENSG00000165219	AAUGCCC
ENSG00000165219	CUAGUAA
ENSG00000165219	GGUGGGC
ENSG00000165219	GUGAAUG
ENSG00000165219	GUGCUUG
ENSG00000165219	UGAAGCU
ENSG00000165219	UGUGCUU
ENSG00000165233	CUUUGGU
ENSG00000165238	UACAAAG
ENSG00000165240	AGCACCA
ENSG00000165240	CCUUCAU
ENSG00000165240	CUUUGGU
ENSG00000165240	GAAACAU
ENSG00000165244	AAAGUGC
ENSG00000165244	AAGUGCA
ENSG00000165244	AGCAGCA
ENSG00000165244	AGCAGCG
ENSG00000165244	AGCAGCU
ENSG00000165244	AGCUUAU
ENSG00000165244	UACAAAG
ENSG00000165246	CAUAUUG
ENSG00000165246	CUCUUCC
ENSG00000165246	CUUUGGU
ENSG00000165246	GGCAGGG
ENSG00000165259	CACACCU
ENSG00000165271	GAGCUGC
ENSG00000165271	GAGUCUU
ENSG00000165271	UCAUACA
ENSG00000165271	UGAGUCU
ENSG00000165275	GUGCCAC
ENSG00000165280	AAGUGCA
ENSG00000165280	ACAAGGU
ENSG00000165280	ACUCAAA
ENSG00000165280	ACUCAGG
ENSG00000165280	AGGGAGG
ENSG00000165280	CCCUGUC
ENSG00000165280	CCCUGUU
ENSG00000165280	CUCCAUU
ENSG00000165280	GCAGCAU
ENSG00000165280	GGUUUAC
ENSG00000165280	GUCCAGA
ENSG00000165280	GUGGCCC
ENSG00000165280	UCCAGAG
ENSG00000165280	UUGGUUC
ENSG00000165280	UUUGCAC
ENSG00000165282	UGGCUGC
ENSG00000165283	UAUUACC
ENSG00000165288	AAGGUGC
ENSG00000165288	CAGUAGA
ENSG00000165300	AAGGCAC
ENSG00000165300	AUGGAUU
ENSG00000165300	CUUCACC
ENSG00000165300	GCUACAU
ENSG00000165300	UGCAUAU
ENSG00000165322	AAAAGUA
ENSG00000165322	AACAAAC
ENSG00000165322	CCAGUGC
ENSG00000165322	CUAUACA
ENSG00000165322	GACCAUG
ENSG00000165322	GUAACAG
ENSG00000165322	GUGCAAA
ENSG00000165322	UUGGUCC
ENSG00000165323	AAAGUGC
ENSG00000165323	AAAGUUU

ENSG00000165323	AGCCAGU
ENSG00000165323	AGUGCUU
ENSG00000165323	AUAAAGU
ENSG00000165323	CAAGAGC
ENSG00000165323	CACUGUU
ENSG00000165323	CAGCUGG
ENSG00000165323	CAGUAGU
ENSG00000165323	CUACAGU
ENSG00000165323	CUGGGGG
ENSG00000165323	GAUCAGA
ENSG00000165323	GCUUCCU
ENSG00000165323	UACAGUU
ENSG00000165323	UAGUGAG
ENSG00000165323	UAUAAAG
ENSG00000165323	UCCCUUU
ENSG00000165323	UGCAAAG
ENSG00000165323	UGCUCAC
ENSG00000165323	UGUGCUU
ENSG00000165323	UUGUGUC
ENSG00000165323	UUUGCAA
ENSG00000165325	AAAAAUC
ENSG00000165338	AAGGCAC
ENSG00000165349	CCAUUAC
ENSG00000165355	AAAACUG
ENSG00000165355	AACCGUU
ENSG00000165355	AAUCUCA
ENSG00000165355	ACACACU
ENSG00000165355	ACAGGUG
ENSG00000165355	AGAUAAA
ENSG00000165355	AGCAGCA
ENSG00000165355	AGCAGCG
ENSG00000165355	AUGUGCC
ENSG00000165355	AUGUGGG
ENSG00000165355	AUUGCAC
ENSG00000165355	CACACCU
ENSG00000165355	CAGGUGA
ENSG00000165355	CAUGGAU
ENSG00000165355	CCAGCAU
ENSG00000165355	CCAGUGC
ENSG00000165355	CCAGUGU
ENSG00000165355	CGUACCG
ENSG00000165355	CUCACAC
ENSG00000165355	CUGGGCA
ENSG00000165355	CUGUUGA
ENSG00000165355	CUUUGGU
ENSG00000165355	GAAACAU
ENSG00000165355	GCCCUGU
ENSG00000165355	GGAUUUC
ENSG00000165355	GGCAGCG
ENSG00000165355	GUGCCAC
ENSG00000165355	UAAUAUC
ENSG00000165355	UAUUGCU
ENSG00000165355	UCACCAC
ENSG00000165355	UCCUGAG
ENSG00000165355	UCUCACC
ENSG00000165355	UGCCAGC
ENSG00000165355	UGCUCAC
ENSG00000165355	UUGGCAA
ENSG00000165355	UUGGCAC
ENSG00000165359	AAUACUG
ENSG00000165359	CAAAGCA
ENSG00000165359	GAGGUAG
ENSG00000165376	AGCAGCA
ENSG00000165376	CAGCAGA

ENSG00000165376	CAGCAGG
ENSG00000165376	CCCCCAG
ENSG00000165376	UCUCCAA
ENSG00000165376	UGUGCUU
ENSG00000165379	AAAAAUC
ENSG00000165379	AAAGUGC
ENSG00000165383	UCCAGAG
ENSG00000165389	AAAGAAU
ENSG00000165389	AAAGCUG
ENSG00000165389	AAAGUAC
ENSG00000165389	AAAGUUU
ENSG00000165389	AACAGUC
ENSG00000165389	ACAAUAU
ENSG00000165389	ACAUUAC
ENSG00000165389	ACCCAUU
ENSG00000165389	AGCAAAA
ENSG00000165389	CAAAACU
ENSG00000165389	CAAAUGC
ENSG00000165389	CCAUUAC
ENSG00000165389	CUAUACA
ENSG00000165389	CUUACAG
ENSG00000165389	CUUUGGU
ENSG00000165389	GAAACAU
ENSG00000165389	GCUACAU
ENSG00000165389	GUGCAAA
ENSG00000165389	UAAUGCU
ENSG00000165389	UCCUGAG
ENSG00000165389	UGAAAGG
ENSG00000165389	UGAAAUG
ENSG00000165389	UUGGUCC
ENSG00000165406	AAGGCAC
ENSG00000165406	AAGUGCU
ENSG00000165406	GGCAGUG
ENSG00000165406	GUAAACA
ENSG00000165409	AAUUUUA
ENSG00000165409	CUAGGCU
ENSG00000165409	GAGAUGA
ENSG00000165410	AGGCAGG
ENSG00000165410	AUGGCAC
ENSG00000165410	CGUGGCC
ENSG00000165410	GUGAGGU
ENSG00000165410	UCCAGUU
ENSG00000165417	GUAGUGU
ENSG00000165424	AAAAAUC
ENSG00000165424	AAAGAAU
ENSG00000165424	AACGGAA
ENSG00000165424	AAGUCUU
ENSG00000165424	AAGUGCA
ENSG00000165424	AAGUGCU
ENSG00000165424	AAUUUUA
ENSG00000165424	ACAAUAU
ENSG00000165424	ACACACC
ENSG00000165424	ACACACU
ENSG00000165424	ACUCAAA
ENSG00000165424	AGUAACA
ENSG00000165424	AUAAUAC
ENSG00000165424	CACAGUG
ENSG00000165424	CCAGGAG
ENSG00000165424	CCCCUGG
ENSG00000165424	CCUGCUG
ENSG00000165424	CCUUCUU
ENSG00000165424	GAAAACA
ENSG00000165424	GAUUGUC
ENSG00000165424	GGCUCAG

ENSG00000165424	GUAAACA
ENSG00000165424	GUAGUGU
ENSG00000165424	GUGGCCC
ENSG00000165424	UAUAAUA
ENSG00000165424	UCACAAG
ENSG00000165424	UCACAGG
ENSG00000165424	UCACAGU
ENSG00000165424	UCAUUUG
ENSG00000165424	UGCAUAG
ENSG00000165424	UGUGUCA
ENSG00000165424	UUCUUGU
ENSG00000165434	AAGGCAC
ENSG00000165434	AGCAGCA
ENSG00000165434	AGCAGCG
ENSG00000165434	AGCAUUC
ENSG00000165434	CACAGUG
ENSG00000165434	CUCACAC
ENSG00000165434	GAAAACA
ENSG00000165434	GAGCUAA
ENSG00000165434	GAGGUAG
ENSG00000165434	GAGGUAU
ENSG00000165434	GGAUUUU
ENSG00000165434	GUGAGGU
ENSG00000165434	UAUUACC
ENSG00000165434	UCAAGUA
ENSG00000165434	UCACAGG
ENSG00000165434	UCACAGU
ENSG00000165434	UUCAAGC
ENSG00000165443	AAGUGCU
ENSG00000165443	AGUGCUG
ENSG00000165443	UGCUGAC
ENSG00000165443	UUGGCAA
ENSG00000165443	UUGGCAC
ENSG00000165449	AAGUGCU
ENSG00000165449	AGUGCUU
ENSG00000165449	CACAAUG
ENSG00000165449	GAGAAGA
ENSG00000165449	GAGGUAG
ENSG00000165449	UUGGCAA
ENSG00000165458	AAAACUG
ENSG00000165458	CCUUCAU
ENSG00000165458	GGACGGA
ENSG00000165458	UCAUUUG
ENSG00000165458	UUCCGGC
ENSG00000165458	UUUCAAC
ENSG00000165462	GGGGCAG
ENSG00000165476	AAAGUGC
ENSG00000165476	AAGUGUG
ENSG00000165476	ACCUGGA
ENSG00000165476	AGUGUGC
ENSG00000165476	CAAAACU
ENSG00000165476	GAGAUGA
ENSG00000165476	UUUCAAC
ENSG00000165478	GAUUGUA
ENSG00000165478	GAUUGUC
ENSG00000165490	AUAAAGU
ENSG00000165490	CAGGUGA
ENSG00000165494	AAAAGUA
ENSG00000165494	UAUAAUA
ENSG00000165495	AAAAAUC
ENSG00000165495	AACAUUC
ENSG00000165495	ACAUCAC
ENSG00000165495	AGCACCA
ENSG00000165495	AUGGCAC

ENSG00000165495	AUUCAUU
ENSG00000165495	CUCAAAC
ENSG00000165495	GAAAACA
ENSG00000165495	GAGAAAU
ENSG00000165495	GAGACCU
ENSG00000165495	GGGGGAA
ENSG00000165495	GUAAACA
ENSG00000165495	UACAAAG
ENSG00000165495	UAUAAAG
ENSG00000165495	UCACAGG
ENSG00000165495	UCACAGU
ENSG00000165495	UCAUUUG
ENSG00000165495	UCUCCAA
ENSG00000165495	UGAAGCU
ENSG00000165495	UGCAAAG
ENSG00000165495	UGCAUAG
ENSG00000165495	UGCAUAU
ENSG00000165525	CUUCUCU
ENSG00000165526	UGAGAAG
ENSG00000165527	ACAUUAC
ENSG00000165527	UAUUGCU
ENSG00000165527	UCACAGU
ENSG00000165548	AAGGCAC
ENSG00000165548	CAGCAGG
ENSG00000165548	CCCCUGG
ENSG00000165548	CUGGAAA
ENSG00000165548	GCUUCCU
ENSG00000165548	GGAGGCA
ENSG00000165555	UCCUGAG
ENSG00000165556	ACAUUCA
ENSG00000165556	GGCAUUG
ENSG00000165556	GGCUCAG
ENSG00000165556	GUGACAG
ENSG00000165556	UCCCUUU
ENSG00000165566	AAAGUGC
ENSG00000165566	AGUGCUU
ENSG00000165566	CCCUGAG
ENSG00000165566	GGCAUUG
ENSG00000165566	UCCCUGU
ENSG00000165566	UGCAAAG
ENSG00000165572	CUAUACA
ENSG00000165572	GCUACAU
ENSG00000165572	UAAUACA
ENSG00000165572	UACAAAG
ENSG00000165572	UUUGCAA
ENSG00000165588	AAAGUAA
ENSG00000165588	AAAGUAC
ENSG00000165588	AAAGUAU
ENSG00000165588	AAGACAU
ENSG00000165588	ACAAUAU
ENSG00000165588	ACACACU
ENSG00000165588	AGGACAC
ENSG00000165588	AGUGCAA
ENSG00000165588	AUAAAGU
ENSG00000165588	CACAGUG
ENSG00000165588	CAUAUUG
ENSG00000165588	GAUUGUA
ENSG00000165588	UCACAGU
ENSG00000165588	UGCAUAG
ENSG00000165617	AAAAACC
ENSG00000165617	AAAUUUC
ENSG00000165617	AAGGCAC
ENSG00000165617	AGCCCUU
ENSG00000165617	AUAUAAC

ENSG00000165617	AUUGCAC
ENSG00000165617	CUGGACU
ENSG00000165617	UAUUACC
ENSG00000165629	UACAAAG
ENSG00000165637	GCUGGUG
ENSG00000165637	GUGACUG
ENSG00000165646	CUUUGGU
ENSG00000165655	AAUUUUA
ENSG00000165655	CUUGGAG
ENSG00000165655	GAGCUAA
ENSG00000165659	AAUACUG
ENSG00000165659	ACUCCAG
ENSG00000165659	ACUGCAG
ENSG00000165659	ACUGUAG
ENSG00000165659	ACUGUGU
ENSG00000165659	AUAAUAC
ENSG00000165659	AUACAAG
ENSG00000165659	AUUCAUU
ENSG00000165659	CACCAGC
ENSG00000165659	CAGGCUC
ENSG00000165659	CUAGGCU
ENSG00000165659	CUCACAC
ENSG00000165659	GCUGGUG
ENSG00000165659	GGAAGAC
ENSG00000165659	GUGACUG
ENSG00000165659	UCACAUU
ENSG00000165659	UCAUAGA
ENSG00000165659	UCUCACC
ENSG00000165659	UGCAUAU
ENSG00000165659	UGGACUG
ENSG00000165659	UUCCUAG
ENSG00000165660	AAGUGCU
ENSG00000165660	AGUAGAC
ENSG00000165660	CAGGCUC
ENSG00000165660	CUAGUAG
ENSG00000165660	CUUGGAG
ENSG00000165660	GGCUCAG
ENSG00000165660	GUGCUUG
ENSG00000165660	UCACAUU
ENSG00000165660	UCCCUUG
ENSG00000165661	AAAUUUC
ENSG00000165661	CAAAUGC
ENSG00000165661	CCCUGAG
ENSG00000165661	UUCAAAU
ENSG00000165669	AAUUGCU
ENSG00000165669	AAUUUUA
ENSG00000165669	CCAUUAC
ENSG00000165669	UAUAAAG
ENSG00000165669	UUAUGCA
ENSG00000165671	UCACAAG
ENSG00000165671	UCCAGAG
ENSG00000165672	UUGAUCA
ENSG00000165675	ACAUUCA
ENSG00000165675	AGGGAGG
ENSG00000165675	CAGUAGU
ENSG00000165675	GUCAACA
ENSG00000165678	AAAGCUG
ENSG00000165685	GGCAGUG
ENSG00000165689	ACUGCAU
ENSG00000165689	UGCAUAU
ENSG00000165699	AAAAACC
ENSG00000165699	AAAACUG
ENSG00000165699	AAACUGU
ENSG00000165699	AAAUUUC

ENSG00000165699	AACAUUC
ENSG00000165699	AAGUCUU
ENSG00000165699	AAGUGCA
ENSG00000165699	ACACACU
ENSG00000165699	ACUAGAU
ENSG00000165699	ACUGGCC
ENSG00000165699	AGCAGCA
ENSG00000165699	AGGUAGU
ENSG00000165699	CACAGUG
ENSG00000165699	CAGCAAA
ENSG00000165699	CAGCUGG
ENSG00000165699	CGUGCAU
ENSG00000165699	CUGGAAA
ENSG00000165699	CUUUGGU
ENSG00000165699	GACCCUG
ENSG00000165699	GAGGUAG
ENSG00000165699	GAUUGUA
ENSG00000165699	UAUAAAG
ENSG00000165699	UCACACA
ENSG00000165699	UCACAGG
ENSG00000165699	UGACAUC
ENSG00000165699	UGGCCCU
ENSG00000165699	UUCCUAG
ENSG00000165699	UUCUAAU
ENSG00000165699	UUGUUCG
ENSG00000165704	GUGCAAA
ENSG00000165724	AAAACUG
ENSG00000165724	AAACUGU
ENSG00000165724	AACACUG
ENSG00000165724	CAGUCUG
ENSG00000165724	UACAAAG
ENSG00000165731	AGUGCUU
ENSG00000165731	CUCCAUU
ENSG00000165731	GGCAGUG
ENSG00000165731	GUGCUUG
ENSG00000165731	UCACAAG
ENSG00000165731	UCACACA
ENSG00000165731	UCACAGU
ENSG00000165731	UGAAAUG
ENSG00000165757	AAAGUGC
ENSG00000165757	ACAUCAC
ENSG00000165757	ACCCUGU
ENSG00000165757	AGUGCAA
ENSG00000165757	CUCAGGG
ENSG00000165757	GCAGGUU
ENSG00000165757	GUGCAAA
ENSG00000165757	UAUAAAG
ENSG00000165757	UCACACA
ENSG00000165775	CUCUUCC
ENSG00000165775	GUAUUCU
ENSG00000165782	AAGUGCA
ENSG00000165782	AGCAGCA
ENSG00000165782	AGCAGCG
ENSG00000165782	AGCAGCU
ENSG00000165782	AGUGCAA
ENSG00000165782	GGAAUGU
ENSG00000165782	GUAGUGU
ENSG00000165782	UGAAGUG
ENSG00000165782	UGCCCUG
ENSG00000165794	GAGAAAU
ENSG00000165795	AGUGCAA
ENSG00000165802	AAAGCUG
ENSG00000165802	AUUGCAC
ENSG00000165802	CUGCCCC

ENSG00000165802	GCUGGUG
ENSG00000165802	GGCUCUG
ENSG00000165804	ACCAGCA
ENSG00000165804	AUCGUAC
ENSG00000165804	CCUGCUG
ENSG00000165804	GGGCCCC
ENSG00000165806	AAAGUGC
ENSG00000165806	AGCACCA
ENSG00000165807	GGAAUGU
ENSG00000165813	AAAGAAU
ENSG00000165813	ACAGUAC
ENSG00000165813	AGCAAAA
ENSG00000165813	AGCCCUU
ENSG00000165813	UACAGUU
ENSG00000165821	AAAGCUG
ENSG00000165821	GUUAGGA
ENSG00000165832	GUCAUUG
ENSG00000165832	UAUAAUA
ENSG00000165832	UUCAAAU
ENSG00000165861	AACAGUC
ENSG00000165861	AAGACAU
ENSG00000165861	GAAGGAA
ENSG00000165861	GAAUUGU
ENSG00000165861	GAGAACU
ENSG00000165861	GAGGCAG
ENSG00000165861	GGAGGCA
ENSG00000165861	GGAUUUC
ENSG00000165861	GGGUGGU
ENSG00000165861	UCCAGUU
ENSG00000165861	UUCUAAU
ENSG00000165868	AAGUCUU
ENSG00000165868	ACAAUAU
ENSG00000165868	ACCUGGA
ENSG00000165868	AUGCAUU
ENSG00000165868	AUUCAUU
ENSG00000165868	CCAGCAU
ENSG00000165868	CUGCAGC
ENSG00000165868	CUGCAGG
ENSG00000165868	GACCUGG
ENSG00000165868	UCACAUU
ENSG00000165868	UGCAAAG
ENSG00000165868	UUCUGCA
ENSG00000165879	CACAAUG
ENSG00000165879	CUGGGGG
ENSG00000165879	GAUCAGA
ENSG00000165879	UACAAAG
ENSG00000165879	UCACAUU
ENSG00000165879	UCCUAUA
ENSG00000165879	UGGAGAU
ENSG00000165879	UUGGAGG
ENSG00000165886	AGCAGCU
ENSG00000165886	CCCUGAG
ENSG00000165886	GACCCUG
ENSG00000165886	GAGGCAG
ENSG00000165886	GUGUGGC
ENSG00000165886	UGUGUGG
ENSG00000165887	AGCAAAA
ENSG00000165891	ACAUUCA
ENSG00000165891	ACCACAG
ENSG00000165891	ACCCUGU
ENSG00000165891	ACUCAGC
ENSG00000165891	AGCACCA
ENSG00000165891	AGGUAGU
ENSG00000165891	AGUGCAA

ENSG00000165891	CACAGUG
ENSG00000165891	UAAUACA
ENSG00000165891	UCACAGG
ENSG00000165891	UUGGUCC
ENSG00000165891	UUUUUGC
ENSG00000165914	GGAAUGU
ENSG00000165915	AUGGCUU
ENSG00000165915	CACAGUG
ENSG00000165915	CCCUGAG
ENSG00000165915	UAUUGCU
ENSG00000165915	UCACAGU
ENSG00000165915	UCCUGAG
ENSG00000165917	AAAGAGG
ENSG00000165917	AAAGUAC
ENSG00000165917	UACAAAG
ENSG00000165934	AGCCCUU
ENSG00000165934	UACAAAG
ENSG00000165934	UGACAUC
ENSG00000165949	CUUCUCU
ENSG00000165983	AAAGGUA
ENSG00000165983	AAGGCAC
ENSG00000165983	GAAACAU
ENSG00000165983	UCAAGUA
ENSG00000165985	AGAUGUG
ENSG00000165985	UGGAUAA
ENSG00000165995	AAACUGU
ENSG00000165995	AAAUUUC
ENSG00000165995	AAGGCAC
ENSG00000165995	CCAGUGU
ENSG00000165995	CUAGUCC
ENSG00000165995	GAUAUGU
ENSG00000165995	GGCAAGA
ENSG00000165995	UCACAGU
ENSG00000165995	UGCUGAC
ENSG00000165996	ACUUGGA
ENSG00000165996	UGGACUG
ENSG00000165997	AUCCCUU
ENSG00000165997	GUGCUUG
ENSG00000165997	UAUAAAG
ENSG00000165997	UCAAGUA
ENSG00000165997	UGUGCUU
ENSG00000165997	UUGUGUC
ENSG00000166002	AAGGCAC
ENSG00000166002	ACAUUCA
ENSG00000166002	UCCCUUU
ENSG00000166002	UGAAAGG
ENSG00000166006	GUGCCUG
ENSG00000166016	AAGGUGC
ENSG00000166016	ACAGUAC
ENSG00000166016	ACAGUAU
ENSG00000166016	AGCAGCA
ENSG00000166016	AGCAGCU
ENSG00000166016	CAGACAG
ENSG00000166016	GACAACU
ENSG00000166016	UCAUACA
ENSG00000166016	UGUCUGG
ENSG00000166025	AGCAGCA
ENSG00000166025	AUGCACC
ENSG00000166025	CAGCAAA
ENSG00000166025	CUUCACC
ENSG00000166025	CUUUGGU
ENSG00000166025	GGCAAGA
ENSG00000166025	GGGGUGC
ENSG00000166025	GGGGUGG

ENSG00000166025	UGGCUGC
ENSG00000166033	UGGCCAC
ENSG00000166037	AAAGUGC
ENSG00000166037	CAAAGUG
ENSG00000166037	CAGCAAA
ENSG00000166037	UACAGUU
ENSG00000166049	CCCCUGG
ENSG00000166068	AAAGUAU
ENSG00000166068	AGUGGUU
ENSG00000166068	CAAAGUG
ENSG00000166068	CAGCAAA
ENSG00000166068	UGUGCUU
ENSG00000166068	UUCAAGC
ENSG00000166068	UUUCAAC
ENSG00000166086	GAGGUAU
ENSG00000166086	GUCAACA
ENSG00000166086	UCACAUU
ENSG00000166086	UCCUUGC
ENSG00000166086	UGGCCAC
ENSG00000166090	AAAGUGC
ENSG00000166090	AGUGCCU
ENSG00000166090	CCUGCUG
ENSG00000166111	CCCUGUU
ENSG00000166111	GAGAACC
ENSG00000166111	GGCAGUG
ENSG00000166111	UUGGUCC
ENSG00000166123	AAAUUUC
ENSG00000166123	AAGUGCA
ENSG00000166123	GAAACAU
ENSG00000166123	GUAAACA
ENSG00000166123	UACAAAG
ENSG00000166123	UAUAAUA
ENSG00000166123	UCCCUUG
ENSG00000166128	AAUACUG
ENSG00000166128	ACAUUCA
ENSG00000166128	AUAUAAC
ENSG00000166128	AUUGCAC
ENSG00000166128	CCCUGAG
ENSG00000166128	GUCAGUU
ENSG00000166128	UGCAAAG
ENSG00000166135	AAAAACC
ENSG00000166135	AAAUUUC
ENSG00000166135	AAUCACU
ENSG00000166135	ACACACU
ENSG00000166135	ACAGGUG
ENSG00000166135	AGCCAGU
ENSG00000166135	AGCUGCC
ENSG00000166135	AGUGCUU
ENSG00000166135	AUGGCUU
ENSG00000166135	CAAGAGC
ENSG00000166135	CACCAGC
ENSG00000166135	CAGCAGG
ENSG00000166135	CAGUCCA
ENSG00000166135	CAGUGCA
ENSG00000166135	CCAUUAC
ENSG00000166135	CCCCCAG
ENSG00000166135	CCUUCAU
ENSG00000166135	CUCUUCC
ENSG00000166135	CUGCUCA
ENSG00000166135	CUGGGCA
ENSG00000166135	GACUUCC
ENSG00000166135	GAGACCU
ENSG00000166135	GAGGCAG
ENSG00000166135	GGACGGA

ENSG00000166135	GGCAGGG
ENSG00000166135	GGGGCAG
ENSG00000166135	GUGACAG
ENSG00000166135	GUGCUUG
ENSG00000166135	UCACAGG
ENSG00000166135	UCCAGAG
ENSG00000166135	UGCCCUG
ENSG00000166135	UUAGAGA
ENSG00000166135	UUGGCAA
ENSG00000166135	UUGGCAC
ENSG00000166147	AAGUCUU
ENSG00000166147	ACACACC
ENSG00000166147	ACAGUAC
ENSG00000166147	AGUGAUC
ENSG00000166147	AGUGCUG
ENSG00000166147	AUGUAAU
ENSG00000166147	CCAUUAC
ENSG00000166147	CCUGUAC
ENSG00000166147	GAGAAAU
ENSG00000166147	GAUAUGU
ENSG00000166147	GAUUGUA
ENSG00000166147	GCACCAU
ENSG00000166147	GGUUGAC
ENSG00000166147	GGUUGUC
ENSG00000166147	GUCUCUG
ENSG00000166147	UCACAUU
ENSG00000166147	UUGGUCC
ENSG00000166159	AACAAAC
ENSG00000166159	AAUUUUA
ENSG00000166159	CAGGCUC
ENSG00000166159	CUGCAGG
ENSG00000166159	GAAACAU
ENSG00000166159	GAAGGAA
ENSG00000166159	GAGAAGA
ENSG00000166159	GGAAGAC
ENSG00000166159	GGGUGUU
ENSG00000166159	GGUUACC
ENSG00000166159	UCCCUUU
ENSG00000166159	UGAGAAG
ENSG00000166165	AACUCUA
ENSG00000166167	AACUGGU
ENSG00000166167	AAGACAU
ENSG00000166167	AAUCACU
ENSG00000166167	ACAUCAC
ENSG00000166167	ACCCUGU
ENSG00000166167	ACUGCAG
ENSG00000166167	AGCAGCG
ENSG00000166167	AGUAGAC
ENSG00000166167	AUGGCUU
ENSG00000166167	CCCCCAG
ENSG00000166167	CUACAAA
ENSG00000166167	CUCCCAA
ENSG00000166167	CUCCCAC
ENSG00000166167	CUCUGGG
ENSG00000166167	CUUCUCU
ENSG00000166167	GAGAAAU
ENSG00000166167	GCAGCAU
ENSG00000166167	GGGUUUA
ENSG00000166167	GGUUUAC
ENSG00000166167	GUUAAUG
ENSG00000166167	UAGUGAG
ENSG00000166167	UAUUACC
ENSG00000166167	UCCCACA
ENSG00000166167	UCCCACC

ENSG00000166167	UCUAGAG
ENSG00000166167	UCUGGAA
ENSG00000166169	GAGGUAG
ENSG00000166170	AAGUGCA
ENSG00000166170	AGCAGCA
ENSG00000166181	AAAAGUA
ENSG00000166181	AAAGGUA
ENSG00000166181	ACAGGUG
ENSG00000166181	CAGUAGU
ENSG00000166181	CUCCAUU
ENSG00000166181	CUUCUCU
ENSG00000166181	GAGAAGA
ENSG00000166181	GAGUCUU
ENSG00000166181	UAUUGCU
ENSG00000166181	UCAUACA
ENSG00000166188	AGCAAAA
ENSG00000166188	AUAUUAU
ENSG00000166188	UUGGUUC
ENSG00000166197	AAAUUUC
ENSG00000166197	AAUUUUA
ENSG00000166197	UAAUAUC
ENSG00000166200	AAAAAUC
ENSG00000166200	ACAGUAU
ENSG00000166200	ACUGCAG
ENSG00000166200	ACUGUUU
ENSG00000166200	AGCAAAA
ENSG00000166200	AGCAGCA
ENSG00000166200	AGGUUAU
ENSG00000166200	AUUGCAC
ENSG00000166200	CUGCAGC
ENSG00000166200	CUUGGGC
ENSG00000166200	GAGAAAU
ENSG00000166200	GGCAAGA
ENSG00000166200	GUCAGUU
ENSG00000166200	GUGACAG
ENSG00000166200	UAUUGCU
ENSG00000166200	UCAAGUA
ENSG00000166200	UCUGGAA
ENSG00000166200	UGCAAAG
ENSG00000166200	UGGAGAU
ENSG00000166200	UGGGAUC
ENSG00000166200	UUCAAGC
ENSG00000166200	UUCUUGU
ENSG00000166200	UUGGUUC
ENSG00000166200	UUUGCAC
ENSG00000166206	AAAAACC
ENSG00000166206	AAAAAUC
ENSG00000166206	AAGUCUU
ENSG00000166206	AGCAAAA
ENSG00000166206	AUGACAC
ENSG00000166206	AUUCAUU
ENSG00000166206	CAAAUGC
ENSG00000166206	CAUGGGU
ENSG00000166206	CUACAAA
ENSG00000166206	GAGAAAU
ENSG00000166206	GAGGCUG
ENSG00000166206	GUGCUUG
ENSG00000166206	UACAAAG
ENSG00000166206	UCAAGUA
ENSG00000166206	UCACAGU
ENSG00000166206	UCACAUU
ENSG00000166206	UCAUUCG
ENSG00000166206	UCCCUUU
ENSG00000166206	UGAAAGG

ENSG00000166206	UUAGGAU
ENSG00000166206	UUAUGCA
ENSG00000166206	UUCAAGC
ENSG00000166224	AAAACUG
ENSG00000166224	AAAAGUA
ENSG00000166224	AAGGCAC
ENSG00000166224	AAGUUGU
ENSG00000166224	ACACACU
ENSG00000166224	ACAGUAC
ENSG00000166224	CCCUGAG
ENSG00000166224	CCUUCAU
ENSG00000166224	CCUUCUG
ENSG00000166224	GCUGUCU
ENSG00000166224	UCCCUUU
ENSG00000166224	UCCUGAG
ENSG00000166224	UCCUUGC
ENSG00000166224	UCUCCAA
ENSG00000166224	UUCAAAU
ENSG00000166225	AAAGUAA
ENSG00000166225	AAGGCAC
ENSG00000166225	AAGGUGC
ENSG00000166225	AAGUGCA
ENSG00000166225	AAGUGCU
ENSG00000166225	AAUGCCC
ENSG00000166225	ACAGUAC
ENSG00000166225	ACCCUGU
ENSG00000166225	ACUGGAU
ENSG00000166225	ACUGUAG
ENSG00000166225	AGCAGCA
ENSG00000166225	AGUGCUG
ENSG00000166225	AUACCUC
ENSG00000166225	AUGUAAU
ENSG00000166225	CACUGUU
ENSG00000166225	CCCUGUU
ENSG00000166225	CUUACAG
ENSG00000166225	GACCUGG
ENSG00000166225	GAGGUAG
ENSG00000166225	GAGGUAU
ENSG00000166225	GCACCAU
ENSG00000166225	GCUGGUG
ENSG00000166225	GUAAACA
ENSG00000166225	GUCUCUG
ENSG00000166225	GUGCAAA
ENSG00000166225	GUGCCAC
ENSG00000166225	UCACAAG
ENSG00000166225	UCCCUUG
ENSG00000166225	UGCAUAG
ENSG00000166225	UGCAUAU
ENSG00000166225	UGCAUUG
ENSG00000166225	UUUUUGC
ENSG00000166228	UUCAAAU
ENSG00000166233	AACAAAC
ENSG00000166233	UAUUACC
ENSG00000166233	UUAUGCA
ENSG00000166257	AAUACUG
ENSG00000166257	AGCACCA
ENSG00000166257	GAAUUGU
ENSG00000166257	GAGAACU
ENSG00000166257	GAGCCCU
ENSG00000166257	GCUGGUG
ENSG00000166257	UCACAGU
ENSG00000166257	UGAGAAG
ENSG00000166257	UGAGAAU
ENSG00000166260	AAAAAUC

ENSG00000166260	AAAAGUA
ENSG00000166260	AAAGUUU
ENSG00000166260	AGUGCCU
ENSG00000166260	CAGGAAC
ENSG00000166260	UCACAGG
ENSG00000166260	UCAUUUG
ENSG00000166260	UUUGCAC
ENSG00000166261	AAAGUGC
ENSG00000166261	AAGUGCA
ENSG00000166261	UGUGCUU
ENSG00000166263	AACUACU
ENSG00000166263	AUGGCAC
ENSG00000166263	CAGCAGG
ENSG00000166263	CAGUCUG
ENSG00000166263	CUCAAAC
ENSG00000166263	CUGGGGG
ENSG00000166263	UGAGUCU
ENSG00000166263	UGGAGAU
ENSG00000166263	UGUGCUU
ENSG00000166265	AAAGGUA
ENSG00000166265	AAUUAUU
ENSG00000166266	AAAGAAU
ENSG00000166266	AAGGCAC
ENSG00000166266	ACAGUAC
ENSG00000166266	ACAGUAU
ENSG00000166266	ACAUUCA
ENSG00000166266	ACUCCAG
ENSG00000166266	AGUGUGC
ENSG00000166266	AUACAAG
ENSG00000166266	GCAUCCC
ENSG00000166266	GCUGGUG
ENSG00000166266	GUGCAAA
ENSG00000166266	UCACAAG
ENSG00000166266	UCAUGAU
ENSG00000166266	UCCAGUU
ENSG00000166266	UCCUUGC
ENSG00000166266	UUUGCAC
ENSG00000166272	AAAGUAU
ENSG00000166272	AAUACUG
ENSG00000166272	ACUGUGU
ENSG00000166272	AGUGGUU
ENSG00000166272	CAAAGUG
ENSG00000166272	CACAGGG
ENSG00000166272	CUCUGGG
ENSG00000166272	CUUCUCU
ENSG00000166272	GAAAACA
ENSG00000166272	GGAAUGU
ENSG00000166272	GGAUUUU
ENSG00000166272	GUAAACA
ENSG00000166272	GUCAGUU
ENSG00000166272	GUGCAAA
ENSG00000166272	UCACAAG
ENSG00000166272	UCACAGG
ENSG00000166272	UCACAGU
ENSG00000166272	UGGCUGC
ENSG00000166272	UUGGUUC
ENSG00000166275	CAGCUGG
ENSG00000166275	UACAAAG
ENSG00000166275	UGCAGCU
ENSG00000166289	AAGGCAC
ENSG00000166289	GGAGAGA
ENSG00000166292	AUAUAAC
ENSG00000166313	CAGACAG
ENSG00000166317	CAAAGCA

ENSG00000166317	CAGCAAA
ENSG00000166317	CUUGUGU
ENSG00000166317	UGAAGCU
ENSG00000166317	UUGGUCC
ENSG00000166326	AAAAAUC
ENSG00000166326	CAAAGCA
ENSG00000166326	GCUUCCU
ENSG00000166326	UCUCCAA
ENSG00000166326	UUGGUUC
ENSG00000166333	GUGACAG
ENSG00000166333	UUGUGAC
ENSG00000166340	AGUGCAA
ENSG00000166340	CAGCAGG
ENSG00000166340	GAGGUAG
ENSG00000166341	CUGGGCA
ENSG00000166342	UCCAGAG
ENSG00000166348	ACAGUAU
ENSG00000166348	GGGUUUA
ENSG00000166348	GGUUUAC
ENSG00000166348	GUAUGUU
ENSG00000166349	AACACUG
ENSG00000166349	ACCUGGA
ENSG00000166349	AUGCAUU
ENSG00000166349	GAGGUAG
ENSG00000166349	GGCAUUG
ENSG00000166349	UCCAGUU
ENSG00000166349	UCCCUGU
ENSG00000166349	UGCCAGC
ENSG00000166387	ACUGUGU
ENSG00000166387	CAGGUGA
ENSG00000166387	GUGAGGU
ENSG00000166387	UUGGCAA
ENSG00000166398	ACCUGGA
ENSG00000166398	AGCACCA
ENSG00000166398	AUAAAGU
ENSG00000166398	AUAUAAC
ENSG00000166398	AUAUUAU
ENSG00000166398	AUGGCAC
ENSG00000166398	CAAAGCA
ENSG00000166398	CCAGUGU
ENSG00000166398	GCACCAU
ENSG00000166398	GGAUUUC
ENSG00000166398	GGCUCAG
ENSG00000166398	GUGAGGU
ENSG00000166398	GUGCAAA
ENSG00000166398	GUGCCUG
ENSG00000166398	UAAUACA
ENSG00000166398	UAUAAAG
ENSG00000166398	UCAUACA
ENSG00000166398	UCUCAAG
ENSG00000166398	UGCAAAG
ENSG00000166398	UUCAAAU
ENSG00000166398	UUUCAAC
ENSG00000166401	GGUGGGC
ENSG00000166402	AAAACUG
ENSG00000166402	ACUGGCU
ENSG00000166402	AGCUGCC
ENSG00000166402	AUGAGCU
ENSG00000166402	AUGGCAC
ENSG00000166402	CAGCAGA
ENSG00000166402	CUAGGCU
ENSG00000166402	CUGCAGC
ENSG00000166402	CUGCAGG
ENSG00000166402	CUUGGAG

ENSG00000166402	GAAAACA
ENSG00000166402	GAAGGAA
ENSG00000166402	GAGGCUG
ENSG00000166402	GCCCUUA
ENSG00000166402	GGGGCAG
ENSG00000166402	GUCUCUG
ENSG00000166402	UCCCUUU
ENSG00000166402	UUUGCAC
ENSG00000166405	AGCACCA
ENSG00000166405	CACAGGG
ENSG00000166405	CUGCUCA
ENSG00000166405	GUGACAG
ENSG00000166407	AACAUUC
ENSG00000166407	ACAUUCA
ENSG00000166407	GAAACAU
ENSG00000166411	AGCAGCA
ENSG00000166411	UGUGCUU
ENSG00000166415	AGCAAAA
ENSG00000166415	GACAACU
ENSG00000166415	GUCAUUG
ENSG00000166428	GGAGGAG
ENSG00000166432	UAAUAUC
ENSG00000166435	AAGUGCA
ENSG00000166435	AAUCUCA
ENSG00000166435	ACAGUAC
ENSG00000166435	ACAGUAU
ENSG00000166435	CAAAGUG
ENSG00000166435	GCUGUCU
ENSG00000166435	GGACUGC
ENSG00000166435	GUGCCAC
ENSG00000166435	UAUUGCU
ENSG00000166435	UGAGAAU
ENSG00000166439	AAUACUG
ENSG00000166439	AAUCACU
ENSG00000166439	ACUGUAG
ENSG00000166439	AGCACCA
ENSG00000166439	AGUGGUU
ENSG00000166439	AUGAGCU
ENSG00000166439	CACCACC
ENSG00000166439	CUUUGGU
ENSG00000166439	GACUUCC
ENSG00000166439	GAGCCCC
ENSG00000166439	GAGCCCU
ENSG00000166439	GCAGAAG
ENSG00000166439	GCAUCCC
ENSG00000166439	GGCAGUG
ENSG00000166439	GUAAACA
ENSG00000166439	UCAUGAU
ENSG00000166439	UCCAGAG
ENSG00000166439	UCCAGUU
ENSG00000166439	UGUCUUU
ENSG00000166439	UUCAAAU
ENSG00000166439	UUCCAUA
ENSG00000166444	AGUGGUU
ENSG00000166444	CUUACAG
ENSG00000166446	AACAAAC
ENSG00000166446	UCACACA
ENSG00000166446	UCUCCAA
ENSG00000166448	AUGAGCU
ENSG00000166448	GGGGUGG
ENSG00000166450	CAGCAAA
ENSG00000166450	GAGGUAU
ENSG00000166450	UCACAUU
ENSG00000166452	AAUCUCU

ENSG00000166452	GUCAACA
ENSG00000166454	ACAUUCA
ENSG00000166454	AUGCAUU
ENSG00000166454	CUUCUCU
ENSG00000166471	GAGAUGA
ENSG00000166471	UCCUGAG
ENSG00000166478	AUGGCUU
ENSG00000166483	AAAGUGC
ENSG00000166483	AAGUGCU
ENSG00000166483	AGCAGCG
ENSG00000166483	CAAAGUG
ENSG00000166483	CUACAAA
ENSG00000166483	GAUUGUA
ENSG00000166483	GAUUGUC
ENSG00000166483	GCUACAU
ENSG00000166483	UACAAAG
ENSG00000166484	AAGGCAC
ENSG00000166484	GGCUCAG
ENSG00000166507	AGGGAGG
ENSG00000166507	GAGGUAG
ENSG00000166507	GAGGUAU
ENSG00000166508	AAGUGCA
ENSG00000166509	AGCACCA
ENSG00000166509	GCAGAAG
ENSG00000166523	AAUUGCU
ENSG00000166526	GUGCAAA
ENSG00000166562	AUAUUAU
ENSG00000166569	UUGGUUC
ENSG00000166575	CCAGUGC
ENSG00000166575	CCAGUGU
ENSG00000166575	CCCUGAG
ENSG00000166575	CUACAGU
ENSG00000166575	CUGGCUC
ENSG00000166575	GGAAUGU
ENSG00000166575	UCCAGUU
ENSG00000166575	UCCCUGU
ENSG00000166579	AAAGUGC
ENSG00000166579	AAGUGCA
ENSG00000166579	AGCUGCC
ENSG00000166579	AGGACAC
ENSG00000166579	AGUGCAA
ENSG00000166579	AUACAAG
ENSG00000166579	AUGAUGA
ENSG00000166579	CUACAAA
ENSG00000166579	GAGAAGA
ENSG00000166579	UAAUACA
ENSG00000166592	AAAAAUC
ENSG00000166595	UACAAAG
ENSG00000166598	AAAAACC
ENSG00000166619	AAAGCUG
ENSG00000166619	AAAGUUU
ENSG00000166619	AAGUCUU
ENSG00000166619	AAUACUG
ENSG00000166619	AAUCUCU
ENSG00000166619	ACAUCAC
ENSG00000166619	AGUGCAA
ENSG00000166619	AUAAAGU
ENSG00000166619	CACAGGG
ENSG00000166619	CAGGAAC
ENSG00000166619	CCAGUGU
ENSG00000166619	GCUGGUG
ENSG00000166619	GGAAUGU
ENSG00000166619	GGAGUCC
ENSG00000166619	GUGACAG

ENSG00000166619	UCACAGG
ENSG00000166619	UGACCUA
ENSG00000166669	AAAGCUG
ENSG00000166669	GGCUGCG
ENSG00000166669	UGGCUGC
ENSG00000166689	GUAAACA
ENSG00000166689	GUGCAAA
ENSG00000166716	AACACUG
ENSG00000166716	AAUUGCU
ENSG00000166716	ACUGUUU
ENSG00000166716	AUACAAG
ENSG00000166716	AUGGCAC
ENSG00000166716	CAAGAGC
ENSG00000166716	CAGCAGA
ENSG00000166716	CAGGCUC
ENSG00000166716	CUCCCAC
ENSG00000166716	GGCAUUG
ENSG00000166716	GGGGUGC
ENSG00000166716	GGGGUGG
ENSG00000166716	UGAAAUG
ENSG00000166716	UUCCUAG
ENSG00000166734	AGCCCUU
ENSG00000166734	CCUUCUG
ENSG00000166734	UGCAAAG
ENSG00000166747	AAGGCAC
ENSG00000166747	CAGGCUC
ENSG00000166747	CCUUCAU
ENSG00000166747	GGGGGAA
ENSG00000166747	UUUCAAC
ENSG00000166780	ACACACC
ENSG00000166780	ACACACU
ENSG00000166780	GCUACAU
ENSG00000166780	UCUUGAG
ENSG00000166780	UGAAAUG
ENSG00000166783	AAGUCAC
ENSG00000166783	GGAAGAC
ENSG00000166783	UGCAUAU
ENSG00000166783	UUAUGCA
ENSG00000166793	AAAAAUC
ENSG00000166793	AAAGUGC
ENSG00000166793	AAAGUUU
ENSG00000166793	AGUGCCU
ENSG00000166793	GCGGAGG
ENSG00000166793	GCUGGUG
ENSG00000166793	GGGGUGG
ENSG00000166793	UGAAAUG
ENSG00000166794	GGAAUGU
ENSG00000166803	CUUACAG
ENSG00000166803	GCACCAU
ENSG00000166803	UAAUACA
ENSG00000166803	UACAAAG
ENSG00000166803	UCAACAG
ENSG00000166813	CAGCAGG
ENSG00000166823	UCACAAG
ENSG00000166825	CCCUGAG
ENSG00000166825	UCCCUGU
ENSG00000166828	GACCAUG
ENSG00000166828	UCACAAG
ENSG00000166831	AACAAAC
ENSG00000166831	AAUCCUU
ENSG00000166831	AUGAUGA
ENSG00000166831	AUUGCAC
ENSG00000166831	CAGCAAA
ENSG00000166831	GAAAACA

ENSG00000166831	UCACAGG
ENSG00000166831	UCACAGU
ENSG00000166831	UGCAUUG
ENSG00000166831	UUUGCAA
ENSG00000166831	UUUGCAC
ENSG00000166833	AAAGUGC
ENSG00000166833	AAGGCAC
ENSG00000166833	AAGUGCA
ENSG00000166833	AAUGUUG
ENSG00000166833	AAUUUUA
ENSG00000166833	ACUAGAU
ENSG00000166833	AGCCCUU
ENSG00000166833	AGUGCAA
ENSG00000166833	CACAGUG
ENSG00000166833	CAGCAAA
ENSG00000166833	CCCUGUU
ENSG00000166833	CUGGAAA
ENSG00000166833	CUGGCUC
ENSG00000166833	GCACCAU
ENSG00000166833	GCAGCAU
ENSG00000166833	GCCCUGU
ENSG00000166833	GCUUCCU
ENSG00000166833	GGAAGCC
ENSG00000166833	GGCAGUG
ENSG00000166833	GGCUCAG
ENSG00000166833	GUCUACU
ENSG00000166833	UAAGACG
ENSG00000166833	UCACAGG
ENSG00000166833	UCACAGU
ENSG00000166833	UCCCUGU
ENSG00000166833	UUGGUUC
ENSG00000166845	AGUAACA
ENSG00000166845	UUCCUCG
ENSG00000166847	AAAGUAA
ENSG00000166847	AGCAGCA
ENSG00000166847	CUGCAGG
ENSG00000166847	GUCAACA
ENSG00000166847	GUGACAG
ENSG00000166847	UUCCUAG
ENSG00000166848	UACAAAG
ENSG00000166860	AAGGCAC
ENSG00000166860	ACACACC
ENSG00000166860	AGCAGCG
ENSG00000166860	AGCAGCU
ENSG00000166860	AGGUAGU
ENSG00000166860	CAAAGUG
ENSG00000166860	CACAGUG
ENSG00000166860	CCAGCAU
ENSG00000166860	CUCCAGC
ENSG00000166860	CUUUGGU
ENSG00000166860	GAGGUAG
ENSG00000166860	GAGGUAU
ENSG00000166860	GCAGCAU
ENSG00000166860	GCAGGAC
ENSG00000166860	GGUUUAC
ENSG00000166860	GUGAGGU
ENSG00000166860	UACAAAG
ENSG00000166860	UCACAGG
ENSG00000166860	UCACAGU
ENSG00000166860	UCCUGAG
ENSG00000166860	UUGGUUC
ENSG00000166863	AAAAAUC
ENSG00000166863	UGUUGAA
ENSG00000166881	AAAUGGU

ENSG00000166881	AACAUUC
ENSG00000166881	AUCCCUU
ENSG00000166881	AUUGCAC
ENSG00000166881	CAGCAAA
ENSG00000166881	CCCUGAG
ENSG00000166881	CUGGCUC
ENSG00000166881	GAGGUAG
ENSG00000166881	GGCUCAG
ENSG00000166881	UCAAGUA
ENSG00000166881	UUCCUCG
ENSG00000166886	AAAUUUC
ENSG00000166886	ACUGCAG
ENSG00000166886	ACUGUAG
ENSG00000166886	CCAGUGU
ENSG00000166886	CUGCAGC
ENSG00000166887	AAAGCUG
ENSG00000166887	GGCAAGA
ENSG00000166887	GUCAGUU
ENSG00000166888	ACAGUAC
ENSG00000166888	ACAGUAU
ENSG00000166888	CAGCUGG
ENSG00000166888	CAGUAGA
ENSG00000166888	GGCCCUG
ENSG00000166888	UCACACA
ENSG00000166888	UUAGGAU
ENSG00000166889	AAAAAUC
ENSG00000166889	ACCAGCA
ENSG00000166889	AGUGUGC
ENSG00000166889	AUACAAG
ENSG00000166889	AUGGCUU
ENSG00000166889	CAGGAAC
ENSG00000166889	CAGUGCA
ENSG00000166889	CUCCAUU
ENSG00000166889	GGAAGAC
ENSG00000166889	GGCAGCG
ENSG00000166889	UCCCACA
ENSG00000166889	UGGAGAU
ENSG00000166889	UGUAUGU
ENSG00000166897	AACAGUC
ENSG00000166897	ACAGUAC
ENSG00000166897	ACAGUAU
ENSG00000166897	AUAUAAC
ENSG00000166897	AUUGCAC
ENSG00000166897	CAAGAGC
ENSG00000166897	CACAGGG
ENSG00000166897	CACAGUG
ENSG00000166897	CACUGUU
ENSG00000166897	CAGGUGA
ENSG00000166897	CUCCAUU
ENSG00000166897	CUCUGGG
ENSG00000166897	CUGGACU
ENSG00000166897	CUGGGCA
ENSG00000166897	GAAAACA
ENSG00000166897	GAGCCCC
ENSG00000166897	GAGCCCU
ENSG00000166897	GAGCUGC
ENSG00000166897	GAGUUGA
ENSG00000166897	GCAGGUU
ENSG00000166897	GCGGGUG
ENSG00000166897	GGAAGAC
ENSG00000166897	GGGGCAG
ENSG00000166897	GGUGGUC
ENSG00000166897	GGUGGUU
ENSG00000166897	GUAAACA

ENSG00000166897	UAUUGCU
ENSG00000166897	UCGUGCA
ENSG00000166897	UGAGGAC
ENSG00000166897	UGCAAAG
ENSG00000166897	UGCUCAC
ENSG00000166897	UGUGCGU
ENSG00000166897	UGUGCUU
ENSG00000166900	ACUCAGG
ENSG00000166900	CAGGAAC
ENSG00000166900	CUCAGGG
ENSG00000166900	GAGAACU
ENSG00000166900	GAGGUAU
ENSG00000166900	GGCAAGA
ENSG00000166900	UCCUGAG
ENSG00000166908	AAGGCAC
ENSG00000166908	AAGUGCA
ENSG00000166908	AUGGCUU
ENSG00000166908	AUUGCAC
ENSG00000166908	CAGUCCA
ENSG00000166908	CCCACCU
ENSG00000166908	CUGACUC
ENSG00000166908	CUGGGGG
ENSG00000166908	GCUGGUG
ENSG00000166908	UAUUGCU
ENSG00000166908	UGCAUUG
ENSG00000166912	AAGGCAC
ENSG00000166912	UGCAUAU
ENSG00000166912	UUGGUUC
ENSG00000166913	AAAGAGC
ENSG00000166913	AAUACUG
ENSG00000166913	ACAUUCA
ENSG00000166913	CAGACAG
ENSG00000166913	UCCUGAG
ENSG00000166920	AAAAAUC
ENSG00000166920	ACUAGAU
ENSG00000166923	AAGUCAC
ENSG00000166923	ACAUUAC
ENSG00000166923	CAGUAGU
ENSG00000166923	GUCAUUG
ENSG00000166923	UCACAGU
ENSG00000166923	UCACAUU
ENSG00000166924	AAAGAGC
ENSG00000166924	ACCCUGU
ENSG00000166924	GGCAGGG
ENSG00000166924	UAAUAUC
ENSG00000166924	UCCUGAG
ENSG00000166924	UGACAUC
ENSG00000166925	AAGGCAC
ENSG00000166928	CUCCAGC
ENSG00000166949	AAUCACU
ENSG00000166949	AAUCUCU
ENSG00000166949	CAGCAAA
ENSG00000166949	CAGCAGA
ENSG00000166949	CUAGGCU
ENSG00000166949	CUAUACA
ENSG00000166949	GAGAUGA
ENSG00000166949	GCUUCCU
ENSG00000166949	GUCUGCC
ENSG00000166949	UCAUUUG
ENSG00000166949	UGAAAUG
ENSG00000166949	UGCCAGC
ENSG00000166949	UUCCAUA
ENSG00000166960	AAAUUUC
ENSG00000166960	AAGUCAC

ENSG00000166960	AAGUGUG
ENSG00000166960	ACAGGUG
ENSG00000166960	ACUGUGU
ENSG00000166960	AGCAGCA
ENSG00000166960	CAGGUGA
ENSG00000166960	CUGGGGG
ENSG00000166960	GAAAACA
ENSG00000166960	GAGGCAG
ENSG00000166960	GCAGCAU
ENSG00000166960	GGAUUUC
ENSG00000166960	GGAUUUU
ENSG00000166960	GGUUGUC
ENSG00000166960	GUAAACA
ENSG00000166960	GUCAACA
ENSG00000166960	GUGAAUG
ENSG00000166960	GUGACUG
ENSG00000166960	UCCUGAG
ENSG00000166960	UGAAAUG
ENSG00000166961	GAGAAAU
ENSG00000166961	UCUGGAA
ENSG00000166961	UCUGGAG
ENSG00000166963	AAUGUUG
ENSG00000166963	ACAUUCA
ENSG00000166963	ACCCAUU
ENSG00000166963	AGCAGCA
ENSG00000166963	AGCAGCU
ENSG00000166963	CAGCUGG
ENSG00000166963	CUGCAGG
ENSG00000166963	CUGCCCC
ENSG00000166963	CUGCUCA
ENSG00000166963	CUUUGGU
ENSG00000166963	GGAAUGU
ENSG00000166963	GGCAGUG
ENSG00000166963	GUGGGGA
ENSG00000166963	UCAAGUA
ENSG00000166963	UCACACA
ENSG00000166974	AACACUG
ENSG00000166974	AGCACCA
ENSG00000166974	CAAAGCA
ENSG00000166974	CCCUGAG
ENSG00000166974	CUCCAGC
ENSG00000166974	GACCCUG
ENSG00000166974	UCUCAAG
ENSG00000166974	UCUCCAA
ENSG00000166974	UGACAUC
ENSG00000166974	UUGGCAA
ENSG00000166987	AAAACUG
ENSG00000166987	AAACUGU
ENSG00000166987	AACCUGG
ENSG00000166987	AAUCCUU
ENSG00000166987	ACAGGUG
ENSG00000166987	ACUCAGC
ENSG00000166987	AGGCAGG
ENSG00000166987	AGGGAGG
ENSG00000166987	AGGGUCU
ENSG00000166987	AGUGCCG
ENSG00000166987	AGUGCCU
ENSG00000166987	AGUGUGC
ENSG00000166987	AUGAUGC
ENSG00000166987	AUGGCUU
ENSG00000166987	CACAGGG
ENSG00000166987	CAGCUGG
ENSG00000166987	CCCCCAG
ENSG00000166987	CCCCUGG

ENSG00000166987	CCGGUUC
ENSG00000166987	CUCCCAC
ENSG00000166987	GACCUGG
ENSG00000166987	GAGGCAG
ENSG00000166987	GAGGCUG
ENSG00000166987	GGAGGAG
ENSG00000166987	GGCAGGG
ENSG00000166987	GGCAGUG
ENSG00000166987	GGGGCAG
ENSG00000166987	GGGUCUU
ENSG00000166987	GGGUGUU
ENSG00000166987	GUAAACA
ENSG00000166987	GUAGUGU
ENSG00000166987	GUCUCUG
ENSG00000166987	GUGCUUG
ENSG00000166987	GUGGGGA
ENSG00000166987	UACAAAG
ENSG00000166987	UCACAGG
ENSG00000166987	UCAUUUG
ENSG00000166987	UGAAAUG
ENSG00000166987	UGUGCUU
ENSG00000166987	UUCAAAU
ENSG00000166997	AAAGUUU
ENSG00000167004	AACUGGU
ENSG00000167004	CAAAGUG
ENSG00000167004	CAGUGCA
ENSG00000167004	UGCAGCU
ENSG00000167005	AAAAGUA
ENSG00000167005	AAAGUAA
ENSG00000167005	AAAGUAU
ENSG00000167005	CAUAUUG
ENSG00000167005	CCUUCUU
ENSG00000167005	CUUUGGU
ENSG00000167005	UGCUCAC
ENSG00000167034	AGAUAAA
ENSG00000167034	CUGGAAA
ENSG00000167034	UGUCUUU
ENSG00000167037	AGGACAC
ENSG00000167037	CUUGGGC
ENSG00000167037	GAUAUGU
ENSG00000167037	UCCUGAG
ENSG00000167037	UGUGCUU
ENSG00000167037	UUGGUCC
ENSG00000167074	AAGUCUU
ENSG00000167074	ACCAGCA
ENSG00000167074	ACCUGGA
ENSG00000167074	AGCCAGU
ENSG00000167074	AGGACAC
ENSG00000167074	CACCAGC
ENSG00000167074	CCCUGAG
ENSG00000167074	GAGAAGA
ENSG00000167074	GCAGGAC
ENSG00000167074	GCUGGAU
ENSG00000167074	UAUUGCU
ENSG00000167074	UCAUGAU
ENSG00000167074	UCUCAAG
ENSG00000167074	UGAAGCU
ENSG00000167074	UGAGAAG
ENSG00000167081	AAUACUG
ENSG00000167081	ACAAUAU
ENSG00000167081	AGCAAAA
ENSG00000167081	AGGUAGU
ENSG00000167081	AGUACUG
ENSG00000167081	AUAAAGU

ENSG00000167081	AUAUAAC
ENSG00000167081	AUUCAUU
ENSG00000167081	CACAGGG
ENSG00000167081	GAGGUAU
ENSG00000167081	GAGUAUU
ENSG00000167081	GAUUGGU
ENSG00000167081	GGAAGAC
ENSG00000167081	UACAGUU
ENSG00000167081	UGUAUAA
ENSG00000167085	ACACACC
ENSG00000167085	CACACCU
ENSG00000167085	CCCACCU
ENSG00000167085	GGGUGUU
ENSG00000167085	UCACAGG
ENSG00000167085	UGAAGUG
ENSG00000167085	UUAGAGA
ENSG00000167088	AAAAACC
ENSG00000167088	AACCUGU
ENSG00000167088	UCUUGAG
ENSG00000167094	AAUUUUA
ENSG00000167105	GGCUCAG
ENSG00000167105	UGAUCCA
ENSG00000167106	AAGUGCA
ENSG00000167106	ACAUUCA
ENSG00000167106	AGCCUGC
ENSG00000167106	AGUGGUU
ENSG00000167106	AUACAAG
ENSG00000167106	AUUCAUU
ENSG00000167106	CAAAGUG
ENSG00000167106	CACAGUG
ENSG00000167106	CACCAGC
ENSG00000167106	CCCCCAG
ENSG00000167106	CCUGCUG
ENSG00000167106	GGAAUGU
ENSG00000167106	GGAGGCA
ENSG00000167106	GGGUUUA
ENSG00000167106	GUCAGUU
ENSG00000167106	GUGCAAA
ENSG00000167106	UCACAGG
ENSG00000167106	UCACAGU
ENSG00000167106	UCAUUUG
ENSG00000167106	UGCAAAG
ENSG00000167106	UGCAUAU
ENSG00000167107	UUAUGCA
ENSG00000167110	AUACAAG
ENSG00000167112	AGCUGCC
ENSG00000167113	AAAGGUA
ENSG00000167114	AAGACAU
ENSG00000167114	CUUUGGU
ENSG00000167114	UUGGUCC
ENSG00000167118	AGCACCA
ENSG00000167118	AGCAGCU
ENSG00000167118	CAGCUGG
ENSG00000167118	GCCUGGA
ENSG00000167118	UCCCUGU
ENSG00000167118	UGGGGGA
ENSG00000167123	AGUGGUU
ENSG00000167123	GAGGUAG
ENSG00000167130	AACACUG
ENSG00000167130	AGCACCA
ENSG00000167130	AGCAGCA
ENSG00000167130	AGCCCUU
ENSG00000167130	AUGGCUU
ENSG00000167130	CAGCAGA

ENSG00000167130	CAGGCUC
ENSG00000167130	CUCUGGG
ENSG00000167130	CUUUGGU
ENSG00000167130	GGCAGGG
ENSG00000167130	GUCUCUG
ENSG00000167130	GUGCUUG
ENSG00000167130	UCUGGAA
ENSG00000167165	CUCAAAC
ENSG00000167165	UCCCACC
ENSG00000167173	GAGGUAG
ENSG00000167173	GAGGUAU
ENSG00000167173	GUGAGGU
ENSG00000167178	AGCACCA
ENSG00000167178	GUGCUUG
ENSG00000167178	UGUGCUU
ENSG00000167178	UUGGUUC
ENSG00000167182	AAAUUUC
ENSG00000167182	AACAAAC
ENSG00000167182	AAGGCAC
ENSG00000167182	AUAAAGU
ENSG00000167182	CACAGUG
ENSG00000167182	CAGUUAC
ENSG00000167182	GGAAUGU
ENSG00000167182	GGAUUUC
ENSG00000167186	AACACUG
ENSG00000167191	AAAUCCC
ENSG00000167191	CAGUGCA
ENSG00000167191	CUCUGGG
ENSG00000167191	CUGGAAA
ENSG00000167191	GGAUUUC
ENSG00000167191	GGCAGGG
ENSG00000167191	GUGUGGC
ENSG00000167191	UCUGGAA
ENSG00000167191	UGUGUGG
ENSG00000167191	UUGGCAC
ENSG00000167193	AAAGCUG
ENSG00000167193	AACAGUC
ENSG00000167193	AAGUGCU
ENSG00000167193	ACCCUGU
ENSG00000167193	AGUAGUU
ENSG00000167193	CAAAUGC
ENSG00000167193	CGUACCG
ENSG00000167193	CUAGUAA
ENSG00000167193	GAUUGGU
ENSG00000167193	GUAGUGU
ENSG00000167193	UGAAAUG
ENSG00000167193	UGGCCCU
ENSG00000167193	UUGACAC
ENSG00000167193	UUGGUCC
ENSG00000167196	AGCAGCU
ENSG00000167202	AAAAAUC
ENSG00000167202	AAAAGUA
ENSG00000167202	AAAGUAU
ENSG00000167202	ACAUUCA
ENSG00000167202	ACUGUAG
ENSG00000167202	CAGCAGA
ENSG00000167202	CUUUGGU
ENSG00000167202	GUAGUGU
ENSG00000167207	CUGGAAA
ENSG00000167207	UCACAGG
ENSG00000167208	CUCCAGC
ENSG00000167230	AGUGCUU
ENSG00000167244	AACACUG
ENSG00000167244	CUGCCCC

ENSG00000167244	GACUUCC
ENSG00000167244	UCCCACA
ENSG00000167244	UUCUGCA
ENSG00000167257	AAAAAUC
ENSG00000167257	ACACACC
ENSG00000167257	ACACACU
ENSG00000167257	ACAUUAC
ENSG00000167257	AGCACCA
ENSG00000167257	AUAAUAC
ENSG00000167257	AUAUAAC
ENSG00000167257	UAUAAUA
ENSG00000167258	AGCAGCA
ENSG00000167264	UAUUGCU
ENSG00000167264	UGUCUUU
ENSG00000167306	AAAAGUA
ENSG00000167306	AAAGUGC
ENSG00000167306	AAGGCAC
ENSG00000167306	AAGUGCA
ENSG00000167306	GAGGUAG
ENSG00000167306	UCAAGUA
ENSG00000167306	UCCCACA
ENSG00000167306	UCCCACC
ENSG00000167306	UUCAAGC
ENSG00000167323	AAGACAU
ENSG00000167323	AGGCAGG
ENSG00000167323	CAAAACU
ENSG00000167323	GGUGGUU
ENSG00000167323	UCCUGAG
ENSG00000167323	UGGACUG
ENSG00000167325	CAGCAAA
ENSG00000167325	CCAGCAU
ENSG00000167332	GAAAACA
ENSG00000167332	UUGGCAC
ENSG00000167371	AACAGUC
ENSG00000167371	AAGUGCU
ENSG00000167371	CAGGCUC
ENSG00000167371	CUCCCAA
ENSG00000167371	CUUUGGU
ENSG00000167371	GAGCCCC
ENSG00000167371	GGGUGGU
ENSG00000167371	GUAAACA
ENSG00000167384	AGUGCUU
ENSG00000167395	AAAUUUC
ENSG00000167395	AGCAAAA
ENSG00000167395	UCCUGAG
ENSG00000167397	CAAAGCA
ENSG00000167419	AAGUCUU
ENSG00000167419	UGAAAUG
ENSG00000167460	ACCCUGU
ENSG00000167460	AGCAUUC
ENSG00000167460	CUUACAG
ENSG00000167460	GACUUCC
ENSG00000167460	UCCCACA
ENSG00000167460	UGACAUC
ENSG00000167460	UUGGUCC
ENSG00000167461	AAGUGCU
ENSG00000167461	CAGCUGG
ENSG00000167461	CUUUGGU
ENSG00000167461	GGCUCAG
ENSG00000167461	UCUCCAA
ENSG00000167468	CACCAGC
ENSG00000167470	AACAAAC
ENSG00000167470	ACCAGCA
ENSG00000167470	AGCAGCG

ENSG00000167470	GUUAAUG
ENSG00000167470	UAAUGCU
ENSG00000167470	UAGGUAU
ENSG00000167470	UCACAUU
ENSG00000167470	UGAAAUG
ENSG00000167491	AAAUCCC
ENSG00000167491	AAGGCAC
ENSG00000167491	ACCCUGU
ENSG00000167491	AUACAAG
ENSG00000167491	CAAAACU
ENSG00000167491	CACAAUG
ENSG00000167491	CACAGUG
ENSG00000167491	GGAAUGU
ENSG00000167491	UCACACA
ENSG00000167491	UGCAUAG
ENSG00000167491	UGCAUAU
ENSG00000167491	UGCCAGC
ENSG00000167491	UUCUGCA
ENSG00000167522	AAAAGUA
ENSG00000167522	AAACUGU
ENSG00000167522	AAAGUAC
ENSG00000167522	ACAGUAU
ENSG00000167522	AGCAAAA
ENSG00000167522	AUAUAAC
ENSG00000167522	CUCCAUU
ENSG00000167522	UACAGUU
ENSG00000167522	UCAAGUA
ENSG00000167522	UGCAAAG
ENSG00000167522	UUCAAGC
ENSG00000167528	AGCACCA
ENSG00000167528	GAGAAAU
ENSG00000167528	UGUGUGG
ENSG00000167528	UUAGGAU
ENSG00000167535	CCAGGAG
ENSG00000167535	CCCUGAG
ENSG00000167535	GAUUGUA
ENSG00000167548	AACUGGU
ENSG00000167548	AAUGCCC
ENSG00000167548	ACACACC
ENSG00000167548	ACACACU
ENSG00000167548	ACACGGG
ENSG00000167548	ACAUCAC
ENSG00000167548	ACCCGGC
ENSG00000167548	ACUCAGG
ENSG00000167548	ACUGCAG
ENSG00000167548	ACUGCAU
ENSG00000167548	AGAUAAA
ENSG00000167548	AGCAGCA
ENSG00000167548	AGCAGCU
ENSG00000167548	AGGCAGG
ENSG00000167548	AGGGUCU
ENSG00000167548	AGUAGUU
ENSG00000167548	AUAUAAC
ENSG00000167548	AUGGAUU
ENSG00000167548	AUGUGCC
ENSG00000167548	CAAAGCA
ENSG00000167548	CACCACC
ENSG00000167548	CAGCAAA
ENSG00000167548	CAGCAGG
ENSG00000167548	CCAGCAU
ENSG00000167548	CCCCUGG
ENSG00000167548	CCUGCUG
ENSG00000167548	CUAUACA
ENSG00000167548	CUCAAAC

ENSG00000167548	CUCACAC
ENSG00000167548	CUCCAGC
ENSG00000167548	CUCUUCC
ENSG00000167548	CUGCAGC
ENSG00000167548	CUGGCUC
ENSG00000167548	CUGGGGG
ENSG00000167548	CUUACAG
ENSG00000167548	CUUGGAG
ENSG00000167548	GAAAACA
ENSG00000167548	GAGAACU
ENSG00000167548	GAGAUGA
ENSG00000167548	GAGGUAG
ENSG00000167548	GAGGUAU
ENSG00000167548	GAGUAUU
ENSG00000167548	GAGUUGG
ENSG00000167548	GAUUGUC
ENSG00000167548	GCAGCAU
ENSG00000167548	GCAGGAC
ENSG00000167548	GCCCUUA
ENSG00000167548	GCCUGGA
ENSG00000167548	GCCUGGG
ENSG00000167548	GGAAGAC
ENSG00000167548	GGCAGUG
ENSG00000167548	GGCUCUG
ENSG00000167548	GGCUGCG
ENSG00000167548	GGGAGCU
ENSG00000167548	GGGGUGG
ENSG00000167548	GGGUCUU
ENSG00000167548	GGUGCGG
ENSG00000167548	GUAAACA
ENSG00000167548	GUCCAGA
ENSG00000167548	GUCUGCC
ENSG00000167548	GUGACUG
ENSG00000167548	GUGAGGU
ENSG00000167548	UACAGUU
ENSG00000167548	UAUAAAG
ENSG00000167548	UCACACA
ENSG00000167548	UCACAUU
ENSG00000167548	UCACCAC
ENSG00000167548	UCCCACC
ENSG00000167548	UCCCUUU
ENSG00000167548	UCCUUGC
ENSG00000167548	UCUCACC
ENSG00000167548	UCUUGAG
ENSG00000167548	UGCAUAU
ENSG00000167548	UGCAUUG
ENSG00000167548	UGCCAGC
ENSG00000167548	UGCCCUG
ENSG00000167548	UGGGAUC
ENSG00000167548	UGGGGGA
ENSG00000167548	UGUGCGG
ENSG00000167548	UGUGCGU
ENSG00000167548	UUCAAAU
ENSG00000167549	AGCACCA
ENSG00000167549	GGAGGCA
ENSG00000167550	GUGCAAA
ENSG00000167552	AGCAGCA
ENSG00000167552	CCCUGUC
ENSG00000167552	CCCUGUU
ENSG00000167565	AAGGCAC
ENSG00000167565	AUUGCAC
ENSG00000167565	CCCUGAG
ENSG00000167565	CGGCCUG
ENSG00000167565	GGGGUCC

ENSG00000167566	AGCACCA
ENSG00000167566	CAGACAG
ENSG00000167566	CCCUGAG
ENSG00000167566	GAGCUGC
ENSG00000167566	GCAACUU
ENSG00000167566	GCACCAU
ENSG00000167566	GCAGAAG
ENSG00000167566	GGCUCAG
ENSG00000167566	UUGGUCC
ENSG00000167578	AGCAGCA
ENSG00000167578	GGCUCAG
ENSG00000167578	GGGCCCC
ENSG00000167578	GUCUUGC
ENSG00000167580	AAUCUCA
ENSG00000167580	AAUCUCU
ENSG00000167580	ACCUGGA
ENSG00000167580	ACUGGCC
ENSG00000167580	AUUGCAC
ENSG00000167580	GCCCUGU
ENSG00000167580	GCCUGGA
ENSG00000167580	GGACCUG
ENSG00000167580	GGGUGGU
ENSG00000167580	UACAGUU
ENSG00000167580	UAUUGCU
ENSG00000167580	UGCCCUG
ENSG00000167580	UGGCCAC
ENSG00000167580	UUCAAGC
ENSG00000167588	AAAGAGG
ENSG00000167588	AACACUG
ENSG00000167588	CACAGGG
ENSG00000167588	CAGCAGA
ENSG00000167588	CAGCAGG
ENSG00000167588	GGAGUCC
ENSG00000167588	GGGGAGC
ENSG00000167588	GUGACAG
ENSG00000167588	GUGGGGA
ENSG00000167588	UGCCAGC
ENSG00000167588	UGCUGAC
ENSG00000167595	CUUUGGU
ENSG00000167601	AUGCAUU
ENSG00000167601	GGCUCAG
ENSG00000167601	UCCUGAG
ENSG00000167601	UGCAUUG
ENSG00000167604	CAGUCCA
ENSG00000167604	CUGGAAA
ENSG00000167604	UCACAGG
ENSG00000167604	UCACAGU
ENSG00000167614	AGUGCCG
ENSG00000167614	AGUGCCU
ENSG00000167614	GGCAGGG
ENSG00000167614	UCCCUUG
ENSG00000167615	CAGUCCA
ENSG00000167615	CAGUUAC
ENSG00000167615	GACAACU
ENSG00000167615	GCAGAAG
ENSG00000167615	GGAGGCA
ENSG00000167615	UCCAGUU
ENSG00000167615	UCUCAAG
ENSG00000167615	UCUGGAA
ENSG00000167615	UGAUCCA
ENSG00000167619	UUCUGCA
ENSG00000167619	UUGGCAC
ENSG00000167635	AAAAGUA
ENSG00000167635	AAGACAU

ENSG00000167635	CUACAAA
ENSG00000167635	GCAACUU
ENSG00000167645	GAGAUGA
ENSG00000167653	GCUUCCU
ENSG00000167654	AAGGCAC
ENSG00000167654	ACCCUGU
ENSG00000167654	AGGGUCU
ENSG00000167654	GAGUGUG
ENSG00000167654	GCUGGUG
ENSG00000167658	GGGCCCC
ENSG00000167670	AAAGUGC
ENSG00000167670	AAGUGCU
ENSG00000167670	CAAAGUG
ENSG00000167670	GCAGGAC
ENSG00000167670	UAAUGCU
ENSG00000167680	AGCCCUU
ENSG00000167680	CAGCUGG
ENSG00000167680	CUGCAGC
ENSG00000167680	GGAAGCC
ENSG00000167680	GUAAACA
ENSG00000167680	GUGCAAA
ENSG00000167680	GUGCUUG
ENSG00000167680	UCACAAG
ENSG00000167680	UCCAGAG
ENSG00000167680	UGUGCUU
ENSG00000167693	AAGGCAC
ENSG00000167693	ACCACAG
ENSG00000167693	GAAGGAA
ENSG00000167693	GCAGAAG
ENSG00000167693	GGAGGAG
ENSG00000167693	GUCCAGA
ENSG00000167693	UCAACAG
ENSG00000167693	UCCAGAG
ENSG00000167693	UCUCCAA
ENSG00000167695	AAAGUGC
ENSG00000167695	AGUGCUU
ENSG00000167695	UGCAAAG
ENSG00000167695	UUGGCAC
ENSG00000167695	UUUGCAC
ENSG00000167702	AGCACCA
ENSG00000167702	CCUGCUG
ENSG00000167702	CUGGGGG
ENSG00000167702	UGGGGGA
ENSG00000167703	AGCACCA
ENSG00000167703	GAGGCAG
ENSG00000167703	GGCAAGA
ENSG00000167703	UUGGCAA
ENSG00000167711	AAUCACU
ENSG00000167711	AUCACUA
ENSG00000167711	AUUCAUU
ENSG00000167711	CAGGUGA
ENSG00000167711	CUACAAA
ENSG00000167711	UACAAAG
ENSG00000167711	UCAUUCG
ENSG00000167716	AAGGCAC
ENSG00000167716	AGCACCA
ENSG00000167716	AUUGCAC
ENSG00000167716	CUCUUCC
ENSG00000167716	GCACCAU
ENSG00000167716	UGCAAAG
ENSG00000167723	CUGCAGC
ENSG00000167723	UGAAAUG
ENSG00000167747	AGUGCUU
ENSG00000167766	UCCCACA

ENSG00000167766	UCCCACC
ENSG00000167767	AAUACUG
ENSG00000167767	AAUCUCA
ENSG00000167767	AGGGAGG
ENSG00000167767	CCUUCUU
ENSG00000167767	GGCAAGA
ENSG00000167767	GGCCCUG
ENSG00000167767	UCCCUUG
ENSG00000167768	GUCUUGC
ENSG00000167768	UUCAAAU
ENSG00000167770	AAAAACC
ENSG00000167770	AGGAGCU
ENSG00000167770	CACCACC
ENSG00000167770	CACCAGC
ENSG00000167770	CUGCCCC
ENSG00000167770	GCCUGGG
ENSG00000167770	GGGGUGC
ENSG00000167770	UCACCAC
ENSG00000167772	CUUCUCU
ENSG00000167772	GAGCCCC
ENSG00000167772	UCCCUCU
ENSG00000167775	CAGCAGG
ENSG00000167775	GGAGUGU
ENSG00000167778	AACACUG
ENSG00000167778	AAGUCAC
ENSG00000167778	AGCAGCU
ENSG00000167778	GUGCAAA
ENSG00000167778	UCACACA
ENSG00000167778	UGUAUAA
ENSG00000167785	CUCCAGC
ENSG00000167797	AGGCAGG
ENSG00000167797	GGCAGGG
ENSG00000167799	CAGCAGG
ENSG00000167799	GCAGAAG
ENSG00000167799	GGAAGAC
ENSG00000167842	GGAAGAC
ENSG00000167851	GUCCAGA
ENSG00000167861	GAUUGUA
ENSG00000167881	AAAAAUC
ENSG00000167889	UAUUGCU
ENSG00000167904	ACUGCAG
ENSG00000167904	AUACAAG
ENSG00000167904	GUGCUUG
ENSG00000167904	UUGGCAA
ENSG00000167930	AAGGCAC
ENSG00000167930	CCAGUGU
ENSG00000167941	GAGAAAU
ENSG00000167941	GAGGUAG
ENSG00000167941	GGCUCAG
ENSG00000167941	UCAUUCG
ENSG00000167941	UGCAUAG
ENSG00000167941	UGCAUAU
ENSG00000167941	UGUGCUU
ENSG00000167941	UUUGCAA
ENSG00000167941	UUUGCAC
ENSG00000167971	AAAGUUU
ENSG00000167971	AAGGCAC
ENSG00000167971	AAUACUG
ENSG00000167971	AGCAGCA
ENSG00000167971	AUUGCAC
ENSG00000167971	CAGACAG
ENSG00000167971	CAGCAAA
ENSG00000167971	GAGCCCU
ENSG00000167971	GAGGUAG

ENSG00000167971	GAGGUAU
ENSG00000167971	GUGCUUG
ENSG00000167971	UAAUACA
ENSG00000167971	UCCUGAG
ENSG00000167971	UGUGCUU
ENSG00000167977	AAGGCAC
ENSG00000167977	CUGGAAA
ENSG00000167977	GCAGGUU
ENSG00000167977	GUAAACA
ENSG00000167977	UAGUGAG
ENSG00000167977	UGCAUAG
ENSG00000167978	AUGCCUU
ENSG00000167978	CAGGAAC
ENSG00000167978	CCCACCG
ENSG00000167978	CCCACCU
ENSG00000167978	CUCCCAC
ENSG00000167978	UCACAGU
ENSG00000167978	UCCCACC
ENSG00000167978	UGGGGGA
ENSG00000167984	CUUCUCU
ENSG00000167987	AGCAGCA
ENSG00000167987	AGCAGCG
ENSG00000167987	AGCAGCU
ENSG00000167987	GAGACCU
ENSG00000167987	GGGGCAG
ENSG00000167987	UAUAAAG
ENSG00000167994	CUUUGGU
ENSG00000167994	GGGGUGC
ENSG00000167995	CAGUGCA
ENSG00000167995	CCUUCAU
ENSG00000167995	UUCAAAU
ENSG00000167996	UGGGUAC
ENSG00000167996	UGGUACC
ENSG00000168000	GGAAUGU
ENSG00000168032	CCAGGAG
ENSG00000168036	AAAAAUC
ENSG00000168036	AACUCUA
ENSG00000168036	AACUGGU
ENSG00000168036	ACAAGGU
ENSG00000168036	ACUCAGC
ENSG00000168036	AGUGUGC
ENSG00000168036	AUACAAG
ENSG00000168036	CUUGUGU
ENSG00000168036	GAGCCCU
ENSG00000168036	GUCACUC
ENSG00000168036	GUGUCAC
ENSG00000168036	UGGGAUC
ENSG00000168036	UGUGUCA
ENSG00000168036	UUCUUGU
ENSG00000168066	AAUCACU
ENSG00000168066	ACAUUCA
ENSG00000168066	CAGCAGA
ENSG00000168066	GAGGCAG
ENSG00000168066	GCAGAAG
ENSG00000168067	AGCAGCU
ENSG00000168067	CUCAGGG
ENSG00000168067	UAUUGCU
ENSG00000168067	UCCAGUU
ENSG00000168071	CAGCUGG
ENSG00000168071	CUUGGGC
ENSG00000168071	GCCUGGG
ENSG00000168077	CUGGAAA
ENSG00000168079	ACCUGGA
ENSG00000168079	AUGUAAU

ENSG00000168081	CAUGGGU
ENSG00000168081	UGCUCAC
ENSG00000168090	GGGGUGC
ENSG00000168090	UCAAGUA
ENSG00000168092	AACACUG
ENSG00000168096	AAUUUUA
ENSG00000168118	ACUGCAU
ENSG00000168135	GAGUGUG
ENSG00000168137	AUUGCAC
ENSG00000168137	CAAAUGC
ENSG00000168137	UAUUACC
ENSG00000168137	UCUCCAA
ENSG00000168137	UUCAAAU
ENSG00000168140	AAAAACC
ENSG00000168140	ACCAGCA
ENSG00000168140	CAGCAGG
ENSG00000168140	CCCCCAG
ENSG00000168140	CCUUCAU
ENSG00000168140	UACAAAG
ENSG00000168148	AAAGAGC
ENSG00000168148	GGCUCUG
ENSG00000168172	AAGACAU
ENSG00000168172	AAUUAUU
ENSG00000168172	CAGCAAA
ENSG00000168172	UCCCUUG
ENSG00000168172	UCCUGAG
ENSG00000168172	UGAAAUG
ENSG00000168172	UGCCAGC
ENSG00000168172	UUGGCAA
ENSG00000168172	UUUGCAA
ENSG00000168175	AAAGCUG
ENSG00000168175	AAAGUUU
ENSG00000168175	AAAUUUC
ENSG00000168175	AAUGCCC
ENSG00000168175	ACAUUCA
ENSG00000168175	AGCAGCA
ENSG00000168175	AGUGCCU
ENSG00000168175	CAGCAGG
ENSG00000168175	CAGGUGA
ENSG00000168175	CCCUGAG
ENSG00000168175	CUCUUCC
ENSG00000168175	CUGGACU
ENSG00000168175	GAGGUAG
ENSG00000168175	GAUUGUC
ENSG00000168175	GGCAUUG
ENSG00000168175	GUCAACA
ENSG00000168175	GUGAAUG
ENSG00000168175	GUGACAG
ENSG00000168175	GUGAGGU
ENSG00000168175	UAUUGCU
ENSG00000168175	UCAUGAU
ENSG00000168175	UCAUUUG
ENSG00000168175	UCCCUGU
ENSG00000168175	UGCAUAU
ENSG00000168175	UUGGCAA
ENSG00000168175	UUGGCAC
ENSG00000168209	AAUACUG
ENSG00000168209	AGUGAUC
ENSG00000168209	AUGGCAC
ENSG00000168209	AUUGCAC
ENSG00000168209	CAGUAGA
ENSG00000168209	CAGUAGU
ENSG00000168209	CUUACAG
ENSG00000168209	GGAAGAC

ENSG00000168209	GUAUUCU
ENSG00000168209	UAAUACA
ENSG00000168209	UCUAGAG
ENSG00000168209	UGCAUAU
ENSG00000168214	UUGGUCC
ENSG00000168228	AAAGAAU
ENSG00000168228	AAGGUGC
ENSG00000168228	CAGCAAA
ENSG00000168228	GUAAACA
ENSG00000168228	GUGUGGC
ENSG00000168228	UCAUUUG
ENSG00000168229	AAGUGCU
ENSG00000168229	AAUGCCC
ENSG00000168229	CACAGUG
ENSG00000168229	CCCACCU
ENSG00000168229	GACCUGG
ENSG00000168229	UCACAGG
ENSG00000168234	AAAGUGC
ENSG00000168242	GGCUCUG
ENSG00000168243	GUUAAUG
ENSG00000168243	UGAAGUG
ENSG00000168246	AAGGCAC
ENSG00000168246	AAUUAUU
ENSG00000168246	AGCACCA
ENSG00000168246	AGCAGCA
ENSG00000168246	CUCCAUU
ENSG00000168246	CUGGCUC
ENSG00000168246	GAGUCUU
ENSG00000168246	GCACCAU
ENSG00000168246	GGCUCUG
ENSG00000168246	UAUUGCU
ENSG00000168246	UCAAGUA
ENSG00000168246	UGAGUCU
ENSG00000168246	UUGUGUC
ENSG00000168255	AUGAUGA
ENSG00000168255	CACCACC
ENSG00000168255	CACCAGC
ENSG00000168255	GAAGGUC
ENSG00000168255	GGCCCUG
ENSG00000168255	UGGCCCU
ENSG00000168256	AGCACCA
ENSG00000168256	CACUCCU
ENSG00000168256	CCCCUGG
ENSG00000168256	CCCUGAG
ENSG00000168256	CUUCACC
ENSG00000168256	GAGGUAG
ENSG00000168256	GAGGUAU
ENSG00000168256	GCUUCCU
ENSG00000168256	UGUGUCA
ENSG00000168259	AUAAAGU
ENSG00000168264	AAAGUGC
ENSG00000168264	AACAAAC
ENSG00000168264	AAGUCAC
ENSG00000168264	AGCAGCG
ENSG00000168264	AGUGCCU
ENSG00000168264	CACCAGC
ENSG00000168264	CAGACAG
ENSG00000168264	CAGCUGG
ENSG00000168264	CAGUCUG
ENSG00000168264	CUCCCAA
ENSG00000168264	CUUGGAG
ENSG00000168264	GGCAGUG
ENSG00000168264	GUAUUCU
ENSG00000168264	GUCAUUG

ENSG00000168264	GUCUUGC
ENSG00000168264	UACAAAG
ENSG00000168264	UCCCUUU
ENSG00000168264	UGGAUAA
ENSG00000168264	UUCUGCA
ENSG00000168267	AUAUAAC
ENSG00000168267	AUUGCAC
ENSG00000168267	UGCAUAG
ENSG00000168269	CUUGGAG
ENSG00000168274	AAAGAGC
ENSG00000168274	GGGUGGU
ENSG00000168280	AAAAAUC
ENSG00000168280	ACUGGCU
ENSG00000168280	AGCAGCG
ENSG00000168280	CAGCAAA
ENSG00000168280	CAGUCUG
ENSG00000168280	CCCCUGG
ENSG00000168280	CUACAGU
ENSG00000168280	CUGCAGC
ENSG00000168280	CUGCAGG
ENSG00000168280	CUUUGGU
ENSG00000168280	GAUCAGA
ENSG00000168280	GUCUGCC
ENSG00000168280	UACAAAG
ENSG00000168282	AGAUAAA
ENSG00000168283	AAAGCUG
ENSG00000168283	AAUACUG
ENSG00000168283	ACUGUUU
ENSG00000168283	AUGGCAC
ENSG00000168283	GAAUUGU
ENSG00000168283	GAUUGUA
ENSG00000168283	GCUACAU
ENSG00000168283	GUGCUUG
ENSG00000168283	UCACAGG
ENSG00000168283	UGAAAUG
ENSG00000168286	AAAAACC
ENSG00000168286	AGCAAAA
ENSG00000168291	GUAACAG
ENSG00000168297	GUAAACA
ENSG00000168298	AAAGAGC
ENSG00000168300	AAAGUGC
ENSG00000168300	AAGUGCA
ENSG00000168300	CAGUAGA
ENSG00000168300	CAGUAGU
ENSG00000168300	GCUGGUG
ENSG00000168300	UAUUGCU
ENSG00000168300	UUCAAGC
ENSG00000168300	UUGGCAA
ENSG00000168300	UUGGCAC
ENSG00000168309	GAAGGAA
ENSG00000168309	GACUUCC
ENSG00000168309	GGCAGUG
ENSG00000168309	GGGUCUU
ENSG00000168309	UUGGCAA
ENSG00000168310	AAAUUUC
ENSG00000168310	AGGAUGU
ENSG00000168310	AUGAGCU
ENSG00000168310	AUGUAAU
ENSG00000168310	GCAGAAG
ENSG00000168310	UCCUGAG
ENSG00000168310	UGUAUAA
ENSG00000168310	UUCAAGC
ENSG00000168310	UUGGUCC
ENSG00000168314	AGGCAGG

ENSG00000168314	CAGCAGA
ENSG00000168314	UGGACUG
ENSG00000168334	UUGGUCC
ENSG00000168348	UCACAGG
ENSG00000168348	UCACAGU
ENSG00000168348	UCUAGAG
ENSG00000168348	UUCCAUA
ENSG00000168356	GAAACAU
ENSG00000168356	GAGGUAU
ENSG00000168385	AAGUGCA
ENSG00000168385	AGCAAAA
ENSG00000168385	CAAAACU
ENSG00000168385	UUCAAAU
ENSG00000168385	UUUCAAC
ENSG00000168394	UAUAAAG
ENSG00000168395	ACAUUAC
ENSG00000168395	ACUGGCC
ENSG00000168395	AGGUAGU
ENSG00000168395	CACAGUG
ENSG00000168395	CUUCACC
ENSG00000168395	GGAUGAG
ENSG00000168395	GGCUCAG
ENSG00000168395	UCACAGG
ENSG00000168395	UCCUUGC
ENSG00000168397	AGCCCUU
ENSG00000168397	CAGGCUC
ENSG00000168397	GAGGUAG
ENSG00000168397	GGCAGUG
ENSG00000168398	GGCAGCG
ENSG00000168398	GGGGCAG
ENSG00000168404	UUCUUGU
ENSG00000168411	ACUUGGA
ENSG00000168411	AGCAGCA
ENSG00000168411	CUCUGGG
ENSG00000168411	GCAGCAU
ENSG00000168411	UCCUGAG
ENSG00000168418	GGGGCAG
ENSG00000168438	AUGGCUU
ENSG00000168439	ACUGGCU
ENSG00000168439	CUGCCCC
ENSG00000168439	UGUAUAA
ENSG00000168447	ACCAGCA
ENSG00000168453	ACCACAG
ENSG00000168453	AGCAGCA
ENSG00000168453	CAGGAAC
ENSG00000168453	UGCCAGC
ENSG00000168453	UGGUACC
ENSG00000168461	UCAAGUA
ENSG00000168476	CUUUGGU
ENSG00000168476	UCAAGUA
ENSG00000168477	CAGUGCA
ENSG00000168477	CUGGCUC
ENSG00000168477	CUUCUCU
ENSG00000168477	GGCUCUG
ENSG00000168477	GUAAACA
ENSG00000168477	UAUUGCU
ENSG00000168481	GCAGAAG
ENSG00000168481	UAUUGCU
ENSG00000168484	UGCUCAC
ENSG00000168487	AACACUG
ENSG00000168487	GGAGUCC
ENSG00000168487	GUAACAG
ENSG00000168487	UGCCCUG
ENSG00000168487	UGGCUGC

ENSG00000168488	AAGGUGC
ENSG00000168488	CAGGAAC
ENSG00000168488	CUCCAGC
ENSG00000168488	GGCCCUG
ENSG00000168488	GUGCUUG
ENSG00000168488	GUUAAUG
ENSG00000168488	UAUAAAG
ENSG00000168490	CCAGGAG
ENSG00000168490	CCCUGAG
ENSG00000168490	CUCAGGG
ENSG00000168490	GGAGAGA
ENSG00000168490	GGCCCUG
ENSG00000168490	GGUUUAC
ENSG00000168491	AAUUUUA
ENSG00000168495	CCUUCUG
ENSG00000168495	GAGGUAG
ENSG00000168495	UCUGGAG
ENSG00000168497	UCACAGG
ENSG00000168497	UCACAGU
ENSG00000168502	ACAUUCA
ENSG00000168505	UACAAAG
ENSG00000168522	AGCAGCA
ENSG00000168528	AAGUCUU
ENSG00000168528	UUAGAGA
ENSG00000168538	CAAAGCA
ENSG00000168539	ACUCAGC
ENSG00000168539	AGGGAGG
ENSG00000168539	GCAGCAU
ENSG00000168539	GCCUGGA
ENSG00000168539	GGGGUGG
ENSG00000168539	UCACAGG
ENSG00000168539	UCACAGU
ENSG00000168539	UGGAGAU
ENSG00000168542	AGGUAGU
ENSG00000168542	GAGGUAU
ENSG00000168546	ACAAGGU
ENSG00000168546	AUAAUAC
ENSG00000168546	GCUUCCU
ENSG00000168546	UAAUACA
ENSG00000168546	UUCCUAG
ENSG00000168546	UUGGUUC
ENSG00000168546	UUUGCAC
ENSG00000168556	AAUUAUU
ENSG00000168556	AUUGCAC
ENSG00000168556	UGCAUAU
ENSG00000168564	AAAACUG
ENSG00000168575	AAAUUUC
ENSG00000168575	AAUUUUA
ENSG00000168575	AGCAGCG
ENSG00000168575	AGCAGCU
ENSG00000168575	AGUAGUU
ENSG00000168575	AUAUAAC
ENSG00000168575	CAGUAGU
ENSG00000168575	CUAGGCU
ENSG00000168575	UAUAAAG
ENSG00000168575	UGGUACC
ENSG00000168591	AAAGUGC
ENSG00000168591	AAGUGCU
ENSG00000168591	AGCAGCA
ENSG00000168591	AGCAGCU
ENSG00000168591	AGUGCUU
ENSG00000168591	AUGCCUU
ENSG00000168591	CAAAGUG
ENSG00000168591	CUGGGGG

ENSG00000168591	GAGCUGC
ENSG00000168591	UGUGCUU
ENSG00000168610	AAAAAUC
ENSG00000168610	AAAGUAA
ENSG00000168610	AAAGUAC
ENSG00000168610	AAAGUAU
ENSG00000168610	AAAUUUC
ENSG00000168610	AAGGCAC
ENSG00000168610	AAGUGCA
ENSG00000168610	AGCAGCU
ENSG00000168610	CCAGGAG
ENSG00000168610	CCCUGAG
ENSG00000168610	CUGGGGG
ENSG00000168610	GGAGGCA
ENSG00000168612	AACAUUC
ENSG00000168612	ACAUUCA
ENSG00000168615	AACAUUC
ENSG00000168615	AAGUGCU
ENSG00000168615	AGUGGUU
ENSG00000168615	CCCUGAG
ENSG00000168615	CGUACCG
ENSG00000168646	AAGUAGC
ENSG00000168646	ACAAUAU
ENSG00000168646	ACAGUAC
ENSG00000168646	ACUGUAG
ENSG00000168646	ACUUGGA
ENSG00000168646	CCUUCAU
ENSG00000168646	CUAUACA
ENSG00000168646	CUCAAAC
ENSG00000168646	CUUGGAG
ENSG00000168646	GCUACAU
ENSG00000168646	UACAGUU
ENSG00000168646	UAUAAUA
ENSG00000168661	UCCCACA
ENSG00000168672	AAAGAGC
ENSG00000168672	AAAGUAU
ENSG00000168672	AAAGUGC
ENSG00000168672	AACACUG
ENSG00000168672	AACAUUC
ENSG00000168672	ACAGUAC
ENSG00000168672	CAAGAGC
ENSG00000168672	CACAGUG
ENSG00000168672	CAGCAGG
ENSG00000168672	CCCCUGG
ENSG00000168672	CCUUCUG
ENSG00000168672	CCUUCUU
ENSG00000168672	CUACAAA
ENSG00000168672	CUCCAUU
ENSG00000168672	GAGUCUU
ENSG00000168672	GCAGGUU
ENSG00000168672	GCCCUUA
ENSG00000168672	GCUUCCU
ENSG00000168672	UCAUUUG
ENSG00000168672	UCUCCAA
ENSG00000168672	UGGUACC
ENSG00000168672	UGUCUGG
ENSG00000168675	AAACUGU
ENSG00000168675	AAAGCUG
ENSG00000168675	AAAUUUC
ENSG00000168675	AAGGCAC
ENSG00000168675	ACAGUAC
ENSG00000168675	ACAUAGA
ENSG00000168675	ACAUUAC
ENSG00000168675	ACCCAUU

ENSG00000168675	ACUCCAG
ENSG00000168675	AGCACCA
ENSG00000168675	AUGCAUU
ENSG00000168675	AUGUGCC
ENSG00000168675	CACACCU
ENSG00000168675	CACUGUU
ENSG00000168675	CAGACAG
ENSG00000168675	CAGCAAA
ENSG00000168675	CAGGUGA
ENSG00000168675	CAUAUUG
ENSG00000168675	CUAUACA
ENSG00000168675	GAGGUAU
ENSG00000168675	GCACCAU
ENSG00000168675	GCAUCCC
ENSG00000168675	GUGCUUG
ENSG00000168675	UAAUAGU
ENSG00000168675	UACAAAG
ENSG00000168675	UAUUGCU
ENSG00000168675	UCACAGG
ENSG00000168675	UCCUGAG
ENSG00000168675	UCCUUGC
ENSG00000168675	UGAAAGG
ENSG00000168675	UGCAUAG
ENSG00000168675	UGCAUAU
ENSG00000168675	UGCUCAC
ENSG00000168675	UGGCUGC
ENSG00000168675	UGGGUAC
ENSG00000168675	UUGGCAC
ENSG00000168675	UUUGCAA
ENSG00000168676	AUGCAUU
ENSG00000168702	AAAUGGU
ENSG00000168702	AAGAACC
ENSG00000168702	AGUGCAA
ENSG00000168702	GAAACAU
ENSG00000168702	GCAGCAU
ENSG00000168710	AGCAGCU
ENSG00000168710	AGCCCUU
ENSG00000168710	AUGCACC
ENSG00000168710	AUGGCUU
ENSG00000168710	AUUCAUU
ENSG00000168710	AUUGCAC
ENSG00000168710	GAGCCCU
ENSG00000168710	GCCCUUA
ENSG00000168710	UAAGACG
ENSG00000168710	UAAUAUC
ENSG00000168710	UCCCUCU
ENSG00000168710	UGCAUUG
ENSG00000168710	UGCUCAC
ENSG00000168710	UUGGCAC
ENSG00000168724	AACUCUA
ENSG00000168724	AAUCACU
ENSG00000168724	ACAUUCA
ENSG00000168724	AUCACUA
ENSG00000168724	AUUCAUU
ENSG00000168724	CACAAUG
ENSG00000168724	CCCUGUC
ENSG00000168724	CCCUGUU
ENSG00000168724	GAGCUAA
ENSG00000168724	UCCCUGU
ENSG00000168724	UCUAGAG
ENSG00000168724	UUGGUUC
ENSG00000168734	AAAAGUA
ENSG00000168743	ACAGUAU
ENSG00000168743	AGCUGCC

ENSG00000168743	AUUGCAC
ENSG00000168743	CUUCUCU
ENSG00000168743	GCAACUU
ENSG00000168743	GCCUGAU
ENSG00000168743	GGCAGUG
ENSG00000168743	UCUGGAG
ENSG00000168743	UUCCUCG
ENSG00000168743	UUCUUGU
ENSG00000168748	AAUUAUU
ENSG00000168748	AGCCCUU
ENSG00000168748	CACAGUG
ENSG00000168748	CCCACCU
ENSG00000168748	CUCCAGC
ENSG00000168748	CUUGGAG
ENSG00000168748	CUUUGGU
ENSG00000168748	GGAAGAC
ENSG00000168748	GGCAGUG
ENSG00000168748	GUCUGCC
ENSG00000168748	GUGAGGU
ENSG00000168748	UCACAGU
ENSG00000168748	UGAGUCU
ENSG00000168748	UGCUGAC
ENSG00000168748	UGUCUGC
ENSG00000168758	AAAAAUC
ENSG00000168758	AACAUUC
ENSG00000168758	ACAUUCA
ENSG00000168758	CCCUGAG
ENSG00000168758	GAAAACA
ENSG00000168758	GAGGUAG
ENSG00000168758	GAGGUAU
ENSG00000168758	GCUGGUG
ENSG00000168758	GGAAGAC
ENSG00000168758	GGCAGUG
ENSG00000168758	GGGGUCC
ENSG00000168758	GUGCCUG
ENSG00000168763	AAAGUGC
ENSG00000168763	AAGUGCU
ENSG00000168763	AGUGCUG
ENSG00000168763	CCCCCAG
ENSG00000168763	CGUGGCC
ENSG00000168763	GAAACAU
ENSG00000168763	GGAAGCC
ENSG00000168763	UCCAGAG
ENSG00000168763	UGCCAGC
ENSG00000168763	UUGGCAA
ENSG00000168763	UUGGCAC
ENSG00000168769	AUACAAG
ENSG00000168769	AUGAUGA
ENSG00000168769	AUGUGCC
ENSG00000168769	CAAAGUG
ENSG00000168769	CCUUCAU
ENSG00000168769	CUCUGGG
ENSG00000168769	CUGCCCC
ENSG00000168769	CUGUUGA
ENSG00000168769	GCCCUGU
ENSG00000168769	GCCUGGA
ENSG00000168769	GCCUGGG
ENSG00000168769	GCUACAU
ENSG00000168769	GGACCUG
ENSG00000168769	GGGUUUA
ENSG00000168769	GUAGUGU
ENSG00000168769	GUGACUG
ENSG00000168769	GUGCCUG
ENSG00000168769	GUGUGGC

ENSG00000168769	UAAUAGU
ENSG00000168769	UAGUGAG
ENSG00000168769	UAUAAAG
ENSG00000168769	UCCUGAG
ENSG00000168769	UGCCAGC
ENSG00000168769	UGCCCUG
ENSG00000168769	UGGACUG
ENSG00000168769	UGUGUGG
ENSG00000168769	UUGGCAA
ENSG00000168772	CUAUACA
ENSG00000168778	UCAUUUG
ENSG00000168779	AAAUUUC
ENSG00000168779	AACCUGU
ENSG00000168779	AAUGUUG
ENSG00000168779	ACUCAAA
ENSG00000168779	AGAUAAA
ENSG00000168779	AGUAACA
ENSG00000168779	AUACAAG
ENSG00000168779	CAAAACU
ENSG00000168779	CAAAGCA
ENSG00000168779	CAAAGUG
ENSG00000168779	CAUGGAU
ENSG00000168779	CUCUUCC
ENSG00000168779	GUCAGUU
ENSG00000168779	UCAACGG
ENSG00000168779	UCAUUCG
ENSG00000168779	UCAUUUG
ENSG00000168779	UUCAAAU
ENSG00000168785	ACAGUAC
ENSG00000168785	ACAGUAU
ENSG00000168785	AGCAGCA
ENSG00000168785	CAGUAGA
ENSG00000168785	CCAGUGC
ENSG00000168785	CCAGUGU
ENSG00000168785	CUACAGU
ENSG00000168795	AAUACUG
ENSG00000168795	ACAGUAU
ENSG00000168795	AUACAAG
ENSG00000168795	CAGACAG
ENSG00000168795	CCAGUGU
ENSG00000168795	CUCCAUU
ENSG00000168795	GAGGUAG
ENSG00000168806	UCAUUUG
ENSG00000168807	AAAGCUG
ENSG00000168807	AAAGUGC
ENSG00000168807	AAGUGCA
ENSG00000168807	ACAGUAC
ENSG00000168807	AGCACCA
ENSG00000168807	AGCAGCG
ENSG00000168807	AGCAGCU
ENSG00000168807	AGCUUAU
ENSG00000168807	AGGUAGU
ENSG00000168807	AGUGCUU
ENSG00000168807	AUAAAGU
ENSG00000168807	CCCUGAG
ENSG00000168807	CCCUGUU
ENSG00000168807	CUGGCUC
ENSG00000168807	GCAGGUU
ENSG00000168807	GGCAAGA
ENSG00000168807	GUCCAGA
ENSG00000168807	GUGUGGC
ENSG00000168807	UCCCUGU
ENSG00000168807	UGCAUAG
ENSG00000168807	UGCAUAU

ENSG00000168807	UGCAUUG
ENSG00000168807	UGCCCUG
ENSG00000168807	UGUGUGG
ENSG00000168807	UUUCAAC
ENSG00000168813	AACAAAC
ENSG00000168813	AACAUUC
ENSG00000168813	AAGGCAC
ENSG00000168813	AAGUCAC
ENSG00000168813	ACAGUAC
ENSG00000168813	ACCUGGA
ENSG00000168813	AGCAAAA
ENSG00000168813	AGCCUGC
ENSG00000168813	AGGUAGU
ENSG00000168813	CCUGUAC
ENSG00000168813	GAAAACA
ENSG00000168813	GAUCAGA
ENSG00000168813	GUAAACA
ENSG00000168813	GUCAGUU
ENSG00000168813	UGCAGCU
ENSG00000168813	UGUCUUU
ENSG00000168830	GAGGUAG
ENSG00000168843	AAGGCAC
ENSG00000168872	GAGGUAU
ENSG00000168872	UCAUUUG
ENSG00000168872	UUUGCAC
ENSG00000168874	ACUGGCC
ENSG00000168874	AGGGAGG
ENSG00000168874	CCCCUGG
ENSG00000168874	CCCUGAG
ENSG00000168874	CUGCCCC
ENSG00000168874	CUUUGGU
ENSG00000168874	GAGGCUG
ENSG00000168874	GCCUGGG
ENSG00000168874	UGAAGCU
ENSG00000168874	UUCUGCA
ENSG00000168874	UUGGCAA
ENSG00000168874	UUGGCAC
ENSG00000168876	GAGGUAG
ENSG00000168876	GGAGGCA
ENSG00000168876	GUGAGGU
ENSG00000168876	UCACACA
ENSG00000168876	UUGACAC
ENSG00000168887	GCUGGAU
ENSG00000168887	GUGAGGU
ENSG00000168887	UCCUGAG
ENSG00000168899	UCUGGAG
ENSG00000168904	UCCUGAG
ENSG00000168906	AAAGCUG
ENSG00000168906	AAGUGUG
ENSG00000168906	AGCUGCC
ENSG00000168906	AGGACAC
ENSG00000168906	AGUGUGC
ENSG00000168906	GGCAUUG
ENSG00000168906	UCACAUU
ENSG00000168906	UGCAUAG
ENSG00000168907	AAGUGUG
ENSG00000168907	CCUUCUG
ENSG00000168916	AAGCGCU
ENSG00000168916	AAUUAUU
ENSG00000168916	AUGCCUU
ENSG00000168916	AUGUCUG
ENSG00000168916	CAGCAGA
ENSG00000168916	UACAAAG
ENSG00000168916	UGAAAUG

ENSG00000168916	UGAUCCA
ENSG00000168916	UGGAUAA
ENSG00000168916	UUUUUGC
ENSG00000168918	UAAUGCU
ENSG00000168924	AUGAUGA
ENSG00000168924	GGAAUGU
ENSG00000168936	AACACUG
ENSG00000168936	AGUAACA
ENSG00000168936	CAGGUGA
ENSG00000168936	CCAGGAG
ENSG00000168936	CUGGACU
ENSG00000168936	GAGGCAG
ENSG00000168936	GCAGAAG
ENSG00000168936	GCAGGUU
ENSG00000168936	GGCAGGG
ENSG00000168936	UCACCAC
ENSG00000168938	AAAGUAU
ENSG00000168939	AAAGUUU
ENSG00000168939	AAGUGUG
ENSG00000168939	ACUCAGC
ENSG00000168939	ACUUGGA
ENSG00000168939	AGAUGUG
ENSG00000168939	AGCAAAA
ENSG00000168939	AGCAGCA
ENSG00000168939	AGCCUGC
ENSG00000168939	AGGAGCU
ENSG00000168939	AGUGAUC
ENSG00000168939	AUACAAG
ENSG00000168939	AUCCUUU
ENSG00000168939	AUGAGCU
ENSG00000168939	AUGGCAC
ENSG00000168939	CAAAGCA
ENSG00000168939	CAGACAG
ENSG00000168939	CAGCAAA
ENSG00000168939	CAGGCUC
ENSG00000168939	CAGUAGA
ENSG00000168939	GAGAUGA
ENSG00000168939	GCACCAU
ENSG00000168939	GCAGCAU
ENSG00000168939	GCCUGAU
ENSG00000168939	GGACUGC
ENSG00000168939	GGCAGUG
ENSG00000168939	GGGGAGC
ENSG00000168939	GGGUUUA
ENSG00000168939	GUCUGCC
ENSG00000168939	GUGACAG
ENSG00000168939	GUGCAAA
ENSG00000168939	UGCAGCU
ENSG00000168939	UUCAAGC
ENSG00000168958	UUUGCAA
ENSG00000168959	CAAAGCA
ENSG00000168959	UUGGCAA
ENSG00000168993	AAUCACU
ENSG00000168993	AUUGCAC
ENSG00000168993	GAAAACA
ENSG00000168993	GCAGGAC
ENSG00000168993	UCAACAG
ENSG00000168993	UGGCUGC
ENSG00000168994	ACAUUAC
ENSG00000168994	AGCAAAA
ENSG00000168994	CAGUCCA
ENSG00000168994	CCUUCUG
ENSG00000168994	UCUCAAG
ENSG00000169016	AAGGCAC

ENSG00000169016	CACAGUG
ENSG00000169016	GAGGUAG
ENSG00000169016	GAGGUAU
ENSG00000169016	GGAGAGA
ENSG00000169016	UCACAGU
ENSG00000169016	UUCUAAU
ENSG00000169031	AUGCACC
ENSG00000169031	UAAGACG
ENSG00000169031	UAAGACU
ENSG00000169031	UCUGGAA
ENSG00000169032	AGCAGCG
ENSG00000169032	AGCAGCU
ENSG00000169032	AGUAGAC
ENSG00000169032	CAGCAAA
ENSG00000169032	CAGUAGA
ENSG00000169032	CUAUACA
ENSG00000169032	GGCUCUG
ENSG00000169032	UUGGCAC
ENSG00000169035	AAAGGUA
ENSG00000169045	AGCUGCC
ENSG00000169045	UACAAAG
ENSG00000169045	UCACAAG
ENSG00000169047	AACAAAC
ENSG00000169047	AGAUGUG
ENSG00000169047	AGCAGCA
ENSG00000169047	AUACCUC
ENSG00000169047	AUCGUAC
ENSG00000169047	CAAAACU
ENSG00000169047	UACAAAG
ENSG00000169047	UCACAGU
ENSG00000169047	UCCUGAG
ENSG00000169047	UGCUGAC
ENSG00000169047	UUGGCAC
ENSG00000169057	AAAGUAC
ENSG00000169057	AAAGUAU
ENSG00000169057	AAAGUGC
ENSG00000169057	AAAUGGU
ENSG00000169057	AAAUUUC
ENSG00000169057	AACACUG
ENSG00000169057	AACAGUC
ENSG00000169057	AACUACU
ENSG00000169057	AACUCUA
ENSG00000169057	AAGAACC
ENSG00000169057	AAGGCAC
ENSG00000169057	AAGUGCA
ENSG00000169057	ACAAUAU
ENSG00000169057	ACAGGUG
ENSG00000169057	ACCACAG
ENSG00000169057	ACCAGCA
ENSG00000169057	ACCCAUU
ENSG00000169057	AGAUAAA
ENSG00000169057	AGCAGCU
ENSG00000169057	AGCCAGU
ENSG00000169057	AGCUGCC
ENSG00000169057	AGGUAGU
ENSG00000169057	AGGUUAU
ENSG00000169057	AGUAACA
ENSG00000169057	AGUGCUU
ENSG00000169057	AUAUAAC
ENSG00000169057	AUCACUA
ENSG00000169057	AUGAUGA
ENSG00000169057	AUGAUGC
ENSG00000169057	AUGGCAC
ENSG00000169057	CAAAACU

ENSG00000169057	CACACCU
ENSG00000169057	CACUCCU
ENSG00000169057	CAGCAAA
ENSG00000169057	CAGCAGA
ENSG00000169057	CAGGAAC
ENSG00000169057	CAGUAGA
ENSG00000169057	CAGUGCA
ENSG00000169057	CAGUUAC
ENSG00000169057	CCCACCG
ENSG00000169057	CCCACCU
ENSG00000169057	CCUUCAU
ENSG00000169057	CUAUACA
ENSG00000169057	CUCCCAC
ENSG00000169057	CUCUAGC
ENSG00000169057	CUCUGGG
ENSG00000169057	CUGGAAA
ENSG00000169057	CUGGGCA
ENSG00000169057	CUUGGAG
ENSG00000169057	GAAGGAA
ENSG00000169057	GACUUCC
ENSG00000169057	GAGGCAG
ENSG00000169057	GAGGUAG
ENSG00000169057	GAUUGGU
ENSG00000169057	GCAGCAU
ENSG00000169057	GCCUGGA
ENSG00000169057	GCUGUCU
ENSG00000169057	GGAAGAC
ENSG00000169057	GGAGGCA
ENSG00000169057	GGCAGCG
ENSG00000169057	GGCAGGG
ENSG00000169057	GGGAACG
ENSG00000169057	GGUUUAC
ENSG00000169057	GUAAACA
ENSG00000169057	GUAACAG
ENSG00000169057	GUCAACA
ENSG00000169057	GUCUCUG
ENSG00000169057	GUUCAUG
ENSG00000169057	UAAGACU
ENSG00000169057	UAAUGCU
ENSG00000169057	UAUAAAG
ENSG00000169057	UAUCAGA
ENSG00000169057	UAUGGUU
ENSG00000169057	UAUUGCU
ENSG00000169057	UCAACAG
ENSG00000169057	UCAUGAU
ENSG00000169057	UCAUUUG
ENSG00000169057	UCCCACC
ENSG00000169057	UCCCUUU
ENSG00000169057	UCCGCUC
ENSG00000169057	UCCUGAG
ENSG00000169057	UCUGGAG
ENSG00000169057	UGAAGUG
ENSG00000169057	UGACUGU
ENSG00000169057	UGCAAAG
ENSG00000169057	UGGCCCU
ENSG00000169057	UGUCUGC
ENSG00000169057	UGUGCUU
ENSG00000169057	UGUGUGG
ENSG00000169057	UUCAAGC
ENSG00000169057	UUCUGCA
ENSG00000169057	UUCUUGU
ENSG00000169057	UUGACAC
ENSG00000169057	UUGGCAC
ENSG00000169057	UUGGUCC

ENSG00000169057	UUGUGUC
ENSG00000169057	UUUGCAC
ENSG00000169057	UUUUUGC
ENSG00000169062	AAAGUGC
ENSG00000169062	AAGUGCU
ENSG00000169062	AGUGCUU
ENSG00000169067	UACAAAG
ENSG00000169071	AACAUUC
ENSG00000169071	AAGGCAC
ENSG00000169071	ACAAUAU
ENSG00000169071	UCCCUUU
ENSG00000169083	AAGGCAC
ENSG00000169083	ACUGCAG
ENSG00000169083	AGUGCAA
ENSG00000169083	GCAGAAG
ENSG00000169085	UGACCUA
ENSG00000169105	AAAAGUA
ENSG00000169105	AAGGCAC
ENSG00000169105	CUUUGGU
ENSG00000169105	GGGUCUU
ENSG00000169118	AAAGGUA
ENSG00000169118	AAAGUGC
ENSG00000169118	AAGUGCA
ENSG00000169118	AGCACCA
ENSG00000169118	AGGAGCU
ENSG00000169118	AGGGAGG
ENSG00000169118	AGUAACA
ENSG00000169118	AGUGCAA
ENSG00000169118	AUAAUAC
ENSG00000169118	AUAUAAC
ENSG00000169118	AUGACAC
ENSG00000169118	AUGCAUU
ENSG00000169118	AUGGCAC
ENSG00000169118	AUGGCUU
ENSG00000169118	CACUGUU
ENSG00000169118	CAGACAG
ENSG00000169118	CAGGAAC
ENSG00000169118	CAGUAGA
ENSG00000169118	CCAGUGC
ENSG00000169118	CUACAGU
ENSG00000169118	CUCCAGC
ENSG00000169118	CUGGGGG
ENSG00000169118	CUUCUCU
ENSG00000169118	CUUGUGU
ENSG00000169118	GAGCUGC
ENSG00000169118	GCAGGUU
ENSG00000169118	GCCUGGG
ENSG00000169118	GGAGGCA
ENSG00000169118	GGCAGGG
ENSG00000169118	GGCAGUG
ENSG00000169118	GGCUCAG
ENSG00000169118	GUCAGUU
ENSG00000169118	GUGCAAA
ENSG00000169118	UAAGACG
ENSG00000169118	UAAGACU
ENSG00000169118	UAUAAUA
ENSG00000169118	UCACAUU
ENSG00000169118	UCAUUUG
ENSG00000169118	UGACAUC
ENSG00000169118	UGAGGAC
ENSG00000169118	UGGCUGC
ENSG00000169118	UUGGUCC
ENSG00000169118	UUGGUUC
ENSG00000169118	UUGUGUC

ENSG00000169122	AAAGUUU
ENSG00000169122	AAGACAU
ENSG00000169122	AAGUCUU
ENSG00000169122	AGGUAGU
ENSG00000169122	AGUGCUG
ENSG00000169122	AGUGCUU
ENSG00000169122	AUGGCUU
ENSG00000169122	CACCACC
ENSG00000169122	CUACAGU
ENSG00000169122	CUUACAG
ENSG00000169122	GAUAUGU
ENSG00000169122	GCACCAU
ENSG00000169122	GGCUCUG
ENSG00000169122	GUUCAUG
ENSG00000169122	UAAGACG
ENSG00000169122	UAAGACU
ENSG00000169122	UACAAAG
ENSG00000169122	UACAGUU
ENSG00000169122	UCACAAG
ENSG00000169122	UUGGCAA
ENSG00000169122	UUGGCAC
ENSG00000169129	GAGUCUU
ENSG00000169129	GAUUGUC
ENSG00000169129	UGAAAUG
ENSG00000169131	GAGGUAG
ENSG00000169139	AGUGCCU
ENSG00000169139	UAUAAAG
ENSG00000169155	AGCACCA
ENSG00000169155	UCCAGUU
ENSG00000169155	UGCCAGC
ENSG00000169174	UACAAAG
ENSG00000169180	AAUCUCU
ENSG00000169180	AUGAUGA
ENSG00000169180	GAAAACA
ENSG00000169180	GAGAAAU
ENSG00000169181	GGCCCUG
ENSG00000169181	GUGCCUG
ENSG00000169181	UAAUACA
ENSG00000169181	UGGCCCU
ENSG00000169184	ACUGUGU
ENSG00000169184	AUCCUUU
ENSG00000169184	CAAAGCA
ENSG00000169184	CACACCU
ENSG00000169184	CACAGUG
ENSG00000169184	CAGCAGA
ENSG00000169184	CCAGUGU
ENSG00000169184	CUCAAAC
ENSG00000169184	GGCCCUG
ENSG00000169184	GUGCAAA
ENSG00000169184	GUGGCCC
ENSG00000169184	GUGGGGA
ENSG00000169184	UCACAGU
ENSG00000169184	UCAUGAU
ENSG00000169184	UGGCCCU
ENSG00000169184	UGUCUUU
ENSG00000169184	UUGAGGC
ENSG00000169193	CUUUGGU
ENSG00000169193	GUGCAAA
ENSG00000169194	AACUCUA
ENSG00000169194	AGUGCUG
ENSG00000169194	AUAUUAU
ENSG00000169194	GAGGUAG
ENSG00000169194	GAGGUAU
ENSG00000169194	GUGAGGU

ENSG00000169213	ACUCAGC
ENSG00000169213	GCCCUGU
ENSG00000169213	GGCCCUG
ENSG00000169218	AUAAAGU
ENSG00000169218	CCUGCUG
ENSG00000169218	UCCAGUU
ENSG00000169218	UCUCCAA
ENSG00000169218	UGAAAUG
ENSG00000169220	AGCUGCC
ENSG00000169220	AUAAUAC
ENSG00000169220	CAGACAG
ENSG00000169220	CUGCCCC
ENSG00000169220	GAGGGGC
ENSG00000169220	UCAUACA
ENSG00000169221	AAAGCGC
ENSG00000169221	GCAGGUU
ENSG00000169223	AGCAAAA
ENSG00000169223	CCAGUGU
ENSG00000169223	CUGGGCA
ENSG00000169223	GGCAAGA
ENSG00000169223	UCACAUU
ENSG00000169228	AGGCAGG
ENSG00000169228	CACAGUG
ENSG00000169228	CAGCAGG
ENSG00000169228	CAGUGCA
ENSG00000169228	GCCUGGG
ENSG00000169230	AGCUGCC
ENSG00000169239	AAUUGCU
ENSG00000169239	ACAUUCA
ENSG00000169239	UAUAUGA
ENSG00000169239	UGUAUAA
ENSG00000169242	CCUGCUG
ENSG00000169242	CUUUGGU
ENSG00000169242	GGCCCUG
ENSG00000169242	UCCAGUU
ENSG00000169245	AGCAGCA
ENSG00000169245	AGCAGCG
ENSG00000169247	AAAUCCC
ENSG00000169247	AACUCUA
ENSG00000169247	CAGUGCA
ENSG00000169247	CAGUUAC
ENSG00000169247	CCAGUGC
ENSG00000169247	CUCAGGG
ENSG00000169247	CUCCCAC
ENSG00000169247	CUCUGGG
ENSG00000169247	GACCUGG
ENSG00000169247	GAGAACU
ENSG00000169247	GAGGCAG
ENSG00000169247	GCUGGUG
ENSG00000169247	GGCUCUG
ENSG00000169247	GGUCUAG
ENSG00000169247	GUCUUGC
ENSG00000169247	UAAGACU
ENSG00000169247	UCUAGAG
ENSG00000169247	UGAAAGG
ENSG00000169252	AAUACUG
ENSG00000169252	ACUGUAG
ENSG00000169252	GAGGUAU
ENSG00000169252	GUAAACA
ENSG00000169252	UAGACUG
ENSG00000169255	AAUACUG
ENSG00000169255	CUGGAAA
ENSG00000169255	UAUUACC
ENSG00000169258	AGCCUGC

ENSG00000169258	CUCAGGG
ENSG00000169258	UCACAAG
ENSG00000169282	AGAUGUG
ENSG00000169282	AGCAGCA
ENSG00000169282	AUGCAUU
ENSG00000169282	AUGGAUU
ENSG00000169282	CACAGUG
ENSG00000169282	CAGCAGA
ENSG00000169282	GAAGGUC
ENSG00000169282	GCAGGUU
ENSG00000169282	GGCAGGG
ENSG00000169282	UCUCAAG
ENSG00000169282	UGCAUUG
ENSG00000169282	UUGUUCG
ENSG00000169291	ACAUUCA
ENSG00000169291	CACAGUG
ENSG00000169291	CAGCUGG
ENSG00000169291	CCUGCUG
ENSG00000169291	CUCACAC
ENSG00000169291	GGAAGCC
ENSG00000169291	GUGCCAC
ENSG00000169291	GUGUCAC
ENSG00000169291	UCACACA
ENSG00000169291	UCACAGG
ENSG00000169291	UCUCACC
ENSG00000169291	UGUGUCA
ENSG00000169291	UUCAAGC
ENSG00000169299	UUCCUAG
ENSG00000169306	ACCCUGU
ENSG00000169306	AGCAAAA
ENSG00000169306	AGCAGCA
ENSG00000169306	AUGUAAU
ENSG00000169306	CCCUGUU
ENSG00000169306	CGAGGAC
ENSG00000169306	CGAGGAG
ENSG00000169306	CUACAAA
ENSG00000169306	GCUACAU
ENSG00000169314	CUGGCUC
ENSG00000169314	GCAACUU
ENSG00000169330	AAAUCCC
ENSG00000169330	ACAAUAU
ENSG00000169330	AGUGUGC
ENSG00000169330	GAGUGUG
ENSG00000169330	GCACCAU
ENSG00000169330	GGAGAGA
ENSG00000169330	GGCAGUG
ENSG00000169330	GUAAACA
ENSG00000169330	GUGCUUG
ENSG00000169330	UAUUGCU
ENSG00000169330	UCCAGAG
ENSG00000169330	UGCAUUG
ENSG00000169330	UGGAGAU
ENSG00000169330	UUGGUCC
ENSG00000169359	GGCAUUG
ENSG00000169359	UUGGCAA
ENSG00000169359	UUGGCAC
ENSG00000169375	AAAGCUG
ENSG00000169375	CUAGUAA
ENSG00000169375	CUUUGGU
ENSG00000169375	UCCCUUU
ENSG00000169398	AAAACUG
ENSG00000169398	CAAAGCA
ENSG00000169398	CUACAAA
ENSG00000169398	UAAUAGU

ENSG00000169398	UAUAAAG
ENSG00000169410	ACACACC
ENSG00000169410	ACACACU
ENSG00000169410	ACAGUAC
ENSG00000169410	AGCUGCC
ENSG00000169410	CAAAACU
ENSG00000169410	CACAGUG
ENSG00000169410	GGCUCUG
ENSG00000169410	GUGCUUG
ENSG00000169410	UACAGUU
ENSG00000169410	UAUUACC
ENSG00000169410	UGGCCCU
ENSG00000169410	UUGGCAC
ENSG00000169410	UUGGUUC
ENSG00000169429	AAGUGCU
ENSG00000169429	AGUGCUU
ENSG00000169432	AACACUG
ENSG00000169432	ACUGCAG
ENSG00000169432	ACUGUGU
ENSG00000169432	CUGCUCA
ENSG00000169432	GAAAACA
ENSG00000169432	UUGGCAA
ENSG00000169432	UUGGCAC
ENSG00000169436	AAAGAGG
ENSG00000169436	CUGUUGA
ENSG00000169436	UCAAGUA
ENSG00000169439	AAUACUG
ENSG00000169439	ACAUAGA
ENSG00000169439	AGUGAUC
ENSG00000169439	AUGCAUU
ENSG00000169439	AUUGCAC
ENSG00000169439	CAAAGUG
ENSG00000169439	GCAACUU
ENSG00000169439	UAAGACU
ENSG00000169439	UGUGCUU
ENSG00000169439	UUCAAAU
ENSG00000169442	CAGCUGG
ENSG00000169490	GAGAUGA
ENSG00000169499	AAUCUCU
ENSG00000169499	ACUCAGC
ENSG00000169499	AUGGCUU
ENSG00000169499	UACAAAG
ENSG00000169499	UAUUACC
ENSG00000169499	UCCAGAG
ENSG00000169499	UGAGAAG
ENSG00000169504	AAAUGGU
ENSG00000169504	CUCUUCC
ENSG00000169504	CUUCUCU
ENSG00000169509	AAGUGCA
ENSG00000169509	GAGGUAG
ENSG00000169519	AAAGAAU
ENSG00000169554	AAAAGUA
ENSG00000169554	AAAUCCC
ENSG00000169554	AAUGUUG
ENSG00000169554	ACAAGGU
ENSG00000169554	ACAGUAC
ENSG00000169554	ACGUCAU
ENSG00000169554	ACUGGAU
ENSG00000169554	ACUGUAG
ENSG00000169554	AGUAGUU
ENSG00000169554	AGUGAUC
ENSG00000169554	AUGGCAC
ENSG00000169554	AUGUAAU
ENSG00000169554	AUUGCAC

ENSG00000169554	CACAAUG
ENSG00000169554	CAGGAAC
ENSG00000169554	CAGUAGU
ENSG00000169554	CAUGGGU
ENSG00000169554	CCAGUAC
ENSG00000169554	CGUGCAU
ENSG00000169554	GCAGAAG
ENSG00000169554	GCUACAU
ENSG00000169554	GCUGGUG
ENSG00000169554	GCUUCCU
ENSG00000169554	GGAAGCC
ENSG00000169554	GGUUACC
ENSG00000169554	GUAAACA
ENSG00000169554	GUCUCUG
ENSG00000169554	GUGCUUG
ENSG00000169554	GUGUGGC
ENSG00000169554	UAAGACG
ENSG00000169554	UAAUAUC
ENSG00000169554	UAGGCCG
ENSG00000169554	UAUAAUA
ENSG00000169554	UCAUACA
ENSG00000169554	UCCAGUU
ENSG00000169554	UGAAGCU
ENSG00000169554	UGCAUAG
ENSG00000169554	UUCUAAU
ENSG00000169554	UUGAGGC
ENSG00000169554	UUUCAAC
ENSG00000169564	ACAUUAC
ENSG00000169564	UAUAAAG
ENSG00000169570	AGCACCA
ENSG00000169594	AUACAAG
ENSG00000169594	GAGAACU
ENSG00000169604	AAGUGUG
ENSG00000169604	ACUGGAU
ENSG00000169604	AGGGUCU
ENSG00000169604	AUAAUAC
ENSG00000169604	AUACAAG
ENSG00000169604	CAAAACU
ENSG00000169604	CAUAUUG
ENSG00000169604	GCUUCCU
ENSG00000169604	GGGGUGG
ENSG00000169604	GUGCAAA
ENSG00000169604	UAAUGCU
ENSG00000169604	UAUAAAG
ENSG00000169604	UCACACA
ENSG00000169604	UCAUUUG
ENSG00000169604	UCUAGAG
ENSG00000169604	UGCCAGC
ENSG00000169635	AAACUGU
ENSG00000169635	AACACUG
ENSG00000169635	AACAUUC
ENSG00000169635	AACCUGG
ENSG00000169635	AAGUGCA
ENSG00000169635	AAGUGCU
ENSG00000169635	ACAAUAU
ENSG00000169635	ACUGGCC
ENSG00000169635	AGAUAAA
ENSG00000169635	AGCAAAA
ENSG00000169635	AGUGCUG
ENSG00000169635	AUAUUAU
ENSG00000169635	AUGAGCU
ENSG00000169635	AUGGCUU
ENSG00000169635	AUGUAAU
ENSG00000169635	CAAGAGC

ENSG00000169635	CACAGGG
ENSG00000169635	CACUCCU
ENSG00000169635	CACUGUU
ENSG00000169635	CAGGAAC
ENSG00000169635	CAGGCUC
ENSG00000169635	CAUAUUG
ENSG00000169635	CCCUGAG
ENSG00000169635	CUCUGGG
ENSG00000169635	CUGGGCA
ENSG00000169635	CUUUGGU
ENSG00000169635	GACCUGG
ENSG00000169635	GAGAAAU
ENSG00000169635	GAGCCCC
ENSG00000169635	GAGCCCU
ENSG00000169635	GAGGCUG
ENSG00000169635	GAGGGGC
ENSG00000169635	GAGGUAU
ENSG00000169635	GGAAUGU
ENSG00000169635	GGAGGAG
ENSG00000169635	GGGGUCC
ENSG00000169635	GGGUCUU
ENSG00000169635	GGUUUAC
ENSG00000169635	GUAAACA
ENSG00000169635	GUCUCUG
ENSG00000169635	GUGAAUG
ENSG00000169635	GUGAGGU
ENSG00000169635	GUGCUUG
ENSG00000169635	UAAGACG
ENSG00000169635	UCACAGU
ENSG00000169635	UGCAGCU
ENSG00000169635	UGGGUAC
ENSG00000169635	UGUGCUU
ENSG00000169635	UGUGUCA
ENSG00000169635	UUCCAUA
ENSG00000169635	UUGGUCC
ENSG00000169635	UUGGUUC
ENSG00000169635	UUGUGAC
ENSG00000169635	UUGUGUC
ENSG00000169635	UUUGCAA
ENSG00000169635	UUUGCAC
ENSG00000169641	AAAGUGC
ENSG00000169641	AAGUGUG
ENSG00000169641	ACAUUCA
ENSG00000169641	AGCACCA
ENSG00000169641	AGCAGCU
ENSG00000169641	AGUGUGC
ENSG00000169641	AUGCACC
ENSG00000169641	AUUGCAC
ENSG00000169641	CAGCAGG
ENSG00000169641	CCCUGAG
ENSG00000169641	CUUGGAG
ENSG00000169641	GAGCCCU
ENSG00000169641	GAGCUGC
ENSG00000169641	GGCAGGG
ENSG00000169641	GGGAGCU
ENSG00000169641	GGUUACC
ENSG00000169641	GUGCAAA
ENSG00000169641	GUGUGGC
ENSG00000169641	UAUUGCU
ENSG00000169641	UGCCAGC
ENSG00000169641	UUGGCAA
ENSG00000169641	UUGGCAC
ENSG00000169682	AAUUUUA
ENSG00000169682	AGCACCA

ENSG00000169682	GGGAGCU
ENSG00000169682	GUAGUGU
ENSG00000169689	GAGAACC
ENSG00000169689	GAGAACU
ENSG00000169689	UUGUGAC
ENSG00000169710	CCCACCG
ENSG00000169710	CUUCACC
ENSG00000169714	AAAGCUG
ENSG00000169714	ACUGGCC
ENSG00000169714	AUAAUAC
ENSG00000169714	AUGCAUU
ENSG00000169714	CAAAGCA
ENSG00000169714	CACCAGC
ENSG00000169714	CUCCAGC
ENSG00000169714	CUCUUCC
ENSG00000169714	GAGGGGC
ENSG00000169714	GCAGAAG
ENSG00000169714	GCCUGAU
ENSG00000169714	GCCUGGG
ENSG00000169714	GGCGACA
ENSG00000169714	UAAUACA
ENSG00000169714	UCACCAC
ENSG00000169714	UGGCCAC
ENSG00000169714	UUCCUCG
ENSG00000169714	UUUUUGC
ENSG00000169718	GCAGCAU
ENSG00000169718	UCCCUGU
ENSG00000169744	AUGGCUU
ENSG00000169750	GACCCUG
ENSG00000169756	UGCAUAU
ENSG00000169758	AAAAAUC
ENSG00000169758	AGUGCUG
ENSG00000169758	CACAGUG
ENSG00000169758	CAGCUGG
ENSG00000169758	CAGUGCA
ENSG00000169758	CCUGCUG
ENSG00000169758	CUGCUCA
ENSG00000169758	GGAGGCA
ENSG00000169758	UCACAGU
ENSG00000169758	UGAAGCU
ENSG00000169758	UGCAGCU
ENSG00000169758	UGCUCAC
ENSG00000169760	AAAAGUA
ENSG00000169760	AGCAGCA
ENSG00000169760	AGCAUUC
ENSG00000169760	CAGCAGA
ENSG00000169760	CAGCAGG
ENSG00000169760	GGAUGAG
ENSG00000169760	UACAAAG
ENSG00000169760	UCCCUUG
ENSG00000169760	UGAGAAG
ENSG00000169760	UGCAUAU
ENSG00000169760	UUGUGUC
ENSG00000169762	AAAGUGC
ENSG00000169762	AACACUG
ENSG00000169762	AAGUGCA
ENSG00000169762	CACAGUG
ENSG00000169762	CUCCCAA
ENSG00000169762	GGUGGUC
ENSG00000169762	GGUGGUU
ENSG00000169762	UAUAAAG
ENSG00000169762	UCACAGG
ENSG00000169762	UCUGGAG
ENSG00000169762	UGCAAAG

ENSG00000169762	UUGGCAC
ENSG00000169764	UAUUGCU
ENSG00000169783	AGGCAGG
ENSG00000169783	CAGUCUG
ENSG00000169783	CUGCCCC
ENSG00000169783	GAGAAGA
ENSG00000169783	GAGGUAG
ENSG00000169783	GAGGUAU
ENSG00000169783	GCCCUGU
ENSG00000169783	GGGGUCC
ENSG00000169783	GUGAGGU
ENSG00000169783	UCAAGUA
ENSG00000169783	UCCUGAG
ENSG00000169783	UGCAUUG
ENSG00000169783	UUCAAGC
ENSG00000169813	AAUUUUA
ENSG00000169813	ACUCAAA
ENSG00000169813	AGCACCA
ENSG00000169813	CACAGUG
ENSG00000169813	CUACAGU
ENSG00000169813	GUCAUUG
ENSG00000169813	GUGCAAA
ENSG00000169813	UACAAAG
ENSG00000169813	UGAGAAG
ENSG00000169813	UGAGAAU
ENSG00000169826	AAAGAGG
ENSG00000169826	ACUGCAU
ENSG00000169826	AGCACCA
ENSG00000169826	AGUGCUU
ENSG00000169851	AAGGCAC
ENSG00000169851	AAGUGCU
ENSG00000169851	ACCACAG
ENSG00000169851	ACUCAGG
ENSG00000169851	AGUGCUU
ENSG00000169851	AUACAAG
ENSG00000169851	AUCGUAC
ENSG00000169851	CAGCAGG
ENSG00000169851	CGUACCG
ENSG00000169851	CGUGCAU
ENSG00000169851	GAGGCAG
ENSG00000169851	GCAGAAG
ENSG00000169851	GCCUGAU
ENSG00000169851	GGCAGGG
ENSG00000169851	GUCUCUG
ENSG00000169851	UAAGACU
ENSG00000169851	UACAAAG
ENSG00000169851	UGCAUAG
ENSG00000169851	UGCAUUG
ENSG00000169855	AGUGAUC
ENSG00000169855	AUUGCAC
ENSG00000169855	CAUGGAU
ENSG00000169855	GAGAACC
ENSG00000169855	GAGAACU
ENSG00000169855	GAUUGUA
ENSG00000169855	GAUUGUC
ENSG00000169855	GUGCUUG
ENSG00000169855	UAGUGAG
ENSG00000169855	UGAGAAG
ENSG00000169855	UGAGAAU
ENSG00000169856	AAAGCUG
ENSG00000169856	CAGGUGA
ENSG00000169856	UUGGUUC
ENSG00000169860	AGAUGUG
ENSG00000169862	AAAAAUC

ENSG00000169862	AAAGAAU
ENSG00000169862	AAAUUUC
ENSG00000169862	AACUGGU
ENSG00000169862	AAUACUG
ENSG00000169862	AAUUUUA
ENSG00000169862	ACAUCAC
ENSG00000169862	AGGCAGG
ENSG00000169862	AUGCAUU
ENSG00000169862	AUGCCUU
ENSG00000169862	AUGGAUU
ENSG00000169862	CUGGAAA
ENSG00000169862	GAAACAU
ENSG00000169862	GAGAAGA
ENSG00000169862	GAGUGUG
ENSG00000169862	GGAGUGU
ENSG00000169862	GGAUGAG
ENSG00000169862	GGCAAGA
ENSG00000169862	GGCAUUG
ENSG00000169862	GUGCUUG
ENSG00000169862	UAUCAGA
ENSG00000169862	UGAGAAG
ENSG00000169862	UGUUGAA
ENSG00000169871	UUGGUCC
ENSG00000169884	CAGUGCA
ENSG00000169884	UUGGAGG
ENSG00000169891	AAAGAGG
ENSG00000169891	AGCACCA
ENSG00000169891	AUGCAUU
ENSG00000169891	CAGUAGU
ENSG00000169891	CUCCCAA
ENSG00000169891	CUGCUCA
ENSG00000169891	CUUCACC
ENSG00000169891	GAGGCAG
ENSG00000169891	GAUUGUC
ENSG00000169891	GGUGGUU
ENSG00000169891	GUCUUGC
ENSG00000169891	GUGAGGU
ENSG00000169891	UAAUGCU
ENSG00000169891	UCACAGG
ENSG00000169891	UCACAGU
ENSG00000169891	UCAUACA
ENSG00000169891	UGAAAGG
ENSG00000169891	UUGGCAA
ENSG00000169891	UUGGCAC
ENSG00000169895	CCUUCUG
ENSG00000169895	UUGGUCC
ENSG00000169896	GCCUGGG
ENSG00000169908	AAAGUUU
ENSG00000169908	GAAACAU
ENSG00000169914	AAAGUGC
ENSG00000169914	AACACUG
ENSG00000169914	AAGGCAC
ENSG00000169914	AAGUCUU
ENSG00000169914	ACAGUAU
ENSG00000169914	AGCAAAA
ENSG00000169914	AUAAAGU
ENSG00000169914	AUACAAG
ENSG00000169914	AUGGCUU
ENSG00000169914	GAGAAAU
ENSG00000169914	GAGGUAG
ENSG00000169914	GAGGUAU
ENSG00000169914	GAGUUGA
ENSG00000169914	GAUUGUC
ENSG00000169914	GCACCAU

ENSG00000169914	GCUGGUG
ENSG00000169914	GGCAAGA
ENSG00000169914	UAAUACA
ENSG00000169914	UGAAGCU
ENSG00000169914	UUGGUUC
ENSG00000169918	GCACCAU
ENSG00000169925	AGGAGCU
ENSG00000169925	GAGGUAG
ENSG00000169925	GAGGUAU
ENSG00000169925	GGAAGAC
ENSG00000169925	UGACCUA
ENSG00000169926	AAAAGUA
ENSG00000169926	AAAUGGU
ENSG00000169926	AAAUUUC
ENSG00000169926	AACAAAC
ENSG00000169926	AACCUGG
ENSG00000169926	AAGGCAC
ENSG00000169926	AAGGCGC
ENSG00000169926	AAGUGCA
ENSG00000169926	AAGUGCU
ENSG00000169926	AAUACUG
ENSG00000169926	AAUCUCA
ENSG00000169926	ACACACU
ENSG00000169926	ACCUGGA
ENSG00000169926	ACUGUAG
ENSG00000169926	AGGACAC
ENSG00000169926	AGUGCAA
ENSG00000169926	AUGGCUU
ENSG00000169926	CACAAUG
ENSG00000169926	CACUGUU
ENSG00000169926	CUUUGGU
ENSG00000169926	GACCCUG
ENSG00000169926	GAGAAAU
ENSG00000169926	GAGAUGA
ENSG00000169926	GAGUGUG
ENSG00000169926	GCCUGGA
ENSG00000169926	GCCUGGG
ENSG00000169926	GGAAUGU
ENSG00000169926	GGCAAGA
ENSG00000169926	GUAAACA
ENSG00000169926	GUAGUGU
ENSG00000169926	GUGAAUG
ENSG00000169926	GUGUGGC
ENSG00000169926	UCAUGAU
ENSG00000169926	UCCCUCU
ENSG00000169926	UCCUAUA
ENSG00000169926	UCCUGAG
ENSG00000169926	UGAUCCA
ENSG00000169926	UGCAUAU
ENSG00000169926	UGGCCAC
ENSG00000169926	UUCAAAU
ENSG00000169926	UUGGCAC
ENSG00000169926	UUUGCAA
ENSG00000169926	UUUGCAC
ENSG00000169933	AAAGGUA
ENSG00000169933	AGUGUGC
ENSG00000169933	CAGGCUC
ENSG00000169933	CUGGACU
ENSG00000169933	GGCUCUG
ENSG00000169933	GGUGGUU
ENSG00000169933	GUGAAUG
ENSG00000169933	GUGCUUG
ENSG00000169933	UGAAAGG
ENSG00000169933	UGUGCUU

ENSG00000169933	UUGGCAC
ENSG00000169933	UUUGCAA
ENSG00000169946	AAAGUAA
ENSG00000169946	AAAGUAC
ENSG00000169946	AACAUUC
ENSG00000169946	AAGUGCA
ENSG00000169946	AAGUGCU
ENSG00000169946	ACUCAGC
ENSG00000169946	AGAUAAA
ENSG00000169946	AGUGCUU
ENSG00000169946	AUAAUAC
ENSG00000169946	AUGUGCC
ENSG00000169946	AUUGCAC
ENSG00000169946	CAAAUGC
ENSG00000169946	CACCAGC
ENSG00000169946	CAGCAAA
ENSG00000169946	CAGUGCA
ENSG00000169946	GCUGGUG
ENSG00000169946	GCUUCCU
ENSG00000169946	UAAUAUC
ENSG00000169946	UACAAAG
ENSG00000169946	UAUCAGA
ENSG00000169946	UAUUGCU
ENSG00000169946	UCAUUUG
ENSG00000169946	UGAGAAG
ENSG00000169946	UGAGAAU
ENSG00000169946	UGCAAAG
ENSG00000169946	UGCAGCU
ENSG00000169946	UGCAUUG
ENSG00000169946	UGUUGAA
ENSG00000169955	CUCUUCC
ENSG00000169955	GAGCGCC
ENSG00000169955	GGAAGCC
ENSG00000169967	AAAAAUC
ENSG00000169967	AAGUGCA
ENSG00000169967	AAGUGCU
ENSG00000169967	ACCCUGU
ENSG00000169967	ACUGGCU
ENSG00000169967	AGCAAAA
ENSG00000169967	AGUGCUU
ENSG00000169967	AUACCUC
ENSG00000169967	CAAGAGC
ENSG00000169967	GAGAAAU
ENSG00000169967	GGUUACC
ENSG00000169992	AAAAAUC
ENSG00000169992	AAGUGCU
ENSG00000169992	AGCAUUC
ENSG00000169992	AGGAGCU
ENSG00000169992	AGGCAGG
ENSG00000169992	AGGGUUG
ENSG00000169992	CAUGGGU
ENSG00000169992	CCUGCUG
ENSG00000169992	CUCUUCC
ENSG00000169992	CUGGGCA
ENSG00000169992	CUGGGGG
ENSG00000169992	CUUUGGU
ENSG00000169992	GCUGGUG
ENSG00000169992	GGAAGAC
ENSG00000169992	GGAGUCC
ENSG00000169992	GGCACGG
ENSG00000169992	GGCAUUG
ENSG00000169992	GGGGUCC
ENSG00000169992	GUCUGCC
ENSG00000169992	UUGGCAA

ENSG00000170004	AAAGUAU
ENSG00000170004	CUUUGGU
ENSG00000170004	GACCCUG
ENSG00000170004	GGAAGAC
ENSG00000170004	GGCAGCG
ENSG00000170004	GUGGGGA
ENSG00000170004	UAUAUGA
ENSG00000170006	AGCAAAA
ENSG00000170006	AGCAGCA
ENSG00000170006	CUUCUCU
ENSG00000170006	CUUGGAG
ENSG00000170006	UUGUGUC
ENSG00000170011	AAAAAUC
ENSG00000170011	AACAUUC
ENSG00000170011	AAGGCAC
ENSG00000170011	AAUCACU
ENSG00000170011	CCCCUGG
ENSG00000170011	GAGGCAG
ENSG00000170011	GAGGUAG
ENSG00000170011	GAGGUAU
ENSG00000170011	GGAGGCA
ENSG00000170011	GGCAGUG
ENSG00000170011	GUAACAG
ENSG00000170011	UUUGCAA
ENSG00000170017	AAGGUGC
ENSG00000170017	ACAUAGA
ENSG00000170017	AUGGCUU
ENSG00000170017	CUUACAG
ENSG00000170017	UACAGUU
ENSG00000170017	UCUCAAG
ENSG00000170017	UGACCUA
ENSG00000170017	UGUAUAA
ENSG00000170027	AAAGAGG
ENSG00000170027	AACAGUC
ENSG00000170027	AAGUGCA
ENSG00000170027	ACAAUAU
ENSG00000170027	ACAUUCA
ENSG00000170027	ACCACAG
ENSG00000170027	ACCAGCA
ENSG00000170027	ACUGCAG
ENSG00000170027	ACUGGAU
ENSG00000170027	CCAGUAC
ENSG00000170027	CCCUGAG
ENSG00000170027	GGAGAGA
ENSG00000170027	UCACAUU
ENSG00000170027	UCCAGUU
ENSG00000170027	UUGGCAC
ENSG00000170035	AUAAAGU
ENSG00000170035	AUAAUAC
ENSG00000170035	GAGCUAA
ENSG00000170035	UAUAAAG
ENSG00000170035	UAUAAUA
ENSG00000170043	AAAACUG
ENSG00000170043	AAAGUUU
ENSG00000170043	GAGGUAG
ENSG00000170044	AAGUGCA
ENSG00000170044	AGUGCAA
ENSG00000170044	CUACAAA
ENSG00000170044	GAGGUAG
ENSG00000170044	UGAAGUG
ENSG00000170044	UUGAGGC
ENSG00000170049	CCGGAGC
ENSG00000170049	GCGGGGC
ENSG00000170049	GGCGGGG

ENSG00000170049	GUUCAUG
ENSG00000170075	CUGGCUC
ENSG00000170085	GAGGUAU
ENSG00000170085	UGUAUAA
ENSG00000170088	ACUGUAG
ENSG00000170088	GGCAGCG
ENSG00000170088	GUAAACA
ENSG00000170091	AAGCGCU
ENSG00000170091	ACUCCAG
ENSG00000170091	AUGCCUU
ENSG00000170091	CAGACAG
ENSG00000170091	CCAGGAG
ENSG00000170091	CUUACAG
ENSG00000170091	GACAACU
ENSG00000170091	UGUCUUU
ENSG00000170091	UUGGCAA
ENSG00000170091	UUGGCAC
ENSG00000170113	AAAGCUG
ENSG00000170113	AAGGCAC
ENSG00000170113	CCCUGAG
ENSG00000170113	CUUCUCU
ENSG00000170113	GAGCUAA
ENSG00000170113	GAGGCAG
ENSG00000170113	GGAAGCC
ENSG00000170113	GUGCAAA
ENSG00000170113	GUUAAUG
ENSG00000170113	UAGAGGA
ENSG00000170113	UAUAAAG
ENSG00000170113	UAUUGCU
ENSG00000170113	UCUGGAA
ENSG00000170113	UGCAUUG
ENSG00000170113	UUGGCAA
ENSG00000170113	UUGGUCC
ENSG00000170142	AACAAAC
ENSG00000170142	ACAGGUG
ENSG00000170142	AGCAGCU
ENSG00000170142	AGUAACA
ENSG00000170142	CUCCCAC
ENSG00000170142	UCCCACC
ENSG00000170144	AAAAACC
ENSG00000170144	AACCUGG
ENSG00000170144	AACCUGU
ENSG00000170144	AAGAACC
ENSG00000170144	AAGACAU
ENSG00000170144	ACAGUAC
ENSG00000170144	ACUCAGG
ENSG00000170144	ACUGCAU
ENSG00000170144	AGAUAAA
ENSG00000170144	AGCUGCC
ENSG00000170144	AUACCUC
ENSG00000170144	AUGACAC
ENSG00000170144	AUGCAUU
ENSG00000170144	AUGCCUU
ENSG00000170144	AUGGAUU
ENSG00000170144	AUGGCUU
ENSG00000170144	AUGUAAU
ENSG00000170144	CAAAGUG
ENSG00000170144	CAGGAAC
ENSG00000170144	CCUGCUG
ENSG00000170144	CCUUCUU
ENSG00000170144	CUCAAAC
ENSG00000170144	CUGACUC
ENSG00000170144	CUGCAGC
ENSG00000170144	CUGGAAA

ENSG00000170144	CUUCUCU
ENSG00000170144	CUUGUAU
ENSG00000170144	GCAGGUU
ENSG00000170144	GGCAUUG
ENSG00000170144	GUCAUUG
ENSG00000170144	UAUUGCU
ENSG00000170144	UCCUGAG
ENSG00000170144	UCCUUGC
ENSG00000170144	UCUGGAA
ENSG00000170144	UGAAGCU
ENSG00000170144	UGAUCCA
ENSG00000170144	UGCAAAG
ENSG00000170144	UGCAGCU
ENSG00000170144	UGCAUUG
ENSG00000170144	UGCCCUG
ENSG00000170144	UGCUGAC
ENSG00000170144	UGGGACA
ENSG00000170144	UGUGUCA
ENSG00000170144	UUGACAC
ENSG00000170144	UUGUGUC
ENSG00000170144	UUUGCAC
ENSG00000170153	AAAGCUG
ENSG00000170153	AGCUGCC
ENSG00000170153	GGAAGCC
ENSG00000170153	UAUUGCU
ENSG00000170153	UCUGGAG
ENSG00000170153	UGGAUAA
ENSG00000170162	UCUGGAA
ENSG00000170180	AAGUCUU
ENSG00000170185	AAGGCAC
ENSG00000170185	ACUGCAG
ENSG00000170185	ACUGCAU
ENSG00000170185	AGUACUG
ENSG00000170185	AUGAUGC
ENSG00000170185	CCCUGAG
ENSG00000170191	AGCACCA
ENSG00000170214	AAAGUGC
ENSG00000170214	AGCAGCU
ENSG00000170214	AGCUGCC
ENSG00000170214	CUGCCCC
ENSG00000170234	AAAAGUA
ENSG00000170234	AUUGCAC
ENSG00000170234	CUCCAUU
ENSG00000170234	GCACCAU
ENSG00000170242	AAAGUUU
ENSG00000170242	AAGGCAC
ENSG00000170242	ACUGCAU
ENSG00000170242	AGUGCUG
ENSG00000170242	AUCCCUU
ENSG00000170242	AUGAGCU
ENSG00000170242	AUGGCAC
ENSG00000170242	AUGUGCC
ENSG00000170242	AUUCAUU
ENSG00000170242	CAAAGCA
ENSG00000170242	CAGUCCA
ENSG00000170242	CAGUGCA
ENSG00000170242	CAGUUAC
ENSG00000170242	CCUGCUG
ENSG00000170242	CUACAAA
ENSG00000170242	GAGCUGC
ENSG00000170242	GAGGCUG
ENSG00000170242	GAGGUAG
ENSG00000170242	GAGGUAU
ENSG00000170242	GAUUGGU

ENSG00000170242	GCAGCAU
ENSG00000170242	GGUUACC
ENSG00000170242	GUAAACA
ENSG00000170242	GUUCAUG
ENSG00000170242	UACAGUU
ENSG00000170242	UAUAAAG
ENSG00000170242	UAUAAUA
ENSG00000170242	UCAUUCG
ENSG00000170242	UCUAGAG
ENSG00000170248	AAAGAAU
ENSG00000170248	AAUCUCU
ENSG00000170248	ACUGCAG
ENSG00000170248	AGCAGCA
ENSG00000170248	AUUGCAC
ENSG00000170248	CCUUCUG
ENSG00000170248	CUCUGGG
ENSG00000170248	CUGCUCA
ENSG00000170248	UAUUGCU
ENSG00000170248	UCCCUUU
ENSG00000170260	CAGCAGG
ENSG00000170260	GAGCCCC
ENSG00000170260	GAGGGGC
ENSG00000170260	GGAAGAC
ENSG00000170265	AGUGCAA
ENSG00000170265	GAGGUAG
ENSG00000170265	GGCGCCU
ENSG00000170266	UGAAGUG
ENSG00000170270	UUAGGAU
ENSG00000170270	UUGACAC
ENSG00000170271	GCCUGGA
ENSG00000170275	AAAUGGU
ENSG00000170275	AAGUAGC
ENSG00000170275	AAUGUUG
ENSG00000170275	ACUGUUU
ENSG00000170275	AUAAUAC
ENSG00000170275	CAAGAGC
ENSG00000170275	CAGACAG
ENSG00000170275	CCAGGAG
ENSG00000170275	CUACAAA
ENSG00000170275	CUAGGCU
ENSG00000170275	CUCUGGG
ENSG00000170275	CUGCAGG
ENSG00000170275	GAAGGAA
ENSG00000170275	GAGGUAG
ENSG00000170275	GGGUUUA
ENSG00000170275	UCACAGG
ENSG00000170275	UCACAUU
ENSG00000170275	UCCCUUU
ENSG00000170275	UGUGCUU
ENSG00000170289	AAUUAUU
ENSG00000170289	CAGCAAA
ENSG00000170289	CUUGGAG
ENSG00000170289	GAUAUGU
ENSG00000170289	GUAAACA
ENSG00000170289	UCUCAAG
ENSG00000170290	GCAGCAU
ENSG00000170296	AUGUGCC
ENSG00000170323	CAGUCCA
ENSG00000170325	AAAGUGC
ENSG00000170325	AACACUG
ENSG00000170325	AUGAUGA
ENSG00000170325	CCUGCUG
ENSG00000170325	CUUUGGU
ENSG00000170340	GAGGUAG

ENSG00000170340	GGCUCAG
ENSG00000170340	GUGAGGU
ENSG00000170345	AAAAAUC
ENSG00000170345	ACAUUCA
ENSG00000170345	ACUAGAU
ENSG00000170345	AGCACCA
ENSG00000170345	AGUAGUU
ENSG00000170345	CAGUAGU
ENSG00000170345	GAUCAGA
ENSG00000170345	GCUACAU
ENSG00000170348	AAGGCAC
ENSG00000170348	AAUUUUA
ENSG00000170348	CACCAGC
ENSG00000170348	CAGCAGA
ENSG00000170348	CAGCAGG
ENSG00000170348	CUCCCAC
ENSG00000170348	CUGGAAA
ENSG00000170348	GAGAAAU
ENSG00000170348	GCUAGCA
ENSG00000170348	GGCAUUG
ENSG00000170348	UACAGUU
ENSG00000170348	UGAGAAG
ENSG00000170348	UGAGAAU
ENSG00000170348	UGCAAAG
ENSG00000170348	UGUAUAA
ENSG00000170348	UUCUGCA
ENSG00000170365	AAAGUAA
ENSG00000170365	CAGUAGA
ENSG00000170365	CCUUCAU
ENSG00000170365	GAGGCAG
ENSG00000170370	AACAAAC
ENSG00000170370	AAGACAU
ENSG00000170370	AGUGCUU
ENSG00000170370	CAGUGCA
ENSG00000170370	CAUAUUG
ENSG00000170370	GCUACAU
ENSG00000170370	GUGCUUG
ENSG00000170374	AAAAGUA
ENSG00000170374	AAGUCAC
ENSG00000170374	AAUUAUU
ENSG00000170374	CCCUGAG
ENSG00000170374	CUAUACA
ENSG00000170374	CUGCCCC
ENSG00000170374	CUGGGGG
ENSG00000170374	GACCCUG
ENSG00000170374	GAUCAGA
ENSG00000170374	GCCUGGG
ENSG00000170374	GGGGCAG
ENSG00000170374	GGGGUCC
ENSG00000170374	GUCUGCC
ENSG00000170374	UAUUACC
ENSG00000170374	UCACAGG
ENSG00000170374	UCACAGU
ENSG00000170374	UCCAGUU
ENSG00000170374	UCCCUCU
ENSG00000170374	UGGGAUC
ENSG00000170374	UUGGCAC
ENSG00000170381	ACCACAG
ENSG00000170381	GAAGGAA
ENSG00000170382	AAAGGUA
ENSG00000170382	UACAAAG
ENSG00000170390	AAAACUG
ENSG00000170390	AAUUAUU
ENSG00000170390	AUUGCAC

ENSG00000170390	UAUUGCU
ENSG00000170390	UCCAGAG
ENSG00000170390	UGCCAGC
ENSG00000170412	AACACUG
ENSG00000170417	AUGGAUU
ENSG00000170417	CUACAAA
ENSG00000170417	GGAUUUC
ENSG00000170417	UCUGGAA
ENSG00000170419	UCCGCUC
ENSG00000170425	CACAGUG
ENSG00000170425	UCACAGG
ENSG00000170430	AGGACAC
ENSG00000170445	UUUUUGC
ENSG00000170448	AGAUAAA
ENSG00000170448	AUGGCUU
ENSG00000170454	UAAUAGU
ENSG00000170464	AAAAACC
ENSG00000170464	CUGCCCC
ENSG00000170464	UAUAAAG
ENSG00000170473	AGCAGCA
ENSG00000170473	CAGGAAC
ENSG00000170473	CAGUCCA
ENSG00000170473	GAGCCCC
ENSG00000170473	UUGUUCG
ENSG00000170482	UCCCUUG
ENSG00000170482	UCCCUUU
ENSG00000170485	AAUCUCA
ENSG00000170485	CCAGUGC
ENSG00000170485	GAAACAU
ENSG00000170485	GAUUGUA
ENSG00000170485	GAUUGUC
ENSG00000170485	GUGCUUG
ENSG00000170486	UAUUGCU
ENSG00000170498	UAUUGCU
ENSG00000170500	AAGUGCU
ENSG00000170500	AAUCACU
ENSG00000170500	GAGAAAU
ENSG00000170500	GAGUAUU
ENSG00000170500	UCACAAG
ENSG00000170500	UUGACAC
ENSG00000170515	AGGCAGG
ENSG00000170515	GAGGCAG
ENSG00000170515	GGAAGCC
ENSG00000170522	AAAGCUG
ENSG00000170522	AGCCCUU
ENSG00000170522	CACAGUG
ENSG00000170522	CCCUGAG
ENSG00000170523	CUGGCUC
ENSG00000170523	GUAUUCU
ENSG00000170525	AAAGUGC
ENSG00000170525	AAGUGCA
ENSG00000170525	AGUGCAA
ENSG00000170525	AUACAAG
ENSG00000170525	CAGACAG
ENSG00000170525	GUGCAAA
ENSG00000170525	UCACACA
ENSG00000170525	UUCAAGC
ENSG00000170525	UUGGCAC
ENSG00000170525	UUGGUCC
ENSG00000170537	AGCCAGU
ENSG00000170537	GAGGUAG
ENSG00000170549	UCAUUUG
ENSG00000170558	AAAGCUG
ENSG00000170558	AAAGGUA

ENSG00000170558	AAAUCCC
ENSG00000170558	AAGAACC
ENSG00000170558	AGCAUUC
ENSG00000170558	AGUGCUU
ENSG00000170558	AUAAUAC
ENSG00000170558	AUGUAAU
ENSG00000170558	CCAGUGC
ENSG00000170558	CUAGUAG
ENSG00000170558	GAUAUGU
ENSG00000170558	GGGGUCC
ENSG00000170558	GUGCCAC
ENSG00000170558	UAAUACA
ENSG00000170558	UACAAAG
ENSG00000170558	UACAGUU
ENSG00000170558	UCCAGUU
ENSG00000170558	UCCCUUU
ENSG00000170558	UGAAGUG
ENSG00000170558	UGGUACC
ENSG00000170558	UGUGCUU
ENSG00000170561	GUGACAG
ENSG00000170561	UGCAUUG
ENSG00000170561	UUGUGAC
ENSG00000170571	UUGGUUC
ENSG00000170577	AACAUUC
ENSG00000170577	ACAUUCA
ENSG00000170577	CUGCAGC
ENSG00000170577	UAUAAAG
ENSG00000170579	ACAGGUG
ENSG00000170579	AGGGUUG
ENSG00000170579	GACAACU
ENSG00000170579	GAGAACU
ENSG00000170579	GAGGCAG
ENSG00000170581	CAGCAGG
ENSG00000170581	CUGGAAA
ENSG00000170581	GUGCAAA
ENSG00000170581	UGGAGAU
ENSG00000170604	AAAGAGG
ENSG00000170604	AGCAGCA
ENSG00000170604	AUAAAGU
ENSG00000170604	GGGGCAG
ENSG00000170606	AAAGCUG
ENSG00000170616	AACUCUA
ENSG00000170616	AGGUAGU
ENSG00000170616	AUGGCUU
ENSG00000170616	CCUUCUG
ENSG00000170616	CCUUCUU
ENSG00000170616	GGCUCUG
ENSG00000170616	GGUAGAC
ENSG00000170616	UAUUGCU
ENSG00000170616	UGUGCGU
ENSG00000170616	UGUGCUU
ENSG00000170624	AAGGCAC
ENSG00000170624	AAUUAUU
ENSG00000170624	AAUUUUA
ENSG00000170624	AGAUAAA
ENSG00000170624	AGCAGCA
ENSG00000170624	CAAGAGC
ENSG00000170624	CACAGUG
ENSG00000170624	CAGCAGG
ENSG00000170624	CCAGUGU
ENSG00000170624	CCCCUGG
ENSG00000170624	CCCUGUC
ENSG00000170624	CUUUGGU
ENSG00000170624	GACCCUG

ENSG00000170624	GAGGUAG
ENSG00000170624	GGCAGCG
ENSG00000170624	GUGCUUG
ENSG00000170624	UCACAUU
ENSG00000170624	UCAUUUG
ENSG00000170624	UCCCUUU
ENSG00000170624	UCCUUGC
ENSG00000170624	UGCAUUG
ENSG00000170624	UGCUGAC
ENSG00000170624	UUGGUCC
ENSG00000170627	AAGUCAC
ENSG00000170633	AGCAUUC
ENSG00000170633	AGCCCUU
ENSG00000170638	AAGGCAC
ENSG00000170638	GAGGUAG
ENSG00000170647	AAAGUAC
ENSG00000170647	AAAGUAU
ENSG00000170647	AAAGUGC
ENSG00000170647	AAGUGCA
ENSG00000170647	AGAUAAA
ENSG00000170647	CAAAGUG
ENSG00000170647	UCCCUGU
ENSG00000170647	UGCAAAG
ENSG00000170647	UGUAUGU
ENSG00000170653	ACAGGUG
ENSG00000170653	CUUGGGC
ENSG00000170653	GUAACAG
ENSG00000170653	UCCUGAG
ENSG00000170653	UGCCCUG
ENSG00000170677	AAGUGCA
ENSG00000170677	AAUACUG
ENSG00000170677	ACUGGCC
ENSG00000170677	AGCAAAA
ENSG00000170677	AGCAGCG
ENSG00000170677	AGCAGCU
ENSG00000170677	AGCUUAU
ENSG00000170677	AGUGCAA
ENSG00000170677	AUUGCAC
ENSG00000170677	CAAAGUG
ENSG00000170677	CUGGCUC
ENSG00000170677	GAUAUGU
ENSG00000170677	GCACCAU
ENSG00000170677	GCUGGUG
ENSG00000170677	GGCAGCG
ENSG00000170677	GGCUCAG
ENSG00000170677	GUAGUGU
ENSG00000170677	GUGCAAA
ENSG00000170677	GUGCUUG
ENSG00000170677	UAAUGCU
ENSG00000170677	UAUUGCU
ENSG00000170677	UCAAGUA
ENSG00000170677	UCACAGU
ENSG00000170677	UUCAAGC
ENSG00000170681	CACUGUU
ENSG00000170681	GCUUCCU
ENSG00000170689	AGCAAAA
ENSG00000170689	CACAAUG
ENSG00000170689	CAGACAG
ENSG00000170689	CCCACCG
ENSG00000170689	CCCACCU
ENSG00000170689	CUCCCAC
ENSG00000170689	UCACAAG
ENSG00000170689	UCCCACC
ENSG00000170703	CAGUCUG

ENSG00000170703	GGGGCAG
ENSG00000170734	ACUCAGC
ENSG00000170734	AUGGAUU
ENSG00000170734	GUGCUUG
ENSG00000170734	UGUGCUU
ENSG00000170734	UUCAAAU
ENSG00000170743	ACCUGGA
ENSG00000170743	AGCACCA
ENSG00000170743	AUGUCUG
ENSG00000170743	CCUUCAU
ENSG00000170743	CUUUGGU
ENSG00000170743	GAGGCUG
ENSG00000170743	GGAGGCA
ENSG00000170743	GGCAGUG
ENSG00000170743	GUGCCUG
ENSG00000170743	UAGGUAU
ENSG00000170743	UGAAAGG
ENSG00000170743	UGCAUAU
ENSG00000170743	UUCCAUA
ENSG00000170743	UUCUAAU
ENSG00000170743	UUGGCAC
ENSG00000170743	UUGGUCC
ENSG00000170745	AUGGCUU
ENSG00000170745	UGAGAAU
ENSG00000170748	UGAAAGG
ENSG00000170759	AUUGCAC
ENSG00000170759	UAAGACG
ENSG00000170759	UAAGACU
ENSG00000170759	UUGGCAC
ENSG00000170776	AAAGUGC
ENSG00000170776	AAGUGCA
ENSG00000170776	ACACACU
ENSG00000170776	AGCACCA
ENSG00000170776	AGCCAGU
ENSG00000170776	CAGCUGG
ENSG00000170776	CCCUGAG
ENSG00000170776	CUCAAAC
ENSG00000170776	CUGGAAA
ENSG00000170776	GAGAUGA
ENSG00000170776	GAGGUAU
ENSG00000170776	GAUUGUA
ENSG00000170776	GAUUGUC
ENSG00000170776	GCAGCAU
ENSG00000170776	GUAUGUU
ENSG00000170776	UAAUAGU
ENSG00000170776	UACAAAG
ENSG00000170776	UGAAAUG
ENSG00000170776	UUCAAAU
ENSG00000170776	UUGACAC
ENSG00000170777	ACAUUCA
ENSG00000170777	CAAAGCA
ENSG00000170779	AAGACAU
ENSG00000170779	AGCACCA
ENSG00000170779	AGCAGCU
ENSG00000170779	UAAGACG
ENSG00000170779	UAAGACU
ENSG00000170779	UUCUAAU
ENSG00000170791	UAAUGCU
ENSG00000170801	CAGCAAA
ENSG00000170801	CAGUAGA
ENSG00000170801	GAAACAU
ENSG00000170801	GGCUCAG
ENSG00000170801	GUAAACA
ENSG00000170801	UGAGGAC

ENSG00000170802	ACUGGCC
ENSG00000170802	ACUGGCU
ENSG00000170802	AGUGGUU
ENSG00000170802	AUGAUGA
ENSG00000170802	AUGAUGC
ENSG00000170802	CCAUUAC
ENSG00000170802	GAAUUGU
ENSG00000170802	GAGUAUU
ENSG00000170802	GGCAGUG
ENSG00000170802	UGAGAAG
ENSG00000170819	CAAAGCA
ENSG00000170832	AAAACUG
ENSG00000170832	AAAUUUC
ENSG00000170832	AACUCUA
ENSG00000170832	AAGUGCA
ENSG00000170832	ACAUCAC
ENSG00000170832	AGUAGAC
ENSG00000170832	AGUGCAA
ENSG00000170832	CAGGAAC
ENSG00000170832	GAGGUAG
ENSG00000170832	GUGCUUG
ENSG00000170832	UGCAUUG
ENSG00000170832	UUCUAAU
ENSG00000170836	UGCAUAG
ENSG00000170852	AGCAGCA
ENSG00000170852	AGUGGUU
ENSG00000170854	ACUGGAU
ENSG00000170871	AAAGUGC
ENSG00000170871	AACAUUC
ENSG00000170871	AAUCCUU
ENSG00000170871	ACAGUAU
ENSG00000170871	ACCACAG
ENSG00000170871	AGCAGCU
ENSG00000170871	AGCCAGU
ENSG00000170871	AGCCCUU
ENSG00000170871	AUCCUUG
ENSG00000170871	CACAGUG
ENSG00000170871	CUAGUCC
ENSG00000170871	CUCAAAC
ENSG00000170871	CUGCAGC
ENSG00000170871	GAGCCCU
ENSG00000170871	GCAGAAG
ENSG00000170871	GCAGCAU
ENSG00000170871	GUCUCUG
ENSG00000170871	UCAGCGG
ENSG00000170871	UCAUACA
ENSG00000170871	UUAGGAU
ENSG00000170871	UUGGUCC
ENSG00000170873	AACACUG
ENSG00000170873	AGCCUGC
ENSG00000170873	AGGAGCU
ENSG00000170873	AUGCCUU
ENSG00000170873	CACAGGG
ENSG00000170873	CCUGCUG
ENSG00000170873	GGAAUGU
ENSG00000170873	UAUUGCU
ENSG00000170881	AACGGAA
ENSG00000170881	ACAAUAU
ENSG00000170881	ACAGUAC
ENSG00000170881	ACAGUAU
ENSG00000170881	ACAUCAC
ENSG00000170881	CACAGUG
ENSG00000170881	UCAACAG
ENSG00000170881	UCACAGG

ENSG00000170881	UUGGCAA
ENSG00000170899	GCACCAU
ENSG00000170899	UGGGAUC
ENSG00000170903	AAAGCUG
ENSG00000170903	AGCCCUU
ENSG00000170915	AAGGCAC
ENSG00000170915	AGCAAAA
ENSG00000170915	GCAGAAG
ENSG00000170915	GGCUCUG
ENSG00000170915	UCAUACA
ENSG00000170915	UCUGGAG
ENSG00000170915	UUGGCAC
ENSG00000170921	AAAGAGG
ENSG00000170921	AAAGUAA
ENSG00000170921	AAAGUAC
ENSG00000170921	AAAGUAU
ENSG00000170921	AAAGUGC
ENSG00000170921	AAAUGGU
ENSG00000170921	AACUACU
ENSG00000170921	AAGUAGC
ENSG00000170921	AAGUGCU
ENSG00000170921	AAUCACU
ENSG00000170921	ACAGUAC
ENSG00000170921	ACAUUCA
ENSG00000170921	ACCCAUU
ENSG00000170921	ACUGUAG
ENSG00000170921	ACUGUGU
ENSG00000170921	AGCCCUU
ENSG00000170921	AGGCAGG
ENSG00000170921	AGUGCAA
ENSG00000170921	AUAAAGU
ENSG00000170921	CACAGUG
ENSG00000170921	CACUCCU
ENSG00000170921	CAGCAAA
ENSG00000170921	CAGCAGA
ENSG00000170921	CAGUAGA
ENSG00000170921	CCAGCAU
ENSG00000170921	CCAGUGU
ENSG00000170921	CUAUACA
ENSG00000170921	CUCAGGG
ENSG00000170921	CUCCAUU
ENSG00000170921	CUGACUC
ENSG00000170921	CUGCAGG
ENSG00000170921	CUGCUCA
ENSG00000170921	CUUCUCU
ENSG00000170921	CUUGUGU
ENSG00000170921	GAGAACU
ENSG00000170921	GAGAUGA
ENSG00000170921	GAUUGUC
ENSG00000170921	GCUGGAU
ENSG00000170921	GGAGUCC
ENSG00000170921	GGCAGUG
ENSG00000170921	GGCAUUG
ENSG00000170921	GGUUACC
ENSG00000170921	GUGCCUG
ENSG00000170921	GUGCUUG
ENSG00000170921	GUGUGGC
ENSG00000170921	UAAUAGU
ENSG00000170921	UACAGUU
ENSG00000170921	UAGACUG
ENSG00000170921	UAUAAAG
ENSG00000170921	UCACAAG
ENSG00000170921	UCACACA
ENSG00000170921	UCACAUU

ENSG00000170921	UCCCUUU
ENSG00000170921	UCCUGAG
ENSG00000170921	UCCUUGC
ENSG00000170921	UCUGGAA
ENSG00000170921	UCUUGAG
ENSG00000170921	UGAUCCA
ENSG00000170921	UGCAAAG
ENSG00000170921	UGCAUUG
ENSG00000170921	UGGGUAC
ENSG00000170921	UGUGCUU
ENSG00000170921	UGUGUCA
ENSG00000170921	UUAGAGA
ENSG00000170921	UUCUGCA
ENSG00000170921	UUCUUGU
ENSG00000170921	UUGUGUC
ENSG00000170925	CUCCCAC
ENSG00000170925	CUUCUCU
ENSG00000170925	UCCCACA
ENSG00000170927	AAAGUAU
ENSG00000170927	GUGCAAA
ENSG00000170927	UUUGCAA
ENSG00000170927	UUUGCAC
ENSG00000170941	GGAGUCC
ENSG00000170941	GGAUUUU
ENSG00000170941	UAUUGCU
ENSG00000170941	UCCCUUG
ENSG00000170941	UCCCUUU
ENSG00000170961	AAAGUGC
ENSG00000170961	AAGUGCA
ENSG00000170961	AGCAGCA
ENSG00000170961	GAGGUAG
ENSG00000170961	GUGAGGU
ENSG00000170962	AACAAAC
ENSG00000170962	UCCAGUU
ENSG00000170967	UAUAAUA
ENSG00000170989	AAAGCUG
ENSG00000170989	AAUGUUG
ENSG00000170989	AAUUGCU
ENSG00000170989	AGUGCAA
ENSG00000170989	AUGUGCC
ENSG00000170989	UAAUGCU
ENSG00000170989	UGCAUUG
ENSG00000171004	ACCCUGU
ENSG00000171017	AAAUCCC
ENSG00000171017	ACAGGUG
ENSG00000171017	GGAGAGA
ENSG00000171033	CAAAGUG
ENSG00000171033	GAUUGGU
ENSG00000171033	GGCAGUG
ENSG00000171033	GGGGCAG
ENSG00000171044	AAGGCAC
ENSG00000171044	AAUACUG
ENSG00000171044	ACAGUAC
ENSG00000171044	ACAUAGA
ENSG00000171044	GUGACAG
ENSG00000171044	GUGCCUG
ENSG00000171055	GUGAAUG
ENSG00000171055	UAAUACA
ENSG00000171055	UAAUGCU
ENSG00000171056	AGCUUAU
ENSG00000171056	AUAUAAC
ENSG00000171056	AUGUCUG
ENSG00000171056	GGCUCAG
ENSG00000171056	UCACAGU

ENSG00000171067	AUUGCAC
ENSG00000171067	GCACCAU
ENSG00000171067	GGAAGCC
ENSG00000171094	UUGGCAC
ENSG00000171105	AGCAAAA
ENSG00000171105	AGCAGCG
ENSG00000171105	AUAUUAU
ENSG00000171105	CACAGUG
ENSG00000171105	CAGACAG
ENSG00000171105	CAGCAAA
ENSG00000171105	CCUUCAU
ENSG00000171105	CUUGUAU
ENSG00000171105	GAAAACA
ENSG00000171105	GAGGUAG
ENSG00000171105	GAGGUAU
ENSG00000171105	GGCUCUG
ENSG00000171105	GGUAGAC
ENSG00000171105	UAGACUG
ENSG00000171105	UCACAGG
ENSG00000171105	UGACAUC
ENSG00000171105	UUGGUCC
ENSG00000171109	AAAAACC
ENSG00000171109	CCCUGAG
ENSG00000171109	CUCAGGG
ENSG00000171119	GAGGUAG
ENSG00000171119	GAGGUAU
ENSG00000171121	UGGCCAC
ENSG00000171126	UGCUCAC
ENSG00000171132	ACAGUAU
ENSG00000171132	ACAUUCA
ENSG00000171132	AGAUAAA
ENSG00000171132	AGCCAGU
ENSG00000171132	AGCUUAU
ENSG00000171132	AUAAUAC
ENSG00000171132	AUACAAG
ENSG00000171132	AUACCUC
ENSG00000171132	AUGAUGC
ENSG00000171132	AUGCCUU
ENSG00000171132	AUGGAUU
ENSG00000171132	AUUGCAC
ENSG00000171132	CACAAUG
ENSG00000171132	CACUGUU
ENSG00000171132	CAGCAAA
ENSG00000171132	CAGCUGG
ENSG00000171132	CUACAGU
ENSG00000171132	GACUUCC
ENSG00000171132	GAGAUGA
ENSG00000171132	GAGUAUU
ENSG00000171132	GCCUGAU
ENSG00000171132	GUCAGUU
ENSG00000171132	GUGCCAC
ENSG00000171132	GUGCUUG
ENSG00000171132	UCACAAG
ENSG00000171132	UCACAUU
ENSG00000171132	UCAUAGA
ENSG00000171132	UGCAGCU
ENSG00000171132	UGUGCUU
ENSG00000171132	UUGGCAA
ENSG00000171150	AAGGCAC
ENSG00000171150	AGUGCAA
ENSG00000171150	AUACAAG
ENSG00000171150	GUGCAAA
ENSG00000171160	ACAGUAU
ENSG00000171160	CUCCAGC

ENSG00000171160	CUGUUGA
ENSG00000171160	GGAGAGA
ENSG00000171160	UCUCCAA
ENSG00000171169	CACAGUG
ENSG00000171169	CCCCUGG
ENSG00000171169	UCCUGAG
ENSG00000171169	UGCCAGC
ENSG00000171204	CAGCAAA
ENSG00000171206	AAAGUGC
ENSG00000171206	AAGUGCU
ENSG00000171206	CAAAGUG
ENSG00000171206	UACAAAG
ENSG00000171208	AUAAAGU
ENSG00000171208	UUGGCAC
ENSG00000171219	CCCUGAG
ENSG00000171219	GGGGUGG
ENSG00000171219	GGUGGGC
ENSG00000171219	UUGGCAC
ENSG00000171223	CCAGUGU
ENSG00000171223	GCGGGGC
ENSG00000171223	GGCGGGG
ENSG00000171224	UCCCUCU
ENSG00000171227	GGCAGGG
ENSG00000171246	AAAGUAU
ENSG00000171246	AACACUG
ENSG00000171246	ACAAUAU
ENSG00000171246	ACAGGUG
ENSG00000171246	ACCCAUU
ENSG00000171246	AGCAAAA
ENSG00000171246	AGUGCUU
ENSG00000171246	AUAAUAC
ENSG00000171246	AUGGCUU
ENSG00000171246	CAAAGUG
ENSG00000171246	CACAGUG
ENSG00000171246	CAGCAAA
ENSG00000171246	CCCACCG
ENSG00000171246	CCCACCU
ENSG00000171246	CUCAGGG
ENSG00000171246	CUCCCAC
ENSG00000171246	CUGCAGC
ENSG00000171246	CUUUGGU
ENSG00000171246	GAGAAGA
ENSG00000171246	GCUGGUG
ENSG00000171246	GGACCUG
ENSG00000171246	GGCAGGG
ENSG00000171246	UCCAGAG
ENSG00000171246	UCCCACC
ENSG00000171246	UCUAGAG
ENSG00000171246	UGCAGCU
ENSG00000171246	UGGGACA
ENSG00000171246	UUGGCAC
ENSG00000171246	UUGGUUC
ENSG00000171282	AAAAAUC
ENSG00000171282	AUGGCUU
ENSG00000171282	CAGUGCA
ENSG00000171282	CCUGUAC
ENSG00000171282	GAGAAAU
ENSG00000171282	GGUGGGC
ENSG00000171282	GUUCAUG
ENSG00000171282	UCCCUGU
ENSG00000171282	UGCAUAG
ENSG00000171282	UUCUGCA
ENSG00000171282	UUUUUGC
ENSG00000171302	AAAGUGC

ENSG00000171302	AGGAGCU
ENSG00000171302	GAGGUAG
ENSG00000171303	CUGGGGG
ENSG00000171303	GAGACCU
ENSG00000171303	GAGGUAU
ENSG00000171303	GCUGGUG
ENSG00000171303	GGACUGC
ENSG00000171303	UGCAAAG
ENSG00000171307	GCCUGAU
ENSG00000171310	UACAAAG
ENSG00000171314	ACAUCAC
ENSG00000171314	AGGGAGG
ENSG00000171314	AUUGCAC
ENSG00000171314	GGAAUGU
ENSG00000171314	UACAGUU
ENSG00000171314	UGCAGCU
ENSG00000171316	AAAGGUA
ENSG00000171316	AACAAAC
ENSG00000171316	AAGAACC
ENSG00000171316	AAGACAU
ENSG00000171316	ACAUCAC
ENSG00000171316	ACAUUCA
ENSG00000171316	ACUGUAG
ENSG00000171316	AGCAAAA
ENSG00000171316	AGCAGCA
ENSG00000171316	AGCUGCC
ENSG00000171316	AGUAACA
ENSG00000171316	CCAGUGC
ENSG00000171316	CUACAGU
ENSG00000171316	CUUACAG
ENSG00000171316	GAGGUAG
ENSG00000171316	GAGGUAU
ENSG00000171316	GAGUGUG
ENSG00000171316	GAUUGUC
ENSG00000171316	GGCAGCG
ENSG00000171316	GUGCCAC
ENSG00000171316	UAAUGCU
ENSG00000171316	UACAGUU
ENSG00000171316	UAUAAAG
ENSG00000171316	UAUUGCU
ENSG00000171316	UCAACAG
ENSG00000171316	UCACAGG
ENSG00000171316	UCACAGU
ENSG00000171316	UCACAUU
ENSG00000171316	UCAUACA
ENSG00000171316	UGACCUA
ENSG00000171316	UGCAUAU
ENSG00000171316	UGGUACC
ENSG00000171316	UGUAUAA
ENSG00000171316	UUAGAGA
ENSG00000171320	GCAGAAG
ENSG00000171320	UAUAAAG
ENSG00000171357	AAUCCUU
ENSG00000171357	ACAUCAC
ENSG00000171357	AGCAGCA
ENSG00000171357	GGGUAAG
ENSG00000171357	UGACAUC
ENSG00000171357	UUCCAUA
ENSG00000171365	AAAGAGG
ENSG00000171365	AAAGCUG
ENSG00000171365	AAAGUAA
ENSG00000171365	AAAGUAU
ENSG00000171365	AACAUUC
ENSG00000171365	AACUGGU

ENSG00000171365	AAGUAGC
ENSG00000171365	ACCCUGU
ENSG00000171365	ACCUGGA
ENSG00000171365	ACUGCAG
ENSG00000171365	ACUGGCU
ENSG00000171365	AGCUGCC
ENSG00000171365	AGGUAGU
ENSG00000171365	AGUACUG
ENSG00000171365	AGUAGAC
ENSG00000171365	AUAAAGU
ENSG00000171365	CAAAGCA
ENSG00000171365	CACAGUG
ENSG00000171365	CAGACAG
ENSG00000171365	CAUAUUG
ENSG00000171365	CCUUCUU
ENSG00000171365	CUACAAA
ENSG00000171365	CUCUUCC
ENSG00000171365	GACCUGG
ENSG00000171365	GAGAAGA
ENSG00000171365	GCAACUU
ENSG00000171365	GCACCAU
ENSG00000171365	GGCUCAG
ENSG00000171365	GGUUGAC
ENSG00000171365	GUAGUGU
ENSG00000171365	GUGCUUG
ENSG00000171365	GUGUCAC
ENSG00000171365	GUUAAUG
ENSG00000171365	UAAUGCU
ENSG00000171365	UAUUACC
ENSG00000171365	UCACAGG
ENSG00000171365	UCCCUUU
ENSG00000171365	UCCUGAG
ENSG00000171365	UCUGGAG
ENSG00000171365	UGAGAAG
ENSG00000171365	UGGAUAA
ENSG00000171365	UGUAUAA
ENSG00000171365	UGUGCUU
ENSG00000171365	UUGGCAC
ENSG00000171365	UUGGUUC
ENSG00000171368	AAAAAUC
ENSG00000171368	AAAGUGC
ENSG00000171368	AAAUUUC
ENSG00000171368	AUAAAGU
ENSG00000171368	AUAUAAC
ENSG00000171368	AUCCCUU
ENSG00000171368	CUAGUAG
ENSG00000171368	UCAAGUA
ENSG00000171368	UCCCUUG
ENSG00000171368	UUCAAGC
ENSG00000171368	UUGACAC
ENSG00000171388	AAGGCAC
ENSG00000171388	AGCAGCA
ENSG00000171388	AGCAGCG
ENSG00000171388	AGCAGCU
ENSG00000171388	AUAAAGU
ENSG00000171388	CAGGCUC
ENSG00000171388	CUGACUC
ENSG00000171388	GAGAAGA
ENSG00000171388	GAGACCU
ENSG00000171388	GGCCCUG
ENSG00000171388	UACAAAG
ENSG00000171388	UCCAGAG
ENSG00000171396	AGCAGCA
ENSG00000171396	AGCAGCU

ENSG00000171396	CAGCAGA
ENSG00000171396	CAGCUGG
ENSG00000171396	GCAGCAU
ENSG00000171403	CAGCUGG
ENSG00000171408	AAAGUUU
ENSG00000171408	AACAGUC
ENSG00000171408	AACUCUA
ENSG00000171408	AAUACUG
ENSG00000171408	ACAAGGU
ENSG00000171408	ACAGGUG
ENSG00000171408	ACAGUAU
ENSG00000171408	AGUGCAA
ENSG00000171408	AUGCAUU
ENSG00000171408	AUGGCUU
ENSG00000171408	CAAAGUG
ENSG00000171408	CAGCUGG
ENSG00000171408	CAGGCUC
ENSG00000171408	CAGUAGA
ENSG00000171408	CAGUAGU
ENSG00000171408	CUAUACA
ENSG00000171408	CUUUGGU
ENSG00000171408	GUGCAAA
ENSG00000171408	UCACAAG
ENSG00000171408	UCACAGG
ENSG00000171408	UCCAGUU
ENSG00000171408	UGCAAAG
ENSG00000171408	UUGGCAA
ENSG00000171408	UUGGCAC
ENSG00000171408	UUGUGAC
ENSG00000171435	AAAGAGC
ENSG00000171435	AACAGUC
ENSG00000171435	AAGUGCU
ENSG00000171435	ACAUAGA
ENSG00000171435	ACCCAUU
ENSG00000171435	ACUGUGU
ENSG00000171435	AGCUGCC
ENSG00000171435	CAGUUAC
ENSG00000171435	CCCUGAG
ENSG00000171435	CUCAAAC
ENSG00000171435	CUCAGGG
ENSG00000171435	CUCUGGG
ENSG00000171435	GAAGGAA
ENSG00000171435	GAGCUAA
ENSG00000171435	GAGCUGC
ENSG00000171435	GCUACAU
ENSG00000171435	GGCAGCG
ENSG00000171435	GGCCCUG
ENSG00000171435	GGUGGUC
ENSG00000171435	UAGACUG
ENSG00000171435	UAUAAAG
ENSG00000171435	UCCUGAG
ENSG00000171435	UCUAGAG
ENSG00000171435	UCUGGAA
ENSG00000171435	UGGCCCU
ENSG00000171435	UUCAAAU
ENSG00000171435	UUGGCAA
ENSG00000171443	AGCACCA
ENSG00000171443	CACCAGC
ENSG00000171444	AAAGUAA
ENSG00000171444	AAUACUG
ENSG00000171444	CUGCAGG
ENSG00000171444	GAAACAU
ENSG00000171444	GUGCUUG
ENSG00000171444	UCAAGUA

ENSG00000171444	UCCCUUU
ENSG00000171444	UCUGGAA
ENSG00000171444	UUCAAAU
ENSG00000171444	UUCAAGC
ENSG00000171450	GGGGUGG
ENSG00000171450	UUGGCAC
ENSG00000171451	AAAUCCC
ENSG00000171451	AACAAAC
ENSG00000171451	AAUCUCU
ENSG00000171451	AGCAGCA
ENSG00000171451	AGCCCUU
ENSG00000171451	AUAAUAC
ENSG00000171451	AUCACUA
ENSG00000171451	CAGGAAC
ENSG00000171451	CCUGCUG
ENSG00000171451	CUCCCAA
ENSG00000171451	CUCCCAC
ENSG00000171451	CUGCAGC
ENSG00000171451	GAAACAU
ENSG00000171451	GACCUGG
ENSG00000171451	GAGAAAU
ENSG00000171451	GCAGCAU
ENSG00000171451	GCAGGAC
ENSG00000171451	GGAGGCA
ENSG00000171451	GGCAGUG
ENSG00000171451	GGGGGAA
ENSG00000171451	GUAAACA
ENSG00000171451	UCUGGAG
ENSG00000171451	UGACAUC
ENSG00000171451	UGAGUCU
ENSG00000171451	UGCUGAC
ENSG00000171451	UUGACAC
ENSG00000171451	UUGAGGC
ENSG00000171451	UUUGCAA
ENSG00000171451	UUUGCAC
ENSG00000171456	AACACUG
ENSG00000171456	ACAAUAU
ENSG00000171456	ACACACC
ENSG00000171456	ACAGGUG
ENSG00000171456	ACCCUGU
ENSG00000171456	ACUGUGU
ENSG00000171456	AGUACUG
ENSG00000171456	AGUGCUU
ENSG00000171456	CAGCAGG
ENSG00000171456	CCAGUAC
ENSG00000171456	CUGGGCA
ENSG00000171456	CUUACAG
ENSG00000171456	GGUGGGC
ENSG00000171456	UAAUGCU
ENSG00000171456	UCACAUU
ENSG00000171456	UGCAUAG
ENSG00000171466	UCCCACA
ENSG00000171471	AUCCCUU
ENSG00000171475	AAAGAGC
ENSG00000171475	AAAGUGC
ENSG00000171475	AAGGCAC
ENSG00000171475	AAGUGCU
ENSG00000171475	ACACACC
ENSG00000171475	AGUGAUC
ENSG00000171475	AGUGCCG
ENSG00000171475	AGUGCCU
ENSG00000171475	CACAGUG
ENSG00000171475	CCCACCU
ENSG00000171475	CCCUGAG

ENSG00000171475	CCUUCUG
ENSG00000171475	CUCUUCC
ENSG00000171475	CUGGGCA
ENSG00000171475	GACCCUG
ENSG00000171475	GAGACCU
ENSG00000171475	GAGUUGG
ENSG00000171475	GAUCAGA
ENSG00000171475	GGAAGAC
ENSG00000171475	GUAGUGU
ENSG00000171475	UCAAGUA
ENSG00000171475	UCACAGG
ENSG00000171475	UCACAGU
ENSG00000171475	UCACAUU
ENSG00000171475	UCCCACA
ENSG00000171475	UCCCUUU
ENSG00000171475	UGAGAAG
ENSG00000171475	UGCAUAG
ENSG00000171475	UGUGCUU
ENSG00000171475	UUCAAGC
ENSG00000171475	UUGGUCC
ENSG00000171492	GCAGCAU
ENSG00000171502	AGCACCA
ENSG00000171502	AGGUAGU
ENSG00000171502	CCAGUAC
ENSG00000171502	GAGGUAG
ENSG00000171502	GUAGUGU
ENSG00000171502	UAUUGCU
ENSG00000171522	AUACCUC
ENSG00000171522	CUCACAC
ENSG00000171522	CUUGUAU
ENSG00000171522	GAGAAGA
ENSG00000171522	UCACAAG
ENSG00000171522	UCUCACC
ENSG00000171532	AAAAGUA
ENSG00000171532	AAAGAGG
ENSG00000171532	AAAGUGC
ENSG00000171532	AAGAACC
ENSG00000171532	AAGUGCA
ENSG00000171532	CAAAGUG
ENSG00000171532	CCCCUGG
ENSG00000171532	CGUCGCC
ENSG00000171532	CUUCACC
ENSG00000171532	GAUUGUA
ENSG00000171532	GGCUCAG
ENSG00000171532	UACAAAG
ENSG00000171532	UCACCAC
ENSG00000171532	UCCGCGC
ENSG00000171532	UCCUUGC
ENSG00000171532	UGCAUAG
ENSG00000171532	UGCAUAU
ENSG00000171532	UGUGCGU
ENSG00000171532	UUCCAUA
ENSG00000171532	UUCCUCG
ENSG00000171533	AAGUGCA
ENSG00000171533	AAUUUUA
ENSG00000171533	AGCAUUC
ENSG00000171533	CACAAUG
ENSG00000171533	CAGCAGG
ENSG00000171533	CUAUACA
ENSG00000171533	GUCAUUG
ENSG00000171533	GUGGCCC
ENSG00000171533	UGGCCAC
ENSG00000171540	AAAGAGC
ENSG00000171540	ACCAGCA

ENSG00000171540	ACUCCAG
ENSG00000171540	AGGGUUG
ENSG00000171540	AUAUUAU
ENSG00000171540	AUGUAAU
ENSG00000171540	CAGUCCA
ENSG00000171540	CUCAAAC
ENSG00000171540	CUCCAGC
ENSG00000171540	CUGCCCC
ENSG00000171540	GAUUGUA
ENSG00000171540	GAUUGUC
ENSG00000171540	GCAGAAG
ENSG00000171540	GUAACAG
ENSG00000171540	GUUCAUG
ENSG00000171540	UCACAUU
ENSG00000171540	UCAUUCG
ENSG00000171540	UCCCUUG
ENSG00000171540	UUCUGCA
ENSG00000171540	UUGUUCG
ENSG00000171551	CUGCCCC
ENSG00000171552	AAAAACC
ENSG00000171552	AAAGAGC
ENSG00000171552	AACAAAC
ENSG00000171552	AGCAGCU
ENSG00000171552	AGUGGUU
ENSG00000171552	CACCAGC
ENSG00000171552	CAGCAGA
ENSG00000171552	CUUCACC
ENSG00000171552	CUUGGGC
ENSG00000171552	GAGGCAG
ENSG00000171552	GAGGUAG
ENSG00000171552	GGAAGAC
ENSG00000171552	GGACGGA
ENSG00000171552	GGCAGGG
ENSG00000171552	GGGUGGU
ENSG00000171552	UAUUACC
ENSG00000171552	UUGGUCC
ENSG00000171560	ACAGUAU
ENSG00000171560	AGCACCA
ENSG00000171560	CUACAGU
ENSG00000171570	AGCAGCA
ENSG00000171570	GAGGUAG
ENSG00000171570	GAGGUAU
ENSG00000171570	UCACAUU
ENSG00000171570	UGCAGCU
ENSG00000171574	UCCCACA
ENSG00000171587	AAAGGUA
ENSG00000171587	GAGUCUU
ENSG00000171587	GUGCAAA
ENSG00000171587	UAUAAAG
ENSG00000171587	UUCUAAU
ENSG00000171587	UUUGCAC
ENSG00000171596	CUGCUCA
ENSG00000171603	AAUUUUA
ENSG00000171603	ACCAGCA
ENSG00000171603	ACUGGCU
ENSG00000171603	AGCAGCU
ENSG00000171603	CUCUGGG
ENSG00000171603	GAGUCUU
ENSG00000171603	GUCAACA
ENSG00000171603	GUCAGUU
ENSG00000171603	UACAAAG
ENSG00000171603	UGACCUA
ENSG00000171604	AUUGCAC
ENSG00000171608	AAAGUUU

ENSG00000171608	ACAAUAU
ENSG00000171608	AGAUGUG
ENSG00000171608	AUAAAGU
ENSG00000171608	AUGGCUU
ENSG00000171608	CAGCUGG
ENSG00000171608	UCACAGG
ENSG00000171608	UCACAGU
ENSG00000171608	UGCAAAG
ENSG00000171617	AAGAACC
ENSG00000171617	AAGUCAC
ENSG00000171617	AAGUGUG
ENSG00000171617	AAUCUCU
ENSG00000171617	ACAGGUG
ENSG00000171617	ACUAGAU
ENSG00000171617	AGCAAAA
ENSG00000171617	CAGCUGG
ENSG00000171617	GAAACAU
ENSG00000171617	GACAACU
ENSG00000171617	GCAGAAG
ENSG00000171617	GCCCUUA
ENSG00000171617	GUGAAUG
ENSG00000171617	GUGCAAA
ENSG00000171617	UACAAAG
ENSG00000171617	UCAAGUA
ENSG00000171617	UCACACA
ENSG00000171617	UGACCUA
ENSG00000171617	UGCAUUG
ENSG00000171617	UGUGUGG
ENSG00000171617	UUGUGUC
ENSG00000171621	AAAACUG
ENSG00000171621	AAACUGU
ENSG00000171621	AAAGAAU
ENSG00000171621	AAAGGUA
ENSG00000171621	AAAUGGU
ENSG00000171621	AGCAGCU
ENSG00000171621	AGGUUAU
ENSG00000171621	AUAAAGU
ENSG00000171621	AUAUUAU
ENSG00000171621	AUUGCAC
ENSG00000171621	GAGUUGG
ENSG00000171621	GGCUCAG
ENSG00000171621	GUGCUUG
ENSG00000171621	UCCUGAG
ENSG00000171621	UUGGCAC
ENSG00000171634	ACAUUCA
ENSG00000171634	AGCAGCA
ENSG00000171634	AGCAGCG
ENSG00000171634	AGCAGCU
ENSG00000171634	CUUCACC
ENSG00000171634	CUUGUAU
ENSG00000171634	GGCAUUG
ENSG00000171634	GUGCAAA
ENSG00000171634	UACAAAG
ENSG00000171634	UGAAAUG
ENSG00000171634	UGCAUAG
ENSG00000171634	UUAUGCA
ENSG00000171634	UUCAAAU
ENSG00000171634	UUCCAUA
ENSG00000171680	AAGUCUU
ENSG00000171680	UAUAAAG
ENSG00000171700	GCCUGGG
ENSG00000171700	UUUUUGC
ENSG00000171703	GAAACAU
ENSG00000171720	AAAAACC

ENSG00000171723	AACACUG
ENSG00000171723	AGCAUUC
ENSG00000171723	AGUAGUU
ENSG00000171723	AUGUGGG
ENSG00000171723	CAGACAG
ENSG00000171723	CAGUCUG
ENSG00000171723	CAGUUAC
ENSG00000171723	GAGAAAU
ENSG00000171723	GCUGGUG
ENSG00000171723	GCUGUCU
ENSG00000171723	UAAUAUC
ENSG00000171723	UAUGGUU
ENSG00000171723	UGCAUAG
ENSG00000171735	AACAGUC
ENSG00000171735	AACUACU
ENSG00000171735	AAGUCUU
ENSG00000171735	ACAGGUG
ENSG00000171735	ACAUUAC
ENSG00000171735	ACUGCAG
ENSG00000171735	ACUGGCC
ENSG00000171735	ACUGUGU
ENSG00000171735	ACUUGGA
ENSG00000171735	AGCAAAA
ENSG00000171735	AGCACCA
ENSG00000171735	AGCAGCU
ENSG00000171735	AGUGCAA
ENSG00000171735	AGUGCUG
ENSG00000171735	CAAAUGC
ENSG00000171735	CAGCAGG
ENSG00000171735	CCAGCAU
ENSG00000171735	CUAUACA
ENSG00000171735	GCACCAU
ENSG00000171735	GUGUGGC
ENSG00000171735	UAAUGCU
ENSG00000171735	UACAGUU
ENSG00000171735	UAUAAAG
ENSG00000171735	UGUGUGG
ENSG00000171735	UUGGCAA
ENSG00000171735	UUGGCAC
ENSG00000171777	AAAUUUC
ENSG00000171777	AGAUAAA
ENSG00000171777	CCUGCUG
ENSG00000171777	CUAGGCU
ENSG00000171786	AAAGUGC
ENSG00000171786	AAGGCAC
ENSG00000171786	AAGUGCA
ENSG00000171786	CAAAGUG
ENSG00000171786	GAUUGUC
ENSG00000171786	UAUAAAG
ENSG00000171791	AAAUGGU
ENSG00000171791	AAGUGUG
ENSG00000171791	AAUACUG
ENSG00000171791	AAUGCCC
ENSG00000171791	ACAUUAC
ENSG00000171791	ACCUGGA
ENSG00000171791	ACUGGAU
ENSG00000171791	ACUGUAG
ENSG00000171791	AGCAGCA
ENSG00000171791	AGCAGCG
ENSG00000171791	AGCAGCU
ENSG00000171791	AGCUUAU
ENSG00000171791	CACACCU
ENSG00000171791	CAGACAG
ENSG00000171791	CAUGGGU

ENSG00000171791	CCCUGAG
ENSG00000171791	CUCGGCG
ENSG00000171791	CUUGGAG
ENSG00000171791	GAAUUGU
ENSG00000171791	GAGAUGA
ENSG00000171791	GCUUCCU
ENSG00000171791	GGAUGAG
ENSG00000171791	GGCAGUG
ENSG00000171791	GGGUUUA
ENSG00000171791	GUAAACA
ENSG00000171791	UAAUACA
ENSG00000171791	UCCCUUG
ENSG00000171791	UGUCUGC
ENSG00000171791	UGUCUGG
ENSG00000171791	UUGACAC
ENSG00000171791	UUGGCAA
ENSG00000171791	UUGGCAC
ENSG00000171791	UUUCAAC
ENSG00000171812	CAAAGCA
ENSG00000171812	GAGGGGC
ENSG00000171812	UGUGUCA
ENSG00000171813	AGCAGCA
ENSG00000171813	UUGGUCC
ENSG00000171823	GGAAGAC
ENSG00000171823	GUGAAUG
ENSG00000171823	UCACAUU
ENSG00000171843	AAGGCAC
ENSG00000171843	ACUGGCU
ENSG00000171843	GAGAUGA
ENSG00000171843	GAGGCUG
ENSG00000171843	GUCAUUG
ENSG00000171843	UGUUGAA
ENSG00000171843	UUCAAGC
ENSG00000171843	UUGGUCC
ENSG00000171848	GAGGUAU
ENSG00000171848	UGAAGCU
ENSG00000171862	AAAGUGC
ENSG00000171862	AAUACUG
ENSG00000171862	ACAGUAU
ENSG00000171862	ACCACAG
ENSG00000171862	AGAUAAA
ENSG00000171862	AUGCCUU
ENSG00000171862	CACAAUG
ENSG00000171862	CCUUCAU
ENSG00000171862	CUGGGCA
ENSG00000171862	GAGGCUG
ENSG00000171862	GAUUGGU
ENSG00000171862	GUUAAUG
ENSG00000171862	UGCAAAG
ENSG00000171862	UGCAUAG
ENSG00000171862	UUCAAGC
ENSG00000171864	CAUAUUG
ENSG00000171864	GAGAAGA
ENSG00000171864	UGAGAAG
ENSG00000171864	UGAGAAU
ENSG00000171867	AGUGCAA
ENSG00000171867	GUCUACU
ENSG00000171867	UGCAAAG
ENSG00000171873	GUAAACA
ENSG00000171877	CUAGGCU
ENSG00000171877	GGAGGAG
ENSG00000171877	UCCCUUU
ENSG00000171885	AAAGGUA
ENSG00000171885	AGCACCA

ENSG00000171885	CAGUGCA
ENSG00000171885	CCUGCUG
ENSG00000171885	GAGAAGA
ENSG00000171885	GUCAUUG
ENSG00000171885	UUCCUAG
ENSG00000171914	AGGAGCU
ENSG00000171914	AUAAUAC
ENSG00000171914	AUACAAG
ENSG00000171914	CCCCUGG
ENSG00000171914	CUGCCCC
ENSG00000171914	GCCCUUA
ENSG00000171914	GGAGUCC
ENSG00000171914	GGCAAGA
ENSG00000171914	GUAACAG
ENSG00000171914	GUGACAG
ENSG00000171914	GUGCAAA
ENSG00000171914	UCACAUU
ENSG00000171928	AGCAGCA
ENSG00000171940	AAAACUG
ENSG00000171940	AAAAGUA
ENSG00000171940	AAAGUGC
ENSG00000171940	AAGUGCA
ENSG00000171940	ACUGUAG
ENSG00000171940	AGCAGCG
ENSG00000171940	AGCUUAU
ENSG00000171940	AUGGCUU
ENSG00000171940	CAGCAGA
ENSG00000171940	CAGCAGG
ENSG00000171940	CAGUAGA
ENSG00000171940	CAGUGCA
ENSG00000171940	GACAACU
ENSG00000171940	GGAAUGU
ENSG00000171940	UAUAAUA
ENSG00000171940	UAUUGCU
ENSG00000171940	UCAAGUA
ENSG00000171940	UCCUGAG
ENSG00000171940	UGCAUAG
ENSG00000171940	UGCAUAU
ENSG00000171940	UGGUACC
ENSG00000171940	UUGGUCC
ENSG00000171951	AACAAAC
ENSG00000171954	CAGCAAA
ENSG00000171954	GAGGGGC
ENSG00000171954	GCGGGGC
ENSG00000171954	GGCGGGG
ENSG00000171988	AAAUUUC
ENSG00000171988	AACAAAC
ENSG00000171988	AACACUG
ENSG00000171988	ACAAUAU
ENSG00000171988	ACAUAGA
ENSG00000171988	AUAAAGU
ENSG00000171988	CACAGUG
ENSG00000171988	GCUGGUG
ENSG00000171988	GGCAGUG
ENSG00000171988	GUAACAG
ENSG00000171988	GUAGUGU
ENSG00000171988	UCACAGU
ENSG00000171988	UUGAUCA
ENSG00000171988	UUGGCAA
ENSG00000171988	UUGUGUC
ENSG00000171992	AAAAAUC
ENSG00000171992	AAAGGUA
ENSG00000171992	ACAAGGU
ENSG00000171992	CCUGCUG

ENSG00000171992	CUGGGCA
ENSG00000171992	GAAAACA
ENSG00000171992	GCAGAAG
ENSG00000171992	GCAGGUU
ENSG00000171992	GGAGGCA
ENSG00000171992	GGCAGUG
ENSG00000171992	UCUCAAG
ENSG00000172007	AUGGCUU
ENSG00000172007	CUUUGGU
ENSG00000172007	GUGCAAA
ENSG00000172007	UCCCUUG
ENSG00000172007	UCCCUUU
ENSG00000172007	UGCAAAG
ENSG00000172007	UUCCUCG
ENSG00000172020	AAAGAGG
ENSG00000172020	AACGGAA
ENSG00000172020	ACACACU
ENSG00000172020	AGUGCAA
ENSG00000172020	AUUGCAC
ENSG00000172020	CACUGUU
ENSG00000172020	CCUGCUG
ENSG00000172053	GAGGUAG
ENSG00000172057	AAGUGCU
ENSG00000172057	AAUACUG
ENSG00000172057	ACUGUAG
ENSG00000172057	AGUGCUU
ENSG00000172057	CAGGCUC
ENSG00000172057	CUCUGGG
ENSG00000172057	GUCCAGA
ENSG00000172057	UACAAAG
ENSG00000172057	UCACACA
ENSG00000172057	UCCAGAG
ENSG00000172059	AAAGUGC
ENSG00000172059	AAGUGCA
ENSG00000172059	AGCAAAA
ENSG00000172059	AGUGCAA
ENSG00000172059	AUAAAGU
ENSG00000172059	CCCUGUC
ENSG00000172059	GUAAACA
ENSG00000172059	UAUUGCU
ENSG00000172061	CAGGAAC
ENSG00000172061	CUCACAC
ENSG00000172061	GGAGAGA
ENSG00000172061	GUCCAGA
ENSG00000172061	GUGACUG
ENSG00000172071	AAAGUAC
ENSG00000172071	ACACACU
ENSG00000172071	AUGGAUU
ENSG00000172071	CACCACC
ENSG00000172071	UAGACUG
ENSG00000172071	UGAAAGG
ENSG00000172081	AGUGGUU
ENSG00000172113	AAAGAGG
ENSG00000172113	GAGAAGA
ENSG00000172113	GAGGUAG
ENSG00000172115	AACACUG
ENSG00000172115	AAGGCAC
ENSG00000172115	GGCAGUG
ENSG00000172115	UUGGCAA
ENSG00000172137	AUCAUAC
ENSG00000172137	UCAUACA
ENSG00000172159	GGCAUUG
ENSG00000172159	UGUAUAA
ENSG00000172175	CAAAGUG

ENSG00000172175	CUCAAAC
ENSG00000172175	UGCCAGC
ENSG00000172175	UUCAAAU
ENSG00000172175	UUCUUGU
ENSG00000172197	AAAACUG
ENSG00000172197	AACAAAC
ENSG00000172197	AAGGCAC
ENSG00000172197	AGGCAGG
ENSG00000172197	AGUGCUG
ENSG00000172197	CUCCCAC
ENSG00000172197	GCACCAU
ENSG00000172197	GCAGAAG
ENSG00000172197	GGAGAGA
ENSG00000172197	GGCAGGG
ENSG00000172197	GGGGUGG
ENSG00000172197	UACAGUU
ENSG00000172197	UGGGGGA
ENSG00000172197	UUCUGCA
ENSG00000172201	AAAAAUC
ENSG00000172201	AAAGCUG
ENSG00000172201	ACAGUAC
ENSG00000172201	AGAUGUG
ENSG00000172201	AUACAAG
ENSG00000172201	AUGUAAU
ENSG00000172201	CUACAAA
ENSG00000172201	CUGGCUC
ENSG00000172201	GUAACAG
ENSG00000172201	UACAGUU
ENSG00000172201	UGAAAUG
ENSG00000172201	UUGGUCC
ENSG00000172209	GGCAGUG
ENSG00000172216	ACUCAGG
ENSG00000172216	CAGUUAC
ENSG00000172239	AUUGCAC
ENSG00000172239	CUACAAA
ENSG00000172239	UAUUGCU
ENSG00000172239	UGCAGCU
ENSG00000172239	UUGGCAA
ENSG00000172239	UUGGCAC
ENSG00000172260	AAAACUG
ENSG00000172260	AAAGCUG
ENSG00000172260	AACUCUA
ENSG00000172260	AAGUUGU
ENSG00000172260	ACAAUAU
ENSG00000172260	ACAGUAC
ENSG00000172260	AGAUAAA
ENSG00000172260	AGCACCA
ENSG00000172260	AUGCAUU
ENSG00000172260	AUUGCAC
ENSG00000172260	CCCACGU
ENSG00000172260	CUACAGU
ENSG00000172260	CUAGUAA
ENSG00000172260	CUCCCAA
ENSG00000172260	GACAACU
ENSG00000172260	GACCAUG
ENSG00000172260	GAGGUAU
ENSG00000172260	GAGUGUG
ENSG00000172260	GGAGUGU
ENSG00000172260	GGGUCUU
ENSG00000172260	GUAAACA
ENSG00000172260	GUCACUC
ENSG00000172260	GUCUACU
ENSG00000172260	GUGCCAC
ENSG00000172260	GUGUCAC

ENSG00000172260	UCACACA
ENSG00000172260	UGCAGCU
ENSG00000172260	UGGAGAU
ENSG00000172260	UUCUAAU
ENSG00000172260	UUGGUUC
ENSG00000172262	CUUUGGU
ENSG00000172264	AAGGCAC
ENSG00000172264	AAUCCUU
ENSG00000172264	AGCUGCC
ENSG00000172264	GGGUAAG
ENSG00000172264	UAGAGGA
ENSG00000172264	UAUAAAG
ENSG00000172264	UGCAUUG
ENSG00000172292	AAAGUGC
ENSG00000172292	AACACUG
ENSG00000172292	AAGUGCU
ENSG00000172292	ACAGUAC
ENSG00000172292	ACUAGAU
ENSG00000172292	ACUGUUU
ENSG00000172292	AUAAAGU
ENSG00000172292	AUGGCAC
ENSG00000172292	CAGACAG
ENSG00000172292	CAGUGCA
ENSG00000172292	CUUUGGU
ENSG00000172292	GUGUCAC
ENSG00000172292	UCACAGG
ENSG00000172292	UCACAGU
ENSG00000172292	UCAUUCG
ENSG00000172292	UUAUGCA
ENSG00000172292	UUGGCAA
ENSG00000172292	UUGUGUC
ENSG00000172296	CAGUGCA
ENSG00000172296	CUGGGCA
ENSG00000172340	AAGGCAC
ENSG00000172340	GAGGUAG
ENSG00000172340	GAGGUAU
ENSG00000172346	CACAGUG
ENSG00000172346	CAGACAG
ENSG00000172346	CCAGUGU
ENSG00000172346	UAUUGCU
ENSG00000172346	UCACAGU
ENSG00000172346	UGCCAGC
ENSG00000172348	AUACAAG
ENSG00000172348	CUUUGGU
ENSG00000172348	GCAGAAG
ENSG00000172348	GGAAUGU
ENSG00000172348	UACAAAG
ENSG00000172348	UAUUGCU
ENSG00000172348	UCACCAC
ENSG00000172348	UGCAUAG
ENSG00000172348	UGCAUAU
ENSG00000172349	GAAGGUC
ENSG00000172349	GACCCUG
ENSG00000172349	UGAGAAU
ENSG00000172350	AAAGUGC
ENSG00000172350	AAAUCCC
ENSG00000172350	AACAGUC
ENSG00000172350	AAGUGCA
ENSG00000172350	ACUGUAG
ENSG00000172350	AGCAGCA
ENSG00000172350	AUAAAGU
ENSG00000172350	AUUGCAC
ENSG00000172350	CUCCAGC
ENSG00000172350	CUGGGGG

ENSG00000172350	CUUGUGU
ENSG00000172350	CUUUGGU
ENSG00000172350	GACUUCC
ENSG00000172350	GCCCUGU
ENSG00000172350	GUCUGCC
ENSG00000172350	GUGCCUG
ENSG00000172350	GUGUGGC
ENSG00000172350	UACAAAG
ENSG00000172350	UAUUGCU
ENSG00000172350	UCCAGAG
ENSG00000172350	UCCAGUU
ENSG00000172350	UCUCCAA
ENSG00000172350	UGUGCUU
ENSG00000172350	UGUGUGG
ENSG00000172350	UUGGUCC
ENSG00000172354	GUAGUGU
ENSG00000172375	CUGCCCC
ENSG00000172375	GAGCUGC
ENSG00000172375	GGAGAGA
ENSG00000172375	UCAAGUA
ENSG00000172375	UCACACA
ENSG00000172375	UGGGACA
ENSG00000172379	ACAAUAU
ENSG00000172379	ACAUUAC
ENSG00000172379	CAGUUAC
ENSG00000172379	UCUAGAG
ENSG00000172380	AAAAAUC
ENSG00000172380	AAGUGCU
ENSG00000172380	AGCAGCU
ENSG00000172380	AGGAGCU
ENSG00000172380	AGUGGUU
ENSG00000172380	CACAGUG
ENSG00000172380	CCAGCAU
ENSG00000172380	GAGCUGC
ENSG00000172380	GCUGGAU
ENSG00000172382	UACAAAG
ENSG00000172399	UGCCAGC
ENSG00000172399	UUCAAAU
ENSG00000172403	AGCAGCU
ENSG00000172403	UCACAUU
ENSG00000172403	UGAGUCU
ENSG00000172404	UAAUGCU
ENSG00000172404	UCACCAC
ENSG00000172404	UUCAAAU
ENSG00000172432	AAAGCUG
ENSG00000172432	AAAUCCC
ENSG00000172432	AACAAAC
ENSG00000172432	AAGUCAC
ENSG00000172432	AGCACCA
ENSG00000172432	GAGAAGA
ENSG00000172432	GAGGUAU
ENSG00000172432	GGGAACG
ENSG00000172432	GUGGGGA
ENSG00000172432	UCCCUUU
ENSG00000172432	UGAUCCA
ENSG00000172432	UGCAGCU
ENSG00000172432	UUCCUCG
ENSG00000172458	GCCCUUA
ENSG00000172458	UUCAAAU
ENSG00000172461	AAAGAAU
ENSG00000172461	AGCCCUU
ENSG00000172461	CACAAUG
ENSG00000172461	CACAGUG
ENSG00000172461	CAGUAGU

ENSG00000172461	CAGUGCA
ENSG00000172461	GGCAUUG
ENSG00000172461	GUGCAAA
ENSG00000172461	GUGCUUG
ENSG00000172461	UCACAGU
ENSG00000172461	UCCAGUU
ENSG00000172461	UUGGCAA
ENSG00000172461	UUGGCAC
ENSG00000172466	CACAGUG
ENSG00000172466	GGAAUGU
ENSG00000172466	UCAUUUG
ENSG00000172469	GAGAAAU
ENSG00000172493	AACACUG
ENSG00000172493	AAGUGCA
ENSG00000172493	AAUACUG
ENSG00000172493	ACAUUCA
ENSG00000172493	CUCAGGG
ENSG00000172493	CUGCUCA
ENSG00000172493	CUUUGGU
ENSG00000172493	GAGGCUG
ENSG00000172493	GAGUUGG
ENSG00000172493	GCUGGUG
ENSG00000172493	GUAACAG
ENSG00000172493	GUAGUGU
ENSG00000172493	UCACAUU
ENSG00000172493	UCUAGAG
ENSG00000172493	UGCAUAG
ENSG00000172497	ACUGCAU
ENSG00000172530	AAAAGUA
ENSG00000172530	AGCAAAA
ENSG00000172530	CAGCAAA
ENSG00000172530	UAUAAAG
ENSG00000172530	UCUUGAG
ENSG00000172530	UUUUUGC
ENSG00000172531	CCCUGAG
ENSG00000172531	CUGCAGC
ENSG00000172531	GAGCUGC
ENSG00000172531	UGCCCUG
ENSG00000172534	AAAAACC
ENSG00000172534	CUACAAA
ENSG00000172534	GCAGCAU
ENSG00000172534	GUGCUUG
ENSG00000172534	UAAUACA
ENSG00000172534	UAUAAUA
ENSG00000172534	UCCUGAG
ENSG00000172534	UCUGGAG
ENSG00000172534	UGAGUCU
ENSG00000172534	UGUGCUU
ENSG00000172534	UUCAAAU
ENSG00000172572	UCACAGU
ENSG00000172572	UGUGCUU
ENSG00000172572	UGUGUCA
ENSG00000172572	UUGUGUC
ENSG00000172575	AUCAUAC
ENSG00000172575	GAGGUAU
ENSG00000172575	UCACAAG
ENSG00000172575	UGUCUUU
ENSG00000172578	AGUGCCU
ENSG00000172578	AUGAUGA
ENSG00000172578	CAGUUAC
ENSG00000172578	CCAGUGC
ENSG00000172578	CCAGUGU
ENSG00000172578	GAGGUAG
ENSG00000172578	GAGGUAU

ENSG00000172578	GUGCCUG
ENSG00000172578	UCCAGUU
ENSG00000172578	UCCCUGU
ENSG00000172578	UCCUGAG
ENSG00000172590	AAAACUG
ENSG00000172590	GGCAGUG
ENSG00000172613	AAAGCUG
ENSG00000172613	AAAGGUA
ENSG00000172613	CAAAGCA
ENSG00000172613	CCCCCAG
ENSG00000172613	CUCAGGG
ENSG00000172613	CUGCAGC
ENSG00000172613	GCUGGAU
ENSG00000172613	GGCAGCG
ENSG00000172613	UCAUGAU
ENSG00000172613	UGCAGCU
ENSG00000172638	CCCACCU
ENSG00000172638	UCCCUUU
ENSG00000172660	AGCAGCA
ENSG00000172660	AUUGCAC
ENSG00000172660	CAGCAGG
ENSG00000172660	CCCACCG
ENSG00000172660	CCCACCU
ENSG00000172660	CUCCCAC
ENSG00000172660	UACAAAG
ENSG00000172660	UAUUGCU
ENSG00000172660	UCCCACC
ENSG00000172660	UUGGCAC
ENSG00000172661	ACCCUGU
ENSG00000172661	GCAGAAG
ENSG00000172663	UCCUGAG
ENSG00000172667	AGAUAAA
ENSG00000172667	AGGCAGG
ENSG00000172667	CAAAGCA
ENSG00000172667	GGAAUGU
ENSG00000172667	UGGUACC
ENSG00000172728	GUCAACA
ENSG00000172728	UUCUAAU
ENSG00000172731	AGGGAGG
ENSG00000172731	AUUGCAC
ENSG00000172731	CUCUGGG
ENSG00000172731	GAGGUAG
ENSG00000172731	UAUUGCU
ENSG00000172733	UGUGCUU
ENSG00000172738	GAGAAGA
ENSG00000172752	CAAAGCA
ENSG00000172757	AACAUUC
ENSG00000172757	AGCCUGC
ENSG00000172757	CCCCCAG
ENSG00000172757	CCUGCUG
ENSG00000172757	GAAGGUC
ENSG00000172757	GGCAGCG
ENSG00000172757	GGCAGGG
ENSG00000172757	GGGGCAG
ENSG00000172757	UUGGCAA
ENSG00000172757	UUGGCAC
ENSG00000172765	AAAGCUG
ENSG00000172765	AAGUGCU
ENSG00000172765	AAUACUG
ENSG00000172765	ACAGUAC
ENSG00000172765	ACAGUAU
ENSG00000172765	ACAUCAC
ENSG00000172765	AGCAGCG
ENSG00000172765	AGUGCUG

ENSG00000172765	AUUGCAC
ENSG00000172765	CACAGUG
ENSG00000172765	CUACAGU
ENSG00000172765	CUAUACA
ENSG00000172765	CUUGGAG
ENSG00000172765	CUUUGGU
ENSG00000172765	GUCAUUG
ENSG00000172765	UCACAGG
ENSG00000172765	UGAUCCA
ENSG00000172765	UUCAAAU
ENSG00000172765	UUCAAGC
ENSG00000172780	AAGGCAC
ENSG00000172780	CAAAUGC
ENSG00000172780	GCAACUU
ENSG00000172780	GGCAGUG
ENSG00000172780	UAUAAAG
ENSG00000172780	UUUGCAA
ENSG00000172782	CCAGUGU
ENSG00000172782	GUCUUGC
ENSG00000172789	AUGACAC
ENSG00000172789	CAGGAAC
ENSG00000172789	GAUAUGU
ENSG00000172794	AAGUGCA
ENSG00000172794	CCCCUGG
ENSG00000172794	GUCCAGA
ENSG00000172794	UCCAGAG
ENSG00000172794	UGGGACA
ENSG00000172795	AUGCAUU
ENSG00000172795	AUGGCUU
ENSG00000172795	AUUGCAC
ENSG00000172795	CAAAGCA
ENSG00000172795	CACAGUG
ENSG00000172795	CAGUAGU
ENSG00000172795	CUACAAA
ENSG00000172795	CUCAGGG
ENSG00000172795	CUUACAG
ENSG00000172795	CUUUGGU
ENSG00000172795	GAAUUGU
ENSG00000172795	GAUUGUA
ENSG00000172795	GAUUGUC
ENSG00000172795	GGAAUGU
ENSG00000172795	GGAGGCA
ENSG00000172795	GUAAACA
ENSG00000172795	GUGCUUG
ENSG00000172795	UACAGUU
ENSG00000172795	UCUGGAA
ENSG00000172795	UGCAUAG
ENSG00000172795	UGCAUAU
ENSG00000172795	UGUGCUU
ENSG00000172795	UUGGUUC
ENSG00000172803	GCCCUGU
ENSG00000172818	AUAUUAU
ENSG00000172818	CCCUGAG
ENSG00000172818	GAUAUGU
ENSG00000172818	GCCCUGU
ENSG00000172818	UGAAAUG
ENSG00000172818	UUUUUGC
ENSG00000172819	AAGGCAC
ENSG00000172819	ACUCAGC
ENSG00000172819	AGGAGCU
ENSG00000172819	CAGUCUG
ENSG00000172819	CCCCUGG
ENSG00000172819	CUCUGGG
ENSG00000172819	GAGAUGA

ENSG00000172819	GCUUCCU
ENSG00000172819	GGAGGAG
ENSG00000172819	GGCAGCG
ENSG00000172819	GGCCCUG
ENSG00000172819	GGCUCAG
ENSG00000172819	GUAGUGU
ENSG00000172819	UGCAAAG
ENSG00000172819	UUUGCAC
ENSG00000172828	CUAGGCU
ENSG00000172830	AGCCCUU
ENSG00000172830	GAGCCCC
ENSG00000172830	UACAAAG
ENSG00000172830	UCUUGAG
ENSG00000172831	GUGAAUG
ENSG00000172840	GAGGUAG
ENSG00000172840	GUAAACA
ENSG00000172840	UUCCAUA
ENSG00000172845	AAAAGUA
ENSG00000172845	AAAGUAA
ENSG00000172845	AAAGUAC
ENSG00000172845	AAGAACC
ENSG00000172845	AAGACAU
ENSG00000172845	AAGGCAC
ENSG00000172845	ACCACAG
ENSG00000172845	CAAAUGC
ENSG00000172845	CAGUUAC
ENSG00000172845	CUACAAA
ENSG00000172845	GGCCCUG
ENSG00000172845	UAAUGCU
ENSG00000172845	UAGACUG
ENSG00000172845	UCAUACA
ENSG00000172845	UCCAGUU
ENSG00000172845	UCUCACC
ENSG00000172845	UUAUGCA
ENSG00000172845	UUGGCAC
ENSG00000172867	UGCAAAG
ENSG00000172869	AAGGCAC
ENSG00000172869	AGCCCUU
ENSG00000172869	AGGUUAU
ENSG00000172869	GAUUGUA
ENSG00000172869	GAUUGUC
ENSG00000172869	GCAACUU
ENSG00000172869	GCUGUCU
ENSG00000172869	UAUUGCU
ENSG00000172869	UCAAGUA
ENSG00000172869	UCCUGAG
ENSG00000172901	AAAGAAU
ENSG00000172915	AAAAGUA
ENSG00000172915	AAAGAGG
ENSG00000172915	AAUCACU
ENSG00000172915	ACAUUCA
ENSG00000172915	AGCCCUU
ENSG00000172915	AGCUUAU
ENSG00000172915	AUGGCUU
ENSG00000172915	CAAAGUG
ENSG00000172915	CAAAUGC
ENSG00000172915	CAGCUGG
ENSG00000172915	CCCCUGG
ENSG00000172915	CUACAAA
ENSG00000172915	CUGGAAA
ENSG00000172915	GAGGCUG
ENSG00000172915	GAGUGUG
ENSG00000172915	GCUGGUG
ENSG00000172915	GGUUACC

ENSG00000172915	UCACACA
ENSG00000172915	UGCAAAG
ENSG00000172915	UGCAUAG
ENSG00000172915	UGUGCUU
ENSG00000172915	UUUGCAA
ENSG00000172932	UAUAAAG
ENSG00000172936	CAGACAG
ENSG00000172936	GACCAUG
ENSG00000172936	GGAGGAG
ENSG00000172936	GGAGGCA
ENSG00000172939	AAAGCUG
ENSG00000172939	AACGGAA
ENSG00000172939	AAGGCAC
ENSG00000172939	AUUGCAC
ENSG00000172939	CACCAGC
ENSG00000172939	CAGUAGU
ENSG00000172939	CAGUGCA
ENSG00000172939	CAUAUUG
ENSG00000172939	CUCUUCC
ENSG00000172939	CUUUGGU
ENSG00000172939	GCAGGAC
ENSG00000172939	GCUAGCA
ENSG00000172939	GGCAAGA
ENSG00000172939	GGCAGUG
ENSG00000172939	GGGGUGC
ENSG00000172939	GUCUACU
ENSG00000172939	GUGCCUG
ENSG00000172939	GUGCUUG
ENSG00000172939	UAUUGCU
ENSG00000172939	UCUGGAG
ENSG00000172939	UGCAUUG
ENSG00000172939	UGGCCAC
ENSG00000172939	UGUGUGG
ENSG00000172939	UUGGCAA
ENSG00000172939	UUUGCAC
ENSG00000172940	GAAGGAA
ENSG00000172943	AAAGCUG
ENSG00000172943	AAUCACU
ENSG00000172943	ACAUUAC
ENSG00000172943	ACUAGAU
ENSG00000172943	AGAUAAA
ENSG00000172943	AGCCAGU
ENSG00000172943	AGGGAGG
ENSG00000172943	AUAUUAU
ENSG00000172943	AUGAUGC
ENSG00000172943	CACAAUG
ENSG00000172943	GAGGUAG
ENSG00000172943	GCAGGAC
ENSG00000172943	GGCAAGA
ENSG00000172943	GGCCCUG
ENSG00000172943	GGGGUCC
ENSG00000172943	GGGUUUA
ENSG00000172943	GUGACUG
ENSG00000172943	UAUAAAG
ENSG00000172943	UCCCUGU
ENSG00000172943	UUGGCAA
ENSG00000172977	AGCUGCC
ENSG00000172977	UAUUGCU
ENSG00000172985	AAAGUAC
ENSG00000172985	AACAAAC
ENSG00000172985	AAGGCAC
ENSG00000172985	AGCACCA
ENSG00000172985	AUAUAAC
ENSG00000172985	GAGGUAG

ENSG00000172985	UAUAAAG
ENSG00000172985	UCUGGAA
ENSG00000172985	UGACCUA
ENSG00000172985	UGCAAAG
ENSG00000172986	GCACCAU
ENSG00000172992	AAGGCAC
ENSG00000172992	GAGAUGA
ENSG00000173020	CACAAUG
ENSG00000173020	CAGCAGG
ENSG00000173020	CCCUGAG
ENSG00000173020	GAAAACA
ENSG00000173020	GAGGCAG
ENSG00000173020	GGAGGCA
ENSG00000173020	GGGCCCC
ENSG00000173020	GGUGGGC
ENSG00000173020	UACAAAG
ENSG00000173020	UAGAGGA
ENSG00000173020	UAUAAAG
ENSG00000173020	UCACAUU
ENSG00000173020	UCCGCUC
ENSG00000173064	AAAGUAC
ENSG00000173064	AAGAACC
ENSG00000173064	AAGUCUU
ENSG00000173064	AGCAGCG
ENSG00000173064	AGCAGCU
ENSG00000173064	AUACAAG
ENSG00000173064	CACAAUG
ENSG00000173064	CAGCAAA
ENSG00000173064	CCAGGAG
ENSG00000173064	CUACAAA
ENSG00000173064	GAAGGUC
ENSG00000173064	GAGGUAG
ENSG00000173064	GGAGGAG
ENSG00000173064	GUGUGGC
ENSG00000173064	UCAAGUA
ENSG00000173064	UCACAAG
ENSG00000173065	AACUGGU
ENSG00000173065	AAGGCAC
ENSG00000173065	ACAGUAC
ENSG00000173065	ACAUUCA
ENSG00000173065	ACCACAG
ENSG00000173065	ACUCAAA
ENSG00000173065	AGCCAGU
ENSG00000173065	AUCACUA
ENSG00000173065	CAAAUGC
ENSG00000173065	CACAGUG
ENSG00000173065	CAGGUGA
ENSG00000173065	CUACAGU
ENSG00000173065	CUCAAAC
ENSG00000173065	CUGGCUC
ENSG00000173065	CUUACAG
ENSG00000173065	GAGAACC
ENSG00000173065	GCAACUU
ENSG00000173065	GCUACAU
ENSG00000173065	GUGGGGA
ENSG00000173065	UACAGUU
ENSG00000173065	UAUUGCU
ENSG00000173065	UCACACA
ENSG00000173065	UCACAGU
ENSG00000173065	UCACAUU
ENSG00000173065	UCAUACA
ENSG00000173065	UCAUUUG
ENSG00000173065	UUGGCAA
ENSG00000173065	UUGGCAC

ENSG00000173065	UUGUGUC
ENSG00000173068	AAAAACC
ENSG00000173068	AAAGGUA
ENSG00000173068	AACAUUC
ENSG00000173068	AACGGAA
ENSG00000173068	AAGACAU
ENSG00000173068	AAGUAGC
ENSG00000173068	AAGUGCA
ENSG00000173068	AAGUGCU
ENSG00000173068	AAUGUUG
ENSG00000173068	ACAAUAU
ENSG00000173068	ACAUUAC
ENSG00000173068	ACAUUCA
ENSG00000173068	ACUGGAU
ENSG00000173068	ACUGUGU
ENSG00000173068	AGAUAAA
ENSG00000173068	AGCCAGU
ENSG00000173068	AGCUUAU
ENSG00000173068	AGGUAGU
ENSG00000173068	AGUGCUG
ENSG00000173068	AGUGUGC
ENSG00000173068	AUAAAGU
ENSG00000173068	AUAUAAC
ENSG00000173068	AUAUUAU
ENSG00000173068	AUGACAC
ENSG00000173068	AUGAUGC
ENSG00000173068	AUGCCUU
ENSG00000173068	AUGGCAC
ENSG00000173068	CAAGAGC
ENSG00000173068	CACACCG
ENSG00000173068	CACACCU
ENSG00000173068	CAGCAGA
ENSG00000173068	CAGCAGG
ENSG00000173068	CCAGGAG
ENSG00000173068	CUGCUCA
ENSG00000173068	CUUUGGU
ENSG00000173068	GAAACAU
ENSG00000173068	GAGAAGA
ENSG00000173068	GAGGCUG
ENSG00000173068	GAGGUAG
ENSG00000173068	GCUGGAU
ENSG00000173068	GGAAGCC
ENSG00000173068	GGCAUUG
ENSG00000173068	GGGUGUU
ENSG00000173068	GUAAACA
ENSG00000173068	GUCUGCC
ENSG00000173068	GUGAAUG
ENSG00000173068	GUGACAG
ENSG00000173068	GUGGGGA
ENSG00000173068	UAAGACG
ENSG00000173068	UAAGACU
ENSG00000173068	UCACACA
ENSG00000173068	UCACAUU
ENSG00000173068	UGAAAGG
ENSG00000173068	UGAAGCU
ENSG00000173068	UGCAUAU
ENSG00000173068	UGCCAGC
ENSG00000173068	UGGCUGC
ENSG00000173068	UGUCUGC
ENSG00000173068	UGUCUGG
ENSG00000173068	UGUGUCA
ENSG00000173068	UUGAUCA
ENSG00000173068	UUGGAGG
ENSG00000173068	UUGGCAC

ENSG00000173068	UUGUGUC
ENSG00000173068	UUGUUCG
ENSG00000173068	UUUGCAC
ENSG00000173068	UUUUUGC
ENSG00000173077	AAGUCUU
ENSG00000173077	UUCCUCG
ENSG00000173114	ACUGUGU
ENSG00000173114	CUACAGU
ENSG00000173114	GAGGUAU
ENSG00000173114	GGAGGAG
ENSG00000173114	UAUUGCU
ENSG00000173114	UUGGUUC
ENSG00000173124	GGAGGCA
ENSG00000173153	AGCAAAA
ENSG00000173153	AGCAGCA
ENSG00000173153	CAGCAGG
ENSG00000173153	CUCUGGG
ENSG00000173153	CUGCAGC
ENSG00000173153	CUGCAGG
ENSG00000173153	CUGGGGG
ENSG00000173153	CUUGGAG
ENSG00000173153	GAGGCAG
ENSG00000173153	GGCAGUG
ENSG00000173153	GGCAUUG
ENSG00000173153	GGCCCUG
ENSG00000173153	GGGAGCU
ENSG00000173153	GGGGCAG
ENSG00000173153	GGGGUCC
ENSG00000173153	GUCUCUG
ENSG00000173153	UCCCUCU
ENSG00000173153	UGCCAGC
ENSG00000173153	UGCCCUG
ENSG00000173153	UGGCCAC
ENSG00000173166	AGCACCA
ENSG00000173166	AGUGCUG
ENSG00000173166	AUGGCAC
ENSG00000173166	CAGUAGA
ENSG00000173166	CCAGCAU
ENSG00000173166	CCAUUAC
ENSG00000173166	CUGCAGG
ENSG00000173166	GGCAGUG
ENSG00000173166	GUAAACA
ENSG00000173166	GUGCAAA
ENSG00000173166	GUUAAUG
ENSG00000173166	UAUUGCU
ENSG00000173166	UCCCACA
ENSG00000173166	UGGAGAU
ENSG00000173166	UGUCUUU
ENSG00000173166	UUCUGCA
ENSG00000173171	GGAAUGU
ENSG00000173171	UCUGGAA
ENSG00000173207	CAAAGCA
ENSG00000173208	AGCUUAU
ENSG00000173208	AGUAGUU
ENSG00000173209	AAAAGUA
ENSG00000173210	AAAAAUC
ENSG00000173210	AAAUCCC
ENSG00000173210	ACAGUAC
ENSG00000173210	ACAUAGA
ENSG00000173210	AGCCCUU
ENSG00000173210	AUAUAAC
ENSG00000173210	AUCCCUU
ENSG00000173210	GAGUUGG
ENSG00000173210	GAUUGUA

ENSG00000173210	GGCAGGG
ENSG00000173214	UCUCCAA
ENSG00000173218	AAGGCAC
ENSG00000173218	AGAUAAA
ENSG00000173218	AGCCAGU
ENSG00000173218	AGUGAUC
ENSG00000173218	AGUGCAA
ENSG00000173218	AUAAAGU
ENSG00000173218	AUGGCAC
ENSG00000173218	AUUCAUU
ENSG00000173218	CAGUAGU
ENSG00000173218	CAGUGCA
ENSG00000173218	CCUGCUG
ENSG00000173218	CCUUCUU
ENSG00000173218	CUUCACC
ENSG00000173218	GAGAAAU
ENSG00000173218	GCACCAU
ENSG00000173218	GCUGGUG
ENSG00000173218	GGCAGGG
ENSG00000173218	GGUGGUC
ENSG00000173218	GUAAACA
ENSG00000173218	GUGCAAA
ENSG00000173218	UCCAGUU
ENSG00000173218	UCCCACA
ENSG00000173218	UCCCACC
ENSG00000173218	UGCAAAG
ENSG00000173218	UUGGUUC
ENSG00000173218	UUUUUGC
ENSG00000173221	GAGGUAG
ENSG00000173226	GAGGUAG
ENSG00000173230	GAGAAAU
ENSG00000173262	AGAUAAA
ENSG00000173262	AGCAGCG
ENSG00000173262	AGCAGCU
ENSG00000173264	CUGGCUC
ENSG00000173264	GAGGUAG
ENSG00000173273	AAAAGUA
ENSG00000173273	AAAGCUG
ENSG00000173273	AAAGUAA
ENSG00000173273	AAAGUAC
ENSG00000173273	AAGGCAC
ENSG00000173273	AAGUGCA
ENSG00000173273	ACUCAAA
ENSG00000173273	AGCUUAU
ENSG00000173273	AGGCAGG
ENSG00000173273	AGUGUGC
ENSG00000173273	AUUCAUU
ENSG00000173273	CAAAGUG
ENSG00000173273	CUUUGGU
ENSG00000173273	GAGUAUU
ENSG00000173273	GAUAUGU
ENSG00000173273	GGCAGUG
ENSG00000173273	GGCUCAG
ENSG00000173273	UUCAAAU
ENSG00000173273	UUCCUCG
ENSG00000173273	UUGGUUC
ENSG00000173273	UUUCAAC
ENSG00000173275	AAAGAAU
ENSG00000173275	CCAUUAC
ENSG00000173275	GCAGCAU
ENSG00000173276	AAAAGUA
ENSG00000173276	AACAAAC
ENSG00000173276	GGAAGCC
ENSG00000173276	GGAAUGU

ENSG00000173276	UGCAUAG
ENSG00000173276	UGCUGAC
ENSG00000173281	AAGUGCA
ENSG00000173281	AGCAAAA
ENSG00000173281	AUCCUUU
ENSG00000173281	CAGCAAA
ENSG00000173281	GGAAUGU
ENSG00000173281	UAUUACC
ENSG00000173281	UCACAGG
ENSG00000173320	AAAACUG
ENSG00000173320	AAACUGU
ENSG00000173327	AAAGUGC
ENSG00000173327	AAGUGCU
ENSG00000173327	AGUGCUG
ENSG00000173327	CCAGUGC
ENSG00000173327	GGGGUCC
ENSG00000173327	GUAGUGU
ENSG00000173327	GUGACAG
ENSG00000173327	UCCAGUU
ENSG00000173334	AAAGUAC
ENSG00000173334	AAGUCAC
ENSG00000173334	ACCCGUA
ENSG00000173334	GAGGUAG
ENSG00000173334	GUGAGGU
ENSG00000173334	UACAAAG
ENSG00000173334	UCUCACC
ENSG00000173338	GCUUCCU
ENSG00000173349	GAGGUAG
ENSG00000173376	GUUCAUG
ENSG00000173376	UAUAAAG
ENSG00000173391	GAGGUAG
ENSG00000173391	GAGGUAU
ENSG00000173391	UCUGGAG
ENSG00000173402	ACAGUAC
ENSG00000173402	AUGGCUU
ENSG00000173402	CUGCCCC
ENSG00000173402	GAAAACA
ENSG00000173402	GUGCAAA
ENSG00000173402	UACAGUU
ENSG00000173402	UAUUGCU
ENSG00000173402	UCCAGUU
ENSG00000173404	AAAGCUG
ENSG00000173404	AAAGUAA
ENSG00000173404	CACAGUG
ENSG00000173404	UCACAAG
ENSG00000173436	AACAUUC
ENSG00000173436	GCUGGAU
ENSG00000173436	GGAAGCC
ENSG00000173436	GGCAGUG
ENSG00000173436	UAAGACG
ENSG00000173436	UAAGACU
ENSG00000173442	UAUUGCU
ENSG00000173451	AAAACUG
ENSG00000173451	AAGUCUU
ENSG00000173451	ACUUGGA
ENSG00000173451	CUUUGGU
ENSG00000173451	GUAUUCU
ENSG00000173451	UGCAUAG
ENSG00000173451	UGCAUAU
ENSG00000173452	AAGUCUU
ENSG00000173452	ACAGUAC
ENSG00000173452	UACAGUU
ENSG00000173452	UCAUUCG
ENSG00000173452	UCUGGAG

ENSG00000173452	UGUCUUU
ENSG00000173456	CACCACC
ENSG00000173456	GGAGAGA
ENSG00000173456	GGCCCUG
ENSG00000173456	GGGGUGC
ENSG00000173456	UCACCAC
ENSG00000173456	UGCAUAU
ENSG00000173457	GGGGCAG
ENSG00000173473	AAAGCUG
ENSG00000173473	AAAGUAC
ENSG00000173473	AAGGCAC
ENSG00000173473	AAGUCUU
ENSG00000173473	ACAGUAU
ENSG00000173473	ACCACAG
ENSG00000173473	ACCUGGA
ENSG00000173473	AGGUAGU
ENSG00000173473	AGUGAUC
ENSG00000173473	AUGCCUU
ENSG00000173473	CAGCAGG
ENSG00000173473	GAGGUAG
ENSG00000173473	UCACAUU
ENSG00000173473	UCCUAUA
ENSG00000173473	UGCAUAG
ENSG00000173473	UUAGGAU
ENSG00000173482	AAAGAGC
ENSG00000173482	AGUGCAA
ENSG00000173482	CAGUGCA
ENSG00000173482	GGCAGUG
ENSG00000173482	UGUGCUU
ENSG00000173486	AACAGUC
ENSG00000173486	CAGUCUG
ENSG00000173511	CUGCAGG
ENSG00000173511	UGUCUGG
ENSG00000173511	UUCCGGC
ENSG00000173540	AAUUAUU
ENSG00000173540	UCUUGAG
ENSG00000173542	AACCUGG
ENSG00000173542	AACCUGU
ENSG00000173542	AAGGCAC
ENSG00000173542	ACAUUAC
ENSG00000173542	AGCACCA
ENSG00000173542	CACCACC
ENSG00000173542	CAGCAAA
ENSG00000173542	CAGUCUG
ENSG00000173542	CUCCAUU
ENSG00000173542	GGAAGAC
ENSG00000173542	GUAUUCU
ENSG00000173542	UCACAGU
ENSG00000173542	UCACAUU
ENSG00000173542	UCCCACA
ENSG00000173542	UGUGCUU
ENSG00000173542	UUCAAGC
ENSG00000173545	AAGUAGC
ENSG00000173545	AGCAGCG
ENSG00000173545	AGCAGCU
ENSG00000173545	CAGCAAA
ENSG00000173546	AUAUUAU
ENSG00000173548	AGCAGCA
ENSG00000173559	AAAAGUA
ENSG00000173559	AAGUGUG
ENSG00000173559	AGCAGCU
ENSG00000173559	CACAGUG
ENSG00000173559	UACAAAG
ENSG00000173559	UCACAGG

ENSG00000173559	UCAUAGA
ENSG00000173567	AUCCUUU
ENSG00000173575	AAAGUAC
ENSG00000173575	AAUACUG
ENSG00000173575	AAUCACU
ENSG00000173575	ACAUCAC
ENSG00000173575	AGGACAC
ENSG00000173575	AGUAACA
ENSG00000173575	AUAUAAC
ENSG00000173575	AUCACUA
ENSG00000173575	AUGGCAC
ENSG00000173575	CAGCAGA
ENSG00000173575	CCUGCUG
ENSG00000173575	GAUCAGA
ENSG00000173575	GCCUGGA
ENSG00000173575	GGCAUUG
ENSG00000173575	UAAUAUC
ENSG00000173575	UGAAAUG
ENSG00000173575	UGCUGAC
ENSG00000173575	UUGGUCC
ENSG00000173588	UUGGCAC
ENSG00000173598	AAGGCAC
ENSG00000173598	ACAUAGA
ENSG00000173598	ACUGUAG
ENSG00000173598	AGCUGCC
ENSG00000173598	AGUAGAC
ENSG00000173598	AUGGCUU
ENSG00000173598	CAAAGCA
ENSG00000173598	CCAGUGC
ENSG00000173598	CUUACAG
ENSG00000173598	GACCAUG
ENSG00000173598	GGAGGCA
ENSG00000173598	GGUUGAC
ENSG00000173598	GUAACAG
ENSG00000173598	UAAUGCU
ENSG00000173598	UAUUACC
ENSG00000173598	UGCAUAG
ENSG00000173598	UGCAUAU
ENSG00000173598	UUGAUCA
ENSG00000173598	UUUGCAA
ENSG00000173599	GAGAUGA
ENSG00000173599	UUGGCAC
ENSG00000173614	CCAUUAC
ENSG00000173614	UUCUGCA
ENSG00000173621	GAGGUAG
ENSG00000173621	GAGGUAU
ENSG00000173653	AAUCUCU
ENSG00000173653	AGCAGCU
ENSG00000173653	CUACAAA
ENSG00000173653	CUGGCUC
ENSG00000173653	GAGAAAU
ENSG00000173653	UACAAAG
ENSG00000173653	UUGGUCC
ENSG00000173674	AACUACU
ENSG00000173674	GCAGCAU
ENSG00000173674	GGAUUUC
ENSG00000173674	GUGCCAC
ENSG00000173674	UAUAAAG
ENSG00000173681	CCCUGAG
ENSG00000173681	UCAACAG
ENSG00000173681	UGCAGCU
ENSG00000173681	UUUGCAC
ENSG00000173681	UUUUUGC
ENSG00000173692	GAAAACA

ENSG00000173698	CUCACAC
ENSG00000173698	GGCAGUG
ENSG00000173698	UACAAAG
ENSG00000173698	UGAGAAG
ENSG00000173705	AGGUUAU
ENSG00000173705	GUUAAUG
ENSG00000173705	UCCUGAG
ENSG00000173705	UUGUGUC
ENSG00000173706	AAGACAU
ENSG00000173706	AAGGUGC
ENSG00000173706	ACAGUAU
ENSG00000173706	AGUAACA
ENSG00000173706	AUUGCAC
ENSG00000173706	CACAGUG
ENSG00000173706	CUACAAA
ENSG00000173706	CUACAGU
ENSG00000173706	CUGGAAA
ENSG00000173706	CUGGGCA
ENSG00000173706	CUUACAG
ENSG00000173706	GCUACAU
ENSG00000173706	GUCACUC
ENSG00000173706	GUGCAAA
ENSG00000173706	UAUUGCU
ENSG00000173706	UCACAGG
ENSG00000173706	UCCAGAG
ENSG00000173706	UCCCUCU
ENSG00000173706	UCUGGAA
ENSG00000173706	UCUGGAG
ENSG00000173726	AGAUAAA
ENSG00000173726	AGCAGCA
ENSG00000173726	AGCCCUU
ENSG00000173726	AGUGUGC
ENSG00000173726	CUGCUCA
ENSG00000173726	GAGAAAU
ENSG00000173726	GGUAGAC
ENSG00000173726	UCAACAG
ENSG00000173726	UGAGAAU
ENSG00000173726	UGCCCUG
ENSG00000173726	UUCAAAU
ENSG00000173757	AAAAAUC
ENSG00000173757	CAUGGAU
ENSG00000173757	CCUUCUG
ENSG00000173757	GACCUGG
ENSG00000173757	GUAACAG
ENSG00000173757	UAUAAAG
ENSG00000173757	UGUAUAA
ENSG00000173786	AGCCAGU
ENSG00000173786	CACUGUU
ENSG00000173786	CAGCAGA
ENSG00000173786	CAGCUGG
ENSG00000173786	CCUUCAU
ENSG00000173786	CUGGGCA
ENSG00000173786	GAGGCAG
ENSG00000173786	GGAAGAC
ENSG00000173786	GGAGGCA
ENSG00000173786	GGCAAGA
ENSG00000173786	GUGAAUG
ENSG00000173786	UAGAGGA
ENSG00000173786	UCCCUCU
ENSG00000173786	UUGGUCC
ENSG00000173801	AAAAACC
ENSG00000173801	AUACAAG
ENSG00000173801	CAAGAGC
ENSG00000173801	CUUUGGU

ENSG00000173801	GAAACAU
ENSG00000173801	GGAGAGA
ENSG00000173801	GGUCUAG
ENSG00000173801	UCACCAC
ENSG00000173801	UUGGUCC
ENSG00000173805	CAGCAAA
ENSG00000173805	CCCUGUC
ENSG00000173805	CUCCAGC
ENSG00000173805	CUGCAGG
ENSG00000173805	GACCCUG
ENSG00000173805	GCUGUCU
ENSG00000173805	GUGGGGA
ENSG00000173805	UGCAAAG
ENSG00000173805	UGGCCCU
ENSG00000173812	CUAGUCC
ENSG00000173821	CCAGUGC
ENSG00000173821	CCUUCAU
ENSG00000173821	GAGCUGC
ENSG00000173821	GGCAGUG
ENSG00000173825	UUCCAUA
ENSG00000173826	UCUCACC
ENSG00000173838	AGGGAGG
ENSG00000173846	AAAGCUG
ENSG00000173846	CCCCCAG
ENSG00000173846	GGCUCAG
ENSG00000173848	AUGGCUU
ENSG00000173848	AUUCAUU
ENSG00000173848	GGAAUGU
ENSG00000173848	GGCUCAG
ENSG00000173852	AAAGAGC
ENSG00000173852	AAAGAGG
ENSG00000173852	AAAGUUU
ENSG00000173852	AAUCUCA
ENSG00000173852	CCAUUAC
ENSG00000173852	UAAUACA
ENSG00000173852	UGCAUAU
ENSG00000173868	AUGGCUU
ENSG00000173868	CACCACC
ENSG00000173868	CUGGCUC
ENSG00000173868	CUUCACC
ENSG00000173868	CUUUGGU
ENSG00000173868	UGCAUAG
ENSG00000173889	AAAGAGC
ENSG00000173889	AAGUCUU
ENSG00000173889	AGCAGCA
ENSG00000173889	AGGUUAU
ENSG00000173889	CAAAGCA
ENSG00000173889	CAGUGCA
ENSG00000173889	CCCCUGG
ENSG00000173889	CCUGUAC
ENSG00000173889	CUACAAA
ENSG00000173889	GAGGCUG
ENSG00000173889	GAGGUAG
ENSG00000173889	GCAGGUU
ENSG00000173889	GGAAGAC
ENSG00000173889	GGCAAGA
ENSG00000173889	GUAAACA
ENSG00000173889	GUUAAUG
ENSG00000173889	UAAUAUC
ENSG00000173889	UAUUGCU
ENSG00000173889	UCAUGAU
ENSG00000173889	UGAAGCU
ENSG00000173889	UGACAUC
ENSG00000173889	UGAUCCA

ENSG00000173894	AAGGCAC
ENSG00000173894	AGCACCA
ENSG00000173894	AGCAGCA
ENSG00000173894	AGGGAGG
ENSG00000173894	CAAAGUG
ENSG00000173894	CAGCAAA
ENSG00000173894	GAGGUAG
ENSG00000173894	GAUUGUC
ENSG00000173894	UCCUGAG
ENSG00000173894	UGGGAUC
ENSG00000173894	UUGGCAA
ENSG00000173894	UUGGCAC
ENSG00000173898	AAUCACU
ENSG00000173898	AGCAGCA
ENSG00000173898	AGCAGCG
ENSG00000173898	AGCAGCU
ENSG00000173898	CACCACC
ENSG00000173898	CAGCAGG
ENSG00000173905	AGCAGCU
ENSG00000173905	AUUCAUU
ENSG00000173905	CAGUAGA
ENSG00000173905	CAGUAGU
ENSG00000173915	AUGAUGA
ENSG00000173917	ACAAUAU
ENSG00000173917	AUAAAGU
ENSG00000173917	UCCUAUA
ENSG00000173918	AAAGAGC
ENSG00000173918	AAUGUUG
ENSG00000173926	AGCAAAA
ENSG00000173926	CCAGUGC
ENSG00000173926	GGCAGCG
ENSG00000173926	UCACACA
ENSG00000173926	UUCAAAU
ENSG00000173926	UUGGCAA
ENSG00000173926	UUGGCAC
ENSG00000173930	AAUUAUU
ENSG00000173930	GUAGUGU
ENSG00000173933	AACUCUA
ENSG00000173933	CCCACCG
ENSG00000173933	CCCACCU
ENSG00000173933	GUAUUCU
ENSG00000173933	UCCCACC
ENSG00000173933	UCCUGAG
ENSG00000173933	UUCUGCA
ENSG00000173947	UAAUAUC
ENSG00000173950	ACCAGCA
ENSG00000173950	CUUCUCU
ENSG00000173991	AGCAGCU
ENSG00000173991	CUACAAA
ENSG00000173991	CUCUGGG
ENSG00000173991	CUGGCUC
ENSG00000173991	UACAAAG
ENSG00000174007	AAAAGUA
ENSG00000174007	AAAGUAA
ENSG00000174007	AGUAACA
ENSG00000174013	AAAGAGG
ENSG00000174013	AAGUGCA
ENSG00000174013	AAUCACU
ENSG00000174013	ACAAUAU
ENSG00000174013	AGCUGCC
ENSG00000174013	AGGAUGU
ENSG00000174013	AGGUAGU
ENSG00000174013	AUCCUUG
ENSG00000174013	CAAAACU

ENSG00000174013	CAGUCCA
ENSG00000174013	CCCUGAG
ENSG00000174013	CUCCAGC
ENSG00000174013	CUUCACC
ENSG00000174013	CUUGGAG
ENSG00000174013	GACCAUG
ENSG00000174013	GAGGAUA
ENSG00000174013	GAGGUAG
ENSG00000174013	GAGGUAU
ENSG00000174013	GUAGUGU
ENSG00000174013	GUGAGGU
ENSG00000174013	UCACAAG
ENSG00000174013	UCCAGUU
ENSG00000174013	UCUCAAG
ENSG00000174013	UCUCACC
ENSG00000174013	UUCUUGU
ENSG00000174016	UCACAUU
ENSG00000174021	GAGGUAG
ENSG00000174059	AUCACUA
ENSG00000174083	GUAGUGU
ENSG00000174099	AAAGAAU
ENSG00000174099	AAAUGGU
ENSG00000174099	ACAAUAU
ENSG00000174099	ACUGUUU
ENSG00000174099	AUGGCUU
ENSG00000174099	CAAAUGC
ENSG00000174099	CACACCG
ENSG00000174099	CACACCU
ENSG00000174099	CAUAUUG
ENSG00000174099	CCCUGAG
ENSG00000174099	CUCAAAC
ENSG00000174099	CUGGCUC
ENSG00000174099	GAAGGAA
ENSG00000174099	UACAGUU
ENSG00000174099	UAUUACC
ENSG00000174099	UCACACA
ENSG00000174099	UCCUAUA
ENSG00000174099	UGCAGCU
ENSG00000174099	UGCAUUG
ENSG00000174099	UGUCUUU
ENSG00000174099	UUCCAUA
ENSG00000174099	UUGAGGC
ENSG00000174106	AAUACUG
ENSG00000174106	AGCUUAU
ENSG00000174106	GUUCAUG
ENSG00000174106	UAUUGCU
ENSG00000174111	AAUGCCC
ENSG00000174111	GUGCUUG
ENSG00000174111	UGUGCUU
ENSG00000174111	UUGGCAA
ENSG00000174123	UAUAAAG
ENSG00000174132	AAGGCAC
ENSG00000174136	AAAGUAC
ENSG00000174136	ACAAUAU
ENSG00000174136	ACUGUUU
ENSG00000174136	AGUGCUU
ENSG00000174136	AUAAAGU
ENSG00000174136	AUGACAC
ENSG00000174136	AUGUAAU
ENSG00000174136	CCAGUGU
ENSG00000174136	CUAUACA
ENSG00000174136	GGCAGGG
ENSG00000174136	GGCAGUG
ENSG00000174136	GGUGGGC

ENSG00000174136	GUGCAAA
ENSG00000174136	UAAUAUC
ENSG00000174136	UAUAAAG
ENSG00000174136	UUCUAAU
ENSG00000174136	UUUGCAA
ENSG00000174137	AAAAAUC
ENSG00000174137	CAAAGCA
ENSG00000174137	UUAGGAU
ENSG00000174151	AAAGUGC
ENSG00000174151	AGGCAGG
ENSG00000174151	CAGCUGG
ENSG00000174151	GAAGGAA
ENSG00000174151	GCAGAAG
ENSG00000174151	GGAGGAG
ENSG00000174151	GGAGGCA
ENSG00000174151	UGCCAGC
ENSG00000174151	UUCUGCA
ENSG00000174165	CUGGCUC
ENSG00000174197	AAAAACC
ENSG00000174197	AAAUCCC
ENSG00000174197	AACAUUC
ENSG00000174197	AAGUGCA
ENSG00000174197	AAUACUG
ENSG00000174197	AAUCUCA
ENSG00000174197	ACAUUCA
ENSG00000174197	ACUAGAU
ENSG00000174197	AGCACCA
ENSG00000174197	AGCAGCU
ENSG00000174197	AUAUAAC
ENSG00000174197	CUGCAGC
ENSG00000174197	CUUUGGU
ENSG00000174197	GAGAAAU
ENSG00000174197	GAGGUAG
ENSG00000174197	GAGGUAU
ENSG00000174197	GCACCAU
ENSG00000174197	GCAGCAU
ENSG00000174197	GGCAUUG
ENSG00000174197	GUGAGGU
ENSG00000174197	UAAUAUC
ENSG00000174197	UACAAAG
ENSG00000174197	UCCCUGU
ENSG00000174197	UCCUGAG
ENSG00000174197	UGUAUAA
ENSG00000174197	UUCAAAU
ENSG00000174197	UUCAAGC
ENSG00000174206	CUUUGGU
ENSG00000174206	GUGCAAA
ENSG00000174225	UCUUGAG
ENSG00000174231	AUACAAG
ENSG00000174233	AACAUUC
ENSG00000174233	AAUGCCC
ENSG00000174233	AAUUUUA
ENSG00000174233	ACAGUAC
ENSG00000174233	ACAUUAC
ENSG00000174233	AGCUGCC
ENSG00000174233	AGUGGUU
ENSG00000174233	AUAUUAU
ENSG00000174233	CACAGUG
ENSG00000174233	CUCCCAC
ENSG00000174233	CUGACUC
ENSG00000174233	CUGCAGG
ENSG00000174233	GAAACAU
ENSG00000174233	GGUUACC
ENSG00000174233	UAUAAAG

ENSG00000174233	UCACAGG
ENSG00000174233	UCACAGU
ENSG00000174233	UUGGUCC
ENSG00000174233	UUGUUCG
ENSG00000174238	ACAUCAC
ENSG00000174238	ACUUGGA
ENSG00000174238	AGCACCA
ENSG00000174238	AUUGCAC
ENSG00000174238	CACAAUG
ENSG00000174238	CACAGGG
ENSG00000174238	CACCACC
ENSG00000174238	CACCAGC
ENSG00000174238	CAGUGCA
ENSG00000174238	CUGUUGA
ENSG00000174238	GCAGCAU
ENSG00000174238	GGAAGAC
ENSG00000174238	GGAGAGA
ENSG00000174238	GUCACUC
ENSG00000174238	UACAAAG
ENSG00000174238	UAUUGCU
ENSG00000174238	UCACAAG
ENSG00000174238	UCACAUU
ENSG00000174238	UCACCAC
ENSG00000174238	UGCAAAG
ENSG00000174238	UGCUGAC
ENSG00000174238	UUUUUGC
ENSG00000174243	CAGUCUG
ENSG00000174243	CUUCUCU
ENSG00000174255	UCCCACC
ENSG00000174282	AACAUUC
ENSG00000174282	AAGGUGC
ENSG00000174282	AAGUGCA
ENSG00000174282	CAGUAGA
ENSG00000174282	CAGUAGU
ENSG00000174282	GAAGGAA
ENSG00000174282	GACCUGG
ENSG00000174282	GGAAUGU
ENSG00000174282	GGCCCUG
ENSG00000174282	UAUUGCU
ENSG00000174282	UGCAAAG
ENSG00000174282	UGGUACC
ENSG00000174282	UUGGUCC
ENSG00000174306	ACUGGCC
ENSG00000174306	AGCAGCG
ENSG00000174306	AGCAGCU
ENSG00000174306	AUAUAAC
ENSG00000174306	CAGUGCA
ENSG00000174306	CCUUCAU
ENSG00000174306	CUCACAC
ENSG00000174306	CUCUGGG
ENSG00000174306	CUGGGCA
ENSG00000174306	CUUUGGU
ENSG00000174306	GAAGGAA
ENSG00000174306	GAGAUGA
ENSG00000174306	GAGUAUU
ENSG00000174306	GCAUCCC
ENSG00000174306	UAUCAGA
ENSG00000174306	UUAGGAU
ENSG00000174306	UUGGCAA
ENSG00000174306	UUGGUCC
ENSG00000174307	AGCAGCA
ENSG00000174307	AGUGUGC
ENSG00000174307	CAGCAGG
ENSG00000174307	GGGGUCC

ENSG00000174307	GUGCAAA
ENSG00000174307	UGAAAUG
ENSG00000174307	UGCAAAG
ENSG00000174332	AAUGUUG
ENSG00000174332	CUUGUAU
ENSG00000174348	AAAAAUC
ENSG00000174348	AAAGAAU
ENSG00000174348	AAGGCAC
ENSG00000174348	AUAUUAU
ENSG00000174348	UAAUGCU
ENSG00000174348	UGUCUGG
ENSG00000174370	AAUUGCU
ENSG00000174370	CUCCAGC
ENSG00000174371	AUACAAG
ENSG00000174437	AUGCAUU
ENSG00000174437	CAGCAAA
ENSG00000174437	GCACCAU
ENSG00000174469	AAAGAGG
ENSG00000174469	AACUCUA
ENSG00000174469	AGGAUGU
ENSG00000174469	AUAAAGU
ENSG00000174469	CAGGAAC
ENSG00000174469	CCCUGUU
ENSG00000174469	CCUUCUG
ENSG00000174469	CUACAAA
ENSG00000174469	GGCUCUG
ENSG00000174469	UAAGACU
ENSG00000174469	UAUAAAG
ENSG00000174469	UCACAAG
ENSG00000174469	UCAUUCG
ENSG00000174469	UCCAGAG
ENSG00000174469	UUGGCAC
ENSG00000174482	GGUAGAC
ENSG00000174482	UUUGCAA
ENSG00000174483	CACCACC
ENSG00000174483	GGUGGUU
ENSG00000174502	AACCUGU
ENSG00000174502	AACUCUA
ENSG00000174502	ACAUUCA
ENSG00000174502	CUGGGCA
ENSG00000174502	GGCAGCG
ENSG00000174502	GUCAUUG
ENSG00000174502	UCAAGUA
ENSG00000174502	UCUCAAG
ENSG00000174502	UCUGGAA
ENSG00000174502	UCUGGAG
ENSG00000174502	UGAAAUG
ENSG00000174502	UUCAAGC
ENSG00000174502	UUGGCAA
ENSG00000174502	UUGGCAC
ENSG00000174514	AAUCCUU
ENSG00000174514	GCUGUCU
ENSG00000174514	UUUCAAC
ENSG00000174516	AGCAGCG
ENSG00000174516	CACAGUG
ENSG00000174516	CAGCAGG
ENSG00000174516	CCCCCAG
ENSG00000174516	GGCUCAG
ENSG00000174564	CAGGCUC
ENSG00000174567	AGGAGCU
ENSG00000174567	CCUUCUG
ENSG00000174574	AAAGUUU
ENSG00000174574	CACUGUU
ENSG00000174574	CCCUGUC

ENSG00000174574	CCCUGUU
ENSG00000174574	CUCAGGG
ENSG00000174574	CUUGGAG
ENSG00000174574	GGAGUCC
ENSG00000174574	GUGCUUG
ENSG00000174574	UCAAGUA
ENSG00000174574	UCACAGG
ENSG00000174574	UCCCUGU
ENSG00000174574	UCGUGCA
ENSG00000174574	UCUUGAG
ENSG00000174574	UGAAAUG
ENSG00000174574	UUCAAGC
ENSG00000174576	AAAGUUU
ENSG00000174576	AAGUCAC
ENSG00000174576	ACAAUAU
ENSG00000174576	AGCACCA
ENSG00000174576	CAAGAGC
ENSG00000174576	CCCACCU
ENSG00000174576	CUACAAA
ENSG00000174576	GAGAACU
ENSG00000174576	GAGCCCC
ENSG00000174576	GGGGUGG
ENSG00000174576	UACAAAG
ENSG00000174595	CAGUCUG
ENSG00000174611	AAAAAUC
ENSG00000174611	AAAUUUC
ENSG00000174611	AAUCACU
ENSG00000174611	AAUCCUU
ENSG00000174611	ACAUAGA
ENSG00000174611	AGCACCA
ENSG00000174611	CACCAGC
ENSG00000174611	CAGCUGG
ENSG00000174611	CCUGCUG
ENSG00000174611	GCAGCAU
ENSG00000174611	GCUGUCU
ENSG00000174611	UCAAGUA
ENSG00000174611	UGUCUUU
ENSG00000174611	UUCAAGC
ENSG00000174611	UUGGCAA
ENSG00000174628	AAAUUUC
ENSG00000174628	AGAUAAA
ENSG00000174628	UGCAAAG
ENSG00000174628	UGCAUAG
ENSG00000174628	UGCAUAU
ENSG00000174628	UUCAAAU
ENSG00000174628	UUCAAGC
ENSG00000174628	UUUCAAC
ENSG00000174640	CCCUGAG
ENSG00000174640	GAGAUGA
ENSG00000174640	UGCUGAC
ENSG00000174652	UCCCACA
ENSG00000174672	AAAAAUC
ENSG00000174672	ACAGUAC
ENSG00000174672	ACAGUAU
ENSG00000174672	AGCUGCC
ENSG00000174672	CCCUGAG
ENSG00000174672	GCAGGAC
ENSG00000174672	UACAGUU
ENSG00000174672	UAUAAAG
ENSG00000174672	UCCCUGU
ENSG00000174697	AGCACCA
ENSG00000174697	CACAGUG
ENSG00000174697	CAGUAGA
ENSG00000174697	GGGCCCC

ENSG00000174697	UCACAGU
ENSG00000174705	AAAGAGC
ENSG00000174705	ACUGCAG
ENSG00000174705	AGCACCA
ENSG00000174705	CUGCAGG
ENSG00000174705	CUGGGCA
ENSG00000174705	CUUUGGU
ENSG00000174705	GCUACAU
ENSG00000174705	UAUUGCU
ENSG00000174705	UGAAGCU
ENSG00000174705	UUGGCAA
ENSG00000174705	UUGGCAC
ENSG00000174705	UUGGUCC
ENSG00000174718	AAAAGUA
ENSG00000174718	UCACAGU
ENSG00000174718	UUCAAGC
ENSG00000174721	UUUGCAA
ENSG00000174721	UUUUUGC
ENSG00000174740	UUAUGCA
ENSG00000174744	UCCAGUU
ENSG00000174748	AAAGCUG
ENSG00000174748	ACUCAAA
ENSG00000174748	AUGCACC
ENSG00000174748	CUGGAAA
ENSG00000174748	GCACCAU
ENSG00000174748	UACAAAG
ENSG00000174748	UAUUGCU
ENSG00000174748	UGACUGU
ENSG00000174748	UUGACAC
ENSG00000174775	ACUGUGU
ENSG00000174780	AACACUG
ENSG00000174780	AAUACUG
ENSG00000174780	AGCAGCA
ENSG00000174780	GUGCUUG
ENSG00000174780	UCACAAG
ENSG00000174780	UGUGCUU
ENSG00000174796	ACUUGGA
ENSG00000174796	CGUACCG
ENSG00000174796	UAAUGCU
ENSG00000174799	ACAUUCA
ENSG00000174799	CACAGUG
ENSG00000174799	UCACAGG
ENSG00000174804	AAAAAUC
ENSG00000174804	AAAGUGC
ENSG00000174804	AAAUCCC
ENSG00000174804	AACCUGU
ENSG00000174804	ACUCCAG
ENSG00000174804	ACUGGCU
ENSG00000174804	AGCACCA
ENSG00000174804	AGUGAUC
ENSG00000174804	AUAUUAU
ENSG00000174804	AUUCAUU
ENSG00000174804	CAGCAGA
ENSG00000174804	CCAGUGC
ENSG00000174804	CUCCAUU
ENSG00000174804	CUCCCAA
ENSG00000174804	CUCUGGG
ENSG00000174804	CUCUUCC
ENSG00000174804	CUGCAGG
ENSG00000174804	CUGCCCC
ENSG00000174804	GAGGUAU
ENSG00000174804	GAGUAUU
ENSG00000174804	GAUUGUA
ENSG00000174804	GCACCAU

ENSG00000174804	GCAGCAU
ENSG00000174804	GCAGGAC
ENSG00000174804	GGCAAGA
ENSG00000174804	GGCAUUG
ENSG00000174804	GGGAACG
ENSG00000174804	GUAACAG
ENSG00000174804	GUCUCUG
ENSG00000174804	GUGAAUG
ENSG00000174804	GUGCUUG
ENSG00000174804	UAAGACG
ENSG00000174804	UAAGACU
ENSG00000174804	UCACACA
ENSG00000174804	UCACAGG
ENSG00000174804	UCACAGU
ENSG00000174804	UCCAGAG
ENSG00000174804	UCCAGUU
ENSG00000174804	UCCCACC
ENSG00000174804	UCCCUGU
ENSG00000174804	UCUGGAA
ENSG00000174804	UCUGGAG
ENSG00000174804	UGAAAGG
ENSG00000174804	UGAGAAU
ENSG00000174804	UGGUACC
ENSG00000174804	UGUCUGG
ENSG00000174804	UGUGCUU
ENSG00000174804	UUGACAC
ENSG00000174807	CAGCUGG
ENSG00000174807	GAGAAGA
ENSG00000174807	UGGAGAU
ENSG00000174839	AAAGUGC
ENSG00000174839	ACUGCAG
ENSG00000174839	AGCACCA
ENSG00000174839	AGCAGCA
ENSG00000174839	AGCAGCU
ENSG00000174839	CAAAGCA
ENSG00000174839	CCAGUGU
ENSG00000174839	GAGGUAU
ENSG00000174839	GAUUGGU
ENSG00000174839	GCUGGUG
ENSG00000174839	UGCCAGC
ENSG00000174839	UUGGCAA
ENSG00000174839	UUGGCAC
ENSG00000174840	AAAGCUG
ENSG00000174840	AUGAGCU
ENSG00000174840	GAGGUAU
ENSG00000174840	UGUGCUU
ENSG00000174871	ACAUAGA
ENSG00000174871	AGCAGCA
ENSG00000174871	AGCCUGC
ENSG00000174871	AGUAGUU
ENSG00000174871	CAGUAGU
ENSG00000174871	CCAGUGC
ENSG00000174871	CCCACCU
ENSG00000174871	CCUGCUG
ENSG00000174871	CUGCAGG
ENSG00000174871	CUGCUCA
ENSG00000174871	CUGGGCA
ENSG00000174871	CUGGGGG
ENSG00000174871	GGGGAGC
ENSG00000174871	GGGGUGC
ENSG00000174871	GGGGUGG
ENSG00000174871	UGGGGGA
ENSG00000174899	CAAAACU
ENSG00000174903	AAUGCCC

ENSG00000174903	ACUCAAA
ENSG00000174903	AGGACAC
ENSG00000174903	AUGGCUU
ENSG00000174903	CAAAUGC
ENSG00000174903	CCCCUGG
ENSG00000174903	GCAGCAU
ENSG00000174903	GGGGAGC
ENSG00000174903	UCCUGAG
ENSG00000174903	UGCAAAG
ENSG00000174915	GGCUCAG
ENSG00000174928	UAUAAAG
ENSG00000174928	UCAUUUG
ENSG00000174938	GCUGGUG
ENSG00000174938	UUGGCAA
ENSG00000174938	UUGGCAC
ENSG00000174939	CCCUGAG
ENSG00000174943	GGAAUGU
ENSG00000174943	UAAUAGU
ENSG00000174950	CAGCUGG
ENSG00000174950	CCUGCUG
ENSG00000174950	CUCUUCC
ENSG00000174950	GCAGCAU
ENSG00000174950	GGCAGCG
ENSG00000174950	GGCUCAG
ENSG00000174951	CCCUGAG
ENSG00000174953	UUCAAAU
ENSG00000174963	AAACGGU
ENSG00000174963	AAAGAAU
ENSG00000174963	AAGAACC
ENSG00000174963	ACAUUCA
ENSG00000174963	ACUGUUU
ENSG00000174963	ACUUGGA
ENSG00000174963	AGUGUGC
ENSG00000174963	AUAUUAU
ENSG00000174963	AUCCUUU
ENSG00000174963	AUGCACC
ENSG00000174963	AUUCAUU
ENSG00000174963	CAGCUGG
ENSG00000174963	CAUGGGU
ENSG00000174963	CUCACAC
ENSG00000174963	CUUGUAU
ENSG00000174963	GCCUGGA
ENSG00000174963	GGAAUGU
ENSG00000174963	GGACGGA
ENSG00000174963	GGGUUUA
ENSG00000174963	GUUAGGA
ENSG00000174963	UAAUAGU
ENSG00000174963	UGGAUAA
ENSG00000174963	UUGACAC
ENSG00000174996	ACCCUGU
ENSG00000174996	CCCUGAG
ENSG00000174996	CUACAGU
ENSG00000174996	CUGCCCC
ENSG00000174996	GAGAUGA
ENSG00000174996	GCGGGGC
ENSG00000174996	GGCGGGG
ENSG00000174996	GGUUACC
ENSG00000175029	AAAACUG
ENSG00000175029	AACACUG
ENSG00000175029	AAUACUG
ENSG00000175029	ACAAUAU
ENSG00000175029	ACAGUAC
ENSG00000175029	ACAGUAU
ENSG00000175029	CUAUACA

ENSG00000175029	GAAAACA
ENSG00000175029	GAGAAAU
ENSG00000175029	GGAAUGU
ENSG00000175029	GGGUUUA
ENSG00000175029	GUCUCUG
ENSG00000175029	GUUCAUG
ENSG00000175029	UCACAAG
ENSG00000175029	UCAUAGA
ENSG00000175029	UCUUGAG
ENSG00000175029	UGAAGUG
ENSG00000175029	UUCCUCG
ENSG00000175029	UUCUUGU
ENSG00000175029	UUGGUCC
ENSG00000175029	UUGUUCG
ENSG00000175029	UUUGCAC
ENSG00000175040	AAGUCUU
ENSG00000175040	AAUACUG
ENSG00000175040	ACUCAAA
ENSG00000175040	CAAAACU
ENSG00000175040	GUCUUGC
ENSG00000175040	UAAUACA
ENSG00000175048	AGCAGCA
ENSG00000175048	UGGCCAC
ENSG00000175066	AAAACUG
ENSG00000175066	AAAUUUC
ENSG00000175066	AGCACCA
ENSG00000175066	AGCAGCU
ENSG00000175066	CUUCUCU
ENSG00000175066	GAAACAU
ENSG00000175066	GCUGGUG
ENSG00000175066	UUCCUAG
ENSG00000175073	ACACACU
ENSG00000175073	AGUGGUU
ENSG00000175073	CACAAUG
ENSG00000175073	CAGACAG
ENSG00000175073	CCCUGAG
ENSG00000175073	CUGGGCA
ENSG00000175073	GCCUGGG
ENSG00000175073	GCUAGCA
ENSG00000175073	GUGCAAA
ENSG00000175073	GUGGCCC
ENSG00000175073	UAAGACG
ENSG00000175073	UCACACA
ENSG00000175073	UCCCUCU
ENSG00000175073	UCCCUGU
ENSG00000175073	UGUCUUU
ENSG00000175073	UUCAAAU
ENSG00000175084	CCUGCUG
ENSG00000175084	CUCCCAC
ENSG00000175084	UCCCUCU
ENSG00000175087	AAUCUCU
ENSG00000175087	ACAAUAU
ENSG00000175087	ACAUUCA
ENSG00000175087	AGGCAGG
ENSG00000175087	CAGCAAA
ENSG00000175087	CCAGCAU
ENSG00000175087	CCAGUGC
ENSG00000175087	CCAGUGU
ENSG00000175087	CUACAGU
ENSG00000175087	CUGGACU
ENSG00000175087	GAAAACA
ENSG00000175087	GACCAUG
ENSG00000175087	GCUACAU
ENSG00000175087	GGCAUUG

ENSG00000175087	GUGCAAA
ENSG00000175087	UAAUACA
ENSG00000175087	UCACCAC
ENSG00000175087	UCCAGUU
ENSG00000175087	UGCAUUG
ENSG00000175087	UUGGUCC
ENSG00000175093	AGCACCA
ENSG00000175093	AGCAGCA
ENSG00000175093	GGCUCUG
ENSG00000175093	GUGACAG
ENSG00000175093	UCAUUCG
ENSG00000175097	UUUGCAA
ENSG00000175105	AAAGUAC
ENSG00000175105	ACAUUCA
ENSG00000175105	AGCAGCA
ENSG00000175105	AUGAUGC
ENSG00000175105	AUUGCAC
ENSG00000175105	CCAGUGU
ENSG00000175105	GCUACAU
ENSG00000175105	GUGCAAA
ENSG00000175105	GUGCUUG
ENSG00000175105	UAUAAAG
ENSG00000175105	UAUUGCU
ENSG00000175105	UCAACAG
ENSG00000175105	UCAUUCG
ENSG00000175105	UGCAUAG
ENSG00000175105	UGCUGAC
ENSG00000175115	GAGGCUG
ENSG00000175130	CUCAGGG
ENSG00000175130	CUGGGCA
ENSG00000175130	GGCUCAG
ENSG00000175130	UAUAAAG
ENSG00000175130	UGGCCAC
ENSG00000175137	AAGGCAC
ENSG00000175137	AGCACCA
ENSG00000175137	CUCAAAC
ENSG00000175137	CUCCCAA
ENSG00000175137	GAGUUGG
ENSG00000175155	AAACUGU
ENSG00000175155	AAAGAAU
ENSG00000175155	AAAGUAU
ENSG00000175155	AACAAAC
ENSG00000175155	AAGUGCU
ENSG00000175155	CAAAGUG
ENSG00000175155	CAAAUGC
ENSG00000175155	CACAGUG
ENSG00000175155	CUCAAAC
ENSG00000175155	CUCAGGG
ENSG00000175155	CUUGUGU
ENSG00000175155	CUUUGGU
ENSG00000175155	GACCUGG
ENSG00000175155	GAGAAAU
ENSG00000175155	GAGGUAG
ENSG00000175155	GGAAUGU
ENSG00000175155	GUCACUC
ENSG00000175155	UAGAGGA
ENSG00000175155	UAUAAAG
ENSG00000175155	UCACAGU
ENSG00000175155	UCCCUUG
ENSG00000175155	UCCCUUU
ENSG00000175155	UCUCAAG
ENSG00000175155	UUAGAGA
ENSG00000175155	UUGGUCC
ENSG00000175155	UUGGUUC

ENSG00000175161	AAAGUGC
ENSG00000175161	AAGGUGC
ENSG00000175161	AAGUGCU
ENSG00000175161	ACUGGCU
ENSG00000175161	AGGACAC
ENSG00000175161	AGUGCUG
ENSG00000175161	AUAUUAU
ENSG00000175161	GAGGCUG
ENSG00000175161	GCUUCCU
ENSG00000175161	GGUUGUC
ENSG00000175161	GUGCUUG
ENSG00000175161	UAUAAAG
ENSG00000175161	UGCAUAG
ENSG00000175161	UGUAUGU
ENSG00000175161	UGUGCUU
ENSG00000175161	UUCAAGC
ENSG00000175161	UUGGCAC
ENSG00000175166	AACAAAC
ENSG00000175166	AGUAACA
ENSG00000175175	AGCACCA
ENSG00000175175	GAGAUGA
ENSG00000175175	GCUGUCU
ENSG00000175182	AUAUUAU
ENSG00000175182	CUGCCCC
ENSG00000175182	UGUCUGC
ENSG00000175182	UUAGAGA
ENSG00000175183	CACAGUG
ENSG00000175193	UACAAAG
ENSG00000175197	UUGGCAC
ENSG00000175203	AAGAACC
ENSG00000175203	ACAGUAC
ENSG00000175203	CAAAGCA
ENSG00000175215	AACGGAA
ENSG00000175215	AACUGGU
ENSG00000175215	AAGGCAC
ENSG00000175215	AGGCAGG
ENSG00000175215	AGGGAGG
ENSG00000175215	AGGUAGU
ENSG00000175215	AUGGCUU
ENSG00000175215	CAGGCUC
ENSG00000175215	CCCUGAG
ENSG00000175215	CUACAAA
ENSG00000175215	CUCUGGG
ENSG00000175215	CUCUUCC
ENSG00000175215	CUGCAGG
ENSG00000175215	CUUUGGU
ENSG00000175215	GACCCUG
ENSG00000175215	GGAAGAC
ENSG00000175215	GGCAGUG
ENSG00000175215	GGCUCUG
ENSG00000175215	UAUUGCU
ENSG00000175215	UCAAGUA
ENSG00000175215	UCACAGU
ENSG00000175215	UCCUGAG
ENSG00000175215	UUCAAGC
ENSG00000175215	UUGGCAA
ENSG00000175215	UUGGCAC
ENSG00000175216	AAAGCUG
ENSG00000175216	GUUAAUG
ENSG00000175220	AAACGGU
ENSG00000175220	AAGUGCA
ENSG00000175220	ACAGUAC
ENSG00000175220	CCAGGAG
ENSG00000175220	GGAGGCA

ENSG00000175220	GGCAGUG
ENSG00000175220	GGCUCUG
ENSG00000175220	GUGCAAA
ENSG00000175220	UGAAAUG
ENSG00000175220	UGAGAAG
ENSG00000175229	AACCUGG
ENSG00000175229	AGCAGCU
ENSG00000175229	AGGCAGG
ENSG00000175229	GGCAGGG
ENSG00000175229	GGCUCAG
ENSG00000175229	GGGAGCU
ENSG00000175229	UUGGUUC
ENSG00000175262	AAAUCCC
ENSG00000175262	UUCAAAU
ENSG00000175264	AAAUUUC
ENSG00000175264	AAGGCAC
ENSG00000175264	AGCAAAA
ENSG00000175264	CAGUGCA
ENSG00000175264	GAAAACA
ENSG00000175264	UCACAGU
ENSG00000175264	UUGGCAA
ENSG00000175265	GUGCAAA
ENSG00000175267	GGCAAGA
ENSG00000175267	UCCUGAG
ENSG00000175274	CUUACAG
ENSG00000175274	UACAAAG
ENSG00000175274	UUUGCAA
ENSG00000175305	CUAUACA
ENSG00000175311	GGCAUUG
ENSG00000175318	AGUGAUC
ENSG00000175318	CACAGUG
ENSG00000175318	UGAAGCU
ENSG00000175329	GCUUCCU
ENSG00000175329	GUCCAGA
ENSG00000175329	UCACACA
ENSG00000175329	UCCAGAG
ENSG00000175344	CCUGCUG
ENSG00000175348	AAAGAGG
ENSG00000175348	ACAGUAC
ENSG00000175348	ACAUCAC
ENSG00000175348	ACAUUCA
ENSG00000175348	AGUGCAA
ENSG00000175348	CACAGUG
ENSG00000175348	UACAAAG
ENSG00000175348	UCACAGU
ENSG00000175348	UGUGCUU
ENSG00000175354	AAAGUAC
ENSG00000175354	AACUACU
ENSG00000175354	CUACAAA
ENSG00000175354	UAUAAAG
ENSG00000175354	UCCUGAG
ENSG00000175356	AACUCUA
ENSG00000175374	AUAUUAU
ENSG00000175376	AAGUCAC
ENSG00000175376	CAGACAG
ENSG00000175376	CCCUGAG
ENSG00000175376	GGAAUGU
ENSG00000175376	UCCUGAG
ENSG00000175387	AAAAGUA
ENSG00000175387	AACUGGU
ENSG00000175387	AAGGUGC
ENSG00000175387	AAGUGCU
ENSG00000175387	AAUACUG
ENSG00000175387	ACAUUCA

ENSG00000175387	ACCCUGU
ENSG00000175387	ACUGUGU
ENSG00000175387	AGUGCUG
ENSG00000175387	AUCCCUU
ENSG00000175387	AUGACAC
ENSG00000175387	AUGCCUU
ENSG00000175387	CACACCU
ENSG00000175387	CAGUGCA
ENSG00000175387	CCCUGAG
ENSG00000175387	CCUGUAC
ENSG00000175387	CUGGCUC
ENSG00000175387	GAGGUAG
ENSG00000175387	GAUUGGU
ENSG00000175387	GCAGAAG
ENSG00000175387	GCAUCCC
ENSG00000175387	GGAAUGU
ENSG00000175387	UCACAGU
ENSG00000175387	UCUAGAG
ENSG00000175387	UCUCAAG
ENSG00000175387	UGGAUAA
ENSG00000175387	UUAUGCA
ENSG00000175387	UUUGCAC
ENSG00000175395	AAGGCAC
ENSG00000175414	AAGGCAC
ENSG00000175414	AGCAGCA
ENSG00000175414	AGGGAGG
ENSG00000175414	GAGGCAG
ENSG00000175414	GUAAACA
ENSG00000175416	AAGUGUG
ENSG00000175416	ACACACC
ENSG00000175426	AAAAAUC
ENSG00000175426	AAAGAAU
ENSG00000175426	AAAGCUG
ENSG00000175426	ACAUCAC
ENSG00000175426	AUGCCUU
ENSG00000175426	UAUAAAG
ENSG00000175426	UCACAAG
ENSG00000175463	AAAGUGC
ENSG00000175470	AAAAACC
ENSG00000175470	GGUCUAG
ENSG00000175482	AGCCAGU
ENSG00000175482	UGCCCUG
ENSG00000175497	AACAAAC
ENSG00000175497	AGCUGCC
ENSG00000175497	GAAAACA
ENSG00000175497	GCCCUUA
ENSG00000175497	GGCAUUG
ENSG00000175497	GUGACAG
ENSG00000175497	UAAUAUC
ENSG00000175497	UGCAAAG
ENSG00000175497	UGCAGCU
ENSG00000175497	UUCUGCA
ENSG00000175505	CAGGAAC
ENSG00000175505	CUGGGGG
ENSG00000175505	GGGUGGU
ENSG00000175505	GUAAACA
ENSG00000175505	GUGGCCC
ENSG00000175505	GUUAAUG
ENSG00000175518	AGGGUUG
ENSG00000175518	GGAAGCC
ENSG00000175538	AGGUAGU
ENSG00000175538	UUCAAAU
ENSG00000175538	UUUCAAC
ENSG00000175544	GGAGAGA

ENSG00000175548	CUACAAA
ENSG00000175548	GACUUCC
ENSG00000175548	UAUCAGA
ENSG00000175550	AAAUUUC
ENSG00000175550	AAGAACC
ENSG00000175550	CUGGGGG
ENSG00000175556	AUUGCAC
ENSG00000175556	UAUUGCU
ENSG00000175564	CCUGCUG
ENSG00000175564	GUCAGUU
ENSG00000175564	GUGCAAA
ENSG00000175564	UAAUAUC
ENSG00000175567	AGCAGCA
ENSG00000175567	CAGCAGA
ENSG00000175567	CAGCAGG
ENSG00000175573	GGAAGCC
ENSG00000175582	AAGUGCU
ENSG00000175582	AAUCUCA
ENSG00000175582	ACUGCAG
ENSG00000175582	AUGAUGA
ENSG00000175582	CACAAUG
ENSG00000175582	CACUCCU
ENSG00000175582	CUAGUAA
ENSG00000175582	CUAUACA
ENSG00000175582	CUCACAC
ENSG00000175582	GGAGGAG
ENSG00000175582	UACAAAG
ENSG00000175582	UAUAAAG
ENSG00000175582	UAUUACC
ENSG00000175582	UCACACA
ENSG00000175582	UCCCUCU
ENSG00000175582	UUCAAGC
ENSG00000175591	CUGGGGG
ENSG00000175591	GACCUGG
ENSG00000175592	AGCAGCA
ENSG00000175592	AGCAGCG
ENSG00000175592	GUGCAAA
ENSG00000175595	GAGGUAG
ENSG00000175595	GAGGUAU
ENSG00000175602	CCUGCUG
ENSG00000175606	AAUACUG
ENSG00000175606	AAUUUUA
ENSG00000175606	AGUGAUC
ENSG00000175606	CUUCACC
ENSG00000175646	CAGGCUC
ENSG00000175662	AAAGGUA
ENSG00000175662	AAGAACC
ENSG00000175662	ACCACAG
ENSG00000175662	AGUGCUG
ENSG00000175662	CCAGUGC
ENSG00000175662	UUCCAUA
ENSG00000175662	UUGACAC
ENSG00000175699	CUUGGAG
ENSG00000175699	GAGAAGA
ENSG00000175707	CAGCUGG
ENSG00000175707	UGAAAUG
ENSG00000175727	AAUUUUA
ENSG00000175727	AGCACCA
ENSG00000175727	CUUUGGU
ENSG00000175727	GUGACUG
ENSG00000175727	UAUUGCU
ENSG00000175727	UCCCUUG
ENSG00000175727	UCCUGAG
ENSG00000175727	UUGGUCC

ENSG00000175746	ACUUGGA
ENSG00000175746	CUUGGAG
ENSG00000175746	GAGCUAA
ENSG00000175746	UUGUUCG
ENSG00000175779	GGCAGUG
ENSG00000175782	CUUGGGC
ENSG00000175785	ACAUUCA
ENSG00000175785	AGAUGUG
ENSG00000175785	AUAAUAC
ENSG00000175785	AUAUAAC
ENSG00000175785	AUGCAUU
ENSG00000175785	AUGGCUU
ENSG00000175785	CAAAACU
ENSG00000175785	CCUUCUG
ENSG00000175785	UGUGUGG
ENSG00000175785	UUGGCAA
ENSG00000175785	UUGGCAC
ENSG00000175793	CACAGGG
ENSG00000175793	GUGUCAC
ENSG00000175793	UCACAGG
ENSG00000175826	CAGUCUG
ENSG00000175826	GAGGCUG
ENSG00000175832	AGCACCA
ENSG00000175832	AGCAGCU
ENSG00000175832	GCCUGGG
ENSG00000175854	CUGGAAA
ENSG00000175854	CUUCUCU
ENSG00000175866	AAAACUG
ENSG00000175866	AACAAAC
ENSG00000175866	AGCACCA
ENSG00000175866	AGCAGCA
ENSG00000175866	CUGGGGG
ENSG00000175866	CUUUGGU
ENSG00000175866	GACCCUG
ENSG00000175866	GCACCAU
ENSG00000175866	GCUUCCU
ENSG00000175866	GGAAUGU
ENSG00000175866	GGAGUCC
ENSG00000175866	GGAUUUC
ENSG00000175866	GGAUUUU
ENSG00000175866	GGGGGAA
ENSG00000175866	GGGGUGG
ENSG00000175866	UGGGGGA
ENSG00000175868	AAGGCAC
ENSG00000175868	ACAAUAU
ENSG00000175868	UCACAAG
ENSG00000175874	CACAAUG
ENSG00000175874	UGAGUCU
ENSG00000175893	AAAACUG
ENSG00000175893	AAAUCCC
ENSG00000175893	AACCUGU
ENSG00000175893	ACAUUAC
ENSG00000175893	AUCAUAC
ENSG00000175893	AUGAUGA
ENSG00000175893	AUUGCAC
ENSG00000175893	CCAGCAU
ENSG00000175893	GCCUGGG
ENSG00000175893	GGAGGAG
ENSG00000175893	GGGGCAG
ENSG00000175893	GGGUGGU
ENSG00000175893	GGUGGGC
ENSG00000175893	GUAAACA
ENSG00000175893	UAUUGCU
ENSG00000175893	UCCCACA

ENSG00000175895	AGCACCA
ENSG00000175895	GAGGCAG
ENSG00000175898	ACUGGCU
ENSG00000175898	AGUGCAA
ENSG00000175898	CCUUCUG
ENSG00000175898	GGGAGCU
ENSG00000175898	GUGCAAA
ENSG00000175898	UCCUUGC
ENSG00000175898	UCUCAAG
ENSG00000175898	UUGGCAA
ENSG00000175898	UUGGCAC
ENSG00000175906	GAGGUAG
ENSG00000175928	AAGUCUU
ENSG00000175928	AUGGCUU
ENSG00000175928	UGUGCUU
ENSG00000175931	AAGGCAC
ENSG00000175931	ACAUAGA
ENSG00000175931	AGCUGCC
ENSG00000175931	GAGCCCC
ENSG00000175931	GUAAACA
ENSG00000175931	UCAACAG
ENSG00000175938	AAGGUGC
ENSG00000175938	ACAAGGU
ENSG00000175938	AGGGAGG
ENSG00000175938	GAAACAU
ENSG00000175938	GAGGCAG
ENSG00000175938	GGAAUGU
ENSG00000175938	GGAGGCA
ENSG00000175938	GGCAGUG
ENSG00000175938	GGGGGAA
ENSG00000175938	UGGCCAC
ENSG00000175938	UGGGGGA
ENSG00000175970	AACAUUC
ENSG00000175970	AAGGCAC
ENSG00000175970	CAGUAGA
ENSG00000175970	CCUUCUU
ENSG00000175970	UCCAGAG
ENSG00000175970	UCCUGAG
ENSG00000175984	UGAGAAU
ENSG00000176018	AAUACUG
ENSG00000176018	AAUGCCC
ENSG00000176018	AGUGCAA
ENSG00000176018	GGAAGAC
ENSG00000176018	GUAAACA
ENSG00000176022	UAUAAAG
ENSG00000176049	AUGGCUU
ENSG00000176049	CAGCAGG
ENSG00000176049	CUUUGGU
ENSG00000176049	UCAAGUA
ENSG00000176058	AGUGCUG
ENSG00000176076	AGCAGCU
ENSG00000176076	AGCUGCC
ENSG00000176076	AUGUCUG
ENSG00000176076	CCUGCUG
ENSG00000176076	GAGGCAG
ENSG00000176076	GGCUCUG
ENSG00000176087	AAGGCAC
ENSG00000176087	AGCAGCA
ENSG00000176087	AGGCAGG
ENSG00000176087	CUCUGGG
ENSG00000176087	GAGAUGA
ENSG00000176087	UCACAAG
ENSG00000176087	UCCCUGU
ENSG00000176102	AGGCAGG

ENSG00000176102	CUACAAA
ENSG00000176102	GCAGGAC
ENSG00000176105	AAAGCUG
ENSG00000176105	AACCUGG
ENSG00000176105	AAGUGCA
ENSG00000176105	ACAGUAC
ENSG00000176105	AGCAAAA
ENSG00000176105	AGUGGUU
ENSG00000176105	CACAGUG
ENSG00000176105	CAGCAAA
ENSG00000176105	CCCUGAG
ENSG00000176105	CUAUACA
ENSG00000176105	CUCCAUU
ENSG00000176105	GACCCUG
ENSG00000176105	GGAAUGU
ENSG00000176105	GUAAACA
ENSG00000176105	GUGCAAA
ENSG00000176105	UCACAGU
ENSG00000176105	UGCCAGC
ENSG00000176105	UUGGCAC
ENSG00000176108	CACCAGC
ENSG00000176142	ACAUCAC
ENSG00000176142	AUACAAG
ENSG00000176142	CAAAACU
ENSG00000176142	GAGGCAG
ENSG00000176142	UAUAAAG
ENSG00000176142	UGUUGAA
ENSG00000176148	GUAAACA
ENSG00000176148	UCCCUUG
ENSG00000176153	UUAGGAU
ENSG00000176165	AAAGUAA
ENSG00000176165	AACAAAC
ENSG00000176165	AAUCACU
ENSG00000176165	ACACGGG
ENSG00000176165	ACAGUAU
ENSG00000176165	ACCACAG
ENSG00000176165	ACUCAAA
ENSG00000176165	ACUGCAG
ENSG00000176165	ACUGCAU
ENSG00000176165	CACAAUG
ENSG00000176165	CUUACAG
ENSG00000176165	GGCAGUG
ENSG00000176165	GGUCUAG
ENSG00000176165	UAGUGAG
ENSG00000176165	UAUAAUA
ENSG00000176165	UCAUAGA
ENSG00000176165	UUGGUCC
ENSG00000176165	UUUGCAA
ENSG00000176170	AAGGCAC
ENSG00000176204	AAGAACC
ENSG00000176204	CCUUCAU
ENSG00000176204	GGUGGUC
ENSG00000176204	GGUUGUC
ENSG00000176204	GGUUUAC
ENSG00000176204	UCAAGUA
ENSG00000176204	UCACACA
ENSG00000176204	UUAGGAU
ENSG00000176204	UUGGUUC
ENSG00000176208	AUACAAG
ENSG00000176225	UACAAAG
ENSG00000176244	AUCACUA
ENSG00000176244	CCUGCUG
ENSG00000176273	AGCAGCG
ENSG00000176273	AGCAGCU

ENSG00000176274	ACAAGGU
ENSG00000176274	ACAUUCA
ENSG00000176274	AGUGCCU
ENSG00000176274	CACAAUG
ENSG00000176274	CCCUGAG
ENSG00000176274	CUACAAA
ENSG00000176274	CUAGUAG
ENSG00000176274	GAGGAUA
ENSG00000176274	GAGGUAU
ENSG00000176274	GAGUUGA
ENSG00000176274	GUCUUGC
ENSG00000176274	UCACAUU
ENSG00000176274	UCCAGAG
ENSG00000176274	UGCUGAC
ENSG00000176274	UGUCUUU
ENSG00000176274	UUCUUGU
ENSG00000176340	UCAUGAU
ENSG00000176358	ACUGUGU
ENSG00000176371	UGCAUAG
ENSG00000176381	CUCUGGG
ENSG00000176381	UCCAGUU
ENSG00000176381	UUCAAAU
ENSG00000176387	AGCCAGU
ENSG00000176387	UCUUGAG
ENSG00000176390	CUUACAG
ENSG00000176390	GAUUGUA
ENSG00000176390	UCAUGAU
ENSG00000176390	UUCAAAU
ENSG00000176406	AUGGCUU
ENSG00000176406	CAGGCUC
ENSG00000176406	GAGAACC
ENSG00000176406	GAGAACU
ENSG00000176406	GCAACUU
ENSG00000176406	GCUGGUG
ENSG00000176406	GGCUCAG
ENSG00000176406	UCAACAG
ENSG00000176406	UGCAAAG
ENSG00000176406	UGCAGCU
ENSG00000176406	UGUCUGC
ENSG00000176406	UUCAAAU
ENSG00000176407	AAAGAAU
ENSG00000176407	AAAGCUG
ENSG00000176407	AAAUCCC
ENSG00000176407	AAGUCUU
ENSG00000176407	AAUACUG
ENSG00000176407	AAUGUUG
ENSG00000176407	ACACACC
ENSG00000176407	ACACACU
ENSG00000176407	ACAGUAC
ENSG00000176407	ACAUUAC
ENSG00000176407	ACAUUCA
ENSG00000176407	ACCACAG
ENSG00000176407	ACUGCAG
ENSG00000176407	ACUGGAU
ENSG00000176407	AGAUGUG
ENSG00000176407	AGGACAC
ENSG00000176407	AGGUUAU
ENSG00000176407	AGUAGUU
ENSG00000176407	AUAAUAC
ENSG00000176407	AUCAUAC
ENSG00000176407	AUGCACC
ENSG00000176407	AUGCAUU
ENSG00000176407	AUGUGCC
ENSG00000176407	CAAAGCA

ENSG00000176407	CACAGGG
ENSG00000176407	CACAGUG
ENSG00000176407	CAGCAAA
ENSG00000176407	CAGCAGA
ENSG00000176407	CAGUAGU
ENSG00000176407	CCAUUAC
ENSG00000176407	CCCCUGG
ENSG00000176407	CUCACAC
ENSG00000176407	CUCUGGG
ENSG00000176407	CUGCAGC
ENSG00000176407	CUUGGAG
ENSG00000176407	CUUGUAU
ENSG00000176407	GACUUCC
ENSG00000176407	GAGAACU
ENSG00000176407	GAGAUGA
ENSG00000176407	GAGGUAG
ENSG00000176407	GAGGUAU
ENSG00000176407	GCAGAAG
ENSG00000176407	GCAGCAU
ENSG00000176407	GGAGGCA
ENSG00000176407	GGAUGAG
ENSG00000176407	GGCGACA
ENSG00000176407	GGGUGUU
ENSG00000176407	GGUUACC
ENSG00000176407	GGUUGUC
ENSG00000176407	GUAACAG
ENSG00000176407	GUAUUCU
ENSG00000176407	GUCAACA
ENSG00000176407	GUGACAG
ENSG00000176407	GUGACUG
ENSG00000176407	GUGCGCA
ENSG00000176407	UAAUACA
ENSG00000176407	UAAUAUC
ENSG00000176407	UACAGUU
ENSG00000176407	UAGGGCC
ENSG00000176407	UAUCAGA
ENSG00000176407	UCAAGUA
ENSG00000176407	UCACACA
ENSG00000176407	UCACAGG
ENSG00000176407	UCACAGU
ENSG00000176407	UCUCAAG
ENSG00000176407	UCUCACC
ENSG00000176407	UGAGAAG
ENSG00000176407	UGAGAAU
ENSG00000176407	UGGGAUC
ENSG00000176407	UGUCUGG
ENSG00000176407	UGUGCGG
ENSG00000176407	UGUGCGU
ENSG00000176407	UGUUGAA
ENSG00000176407	UUAUGCA
ENSG00000176407	UUCCUCG
ENSG00000176407	UUGGCAA
ENSG00000176407	UUGGUUC
ENSG00000176407	UUUGCAA
ENSG00000176407	UUUGCAC
ENSG00000176407	UUUUUGC
ENSG00000176410	GGUAGAC
ENSG00000176410	UCUCACC
ENSG00000176422	AACUGGU
ENSG00000176422	AAUACUG
ENSG00000176422	CACUCCU
ENSG00000176422	CUCUUCC
ENSG00000176422	CUUUGGU
ENSG00000176422	GAGCCCC

ENSG00000176422	GAGGUAG
ENSG00000176428	CUGCCCC
ENSG00000176428	GCGGGGC
ENSG00000176428	GGCGGGG
ENSG00000176438	UCCAGUU
ENSG00000176438	UGUAUAA
ENSG00000176444	AGCACCA
ENSG00000176444	UAUAAAG
ENSG00000176454	AUAUAAC
ENSG00000176454	UACAAAG
ENSG00000176463	GAGAAAU
ENSG00000176463	UGUCUGG
ENSG00000176490	AGCACCA
ENSG00000176490	AGUGAUC
ENSG00000176490	CCCUGAG
ENSG00000176490	GGAAGAC
ENSG00000176490	UAUUGCU
ENSG00000176490	UCACAGU
ENSG00000176490	UGUGCUU
ENSG00000176531	GCGGGGC
ENSG00000176531	GGCGGGG
ENSG00000176531	UCACAGG
ENSG00000176532	AAGUGCA
ENSG00000176532	UACAGUU
ENSG00000176533	AACACUG
ENSG00000176533	AUGGCUU
ENSG00000176533	GCCUGGA
ENSG00000176542	AAAGUGC
ENSG00000176542	AAGGCAC
ENSG00000176542	AAGUGCA
ENSG00000176542	ACUGCAG
ENSG00000176542	AGCACCA
ENSG00000176542	AGUAACA
ENSG00000176542	AUGGCAC
ENSG00000176542	CACACCG
ENSG00000176542	CACACCU
ENSG00000176542	CACAGUG
ENSG00000176542	CAGCAGA
ENSG00000176542	CAGCUGG
ENSG00000176542	CCAGUGU
ENSG00000176542	CUACAGU
ENSG00000176542	CUCCCAC
ENSG00000176542	CUGCAGC
ENSG00000176542	CUGCUCA
ENSG00000176542	GAAACAU
ENSG00000176542	GAAGGUC
ENSG00000176542	GAGAAAU
ENSG00000176542	GAGGUAU
ENSG00000176542	GAGUCUU
ENSG00000176542	GCACCAU
ENSG00000176542	GGAAGCC
ENSG00000176542	GGCAAGA
ENSG00000176542	GUGACUG
ENSG00000176542	UAAUACA
ENSG00000176542	UAUAAAG
ENSG00000176542	UAUUACC
ENSG00000176542	UCAAGUA
ENSG00000176542	UCACACA
ENSG00000176542	UGAGUCU
ENSG00000176563	CAGUCUG
ENSG00000176563	GUAUUCU
ENSG00000176595	GUCACUC
ENSG00000176595	UCUAGAG
ENSG00000176597	AGCACCA

ENSG00000176597	CAUAUUG
ENSG00000176597	GACCAUG
ENSG00000176597	GUGACAG
ENSG00000176619	AAAGUUU
ENSG00000176619	AUAUUAU
ENSG00000176619	GAAACAU
ENSG00000176619	GAGCUGC
ENSG00000176619	GGCAGUG
ENSG00000176619	GGCUCAG
ENSG00000176619	UAAGACU
ENSG00000176619	UACAAAG
ENSG00000176619	UCCAGUU
ENSG00000176619	UGAAAUG
ENSG00000176623	AUCCUUU
ENSG00000176623	CUUCUCU
ENSG00000176624	AACAUUC
ENSG00000176624	AAGUCUU
ENSG00000176624	AAUACUG
ENSG00000176624	AGUGCUU
ENSG00000176624	AUAAUAC
ENSG00000176624	AUCAUAC
ENSG00000176624	CAAAACU
ENSG00000176624	CAGUGCA
ENSG00000176624	GUUAAUG
ENSG00000176641	AUGGCUU
ENSG00000176641	UAUUGCU
ENSG00000176658	AAAGUGC
ENSG00000176658	AAAGUUU
ENSG00000176658	AAGUGCU
ENSG00000176658	AGGGUCU
ENSG00000176658	AGUGCAA
ENSG00000176658	AGUGCUU
ENSG00000176658	CAAGAGC
ENSG00000176658	CUGCUCA
ENSG00000176658	CUUUGGU
ENSG00000176658	GAGGGGC
ENSG00000176658	GGCAAGA
ENSG00000176658	GGGUCUU
ENSG00000176658	GUGCUUG
ENSG00000176658	UCAUUUG
ENSG00000176658	UGCAAAG
ENSG00000176658	UGCUCAC
ENSG00000176658	UUGGCAA
ENSG00000176658	UUGGCAC
ENSG00000176678	GAGGCUG
ENSG00000176678	GUUAAUG
ENSG00000176697	AAAAACC
ENSG00000176697	AAACGGU
ENSG00000176697	AAAUCCC
ENSG00000176697	AACGGAA
ENSG00000176697	AAUGCCC
ENSG00000176697	ACCACAG
ENSG00000176697	ACUGUAG
ENSG00000176697	ACUGUUU
ENSG00000176697	AUACAAG
ENSG00000176697	AUAUAAC
ENSG00000176697	AUGUAAU
ENSG00000176697	CACUGUU
ENSG00000176697	CAUGGAU
ENSG00000176697	CCUGCUG
ENSG00000176697	CUAUACA
ENSG00000176697	CUUGUAU
ENSG00000176697	GACAACU
ENSG00000176697	GAGAACC

ENSG00000176697	GAUUGUA
ENSG00000176697	GUAAACA
ENSG00000176697	GUUAAUG
ENSG00000176697	UAAUGCU
ENSG00000176697	UCAUUUG
ENSG00000176697	UCCAGAG
ENSG00000176697	UCCCUUG
ENSG00000176697	UGACAUC
ENSG00000176697	UGCAAAG
ENSG00000176697	UGUAUAA
ENSG00000176697	UGUGCGG
ENSG00000176697	UGUGCGU
ENSG00000176697	UUGGCAA
ENSG00000176731	UGAAGUG
ENSG00000176749	AAAAGUA
ENSG00000176749	AAUCCUU
ENSG00000176749	ACAAUAU
ENSG00000176749	ACACACU
ENSG00000176749	ACUGCAU
ENSG00000176749	AGCAGCG
ENSG00000176749	AGCAGCU
ENSG00000176749	AUCCUUG
ENSG00000176749	CAGUAGA
ENSG00000176749	CAGUAGU
ENSG00000176749	GAAGGAA
ENSG00000176749	GCAGCAU
ENSG00000176749	UAAUAUC
ENSG00000176749	UAUAAAG
ENSG00000176749	UCAAGUA
ENSG00000176749	UCACAGG
ENSG00000176749	UCACAGU
ENSG00000176749	UCACAUU
ENSG00000176749	UCAUAGA
ENSG00000176749	UCAUGAU
ENSG00000176749	UUGGUCC
ENSG00000176749	UUGUUCG
ENSG00000176749	UUUUUGC
ENSG00000176753	AAAGAAU
ENSG00000176753	GCUUCCU
ENSG00000176769	ACAAUAU
ENSG00000176769	AUACAAG
ENSG00000176769	AUAUUAU
ENSG00000176769	GAAACAU
ENSG00000176769	GAGAAAU
ENSG00000176769	GGUUUAC
ENSG00000176769	UACAAAG
ENSG00000176769	UGCAUAG
ENSG00000176769	UGCAUAU
ENSG00000176783	AAGGCAC
ENSG00000176783	GAGGCUG
ENSG00000176783	UUGGCAC
ENSG00000176788	AAGUAGC
ENSG00000176788	AAUACUG
ENSG00000176788	ACAGGUG
ENSG00000176788	ACUGUUU
ENSG00000176788	AUACAAG
ENSG00000176788	AUGCAUU
ENSG00000176788	GCAUCCC
ENSG00000176788	GGAAGAC
ENSG00000176788	GGAUGAG
ENSG00000176788	UAUUACC
ENSG00000176788	UUGGCAC
ENSG00000176845	AUAAUAC
ENSG00000176853	AGUGCUG

ENSG00000176853	CUGCAGC
ENSG00000176853	CUUUGGU
ENSG00000176853	GAUUGUC
ENSG00000176853	GGAAUGU
ENSG00000176853	GUGACUG
ENSG00000176853	GUGCCAC
ENSG00000176853	UCUCAAG
ENSG00000176853	UUGAUCA
ENSG00000176871	AUAUUAU
ENSG00000176871	GAGAAAU
ENSG00000176871	GCAGAAG
ENSG00000176871	UAUGGUU
ENSG00000176884	ACAAGGU
ENSG00000176884	AGCAGCA
ENSG00000176884	CAGCAGG
ENSG00000176884	CCAGCAU
ENSG00000176884	CUCUGGG
ENSG00000176884	GCAGGAC
ENSG00000176884	GGCCCUG
ENSG00000176884	GGUGGGC
ENSG00000176884	UCCCUCU
ENSG00000176887	AAACUGU
ENSG00000176887	AACAUUC
ENSG00000176887	AAGACAU
ENSG00000176887	ACAGUAU
ENSG00000176887	ACCAGCA
ENSG00000176887	ACCUGGA
ENSG00000176887	ACUGCAU
ENSG00000176887	AGAUAAA
ENSG00000176887	AGGCAGG
ENSG00000176887	AGGUAGU
ENSG00000176887	AGUAACA
ENSG00000176887	AGUGCCU
ENSG00000176887	AGUGCUG
ENSG00000176887	AGUGCUU
ENSG00000176887	AGUGGUU
ENSG00000176887	AUAAAGU
ENSG00000176887	AUACAAG
ENSG00000176887	AUCCUUU
ENSG00000176887	CAAAGCA
ENSG00000176887	CACAGUG
ENSG00000176887	CACUGUU
ENSG00000176887	CAGCAGG
ENSG00000176887	CAGUCUG
ENSG00000176887	CAGUGCA
ENSG00000176887	CCGGAGC
ENSG00000176887	CUAGGCU
ENSG00000176887	CUGGGGG
ENSG00000176887	GAGGUAU
ENSG00000176887	GAGUAUU
ENSG00000176887	GAUCAGA
ENSG00000176887	GCAUCCC
ENSG00000176887	GCCUGAU
ENSG00000176887	GCUGUCU
ENSG00000176887	GGGCCCC
ENSG00000176887	GGGGCAG
ENSG00000176887	GGUCUAG
ENSG00000176887	GUAAACA
ENSG00000176887	GUAACAG
ENSG00000176887	GUAGUGU
ENSG00000176887	GUCAGUU
ENSG00000176887	GUGCUUG
ENSG00000176887	GUGGCCC
ENSG00000176887	UAUAAAG

ENSG00000176887	UAUUACC
ENSG00000176887	UCACACA
ENSG00000176887	UCACAUU
ENSG00000176887	UCAUACA
ENSG00000176887	UCCAGAG
ENSG00000176887	UCCCUUG
ENSG00000176887	UGCAAAG
ENSG00000176887	UGCAUAG
ENSG00000176887	UGCAUAU
ENSG00000176887	UGGCCCU
ENSG00000176887	UGUCUGG
ENSG00000176887	UUCUGCA
ENSG00000176887	UUGGCAA
ENSG00000176887	UUUGCAA
ENSG00000176903	UGAAAUG
ENSG00000176907	ACAGUAC
ENSG00000176907	AGCAUUC
ENSG00000176907	CACAGUG
ENSG00000176907	GUAAACA
ENSG00000176907	UCACAGG
ENSG00000176915	AGCAAAA
ENSG00000176928	AGGUAGU
ENSG00000176928	GAGGUAU
ENSG00000176928	UUGGCAA
ENSG00000176946	AAAAAUC
ENSG00000176946	UUGGUUC
ENSG00000176971	ACUGGCU
ENSG00000176971	AGCCAGU
ENSG00000176971	AGUGCAA
ENSG00000176971	GUCUCUG
ENSG00000176973	AGCAGCU
ENSG00000176974	AAAGCUG
ENSG00000176974	AAAUGGU
ENSG00000176974	GGUUGUC
ENSG00000176974	GUGCUUG
ENSG00000176974	UGAAAUG
ENSG00000176974	UUCUUGU
ENSG00000176974	UUUGCAA
ENSG00000176986	AGCCCUU
ENSG00000176986	AUGCAUU
ENSG00000176986	AUUCAUU
ENSG00000176986	AUUGCAC
ENSG00000176986	CAGGCUC
ENSG00000176986	CCAGUGU
ENSG00000176986	CUGGCUC
ENSG00000176986	GAGGUAG
ENSG00000176986	GGGAGCU
ENSG00000176986	UACAAAG
ENSG00000176994	AAAAGUA
ENSG00000176994	AAGGCAC
ENSG00000176994	AAGUGCA
ENSG00000176994	AAUCCUU
ENSG00000176994	ACCCUGU
ENSG00000176994	AGGUAGU
ENSG00000176994	AGUAGAC
ENSG00000176994	AGUGCAA
ENSG00000176994	AUCCUUU
ENSG00000176994	CACAGGG
ENSG00000176994	CACAGUG
ENSG00000176994	CCCUGAG
ENSG00000176994	CUACAAA
ENSG00000176994	CUUGUGU
ENSG00000176994	GACCAUG
ENSG00000176994	GAGGUAG

ENSG00000176994	GUCUACU
ENSG00000176994	GUGCAAA
ENSG00000176994	UCACAGG
ENSG00000176994	UCACAGU
ENSG00000176994	UCCAGUU
ENSG00000176994	UCUAGAG
ENSG00000176994	UGCAAAG
ENSG00000176994	UUCAAAU
ENSG00000176994	UUGGCAA
ENSG00000176994	UUGGCAC
ENSG00000177000	AGCAGCU
ENSG00000177000	AGGCAGG
ENSG00000177000	CAGCAGG
ENSG00000177000	CAGCUGG
ENSG00000177000	CUGCCCC
ENSG00000177000	GGAGGCA
ENSG00000177000	GGCAGCG
ENSG00000177000	GGCAGGG
ENSG00000177000	GGGCCCC
ENSG00000177000	GGGGCAG
ENSG00000177034	AAAAACC
ENSG00000177034	AAGGCAC
ENSG00000177034	AGCACCA
ENSG00000177034	AUAAAGU
ENSG00000177034	AUGUCUG
ENSG00000177034	GAGAAAU
ENSG00000177034	GGAUUUC
ENSG00000177034	UGAGAAG
ENSG00000177034	UGAGAAU
ENSG00000177034	UUGGUCC
ENSG00000177045	AGCACCA
ENSG00000177045	CUUUGGU
ENSG00000177045	GUAACAG
ENSG00000177045	UAAUAGU
ENSG00000177045	UUGGUCC
ENSG00000177051	AGCUGCC
ENSG00000177051	CUGCUCA
ENSG00000177051	GAGAUGA
ENSG00000177051	GCUGGUG
ENSG00000177051	GGUGGGC
ENSG00000177051	UAUAAAG
ENSG00000177051	UCACAGG
ENSG00000177051	UCACAGU
ENSG00000177058	UGAGAAU
ENSG00000177082	CAGCUGG
ENSG00000177096	AACCUGG
ENSG00000177098	AACCUGG
ENSG00000177098	AAGGCAC
ENSG00000177098	AAUUUUA
ENSG00000177098	AGCAGCA
ENSG00000177098	CCCUGAG
ENSG00000177098	CUACAAA
ENSG00000177098	GAGGUAG
ENSG00000177098	GUGCAAA
ENSG00000177103	AAAGGUA
ENSG00000177103	GAAACAU
ENSG00000177103	GAGAAAU
ENSG00000177103	GAGAAGA
ENSG00000177103	GCCCUGU
ENSG00000177103	GUGCCUG
ENSG00000177103	UAUUGCU
ENSG00000177103	UGCCCUG
ENSG00000177105	CUCAGGG
ENSG00000177106	AUAAUAC

ENSG00000177106	GAGCCCC
ENSG00000177108	AUACCUC
ENSG00000177108	CAAAUGC
ENSG00000177108	CAGUGCA
ENSG00000177108	CCUUCUU
ENSG00000177108	GCUGUCU
ENSG00000177108	GGGGCAG
ENSG00000177108	UGGCUGC
ENSG00000177125	AACAGUC
ENSG00000177125	AAGGCAC
ENSG00000177125	AAGUGUG
ENSG00000177125	ACAUUCA
ENSG00000177125	ACUCAAA
ENSG00000177125	AGUACUG
ENSG00000177125	AUAAAGU
ENSG00000177125	AUCCUUU
ENSG00000177125	AUGACAC
ENSG00000177125	AUGGCUU
ENSG00000177125	AUUGCAC
ENSG00000177125	CACAGUG
ENSG00000177125	CCCUGAG
ENSG00000177125	CUUUGGU
ENSG00000177125	GAAGGUC
ENSG00000177125	GCACCAU
ENSG00000177125	GCAGCAU
ENSG00000177125	GGCAAGA
ENSG00000177125	GUAAACA
ENSG00000177125	GUCACUC
ENSG00000177125	GUGCUUG
ENSG00000177125	UAUAAAG
ENSG00000177125	UAUUGCU
ENSG00000177125	UCCCUUU
ENSG00000177125	UGAAGUG
ENSG00000177125	UGACCUA
ENSG00000177125	UGAGAAU
ENSG00000177125	UGAGUCU
ENSG00000177125	UGCAUAG
ENSG00000177125	UGCAUAU
ENSG00000177125	UGCCAGC
ENSG00000177125	UGGAUAA
ENSG00000177125	UGUGCUU
ENSG00000177125	UUCAAGC
ENSG00000177125	UUCUUGU
ENSG00000177125	UUUUUGC
ENSG00000177150	AAGGCAC
ENSG00000177169	AAAGCUG
ENSG00000177169	AAGUGCU
ENSG00000177169	AAGUGUG
ENSG00000177169	AUAAAGU
ENSG00000177169	CACAGUG
ENSG00000177169	UUCAAGC
ENSG00000177189	AAACGGU
ENSG00000177189	AAAGAGG
ENSG00000177189	AAAGCGC
ENSG00000177189	AAAGGUA
ENSG00000177189	AAAGUGC
ENSG00000177189	AAGUCAC
ENSG00000177189	AAUGUUG
ENSG00000177189	ACCAGCA
ENSG00000177189	ACUGUAG
ENSG00000177189	AGAUAAA
ENSG00000177189	AGCAAAA
ENSG00000177189	AGCACCA
ENSG00000177189	AGCUUAU

ENSG00000177189	AGUGCAA
ENSG00000177189	AGUGCCU
ENSG00000177189	AGUGCUU
ENSG00000177189	AGUGGUU
ENSG00000177189	AUAAAGU
ENSG00000177189	AUACAAG
ENSG00000177189	AUGCCUU
ENSG00000177189	CAGCAAA
ENSG00000177189	CAGCAGG
ENSG00000177189	CAGCUGG
ENSG00000177189	CAGGUGA
ENSG00000177189	CCCACCG
ENSG00000177189	CCCACCU
ENSG00000177189	CUCUGGG
ENSG00000177189	CUCUUCC
ENSG00000177189	GAGAAGA
ENSG00000177189	GAGGUAG
ENSG00000177189	GCAGCAU
ENSG00000177189	GCAGGUU
ENSG00000177189	GCCCUGU
ENSG00000177189	GGCAGCG
ENSG00000177189	GGCUCUG
ENSG00000177189	GGUUGAC
ENSG00000177189	GUGUCAC
ENSG00000177189	GUUCAUG
ENSG00000177189	UACAAAG
ENSG00000177189	UACAGUU
ENSG00000177189	UAGACUG
ENSG00000177189	UAUUGCU
ENSG00000177189	UCAACAG
ENSG00000177189	UCAUUCG
ENSG00000177189	UCCCACC
ENSG00000177189	UCCCUUG
ENSG00000177189	UCCUGAG
ENSG00000177189	UGGGACA
ENSG00000177189	UGGGUAC
ENSG00000177189	UGGUACC
ENSG00000177189	UGUGCUU
ENSG00000177189	UGUGUCA
ENSG00000177189	UUGUGUC
ENSG00000177200	AAGACAU
ENSG00000177200	AAUCACU
ENSG00000177200	ACAUUCA
ENSG00000177200	ACUCAGC
ENSG00000177200	AGCAGCA
ENSG00000177200	AGCAGCG
ENSG00000177200	AGCAGCU
ENSG00000177200	AUAUUAU
ENSG00000177200	AUCACUA
ENSG00000177200	AUGAUGA
ENSG00000177200	AUUCAUU
ENSG00000177200	AUUGCAC
ENSG00000177200	CAGUGCA
ENSG00000177200	CAUGGGU
ENSG00000177200	CUGGGCA
ENSG00000177200	GAGGCUG
ENSG00000177200	GUAACAG
ENSG00000177200	UACAAAG
ENSG00000177200	UAUAAUA
ENSG00000177200	UGAAAUG
ENSG00000177200	UGCUGAC
ENSG00000177239	GUAAACA
ENSG00000177272	ACUGCAU
ENSG00000177283	AACAAAC

ENSG00000177283	AAGGCAC
ENSG00000177283	AAUUAUU
ENSG00000177283	ACCCGUA
ENSG00000177283	AUAUAAC
ENSG00000177283	GCCUGGA
ENSG00000177283	GCCUGGG
ENSG00000177283	GGGGUGC
ENSG00000177283	UAAGACG
ENSG00000177283	UAAGACU
ENSG00000177283	UACAAAG
ENSG00000177283	UGUAUAA
ENSG00000177300	ACAGGUG
ENSG00000177300	AUGUAAU
ENSG00000177300	GUCUUGC
ENSG00000177301	UAUUGCU
ENSG00000177303	AACAUUC
ENSG00000177303	GGAAUGU
ENSG00000177303	GUUAAUG
ENSG00000177311	AAAAAUC
ENSG00000177311	AAUACUG
ENSG00000177311	ACAGUAC
ENSG00000177311	ACUGUAG
ENSG00000177311	AGAUAAA
ENSG00000177311	AGCAAAA
ENSG00000177311	AGCCCUU
ENSG00000177311	AUACAAG
ENSG00000177311	CACUGUU
ENSG00000177311	CAGGAAC
ENSG00000177311	CCCUGAG
ENSG00000177311	CUUUGGU
ENSG00000177311	GCUGUCU
ENSG00000177311	GGAAGAC
ENSG00000177311	GGCUCUG
ENSG00000177311	UACAAAG
ENSG00000177311	UAGACUG
ENSG00000177311	UAUAAUA
ENSG00000177311	UAUCAGA
ENSG00000177311	UAUUGCU
ENSG00000177311	UCAAGUA
ENSG00000177311	UGUAUGU
ENSG00000177311	UUGGUCC
ENSG00000177311	UUGUGAC
ENSG00000177352	AAAGGUA
ENSG00000177352	AAGUCAC
ENSG00000177352	CAGUGCA
ENSG00000177352	UCACAGU
ENSG00000177352	UGAAAGG
ENSG00000177354	ACCCAUU
ENSG00000177370	CCAGGAG
ENSG00000177370	UGCCCUG
ENSG00000177374	AAGGCAC
ENSG00000177374	AUACAAG
ENSG00000177374	CACAGUG
ENSG00000177374	GAGGUAG
ENSG00000177374	GGCCCUG
ENSG00000177374	GUGCCAC
ENSG00000177374	UCACAGU
ENSG00000177374	UGGCCCU
ENSG00000177380	ACCCGUA
ENSG00000177380	AGCAGCA
ENSG00000177380	AGCAGCG
ENSG00000177380	AGCAGCU
ENSG00000177380	CAGUCCA
ENSG00000177380	CUUGGGC

ENSG00000177380	GAAGGAA
ENSG00000177380	GCGGGGC
ENSG00000177380	GGAAGAC
ENSG00000177380	GGAAGCC
ENSG00000177380	GGCACGG
ENSG00000177380	GGCGGGG
ENSG00000177380	GGGCCCC
ENSG00000177380	UUGGUCC
ENSG00000177426	ACAGUAC
ENSG00000177426	CAGUCUG
ENSG00000177426	UCACAUU
ENSG00000177427	CUCUGGG
ENSG00000177432	AAGGCAC
ENSG00000177432	AUACAAG
ENSG00000177432	GGGUGUU
ENSG00000177432	UCACAAG
ENSG00000177432	UCACACA
ENSG00000177432	UCACAGG
ENSG00000177432	UCACAGU
ENSG00000177432	UUGGUUC
ENSG00000177463	AAAACUG
ENSG00000177463	AAACGGU
ENSG00000177463	AACCUGG
ENSG00000177463	AAGGCAC
ENSG00000177463	ACUGUAG
ENSG00000177463	AGCAUUC
ENSG00000177463	AGCUUAU
ENSG00000177463	AGUAGUU
ENSG00000177463	AGUGCUU
ENSG00000177463	AUACAAG
ENSG00000177463	CCCACCU
ENSG00000177463	GAAACAU
ENSG00000177463	GAGUUGA
ENSG00000177463	GAGUUGG
ENSG00000177463	GAUUGUA
ENSG00000177463	GCAGCAU
ENSG00000177463	GCCCUGU
ENSG00000177463	GCCCUUA
ENSG00000177463	GGAGGCA
ENSG00000177463	GGCAGCG
ENSG00000177463	GGCCCUG
ENSG00000177463	GUCUCUG
ENSG00000177463	UAAUACA
ENSG00000177463	UAGGUAU
ENSG00000177463	UCAUUCG
ENSG00000177463	UGAAAUG
ENSG00000177463	UGAGAAG
ENSG00000177463	UGCUGAC
ENSG00000177463	UUCUAAU
ENSG00000177464	UGCAUAG
ENSG00000177464	UGCAUAU
ENSG00000177465	GUCAACA
ENSG00000177468	AACAAAC
ENSG00000177468	AACACUG
ENSG00000177468	AAUCCUU
ENSG00000177468	AAUCUCA
ENSG00000177468	ACAAGGU
ENSG00000177468	AUAUAAC
ENSG00000177468	AUCCUUG
ENSG00000177468	AUGCAUU
ENSG00000177468	AUUCAUU
ENSG00000177468	GAGUGUG
ENSG00000177468	UGAAAUG
ENSG00000177468	UGACCUA

ENSG00000177469	AGCACCA
ENSG00000177469	AGGACAC
ENSG00000177469	CACCAGC
ENSG00000177469	CCUGCUG
ENSG00000177469	CUCACAC
ENSG00000177469	CUGCCCC
ENSG00000177469	CUGCUCA
ENSG00000177469	GCAGGUU
ENSG00000177469	UGCUCAC
ENSG00000177469	UUGUGUC
ENSG00000177479	ACCCUGU
ENSG00000177479	AGCCUGC
ENSG00000177479	CCAGUGU
ENSG00000177479	GGCCCUG
ENSG00000177479	GGGGGAA
ENSG00000177479	UCCCUCU
ENSG00000177485	AAAAACC
ENSG00000177485	AAGUGCU
ENSG00000177485	AGCAGCA
ENSG00000177485	AUGAGCU
ENSG00000177485	CUAGGCU
ENSG00000177485	GAAAACA
ENSG00000177485	GACCCUG
ENSG00000177508	CAAAGUG
ENSG00000177508	CUAUACA
ENSG00000177511	AAGUGCU
ENSG00000177511	ACAUUCA
ENSG00000177511	AGUACUG
ENSG00000177511	AGUGCUG
ENSG00000177511	CUACAGU
ENSG00000177511	CUGUUGA
ENSG00000177511	GCAGCAU
ENSG00000177511	GGCAGUG
ENSG00000177511	GGCAUUG
ENSG00000177511	GGGGCAG
ENSG00000177511	GGUCUAG
ENSG00000177511	UCACACA
ENSG00000177511	UCAUUUG
ENSG00000177511	UGGCCAC
ENSG00000177511	UGUCUUU
ENSG00000177511	UUCUGCA
ENSG00000177519	CCCACCU
ENSG00000177519	GUGUCAC
ENSG00000177542	AAAACUG
ENSG00000177542	AGCACCA
ENSG00000177542	AGCAGCA
ENSG00000177542	AGCAGCU
ENSG00000177542	AGGUAGU
ENSG00000177542	GAGCCCC
ENSG00000177542	GGAAUGU
ENSG00000177542	GUAGUGU
ENSG00000177548	UUGGUUC
ENSG00000177551	AAAAACC
ENSG00000177551	AAGUGCU
ENSG00000177551	AAUUAUU
ENSG00000177551	AGUGCAA
ENSG00000177551	UCACCAC
ENSG00000177551	UGAAGUG
ENSG00000177551	UUGGCAC
ENSG00000177556	UUGGUCC
ENSG00000177565	AAAGCUG
ENSG00000177565	AAUACUG
ENSG00000177565	AAUUAUU
ENSG00000177565	ACUCAAA

ENSG00000177565	ACUCAGC
ENSG00000177565	ACUGGCC
ENSG00000177565	AGUGCUU
ENSG00000177565	AUGGCUU
ENSG00000177565	AUGUGCC
ENSG00000177565	CAGCAGA
ENSG00000177565	CAGCAGG
ENSG00000177565	CAGUGCA
ENSG00000177565	CCAGGAG
ENSG00000177565	CCAGUGU
ENSG00000177565	CUAGUAG
ENSG00000177565	CUGGACU
ENSG00000177565	GGACCUG
ENSG00000177565	GGACUGC
ENSG00000177565	GGGAGCU
ENSG00000177565	UAUUGCU
ENSG00000177565	UCAUAGA
ENSG00000177565	UCUGGAA
ENSG00000177565	UCUGGAG
ENSG00000177565	UGCAAAG
ENSG00000177565	UGGACUG
ENSG00000177565	UGGCUGC
ENSG00000177565	UUCAAAU
ENSG00000177565	UUGUGUC
ENSG00000177565	UUUGCAC
ENSG00000177570	AACAGUC
ENSG00000177570	AAGACAU
ENSG00000177570	AAUUAUU
ENSG00000177570	ACAUUCA
ENSG00000177570	AGCAAAA
ENSG00000177570	AGCAGCU
ENSG00000177570	AGCUGCC
ENSG00000177570	AGUGCUU
ENSG00000177570	AUGUGCC
ENSG00000177570	AUUCAUU
ENSG00000177570	CACAGUG
ENSG00000177570	CAGCAGA
ENSG00000177570	CAGUCUG
ENSG00000177570	CCAGUAC
ENSG00000177570	CUGCAGC
ENSG00000177570	GAGGUAG
ENSG00000177570	GCAGAAG
ENSG00000177570	GCUGGUG
ENSG00000177570	GGAGAGA
ENSG00000177570	GGCAGGG
ENSG00000177570	GUCAUUG
ENSG00000177570	GUGACAG
ENSG00000177570	GUGGCCC
ENSG00000177570	UAGAGGA
ENSG00000177570	UCAACAG
ENSG00000177570	UCAUUUG
ENSG00000177570	UCCUGAG
ENSG00000177570	UGAAGCU
ENSG00000177570	UGCAAAG
ENSG00000177570	UGCUGAC
ENSG00000177570	UUGGCAA
ENSG00000177570	UUGGCAC
ENSG00000177570	UUUGCAA
ENSG00000177570	UUUGCAC
ENSG00000177575	ACAUUCA
ENSG00000177575	AGCAGCA
ENSG00000177575	UCACAUU
ENSG00000177606	AAUCUCU
ENSG00000177606	AGGCAGG

ENSG00000177606	CUGGAAA
ENSG00000177606	CUGGGCA
ENSG00000177606	GUUAGGA
ENSG00000177606	UAAUACA
ENSG00000177606	UAUUACC
ENSG00000177606	UUGUGAC
ENSG00000177613	AAGUGUG
ENSG00000177613	UUUUUGC
ENSG00000177614	AAAAGUA
ENSG00000177614	AACCUGG
ENSG00000177614	AACCUGU
ENSG00000177614	AGUGCUU
ENSG00000177614	CAGGCUC
ENSG00000177614	CUCCAUU
ENSG00000177614	GUGGGGA
ENSG00000177614	UCUCCAA
ENSG00000177666	AAGGCAC
ENSG00000177674	GGCAGUG
ENSG00000177685	AGCAGCA
ENSG00000177685	CUGGGGG
ENSG00000177688	AGCAAAA
ENSG00000177694	AACAUUC
ENSG00000177694	ACAUUCA
ENSG00000177694	AGUGGUU
ENSG00000177694	AUGGCUU
ENSG00000177694	CAAAGCA
ENSG00000177694	GGAAUGU
ENSG00000177694	GGCUCUG
ENSG00000177694	GUCCAGA
ENSG00000177697	AAGGCAC
ENSG00000177697	CAGUAGU
ENSG00000177697	UGAAGCU
ENSG00000177728	AAAUGGU
ENSG00000177728	CCAGUAC
ENSG00000177728	UCCAGUU
ENSG00000177732	ACAGGUG
ENSG00000177732	ACCACAG
ENSG00000177732	AGCACCA
ENSG00000177732	AGGUAGU
ENSG00000177732	AUGUAAU
ENSG00000177732	AUGUCUG
ENSG00000177732	CACAGGG
ENSG00000177732	CAGACAG
ENSG00000177732	CAGUCCA
ENSG00000177732	CCAGUGC
ENSG00000177732	CCUGCUG
ENSG00000177732	CCUGUAC
ENSG00000177732	CUCUGGG
ENSG00000177732	CUUCACC
ENSG00000177732	GAGACCU
ENSG00000177732	GAGGCAG
ENSG00000177732	GAGUGUG
ENSG00000177732	GCUGGUG
ENSG00000177732	GGAGGCA
ENSG00000177732	GGCAGGG
ENSG00000177732	GGCGGGG
ENSG00000177732	GGGAACG
ENSG00000177732	GGGCGUG
ENSG00000177732	GGGGCAG
ENSG00000177732	GGGGUCC
ENSG00000177732	GGGGUGG
ENSG00000177732	GUAAACA
ENSG00000177732	GUAUGUU
ENSG00000177732	GUGAAUG

ENSG00000177732	GUGCAAA
ENSG00000177732	GUGGCCC
ENSG00000177732	UAGUGAG
ENSG00000177732	UCCCACA
ENSG00000177732	UCCUGAG
ENSG00000177732	UCUCCAA
ENSG00000177732	UGCCAGC
ENSG00000177732	UGGGGGA
ENSG00000177732	UGUAUGU
ENSG00000177732	UGUCUGC
ENSG00000177732	UGUCUGG
ENSG00000177732	UGUCUUU
ENSG00000177732	UGUGUGG
ENSG00000177732	UUGUGUC
ENSG00000177732	UUGUUCG
ENSG00000177733	CACAAUG
ENSG00000177733	CUACAAA
ENSG00000177733	UGGGAUC
ENSG00000177764	AAAGUUU
ENSG00000177764	AAGUGUG
ENSG00000177764	ACAUUCA
ENSG00000177764	AGCUUAU
ENSG00000177764	AGGUAGU
ENSG00000177764	AUGGCUU
ENSG00000177764	GAGGUAG
ENSG00000177764	GGGGUCC
ENSG00000177764	UGAAGUG
ENSG00000177764	UGGCCAC
ENSG00000177764	UUGGCAA
ENSG00000177807	AAGUGCA
ENSG00000177807	CCUUCUG
ENSG00000177807	GAGUCUU
ENSG00000177807	GCCCUGU
ENSG00000177807	GUCUUGC
ENSG00000177807	UGCCCUG
ENSG00000177807	UUGAUCA
ENSG00000177853	AAGUGCU
ENSG00000177853	ACAGUAC
ENSG00000177853	ACAUUAC
ENSG00000177853	AGUAGAC
ENSG00000177853	AGUGCAA
ENSG00000177853	CCCUGAG
ENSG00000177853	UACAGUU
ENSG00000177853	UCACAUU
ENSG00000177853	UGACAUC
ENSG00000177853	UGAUCCA
ENSG00000177853	UGCAUAG
ENSG00000177853	UUAUGCA
ENSG00000177879	GUAAACA
ENSG00000177885	AAAAACC
ENSG00000177885	AAAGCUG
ENSG00000177885	AAAUGGU
ENSG00000177885	AAGGCAC
ENSG00000177885	ACACACC
ENSG00000177885	ACACACU
ENSG00000177885	ACAUAGA
ENSG00000177885	ACCAGCA
ENSG00000177885	AUCACUA
ENSG00000177885	AUUCAUU
ENSG00000177885	CACAGUG
ENSG00000177885	CAGCAAA
ENSG00000177885	CCCACCG
ENSG00000177885	CCCACCU
ENSG00000177885	CUGGACU

ENSG00000177885	GGACUGC
ENSG00000177885	GGCAUUG
ENSG00000177885	UCCCACC
ENSG00000177885	UGCAUAU
ENSG00000177885	UGGACUG
ENSG00000177885	UUGGCAA
ENSG00000177885	UUGGCAC
ENSG00000177885	UUUGCAA
ENSG00000177885	UUUGCAC
ENSG00000177888	AAGAACC
ENSG00000177888	AAGGCAC
ENSG00000177888	ACAUUCA
ENSG00000177888	AGCACCA
ENSG00000177888	AGCAGCA
ENSG00000177888	AGCAGCG
ENSG00000177888	AGGACAC
ENSG00000177888	AGGCAGG
ENSG00000177888	AGUGGUU
ENSG00000177888	GAGUAUU
ENSG00000177888	GGAAUGU
ENSG00000177888	GGAGUGU
ENSG00000177888	GUGCCAC
ENSG00000177888	GUGCUUG
ENSG00000177888	UGUGCUU
ENSG00000177888	UUGACAC
ENSG00000177888	UUGGCAA
ENSG00000177888	UUGGUUC
ENSG00000177889	AAAUGGU
ENSG00000177889	AACAAAC
ENSG00000177889	AAUCACU
ENSG00000177889	AAUGUUG
ENSG00000177889	ACUGUGU
ENSG00000177889	AGCUGCC
ENSG00000177889	AUGUCUG
ENSG00000177889	CCUUCAU
ENSG00000177889	CUGCAGC
ENSG00000177889	GAUUGUC
ENSG00000177889	UAAGACU
ENSG00000177889	UACAAAG
ENSG00000177889	UAGUGAG
ENSG00000177889	UCACAAG
ENSG00000177889	UCACAGG
ENSG00000177889	UCAUUCG
ENSG00000177889	UGCAGCU
ENSG00000177889	UGUCUGG
ENSG00000177917	CCAGUGU
ENSG00000177917	GUAAACA
ENSG00000177917	UCAAGUA
ENSG00000177951	CUUUGGU
ENSG00000177963	AGCACCA
ENSG00000177963	AGCAGCA
ENSG00000177963	CUGGAAA
ENSG00000177963	GGAAGCC
ENSG00000177963	UCUGGAA
ENSG00000177981	ACUGCAG
ENSG00000177981	GAAAACA
ENSG00000177981	GAGAAAU
ENSG00000177981	GGAUUUU
ENSG00000177981	UGCAUAG
ENSG00000177981	UGCAUAU
ENSG00000177981	UGUGUCA
ENSG00000177981	UUCCUAG
ENSG00000177981	UUGUGAC
ENSG00000177981	UUUGCAC

ENSG00000177981	UUUUUGC
ENSG00000177994	AAGUUGU
ENSG00000178031	AACAAAC
ENSG00000178031	AACAUUC
ENSG00000178031	AAUUAUU
ENSG00000178031	CAGGAAC
ENSG00000178031	CUGCAGG
ENSG00000178031	UAAUAUC
ENSG00000178031	UACAGUU
ENSG00000178031	UCACAAG
ENSG00000178031	UCACAGG
ENSG00000178031	UCACAGU
ENSG00000178031	UCUGGAA
ENSG00000178031	UCUGGAG
ENSG00000178031	UUAGAGA
ENSG00000178031	UUGUGUC
ENSG00000178033	AAUUUUA
ENSG00000178033	UGAGUCU
ENSG00000178033	UGUCUUU
ENSG00000178053	AGCACCA
ENSG00000178053	CAAAUGC
ENSG00000178053	GAUUGGU
ENSG00000178053	GCACCAU
ENSG00000178074	AAAGAAU
ENSG00000178074	CUCAAAC
ENSG00000178074	GCAGAAG
ENSG00000178074	UCUCAAG
ENSG00000178074	UCUGGAG
ENSG00000178074	UGCAUUG
ENSG00000178074	UGGCCAC
ENSG00000178074	UUGGUCC
ENSG00000178074	UUUGCAA
ENSG00000178074	UUUGCAC
ENSG00000178075	UCACAGG
ENSG00000178075	UCACAGU
ENSG00000178104	CUCAAAC
ENSG00000178104	UCCCUGU
ENSG00000178149	AACAUUC
ENSG00000178149	CAGACAG
ENSG00000178163	AGCAGCA
ENSG00000178163	CCUUCAU
ENSG00000178163	GUGCUUG
ENSG00000178163	UAUAAAG
ENSG00000178163	UCUAGAG
ENSG00000178163	UGUGCUU
ENSG00000178163	UUCUGCA
ENSG00000178171	GAGGUAG
ENSG00000178177	AAGUCUU
ENSG00000178177	AGCACCA
ENSG00000178177	AGGUAGU
ENSG00000178177	AGUGCAA
ENSG00000178177	AUAUUAU
ENSG00000178177	CACAGGG
ENSG00000178177	CAUGGGU
ENSG00000178177	GAGGUAG
ENSG00000178177	GGCUCUG
ENSG00000178177	GUAAACA
ENSG00000178177	GUGCUUG
ENSG00000178177	UAAUAUC
ENSG00000178177	UCACAGG
ENSG00000178177	UGCUGAC
ENSG00000178184	ACCAGCA
ENSG00000178184	AGCAGCU
ENSG00000178184	CAGCAGG

ENSG00000178184	UCACAUU
ENSG00000178184	UUGGUCC
ENSG00000178188	AGAUAAA
ENSG00000178188	AGUACUG
ENSG00000178188	CUCUUCC
ENSG00000178188	GGGGUGG
ENSG00000178188	GUAGUGU
ENSG00000178188	UGGCUGC
ENSG00000178202	GAGUGUG
ENSG00000178202	GGAGUGU
ENSG00000178202	UAUCAGA
ENSG00000178202	UCACAGG
ENSG00000178202	UCACAGU
ENSG00000178217	AAAACUG
ENSG00000178217	AAAGCUG
ENSG00000178217	ACAAGGU
ENSG00000178217	AUUCAUU
ENSG00000178217	CAAAGCA
ENSG00000178217	CAGACAG
ENSG00000178217	CAGGAAC
ENSG00000178217	CCAGGAG
ENSG00000178217	CUCACAC
ENSG00000178217	GACAACU
ENSG00000178217	GUGACAG
ENSG00000178217	UAUAUGA
ENSG00000178217	UCAUUCG
ENSG00000178217	UCCAGAG
ENSG00000178217	UCUCAAG
ENSG00000178217	UGAGAAU
ENSG00000178217	UUCAAAU
ENSG00000178235	AGUGCUG
ENSG00000178235	AUGCCUU
ENSG00000178235	AUGGCAC
ENSG00000178235	CUGGAAA
ENSG00000178235	GAAAACA
ENSG00000178235	GCAGCAU
ENSG00000178235	GCUUCCU
ENSG00000178235	UCGUGCA
ENSG00000178235	UGCUGAC
ENSG00000178252	CAGACAG
ENSG00000178252	CAGGAAC
ENSG00000178252	GGAAUGU
ENSG00000178301	GAGUCUU
ENSG00000178403	AAAGUGC
ENSG00000178403	AACCUGG
ENSG00000178403	AACCUGU
ENSG00000178403	AAGUGCA
ENSG00000178403	CUACAAA
ENSG00000178403	GACUUCC
ENSG00000178403	UCCCACA
ENSG00000178425	CCCUGAG
ENSG00000178449	CAGGUGA
ENSG00000178449	CUGCUCA
ENSG00000178537	UCAAGUA
ENSG00000178538	AAAGUUU
ENSG00000178538	UGUCUUU
ENSG00000178562	AAGUGUG
ENSG00000178562	GACUUCC
ENSG00000178562	GAGCCCU
ENSG00000178562	GGCAAGA
ENSG00000178562	GGCUCAG
ENSG00000178562	UCCAGAG
ENSG00000178562	UCCAGUU
ENSG00000178562	UCCUAUA

ENSG00000178562	UCUGGAA
ENSG00000178562	UUCCAUA
ENSG00000178562	UUGGUUC
ENSG00000178567	AAGUGUG
ENSG00000178567	AAUCCUU
ENSG00000178567	AGCAAAA
ENSG00000178567	AGUGUGC
ENSG00000178567	AUCCUUG
ENSG00000178567	CAGUCCA
ENSG00000178567	CUAUACA
ENSG00000178568	AACAAAC
ENSG00000178568	AAGUGCU
ENSG00000178568	ACCAGCA
ENSG00000178568	ACCCAUU
ENSG00000178568	AGCUGCC
ENSG00000178568	AGUAGAC
ENSG00000178568	AGUAGUU
ENSG00000178568	AGUGCAA
ENSG00000178568	AGUGCUU
ENSG00000178568	AUACAAG
ENSG00000178568	AUCCUUG
ENSG00000178568	AUGUGCC
ENSG00000178568	CAAAGUG
ENSG00000178568	CAAAUGC
ENSG00000178568	CAAGAGC
ENSG00000178568	CACAAUG
ENSG00000178568	CACUCCU
ENSG00000178568	CCAGUGC
ENSG00000178568	CCAGUGU
ENSG00000178568	CCCUGAG
ENSG00000178568	CCCUGUC
ENSG00000178568	CGACCCA
ENSG00000178568	GAGGCAG
ENSG00000178568	GCAACUU
ENSG00000178568	GCUGGUG
ENSG00000178568	GGCAUUG
ENSG00000178568	GUAACAG
ENSG00000178568	UACAAAG
ENSG00000178568	UAUUACC
ENSG00000178568	UAUUGCU
ENSG00000178568	UCACACA
ENSG00000178568	UCAUGAU
ENSG00000178568	UCCCUGU
ENSG00000178568	UCUCAAG
ENSG00000178568	UGAGAAG
ENSG00000178568	UGAGAAU
ENSG00000178568	UGUGCUU
ENSG00000178568	UUAUGCA
ENSG00000178568	UUCCAUA
ENSG00000178568	UUGGCAA
ENSG00000178568	UUGGCAC
ENSG00000178573	AACACUG
ENSG00000178573	AACAGUC
ENSG00000178573	AACUCUA
ENSG00000178573	AAGACAU
ENSG00000178573	AAGUUGU
ENSG00000178573	AAUACUG
ENSG00000178573	AAUGCCC
ENSG00000178573	AAUUAUU
ENSG00000178573	ACAUCAC
ENSG00000178573	AGGAUGU
ENSG00000178573	AGGUUAU
ENSG00000178573	AGUAACA
ENSG00000178573	AUAAAGU

ENSG00000178573	AUAUAAC
ENSG00000178573	AUGUAAU
ENSG00000178573	CAGCAAA
ENSG00000178573	CAGUCCA
ENSG00000178573	CAGUGCA
ENSG00000178573	CCAGCAU
ENSG00000178573	CCCUGAG
ENSG00000178573	CUACAGU
ENSG00000178573	CUUUGGU
ENSG00000178573	GAAACAU
ENSG00000178573	GAAGGUC
ENSG00000178573	GAGAUGA
ENSG00000178573	GAGGCAG
ENSG00000178573	GCACCAU
ENSG00000178573	GCAGGUU
ENSG00000178573	GGAUUUC
ENSG00000178573	GUAAACA
ENSG00000178573	GUCCAGA
ENSG00000178573	GUCUCUG
ENSG00000178573	UAAUACA
ENSG00000178573	UAAUAUC
ENSG00000178573	UAGACUG
ENSG00000178573	UAUAAAG
ENSG00000178573	UAUAUGA
ENSG00000178573	UCCAGUU
ENSG00000178573	UUCUGCA
ENSG00000178573	UUGACAC
ENSG00000178573	UUGGCAA
ENSG00000178573	UUGGCAC
ENSG00000178573	UUGGUUC
ENSG00000178573	UUUCAAC
ENSG00000178585	AAAAAUC
ENSG00000178585	ACCCUGU
ENSG00000178585	AGCACCA
ENSG00000178585	AGCAGCA
ENSG00000178585	AUAUAAC
ENSG00000178585	AUCCCUU
ENSG00000178585	AUUGCAC
ENSG00000178585	CCCUGUU
ENSG00000178585	GCUGGUG
ENSG00000178585	GGAAGCC
ENSG00000178585	GGGGUGG
ENSG00000178585	GUUAAUG
ENSG00000178585	UCAAGUA
ENSG00000178585	UCCCUUU
ENSG00000178585	UGACCUA
ENSG00000178585	UGCAAAG
ENSG00000178585	UUCAAGC
ENSG00000178585	UUGUGUC
ENSG00000178585	UUUCAAC
ENSG00000178607	AAGGCAC
ENSG00000178645	CCUUCAU
ENSG00000178685	CCCCUGG
ENSG00000178685	UGCUGAC
ENSG00000178691	AAAGCUG
ENSG00000178691	ACCACAG
ENSG00000178691	AGCAGCA
ENSG00000178691	CACAGUG
ENSG00000178691	CAGUAGU
ENSG00000178691	GCAGCAU
ENSG00000178691	GCUAGCA
ENSG00000178691	UCACAGU
ENSG00000178695	AAGGCAC
ENSG00000178695	AAGUCAC

ENSG00000178695	ACAGUAC
ENSG00000178695	AUACAAG
ENSG00000178695	CCUUCUG
ENSG00000178695	CUACAGU
ENSG00000178695	CUUUGGU
ENSG00000178695	UACAAAG
ENSG00000178695	UAUUGCU
ENSG00000178695	UCCCACC
ENSG00000178695	UGCUCAC
ENSG00000178695	UUGGUUC
ENSG00000178718	CUCAGGG
ENSG00000178718	UGAAAGG
ENSG00000178726	AGCUUAU
ENSG00000178726	UUCCUAG
ENSG00000178726	UUCUUGU
ENSG00000178732	UGAAAUG
ENSG00000178741	AAGUAGC
ENSG00000178741	UCAAGUA
ENSG00000178741	UUCAAAU
ENSG00000178761	AAGGCAC
ENSG00000178761	AUAAAGU
ENSG00000178761	GCUGGUG
ENSG00000178761	UCCCUCU
ENSG00000178761	UGAGAAG
ENSG00000178764	AAAGGUA
ENSG00000178764	AAAGUGC
ENSG00000178764	AAGUGCA
ENSG00000178764	ACUCAAA
ENSG00000178764	AGCCCUU
ENSG00000178764	AGGUUAU
ENSG00000178764	CAGUAGU
ENSG00000178764	CCAGUAC
ENSG00000178764	GGAGUCC
ENSG00000178764	GUUAGGA
ENSG00000178764	UAUAAAG
ENSG00000178764	UCCAGUU
ENSG00000178764	UUGGCAA
ENSG00000178772	UGCAAAG
ENSG00000178772	UUUGCAA
ENSG00000178776	CUGGAAA
ENSG00000178776	GAGAACU
ENSG00000178802	AGUGAUC
ENSG00000178802	CUGGCUC
ENSG00000178802	GGCUCAG
ENSG00000178826	ACUCAAA
ENSG00000178826	GAAAACA
ENSG00000178860	AAAUUUC
ENSG00000178860	AUUCAUU
ENSG00000178860	CUGGCUC
ENSG00000178878	AACACUG
ENSG00000178878	AGUAGAC
ENSG00000178878	AUCCUUU
ENSG00000178878	CAAAGCA
ENSG00000178878	CAGACAG
ENSG00000178878	CCUUCAU
ENSG00000178878	CUUACAG
ENSG00000178878	GGGCCCC
ENSG00000178878	GUGCAAA
ENSG00000178904	GGCAAGA
ENSG00000178904	UCACAGU
ENSG00000178904	UGUGCUU
ENSG00000178904	UUGGCAA
ENSG00000178919	AAAGAGG
ENSG00000178919	ACUAGAU

ENSG00000178951	AAGUGCA
ENSG00000178951	ACCCGUA
ENSG00000178951	AGAUAAA
ENSG00000178951	AGUGCCG
ENSG00000178951	AGUGCCU
ENSG00000178951	AUAAAGU
ENSG00000178951	CAAAGUG
ENSG00000178951	CAGUGCA
ENSG00000178951	CCAGGAG
ENSG00000178951	CCCUGAG
ENSG00000178951	CUCAGGG
ENSG00000178951	GCCCUGU
ENSG00000178951	UACAAAG
ENSG00000178951	UGCAUAU
ENSG00000178951	UGCCCUG
ENSG00000178951	UUGGUUC
ENSG00000178965	AAAGUGC
ENSG00000178965	AAGUGCU
ENSG00000178965	CACAGUG
ENSG00000178966	AAUACUG
ENSG00000178966	GAAAACA
ENSG00000178971	AGCACCA
ENSG00000178971	GAGCUAA
ENSG00000178974	AAGGCGC
ENSG00000178974	GGUUGUC
ENSG00000178974	GUAAACA
ENSG00000178974	GUCCAGA
ENSG00000178974	UCCAGUU
ENSG00000178974	UGCAUUG
ENSG00000178996	AAAGUAU
ENSG00000178996	AACAGUC
ENSG00000178996	ACAUAGA
ENSG00000178996	ACCCAUU
ENSG00000178996	AGCUGCC
ENSG00000178996	AUGCACC
ENSG00000178996	AUGGCUU
ENSG00000178996	CACAGUG
ENSG00000178996	CCCUGAG
ENSG00000178996	CUGGCUC
ENSG00000178996	GAGGCAG
ENSG00000178996	GGAGUCC
ENSG00000178996	GUAGUGU
ENSG00000178996	GUGCUUG
ENSG00000178996	UCACAGU
ENSG00000178996	UGUGCUU
ENSG00000178996	UUGAGGC
ENSG00000179008	UUCUUGU
ENSG00000179010	CUUUGGU
ENSG00000179023	AACAAAC
ENSG00000179023	GCAACUU
ENSG00000179023	GUAACAG
ENSG00000179029	AAUCACU
ENSG00000179029	GGGUGUU
ENSG00000179041	AAAAAUC
ENSG00000179051	AAAGGUA
ENSG00000179051	AAAGUUU
ENSG00000179051	AACGGAA
ENSG00000179051	AAGGCAC
ENSG00000179051	ACUCCAG
ENSG00000179051	AGCACCA
ENSG00000179051	AGCUGCC
ENSG00000179051	AUAUAAC
ENSG00000179051	AUGAGCU
ENSG00000179051	CAAAGUG

ENSG00000179051	CAGUCCA
ENSG00000179051	CAGUGCA
ENSG00000179051	CCAGUGC
ENSG00000179051	CUCAGGG
ENSG00000179051	CUGGACU
ENSG00000179051	GGAAGAC
ENSG00000179051	GGCAUUG
ENSG00000179051	GUCCAGA
ENSG00000179051	UGCUCAC
ENSG00000179051	UGGUACC
ENSG00000179051	UUGGCAA
ENSG00000179051	UUGGCAC
ENSG00000179057	CUCAAAC
ENSG00000179057	GUGGGGA
ENSG00000179057	UCAUGAU
ENSG00000179057	UCCUUGC
ENSG00000179059	UUCCUAG
ENSG00000179071	AAAGUAA
ENSG00000179071	AAAGUAC
ENSG00000179071	UAUAAUA
ENSG00000179083	GGCAGUG
ENSG00000179087	AAUCUCA
ENSG00000179088	UGACAUC
ENSG00000179088	UUGACAC
ENSG00000179094	AGCACCA
ENSG00000179094	AGCAGCU
ENSG00000179094	CAGCAGA
ENSG00000179094	CAGCAGG
ENSG00000179094	CCCACCG
ENSG00000179094	CCCACCU
ENSG00000179094	GAGGGGC
ENSG00000179094	GGCUCAG
ENSG00000179094	UCCCACC
ENSG00000179097	UGCAUAU
ENSG00000179104	AAGUGCA
ENSG00000179104	UCACAGU
ENSG00000179111	AGGCAGG
ENSG00000179111	GUCUGCC
ENSG00000179111	UCCCACC
ENSG00000179119	AAAAGUA
ENSG00000179119	AAAGUGC
ENSG00000179119	AACUACU
ENSG00000179119	AAGGCAC
ENSG00000179119	AAGUGCA
ENSG00000179119	AAUGCCC
ENSG00000179119	ACAUUCA
ENSG00000179119	ACCCUGU
ENSG00000179119	AGCACCA
ENSG00000179119	AGCAGCA
ENSG00000179119	AGCCUGC
ENSG00000179119	AGCUGCC
ENSG00000179119	AGUGCAA
ENSG00000179119	CAGUGCA
ENSG00000179119	CCUGCUG
ENSG00000179119	CUCUAGC
ENSG00000179119	CUCUUCC
ENSG00000179119	CUUCACC
ENSG00000179119	GAUUGUA
ENSG00000179119	GGAAGAC
ENSG00000179119	GUCAACA
ENSG00000179119	GUGCAAA
ENSG00000179119	UAAUGCU
ENSG00000179119	UAUUGCU
ENSG00000179119	UCAACAG

ENSG00000179119	UCACAGG
ENSG00000179119	UCACAGU
ENSG00000179119	UCCAGUU
ENSG00000179119	UGGUACC
ENSG00000179119	UUGGUCC
ENSG00000179119	UUGGUUC
ENSG00000179134	AAAAAUC
ENSG00000179134	AACAAAC
ENSG00000179134	AACCGUU
ENSG00000179134	AAGAACC
ENSG00000179134	AAGGCAC
ENSG00000179134	ACCAGCA
ENSG00000179134	AUAUAAC
ENSG00000179134	CAGCAGA
ENSG00000179134	CUCGCUG
ENSG00000179134	GACCCUG
ENSG00000179134	GGCUCUG
ENSG00000179134	GUGAGGU
ENSG00000179134	UGAGAAU
ENSG00000179151	AAGGCGC
ENSG00000179151	ACAGUAC
ENSG00000179151	ACAGUAU
ENSG00000179151	AGCACCA
ENSG00000179151	AGCUGCC
ENSG00000179151	AGUGCUG
ENSG00000179151	CAGGCUC
ENSG00000179151	CCCCCAG
ENSG00000179151	CUCACAC
ENSG00000179151	GCCUGGA
ENSG00000179151	GCUGGUG
ENSG00000179152	UGGCUGC
ENSG00000179163	UCACAUU
ENSG00000179165	GAGGUAU
ENSG00000179165	GGUAGAC
ENSG00000179195	AAAACUG
ENSG00000179195	AACUCUA
ENSG00000179195	AAUCACU
ENSG00000179195	AUCACUA
ENSG00000179195	CUGGAAA
ENSG00000179195	GACUUCC
ENSG00000179195	GCCUGGA
ENSG00000179195	GUCAUUG
ENSG00000179195	UAAUAGU
ENSG00000179195	UCCUGAG
ENSG00000179195	UUGGUCC
ENSG00000179218	ACAUUCA
ENSG00000179218	CAGUCCA
ENSG00000179218	CUUACAG
ENSG00000179218	GGAGGCA
ENSG00000179218	UAAUAGU
ENSG00000179222	CAAAACU
ENSG00000179222	GUCAACA
ENSG00000179222	UCAACAG
ENSG00000179241	AAAGUGC
ENSG00000179241	AACAAAC
ENSG00000179241	AAGUGCA
ENSG00000179241	ACACACU
ENSG00000179241	AGGAGCU
ENSG00000179241	AUGCAUU
ENSG00000179241	CAGGCUC
ENSG00000179241	CCUUCAU
ENSG00000179241	CUAUACA
ENSG00000179241	CUGCAGG
ENSG00000179241	CUUUGGU

ENSG00000179241	UUCAAAU
ENSG00000179242	AAAAACC
ENSG00000179242	AGUGCCU
ENSG00000179242	AGUGCUU
ENSG00000179242	UCAAGUA
ENSG00000179262	CAAAACU
ENSG00000179262	GAGGGGC
ENSG00000179262	UGGGACA
ENSG00000179270	ACUGCAG
ENSG00000179270	ACUGCAU
ENSG00000179270	GCAGAAG
ENSG00000179270	GCAGCAU
ENSG00000179270	GUGGCCC
ENSG00000179270	UCUGGAG
ENSG00000179270	UGCAGCU
ENSG00000179270	UGUCUUU
ENSG00000179270	UUCUUGU
ENSG00000179284	AGCAGCU
ENSG00000179292	GAGGCUG
ENSG00000179292	UCUGGAA
ENSG00000179292	UGUGCUU
ENSG00000179295	AAAGGUA
ENSG00000179295	AAGGCAC
ENSG00000179295	ACAAUAU
ENSG00000179295	ACACACU
ENSG00000179295	AUAUUAU
ENSG00000179295	AUCCUUU
ENSG00000179295	AUGAUGA
ENSG00000179295	CAAAGCA
ENSG00000179295	CACAAUG
ENSG00000179295	CUAGUCC
ENSG00000179295	CUUCACC
ENSG00000179295	CUUGUGU
ENSG00000179295	GAAACAU
ENSG00000179295	GAGCUGC
ENSG00000179295	GCAGGAC
ENSG00000179295	GCUGGUG
ENSG00000179295	GGCCCUG
ENSG00000179295	GGUCUAG
ENSG00000179295	GUCUACU
ENSG00000179295	GUGCUUG
ENSG00000179295	GUGGGGA
ENSG00000179295	UACAAAG
ENSG00000179295	UACAGUU
ENSG00000179295	UCCCUUG
ENSG00000179295	UCCCUUU
ENSG00000179295	UCUUGAG
ENSG00000179295	UGACAUC
ENSG00000179295	UUAGGAU
ENSG00000179295	UUUUUGC
ENSG00000179299	CUUCUCU
ENSG00000179300	CUUCUCU
ENSG00000179314	AAGAACC
ENSG00000179314	AAUUUUA
ENSG00000179314	ACAUCAC
ENSG00000179314	AGCAGCU
ENSG00000179314	AGCUGCC
ENSG00000179314	CUUCUCU
ENSG00000179314	GAGAUGA
ENSG00000179314	GGCUCAG
ENSG00000179314	UUGGUUC
ENSG00000179335	GGCUCUG
ENSG00000179335	GUGCUUG
ENSG00000179335	UCACAUU

ENSG00000179335	UGUGCUU
ENSG00000179348	AAAAACC
ENSG00000179348	AAAAGUA
ENSG00000179348	AAAGUAU
ENSG00000179348	AACAGUC
ENSG00000179348	AAUACUG
ENSG00000179348	AAUUAUU
ENSG00000179348	AGCAAAA
ENSG00000179348	AUAUAAC
ENSG00000179348	CACAGUG
ENSG00000179348	CAUAUUG
ENSG00000179348	GAGGAUA
ENSG00000179348	GAUUGUA
ENSG00000179348	GAUUGUC
ENSG00000179348	GCCUGGG
ENSG00000179348	GUCUCUG
ENSG00000179348	UCACAGG
ENSG00000179361	AACAUUC
ENSG00000179361	AGCAAAA
ENSG00000179361	AGCUGCC
ENSG00000179361	CAGCAGG
ENSG00000179361	CUAGGCU
ENSG00000179361	CUUUGGU
ENSG00000179361	GAUUGGU
ENSG00000179361	GAUUGUC
ENSG00000179361	GCCUGGG
ENSG00000179361	GGACCUG
ENSG00000179361	UACAAAG
ENSG00000179361	UCACAUU
ENSG00000179361	UCCCUUU
ENSG00000179361	UCCUGAG
ENSG00000179361	UCUCCAA
ENSG00000179361	UGAAAUG
ENSG00000179361	UGCAGCU
ENSG00000179362	AAAACUG
ENSG00000179362	AAACUGU
ENSG00000179362	AAAGUUU
ENSG00000179362	UAUAAAG
ENSG00000179362	UUCAAAU
ENSG00000179364	ACAUUCA
ENSG00000179364	ACUGUUU
ENSG00000179364	CCCUGAG
ENSG00000179364	GAGGUAG
ENSG00000179364	UAAGACG
ENSG00000179387	GAUUGUA
ENSG00000179388	AAAAGUA
ENSG00000179388	AAAGUAU
ENSG00000179388	AAAGUGC
ENSG00000179388	AAAUCCC
ENSG00000179388	AAGUCUU
ENSG00000179388	AAUACUG
ENSG00000179388	AAUGUUG
ENSG00000179388	ACAGUAC
ENSG00000179388	ACAUUAC
ENSG00000179388	ACAUUCA
ENSG00000179388	ACUCAAA
ENSG00000179388	ACUCCAG
ENSG00000179388	ACUGUAG
ENSG00000179388	AGCAAAA
ENSG00000179388	AGCAUUC
ENSG00000179388	AGCUUAU
ENSG00000179388	AGUGCAA
ENSG00000179388	AUAAAGU
ENSG00000179388	AUACAAG

ENSG00000179388	AUGUCUG
ENSG00000179388	CACAAUG
ENSG00000179388	CACCACC
ENSG00000179388	CAGUGCA
ENSG00000179388	CUCUGGG
ENSG00000179388	CUGGGCA
ENSG00000179388	CUUUGGU
ENSG00000179388	GAGAAGA
ENSG00000179388	GAGGUAG
ENSG00000179388	GAUUGUA
ENSG00000179388	GGAAGAC
ENSG00000179388	GUGCAAA
ENSG00000179388	UAUAAAG
ENSG00000179388	UCACAAG
ENSG00000179388	UCACAGU
ENSG00000179388	UGAAAUG
ENSG00000179388	UGCAAAG
ENSG00000179388	UGCAUAU
ENSG00000179388	UUCAAAU
ENSG00000179388	UUCUAAU
ENSG00000179388	UUGGCAC
ENSG00000179397	AAGUAGC
ENSG00000179431	AAUUUUA
ENSG00000179431	AUGCACC
ENSG00000179431	CGUUGGC
ENSG00000179431	GGGCGUA
ENSG00000179431	GGGCGUG
ENSG00000179431	UCUCACC
ENSG00000179431	UGAAAUG
ENSG00000179449	AAAGUAU
ENSG00000179449	UUGGCAA
ENSG00000179449	UUGGCAC
ENSG00000179454	AAGUGCU
ENSG00000179454	ACUUGGA
ENSG00000179454	AUGGCUU
ENSG00000179454	GGAAGAC
ENSG00000179454	GUGCAAA
ENSG00000179454	UACAGUU
ENSG00000179454	UGCAGCU
ENSG00000179455	AAAAACC
ENSG00000179455	UCUUGAG
ENSG00000179456	AACAAAC
ENSG00000179456	AAGGCGC
ENSG00000179456	AAGUGCU
ENSG00000179456	AAUCACU
ENSG00000179456	AGAUGUG
ENSG00000179456	AGCAUUC
ENSG00000179456	AUACAAG
ENSG00000179456	AUGCAUU
ENSG00000179456	AUUGCAC
ENSG00000179456	CAAAGCA
ENSG00000179456	CAGUGCA
ENSG00000179456	CCAGUGU
ENSG00000179456	CCAUUAC
ENSG00000179456	CCCCCAG
ENSG00000179456	GAAAACA
ENSG00000179456	GCCUGGG
ENSG00000179456	UAAUGCU
ENSG00000179456	UACAGUU
ENSG00000179456	UAUUGCU
ENSG00000179456	UCACAGG
ENSG00000179456	UCACAGU
ENSG00000179456	UGAAGCU
ENSG00000179456	UGUCUUU

ENSG00000179456	UUCAAGC
ENSG00000179456	UUGACAC
ENSG00000179456	UUUUUGC
ENSG00000179476	ACCCUGU
ENSG00000179476	ACCUGGA
ENSG00000179476	AGCCCUU
ENSG00000179476	UGGAUAA
ENSG00000179476	UUUGCAA
ENSG00000179520	AUGGAUU
ENSG00000179520	CAGCAGA
ENSG00000179520	GGCAAGA
ENSG00000179528	CUGGACU
ENSG00000179542	UAUAAAG
ENSG00000179546	GGUAGAC
ENSG00000179562	CACAGUG
ENSG00000179562	GAGAAGA
ENSG00000179562	UCACAUU
ENSG00000179564	GGAAUGU
ENSG00000179583	GCCUGGA
ENSG00000179583	GUAGUGU
ENSG00000179588	AAGUGCA
ENSG00000179588	AAGUGCU
ENSG00000179593	GGUUGUC
ENSG00000179600	CUCACAC
ENSG00000179603	AACAUUC
ENSG00000179603	CCAUUAC
ENSG00000179603	UGCAAAG
ENSG00000179603	UGCAUUG
ENSG00000179603	UUUCAAC
ENSG00000179604	AAUGUUG
ENSG00000179604	UCUGGAA
ENSG00000179604	UUCCUAG
ENSG00000179632	CAAAGUG
ENSG00000179632	CAGCAAA
ENSG00000179632	CAGUGCA
ENSG00000179632	CUGCAGC
ENSG00000179632	GAGCUGC
ENSG00000179632	GCAGCAU
ENSG00000179632	GGAGUGU
ENSG00000179639	UCUUGAG
ENSG00000179674	AAAGUUU
ENSG00000179761	UCCUUGC
ENSG00000179774	AAAGUUU
ENSG00000179774	UGAGGAC
ENSG00000179776	ACAGUAU
ENSG00000179776	AUGCACC
ENSG00000179776	CACAGUG
ENSG00000179776	GAGCUGC
ENSG00000179776	GCACCAU
ENSG00000179776	UCACAGG
ENSG00000179776	UCCCUGU
ENSG00000179813	AAGGUGC
ENSG00000179813	ACAUUCA
ENSG00000179813	AGAUAAA
ENSG00000179813	AUCACUA
ENSG00000179813	CAAAGUG
ENSG00000179813	CAGCAAA
ENSG00000179813	CCUUCUU
ENSG00000179813	GCACCAU
ENSG00000179813	UUCUGCA
ENSG00000179820	AAGGCAC
ENSG00000179820	AGCAGCA
ENSG00000179820	CUCCCAA
ENSG00000179820	GGCAGUG

ENSG00000179820	UCUGGAG
ENSG00000179820	UUGGAGG
ENSG00000179820	UUGGCAA
ENSG00000179820	UUGGCAC
ENSG00000179833	AAAGAAU
ENSG00000179833	AAUGCCC
ENSG00000179833	AUAAUAC
ENSG00000179833	AUGGCUU
ENSG00000179833	AUUGCAC
ENSG00000179833	CACAGUG
ENSG00000179833	CAGGAAC
ENSG00000179833	CCUUCAU
ENSG00000179833	GGCAAGA
ENSG00000179833	GUGCUUG
ENSG00000179833	UAAUACA
ENSG00000179833	UCACAGU
ENSG00000179833	UUCCAUA
ENSG00000179837	AAAGUGC
ENSG00000179837	CAGACAG
ENSG00000179837	CAGCAGA
ENSG00000179837	UACAGUU
ENSG00000179837	UUGGCAA
ENSG00000179841	AGCACCA
ENSG00000179841	AGCCCUU
ENSG00000179841	AUGUAAU
ENSG00000179841	CAGACAG
ENSG00000179841	CUGGGGG
ENSG00000179841	GAGGUAG
ENSG00000179841	GGAGGAG
ENSG00000179841	UUAUGCA
ENSG00000179862	UUGGAGG
ENSG00000179862	UUGGUUC
ENSG00000179869	UCAACAG
ENSG00000179886	AGCACCA
ENSG00000179886	CAGUGCA
ENSG00000179886	GUGACUG
ENSG00000179889	AACAUUC
ENSG00000179889	ACAUUCA
ENSG00000179889	AGCAAAA
ENSG00000179889	AUUGCAC
ENSG00000179889	GAGUUGG
ENSG00000179889	GCACCAU
ENSG00000179889	GGAAGAC
ENSG00000179889	UCUCAAG
ENSG00000179889	UUCAAGC
ENSG00000179889	UUGGCAC
ENSG00000179912	AAAAACC
ENSG00000179912	AACUCUA
ENSG00000179912	AAUACUG
ENSG00000179912	ACAAUAU
ENSG00000179912	AUGUGCC
ENSG00000179912	AUUCAUU
ENSG00000179912	CAGCAAA
ENSG00000179912	CCAGUGU
ENSG00000179912	CUCAAAC
ENSG00000179912	GAGAAGA
ENSG00000179912	GGAGUCC
ENSG00000179912	GUAUGUU
ENSG00000179912	GUGCAAA
ENSG00000179912	UACAGUU
ENSG00000179912	UGUAUGU
ENSG00000179915	AACACUG
ENSG00000179915	AGAUAAA
ENSG00000179915	CGUGCAU

ENSG00000179915	GUGCUUG
ENSG00000179915	UGUGCUU
ENSG00000179918	GGCUCUG
ENSG00000179918	GUCCAGA
ENSG00000179918	UUCUUGU
ENSG00000179922	AGUGCAA
ENSG00000179922	CAGUGCA
ENSG00000179922	GAGGUAG
ENSG00000179922	GAGGUAU
ENSG00000179922	GGCUCUG
ENSG00000179922	GUGAGGU
ENSG00000179922	UAAUAGU
ENSG00000179933	CUUACAG
ENSG00000179933	GACAACU
ENSG00000179933	GGGAACG
ENSG00000179933	UCCAGUU
ENSG00000179933	UUCCUAG
ENSG00000179965	CCCACCU
ENSG00000179965	UCACAGG
ENSG00000179981	AAGGCAC
ENSG00000179981	AAUUUUA
ENSG00000179981	AUGCCUU
ENSG00000179981	CAGGAAC
ENSG00000179981	GAAAACA
ENSG00000179981	GCAACUU
ENSG00000179981	GGCUCAG
ENSG00000179981	GUCCAGA
ENSG00000179981	UGCAUAG
ENSG00000180008	ACCACAG
ENSG00000180008	AGGUAGU
ENSG00000180008	AGUGAUC
ENSG00000180008	AUGGCUU
ENSG00000180008	CAGGAAC
ENSG00000180008	CCCUGAG
ENSG00000180008	CUACAAA
ENSG00000180008	GAAGGAA
ENSG00000180008	GGCAGUG
ENSG00000180008	GUCCAGA
ENSG00000180008	UAUAUGA
ENSG00000180008	UCACAGG
ENSG00000180008	UCACAGU
ENSG00000180008	UCAUAGA
ENSG00000180008	UCCUAUA
ENSG00000180008	UUCUAAU
ENSG00000180008	UUGAUCA
ENSG00000180011	AACAUUC
ENSG00000180011	AAGUCUU
ENSG00000180011	ACAUUAC
ENSG00000180011	ACUCAAA
ENSG00000180011	AGCAGCA
ENSG00000180011	AGCAGCG
ENSG00000180011	AGCAGCU
ENSG00000180011	AGCUUAU
ENSG00000180011	AGGACAC
ENSG00000180011	AGUGCAA
ENSG00000180011	AGUGCUG
ENSG00000180011	AGUGCUU
ENSG00000180011	CACAGUG
ENSG00000180011	CAGUGCA
ENSG00000180011	CUUGUAU
ENSG00000180011	GAAAACA
ENSG00000180011	GCUACAU
ENSG00000180011	GCUAGCA
ENSG00000180011	GGCAGCG

ENSG00000180011	GUCAUUG
ENSG00000180011	GUGACAG
ENSG00000180011	UAGGUAU
ENSG00000180011	UCACAGU
ENSG00000180011	UCCCUUU
ENSG00000180011	UGAGGAC
ENSG00000180011	UGAUCCA
ENSG00000180011	UGUCUUU
ENSG00000180011	UUCCAUA
ENSG00000180011	UUGGCAA
ENSG00000180011	UUGGCAC
ENSG00000180011	UUGGUUC
ENSG00000180138	AAAACUG
ENSG00000180138	AAAUCCC
ENSG00000180138	CACAAUG
ENSG00000180138	GUUAAUG
ENSG00000180138	UCACAAG
ENSG00000180138	UGUGUCA
ENSG00000180155	AGGGAGG
ENSG00000180155	UGGGGGA
ENSG00000180182	AAGUGCA
ENSG00000180182	ACACACC
ENSG00000180182	ACAGUAC
ENSG00000180182	ACUGGCC
ENSG00000180182	CAAAGCA
ENSG00000180182	CACAGUG
ENSG00000180182	CAUAUUG
ENSG00000180182	CUAUACA
ENSG00000180182	GGCAUUG
ENSG00000180182	UACAGUU
ENSG00000180182	UAUUGCU
ENSG00000180182	UGCCAGC
ENSG00000180182	UGGGUGA
ENSG00000180182	UUGGCAA
ENSG00000180190	AAGGCAC
ENSG00000180190	GGAAGAC
ENSG00000180198	CCCUGUU
ENSG00000180198	GGCUGCG
ENSG00000180198	GUGCUUG
ENSG00000180198	UGGCUGC
ENSG00000180198	UGUGCUU
ENSG00000180219	AUGUGGG
ENSG00000180228	AAAGAGC
ENSG00000180228	AGCACCA
ENSG00000180228	GCUACAU
ENSG00000180228	UGAAGCU
ENSG00000180233	UCCCUGU
ENSG00000180233	UGCAUAG
ENSG00000180251	GUAAACA
ENSG00000180259	UCCAGAG
ENSG00000180259	UUCCUCG
ENSG00000180263	AAGACAU
ENSG00000180263	AAGUGCA
ENSG00000180263	AUACCUC
ENSG00000180263	CAGCAGA
ENSG00000180263	CUGGAAA
ENSG00000180263	GACCAUG
ENSG00000180263	GAGAUGA
ENSG00000180263	GAGGUAU
ENSG00000180263	GCACCAU
ENSG00000180263	GUAAACA
ENSG00000180263	UCACAGG
ENSG00000180263	UCCAGUU
ENSG00000180263	UCUGGAA

ENSG00000180287	GUGCUUG
ENSG00000180304	ACACACU
ENSG00000180304	ACUGUUU
ENSG00000180304	AGUAACA
ENSG00000180304	CUAGGCU
ENSG00000180304	UUCUAAU
ENSG00000180304	UUGACAC
ENSG00000180304	UUUCAAC
ENSG00000180305	CUCCCAC
ENSG00000180318	AGCAGCU
ENSG00000180318	AGCUUAU
ENSG00000180329	GGAAGAC
ENSG00000180329	GGAGAGA
ENSG00000180332	CACAGUG
ENSG00000180332	GGCUGCG
ENSG00000180332	UACAGUU
ENSG00000180332	UCACAGU
ENSG00000180332	UGGCUGC
ENSG00000180353	GGAGGCA
ENSG00000180354	AAGUGCA
ENSG00000180354	AAGUGCU
ENSG00000180354	ACUAGAU
ENSG00000180354	AUGGCUU
ENSG00000180354	CACAGUG
ENSG00000180354	CCAGGAG
ENSG00000180354	CCCACCU
ENSG00000180354	GACAACU
ENSG00000180354	GCCUGGA
ENSG00000180354	GGAUGAG
ENSG00000180354	UAAUAGU
ENSG00000180354	UCACAGG
ENSG00000180354	UGUGCUU
ENSG00000180354	UUGGCAC
ENSG00000180354	UUUGCAC
ENSG00000180357	AAAAAUC
ENSG00000180357	AAAGAGC
ENSG00000180357	AACACUG
ENSG00000180357	ACCAGCA
ENSG00000180357	AGCACCA
ENSG00000180357	AGCAGCA
ENSG00000180357	AGCCAGU
ENSG00000180357	AGCUGCC
ENSG00000180357	AGGGUCU
ENSG00000180357	AUAUAAC
ENSG00000180357	CAGCAAA
ENSG00000180357	CAGCAGA
ENSG00000180357	CAGCAGG
ENSG00000180357	CAGGAAC
ENSG00000180357	CCUUCAU
ENSG00000180357	CUCUAGC
ENSG00000180357	CUCUGGG
ENSG00000180357	CUCUUCC
ENSG00000180357	GAGAAAU
ENSG00000180357	GAGGGGC
ENSG00000180357	GAUUGUA
ENSG00000180357	GCAGGAC
ENSG00000180357	GCCUGAU
ENSG00000180357	GCGGGUG
ENSG00000180357	GCUGGUG
ENSG00000180357	GCUGUCU
ENSG00000180357	GGCUGCG
ENSG00000180357	GGGGCAG
ENSG00000180357	GGUGGGC
ENSG00000180357	GGUGGUC

ENSG00000180357	GUCUCUG
ENSG00000180357	GUCUGCC
ENSG00000180357	GUGCAAA
ENSG00000180357	GUGCUUG
ENSG00000180357	UACAAAG
ENSG00000180357	UACAGUU
ENSG00000180357	UCACAAG
ENSG00000180357	UCACAGU
ENSG00000180357	UGCAUAG
ENSG00000180357	UGUAUAA
ENSG00000180357	UGUGCUU
ENSG00000180357	UUGGCAC
ENSG00000180357	UUGUGAC
ENSG00000180370	AAAGGUA
ENSG00000180370	AAAGUAU
ENSG00000180370	AAAGUUU
ENSG00000180370	AAGUGCA
ENSG00000180370	AAGUGCU
ENSG00000180370	CACAAUG
ENSG00000180370	CUUUGGU
ENSG00000180370	GGGAUCG
ENSG00000180370	GUUAAUG
ENSG00000180370	UAUAAAG
ENSG00000180370	UGCCAGC
ENSG00000180370	UGGGAUC
ENSG00000180370	UUGGUUC
ENSG00000180370	UUUCAAC
ENSG00000180398	ACUCAGC
ENSG00000180398	AGGAUGU
ENSG00000180398	AGUAGUU
ENSG00000180398	CCAGUGU
ENSG00000180398	GGCAGUG
ENSG00000180398	UACAAAG
ENSG00000180398	UUGACAC
ENSG00000180398	UUGGCAA
ENSG00000180432	AAAGAGG
ENSG00000180440	AAGUUGU
ENSG00000180447	AAAGUAC
ENSG00000180447	AACUGGU
ENSG00000180447	AAGGCAC
ENSG00000180447	AAUUAUU
ENSG00000180447	AUACAAG
ENSG00000180447	AUGCAUU
ENSG00000180447	CAGUGCA
ENSG00000180447	CUACAAA
ENSG00000180447	UAUAAAG
ENSG00000180483	AAUUUUA
ENSG00000180488	AAGGUGC
ENSG00000180488	ACAGUAU
ENSG00000180488	AGCAGCG
ENSG00000180488	CAAAGCA
ENSG00000180488	CCCUGAG
ENSG00000180488	GCAGCAU
ENSG00000180488	UUGGUCC
ENSG00000180509	ACUGGCC
ENSG00000180530	AACCUGG
ENSG00000180530	ACAAGGU
ENSG00000180530	AUAUAAC
ENSG00000180530	CAAAUGC
ENSG00000180530	CACAGGG
ENSG00000180530	CAGCAGG
ENSG00000180530	CCCCCAG
ENSG00000180530	CUGCAGG
ENSG00000180530	GCAACUU

ENSG00000180530	GCAGCAU
ENSG00000180530	GCAGGAC
ENSG00000180530	GGCCCUG
ENSG00000180530	GGUUGUC
ENSG00000180530	GUAACAG
ENSG00000180530	GUGGCCC
ENSG00000180530	UAAGACU
ENSG00000180530	UCAAGUA
ENSG00000180530	UCACACA
ENSG00000180530	UCCUGAG
ENSG00000180530	UGCAUUG
ENSG00000180530	UGGCCCU
ENSG00000180543	AAAGCUG
ENSG00000180543	AAGUCAC
ENSG00000180543	ACUGGCC
ENSG00000180543	ACUGGCU
ENSG00000180543	CAGCAGG
ENSG00000180543	CCAGCAU
ENSG00000180543	CUGGCUC
ENSG00000180543	UCACAGG
ENSG00000180543	UCACAGU
ENSG00000180543	UCUUGAG
ENSG00000180573	AAAGAGC
ENSG00000180573	GGCUCUG
ENSG00000180592	AAAUUUC
ENSG00000180592	CAGUAGA
ENSG00000180592	GAGGUAG
ENSG00000180592	GAGGUAU
ENSG00000180592	GAUUGUA
ENSG00000180592	GGCUCAG
ENSG00000180592	UGGGUGA
ENSG00000180596	AAAGAGC
ENSG00000180596	GGCUCUG
ENSG00000180611	AUGUAAU
ENSG00000180611	GAUAUGU
ENSG00000180611	GCAGAAG
ENSG00000180611	GCUGGAU
ENSG00000180611	GUGCAAA
ENSG00000180616	CAGACAG
ENSG00000180616	UCUAGAG
ENSG00000180626	CCAGUGC
ENSG00000180626	CCAGUGU
ENSG00000180628	AACAUUC
ENSG00000180628	AAGGCAC
ENSG00000180628	AAGUCAC
ENSG00000180628	AGCAAAA
ENSG00000180628	AGCUGCC
ENSG00000180628	AGGACAC
ENSG00000180628	AGUGCAA
ENSG00000180628	CAGCUGG
ENSG00000180628	CAGUAGA
ENSG00000180628	CAGUAGU
ENSG00000180628	GAGGCAG
ENSG00000180628	GCAGCAU
ENSG00000180628	GCAGGAC
ENSG00000180628	GUGCAAA
ENSG00000180628	UAAUAGU
ENSG00000180628	UAGGCCG
ENSG00000180628	UGUCUUU
ENSG00000180628	UUGACAC
ENSG00000180660	AAAGUAC
ENSG00000180660	AACAUUC
ENSG00000180660	CCAGUGC
ENSG00000180660	GUAAACA

ENSG00000180667	AAGGCAC
ENSG00000180667	AGCAGCG
ENSG00000180667	AGCAGCU
ENSG00000180667	AGGUAGU
ENSG00000180667	AGUGCUG
ENSG00000180667	AGUGCUU
ENSG00000180667	AUGGCAC
ENSG00000180667	CCCUGAG
ENSG00000180667	CCUUCUG
ENSG00000180667	GAGGUAU
ENSG00000180667	GCAUCCC
ENSG00000180667	GGCUCAG
ENSG00000180667	UCCCUGU
ENSG00000180667	UCUCAAG
ENSG00000180667	UCUGGAG
ENSG00000180667	UGCAUAG
ENSG00000180694	AGCCCUU
ENSG00000180694	CACAGUG
ENSG00000180694	GAGUCUU
ENSG00000180694	UCACAUU
ENSG00000180694	UCCCACA
ENSG00000180772	GAGAAAU
ENSG00000180776	AAGGCAC
ENSG00000180787	UUGGCAA
ENSG00000180801	AAAGUGC
ENSG00000180801	AGUGCUU
ENSG00000180801	GGCAGUG
ENSG00000180801	UACAGUU
ENSG00000180801	UUGGCAC
ENSG00000180801	UUGGUCC
ENSG00000180818	UCUCCAA
ENSG00000180818	UGCAUUG
ENSG00000180834	AGCACCA
ENSG00000180834	GAUUGUA
ENSG00000180834	GCUAGCA
ENSG00000180834	GUGCCUG
ENSG00000180861	AGCAAAA
ENSG00000180861	AUGGCUU
ENSG00000180861	CAGCAAA
ENSG00000180861	CUCCCAA
ENSG00000180861	UCUUGAG
ENSG00000180861	UGAGAAU
ENSG00000180861	UGAUCCA
ENSG00000180875	AAUACUG
ENSG00000180875	AAUCCUU
ENSG00000180875	ACAUUAC
ENSG00000180875	ACUGCAG
ENSG00000180875	AUGGCAC
ENSG00000180875	CACAGGG
ENSG00000180875	CAUAUUG
ENSG00000180875	GAAACAU
ENSG00000180875	GAGACCU
ENSG00000180875	GGAGUGU
ENSG00000180875	GGCAGUG
ENSG00000180875	UAUAAAG
ENSG00000180875	UAUUGCU
ENSG00000180875	UCACAUU
ENSG00000180875	UCAUUUG
ENSG00000180875	UCCCACC
ENSG00000180875	UGUGUGG
ENSG00000180875	UUCUGCA
ENSG00000180878	AGCAGCU
ENSG00000180891	AGCACCA
ENSG00000180891	CAGCUGG

ENSG00000180891	CUGCAGC
ENSG00000180891	GCACCAU
ENSG00000180891	GGCAGUG
ENSG00000180891	UGCAGCU
ENSG00000180900	GGUUGUC
ENSG00000180900	UACAGUU
ENSG00000180900	UUGGUUC
ENSG00000180901	AAAGCUG
ENSG00000180901	CUUUGGU
ENSG00000180901	GCAACUU
ENSG00000180901	GCAGGUU
ENSG00000180901	UUGGCAA
ENSG00000180901	UUGGCAC
ENSG00000180914	AGCACCA
ENSG00000180929	UACAGUU
ENSG00000180929	UCCCUGU
ENSG00000180929	UGCCCUG
ENSG00000180929	UUUCAAC
ENSG00000180957	AACACUG
ENSG00000180957	ACAGUAU
ENSG00000180957	GAGAAAU
ENSG00000180957	GUAUGUU
ENSG00000180957	UAUAAAG
ENSG00000180957	UGACCUA
ENSG00000180957	UGCAUAG
ENSG00000180957	UGCAUAU
ENSG00000180957	UGUAUGU
ENSG00000180957	UUGGUCC
ENSG00000180964	ACACACC
ENSG00000180964	ACACACU
ENSG00000180964	UCCAGAG
ENSG00000180979	AACAAAC
ENSG00000180979	CUUCUCU
ENSG00000180979	UAUAAAG
ENSG00000180979	UGCAUAG
ENSG00000180998	AAGUGCU
ENSG00000180998	AGAUAAA
ENSG00000180998	AGUGCUG
ENSG00000180998	AUUCAUU
ENSG00000180998	CAGUGCA
ENSG00000180998	GAGGUAU
ENSG00000180998	GUGCUUG
ENSG00000180998	UAGUGAG
ENSG00000180998	UCCCUUG
ENSG00000180998	UGUGCUU
ENSG00000181026	GAGGUAG
ENSG00000181031	AACACUG
ENSG00000181031	AAGGCAC
ENSG00000181031	UCCAGAG
ENSG00000181035	ACUCCAG
ENSG00000181039	AGCAGCA
ENSG00000181039	CAGCAGA
ENSG00000181039	GAGGCUG
ENSG00000181039	UGUGCUU
ENSG00000181045	UACAAAG
ENSG00000181061	AGCAAAA
ENSG00000181061	AGCCUGC
ENSG00000181061	GCAGCAU
ENSG00000181061	UGACCUA
ENSG00000181072	AAGUGCA
ENSG00000181072	AGCCCUU
ENSG00000181072	AGUGCAA
ENSG00000181072	AUUGCAC
ENSG00000181090	AAACUGU

ENSG00000181090	AAAUGGU
ENSG00000181090	AAGGUGC
ENSG00000181090	AAUCACU
ENSG00000181090	ACUGCAG
ENSG00000181090	GACCUGG
ENSG00000181090	UUGUUCG
ENSG00000181104	GGCCCUG
ENSG00000181104	UGCAAAG
ENSG00000181143	GGUUUAC
ENSG00000181163	AAGUGCU
ENSG00000181163	AGUGCCG
ENSG00000181163	AGUGCCU
ENSG00000181163	UUCAAAU
ENSG00000181192	AAAAAUC
ENSG00000181192	AAAACUG
ENSG00000181192	AAAUCCC
ENSG00000181192	AGCACCA
ENSG00000181192	AGCAGCA
ENSG00000181192	CUGGAAA
ENSG00000181218	AAAGAGC
ENSG00000181220	ACAGUAU
ENSG00000181220	AGUGAUC
ENSG00000181220	CAGUUAC
ENSG00000181220	GCUGGUG
ENSG00000181222	CAGGAAC
ENSG00000181274	AAAGUUU
ENSG00000181274	GAGAAAU
ENSG00000181274	UCACAUU
ENSG00000181274	UUCAAGC
ENSG00000181274	UUUCAAC
ENSG00000181291	AAAAAUC
ENSG00000181291	AAAUCCC
ENSG00000181291	AAGGCGC
ENSG00000181291	AAGUCUU
ENSG00000181291	AUCCUUG
ENSG00000181291	CACAGUG
ENSG00000181291	CCCUGAG
ENSG00000181291	GAGCUGC
ENSG00000181291	GCAGGAC
ENSG00000181291	GGGAGCU
ENSG00000181291	GGUGGGC
ENSG00000181315	AGUAACA
ENSG00000181315	AUGGCUU
ENSG00000181315	GAGGUAU
ENSG00000181315	UCUCCAA
ENSG00000181322	UCCUGAG
ENSG00000181333	AACAUUC
ENSG00000181333	AACUACU
ENSG00000181333	AGCAGCA
ENSG00000181333	GAAACAU
ENSG00000181392	UAUUACC
ENSG00000181392	UGUGUCA
ENSG00000181409	AGCAGCA
ENSG00000181409	AGCAGCG
ENSG00000181409	AGCAGCU
ENSG00000181409	AUUGCAC
ENSG00000181409	CACCAGC
ENSG00000181409	CUUUGGU
ENSG00000181409	GCACCAU
ENSG00000181409	GCUAGCA
ENSG00000181409	UUGGCAC
ENSG00000181418	ACUGUGU
ENSG00000181418	GAGGUAG
ENSG00000181418	UCUGGAA

ENSG00000181418	UGAAGUG
ENSG00000181449	AAACUGU
ENSG00000181449	AAUACUG
ENSG00000181449	ACUGUAG
ENSG00000181449	AGCUUAU
ENSG00000181449	AUACAAG
ENSG00000181449	CUAUACA
ENSG00000181449	UACAAAG
ENSG00000181449	UUGGCAA
ENSG00000181467	AAAAGUA
ENSG00000181467	AAAGCUG
ENSG00000181467	AACAUUC
ENSG00000181467	AAGGCAC
ENSG00000181467	ACAGGUG
ENSG00000181467	AGAUGUG
ENSG00000181467	CAAAUGC
ENSG00000181467	CACAGGG
ENSG00000181467	CAGCAGA
ENSG00000181467	CAGCAGG
ENSG00000181467	CCCCCAG
ENSG00000181467	CUGGAAA
ENSG00000181467	GGAGGAG
ENSG00000181467	GGCCCUG
ENSG00000181467	GGGGUGC
ENSG00000181467	GUAAACA
ENSG00000181467	GUAACAG
ENSG00000181467	GUGCAAA
ENSG00000181467	GUGCGCA
ENSG00000181467	UCUGGAG
ENSG00000181467	UGCAUAU
ENSG00000181467	UGGAUAA
ENSG00000181467	UGGUACC
ENSG00000181467	UUGACAC
ENSG00000181467	UUGGCAC
ENSG00000181472	AAUCUCU
ENSG00000181472	CUAUACA
ENSG00000181472	GAGAACC
ENSG00000181472	UAUUACC
ENSG00000181472	UCACAUU
ENSG00000181481	AAGGCAC
ENSG00000181495	ACAUUAC
ENSG00000181495	GUUAAUG
ENSG00000181495	UAUAUGA
ENSG00000181495	UGCAGCU
ENSG00000181513	CAAGAGC
ENSG00000181513	GGCUCAG
ENSG00000181541	AACAUUC
ENSG00000181541	ACAUUCA
ENSG00000181541	UUGGCAC
ENSG00000181555	AAAAAUC
ENSG00000181555	UAAUACA
ENSG00000181555	UAUAAUA
ENSG00000181555	UCACAUU
ENSG00000181577	GCCUGGG
ENSG00000181585	GCCUGGA
ENSG00000181585	UGAGAAG
ENSG00000181588	AAAACUG
ENSG00000181588	AAAGCUG
ENSG00000181588	AAAGGUA
ENSG00000181588	AAAGUAU
ENSG00000181588	AAGUGCA
ENSG00000181588	AAUACUG
ENSG00000181588	AGUGCAA
ENSG00000181588	AUAAAGU

ENSG00000181588	AUAUAAC
ENSG00000181588	GUAAACA
ENSG00000181588	UGAAGUG
ENSG00000181619	UGCAUUG
ENSG00000181619	UUUUUGC
ENSG00000181649	UAAGACG
ENSG00000181652	CCUGCUG
ENSG00000181652	GAGGCUG
ENSG00000181652	GGGAGCU
ENSG00000181656	AAAGCUG
ENSG00000181656	AGGAGCU
ENSG00000181656	CAAAGCA
ENSG00000181656	UACAAAG
ENSG00000181656	UAUUGCU
ENSG00000181666	AAAGUUU
ENSG00000181690	AAAGCUG
ENSG00000181690	AAAGUGC
ENSG00000181690	AAGGCAC
ENSG00000181690	ACAGUAU
ENSG00000181690	ACUCAAA
ENSG00000181690	ACUGUAG
ENSG00000181690	AGAUAAA
ENSG00000181690	AGCAAAA
ENSG00000181690	AGCACCA
ENSG00000181690	AGUAGUU
ENSG00000181690	AUGCCUU
ENSG00000181690	AUGGCAC
ENSG00000181690	CAAAGCA
ENSG00000181690	CACAGUG
ENSG00000181690	CACUCCU
ENSG00000181690	CUGGCUC
ENSG00000181690	CUUCACC
ENSG00000181690	CUUGUAU
ENSG00000181690	CUUUGGU
ENSG00000181690	GAAAACA
ENSG00000181690	GCACCAU
ENSG00000181690	GCAGCAU
ENSG00000181690	GGAAUGU
ENSG00000181690	GGAGUGU
ENSG00000181690	GGCUCAG
ENSG00000181690	GUGCAAA
ENSG00000181690	UAUGGUU
ENSG00000181690	UCACAGU
ENSG00000181690	UCAUUCG
ENSG00000181690	UGAGAAG
ENSG00000181690	UGAUCCA
ENSG00000181690	UGCAGCU
ENSG00000181690	UUGUGAC
ENSG00000181704	AAGGCAC
ENSG00000181722	AAAGAAU
ENSG00000181722	AGUGCCG
ENSG00000181722	AGUGCCU
ENSG00000181722	AUGUAAU
ENSG00000181722	CAGCAGG
ENSG00000181722	UAGAGGA
ENSG00000181722	UUAGAGA
ENSG00000181722	UUGUUCG
ENSG00000181744	AAGGCAC
ENSG00000181744	AAGUUGU
ENSG00000181744	AUAAAGU
ENSG00000181744	CACAGGG
ENSG00000181744	CUUUGGU
ENSG00000181751	AAGGUGC
ENSG00000181751	ACCCAUU

ENSG00000181751	AUAUAAC
ENSG00000181751	AUUCAUU
ENSG00000181751	CCAGUGC
ENSG00000181751	CUUUGGU
ENSG00000181751	GUGCAAA
ENSG00000181751	UACAGUU
ENSG00000181751	UGAAAUG
ENSG00000181751	UGACAUC
ENSG00000181751	UUGGCAA
ENSG00000181751	UUGGCAC
ENSG00000181754	AGGAGCU
ENSG00000181754	GUCUUGC
ENSG00000181773	AAAAACC
ENSG00000181773	AACAAAC
ENSG00000181773	ACUCCAG
ENSG00000181773	ACUUGGA
ENSG00000181773	AUGGCUU
ENSG00000181773	CCCCCAG
ENSG00000181773	GAGGCUG
ENSG00000181773	GCCUGGG
ENSG00000181773	GCUGGUG
ENSG00000181773	GGCUCUG
ENSG00000181781	UCCCUUU
ENSG00000181788	AAAGCUG
ENSG00000181788	AACUCUA
ENSG00000181788	AAGACAU
ENSG00000181788	CAGUAGA
ENSG00000181788	GGAAGCC
ENSG00000181788	UGUGCUU
ENSG00000181788	UUCCUAG
ENSG00000181789	GUAGUGU
ENSG00000181790	CUGGCUC
ENSG00000181790	GGCUCUG
ENSG00000181790	UGGCUGC
ENSG00000181804	AAGGCAC
ENSG00000181804	GAGGUAG
ENSG00000181804	UGCAGCU
ENSG00000181817	UGAAAGG
ENSG00000181826	AAAGCUG
ENSG00000181826	AAAGUAC
ENSG00000181826	AAAGUGC
ENSG00000181826	ACAUCAC
ENSG00000181826	CUUACAG
ENSG00000181826	CUUGGAG
ENSG00000181826	CUUUGGU
ENSG00000181826	GCAACUU
ENSG00000181826	GGGAACG
ENSG00000181826	GUAUUCU
ENSG00000181826	UACAAAG
ENSG00000181826	UCAAGUA
ENSG00000181826	UGUUGAA
ENSG00000181826	UUCCAUA
ENSG00000181826	UUGACAC
ENSG00000181826	UUGGCAC
ENSG00000181830	AAAGCUG
ENSG00000181830	CCCCUGG
ENSG00000181830	CCCUGAG
ENSG00000181830	GCAACUU
ENSG00000181830	UUGGCAC
ENSG00000181852	ACCAGCA
ENSG00000181852	AGCAGCA
ENSG00000181852	AGCAGCU
ENSG00000181852	AGGCAGG
ENSG00000181852	CCCUGUC

ENSG00000181852	CUGGCUC
ENSG00000181852	GCUUCCU
ENSG00000181852	GGCAGUG
ENSG00000181852	GUGCUUG
ENSG00000181852	UGAAAGG
ENSG00000181852	UGCAAAG
ENSG00000181852	UUGGCAC
ENSG00000181852	UUUCAAC
ENSG00000181856	AAGUGCU
ENSG00000181856	AGUGCUG
ENSG00000181856	CUCCCAC
ENSG00000181856	GAGGGGC
ENSG00000181856	GGAGGAG
ENSG00000181856	GGGGUGC
ENSG00000181856	GUAUUCU
ENSG00000181856	UCCCACC
ENSG00000181856	UCUGGAG
ENSG00000181856	UGGCCAC
ENSG00000181856	UUCAAAU
ENSG00000181885	ACUGUAG
ENSG00000181885	UAUUACC
ENSG00000181904	AAAGGUA
ENSG00000181904	AGCACCA
ENSG00000181904	UGACAUC
ENSG00000181904	UGCAUAG
ENSG00000181915	AAGCGCU
ENSG00000181915	AAUUAUU
ENSG00000181915	AGCCCUU
ENSG00000181915	AUUCAUU
ENSG00000181915	AUUGCAC
ENSG00000181915	CAGCUGG
ENSG00000181915	GGAAGCC
ENSG00000181915	GUAAACA
ENSG00000181915	UGCAUUG
ENSG00000181915	UGUGCUU
ENSG00000181915	UGUGUCA
ENSG00000181915	UUAGGAU
ENSG00000181915	UUAUGCA
ENSG00000181924	AUCCCUU
ENSG00000181924	UUGGCAC
ENSG00000181929	CUGGGGG
ENSG00000181929	GAUCAGA
ENSG00000181929	GUCAUUG
ENSG00000181929	GUGAGGU
ENSG00000181929	UUAGAGA
ENSG00000181938	GGAAGAC
ENSG00000181965	AAAGUGC
ENSG00000181965	AAGUGCA
ENSG00000181965	AGUGCAA
ENSG00000181965	GUGCAAA
ENSG00000181982	CCCCUGG
ENSG00000181982	CCCUGAG
ENSG00000181982	UCAUUCG
ENSG00000182010	AAAGCUG
ENSG00000182010	ACAGGUG
ENSG00000182010	CAGGUGA
ENSG00000182010	GCAGCAU
ENSG00000182010	GGCAUUG
ENSG00000182010	GGGUGGU
ENSG00000182010	UGACUGU
ENSG00000182010	UGGACUG
ENSG00000182010	UGGGUGA
ENSG00000182010	UUGACAC
ENSG00000182010	UUGUUCG

ENSG00000182013	UCUAGAG
ENSG00000182035	CUGACUC
ENSG00000182040	AAUACUG
ENSG00000182040	ACUGCAG
ENSG00000182040	AUUCAUU
ENSG00000182040	GCAACUU
ENSG00000182040	UGGCUGC
ENSG00000182087	AAGGCAC
ENSG00000182087	AGCACCA
ENSG00000182087	AUACAAG
ENSG00000182087	GGAAUGU
ENSG00000182093	AGUGGUU
ENSG00000182095	AAACUGU
ENSG00000182095	AAGUGCA
ENSG00000182095	ACAGGUG
ENSG00000182095	ACAGUAC
ENSG00000182095	ACAGUAU
ENSG00000182095	AGCACCA
ENSG00000182095	AGUGCUU
ENSG00000182095	AUAUAAC
ENSG00000182095	CACAGUG
ENSG00000182095	CUCCAUU
ENSG00000182095	CUGGCUC
ENSG00000182095	GAUUGUC
ENSG00000182095	GCACCAU
ENSG00000182095	GUAGUGU
ENSG00000182095	UAUAAUA
ENSG00000182095	UCACACA
ENSG00000182095	UCACAGG
ENSG00000182095	UGUCUGC
ENSG00000182107	AAAGAAU
ENSG00000182107	GCUUCCU
ENSG00000182107	GUGCCUG
ENSG00000182108	GGCACGG
ENSG00000182108	GUAAACA
ENSG00000182108	UAUUGCU
ENSG00000182108	UUGGCAC
ENSG00000182132	GGAGGCA
ENSG00000182149	AAGGCAC
ENSG00000182149	ACUGGAU
ENSG00000182149	CCCUGAG
ENSG00000182149	GGAAGCC
ENSG00000182149	UGAGAAU
ENSG00000182150	AUUCAUU
ENSG00000182150	CUACAAA
ENSG00000182150	CUCCAUU
ENSG00000182150	GAAACAU
ENSG00000182150	GAGUAUU
ENSG00000182150	GGGAACG
ENSG00000182150	UACAAAG
ENSG00000182150	UCAUUUG
ENSG00000182150	UUAGGAU
ENSG00000182158	AAAGGUA
ENSG00000182158	AGUGCUU
ENSG00000182158	AUAAUAC
ENSG00000182158	AUUGCAC
ENSG00000182158	CGGCCUG
ENSG00000182158	CUCCAUU
ENSG00000182158	CUUUGGU
ENSG00000182158	GAGCUGC
ENSG00000182158	GGAAGCC
ENSG00000182158	GGAGGAG
ENSG00000182158	GGAUUUU
ENSG00000182158	UAUAAUA

ENSG00000182158	UGCAAAG
ENSG00000182168	AAACUGU
ENSG00000182168	AAAGUUU
ENSG00000182173	AAGGCAC
ENSG00000182173	AUGGCUU
ENSG00000182175	AACAAAC
ENSG00000182175	ACAGUAC
ENSG00000182175	ACAUUCA
ENSG00000182175	ACCAGCA
ENSG00000182175	AGUGCUU
ENSG00000182175	AUAAAGU
ENSG00000182175	CAGCAGG
ENSG00000182175	CAGCUGG
ENSG00000182175	CAGUGCA
ENSG00000182175	CCAGUGU
ENSG00000182175	CUUGGAG
ENSG00000182175	GCCUGGG
ENSG00000182175	UACAGUU
ENSG00000182175	UGCAUUG
ENSG00000182185	CUGACUC
ENSG00000182195	CAAAUGC
ENSG00000182195	CAGCAGG
ENSG00000182195	CUGCAGC
ENSG00000182197	AAUGUUG
ENSG00000182197	GUGAGGU
ENSG00000182197	UGCAUAG
ENSG00000182197	UUCAAAU
ENSG00000182199	AAAAACC
ENSG00000182199	AACAAAC
ENSG00000182199	GAUCAGA
ENSG00000182199	GGGGUCC
ENSG00000182199	GGUGGGC
ENSG00000182199	UCAUUUG
ENSG00000182240	GAGGUAG
ENSG00000182247	ACAUCAC
ENSG00000182247	AUAAUAC
ENSG00000182247	AUACAAG
ENSG00000182247	AUGCACC
ENSG00000182247	GUGAAUG
ENSG00000182247	GUGCUUG
ENSG00000182247	UCAAGUA
ENSG00000182247	UGAUCCA
ENSG00000182247	UGUGCUU
ENSG00000182255	UGCAUAG
ENSG00000182263	AAAGCUG
ENSG00000182263	AAAGUAC
ENSG00000182263	AAAGUAU
ENSG00000182263	AAUCACU
ENSG00000182263	ACAUAGA
ENSG00000182263	ACUGUAG
ENSG00000182263	ACUGUUU
ENSG00000182263	AGCACCA
ENSG00000182263	AGUGCUG
ENSG00000182263	AUAUUAU
ENSG00000182263	CAGACAG
ENSG00000182263	CAGCAGG
ENSG00000182263	CCAGUAC
ENSG00000182263	CCAGUGC
ENSG00000182263	CCCUGUU
ENSG00000182263	GAGGCAG
ENSG00000182263	GCAGAAG
ENSG00000182263	GCAGGUU
ENSG00000182263	GUAAACA
ENSG00000182263	GUCCAGA

ENSG00000182263	GUGAGGU
ENSG00000182263	GUGCCAC
ENSG00000182263	UAAUAUC
ENSG00000182263	UAUAAAG
ENSG00000182263	UGAAGUG
ENSG00000182263	UUGAGGC
ENSG00000182263	UUGGCAA
ENSG00000182263	UUUCAAC
ENSG00000182263	UUUGCAC
ENSG00000182263	UUUUUGC
ENSG00000182287	AGCAGCU
ENSG00000182287	CACAAUG
ENSG00000182287	CUUACAG
ENSG00000182287	UAUUACC
ENSG00000182287	UGAAAUG
ENSG00000182287	UUGGUUC
ENSG00000182318	AAGGCAC
ENSG00000182318	GUAAACA
ENSG00000182318	UCUGGAA
ENSG00000182324	AUGGCAC
ENSG00000182324	CAGCAGG
ENSG00000182324	GCCCUGU
ENSG00000182324	GGCAUUG
ENSG00000182324	UUGGCAA
ENSG00000182324	UUGGCAC
ENSG00000182346	AUCACUA
ENSG00000182359	GAGAACC
ENSG00000182359	GAGAACU
ENSG00000182359	UGUGCUU
ENSG00000182379	UCCCUUG
ENSG00000182389	AACUGGU
ENSG00000182389	GGUUUAC
ENSG00000182389	UACAAAG
ENSG00000182389	UAUAAAG
ENSG00000182389	UUGGUUC
ENSG00000182400	AAUCCUU
ENSG00000182400	CCCUGAG
ENSG00000182400	CUUUGGU
ENSG00000182400	UGAAAUG
ENSG00000182446	AAAAGUA
ENSG00000182446	AAGGCAC
ENSG00000182446	AAGUCUU
ENSG00000182446	ACUCAAA
ENSG00000182446	CACUCCU
ENSG00000182446	CUCAGGG
ENSG00000182446	CUUUGGU
ENSG00000182446	GGUUUAC
ENSG00000182446	GUGUGGC
ENSG00000182450	CUUUGGU
ENSG00000182472	AUAUAAC
ENSG00000182472	CUCUGGG
ENSG00000182472	GUCUCUG
ENSG00000182472	UCUGGAA
ENSG00000182472	UCUGGAG
ENSG00000182472	UGGAUAA
ENSG00000182472	UGGCUGC
ENSG00000182473	GGUUUAC
ENSG00000182481	AAGACAU
ENSG00000182481	AAGUGCU
ENSG00000182481	ACAUAGA
ENSG00000182481	GCUACAU
ENSG00000182481	UACAGUU
ENSG00000182489	AAAGCUG
ENSG00000182489	UCAAGUA

ENSG00000182489	UUGGAGG
ENSG00000182500	AAAGUGC
ENSG00000182500	AAGUGCA
ENSG00000182500	CUCUUCC
ENSG00000182504	AGCACCA
ENSG00000182504	AUAAAGU
ENSG00000182508	UCCCACC
ENSG00000182512	AGUGGUU
ENSG00000182512	UACAGUU
ENSG00000182533	ACAGUAU
ENSG00000182533	AGGACAC
ENSG00000182533	UACAGUU
ENSG00000182534	ACCAGCA
ENSG00000182534	UCCAGAG
ENSG00000182541	AACCUGU
ENSG00000182541	ACCCAUU
ENSG00000182541	AGUAACA
ENSG00000182541	CAGCAGG
ENSG00000182541	CUGCAGG
ENSG00000182541	CUUCACC
ENSG00000182541	GAGAUGA
ENSG00000182541	UCACAGU
ENSG00000182541	UCACAUU
ENSG00000182552	AGAUAAA
ENSG00000182552	UUGGCAA
ENSG00000182552	UUGUGAC
ENSG00000182568	AAAACUG
ENSG00000182568	AAGACAU
ENSG00000182568	AAGGUGC
ENSG00000182568	ACAUAGA
ENSG00000182568	ACUGCAU
ENSG00000182568	ACUGUGU
ENSG00000182568	AGUAACA
ENSG00000182568	AUACAAG
ENSG00000182568	CACUCCU
ENSG00000182568	CAGUCUG
ENSG00000182568	GAAUUGU
ENSG00000182568	GAUUGUC
ENSG00000182568	GGCAGUG
ENSG00000182568	GUAAACA
ENSG00000182568	GUGCAAA
ENSG00000182568	UAAGACG
ENSG00000182568	UAAGACU
ENSG00000182568	UAAUGCU
ENSG00000182568	UACAGUU
ENSG00000182568	UAUAAUA
ENSG00000182568	UCAUAGA
ENSG00000182568	UGAAGCU
ENSG00000182568	UGCAAAG
ENSG00000182568	UUCUAAU
ENSG00000182572	AAAGAGC
ENSG00000182572	GGCUCUG
ENSG00000182575	CAGACAG
ENSG00000182575	GGCCCUG
ENSG00000182578	GAGAACC
ENSG00000182578	GAGAACU
ENSG00000182578	GGCAGUG
ENSG00000182578	UGACUGU
ENSG00000182580	AGCACCA
ENSG00000182580	CCCUGUC
ENSG00000182580	CCCUGUU
ENSG00000182580	GGCGGGG
ENSG00000182580	GUCUCUG
ENSG00000182580	GUGCAAA

ENSG00000182580	UAUUGCU
ENSG00000182580	UCCCUGU
ENSG00000182580	UCCUGAG
ENSG00000182591	CUGGAAA
ENSG00000182591	GCAGCAU
ENSG00000182601	GAGAAAU
ENSG00000182601	UCAUACA
ENSG00000182601	UCAUUUG
ENSG00000182601	UGCAUUG
ENSG00000182606	AGCAGCA
ENSG00000182606	AGCAGCG
ENSG00000182606	AGCAGCU
ENSG00000182606	AUGCACC
ENSG00000182611	AAAGAGC
ENSG00000182621	AAAGUGC
ENSG00000182621	AAGGCAC
ENSG00000182621	ACACACU
ENSG00000182621	CCAGUGC
ENSG00000182621	UGCAUAU
ENSG00000182636	AAGUGUG
ENSG00000182636	AAUACUG
ENSG00000182636	AAUGUUG
ENSG00000182636	ACUGCAG
ENSG00000182636	ACUGCAU
ENSG00000182636	AGUGUGC
ENSG00000182674	UAUUGCU
ENSG00000182676	AAAAGUA
ENSG00000182704	AAGGCAC
ENSG00000182704	CAGUCUG
ENSG00000182704	UCCAGAG
ENSG00000182704	UUGGCAC
ENSG00000182718	AAAGUAA
ENSG00000182718	AAAGUAU
ENSG00000182718	ACAGUAC
ENSG00000182718	CUUUGGU
ENSG00000182718	UCACAUU
ENSG00000182732	AGCAGCU
ENSG00000182732	AGGUAGU
ENSG00000182732	CACCAGC
ENSG00000182732	CAGCUGG
ENSG00000182732	GAGCGCC
ENSG00000182732	GAGGCUG
ENSG00000182732	GAGGUAG
ENSG00000182732	GAGGUAU
ENSG00000182732	GCAGGUU
ENSG00000182732	GCUGGUG
ENSG00000182732	GGUUACC
ENSG00000182732	GUAAACA
ENSG00000182732	UCACAGG
ENSG00000182732	UCACAGU
ENSG00000182732	UCCAGAG
ENSG00000182742	UCAUUUG
ENSG00000182747	GUAAACA
ENSG00000182752	AAAAACC
ENSG00000182752	AACCUGG
ENSG00000182752	AACCUGU
ENSG00000182752	AAGGCAC
ENSG00000182752	AAGGUGC
ENSG00000182752	AAGUAGC
ENSG00000182752	ACACACC
ENSG00000182752	ACACACU
ENSG00000182752	ACAGUAU
ENSG00000182752	ACUGGCU
ENSG00000182752	AGCAAAA

ENSG00000182752	AGCAGCG
ENSG00000182752	AGGGAGG
ENSG00000182752	AGGUUAU
ENSG00000182752	AUAUAAC
ENSG00000182752	AUGUAAU
ENSG00000182752	CACAAUG
ENSG00000182752	CACAGUG
ENSG00000182752	CACUGUU
ENSG00000182752	CAGCAGG
ENSG00000182752	CUCUAGC
ENSG00000182752	CUGCAGC
ENSG00000182752	CUGCAGG
ENSG00000182752	GAGCUGC
ENSG00000182752	GAGGUAG
ENSG00000182752	GAGGUAU
ENSG00000182752	GCUGGUG
ENSG00000182752	GGAAGAC
ENSG00000182752	UAUAAAG
ENSG00000182752	UCACAGU
ENSG00000182752	UCCAGAG
ENSG00000182752	UGCAGCU
ENSG00000182752	UUGGCAC
ENSG00000182771	AAAGAGC
ENSG00000182771	AAAUCCC
ENSG00000182771	AAUUAUU
ENSG00000182771	ACAUUCA
ENSG00000182771	AUACCUC
ENSG00000182771	AUUCAUU
ENSG00000182771	CAAAUGC
ENSG00000182771	CUCCAUU
ENSG00000182771	CUGCUCA
ENSG00000182771	GACUUCC
ENSG00000182771	GAGACCU
ENSG00000182771	GAGCUGC
ENSG00000182771	GCUGGUG
ENSG00000182771	GGCAGUG
ENSG00000182771	GGGUGGU
ENSG00000182771	GGUGGGC
ENSG00000182771	GUCAUUG
ENSG00000182771	GUCUCUG
ENSG00000182771	GUGUGGC
ENSG00000182771	UACAGUU
ENSG00000182771	UAUAAAG
ENSG00000182771	UCACAAG
ENSG00000182771	UCACACA
ENSG00000182771	UGUGUGG
ENSG00000182771	UUGGCAA
ENSG00000182782	AUGGAUU
ENSG00000182782	UCCAGAG
ENSG00000182791	AGGGAGG
ENSG00000182795	UAUAAAG
ENSG00000182827	AGUGCCG
ENSG00000182827	CUCCCAA
ENSG00000182827	GUCUCUG
ENSG00000182831	AAAGUGC
ENSG00000182831	AAGUGCU
ENSG00000182831	AAUUGCU
ENSG00000182831	ACAAUAU
ENSG00000182831	ACAGUAC
ENSG00000182831	ACUGUUU
ENSG00000182831	AGCAAAA
ENSG00000182831	AGCAGCA
ENSG00000182831	AUGAUGC
ENSG00000182831	CACCAGC

ENSG00000182831	CAGCAGG
ENSG00000182831	CAGUCCA
ENSG00000182831	CUGUUGA
ENSG00000182831	GACCAUG
ENSG00000182831	GAGGAUA
ENSG00000182831	GCAGCAU
ENSG00000182831	GGCAGGG
ENSG00000182831	GGUGGGC
ENSG00000182831	UCCCUUG
ENSG00000182831	UGAAGCU
ENSG00000182831	UGACAUC
ENSG00000182831	UGAGGAC
ENSG00000182831	UGUCUUU
ENSG00000182831	UGUUGAA
ENSG00000182831	UUGACAC
ENSG00000182836	AAAGUAC
ENSG00000182836	AACACUG
ENSG00000182836	AAGAACC
ENSG00000182836	AAGUGCA
ENSG00000182836	ACAAGGU
ENSG00000182836	ACACACC
ENSG00000182836	ACAUUCA
ENSG00000182836	ACUGUAG
ENSG00000182836	AGCCCUU
ENSG00000182836	AUGGCAC
ENSG00000182836	AUGGCUU
ENSG00000182836	AUGUGCC
ENSG00000182836	CCCACCG
ENSG00000182836	CCCACCU
ENSG00000182836	CCUGCUG
ENSG00000182836	CUACAAA
ENSG00000182836	CUCCAUU
ENSG00000182836	CUUCUCU
ENSG00000182836	GACUUCC
ENSG00000182836	GAGAAGA
ENSG00000182836	GCCCUUA
ENSG00000182836	GUGUCAC
ENSG00000182836	UCAUACA
ENSG00000182836	UGUGCUU
ENSG00000182866	GAGGUAU
ENSG00000182870	AAGGCAC
ENSG00000182871	CUUUGGU
ENSG00000182872	UACAAAG
ENSG00000182872	UAUUACC
ENSG00000182890	GUAAACA
ENSG00000182890	UGAGAAG
ENSG00000182896	GCCUGGG
ENSG00000182901	ACAAUAU
ENSG00000182901	AGAUAAA
ENSG00000182902	CAGUGCA
ENSG00000182902	GAGGUAU
ENSG00000182909	CAGCUGG
ENSG00000182909	CAGGCUC
ENSG00000182909	CUGGACU
ENSG00000182909	GGACUGC
ENSG00000182909	UCCAGAG
ENSG00000182909	UGGACUG
ENSG00000182923	AACAAAC
ENSG00000182923	AAGUCAC
ENSG00000182923	GAGAAAU
ENSG00000182923	GGAUUUU
ENSG00000182931	GGUUUAC
ENSG00000182934	AGCAGCG
ENSG00000182934	AGCAGCU

ENSG00000182934	AUAUAAC
ENSG00000182934	CAAGAGC
ENSG00000182934	CAGACAG
ENSG00000182934	CUGCAGC
ENSG00000182934	CUUCACC
ENSG00000182934	GGCAGUG
ENSG00000182934	UAUUGCU
ENSG00000182944	AAAUUUC
ENSG00000182944	GGUUUAC
ENSG00000182952	AAAAAUC
ENSG00000182952	AAAACUG
ENSG00000182957	CAGCUGG
ENSG00000182963	AAAGUUU
ENSG00000182963	AACAUUC
ENSG00000182963	AACUACU
ENSG00000182963	AAGGCAC
ENSG00000182963	AAUGUUG
ENSG00000182963	AAUUAUU
ENSG00000182963	ACUCAAA
ENSG00000182963	ACUGUAG
ENSG00000182963	AGUGCCG
ENSG00000182963	AUGUGCC
ENSG00000182963	CCCCUGG
ENSG00000182963	CUUUGGU
ENSG00000182963	GAGGUAU
ENSG00000182963	GAGUAUU
ENSG00000182963	GCAGGUU
ENSG00000182963	GCAUCCC
ENSG00000182963	GCCUGAU
ENSG00000182963	GGCUCUG
ENSG00000182963	GUAUUCU
ENSG00000182963	UCUGGAA
ENSG00000182963	UUCAAAU
ENSG00000182963	UUCUGCA
ENSG00000182968	AAUACUG
ENSG00000182968	ACUGUAG
ENSG00000182968	AGCAAAA
ENSG00000182968	AUGCAUU
ENSG00000182968	CAGUCUG
ENSG00000182968	CUACAGU
ENSG00000182968	GCAUCCC
ENSG00000182968	GCUGUCU
ENSG00000182968	UAAUGCU
ENSG00000182968	UACAAAG
ENSG00000182968	UUGGUUC
ENSG00000182973	GUAACAG
ENSG00000182979	AAAAAUC
ENSG00000182979	AACAUUC
ENSG00000182979	GUAAACA
ENSG00000182979	UAAUAUC
ENSG00000182985	AAGGCAC
ENSG00000182985	CAGCAGG
ENSG00000182985	CAGUGCA
ENSG00000182985	UACAAAG
ENSG00000182985	UAGUGAG
ENSG00000182985	UUUGCAA
ENSG00000183020	UCCCUUG
ENSG00000183023	AAAACUG
ENSG00000183023	ACACACC
ENSG00000183023	ACACACU
ENSG00000183023	AUAUAAC
ENSG00000183023	CAGACAG
ENSG00000183023	CAGCAGA
ENSG00000183023	CAGGUGA

ENSG00000183023	GGAAUGU
ENSG00000183023	GGAGGCA
ENSG00000183023	GGCCCUG
ENSG00000183023	GGGUUUA
ENSG00000183023	UAUUGCU
ENSG00000183023	UGAAGCU
ENSG00000183023	UGCAAAG
ENSG00000183023	UGUGUCA
ENSG00000183023	UUCCUCG
ENSG00000183044	AAAAAUC
ENSG00000183044	AGGAUGU
ENSG00000183044	AUGGCUU
ENSG00000183044	CAUGGAU
ENSG00000183044	CCAGGAG
ENSG00000183044	CUUUGGU
ENSG00000183044	GAGGAUA
ENSG00000183044	GGACCUG
ENSG00000183044	GGUUACC
ENSG00000183044	UAAGACU
ENSG00000183044	UAUAAAG
ENSG00000183044	UGCAUAG
ENSG00000183044	UGUGCUU
ENSG00000183049	AGCACCA
ENSG00000183049	GAAGGUC
ENSG00000183049	GAGAAGA
ENSG00000183049	GCACCAU
ENSG00000183049	UUGUGAC
ENSG00000183072	AAAAACC
ENSG00000183087	AAAAAUC
ENSG00000183087	AAUUAUU
ENSG00000183092	AAAAAUC
ENSG00000183092	ACAGUAU
ENSG00000183092	GAGGUAU
ENSG00000183092	UAUAAAG
ENSG00000183092	UGUCUUU
ENSG00000183114	ACAUAGA
ENSG00000183114	CUUUGGU
ENSG00000183114	GAUCAGA
ENSG00000183114	UUGGUCC
ENSG00000183117	AAAAACC
ENSG00000183117	AAUGCCC
ENSG00000183117	AAUUAUU
ENSG00000183117	ACACACU
ENSG00000183117	ACUGUUU
ENSG00000183117	AGAUGUG
ENSG00000183117	AGCAGCA
ENSG00000183117	AGCCUGC
ENSG00000183117	AGUGUGC
ENSG00000183117	AUAAUAC
ENSG00000183117	AUAUUAU
ENSG00000183117	AUGGCUU
ENSG00000183117	GAGGCAG
ENSG00000183117	GAGGUAU
ENSG00000183117	GGCAAGA
ENSG00000183117	GGCAGUG
ENSG00000183117	UAAUAUC
ENSG00000183117	UAUUGCU
ENSG00000183117	UGAAAGG
ENSG00000183117	UGCCAGC
ENSG00000183117	UUUUUGC
ENSG00000183137	GAAACAU
ENSG00000183137	GUGACAG
ENSG00000183155	GAAAACA
ENSG00000183155	UUCUGCA

ENSG00000183155	UUGGCAA
ENSG00000183155	UUGGCAC
ENSG00000183160	CCCUGUC
ENSG00000183160	GGAUGAG
ENSG00000183161	GUAAACA
ENSG00000183166	AAGUUGU
ENSG00000183166	ACUUGGA
ENSG00000183166	AGCCCUU
ENSG00000183166	AGGGUUG
ENSG00000183166	AUAAAGU
ENSG00000183166	AUAUAAC
ENSG00000183166	AUAUUAU
ENSG00000183166	AUGGCAC
ENSG00000183166	AUGGCUU
ENSG00000183166	CACAGUG
ENSG00000183166	CAGCAAA
ENSG00000183166	CAGCAGG
ENSG00000183166	CUCAGGG
ENSG00000183166	CUGCCCC
ENSG00000183166	CUGGACU
ENSG00000183166	GAGGUAG
ENSG00000183166	GAGGUAU
ENSG00000183166	GAGUCUU
ENSG00000183166	GGAGAGA
ENSG00000183166	GGGAGCU
ENSG00000183166	GGUGCGG
ENSG00000183166	GUCUGCC
ENSG00000183166	GUGCCUG
ENSG00000183166	GUGCUUG
ENSG00000183166	UAAUAGU
ENSG00000183166	UACAAAG
ENSG00000183166	UAGACUG
ENSG00000183166	UAUUGCU
ENSG00000183166	UCAACAG
ENSG00000183166	UCCUGAG
ENSG00000183166	UCUGGAA
ENSG00000183166	UGAGUCU
ENSG00000183166	UGCAUAU
ENSG00000183166	UGCUGAC
ENSG00000183166	UUAGAGA
ENSG00000183166	UUGGCAA
ENSG00000183172	GAGGCUG
ENSG00000183207	UCACAGU
ENSG00000183242	ACAGGUG
ENSG00000183242	AUAUAAC
ENSG00000183242	CAUGGGU
ENSG00000183242	CUGCUCA
ENSG00000183242	CUGGGGG
ENSG00000183242	GCAGAAG
ENSG00000183242	GGAAGCC
ENSG00000183242	GGCAUUG
ENSG00000183242	GGGAGCU
ENSG00000183242	GGGGUCC
ENSG00000183242	GGUGCGG
ENSG00000183242	UAAUGCU
ENSG00000183242	UCCAGAG
ENSG00000183242	UGGCCAC
ENSG00000183242	UUCUAAU
ENSG00000183242	UUUGCAA
ENSG00000183255	AAUGCCC
ENSG00000183255	CAAAUGC
ENSG00000183283	AAACUGU
ENSG00000183283	AAAGUGC
ENSG00000183283	ACCACAG

ENSG00000183283	AGUGCUG
ENSG00000183283	AUUCAUU
ENSG00000183283	CACAGUG
ENSG00000183283	CAGUUAC
ENSG00000183283	CCCUGAG
ENSG00000183283	GAGAAGA
ENSG00000183283	GCCUGAU
ENSG00000183283	UAAUAUC
ENSG00000183283	UGAGAAG
ENSG00000183283	UUCAAAU
ENSG00000183283	UUCCAUA
ENSG00000183287	CUGGGCA
ENSG00000183307	AAAGCUG
ENSG00000183307	ACCCUGU
ENSG00000183307	AGCAGCA
ENSG00000183307	AGGUAGU
ENSG00000183307	CAGCAAA
ENSG00000183307	CAGGCUC
ENSG00000183307	CAGUGCA
ENSG00000183307	GAGGUAG
ENSG00000183307	GCAACUU
ENSG00000183307	GCAGCAU
ENSG00000183307	GGAGAGA
ENSG00000183307	GGCUCAG
ENSG00000183307	GUAAACA
ENSG00000183307	GUGAGGU
ENSG00000183307	UAGGUAU
ENSG00000183307	UAGUGAG
ENSG00000183307	UCACAGU
ENSG00000183307	UCUCAAG
ENSG00000183307	UGCUCAC
ENSG00000183307	UUGGUCC
ENSG00000183307	UUUGCAA
ENSG00000183317	AAAAAUC
ENSG00000183317	AAGGCAC
ENSG00000183317	ACCCUGU
ENSG00000183317	AGCACCA
ENSG00000183317	CACUCCU
ENSG00000183317	CCCUGAG
ENSG00000183317	UUCCAUA
ENSG00000183337	AAGUCUU
ENSG00000183337	AGUGCCU
ENSG00000183337	UAAUAGU
ENSG00000183337	UAUGGUU
ENSG00000183354	AAUGCCC
ENSG00000183354	AAUUUUA
ENSG00000183354	AUGUAAU
ENSG00000183379	CUGCAGG
ENSG00000183386	AUUGCAC
ENSG00000183386	GCAGAAG
ENSG00000183386	UAUUGCU
ENSG00000183386	UCUGGAA
ENSG00000183423	AAUUAUU
ENSG00000183431	AGAUAAA
ENSG00000183431	AGCAAAA
ENSG00000183431	AUGCCUU
ENSG00000183431	CCAGCAU
ENSG00000183431	GAGAAAU
ENSG00000183431	UGCCAGC
ENSG00000183434	UCCUGAG
ENSG00000183454	AAUCUCA
ENSG00000183454	AAUCUCU
ENSG00000183454	AUGCAUU
ENSG00000183454	CACCAGC

ENSG00000183454	CAUAUUG
ENSG00000183454	CCCUGAG
ENSG00000183454	CUAUACA
ENSG00000183454	UGCAGCU
ENSG00000183454	UGUGUCA
ENSG00000183454	UUAUGCA
ENSG00000183475	GUGCAAA
ENSG00000183475	UGCAUAU
ENSG00000183484	AUAAAGU
ENSG00000183496	AAAGUAA
ENSG00000183496	AAAGUAU
ENSG00000183496	AACAUUC
ENSG00000183496	AAUACUG
ENSG00000183496	AUACAAG
ENSG00000183496	AUCAUAC
ENSG00000183496	CCAUUAC
ENSG00000183496	CUACAAA
ENSG00000183496	CUCCAUU
ENSG00000183496	GAUAUGU
ENSG00000183496	UAAUACA
ENSG00000183496	UGCUCAC
ENSG00000183496	UGGGUGA
ENSG00000183496	UUCAAGC
ENSG00000183496	UUUCAAC
ENSG00000183496	UUUGCAA
ENSG00000183508	AAAGAAU
ENSG00000183508	AACAUUC
ENSG00000183508	AAGUGCA
ENSG00000183508	AAUACUG
ENSG00000183508	ACAUUCA
ENSG00000183508	ACCCAUU
ENSG00000183508	ACUGUUU
ENSG00000183508	ACUUGGA
ENSG00000183508	AGUGCUU
ENSG00000183508	CACAGGG
ENSG00000183508	CACCACC
ENSG00000183508	CACUGUU
ENSG00000183508	CAGUUAC
ENSG00000183508	GGAAGAC
ENSG00000183508	GGCUCAG
ENSG00000183508	GUCAGUU
ENSG00000183508	GUGCAAA
ENSG00000183508	UCUCAAG
ENSG00000183508	UGGGUAC
ENSG00000183508	UUCCUAG
ENSG00000183508	UUGGUCC
ENSG00000183513	AGGAUGU
ENSG00000183520	UACAAAG
ENSG00000183527	AAAGUAA
ENSG00000183558	AAAGAGC
ENSG00000183570	AAGUGCU
ENSG00000183570	AGUGCUG
ENSG00000183576	AAUCACU
ENSG00000183576	AAUCUCU
ENSG00000183576	AGCAAAA
ENSG00000183576	AUAAAGU
ENSG00000183576	AUCACUA
ENSG00000183576	CUGCAGC
ENSG00000183576	CUGCAGG
ENSG00000183576	GCAGAAG
ENSG00000183576	UAUAAAG
ENSG00000183578	ACACACC
ENSG00000183578	AGGCAGG
ENSG00000183578	GAAAACA

ENSG00000183578	UCCCUCU
ENSG00000183579	AAGGCAC
ENSG00000183579	AAGUGCA
ENSG00000183579	ACAGGUG
ENSG00000183579	ACAGUAU
ENSG00000183579	AGCAGCA
ENSG00000183579	AGCAGCU
ENSG00000183579	AGCCCUU
ENSG00000183579	AGGCAGG
ENSG00000183579	AGGGUUG
ENSG00000183579	AGUAGAC
ENSG00000183579	AGUGCAA
ENSG00000183579	AGUGCCG
ENSG00000183579	AUAUUAU
ENSG00000183579	AUGUAAU
ENSG00000183579	CACACCU
ENSG00000183579	CCCCCAG
ENSG00000183579	CCCUGAG
ENSG00000183579	CUAUACA
ENSG00000183579	CUCACAC
ENSG00000183579	CUGCAGG
ENSG00000183579	CUGGAAA
ENSG00000183579	GAGAACC
ENSG00000183579	GAGCCCU
ENSG00000183579	GCAGCAU
ENSG00000183579	GGAGUCC
ENSG00000183579	GUGCAAA
ENSG00000183579	GUUAAUG
ENSG00000183579	UAAUAGU
ENSG00000183579	UAAUGCU
ENSG00000183579	UCAAGUA
ENSG00000183579	UCACAUU
ENSG00000183579	UCAUUUG
ENSG00000183579	UCCCACA
ENSG00000183579	UCCCUUU
ENSG00000183579	UGAAAUG
ENSG00000183579	UGAGAAG
ENSG00000183579	UUCUAAU
ENSG00000183579	UUCUGCA
ENSG00000183579	UUGGAGG
ENSG00000183579	UUGGUCC
ENSG00000183579	UUGUGUC
ENSG00000183579	UUUCAAC
ENSG00000183580	AGAUGUG
ENSG00000183580	AGCAUUC
ENSG00000183580	AUGUGCC
ENSG00000183580	CAUAUUG
ENSG00000183580	CUACAGU
ENSG00000183580	CUUGGAG
ENSG00000183580	GAAGGAA
ENSG00000183580	GUGCCUG
ENSG00000183580	UAUUGCU
ENSG00000183580	UCUAGAG
ENSG00000183580	UUCUUGU
ENSG00000183615	CUCUGGG
ENSG00000183638	CAAAGUG
ENSG00000183638	CUGCAGC
ENSG00000183638	CUGCAGG
ENSG00000183643	ACUUGGA
ENSG00000183643	CUCUGGG
ENSG00000183643	GCUGUCU
ENSG00000183643	UGGAUAA
ENSG00000183655	AGCACCA
ENSG00000183662	AAAGUGC

ENSG00000183662	AAGUGCA
ENSG00000183662	AGUGCAA
ENSG00000183682	AGCAGCA
ENSG00000183682	GUCACUC
ENSG00000183688	AAAGUAU
ENSG00000183688	AGCAAAA
ENSG00000183688	AGCAGCA
ENSG00000183688	GGAAUGU
ENSG00000183691	ACACACC
ENSG00000183691	ACACACU
ENSG00000183691	ACAGUAC
ENSG00000183691	ACAGUAU
ENSG00000183691	CAGUAGA
ENSG00000183691	UAUAAUA
ENSG00000183715	AAAAAUC
ENSG00000183715	AAAGUGC
ENSG00000183715	AACUACU
ENSG00000183715	AAUUAUU
ENSG00000183715	ACCCAUU
ENSG00000183715	AGGUAGU
ENSG00000183715	AGUGCUU
ENSG00000183715	AUUCAUU
ENSG00000183715	CACACCU
ENSG00000183715	CUCUUCC
ENSG00000183715	CUUUGGU
ENSG00000183715	GUAACAG
ENSG00000183715	GUUAAUG
ENSG00000183715	UAUUGCU
ENSG00000183715	UCAUUUG
ENSG00000183715	UGACAUC
ENSG00000183715	UGAGAAU
ENSG00000183715	UUAGAGA
ENSG00000183718	ACUGUUU
ENSG00000183718	CACUGUU
ENSG00000183722	CUUUGGU
ENSG00000183722	UCACAGG
ENSG00000183722	UCACAGU
ENSG00000183722	UGUGCUU
ENSG00000183722	UUGAUCA
ENSG00000183722	UUUCAAC
ENSG00000183723	AGUAACA
ENSG00000183723	CAGCAAA
ENSG00000183723	GGGCCCC
ENSG00000183723	UCACCAC
ENSG00000183723	UCAUUUG
ENSG00000183723	UGAGAAG
ENSG00000183723	UGCAGCU
ENSG00000183723	UUCAAGC
ENSG00000183723	UUUCAAC
ENSG00000183726	AGUGGUU
ENSG00000183726	GGACCUG
ENSG00000183735	AUGGCUU
ENSG00000183741	ACUUGGA
ENSG00000183741	AGCACCA
ENSG00000183741	AGCCCUU
ENSG00000183741	AGCUGCC
ENSG00000183741	AUGACAC
ENSG00000183741	CAAAACU
ENSG00000183741	CAAAGCA
ENSG00000183741	CUGGAAA
ENSG00000183741	CUUUGGU
ENSG00000183741	GCCCUUA
ENSG00000183741	GGACGGA
ENSG00000183741	GGGGGAA

ENSG00000183741	GUCUCUG
ENSG00000183741	UACAAAG
ENSG00000183741	UGCAAAG
ENSG00000183741	UUGGCAC
ENSG00000183741	UUUGCAC
ENSG00000183748	UGCCAGC
ENSG00000183762	AACAAAC
ENSG00000183762	AACAUUC
ENSG00000183762	ACAUUCA
ENSG00000183762	AUAUUAU
ENSG00000183762	CAAAGUG
ENSG00000183762	CACACCU
ENSG00000183762	CACCACC
ENSG00000183762	CAGCAGG
ENSG00000183762	CAGCUGG
ENSG00000183762	CUCAAAC
ENSG00000183762	CUGCAGG
ENSG00000183762	GAAGGUC
ENSG00000183762	GACCUGG
ENSG00000183762	GAGGUAG
ENSG00000183762	GUAAACA
ENSG00000183762	UACAAAG
ENSG00000183762	UCCCUUU
ENSG00000183762	UCUCAAG
ENSG00000183762	UCUUGAG
ENSG00000183762	UGAGAAU
ENSG00000183762	UGCAUAG
ENSG00000183762	UGUAUAA
ENSG00000183762	UUGAUCA
ENSG00000183763	CCUGCUG
ENSG00000183763	GGCAGGG
ENSG00000183763	GGGGCAG
ENSG00000183770	AAGUGCU
ENSG00000183770	UCACAUU
ENSG00000183773	CAGCAGA
ENSG00000183773	UUGGCAC
ENSG00000183775	AAACUGU
ENSG00000183775	AAGGUGC
ENSG00000183775	ACUGUGU
ENSG00000183775	AGGCAGG
ENSG00000183775	AGGGAGG
ENSG00000183775	CAGUAGU
ENSG00000183775	CUAUACA
ENSG00000183775	CUUGUGU
ENSG00000183775	GAGGUAG
ENSG00000183775	UAUUACC
ENSG00000183775	UCAAGUA
ENSG00000183775	UGGGAUC
ENSG00000183775	UGUGCUU
ENSG00000183775	UUGGCAC
ENSG00000183778	CAGGCUC
ENSG00000183779	GUUAAUG
ENSG00000183779	UAAUGCU
ENSG00000183780	AAGGCAC
ENSG00000183780	UCACAGU
ENSG00000183783	AGCAGCA
ENSG00000183785	CAGCAGG
ENSG00000183798	CAGCAGA
ENSG00000183798	GGCUCUG
ENSG00000183798	UGAAGCU
ENSG00000183808	AAUACUG
ENSG00000183808	CAGUCUG
ENSG00000183808	UCAAGUA
ENSG00000183808	UCACAGU

ENSG00000183814	AUACAAG
ENSG00000183826	AAAGAGG
ENSG00000183826	AACAAAC
ENSG00000183826	AAUUUUA
ENSG00000183826	ACCCAUU
ENSG00000183826	ACUCAGC
ENSG00000183826	ACUCCAG
ENSG00000183826	AUAAUAC
ENSG00000183826	CAGGAAC
ENSG00000183826	CCAGUGC
ENSG00000183826	CCAGUGU
ENSG00000183826	CUGCAGC
ENSG00000183826	GAGCUGC
ENSG00000183826	GAGGUAG
ENSG00000183826	GGGUGUU
ENSG00000183826	GUUAAUG
ENSG00000183826	UAUAAUA
ENSG00000183826	UCAUUUG
ENSG00000183826	UGAGAAU
ENSG00000183826	UGCCAGC
ENSG00000183826	UGCUGAC
ENSG00000183826	UUAGAGA
ENSG00000183826	UUGACAC
ENSG00000183826	UUGGCAA
ENSG00000183831	CUUGGAG
ENSG00000183831	UAAUAUC
ENSG00000183833	AAAAAUC
ENSG00000183833	UCCAGUU
ENSG00000183840	AAAGAGC
ENSG00000183853	AGCACCA
ENSG00000183853	CUGCCCC
ENSG00000183853	GAGACCU
ENSG00000183853	GCACCAU
ENSG00000183853	GGGUGUU
ENSG00000183856	CUGCAGG
ENSG00000183856	GGCCCUG
ENSG00000183862	GAGUUGA
ENSG00000183864	AAACUGU
ENSG00000183864	AAAGAAU
ENSG00000183864	AACAUUC
ENSG00000183864	AAGGCAC
ENSG00000183864	AAUUAUU
ENSG00000183864	AGUGCAA
ENSG00000183864	CCUUCAU
ENSG00000183864	CUGGAAA
ENSG00000183864	CUUACAG
ENSG00000183864	GAGAUGA
ENSG00000183864	GAGGUAG
ENSG00000183864	GAUUGUC
ENSG00000183864	GGCAGGG
ENSG00000183864	GGCAGUG
ENSG00000183864	UACAAAG
ENSG00000183864	UCCUGAG
ENSG00000183864	UGAAGCU
ENSG00000183864	UUUUUGC
ENSG00000183873	AAAUUUC
ENSG00000183873	AAGUGCU
ENSG00000183873	AAUACUG
ENSG00000183873	AGCAGCU
ENSG00000183873	CCCUGAG
ENSG00000183873	CUCUUCC
ENSG00000183873	GAGGUAG
ENSG00000183873	GAUUGUC
ENSG00000183873	UCCCUGU

ENSG00000183873	UGCAUAU
ENSG00000183878	ACUCAAA
ENSG00000183878	AGAUGUG
ENSG00000183878	AUGUGCC
ENSG00000183878	CAAAACU
ENSG00000183878	CUACAAA
ENSG00000183878	GCAGAAG
ENSG00000183878	GUAGUGU
ENSG00000183878	GUCCAGA
ENSG00000183878	GUGCAAA
ENSG00000183878	UCACAAG
ENSG00000183878	UCCAGUU
ENSG00000183878	UCCUGAG
ENSG00000183878	UCUGGAA
ENSG00000183878	UGCAUAU
ENSG00000183900	AAAAGUA
ENSG00000183900	AAAGUAC
ENSG00000183900	AAUCUCU
ENSG00000183900	CUUACAG
ENSG00000183900	UGAGAAU
ENSG00000183900	UGCCAGC
ENSG00000183908	CUGGAAA
ENSG00000183908	GCAGCAU
ENSG00000183908	GGCAGUG
ENSG00000183908	UGUGCUU
ENSG00000183918	GAGCCCU
ENSG00000183918	GUCUCUG
ENSG00000183943	AAAGUUU
ENSG00000183943	GUGCAAA
ENSG00000183943	UACAAAG
ENSG00000183943	UAUAAAG
ENSG00000183943	UGUCUUU
ENSG00000183955	AAGUUGU
ENSG00000183955	ACUGCAU
ENSG00000183955	AGCACCA
ENSG00000183955	AGGUAGU
ENSG00000183955	CAAGAGC
ENSG00000183955	CUACAGU
ENSG00000183955	CUUGUAU
ENSG00000183955	GUUCAUG
ENSG00000183955	UAAUAUC
ENSG00000183955	UAGGUAU
ENSG00000183955	UAUAAAG
ENSG00000183955	UCCCUUG
ENSG00000183955	UGUAUGU
ENSG00000183955	UUCCUAG
ENSG00000183955	UUGAGGC
ENSG00000183960	AAAAAUC
ENSG00000183960	AACAUUC
ENSG00000183960	ACAUUCA
ENSG00000183960	ACUGCAU
ENSG00000183960	CAGCAGA
ENSG00000183960	CUAUACA
ENSG00000183960	CUGCUCA
ENSG00000183960	GAGAUGA
ENSG00000183960	GAUUGUA
ENSG00000183960	UAUAAAG
ENSG00000183960	UCAUACA
ENSG00000183960	UCCCUUU
ENSG00000183960	UGCAAAG
ENSG00000183960	UUGAUCA
ENSG00000184005	ACAGUAU
ENSG00000184005	ACUGGCC
ENSG00000184005	AUAAAGU

ENSG00000184005	UACAGUU
ENSG00000184005	UAUUACC
ENSG00000184007	AAAGUGC
ENSG00000184007	CCUUCAU
ENSG00000184007	GCUGGUG
ENSG00000184007	UCCAGAG
ENSG00000184009	ACCCUGU
ENSG00000184009	AGUGCUG
ENSG00000184009	CAAAUGC
ENSG00000184009	CACAAUG
ENSG00000184009	GAGGCUG
ENSG00000184009	GCAGCAU
ENSG00000184009	GCUAGCA
ENSG00000184012	GAGGUAU
ENSG00000184047	AGCACCA
ENSG00000184058	AAAGAGG
ENSG00000184058	UUGGCAC
ENSG00000184083	AAACUGU
ENSG00000184083	AAAGAGC
ENSG00000184083	AAAGAGG
ENSG00000184083	AAAGCUG
ENSG00000184083	AAUACUG
ENSG00000184083	ACAUUCA
ENSG00000184083	ACCAGCA
ENSG00000184083	ACUGGCU
ENSG00000184083	AUAAAGU
ENSG00000184083	AUAUAAC
ENSG00000184083	CAAAGCA
ENSG00000184083	CACAGUG
ENSG00000184083	CAGCAGA
ENSG00000184083	CCUUCAU
ENSG00000184083	CUAGGCU
ENSG00000184083	CUGCCCC
ENSG00000184083	CUGGGCA
ENSG00000184083	GAGGCAG
ENSG00000184083	GGCAAGA
ENSG00000184083	GGCUCAG
ENSG00000184083	GGUUGUC
ENSG00000184083	GUCAGUU
ENSG00000184083	GUCUACU
ENSG00000184083	GUCUCUG
ENSG00000184083	UACAAAG
ENSG00000184083	UAUAAAG
ENSG00000184083	UCACAGU
ENSG00000184083	UCUGGAA
ENSG00000184083	UGCAAAG
ENSG00000184083	UGGGACA
ENSG00000184083	UUAGGAU
ENSG00000184083	UUCAAAU
ENSG00000184083	UUCCAUA
ENSG00000184083	UUGGCAA
ENSG00000184113	GGCAGGG
ENSG00000184117	GAGGUAU
ENSG00000184117	GGCUCAG
ENSG00000184144	AAAAACC
ENSG00000184144	AAGAACC
ENSG00000184144	AGCCCUU
ENSG00000184144	CACAGUG
ENSG00000184144	GAGGGGC
ENSG00000184144	GCAGGAC
ENSG00000184144	GGCAGUG
ENSG00000184144	GGGUCUU
ENSG00000184156	UACAAAG
ENSG00000184160	UUGGCAC

ENSG00000184162	ACUCAGG
ENSG00000184162	CUCAGGG
ENSG00000184162	GCUUCCU
ENSG00000184182	ACAUUAC
ENSG00000184185	AAAAACC
ENSG00000184185	AAAGAGG
ENSG00000184185	AACAGUC
ENSG00000184185	AAGGCAC
ENSG00000184185	AAGUGCA
ENSG00000184185	ACCAGCA
ENSG00000184185	AGCACCA
ENSG00000184185	AUGCAUU
ENSG00000184185	CACCAGC
ENSG00000184185	CCCUGAG
ENSG00000184185	CUAGUCC
ENSG00000184185	CUGCUCA
ENSG00000184185	GAGUCUU
ENSG00000184185	UCCAGUU
ENSG00000184185	UGAGUCU
ENSG00000184185	UGCAGCU
ENSG00000184185	UUGGUCC
ENSG00000184194	GUCUGCC
ENSG00000184203	AAAAGUA
ENSG00000184203	AAGUCAC
ENSG00000184203	AUACAAG
ENSG00000184203	GAGAAAU
ENSG00000184203	UACAAAG
ENSG00000184203	UUCCAUA
ENSG00000184205	AAAAAUC
ENSG00000184205	AAGUGCA
ENSG00000184205	AGCAGCA
ENSG00000184205	AGUGCAA
ENSG00000184205	CCCCCAG
ENSG00000184205	GGCCCUG
ENSG00000184205	UGCAUAG
ENSG00000184216	AGUGUGC
ENSG00000184216	GAGAACC
ENSG00000184216	GUAGUGU
ENSG00000184216	GUGACUG
ENSG00000184216	UAUUACC
ENSG00000184226	AAGGCAC
ENSG00000184226	ACCUGGA
ENSG00000184226	AGCAGCG
ENSG00000184226	AGCAGCU
ENSG00000184226	AGUAACA
ENSG00000184226	AUGCACC
ENSG00000184226	AUGCCUU
ENSG00000184226	AUUGCAC
ENSG00000184226	CAAAGCA
ENSG00000184226	CAGUCCA
ENSG00000184226	CUUGUGU
ENSG00000184226	GCAACUU
ENSG00000184226	GGCAUUG
ENSG00000184226	UAUUGCU
ENSG00000184226	UCAAGUA
ENSG00000184226	UGGUACC
ENSG00000184226	UUAGAGA
ENSG00000184232	AAGGCAC
ENSG00000184232	CUGCCCC
ENSG00000184232	CUGGAAA
ENSG00000184232	UCAAGUA
ENSG00000184232	UCACAGG
ENSG00000184232	UCACAGU
ENSG00000184254	ACAGUAC

ENSG00000184254	ACCACAG
ENSG00000184254	AGAUAAA
ENSG00000184254	AGCAUUC
ENSG00000184254	CCCUGAG
ENSG00000184254	UAUAAAG
ENSG00000184254	UUCCAUA
ENSG00000184254	UUCUAAU
ENSG00000184260	AAAGAGC
ENSG00000184260	GGCUCUG
ENSG00000184261	AAAGCGC
ENSG00000184270	AAAGAGC
ENSG00000184270	GGCUCUG
ENSG00000184271	AAAGUGC
ENSG00000184271	AAGUGCU
ENSG00000184271	CAAAGUG
ENSG00000184271	CUGCCCC
ENSG00000184271	GAGGCUG
ENSG00000184277	AAAGUAC
ENSG00000184277	ACAUUAC
ENSG00000184277	UCACAGG
ENSG00000184277	UCACAGU
ENSG00000184292	UACAAAG
ENSG00000184304	UCAACAG
ENSG00000184304	UGCAAAG
ENSG00000184304	UGUCUUU
ENSG00000184304	UUCUGCA
ENSG00000184304	UUGGCAA
ENSG00000184304	UUGUUCG
ENSG00000184307	AAAAAUC
ENSG00000184307	AAAGAAU
ENSG00000184307	AAAGUUU
ENSG00000184307	AGUGCAA
ENSG00000184307	AUACAAG
ENSG00000184307	AUGGCUU
ENSG00000184307	GGCAGUG
ENSG00000184307	GUGCUUG
ENSG00000184307	UGUGCUU
ENSG00000184343	AAGGCAC
ENSG00000184347	AAAAAUC
ENSG00000184347	CUCUUCC
ENSG00000184347	UUUUUGC
ENSG00000184349	GGAUGAG
ENSG00000184349	UGACAUC
ENSG00000184349	UUGGCAA
ENSG00000184349	UUGGCAC
ENSG00000184357	AAAGAGC
ENSG00000184368	AUGGCAC
ENSG00000184371	AGGAGCU
ENSG00000184371	AGUGCAA
ENSG00000184371	CCUUCAU
ENSG00000184371	GCUUCCU
ENSG00000184371	GGCAGGG
ENSG00000184371	UCACAGG
ENSG00000184371	UCACAGU
ENSG00000184371	UCCUGAG
ENSG00000184381	GUCAGUU
ENSG00000184388	AAGUCUU
ENSG00000184388	CUCUGGG
ENSG00000184388	CUGCAGG
ENSG00000184388	CUGUUGA
ENSG00000184388	UUGGCAA
ENSG00000184402	AAAGUAU
ENSG00000184402	AAAGUGC
ENSG00000184402	ACAAUAU

ENSG00000184402	AGUGCUU
ENSG00000184402	CAAAGUG
ENSG00000184402	CACAGUG
ENSG00000184402	CCAGCAU
ENSG00000184402	CUAUACA
ENSG00000184402	CUGGGCA
ENSG00000184402	GAAACAU
ENSG00000184402	GGCAAGA
ENSG00000184402	UCACAGG
ENSG00000184402	UCACAUU
ENSG00000184402	UGUAUAA
ENSG00000184402	UUGGCAA
ENSG00000184408	AAGUGCA
ENSG00000184408	AUGCAUU
ENSG00000184408	AUUGCAC
ENSG00000184408	GAGAAAU
ENSG00000184408	UCCUGAG
ENSG00000184408	UGUGCUU
ENSG00000184408	UUCAAGC
ENSG00000184454	AGCACCA
ENSG00000184454	AGCUGCC
ENSG00000184454	AGUACUG
ENSG00000184454	GACCCUG
ENSG00000184454	GAGUCUU
ENSG00000184454	UAUUACC
ENSG00000184454	UGAAGCU
ENSG00000184454	UGAGUCU
ENSG00000184481	ACUCCAG
ENSG00000184481	ACUGCAU
ENSG00000184481	AGGCAGG
ENSG00000184481	AGGGAGG
ENSG00000184481	CACAGUG
ENSG00000184481	CAGCAGG
ENSG00000184481	CAGGCUC
ENSG00000184481	CCAGUGC
ENSG00000184481	CUAGUAA
ENSG00000184481	CUCCCAC
ENSG00000184481	CUGGGGG
ENSG00000184481	GACUUCC
ENSG00000184481	GAGCCCC
ENSG00000184481	GAGUGUG
ENSG00000184481	GGCCCUG
ENSG00000184481	GGGAACG
ENSG00000184481	GGGUAAG
ENSG00000184481	GUCUCUG
ENSG00000184481	UAAGACU
ENSG00000184481	UAUAAAG
ENSG00000184481	UGAUCCA
ENSG00000184481	UGGCCCU
ENSG00000184481	UGGGGGA
ENSG00000184481	UUGGCAC
ENSG00000184481	UUGUGUC
ENSG00000184486	AGGCAGG
ENSG00000184486	GUGCAAA
ENSG00000184486	UCCCUGU
ENSG00000184486	UUGAGGC
ENSG00000184489	AGUGGUU
ENSG00000184489	UAUUGCU
ENSG00000184508	CACCACC
ENSG00000184508	CUCUUCC
ENSG00000184508	CUUCACC
ENSG00000184508	UCACCAC
ENSG00000184508	UUGUUCG
ENSG00000184517	GGAGAGA

ENSG00000184545	AAAGUGC
ENSG00000184545	AAGUGCA
ENSG00000184545	AUAAUAC
ENSG00000184545	AUAUAAC
ENSG00000184545	AUGGCUU
ENSG00000184545	AUGUAAU
ENSG00000184545	GGCUCAG
ENSG00000184545	UAUUACC
ENSG00000184545	UAUUGCU
ENSG00000184545	UGCCAGC
ENSG00000184557	ACAUUCA
ENSG00000184557	AGUGCUG
ENSG00000184557	AGUGCUU
ENSG00000184557	CAGUGCA
ENSG00000184557	GAUCAGA
ENSG00000184557	GAUUGUA
ENSG00000184557	GUGCCAC
ENSG00000184557	UGGGACA
ENSG00000184557	UGUGCUU
ENSG00000184557	UUCAAGC
ENSG00000184557	UUGAGGC
ENSG00000184560	CUGGACU
ENSG00000184564	AGCAGCA
ENSG00000184564	AUGGCUU
ENSG00000184564	UGUGCUU
ENSG00000184571	GUCAGUU
ENSG00000184574	GGAAGCC
ENSG00000184575	AUGGCAC
ENSG00000184575	GCAACUU
ENSG00000184584	GGCUCAG
ENSG00000184588	AAGGCAC
ENSG00000184588	ACAAUAU
ENSG00000184588	AGUAGUU
ENSG00000184588	AGUGCAA
ENSG00000184588	CACCACC
ENSG00000184588	CAGUAGA
ENSG00000184588	CAGUAGU
ENSG00000184588	CAGUGCA
ENSG00000184588	GAGAAAU
ENSG00000184588	GGAAGAC
ENSG00000184588	GGCAGUG
ENSG00000184588	UAUAAAG
ENSG00000184588	UCAAGUA
ENSG00000184602	AAAGCGC
ENSG00000184602	AACAAAC
ENSG00000184602	AAGCGCU
ENSG00000184602	AAUUUUA
ENSG00000184602	ACAGUAC
ENSG00000184602	ACAGUAU
ENSG00000184602	ACAUUCA
ENSG00000184602	AGCAAAA
ENSG00000184602	AGUAACA
ENSG00000184602	CACAGGG
ENSG00000184602	CAGUAGU
ENSG00000184602	CCAGUGC
ENSG00000184602	CCAGUGU
ENSG00000184602	GAGGUAG
ENSG00000184602	GCAGAAG
ENSG00000184602	GGCAAGA
ENSG00000184602	UAUUGCU
ENSG00000184602	UCACAGU
ENSG00000184602	UGCCAGC
ENSG00000184611	ACAGUAU
ENSG00000184611	CCCUGAG

ENSG00000184611	GAAAACA
ENSG00000184611	GGCAGUG
ENSG00000184611	UCAAGUA
ENSG00000184611	UGCAUAG
ENSG00000184611	UUUGCAA
ENSG00000184611	UUUGCAC
ENSG00000184613	GAGAAAU
ENSG00000184634	AAAAACC
ENSG00000184634	AAAAAUC
ENSG00000184634	AACAAAC
ENSG00000184634	UUUGCAA
ENSG00000184640	AAGGCAC
ENSG00000184640	AGCAAAA
ENSG00000184640	AUACAAG
ENSG00000184640	CAGACAG
ENSG00000184640	CAGGAAC
ENSG00000184640	CUUGGGC
ENSG00000184640	GAAGGAA
ENSG00000184640	GGAGGCA
ENSG00000184640	UUCAAAU
ENSG00000184640	UUGGCAC
ENSG00000184672	AAAAAUC
ENSG00000184672	UACAAAG
ENSG00000184672	UCACAGU
ENSG00000184672	UGUAUAA
ENSG00000184672	UUCUAAU
ENSG00000184675	AGAUAAA
ENSG00000184675	AGGAGCU
ENSG00000184675	AGUGGUU
ENSG00000184675	CACUGUU
ENSG00000184675	CAUGGGU
ENSG00000184675	CUGCCCC
ENSG00000184675	CUUUGGU
ENSG00000184675	GAGAAAU
ENSG00000184675	GAGGCUG
ENSG00000184675	GCAUCCC
ENSG00000184675	GCUUCCU
ENSG00000184675	GGAUUUU
ENSG00000184675	GGGUAAG
ENSG00000184675	GUAAACA
ENSG00000184675	GUGCUUG
ENSG00000184675	UCACAAG
ENSG00000184675	UCCUGAG
ENSG00000184675	UCCUUGC
ENSG00000184675	UGAAGUG
ENSG00000184675	UGGAGAU
ENSG00000184675	UUCUGCA
ENSG00000184675	UUGGAGG
ENSG00000184675	UUUCAAC
ENSG00000184677	AAGUCUU
ENSG00000184677	AAUUAUU
ENSG00000184677	AAUUGCU
ENSG00000184677	AGAUAAA
ENSG00000184677	AGCACCA
ENSG00000184677	AUGGAUU
ENSG00000184677	AUUGCAC
ENSG00000184677	CAAGAGC
ENSG00000184677	CAGGAAC
ENSG00000184677	CUUGGGC
ENSG00000184677	GCACCAU
ENSG00000184677	GUAAACA
ENSG00000184677	GUGGGGA
ENSG00000184677	UCAUUUG
ENSG00000184677	UUAGGAU

ENSG00000184677	UUCCAUA
ENSG00000184677	UUGGCAA
ENSG00000184677	UUGGCAC
ENSG00000184678	AAAGAGC
ENSG00000184678	GGCAGGG
ENSG00000184678	GGGUGGU
ENSG00000184702	GCGGGUG
ENSG00000184743	AAAGCUG
ENSG00000184743	AAGGCAC
ENSG00000184743	AAGUGCA
ENSG00000184787	AAAAAUC
ENSG00000184787	AAGGCAC
ENSG00000184787	ACCAGCA
ENSG00000184787	GAGGUAG
ENSG00000184787	UAUUGCU
ENSG00000184787	UUCUGCA
ENSG00000184788	UGCCAGC
ENSG00000184792	AAAAAUC
ENSG00000184792	AAGGCAC
ENSG00000184792	ACUUGGA
ENSG00000184792	CACCAGC
ENSG00000184792	CAGACAG
ENSG00000184792	CUGGAAA
ENSG00000184792	UAUUGCU
ENSG00000184792	UGCUGAC
ENSG00000184811	GGCAGUG
ENSG00000184811	GUUCAUG
ENSG00000184811	UCCAGAG
ENSG00000184811	UUCCAUA
ENSG00000184825	AAAGAGC
ENSG00000184828	AAGGCAC
ENSG00000184828	CUUCUCU
ENSG00000184838	AAGUGCU
ENSG00000184838	UUAUGCA
ENSG00000184840	AGUAACA
ENSG00000184840	CCCCCAG
ENSG00000184840	CCCUGAG
ENSG00000184840	GGAAGAC
ENSG00000184840	GUAACAG
ENSG00000184840	UCCCACA
ENSG00000184845	AAGUGCU
ENSG00000184845	AGCACCA
ENSG00000184845	AUAAAGU
ENSG00000184863	AAGACAU
ENSG00000184863	AAGGCAC
ENSG00000184863	AAUCCUU
ENSG00000184863	ACACACC
ENSG00000184863	ACACACU
ENSG00000184863	AGUGCAA
ENSG00000184863	AUAAUAC
ENSG00000184863	AUGCAUU
ENSG00000184863	CACUGUU
ENSG00000184863	CCAUUAC
ENSG00000184863	CUUCACC
ENSG00000184863	GCCCUUA
ENSG00000184863	GGAGAGA
ENSG00000184863	UAAUACA
ENSG00000184863	UAAUAUC
ENSG00000184863	UAUAAAG
ENSG00000184863	UGCAAAG
ENSG00000184863	UGCAUUG
ENSG00000184863	UGGAGAU
ENSG00000184863	UUAGGAU
ENSG00000184863	UUGGAGG

ENSG00000184867	AAAUGGU
ENSG00000184867	ACAUCAC
ENSG00000184886	AAAGUAA
ENSG00000184897	CGUCGCC
ENSG00000184897	UUCAAAU
ENSG00000184900	ACAGUAC
ENSG00000184900	ACAGUAU
ENSG00000184900	UGGCCCU
ENSG00000184916	AAAGAAU
ENSG00000184916	AAGGCAC
ENSG00000184916	AAUACUG
ENSG00000184916	GUAAACA
ENSG00000184916	UAAUAUC
ENSG00000184939	CUUUGGU
ENSG00000184939	UUAGGAU
ENSG00000184979	UAUAAAG
ENSG00000184983	ACAGGUG
ENSG00000184986	GAGAUGA
ENSG00000184988	GCCUGGA
ENSG00000184990	AUAAAGU
ENSG00000184990	UAUAAAG
ENSG00000184992	UACAAAG
ENSG00000185000	CCCUGAG
ENSG00000185000	GGGGCAG
ENSG00000185008	AAGGUGC
ENSG00000185008	ACAGUAU
ENSG00000185008	ACAUUCA
ENSG00000185008	ACCACAG
ENSG00000185008	AGCAGCU
ENSG00000185008	GAGAAGA
ENSG00000185008	GAUAUGU
ENSG00000185008	GUGCAAA
ENSG00000185008	GUGCUUG
ENSG00000185008	GUUCAUG
ENSG00000185008	UGCAUUG
ENSG00000185008	UGUGUGG
ENSG00000185009	AACAAAC
ENSG00000185009	AAGGCAC
ENSG00000185009	ACAAUAU
ENSG00000185009	ACUGCAG
ENSG00000185009	ACUGCAU
ENSG00000185009	ACUGGAU
ENSG00000185009	ACUGUGU
ENSG00000185009	AGCACCA
ENSG00000185009	AGCUUAU
ENSG00000185009	AGGUUAU
ENSG00000185009	AGUGCUU
ENSG00000185009	AUGGCAC
ENSG00000185009	CUACAGU
ENSG00000185009	CUGCAGC
ENSG00000185009	GAAAACA
ENSG00000185009	GAGAUGA
ENSG00000185009	GAUCAGA
ENSG00000185009	GCAGCAU
ENSG00000185009	GCUACAU
ENSG00000185009	GUAAACA
ENSG00000185009	UCCCACA
ENSG00000185009	UCCCACC
ENSG00000185009	UCCCUGU
ENSG00000185009	UCCCUUG
ENSG00000185009	UGAGAAU
ENSG00000185009	UUGAUCA
ENSG00000185009	UUGGCAA
ENSG00000185009	UUGGCAC

ENSG00000185010	GCUUCCU
ENSG00000185015	UAAUGCU
ENSG00000185015	UAUAUGA
ENSG00000185019	AUGGCUU
ENSG00000185019	CAGCAGG
ENSG00000185019	CUGGCUC
ENSG00000185019	GCCUGGG
ENSG00000185019	GUGCCUG
ENSG00000185022	AGAUAAA
ENSG00000185022	AUAAAGU
ENSG00000185022	UAUAAAG
ENSG00000185033	CCCUGAG
ENSG00000185033	CUGGGCA
ENSG00000185033	GCCUGAU
ENSG00000185033	GGCAGUG
ENSG00000185046	AAGGCAC
ENSG00000185046	CACAAUG
ENSG00000185046	UAUUACC
ENSG00000185049	GAGAAAU
ENSG00000185052	AAAGUAC
ENSG00000185052	AAAGUAU
ENSG00000185052	AAUCUCA
ENSG00000185052	ACAUUCA
ENSG00000185052	AGCAGCA
ENSG00000185052	AGCAGCG
ENSG00000185052	AGCAGCU
ENSG00000185052	AGUGAUC
ENSG00000185052	AGUGCAA
ENSG00000185052	AGUGCUU
ENSG00000185052	CACAAUG
ENSG00000185052	CCAGUGC
ENSG00000185052	CUGGCUC
ENSG00000185052	GAUCAGA
ENSG00000185052	GCCUGGA
ENSG00000185052	GCCUGGG
ENSG00000185052	GGCAAGA
ENSG00000185052	GGCUCAG
ENSG00000185052	UAUUGCU
ENSG00000185052	UCACAAG
ENSG00000185053	AAGGCAC
ENSG00000185070	AAAGAAU
ENSG00000185070	AAAGUAC
ENSG00000185070	AACAUUC
ENSG00000185070	AAGUGUG
ENSG00000185070	AAUCACU
ENSG00000185070	ACAGUAU
ENSG00000185070	ACUGUAG
ENSG00000185070	AGCCCUU
ENSG00000185070	AGUGGUU
ENSG00000185070	AUAAAGU
ENSG00000185070	CACAGUG
ENSG00000185070	CCUGUAC
ENSG00000185070	GUAACAG
ENSG00000185070	UAUAAAG
ENSG00000185070	UCACAGU
ENSG00000185070	UGCAUAG
ENSG00000185070	UGCAUAU
ENSG00000185070	UGUGCUU
ENSG00000185085	AAUUUUA
ENSG00000185088	AAAACUG
ENSG00000185088	UACAAAG
ENSG00000185090	ACAAGGU
ENSG00000185090	CACAGUG
ENSG00000185090	GAGGUAU

ENSG00000185090	UCACAGU
ENSG00000185112	AACAAAC
ENSG00000185112	AGCAAAA
ENSG00000185112	CAGCAAA
ENSG00000185112	GAGGUAG
ENSG00000185112	GAGGUAU
ENSG00000185112	UCACAAG
ENSG00000185112	UUGGCAC
ENSG00000185112	UUUGCAA
ENSG00000185122	CAGUCCA
ENSG00000185127	CAAAACU
ENSG00000185127	CAGCAAA
ENSG00000185127	UCACAGG
ENSG00000185127	UCACAGU
ENSG00000185127	UGUGCUU
ENSG00000185129	AACAAAC
ENSG00000185129	AACAUUC
ENSG00000185129	AAUGUUG
ENSG00000185129	AGCAGCG
ENSG00000185129	AGCCCUU
ENSG00000185129	CAAAUGC
ENSG00000185129	CACAGGG
ENSG00000185129	CAGACAG
ENSG00000185129	CAGCAAA
ENSG00000185129	CUACAAA
ENSG00000185129	CUUUGGU
ENSG00000185129	GUCAGUU
ENSG00000185129	GUCCAGA
ENSG00000185129	UAUUACC
ENSG00000185129	UCAAGUA
ENSG00000185129	UGCAAAG
ENSG00000185129	UGCAUAU
ENSG00000185129	UGUGCUU
ENSG00000185129	UUCUGCA
ENSG00000185149	CAGCAGA
ENSG00000185164	UCUCACC
ENSG00000185219	AUGCAUU
ENSG00000185219	CAGCAGG
ENSG00000185219	CCAGGAG
ENSG00000185219	CUGGCUC
ENSG00000185219	CUUCUCU
ENSG00000185250	CUGGGCA
ENSG00000185250	GCAACUU
ENSG00000185250	GGCUCAG
ENSG00000185250	UUGGCAA
ENSG00000185262	CCAGUGU
ENSG00000185262	CUCCAGC
ENSG00000185262	UAUAAAG
ENSG00000185262	UCCAGUU
ENSG00000185269	ACAAUAU
ENSG00000185269	AGUGCUU
ENSG00000185269	CCAGUGU
ENSG00000185269	UACAAAG
ENSG00000185269	UAUAAAG
ENSG00000185272	CUUUGGU
ENSG00000185274	AACUCUA
ENSG00000185274	AUAUUAU
ENSG00000185274	CUGGGCA
ENSG00000185274	GCUGUCU
ENSG00000185274	GGCAGGG
ENSG00000185278	CCCUGAG
ENSG00000185305	AAUUAUU
ENSG00000185305	AGUGGUU
ENSG00000185305	CAGUAGA

ENSG00000185305	CUGCUCA
ENSG00000185305	GAGAUGA
ENSG00000185305	GUAAACA
ENSG00000185305	GUAGUGU
ENSG00000185305	UUAUGCA
ENSG00000185332	CUGCAGG
ENSG00000185332	UGUGUGG
ENSG00000185338	CAGCAGA
ENSG00000185338	CCCCUGG
ENSG00000185338	CUCCAGC
ENSG00000185338	GCUACAU
ENSG00000185338	GUUAAUG
ENSG00000185338	UAAUGCU
ENSG00000185338	UACAGUU
ENSG00000185338	UGCAAAG
ENSG00000185338	UUCUGCA
ENSG00000185340	AAGGCAC
ENSG00000185340	GGAAUGU
ENSG00000185344	AAGGCAC
ENSG00000185344	AAUUAUU
ENSG00000185344	AUAUUAU
ENSG00000185344	GAGGUAU
ENSG00000185344	GGCUCAG
ENSG00000185344	UGCUCAC
ENSG00000185345	UCUCACC
ENSG00000185352	AUAUUAU
ENSG00000185352	CACAAUG
ENSG00000185352	CUUGGGC
ENSG00000185352	GAUCAGA
ENSG00000185352	GUGCUUG
ENSG00000185352	UCUCAAG
ENSG00000185352	UUCAAAU
ENSG00000185386	GAGGUAG
ENSG00000185414	GGAUUUC
ENSG00000185418	AAAUUUC
ENSG00000185432	AAAGUAU
ENSG00000185432	UCCCUUU
ENSG00000185436	AAAAAUC
ENSG00000185442	GAAGGUC
ENSG00000185477	AUAUUAU
ENSG00000185477	UCUGGAG
ENSG00000185477	UGAGUCU
ENSG00000185477	UUGAGGC
ENSG00000185477	UUGUGUC
ENSG00000185477	UUUUUGC
ENSG00000185483	UUCUGCA
ENSG00000185499	CACCAGC
ENSG00000185504	AGCACCA
ENSG00000185504	CACAGUG
ENSG00000185515	GUGCUUG
ENSG00000185515	UUUGCAA
ENSG00000185518	AAAACUG
ENSG00000185518	AAAGUGC
ENSG00000185518	AAAUGGU
ENSG00000185518	AUGGCUU
ENSG00000185518	GCCCUUA
ENSG00000185518	UGCUCAC
ENSG00000185518	UGUGCUU
ENSG00000185519	CCCACCU
ENSG00000185532	AAAGCUG
ENSG00000185532	AAUGCCC
ENSG00000185532	ACAUUCA
ENSG00000185532	ACUGUAG
ENSG00000185532	AGCACCA

ENSG00000185532	AGCAGCA
ENSG00000185532	CAGCAAA
ENSG00000185532	GAGAAAU
ENSG00000185532	GCAGCAU
ENSG00000185532	GCUGUCU
ENSG00000185532	GGCAGCG
ENSG00000185532	GUGCUUG
ENSG00000185551	AACUCUA
ENSG00000185551	AACUGGU
ENSG00000185551	AAGUUGU
ENSG00000185551	AAUGUUG
ENSG00000185551	ACAGUAC
ENSG00000185551	ACAUAGA
ENSG00000185551	AUAUAAC
ENSG00000185551	AUGCAUU
ENSG00000185551	CACAGUG
ENSG00000185551	CAGUUAC
ENSG00000185551	GUAAACA
ENSG00000185551	GUAACAG
ENSG00000185551	UACAGUU
ENSG00000185551	UCACAGG
ENSG00000185551	UCCUAUA
ENSG00000185551	UGCAAAG
ENSG00000185567	AGCAGCA
ENSG00000185585	AGCUGCC
ENSG00000185585	CAGCUGG
ENSG00000185585	CUCACAC
ENSG00000185585	GACCCUG
ENSG00000185585	GAGUGUG
ENSG00000185585	GCAGGAC
ENSG00000185585	GCAGGUU
ENSG00000185585	GCCUGAU
ENSG00000185585	GGAAUGU
ENSG00000185585	GGGGUGG
ENSG00000185585	UACAAAG
ENSG00000185585	UCUGGAA
ENSG00000185591	AAAGCUG
ENSG00000185591	AAAUUUC
ENSG00000185591	AAGACAU
ENSG00000185591	AAUACUG
ENSG00000185591	AAUCUCA
ENSG00000185591	AAUUUUA
ENSG00000185591	ACACACC
ENSG00000185591	ACACACU
ENSG00000185591	ACAUUCA
ENSG00000185591	ACCAGCA
ENSG00000185591	ACUGGCU
ENSG00000185591	AGCACCA
ENSG00000185591	AGCUGCC
ENSG00000185591	AGUAGAC
ENSG00000185591	AGUGCAA
ENSG00000185591	AGUGCCU
ENSG00000185591	AGUGCUG
ENSG00000185591	AGUGCUU
ENSG00000185591	AUACAAG
ENSG00000185591	AUCCUUG
ENSG00000185591	AUGGCUU
ENSG00000185591	AUUGCAC
ENSG00000185591	CAAAGCA
ENSG00000185591	CAGCAGA
ENSG00000185591	CAGCAGG
ENSG00000185591	CAGUAGA
ENSG00000185591	CAGUAGU
ENSG00000185591	CAGUCUG

ENSG00000185591	CAGUGCA
ENSG00000185591	CCAGUAC
ENSG00000185591	CCAGUGU
ENSG00000185591	CUCCAGC
ENSG00000185591	CUGCAGC
ENSG00000185591	CUGCAGG
ENSG00000185591	CUGGCUC
ENSG00000185591	CUGGGCA
ENSG00000185591	CUUGGAG
ENSG00000185591	GAAGGAA
ENSG00000185591	GAGGCAG
ENSG00000185591	GCAGGUU
ENSG00000185591	GCAUCCC
ENSG00000185591	GCCUGAU
ENSG00000185591	GCUUCCU
ENSG00000185591	GGAAGCC
ENSG00000185591	GGAAUGU
ENSG00000185591	GGCUCAG
ENSG00000185591	GGCUCUG
ENSG00000185591	GGGGCAG
ENSG00000185591	GGGUAAG
ENSG00000185591	GGUGGGC
ENSG00000185591	GUCAGUU
ENSG00000185591	GUCUCUG
ENSG00000185591	GUGAAUG
ENSG00000185591	GUGCCUG
ENSG00000185591	GUGCUUG
ENSG00000185591	UAUCAGA
ENSG00000185591	UAUUGCU
ENSG00000185591	UCACACA
ENSG00000185591	UCACAGU
ENSG00000185591	UCAUACA
ENSG00000185591	UCAUAGA
ENSG00000185591	UCCAGUU
ENSG00000185591	UCCCUGU
ENSG00000185591	UCCCUUG
ENSG00000185591	UCCCUUU
ENSG00000185591	UCCUGAG
ENSG00000185591	UGAAAGG
ENSG00000185591	UGAAAUG
ENSG00000185591	UGAGGAC
ENSG00000185591	UGCAGCU
ENSG00000185591	UGGUACC
ENSG00000185591	UGUGCUU
ENSG00000185591	UGUGUCA
ENSG00000185591	UUAGGAU
ENSG00000185591	UUCCGGC
ENSG00000185591	UUGAUCA
ENSG00000185591	UUGGUCC
ENSG00000185591	UUGUUCG
ENSG00000185610	GCUGGAU
ENSG00000185619	AAAGCUG
ENSG00000185619	AACAGUC
ENSG00000185619	AAUCCUU
ENSG00000185619	ACACACU
ENSG00000185619	ACCUGGA
ENSG00000185619	ACUCAAA
ENSG00000185619	AGCACCA
ENSG00000185619	AGCAUUC
ENSG00000185619	AUAAUAC
ENSG00000185619	AUACAAG
ENSG00000185619	AUCAUAC
ENSG00000185619	AUGCCUU
ENSG00000185619	AUUGCAC

ENSG00000185619	CAAAACU
ENSG00000185619	CAAAGCA
ENSG00000185619	CACAAUG
ENSG00000185619	CACAGUG
ENSG00000185619	CAGCAGA
ENSG00000185619	CAGCAGG
ENSG00000185619	CUACAAA
ENSG00000185619	GACCUGG
ENSG00000185619	GAGGUAG
ENSG00000185619	GAGGUAU
ENSG00000185619	GCACCAU
ENSG00000185619	GCUACAU
ENSG00000185619	GCUGGAU
ENSG00000185619	GCUGGUG
ENSG00000185619	GGUAGAC
ENSG00000185619	GUAAACA
ENSG00000185619	GUAGUGU
ENSG00000185619	GUCUUGC
ENSG00000185619	UAUAAUA
ENSG00000185619	UAUAUGA
ENSG00000185619	UAUUACC
ENSG00000185619	UCACAGG
ENSG00000185619	UCACAGU
ENSG00000185619	UCCCUUU
ENSG00000185619	UCCUGAG
ENSG00000185619	UGCAAAG
ENSG00000185619	UUGGAGG
ENSG00000185621	UUCCAUA
ENSG00000185624	AAAAGUA
ENSG00000185624	AAAGUAC
ENSG00000185624	ACAUCAC
ENSG00000185624	AUCCCUU
ENSG00000185624	UCCCUGU
ENSG00000185630	GGAGGCA
ENSG00000185633	ACGCUCA
ENSG00000185633	AGGGAGG
ENSG00000185633	CUUGGGC
ENSG00000185633	GGGGAGC
ENSG00000185633	UGAUCCA
ENSG00000185633	UUGAUCA
ENSG00000185634	AAAUGGU
ENSG00000185634	AAGGCAC
ENSG00000185634	AUAAAGU
ENSG00000185634	CAAAGCA
ENSG00000185634	CUGCUCA
ENSG00000185634	GUGCUUG
ENSG00000185634	UCAAGUA
ENSG00000185634	UGACAUC
ENSG00000185634	UUGGCAC
ENSG00000185650	AAAGAAU
ENSG00000185650	AAAGCUG
ENSG00000185650	AAGGCAC
ENSG00000185650	AUAUUAU
ENSG00000185650	CUAUACA
ENSG00000185650	GGCAAGA
ENSG00000185650	GGGUUUA
ENSG00000185650	UAAUAUC
ENSG00000185650	UCUAGAG
ENSG00000185650	UCUCCAA
ENSG00000185650	UGCAUUG
ENSG00000185650	UGUAUGU
ENSG00000185651	AAAACUG
ENSG00000185651	AAAUGGU
ENSG00000185651	AAGACAU

ENSG00000185651	ACAAUAU
ENSG00000185651	ACAGGUG
ENSG00000185651	ACUGUAG
ENSG00000185651	AGUAACA
ENSG00000185651	AUCCUUU
ENSG00000185651	CAAAACU
ENSG00000185651	CAGCAGA
ENSG00000185651	CCCUGAG
ENSG00000185651	CCUGCUG
ENSG00000185651	CUAGUCC
ENSG00000185651	CUCACAC
ENSG00000185651	CUGCUCA
ENSG00000185651	GCGGGGC
ENSG00000185651	GCUUCCU
ENSG00000185651	GGCAUUG
ENSG00000185651	GUCUCUG
ENSG00000185651	UCCCACA
ENSG00000185651	UGAAGCU
ENSG00000185651	UUCAAAU
ENSG00000185651	UUGGCAA
ENSG00000185651	UUGGCAC
ENSG00000185652	AUAAUAC
ENSG00000185652	AUAUAAC
ENSG00000185652	AUCAUAC
ENSG00000185652	UGCAUAU
ENSG00000185658	AAAAGUA
ENSG00000185658	AAAGUAC
ENSG00000185658	AAAGUAU
ENSG00000185658	AACACUG
ENSG00000185658	AAGUAGC
ENSG00000185658	AAGUGCA
ENSG00000185658	AAUACUG
ENSG00000185658	ACACACU
ENSG00000185658	AGCACCA
ENSG00000185658	AGCCCUU
ENSG00000185658	AGUGCUG
ENSG00000185658	AGUGCUU
ENSG00000185658	CCCUGAG
ENSG00000185658	CCUGUAC
ENSG00000185658	CUUGGAG
ENSG00000185658	GAGUGUG
ENSG00000185658	GCACCAU
ENSG00000185658	GCCCUGU
ENSG00000185658	GCUACAU
ENSG00000185658	GGAAGAC
ENSG00000185658	GGAGUGU
ENSG00000185658	GGAUUUC
ENSG00000185658	GUCAGUU
ENSG00000185658	GUGCAAA
ENSG00000185658	GUGUCAC
ENSG00000185658	UAAUGCU
ENSG00000185658	UCACAGG
ENSG00000185658	UCACCAC
ENSG00000185658	UCCCUGU
ENSG00000185658	UGUGUCA
ENSG00000185658	UUGGCAA
ENSG00000185658	UUGGUUC
ENSG00000185658	UUUGCAC
ENSG00000185666	CUCCAUU
ENSG00000185666	GAGGCAG
ENSG00000185666	UAUUGCU
ENSG00000185668	AAAGUUU
ENSG00000185668	ACAGUAC
ENSG00000185668	ACAGUAU

ENSG00000185668	AUAAAGU
ENSG00000185668	CCCACCU
ENSG00000185668	CUCACAC
ENSG00000185668	CUCAGGG
ENSG00000185668	GAGUUGG
ENSG00000185668	UAUAAAG
ENSG00000185670	CUGGCUC
ENSG00000185670	CUUGGAG
ENSG00000185697	AAGUGCU
ENSG00000185697	AGCAGCG
ENSG00000185697	AGCAGCU
ENSG00000185697	CAGUGCA
ENSG00000185697	CUACAGU
ENSG00000185697	GAGAACU
ENSG00000185697	GGAGAGA
ENSG00000185697	GUGCAAA
ENSG00000185722	AAAGCUG
ENSG00000185722	AAGGCAC
ENSG00000185722	AAGUCUU
ENSG00000185722	AAUUUUA
ENSG00000185722	ACAUUCA
ENSG00000185722	ACCCUGU
ENSG00000185722	ACUGGCC
ENSG00000185722	AGCAGCU
ENSG00000185722	AGUGGUU
ENSG00000185722	CCCUGUU
ENSG00000185722	GAAUUGU
ENSG00000185722	GAGGUAG
ENSG00000185722	GAGGUAU
ENSG00000185722	GCAGCAU
ENSG00000185722	GCUGGUG
ENSG00000185722	GGAAGAC
ENSG00000185722	GGAGAGA
ENSG00000185722	GGCAAGA
ENSG00000185722	GGCUCUG
ENSG00000185722	GUCAGUU
ENSG00000185722	GUGCCUG
ENSG00000185722	UAGAGGA
ENSG00000185722	UCCCUUU
ENSG00000185728	AAAGUUU
ENSG00000185728	AACUGGU
ENSG00000185728	AAGUGCU
ENSG00000185728	AAGUGUG
ENSG00000185728	AAUCCUU
ENSG00000185728	AAUUUUA
ENSG00000185728	ACAGUAU
ENSG00000185728	ACUCCAG
ENSG00000185728	AGGAUGU
ENSG00000185728	AGUGCUU
ENSG00000185728	AUCCUUG
ENSG00000185728	CAGCAGA
ENSG00000185728	CAUGGAU
ENSG00000185728	CCAUUAC
ENSG00000185728	GAGGUAG
ENSG00000185728	GAGGUAU
ENSG00000185728	GUGCCUG
ENSG00000185728	UACAAAG
ENSG00000185728	UCAAGUA
ENSG00000185728	UCAUGAU
ENSG00000185728	UCCUUGC
ENSG00000185728	UGAAGCU
ENSG00000185728	UGCAUAU
ENSG00000185728	UGCAUUG
ENSG00000185739	AAGACAU

ENSG00000185739	AGCCCUU
ENSG00000185739	CACAGUG
ENSG00000185739	CCAGUGU
ENSG00000185739	CUGACUC
ENSG00000185739	GAGCCCU
ENSG00000185739	GGCUCAG
ENSG00000185739	GUGUGGC
ENSG00000185739	UCACAGG
ENSG00000185739	UCACCAC
ENSG00000185760	AGCAGCA
ENSG00000185760	AGCAGCG
ENSG00000185760	AUGCAUU
ENSG00000185760	GUUCAUG
ENSG00000185774	UCAACAG
ENSG00000185774	UGCAUAG
ENSG00000185787	AGCAGCU
ENSG00000185787	CACCAGC
ENSG00000185787	UUGGCAA
ENSG00000185800	ACUUGGA
ENSG00000185800	CAGCAGG
ENSG00000185800	CCAGGAG
ENSG00000185800	GGCAGUG
ENSG00000185811	AUUGCAC
ENSG00000185811	CAGGUGA
ENSG00000185811	CAGUGCA
ENSG00000185811	GAGGCAG
ENSG00000185811	GGCAAGA
ENSG00000185811	GUGCUUG
ENSG00000185811	UACAGUU
ENSG00000185811	UAUUGCU
ENSG00000185811	UCACAGG
ENSG00000185811	UCACCAC
ENSG00000185811	UGCAUAG
ENSG00000185811	UUGGCAA
ENSG00000185818	AACAAAC
ENSG00000185818	CAAAGCA
ENSG00000185818	CACAGGG
ENSG00000185818	CAGGCUC
ENSG00000185818	GUGCUUG
ENSG00000185818	UCACAGG
ENSG00000185825	GGCAGGG
ENSG00000185860	CAGCAGG
ENSG00000185875	UAUUGCU
ENSG00000185875	UCACAAG
ENSG00000185883	UAUAAAG
ENSG00000185896	AAAGAAU
ENSG00000185896	AAGUGUG
ENSG00000185896	AGUGUGC
ENSG00000185896	CUUUGGU
ENSG00000185896	UACAAAG
ENSG00000185896	UCCCUUU
ENSG00000185896	UGCAUAG
ENSG00000185905	GGCUCUG
ENSG00000185909	ACUCCAG
ENSG00000185909	GAGGAUA
ENSG00000185909	GAGGCAG
ENSG00000185909	GAGGUAG
ENSG00000185909	GAGGUAU
ENSG00000185909	GCCCUGU
ENSG00000185920	AAAAGUA
ENSG00000185920	AAACUGU
ENSG00000185920	AAAGAGG
ENSG00000185920	AAAGUAA
ENSG00000185920	AAAUCCC

ENSG00000185920	AACAGUC
ENSG00000185920	AAUACUG
ENSG00000185920	AAUCCUU
ENSG00000185920	ACACACC
ENSG00000185920	ACAGUAU
ENSG00000185920	ACCAGCA
ENSG00000185920	AGCAGCA
ENSG00000185920	AGCUUAU
ENSG00000185920	AGGGUUG
ENSG00000185920	AUGGCAC
ENSG00000185920	AUUCAUU
ENSG00000185920	CACAGUG
ENSG00000185920	CACUGUU
ENSG00000185920	CCAGCAU
ENSG00000185920	CUCUUCC
ENSG00000185920	CUGGAAA
ENSG00000185920	CUUUGGU
ENSG00000185920	GAGACCU
ENSG00000185920	GAUAUGU
ENSG00000185920	GCAGAAG
ENSG00000185920	GUGACUG
ENSG00000185920	UACAAAG
ENSG00000185920	UACAGUU
ENSG00000185920	UAUAAAG
ENSG00000185920	UAUAUGA
ENSG00000185920	UCACAGU
ENSG00000185920	UCAUACA
ENSG00000185920	UCCCUUU
ENSG00000185920	UCUGGAG
ENSG00000185920	UGCAUAU
ENSG00000185920	UGCCAGC
ENSG00000185920	UUCAAGC
ENSG00000185924	AAAACUG
ENSG00000185924	AACACUG
ENSG00000185924	AUAUAAC
ENSG00000185924	CUUUGGU
ENSG00000185924	GCCUGAU
ENSG00000185924	GGCAGUG
ENSG00000185924	GUGCAAA
ENSG00000185924	UAUAAAG
ENSG00000185924	UAUAAUA
ENSG00000185924	UGCAUAG
ENSG00000185924	UUGGUCC
ENSG00000185928	GGGGUGG
ENSG00000185933	AAUACUG
ENSG00000185933	GCCUGGG
ENSG00000185946	ACGCACU
ENSG00000185950	AAGUCAC
ENSG00000185950	AAGUCUU
ENSG00000185950	ACUGUUU
ENSG00000185950	AGCAGCA
ENSG00000185950	AUGCAUU
ENSG00000185950	AUGGCUU
ENSG00000185950	CCAGCAU
ENSG00000185950	GAGGUAG
ENSG00000185950	GAGGUAU
ENSG00000185950	GCAGCAU
ENSG00000185950	GUAAACA
ENSG00000185950	UAUUGCU
ENSG00000185950	UCACAUU
ENSG00000185950	UGAAAUG
ENSG00000185950	UGCAUUG
ENSG00000185950	UGUCUUU
ENSG00000185950	UUUGCAA

ENSG00000185960	AUAAUAC
ENSG00000185960	UAAUGCU
ENSG00000185960	UUGGCAA
ENSG00000185963	AAAGUGC
ENSG00000185963	AAGGCAC
ENSG00000185963	AAGUGCA
ENSG00000185963	AAUACUG
ENSG00000185963	AAUCACU
ENSG00000185963	ACAGUAU
ENSG00000185963	AGUGCCU
ENSG00000185963	CAGCAAA
ENSG00000185963	GGAUUUU
ENSG00000185963	UACAGUU
ENSG00000185963	UCAAGUA
ENSG00000185963	UCCUGAG
ENSG00000185963	UUCAAGC
ENSG00000185963	UUCUUGU
ENSG00000185963	UUGGCAA
ENSG00000185963	UUGGUUC
ENSG00000185985	AAGGCAC
ENSG00000185985	CAAAGCA
ENSG00000185985	CUUUGGU
ENSG00000185985	GAGAAAU
ENSG00000185985	UUGGAGG
ENSG00000185989	AAAAAUC
ENSG00000186007	CACAAUG
ENSG00000186017	AAGUGCU
ENSG00000186020	UCAUUUG
ENSG00000186047	ACAAUAU
ENSG00000186047	UUGUGUC
ENSG00000186063	AAAUGGU
ENSG00000186063	CACAAUG
ENSG00000186063	CACAGGG
ENSG00000186063	CUGGAAA
ENSG00000186063	GACCUGG
ENSG00000186063	UCACAGG
ENSG00000186073	AAAGUAA
ENSG00000186073	CAGCUGG
ENSG00000186073	CAGGUGA
ENSG00000186073	CCAGGAG
ENSG00000186073	CUCUUCC
ENSG00000186073	CUUCUCU
ENSG00000186073	GAAAACA
ENSG00000186073	GCAGGAC
ENSG00000186073	GCUUCCU
ENSG00000186073	GGAGGAG
ENSG00000186073	GGGUUUA
ENSG00000186073	UGCCAGC
ENSG00000186073	UGGCUGC
ENSG00000186073	UGUGUCA
ENSG00000186073	UUGACAC
ENSG00000186074	CCCUGAG
ENSG00000186075	UGACCUA
ENSG00000186075	UUCUAAU
ENSG00000186081	UAUAAAG
ENSG00000186106	GAGGUAG
ENSG00000186106	UUGGUCC
ENSG00000186111	AAGACAU
ENSG00000186111	ACAGUAU
ENSG00000186111	AUUGCAC
ENSG00000186111	GAUUGUC
ENSG00000186111	UAAGACG
ENSG00000186111	UAAGACU
ENSG00000186111	UGCAUAG

ENSG00000186111	UUAGAGA
ENSG00000186130	AAGUGCU
ENSG00000186130	ACAAGGU
ENSG00000186130	CUUACAG
ENSG00000186130	GGAAUGU
ENSG00000186130	UGCCCUG
ENSG00000186143	AGCCCUU
ENSG00000186143	GGCUCUG
ENSG00000186150	CCCUGAG
ENSG00000186153	UUGUGAC
ENSG00000186174	AAAAAUC
ENSG00000186174	AAAGCGC
ENSG00000186174	AAGGCAC
ENSG00000186174	ACAGUAU
ENSG00000186174	AGCACCA
ENSG00000186174	AGCAGCA
ENSG00000186174	AGGCAGG
ENSG00000186174	AUAUAAC
ENSG00000186174	AUGGCUU
ENSG00000186174	CAGCAGA
ENSG00000186174	CCUUCAU
ENSG00000186174	CUGGGGG
ENSG00000186174	CUUCACC
ENSG00000186174	GCCUGGG
ENSG00000186174	GGAAGCC
ENSG00000186174	GUGCCUG
ENSG00000186174	UCACAUU
ENSG00000186174	UCCCUUU
ENSG00000186174	UGGCCCU
ENSG00000186174	UUCAAAU
ENSG00000186184	AGCACCA
ENSG00000186184	UGGCUGC
ENSG00000186184	UUCUUGU
ENSG00000186187	AAAAAUC
ENSG00000186187	AAUGCCC
ENSG00000186187	AAUUUUA
ENSG00000186187	ACAUCAC
ENSG00000186187	AGCCUGC
ENSG00000186187	AGGACAC
ENSG00000186187	AUGCCUU
ENSG00000186187	AUGUGCC
ENSG00000186187	CAAAACU
ENSG00000186187	CACAAUG
ENSG00000186187	CACCAGC
ENSG00000186187	CAGUGCA
ENSG00000186187	CUGCCCC
ENSG00000186187	CUGGGCA
ENSG00000186187	GAAAACA
ENSG00000186187	GAGCUGC
ENSG00000186187	GAGGGGC
ENSG00000186187	GAUUGUC
ENSG00000186187	GCCCUUA
ENSG00000186187	GGCUCUG
ENSG00000186187	GUCACUC
ENSG00000186187	GUGCCAC
ENSG00000186187	UAAUACA
ENSG00000186187	UAAUAUC
ENSG00000186187	UCCCUCU
ENSG00000186187	UCCUUGC
ENSG00000186187	UCUCAAG
ENSG00000186187	UCUGGAA
ENSG00000186187	UGCCAGC
ENSG00000186187	UGCCCUG
ENSG00000186187	UUCAAAU

ENSG00000186187	UUGGCAC
ENSG00000186197	ACAGGUG
ENSG00000186197	ACCUGGA
ENSG00000186197	AGCAGCU
ENSG00000186197	AGCUGCC
ENSG00000186197	AGGAUGU
ENSG00000186197	CCAGGAG
ENSG00000186197	CUGGGCA
ENSG00000186197	GGCAUUG
ENSG00000186197	GGUUGUC
ENSG00000186197	UCAUAGA
ENSG00000186197	UCUCCAA
ENSG00000186212	AACCUGG
ENSG00000186212	UCCCUCU
ENSG00000186231	GUUCAUG
ENSG00000186231	UGACAUC
ENSG00000186260	AAGUGCA
ENSG00000186260	AAUCACU
ENSG00000186260	AAUUGCU
ENSG00000186260	ACUUGGA
ENSG00000186260	AGUGCUG
ENSG00000186260	AUGGCAC
ENSG00000186260	CAGGAAC
ENSG00000186260	CCAGUGU
ENSG00000186260	CUAUACA
ENSG00000186260	CUCAAAC
ENSG00000186260	CUGGACU
ENSG00000186260	CUUACAG
ENSG00000186260	CUUUGGU
ENSG00000186260	GAGAUGA
ENSG00000186260	GAUAUGU
ENSG00000186260	GGAAUGU
ENSG00000186260	GGCAUUG
ENSG00000186260	GGCUCAG
ENSG00000186260	GGGGUGC
ENSG00000186260	GUAAACA
ENSG00000186260	UGCAGCU
ENSG00000186260	UUCUUGU
ENSG00000186260	UUGGCAC
ENSG00000186260	UUGGUCC
ENSG00000186265	AAUUUUA
ENSG00000186283	AAUUUUA
ENSG00000186288	CUCUGGG
ENSG00000186288	CUGCAGG
ENSG00000186288	GCUGGUG
ENSG00000186288	UUGGCAA
ENSG00000186298	ACCAGCA
ENSG00000186298	AGUGGUU
ENSG00000186298	AUCCCUU
ENSG00000186298	AUGGCUU
ENSG00000186298	UCACAGG
ENSG00000186298	UCCCUUU
ENSG00000186298	UGUGCUU
ENSG00000186310	AAGGCAC
ENSG00000186310	AGUGCAA
ENSG00000186310	UAAUAGU
ENSG00000186310	UCACAGG
ENSG00000186310	UCACAGU
ENSG00000186314	AACUACU
ENSG00000186314	AAUUAUU
ENSG00000186314	AUGGCUU
ENSG00000186318	AACACUG
ENSG00000186318	AAGGCAC
ENSG00000186318	ACCUGGA

ENSG00000186318	AGCACCA
ENSG00000186318	AGCAGCA
ENSG00000186318	AGCCAGU
ENSG00000186318	AUAAAGU
ENSG00000186318	AUAAUAC
ENSG00000186318	AUGGCUU
ENSG00000186318	AUGUCUG
ENSG00000186318	CACAGUG
ENSG00000186318	CAGCAGG
ENSG00000186318	GCCCUGU
ENSG00000186318	GCCUGGG
ENSG00000186318	GGAAGAC
ENSG00000186318	GGGGUGC
ENSG00000186318	GGGGUGG
ENSG00000186318	GGUUUAC
ENSG00000186318	UAAUACA
ENSG00000186318	UAUAAAG
ENSG00000186318	UAUAAUA
ENSG00000186318	UCACAGG
ENSG00000186318	UCCUGAG
ENSG00000186318	UGCCAGC
ENSG00000186318	UGCCCUG
ENSG00000186318	UGGGACA
ENSG00000186318	UGGGUGA
ENSG00000186318	UUGGUUC
ENSG00000186326	UAAUAGU
ENSG00000186326	UUCUUGU
ENSG00000186335	UGCAAAG
ENSG00000186340	AAGGCAC
ENSG00000186340	ACGUCAU
ENSG00000186340	CAGCAGA
ENSG00000186340	CUUUGGU
ENSG00000186340	GUAAACA
ENSG00000186340	UACAAAG
ENSG00000186350	AAAGCUG
ENSG00000186350	AAGGCAC
ENSG00000186350	ACGCUCA
ENSG00000186350	AGUGCUG
ENSG00000186350	AGUGCUU
ENSG00000186350	AUUCAUU
ENSG00000186350	CAAAGCA
ENSG00000186350	CACAGUG
ENSG00000186350	CCCACCG
ENSG00000186350	CCCACCU
ENSG00000186350	CUUUGGU
ENSG00000186350	GAUUGUC
ENSG00000186350	GCAGAAG
ENSG00000186350	GGAUUUC
ENSG00000186350	GGCAUUG
ENSG00000186350	UCACAGG
ENSG00000186350	UCACAGU
ENSG00000186350	UCACCAC
ENSG00000186350	UCCCACC
ENSG00000186352	CUUCACC
ENSG00000186354	AACAAAC
ENSG00000186354	AGAUGUG
ENSG00000186354	AUGGAUU
ENSG00000186354	CACCACC
ENSG00000186354	CUUGGAG
ENSG00000186354	UCACACA
ENSG00000186354	UUCUUGU
ENSG00000186364	AAGUUGU
ENSG00000186416	AAGGCAC
ENSG00000186416	ACCACAG

ENSG00000186416	AGCACCA
ENSG00000186417	AGCUGCC
ENSG00000186417	CUUUGGU
ENSG00000186417	GCAACUU
ENSG00000186417	GCCUGGG
ENSG00000186432	AAACUGU
ENSG00000186432	AAAGAGC
ENSG00000186432	AAAGAGG
ENSG00000186432	AAAGGUA
ENSG00000186432	AAAGUGC
ENSG00000186432	AACACUG
ENSG00000186432	AAGGCAC
ENSG00000186432	ACCACAG
ENSG00000186432	AGAUAAA
ENSG00000186432	AGCAUUC
ENSG00000186432	AGUGCUG
ENSG00000186432	AUAAAGU
ENSG00000186432	CAAAUGC
ENSG00000186432	CAGUGCA
ENSG00000186432	CCAGUGC
ENSG00000186432	GAUAUGU
ENSG00000186432	GCACCAU
ENSG00000186432	GCAGCAU
ENSG00000186432	GCCUGGG
ENSG00000186432	GGCUCAG
ENSG00000186432	GGCUGCG
ENSG00000186432	GUAUGUU
ENSG00000186432	GUCCAGA
ENSG00000186432	GUGCAAA
ENSG00000186432	UAAUGCU
ENSG00000186432	UCAUUUG
ENSG00000186432	UGCAUUG
ENSG00000186432	UGGCUGC
ENSG00000186432	UGUGCUU
ENSG00000186432	UUCCUAG
ENSG00000186432	UUCUGCA
ENSG00000186439	GUAAACA
ENSG00000186442	ACAUCAC
ENSG00000186442	GGCGACA
ENSG00000186469	AACAUUC
ENSG00000186469	AACUCUA
ENSG00000186469	AAGGCAC
ENSG00000186469	ACAUUCA
ENSG00000186469	AGCACCA
ENSG00000186469	AGCAGCU
ENSG00000186469	CCAGGAG
ENSG00000186469	CUGGAAA
ENSG00000186469	GAGGUAU
ENSG00000186469	GCAGAAG
ENSG00000186469	GCUGUCU
ENSG00000186469	GUAACAG
ENSG00000186469	UCACAUU
ENSG00000186469	UCAUUCG
ENSG00000186469	UCUGGAA
ENSG00000186469	UGCCCUG
ENSG00000186470	AAUCUCU
ENSG00000186472	AAAAGUA
ENSG00000186472	ACCAGCA
ENSG00000186472	AUACAAG
ENSG00000186472	CAGCAGA
ENSG00000186472	CAGUAGU
ENSG00000186472	CCAGUAC
ENSG00000186472	GAGCCCU
ENSG00000186472	GCUGGUG

ENSG00000186472	GGUUACC
ENSG00000186472	GUGCUUG
ENSG00000186472	UCCAGUU
ENSG00000186472	UGCAUAU
ENSG00000186472	UUGGCAC
ENSG00000186472	UUGGUCC
ENSG00000186479	AAAGUAA
ENSG00000186479	AACACUG
ENSG00000186479	AAGACAU
ENSG00000186479	ACCAGCA
ENSG00000186479	ACUGGAU
ENSG00000186479	AGCUUAU
ENSG00000186479	CACAAUG
ENSG00000186479	CAGUAGA
ENSG00000186479	CCAGCAU
ENSG00000186479	CCUGCUG
ENSG00000186479	UAUAAAG
ENSG00000186479	UCUCCAA
ENSG00000186479	UCUGGAG
ENSG00000186479	UGCAUAG
ENSG00000186479	UUCCUAG
ENSG00000186479	UUGAGGC
ENSG00000186480	AAAACUG
ENSG00000186480	ACAAGGU
ENSG00000186480	AGCACCA
ENSG00000186480	AUGGCAC
ENSG00000186480	AUUGCAC
ENSG00000186480	GAGUAUU
ENSG00000186480	GAUUGGU
ENSG00000186480	GAUUGUC
ENSG00000186480	GCACCAU
ENSG00000186480	GUGCAAA
ENSG00000186480	UCCUAUA
ENSG00000186480	UGCUCAC
ENSG00000186480	UGUGCUU
ENSG00000186480	UUCAAAU
ENSG00000186487	AAAAAUC
ENSG00000186487	AAACUGU
ENSG00000186487	AAAGCUG
ENSG00000186487	AAAGUAA
ENSG00000186487	AAAGUAC
ENSG00000186487	AAAGUAU
ENSG00000186487	AAAUCCC
ENSG00000186487	AAGUCUU
ENSG00000186487	AAGUGCA
ENSG00000186487	ACAAGGU
ENSG00000186487	ACAAUAU
ENSG00000186487	ACAUAGA
ENSG00000186487	ACAUUAC
ENSG00000186487	ACCCUGU
ENSG00000186487	ACUGUUU
ENSG00000186487	AGCAGCU
ENSG00000186487	AGCAUUC
ENSG00000186487	AGUGCAA
ENSG00000186487	AGUGCCG
ENSG00000186487	AUAAAGU
ENSG00000186487	AUACAAG
ENSG00000186487	AUAUAAC
ENSG00000186487	AUGCAUU
ENSG00000186487	AUGGCUU
ENSG00000186487	AUUGCAC
ENSG00000186487	CAAAGCA
ENSG00000186487	CACACCU
ENSG00000186487	CACUGUU

ENSG00000186487	CAGCAAA
ENSG00000186487	CCCUGUC
ENSG00000186487	CCCUGUU
ENSG00000186487	CUACAAA
ENSG00000186487	CUUGUAU
ENSG00000186487	GAAACAU
ENSG00000186487	GAAGGAA
ENSG00000186487	GACCCUG
ENSG00000186487	GAGUAUU
ENSG00000186487	GCAACUU
ENSG00000186487	GGCAGCG
ENSG00000186487	GUAUUCU
ENSG00000186487	GUGACAG
ENSG00000186487	GUGCAAA
ENSG00000186487	GUGCUUG
ENSG00000186487	GUGUCAC
ENSG00000186487	UAAUAGU
ENSG00000186487	UAUAAAG
ENSG00000186487	UAUCAGA
ENSG00000186487	UCCCUGU
ENSG00000186487	UCCCUUU
ENSG00000186487	UGCAUAU
ENSG00000186487	UUAGAGA
ENSG00000186487	UUCUAAU
ENSG00000186487	UUGGCAA
ENSG00000186487	UUGUGAC
ENSG00000186487	UUUUUGC
ENSG00000186493	CGCCUCC
ENSG00000186501	CUGGAAA
ENSG00000186501	GAGGCAG
ENSG00000186501	UCUGGAA
ENSG00000186517	AGUGCUU
ENSG00000186522	CAAGAGC
ENSG00000186522	CUGGAAA
ENSG00000186522	UCUGGAA
ENSG00000186523	GGAUGAG
ENSG00000186530	AGCCAGU
ENSG00000186530	CUGGGGG
ENSG00000186530	GGGGCAG
ENSG00000186530	GGGGUGG
ENSG00000186530	UCUCCAA
ENSG00000186530	UUCCUAG
ENSG00000186532	GGAAUGU
ENSG00000186532	GGAGUGU
ENSG00000186532	UCCCACA
ENSG00000186532	UCCCACC
ENSG00000186564	GCCUGGG
ENSG00000186564	UCACAUU
ENSG00000186564	UCUGGAG
ENSG00000186564	UGUCUUU
ENSG00000186566	AAAGAGC
ENSG00000186566	AAAGAGG
ENSG00000186566	AAAGUUU
ENSG00000186566	AAGGCAC
ENSG00000186566	ACAUUAC
ENSG00000186566	AGUGCAA
ENSG00000186566	AUAUUAU
ENSG00000186566	AUCACUA
ENSG00000186566	AUUGCAC
ENSG00000186566	CCAGUGU
ENSG00000186566	CCCUGAG
ENSG00000186566	CUUUGGU
ENSG00000186566	GAAAACA
ENSG00000186566	GAAACAU

ENSG00000186566	GAGCUAA
ENSG00000186566	GCAGCAU
ENSG00000186566	GGUUUAC
ENSG00000186566	GUUAAUG
ENSG00000186566	UAAUACA
ENSG00000186566	UAUAUGA
ENSG00000186566	UAUUGCU
ENSG00000186566	UCCAGUU
ENSG00000186566	UCCUAUA
ENSG00000186566	UGCUCAC
ENSG00000186566	UGGCUGC
ENSG00000186566	UUUGCAA
ENSG00000186566	UUUGCAC
ENSG00000186575	AUGUAAU
ENSG00000186575	AUUGCAC
ENSG00000186575	CCCUGUC
ENSG00000186575	CCUGCUG
ENSG00000186575	GAGAACU
ENSG00000186575	UGGCCCU
ENSG00000186575	UUGAGGC
ENSG00000186577	UACAAAG
ENSG00000186591	AUAAUAC
ENSG00000186591	AUAUAAC
ENSG00000186591	AUGCAUU
ENSG00000186591	AUGUGGG
ENSG00000186591	AUUCAUU
ENSG00000186591	CAGUAGA
ENSG00000186591	CAGUAGU
ENSG00000186591	CAUAUUG
ENSG00000186591	CUUUGGU
ENSG00000186591	GAUUGUA
ENSG00000186591	GAUUGUC
ENSG00000186591	GUGCUUG
ENSG00000186591	UAUCAGA
ENSG00000186591	UAUUGCU
ENSG00000186591	UGAGAAG
ENSG00000186591	UGAGAAU
ENSG00000186594	UAAUAUC
ENSG00000186603	CACACCU
ENSG00000186629	AAAGCUG
ENSG00000186629	AGCCCUU
ENSG00000186629	CAGGUGA
ENSG00000186629	CCAGGAG
ENSG00000186629	UCCUGAG
ENSG00000186629	UCUGGAA
ENSG00000186629	UCUGGAG
ENSG00000186629	UCUUGAG
ENSG00000186629	UGGCCAC
ENSG00000186629	UUCCAUA
ENSG00000186638	GAGAACU
ENSG00000186638	UACAAAG
ENSG00000186638	UGCAAAG
ENSG00000186642	AAGGCAC
ENSG00000186648	GGAGAGA
ENSG00000186652	GAGGCAG
ENSG00000186660	AAAGUAC
ENSG00000186660	AAAUUUC
ENSG00000186660	AAGUGCA
ENSG00000186660	AGCACCA
ENSG00000186660	AGUGAUC
ENSG00000186660	AGUGCAA
ENSG00000186660	AGUGCUG
ENSG00000186660	CAAAGUG
ENSG00000186660	CUAGGCU

ENSG00000186660	GAGGCUG
ENSG00000186660	GCACCAU
ENSG00000186660	GGAAUGU
ENSG00000186660	GUAGUGU
ENSG00000186660	GUGCAAA
ENSG00000186660	UGAGGAC
ENSG00000186675	AUUCAUU
ENSG00000186687	AGAUAAA
ENSG00000186687	CCAGGAG
ENSG00000186687	UACAAAG
ENSG00000186714	UAUAAAG
ENSG00000186714	UUGUUCG
ENSG00000186716	AAAGUGC
ENSG00000186716	AGCAGCA
ENSG00000186716	GGAAGAC
ENSG00000186716	GUCUCUG
ENSG00000186732	UCCUGAG
ENSG00000186766	ACACACC
ENSG00000186767	AAAGUAC
ENSG00000186787	GUCUACU
ENSG00000186815	UGCAUAU
ENSG00000186834	AAAGUUU
ENSG00000186834	AAGGCAC
ENSG00000186834	AAUGUUG
ENSG00000186834	ACAUUCA
ENSG00000186834	AUAUAAC
ENSG00000186834	CCAGUGC
ENSG00000186834	CCAGUGU
ENSG00000186834	UACAAAG
ENSG00000186834	UUCAAAU
ENSG00000186868	AACAGUC
ENSG00000186868	ACAUUCA
ENSG00000186868	CAUGGGU
ENSG00000186868	GAUUGUC
ENSG00000186868	GCAGGUU
ENSG00000186868	GGCUCUG
ENSG00000186868	GUCACUC
ENSG00000186868	UACAAAG
ENSG00000186868	UCACAGG
ENSG00000186868	UCACAGU
ENSG00000186897	GGCGCCU
ENSG00000186908	AAGGCAC
ENSG00000186908	AGCAGCU
ENSG00000186908	GGUCUAG
ENSG00000186908	GUAAACA
ENSG00000186908	UACAAAG
ENSG00000186918	AAAAAUC
ENSG00000186918	AACAGUC
ENSG00000186918	AACCUGG
ENSG00000186918	AAGACAU
ENSG00000186918	AAGGCGC
ENSG00000186918	AAUACUG
ENSG00000186918	ACUCAGC
ENSG00000186918	AGUAACA
ENSG00000186918	AUAAUAC
ENSG00000186918	CAGACAG
ENSG00000186918	CCCUGAG
ENSG00000186918	GGCUCUG
ENSG00000186918	GUAACAG
ENSG00000186918	GUAGUGU
ENSG00000186918	GUCAGUU
ENSG00000186918	UAUAAAG
ENSG00000186918	UCCCUCU
ENSG00000186918	UUGGUCC

ENSG00000186951	AAACUGU
ENSG00000186951	AAUUGCU
ENSG00000186951	ACAGUAC
ENSG00000186951	ACCCUGU
ENSG00000186951	AGCAAAA
ENSG00000186951	AGCUGCC
ENSG00000186951	CAAAACU
ENSG00000186951	CAAGAGC
ENSG00000186951	CACAGUG
ENSG00000186951	GAGCUGC
ENSG00000186951	GAGGUAG
ENSG00000186951	GGCUCAG
ENSG00000186951	GUGAGGU
ENSG00000186951	UACAGUU
ENSG00000186951	UCCCUGU
ENSG00000186998	CCCUGAG
ENSG00000186998	GACCCUG
ENSG00000187017	CACCACC
ENSG00000187033	AAAACUG
ENSG00000187049	UCAUGAU
ENSG00000187068	AGCAUUC
ENSG00000187068	AUGGCUU
ENSG00000187068	CCCCUGG
ENSG00000187068	GAGCUGC
ENSG00000187068	GGAAUGU
ENSG00000187068	GGCAGUG
ENSG00000187068	GGCUGCG
ENSG00000187068	UCAUACA
ENSG00000187068	UGAAGCU
ENSG00000187068	UGGCUGC
ENSG00000187068	UUAGGAU
ENSG00000187068	UUCCAUA
ENSG00000187068	UUCUAAU
ENSG00000187068	UUUCAAC
ENSG00000187079	AAAACUG
ENSG00000187079	AAAGAGC
ENSG00000187079	AAAGGUA
ENSG00000187079	AAAGUAA
ENSG00000187079	AACAGUC
ENSG00000187079	AACAUUC
ENSG00000187079	AAGACAU
ENSG00000187079	AAGUCAC
ENSG00000187079	AAUACUG
ENSG00000187079	AAUCUCA
ENSG00000187079	ACAUUCA
ENSG00000187079	ACCUGGA
ENSG00000187079	ACUGUAG
ENSG00000187079	AGCAGCA
ENSG00000187079	AGCAUUC
ENSG00000187079	AGUAACA
ENSG00000187079	AGUGGUU
ENSG00000187079	AUGGAUU
ENSG00000187079	AUGGCAC
ENSG00000187079	AUUCAUU
ENSG00000187079	CAGGAAC
ENSG00000187079	CAGUGCA
ENSG00000187079	CCAGUAC
ENSG00000187079	CCUUCAU
ENSG00000187079	CUGCAGC
ENSG00000187079	CUUGUAU
ENSG00000187079	CUUUGGU
ENSG00000187079	GCUGGUG
ENSG00000187079	GCUUCCU
ENSG00000187079	GGCAGCG

ENSG00000187079	GGGGAGC
ENSG00000187079	GGUUUAC
ENSG00000187079	GUAAACA
ENSG00000187079	GUAACAG
ENSG00000187079	GUAGUGU
ENSG00000187079	GUCACUC
ENSG00000187079	GUCAUUG
ENSG00000187079	GUCUACU
ENSG00000187079	GUCUGCC
ENSG00000187079	GUGAAUG
ENSG00000187079	UACAGUU
ENSG00000187079	UAGUGAG
ENSG00000187079	UAUUGCU
ENSG00000187079	UCACAAG
ENSG00000187079	UCACACA
ENSG00000187079	UCACAUU
ENSG00000187079	UCUAGAG
ENSG00000187079	UGUCUGC
ENSG00000187079	UGUCUGG
ENSG00000187079	UGUGUCA
ENSG00000187079	UUAGAGA
ENSG00000187079	UUCUAAU
ENSG00000187079	UUGAGGC
ENSG00000187079	UUGGCAA
ENSG00000187079	UUGGCAC
ENSG00000187079	UUGGUUC
ENSG00000187079	UUGUUCG
ENSG00000187079	UUUGCAA
ENSG00000187091	AGCAGCA
ENSG00000187091	GAGGCAG
ENSG00000187097	GAGGCAG
ENSG00000187098	AGUAACA
ENSG00000187098	AUUGCAC
ENSG00000187098	CAGCAGG
ENSG00000187098	GAGAAAU
ENSG00000187098	GUGCUUG
ENSG00000187098	UCACAUU
ENSG00000187098	UCAUUCG
ENSG00000187098	UGCAUAG
ENSG00000187109	AAAGUAA
ENSG00000187109	AAGGCAC
ENSG00000187109	ACAGUAC
ENSG00000187109	ACUGCAU
ENSG00000187109	ACUGGAU
ENSG00000187109	AGCCUGC
ENSG00000187109	AGGUUAU
ENSG00000187109	CAAAUGC
ENSG00000187109	CAGUAGU
ENSG00000187109	CAGUGCA
ENSG00000187109	CUUUGGU
ENSG00000187109	GCAGAAG
ENSG00000187109	GCUACAU
ENSG00000187109	GGCUCUG
ENSG00000187109	UAAGACG
ENSG00000187109	UAAGACU
ENSG00000187109	UCACAUU
ENSG00000187122	AAGGCAC
ENSG00000187122	GUGCAAA
ENSG00000187123	AAAAAUC
ENSG00000187123	AAGUGCU
ENSG00000187123	AGUGCCU
ENSG00000187123	AGUGCUU
ENSG00000187123	AUAUUAU
ENSG00000187123	CACCACC

ENSG00000187123	CCCUGAG
ENSG00000187123	CCUUCAU
ENSG00000187123	CUGUUGA
ENSG00000187123	GAAGGAA
ENSG00000187123	UCCCUCU
ENSG00000187123	UGUUGAA
ENSG00000187123	UUUCAAC
ENSG00000187140	AAAAAUC
ENSG00000187140	AAAACUG
ENSG00000187140	AACAUUC
ENSG00000187140	AAGUUGU
ENSG00000187140	AGAUAAA
ENSG00000187140	AUAAUAC
ENSG00000187140	AUAUAAC
ENSG00000187140	UCAUUCG
ENSG00000187145	CCUUCUU
ENSG00000187147	AGGCAGG
ENSG00000187147	AUAUUAU
ENSG00000187147	CAAAUGC
ENSG00000187147	CACACCG
ENSG00000187147	CACACCU
ENSG00000187147	CUGGCUC
ENSG00000187147	GAGCUAA
ENSG00000187147	GAGGCAG
ENSG00000187147	GUGCUUG
ENSG00000187147	UAAGACU
ENSG00000187147	UCCAGAG
ENSG00000187147	UGUAUAA
ENSG00000187147	UGUGCUU
ENSG00000187164	AACAAAC
ENSG00000187164	AGCCCUU
ENSG00000187164	CAAAACU
ENSG00000187164	CCAUUAC
ENSG00000187164	CCCUGAG
ENSG00000187164	GUGCAAA
ENSG00000187164	UUAGGAU
ENSG00000187164	UUGGUUC
ENSG00000187172	AAAAGUA
ENSG00000187172	AAAGUAU
ENSG00000187172	ACUCCAG
ENSG00000187172	AUCCCUU
ENSG00000187172	AUGGCAC
ENSG00000187172	CACUGUU
ENSG00000187172	CCCUGAG
ENSG00000187172	CUGGGGG
ENSG00000187172	CUUCUCU
ENSG00000187172	GGAGUGU
ENSG00000187172	GGUGGGC
ENSG00000187172	GGUUUAC
ENSG00000187172	GUCACUC
ENSG00000187172	GUGACUG
ENSG00000187172	UGAAAUG
ENSG00000187172	UGGUACC
ENSG00000187172	UGUCUGG
ENSG00000187175	GUGGCCC
ENSG00000187175	GUGUGGC
ENSG00000187189	AAAAAUC
ENSG00000187189	CCAGCAU
ENSG00000187189	GAGGCAG
ENSG00000187189	GGAAUGU
ENSG00000187189	GUUAGGA
ENSG00000187189	UCCAGUU
ENSG00000187189	UUUGCAA
ENSG00000187191	GAUUGGU

ENSG00000187191	UCUUGAG
ENSG00000187210	AGCAAAA
ENSG00000187210	GAGAUGA
ENSG00000187210	UUGGCAA
ENSG00000187231	GCACCAU
ENSG00000187231	UGAAAUG
ENSG00000187239	AAGGCAC
ENSG00000187239	AAGGUGC
ENSG00000187239	AUGCAUU
ENSG00000187239	CCCUGUC
ENSG00000187239	CUCCAGC
ENSG00000187239	CUGCAGC
ENSG00000187239	CUUUGGU
ENSG00000187239	GGAAGCC
ENSG00000187239	GGACUGC
ENSG00000187239	UGACUGU
ENSG00000187239	UGCAGCU
ENSG00000187239	UGGACUG
ENSG00000187239	UGUGUGG
ENSG00000187239	UUCUGCA
ENSG00000187239	UUGGUUC
ENSG00000187257	AGUGCUG
ENSG00000187257	AGUGCUU
ENSG00000187257	CAGUGCA
ENSG00000187257	CUACAGU
ENSG00000187257	GGAAUGU
ENSG00000187257	UGGCCCU
ENSG00000187288	GUGGGGA
ENSG00000187323	AAUCCUU
ENSG00000187323	AUCCUUU
ENSG00000187323	CCUGUAC
ENSG00000187323	CUUUGGU
ENSG00000187323	UAAGACU
ENSG00000187323	UGCAUAG
ENSG00000187323	UGUGCUU
ENSG00000187325	AACAUUC
ENSG00000187325	ACAUUCA
ENSG00000187325	AGGCAGG
ENSG00000187325	AGUGGUU
ENSG00000187325	GAGAACC
ENSG00000187325	GAGAACU
ENSG00000187325	GAGGUAG
ENSG00000187325	GAGGUAU
ENSG00000187391	AAAAAUC
ENSG00000187391	AAAAGUA
ENSG00000187391	AACACUG
ENSG00000187391	AGUGGUU
ENSG00000187391	CCCUGUC
ENSG00000187391	CUGCAGC
ENSG00000187391	CUUACAG
ENSG00000187391	GAUAUGU
ENSG00000187391	GGAAUGU
ENSG00000187391	GUAAACA
ENSG00000187391	GUGACUG
ENSG00000187391	GUGCUUG
ENSG00000187391	UCAUACA
ENSG00000187391	UUAUGCA
ENSG00000187391	UUCCAUA
ENSG00000187391	UUGACAC
ENSG00000187398	AGGUUAU
ENSG00000187416	AAAUGGU
ENSG00000187416	AACAAAC
ENSG00000187416	AACUACU
ENSG00000187416	AAGUUGU

ENSG00000187416	ACACACC
ENSG00000187416	ACACACU
ENSG00000187416	AGUAACA
ENSG00000187416	AUGAGCU
ENSG00000187416	GAGCUGC
ENSG00000187416	GGACCUG
ENSG00000187416	GGGAACG
ENSG00000187416	UACAAAG
ENSG00000187416	UAGUGAG
ENSG00000187416	UGUGUGG
ENSG00000187475	AAAGAGC
ENSG00000187486	AGCCAGU
ENSG00000187486	CUCACAC
ENSG00000187486	GAGGCUG
ENSG00000187486	GUAACAG
ENSG00000187498	AAUUGCU
ENSG00000187498	AGCUUAU
ENSG00000187498	AUAUAAC
ENSG00000187498	CAGCAAA
ENSG00000187498	GAGGUAG
ENSG00000187498	GAGGUAU
ENSG00000187498	GUGAGGU
ENSG00000187498	UAUUACC
ENSG00000187510	GAGGUAU
ENSG00000187531	AAAGUGC
ENSG00000187531	CAAAGUG
ENSG00000187555	AAAAACC
ENSG00000187555	AACUCUA
ENSG00000187555	ACUCAAA
ENSG00000187555	ACUGCAG
ENSG00000187555	ACUGCAU
ENSG00000187555	AGCAAAA
ENSG00000187555	AUACAAG
ENSG00000187555	CAAAACU
ENSG00000187555	CAGCAAA
ENSG00000187555	CAGUGCA
ENSG00000187555	CCAGCAU
ENSG00000187555	GGAGGAG
ENSG00000187555	UAGUGAG
ENSG00000187555	UAUAAAG
ENSG00000187555	UCAACAG
ENSG00000187555	UGCUCAC
ENSG00000187555	UGCUGAC
ENSG00000187566	AAAUUUC
ENSG00000187595	AAAGUUU
ENSG00000187595	ACUGGCC
ENSG00000187595	ACUGGCU
ENSG00000187595	AGCACCA
ENSG00000187595	CACAGUG
ENSG00000187595	UCAUUUG
ENSG00000187595	UCCCUCU
ENSG00000187595	UGCAUAU
ENSG00000187605	AAAAGUA
ENSG00000187605	AAAUGGU
ENSG00000187605	AACACUG
ENSG00000187605	AACCUGG
ENSG00000187605	AAGGCAC
ENSG00000187605	AAGUAGC
ENSG00000187605	ACAAUAU
ENSG00000187605	ACAGUAC
ENSG00000187605	ACAGUAU
ENSG00000187605	ACCACAG
ENSG00000187605	ACUGUAG
ENSG00000187605	AGCAGCA

ENSG00000187605	AGCAUUC
ENSG00000187605	AGGUUAU
ENSG00000187605	AGUGCAA
ENSG00000187605	AGUGCUG
ENSG00000187605	AGUGCUU
ENSG00000187605	AUAAUAC
ENSG00000187605	AUGGCAC
ENSG00000187605	CAAAGCA
ENSG00000187605	CAAAGUG
ENSG00000187605	CAGCAGA
ENSG00000187605	CAGCAGG
ENSG00000187605	CAGGAAC
ENSG00000187605	CCAGCAU
ENSG00000187605	CCCUGAG
ENSG00000187605	CCUGUAC
ENSG00000187605	CUAGUCC
ENSG00000187605	CUCCAUU
ENSG00000187605	CUCCCAA
ENSG00000187605	CUGCUCA
ENSG00000187605	CUGGCUC
ENSG00000187605	CUUGGGC
ENSG00000187605	GAAACAU
ENSG00000187605	GAAGGUC
ENSG00000187605	GAGACCU
ENSG00000187605	GAGGCAG
ENSG00000187605	GCAUCCC
ENSG00000187605	GCUAGCA
ENSG00000187605	GGAGGCA
ENSG00000187605	GUAAACA
ENSG00000187605	GUAGUGU
ENSG00000187605	GUGACAG
ENSG00000187605	GUGCAAA
ENSG00000187605	UAUGGUU
ENSG00000187605	UCACAGG
ENSG00000187605	UCACAGU
ENSG00000187605	UCACCAC
ENSG00000187605	UCAUUUG
ENSG00000187605	UCCCACA
ENSG00000187605	UCCUAUA
ENSG00000187605	UCUCCAA
ENSG00000187605	UCUUGAG
ENSG00000187605	UGAAAGG
ENSG00000187605	UGCAAAG
ENSG00000187605	UGCAUAG
ENSG00000187605	UGGCCAC
ENSG00000187605	UGGGUAC
ENSG00000187605	UUCAAAU
ENSG00000187605	UUCAAGC
ENSG00000187605	UUCCUAG
ENSG00000187605	UUGAGGC
ENSG00000187605	UUGGUCC
ENSG00000187605	UUGUGUC
ENSG00000187605	UUUCAAC
ENSG00000187634	GACCCUG
ENSG00000187650	AAAGAGG
ENSG00000187650	CAGACAG
ENSG00000187653	ACUGCAU
ENSG00000187653	AGUAGUU
ENSG00000187653	CAGUAGA
ENSG00000187653	CAGUGCA
ENSG00000187653	CUUACAG
ENSG00000187664	ACAGGUG
ENSG00000187664	GGGGUCC
ENSG00000187664	GGUUACC

ENSG00000187664	UUCCAUA
ENSG00000187672	AAAACUG
ENSG00000187672	AACCUGG
ENSG00000187672	ACACACC
ENSG00000187672	AGCAGCA
ENSG00000187672	CAAAACU
ENSG00000187672	CUUUGGU
ENSG00000187672	UCACAGU
ENSG00000187672	UCAUACA
ENSG00000187672	UGUGCUU
ENSG00000187672	UUGGCAC
ENSG00000187676	AAGGCAC
ENSG00000187676	GUUAAUG
ENSG00000187678	AAAAACC
ENSG00000187678	AAAGUGC
ENSG00000187678	AACACUG
ENSG00000187678	AACCUGU
ENSG00000187678	AAGAACC
ENSG00000187678	AAGUGCA
ENSG00000187678	ACCACAG
ENSG00000187678	AGCACCA
ENSG00000187678	AGCAGCA
ENSG00000187678	AGCCCUU
ENSG00000187678	AGUGGUU
ENSG00000187678	AUACAAG
ENSG00000187678	CAAAGUG
ENSG00000187678	CUUACAG
ENSG00000187678	GCUAGCA
ENSG00000187678	GGCAAGA
ENSG00000187678	GGUUACC
ENSG00000187678	UAUGGUU
ENSG00000187678	UCACAAG
ENSG00000187678	UCCAGAG
ENSG00000187678	UCUGGAA
ENSG00000187678	UGAGAAG
ENSG00000187678	UUGGCAA
ENSG00000187678	UUGGUCC
ENSG00000187678	UUUGCAA
ENSG00000187678	UUUGCAC
ENSG00000187689	AAAUUUC
ENSG00000187714	GGAGGAG
ENSG00000187714	UUGGCAC
ENSG00000187715	AUAAAGU
ENSG00000187720	AAAGUAU
ENSG00000187720	AGCACCA
ENSG00000187720	AUCCUUG
ENSG00000187720	AUCCUUU
ENSG00000187720	CCUUCUU
ENSG00000187720	UGUCUUU
ENSG00000187720	UUCCUAG
ENSG00000187735	AAAGCUG
ENSG00000187735	GCAGGAC
ENSG00000187735	GUCCAGA
ENSG00000187736	GGGGUGG
ENSG00000187736	GUCUUGC
ENSG00000187742	ACAUUCA
ENSG00000187764	AUCCCUU
ENSG00000187764	UAUAAAG
ENSG00000187764	UGAGAAU
ENSG00000187764	UGGCUGC
ENSG00000187772	AAGGCAC
ENSG00000187772	AAUCUCA
ENSG00000187772	AGGUAGU
ENSG00000187772	AUUCAUU

ENSG00000187772	CAGUAGU
ENSG00000187772	CAGUGCA
ENSG00000187772	CCCUGAG
ENSG00000187772	GGUAGAC
ENSG00000187772	UCCCUGU
ENSG00000187772	UCCUGAG
ENSG00000187772	UGAAAUG
ENSG00000187772	UUGGUCC
ENSG00000187778	CCAGGAG
ENSG00000187778	GGCAGGG
ENSG00000187778	UACAAAG
ENSG00000187792	AAAGUGC
ENSG00000187792	AGGGAGG
ENSG00000187792	GGUUGUC
ENSG00000187800	GAGGCUG
ENSG00000187800	GGCAGGG
ENSG00000187823	GCUACAU
ENSG00000187823	GGAUUUU
ENSG00000187824	UUGGUCC
ENSG00000187837	AAAGAGC
ENSG00000187838	AAAGUUU
ENSG00000187838	AGCACCA
ENSG00000187838	CACCAGC
ENSG00000187838	CUUUGGU
ENSG00000187840	CCCUGAG
ENSG00000187840	GAGGUAU
ENSG00000187840	GGAGGCA
ENSG00000187840	UGCCCUG
ENSG00000187840	UUCCUCG
ENSG00000187855	UCCAGUU
ENSG00000187866	AAGGCAC
ENSG00000187866	ACACACU
ENSG00000187866	AGCAGCA
ENSG00000187866	AGCAGCU
ENSG00000187866	AGUGUGC
ENSG00000187866	GGCAAGA
ENSG00000187866	GGGUAAG
ENSG00000187866	UCAACAG
ENSG00000187889	GGCUCAG
ENSG00000187939	GGCUCUG
ENSG00000187942	CGGUGCU
ENSG00000187957	AACUCUA
ENSG00000187961	AAGGCAC
ENSG00000187961	AUGUGGG
ENSG00000187961	UAUUGCU
ENSG00000187969	AUAAUAC
ENSG00000187969	UUCCUCG
ENSG00000187990	AAAGAGC
ENSG00000188001	ACAAUAU
ENSG00000188001	CUGUUGA
ENSG00000188001	UACAGUU
ENSG00000188001	UCAACAG
ENSG00000188001	UCAUUCG
ENSG00000188001	UCUUGAG
ENSG00000188001	UUGAGGC
ENSG00000188015	UGAAAGG
ENSG00000188021	AAGUCAC
ENSG00000188026	AAGGCAC
ENSG00000188026	CUGCAGG
ENSG00000188026	GAGAAGA
ENSG00000188026	UCCUUGC
ENSG00000188042	AAACUGU
ENSG00000188042	AAAGUGC
ENSG00000188042	AAGUGCU

ENSG00000188042	ACCACAG
ENSG00000188042	ACUGUGU
ENSG00000188042	AGUGCUU
ENSG00000188042	AGUGUGC
ENSG00000188042	CACAGGG
ENSG00000188042	CACCACC
ENSG00000188042	CAGCUGG
ENSG00000188042	CUACAAA
ENSG00000188042	CUGGAAA
ENSG00000188042	CUUUGGU
ENSG00000188042	GCCCUGU
ENSG00000188042	GCGGGGC
ENSG00000188042	GCUGGUG
ENSG00000188042	GGAAGAC
ENSG00000188042	GGCCCUG
ENSG00000188042	GGCGGGG
ENSG00000188042	GUAAACA
ENSG00000188042	UCAACAG
ENSG00000188042	UCAAGUA
ENSG00000188042	UCACAGU
ENSG00000188042	UCCCUUU
ENSG00000188042	UCUGGAA
ENSG00000188042	UGCCCUG
ENSG00000188042	UUCAAGC
ENSG00000188042	UUGGCAA
ENSG00000188042	UUGGCAC
ENSG00000188042	UUGGUUC
ENSG00000188042	UUGUUCG
ENSG00000188120	GAUUGGU
ENSG00000188120	UCUUGAG
ENSG00000188130	CUACAAA
ENSG00000188133	CUACAAA
ENSG00000188133	UUCUAAU
ENSG00000188153	ACAAUAU
ENSG00000188153	ACUGCAG
ENSG00000188153	GAGGUAG
ENSG00000188153	GAGGUAU
ENSG00000188153	GCACCAU
ENSG00000188157	AAGUCAC
ENSG00000188157	ACAGUAU
ENSG00000188157	AGGGAGG
ENSG00000188157	CACAGUG
ENSG00000188157	UACAAAG
ENSG00000188157	UCACAGU
ENSG00000188158	AAAGUGC
ENSG00000188158	AACUGGU
ENSG00000188158	ACACACC
ENSG00000188158	AGUGCAA
ENSG00000188158	CCAGCAU
ENSG00000188158	GUGACUG
ENSG00000188158	GUGCAAA
ENSG00000188158	UAUCAGA
ENSG00000188158	UUGGCAA
ENSG00000188176	UAUAAAG
ENSG00000188177	AGCAGCA
ENSG00000188177	AGCAGCG
ENSG00000188177	AUAUAAC
ENSG00000188177	AUGACAC
ENSG00000188177	CUACAGU
ENSG00000188177	GGCAAGA
ENSG00000188177	GUCACUC
ENSG00000188177	UCACAUU
ENSG00000188177	UCCCUCU
ENSG00000188177	UUCAAAU

ENSG00000188177	UUGGCAC
ENSG00000188215	AAAACUG
ENSG00000188215	AAAAGUA
ENSG00000188215	AAAGUAA
ENSG00000188215	AAAGUAC
ENSG00000188215	AAAGUGC
ENSG00000188215	ACAAGGU
ENSG00000188215	ACACACC
ENSG00000188215	ACACACU
ENSG00000188215	ACAGUAC
ENSG00000188215	AGCAAAA
ENSG00000188215	AGCCCUU
ENSG00000188215	AGCUUAU
ENSG00000188215	AGUGGUU
ENSG00000188215	CAAAACU
ENSG00000188215	CAGCAAA
ENSG00000188215	CCAGCAU
ENSG00000188215	GAAGGAA
ENSG00000188215	GAGCCCU
ENSG00000188215	GAGGUAG
ENSG00000188215	GAGGUAU
ENSG00000188215	GCCCUGU
ENSG00000188215	GUCUACU
ENSG00000188215	GUGCAAA
ENSG00000188215	GUUAAUG
ENSG00000188215	UAAUGCU
ENSG00000188215	UGACUGU
ENSG00000188215	UGCCAGC
ENSG00000188215	UGCCCUG
ENSG00000188215	UUAGAGA
ENSG00000188215	UUCAAGC
ENSG00000188227	GAAACAU
ENSG00000188227	UGCAUAU
ENSG00000188312	AAAUUUC
ENSG00000188312	AAUUGCU
ENSG00000188312	AGUGUGC
ENSG00000188312	GAUUGUC
ENSG00000188312	GGCAUUG
ENSG00000188312	GGGGUCC
ENSG00000188312	GUAACAG
ENSG00000188312	GUGCCAC
ENSG00000188312	UGAGAAG
ENSG00000188312	UGCUGAC
ENSG00000188312	UUCUAAU
ENSG00000188312	UUGGCAA
ENSG00000188312	UUGGCAC
ENSG00000188312	UUGUGAC
ENSG00000188315	AAAACUG
ENSG00000188315	AGGCAGG
ENSG00000188315	GAAACAU
ENSG00000188315	UUCAAAU
ENSG00000188322	AAACUGU
ENSG00000188322	AAGGCAC
ENSG00000188322	AGCCCUU
ENSG00000188322	AUUGCAC
ENSG00000188322	GAGGUAG
ENSG00000188322	GAGGUAU
ENSG00000188322	GCAGGAC
ENSG00000188322	GGCAGGG
ENSG00000188322	GGUGGGC
ENSG00000188322	GUAAACA
ENSG00000188322	GUCUACU
ENSG00000188322	UACAAAG
ENSG00000188322	UAUUGCU

ENSG00000188338	CUGGGCA
ENSG00000188338	GGAAUGU
ENSG00000188386	UGGGUGA
ENSG00000188419	AACUCUA
ENSG00000188419	AGGUUAU
ENSG00000188419	GUAUUCU
ENSG00000188419	UUGGUUC
ENSG00000188428	AAUCACU
ENSG00000188428	GAGGUAG
ENSG00000188428	GAGGUAU
ENSG00000188428	UCACAGU
ENSG00000188428	UCACAUU
ENSG00000188428	UUGACAC
ENSG00000188483	AAAUUUC
ENSG00000188483	ACCCGGC
ENSG00000188483	AUAAAGU
ENSG00000188483	AUGCACC
ENSG00000188483	CAGGUGA
ENSG00000188483	GACAACU
ENSG00000188483	GCUGGUG
ENSG00000188483	GGAGGAG
ENSG00000188483	UCCCUUU
ENSG00000188483	UGUCUGG
ENSG00000188483	UUCUGCA
ENSG00000188483	UUGGCAA
ENSG00000188486	CAGUCCA
ENSG00000188486	GCCCUUA
ENSG00000188486	GGCCCUG
ENSG00000188486	GUGGCCC
ENSG00000188486	UGGCCCU
ENSG00000188487	GGCAAGA
ENSG00000188487	UCAACAG
ENSG00000188488	GCUUCCU
ENSG00000188488	GUCCAGA
ENSG00000188488	UCCAGAG
ENSG00000188542	UCUCCAA
ENSG00000188549	CAGGAAC
ENSG00000188549	GACCUGG
ENSG00000188549	GAGGCUG
ENSG00000188549	GCGGAGG
ENSG00000188549	GGAAGCC
ENSG00000188549	GUGCCUG
ENSG00000188549	UCAUUUG
ENSG00000188549	UGUGCGG
ENSG00000188549	UGUGCGU
ENSG00000188549	UGUUGAA
ENSG00000188554	AGCAGCA
ENSG00000188554	AUACAAG
ENSG00000188554	CAGCAGG
ENSG00000188554	UAAUACA
ENSG00000188580	CUGUUGA
ENSG00000188580	GAGGAUA
ENSG00000188580	GUAAACA
ENSG00000188580	UGACCUA
ENSG00000188580	UUCCUAG
ENSG00000188582	AAGGCAC
ENSG00000188582	AGCAGCU
ENSG00000188582	AUAUUAU
ENSG00000188582	AUGCAUU
ENSG00000188582	CACUGUU
ENSG00000188582	CCAGUGC
ENSG00000188582	CCAGUGU
ENSG00000188582	UCACAGG
ENSG00000188582	UCACAUU

ENSG00000188582	UCCAGUU
ENSG00000188612	ACUGGAU
ENSG00000188612	AGCAAAA
ENSG00000188612	AGGAUGU
ENSG00000188612	CAGUUAC
ENSG00000188613	ACAGUAC
ENSG00000188613	ACAGUAU
ENSG00000188613	ACUGUAG
ENSG00000188613	AGCAAAA
ENSG00000188613	AGCACCA
ENSG00000188613	AGUGCCU
ENSG00000188613	CUAGUAA
ENSG00000188613	CUCCAUU
ENSG00000188613	GCACCAU
ENSG00000188613	GUAUUCU
ENSG00000188636	AGCACCA
ENSG00000188636	CAGCAGG
ENSG00000188636	GCACCAU
ENSG00000188636	GCUGGUG
ENSG00000188636	GGCAUUG
ENSG00000188636	UACAAAG
ENSG00000188636	UCAUUUG
ENSG00000188636	UCCCACC
ENSG00000188636	UGGCUGC
ENSG00000188636	UUGGCAA
ENSG00000188636	UUGGCAC
ENSG00000188647	AAGGCAC
ENSG00000188647	ACACACU
ENSG00000188647	AUCCUUU
ENSG00000188647	AUGAUGC
ENSG00000188647	CCCUGAG
ENSG00000188647	CUUGGAG
ENSG00000188647	GAGAAAU
ENSG00000188647	GAGGCAG
ENSG00000188647	GGAAUGU
ENSG00000188647	GUGAAUG
ENSG00000188647	UGUGCUU
ENSG00000188687	AAAGUAA
ENSG00000188687	AAAGUAC
ENSG00000188687	AAGUGCA
ENSG00000188687	ACUGGCU
ENSG00000188687	AUGCCUU
ENSG00000188687	CAGGUGA
ENSG00000188687	GAAGGAA
ENSG00000188687	GCACCAU
ENSG00000188687	GGGUGGU
ENSG00000188687	GGUGGUC
ENSG00000188687	UCCAGUU
ENSG00000188706	AAAGUGC
ENSG00000188706	AAGUGCA
ENSG00000188706	AAGUGCU
ENSG00000188706	CAAAGUG
ENSG00000188706	CCCUGAG
ENSG00000188706	CUCUUCC
ENSG00000188706	CUGGACU
ENSG00000188706	GGUUACC
ENSG00000188706	UCCCUGU
ENSG00000188725	AAAGCUG
ENSG00000188725	UCCAGUU
ENSG00000188760	CCCUGAG
ENSG00000188760	GCUGGUG
ENSG00000188760	UAUAAAG
ENSG00000188760	UUGGCAA
ENSG00000188761	AGAUGUG

ENSG00000188761	CCAGCAU
ENSG00000188761	UUAGAGA
ENSG00000188761	UUCAAAU
ENSG00000188763	ACAUCAC
ENSG00000188763	CAGCUGG
ENSG00000188763	GGGUUUA
ENSG00000188763	UCCAGUU
ENSG00000188778	AAGACAU
ENSG00000188778	GAGGUAG
ENSG00000188778	GAGGUAU
ENSG00000188778	GUGAGGU
ENSG00000188778	UCUGGAG
ENSG00000188783	AGCACCA
ENSG00000188783	CACCAGC
ENSG00000188783	GCAGGAC
ENSG00000188783	GGCGGGG
ENSG00000188783	UCAAGUA
ENSG00000188783	UCUAGAG
ENSG00000188783	UGAAAUG
ENSG00000188783	UGUCUUU
ENSG00000188786	AAAGUAA
ENSG00000188786	AACAGUC
ENSG00000188786	AACAUUC
ENSG00000188786	AACCUGU
ENSG00000188786	AAGGCAC
ENSG00000188786	AAGGUGC
ENSG00000188786	AAGUGCA
ENSG00000188786	AAUGCCC
ENSG00000188786	ACAUUCA
ENSG00000188786	ACCCUGU
ENSG00000188786	ACUGCAU
ENSG00000188786	AGUACUG
ENSG00000188786	AGUGCAA
ENSG00000188786	AUACAAG
ENSG00000188786	CACACCG
ENSG00000188786	CACACCU
ENSG00000188786	CAGGCUC
ENSG00000188786	CCAGUGC
ENSG00000188786	CCUUCAU
ENSG00000188786	CUCAGGG
ENSG00000188786	CUGCUCA
ENSG00000188786	CUGGACU
ENSG00000188786	GAAACAU
ENSG00000188786	GACCCUG
ENSG00000188786	GAUUGGU
ENSG00000188786	GCACCAU
ENSG00000188786	GCAGCAU
ENSG00000188786	UAAUAGU
ENSG00000188786	UACAAAG
ENSG00000188786	UGAGAAG
ENSG00000188786	UGCAGCU
ENSG00000188786	UGCAUAG
ENSG00000188786	UGCAUAU
ENSG00000188786	UGGGACA
ENSG00000188786	UUCUGCA
ENSG00000188786	UUGGCAC
ENSG00000188786	UUUCAAC
ENSG00000188786	UUUGCAC
ENSG00000188800	GGAGUCC
ENSG00000188807	GGCUCUG
ENSG00000188807	GGUUACC
ENSG00000188807	UCACACA
ENSG00000188811	AAGUGCU
ENSG00000188811	AGUGCUU

ENSG00000188811	UUGGCAA
ENSG00000188816	AAAAAUC
ENSG00000188827	GCUGGUG
ENSG00000188827	UAUUGCU
ENSG00000188906	GUGCAAA
ENSG00000188906	UUCAAAU
ENSG00000188984	UCUCCAA
ENSG00000188987	UGGCCCU
ENSG00000188994	AAGGCAC
ENSG00000188994	ACAUUCA
ENSG00000188994	AUACAAG
ENSG00000188994	AUGGCAC
ENSG00000188994	AUGGCUU
ENSG00000188994	CUAUACA
ENSG00000188994	CUUACAG
ENSG00000188994	GCUGGUG
ENSG00000188994	GCUUCCU
ENSG00000188994	GGCAGCG
ENSG00000188994	GUCAUUG
ENSG00000188994	GUGUCAC
ENSG00000188994	GUUCAUG
ENSG00000188994	UACAAAG
ENSG00000188994	UAGACUG
ENSG00000188994	UCACCAC
ENSG00000188994	UCCUAUA
ENSG00000188994	UGUAUAA
ENSG00000188994	UUCCAUA
ENSG00000188994	UUUUUGC
ENSG00000188997	AAGUCAC
ENSG00000188997	AGGGUUG
ENSG00000188997	AGGUAGU
ENSG00000188997	CCCUGAG
ENSG00000188997	CCUGCUG
ENSG00000188997	GAGGUAU
ENSG00000188997	GCCCUGU
ENSG00000188997	UGAUCCA
ENSG00000188997	UUGAUCA
ENSG00000189007	AAGGUGC
ENSG00000189007	CAAAACU
ENSG00000189007	CAAAGCA
ENSG00000189007	CAAAUGC
ENSG00000189007	CCUUCUG
ENSG00000189007	CUCCAUU
ENSG00000189007	GCCUGGA
ENSG00000189007	GGCAGUG
ENSG00000189007	GUCUCUG
ENSG00000189007	UAUCAGA
ENSG00000189007	UGAGAAG
ENSG00000189007	UGCAUAU
ENSG00000189007	UUGGCAA
ENSG00000189007	UUGGCAC
ENSG00000189007	UUGUUCG
ENSG00000189043	CCUUCAU
ENSG00000189043	UGUGCGU
ENSG00000189050	AAAAAUC
ENSG00000189056	CACUGUU
ENSG00000189056	GUGCUUG
ENSG00000189060	GUCUCUG
ENSG00000189067	AAAGUAC
ENSG00000189067	AAAGUGC
ENSG00000189067	AAGGCAC
ENSG00000189067	AGCAGCA
ENSG00000189067	UCACAGU
ENSG00000189079	AAAACUG

ENSG00000189079	AAAAGUA
ENSG00000189079	AAAUGGU
ENSG00000189079	AAAUUUC
ENSG00000189079	AACCUGU
ENSG00000189079	ACAAGGU
ENSG00000189079	ACAGUAC
ENSG00000189079	ACAUUCA
ENSG00000189079	ACCACAG
ENSG00000189079	ACUGUUU
ENSG00000189079	AGCAAAA
ENSG00000189079	AGUAACA
ENSG00000189079	CAAAACU
ENSG00000189079	CACAGUG
ENSG00000189079	CAGCAGA
ENSG00000189079	CAGUCUG
ENSG00000189079	CCAGUGC
ENSG00000189079	CCAGUGU
ENSG00000189079	CUACAAA
ENSG00000189079	GGAAUGU
ENSG00000189079	GGAGGAG
ENSG00000189079	GGCUCAG
ENSG00000189079	GGCUGCG
ENSG00000189079	GGUGGUU
ENSG00000189079	UAAGACG
ENSG00000189079	UACAAAG
ENSG00000189079	UACAGUU
ENSG00000189079	UAUAAUA
ENSG00000189079	UCAAGUA
ENSG00000189079	UCACAAG
ENSG00000189079	UCACAGU
ENSG00000189079	UCCAGUU
ENSG00000189079	UGAAAGG
ENSG00000189079	UGAAAUG
ENSG00000189079	UGGCUGC
ENSG00000189079	UUCCAUA
ENSG00000189079	UUCUAAU
ENSG00000189091	AAAAGUA
ENSG00000189091	AACAUUC
ENSG00000189091	AGUAGAC
ENSG00000189120	CAGGCUC
ENSG00000189120	CAGUAGA
ENSG00000189120	CCAGCAU
ENSG00000189120	GGCUCAG
ENSG00000189120	UCACAGG
ENSG00000189120	UCACAGU
ENSG00000189120	UCCCUUU
ENSG00000189120	UGAAAGG
ENSG00000189143	UACAAAG
ENSG00000189159	AAAGUGC
ENSG00000189159	AACAGUC
ENSG00000189159	AAGUGCU
ENSG00000189159	ACCAGCA
ENSG00000189159	AGCACCA
ENSG00000189159	AGUGCUU
ENSG00000189159	AUAAAGU
ENSG00000189159	CACCAGC
ENSG00000189159	CAGUCCA
ENSG00000189159	CCAGCAU
ENSG00000189159	GAAAACA
ENSG00000189182	CCAGCAU
ENSG00000189184	AAUCACU
ENSG00000189184	ACAGUAC
ENSG00000189184	GCAACUU
ENSG00000189184	UAAUAUC

ENSG00000189212	ACAUUCA
ENSG00000189221	CUGCUCA
ENSG00000189221	GAGGCAG
ENSG00000189241	CCAGCAU
ENSG00000189266	AGCAGCA
ENSG00000189266	AGCAGCG
ENSG00000189266	CUUUGGU
ENSG00000189266	GCUACAU
ENSG00000189266	GGCUCUG
ENSG00000189292	AAGGCAC
ENSG00000189292	AAUCUCU
ENSG00000189292	CAUAUUG
ENSG00000189298	AAGGCAC
ENSG00000189308	AAAGUUU
ENSG00000189308	AACGGAA
ENSG00000189308	AAGGUGC
ENSG00000189308	ACAAUAU
ENSG00000189308	ACACACU
ENSG00000189308	ACAUCAC
ENSG00000189308	ACUAGAU
ENSG00000189308	ACUGCAG
ENSG00000189308	ACUGCAU
ENSG00000189308	AUACAAG
ENSG00000189308	AUCAUAC
ENSG00000189308	AUUGCAC
ENSG00000189308	CAAAUGC
ENSG00000189308	CAGCAAA
ENSG00000189308	CUACAAA
ENSG00000189308	CUAGUAG
ENSG00000189308	GAGCUAA
ENSG00000189308	GUGCAAA
ENSG00000189308	GUUAGGA
ENSG00000189308	GUUCAUG
ENSG00000189308	UAAUACA
ENSG00000189308	UGGGAUC
ENSG00000189308	UUCUGCA
ENSG00000189319	AAAGUAA
ENSG00000189319	AAAUGGU
ENSG00000189319	AAGGCAC
ENSG00000189319	AGUAACA
ENSG00000189319	AGUGCAA
ENSG00000189319	CUACAAA
ENSG00000189319	GACCUGG
ENSG00000189319	GAGACCU
ENSG00000189319	GCAGAAG
ENSG00000189319	GGAGAGA
ENSG00000189319	GGCAAGA
ENSG00000189319	UGAGAAU
ENSG00000189319	UGUCUGC
ENSG00000189325	CAGUGCA
ENSG00000189325	CCAGCAU
ENSG00000189325	UGCCAGC
ENSG00000189334	AAUGCCC
ENSG00000189334	CAAAUGC
ENSG00000189337	GAGGCUG
ENSG00000189337	GGGUGUU
ENSG00000189337	GUUAAUG
ENSG00000189337	UCCUGAG
ENSG00000189369	AAAGCUG
ENSG00000189369	ACAGUAU
ENSG00000189369	GAGAAAU
ENSG00000189369	UCACAGU
ENSG00000189369	UUCAAAU
ENSG00000189377	GGGUAAG

ENSG00000189403	AAAAACC
ENSG00000189403	AAGAACC
ENSG00000189403	ACUGUAG
ENSG00000189403	AGCAAAA
ENSG00000189403	AGCAUUC
ENSG00000189403	AGUGUGC
ENSG00000189403	AUACAAG
ENSG00000189403	CAAAUGC
ENSG00000189403	CCAGGAG
ENSG00000189403	CUUACAG
ENSG00000189403	GACAACU
ENSG00000189403	GAGUUGA
ENSG00000189403	GAGUUGG
ENSG00000189403	GCACCAU
ENSG00000189403	GUAGUGU
ENSG00000189403	UAUAUGA
ENSG00000189403	UGUGCUU
ENSG00000189403	UUCCAUA
ENSG00000189410	AAGGCAC
ENSG00000189410	AGUGCUU
ENSG00000189410	CUCAGGG
ENSG00000189410	GGAAGAC
ENSG00000189410	UCAAGUA
ENSG00000189410	UUCAAGC
ENSG00000196072	AAAGUAA
ENSG00000196072	UCCAGUU
ENSG00000196083	AAAGUGC
ENSG00000196083	AAGUGCA
ENSG00000196083	AGUGCAA
ENSG00000196083	CAAAUGC
ENSG00000196083	GACCAUG
ENSG00000196083	GAGAUGA
ENSG00000196083	GCACCAU
ENSG00000196083	GGUUUAC
ENSG00000196083	UACAAAG
ENSG00000196083	UGCAAAG
ENSG00000196090	AAACUGU
ENSG00000196090	AAAUUUC
ENSG00000196090	AAGUGCA
ENSG00000196090	AAUCUCA
ENSG00000196090	AAUUGCU
ENSG00000196090	AGUGCUG
ENSG00000196090	AUGGCUU
ENSG00000196090	CACUGUU
ENSG00000196090	CAGCAGA
ENSG00000196090	GAAAACA
ENSG00000196090	GAAUUGU
ENSG00000196090	GGGGAGC
ENSG00000196090	GGGGUGG
ENSG00000196090	GUGCUUG
ENSG00000196090	UCACAGG
ENSG00000196090	UCACAGU
ENSG00000196090	UCCCACA
ENSG00000196090	UCUGGAG
ENSG00000196090	UGACCUA
ENSG00000196090	UGUGCUU
ENSG00000196090	UUGAUCA
ENSG00000196090	UUGGUCC
ENSG00000196092	CUGGGGG
ENSG00000196092	GGGGUCC
ENSG00000196092	UCUCCAA
ENSG00000196104	AAGGCAC
ENSG00000196104	AGCAGCA
ENSG00000196104	UUUGCAA

ENSG00000196132	AAAGAAU
ENSG00000196132	AACAAAC
ENSG00000196132	AAGUGCA
ENSG00000196132	AAUACUG
ENSG00000196132	ACAGUAU
ENSG00000196132	AGUGCAA
ENSG00000196132	AUAUAAC
ENSG00000196132	CACAAUG
ENSG00000196132	CACAGUG
ENSG00000196132	CCAGGAG
ENSG00000196132	GAGAACU
ENSG00000196132	UCACAGU
ENSG00000196132	UCCCUGU
ENSG00000196132	UCCUGAG
ENSG00000196132	UGCAAAG
ENSG00000196150	AAAGAGG
ENSG00000196155	GCUGGAU
ENSG00000196155	UAUAAAG
ENSG00000196169	GGAUUUU
ENSG00000196169	UUGGCAA
ENSG00000196169	UUGGCAC
ENSG00000196176	GGCCCUG
ENSG00000196177	AAAUUUC
ENSG00000196177	AAUCCUU
ENSG00000196177	AGGGAGG
ENSG00000196177	AUGACAC
ENSG00000196177	GUGCAAA
ENSG00000196177	UGAAAGG
ENSG00000196177	UGACAUC
ENSG00000196177	UUGGCAA
ENSG00000196177	UUGGCAC
ENSG00000196182	AGCUUAU
ENSG00000196182	AUACAAG
ENSG00000196182	CACAGUG
ENSG00000196182	CAGUCCA
ENSG00000196182	CUCACAC
ENSG00000196182	GACCUGG
ENSG00000196182	GAGCCCU
ENSG00000196182	GAGGCAG
ENSG00000196182	GAGGUAG
ENSG00000196182	GCAGGAC
ENSG00000196182	GGCAAGA
ENSG00000196182	GUCACUC
ENSG00000196182	GUCCAGA
ENSG00000196182	GUGACUG
ENSG00000196182	UCACAGU
ENSG00000196182	UCCAGAG
ENSG00000196182	UCUCAAG
ENSG00000196182	UGGUACC
ENSG00000196182	UUUUUGC
ENSG00000196187	GGAGGCA
ENSG00000196188	GGAGAGA
ENSG00000196189	GGCUCAG
ENSG00000196199	AAAUUUC
ENSG00000196199	AAUUUUA
ENSG00000196199	CAGUCUG
ENSG00000196199	CUAGUAG
ENSG00000196199	CUGGACU
ENSG00000196199	GGUUUAC
ENSG00000196208	AGUGCUU
ENSG00000196208	AUACAAG
ENSG00000196208	AUGCCUU
ENSG00000196208	CUUUGGU
ENSG00000196208	GAGGUAG

ENSG00000196208	GGAAGCC
ENSG00000196208	UAAUAGU
ENSG00000196208	UAUUGCU
ENSG00000196208	UUGGUUC
ENSG00000196220	AAAGUGC
ENSG00000196220	AAGGCAC
ENSG00000196220	AAGGCGC
ENSG00000196220	AAGUGCA
ENSG00000196220	AAUGCCC
ENSG00000196220	AGCAGCU
ENSG00000196220	AGCCUGC
ENSG00000196220	AGGCAGG
ENSG00000196220	CACACCU
ENSG00000196220	CACAGGG
ENSG00000196220	CAGACAG
ENSG00000196220	CAGCAGA
ENSG00000196220	CCAGUGC
ENSG00000196220	CCAGUGU
ENSG00000196220	CCCCCAG
ENSG00000196220	CCCCUGG
ENSG00000196220	CUCCCAA
ENSG00000196220	CUCCCAC
ENSG00000196220	CUCUGGG
ENSG00000196220	CUCUUCC
ENSG00000196220	CUGCAGC
ENSG00000196220	CUUUGGU
ENSG00000196220	GAGAAAU
ENSG00000196220	GAGCUGC
ENSG00000196220	GAGGUAG
ENSG00000196220	GAGGUAU
ENSG00000196220	GCACCAU
ENSG00000196220	GCUGGUG
ENSG00000196220	GGCAAGA
ENSG00000196220	GGCAGGG
ENSG00000196220	GGCAUUG
ENSG00000196220	GGGGCAG
ENSG00000196220	GUCAGUU
ENSG00000196220	GUCUCUG
ENSG00000196220	GUGCAAA
ENSG00000196220	UAUUGCU
ENSG00000196220	UCACAGU
ENSG00000196220	UCACAUU
ENSG00000196220	UCAUAGA
ENSG00000196220	UCCAGUU
ENSG00000196220	UCCCACA
ENSG00000196220	UCCCACC
ENSG00000196220	UCCCUGU
ENSG00000196220	UCCCUUG
ENSG00000196220	UCCUUGC
ENSG00000196220	UCUGGAA
ENSG00000196220	UGACCUA
ENSG00000196220	UGCAAAG
ENSG00000196220	UGUCUUU
ENSG00000196220	UUCUGCA
ENSG00000196220	UUGGUCC
ENSG00000196226	AAAGAGC
ENSG00000196230	AAUACUG
ENSG00000196230	AUAAUAC
ENSG00000196230	GAGGCAG
ENSG00000196230	UUUCAAC
ENSG00000196233	ACAGUAU
ENSG00000196233	CUACAGU
ENSG00000196233	CUGCAGC
ENSG00000196233	GAGGUAG

ENSG00000196233	GAGGUAU
ENSG00000196233	GAUUGUC
ENSG00000196233	GCAACUU
ENSG00000196233	UGAAAUG
ENSG00000196233	UGCUCAC
ENSG00000196233	UUGGCAC
ENSG00000196235	UGAAAGG
ENSG00000196236	AGCAAAA
ENSG00000196262	AACUCUA
ENSG00000196262	GAGCUAA
ENSG00000196268	UAAUAUC
ENSG00000196277	AAACUGU
ENSG00000196277	ACAAUAU
ENSG00000196277	ACAUUCA
ENSG00000196277	AGCAGCA
ENSG00000196277	AGCAGCU
ENSG00000196277	AUGCAUU
ENSG00000196277	GGCAGUG
ENSG00000196277	UACAAAG
ENSG00000196277	UUGGUCC
ENSG00000196284	AUAUAAC
ENSG00000196284	CUGGAAA
ENSG00000196284	GUAAACA
ENSG00000196284	UAAUAUC
ENSG00000196284	UUCUUGU
ENSG00000196296	AGCAGCU
ENSG00000196296	GGAGGAG
ENSG00000196296	GUGACAG
ENSG00000196305	ACUGCAU
ENSG00000196305	UGCAUAG
ENSG00000196313	AAAUUUC
ENSG00000196313	AACAGUC
ENSG00000196313	AGCUUAU
ENSG00000196313	CACAGUG
ENSG00000196313	CUCCAGC
ENSG00000196313	CUCCCAA
ENSG00000196313	CUCCCAC
ENSG00000196313	UCAAGUA
ENSG00000196313	UCACAGU
ENSG00000196323	AAAGAGG
ENSG00000196323	AAAGUGC
ENSG00000196323	AAGGUGC
ENSG00000196323	AAGUAGC
ENSG00000196323	AAGUGCU
ENSG00000196323	ACUCAGG
ENSG00000196323	ACUGGAU
ENSG00000196323	ACUGGCU
ENSG00000196323	AGCCAGU
ENSG00000196323	AGGCAGG
ENSG00000196323	AGGGUCU
ENSG00000196323	AGUGCAA
ENSG00000196323	AGUGCUU
ENSG00000196323	AUGGCUU
ENSG00000196323	CAAAGCA
ENSG00000196323	CAGUGCA
ENSG00000196323	CUAUACA
ENSG00000196323	CUCUGGG
ENSG00000196323	CUCUUCC
ENSG00000196323	CUUUGGU
ENSG00000196323	GAGACCU
ENSG00000196323	GAGGCAG
ENSG00000196323	GAUUGUC
ENSG00000196323	GCUAGCA
ENSG00000196323	GCUGGUG

ENSG00000196323	GGAAGAC
ENSG00000196323	GGCUGCG
ENSG00000196323	GGUUUAC
ENSG00000196323	GUGCCAC
ENSG00000196323	UAUGGUU
ENSG00000196323	UCCCUGU
ENSG00000196323	UGUAUAA
ENSG00000196331	AAAGAGC
ENSG00000196338	AAGAACC
ENSG00000196338	ACUGUGU
ENSG00000196338	AGCACCA
ENSG00000196338	AGGGAGG
ENSG00000196338	GAGAAAU
ENSG00000196338	UCCAGAG
ENSG00000196338	UGAAAGG
ENSG00000196353	AAAUUUC
ENSG00000196353	AACAAAC
ENSG00000196353	GGCGACA
ENSG00000196353	UCAACAG
ENSG00000196353	UUCAAAU
ENSG00000196358	CCCCCAG
ENSG00000196361	AAAAAUC
ENSG00000196361	AAAACUG
ENSG00000196361	AACCUGU
ENSG00000196361	AACUGGU
ENSG00000196361	CAGCAGA
ENSG00000196361	CUCCCAC
ENSG00000196361	GAGCCCU
ENSG00000196361	GAGGUAU
ENSG00000196361	GCAGAAG
ENSG00000196361	GCCCUUA
ENSG00000196361	GGAGAGA
ENSG00000196361	GGAUUUU
ENSG00000196361	GUAAACA
ENSG00000196361	UACAAAG
ENSG00000196361	UAUUACC
ENSG00000196361	UAUUGCU
ENSG00000196361	UCACCAC
ENSG00000196361	UCCCACC
ENSG00000196361	UCCCUUG
ENSG00000196361	UCCUUGC
ENSG00000196361	UCUCCAA
ENSG00000196361	UGCUCAC
ENSG00000196363	AAGACAU
ENSG00000196363	CAAAUGC
ENSG00000196363	CCCACCU
ENSG00000196363	CCUUCUG
ENSG00000196363	CUACAAA
ENSG00000196363	UAUUACC
ENSG00000196363	UUGGCAA
ENSG00000196367	ACAUUCA
ENSG00000196367	UAUAAAG
ENSG00000196367	UCACAUU
ENSG00000196367	UGUGCUU
ENSG00000196368	AAAGUAU
ENSG00000196368	AGCACCA
ENSG00000196368	GGCUCUG
ENSG00000196368	GUAGUGU
ENSG00000196368	UACAAAG
ENSG00000196368	UAUAAAG
ENSG00000196368	UUCAAGC
ENSG00000196368	UUCUUGU
ENSG00000196371	GUAAACA
ENSG00000196371	UUCUAAU

ENSG00000196374	AAAGAGC
ENSG00000196374	GGCUCUG
ENSG00000196376	AAAAAUC
ENSG00000196376	ACAAUAU
ENSG00000196376	ACAUUCA
ENSG00000196376	ACCCAUU
ENSG00000196376	AGCCAGU
ENSG00000196376	CACAGUG
ENSG00000196376	GCCUGGA
ENSG00000196376	GGAAUGU
ENSG00000196376	GGAGUCC
ENSG00000196376	GUCAGUU
ENSG00000196376	GUGCUUG
ENSG00000196376	UAAUAGU
ENSG00000196376	UGUGCUU
ENSG00000196396	AACUACU
ENSG00000196396	AAGGCAC
ENSG00000196396	ACACACU
ENSG00000196396	ACCACAG
ENSG00000196396	AGCACCA
ENSG00000196396	AGCAGCU
ENSG00000196396	AGCCCUU
ENSG00000196396	AUGCAUU
ENSG00000196396	AUGUCUG
ENSG00000196396	CACACCU
ENSG00000196396	GGAAUGU
ENSG00000196405	UAUUACC
ENSG00000196407	UGCUGAC
ENSG00000196411	AAAGUGC
ENSG00000196411	AAGUGCA
ENSG00000196411	AGUGCAA
ENSG00000196411	AUACAAG
ENSG00000196411	CAAAGUG
ENSG00000196411	CUGCCCC
ENSG00000196411	CUUUGGU
ENSG00000196411	UUGGUCC
ENSG00000196422	AGGACAC
ENSG00000196422	GUGCUUG
ENSG00000196422	UACAAAG
ENSG00000196422	UGUGCUU
ENSG00000196428	AAAGUGC
ENSG00000196428	ACAAUAU
ENSG00000196428	AGUGCUG
ENSG00000196428	AGUGGUU
ENSG00000196428	GAGGUAG
ENSG00000196428	GAGGUAU
ENSG00000196428	GAUUGUA
ENSG00000196428	GCCCUGU
ENSG00000196428	UGGAUAA
ENSG00000196428	UUCAAGC
ENSG00000196449	CCAGCAU
ENSG00000196449	UUCAAAU
ENSG00000196453	AAAAAUC
ENSG00000196453	ACAGUAU
ENSG00000196458	CCUGCUG
ENSG00000196458	CUUCACC
ENSG00000196458	GUGCUUG
ENSG00000196459	UUCUGCA
ENSG00000196470	AACACUG
ENSG00000196470	AAUUAUU
ENSG00000196470	ACUGGCC
ENSG00000196470	AGCAGCA
ENSG00000196470	AGUGGUU
ENSG00000196470	AUGGCUU

ENSG00000196470	CCUGUAC
ENSG00000196470	CUGUUGA
ENSG00000196470	GCAACUU
ENSG00000196476	AGGGUCU
ENSG00000196482	AACACUG
ENSG00000196482	AACAGUC
ENSG00000196482	AAUUGCU
ENSG00000196482	AAUUUUA
ENSG00000196482	ACAGUAU
ENSG00000196482	ACCCAUU
ENSG00000196482	ACCCUGU
ENSG00000196482	ACUGUUU
ENSG00000196482	AGAUGUG
ENSG00000196482	AGCAGCA
ENSG00000196482	AGCAGCU
ENSG00000196482	AUAUUAU
ENSG00000196482	AUGAUGA
ENSG00000196482	AUGCACC
ENSG00000196482	CAGCAGG
ENSG00000196482	CCAGGAG
ENSG00000196482	CCCUGAG
ENSG00000196482	CUACAGU
ENSG00000196482	CUAUACA
ENSG00000196482	CUUCUCU
ENSG00000196482	GAGAUGA
ENSG00000196482	GCAUCCC
ENSG00000196482	GGAAGCC
ENSG00000196482	GGCAGGG
ENSG00000196482	GUAAACA
ENSG00000196482	GUUAGGA
ENSG00000196482	UAAUAUC
ENSG00000196482	UAUAAUA
ENSG00000196482	UCACACA
ENSG00000196482	UCAUUUG
ENSG00000196482	UCCCUGU
ENSG00000196482	UCCCUUG
ENSG00000196482	UCCUGAG
ENSG00000196482	UGAAAGG
ENSG00000196482	UGCAUUG
ENSG00000196482	UUAGGAU
ENSG00000196482	UUGGCAA
ENSG00000196482	UUGGCAC
ENSG00000196498	AAUACUG
ENSG00000196498	AGCACCA
ENSG00000196498	AGCAGCA
ENSG00000196498	CAAAUGC
ENSG00000196498	CCCACCG
ENSG00000196498	CCCCUGG
ENSG00000196498	CCCUGAG
ENSG00000196498	CUUUGGU
ENSG00000196498	GAAGGUC
ENSG00000196498	GGACGGA
ENSG00000196498	GUCCAGA
ENSG00000196498	UCCCACC
ENSG00000196498	UUGGUUC
ENSG00000196504	AAAUUUC
ENSG00000196504	AAGGCAC
ENSG00000196504	AAUCUCA
ENSG00000196504	ACUGUAG
ENSG00000196504	AGGGUCU
ENSG00000196504	AUUGCAC
ENSG00000196504	CAUGGAU
ENSG00000196504	CCAGUGU
ENSG00000196504	GAAACAU

ENSG00000196504	GACAACU
ENSG00000196504	GCAACUU
ENSG00000196504	UCCCUUG
ENSG00000196504	UCCCUUU
ENSG00000196504	UCUUGAG
ENSG00000196504	UGAAAGG
ENSG00000196504	UGAGGAC
ENSG00000196504	UUCAAAU
ENSG00000196504	UUUUUGC
ENSG00000196505	AUACAAG
ENSG00000196505	UUCCAUA
ENSG00000196507	GCUGGUG
ENSG00000196507	UGGGUGA
ENSG00000196517	AAAGUGC
ENSG00000196517	AACACUG
ENSG00000196517	CAAAGUG
ENSG00000196517	GUAAACA
ENSG00000196517	GUCUCUG
ENSG00000196517	UAUUGCU
ENSG00000196517	UUGGCAA
ENSG00000196526	AAUUUUA
ENSG00000196526	ACAAUAU
ENSG00000196526	ACAGUAC
ENSG00000196526	ACAGUAU
ENSG00000196526	ACAUUCA
ENSG00000196526	CACAGUG
ENSG00000196526	CUUUGGU
ENSG00000196526	GAGAAAU
ENSG00000196526	GGCAGCG
ENSG00000196526	UCACAGG
ENSG00000196526	UCUGGAA
ENSG00000196526	UGAGAAG
ENSG00000196526	UGAGAAU
ENSG00000196532	AAAGAGC
ENSG00000196533	GGGUCUU
ENSG00000196535	CAGCAGG
ENSG00000196535	CCCUGAG
ENSG00000196542	UGCAUAG
ENSG00000196542	UUGGCAA
ENSG00000196544	AGCAGCA
ENSG00000196544	CCCUGAG
ENSG00000196547	ACUCAGC
ENSG00000196547	CACAGUG
ENSG00000196547	CUGCCCC
ENSG00000196547	UCACAGU
ENSG00000196547	UGAAGCU
ENSG00000196547	UGAAGUG
ENSG00000196549	AAUCUCU
ENSG00000196549	CUCAAAC
ENSG00000196549	UCAUUUG
ENSG00000196549	UGCAAAG
ENSG00000196549	UGUAUAA
ENSG00000196549	UUCAAGC
ENSG00000196549	UUUCAAC
ENSG00000196557	AACCUGU
ENSG00000196557	AUUGCAC
ENSG00000196557	UAUUGCU
ENSG00000196562	CAAAGCA
ENSG00000196562	GUGAGGU
ENSG00000196576	CGUACCG
ENSG00000196576	GCUGGUG
ENSG00000196576	GUGAGGU
ENSG00000196576	UACAAAG
ENSG00000196581	AACGGAA

ENSG00000196581	AAUUGCU
ENSG00000196581	ACAGUAU
ENSG00000196581	AUGGCAC
ENSG00000196581	CCCCCAG
ENSG00000196581	CUACAGU
ENSG00000196581	CUGCCCC
ENSG00000196581	GAAAACA
ENSG00000196581	GCCCUGU
ENSG00000196581	GCUACAU
ENSG00000196581	GUCUGCC
ENSG00000196581	UCACCAC
ENSG00000196581	UGCCCUG
ENSG00000196581	UUGGCAC
ENSG00000196586	ACUCAGG
ENSG00000196586	ACUUGGA
ENSG00000196586	AGGUUAU
ENSG00000196586	CAGCUGG
ENSG00000196586	GAGAACC
ENSG00000196586	GAGAACU
ENSG00000196586	GUGAAUG
ENSG00000196586	UCACAUU
ENSG00000196586	UCAUUUG
ENSG00000196586	UCUAGAG
ENSG00000196588	AUAAUAC
ENSG00000196588	AUAUAAC
ENSG00000196588	CCUGUAC
ENSG00000196588	CUGCCCC
ENSG00000196588	GAGGUAU
ENSG00000196588	GAUUGUA
ENSG00000196588	GGAAUGU
ENSG00000196588	UAUAAUA
ENSG00000196591	AAAACUG
ENSG00000196591	CAGUCCA
ENSG00000196591	GCUUCCU
ENSG00000196591	UAAUAGU
ENSG00000196591	UUGACAC
ENSG00000196591	UUGGCAA
ENSG00000196591	UUUUUGC
ENSG00000196628	AAAGUAA
ENSG00000196628	AACCUGU
ENSG00000196628	AAGGCAC
ENSG00000196628	AAGUGCA
ENSG00000196628	AAUACUG
ENSG00000196628	AAUCUCU
ENSG00000196628	ACAAGGU
ENSG00000196628	ACUCAGC
ENSG00000196628	ACUGGCC
ENSG00000196628	ACUGUUU
ENSG00000196628	ACUUGGA
ENSG00000196628	AGCAUUC
ENSG00000196628	AGUGCUG
ENSG00000196628	AUGCAUU
ENSG00000196628	AUUGCAC
ENSG00000196628	CACAGUG
ENSG00000196628	CCAUUAC
ENSG00000196628	CUCUUCC
ENSG00000196628	CUUCACC
ENSG00000196628	GAAAACA
ENSG00000196628	GAAGGAA
ENSG00000196628	GAGGCAG
ENSG00000196628	GAGUUGG
ENSG00000196628	GCAACUU
ENSG00000196628	GCUACAU
ENSG00000196628	GCUUCCU

ENSG00000196628	GGAAGAC
ENSG00000196628	GGAGGAG
ENSG00000196628	GGAGGCA
ENSG00000196628	GUGUGGC
ENSG00000196628	UAUAAUA
ENSG00000196628	UCACAAG
ENSG00000196628	UCACCAC
ENSG00000196628	UCCAGUU
ENSG00000196628	UCCUAUA
ENSG00000196628	UCCUUGC
ENSG00000196628	UGCUGAC
ENSG00000196628	UGUAUAA
ENSG00000196628	UGUGUGG
ENSG00000196628	UUUCAAC
ENSG00000196632	AAAACUG
ENSG00000196632	AAAAGUA
ENSG00000196632	AAAGAAU
ENSG00000196632	AAGUGCA
ENSG00000196632	ACUGCAG
ENSG00000196632	ACUGGAU
ENSG00000196632	ACUGUGU
ENSG00000196632	AGCAUUC
ENSG00000196632	AGCCAGU
ENSG00000196632	AGCUUAU
ENSG00000196632	AGUGCAA
ENSG00000196632	CCAGUGU
ENSG00000196632	CUGCAGG
ENSG00000196632	GCAACUU
ENSG00000196632	UCACAUU
ENSG00000196632	UGGAUAA
ENSG00000196632	UGUGUCA
ENSG00000196639	AAAAAUC
ENSG00000196639	CUGCAGC
ENSG00000196639	GGCUCAG
ENSG00000196639	UGAGAAG
ENSG00000196639	UGCAGCU
ENSG00000196642	GUAAACA
ENSG00000196644	CAGUCUG
ENSG00000196652	CCCUGAG
ENSG00000196652	CUACAAA
ENSG00000196652	CUUCUCU
ENSG00000196652	UCCCUGU
ENSG00000196652	UGCUGAC
ENSG00000196652	UUCAAAU
ENSG00000196664	AGAUAAA
ENSG00000196664	UGCAAAG
ENSG00000196664	UUUGCAA
ENSG00000196700	AAAAACC
ENSG00000196700	AAAAAUC
ENSG00000196700	AAGUGCA
ENSG00000196700	AAGUGCU
ENSG00000196700	AGCACCA
ENSG00000196700	AUAAAGU
ENSG00000196700	AUUGCAC
ENSG00000196700	CCAGUGC
ENSG00000196700	CCAGUGU
ENSG00000196700	CCCUGAG
ENSG00000196700	CUGGAAA
ENSG00000196700	GGUUACC
ENSG00000196700	UAUGGUU
ENSG00000196700	UCACAAG
ENSG00000196700	UCCAGUU
ENSG00000196700	UGUAUAA
ENSG00000196704	GAAGGUC

ENSG00000196711	AGAUAAA
ENSG00000196712	AACAUUC
ENSG00000196712	AAGUGCA
ENSG00000196712	ACAUUAC
ENSG00000196712	ACUGUGU
ENSG00000196712	AGCAGCA
ENSG00000196712	AUAUUAU
ENSG00000196712	AUCAUAC
ENSG00000196712	AUGGCUU
ENSG00000196712	AUUGCAC
ENSG00000196712	GAGUUGA
ENSG00000196712	UCACAGG
ENSG00000196712	UCACAGU
ENSG00000196712	UGCAUAG
ENSG00000196712	UGCAUAU
ENSG00000196712	UUGAUCA
ENSG00000196712	UUGGCAA
ENSG00000196712	UUGGCAC
ENSG00000196715	AAAGCUG
ENSG00000196715	UCAUGAU
ENSG00000196724	ACAUUCA
ENSG00000196730	AAGGCAC
ENSG00000196730	AGGGAGG
ENSG00000196730	GAGAUGA
ENSG00000196730	GAGGUAG
ENSG00000196730	UAAUAUC
ENSG00000196743	AAGUUGU
ENSG00000196743	UAAUACA
ENSG00000196747	AAAGAGC
ENSG00000196747	CUUUGGU
ENSG00000196767	CCAGUGC
ENSG00000196776	AAAGAGG
ENSG00000196776	AAUCACU
ENSG00000196776	ACUCCAG
ENSG00000196776	ACUGUAG
ENSG00000196776	AGGCAGG
ENSG00000196776	AUAAUAC
ENSG00000196776	AUAUAAC
ENSG00000196776	AUGGCUU
ENSG00000196776	AUGUGCC
ENSG00000196776	CAAAACU
ENSG00000196776	CACACCU
ENSG00000196776	CAGCAGG
ENSG00000196776	CUGGCUC
ENSG00000196776	CUUUGGU
ENSG00000196776	GACAACU
ENSG00000196776	GAGGCAG
ENSG00000196776	GCUACAU
ENSG00000196776	UUGGUCC
ENSG00000196781	ACUGUAG
ENSG00000196781	AUACAAG
ENSG00000196782	AAAAGUA
ENSG00000196782	AAAGUAA
ENSG00000196782	AACAAAC
ENSG00000196782	AAGUGCA
ENSG00000196782	ACAUUAC
ENSG00000196782	AGUAGAC
ENSG00000196782	AGUGCAA
ENSG00000196782	AUGAUGA
ENSG00000196782	CCAGUAC
ENSG00000196782	CCCACCU
ENSG00000196782	CUGCAGC
ENSG00000196782	CUGCAGG
ENSG00000196782	GGAGGAG

ENSG00000196782	UACAAAG
ENSG00000196782	UAUAUGA
ENSG00000196782	UCCCACC
ENSG00000196782	UGAAGUG
ENSG00000196782	UUGGUCC
ENSG00000196782	UUGGUUC
ENSG00000196782	UUUUUGC
ENSG00000196787	AAAAAUC
ENSG00000196787	AAGUCAC
ENSG00000196787	CUAGUAA
ENSG00000196787	CUAGUAG
ENSG00000196787	GAGACCU
ENSG00000196787	GCAGAAG
ENSG00000196787	GUGCUUG
ENSG00000196787	UACAAAG
ENSG00000196787	UCACAGG
ENSG00000196787	UCACAGU
ENSG00000196787	UGGGACA
ENSG00000196787	UGUGCUU
ENSG00000196787	UUCCUCG
ENSG00000196787	UUUGCAA
ENSG00000196787	UUUGCAC
ENSG00000196792	AAAAAUC
ENSG00000196792	AAAGUUU
ENSG00000196792	AAUUUUA
ENSG00000196792	AGCACCA
ENSG00000196792	AUAUAAC
ENSG00000196792	AUCCUUG
ENSG00000196792	CUUGGAG
ENSG00000196792	GAGAUGA
ENSG00000196792	GGAAGAC
ENSG00000196792	UAAUGCU
ENSG00000196792	UACAAAG
ENSG00000196792	UCUUGAG
ENSG00000196792	UGUCUUU
ENSG00000196814	AACACUG
ENSG00000196814	ACCACAG
ENSG00000196814	AGCCCUU
ENSG00000196814	AGGGAGG
ENSG00000196814	AUAUUAU
ENSG00000196814	CAGUAGA
ENSG00000196814	CAGUAGU
ENSG00000196814	GAGCCCU
ENSG00000196814	GAGGUAG
ENSG00000196814	GAGGUAU
ENSG00000196814	GUGCUUG
ENSG00000196814	UCACAGU
ENSG00000196814	UCACAUU
ENSG00000196814	UCCCUUU
ENSG00000196814	UGUGCUU
ENSG00000196821	AGCAUUC
ENSG00000196821	CAGACAG
ENSG00000196821	CCUGCUG
ENSG00000196821	CCUUCUG
ENSG00000196821	CUUGUAU
ENSG00000196821	CUUUGGU
ENSG00000196821	GCUGGUG
ENSG00000196821	GCUUCCU
ENSG00000196821	GUUAAUG
ENSG00000196821	UCAUUUG
ENSG00000196821	UGCAUAG
ENSG00000196821	UGCAUAU
ENSG00000196821	UGCUGAC
ENSG00000196821	UGUCUUU

ENSG00000196821	UUCUUGU
ENSG00000196821	UUGGCAA
ENSG00000196821	UUGGCAC
ENSG00000196850	AUGGCUU
ENSG00000196850	CAGCAGG
ENSG00000196850	GAGGUAG
ENSG00000196850	GAGGUAU
ENSG00000196850	GUAAACA
ENSG00000196850	UGAAAUG
ENSG00000196850	UGCCAGC
ENSG00000196866	AAAGAGC
ENSG00000196866	GGGUGGU
ENSG00000196872	AAAAAUC
ENSG00000196872	AAUUAUU
ENSG00000196872	AGCUGCC
ENSG00000196872	AUGCAUU
ENSG00000196872	GUAAACA
ENSG00000196872	UGCAUUG
ENSG00000196876	AACAAAC
ENSG00000196876	AAGUUGU
ENSG00000196878	UUGGUCC
ENSG00000196890	AAAGAGC
ENSG00000196914	AAAACUG
ENSG00000196914	AAAGAGG
ENSG00000196914	AAAGGUA
ENSG00000196914	AAGUCUU
ENSG00000196914	ACCUGGA
ENSG00000196914	ACUGGCU
ENSG00000196914	AGCUUAU
ENSG00000196914	AGUGAUC
ENSG00000196914	AGUGCAA
ENSG00000196914	AGUGCCU
ENSG00000196914	AGUGUGC
ENSG00000196914	AUCCUUG
ENSG00000196914	AUGUGCC
ENSG00000196914	CAGCAGA
ENSG00000196914	CAGCAGG
ENSG00000196914	CAGUAGA
ENSG00000196914	CAGUAGU
ENSG00000196914	CAGUGCA
ENSG00000196914	CCAGUGC
ENSG00000196914	CCAGUGU
ENSG00000196914	CCCACCG
ENSG00000196914	CCCACCU
ENSG00000196914	CCUUCUG
ENSG00000196914	CCUUCUU
ENSG00000196914	CUAGUAG
ENSG00000196914	CUGCUCA
ENSG00000196914	CUGGAAA
ENSG00000196914	GAAACAU
ENSG00000196914	GAUCAGA
ENSG00000196914	GAUUGUC
ENSG00000196914	GCACCAU
ENSG00000196914	GCUUCCU
ENSG00000196914	GUAGUGU
ENSG00000196914	GUGCAAA
ENSG00000196914	GUGCCUG
ENSG00000196914	GUGCUUG
ENSG00000196914	UAAUAUC
ENSG00000196914	UACAAAG
ENSG00000196914	UAGUGAG
ENSG00000196914	UAUCAGA
ENSG00000196914	UCAAGUA
ENSG00000196914	UCCAGAG

ENSG00000196914	UCCAGUU
ENSG00000196914	UCCCACC
ENSG00000196914	UGCAGCU
ENSG00000196914	UGGAUAA
ENSG00000196914	UGGGUGA
ENSG00000196914	UGUAUGU
ENSG00000196914	UUCAAGC
ENSG00000196914	UUGGCAA
ENSG00000196914	UUGGCAC
ENSG00000196914	UUGUUCG
ENSG00000196914	UUUCAAC
ENSG00000196917	AAAGAGC
ENSG00000196917	CCUUCUU
ENSG00000196917	UGAAAUG
ENSG00000196917	UUUCAAC
ENSG00000196924	AGGCAGG
ENSG00000196924	CAGGUGA
ENSG00000196932	AAGGCAC
ENSG00000196932	AUCCUUG
ENSG00000196932	CCCUGAG
ENSG00000196932	CCUUCAU
ENSG00000196932	CUGGAAA
ENSG00000196932	GACUUCC
ENSG00000196932	GAGGUAG
ENSG00000196932	GAGGUAU
ENSG00000196932	GAUCAGA
ENSG00000196932	GCCCUUA
ENSG00000196932	GUGAGGU
ENSG00000196932	UCCCUUG
ENSG00000196932	UCCCUUU
ENSG00000196932	UUCUGCA
ENSG00000196935	AAGUGUG
ENSG00000196935	AAUGCCC
ENSG00000196935	ACAUAGA
ENSG00000196935	ACAUUCA
ENSG00000196935	CAAAUGC
ENSG00000196935	CAGACAG
ENSG00000196935	CUCCAGC
ENSG00000196935	GUCCAGA
ENSG00000196935	GUUAAUG
ENSG00000196935	UACAAAG
ENSG00000196935	UUUCAAC
ENSG00000196937	AAAGUGC
ENSG00000196937	AAUCCUU
ENSG00000196937	GGCUGCG
ENSG00000196937	GUGCUUG
ENSG00000196937	UGGCUGC
ENSG00000196950	AAGAACC
ENSG00000196950	AAUGUUG
ENSG00000196950	ACACACC
ENSG00000196950	ACAGUAU
ENSG00000196950	ACAUUAC
ENSG00000196950	ACUGCAU
ENSG00000196950	AGCAGCU
ENSG00000196950	AGUAGUU
ENSG00000196950	CACAGUG
ENSG00000196950	CACUGUU
ENSG00000196950	CAGUAGA
ENSG00000196950	CUGUUGA
ENSG00000196950	GAGAUGA
ENSG00000196950	GAUUGUC
ENSG00000196950	GUAGUGU
ENSG00000196950	GUGACAG
ENSG00000196950	UAAUGCU

ENSG00000196950	UCACAUU
ENSG00000196950	UUCUAAU
ENSG00000196950	UUGGCAC
ENSG00000196961	AGCAGCA
ENSG00000196961	AGGGAGG
ENSG00000196961	AUAAUAC
ENSG00000196961	CAGCAGA
ENSG00000196961	GGGGCAG
ENSG00000196961	GUAAACA
ENSG00000196961	UAAUACA
ENSG00000196981	AGGCAGG
ENSG00000196998	AAGUGCU
ENSG00000196998	UAAUGCU
ENSG00000197006	ACCUGGA
ENSG00000197006	CUACAAA
ENSG00000197006	GGAGGCA
ENSG00000197006	UACAAAG
ENSG00000197006	UGCCAGC
ENSG00000197019	GGCUCAG
ENSG00000197019	UCUCCAA
ENSG00000197024	CUGCAGC
ENSG00000197024	GAUUGUC
ENSG00000197024	UUGGCAC
ENSG00000197037	AAGGCAC
ENSG00000197037	CAAAGUG
ENSG00000197037	GGCAAGA
ENSG00000197037	UAAUACA
ENSG00000197037	UUCAAAU
ENSG00000197045	AAAGCUG
ENSG00000197045	AAAGUAU
ENSG00000197045	AACAUUC
ENSG00000197045	AAGGCAC
ENSG00000197045	ACAGUAU
ENSG00000197045	ACAUUCA
ENSG00000197045	ACUGCAU
ENSG00000197045	AGCACCA
ENSG00000197045	AGCCCUU
ENSG00000197045	AGUGCAA
ENSG00000197045	AUGGCUU
ENSG00000197045	CCUUCAU
ENSG00000197045	CUAGUCC
ENSG00000197045	GCACCAU
ENSG00000197045	GCAGGAC
ENSG00000197045	GGAAUGU
ENSG00000197045	GUGCAAA
ENSG00000197045	UAAGACU
ENSG00000197061	GGCCCUG
ENSG00000197063	AAAAACC
ENSG00000197063	AAAAGUA
ENSG00000197063	AAAGUAA
ENSG00000197063	AAAGUUU
ENSG00000197063	AACUCUA
ENSG00000197063	AAUACUG
ENSG00000197063	ACCACAG
ENSG00000197063	ACCAGCA
ENSG00000197063	AGCAAAA
ENSG00000197063	AGCACCA
ENSG00000197063	AGUGCAA
ENSG00000197063	AUACAAG
ENSG00000197063	AUGUCUG
ENSG00000197063	CACCAGC
ENSG00000197063	CAGCAGA
ENSG00000197063	CAGUGCA
ENSG00000197063	CUGCAGG

ENSG00000197063	CUGGACU
ENSG00000197063	GGAAGAC
ENSG00000197063	GGCACGG
ENSG00000197063	GGUUUAC
ENSG00000197063	GUAAACA
ENSG00000197063	GUGCUUG
ENSG00000197063	GUUAAUG
ENSG00000197063	UAUAAAG
ENSG00000197063	UGCAAAG
ENSG00000197063	UGCAGCU
ENSG00000197063	UUGGUCC
ENSG00000197063	UUUUUGC
ENSG00000197070	AAGGCAC
ENSG00000197070	UCCUGAG
ENSG00000197081	AAGUCUU
ENSG00000197081	AAUACUG
ENSG00000197081	ACAGUAC
ENSG00000197081	ACAGUAU
ENSG00000197081	AGCAGCA
ENSG00000197081	AUAUUAU
ENSG00000197081	CUUUGGU
ENSG00000197081	GGAGAGA
ENSG00000197081	UACAGUU
ENSG00000197081	UCCCUUG
ENSG00000197081	UUGGUUC
ENSG00000197106	AAAAAUC
ENSG00000197106	AACACUG
ENSG00000197106	AAGAACC
ENSG00000197106	AAUACUG
ENSG00000197106	ACAAUAU
ENSG00000197106	ACAGUAU
ENSG00000197106	AGCUGCC
ENSG00000197106	AUAAUAC
ENSG00000197106	AUACAAG
ENSG00000197106	CACCACC
ENSG00000197106	CACCAGC
ENSG00000197106	CCCCUGG
ENSG00000197106	CCCUGAG
ENSG00000197106	CCCUGUU
ENSG00000197106	CUACAAA
ENSG00000197106	CUGGAAA
ENSG00000197106	GAGGAUA
ENSG00000197106	GCUGGUG
ENSG00000197106	GGCAGUG
ENSG00000197106	GGGUUUA
ENSG00000197106	GUCAUUG
ENSG00000197106	GUGCUUG
ENSG00000197106	UAUAAUA
ENSG00000197106	UAUUGCU
ENSG00000197106	UCUGGAA
ENSG00000197111	AAAAAUC
ENSG00000197111	ACAUUAC
ENSG00000197111	ACCUGGA
ENSG00000197111	CAGUCCA
ENSG00000197111	UAUAAAG
ENSG00000197114	AAUCACU
ENSG00000197121	AAGGCAC
ENSG00000197121	AAGUAGC
ENSG00000197121	AAUCACU
ENSG00000197121	CACAGUG
ENSG00000197121	CAUGGGU
ENSG00000197121	CUGACUC
ENSG00000197121	CUUCUCU
ENSG00000197121	CUUUGGU

ENSG00000197121	GAGGUAU
ENSG00000197121	GGCAGCG
ENSG00000197121	GGGUUUA
ENSG00000197121	UCACAGU
ENSG00000197121	UCAUUUG
ENSG00000197121	UCCAGAG
ENSG00000197121	UCCCUUU
ENSG00000197121	UGCAAAG
ENSG00000197121	UUGGUCC
ENSG00000197121	UUUGCAA
ENSG00000197122	AAAGUAU
ENSG00000197122	AAAUUUC
ENSG00000197122	ACAAUAU
ENSG00000197122	ACAGUAU
ENSG00000197122	CACUGUU
ENSG00000197122	CAGACAG
ENSG00000197122	CUGGCUC
ENSG00000197122	CUUUGGU
ENSG00000197122	GGCAAGA
ENSG00000197122	GGCGGGG
ENSG00000197122	UCCUGAG
ENSG00000197122	UGCAUAG
ENSG00000197122	UGGGGGA
ENSG00000197122	UUGUGUC
ENSG00000197128	AUGGCUU
ENSG00000197136	UAGGGCC
ENSG00000197136	UGGCCAC
ENSG00000197140	GAAGGAA
ENSG00000197142	AAACUGU
ENSG00000197142	CUGGAAA
ENSG00000197147	UCACAAG
ENSG00000197153	AAAGAGC
ENSG00000197157	AAAGCUG
ENSG00000197157	CAGACAG
ENSG00000197157	CUGGGGG
ENSG00000197170	AAAAACC
ENSG00000197177	CAUGGAU
ENSG00000197177	GUUCAUG
ENSG00000197183	GGAGUGU
ENSG00000197217	CCCUGAG
ENSG00000197217	GAGGCAG
ENSG00000197226	AAGGCAC
ENSG00000197226	AUGAGCU
ENSG00000197226	CUGCAGG
ENSG00000197226	GGACCUG
ENSG00000197226	UGAAAUG
ENSG00000197245	CUCCCAC
ENSG00000197245	UCCCACC
ENSG00000197256	AAGUGUG
ENSG00000197256	CGUACCG
ENSG00000197256	GAGAAGA
ENSG00000197256	GGAGGAG
ENSG00000197256	UCAUUUG
ENSG00000197256	UCUCAAG
ENSG00000197273	CUGGAAA
ENSG00000197273	GGUUUAC
ENSG00000197273	UCUGGAA
ENSG00000197283	ACACACC
ENSG00000197283	ACACACU
ENSG00000197283	ACAGUAU
ENSG00000197283	ACUCCAG
ENSG00000197283	AUGGCUU
ENSG00000197283	CACAGGG
ENSG00000197283	CACCACC

ENSG00000197283	CUCUGGG
ENSG00000197283	GAGAAAU
ENSG00000197283	GAGCCCC
ENSG00000197283	GAGGCAG
ENSG00000197283	GAUAUGU
ENSG00000197283	GGAAGCC
ENSG00000197283	GGAGAGA
ENSG00000197283	GGGCCCC
ENSG00000197283	GGUGGUU
ENSG00000197283	UCACAUU
ENSG00000197283	UGUGCGU
ENSG00000197283	UUCCUAG
ENSG00000197283	UUGGAGG
ENSG00000197312	GAGGUAU
ENSG00000197312	GUGAGGU
ENSG00000197321	AAGGCAC
ENSG00000197321	CUAUACA
ENSG00000197321	GAGAUGA
ENSG00000197323	AAGACAU
ENSG00000197323	AAUCACU
ENSG00000197323	AAUGUUG
ENSG00000197323	AGCUUAU
ENSG00000197323	CAAAGUG
ENSG00000197323	UAUUGCU
ENSG00000197323	UCCUAUA
ENSG00000197324	GGAGUGU
ENSG00000197324	UUGGCAA
ENSG00000197329	GUAAACA
ENSG00000197329	UGCAUAU
ENSG00000197343	ACAAGGU
ENSG00000197343	CACACCU
ENSG00000197343	GCUGGUG
ENSG00000197343	UCAACAG
ENSG00000197343	UCAAGUA
ENSG00000197375	AAGGCAC
ENSG00000197375	AAGUGCU
ENSG00000197375	CAGGUGA
ENSG00000197375	GUAAACA
ENSG00000197375	UUGAUCA
ENSG00000197375	UUGGCAA
ENSG00000197380	AAAGAGG
ENSG00000197380	CUGCAGG
ENSG00000197380	CUUUGGU
ENSG00000197380	GACCCUG
ENSG00000197380	GGGUCUU
ENSG00000197380	GUGAAUG
ENSG00000197381	AAUUAUU
ENSG00000197381	ACUCAGC
ENSG00000197381	ACUCAGG
ENSG00000197381	CAGUAGU
ENSG00000197381	GACCUGG
ENSG00000197381	GGAAGCC
ENSG00000197381	GUGCUUG
ENSG00000197381	UCCUGAG
ENSG00000197381	UGUGCUU
ENSG00000197386	AAAUCCC
ENSG00000197386	AAUCUCA
ENSG00000197386	AAUUUUA
ENSG00000197386	AGGCAGG
ENSG00000197386	AGGGAGG
ENSG00000197386	CACAGUG
ENSG00000197386	CAGGCUC
ENSG00000197386	GCAGCAU
ENSG00000197386	GGAGGCA

ENSG00000197386	GGCAGGG
ENSG00000197386	UAUUGCU
ENSG00000197386	UCACAGU
ENSG00000197386	UGAGAAG
ENSG00000197406	ACAGGUG
ENSG00000197406	CAGCAGA
ENSG00000197406	CAGCAGG
ENSG00000197406	GGCUCAG
ENSG00000197409	AAAGAGC
ENSG00000197417	GGAAGCC
ENSG00000197417	GGGAUCG
ENSG00000197429	AAAAAUC
ENSG00000197429	UGCAAAG
ENSG00000197429	UUCAAAU
ENSG00000197429	UUUCAAC
ENSG00000197429	UUUGCAA
ENSG00000197430	UCCCUGU
ENSG00000197430	UUCUAAU
ENSG00000197442	AACUGGU
ENSG00000197442	AAUACUG
ENSG00000197442	UCACAUU
ENSG00000197444	CUUCACC
ENSG00000197444	UGGUACC
ENSG00000197448	AAGGCAC
ENSG00000197451	AAAAGUA
ENSG00000197451	AAAGGUA
ENSG00000197451	AAAGUAC
ENSG00000197451	AACACUG
ENSG00000197451	ACAGUAC
ENSG00000197451	ACAGUAU
ENSG00000197451	ACAUUAC
ENSG00000197457	CCCUGAG
ENSG00000197457	CUGGGGG
ENSG00000197457	GAGAUGA
ENSG00000197457	GGCUCUG
ENSG00000197461	ACAAUAU
ENSG00000197461	CAGGAAC
ENSG00000197461	GCUACAU
ENSG00000197461	GGAAUGU
ENSG00000197461	GUAACAG
ENSG00000197461	GUCUCUG
ENSG00000197461	UACAAAG
ENSG00000197461	UAUGGUU
ENSG00000197461	UCACAUU
ENSG00000197461	UGCAUAG
ENSG00000197461	UUGUUCG
ENSG00000197467	AAAAAUC
ENSG00000197467	UUGGCAC
ENSG00000197471	AAAUUUC
ENSG00000197471	CCUUCUU
ENSG00000197471	UUCCUCG
ENSG00000197520	GAGAAAU
ENSG00000197535	AAGGCAC
ENSG00000197535	AGUGCCG
ENSG00000197535	CAGGUGA
ENSG00000197535	CCAGUGC
ENSG00000197535	CCAGUGU
ENSG00000197535	GAGAAAU
ENSG00000197535	GGGUCUU
ENSG00000197535	GUCUGCC
ENSG00000197535	UGCAUAG
ENSG00000197535	UGUCUGC
ENSG00000197535	UUAGAGA
ENSG00000197535	UUGGUUC

ENSG00000197557	CUUCACC
ENSG00000197557	CUUGUGU
ENSG00000197557	GUUAAUG
ENSG00000197557	UCAUUUG
ENSG00000197557	UUCAAGC
ENSG00000197557	UUCUAAU
ENSG00000197562	AAAAAUC
ENSG00000197562	ACUGUAG
ENSG00000197562	AGCACCA
ENSG00000197562	GAGGUAG
ENSG00000197562	GUAGUGU
ENSG00000197562	UACAAAG
ENSG00000197565	ACCUGGA
ENSG00000197565	CCUUCUU
ENSG00000197565	GAGGUAU
ENSG00000197565	GUCUCUG
ENSG00000197576	AGCUGCC
ENSG00000197576	UACAGUU
ENSG00000197584	GAGCCCC
ENSG00000197584	GAUAUGU
ENSG00000197584	GGCUCAG
ENSG00000197584	UACAGUU
ENSG00000197584	UGCAUAG
ENSG00000197584	UGGCUGC
ENSG00000197584	UUGGCAA
ENSG00000197587	GCCUGGG
ENSG00000197587	GGAGGAG
ENSG00000197587	GGGGUCC
ENSG00000197587	UGAAAGG
ENSG00000197594	CAAAGUG
ENSG00000197594	CCUUCUG
ENSG00000197594	CCUUCUU
ENSG00000197594	CUUUGGU
ENSG00000197594	UCCCUGU
ENSG00000197594	UGUCUUU
ENSG00000197601	AAAGAAU
ENSG00000197601	AGAUGUG
ENSG00000197601	UACAAAG
ENSG00000197601	UCCAGAG
ENSG00000197601	UUGGUUC
ENSG00000197614	GACAACU
ENSG00000197614	UGGACUG
ENSG00000197622	AAAAGUA
ENSG00000197622	AGGAGCU
ENSG00000197622	AGGGAGG
ENSG00000197622	CAGCAAA
ENSG00000197622	CAGUCCA
ENSG00000197622	CCAGGAG
ENSG00000197622	CCCUGAG
ENSG00000197622	CCUGCUG
ENSG00000197622	CUCCAGC
ENSG00000197622	CUCUUCC
ENSG00000197622	GAGGUAG
ENSG00000197622	GGAGUCC
ENSG00000197622	UGAGUCU
ENSG00000197629	UCCCACA
ENSG00000197635	AGCACCA
ENSG00000197646	AGUGCUU
ENSG00000197646	CUGGAAA
ENSG00000197651	ACUAGAU
ENSG00000197651	AGAUGUG
ENSG00000197651	CAGUUAC
ENSG00000197651	CUCCAUU
ENSG00000197651	GACCAUG

ENSG00000197651	GCAACUU
ENSG00000197651	GUGCAAA
ENSG00000197651	UCAUACA
ENSG00000197694	AGCACCA
ENSG00000197694	GCACCAU
ENSG00000197697	AAAGAGC
ENSG00000197702	AUGUGCC
ENSG00000197702	AUGUGGG
ENSG00000197702	CCCUGUU
ENSG00000197702	CUCCAUU
ENSG00000197702	CUGUUGA
ENSG00000197702	GAGCCCU
ENSG00000197702	GCAGGAC
ENSG00000197702	GGACCUG
ENSG00000197702	GUGCCUG
ENSG00000197702	UACAAAG
ENSG00000197702	UCCCUGU
ENSG00000197702	UUUGCAA
ENSG00000197702	UUUGCAC
ENSG00000197705	AUAAAGU
ENSG00000197705	AUCCUUU
ENSG00000197705	CAGUCUG
ENSG00000197705	GCAGGUU
ENSG00000197705	GUGACAG
ENSG00000197705	UCAUUUG
ENSG00000197705	UUCUAAU
ENSG00000197712	GAGAAAU
ENSG00000197713	AAAGUUU
ENSG00000197713	AAGUUGU
ENSG00000197713	AUAUUAU
ENSG00000197713	AUGUAAU
ENSG00000197713	GUCCAGA
ENSG00000197724	AAGGUGC
ENSG00000197724	ACAAGGU
ENSG00000197724	ACAUUCA
ENSG00000197724	CUACAAA
ENSG00000197724	GAGAAAU
ENSG00000197724	GCCUGAU
ENSG00000197724	GCUACAU
ENSG00000197724	UCACAAG
ENSG00000197746	ACAUUCA
ENSG00000197746	AGGAGCU
ENSG00000197757	AGGGAGG
ENSG00000197757	CACAGUG
ENSG00000197757	CCCCCAG
ENSG00000197757	GGAGGAG
ENSG00000197757	GGGGCAG
ENSG00000197757	GUGCCAC
ENSG00000197757	UAAUAUC
ENSG00000197757	UCACAGU
ENSG00000197757	UGUGCGG
ENSG00000197771	ACCCUGU
ENSG00000197771	AGCACCA
ENSG00000197771	CCCUGUU
ENSG00000197771	CUAGUCC
ENSG00000197771	GGUCUAG
ENSG00000197771	UUUCAAC
ENSG00000197776	AUAUAAC
ENSG00000197779	AUGCAUU
ENSG00000197779	CACAAUG
ENSG00000197779	GAAGGUC
ENSG00000197798	AAAAACC
ENSG00000197798	AAAACUG
ENSG00000197798	ACUGUAG

ENSG00000197798	CAAAACU
ENSG00000197798	CCUUCAU
ENSG00000197798	UCAAGUA
ENSG00000197808	UCAUUUG
ENSG00000197818	AAAGGUA
ENSG00000197818	AACAUUC
ENSG00000197818	ACAUUCA
ENSG00000197818	UCCCUCU
ENSG00000197846	AAAGAGC
ENSG00000197846	GGCUCUG
ENSG00000197859	AAAGUGC
ENSG00000197860	AAGACAU
ENSG00000197860	AAGUGCA
ENSG00000197860	ACAUUCA
ENSG00000197860	AGUGCAA
ENSG00000197860	AUAAAGU
ENSG00000197860	CCAGUAC
ENSG00000197860	UGGUACC
ENSG00000197860	UUCUAAU
ENSG00000197879	AAGGUGC
ENSG00000197879	ACAAGGU
ENSG00000197879	AGCAGCA
ENSG00000197879	AUAAAGU
ENSG00000197879	AUGGCUU
ENSG00000197879	CAGACAG
ENSG00000197879	CAGCAGA
ENSG00000197879	CAGCAGG
ENSG00000197879	CCCACCU
ENSG00000197879	CCCUGUU
ENSG00000197879	CGGCCUG
ENSG00000197879	CUCUGGG
ENSG00000197879	GAGUCUU
ENSG00000197879	GGCAGUG
ENSG00000197879	GUGGCCC
ENSG00000197879	UAUAAAG
ENSG00000197879	UAUUGCU
ENSG00000197879	UCCCUGU
ENSG00000197879	UCUGGAA
ENSG00000197879	UGUCUGG
ENSG00000197879	UUGGCAA
ENSG00000197879	UUGGCAC
ENSG00000197892	CACCAGC
ENSG00000197892	CUUUGGU
ENSG00000197892	GGAUUUC
ENSG00000197892	GUGCAAA
ENSG00000197894	GAAAACA
ENSG00000197921	AUCCUUU
ENSG00000197921	CUACAAA
ENSG00000197921	GACUUCC
ENSG00000197921	GAGGAUA
ENSG00000197921	UCACAAG
ENSG00000197921	UCAUAGA
ENSG00000197930	GAGGUAG
ENSG00000197930	UACAAAG
ENSG00000197930	UUGAGGC
ENSG00000197930	UUGGCAC
ENSG00000197933	UCCCACA
ENSG00000197948	AGCACCA
ENSG00000197948	CAGCUGG
ENSG00000197948	CCAUUAC
ENSG00000197948	CUGGCUC
ENSG00000197948	GACCCUG
ENSG00000197948	GCAGCAU
ENSG00000197948	GGGAGCU

ENSG00000197948	GGGCCCC
ENSG00000197948	UGAGUCU
ENSG00000197951	AACUCUA
ENSG00000197959	ACAAUAU
ENSG00000197959	AGCACCA
ENSG00000197959	AGCUGCC
ENSG00000197959	CAAAUGC
ENSG00000197959	CACAAUG
ENSG00000197959	GCUGUCU
ENSG00000197959	GGUUACC
ENSG00000197959	UCACAAG
ENSG00000197959	UCCAGUU
ENSG00000197959	UCUCACC
ENSG00000197959	UGACAUC
ENSG00000197959	UGAGAAG
ENSG00000197959	UGAGAAU
ENSG00000197959	UGCAUAG
ENSG00000197959	UUGACAC
ENSG00000197965	AAAGUAC
ENSG00000197965	AAGGCAC
ENSG00000197965	UCACAAG
ENSG00000197965	UCCAGUU
ENSG00000197965	UCCCUCU
ENSG00000197965	UCCUUGC
ENSG00000197971	AUGCCUU
ENSG00000197971	CAAAACU
ENSG00000197971	CACAAUG
ENSG00000197971	GGGUGUU
ENSG00000197977	AUACAAG
ENSG00000197977	GGUUUAC
ENSG00000197982	ACCUGGA
ENSG00000197982	CUGCAGC
ENSG00000197982	UGAAAUG
ENSG00000197991	AUGCCUU
ENSG00000198000	ACAUUCA
ENSG00000198010	AAAGUGC
ENSG00000198010	AACAAAC
ENSG00000198010	AACUACU
ENSG00000198010	AAGAACC
ENSG00000198010	AAUGCCC
ENSG00000198010	AAUUGCU
ENSG00000198010	ACCCUGU
ENSG00000198010	ACUCAGG
ENSG00000198010	ACUGCAG
ENSG00000198010	ACUGCAU
ENSG00000198010	AGCAGCA
ENSG00000198010	AGCAGCG
ENSG00000198010	AGCAGCU
ENSG00000198010	AGCCCUU
ENSG00000198010	AGCUGCC
ENSG00000198010	AGGACAC
ENSG00000198010	AGGUAGU
ENSG00000198010	AUACAAG
ENSG00000198010	AUGCACC
ENSG00000198010	AUGGCUU
ENSG00000198010	CAAAGUG
ENSG00000198010	CAGCAAA
ENSG00000198010	CAGCAGG
ENSG00000198010	CUCAAAC
ENSG00000198010	CUCUGGG
ENSG00000198010	CUGGGGG
ENSG00000198010	CUUGUGU
ENSG00000198010	GAAAACA
ENSG00000198010	GAGAACC

ENSG00000198010	GAGCCCC
ENSG00000198010	GAGCCCU
ENSG00000198010	GAGGCUG
ENSG00000198010	GCUUCCU
ENSG00000198010	UAGGCCG
ENSG00000198010	UAUAAAG
ENSG00000198010	UCACAUU
ENSG00000198010	UCAUUUG
ENSG00000198010	UCCUUGC
ENSG00000198010	UCUCAAG
ENSG00000198010	UGCAUUG
ENSG00000198010	UUGGCAA
ENSG00000198018	GAGAAGA
ENSG00000198018	GUCUCUG
ENSG00000198026	AGCCUGC
ENSG00000198026	CUGGCUC
ENSG00000198046	CUUUGGU
ENSG00000198053	AAAGUGC
ENSG00000198053	AAAGUUU
ENSG00000198053	AAGUGCU
ENSG00000198053	AAGUUGU
ENSG00000198053	ACCACAG
ENSG00000198053	AGUGCUU
ENSG00000198053	AUACAAG
ENSG00000198053	AUUGCAC
ENSG00000198053	CUCUUCC
ENSG00000198053	GCAACUU
ENSG00000198053	GGAGAGA
ENSG00000198053	UACAAAG
ENSG00000198053	UCAACAG
ENSG00000198053	UCACAUU
ENSG00000198053	UCCAGUU
ENSG00000198053	UUCAAGC
ENSG00000198053	UUCUAAU
ENSG00000198053	UUUGCAC
ENSG00000198055	AAAAAUC
ENSG00000198055	AAAACUG
ENSG00000198055	AAACUGU
ENSG00000198055	AACACUG
ENSG00000198055	ACUCAGC
ENSG00000198055	CAAAACU
ENSG00000198055	CACAGUG
ENSG00000198055	GGAAUGU
ENSG00000198055	GUAAACA
ENSG00000198055	UAUUGCU
ENSG00000198055	UCACAGU
ENSG00000198055	UUGGCAA
ENSG00000198055	UUGGCAC
ENSG00000198060	AAGGUGC
ENSG00000198060	AGCAGCA
ENSG00000198060	AGCCAGU
ENSG00000198060	AUGCAUU
ENSG00000198060	CACAGUG
ENSG00000198060	CUUCACC
ENSG00000198060	UCACAAG
ENSG00000198060	UCACAGG
ENSG00000198081	AAAGUGC
ENSG00000198081	AAGUGCA
ENSG00000198081	CAAAGCA
ENSG00000198081	CAGCAAA
ENSG00000198081	UGAAAUG
ENSG00000198087	AGCAGCG
ENSG00000198087	AGCAGCU
ENSG00000198087	AGUGCAA

ENSG00000198087	AUUGCAC
ENSG00000198087	CACAGUG
ENSG00000198087	CAGCAAA
ENSG00000198087	CAGUAGA
ENSG00000198087	GAUUGUA
ENSG00000198087	GCAACUU
ENSG00000198087	GCUACAU
ENSG00000198087	GUCAUUG
ENSG00000198087	UCACAGU
ENSG00000198087	UCCUAUA
ENSG00000198088	ACUGCAG
ENSG00000198089	AAUUGCU
ENSG00000198089	AUUCAUU
ENSG00000198089	UCAUUUG
ENSG00000198089	UGAAAUG
ENSG00000198105	AGGUAGU
ENSG00000198105	CUUUGGU
ENSG00000198105	GGCAGUG
ENSG00000198108	CCAGUAC
ENSG00000198108	GGAAGAC
ENSG00000198108	UCACAUU
ENSG00000198108	UGGUACC
ENSG00000198121	AAGUCUU
ENSG00000198121	UGCCCUG
ENSG00000198121	UUCUAAU
ENSG00000198131	CACAAUG
ENSG00000198131	CUCCAGC
ENSG00000198142	AAAGUGC
ENSG00000198142	AGCACCA
ENSG00000198142	AGCAGCA
ENSG00000198142	AGUGCUU
ENSG00000198142	AUAAAGU
ENSG00000198142	CAGUAGA
ENSG00000198142	GCACCAU
ENSG00000198142	GCAGCAU
ENSG00000198142	UAAUAUC
ENSG00000198142	UUCAAGC
ENSG00000198142	UUCCAUA
ENSG00000198142	UUCUGCA
ENSG00000198142	UUUCAAC
ENSG00000198146	ACAGGUG
ENSG00000198146	AGUGGUU
ENSG00000198146	AUCACUA
ENSG00000198146	CCCUGUU
ENSG00000198146	GUAAACA
ENSG00000198146	UAUUGCU
ENSG00000198146	UCAUACA
ENSG00000198146	UUGACAC
ENSG00000198160	AAAGUAA
ENSG00000198160	AAAUUUC
ENSG00000198160	AAUACUG
ENSG00000198160	ACUGUUU
ENSG00000198160	AGUGCUU
ENSG00000198160	AUGCAUU
ENSG00000198160	CACUGUU
ENSG00000198160	GCCUGAU
ENSG00000198160	GGCUGCG
ENSG00000198160	UACAGUU
ENSG00000198160	UCUGGAA
ENSG00000198160	UCUGGAG
ENSG00000198160	UGGAUAA
ENSG00000198160	UGGCUGC
ENSG00000198160	UGUCUUU
ENSG00000198160	UGUUGAA

ENSG00000198160	UUCAAAU
ENSG00000198162	ACAGGUG
ENSG00000198162	CAAAGUG
ENSG00000198162	CAGGUGA
ENSG00000198162	CAGUCUG
ENSG00000198162	GUCUCUG
ENSG00000198162	UGAAAUG
ENSG00000198162	UUCAAGC
ENSG00000198162	UUGGUCC
ENSG00000198168	AAGGCAC
ENSG00000198168	AGCCCUU
ENSG00000198168	GGCAGUG
ENSG00000198176	AACAUUC
ENSG00000198176	ACCAGCA
ENSG00000198176	AUAUAAC
ENSG00000198176	AUGUAAU
ENSG00000198176	CACCAGC
ENSG00000198176	UCCUGAG
ENSG00000198182	CUCUGGG
ENSG00000198182	GAGUUGG
ENSG00000198182	GCUUCCU
ENSG00000198182	GGAGGAG
ENSG00000198182	GGAUGAG
ENSG00000198182	UCUCCAA
ENSG00000198183	CAGCAGA
ENSG00000198198	UGAGAAG
ENSG00000198211	AAUACUG
ENSG00000198216	AGCAGCA
ENSG00000198216	AGCCCUU
ENSG00000198216	AGGGAGG
ENSG00000198216	AUGGCAC
ENSG00000198216	AUGGCUU
ENSG00000198216	CAGCAAA
ENSG00000198216	CUCUUCC
ENSG00000198216	CUUUGGU
ENSG00000198216	GAGAAGA
ENSG00000198216	GAGGCAG
ENSG00000198216	GAGGUAG
ENSG00000198216	GAGGUAU
ENSG00000198216	GGGGAGC
ENSG00000198216	GGGGGAA
ENSG00000198216	UGCAAAG
ENSG00000198216	UGCCCUG
ENSG00000198216	UUGGUUC
ENSG00000198218	UGUAUGU
ENSG00000198221	UUUGCAA
ENSG00000198231	AAAGAAU
ENSG00000198231	AGCAAAA
ENSG00000198231	CCCUGAG
ENSG00000198231	UCACAAG
ENSG00000198242	CAGCUGG
ENSG00000198242	CUGGACU
ENSG00000198252	AAAACUG
ENSG00000198252	AAAGUAC
ENSG00000198252	AAAGUAU
ENSG00000198252	AAGGCAC
ENSG00000198252	AUACCUC
ENSG00000198252	CAAAGCA
ENSG00000198252	GGAAGCC
ENSG00000198252	UAUAAAG
ENSG00000198252	UAUUGCU
ENSG00000198252	UGCAUUG
ENSG00000198252	UUCAAGC
ENSG00000198252	UUUGCAA

ENSG00000198265	AAAUGGU
ENSG00000198265	ACAGUAU
ENSG00000198265	ACAUCAC
ENSG00000198265	ACUGGAU
ENSG00000198265	AGGCAGG
ENSG00000198265	AUCAUAC
ENSG00000198265	CAGCAAA
ENSG00000198265	CCAGCAU
ENSG00000198265	CCAGUAC
ENSG00000198265	CUGUUGA
ENSG00000198265	GAGAAAU
ENSG00000198265	GGCAAGA
ENSG00000198265	GUAACAG
ENSG00000198265	GUCUACU
ENSG00000198265	UGCAUAG
ENSG00000198265	UGGAGAU
ENSG00000198265	UGUUGAA
ENSG00000198265	UUUGCAA
ENSG00000198286	UAAUGCU
ENSG00000198301	CUUGUAU
ENSG00000198301	GUAACAG
ENSG00000198315	AAAAAUC
ENSG00000198315	AAGUCAC
ENSG00000198315	AGUGCCU
ENSG00000198315	AGUGCUG
ENSG00000198315	CAAGAGC
ENSG00000198315	CACCAGC
ENSG00000198315	GAGUCUU
ENSG00000198315	GGAGUCC
ENSG00000198315	GGGGUGG
ENSG00000198315	GGGUGGU
ENSG00000198315	GUGCCUG
ENSG00000198315	UCAAGUA
ENSG00000198315	UCUCACC
ENSG00000198315	UGAGUCU
ENSG00000198324	GUAAACA
ENSG00000198324	UGAGGAC
ENSG00000198327	GGCCCUG
ENSG00000198353	AAGAACC
ENSG00000198353	AUAUUAU
ENSG00000198353	GACUUCC
ENSG00000198353	GGUUUAC
ENSG00000198355	AAGGCAC
ENSG00000198355	ACUCAAA
ENSG00000198355	AGCAGCA
ENSG00000198355	UGAAAUG
ENSG00000198355	UGCAUUG
ENSG00000198356	CUGCCCC
ENSG00000198356	GGGGGAA
ENSG00000198356	GUGCAAA
ENSG00000198363	AAAAGUA
ENSG00000198363	AGCAGCA
ENSG00000198363	GGAAUGU
ENSG00000198363	GGGGCAG
ENSG00000198363	UAUAAAG
ENSG00000198363	UAUGGUU
ENSG00000198363	UCACAGG
ENSG00000198363	UCACAGU
ENSG00000198363	UGAAGCU
ENSG00000198366	AAAGAGC
ENSG00000198369	AAAGCUG
ENSG00000198369	AACACUG
ENSG00000198369	AAGAACC
ENSG00000198369	AAUGUUG

ENSG00000198369	ACACACC
ENSG00000198369	ACACACU
ENSG00000198369	ACAGGUG
ENSG00000198369	ACAUCAC
ENSG00000198369	AUCAUAC
ENSG00000198369	CACUGUU
ENSG00000198369	CUGCAGC
ENSG00000198369	CUUGUAU
ENSG00000198369	GAGGCUG
ENSG00000198369	GAUUGUA
ENSG00000198369	GAUUGUC
ENSG00000198369	GCUACAU
ENSG00000198369	GUGCAAA
ENSG00000198369	GUGCUUG
ENSG00000198369	UAUAAAG
ENSG00000198369	UGCAGCU
ENSG00000198369	UGUAUAA
ENSG00000198369	UUGGUUC
ENSG00000198373	CUGGGGG
ENSG00000198373	GAUUGUA
ENSG00000198373	GAUUGUC
ENSG00000198373	GGCAGGG
ENSG00000198373	UACAAAG
ENSG00000198373	UAUUGCU
ENSG00000198374	AAAGAGC
ENSG00000198380	AUAUAAC
ENSG00000198380	UCACAAG
ENSG00000198382	AAAGAGC
ENSG00000198382	AAAGAGG
ENSG00000198382	AAAUGGU
ENSG00000198382	AAGUGCU
ENSG00000198382	AAUCUCU
ENSG00000198382	AUUGCAC
ENSG00000198382	CAUGGAU
ENSG00000198382	CCCUGAG
ENSG00000198382	CUCUGGG
ENSG00000198382	CUGGAAA
ENSG00000198382	GCACCAU
ENSG00000198382	UCACAUU
ENSG00000198382	UCUGGAA
ENSG00000198382	UGCAGCU
ENSG00000198382	UGGAUAA
ENSG00000198382	UGGGUAC
ENSG00000198393	UCUGGAA
ENSG00000198399	AAGGCAC
ENSG00000198399	AAUACUG
ENSG00000198399	ACAGUAU
ENSG00000198399	CACAGUG
ENSG00000198399	UCCUGAG
ENSG00000198399	UGCAUAU
ENSG00000198408	AAAGUGC
ENSG00000198408	CUACAAA
ENSG00000198408	GGAGGCA
ENSG00000198408	UGAAGCU
ENSG00000198420	CUUUGGU
ENSG00000198420	UCUUGAG
ENSG00000198420	UUCUUGU
ENSG00000198431	CCCUGAG
ENSG00000198431	CCUUCAU
ENSG00000198431	GACUUCC
ENSG00000198431	UUCUUGU
ENSG00000198435	AAAGGUA
ENSG00000198435	AAGUGUG
ENSG00000198435	AGUGCAA

ENSG00000198435	AUGACAC
ENSG00000198435	GAGGUAG
ENSG00000198435	GUGCAAA
ENSG00000198435	GUUAAUG
ENSG00000198435	UAAUAUC
ENSG00000198440	GAGGUAU
ENSG00000198455	AACUCUA
ENSG00000198455	GGCUCAG
ENSG00000198477	ACAUUCA
ENSG00000198477	AGUGCCU
ENSG00000198477	CAGGAAC
ENSG00000198477	UUGGCAC
ENSG00000198478	AGCAGCU
ENSG00000198478	AUGCAUU
ENSG00000198478	CCUGCUG
ENSG00000198478	CUGCUCA
ENSG00000198478	CUUUGGU
ENSG00000198478	GACCUGG
ENSG00000198478	GAGGCAG
ENSG00000198478	GGGAGCU
ENSG00000198478	GGGGAGC
ENSG00000198478	GUAACAG
ENSG00000198478	UAAGACU
ENSG00000198478	UAAUAUC
ENSG00000198478	UCACAGU
ENSG00000198478	UCCAGUU
ENSG00000198478	UGAAAUG
ENSG00000198478	UGGUACC
ENSG00000198478	UUGGCAA
ENSG00000198492	AACAUUC
ENSG00000198492	ACAUUCA
ENSG00000198492	GUGCAAA
ENSG00000198492	UCAACAG
ENSG00000198492	UCUCCAA
ENSG00000198498	AAUUAUU
ENSG00000198517	AAAGGUA
ENSG00000198517	AACAAAC
ENSG00000198517	ACAGUAC
ENSG00000198517	ACAGUAU
ENSG00000198517	AGAUAAA
ENSG00000198517	AUGGAUU
ENSG00000198517	AUUGCAC
ENSG00000198517	GAAAACA
ENSG00000198517	UACAGUU
ENSG00000198517	UAUUGCU
ENSG00000198517	UGAAAUG
ENSG00000198518	GGCCCUG
ENSG00000198522	AGCAGCA
ENSG00000198522	CAGCAGA
ENSG00000198522	CAGCAGG
ENSG00000198554	AAAUUUC
ENSG00000198558	GGCCCUG
ENSG00000198561	AAAAACC
ENSG00000198561	AAGGCAC
ENSG00000198561	ACACACC
ENSG00000198561	AGCACCA
ENSG00000198561	AUAUAAC
ENSG00000198561	AUGUGCC
ENSG00000198561	CAGCAGA
ENSG00000198561	CCCACCG
ENSG00000198561	CCUUCUU
ENSG00000198561	CUGCAGG
ENSG00000198561	GAGAAAU
ENSG00000198561	GAGAUGA

ENSG00000198561	GAGGCAG
ENSG00000198561	GCAGCAU
ENSG00000198561	GGAGGCA
ENSG00000198561	GGAUUUU
ENSG00000198561	GGCAGUG
ENSG00000198561	GUAGUGU
ENSG00000198561	UAUCAGA
ENSG00000198561	UAUUACC
ENSG00000198561	UCCAGUU
ENSG00000198561	UCUCAAG
ENSG00000198561	UUGGCAC
ENSG00000198561	UUGGUUC
ENSG00000198563	CAGUAGU
ENSG00000198563	CCUUCAU
ENSG00000198563	CUUCACC
ENSG00000198563	GGUGGUC
ENSG00000198563	GGUGGUU
ENSG00000198563	GUAGUGU
ENSG00000198570	GGACCUG
ENSG00000198570	GUGGGGA
ENSG00000198574	GCUGGUG
ENSG00000198576	AGGGAGG
ENSG00000198576	GACCCUG
ENSG00000198576	UGCCAGC
ENSG00000198586	AGCAGCG
ENSG00000198586	AUGGCUU
ENSG00000198586	CUUUGGU
ENSG00000198586	GAUUGUA
ENSG00000198586	GAUUGUC
ENSG00000198586	GCAGCAU
ENSG00000198586	UAUUACC
ENSG00000198586	UGCAUAG
ENSG00000198586	UUGGUUC
ENSG00000198589	AAAAAUC
ENSG00000198589	AAAAGUA
ENSG00000198589	UACAAAG
ENSG00000198597	AUGCCUU
ENSG00000198597	CAAAGCA
ENSG00000198597	CCUUCAU
ENSG00000198597	UUCUAAU
ENSG00000198604	AGUACUG
ENSG00000198604	UCCUGAG
ENSG00000198612	CAAAUGC
ENSG00000198624	CCUUCUU
ENSG00000198624	CUGCAGC
ENSG00000198624	UCCUGAG
ENSG00000198626	UACAAAG
ENSG00000198626	UAUUGCU
ENSG00000198626	UUCUGCA
ENSG00000198626	UUGAUCA
ENSG00000198642	AGCACCA
ENSG00000198642	AGGGAGG
ENSG00000198642	GAGGCAG
ENSG00000198642	GCCCUUA
ENSG00000198642	UUGGUCC
ENSG00000198642	UUGUUCG
ENSG00000198643	AGCAAAA
ENSG00000198643	CAGCAAA
ENSG00000198648	AAGGCAC
ENSG00000198648	AGCUGCC
ENSG00000198648	AUAUUAU
ENSG00000198648	AUUGCAC
ENSG00000198648	CACAGUG
ENSG00000198648	GAGCUGC

ENSG00000198648	GGAAUGU
ENSG00000198648	GUGCAAA
ENSG00000198648	UAUAAAG
ENSG00000198648	UCACAGG
ENSG00000198650	CCCUGAG
ENSG00000198650	GAGUGUG
ENSG00000198650	GUGAGGU
ENSG00000198650	UUAUGCA
ENSG00000198663	ACAUUAC
ENSG00000198663	AGGGAGG
ENSG00000198663	AGUGCUG
ENSG00000198663	CUGGACU
ENSG00000198663	GAGGCUG
ENSG00000198663	UCUGGAA
ENSG00000198663	UGCUGAC
ENSG00000198663	UUAUGCA
ENSG00000198663	UUCAAAU
ENSG00000198663	UUCUGCA
ENSG00000198663	UUGACAC
ENSG00000198668	AAGUGUG
ENSG00000198668	AAUGUUG
ENSG00000198668	AAUUUUA
ENSG00000198668	ACUUGGA
ENSG00000198668	AGCAAAA
ENSG00000198668	AGCAUUC
ENSG00000198668	AUGACAC
ENSG00000198668	CAAAGCA
ENSG00000198668	CCUUCAU
ENSG00000198668	GAGAUGA
ENSG00000198668	GAGGUAG
ENSG00000198668	GAGGUAU
ENSG00000198668	GGAAUGU
ENSG00000198668	GUUAAUG
ENSG00000198668	UCCCUUG
ENSG00000198668	UCCUGAG
ENSG00000198668	UGAAAGG
ENSG00000198668	UGACAUC
ENSG00000198668	UGACUGU
ENSG00000198668	UGUGCUU
ENSG00000198668	UUGACAC
ENSG00000198668	UUGGUCC
ENSG00000198668	UUUGCAC
ENSG00000198673	UAAUACA
ENSG00000198680	AAUGUUG
ENSG00000198680	ACACACU
ENSG00000198680	AUAUUAU
ENSG00000198682	ACCACAG
ENSG00000198682	CAGCAAA
ENSG00000198682	CUCAAAC
ENSG00000198682	UCAUUUG
ENSG00000198689	AAAGAGC
ENSG00000198689	AAGGCAC
ENSG00000198689	ACAUUCA
ENSG00000198689	CACUGUU
ENSG00000198689	CUCUUCC
ENSG00000198689	CUUCACC
ENSG00000198689	UGCAUAG
ENSG00000198691	CAGGUGA
ENSG00000198700	AGCAGCA
ENSG00000198700	AGCAGCG
ENSG00000198700	AUUCAUU
ENSG00000198700	CAGCAGA
ENSG00000198700	CAGCAGG
ENSG00000198700	CUGGACU

ENSG00000198700	CUGGCUC
ENSG00000198700	GAAUUGU
ENSG00000198700	GAGGUAG
ENSG00000198700	GCAGCAU
ENSG00000198700	GUCACUC
ENSG00000198700	GUGUCAC
ENSG00000198700	UAUCAGA
ENSG00000198700	UCCUAUA
ENSG00000198700	UCUCAAG
ENSG00000198700	UGUCUGG
ENSG00000198700	UUGGCAA
ENSG00000198700	UUGGCAC
ENSG00000198700	UUGUUCG
ENSG00000198704	CAUGGAU
ENSG00000198704	CUGCUCA
ENSG00000198704	GACCUGG
ENSG00000198704	UGGAGAU
ENSG00000198719	AACAAAC
ENSG00000198719	ACAAUAU
ENSG00000198719	ACAUAGA
ENSG00000198719	AGCAUUC
ENSG00000198719	AGUGCAA
ENSG00000198719	AGUGCCU
ENSG00000198719	AUACAAG
ENSG00000198719	GAGAACC
ENSG00000198719	GCAGCAU
ENSG00000198719	UAUAAAG
ENSG00000198720	AAGUGCA
ENSG00000198720	AAGUGCU
ENSG00000198720	AGCAGCA
ENSG00000198720	AGCAGCG
ENSG00000198720	AGCAGCU
ENSG00000198720	CAGCUGG
ENSG00000198720	CCCUGAG
ENSG00000198720	GCACCAU
ENSG00000198720	GCUAGCA
ENSG00000198720	GCUGGUG
ENSG00000198720	GGGGAGC
ENSG00000198720	GUGCUUG
ENSG00000198720	UGAAAUG
ENSG00000198720	UGUGCUU
ENSG00000198720	UGUGUCA
ENSG00000198722	AGCACCA
ENSG00000198722	CAGCAAA
ENSG00000198722	UAUUACC
ENSG00000198728	GGGGCAG
ENSG00000198729	AGCCCUU
ENSG00000198729	CAAAGCA
ENSG00000198729	CUUUGGU
ENSG00000198729	GACCUGG
ENSG00000198729	GCCUGAU
ENSG00000198729	UGCCAGC
ENSG00000198732	AAAAGUA
ENSG00000198732	AAGUGCA
ENSG00000198732	AAGUUGU
ENSG00000198732	AAUCUCU
ENSG00000198732	AGUGCAA
ENSG00000198732	CAGUAGA
ENSG00000198732	CAGUAGU
ENSG00000198732	CCUGUAC
ENSG00000198732	CUACAAA
ENSG00000198732	CUAUACA
ENSG00000198732	CUUCUCU
ENSG00000198732	GUCAGUU

ENSG00000198732	GUGCAAA
ENSG00000198732	UCCCUUG
ENSG00000198736	CAGCAGG
ENSG00000198739	AAAGAAU
ENSG00000198739	AACAAAC
ENSG00000198739	AAUACUG
ENSG00000198739	ACAGGUG
ENSG00000198739	ACAUUAC
ENSG00000198739	ACUGCAG
ENSG00000198739	ACUGUAG
ENSG00000198739	AGGUAGU
ENSG00000198739	AUCCCUU
ENSG00000198739	AUUCAUU
ENSG00000198739	CCCACCU
ENSG00000198739	CUAGGCU
ENSG00000198739	CUGUUGA
ENSG00000198739	GGUUACC
ENSG00000198739	UACAGUU
ENSG00000198739	UCCCUCU
ENSG00000198739	UCGUGCA
ENSG00000198739	UCUGGAG
ENSG00000198740	AAAGAGC
ENSG00000198740	AAAGAGG
ENSG00000198740	AAAGCUG
ENSG00000198740	AAAUGGU
ENSG00000198740	AAUCUCU
ENSG00000198740	AGCCCUU
ENSG00000198740	AUACCUC
ENSG00000198740	AUGGCUU
ENSG00000198740	CACAGUG
ENSG00000198740	CAGCUGG
ENSG00000198740	CUCACAC
ENSG00000198740	GGAAUGU
ENSG00000198740	GUUAGGA
ENSG00000198740	UACAAAG
ENSG00000198740	UAUAUGA
ENSG00000198740	UCAUUUG
ENSG00000198740	UGAGGAC
ENSG00000198740	UUGAGGC
ENSG00000198740	UUGUGAC
ENSG00000198742	AAAAACC
ENSG00000198742	AAAGUGC
ENSG00000198742	AACAAAC
ENSG00000198742	AAUACUG
ENSG00000198742	AAUCACU
ENSG00000198742	ACUGUUU
ENSG00000198742	AGCAGCG
ENSG00000198742	AGCCUGC
ENSG00000198742	AGUGGUU
ENSG00000198742	AUGAGCU
ENSG00000198742	AUGUAAU
ENSG00000198742	AUUGCAC
ENSG00000198742	CAGCUGG
ENSG00000198742	CCCUGAG
ENSG00000198742	CUUUGGU
ENSG00000198742	GCAGAAG
ENSG00000198742	GCUUCCU
ENSG00000198742	GGCUGCG
ENSG00000198742	GGGAGCU
ENSG00000198742	GGUUGAC
ENSG00000198742	GUAACAG
ENSG00000198742	GUGCAAA
ENSG00000198742	UACAAAG
ENSG00000198742	UAUUGCU

ENSG00000198742	UCACAAG
ENSG00000198742	UGACAUC
ENSG00000198742	UGCAUAG
ENSG00000198742	UGGCUGC
ENSG00000198742	UUCCAUA
ENSG00000198742	UUGACAC
ENSG00000198742	UUGGUUC
ENSG00000198743	AAAGUGC
ENSG00000198743	AAGGCAC
ENSG00000198743	ACACACC
ENSG00000198743	ACACACU
ENSG00000198743	ACAGUAC
ENSG00000198743	ACUCAAA
ENSG00000198743	AGCAGCA
ENSG00000198743	AGCAGCU
ENSG00000198743	AGGACAC
ENSG00000198743	CACAGGG
ENSG00000198743	CAGCUGG
ENSG00000198743	CAGUGCA
ENSG00000198743	CCUUCAU
ENSG00000198743	CUCACAC
ENSG00000198743	GCAGCAU
ENSG00000198743	GUGCUUG
ENSG00000198743	UAAUACA
ENSG00000198743	UAUGGUU
ENSG00000198743	UCUCACC
ENSG00000198743	UGACCUA
ENSG00000198743	UGCUGAC
ENSG00000198743	UGGUACC
ENSG00000198746	GAGGUAG
ENSG00000198753	CCAGCAU
ENSG00000198756	AUAUUAU
ENSG00000198756	CACACCU
ENSG00000198756	CAGUAGA
ENSG00000198756	UAUAAAG
ENSG00000198756	UCACAGG
ENSG00000198758	CUUGGAG
ENSG00000198768	AGCCCUU
ENSG00000198771	CAGGCUC
ENSG00000198771	CCAGCAU
ENSG00000198771	GAGAACU
ENSG00000198783	GGGUCUU
ENSG00000198785	AAAGAAU
ENSG00000198785	AAAGAGC
ENSG00000198785	AAGGCAC
ENSG00000198785	ACCCUGU
ENSG00000198785	ACUGUUU
ENSG00000198785	AGCAAAA
ENSG00000198785	CCUGUAC
ENSG00000198785	CUGCUCA
ENSG00000198785	GAGGUAG
ENSG00000198785	GAGGUAU
ENSG00000198785	UCAUAGA
ENSG00000198785	UGAGGAC
ENSG00000198785	UGCAUUG
ENSG00000198785	UGCUCAC
ENSG00000198785	UUCAAGC
ENSG00000198791	AAAAACC
ENSG00000198791	AAAGCUG
ENSG00000198791	AAGUGCA
ENSG00000198791	AGAUAAA
ENSG00000198791	AGUGCAA
ENSG00000198791	GAAAACA
ENSG00000198791	GUGCAAA

ENSG00000198791	UACAAAG
ENSG00000198792	AAAGAGC
ENSG00000198792	AAGGCAC
ENSG00000198792	ACAUCAC
ENSG00000198792	AUCAUAC
ENSG00000198792	AUUGCAC
ENSG00000198792	CAAAACU
ENSG00000198792	CUGGCUC
ENSG00000198792	GCAGCAU
ENSG00000198792	GCCUGGA
ENSG00000198792	GGAGGAG
ENSG00000198792	GGCUCUG
ENSG00000198792	GGUGGUC
ENSG00000198792	UACAAAG
ENSG00000198792	UGCCAGC
ENSG00000198792	UUCAAGC
ENSG00000198792	UUUCAAC
ENSG00000198794	AAAGUGC
ENSG00000198794	AAGUGCA
ENSG00000198794	ACUCAGC
ENSG00000198794	AGCACCA
ENSG00000198794	GAGACCU
ENSG00000198794	GAGCCCC
ENSG00000198794	GCAGCAU
ENSG00000198794	GCAUCCC
ENSG00000198794	GGAGGAG
ENSG00000198794	GGGGGAA
ENSG00000198794	GUGCUUG
ENSG00000198794	UGCAUAG
ENSG00000198794	UGGAGAU
ENSG00000198794	UGUGCUU
ENSG00000198795	ACUCAAA
ENSG00000198795	AGUAACA
ENSG00000198795	GAAAACA
ENSG00000198795	UAGGGCC
ENSG00000198795	UAUCAGA
ENSG00000198795	UGCAAAG
ENSG00000198797	AAAGAAU
ENSG00000198797	AAUCACU
ENSG00000198797	GAAAACA
ENSG00000198797	UUGGCAC
ENSG00000198799	AGCAGCA
ENSG00000198799	AGGUAGU
ENSG00000198799	GAGGUAU
ENSG00000198799	UAUAAAG
ENSG00000198807	AGUGGUU
ENSG00000198807	CCUUCAU
ENSG00000198812	CAGCUGG
ENSG00000198814	AACCGUU
ENSG00000198814	ACUUGGA
ENSG00000198814	GAGUUGG
ENSG00000198814	GCAGCAU
ENSG00000198815	AAGUGCU
ENSG00000198815	ACUGCAU
ENSG00000198815	ACUGUAG
ENSG00000198815	ACUUGGA
ENSG00000198815	AGUACUG
ENSG00000198815	CAAAUGC
ENSG00000198815	CAGCAAA
ENSG00000198815	CAGCUGG
ENSG00000198815	CUCCCAC
ENSG00000198815	CUGCAGG
ENSG00000198815	GAUUGUA
ENSG00000198815	GCAGAAG

ENSG00000198815	GCAGCAU
ENSG00000198815	GGCAGCG
ENSG00000198815	GUUAGGA
ENSG00000198815	UACAAAG
ENSG00000198815	UCAAGUA
ENSG00000198815	UCAUAGA
ENSG00000198815	UCCCUCU
ENSG00000198815	UCGUGCA
ENSG00000198815	UGAAAUG
ENSG00000198815	UGACUGU
ENSG00000198815	UGCAGCU
ENSG00000198815	UGCAUAU
ENSG00000198815	UGUCUUU
ENSG00000198821	CAGCAGG
ENSG00000198822	AUCACUA
ENSG00000198822	CUACAAA
ENSG00000198822	UUCUAAU
ENSG00000198824	UCUGGAA
ENSG00000198825	AACAAAC
ENSG00000198825	UGUAUAA
ENSG00000198826	AACAUUC
ENSG00000198826	GAGCUAA
ENSG00000198826	UCCUGAG
ENSG00000198826	UUGGCAA
ENSG00000198830	AAAACUG
ENSG00000198830	AAAGUUU
ENSG00000198830	ACACACU
ENSG00000198830	ACAGUAU
ENSG00000198830	AGGCAGG
ENSG00000198830	AUACAAG
ENSG00000198830	CACCAGC
ENSG00000198830	CCUUCUU
ENSG00000198830	GACCCUG
ENSG00000198830	GCAGGAC
ENSG00000198830	GCCUGAU
ENSG00000198830	GUCCAGA
ENSG00000198830	UAUAAAG
ENSG00000198830	UCACAAG
ENSG00000198836	AAUUUUA
ENSG00000198836	CACAGGG
ENSG00000198836	CACUGUU
ENSG00000198836	GGGUUUA
ENSG00000198836	UCACAGG
ENSG00000198836	UCAUUCG
ENSG00000198837	AAUCCUU
ENSG00000198837	AGCAGCU
ENSG00000198837	CAAAACU
ENSG00000198837	CUACAAA
ENSG00000198837	GGCUCAG
ENSG00000198837	UAUUGCU
ENSG00000198838	UAUUGCU
ENSG00000198838	UGCAUAU
ENSG00000198844	AGGUAGU
ENSG00000198844	CUCCAUU
ENSG00000198844	CUUCACC
ENSG00000198844	GAGGCUG
ENSG00000198844	GAGGUAG
ENSG00000198844	GGCAAGA
ENSG00000198844	GUCUGCC
ENSG00000198844	UGUCUGC
ENSG00000198846	AACAAAC
ENSG00000198846	AACAUUC
ENSG00000198846	AAGGCAC
ENSG00000198846	AAGUCUU

ENSG00000198846	AAGUGCA
ENSG00000198846	ACAGUAU
ENSG00000198846	ACAUAGA
ENSG00000198846	ACAUUCA
ENSG00000198846	ACCCAUU
ENSG00000198846	AGCCAGU
ENSG00000198846	CAGUUAC
ENSG00000198846	CUACAAA
ENSG00000198846	CUUGUAU
ENSG00000198846	GCAACUU
ENSG00000198846	GGCAGUG
ENSG00000198846	UCACAGG
ENSG00000198846	UCACAGU
ENSG00000198846	UUCCAUA
ENSG00000198846	UUGGCAA
ENSG00000198846	UUGGCAC
ENSG00000198853	CCAGCAU
ENSG00000198853	CCCUGAG
ENSG00000198853	GCCUGGG
ENSG00000198855	UCUAGAG
ENSG00000198858	AAAAACC
ENSG00000198858	AGCACCA
ENSG00000198863	AAGUGCA
ENSG00000198863	CAAAGUG
ENSG00000198863	UAAUAGU
ENSG00000198873	GGCUCUG
ENSG00000198879	AGCUGCC
ENSG00000198879	GUGCUUG
ENSG00000198879	UGUGCUU
ENSG00000198881	GAGGAUA
ENSG00000198887	UAUAUGA
ENSG00000198887	UGACCUA
ENSG00000198890	AAGUGCU
ENSG00000198890	AGUGCUU
ENSG00000198890	GAAACAU
ENSG00000198890	UGGCUGC
ENSG00000198892	CUCCCAA
ENSG00000198894	CACAGUG
ENSG00000198894	CAGCAGA
ENSG00000198894	UACAAAG
ENSG00000198894	UCACAGG
ENSG00000198894	UUCCUAG
ENSG00000198898	AAAGUAU
ENSG00000198898	AUGCAUU
ENSG00000198898	GCAGCAU
ENSG00000198898	GGUAGAC
ENSG00000198898	UAAUACA
ENSG00000198898	UCACAUU
ENSG00000198898	UGUGCUU
ENSG00000198900	ACUGGCU
ENSG00000198900	CUACAGU
ENSG00000198900	CUGGCUC
ENSG00000198900	GACAACU
ENSG00000198900	UACAGUU
ENSG00000198901	AGUGCUG
ENSG00000198901	GUGCAAA
ENSG00000198901	UGCAAAG
ENSG00000198901	UUGGCAA
ENSG00000198901	UUGGCAC
ENSG00000198908	CUGGCUC
ENSG00000198909	AAAAGUA
ENSG00000198909	AAAGUGC
ENSG00000198909	AACAGUC
ENSG00000198909	AAGGCAC

ENSG00000198909	AAGUGCA
ENSG00000198909	ACAUUAC
ENSG00000198909	ACAUUCA
ENSG00000198909	ACUGGCC
ENSG00000198909	AUAUAAC
ENSG00000198909	AUCCCUU
ENSG00000198909	AUGGCUU
ENSG00000198909	CUUCUCU
ENSG00000198909	GAGGUAG
ENSG00000198909	GAGGUAU
ENSG00000198909	GCAGGUU
ENSG00000198909	GGAGGAG
ENSG00000198909	GGCUGCG
ENSG00000198909	GUAACAG
ENSG00000198909	GUCAACA
ENSG00000198909	UCACAUU
ENSG00000198909	UCCAGUU
ENSG00000198909	UCCCACA
ENSG00000198909	UCCCUUU
ENSG00000198909	UCCUGAG
ENSG00000198909	UGCAUUG
ENSG00000198909	UUGGAGG
ENSG00000198909	UUGGCAA
ENSG00000198909	UUGGUCC
ENSG00000198909	UUUGCAC
ENSG00000198910	ACUUGGA
ENSG00000198910	CAGCUGG
ENSG00000198910	GGGUUUA
ENSG00000198910	GGUGGGC
ENSG00000198910	UGCAGCU
ENSG00000198910	UUGGCAC
ENSG00000198911	CUCCCAC
ENSG00000198911	CUGCCCC
ENSG00000198911	UCCCACC
ENSG00000198912	GUAAACA
ENSG00000198915	AAAGUGC
ENSG00000198915	AAAUCCC
ENSG00000198915	AAGGCAC
ENSG00000198915	AAGUGCU
ENSG00000198915	ACUAGAU
ENSG00000198915	ACUCAAA
ENSG00000198915	CCCUGAG
ENSG00000198915	CUCCCAA
ENSG00000198915	CUCCCAC
ENSG00000198915	GGCCCUG
ENSG00000198915	GUAAACA
ENSG00000198915	GUGCAAA
ENSG00000198915	UAUGGUU
ENSG00000198915	UGAAGUG
ENSG00000198915	UGCCCUG
ENSG00000198919	AAUUAUU
ENSG00000198920	CCAGUGU
ENSG00000198920	GGCAGUG
ENSG00000198925	AGCACCA
ENSG00000198925	AGCAGCA
ENSG00000198925	CCAGCAU
ENSG00000198925	CUGCAGC
ENSG00000198925	GGCAGCG
ENSG00000198925	GGCAGUG
ENSG00000198925	GGGAGCU
ENSG00000198925	GUGAGGU
ENSG00000198925	UCCCUUG
ENSG00000198925	UUGGCAC
ENSG00000198929	AUGGCUU

ENSG00000198929	CAAAGCA
ENSG00000198929	GAAGGAA
ENSG00000198929	UCCCUUG
ENSG00000198929	UCCCUUU
ENSG00000198929	UGAGAAG
ENSG00000198933	AGCAUUC
ENSG00000198933	CCUUCUG
ENSG00000198933	GAGGUAU
ENSG00000198933	GUGCCUG
ENSG00000198933	UCACAGG
ENSG00000198933	UCACAGU
ENSG00000198933	UGGACUG
ENSG00000198937	UAUUGCU
ENSG00000198939	UCACAUU
ENSG00000198939	UCCAGUU
ENSG00000198944	AACAUUC
ENSG00000198944	CACAGUG
ENSG00000198944	GAGGUAG
ENSG00000198944	UCACAGG
ENSG00000198945	AAGUGCA
ENSG00000198945	GUGCAAA
ENSG00000198946	CAGUAGU
ENSG00000198947	AAAGCUG
ENSG00000198947	AAAUUUC
ENSG00000198947	AACAAAC
ENSG00000198947	AAGGCAC
ENSG00000198947	AAUACUG
ENSG00000198947	ACAGUAC
ENSG00000198947	ACUCCAG
ENSG00000198947	CAAAGUG
ENSG00000198947	CACUCCU
ENSG00000198947	CAGGAAC
ENSG00000198947	CUUGUAU
ENSG00000198947	GAGGUAU
ENSG00000198947	GGCAAGA
ENSG00000198947	GGGUUUA
ENSG00000198947	GUAAACA
ENSG00000198947	GUGAGGU
ENSG00000198947	UCAAGUA
ENSG00000198947	UGCAAAG
ENSG00000198947	UGCAUAU
ENSG00000198947	UUCAAAU
ENSG00000198947	UUCCAUA
ENSG00000198947	UUUGCAA
ENSG00000198948	AAAGAAU
ENSG00000198948	AACAUUC
ENSG00000198948	ACAUUCA
ENSG00000198948	AGAUGUG
ENSG00000198948	AUAAUAC
ENSG00000198948	AUAUAAC
ENSG00000198948	AUGCAUU
ENSG00000198948	AUGUGCC
ENSG00000198948	CUUUGGU
ENSG00000198948	GAGAAAU
ENSG00000198948	GAGGUAG
ENSG00000198948	GAGGUAU
ENSG00000198948	GAUAUGU
ENSG00000198948	GUAAACA
ENSG00000198948	GUGCAAA
ENSG00000198948	UCAAGUA
ENSG00000198948	UGACAUC
ENSG00000198948	UGCAUAG
ENSG00000198948	UUCCUAG
ENSG00000198951	CACUCCU

ENSG00000198951	GAGGUAG
ENSG00000198952	ACAGGUG
ENSG00000198952	AGCUGCC
ENSG00000198952	AGGGAGG
ENSG00000198952	AUUCAUU
ENSG00000198952	GAGCUGC
ENSG00000198952	GGAGUCC
ENSG00000198952	GGUGGGC
ENSG00000198952	UCAUGAU
ENSG00000198952	UCCAGAG
ENSG00000198952	UCUGGAG
ENSG00000198952	UGAGUCU
ENSG00000198952	UGGCUGC
ENSG00000198959	CAAAGCA
ENSG00000198959	UGGAGAU
ENSG00000198960	ACCACAG
ENSG00000198961	AAAAGUA
ENSG00000198961	AAAGUAC
ENSG00000198961	AAAUCCC
ENSG00000198961	AACAUUC
ENSG00000198961	ACAAUAU
ENSG00000198961	ACAGUAU
ENSG00000198961	ACAUUAC
ENSG00000198961	ACAUUCA
ENSG00000198961	AGCAGCU
ENSG00000198961	CUUUGGU
ENSG00000198961	GAAAACA
ENSG00000198961	GACCUGG
ENSG00000198961	UGCAUUG
ENSG00000198961	UGGGACA
ENSG00000198963	AAACUGU
ENSG00000198963	AAGUGCU
ENSG00000198963	ACAAUAU
ENSG00000198963	ACAUAGA
ENSG00000198963	ACAUUCA
ENSG00000198963	ACUGUAG
ENSG00000198963	AUGCAUU
ENSG00000198963	CAGUAGU
ENSG00000198963	CCAGUGU
ENSG00000198963	GAGCCCC
ENSG00000198963	UCCUAUA
ENSG00000198963	UGCAUAU
ENSG00000198963	UGUCUUU
ENSG00000198964	AAAGUAU
ENSG00000198964	AAGGCAC
ENSG00000198964	AAUCACU
ENSG00000198964	AGUGCUG
ENSG00000198964	AUAAAGU
ENSG00000198964	AUGGCUU
ENSG00000198964	CUUACAG
ENSG00000198964	CUUCACC
ENSG00000198964	UCACAGU
ENSG00000198964	UGGAGAU
ENSG00000198964	UUGGAGG
ENSG00000198964	UUGGCAA
ENSG00000203710	UCUAGAG
ENSG00000203710	UGUUGAA
ENSG00000203727	AAGUUGU
ENSG00000203727	ACCUGGA
ENSG00000203727	AUAUUAU
ENSG00000203727	AUCCUUU
ENSG00000203727	CACAAUG
ENSG00000203727	CAGUCCA
ENSG00000203727	CUAUACA

ENSG00000203727	CUGGAAA
ENSG00000203727	GCCCUUA
ENSG00000203727	GGAAGCC
ENSG00000203727	UCCAGAG
ENSG00000203727	UCCAGUU
ENSG00000203727	UCCCUUG
ENSG00000203727	UGCAAAG
ENSG00000203727	UUCAAAU
ENSG00000203727	UUUGCAA
ENSG00000203759	AAAAACC
ENSG00000203759	AAAGCUG
ENSG00000203759	AAAUUUC
ENSG00000203759	AACAGUC
ENSG00000203759	AACGCCU
ENSG00000203759	AAGUGCU
ENSG00000203759	AAUCUCA
ENSG00000203759	AAUGCCC
ENSG00000203759	ACAUCAC
ENSG00000203759	ACAUUAC
ENSG00000203759	ACAUUCA
ENSG00000203759	AGCUGCC
ENSG00000203759	AGUGCUU
ENSG00000203759	AUAAAGU
ENSG00000203759	AUAAUAC
ENSG00000203759	AUAUUAU
ENSG00000203759	AUCAUAC
ENSG00000203759	AUCCCUU
ENSG00000203759	AUCCUUG
ENSG00000203759	CAGACAG
ENSG00000203759	CCAGGAG
ENSG00000203759	CCCACCG
ENSG00000203759	CCCACCU
ENSG00000203759	CCCCCAG
ENSG00000203759	CUACAGU
ENSG00000203759	CUAGUAG
ENSG00000203759	CUGCCCC
ENSG00000203759	CUUUGGU
ENSG00000203759	GAAAACA
ENSG00000203759	GACUUCC
ENSG00000203759	GAGAAGA
ENSG00000203759	GAGCCCC
ENSG00000203759	GAGGUAG
ENSG00000203759	GCUACAU
ENSG00000203759	GCUGGUG
ENSG00000203759	GCUUCCU
ENSG00000203759	GGAGAGA
ENSG00000203759	GGCUCAG
ENSG00000203759	GGCUCUG
ENSG00000203759	GGUGGGC
ENSG00000203759	GUAACAG
ENSG00000203759	GUAUUCU
ENSG00000203759	GUCUACU
ENSG00000203759	GUGCAAA
ENSG00000203759	UAUAAAG
ENSG00000203759	UAUAAUA
ENSG00000203759	UAUCAGA
ENSG00000203759	UCCCACA
ENSG00000203759	UCCCACC
ENSG00000203759	UCCCUUU
ENSG00000203759	UCUGGAG
ENSG00000203759	UGGGGGA
ENSG00000203759	UUCUUGU
ENSG00000203759	UUGGAGG
ENSG00000203759	UUGGUUC

ENSG00000203772	GGCAGUG
ENSG00000203778	AAGGUGC
ENSG00000203778	GUGCCAC
ENSG00000203782	AGGUAGU
ENSG00000203782	GAGGUAG
ENSG00000203784	CAGCAGA
ENSG00000203784	GUUCAUG
ENSG00000203791	CACUGUU
ENSG00000203791	UCCAGAG
ENSG00000203791	UGGGUGA
ENSG00000203811	AAAGAGC
ENSG00000203812	AAAGAGC
ENSG00000203813	AAAGAGC
ENSG00000203814	AAAGAGC
ENSG00000203852	AAAGAGC
ENSG00000203879	ACAUUCA
ENSG00000203879	CCUUCUG
ENSG00000203879	CUCCCAA
ENSG00000203879	CUCCCAC
ENSG00000203879	GGGCCCC
ENSG00000203879	UCUCCAA
ENSG00000203879	UGUGCUU
ENSG00000203879	UUGGUCC
ENSG00000203879	UUUGCAA
ENSG00000203880	AAGGUGC
ENSG00000203883	AAAGUAA
ENSG00000203950	CUCUGGG
ENSG00000204003	CAGGUGA
ENSG00000204003	CUGCAGC
ENSG00000204033	CCUUCUG
ENSG00000204052	UCCAGAG
ENSG00000204065	CAUGGGU
ENSG00000204065	UGGGUGA
ENSG00000204070	AAUACUG
ENSG00000204070	AGUGGUU
ENSG00000204070	CCUUCUU
ENSG00000204070	CUUCUCU
ENSG00000204070	GCUGGUG
ENSG00000204070	GGCUCAG
ENSG00000204070	UGUGCUU
ENSG00000204084	AAAGUUU
ENSG00000204084	AAAUCCC
ENSG00000204084	AAGGCAC
ENSG00000204084	ACAGGUG
ENSG00000204084	AUAUUAU
ENSG00000204084	CUUCACC
ENSG00000204084	GAAACAU
ENSG00000204084	GAGCUGC
ENSG00000204084	GAGUGUG
ENSG00000204084	GCCCUGU
ENSG00000204084	GGCUCAG
ENSG00000204084	UGCCCUG
ENSG00000204086	UUCAAAU
ENSG00000204103	AAAGUUU
ENSG00000204103	AAAUCCC
ENSG00000204103	AAAUGGU
ENSG00000204103	AAUGCCC
ENSG00000204103	AAUUAUU
ENSG00000204103	ACUGUUU
ENSG00000204103	AGCAAAA
ENSG00000204103	AGUGCAA
ENSG00000204103	AUAUAAC
ENSG00000204103	AUCAUAC
ENSG00000204103	AUCCUUU

ENSG00000204103	CAGUCUG
ENSG00000204103	CCAGCAU
ENSG00000204103	GACCUGG
ENSG00000204103	GCACCAU
ENSG00000204103	GCAUCCC
ENSG00000204103	GUCAGUU
ENSG00000204103	GUCCAGA
ENSG00000204103	GUUAAUG
ENSG00000204103	UAAUACA
ENSG00000204103	UAAUGCU
ENSG00000204103	UCAACAG
ENSG00000204103	UGAAAUG
ENSG00000204103	UUCAAAU
ENSG00000204103	UUUCAAC
ENSG00000204116	AACUACU
ENSG00000204116	AAGACAU
ENSG00000204116	AAGUGCA
ENSG00000204116	AAGUGUG
ENSG00000204116	AAUUGCU
ENSG00000204116	ACAAGGU
ENSG00000204116	ACAUAGA
ENSG00000204116	AGGCAGG
ENSG00000204116	AUGAUGC
ENSG00000204116	CAGCAAA
ENSG00000204116	CCAGUGC
ENSG00000204116	GAGGCAG
ENSG00000204116	GAGGUAG
ENSG00000204116	GCAUCCC
ENSG00000204116	GCUUCCU
ENSG00000204116	GGCUCAG
ENSG00000204116	GUGACUG
ENSG00000204116	UCAAGUA
ENSG00000204116	UGUCUUU
ENSG00000204116	UUAUGCA
ENSG00000204120	AAGGUGC
ENSG00000204120	AAGUCAC
ENSG00000204120	ACUCAGC
ENSG00000204120	ACUCCAG
ENSG00000204120	AGCAAAA
ENSG00000204120	AUCCCUU
ENSG00000204120	AUUGCAC
ENSG00000204120	CAAAGCA
ENSG00000204120	CAGCAGA
ENSG00000204120	CAGCAGG
ENSG00000204120	CAGUAGA
ENSG00000204120	CUAGUCC
ENSG00000204120	CUCCAGC
ENSG00000204120	CUCUUCC
ENSG00000204120	GAAGGUC
ENSG00000204120	GCACCAU
ENSG00000204120	GCAGAAG
ENSG00000204120	GGGGAGC
ENSG00000204120	GUAAACA
ENSG00000204120	GUAUGUU
ENSG00000204120	GUCUCUG
ENSG00000204120	UACAAAG
ENSG00000204120	UCCCUCU
ENSG00000204120	UUCCUCG
ENSG00000204120	UUGUGUC
ENSG00000204130	AAGGCAC
ENSG00000204130	GAAGGAA
ENSG00000204130	UCUGGAG
ENSG00000204138	AAUUAUU
ENSG00000204138	ACAAGGU

ENSG00000204138	ACAUCAC
ENSG00000204138	ACAUUAC
ENSG00000204138	CCAGUGC
ENSG00000204138	CCAGUGU
ENSG00000204138	CUGGCUC
ENSG00000204138	GUGAGGU
ENSG00000204138	UAUGGUU
ENSG00000204138	UAUUGCU
ENSG00000204138	UCCAGUU
ENSG00000204138	UGAAAUG
ENSG00000204138	UUCUGCA
ENSG00000204138	UUGGCAC
ENSG00000204160	AAUACUG
ENSG00000204160	CUGGGCA
ENSG00000204160	CUUUGGU
ENSG00000204160	GGAAGCC
ENSG00000204160	GGCAAGA
ENSG00000204160	UAUUACC
ENSG00000204160	UCAAGUA
ENSG00000204160	UUCAAGC
ENSG00000204175	AGCCCUU
ENSG00000204175	GCCCUUA
ENSG00000204176	CUUCUCU
ENSG00000204186	AUAUAAC
ENSG00000204186	CAAAUGC
ENSG00000204217	AAAGAGG
ENSG00000204217	AACCUGU
ENSG00000204217	ACAUUCA
ENSG00000204217	ACCCGUA
ENSG00000204217	ACUGCAG
ENSG00000204217	AGCCCUU
ENSG00000204217	AUGGCUU
ENSG00000204217	CAUGGAU
ENSG00000204217	CCCUGAG
ENSG00000204217	CUCCAUU
ENSG00000204217	GAAAACA
ENSG00000204217	GAGACCU
ENSG00000204217	GAGCCCU
ENSG00000204217	GCACCAU
ENSG00000204217	GGAAGCC
ENSG00000204217	GGCCCUG
ENSG00000204217	GGCUCAG
ENSG00000204217	GUCAUUG
ENSG00000204217	UAAUACA
ENSG00000204217	UGCAUAG
ENSG00000204217	UGCUGAC
ENSG00000204217	UUGACAC
ENSG00000204217	UUGUUCG
ENSG00000204228	AGGGUCU
ENSG00000204231	AACCUGG
ENSG00000204231	ACAGUAU
ENSG00000204231	ACUGCAG
ENSG00000204231	AGUGCUU
ENSG00000204231	CCAGUGC
ENSG00000204231	CUGACUC
ENSG00000204231	CUGCCCC
ENSG00000204231	GAGUCUU
ENSG00000204231	UCCAGAG
ENSG00000204231	UGAAAGG
ENSG00000204248	CACAGUG
ENSG00000204248	UCACAGU
ENSG00000204252	AGACGGG
ENSG00000204252	CAUAUUG
ENSG00000204252	CCGGAGC

ENSG00000204252	GGAAGCC
ENSG00000204252	GGACGGA
ENSG00000204252	GGCAGCG
ENSG00000204252	UGAGAAU
ENSG00000204256	AAAGAGC
ENSG00000204256	AACAAAC
ENSG00000204262	ACAGUAU
ENSG00000204262	AGCAAAA
ENSG00000204262	CAAGAGC
ENSG00000204262	CCAUUAC
ENSG00000204262	CUCAGGG
ENSG00000204262	GAAGGAA
ENSG00000204262	GAGAUGA
ENSG00000204262	GAGGUAG
ENSG00000204262	GAGGUAU
ENSG00000204262	GCACCAU
ENSG00000204262	UUCUUGU
ENSG00000204267	CAAAGCA
ENSG00000204267	CCUGCUG
ENSG00000204267	UUCCUAG
ENSG00000204271	AUGCCUU
ENSG00000204271	CUGGACU
ENSG00000204271	GAUUGUA
ENSG00000204271	GUCUACU
ENSG00000204271	UAAUGCU
ENSG00000204271	UAGGGCC
ENSG00000204271	UUGGCAC
ENSG00000204291	GAGGUAG
ENSG00000204291	GUAUGUU
ENSG00000204291	GUCAACA
ENSG00000204291	UUGGUUC
ENSG00000204301	UUCAAAU
ENSG00000204304	AAAAAUC
ENSG00000204304	AAAGUAC
ENSG00000204304	AAAGUAU
ENSG00000204304	AAGUAGC
ENSG00000204304	AUGAGCU
ENSG00000204304	CAGACAG
ENSG00000204304	CUCCCAC
ENSG00000204304	CUGCCCC
ENSG00000204304	GAGAACU
ENSG00000204304	GAGCUGC
ENSG00000204304	GAGGGGC
ENSG00000204304	GAGGUAG
ENSG00000204304	GAGGUAU
ENSG00000204304	GGCAGGG
ENSG00000204304	GGGGUGC
ENSG00000204304	GGGGUGG
ENSG00000204304	GGUUUAC
ENSG00000204304	GUCCAGA
ENSG00000204304	GUGCUUG
ENSG00000204304	GUGGGGA
ENSG00000204304	UCCCACC
ENSG00000204304	UCCUGAG
ENSG00000204304	UGGGACA
ENSG00000204304	UGUGCUU
ENSG00000204304	UUGGCAC
ENSG00000204308	AAUACUG
ENSG00000204308	AGGUAGU
ENSG00000204308	CACUCCU
ENSG00000204308	CCCACCU
ENSG00000204308	GAGGUAG
ENSG00000204308	GGUGGGC
ENSG00000204310	AAAACUG

ENSG00000204310	ACAUUCA
ENSG00000204310	ACUGGCU
ENSG00000204310	AGGCAGG
ENSG00000204310	CUCCAGC
ENSG00000204310	CUGCAGG
ENSG00000204310	CUGGCUC
ENSG00000204310	GAGACCU
ENSG00000204310	GAGAUGA
ENSG00000204310	GAGGCAG
ENSG00000204310	GCAGCAU
ENSG00000204310	GGAAUGU
ENSG00000204310	GGCAGGG
ENSG00000204310	GGCCCUG
ENSG00000204310	GGCUCAG
ENSG00000204310	UCCCACA
ENSG00000204335	AACCUGG
ENSG00000204352	UGAAGCU
ENSG00000204371	CUCUGGG
ENSG00000204371	GGAAUGU
ENSG00000204381	CCUGCUG
ENSG00000204388	AACAUUC
ENSG00000204392	AAUUUUA
ENSG00000204394	UAUUGCU
ENSG00000204406	AAAGAAU
ENSG00000204406	AAAGUGC
ENSG00000204406	AAGUGUG
ENSG00000204406	AGAUAAA
ENSG00000204406	AUAAAGU
ENSG00000204406	GAAACAU
ENSG00000204435	CCCACCG
ENSG00000204435	CCCACCU
ENSG00000204435	UCCCACA
ENSG00000204435	UCCUGAG
ENSG00000204435	UGAAAGG
ENSG00000204439	AGUGGUU
ENSG00000204439	CAGCUGG
ENSG00000204439	CCCUGAG
ENSG00000204439	CUGGCUC
ENSG00000204439	GCUGGUG
ENSG00000204439	UAUUGCU
ENSG00000204439	UGCUGAC
ENSG00000204442	AAAGCUG
ENSG00000204442	AAAUGGU
ENSG00000204442	ACUCAGC
ENSG00000204442	ACUGUAG
ENSG00000204442	AGCCCUU
ENSG00000204442	CAGACAG
ENSG00000204442	GCCCUUA
ENSG00000204442	GUAAACA
ENSG00000204442	UGAAAUG
ENSG00000204442	UGUAUAA
ENSG00000204463	AAUACUG
ENSG00000204463	ACUGUGU
ENSG00000204463	GGGGGAA
ENSG00000204469	AUACAAG
ENSG00000204469	UAUAAUA
ENSG00000204531	ACUGUGU
ENSG00000204539	GAGCUAA
ENSG00000204539	GGAGGAG
ENSG00000204564	AGCAGCU
ENSG00000204564	CAGUGCA
ENSG00000204564	CUGGCUC
ENSG00000204569	AAGUGCA
ENSG00000204569	AAGUGCU

ENSG00000204569	AAUACUG
ENSG00000204569	ACUGGAU
ENSG00000204569	AUACAAG
ENSG00000204569	CAGCUGG
ENSG00000204569	CAGUCCA
ENSG00000204569	CUUCUCU
ENSG00000204569	GAAAACA
ENSG00000204569	GAGCCCU
ENSG00000204569	GGCAGUG
ENSG00000204569	GUAGUGU
ENSG00000204569	UCAUAGA
ENSG00000204576	AAGGCAC
ENSG00000204576	AAUACUG
ENSG00000204576	ACAGGUG
ENSG00000204576	ACAGUAC
ENSG00000204576	AGCACCA
ENSG00000204576	AGCAGCU
ENSG00000204576	CAAAGCA
ENSG00000204576	CACAAUG
ENSG00000204576	CACAGGG
ENSG00000204576	CCAGGAG
ENSG00000204576	CCAGUGC
ENSG00000204576	CCCACCU
ENSG00000204576	CUCAGGG
ENSG00000204576	GAAAACA
ENSG00000204576	GAUCAGA
ENSG00000204576	GGAGGCA
ENSG00000204576	UCACAAG
ENSG00000204576	UCACAGG
ENSG00000204576	UCACAGU
ENSG00000204576	UCCCACC
ENSG00000204576	UCUCCAA
ENSG00000204576	UGGAGAU
ENSG00000204576	UUGGCAA
ENSG00000204576	UUGGCAC
ENSG00000204580	AGCCAGU
ENSG00000204580	AUAUUAU
ENSG00000204580	CAGCUGG
ENSG00000204580	CCAGUGC
ENSG00000204580	CCCCUGG
ENSG00000204580	GGGUGGU
ENSG00000204580	GUCUCUG
ENSG00000204590	CAAAGCA
ENSG00000204599	AAUCUCU
ENSG00000204599	ACCACAG
ENSG00000204599	CAGCAGG
ENSG00000204599	CUGGAAA
ENSG00000204599	CUGUUGA
ENSG00000204599	GACCCUG
ENSG00000204599	GUCUUGC
ENSG00000204599	UCUGGAA
ENSG00000204618	AGCACCA
ENSG00000204618	GCACCAU
ENSG00000204618	UAUUGCU
ENSG00000204619	AAAGCUG
ENSG00000204619	AAGUAGC
ENSG00000204619	AGCAGCA
ENSG00000204619	AGCAGCG
ENSG00000204619	AGCAGCU
ENSG00000204619	CAGACAG
ENSG00000204619	CUGGAAA
ENSG00000204619	GACCCUG
ENSG00000204619	GAGAACC
ENSG00000204619	GAGAACU

ENSG00000204619	UCUGGAA
ENSG00000204619	UCUGGAG
ENSG00000204619	UUGGCAA
ENSG00000204619	UUGGCAC
ENSG00000204624	CUCUUCC
ENSG00000204624	CUGGCUC
ENSG00000204624	GGCUCUG
ENSG00000204634	AUAUUAU
ENSG00000204634	AUUGCAC
ENSG00000204634	CCAGUGC
ENSG00000204634	CCAGUGU
ENSG00000204634	CUUUGGU
ENSG00000204634	GAAACAU
ENSG00000204645	CAGUAGU
ENSG00000204673	AAAACUG
ENSG00000204673	AAGGCAC
ENSG00000204673	AAUCUCA
ENSG00000204673	CCCACCU
ENSG00000204673	CCCUGAG
ENSG00000204673	CUGCCCC
ENSG00000204673	GUAGUGU
ENSG00000204673	UGGCCAC
ENSG00000204681	AACCUGU
ENSG00000204681	AACUGGU
ENSG00000204681	CCUUCUU
ENSG00000204681	GCAGGUU
ENSG00000204681	UGCCAGC
ENSG00000204681	UUCUUGU
ENSG00000204682	AAAGUUU
ENSG00000204682	GGCGACA
ENSG00000204682	UCACAGG
ENSG00000204682	UCCCACA
ENSG00000204682	UCCCACC
ENSG00000204682	UUGGUUC
ENSG00000204682	UUUGCAA
ENSG00000204688	CUGCAGG
ENSG00000204688	GGAAUGU
ENSG00000204688	GGGGAGC
ENSG00000204688	GUGGGGA
ENSG00000204694	CCUGCUG
ENSG00000204713	GUGCCUG
ENSG00000204764	AACCUGG
ENSG00000204764	AAGUCAC
ENSG00000204764	GGCAUUG
ENSG00000204764	GUCUCUG
ENSG00000204764	UAGACUG
ENSG00000204764	UCUAGAG
ENSG00000204764	UUAUGCA
ENSG00000204764	UUGGUUC
ENSG00000204839	AGCACCA
ENSG00000204839	GGGGUGG
ENSG00000204842	AAAACUG
ENSG00000204842	AAAAGUA
ENSG00000204842	AAGUCUU
ENSG00000204842	AAUCACU
ENSG00000204842	AGCAGCG
ENSG00000204842	AGCAGCU
ENSG00000204842	GCUAGCA
ENSG00000204842	UAUAAAG
ENSG00000204842	UCCUAUA
ENSG00000204842	UGUGCUU
ENSG00000204843	CCCUGAG
ENSG00000204843	CUUUGGU
ENSG00000204843	GCAGAAG

ENSG00000204856	UUGGCAA
ENSG00000204899	AUAAAGU
ENSG00000204899	UAUGGUU
ENSG00000204923	ACUAGAU
ENSG00000204946	CAGGAAC
ENSG00000204946	UCCUGAG
ENSG00000204952	AAAAGUA
ENSG00000204956	AAAACUG
ENSG00000204956	CACCAGC
ENSG00000204956	GGAGGCA
ENSG00000204956	UGCAUAG
ENSG00000204956	UGGCCAC
ENSG00000204962	AAAGAAU
ENSG00000204962	AAGUGCA
ENSG00000204962	AGCAGCG
ENSG00000204962	AGCAGCU
ENSG00000204962	AUAAUAC
ENSG00000204962	CAAAGUG
ENSG00000204962	GAGGCAG
ENSG00000204962	UAAUACA
ENSG00000204962	UAAUAGU
ENSG00000204962	UACAAAG
ENSG00000204962	UAUAAUA
ENSG00000204962	UCAACAG
ENSG00000204962	UCACAUU
ENSG00000204962	UCAUACA
ENSG00000204962	UGCAUAG
ENSG00000204962	UGCAUAU
ENSG00000204962	UGUGCUU
ENSG00000204962	UUUGCAA
ENSG00000204962	UUUGCAC
ENSG00000204963	AAAGAAU
ENSG00000204963	AAGUGCA
ENSG00000204963	AGCAGCG
ENSG00000204963	AGCAGCU
ENSG00000204963	AUAAUAC
ENSG00000204963	CAAAGUG
ENSG00000204963	GAGGCAG
ENSG00000204963	UAAUACA
ENSG00000204963	UAAUAGU
ENSG00000204963	UACAAAG
ENSG00000204963	UAUAAUA
ENSG00000204963	UCAACAG
ENSG00000204963	UCACAUU
ENSG00000204963	UCAUACA
ENSG00000204963	UGCAUAG
ENSG00000204963	UGCAUAU
ENSG00000204963	UGUGCUU
ENSG00000204963	UUUGCAA
ENSG00000204963	UUUGCAC
ENSG00000204965	AAAGAAU
ENSG00000204965	AAGUGCA
ENSG00000204965	AGCAGCG
ENSG00000204965	AGCAGCU
ENSG00000204965	AUAAUAC
ENSG00000204965	CAAAGUG
ENSG00000204965	GAGGCAG
ENSG00000204965	UAAUACA
ENSG00000204965	UAAUAGU
ENSG00000204965	UACAAAG
ENSG00000204965	UAUAAUA
ENSG00000204965	UCAACAG
ENSG00000204965	UCACAUU
ENSG00000204965	UCAUACA

ENSG00000204965	UGCAUAG
ENSG00000204965	UGCAUAU
ENSG00000204965	UGUGCUU
ENSG00000204965	UUUGCAA
ENSG00000204965	UUUGCAC
ENSG00000204967	AAAGAAU
ENSG00000204967	AAGUGCA
ENSG00000204967	AGCAGCG
ENSG00000204967	AGCAGCU
ENSG00000204967	AUAAUAC
ENSG00000204967	CAAAGUG
ENSG00000204967	GAGGCAG
ENSG00000204967	UAAUACA
ENSG00000204967	UAAUAGU
ENSG00000204967	UACAAAG
ENSG00000204967	UAUAAUA
ENSG00000204967	UCAACAG
ENSG00000204967	UCACAUU
ENSG00000204967	UCAUACA
ENSG00000204967	UGCAUAG
ENSG00000204967	UGCAUAU
ENSG00000204967	UGUGCUU
ENSG00000204967	UUUGCAA
ENSG00000204967	UUUGCAC
ENSG00000204969	AAAGAAU
ENSG00000204969	AAGUGCA
ENSG00000204969	AGCAGCG
ENSG00000204969	AGCAGCU
ENSG00000204969	AUAAUAC
ENSG00000204969	CAAAGUG
ENSG00000204969	GAGGCAG
ENSG00000204969	UAAUACA
ENSG00000204969	UAAUAGU
ENSG00000204969	UACAAAG
ENSG00000204969	UAUAAUA
ENSG00000204969	UCAACAG
ENSG00000204969	UCACAUU
ENSG00000204969	UCAUACA
ENSG00000204969	UGCAUAG
ENSG00000204969	UGCAUAU
ENSG00000204969	UGUGCUU
ENSG00000204969	UUUGCAA
ENSG00000204969	UUUGCAC
ENSG00000204970	AAAGAAU
ENSG00000204970	AAGUGCA
ENSG00000204970	AGCAGCG
ENSG00000204970	AGCAGCU
ENSG00000204970	AUAAUAC
ENSG00000204970	CAAAGUG
ENSG00000204970	GAGGCAG
ENSG00000204970	UAAUACA
ENSG00000204970	UAAUAGU
ENSG00000204970	UACAAAG
ENSG00000204970	UAUAAUA
ENSG00000204970	UCAACAG
ENSG00000204970	UCACAUU
ENSG00000204970	UCAUACA
ENSG00000204970	UGCAUAG
ENSG00000204970	UGCAUAU
ENSG00000204970	UGUGCUU
ENSG00000204970	UUUGCAA
ENSG00000204970	UUUGCAC
ENSG00000204977	GGGAACG
ENSG00000204977	UUUGCAA

ENSG00000204991	UAGGCCG
ENSG00000204991	UCCUGAG
ENSG00000205056	AAAUUUC
ENSG00000205056	ACAUUAC
ENSG00000205056	AGUAACA
ENSG00000205056	AUGGAUU
ENSG00000205056	GAGAAAU
ENSG00000205056	GAGCUAA
ENSG00000205056	GAGGCAG
ENSG00000205056	GUAACAG
ENSG00000205056	UCAUUUG
ENSG00000205056	UGAAAGG
ENSG00000205056	UGAGAAU
ENSG00000205056	UGCUGAC
ENSG00000205056	UGGAGAU
ENSG00000205056	UUUUUGC
ENSG00000205060	CUCAGGG
ENSG00000205089	CAGCUGG
ENSG00000205089	CUGCUCA
ENSG00000205089	GAGGCAG
ENSG00000205155	AAGUCUU
ENSG00000205189	AGCACCA
ENSG00000205189	CUACAGU
ENSG00000205189	GAGGUAU
ENSG00000205189	GUAGUGU
ENSG00000205189	UCACAGG
ENSG00000205189	UCACAGU
ENSG00000205189	UUGGUCC
ENSG00000205208	GUAUUCU
ENSG00000205208	UACAAAG
ENSG00000205213	ACUGGCC
ENSG00000205213	ACUGGCU
ENSG00000205213	GAGGUAG
ENSG00000205213	GGCAGCG
ENSG00000205213	UCCUGAG
ENSG00000205213	UGGCCCU
ENSG00000205250	AAAAAUC
ENSG00000205250	AAAAGUA
ENSG00000205250	AAUCUCU
ENSG00000205250	CAGUCUG
ENSG00000205250	UUCUAAU
ENSG00000205269	AAAACUG
ENSG00000205269	AACCUGG
ENSG00000205269	AACCUGU
ENSG00000205269	AAGGCAC
ENSG00000205269	ACCCUGU
ENSG00000205269	AGCAGCA
ENSG00000205269	AGCAGCG
ENSG00000205269	AGCAGCU
ENSG00000205269	AGUGCUG
ENSG00000205269	AUGCAUU
ENSG00000205269	CACAGUG
ENSG00000205269	CAGUAGU
ENSG00000205269	CCCUGAG
ENSG00000205269	CCCUGUC
ENSG00000205269	CCCUGUU
ENSG00000205269	CUUGUAU
ENSG00000205269	CUUUGGU
ENSG00000205269	GCCCUUA
ENSG00000205269	UAGGCCG
ENSG00000205269	UCACAGG
ENSG00000205269	UCACAUU
ENSG00000205269	UCCCUGU
ENSG00000205269	UGCAAAG

ENSG00000205269	UGCAUAG
ENSG00000205269	UUGGCAA
ENSG00000205279	AAGUCUU
ENSG00000205279	AUUCAUU
ENSG00000205302	AAGACAU
ENSG00000205302	CAGUCCA
ENSG00000205302	GGUUUAC
ENSG00000205302	UGCAGCU
ENSG00000205336	AAGUCUU
ENSG00000205336	UACAGUU
ENSG00000205339	AGCUGCC
ENSG00000205339	CCUUCAU
ENSG00000205339	CUGGAAA
ENSG00000205352	UGAAGCU
ENSG00000205420	CUGCAGC
ENSG00000205423	AAAAACC
ENSG00000205423	AAAGAAU
ENSG00000205423	AACACUG
ENSG00000205423	ACCACAG
ENSG00000205423	UACAGUU
ENSG00000205423	UUCAAAU
ENSG00000205426	AAAGUGC
ENSG00000205426	CAAAGUG
ENSG00000205445	GGCAGGG
ENSG00000205531	AUGCAUU
ENSG00000205531	UAAUAGU
ENSG00000205531	UACAAAG
ENSG00000205531	UUCCUAG
ENSG00000205542	ACUGCAU
ENSG00000205542	AGUAGUU
ENSG00000205542	CAGUAGA
ENSG00000205542	CAGUGCA
ENSG00000205542	CUUACAG
ENSG00000205560	CAGCUGG
ENSG00000205581	UACAAAG
ENSG00000205629	CUGGCUC
ENSG00000205683	CUUUGGU
ENSG00000205726	AAGUCAC
ENSG00000205726	AGCAUUC
ENSG00000205726	CUCCCAA
ENSG00000205726	CUCCCAC
ENSG00000205726	GCUGGAU
ENSG00000205726	GGCAGUG
ENSG00000205726	UCCCUCU
ENSG00000205726	UGUGCUU
ENSG00000205726	UUCAAGC
ENSG00000205726	UUGAGGC
ENSG00000205808	AGGUAGU
ENSG00000205808	CUUUGGU
ENSG00000205808	GAGGUAG
ENSG00000205808	GUGCCUG
ENSG00000205838	ACAAGGU
ENSG00000205838	ACCCAUU
ENSG00000205916	GAUUGGU
ENSG00000205916	UCUUGAG
ENSG00000205927	AAUCUCA
ENSG00000205927	UACAAAG
ENSG00000205927	UUUGCAC
ENSG00000205927	UUUUUGC
ENSG00000205929	AAAUCCC
ENSG00000205937	AAAGUAC
ENSG00000205937	AACCUGG
ENSG00000205937	AACCUGU
ENSG00000205937	ACUCAAA

ENSG00000205937	CUACAAA
ENSG00000205937	GCUACAU
ENSG00000205937	GGCUCUG
ENSG00000205937	UACAAAG
ENSG00000205937	UGACUGU
ENSG00000205944	GAUUGGU
ENSG00000205944	UCUUGAG
ENSG00000205981	GAGGCAG
ENSG00000206026	ACAAGGU
ENSG00000206026	ACAGUAC
ENSG00000206026	ACAGUAU
ENSG00000206026	ACAUCAC
ENSG00000206026	AGCACCA
ENSG00000206026	CAGCUGG
ENSG00000206026	GGUGGUC
ENSG00000206026	GUUAAUG
ENSG00000206026	UACAGUU
ENSG00000206026	UAUAUGA
ENSG00000206026	UAUUACC
ENSG00000206026	UGAGGAC
ENSG00000206026	UUGACAC
ENSG00000206026	UUGAUCA
ENSG00000206053	AAAUGGU
ENSG00000206053	AAUGUUG
ENSG00000206053	ACUCAAA
ENSG00000206053	CCCACCG
ENSG00000206053	CUGCAGG
ENSG00000206053	CUUUGGU
ENSG00000206053	GGUUGAC
ENSG00000206053	UUGGCAC
ENSG00000206053	UUGGUUC
ENSG00000206190	GAAACAU
ENSG00000206190	UCAAGUA
ENSG00000206190	UUCAAGC
ENSG00000206384	AGCACCA
ENSG00000206384	UUUCAAC
ENSG00000206418	AAAGUGC
ENSG00000206418	AAUUUUA
ENSG00000206527	ACUCAAA
ENSG00000206527	ACUGGAU
ENSG00000206527	CUGCAGG
ENSG00000206538	AAGUGCA
ENSG00000206538	ACACACU
ENSG00000206538	AGCAAAA
ENSG00000206538	AGCAUUC
ENSG00000206538	AGUGCAA
ENSG00000206538	AGUGCUU
ENSG00000206538	GAAAACA
ENSG00000206538	GAGGUAG
ENSG00000206538	GAGGUAU
ENSG00000206538	GGCUCAG
ENSG00000206538	GUAUUCU
ENSG00000206538	UCAUUCG
ENSG00000206538	UGCCAGC
ENSG00000206538	UGCUGAC
ENSG00000206538	UUGGCAC
ENSG00000206560	AAAGAAU
ENSG00000206560	AAAGGUA
ENSG00000206560	AAAGUUU
ENSG00000206560	ACAGUAU
ENSG00000206560	ACAUUAC
ENSG00000206560	ACUGUUU
ENSG00000206560	AGGGAGG
ENSG00000206560	AGUGCAA

ENSG00000206560	CACAAUG
ENSG00000206560	CUAGUAG
ENSG00000206560	CUGGAAA
ENSG00000206560	GAGGUAG
ENSG00000206560	GGACUGC
ENSG00000206560	UAUUGCU
ENSG00000206560	UCACAAG
ENSG00000206560	UCACACA
ENSG00000206560	UGGACUG
ENSG00000206561	AACAGUC
ENSG00000206561	AAGACAU
ENSG00000206561	CAGCAGG
ENSG00000206561	UAAGACG
ENSG00000206561	UAAGACU
ENSG00000206561	UCCAGUU
ENSG00000206579	AGCACCA
ENSG00000206579	GAGGUAU
ENSG00000206579	GCACCAU
ENSG00000206579	UGCAUAG
ENSG00000206579	UUGGCAA
ENSG00000206579	UUGGCAC
ENSG00000211445	CAGGAAC
ENSG00000211445	CAGGCUC
ENSG00000211445	GCAGAAG
ENSG00000211445	GGAGGCA
ENSG00000211448	AAAACUG
ENSG00000211448	AAUCUCA
ENSG00000211448	ACAUCAC
ENSG00000211448	ACUGGCC
ENSG00000211448	AGUACUG
ENSG00000211448	CUUUGGU
ENSG00000211448	GACUUCC
ENSG00000211448	GAGACCU
ENSG00000211448	GUAAACA
ENSG00000211448	UCACAAG
ENSG00000211448	UCCUGAG
ENSG00000211448	UGAAAUG
ENSG00000211450	ACAUCAC
ENSG00000211450	UGGCCAC
ENSG00000211452	AAGUCAC
ENSG00000211452	GAGCUAA
ENSG00000211455	AAAAACC
ENSG00000211455	AAAGUUU
ENSG00000211455	AGCACCA
ENSG00000211455	AGGCAGG
ENSG00000211455	AGUAGUU
ENSG00000211455	AUGGCAC
ENSG00000211455	AUGGCUU
ENSG00000211455	CACAGUG
ENSG00000211455	CAGCUGG
ENSG00000211455	CAGUGCA
ENSG00000211455	CUACAAA
ENSG00000211455	CUUUGGU
ENSG00000211455	GGCAGUG
ENSG00000211455	UACAGUU
ENSG00000211455	UCCAGUU
ENSG00000211455	UGCAGCU
ENSG00000211455	UGGUACC
ENSG00000211455	UUCCUCG
ENSG00000211456	AAAGAAU
ENSG00000211456	ACUCCAG
ENSG00000211460	AAGUCAC
ENSG00000211460	AUGACAC
ENSG00000212747	AGGUAGU

ENSG00000212864	GGCUGCG
ENSG00000212864	UUGGCAC
ENSG00000212916	AGCAAAA
ENSG00000213015	GAGGGGC
ENSG00000213015	GGAAUGU
ENSG00000213023	AGCAGCA
ENSG00000213023	AUGGCUU
ENSG00000213023	CCCCCAG
ENSG00000213047	AAGGCAC
ENSG00000213057	AAGACAU
ENSG00000213064	CAGGCUC
ENSG00000213066	AAGGCAC
ENSG00000213066	CUGCAGC
ENSG00000213066	CUGCAGG
ENSG00000213066	GAUUGUA
ENSG00000213071	AGUAGUU
ENSG00000213071	CCCACCG
ENSG00000213071	CUGGGGG
ENSG00000213071	UCAUAGA
ENSG00000213079	AAAAACC
ENSG00000213079	AAAGGUA
ENSG00000213079	AAUCUCU
ENSG00000213079	ACUGCAG
ENSG00000213079	GAGAUGA
ENSG00000213079	GUCAGUU
ENSG00000213079	UUCCAUA
ENSG00000213171	CAGCAGG
ENSG00000213171	GCAGGUU
ENSG00000213186	CAAGAGC
ENSG00000213186	CUGGAAA
ENSG00000213186	UCUGGAA
ENSG00000213190	AGCACCA
ENSG00000213190	CACCACC
ENSG00000213190	GGAGAGA
ENSG00000213190	UGUCUUU
ENSG00000213204	AAUUAUU
ENSG00000213213	CUGGAAA
ENSG00000213240	AAAAACC
ENSG00000213240	AAUCACU
ENSG00000213240	ACACACU
ENSG00000213240	GACUUCC
ENSG00000213240	GAUAUGU
ENSG00000213240	GGAGGAG
ENSG00000213240	UCUCAAG
ENSG00000213240	UGGAUAA
ENSG00000213265	CCCACCG
ENSG00000213281	AGGAGCU
ENSG00000213281	CAGUGCA
ENSG00000213281	CUCUGGG
ENSG00000213281	GAGGUAG
ENSG00000213281	GGCUCUG
ENSG00000213341	AGCAAAA
ENSG00000213341	AUAUUAU
ENSG00000213341	CAGCAAA
ENSG00000213347	AAUACUG
ENSG00000213390	AAGGCAC
ENSG00000213390	ACUGGCC
ENSG00000213390	AUGGCUU
ENSG00000213390	CCAGUGC
ENSG00000213390	CCAGUGU
ENSG00000213390	CUGGCUC
ENSG00000213390	CUGGGCA
ENSG00000213390	GAGGUAU
ENSG00000213390	GGCUCAG

ENSG00000213390	UAGAGGA
ENSG00000213390	UCCCUCU
ENSG00000213390	UGACCUA
ENSG00000213420	AACACUG
ENSG00000213420	GGGCCCC
ENSG00000213445	GGCAGUG
ENSG00000213445	UACAAAG
ENSG00000213445	UCCUGAG
ENSG00000213463	AAGUCUU
ENSG00000213463	CCCACCU
ENSG00000213463	CUAGUAA
ENSG00000213463	CUUUGGU
ENSG00000213463	GCCUGAU
ENSG00000213463	UGCAAAG
ENSG00000213463	UGCCAGC
ENSG00000213465	CAGCAGG
ENSG00000213465	GGAGGAG
ENSG00000213465	GGGGUGG
ENSG00000213533	AGUGCAA
ENSG00000213533	GAGGUAG
ENSG00000213533	GCAACUU
ENSG00000213533	GGAAGAC
ENSG00000213533	GUAGUGU
ENSG00000213533	UCACAGU
ENSG00000213533	UGCAUAG
ENSG00000213533	UUGGUCC
ENSG00000213551	UACAAAG
ENSG00000213578	GCCUGGA
ENSG00000213578	UGAAGCU
ENSG00000213578	UGCCAGC
ENSG00000213588	AAAGGUA
ENSG00000213588	AAGUGCU
ENSG00000213588	AGCAGCA
ENSG00000213588	UCCUGAG
ENSG00000213614	CACAGGG
ENSG00000213614	GCCUGGG
ENSG00000213614	GGAAGCC
ENSG00000213625	UAUAAAG
ENSG00000213626	AAAAGUA
ENSG00000213626	AGGAGCU
ENSG00000213626	AGUAGUU
ENSG00000213626	CAGUUAC
ENSG00000213626	GAGAUGA
ENSG00000213626	GAGGUAG
ENSG00000213626	GCAGGUU
ENSG00000213626	GUGCAAA
ENSG00000213626	UAUUGCU
ENSG00000213626	UCACAGU
ENSG00000213639	AAAGCUG
ENSG00000213639	AACUGGU
ENSG00000213639	AAGACAU
ENSG00000213639	AAUUAUU
ENSG00000213639	ACAUUCA
ENSG00000213639	ACCACAG
ENSG00000213639	ACCAGCA
ENSG00000213639	ACCCGUA
ENSG00000213639	ACUGCAG
ENSG00000213639	ACUGCAU
ENSG00000213639	ACUGGAU
ENSG00000213639	AGCAAAA
ENSG00000213639	AGCAUUC
ENSG00000213639	AUGAGCU
ENSG00000213639	AUGCAUU
ENSG00000213639	CAGGCUC

ENSG00000213639	CAGGUGA
ENSG00000213639	CAGUGCA
ENSG00000213639	CUACAAA
ENSG00000213639	CUAUACA
ENSG00000213639	CUCUUCC
ENSG00000213639	GACAACU
ENSG00000213639	GCAGGUU
ENSG00000213639	GCUGGAU
ENSG00000213639	GGAUUUC
ENSG00000213639	GGAUUUU
ENSG00000213639	GGUUUAC
ENSG00000213639	GUGACAG
ENSG00000213639	GUUAAUG
ENSG00000213639	UACAAAG
ENSG00000213639	UACAGUU
ENSG00000213639	UAGUGAG
ENSG00000213639	UAUUACC
ENSG00000213639	UAUUGCU
ENSG00000213639	UGAAAUG
ENSG00000213639	UGAAGCU
ENSG00000213639	UGACAUC
ENSG00000213639	UGUUGAA
ENSG00000213639	UUGACAC
ENSG00000213639	UUGAUCA
ENSG00000213654	GUCUCUG
ENSG00000213658	AAGGUGC
ENSG00000213658	CAAAGUG
ENSG00000213658	CAGGCUC
ENSG00000213658	CUCAGGG
ENSG00000213672	AAGGCAC
ENSG00000213672	AGUGCAA
ENSG00000213672	AUGGCUU
ENSG00000213672	CUGCAGG
ENSG00000213672	GUGCAAA
ENSG00000213676	AGUGCUU
ENSG00000213694	UCCCUCU
ENSG00000213699	CACCAGC
ENSG00000213699	CCUGCUG
ENSG00000213699	GCCUGGA
ENSG00000213741	AAUGUUG
ENSG00000213741	AUACCUC
ENSG00000213741	UGGCCCU
ENSG00000213760	CCCUGUU
ENSG00000213853	AAAAACC
ENSG00000213853	AUACAAG
ENSG00000213859	ACACACC
ENSG00000213859	CAGGUGA
ENSG00000213859	CUUGGGC
ENSG00000213865	AAAUUUC
ENSG00000213865	GGAUGAG
ENSG00000213901	ACCCUGU
ENSG00000213901	GACCCUG
ENSG00000213923	AAAAGUA
ENSG00000213923	AAAGCUG
ENSG00000213923	AAAGUAC
ENSG00000213923	AACACUG
ENSG00000213923	UAUUACC
ENSG00000213923	UCUGGAA
ENSG00000213923	UUCAAAU
ENSG00000213928	GUGCUUG
ENSG00000213928	UAUAAAG
ENSG00000213977	CAGCAGG
ENSG00000213977	GCAGAAG
ENSG00000213977	UCCUGAG

ENSG00000213983	CCUGCUG
ENSG00000214013	UGAGAAU
ENSG00000214014	GAGGCUG
ENSG00000214021	UCACAGG
ENSG00000214022	UAUUGCU
ENSG00000214046	GGAUUUC
ENSG00000214046	GGUUUAC
ENSG00000214063	AGCACCA
ENSG00000214063	GGAAUGU
ENSG00000214078	AAAGUAU
ENSG00000214113	GGUGGUC
ENSG00000214114	AAGGCAC
ENSG00000214114	ACAUUCA
ENSG00000214114	ACCCUGU
ENSG00000214114	AGAUAAA
ENSG00000214114	AGCUGCC
ENSG00000214114	AGGCAGG
ENSG00000214114	AUACAAG
ENSG00000214114	CAAAGUG
ENSG00000214114	GAAGGAA
ENSG00000214114	GAGAAGA
ENSG00000214114	GAGGUAG
ENSG00000214114	GAGGUAU
ENSG00000214114	GGGAACG
ENSG00000214114	GUGAGGU
ENSG00000214114	UAUCAGA
ENSG00000214114	UCACAGG
ENSG00000214114	UCACAGU
ENSG00000214114	UCAUACA
ENSG00000214114	UGAGAAG
ENSG00000214114	UGCAUAU
ENSG00000214128	UUCUUGU
ENSG00000214140	AGCAGCU
ENSG00000214140	AGCUGCC
ENSG00000214140	CAGCUGG
ENSG00000214140	CUGGGGG
ENSG00000214140	GAGAAAU
ENSG00000214193	AUGUCUG
ENSG00000214193	CUGGCUC
ENSG00000214193	GGCAGCG
ENSG00000214193	UUCCUCG
ENSG00000214435	GAAGGAA
ENSG00000214517	CCCUGAG
ENSG00000214517	CUGGGCA
ENSG00000214517	GUGCUUG
ENSG00000214517	UCACAGU
ENSG00000214517	UGUGCUU
ENSG00000214575	AAAGCUG
ENSG00000214575	AAGGCAC
ENSG00000214575	ACAGUAC
ENSG00000214575	ACAGUAU
ENSG00000214575	ACCAGCA
ENSG00000214575	AGCCCUU
ENSG00000214575	AUGGCAC
ENSG00000214575	AUUGCAC
ENSG00000214575	CUUUGGU
ENSG00000214575	GAGACCU
ENSG00000214575	GAGGUAU
ENSG00000214575	GGAAUGU
ENSG00000214575	GUCUUGC
ENSG00000214575	GUGCAAA
ENSG00000214575	UACAGUU
ENSG00000214575	UUGGCAA
ENSG00000214655	GCUGUCU

ENSG00000214655	UAUAAAG
ENSG00000214706	UUGGUUC
ENSG00000214717	GGUUUAC
ENSG00000214753	AACAAAC
ENSG00000214753	AAGACAU
ENSG00000214753	AAGUGCU
ENSG00000214753	ACAAUAU
ENSG00000214753	GGUUGUC
ENSG00000214753	UACAAAG
ENSG00000214753	UACAGUU
ENSG00000214753	UCAACAG
ENSG00000214827	AGCAGCG
ENSG00000214827	AGGAGCU
ENSG00000214827	CUUCUCU
ENSG00000214827	GAAGGAA
ENSG00000214872	UAAUGCU
ENSG00000214882	CUGCUCA
ENSG00000214882	GCCUGGG
ENSG00000214882	GUAAACA
ENSG00000214882	UCCCUUG
ENSG00000214882	UUAUGCA
ENSG00000214882	UUCAAGC
ENSG00000214882	UUUCAAC
ENSG00000215012	CAAAGUG
ENSG00000215012	CUCACAC
ENSG00000215012	GCUUCCU
ENSG00000215012	UCACACA
ENSG00000215012	UCAUUCG
ENSG00000215012	UCCAGUU
ENSG00000215021	AAAAAUC
ENSG00000215021	AAUCACU
ENSG00000215021	GAGAAAU
ENSG00000215021	UGAGAAG
ENSG00000215193	CUGGAAA
ENSG00000215193	GUAACAG
ENSG00000215252	GUGCAAA
ENSG00000215262	CCAGUGC
ENSG00000215262	CCAGUGU
ENSG00000215271	AAGUUGU
ENSG00000215271	AGUAACA
ENSG00000215271	CAAAGUG
ENSG00000215301	AAAAGUA
ENSG00000215301	AAAGAAU
ENSG00000215301	AAAGUAU
ENSG00000215301	AACAUUC
ENSG00000215301	AAGUCAC
ENSG00000215301	AAGUGCA
ENSG00000215301	AAUACUG
ENSG00000215301	AAUCACU
ENSG00000215301	AAUUGCU
ENSG00000215301	ACAAGGU
ENSG00000215301	ACAAUAU
ENSG00000215301	ACAGUAC
ENSG00000215301	ACAUCAC
ENSG00000215301	ACAUUAC
ENSG00000215301	AGCAAAA
ENSG00000215301	AGCAGCG
ENSG00000215301	AGCAGCU
ENSG00000215301	AGCCAGU
ENSG00000215301	AGCCUGC
ENSG00000215301	AGGACAC
ENSG00000215301	AGUGCUU
ENSG00000215301	AUAUAAC
ENSG00000215301	AUGGCUU

ENSG00000215301	AUGUGCC
ENSG00000215301	CACACCG
ENSG00000215301	CAGCUGG
ENSG00000215301	CCAGGAG
ENSG00000215301	CCAGUGU
ENSG00000215301	CCUGCUG
ENSG00000215301	CUACAGU
ENSG00000215301	CUCACAC
ENSG00000215301	CUGGACU
ENSG00000215301	CUGGCUC
ENSG00000215301	CUUUGGU
ENSG00000215301	GAAAACA
ENSG00000215301	GAGGAUA
ENSG00000215301	GCAACUU
ENSG00000215301	GCACCAU
ENSG00000215301	GGAAUGU
ENSG00000215301	GGAUGAG
ENSG00000215301	GUGAAUG
ENSG00000215301	GUGCCAC
ENSG00000215301	GUUCAUG
ENSG00000215301	UAAGACG
ENSG00000215301	UAAGACU
ENSG00000215301	UACAAAG
ENSG00000215301	UACAGUU
ENSG00000215301	UAUCAGA
ENSG00000215301	UCAACAG
ENSG00000215301	UCUGGAG
ENSG00000215301	UGAGAAU
ENSG00000215301	UGAGGAC
ENSG00000215301	UGCAUUG
ENSG00000215301	UGGCCAC
ENSG00000215301	UGUCUUU
ENSG00000215301	UUCAAAU
ENSG00000215301	UUGAUCA
ENSG00000215301	UUGGUCC
ENSG00000215301	UUUUUGC
ENSG00000215386	GAUUGGU
ENSG00000215386	GUCACUC
ENSG00000215386	UGGCUGC
ENSG00000215397	AAAGUGC
ENSG00000215397	AAGUGCU
ENSG00000215397	AAUACUG
ENSG00000215397	AAUUAUU
ENSG00000215397	AGGUAGU
ENSG00000215397	CAGCUGG
ENSG00000215397	CAGUCCA
ENSG00000215397	GAGGUAG
ENSG00000215397	GGCUCAG
ENSG00000215397	GGGGUCC
ENSG00000215397	GUGCUUG
ENSG00000215397	UAAUAUC
ENSG00000215397	UGCAUAG
ENSG00000215397	UGCAUAU
ENSG00000215397	UGUGCUU
ENSG00000215421	CUUUGGU
ENSG00000215421	UUCAAAU
ENSG00000215440	AAUUGCU
ENSG00000215440	CAAAGUG
ENSG00000215440	GAGGUAG
ENSG00000215440	GAGGUAU
ENSG00000215440	GUGAGGU
ENSG00000215440	GUGCAAA
ENSG00000215440	UGCAAAG
ENSG00000215454	CAGCAGG

ENSG00000215455	CAGCAGA
ENSG00000215455	CAGCAGG
ENSG00000215568	CUCCCAC
ENSG00000215712	AAAGAAU
ENSG00000215712	AACAUUC
ENSG00000215712	AAGUCUU
ENSG00000215712	ACAAUAU
ENSG00000215712	ACAUUAC
ENSG00000215712	AGAUAAA
ENSG00000215712	AGUGCUG
ENSG00000215712	AGUGCUU
ENSG00000215712	AUGGAUU
ENSG00000215712	CAAAUGC
ENSG00000215712	CAGCAAA
ENSG00000215712	CCAGGAG
ENSG00000215712	CCAUUAC
ENSG00000215712	GGAUUUC
ENSG00000215712	GGUCUAG
ENSG00000215712	UAAUAGU
ENSG00000215712	UGAAAUG
ENSG00000215712	UUAUGCA
ENSG00000215712	UUCAAAU
ENSG00000215712	UUCCAUA
ENSG00000215717	ACAAGGU
ENSG00000215717	ACCCUGU
ENSG00000215717	GUGCAAA
ENSG00000215788	GUCUACU
ENSG00000216490	GCACCAU
ENSG00000216937	AAAGUAU
ENSG00000217128	AAACUGU
ENSG00000217128	AGGUAGU
ENSG00000217128	AGGUUAU
ENSG00000217128	AGUAGAC
ENSG00000217128	AUCCCUU
ENSG00000217128	CUUACAG
ENSG00000217128	CUUGGGC
ENSG00000217128	CUUUGGU
ENSG00000217128	GAAAACA
ENSG00000217128	GAAACAU
ENSG00000217128	GAGGUAU
ENSG00000217128	UAAGACG
ENSG00000217128	UAAGACU
ENSG00000217128	UAAUACA
ENSG00000217128	UCAAGUA
ENSG00000217128	UCCUAUA
ENSG00000217128	UGACAUC
ENSG00000217128	UUGGUUC
ENSG00000217128	UUUUUGC
ENSG00000218336	AAGGUGC
ENSG00000218336	ACAGUAC
ENSG00000218336	ACAGUAU
ENSG00000218336	ACUGUAG
ENSG00000218336	AGUGCCU
ENSG00000218336	AUGCAUU
ENSG00000218336	AUGUAAU
ENSG00000218336	AUUCAUU
ENSG00000218336	CAAAGCA
ENSG00000218336	UCACACA
ENSG00000218336	UGCAUAU
ENSG00000218336	UGUGCUU
ENSG00000218823	AAAUCCC
ENSG00000218823	AAAUUUC
ENSG00000218823	AAGUGCU
ENSG00000218823	CAAAGCA

ENSG00000218823	CAGUUAC
ENSG00000218823	GCCUGGA
ENSG00000218823	GCCUGGG
ENSG00000218823	GUGCCAC
ENSG00000218891	CCAGUGC
ENSG00000218891	CCAGUGU
ENSG00000218891	UCACAUU
ENSG00000219438	AAAAACC
ENSG00000219438	AAAACUG
ENSG00000219438	AAAGUAU
ENSG00000219438	AAAUGGU
ENSG00000219438	ACUGUAG
ENSG00000219438	AUACAAG
ENSG00000219438	GGUCUAG
ENSG00000219438	GUAACAG
ENSG00000219438	GUUAAUG
ENSG00000219438	UAUGGUU
ENSG00000219438	UAUUGCU
ENSG00000219438	UCUGGAG
ENSG00000219438	UUCAAAU
ENSG00000219438	UUGGUCC
ENSG00000220205	AAAAAUC
ENSG00000220205	AAAACUG
ENSG00000220205	AAUUAUU
ENSG00000220205	ACACACU
ENSG00000220205	ACCACAG
ENSG00000220205	AGGAGCU
ENSG00000220205	AUAAUAC
ENSG00000220205	AUAUAAC
ENSG00000220205	AUGGCUU
ENSG00000220205	CACAAUG
ENSG00000220205	CACCACC
ENSG00000220205	CAGUCCA
ENSG00000220205	CUACAAA
ENSG00000220205	CUAGUAG
ENSG00000220205	CUCUGGG
ENSG00000220205	CUGGGCA
ENSG00000220205	CUGGGGG
ENSG00000220205	GGAAUGU
ENSG00000220205	GGAGGAG
ENSG00000220205	GGGUAAG
ENSG00000220205	GUCAGUU
ENSG00000220205	GUGCCAC
ENSG00000220205	UCACAAG
ENSG00000220205	UGCAUAG
ENSG00000220205	UGCAUAU
ENSG00000220205	UUGGUCC
ENSG00000221818	AAGUCUU
ENSG00000221818	AGUAGUU
ENSG00000221818	CCCUGUU
ENSG00000221818	CUACAAA
ENSG00000221818	UGAAAGG
ENSG00000221818	UGGUACC
ENSG00000221823	AAACUGU
ENSG00000221823	AAAGUAA
ENSG00000221823	AACACUG
ENSG00000221823	AAGUGCU
ENSG00000221823	ACAUUCA
ENSG00000221823	ACUGUGU
ENSG00000221823	AGCAAAA
ENSG00000221823	AGGACAC
ENSG00000221823	AUAUUAU
ENSG00000221823	AUGCACC
ENSG00000221823	CAAAACU

ENSG00000221823	CCUUCAU
ENSG00000221823	GAAGGAA
ENSG00000221823	GAGGCUG
ENSG00000221823	GCAGCAU
ENSG00000221823	GCUGGAU
ENSG00000221823	GUAGUGU
ENSG00000221823	UCAAGUA
ENSG00000221823	UCACAAG
ENSG00000221823	UCACACA
ENSG00000221823	UCACAGG
ENSG00000221823	UCACAGU
ENSG00000221823	UCCAGAG
ENSG00000221823	UCCCUUU
ENSG00000221823	UCUUGAG
ENSG00000221823	UGAUCCA
ENSG00000221823	UGGAGAU
ENSG00000221823	UGGGUGA
ENSG00000221823	UUGACAC
ENSG00000221837	GGCAGGG
ENSG00000221866	AAAAGUA
ENSG00000221866	AAAGGUA
ENSG00000221866	AAGUAGC
ENSG00000221866	AAUUAUU
ENSG00000221866	ACUCAAA
ENSG00000221866	AGCACCA
ENSG00000221866	AGCAGCA
ENSG00000221866	AGCCCUU
ENSG00000221866	AGGGAGG
ENSG00000221866	AUAAUAC
ENSG00000221866	AUACAAG
ENSG00000221866	AUAUUAU
ENSG00000221866	AUGCCUU
ENSG00000221866	AUGGCAC
ENSG00000221866	CACUGUU
ENSG00000221866	CUCUUCC
ENSG00000221866	CUGCAGC
ENSG00000221866	CUGCAGG
ENSG00000221866	CUUUGGU
ENSG00000221866	GAGCCCU
ENSG00000221866	GAGGUAG
ENSG00000221866	GAGGUAU
ENSG00000221866	GCACCAU
ENSG00000221866	GCUGGUG
ENSG00000221866	GGCAAGA
ENSG00000221866	GGCACGG
ENSG00000221866	GGCCCUG
ENSG00000221866	GUGCUUG
ENSG00000221866	UACAAAG
ENSG00000221866	UAGACUG
ENSG00000221866	UCCUGAG
ENSG00000221866	UGCAUAG
ENSG00000221866	UGGUACC
ENSG00000221866	UGUGCUU
ENSG00000221866	UUCUGCA
ENSG00000221866	UUGGUUC
ENSG00000221866	UUGUGUC
ENSG00000221866	UUUUUGC
ENSG00000221869	AAAAAUC
ENSG00000221869	AGCUGCC
ENSG00000221869	CUACAAA
ENSG00000221869	GAGGUAG
ENSG00000221869	GAGGUAU
ENSG00000221870	AACAGUC
ENSG00000221870	AACCUGU

ENSG00000221870	AAGGCAC
ENSG00000221870	GCAGGUU
ENSG00000221870	GGUGGGC
ENSG00000221870	UGAAAUG
ENSG00000221890	AACAAAC
ENSG00000221890	CAAAGCA
ENSG00000221890	CAGCAGG
ENSG00000221890	CCAGGAG
ENSG00000221890	CUAGUCC
ENSG00000221890	CUCUGGG
ENSG00000221890	CUGGCUC
ENSG00000221890	GGCUCUG
ENSG00000221890	GGGGUGG
ENSG00000221890	UCACAGU
ENSG00000221899	ACAAGGU
ENSG00000221899	GGCAAGA
ENSG00000221899	UCCAGAG
ENSG00000221899	UCGUGCA
ENSG00000221899	UUGUUCG
ENSG00000221914	AAAGUGC
ENSG00000221914	AACACUG
ENSG00000221914	AUGAGCU
ENSG00000221914	AUGGCAC
ENSG00000221914	CACCACC
ENSG00000221914	CCAGGAG
ENSG00000221914	CUGGAAA
ENSG00000221914	GCUAGCA
ENSG00000221914	GGCAAGA
ENSG00000221914	GUGCUUG
ENSG00000221914	UGUGCUU
ENSG00000221946	CCCACCG
ENSG00000221949	AGUGAUC
ENSG00000221949	GGAUUUU
ENSG00000221968	AAAAAUC
ENSG00000221978	AAAGAGC
ENSG00000221978	AAAGCGC
ENSG00000221978	AAAGCUG
ENSG00000221978	AAAGGUA
ENSG00000221978	AACACUG
ENSG00000221978	AACAUUC
ENSG00000221978	AAGCGCU
ENSG00000221978	AAGUCUU
ENSG00000221978	ACUCAGC
ENSG00000221978	AGAUAAA
ENSG00000221978	AGCACCA
ENSG00000221978	AGCCUGC
ENSG00000221978	AGGAGCU
ENSG00000221978	AGUGCAA
ENSG00000221978	CAAAGCA
ENSG00000221978	CACACCU
ENSG00000221978	CAGCAGA
ENSG00000221978	CUUGUAU
ENSG00000221978	GCAACUU
ENSG00000221978	GCACCAU
ENSG00000221978	GCUGGUG
ENSG00000221978	GGAGUCC
ENSG00000221978	UCAACAG
ENSG00000221978	UCACAUU
ENSG00000221978	UCCUGAG
ENSG00000221978	UGCAAAG
ENSG00000221978	UUCUUGU
ENSG00000221986	GCUUCCU
ENSG00000221988	AGCAGCA
ENSG00000221988	UCCUGAG

ENSG00000221994	UCCCACA
ENSG00000222014	GUGCUUG
ENSG00000222028	AAAGAGG
ENSG00000222040	AAUCACU
ENSG00000222040	ACACACC
ENSG00000222040	CAAAGCA
ENSG00000222040	CAGCAGA
ENSG00000222040	GGAAGAC
ENSG00000222040	GUAAACA
ENSG00000222040	GUGAAUG
ENSG00000222040	UACAAAG
ENSG00000222040	UCACACA
ENSG00000222040	UCACAUU
ENSG00000222040	UCCAGUU
ENSG00000222047	GACUUCC
ENSG00000223380	AAUCUCA
ENSG00000223380	AUGGCUU
ENSG00000223380	CUGCCCC
ENSG00000223380	GAGGCAG
ENSG00000223380	GAGUCUU
ENSG00000223380	GGAAUGU
ENSG00000223380	GUGCAAA
ENSG00000223380	UAAUACA
ENSG00000223380	UUCUAAU
ENSG00000223501	CAGGUGA
ENSG00000225190	AAAGUGC
ENSG00000225190	AAGGCAC
ENSG00000225190	AAGUGCU
ENSG00000225190	ACUCAGC
ENSG00000225190	ACUGCAG
ENSG00000225190	ACUGCAU
ENSG00000225190	AGUGCUG
ENSG00000225190	AUGGCUU
ENSG00000225190	AUUGCAC
ENSG00000225190	CAAAGUG
ENSG00000225190	CUCAGGG
ENSG00000225190	GAAAACA
ENSG00000225190	GCUGGUG
ENSG00000225190	GGAAUGU
ENSG00000225190	GGCUCUG
ENSG00000225190	UGCAUAG
ENSG00000225190	UGCAUAU
ENSG00000225190	UGCUGAC
ENSG00000225190	UGGGUGA
ENSG00000225190	UUGGCAA
ENSG00000225190	UUGGCAC
ENSG00000225697	GGAGUCC
ENSG00000225830	GAGGUAU
ENSG00000226979	AGGCAGG
ENSG00000226979	GGAUUUU
ENSG00000227345	AGCACCA
ENSG00000227345	CUUUGGU
ENSG00000227345	UUGGUUC
ENSG00000227372	AGUGCUG
ENSG00000227372	CUUUGGU
ENSG00000227372	GUAACAG
ENSG00000227500	AACAAAC
ENSG00000227500	GAGAUGA
ENSG00000227802	UAUAAAG
ENSG00000229117	GUCCAGA
ENSG00000230989	AAAAACC
ENSG00000230989	AAAAGUA
ENSG00000230989	AACCUGU
ENSG00000230989	AAUCUCA

ENSG00000230989	CAAAACU
ENSG00000230989	GGAUUUC
ENSG00000230989	UUGGCAA
ENSG00000231852	GAGAAGA
ENSG00000231925	GAGCGCC
ENSG00000232810	CCCCUGG
ENSG00000232810	CUGGAAA
ENSG00000232810	GAAAACA
ENSG00000232810	GUGCAAA
ENSG00000233034	CUUUGGU
ENSG00000233224	AAAGAGC
ENSG00000233822	AAAGAGC
ENSG00000234127	ACUCAGG
ENSG00000234127	GCAGCAU
ENSG00000234186	UGUCUUU
ENSG00000234545	AAAGUAC
ENSG00000234545	AAAUUUC
ENSG00000234545	AAGGCAC
ENSG00000234545	ACUGUGU
ENSG00000234545	CAAAGUG
ENSG00000234545	UCACACA
ENSG00000234545	UCACAGG
ENSG00000234545	UUCAAAU
ENSG00000235568	CCCACCU
ENSG00000235718	UCUAGAG
ENSG00000236279	AGCACCA
ENSG00000237190	AUGAUGA
ENSG00000237190	UAUAAAG
ENSG00000237190	UCACAUU
ENSG00000237441	CUGCCCC
ENSG00000238227	ACCAGCA
ENSG00000238227	AGCACCA
ENSG00000238227	AGCAGCA
ENSG00000238227	AGCAGCU
ENSG00000238227	AGCCCUU
ENSG00000238227	CACCAGC
ENSG00000238227	GACCAUG
ENSG00000238227	GCAGCAU
ENSG00000238227	GCAGGUU
ENSG00000238227	GGCAGUG
ENSG00000238227	GUGCAAA
ENSG00000238227	UAAUGCU
ENSG00000239264	AAAGUAU
ENSG00000239264	AAGGCAC
ENSG00000239264	AUACAAG
ENSG00000239264	AUGGCAC
ENSG00000239264	CACAGUG
ENSG00000239264	CUCAAAC
ENSG00000239264	CUUCUCU
ENSG00000239264	CUUUGGU
ENSG00000239264	UUGGUCC
ENSG00000239306	AAAUGGU
ENSG00000239306	AAGGCGC
ENSG00000239306	ACUUGGA
ENSG00000239306	CACCACC
ENSG00000239306	CUGGACU
ENSG00000239306	GCAGGUU
ENSG00000239306	GGACUGC
ENSG00000239306	UGGACUG
ENSG00000239306	UUCUAAU
ENSG00000239388	UCUCAAG
ENSG00000239389	AAAGAAU
ENSG00000239389	AAGUGCA
ENSG00000239389	AGCAGCG

ENSG00000239389	AGCAGCU
ENSG00000239389	AUAAUAC
ENSG00000239389	CAAAGUG
ENSG00000239389	GAGGCAG
ENSG00000239389	UAAUACA
ENSG00000239389	UAAUAGU
ENSG00000239389	UACAAAG
ENSG00000239389	UAUAAUA
ENSG00000239389	UCAACAG
ENSG00000239389	UCACAUU
ENSG00000239389	UCAUACA
ENSG00000239389	UGCAUAG
ENSG00000239389	UGCAUAU
ENSG00000239389	UGUGCUU
ENSG00000239389	UUUGCAA
ENSG00000239389	UUUGCAC
ENSG00000239697	CACAGGG
ENSG00000239704	GCCUGGA
ENSG00000239779	UGUAUGU
ENSG00000240021	UUCAAGC
ENSG00000240065	UACAAAG
ENSG00000240065	UAUUACC
ENSG00000240184	AAAACUG
ENSG00000240184	CACCAGC
ENSG00000240184	GGAGGCA
ENSG00000240184	UGCAUAG
ENSG00000240184	UGGCCAC
ENSG00000240224	CUCAAAC
ENSG00000240224	UCCCACC
ENSG00000240428	UCCCUCU
ENSG00000240583	AUGGAUU
ENSG00000240583	CAGGUGA
ENSG00000240583	CUGCCCC
ENSG00000240583	CUUGGGC
ENSG00000240583	GGUCUAG
ENSG00000240583	UUGGUCC
ENSG00000240694	AUGCAUU
ENSG00000240694	UGCAUUG
ENSG00000240764	AAAACUG
ENSG00000240764	CACCAGC
ENSG00000240764	GGAGGCA
ENSG00000240764	UGCAUAG
ENSG00000240764	UGGCCAC
ENSG00000240849	ACAUUAC
ENSG00000240849	AGUAGAC
ENSG00000240849	AUGGCUU
ENSG00000240849	GGCUCAG
ENSG00000240849	GGGGUGG
ENSG00000240849	GUAUUCU
ENSG00000240849	UAUAAAG
ENSG00000240849	UCAAGUA
ENSG00000240849	UGUGUCA
ENSG00000240849	UUGGCAA
ENSG00000240849	UUGGCAC
ENSG00000241058	CAGCUGG
ENSG00000241119	CUCAAAC
ENSG00000241119	UCCCACC
ENSG00000241322	CCUGUAC
ENSG00000241322	UCAACAG
ENSG00000241360	AACACUG
ENSG00000241553	AAUCACU
ENSG00000241553	CUGGCUC
ENSG00000241553	GCUAGCA
ENSG00000241553	GGGGCAG

ENSG00000241553	UACAGUU
ENSG00000241553	UUCAAGC
ENSG00000241635	CUCAAAC
ENSG00000241635	UCCCACC
ENSG00000241685	CUUUGGU
ENSG00000241697	AAAAGUA
ENSG00000241697	AACAGUC
ENSG00000241839	AAAGUGC
ENSG00000241839	AAAUCCC
ENSG00000241839	AAGGCGC
ENSG00000241839	AAGUGCA
ENSG00000241839	CAGCUGG
ENSG00000241839	CAGGAAC
ENSG00000241839	GAGAUGA
ENSG00000241839	GCUGGUG
ENSG00000241839	GGAAUGU
ENSG00000241839	GGAGGCA
ENSG00000241839	GGCUCAG
ENSG00000241839	GUAAACA
ENSG00000241839	UAUUGCU
ENSG00000241852	AGCAGCA
ENSG00000241852	GAGGUAU
ENSG00000241852	GGAUUUC
ENSG00000241852	UCACAUU
ENSG00000241878	AAUACUG
ENSG00000241878	ACUGUGU
ENSG00000241878	ACUGUUU
ENSG00000241878	AGCAGCG
ENSG00000241878	AGCAGCU
ENSG00000241878	CUGCAGG
ENSG00000241878	GAGAAAU
ENSG00000241878	GCAGGAC
ENSG00000241878	GGCCCUG
ENSG00000241878	UACAGUU
ENSG00000241878	UCACAGG
ENSG00000241878	UCACAGU
ENSG00000241878	UUCAAAU
ENSG00000241878	UUUGCAA
ENSG00000241973	CCAGUGU
ENSG00000241973	GAUUGUC
ENSG00000241978	AAAAAUC
ENSG00000241978	AAAGCUG
ENSG00000241978	AACAUUC
ENSG00000241978	AAUACUG
ENSG00000241978	ACACACC
ENSG00000241978	ACUGCAU
ENSG00000241978	AUCCCUU
ENSG00000241978	AUGGCUU
ENSG00000241978	CAAAACU
ENSG00000241978	CACUCCU
ENSG00000241978	CAGCAAA
ENSG00000241978	CAUAUUG
ENSG00000241978	CCAUUAC
ENSG00000241978	GAGAAAU
ENSG00000241978	GCAGCAU
ENSG00000241978	GGCAGCG
ENSG00000241978	GGUCUAG
ENSG00000241978	GUGACUG
ENSG00000241978	UACAAAG
ENSG00000241978	UAGUGAG
ENSG00000241978	UCUGGAA
ENSG00000241978	UGGCUGC
ENSG00000241978	UUAGGAU
ENSG00000241994	CAAAUGC

ENSG00000241994	GCAGAAG
ENSG00000241994	UGAAGUG
ENSG00000242028	UAUUGCU
ENSG00000242108	AAUUAUU
ENSG00000242108	AAUUUUA
ENSG00000242108	ACAAUAU
ENSG00000242108	AGCAAAA
ENSG00000242108	AGGCAGG
ENSG00000242108	AUAAUAC
ENSG00000242108	GCAGCAU
ENSG00000242108	UCAUUUG
ENSG00000242265	AAAGAAU
ENSG00000242265	AGCAAAA
ENSG00000242265	AGGUAGU
ENSG00000242265	AUACCUC
ENSG00000242265	AUCCUUG
ENSG00000242265	AUGAUGA
ENSG00000242265	AUGCACC
ENSG00000242265	CACAGUG
ENSG00000242265	CAGCAAA
ENSG00000242265	CAGCAGG
ENSG00000242265	CUCAAAC
ENSG00000242265	CUGCAGC
ENSG00000242265	CUGCUCA
ENSG00000242265	CUUCACC
ENSG00000242265	CUUCUCU
ENSG00000242265	GAGAAGA
ENSG00000242265	GAGGUAG
ENSG00000242265	GAGGUAU
ENSG00000242265	GAGUGUG
ENSG00000242265	GCAGCAU
ENSG00000242265	GCUUCCU
ENSG00000242265	GGAGGCA
ENSG00000242265	GGAGUGU
ENSG00000242265	GGCAGUG
ENSG00000242265	GGGAGCU
ENSG00000242265	UAAUAGU
ENSG00000242265	UCACAGG
ENSG00000242265	UCACCAC
ENSG00000242265	UCCAGUU
ENSG00000242265	UCCCUUU
ENSG00000242265	UGAAAGG
ENSG00000242265	UGCAGCU
ENSG00000242265	UUUGCAC
ENSG00000242366	CUCAAAC
ENSG00000242366	UCCCACC
ENSG00000242372	GUCCAGA
ENSG00000242372	UCCAGAG
ENSG00000242419	AAAACUG
ENSG00000242419	CACCAGC
ENSG00000242419	GGAGGCA
ENSG00000242419	UGCAUAG
ENSG00000242419	UGGCCAC
ENSG00000242441	CAGUGCA
ENSG00000242515	CUCAAAC
ENSG00000242515	UCCCACC
ENSG00000242616	AAGGCAC
ENSG00000242689	GUGAGGU
ENSG00000242732	ACACACC
ENSG00000242732	CUUACAG
ENSG00000242732	UGAGGAC
ENSG00000243056	GGUCUAG
ENSG00000243135	CUCAAAC
ENSG00000243135	UCCCACC

ENSG00000243156	AAAGUGC
ENSG00000243156	CCAGUGC
ENSG00000243156	CUAGUAA
ENSG00000243156	CUUUGGU
ENSG00000243156	GGAGGAG
ENSG00000243156	GUGCAAA
ENSG00000243156	UCAUUUG
ENSG00000243156	UCCAGUU
ENSG00000243156	UUCAAGC
ENSG00000243232	AAAGAAU
ENSG00000243232	AAGUGCA
ENSG00000243232	AGCAGCG
ENSG00000243232	AGCAGCU
ENSG00000243232	AUAAUAC
ENSG00000243232	CAAAGUG
ENSG00000243232	GAGGCAG
ENSG00000243232	UAAUACA
ENSG00000243232	UAAUAGU
ENSG00000243232	UACAAAG
ENSG00000243232	UAUAAUA
ENSG00000243232	UCAACAG
ENSG00000243232	UCACAUU
ENSG00000243232	UCAUACA
ENSG00000243232	UGCAUAG
ENSG00000243232	UGCAUAU
ENSG00000243232	UGUGCUU
ENSG00000243232	UUUGCAA
ENSG00000243232	UUUGCAC
ENSG00000243244	AGAUGUG
ENSG00000243244	GUUAAUG
ENSG00000243244	UAAGACU
ENSG00000243279	GGCUCUG
ENSG00000243284	AACACUG
ENSG00000243284	GAGCCCC
ENSG00000243335	GUAAACA
ENSG00000243335	UUCUAAU
ENSG00000243335	UUGGCAA
ENSG00000243335	UUGGCAC
ENSG00000243364	AAAAAUC
ENSG00000243364	GGUUUAC
ENSG00000243364	UAUAAAG
ENSG00000243364	UUGGUCC
ENSG00000243414	UUAGAGA
ENSG00000243444	AAAGGUA
ENSG00000243444	AACAGUC
ENSG00000243444	AAGGCAC
ENSG00000243444	AAUCACU
ENSG00000243444	AAUGUUG
ENSG00000243444	ACAGUAC
ENSG00000243444	ACAGUAU
ENSG00000243444	ACAUUAC
ENSG00000243444	ACCCUGU
ENSG00000243444	ACUGUUU
ENSG00000243444	AGCACCA
ENSG00000243444	AGCAGCU
ENSG00000243444	AGGCAGG
ENSG00000243444	AGUGGUU
ENSG00000243444	AUGGCAC
ENSG00000243444	AUUCAUU
ENSG00000243444	CAAAGUG
ENSG00000243444	CAAAUGC
ENSG00000243444	CACAGUG
ENSG00000243444	CAGACAG
ENSG00000243444	CAGCAAA

ENSG00000243444	CAGGCUC
ENSG00000243444	CUUUGGU
ENSG00000243444	GAUUGUC
ENSG00000243444	GCACCAU
ENSG00000243444	GCUUCCU
ENSG00000243444	GGAAGCC
ENSG00000243444	GGAGGCA
ENSG00000243444	GGGUGUU
ENSG00000243444	GUCCAGA
ENSG00000243444	GUGAGGU
ENSG00000243444	GUGCUUG
ENSG00000243444	UAAUACA
ENSG00000243444	UAAUGCU
ENSG00000243444	UCACAAG
ENSG00000243444	UCACAGG
ENSG00000243444	UCAUUUG
ENSG00000243444	UCCCUUU
ENSG00000243444	UGCAUAU
ENSG00000243646	CACCAGC
ENSG00000243667	AAUCUCU
ENSG00000243709	AAGUGCA
ENSG00000243709	AGUGCUU
ENSG00000243725	AGGGAGG
ENSG00000243789	GCAACUU
ENSG00000243943	GAAGGAA
ENSG00000243943	UAUUGCU
ENSG00000243978	AACACUG
ENSG00000243978	GAGAUGA
ENSG00000243978	GCCUGGA
ENSG00000243978	GUGCCUG
ENSG00000244005	AGCAGCA
ENSG00000244038	GUGCCAC
ENSG00000244122	CUCAAAC
ENSG00000244122	UCCCACC
ENSG00000244405	ACUGUAG
ENSG00000244405	AGCCCUU
ENSG00000244405	AUGCAUU
ENSG00000244405	CAAAACU
ENSG00000244405	CAGUUAC
ENSG00000244405	CCCUGUU
ENSG00000244405	CUACAAA
ENSG00000244405	GAGAACC
ENSG00000244405	GAUUGUC
ENSG00000244405	GCCCUGU
ENSG00000244405	GGCAGGG
ENSG00000244405	GGCCCUG
ENSG00000244405	UAUAAAG
ENSG00000244405	UCUGGAA
ENSG00000244405	UGCCCUG
ENSG00000244405	UGUCUUU
ENSG00000244462	AAGGCAC
ENSG00000244462	ACCUGGA
ENSG00000244462	AUGCAUU
ENSG00000244462	CAAGAGC
ENSG00000244462	CUGGAAA
ENSG00000244462	GAAAACA
ENSG00000244462	GGCAAGA
ENSG00000244462	UAUUGCU
ENSG00000244474	CUCAAAC
ENSG00000244474	UCCCACC
ENSG00000244486	CAAAACU
ENSG00000244486	GAGGCAG
ENSG00000244617	AAUUAUU
ENSG00000244617	GGGUCUU

ENSG00000244687	ACUGUGU
ENSG00000244687	AGAUGUG
ENSG00000244687	AGUGGUU
ENSG00000244687	AUGUGCC
ENSG00000244687	CACAGUG
ENSG00000244687	UCACAGG
ENSG00000244754	CAGGCUC
ENSG00000244754	GUGAGGU
ENSG00000245848	ACAGUAU
ENSG00000245848	ACAUUCA
ENSG00000245848	AUACAAG
ENSG00000245848	AUUGCAC
ENSG00000245848	GGCAAGA
ENSG00000245848	UCACAUU
ENSG00000247596	AAAAAUC
ENSG00000247596	AGCAUUC
ENSG00000248383	AAAGAAU
ENSG00000248383	AAGUGCA
ENSG00000248383	AGCAGCG
ENSG00000248383	AGCAGCU
ENSG00000248383	AUAAUAC
ENSG00000248383	CAAAGUG
ENSG00000248383	GAGGCAG
ENSG00000248383	UAAUACA
ENSG00000248383	UAAUAGU
ENSG00000248383	UACAAAG
ENSG00000248383	UAUAAUA
ENSG00000248383	UCAACAG
ENSG00000248383	UCACAUU
ENSG00000248383	UCAUACA
ENSG00000248383	UGCAUAG
ENSG00000248383	UGCAUAU
ENSG00000248383	UGUGCUU
ENSG00000248383	UUUGCAA
ENSG00000248383	UUUGCAC
ENSG00000248485	GGAGAGA
ENSG00000248541	AAAGAGC
ENSG00000248857	AAAGCUG
ENSG00000248857	AAGUGCU
ENSG00000248857	CUUGGAG
ENSG00000248857	UGCAAAG
ENSG00000248871	UCCAGUU
ENSG00000249158	AAAGAAU
ENSG00000249158	AAGUGCA
ENSG00000249158	AGCAGCG
ENSG00000249158	AGCAGCU
ENSG00000249158	AUAAUAC
ENSG00000249158	CAAAGUG
ENSG00000249158	GAGGCAG
ENSG00000249158	UAAUACA
ENSG00000249158	UAAUAGU
ENSG00000249158	UACAAAG
ENSG00000249158	UAUAAUA
ENSG00000249158	UCAACAG
ENSG00000249158	UCACAUU
ENSG00000249158	UCAUACA
ENSG00000249158	UGCAUAG
ENSG00000249158	UGCAUAU
ENSG00000249158	UGUGCUU
ENSG00000249158	UUUGCAA
ENSG00000249158	UUUGCAC
ENSG00000249915	AAAAGUA
ENSG00000249915	AAAGUAA
ENSG00000249915	AUGGCAC

ENSG00000249992	AAAGAGC
ENSG00000249992	AUGUCUG
ENSG00000249992	GGAGGAG
ENSG00000249992	UACAAAG
ENSG00000249992	UUCUGCA
ENSG00000250120	AAAGAAU
ENSG00000250120	AAGUGCA
ENSG00000250120	AGCAGCG
ENSG00000250120	AGCAGCU
ENSG00000250120	AUAAUAC
ENSG00000250120	CAAAGUG
ENSG00000250120	GAGGCAG
ENSG00000250120	UAAUACA
ENSG00000250120	UAAUAGU
ENSG00000250120	UACAAAG
ENSG00000250120	UAUAAUA
ENSG00000250120	UCAACAG
ENSG00000250120	UCACAUU
ENSG00000250120	UCAUACA
ENSG00000250120	UGCAUAG
ENSG00000250120	UGCAUAU
ENSG00000250120	UGUGCUU
ENSG00000250120	UUUGCAA
ENSG00000250120	UUUGCAC
ENSG00000250510	AGCUGCC
ENSG00000250510	GAGGCAG
ENSG00000250510	GGAGGCA
ENSG00000251322	AAAUUUC
ENSG00000251322	ACAGGUG
ENSG00000251322	ACAUAGA
ENSG00000251322	ACCCUGU
ENSG00000251322	AUAAUAC
ENSG00000251322	CACAGUG
ENSG00000251322	CAGCAAA
ENSG00000251322	CAGCUGG
ENSG00000251322	CCCCUGG
ENSG00000251322	CCGAGCC
ENSG00000251322	CGCCCUU
ENSG00000251322	GAAACAU
ENSG00000251322	GAGGGGC
ENSG00000251322	GGAAGAC
ENSG00000251322	GGCCCUG
ENSG00000251322	GGGGUGG
ENSG00000251322	GUCCAGA
ENSG00000251322	GUGGGGA
ENSG00000251322	UAUAAAG
ENSG00000251322	UAUUACC
ENSG00000251322	UCACAGU
ENSG00000251380	AAAUGGU
ENSG00000251380	AAGUGCU
ENSG00000251380	ACUCAAA
ENSG00000251380	UCCAGAG
ENSG00000251664	AAAGAAU
ENSG00000251664	AAGUGCA
ENSG00000251664	AGCAGCG
ENSG00000251664	AGCAGCU
ENSG00000251664	AUAAUAC
ENSG00000251664	CAAAGUG
ENSG00000251664	GAGGCAG
ENSG00000251664	UAAUACA
ENSG00000251664	UAAUAGU
ENSG00000251664	UACAAAG
ENSG00000251664	UAUAAUA
ENSG00000251664	UCAACAG

ENSG00000251664	UCACAUU
ENSG00000251664	UCAUACA
ENSG00000251664	UGCAUAG
ENSG00000251664	UGCAUAU
ENSG00000251664	UGUGCUU
ENSG00000251664	UUUGCAA
ENSG00000251664	UUUGCAC
ENSG00000253148	CAAAUGC
ENSG00000253148	UUCUAAU
ENSG00000253159	AAAACUG
ENSG00000253159	CACCAGC
ENSG00000253159	GGAGGCA
ENSG00000253159	UGCAUAG
ENSG00000253159	UGGCCAC
ENSG00000253293	AACAUUC
ENSG00000253293	ACAGGUG
ENSG00000253293	ACAGUAU
ENSG00000253293	CACAGUG
ENSG00000253293	UAUAAAG
ENSG00000253293	UCACAGG
ENSG00000253293	UCAUUCG
ENSG00000253293	UGUGCUU
ENSG00000253304	AAUCUCU
ENSG00000253304	AGUAGUU
ENSG00000253304	AGUGGUU
ENSG00000253304	AUUGCAC
ENSG00000253304	CAGCAGA
ENSG00000253304	CAGUAGU
ENSG00000253304	GAAGGAA
ENSG00000253304	GGAUUUC
ENSG00000253304	GUCUUGC
ENSG00000253304	UCAAGUA
ENSG00000253304	UGCAUUG
ENSG00000253304	UUUCAAC
ENSG00000253305	AAAACUG
ENSG00000253305	CACCAGC
ENSG00000253305	GGAGGCA
ENSG00000253305	UGCAUAG
ENSG00000253305	UGGCCAC
ENSG00000253313	CCCUGAG
ENSG00000253313	UUGGCAC
ENSG00000253485	AAAACUG
ENSG00000253485	CACCAGC
ENSG00000253485	GGAGGCA
ENSG00000253485	UGCAUAG
ENSG00000253485	UGGCCAC
ENSG00000253537	AAAACUG
ENSG00000253537	CACCAGC
ENSG00000253537	GGAGGCA
ENSG00000253537	UGCAUAG
ENSG00000253537	UGGCCAC
ENSG00000253731	AAAACUG
ENSG00000253731	CACCAGC
ENSG00000253731	GGAGGCA
ENSG00000253731	UGCAUAG
ENSG00000253731	UGGCCAC
ENSG00000253767	AAAACUG
ENSG00000253767	CACCAGC
ENSG00000253767	GGAGGCA
ENSG00000253767	UGCAUAG
ENSG00000253767	UGGCCAC
ENSG00000253797	AAAACUG
ENSG00000253797	AAUCACU
ENSG00000253797	UGUCUGC

ENSG00000253797	UGUCUGG
ENSG00000253797	UUCUUGU
ENSG00000253846	AAAACUG
ENSG00000253846	CACCAGC
ENSG00000253846	GGAGGCA
ENSG00000253846	UGCAUAG
ENSG00000253846	UGGCCAC
ENSG00000253873	AAAACUG
ENSG00000253873	CACCAGC
ENSG00000253873	GGAGGCA
ENSG00000253873	UGCAUAG
ENSG00000253873	UGGCCAC
ENSG00000253910	AAAACUG
ENSG00000253910	CACCAGC
ENSG00000253910	GGAGGCA
ENSG00000253910	UGCAUAG
ENSG00000253910	UGGCCAC
ENSG00000253928	AUAAAGU
ENSG00000253928	UAUAUGA
ENSG00000253928	UGAAAGG
ENSG00000253950	AAGUGCA
ENSG00000253950	ACAUUAC
ENSG00000253950	CACACCG
ENSG00000253950	UAUAAUA
ENSG00000253950	UGCCAGC
ENSG00000253953	AAAACUG
ENSG00000253953	CACCAGC
ENSG00000253953	GGAGGCA
ENSG00000253953	UGCAUAG
ENSG00000253953	UGGCCAC
ENSG00000253958	AGAUAAA
ENSG00000253958	UAUAAAG
ENSG00000254108	AAAGAAU
ENSG00000254108	AAGUGCA
ENSG00000254108	AGCAGCG
ENSG00000254108	AGCAGCU
ENSG00000254108	AUAAUAC
ENSG00000254108	CAAAGUG
ENSG00000254108	GAGGCAG
ENSG00000254108	UAAUACA
ENSG00000254108	UAAUAGU
ENSG00000254108	UACAAAG
ENSG00000254108	UAUAAUA
ENSG00000254108	UCAACAG
ENSG00000254108	UCACAUU
ENSG00000254108	UCAUACA
ENSG00000254108	UGCAUAG
ENSG00000254108	UGCAUAU
ENSG00000254108	UGUGCUU
ENSG00000254108	UUUGCAA
ENSG00000254108	UUUGCAC
ENSG00000254122	AAAACUG
ENSG00000254122	CACCAGC
ENSG00000254122	GGAGGCA
ENSG00000254122	UGCAUAG
ENSG00000254122	UGGCCAC
ENSG00000254147	ACCAGCA
ENSG00000254147	CCUGCUG
ENSG00000254147	GGCUCAG
ENSG00000254147	UAAUAUC
ENSG00000254221	AAAACUG
ENSG00000254221	CACCAGC
ENSG00000254221	GGAGGCA
ENSG00000254221	UGCAUAG

ENSG00000254221	UGGCCAC
ENSG00000254245	AAAACUG
ENSG00000254245	CACCAGC
ENSG00000254245	GGAGGCA
ENSG00000254245	UGCAUAG
ENSG00000254245	UGGCCAC
